# Author Correction: Deep learning boosts sensitivity of mass spectrometry-based immunopeptidomics

**DOI:** 10.1038/s41467-021-24263-w

**Published:** 2021-06-23

**Authors:** Mathias Wilhelm, Daniel P. Zolg, Michael Graber, Siegfried Gessulat, Tobias Schmidt, Karsten Schnatbaum, Celina Schwencke-Westphal, Philipp Seifert, Niklas de Andrade Krätzig, Johannes Zerweck, Tobias Knaute, Eva Bräunlein, Patroklos Samaras, Ludwig Lautenbacher, Susan Klaeger, Holger Wenschuh, Roland Rad, Bernard Delanghe, Andreas Huhmer, Steven A. Carr, Karl R. Clauser, Angela M. Krackhardt, Ulf Reimer, Bernhard Kuster

**Affiliations:** 1 Computational Mass Spectrometry, Technical University of Munich (TUM), Freising, Germany; 2 Chair of Proteomics and Bioanalytics, Technical University of Munich (TUM), Freising, Germany; 3 JPT Peptide Technologies GmbH, Berlin, Germany; 4 Klinik und Poliklinik für Innere Medizin III, Klinikum rechts der Isar, School of Medicine, Technical University of Munich (TUM), Munich, Germany; 5 German Cancer Consortium (DKTK), partner site Munich; and German Cancer Research Center (DKFZ), Heidelberg, Germany; 6 Center for Translational Cancer Research (TranslaTUM), TUM School of Medicine, Technical University of Munich (TUM), Munich, Germany; 7 Institute of Molecular Oncology and Functional Genomics, TUM School of Medicine, Technical University of Munich (TUM), Munich, Germany; 8 Klinik und Poliklinik für Innere Medizin II, Klinikum rechts der Isar, School of Medicine, Technical University of Munich (TUM), Munich, Germany; 9 Broad Institute of MIT and Harvard, Cambridge, MA USA; 10 Thermo Fisher Scientific, Bremen, Germany; 11 Thermo Fisher Scientific, San Jose, CA USA; 12 Bavarian Biomolecular Mass Spectrometry Center (BayBioMS), Technical University of Munich (TUM), Freising, Germany

**Keywords:** Proteomics, Mass spectrometry, Machine learning, MHC, Molecular medicine

Correction to: *Nature Communications* 10.1038/s41467-021-23713-9, published online 7 June 2021.

In the original version of this Article, Fig. 5 was incorrectly referenced in place for Fig. 4 on three occasions in the Results subsection “Prosit rescoring questions the prevalence of proteasomal splicing of peptides”. The second sentence of the third paragraph reads “Fig. 5c shows an example where the spliced peptide proposed in the original study using Mascot (top panel, splice position indicated by the pipe symbol) and the canonical peptide proposed by rescored MaxQuant and rescored MSFragger (bottom panel) only differ by the isobaric amino acid combination of GVA and NL in the 3 C-terminal amino acids (Levenshtein distance of 3)”; it should read “Fig. 4c shows an example where the spliced peptide proposed in the original study using Mascot (top panel, splice position indicated by the pipe symbol) and the canonical peptide proposed by rescored MaxQuant and rescored MSFragger (bottom panel) only differ by the isobaric amino acid combination of GVA and NL in the 3 C-terminal amino acids (Levenshtein distance of 3)”. The first sentence in the fourth paragraph reads “In total, the re-analysis indicates that 1067 of the 1230 (87%) proposed spliced peptides are not conclusively supported by the mass spectrometry data (Fig. 5d)”; it should read “In total, the re-analysis indicates that 1067 of the 1230 (87%) proposed spliced peptides are not conclusively supported by the mass spectrometry data (Fig. 4d)”. The second sentence in the fourth paragraph reads “This is because either (i) they did not remain confident when using the Prosit-based rescoring pipeline (rejecting 596 spliced peptides, 48%), or (ii) the spliced and canonical peptides are I/L isomers (90 spliced peptides, 7%) that cannot be distinguished by mass spectrometry or (iii) a more confident canonical PSM was identified by MaxQuant and/or MSFragger (315 spliced peptides, 26%), or (iv) the proposed spliced peptide did not have a substantially better percolator score supporting its identification over a canonical peptide (66 peptides, 5%; also see the example in Fig. 5c, Supplementary Fig. S14, and Supplementary Notes)”; this should read “This is because either (i) they did not remain confident when using the Prosit-based rescoring pipeline (rejecting 596 spliced peptides, 48%), or (ii) the spliced and canonical peptides are I/L isomers (90 spliced peptides, 7%) that cannot be distinguished by mass spectrometry or (iii) a more confident canonical PSM was identified by MaxQuant and/or MSFragger (315 spliced peptides, 26%), or (iv) the proposed spliced peptide did not have a substantially better percolator score supporting its identification over a canonical peptide (66 peptides, 5%; also see the example in Fig. 4c, Supplementary Fig. S14, and Supplementary Notes)”.

This has been corrected in both the HTML and PDF versions of the Article.

