# Supplementary material for: Deep learning boosts sensitivity of mass spectrometry-based immunopeptidomics
Source: Nat Commun. 2021 Jun 7;12:3346. doi: 10.1038/s41467-021-23713-9 (PMC8184761; doi:10.1038/s41467-021-23713-9)
Supplement: Supplementary file 1 — Supplementary Information [file 41467_2021_23713_MOESM1_ESM.pdf]

Supplementary Figures and Notes

Deep learning boosts sensitivity of mass spectrometry-based immunopeptidomics

Contents

Supplementary Figures ... 3

Supplementary Figure S1 ... 3

Supplementary Figure S2 ... 4

Supplementary Figure S3 ... 5

Supplementary Figure S4 ... 6

Supplementary Figure S5 ... 7

Supplementary Figure S6 ... 8

Supplementary Figure S7 ... 9

Supplementary Figure S8 ... 10

Supplementary Figure S9 ... 11

Supplementary Figure S10 ... 12

Supplementary Figure S11 ... 13

Supplementary Figure S12 ... 14

Supplementary Figure S13 ... 15

Supplementary Figure S14 ... 17

Supplementary Figure S15 ... 19

Supplementary Notes ... 20

Characterization of the synthetic non-tryptic peptide standards ... 20

Comparison of SysteMHC to ProteomeTools ... 22

Prosit 2020 model training ... 23

Prediction quality for peptides dominantly fragmenting into neutral losses or internal ion series ... 24

Addition to Prosit boosts the number of identified HLA peptides from human cell lines ... 25

Addition to Prosit rescoring questions prior claims of proteasomal splicing of peptides ... 26

Supplementary References ... 30

Supplementary Figures

Supplementary Figure S1

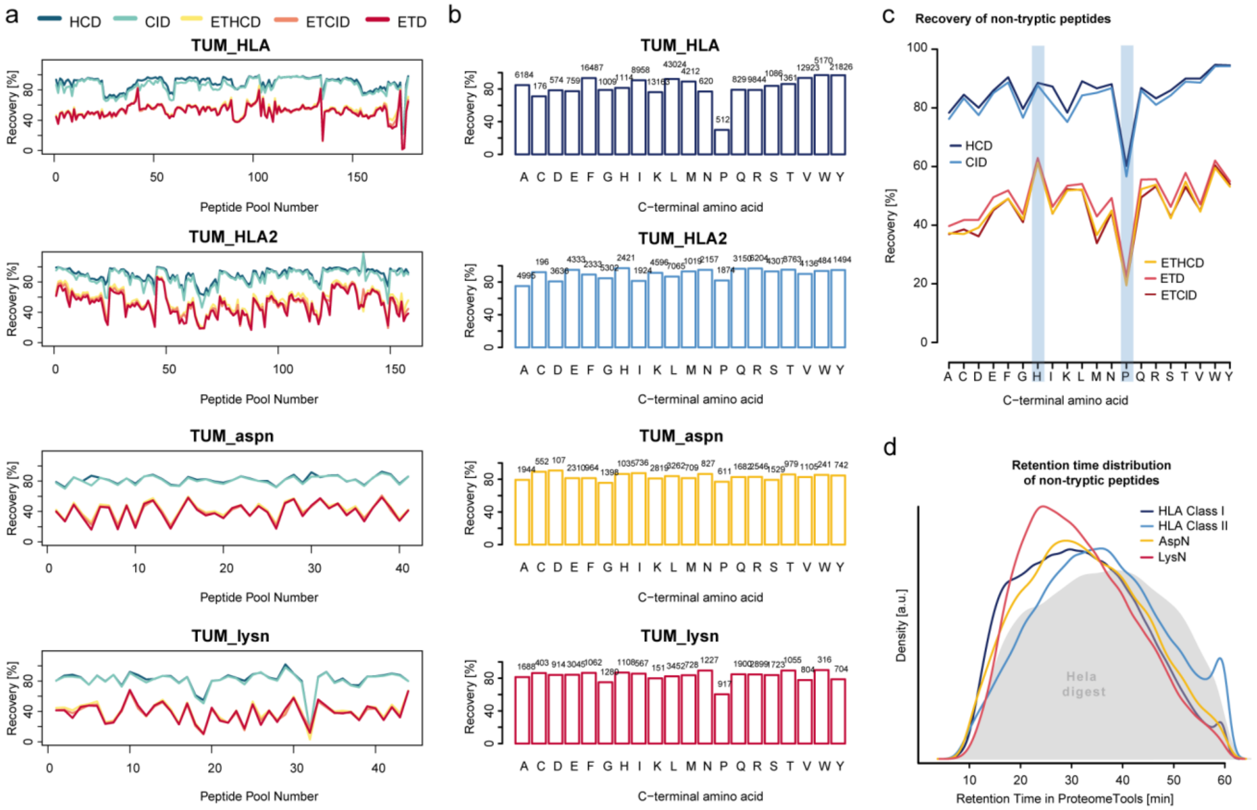

Peptide synthesis recovery and iRT.

(a) Percentage of synthesized peptides successfully identified by fragmentation method over peptide sets and peptide pools (of usually 1000 peptides).

(b) Percentage of synthesized peptides successfully identified over C-terminal amino acid, split by peptide set.

(c) Overall percentage of synthesized peptides successfully identified, split by fragmentation.

(d) Density of recorded peptide retention time split by different peptide sets. Trypsin HeLa digest on identical gradient for reference.

Supplementary Figure S2

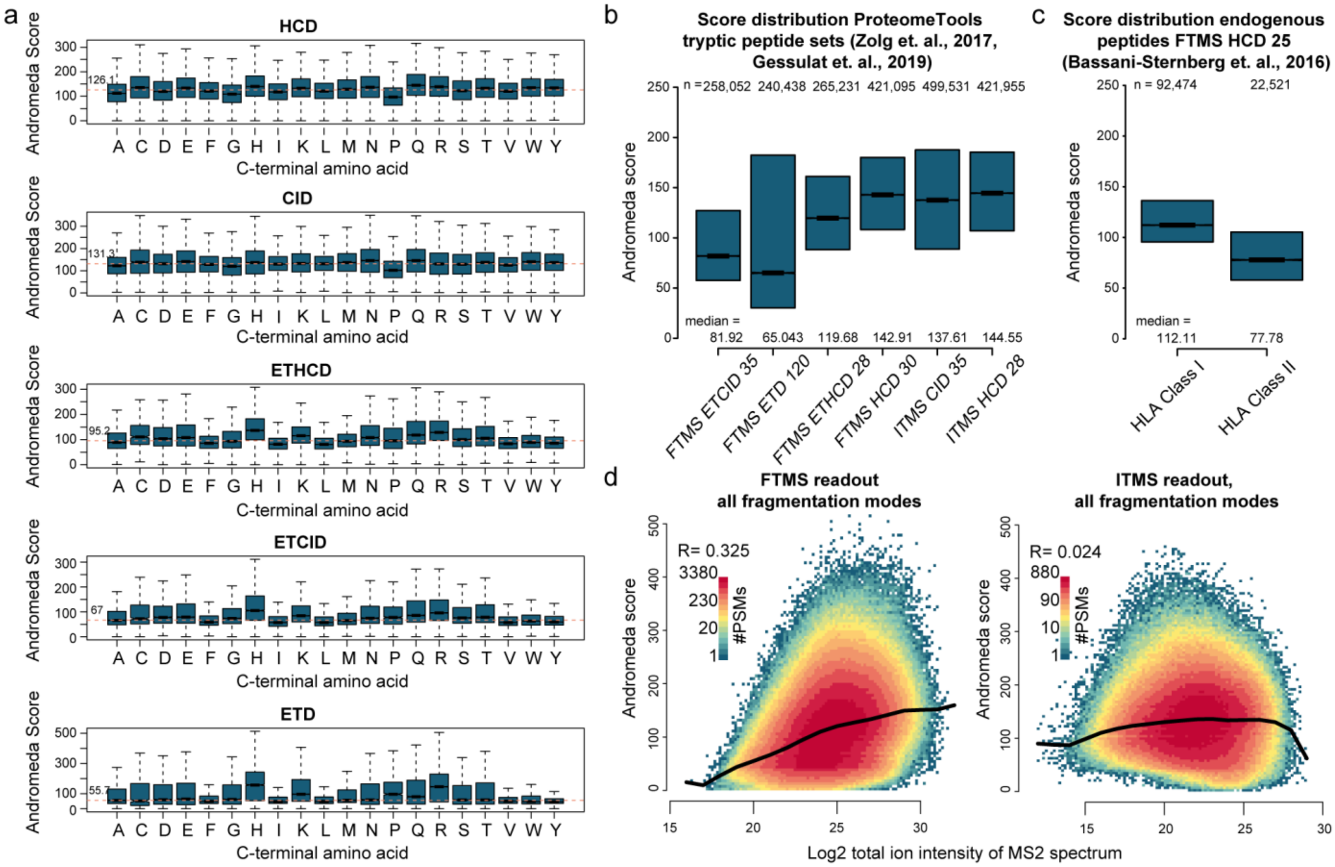

Scores over fragmentation methods.

(a) Boxplot of Andromeda scores achieved split by fragmentation method and peptide C-terminal amino acid. The highest scoring PSM per peptide sequence and fragmentation method were used as input. Whiskers indicate quantiles.

(b) Boxplot of Andromeda scores achieved split by fragmentation method for tryptic peptides contained in the ProteomeTools dataset. The box indicates the interquartile range (IQR). The black line marks the median, notches extend to 1.58 * IQR / sqrt(n), no whiskers or outliers outside IQR shown.

(c) Boxplot of Andromeda scores achieved split by HLA peptide class for endogenous peptides contained in the Bassani-Sternberg et. Al., 2016 publication.

(d) Dependency of achieved search engine scores and MS2 TIC for the Orbitrap mass analyzer (FTMS, top) and ion trap mass analyzer (ITMS, bottom). All PSMs from all fragmentation techniques were accounted for. The color scale reflects the logarithm of the underlying number of PSMs. The black line connects the median Andromeda score of 100 equal sized score bins along the intensity scale. The Pearson's R is indicated.

Supplementary Figure S3

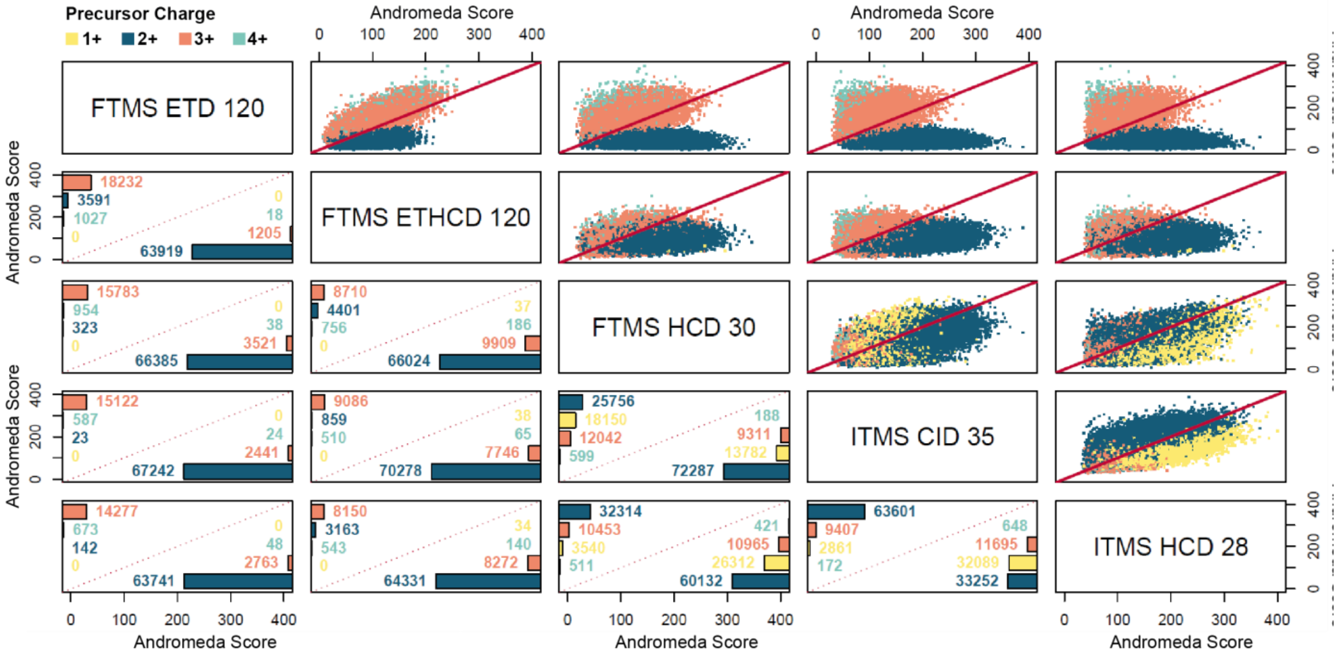

Fragmentation characteristics of HLA Class I peptides

Head-to-head comparison of different fragmentation methods for peptides from the HLA Class I set. For every fragmentation method, the highest Andromeda score of a modified sequence and precursor charge combination is compared as scatter plot and bar plot. Colors indicate precursor charge. Barplots are normalized within one comparison and indicate the amount of datapoints that are not clearly distinguishable in the scatter plot

Supplementary Figure S4

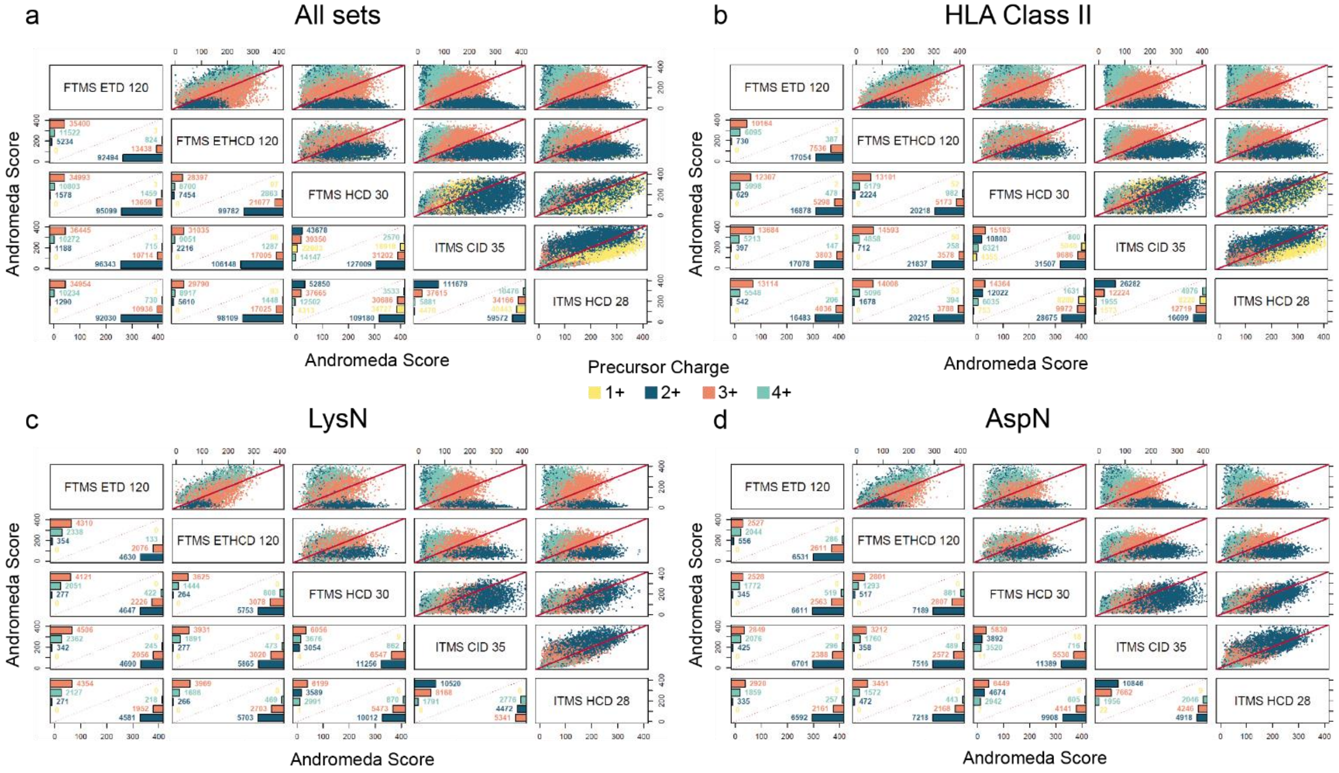

Fragmentation characteristics of peptide sets

(a-d) Head-to-head comparison of different fragmentation methods for different peptide sets. For every fragmentation method, the highest Andromeda score of a modified sequence and precursor charge combination is compared as scatter plot and bar plot. Colors indicate precursor charge. Barplots are normalized within one comparison and indicate the amount of datapoints that are not clearly distinguishable in the scatter plot.

Supplementary Figure S5

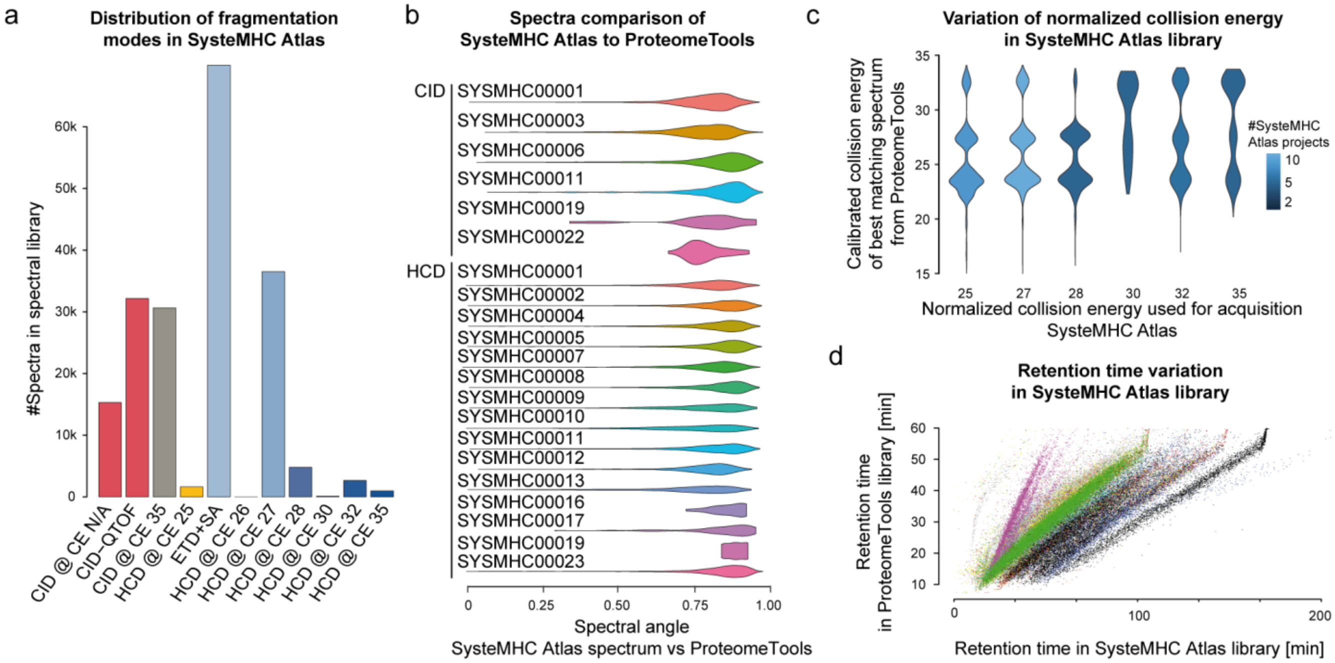

Comparison of the ProteomeTools resource to the SysteMHC Atlas resource

(a) Barchart of the number of spectra of HLA peptides available in the SysteMHC Atlas resource for the listed fragmentation settings, covering HCD (synonym CID-QTOF), CID, ETD + supplemental activation (SA).

(b) Beanplot of spectral angles when comparing b- and y-ions annotated in spectra from projects listed in the SysteMHC Atlas resource to the best matching spectra from the ProteomeTools resource for CID (top) and HCD (bottom).

(c) Beanplot of the calibrated collision energy (CE) from ProteomeTools of the best matching (highest spectral angle) spectra for each spectrum and collision energy available in the SysteMHC Atlas resource. The color of the beans indicate the number of projects which contain spectra annotated with collision energies of 25, 27, 28, 30, 32 or 35.

(d) Scatterplot of retention times of HLA peptides available in the SysteMHC Atlas resource (x-axis) and the ProteomeTools resource (y-axis) colored by the SysteMHC Atlas project the HLA peptide originates from.

Supplementary Figure S6

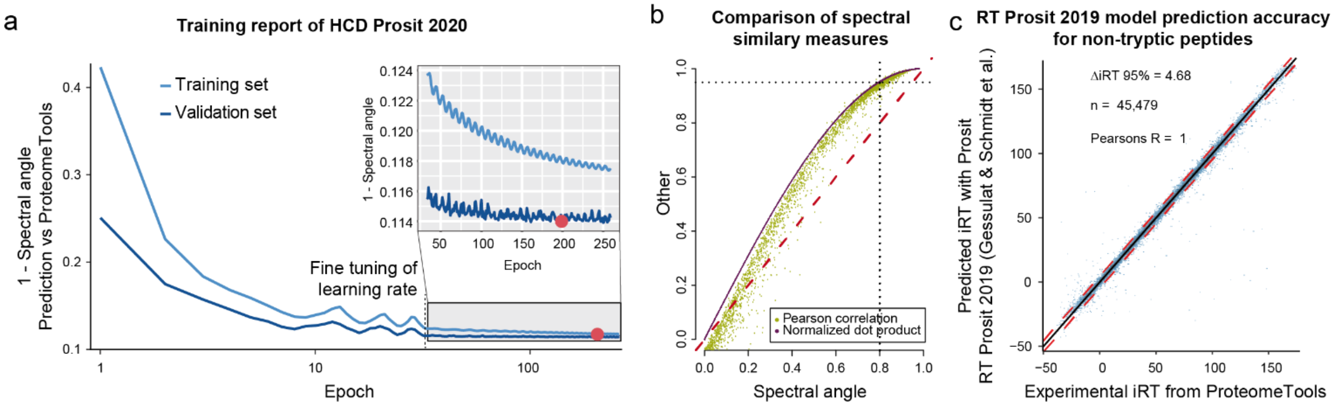

Deep learning framework Prosit for high quality non-tryptic peptide fragment intensity and retention time prediction

(a) The deep learning framework Prosit was trained on the combination of data available prior to this study (Tryptic peptides, top left panel; Tryptic extension, top middle panel) and non-tryptic peptides (top right panel) from the ProteomeTools resource. Bottom panel depicts the spectrum loss (1 – spectral angle of predictions compared to spectra from ProteomeTools) for the training (light blue) and validation (dark blue) set when learning the new non-tryptic HCD model for Prosit for ~250 epochs. Early stopping was used to stop training and the model with the lowest validation loss (red dot) was used as the final model. After ~30 epochs, the learning rate was adjusted (dashed line). The inset shows the train and validation loss of Prosit from this point onwards.

(b) Comparison of spectral angle (x-axis) against Pearson correlation and normalized dot product (y-axis). A spectral angle of 0.8 (20% left to maximum) corresponds to a Pearson correlation and normalized dot product of about ~0.95 (5% left to maximum). The red dashed line shows the diagonal.

(c) Scatter plot of predicted indexed retention times (iRT) with the 2019 iRT Prosit model compared to experimentally determined iRTs of HLA class I, HLA class II, AspN and LysN peptides contained in the holdout set of the 2020 HCD Prosit model. The solid black line indicates the diagonal. The dashed red lines show the delta iRT necessary to encompass 95% of the peptide. The Pearsons R is indicated in the top.

Supplementary Figure S7

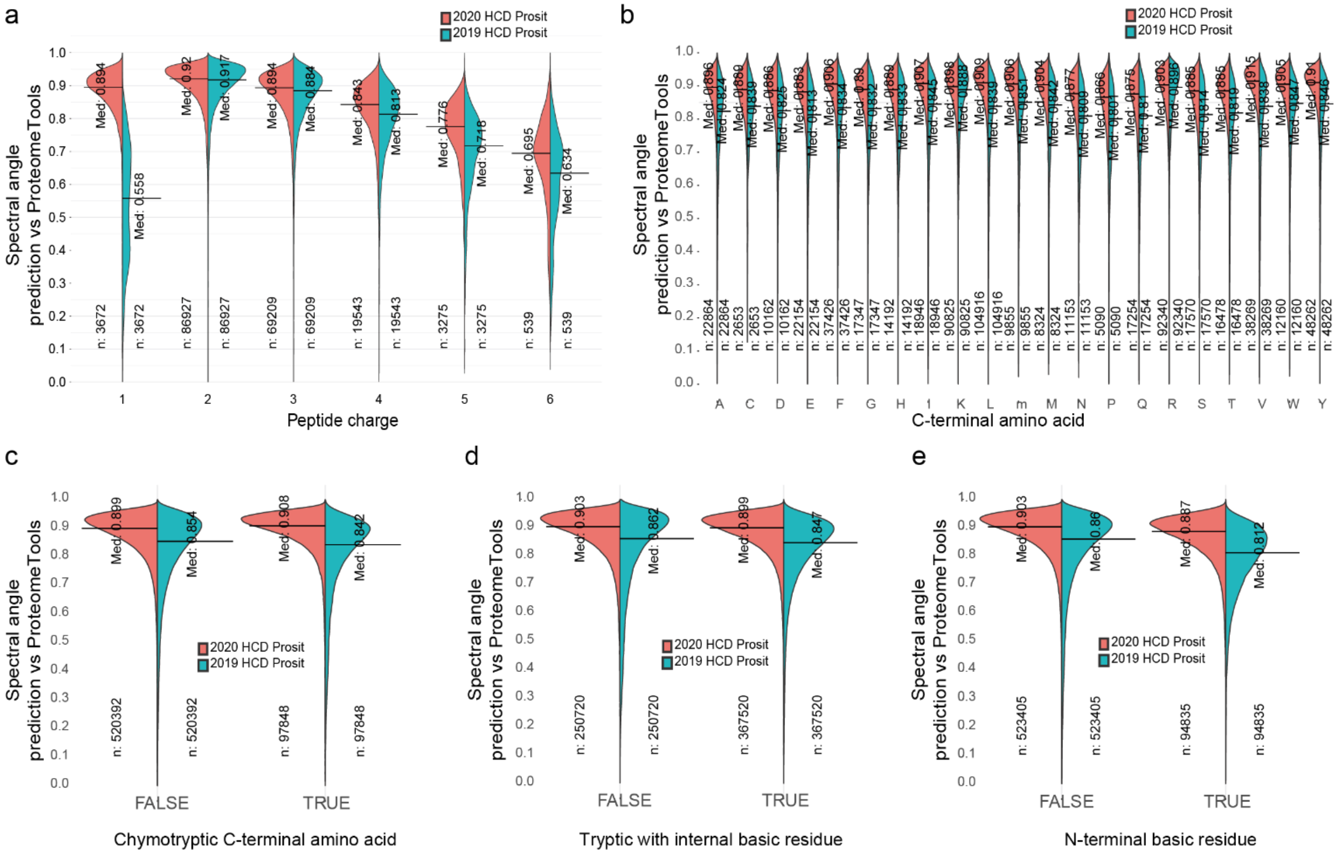

Performance differences between the 2020 HCD Prosit vs 2019 HCD Prosit

(a) Beanplot comparing the prediction accuracy of the new non-tryptic 2020 HCD Prosit model (red, this study) against the the 2019 HCD Prosit model (green, Gessulat et. al, 2019) trained on the tryptic datasets for different precursor charge states. The number of underlying spectra is indicated at the bottom. The black solid line and corresponding numbers indicate the median spectral angle for each bean.

(b) Beanplot comparing the prediction accuracy of the 2020 HCD Prosit against the 2019 HCD Prosit model for all C-terminal amino acids.

(c) Beanplot comparing the prediction accuracy of the 2020 HCD Prosit against the 2019 HCD Prosit model for peptides classified from chymotryptic origin (C-terminal amino acid Y, W, or F)

(d) Beanplot comparing the prediction accuracy of the 2020 HCD Prosit against the 2019 HCD Prosit model for peptides containing both a tryptic C-terminus (K or R at C-terminus) and at least one additional basic amino acid residue (H, K or R) within the peptide.

(e) Beanplot comparing the prediction accuracy of the 2020 HCD Prosit against the 2019 HCD Prosit model for peptides containing a basic amino acid residue (H, K or R) as N-terminal amino acid and no basic residue at the C-terminus.

Supplementary Figure S8

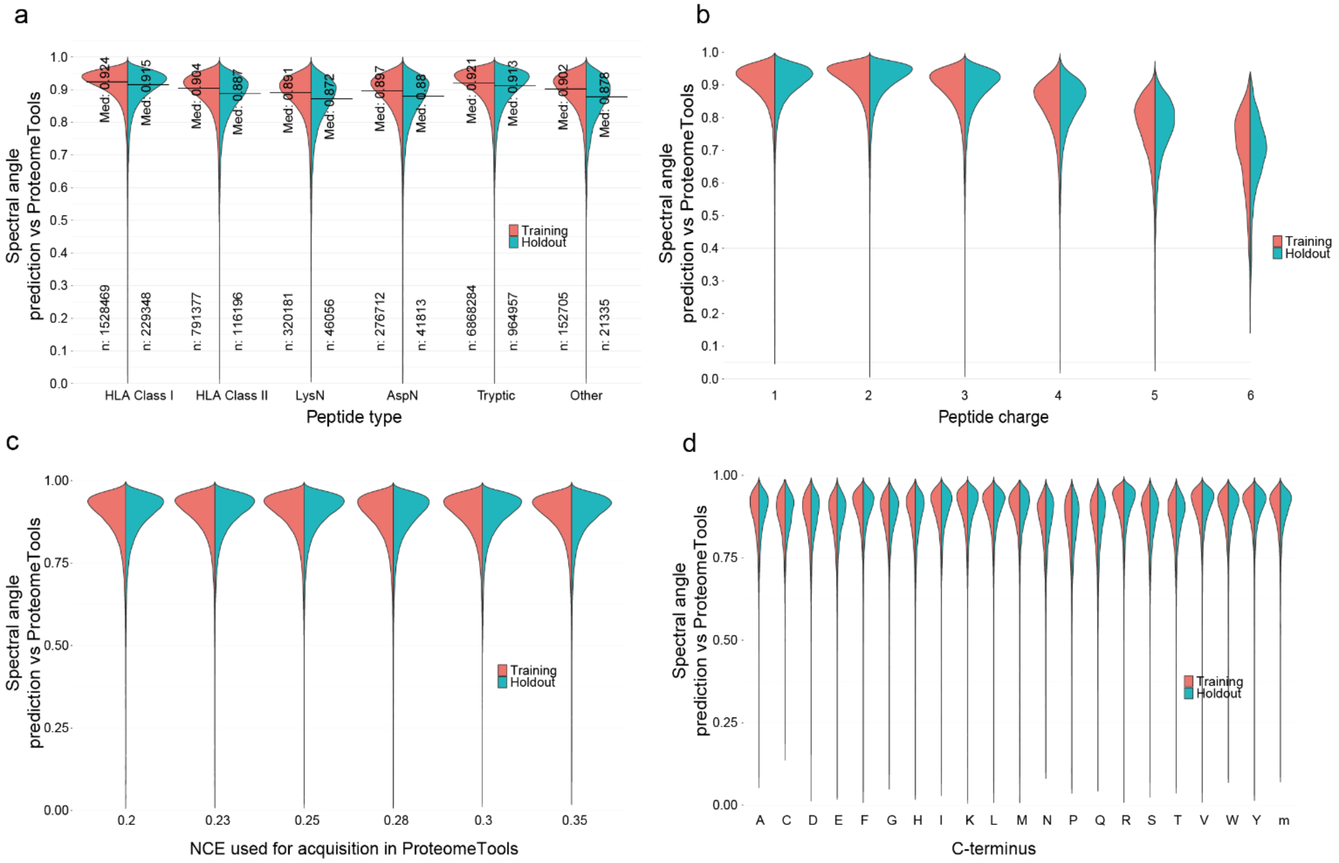

Performance deep learning framework HCD Prosit 2020 model

(a) Beanplot comparing the prediction accuracy of the new non-tryptic 2020HCD Prosit model (red, this study) against the training and holdout dataset for the four newly introduced peptides sets (HLA class I, HLA class II, LysN, and AspN) and the previously published tryptic peptides. The number of underlying spectra is indicated at the bottom. The black solid line and corresponding numbers indicate the median spectral angle for each bean.

(b) Beanplot comparing the prediction accuracy of the new non-tryptic 2020 HCD Prosit model (red, this study) against the training and holdout dataset for different precursor charge states.

(c) Beanplot comparing the prediction accuracy of the new non-tryptic 2020 HCD Prosit model (red, this study) against the training and holdout dataset across normalized collision energies.

(d) Beanplot comparing the prediction accuracy of the new non-tryptic 2020 HCD Prosit model (red, this study) against the training and holdout dataset across the peptide n-terminal amino acid.

Supplementary Figure S9

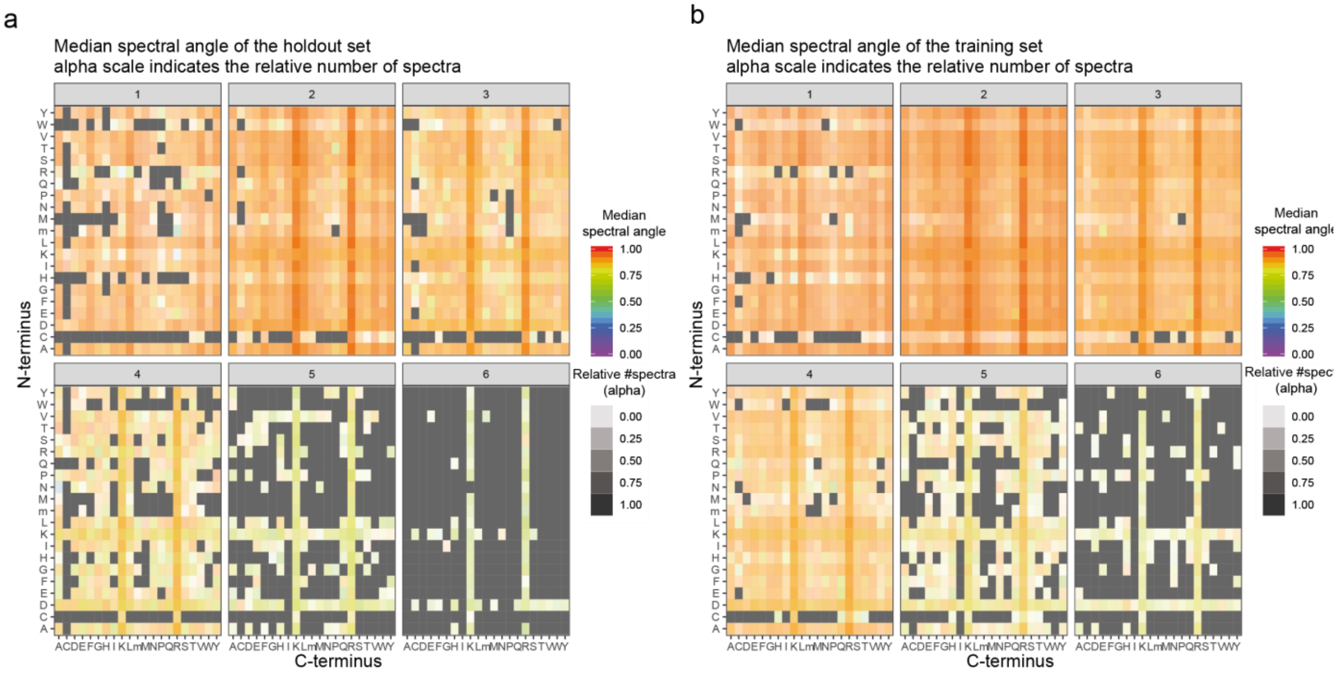

Performance deep learning framework Prosit 2020 HCD model across terminal amino acids.
(a-b) Heatmaps of median spectral angles of spectra separated by N- (y-axis) and C-terminal (x-axis) amino acid for singly, doubly and triply charged precursors achieved by the 2020 HCD Prosit model compared to peptides contained in the holdout set. A small "m" indicates oxidized methionine. The alpha value of the color was used to indicate the relative number of spectra contained in each precursor charge state.

Supplementary Figure S10

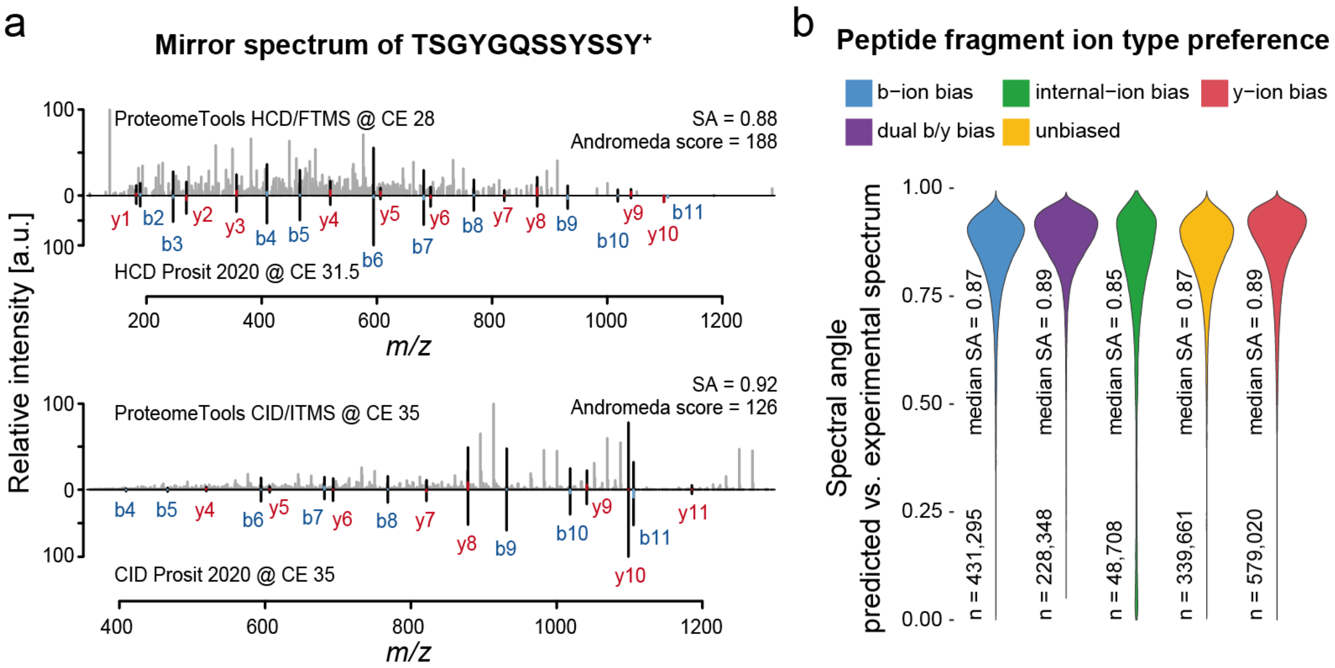

Performance deep learning framework Prosit 2020 HCD model for complicated peptides.

(a) Mirror spectrum of the singly charged peptide TSGYGQSSYSSY between the HCD ProteomeTools spectrum (top panel, top spectrum) to its prediction by the 2020 HCD Prosit model (top panel, bottom spectrum) and the CID ProteomeTools spectrum (bottom panel, top spectrum) and its prediction by the 2020 CID Prosit model (bottom panel, bottom spectrum). Fragment ions are annotated in blue and red for b- and y-ions, respectively. Matching peaks (present in both spectra) are visualized in black whereas peaks only present in the top (experimental) spectrum are annotated in grey.

(b) Beanplot showing the spectral angle distribution (prediction vs. experimental spectra) of 2020 HCD Prosit predicted spectra compared to spectra confidently identified by Sarkizova & Klaeger et al. The annotation and identified peptides from SpectrumMill HLA v2 was used to group peptides based on their fragmentation preference.

Supplementary Figure S11

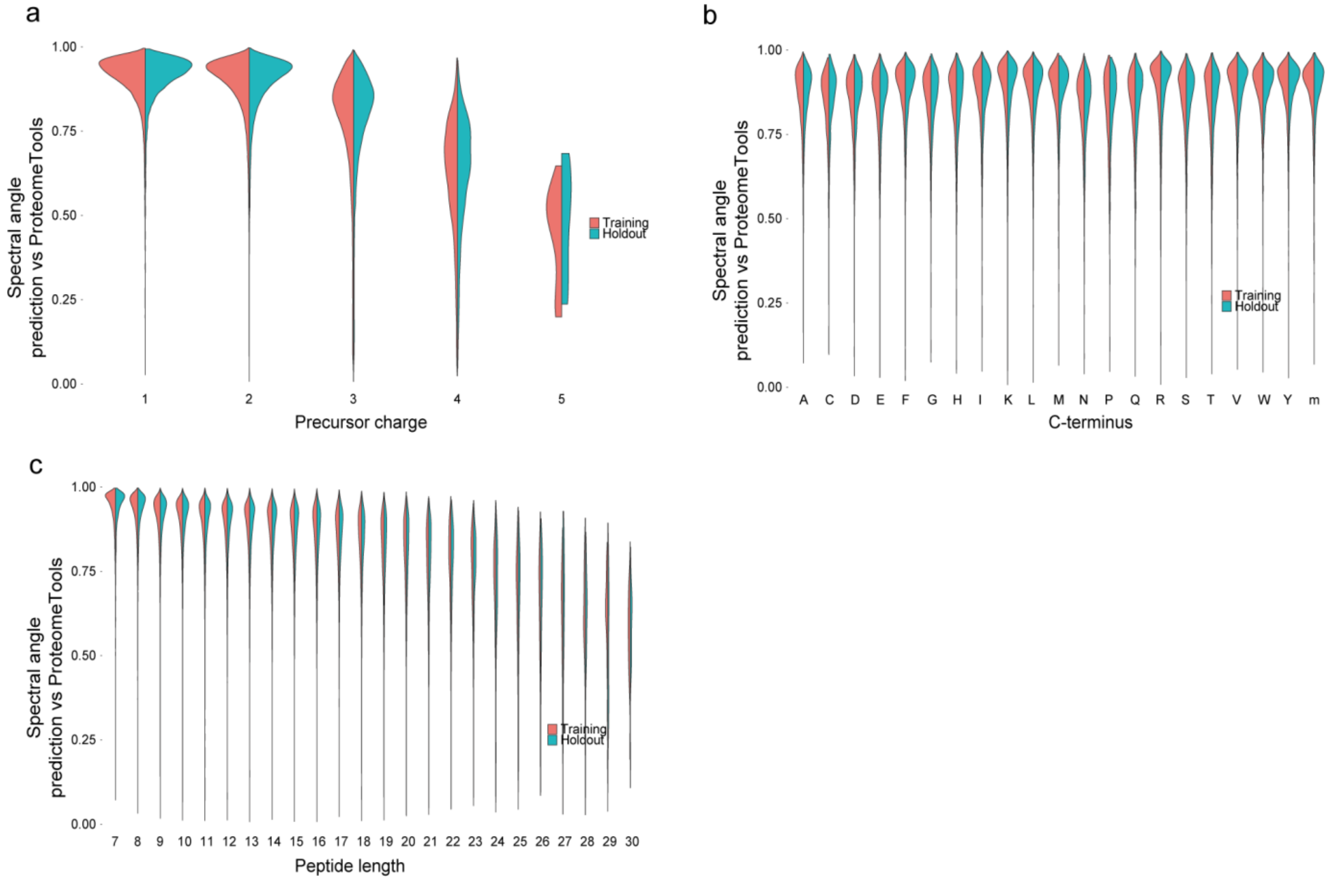

Performance deep learning framework Prosit 2020 CID ITMS model.

(a) Beanplot comparing the prediction accuracy of the new non-tryptic 2020 CID ITMS Prosit model (red, this study) against the training and holdout dataset across peptide precursor charge state. The number of underlying spectra is indicated at the bottom. The black solid line and corresponding numbers indicate the median spectral angle for each bean.

(b) Beanplot comparing the prediction accuracy of the new non-tryptic 2020 CID ITMS Prosit model (red, this study) against the training and holdout dataset across the peptide n-terminal amino acid.

(c) Beanplot comparing the prediction accuracy of the new non-tryptic 2020 CID ITMS Prosit model (red, this study) against the training and holdout dataset across peptide length

Supplementary Figure S12

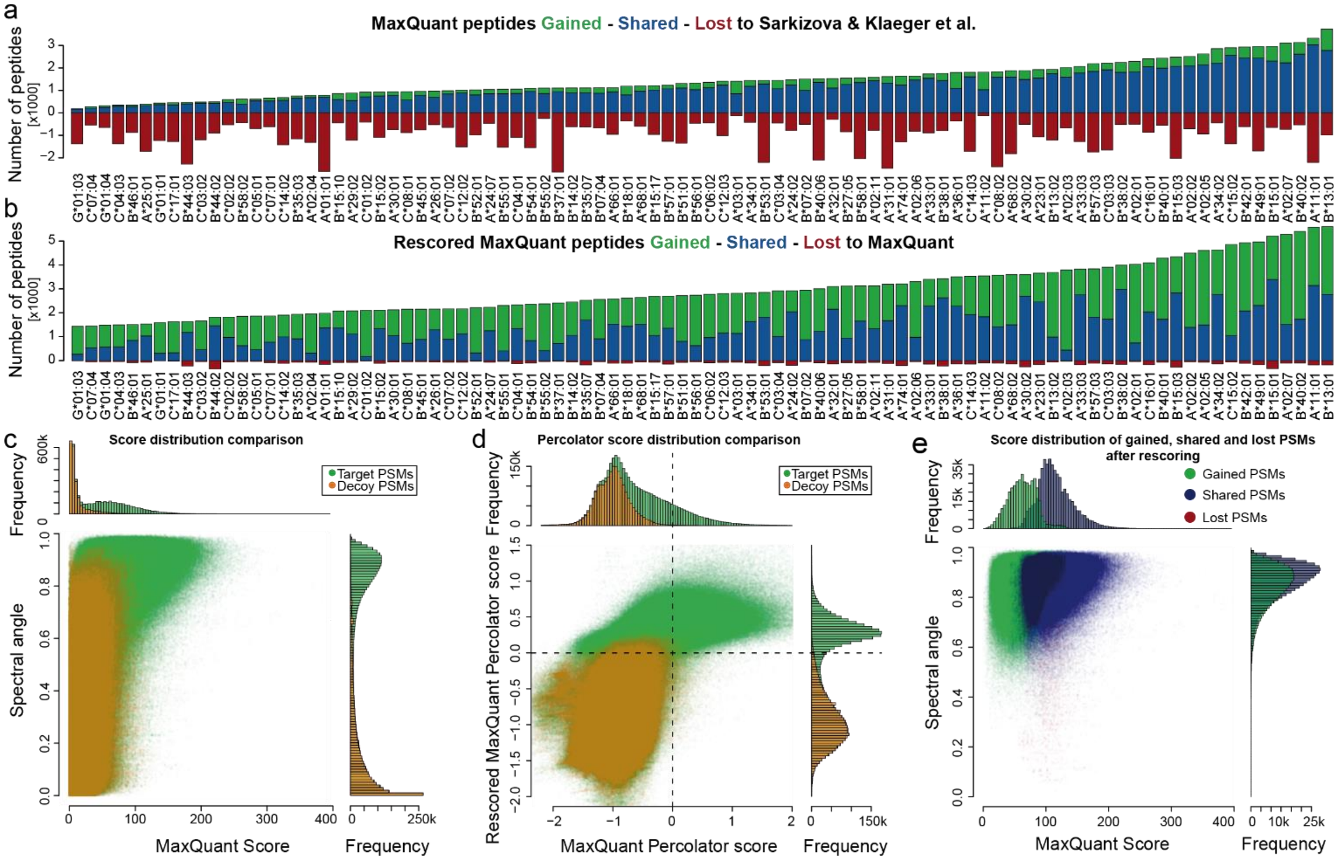

Rescoring Sarkizova & Klaeger et al. dataset.

(a) Vennbar showing the number of peptides lost (red), shared (blue) and gained (green) when comparing results obtained from the MaxQuant without rescoring to the result published by Sarkizova & Klaeger et al. using Spectrum Mill HLA v2.

(b) Vennbar showing the number of peptides lost (red), shared (blue) and gained (green) when comparing results obtained from the results obtained from rescored MaxQuant to MaxQuant without rescoring.

(c) Correlation of the MaxQuant score (Andromeda) to spectral angle (prediction vs experimental spectrum) for target (green) and decoy (orange) PSMs during the rescoring process. The marginal histograms show the separation of target and decoy PSMs for each score.

(d) Correlation of Percolator scores for all target (green) and decoy (orange) PSMs from either the Andromeda or Prosit set of scores. The marginal histograms show the separation power of the different feature sets.

(e) Correlation of MaxQuant score (Andromeda) to spectral angle (prediction vs experimental spectrum) for all confident PSMs identified by either MaxQuant or rescored MaxQuant. The PSMs gained (green), shared (blue) and lost (red) by rescoring with the Prosit 2020 model are shows separately.

Supplementary Figure S13

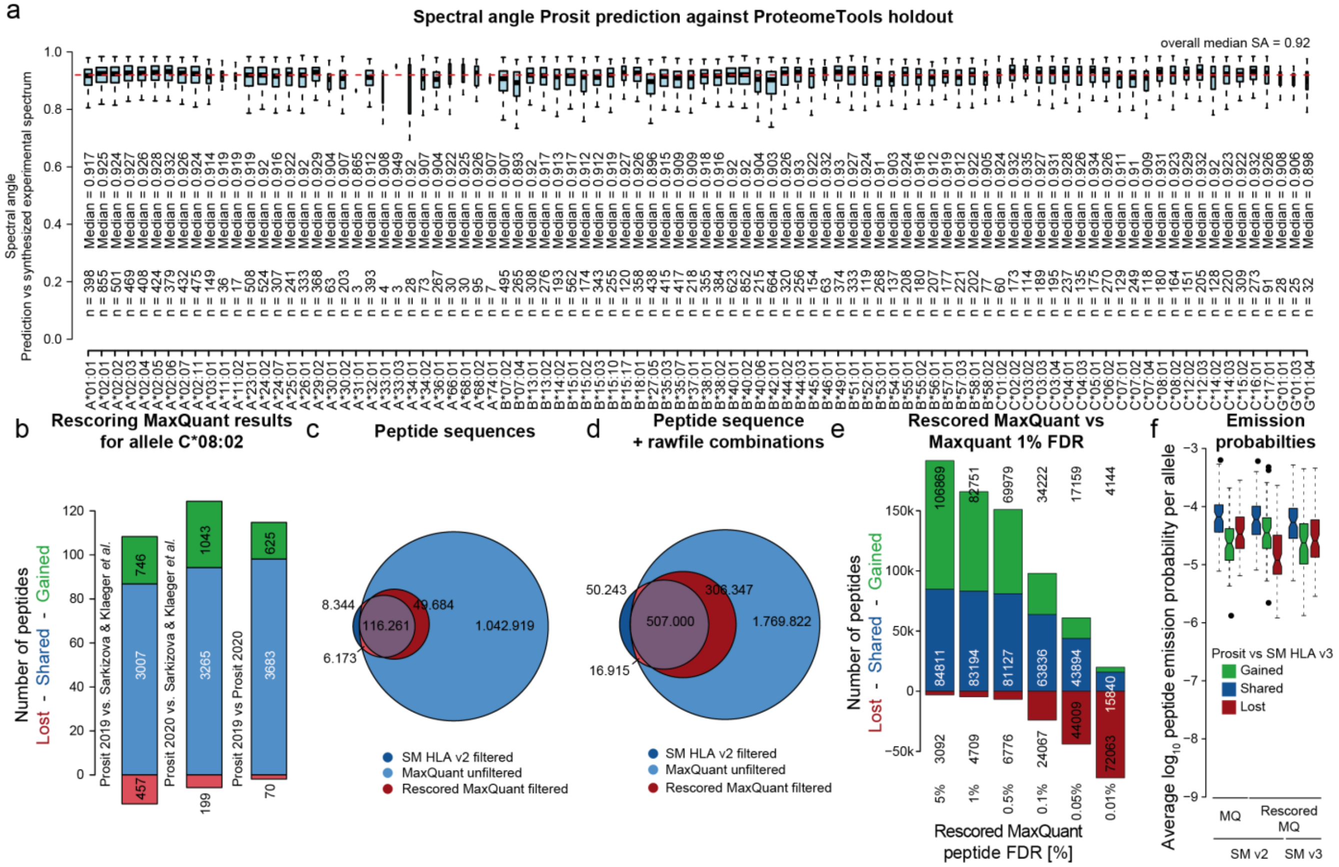

Rescoring Sarkizova & Klaeger et al. dataset.

(a) Spectral angle distribution comparing spectra generated by the Prosit 2020 HCD model against experimental spectra in the holdout dataset. Peptides in the holdout set were assigned to a specific allele if that peptide sequence was confidently identified by Sarkizova & Klaeger et al. The box indicates the interquartile range (IQR). The black line marks the median, whiskers extend to 1.5 * IQR from the hinge. Data outside whiskers are not plotted.

(b) Vennbar comparing the result obtained when rescoring the unfiltered MaxQuant results of allele C*08:02 with either the Prosit 2019 HCD model (left bar) or with the Prosit 2020 HCD model (middle bar). With the Prosit 2020 HCD model, a larger overlap (blue), increase (green) and smaller loss (red) of peptide is visible. Overall, the Prosit 2020 HCD model increase the number of confidently identified HLA peptides by >15% in comparison to the Prosit 2019 HCD model (right bar).

(c) Venn diagram of peptide sequences from Spectrum Mill (SM) HLA v2 (Sarkizova & Klaeger et al.) in dark blue, the input to Prosit rescoring (MaxQuant unfiltered) in light blue and 1% FDR filtered results obtained by rescoring the MaxQuant results in red. 8344 cannot be confidently identified by Rescoring MaxQuant.

(d) Venn diagram of the combination of peptide sequence and rawfile from Spectrum Mill HLA v2 (Sarkizova & Klaeger et al.) in dark blue, the input to Prosit rescoring (MaxQuant unfiltered) in light blue and the 1% FDR filtered results by rescoring in red. The fraction of peptides not shared between SM and MaxQuant increased in comparison to (d).

(e) Vennbar of comparing the number of HLA peptides gained, shared and lost at varying q-value cutoffs (x-axis) from the rescored MaxQuant results compared to the result obtained from a 1% MaxQuant search.
(f) Boxplot of the average emission probabilities per allele of peptides shared (blue), gained (green) and lost (red) when comparing MaxQuant to Spectrum Mill HLA v2 (first three boxes), Rescored MaxQuant to Spectrum Mill HLA v2 (middle three boxes) and Rescored MaxQuant to Spectrum Mill HLA v3 (last three boxes). The number of alleles (n) for which an average emission probability was calculated is depicted at the bottom. The box indicates the interquartile range (IQR). The black line marks the median, notches extend to 1.58 * IQR / sqrt(n), whiskers to 1.5 * IQR from the hinge. Data outside whiskers are plotted individually as black dots.

Supplementary Figure S14

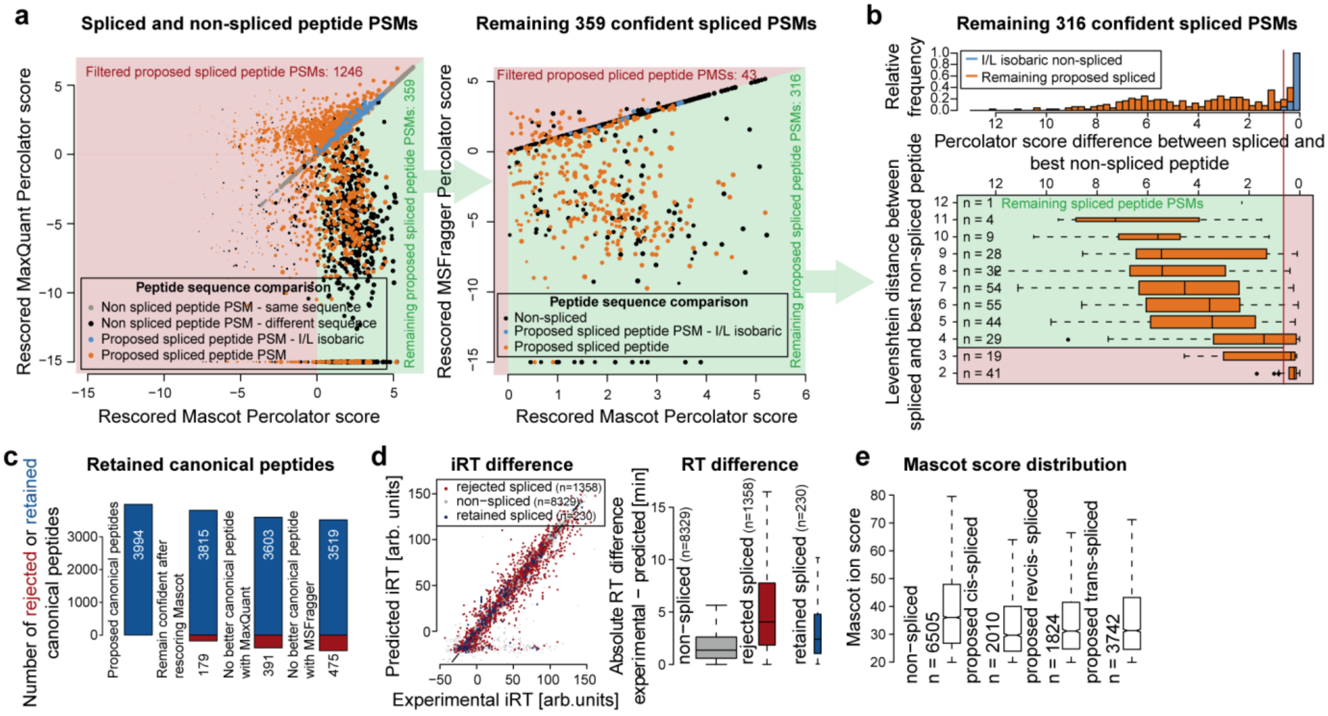

Re-assessment of proposed spliced peptide events by deep learning.

(a) Scatter plots of spectral confidence scores (discrimination score) estimated by Percolator for peptide spectrum matches (PSMs) derived from the rescored MaxQuant (y-axis) and rescored Mascot (x-axis) results for PSMs reported in the original study (left panel) and rescored MSFragger (y-axis) and rescored Mascot (x-axis) results for PSMs in the green shaded area in the Mascot/MaxQuant comparison (right panel). Non spliced peptides are shown in grey if the peptide sequence proposed by MaxQuant is equal (replacement of I/L allowed, I/L isobaric) to the one proposed by Mascot, or black in case they differ. Proposed spliced peptides are shown in light blue if their proposed peptide sequence is I/L isobaric with the peptide sequence proposed by MaxQuant (left panel) or MSFragger (right panel), or orange in case the peptide sequence differ. A discrimination score of 0 corresponds to an estimated PSM FDR of 1%. The red shaded region marks the area where the discrimination score suggests non-confidence (<0) or the discrimination score of the MaxQuant rescoring pipeline (left panel) or MSFragger rescoring pipeline (right panel) is higher (diagonal) than that of the Mascot rescoring pipeline, respectively. Spectra not identified by either MaxQuant or MSFragger were set to a discrimination score of -15. Note that the MaxQuant and MSFragger search was performed without the addition of spliced peptides and that the three workflows shared the same Percolator model to enable comparison. The size of the dots is proportional to the spectral angle comparing the experimental spectrum to the HCD Prosit 2020 predicted spectrum of the peptide sequence proposed by the original Mascot search.

(b) The top panel shows the normalized marginal discrimination score difference of the boxplot below (orange) and the distribution of the distribution of the discrimination score difference for spectra where MaxQuant and MSFragger differ in their proposed peptide sequence exactly only by replacing I to L or vice versa (I/L isobaric, light blue). The red line indicates the 99%ile of the light blue distribution marking the discrimination score difference threshold under which no significant difference between any two PSMs is deemed possible. The red shaded region in the boxplot marks the are where proposed spliced peptide PSMs cannot be “significantly”

differentiated to their non-spliced peptide PSMs counterpart. The bottom panel shows a boxplot of the distribution of the discrimination score difference between a proposed spliced PSM (Mascot) and its highest scoring non-spliced peptide PSMs (MaxQuant and MSFragger) for different Levenshtein distance values. The Levenshtein distance measures the number of substituted, removed or added amino acids when comparing the proposed spliced peptide sequence to the highest scoring non-spliced peptide sequence. The box indicates the interquartile range (IQR), its whiskers 1.5× IQR values, and the black line the median.

(c) Barplot of the number of peptides rejected (red) and retained (blue) after various consecutive quality assurance filtering steps on non-spliced proposed peptides by Liepe et al.

(d) Analysis of the difference in predicted and experimental (observed) retention time of non-spliced and proposed spliced peptides. Scatter plot (left side) shows the experimental indexed retention time (iRT) in comparison to the predicted iRT for non-spliced (gray), rejected proposed spliced petides (red, see Main Figure 4) and retained proposed spliced peptides (blue, see Main Figure 4). Boxplot (right side) shows the diference between predicted and experimental retention time (RT) for the same peptide classes. N=X underlying PSMs. The box indicates the interquartile range (IQR). The black line marks the median, whiskers extend to 1.5 * IQR from the hinge. Data outside whiskers are not plotted.

(e) Boxplot of the Mascot ion score distribution of non-spliced and proposed cis, reverse-cid and trans spliced peptides from Specht et al. 2020 acquired on a Q Exactive Orbitrap (HCD fragmentation) at the Max Plank Institute. N=X underlying PSMs. The box indicates the interquartile range (IQR). The black line marks the median, notches extend to 1.58 * IQR / sqrt(n), whiskers to 1.5 * IQR from the hinge. Data outside whiskers are not plotted.

Supplementary Figure S15

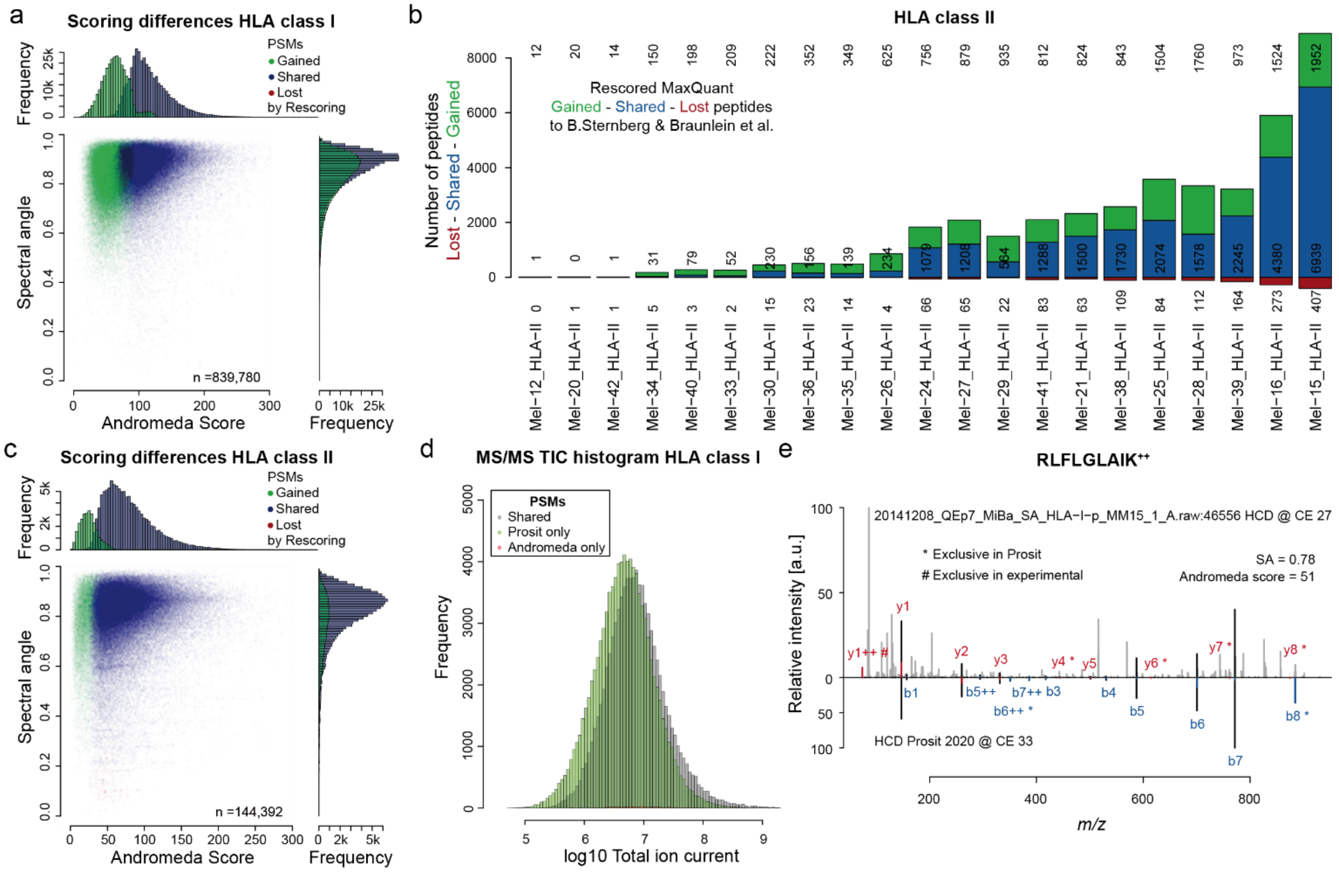

Prosit rescoring of 25 patient melanoma samples.

(a) Correlation of MaxQuant score (Andromeda) to spectral angle (prediction vs experimental spectrum) for all confident PSMs identified by either MaxQuant or rescored MaxQuant for the HLA class I dataset. The PSMs gained (green), shared (blue) and lost (red) by rescoring with the Prosit 2020 model are shows separately.

(b) Vennbar showing the number of peptides lost (red bar, top numbers), shared (blue, middle numbers) and gained (green, bottom numbers) when comparing results for HLA Class II peptides obtained from the MaxQuant rescoring pipeline to the results obtained from plain MaxQuant (top panel) for each patient published in B.-Sternberg & Bräunlein et al.

(c) Correlation of MaxQuant score (Andromeda) to spectral angle (prediction vs experimental spectrum) for all confident PSMs identified by either MaxQuant or rescored MaxQuant for the HLA class II dataset. The PSMs gained (green), shared (blue) and lost (red) by rescoring with the Prosit 2020 model are shows separately.

(d) Histogram showing the total ion current (TIC) of tandem mass spectra (MS/MS) confidently identified exclusively by MaxQuant (red) or Prosit (green) in comparison to MS/MS spectra identified by both (gray).

(e) Mirror spectrum of the experimental spectrum matched to the mutated peptide RLFLGLAIK (gene KIF2C) from patient Mel15 that exhibited strong responses in the immunogenicity assay and the respective Prosit prediction. Fragment ions are annotated in blue and red for b- and y-ions, respectively. Matching peaks (present in both spectra) are visualized in black whereas peaks only present in the top (experimental) spectrum are annotated in grey.

Supplementary Notes

Characterization of the synthetic non-tryptic peptide standards

Figure 1c demonstrates that only 0.2 % of peptides within ProteomeTools have a C-terminal cysteine residue and only 0.9 % of peptides have an N-terminal cysteine residue. These numbers however are not due to peptide synthesis bias or mass spectrometric bias as Supplemental Figure S1c demonstrate a >80% recovery rate for synthesized peptides containing a C-terminal cysteine but were underrepresented in the resources that ProteomeTools peptide sequences were selected from. In brief, we assembled peptides from ProteomicsDB (proteotypic dataset); *insilico* generated peptides for genes lacking mass spectrometric evidence (missing gene dataset) as well as the HLA peptide resources IEDB, SysteMHC as well as important publications (HLA peptides and other non-tryptic proteases). Here, peptides with cysteine as C-terminus or N-terminus seem to be gravely underrepresented. This is partially due to the immunopeptidomics sample workflow that often skips alkylating cysteine residues during sample processing. Under non-reducing conditions, such unmodified cysteine residues are prone to form intramolecular disulfide bridges and are not identified in subsequent LC-MS analysis. Even studies that used an alkylation reagent in the lysis buffer identified few peptides with cysteine residues on N- or C-term, e.g. in the 2016 Bassani-Sternberg study[2] only contained 13 HLA peptides with N-terminal cysteine as well as 69 HLA peptides with C-terminal cysteine are identified. Taken together, the ProteomeTools resource reflects the overall underrepresentation of such peptides in proteomic datasets.

The elution profiles of the peptide over the chromatographic gradient were compared to a classical HeLa cell line digest. Synthesized peptide sets contained overall more hydrophilic peptides then the cell line digest (Supplementary Figure S1d). This calls for more shallow gradients compared to tryptic digests except the for-HLA Class II peptides, which showed a comparable elution profile while containing mostly peptides with length >=15 and a small fraction of very hydrophobic peptides that was not properly chromatographically resolved.

When comparing the Andromeda scores across 11 different fragmentation settings (including 6 HCD collision energies) the results corroborated previous findings: CID and HCD (both FTMS and ITMS) achieved the highest overall scores, with a flat bell-shaped curve of scores over HCD collision energy. ETD and combined scan modes did not work well for HLC Class I, as most precursors were only doubly charged (data not shown), while more than doubly charged precursors from the AspN and LysN set resulted in high scores. In terms of the dependency of scores and C-terminal amino acid, the only observable bias concerned peptides with a c-terminal proline residue which resulted in considerably lower scores for CID/HCD fragmentation. Obviously, peptides with c-terminal basic amino acid residues scores well in ETD based scan types (Supplemental Figure S2a).

Over the years, studies have been employed several different fragmentation methods for non-tryptic peptides and HLA peptides, also driven by new instrument generations and the development of combined fragmentation modes which have been suggested to extend the coverage. Using the synthetic dataset, we assessed different fragmentation modes and analyzers by pairwise comparing the highest obtained score by modified-sequence-charge-fragmentation-analyzer combination (Supplemental Figure S3 for HLA Class I peptides, Supplemental Figure S4 for global analysis and other sets). Here, only in a small, charge state 3 and more subset of HLA Class I peptides worked well in ETD and ETHCD, while singly charged precursors were not identified at all. In addition, the slow scan speed of resulted in a much more shallow sampling of the sample and is not recommended for the analysis of HLA samples, unless used as additional fragmentation technique in a multiplexed LC-MS run also employing either HCD or CID[1]. Between CID and HCD, the score distributions are relatively wide with CID yielding slightly higher scores. Overall, both HCD and CID present a good compromise for all charge states (Supplemental Figure S4c, right column). Again, the mass analyzer makes a difference with ITMS readout working especially well for singly charged peptides where fewer and less intense fragments were observed.

A much stronger effect on the search engine scores achieved had the choice of the mass analyzer. Here, the sensitive but low accuracy ion trap (ITMS) showed no correlation (R=0.02) of scores and MS2 TIC and seems better

suited for the analysis low abundant analytes in comparison to the Orbitrap which showed a much stronger dependency between MS2 TIC and achieved search engine scores (FTMS ; Supplemental Figure S2d). This suggests that even peptides very close to the detection limit can achieve reasonably high search engine scores using the sensitivity of the ion trap, a clear advantage for the analyses of substoichiometric HLA peptide pulldowns.

Comparison of SysteMHC to ProteomeTools

The peptides sequences selected for synthesis originated from publicly available data or HLA repositories aggregating several studies [2-4]. The overlaps of the individual resources reveal quite some non-overlap of stored sequences, due to the vast diversity of HLA sequences, applied wet lab workflows, readout and data analysis pipeline (Figure 1b). Hence, the synthetic standards generated only cover a subset a proportion of the ever-identified HLA sequences, particularly due to recent updates to the resources past sampling the peptide sequences back in 2017. However, the overlapping proportion of peptides and spectra can be used to assess the suitability of gathered community data for the analysis of immunopeptidomes. The SysteMHC Atlas[4] aims provide a baseline of the immunopeptidome by reanalyzing 23 studies and providing a spectral library from the combined results. By nature, such a collection of spectra will be of heterogeneous nature, as multiple fragmentation methods, collision energies, setting, and instruments were used for data collection (Supplementary Figure S5a).

We tested the spectral quality of the spectra library against the respective synthetic peptide spectra to get a feeling for the quality of the spectral library. Supplementary Figure S5b demonstrates a generally good agreement of the spectral angle distributions between the spectra originating from the different studies, however the median spectral angles observed is around 0.86 in HCD with 2 outlier studies.

As reported previously, we observed differences in the generated HCD tandem mass spectra for instruments of the same make and type and drifting CE calibrations over time[5]. Hence, we investigated, how similar the library spectra that were acquired using the same nominal CE setting in fact are. To remove the bias of drifting collision

energies for a single instrument, we calibrated all ProteomeTools data to a synthetic standard run we obtained at the beginning of the project. We then compared the experimental spectra to the synthetic peptide collection (Supplementary Figure S6c). It becomes clear, that within the spectra acquired with identical settings on different instruments over long periods of time vary quite a bit, as the respective best matching ProteomeTools spectra are spread over several CE.

Further, we compared the retention times recorded in the spectral library to the retention times observed in ProteomeTools (Supplementary Figure S5d). As no common internal standards were used in the aggregated studies, no indexed retention time values are reported, hence the different studies can be clearly distinguished based on the LC-MS method applied. This renders the recorded retention time information from the spectral library unsuited as criterion for data analysis.

We conclude, that the SysteMHC library contains a reasonable amount of high quality scans that well resemble synthetic spectra, however the resource is quite heterogeneous in the spectral appearance with no resolution on collision energy and retention time information that cannot be readily applied to one's LC system. When applied to the analysis using spectral library searches or DIA and targeted measurements, such variance will bias the result as both spectral appearance and iRT are crucial identification criteria. Hence, such resource library collected from multiple studies will always suffer from inconsistently acquired data and in-house generated spectral libraries clearly preferred for in-depth data analysis.

Prosit 2020 model training

Prosit uses the normalized spectral angle as an objective function because of its higher sensitivity as a similarity measure[6]. Prosit's ability to predict fragment ion intensity quickly increased during the first few epochs (Supplemental Figure S6a) and reached decent accuracy by epoch ~30. At this point, the learning rate was adjusted to allow a more fine-tuned learning of Prosit. The oscillation of the spectral angle is due to the use of a cyclic

learning rate which was employed to avoid the convergence to a local minimum. Learning stopped at epoch ~250 at which no progress was monitored anymore (early stopping) and the model with the highest overall spectral angle (red dot) was used for further analysis. The fact that spectral loss on the validation set did not increase at later epochs, and in fact is lower than the spectral loss of the training, suggests that no major overfitting has occurred. This notion is corroborated by the marginal difference between the median spectral angle observed between training and holdout set across different peptide properties (Supplementary Figure S8). In addition, no major bias is visible when comparing the median spectral angles of peptides separated by all observed N- and C-termini combination between the training and holdout set (Supplementary Figure S9). The same learning procedure (data not shown) as well as general observations with regard to overfitting were observed for the CID Prosit 2020 model.

Prediction quality for peptides dominantly fragmenting into neutral losses or internal ion series

As expected, a large proportion of the intensity in the spectrum of HLA peptides can be attributed to internal ion and neutral loss peaks (see also Figure 1d). Astonishingly, while Prosit was not trained on these peaks, it had learned to account for unknown fragmentation channels and their effect on the b- and y-ions into the predicted b/y-fragment ion intensities, resulting in well matching predictions for these fragment ions. One such case is exemplified in a mirror plot of the singly charged peptide TSGYGQSSYSSY in Supplementary Figure S10a. The experimental and predicted fragment ion intensities of the annotated b- and y-ions were in very good agreement (SA 0.88) for both the HCD scan (top) and CID scan (bottom).

To demonstrate Prosit's prediction accuracy on different peptides and its ability on an external dataset, we retrieved the raw data and identification results from a recent mono allelic HLA Class I cell line study by Sarkizova & Klaeger *et al.*[7]. Spectrum Mill HLA v2, a search engine partially optimized for the identification of HLA peptides by e.g. utilizing knowledge on internal ions for scoring, was used in this stud and provides information on the

preference for internal ions of any identified peptide. This allowed the investigation whether Prosit, exclusively trained on b/y-ions, shows a bias towards HLA peptides which pre-dominantly fragment into such ions or other fragment ions. For this purpose, we extracted all confidently identified spectra from the original study and plotted the distribution of spectral angles (comparing Prosit predicted b- and y-ion intensities only to the experimental spectra) separated by the inferred peptide fragmentation category of Spectrum Mill HLA v2. Supplementary Figure S10b demonstrates that although the spectral angle distribution of peptides which have an internal-ion bias appears slightly broader, no major bias of Prosit is visible, supporting that Prosit has learned to adjust its predictions accordingly and can be readily used for any peptide of interest.

Additionally, the mono allelic cell line data was acquired over the last 5 years on at least 3 different mass spectrometers (QE, HF, Lumos Orbitraps). Because of Prosit's unique ability to predict spectra at varying collision energies, it was not required to train different prediction models (as the collision energy varied by up to 15 points across the dataset) but instead we calibrated the collision energy used for prediction to match the fragmentation spectra for every allele separately.

Addition to Prosit boosts the number of identified HLA peptides from human cell lines

Comparing the results of reprocessing the raw MS data using MaxQuant/Andromeda to those obtained by Spectrum Mill HLA v2, showed that MaxQuant identified far fewer HLA peptides (Supplementary Figure S12a). Rescoring the MaxQuant results using the newly developed Prosit model led to a drastic increase of peptide identifications (Supplementary Figure S12b, top panel, green bars). For all cell lines, almost all confident identifications by MaxQuant were retained (blue bars) and only few peptides were rejected (red bar), suggesting that the Andromeda score used by MaxQuant lacks the necessary sensitivity to confidently identify a large fraction of HLA peptides that are present in the data. On average, Prosit rescoring increased the number of confidently identified HLA peptides by a factor of 2.6, exemplified on C*12:03, and some alleles showed gains of up to 10-fold

(HLA allele C*01:02, 181 vs 1937 peptides). This strong increase can be attributed to the additional sensitivity provided by the intensity-based Prosit scores, as indicated by the strong separation of true and false positive in comparison to MaxQuant (Supplemental Figure S12c-e).

As expected, the large majority of peptide not confidently identified by MaxQuant but Spectrum Mill HLA v2 appear to be true binders when compared to expected binding motif (Supplementary Figure S12f, MQ vs SM HLA v2). Somewhat surprisingly, peptides added by MaxQuant in comparison to Spectrum Mill HLA v2 show an even decreased average emission probability, suggesting that a large majority of these peptides are likely non-binders (false positives). Rescoring the MaxQuant results drastically changed this observation as peptide added by rescored MaxQuant in comparison to Spectrum Mill HLA v2 show a generally similar emission probability distribution in comparison to peptide lost (Supplementary Figure S12f, Rescored MaxQuant vs SM HLA v2). When comparing the rescored MaxQuant results to the results obtained from Spectrum Mill HLA v3, peptides not confidently identified by rescored MaxQuant exhibit a similar emission probability as peptides added by this workflow (Supplementary Figure S12f, Rescored MaxQuant vs SM HLA v3), suggesting that this workflow failed to identify many potential likely true binders. This is due to the lack of these sequences in the MaxQuant results and the resulting inability of Prosit to rescue such peptides.

Addition to Prosit rescoring questions prior claims of proteasomal splicing of peptides

In order the check the validity of the proposed spliced peptide events, we reprocessed the raw data used in Liepe *et al.*[8] with MaxQuant and MSFragger using a fasta database consisting of only canonical proteins (and thus peptides) followed by the Prosit rescoring toolchain. The rational for not incorporating spliced peptide hypothesis in the fasta file was that their presence significantly increases the search space (~40 MB canonical vs 1.5 TB spliced fasta) and thus the best non-spliced peptide might simply not appear in the top 10 ranking PSMs in the original Mascot results. To allow a comparison of matches across the three workflows (Mascot, MaxQuant and MSFragger

– all rescored using Prosit), we trained a single Percolator model based on the MaxQuant results and re-asses the confidence of each PSM using this model. Because of this, we are able to compare a PSM from Mascot to a PSM from MaxQuant for the same spectrum. Without Prosit rescoring, this would not be possible due to the different scoring schemas employed by each search engine. In addition, the pre-trained Percolator model allows us to assess the overall false discovery rate of the Mascot PSMs.

The rescored Mascot Percolator scores of all PSMs reported by Liepe *et al.* were then plotted against the rescored MaxQuant Percolator scores (Supplementary Figure S14a, left panel). As expected, PSMs where both search engines agree in the identified peptide sequence appear on the diagonal (grey points). Because the Percolator model was trained to separate PSMs at 1% FDR, every point below 0 on either the horizontal or vertical axis are considered non-confident (<1% PSM FDR) in the respective results. Because of this, about half of the proposed spliced peptide PSMs do not survive this estimated FDR cutoff after rescoring. Surprisingly, 126 proposed spliced peptide PSMs match into the canonical search space when using I/L ambiguity (marked in blue). This supports the notion that the original results did not contain (the best) relevant canonical peptide matches in the top 10 ranked list. Further 10 PSMs mapped into the canonical space, very likely due to updates and differences in human Swissprot databases used for re-processing with MaxQuant.

In the projected confidence space (Supplementary Figure S14a), the diagonal separates spectra which can be either better explained by the peptide sequence retrieved from the Mascot search (below diagonal) or MaxQuant search (above diagonal). A large fraction of proposed spliced peptide PSMs appear above the diagonal. This is likely caused by the previous observation that classical search engine scores based on ion counting only provide limited discrimination power to separate PSMs of peptide sequences which produce a very similar fragment ion pattern. In total, 1,246 proposed spliced peptide PSMs were considered incorrect in this analysis (Supplementary Figure S14a, red area) either because they do not survive the projected FDR cutoff, have an I/L isobaric peptide sequence in the canonical peptide space, or can be better explained by a canonical peptide.

A large fraction of canonical peptide PSMs is located below the diagonal and above the rescored Mascot Percolator score of 0 (Supplementary Figure 14a, left panel, black points), which shows that MaxQuant failed to identify the correct peptide sequence due to known search engines differences. Given these results, we hypothesized that the remaining 359 proposed spliced PSMs (Supplementary Figure S14a, left panel, green area), might also have better explanations in the canonical search engine. To test this, we repeated the rescoring analysis with the remaining spectra using MSFragger[9], again employing the pre-trained percolator model from MaxQuant. When comparing the Percolator scores of PSMs from the Mascot and MSFragger search (Supplementary Figure S14a, right panel), it becomes apparent that the large majority of spectra previously located below the diagonal of canonical peptides (black points) are now shifted to the diagonal, supporting the hypothesis that MaxQuant failed to report the correct peptide sequence. While some of the remaining proposed spliced peptide PSMs (orange points) where shifted on top or even above the diagonal (blue points again I/L isobaric with the MSFragger results), a larger proportion of spectra annotated as spliced peptides remain below the diagonal as MSFragger did not report a peptide sequence from the canonical space with better quality. In total, MSFragger reduced the number of proposed spliced peptide PSMs by 43, leaving 316 confident PSMs for splices peptides.

When investigating the population of orange data points in the green shaded areas (Supplementary Figure S14a left and right panel), it becomes apparent that a sizable proportion is located close the diagonal, with a similar spread around the diagonal as obverse for peptides which are I/L isobaric. Apart from the I to L conversion (or vice versa), especially when considering the very large space of proposed spliced peptides, other amino acid conversions are expected and may result in higher scores by chance. Supplementary Figure S14b shows that a large fraction of the remaining proposed spliced peptide PSMs have either a low confidence score difference or low Levenshtein distance (simplified: the number of amino acids different between two sequences) to the best canonical peptide PSM proposed by MaxQuant and/or MSFragger. In order to estimate a confidence score difference cutoff which is necessary to differentiate two hypothesis, we investigated the absolute score differences exhibited by canonical peptide PSMs proposed by MaxQuant and MSFragger which are I/L isobaric

(Supplementary Figure 14b top panel, blue distribution), as such conversions are considered (largely) non-differentiable using mass spectrometry and HCD fragmentation. The 99% of these PSMs are covered when using a confidence score difference of 0.64 (red line), which suggests that spectra which are annotated with a spliced and canonical peptide exhibiting a score difference smaller than that (Supplementary Figure S14b, bottom panel red line) should not be labeled as a spliced peptide but rather as a non-spliced peptide (Occam's razor). The median Percolator score difference of spliced peptides with a Levenshtein difference of 2 or 3 is below this estimated cutoff and proposed spliced peptide PSMs of these two categories as well as PSM whose Percolator score difference is below the estimated cutoff are excluded from further analysis. This last filtering removes 75 additional PSMs with only 241 of the originally 1,605 proposed spliced peptide PSMs remaining confident.

Supplementary References

1. Davis, S. et al. Expanding Proteome Coverage with CHarge Ordered Parallel Ion aNalysis (CHOPIN) Combined with Broad Specificity Proteolysis. *Journal of Proteome Research* **16**, 1288–1299 (2017).
2. Bassani-Sternberg, M. et al. Direct identification of clinically relevant neoepitopes presented on native human melanoma tissue by mass spectrometry. *Nature Communications* **7**, 1–16 (2016).
3. Vita, R. et al. The Immune Epitope Database (IEDB): 2018 update. *Nucleic acids research* **47**, 339–343 (2019).
4. Shao, W. et al. The SysteMHC Atlas project. *Nucleic Acids Research* **46**, D1237–D1247 (2018).
5. Zolg, D.P. et al. PROCAL: A Set of 40 Peptide Standards for Retention Time Indexing, Column Performance Monitoring, and Collision Energy Calibration. *PROTEOMICS* **17**, 1700263 (2017).
6. Toprak, U.H. et al. Conserved Peptide Fragmentation as a Benchmarking Tool for Mass Spectrometers and a Discriminating Feature for Targeted Proteomics. *Molecular & Cellular Proteomics : MCP* **13**, 2056–2071 (2014).
7. Sarkizova, S. et al. A large peptidome dataset improves HLA class I epitope prediction across most of the human population. *Nature Biotechnology* **38**, 199–209 (2019).
8. Liepe, J., Sidney, J., Lorenz, F.K.M., Sette, A. & Mishto, M. Mapping the MHC Class I–Spliced Immunopeptidome of Cancer Cells. *Cancer Immunology Research* **7**, 62–76 (2019).
9. Kong, A.T., Leprevost, F.V., Avtonomov, D.M., Mellacheruvu, D. & Nesvizhskii, A.I. MSFragger: ultrafast and comprehensive peptide identification in mass spectrometry–based proteomics. *Nature Methods* **14**, 513–520 (2017).
