# Supplementary material for: Deep learning boosts sensitivity of mass spectrometry-based immunopeptidomics
Source: Nat Commun. 2021 Jun 7;12:3346. doi: 10.1038/s41467-021-23713-9 (PMC8184761; doi:10.1038/s41467-021-23713-9)
Supplement: Supplementary file 3 — Description of Additional Supplementary Files [file 41467_2021_23713_MOESM3_ESM.pdf]

Description of Additional Supplementary Files

File Name: Supplementary Data 1
Description: Non-tryptic ProteomeTools resource

File Name: Supplementary Data 2
Description: SysteMHC spectrum comparison to ProteomeTools

File Name: Supplementary Data 3
Description: Results of the re-analysis of a large monoallelic HLA Class I cell line study

File Name: Supplementary Data 4
Description: Peptide binding motifs of monoallelic dataset

File Name: Supplementary Data 5
Description: Results of the re-analysis of proposed proteasomal splicing events

File Name: Supplementary Data 6
Description: All proposed spliced peptide spectrum matches and alternative hypothesis by MaxQuant and/or MSFragger

File Name: Supplementary Data 7
Description: Results of the re-analysis of the melanoma patient HLA dataset
