# Supplementary material for: Deep learning boosts sensitivity of mass spectrometry-based immunopeptidomics
Source: Nat Commun. 2021 Jun 7;12:3346. doi: 10.1038/s41467-021-23713-9 (PMC8184761; doi:10.1038/s41467-021-23713-9)
Supplement: Supplementary file 11 — Reporting Summary [file 41467_2021_23713_MOESM11_ESM.pdf]

nature research

Corresponding author(s): Mathias Wilhelm, Bernhard Kuster

Last updated by author(s): 29/05/03

Reporting Summary

Nature Research wishes to improve the reproducibility of the work that we publish. This form provides structure for consistency and transparency in reporting. For further information on Nature Research policies, see our Editorial Policies and the Editorial Policy Checklist.

Statistics

For all statistical analyses, confirm that the following items are present in the figure legend, table legend, main text, or Methods section.

n/a	Confirmed	
☐	☒	The exact sample size (*n*) for each experimental group/condition, given as a discrete number and unit of measurement
☐	☒	A statement on whether measurements were taken from distinct samples or whether the same sample was measured repeatedly
☐	☒	The statistical test(s) used AND whether they are one- or two-sided<br>*Only common tests should be described solely by name; describe more complex techniques in the Methods section.*
☐	☒	A description of all covariates tested
☐	☒	A description of any assumptions or corrections, such as tests of normality and adjustment for multiple comparisons
☐	☒	A full description of the statistical parameters including central tendency (e.g. means) or other basic estimates (e.g. regression coefficient) AND variation (e.g. standard deviation) or associated estimates of uncertainty (e.g. confidence intervals)
☐	☒	For null hypothesis testing, the test statistic (e.g. *F*, *t*, *r*) with confidence intervals, effect sizes, degrees of freedom and *P* value noted<br>*Give P values as exact values whenever suitable.*
☐	☒	For Bayesian analysis, information on the choice of priors and Markov chain Monte Carlo settings
☐	☒	For hierarchical and complex designs, identification of the appropriate level for tests and full reporting of outcomes
☐	☒	Estimates of effect sizes (e.g. Cohen's *d*, Pearson's *r*), indicating how they were calculated

Our web collection on statistics for biologists contains articles on many of the points above.

Software and code

Policy information about availability of computer code

Data collection	For data acquisition, Xcalibur v4.2 and Orbitrap Fusion Lumos Tune v3.0 were used. After acquisition, data was processed with MaxQuant (1.5.3.30) and stored in a custom PostgreSQL (9.5.7) database. Data extraction was performed using custom bash (4.3.48), R (3.4.0) and SQL (9.5.7) scripts.
Data analysis	The machine learning model was implemented in Python with keras (2.1.1), tensorflow (1.4.0), numpy (1.14.5) and scipy (1.1.0) and compiled to use graphic processing units (GPU). Source code is available at www.github.com/kusterlab/prosit/.<br>Further software used: Thermo RawFileReader (3.0.54), MaxQuant (v1.5.3.30, v1.6.0.1), R (v3.5, v3.6), python (v3.6), SpectrumMill (v6.1 pre-release with HLA v2 scoring, v7.0 pre-release with the HLA v3 scoring), MSFragger (v3.0), Percolator (v3.00, v3.01, v3.05), MuTect2 (v4.1.0.038) for WES, Strelka2 (v2.9.1039) for RNA-Seq data, NetMHC (v4.0), HLAthena (v1.0 2019), Immunospot (v5.4.0.1)

For manuscripts utilizing custom algorithms or software that are central to the research but not yet described in published literature, software must be made available to editors and reviewers. We strongly encourage code deposition in a community repository (e.g. GitHub). See the Nature Research guidelines for submitting code & software for further information.

Data

Policy information about availability of data

All manuscripts must include a data availability statement. This statement should provide the following information, where applicable:

- Accession codes, unique identifiers, or web links for publicly available datasets
- A list of figures that have associated raw data
- A description of any restrictions on data availability

Reference spectra for synthetic peptide originating from the proteases LysN and AspN are available at https://www.proteomicsdb.org, and updates to the resource are available at https://www.proteometools.org. Updated models of Prosit (Prosit_2020_intensity_HCD and Prosit_2020_intensity_CID) and the presented

rescoring functionality are freely available through the web interface at https://www.proteomicsdb.org/prosit. Trained model files (https://figshare.com/articles/dataset/Prosit_Non_tryptic_-_Model_-_Fragmentation/12936947) and training data (https://figshare.com/articles/dataset/ProteomeTools_non_tryptic_-_Prosit_fragmentation_-_Data/12937092) are available on figshare. The mass spectrometric raw and search data of the synthetic ProteomeTools peptides have been deposited with the ProteomeXchange Consortium via the PRIDE repository with the dataset identifier PXD021013 [https://www.ebi.ac.uk/pride/archive/projects/PXD021013]. The search data including intermediate results underlying the presented analysis have been deposited under the dataset identifier PXD021398 [https://www.ebi.ac.uk/pride/archive/projects/PXD021398].

Field-specific reporting

Please select the one below that is the best fit for your research. If you are not sure, read the appropriate sections before making your selection.

☒ Life sciences ☐ Behavioural & social sciences ☐ Ecological, evolutionary & environmental sciences

For a reference copy of the document with all sections, see nature.com/documents/nr-reporting-summary-flat.pdf

Life sciences study design

All studies must disclose on these points even when the disclosure is negative.

Sample size	Synthetic peptide training data for the deep neural network was split into three distinct sets with each peptide sequence only included in one of the three: “Training” (70%, HCD n=9.9 million, CID n=2.9 million), “Test” (20%, HCD n=2.8 million, CID=0.8 million) and “Holdout” (10%, HCD n=1.4 million, CID=0.4 million). Prior experiments have shown that these are sufficient to train accurate deep learning models.
Data exclusions	For training, the data was restricted to maximum 3 PSMs per precursor (peptide sequence, modifications, charge combination) with an Andromeda score >40 and decoy hits were excluded to 1) allow training data to fit into main memory of the computer and 2) filter for high quality spectra.
Replication	In deep learning, replication is not common and thus was not performed here.
Randomization	"Training", "Test" and "Holdout" peptide sets for deep learning were generated using random splits of the available training data, with the constraint that a peptide sequence was only allowed to be present in either of the three groups.
Blinding	To avoid over-fitting of the deep learning model we applied three regularization techniques: early stopping, dropout and the use of noisy data.

Reporting for specific materials, systems and methods

We require information from authors about some types of materials, experimental systems and methods used in many studies. Here, indicate whether each material, system or method listed is relevant to your study. If you are not sure if a list item applies to your research, read the appropriate section before selecting a response.

Materials & experimental systems

n/a	Involved in the study	
☐	☒	Antibodies
☒	☐	Eukaryotic cell lines
☒	☐	Palaeontology and archaeology
☒	☐	Animals and other organisms
☐	☒	Human research participants
☒	☐	Clinical data
☒	☐	Dual use research of concern

Methods

n/a	Involved in the study	
☒	☐	ChIP-seq
☒	☐	Flow cytometry
☒	☐	MRI-based neuroimaging

Antibodies

Antibodies used	IFN-γ capture antibody (1-D1K, Mabtech), IFN-γ-detection antibody (7-B6-1-biotin, Mabtech)
Validation	Antibodies used in this study were commercially available and validated by the manufacturer for ELISpot and ELISA. In addition, we used Phorbol-12-myristat-13-acetat (PMA)/Ionomycin-stimulated cells as positive control for IFN-γ secretion and T cells without stimulus as negative control to discriminate antigen-specific staining.<br>Links to the product webpages of the manufacturer are listed where more details and references are provided: https://www.mabtech.com/products/anti-human-ifn-gamma-antibody-1-d1k-purified-3420-3, https://www.mabtech.com/products/anti-human-ifn-gamma-antibody-7-b6-1-biotinylated-3420-6. Publications using the above mentioned antibodies can be found as follows: https://www.mabtech.com/knowledge-center/references/citations-database?analyte=ifn-%CE%B3&reactivity=human&application=elispot.

Human research participants

Policy information about studies involving human research participants

Population characteristics	Leftover Peripheral Blood Mononuclear Cells (PBMCs) from Mel15 (original study Bassani-Sternberg, M. et al. "Direct identification of clinically relevant neoepitopes presented on native human melanoma tissue by mass spectrometry", Nature Communications 7, 1–16 2016) were used for the immunogenicity assay.
Recruitment	In this study, no participants were recruited.
Ethics oversight	Application 193/17S; Ethics Commission, Faculty of Medicine, Technical University of Munich, Germany

Note that full information on the approval of the study protocol must also be provided in the manuscript.
