# Supplementary material for: Deep learning boosts sensitivity of mass spectrometry-based immunopeptidomics
Source: Nat Commun. 2021 Jun 7;12:3346. doi: 10.1038/s41467-021-23713-9 (PMC8184761; doi:10.1038/s41467-021-23713-9)
Supplement: Supplementary file 7 — Supplementary Data 4 [file 41467_2021_23713_MOESM7_ESM.pdf]

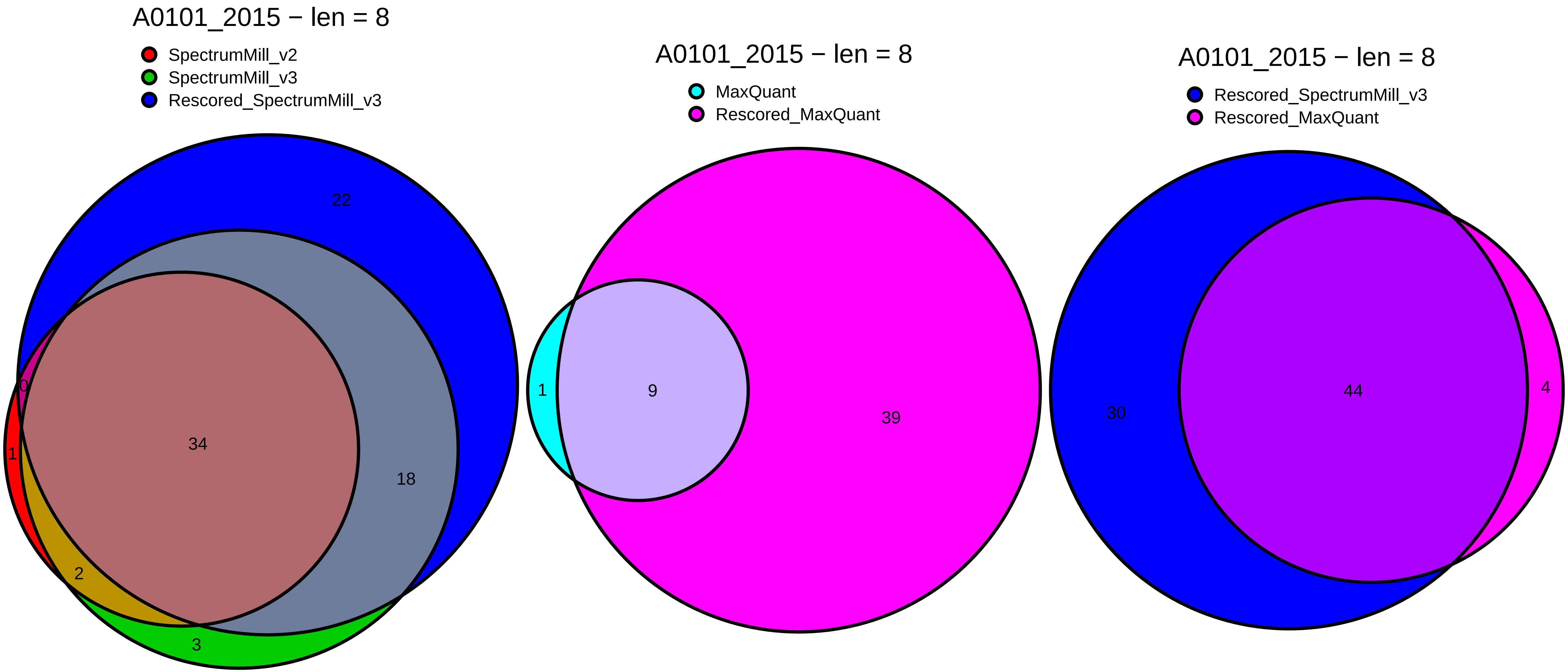
A0101_2015 − len = 8
SpectrumMill_v2
SpectrumMill_v3
Rescored_SpectrumMill_v3
22
0
1
34
18
2
3
A0101_2015 − len = 8
MaxQuant
Rescored_MaxQuant
1
9
39
A0101_2015 − len = 8
Rescored_SpectrumMill_v3
Rescored_MaxQuant
30
44
4

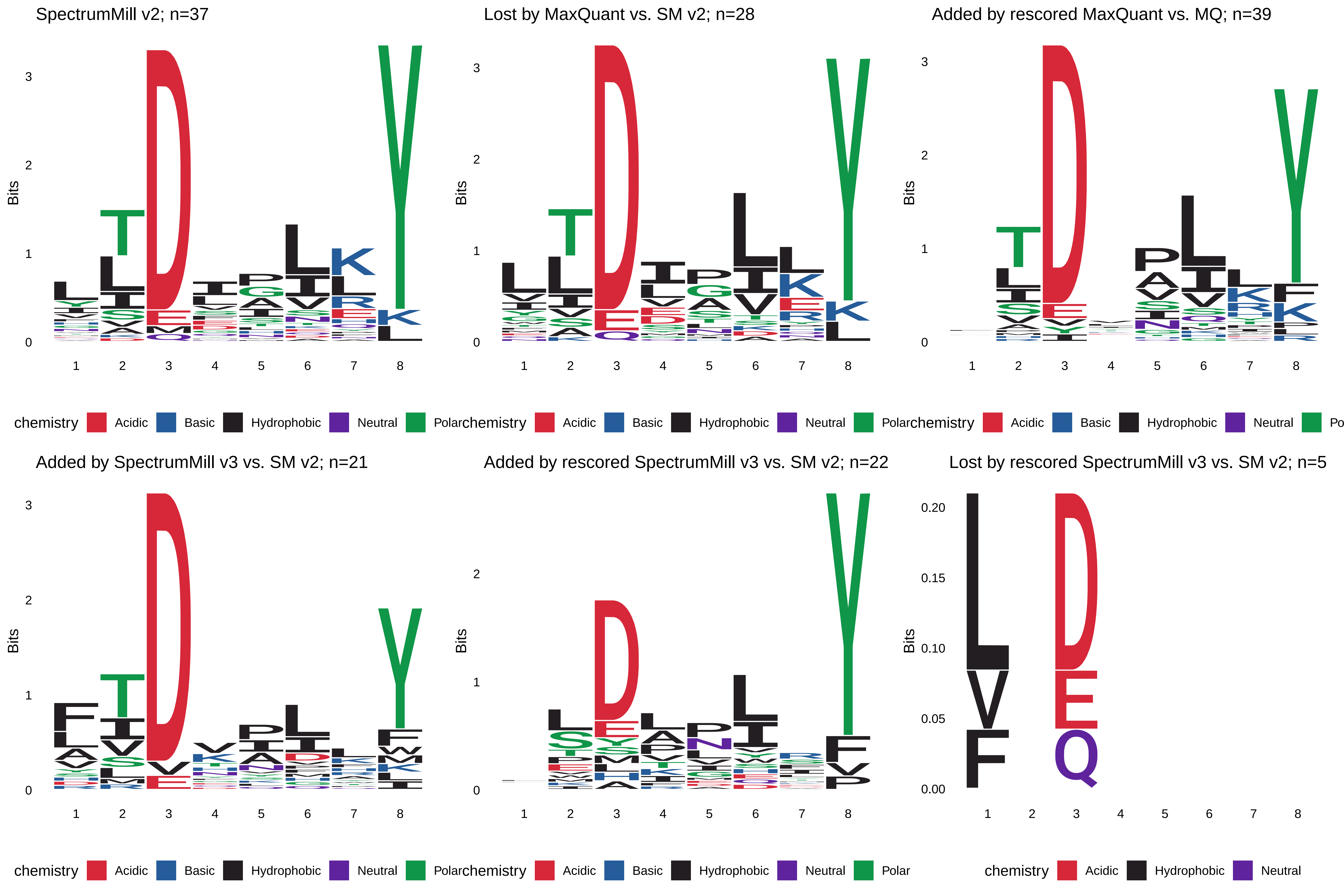
SpectrumMill v2; n=37
Lost by MaxQuant vs. SM v2; n=28
Added by rescored MaxQuant vs. MQ; n=39
Bits
chemistry Acidic Basic Hydrophobic Neutral Polar
Added by SpectrumMill v3 vs. SM v2; n=21
Added by rescored SpectrumMill v3 vs. SM v2; n=22
Lost by rescored SpectrumMill v3 vs. SM v2; n=5
chemistry Acidic Hydrophobic Neutral

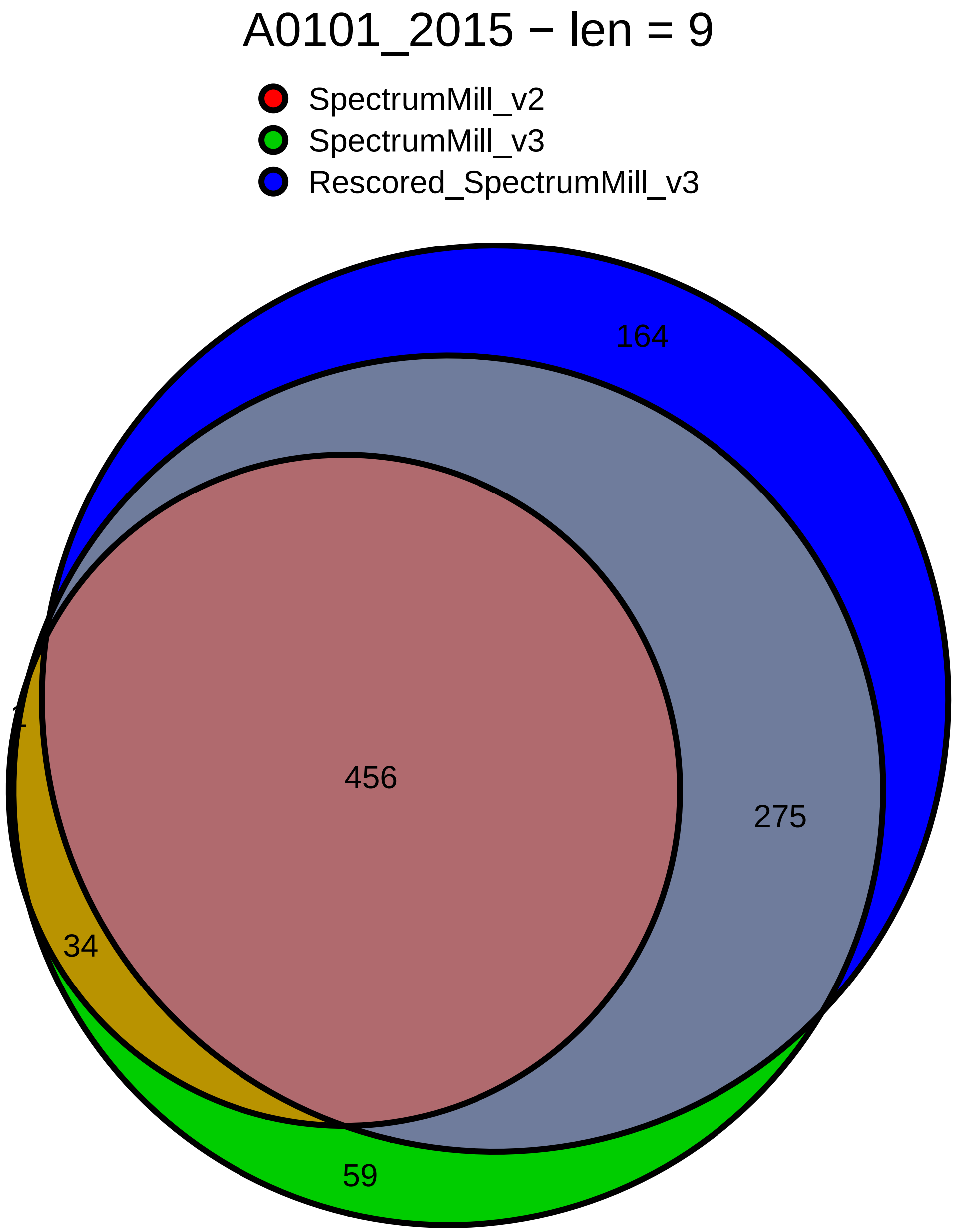
A0101_2015 − len = 9
SpectrumMill_v2
SpectrumMill_v3
Rescored_SpectrumMill_v3
164
456
275
34
59

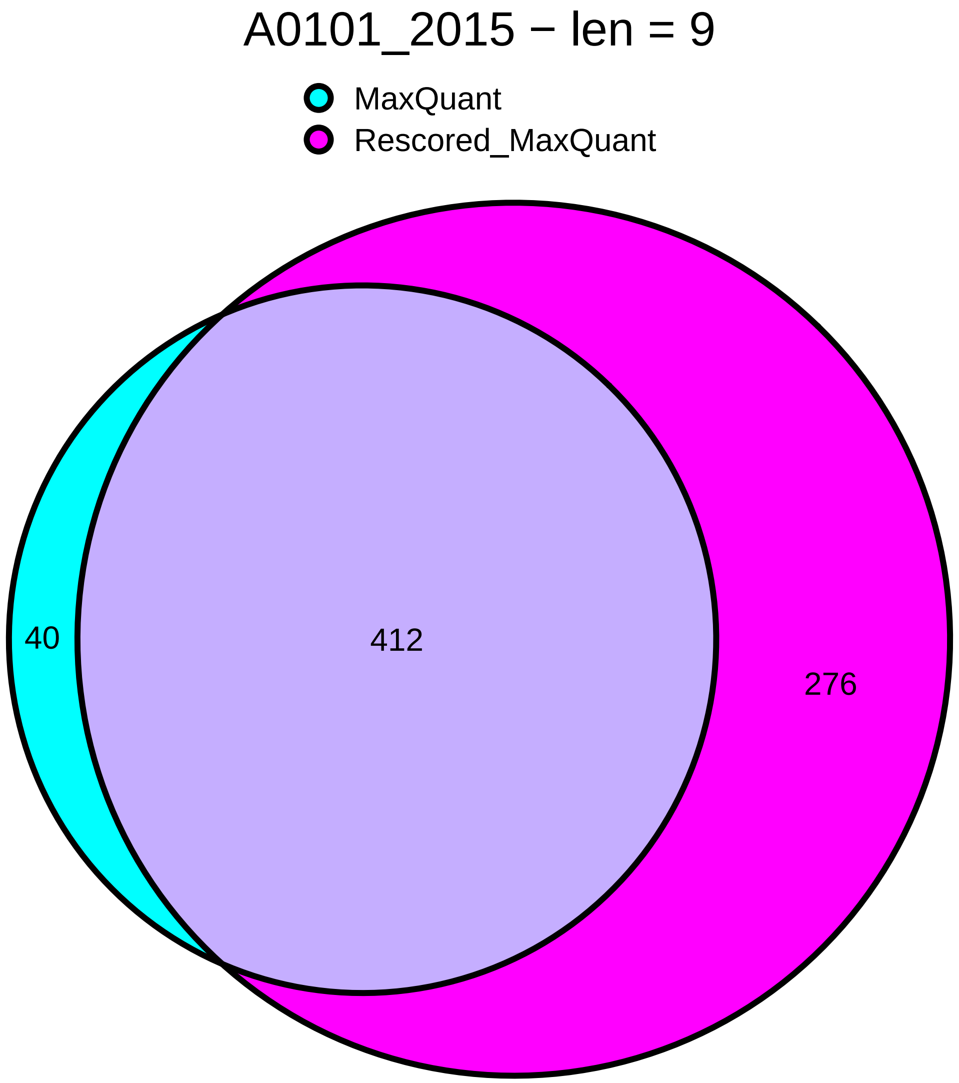
A0101_2015 − len = 9
MaxQuant
Rescored_MaxQuant
40
412
276

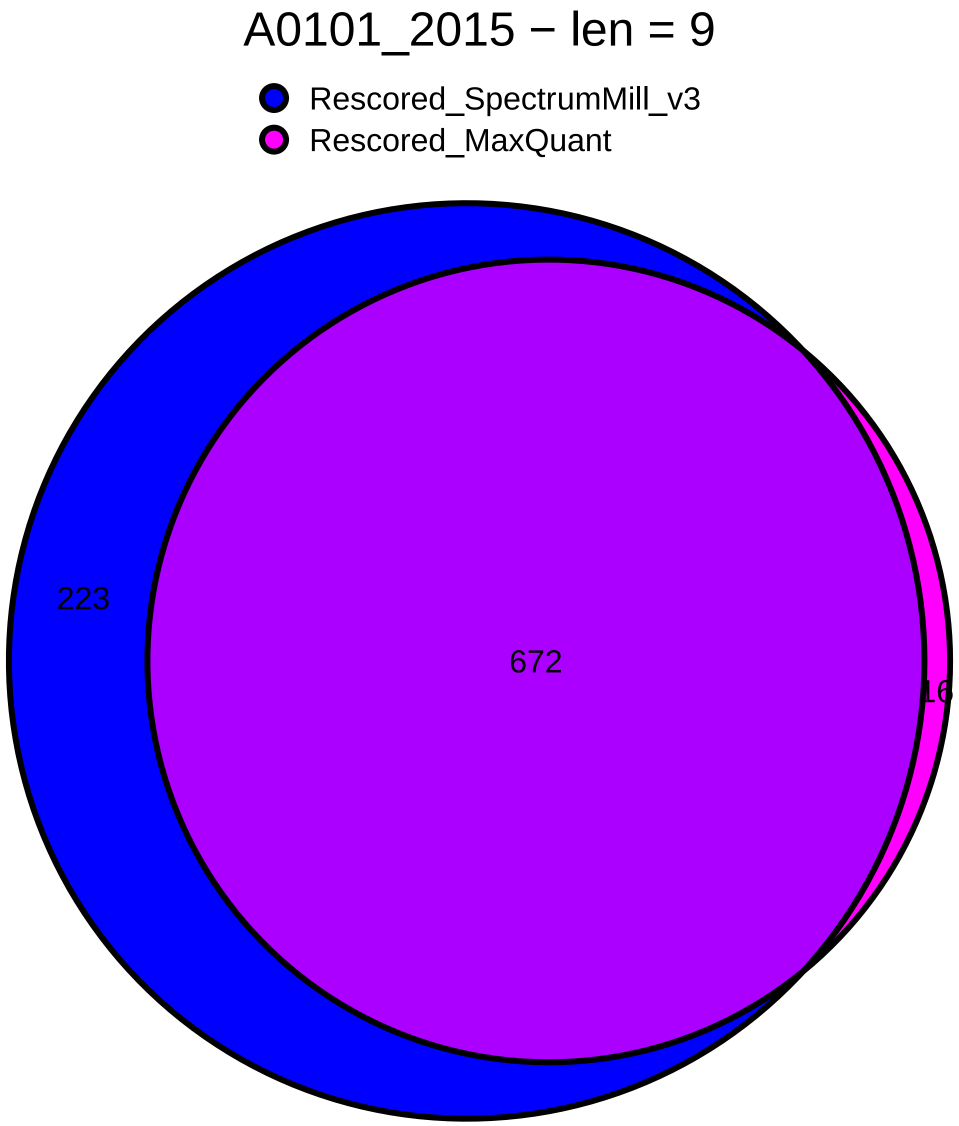
A0101_2015 − len = 9
Rescored_SpectrumMill_v3
Rescored_MaxQuant
223
672

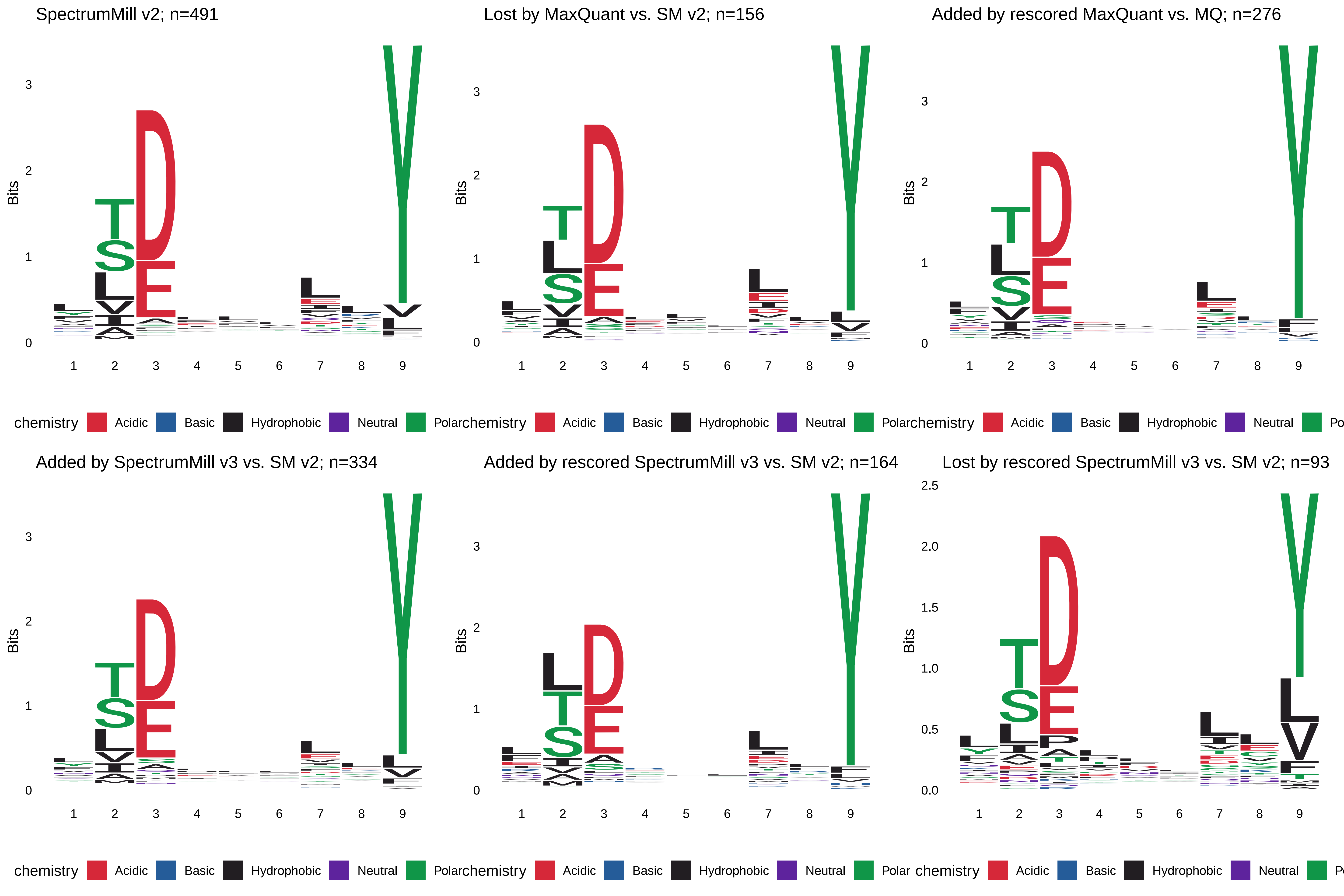
SpectrumMill v2; n=491
Lost by MaxQuant vs. SM v2; n=156
Added by rescored MaxQuant vs. MQ; n=276
Added by SpectrumMill v3 vs. SM v2; n=334
Added by rescored SpectrumMill v3 vs. SM v2; n=164
Lost by rescored SpectrumMill v3 vs. SM v2; n=93
Bits
0
1
2
3
0.0
0.5
1.0
1.5
2.0
2.5
1 2 3 4 5 6 7 8 9
chemistry
Acidic
Basic
Hydrophobic
Neutral
Polar

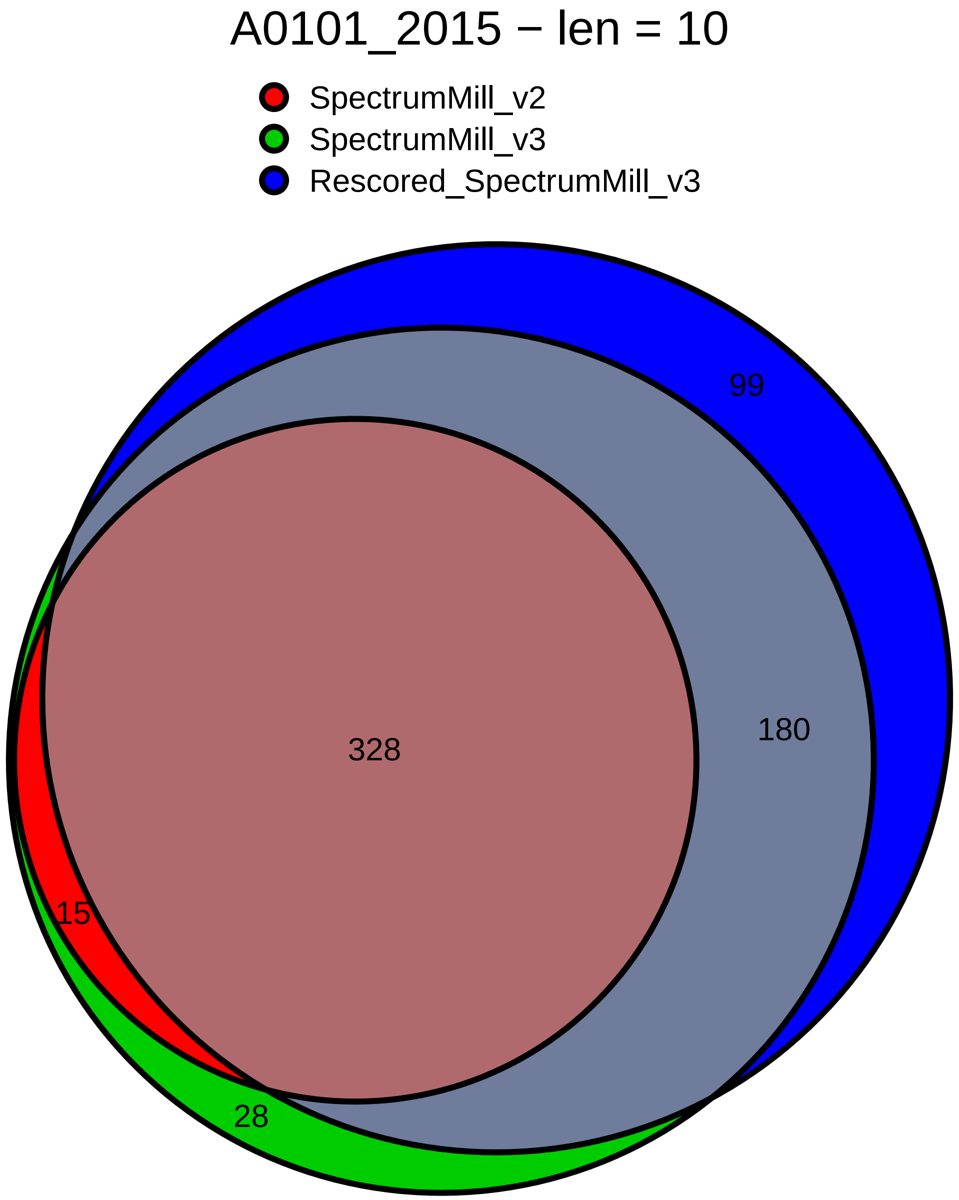
A0101_2015 − len = 10
SpectrumMill_v2
SpectrumMill_v3
Rescored_SpectrumMill_v3
99
180
328
15
28

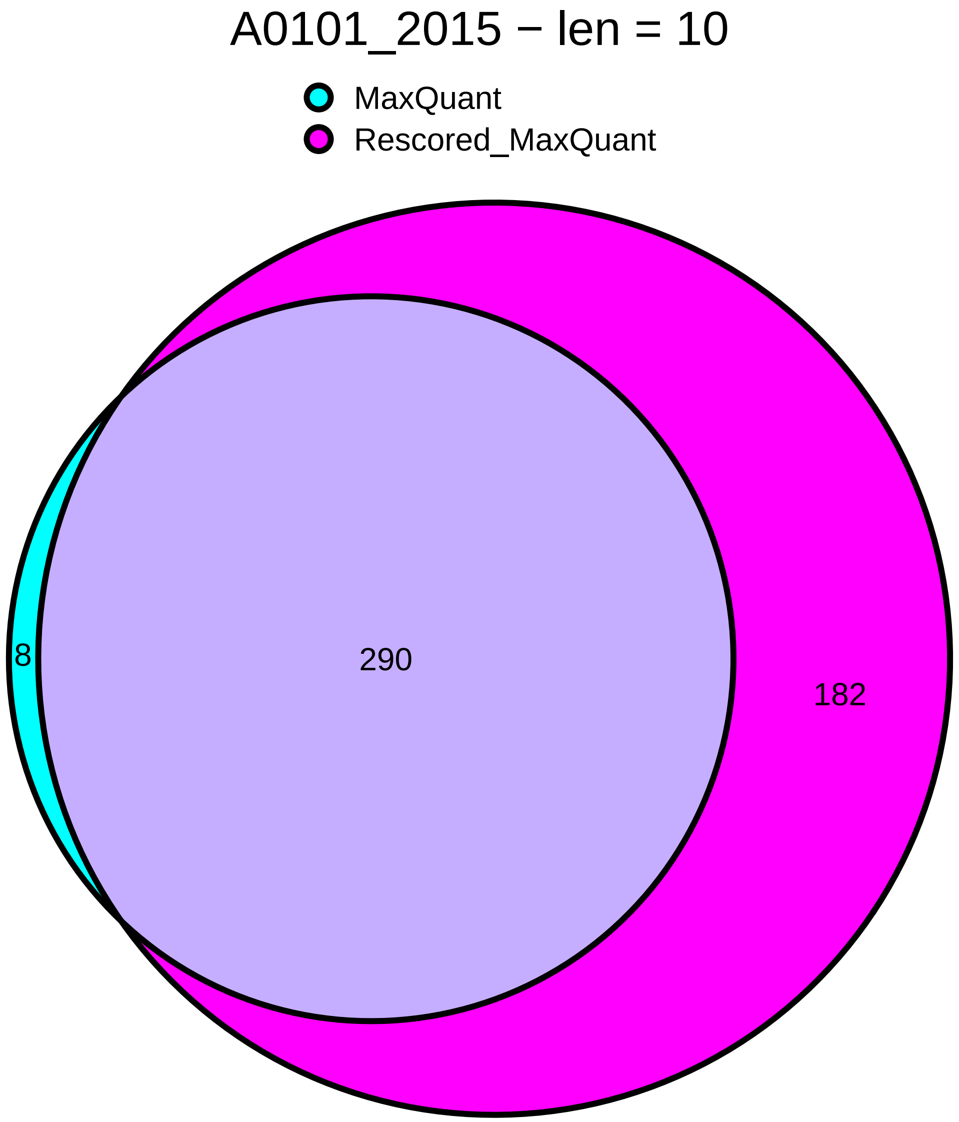
A0101_2015 − len = 10
MaxQuant
Rescored_MaxQuant
8
290
182

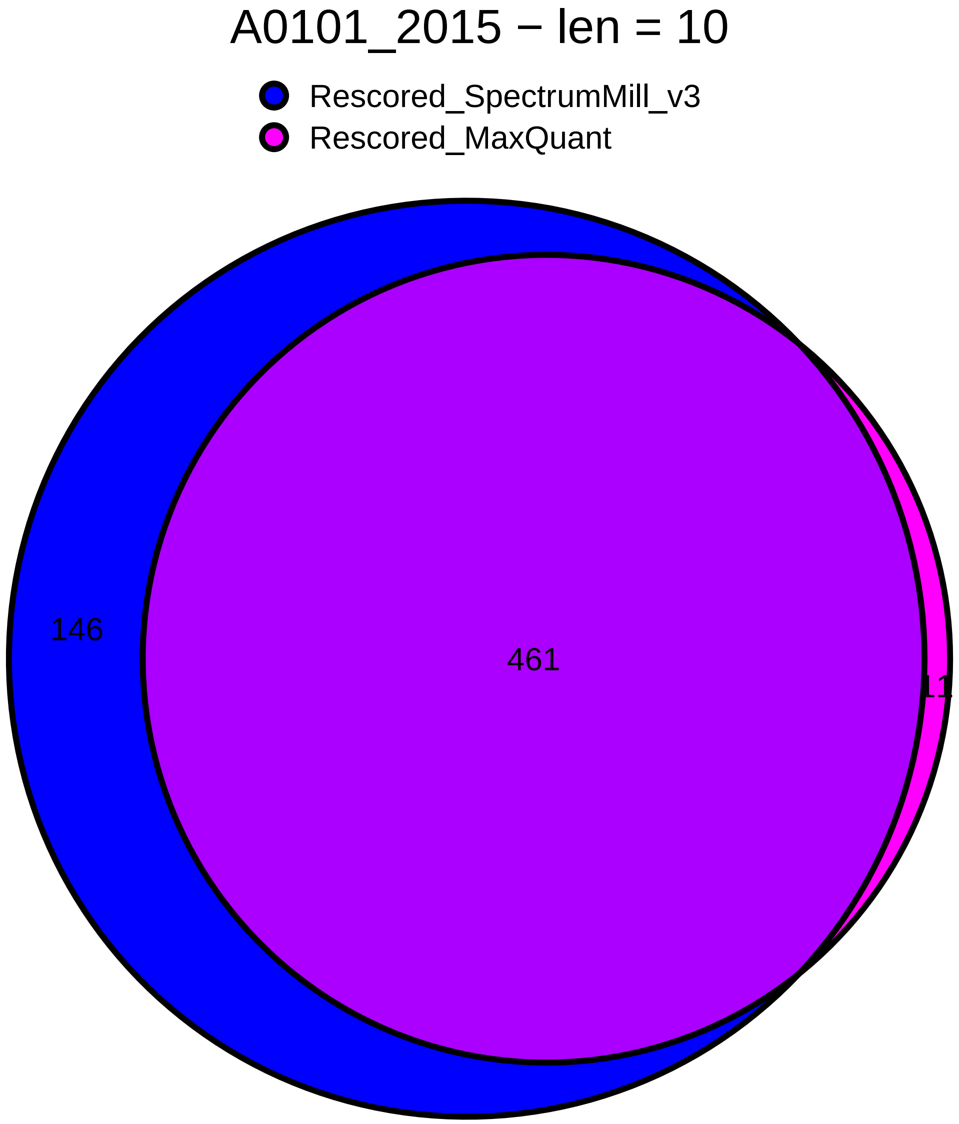
A0101_2015 − len = 10
Rescored_SpectrumMill_v3
Rescored_MaxQuant
146
461

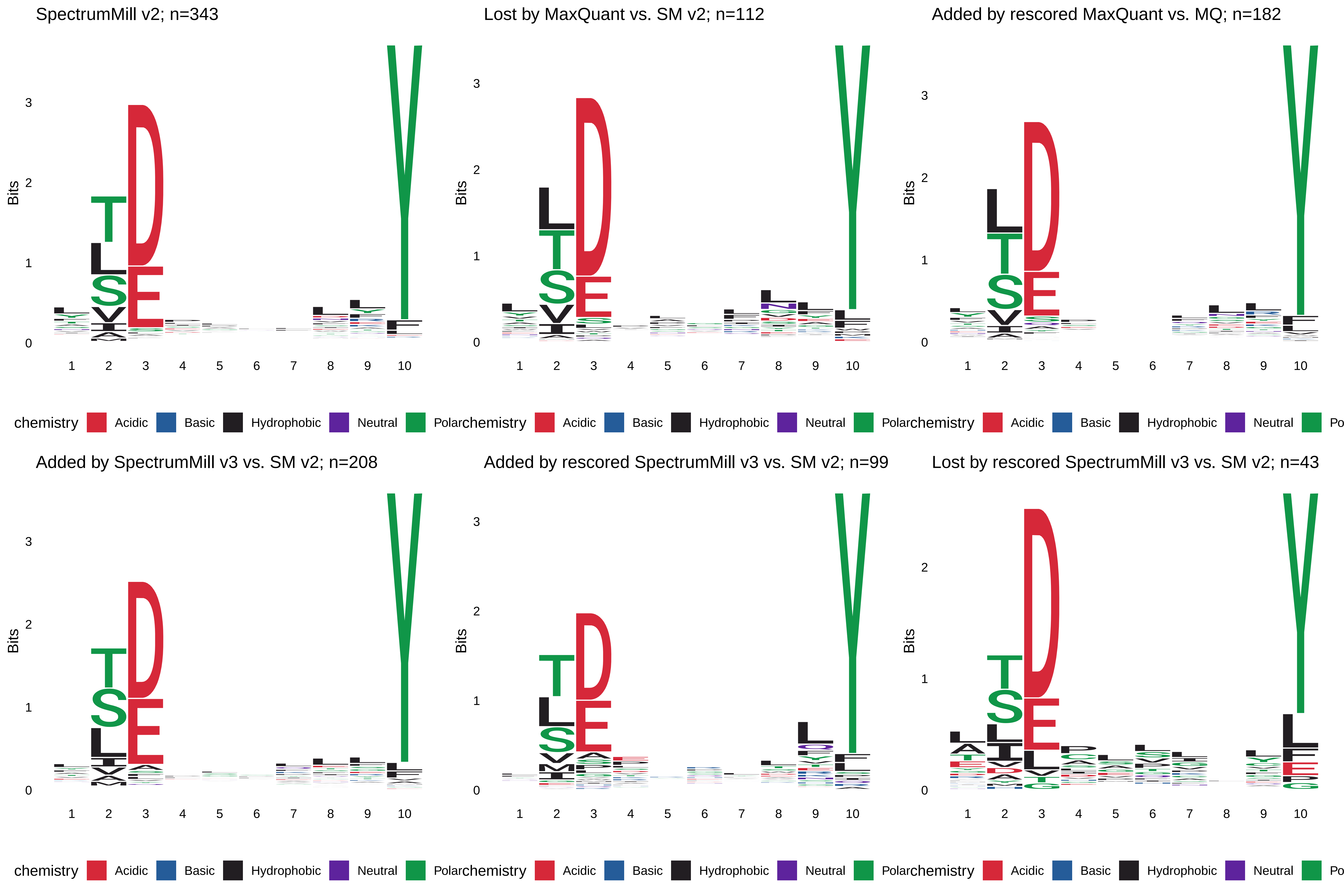
SpectrumMill v2; n=343
Lost by MaxQuant vs. SM v2; n=112
Added by rescored MaxQuant vs. MQ; n=182
Added by SpectrumMill v3 vs. SM v2; n=208
Added by rescored SpectrumMill v3 vs. SM v2; n=99
Lost by rescored SpectrumMill v3 vs. SM v2; n=43
Bits
0
1
2
3
1 2 3 4 5 6 7 8 9 10
chemistry Acidic Basic Hydrophobic Neutral Polarchemistry Acidic Basic Hydrophobic Neutral Polarchemistry Acidic Basic Hydrophobic Neutral Po
chemistry Acidic Basic Hydrophobic Neutral Polarchemistry Acidic Basic Hydrophobic Neutral Polarchemistry Acidic Basic Hydrophobic Neutral Po

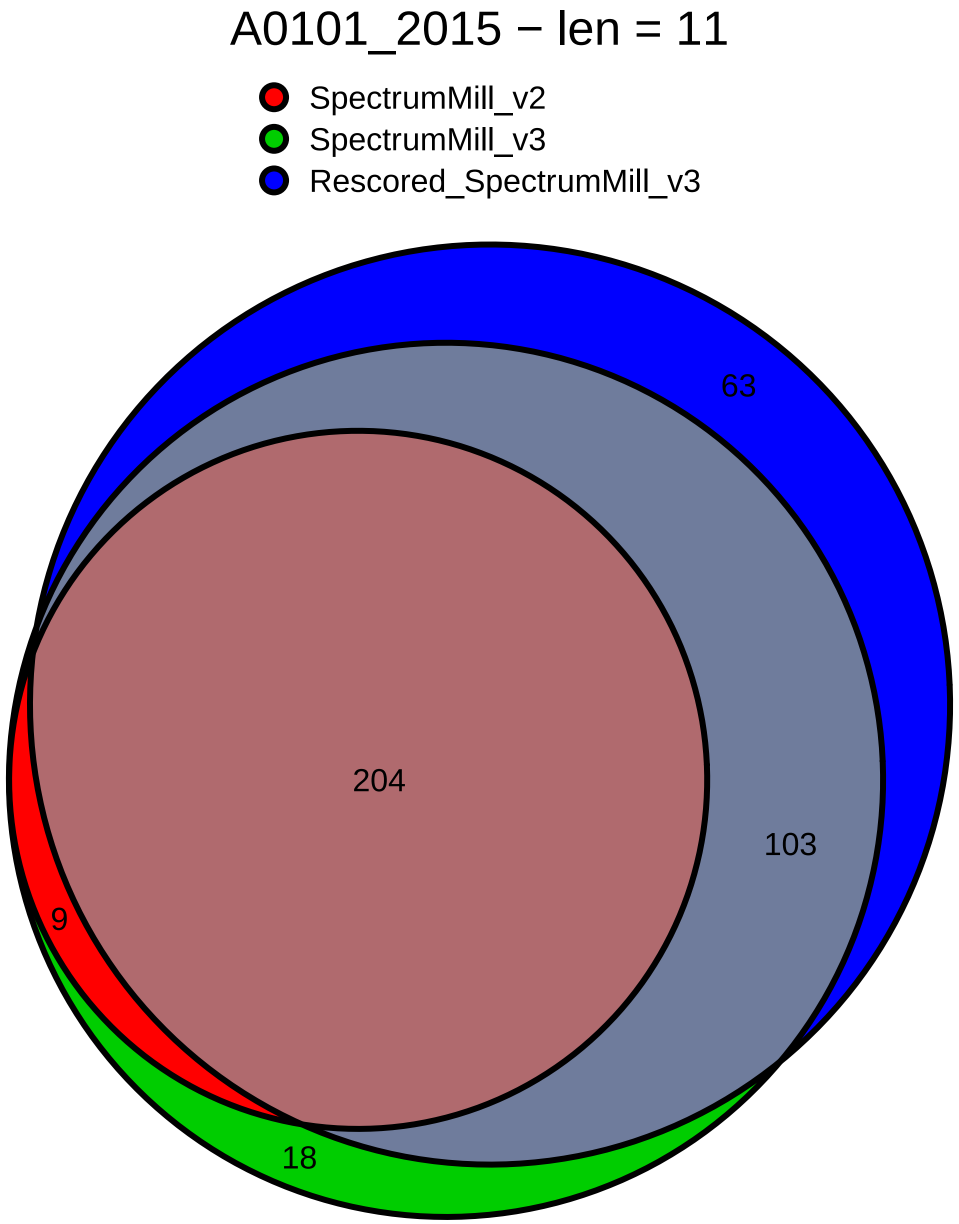
A0101_2015 − len = 11
SpectrumMill_v2
SpectrumMill_v3
Rescored_SpectrumMill_v3
63
204
103
9
18

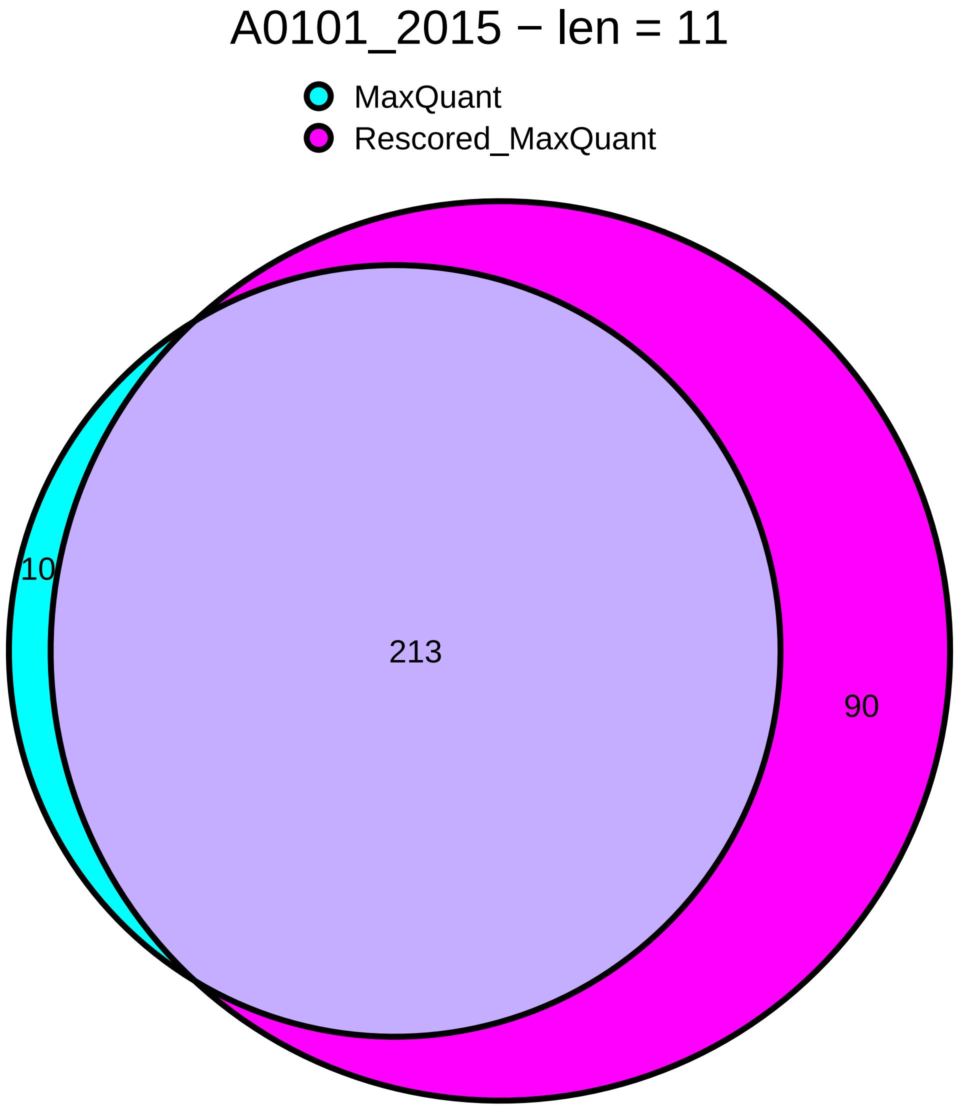
A0101_2015 − len = 11
MaxQuant
Rescored_MaxQuant
10
213
90

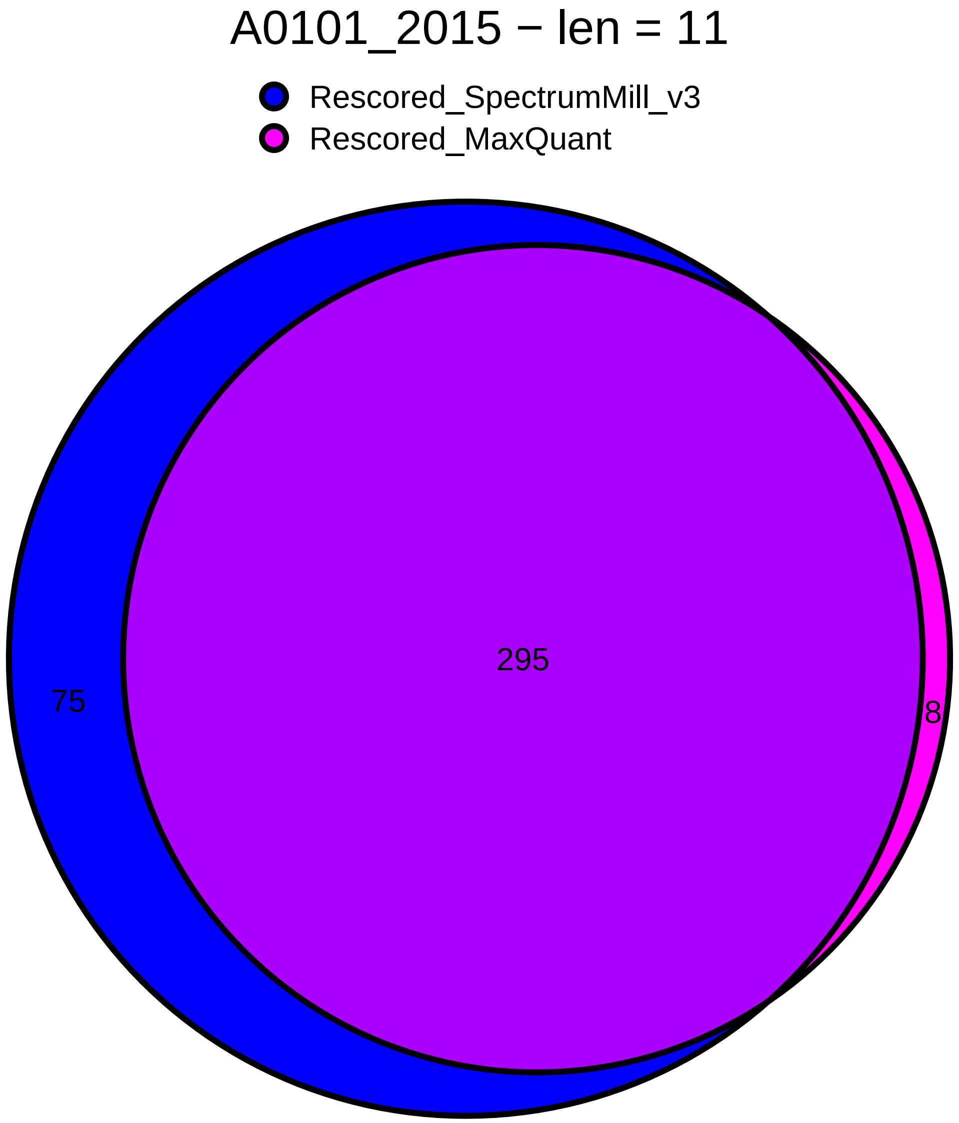
A0101_2015 − len = 11
Rescored_SpectrumMill_v3
Rescored_MaxQuant
75
295
8

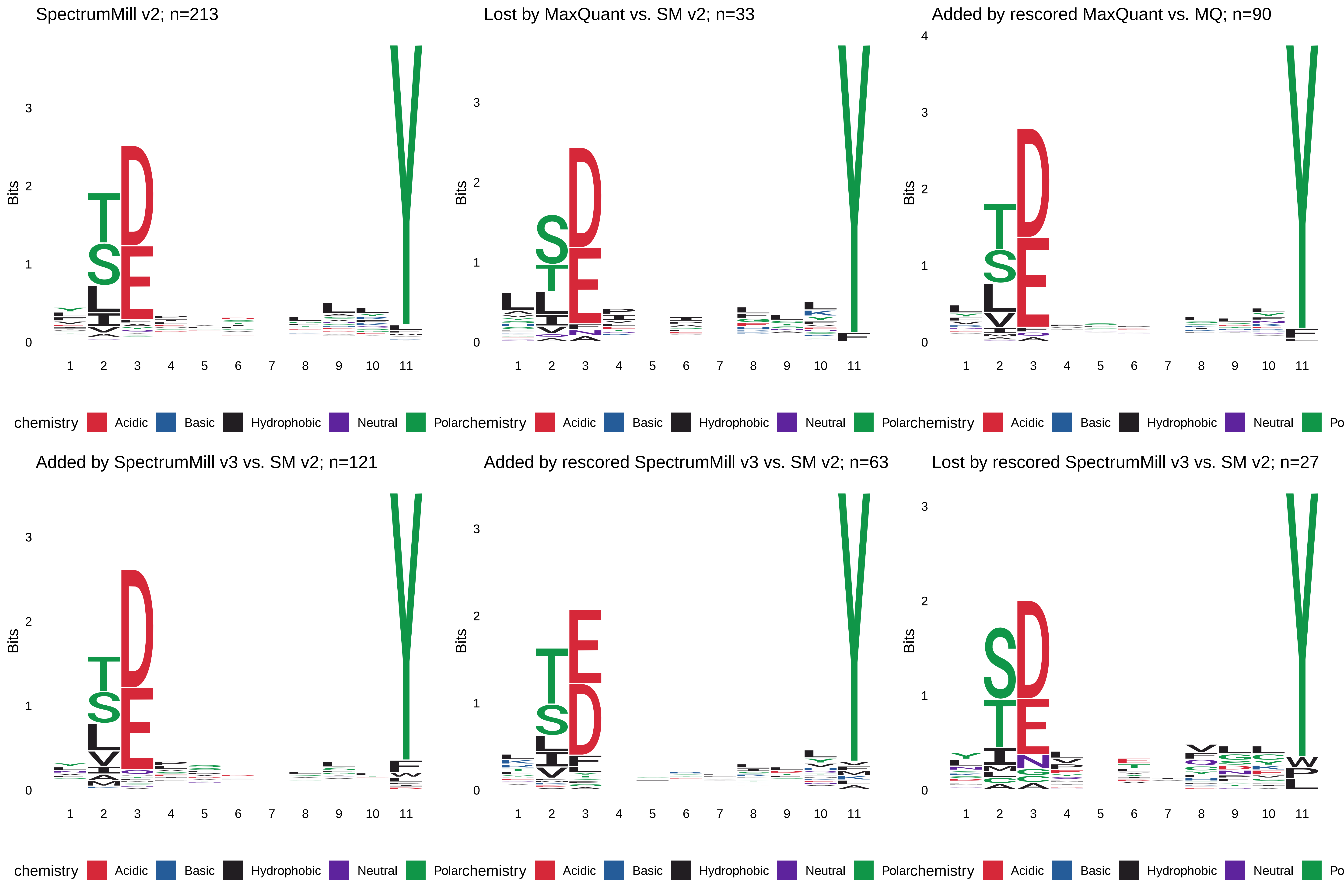

SpectrumMill v2; n=213
Lost by MaxQuant vs. SM v2; n=33
Added by rescored MaxQuant vs. MQ; n=90
Added by SpectrumMill v3 vs. SM v2; n=121
Added by rescored SpectrumMill v3 vs. SM v2; n=63
Lost by rescored SpectrumMill v3 vs. SM v2; n=27
Bits
1 2 3 4 5 6 7 8 9 10 11
chemistry
Acidic
Basic
Hydrophobic
Neutral
Polar

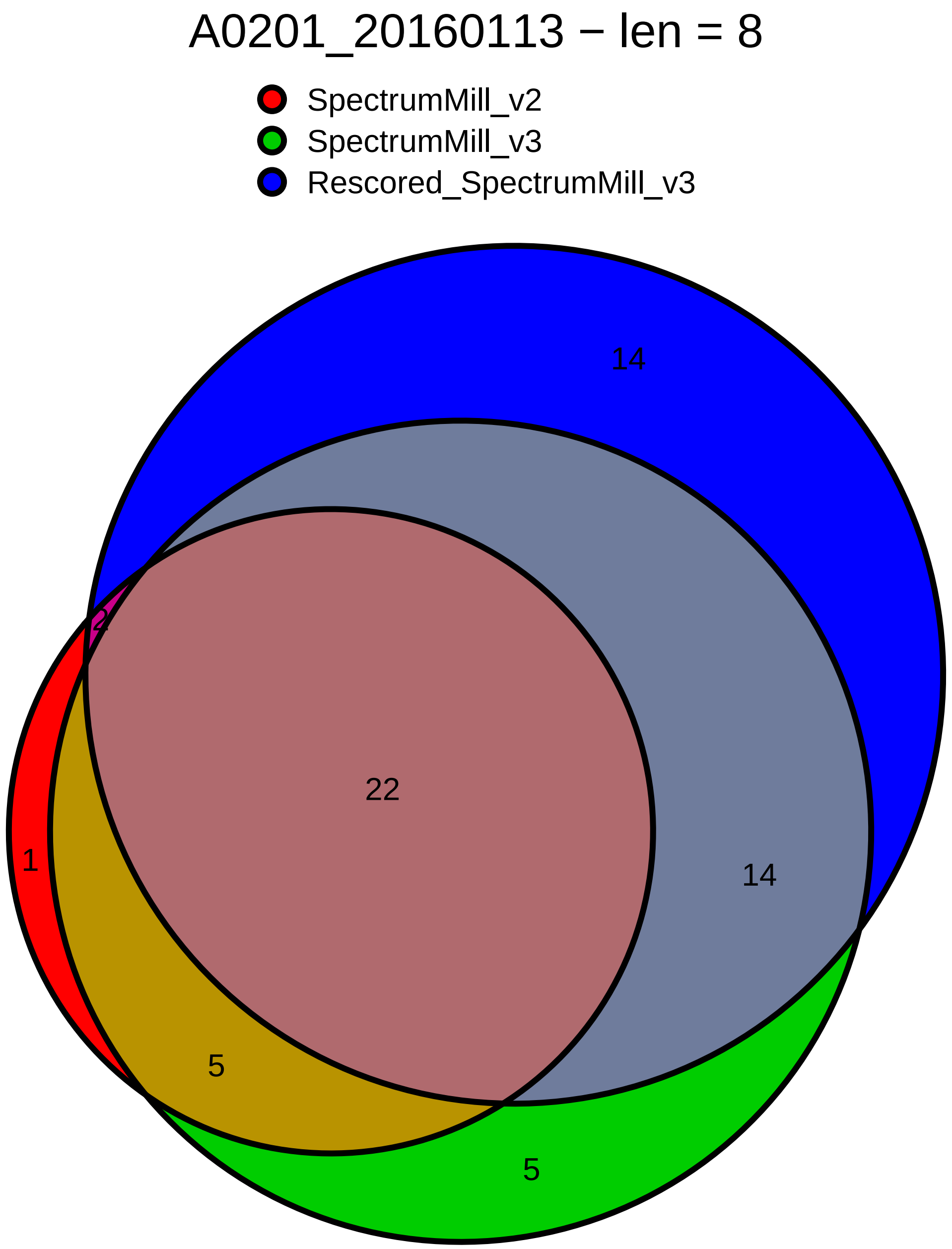
A0201_20160113 − len = 8
SpectrumMill_v2
SpectrumMill_v3
Rescored_SpectrumMill_v3
14
2
22
1
14
5
5

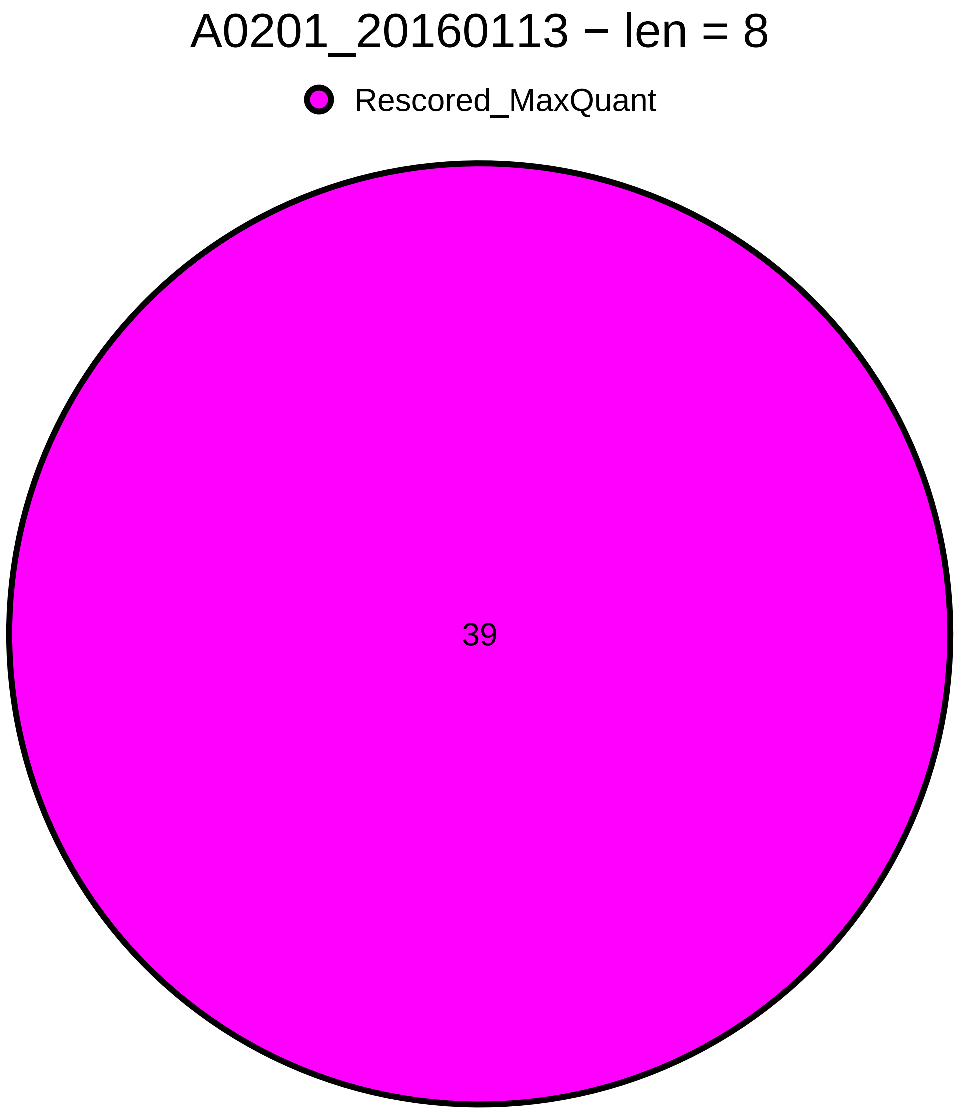
A0201_20160113 − len = 8
Rescored_MaxQuant
39

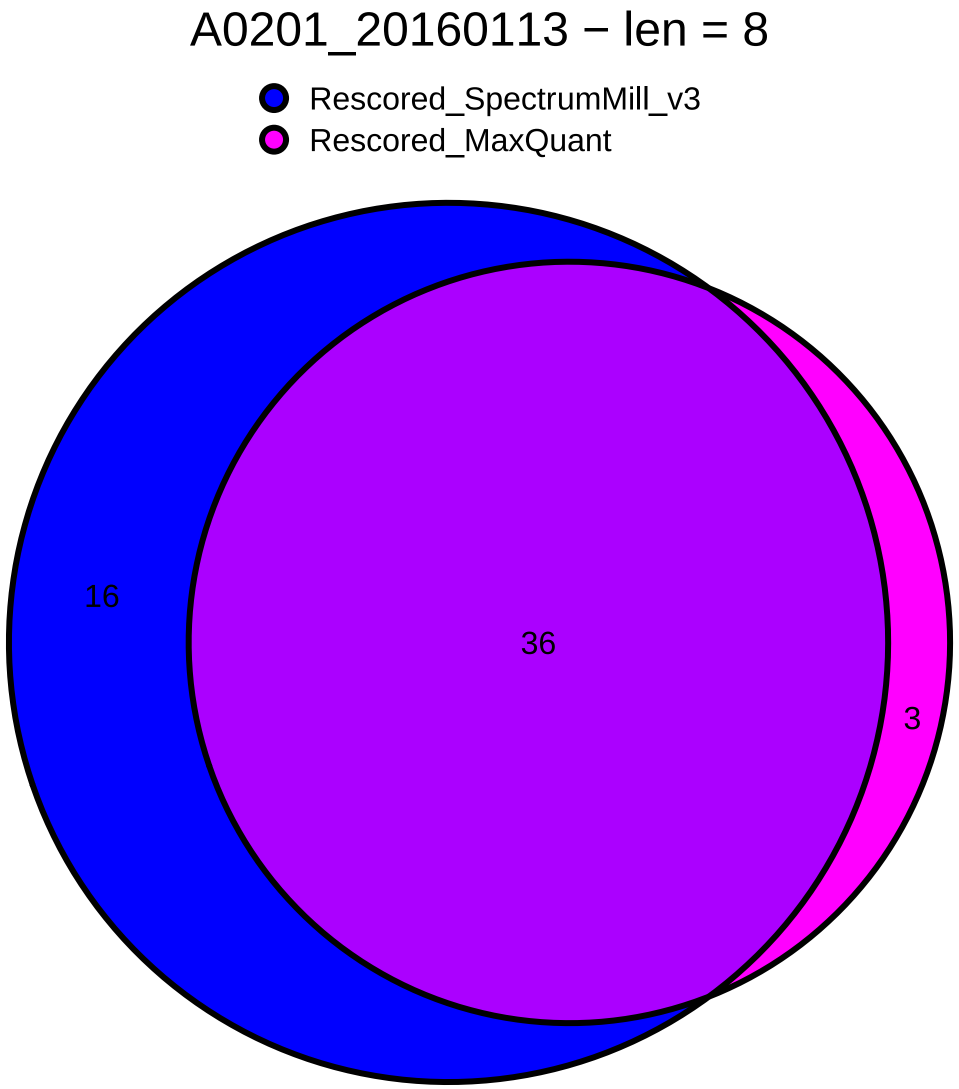
A0201_20160113 − len = 8
Rescored_SpectrumMill_v3
Rescored_MaxQuant
16
36
3

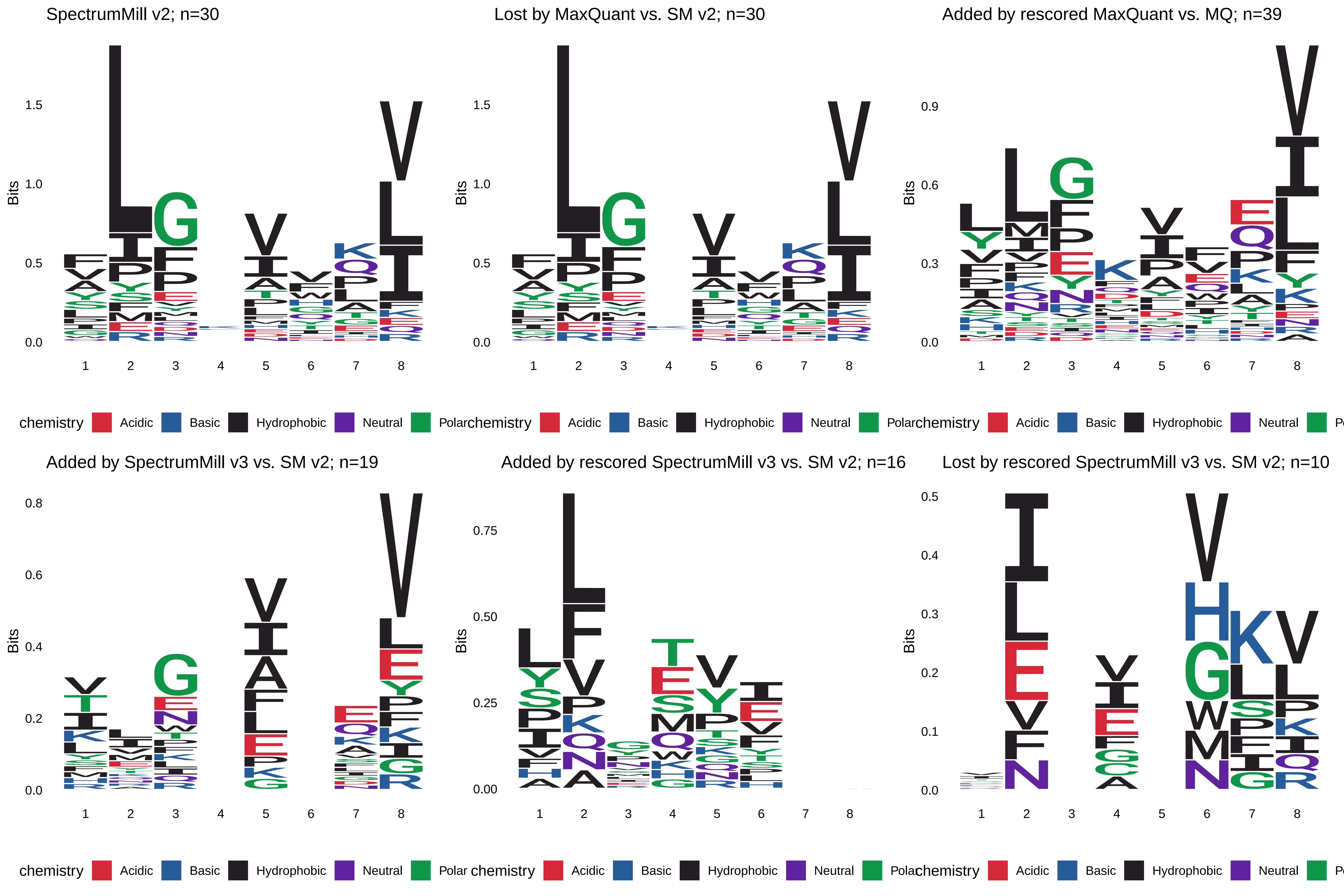
SpectrumMill v2; n=30
Lost by MaxQuant vs. SM v2; n=30
Added by rescored MaxQuant vs. MQ; n=39
Bits
1 2 3 4 5 6 7 8
chemistry Acidic Basic Hydrophobic Neutral Polar
Added by SpectrumMill v3 vs. SM v2; n=19
Added by rescored SpectrumMill v3 vs. SM v2; n=16
Lost by rescored SpectrumMill v3 vs. SM v2; n=10
Bits
1 2 3 4 5 6 7 8
chemistry Acidic Basic Hydrophobic Neutral Polar

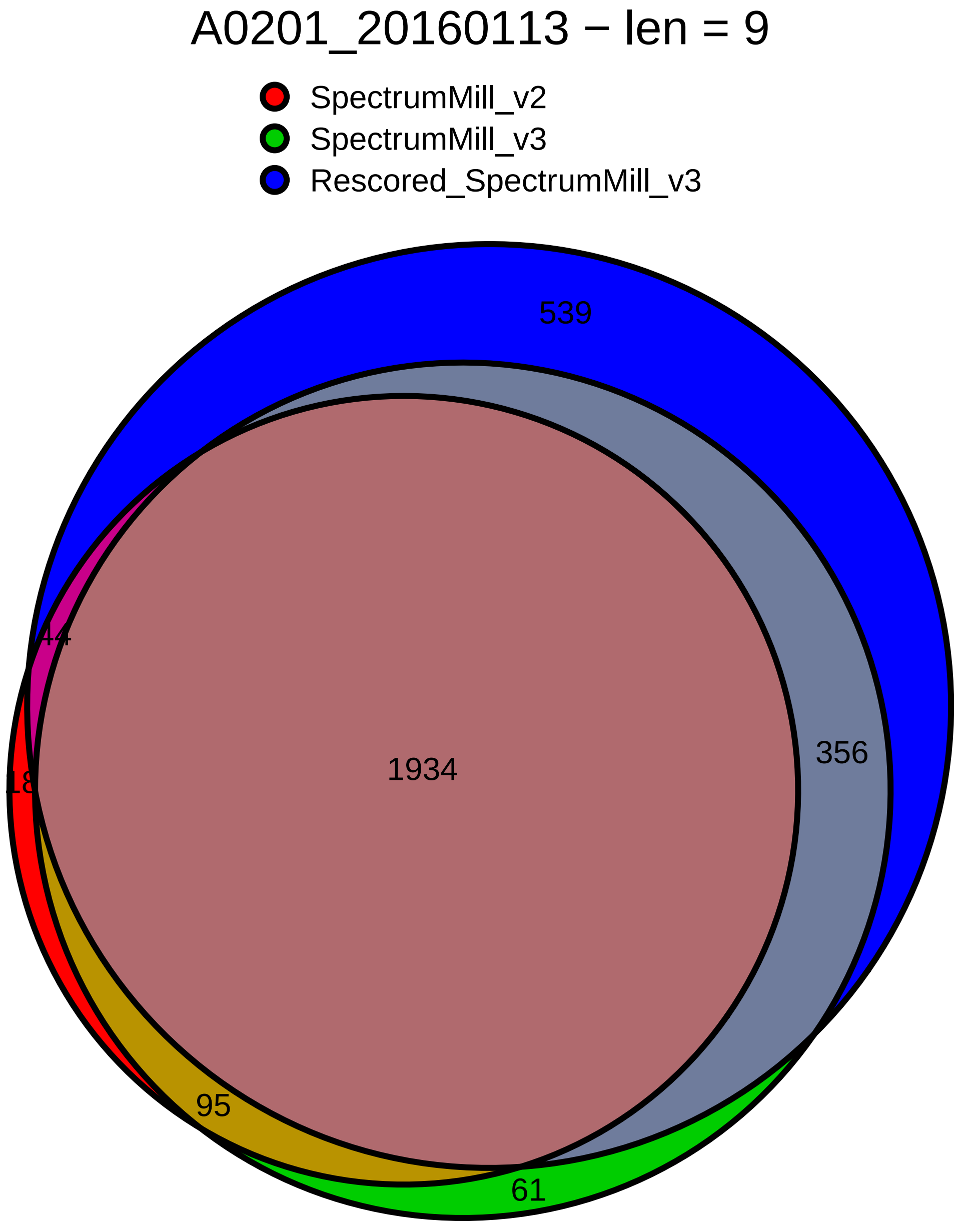
A0201_20160113 − len = 9
SpectrumMill_v2
SpectrumMill_v3
Rescored_SpectrumMill_v3
539
44
18
1934
356
95
61

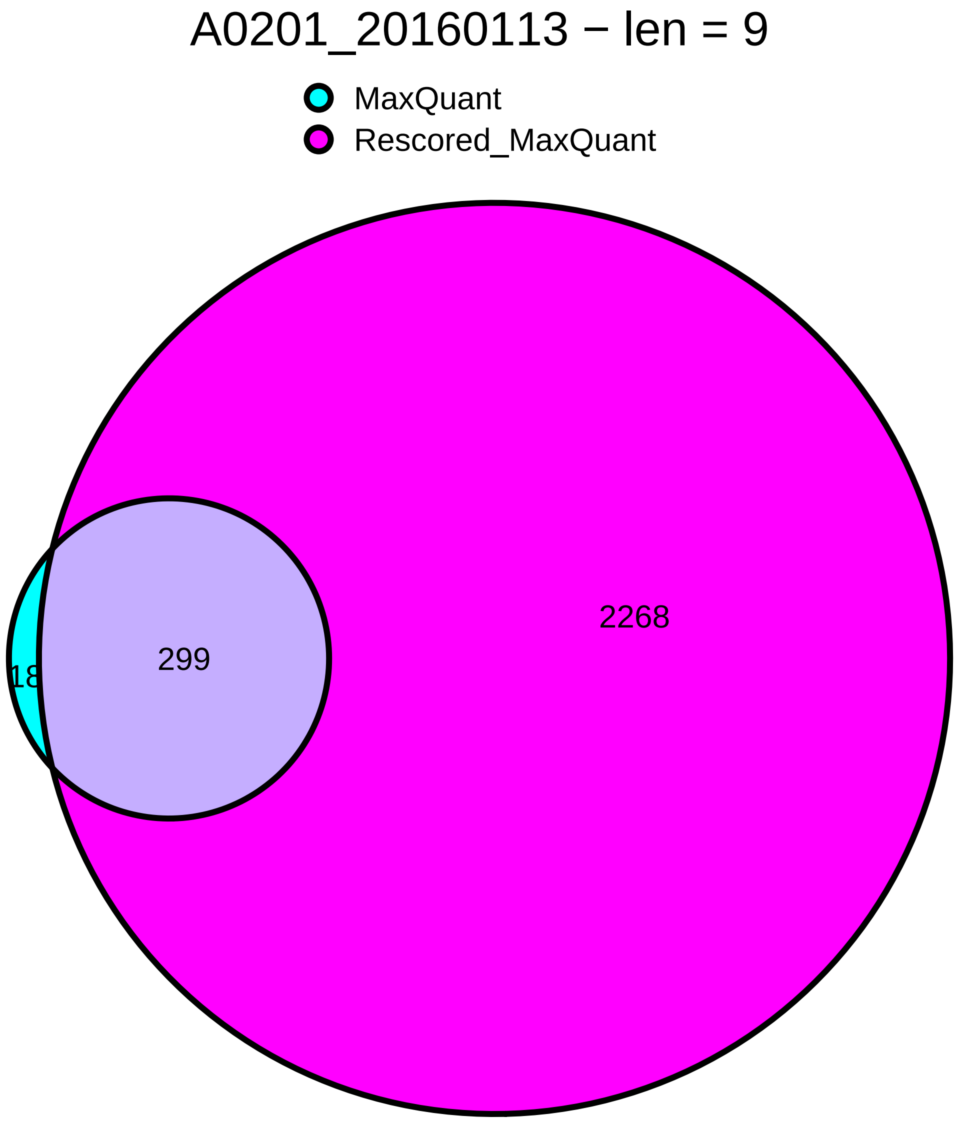
A0201_20160113 − len = 9
MaxQuant
Rescored_MaxQuant
18
299
2268

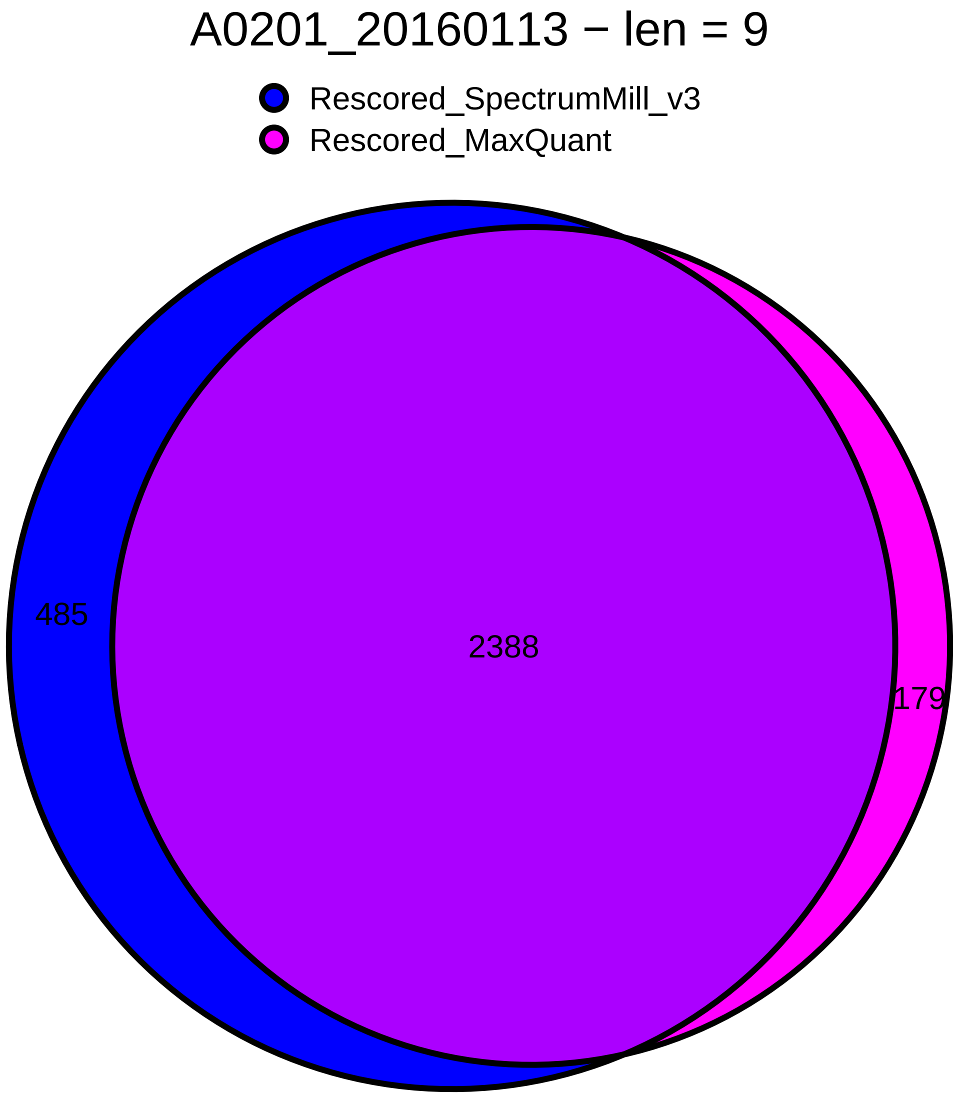
A0201_20160113 − len = 9
Rescored_SpectrumMill_v3
Rescored_MaxQuant
485
2388
179

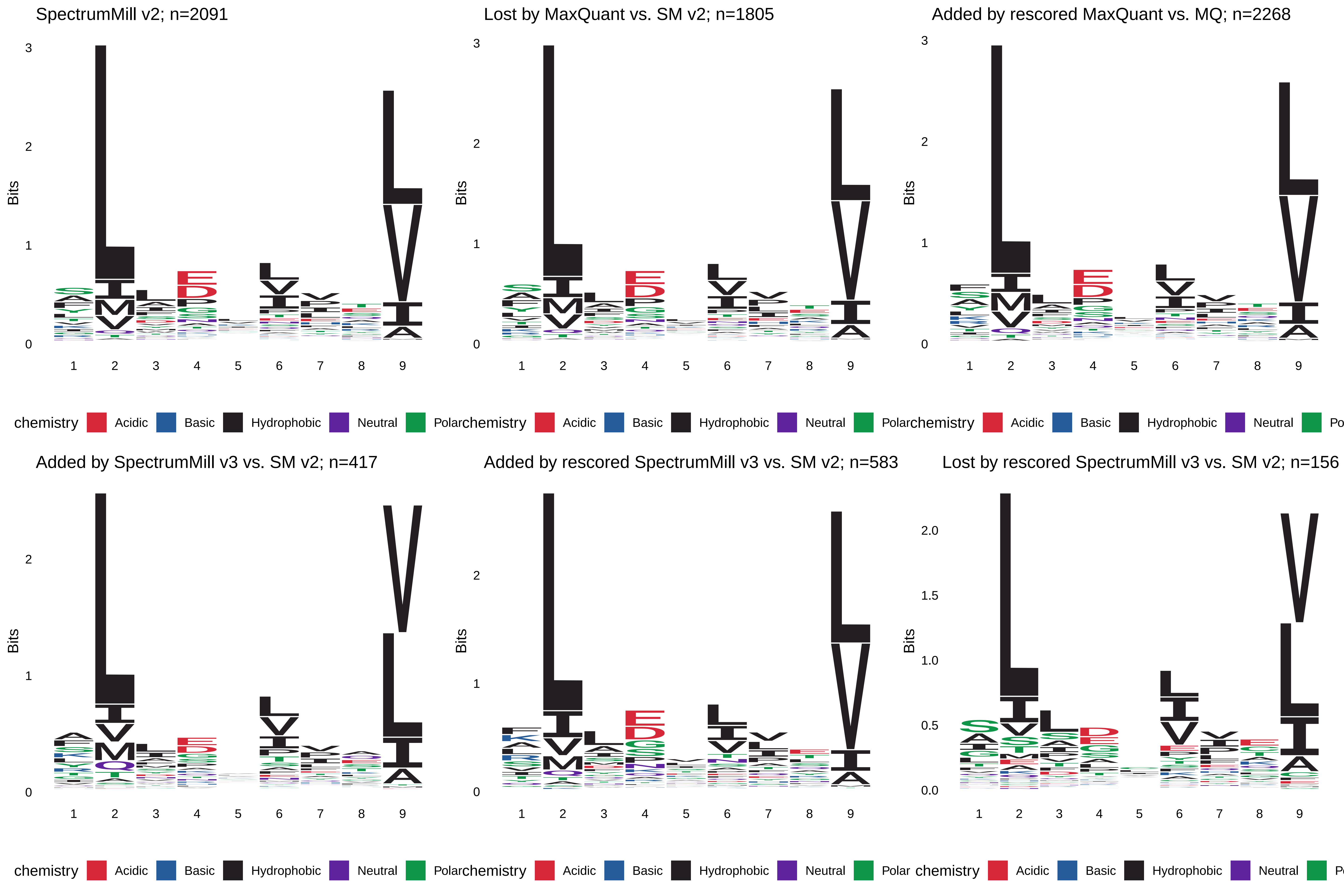
SpectrumMill v2; n=2091
Lost by MaxQuant vs. SM v2; n=1805
Added by rescored MaxQuant vs. MQ; n=2268
Added by SpectrumMill v3 vs. SM v2; n=417
Added by rescored SpectrumMill v3 vs. SM v2; n=583
Lost by rescored SpectrumMill v3 vs. SM v2; n=156
Bits
1 2 3 4 5 6 7 8 9
chemistry
Acidic
Basic
Hydrophobic
Neutral
Polar

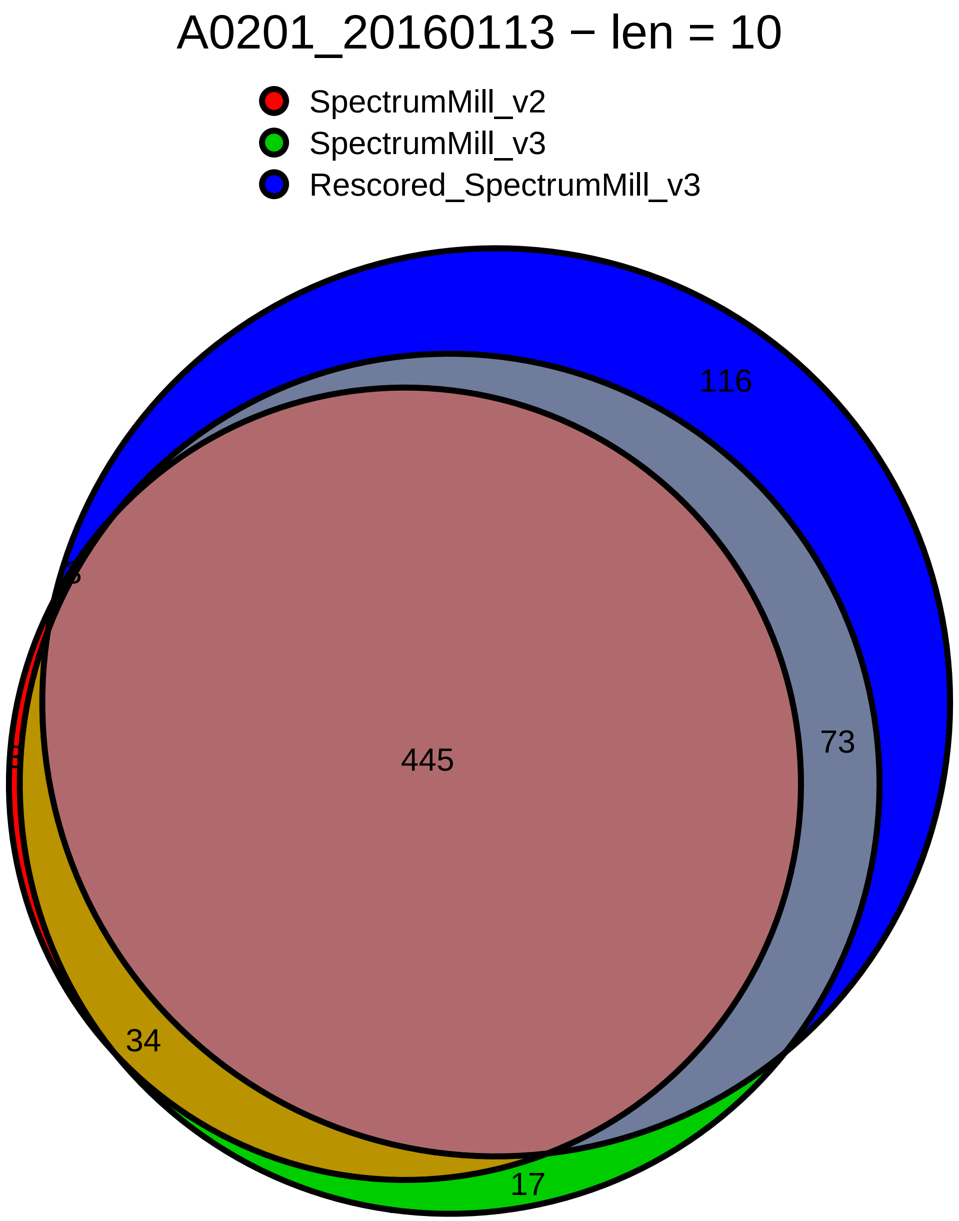
A0201_20160113 − len = 10
SpectrumMill_v2
SpectrumMill_v3
Rescored_SpectrumMill_v3
116
73
445
34
17

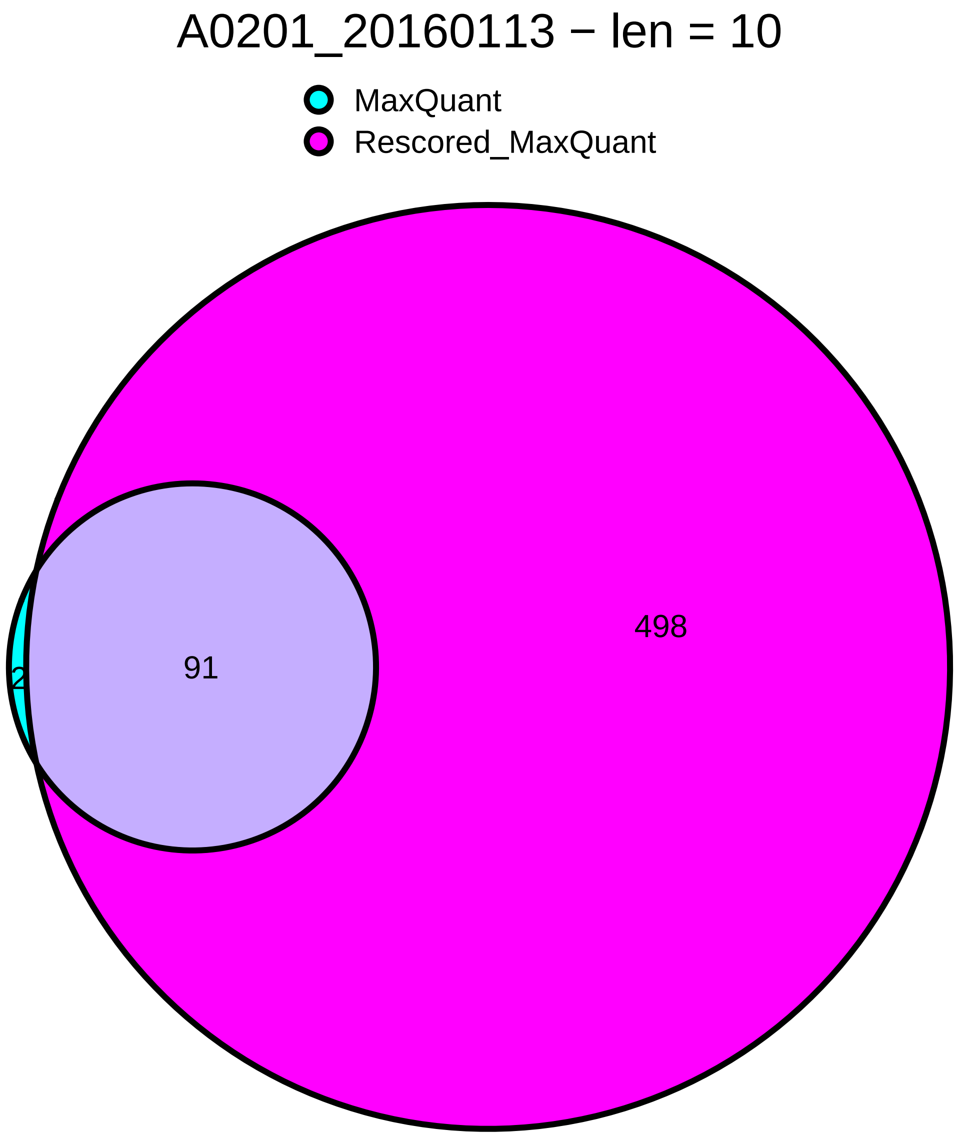
A0201_20160113 − len = 10
MaxQuant
Rescored_MaxQuant
91
498

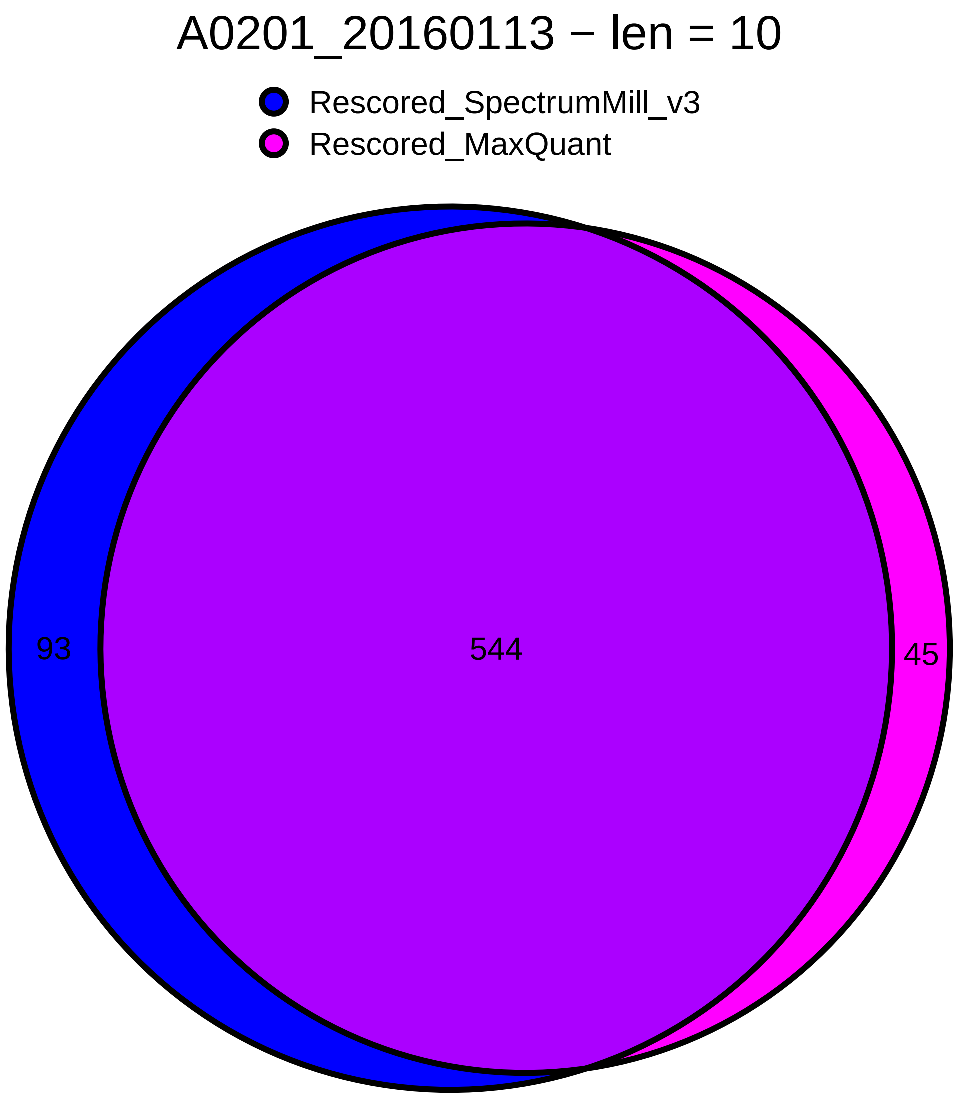
A0201_20160113 − len = 10
Rescored_SpectrumMill_v3
Rescored_MaxQuant
93
544
45

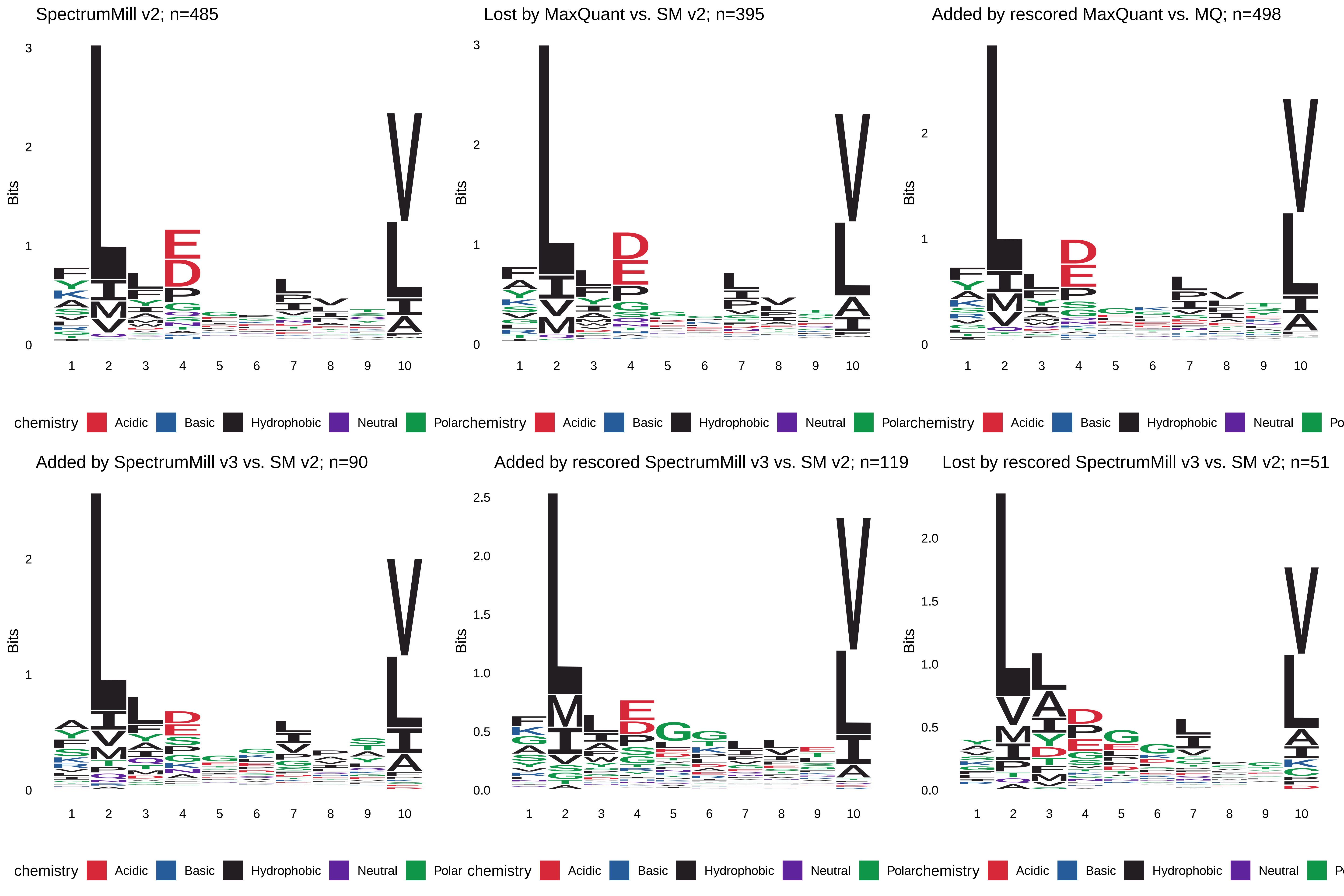
SpectrumMill v2; n=485
Lost by MaxQuant vs. SM v2; n=395
Added by rescored MaxQuant vs. MQ; n=498
Bits
1 2 3 4 5 6 7 8 9 10
chemistry Acidic Basic Hydrophobic Neutral Polar
Added by SpectrumMill v3 vs. SM v2; n=90
Added by rescored SpectrumMill v3 vs. SM v2; n=119
Lost by rescored SpectrumMill v3 vs. SM v2; n=51
Bits
1 2 3 4 5 6 7 8 9 10
chemistry Acidic Basic Hydrophobic Neutral Polar

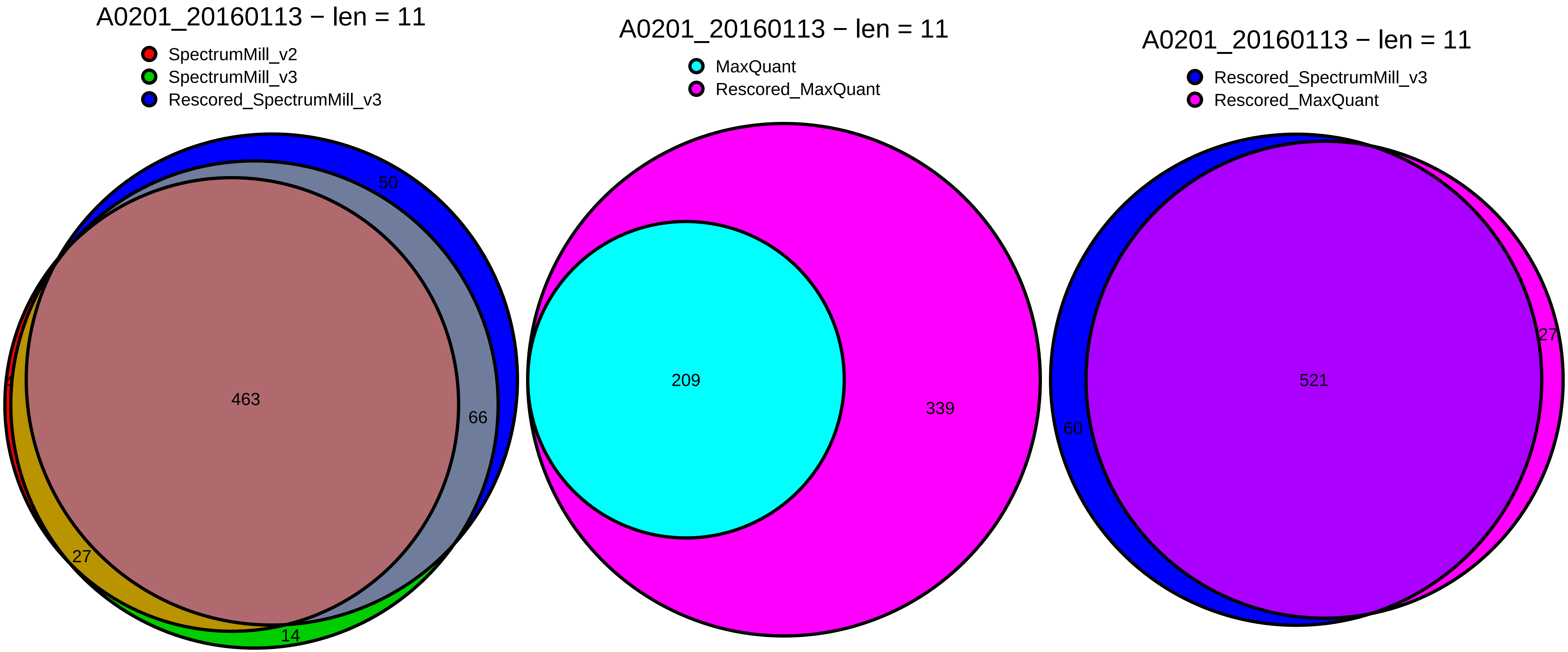
A0201_20160113 − len = 11
SpectrumMill_v2
SpectrumMill_v3
Rescored_SpectrumMill_v3
50
463
66
27
14
A0201_20160113 − len = 11
MaxQuant
Rescored_MaxQuant
209
339
A0201_20160113 − len = 11
Rescored_SpectrumMill_v3
Rescored_MaxQuant
521
60
27

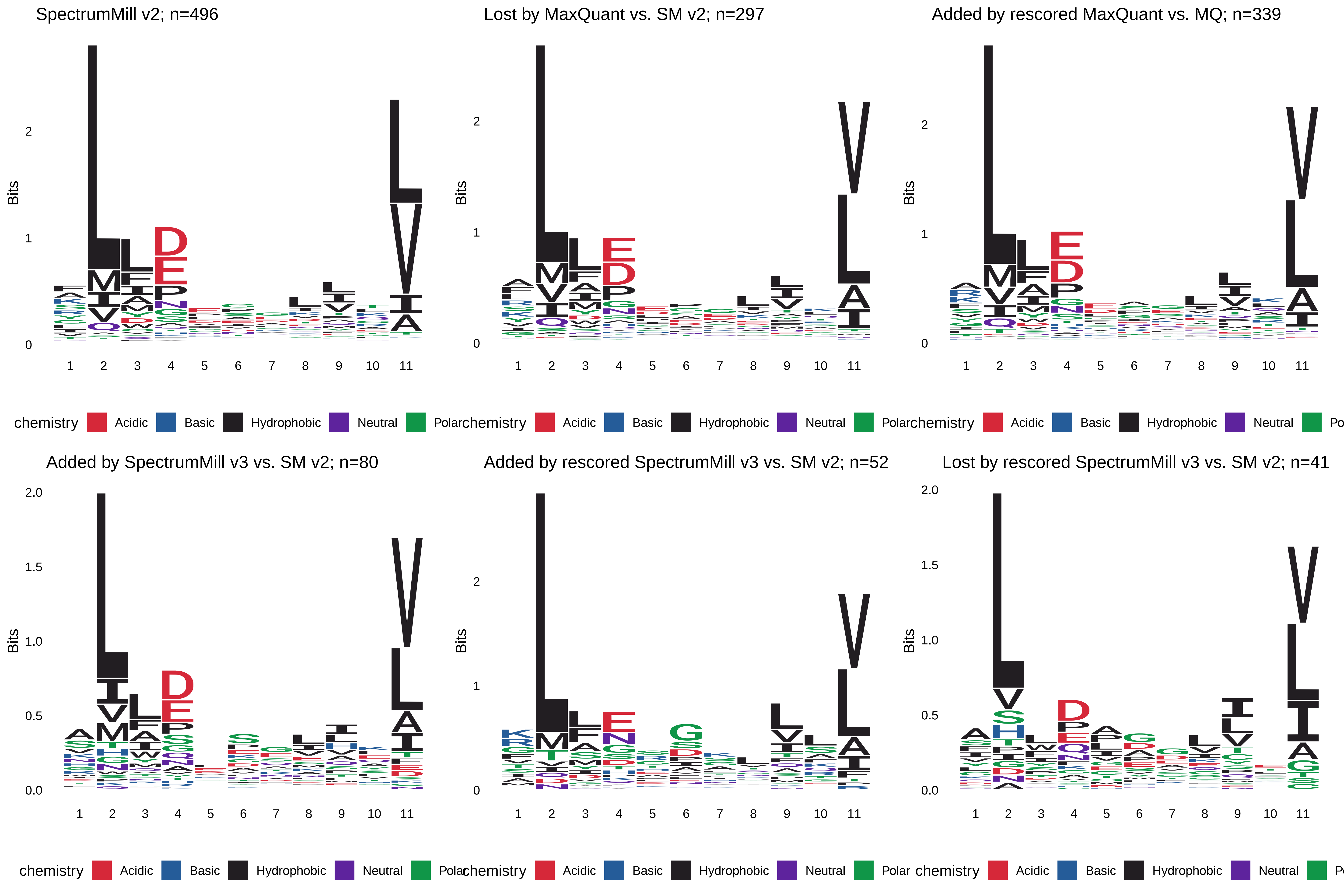
SpectrumMill v2; n=496
Lost by MaxQuant vs. SM v2; n=297
Added by rescored MaxQuant vs. MQ; n=339
Added by SpectrumMill v3 vs. SM v2; n=80
Added by rescored SpectrumMill v3 vs. SM v2; n=52
Lost by rescored SpectrumMill v3 vs. SM v2; n=41
Bits
1 2 3 4 5 6 7 8 9 10 11
chemistry
Acidic
Basic
Hydrophobic
Neutral
Polar

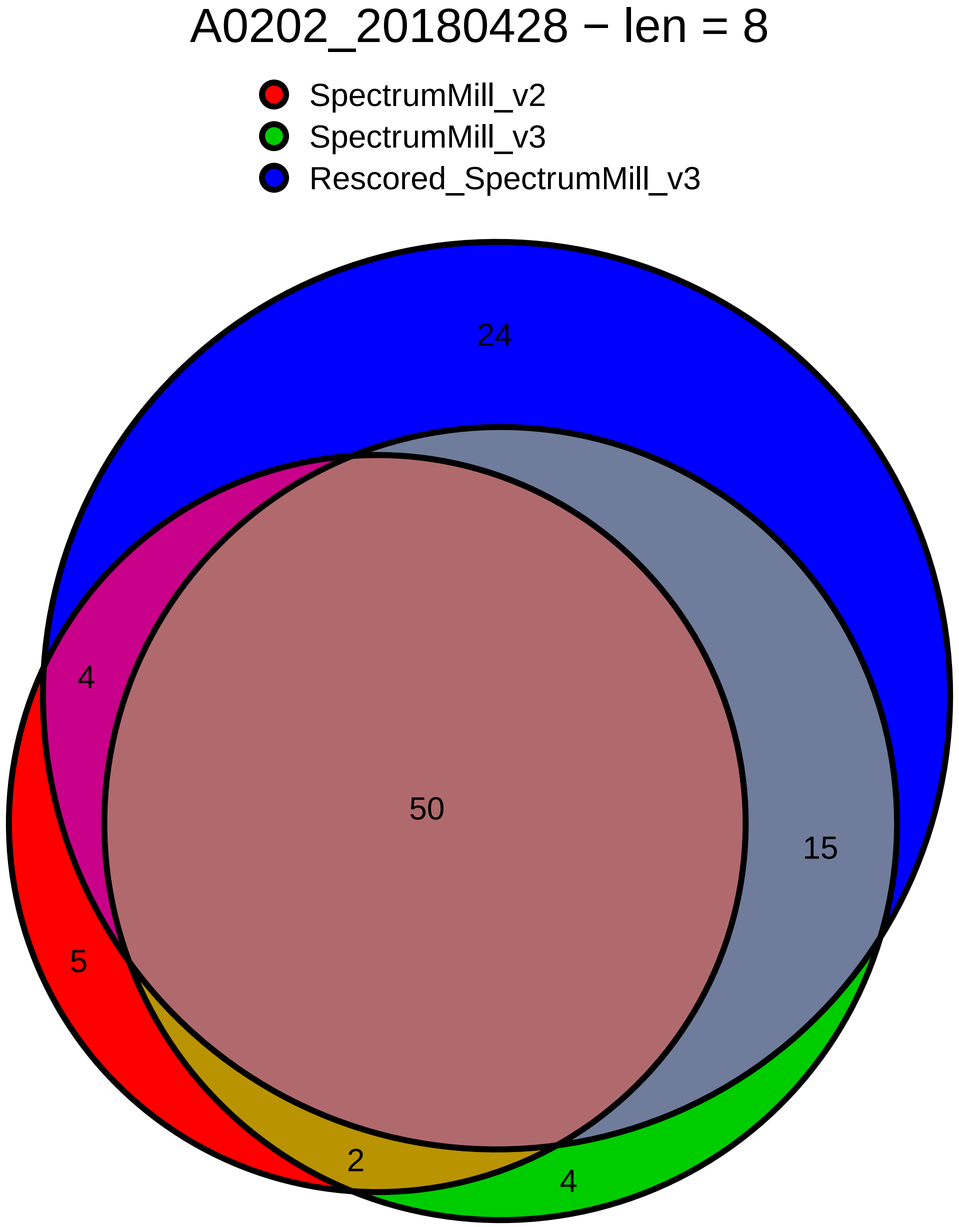

A0202_20180428 − len = 8
SpectrumMill_v2
SpectrumMill_v3
Rescored_SpectrumMill_v3
24
4
50
15
5
2
4

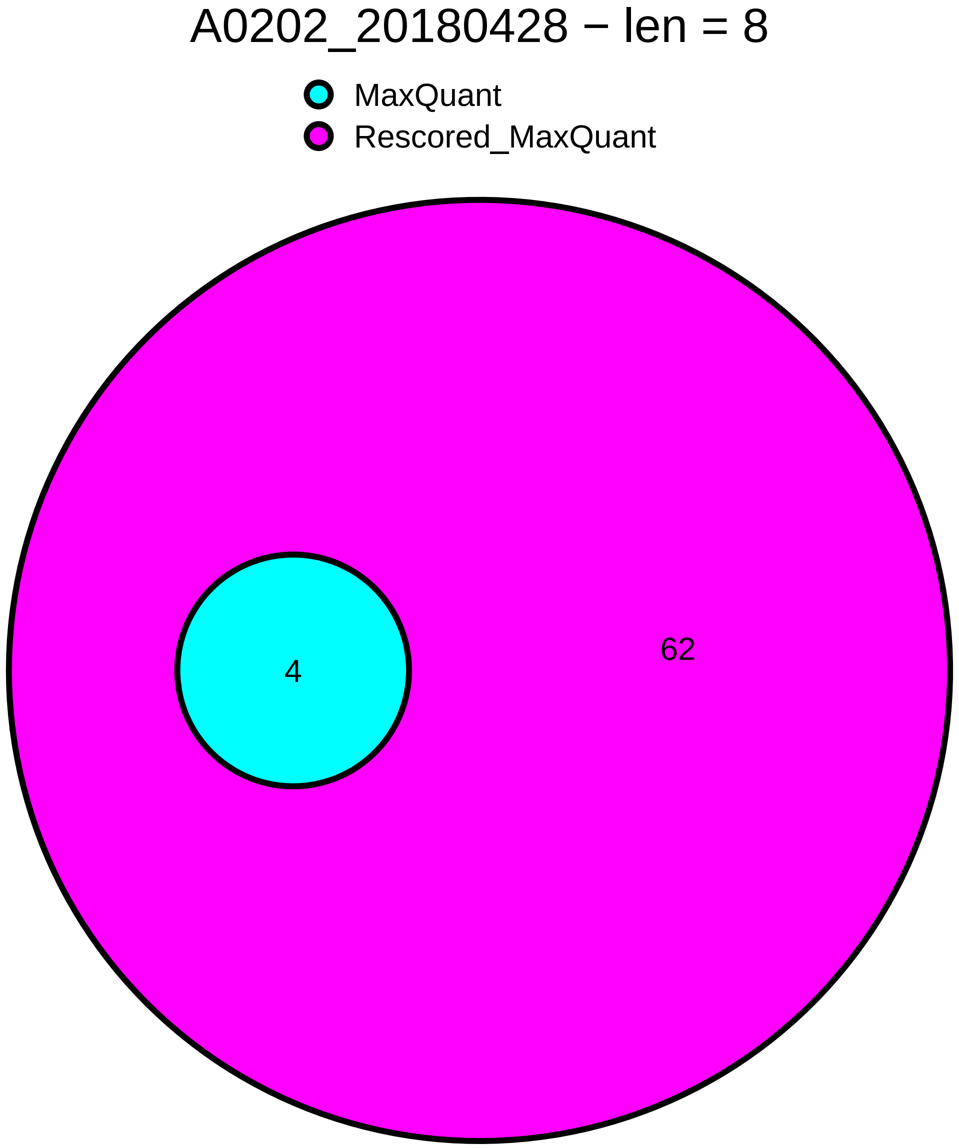

A0202_20180428 − len = 8
MaxQuant
Rescored_MaxQuant
4
62

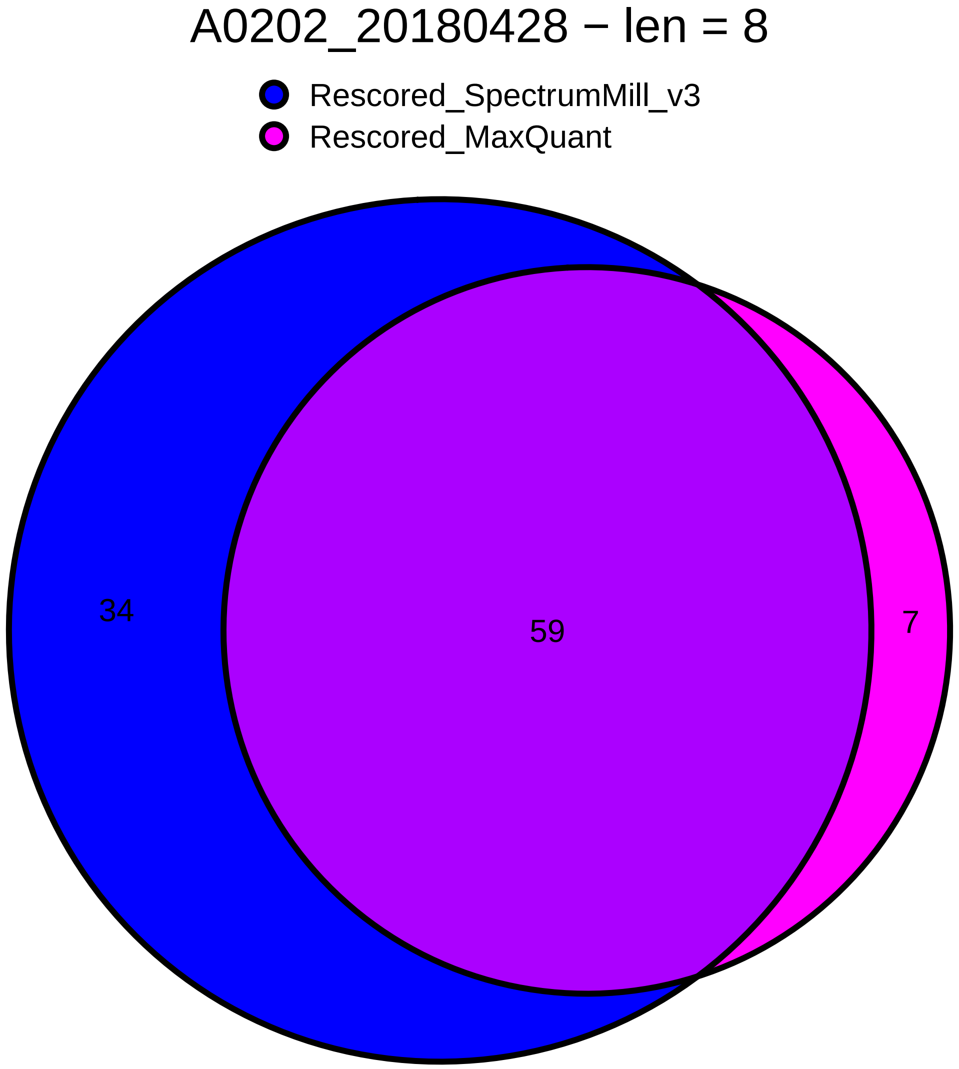

A0202_20180428 − len = 8
Rescored_SpectrumMill_v3
Rescored_MaxQuant
34
59
7

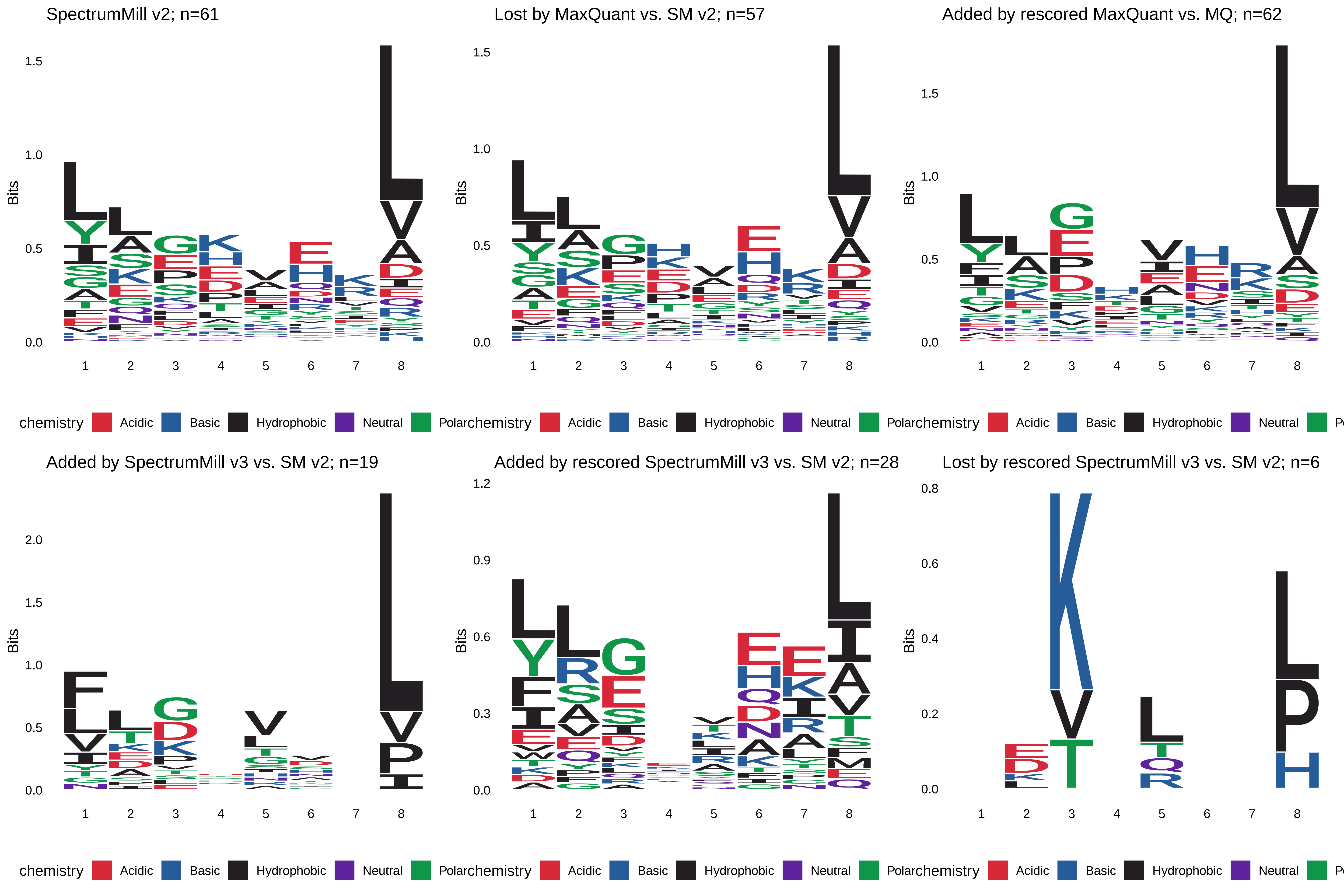
SpectrumMill v2; n=61
Lost by MaxQuant vs. SM v2; n=57
Added by rescored MaxQuant vs. MQ; n=62
Added by SpectrumMill v3 vs. SM v2; n=19
Added by rescored SpectrumMill v3 vs. SM v2; n=28
Lost by rescored SpectrumMill v3 vs. SM v2; n=6
Bits
1 2 3 4 5 6 7 8
chemistry Acidic Basic Hydrophobic Neutral Polar

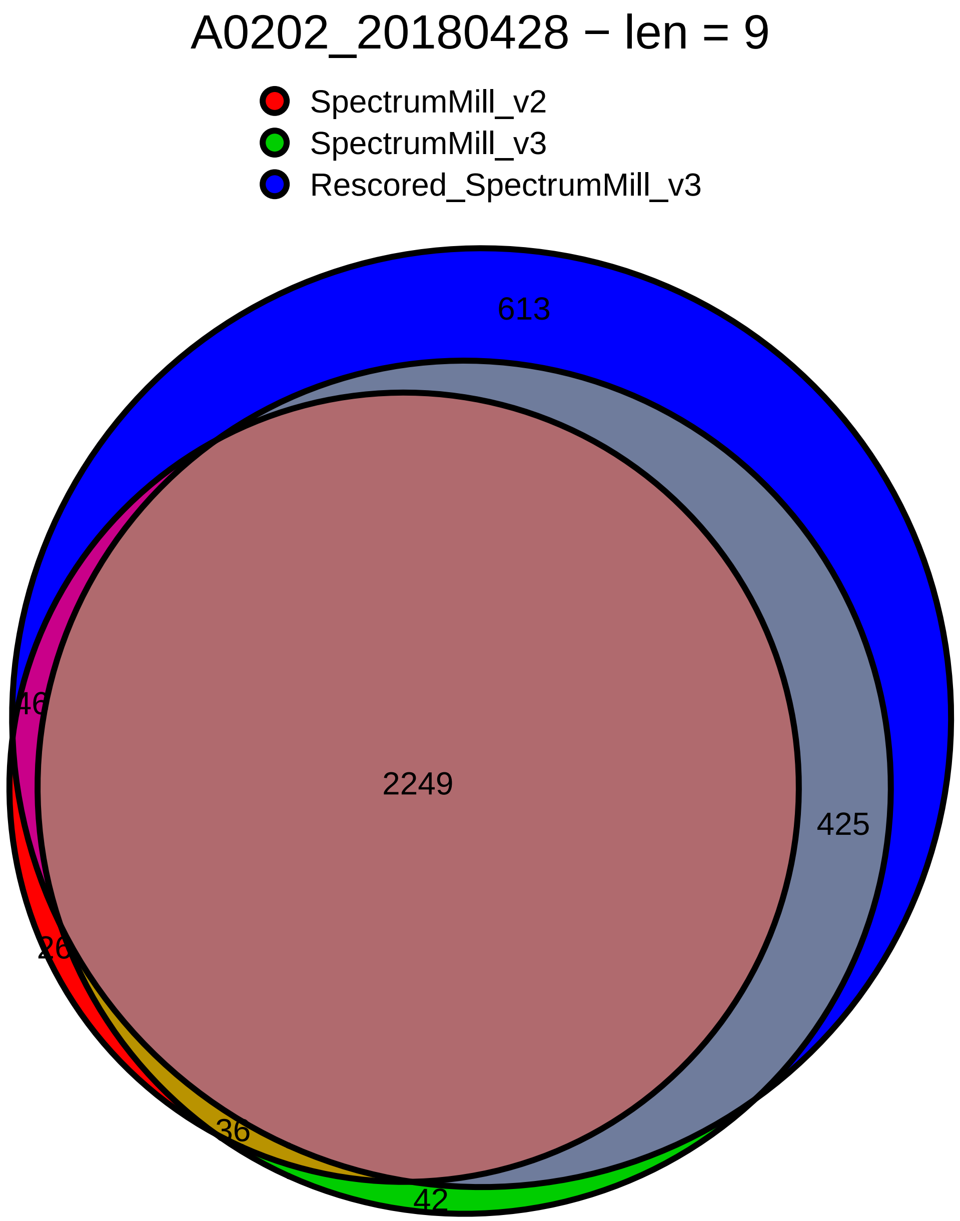
A0202_20180428 − len = 9
SpectrumMill_v2
SpectrumMill_v3
Rescored_SpectrumMill_v3
613
46
2249
425
26
36
42

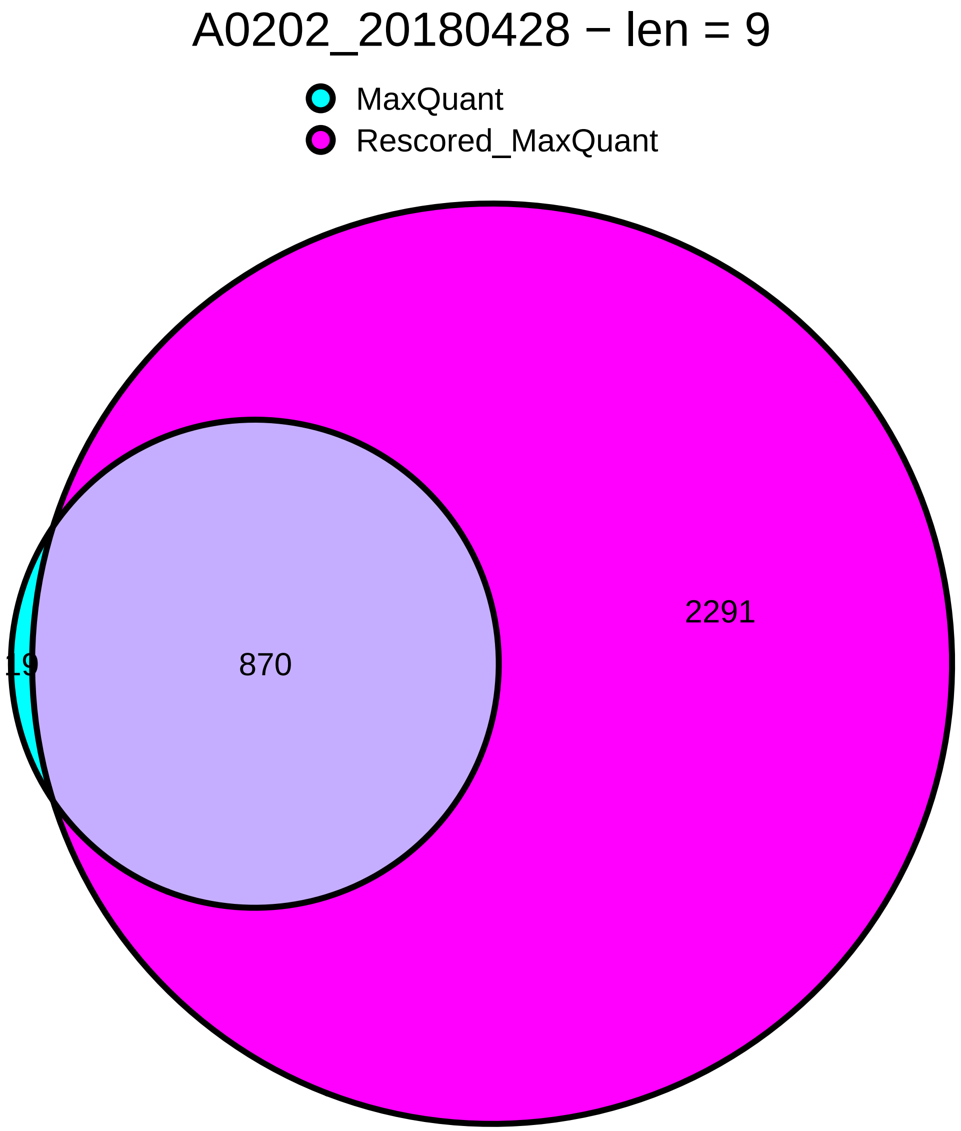
A0202_20180428 − len = 9
MaxQuant
Rescored_MaxQuant
19
870
2291

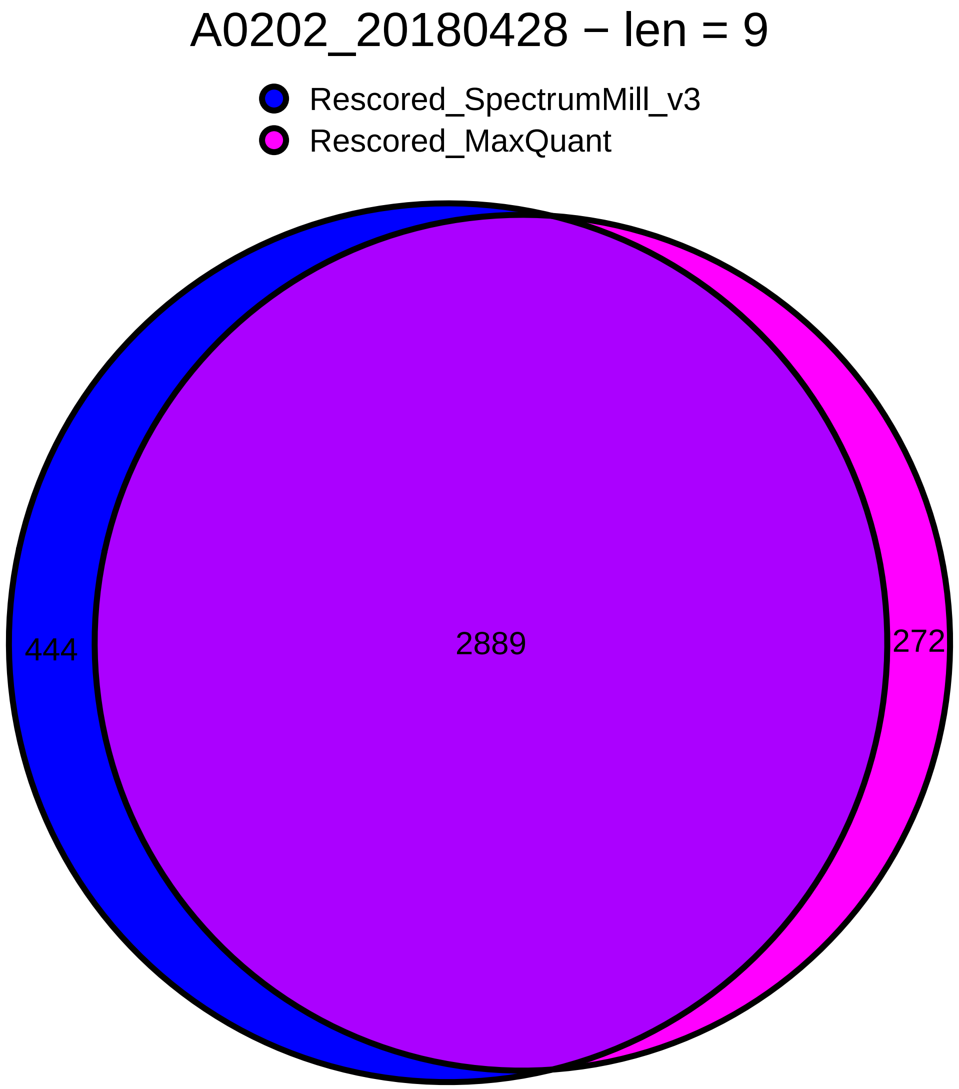
A0202_20180428 − len = 9
Rescored_SpectrumMill_v3
Rescored_MaxQuant
444
2889
272

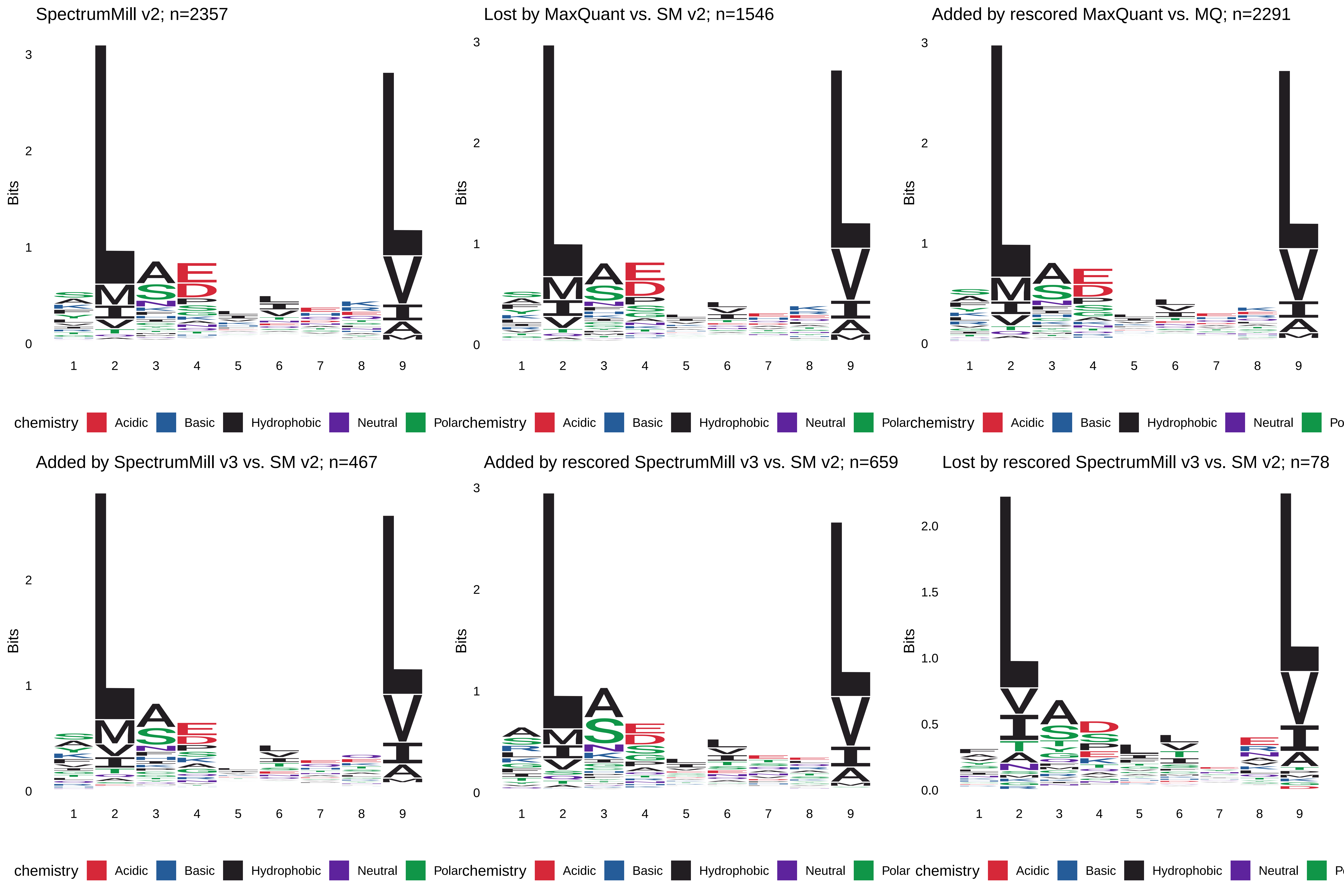

SpectrumMill v2; n=2357
Lost by MaxQuant vs. SM v2; n=1546
Added by rescored MaxQuant vs. MQ; n=2291
Added by SpectrumMill v3 vs. SM v2; n=467
Added by rescored SpectrumMill v3 vs. SM v2; n=659
Lost by rescored SpectrumMill v3 vs. SM v2; n=78
Bits
1 2 3 4 5 6 7 8 9
chemistry
Acidic
Basic
Hydrophobic
Neutral
Polar

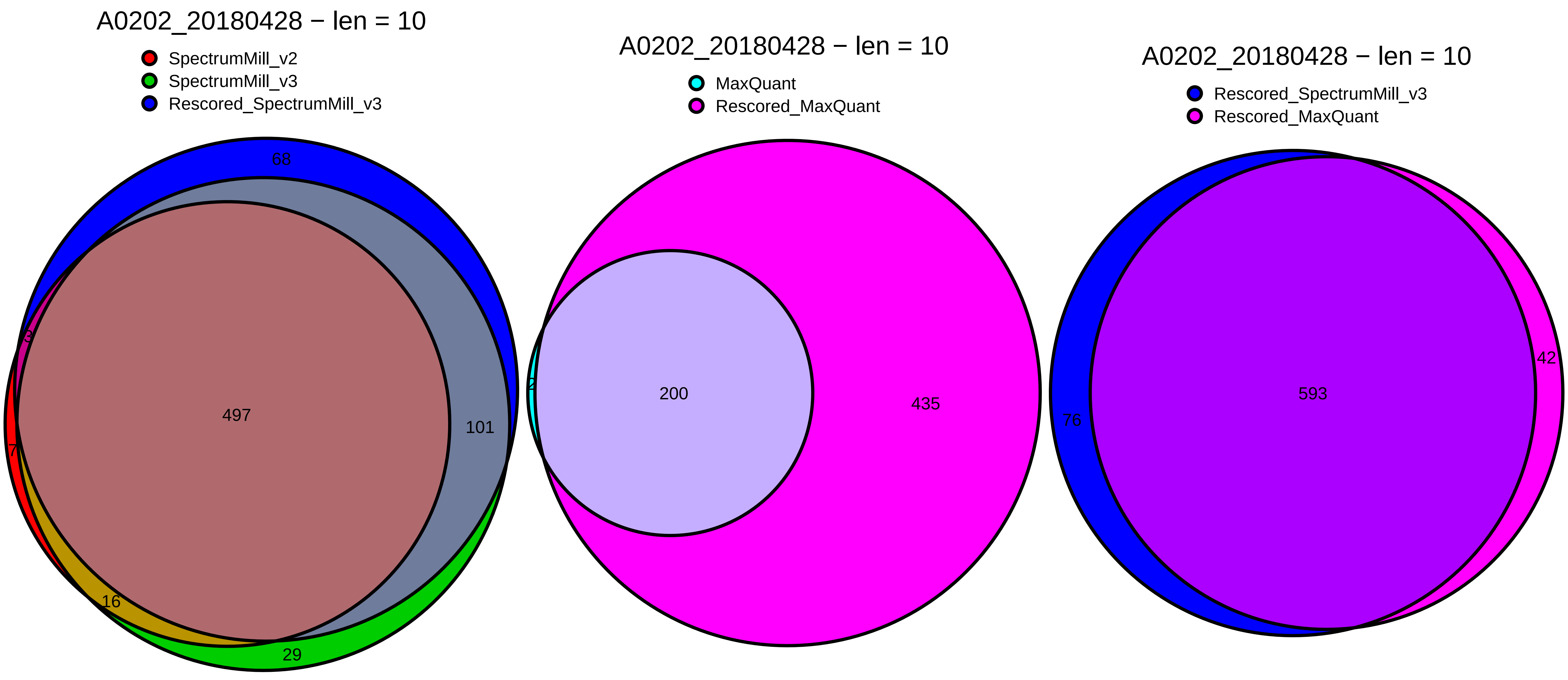
A0202_20180428 − len = 10
SpectrumMill_v2
SpectrumMill_v3
Rescored_SpectrumMill_v3
68
497
101
16
29
A0202_20180428 − len = 10
MaxQuant
Rescored_MaxQuant
200
435
A0202_20180428 − len = 10
Rescored_SpectrumMill_v3
Rescored_MaxQuant
593
76
42

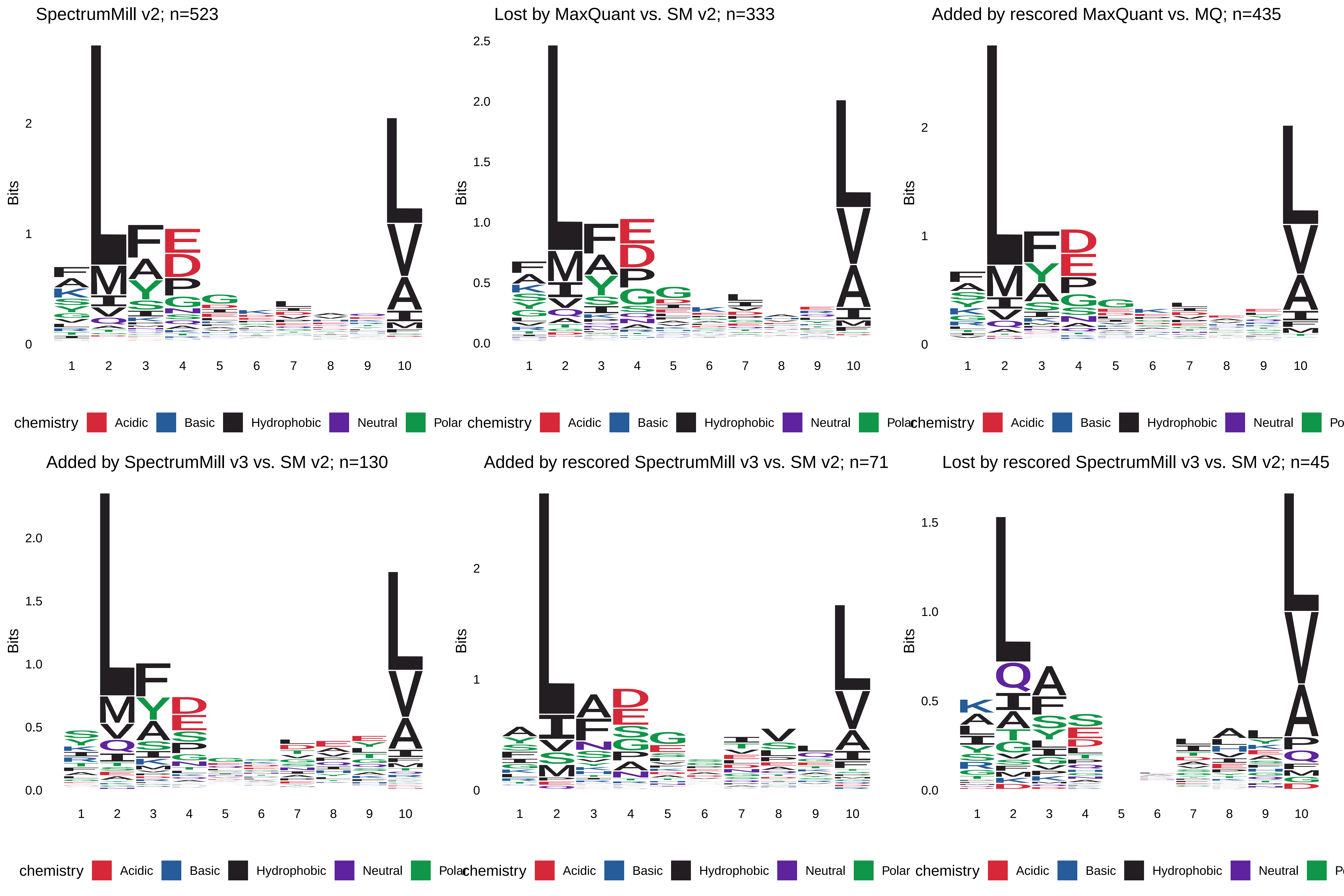
SpectrumMill v2; n=523
Lost by MaxQuant vs. SM v2; n=333
Added by rescored MaxQuant vs. MQ; n=435
Added by SpectrumMill v3 vs. SM v2; n=130
Added by rescored SpectrumMill v3 vs. SM v2; n=71
Lost by rescored SpectrumMill v3 vs. SM v2; n=45
Bits
chemistry
Acidic
Basic
Hydrophobic
Neutral
Polar

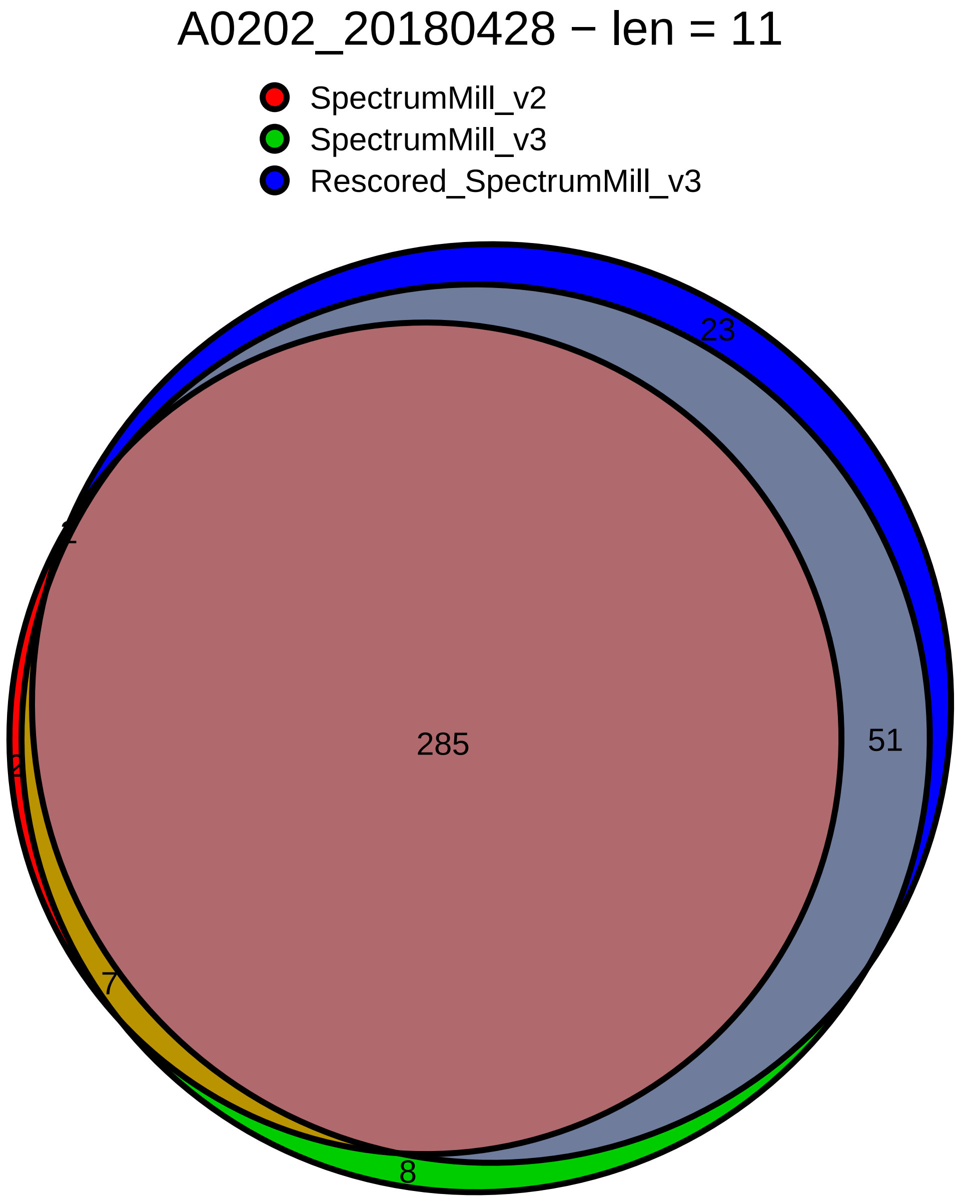

A0202_20180428 − len = 11
SpectrumMill_v2
SpectrumMill_v3
Rescored_SpectrumMill_v3
23
285
51
7
8

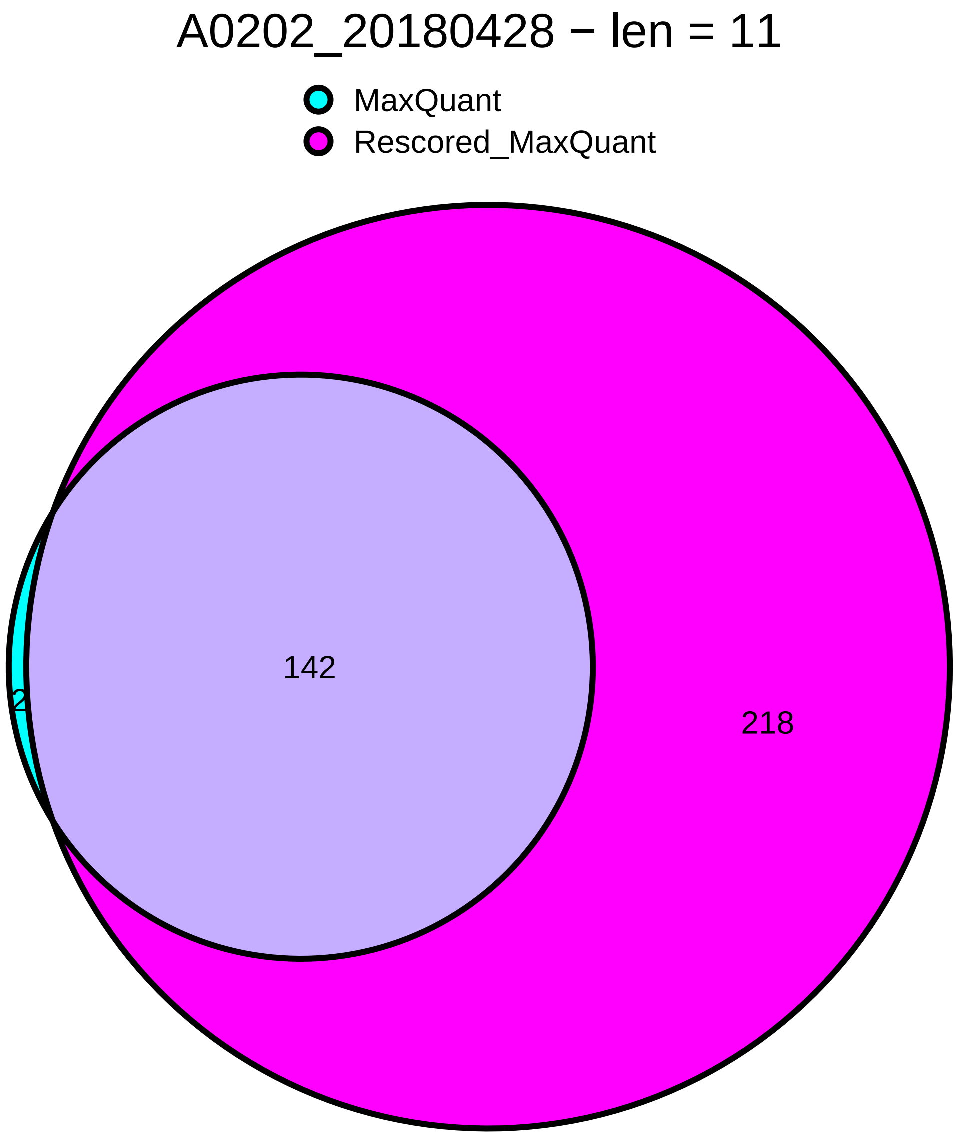

A0202_20180428 − len = 11
MaxQuant
Rescored_MaxQuant
142
218

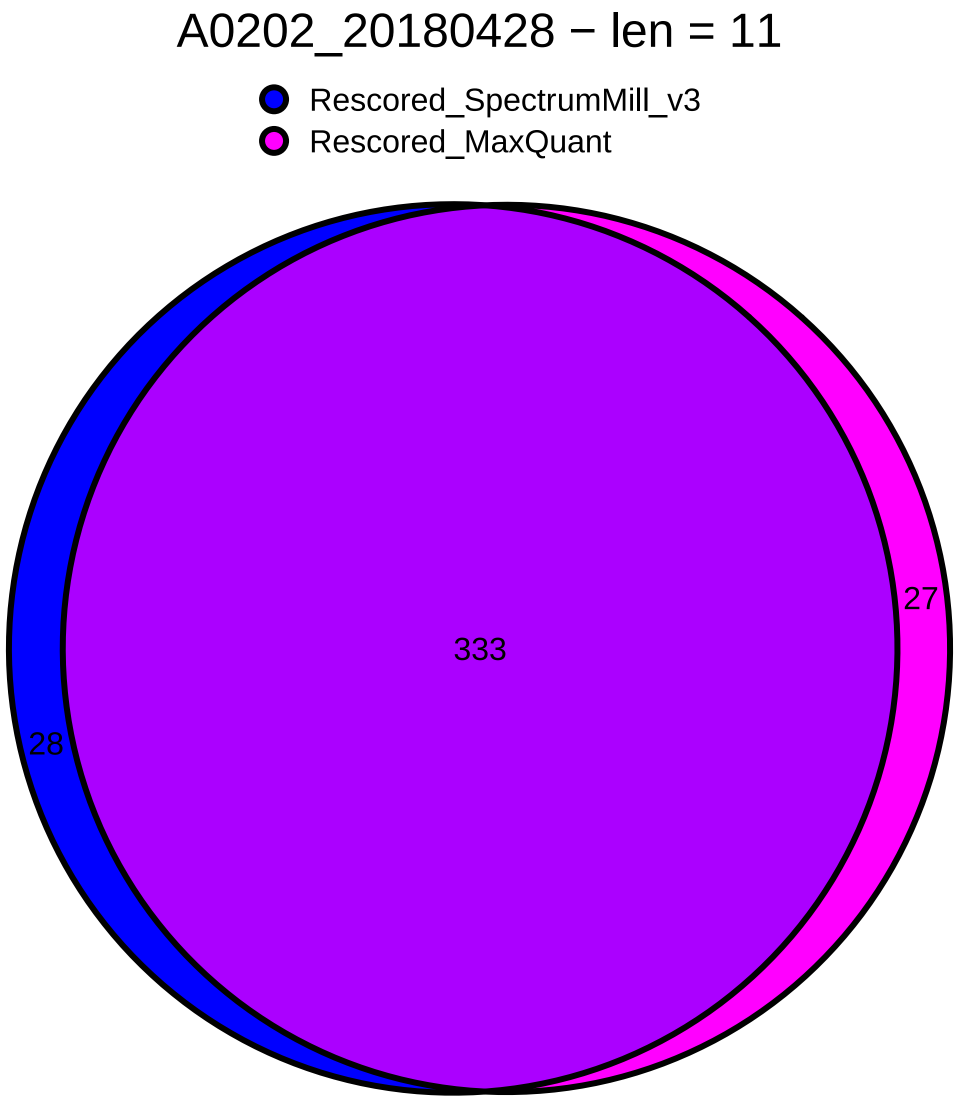

A0202_20180428 − len = 11
Rescored_SpectrumMill_v3
Rescored_MaxQuant
333
28
27

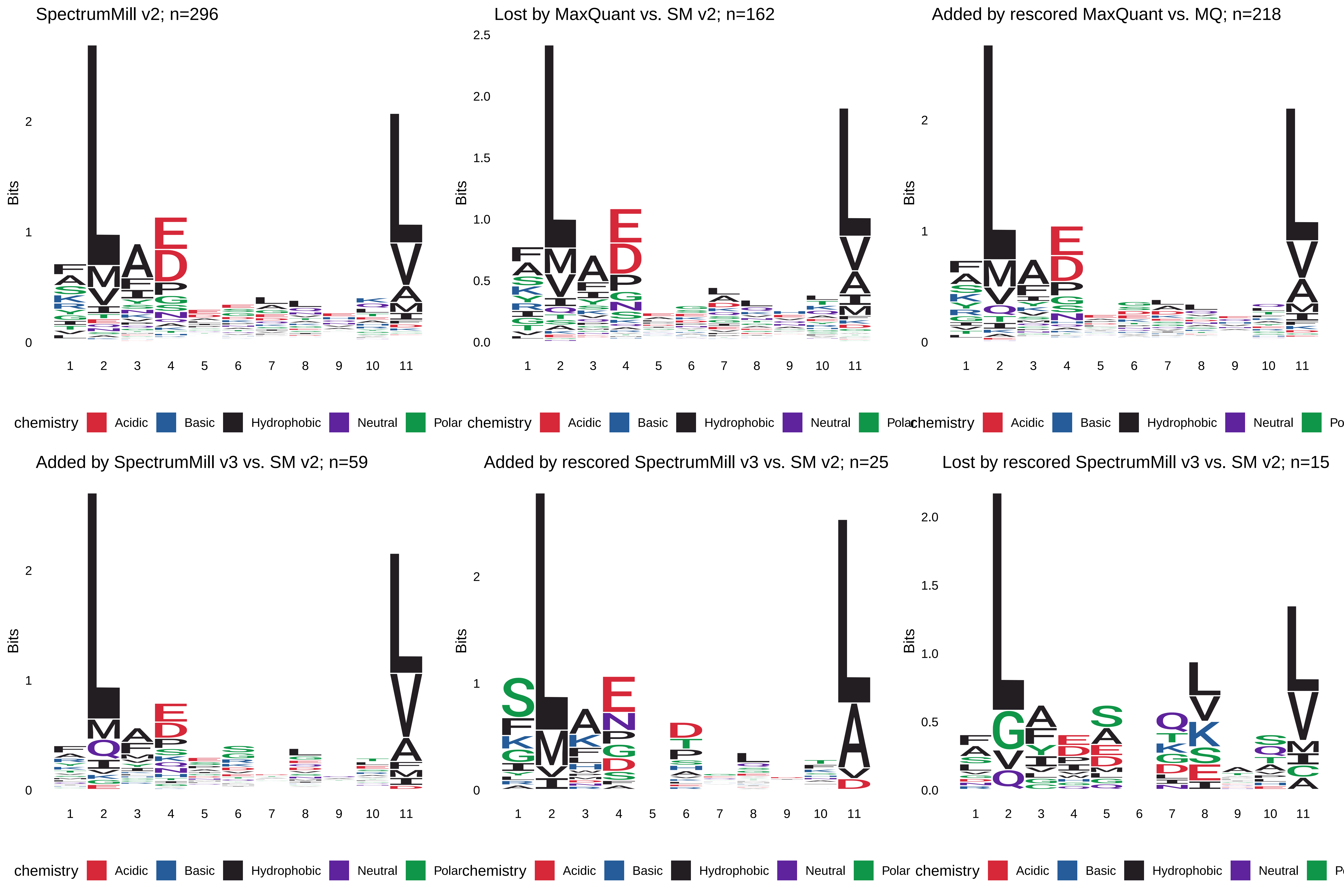

SpectrumMill v2; n=296
Lost by MaxQuant vs. SM v2; n=162
Added by rescored MaxQuant vs. MQ; n=218
Added by SpectrumMill v3 vs. SM v2; n=59
Added by rescored SpectrumMill v3 vs. SM v2; n=25
Lost by rescored SpectrumMill v3 vs. SM v2; n=15
Bits
1 2 3 4 5 6 7 8 9 10 11
chemistry
Acidic
Basic
Hydrophobic
Neutral
Polar

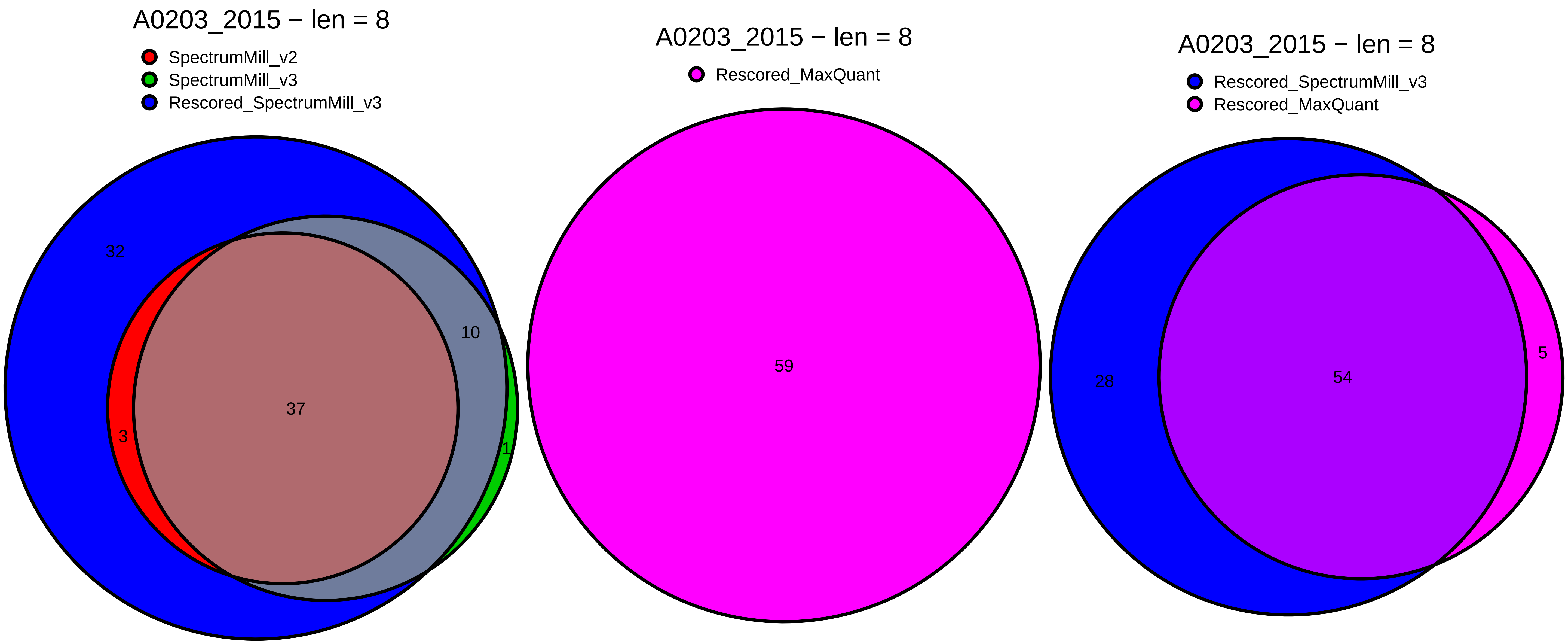

A0203_2015 − len = 8
SpectrumMill_v2
SpectrumMill_v3
Rescored_SpectrumMill_v3
32
10
37
3
1
A0203_2015 − len = 8
Rescored_MaxQuant
59
A0203_2015 − len = 8
Rescored_SpectrumMill_v3
Rescored_MaxQuant
28
54
5

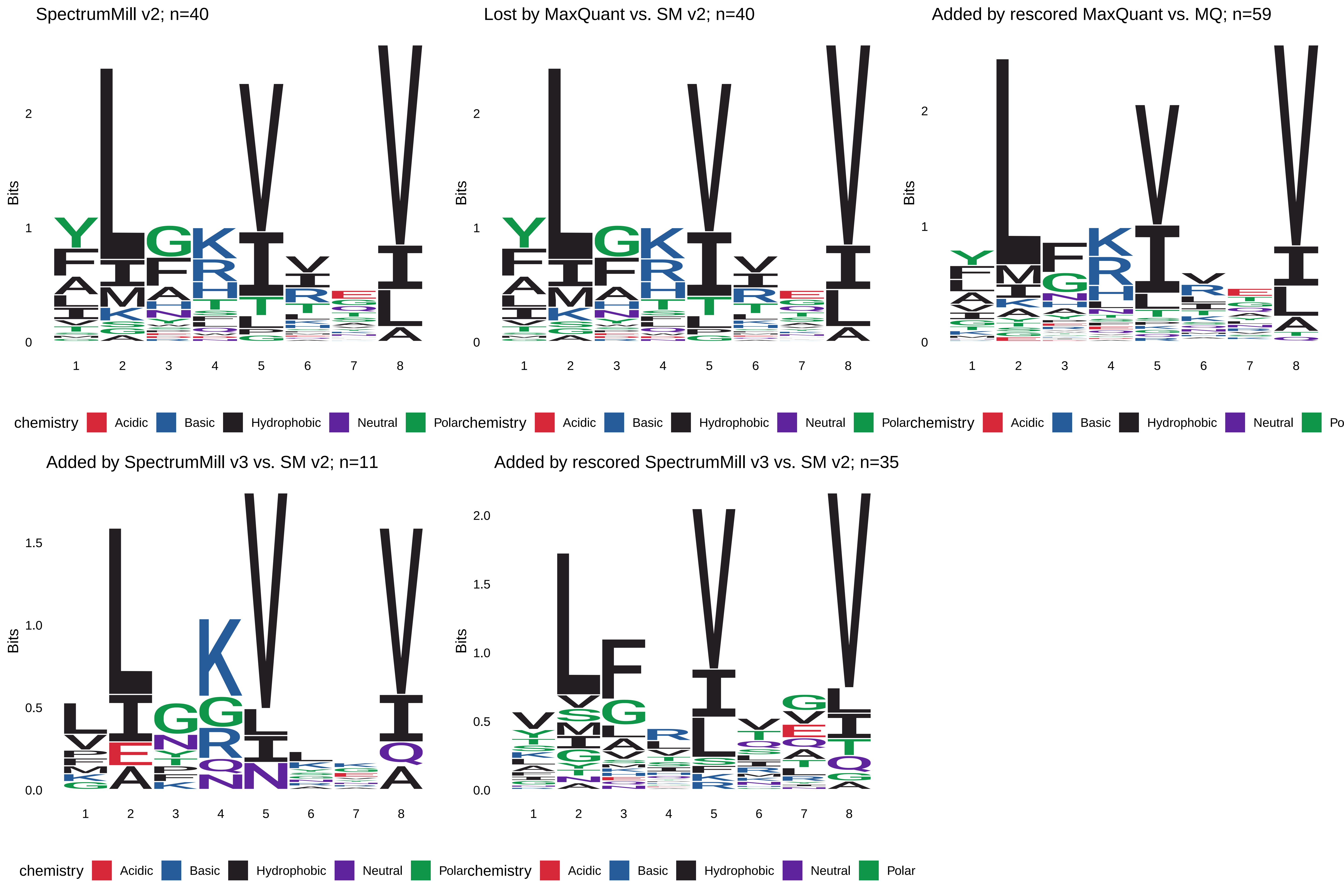
SpectrumMill v2; n=40
Lost by MaxQuant vs. SM v2; n=40
Added by rescored MaxQuant vs. MQ; n=59
Added by SpectrumMill v3 vs. SM v2; n=11
Added by rescored SpectrumMill v3 vs. SM v2; n=35
Bits
chemistry
Acidic
Basic
Hydrophobic
Neutral
Polar

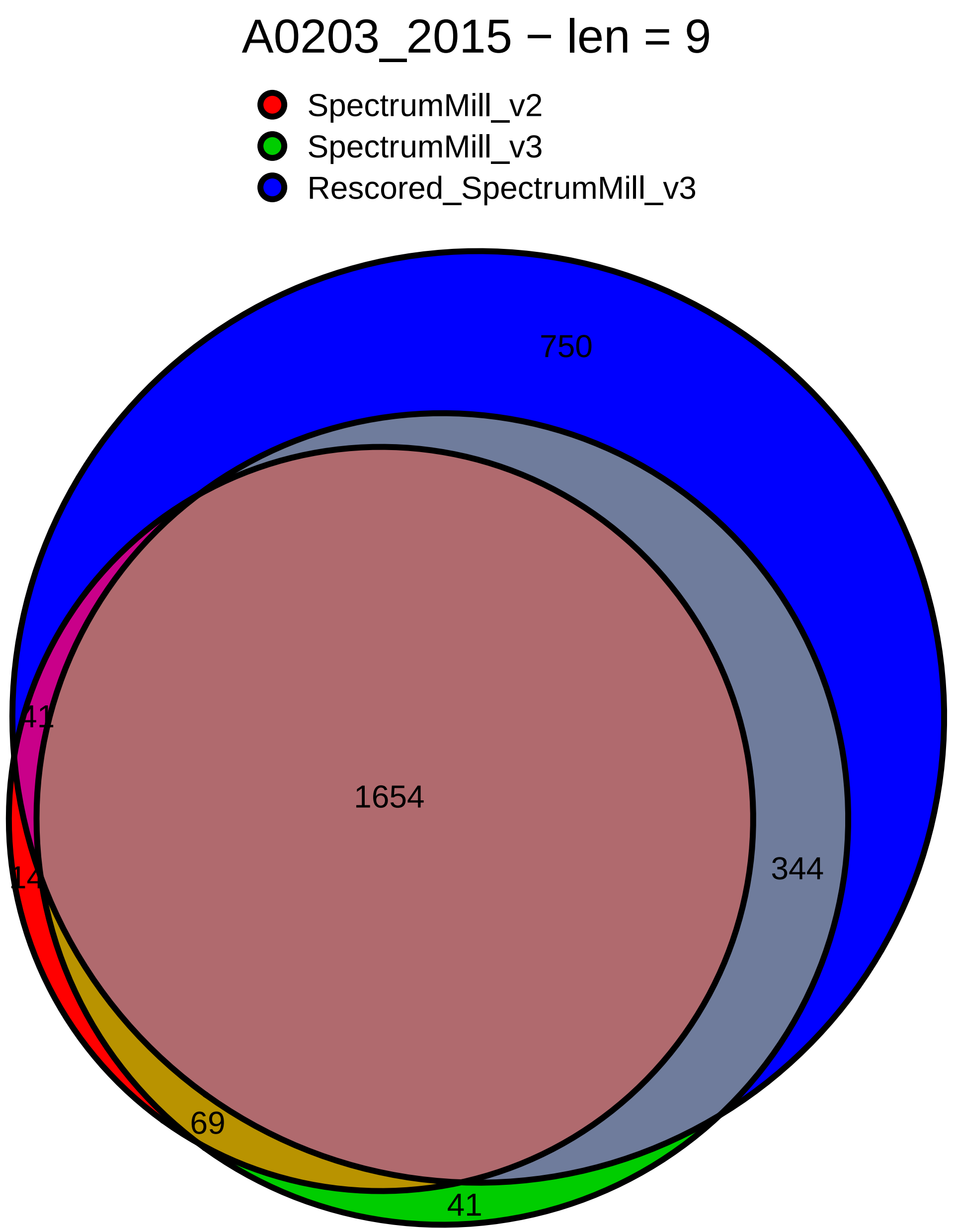
A0203_2015 − len = 9
SpectrumMill_v2
SpectrumMill_v3
Rescored_SpectrumMill_v3
750
41
1654
14
344
69
41

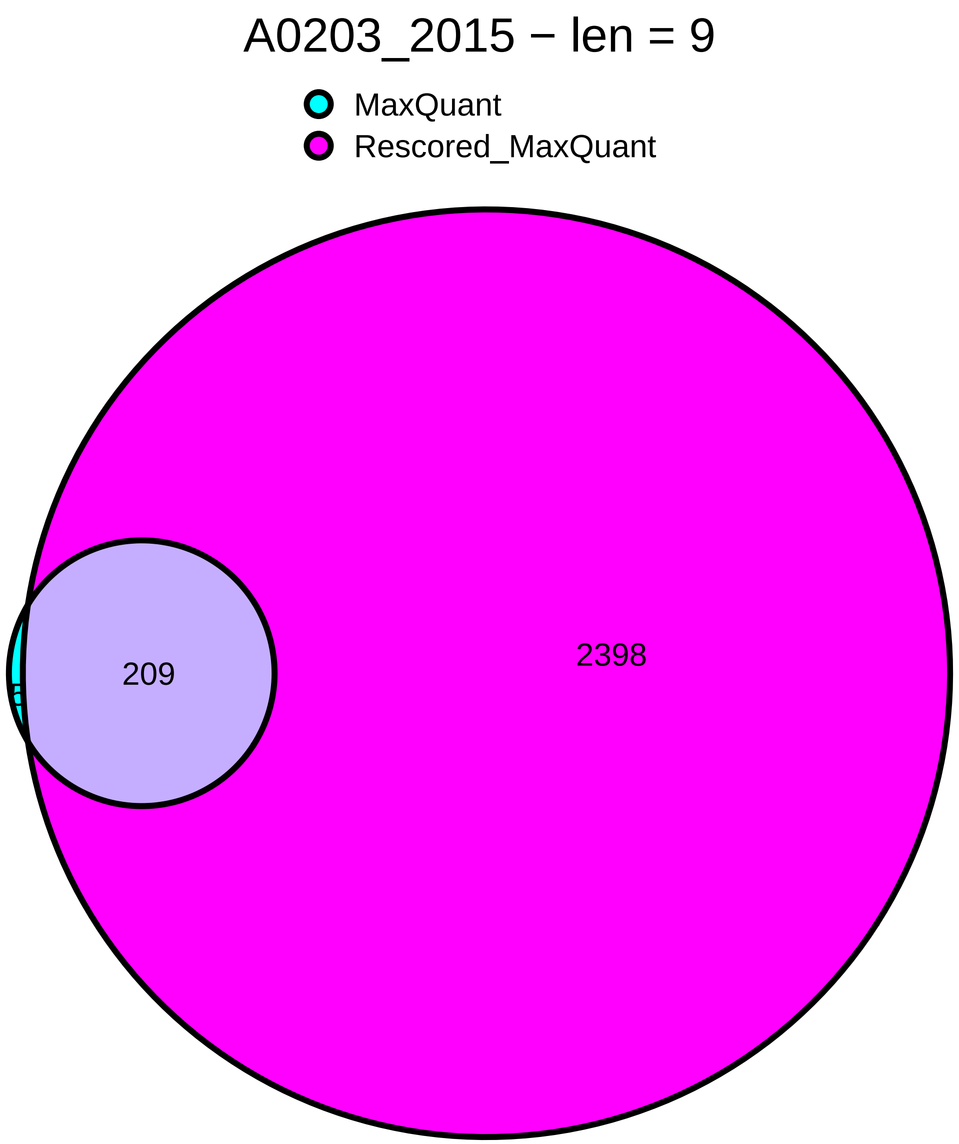
A0203_2015 − len = 9
MaxQuant
Rescored_MaxQuant
209
2398

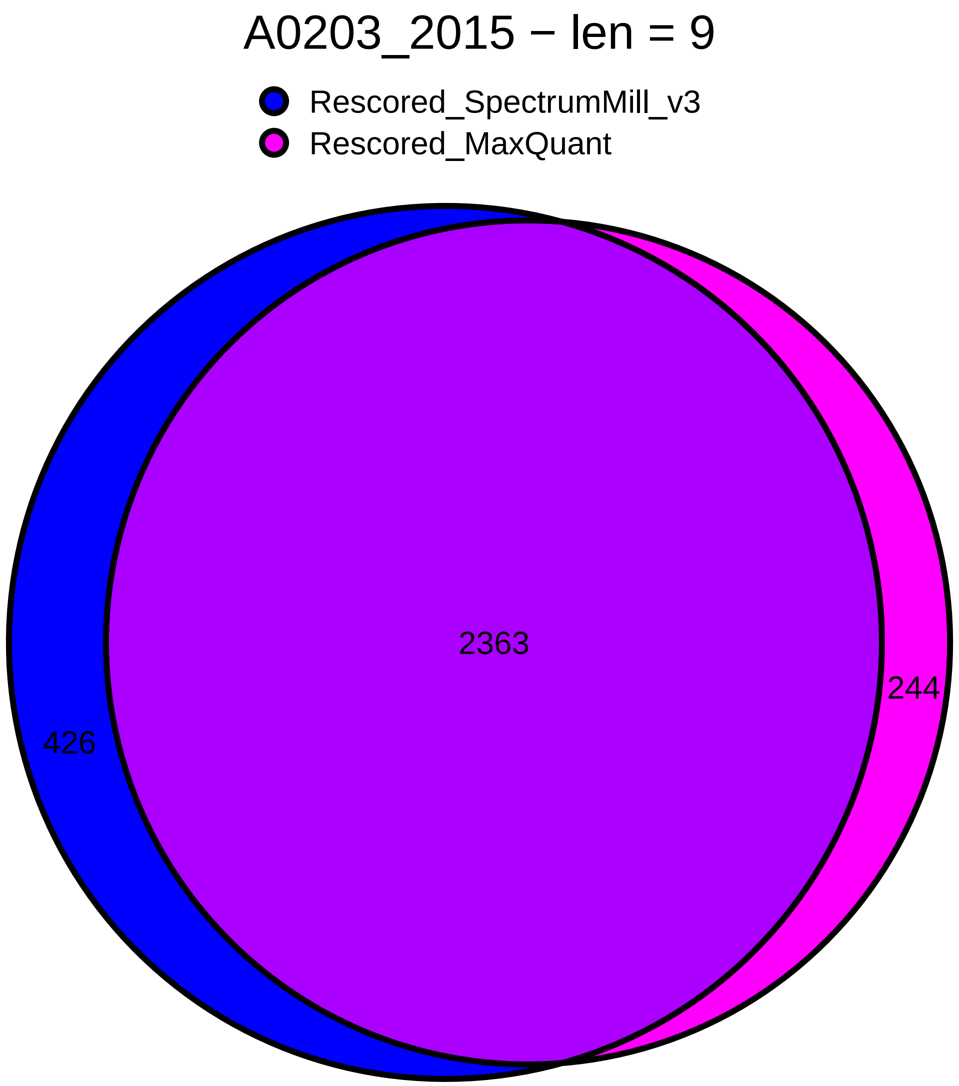
A0203_2015 − len = 9
Rescored_SpectrumMill_v3
Rescored_MaxQuant
2363
426
244

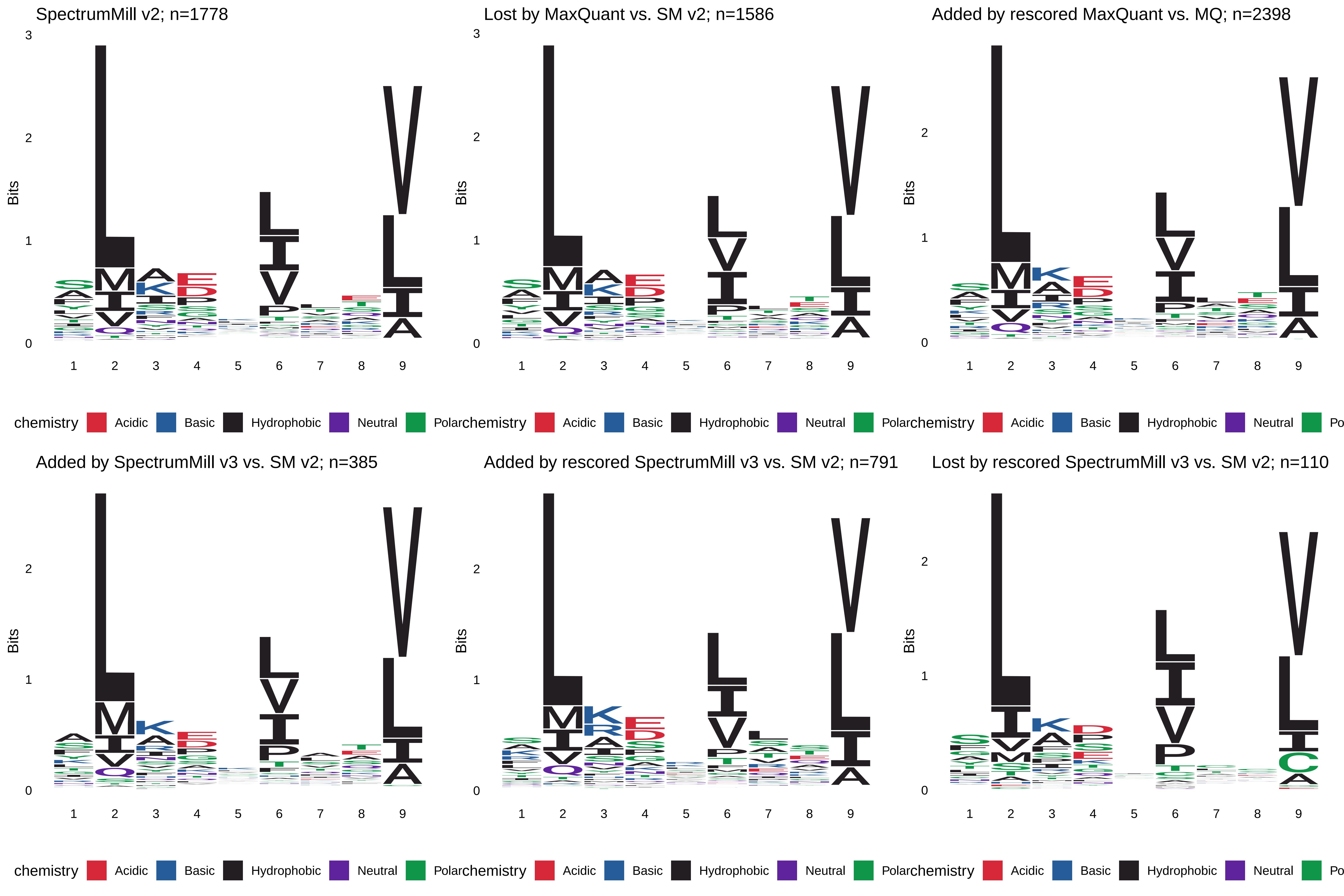
SpectrumMill v2; n=1778
Lost by MaxQuant vs. SM v2; n=1586
Added by rescored MaxQuant vs. MQ; n=2398
Added by SpectrumMill v3 vs. SM v2; n=385
Added by rescored SpectrumMill v3 vs. SM v2; n=791
Lost by rescored SpectrumMill v3 vs. SM v2; n=110
Bits
0
1
2
3
1 2 3 4 5 6 7 8 9
chemistry
Acidic
Basic
Hydrophobic
Neutral
Polar

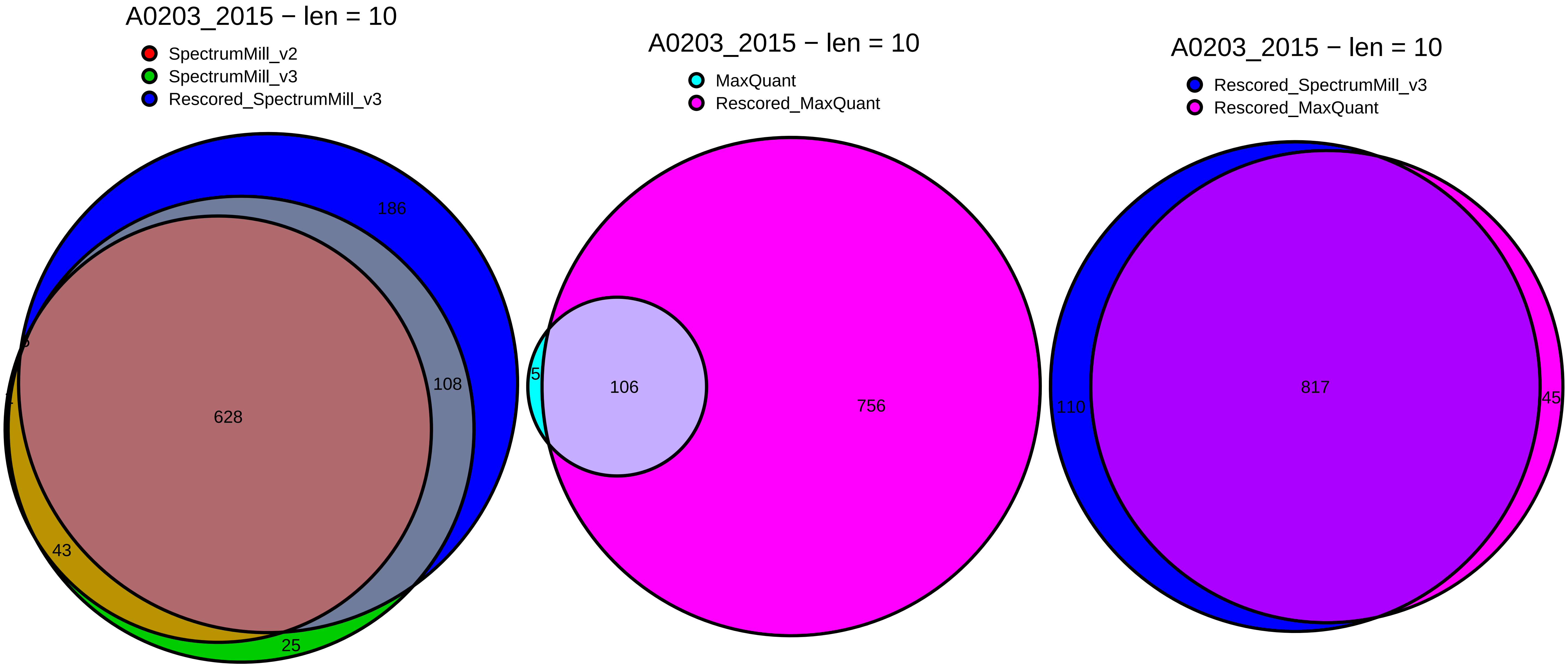
A0203_2015 − len = 10
SpectrumMill_v2
SpectrumMill_v3
Rescored_SpectrumMill_v3
186
108
628
43
25
A0203_2015 − len = 10
MaxQuant
Rescored_MaxQuant
5
106
756
A0203_2015 − len = 10
Rescored_SpectrumMill_v3
Rescored_MaxQuant
110
817
45

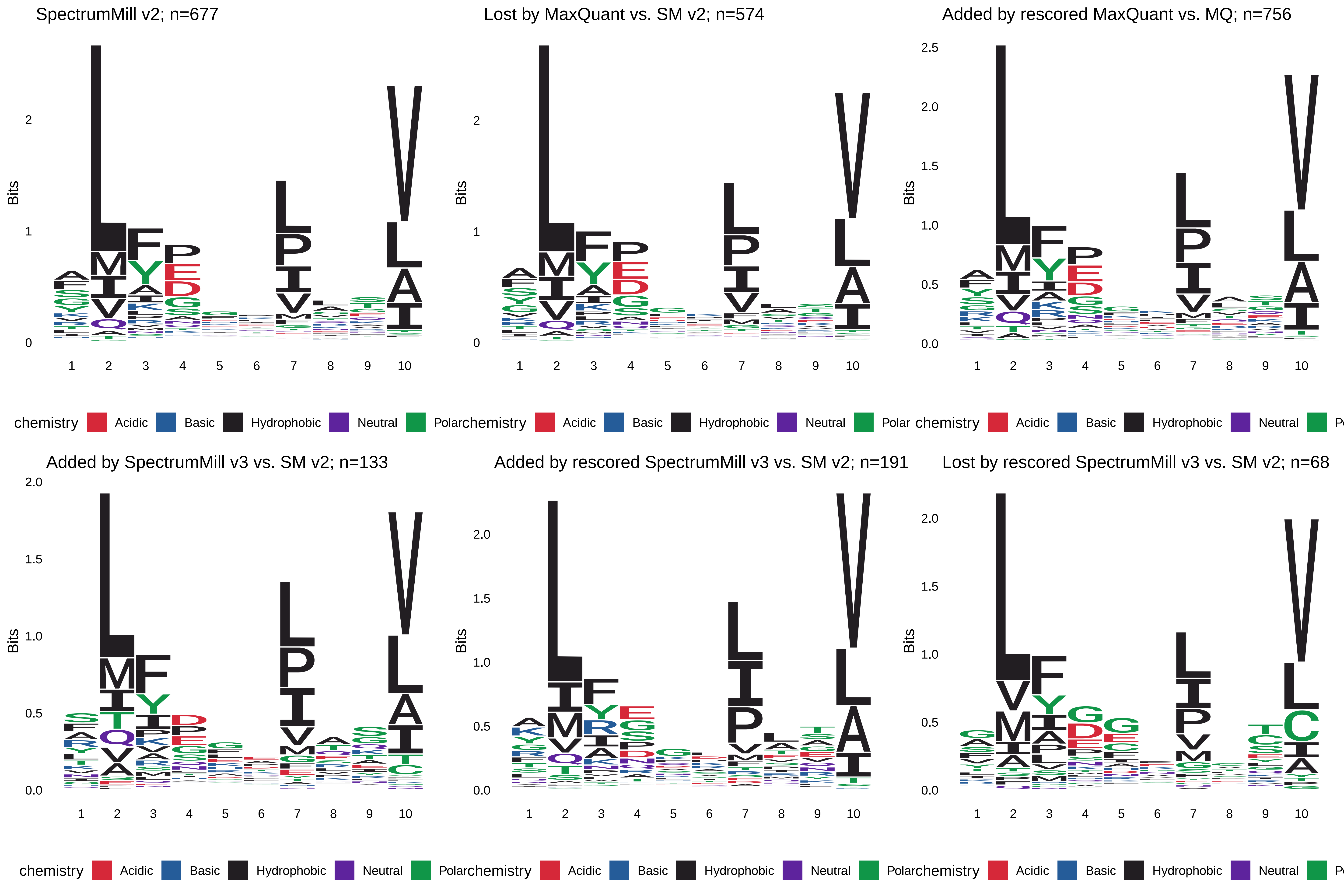

SpectrumMill v2; n=677
Lost by MaxQuant vs. SM v2; n=574
Added by rescored MaxQuant vs. MQ; n=756
Added by SpectrumMill v3 vs. SM v2; n=133
Added by rescored SpectrumMill v3 vs. SM v2; n=191
Lost by rescored SpectrumMill v3 vs. SM v2; n=68
Bits
0
1
2
0.0
0.5
1.0
1.5
2.0
2.5
1 2 3 4 5 6 7 8 9 10
chemistry
Acidic
Basic
Hydrophobic
Neutral
Polar

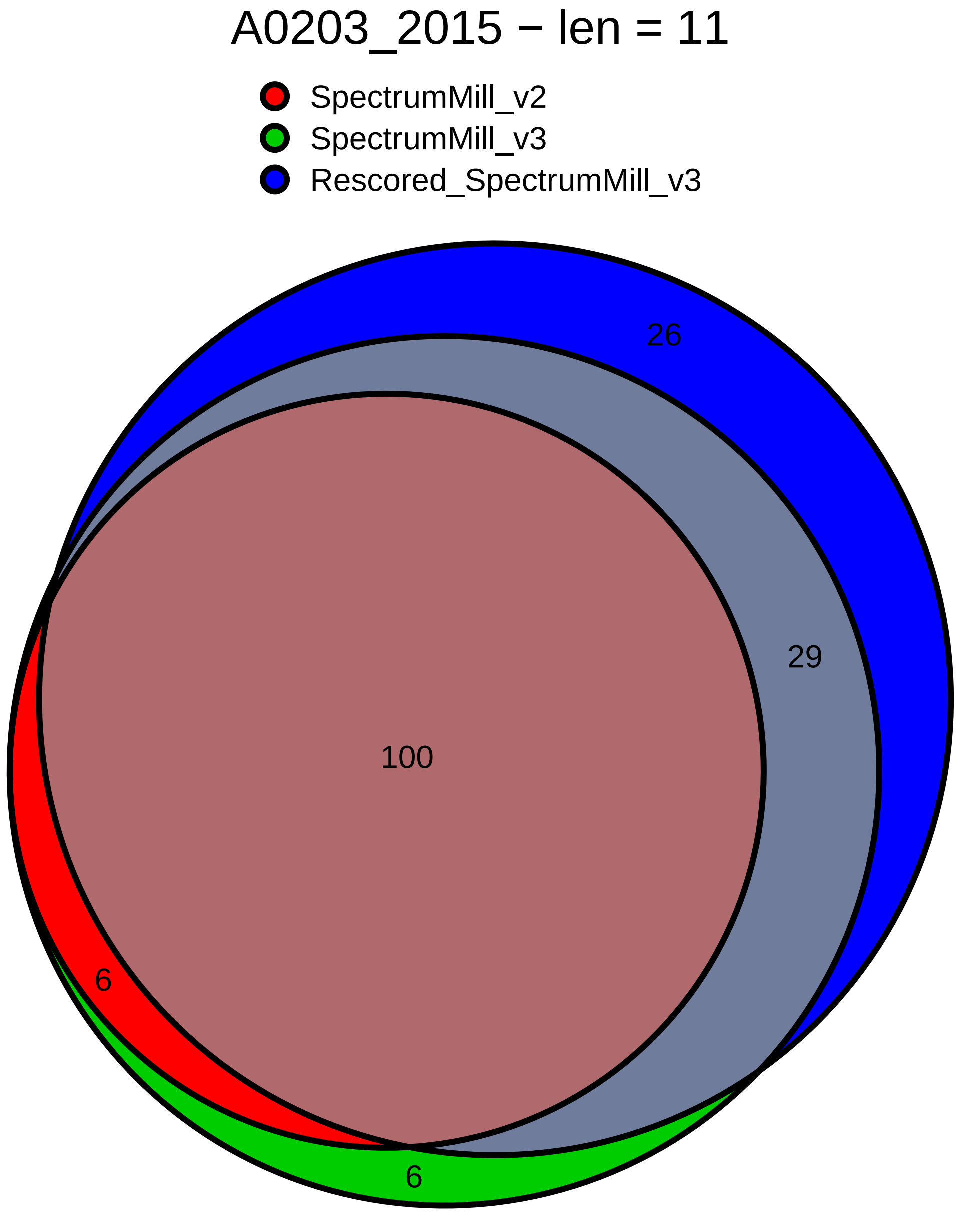
A0203_2015 − len = 11
SpectrumMill_v2
SpectrumMill_v3
Rescored_SpectrumMill_v3
26
29
100
6
6

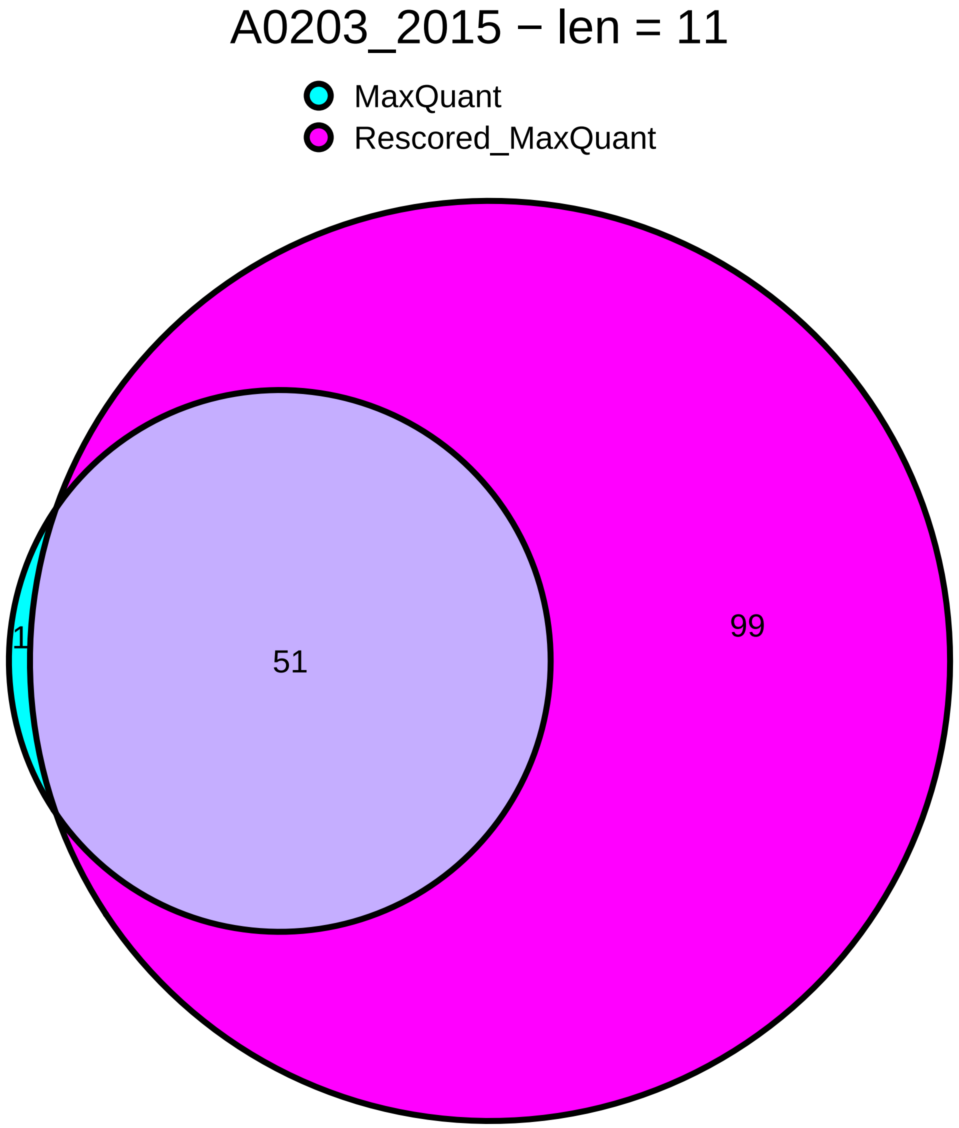
A0203_2015 − len = 11
MaxQuant
Rescored_MaxQuant
1
51
99

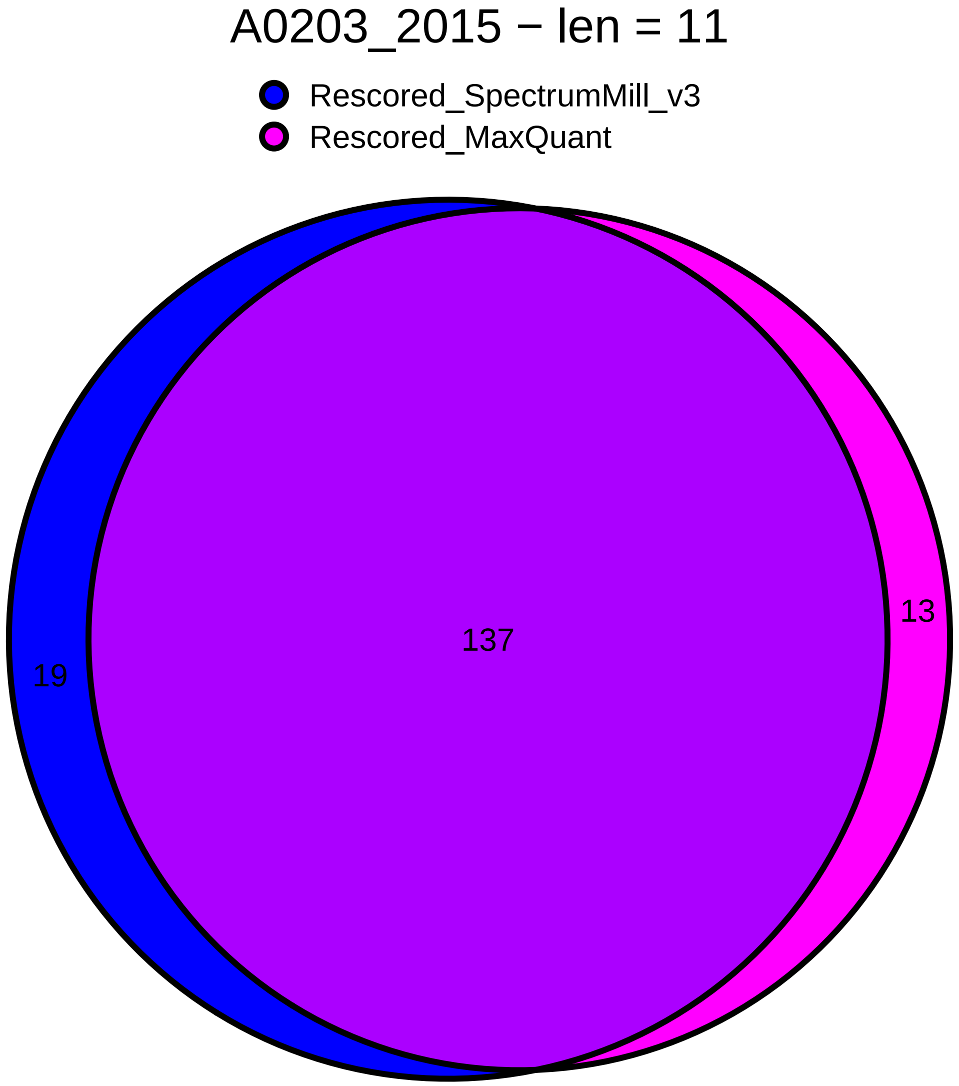
A0203_2015 − len = 11
Rescored_SpectrumMill_v3
Rescored_MaxQuant
19
137
13

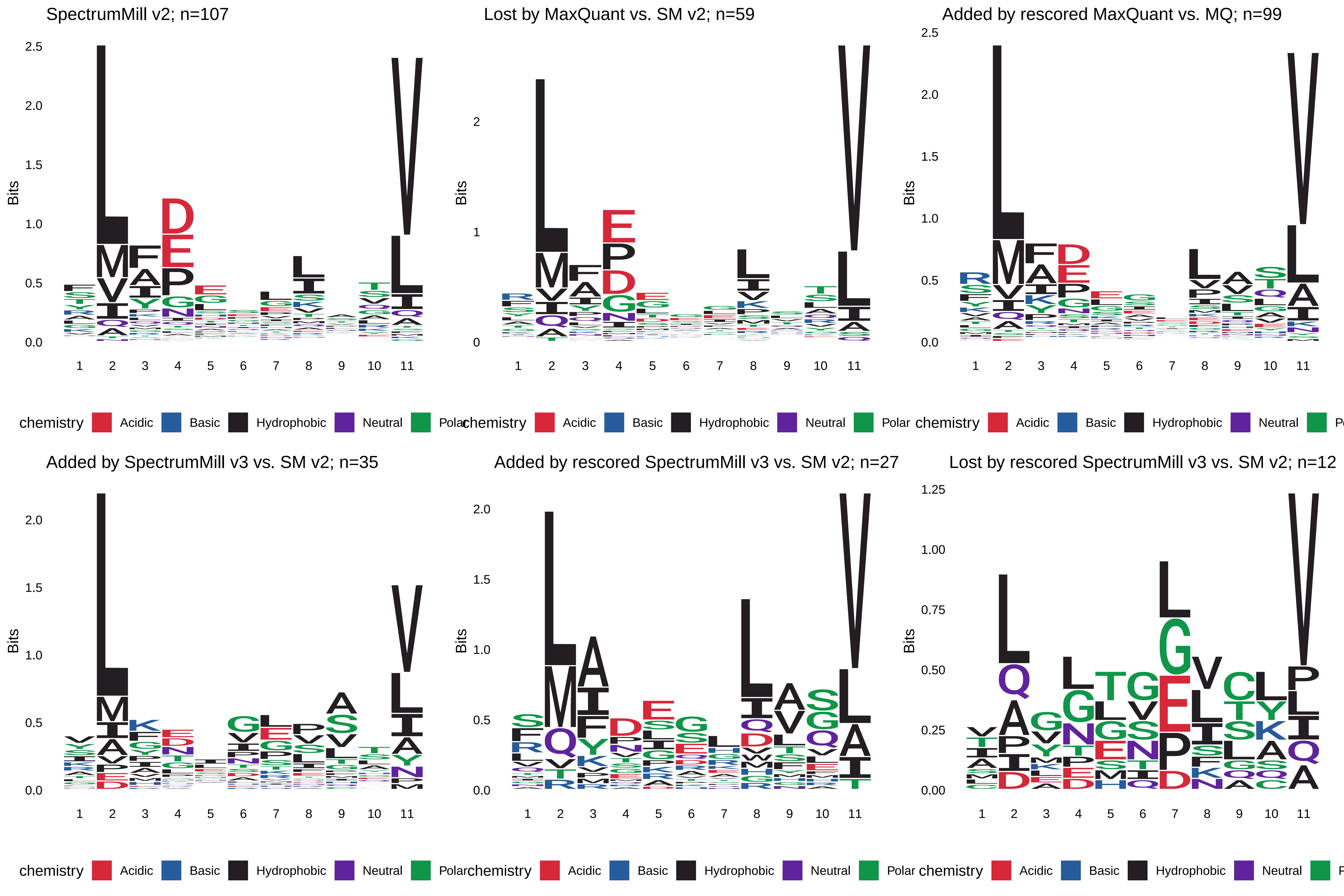
SpectrumMill v2; n=107
Lost by MaxQuant vs. SM v2; n=59
Added by rescored MaxQuant vs. MQ; n=99
Added by SpectrumMill v3 vs. SM v2; n=35
Added by rescored SpectrumMill v3 vs. SM v2; n=27
Lost by rescored SpectrumMill v3 vs. SM v2; n=12
Bits
1 2 3 4 5 6 7 8 9 10 11
chemistry
Acidic
Basic
Hydrophobic
Neutral
Polar

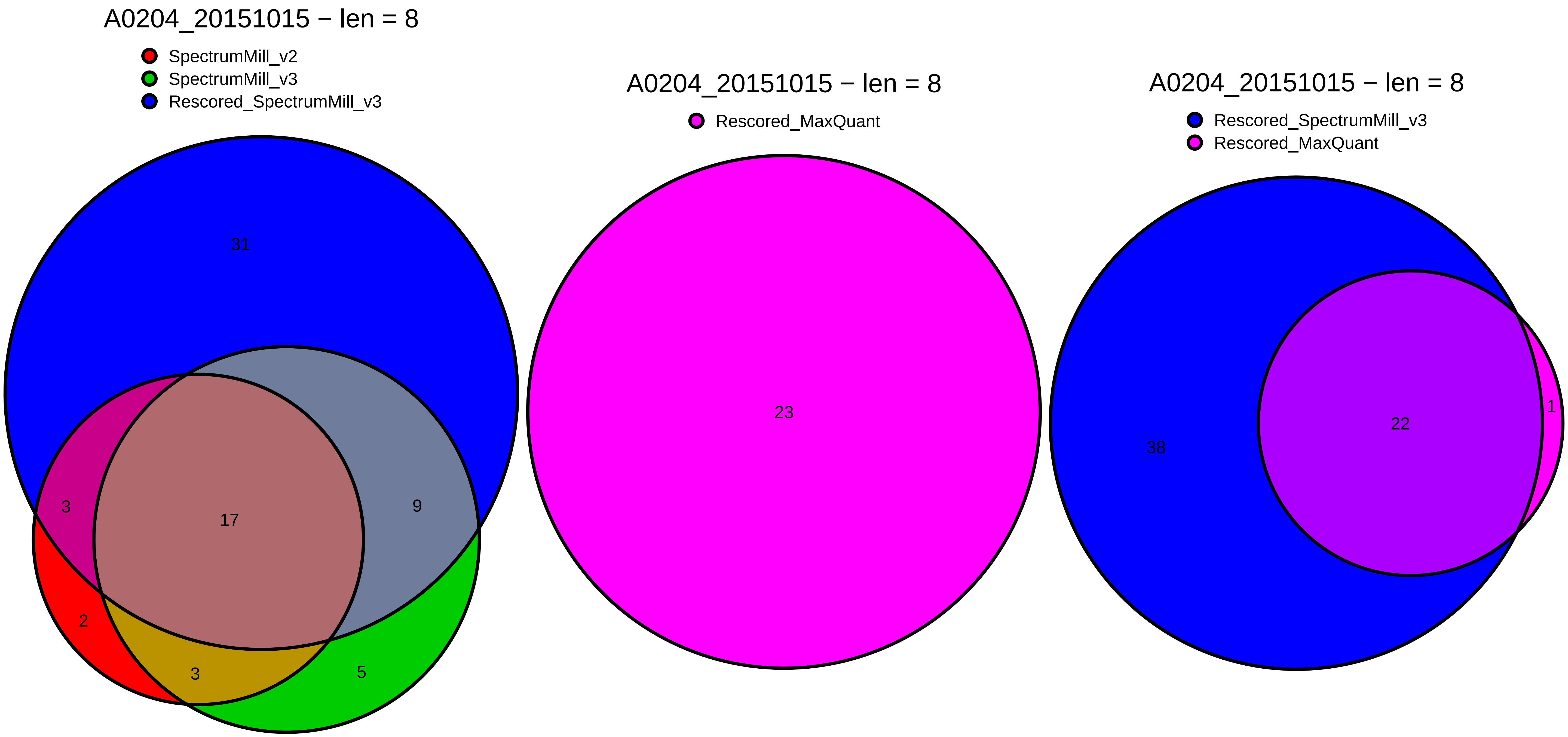
A0204_20151015 − len = 8
SpectrumMill_v2
SpectrumMill_v3
Rescored_SpectrumMill_v3
31
3
17
9
2
3
5
A0204_20151015 − len = 8
Rescored_MaxQuant
23
A0204_20151015 − len = 8
Rescored_SpectrumMill_v3
Rescored_MaxQuant
38
22
1

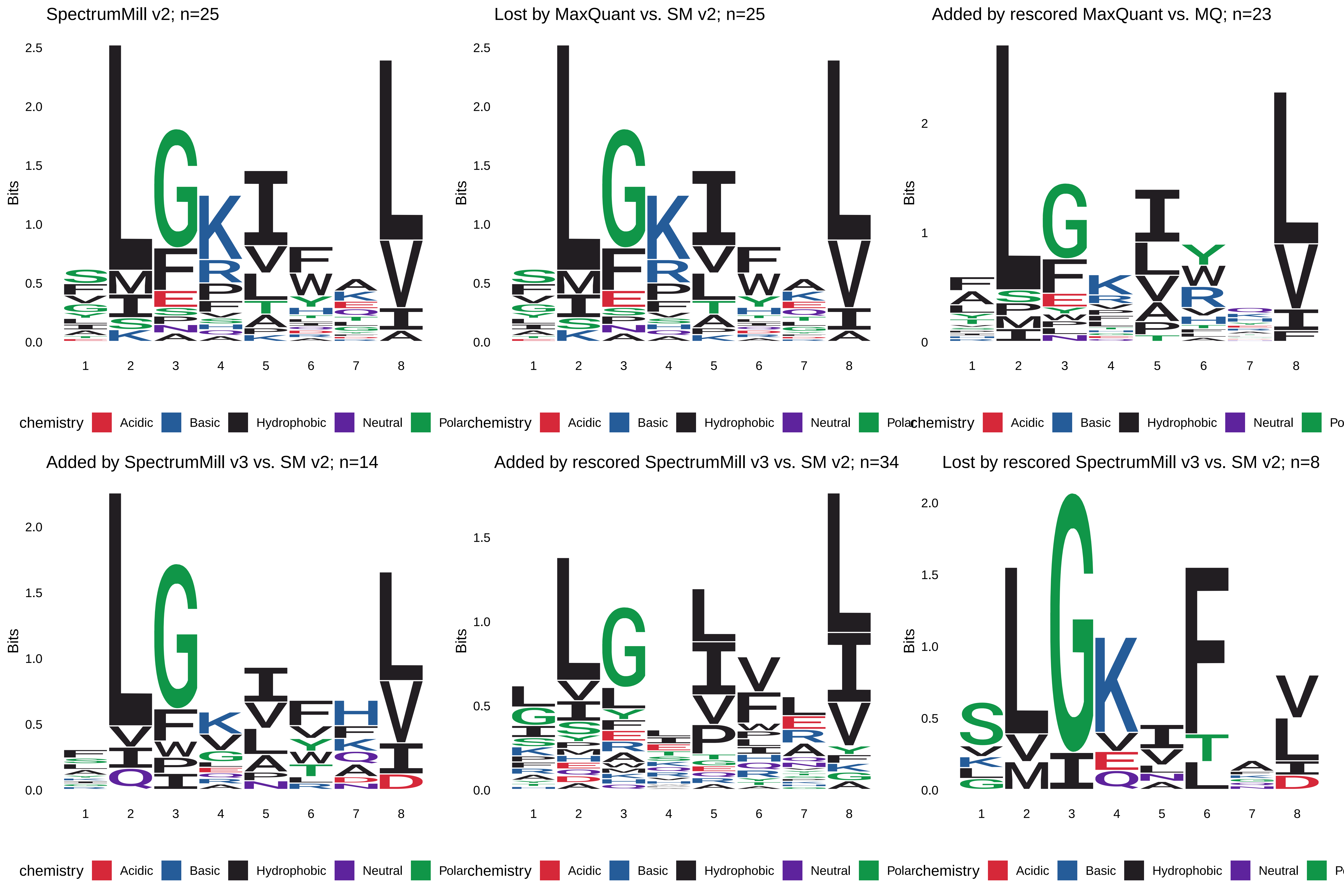
SpectrumMill v2; n=25
Lost by MaxQuant vs. SM v2; n=25
Added by rescored MaxQuant vs. MQ; n=23
Added by SpectrumMill v3 vs. SM v2; n=14
Added by rescored SpectrumMill v3 vs. SM v2; n=34
Lost by rescored SpectrumMill v3 vs. SM v2; n=8
Bits
1 2 3 4 5 6 7 8
chemistry
Acidic
Basic
Hydrophobic
Neutral
Polar

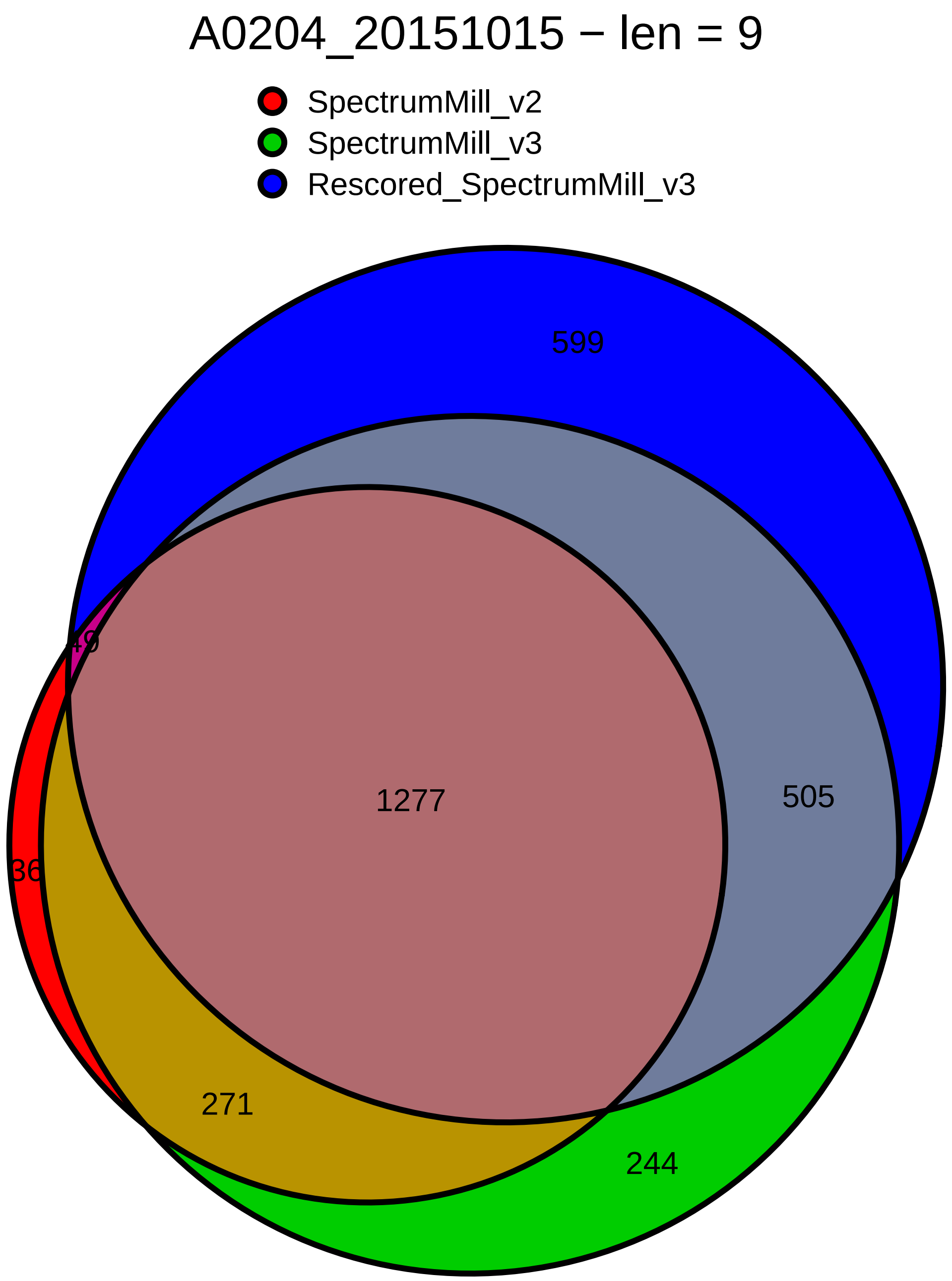
A0204_20151015 − len = 9
SpectrumMill_v2
SpectrumMill_v3
Rescored_SpectrumMill_v3
599
49
1277
505
36
271
244

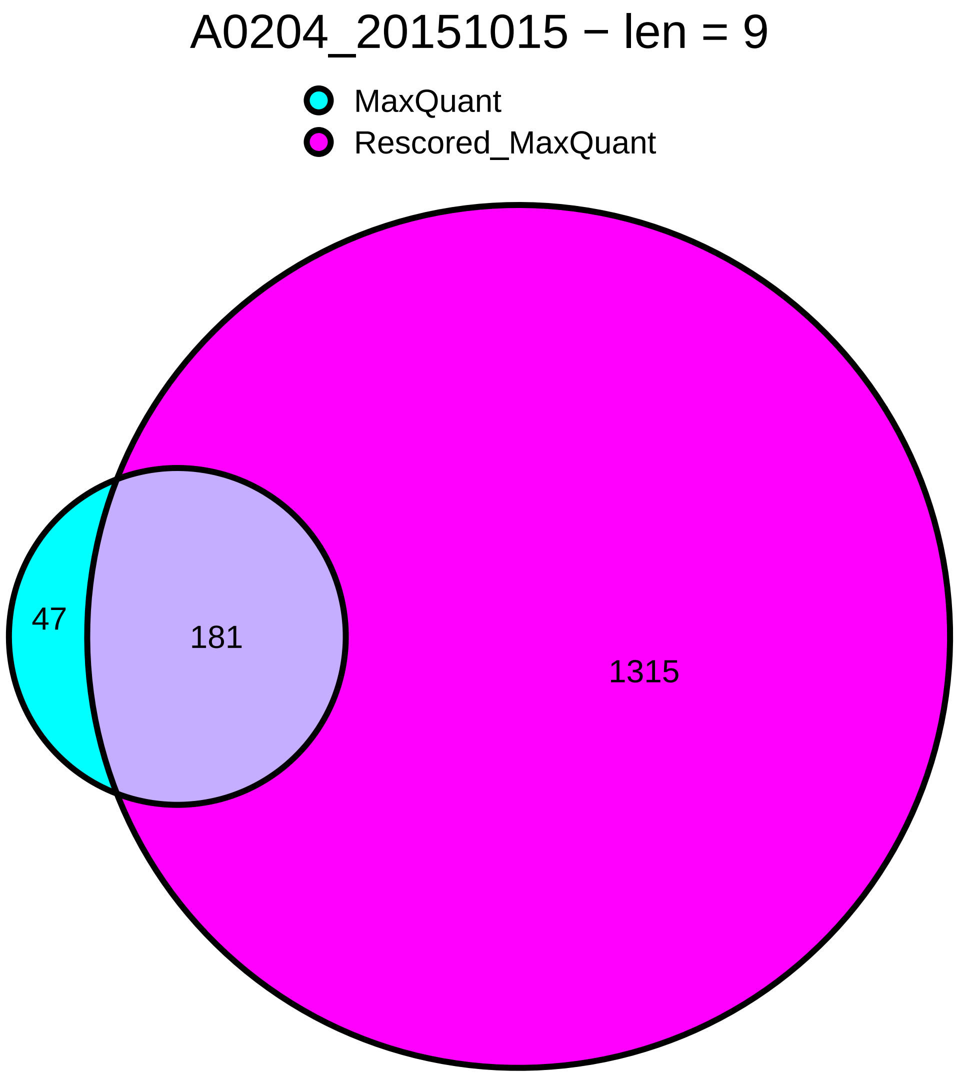
A0204_20151015 − len = 9
MaxQuant
Rescored_MaxQuant
47
181
1315

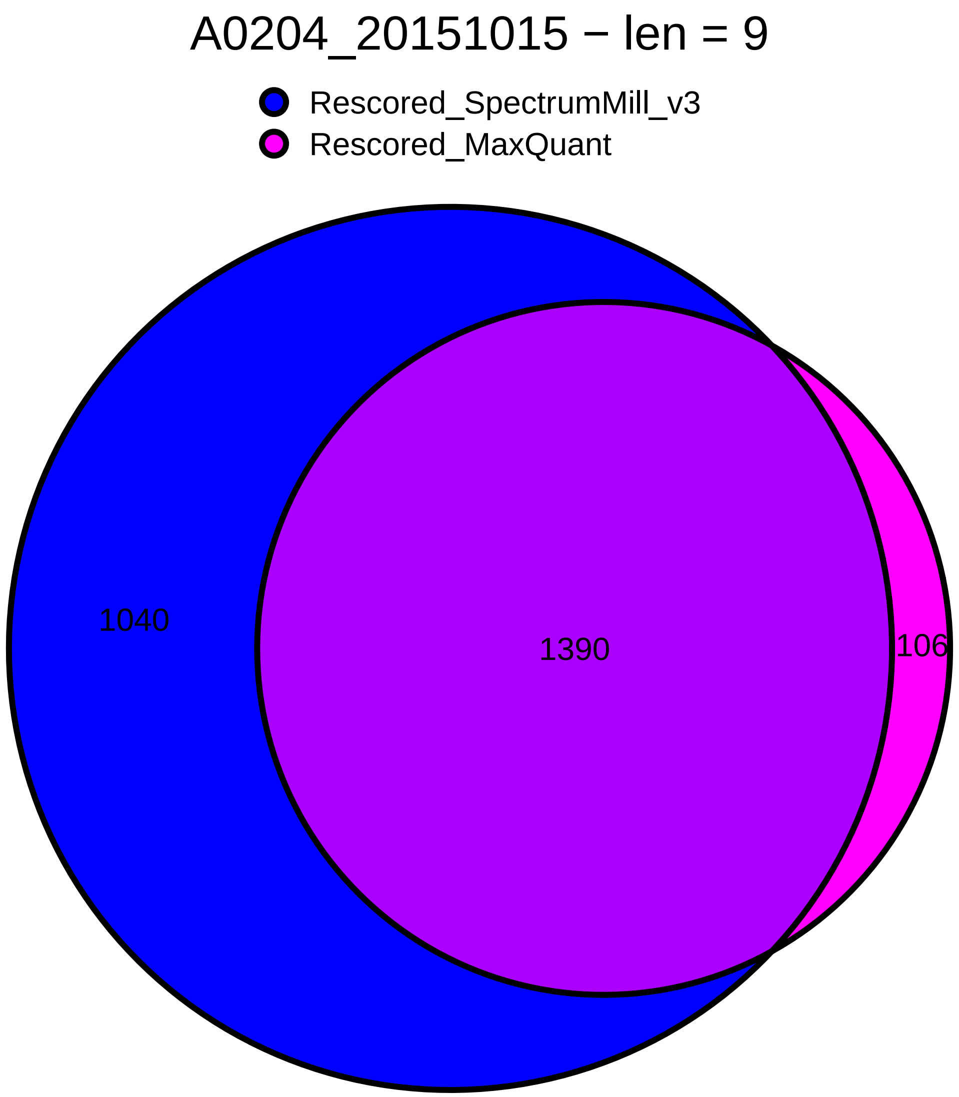
A0204_20151015 − len = 9
Rescored_SpectrumMill_v3
Rescored_MaxQuant
1040
1390
106

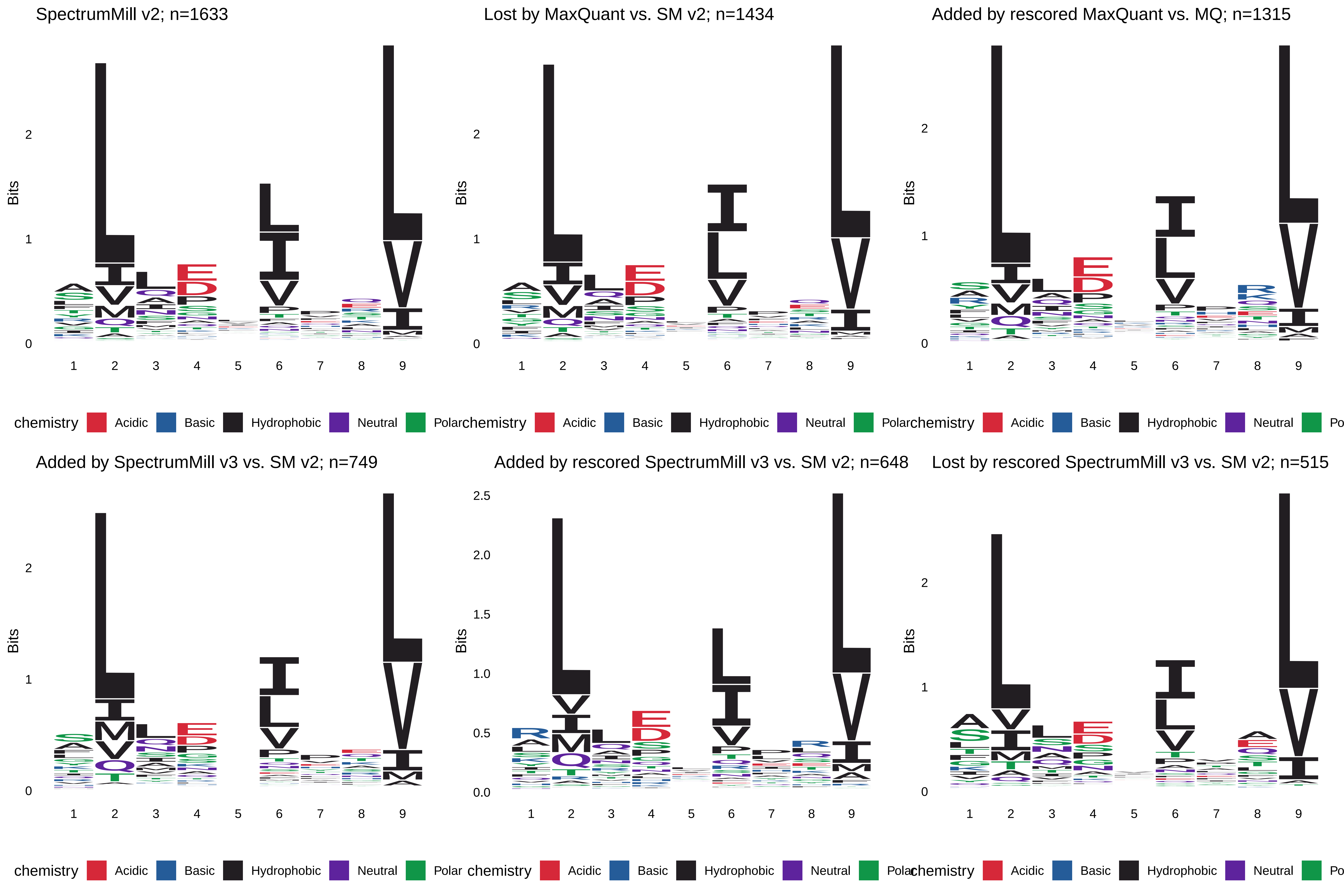
SpectrumMill v2; n=1633
Lost by MaxQuant vs. SM v2; n=1434
Added by rescored MaxQuant vs. MQ; n=1315
Added by SpectrumMill v3 vs. SM v2; n=749
Added by rescored SpectrumMill v3 vs. SM v2; n=648
Lost by rescored SpectrumMill v3 vs. SM v2; n=515
Bits
chemistry
Acidic
Basic
Hydrophobic
Neutral
Polar

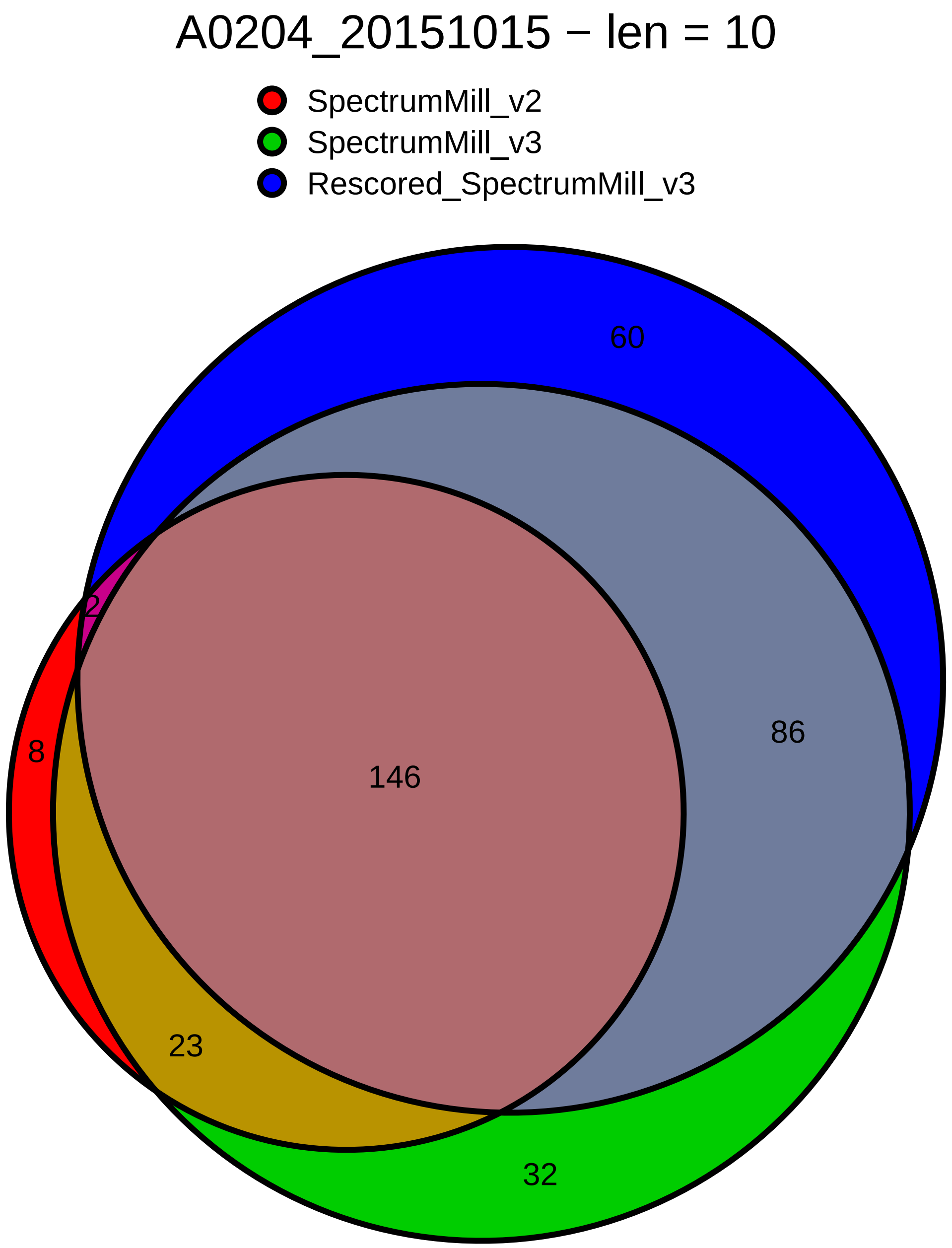
A0204_20151015 − len = 10
SpectrumMill_v2
SpectrumMill_v3
Rescored_SpectrumMill_v3
60
2
8
146
86
23
32

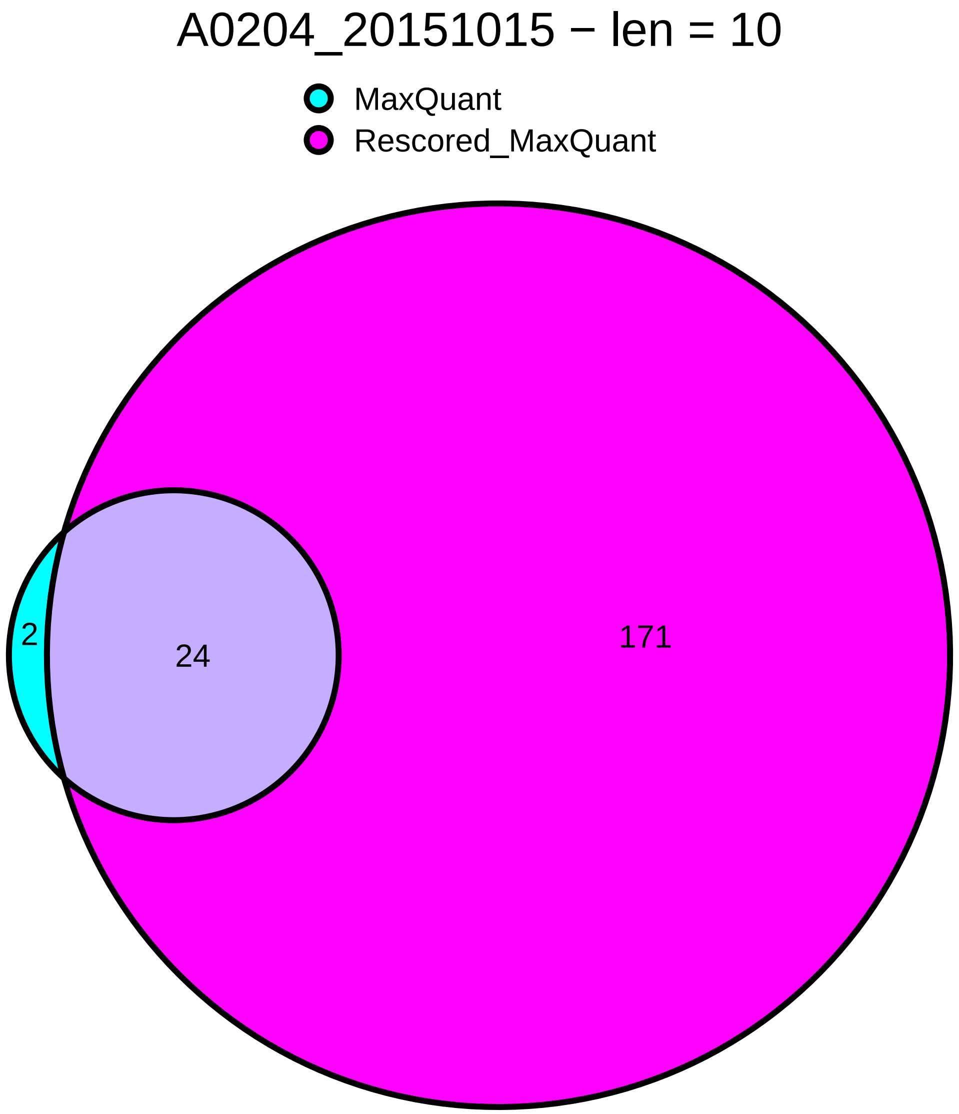
A0204_20151015 − len = 10
MaxQuant
Rescored_MaxQuant
2
24
171

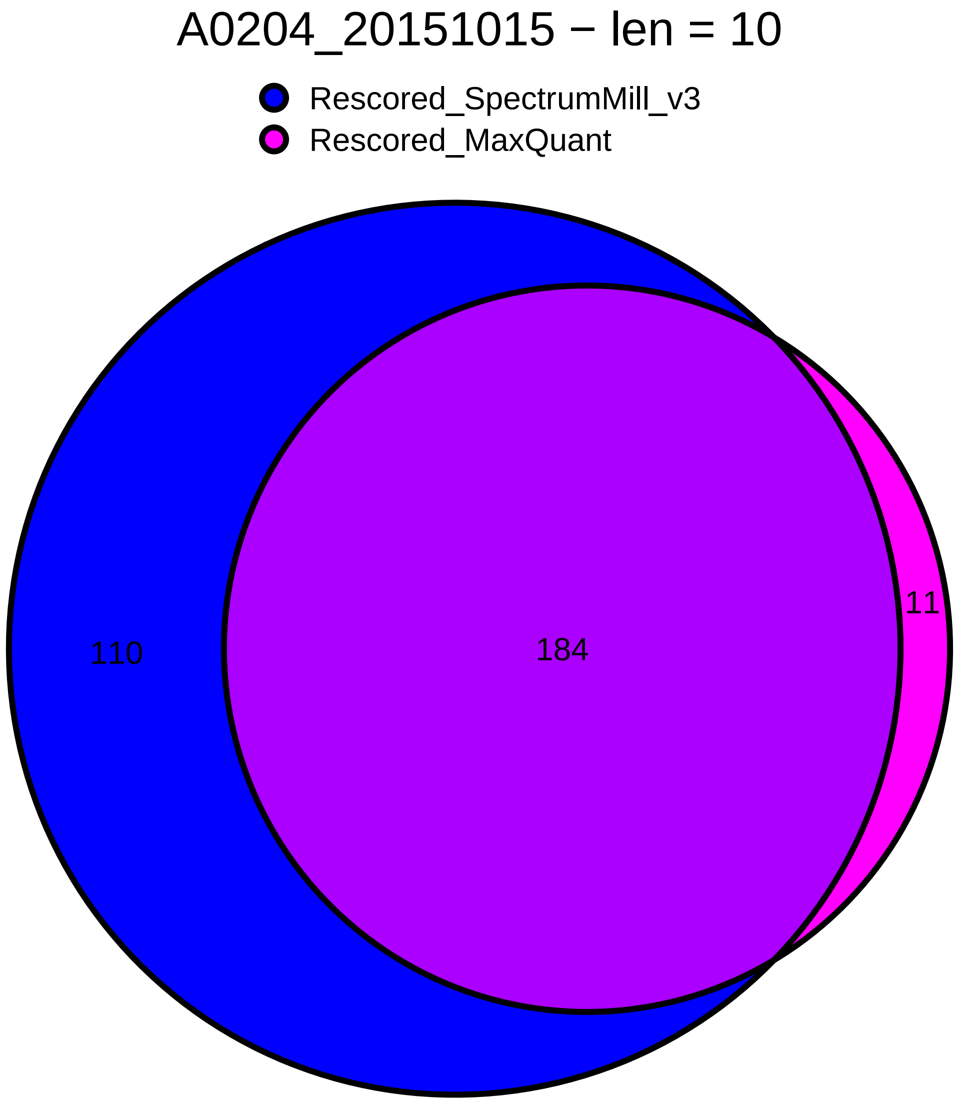
A0204_20151015 − len = 10
Rescored_SpectrumMill_v3
Rescored_MaxQuant
110
184
11

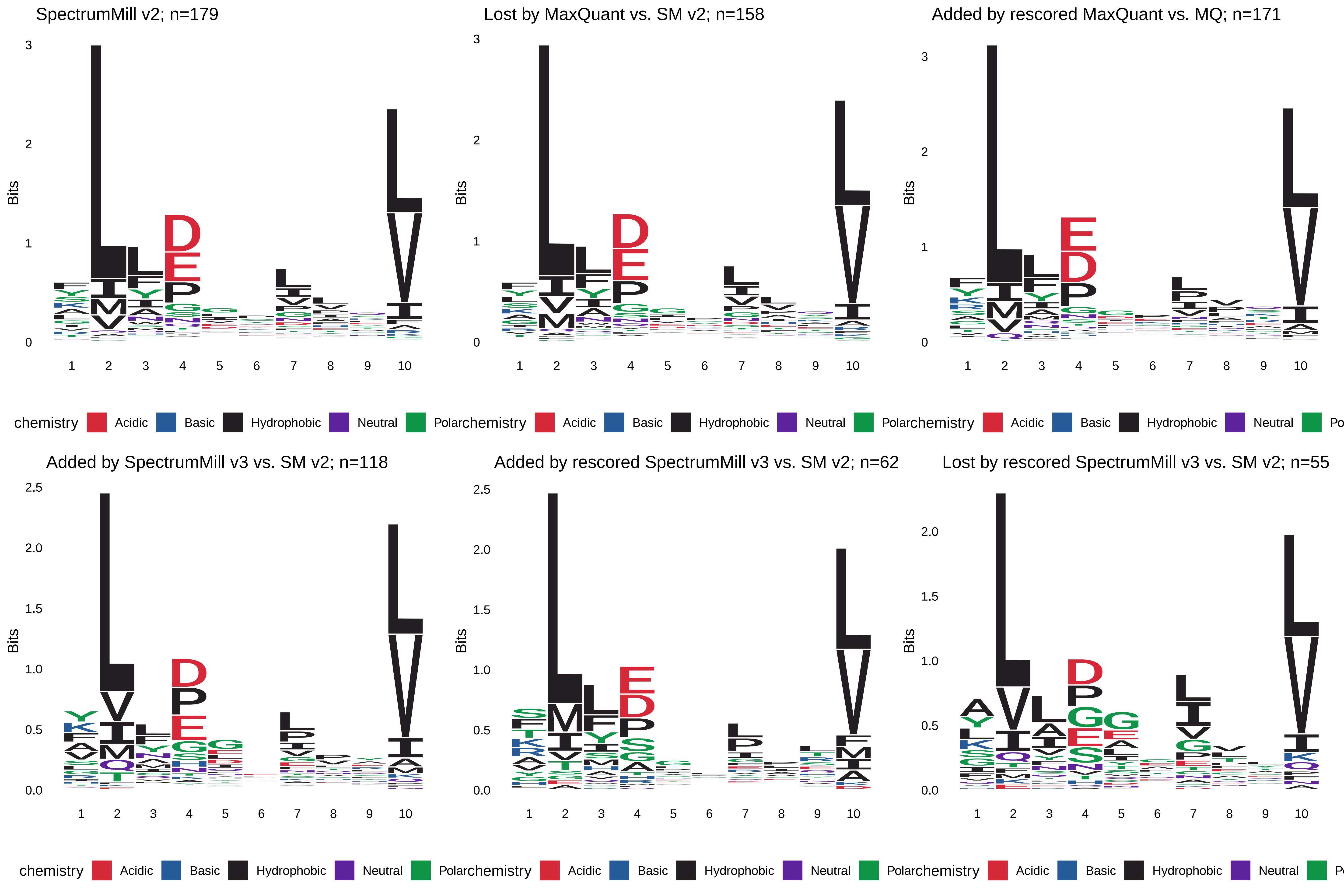
SpectrumMill v2; n=179
Lost by MaxQuant vs. SM v2; n=158
Added by rescored MaxQuant vs. MQ; n=171
Added by SpectrumMill v3 vs. SM v2; n=118
Added by rescored SpectrumMill v3 vs. SM v2; n=62
Lost by rescored SpectrumMill v3 vs. SM v2; n=55
Bits
1 2 3 4 5 6 7 8 9 10
chemistry Acidic Basic Hydrophobic Neutral Polarchemistry Acidic Basic Hydrophobic Neutral Polarchemistry Acidic Basic Hydrophobic Neutral Po
chemistry Acidic Basic Hydrophobic Neutral Polarchemistry Acidic Basic Hydrophobic Neutral Polarchemistry Acidic Basic Hydrophobic Neutral P

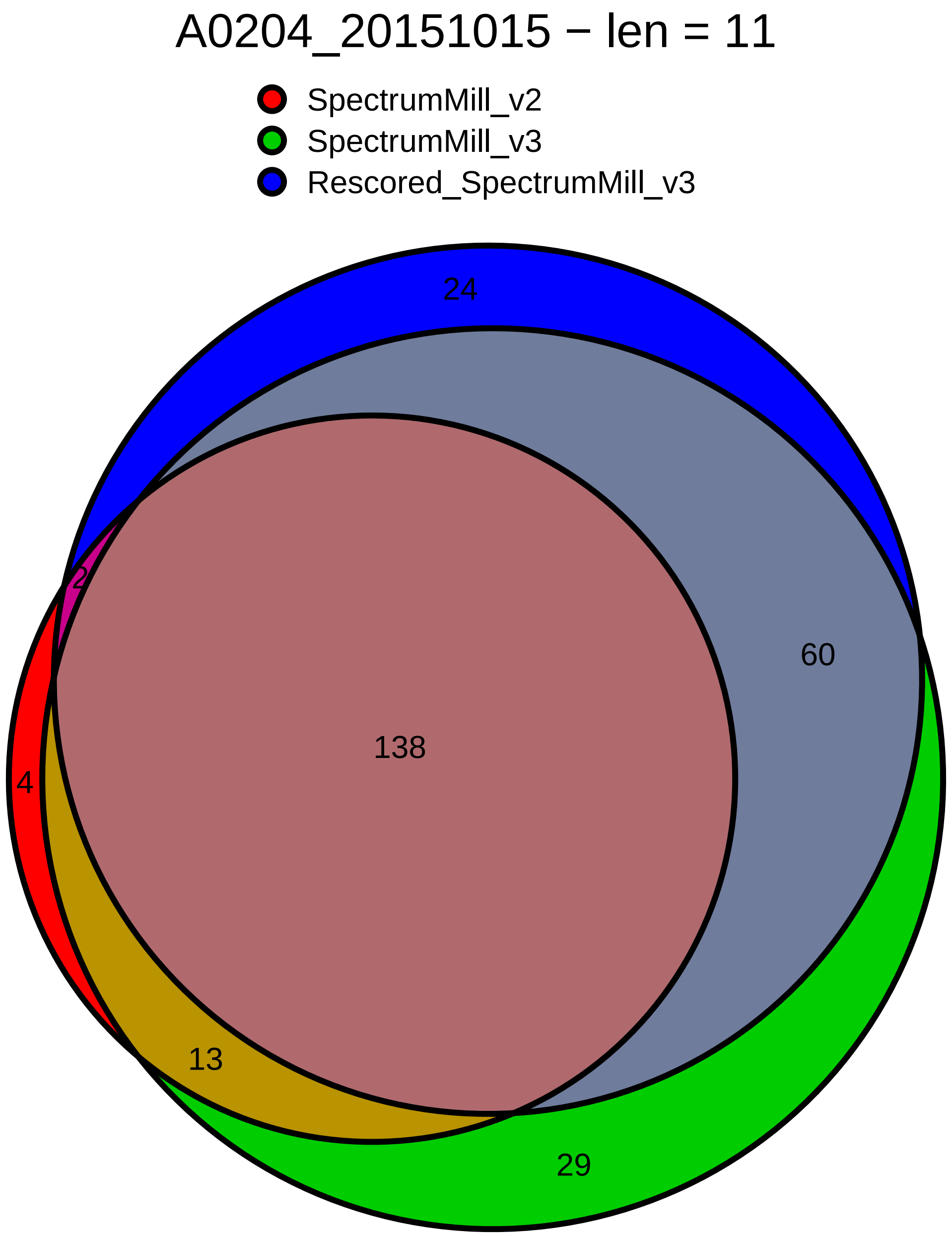
A0204_20151015 − len = 11
SpectrumMill_v2
SpectrumMill_v3
Rescored_SpectrumMill_v3
24
2
60
4
138
13
29

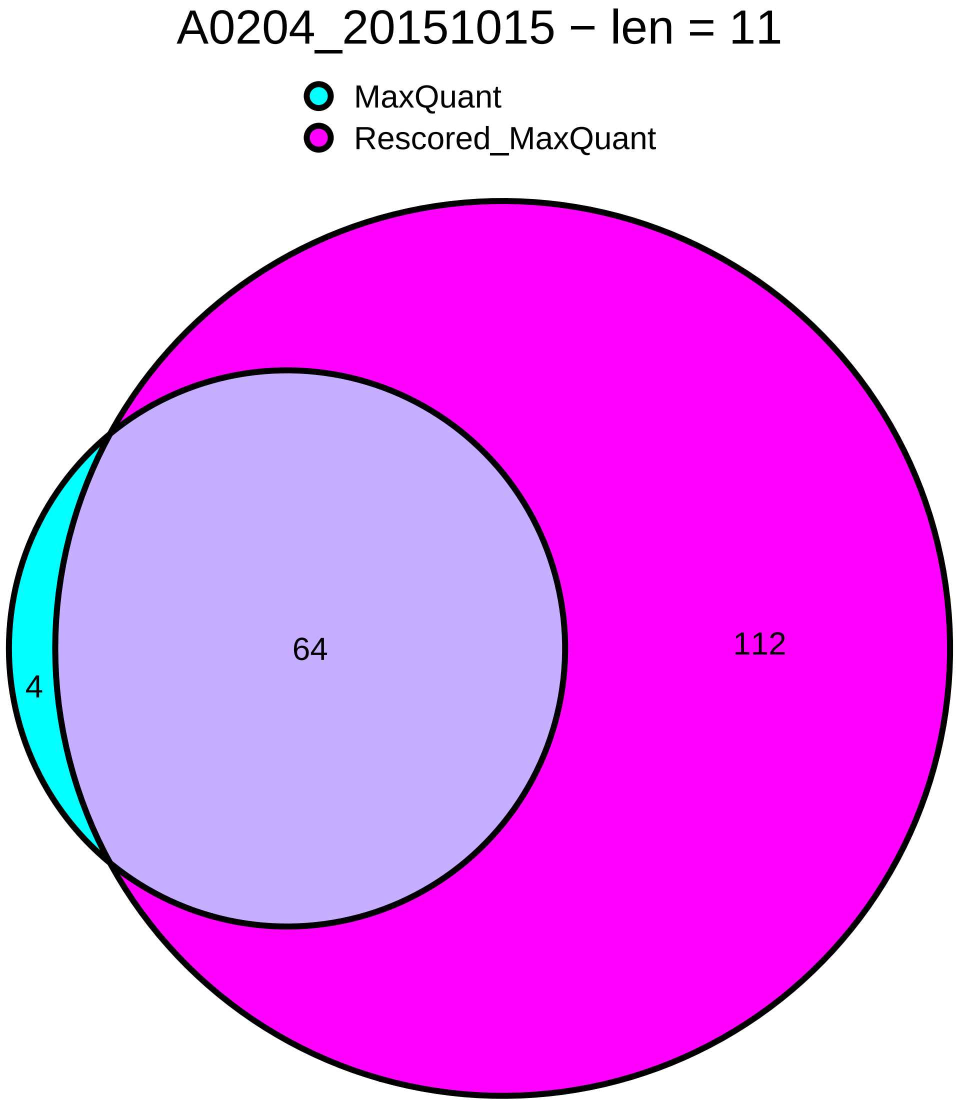
A0204_20151015 − len = 11
MaxQuant
Rescored_MaxQuant
4
64
112

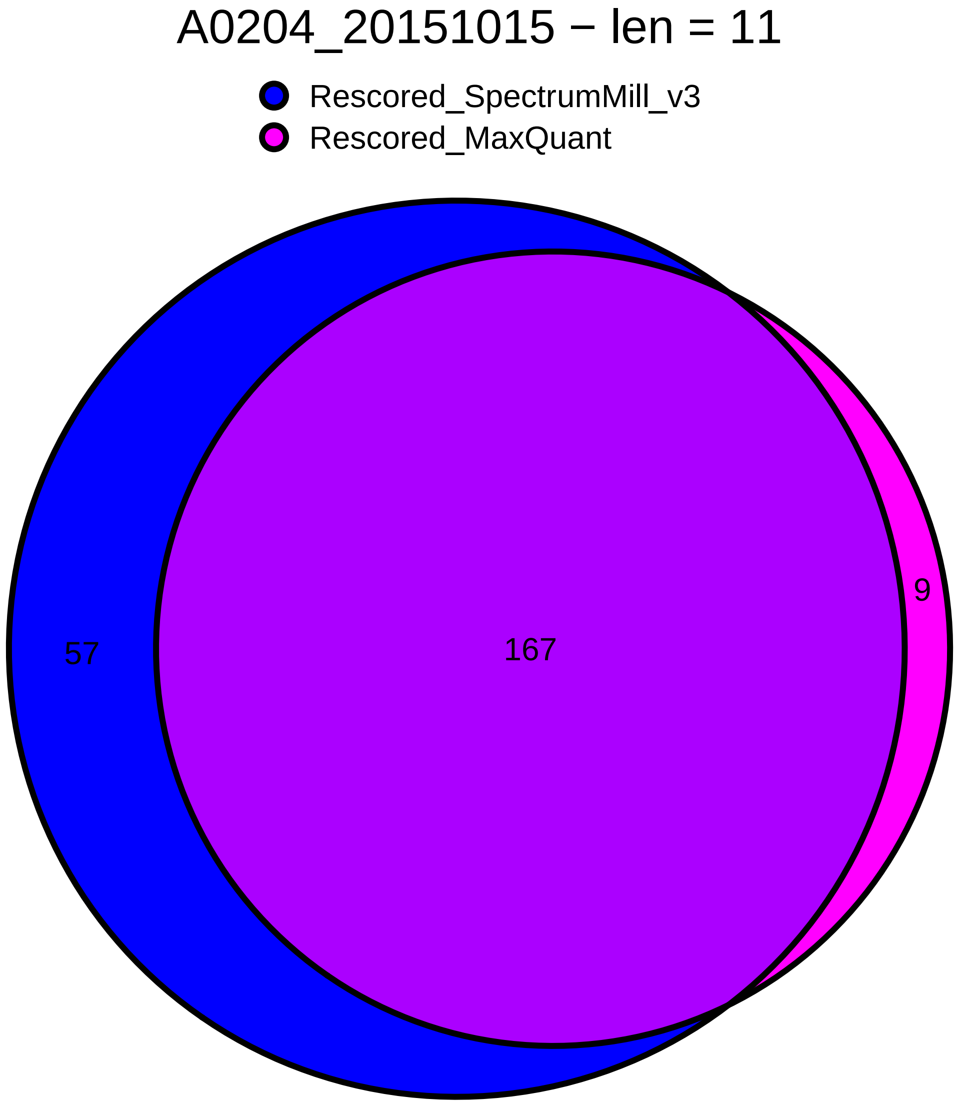
A0204_20151015 − len = 11
Rescored_SpectrumMill_v3
Rescored_MaxQuant
57
167
9

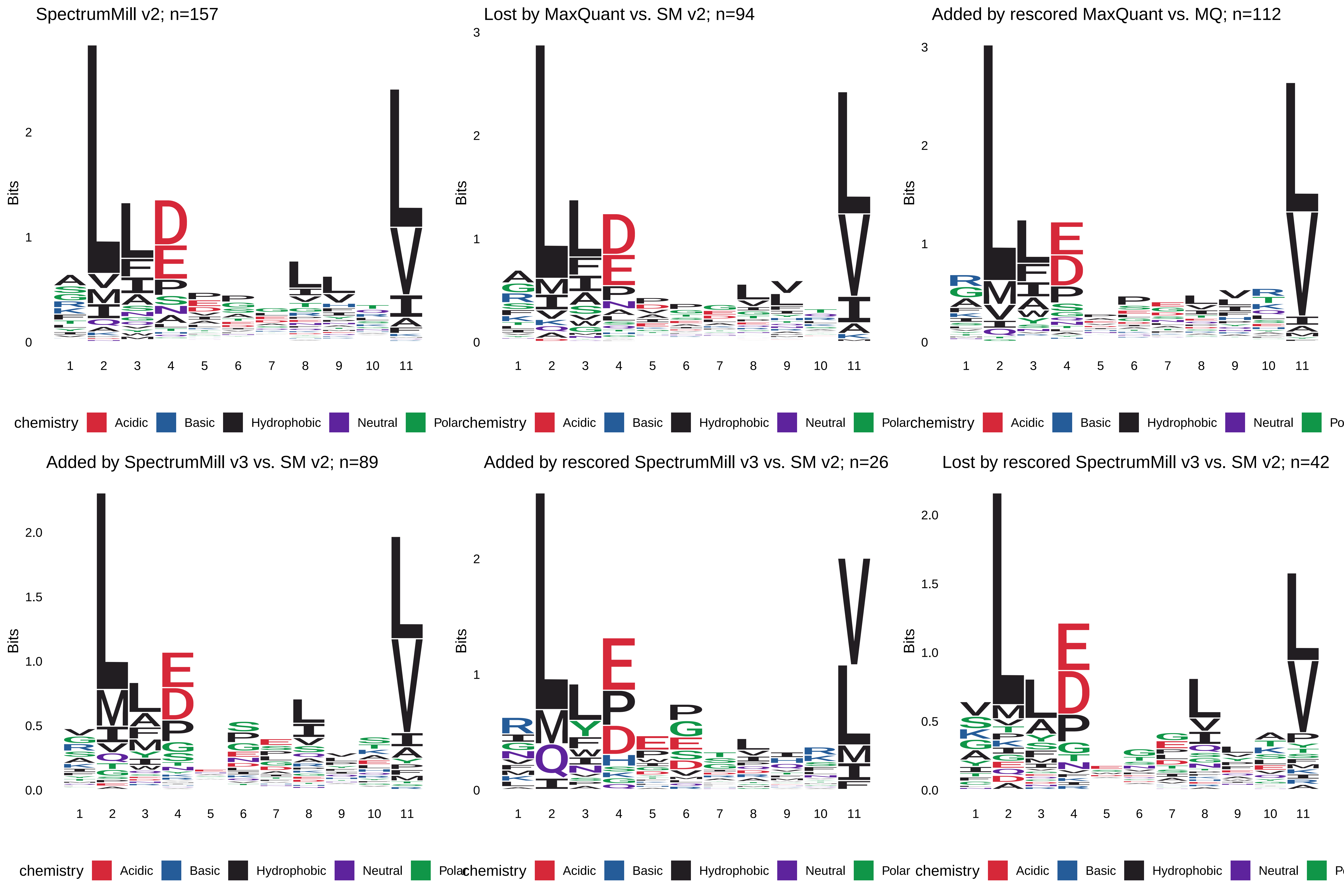

SpectrumMill v2; n=157
Lost by MaxQuant vs. SM v2; n=94
Added by rescored MaxQuant vs. MQ; n=112
Added by SpectrumMill v3 vs. SM v2; n=89
Added by rescored SpectrumMill v3 vs. SM v2; n=26
Lost by rescored SpectrumMill v3 vs. SM v2; n=42
Bits
1 2 3 4 5 6 7 8 9 10 11
chemistry
Acidic
Basic
Hydrophobic
Neutral
Polar

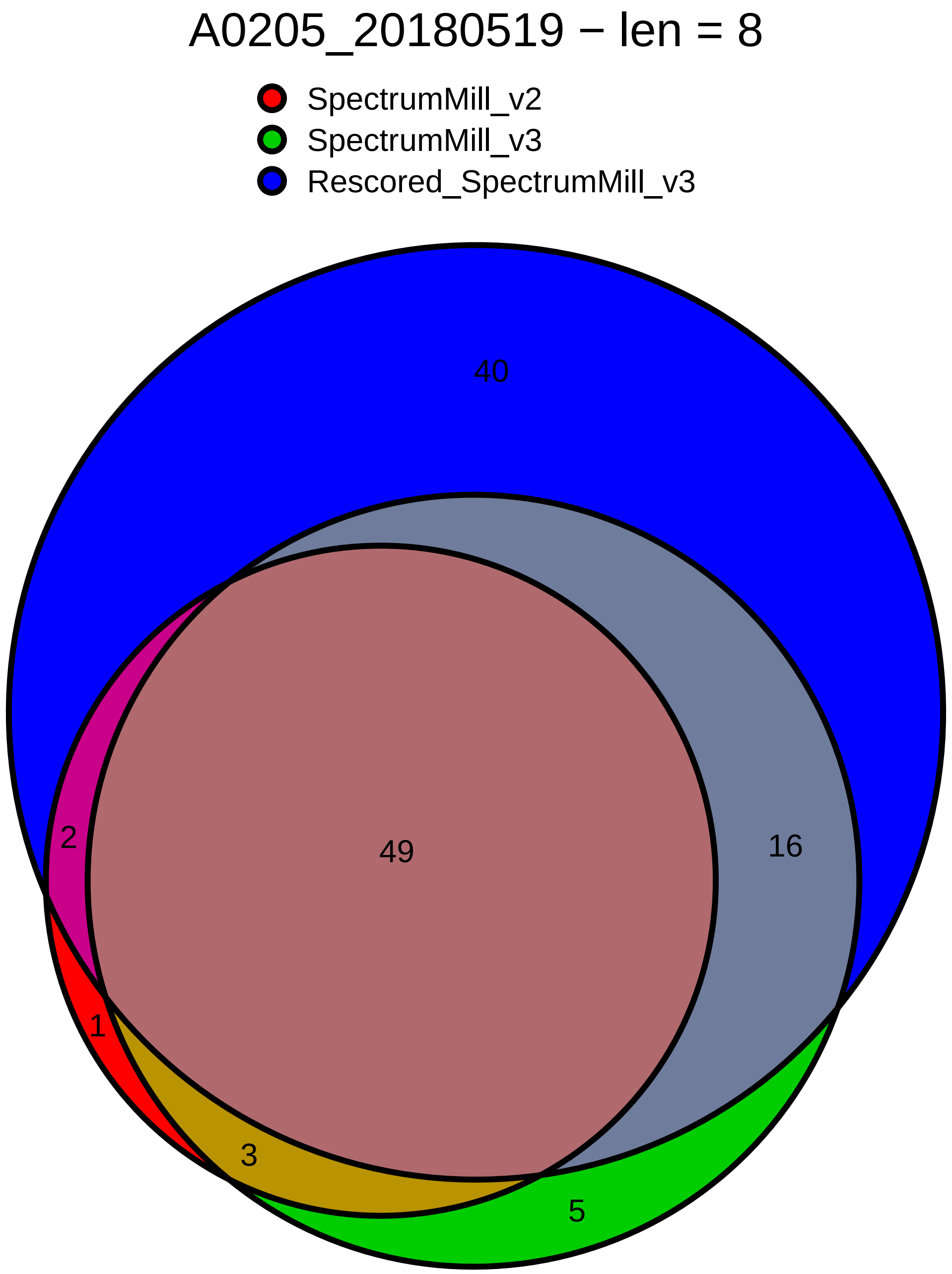
A0205_20180519 − len = 8
SpectrumMill_v2
SpectrumMill_v3
Rescored_SpectrumMill_v3
40
2
49
16
1
3
5

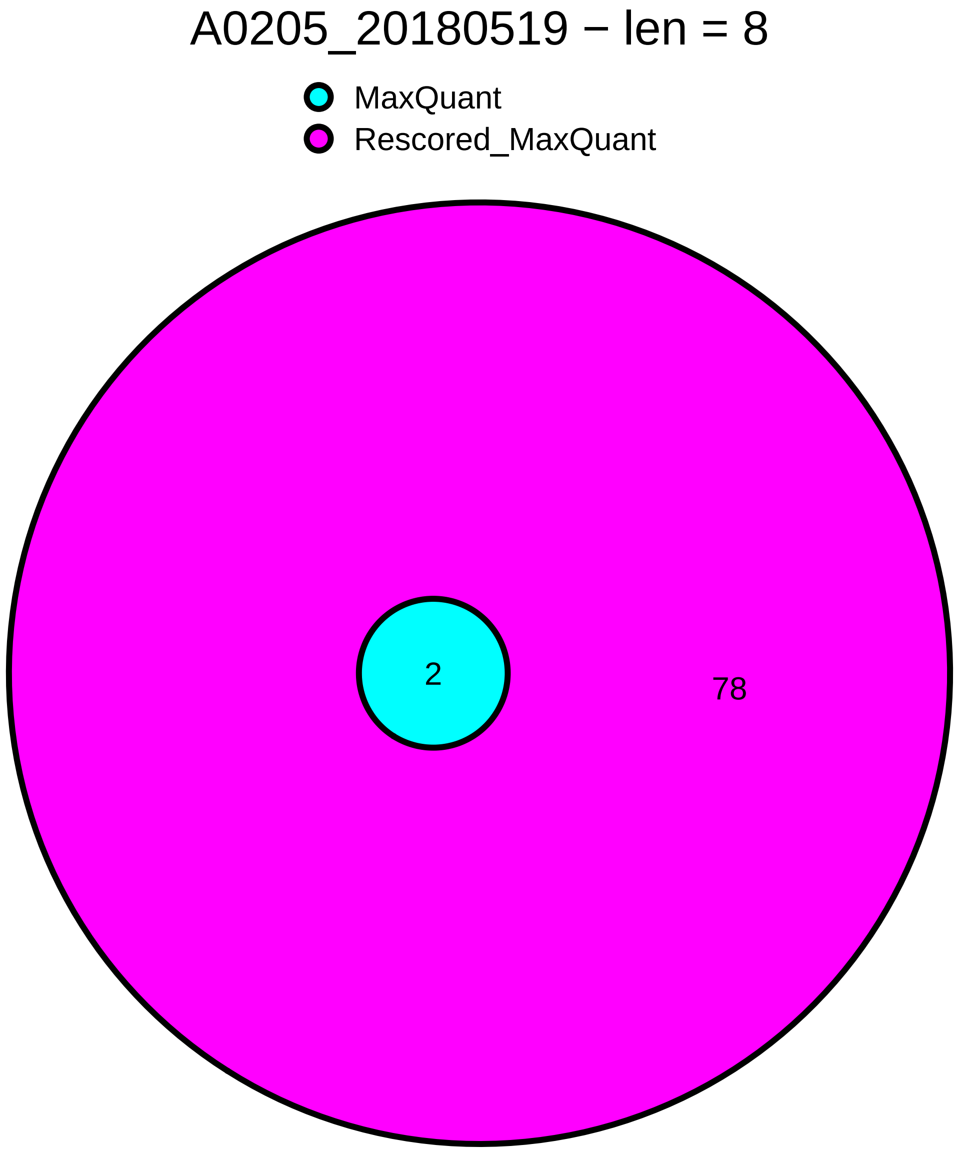
A0205_20180519 − len = 8
MaxQuant
Rescored_MaxQuant
2
78

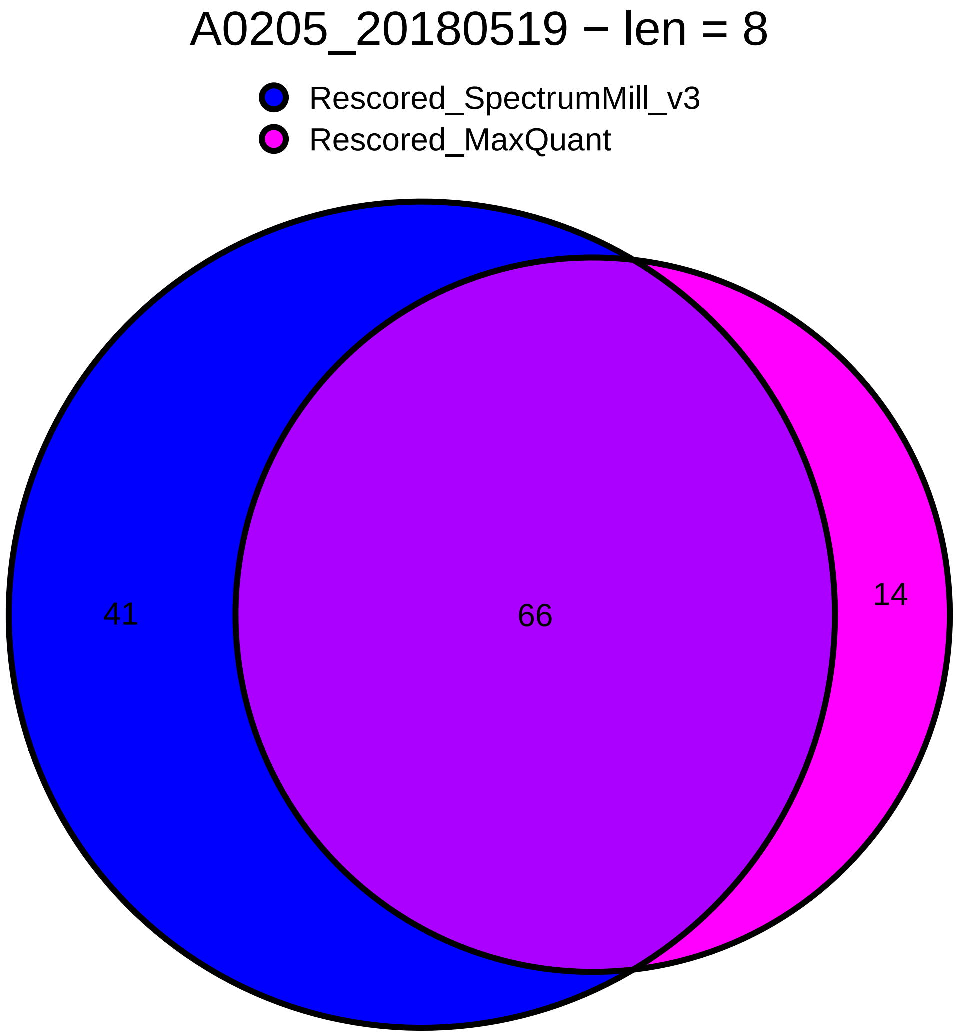
A0205_20180519 − len = 8
Rescored_SpectrumMill_v3
Rescored_MaxQuant
41
66
14

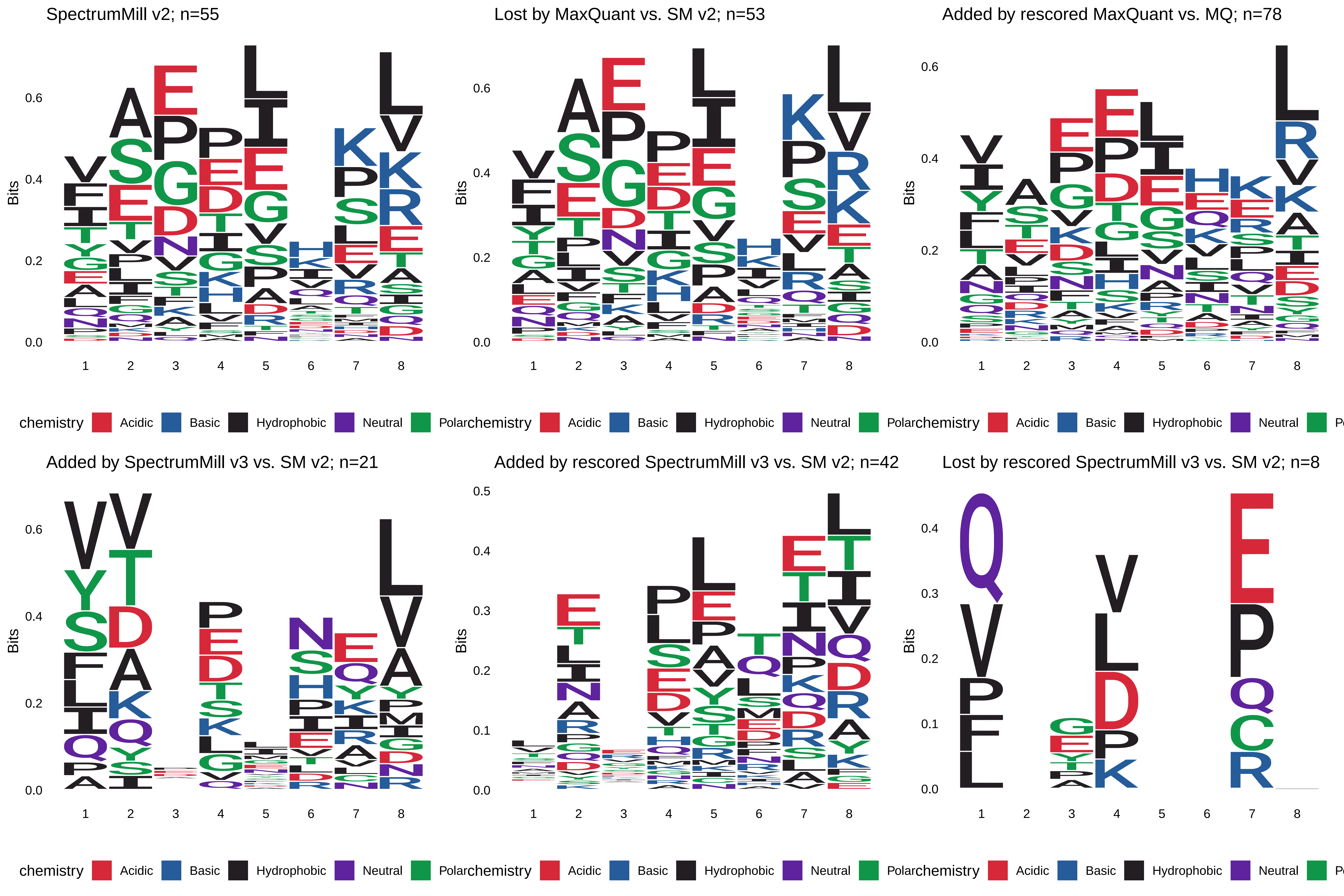
SpectrumMill v2; n=55
Lost by MaxQuant vs. SM v2; n=53
Added by rescored MaxQuant vs. MQ; n=78
Bits
chemistry Acidic Basic Hydrophobic Neutral Polarchemistry Acidic Basic Hydrophobic Neutral Polarchemistry Acidic Basic Hydrophobic Neutral
Added by SpectrumMill v3 vs. SM v2; n=21
Added by rescored SpectrumMill v3 vs. SM v2; n=42
Lost by rescored SpectrumMill v3 vs. SM v2; n=8
Bits
chemistry Acidic Basic Hydrophobic Neutral Polarchemistry Acidic Basic Hydrophobic Neutral Polarchemistry Acidic Basic Hydrophobic Neutral

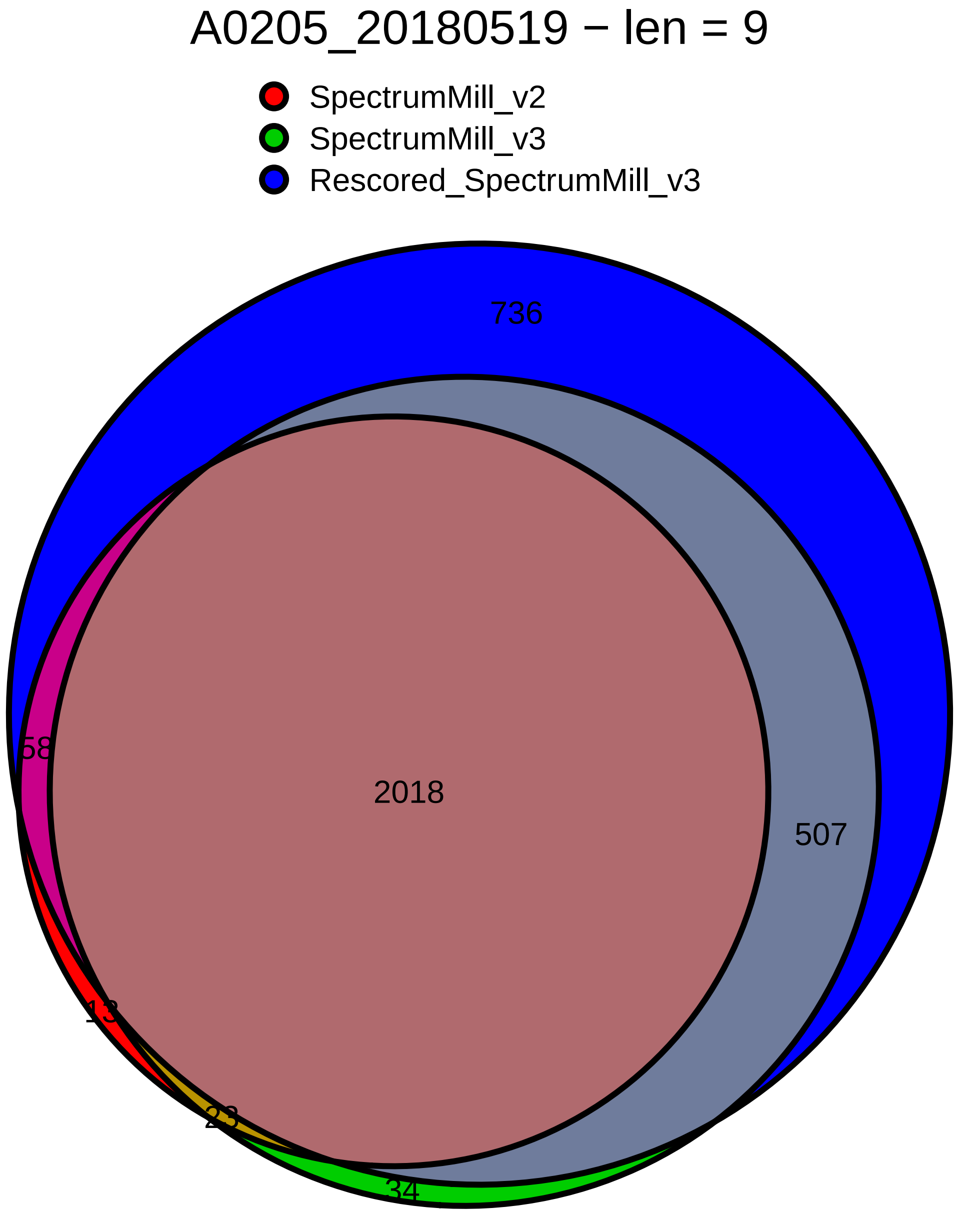
A0205_20180519 − len = 9
SpectrumMill_v2
SpectrumMill_v3
Rescored_SpectrumMill_v3
736
58
2018
507
13
23
34

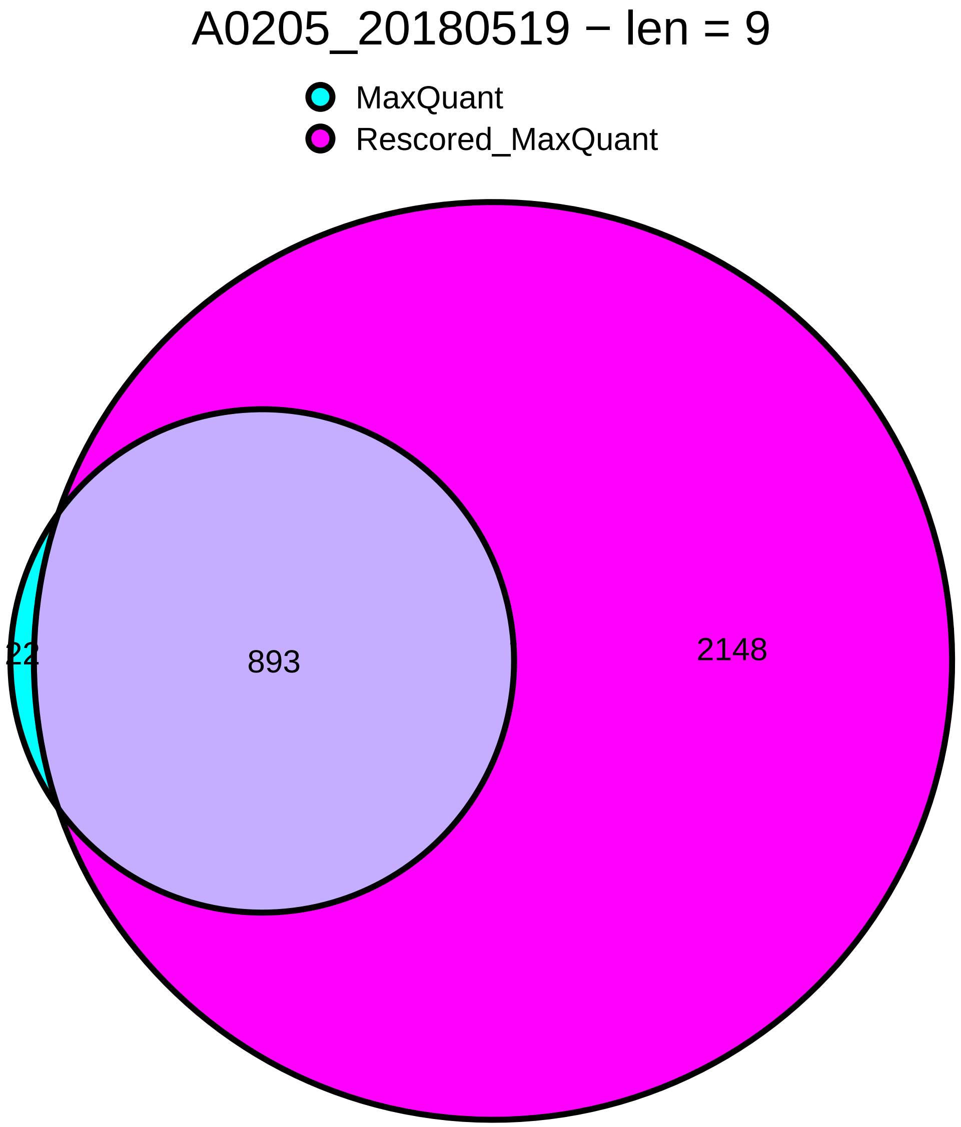
A0205_20180519 − len = 9
MaxQuant
Rescored_MaxQuant
22
893
2148

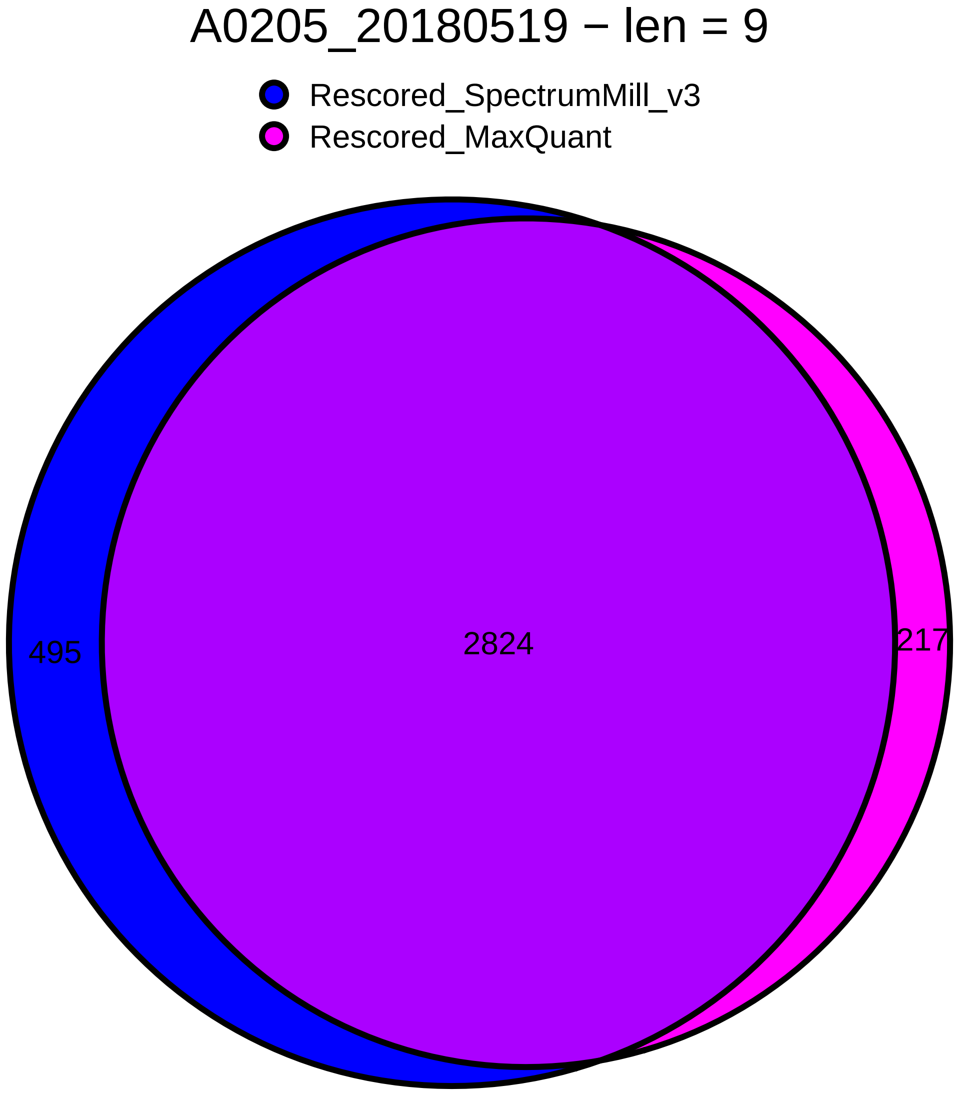
A0205_20180519 − len = 9
Rescored_SpectrumMill_v3
Rescored_MaxQuant
495
2824
217

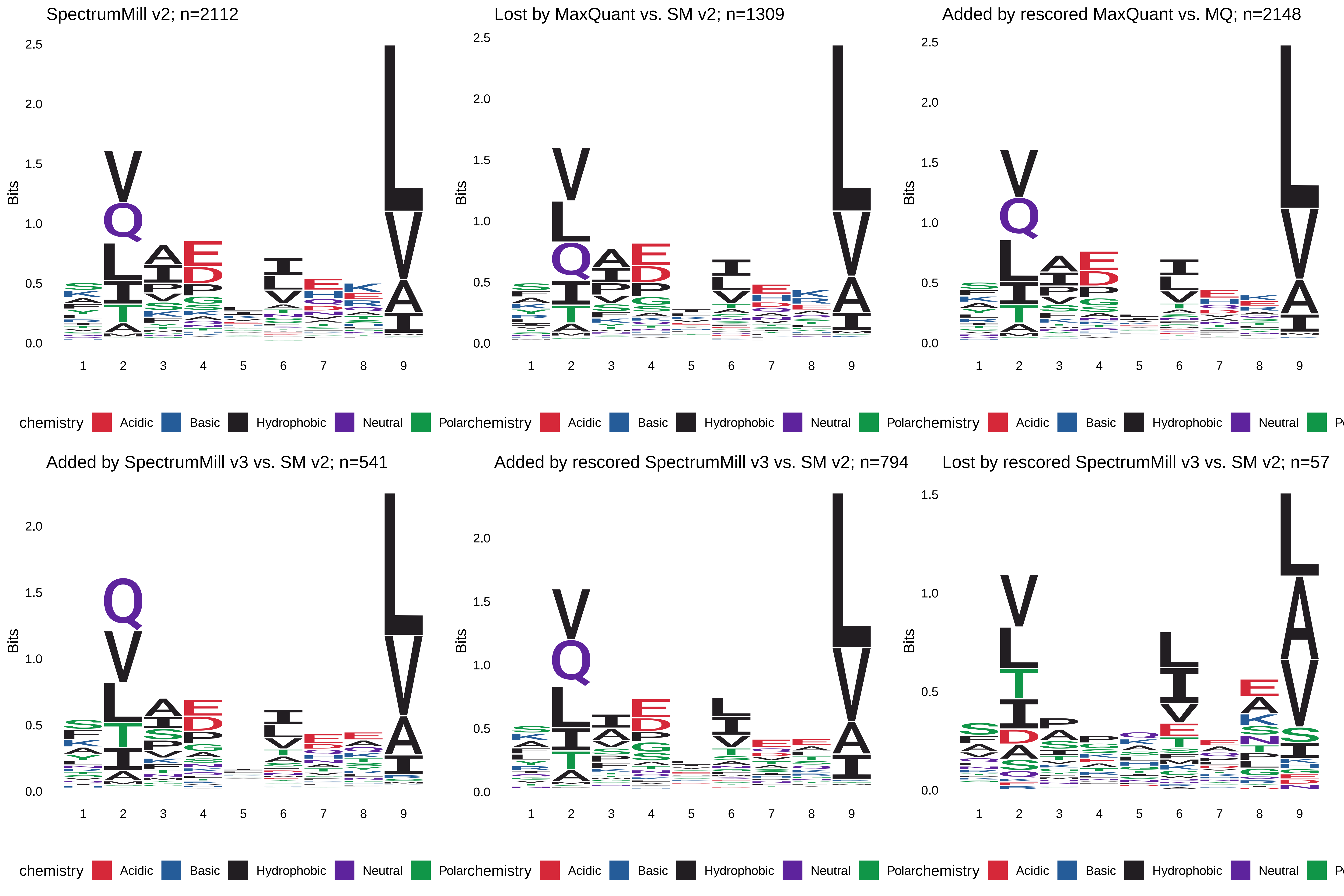
SpectrumMill v2; n=2112
Lost by MaxQuant vs. SM v2; n=1309
Added by rescored MaxQuant vs. MQ; n=2148
Added by SpectrumMill v3 vs. SM v2; n=541
Added by rescored SpectrumMill v3 vs. SM v2; n=794
Lost by rescored SpectrumMill v3 vs. SM v2; n=57
Bits
1 2 3 4 5 6 7 8 9
chemistry Acidic Basic Hydrophobic Neutral Polarchemistry Acidic Basic Hydrophobic Neutral Polarchemistry Acidic Basic Hydrophobic Neutral
chemistry Acidic Basic Hydrophobic Neutral Polarchemistry Acidic Basic Hydrophobic Neutral Polarchemistry Acidic Basic Hydrophobic Neutral

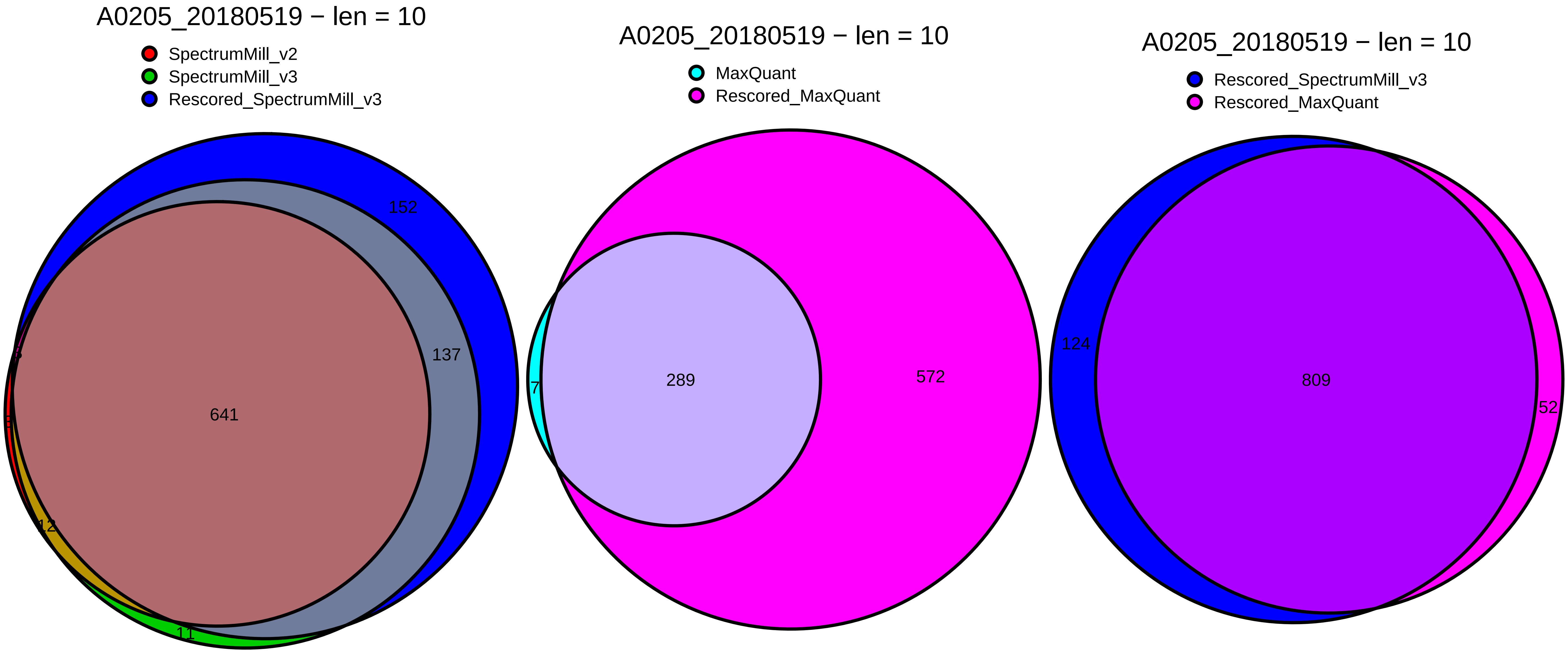
A0205_20180519 − len = 10
SpectrumMill_v2
SpectrumMill_v3
Rescored_SpectrumMill_v3
152
137
641
12
11
A0205_20180519 − len = 10
MaxQuant
Rescored_MaxQuant
7
289
572
A0205_20180519 − len = 10
Rescored_SpectrumMill_v3
Rescored_MaxQuant
124
809
52

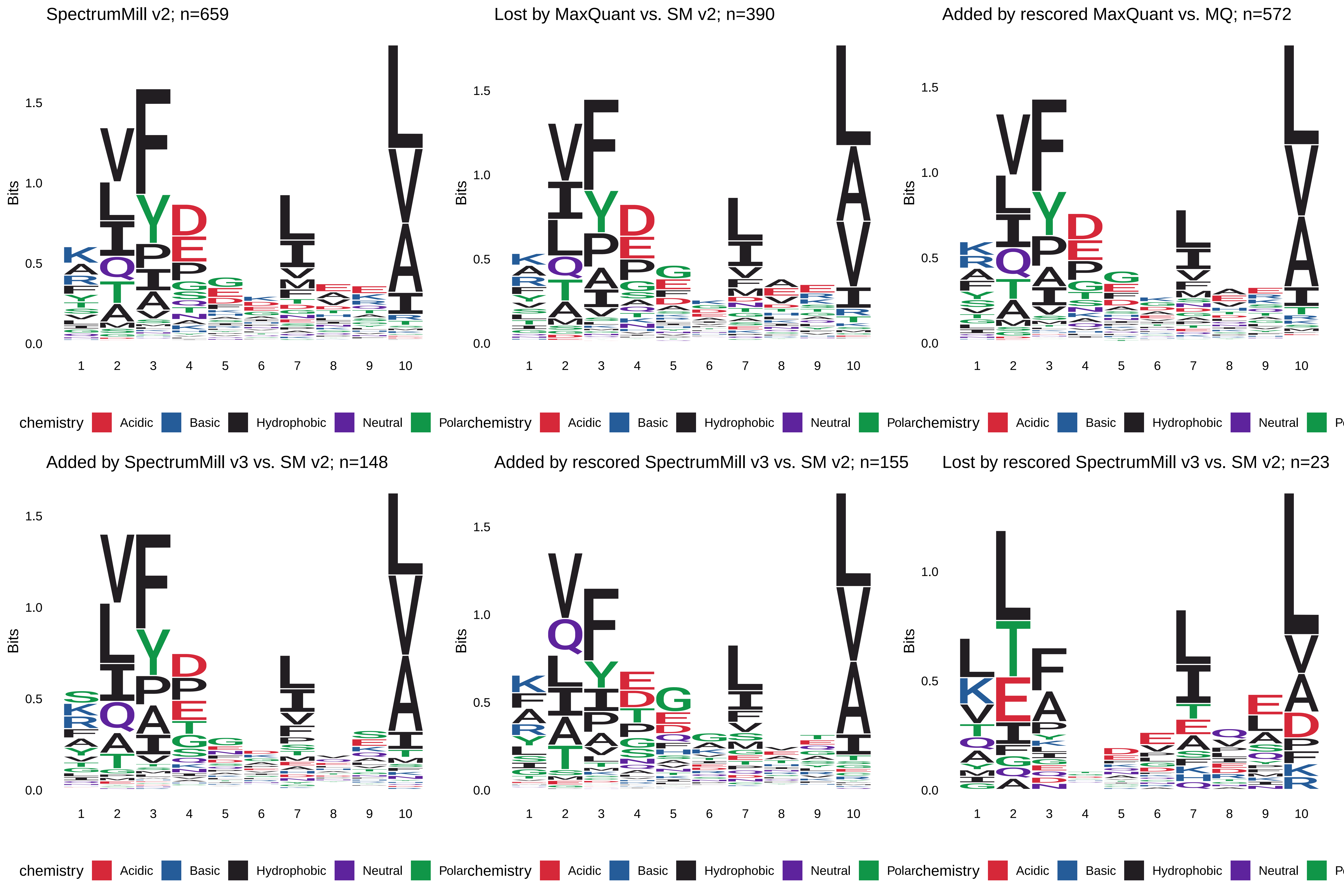
SpectrumMill v2; n=659
Lost by MaxQuant vs. SM v2; n=390
Added by rescored MaxQuant vs. MQ; n=572
Added by SpectrumMill v3 vs. SM v2; n=148
Added by rescored SpectrumMill v3 vs. SM v2; n=155
Lost by rescored SpectrumMill v3 vs. SM v2; n=23
Bits
0.0
0.5
1.0
1.5
1 2 3 4 5 6 7 8 9 10
chemistry
Acidic
Basic
Hydrophobic
Neutral
Polar

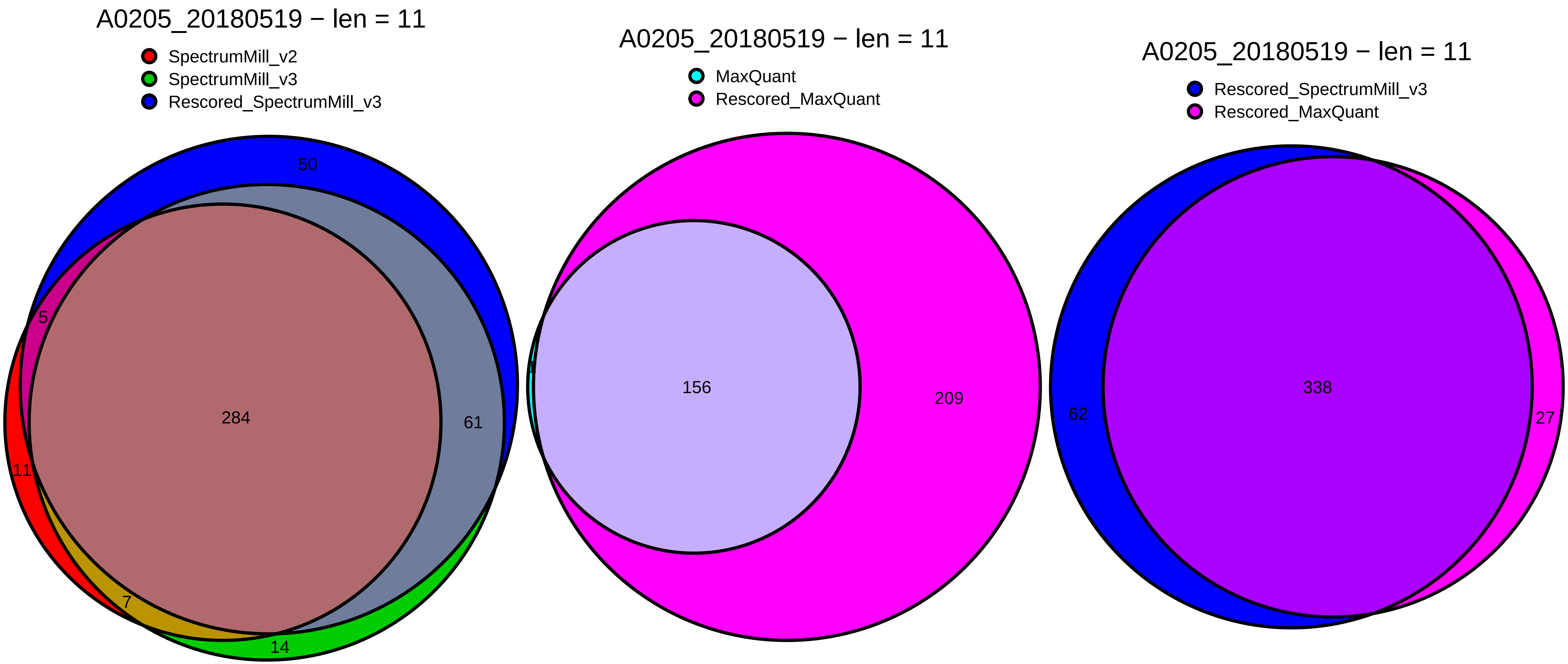
A0205_20180519 − len = 11
SpectrumMill_v2
SpectrumMill_v3
Rescored_SpectrumMill_v3
50
5
284
61
11
7
14
A0205_20180519 − len = 11
MaxQuant
Rescored_MaxQuant
156
209
A0205_20180519 − len = 11
Rescored_SpectrumMill_v3
Rescored_MaxQuant
338
62
27

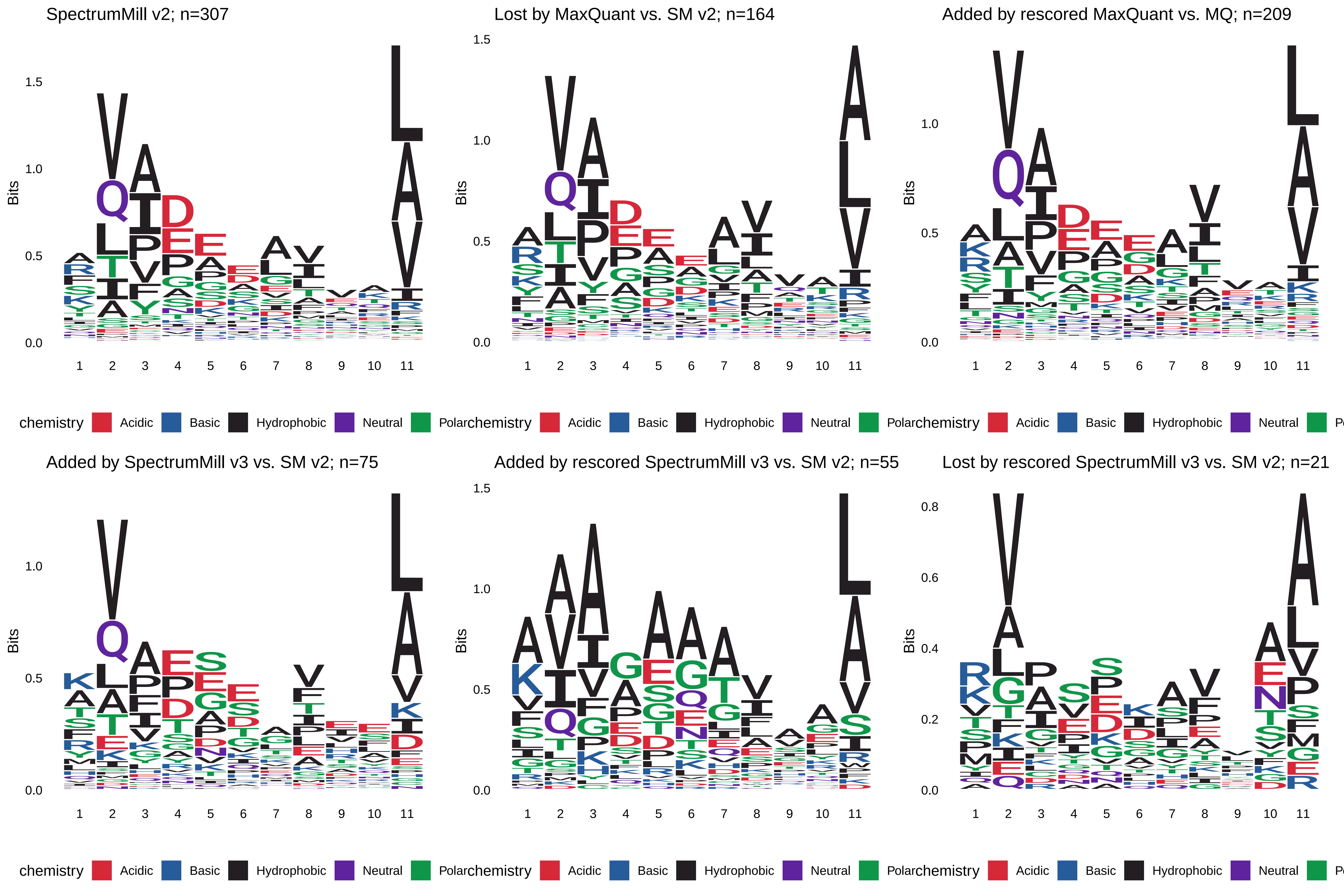
SpectrumMill v2; n=307
Lost by MaxQuant vs. SM v2; n=164
Added by rescored MaxQuant vs. MQ; n=209
Added by SpectrumMill v3 vs. SM v2; n=75
Added by rescored SpectrumMill v3 vs. SM v2; n=55
Lost by rescored SpectrumMill v3 vs. SM v2; n=21
Bits
1 2 3 4 5 6 7 8 9 10 11
chemistry Acidic Basic Hydrophobic Neutral Polarchemistry Acidic Basic Hydrophobic Neutral Polarchemistry Acidic Basic Hydrophobic Neutral
chemistry Acidic Basic Hydrophobic Neutral Polarchemistry Acidic Basic Hydrophobic Neutral Polarchemistry Acidic Basic Hydrophobic Neutral

A0206_20180724 − len = 8

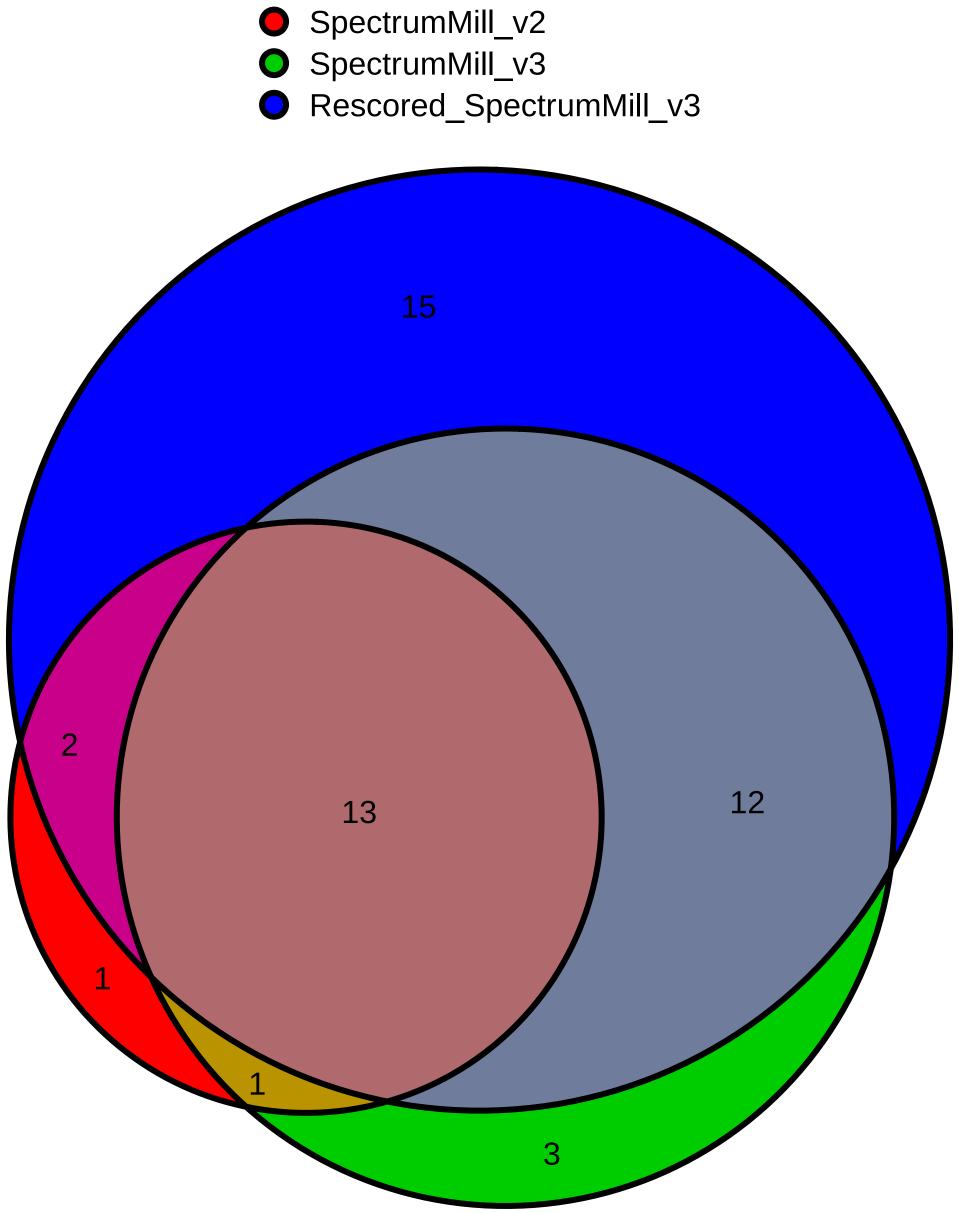

A0206_20180724 − len = 8

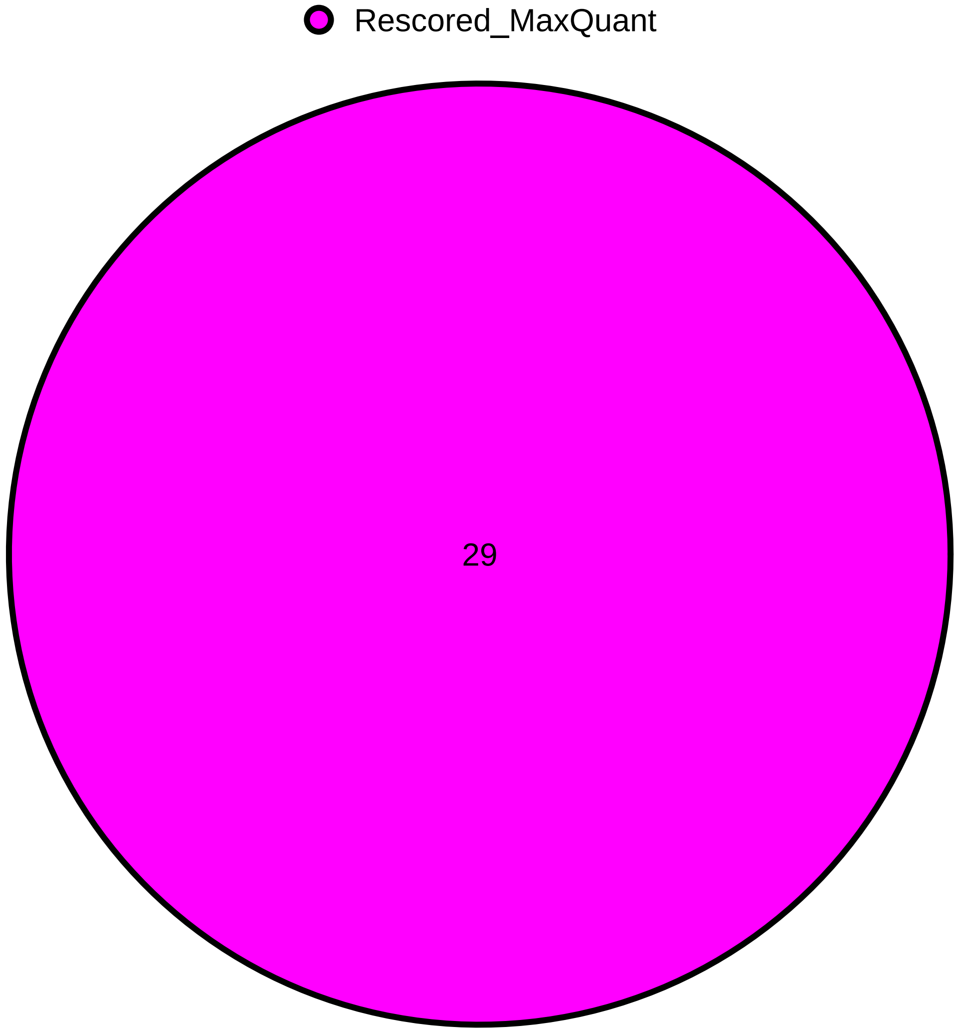

A0206_20180724 − len = 8

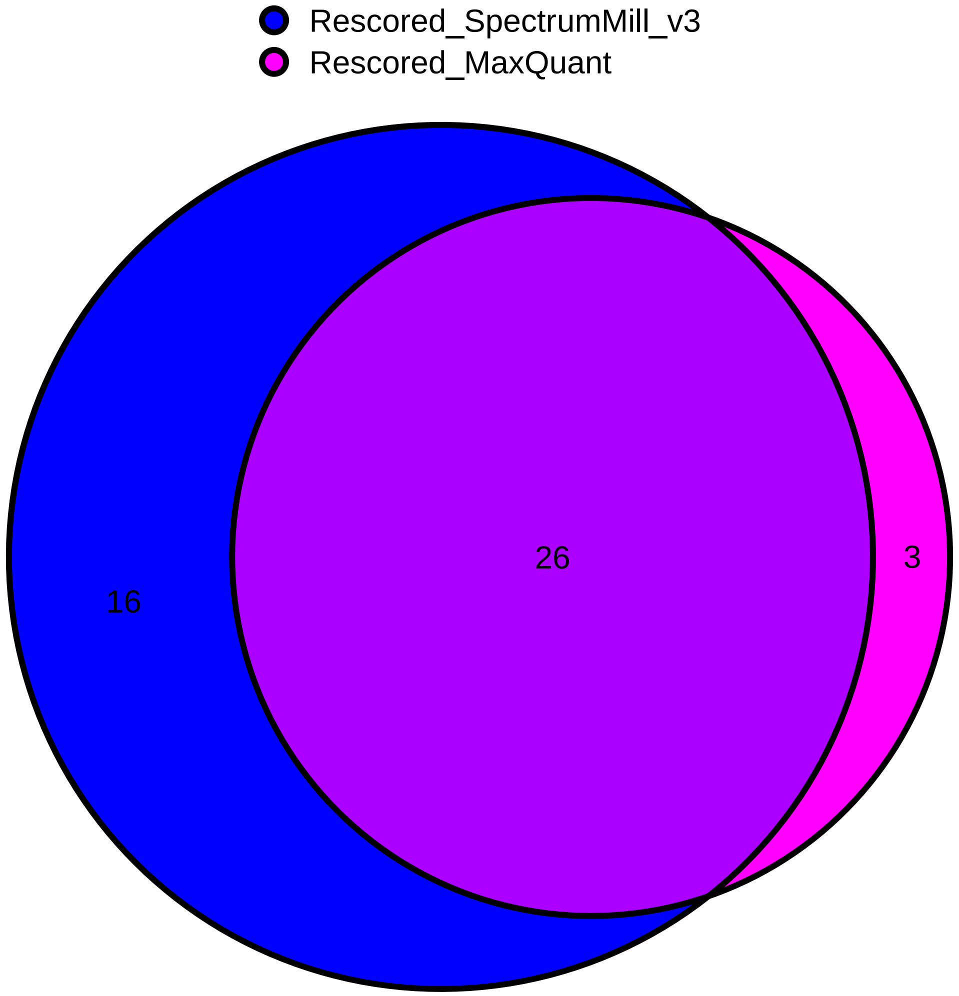

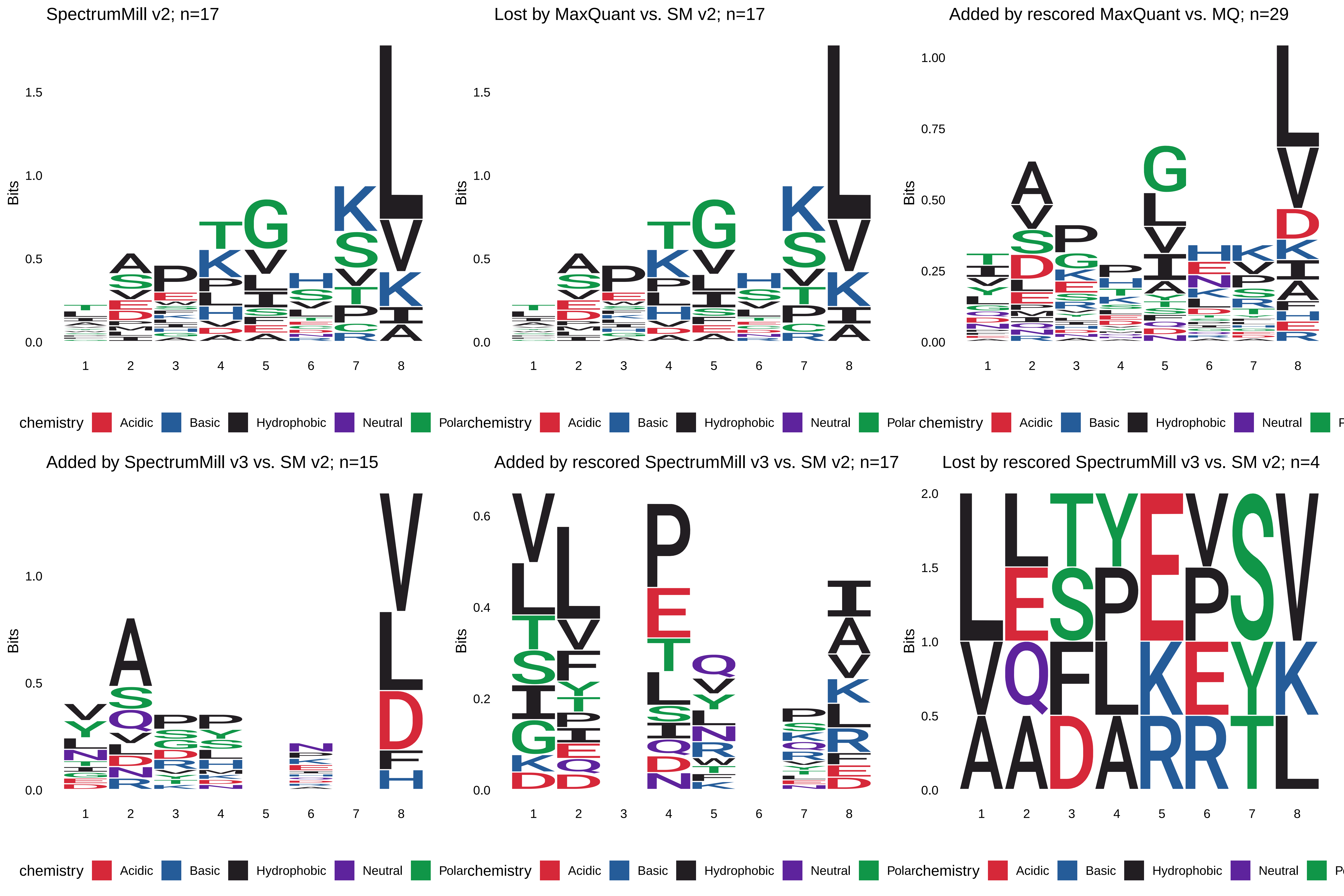
SpectrumMill v2; n=17
Lost by MaxQuant vs. SM v2; n=17
Added by rescored MaxQuant vs. MQ; n=29
Added by SpectrumMill v3 vs. SM v2; n=15
Added by rescored SpectrumMill v3 vs. SM v2; n=17
Lost by rescored SpectrumMill v3 vs. SM v2; n=4
Bits
chemistry
Acidic
Basic
Hydrophobic
Neutral
Polar

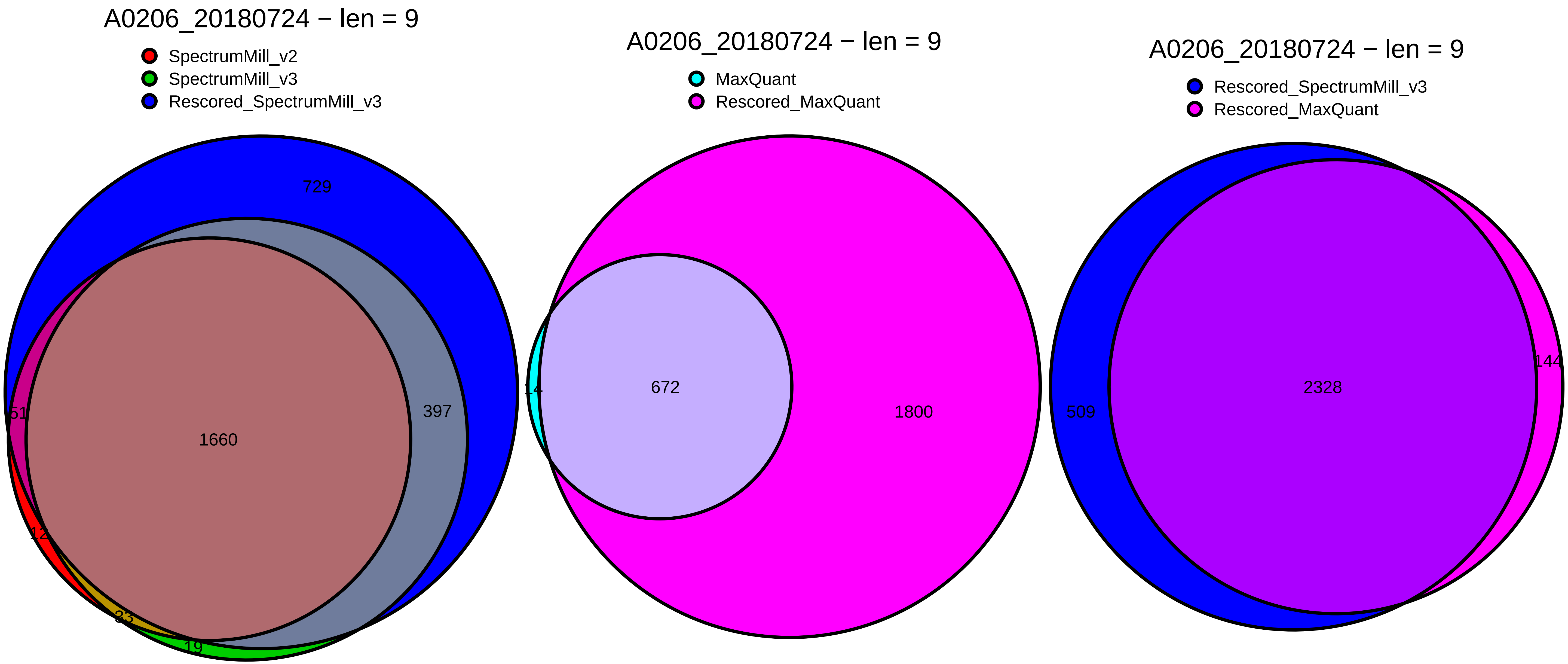

A0206_20180724 − len = 9
SpectrumMill_v2
SpectrumMill_v3
Rescored_SpectrumMill_v3
729
397
1660
51
12
19
A0206_20180724 − len = 9
MaxQuant
Rescored_MaxQuant
14
672
1800
A0206_20180724 − len = 9
Rescored_SpectrumMill_v3
Rescored_MaxQuant
509
2328
144

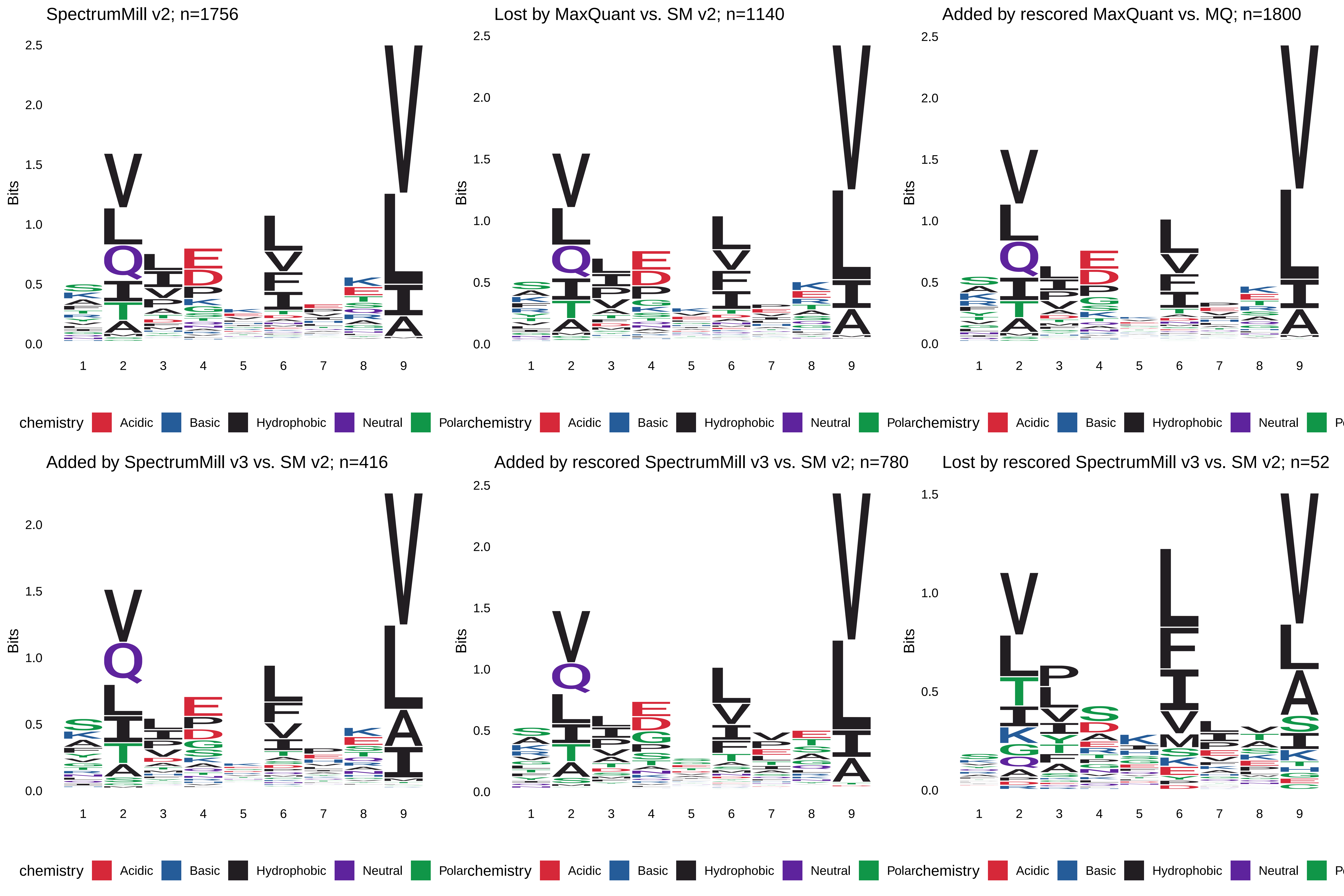

SpectrumMill v2; n=1756
Lost by MaxQuant vs. SM v2; n=1140
Added by rescored MaxQuant vs. MQ; n=1800
Added by SpectrumMill v3 vs. SM v2; n=416
Added by rescored SpectrumMill v3 vs. SM v2; n=780
Lost by rescored SpectrumMill v3 vs. SM v2; n=52
Bits
0.0
0.5
1.0
1.5
2.0
2.5
1 2 3 4 5 6 7 8 9
chemistry
Acidic
Basic
Hydrophobic
Neutral
Polarchemistry
Acidic
Basic
Hydrophobic
Neutral
Polarchemistry
Acidic
Basic
Hydrophobic
Neutral

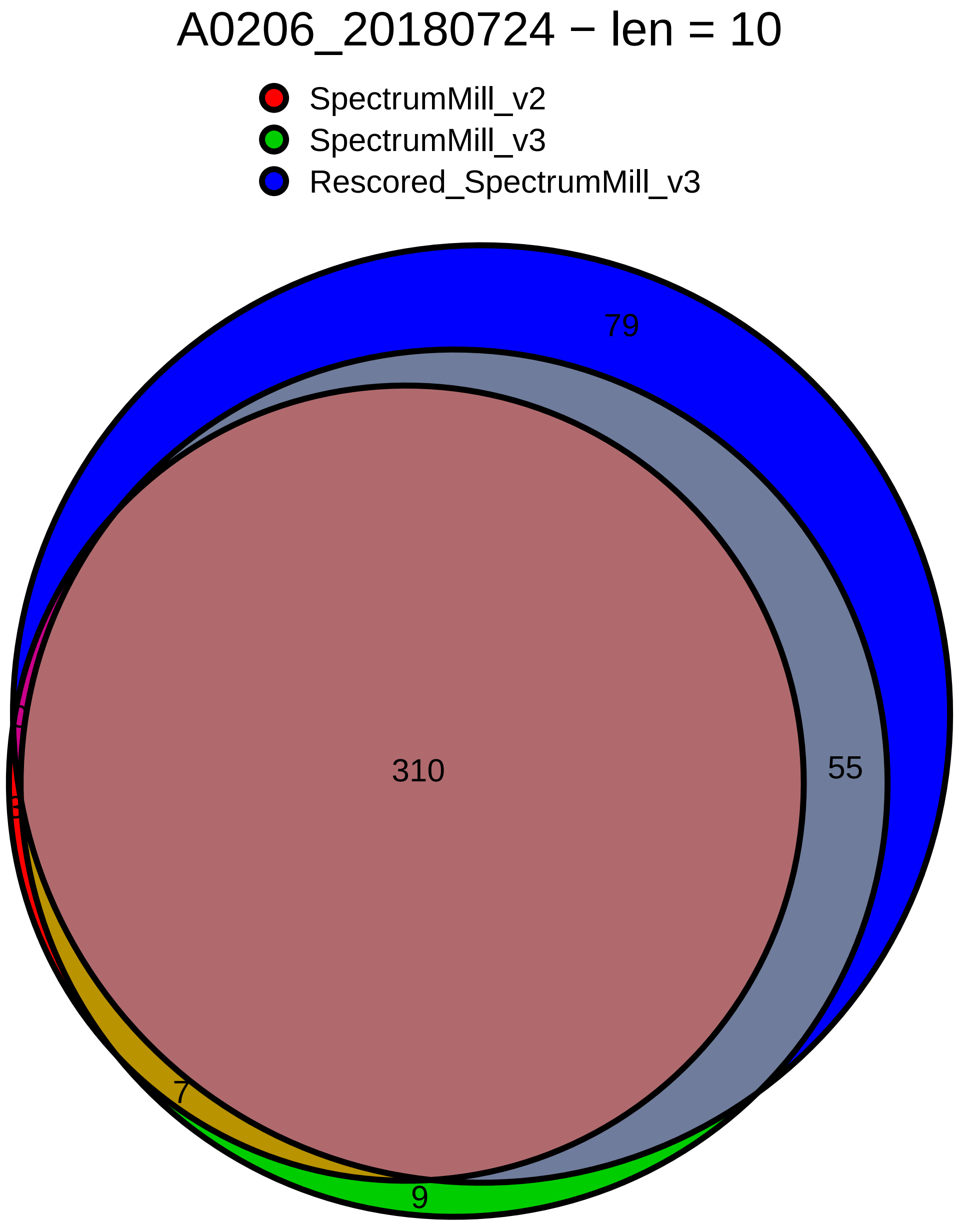
A0206_20180724 − len = 10
SpectrumMill_v2
SpectrumMill_v3
Rescored_SpectrumMill_v3
79
310
55
7
9

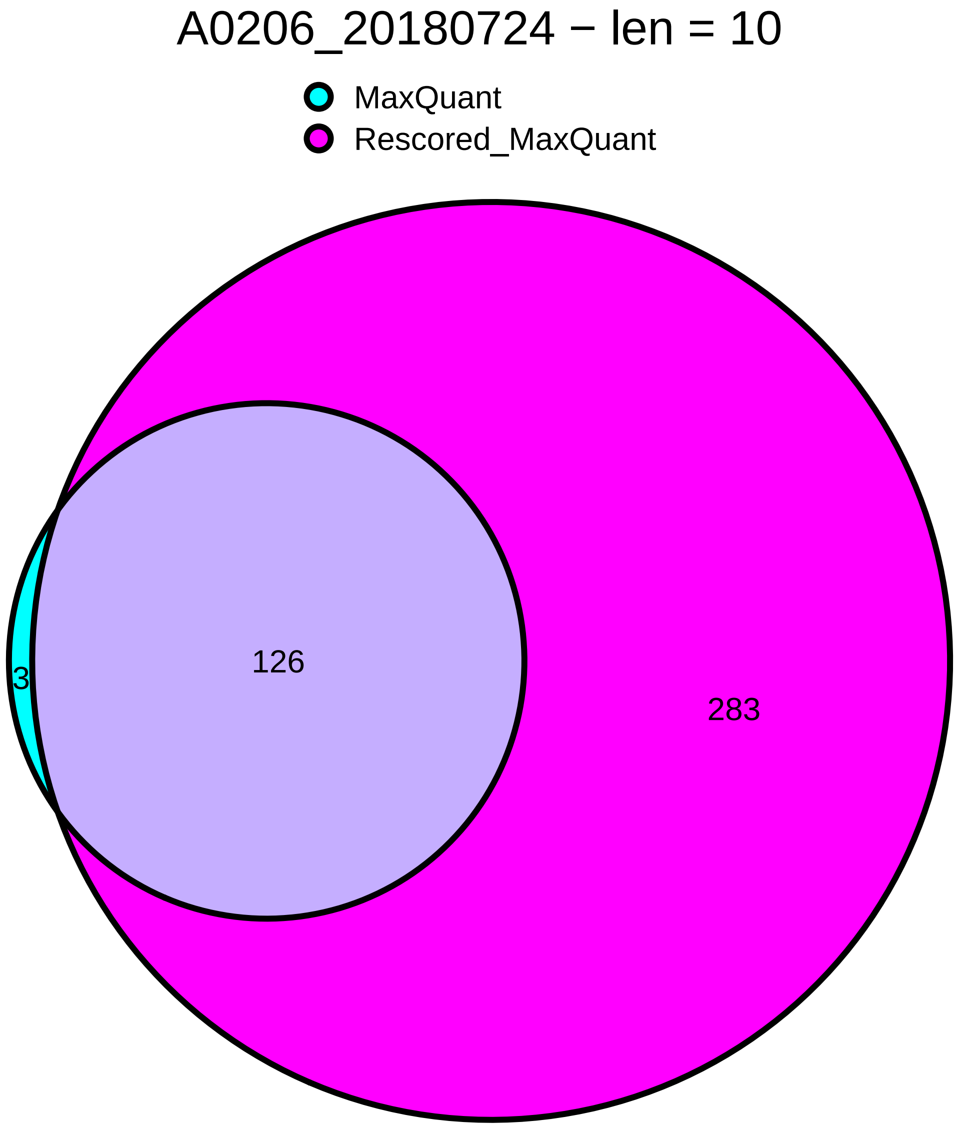
A0206_20180724 − len = 10
MaxQuant
Rescored_MaxQuant
3
126
283

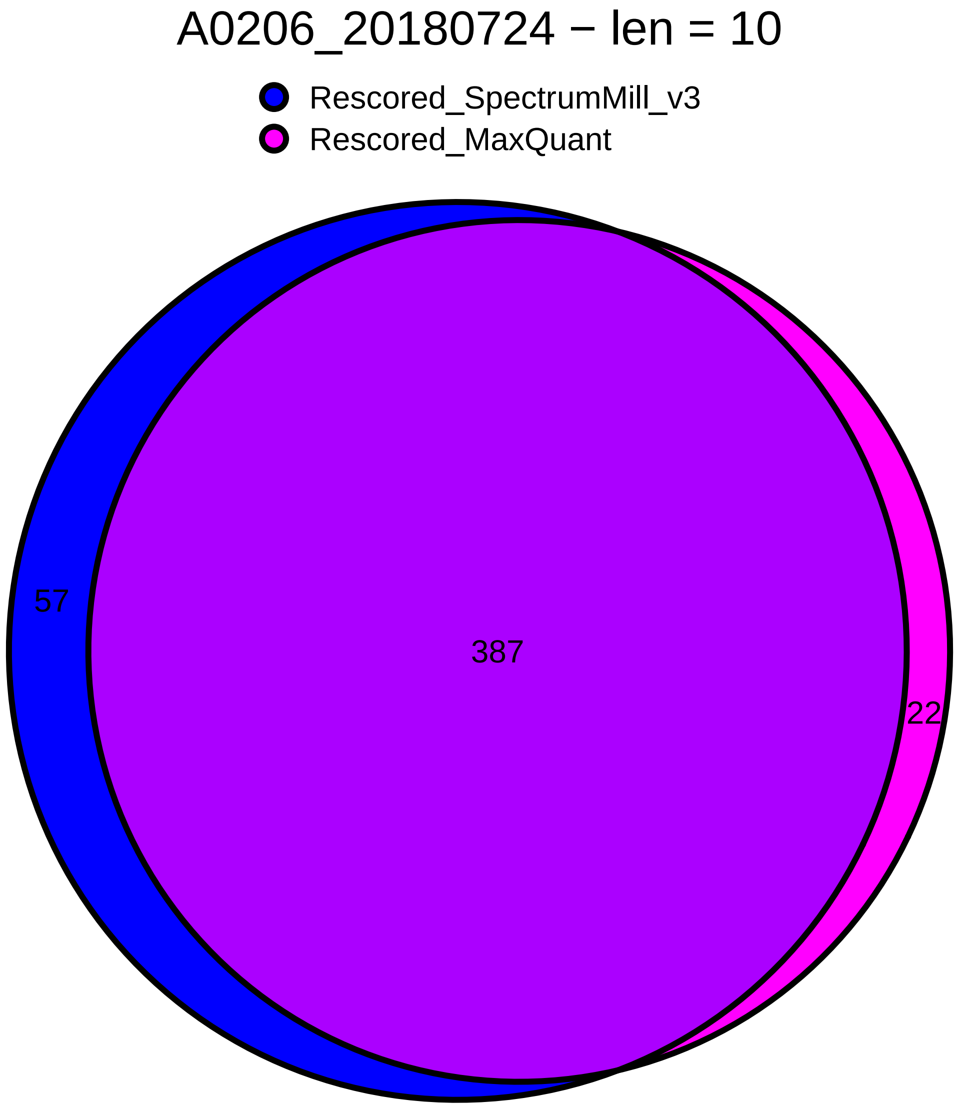
A0206_20180724 − len = 10
Rescored_SpectrumMill_v3
Rescored_MaxQuant
57
387
22

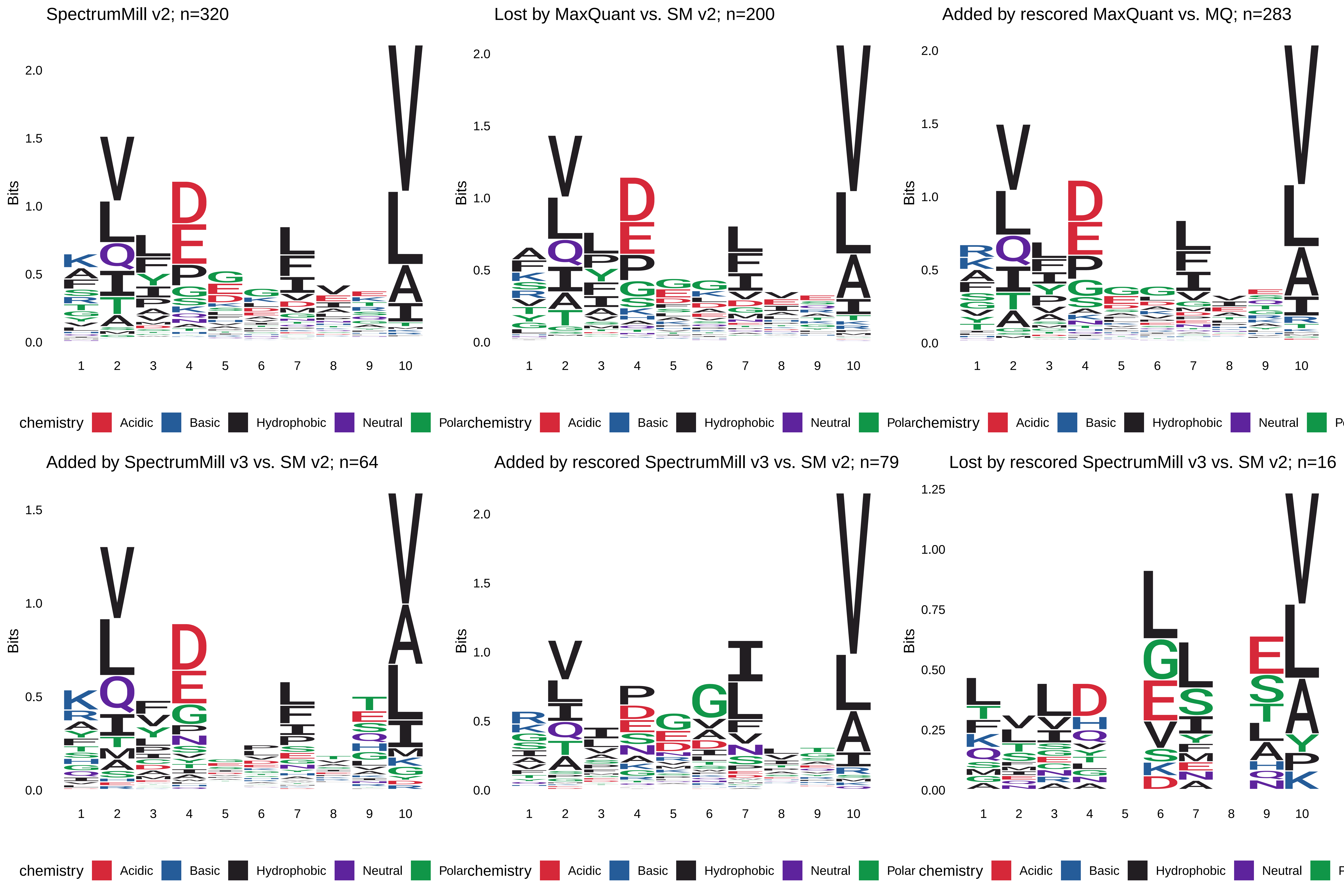
SpectrumMill v2; n=320
Lost by MaxQuant vs. SM v2; n=200
Added by rescored MaxQuant vs. MQ; n=283
Added by SpectrumMill v3 vs. SM v2; n=64
Added by rescored SpectrumMill v3 vs. SM v2; n=79
Lost by rescored SpectrumMill v3 vs. SM v2; n=16
Bits
chemistry
Acidic
Basic
Hydrophobic
Neutral
Polar

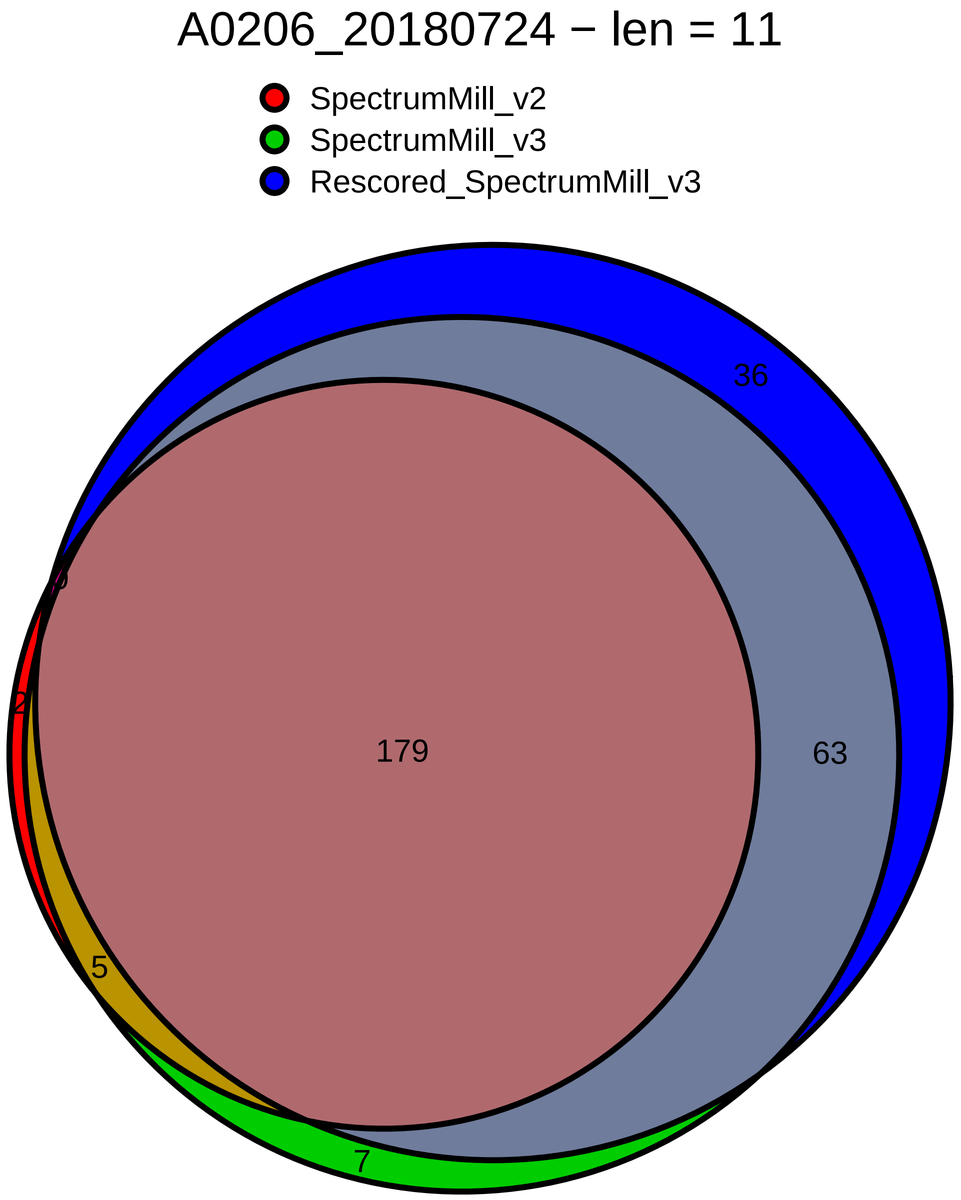
A0206_20180724 − len = 11
SpectrumMill_v2
SpectrumMill_v3
Rescored_SpectrumMill_v3
36
2
179
63
5
7

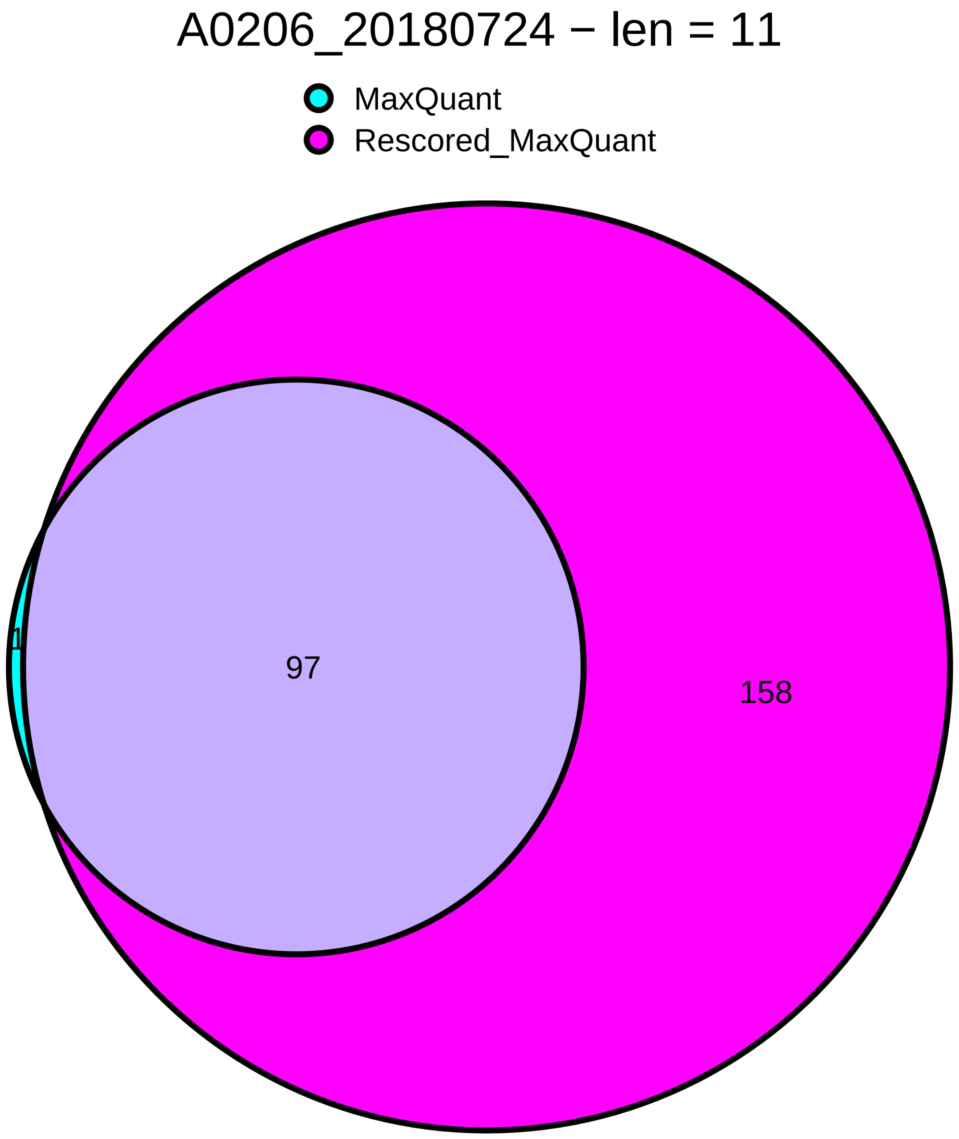
A0206_20180724 − len = 11
MaxQuant
Rescored_MaxQuant
97
158

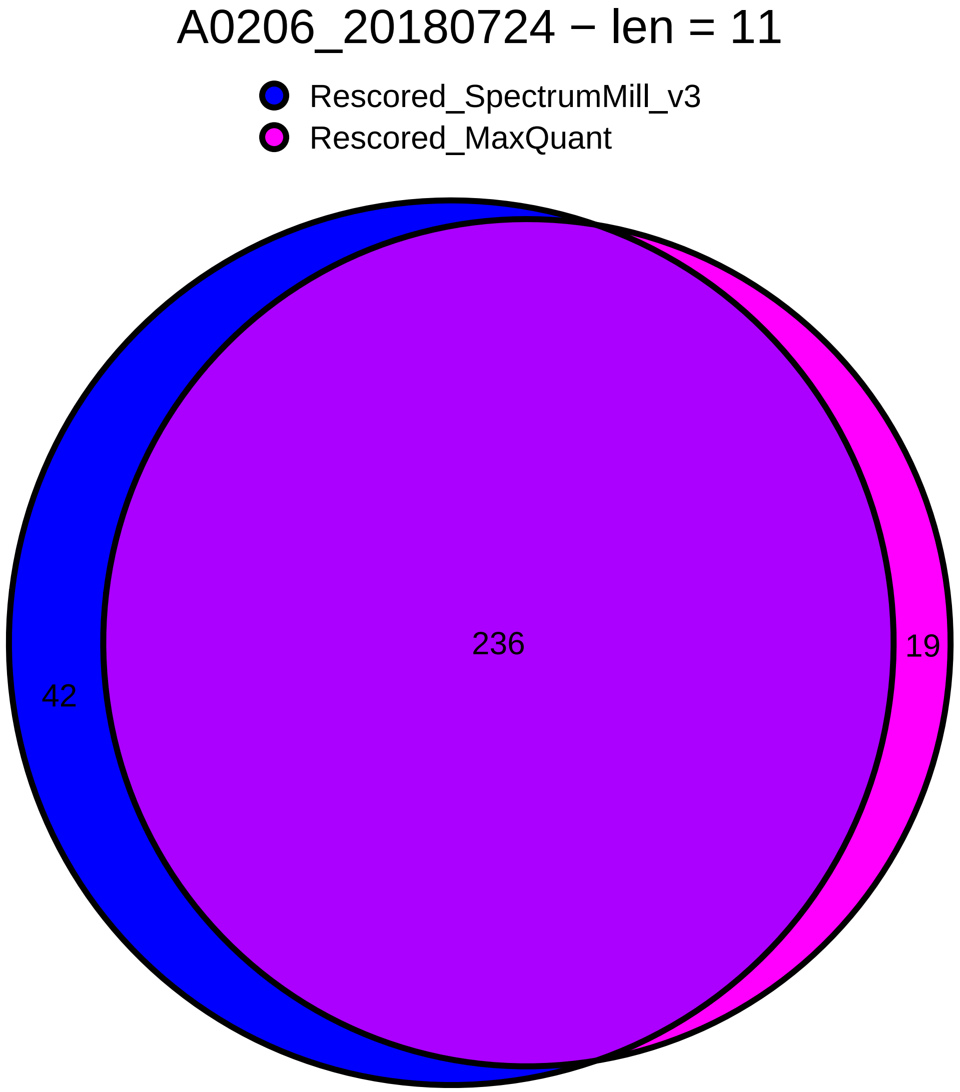
A0206_20180724 − len = 11
Rescored_SpectrumMill_v3
Rescored_MaxQuant
42
236
19

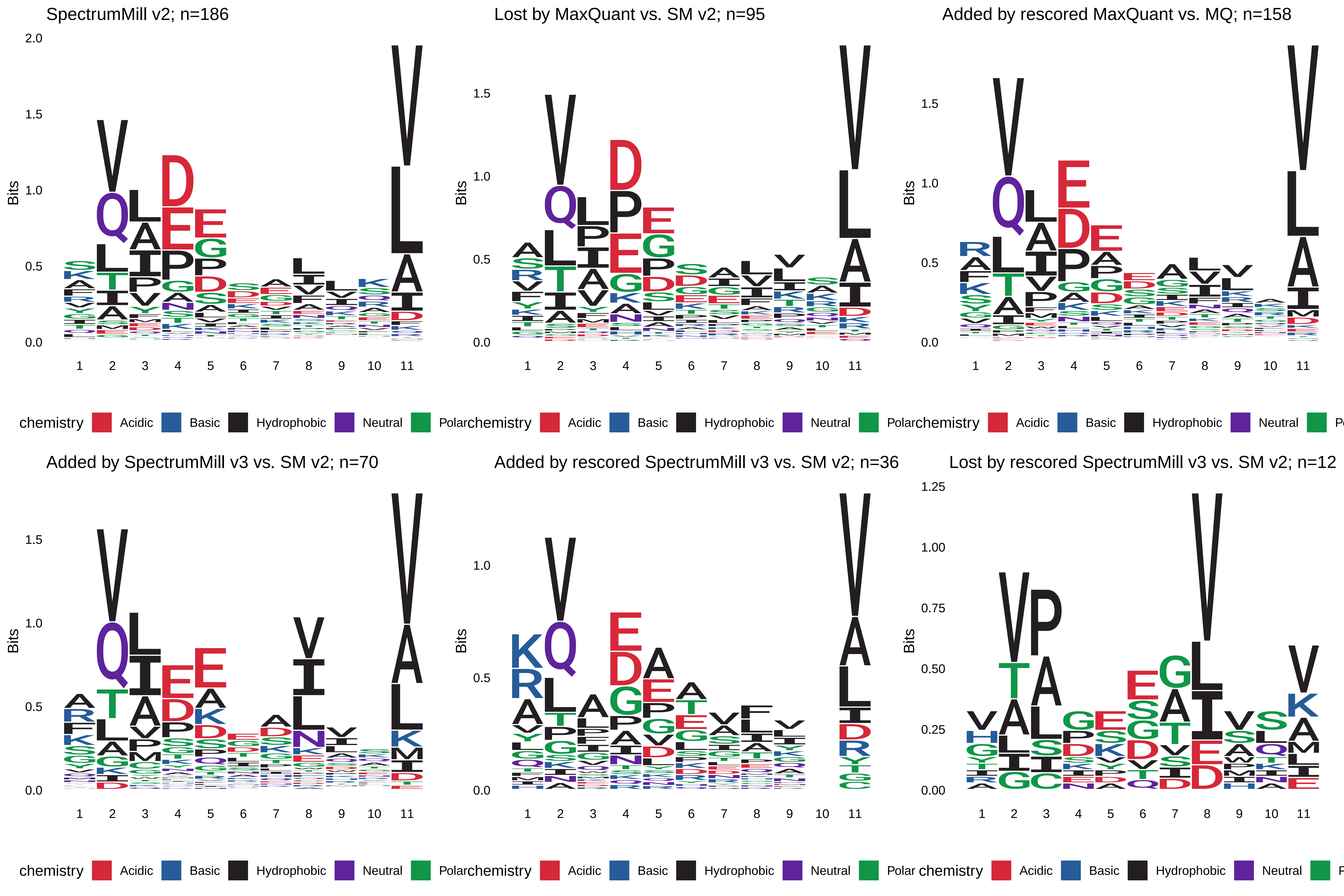

SpectrumMill v2; n=186
Lost by MaxQuant vs. SM v2; n=95
Added by rescored MaxQuant vs. MQ; n=158
Added by SpectrumMill v3 vs. SM v2; n=70
Added by rescored SpectrumMill v3 vs. SM v2; n=36
Lost by rescored SpectrumMill v3 vs. SM v2; n=12
Bits
chemistry Acidic Basic Hydrophobic Neutral Polar

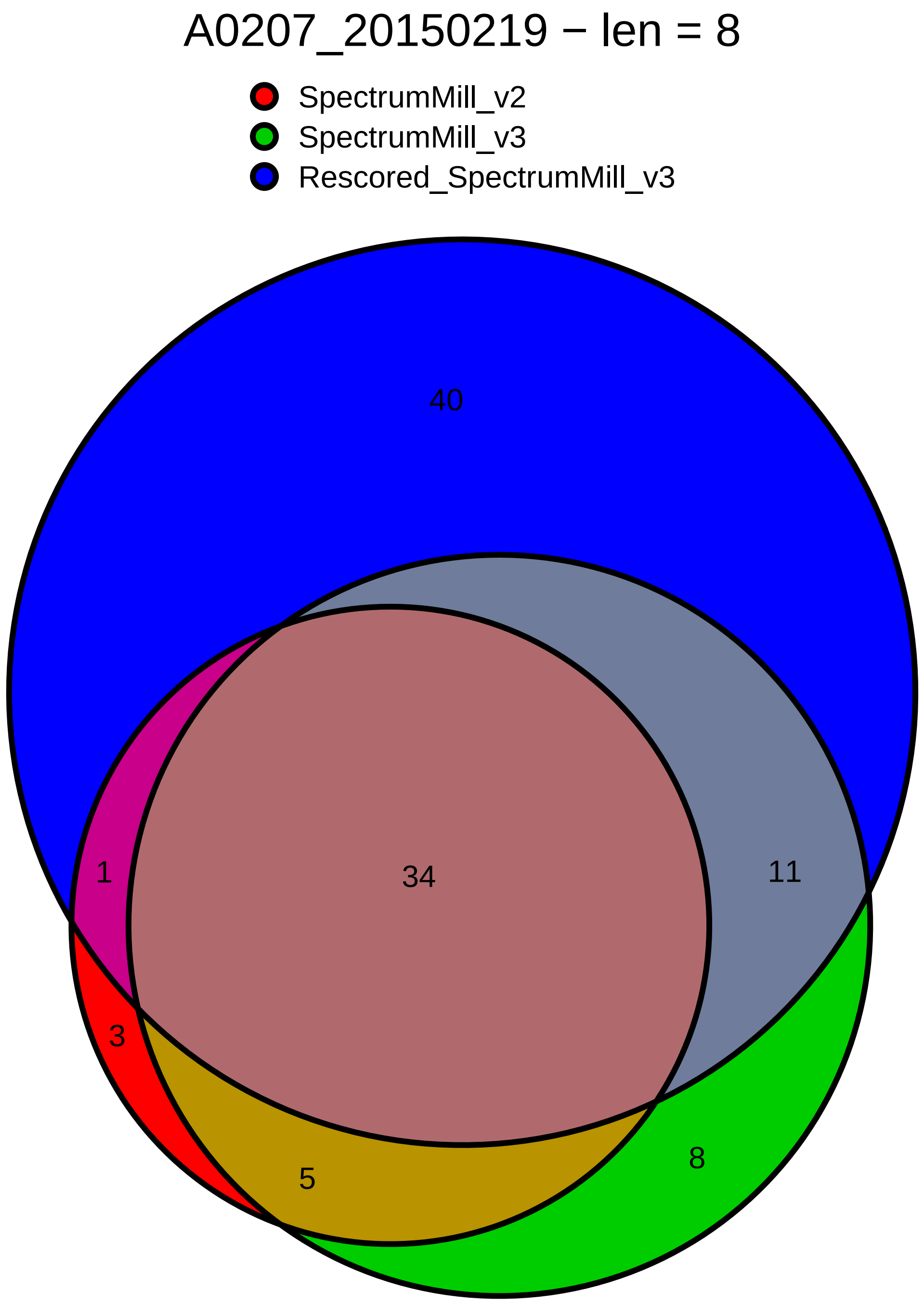
A0207_20150219 − len = 8
SpectrumMill_v2
SpectrumMill_v3
Rescored_SpectrumMill_v3
40
1
34
11
3
5
8

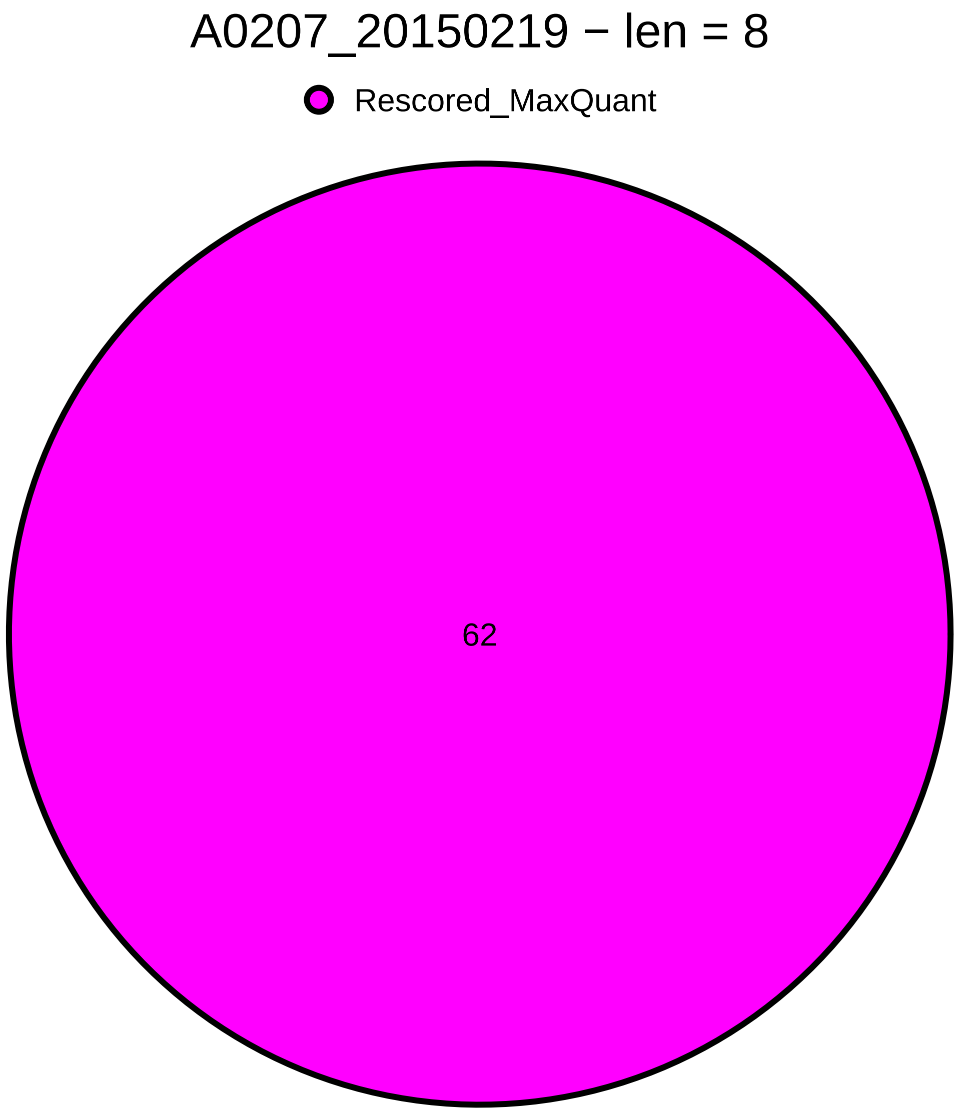
A0207_20150219 − len = 8
Rescored_MaxQuant
62

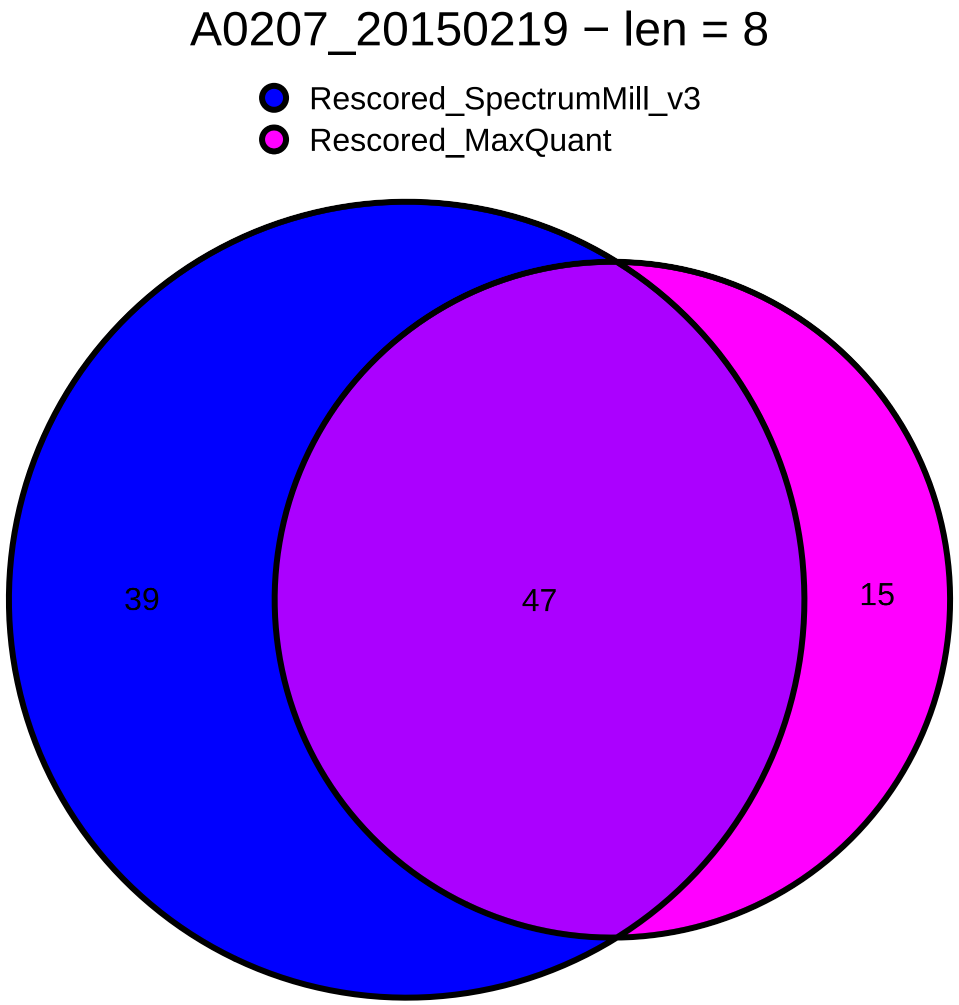
A0207_20150219 − len = 8
Rescored_SpectrumMill_v3
Rescored_MaxQuant
39
47
15

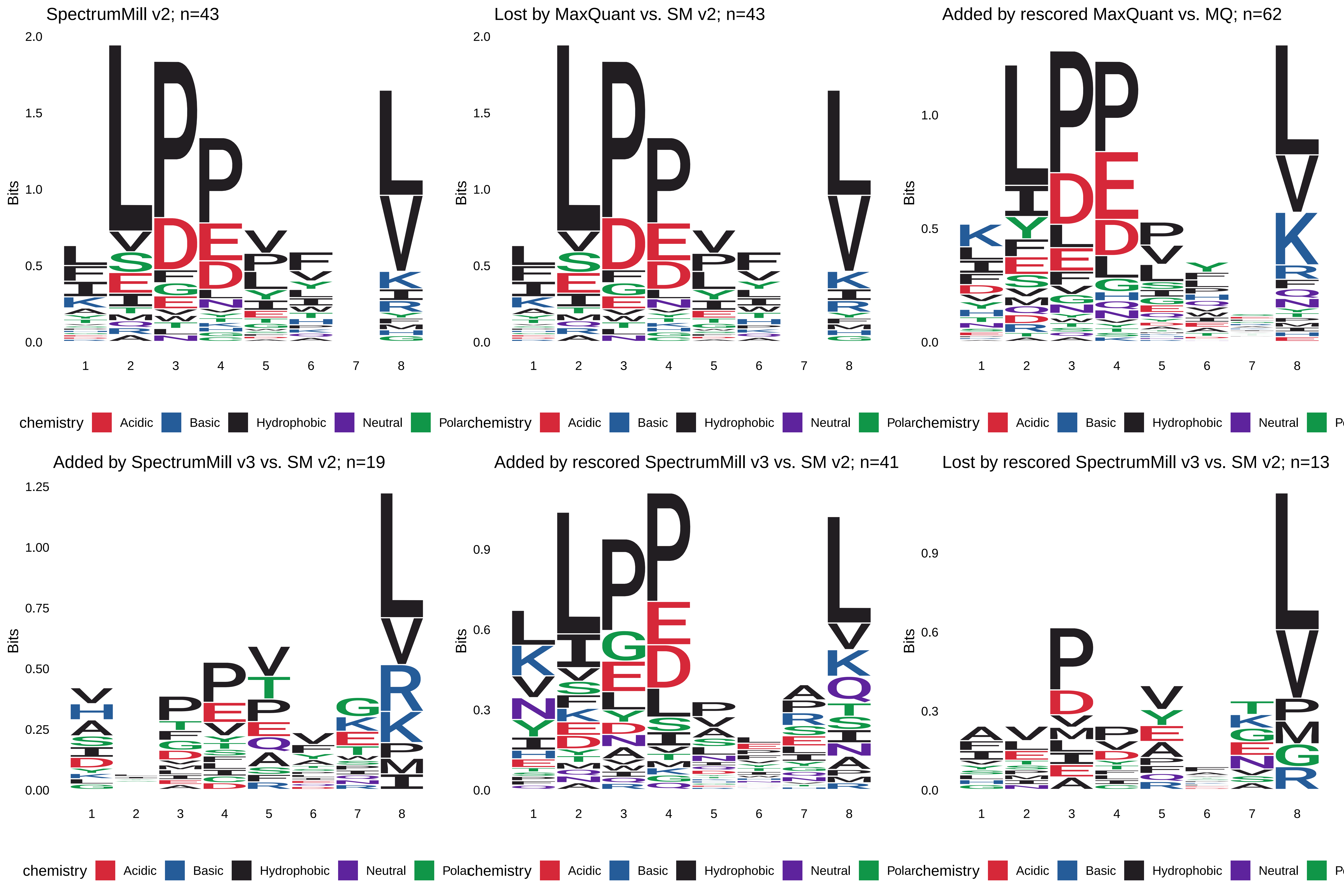
SpectrumMill v2; n=43
Lost by MaxQuant vs. SM v2; n=43
Added by rescored MaxQuant vs. MQ; n=62
Added by SpectrumMill v3 vs. SM v2; n=19
Added by rescored SpectrumMill v3 vs. SM v2; n=41
Lost by rescored SpectrumMill v3 vs. SM v2; n=13
Bits
1 2 3 4 5 6 7 8
chemistry
Acidic
Basic
Hydrophobic
Neutral
Polar

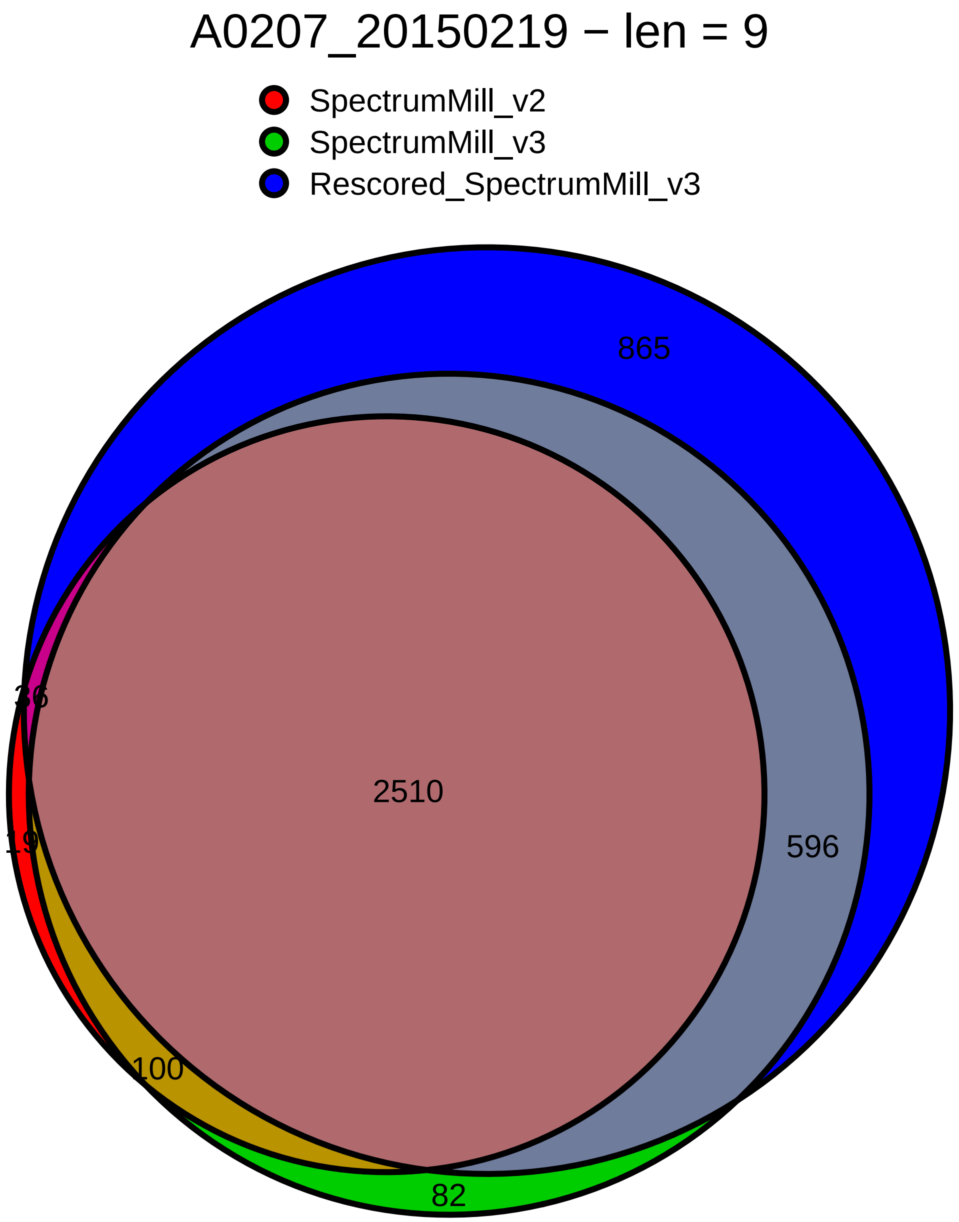
A0207_20150219 − len = 9
SpectrumMill_v2
SpectrumMill_v3
Rescored_SpectrumMill_v3
865
36
2510
19
596
100
82

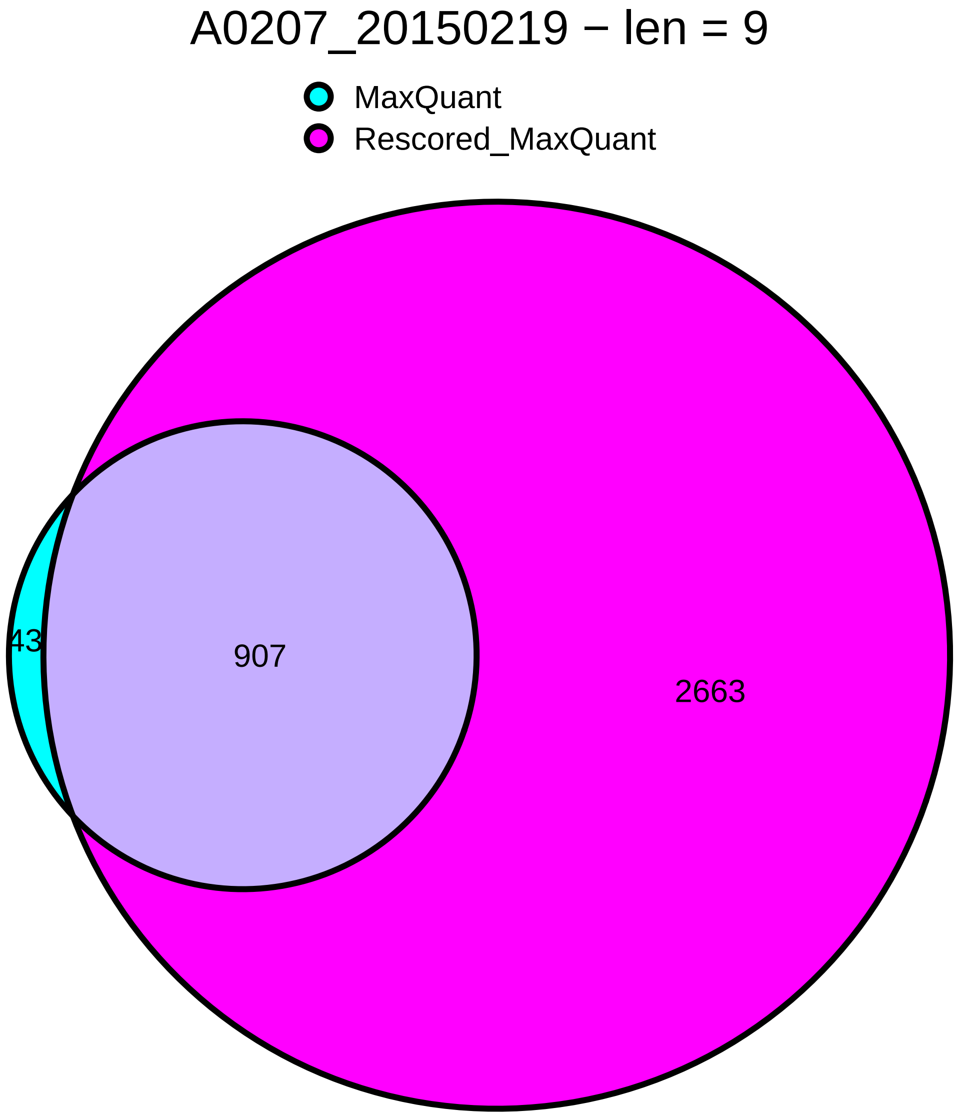
A0207_20150219 − len = 9
MaxQuant
Rescored_MaxQuant
43
907
2663

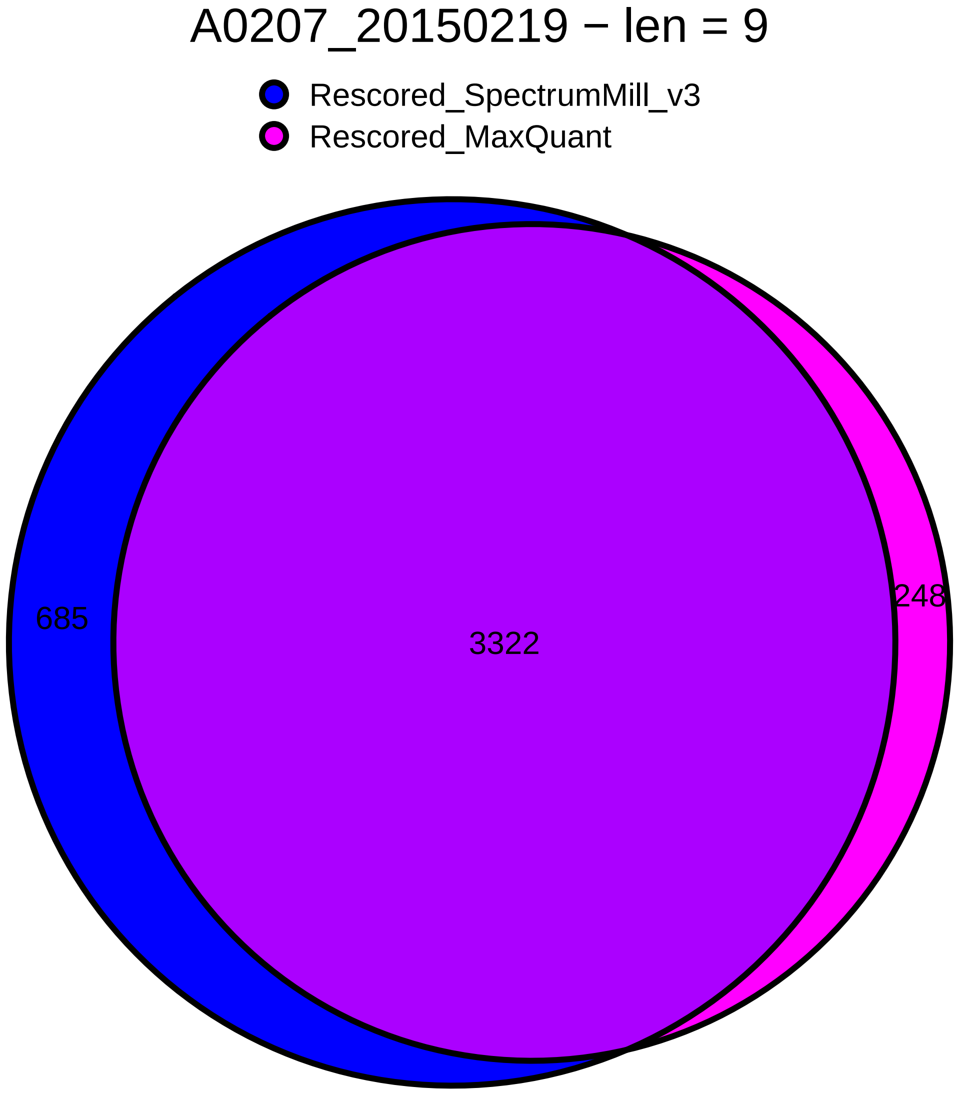
A0207_20150219 − len = 9
Rescored_SpectrumMill_v3
Rescored_MaxQuant
685
3322
248

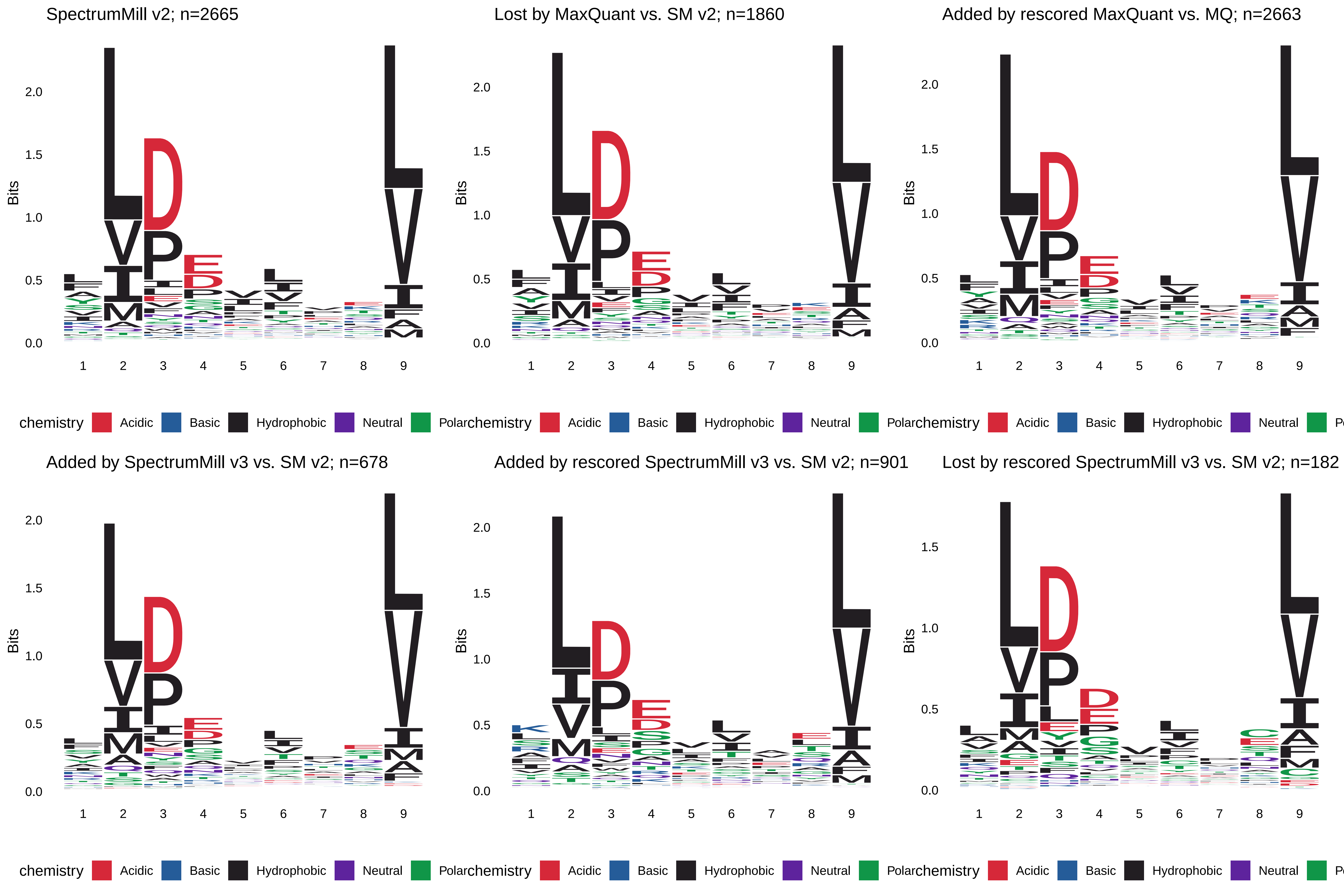
SpectrumMill v2; n=2665
Lost by MaxQuant vs. SM v2; n=1860
Added by rescored MaxQuant vs. MQ; n=2663
Added by SpectrumMill v3 vs. SM v2; n=678
Added by rescored SpectrumMill v3 vs. SM v2; n=901
Lost by rescored SpectrumMill v3 vs. SM v2; n=182
Bits
0.0
0.5
1.0
1.5
2.0
1 2 3 4 5 6 7 8 9
chemistry
Acidic
Basic
Hydrophobic
Neutral
Polar

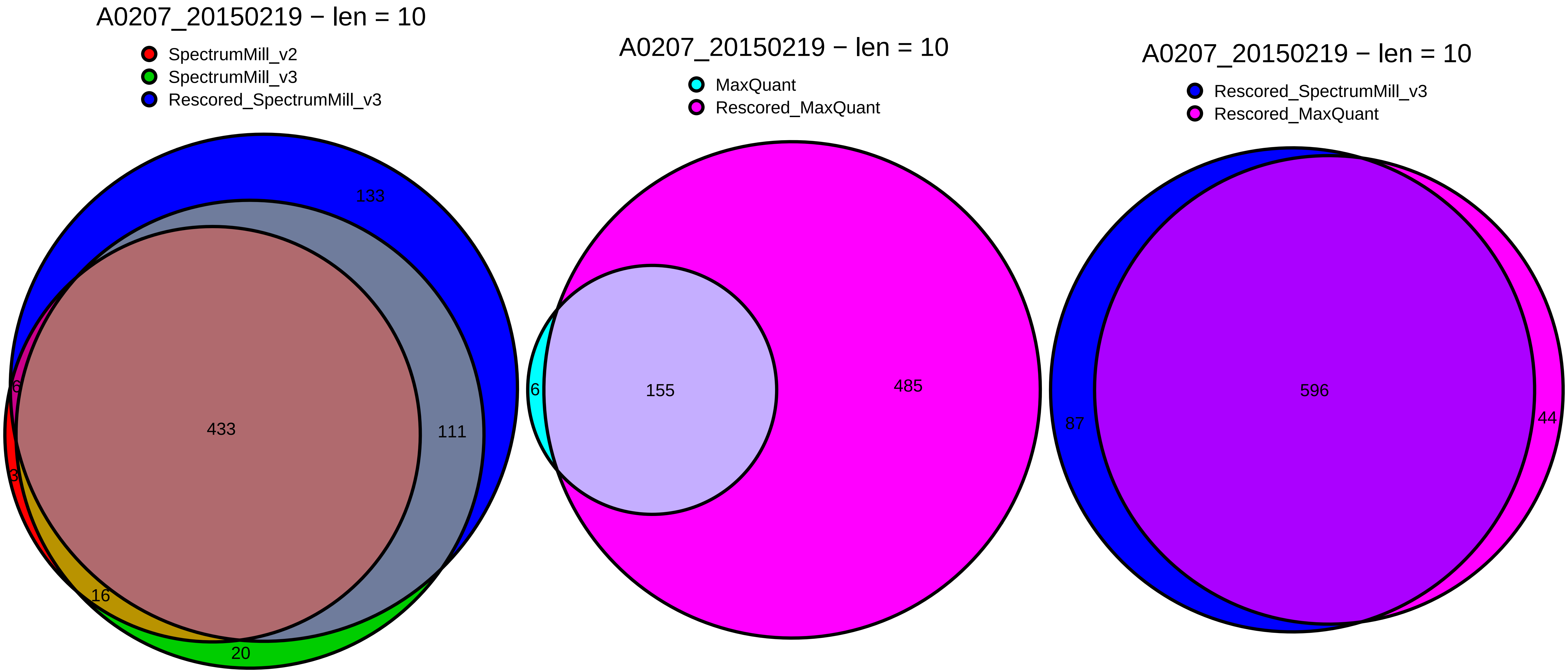
A0207_20150219 − len = 10
SpectrumMill_v2
SpectrumMill_v3
Rescored_SpectrumMill_v3
133
6
433
111
3
16
20
A0207_20150219 − len = 10
MaxQuant
Rescored_MaxQuant
6
155
485
A0207_20150219 − len = 10
Rescored_SpectrumMill_v3
Rescored_MaxQuant
596
87
44

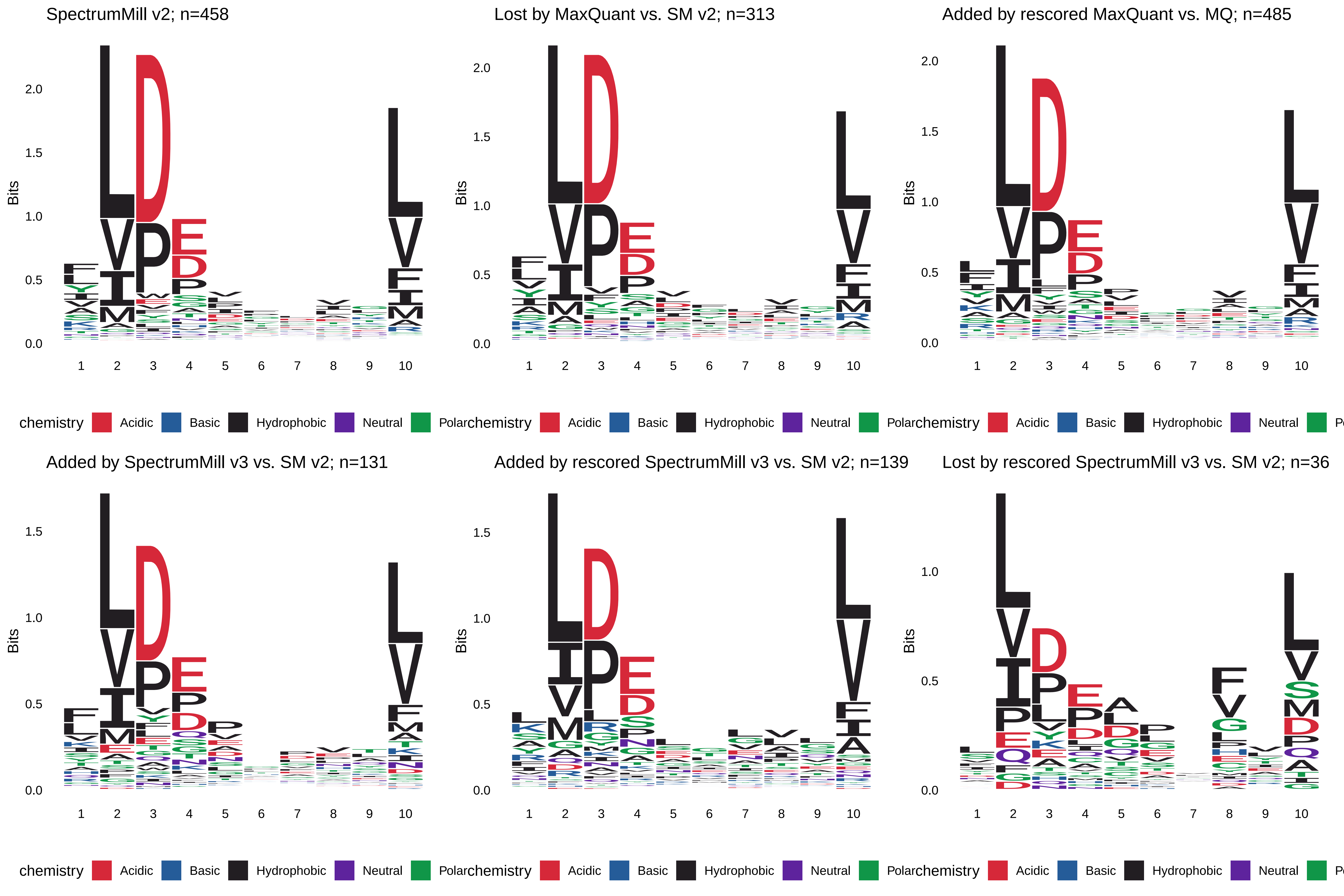
SpectrumMill v2; n=458
Lost by MaxQuant vs. SM v2; n=313
Added by rescored MaxQuant vs. MQ; n=485
Added by SpectrumMill v3 vs. SM v2; n=131
Added by rescored SpectrumMill v3 vs. SM v2; n=139
Lost by rescored SpectrumMill v3 vs. SM v2; n=36
Bits
chemistry
Acidic
Basic
Hydrophobic
Neutral
Polar

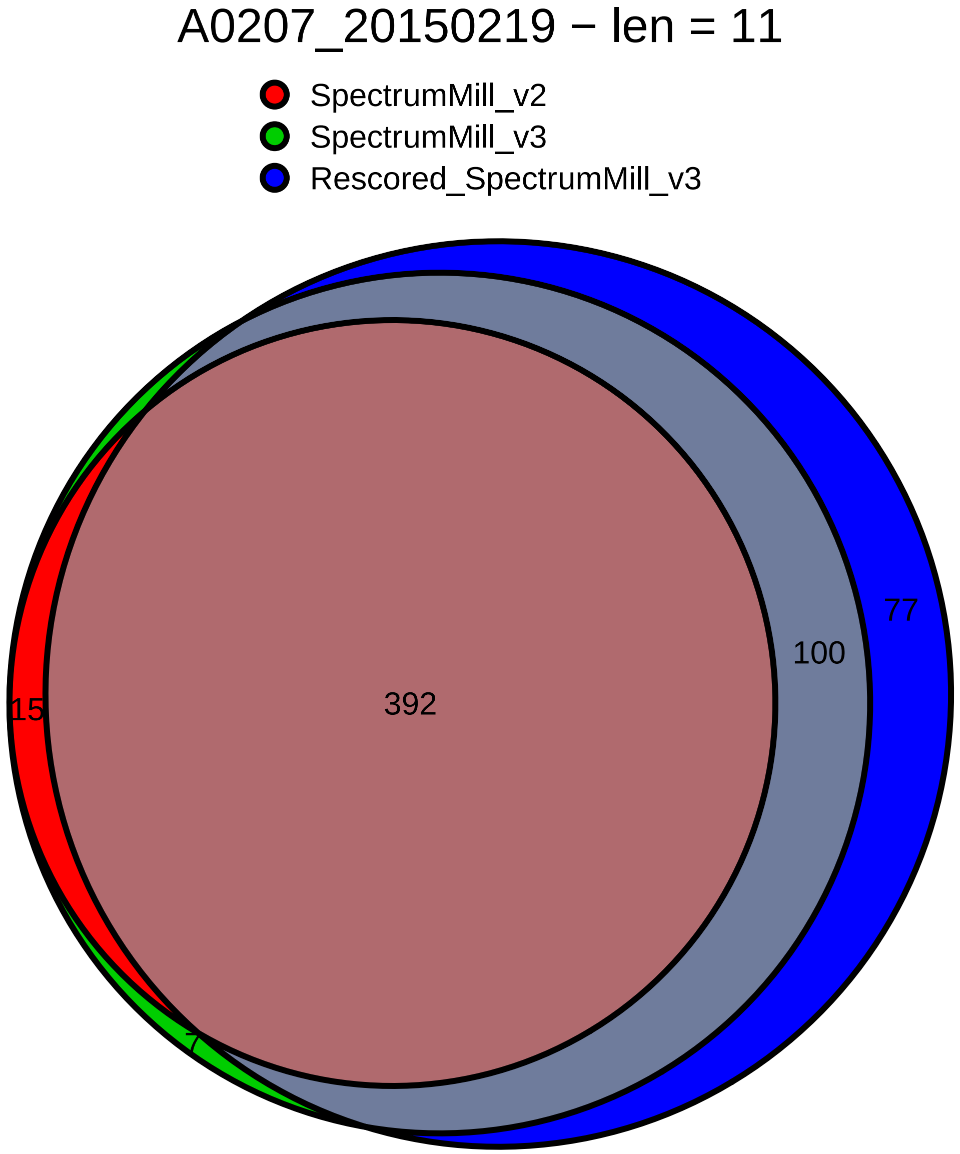
A0207_20150219 − len = 11
SpectrumMill_v2
SpectrumMill_v3
Rescored_SpectrumMill_v3
15
392
100
77
7

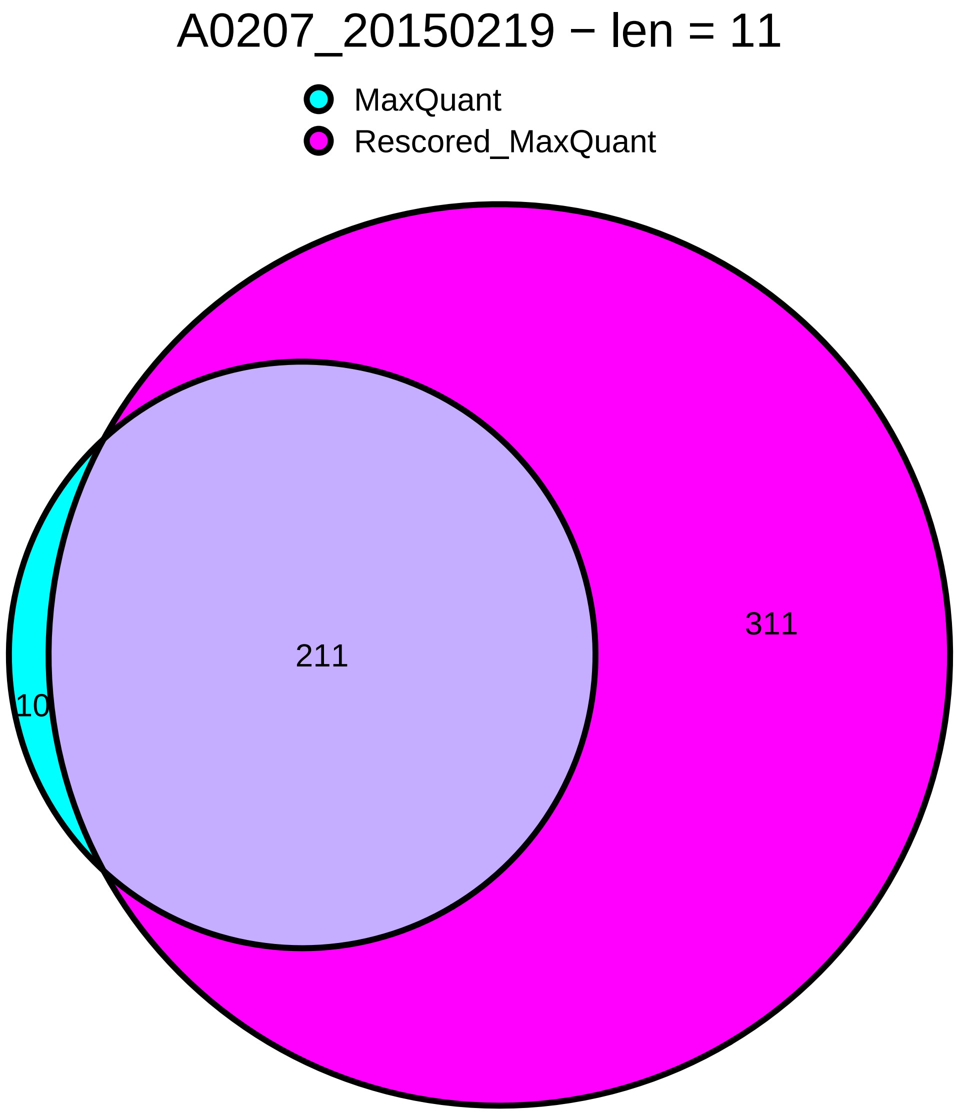
A0207_20150219 − len = 11
MaxQuant
Rescored_MaxQuant
10
211
311

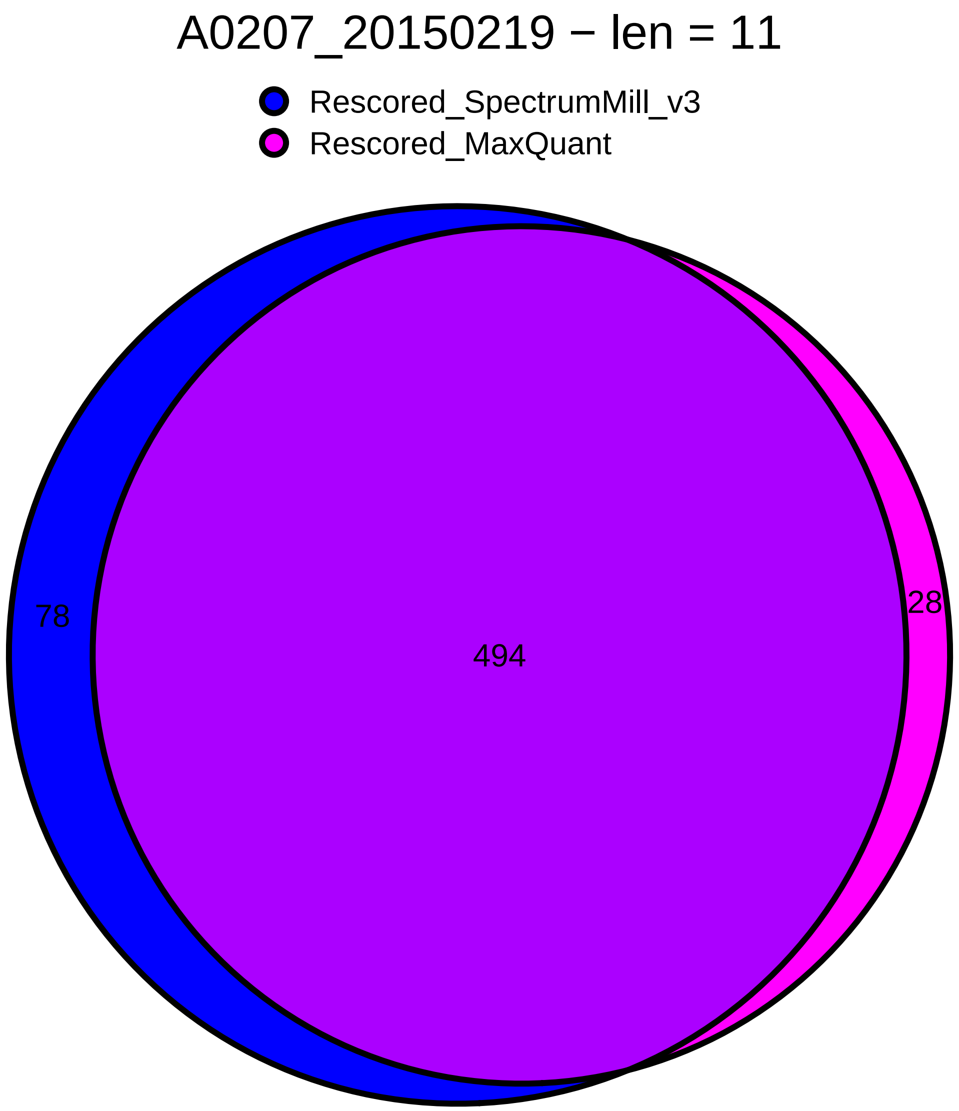
A0207_20150219 − len = 11
Rescored_SpectrumMill_v3
Rescored_MaxQuant
78
494
28

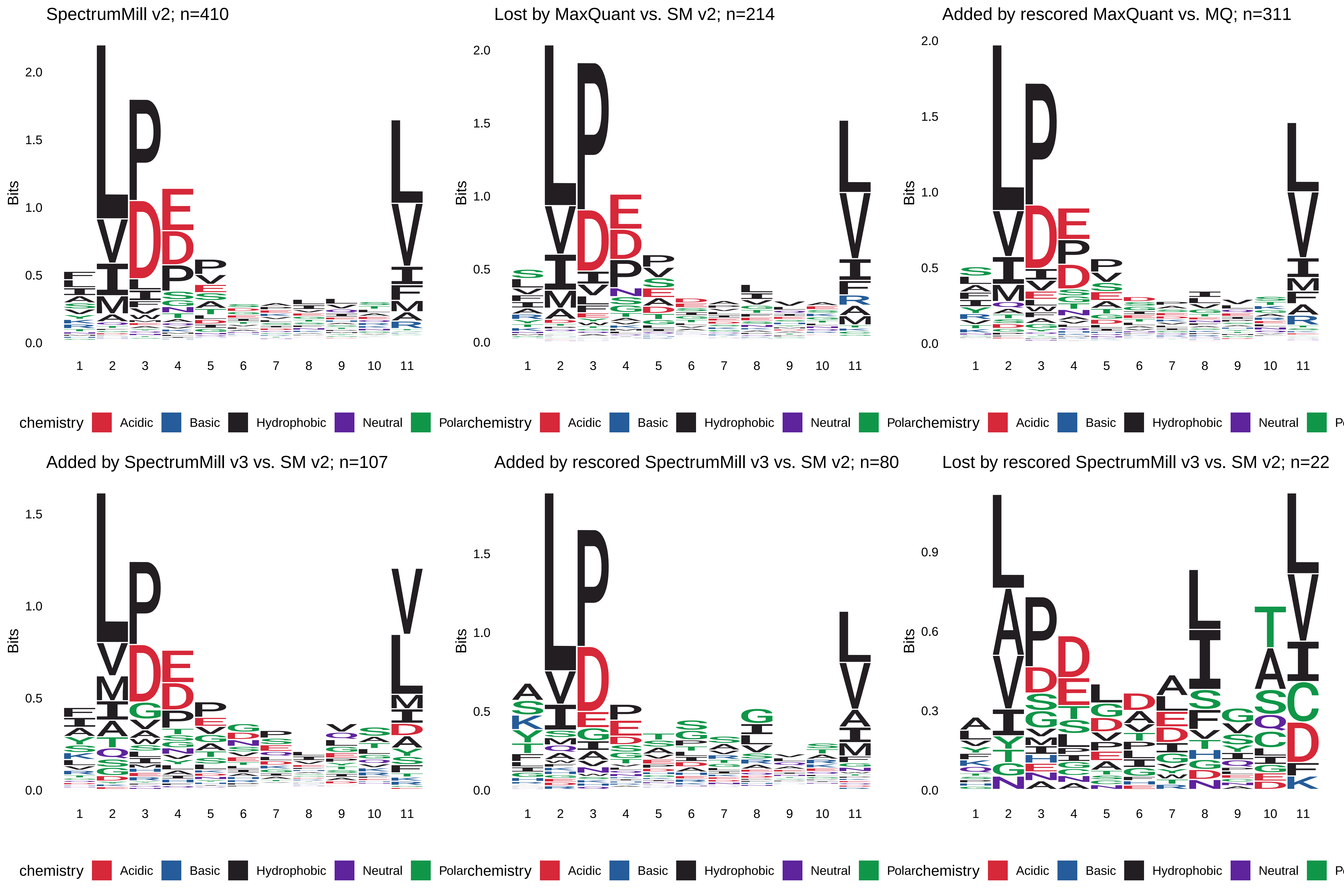
SpectrumMill v2; n=410
Lost by MaxQuant vs. SM v2; n=214
Added by rescored MaxQuant vs. MQ; n=311
Added by SpectrumMill v3 vs. SM v2; n=107
Added by rescored SpectrumMill v3 vs. SM v2; n=80
Lost by rescored SpectrumMill v3 vs. SM v2; n=22
Bits
0.0
0.5
1.0
1.5
2.0
0.3
0.6
0.9
1 2 3 4 5 6 7 8 9 10 11
chemistry
Acidic
Basic
Hydrophobic
Neutral
Polar

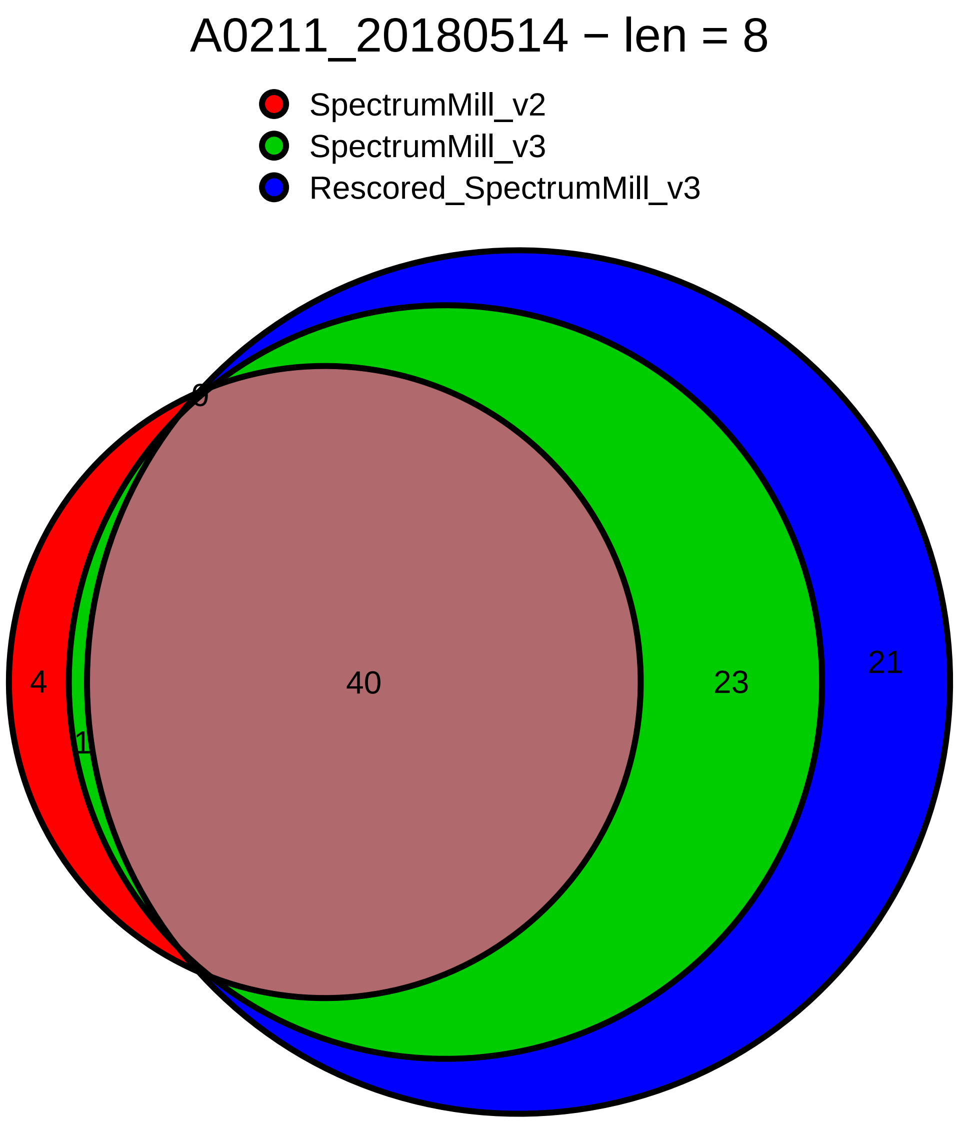
A0211_20180514 − len = 8
SpectrumMill_v2
SpectrumMill_v3
Rescored_SpectrumMill_v3
0
4
1
40
23
21

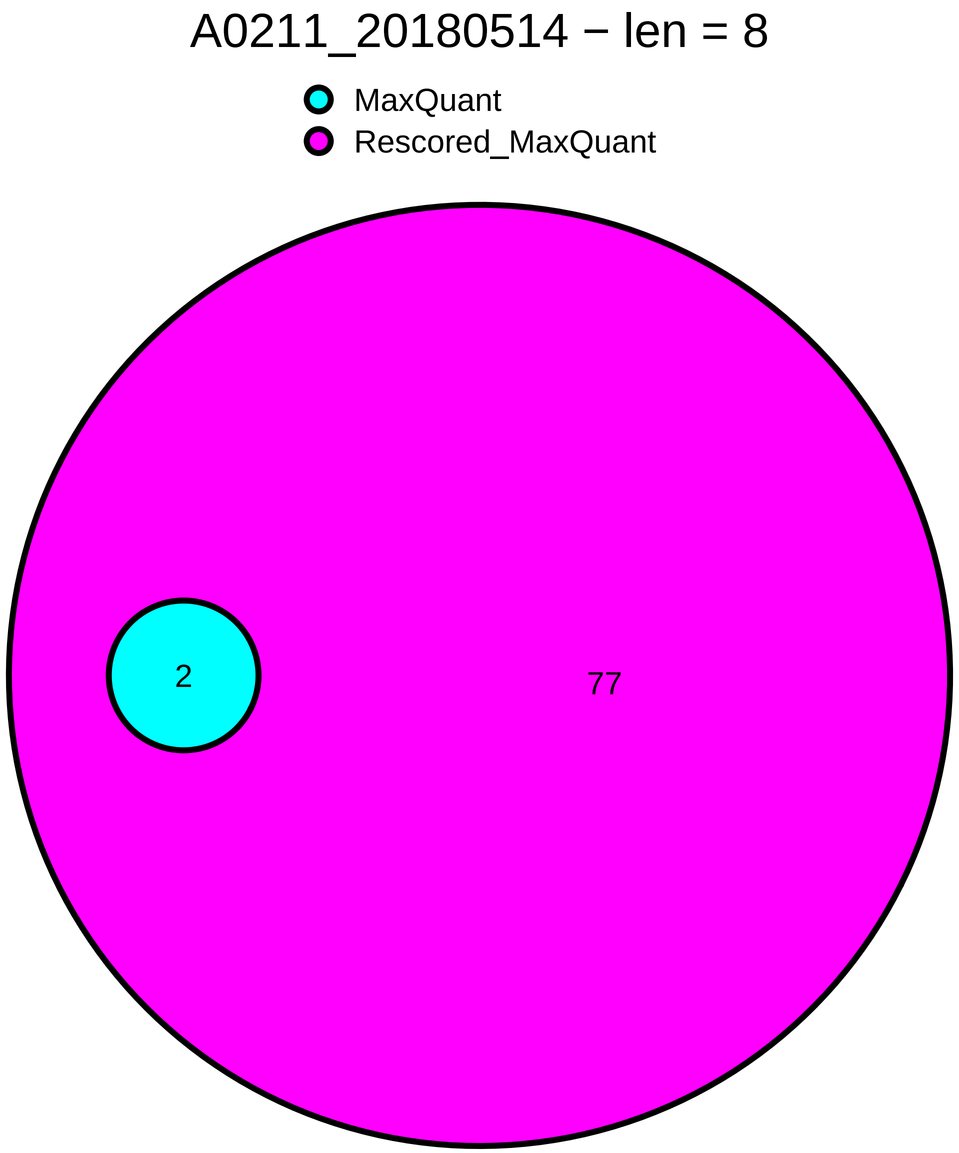
A0211_20180514 − len = 8
MaxQuant
Rescored_MaxQuant
2
77

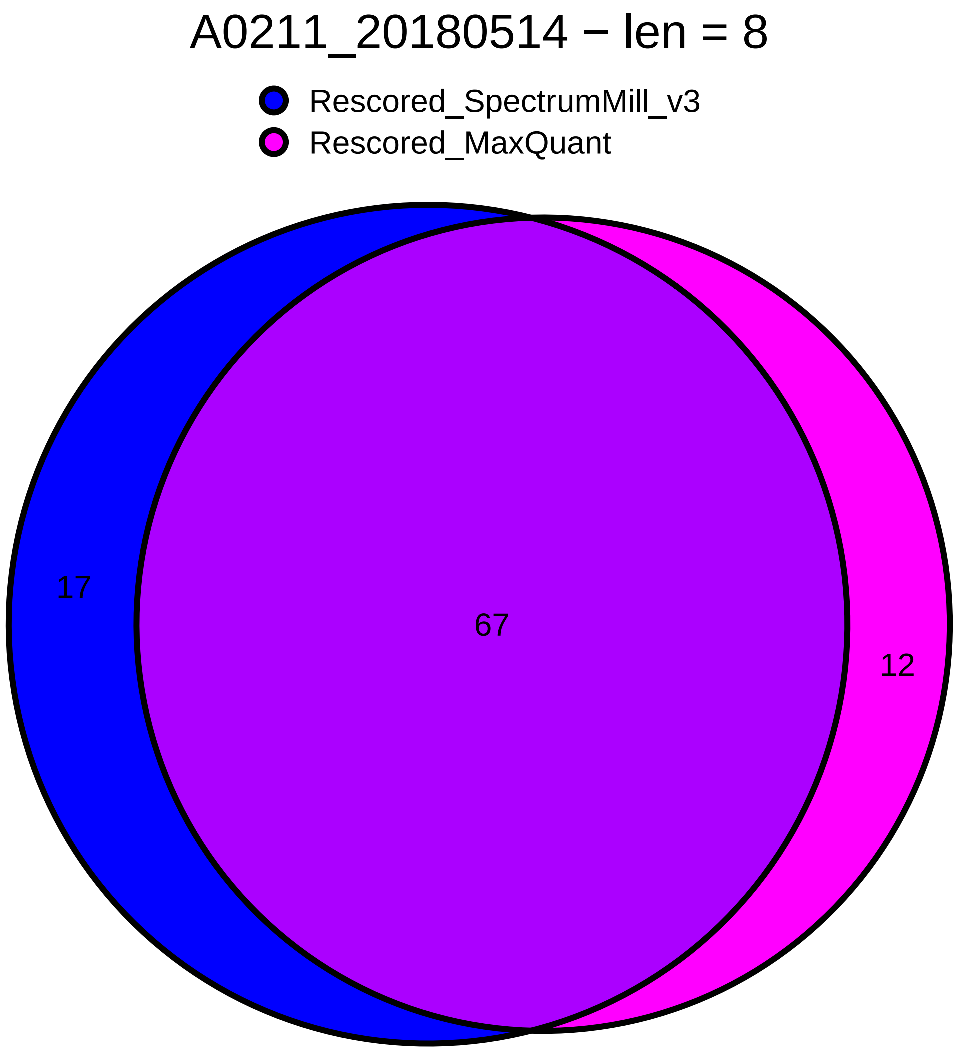
A0211_20180514 − len = 8
Rescored_SpectrumMill_v3
Rescored_MaxQuant
17
67
12

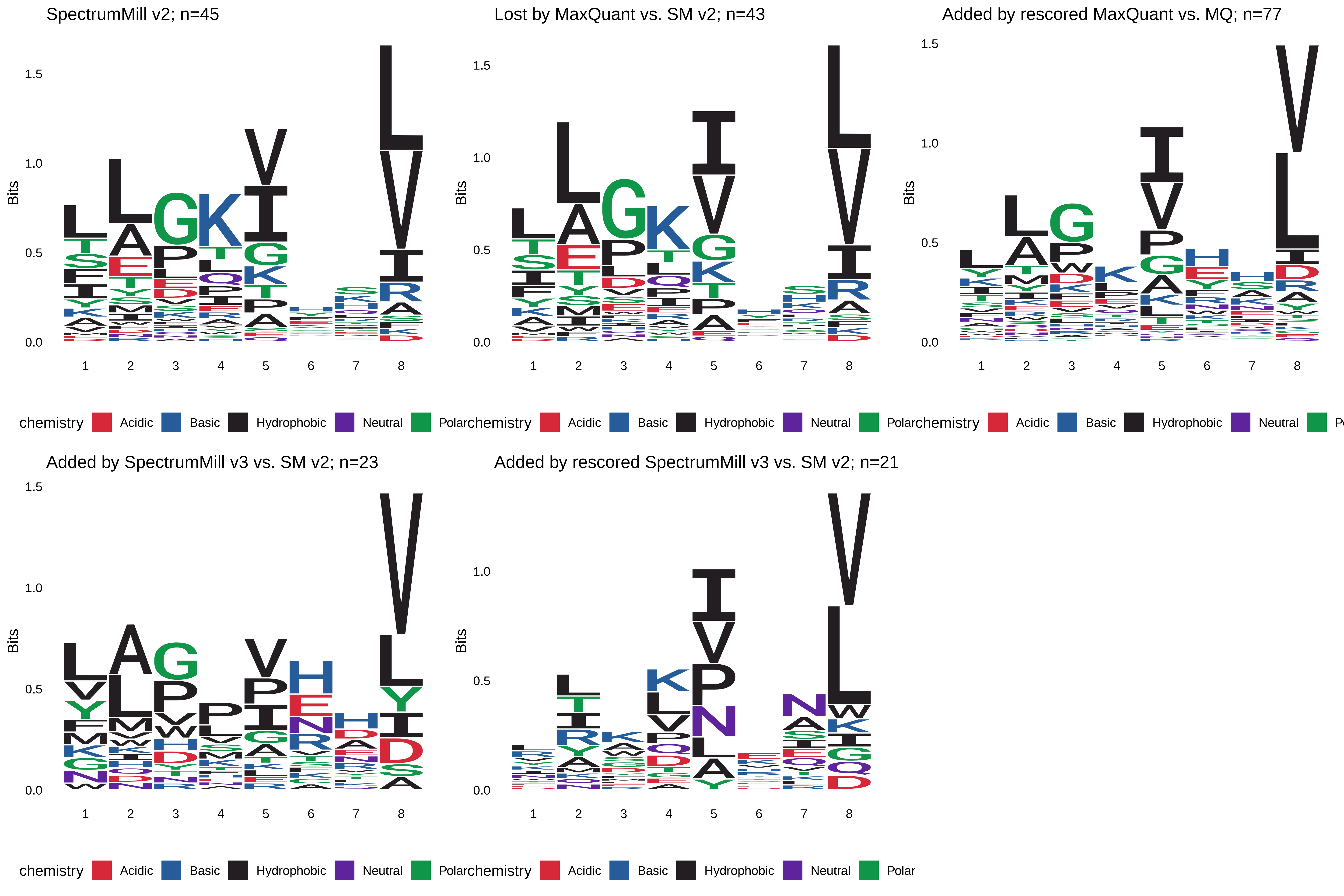
SpectrumMill v2; n=45
Lost by MaxQuant vs. SM v2; n=43
Added by rescored MaxQuant vs. MQ; n=77
Added by SpectrumMill v3 vs. SM v2; n=23
Added by rescored SpectrumMill v3 vs. SM v2; n=21
Bits
1.5
1.0
0.5
0.0
1 2 3 4 5 6 7 8
chemistry Acidic Basic Hydrophobic Neutral Polar

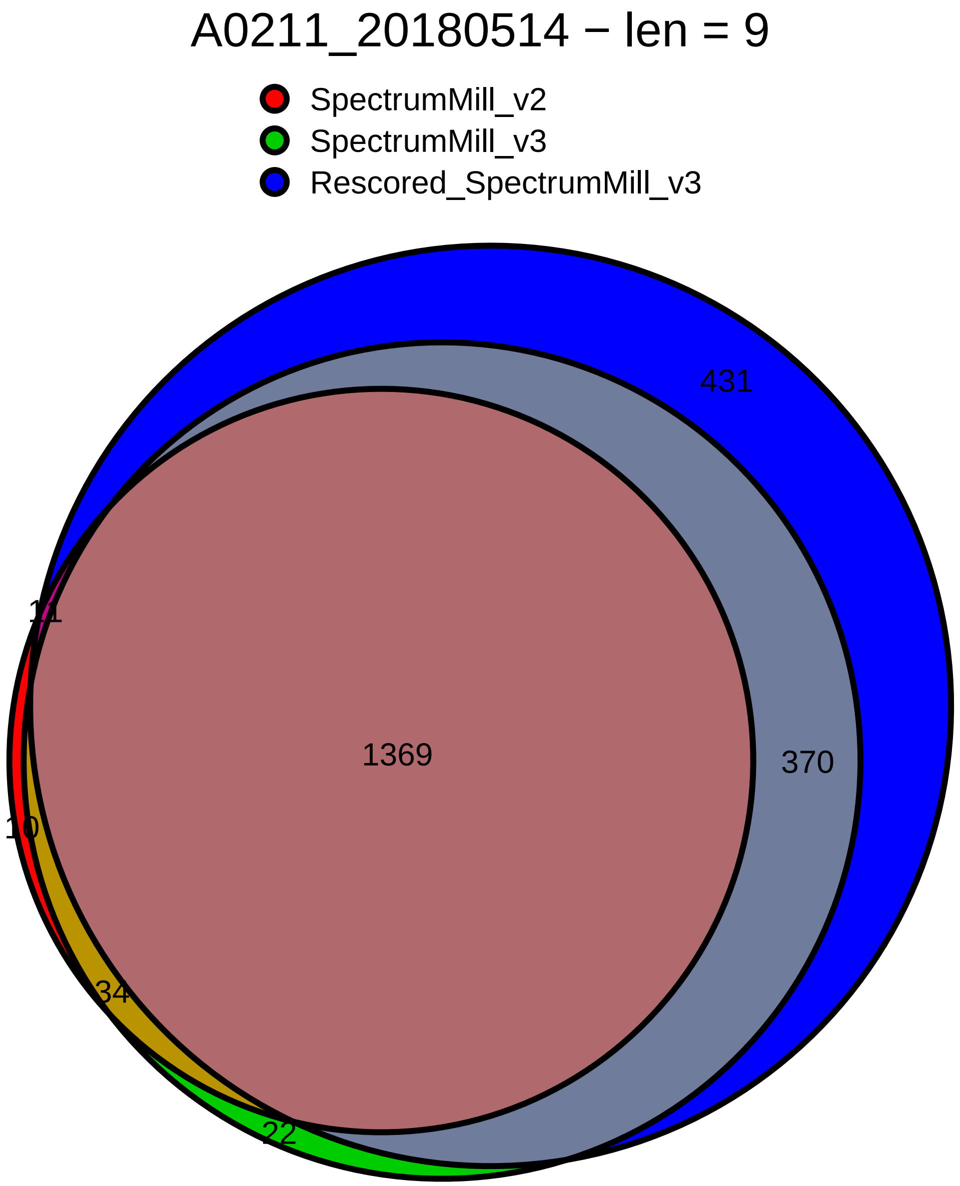
A0211_20180514 − len = 9
SpectrumMill_v2
SpectrumMill_v3
Rescored_SpectrumMill_v3
431
11
1369
370
10
34
22

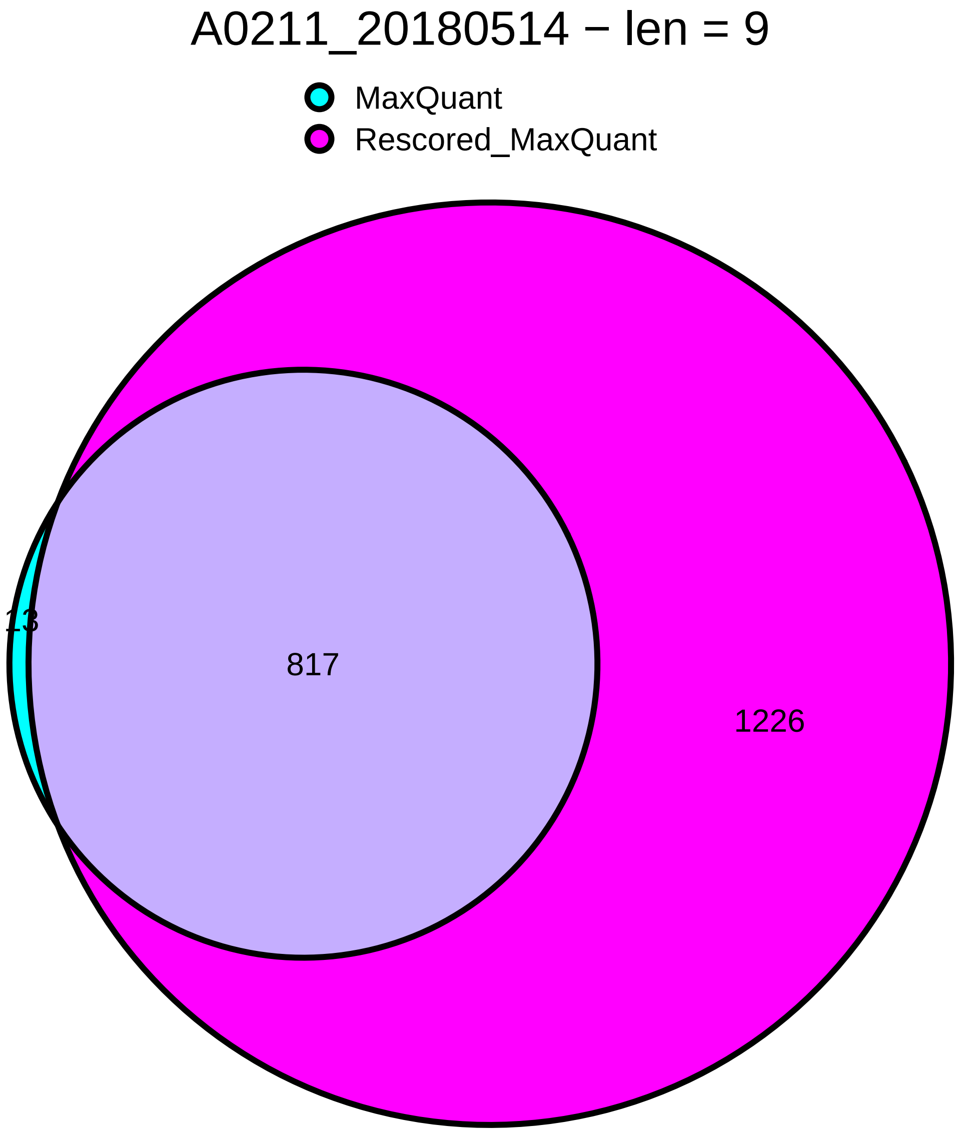
A0211_20180514 − len = 9
MaxQuant
Rescored_MaxQuant
13
817
1226

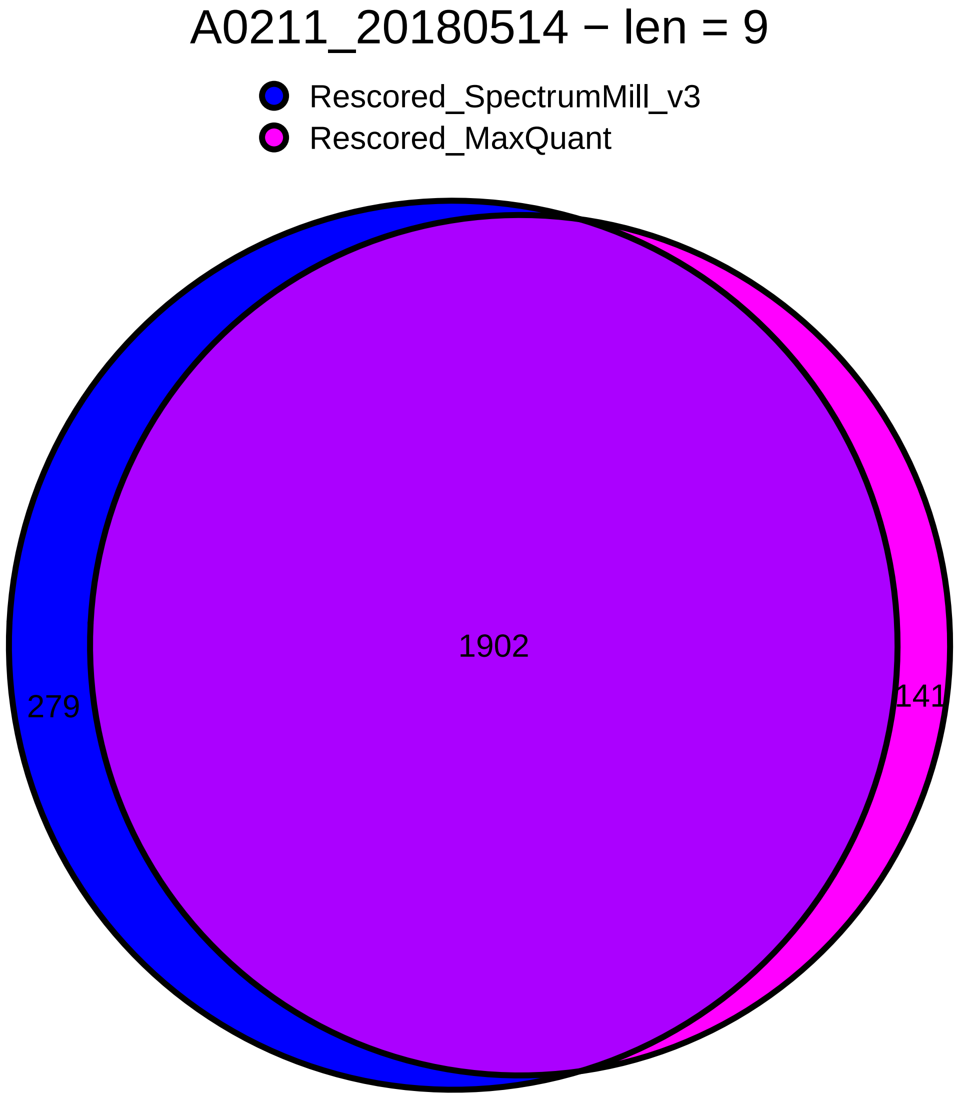
A0211_20180514 − len = 9
Rescored_SpectrumMill_v3
Rescored_MaxQuant
1902
279
141

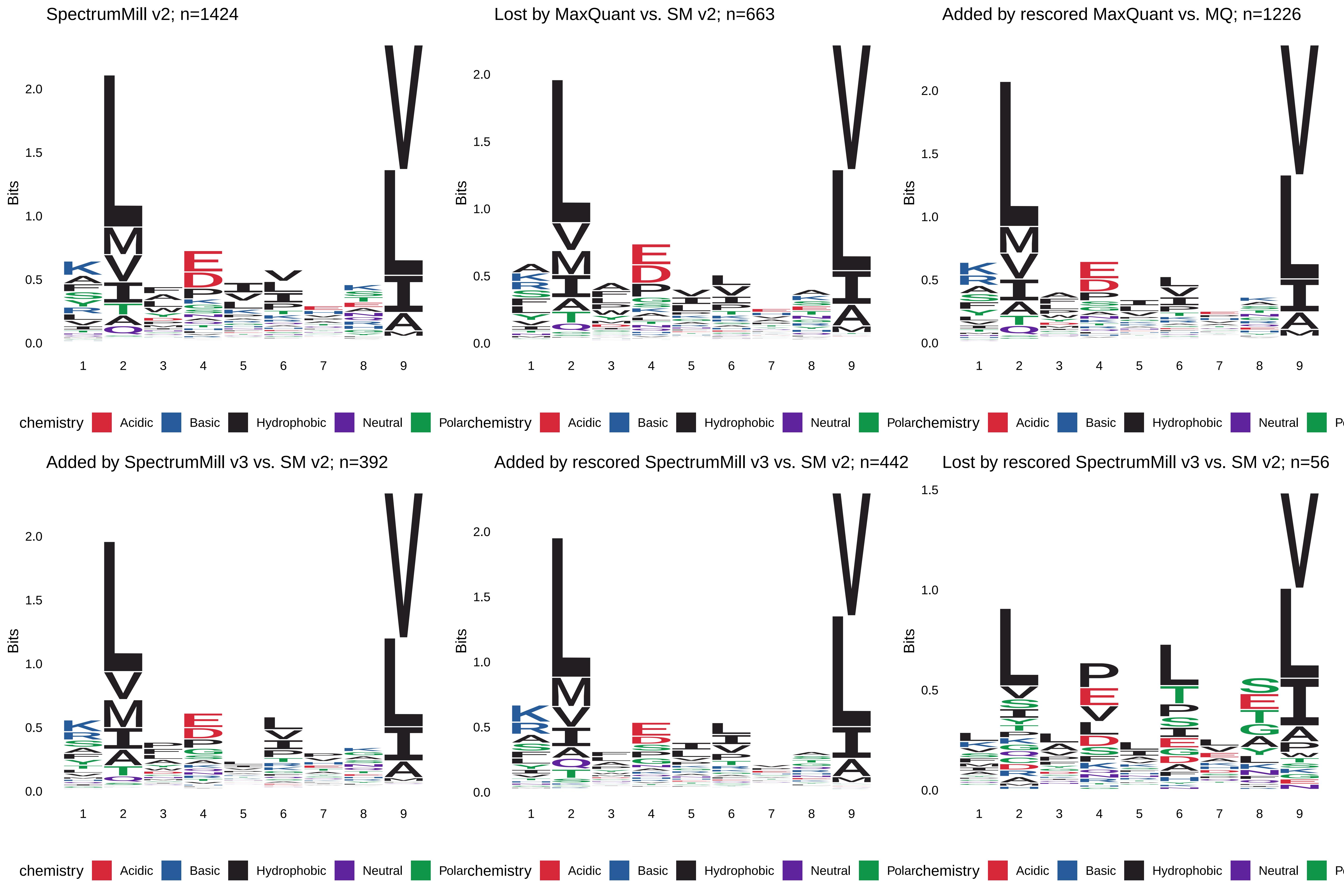

SpectrumMill v2; n=1424
Lost by MaxQuant vs. SM v2; n=663
Added by rescored MaxQuant vs. MQ; n=1226
Added by SpectrumMill v3 vs. SM v2; n=392
Added by rescored SpectrumMill v3 vs. SM v2; n=442
Lost by rescored SpectrumMill v3 vs. SM v2; n=56
Bits
0.0
0.5
1.0
1.5
2.0
1 2 3 4 5 6 7 8 9
chemistry
Acidic
Basic
Hydrophobic
Neutral
Polar

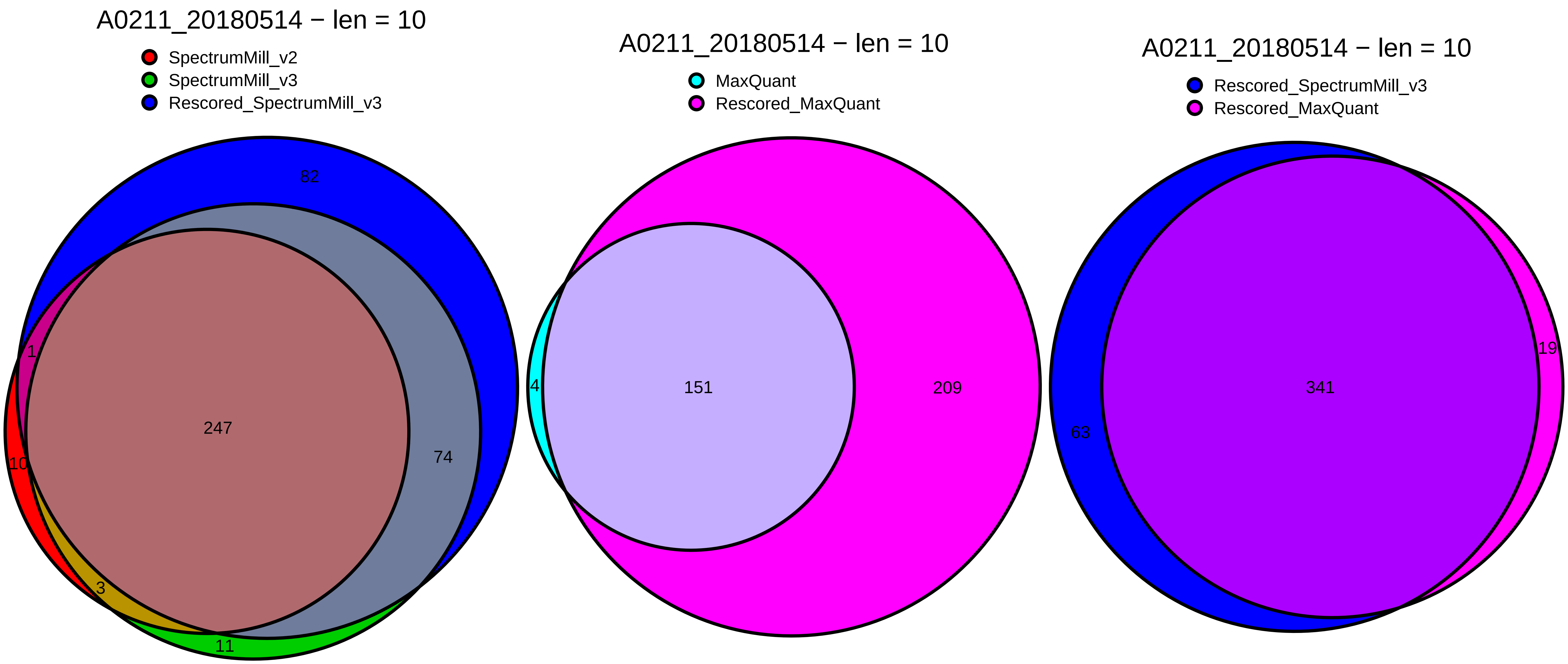

A0211_20180514 − len = 10
SpectrumMill_v2
SpectrumMill_v3
Rescored_SpectrumMill_v3
82
1
247
74
10
3
11
A0211_20180514 − len = 10
MaxQuant
Rescored_MaxQuant
4
151
209
A0211_20180514 − len = 10
Rescored_SpectrumMill_v3
Rescored_MaxQuant
19
341
63

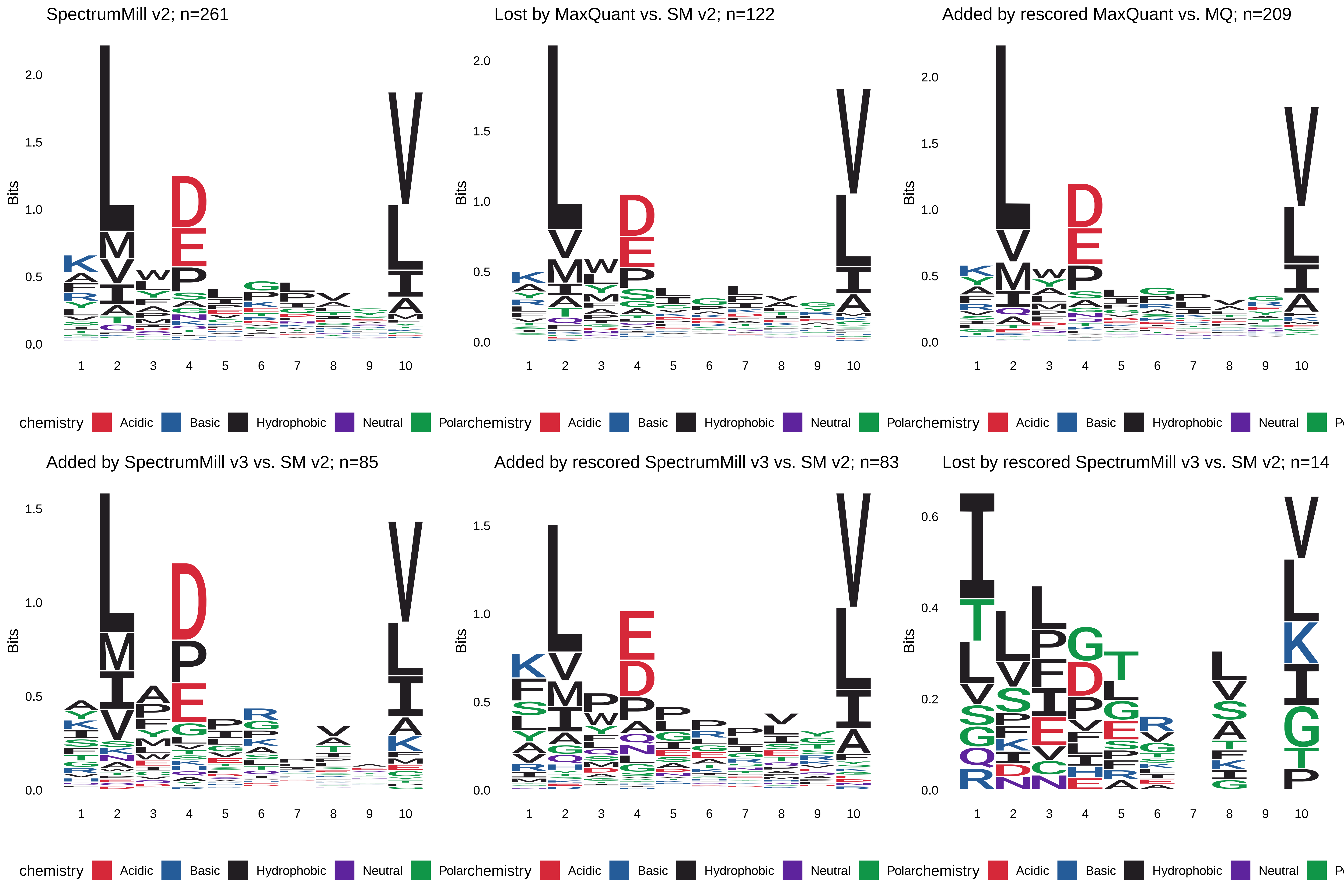
SpectrumMill v2; n=261
Lost by MaxQuant vs. SM v2; n=122
Added by rescored MaxQuant vs. MQ; n=209
Added by SpectrumMill v3 vs. SM v2; n=85
Added by rescored SpectrumMill v3 vs. SM v2; n=83
Lost by rescored SpectrumMill v3 vs. SM v2; n=14
Bits
1 2 3 4 5 6 7 8 9 10
chemistry Acidic Basic Hydrophobic Neutral Polar

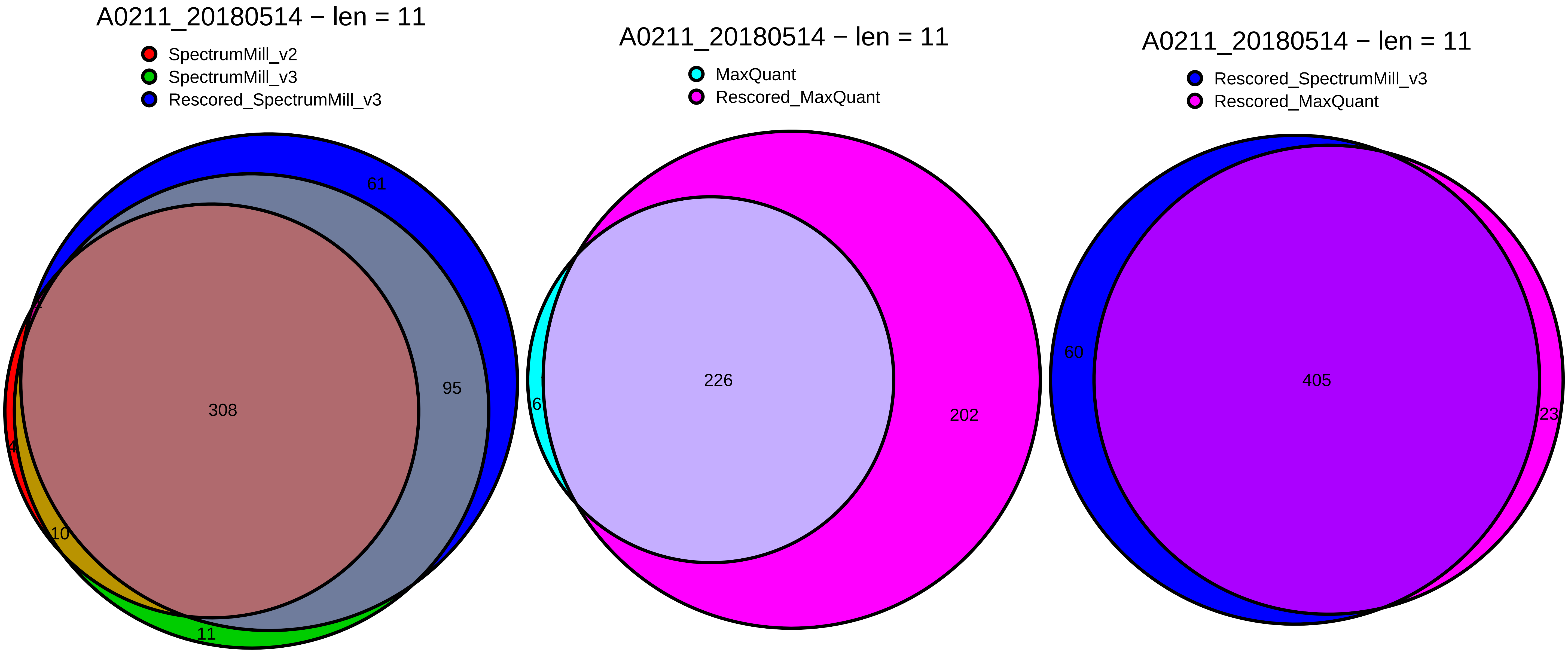
A0211_20180514 − len = 11
SpectrumMill_v2
SpectrumMill_v3
Rescored_SpectrumMill_v3
61
95
308
4
10
11
A0211_20180514 − len = 11
MaxQuant
Rescored_MaxQuant
6
226
202
A0211_20180514 − len = 11
Rescored_SpectrumMill_v3
Rescored_MaxQuant
60
405
23

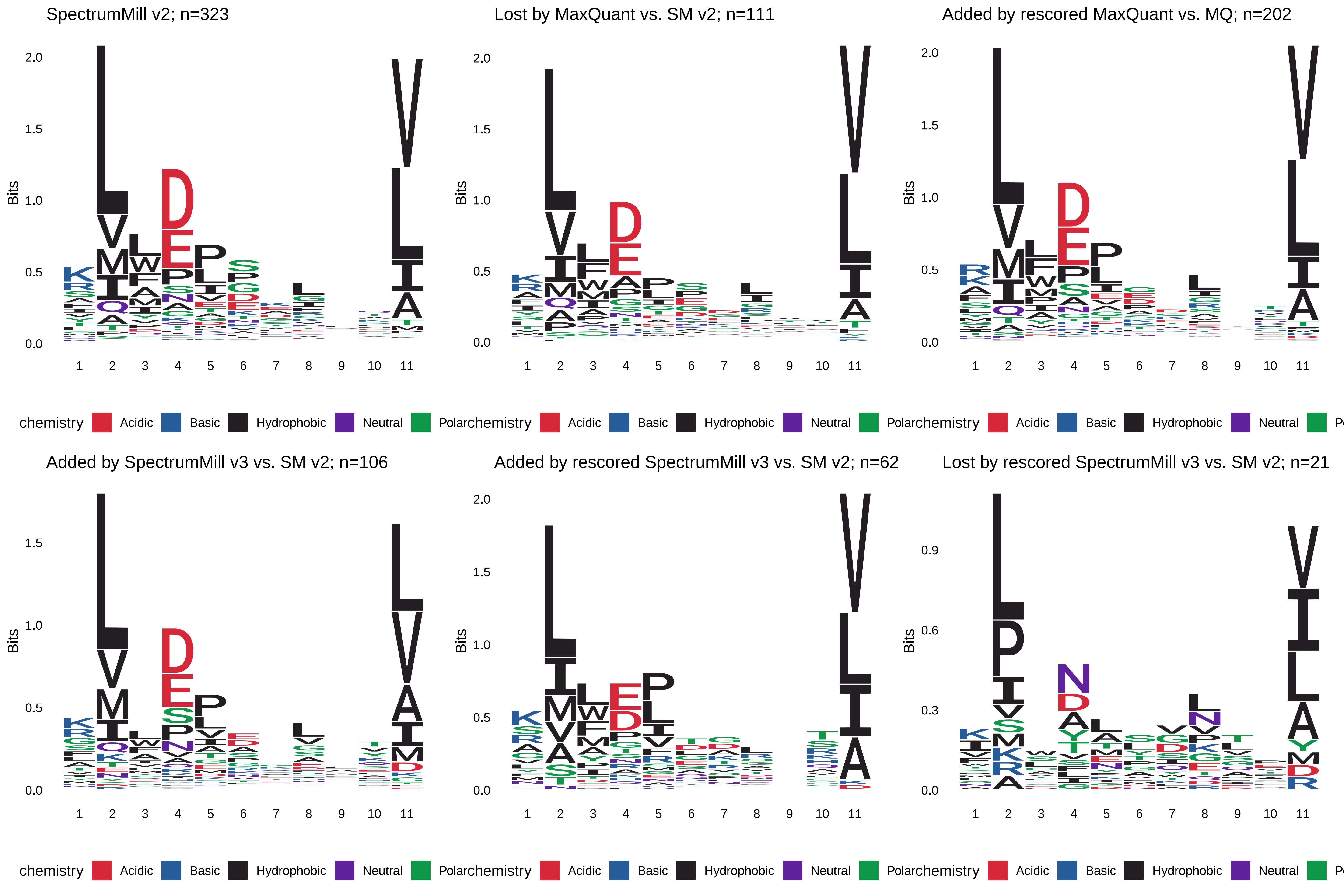

SpectrumMill v2; n=323
Lost by MaxQuant vs. SM v2; n=111
Added by rescored MaxQuant vs. MQ; n=202
Added by SpectrumMill v3 vs. SM v2; n=106
Added by rescored SpectrumMill v3 vs. SM v2; n=62
Lost by rescored SpectrumMill v3 vs. SM v2; n=21
Bits
0.0
0.5
1.0
1.5
2.0
1 2 3 4 5 6 7 8 9 10 11
chemistry
Acidic
Basic
Hydrophobic
Neutral
Polar

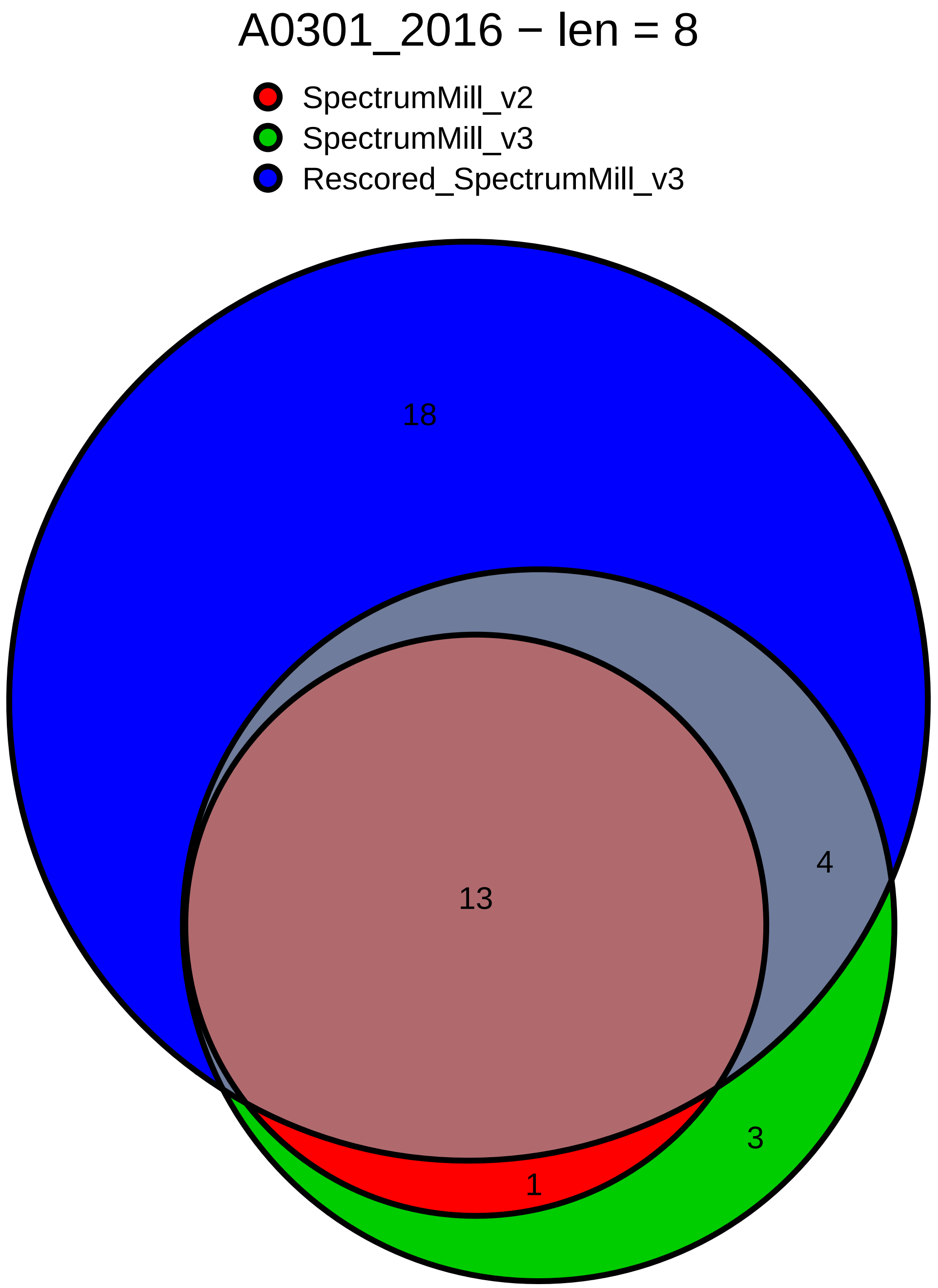
A0301_2016 − len = 8
SpectrumMill_v2
SpectrumMill_v3
Rescored_SpectrumMill_v3
18
13
4
3
1

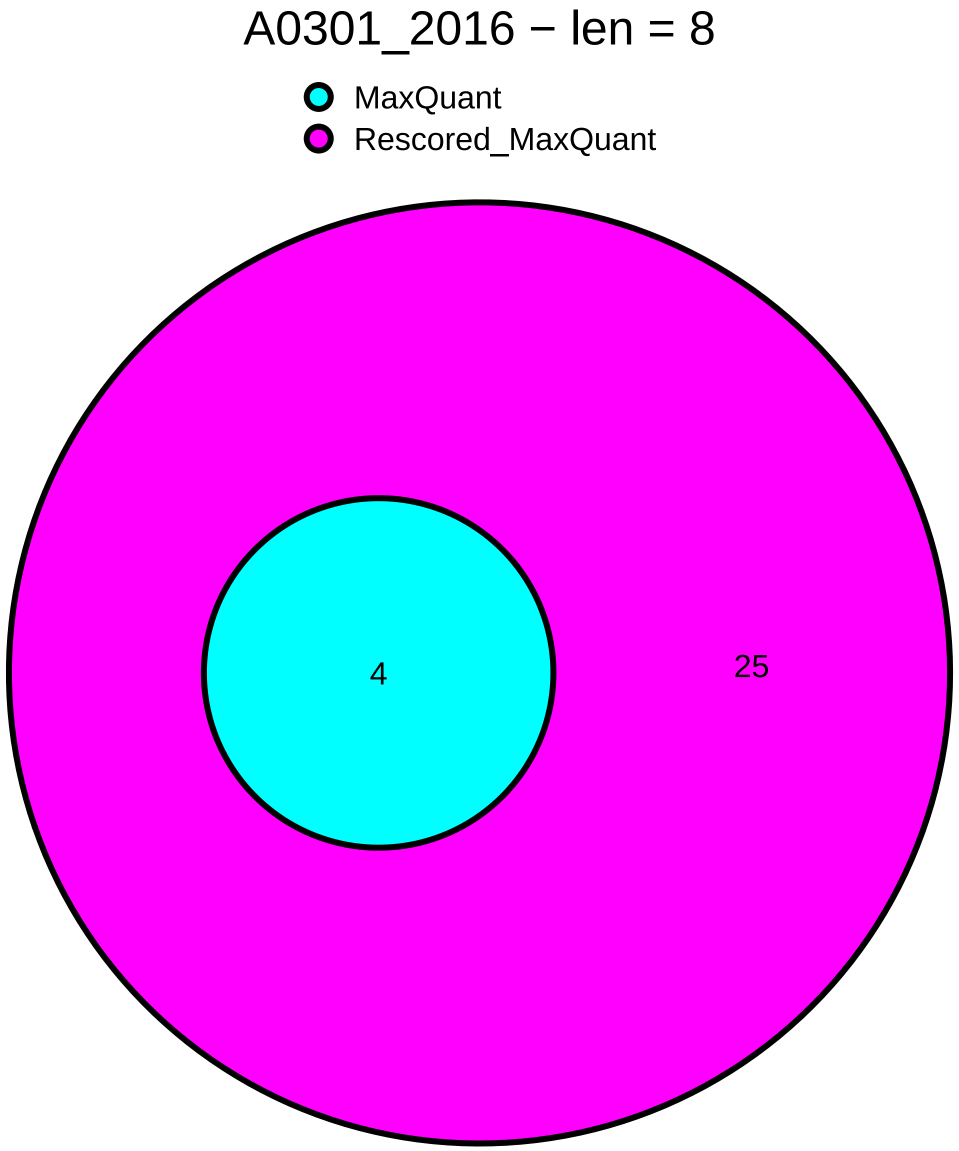
A0301_2016 − len = 8
MaxQuant
Rescored_MaxQuant
4
25

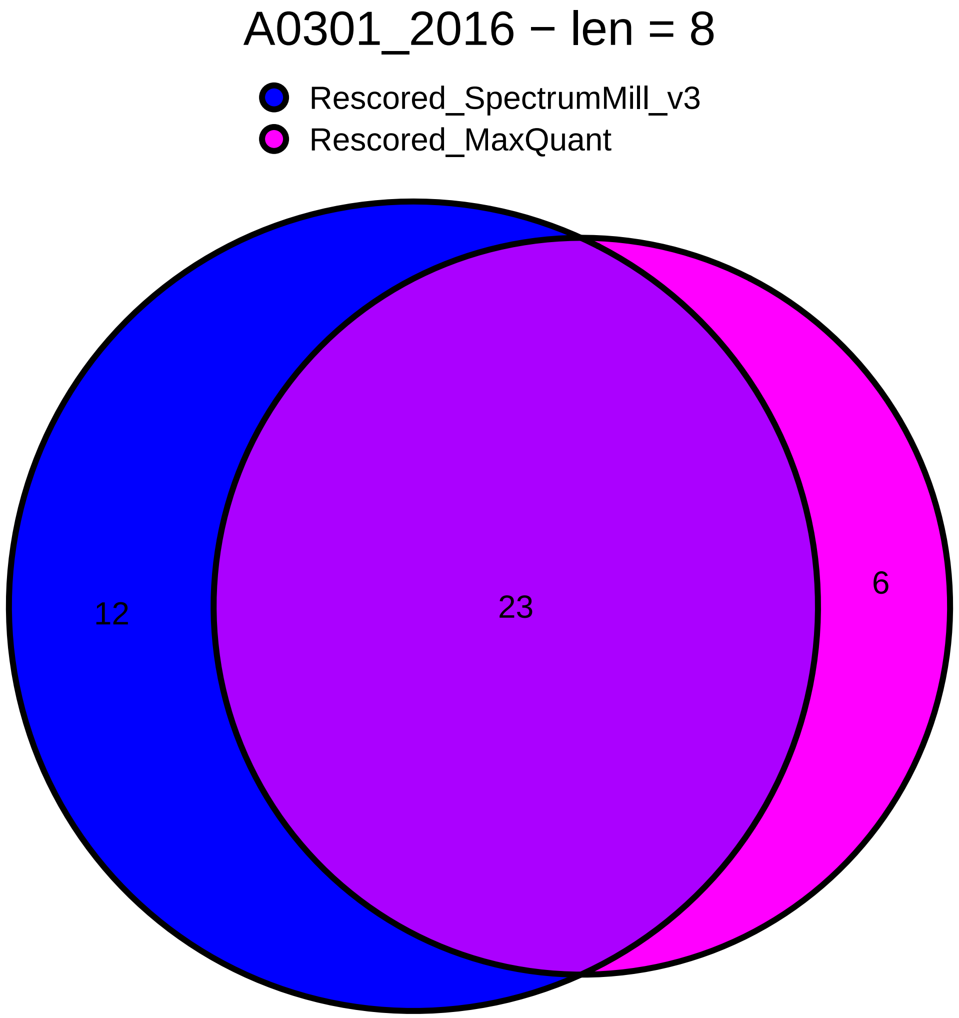
A0301_2016 − len = 8
Rescored_SpectrumMill_v3
Rescored_MaxQuant
12
23
6

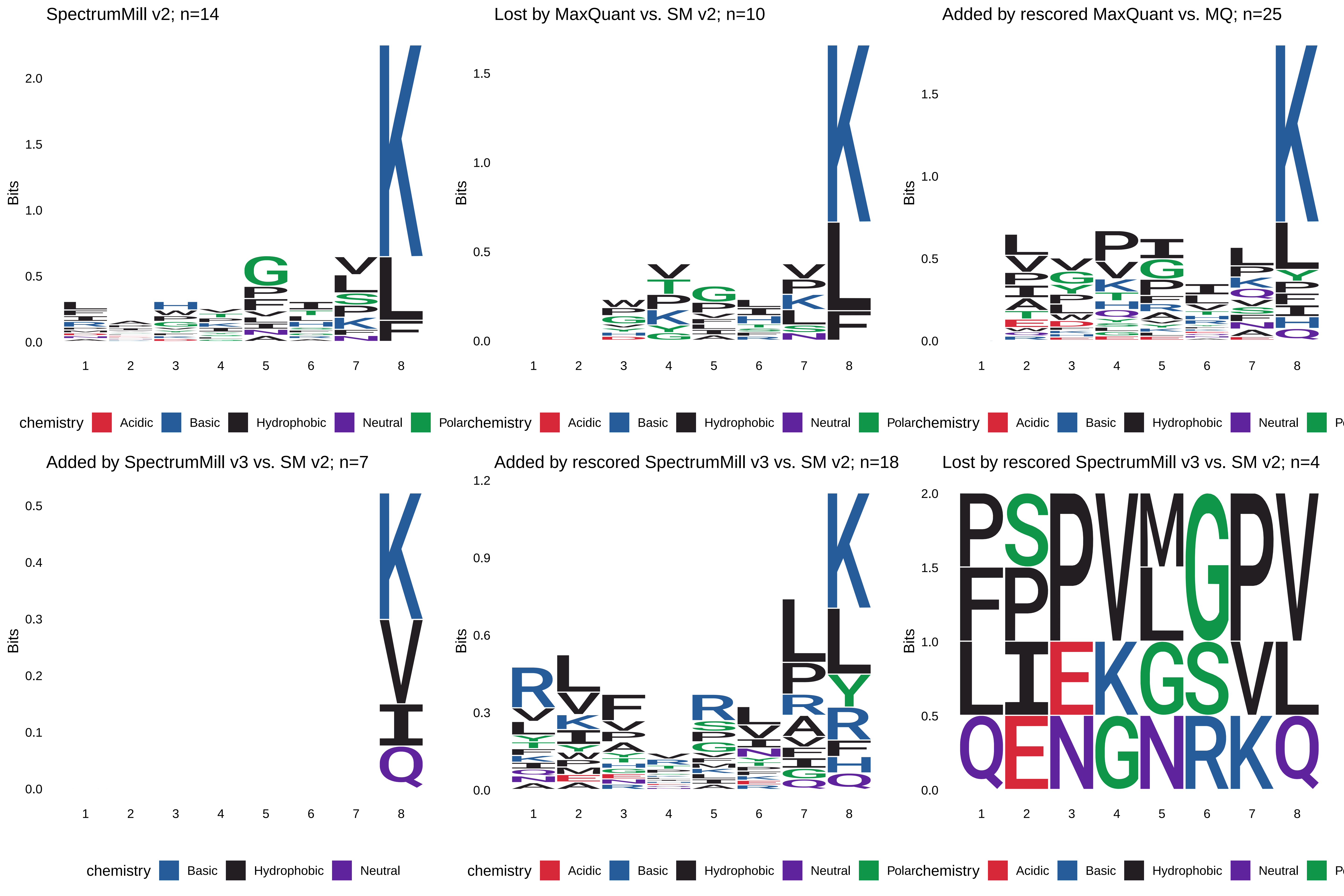
SpectrumMill v2; n=14
Lost by MaxQuant vs. SM v2; n=10
Added by rescored MaxQuant vs. MQ; n=25
Bits
1 2 3 4 5 6 7 8
chemistry Acidic Basic Hydrophobic Neutral Polar
Added by SpectrumMill v3 vs. SM v2; n=7
Added by rescored SpectrumMill v3 vs. SM v2; n=18
Lost by rescored SpectrumMill v3 vs. SM v2; n=4
chemistry Basic Hydrophobic Neutral

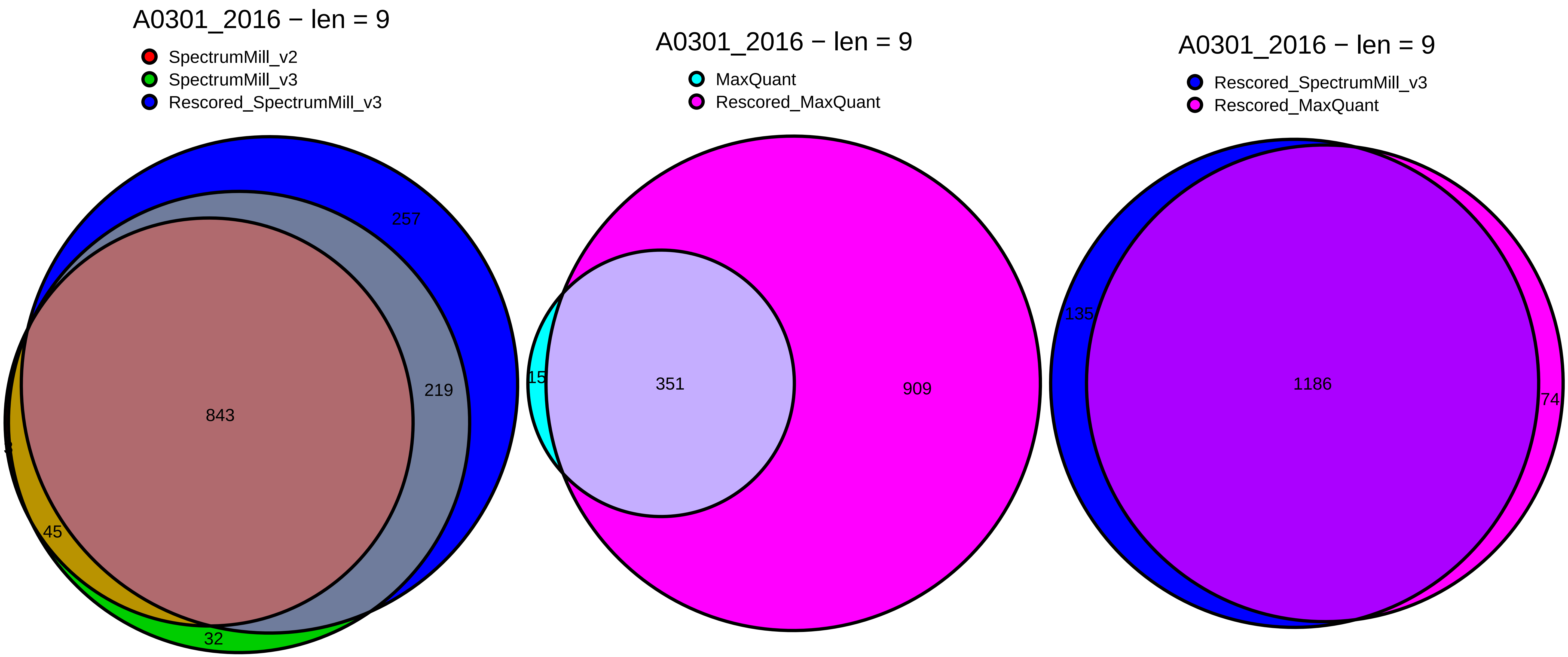
A0301_2016 − len = 9
SpectrumMill_v2
SpectrumMill_v3
Rescored_SpectrumMill_v3
257
219
843
45
32
A0301_2016 − len = 9
MaxQuant
Rescored_MaxQuant
15
351
909
A0301_2016 − len = 9
Rescored_SpectrumMill_v3
Rescored_MaxQuant
135
1186
74

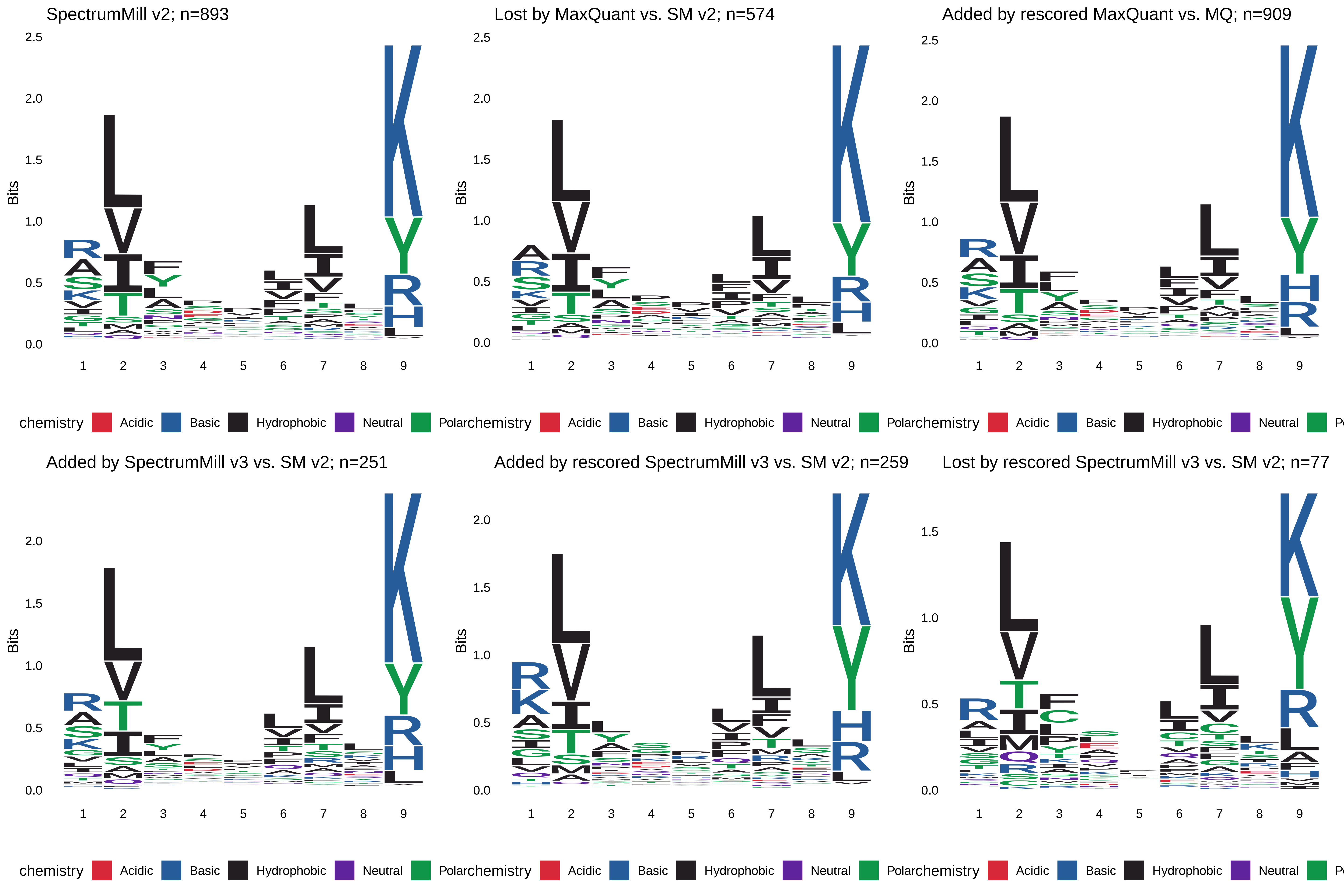
SpectrumMill v2; n=893
Lost by MaxQuant vs. SM v2; n=574
Added by rescored MaxQuant vs. MQ; n=909
Added by SpectrumMill v3 vs. SM v2; n=251
Added by rescored SpectrumMill v3 vs. SM v2; n=259
Lost by rescored SpectrumMill v3 vs. SM v2; n=77
Bits
0.0
0.5
1.0
1.5
2.0
2.5
1 2 3 4 5 6 7 8 9
chemistry Acidic Basic Hydrophobic Neutral Polarchemistry Acidic Basic Hydrophobic Neutral Polarchemistry Acidic Basic Hydrophobic Neutral Po
chemistry Acidic Basic Hydrophobic Neutral Polarchemistry Acidic Basic Hydrophobic Neutral Polarchemistry Acidic Basic Hydrophobic Neutral Po

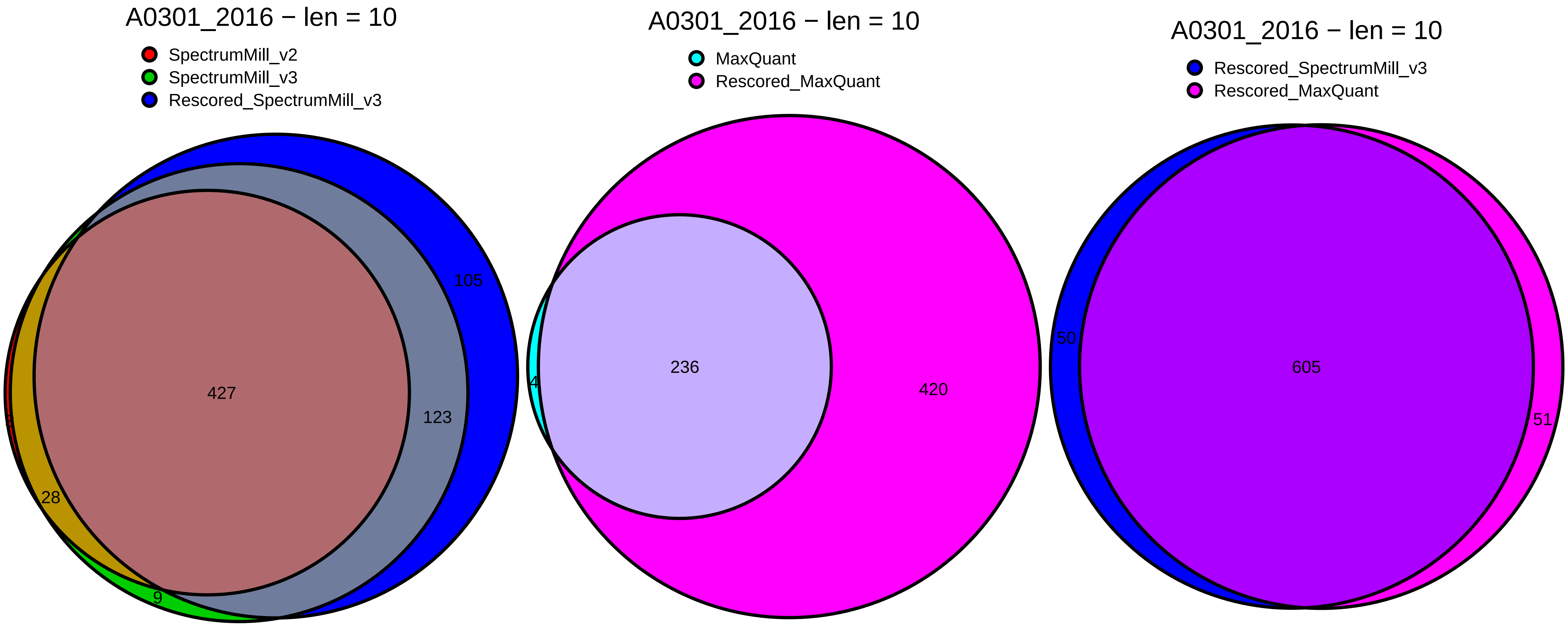
A0301_2016 − len = 10
SpectrumMill_v2
SpectrumMill_v3
Rescored_SpectrumMill_v3
105
427
123
28
9
A0301_2016 − len = 10
MaxQuant
Rescored_MaxQuant
4
236
420
A0301_2016 − len = 10
Rescored_SpectrumMill_v3
Rescored_MaxQuant
50
605
51

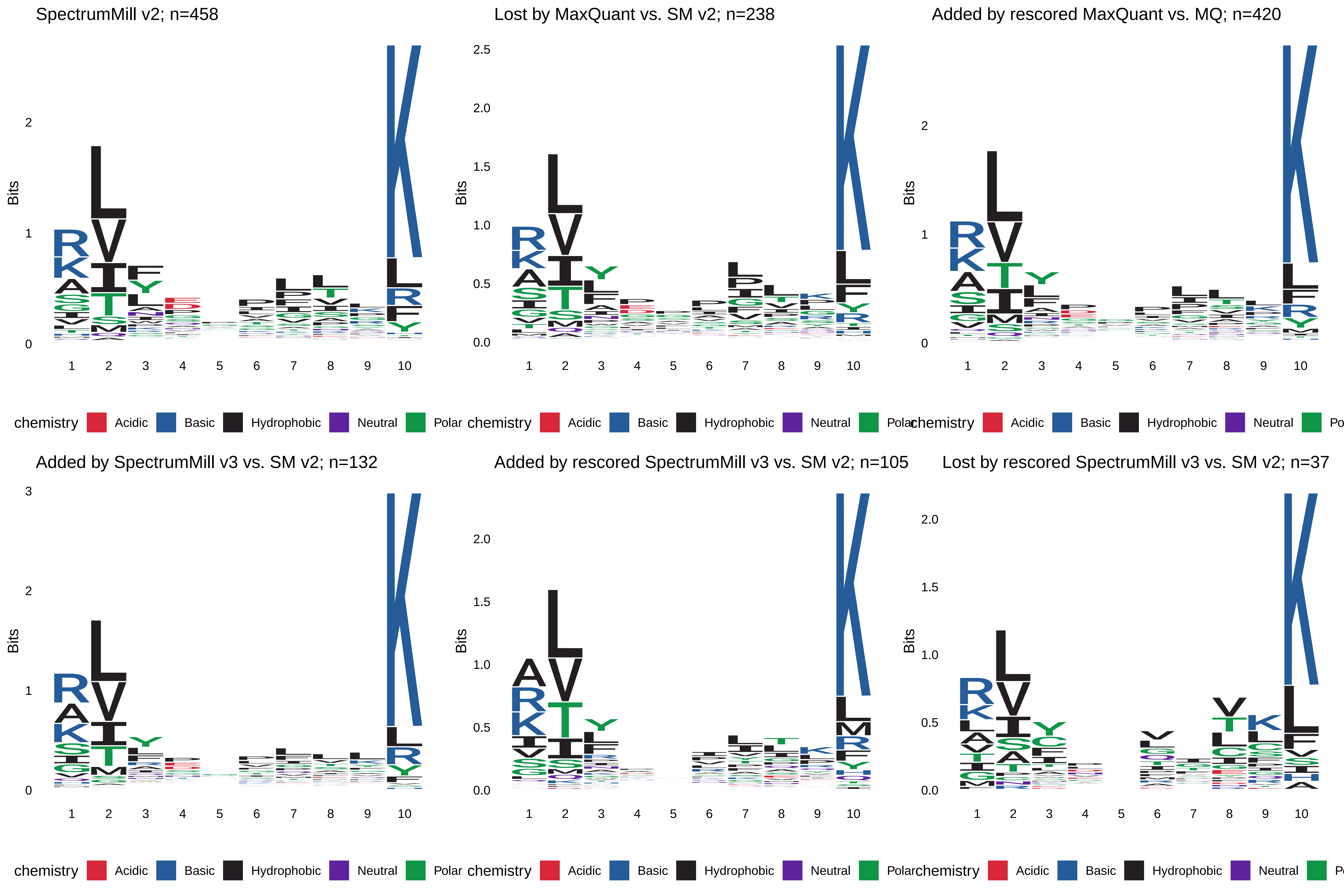
SpectrumMill v2; n=458
Lost by MaxQuant vs. SM v2; n=238
Added by rescored MaxQuant vs. MQ; n=420
Added by SpectrumMill v3 vs. SM v2; n=132
Added by rescored SpectrumMill v3 vs. SM v2; n=105
Lost by rescored SpectrumMill v3 vs. SM v2; n=37
Bits
1 2 3 4 5 6 7 8 9 10
chemistry
Acidic
Basic
Hydrophobic
Neutral
Polar

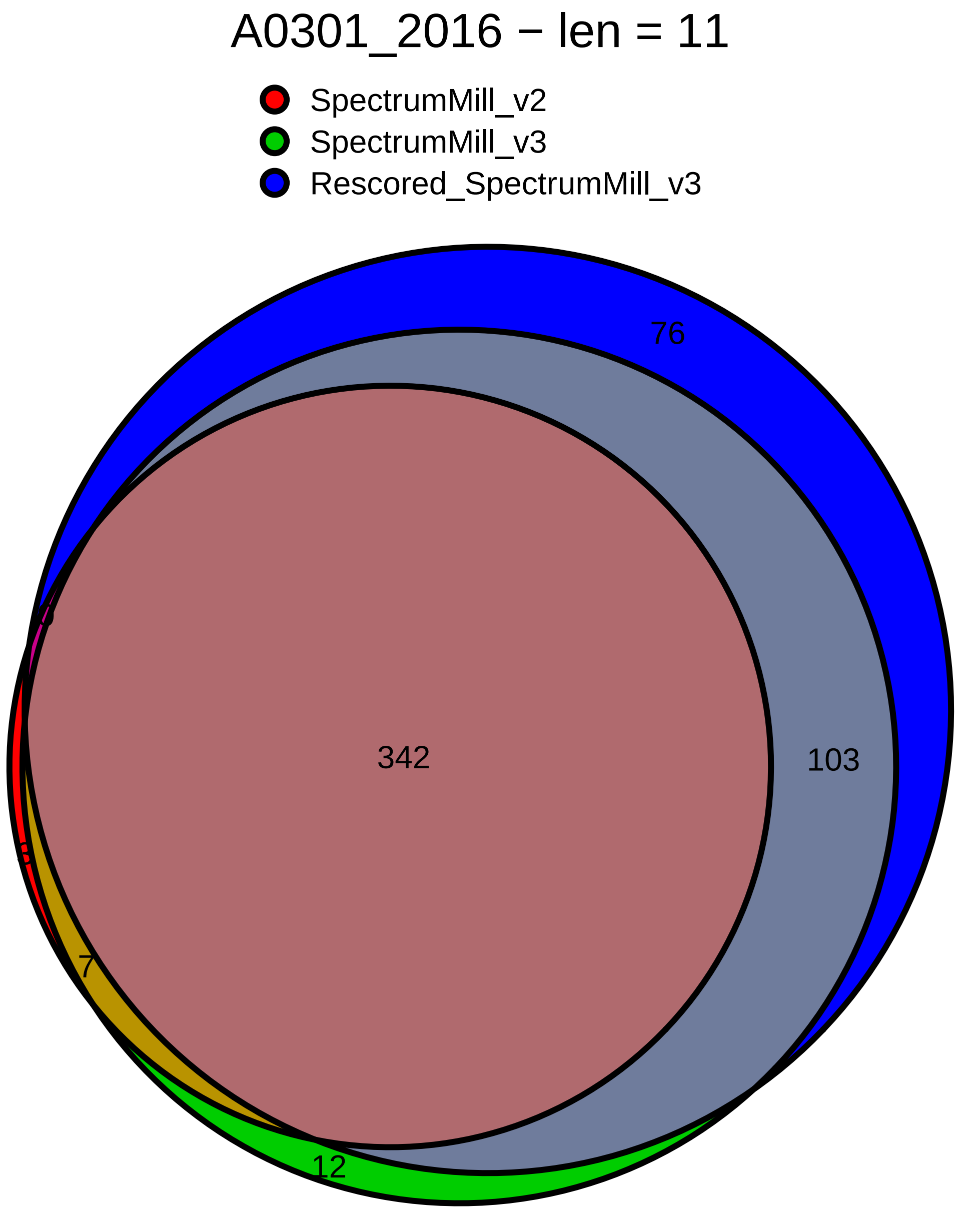
A0301_2016 − len = 11
SpectrumMill_v2
SpectrumMill_v3
Rescored_SpectrumMill_v3
76
342
103
7
12

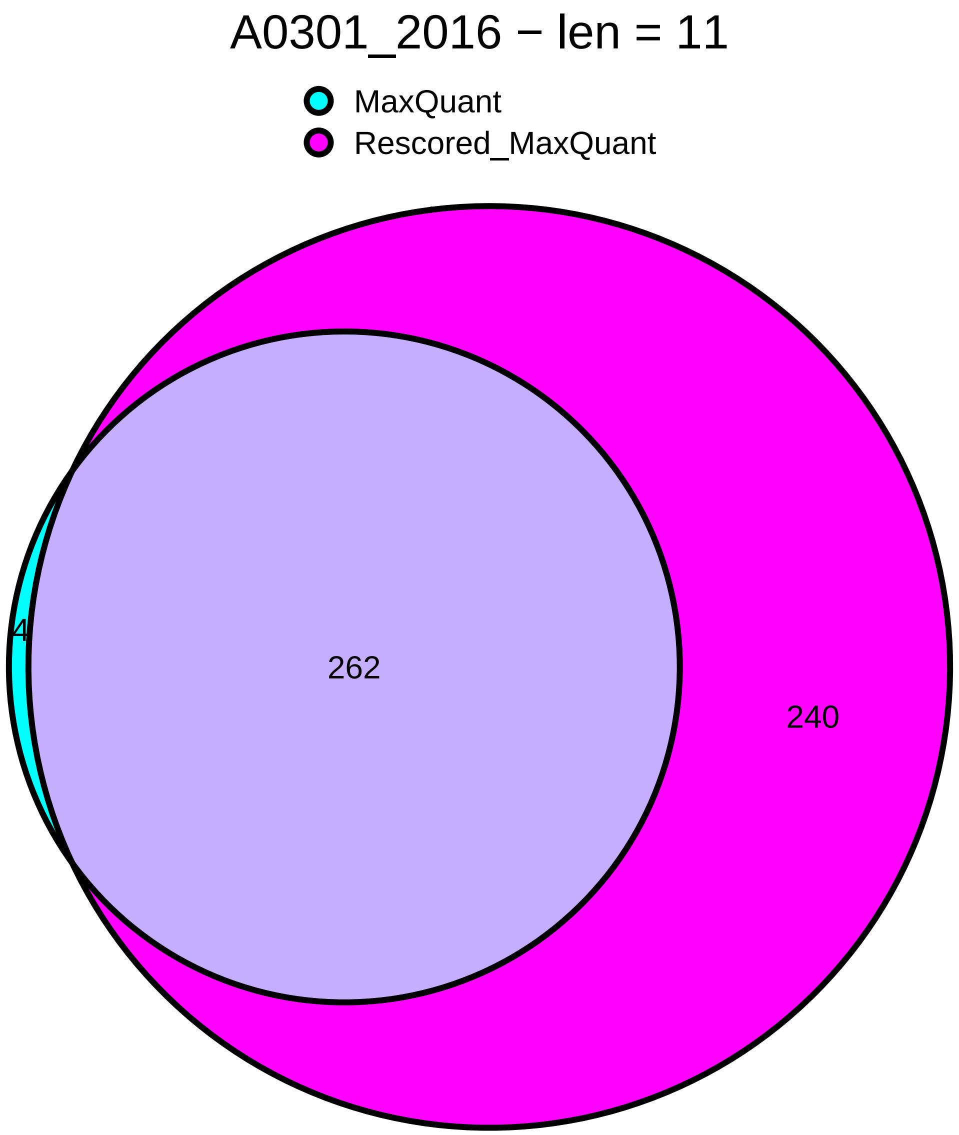
A0301_2016 − len = 11
MaxQuant
Rescored_MaxQuant
262
240

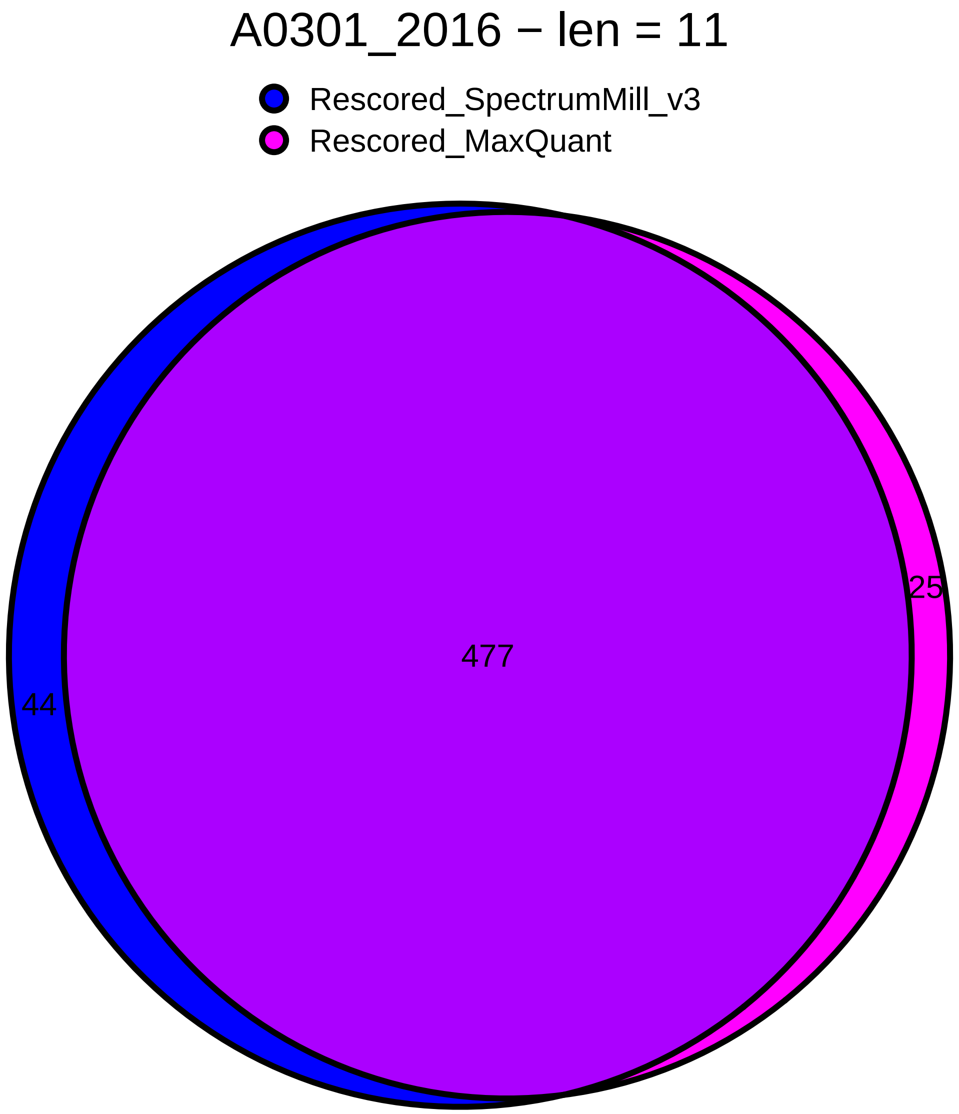
A0301_2016 − len = 11
Rescored_SpectrumMill_v3
Rescored_MaxQuant
477
44
25

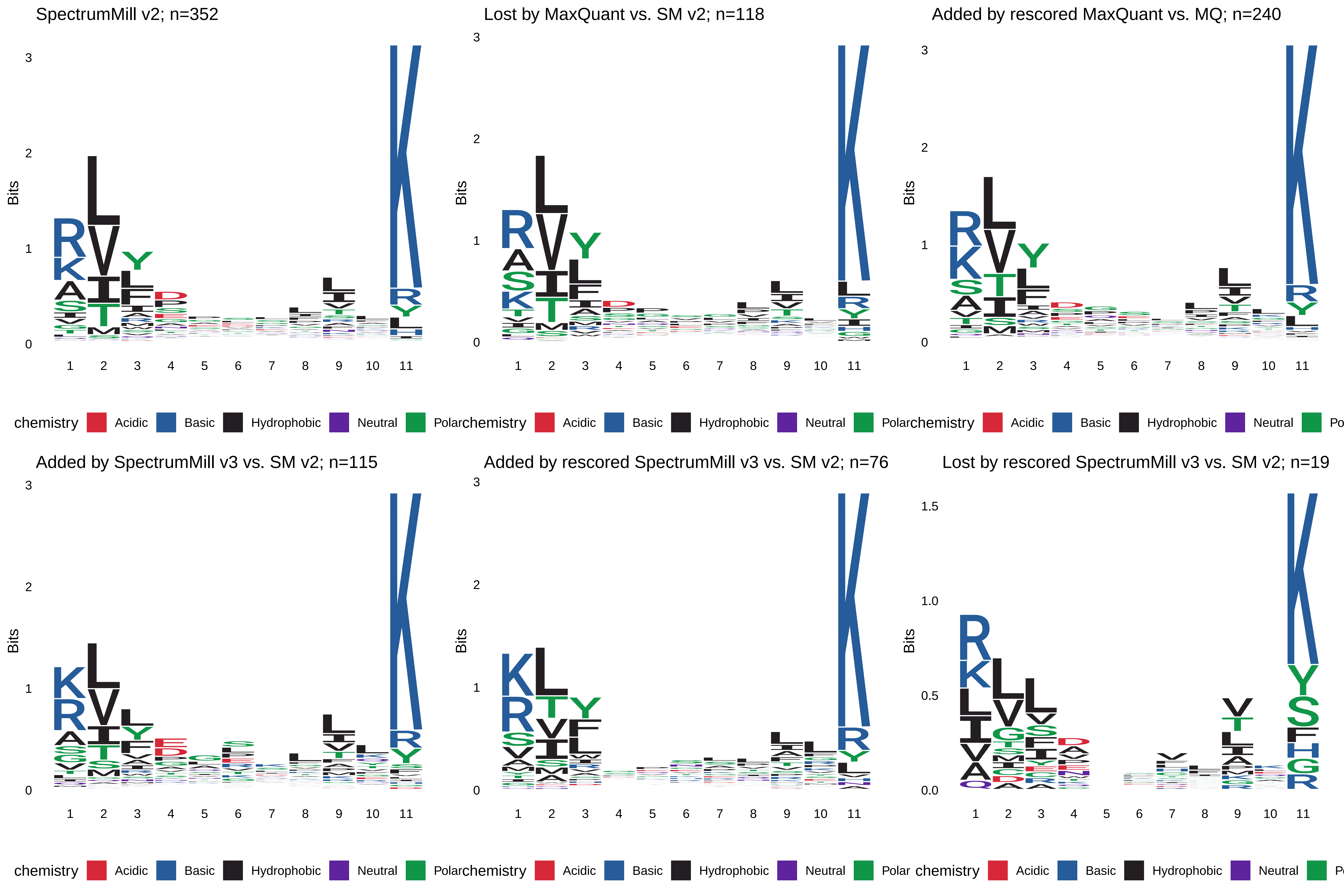
SpectrumMill v2; n=352
Lost by MaxQuant vs. SM v2; n=118
Added by rescored MaxQuant vs. MQ; n=240
Added by SpectrumMill v3 vs. SM v2; n=115
Added by rescored SpectrumMill v3 vs. SM v2; n=76
Lost by rescored SpectrumMill v3 vs. SM v2; n=19
Bits
1 2 3 4 5 6 7 8 9 10 11
chemistry
Acidic
Basic
Hydrophobic
Neutral
Polar

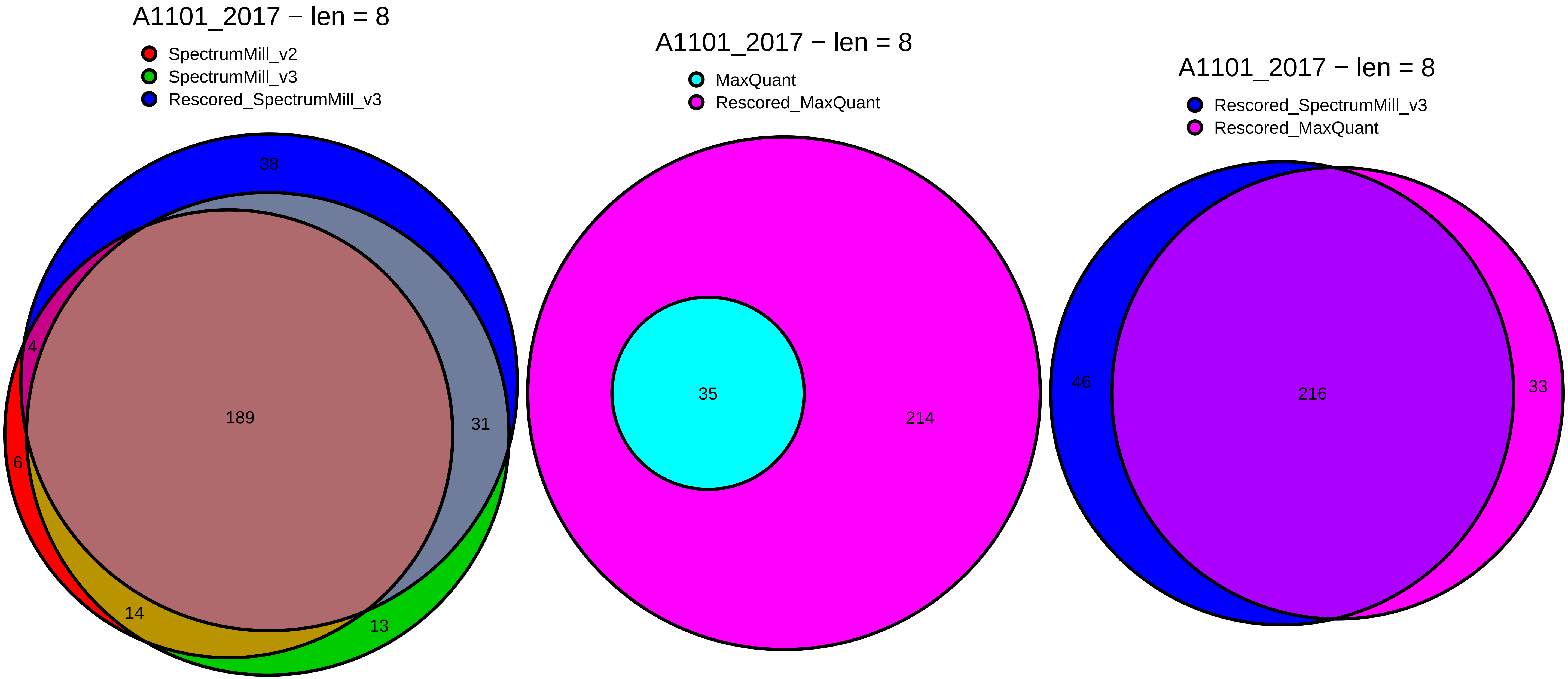

A1101_2017 − len = 8
SpectrumMill_v2
SpectrumMill_v3
Rescored_SpectrumMill_v3
38
4
189
31
6
14
13
A1101_2017 − len = 8
MaxQuant
Rescored_MaxQuant
35
214
A1101_2017 − len = 8
Rescored_SpectrumMill_v3
Rescored_MaxQuant
46
216
33

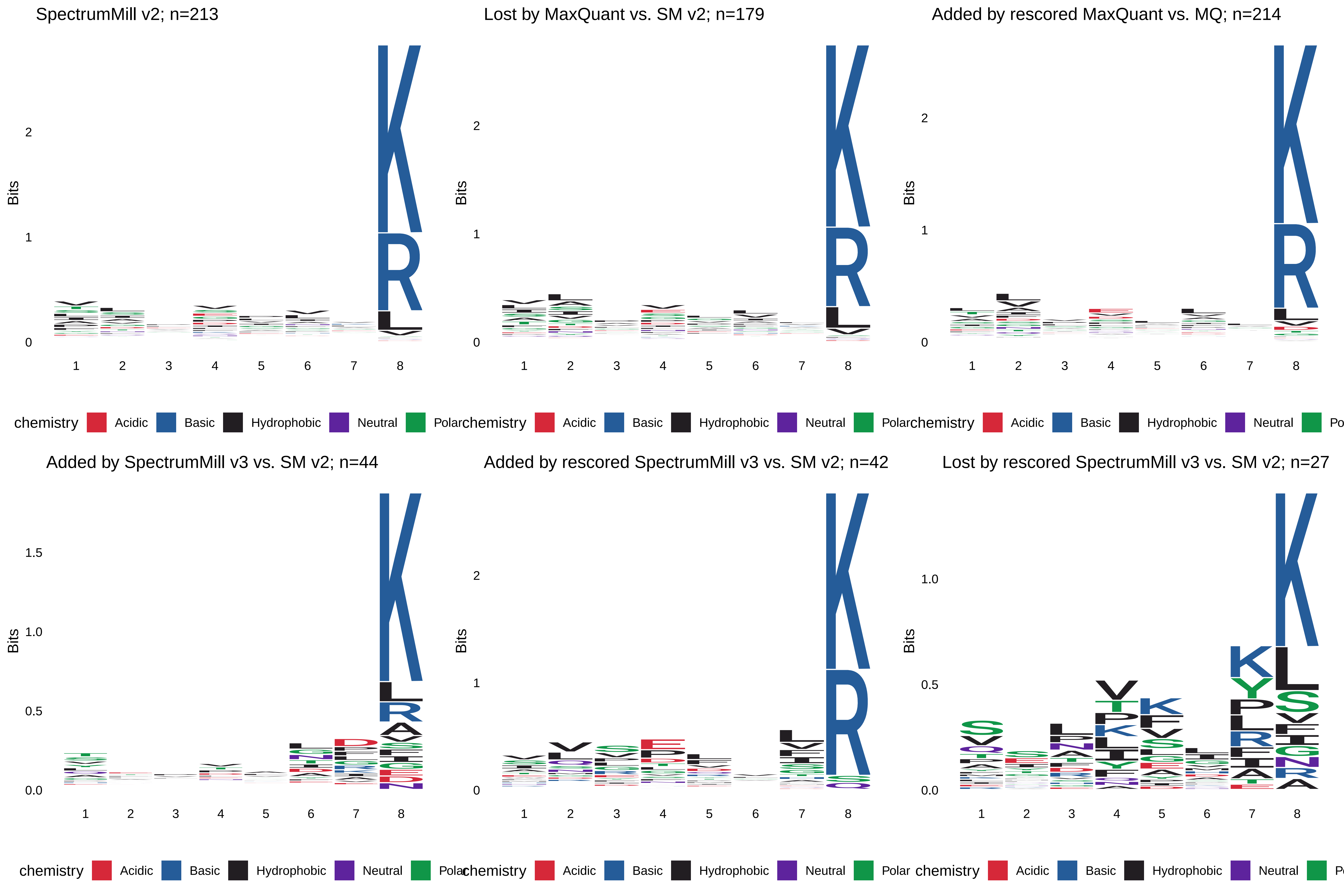
SpectrumMill v2; n=213
Lost by MaxQuant vs. SM v2; n=179
Added by rescored MaxQuant vs. MQ; n=214
Added by SpectrumMill v3 vs. SM v2; n=44
Added by rescored SpectrumMill v3 vs. SM v2; n=42
Lost by rescored SpectrumMill v3 vs. SM v2; n=27
Bits
1 2 3 4 5 6 7 8
chemistry Acidic Basic Hydrophobic Neutral Polar

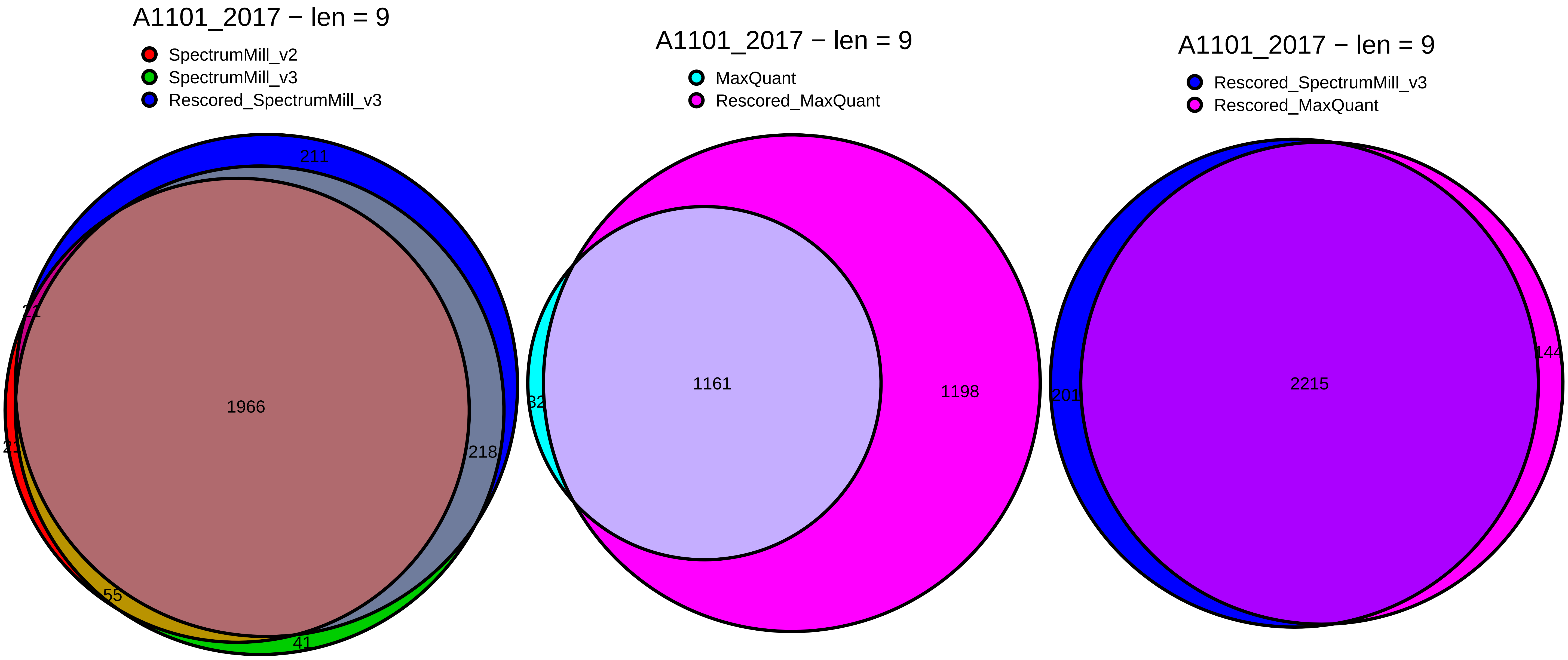

A1101_2017 − len = 9
SpectrumMill_v2
SpectrumMill_v3
Rescored_SpectrumMill_v3
211
21
21
1966
218
55
41
A1101_2017 − len = 9
MaxQuant
Rescored_MaxQuant
32
1161
1198
A1101_2017 − len = 9
Rescored_SpectrumMill_v3
Rescored_MaxQuant
201
2215
144

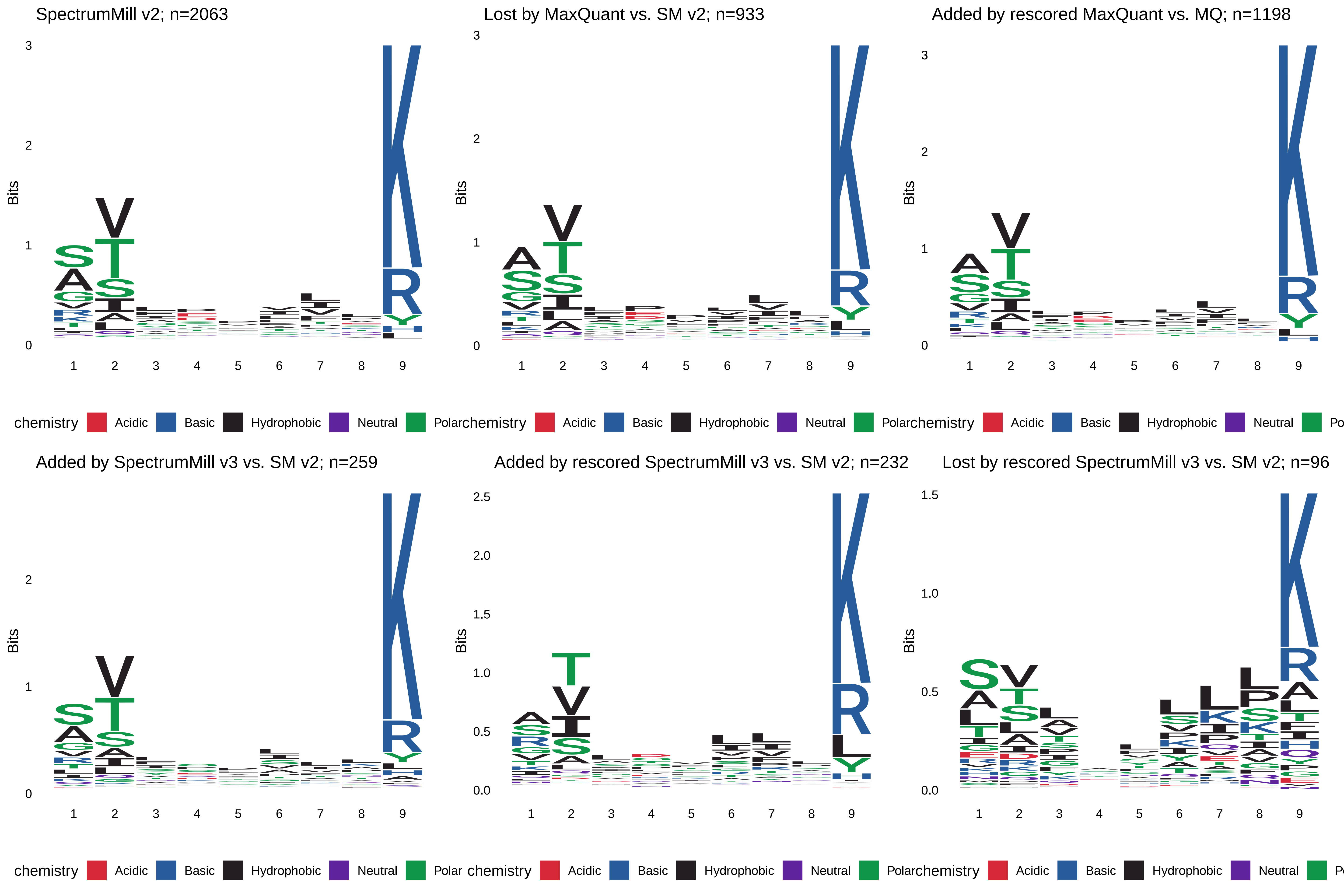
SpectrumMill v2; n=2063
Lost by MaxQuant vs. SM v2; n=933
Added by rescored MaxQuant vs. MQ; n=1198
Added by SpectrumMill v3 vs. SM v2; n=259
Added by rescored SpectrumMill v3 vs. SM v2; n=232
Lost by rescored SpectrumMill v3 vs. SM v2; n=96
Bits
1 2 3 4 5 6 7 8 9
chemistry
Acidic
Basic
Hydrophobic
Neutral
Polar

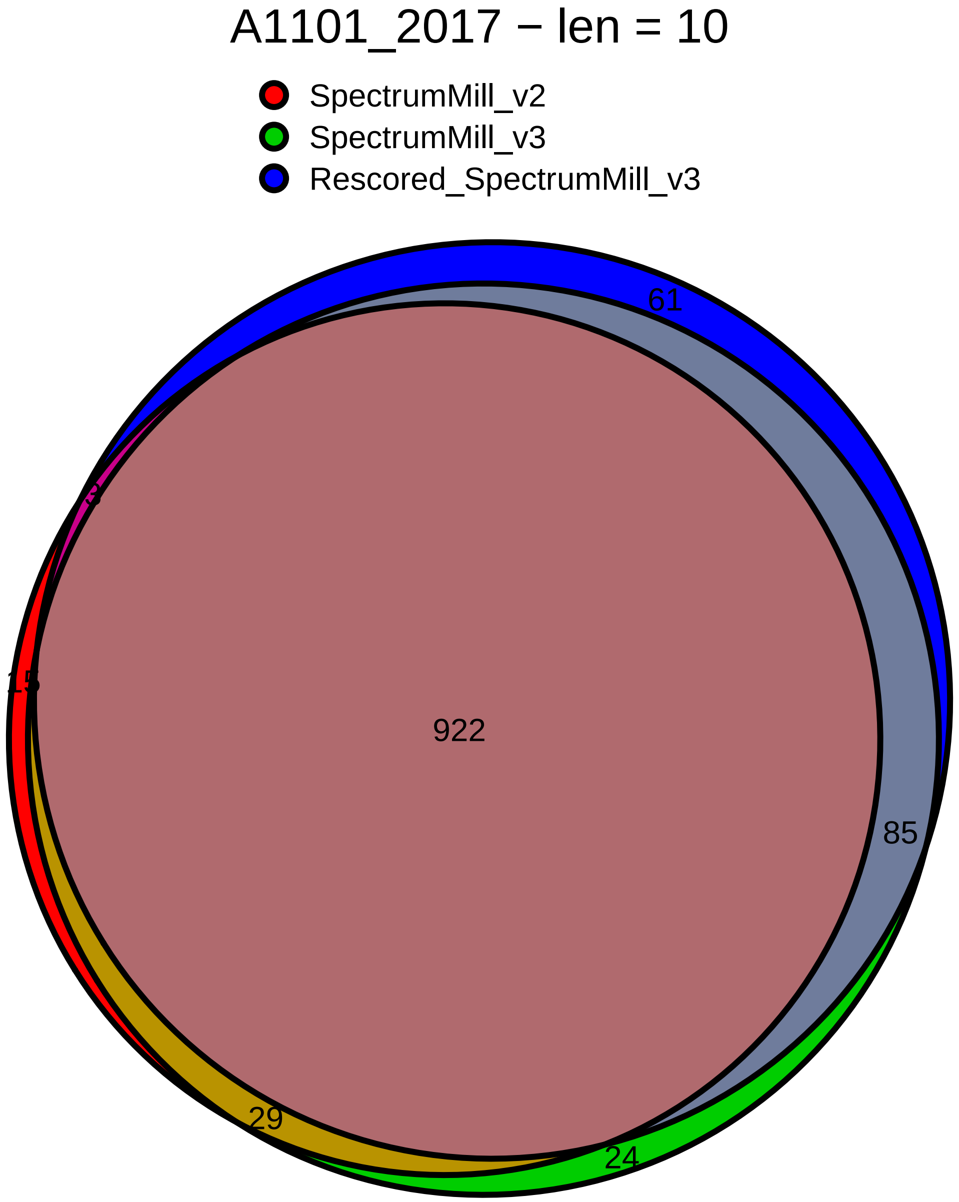
A1101_2017 − len = 10
SpectrumMill_v2
SpectrumMill_v3
Rescored_SpectrumMill_v3
61
3
15
922
85
29
24

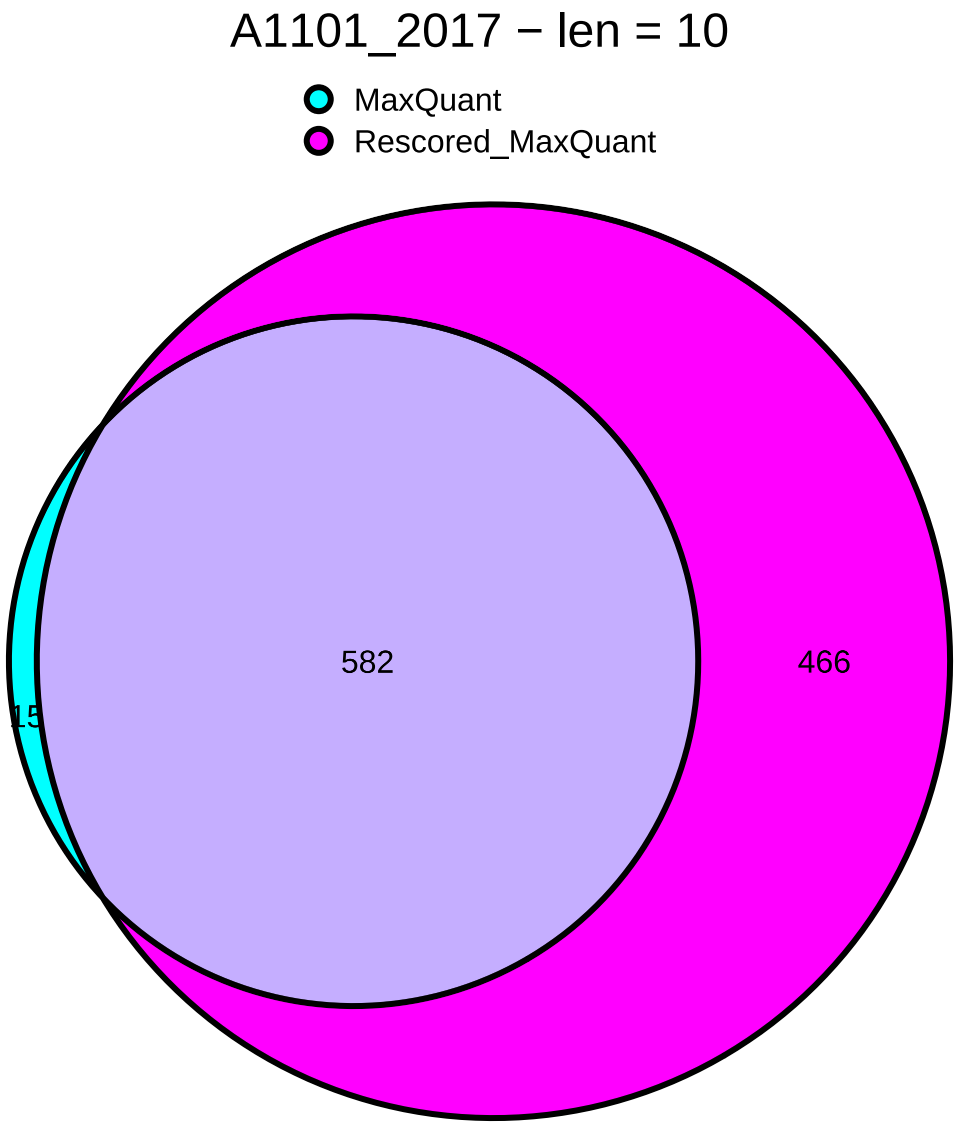
A1101_2017 − len = 10
MaxQuant
Rescored_MaxQuant
582
466
15

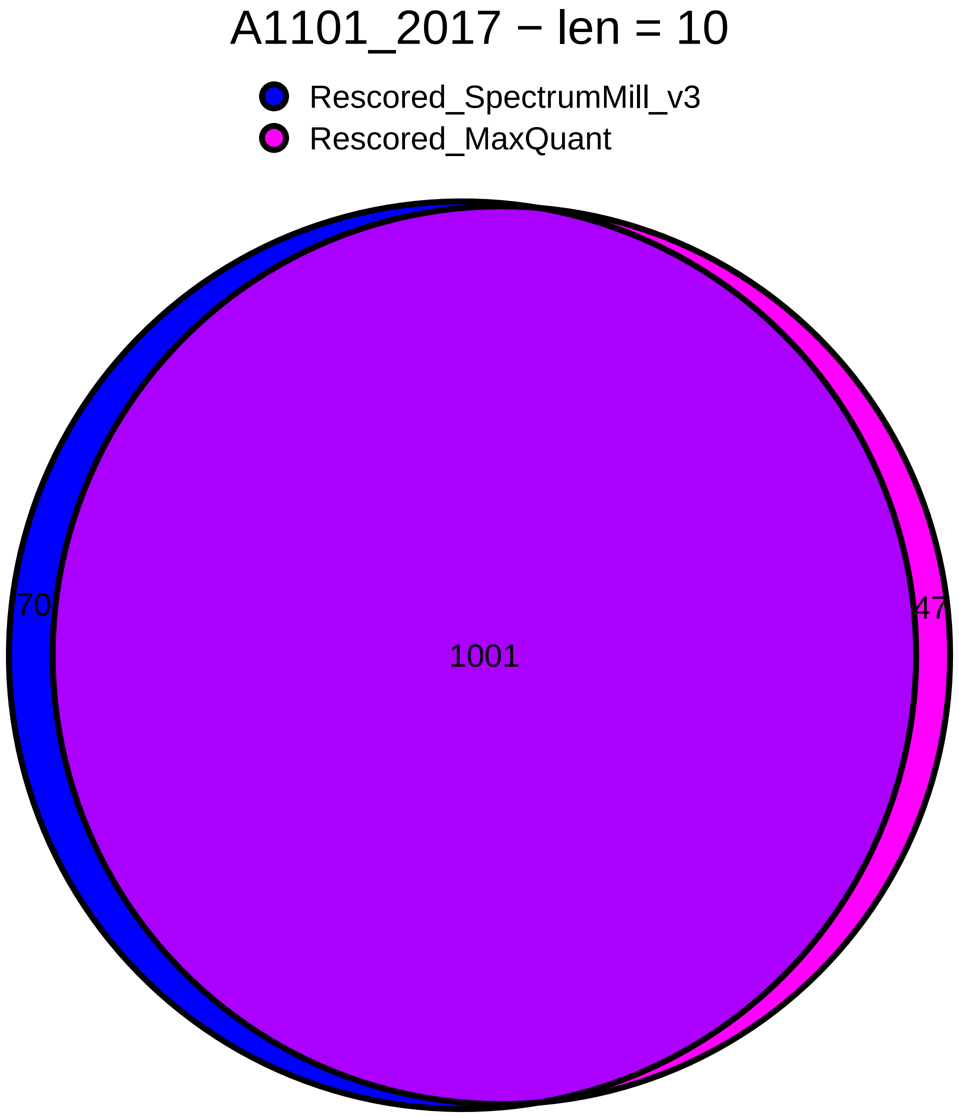
A1101_2017 − len = 10
Rescored_SpectrumMill_v3
Rescored_MaxQuant
70
1001
47

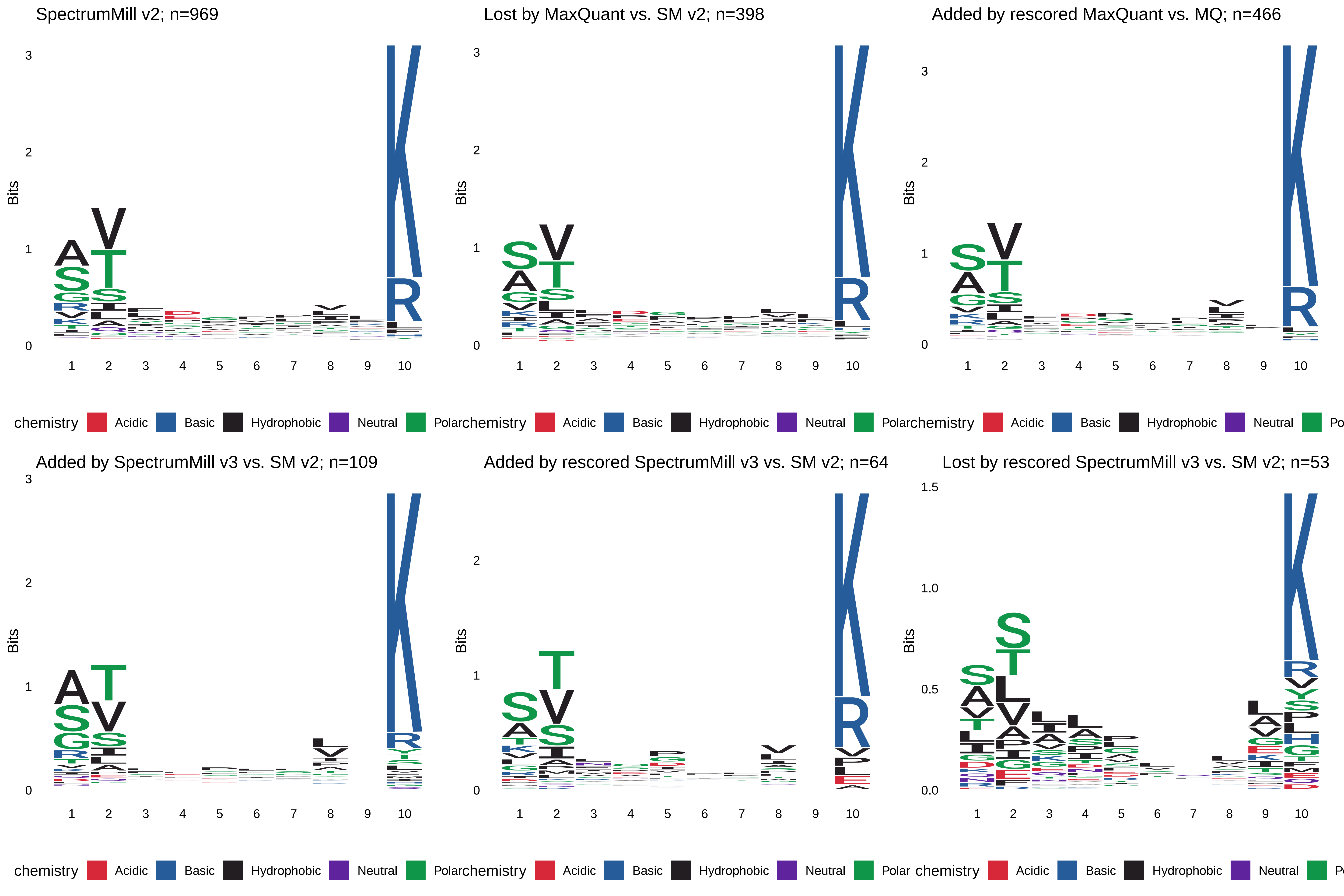
SpectrumMill v2; n=969
Lost by MaxQuant vs. SM v2; n=398
Added by rescored MaxQuant vs. MQ; n=466
Added by SpectrumMill v3 vs. SM v2; n=109
Added by rescored SpectrumMill v3 vs. SM v2; n=64
Lost by rescored SpectrumMill v3 vs. SM v2; n=53
Bits
1 2 3 4 5 6 7 8 9 10
chemistry Acidic Basic Hydrophobic Neutral Polar

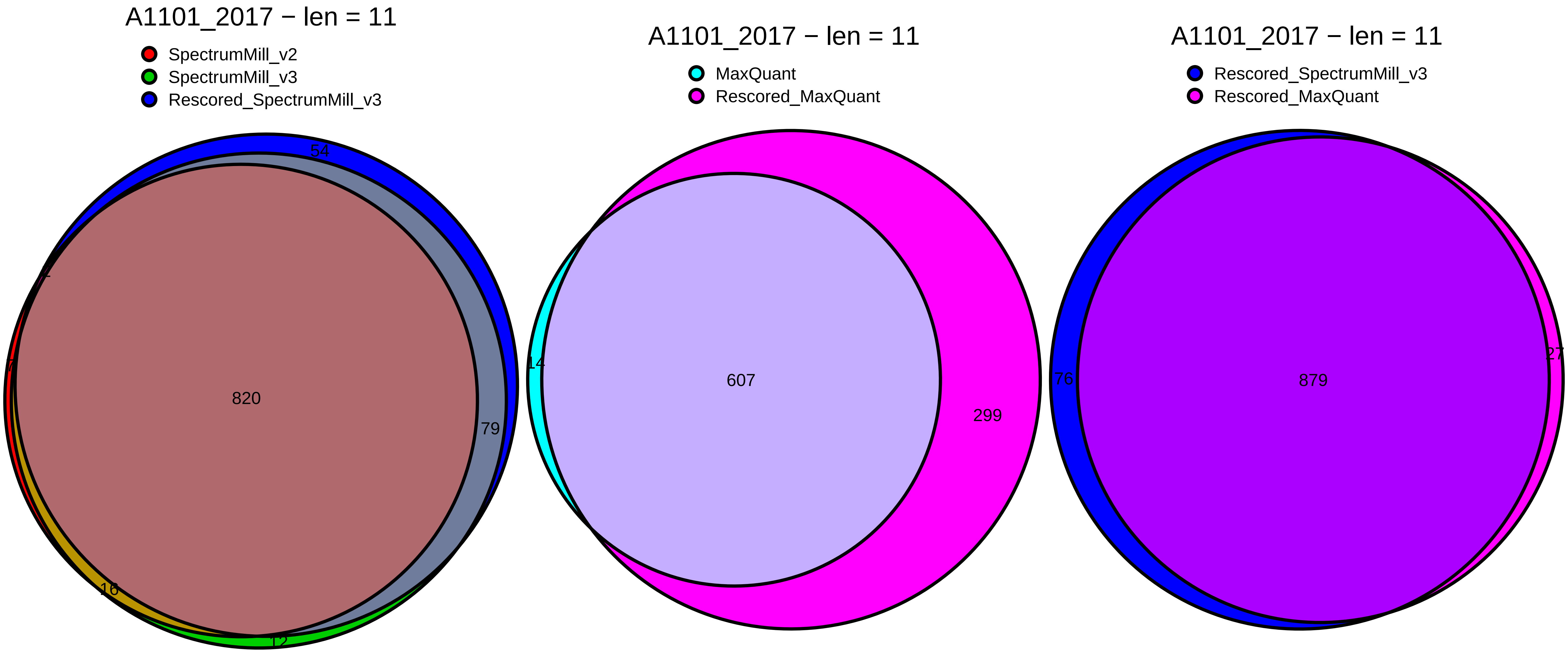
A1101_2017 − len = 11
SpectrumMill_v2
SpectrumMill_v3
Rescored_SpectrumMill_v3
54
820
79
16
12
A1101_2017 − len = 11
MaxQuant
Rescored_MaxQuant
14
607
299
A1101_2017 − len = 11
Rescored_SpectrumMill_v3
Rescored_MaxQuant
76
879

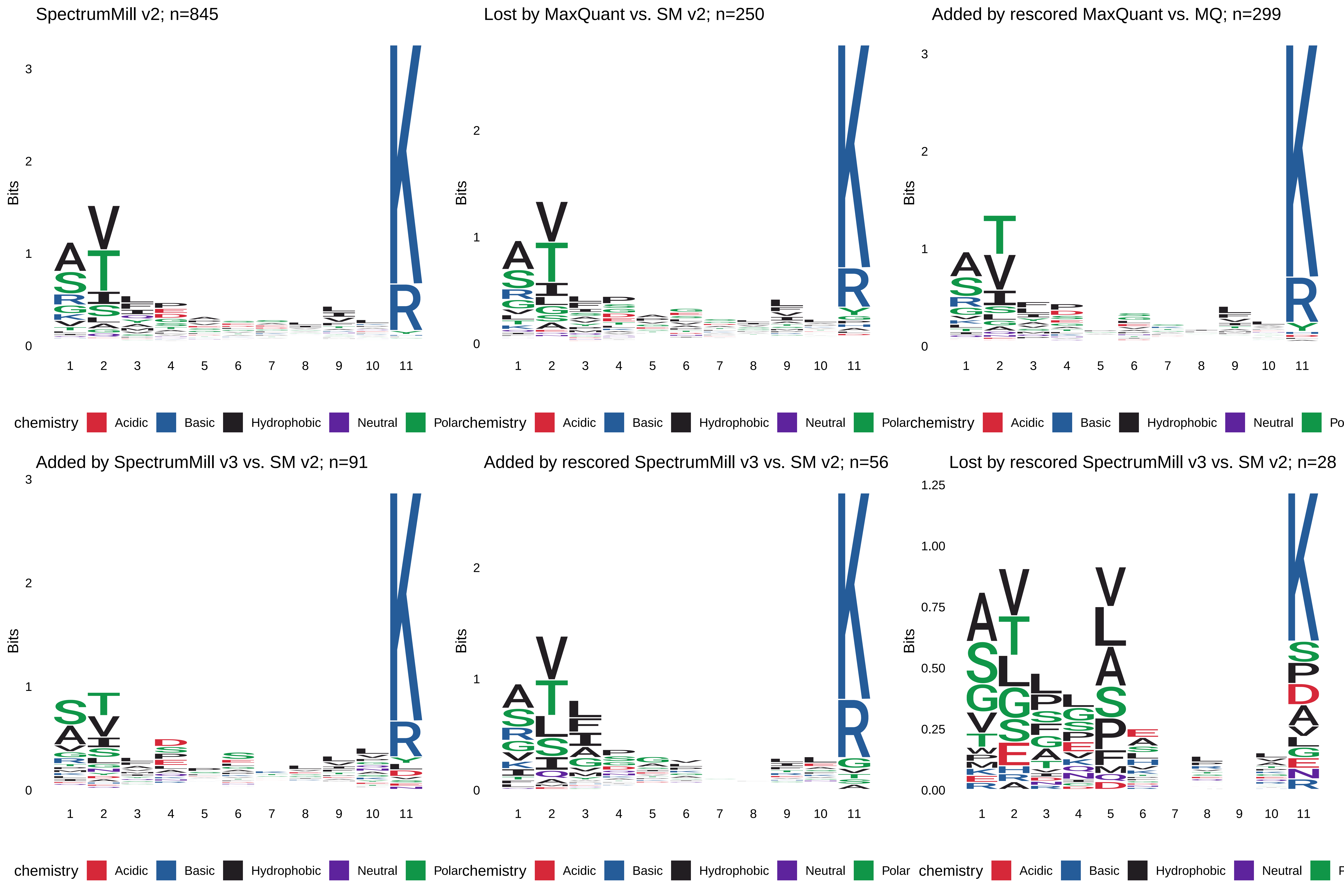

SpectrumMill v2; n=845
Lost by MaxQuant vs. SM v2; n=250
Added by rescored MaxQuant vs. MQ; n=299
Added by SpectrumMill v3 vs. SM v2; n=91
Added by rescored SpectrumMill v3 vs. SM v2; n=56
Lost by rescored SpectrumMill v3 vs. SM v2; n=28
Bits
1 2 3 4 5 6 7 8 9 10 11
chemistry
Acidic
Basic
Hydrophobic
Neutral
Polar

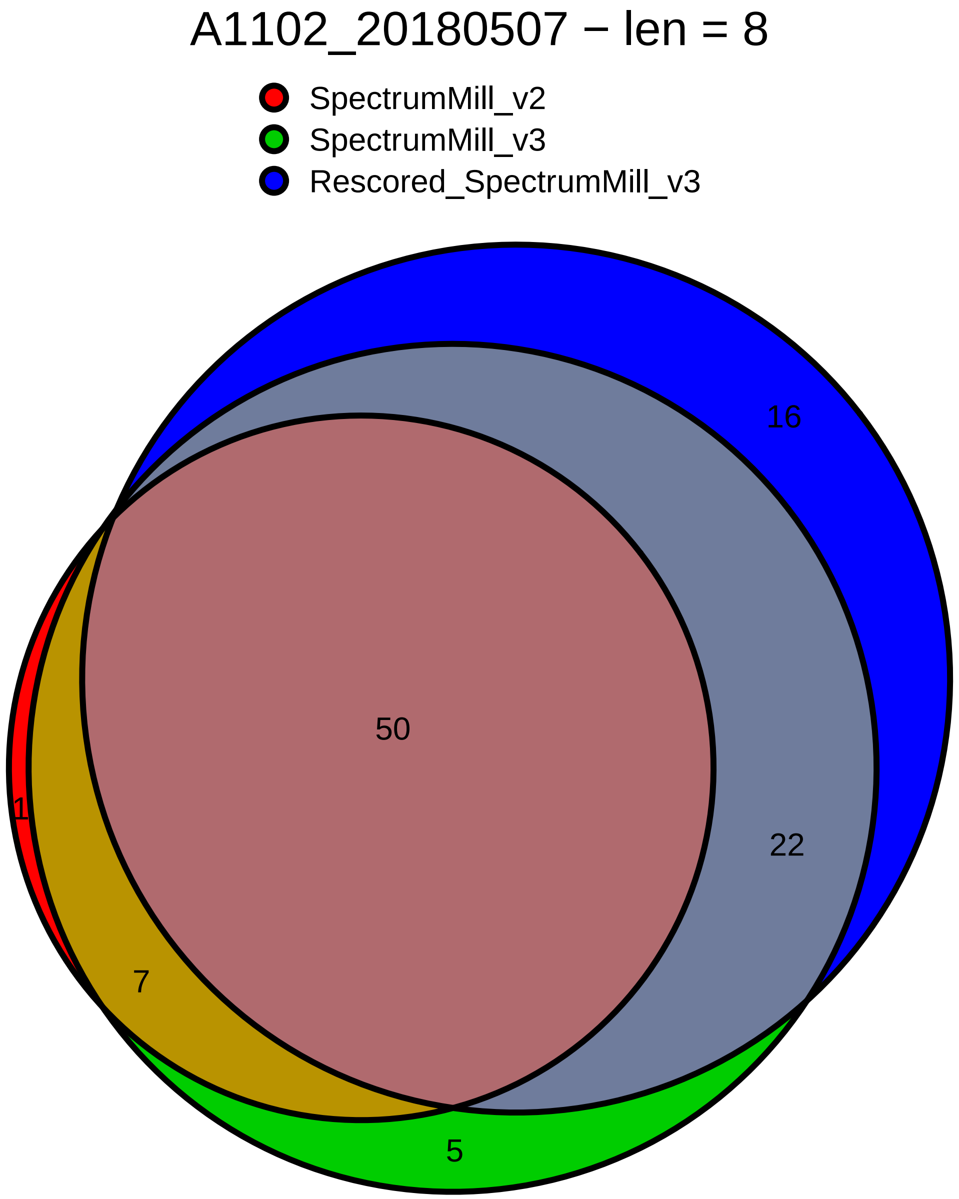
A1102_20180507 − len = 8
SpectrumMill_v2
SpectrumMill_v3
Rescored_SpectrumMill_v3
16
50
1
22
7
5

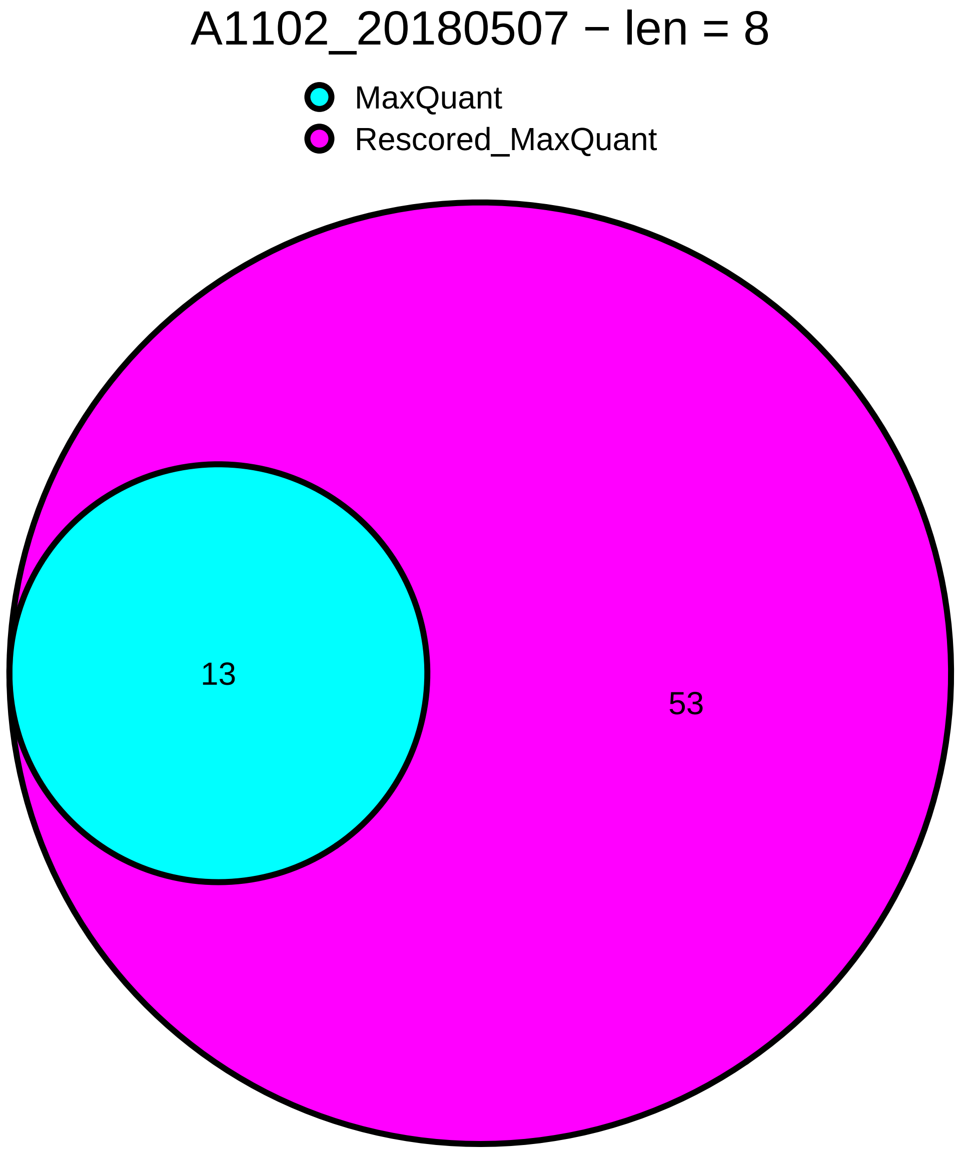
A1102_20180507 − len = 8
MaxQuant
Rescored_MaxQuant
13
53

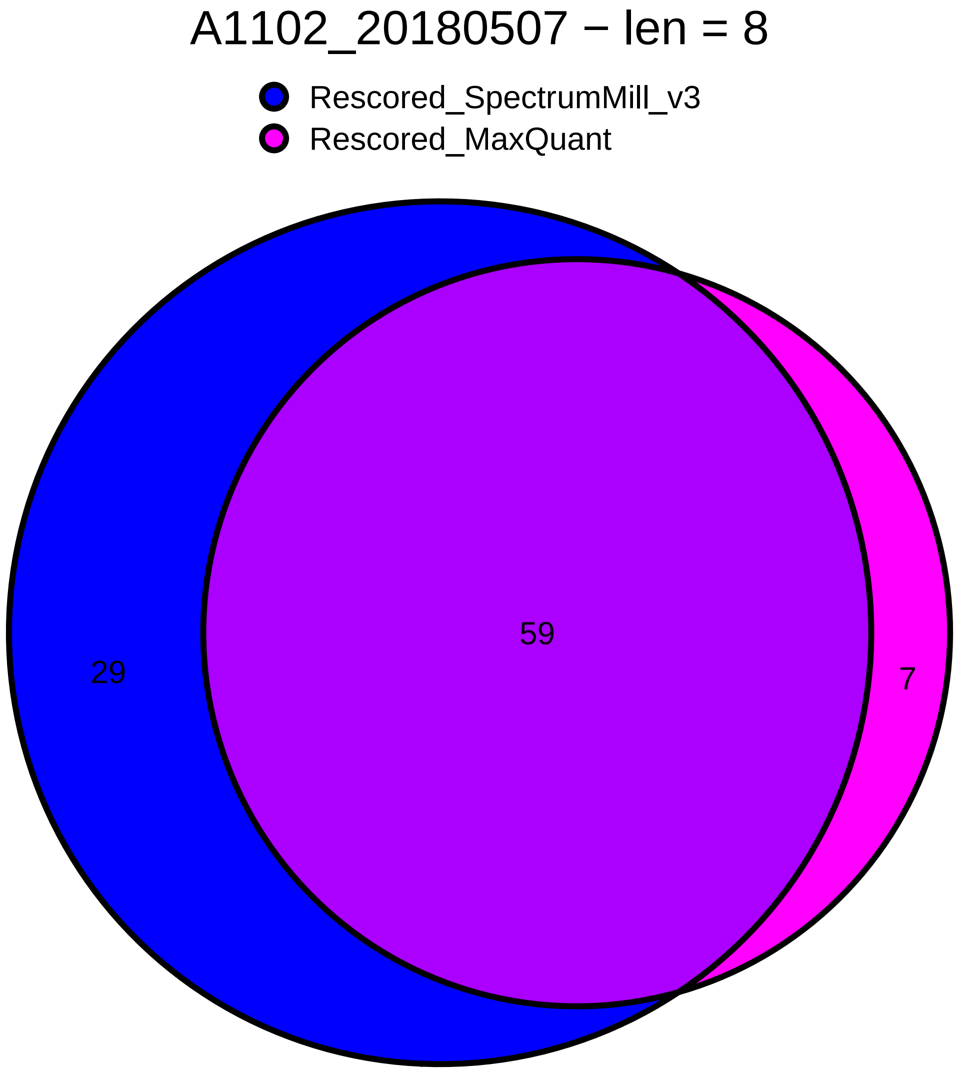
A1102_20180507 − len = 8
Rescored_SpectrumMill_v3
Rescored_MaxQuant
29
59
7

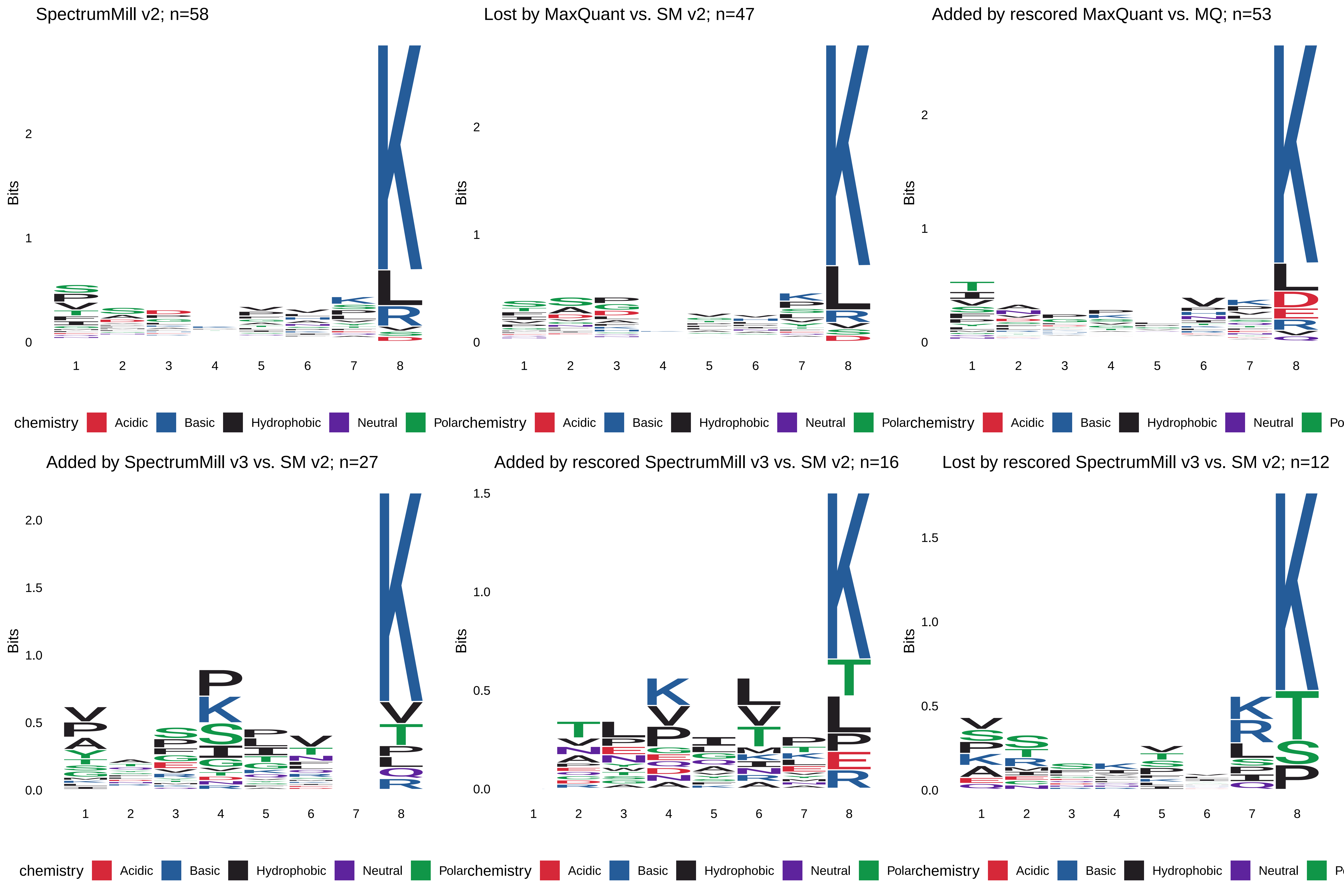

SpectrumMill v2; n=58
Lost by MaxQuant vs. SM v2; n=47
Added by rescored MaxQuant vs. MQ; n=53
Added by SpectrumMill v3 vs. SM v2; n=27
Added by rescored SpectrumMill v3 vs. SM v2; n=16
Lost by rescored SpectrumMill v3 vs. SM v2; n=12
Bits
chemistry
Acidic
Basic
Hydrophobic
Neutral
Polar

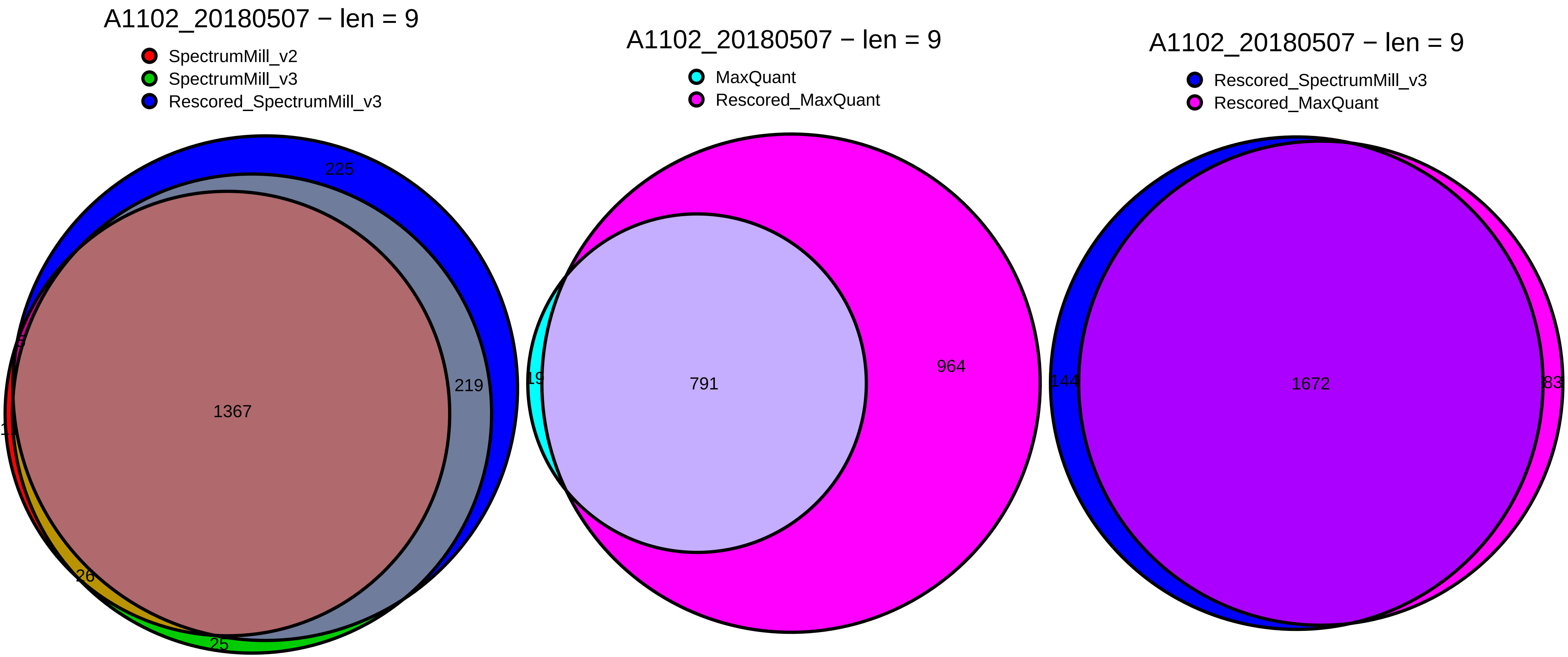
A1102_20180507 − len = 9
SpectrumMill_v2
SpectrumMill_v3
Rescored_SpectrumMill_v3
225
219
1367
26
25
A1102_20180507 − len = 9
MaxQuant
Rescored_MaxQuant
19
791
964
A1102_20180507 − len = 9
Rescored_SpectrumMill_v3
Rescored_MaxQuant
144
1672

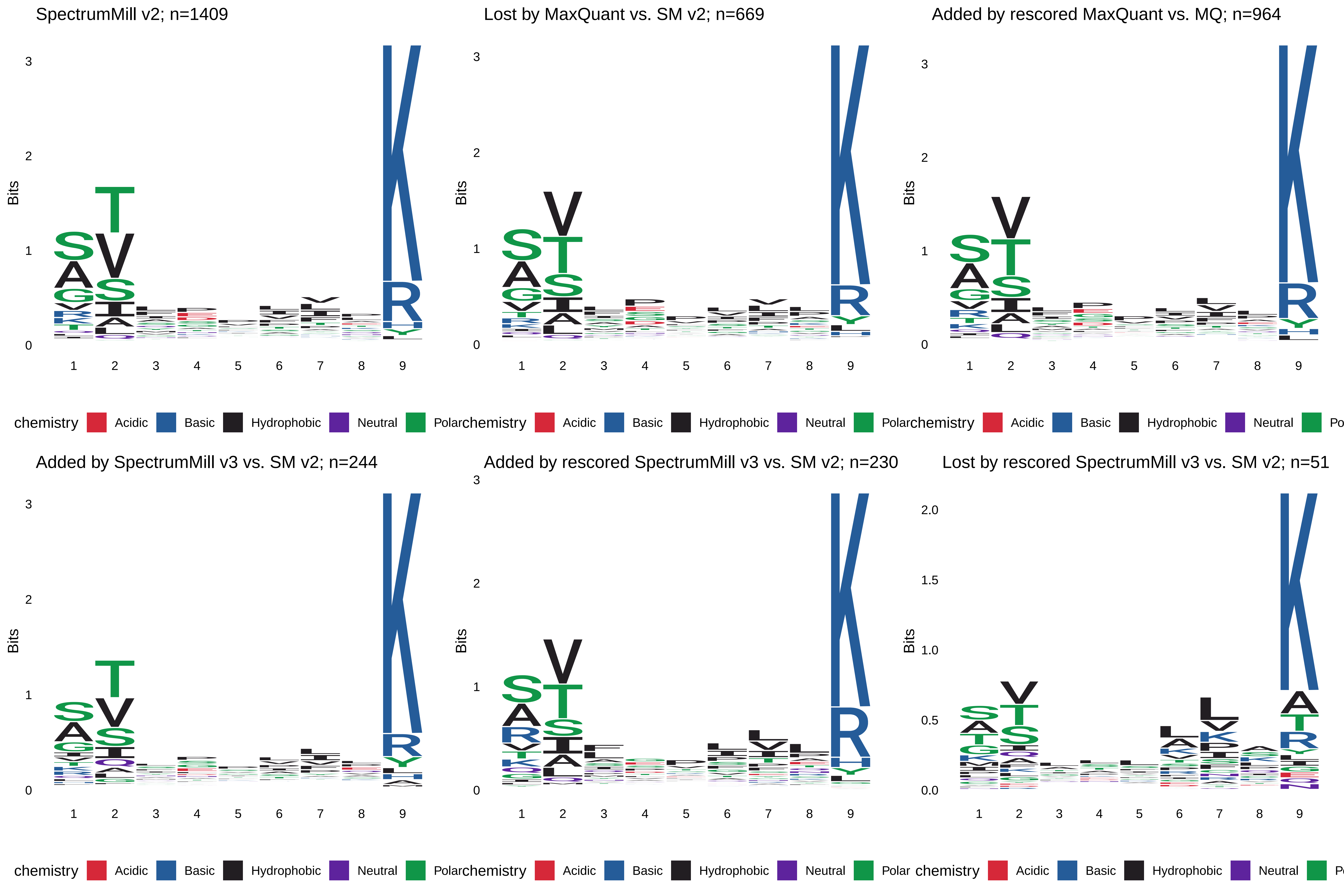
SpectrumMill v2; n=1409
Lost by MaxQuant vs. SM v2; n=669
Added by rescored MaxQuant vs. MQ; n=964
Added by SpectrumMill v3 vs. SM v2; n=244
Added by rescored SpectrumMill v3 vs. SM v2; n=230
Lost by rescored SpectrumMill v3 vs. SM v2; n=51
Bits
1 2 3 4 5 6 7 8 9
chemistry
Acidic
Basic
Hydrophobic
Neutral
Polar

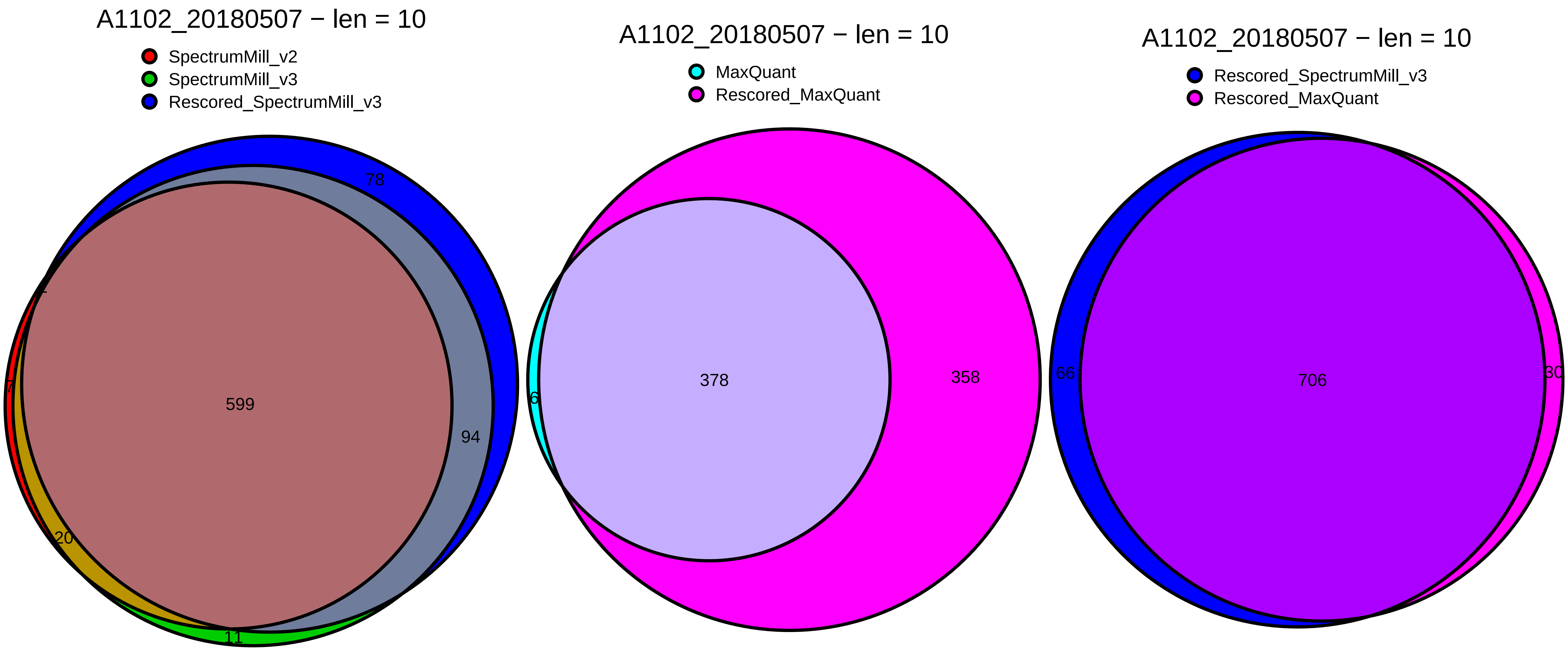
A1102_20180507 − len = 10
SpectrumMill_v2
SpectrumMill_v3
Rescored_SpectrumMill_v3
78
599
94
20
11
A1102_20180507 − len = 10
MaxQuant
Rescored_MaxQuant
6
378
358
A1102_20180507 − len = 10
Rescored_SpectrumMill_v3
Rescored_MaxQuant
66
706
30

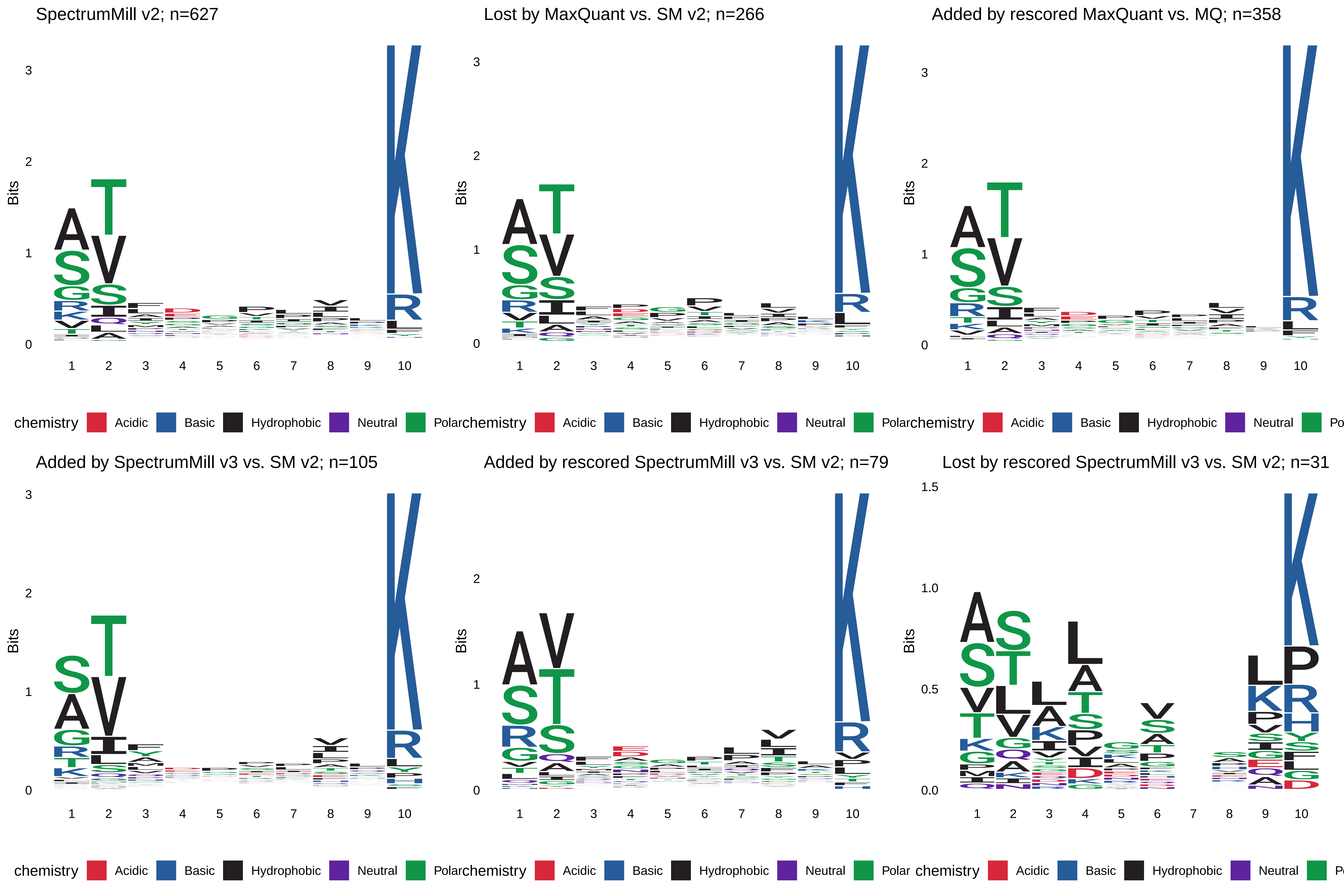

SpectrumMill v2; n=627
Lost by MaxQuant vs. SM v2; n=266
Added by rescored MaxQuant vs. MQ; n=358
Added by SpectrumMill v3 vs. SM v2; n=105
Added by rescored SpectrumMill v3 vs. SM v2; n=79
Lost by rescored SpectrumMill v3 vs. SM v2; n=31
Bits
1 2 3 4 5 6 7 8 9 10
chemistry Acidic Basic Hydrophobic Neutral Polar

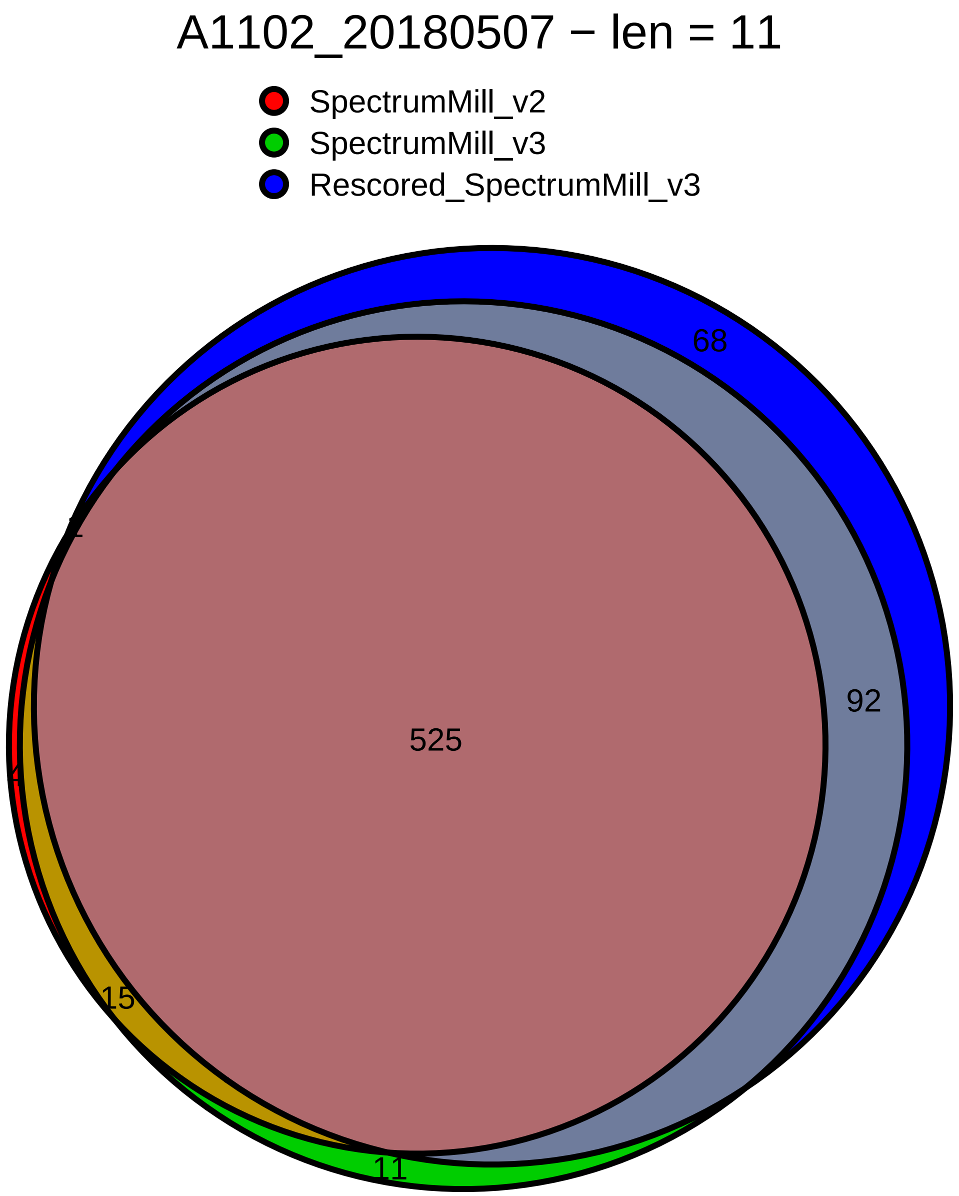
A1102_20180507 − len = 11
SpectrumMill_v2
SpectrumMill_v3
Rescored_SpectrumMill_v3
68
92
525
15
11

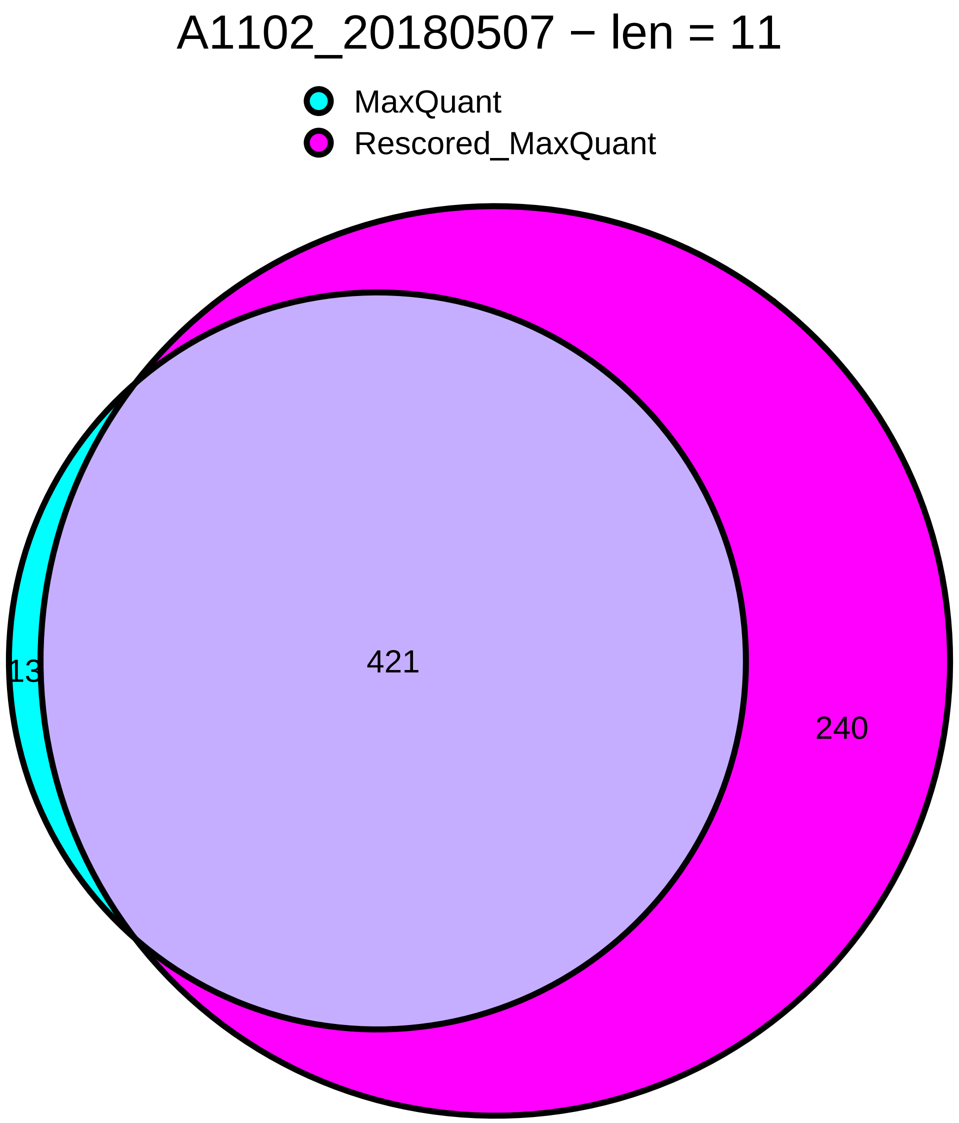
A1102_20180507 − len = 11
MaxQuant
Rescored_MaxQuant
13
421
240

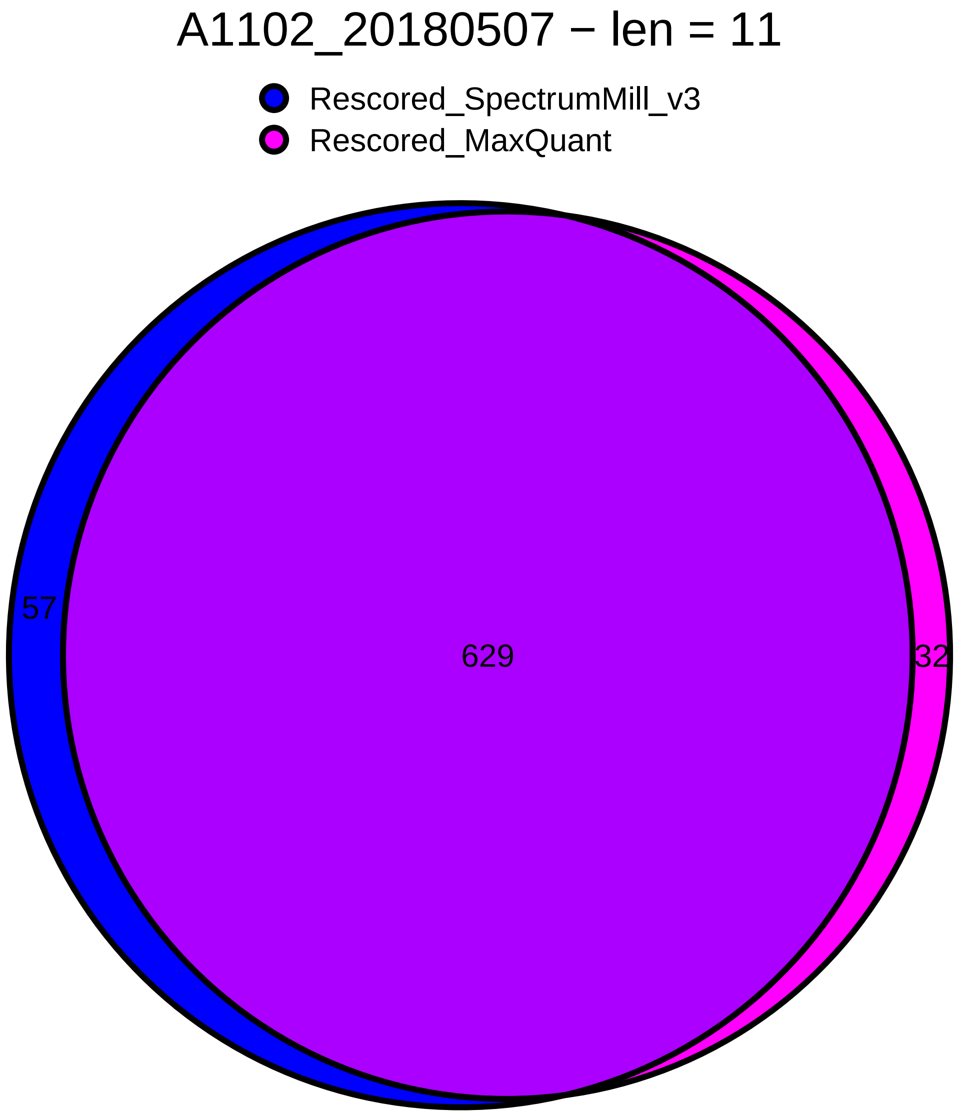
A1102_20180507 − len = 11
Rescored_SpectrumMill_v3
Rescored_MaxQuant
57
629
32

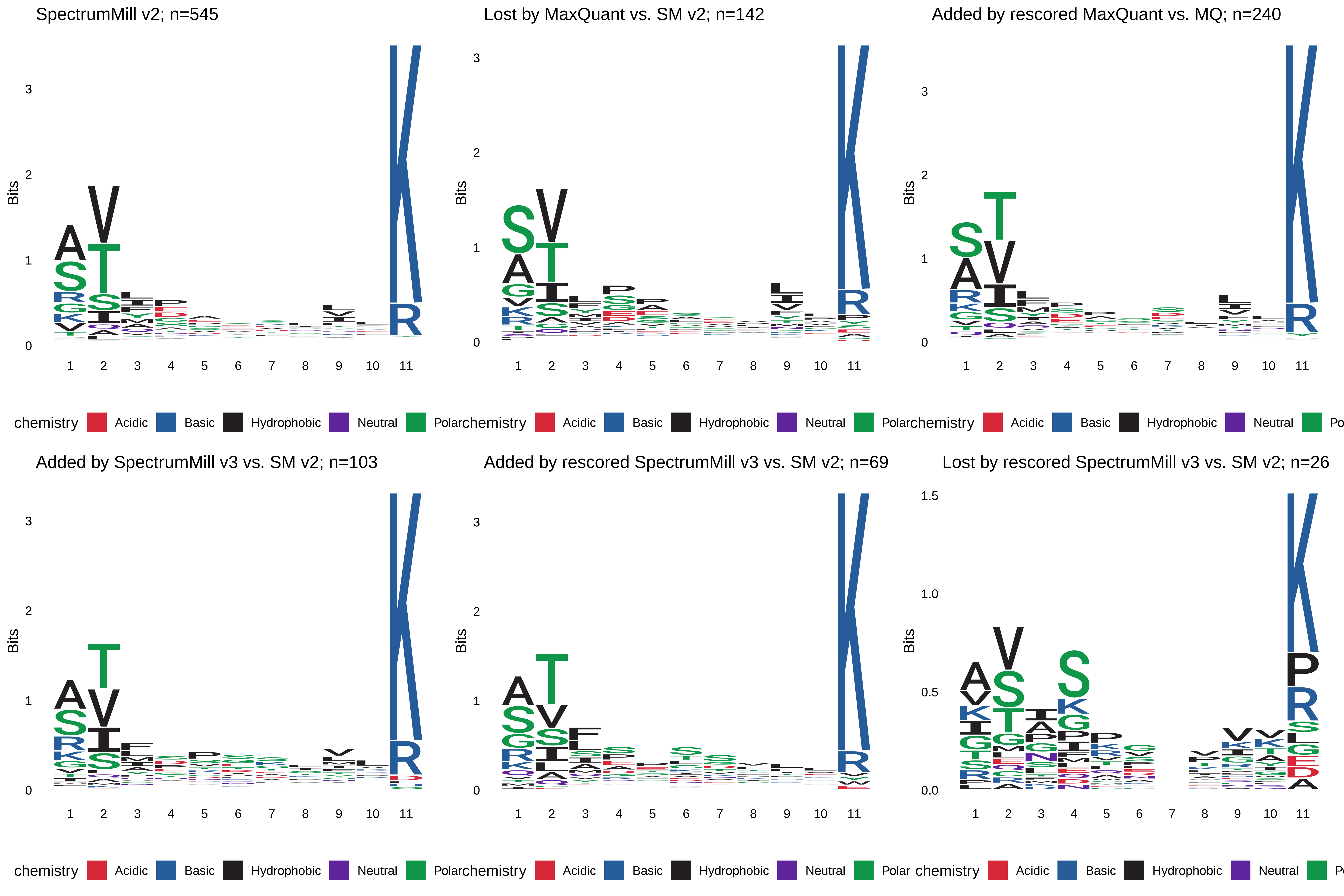
SpectrumMill v2; n=545
Lost by MaxQuant vs. SM v2; n=142
Added by rescored MaxQuant vs. MQ; n=240
Added by SpectrumMill v3 vs. SM v2; n=103
Added by rescored SpectrumMill v3 vs. SM v2; n=69
Lost by rescored SpectrumMill v3 vs. SM v2; n=26
Bits
1 2 3 4 5 6 7 8 9 10 11
chemistry
Acidic
Basic
Hydrophobic
Neutral
Polar

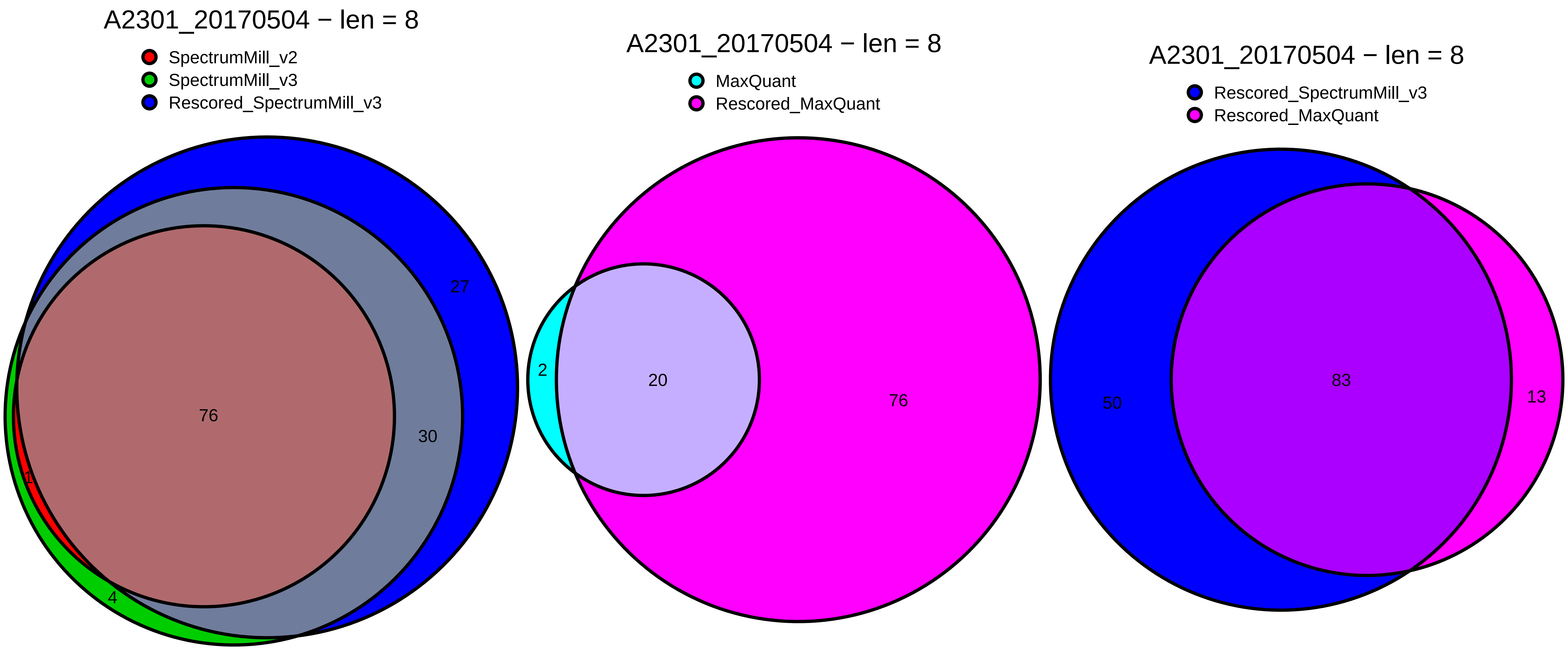
A2301_20170504 − len = 8
SpectrumMill_v2
SpectrumMill_v3
Rescored_SpectrumMill_v3
27
76
30
1
4
A2301_20170504 − len = 8
MaxQuant
Rescored_MaxQuant
2
20
76
A2301_20170504 − len = 8
Rescored_SpectrumMill_v3
Rescored_MaxQuant
50
83
13

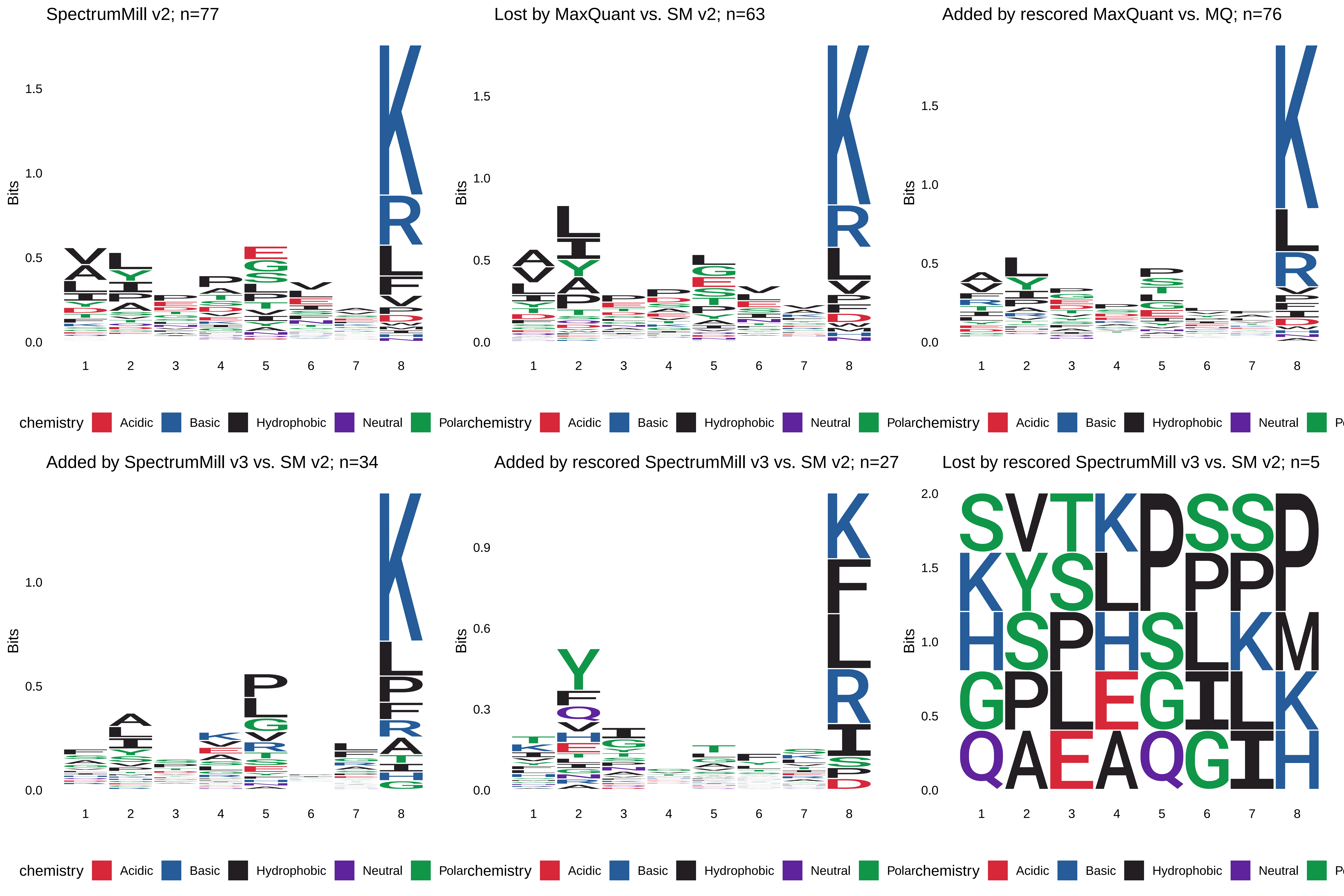
SpectrumMill v2; n=77
Lost by MaxQuant vs. SM v2; n=63
Added by rescored MaxQuant vs. MQ; n=76
Added by SpectrumMill v3 vs. SM v2; n=34
Added by rescored SpectrumMill v3 vs. SM v2; n=27
Lost by rescored SpectrumMill v3 vs. SM v2; n=5
Bits
chemistry
Acidic
Basic
Hydrophobic
Neutral
Polar

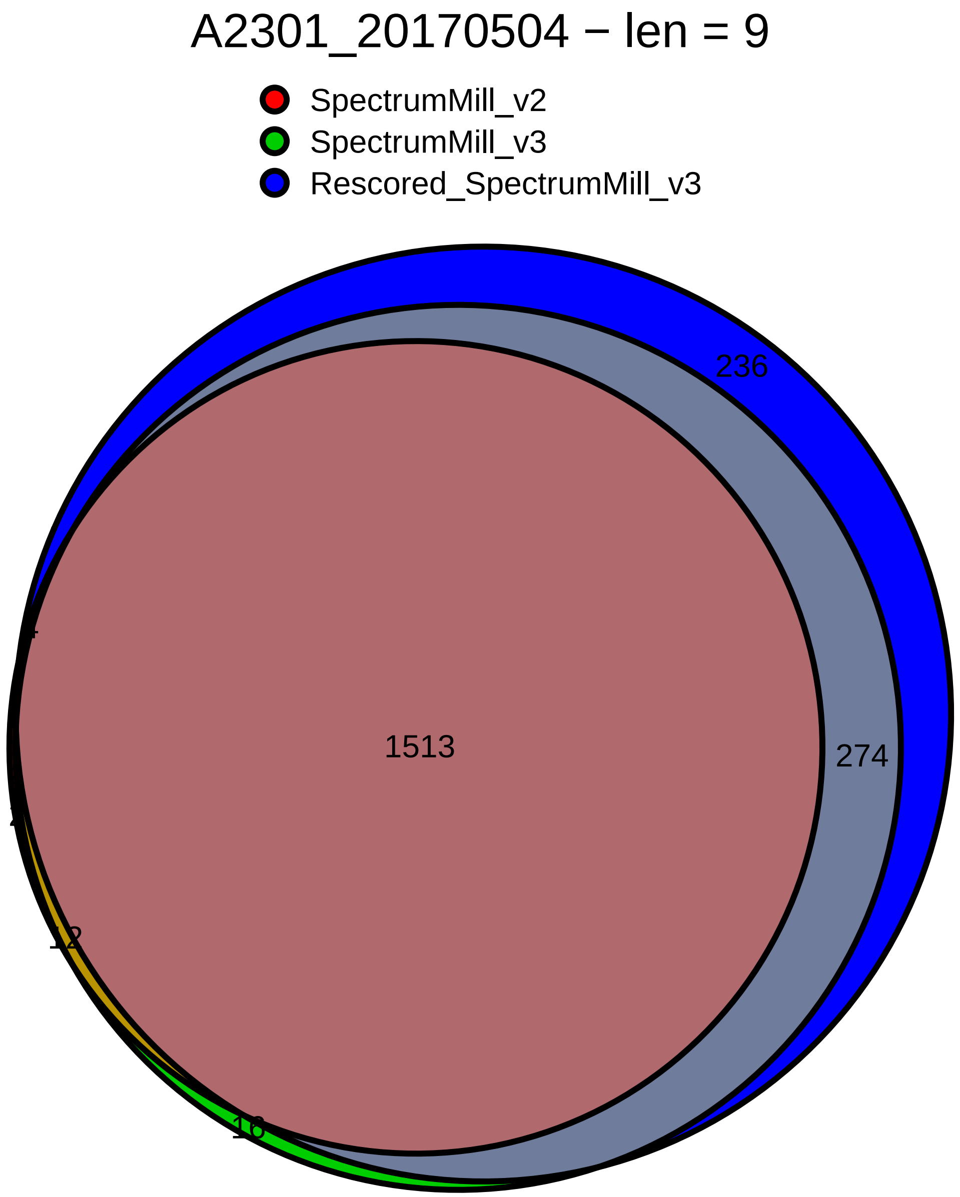
A2301_20170504 − len = 9
SpectrumMill_v2
SpectrumMill_v3
Rescored_SpectrumMill_v3
236
274
1513
12
16

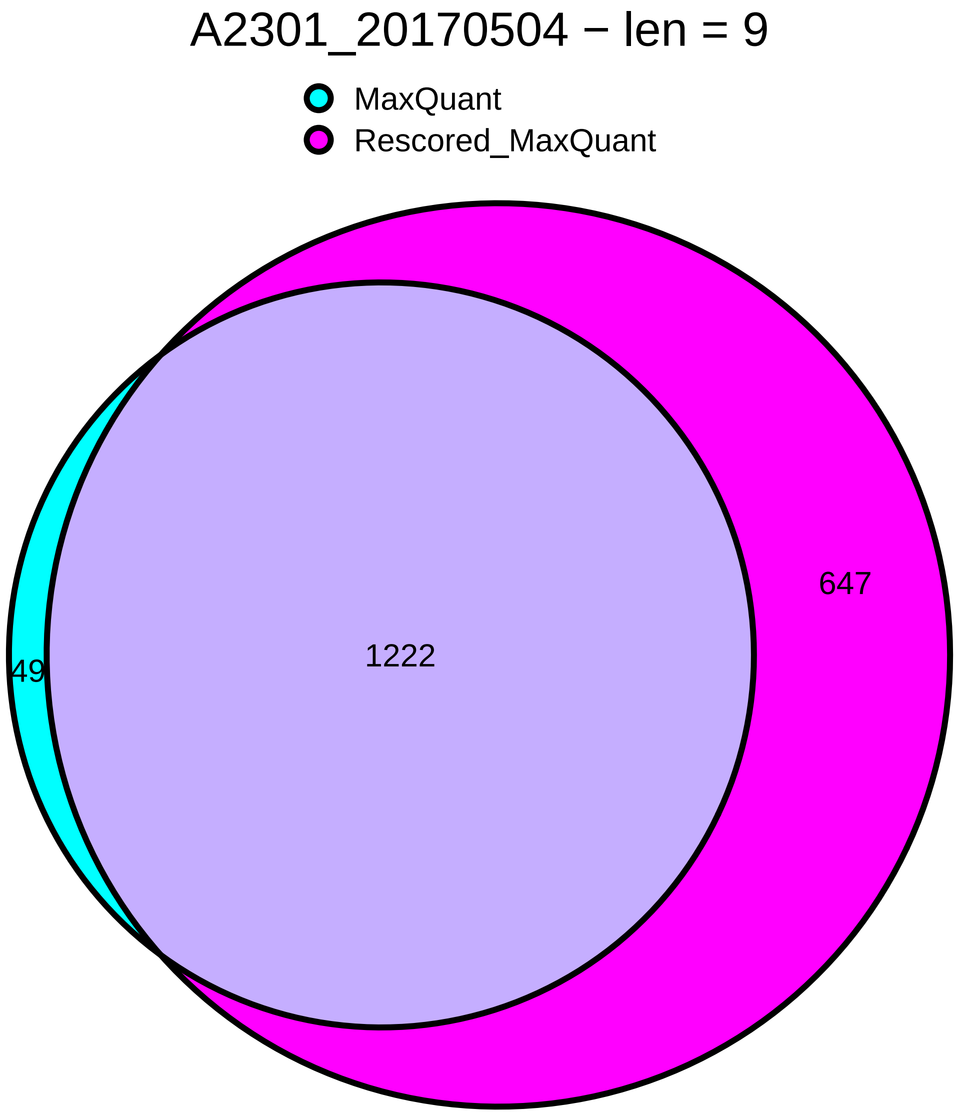
A2301_20170504 − len = 9
MaxQuant
Rescored_MaxQuant
49
1222
647

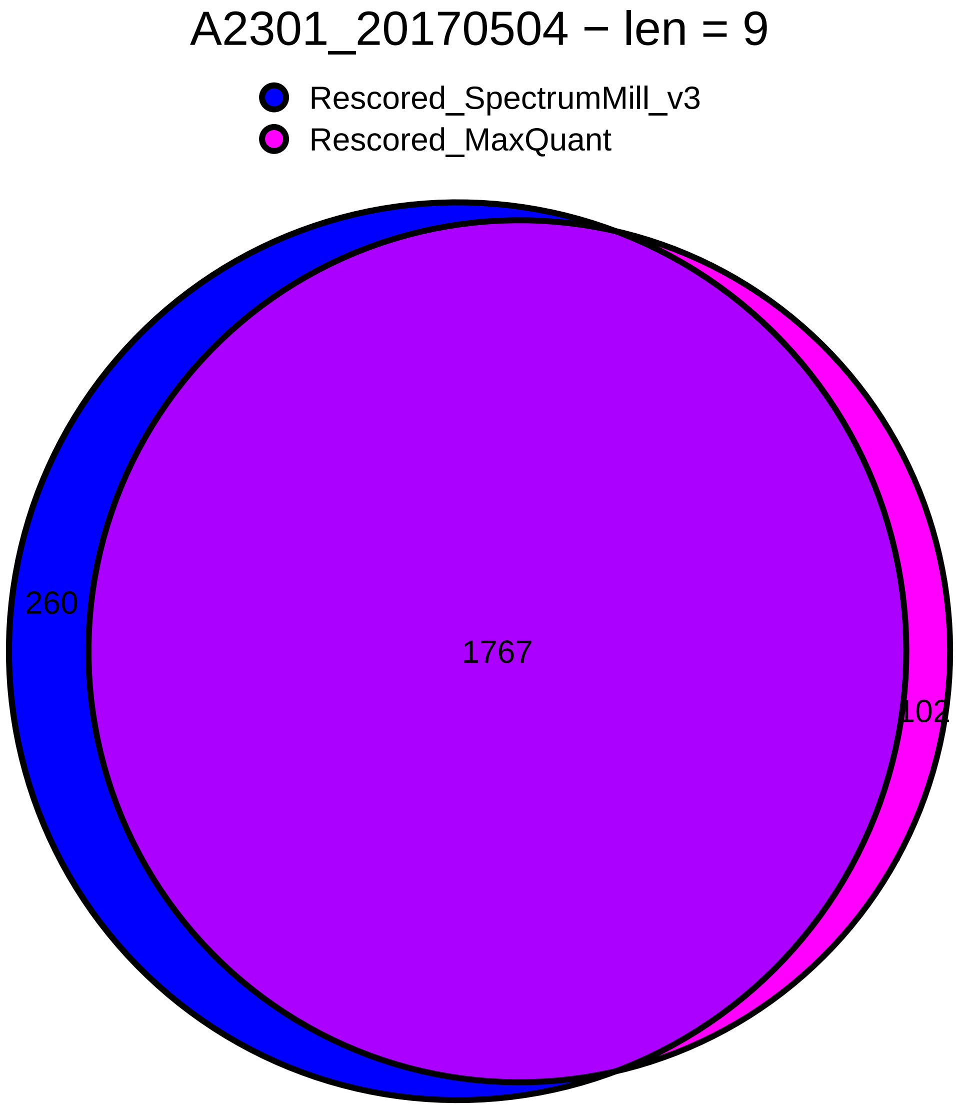
A2301_20170504 − len = 9
Rescored_SpectrumMill_v3
Rescored_MaxQuant
260
1767

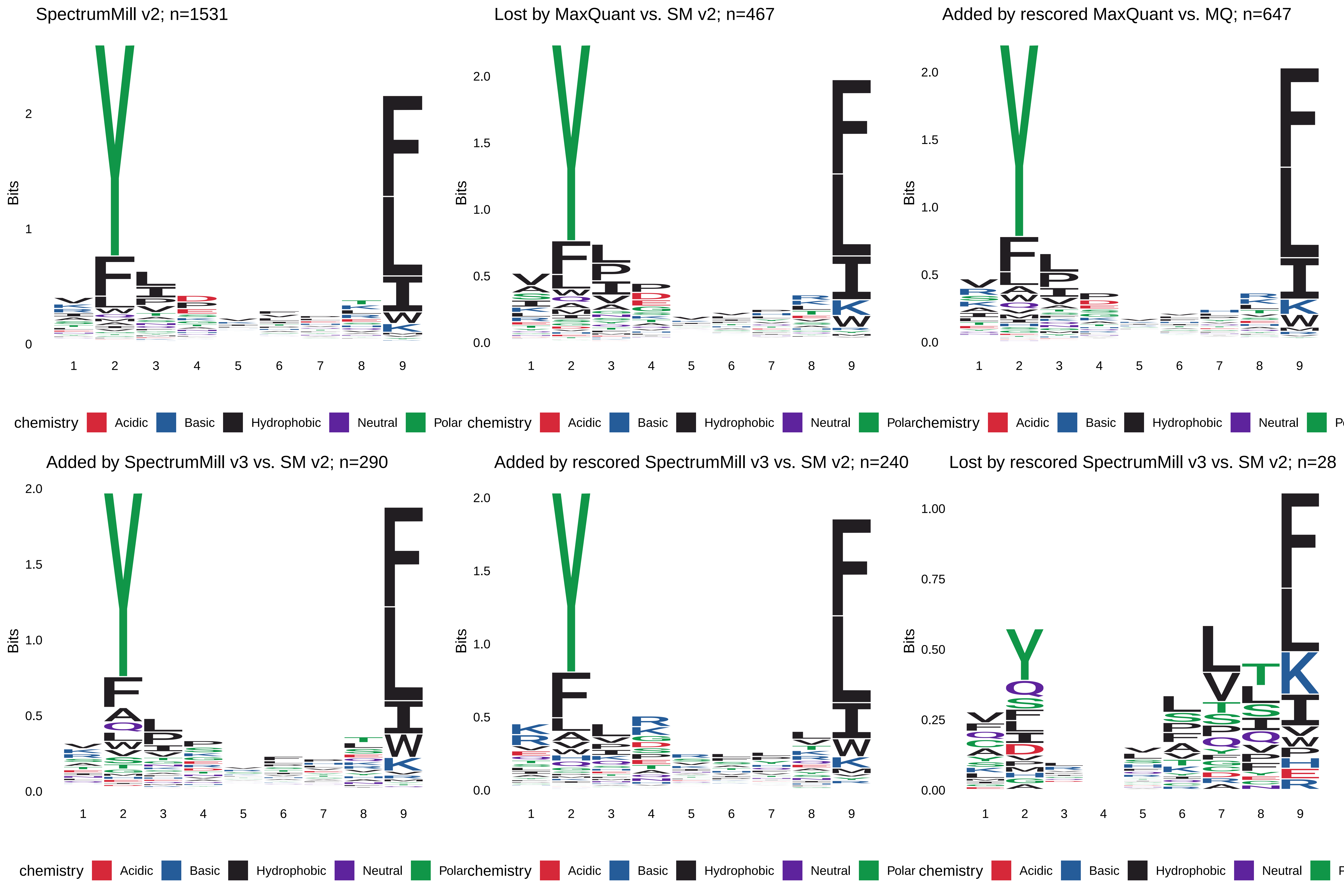
SpectrumMill v2; n=1531
Lost by MaxQuant vs. SM v2; n=467
Added by rescored MaxQuant vs. MQ; n=647
Added by SpectrumMill v3 vs. SM v2; n=290
Added by rescored SpectrumMill v3 vs. SM v2; n=240
Lost by rescored SpectrumMill v3 vs. SM v2; n=28
Bits
1 2 3 4 5 6 7 8 9
chemistry
Acidic
Basic
Hydrophobic
Neutral
Polar

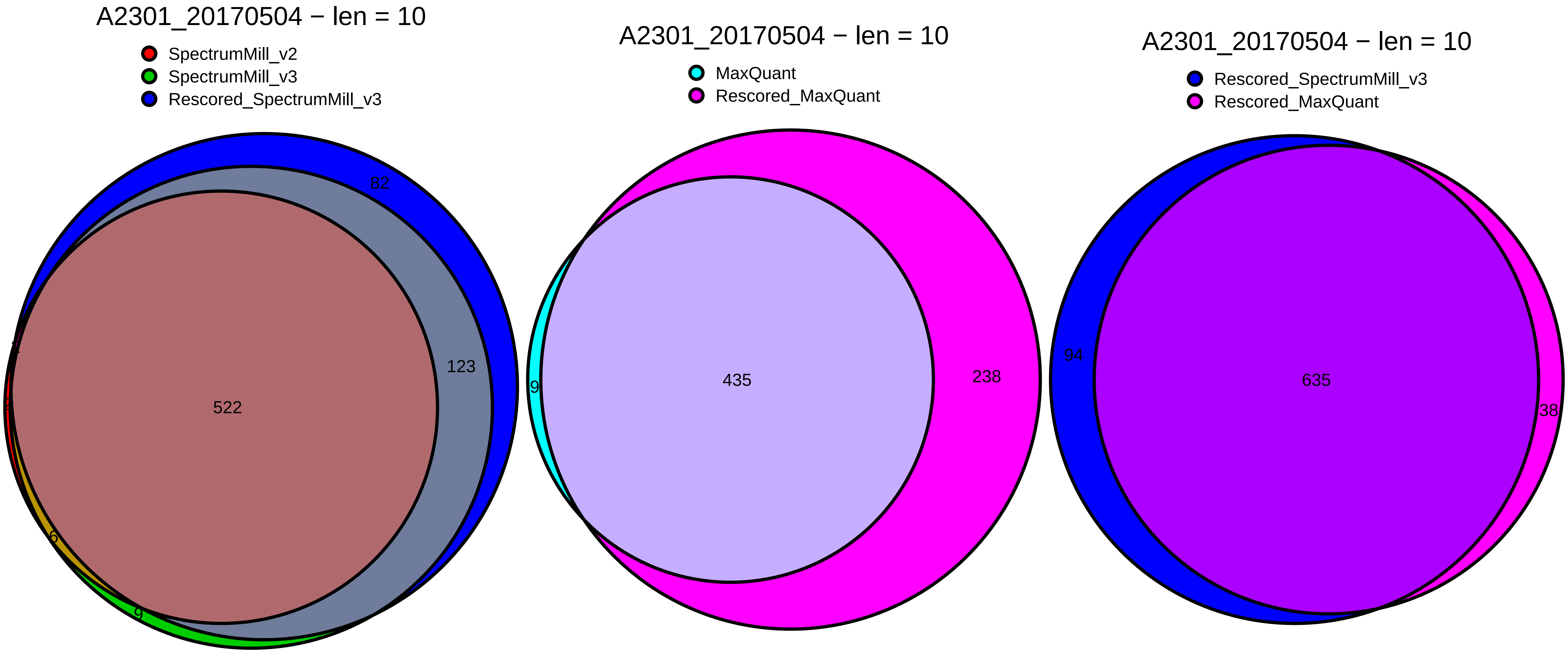
A2301_20170504 − len = 10
SpectrumMill_v2
SpectrumMill_v3
Rescored_SpectrumMill_v3
82
123
522
6
9
A2301_20170504 − len = 10
MaxQuant
Rescored_MaxQuant
9
435
238
A2301_20170504 − len = 10
Rescored_SpectrumMill_v3
Rescored_MaxQuant
94
635
38

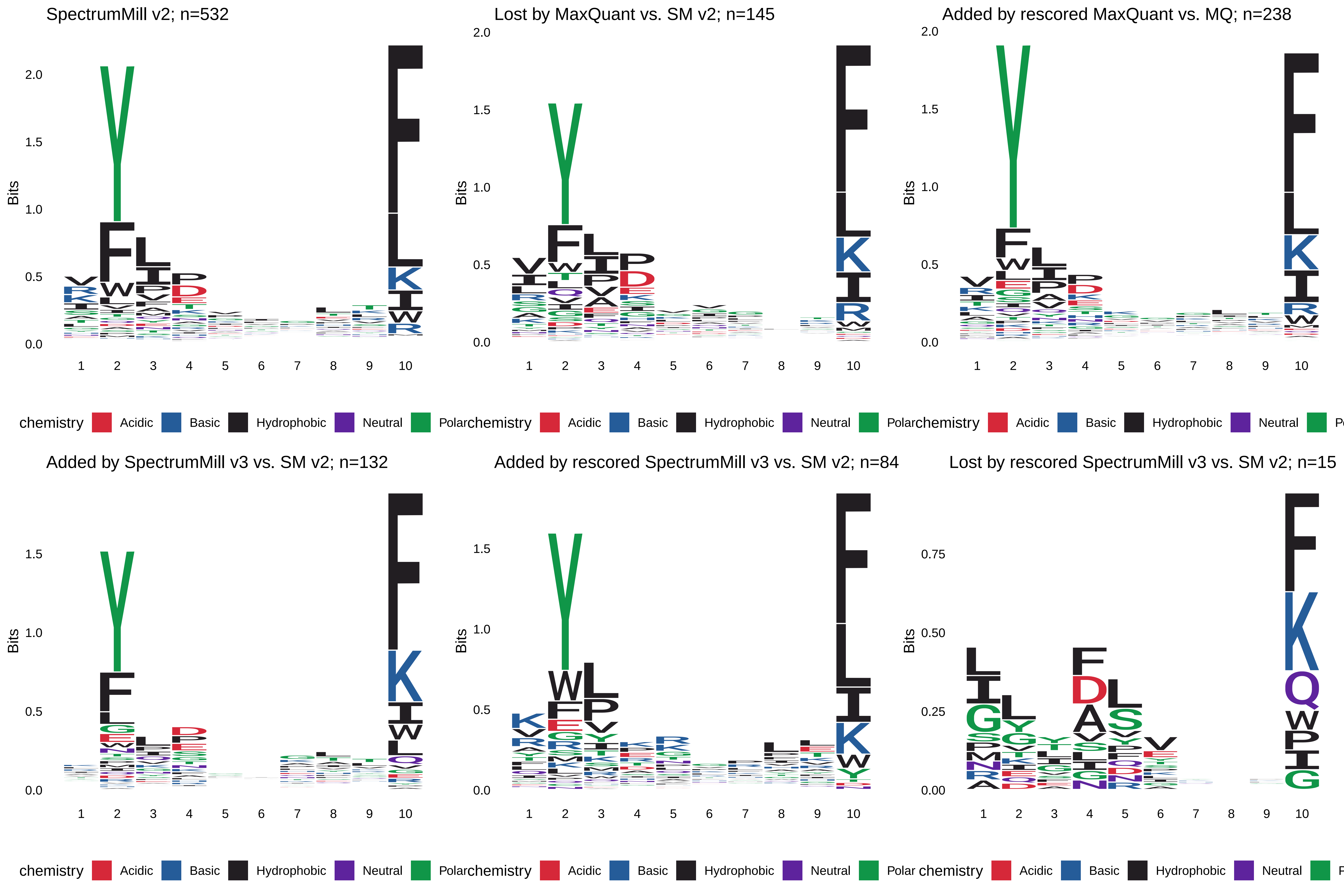

SpectrumMill v2; n=532
Lost by MaxQuant vs. SM v2; n=145
Added by rescored MaxQuant vs. MQ; n=238
Added by SpectrumMill v3 vs. SM v2; n=132
Added by rescored SpectrumMill v3 vs. SM v2; n=84
Lost by rescored SpectrumMill v3 vs. SM v2; n=15
Bits
1 2 3 4 5 6 7 8 9 10
chemistry
Acidic
Basic
Hydrophobic
Neutral
Polar

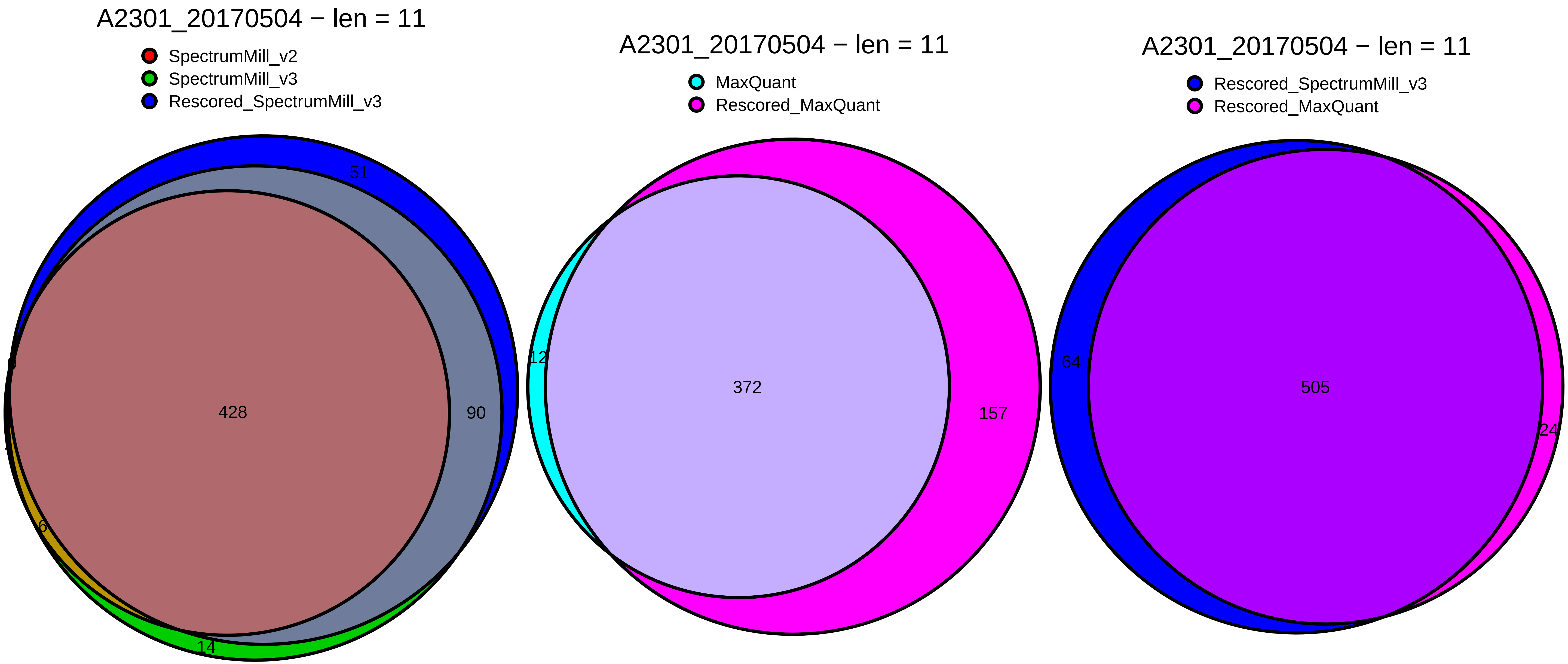
A2301_20170504 − len = 11
SpectrumMill_v2
SpectrumMill_v3
Rescored_SpectrumMill_v3
51
428
90
6
14
A2301_20170504 − len = 11
MaxQuant
Rescored_MaxQuant
12
372
157
A2301_20170504 − len = 11
Rescored_SpectrumMill_v3
Rescored_MaxQuant
64
505
24

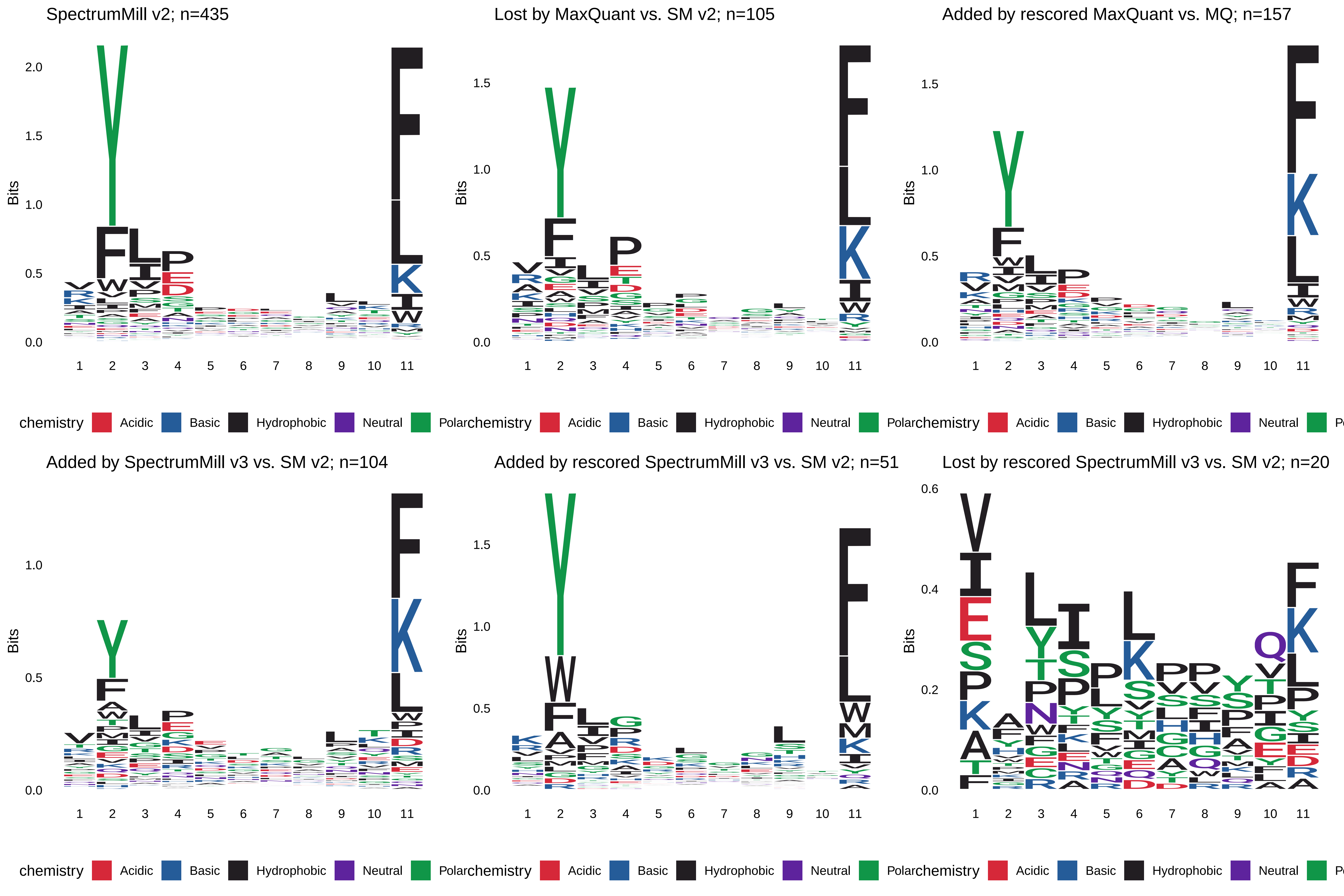
SpectrumMill v2; n=435
Lost by MaxQuant vs. SM v2; n=105
Added by rescored MaxQuant vs. MQ; n=157
Added by SpectrumMill v3 vs. SM v2; n=104
Added by rescored SpectrumMill v3 vs. SM v2; n=51
Lost by rescored SpectrumMill v3 vs. SM v2; n=20
Bits
1 2 3 4 5 6 7 8 9 10 11
chemistry
Acidic
Basic
Hydrophobic
Neutral
Polar

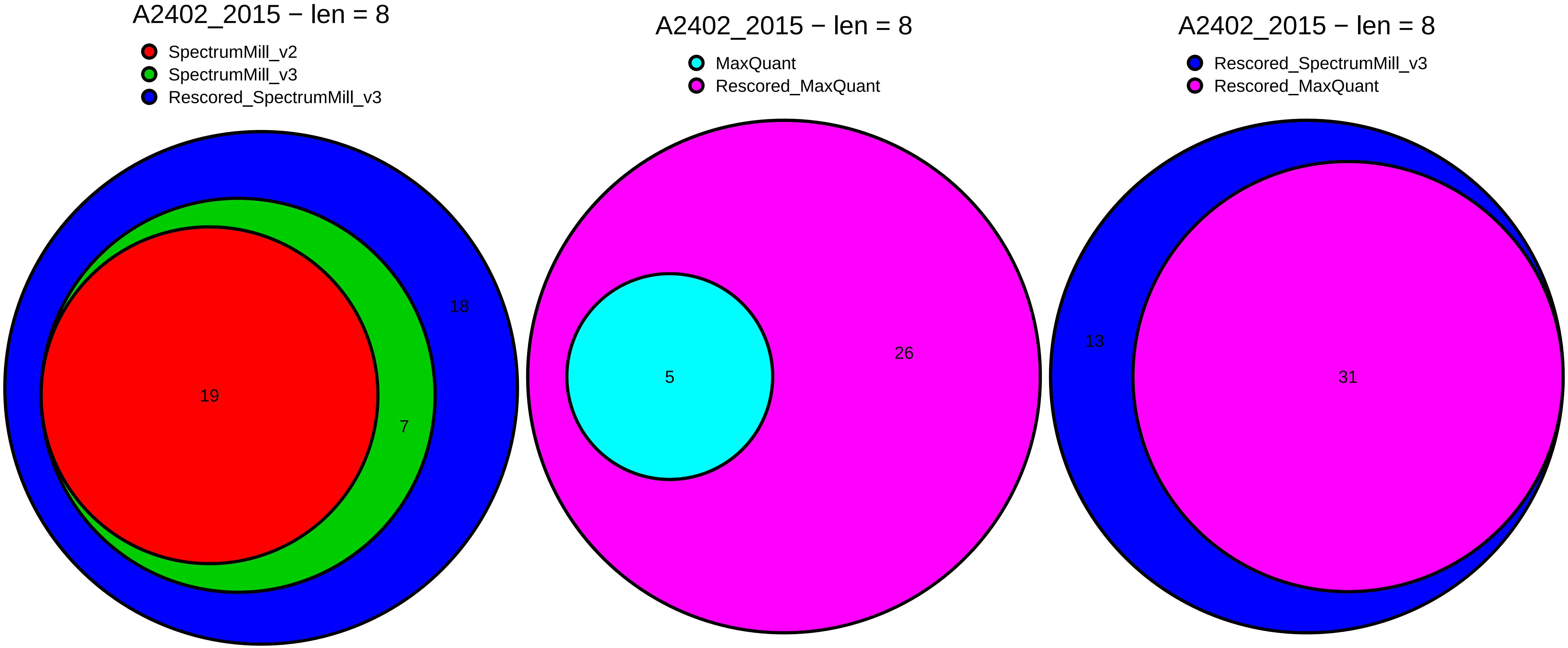
A2402_2015 − len = 8
SpectrumMill_v2
SpectrumMill_v3
Rescored_SpectrumMill_v3
18
19
7
A2402_2015 − len = 8
MaxQuant
Rescored_MaxQuant
5
26
A2402_2015 − len = 8
Rescored_SpectrumMill_v3
Rescored_MaxQuant
13
31

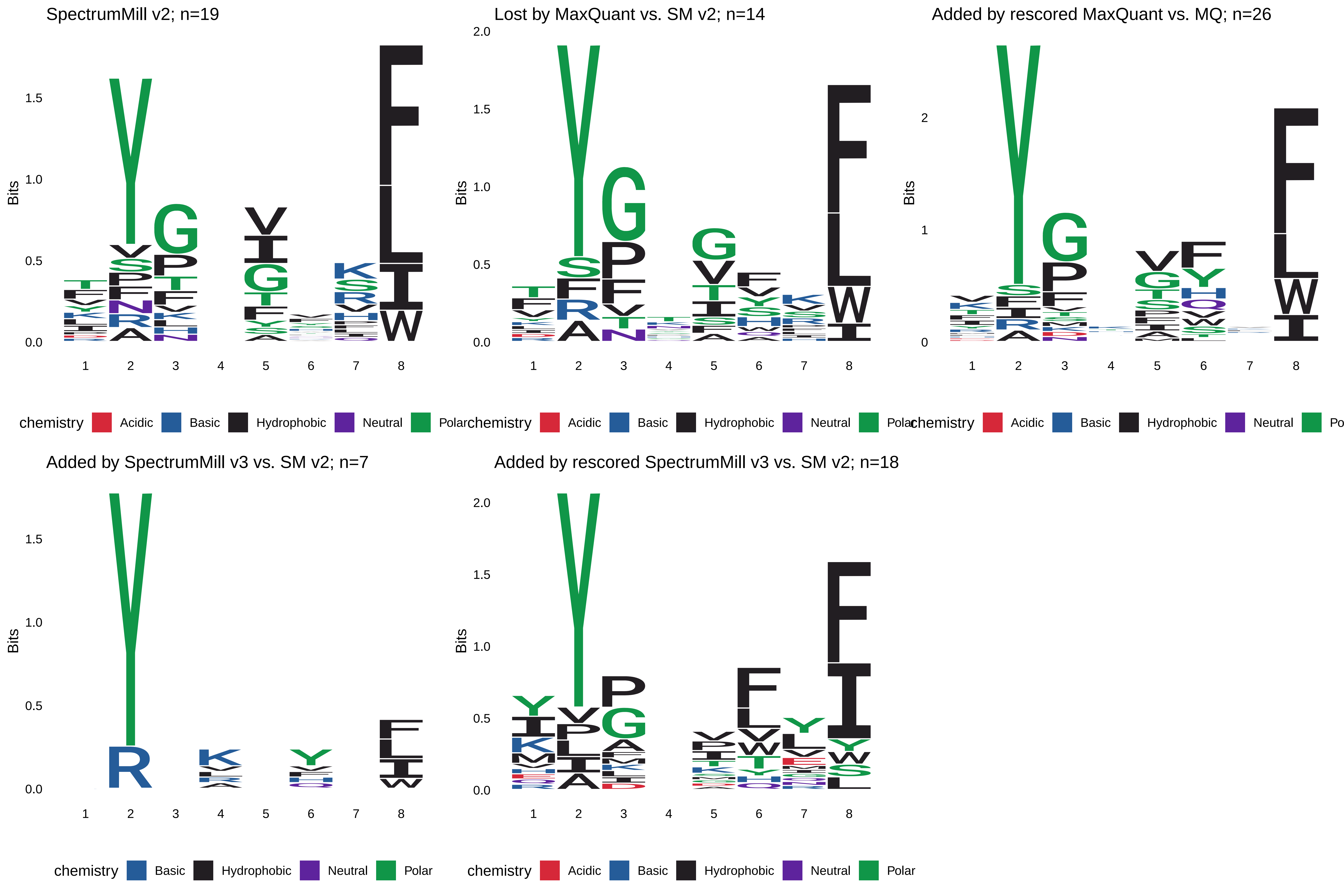
SpectrumMill v2; n=19
Lost by MaxQuant vs. SM v2; n=14
Added by rescored MaxQuant vs. MQ; n=26
Added by SpectrumMill v3 vs. SM v2; n=7
Added by rescored SpectrumMill v3 vs. SM v2; n=18
Bits
0.0
0.5
1.0
1.5
2.0
0
1
2
1 2 3 4 5 6 7 8
chemistry Acidic Basic Hydrophobic Neutral Polar
chemistry Basic Hydrophobic Neutral Polar

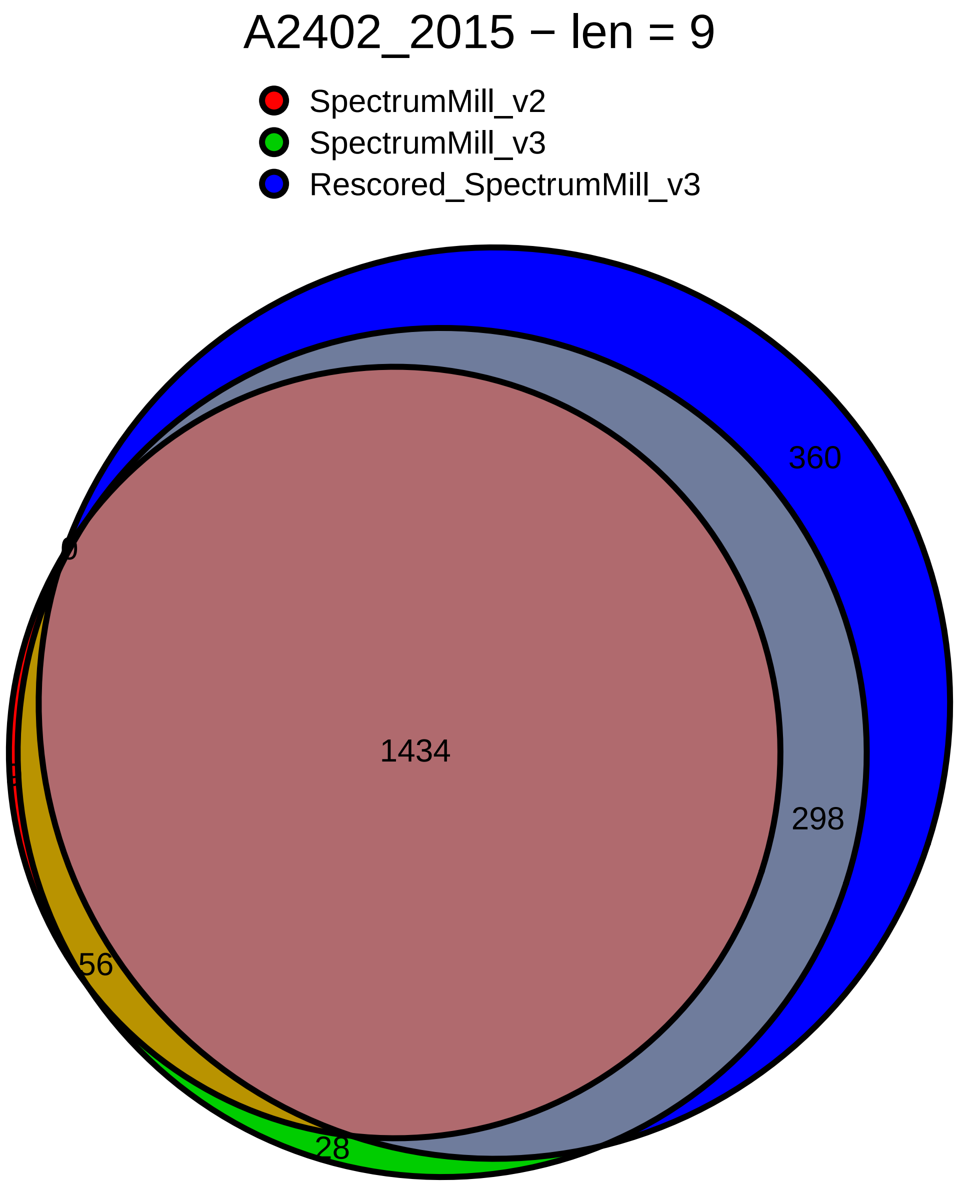
A2402_2015 − len = 9
SpectrumMill_v2
SpectrumMill_v3
Rescored_SpectrumMill_v3
360
0
1434
298
56
28

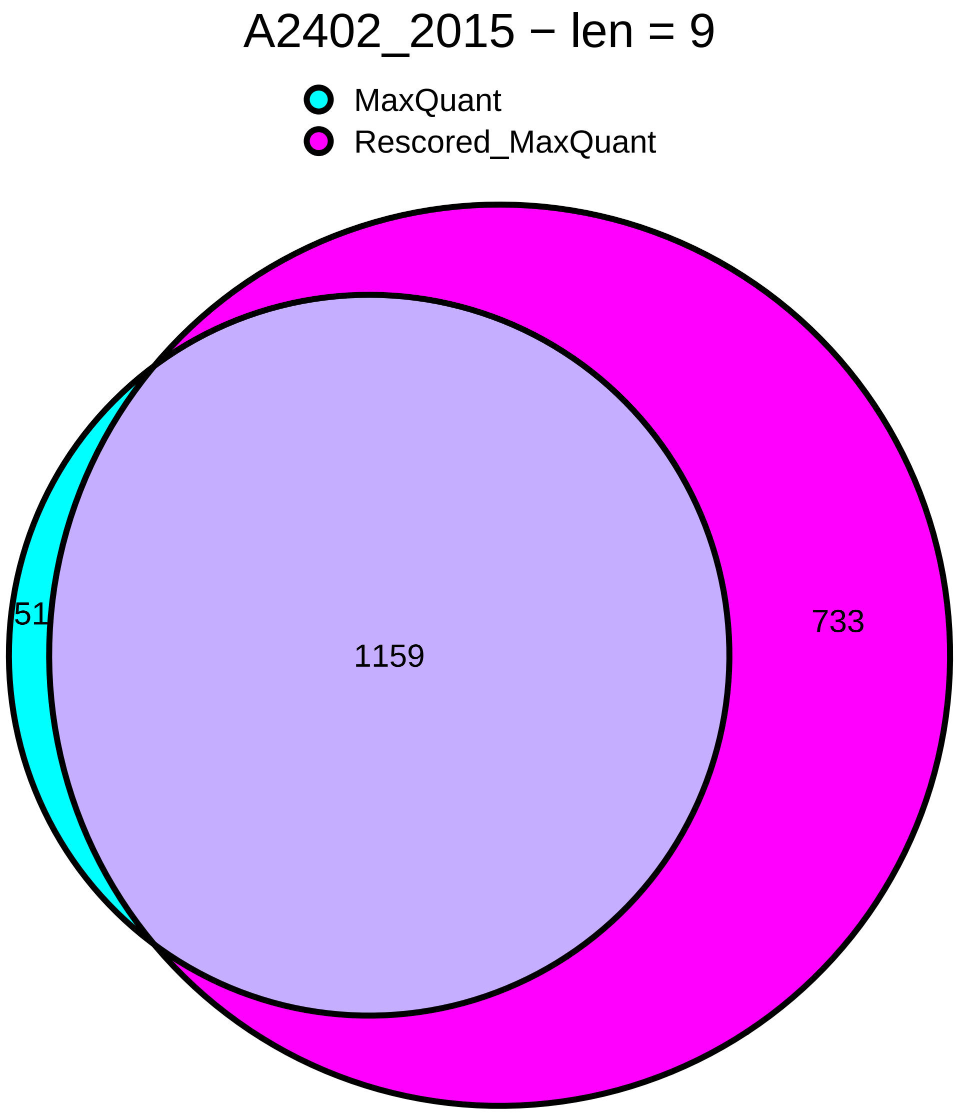
A2402_2015 − len = 9
MaxQuant
Rescored_MaxQuant
51
1159
733

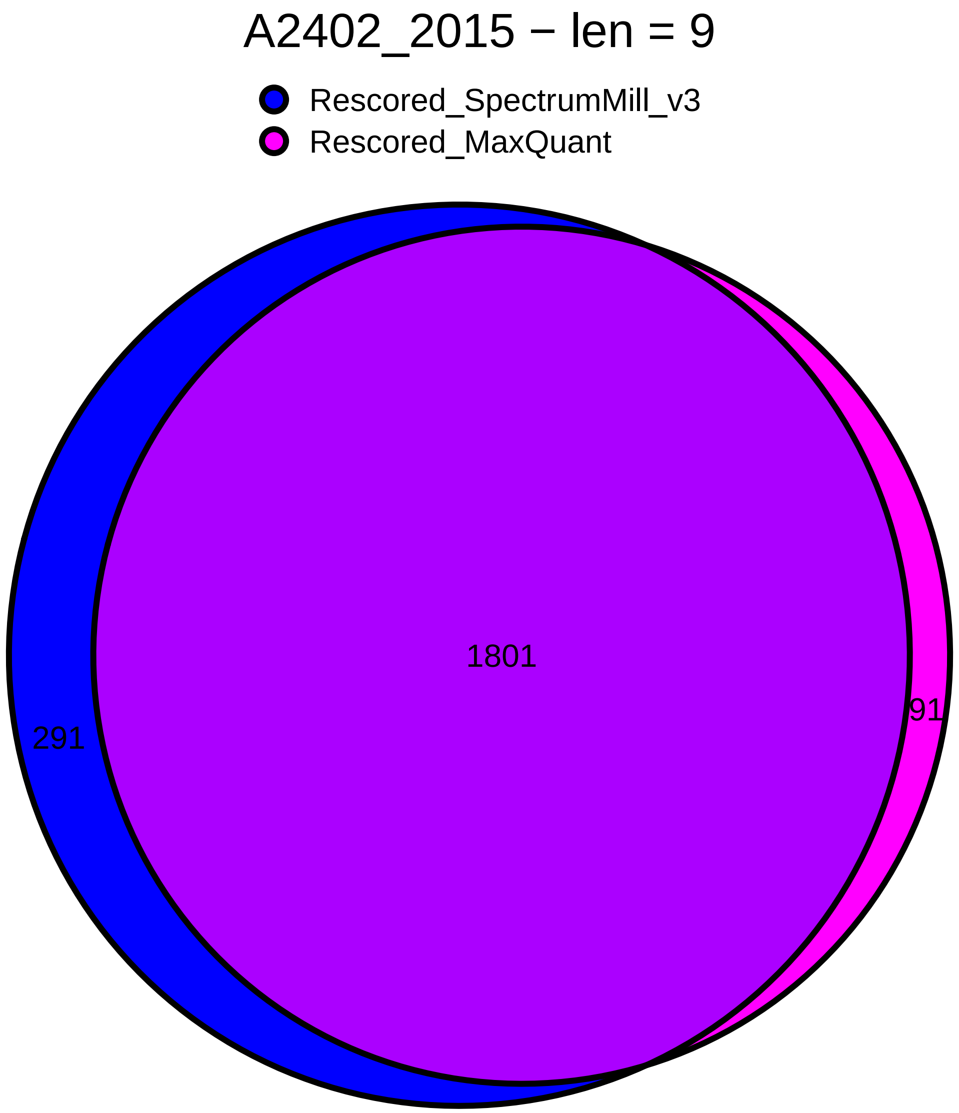
A2402_2015 − len = 9
Rescored_SpectrumMill_v3
Rescored_MaxQuant
1801
291
91

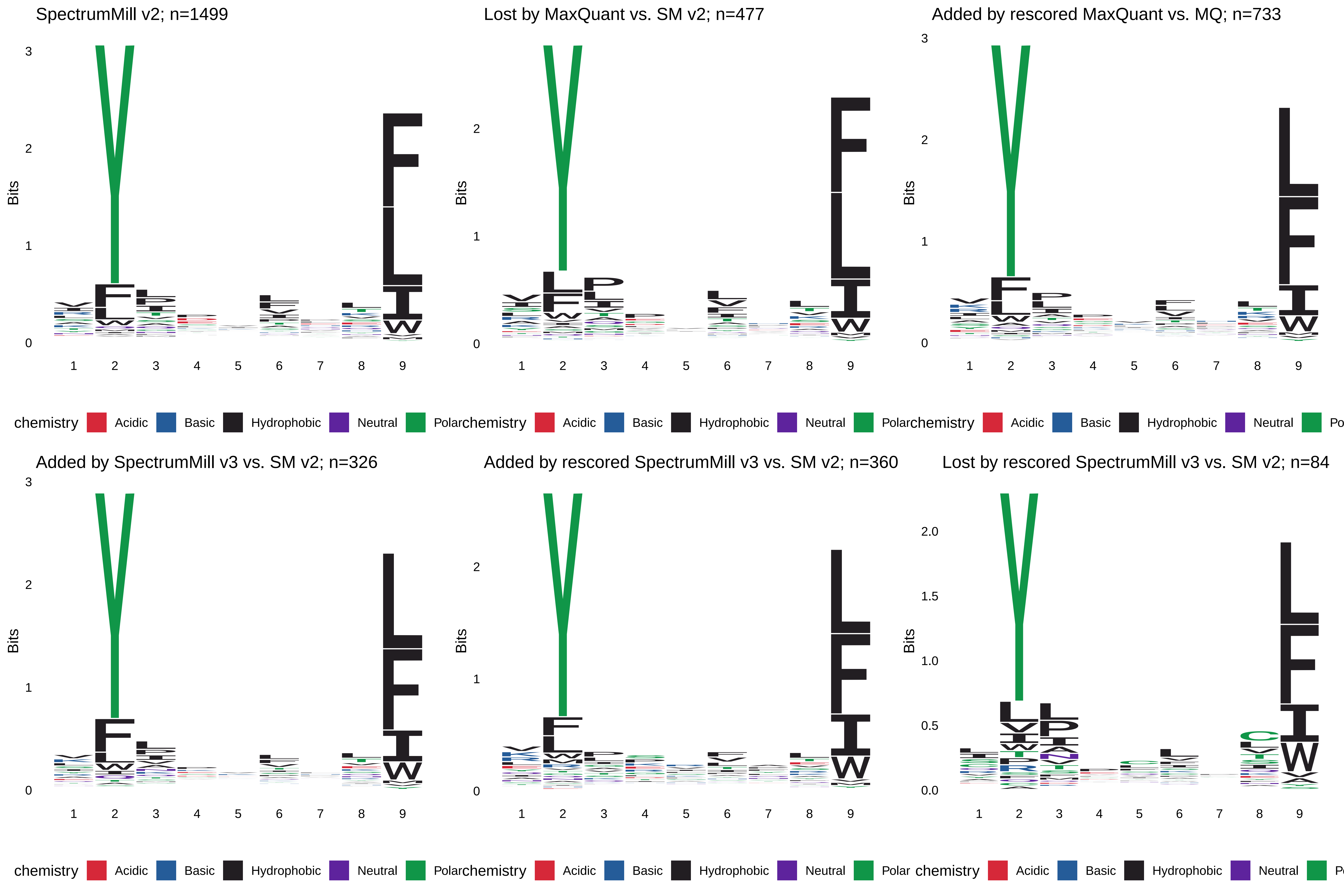
SpectrumMill v2; n=1499
Lost by MaxQuant vs. SM v2; n=477
Added by rescored MaxQuant vs. MQ; n=733
Bits
0
1
2
3
1 2 3 4 5 6 7 8 9
chemistry
Acidic
Basic
Hydrophobic
Neutral
Polar
Added by SpectrumMill v3 vs. SM v2; n=326
Added by rescored SpectrumMill v3 vs. SM v2; n=360
Lost by rescored SpectrumMill v3 vs. SM v2; n=84
0.0
0.5
1.0
1.5
2.0

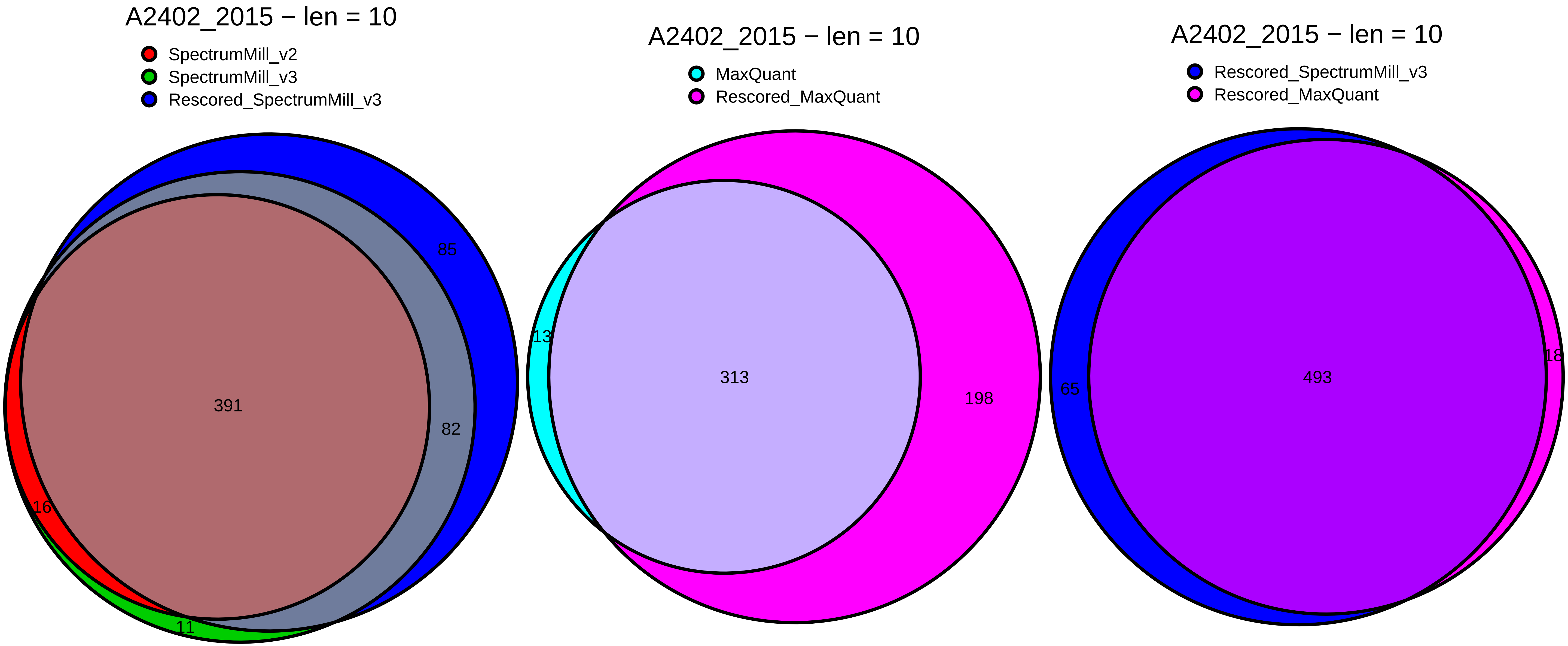
A2402_2015 − len = 10
SpectrumMill_v2
SpectrumMill_v3
Rescored_SpectrumMill_v3
85
391
82
16
11
A2402_2015 − len = 10
MaxQuant
Rescored_MaxQuant
13
313
198
A2402_2015 − len = 10
Rescored_SpectrumMill_v3
Rescored_MaxQuant
65
493
18

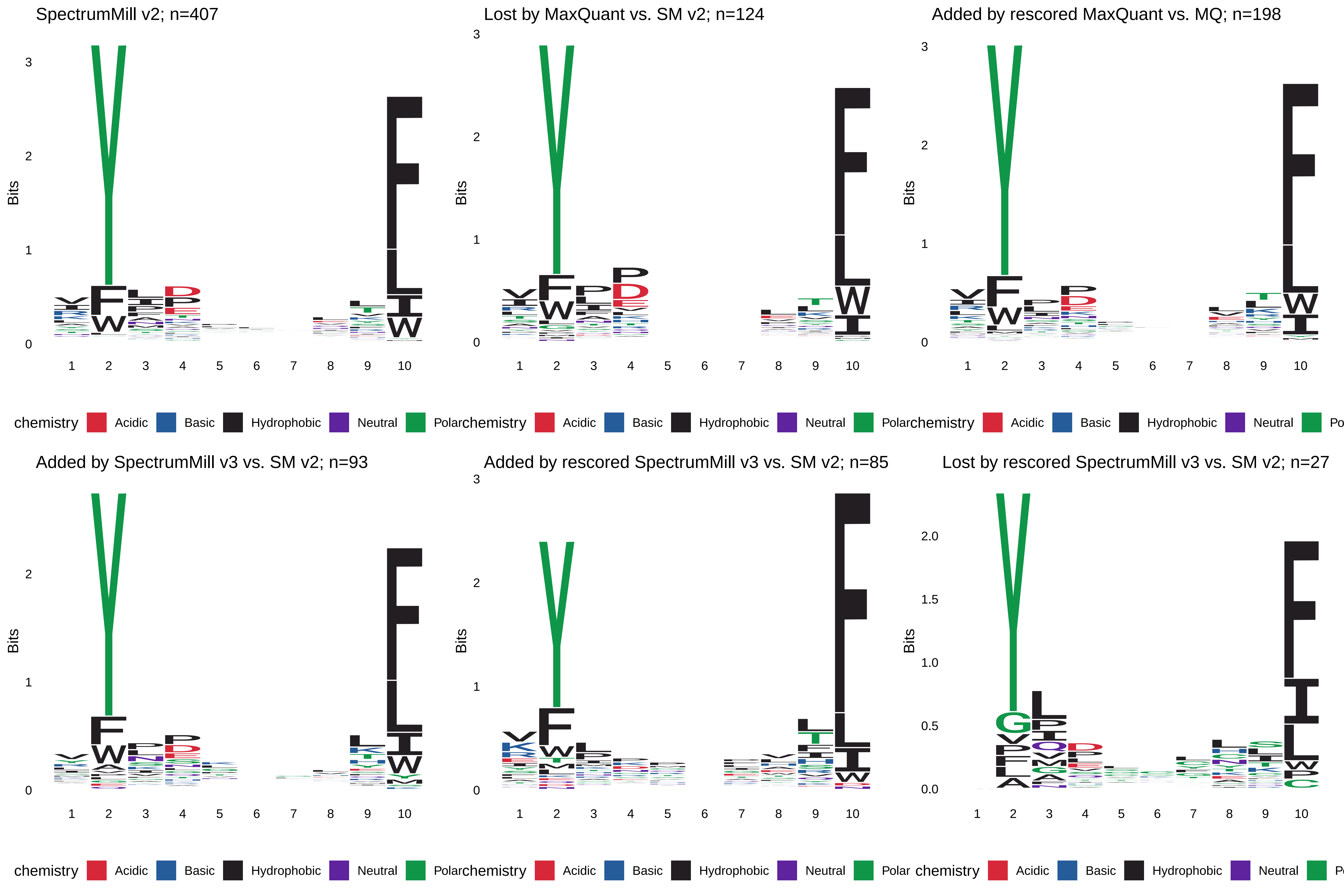
SpectrumMill v2; n=407
Lost by MaxQuant vs. SM v2; n=124
Added by rescored MaxQuant vs. MQ; n=198
Added by SpectrumMill v3 vs. SM v2; n=93
Added by rescored SpectrumMill v3 vs. SM v2; n=85
Lost by rescored SpectrumMill v3 vs. SM v2; n=27
Bits
1 2 3 4 5 6 7 8 9 10
chemistry
Acidic
Basic
Hydrophobic
Neutral
Polar

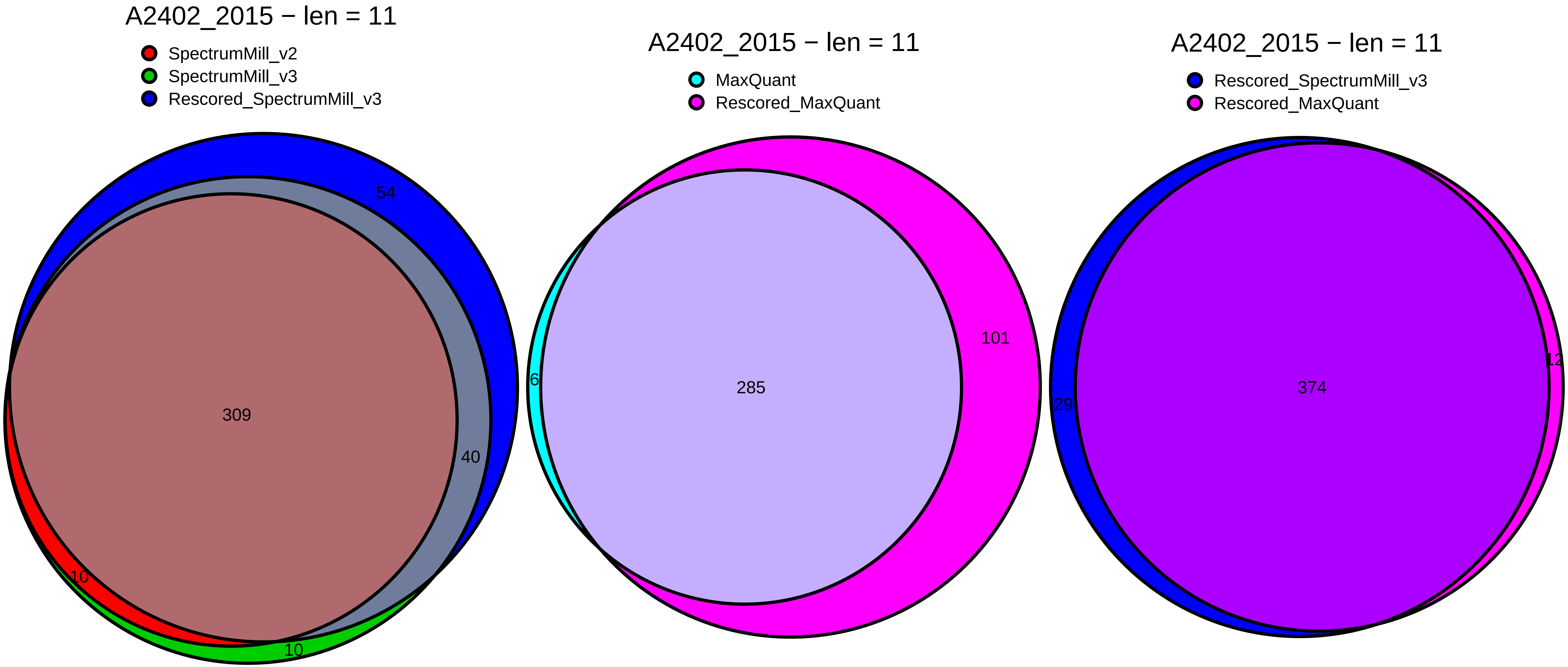

A2402_2015 − len = 11
SpectrumMill_v2
SpectrumMill_v3
Rescored_SpectrumMill_v3
54
309
40
10
10
A2402_2015 − len = 11
MaxQuant
Rescored_MaxQuant
6
285
101
A2402_2015 − len = 11
Rescored_SpectrumMill_v3
Rescored_MaxQuant
29
374
12

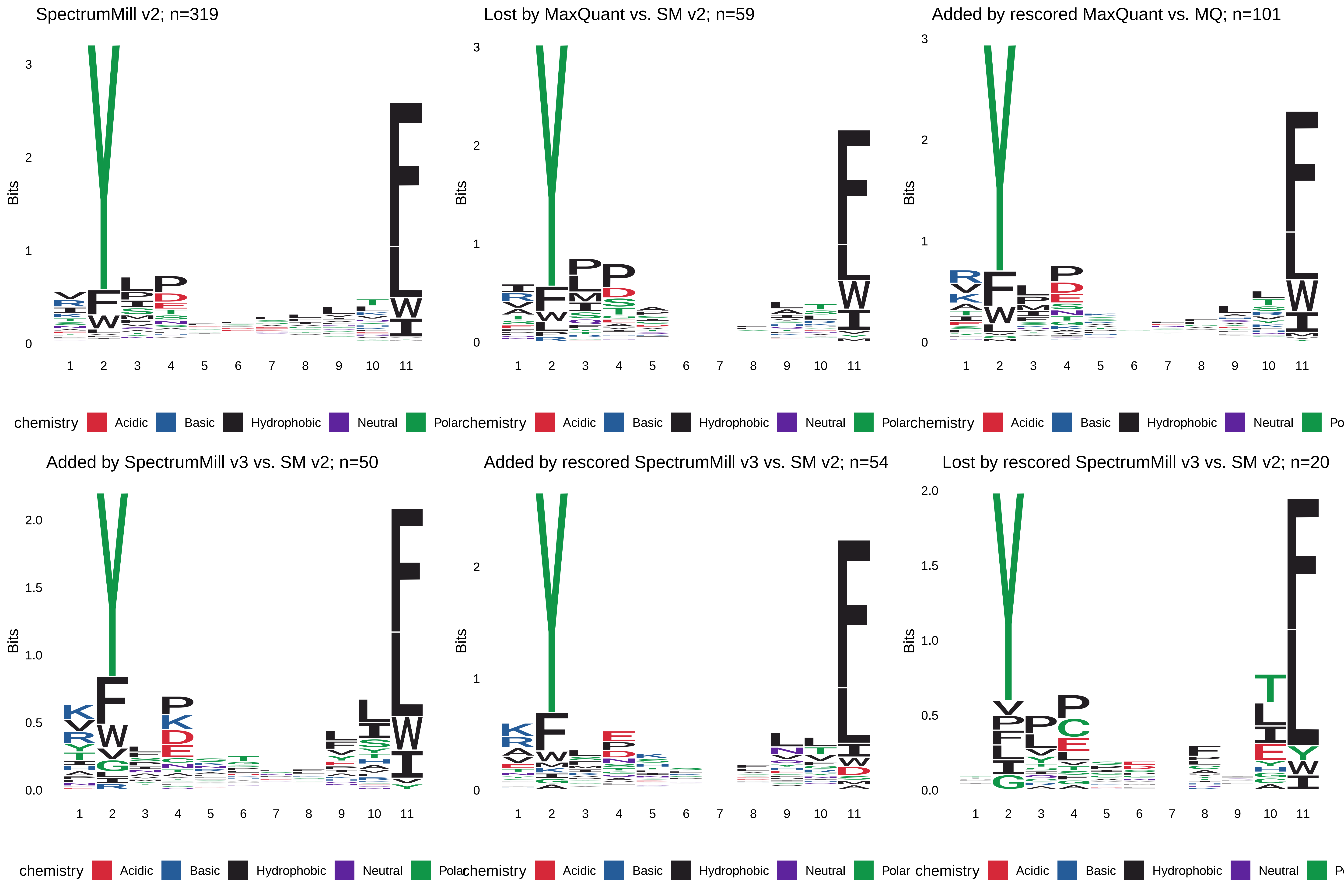

SpectrumMill v2; n=319
Lost by MaxQuant vs. SM v2; n=59
Added by rescored MaxQuant vs. MQ; n=101
Added by SpectrumMill v3 vs. SM v2; n=50
Added by rescored SpectrumMill v3 vs. SM v2; n=54
Lost by rescored SpectrumMill v3 vs. SM v2; n=20
Bits
chemistry
Acidic
Basic
Hydrophobic
Neutral
Polar

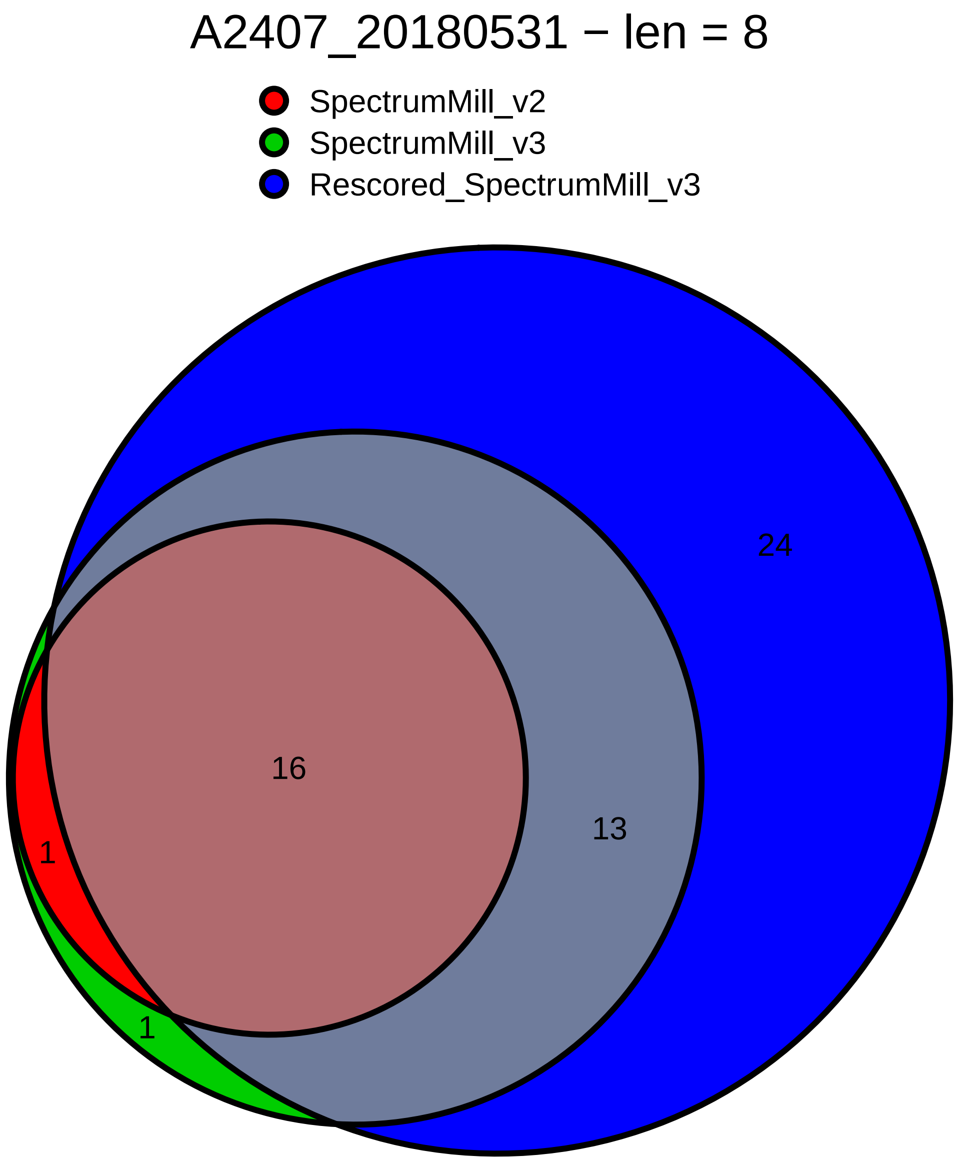
A2407_20180531 − len = 8
SpectrumMill_v2
SpectrumMill_v3
Rescored_SpectrumMill_v3
24
16
13
1
1

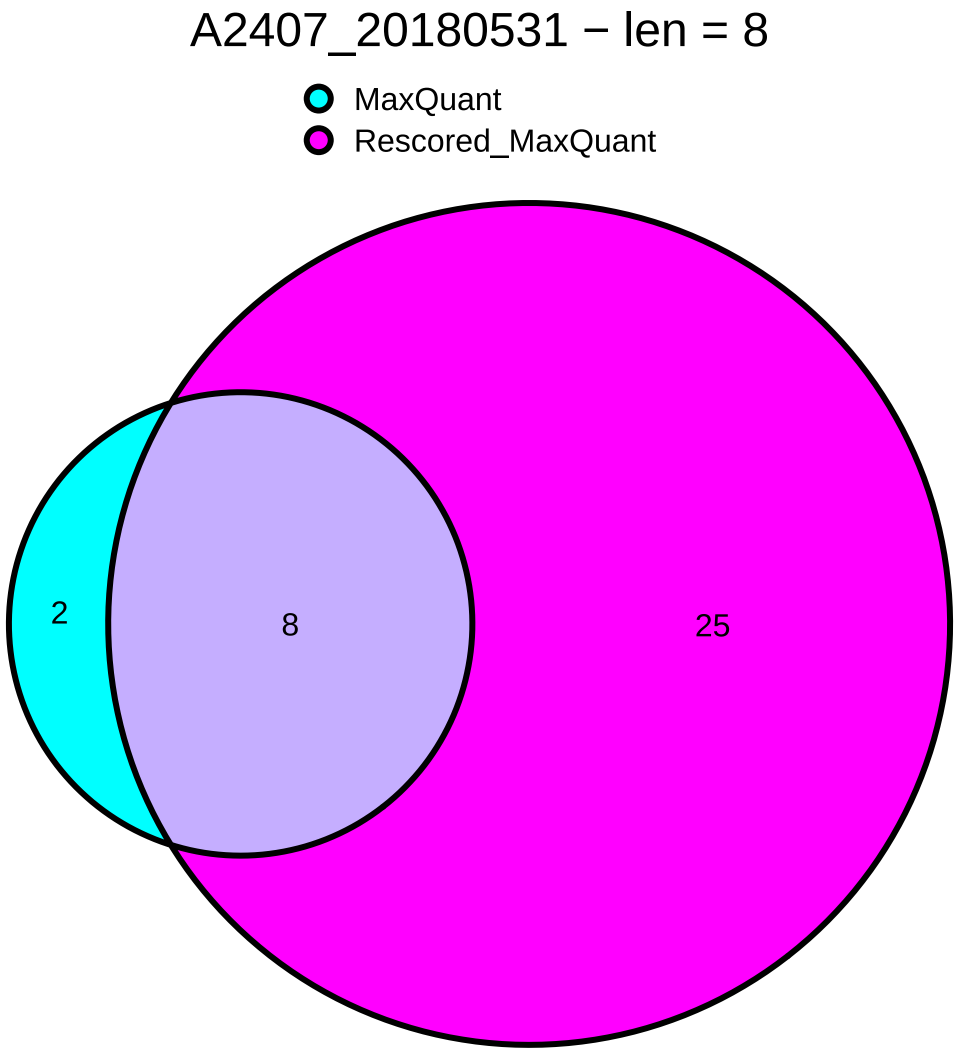
A2407_20180531 − len = 8
MaxQuant
Rescored_MaxQuant
2
8
25

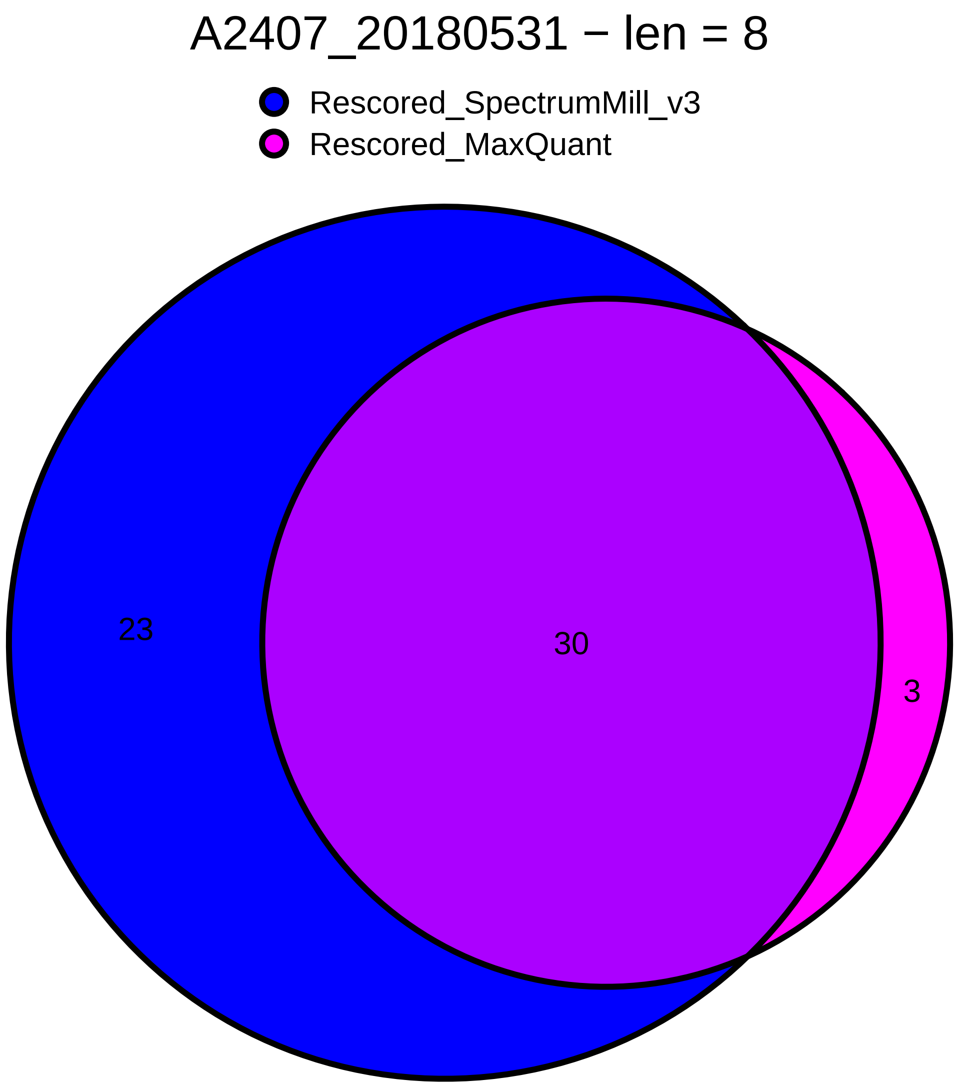
A2407_20180531 − len = 8
Rescored_SpectrumMill_v3
Rescored_MaxQuant
23
30
3

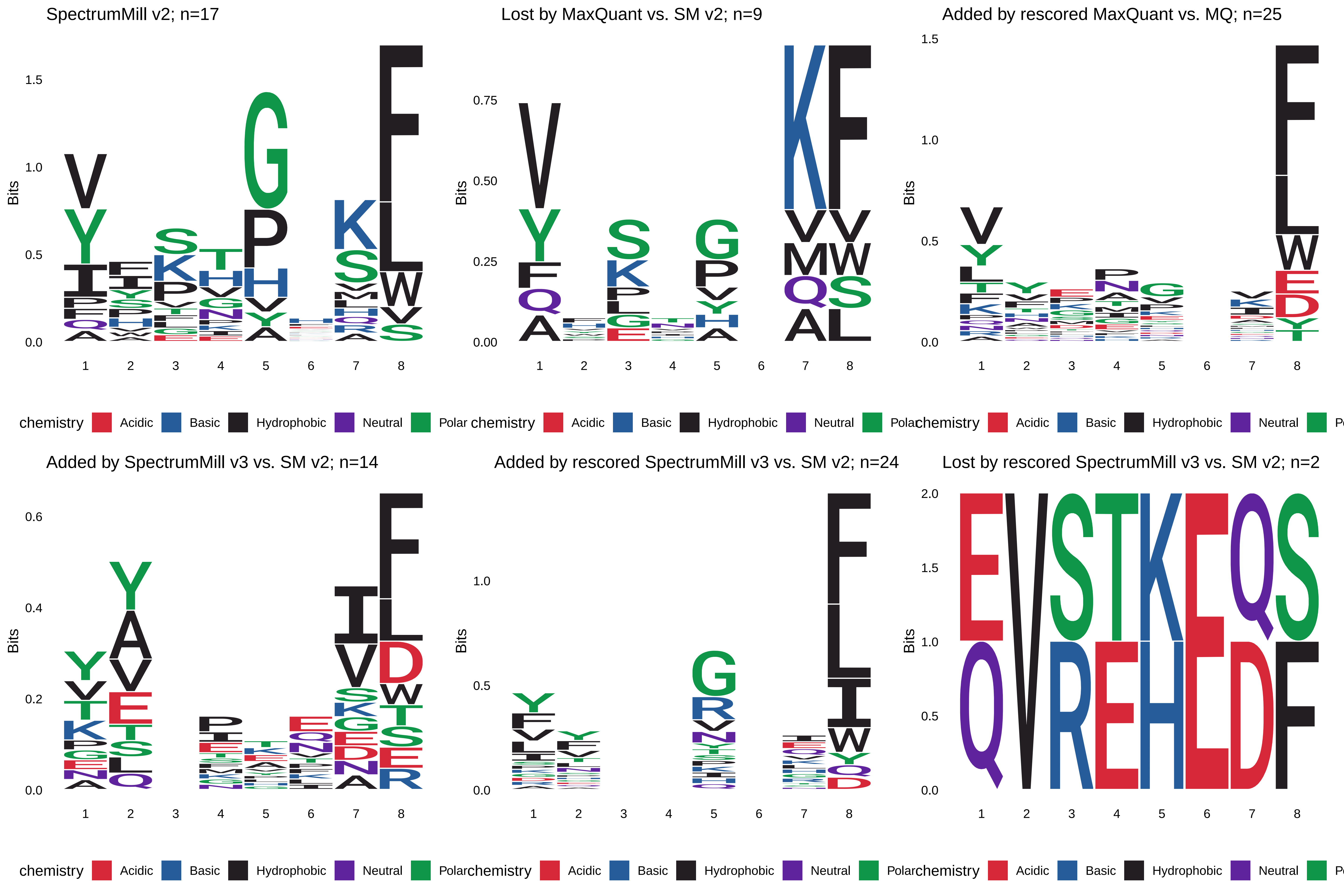
SpectrumMill v2; n=17
Lost by MaxQuant vs. SM v2; n=9
Added by rescored MaxQuant vs. MQ; n=25
Added by SpectrumMill v3 vs. SM v2; n=14
Added by rescored SpectrumMill v3 vs. SM v2; n=24
Lost by rescored SpectrumMill v3 vs. SM v2; n=2
Bits
1 2 3 4 5 6 7 8
chemistry Acidic Basic Hydrophobic Neutral Polar

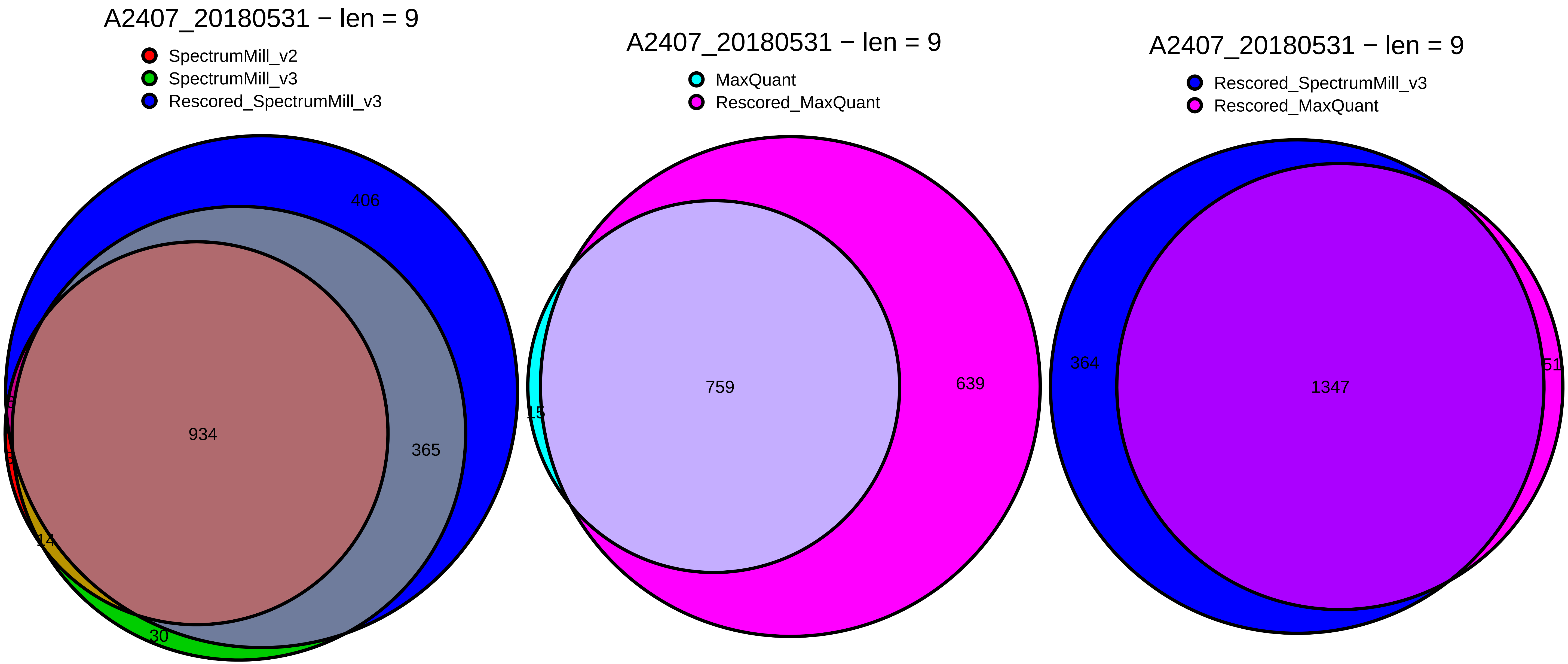

A2407_20180531 − len = 9
SpectrumMill_v2
SpectrumMill_v3
Rescored_SpectrumMill_v3
406
934
365
14
30
A2407_20180531 − len = 9
MaxQuant
Rescored_MaxQuant
759
639
15
A2407_20180531 − len = 9
Rescored_SpectrumMill_v3
Rescored_MaxQuant
364
1347
51

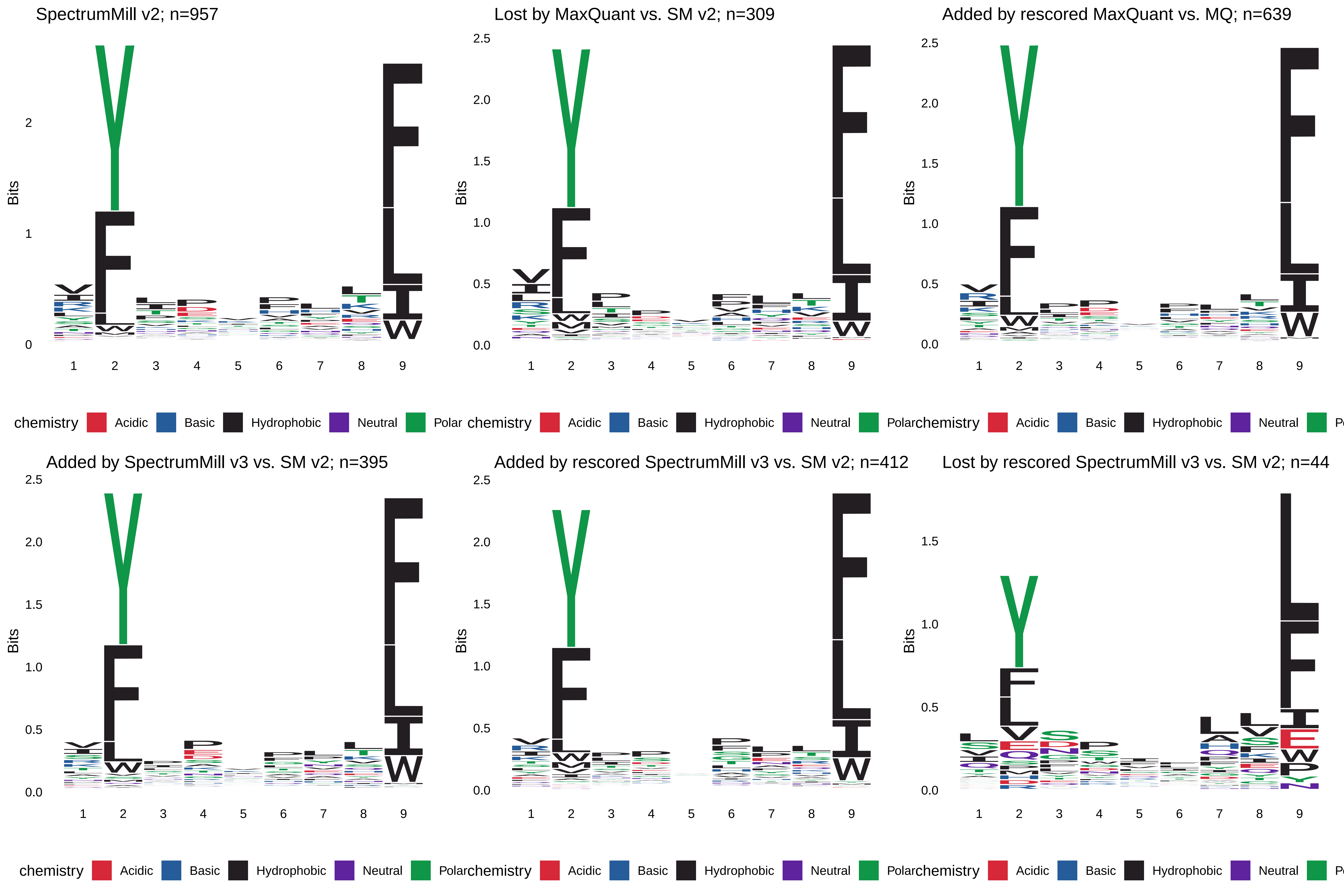
SpectrumMill v2; n=957
Lost by MaxQuant vs. SM v2; n=309
Added by rescored MaxQuant vs. MQ; n=639
Added by SpectrumMill v3 vs. SM v2; n=395
Added by rescored SpectrumMill v3 vs. SM v2; n=412
Lost by rescored SpectrumMill v3 vs. SM v2; n=44
Bits
1 2 3 4 5 6 7 8 9
chemistry
Acidic
Basic
Hydrophobic
Neutral
Polar

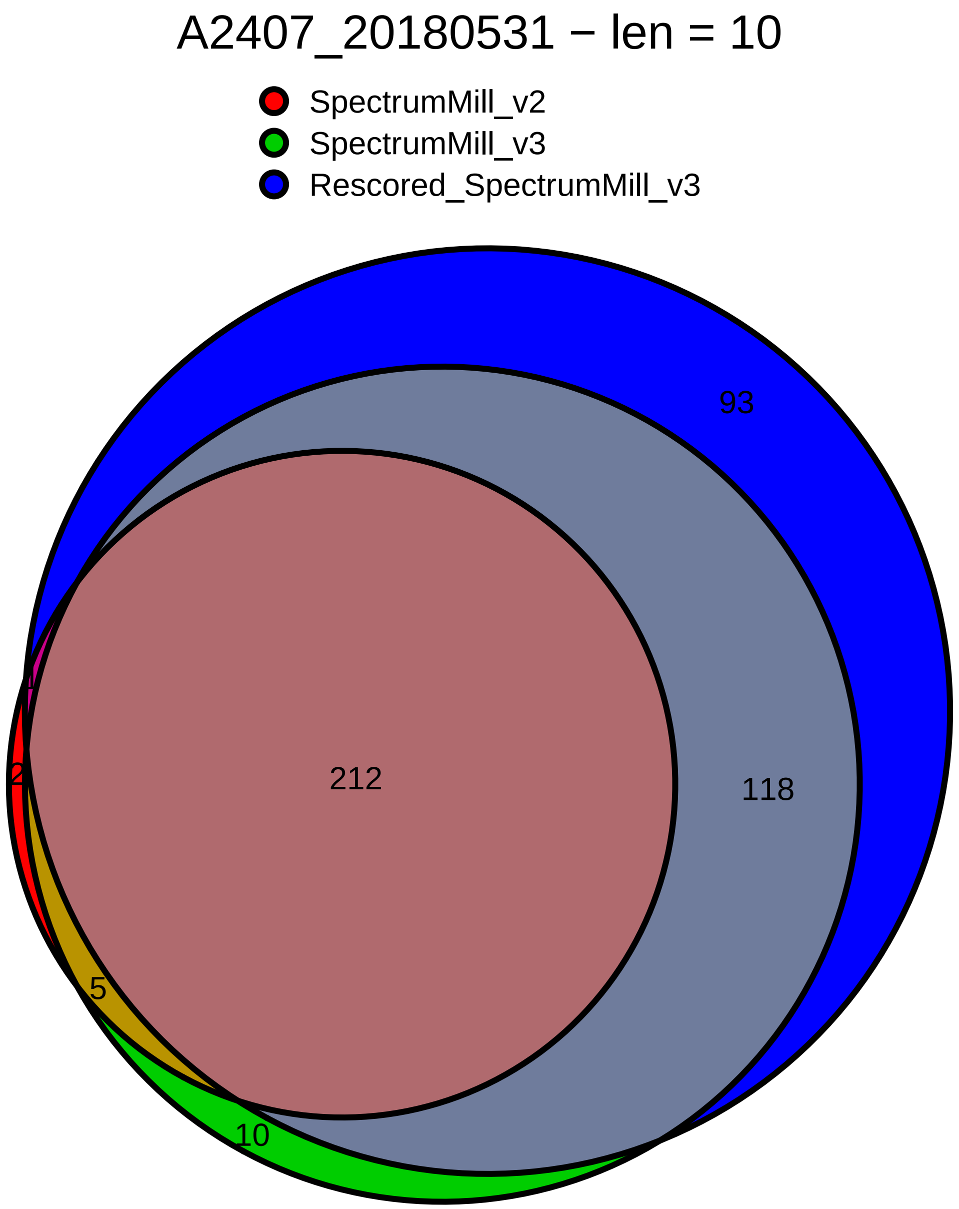
A2407_20180531 − len = 10
SpectrumMill_v2
SpectrumMill_v3
Rescored_SpectrumMill_v3
93
212
118
2
5
10

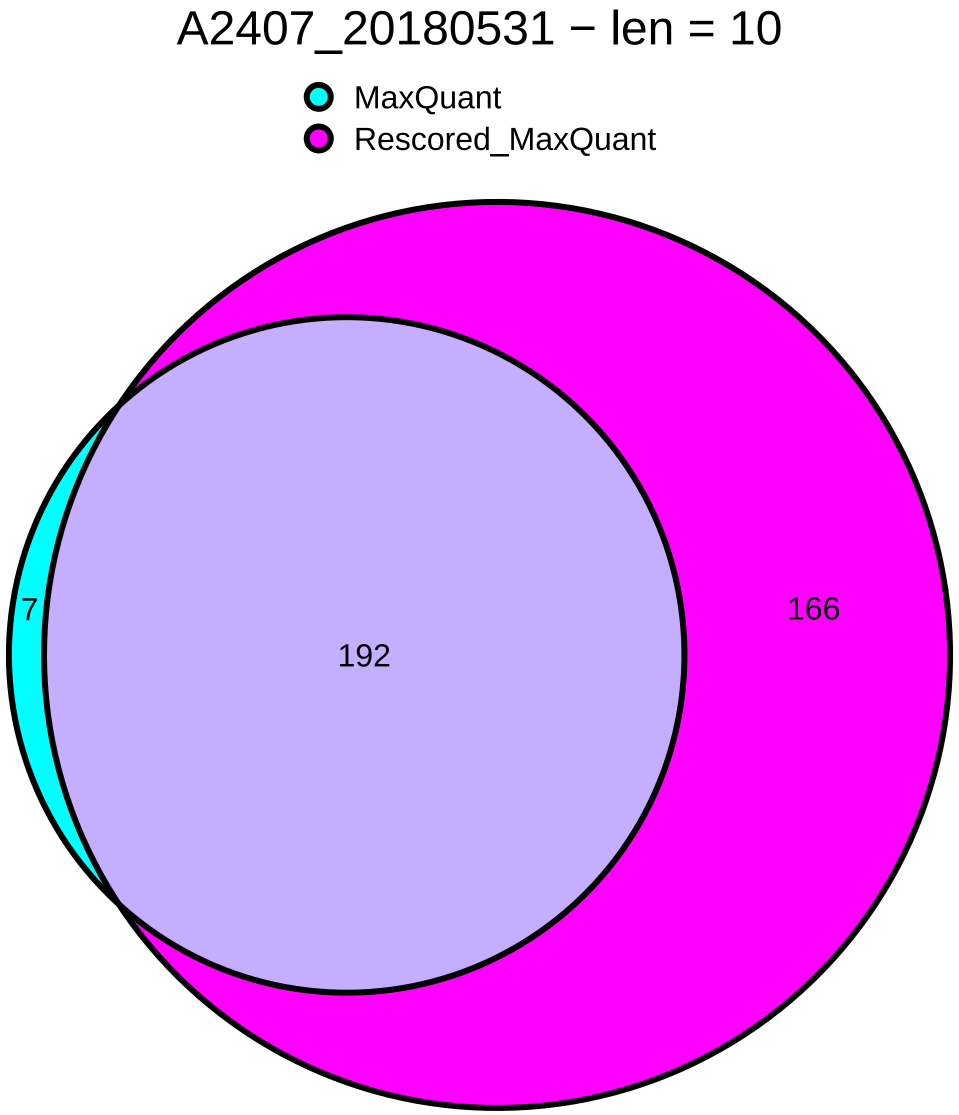
A2407_20180531 − len = 10
MaxQuant
Rescored_MaxQuant
7
192
166

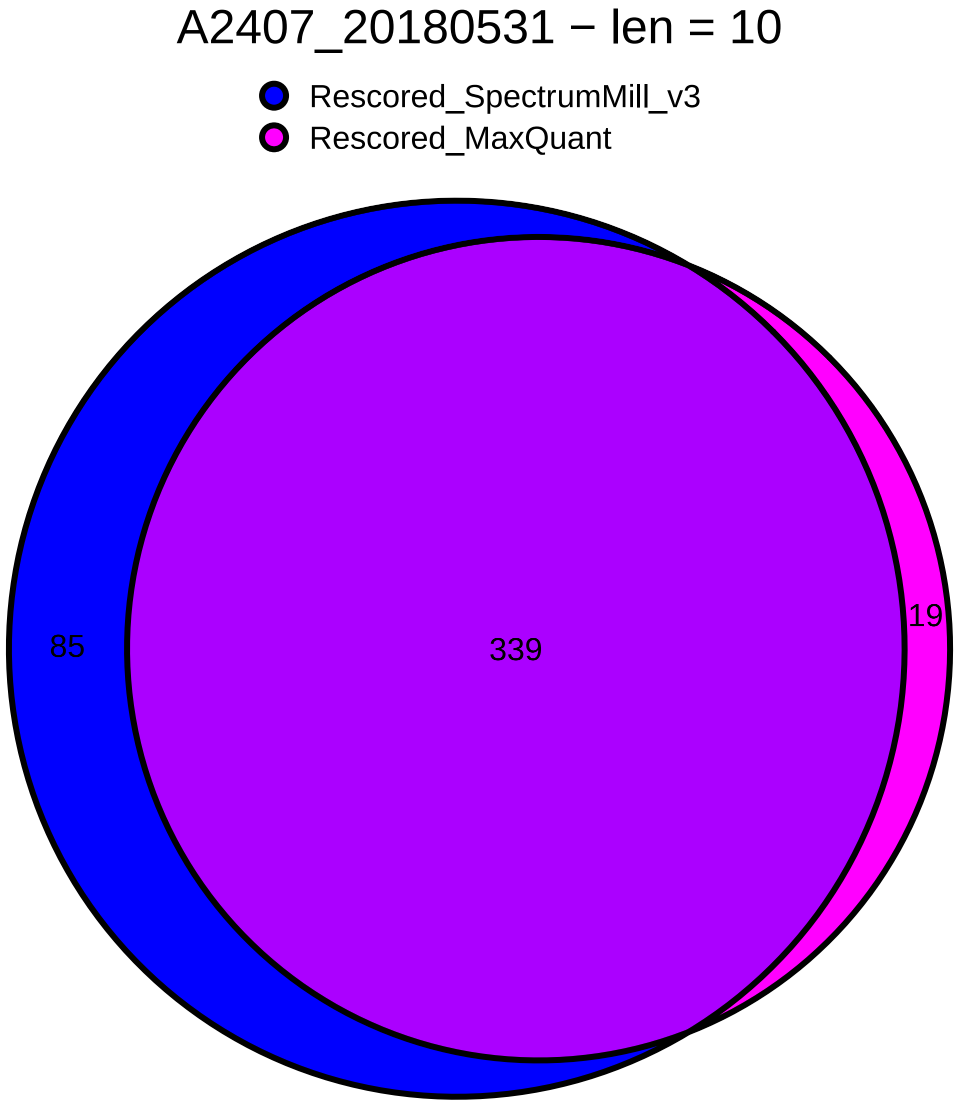
A2407_20180531 − len = 10
Rescored_SpectrumMill_v3
Rescored_MaxQuant
85
339
19

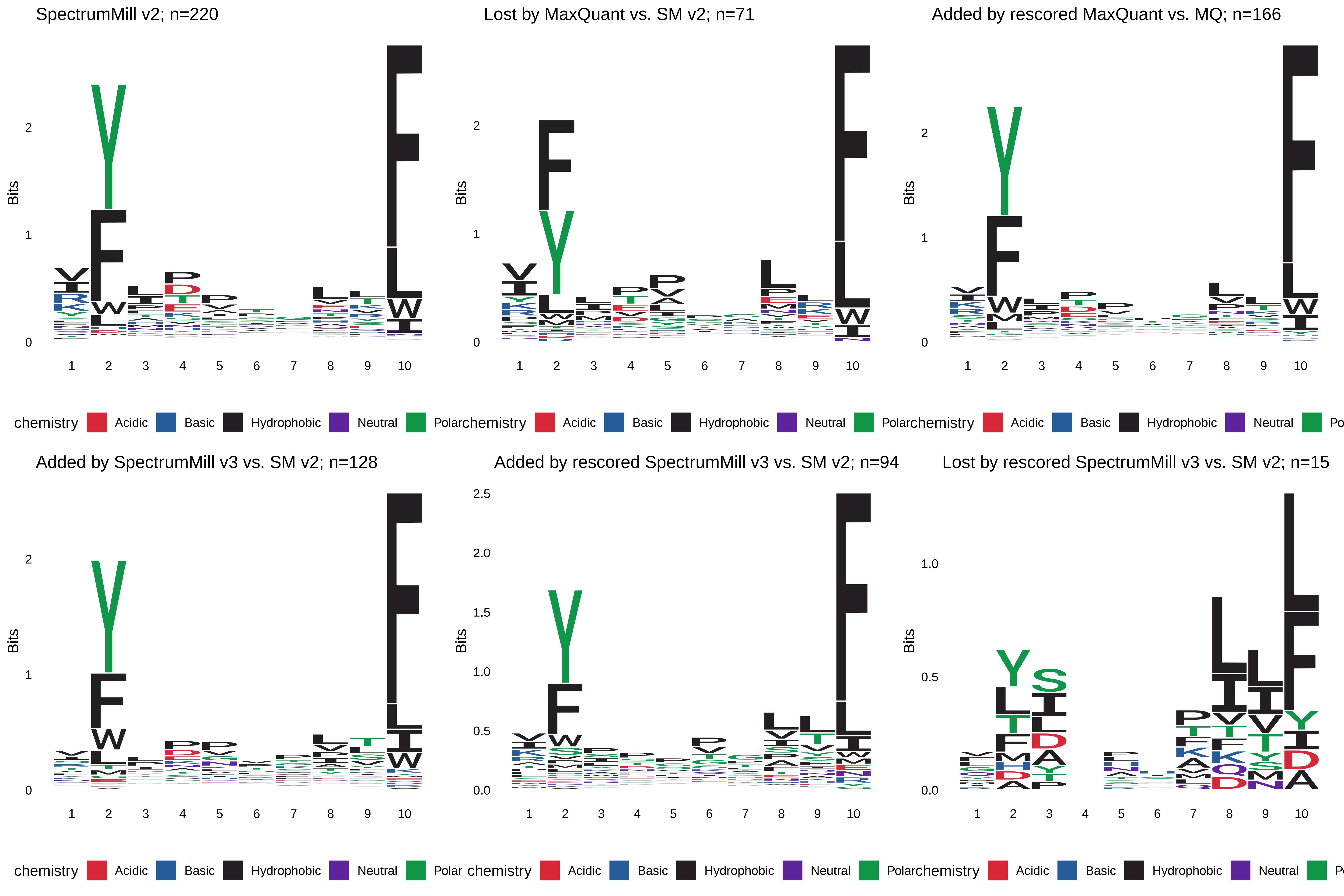
SpectrumMill v2; n=220
Lost by MaxQuant vs. SM v2; n=71
Added by rescored MaxQuant vs. MQ; n=166
Added by SpectrumMill v3 vs. SM v2; n=128
Added by rescored SpectrumMill v3 vs. SM v2; n=94
Lost by rescored SpectrumMill v3 vs. SM v2; n=15
Bits
1 2 3 4 5 6 7 8 9 10
chemistry
Acidic
Basic
Hydrophobic
Neutral
Polar

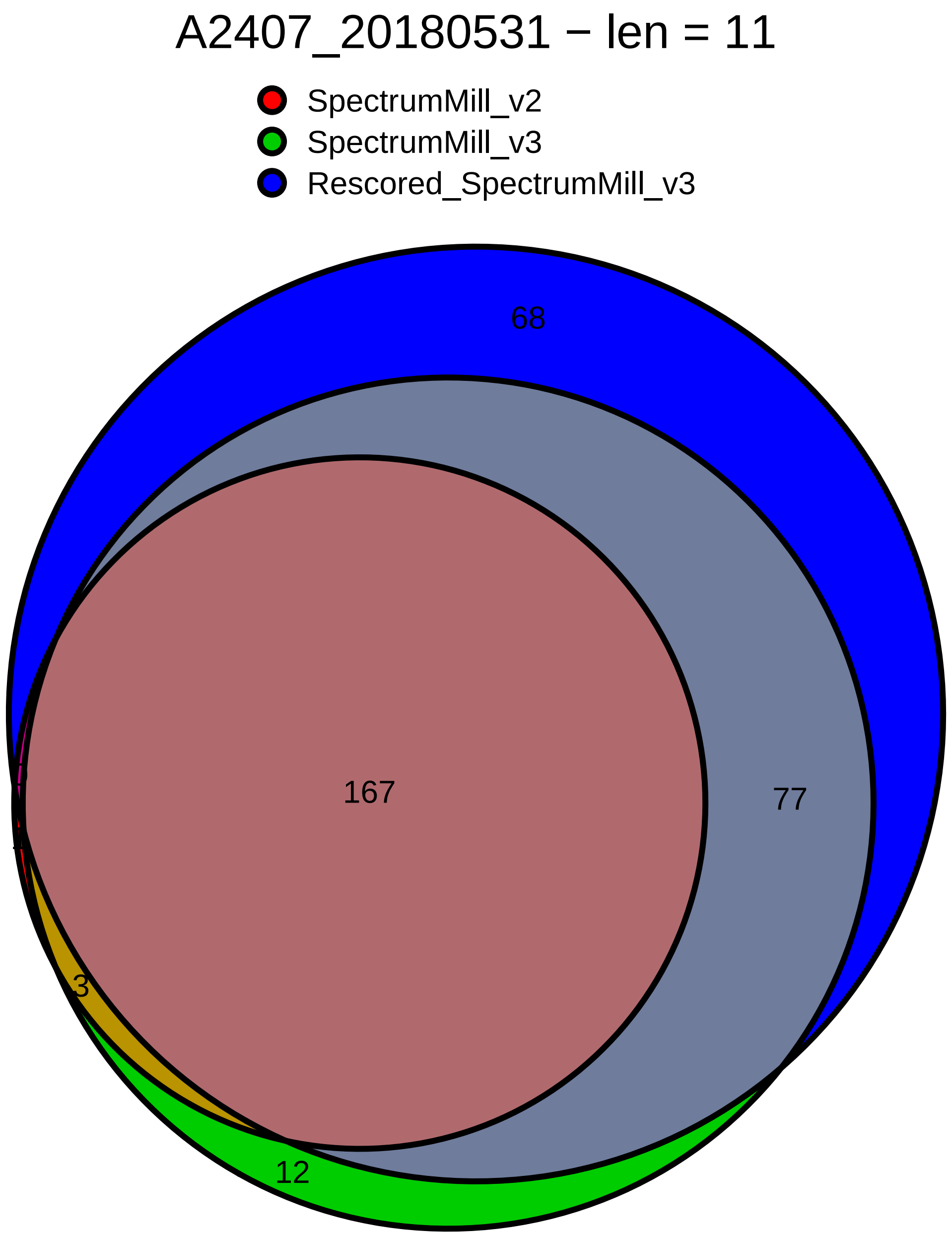

A2407_20180531 − len = 11
SpectrumMill_v2
SpectrumMill_v3
Rescored_SpectrumMill_v3
68
167
77
3
12

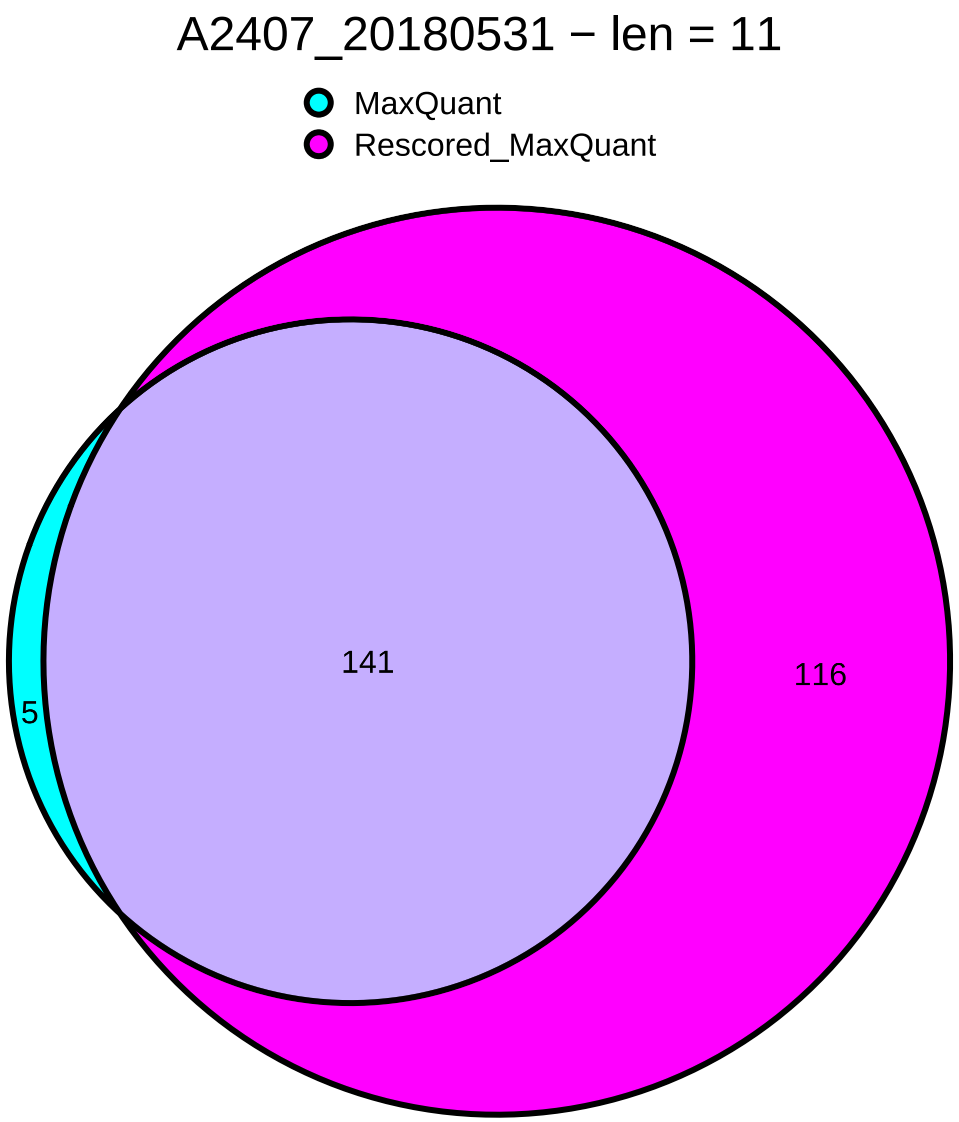

A2407_20180531 − len = 11
MaxQuant
Rescored_MaxQuant
141
116
5

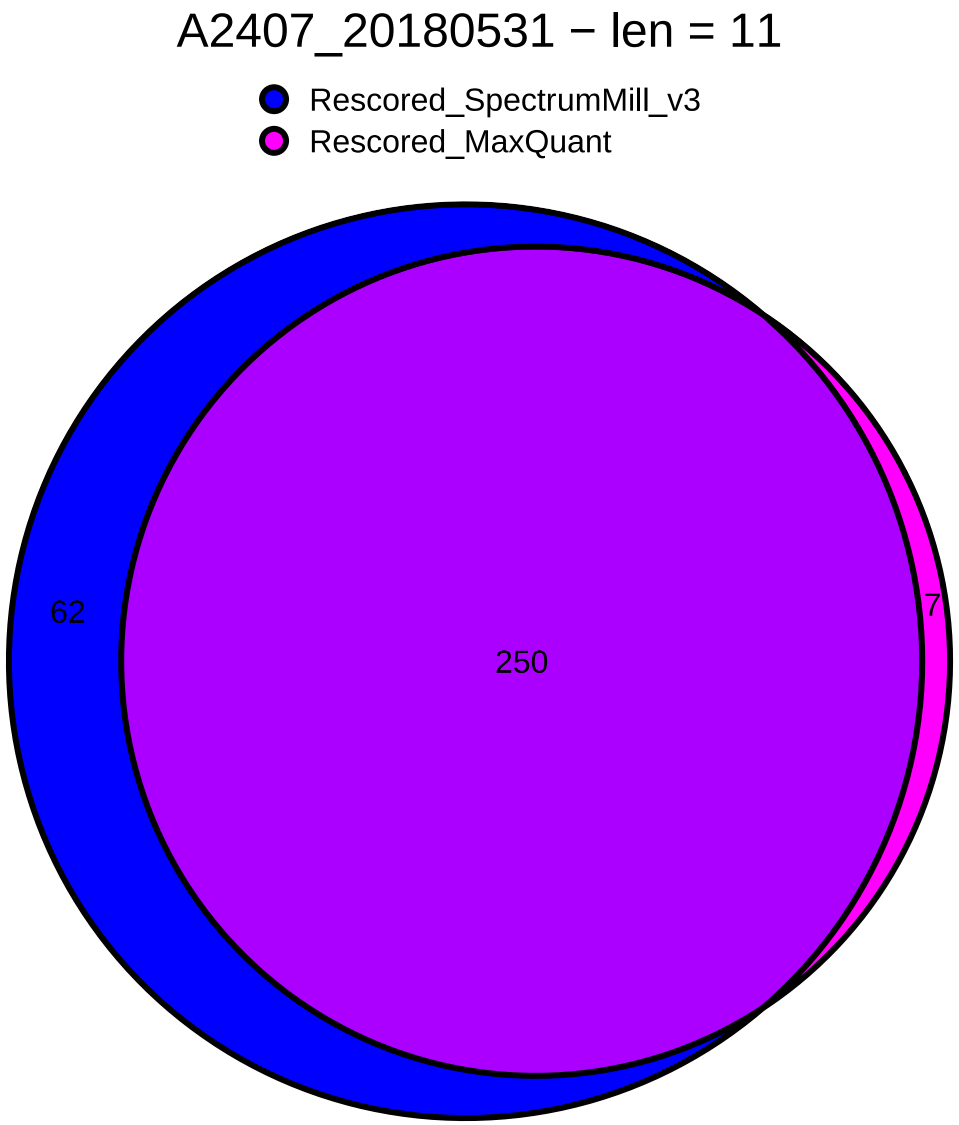

A2407_20180531 − len = 11
Rescored_SpectrumMill_v3
Rescored_MaxQuant
62
250
7

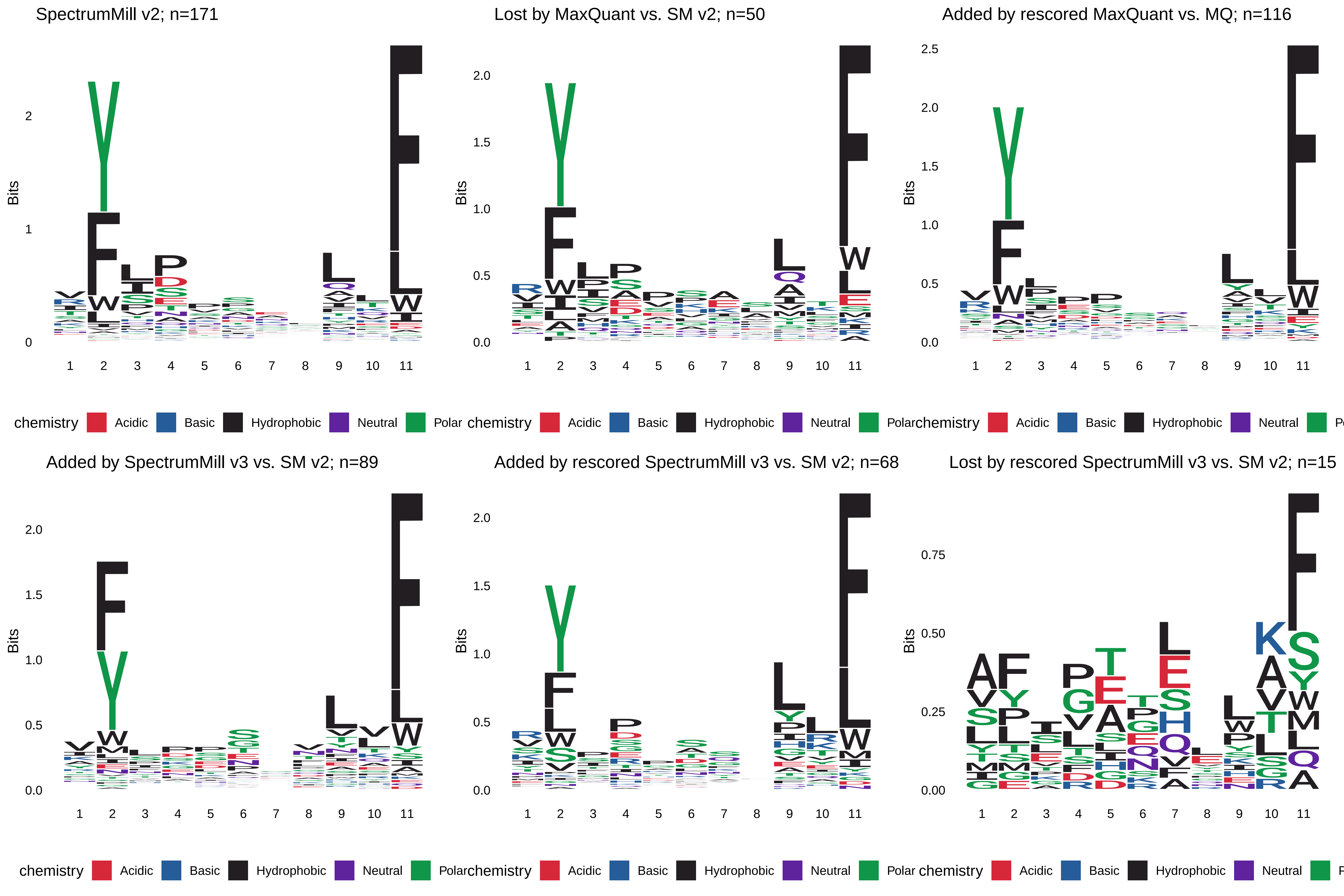
SpectrumMill v2; n=171
Lost by MaxQuant vs. SM v2; n=50
Added by rescored MaxQuant vs. MQ; n=116
Added by SpectrumMill v3 vs. SM v2; n=89
Added by rescored SpectrumMill v3 vs. SM v2; n=68
Lost by rescored SpectrumMill v3 vs. SM v2; n=15
Bits
1 2 3 4 5 6 7 8 9 10 11
chemistry
Acidic
Basic
Hydrophobic
Neutral
Polar

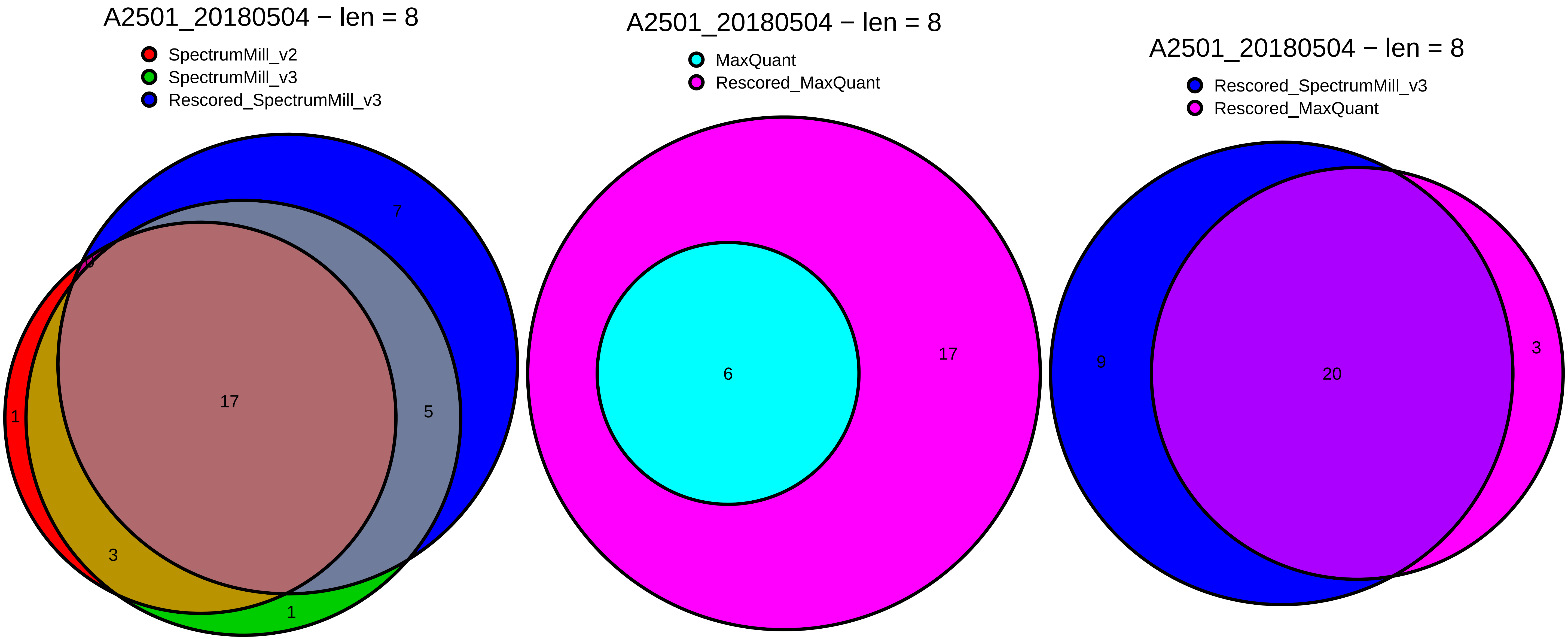
A2501_20180504 − len = 8
SpectrumMill_v2
SpectrumMill_v3
Rescored_SpectrumMill_v3
7
0
1
17
5
3
1
A2501_20180504 − len = 8
MaxQuant
Rescored_MaxQuant
6
17
A2501_20180504 − len = 8
Rescored_SpectrumMill_v3
Rescored_MaxQuant
9
20
3

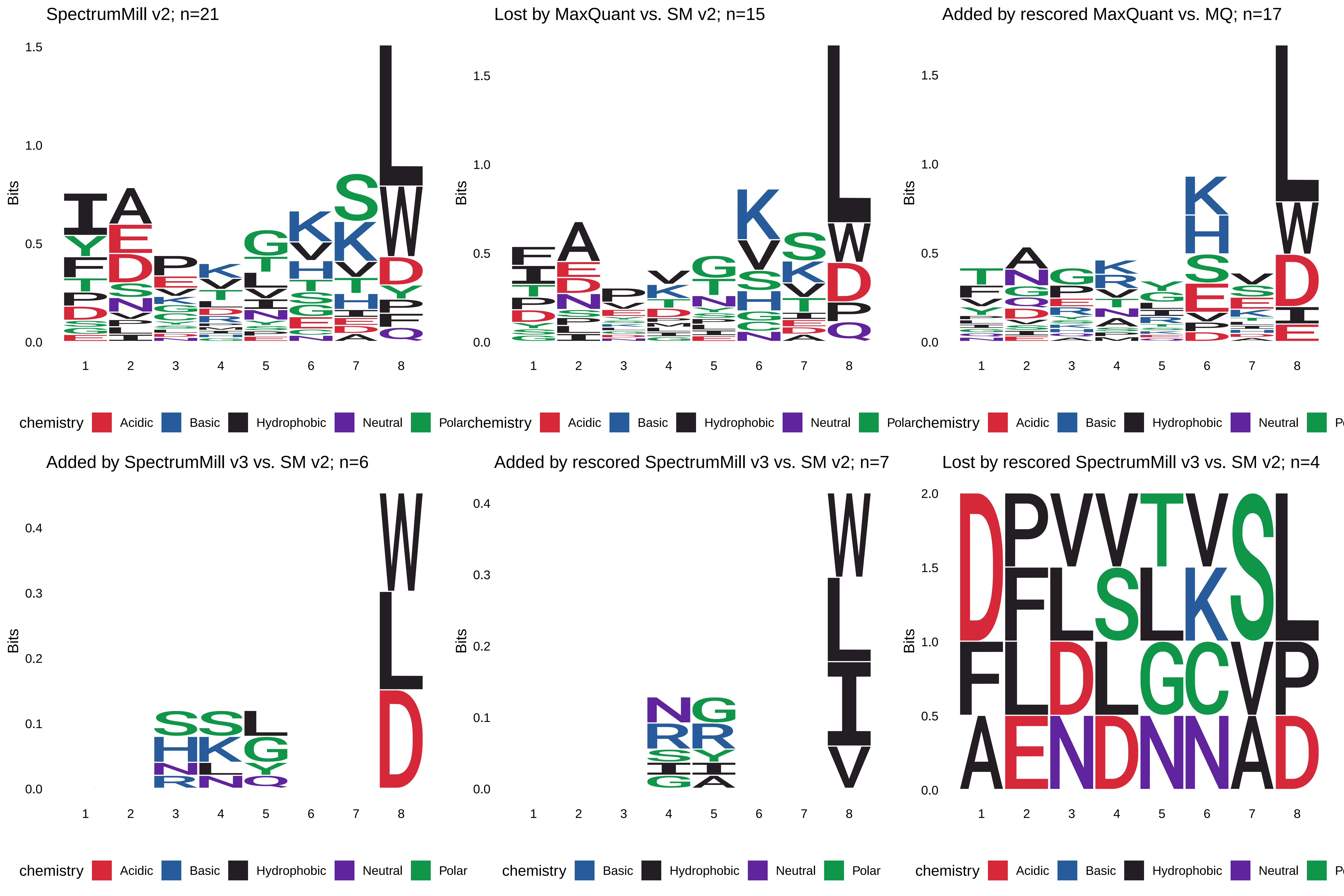
SpectrumMill v2; n=21
Lost by MaxQuant vs. SM v2; n=15
Added by rescored MaxQuant vs. MQ; n=17
Added by SpectrumMill v3 vs. SM v2; n=6
Added by rescored SpectrumMill v3 vs. SM v2; n=7
Lost by rescored SpectrumMill v3 vs. SM v2; n=4
Bits
1 2 3 4 5 6 7 8
chemistry Acidic Basic Hydrophobic Neutral Polar

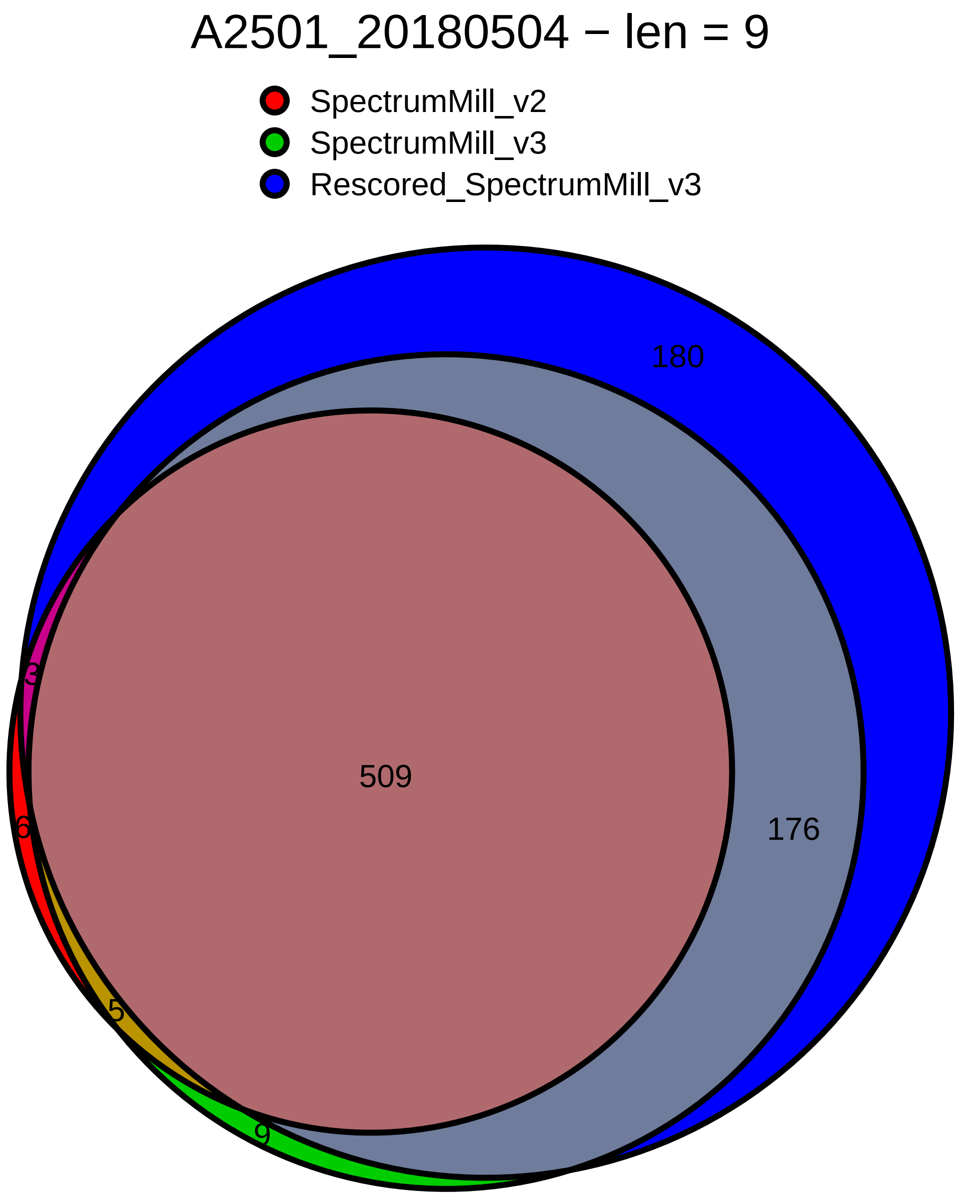
A2501_20180504 − len = 9
SpectrumMill_v2
SpectrumMill_v3
Rescored_SpectrumMill_v3
180
3
509
176
6
5
9

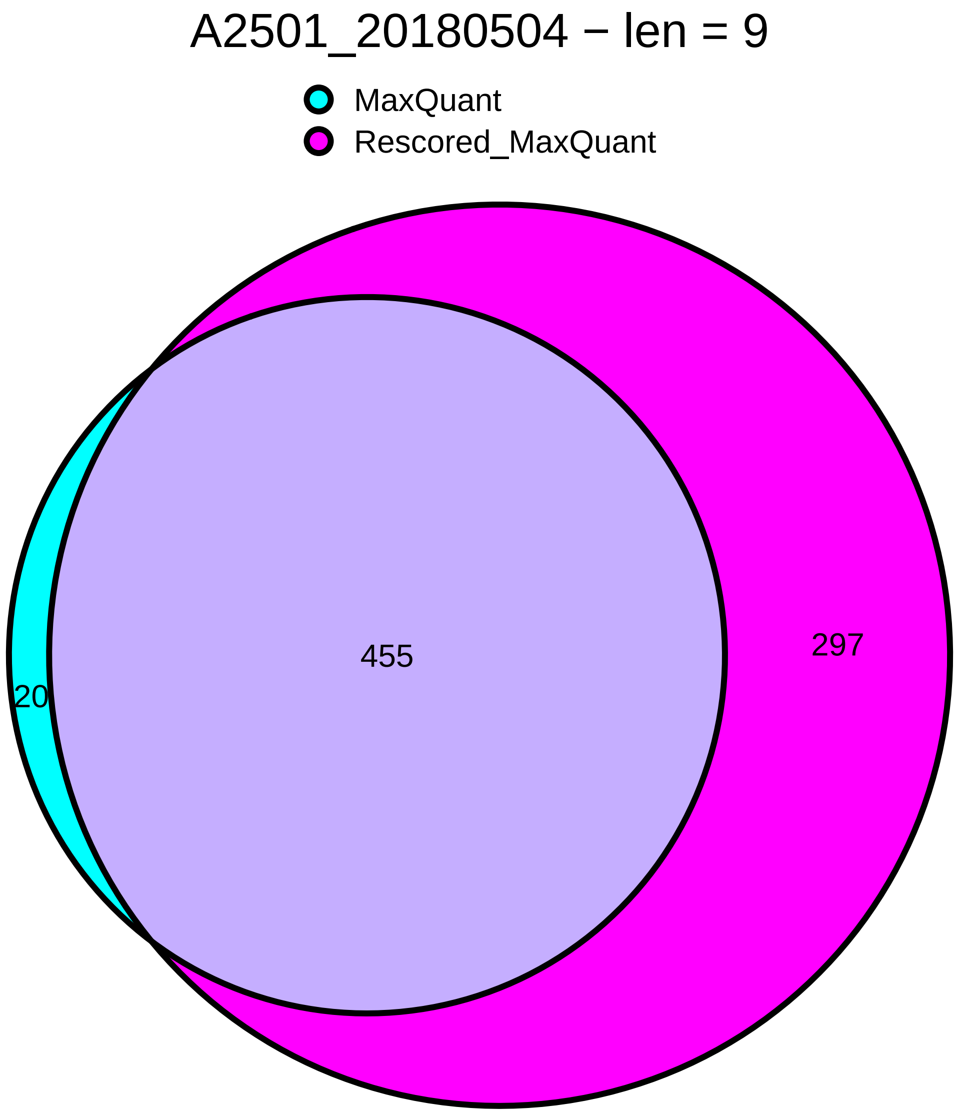
A2501_20180504 − len = 9
MaxQuant
Rescored_MaxQuant
455
297
20

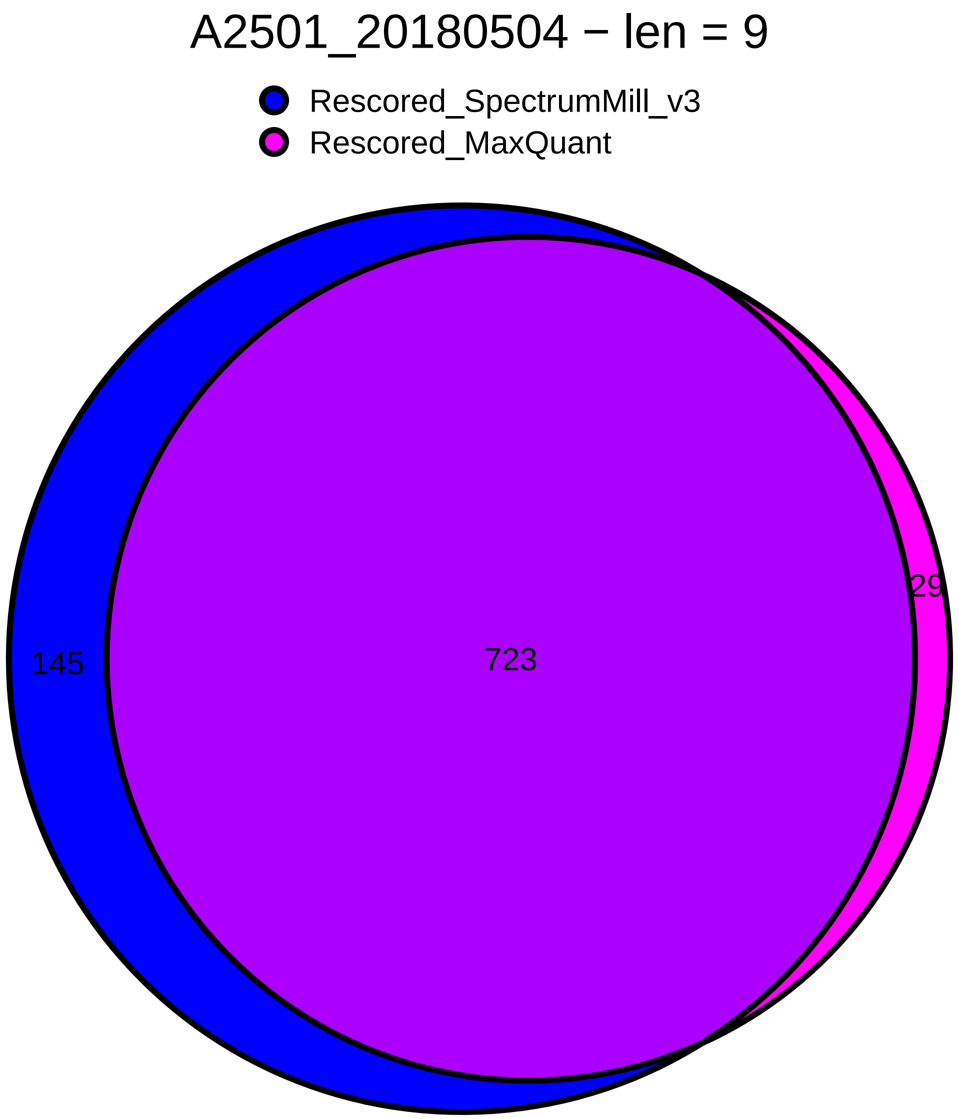
A2501_20180504 − len = 9
Rescored_SpectrumMill_v3
Rescored_MaxQuant
145
723
29

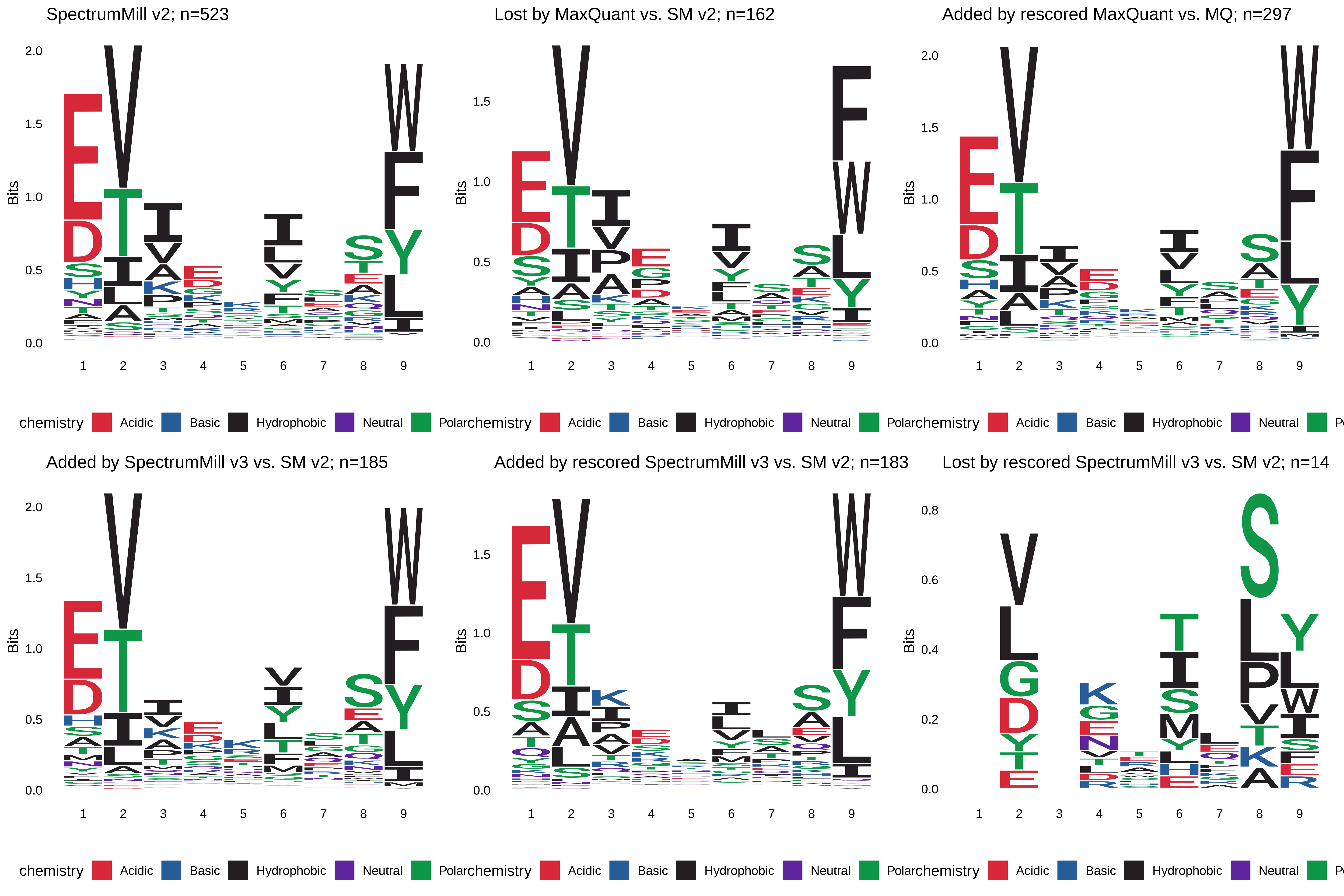
SpectrumMill v2; n=523
Lost by MaxQuant vs. SM v2; n=162
Added by rescored MaxQuant vs. MQ; n=297
Added by SpectrumMill v3 vs. SM v2; n=185
Added by rescored SpectrumMill v3 vs. SM v2; n=183
Lost by rescored SpectrumMill v3 vs. SM v2; n=14
Bits
1 2 3 4 5 6 7 8 9
chemistry Acidic Basic Hydrophobic Neutral Polarchemistry Acidic Basic Hydrophobic Neutral Polarchemistry Acidic Basic Hydrophobic Neutral
chemistry Acidic Basic Hydrophobic Neutral Polarchemistry Acidic Basic Hydrophobic Neutral Polarchemistry Acidic Basic Hydrophobic Neutral

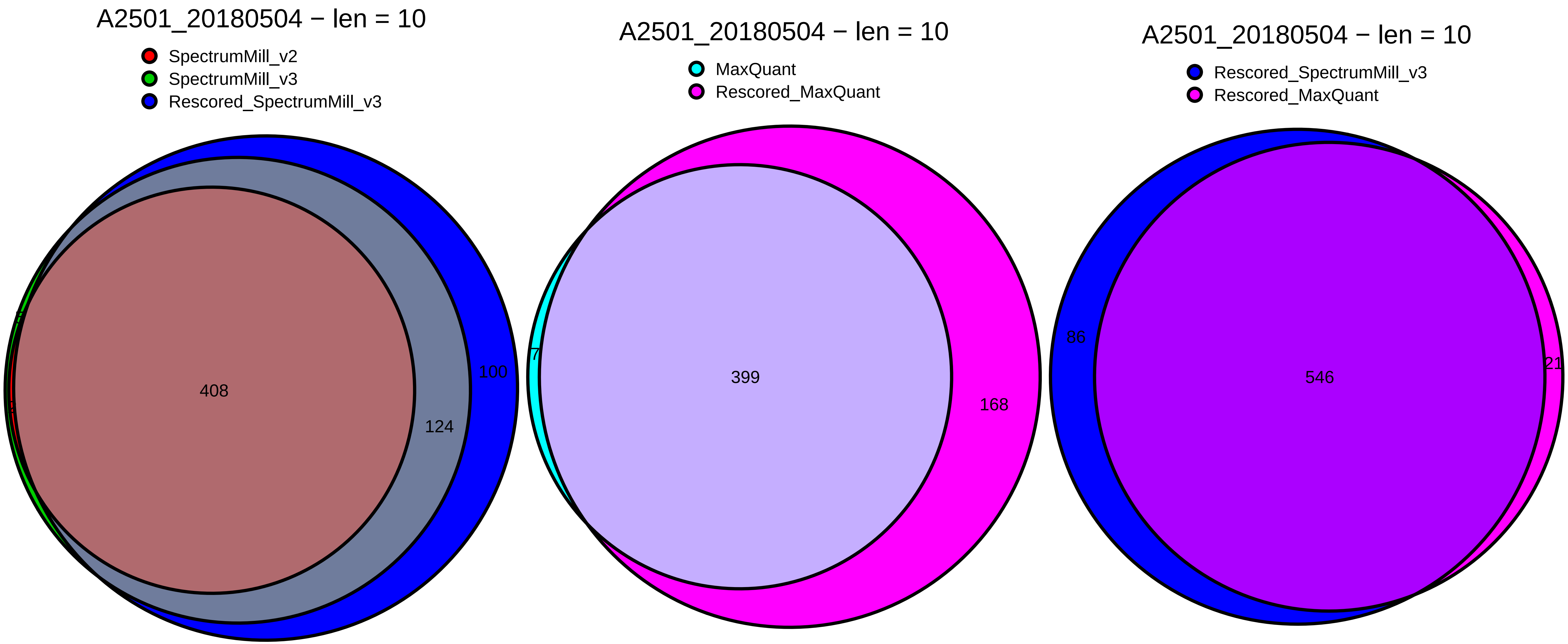
A2501_20180504 − len = 10
SpectrumMill_v2
SpectrumMill_v3
Rescored_SpectrumMill_v3
408
124
100
A2501_20180504 − len = 10
MaxQuant
Rescored_MaxQuant
7
399
168
A2501_20180504 − len = 10
Rescored_SpectrumMill_v3
Rescored_MaxQuant
86
546
21

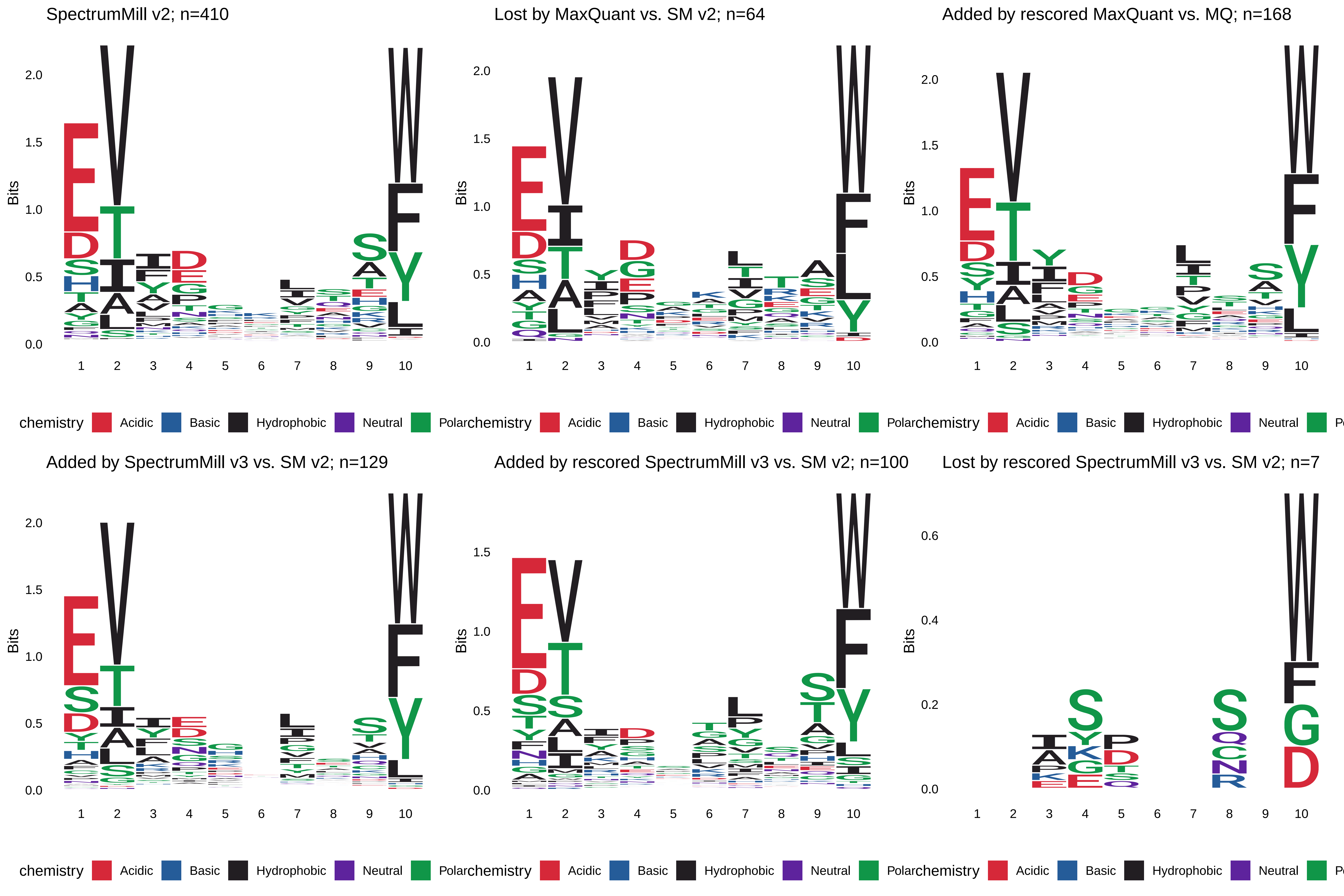
SpectrumMill v2; n=410
Lost by MaxQuant vs. SM v2; n=64
Added by rescored MaxQuant vs. MQ; n=168
Added by SpectrumMill v3 vs. SM v2; n=129
Added by rescored SpectrumMill v3 vs. SM v2; n=100
Lost by rescored SpectrumMill v3 vs. SM v2; n=7
Bits
chemistry
Acidic
Basic
Hydrophobic
Neutral
Polar

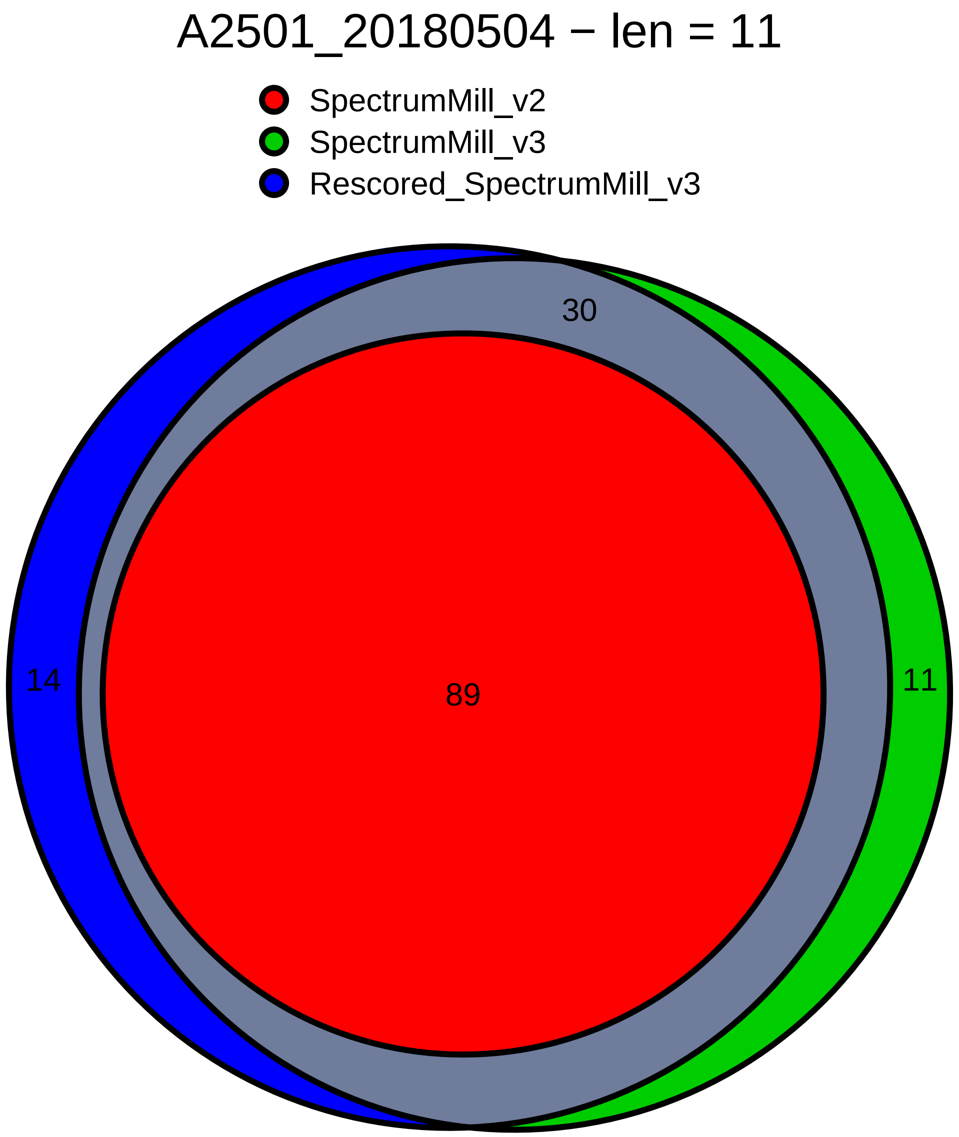
A2501_20180504 − len = 11
SpectrumMill_v2
SpectrumMill_v3
Rescored_SpectrumMill_v3
30
14
89
11

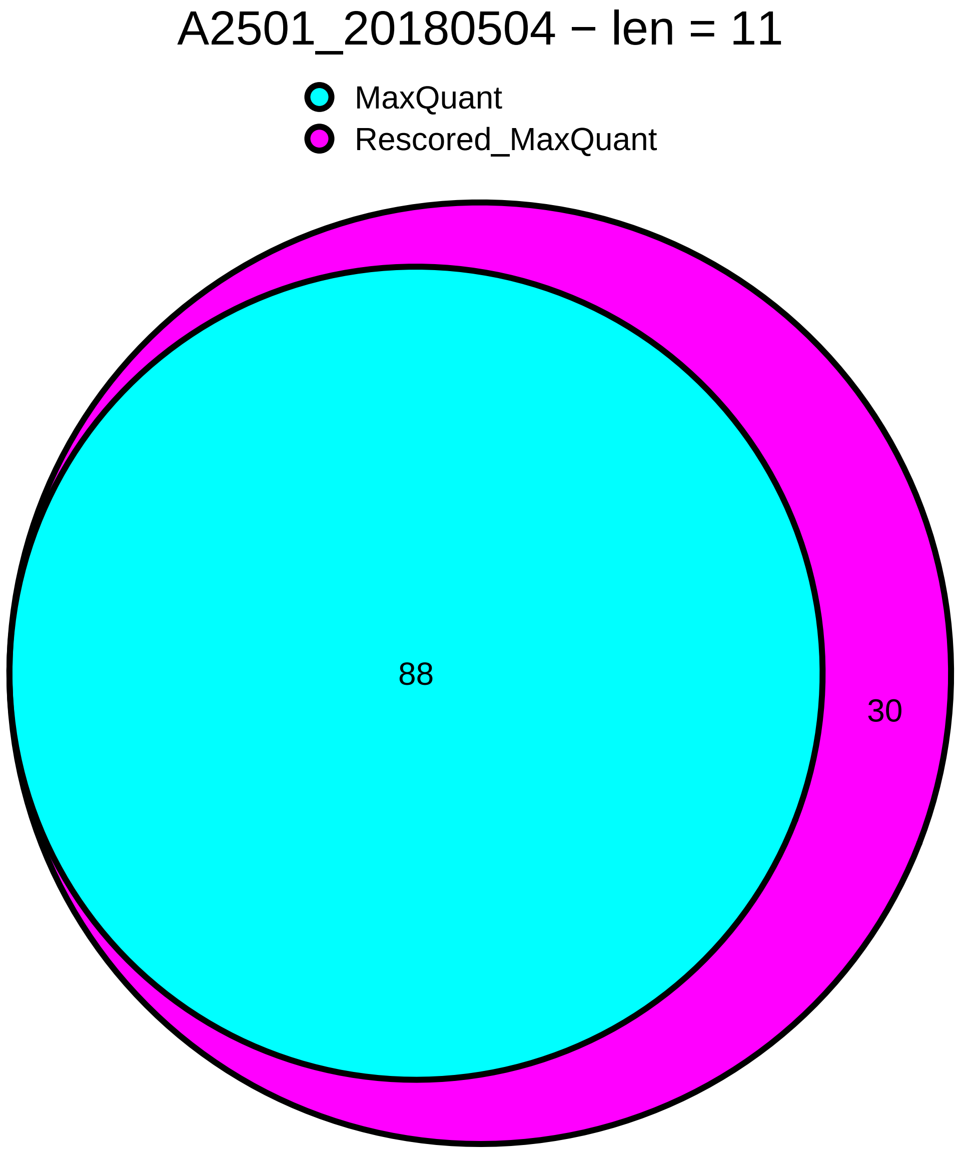
A2501_20180504 − len = 11
MaxQuant
Rescored_MaxQuant
88
30

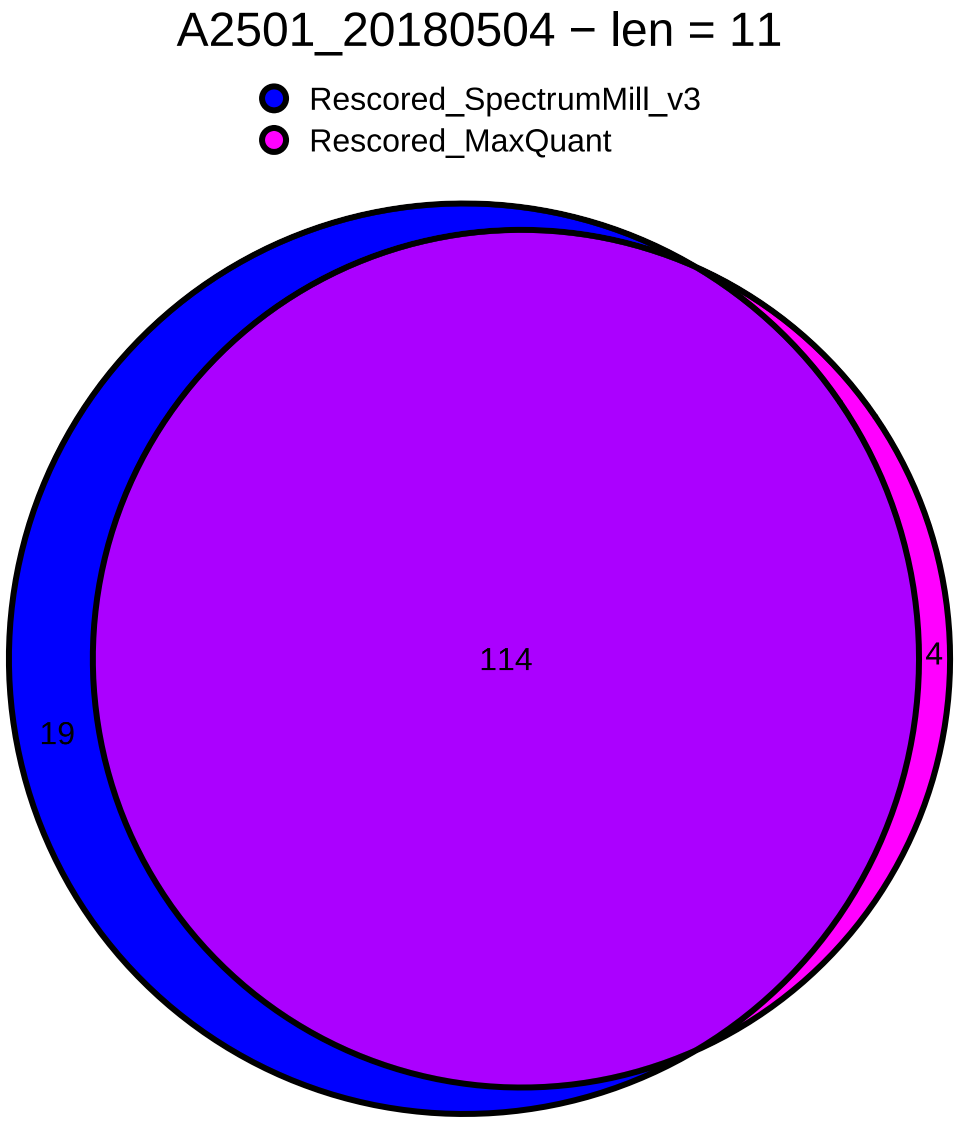
A2501_20180504 − len = 11
Rescored_SpectrumMill_v3
Rescored_MaxQuant
114
19
4

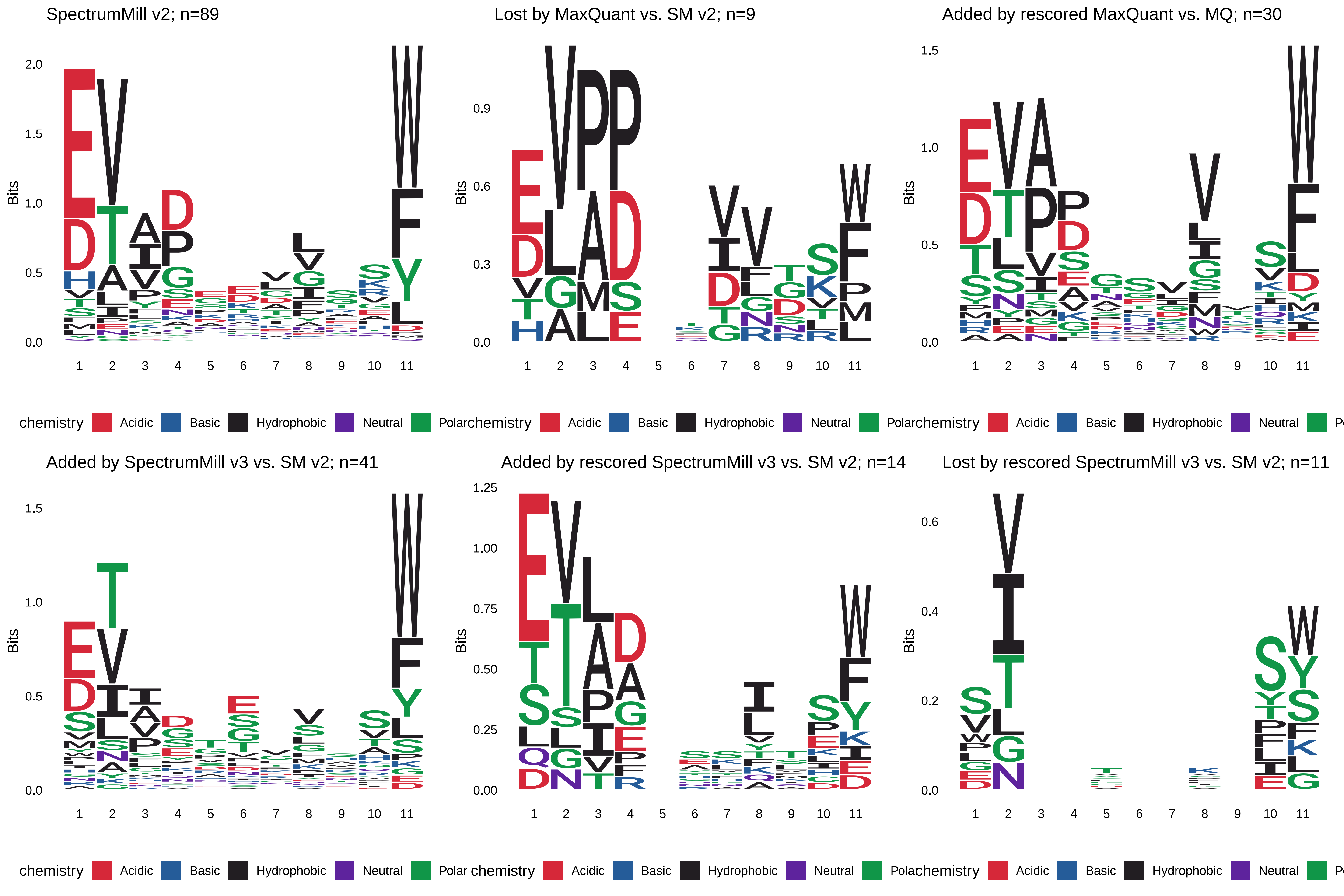
SpectrumMill v2; n=89
Lost by MaxQuant vs. SM v2; n=9
Added by rescored MaxQuant vs. MQ; n=30
Added by SpectrumMill v3 vs. SM v2; n=41
Added by rescored SpectrumMill v3 vs. SM v2; n=14
Lost by rescored SpectrumMill v3 vs. SM v2; n=11
Bits
1 2 3 4 5 6 7 8 9 10 11
chemistry Acidic Basic Hydrophobic Neutral Polar

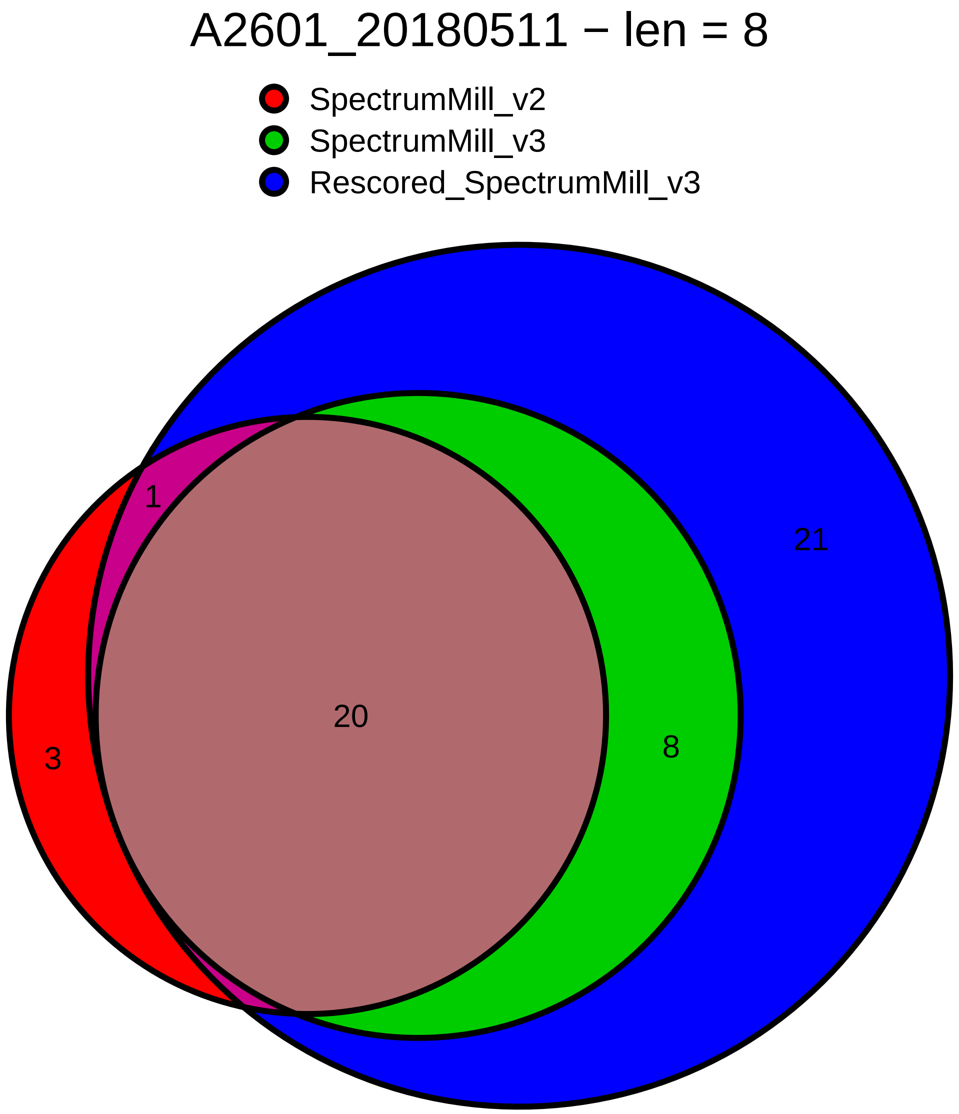
A2601_20180511 − len = 8
SpectrumMill_v2
SpectrumMill_v3
Rescored_SpectrumMill_v3
1
21
3
20
8

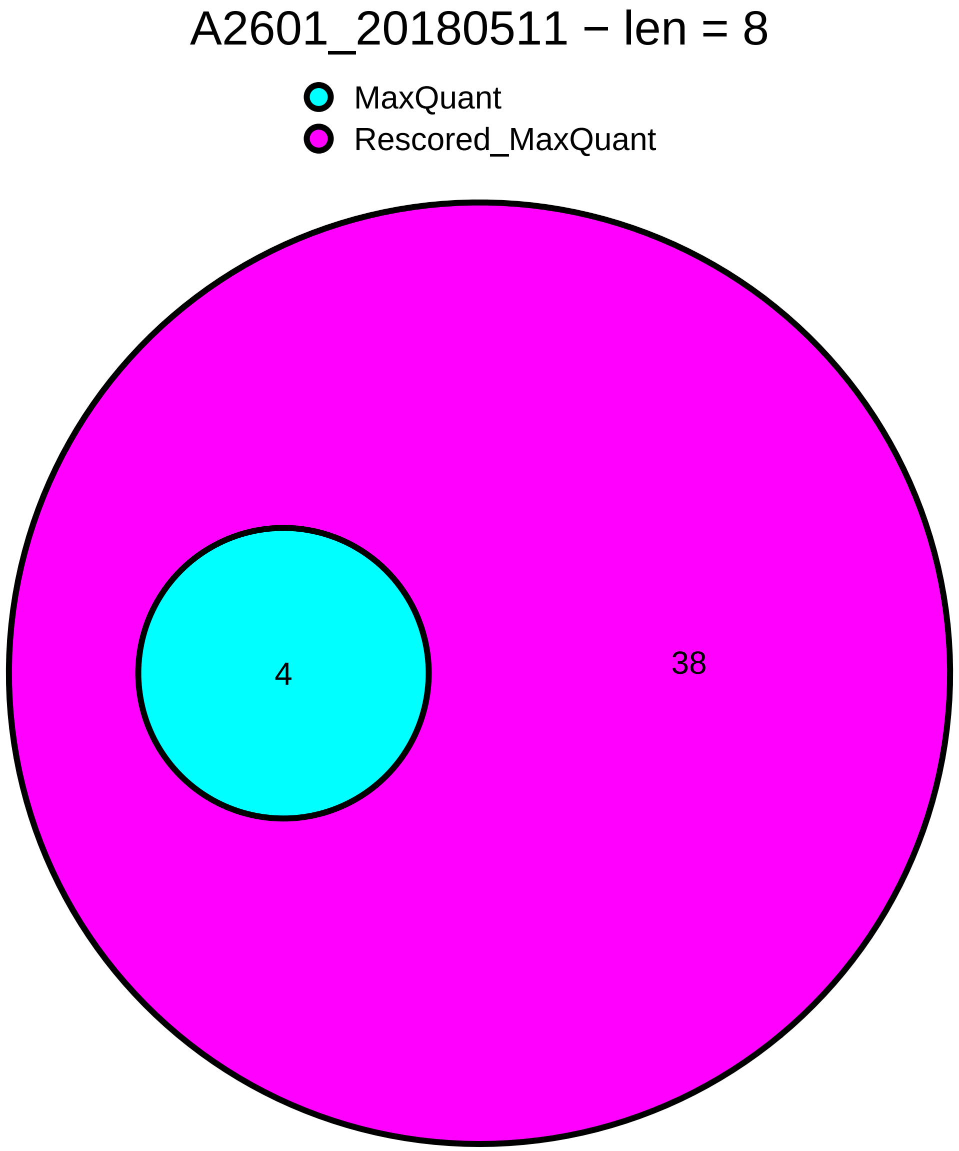
A2601_20180511 − len = 8
MaxQuant
Rescored_MaxQuant
4
38

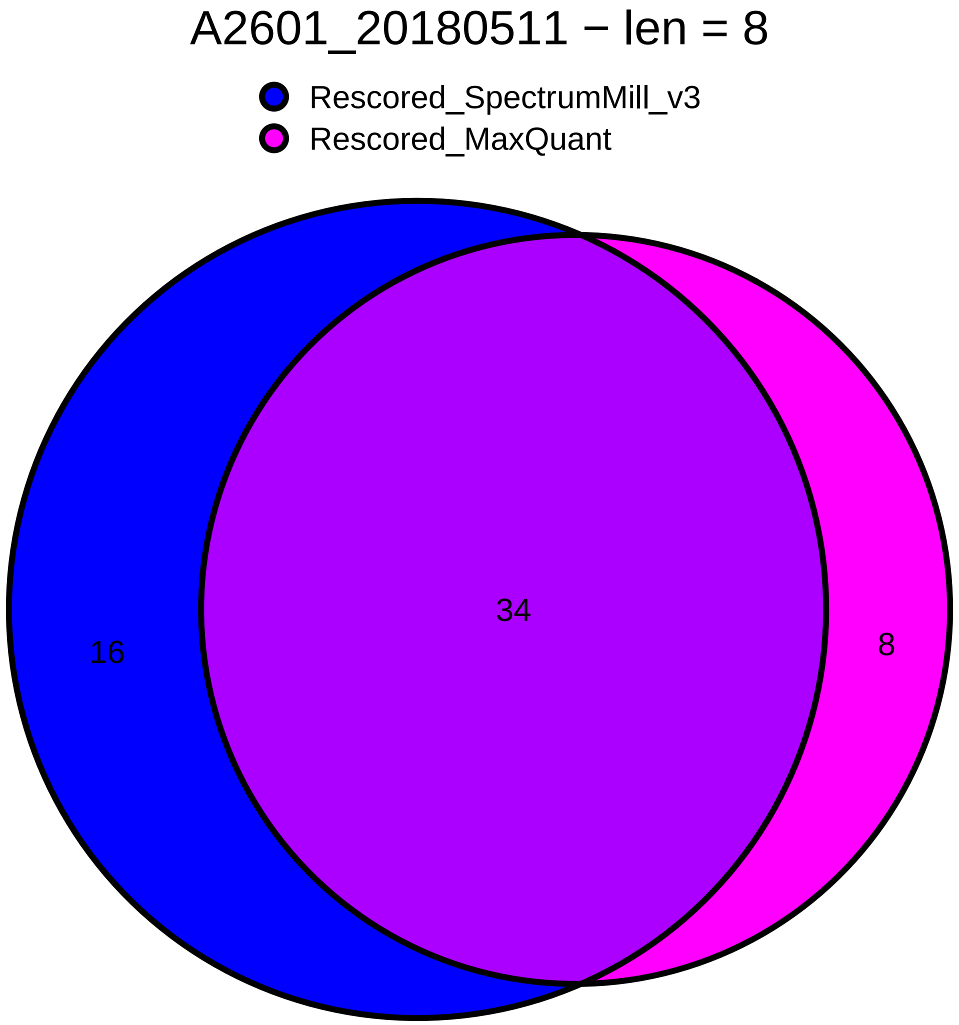
A2601_20180511 − len = 8
Rescored_SpectrumMill_v3
Rescored_MaxQuant
16
34
8

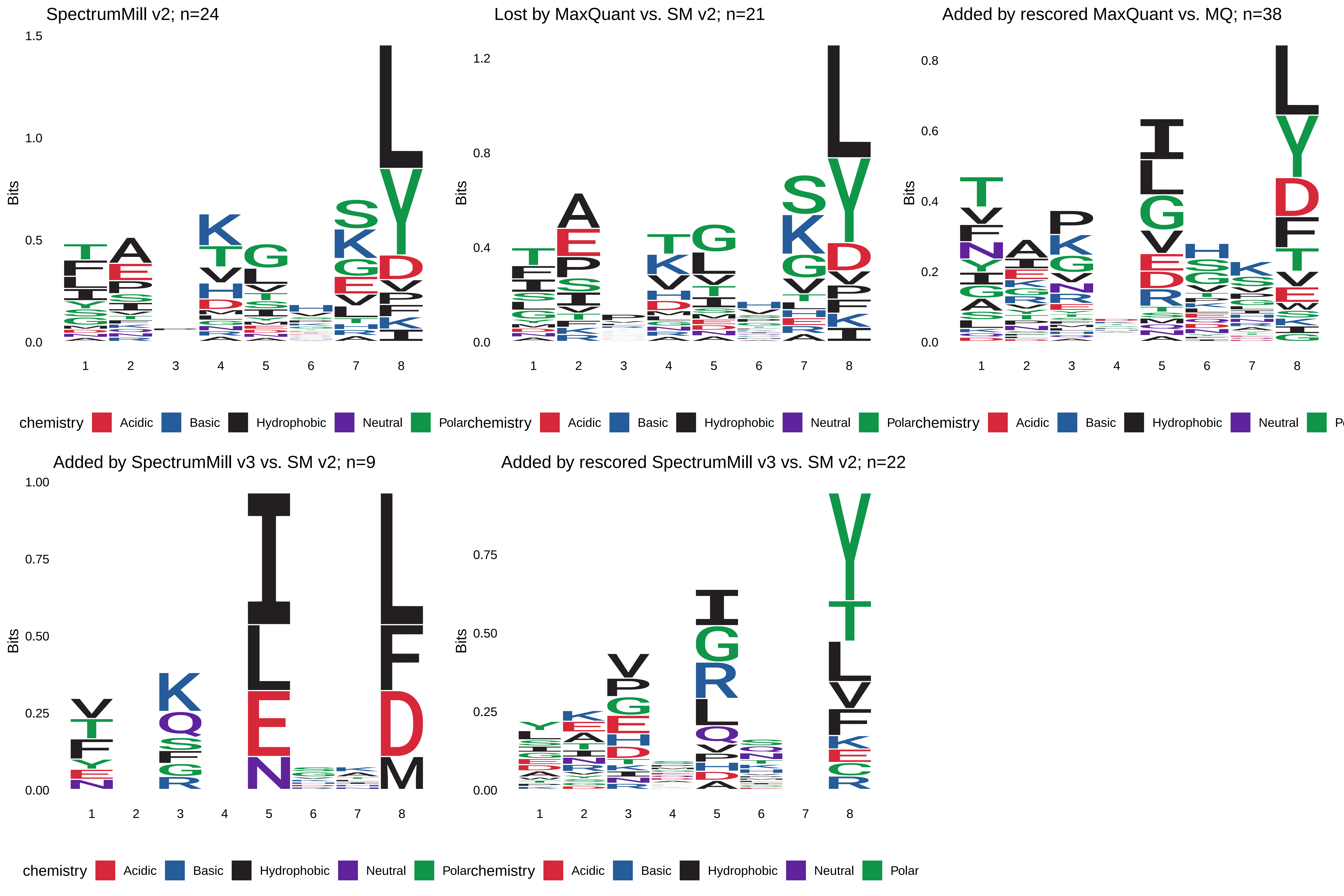
SpectrumMill v2; n=24
Lost by MaxQuant vs. SM v2; n=21
Added by rescored MaxQuant vs. MQ; n=38
Added by SpectrumMill v3 vs. SM v2; n=9
Added by rescored SpectrumMill v3 vs. SM v2; n=22
Bits
1 2 3 4 5 6 7 8
chemistry Acidic Basic Hydrophobic Neutral Polar

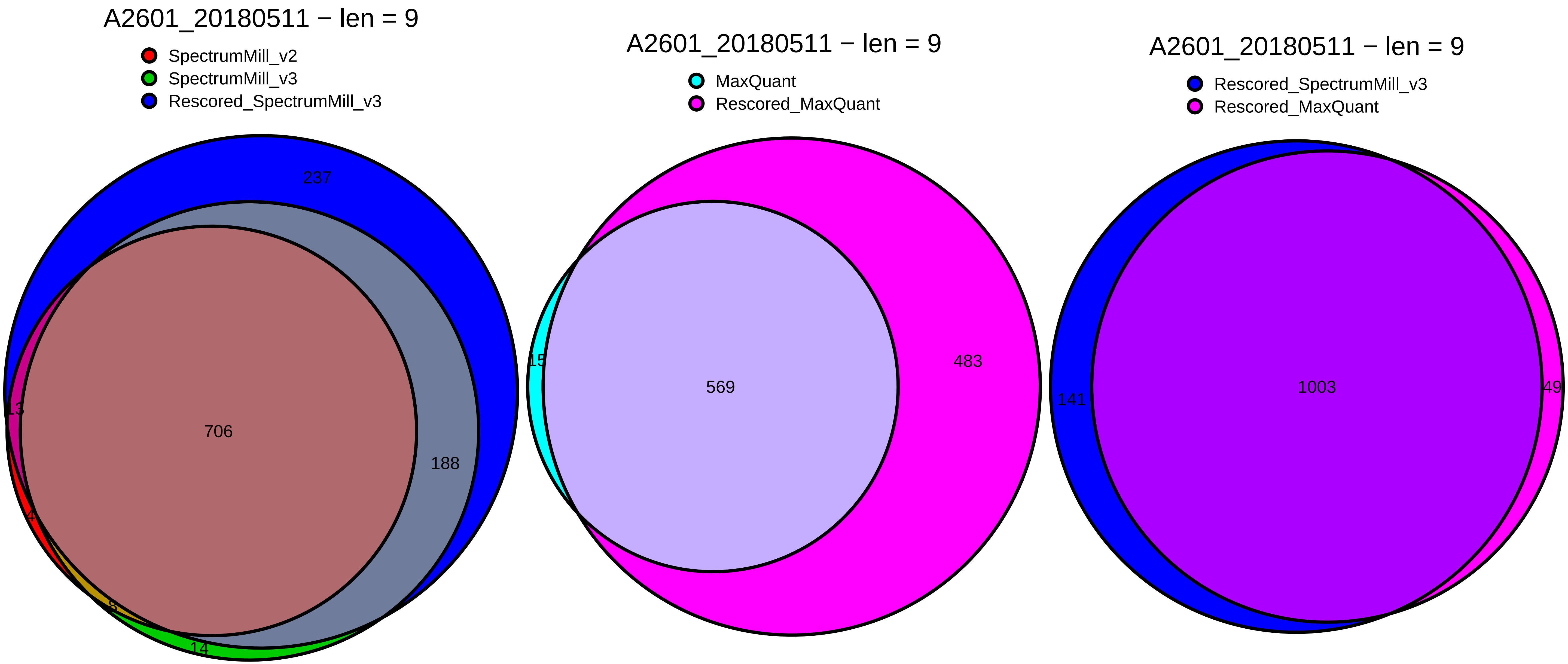

A2601_20180511 − len = 9
SpectrumMill_v2
SpectrumMill_v3
Rescored_SpectrumMill_v3
237
13
706
188
4
8
14
A2601_20180511 − len = 9
MaxQuant
Rescored_MaxQuant
15
569
483
A2601_20180511 − len = 9
Rescored_SpectrumMill_v3
Rescored_MaxQuant
141
1003
49

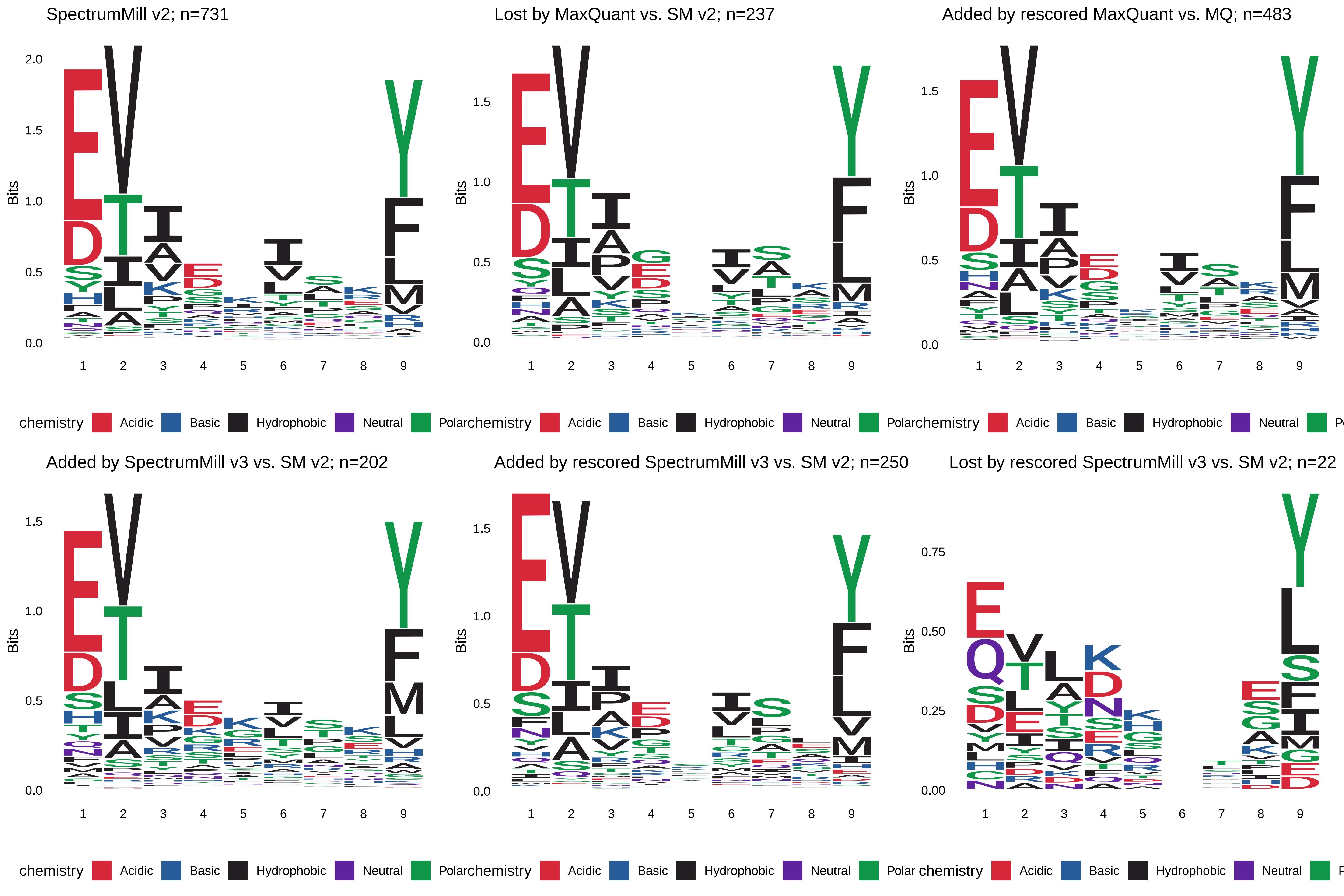

SpectrumMill v2; n=731
Lost by MaxQuant vs. SM v2; n=237
Added by rescored MaxQuant vs. MQ; n=483
Added by SpectrumMill v3 vs. SM v2; n=202
Added by rescored SpectrumMill v3 vs. SM v2; n=250
Lost by rescored SpectrumMill v3 vs. SM v2; n=22
Bits
1 2 3 4 5 6 7 8 9
chemistry Acidic Basic Hydrophobic Neutral Polar

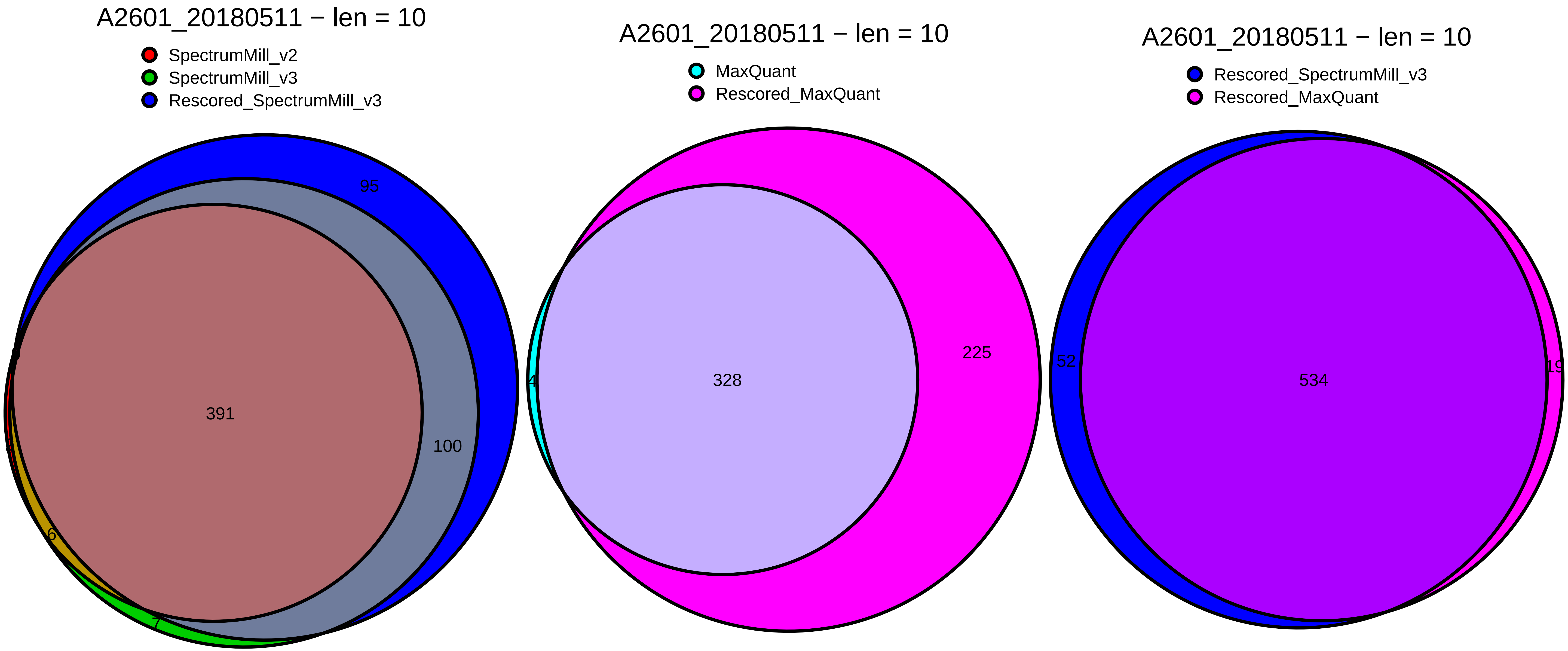

A2601_20180511 − len = 10
SpectrumMill_v2
SpectrumMill_v3
Rescored_SpectrumMill_v3
95
391
100
6
7
A2601_20180511 − len = 10
MaxQuant
Rescored_MaxQuant
328
225
A2601_20180511 − len = 10
Rescored_SpectrumMill_v3
Rescored_MaxQuant
52
534

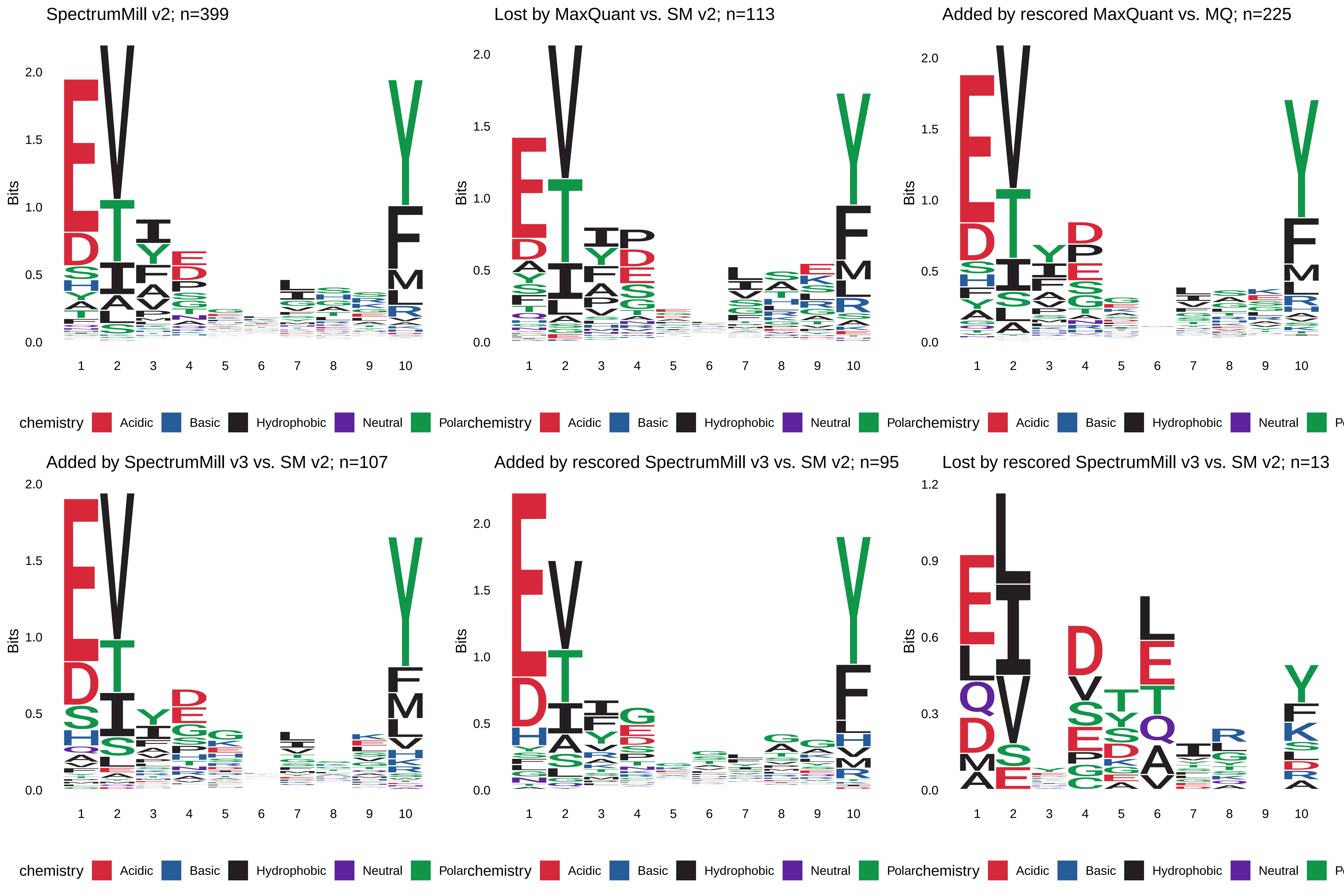

SpectrumMill v2; n=399
Lost by MaxQuant vs. SM v2; n=113
Added by rescored MaxQuant vs. MQ; n=225
Added by SpectrumMill v3 vs. SM v2; n=107
Added by rescored SpectrumMill v3 vs. SM v2; n=95
Lost by rescored SpectrumMill v3 vs. SM v2; n=13
Bits
1 2 3 4 5 6 7 8 9 10
chemistry
Acidic
Basic
Hydrophobic
Neutral
Polar

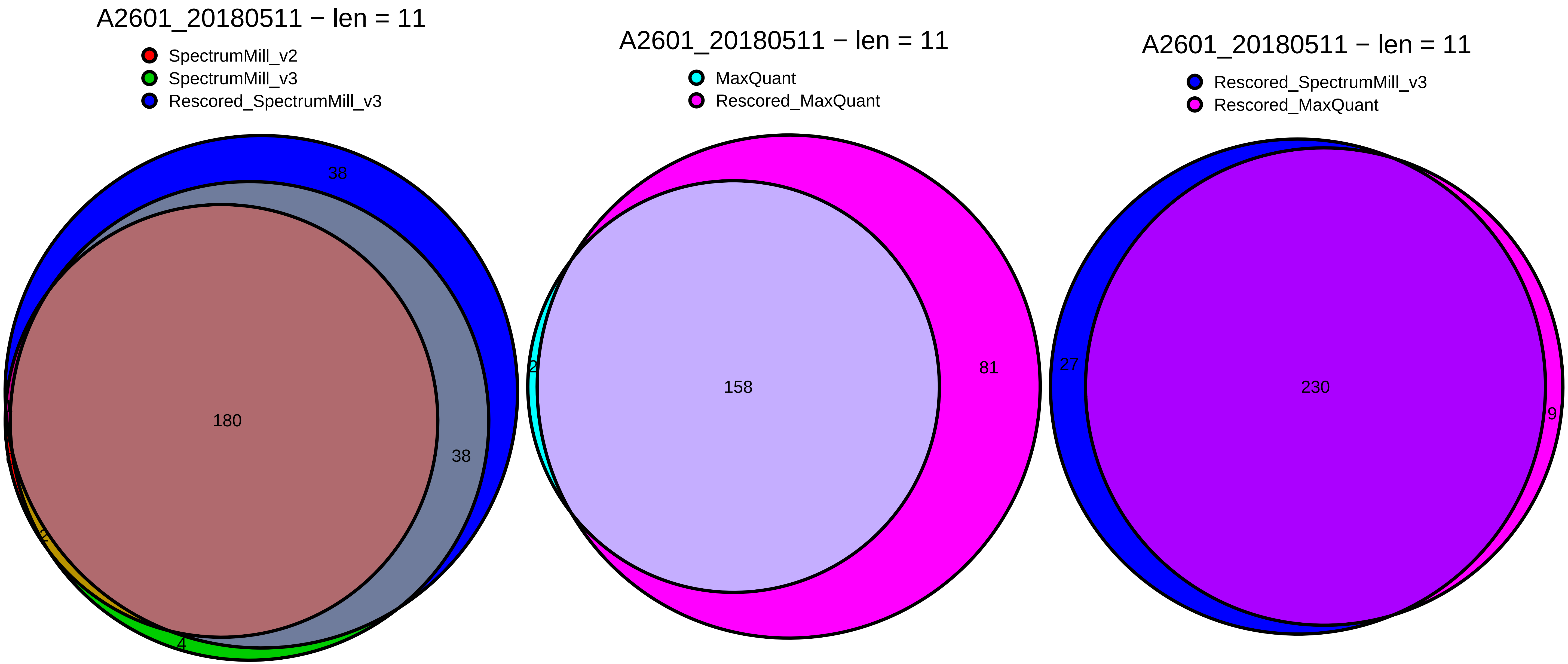
A2601_20180511 − len = 11
SpectrumMill_v2
SpectrumMill_v3
Rescored_SpectrumMill_v3
38
180
38
4
A2601_20180511 − len = 11
MaxQuant
Rescored_MaxQuant
158
81
A2601_20180511 − len = 11
Rescored_SpectrumMill_v3
Rescored_MaxQuant
27
230
9

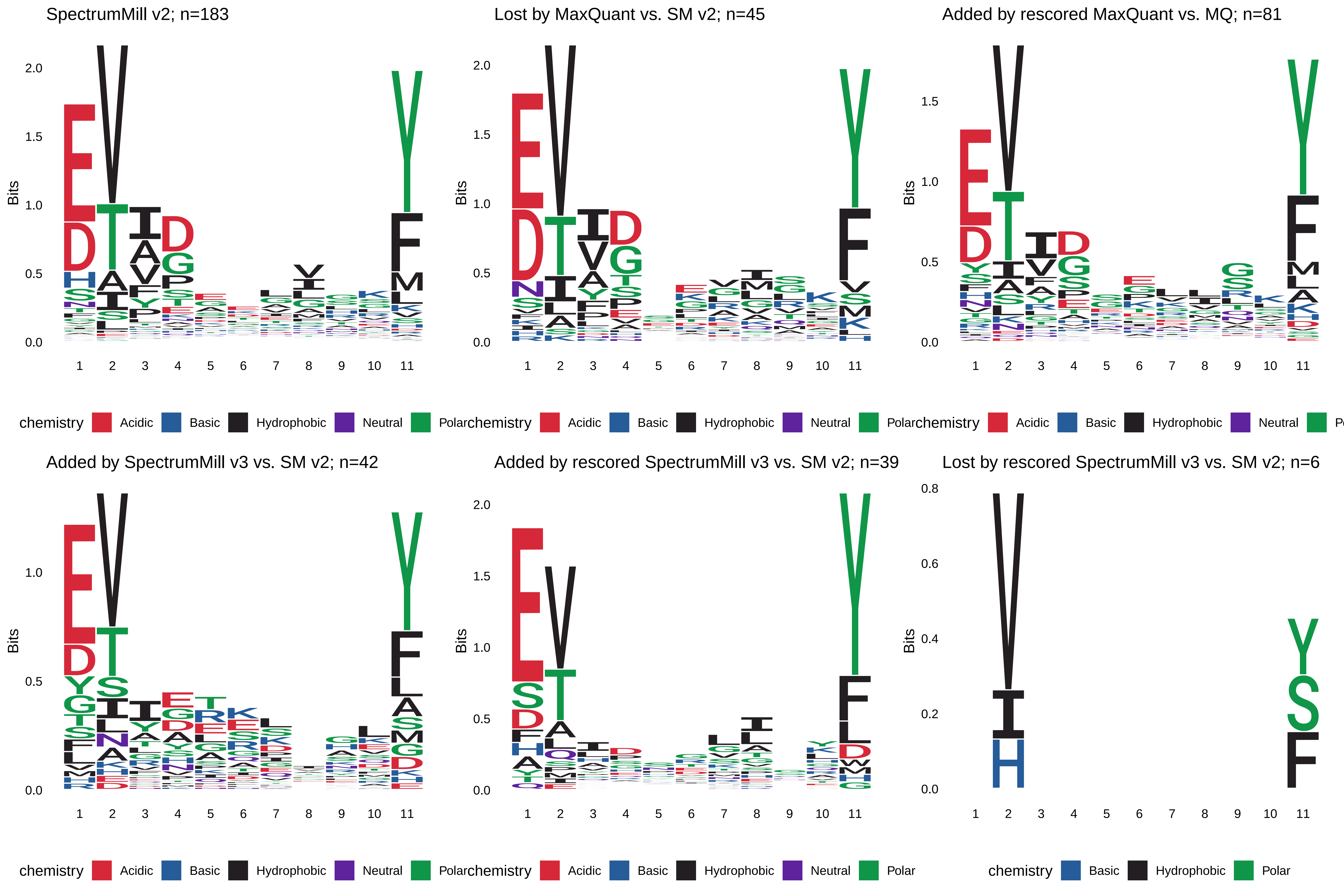

SpectrumMill v2; n=183
Lost by MaxQuant vs. SM v2; n=45
Added by rescored MaxQuant vs. MQ; n=81
Bits
0.0
0.5
1.0
1.5
2.0
1 2 3 4 5 6 7 8 9 10 11
chemistry Acidic Basic Hydrophobic Neutral Polar
Added by SpectrumMill v3 vs. SM v2; n=42
Added by rescored SpectrumMill v3 vs. SM v2; n=39
Lost by rescored SpectrumMill v3 vs. SM v2; n=6
0.2
0.4
0.6
0.8
chemistry Basic Hydrophobic Polar

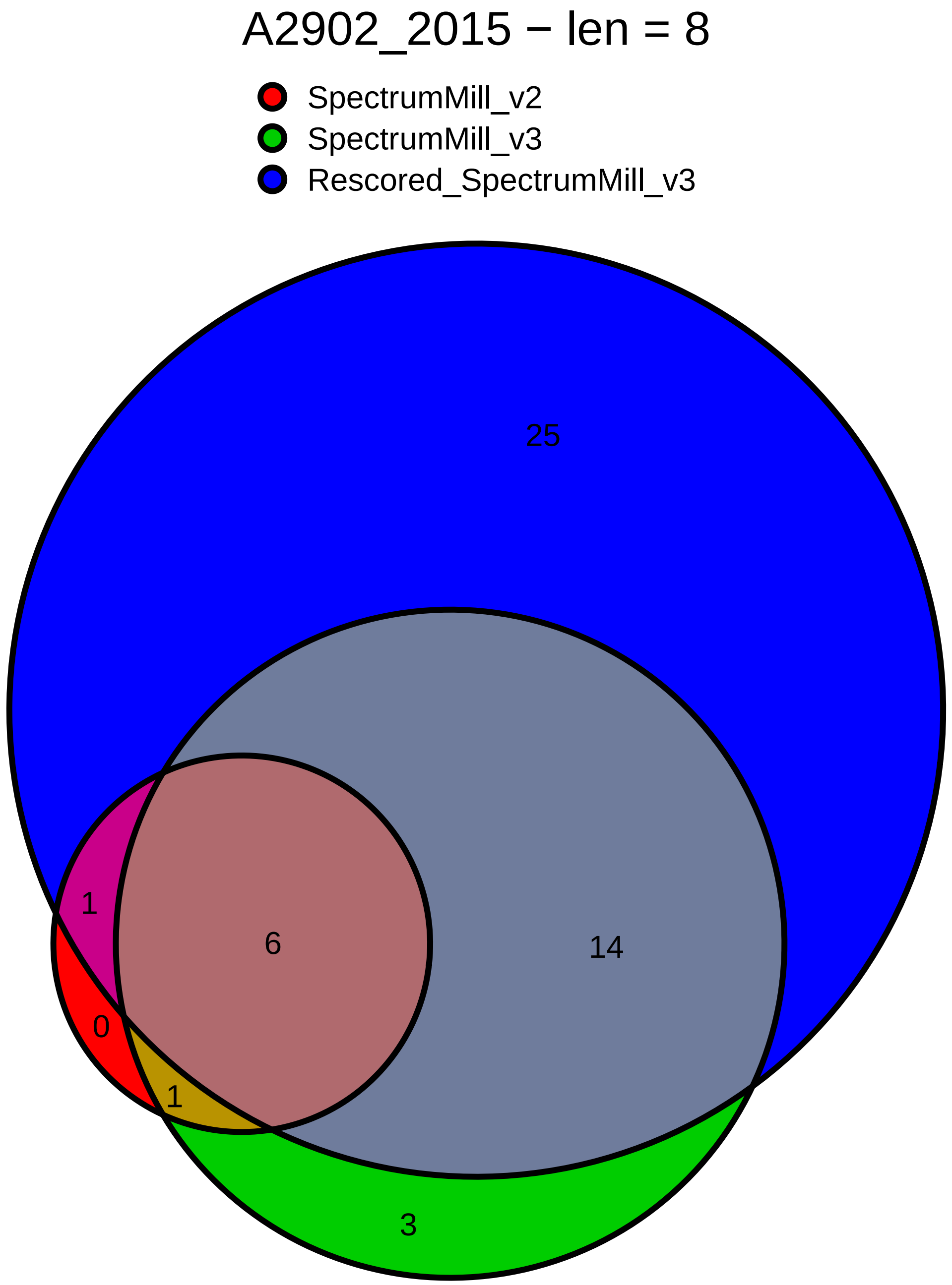
A2902_2015 − len = 8
SpectrumMill_v2
SpectrumMill_v3
Rescored_SpectrumMill_v3
25
1
6
14
0
1
3

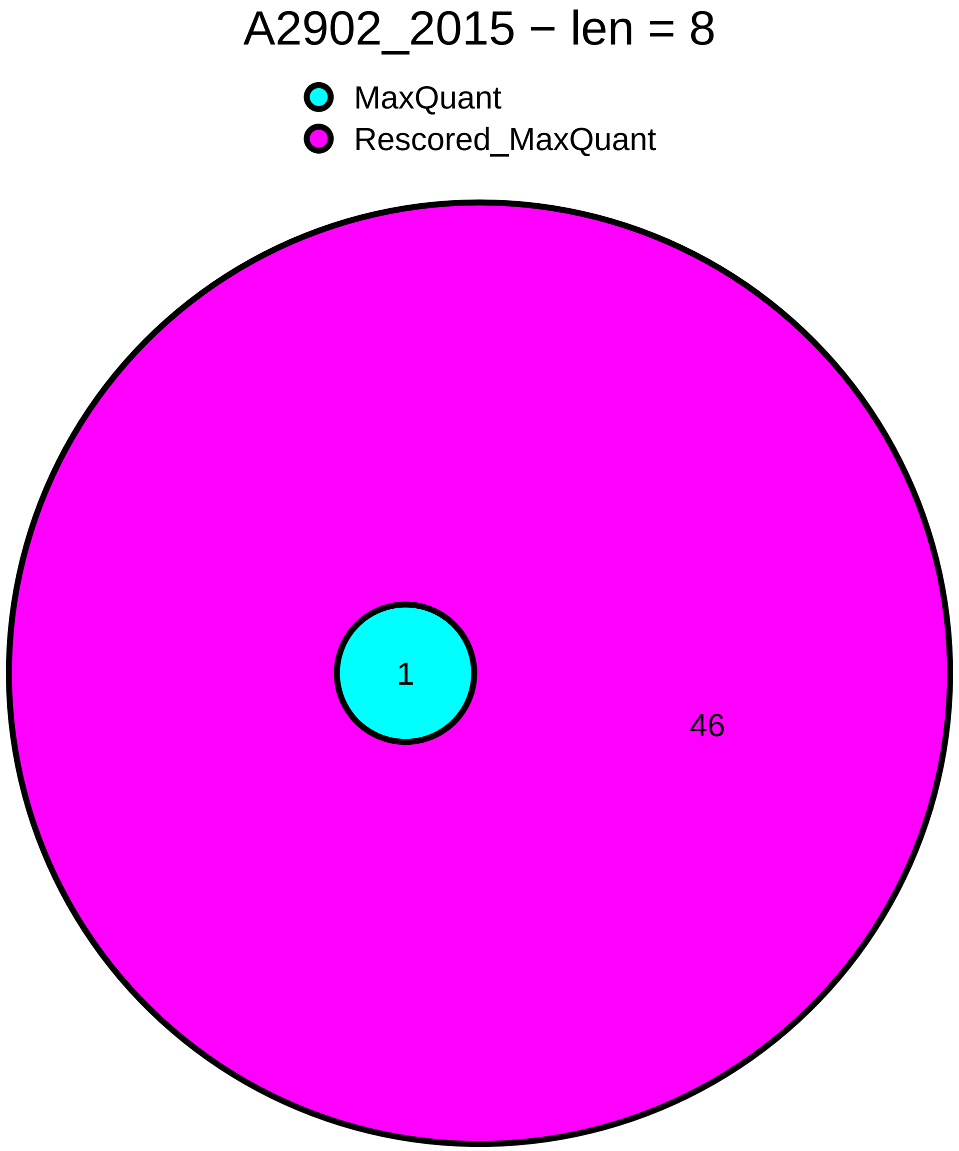
A2902_2015 − len = 8
MaxQuant
Rescored_MaxQuant
1
46

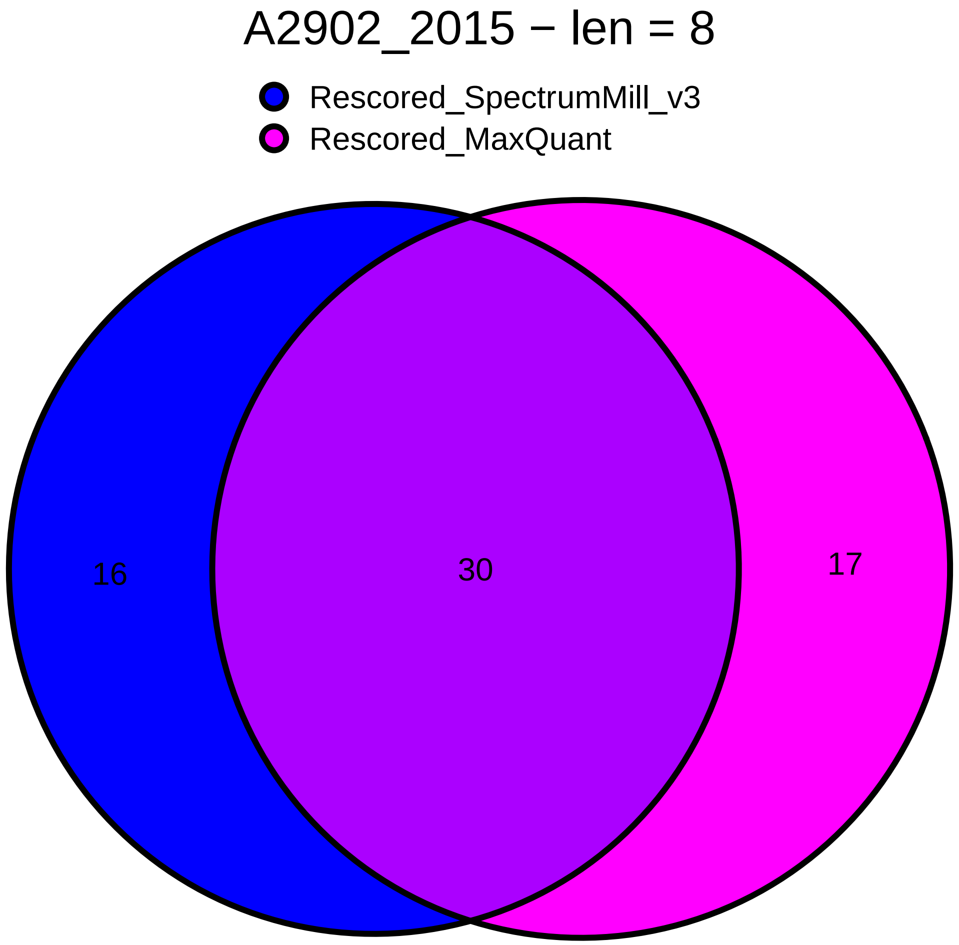
A2902_2015 − len = 8
Rescored_SpectrumMill_v3
Rescored_MaxQuant
16
30
17

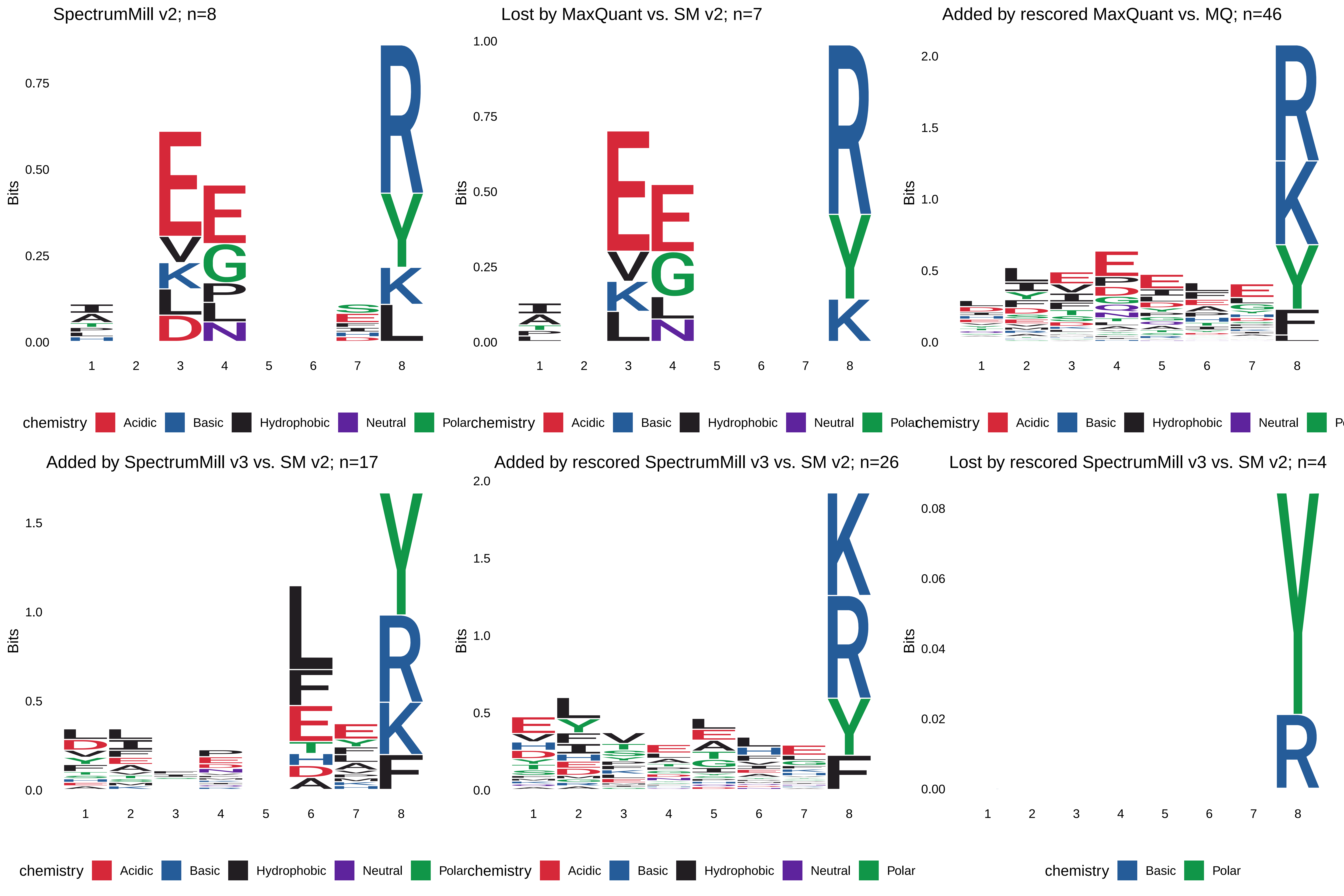

SpectrumMill v2; n=8
Lost by MaxQuant vs. SM v2; n=7
Added by rescored MaxQuant vs. MQ; n=46
Added by SpectrumMill v3 vs. SM v2; n=17
Added by rescored SpectrumMill v3 vs. SM v2; n=26
Lost by rescored SpectrumMill v3 vs. SM v2; n=4
Bits
1 2 3 4 5 6 7 8
chemistry Acidic Basic Hydrophobic Neutral Polar
chemistry Basic Polar

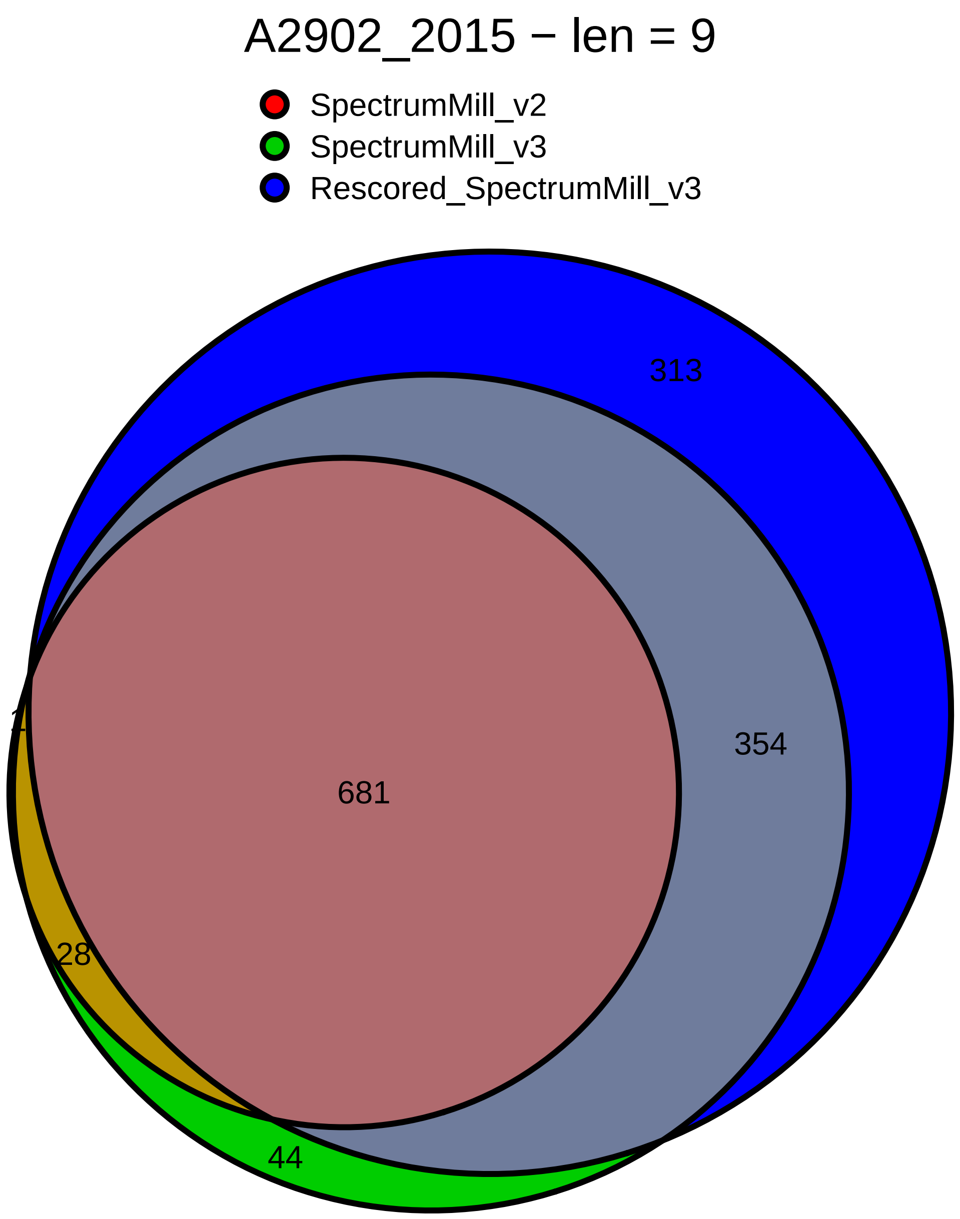
A2902_2015 − len = 9
SpectrumMill_v2
SpectrumMill_v3
Rescored_SpectrumMill_v3
313
354
681
28
44

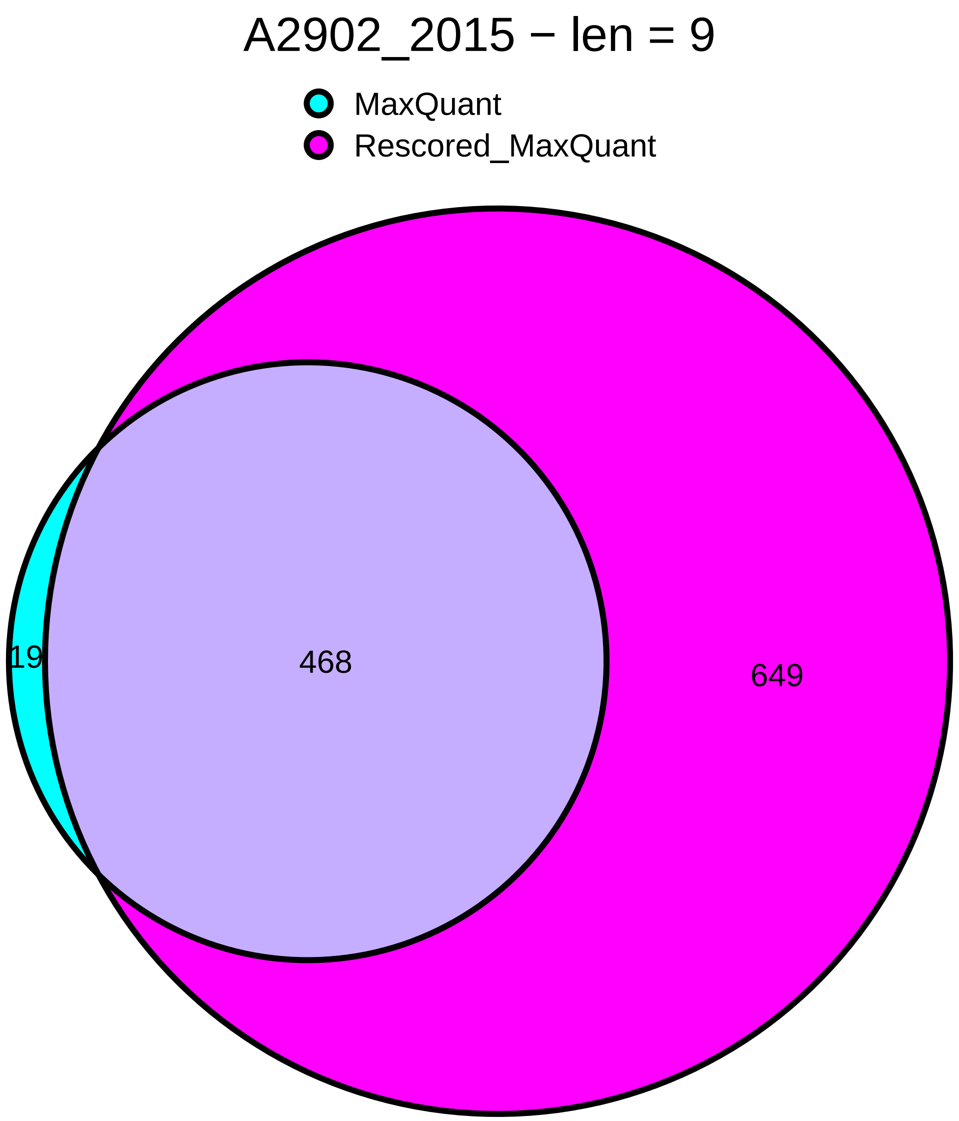
A2902_2015 − len = 9
MaxQuant
Rescored_MaxQuant
19
468
649

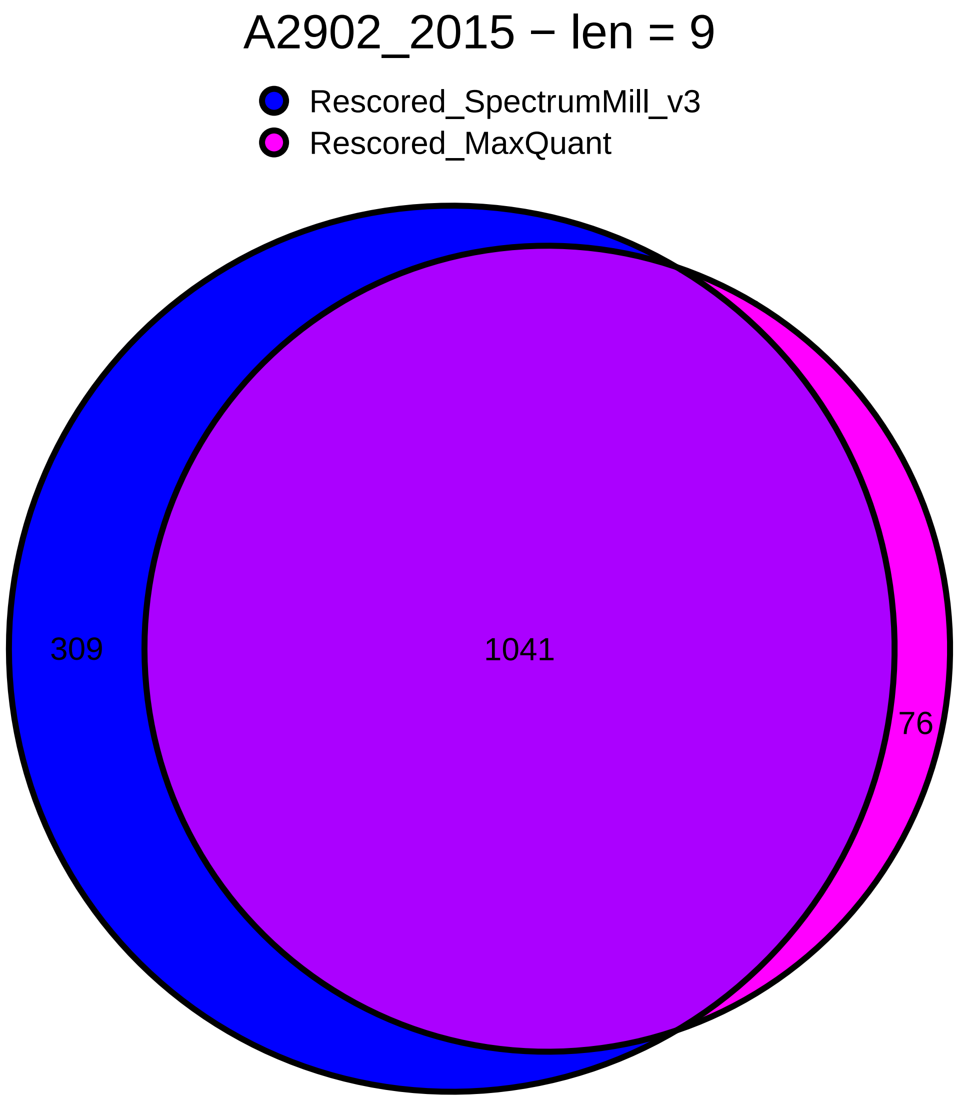
A2902_2015 − len = 9
Rescored_SpectrumMill_v3
Rescored_MaxQuant
309
1041
76

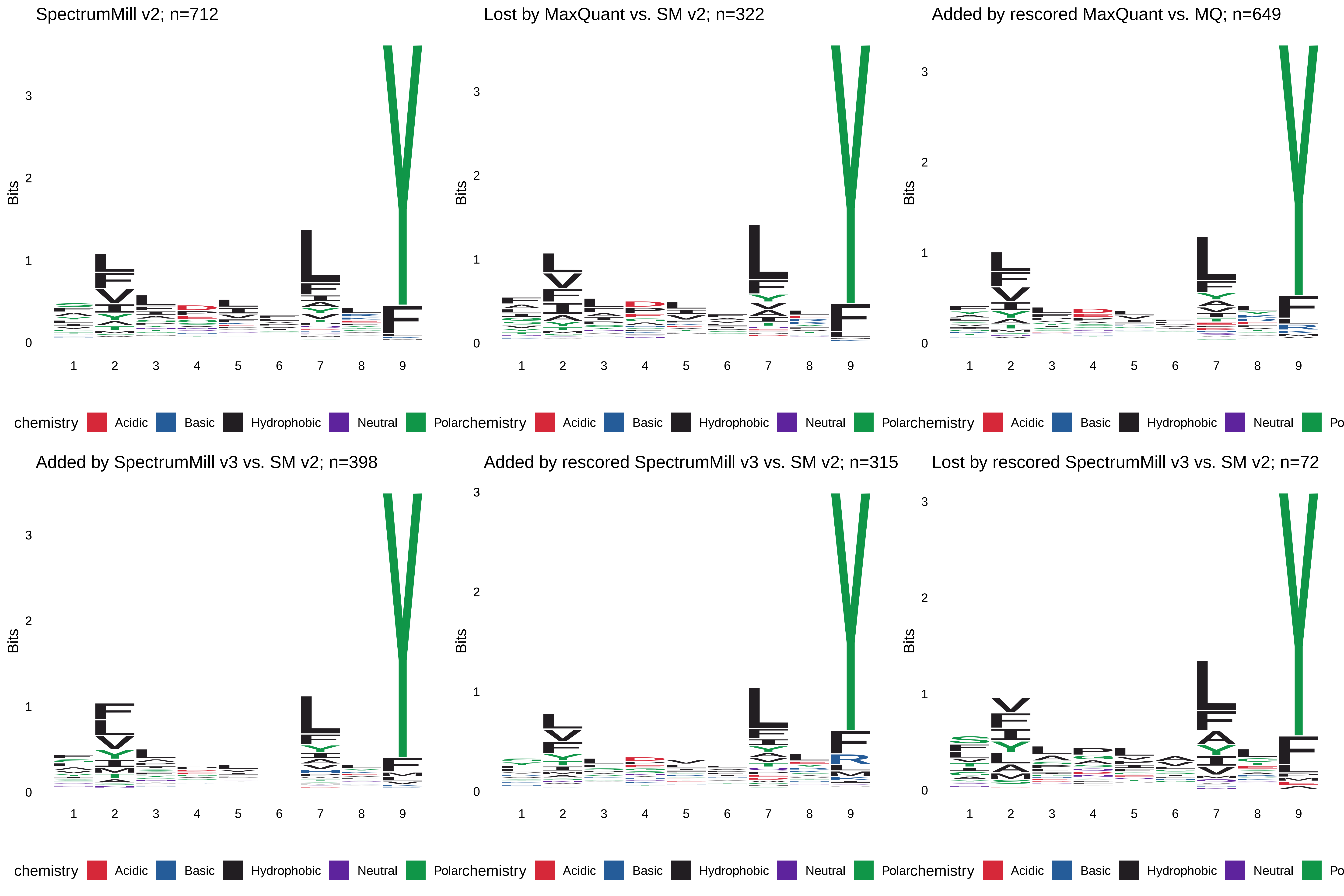
SpectrumMill v2; n=712
Lost by MaxQuant vs. SM v2; n=322
Added by rescored MaxQuant vs. MQ; n=649
Bits
0
1
2
3
1 2 3 4 5 6 7 8 9
chemistry
Acidic
Basic
Hydrophobic
Neutral
Polar
Added by SpectrumMill v3 vs. SM v2; n=398
Added by rescored SpectrumMill v3 vs. SM v2; n=315
Lost by rescored SpectrumMill v3 vs. SM v2; n=72

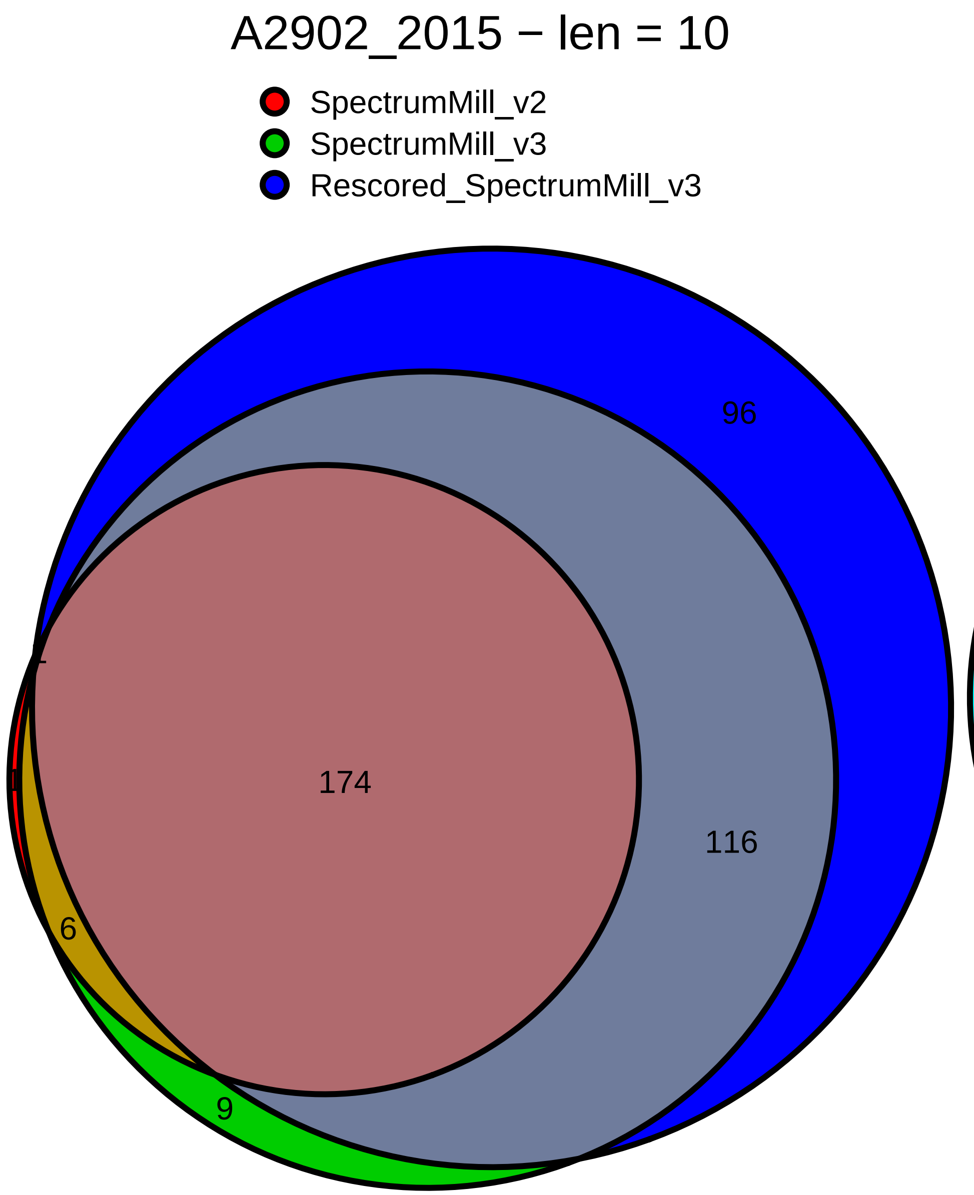
A2902_2015 − len = 10
SpectrumMill_v2
SpectrumMill_v3
Rescored_SpectrumMill_v3
96
174
116
6
9

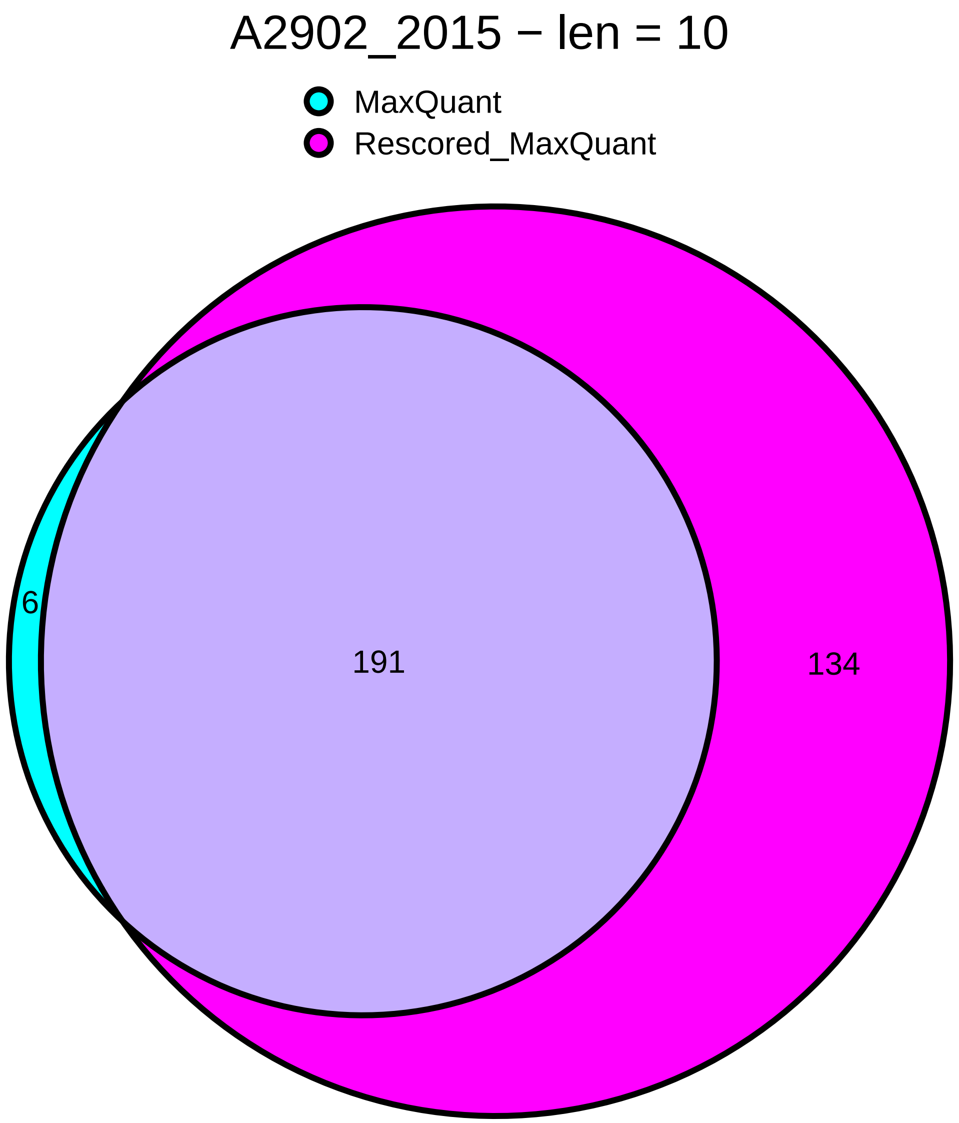
A2902_2015 − len = 10
MaxQuant
Rescored_MaxQuant
6
191
134

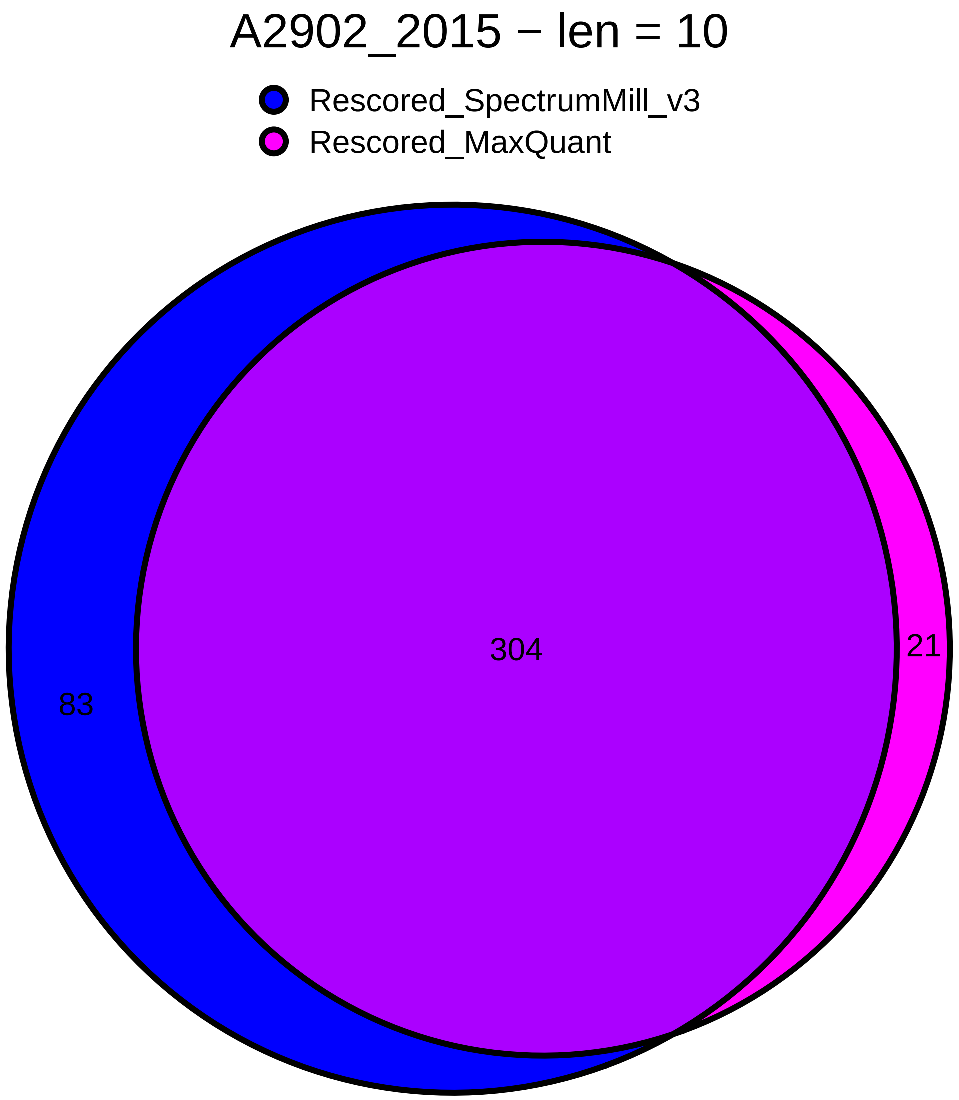
A2902_2015 − len = 10
Rescored_SpectrumMill_v3
Rescored_MaxQuant
83
304
21

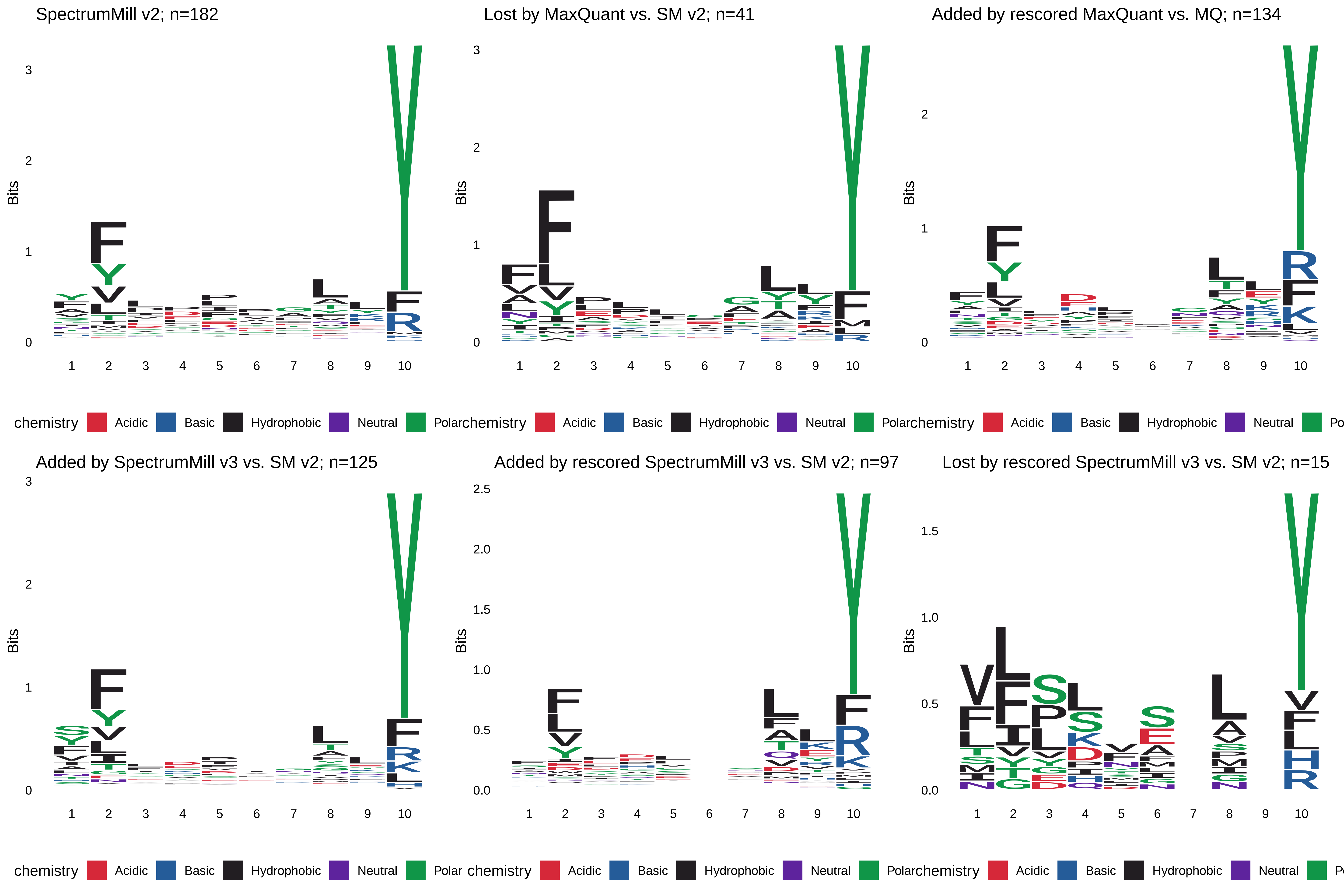
SpectrumMill v2; n=182
Lost by MaxQuant vs. SM v2; n=41
Added by rescored MaxQuant vs. MQ; n=134
Added by SpectrumMill v3 vs. SM v2; n=125
Added by rescored SpectrumMill v3 vs. SM v2; n=97
Lost by rescored SpectrumMill v3 vs. SM v2; n=15
Bits
1 2 3 4 5 6 7 8 9 10
chemistry
Acidic
Basic
Hydrophobic
Neutral
Polar

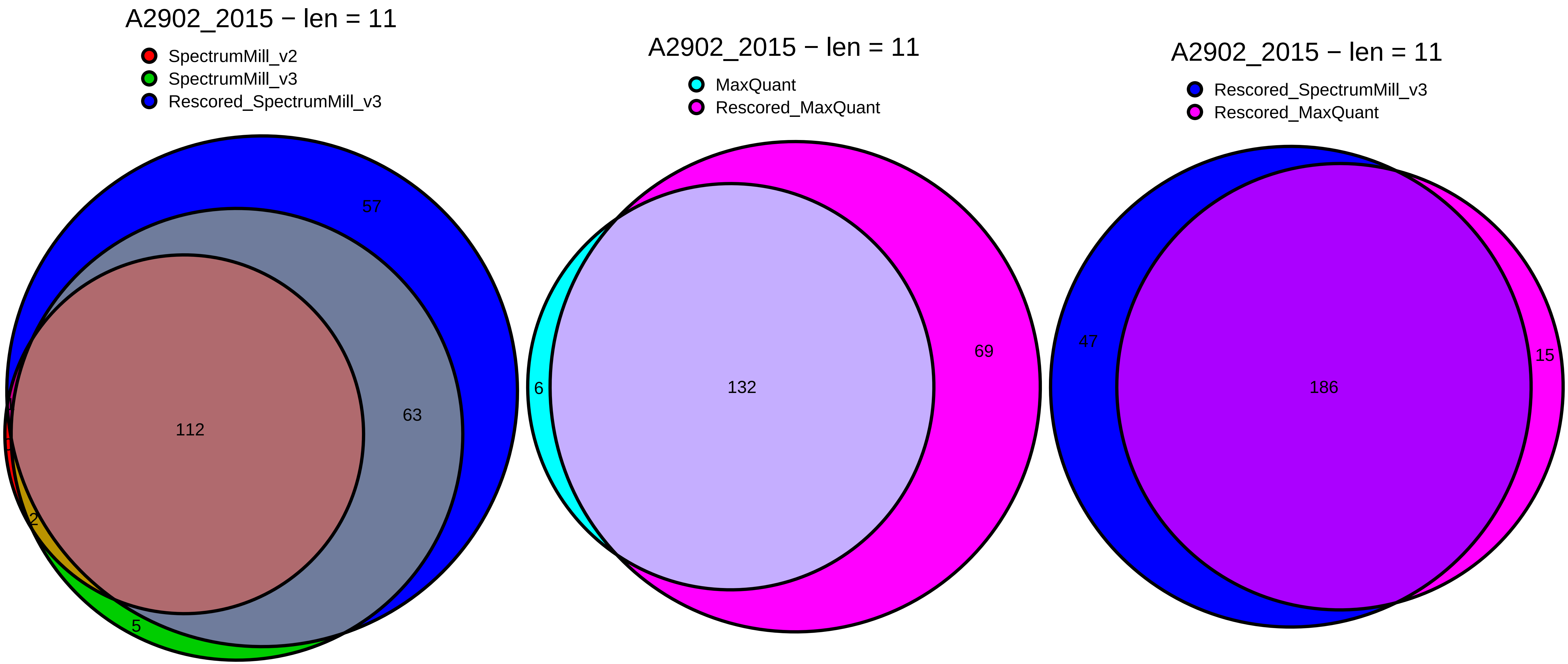
A2902_2015 − len = 11
SpectrumMill_v2
SpectrumMill_v3
Rescored_SpectrumMill_v3
57
63
112
2
5
A2902_2015 − len = 11
MaxQuant
Rescored_MaxQuant
6
132
69
A2902_2015 − len = 11
Rescored_SpectrumMill_v3
Rescored_MaxQuant
47
186
15

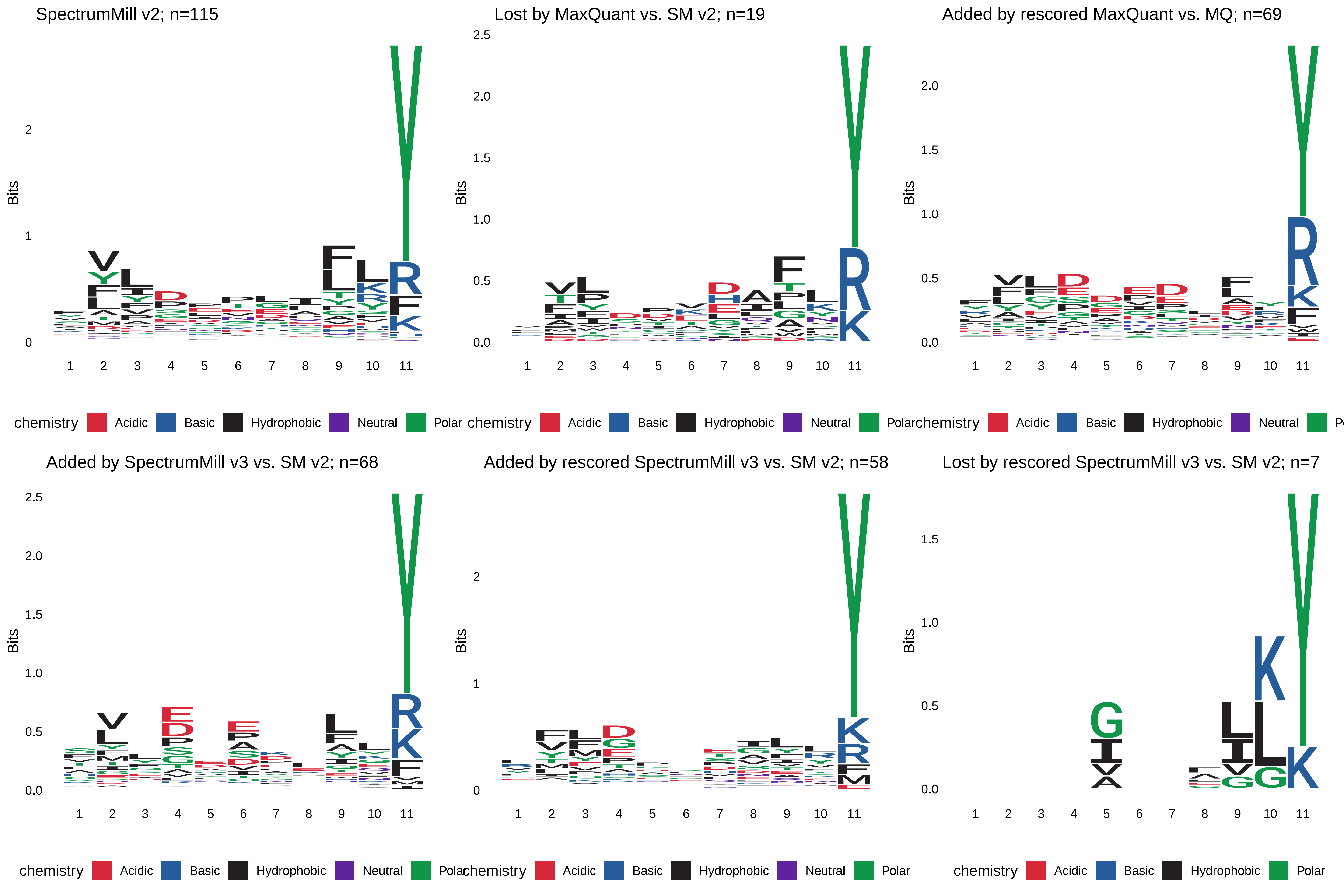

SpectrumMill v2; n=115
Lost by MaxQuant vs. SM v2; n=19
Added by rescored MaxQuant vs. MQ; n=69
Added by SpectrumMill v3 vs. SM v2; n=68
Added by rescored SpectrumMill v3 vs. SM v2; n=58
Lost by rescored SpectrumMill v3 vs. SM v2; n=7
Bits
1 2 3 4 5 6 7 8 9 10 11
chemistry
Acidic
Basic
Hydrophobic
Neutral
Polar

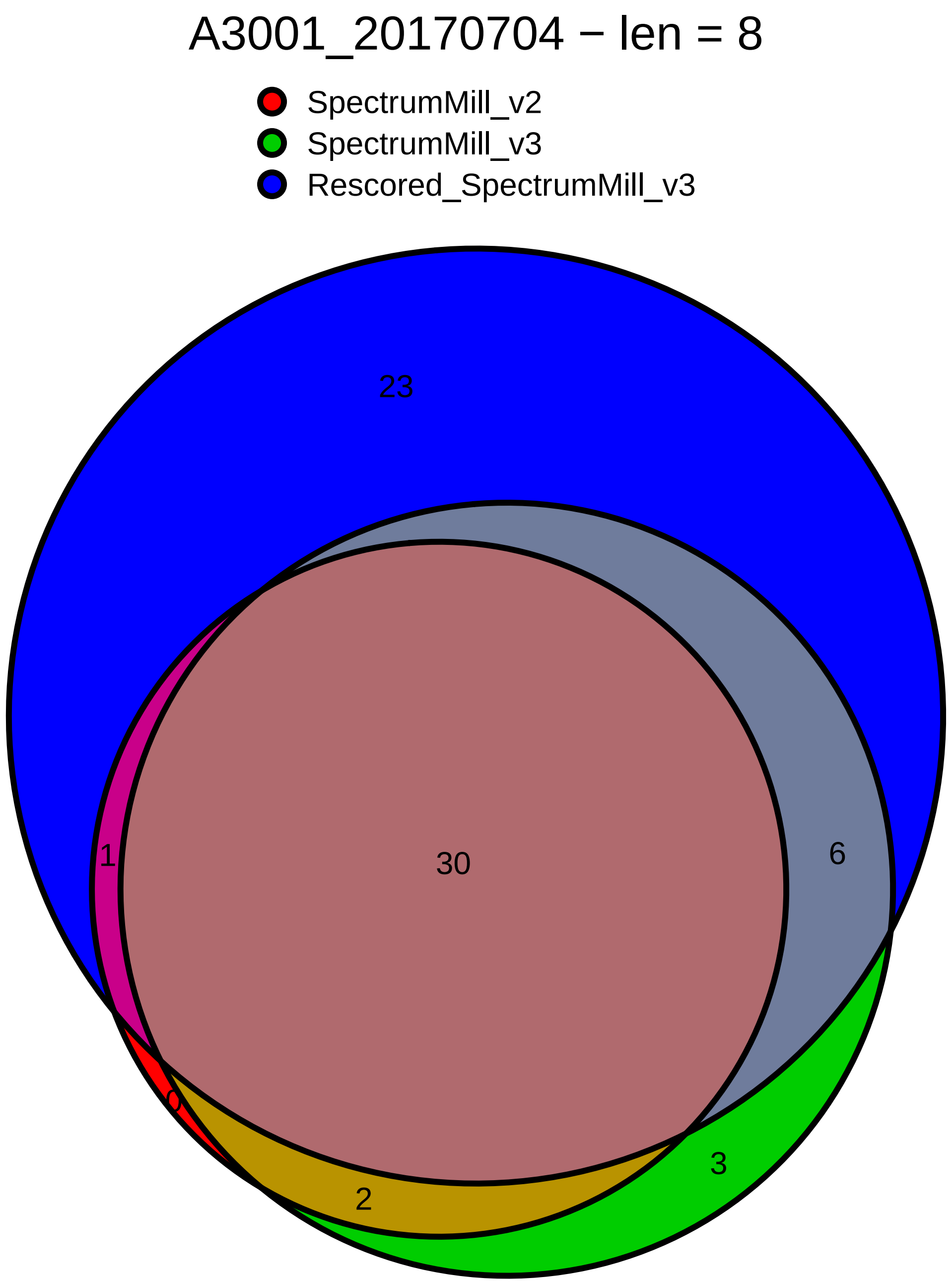
A3001_20170704 − len = 8
SpectrumMill_v2
SpectrumMill_v3
Rescored_SpectrumMill_v3
23
1
30
6
0
2
3

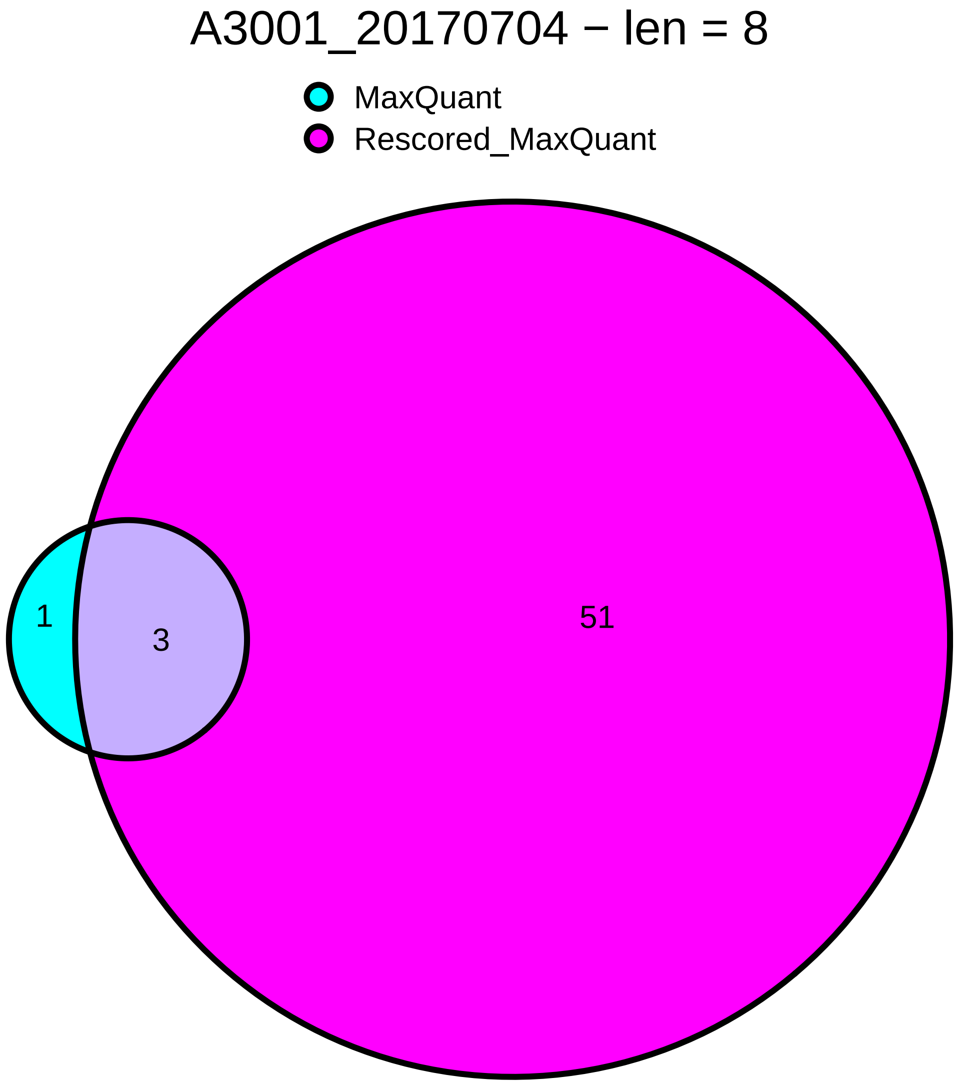
A3001_20170704 − len = 8
MaxQuant
Rescored_MaxQuant
1
3
51

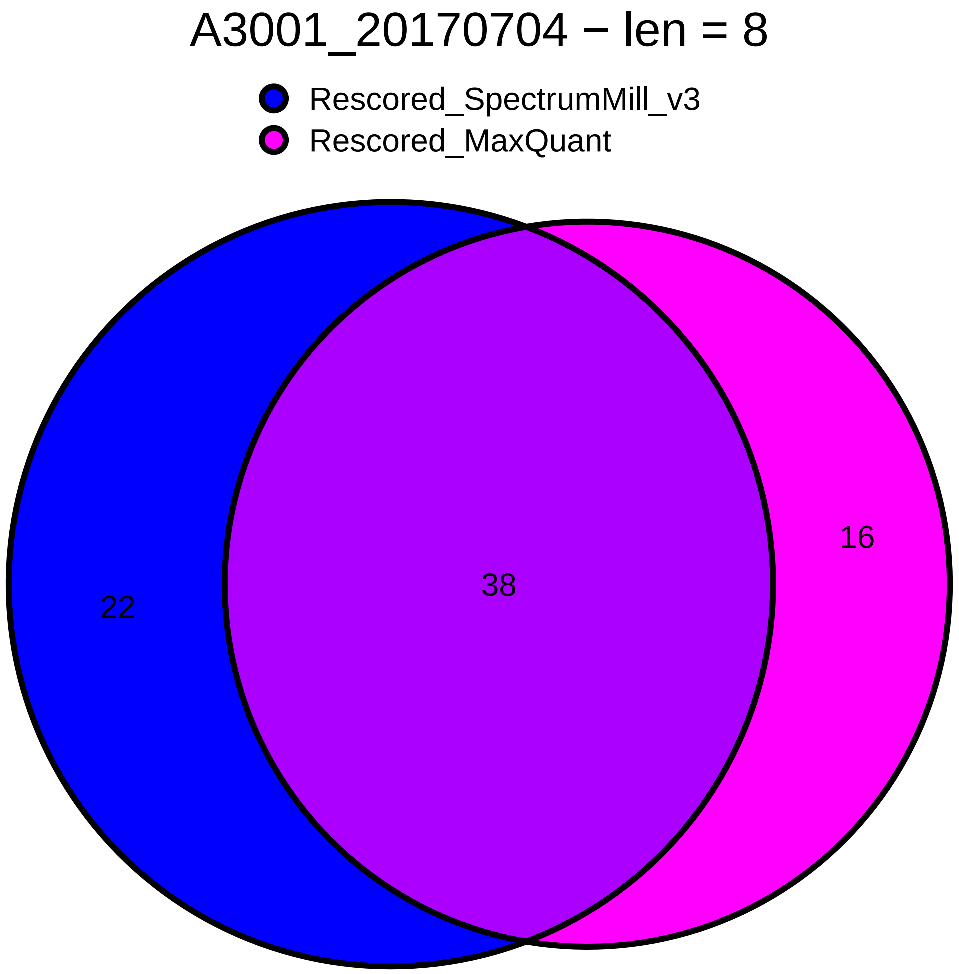
A3001_20170704 − len = 8
Rescored_SpectrumMill_v3
Rescored_MaxQuant
22
38
16

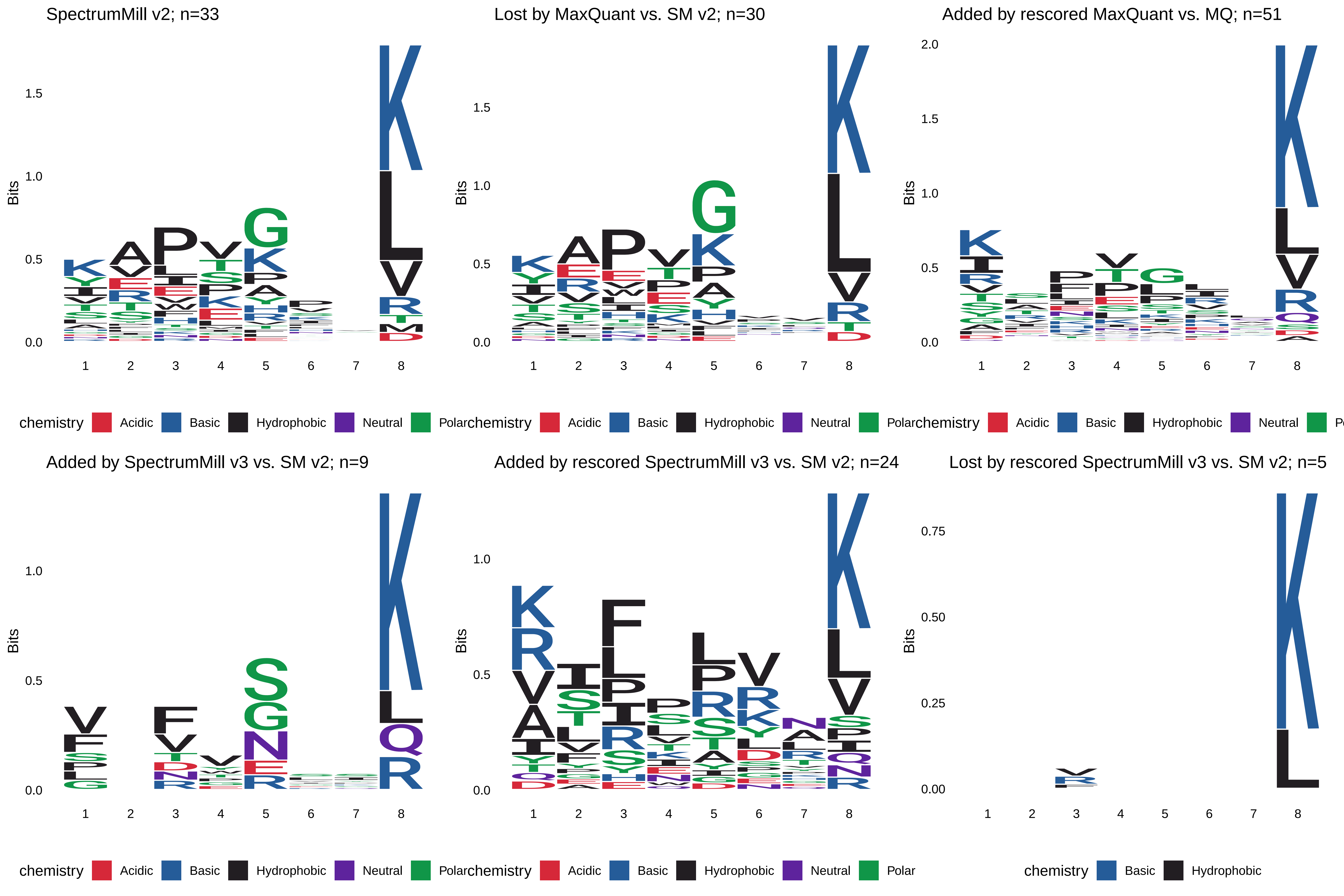

SpectrumMill v2; n=33
Lost by MaxQuant vs. SM v2; n=30
Added by rescored MaxQuant vs. MQ; n=51
Added by SpectrumMill v3 vs. SM v2; n=9
Added by rescored SpectrumMill v3 vs. SM v2; n=24
Lost by rescored SpectrumMill v3 vs. SM v2; n=5
Bits
1 2 3 4 5 6 7 8
chemistry Acidic Basic Hydrophobic Neutral Polar
chemistry Basic Hydrophobic

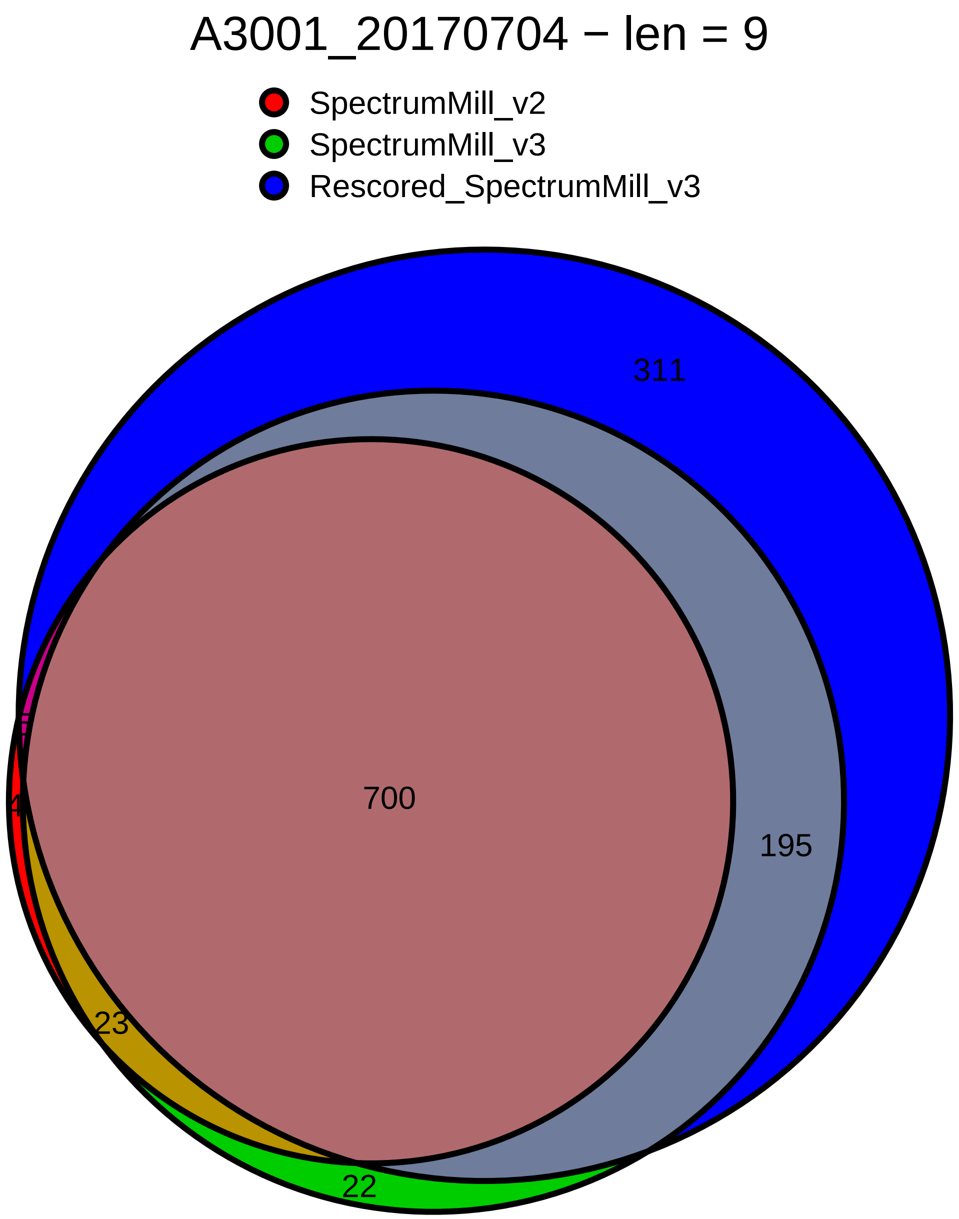
A3001_20170704 − len = 9
SpectrumMill_v2
SpectrumMill_v3
Rescored_SpectrumMill_v3
311
700
195
23
22

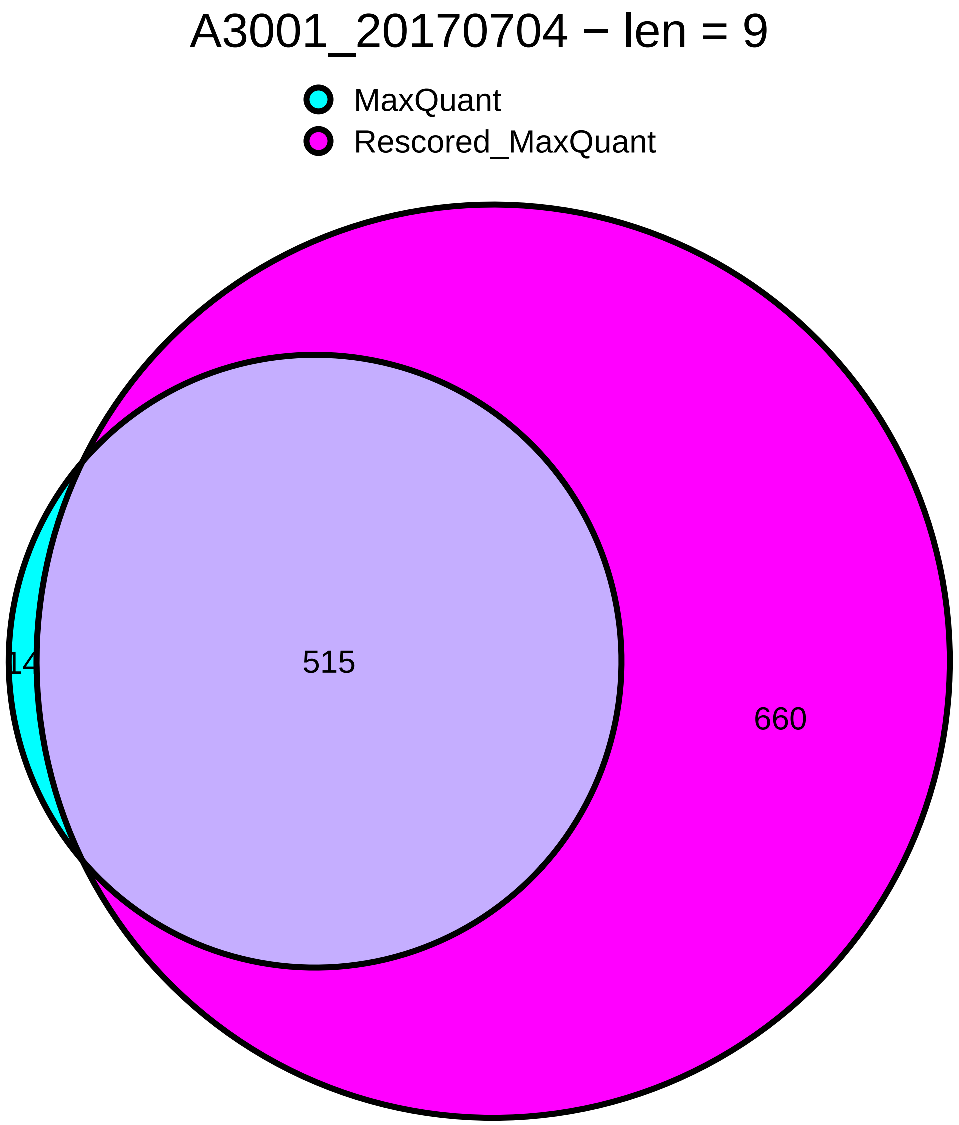
A3001_20170704 − len = 9
MaxQuant
Rescored_MaxQuant
515
660

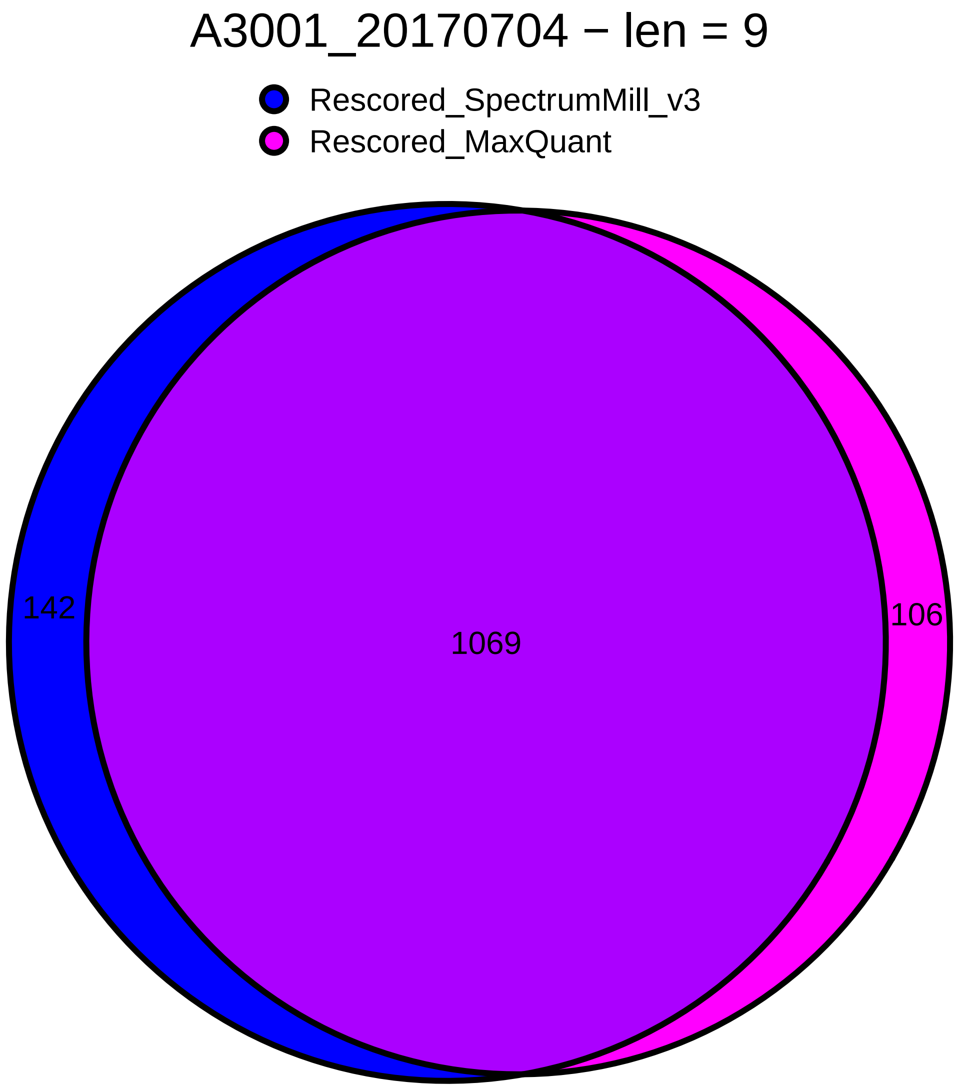
A3001_20170704 − len = 9
Rescored_SpectrumMill_v3
Rescored_MaxQuant
142
1069
106

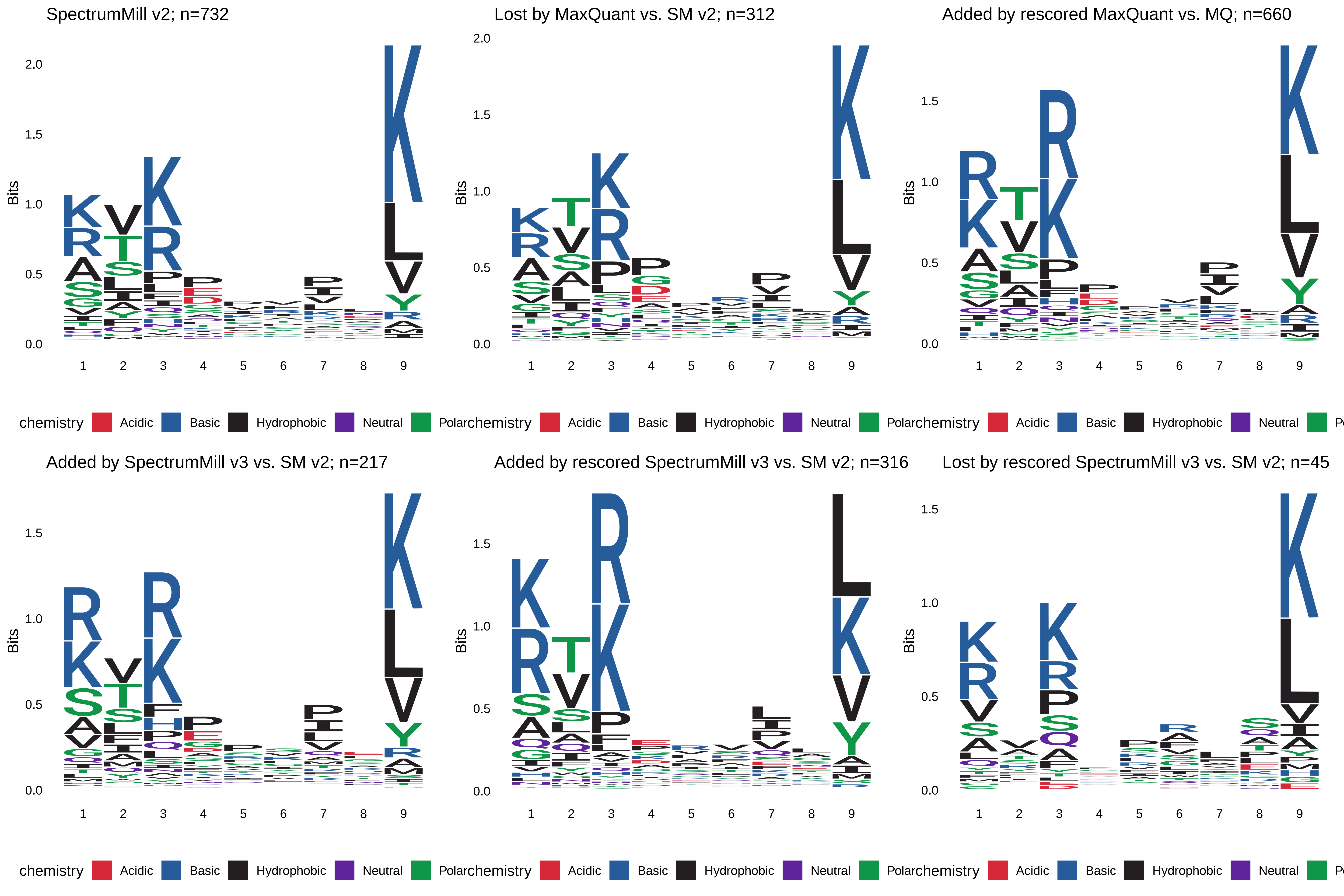
SpectrumMill v2; n=732
Lost by MaxQuant vs. SM v2; n=312
Added by rescored MaxQuant vs. MQ; n=660
Bits
0.0
0.5
1.0
1.5
2.0
1 2 3 4 5 6 7 8 9
chemistry Acidic Basic Hydrophobic Neutral Polar
Added by SpectrumMill v3 vs. SM v2; n=217
Added by rescored SpectrumMill v3 vs. SM v2; n=316
Lost by rescored SpectrumMill v3 vs. SM v2; n=45
Bits
0.0
0.5
1.0
1.5
1 2 3 4 5 6 7 8 9
chemistry Acidic Basic Hydrophobic Neutral Polar

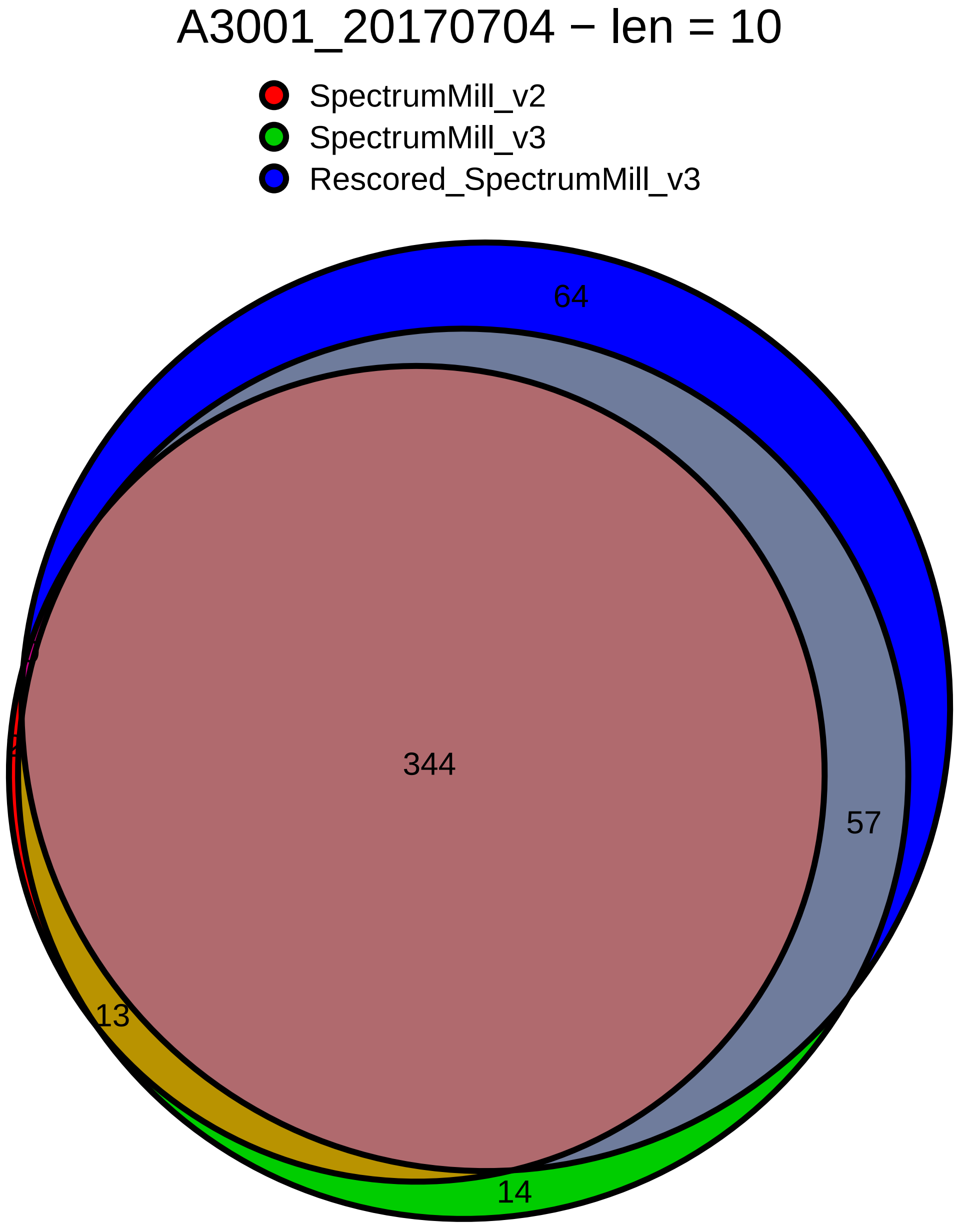

A3001_20170704 − len = 10
SpectrumMill_v2
SpectrumMill_v3
Rescored_SpectrumMill_v3
64
344
57
13
14

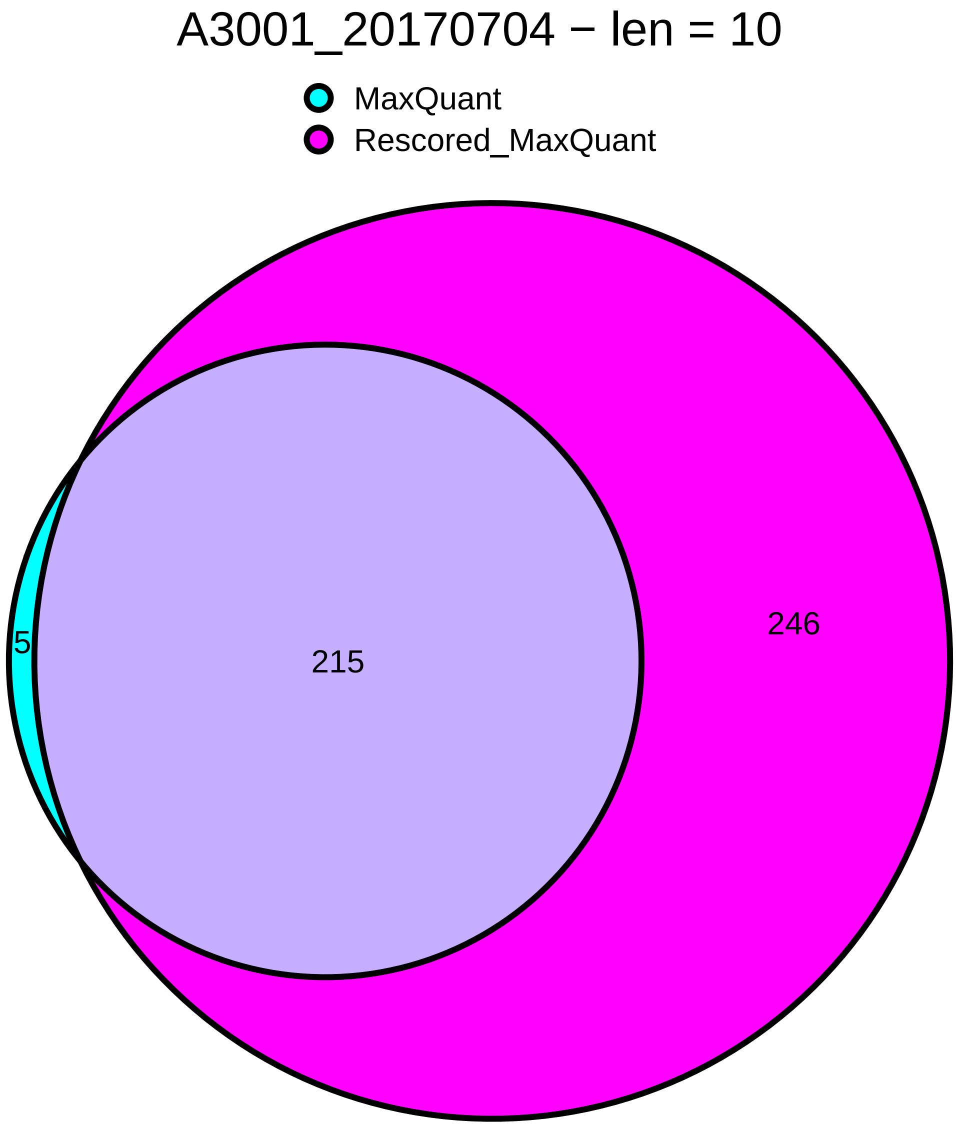

A3001_20170704 − len = 10
MaxQuant
Rescored_MaxQuant
5
215
246

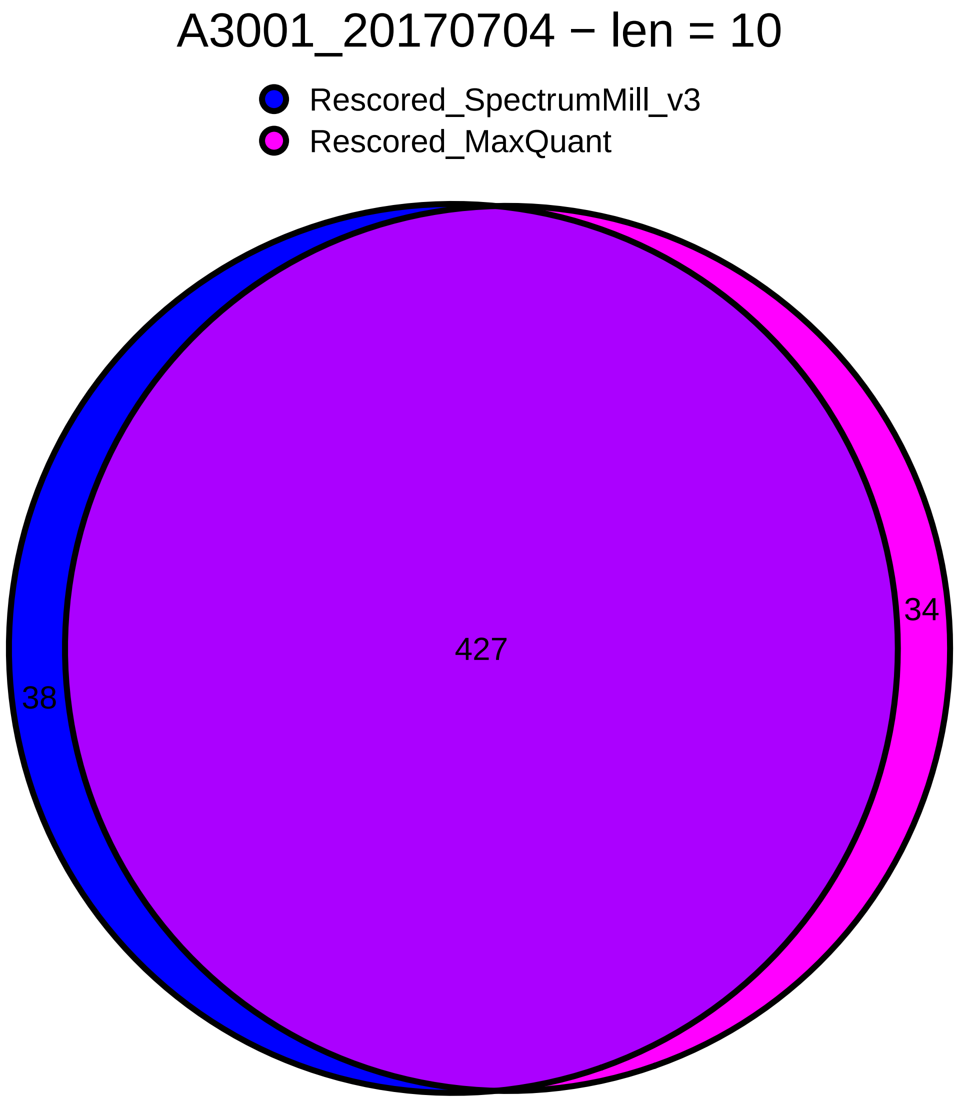

A3001_20170704 − len = 10
Rescored_SpectrumMill_v3
Rescored_MaxQuant
38
427
34

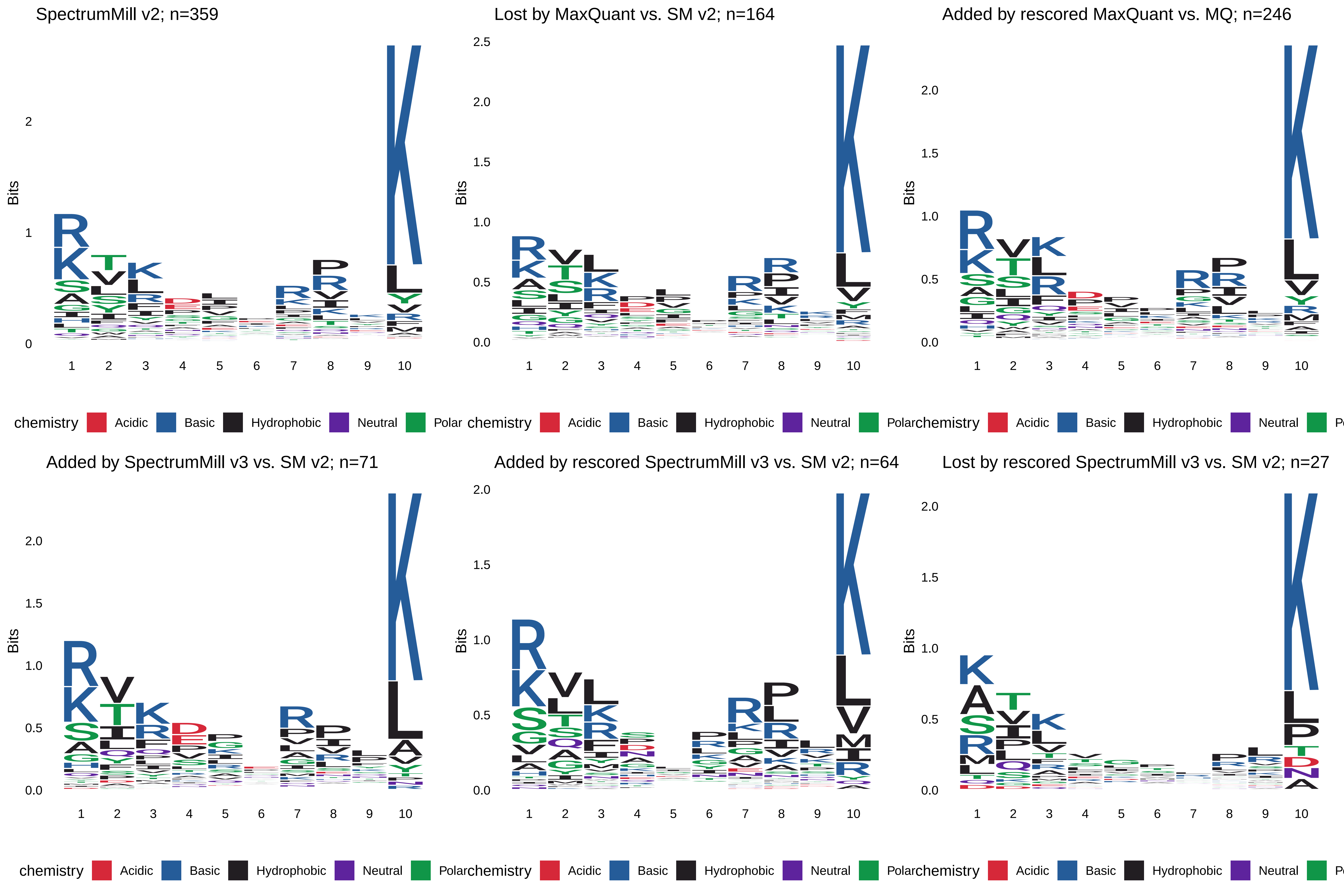

SpectrumMill v2; n=359
Lost by MaxQuant vs. SM v2; n=164
Added by rescored MaxQuant vs. MQ; n=246
Added by SpectrumMill v3 vs. SM v2; n=71
Added by rescored SpectrumMill v3 vs. SM v2; n=64
Lost by rescored SpectrumMill v3 vs. SM v2; n=27
Bits
1 2 3 4 5 6 7 8 9 10
chemistry Acidic Basic Hydrophobic Neutral Polar
chemistry Acidic Basic Hydrophobic Neutral Polar
chemistry Acidic Basic Hydrophobic Neutral Polar
chemistry Acidic Basic Hydrophobic Neutral Polar

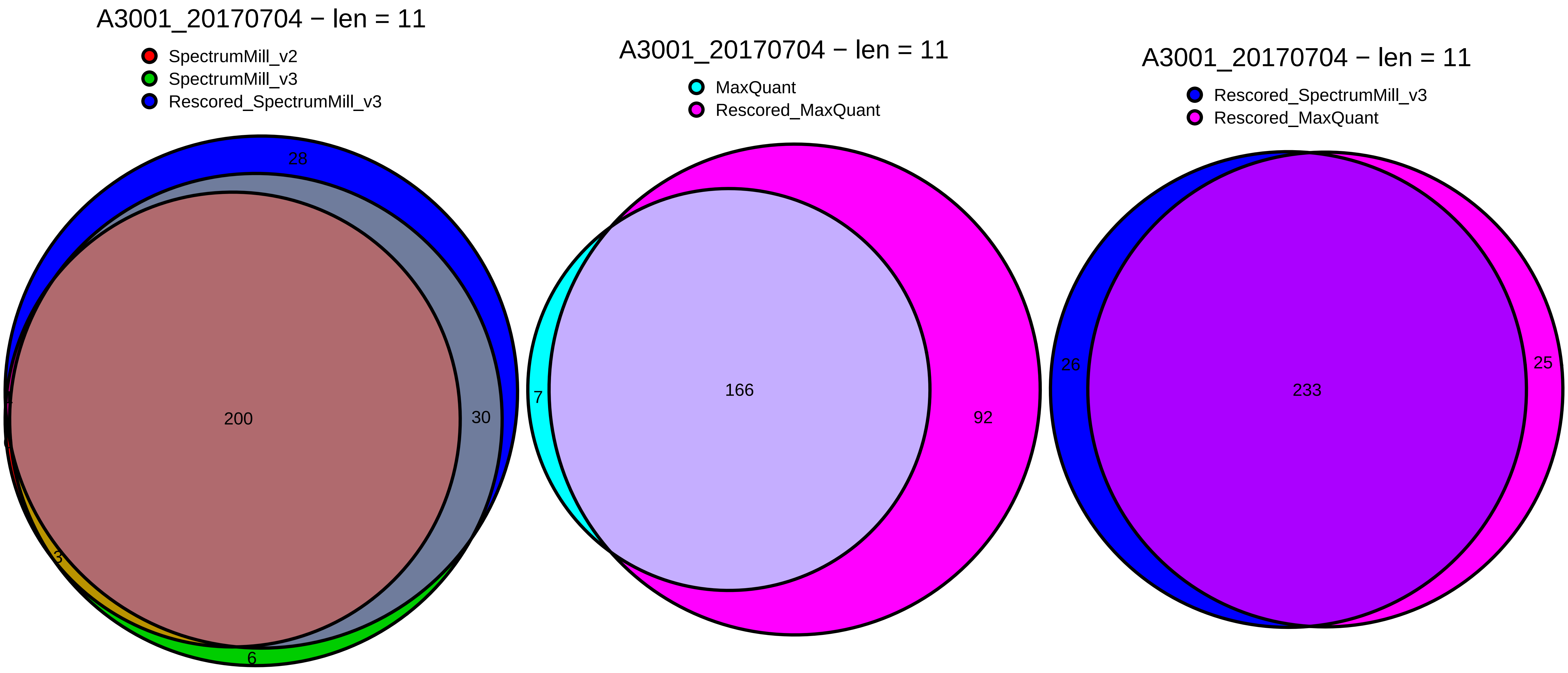
A3001_20170704 − len = 11
SpectrumMill_v2
SpectrumMill_v3
Rescored_SpectrumMill_v3
28
200
30
3
6
A3001_20170704 − len = 11
MaxQuant
Rescored_MaxQuant
7
166
92
A3001_20170704 − len = 11
Rescored_SpectrumMill_v3
Rescored_MaxQuant
26
233
25

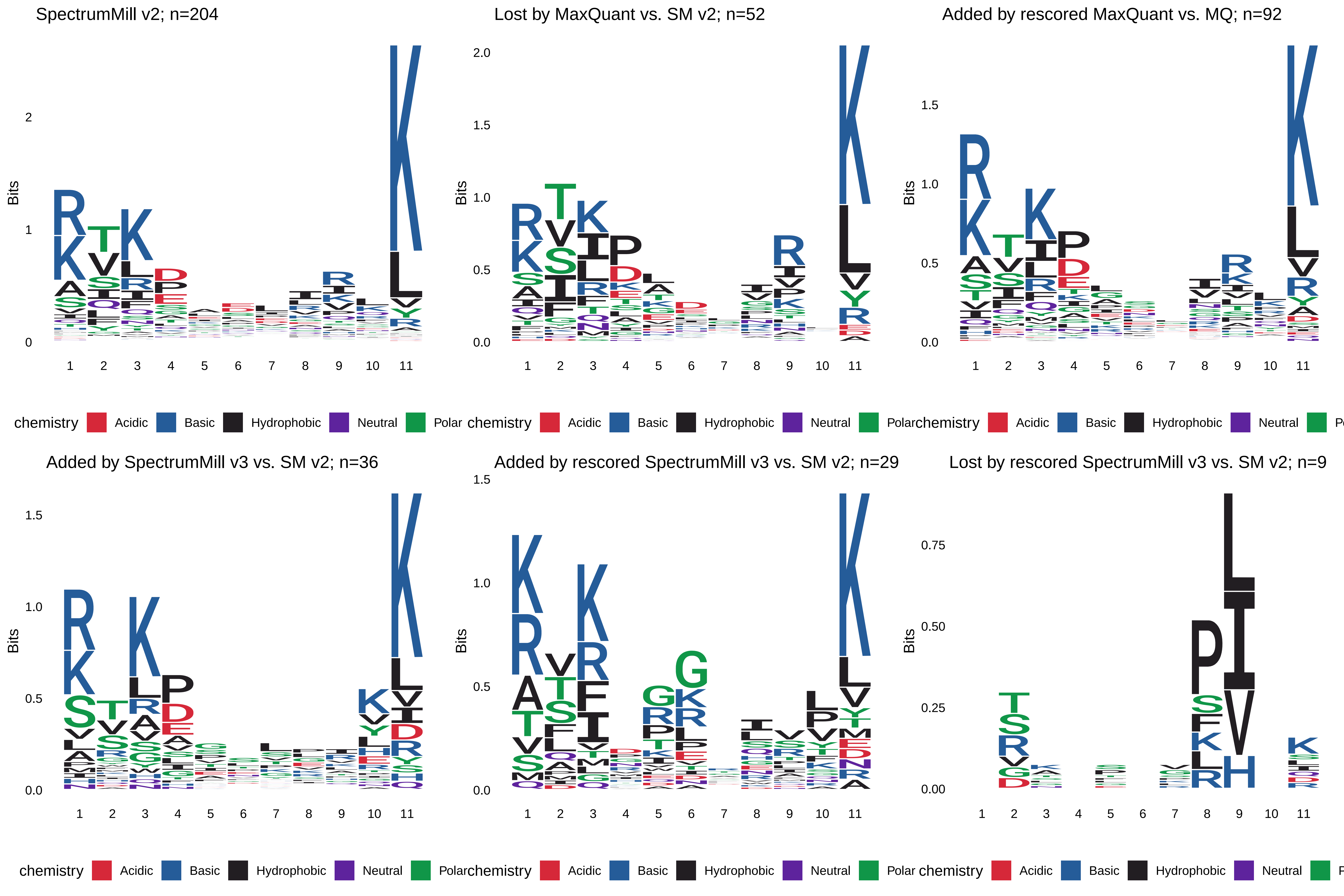

SpectrumMill v2; n=204
Lost by MaxQuant vs. SM v2; n=52
Added by rescored MaxQuant vs. MQ; n=92
Bits
1 2 3 4 5 6 7 8 9 10 11
chemistry Acidic Basic Hydrophobic Neutral Polar
Added by SpectrumMill v3 vs. SM v2; n=36
Added by rescored SpectrumMill v3 vs. SM v2; n=29
Lost by rescored SpectrumMill v3 vs. SM v2; n=9
Bits
1 2 3 4 5 6 7 8 9 10 11
chemistry Acidic Basic Hydrophobic Neutral Polar

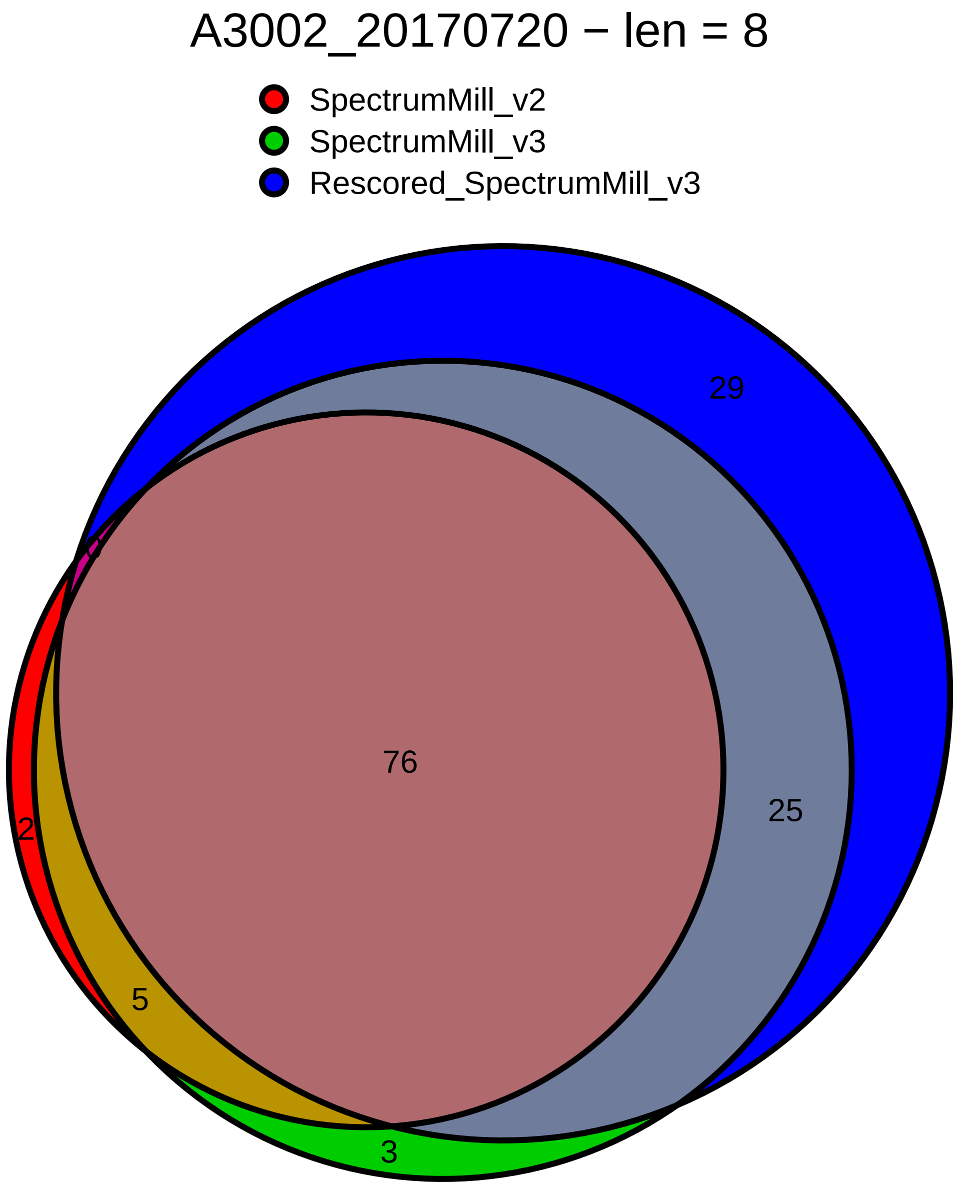
A3002_20170720 − len = 8
SpectrumMill_v2
SpectrumMill_v3
Rescored_SpectrumMill_v3
29
2
76
25
5
3

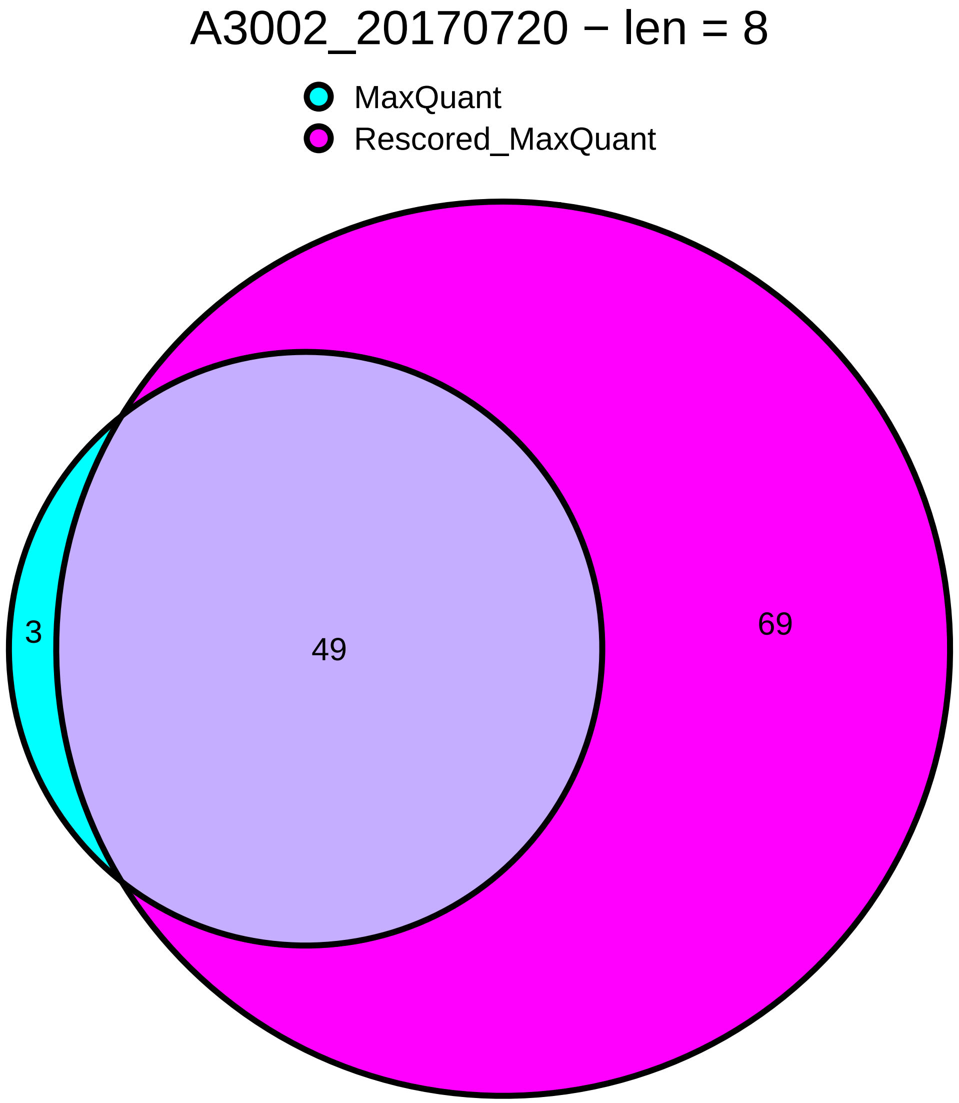
A3002_20170720 − len = 8
MaxQuant
Rescored_MaxQuant
3
49
69

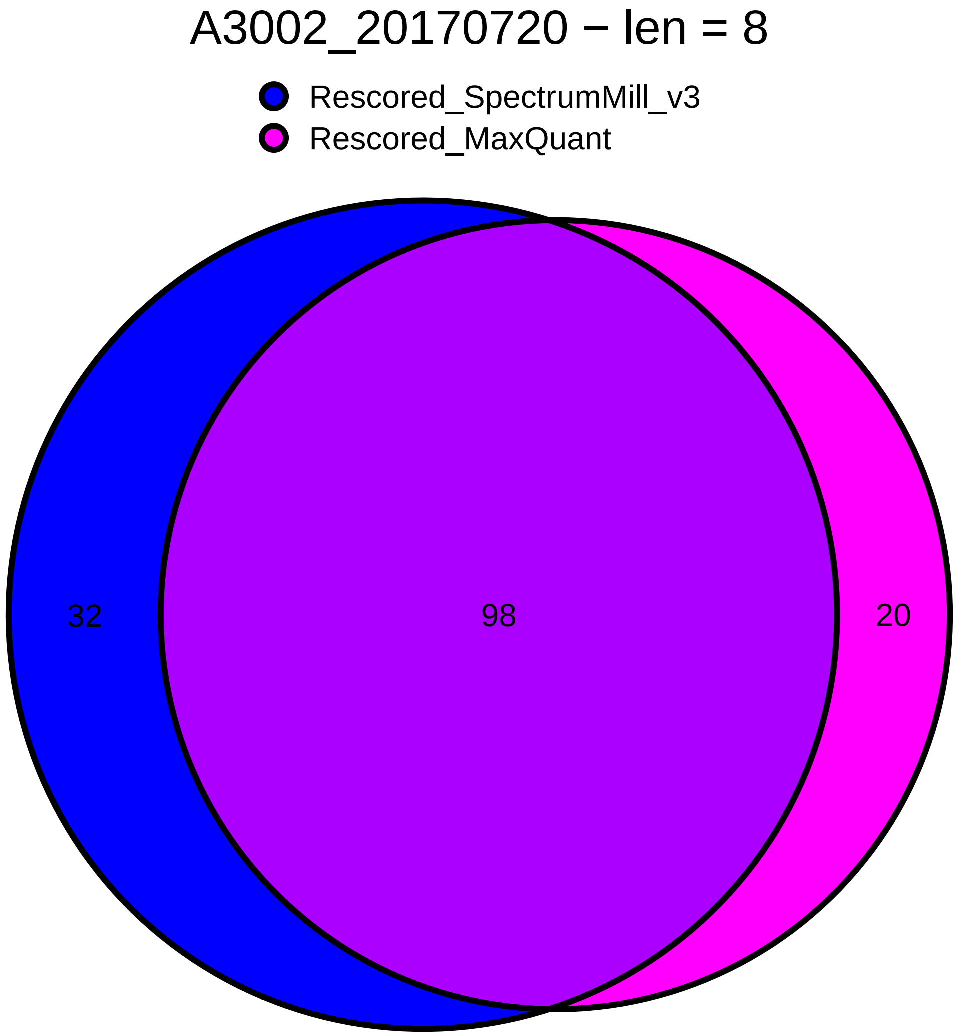
A3002_20170720 − len = 8
Rescored_SpectrumMill_v3
Rescored_MaxQuant
32
98
20

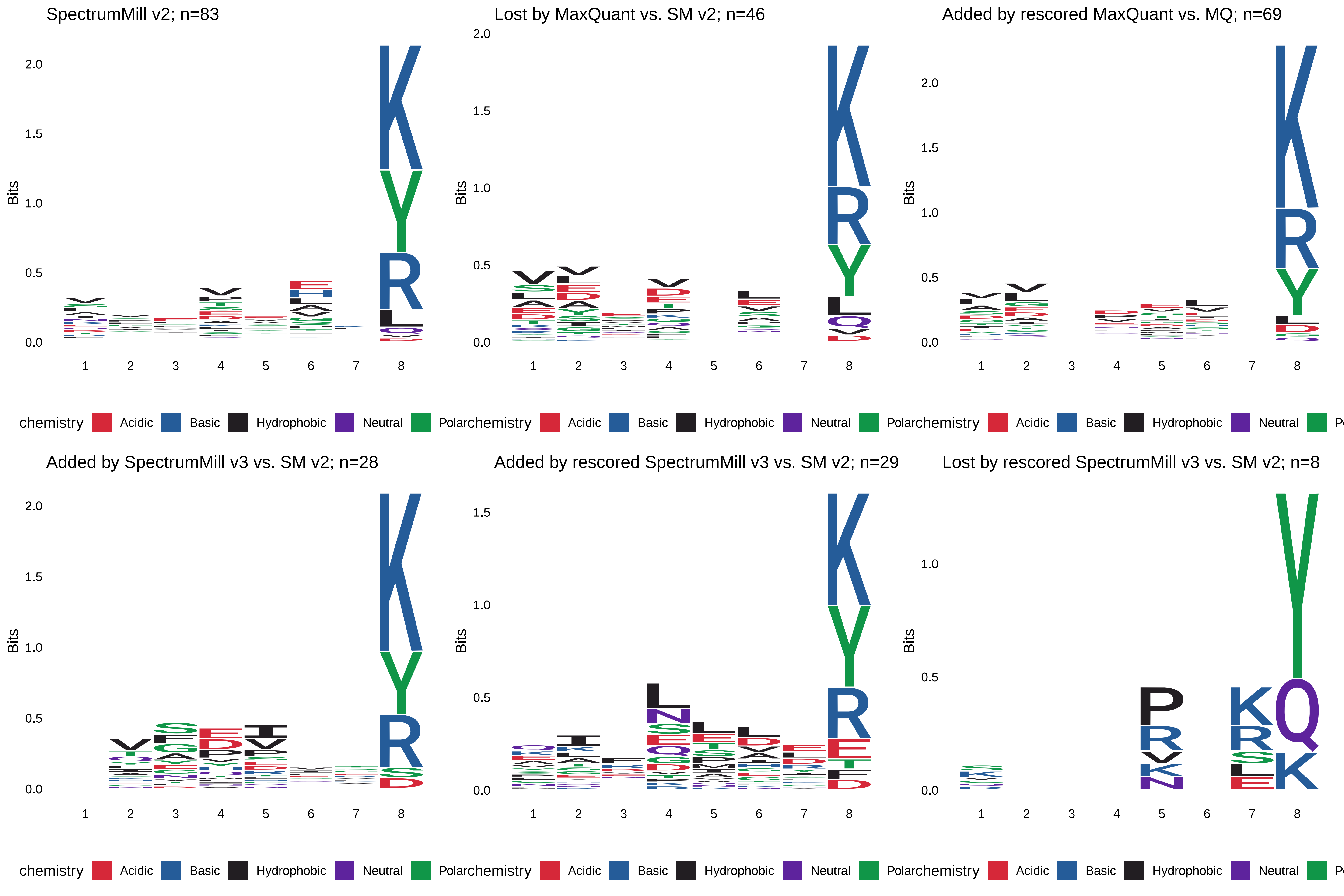
SpectrumMill v2; n=83
Lost by MaxQuant vs. SM v2; n=46
Added by rescored MaxQuant vs. MQ; n=69
Added by SpectrumMill v3 vs. SM v2; n=28
Added by rescored SpectrumMill v3 vs. SM v2; n=29
Lost by rescored SpectrumMill v3 vs. SM v2; n=8
Bits
0.0
0.5
1.0
1.5
2.0
1
2
3
4
5
6
7
8
chemistry
Acidic
Basic
Hydrophobic
Neutral
Polar

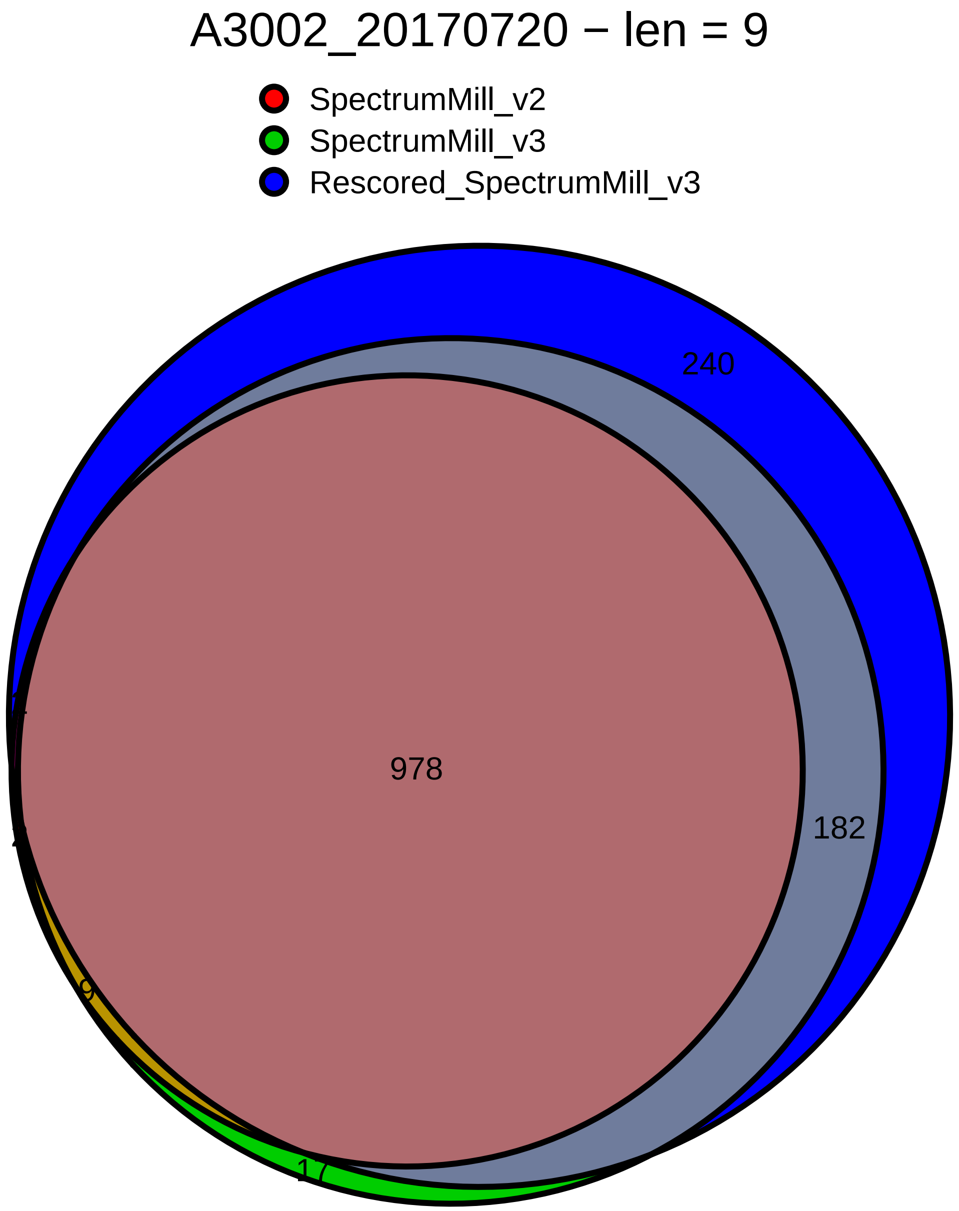
A3002_20170720 − len = 9
SpectrumMill_v2
SpectrumMill_v3
Rescored_SpectrumMill_v3
240
978
182
9
17

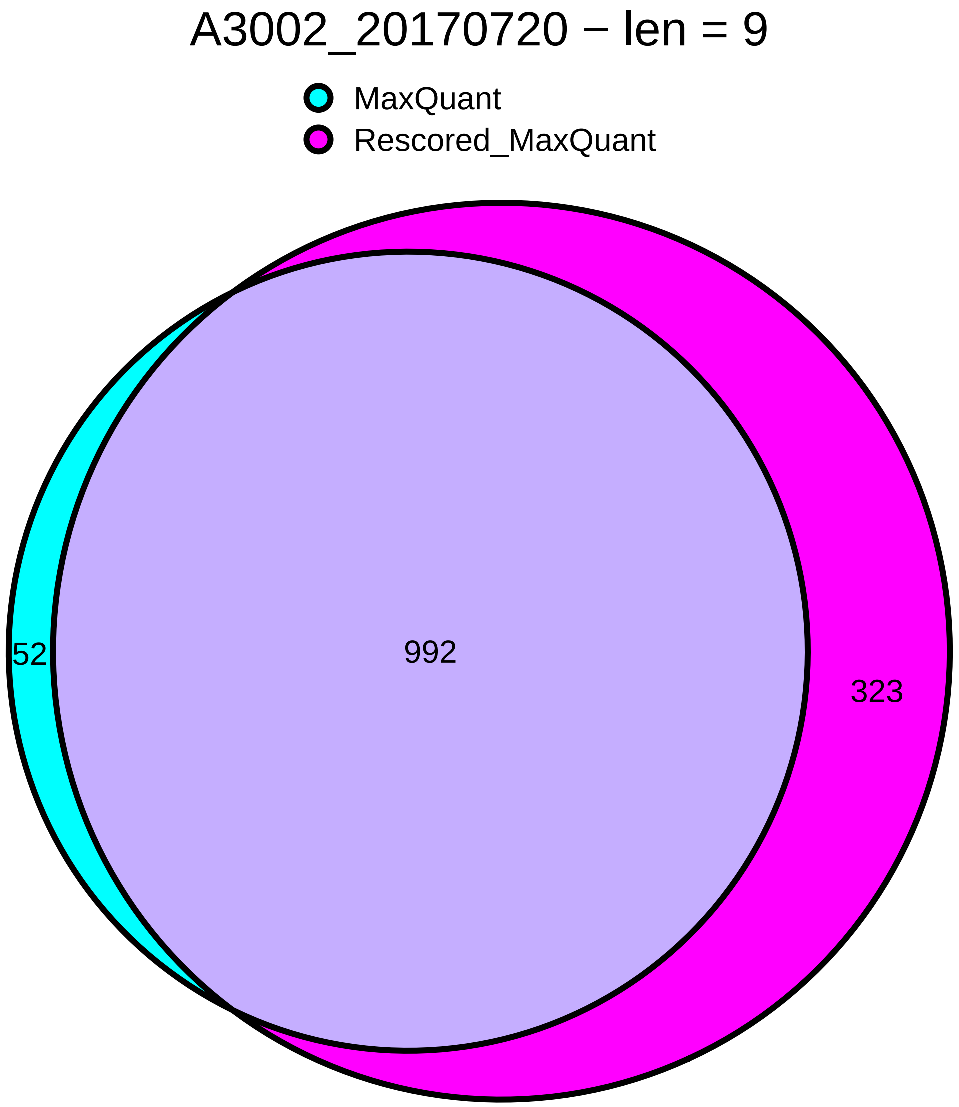
A3002_20170720 − len = 9
MaxQuant
Rescored_MaxQuant
52
992
323

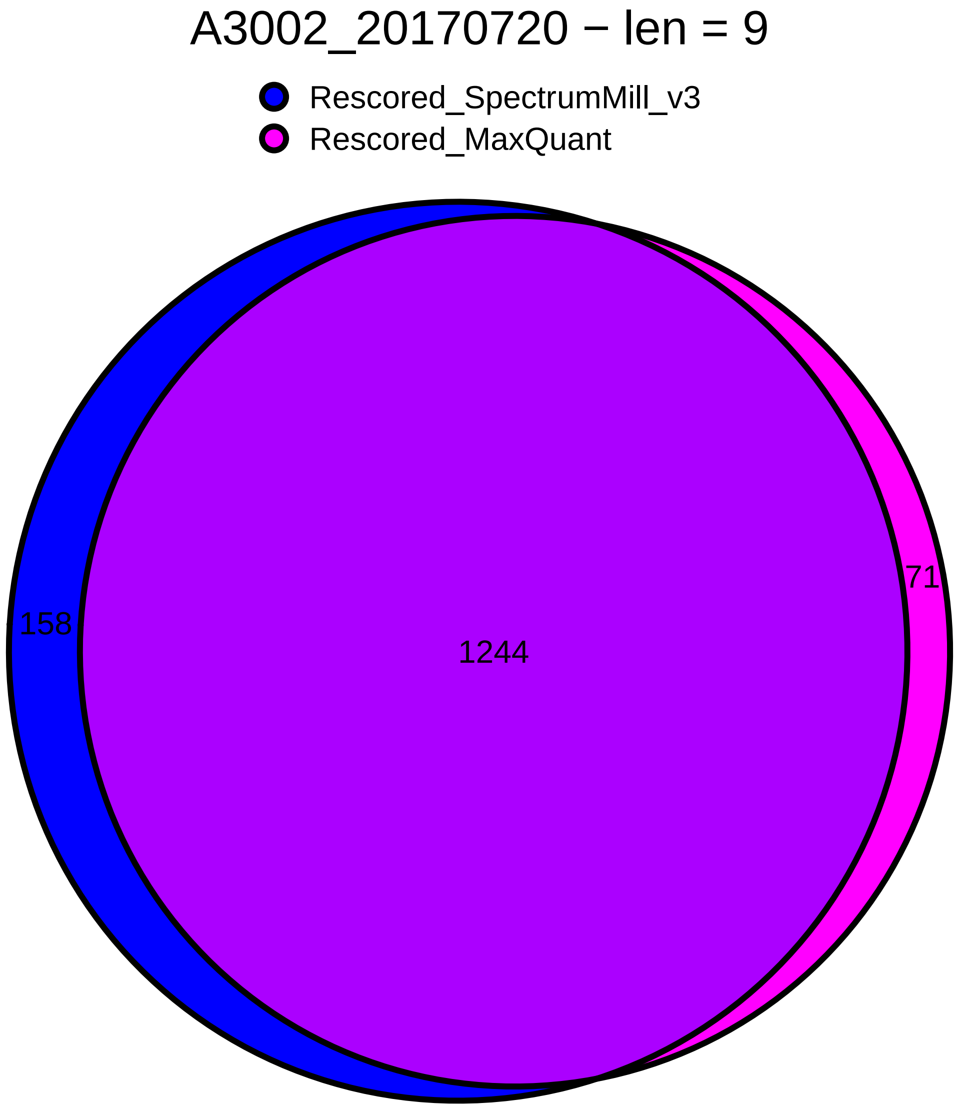
A3002_20170720 − len = 9
Rescored_SpectrumMill_v3
Rescored_MaxQuant
158
1244
71

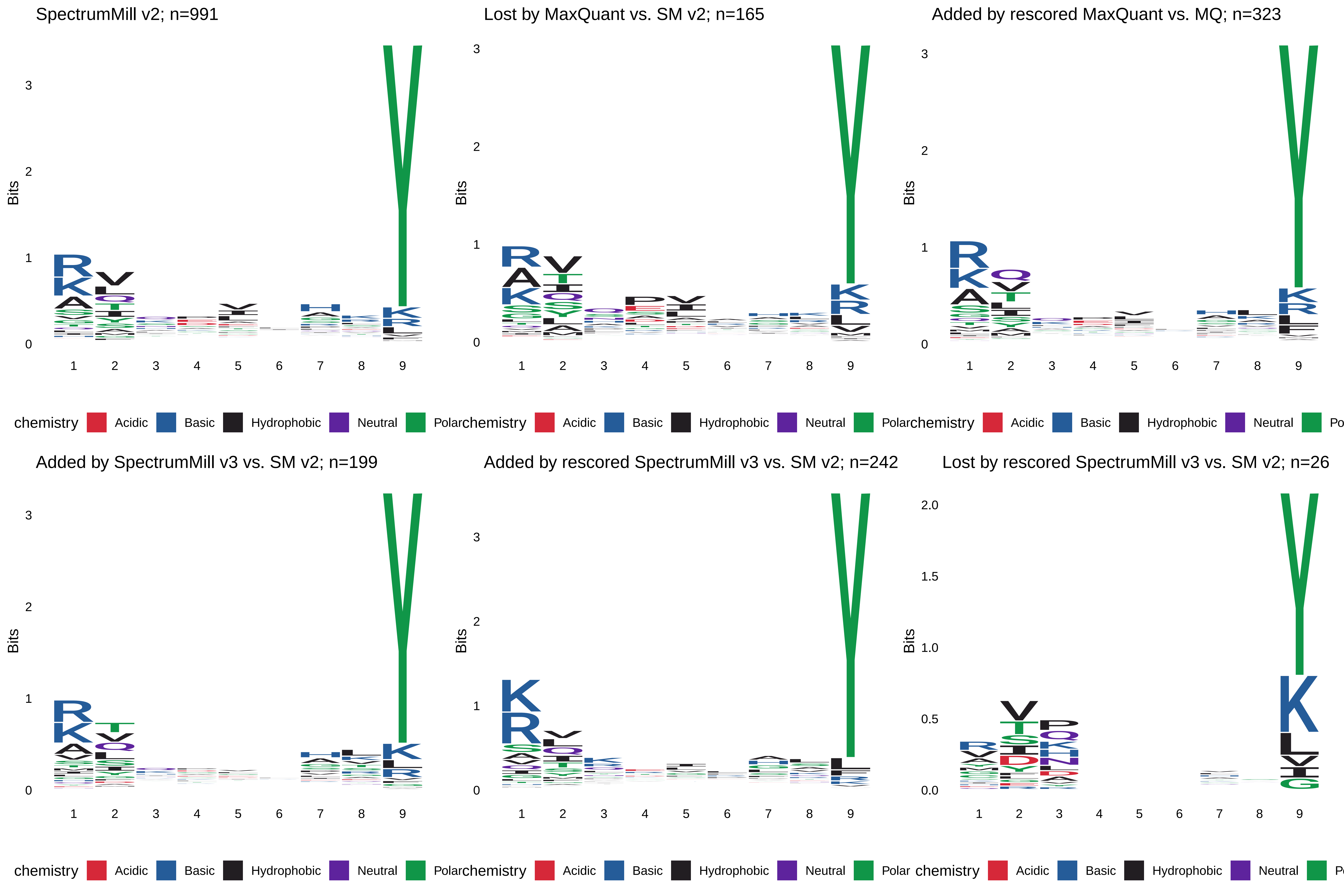
SpectrumMill v2; n=991
Lost by MaxQuant vs. SM v2; n=165
Added by rescored MaxQuant vs. MQ; n=323
Added by SpectrumMill v3 vs. SM v2; n=199
Added by rescored SpectrumMill v3 vs. SM v2; n=242
Lost by rescored SpectrumMill v3 vs. SM v2; n=26
Bits
1 2 3 4 5 6 7 8 9
chemistry
Acidic
Basic
Hydrophobic
Neutral
Polar

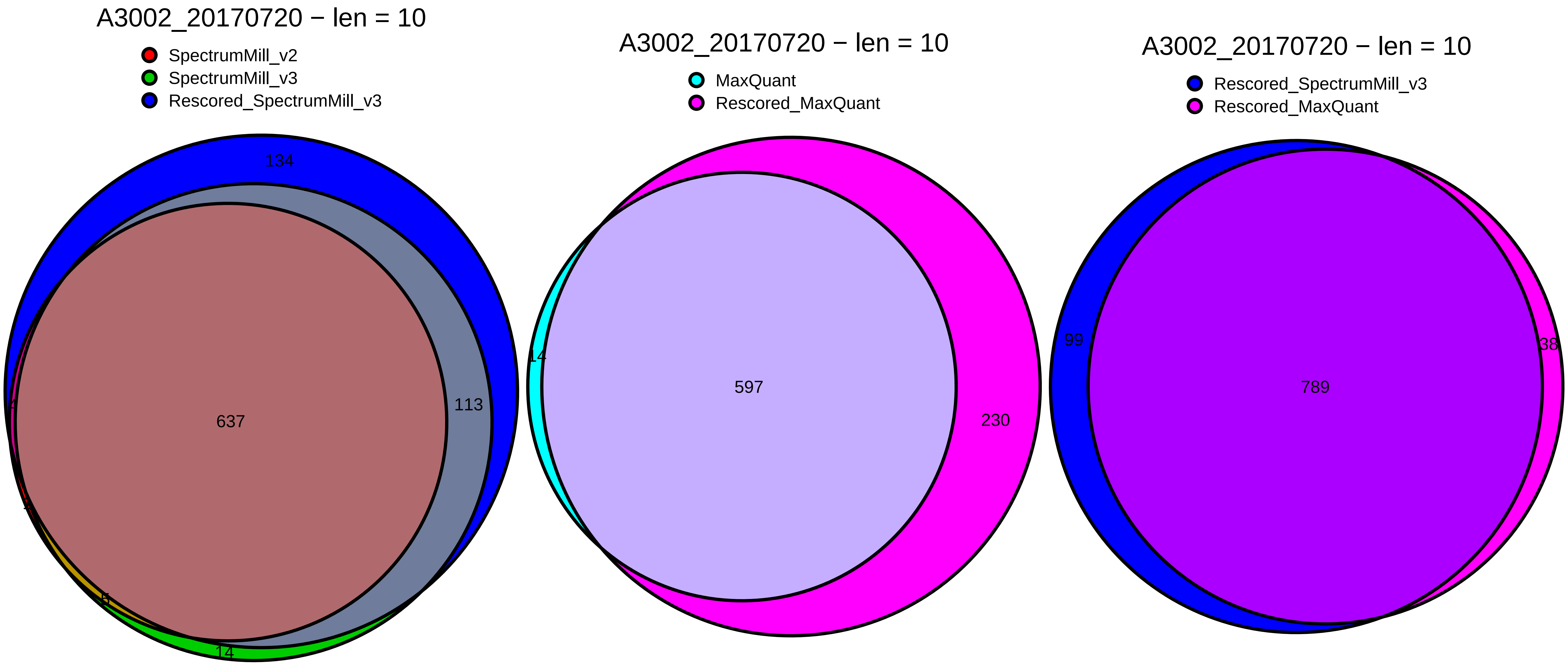

A3002_20170720 − len = 10
SpectrumMill_v2
SpectrumMill_v3
Rescored_SpectrumMill_v3
134
113
637
14
A3002_20170720 − len = 10
MaxQuant
Rescored_MaxQuant
597
230
A3002_20170720 − len = 10
Rescored_SpectrumMill_v3
Rescored_MaxQuant
99
789
38

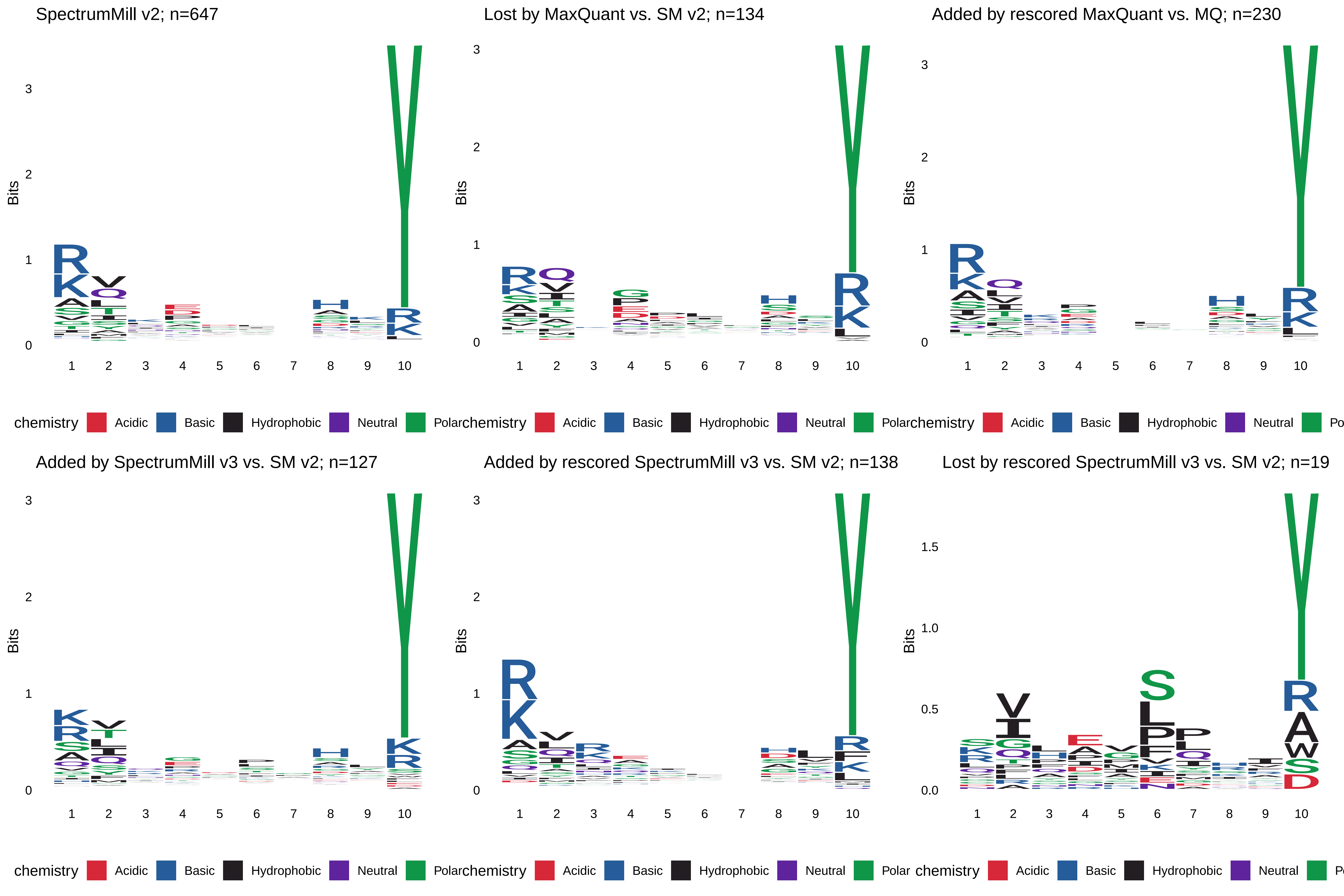
SpectrumMill v2; n=647
Lost by MaxQuant vs. SM v2; n=134
Added by rescored MaxQuant vs. MQ; n=230
Added by SpectrumMill v3 vs. SM v2; n=127
Added by rescored SpectrumMill v3 vs. SM v2; n=138
Lost by rescored SpectrumMill v3 vs. SM v2; n=19
Bits
0
1
2
3
0.0
0.5
1.0
1.5
1 2 3 4 5 6 7 8 9 10
chemistry
Acidic
Basic
Hydrophobic
Neutral
Polar

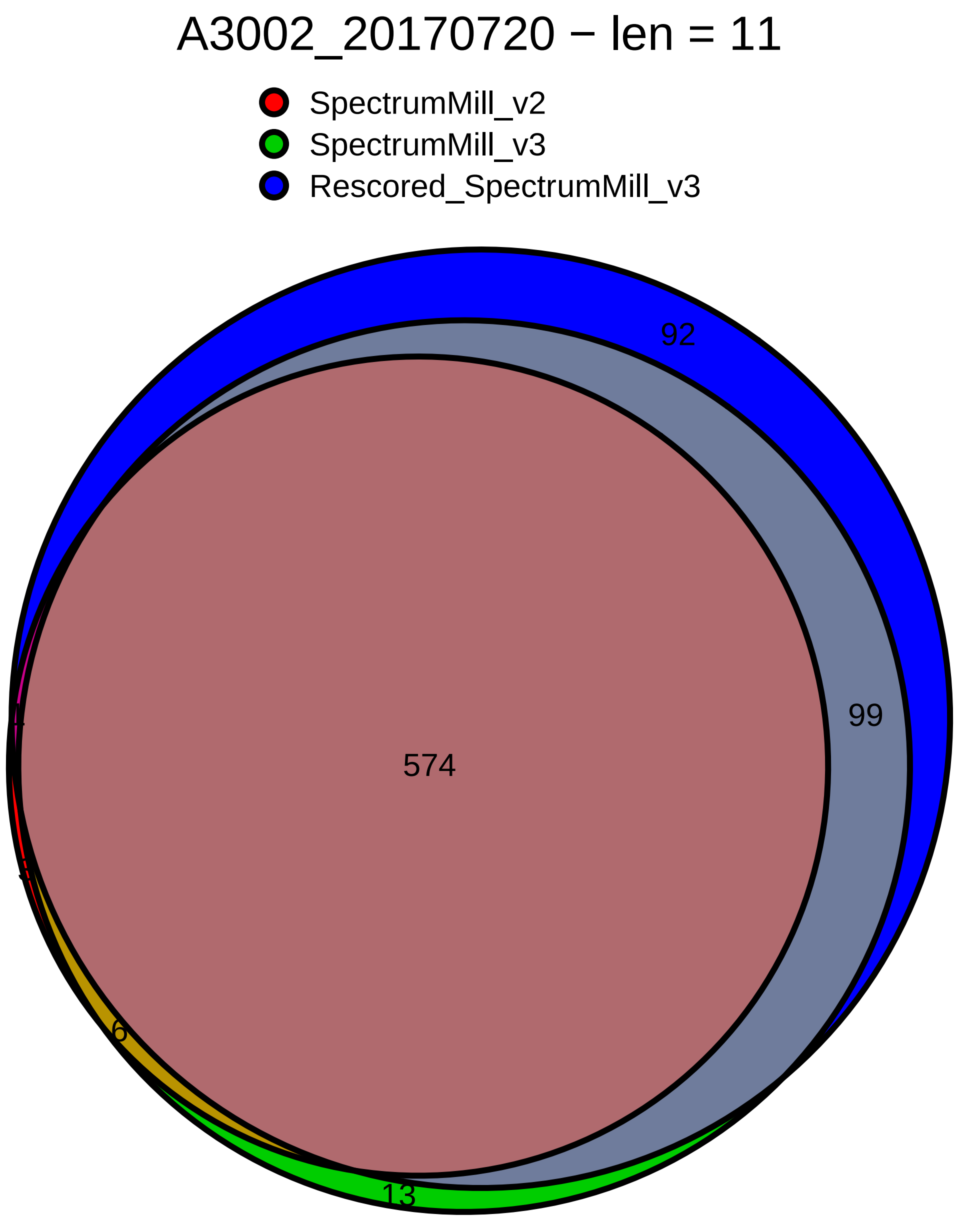
A3002_20170720 − len = 11
SpectrumMill_v2
SpectrumMill_v3
Rescored_SpectrumMill_v3
92
99
574
6
13

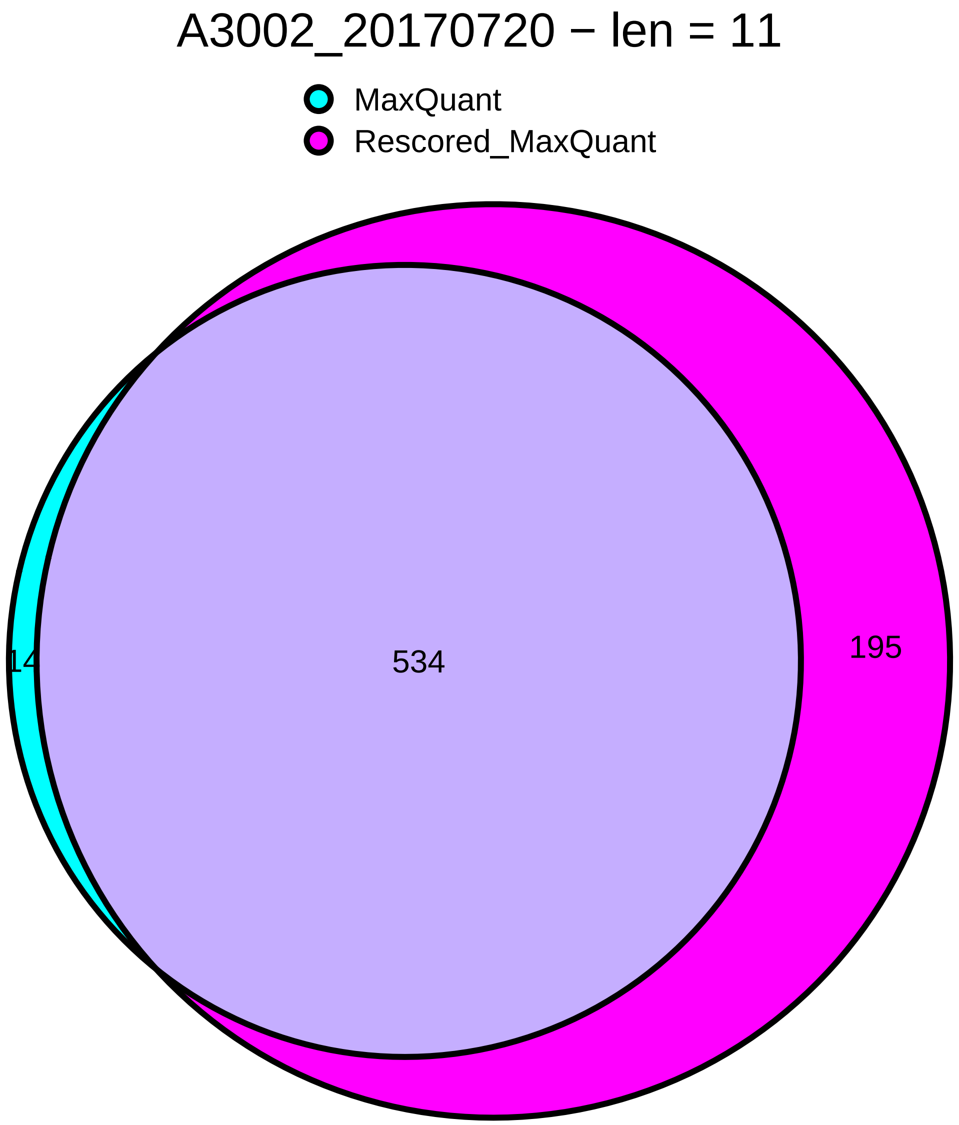
A3002_20170720 − len = 11
MaxQuant
Rescored_MaxQuant
14
534
195

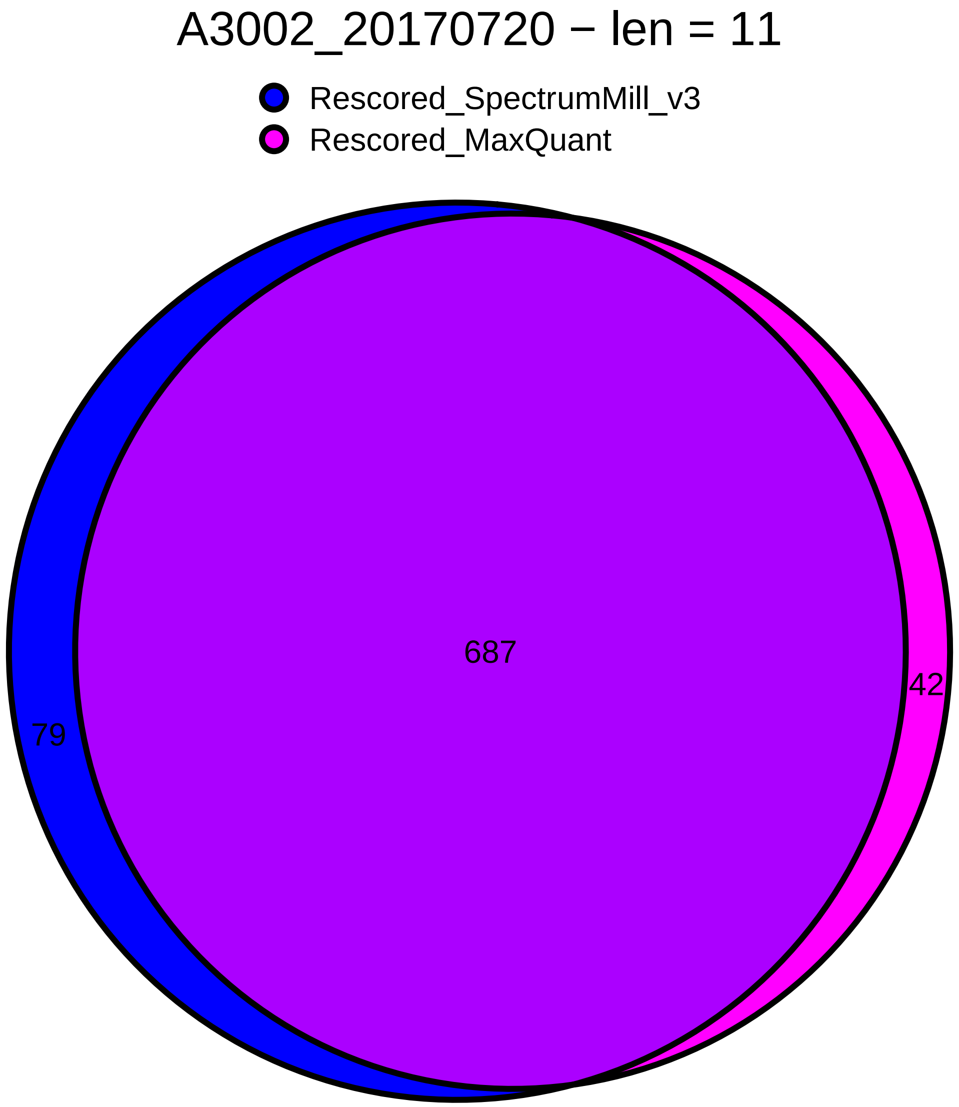
A3002_20170720 − len = 11
Rescored_SpectrumMill_v3
Rescored_MaxQuant
79
687
42

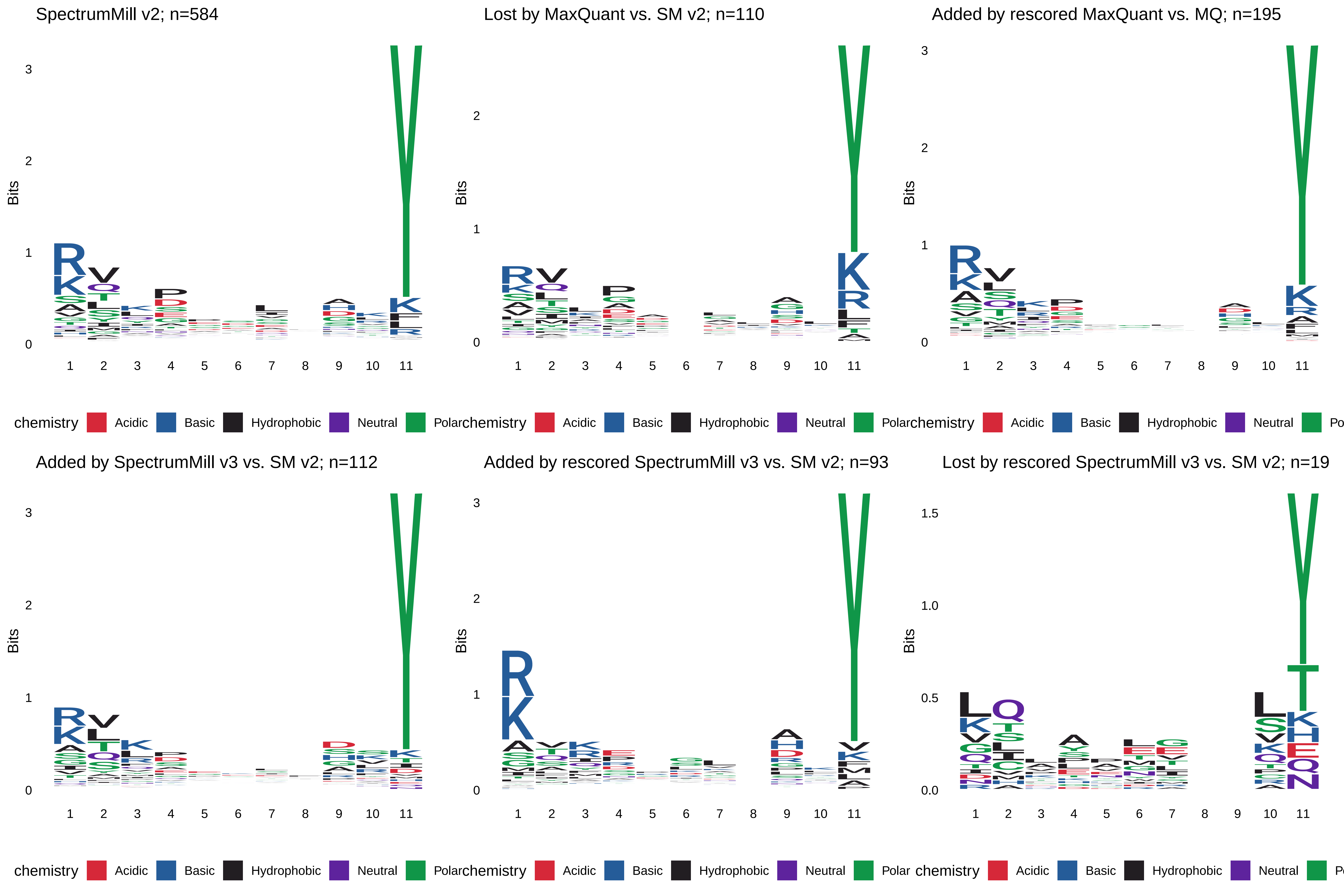
SpectrumMill v2; n=584
Lost by MaxQuant vs. SM v2; n=110
Added by rescored MaxQuant vs. MQ; n=195
Added by SpectrumMill v3 vs. SM v2; n=112
Added by rescored SpectrumMill v3 vs. SM v2; n=93
Lost by rescored SpectrumMill v3 vs. SM v2; n=19
Bits
1 2 3 4 5 6 7 8 9 10 11
chemistry Acidic Basic Hydrophobic Neutral Polar

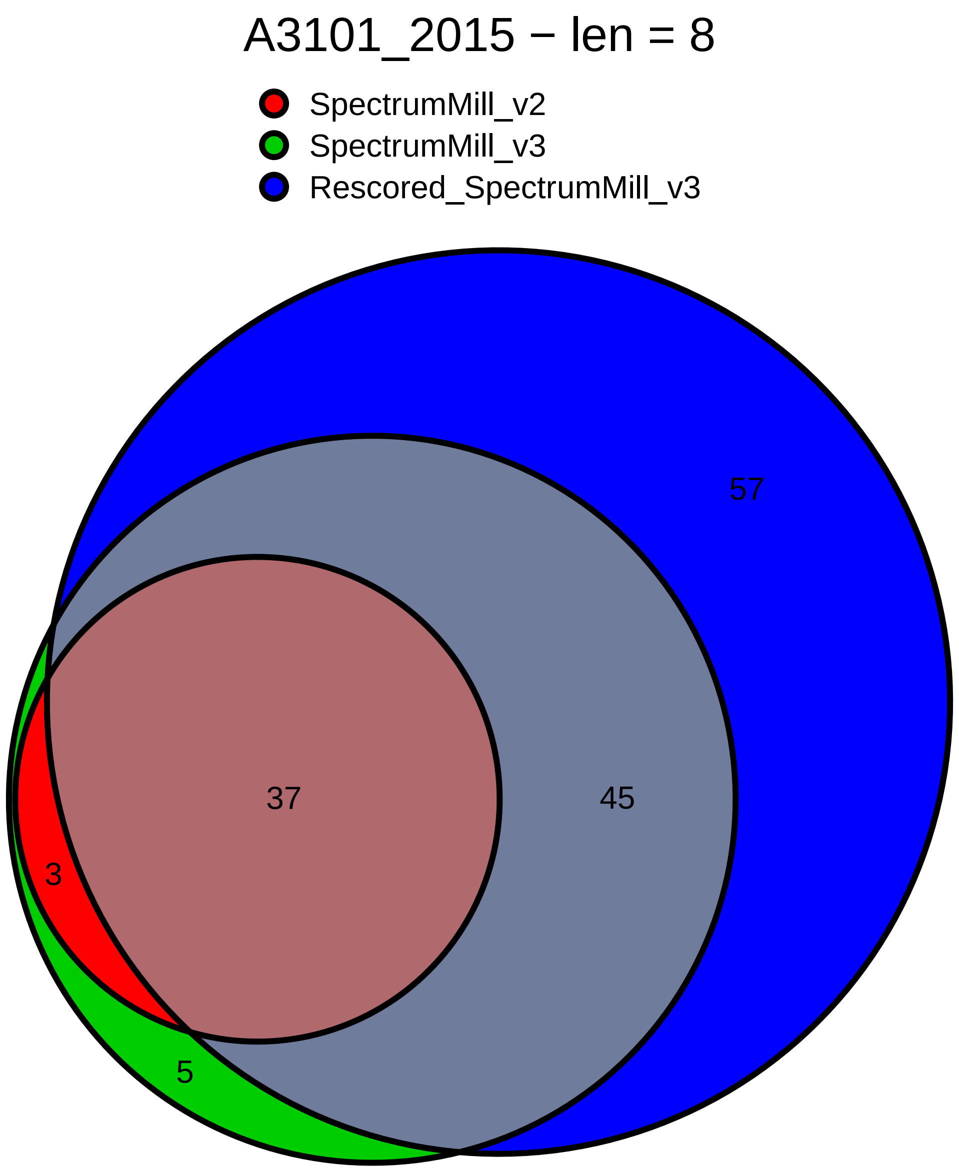
A3101_2015 − len = 8
SpectrumMill_v2
SpectrumMill_v3
Rescored_SpectrumMill_v3
57
37
45
3
5

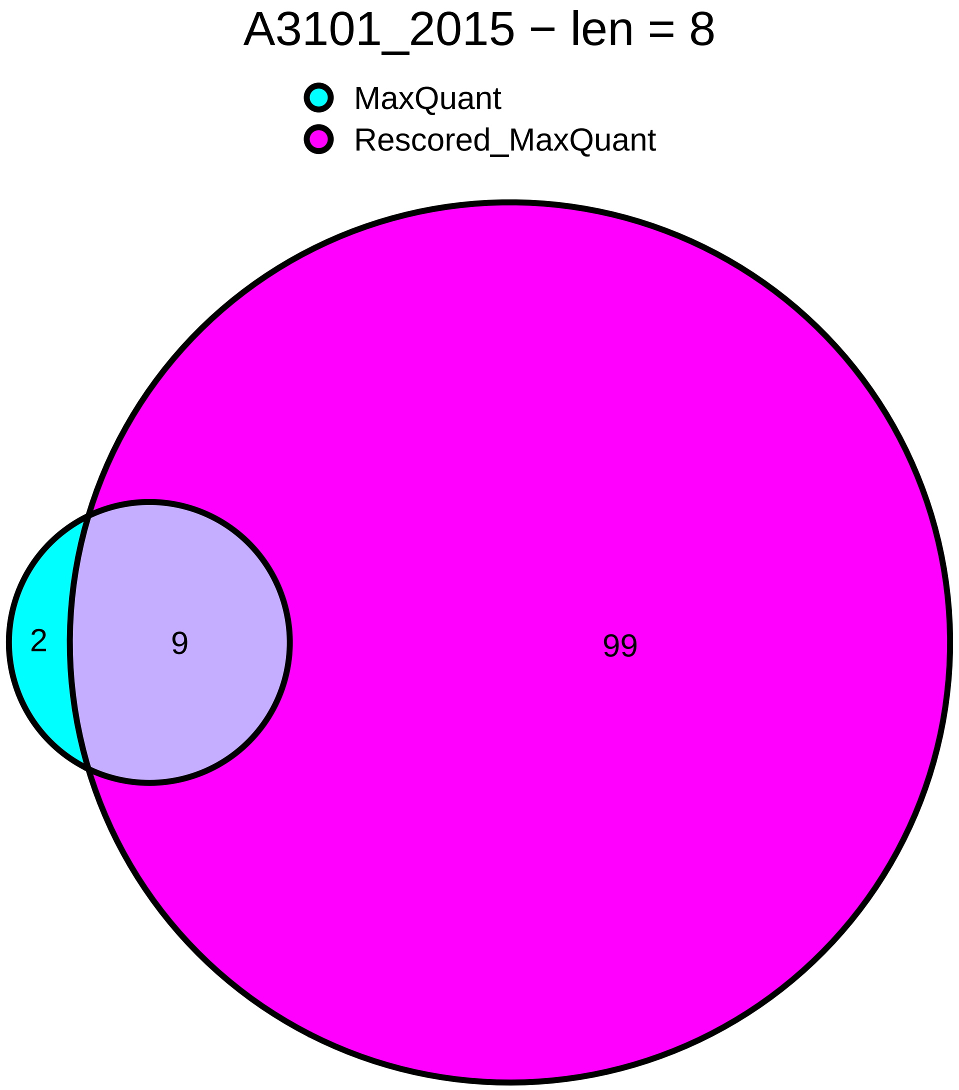
A3101_2015 − len = 8
MaxQuant
Rescored_MaxQuant
2
9
99

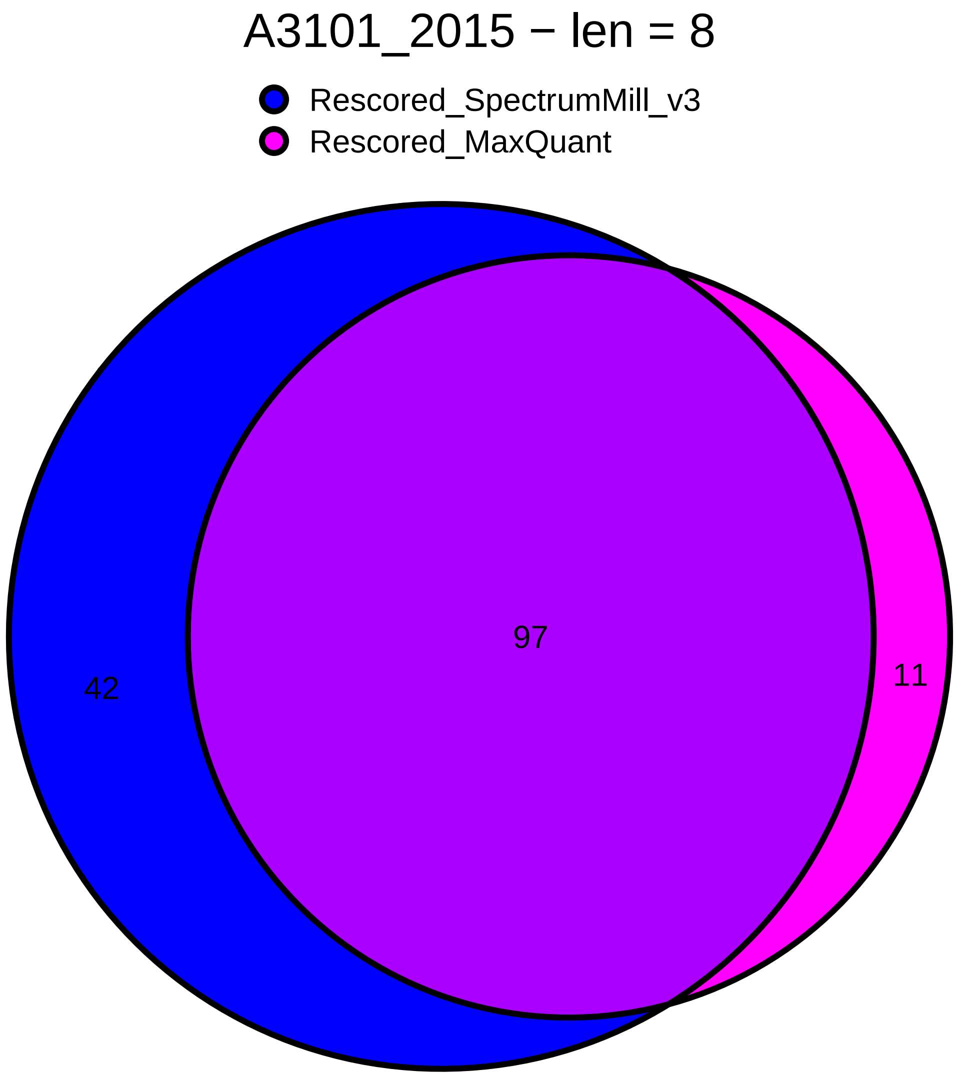
A3101_2015 − len = 8
Rescored_SpectrumMill_v3
Rescored_MaxQuant
42
97
11

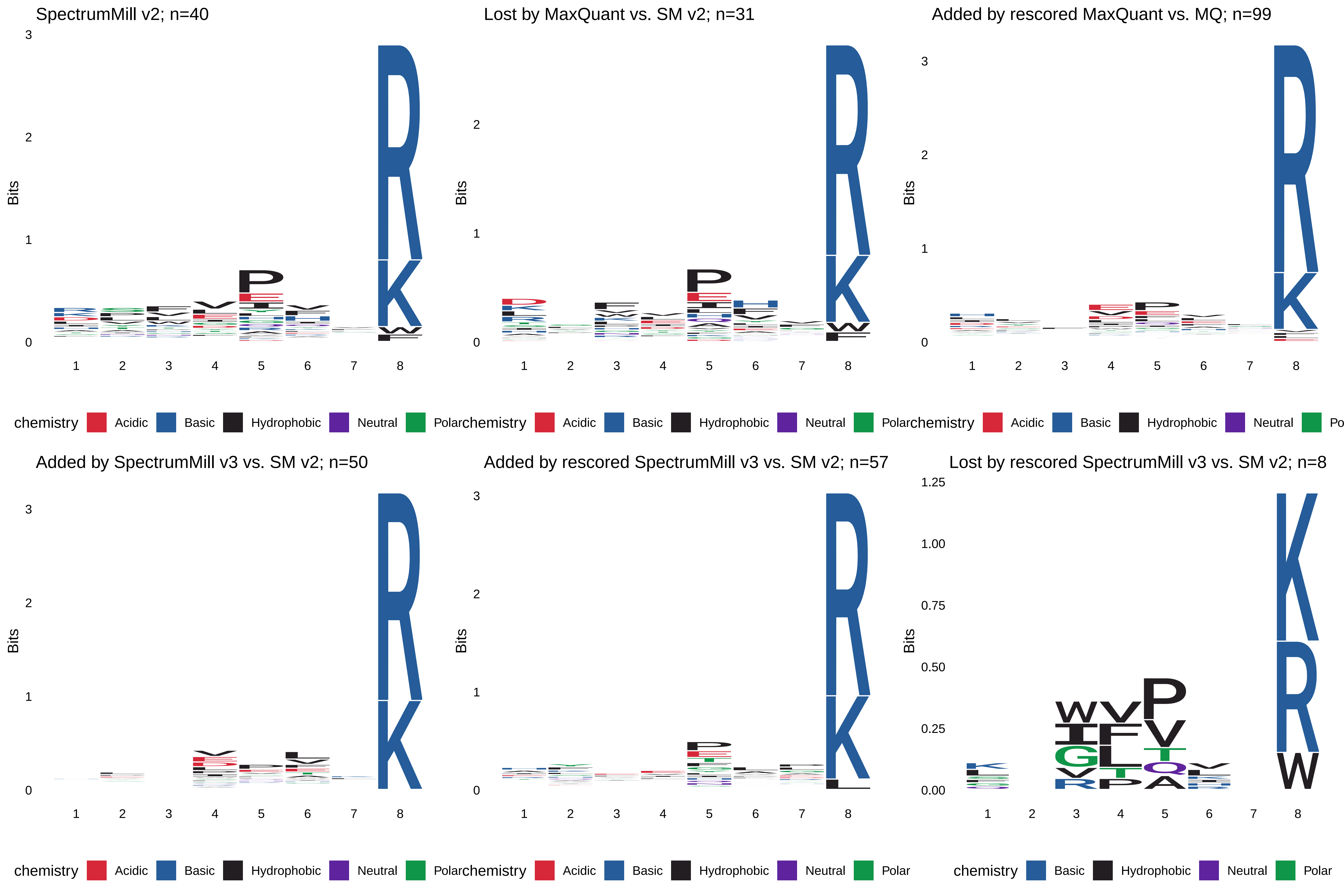
SpectrumMill v2; n=40
Lost by MaxQuant vs. SM v2; n=31
Added by rescored MaxQuant vs. MQ; n=99
Added by SpectrumMill v3 vs. SM v2; n=50
Added by rescored SpectrumMill v3 vs. SM v2; n=57
Lost by rescored SpectrumMill v3 vs. SM v2; n=8
Bits
1 2 3 4 5 6 7 8
chemistry
Acidic
Basic
Hydrophobic
Neutral
Polar

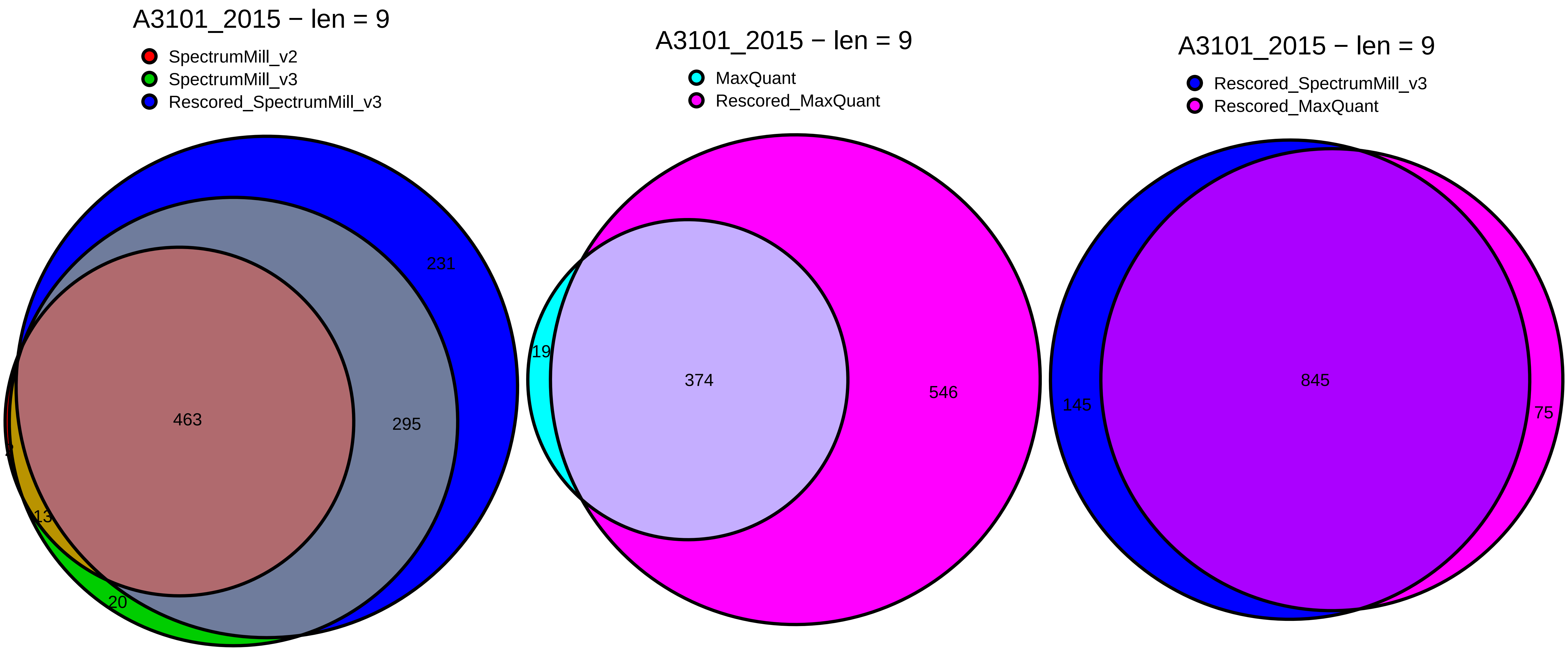
A3101_2015 − len = 9
SpectrumMill_v2
SpectrumMill_v3
Rescored_SpectrumMill_v3
231
463
295
13
20
A3101_2015 − len = 9
MaxQuant
Rescored_MaxQuant
19
374
546
A3101_2015 − len = 9
Rescored_SpectrumMill_v3
Rescored_MaxQuant
845
145
75

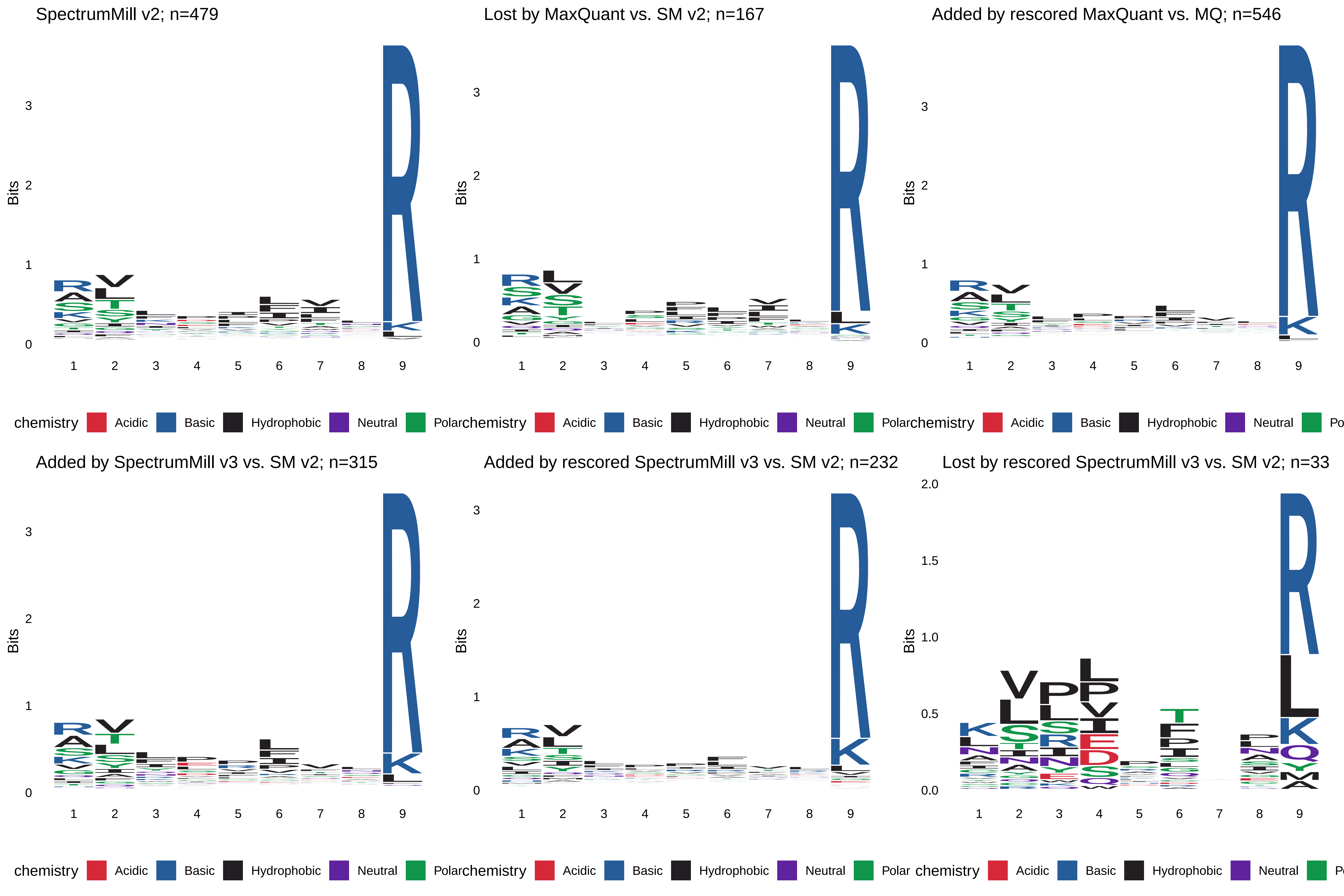
SpectrumMill v2; n=479
Lost by MaxQuant vs. SM v2; n=167
Added by rescored MaxQuant vs. MQ; n=546
Added by SpectrumMill v3 vs. SM v2; n=315
Added by rescored SpectrumMill v3 vs. SM v2; n=232
Lost by rescored SpectrumMill v3 vs. SM v2; n=33
Bits
0
1
2
3
0.0
0.5
1.0
1.5
2.0
1
2
3
4
5
6
7
8
9
chemistry
Acidic
Basic
Hydrophobic
Neutral
Polar

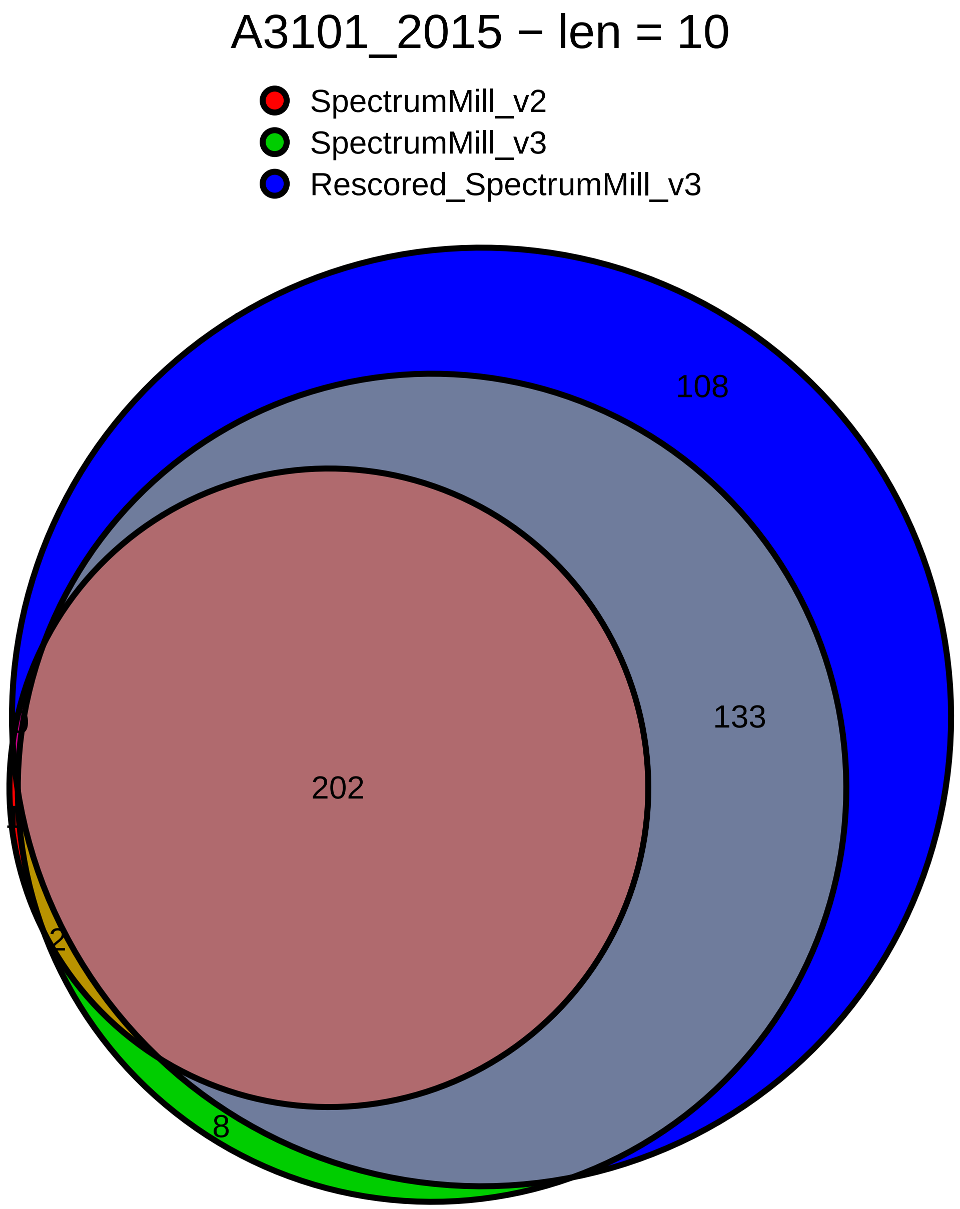
A3101_2015 − len = 10
SpectrumMill_v2
SpectrumMill_v3
Rescored_SpectrumMill_v3
108
133
202
2
8

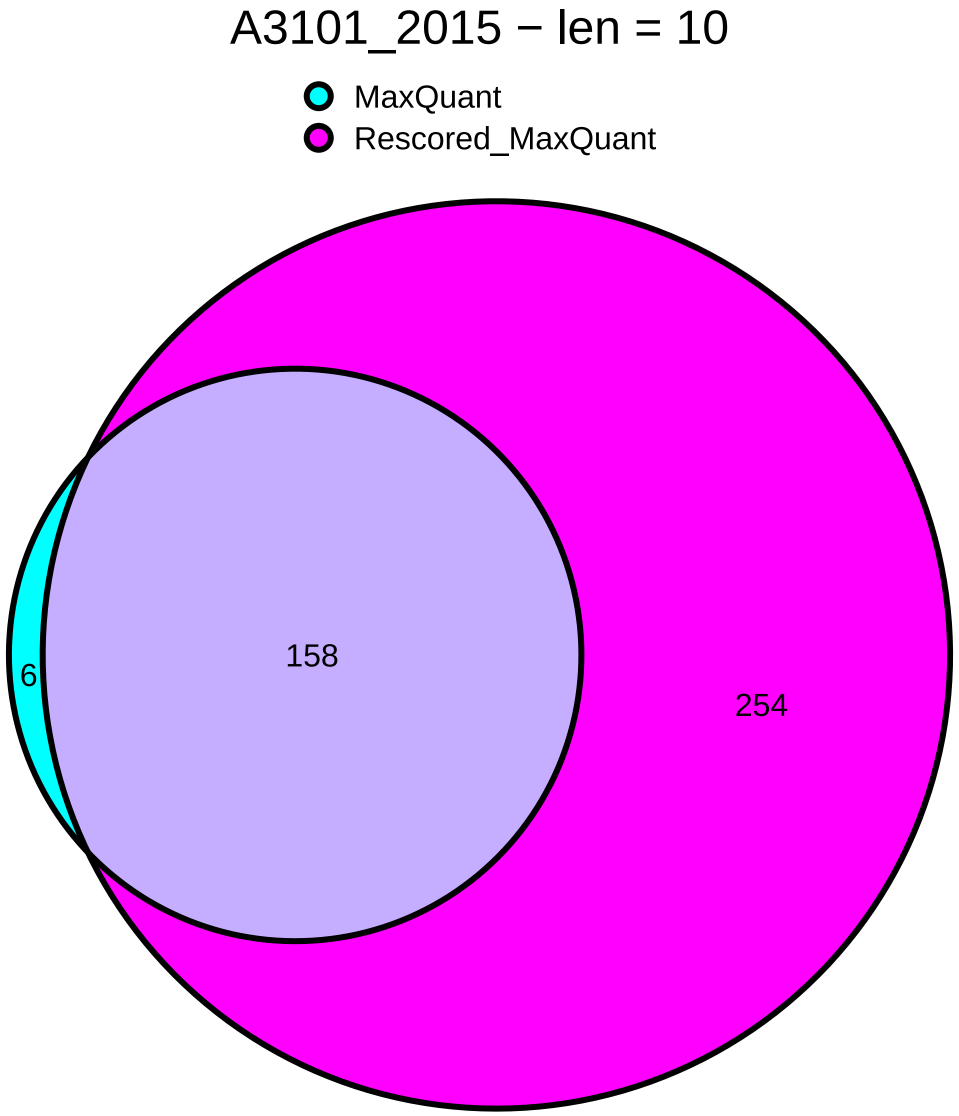
A3101_2015 − len = 10
MaxQuant
Rescored_MaxQuant
6
158
254

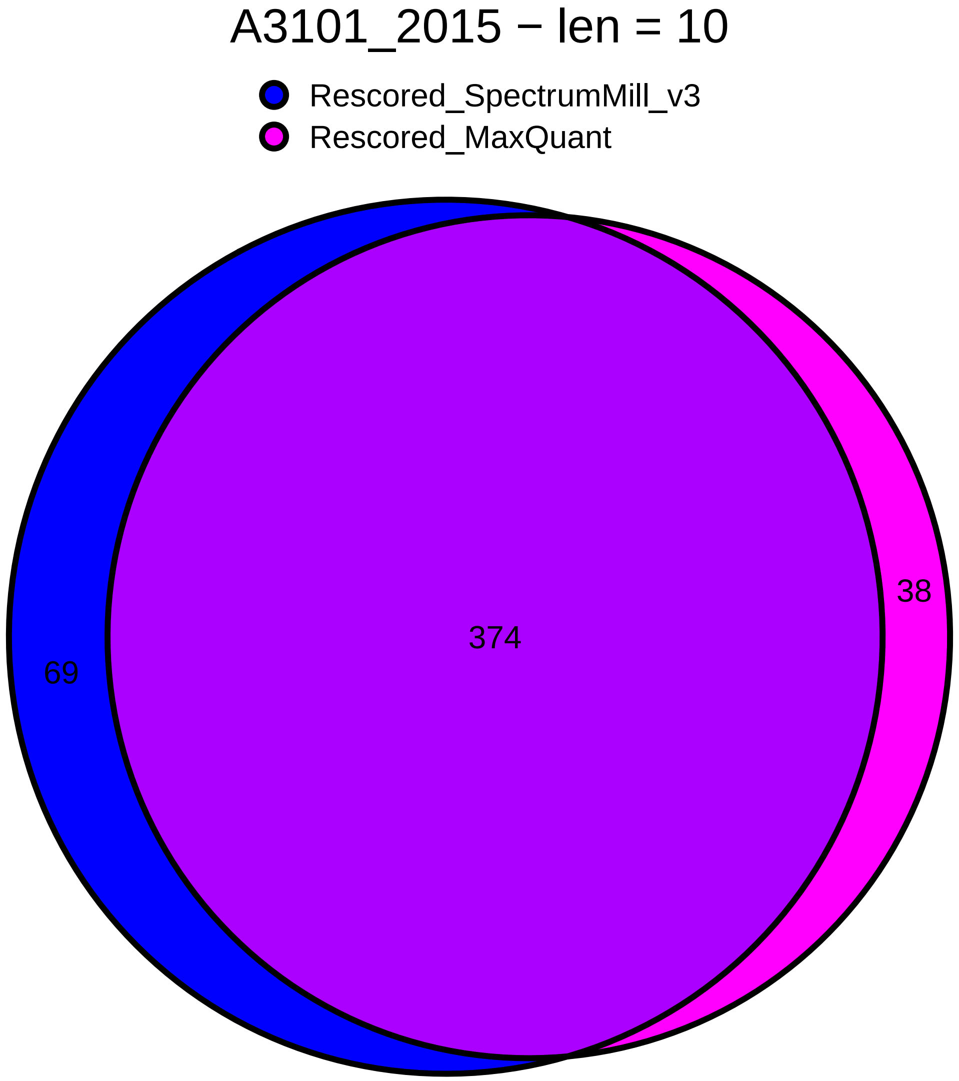
A3101_2015 − len = 10
Rescored_SpectrumMill_v3
Rescored_MaxQuant
69
374
38

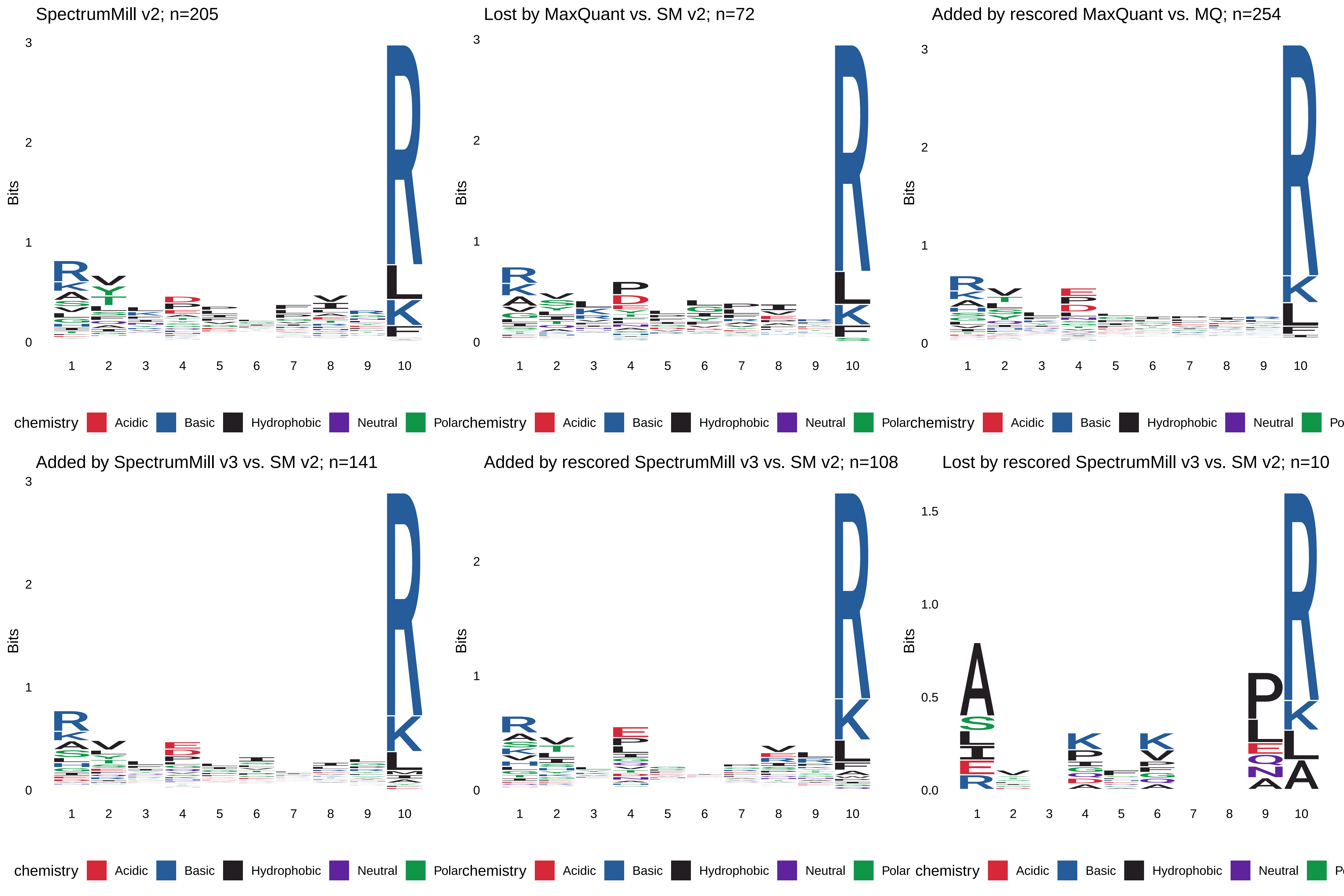
SpectrumMill v2; n=205
Lost by MaxQuant vs. SM v2; n=72
Added by rescored MaxQuant vs. MQ; n=254
Added by SpectrumMill v3 vs. SM v2; n=141
Added by rescored SpectrumMill v3 vs. SM v2; n=108
Lost by rescored SpectrumMill v3 vs. SM v2; n=10
Bits
1 2 3 4 5 6 7 8 9 10
chemistry
Acidic
Basic
Hydrophobic
Neutral
Polar

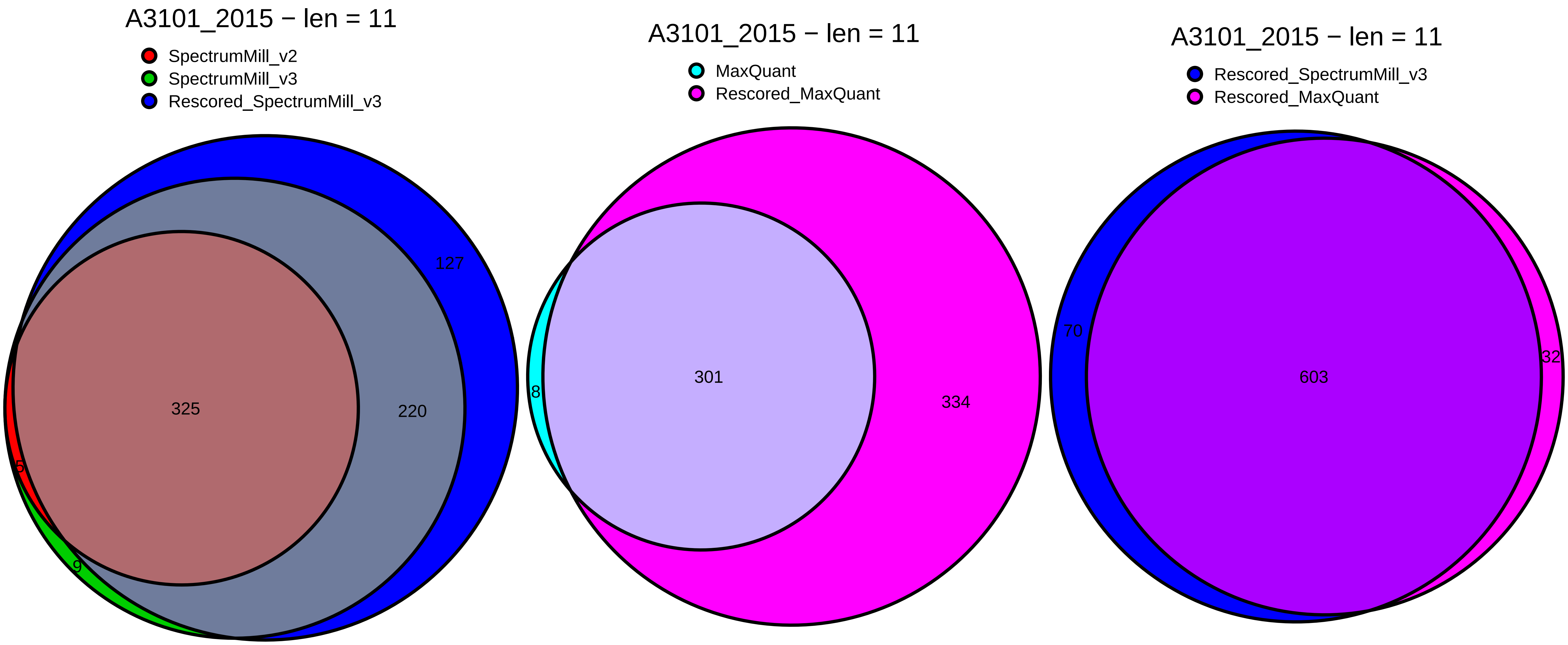
A3101_2015 − len = 11
SpectrumMill_v2
SpectrumMill_v3
Rescored_SpectrumMill_v3
127
325
220
5
9
A3101_2015 − len = 11
MaxQuant
Rescored_MaxQuant
8
301
334
A3101_2015 − len = 11
Rescored_SpectrumMill_v3
Rescored_MaxQuant
70
603
32

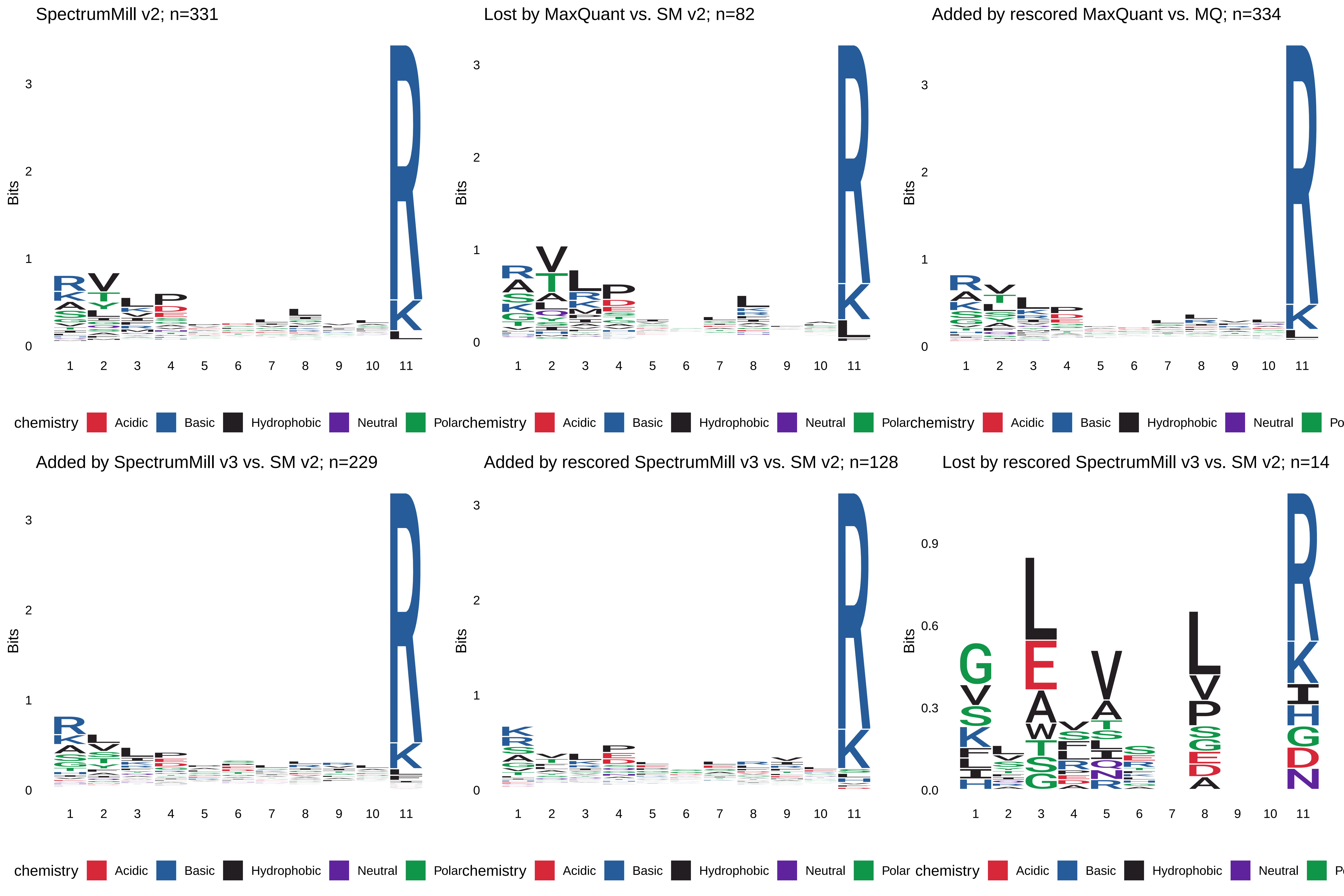
SpectrumMill v2; n=331
Lost by MaxQuant vs. SM v2; n=82
Added by rescored MaxQuant vs. MQ; n=334
Added by SpectrumMill v3 vs. SM v2; n=229
Added by rescored SpectrumMill v3 vs. SM v2; n=128
Lost by rescored SpectrumMill v3 vs. SM v2; n=14
Bits
1 2 3 4 5 6 7 8 9 10 11
chemistry
Acidic
Basic
Hydrophobic
Neutral
Polar

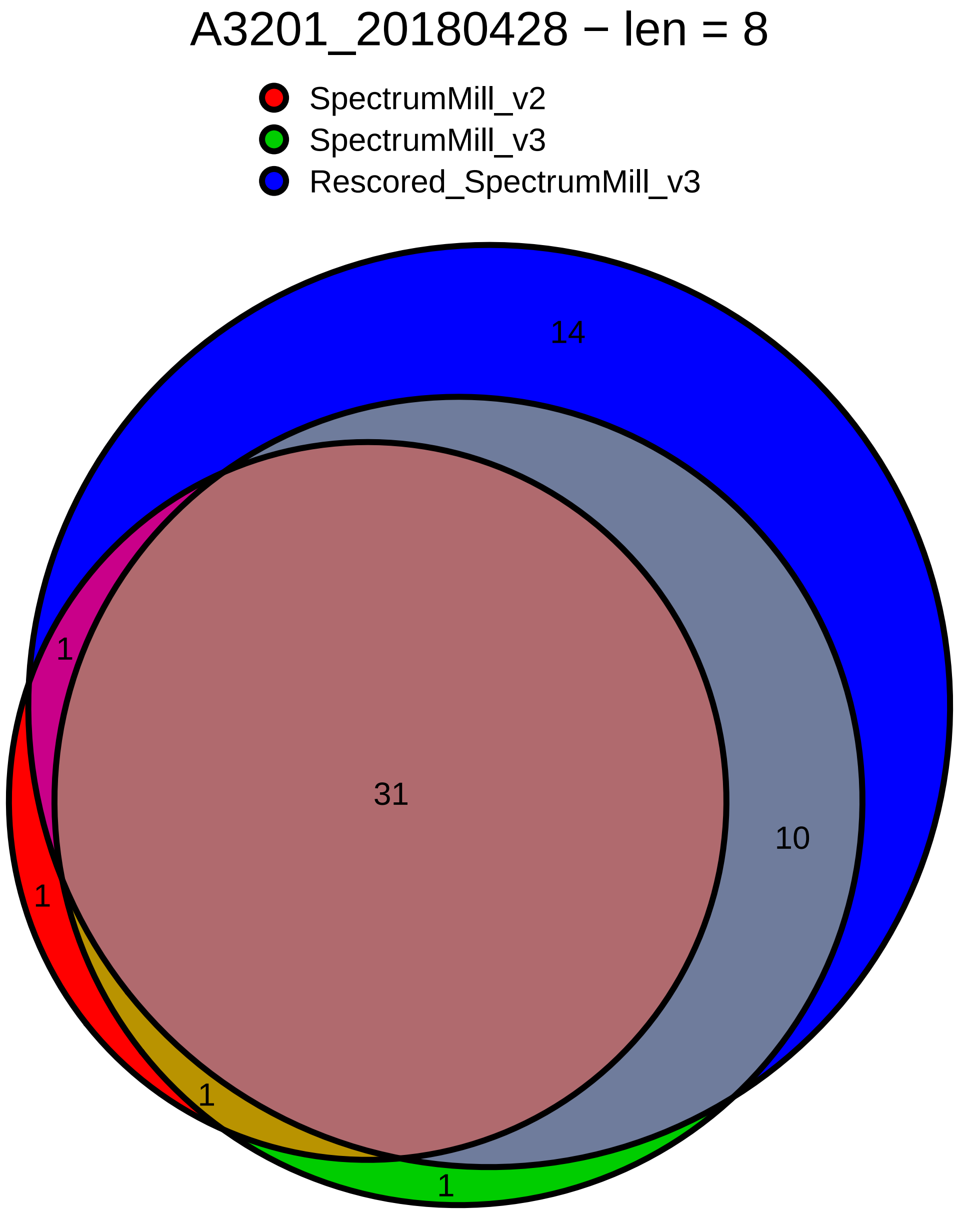
A3201_20180428 − len = 8
SpectrumMill_v2
SpectrumMill_v3
Rescored_SpectrumMill_v3
14
1
31
10
1
1
1

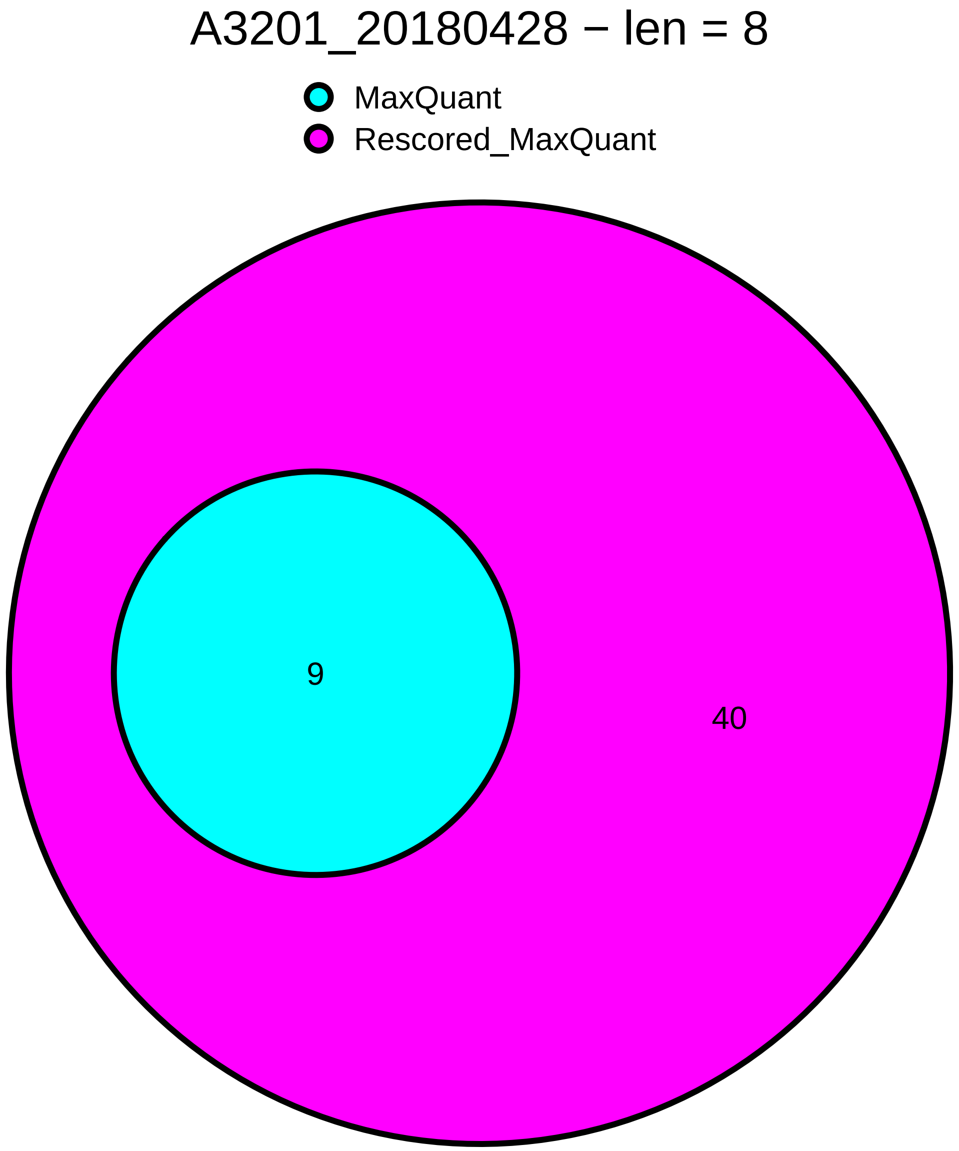
A3201_20180428 − len = 8
MaxQuant
Rescored_MaxQuant
9
40

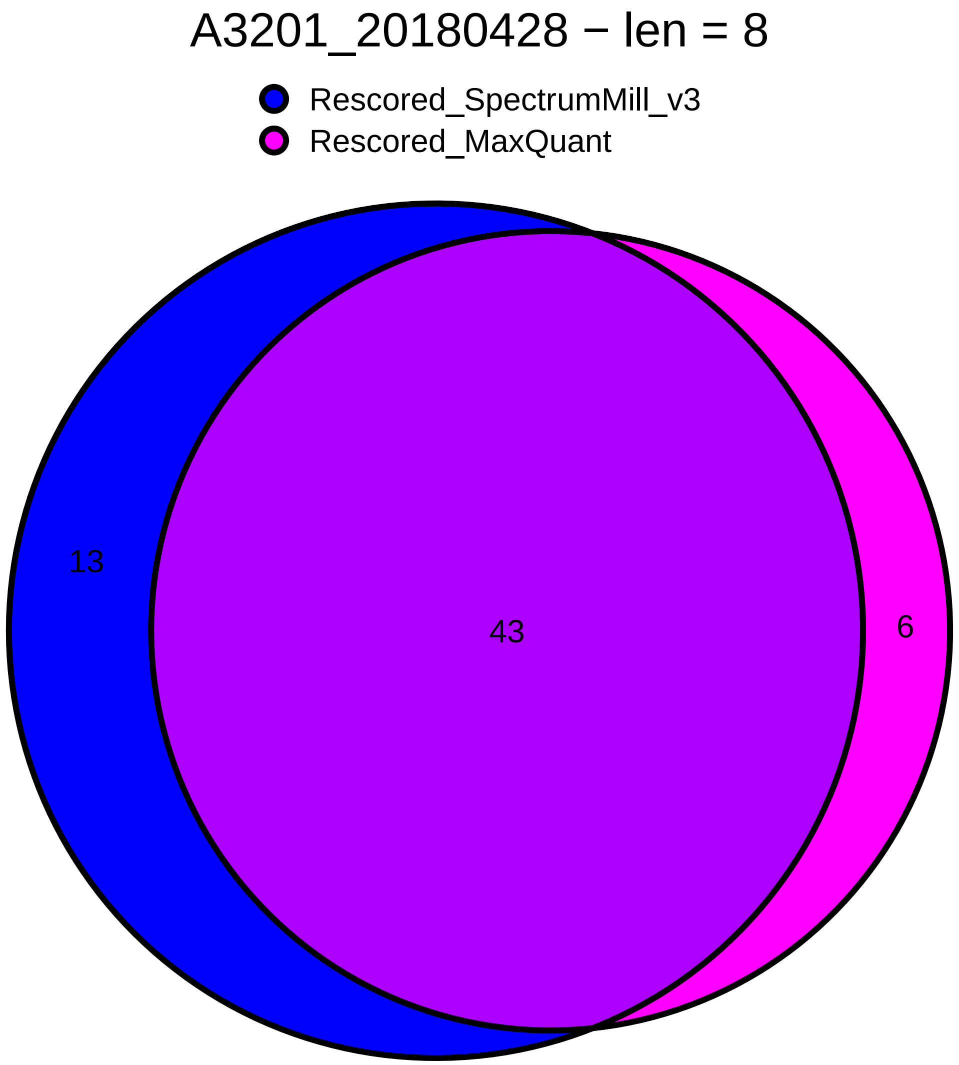
A3201_20180428 − len = 8
Rescored_SpectrumMill_v3
Rescored_MaxQuant
13
43
6

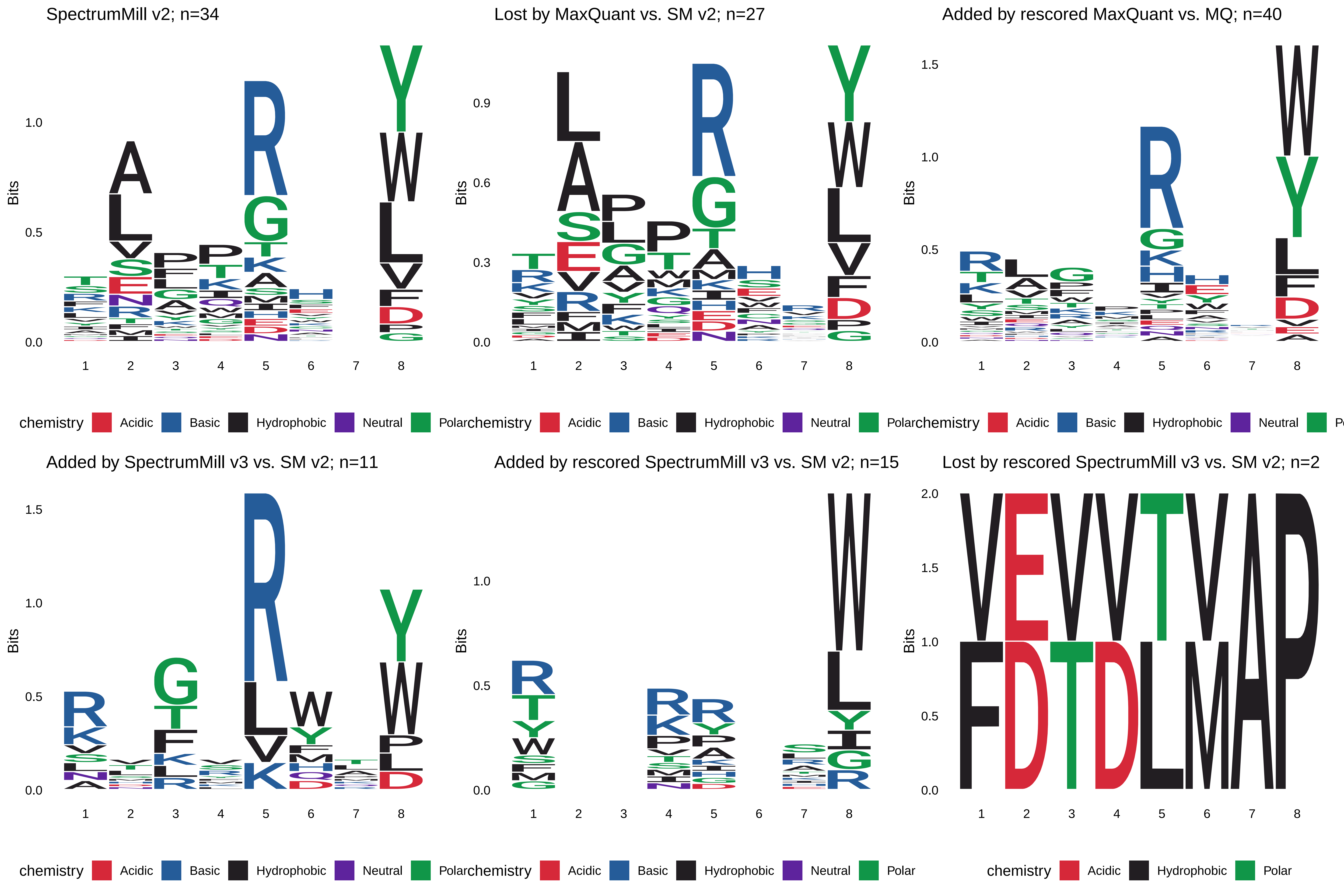
SpectrumMill v2; n=34
Lost by MaxQuant vs. SM v2; n=27
Added by rescored MaxQuant vs. MQ; n=40
Added by SpectrumMill v3 vs. SM v2; n=11
Added by rescored SpectrumMill v3 vs. SM v2; n=15
Lost by rescored SpectrumMill v3 vs. SM v2; n=2
Bits
1 2 3 4 5 6 7 8
chemistry Acidic Basic Hydrophobic Neutral Polar
chemistry Acidic Hydrophobic Polar

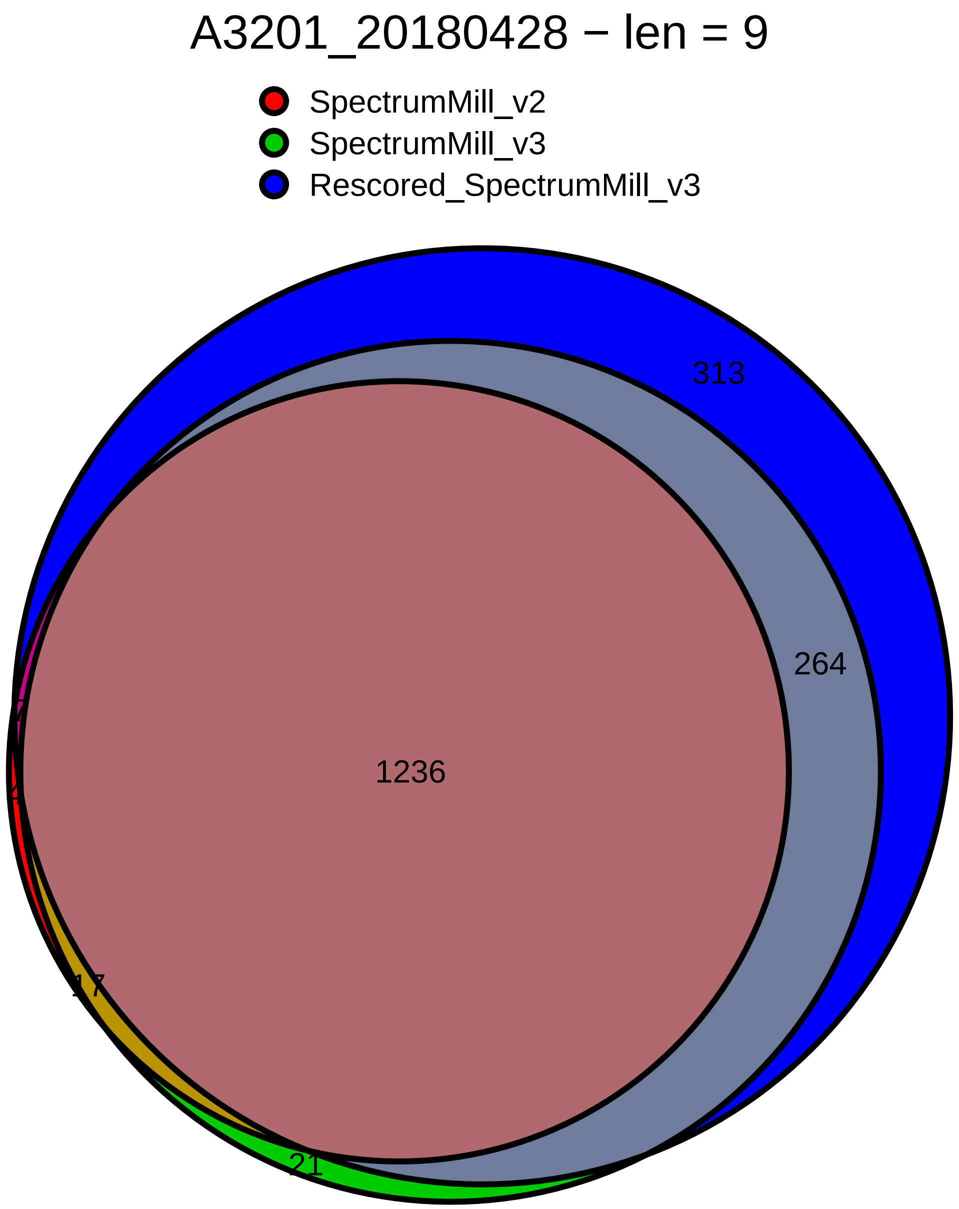
A3201_20180428 − len = 9
SpectrumMill_v2
SpectrumMill_v3
Rescored_SpectrumMill_v3
313
264
1236
17
21

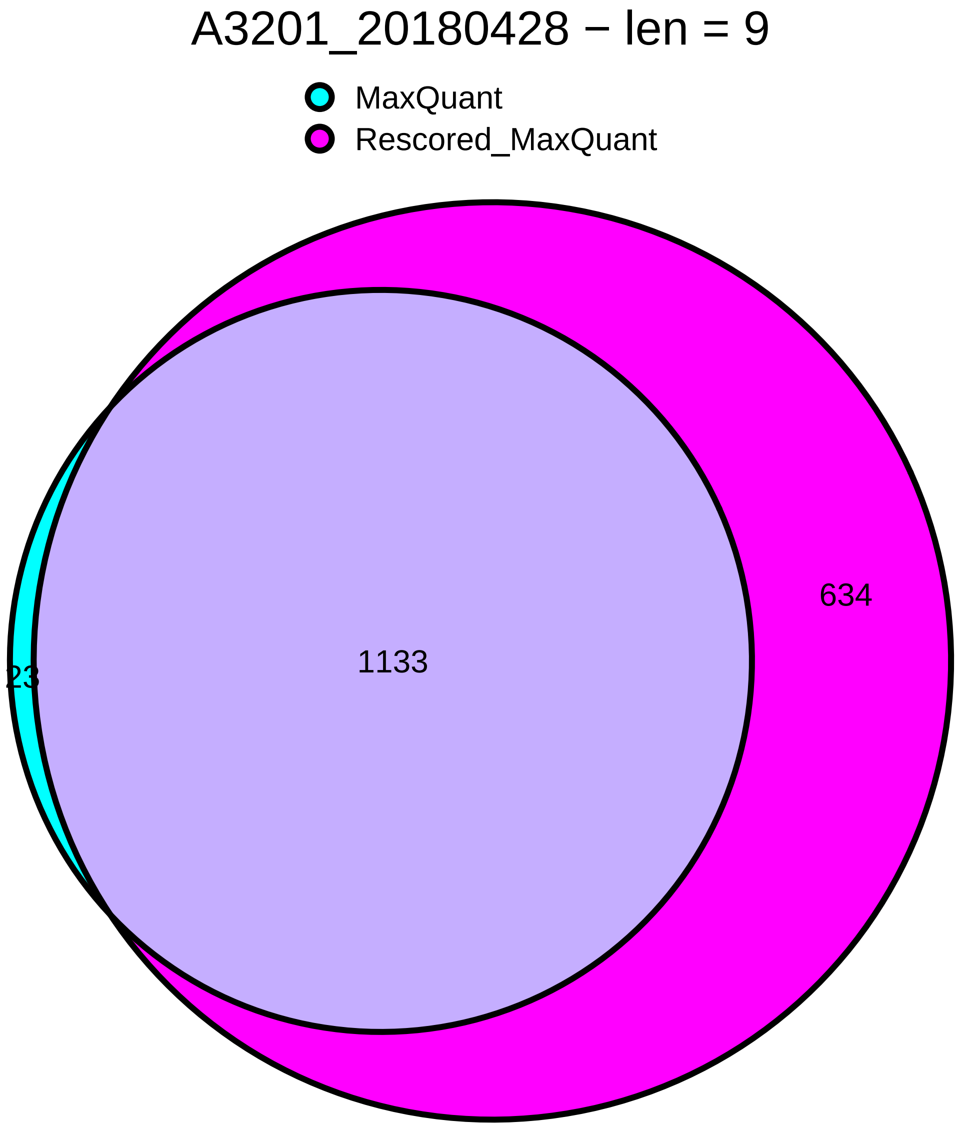
A3201_20180428 − len = 9
MaxQuant
Rescored_MaxQuant
634
1133
23

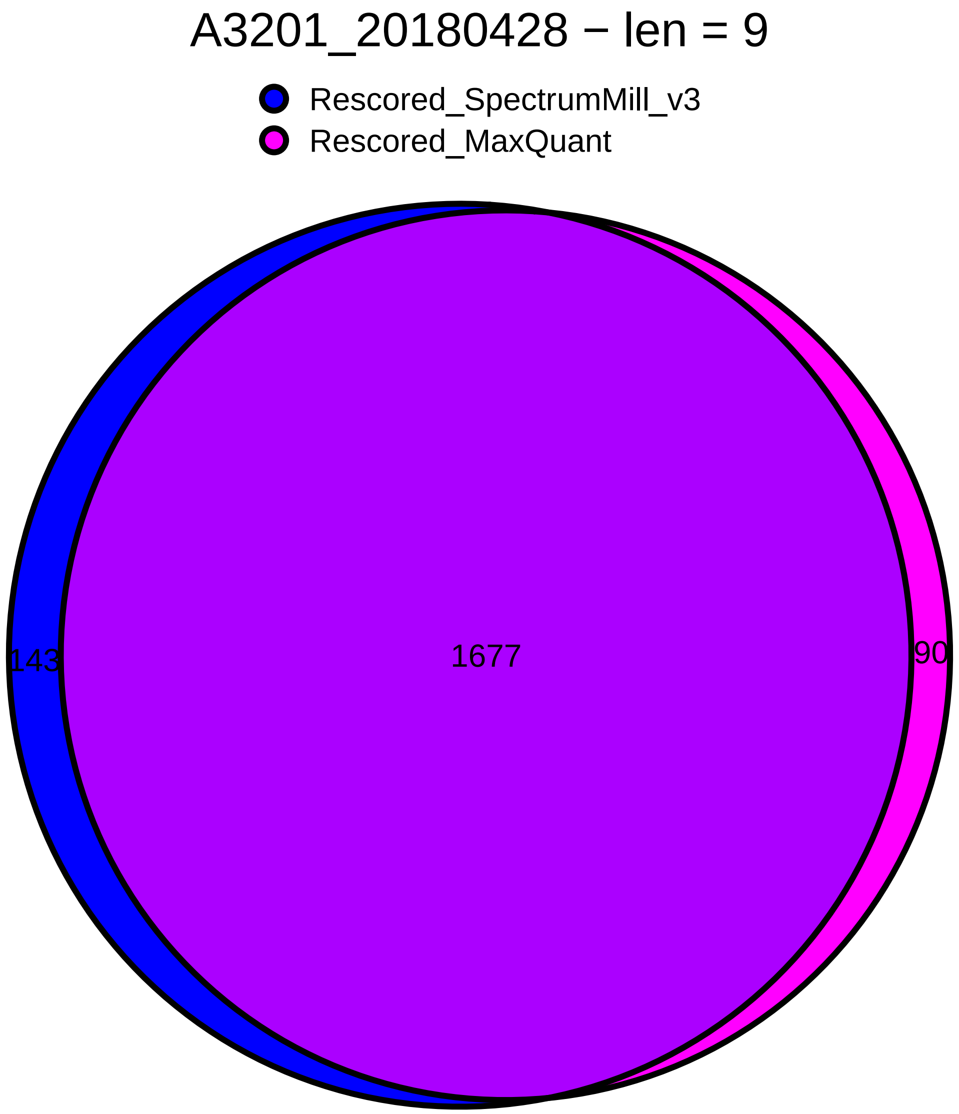
A3201_20180428 − len = 9
Rescored_SpectrumMill_v3
Rescored_MaxQuant
143
1677
90

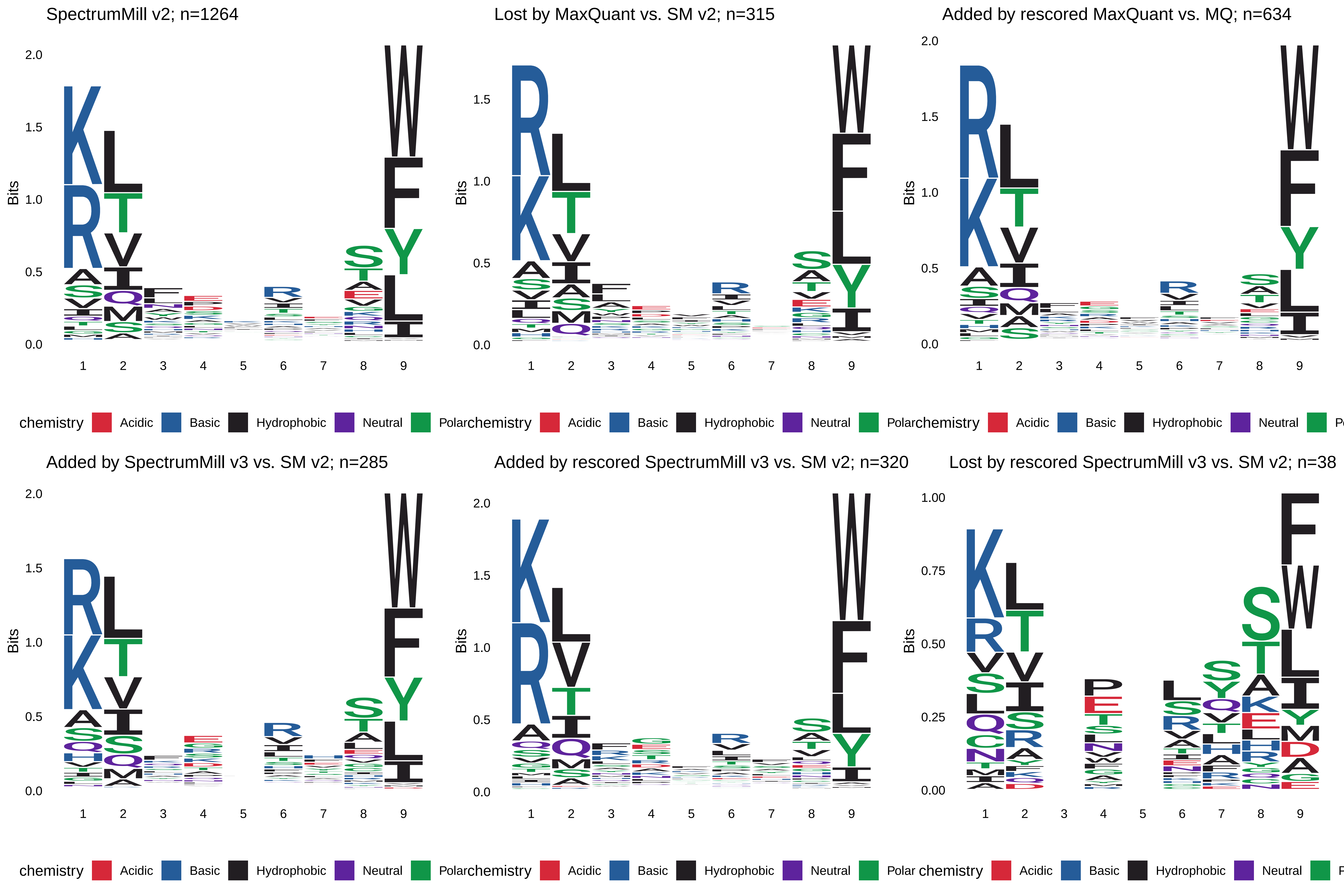
SpectrumMill v2; n=1264
Lost by MaxQuant vs. SM v2; n=315
Added by rescored MaxQuant vs. MQ; n=634
Added by SpectrumMill v3 vs. SM v2; n=285
Added by rescored SpectrumMill v3 vs. SM v2; n=320
Lost by rescored SpectrumMill v3 vs. SM v2; n=38
Bits
1 2 3 4 5 6 7 8 9
chemistry Acidic Basic Hydrophobic Neutral Polar

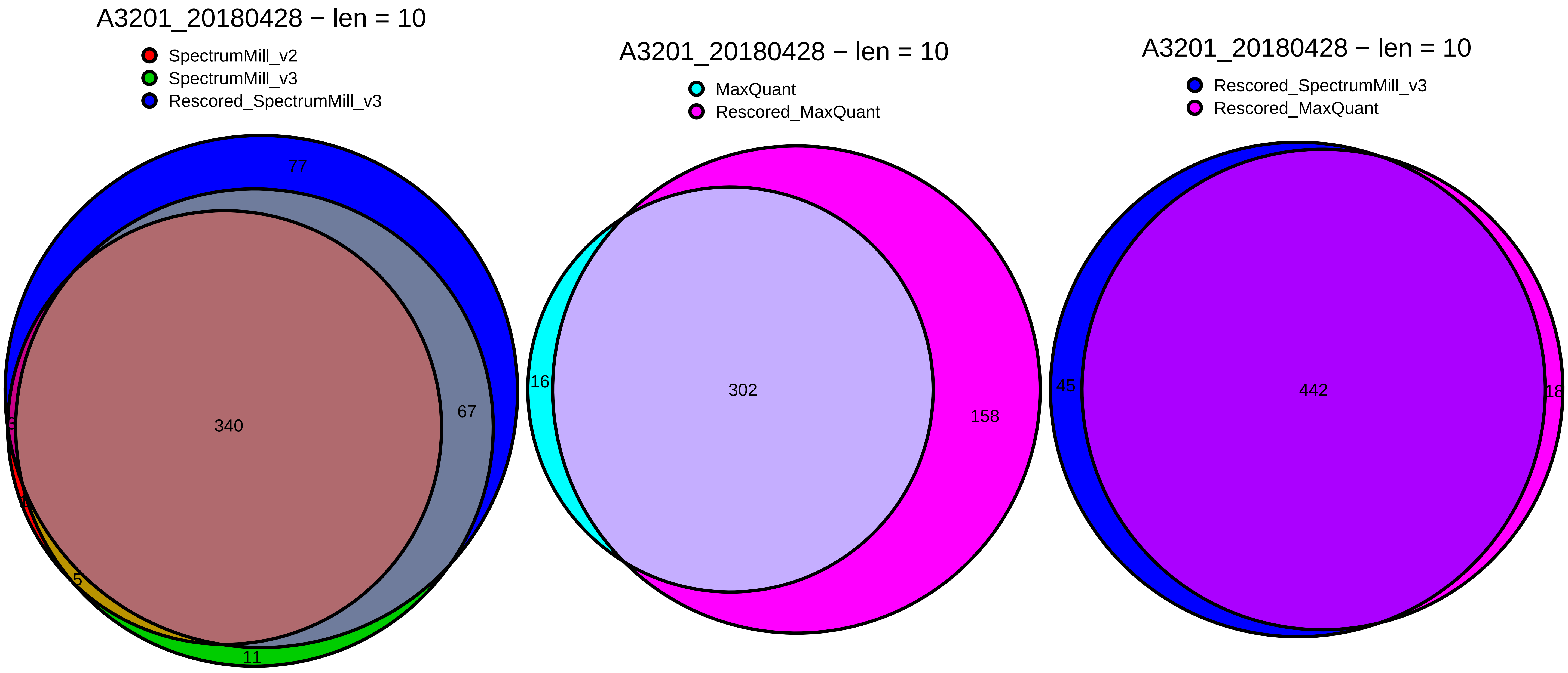
A3201_20180428 − len = 10
SpectrumMill_v2
SpectrumMill_v3
Rescored_SpectrumMill_v3
77
67
340
5
11
A3201_20180428 − len = 10
MaxQuant
Rescored_MaxQuant
16
302
158
A3201_20180428 − len = 10
Rescored_SpectrumMill_v3
Rescored_MaxQuant
45
442
18

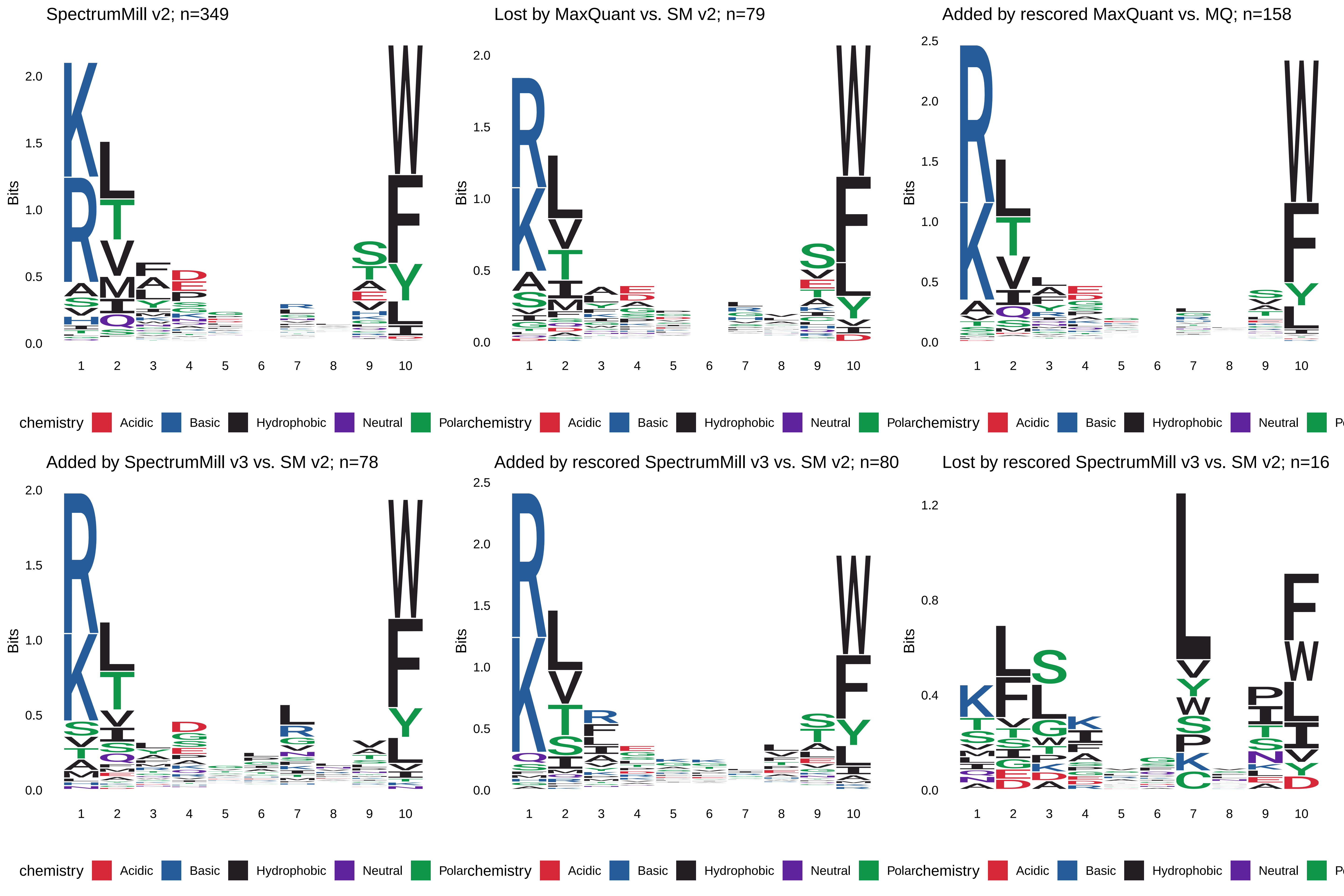
SpectrumMill v2; n=349
Lost by MaxQuant vs. SM v2; n=79
Added by rescored MaxQuant vs. MQ; n=158
Bits
1 2 3 4 5 6 7 8 9 10
chemistry Acidic Basic Hydrophobic Neutral Polarchemistry Acidic Basic Hydrophobic Neutral Polarchemistry Acidic Basic Hydrophobic Neutral Po
Added by SpectrumMill v3 vs. SM v2; n=78
Added by rescored SpectrumMill v3 vs. SM v2; n=80
Lost by rescored SpectrumMill v3 vs. SM v2; n=16
Bits
1 2 3 4 5 6 7 8 9 10
chemistry Acidic Basic Hydrophobic Neutral Polarchemistry Acidic Basic Hydrophobic Neutral Polarchemistry Acidic Basic Hydrophobic Neutral Po

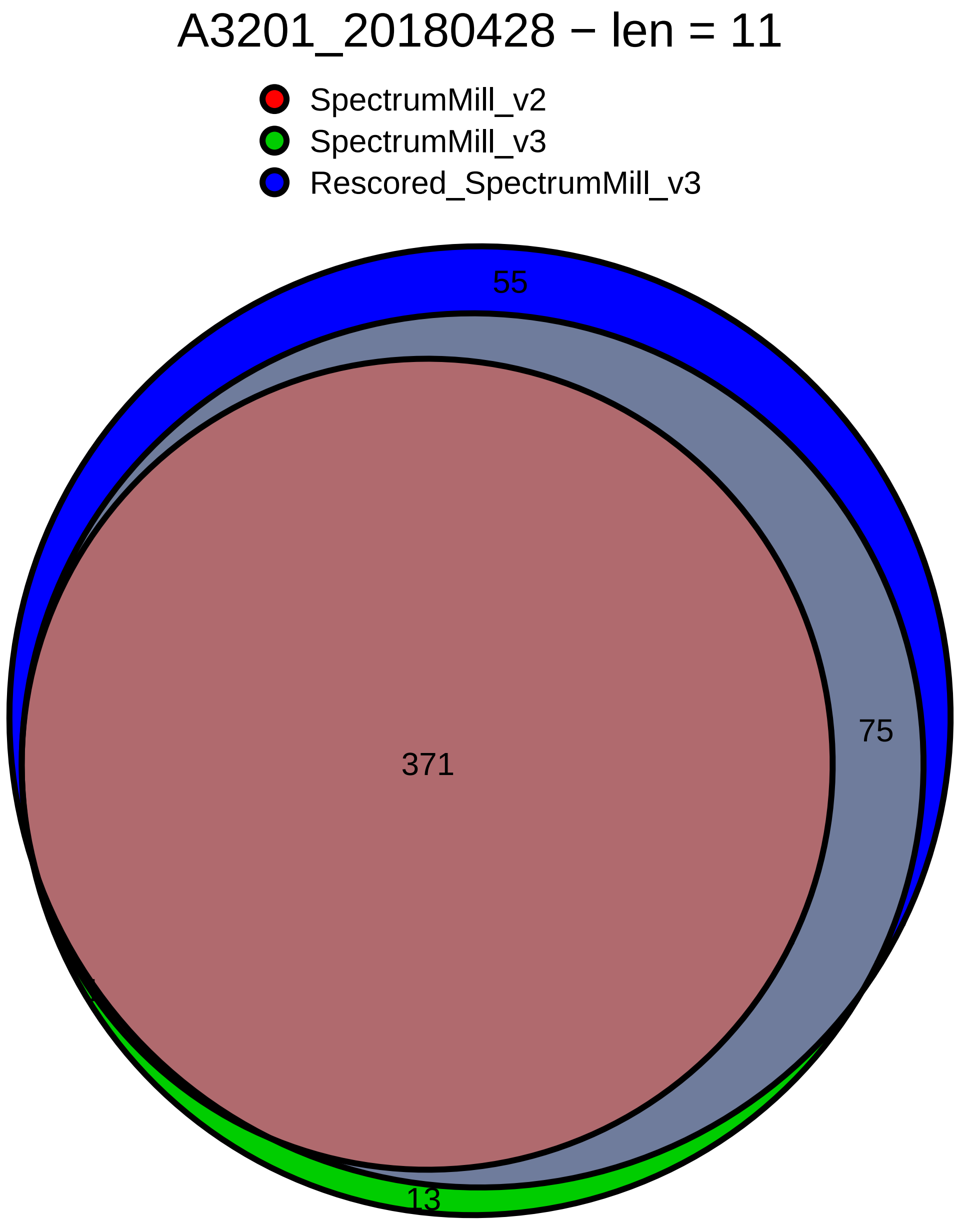

A3201_20180428 − len = 11
SpectrumMill_v2
SpectrumMill_v3
Rescored_SpectrumMill_v3
55
75
371
13

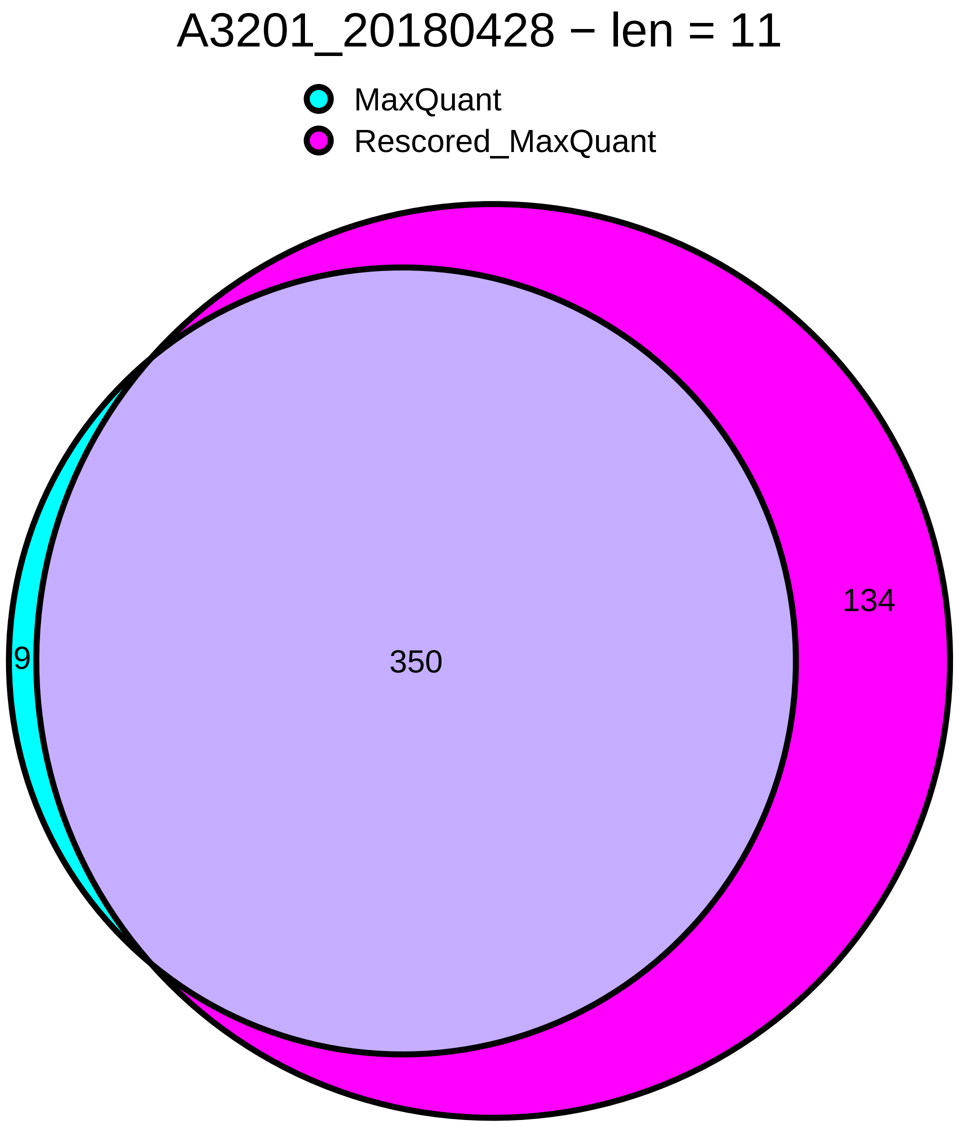

A3201_20180428 − len = 11
MaxQuant
Rescored_MaxQuant
9
350
134

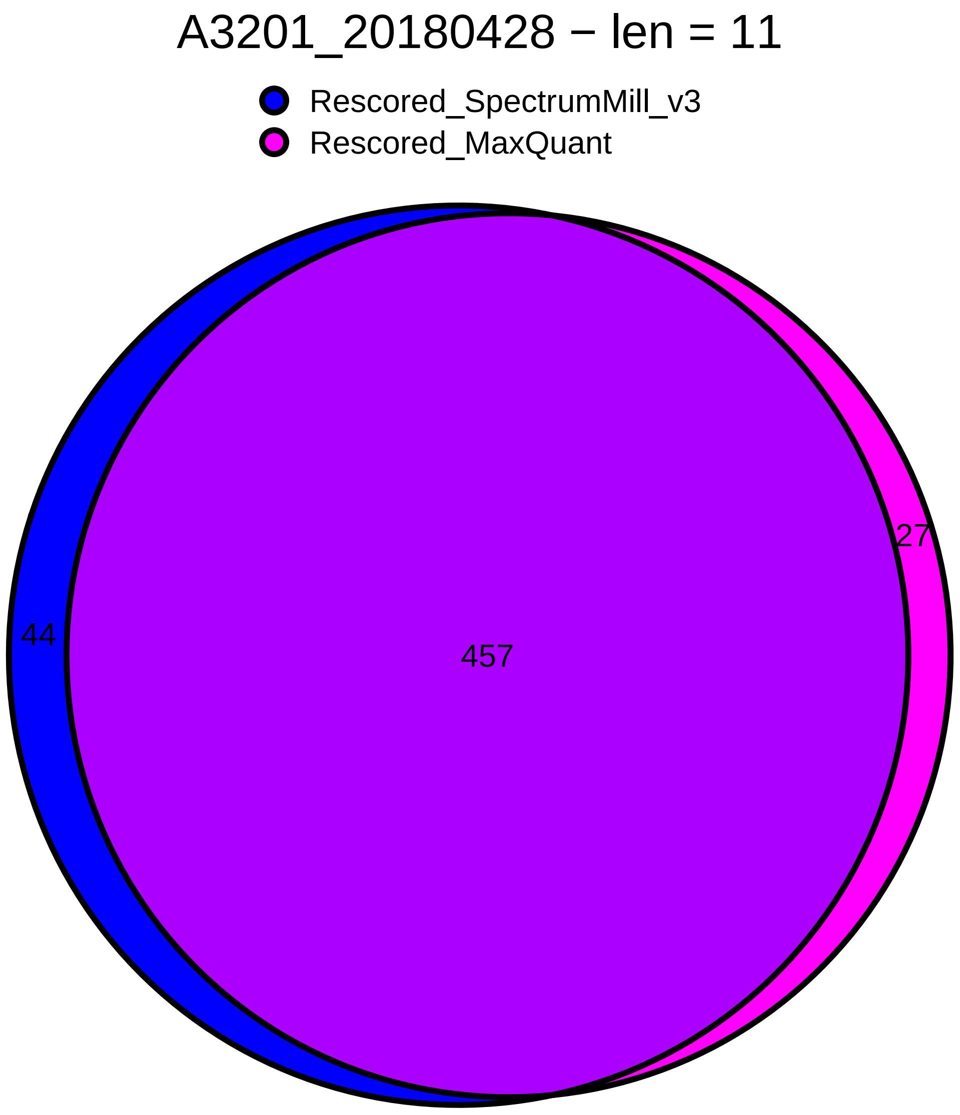

A3201_20180428 − len = 11
Rescored_SpectrumMill_v3
Rescored_MaxQuant
44
457
27

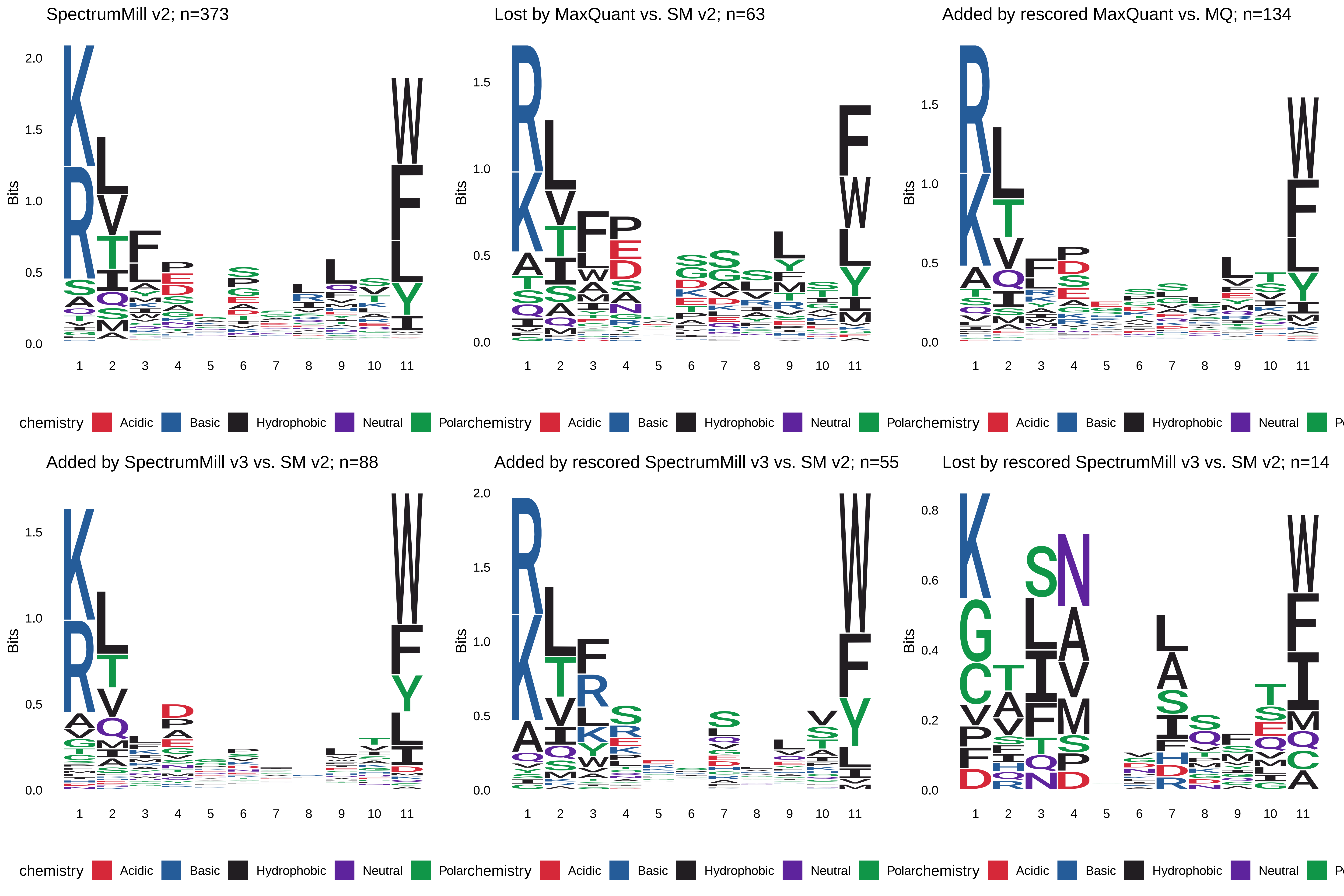
SpectrumMill v2; n=373
Lost by MaxQuant vs. SM v2; n=63
Added by rescored MaxQuant vs. MQ; n=134
Added by SpectrumMill v3 vs. SM v2; n=88
Added by rescored SpectrumMill v3 vs. SM v2; n=55
Lost by rescored SpectrumMill v3 vs. SM v2; n=14
Bits
chemistry Acidic Basic Hydrophobic Neutral Polar
chemistry Acidic Basic Hydrophobic Neutral Polar
chemistry Acidic Basic Hydrophobic Neutral
chemistry Acidic Basic Hydrophobic Neutral Polar
chemistry Acidic Basic Hydrophobic Neutral Polar
chemistry Acidic Basic Hydrophobic Neutral

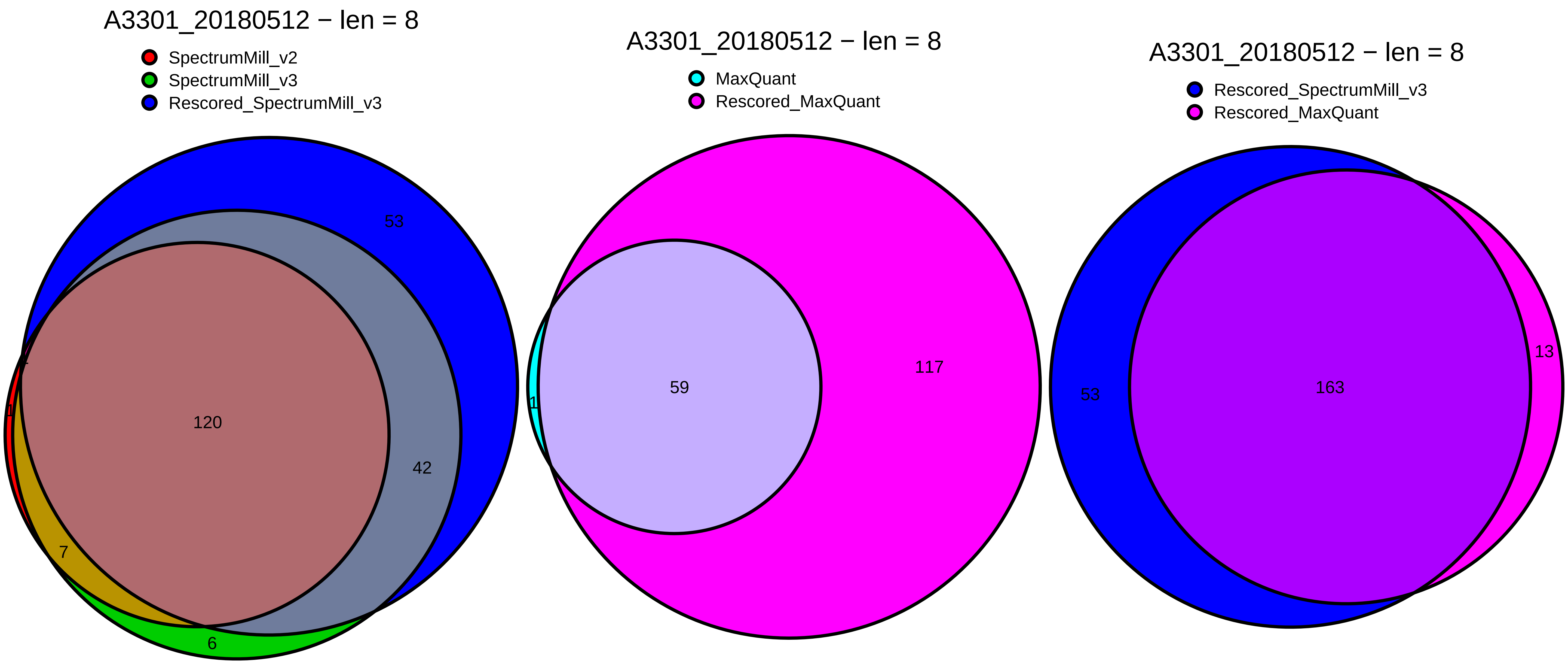
A3301_20180512 − len = 8
SpectrumMill_v2
SpectrumMill_v3
Rescored_SpectrumMill_v3
53
1
1
120
42
7
6
A3301_20180512 − len = 8
MaxQuant
Rescored_MaxQuant
1
59
117
A3301_20180512 − len = 8
Rescored_SpectrumMill_v3
Rescored_MaxQuant
53
163
13

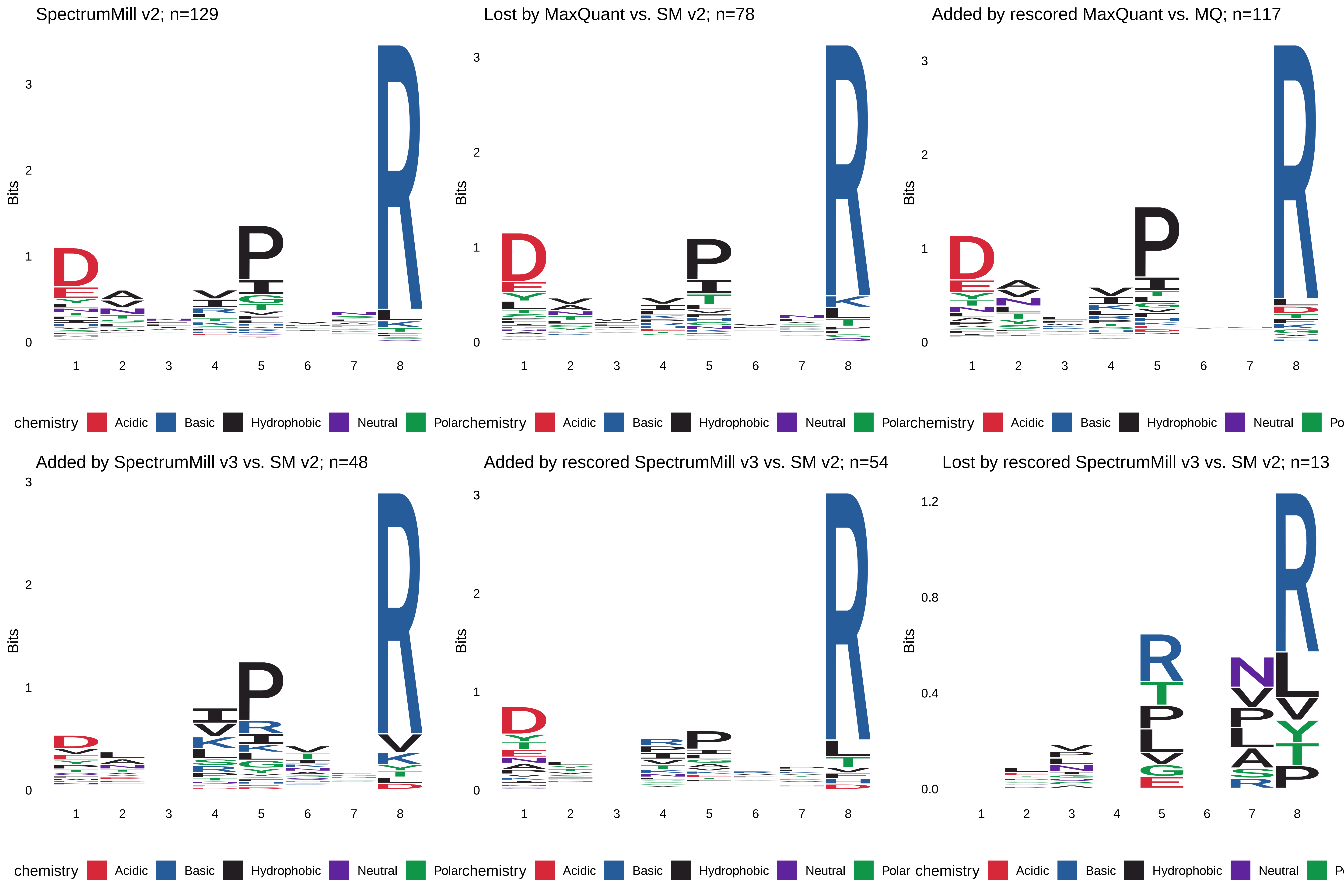
SpectrumMill v2; n=129
Lost by MaxQuant vs. SM v2; n=78
Added by rescored MaxQuant vs. MQ; n=117
Added by SpectrumMill v3 vs. SM v2; n=48
Added by rescored SpectrumMill v3 vs. SM v2; n=54
Lost by rescored SpectrumMill v3 vs. SM v2; n=13
Bits
chemistry
Acidic
Basic
Hydrophobic
Neutral
Polar

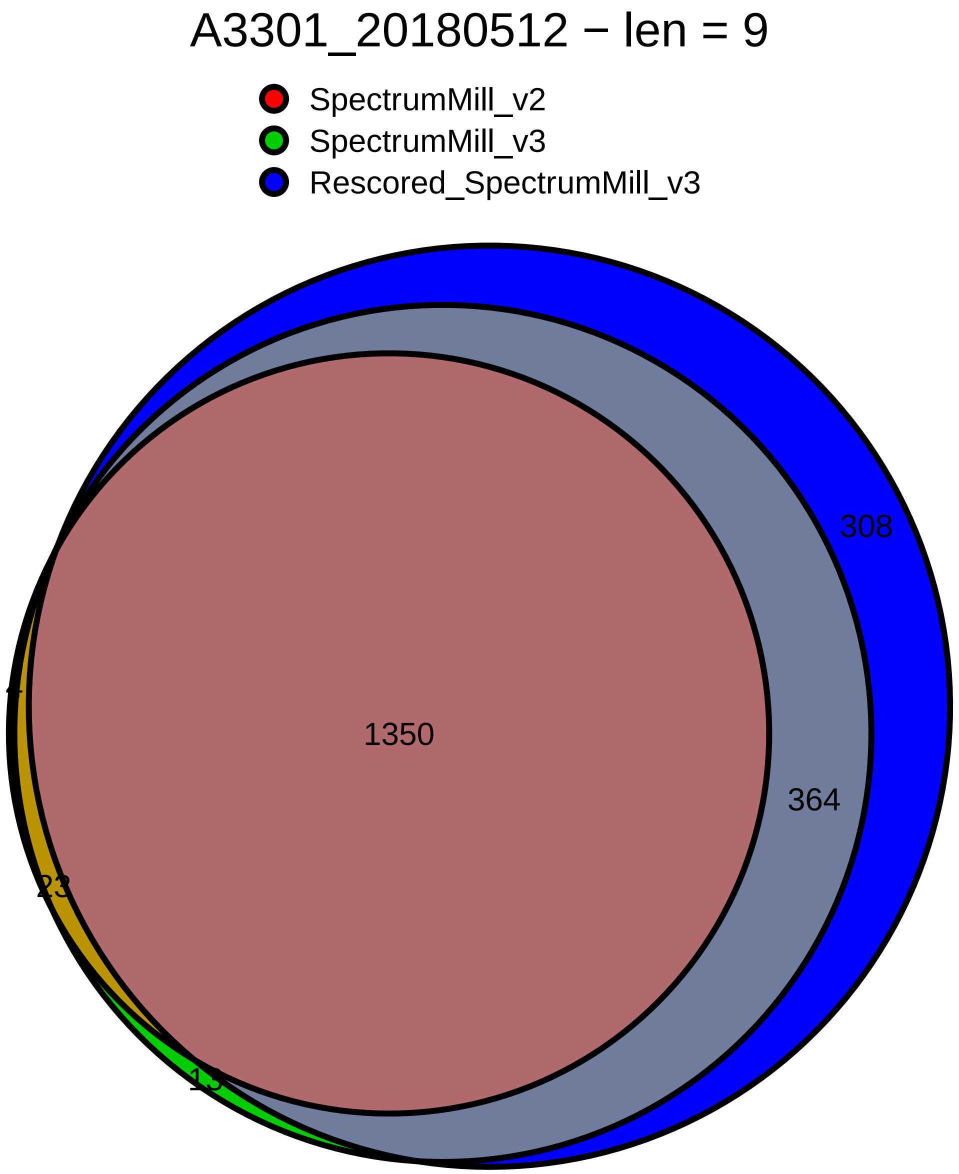
A3301_20180512 − len = 9
SpectrumMill_v2
SpectrumMill_v3
Rescored_SpectrumMill_v3
308
1350
364
23
13

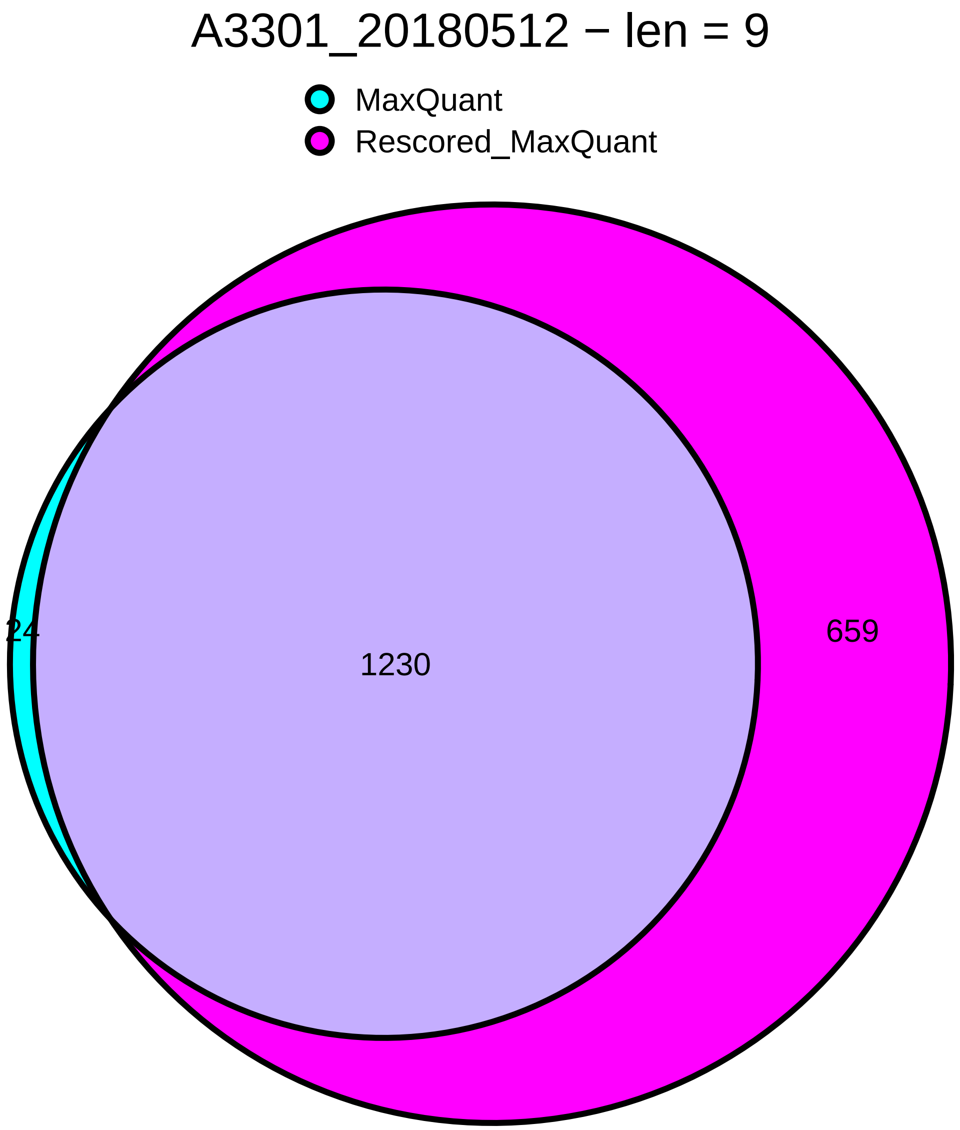
A3301_20180512 − len = 9
MaxQuant
Rescored_MaxQuant
24
1230
659

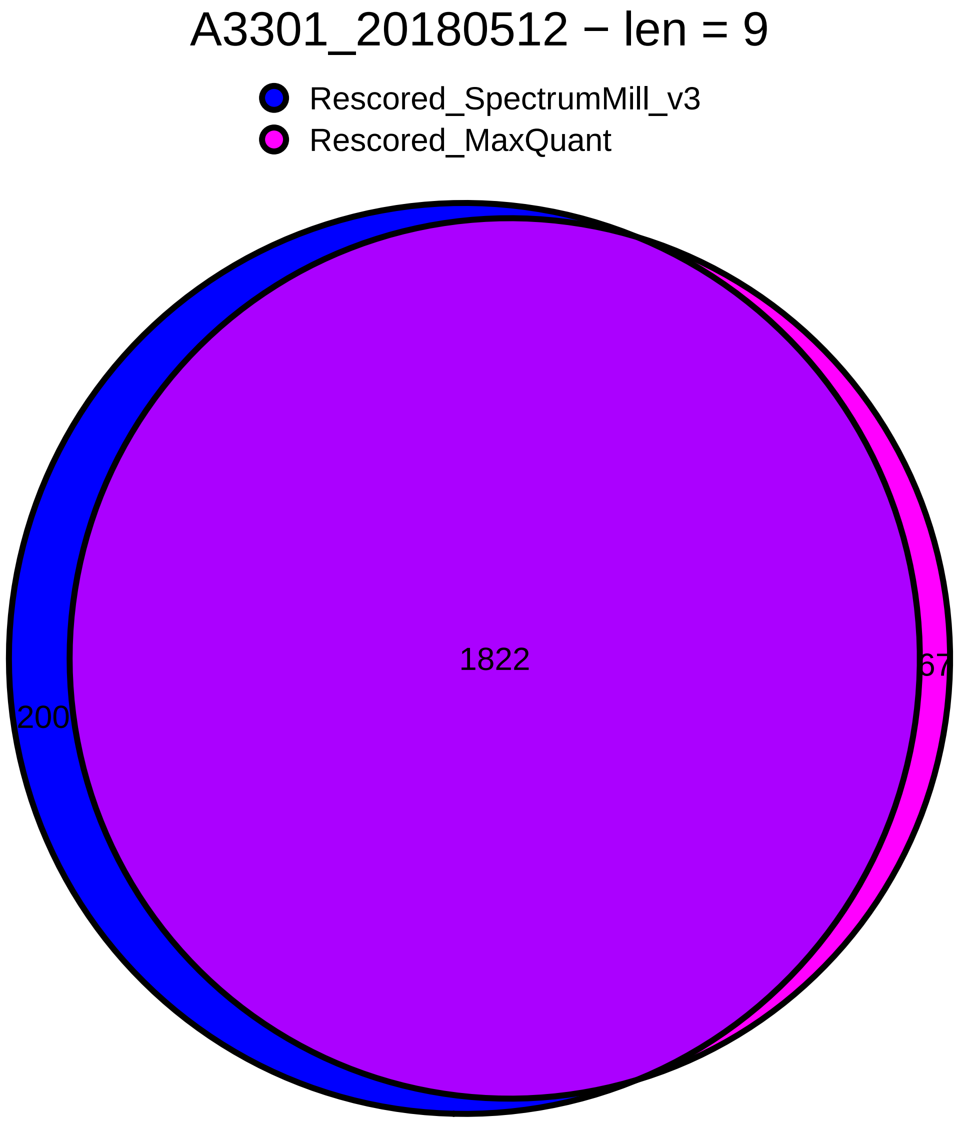
A3301_20180512 − len = 9
Rescored_SpectrumMill_v3
Rescored_MaxQuant
1822
200
67

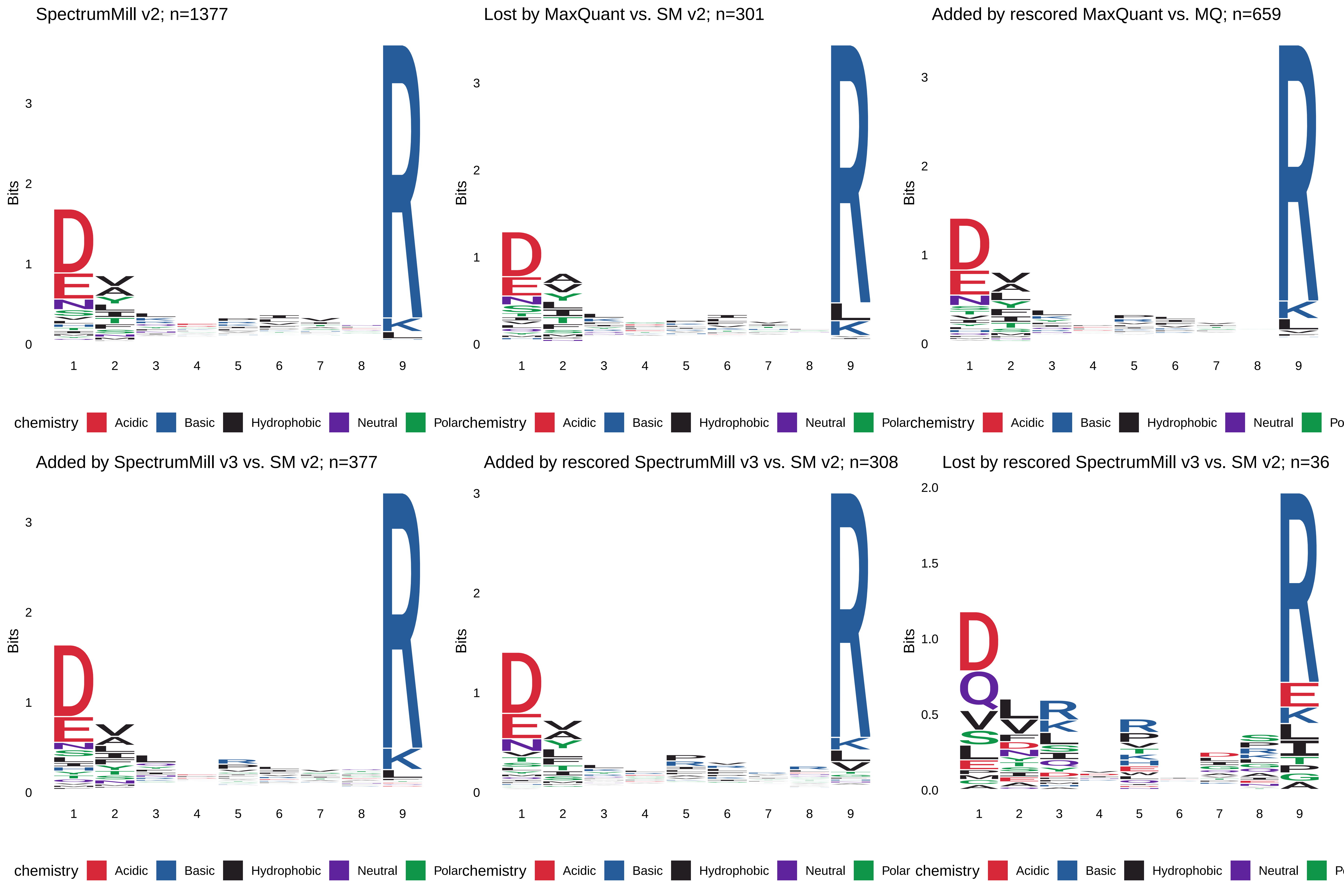
SpectrumMill v2; n=1377
Lost by MaxQuant vs. SM v2; n=301
Added by rescored MaxQuant vs. MQ; n=659
Added by SpectrumMill v3 vs. SM v2; n=377
Added by rescored SpectrumMill v3 vs. SM v2; n=308
Lost by rescored SpectrumMill v3 vs. SM v2; n=36
Bits
0
1
2
3
0.0
0.5
1.0
1.5
2.0
1 2 3 4 5 6 7 8 9
chemistry Acidic Basic Hydrophobic Neutral Polar

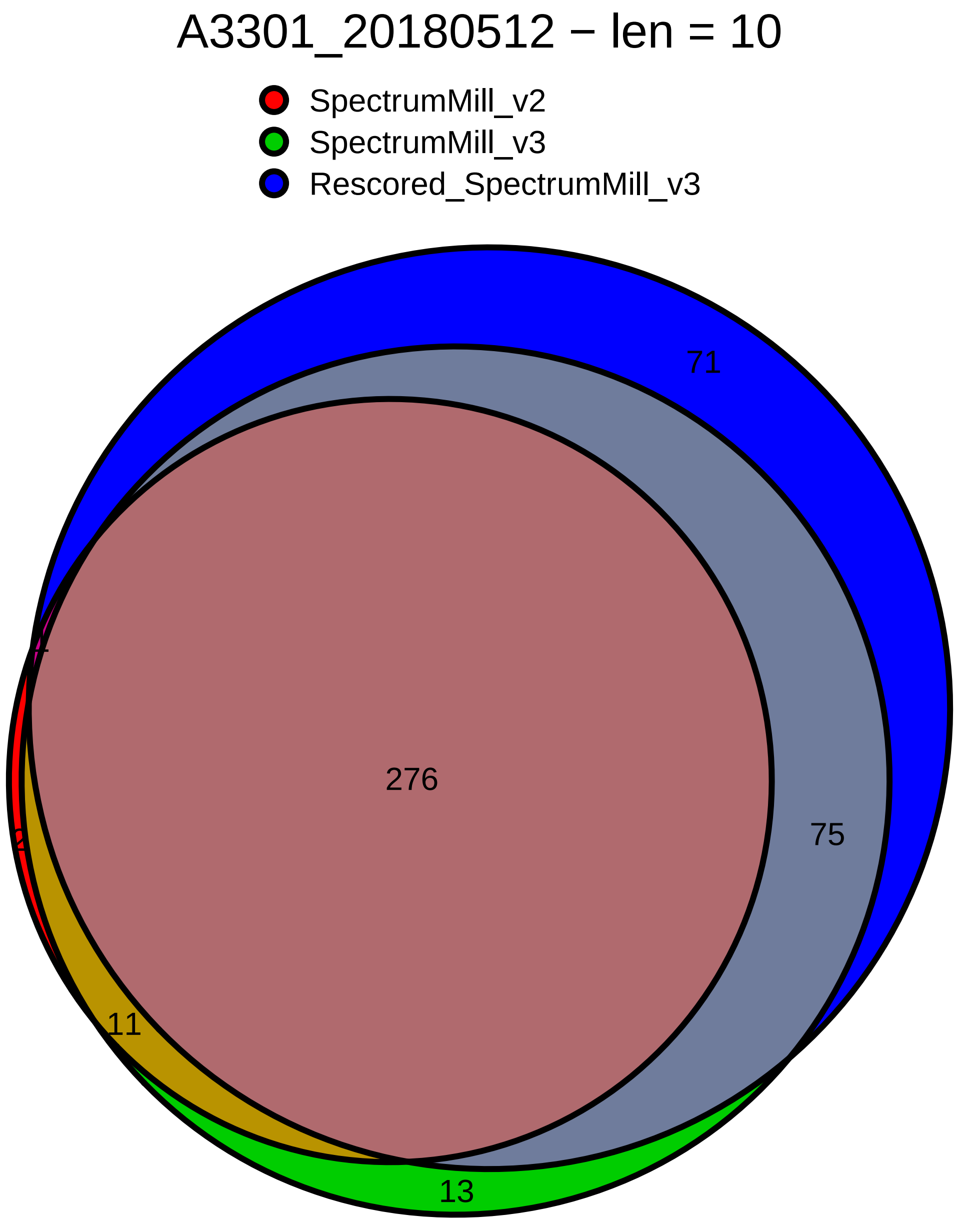
A3301_20180512 − len = 10
SpectrumMill_v2
SpectrumMill_v3
Rescored_SpectrumMill_v3
71
276
75
11
13
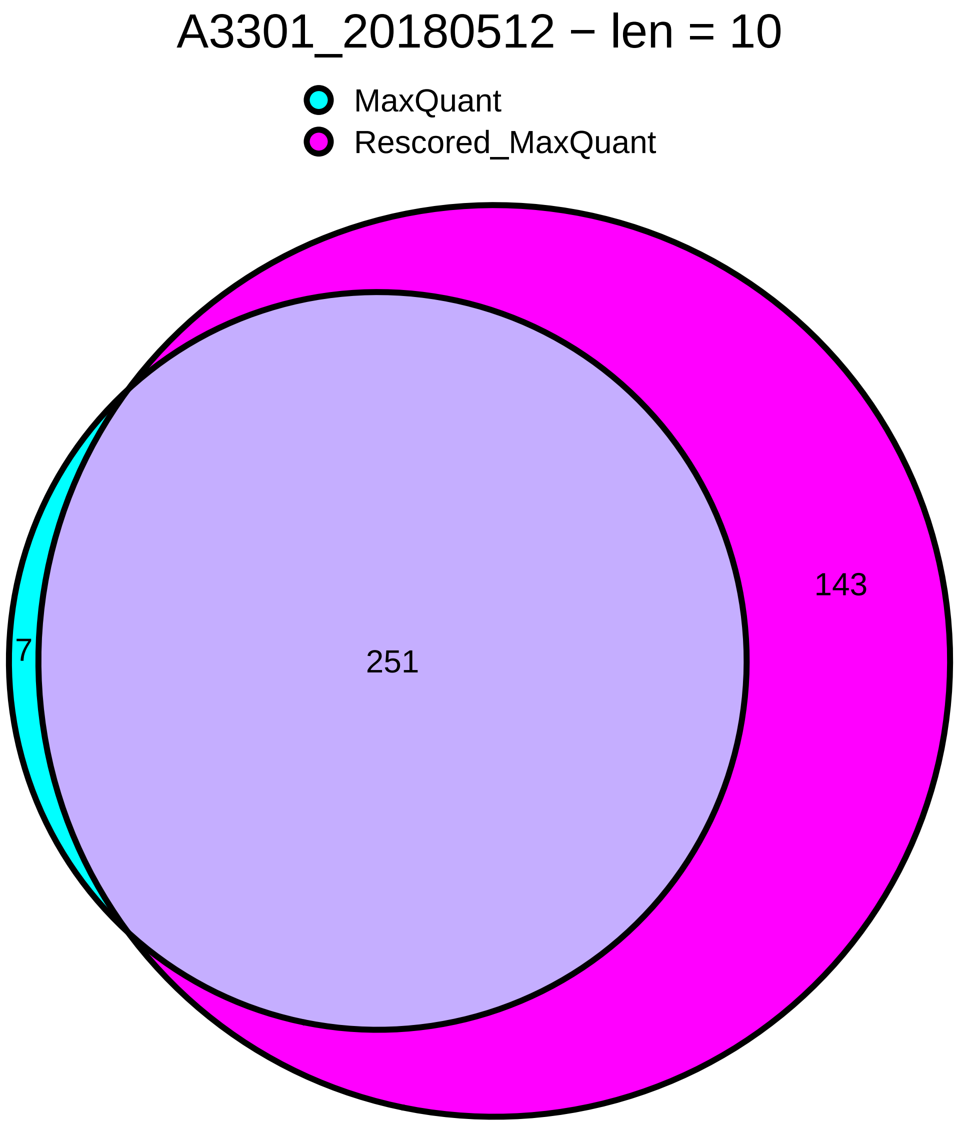
A3301_20180512 − len = 10
MaxQuant
Rescored_MaxQuant
7
251
143
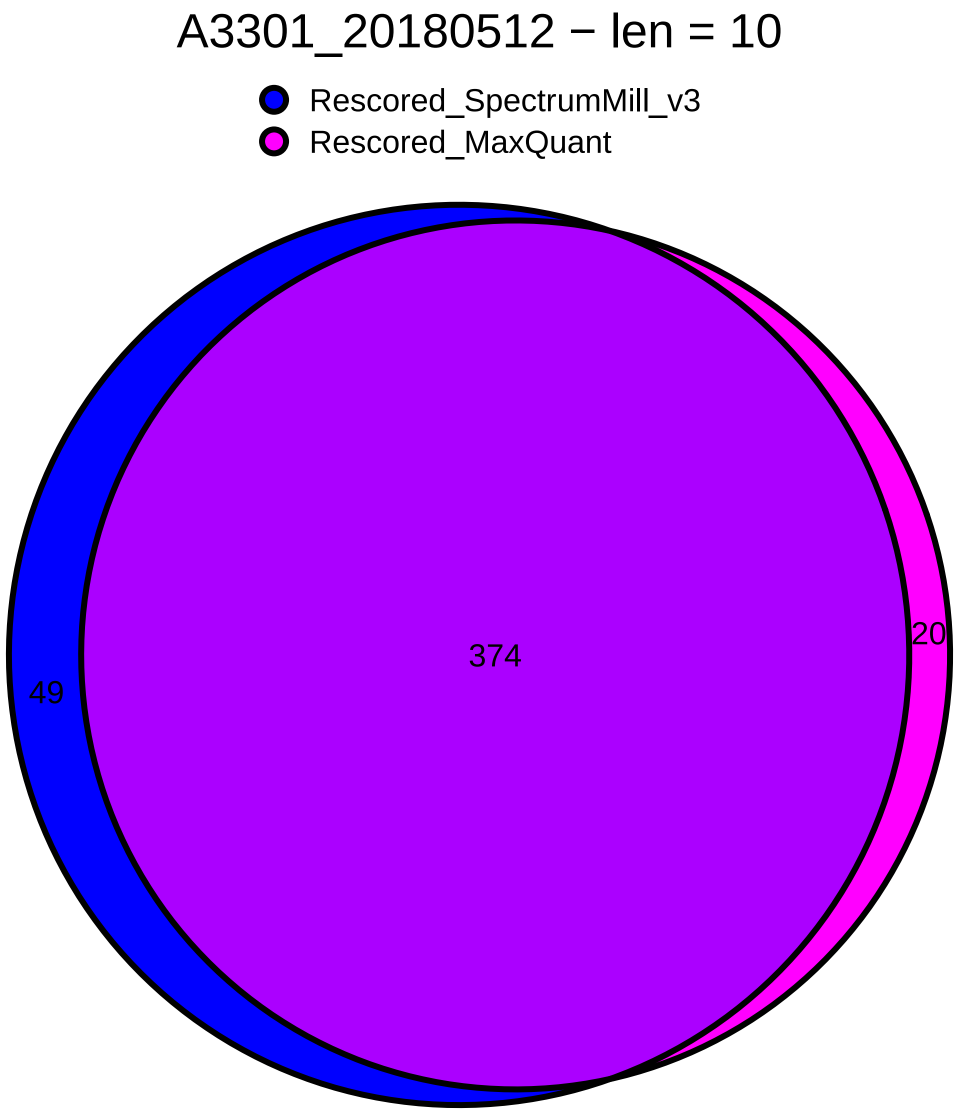
A3301_20180512 − len = 10
Rescored_SpectrumMill_v3
Rescored_MaxQuant
49
374
20

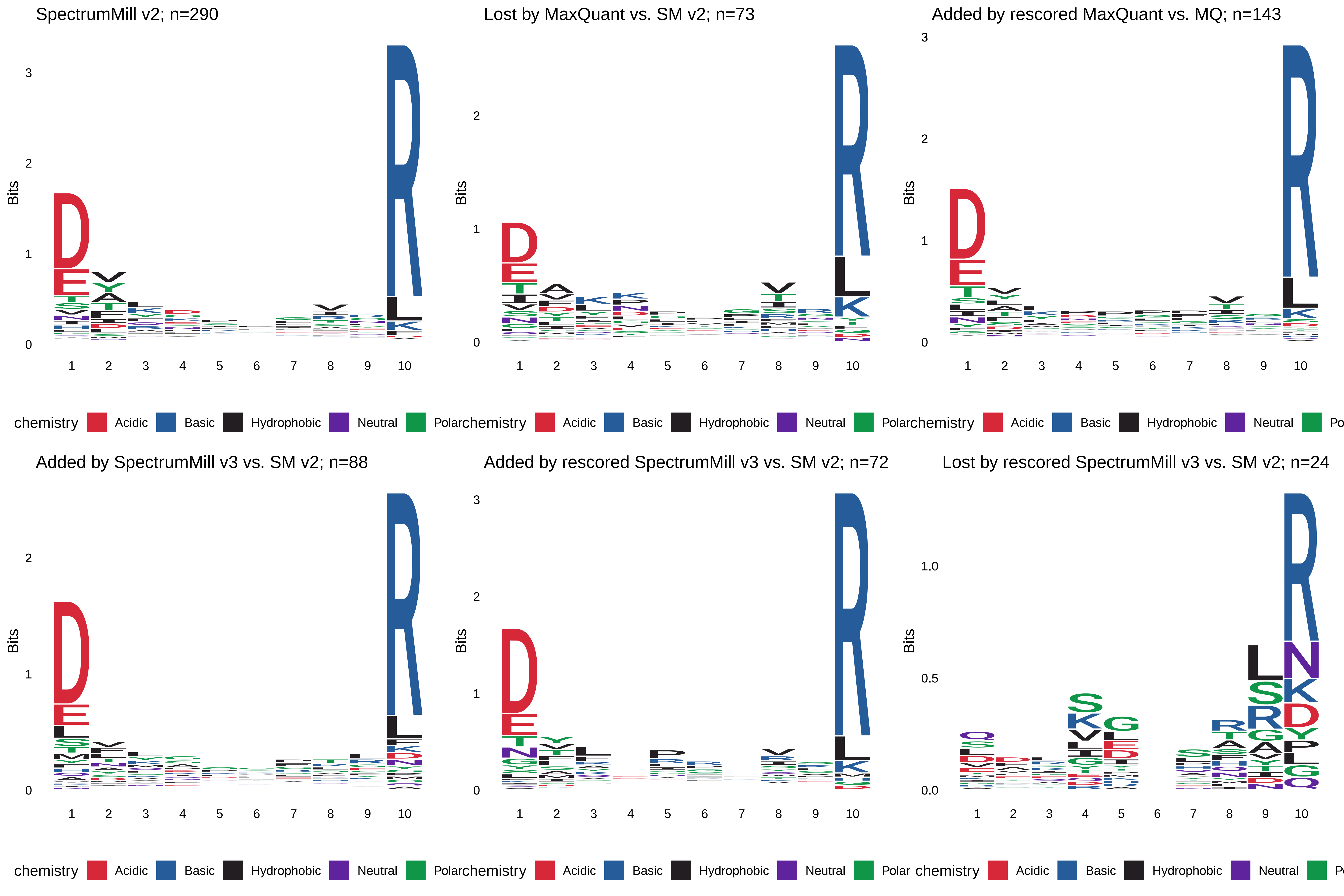

SpectrumMill v2; n=290
Lost by MaxQuant vs. SM v2; n=73
Added by rescored MaxQuant vs. MQ; n=143
Added by SpectrumMill v3 vs. SM v2; n=88
Added by rescored SpectrumMill v3 vs. SM v2; n=72
Lost by rescored SpectrumMill v3 vs. SM v2; n=24
Bits
1 2 3 4 5 6 7 8 9 10
chemistry Acidic Basic Hydrophobic Neutral Polar

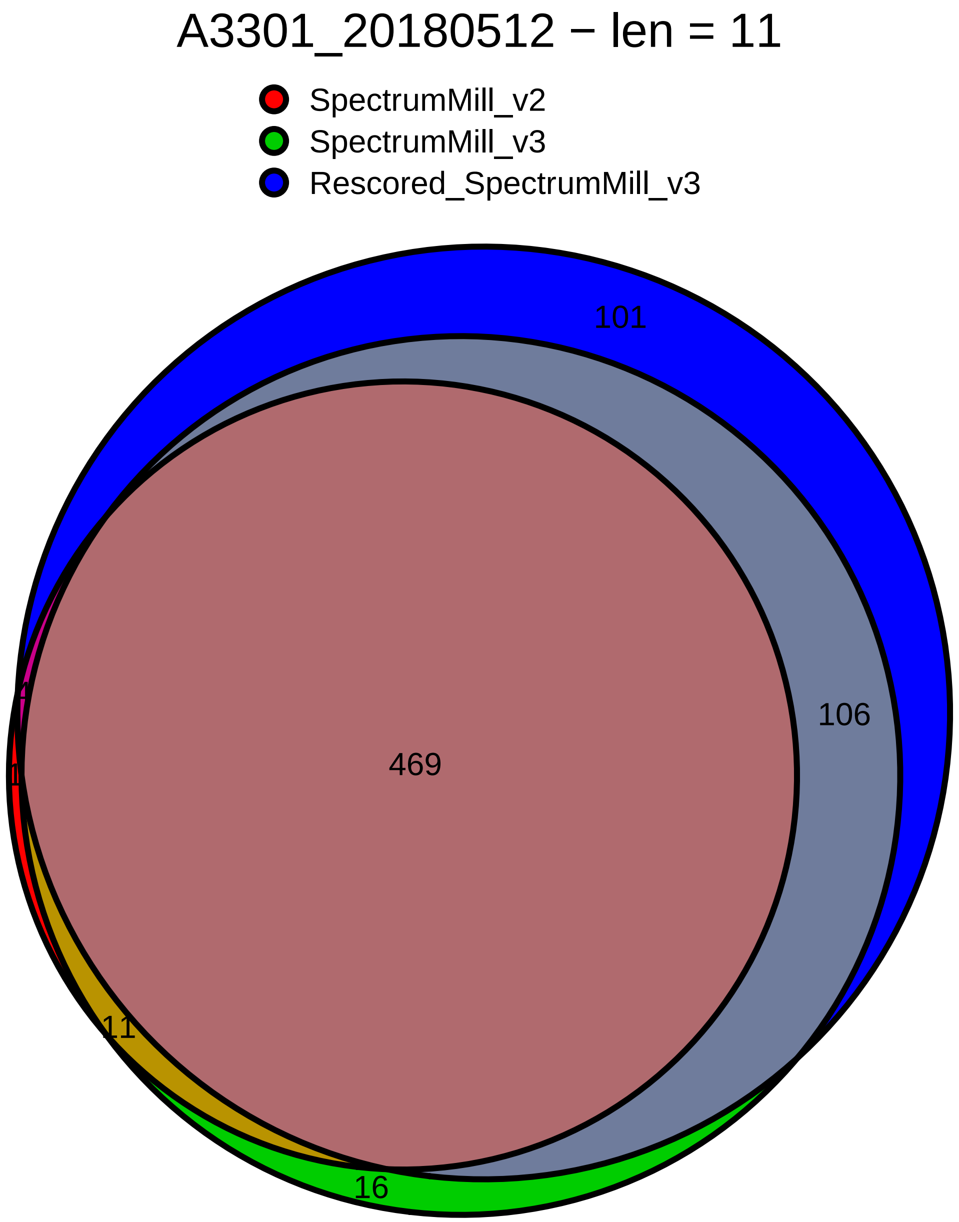
A3301_20180512 − len = 11
SpectrumMill_v2
SpectrumMill_v3
Rescored_SpectrumMill_v3
101
106
469
11
16

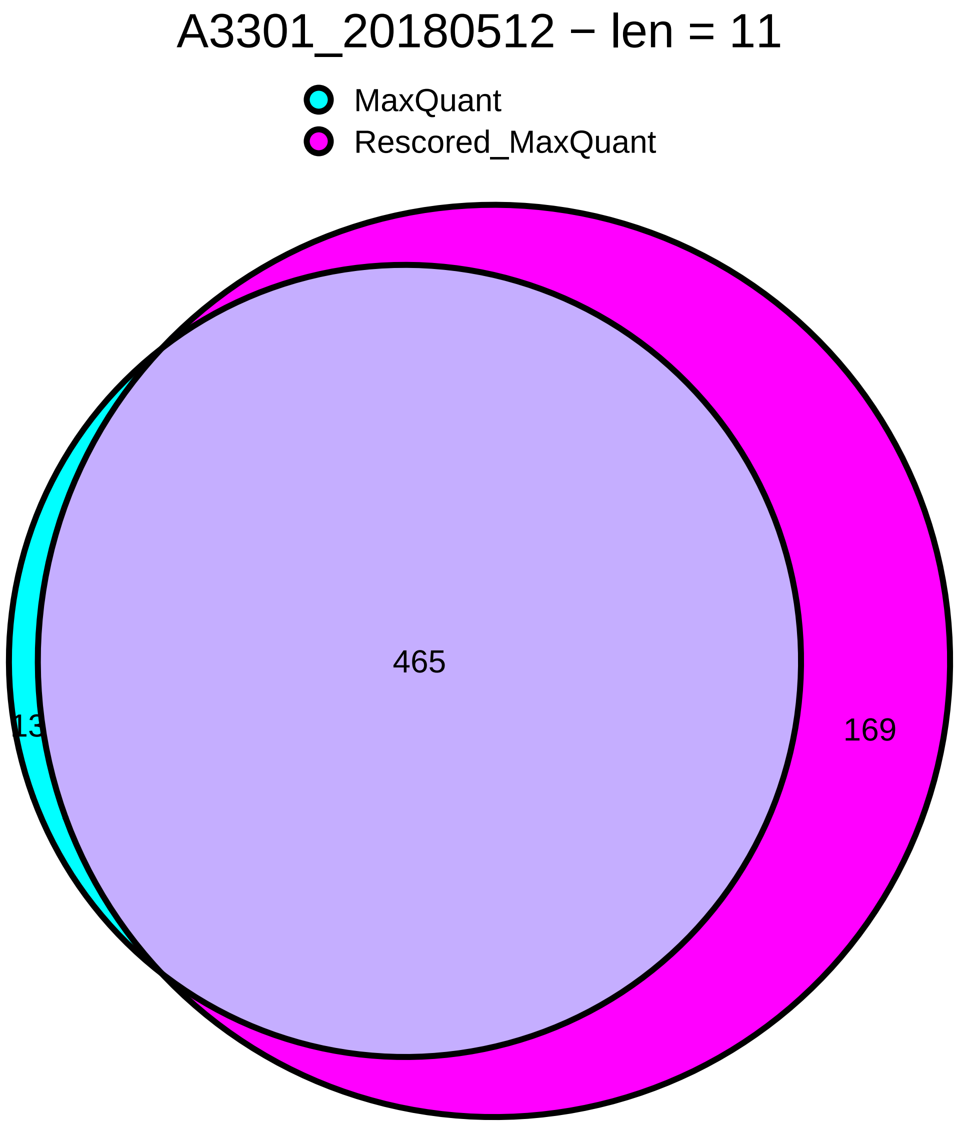
A3301_20180512 − len = 11
MaxQuant
Rescored_MaxQuant
465
169
13

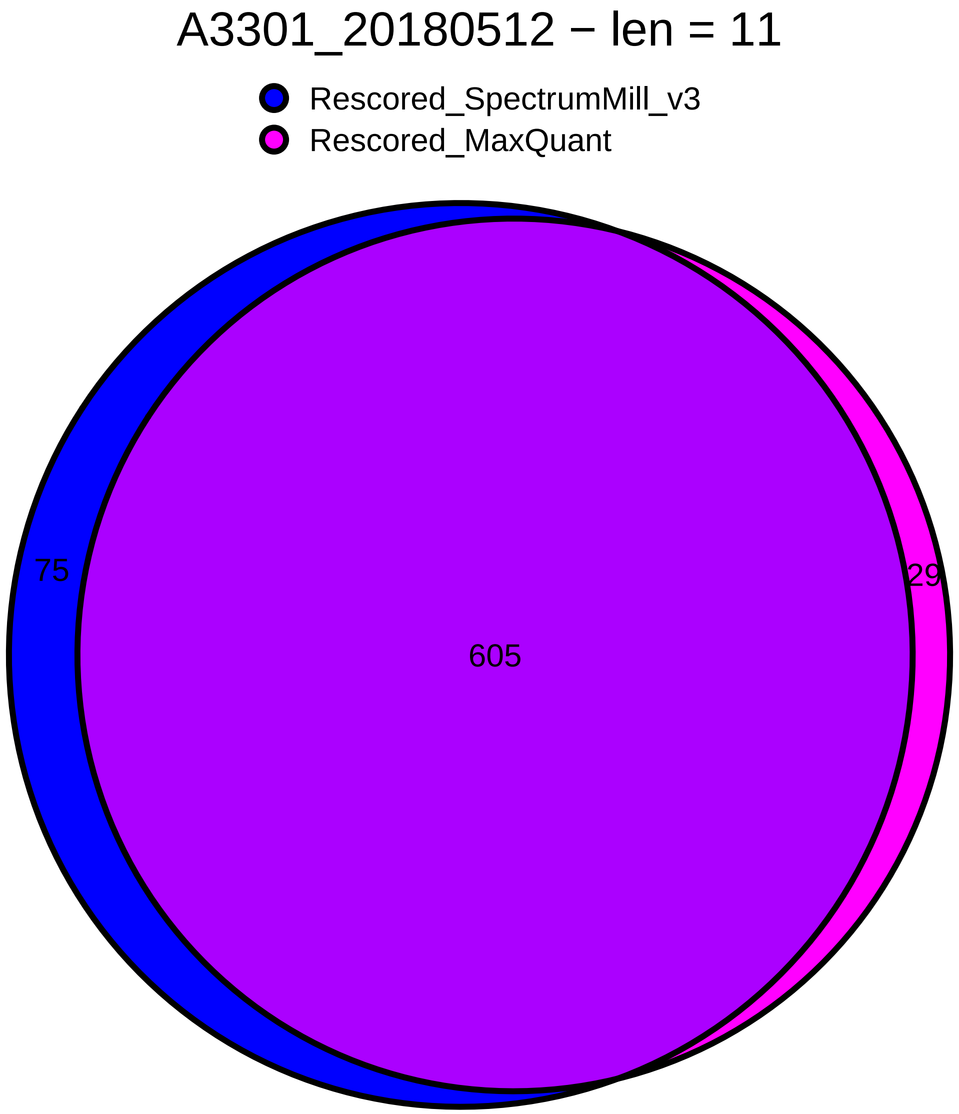
A3301_20180512 − len = 11
Rescored_SpectrumMill_v3
Rescored_MaxQuant
75
605
29

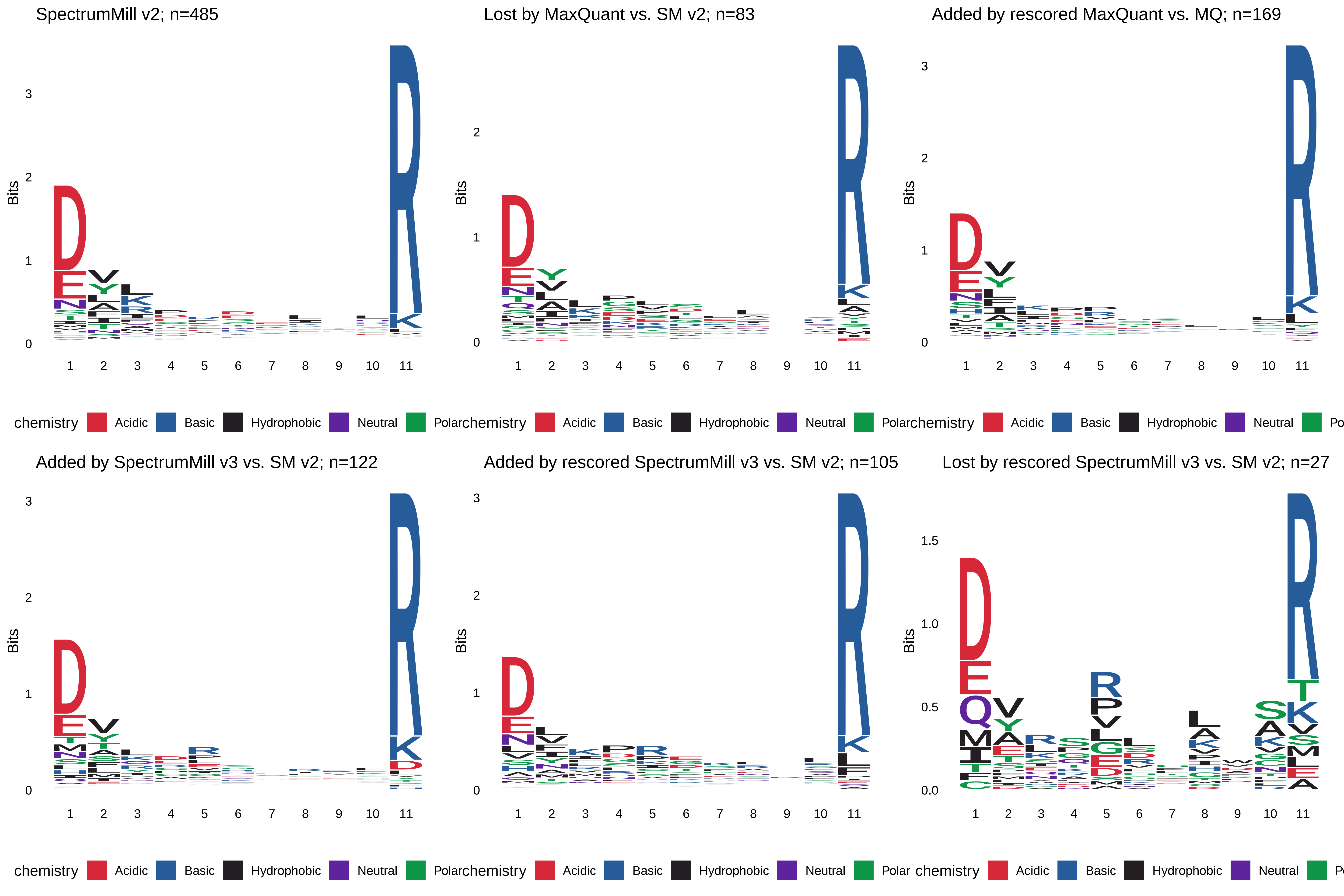

SpectrumMill v2; n=485
Lost by MaxQuant vs. SM v2; n=83
Added by rescored MaxQuant vs. MQ; n=169
Bits
1 2 3 4 5 6 7 8 9 10 11
chemistry Acidic Basic Hydrophobic Neutral Polar
Added by SpectrumMill v3 vs. SM v2; n=122
Added by rescored SpectrumMill v3 vs. SM v2; n=105
Lost by rescored SpectrumMill v3 vs. SM v2; n=27
Bits
1 2 3 4 5 6 7 8 9 10 11
chemistry Acidic Basic Hydrophobic Neutral Polar

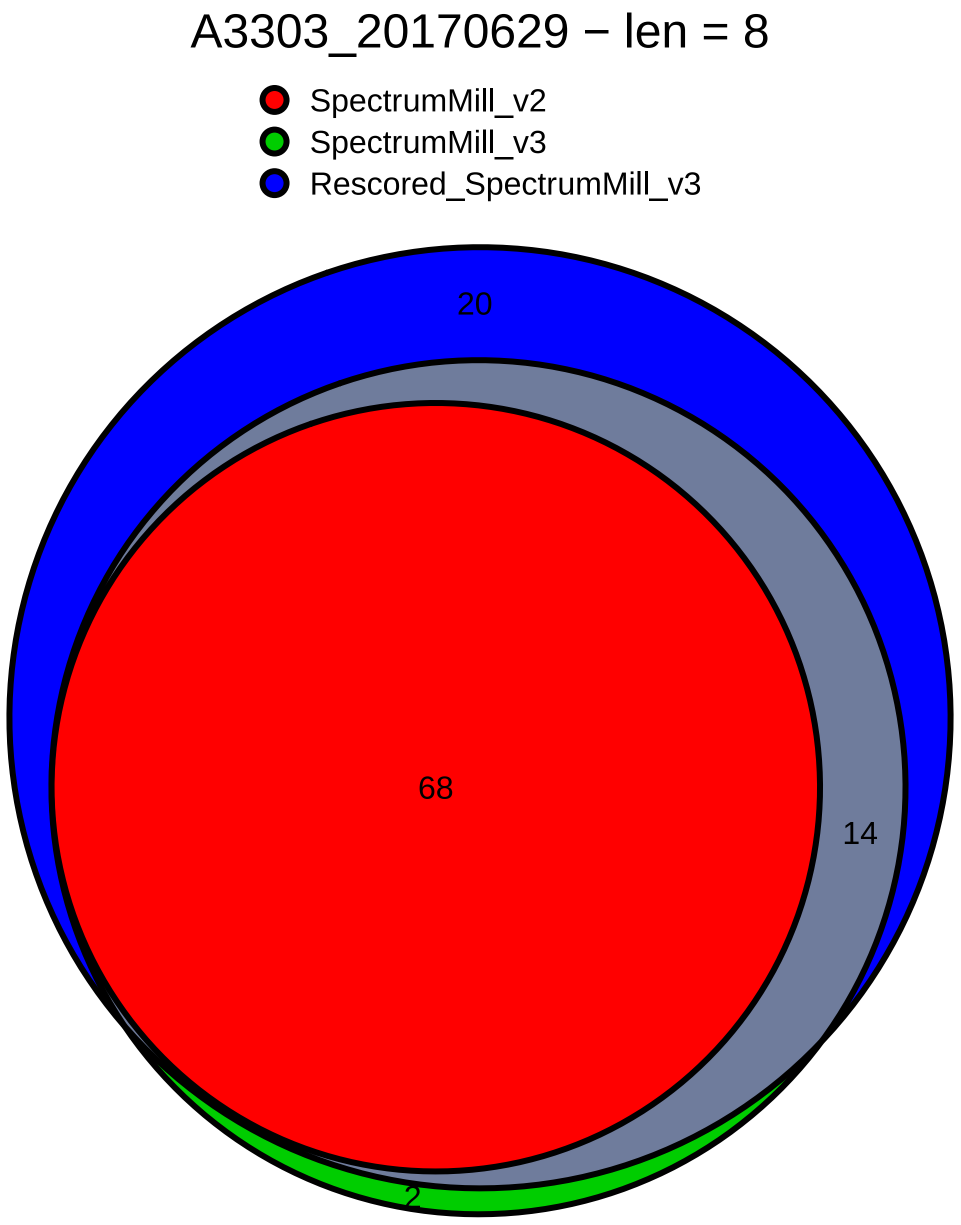
A3303_20170629 − len = 8
SpectrumMill_v2
SpectrumMill_v3
Rescored_SpectrumMill_v3
20
68
14
2

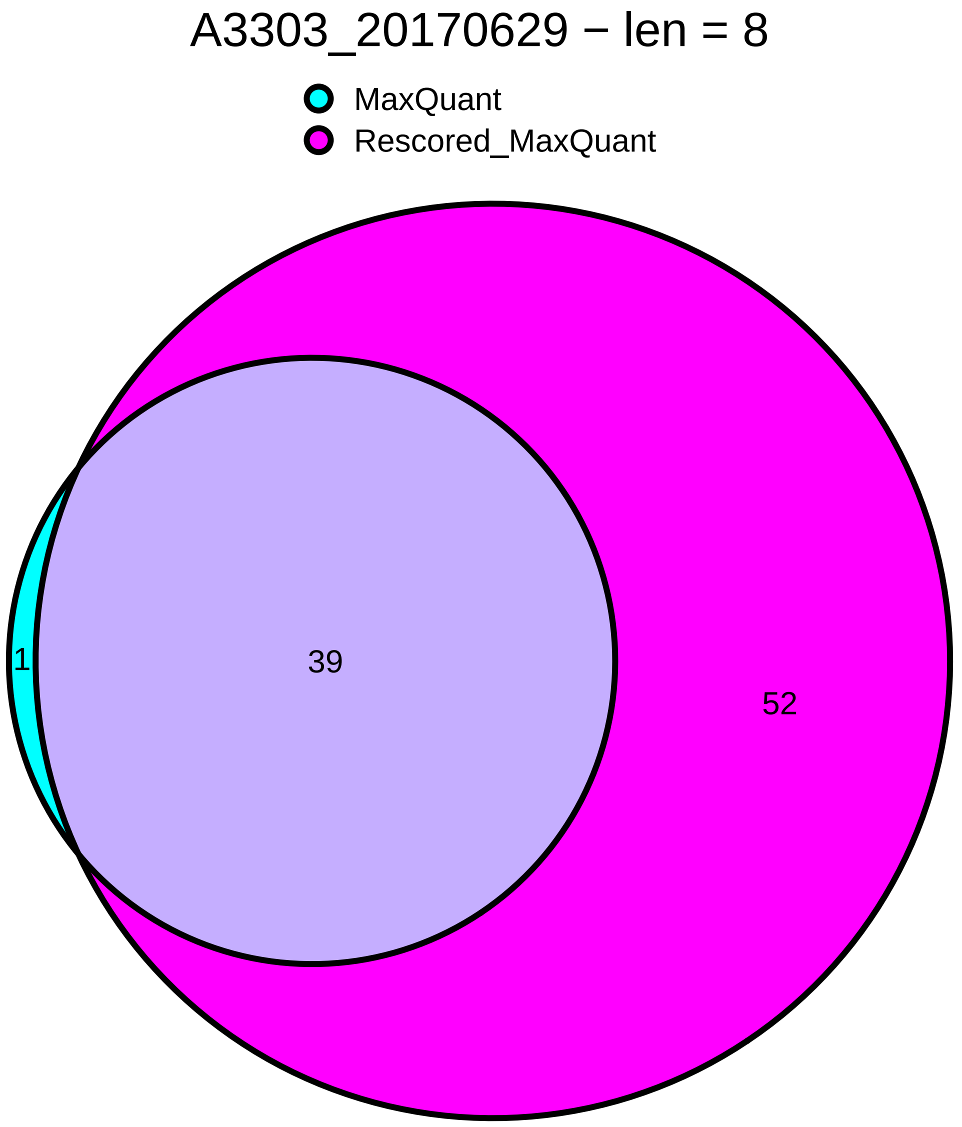
A3303_20170629 − len = 8
MaxQuant
Rescored_MaxQuant
1
39
52

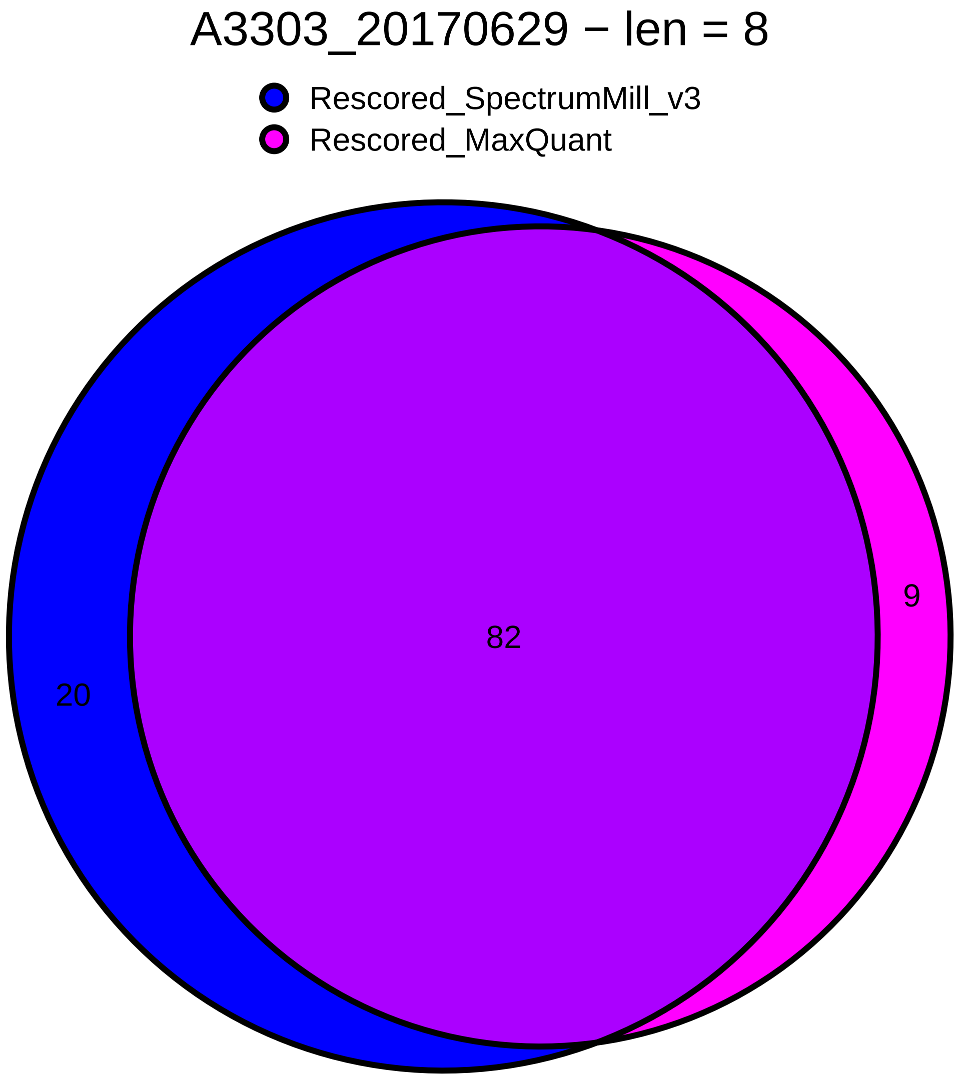
A3303_20170629 − len = 8
Rescored_SpectrumMill_v3
Rescored_MaxQuant
20
82
9

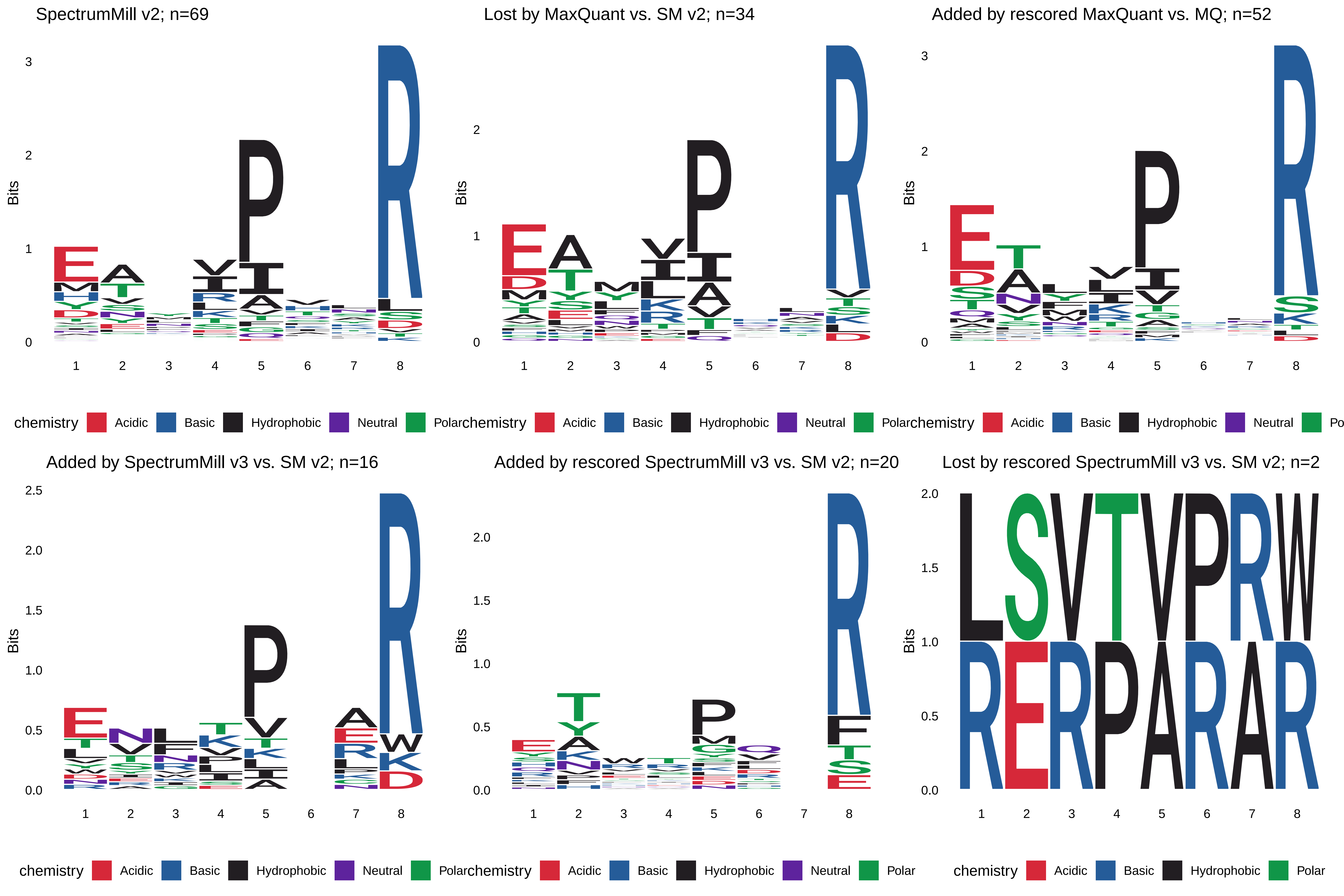
SpectrumMill v2; n=69
Lost by MaxQuant vs. SM v2; n=34
Added by rescored MaxQuant vs. MQ; n=52
Added by SpectrumMill v3 vs. SM v2; n=16
Added by rescored SpectrumMill v3 vs. SM v2; n=20
Lost by rescored SpectrumMill v3 vs. SM v2; n=2
Bits
1 2 3 4 5 6 7 8
LSVTVPRW
RERPARAR
chemistry Acidic Basic Hydrophobic Neutral Polar

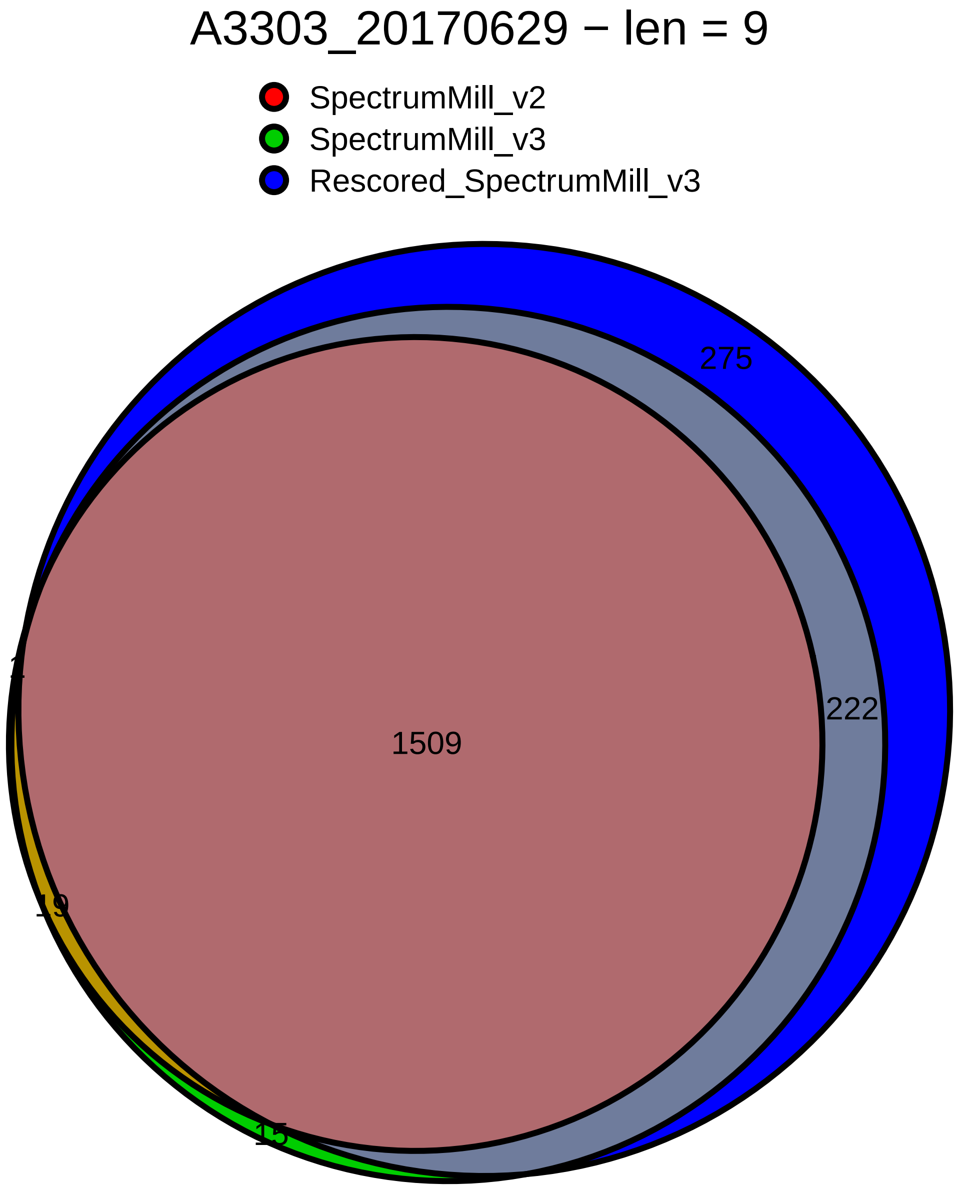
A3303_20170629 − len = 9
SpectrumMill_v2
SpectrumMill_v3
Rescored_SpectrumMill_v3
275
222
1509
19
15

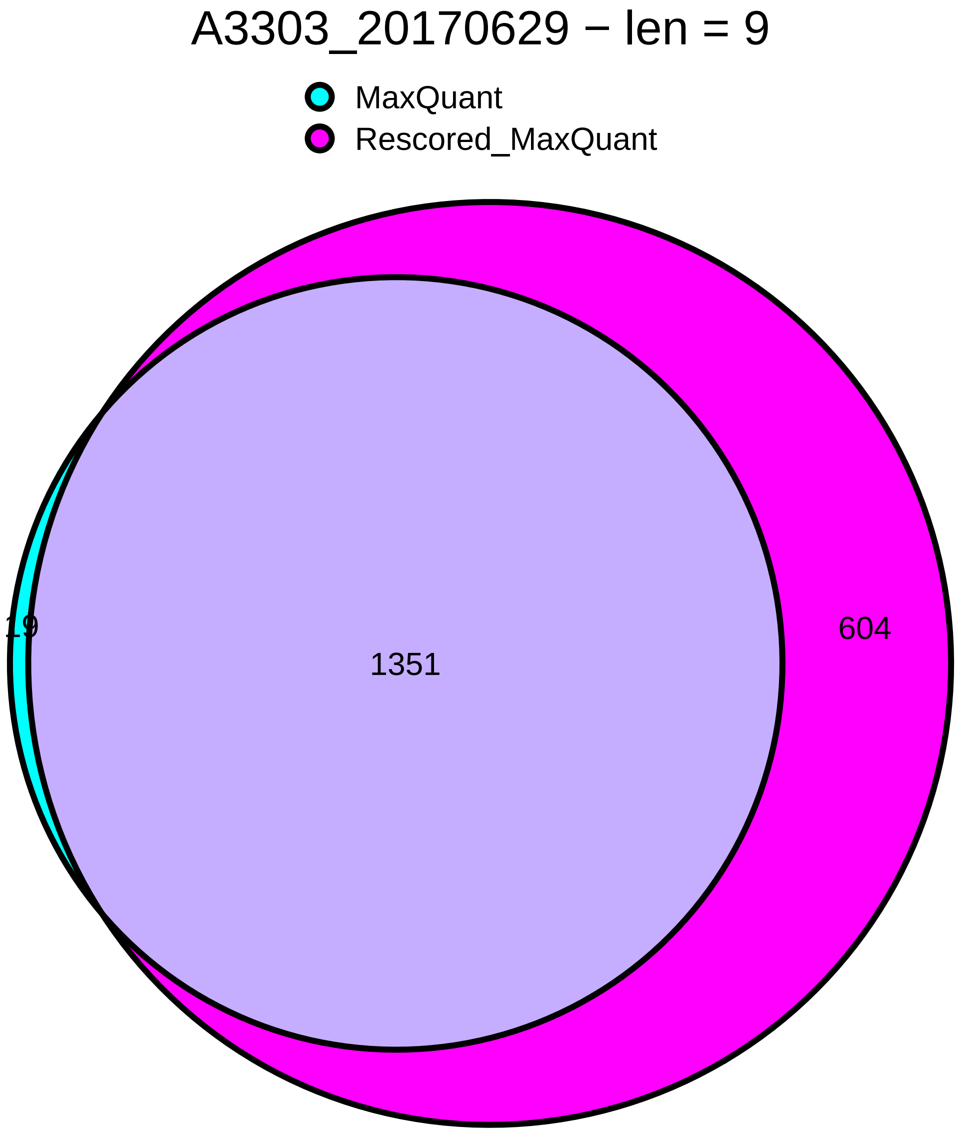
A3303_20170629 − len = 9
MaxQuant
Rescored_MaxQuant
19
1351
604

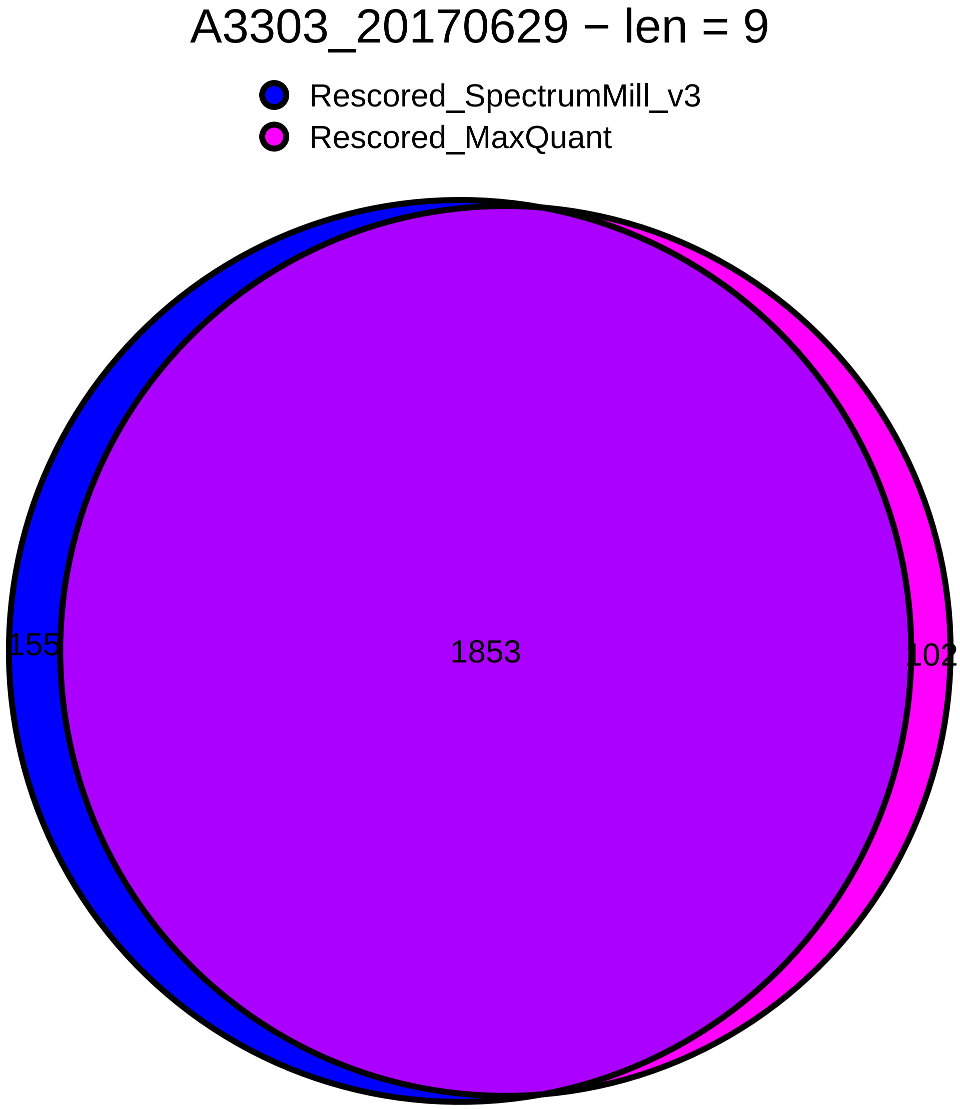
A3303_20170629 − len = 9
Rescored_SpectrumMill_v3
Rescored_MaxQuant
155
1853

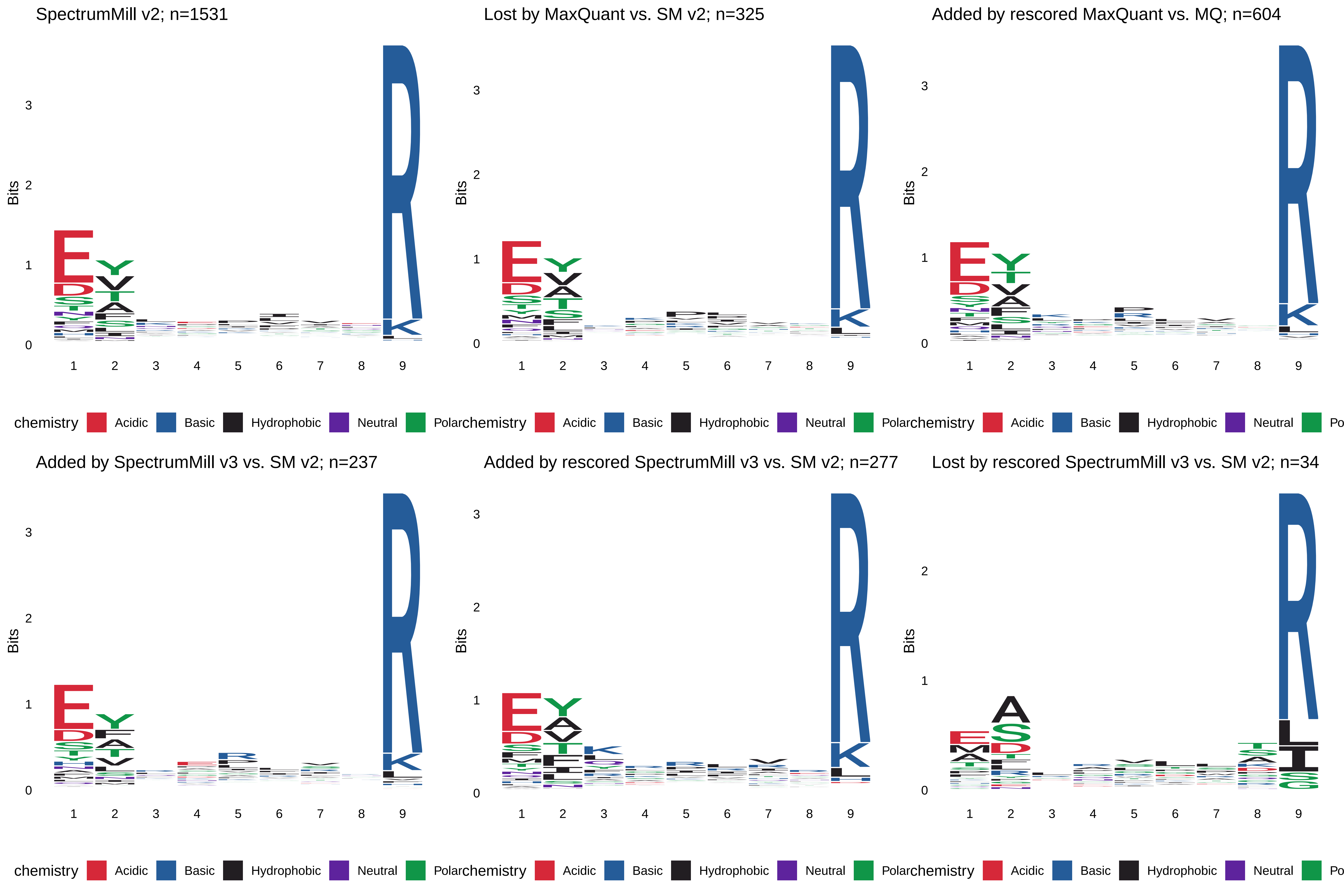
SpectrumMill v2; n=1531
Lost by MaxQuant vs. SM v2; n=325
Added by rescored MaxQuant vs. MQ; n=604
Added by SpectrumMill v3 vs. SM v2; n=237
Added by rescored SpectrumMill v3 vs. SM v2; n=277
Lost by rescored SpectrumMill v3 vs. SM v2; n=34
Bits
1 2 3 4 5 6 7 8 9
chemistry
Acidic
Basic
Hydrophobic
Neutral
Polar

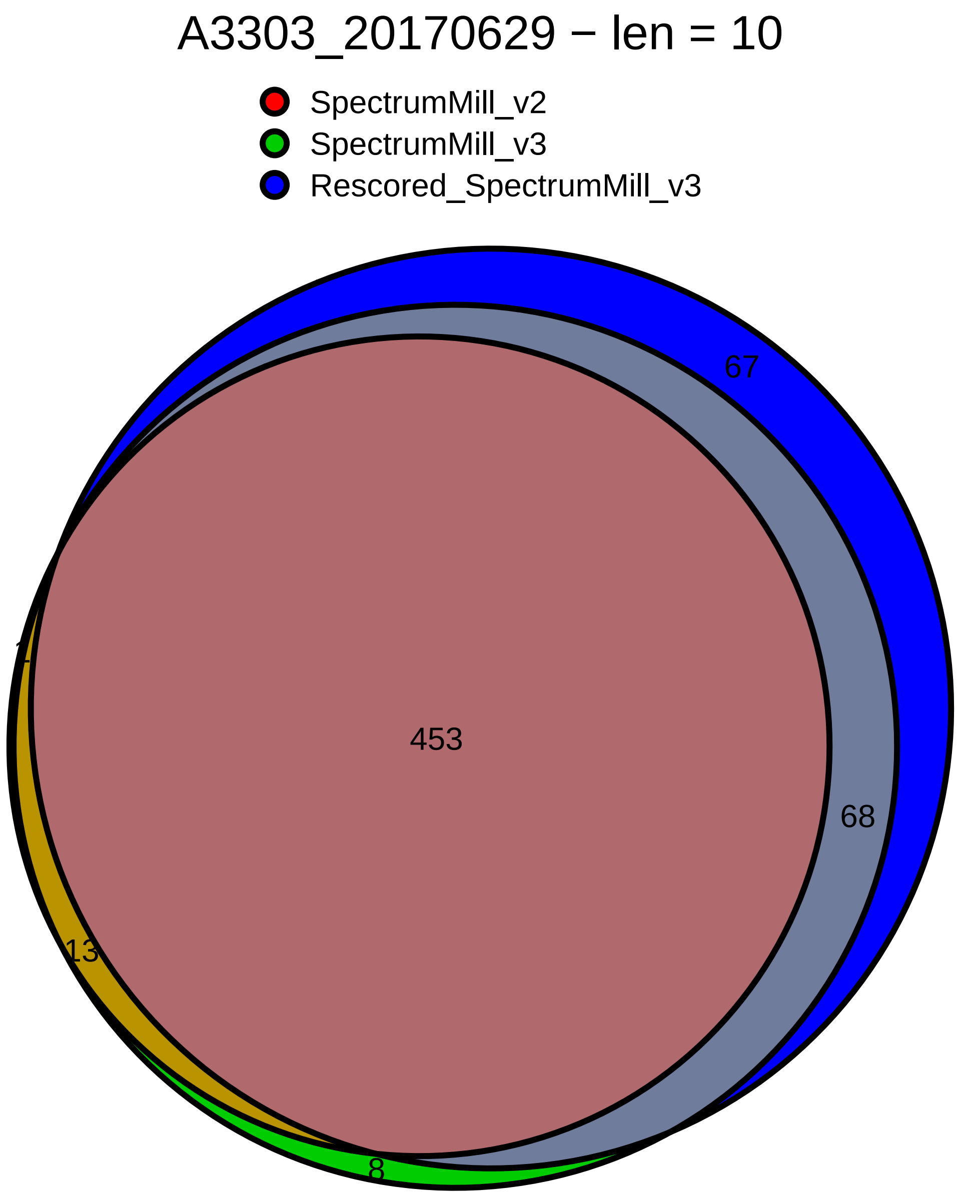
A3303_20170629 − len = 10
SpectrumMill_v2
SpectrumMill_v3
Rescored_SpectrumMill_v3
67
453
68
13
8

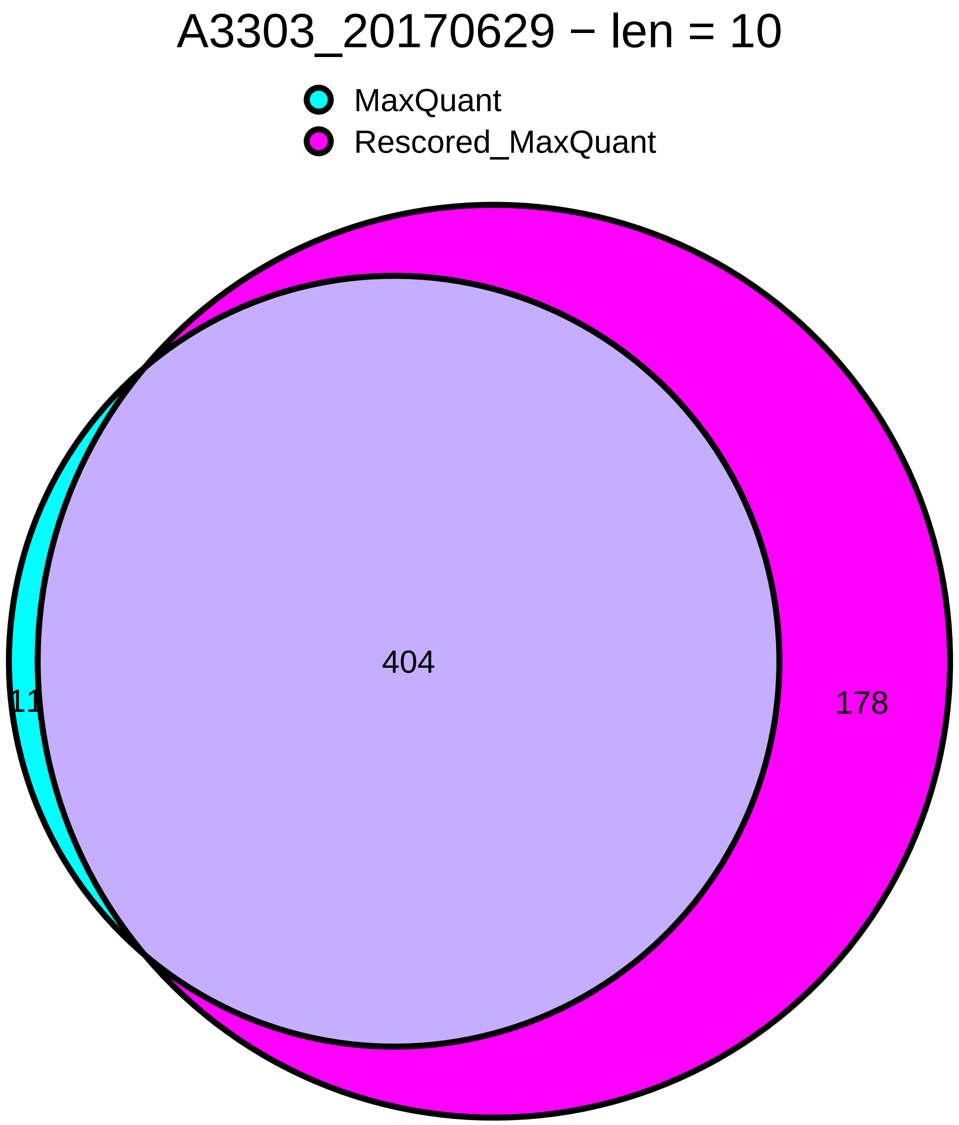
A3303_20170629 − len = 10
MaxQuant
Rescored_MaxQuant
404
178

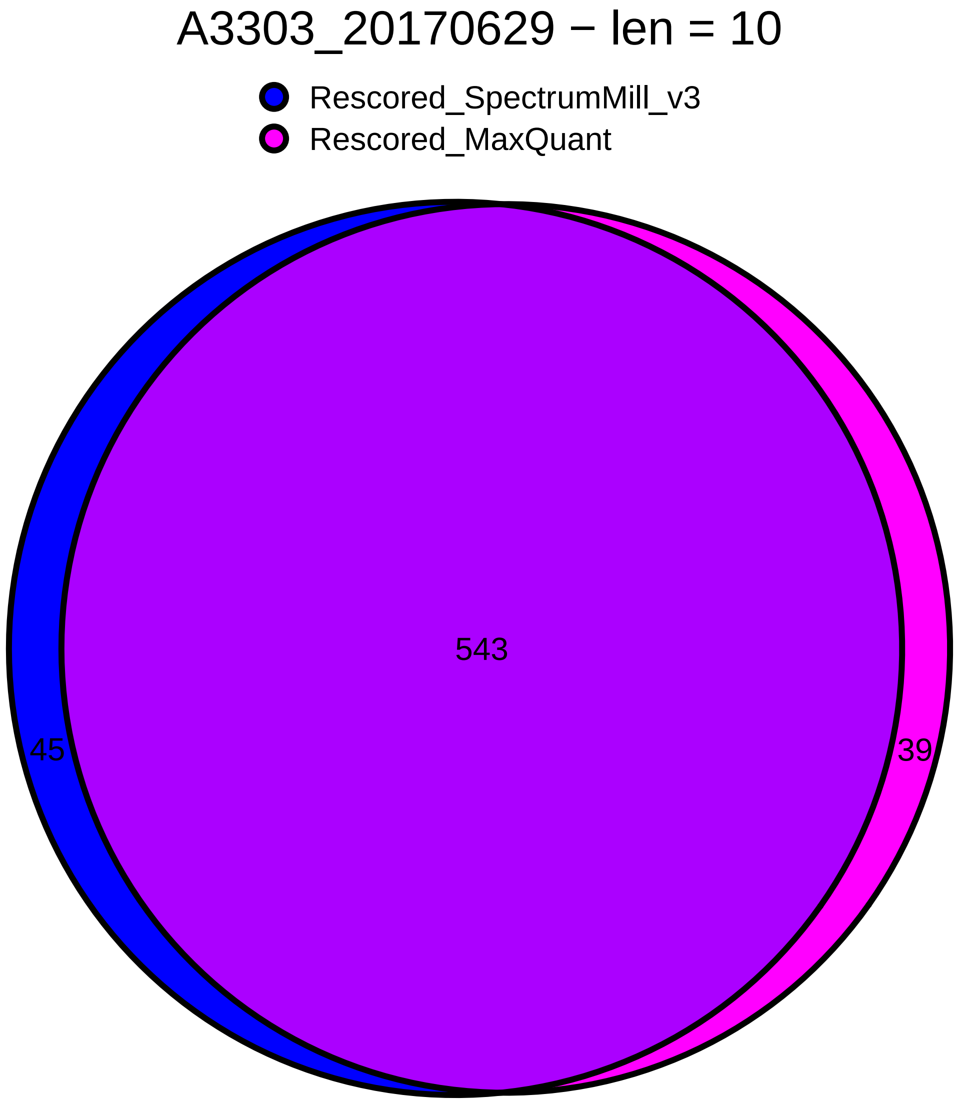
A3303_20170629 − len = 10
Rescored_SpectrumMill_v3
Rescored_MaxQuant
543
45
39

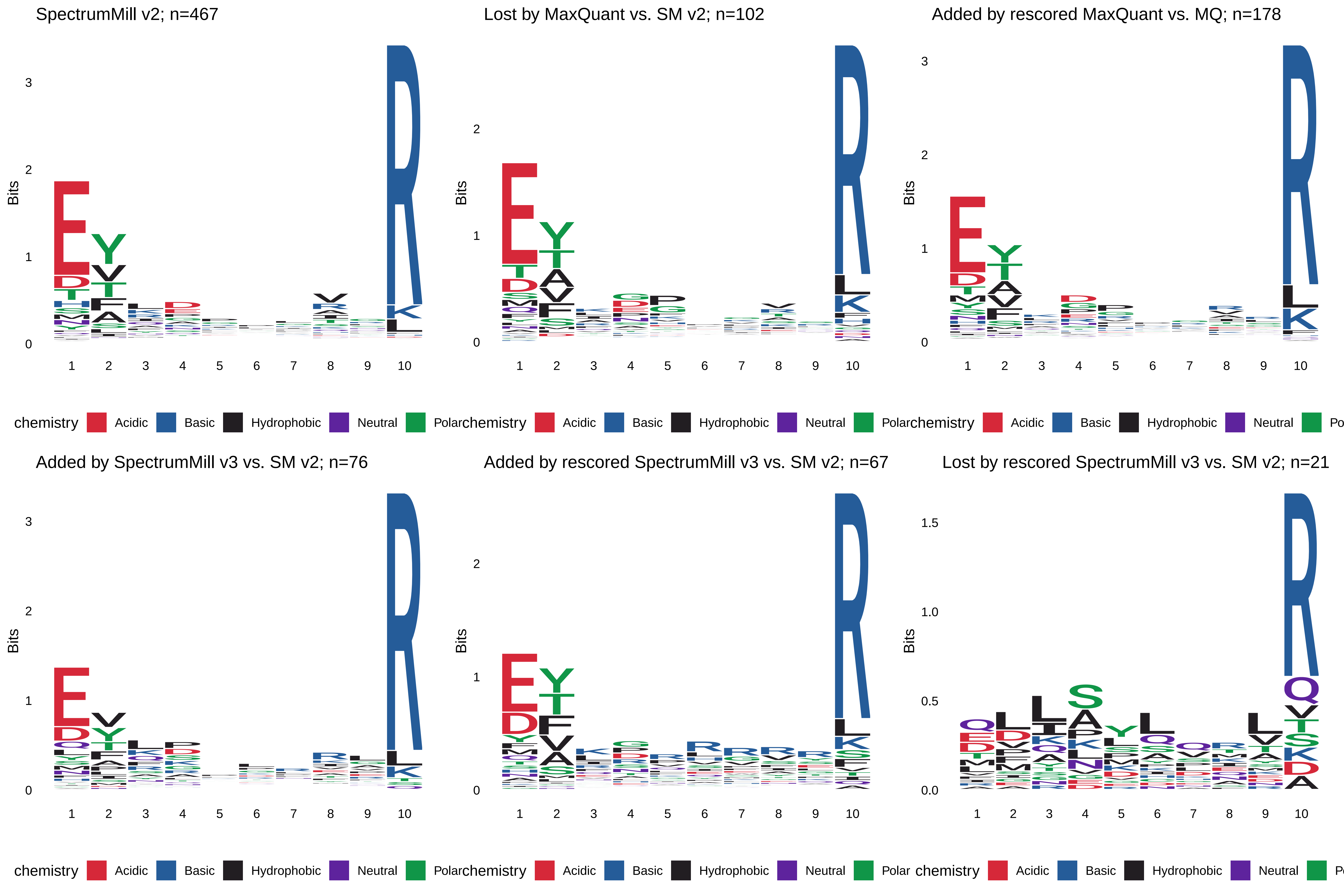
SpectrumMill v2; n=467
Lost by MaxQuant vs. SM v2; n=102
Added by rescored MaxQuant vs. MQ; n=178
Added by SpectrumMill v3 vs. SM v2; n=76
Added by rescored SpectrumMill v3 vs. SM v2; n=67
Lost by rescored SpectrumMill v3 vs. SM v2; n=21
Bits
1 2 3 4 5 6 7 8 9 10
chemistry
Acidic
Basic
Hydrophobic
Neutral
Polar

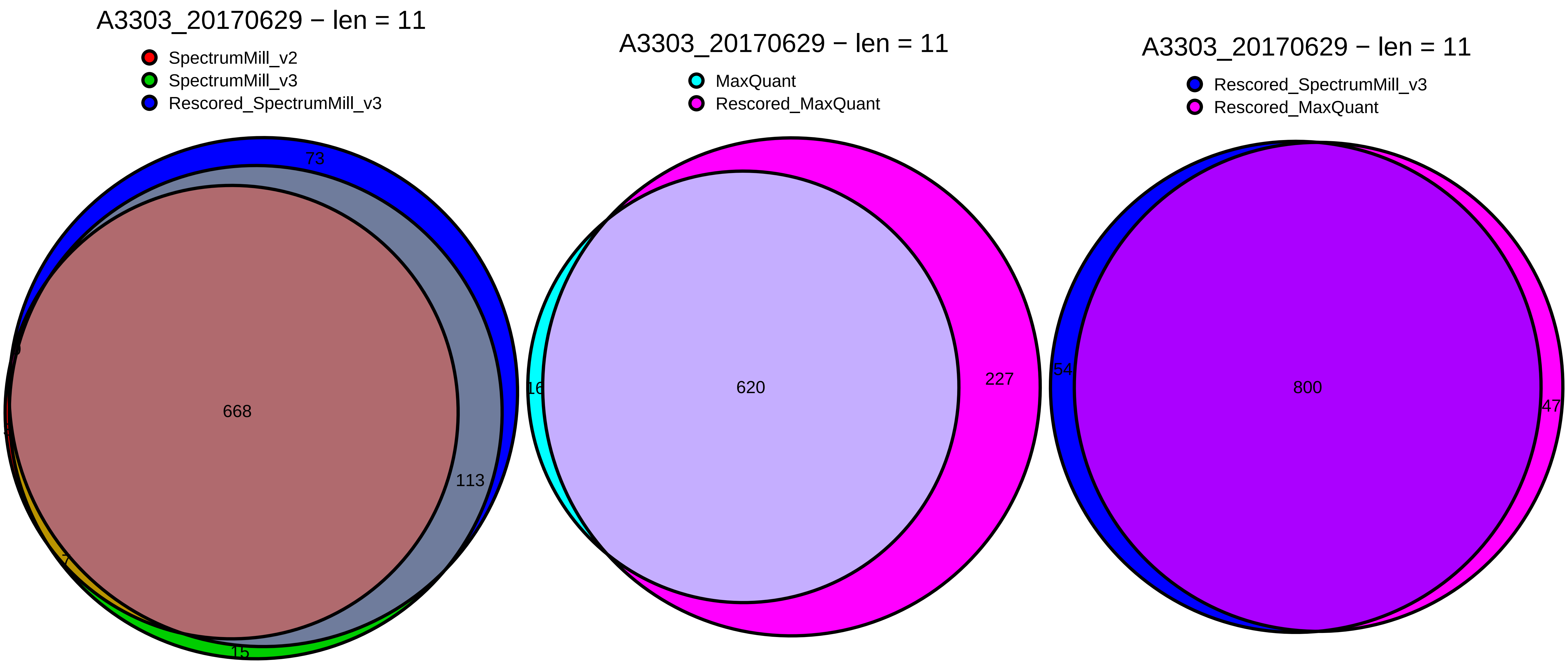
A3303_20170629 − len = 11
SpectrumMill_v2
SpectrumMill_v3
Rescored_SpectrumMill_v3
73
668
113
15
A3303_20170629 − len = 11
MaxQuant
Rescored_MaxQuant
16
620
227
A3303_20170629 − len = 11
Rescored_SpectrumMill_v3
Rescored_MaxQuant
54
800
47

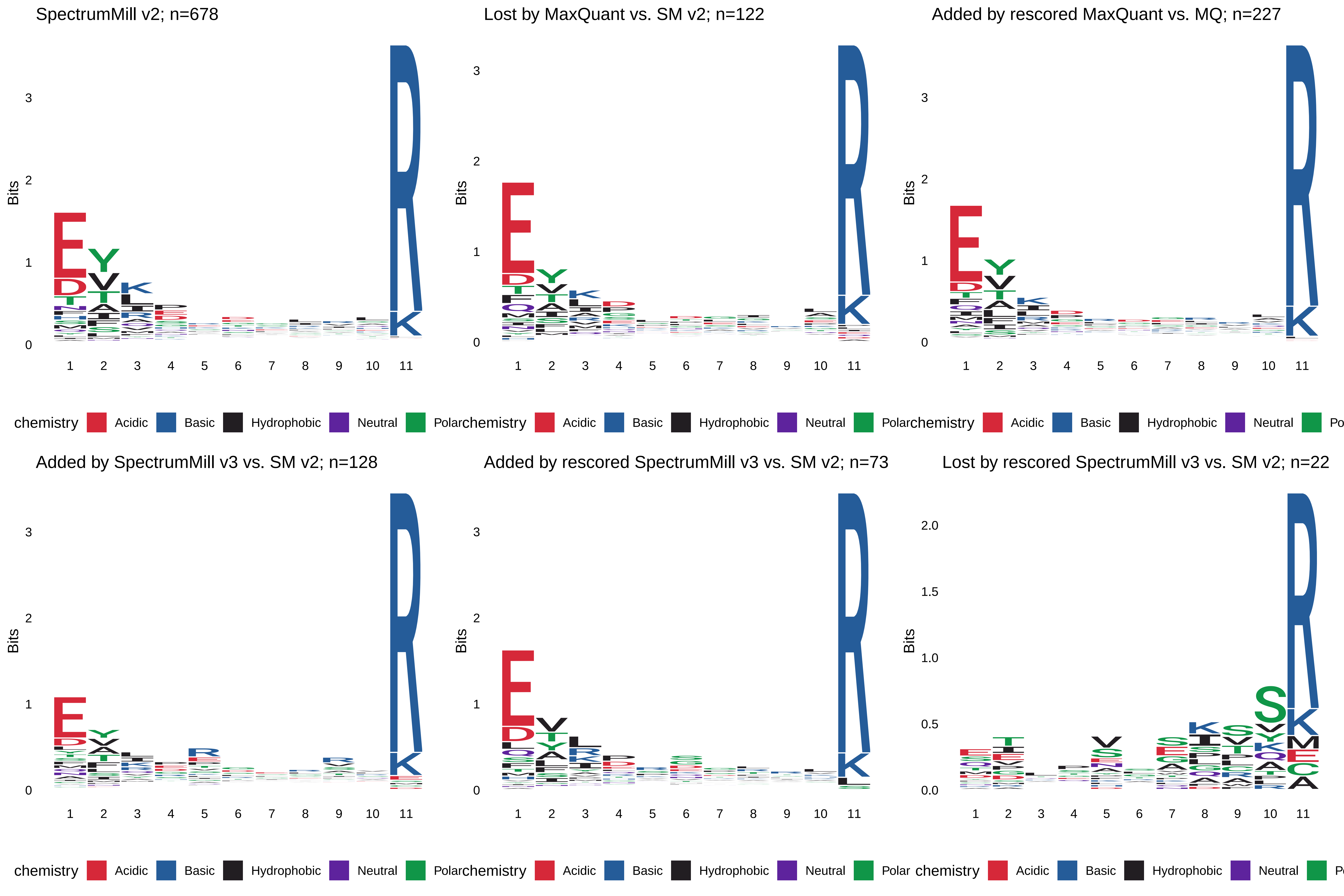
SpectrumMill v2; n=678
Lost by MaxQuant vs. SM v2; n=122
Added by rescored MaxQuant vs. MQ; n=227
Added by SpectrumMill v3 vs. SM v2; n=128
Added by rescored SpectrumMill v3 vs. SM v2; n=73
Lost by rescored SpectrumMill v3 vs. SM v2; n=22
Bits
0
1
2
3
0.0
0.5
1.0
1.5
2.0
1 2 3 4 5 6 7 8 9 10 11
chemistry
Acidic
Basic
Hydrophobic
Neutral
Polar

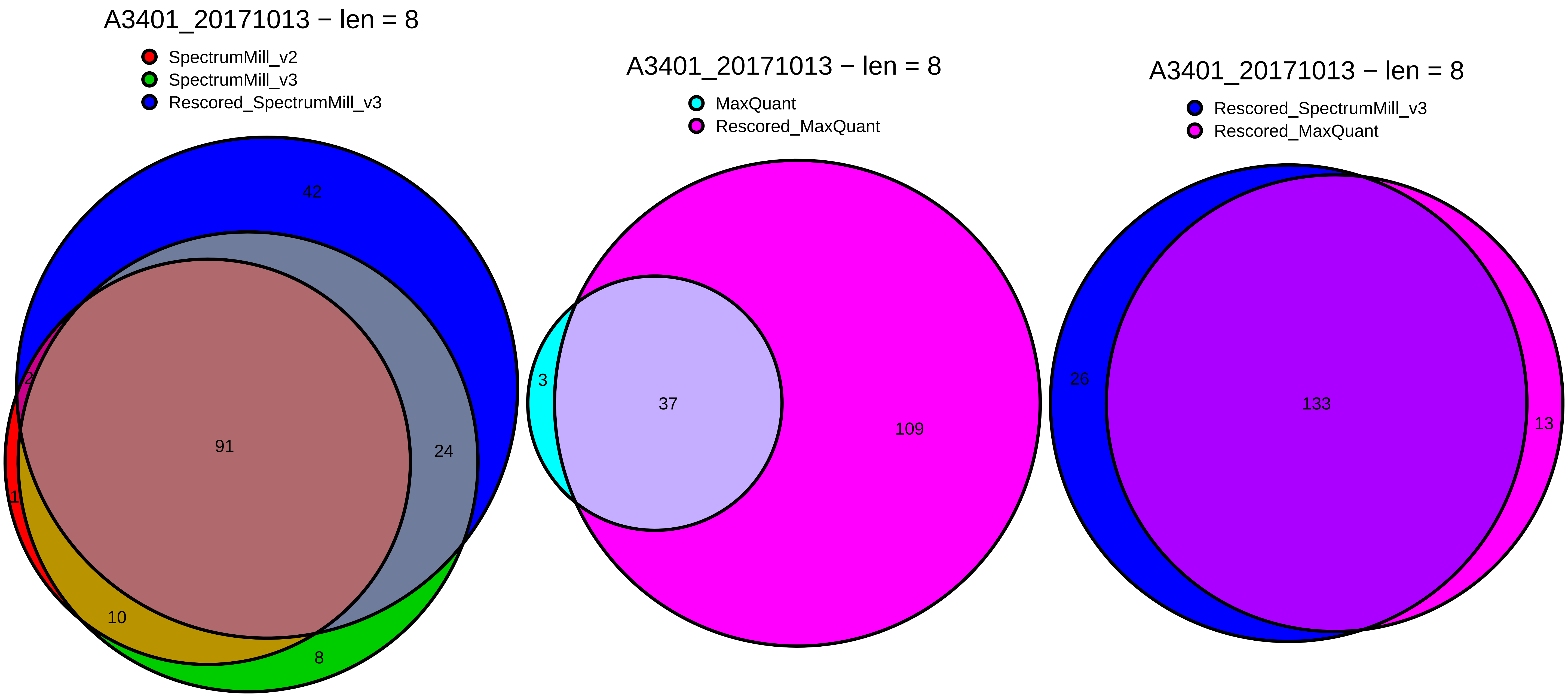
A3401_20171013 − len = 8
SpectrumMill_v2
SpectrumMill_v3
Rescored_SpectrumMill_v3
42
2
91
24
1
10
8
A3401_20171013 − len = 8
MaxQuant
Rescored_MaxQuant
3
37
109
A3401_20171013 − len = 8
Rescored_SpectrumMill_v3
Rescored_MaxQuant
26
133
13

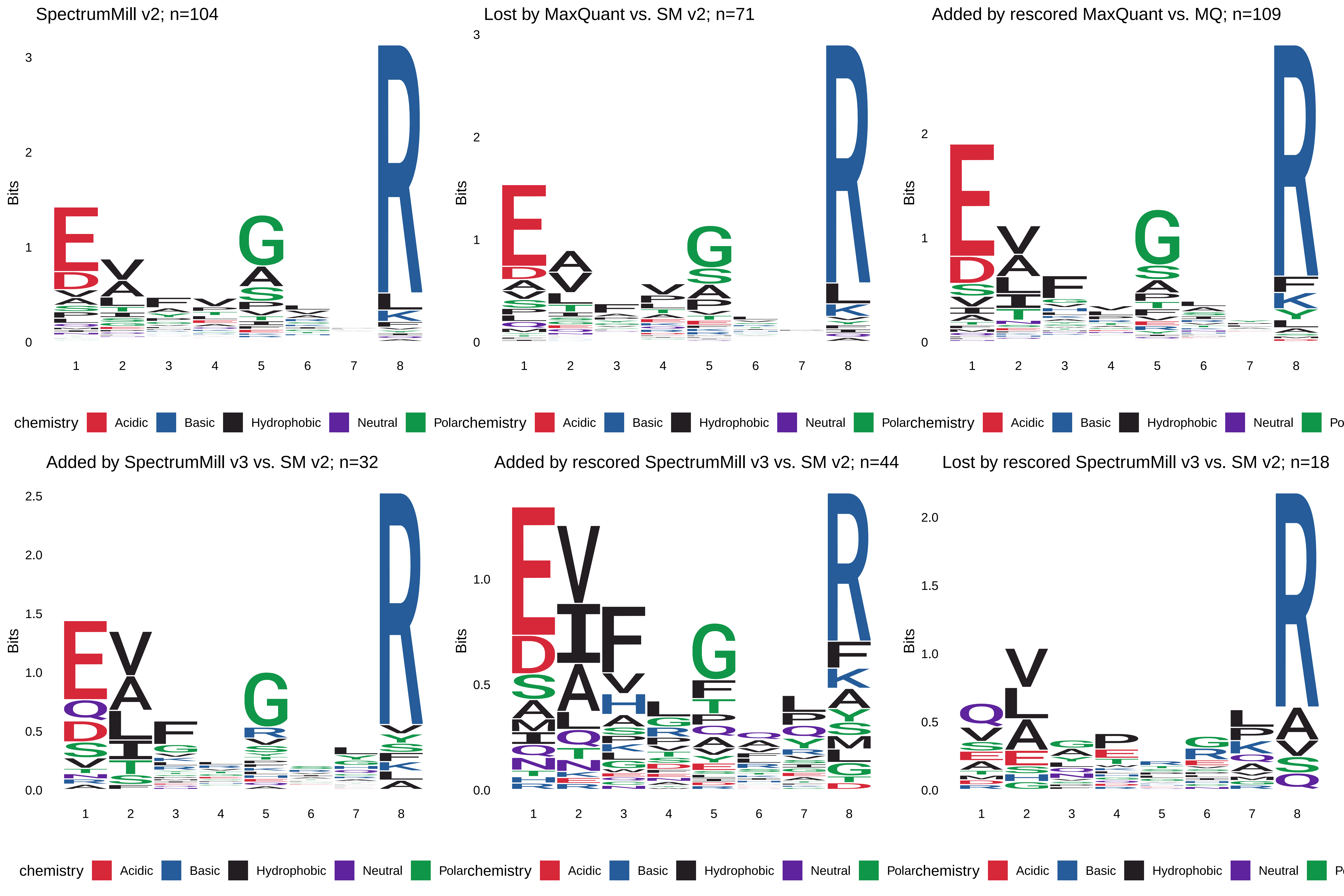
SpectrumMill v2; n=104
Lost by MaxQuant vs. SM v2; n=71
Added by rescored MaxQuant vs. MQ; n=109
Bits
0
1
2
3
1 2 3 4 5 6 7 8
chemistry Acidic Basic Hydrophobic Neutral Polarchemistry Acidic Basic Hydrophobic Neutral Polarchemistry Acidic Basic Hydrophobic Neutral Po
Added by SpectrumMill v3 vs. SM v2; n=32
Added by rescored SpectrumMill v3 vs. SM v2; n=44
Lost by rescored SpectrumMill v3 vs. SM v2; n=18
0.0
0.5
1.0
1.5
2.0
2.5
chemistry Acidic Basic Hydrophobic Neutral Polarchemistry Acidic Basic Hydrophobic Neutral Polarchemistry Acidic Basic Hydrophobic Neutral Po

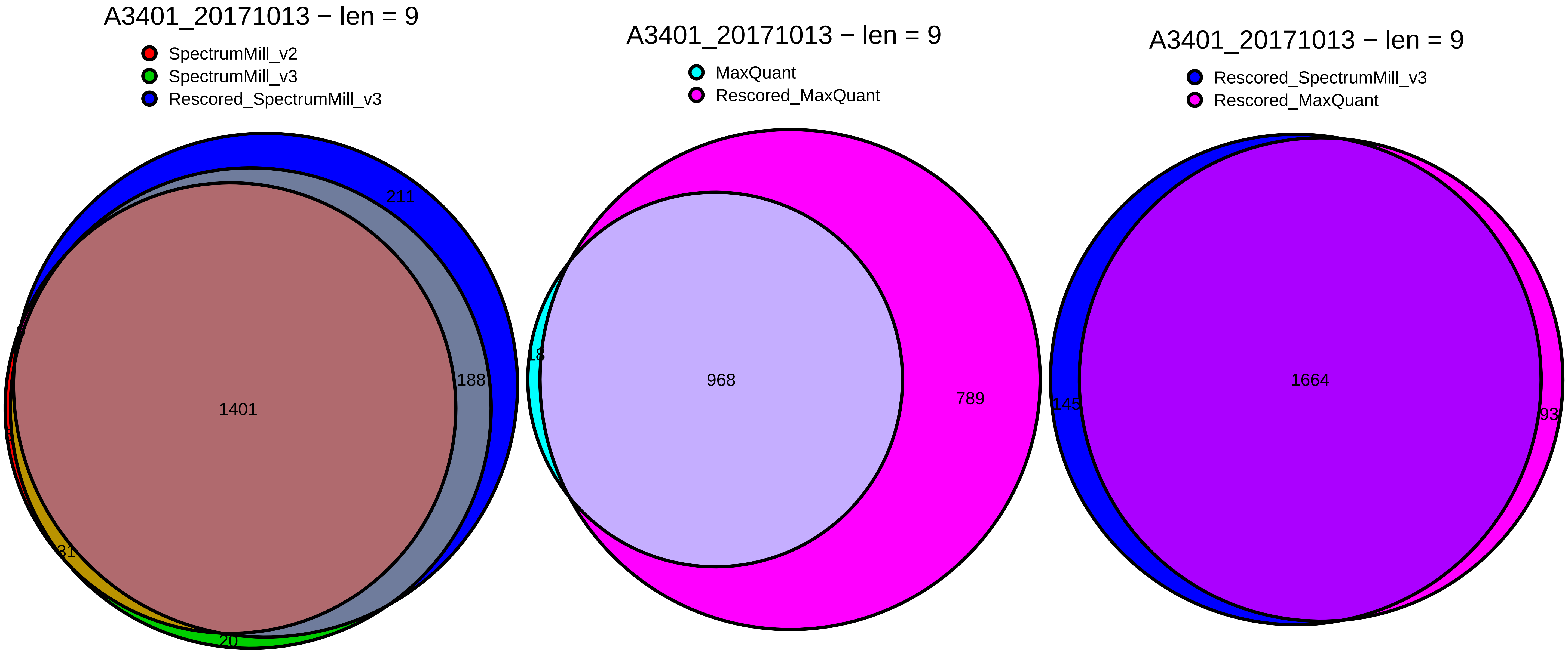
A3401_20171013 − len = 9
SpectrumMill_v2
SpectrumMill_v3
Rescored_SpectrumMill_v3
211
188
1401
31
20
A3401_20171013 − len = 9
MaxQuant
Rescored_MaxQuant
18
968
789
A3401_20171013 − len = 9
Rescored_SpectrumMill_v3
Rescored_MaxQuant
145
1664
93

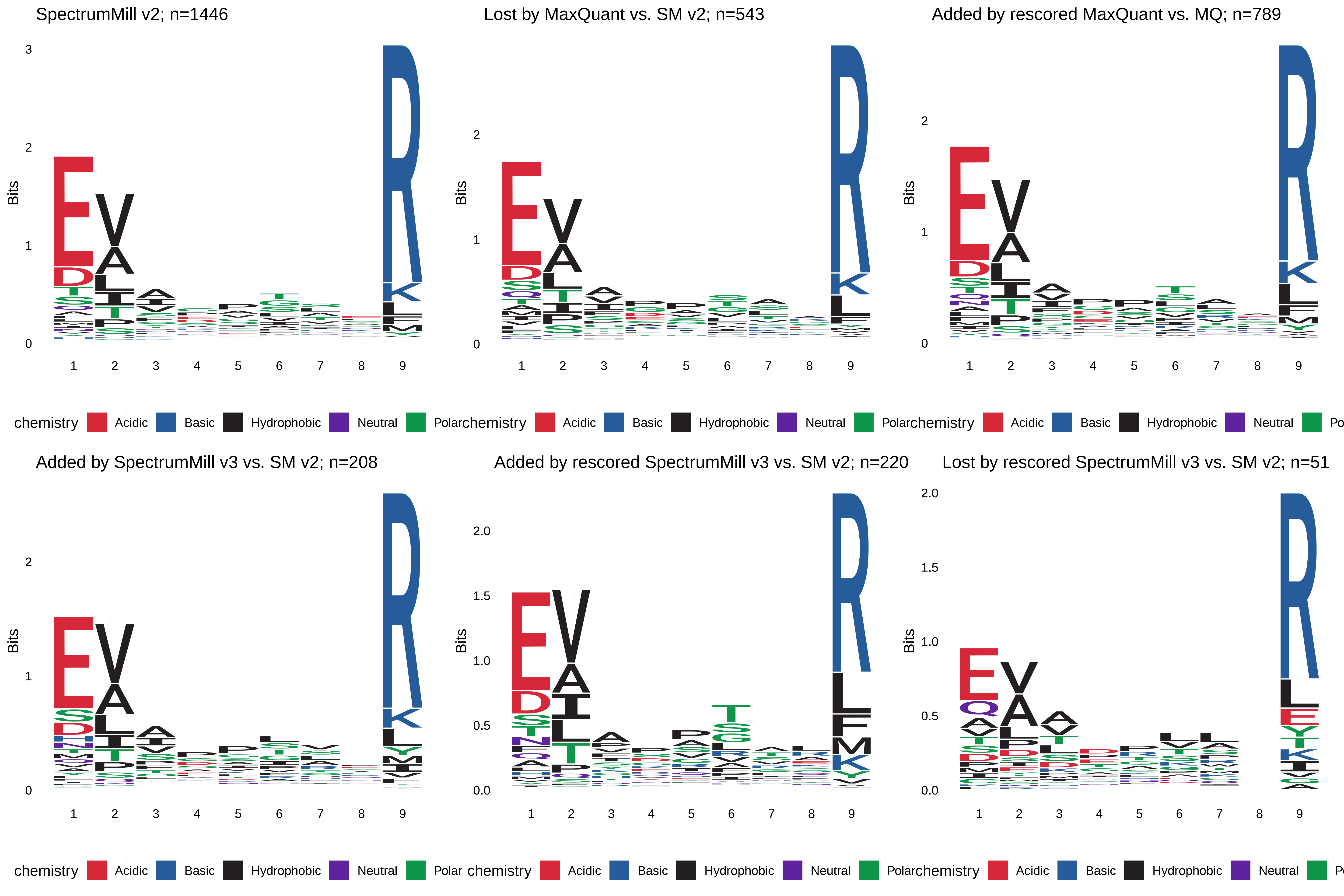
SpectrumMill v2; n=1446
Lost by MaxQuant vs. SM v2; n=543
Added by rescored MaxQuant vs. MQ; n=789
Added by SpectrumMill v3 vs. SM v2; n=208
Added by rescored SpectrumMill v3 vs. SM v2; n=220
Lost by rescored SpectrumMill v3 vs. SM v2; n=51
Bits
1 2 3 4 5 6 7 8 9
chemistry Acidic Basic Hydrophobic Neutral Polar

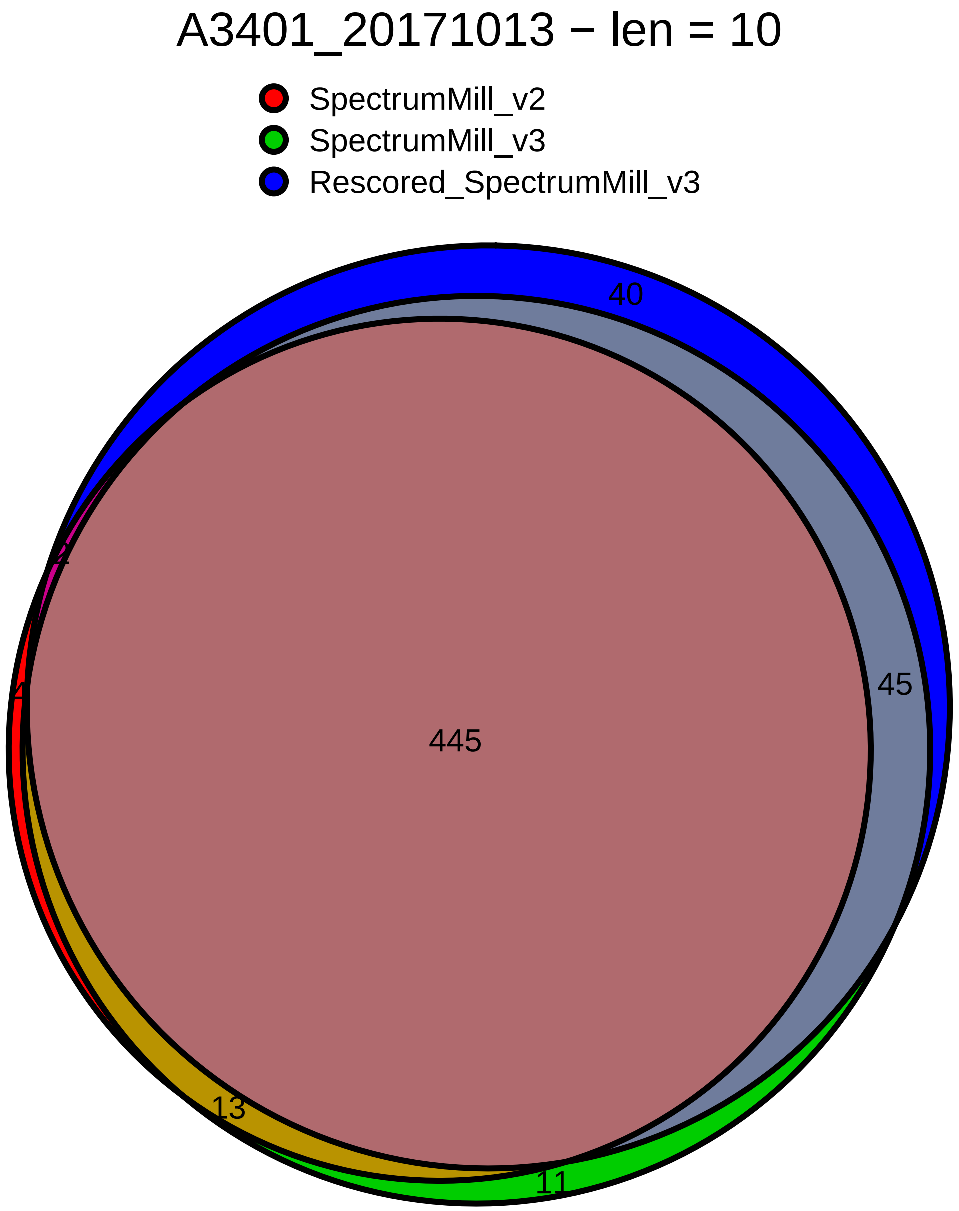
A3401_20171013 − len = 10
SpectrumMill_v2
SpectrumMill_v3
Rescored_SpectrumMill_v3
40
45
445
13
11

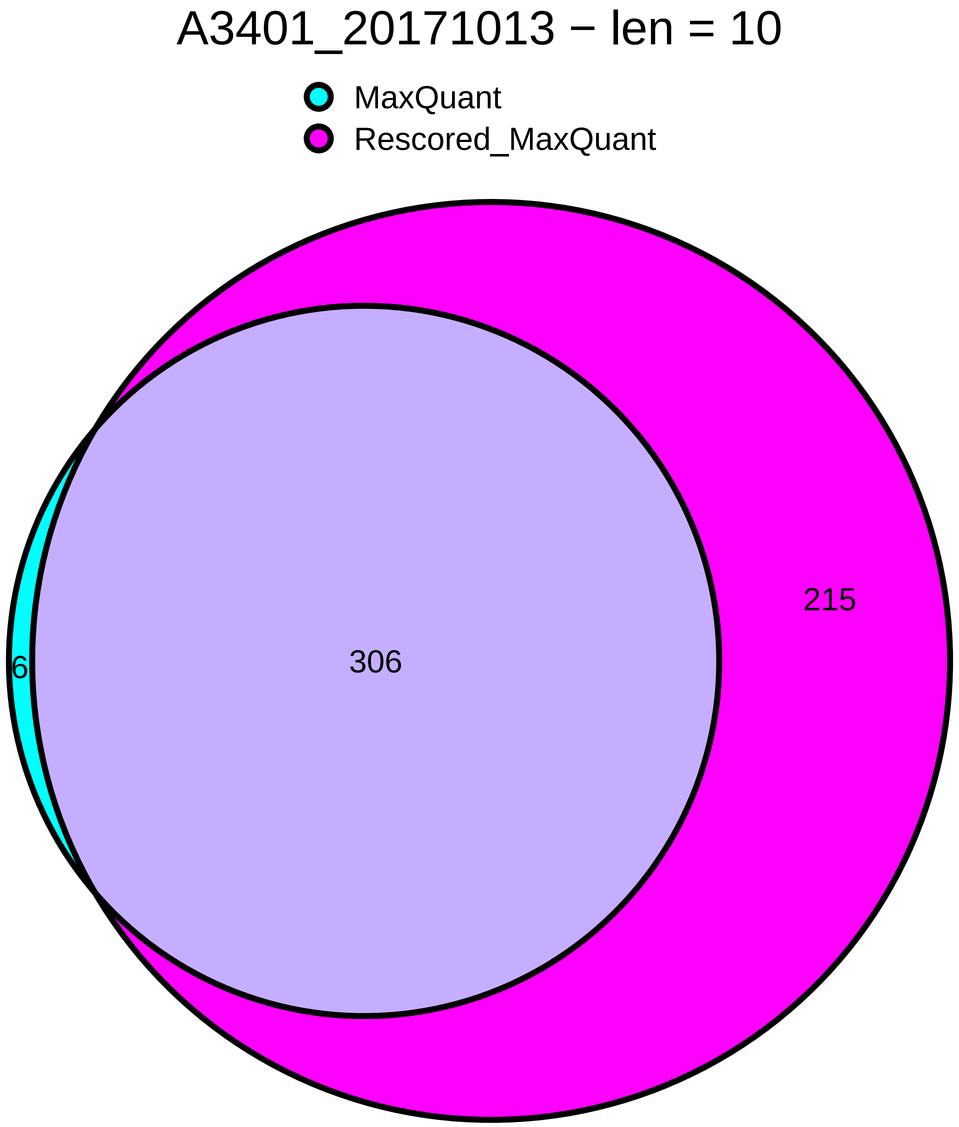
A3401_20171013 − len = 10
MaxQuant
Rescored_MaxQuant
6
306
215

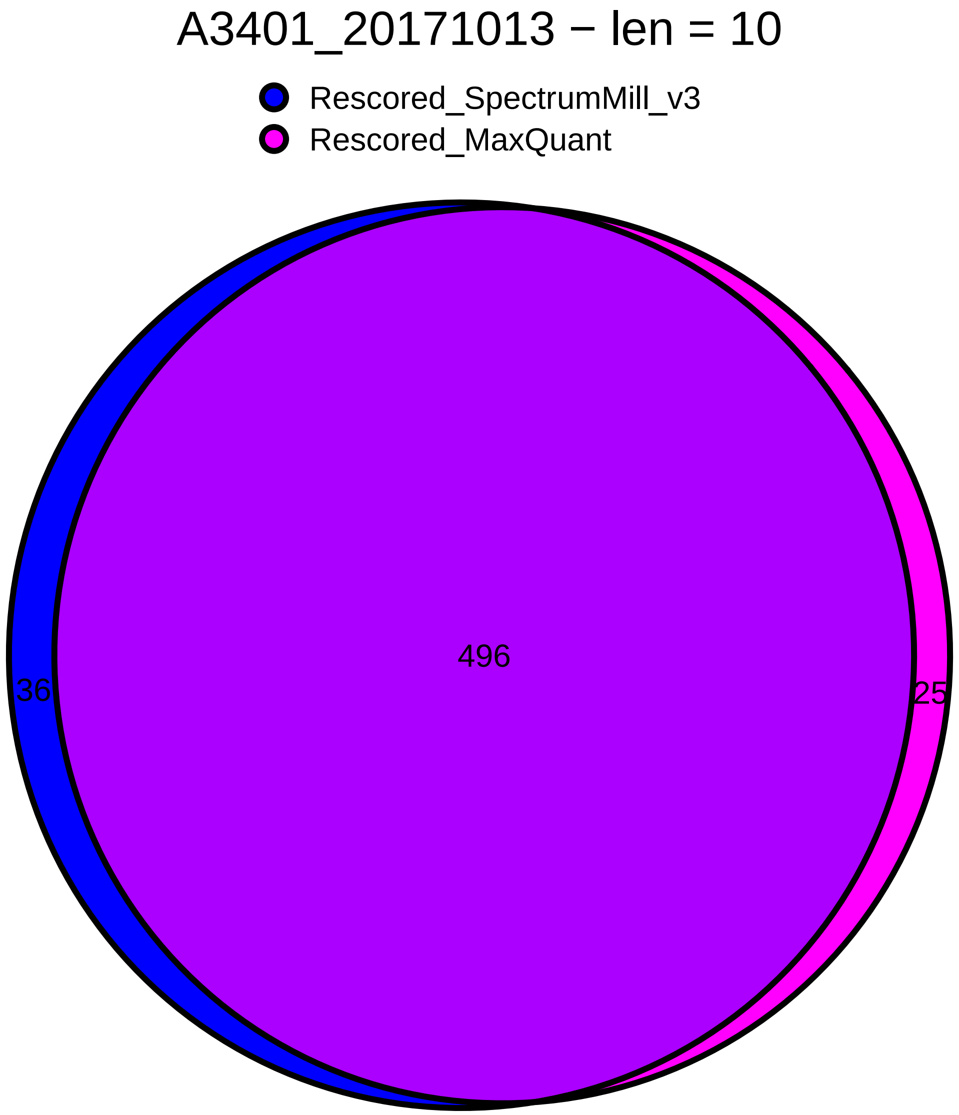
A3401_20171013 − len = 10
Rescored_SpectrumMill_v3
Rescored_MaxQuant
36
496
25

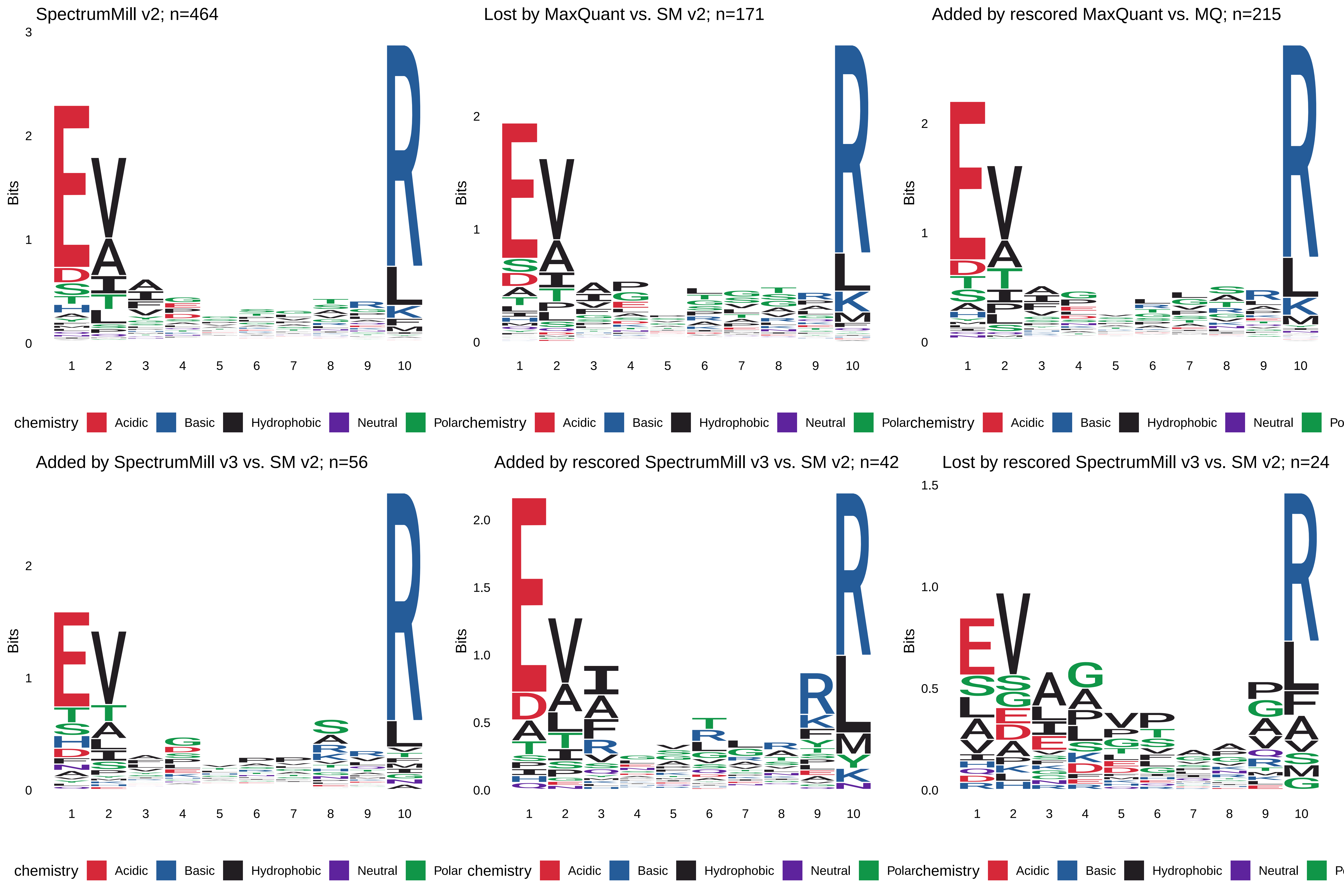
SpectrumMill v2; n=464
Lost by MaxQuant vs. SM v2; n=171
Added by rescored MaxQuant vs. MQ; n=215
Added by SpectrumMill v3 vs. SM v2; n=56
Added by rescored SpectrumMill v3 vs. SM v2; n=42
Lost by rescored SpectrumMill v3 vs. SM v2; n=24
Bits
1 2 3 4 5 6 7 8 9 10
chemistry
Acidic
Basic
Hydrophobic
Neutral
Polar

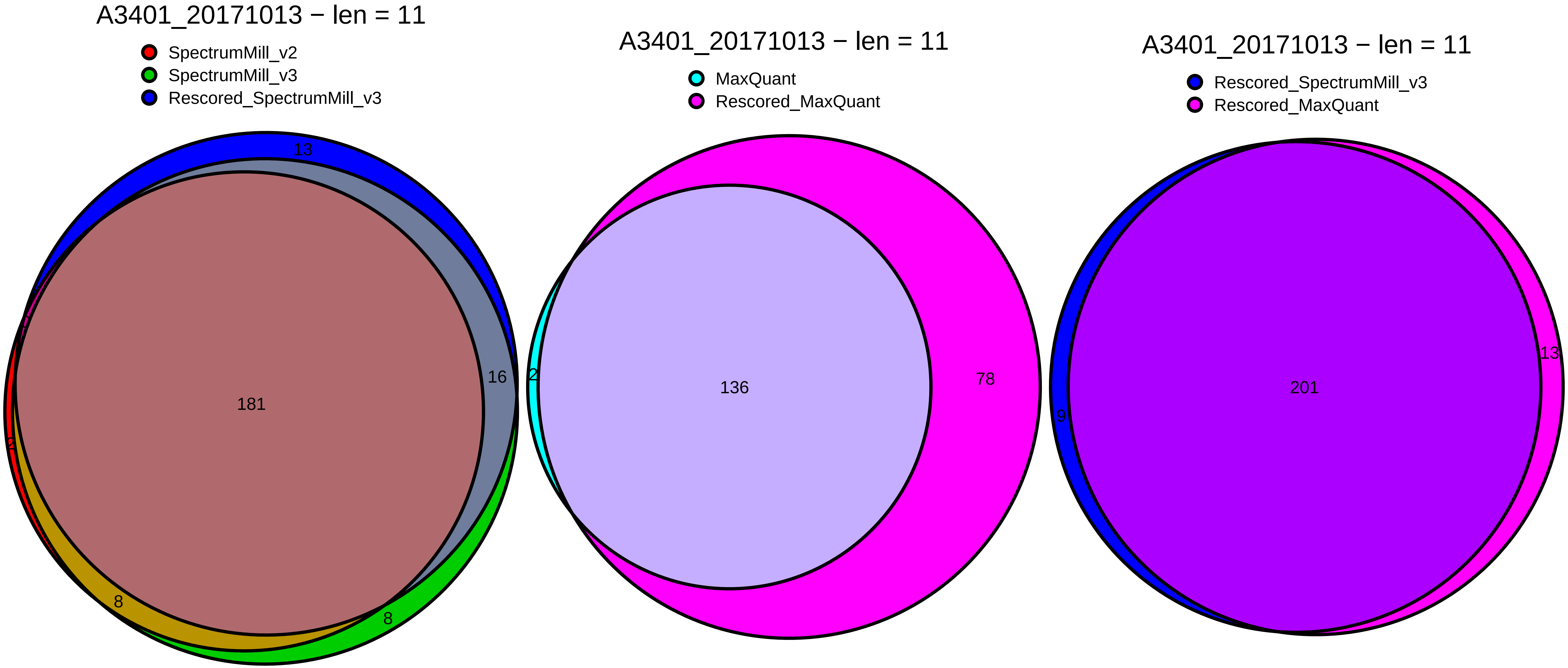
A3401_20171013 − len = 11
SpectrumMill_v2
SpectrumMill_v3
Rescored_SpectrumMill_v3
13
16
181
8
8
A3401_20171013 − len = 11
MaxQuant
Rescored_MaxQuant
2
136
78
A3401_20171013 − len = 11
Rescored_SpectrumMill_v3
Rescored_MaxQuant
201
13
9

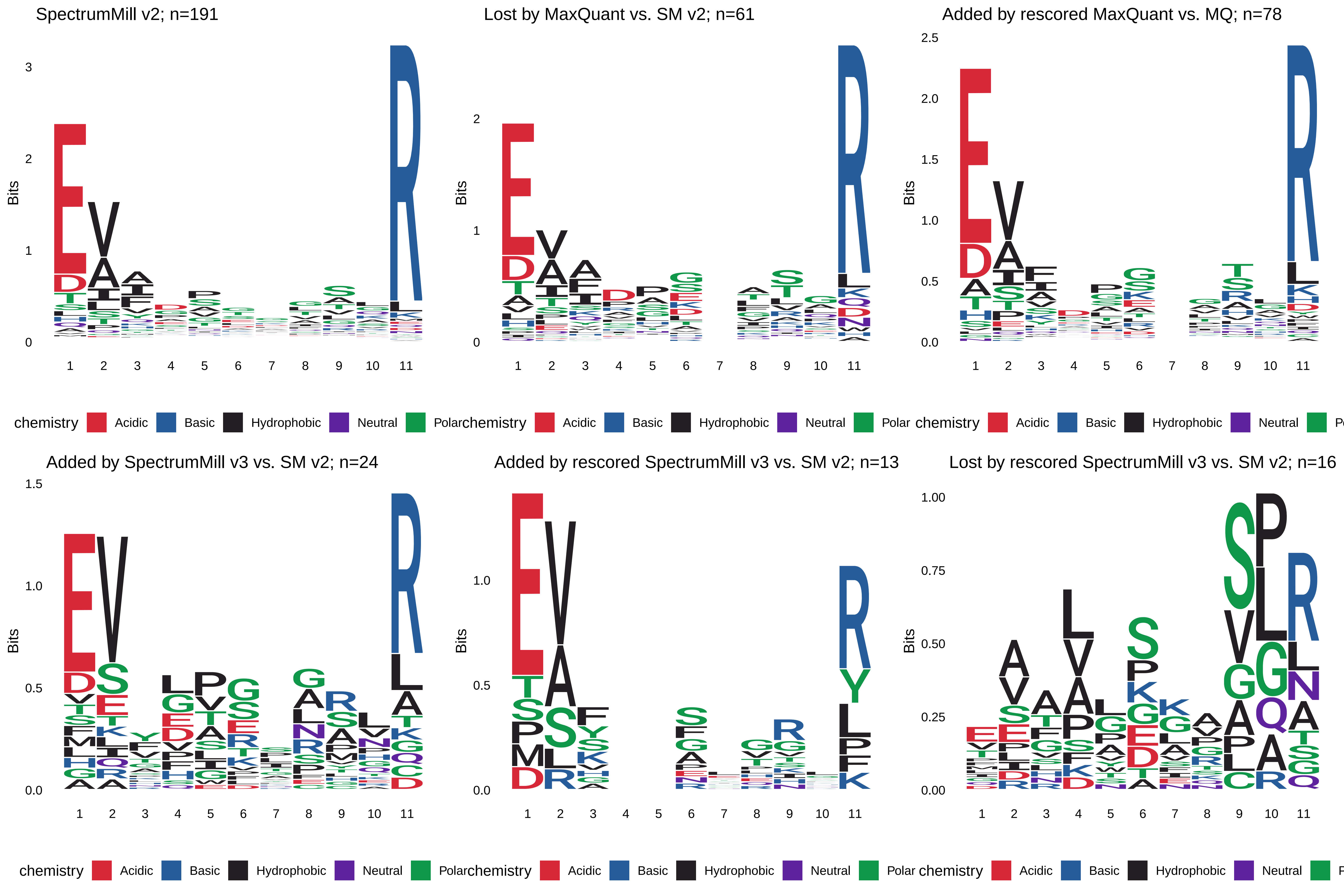
SpectrumMill v2; n=191
Lost by MaxQuant vs. SM v2; n=61
Added by rescored MaxQuant vs. MQ; n=78
Added by SpectrumMill v3 vs. SM v2; n=24
Added by rescored SpectrumMill v3 vs. SM v2; n=13
Lost by rescored SpectrumMill v3 vs. SM v2; n=16
Bits
1 2 3 4 5 6 7 8 9 10 11
chemistry
Acidic
Basic
Hydrophobic
Neutral
Polar

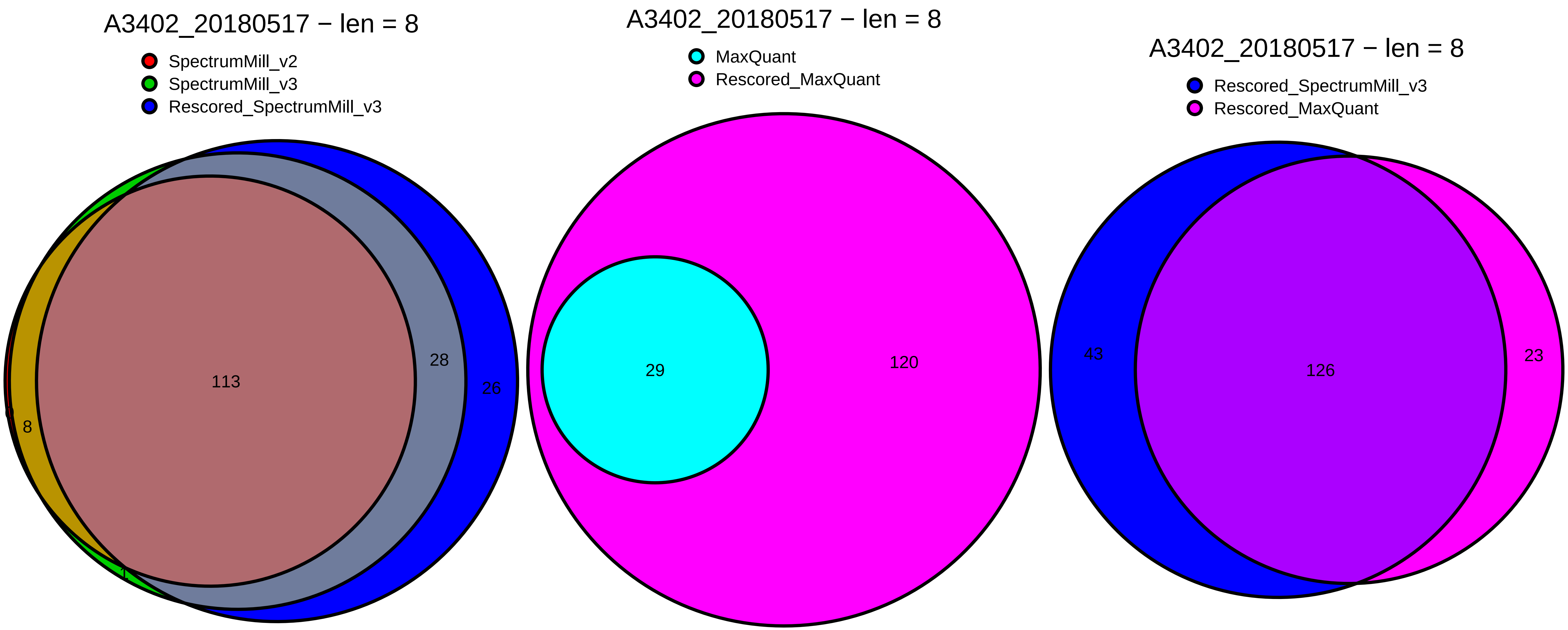
A3402_20180517 − len = 8
SpectrumMill_v2
SpectrumMill_v3
Rescored_SpectrumMill_v3
113
28
26
8
1
A3402_20180517 − len = 8
MaxQuant
Rescored_MaxQuant
29
120
A3402_20180517 − len = 8
Rescored_SpectrumMill_v3
Rescored_MaxQuant
43
126
23

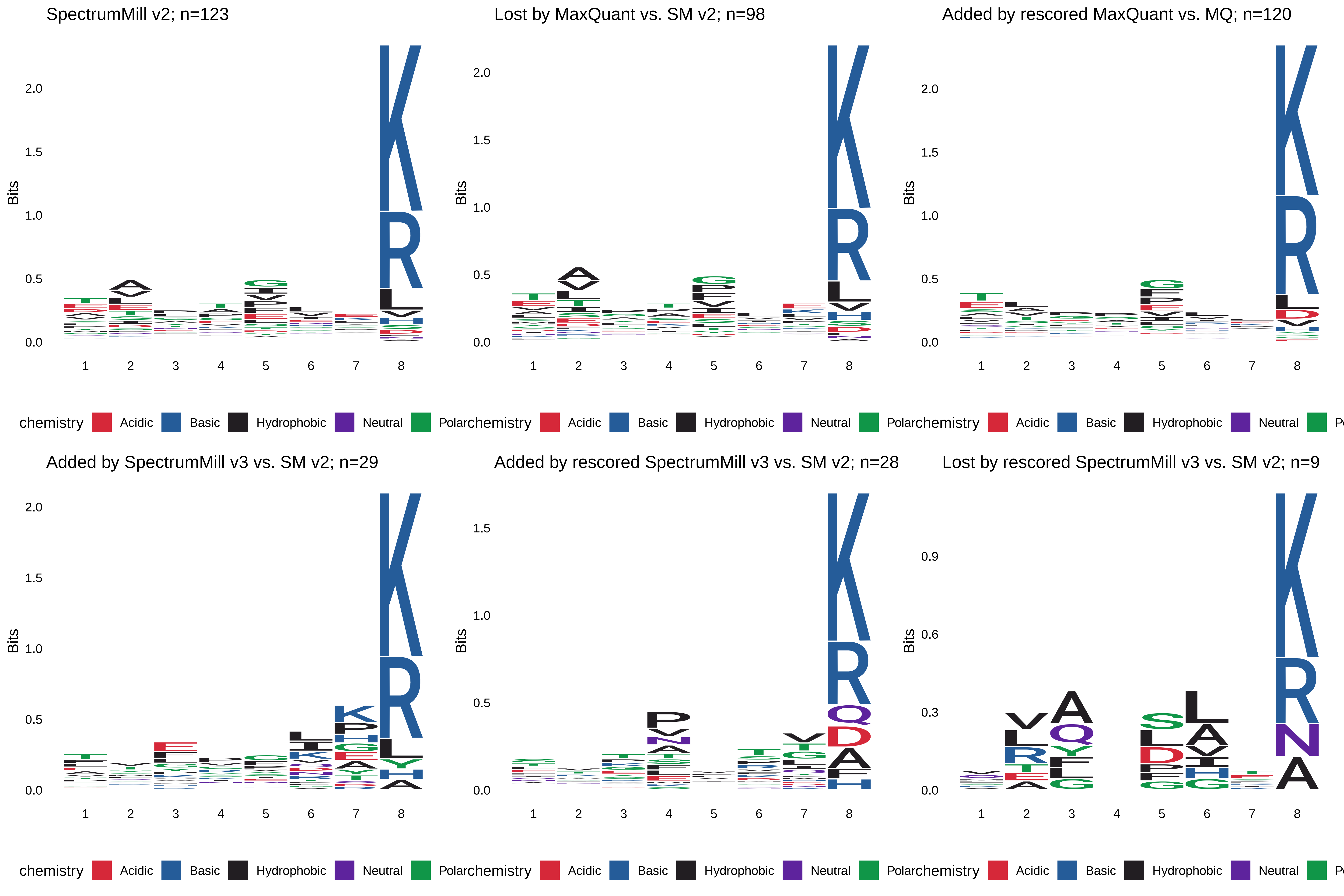

SpectrumMill v2; n=123
Lost by MaxQuant vs. SM v2; n=98
Added by rescored MaxQuant vs. MQ; n=120
Added by SpectrumMill v3 vs. SM v2; n=29
Added by rescored SpectrumMill v3 vs. SM v2; n=28
Lost by rescored SpectrumMill v3 vs. SM v2; n=9
Bits
chemistry
Acidic
Basic
Hydrophobic
Neutral
Polar

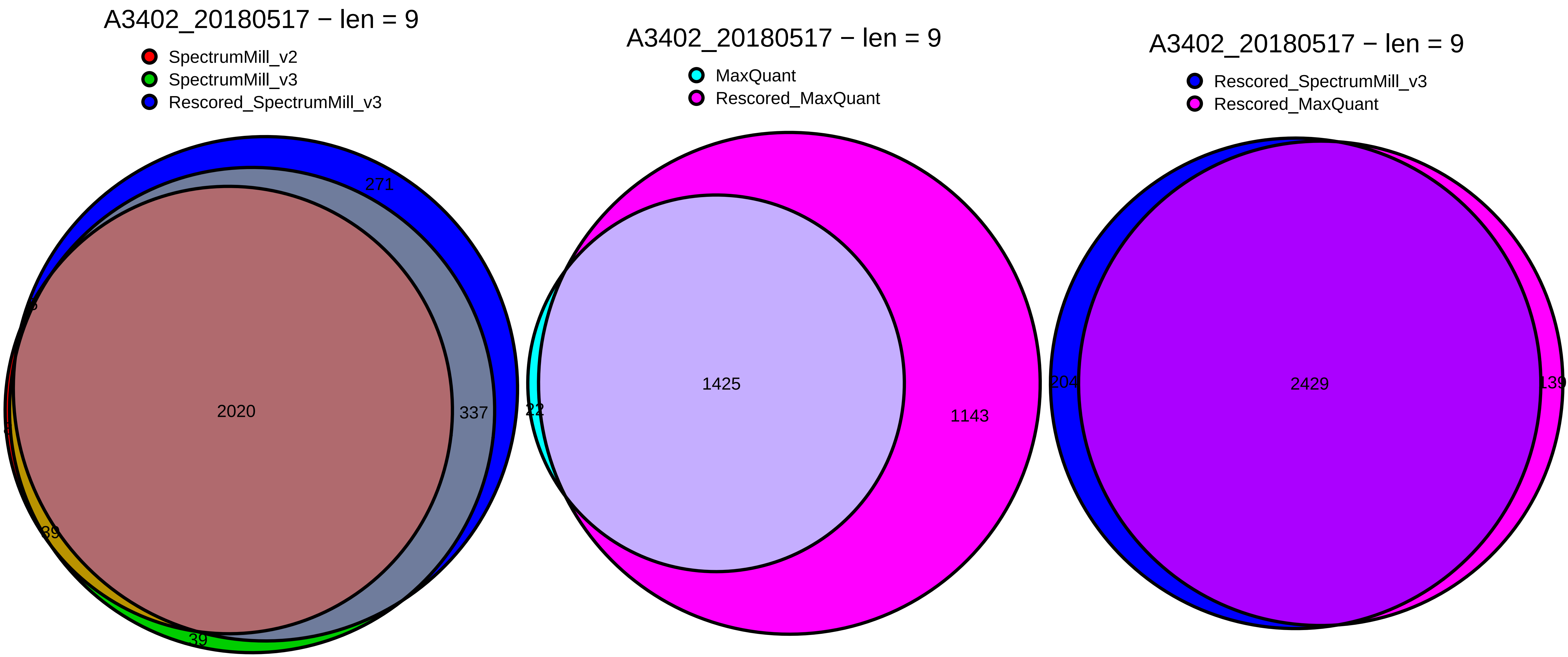
A3402_20180517 − len = 9
SpectrumMill_v2
SpectrumMill_v3
Rescored_SpectrumMill_v3
271
2020
337
39
39
A3402_20180517 − len = 9
MaxQuant
Rescored_MaxQuant
1425
22
1143
A3402_20180517 − len = 9
Rescored_SpectrumMill_v3
Rescored_MaxQuant
204
2429
139

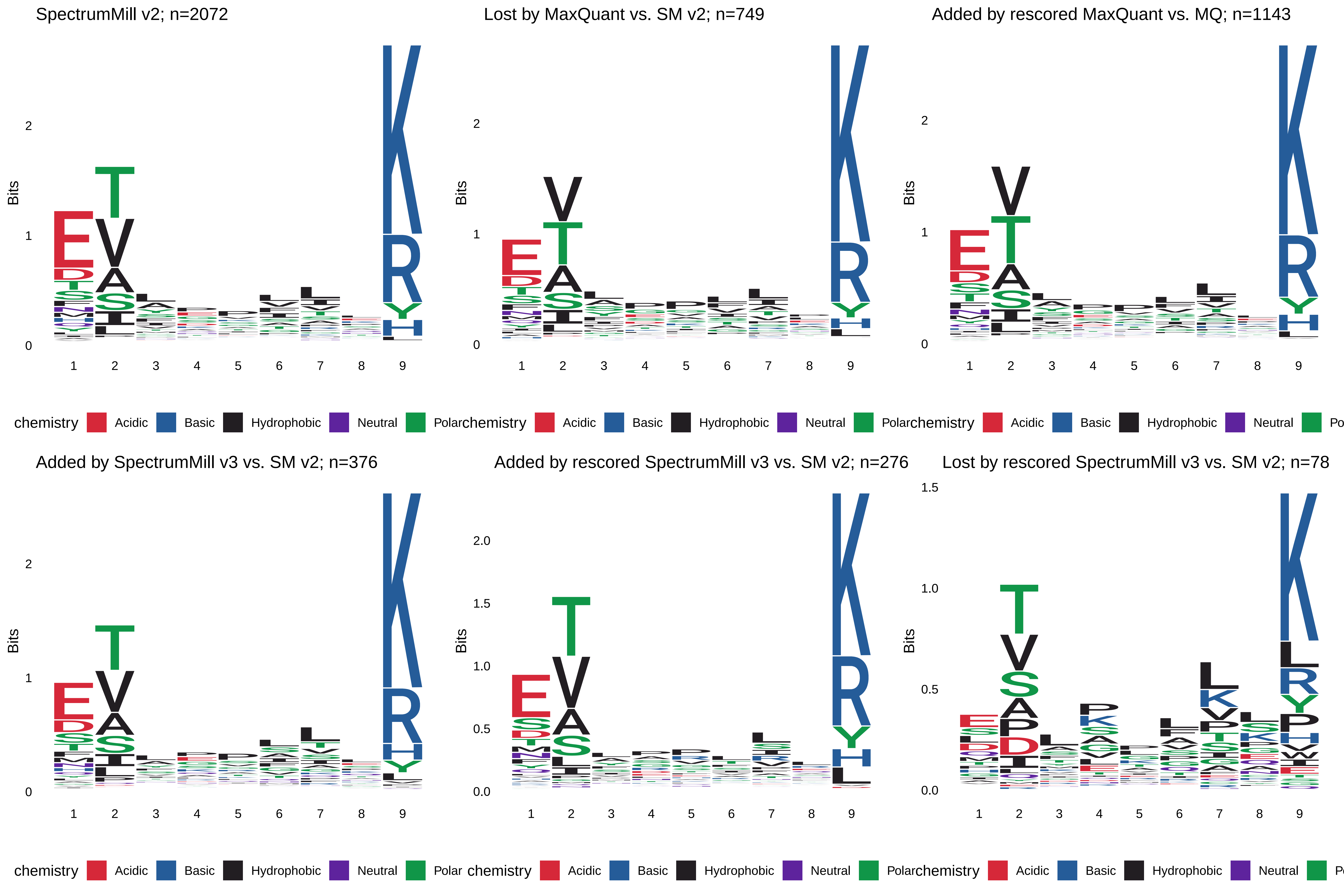
SpectrumMill v2; n=2072
Lost by MaxQuant vs. SM v2; n=749
Added by rescored MaxQuant vs. MQ; n=1143
Added by SpectrumMill v3 vs. SM v2; n=376
Added by rescored SpectrumMill v3 vs. SM v2; n=276
Lost by rescored SpectrumMill v3 vs. SM v2; n=78
Bits
1 2 3 4 5 6 7 8 9
chemistry
Acidic
Basic
Hydrophobic
Neutral
Polar

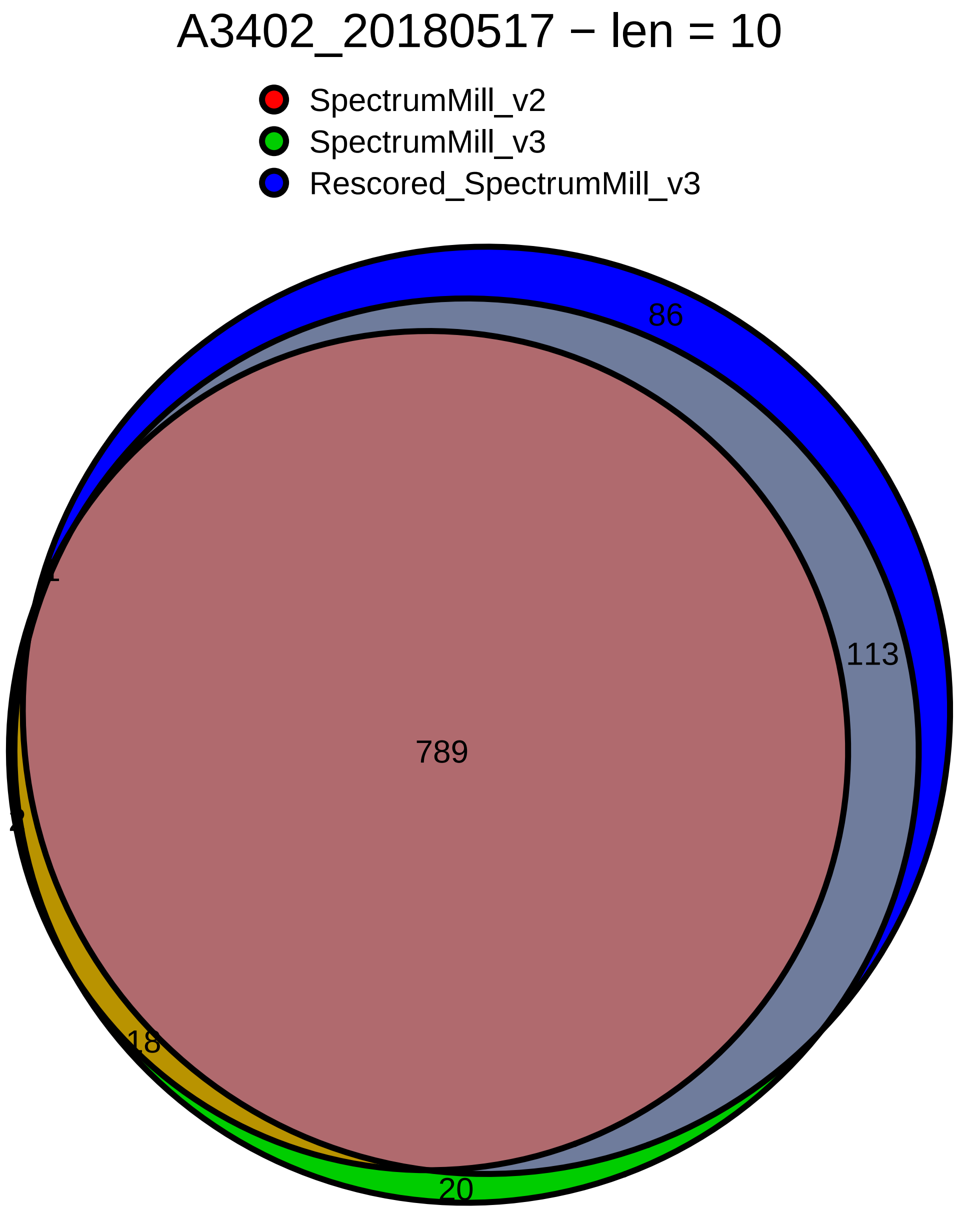
A3402_20180517 − len = 10
SpectrumMill_v2
SpectrumMill_v3
Rescored_SpectrumMill_v3
86
113
789
18
20

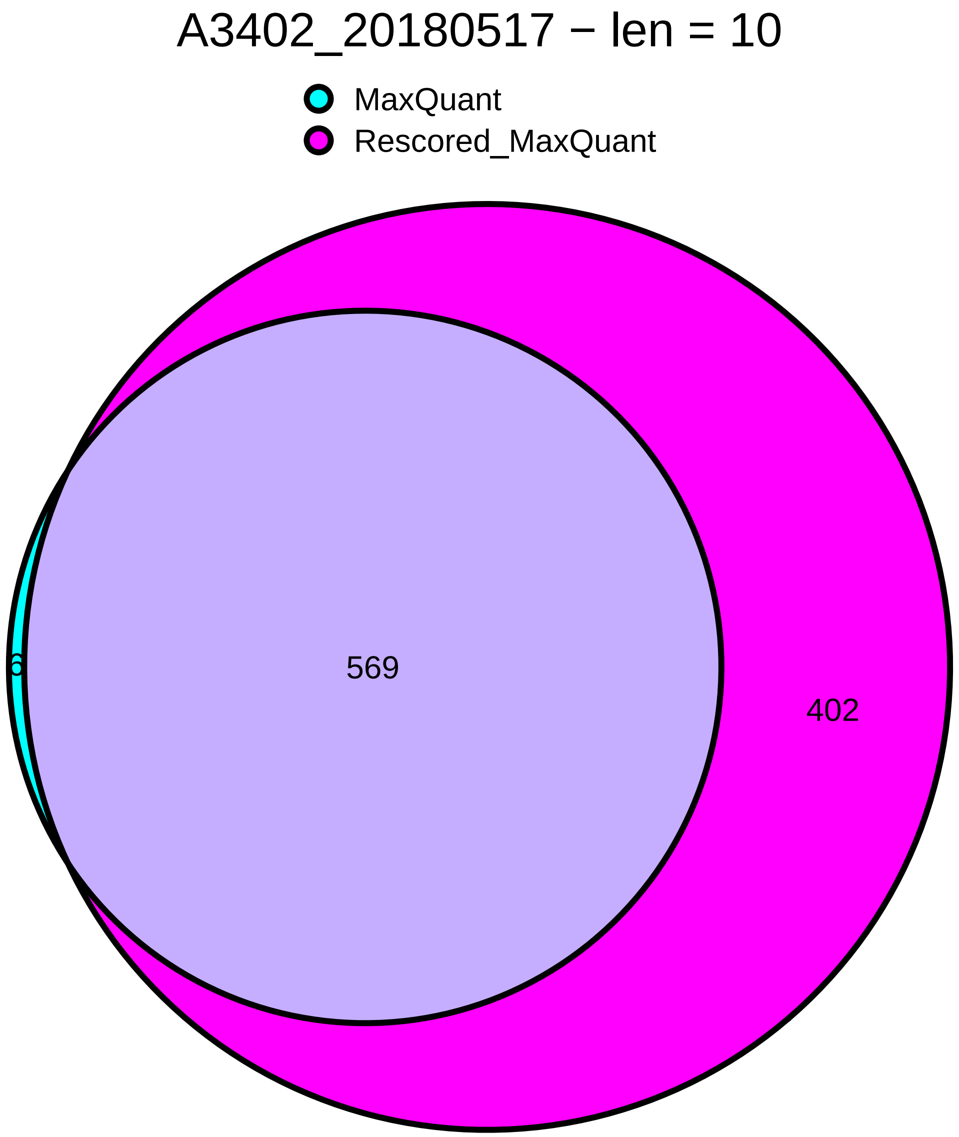
A3402_20180517 − len = 10
MaxQuant
Rescored_MaxQuant
569
402

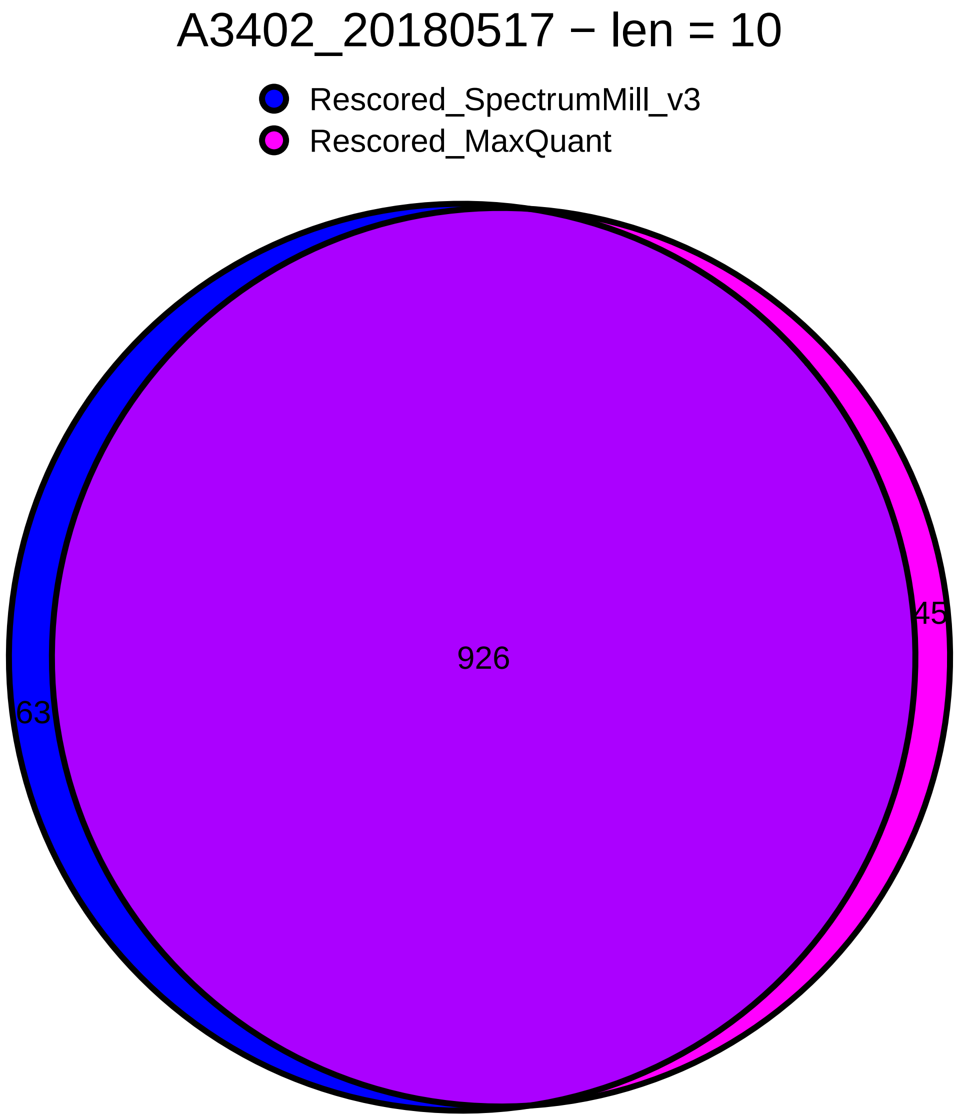
A3402_20180517 − len = 10
Rescored_SpectrumMill_v3
Rescored_MaxQuant
926
63
45

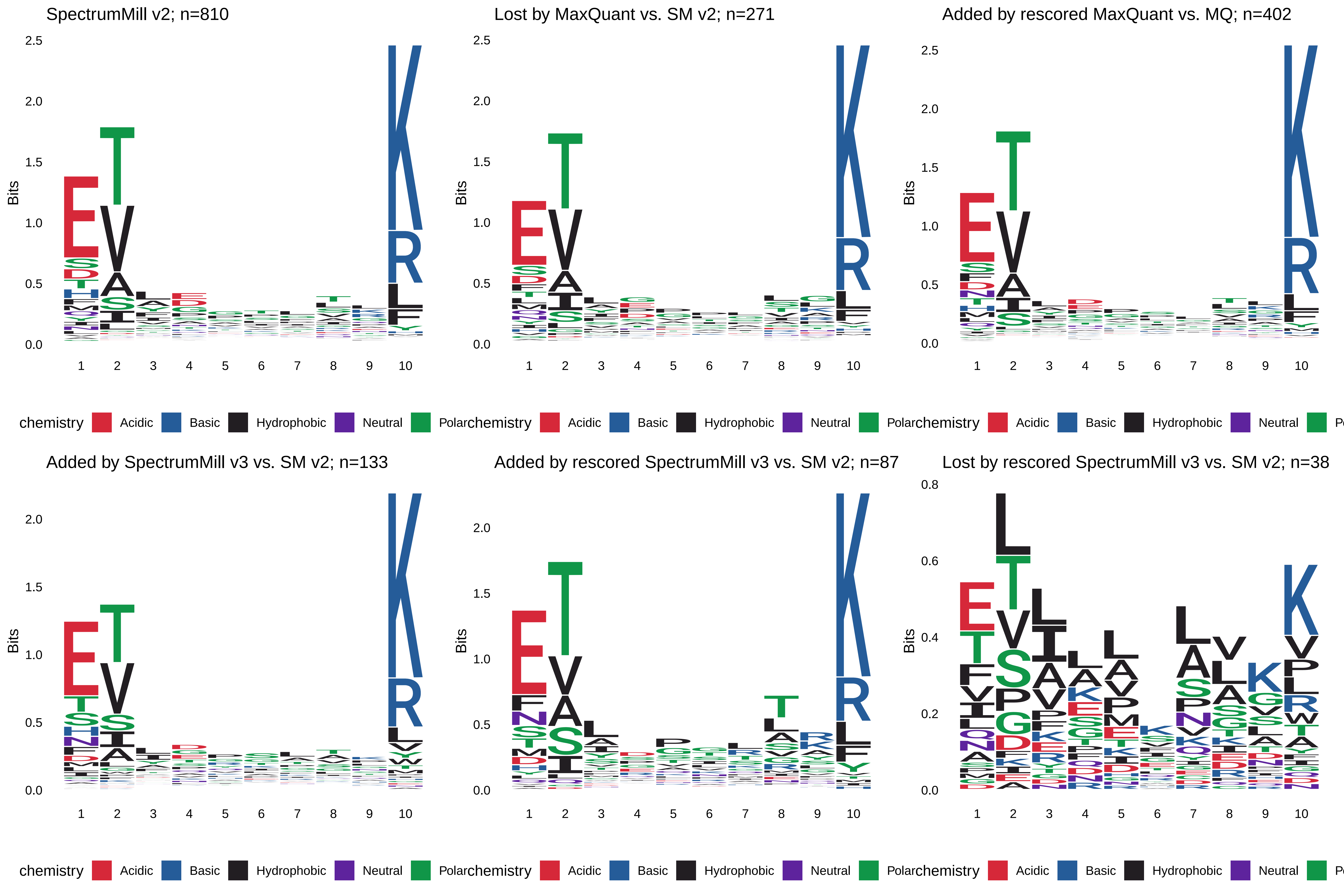
SpectrumMill v2; n=810
Lost by MaxQuant vs. SM v2; n=271
Added by rescored MaxQuant vs. MQ; n=402
Added by SpectrumMill v3 vs. SM v2; n=133
Added by rescored SpectrumMill v3 vs. SM v2; n=87
Lost by rescored SpectrumMill v3 vs. SM v2; n=38
Bits
chemistry
Acidic
Basic
Hydrophobic
Neutral
Polar

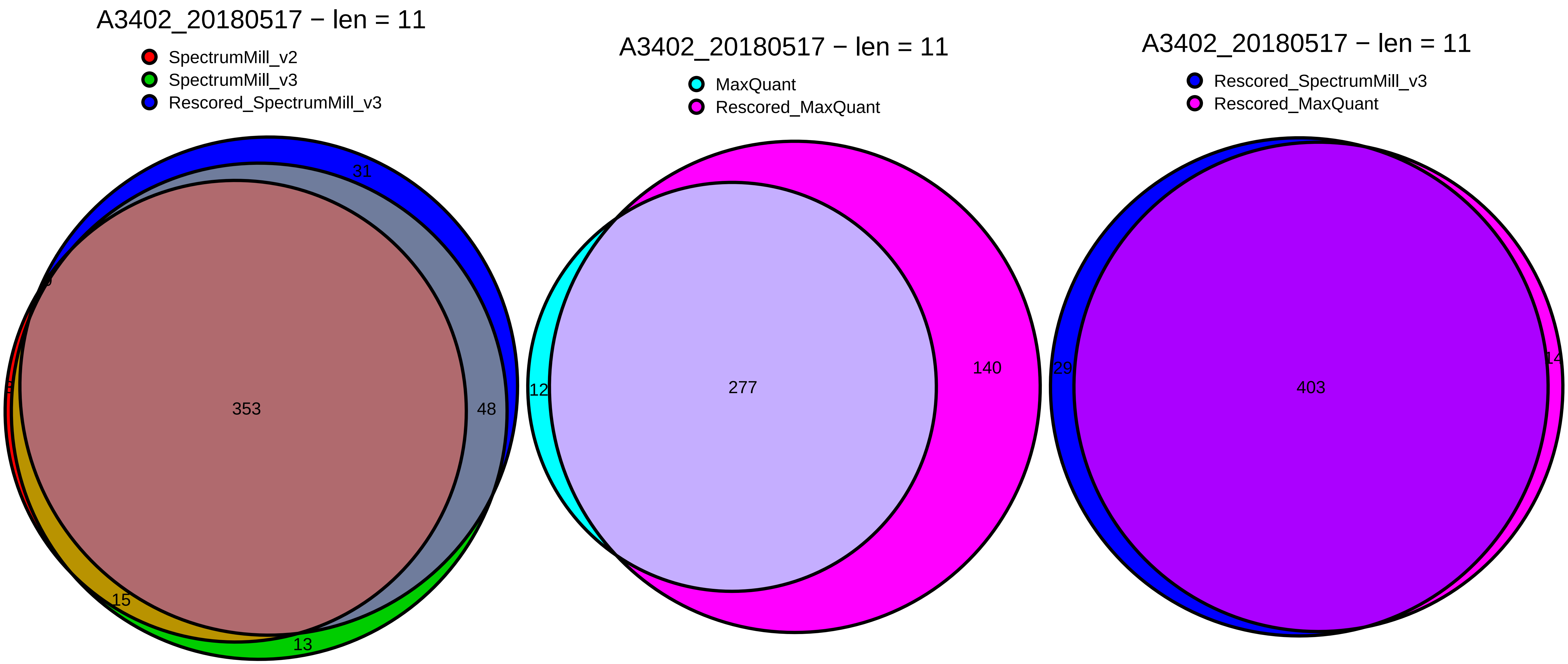
A3402_20180517 − len = 11
SpectrumMill_v2
SpectrumMill_v3
Rescored_SpectrumMill_v3
31
353
48
15
13
A3402_20180517 − len = 11
MaxQuant
Rescored_MaxQuant
12
277
140
A3402_20180517 − len = 11
Rescored_SpectrumMill_v3
Rescored_MaxQuant
29
403

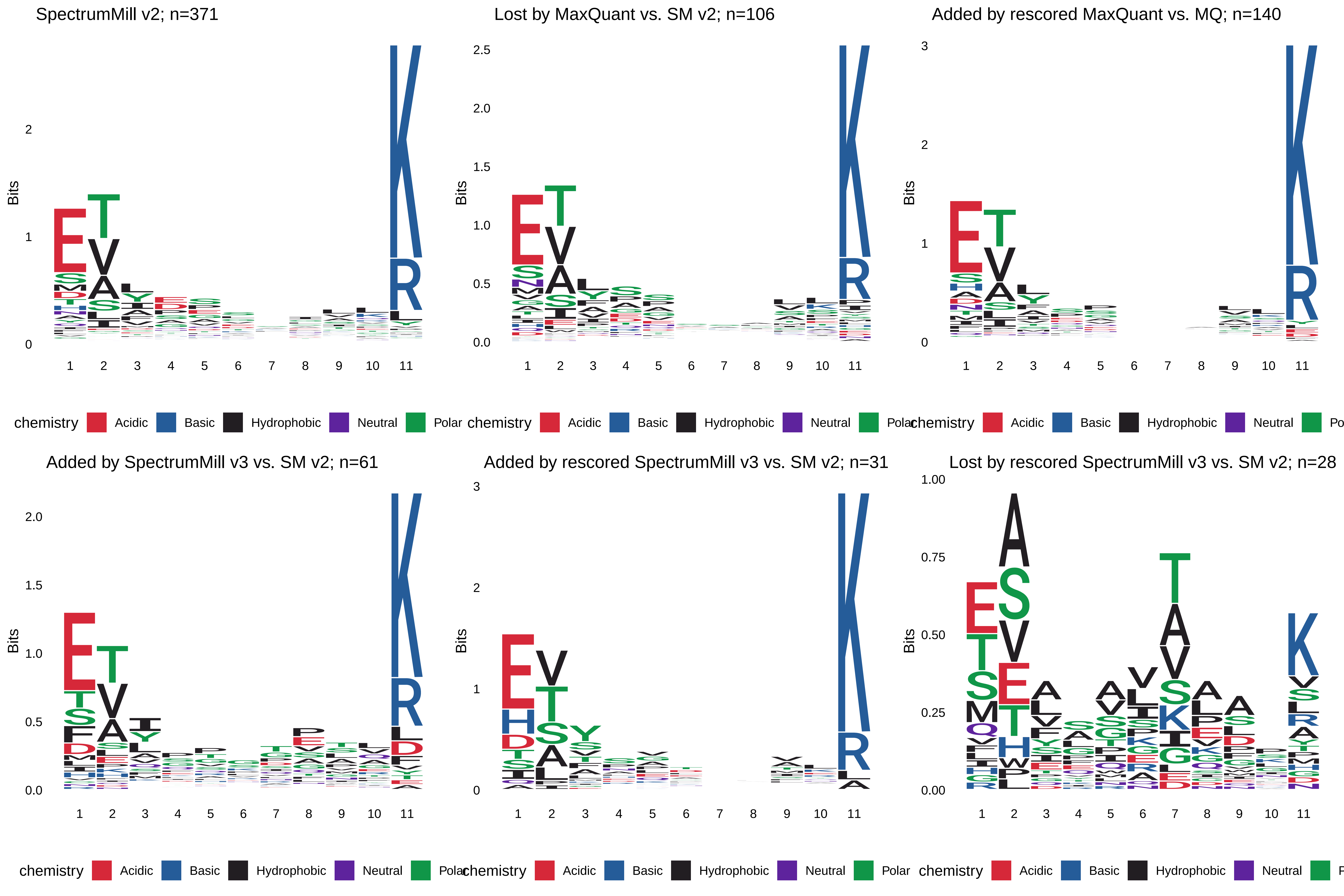
SpectrumMill v2; n=371
Lost by MaxQuant vs. SM v2; n=106
Added by rescored MaxQuant vs. MQ; n=140
Added by SpectrumMill v3 vs. SM v2; n=61
Added by rescored SpectrumMill v3 vs. SM v2; n=31
Lost by rescored SpectrumMill v3 vs. SM v2; n=28
Bits
1 2 3 4 5 6 7 8 9 10 11
chemistry
Acidic
Basic
Hydrophobic
Neutral
Polar

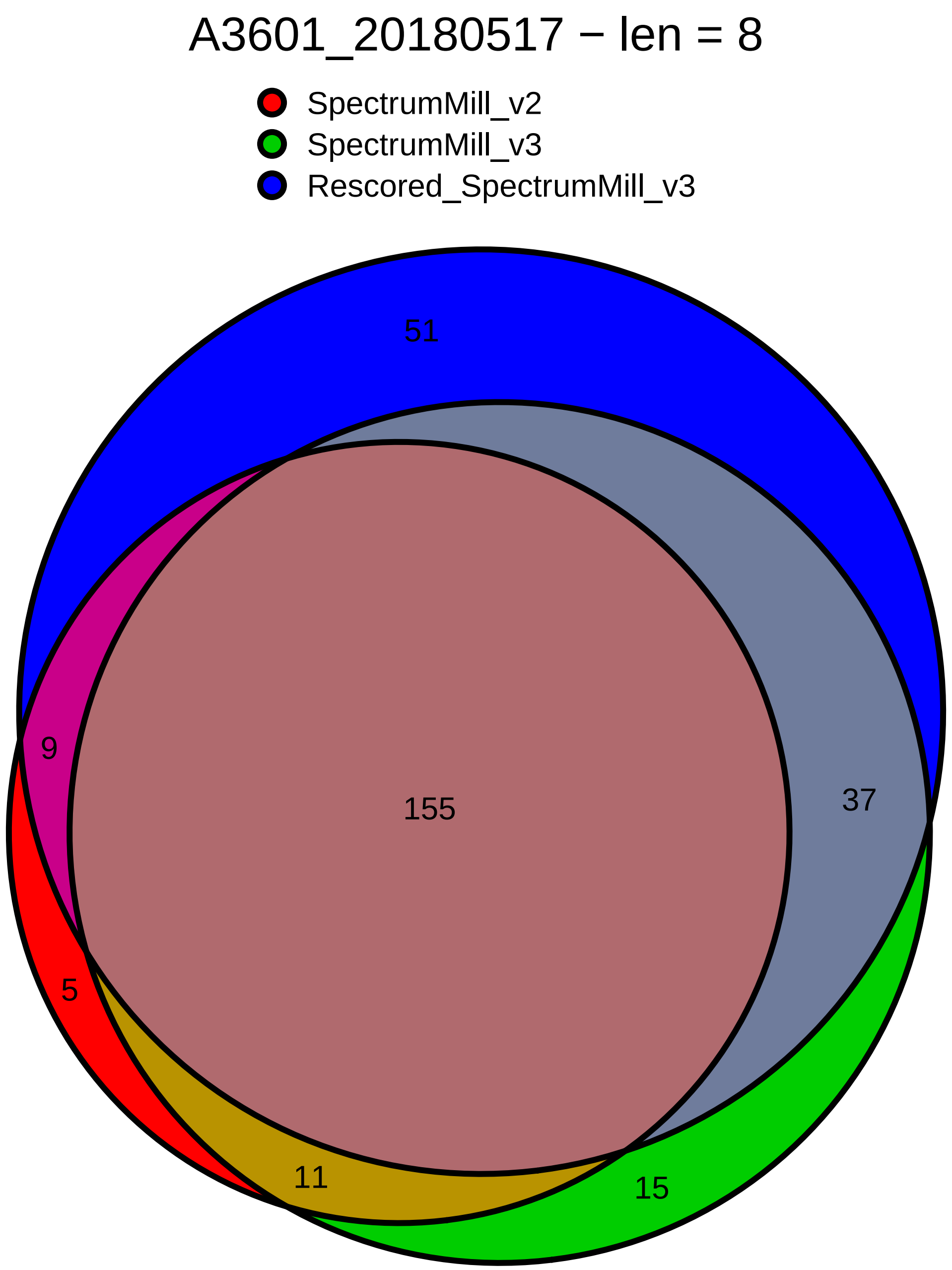
A3601_20180517 − len = 8
SpectrumMill_v2
SpectrumMill_v3
Rescored_SpectrumMill_v3
51
9
155
37
5
11
15

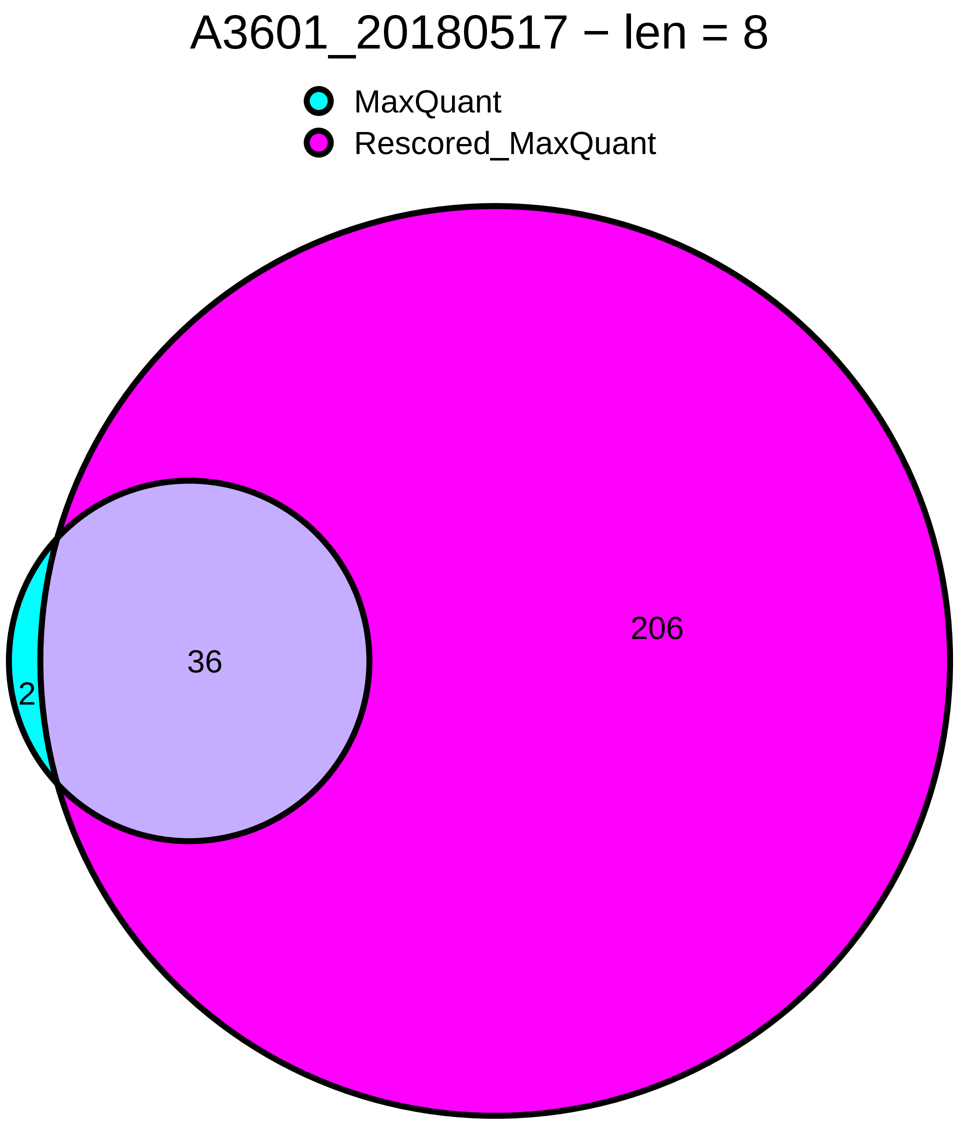
A3601_20180517 − len = 8
MaxQuant
Rescored_MaxQuant
2
36
206

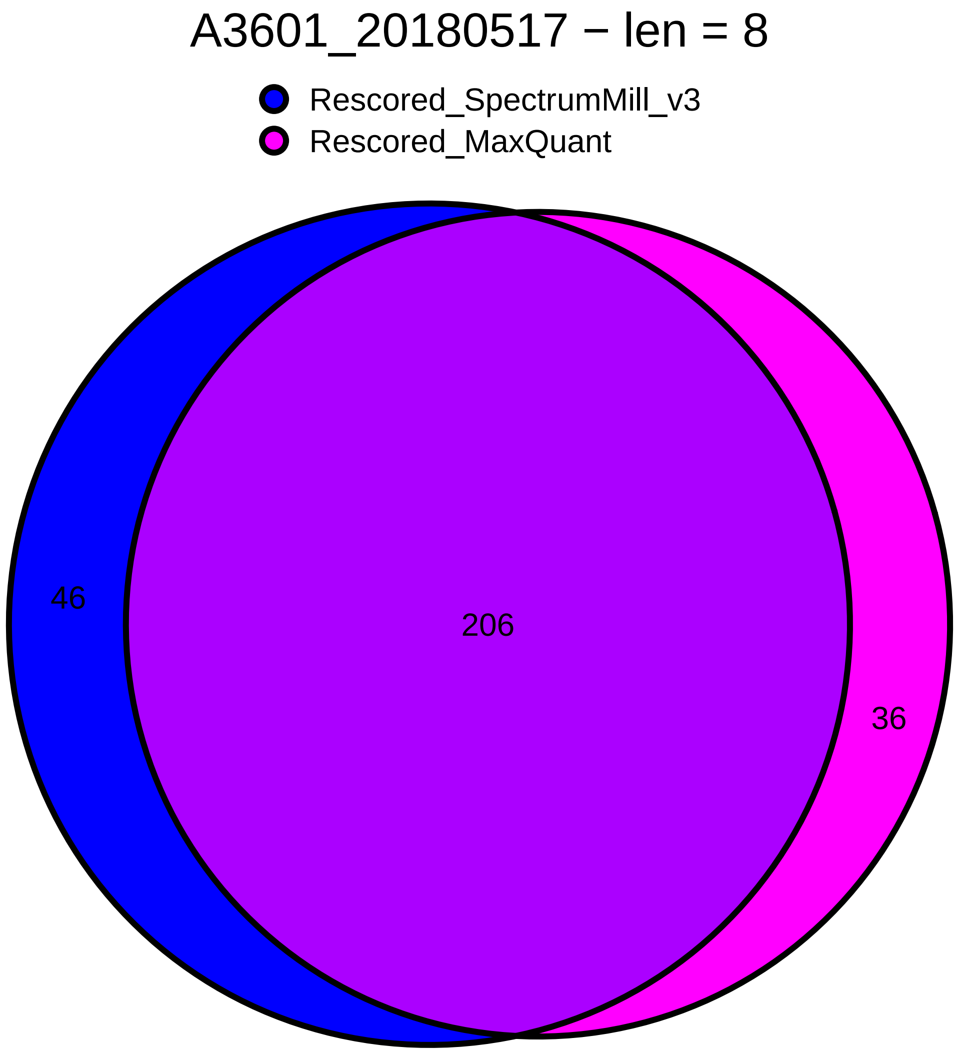
A3601_20180517 − len = 8
Rescored_SpectrumMill_v3
Rescored_MaxQuant
46
206
36

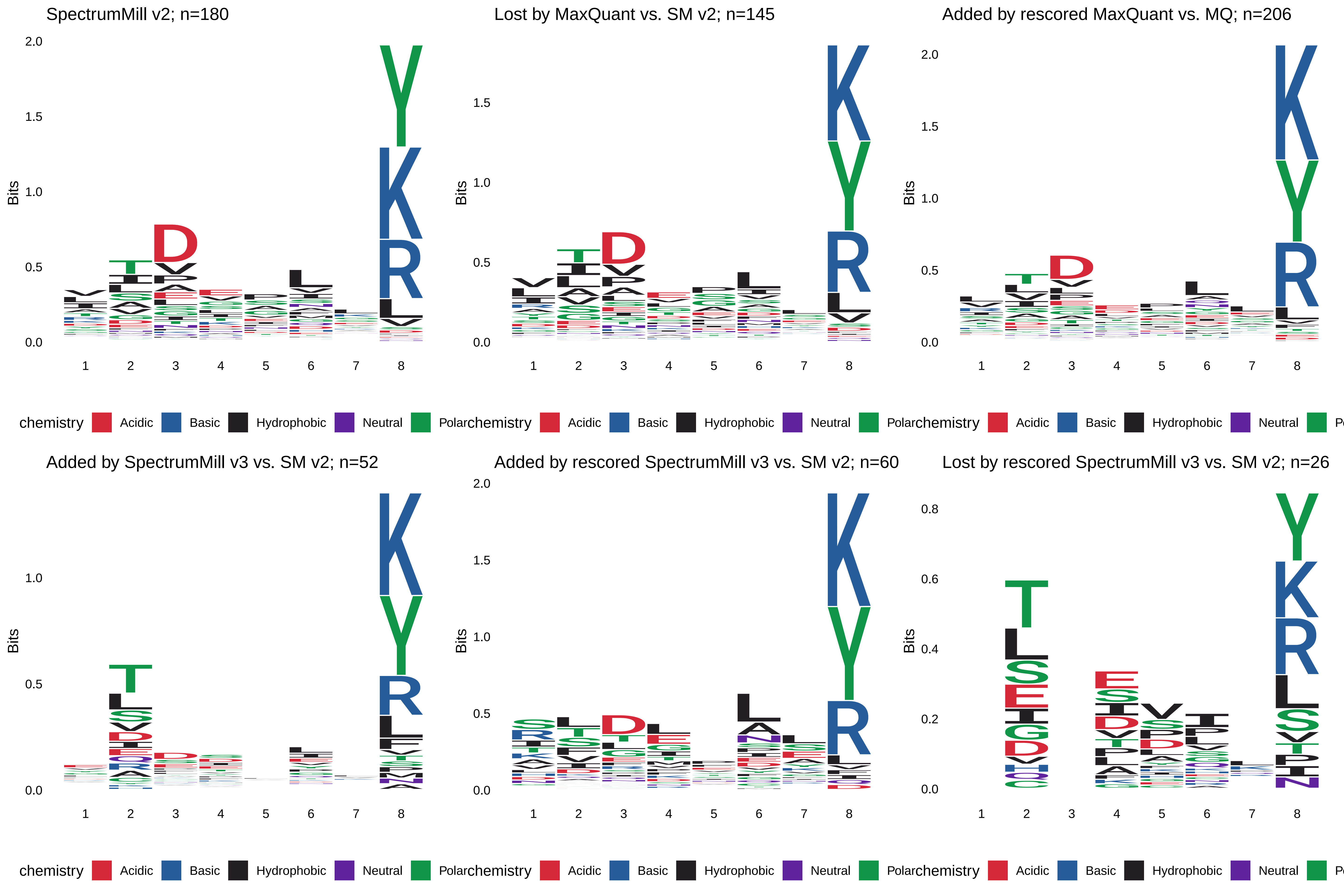
SpectrumMill v2; n=180
Lost by MaxQuant vs. SM v2; n=145
Added by rescored MaxQuant vs. MQ; n=206
Added by SpectrumMill v3 vs. SM v2; n=52
Added by rescored SpectrumMill v3 vs. SM v2; n=60
Lost by rescored SpectrumMill v3 vs. SM v2; n=26
Bits
chemistry
Acidic
Basic
Hydrophobic
Neutral
Polar

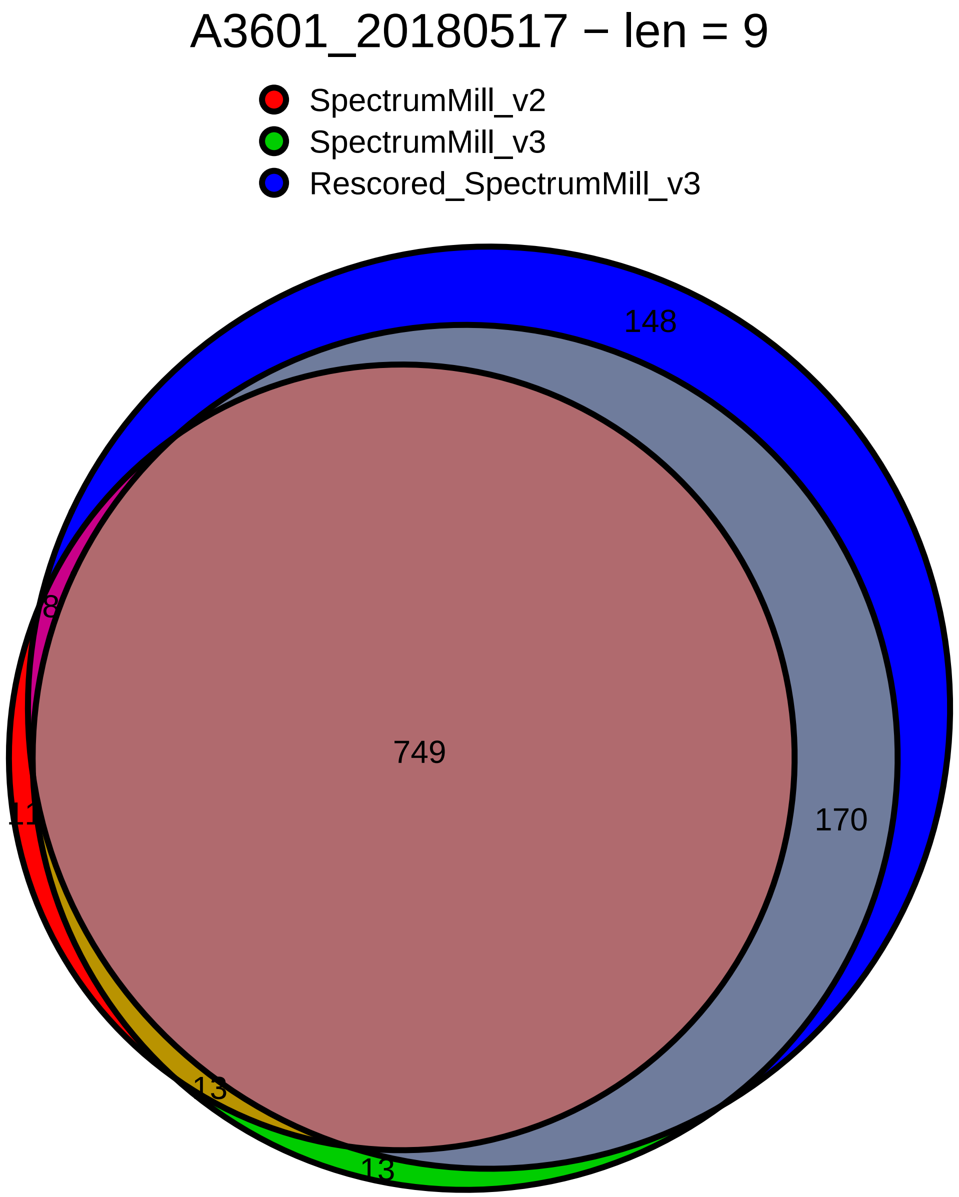
A3601_20180517 − len = 9
SpectrumMill_v2
SpectrumMill_v3
Rescored_SpectrumMill_v3
148
8
749
170
13
13

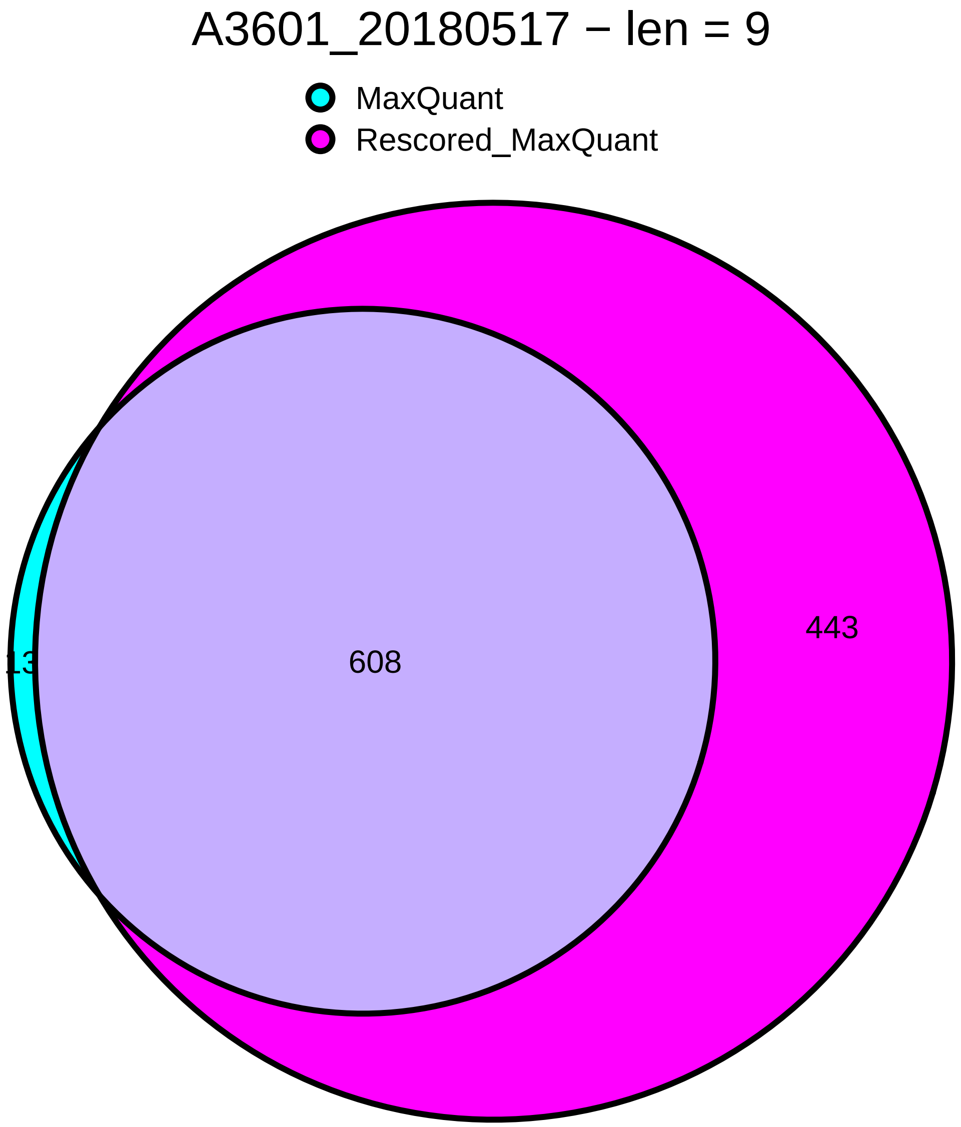
A3601_20180517 − len = 9
MaxQuant
Rescored_MaxQuant
608
443

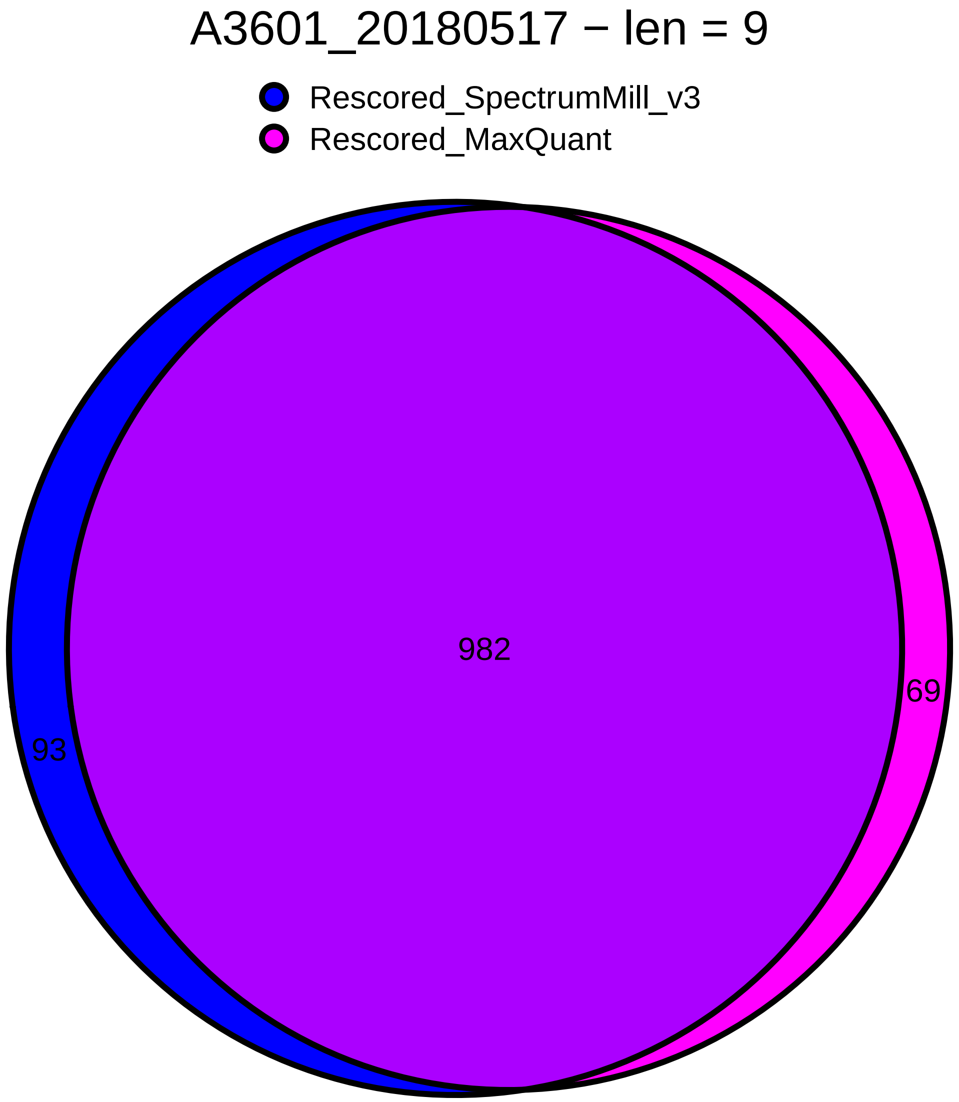
A3601_20180517 − len = 9
Rescored_SpectrumMill_v3
Rescored_MaxQuant
982
93
69

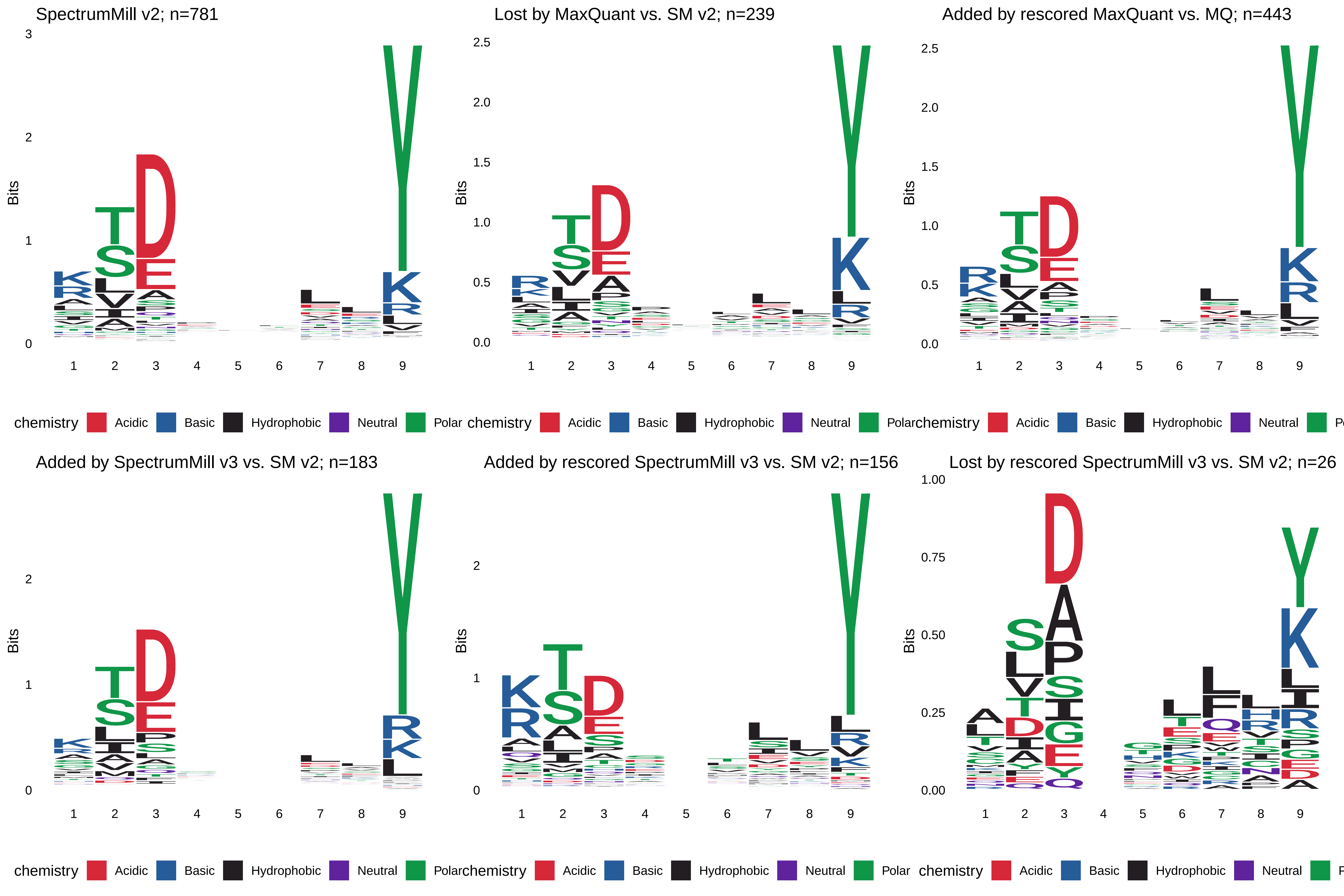
SpectrumMill v2; n=781
Lost by MaxQuant vs. SM v2; n=239
Added by rescored MaxQuant vs. MQ; n=443
Added by SpectrumMill v3 vs. SM v2; n=183
Added by rescored SpectrumMill v3 vs. SM v2; n=156
Lost by rescored SpectrumMill v3 vs. SM v2; n=26
Bits
1 2 3 4 5 6 7 8 9
chemistry
Acidic
Basic
Hydrophobic
Neutral
Polar

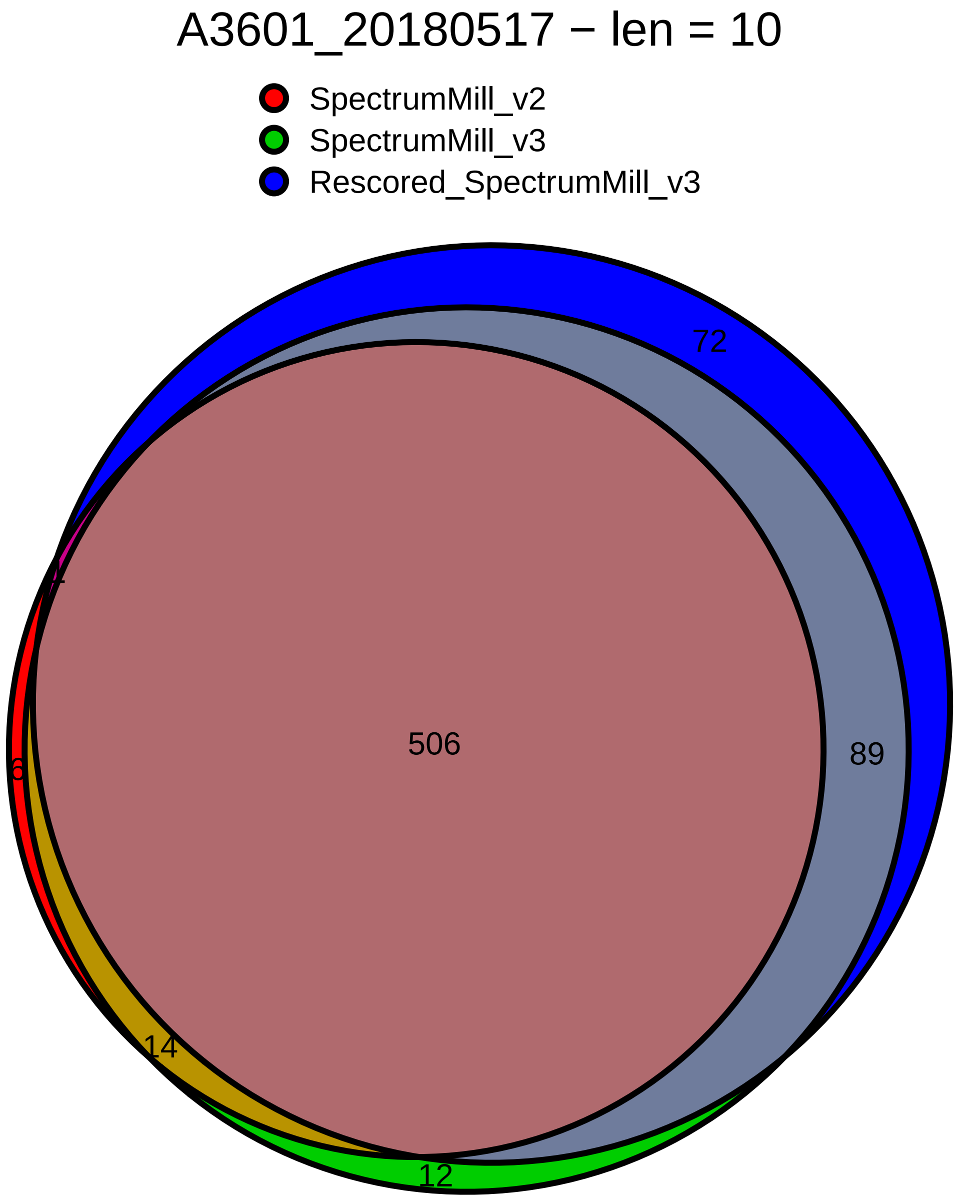
A3601_20180517 − len = 10
SpectrumMill_v2
SpectrumMill_v3
Rescored_SpectrumMill_v3
72
506
89
14
12

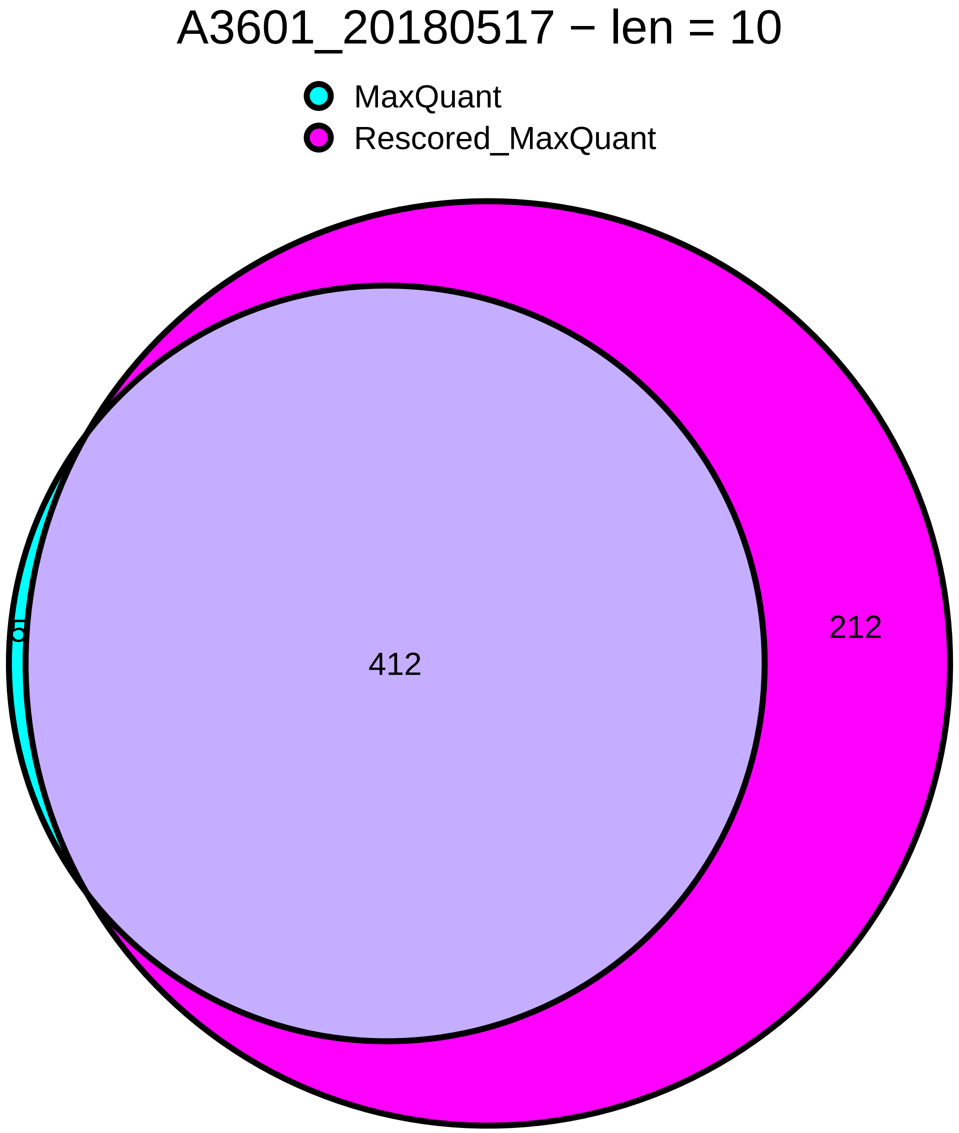
A3601_20180517 − len = 10
MaxQuant
Rescored_MaxQuant
412
212

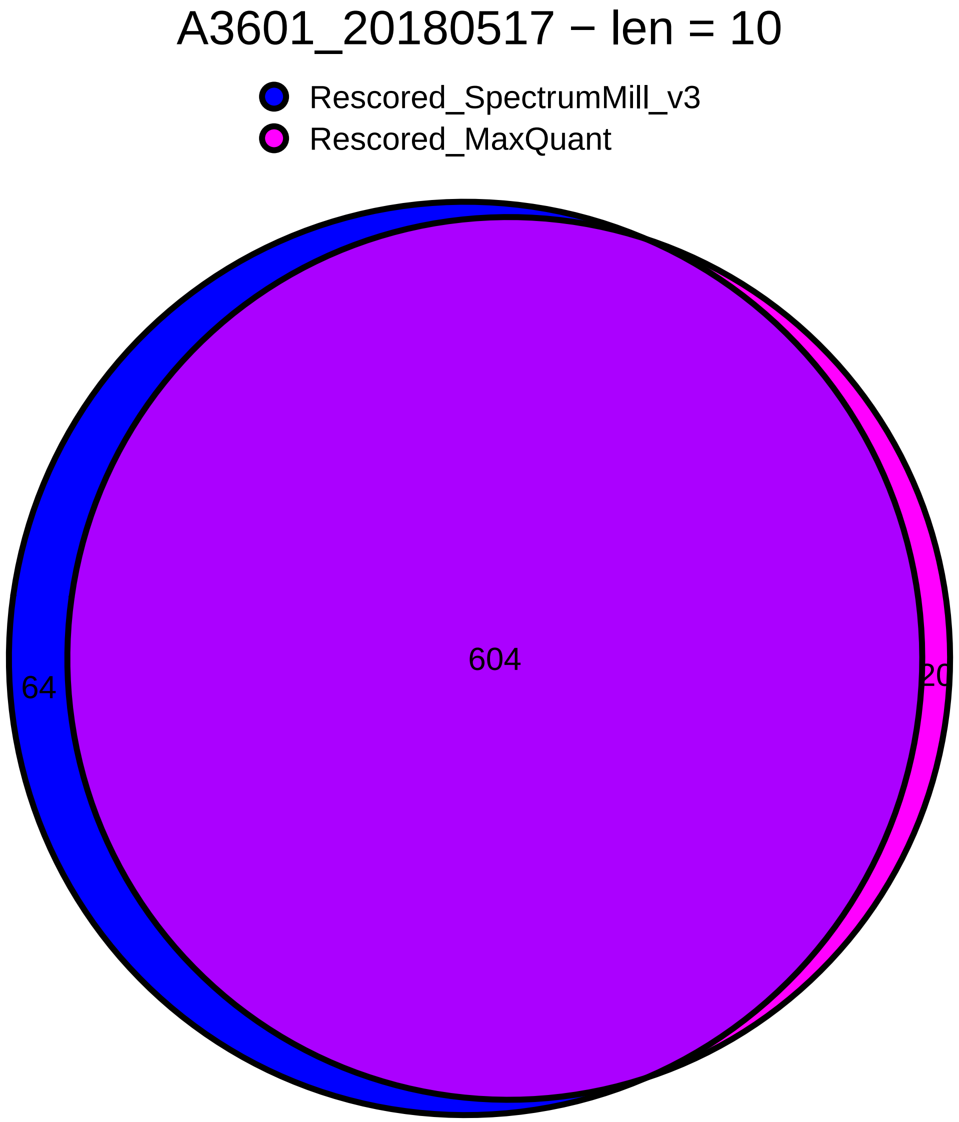
A3601_20180517 − len = 10
Rescored_SpectrumMill_v3
Rescored_MaxQuant
64
604

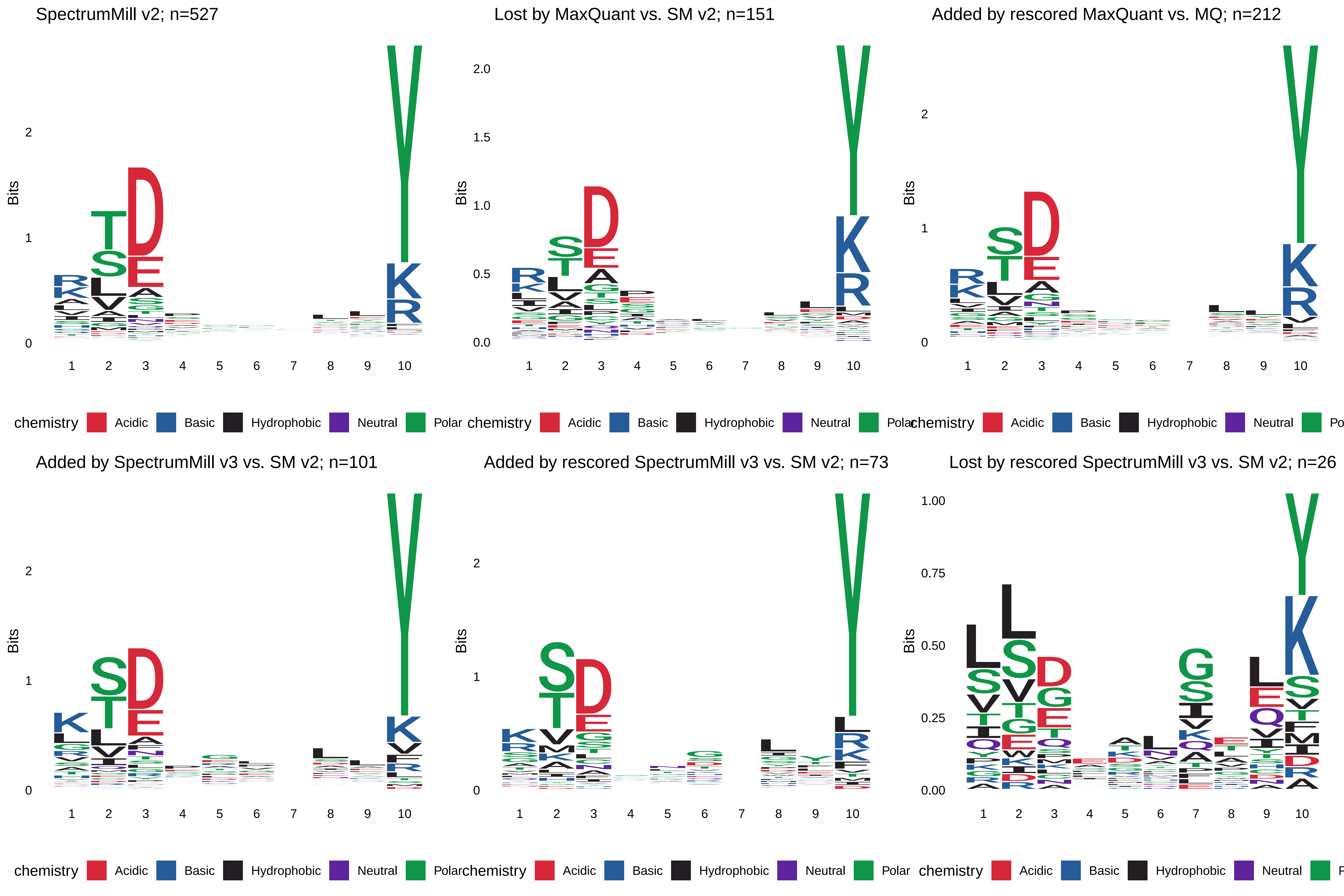
SpectrumMill v2; n=527
Lost by MaxQuant vs. SM v2; n=151
Added by rescored MaxQuant vs. MQ; n=212
Added by SpectrumMill v3 vs. SM v2; n=101
Added by rescored SpectrumMill v3 vs. SM v2; n=73
Lost by rescored SpectrumMill v3 vs. SM v2; n=26
Bits
0
1
2
0.0
0.5
1.0
1.5
2.0
0.00
0.25
0.50
0.75
1.00
1 2 3 4 5 6 7 8 9 10
chemistry
Acidic
Basic
Hydrophobic
Neutral
Polar

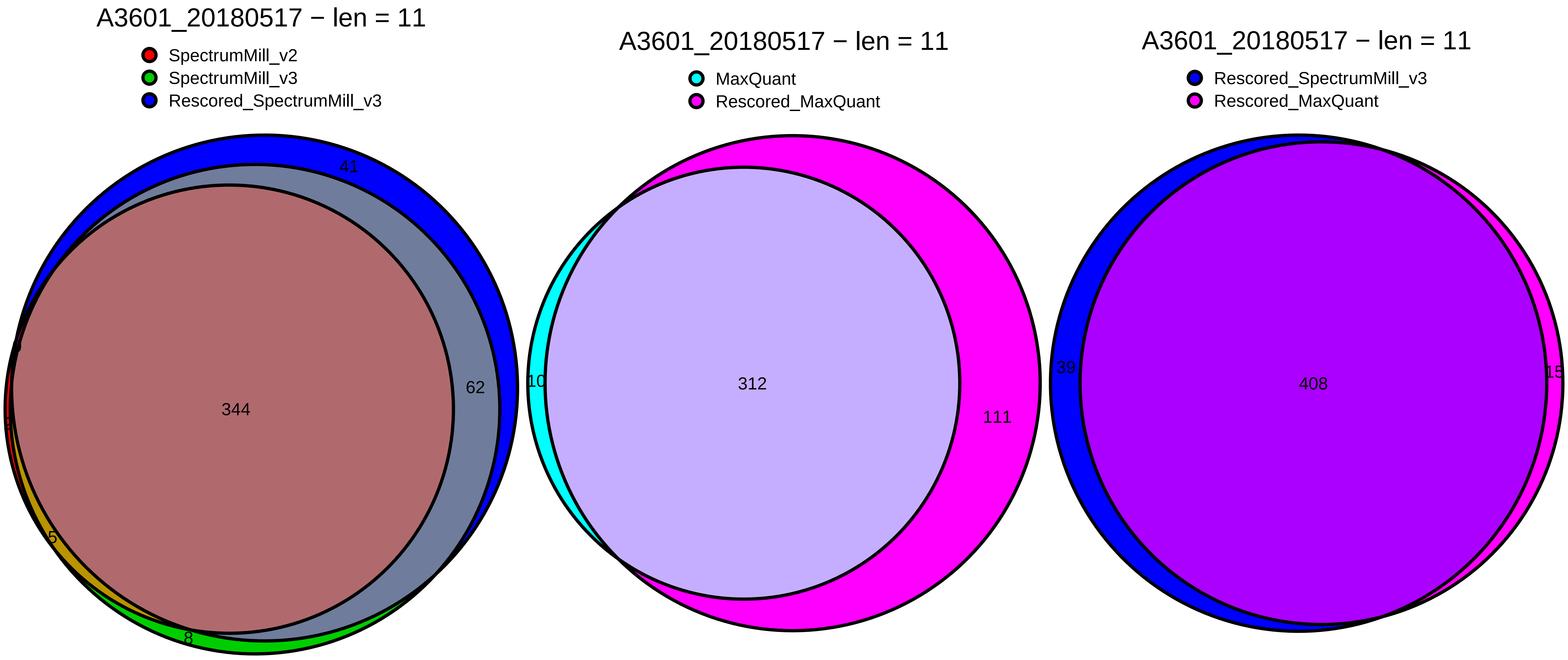
A3601_20180517 − len = 11
SpectrumMill_v2
SpectrumMill_v3
Rescored_SpectrumMill_v3
41
62
344
5
8
A3601_20180517 − len = 11
MaxQuant
Rescored_MaxQuant
10
312
111
A3601_20180517 − len = 11
Rescored_SpectrumMill_v3
Rescored_MaxQuant
39
408
15

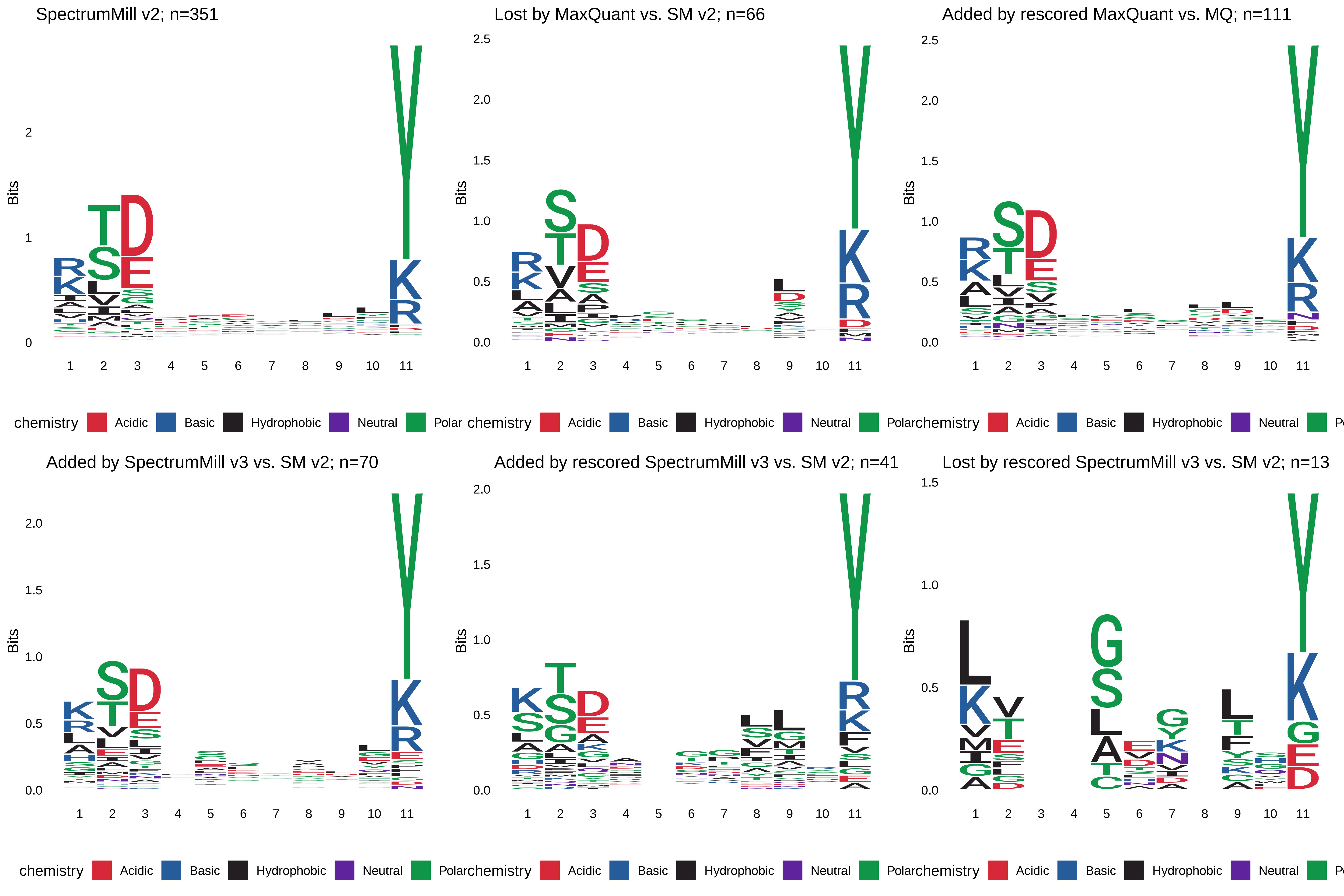

SpectrumMill v2; n=351
Lost by MaxQuant vs. SM v2; n=66
Added by rescored MaxQuant vs. MQ; n=111
Added by SpectrumMill v3 vs. SM v2; n=70
Added by rescored SpectrumMill v3 vs. SM v2; n=41
Lost by rescored SpectrumMill v3 vs. SM v2; n=13
Bits
1 2 3 4 5 6 7 8 9 10 11
chemistry
Acidic
Basic
Hydrophobic
Neutral
Polar

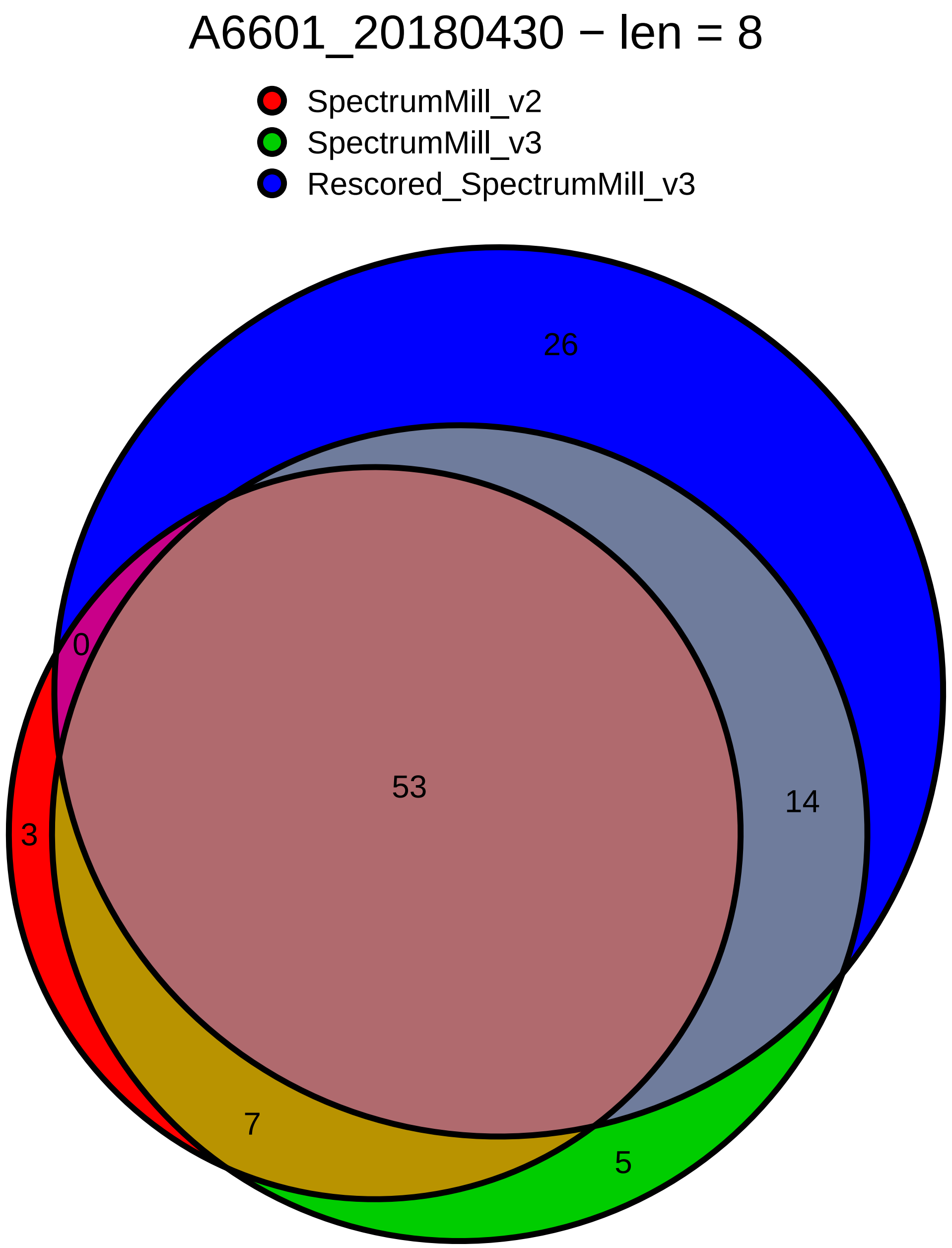

A6601_20180430 − len = 8
SpectrumMill_v2
SpectrumMill_v3
Rescored_SpectrumMill_v3
26
0
53
14
3
7
5

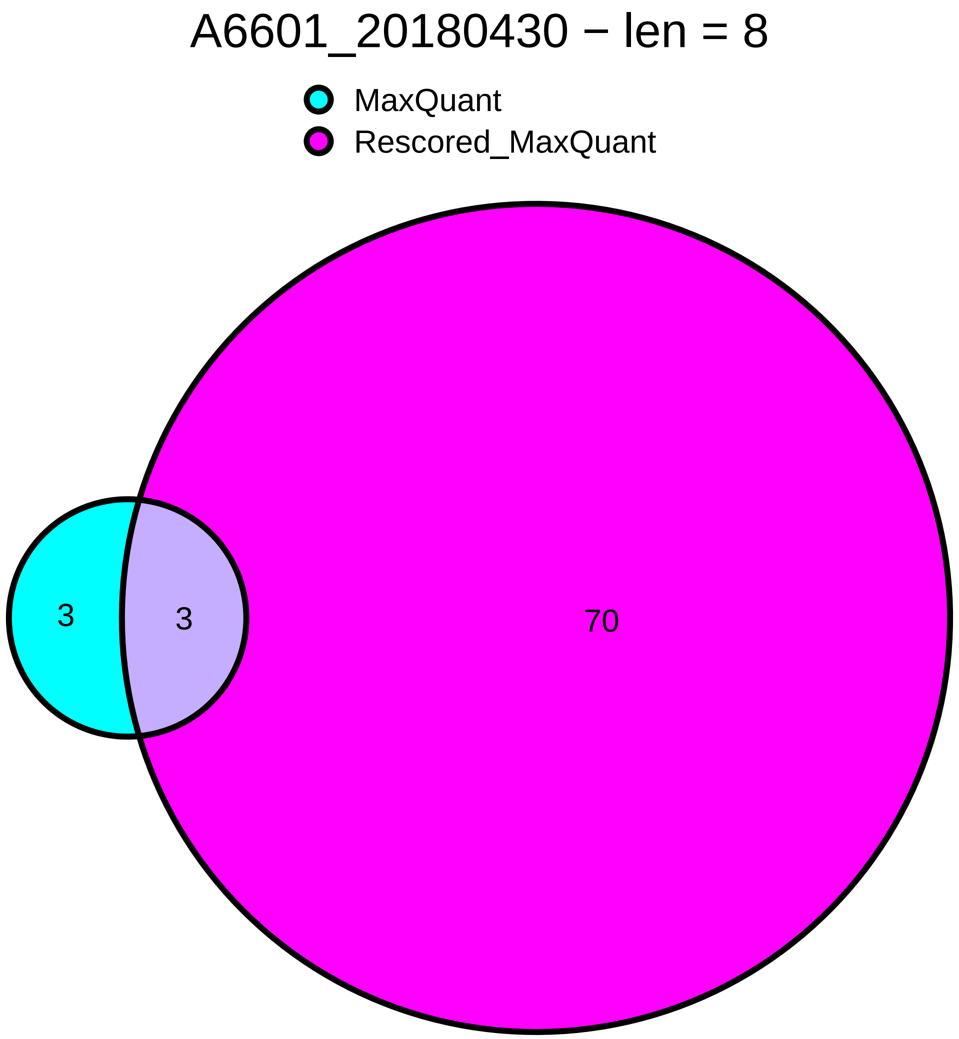

A6601_20180430 − len = 8
MaxQuant
Rescored_MaxQuant
3
3
70

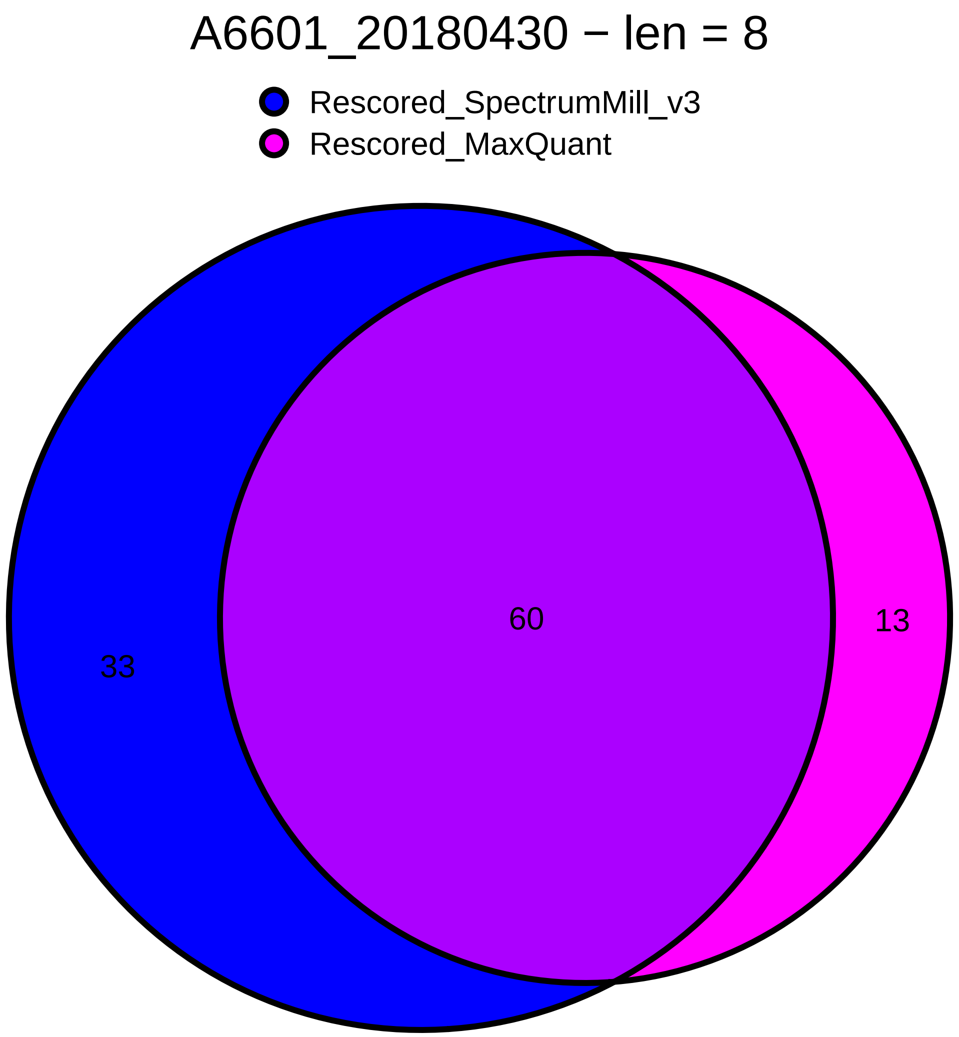

A6601_20180430 − len = 8
Rescored_SpectrumMill_v3
Rescored_MaxQuant
33
60
13

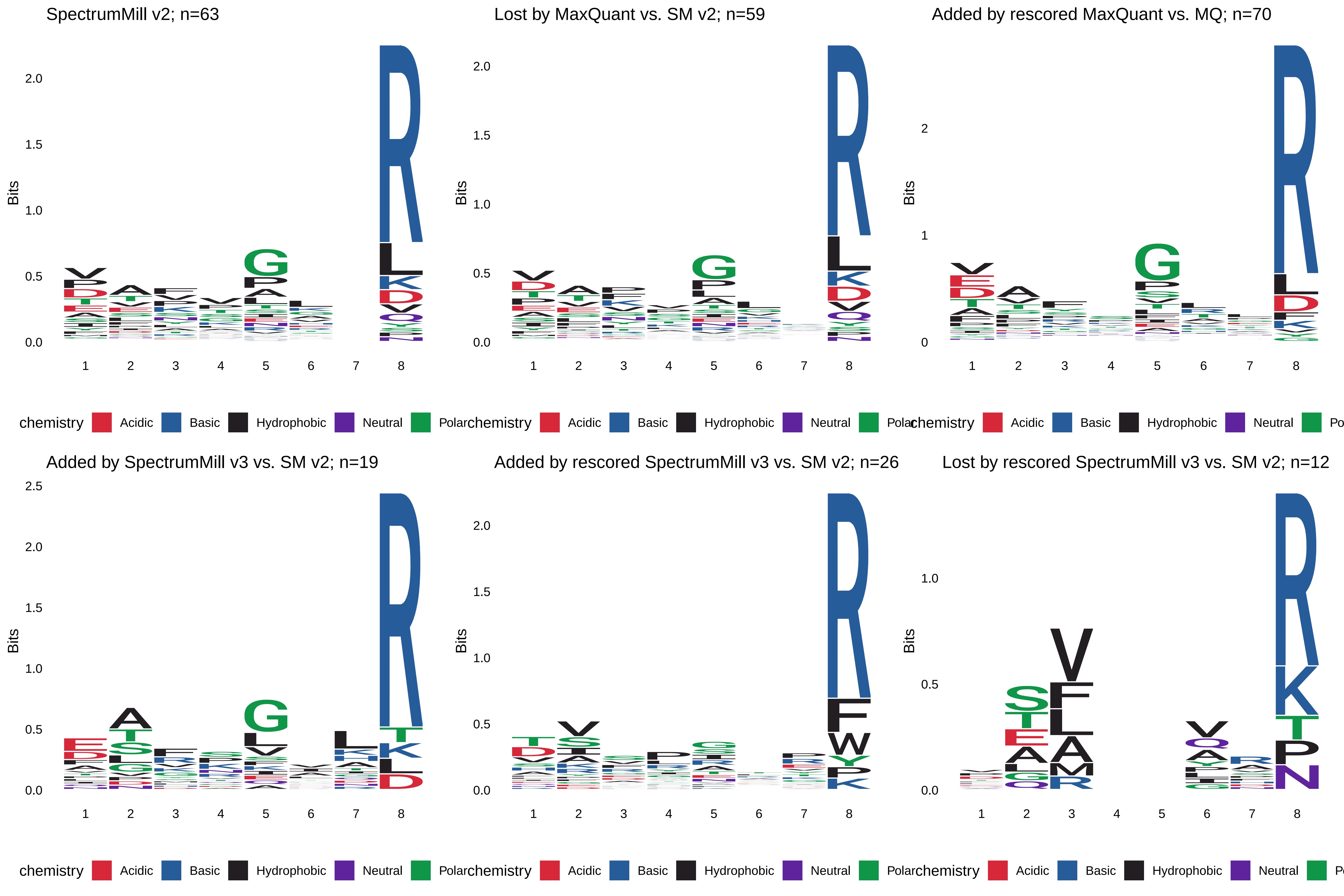
SpectrumMill v2; n=63
Lost by MaxQuant vs. SM v2; n=59
Added by rescored MaxQuant vs. MQ; n=70
Added by SpectrumMill v3 vs. SM v2; n=19
Added by rescored SpectrumMill v3 vs. SM v2; n=26
Lost by rescored SpectrumMill v3 vs. SM v2; n=12
Bits
chemistry
Acidic
Basic
Hydrophobic
Neutral
Polar

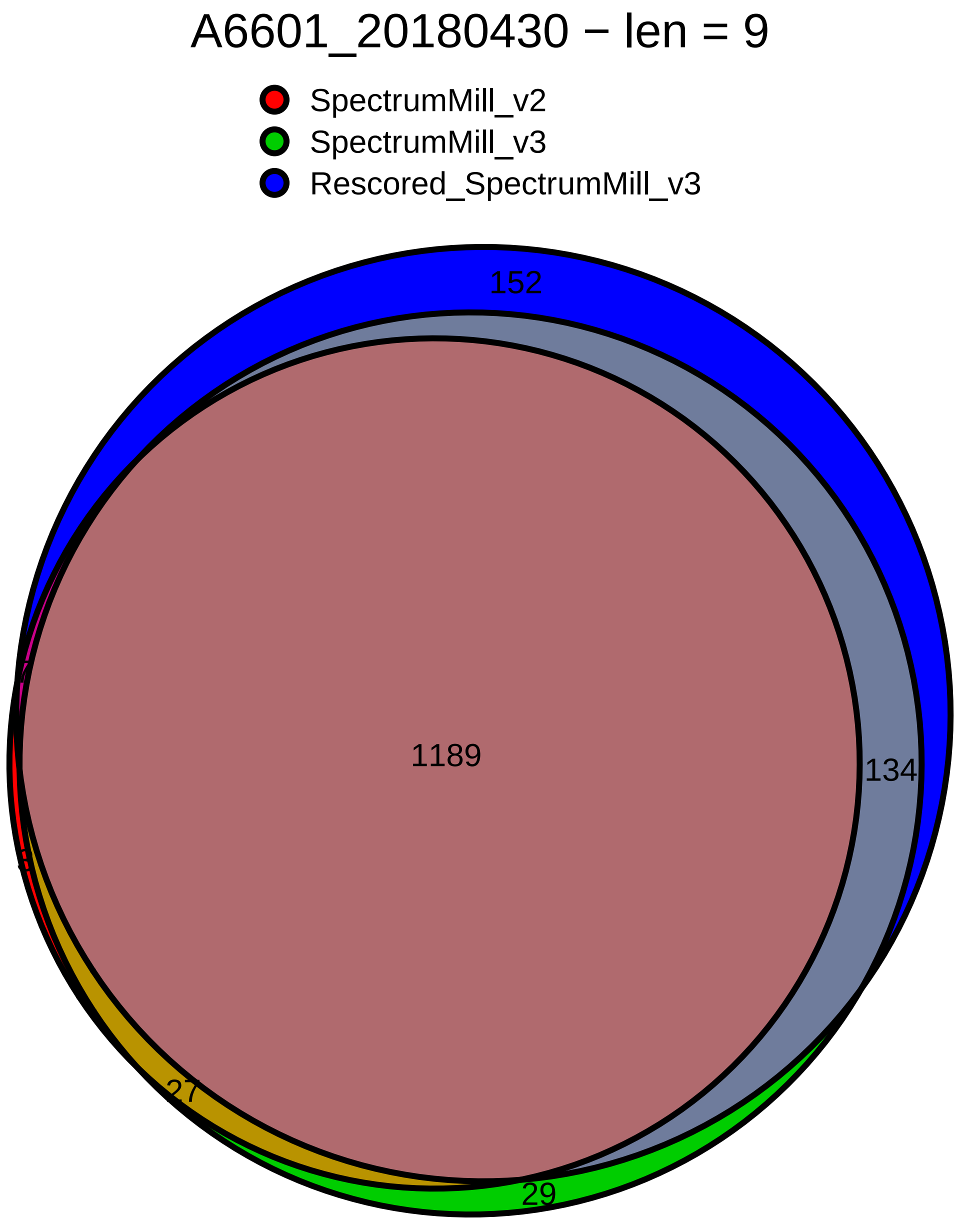
A6601_20180430 − len = 9
SpectrumMill_v2
SpectrumMill_v3
Rescored_SpectrumMill_v3
152
1189
134
27
29

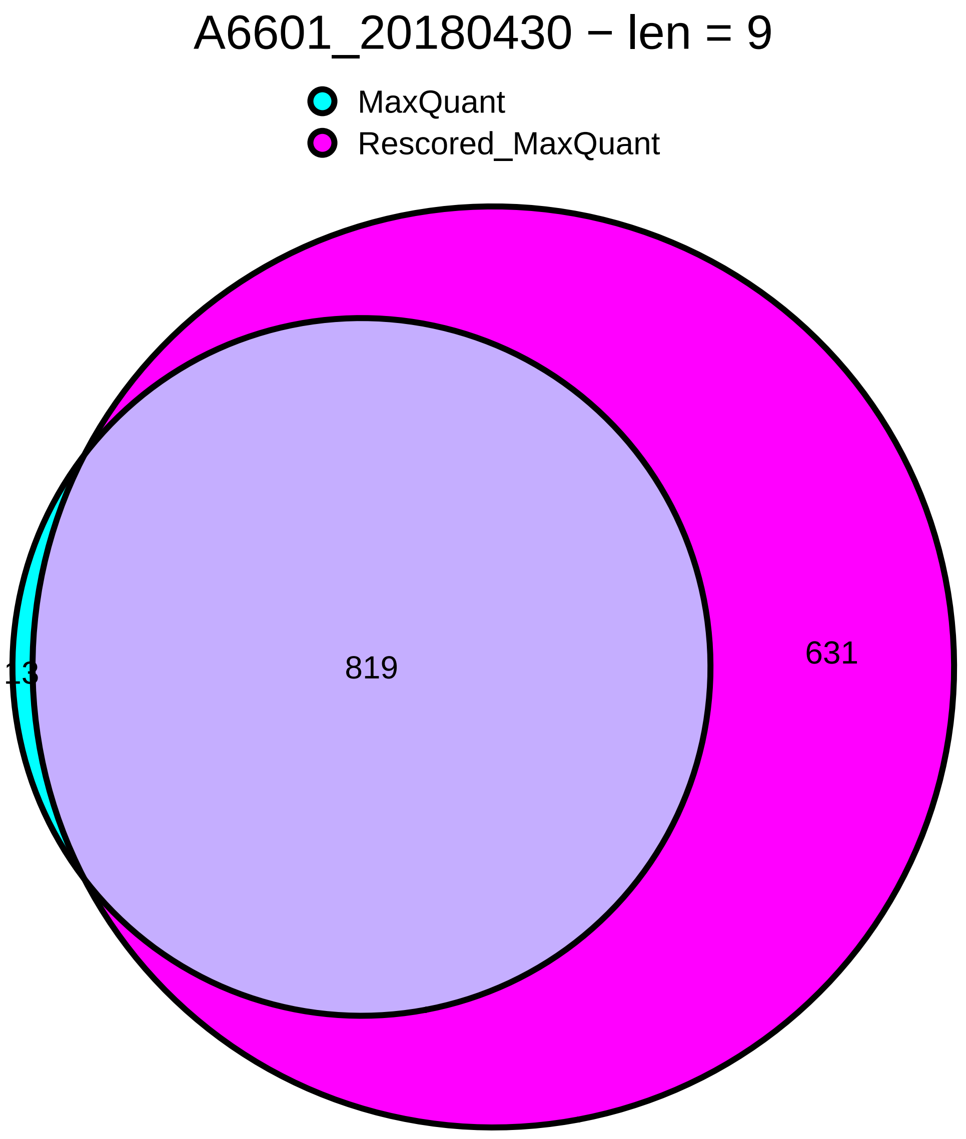
A6601_20180430 − len = 9
MaxQuant
Rescored_MaxQuant
819
631

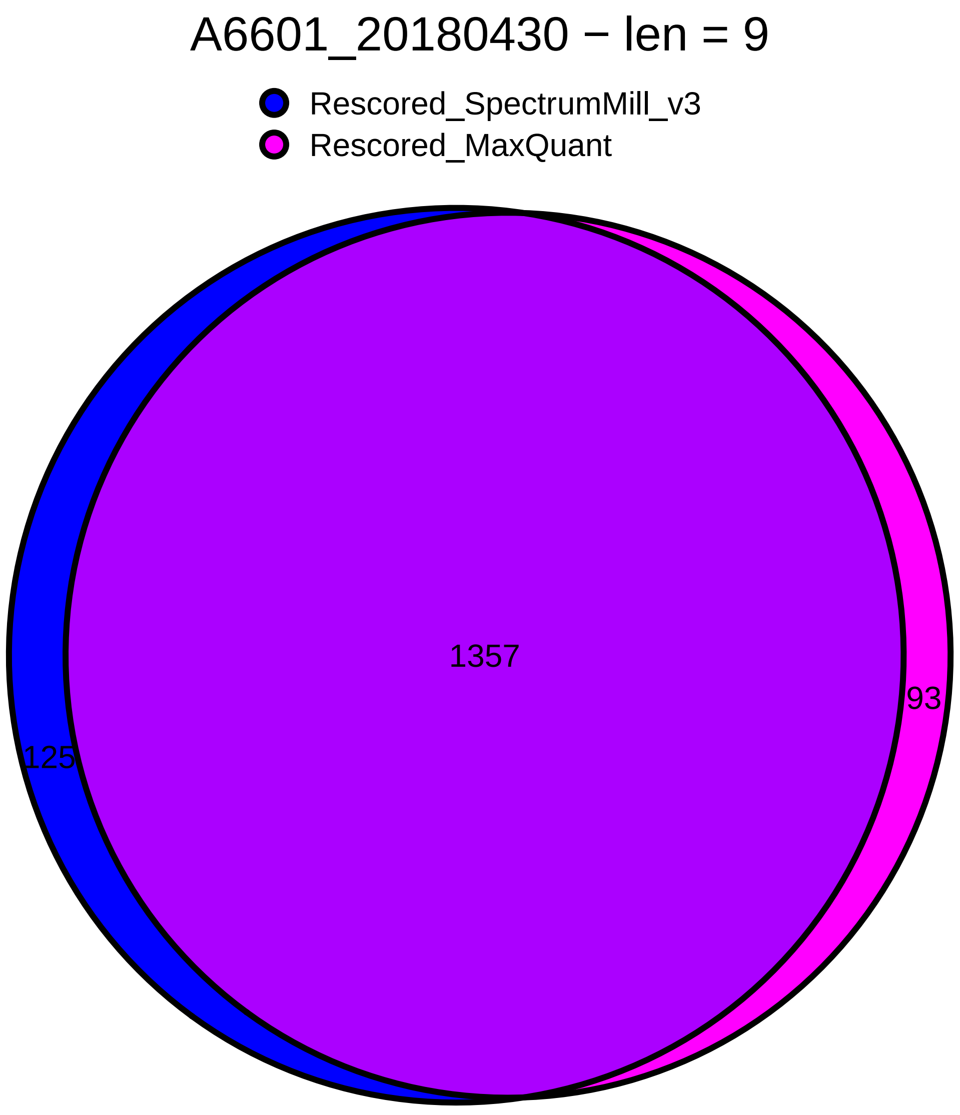
A6601_20180430 − len = 9
Rescored_SpectrumMill_v3
Rescored_MaxQuant
1357
125
93

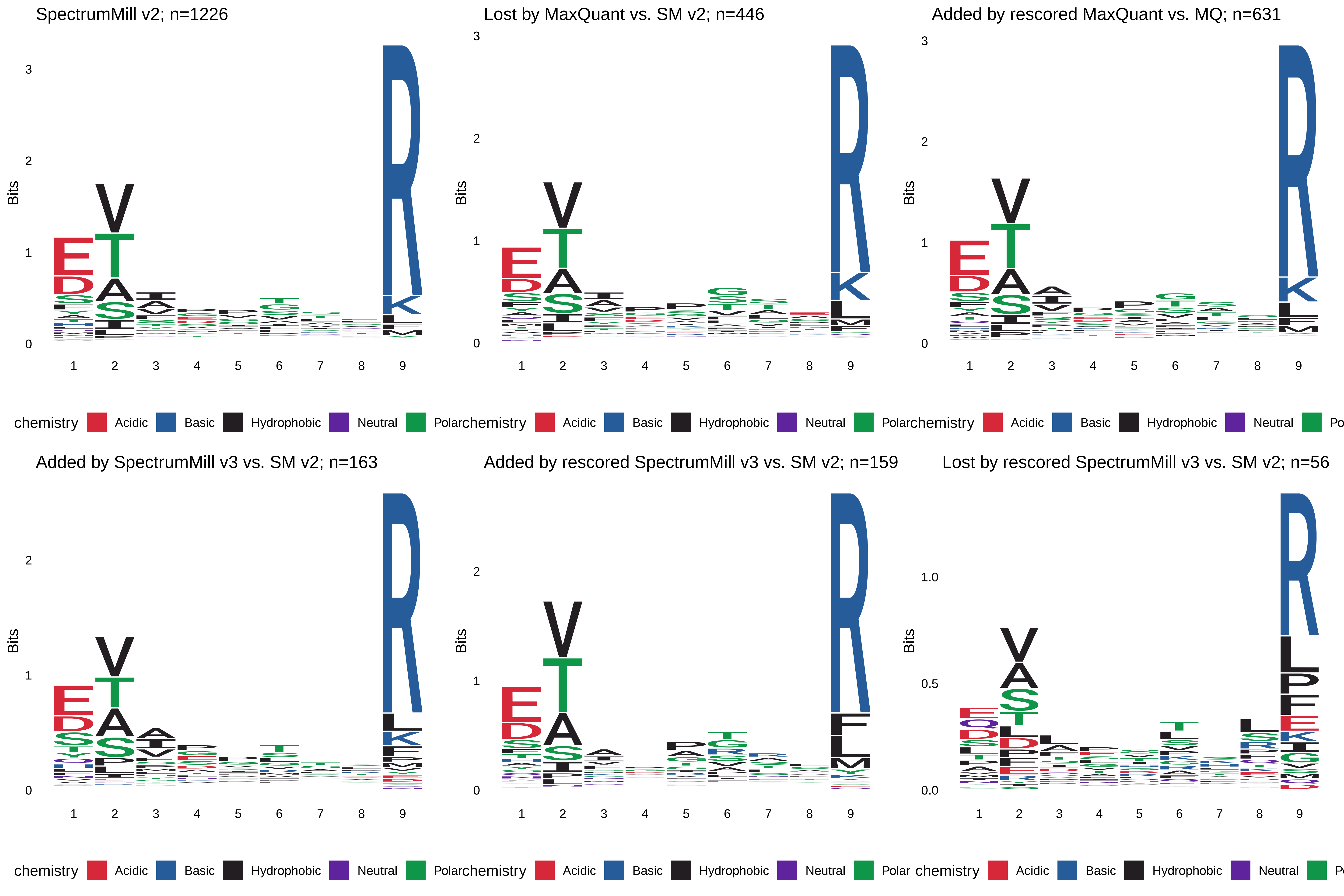
SpectrumMill v2; n=1226
Lost by MaxQuant vs. SM v2; n=446
Added by rescored MaxQuant vs. MQ; n=631
Added by SpectrumMill v3 vs. SM v2; n=163
Added by rescored SpectrumMill v3 vs. SM v2; n=159
Lost by rescored SpectrumMill v3 vs. SM v2; n=56
Bits
1 2 3 4 5 6 7 8 9
chemistry Acidic Basic Hydrophobic Neutral Polar

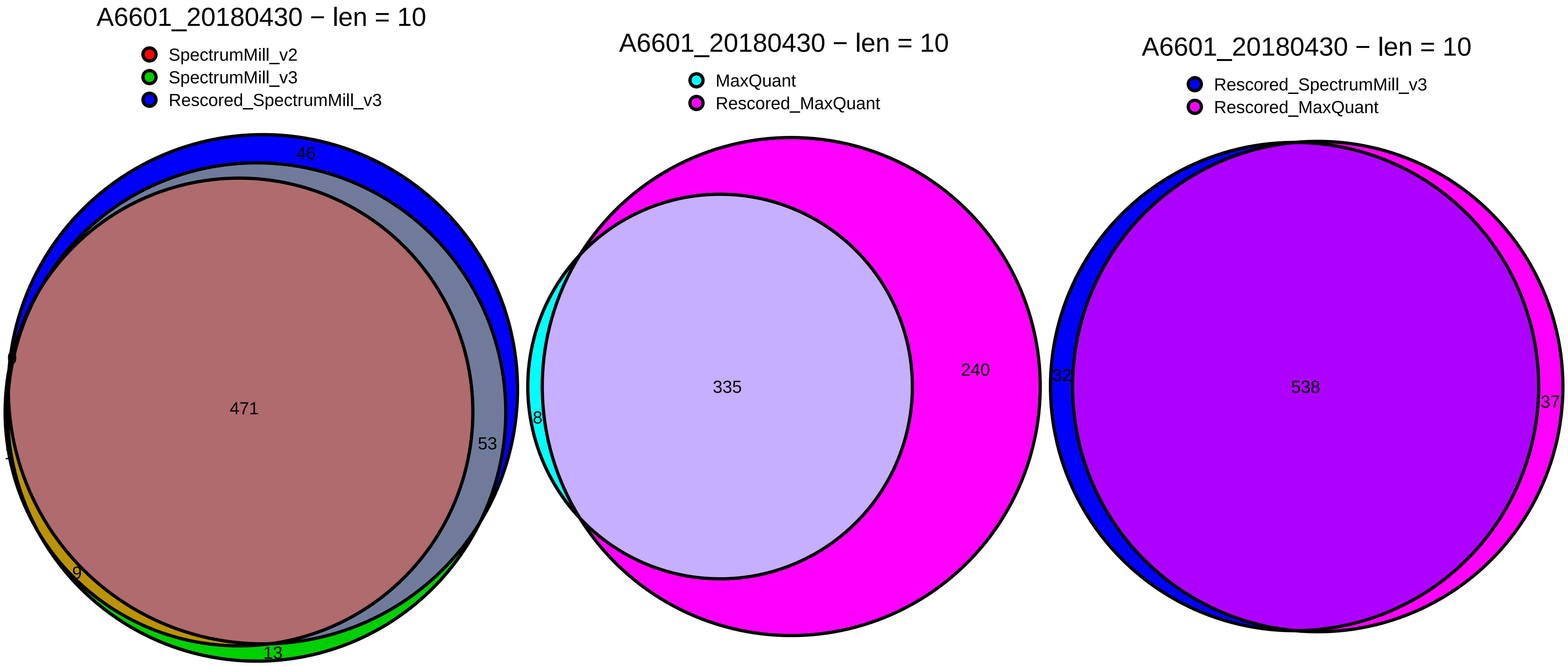

A6601_20180430 − len = 10
SpectrumMill_v2
SpectrumMill_v3
Rescored_SpectrumMill_v3
46
471
53
9
13
A6601_20180430 − len = 10
MaxQuant
Rescored_MaxQuant
335
240
8
A6601_20180430 − len = 10
Rescored_SpectrumMill_v3
Rescored_MaxQuant
32
538
37

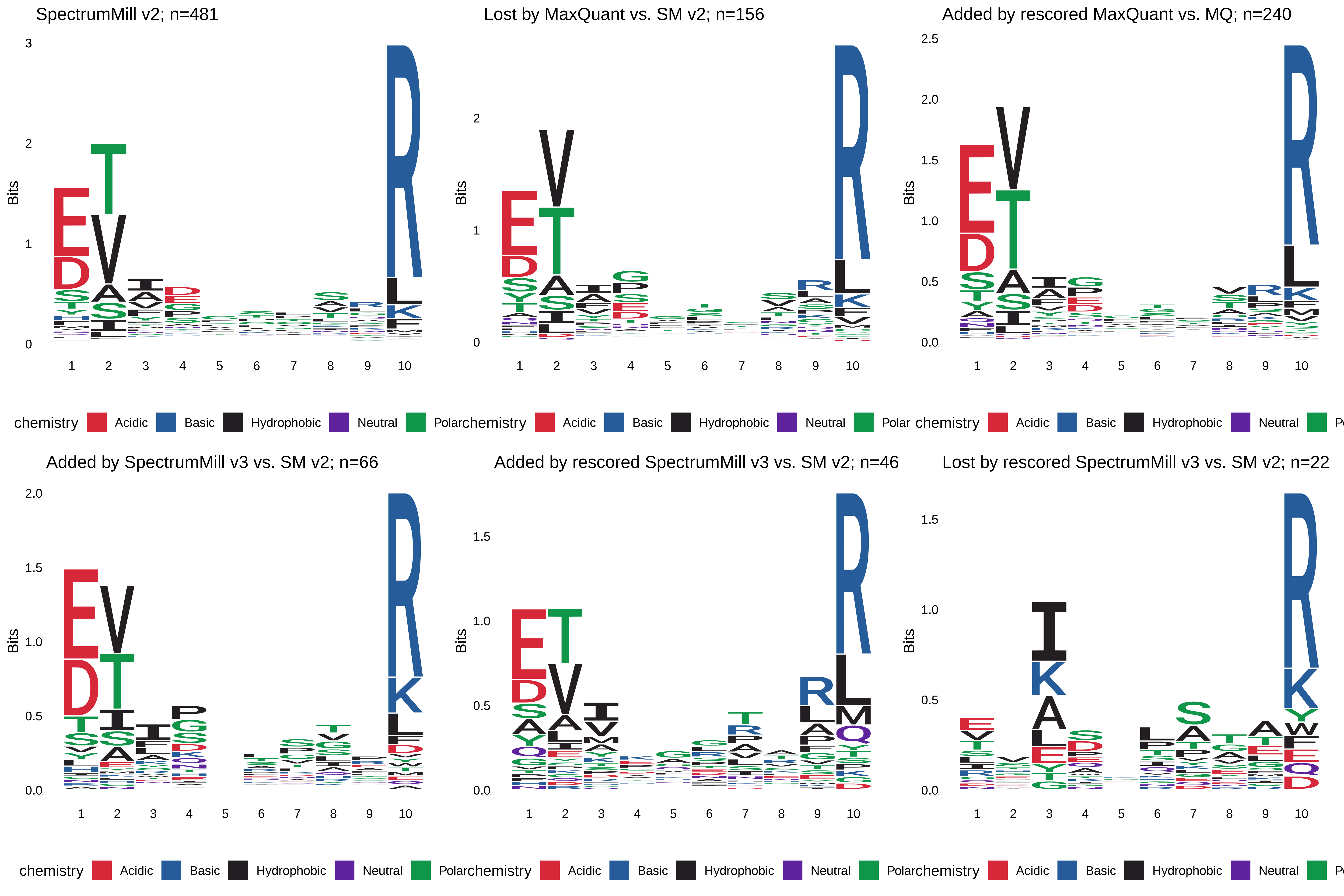
SpectrumMill v2; n=481
Lost by MaxQuant vs. SM v2; n=156
Added by rescored MaxQuant vs. MQ; n=240
Bits
1 2 3 4 5 6 7 8 9 10
chemistry Acidic Basic Hydrophobic Neutral Polar
Added by SpectrumMill v3 vs. SM v2; n=66
Added by rescored SpectrumMill v3 vs. SM v2; n=46
Lost by rescored SpectrumMill v3 vs. SM v2; n=22
Bits
1 2 3 4 5 6 7 8 9 10
chemistry Acidic Basic Hydrophobic Neutral Polar

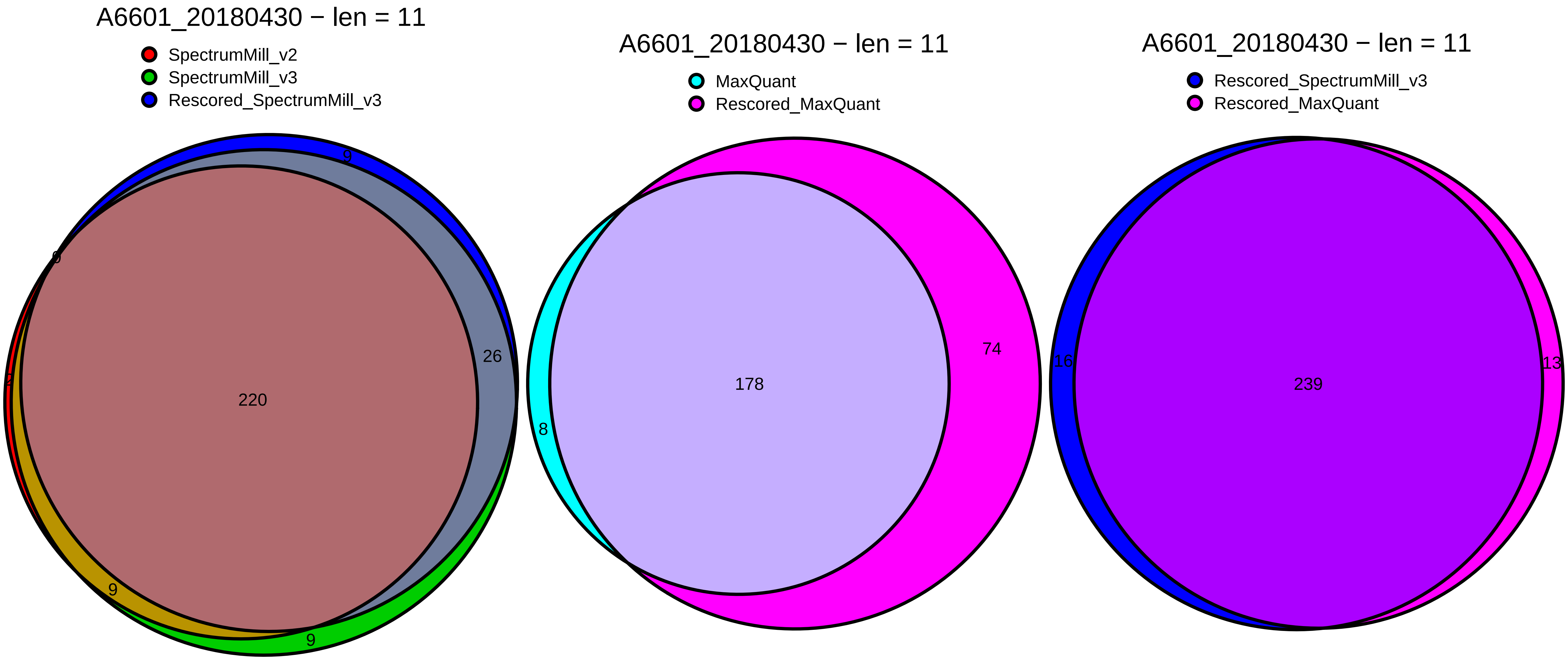
A6601_20180430 − len = 11
SpectrumMill_v2
SpectrumMill_v3
Rescored_SpectrumMill_v3
9
0
26
220
9
9
A6601_20180430 − len = 11
MaxQuant
Rescored_MaxQuant
178
74
8
A6601_20180430 − len = 11
Rescored_SpectrumMill_v3
Rescored_MaxQuant
16
239
13

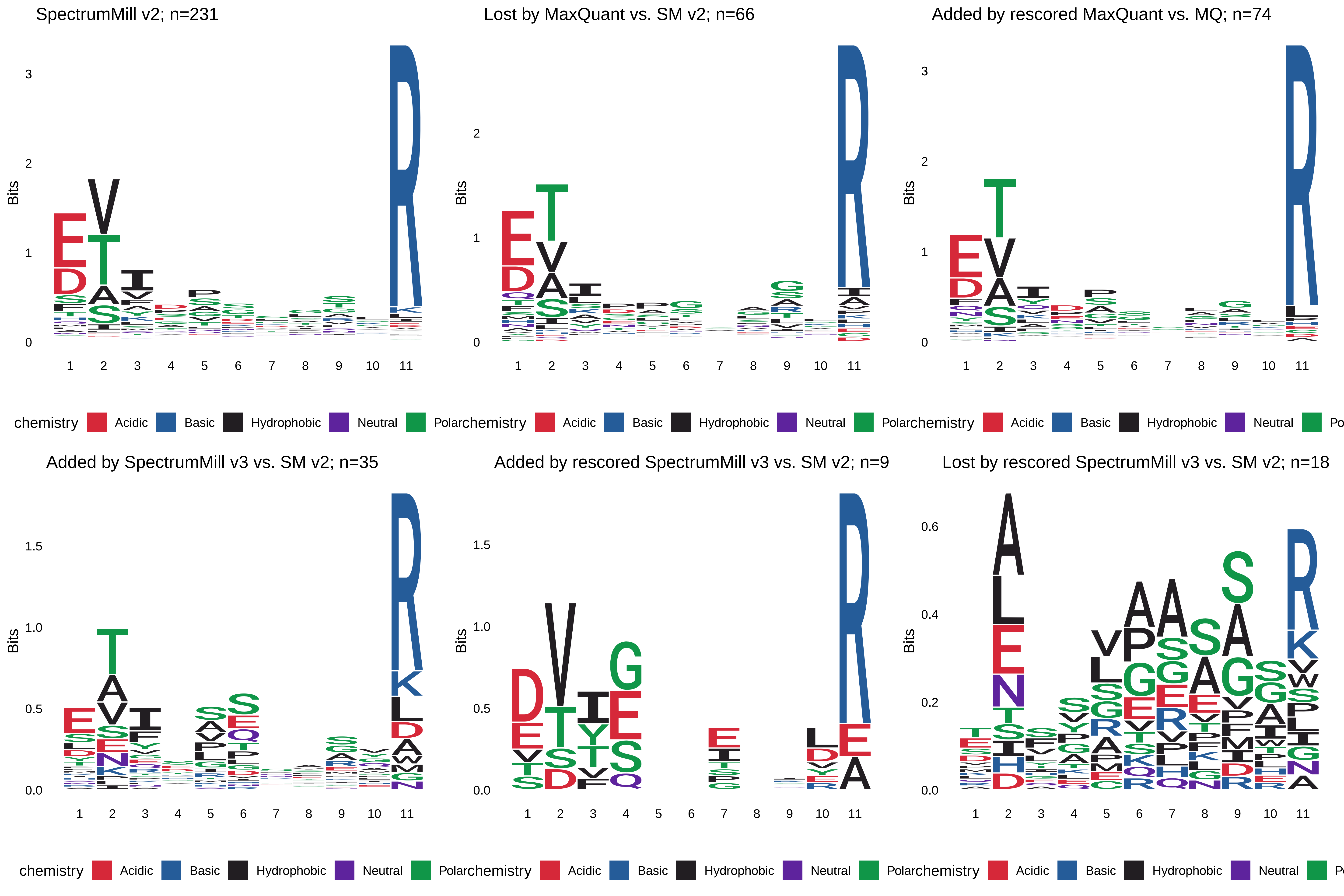
SpectrumMill v2; n=231
Lost by MaxQuant vs. SM v2; n=66
Added by rescored MaxQuant vs. MQ; n=74
Added by SpectrumMill v3 vs. SM v2; n=35
Added by rescored SpectrumMill v3 vs. SM v2; n=9
Lost by rescored SpectrumMill v3 vs. SM v2; n=18
Bits
1 2 3 4 5 6 7 8 9 10 11
chemistry
Acidic
Basic
Hydrophobic
Neutral
Polar

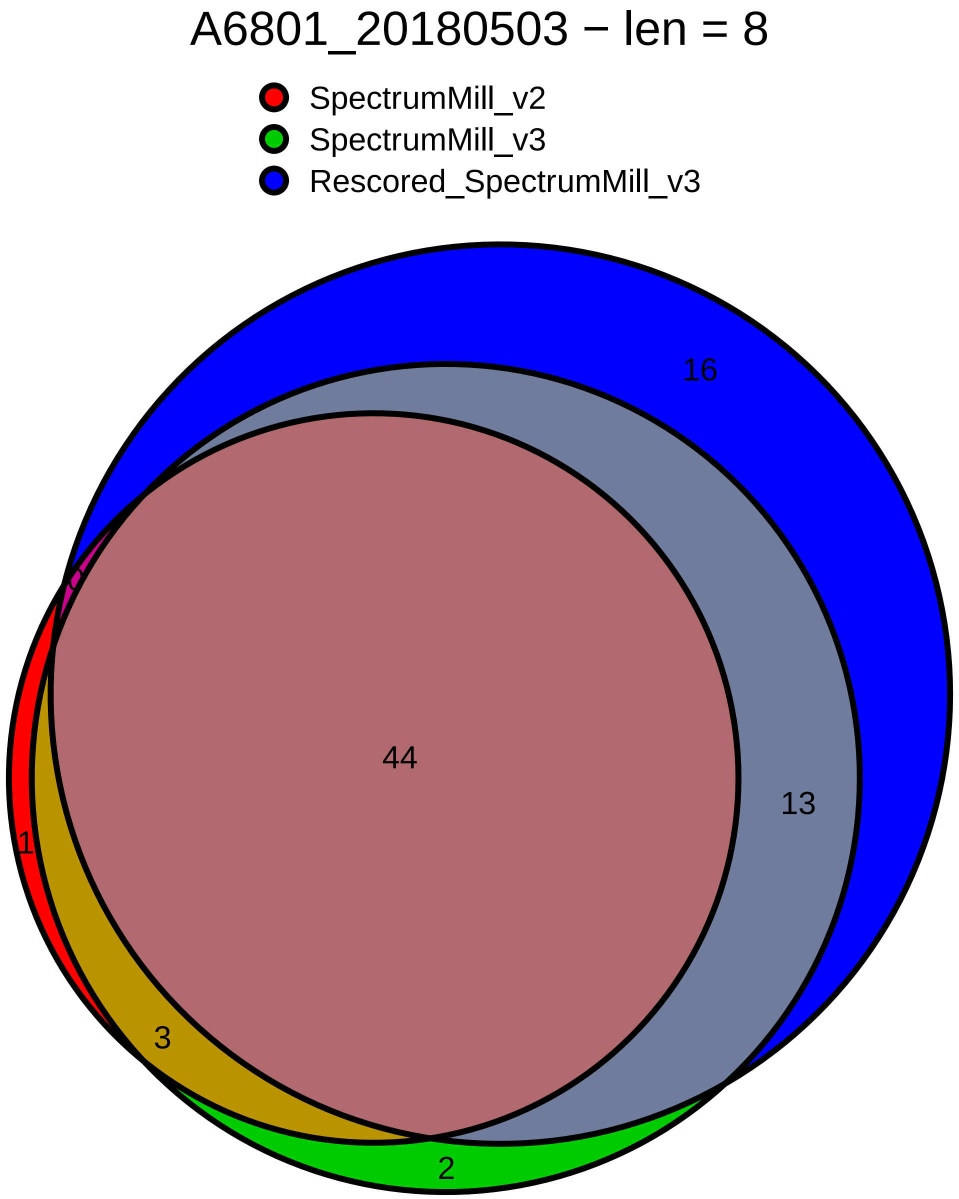
A6801_20180503 − len = 8
SpectrumMill_v2
SpectrumMill_v3
Rescored_SpectrumMill_v3
16
44
13
1
3
2

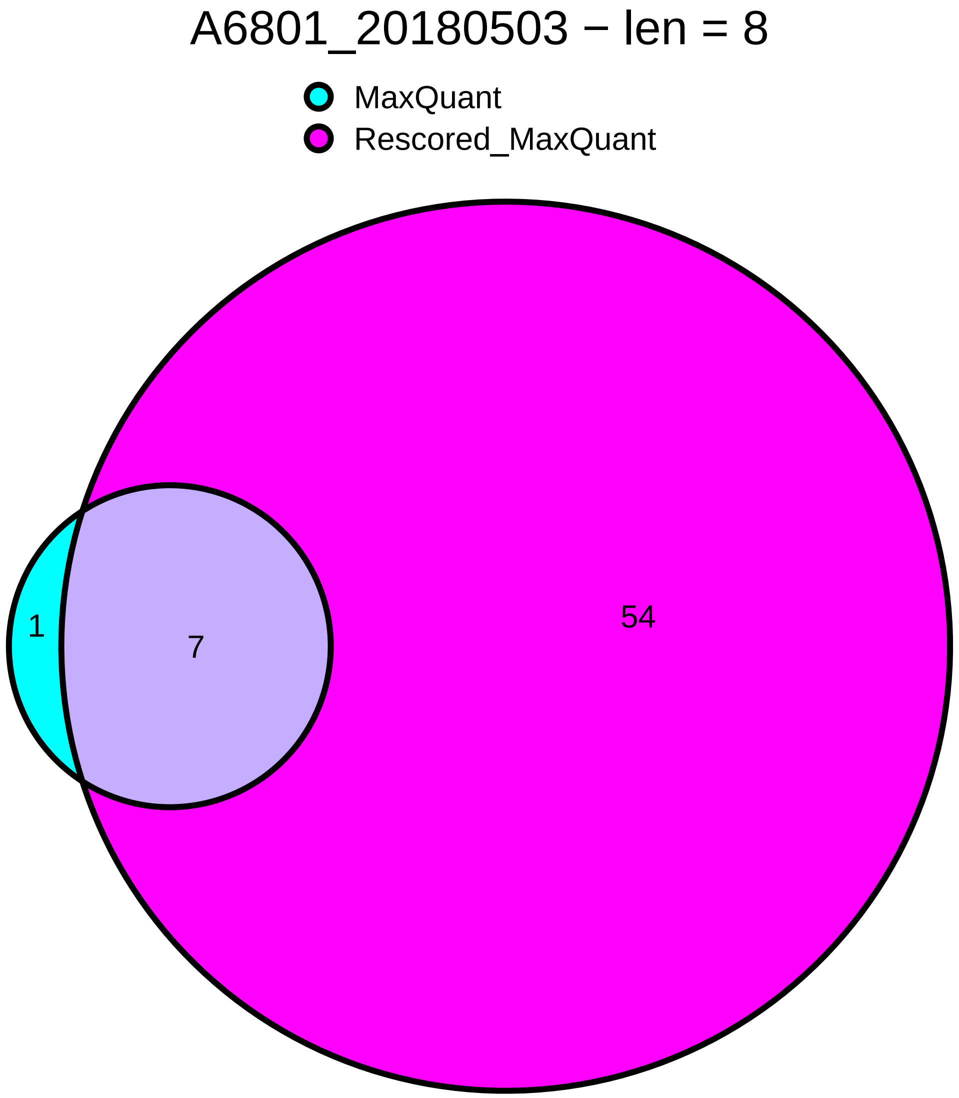
A6801_20180503 − len = 8
MaxQuant
Rescored_MaxQuant
1
7
54

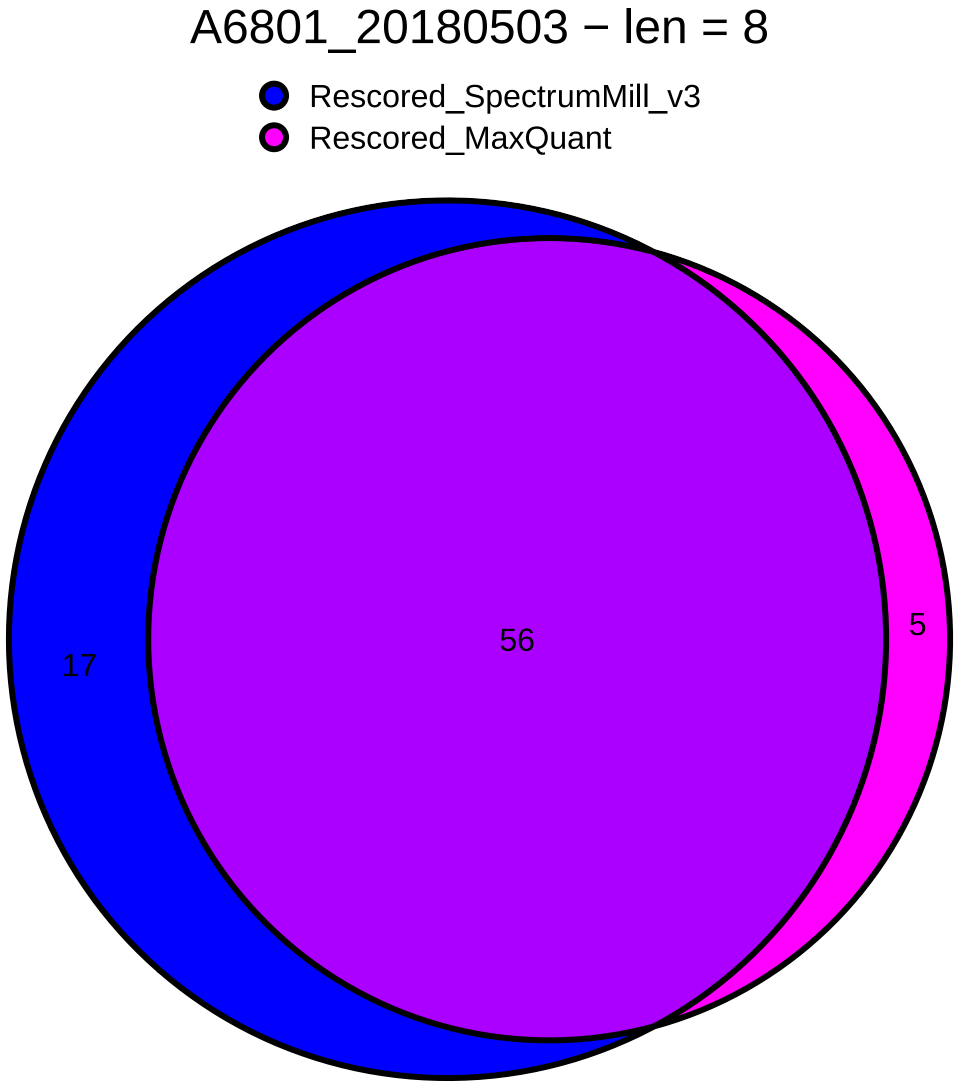
A6801_20180503 − len = 8
Rescored_SpectrumMill_v3
Rescored_MaxQuant
17
56
5

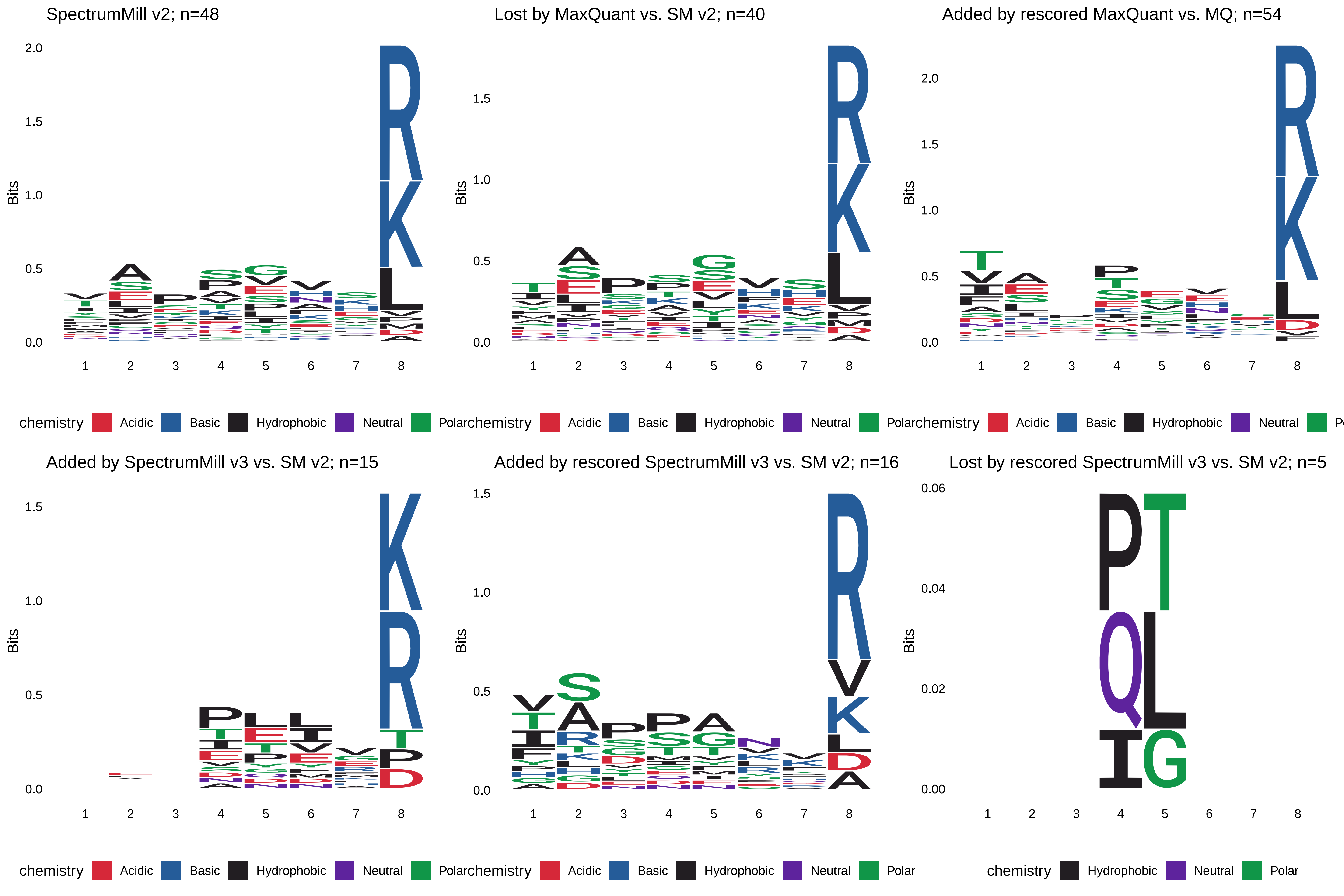
SpectrumMill v2; n=48
Lost by MaxQuant vs. SM v2; n=40
Added by rescored MaxQuant vs. MQ; n=54
Added by SpectrumMill v3 vs. SM v2; n=15
Added by rescored SpectrumMill v3 vs. SM v2; n=16
Lost by rescored SpectrumMill v3 vs. SM v2; n=5
Bits
1 2 3 4 5 6 7 8
chemistry Acidic Basic Hydrophobic Neutral Polar
chemistry Hydrophobic Neutral Polar

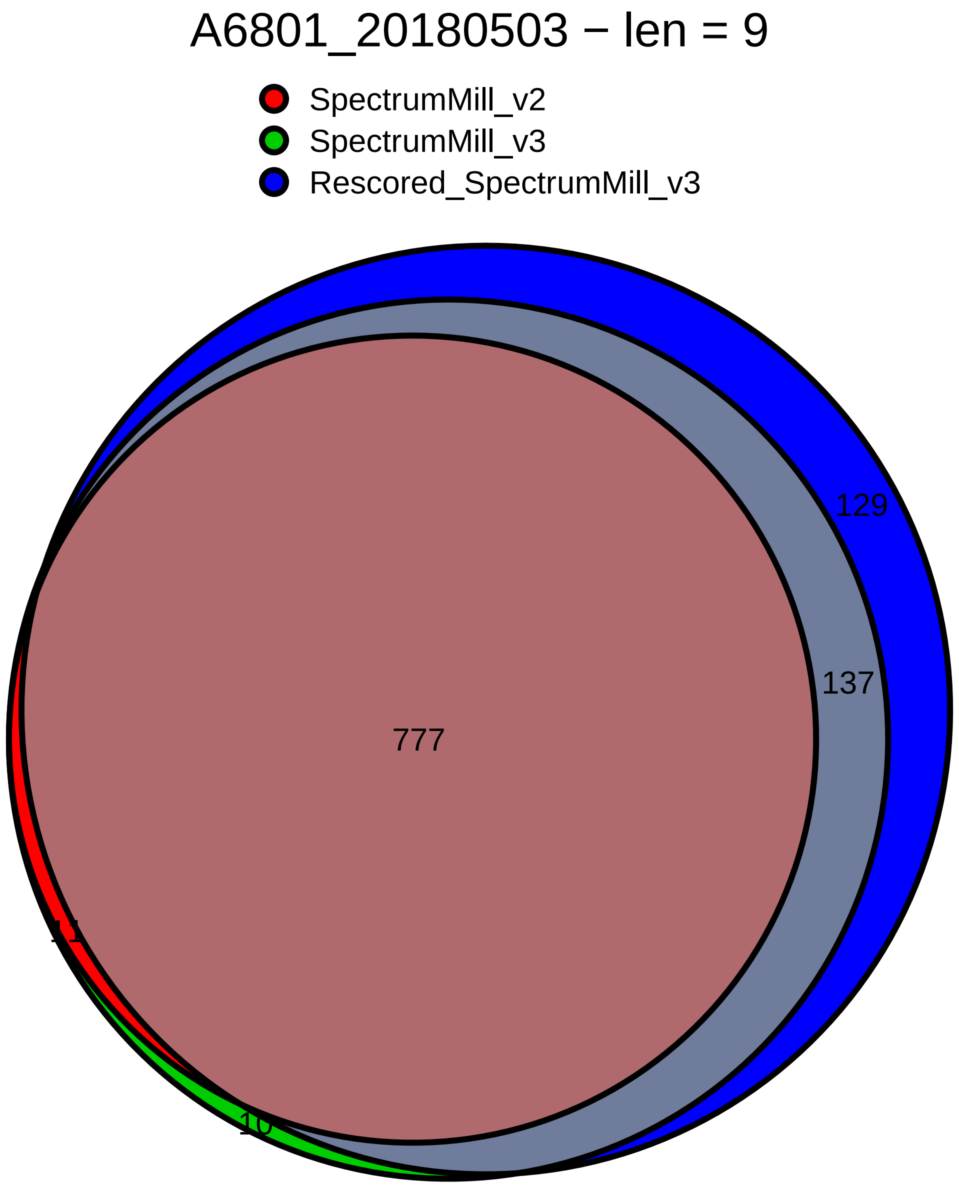
A6801_20180503 − len = 9
SpectrumMill_v2
SpectrumMill_v3
Rescored_SpectrumMill_v3
129
137
777
11
10

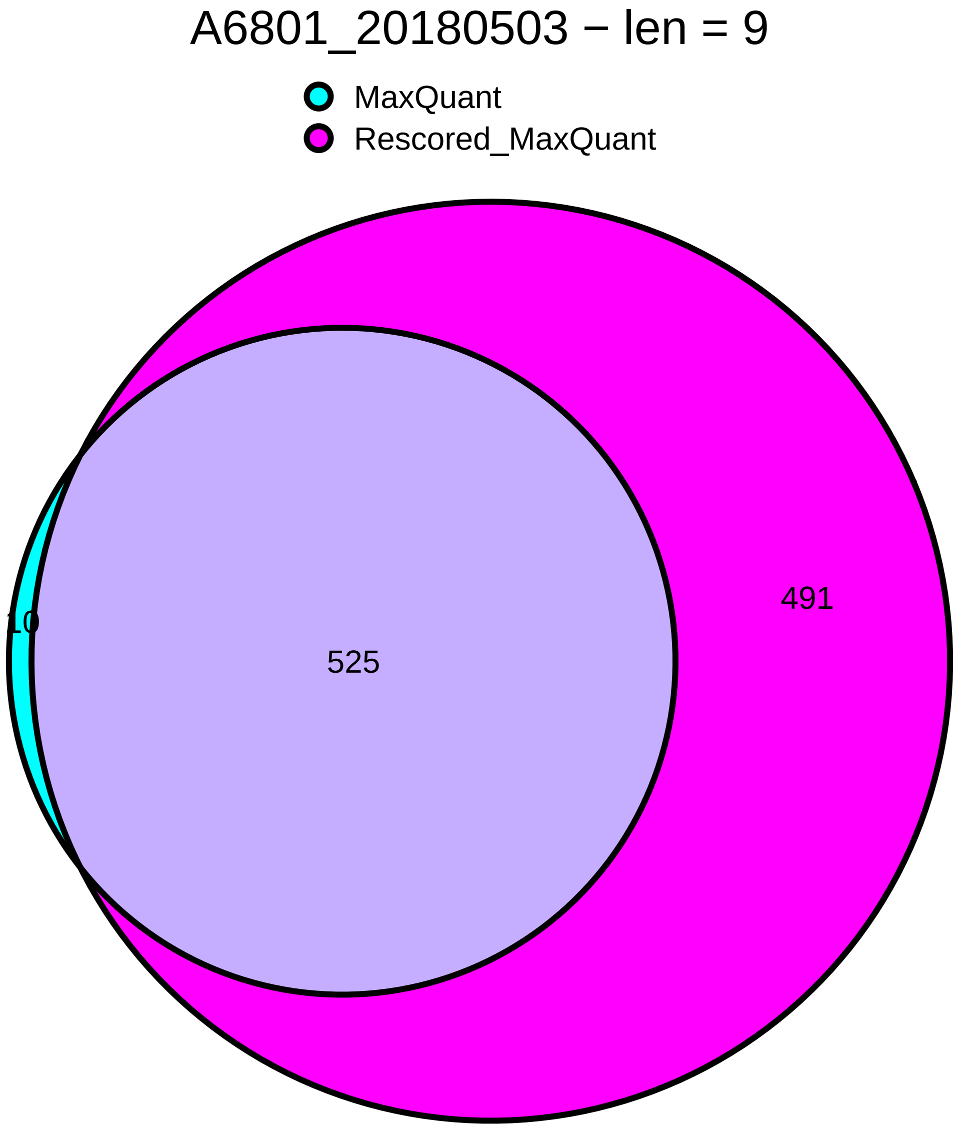
A6801_20180503 − len = 9
MaxQuant
Rescored_MaxQuant
10
525
491

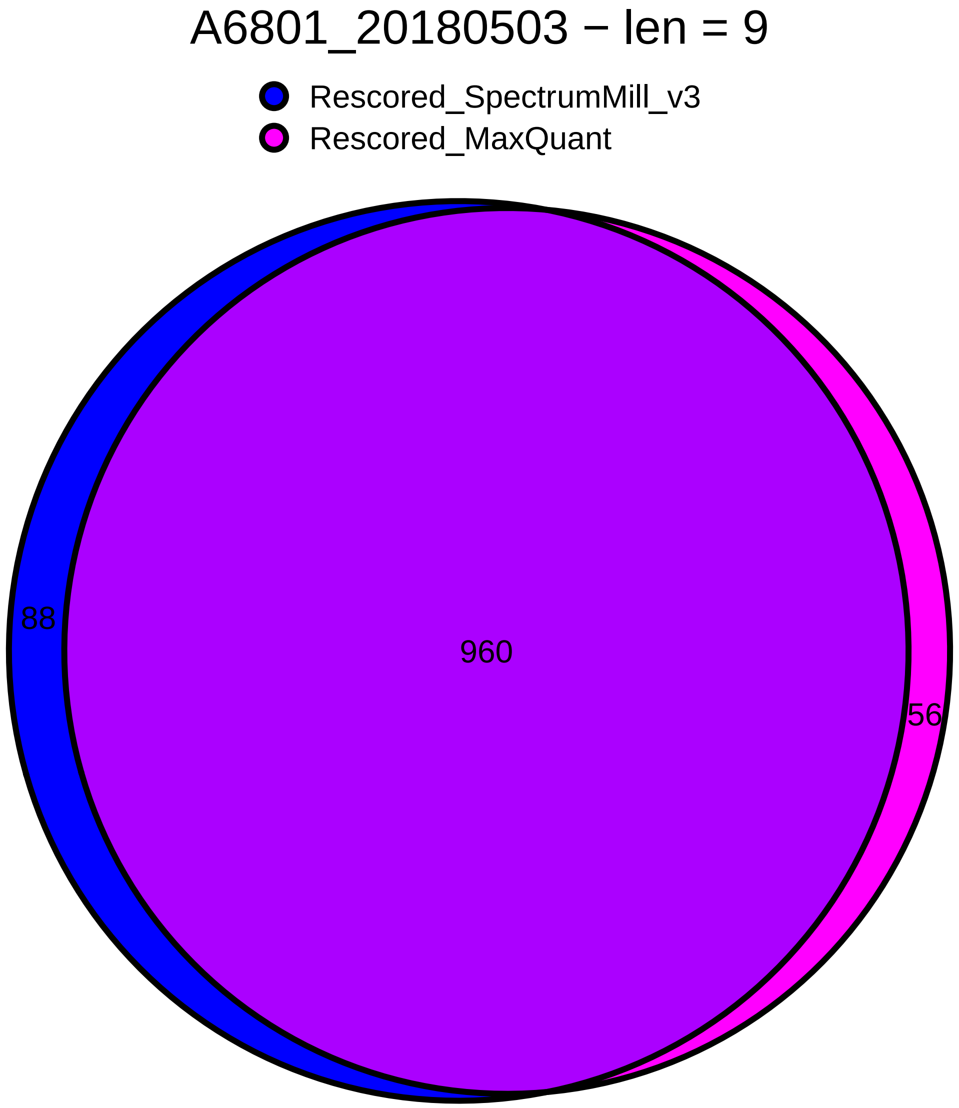
A6801_20180503 − len = 9
Rescored_SpectrumMill_v3
Rescored_MaxQuant
88
960
56

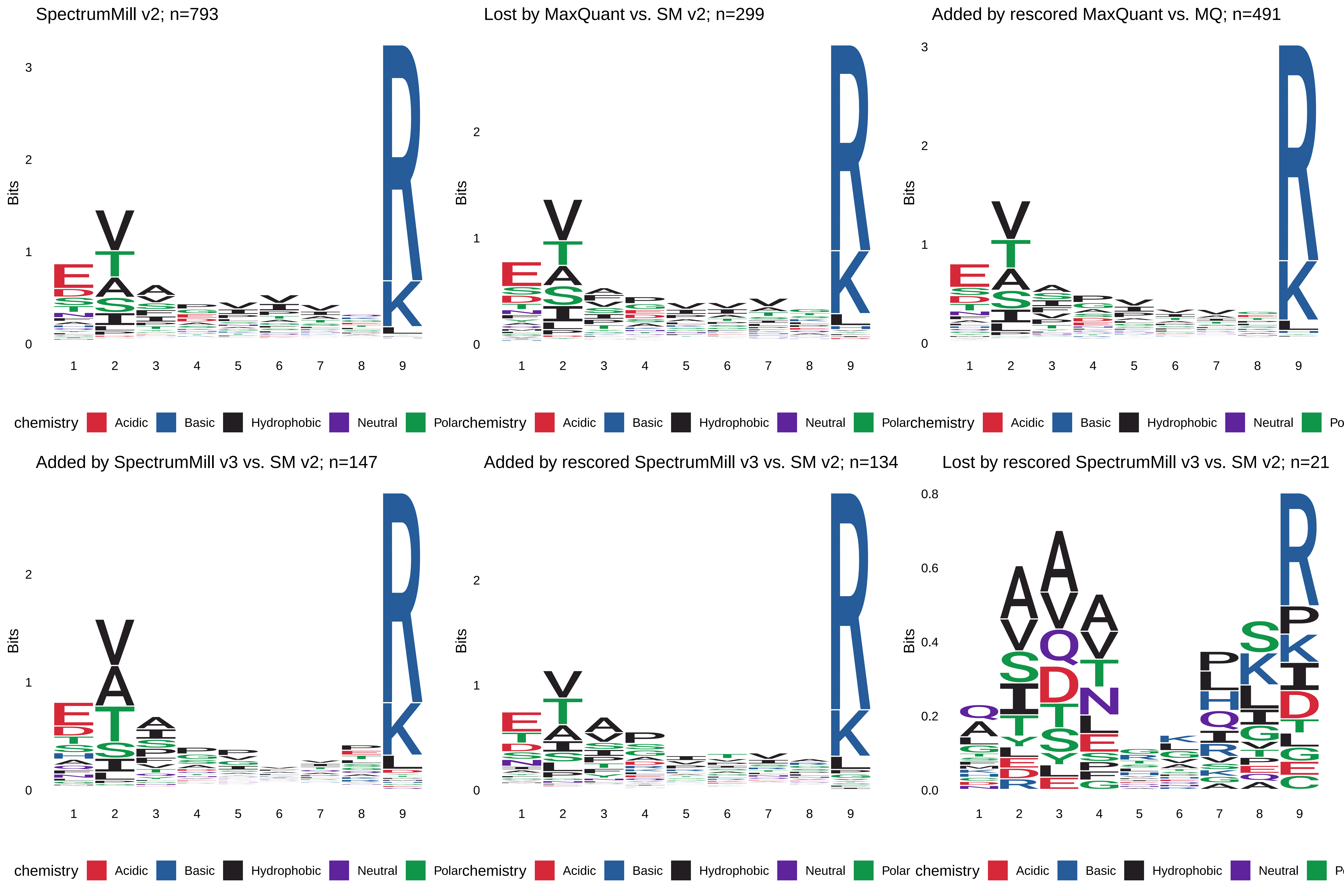

SpectrumMill v2; n=793
Lost by MaxQuant vs. SM v2; n=299
Added by rescored MaxQuant vs. MQ; n=491
Added by SpectrumMill v3 vs. SM v2; n=147
Added by rescored SpectrumMill v3 vs. SM v2; n=134
Lost by rescored SpectrumMill v3 vs. SM v2; n=21
Bits
chemistry Acidic Basic Hydrophobic Neutral Polar

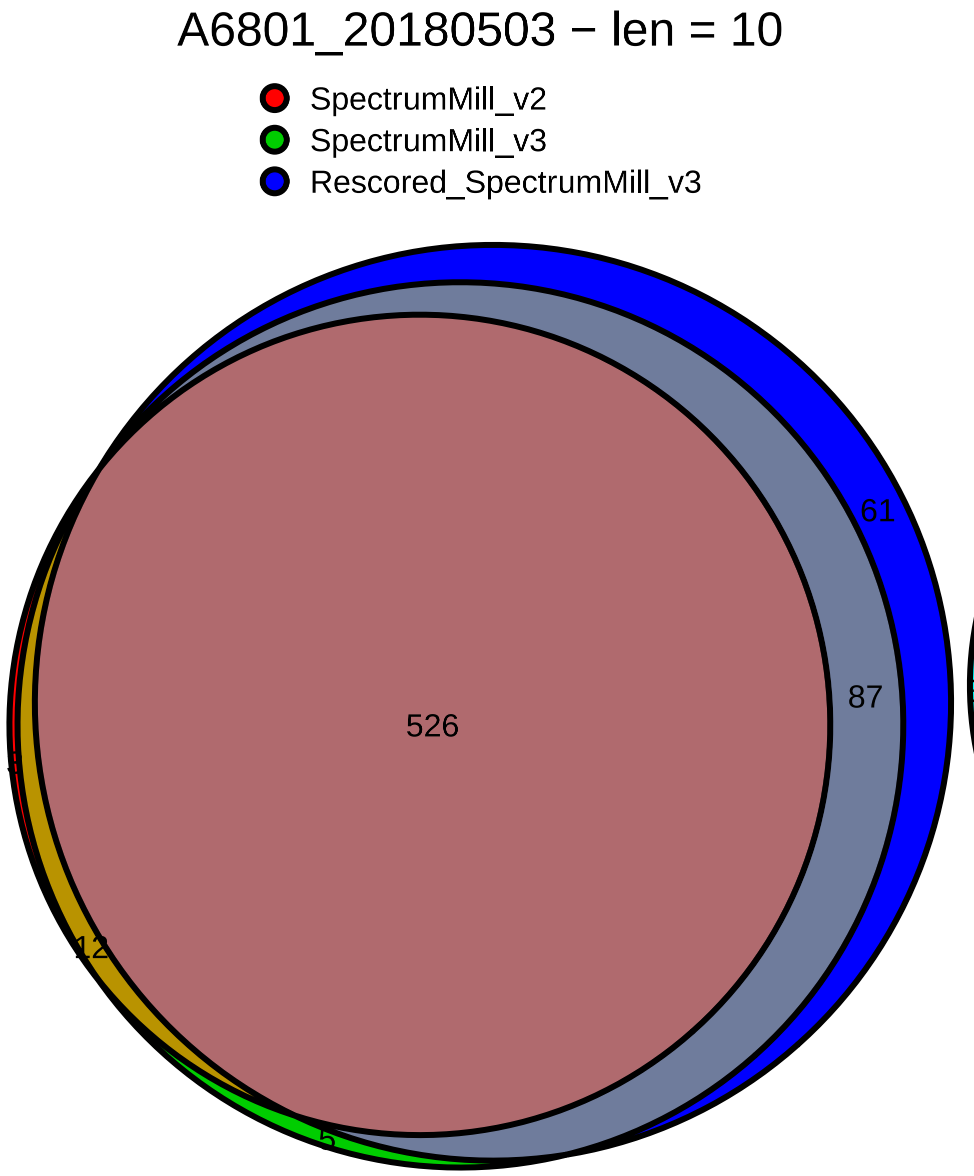
A6801_20180503 − len = 10
SpectrumMill_v2
SpectrumMill_v3
Rescored_SpectrumMill_v3
61
87
526
12
5

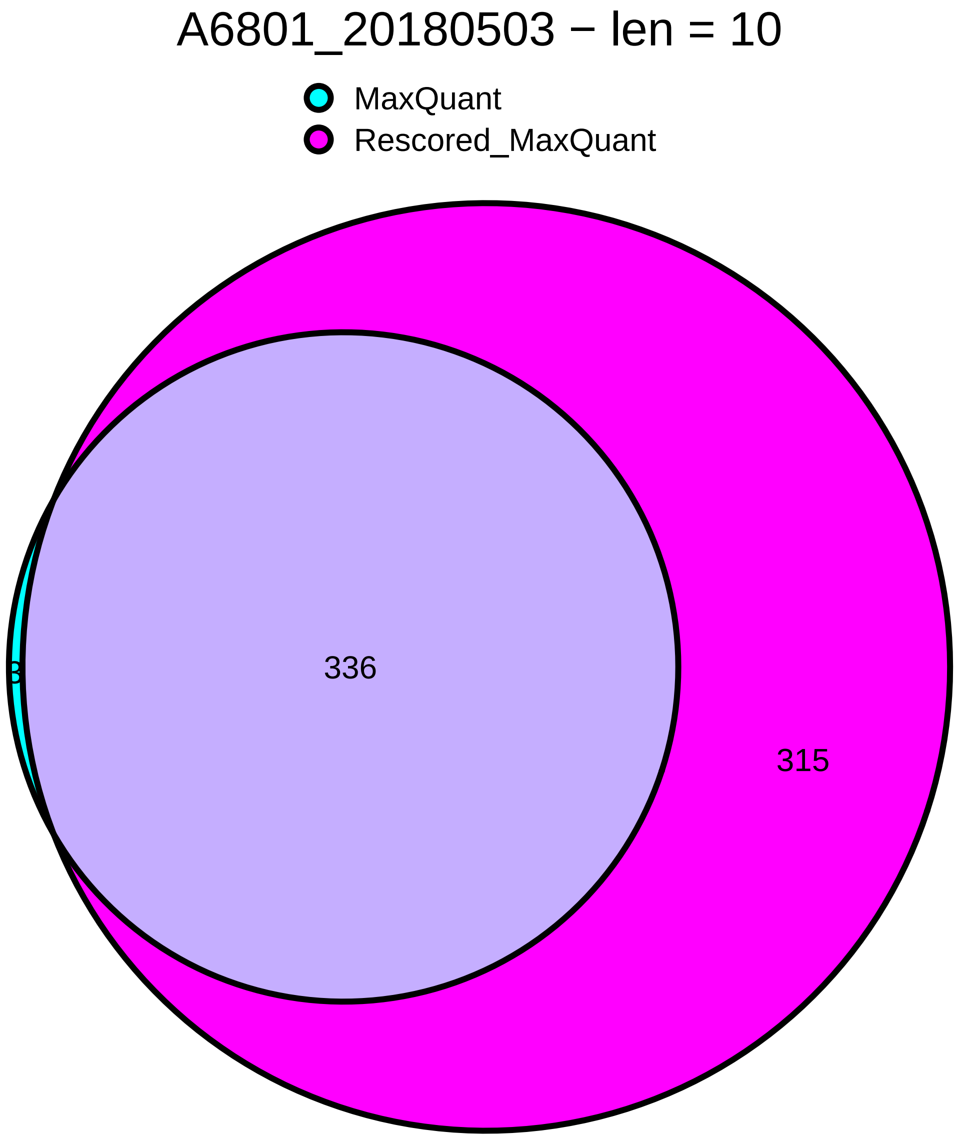
A6801_20180503 − len = 10
MaxQuant
Rescored_MaxQuant
336
315

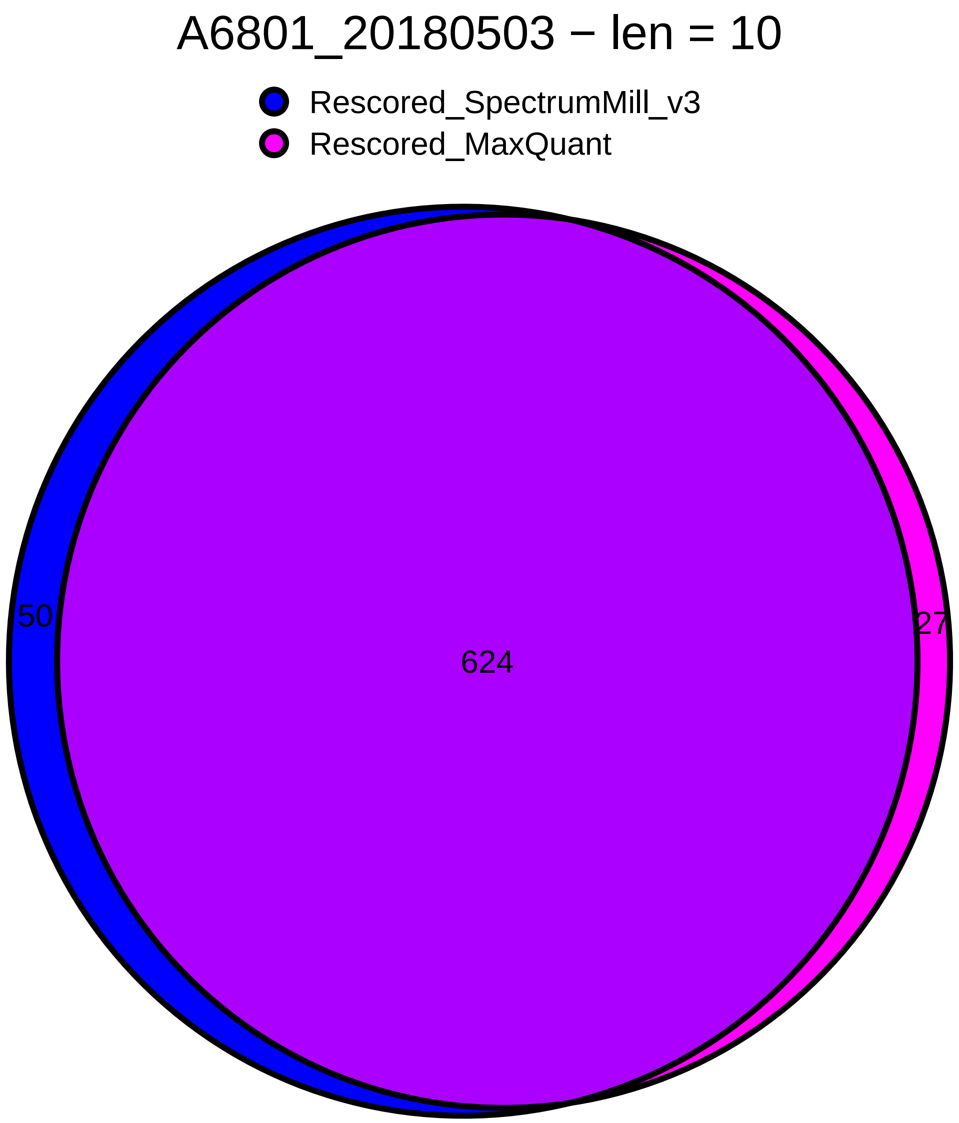
A6801_20180503 − len = 10
Rescored_SpectrumMill_v3
Rescored_MaxQuant
50
624
27

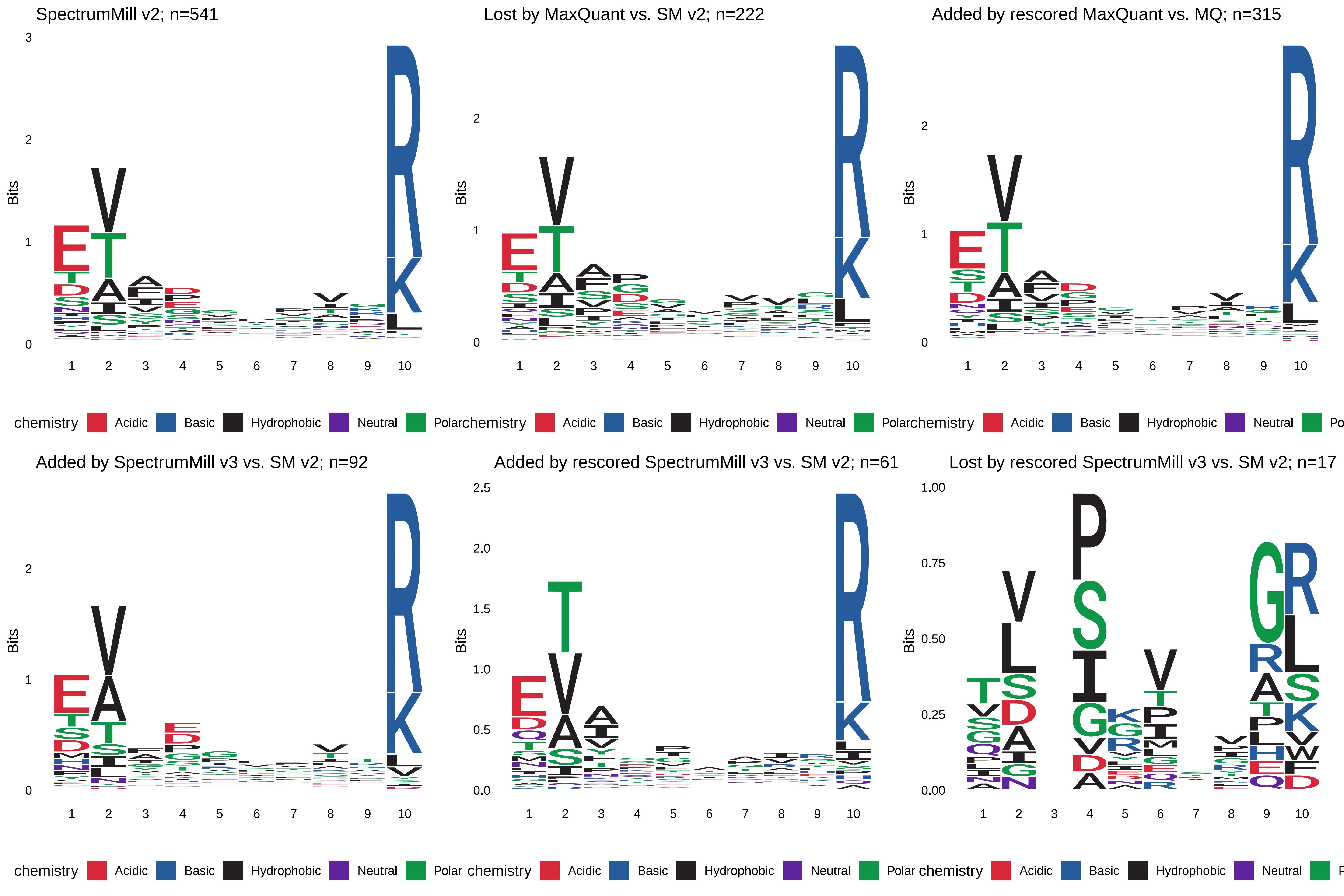
SpectrumMill v2; n=541
Lost by MaxQuant vs. SM v2; n=222
Added by rescored MaxQuant vs. MQ; n=315
Bits
1 2 3 4 5 6 7 8 9 10
chemistry Acidic Basic Hydrophobic Neutral Polarchemistry Acidic Basic Hydrophobic Neutral Polarchemistry Acidic Basic Hydrophobic Neutral Po
Added by SpectrumMill v3 vs. SM v2; n=92
Added by rescored SpectrumMill v3 vs. SM v2; n=61
Lost by rescored SpectrumMill v3 vs. SM v2; n=17
Bits
1 2 3 4 5 6 7 8 9 10
chemistry Acidic Basic Hydrophobic Neutral Polar chemistry Acidic Basic Hydrophobic Neutral Polar chemistry Acidic Basic Hydrophobic Neutral P

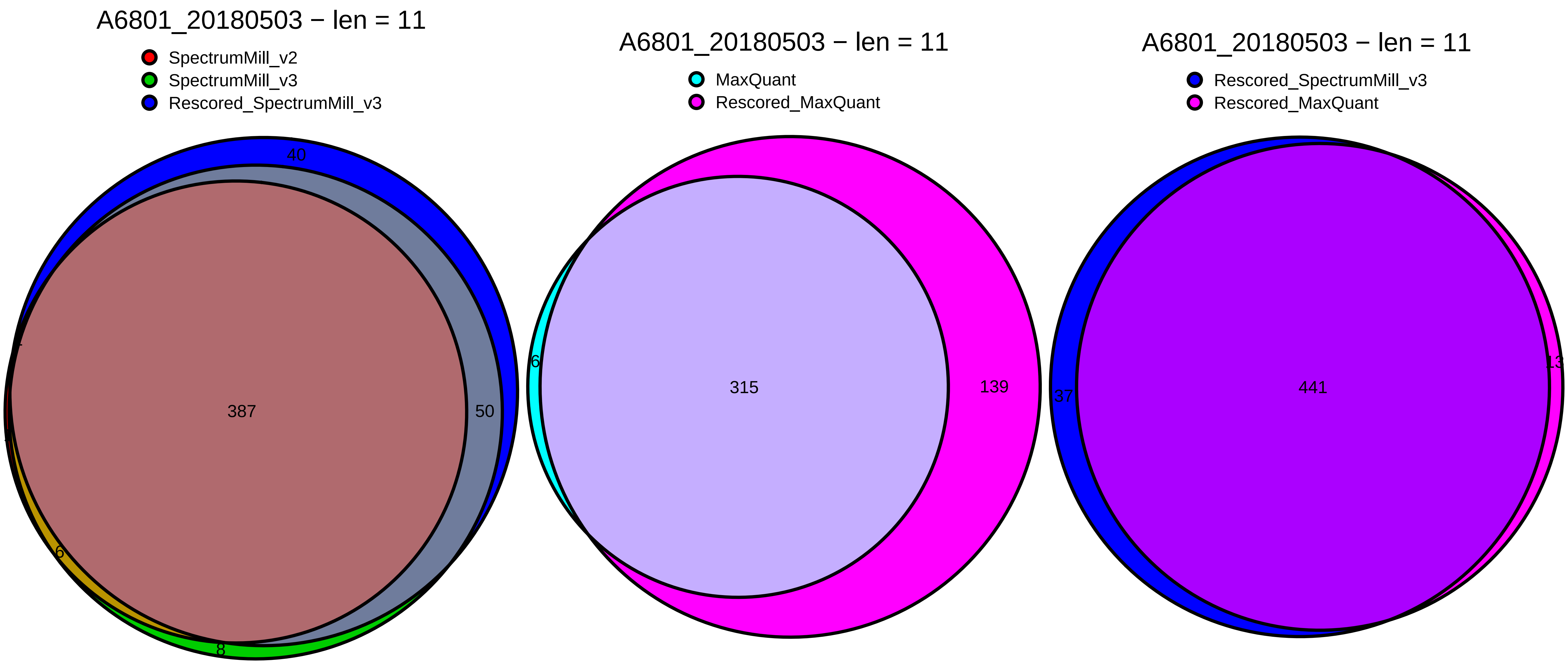
A6801_20180503 − len = 11
SpectrumMill_v2
SpectrumMill_v3
Rescored_SpectrumMill_v3
40
387
50
6
8
A6801_20180503 − len = 11
MaxQuant
Rescored_MaxQuant
6
315
139
A6801_20180503 − len = 11
Rescored_SpectrumMill_v3
Rescored_MaxQuant
37
441

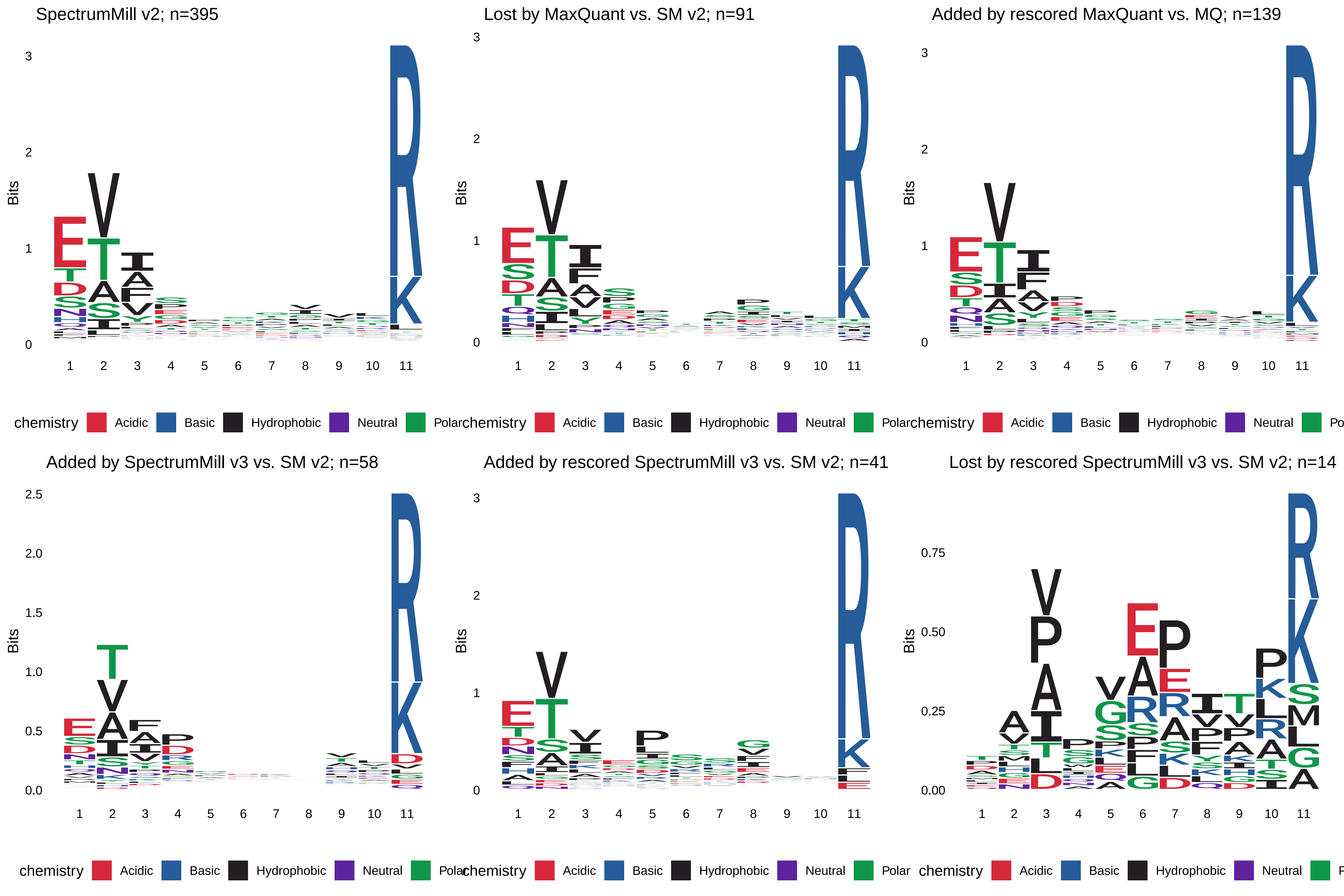
SpectrumMill v2; n=395
Lost by MaxQuant vs. SM v2; n=91
Added by rescored MaxQuant vs. MQ; n=139
Added by SpectrumMill v3 vs. SM v2; n=58
Added by rescored SpectrumMill v3 vs. SM v2; n=41
Lost by rescored SpectrumMill v3 vs. SM v2; n=14
Bits
1 2 3 4 5 6 7 8 9 10 11
chemistry
Acidic
Basic
Hydrophobic
Neutral
Polar

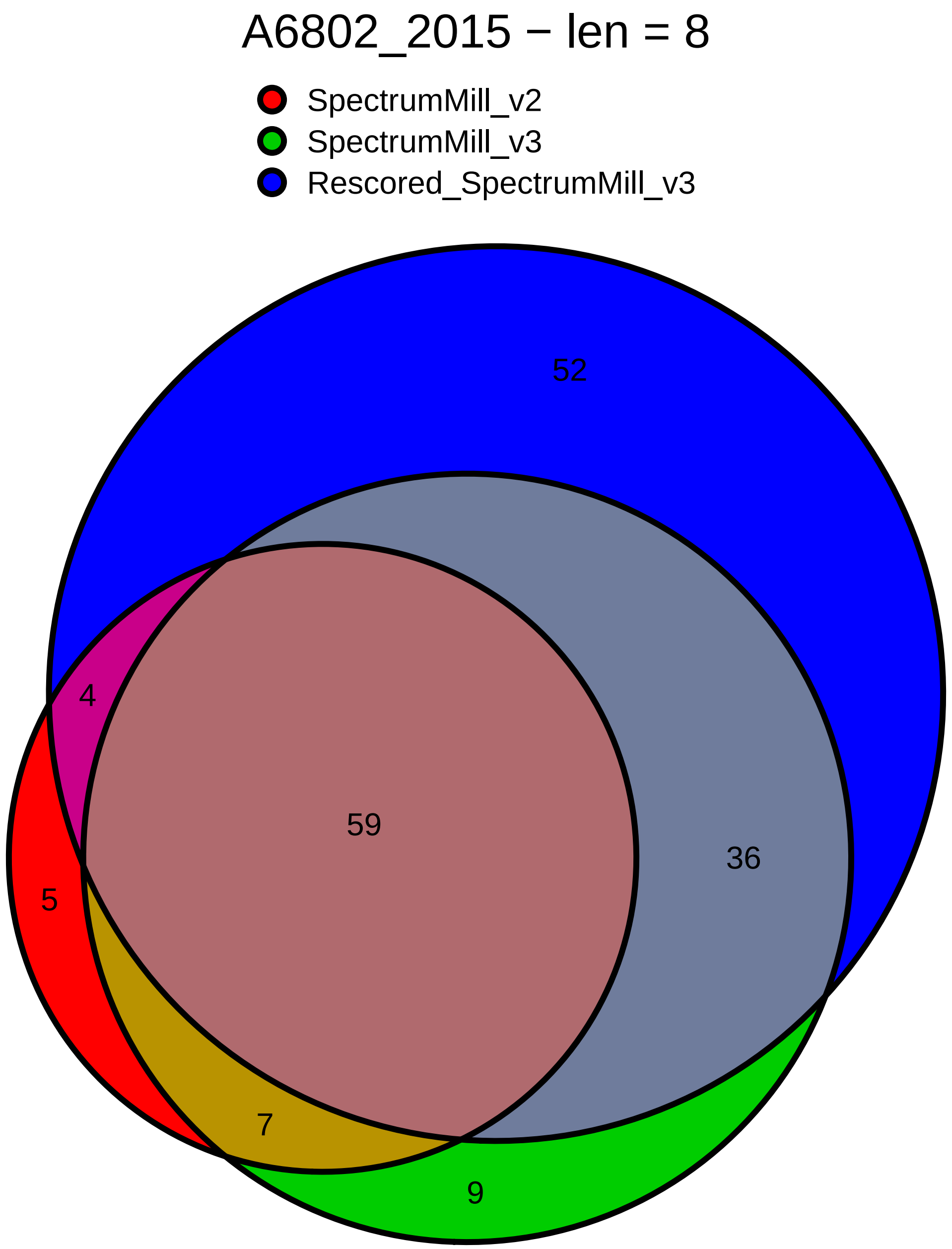
A6802_2015 − len = 8
SpectrumMill_v2
SpectrumMill_v3
Rescored_SpectrumMill_v3
52
4
59
36
5
7
9

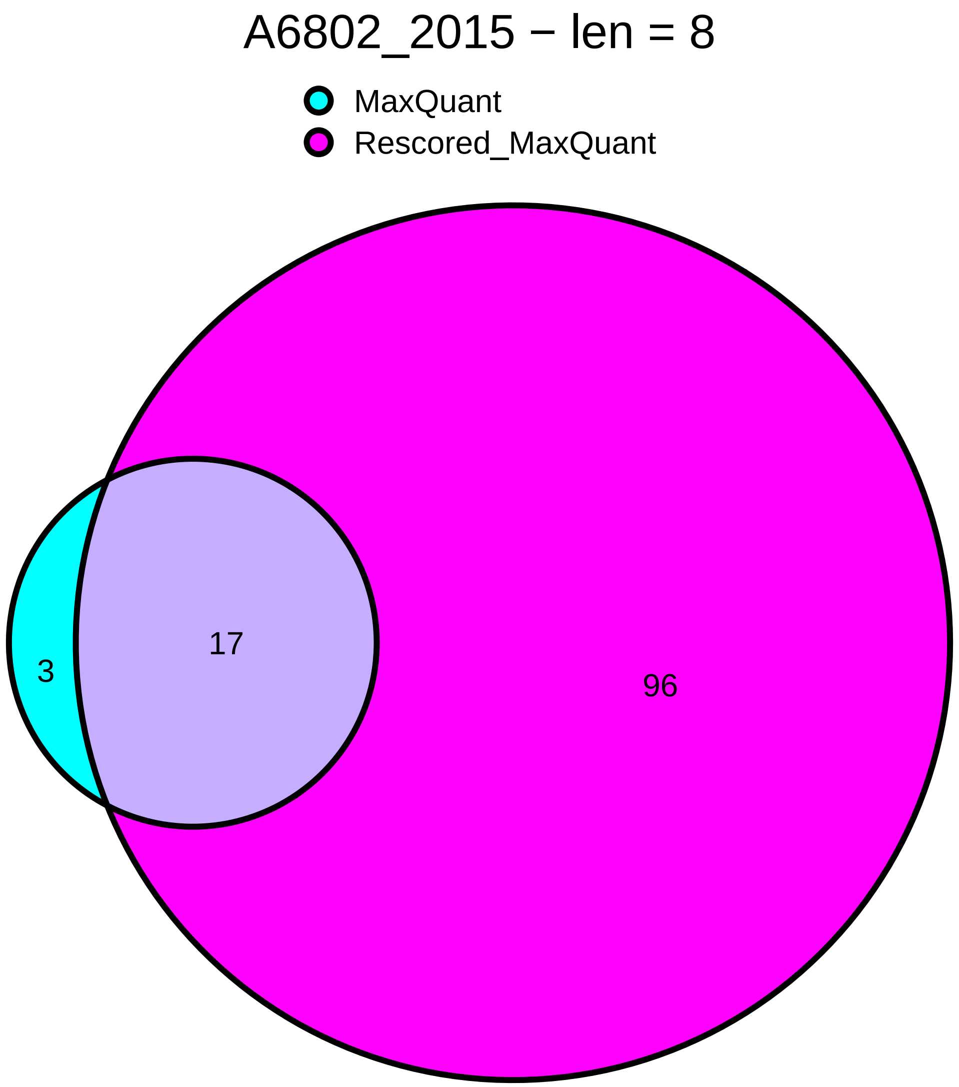
A6802_2015 − len = 8
MaxQuant
Rescored_MaxQuant
3
17
96

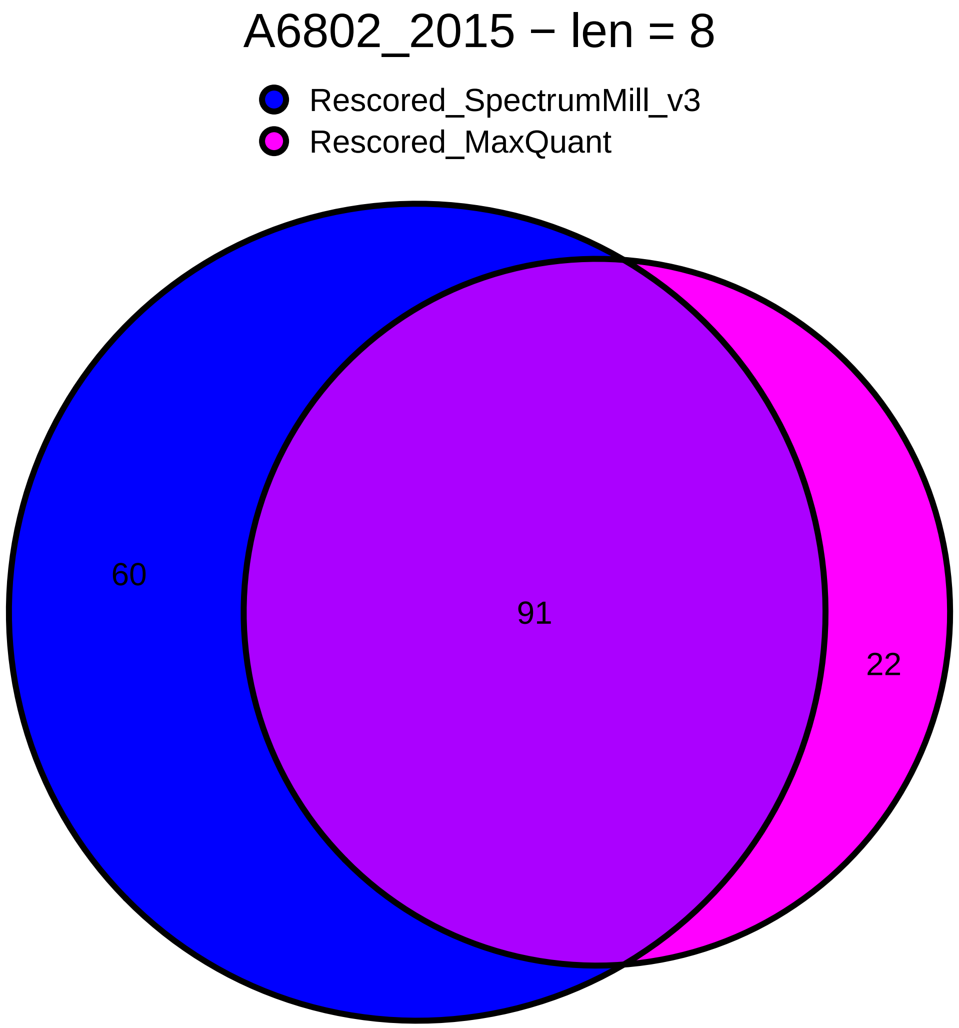
A6802_2015 − len = 8
Rescored_SpectrumMill_v3
Rescored_MaxQuant
60
91
22

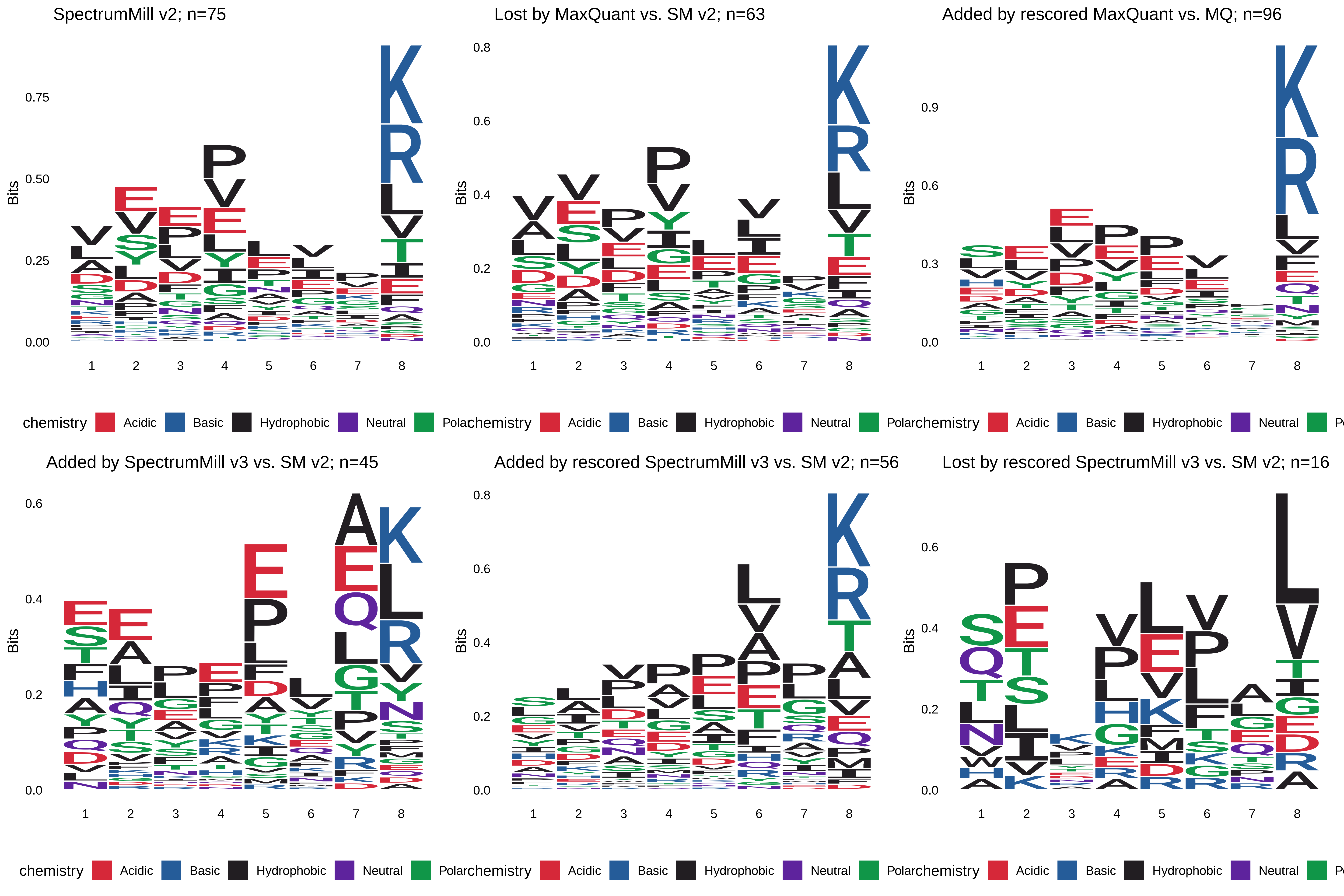

SpectrumMill v2; n=75
Lost by MaxQuant vs. SM v2; n=63
Added by rescored MaxQuant vs. MQ; n=96
Added by SpectrumMill v3 vs. SM v2; n=45
Added by rescored SpectrumMill v3 vs. SM v2; n=56
Lost by rescored SpectrumMill v3 vs. SM v2; n=16
Bits
1 2 3 4 5 6 7 8
chemistry
Acidic
Basic
Hydrophobic
Neutral
Polar

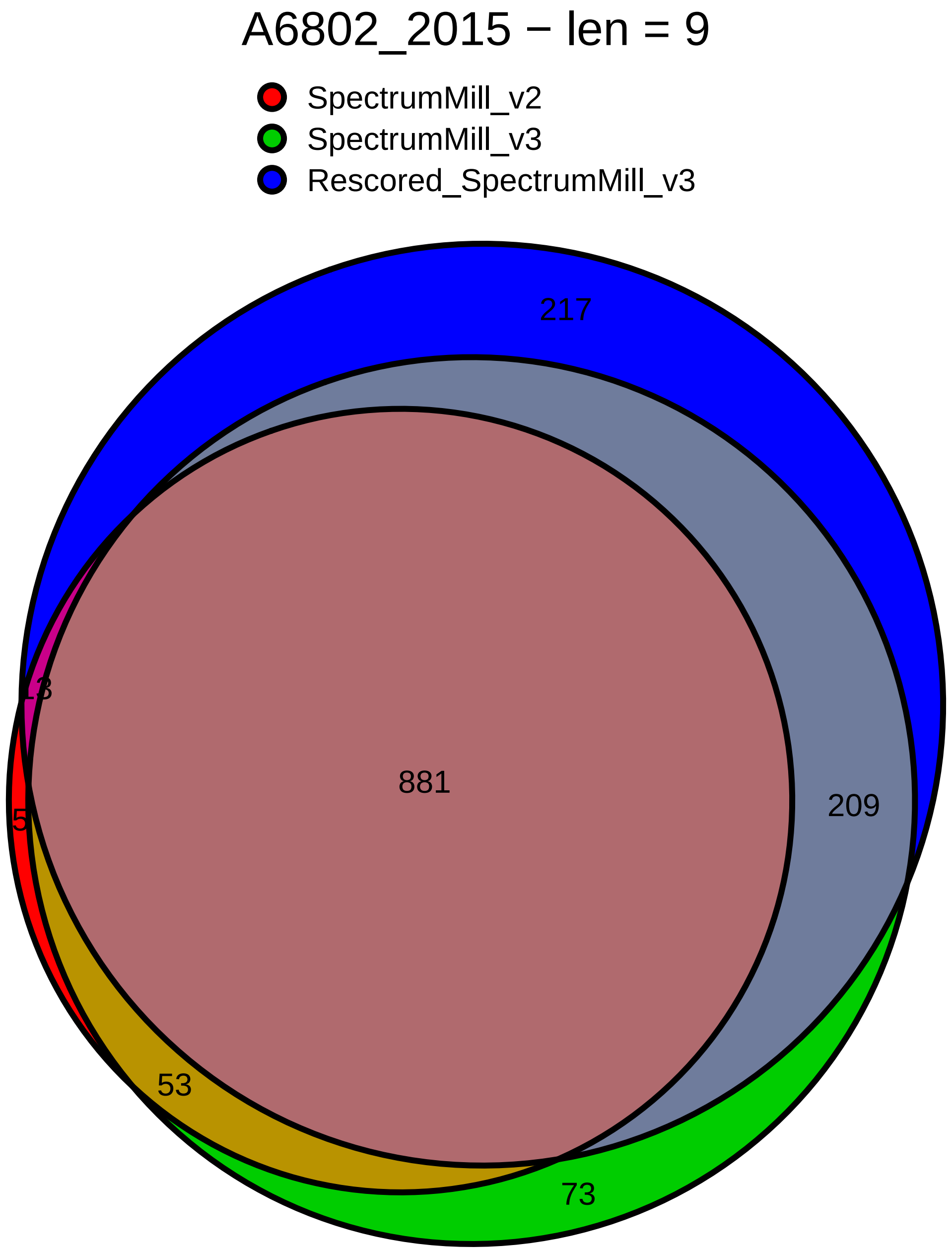
A6802_2015 − len = 9
SpectrumMill_v2
SpectrumMill_v3
Rescored_SpectrumMill_v3
217
13
881
209
5
53
73

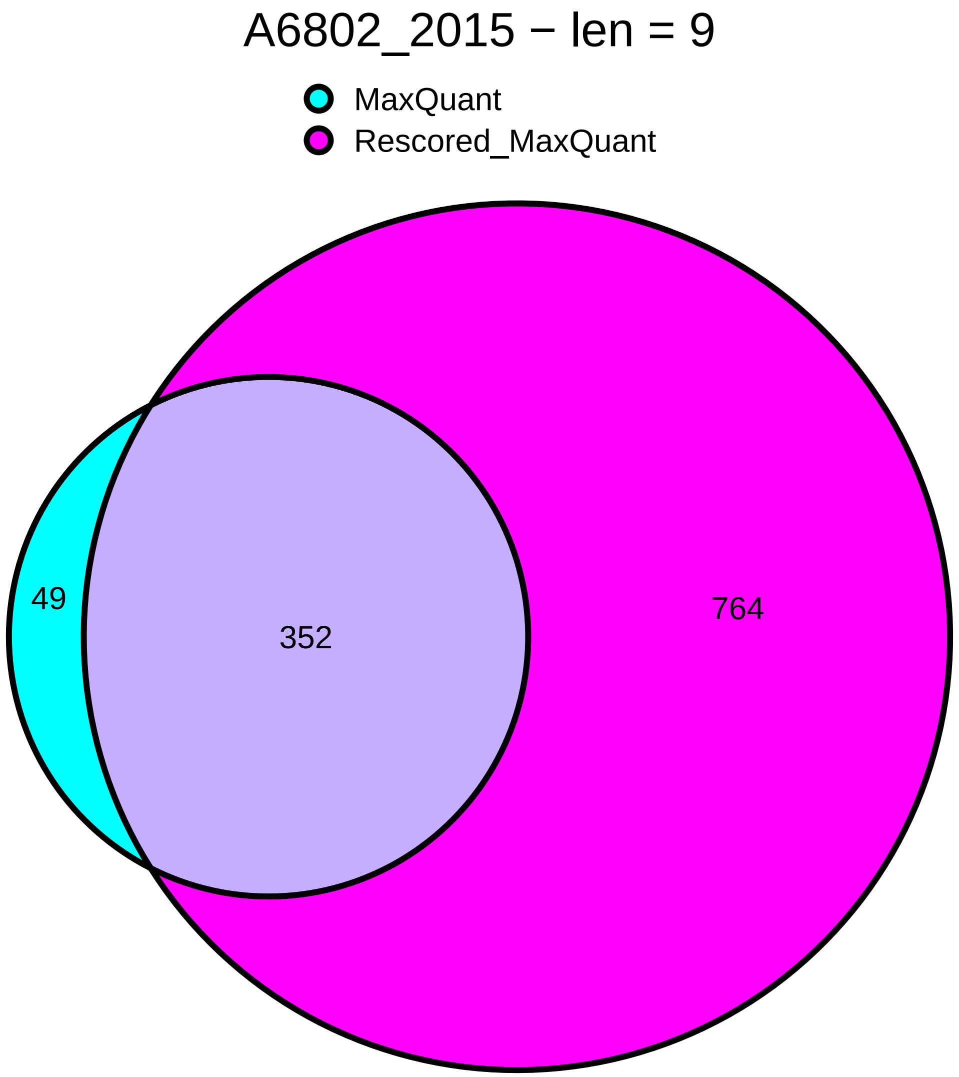
A6802_2015 − len = 9
MaxQuant
Rescored_MaxQuant
49
352
764

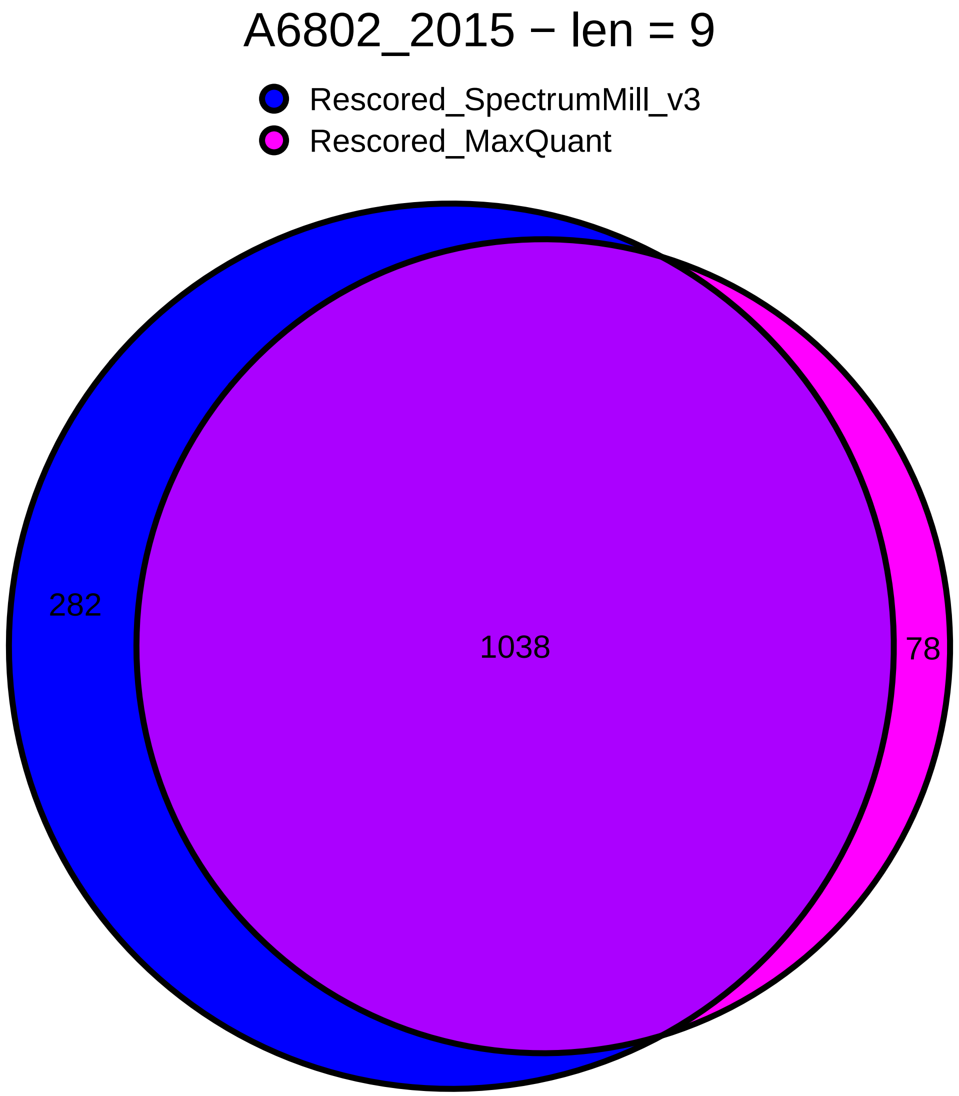
A6802_2015 − len = 9
Rescored_SpectrumMill_v3
Rescored_MaxQuant
282
1038
78

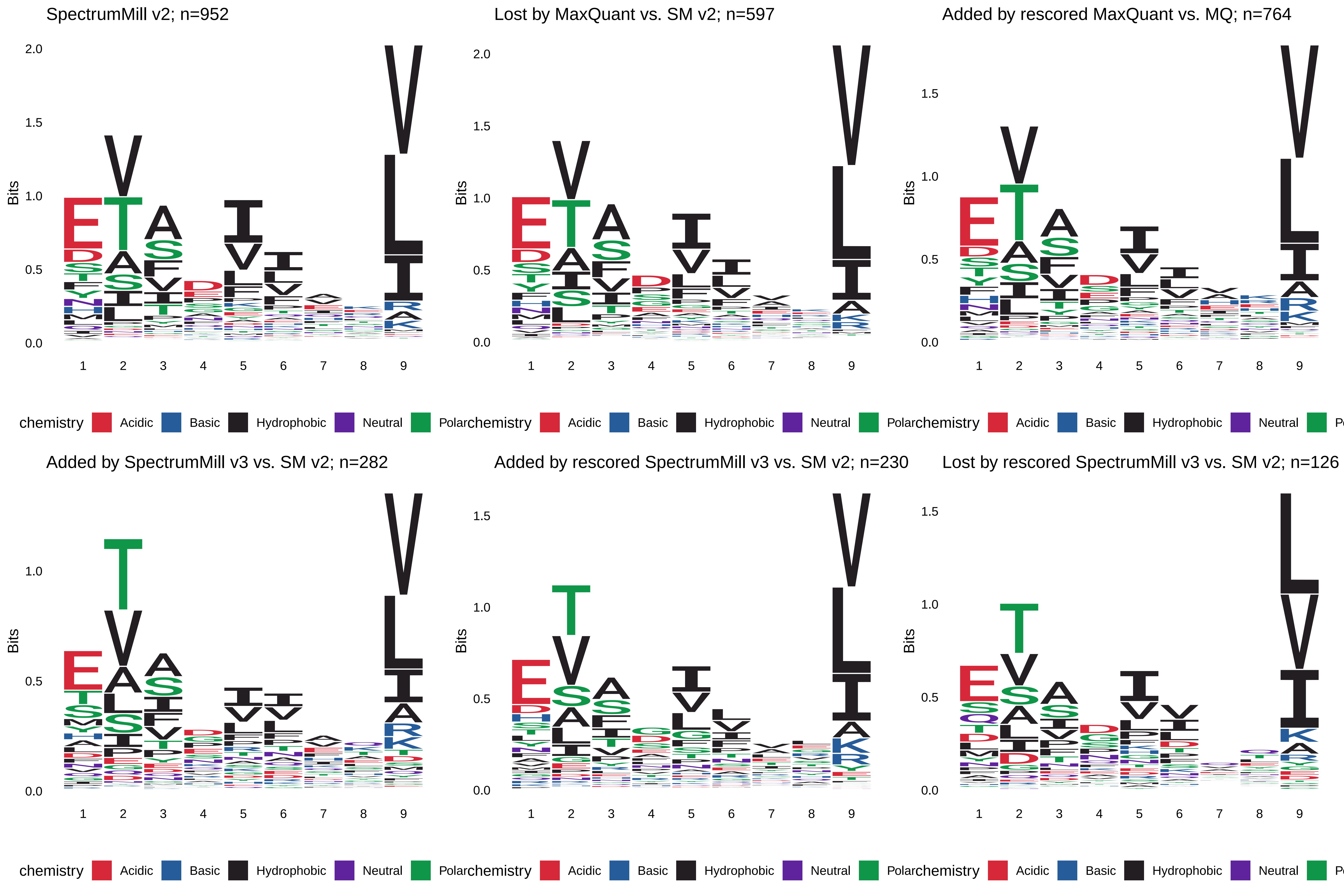

SpectrumMill v2; n=952
Lost by MaxQuant vs. SM v2; n=597
Added by rescored MaxQuant vs. MQ; n=764
Added by SpectrumMill v3 vs. SM v2; n=282
Added by rescored SpectrumMill v3 vs. SM v2; n=230
Lost by rescored SpectrumMill v3 vs. SM v2; n=126
Bits
1 2 3 4 5 6 7 8 9
chemistry
Acidic
Basic
Hydrophobic
Neutral
Polar

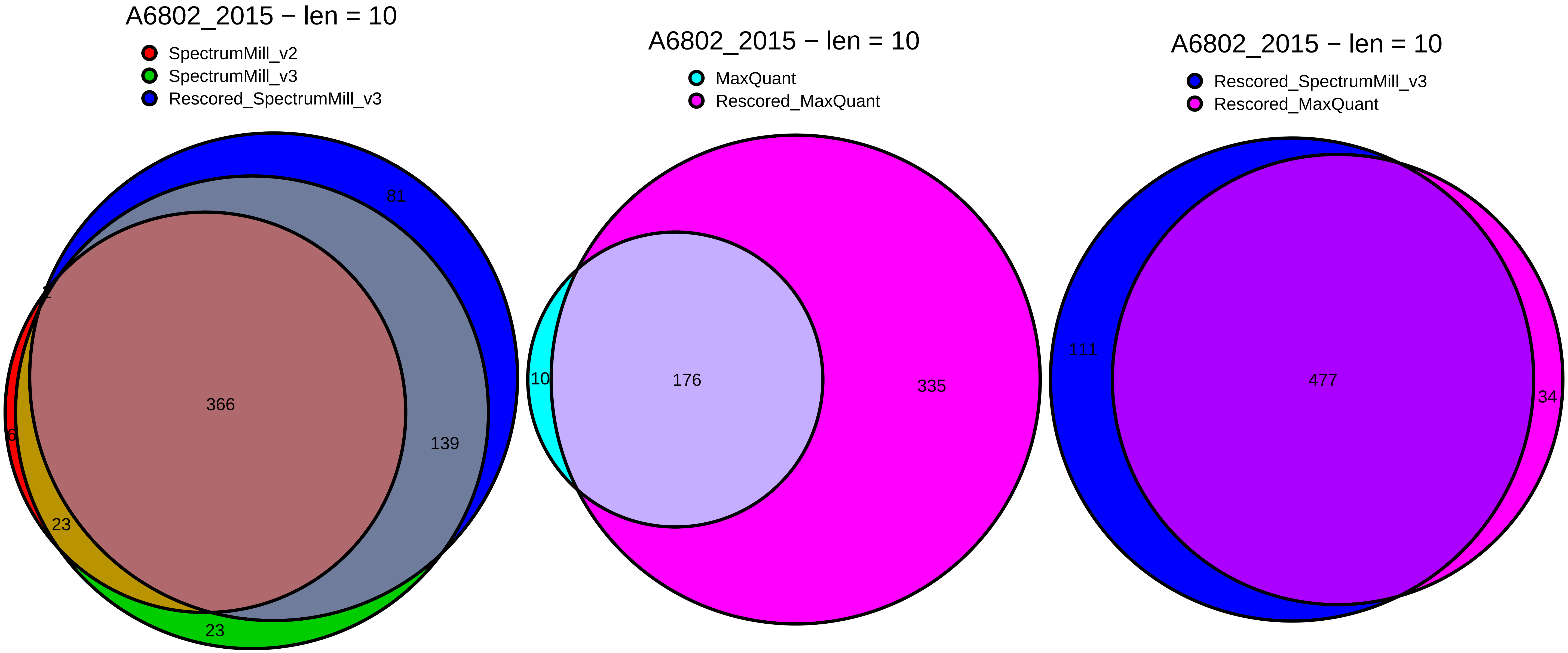
A6802_2015 − len = 10
SpectrumMill_v2
SpectrumMill_v3
Rescored_SpectrumMill_v3
81
1
6
366
139
23
23
A6802_2015 − len = 10
MaxQuant
Rescored_MaxQuant
10
176
335
A6802_2015 − len = 10
Rescored_SpectrumMill_v3
Rescored_MaxQuant
111
477
34

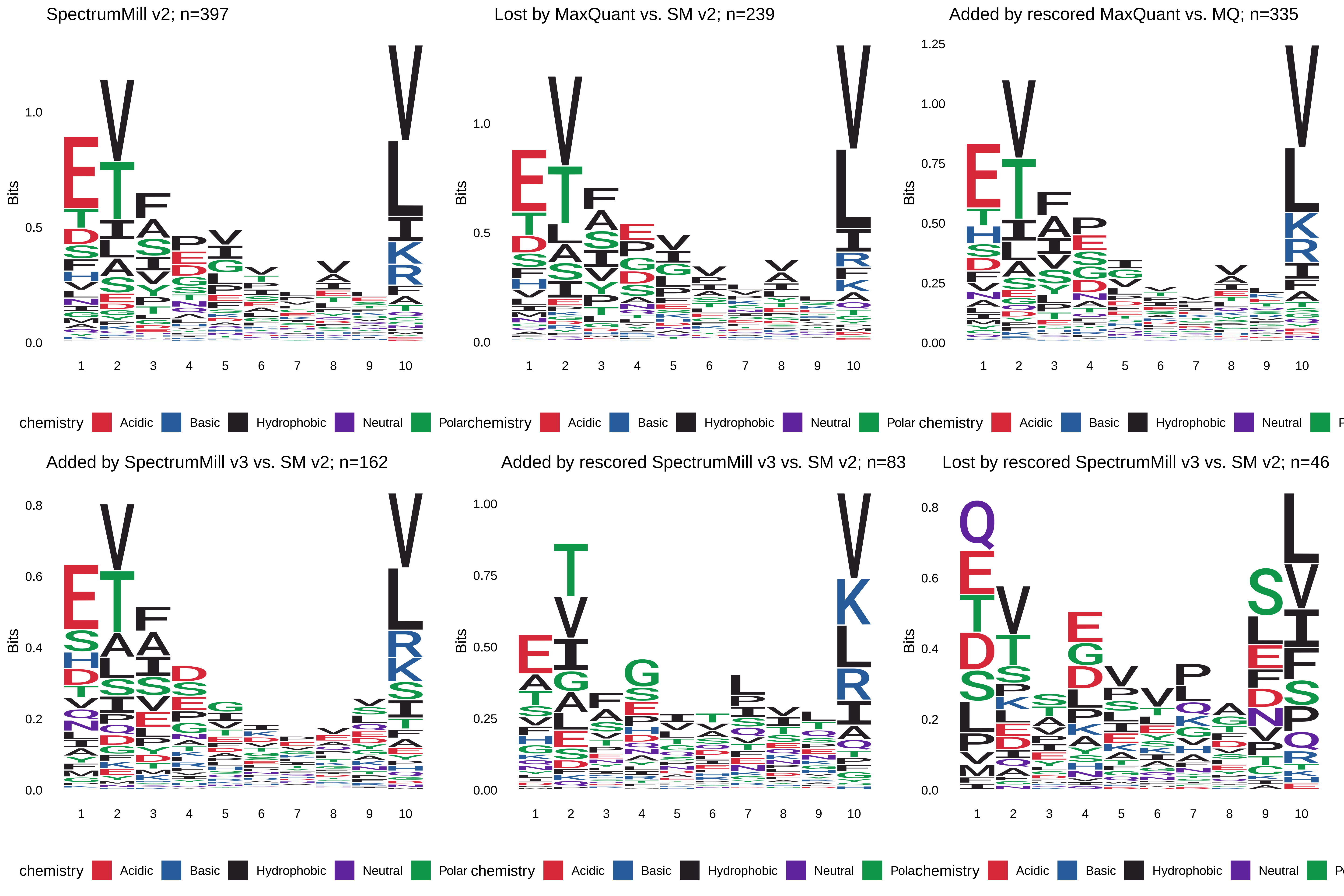
SpectrumMill v2; n=397
Lost by MaxQuant vs. SM v2; n=239
Added by rescored MaxQuant vs. MQ; n=335
Added by SpectrumMill v3 vs. SM v2; n=162
Added by rescored SpectrumMill v3 vs. SM v2; n=83
Lost by rescored SpectrumMill v3 vs. SM v2; n=46
Bits
1 2 3 4 5 6 7 8 9 10
chemistry
Acidic
Basic
Hydrophobic
Neutral
Polar

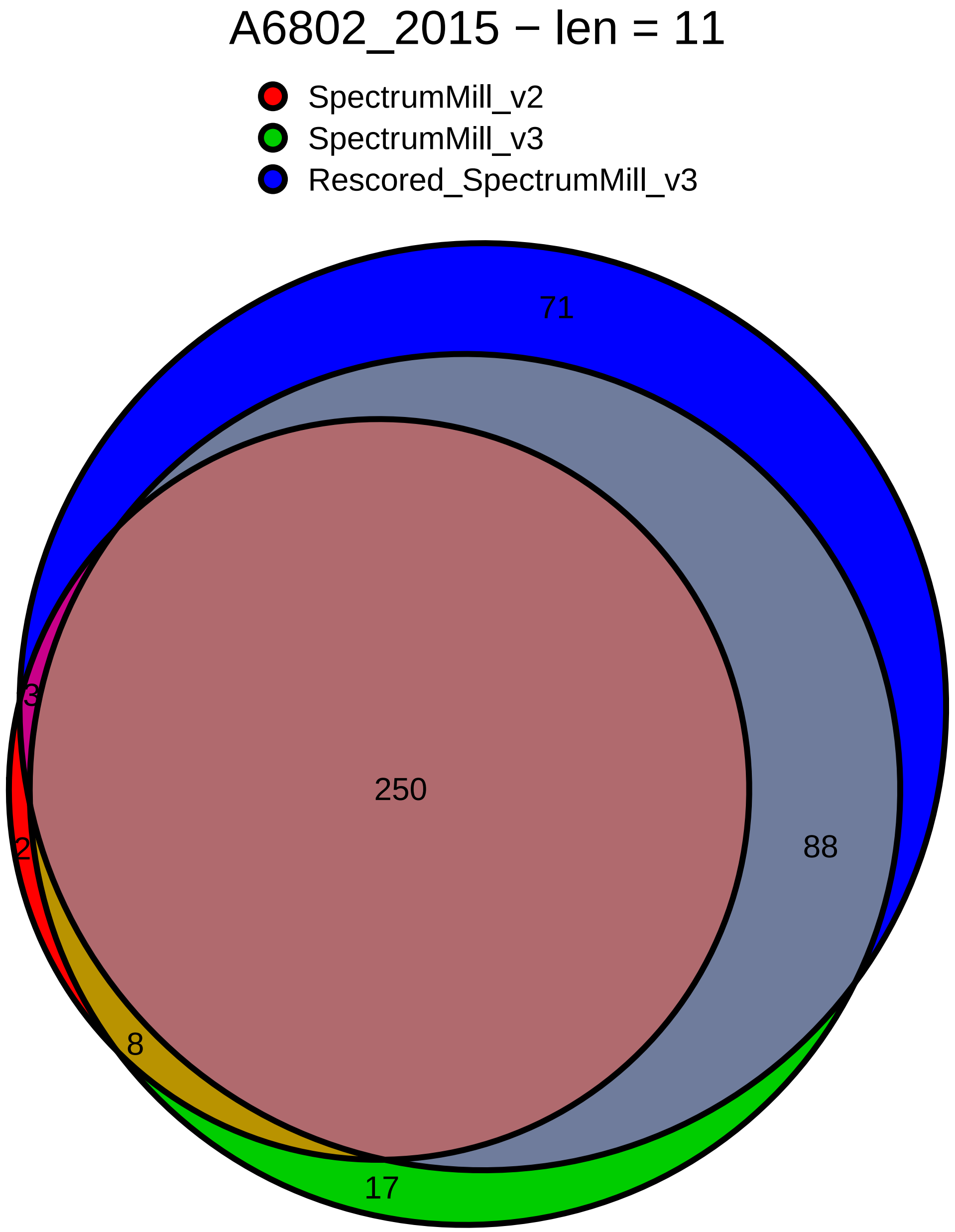

A6802_2015 − len = 11
SpectrumMill_v2
SpectrumMill_v3
Rescored_SpectrumMill_v3
71
3
250
88
2
8
17

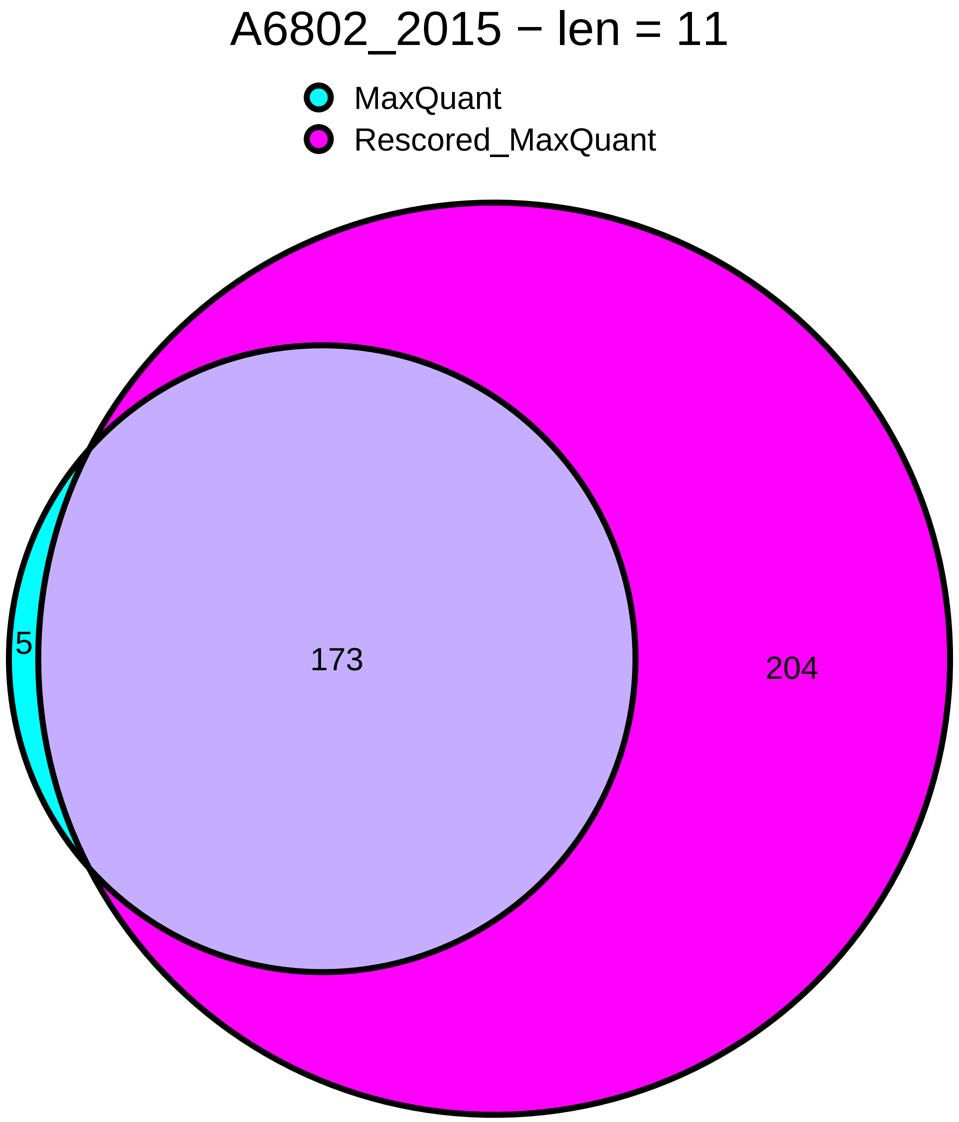

A6802_2015 − len = 11
MaxQuant
Rescored_MaxQuant
5
173
204

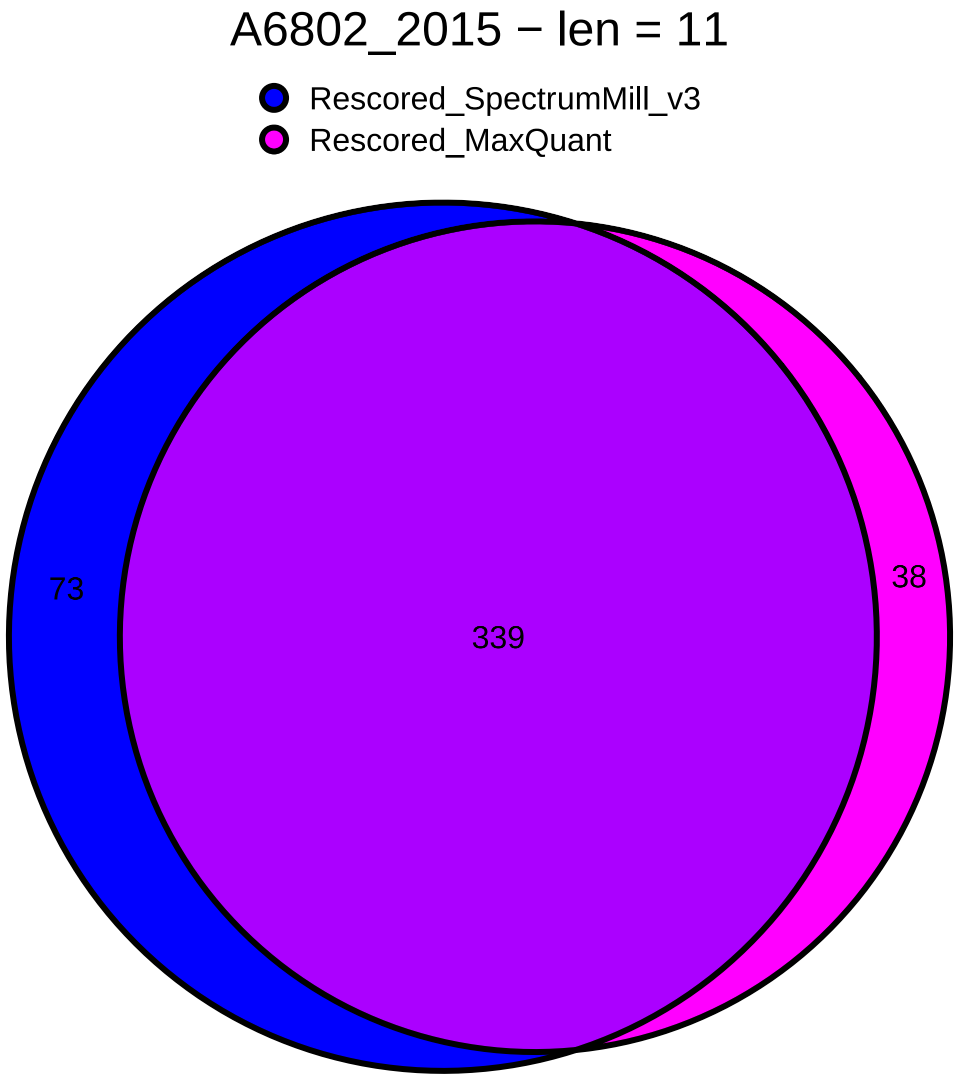

A6802_2015 − len = 11
Rescored_SpectrumMill_v3
Rescored_MaxQuant
73
339
38

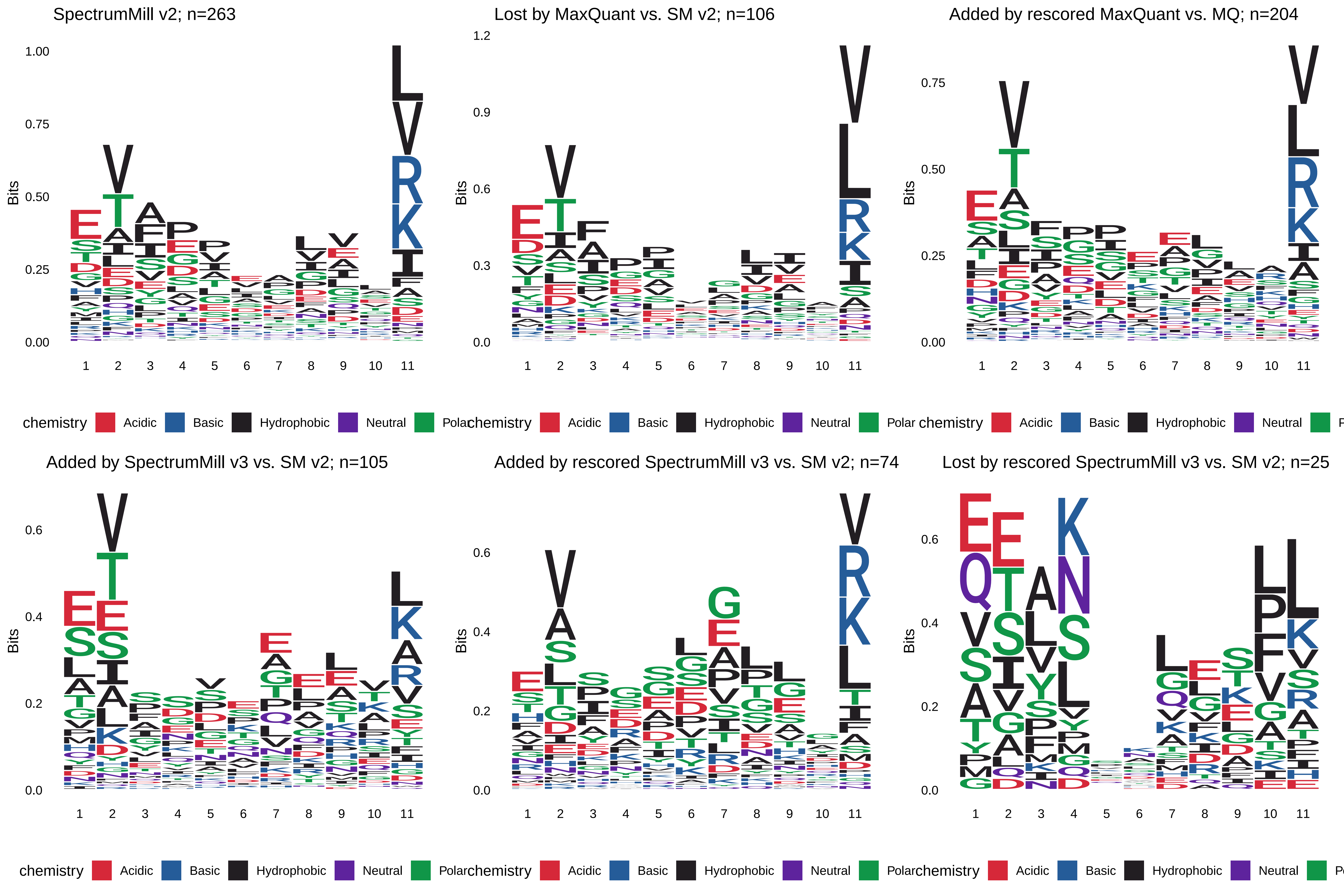
SpectrumMill v2; n=263
Lost by MaxQuant vs. SM v2; n=106
Added by rescored MaxQuant vs. MQ; n=204
Added by SpectrumMill v3 vs. SM v2; n=105
Added by rescored SpectrumMill v3 vs. SM v2; n=74
Lost by rescored SpectrumMill v3 vs. SM v2; n=25
Bits
1 2 3 4 5 6 7 8 9 10 11
chemistry Acidic Basic Hydrophobic Neutral Polar

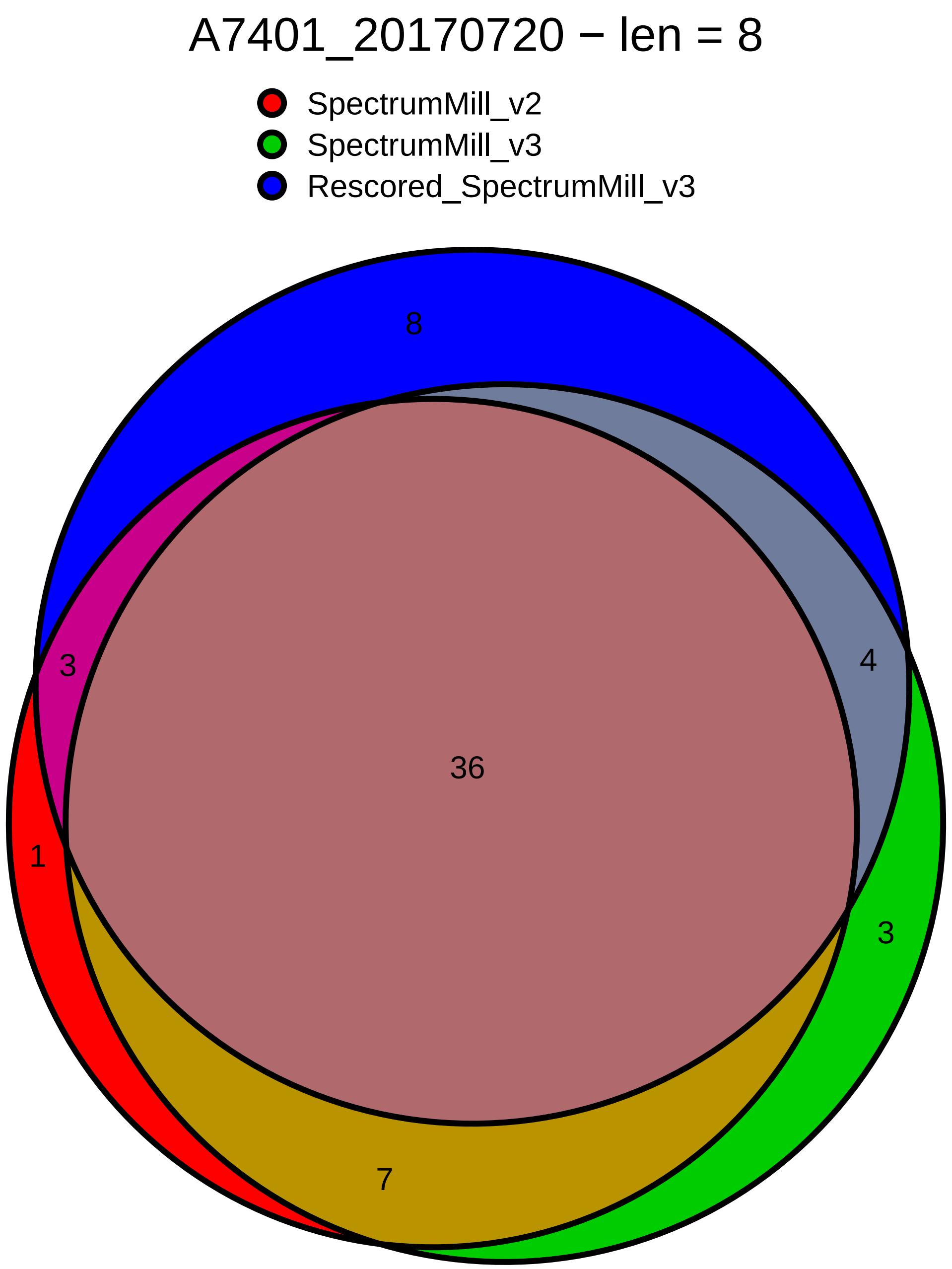
A7401_20170720 − len = 8
SpectrumMill_v2
SpectrumMill_v3
Rescored_SpectrumMill_v3
8
3
4
36
1
3
7

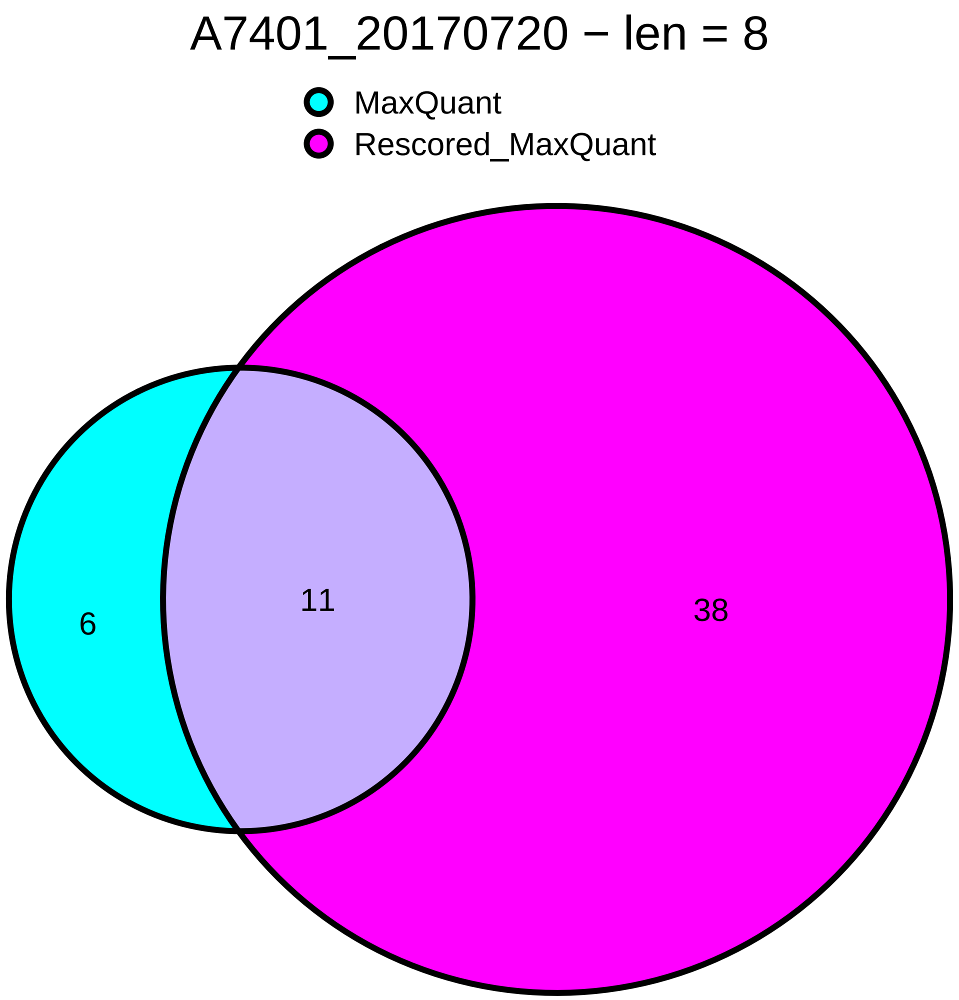
A7401_20170720 − len = 8
MaxQuant
Rescored_MaxQuant
6
11
38

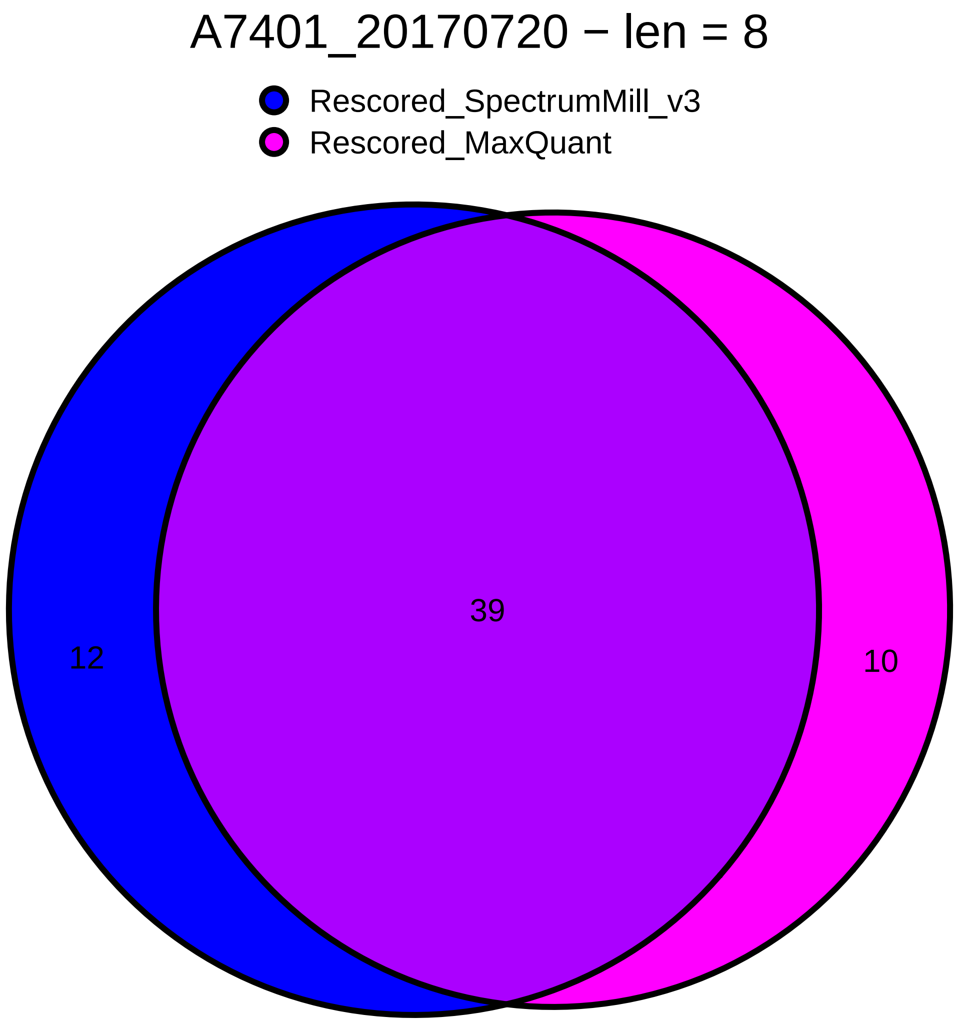
A7401_20170720 − len = 8
Rescored_SpectrumMill_v3
Rescored_MaxQuant
12
39
10

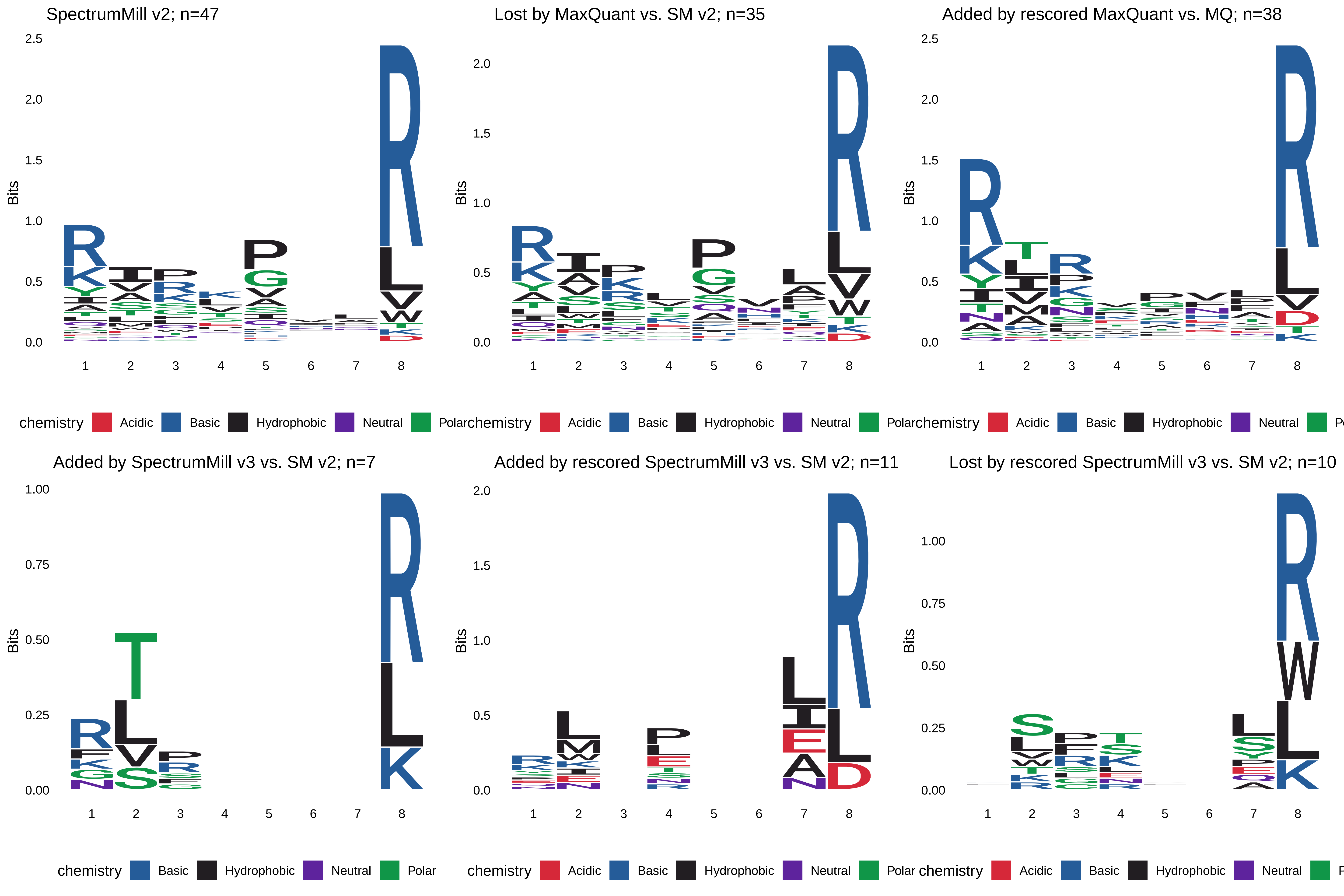

SpectrumMill v2; n=47
Lost by MaxQuant vs. SM v2; n=35
Added by rescored MaxQuant vs. MQ; n=38
Added by SpectrumMill v3 vs. SM v2; n=7
Added by rescored SpectrumMill v3 vs. SM v2; n=11
Lost by rescored SpectrumMill v3 vs. SM v2; n=10
Bits
1
2
3
4
5
6
7
8
chemistry
Acidic
Basic
Hydrophobic
Neutral
Polar

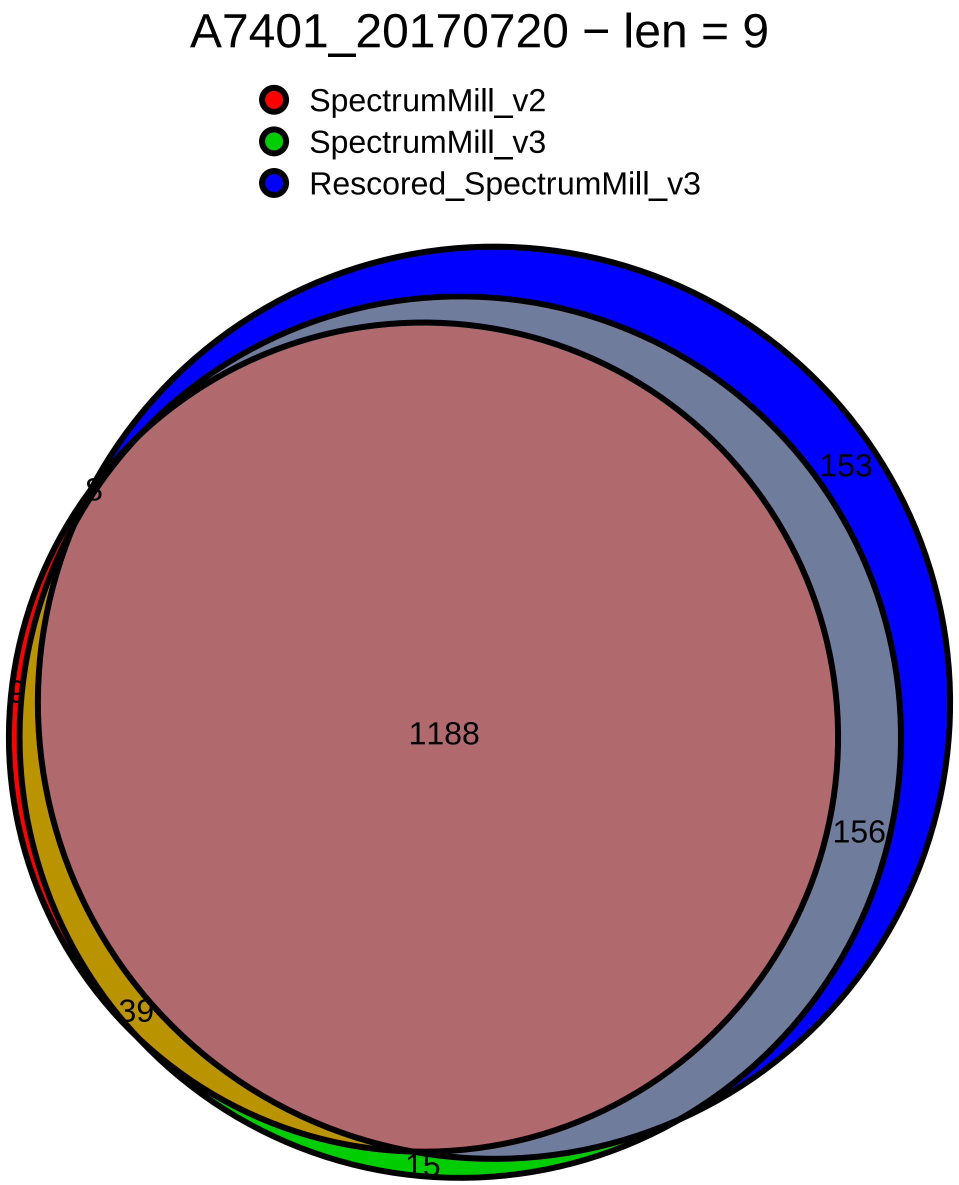
A7401_20170720 − len = 9
SpectrumMill_v2
SpectrumMill_v3
Rescored_SpectrumMill_v3
8
153
1188
156
39
15

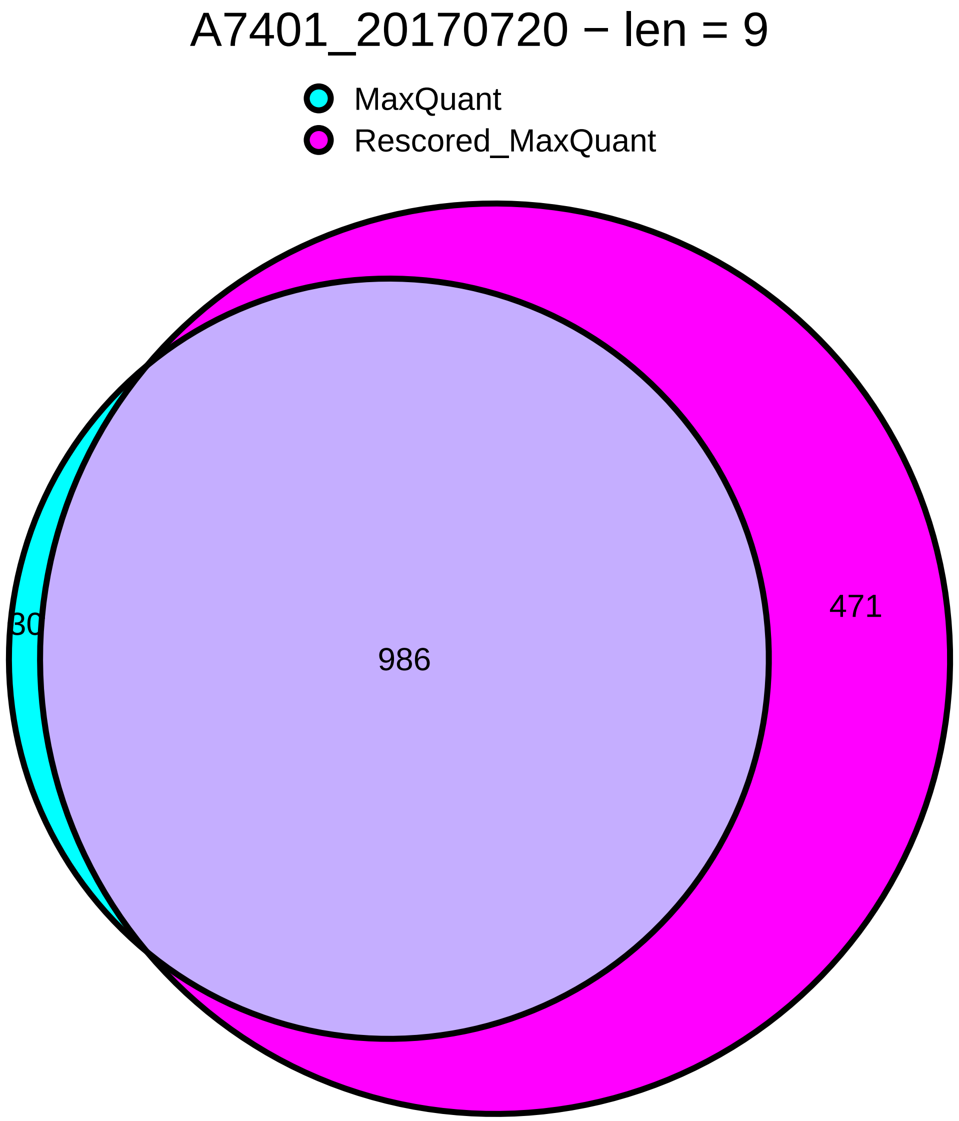
A7401_20170720 − len = 9
MaxQuant
Rescored_MaxQuant
30
986
471

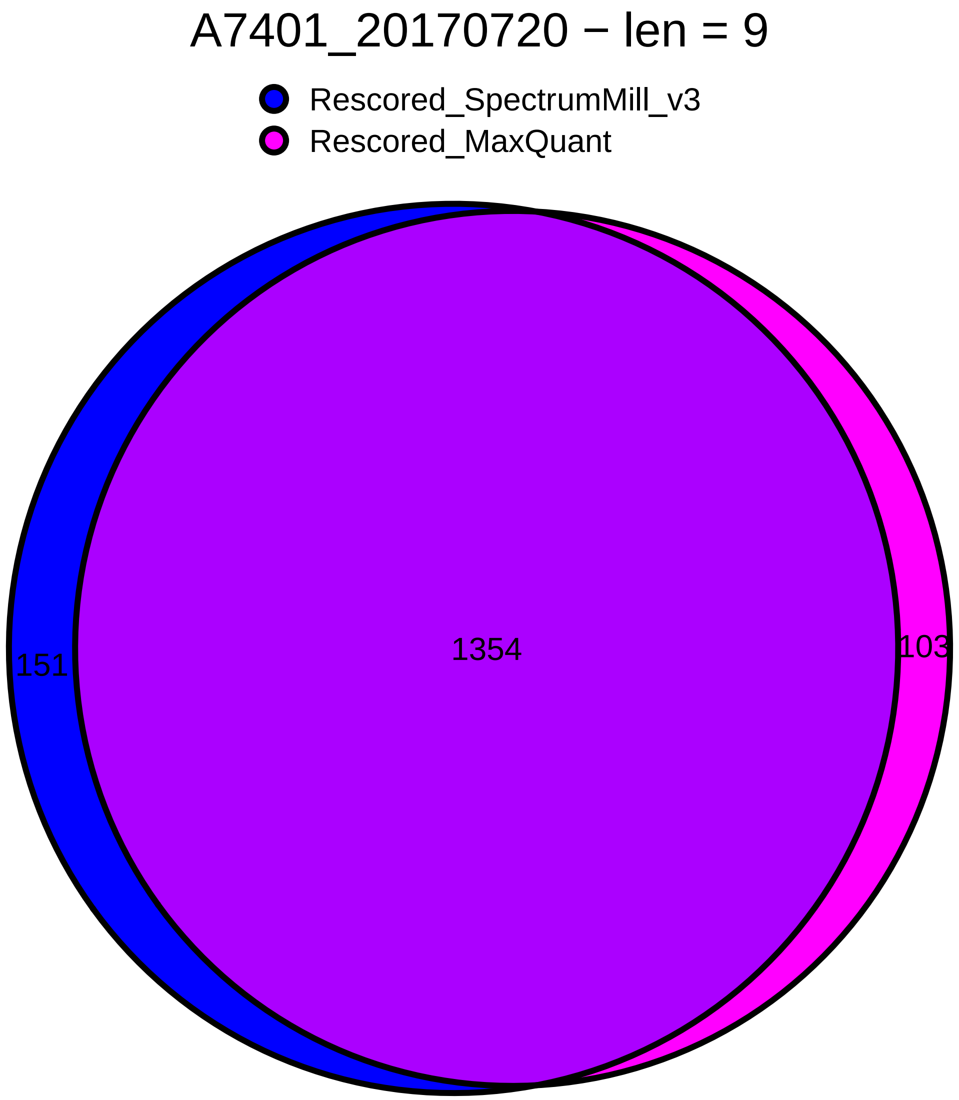
A7401_20170720 − len = 9
Rescored_SpectrumMill_v3
Rescored_MaxQuant
151
1354
103

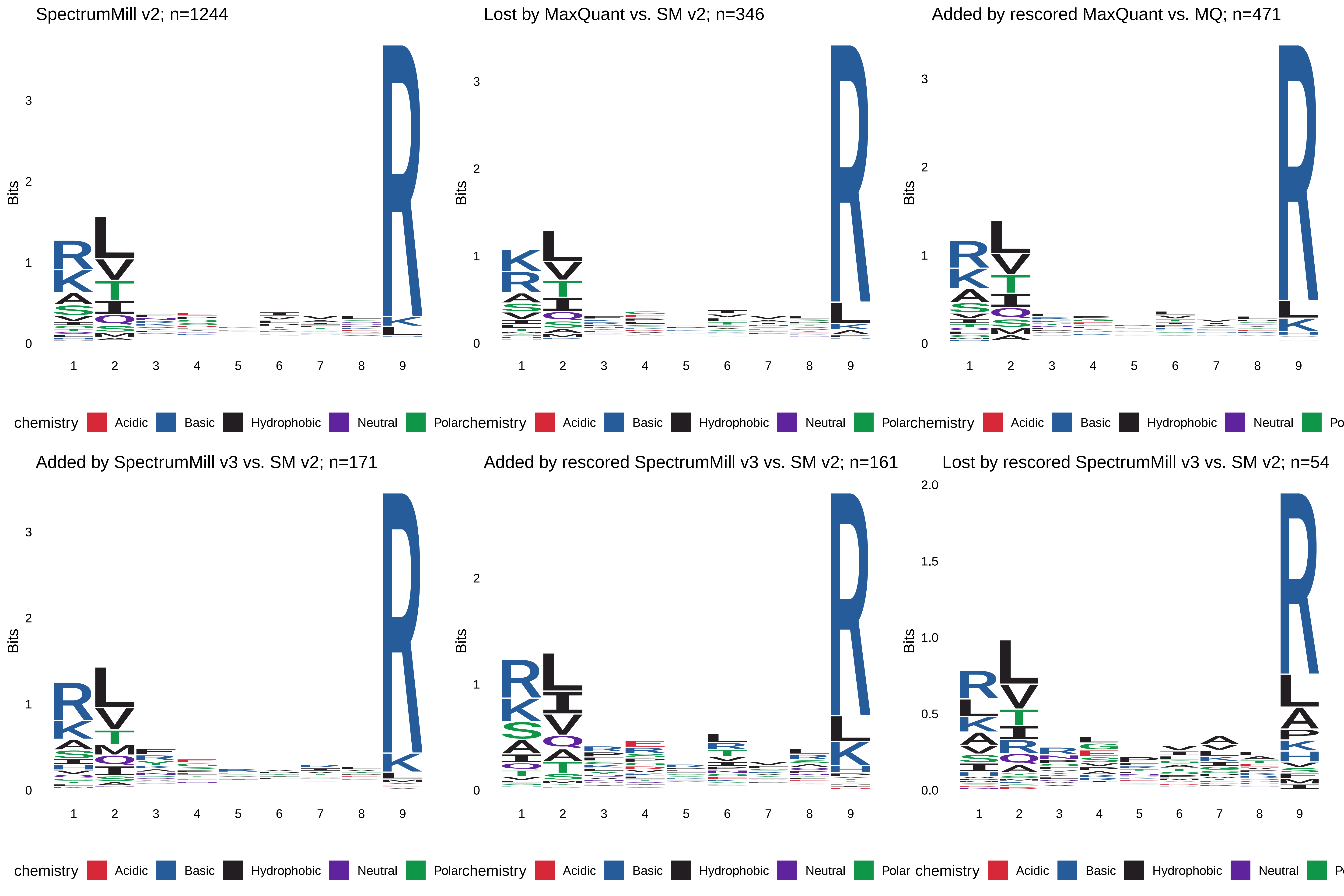
SpectrumMill v2; n=1244
Lost by MaxQuant vs. SM v2; n=346
Added by rescored MaxQuant vs. MQ; n=471
Bits
1 2 3 4 5 6 7 8 9
chemistry Acidic Basic Hydrophobic Neutral Polar
Added by SpectrumMill v3 vs. SM v2; n=171
Added by rescored SpectrumMill v3 vs. SM v2; n=161
Lost by rescored SpectrumMill v3 vs. SM v2; n=54
Bits
1 2 3 4 5 6 7 8 9
chemistry Acidic Basic Hydrophobic Neutral Polar

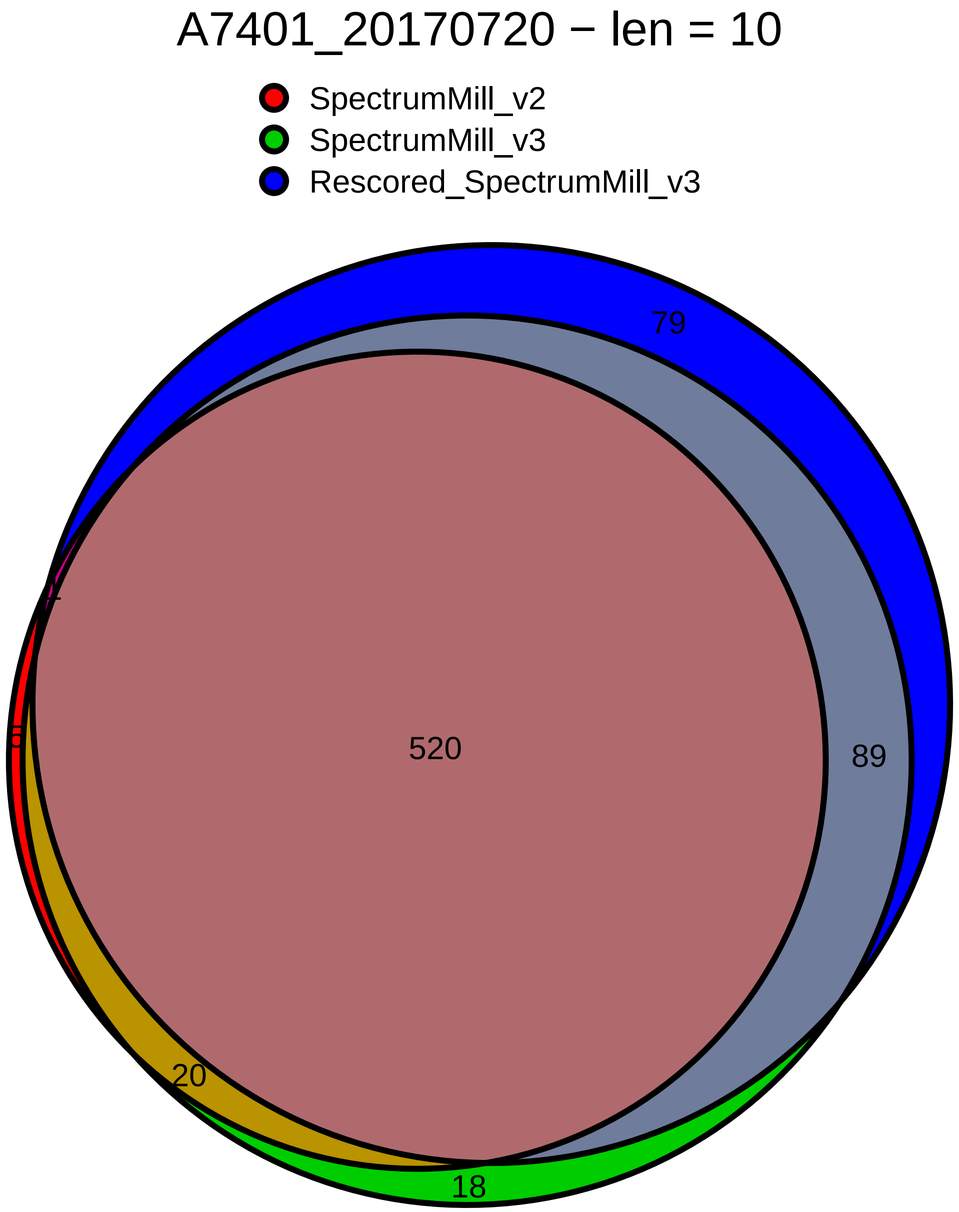
A7401_20170720 − len = 10
SpectrumMill_v2
SpectrumMill_v3
Rescored_SpectrumMill_v3
79
520
89
20
18

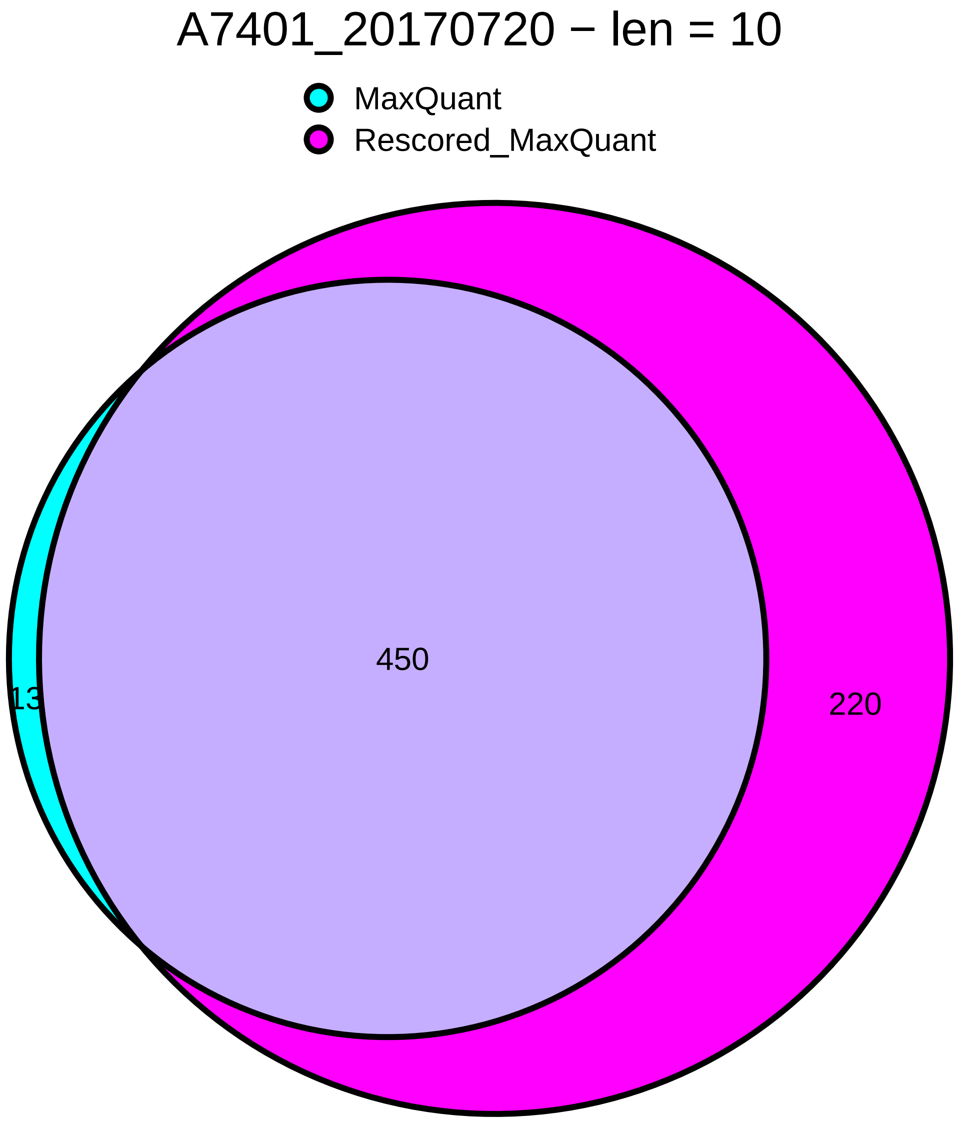
A7401_20170720 − len = 10
MaxQuant
Rescored_MaxQuant
450
13
220

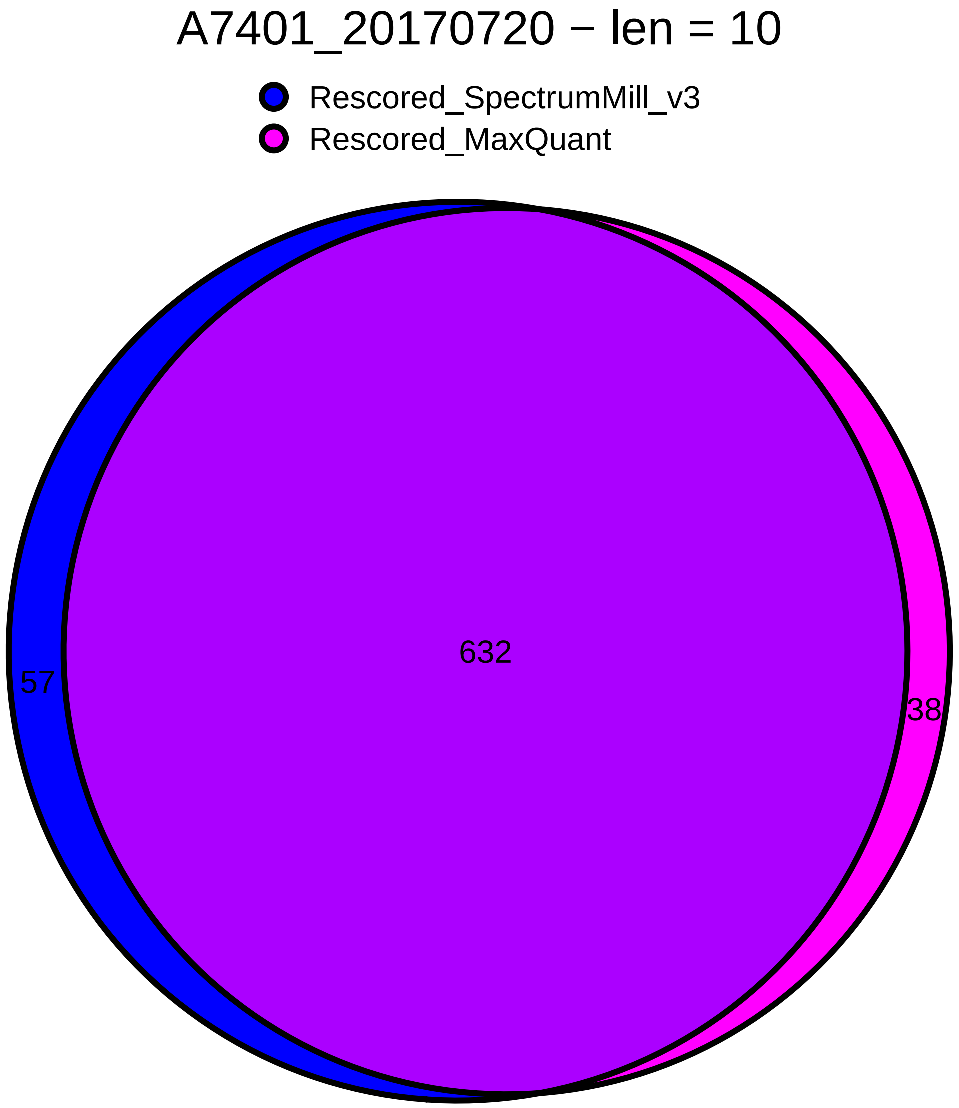
A7401_20170720 − len = 10
Rescored_SpectrumMill_v3
Rescored_MaxQuant
632
57
38

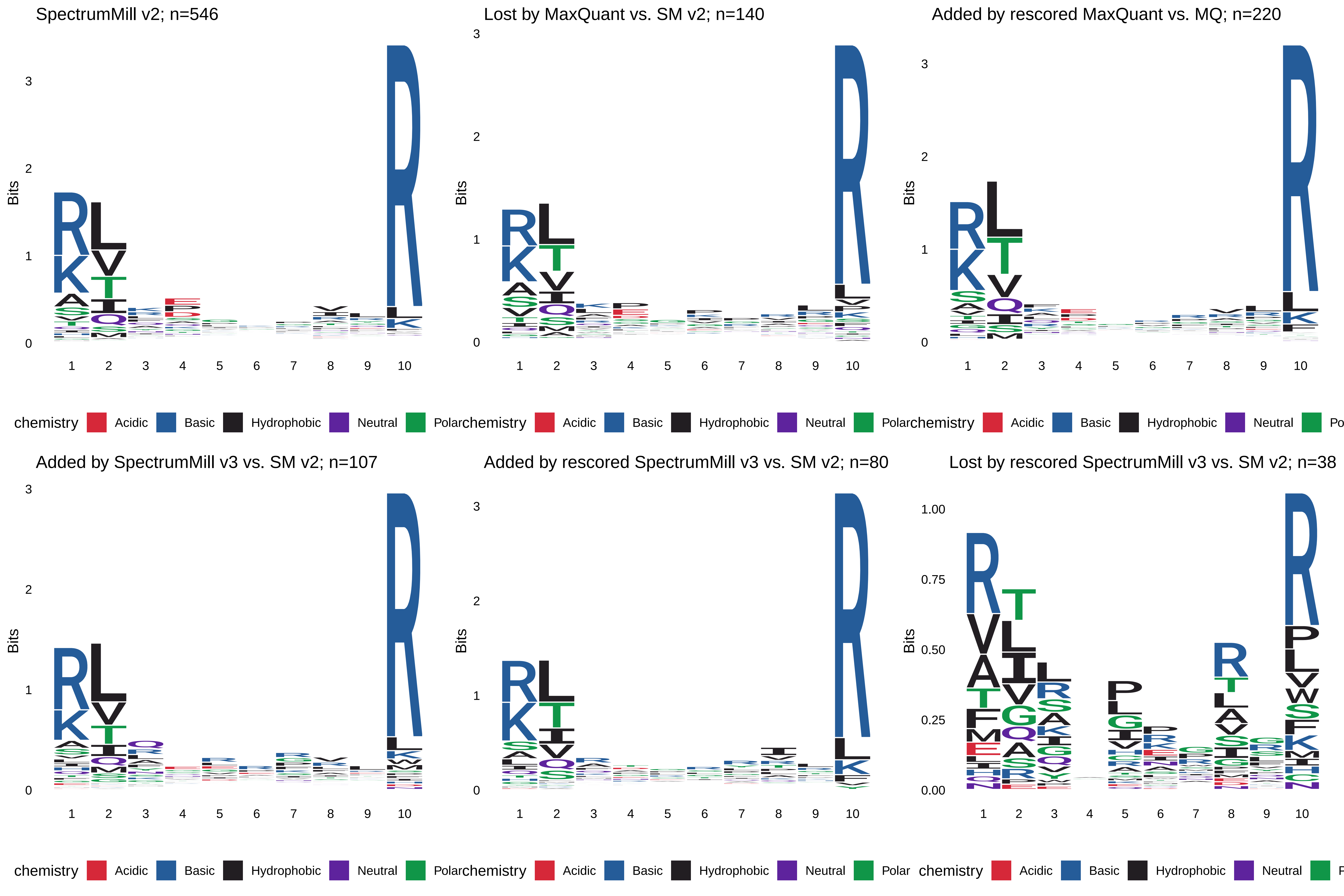
SpectrumMill v2; n=546
Lost by MaxQuant vs. SM v2; n=140
Added by rescored MaxQuant vs. MQ; n=220
Added by SpectrumMill v3 vs. SM v2; n=107
Added by rescored SpectrumMill v3 vs. SM v2; n=80
Lost by rescored SpectrumMill v3 vs. SM v2; n=38
Bits
1 2 3 4 5 6 7 8 9 10
0 1 2 3
0.00 0.25 0.50 0.75 1.00
chemistry
Acidic
Basic
Hydrophobic
Neutral
Polar

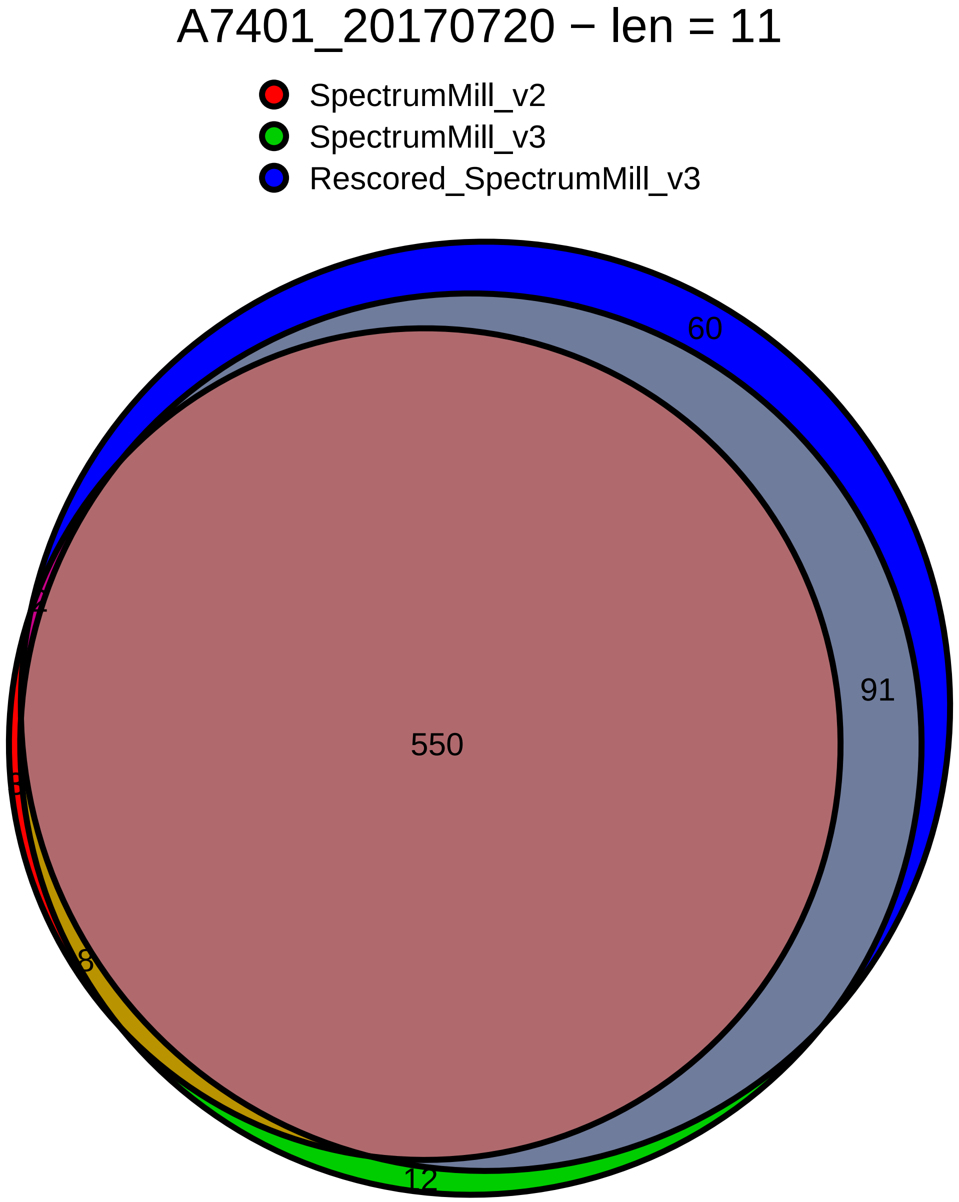
A7401_20170720 − len = 11
SpectrumMill_v2
SpectrumMill_v3
Rescored_SpectrumMill_v3
60
91
550
8
12

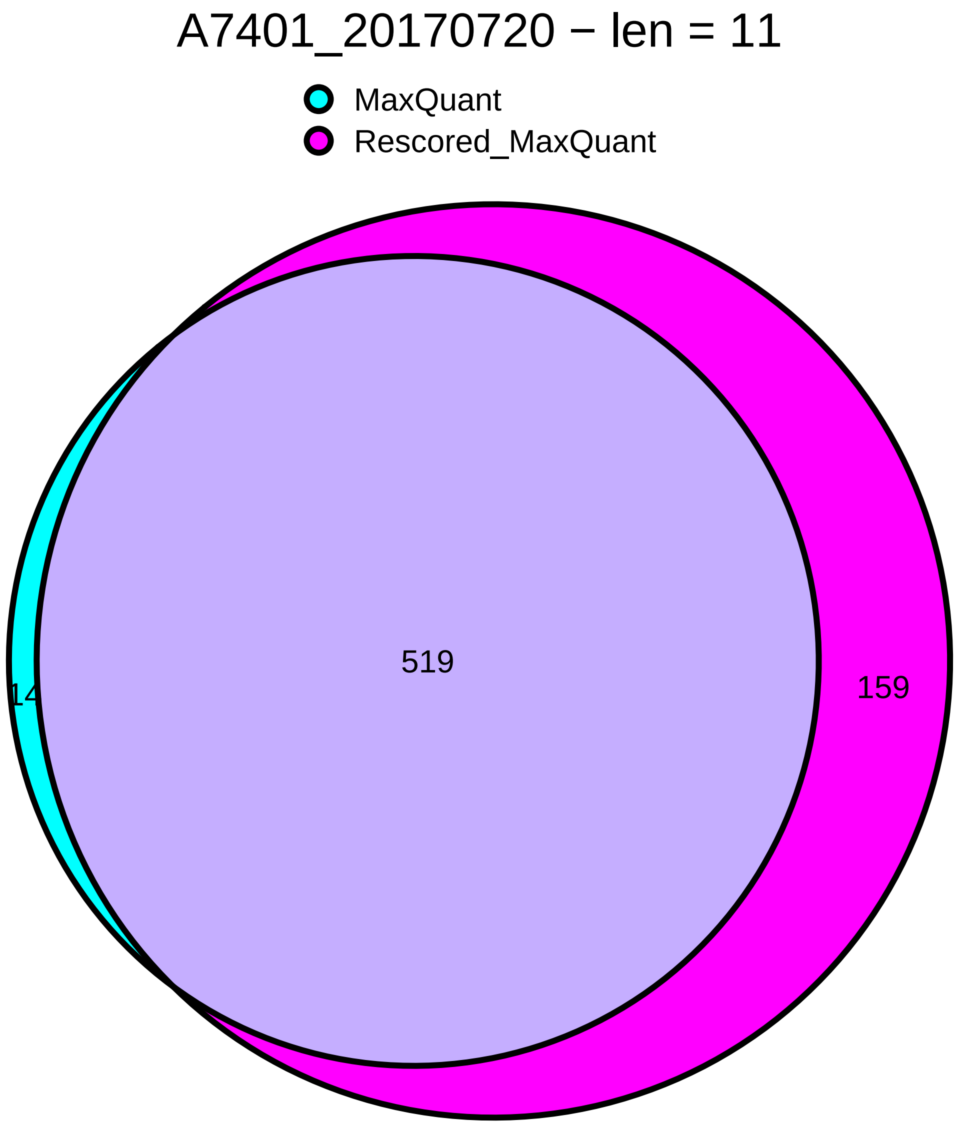
A7401_20170720 − len = 11
MaxQuant
Rescored_MaxQuant
14
519
159

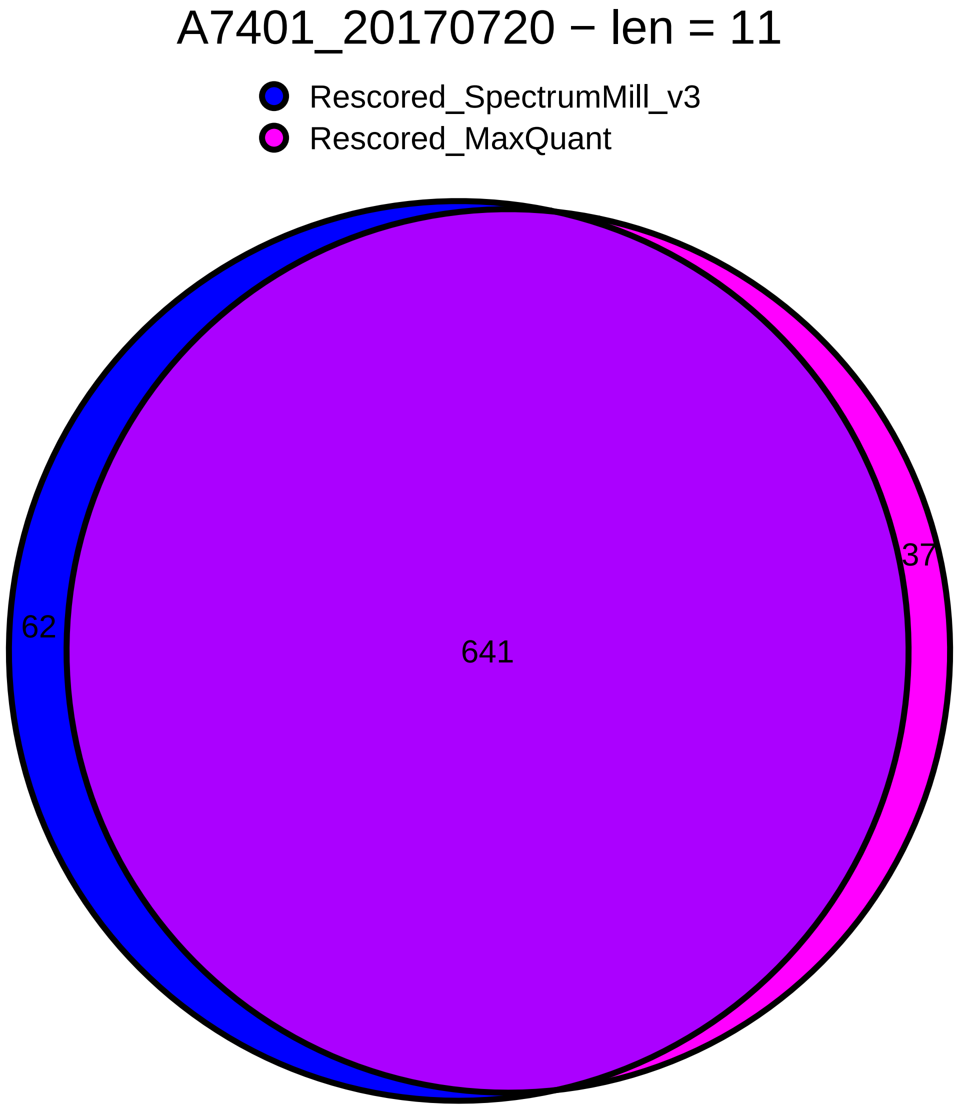
A7401_20170720 − len = 11
Rescored_SpectrumMill_v3
Rescored_MaxQuant
62
641
37

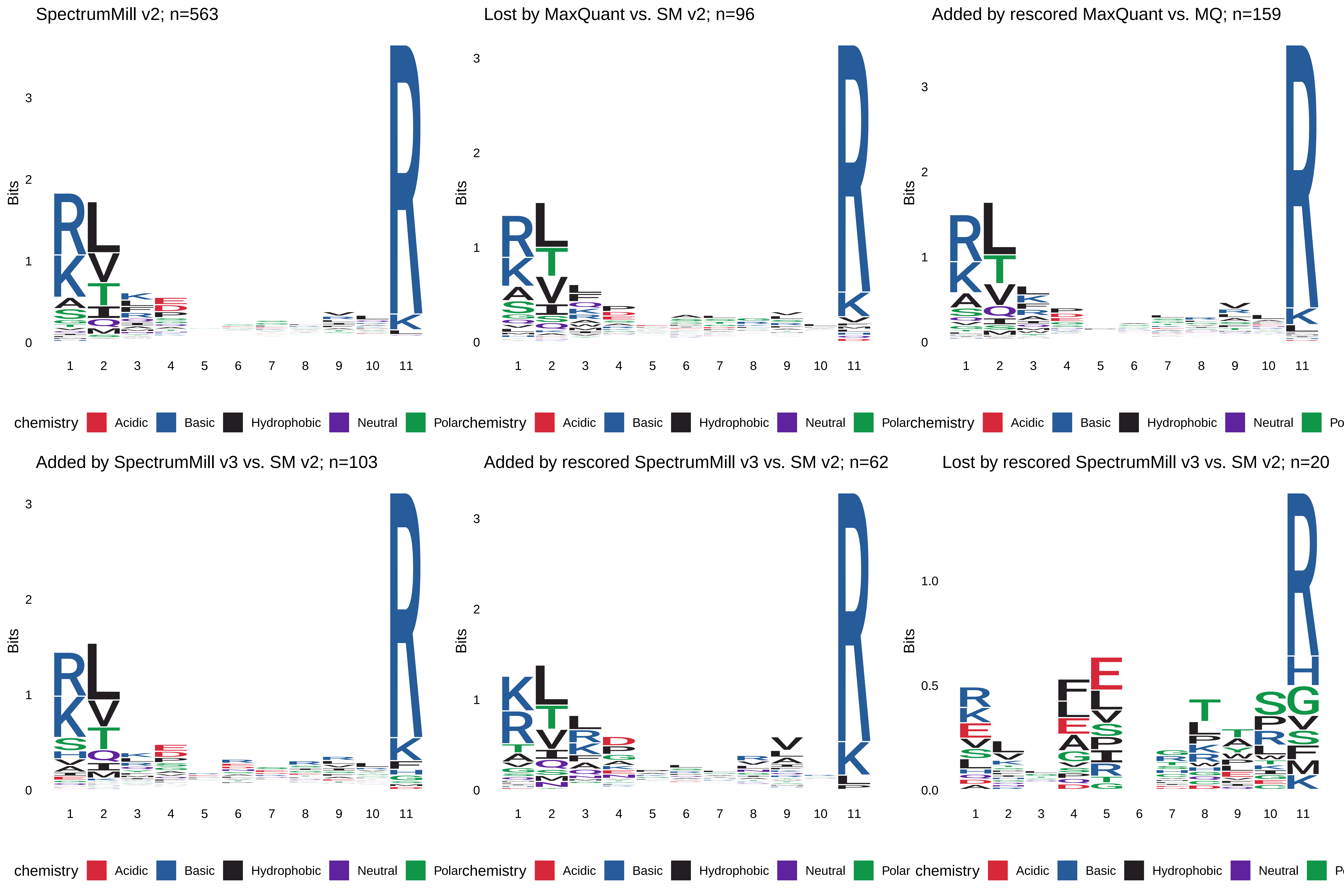
SpectrumMill v2; n=563
Lost by MaxQuant vs. SM v2; n=96
Added by rescored MaxQuant vs. MQ; n=159
Added by SpectrumMill v3 vs. SM v2; n=103
Added by rescored SpectrumMill v3 vs. SM v2; n=62
Lost by rescored SpectrumMill v3 vs. SM v2; n=20
Bits
1 2 3 4 5 6 7 8 9 10 11
chemistry Acidic Basic Hydrophobic Neutral Polar

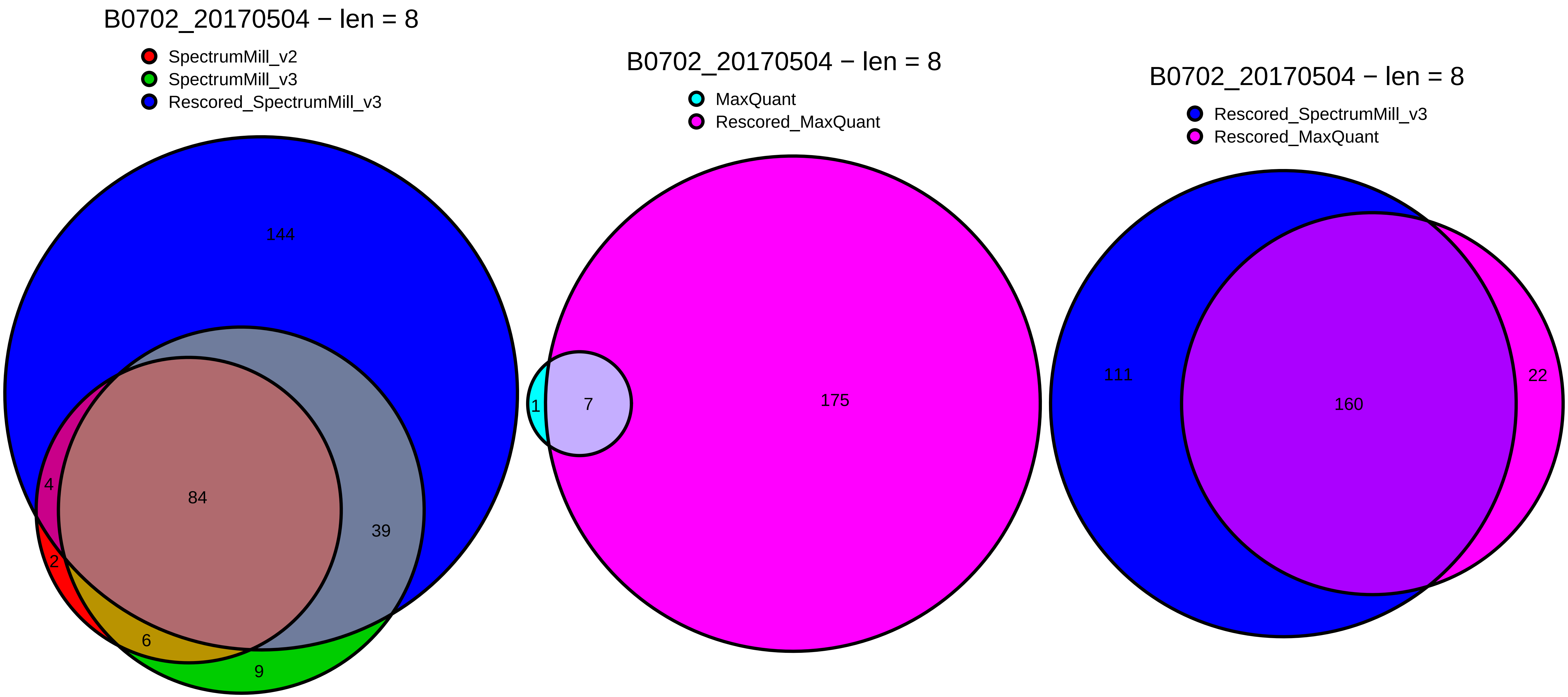

B0702_20170504 − len = 8
SpectrumMill_v2
SpectrumMill_v3
Rescored_SpectrumMill_v3
144
4
84
39
2
6
9
B0702_20170504 − len = 8
MaxQuant
Rescored_MaxQuant
1
7
175
B0702_20170504 − len = 8
Rescored_SpectrumMill_v3
Rescored_MaxQuant
111
160
22

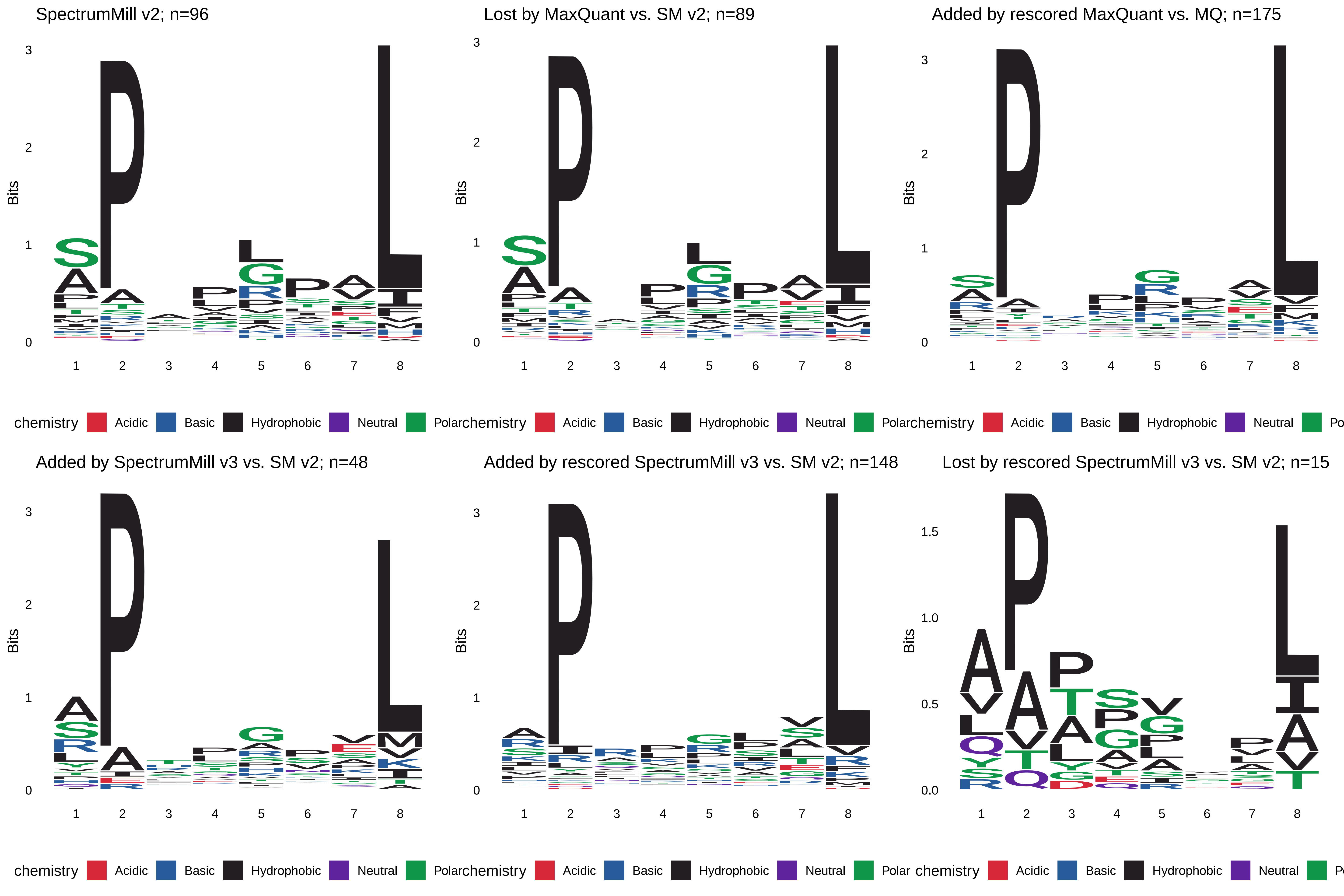
SpectrumMill v2; n=96
Lost by MaxQuant vs. SM v2; n=89
Added by rescored MaxQuant vs. MQ; n=175
Added by SpectrumMill v3 vs. SM v2; n=48
Added by rescored SpectrumMill v3 vs. SM v2; n=148
Lost by rescored SpectrumMill v3 vs. SM v2; n=15
Bits
chemistry
Acidic
Basic
Hydrophobic
Neutral
Polar

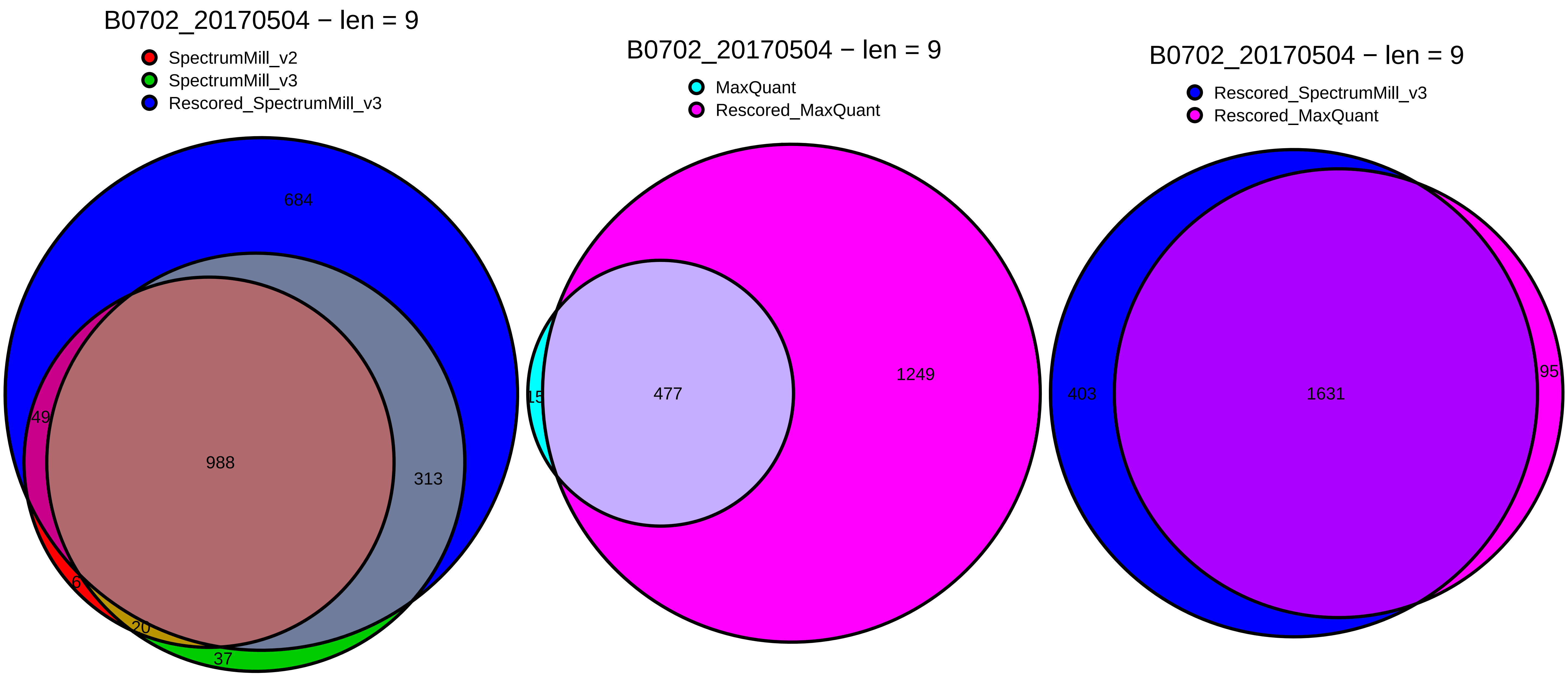

B0702_20170504 − len = 9
SpectrumMill_v2
SpectrumMill_v3
Rescored_SpectrumMill_v3
684
49
988
313
6
20
37
B0702_20170504 − len = 9
MaxQuant
Rescored_MaxQuant
15
477
1249
B0702_20170504 − len = 9
Rescored_SpectrumMill_v3
Rescored_MaxQuant
403
1631
95

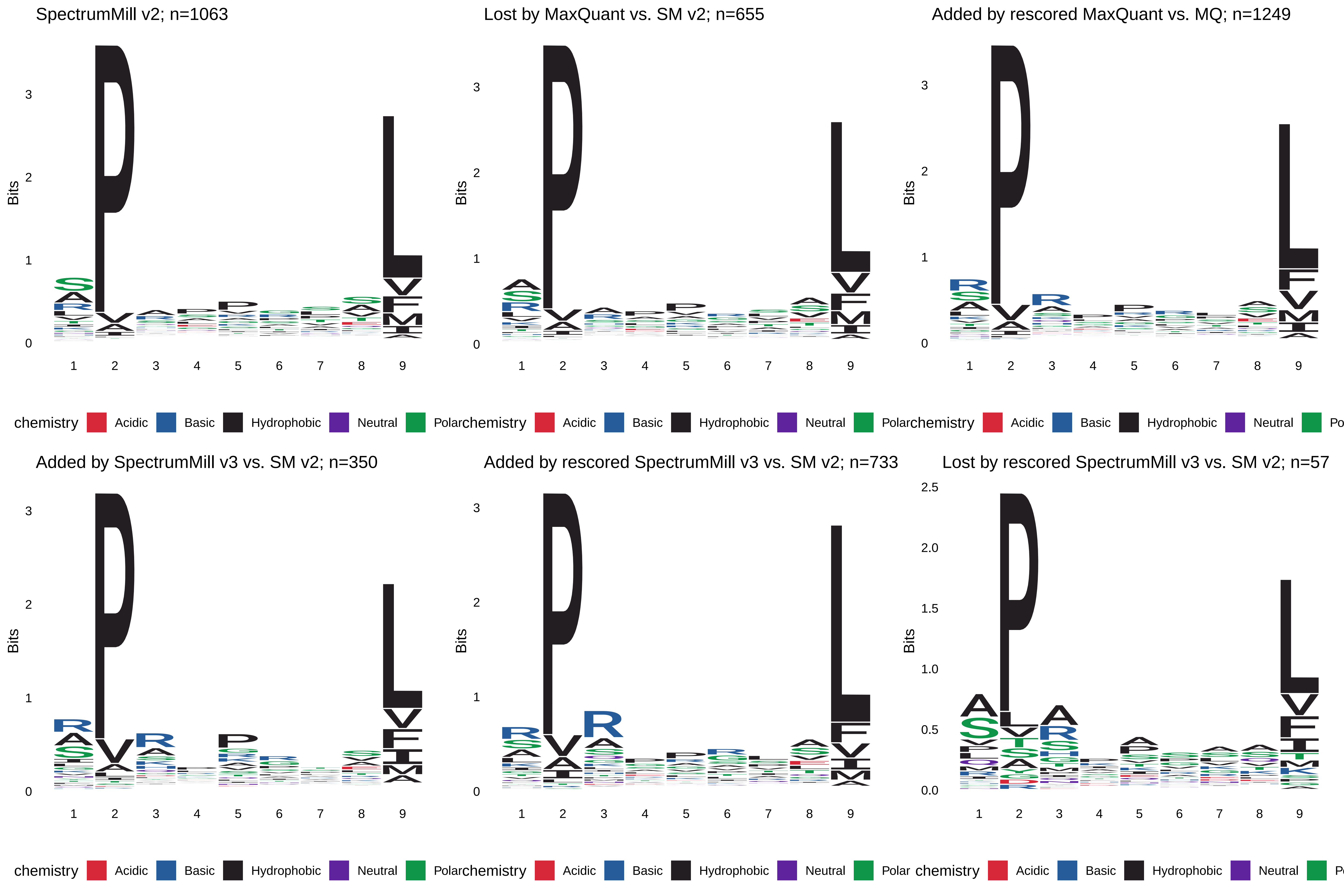
SpectrumMill v2; n=1063
Lost by MaxQuant vs. SM v2; n=655
Added by rescored MaxQuant vs. MQ; n=1249
Added by SpectrumMill v3 vs. SM v2; n=350
Added by rescored SpectrumMill v3 vs. SM v2; n=733
Lost by rescored SpectrumMill v3 vs. SM v2; n=57
Bits
0
1
2
3
0.0
0.5
1.0
1.5
2.0
2.5
1 2 3 4 5 6 7 8 9
chemistry
Acidic
Basic
Hydrophobic
Neutral
Polar

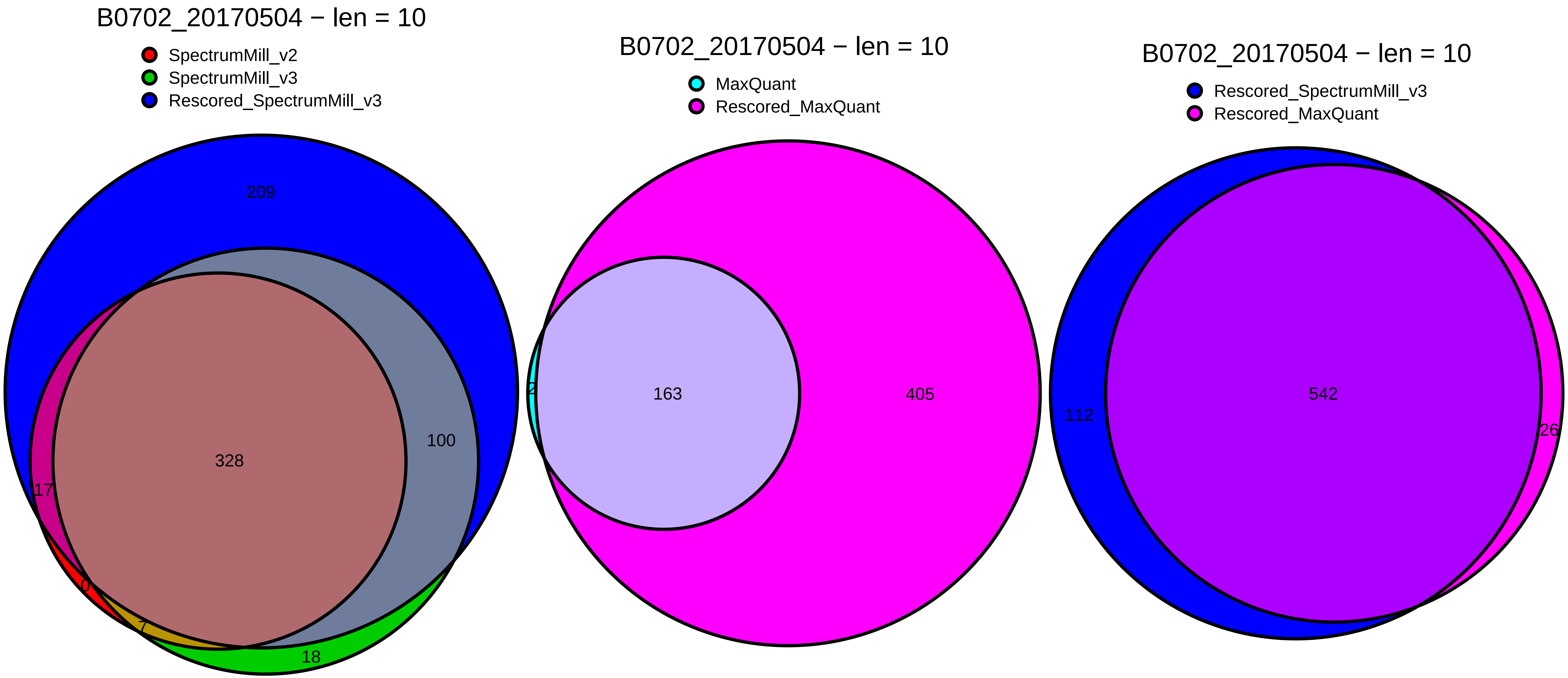

B0702_20170504 − len = 10
SpectrumMill_v2
SpectrumMill_v3
Rescored_SpectrumMill_v3
209
328
100
17
0
7
18
B0702_20170504 − len = 10
MaxQuant
Rescored_MaxQuant
163
405
B0702_20170504 − len = 10
Rescored_SpectrumMill_v3
Rescored_MaxQuant
112
542
26

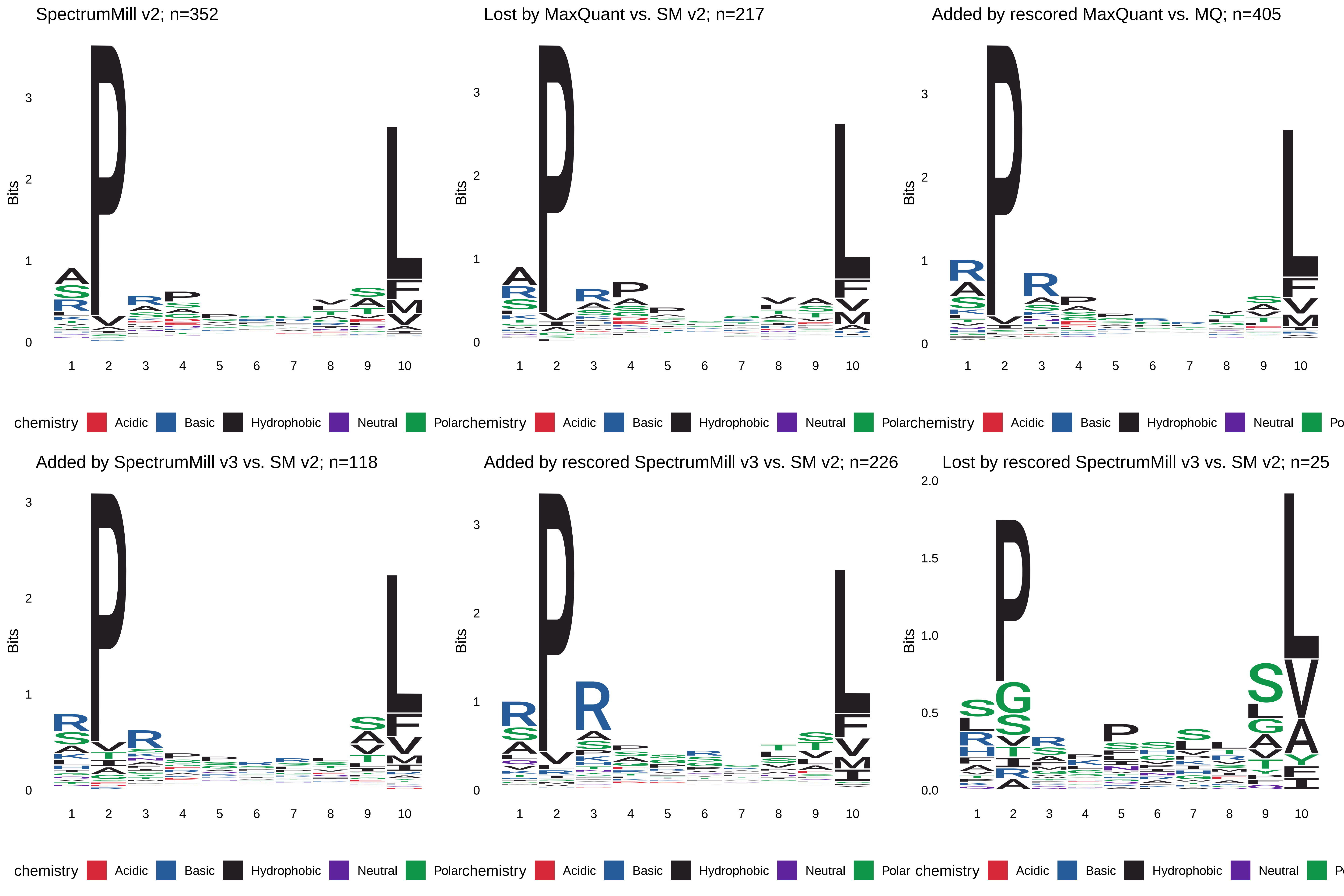

SpectrumMill v2; n=352
Lost by MaxQuant vs. SM v2; n=217
Added by rescored MaxQuant vs. MQ; n=405
Added by SpectrumMill v3 vs. SM v2; n=118
Added by rescored SpectrumMill v3 vs. SM v2; n=226
Lost by rescored SpectrumMill v3 vs. SM v2; n=25
Bits
chemistry
Acidic
Basic
Hydrophobic
Neutral
Polar

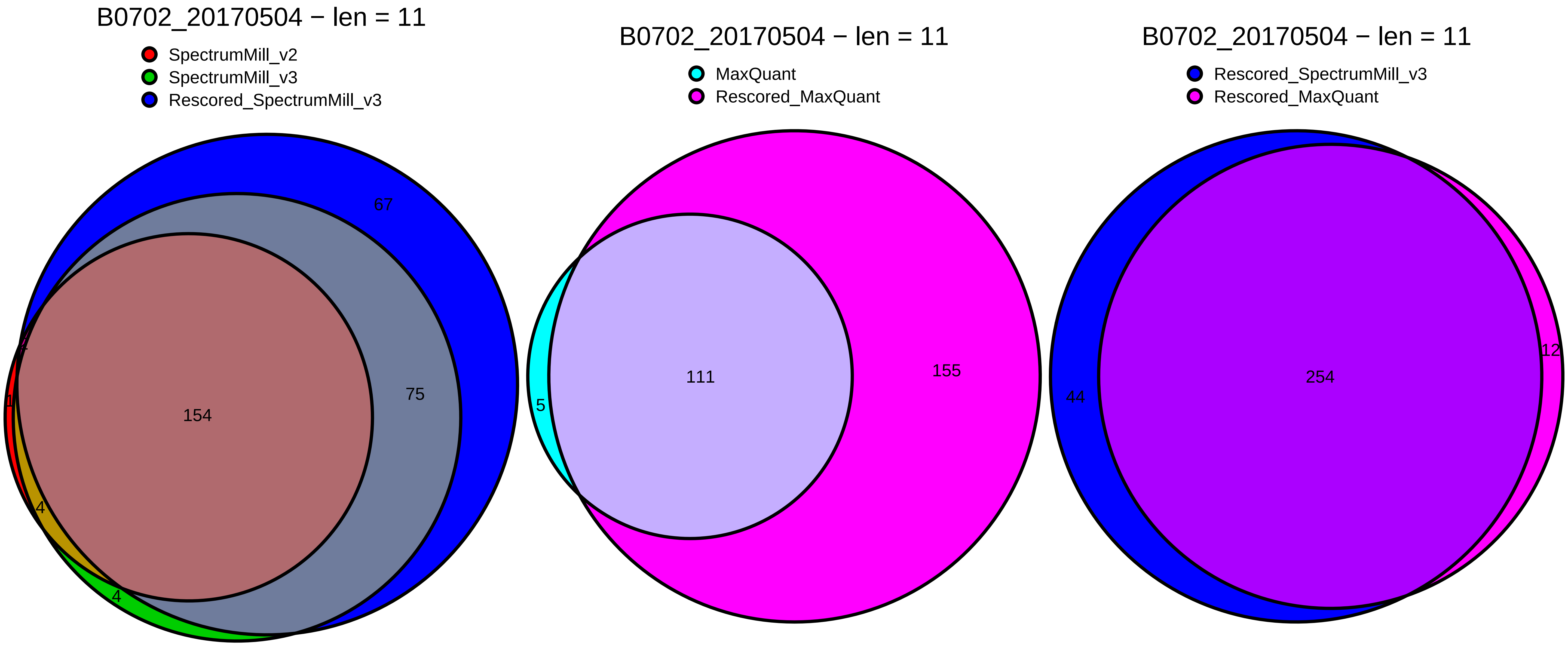

B0702_20170504 − len = 11
SpectrumMill_v2
SpectrumMill_v3
Rescored_SpectrumMill_v3
67
75
154
4
4
B0702_20170504 − len = 11
MaxQuant
Rescored_MaxQuant
5
111
155
B0702_20170504 − len = 11
Rescored_SpectrumMill_v3
Rescored_MaxQuant
44
254
12

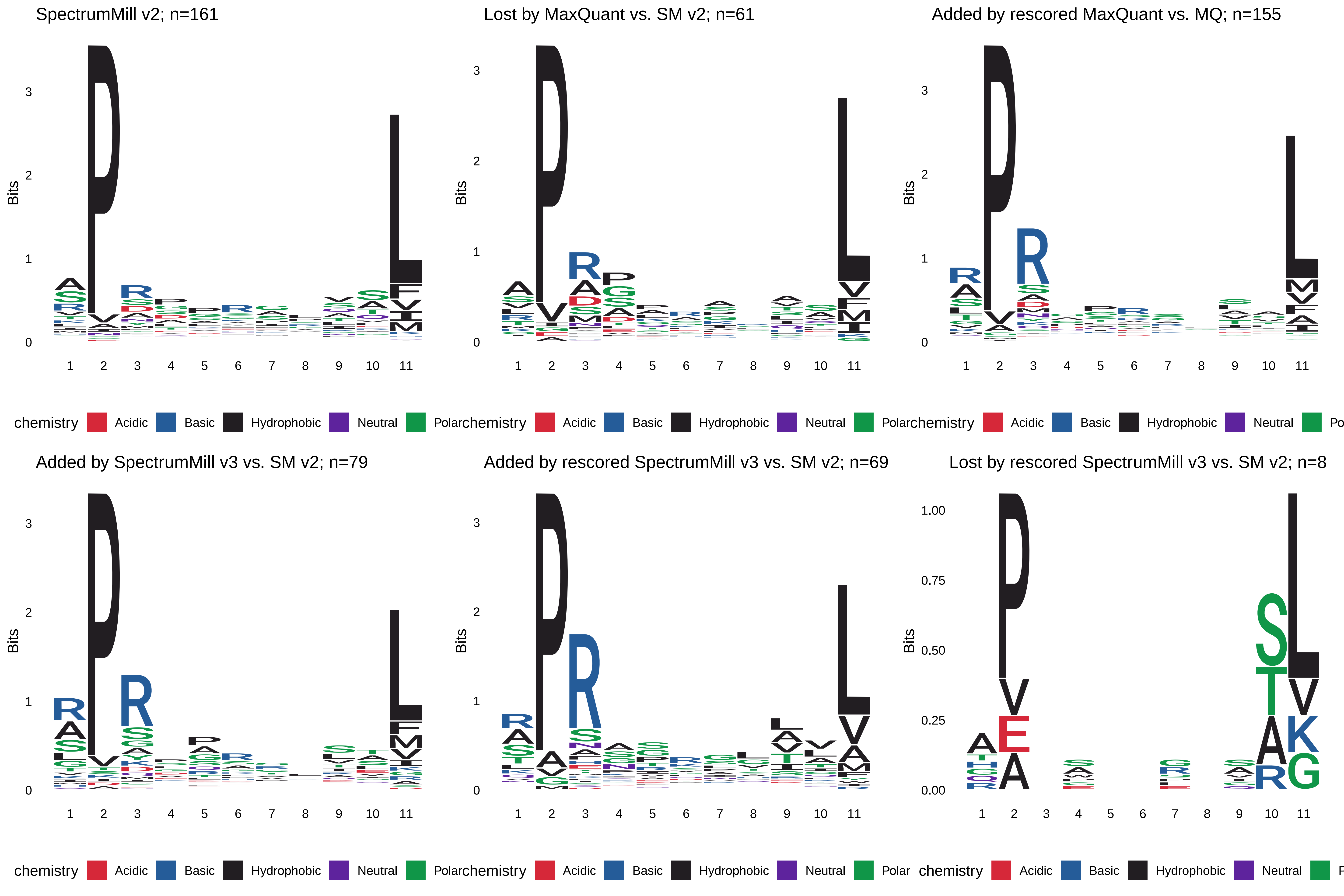
SpectrumMill v2; n=161
Lost by MaxQuant vs. SM v2; n=61
Added by rescored MaxQuant vs. MQ; n=155
Added by SpectrumMill v3 vs. SM v2; n=79
Added by rescored SpectrumMill v3 vs. SM v2; n=69
Lost by rescored SpectrumMill v3 vs. SM v2; n=8
Bits
1 2 3 4 5 6 7 8 9 10 11
chemistry
Acidic
Basic
Hydrophobic
Neutral
Polar

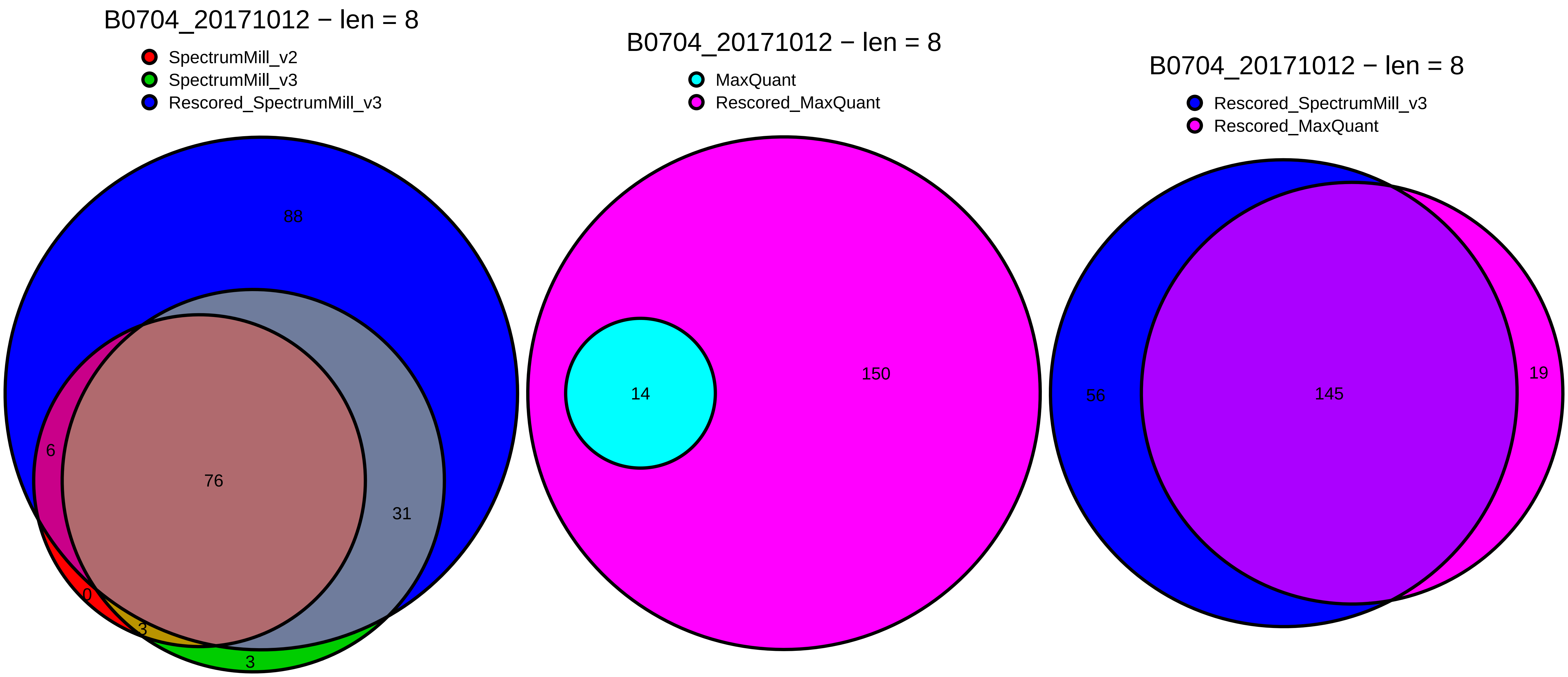
B0704_20171012 − len = 8
SpectrumMill_v2
SpectrumMill_v3
Rescored_SpectrumMill_v3
88
6
76
31
0
3
3
B0704_20171012 − len = 8
MaxQuant
Rescored_MaxQuant
14
150
B0704_20171012 − len = 8
Rescored_SpectrumMill_v3
Rescored_MaxQuant
56
145
19

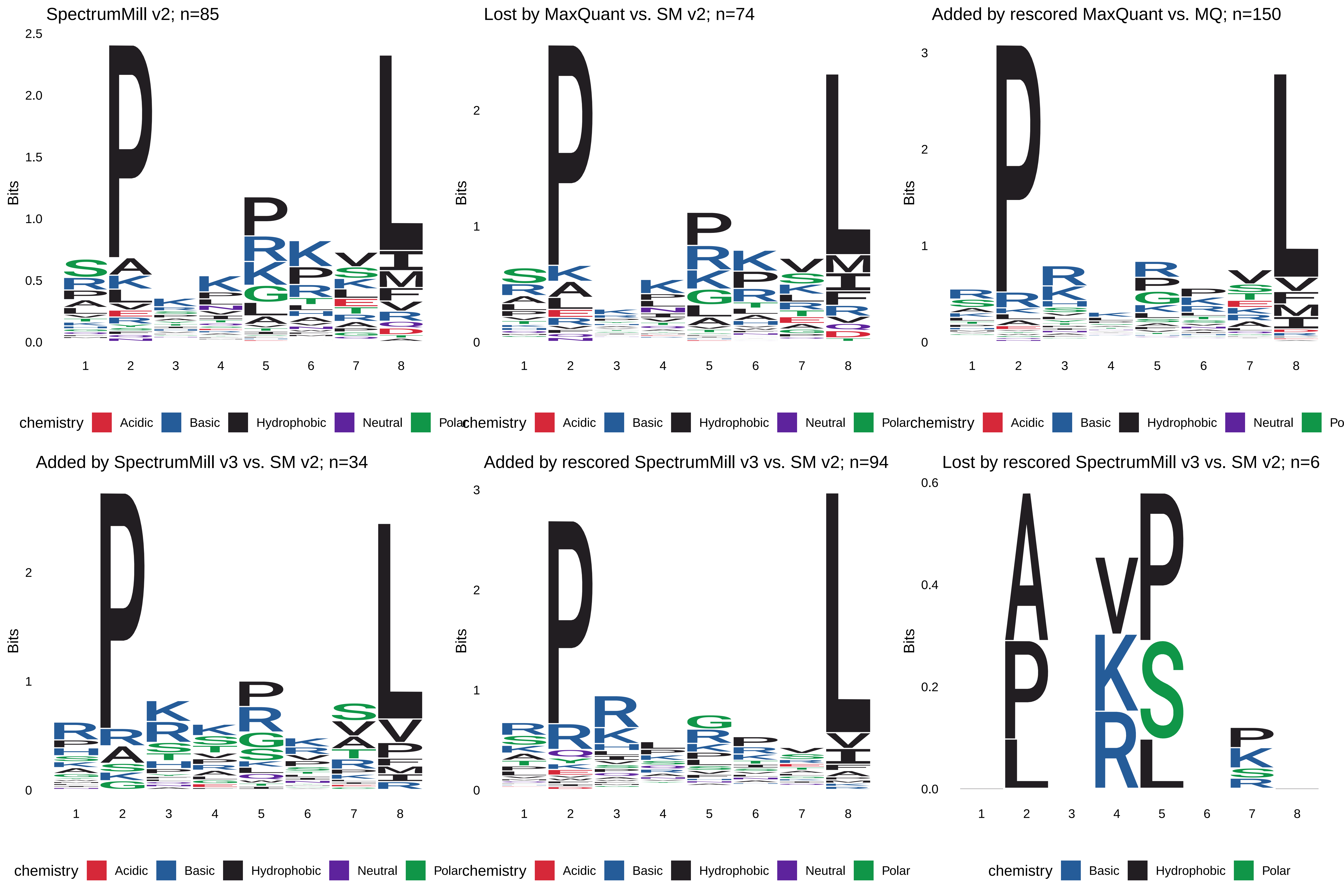
SpectrumMill v2; n=85
Lost by MaxQuant vs. SM v2; n=74
Added by rescored MaxQuant vs. MQ; n=150
Added by SpectrumMill v3 vs. SM v2; n=34
Added by rescored SpectrumMill v3 vs. SM v2; n=94
Lost by rescored SpectrumMill v3 vs. SM v2; n=6
Bits
1 2 3 4 5 6 7 8
chemistry
Acidic
Basic
Hydrophobic
Neutral
Polar

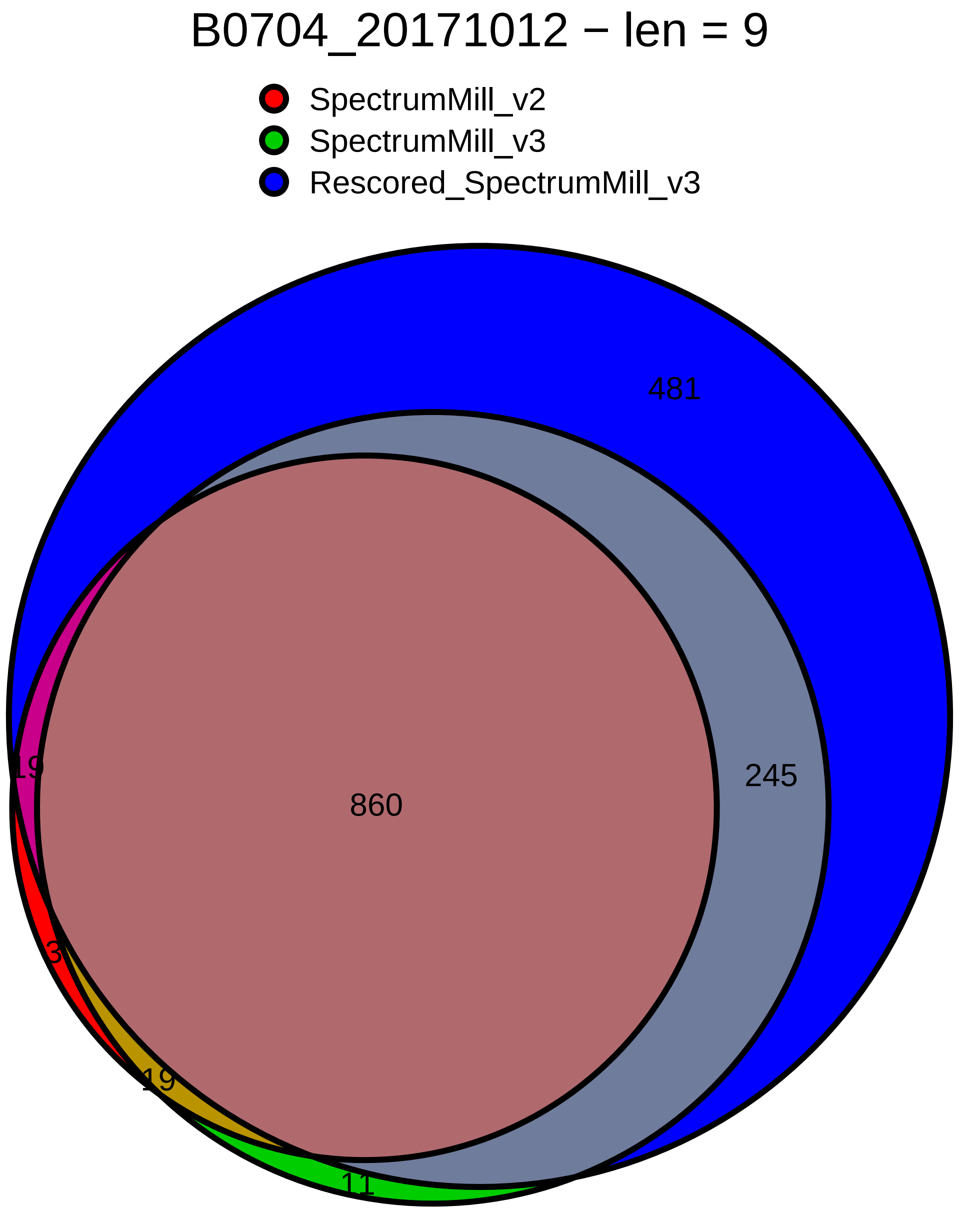
B0704_20171012 − len = 9
SpectrumMill_v2
SpectrumMill_v3
Rescored_SpectrumMill_v3
481
245
860
19
3
19
11

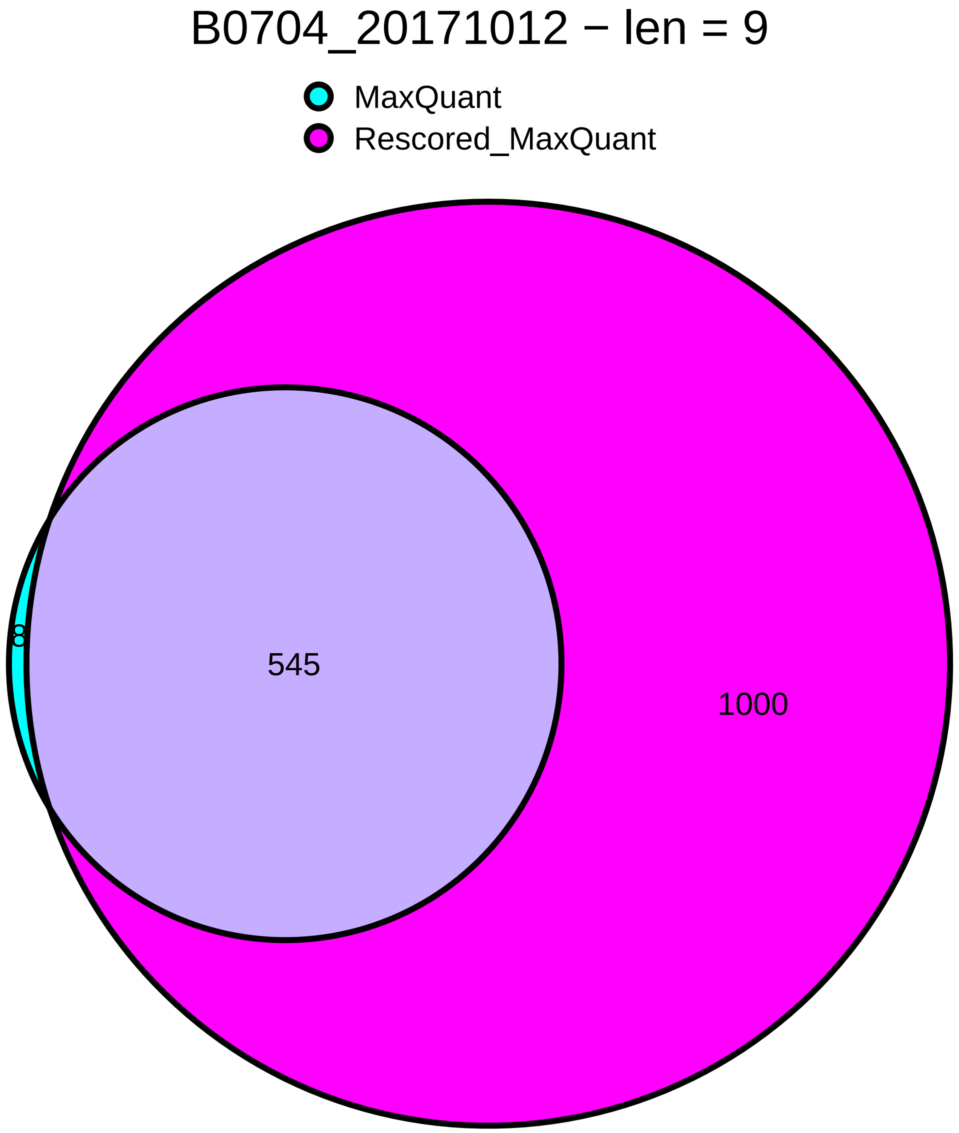
B0704_20171012 − len = 9
MaxQuant
Rescored_MaxQuant
8
545
1000

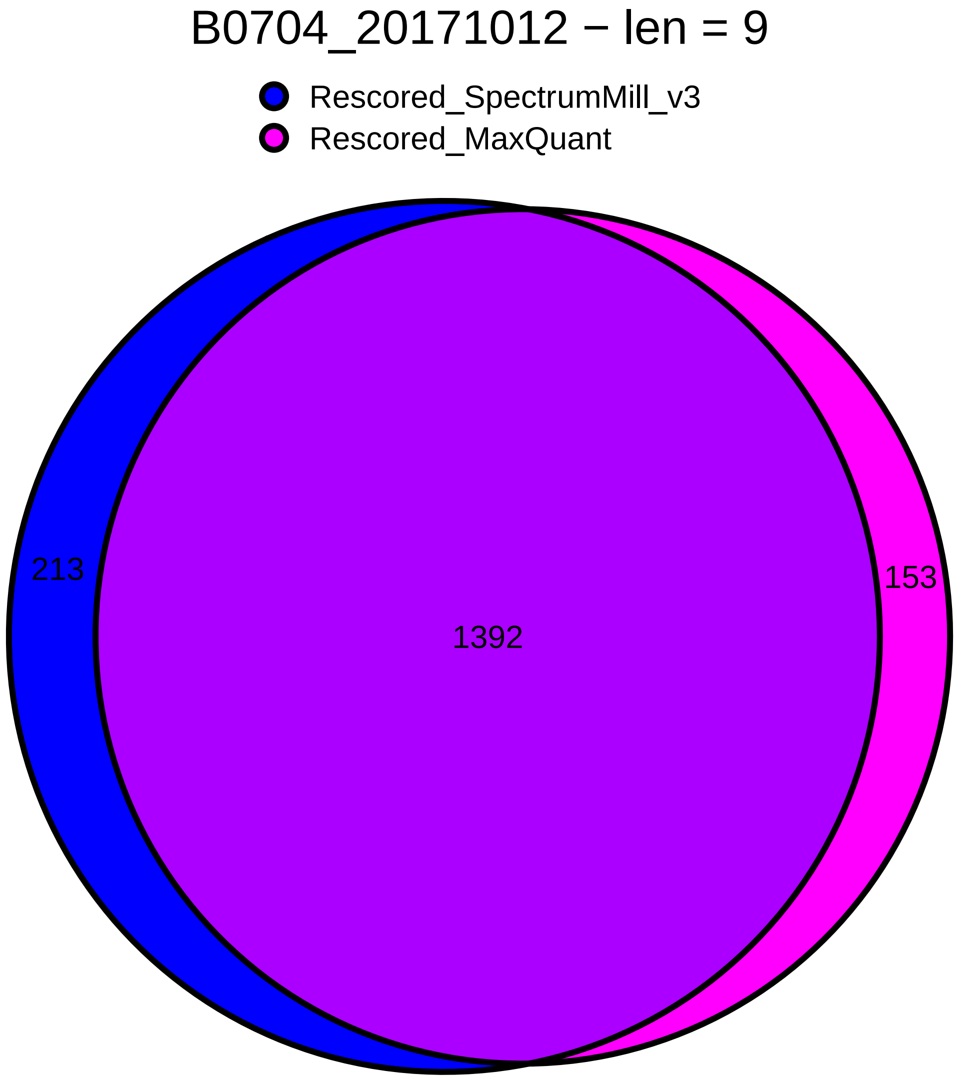
B0704_20171012 − len = 9
Rescored_SpectrumMill_v3
Rescored_MaxQuant
213
1392
153

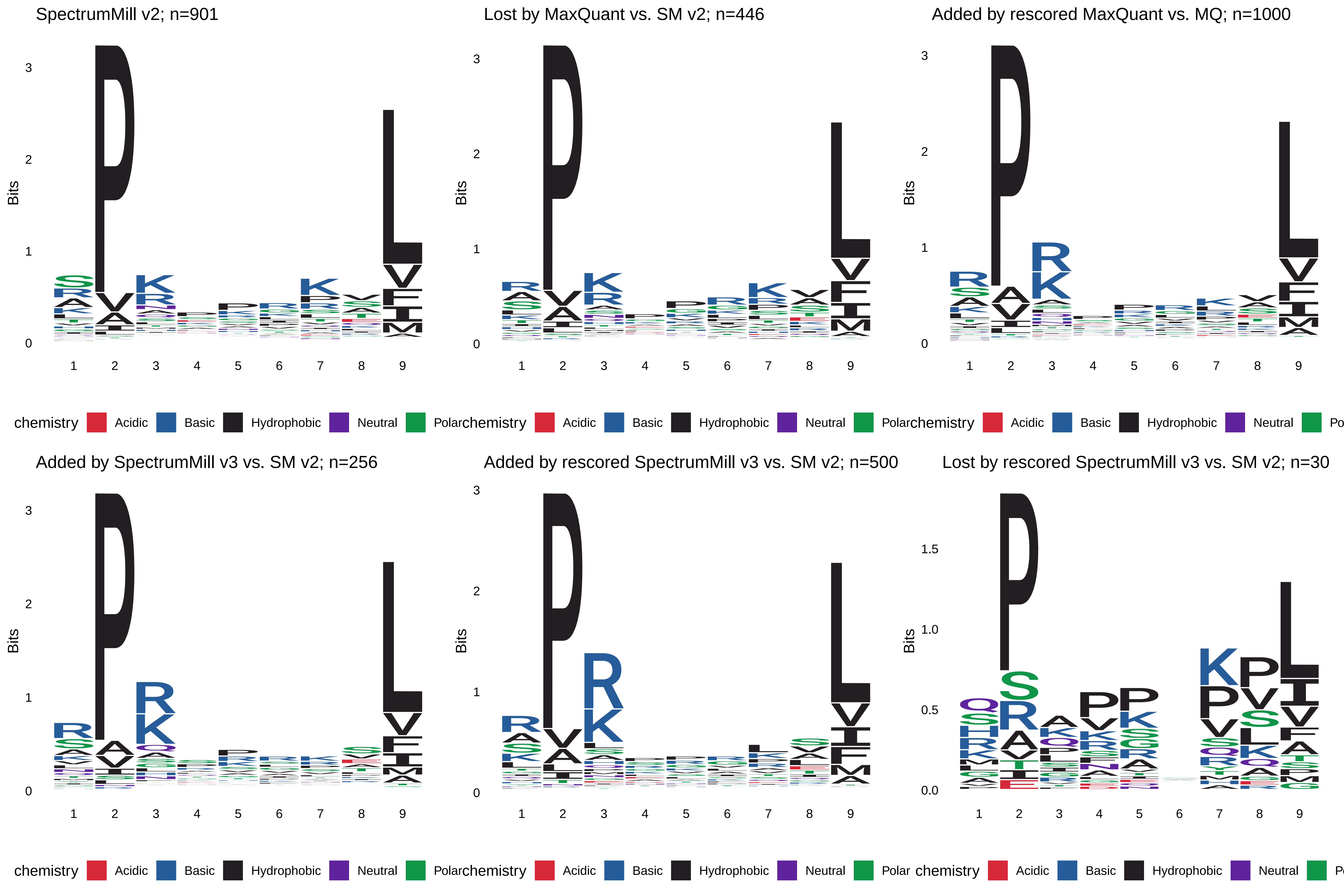
SpectrumMill v2; n=901
Lost by MaxQuant vs. SM v2; n=446
Added by rescored MaxQuant vs. MQ; n=1000
Added by SpectrumMill v3 vs. SM v2; n=256
Added by rescored SpectrumMill v3 vs. SM v2; n=500
Lost by rescored SpectrumMill v3 vs. SM v2; n=30
Bits
1 2 3 4 5 6 7 8 9
chemistry
Acidic
Basic
Hydrophobic
Neutral
Polar

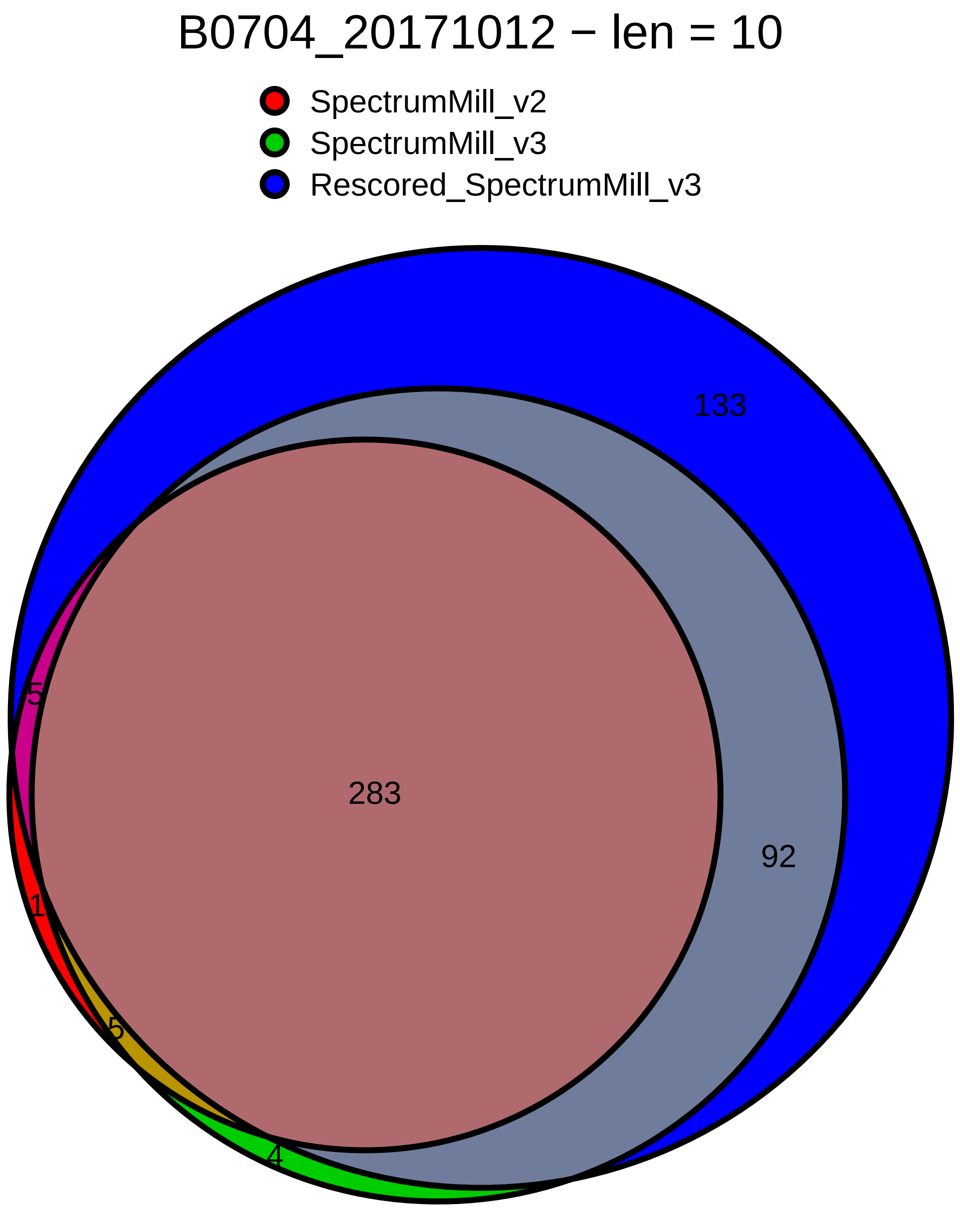
B0704_20171012 − len = 10
SpectrumMill_v2
SpectrumMill_v3
Rescored_SpectrumMill_v3
133
5
283
92
1
5
4

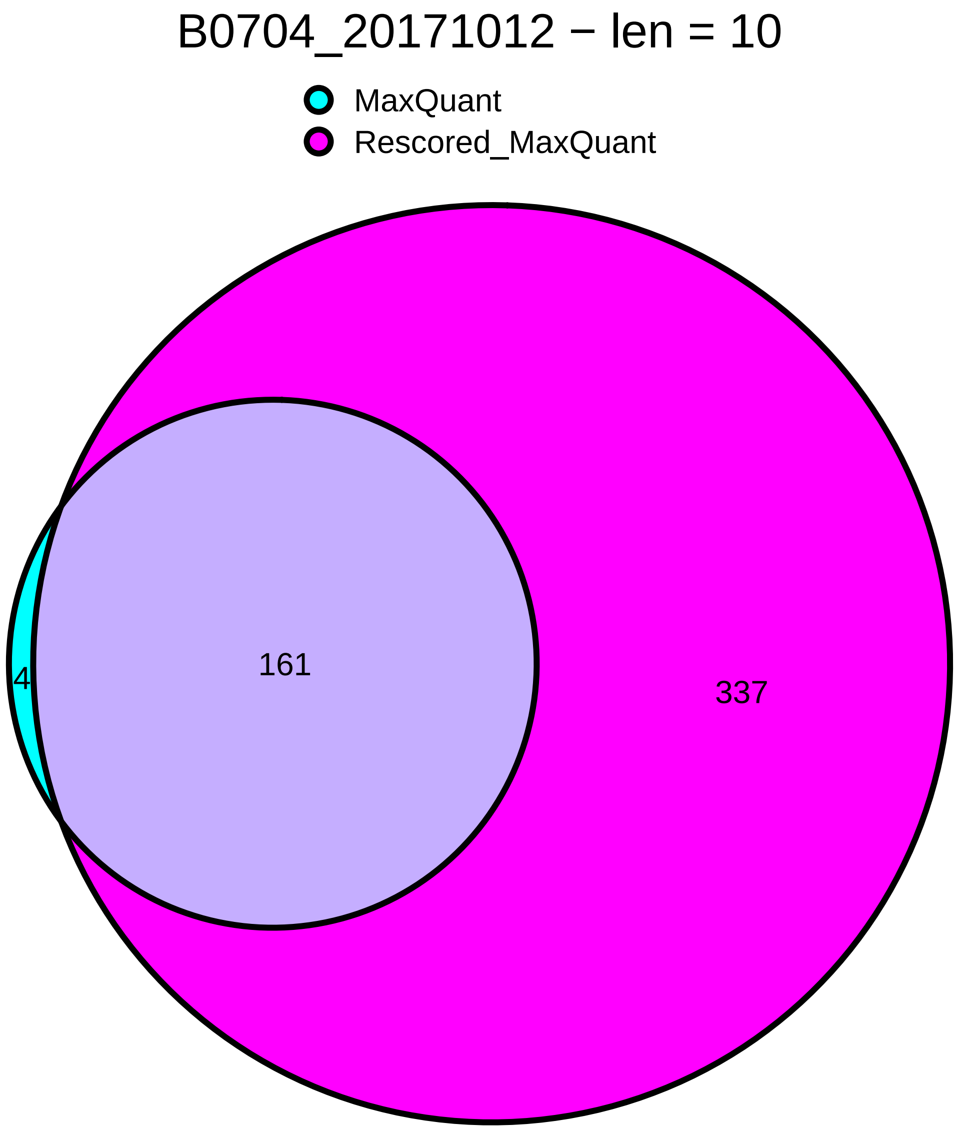
B0704_20171012 − len = 10
MaxQuant
Rescored_MaxQuant
4
161
337

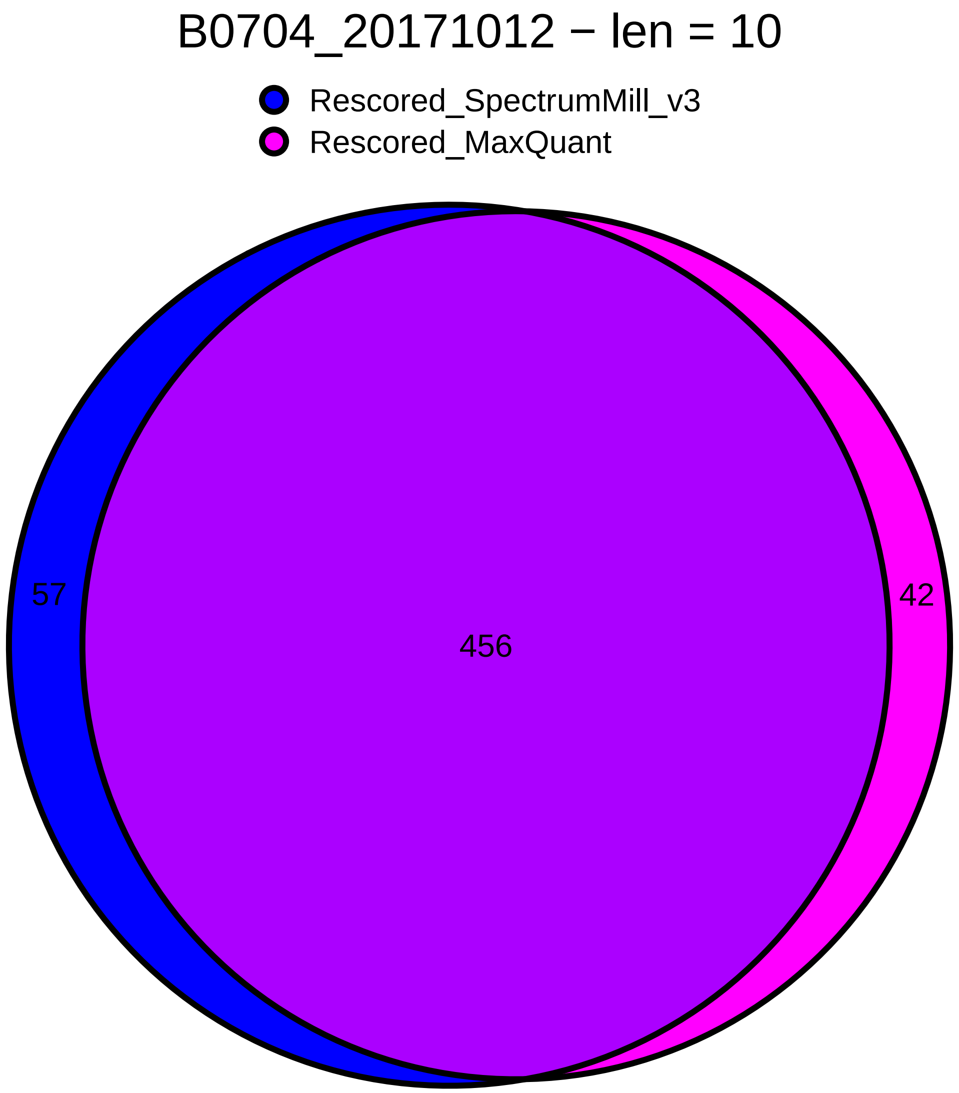
B0704_20171012 − len = 10
Rescored_SpectrumMill_v3
Rescored_MaxQuant
57
456
42

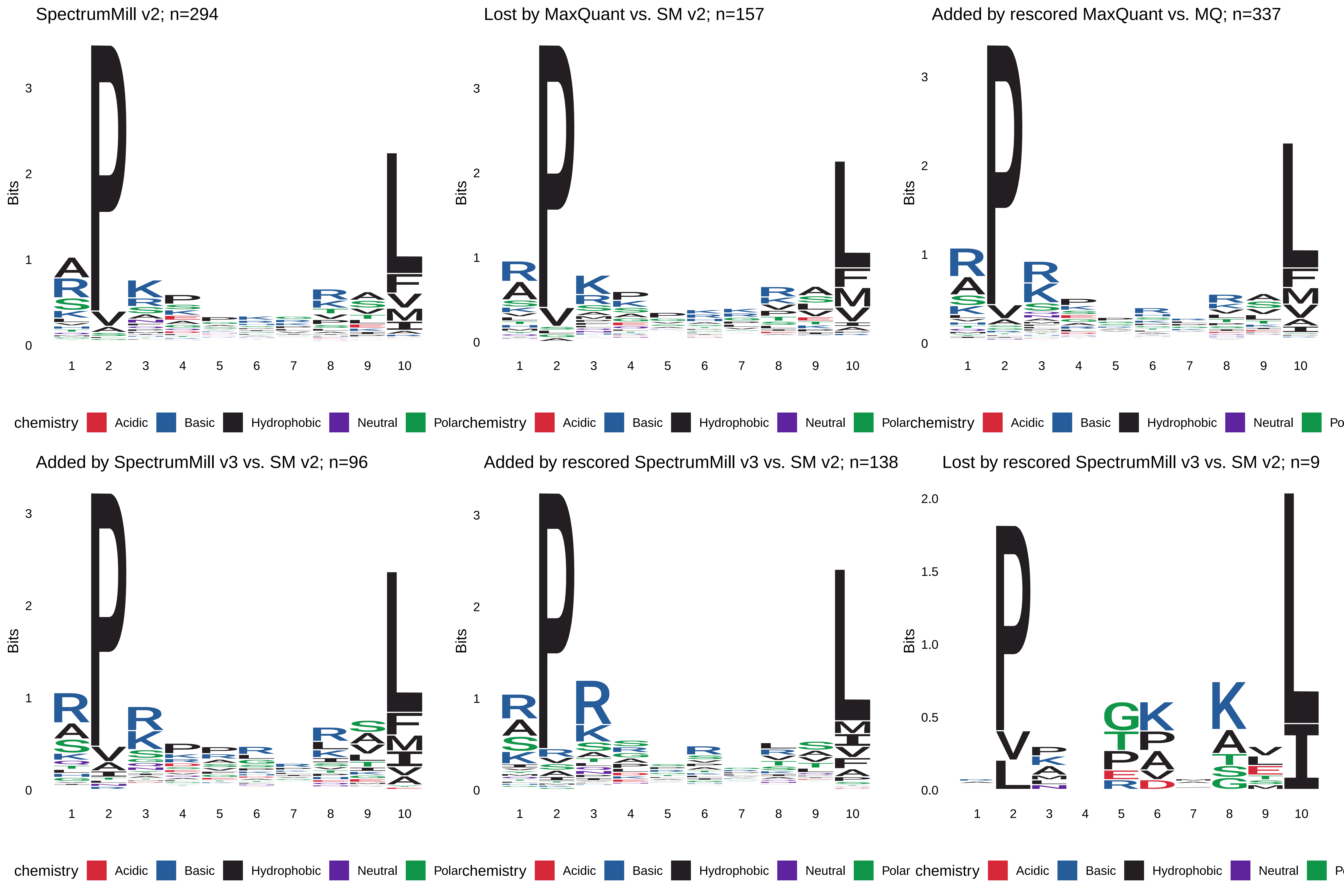
SpectrumMill v2; n=294
Lost by MaxQuant vs. SM v2; n=157
Added by rescored MaxQuant vs. MQ; n=337
Added by SpectrumMill v3 vs. SM v2; n=96
Added by rescored SpectrumMill v3 vs. SM v2; n=138
Lost by rescored SpectrumMill v3 vs. SM v2; n=9
Bits
1 2 3 4 5 6 7 8 9 10
chemistry Acidic Basic Hydrophobic Neutral Polar

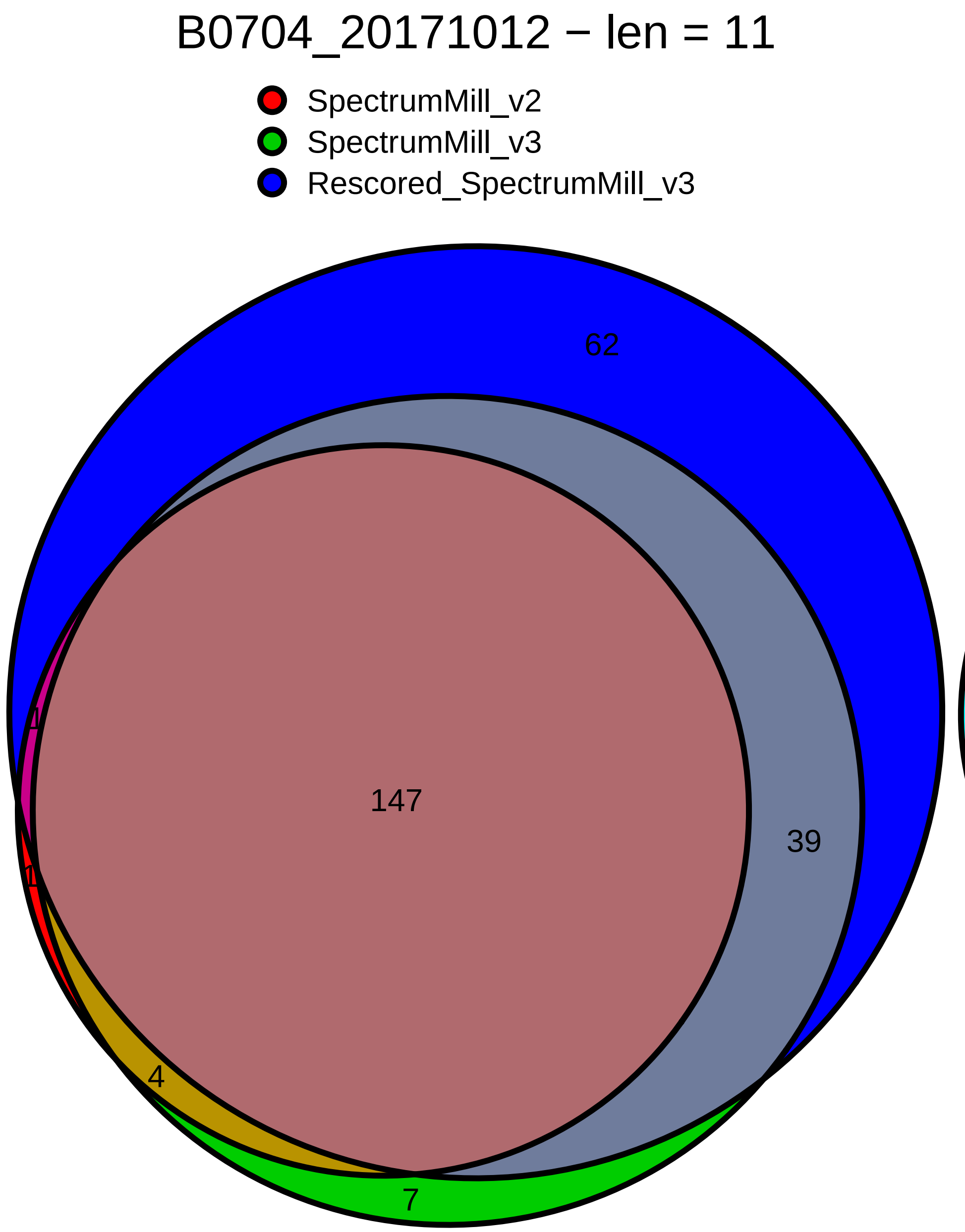
B0704_20171012 − len = 11
SpectrumMill_v2
SpectrumMill_v3
Rescored_SpectrumMill_v3
62
147
39
4
7

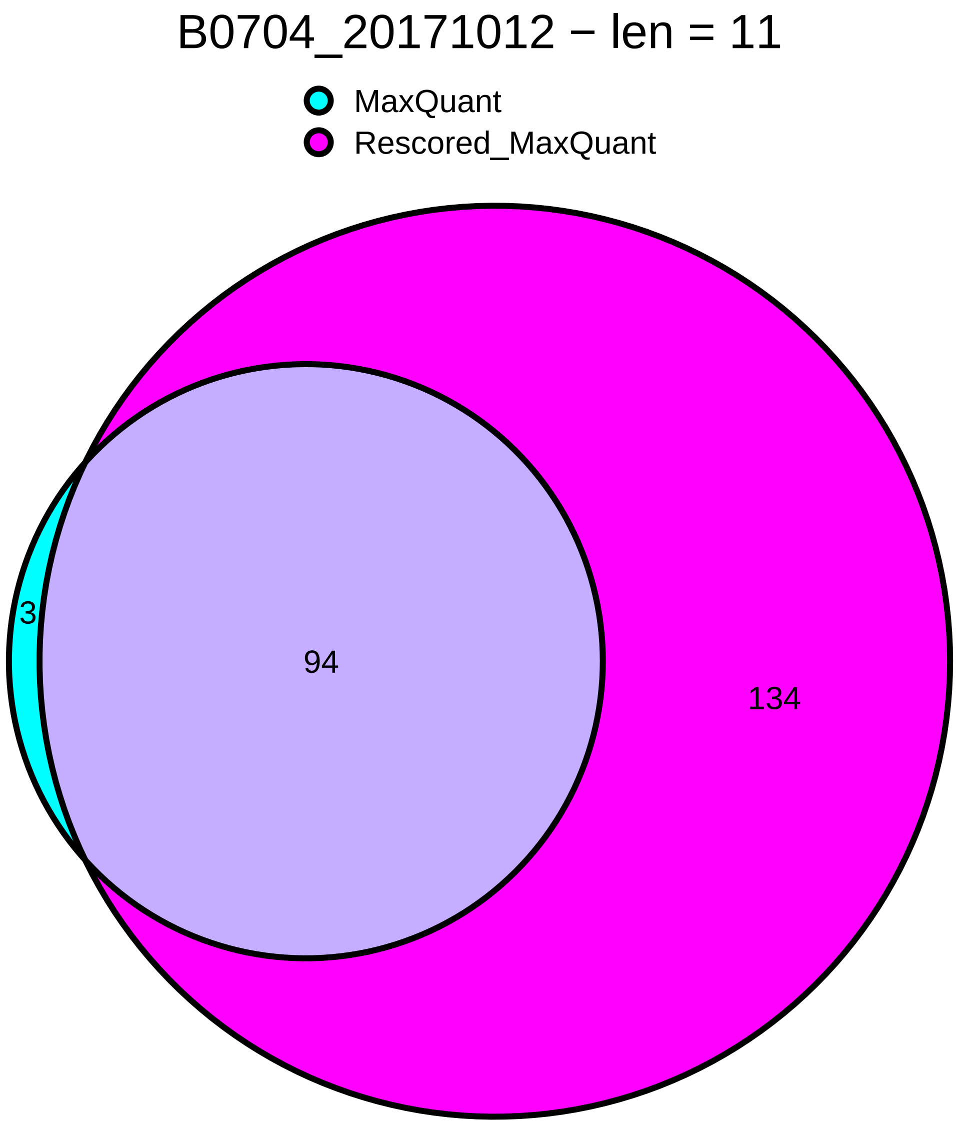
B0704_20171012 − len = 11
MaxQuant
Rescored_MaxQuant
3
94
134

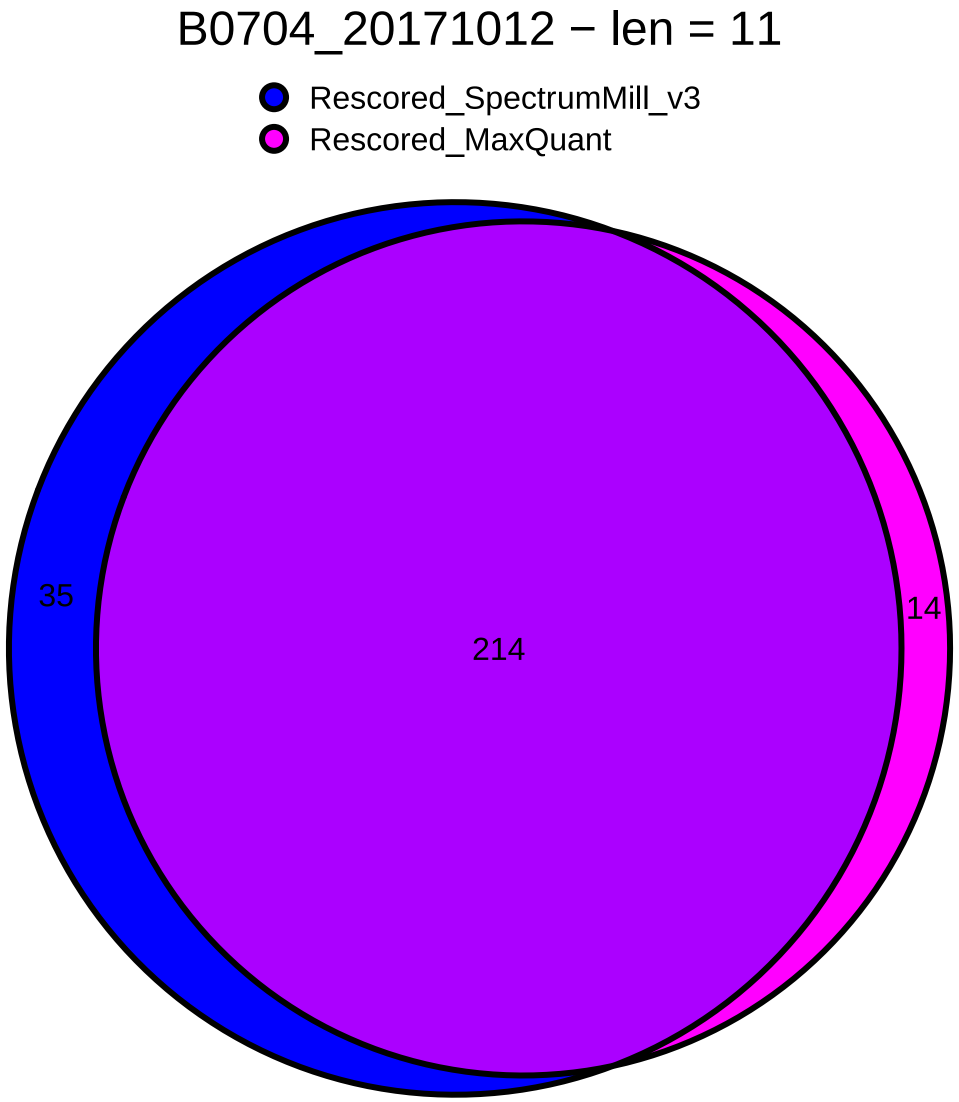
B0704_20171012 − len = 11
Rescored_SpectrumMill_v3
Rescored_MaxQuant
35
214
14

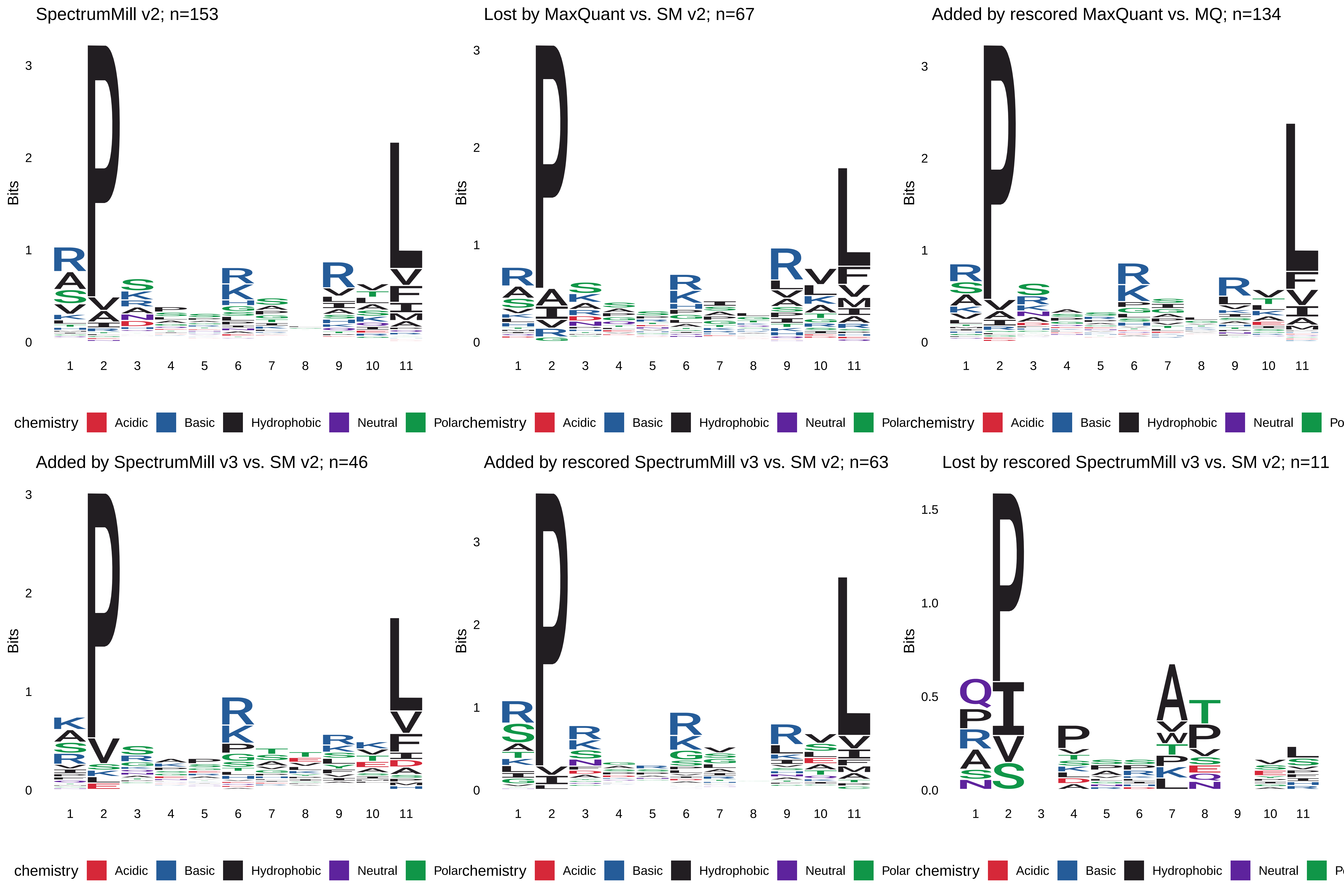

SpectrumMill v2; n=153
Lost by MaxQuant vs. SM v2; n=67
Added by rescored MaxQuant vs. MQ; n=134
Added by SpectrumMill v3 vs. SM v2; n=46
Added by rescored SpectrumMill v3 vs. SM v2; n=63
Lost by rescored SpectrumMill v3 vs. SM v2; n=11
Bits
1 2 3 4 5 6 7 8 9 10 11
chemistry
Acidic
Basic
Hydrophobic
Neutral
Polar

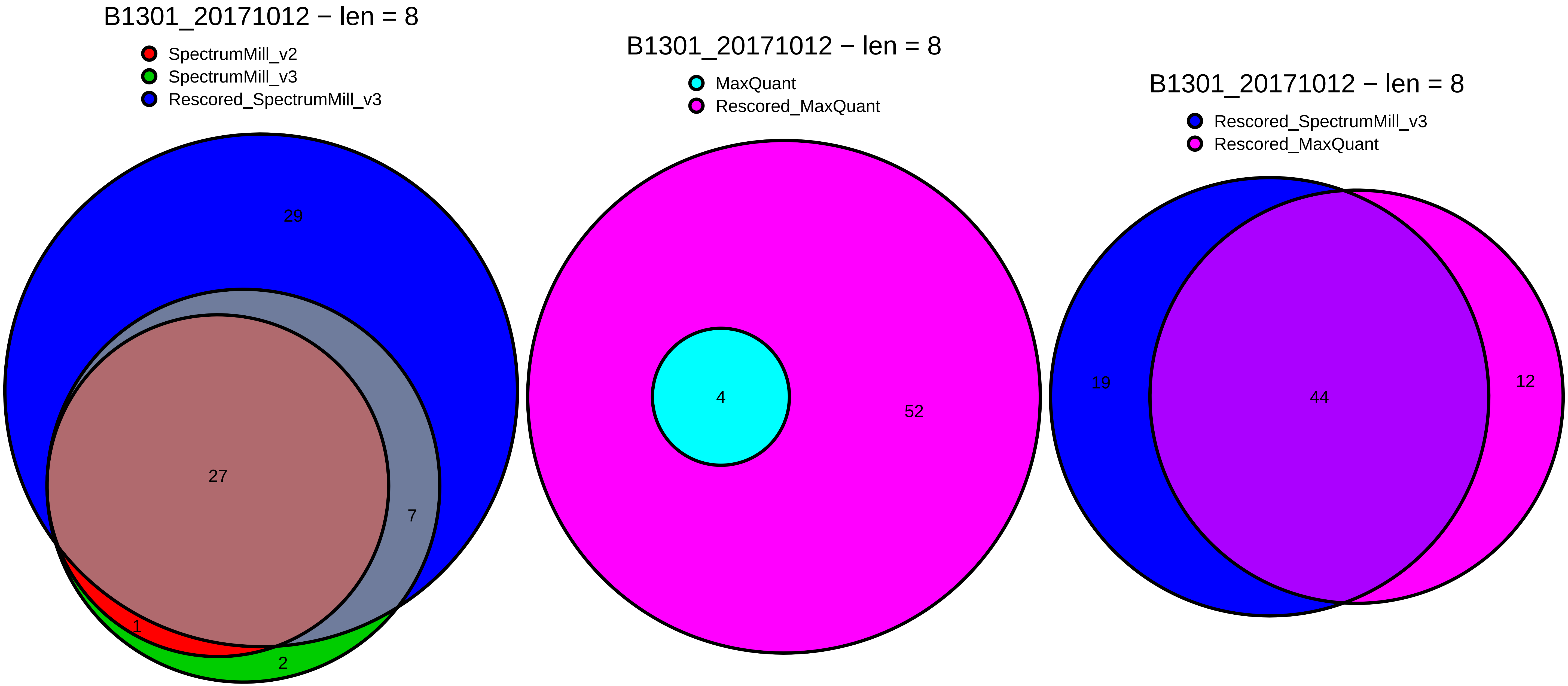
B1301_20171012 − len = 8
SpectrumMill_v2
SpectrumMill_v3
Rescored_SpectrumMill_v3
29
27
7
1
2
B1301_20171012 − len = 8
MaxQuant
Rescored_MaxQuant
4
52
B1301_20171012 − len = 8
Rescored_SpectrumMill_v3
Rescored_MaxQuant
19
44
12

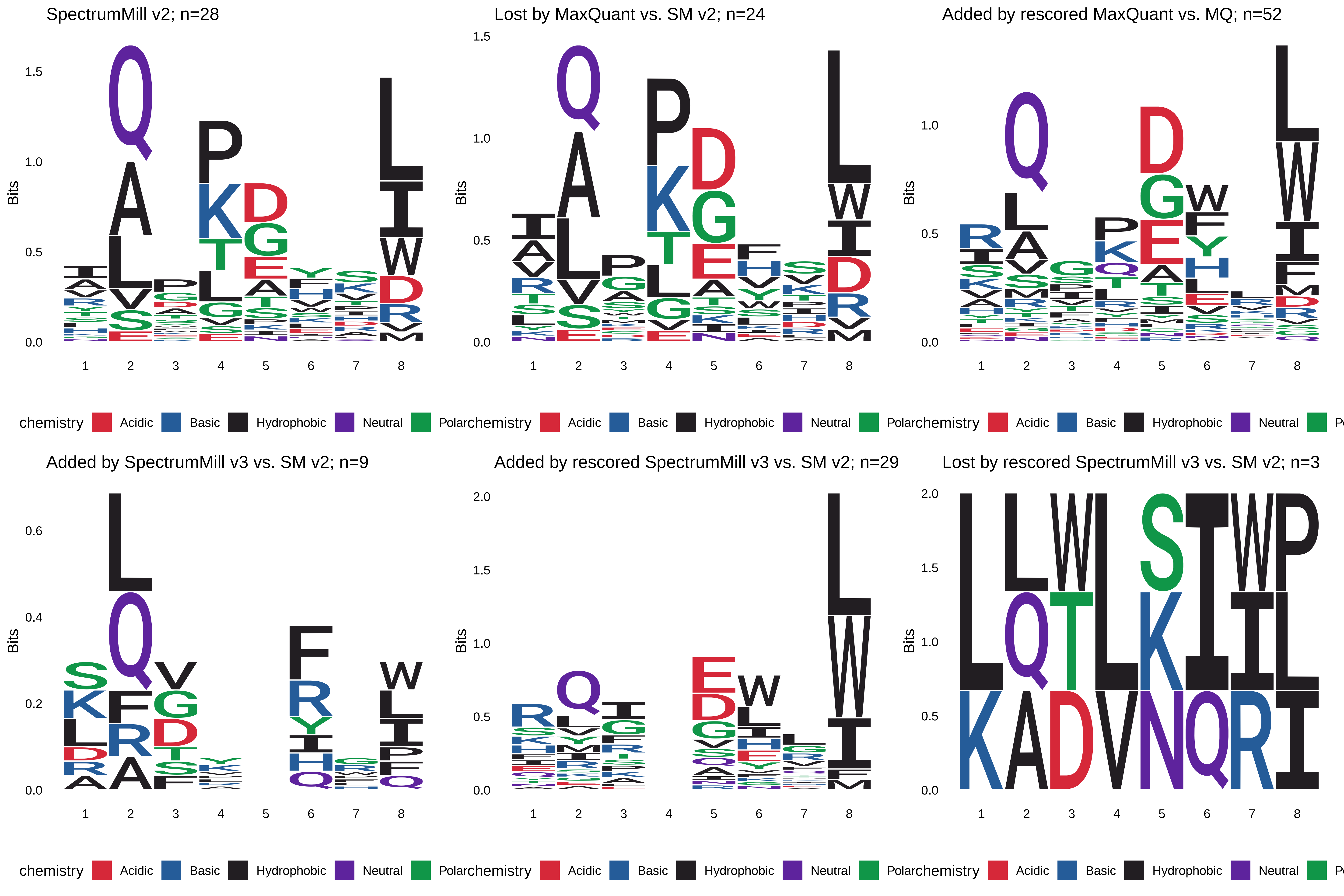

SpectrumMill v2; n=28
Lost by MaxQuant vs. SM v2; n=24
Added by rescored MaxQuant vs. MQ; n=52
Bits
chemistry Acidic Basic Hydrophobic Neutral Polar
Added by SpectrumMill v3 vs. SM v2; n=9
Added by rescored SpectrumMill v3 vs. SM v2; n=29
Lost by rescored SpectrumMill v3 vs. SM v2; n=3

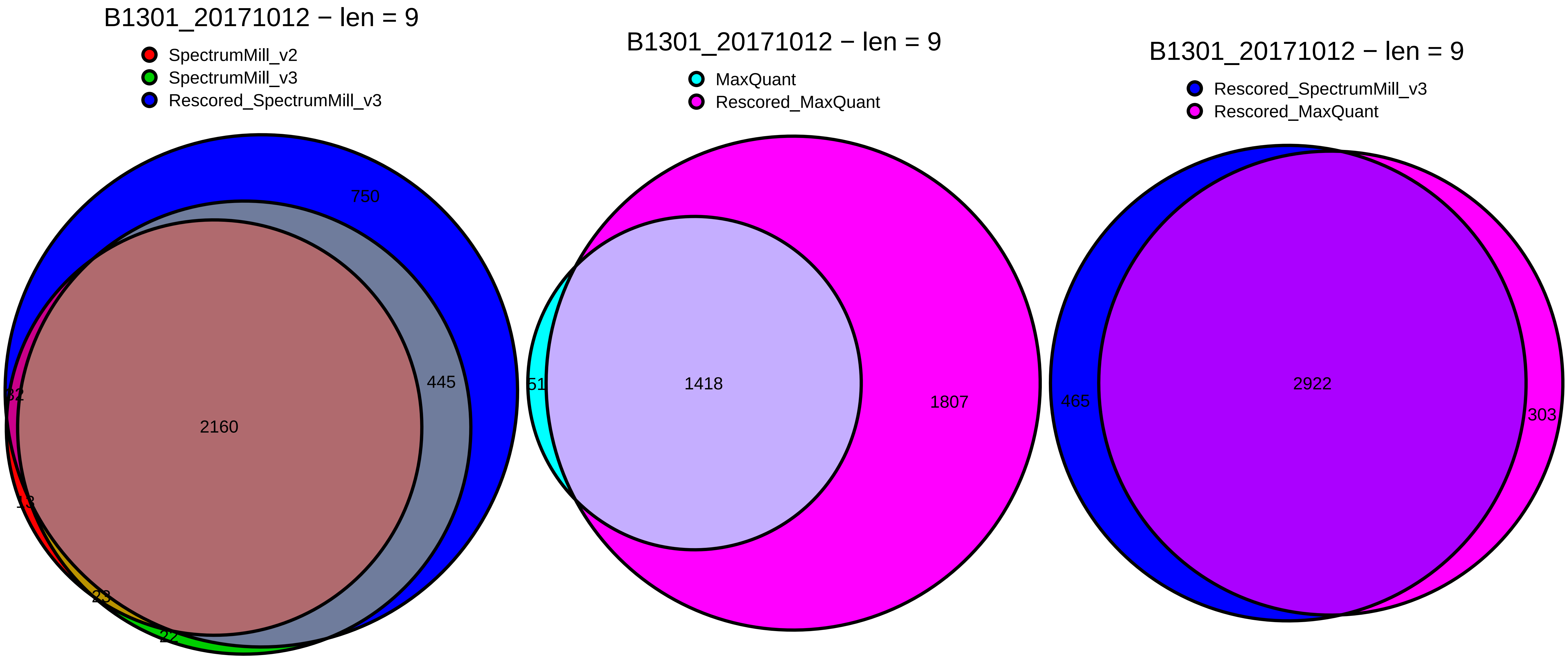
B1301_20171012 − len = 9
SpectrumMill_v2
SpectrumMill_v3
Rescored_SpectrumMill_v3
750
445
32
2160
13
23
22
B1301_20171012 − len = 9
MaxQuant
Rescored_MaxQuant
51
1418
1807
B1301_20171012 − len = 9
Rescored_SpectrumMill_v3
Rescored_MaxQuant
465
2922
303

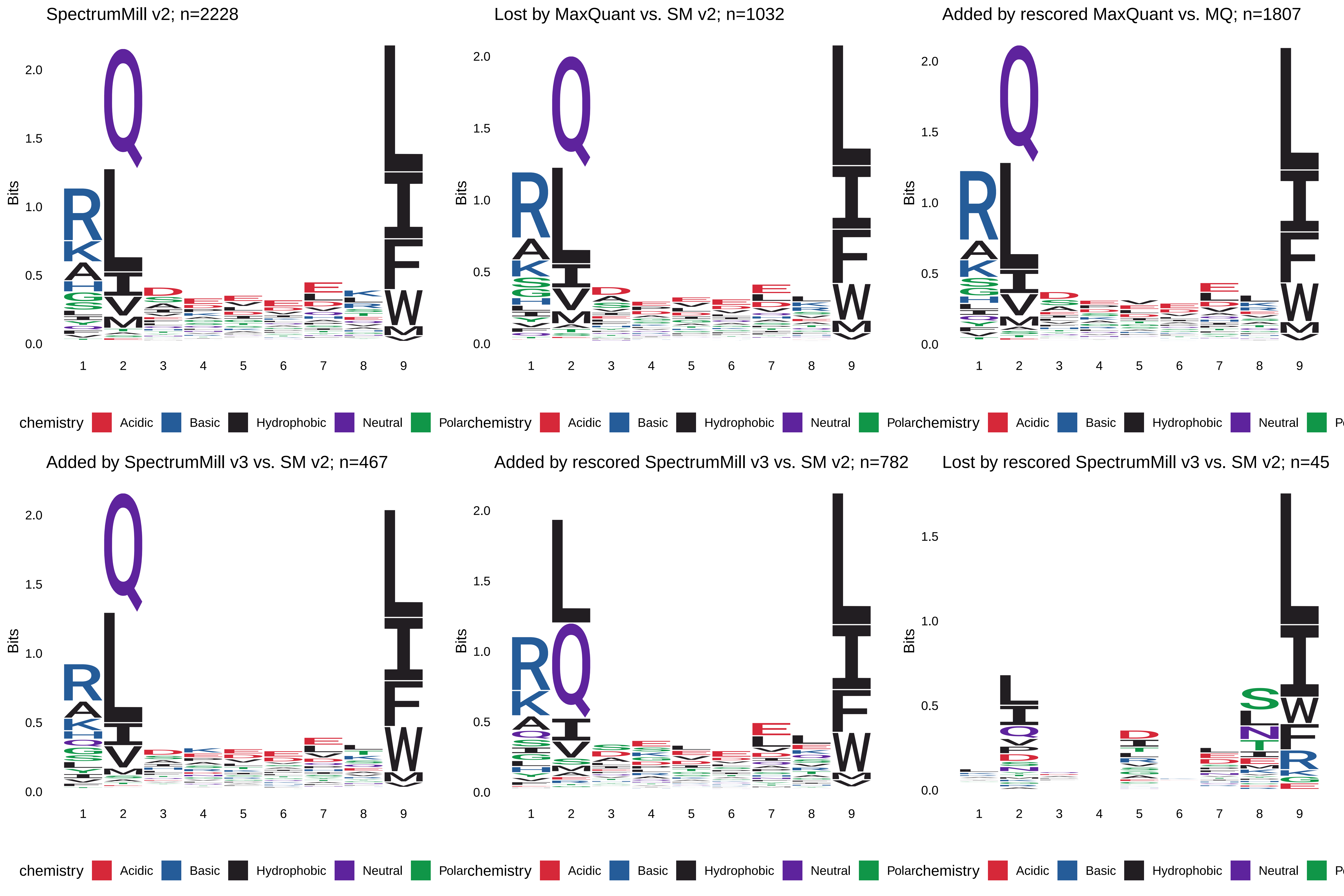
SpectrumMill v2; n=2228
Lost by MaxQuant vs. SM v2; n=1032
Added by rescored MaxQuant vs. MQ; n=1807
Added by SpectrumMill v3 vs. SM v2; n=467
Added by rescored SpectrumMill v3 vs. SM v2; n=782
Lost by rescored SpectrumMill v3 vs. SM v2; n=45
Bits
0.0
0.5
1.0
1.5
2.0
1
2
3
4
5
6
7
8
9
chemistry
Acidic
Basic
Hydrophobic
Neutral
Polar

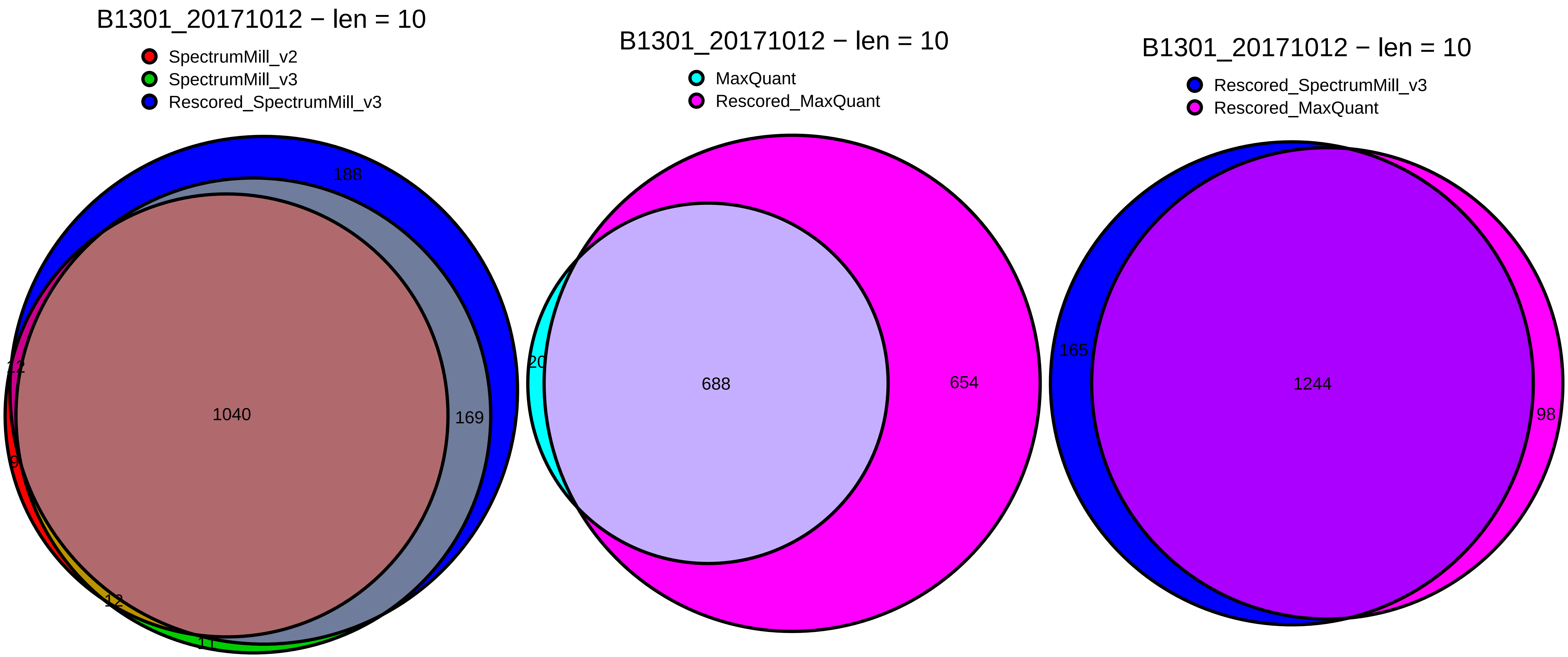
B1301_20171012 − len = 10
SpectrumMill_v2
SpectrumMill_v3
Rescored_SpectrumMill_v3
188
1040
169
12
11
B1301_20171012 − len = 10
MaxQuant
Rescored_MaxQuant
20
688
654
B1301_20171012 − len = 10
Rescored_SpectrumMill_v3
Rescored_MaxQuant
165
1244
98

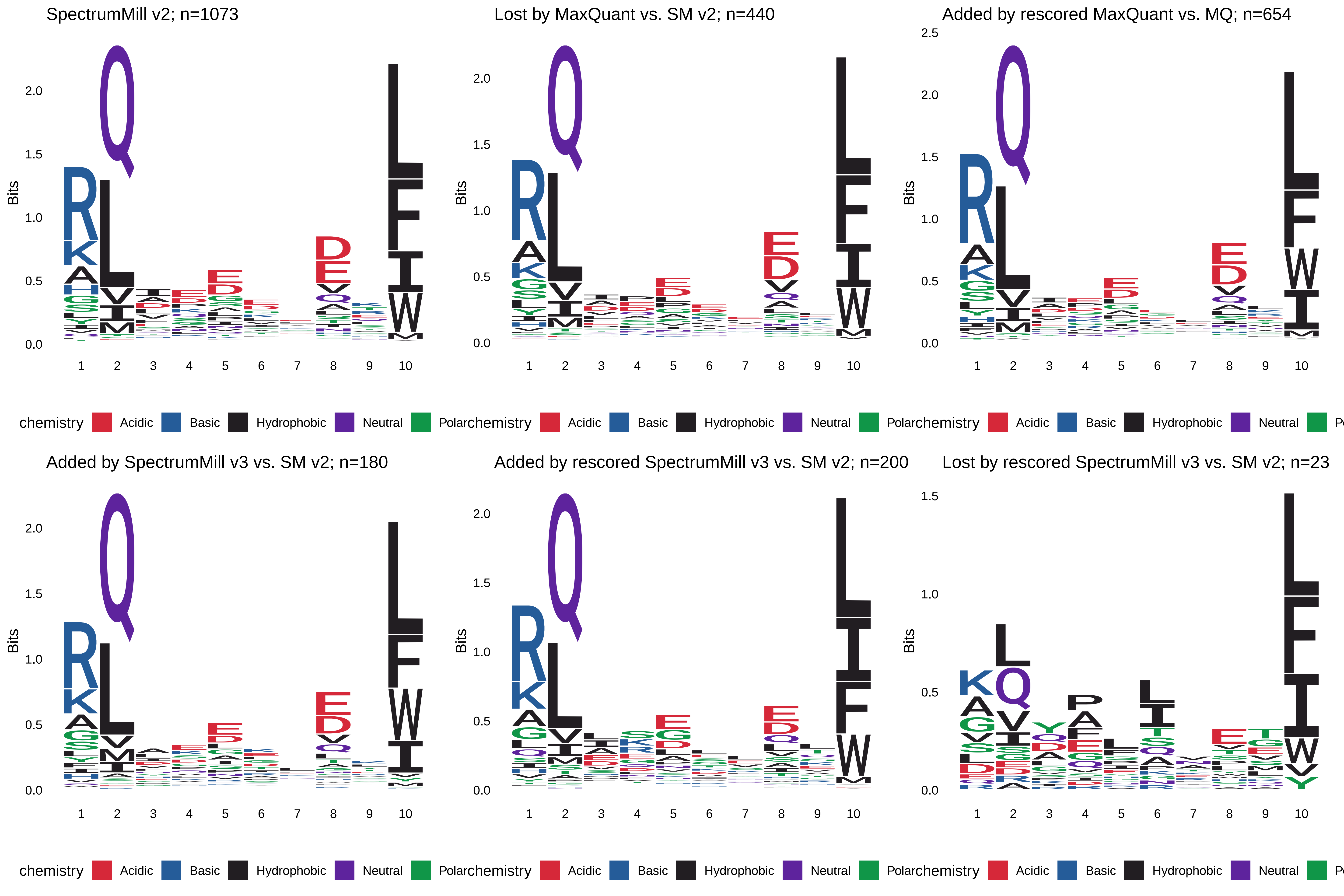
SpectrumMill v2; n=1073
Lost by MaxQuant vs. SM v2; n=440
Added by rescored MaxQuant vs. MQ; n=654
Added by SpectrumMill v3 vs. SM v2; n=180
Added by rescored SpectrumMill v3 vs. SM v2; n=200
Lost by rescored SpectrumMill v3 vs. SM v2; n=23
Bits
0.0
0.5
1.0
1.5
2.0
2.5
1 2 3 4 5 6 7 8 9 10
chemistry Acidic Basic Hydrophobic Neutral Polarchemistry Acidic Basic Hydrophobic Neutral Polarchemistry Acidic Basic Hydrophobic Neutral Po

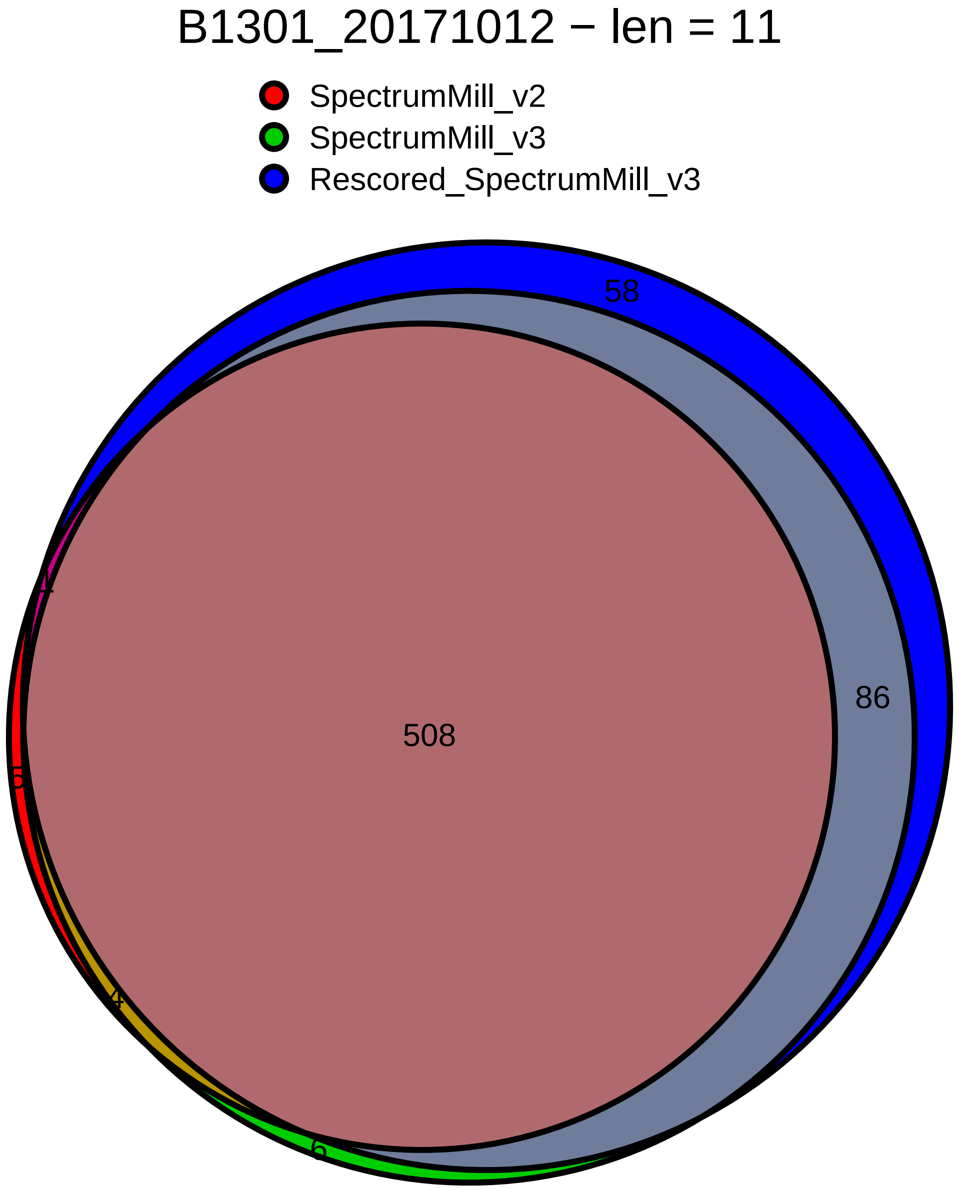
B1301_20171012 − len = 11
SpectrumMill_v2
SpectrumMill_v3
Rescored_SpectrumMill_v3
58
86
508

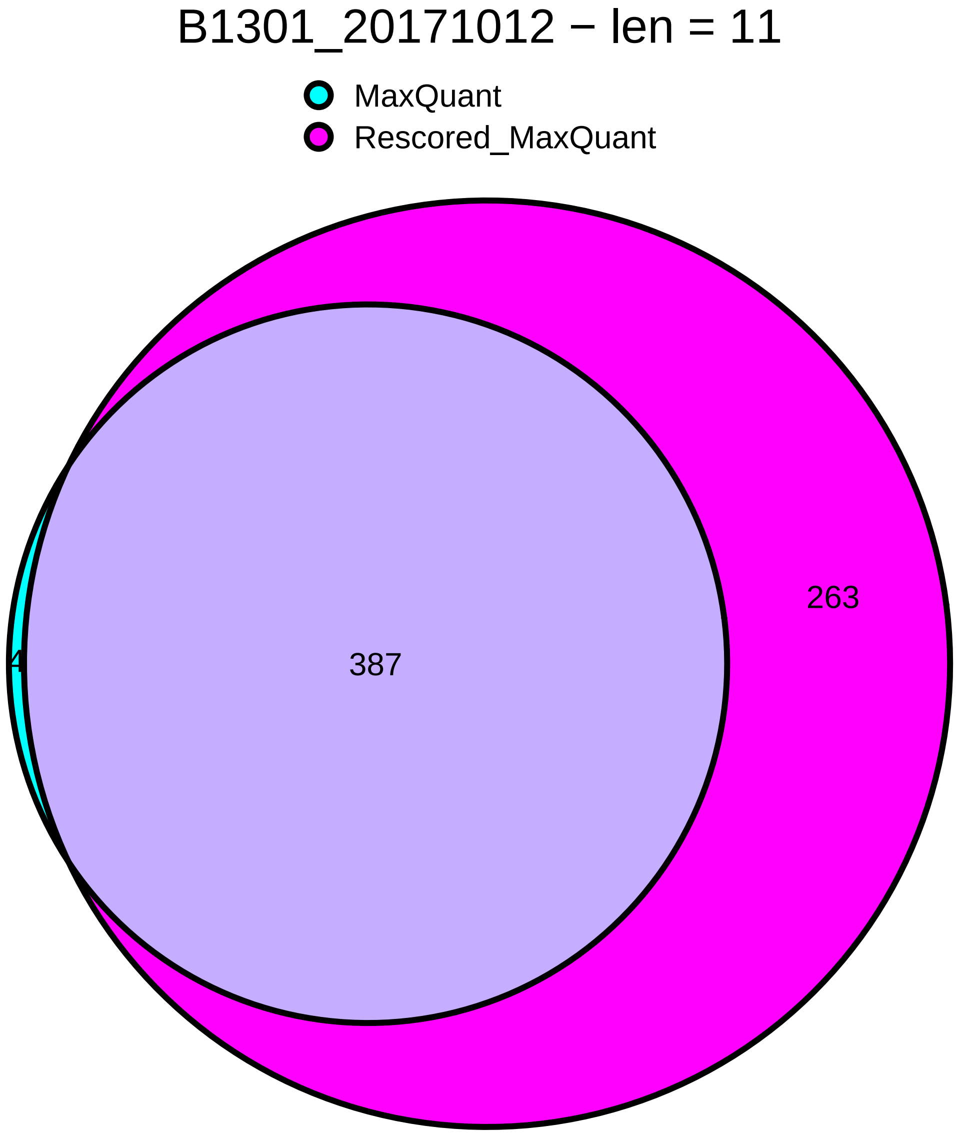
B1301_20171012 − len = 11
MaxQuant
Rescored_MaxQuant
387
263

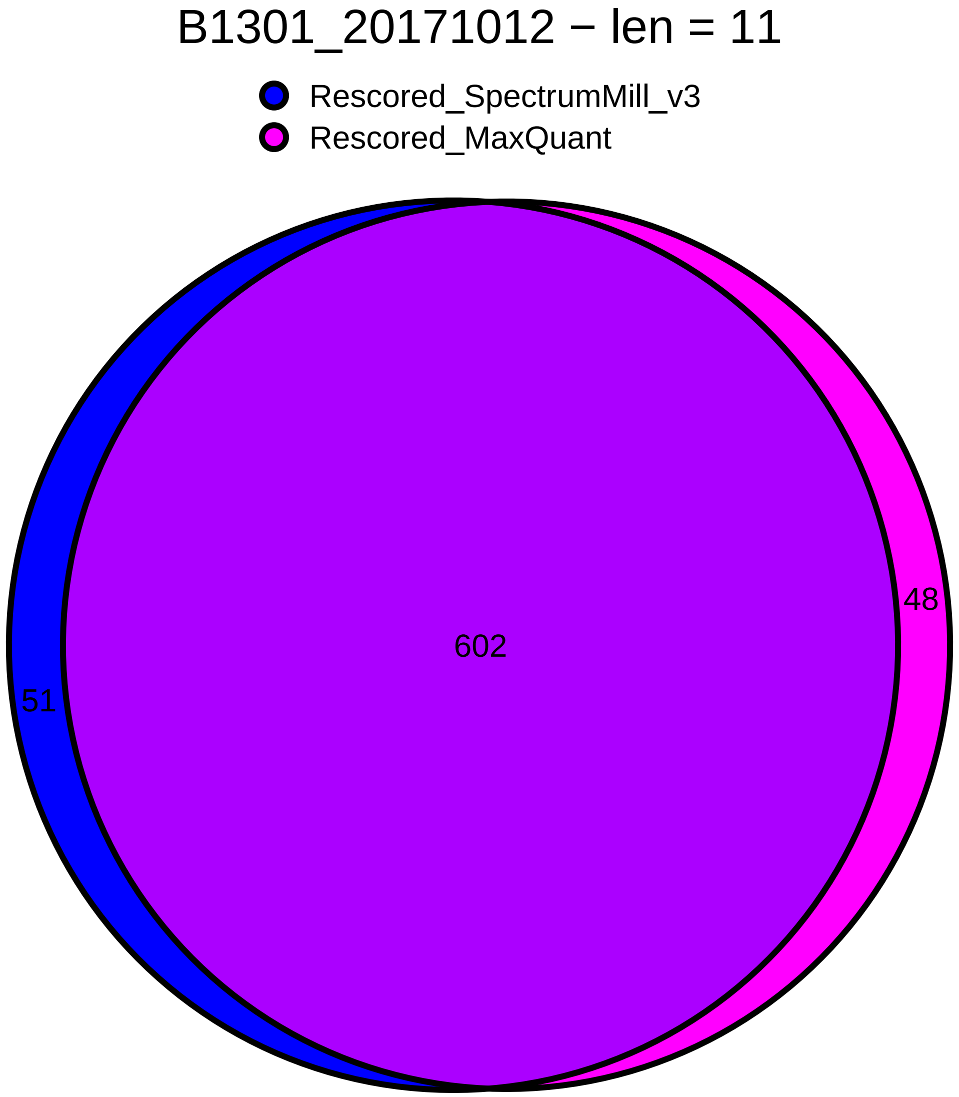
B1301_20171012 − len = 11
Rescored_SpectrumMill_v3
Rescored_MaxQuant
602
51
48

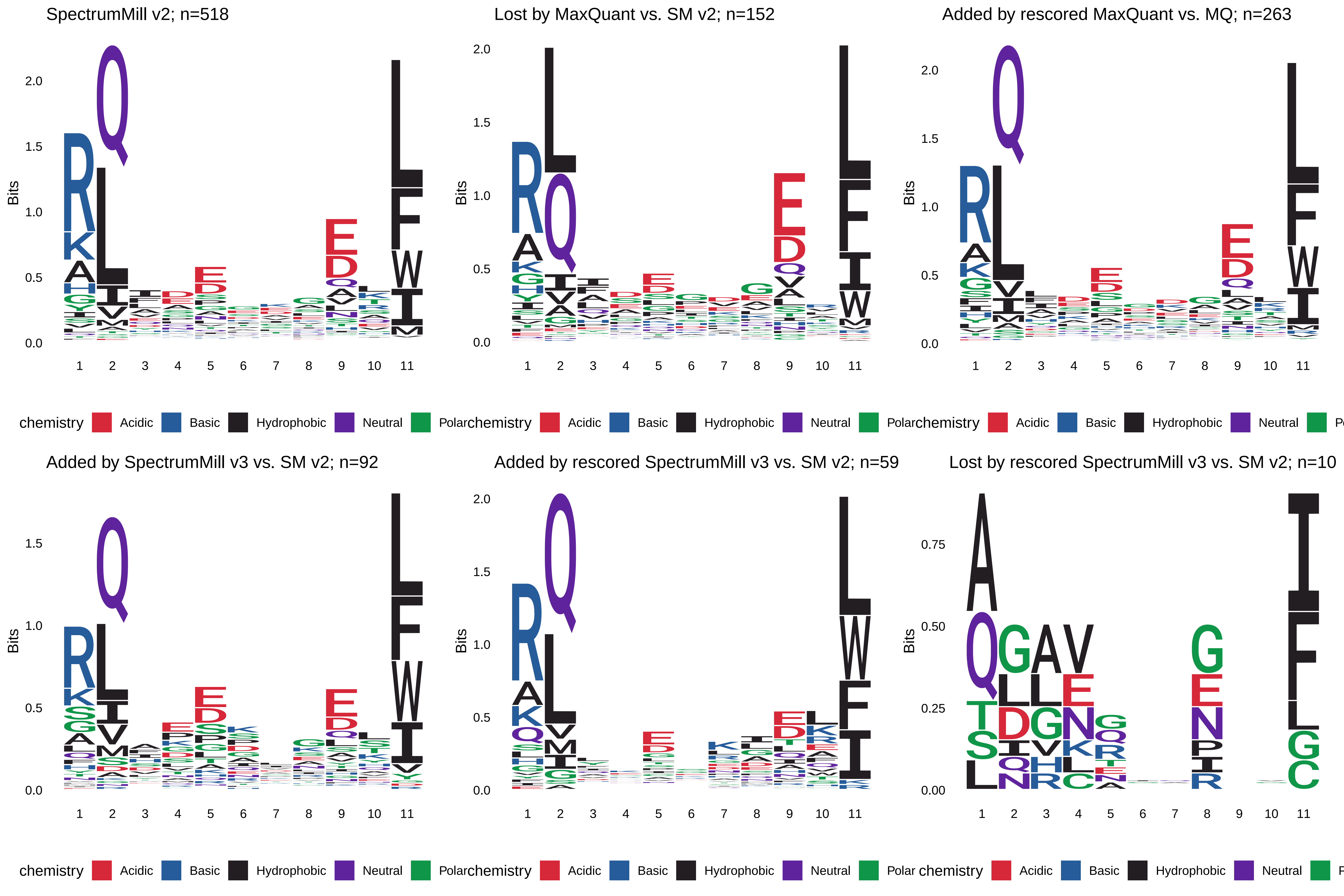

SpectrumMill v2; n=518
Lost by MaxQuant vs. SM v2; n=152
Added by rescored MaxQuant vs. MQ; n=263
Added by SpectrumMill v3 vs. SM v2; n=92
Added by rescored SpectrumMill v3 vs. SM v2; n=59
Lost by rescored SpectrumMill v3 vs. SM v2; n=10
Bits
1 2 3 4 5 6 7 8 9 10 11
chemistry Acidic Basic Hydrophobic Neutral Polar

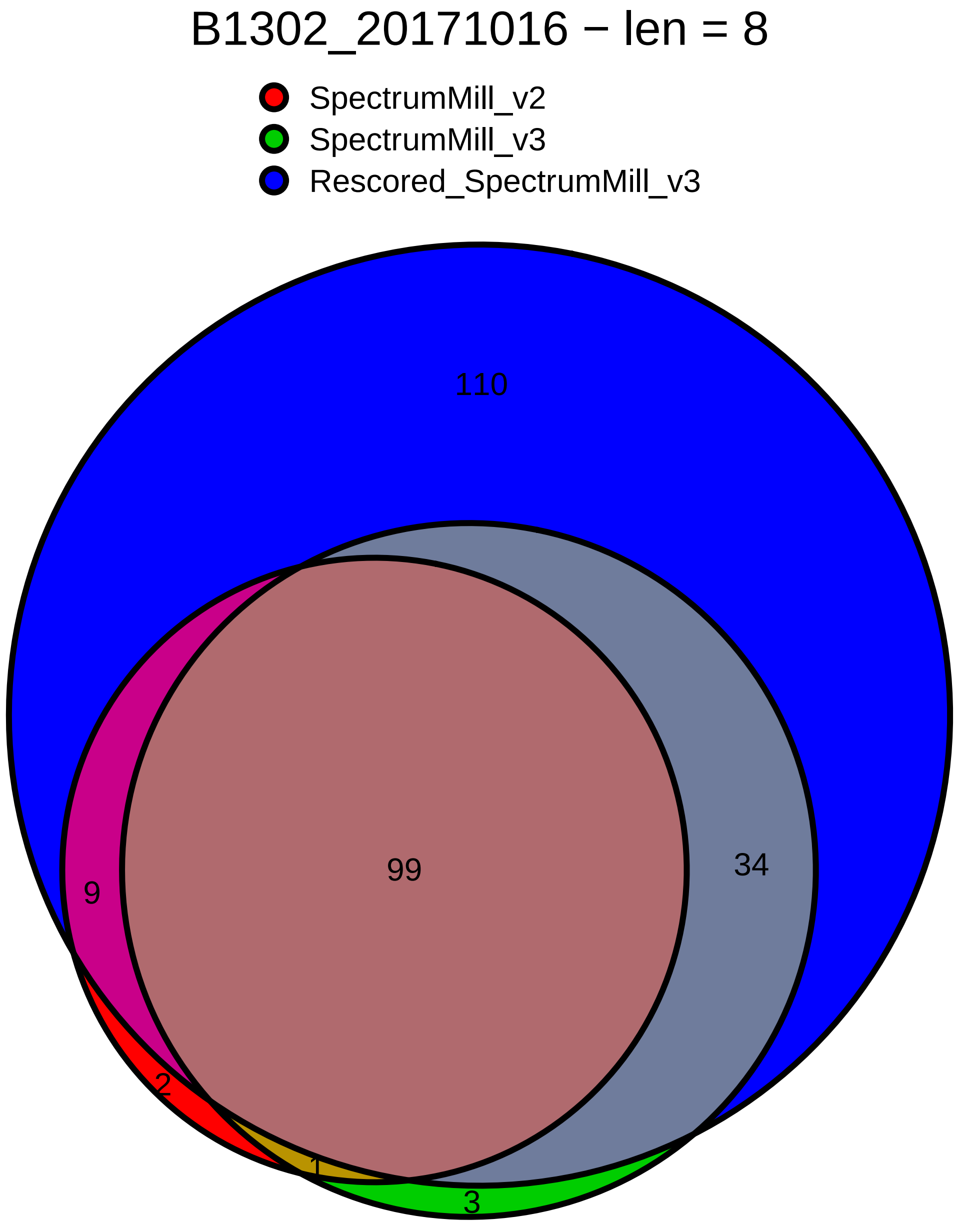
B1302_20171016 − len = 8
SpectrumMill_v2
SpectrumMill_v3
Rescored_SpectrumMill_v3
110
9
99
34
2
1
3

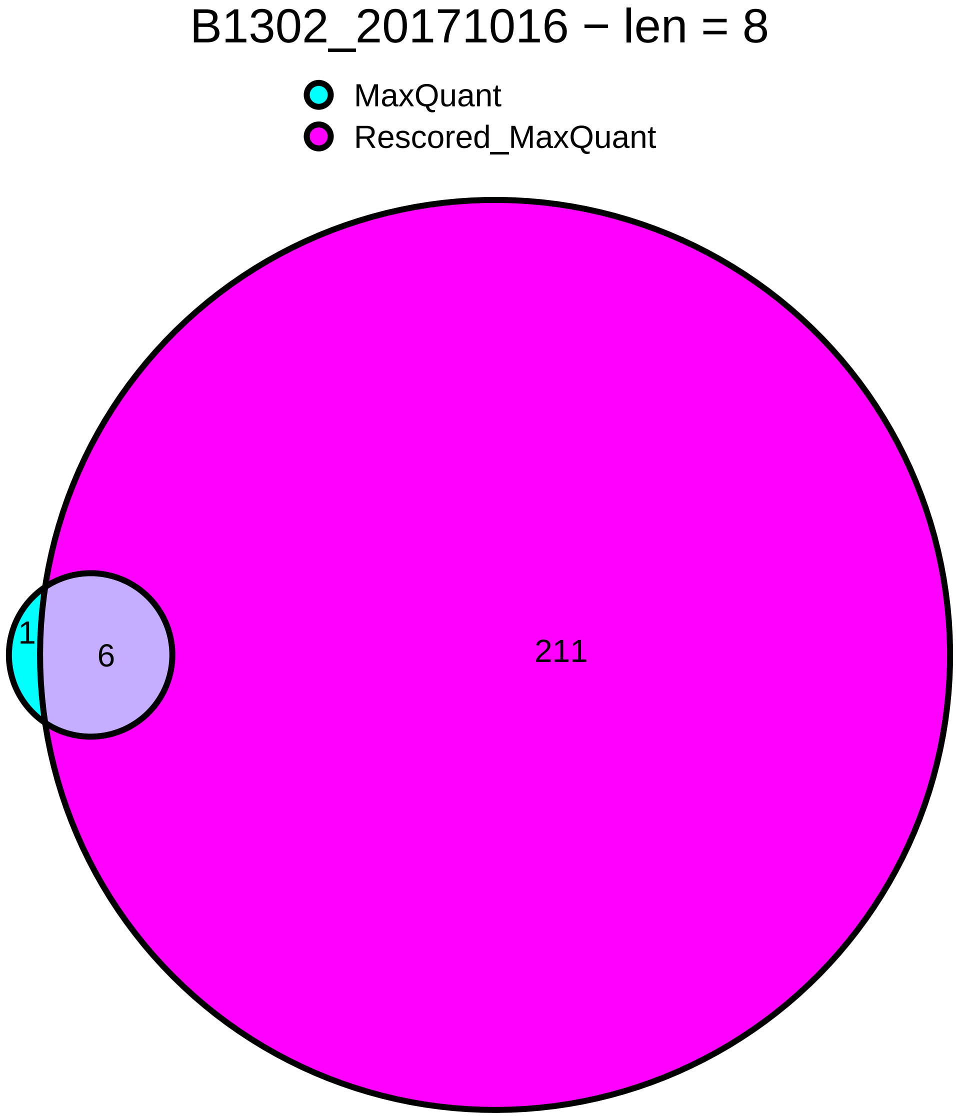
B1302_20171016 − len = 8
MaxQuant
Rescored_MaxQuant
1
6
211

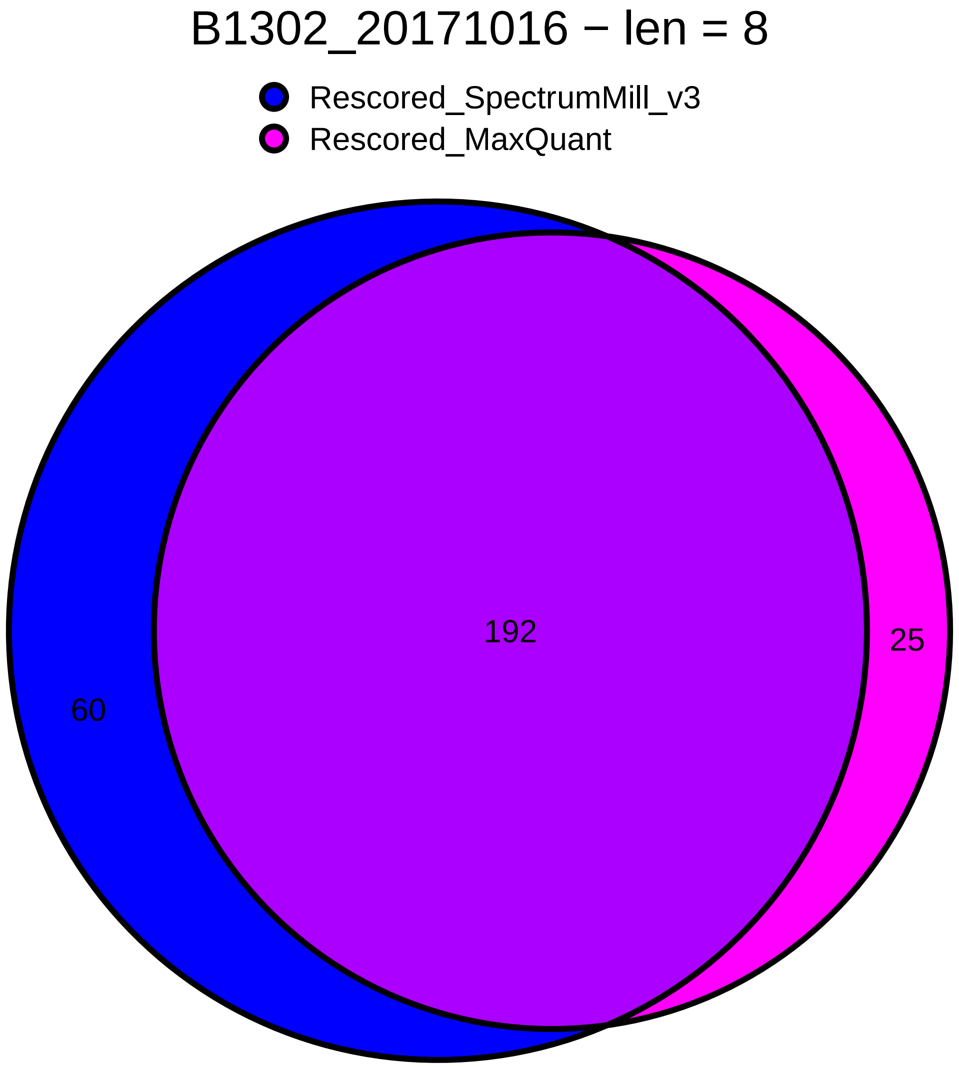
B1302_20171016 − len = 8
Rescored_SpectrumMill_v3
Rescored_MaxQuant
60
192
25

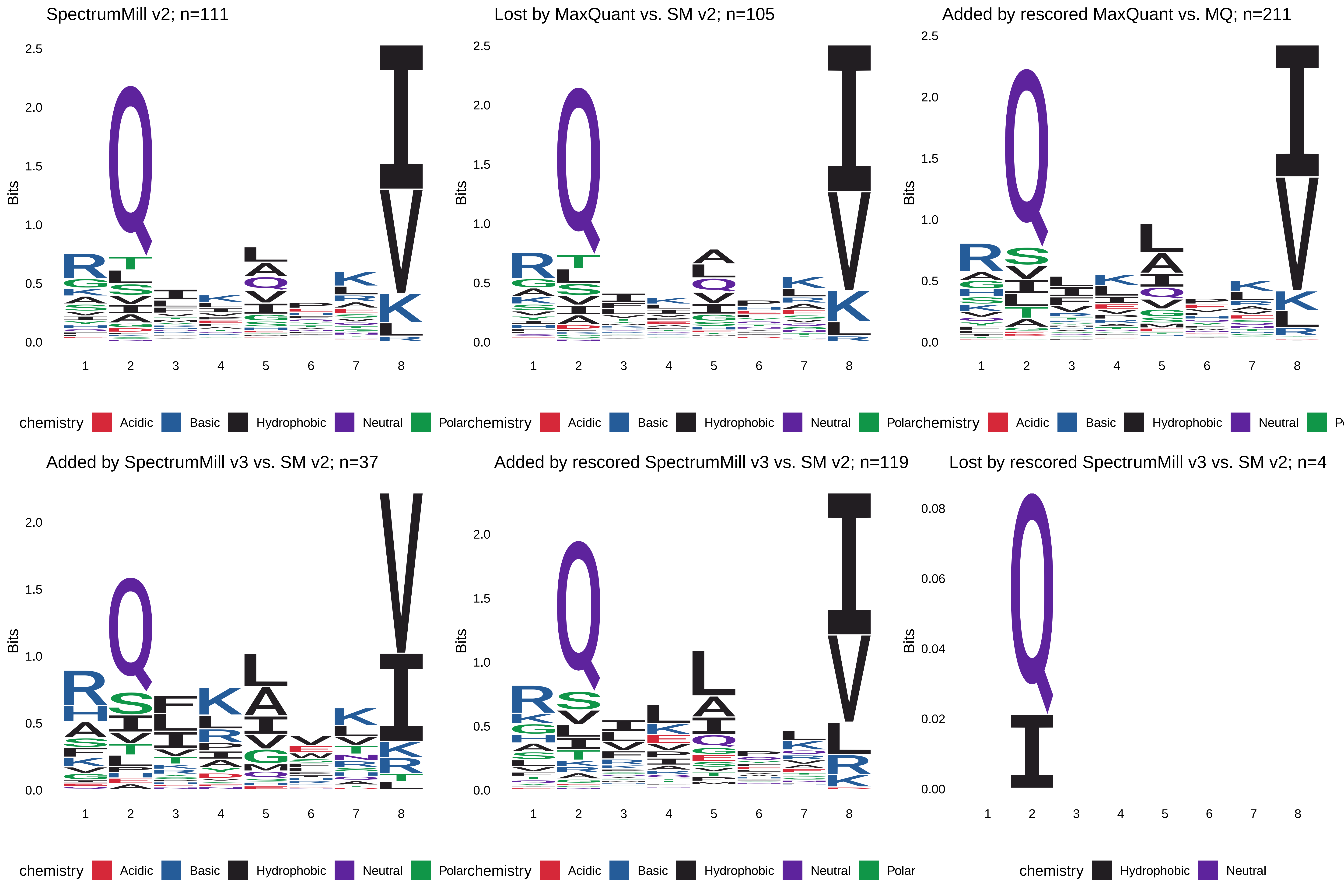

SpectrumMill v2; n=111
Lost by MaxQuant vs. SM v2; n=105
Added by rescored MaxQuant vs. MQ; n=211
Bits
0.0
0.5
1.0
1.5
2.0
2.5
1 2 3 4 5 6 7 8
chemistry Acidic Basic Hydrophobic Neutral Polar
chemistry Acidic Basic Hydrophobic Neutral Polar
chemistry Acidic Basic Hydrophobic Neutral
Added by SpectrumMill v3 vs. SM v2; n=37
Added by rescored SpectrumMill v3 vs. SM v2; n=119
Lost by rescored SpectrumMill v3 vs. SM v2; n=4
0.00
0.02
0.04
0.06
0.08
chemistry Acidic Basic Hydrophobic Neutral Polar
chemistry Acidic Basic Hydrophobic Neutral Polar
chemistry Hydrophobic Neutral

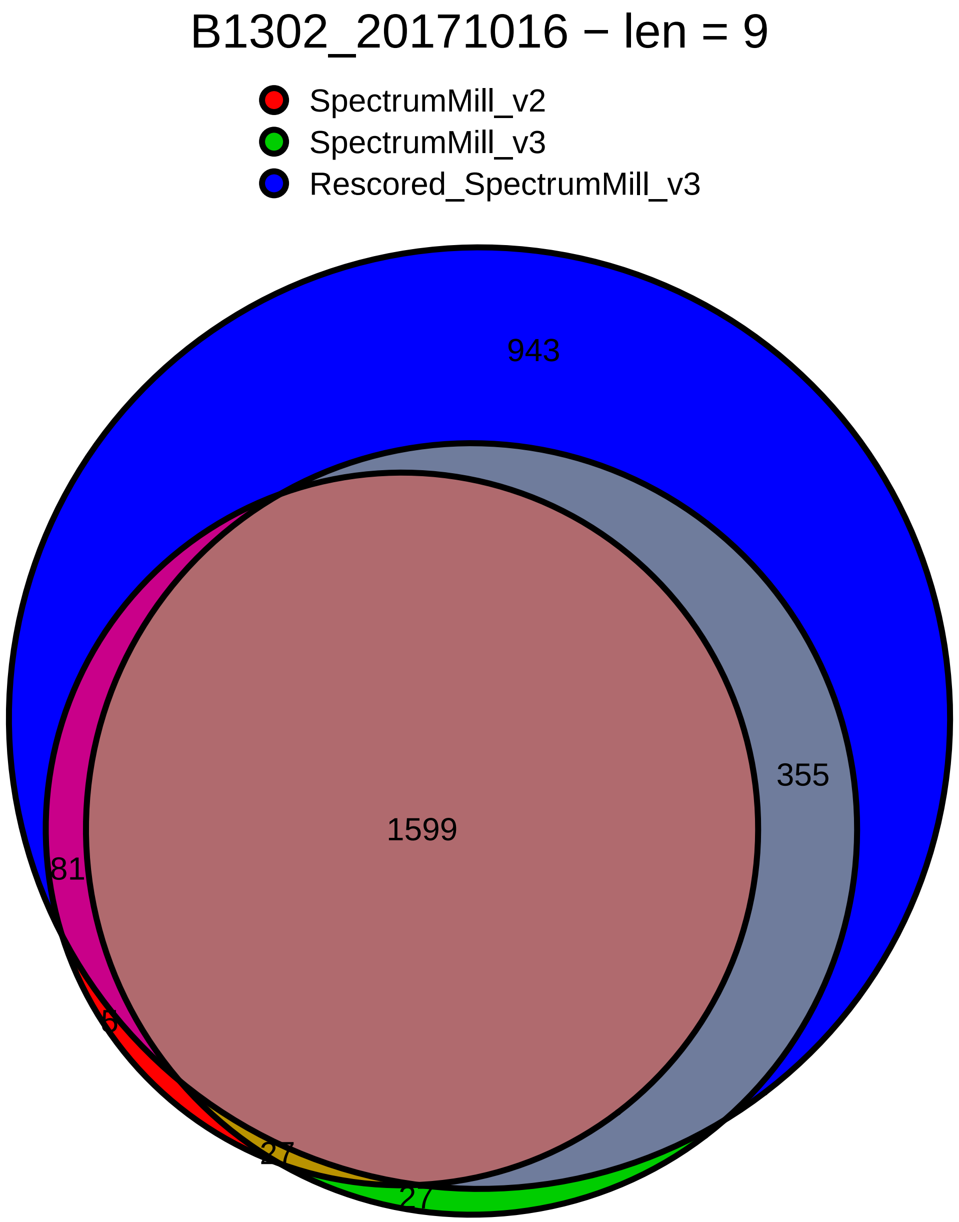
B1302_20171016 − len = 9
SpectrumMill_v2
SpectrumMill_v3
Rescored_SpectrumMill_v3
943
355
1599
81
6
27
27

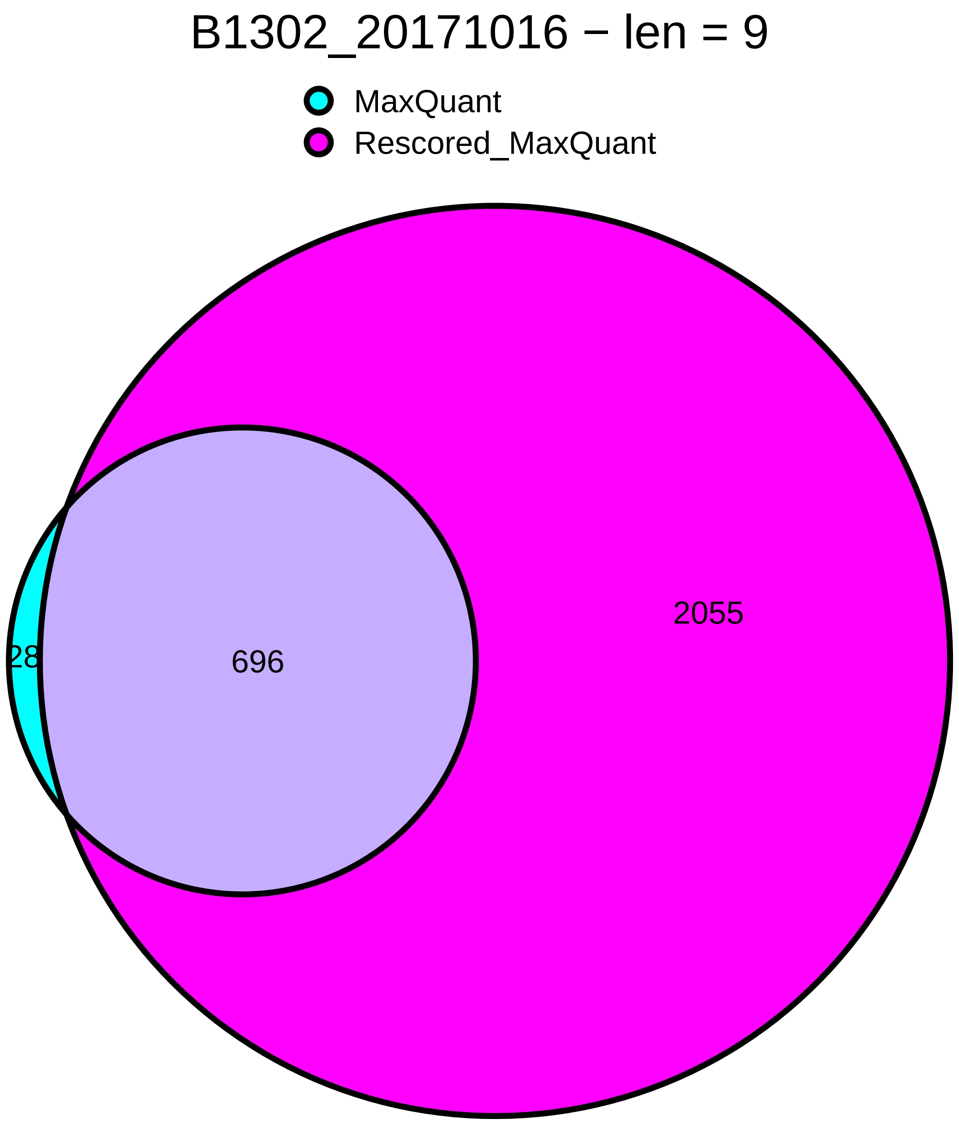
B1302_20171016 − len = 9
MaxQuant
Rescored_MaxQuant
28
696
2055

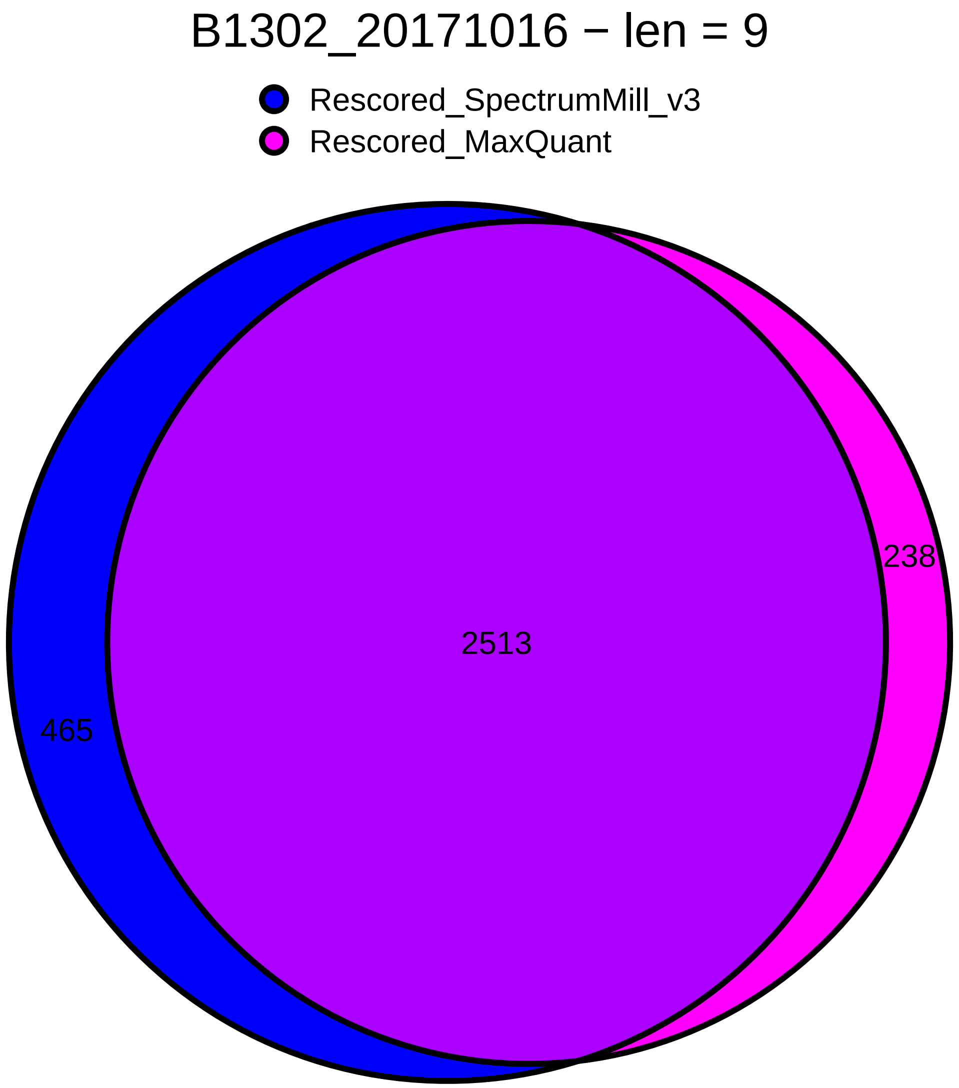
B1302_20171016 − len = 9
Rescored_SpectrumMill_v3
Rescored_MaxQuant
465
2513
238

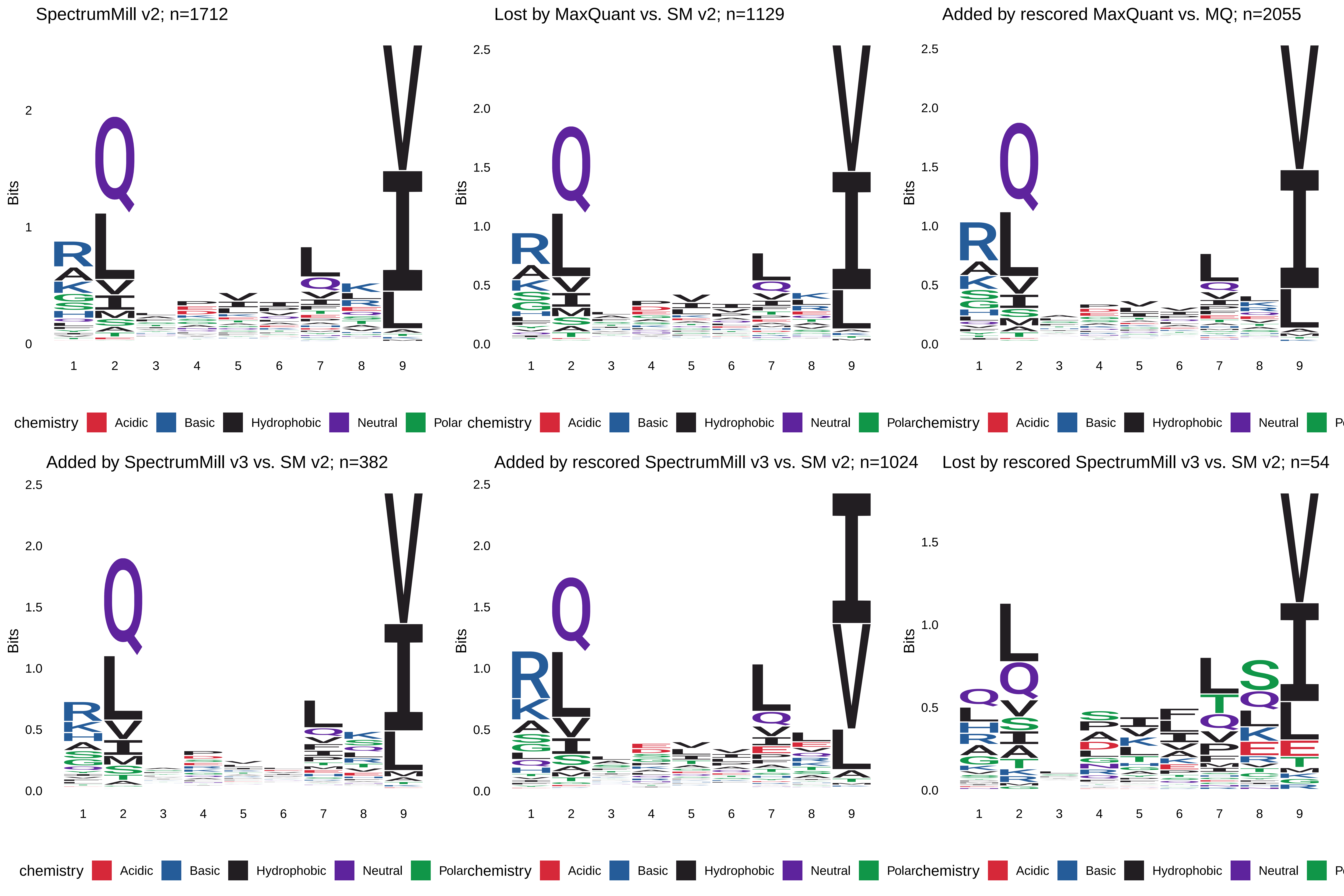
SpectrumMill v2; n=1712
Lost by MaxQuant vs. SM v2; n=1129
Added by rescored MaxQuant vs. MQ; n=2055
Added by SpectrumMill v3 vs. SM v2; n=382
Added by rescored SpectrumMill v3 vs. SM v2; n=1024
Lost by rescored SpectrumMill v3 vs. SM v2; n=54
Bits
1 2 3 4 5 6 7 8 9
chemistry
Acidic
Basic
Hydrophobic
Neutral
Polar

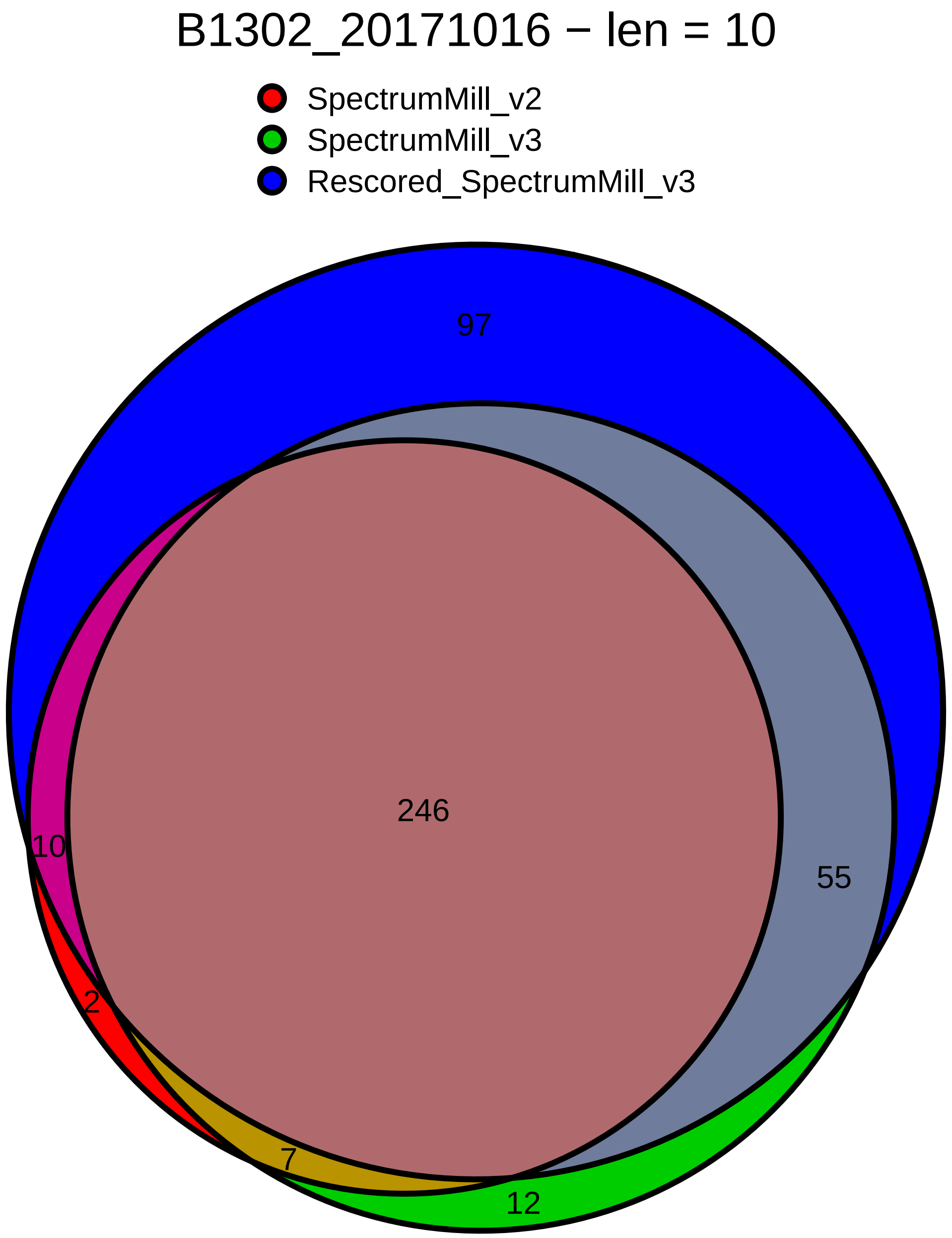
B1302_20171016 − len = 10
SpectrumMill_v2
SpectrumMill_v3
Rescored_SpectrumMill_v3
97
246
10
55
2
7
12

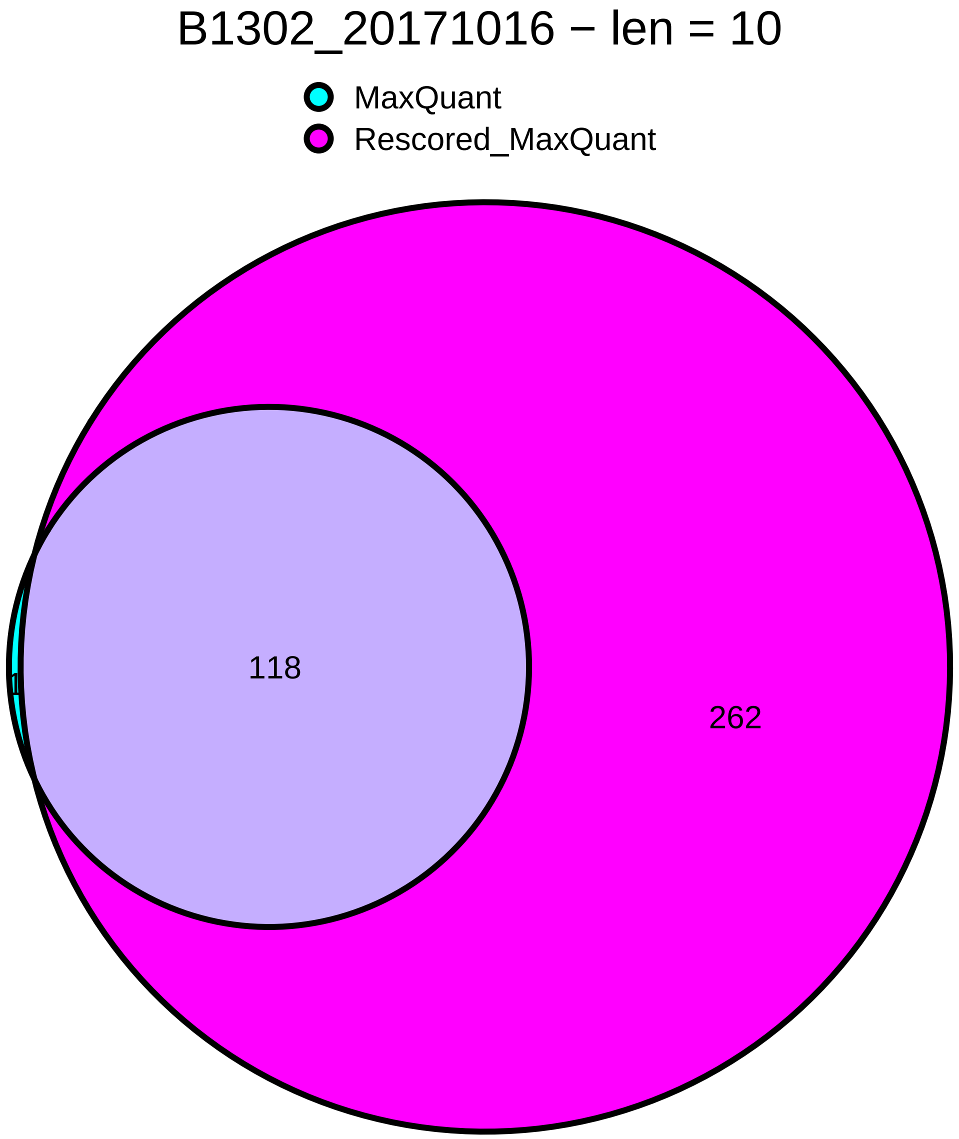
B1302_20171016 − len = 10
MaxQuant
Rescored_MaxQuant
118
262

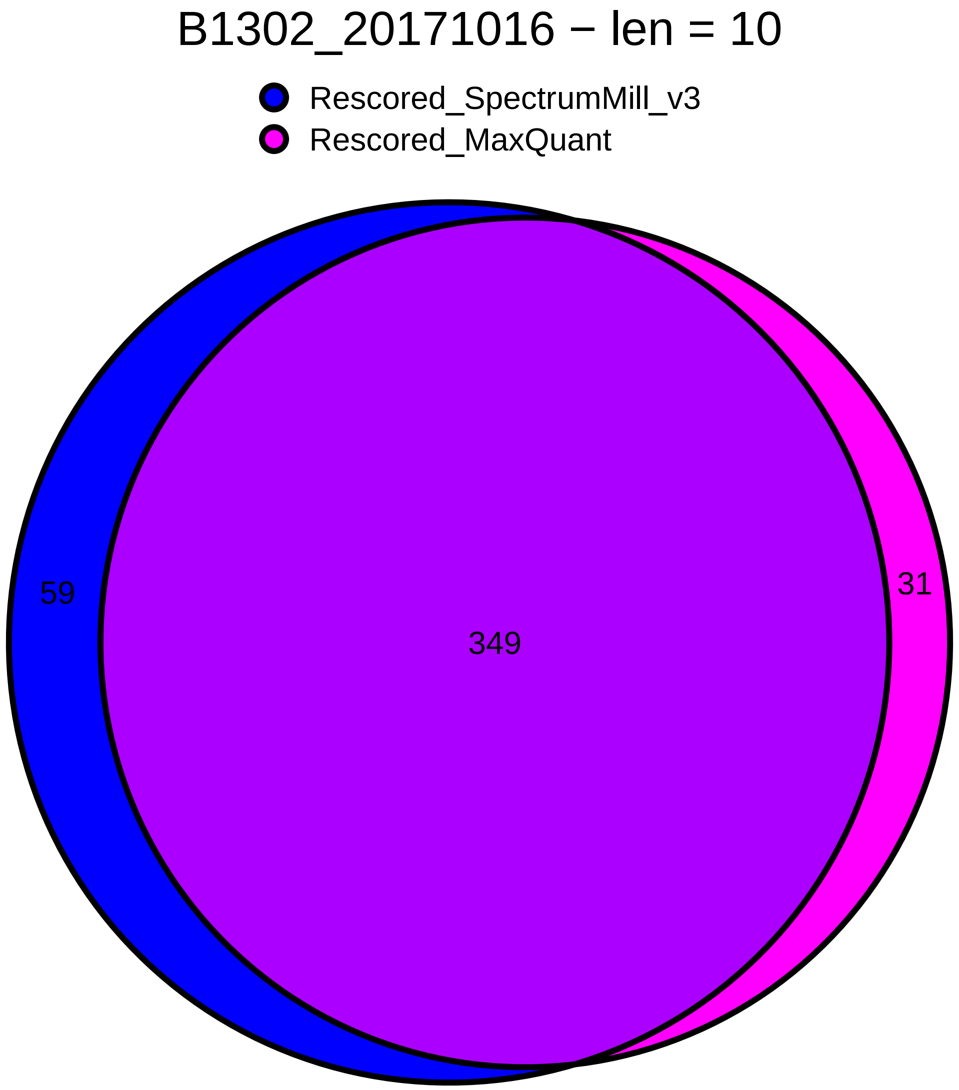
B1302_20171016 − len = 10
Rescored_SpectrumMill_v3
Rescored_MaxQuant
59
349
31

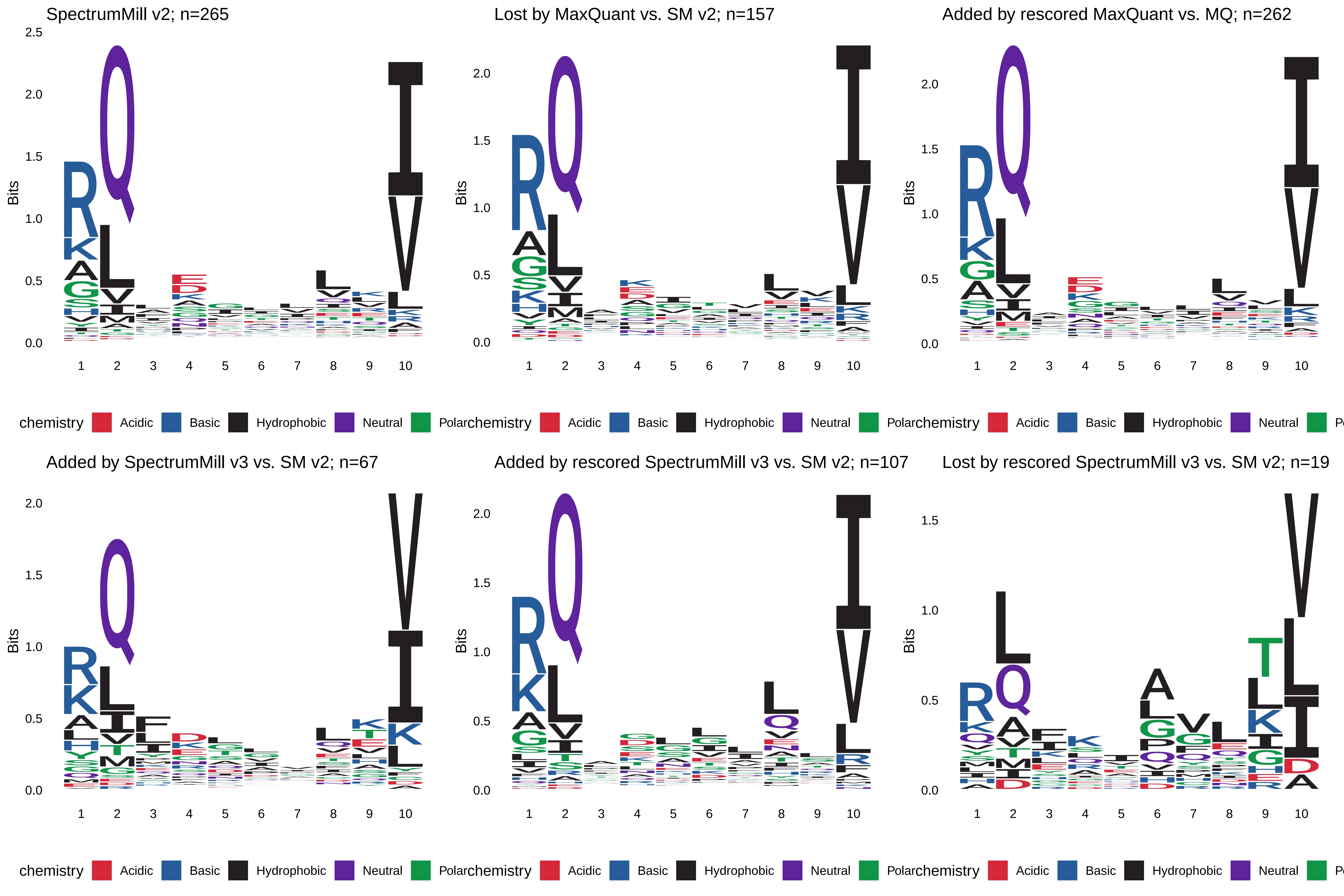
SpectrumMill v2; n=265
Lost by MaxQuant vs. SM v2; n=157
Added by rescored MaxQuant vs. MQ; n=262
Added by SpectrumMill v3 vs. SM v2; n=67
Added by rescored SpectrumMill v3 vs. SM v2; n=107
Lost by rescored SpectrumMill v3 vs. SM v2; n=19
Bits
1 2 3 4 5 6 7 8 9 10
chemistry Acidic Basic Hydrophobic Neutral Polar

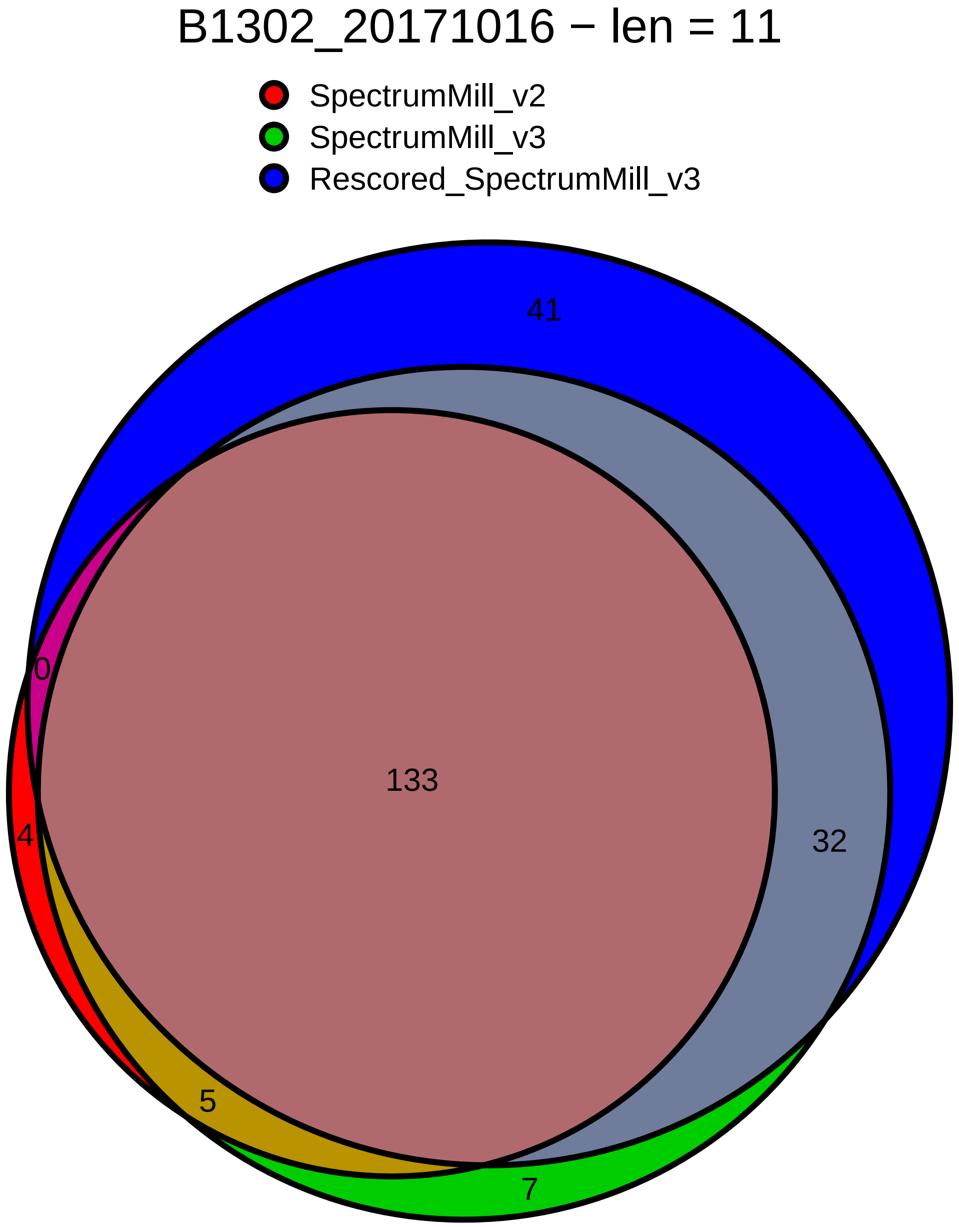
B1302_20171016 − len = 11
SpectrumMill_v2
SpectrumMill_v3
Rescored_SpectrumMill_v3
41
0
133
4
32
5
7

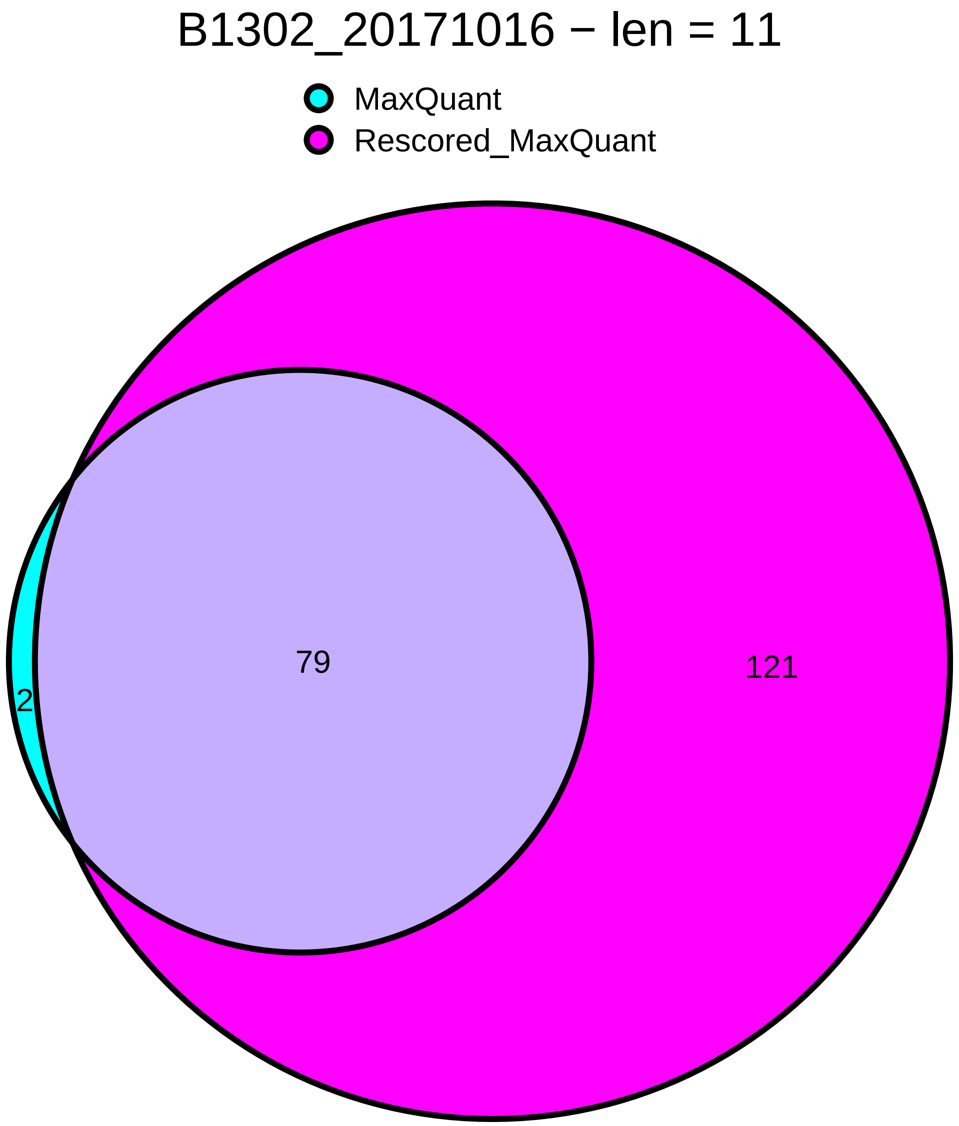
B1302_20171016 − len = 11
MaxQuant
Rescored_MaxQuant
79
2
121

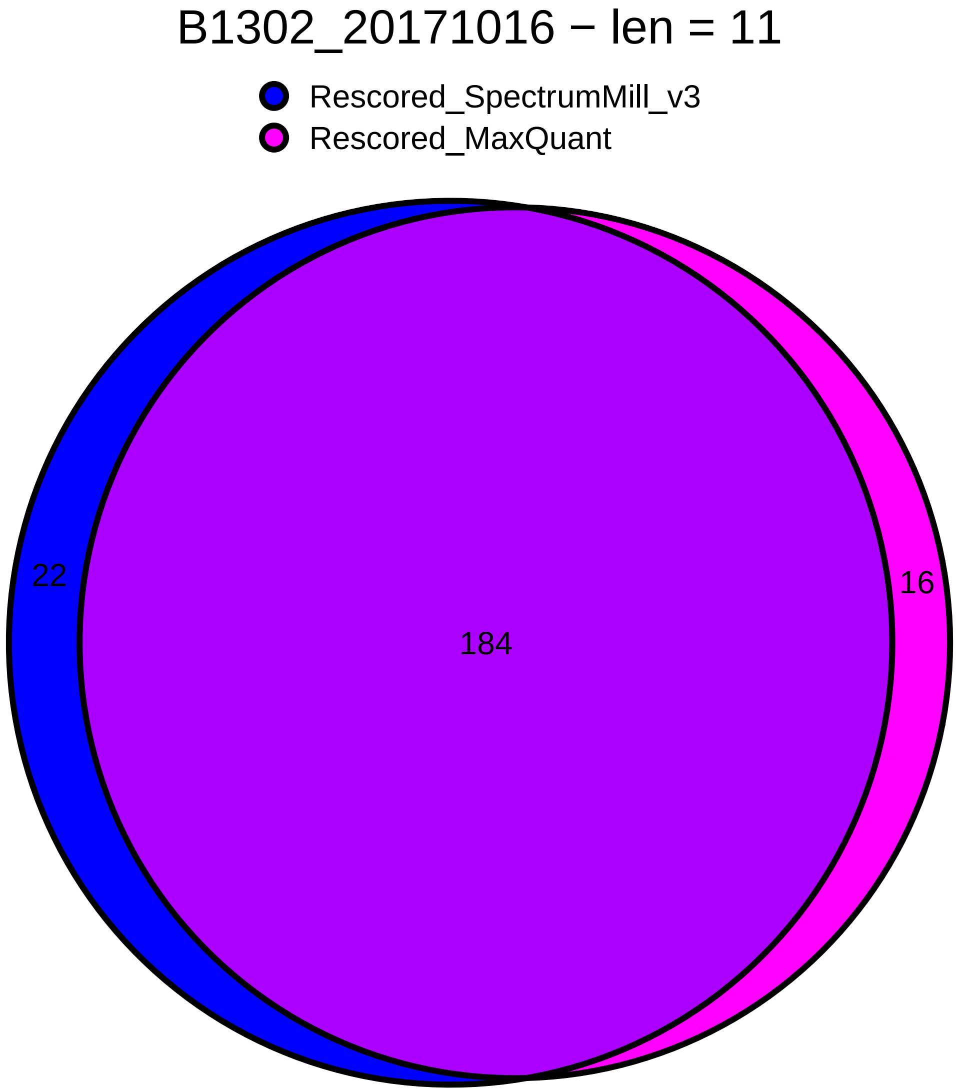
B1302_20171016 − len = 11
Rescored_SpectrumMill_v3
Rescored_MaxQuant
22
184
16

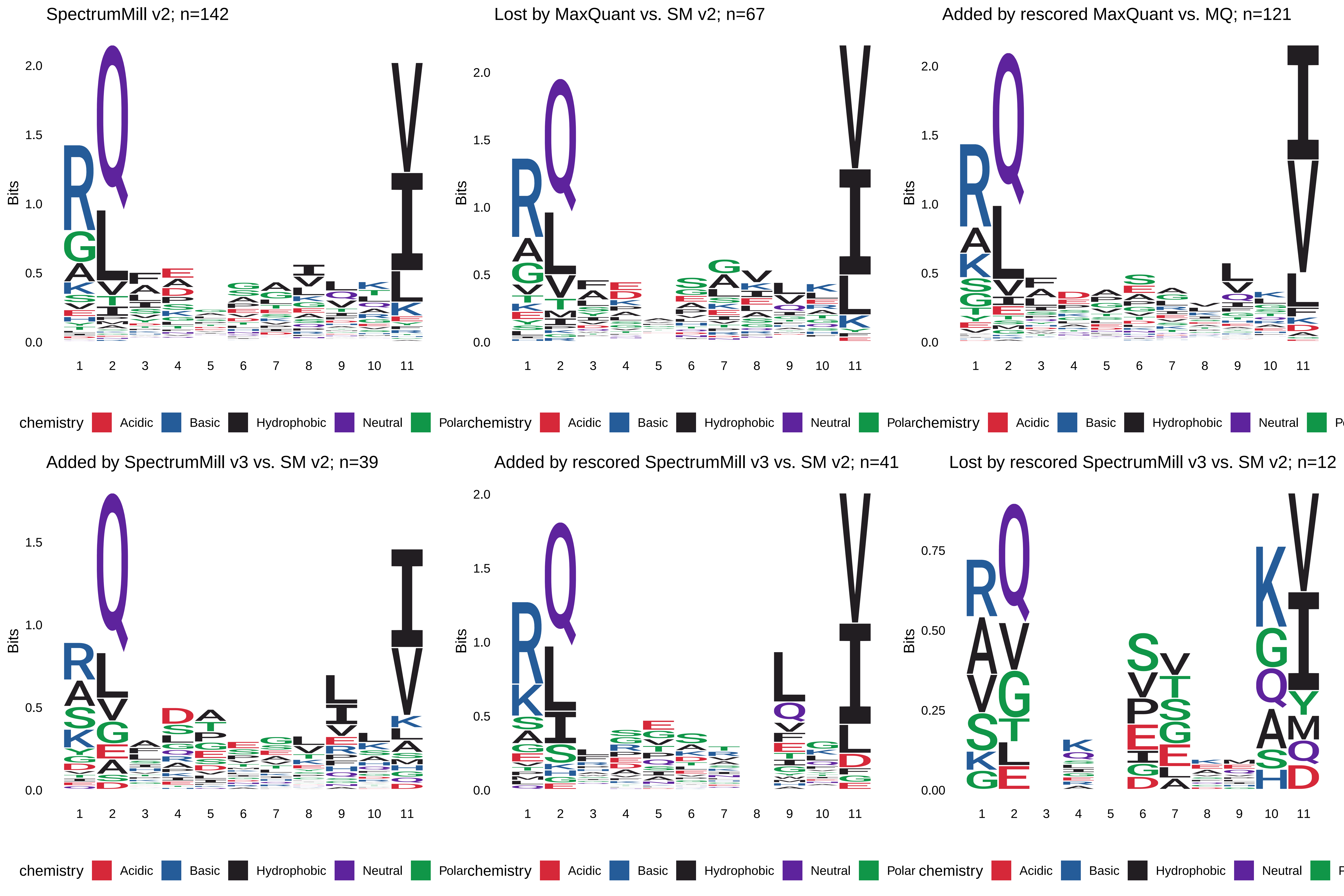
SpectrumMill v2; n=142
Lost by MaxQuant vs. SM v2; n=67
Added by rescored MaxQuant vs. MQ; n=121
Added by SpectrumMill v3 vs. SM v2; n=39
Added by rescored SpectrumMill v3 vs. SM v2; n=41
Lost by rescored SpectrumMill v3 vs. SM v2; n=12
Bits
1 2 3 4 5 6 7 8 9 10 11
chemistry
Acidic
Basic
Hydrophobic
Neutral
Polar

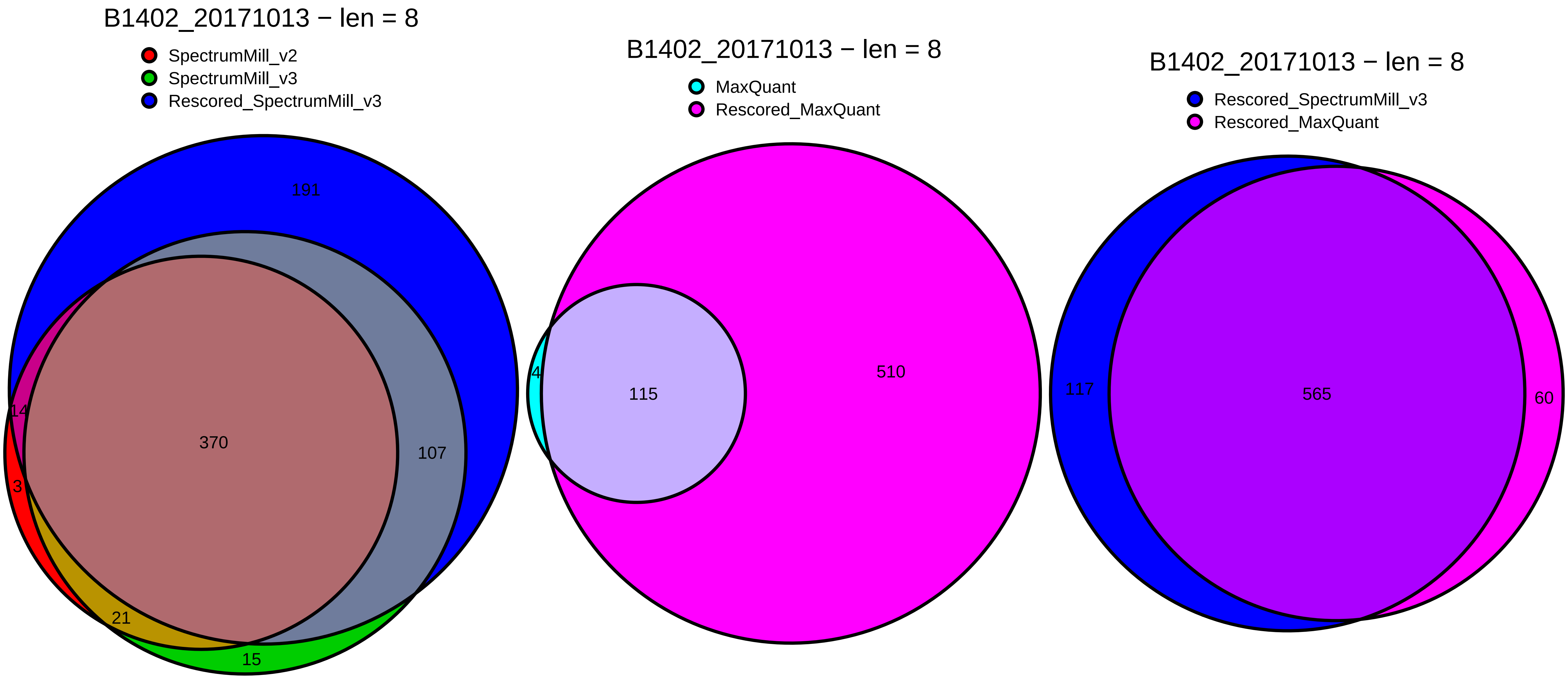
B1402_20171013 − len = 8
SpectrumMill_v2
SpectrumMill_v3
Rescored_SpectrumMill_v3
191
14
370
107
3
21
15
B1402_20171013 − len = 8
MaxQuant
Rescored_MaxQuant
4
115
510
B1402_20171013 − len = 8
Rescored_SpectrumMill_v3
Rescored_MaxQuant
117
565
60

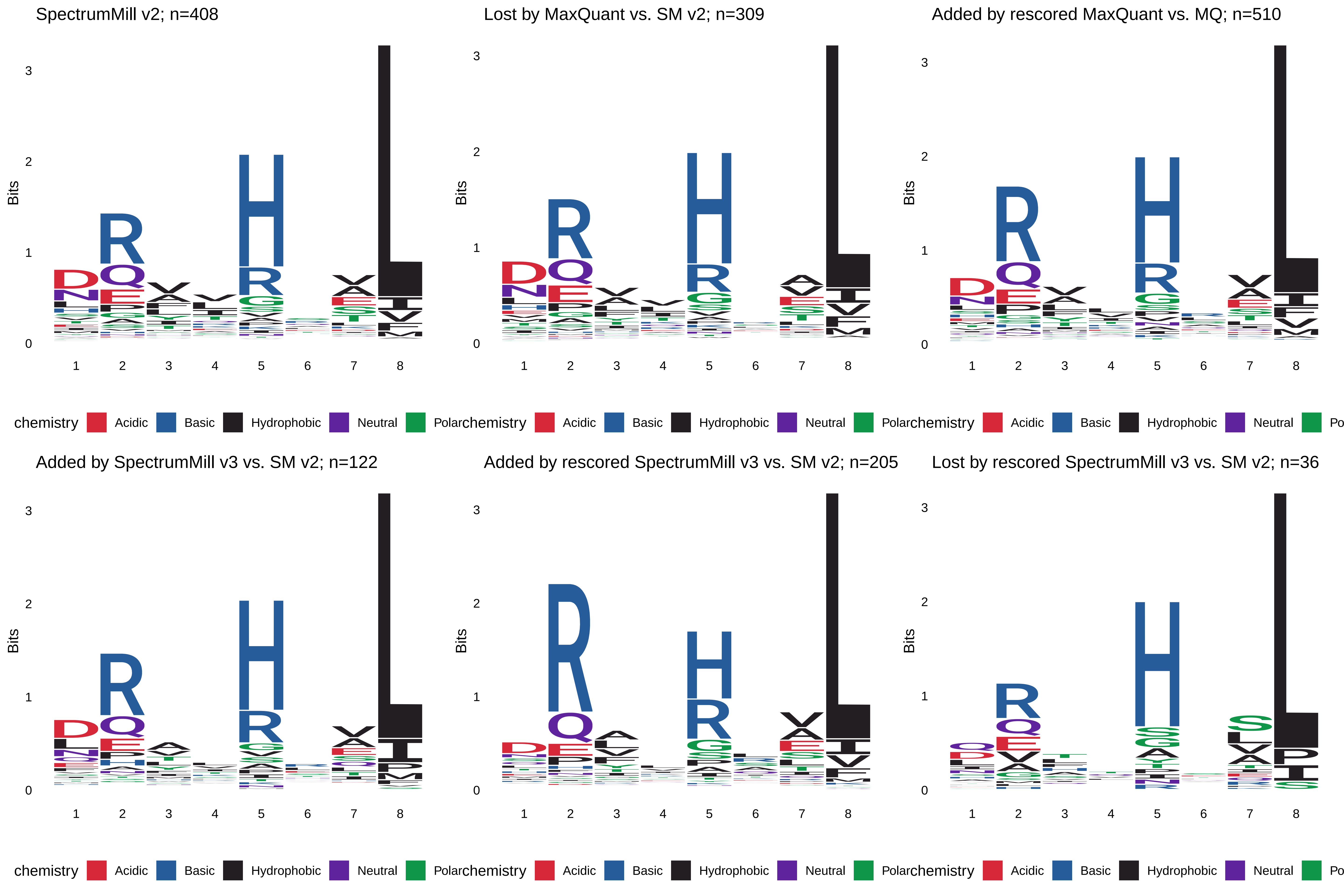
SpectrumMill v2; n=408
Lost by MaxQuant vs. SM v2; n=309
Added by rescored MaxQuant vs. MQ; n=510
Added by SpectrumMill v3 vs. SM v2; n=122
Added by rescored SpectrumMill v3 vs. SM v2; n=205
Lost by rescored SpectrumMill v3 vs. SM v2; n=36
Bits
0
1
2
3
1 2 3 4 5 6 7 8
chemistry Acidic Basic Hydrophobic Neutral Polar

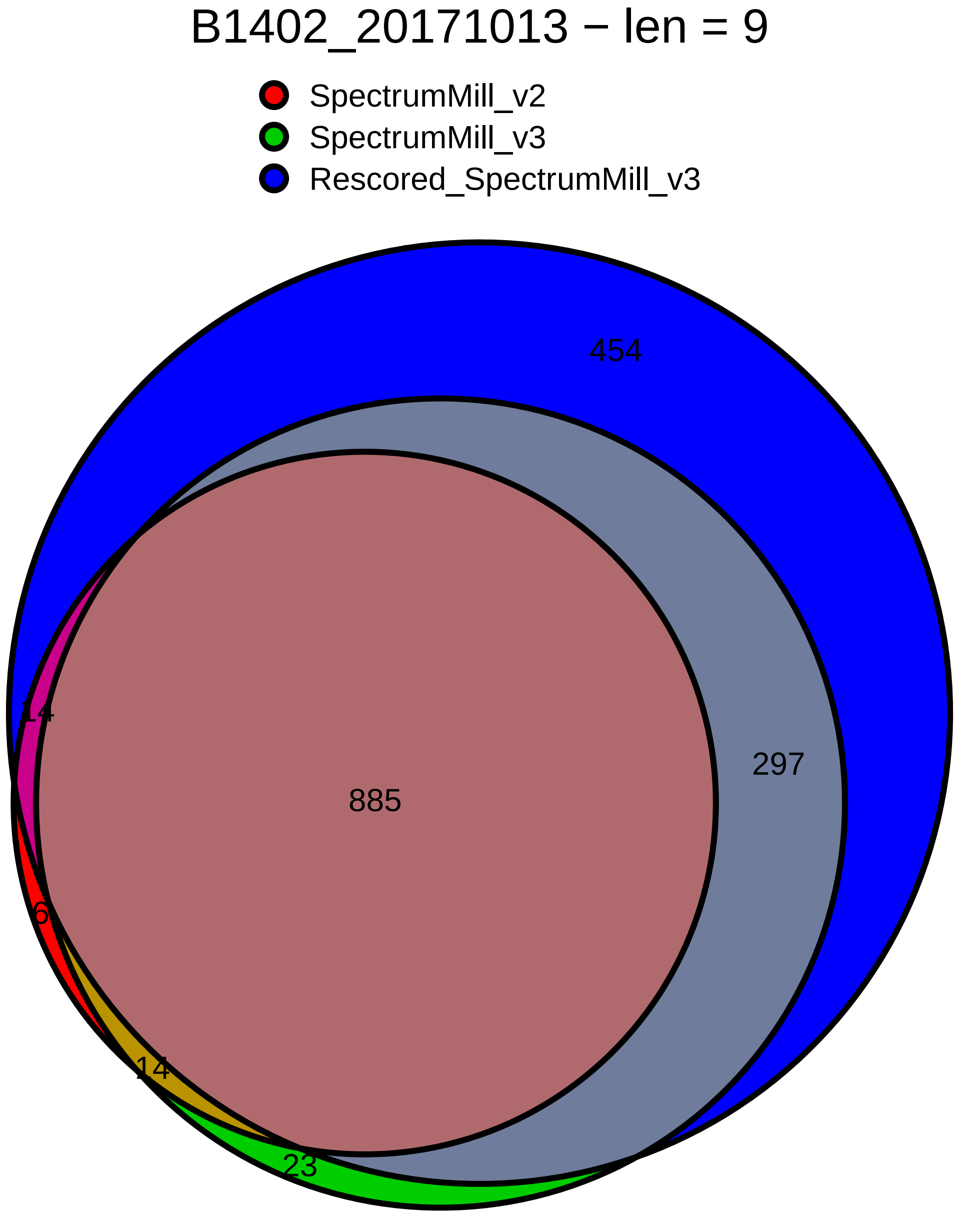
B1402_20171013 − len = 9
SpectrumMill_v2
SpectrumMill_v3
Rescored_SpectrumMill_v3
454
14
297
885
6
14
23

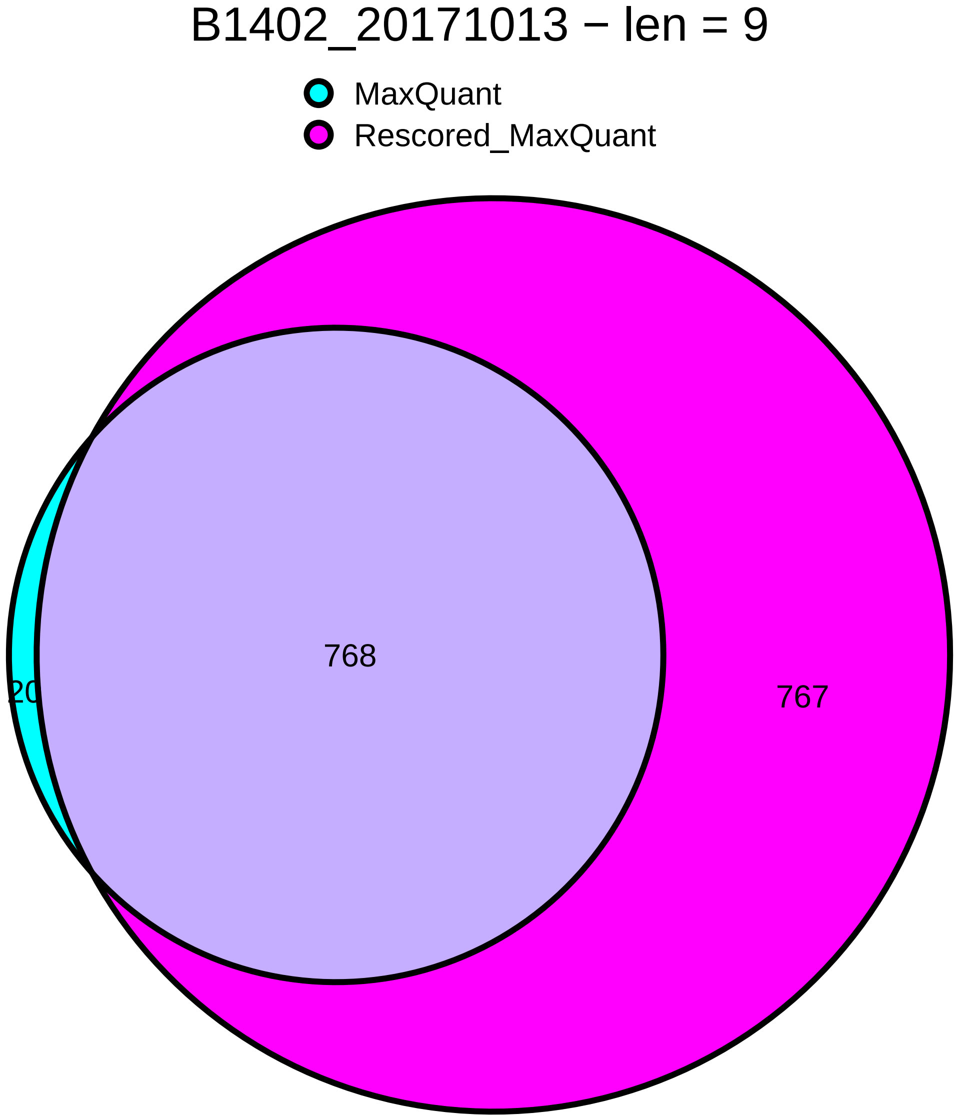
B1402_20171013 − len = 9
MaxQuant
Rescored_MaxQuant
20
768
767

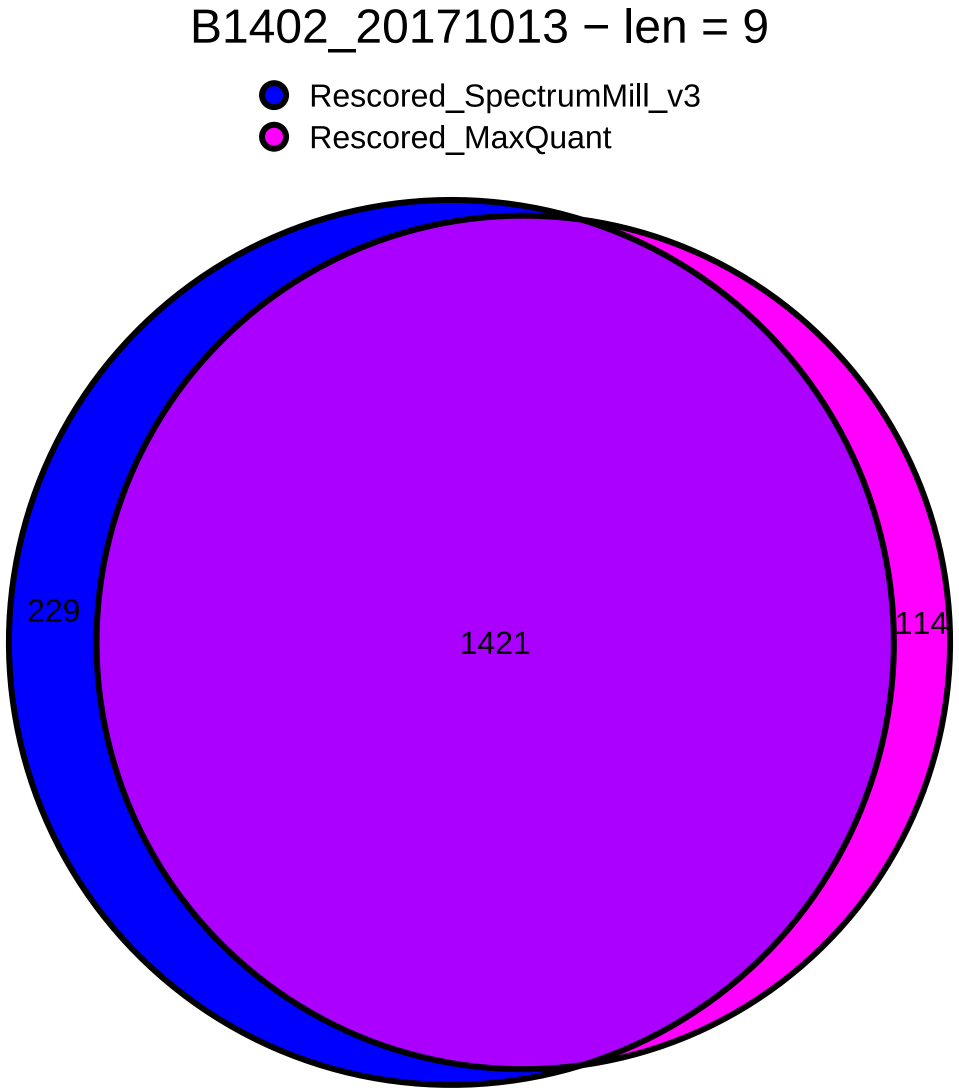
B1402_20171013 − len = 9
Rescored_SpectrumMill_v3
Rescored_MaxQuant
229
1421
114

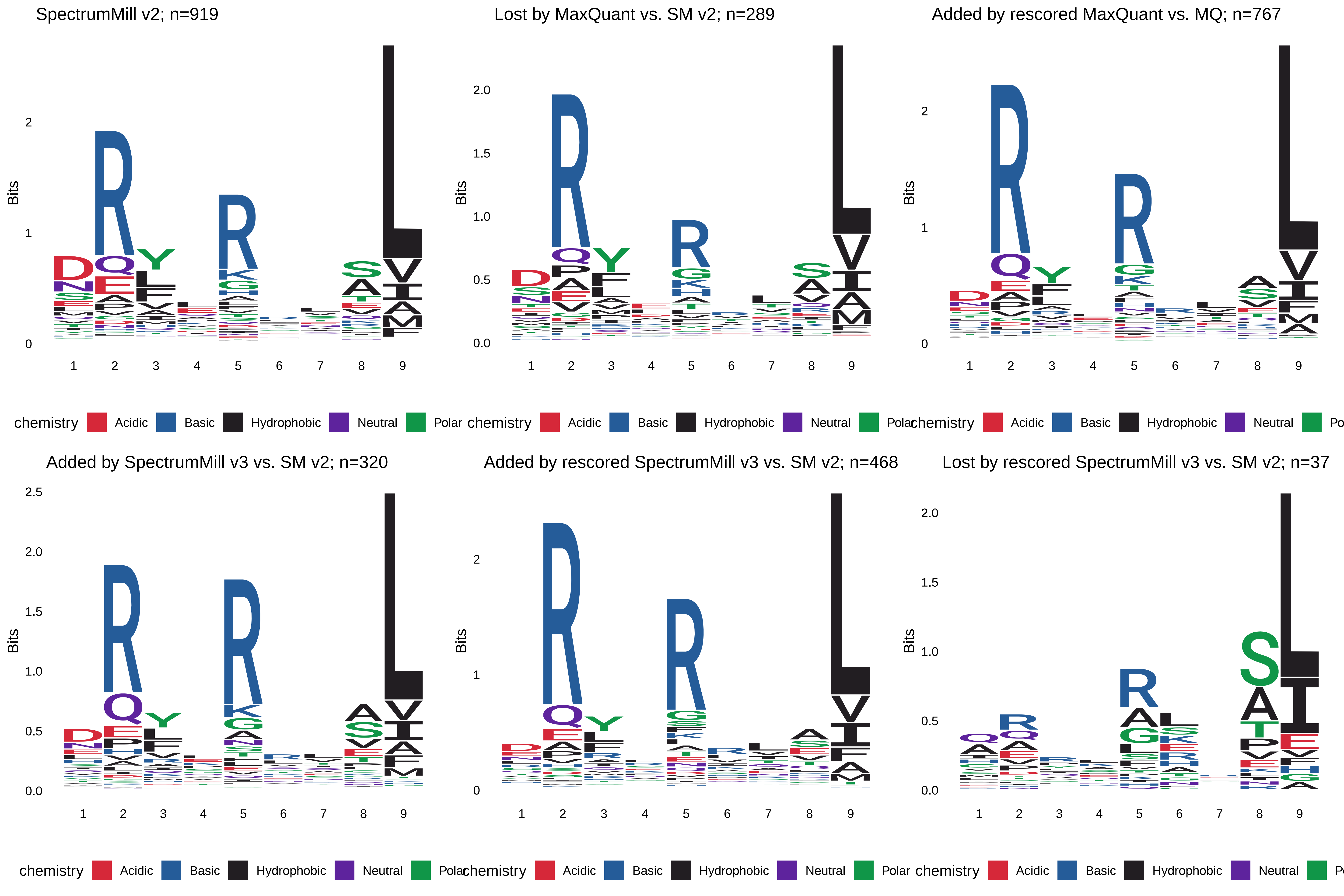
SpectrumMill v2; n=919
Lost by MaxQuant vs. SM v2; n=289
Added by rescored MaxQuant vs. MQ; n=767
Added by SpectrumMill v3 vs. SM v2; n=320
Added by rescored SpectrumMill v3 vs. SM v2; n=468
Lost by rescored SpectrumMill v3 vs. SM v2; n=37
Bits
1 2 3 4 5 6 7 8 9
chemistry
Acidic
Basic
Hydrophobic
Neutral
Polar

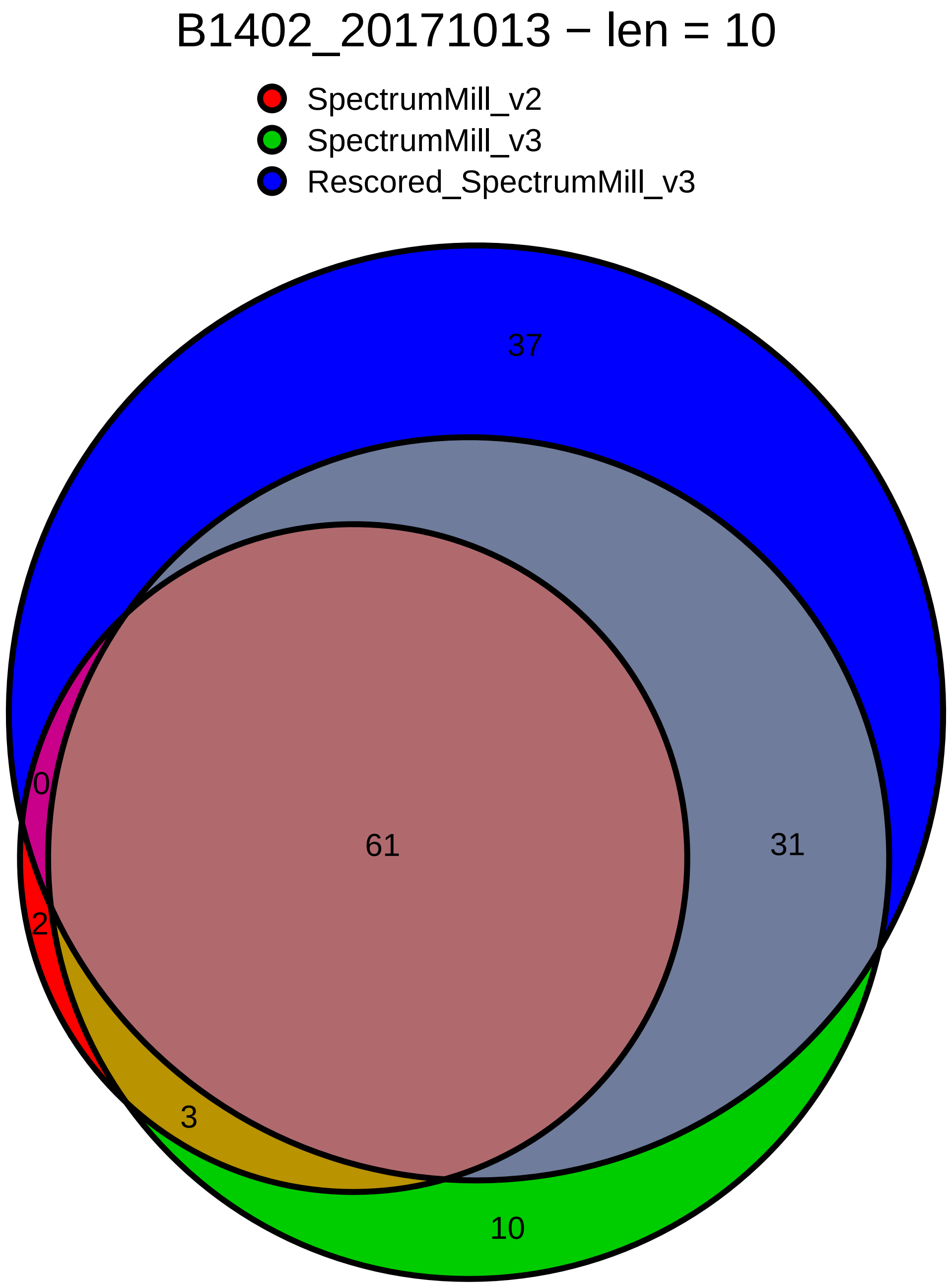
B1402_20171013 − len = 10
SpectrumMill_v2
SpectrumMill_v3
Rescored_SpectrumMill_v3
37
0
61
31
2
3
10

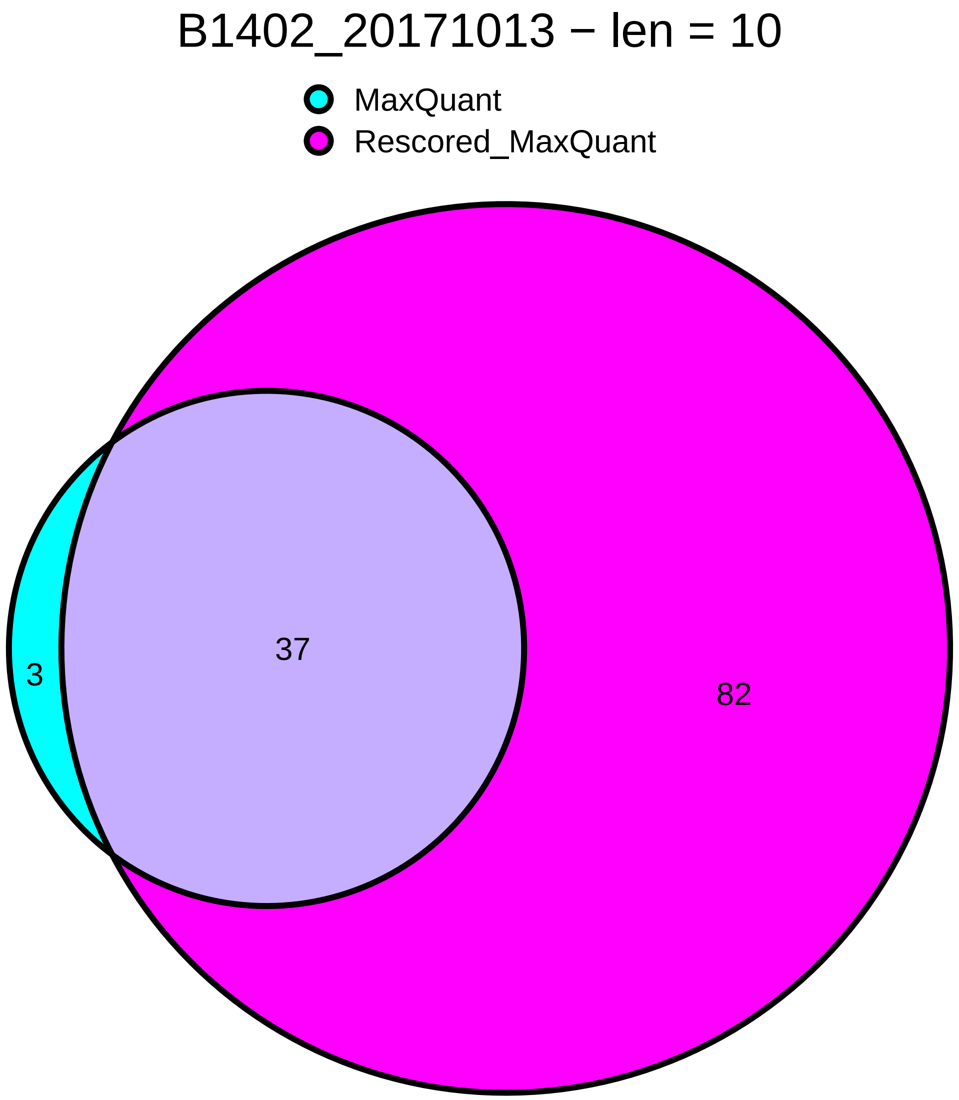
B1402_20171013 − len = 10
MaxQuant
Rescored_MaxQuant
3
37
82

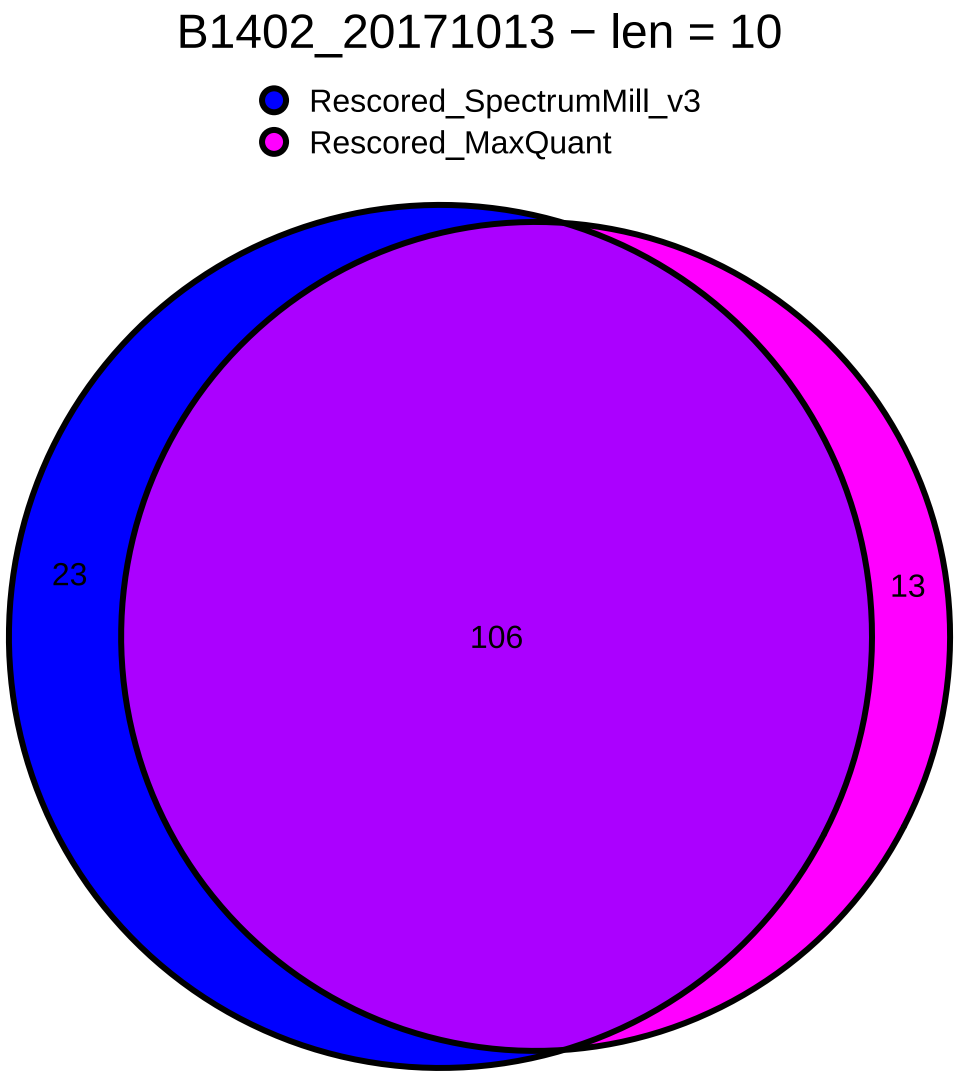
B1402_20171013 − len = 10
Rescored_SpectrumMill_v3
Rescored_MaxQuant
23
106
13

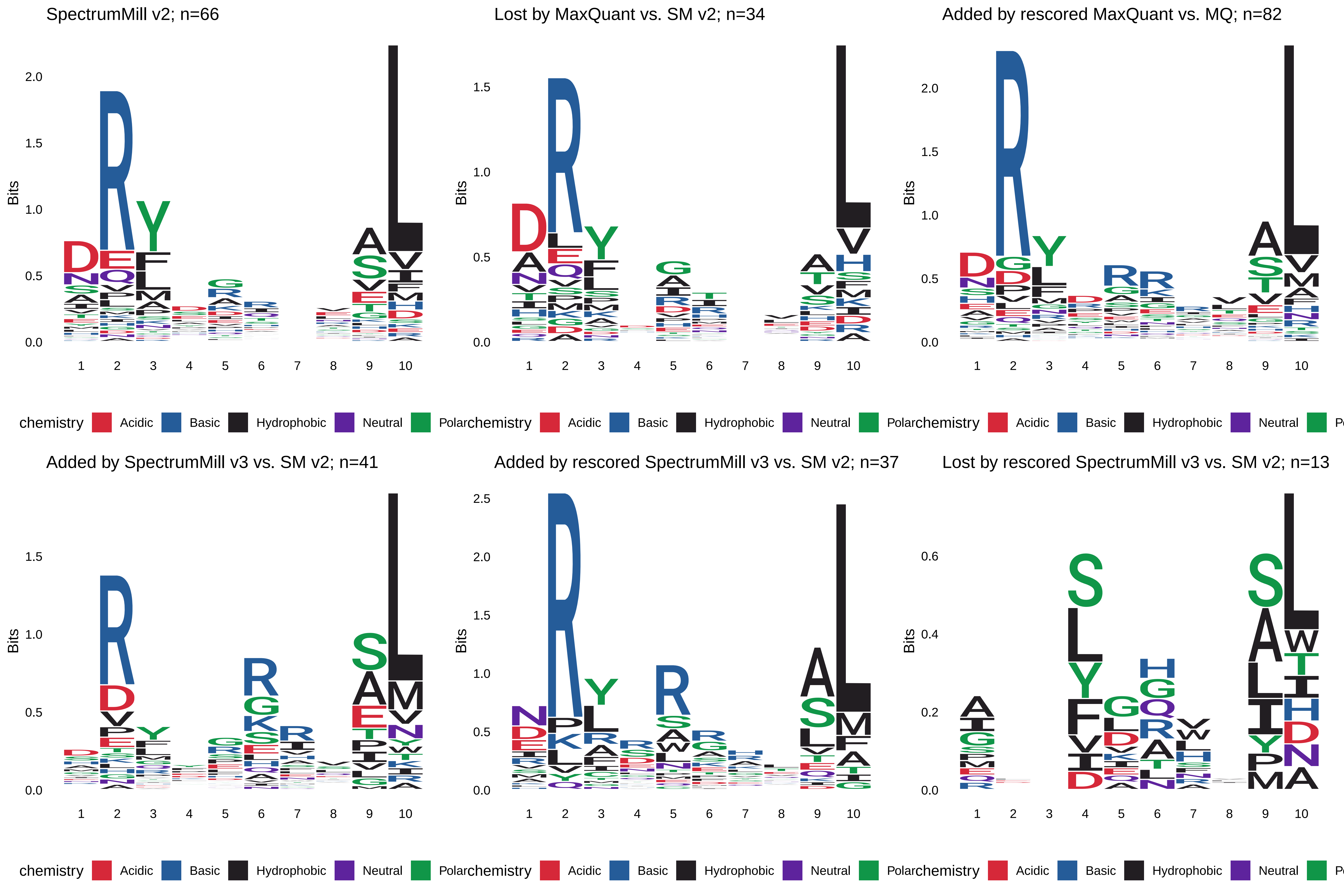

SpectrumMill v2; n=66
Lost by MaxQuant vs. SM v2; n=34
Added by rescored MaxQuant vs. MQ; n=82
Added by SpectrumMill v3 vs. SM v2; n=41
Added by rescored SpectrumMill v3 vs. SM v2; n=37
Lost by rescored SpectrumMill v3 vs. SM v2; n=13
Bits
1 2 3 4 5 6 7 8 9 10
chemistry Acidic Basic Hydrophobic Neutral Polar

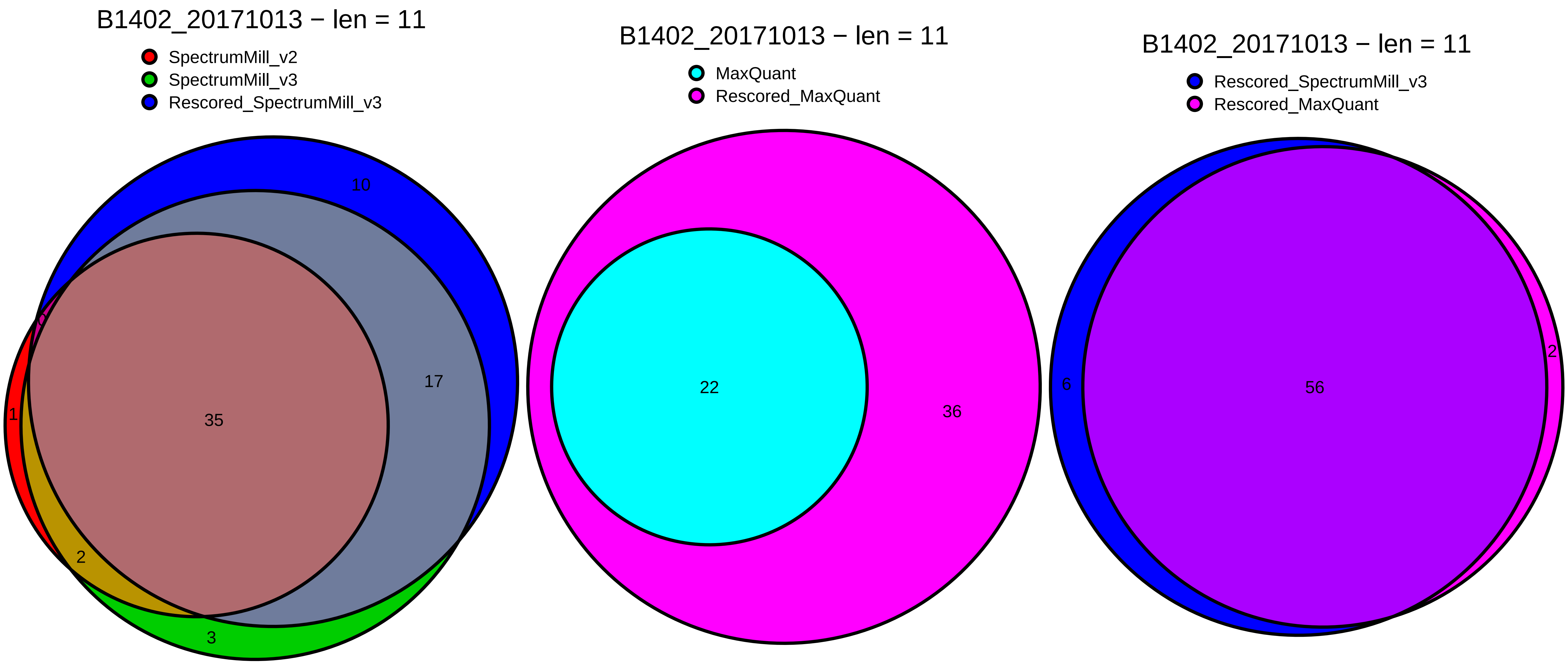
B1402_20171013 − len = 11
SpectrumMill_v2
SpectrumMill_v3
Rescored_SpectrumMill_v3
10
0
17
1
35
2
3
B1402_20171013 − len = 11
MaxQuant
Rescored_MaxQuant
22
36
B1402_20171013 − len = 11
Rescored_SpectrumMill_v3
Rescored_MaxQuant
6
56
2

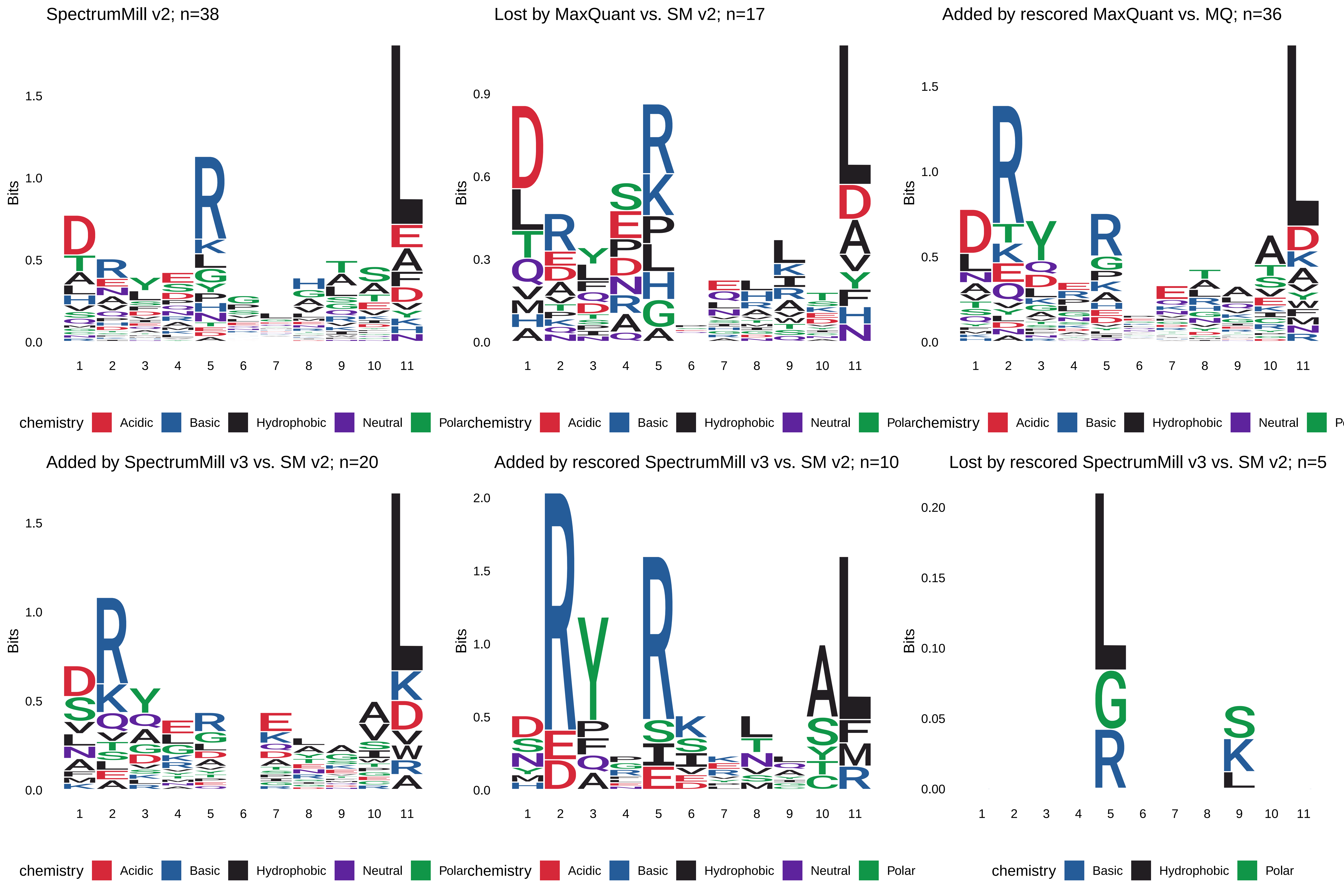

SpectrumMill v2; n=38
Lost by MaxQuant vs. SM v2; n=17
Added by rescored MaxQuant vs. MQ; n=36
Added by SpectrumMill v3 vs. SM v2; n=20
Added by rescored SpectrumMill v3 vs. SM v2; n=10
Lost by rescored SpectrumMill v3 vs. SM v2; n=5
Bits
chemistry
Acidic
Basic
Hydrophobic
Neutral
Polar

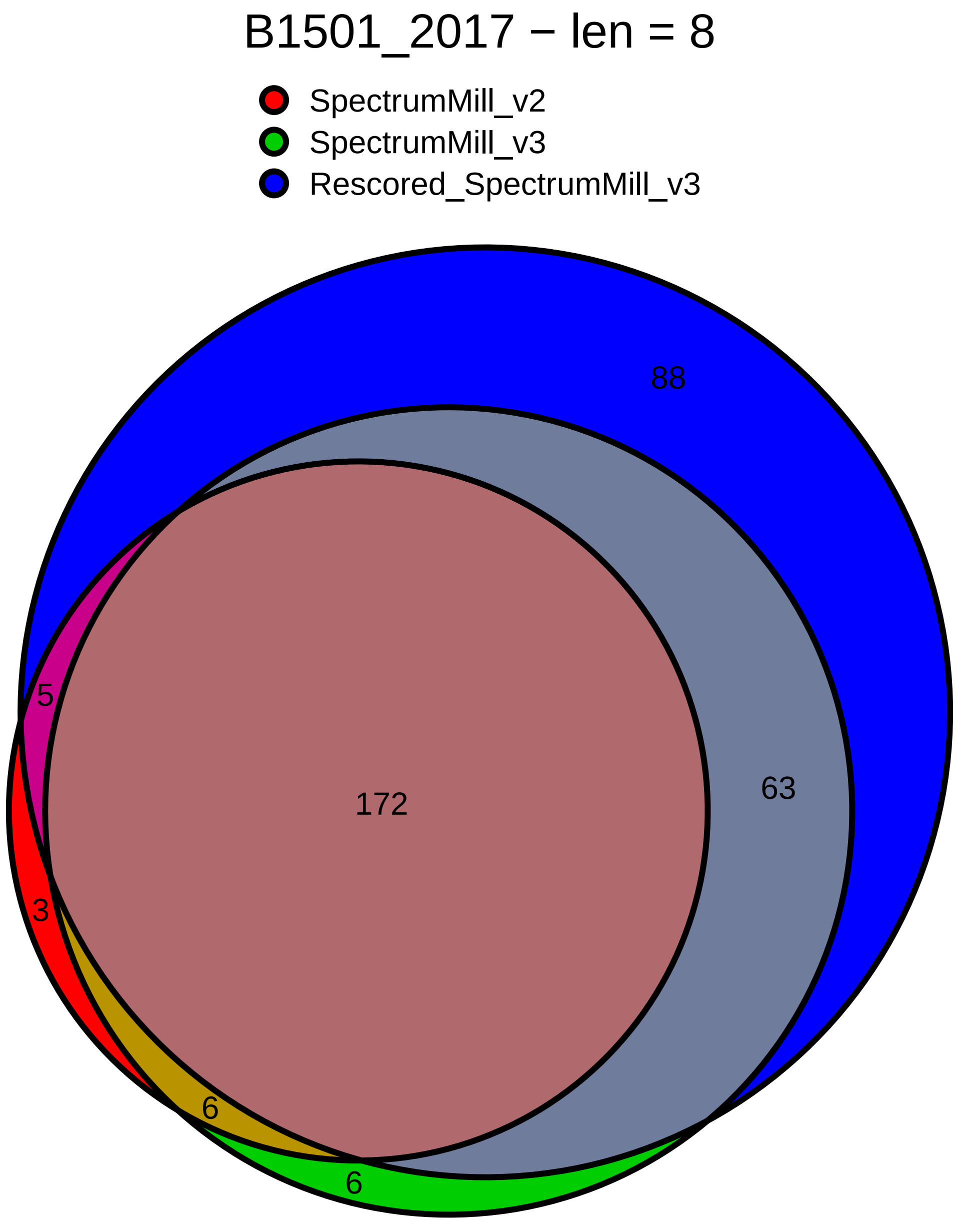

B1501_2017 − len = 8
SpectrumMill_v2
SpectrumMill_v3
Rescored_SpectrumMill_v3
88
5
172
63
3
6
6

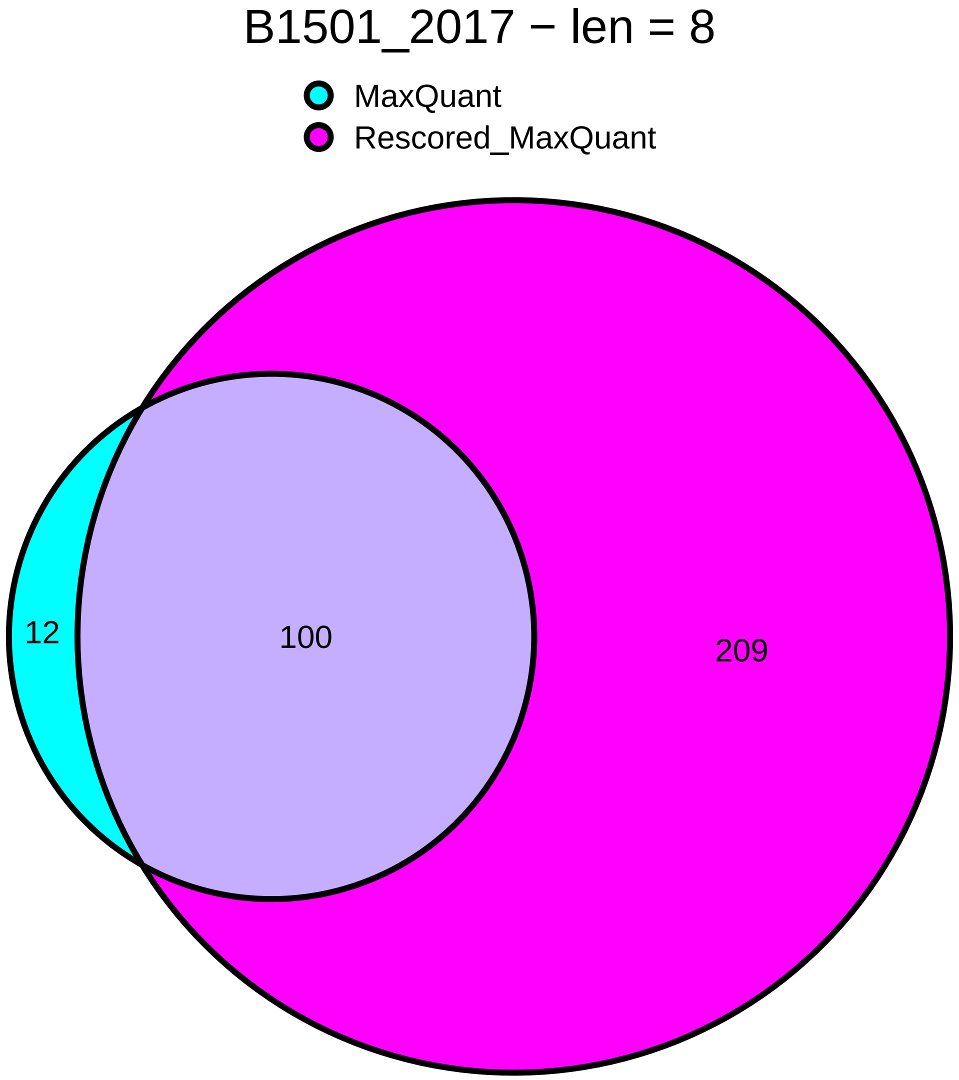

B1501_2017 − len = 8
MaxQuant
Rescored_MaxQuant
12
100
209

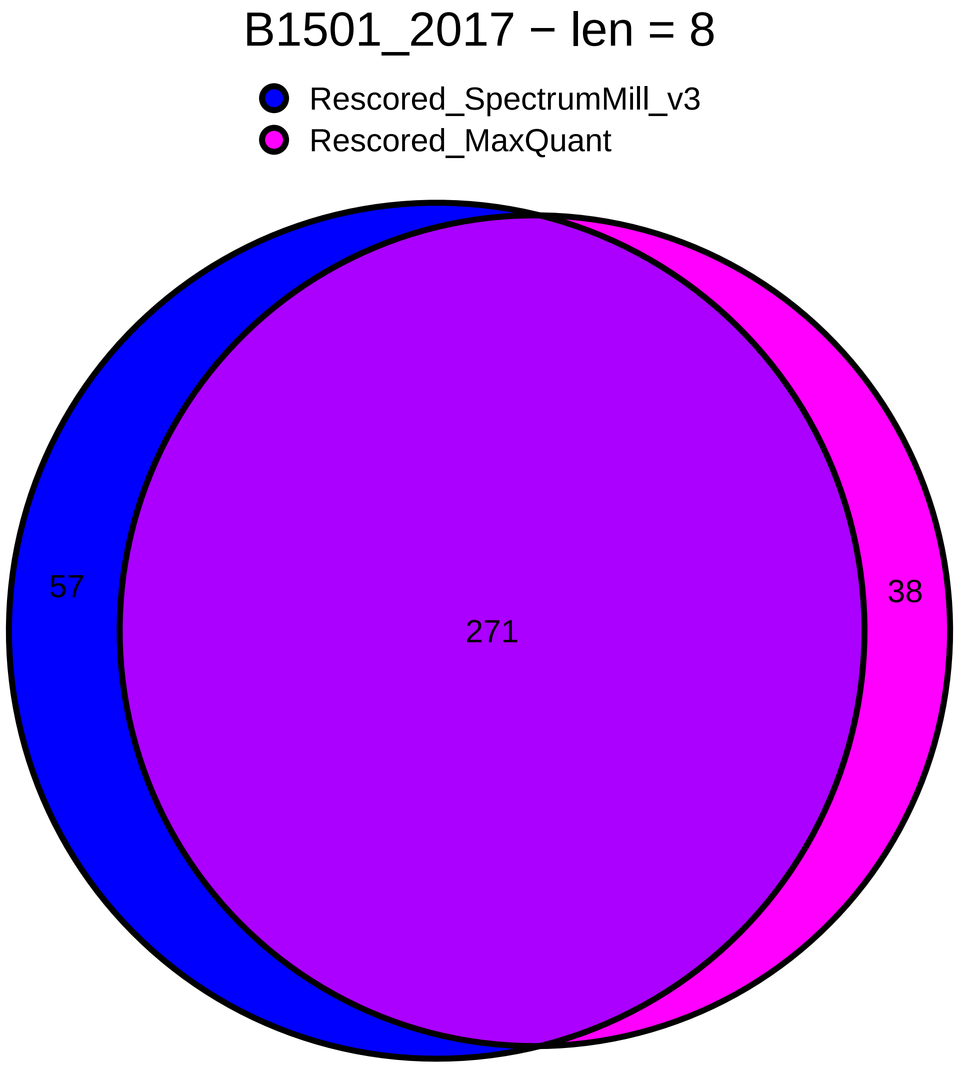

B1501_2017 − len = 8
Rescored_SpectrumMill_v3
Rescored_MaxQuant
57
271
38

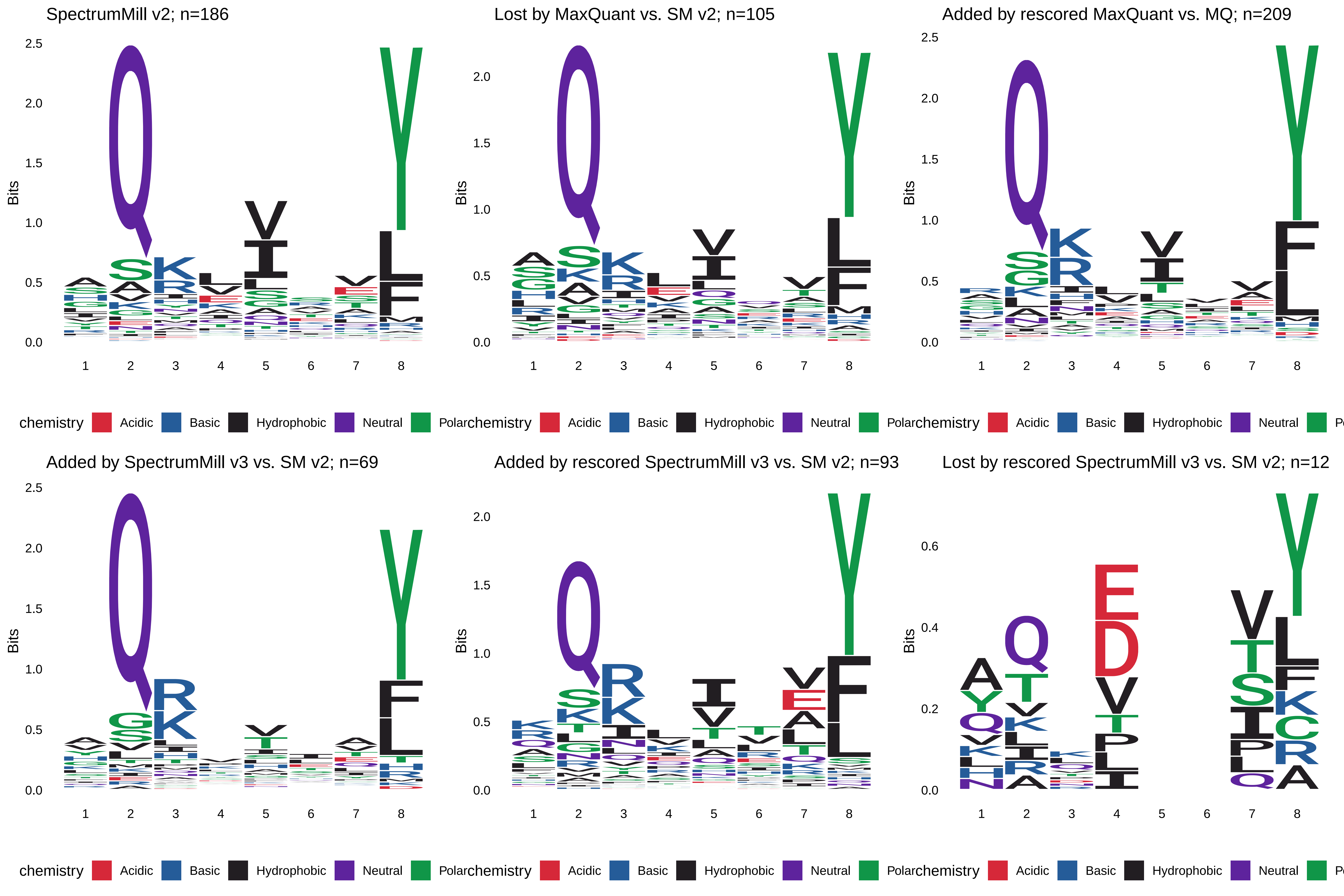

SpectrumMill v2; n=186
Lost by MaxQuant vs. SM v2; n=105
Added by rescored MaxQuant vs. MQ; n=209
Added by SpectrumMill v3 vs. SM v2; n=69
Added by rescored SpectrumMill v3 vs. SM v2; n=93
Lost by rescored SpectrumMill v3 vs. SM v2; n=12
Bits
1 2 3 4 5 6 7 8
chemistry Acidic Basic Hydrophobic Neutral Polar

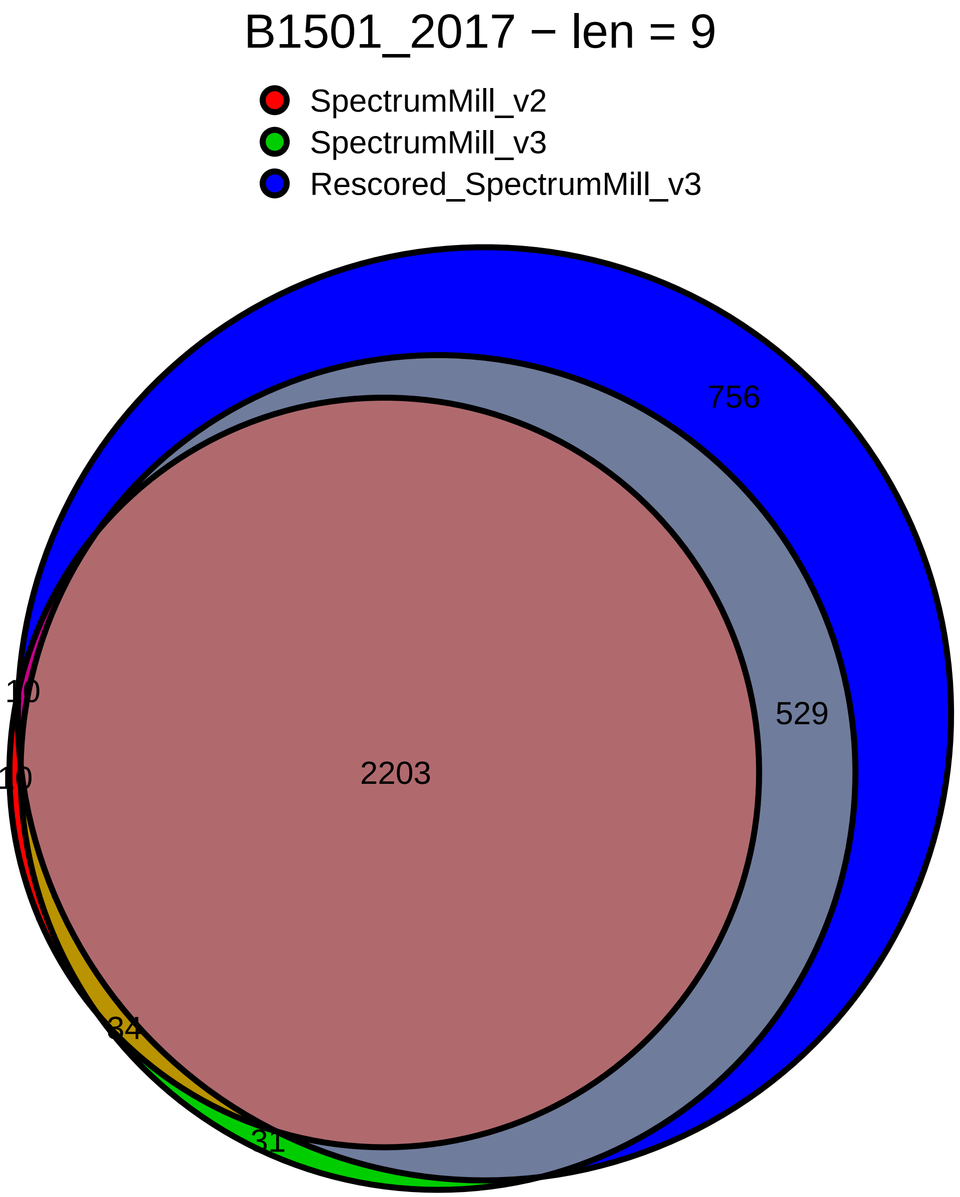
B1501_2017 − len = 9
SpectrumMill_v2
SpectrumMill_v3
Rescored_SpectrumMill_v3
756
529
10
10
2203
84
31

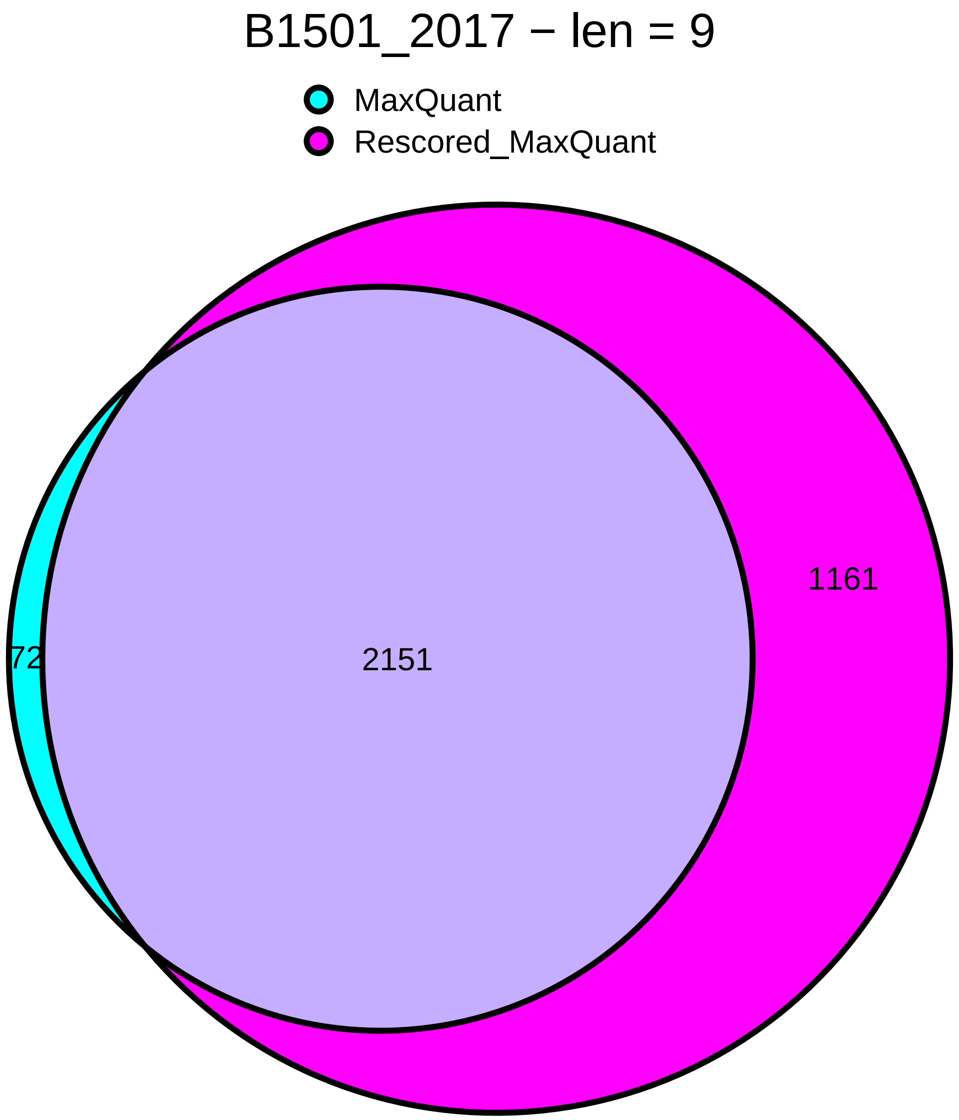
B1501_2017 − len = 9
MaxQuant
Rescored_MaxQuant
72
2151
1161

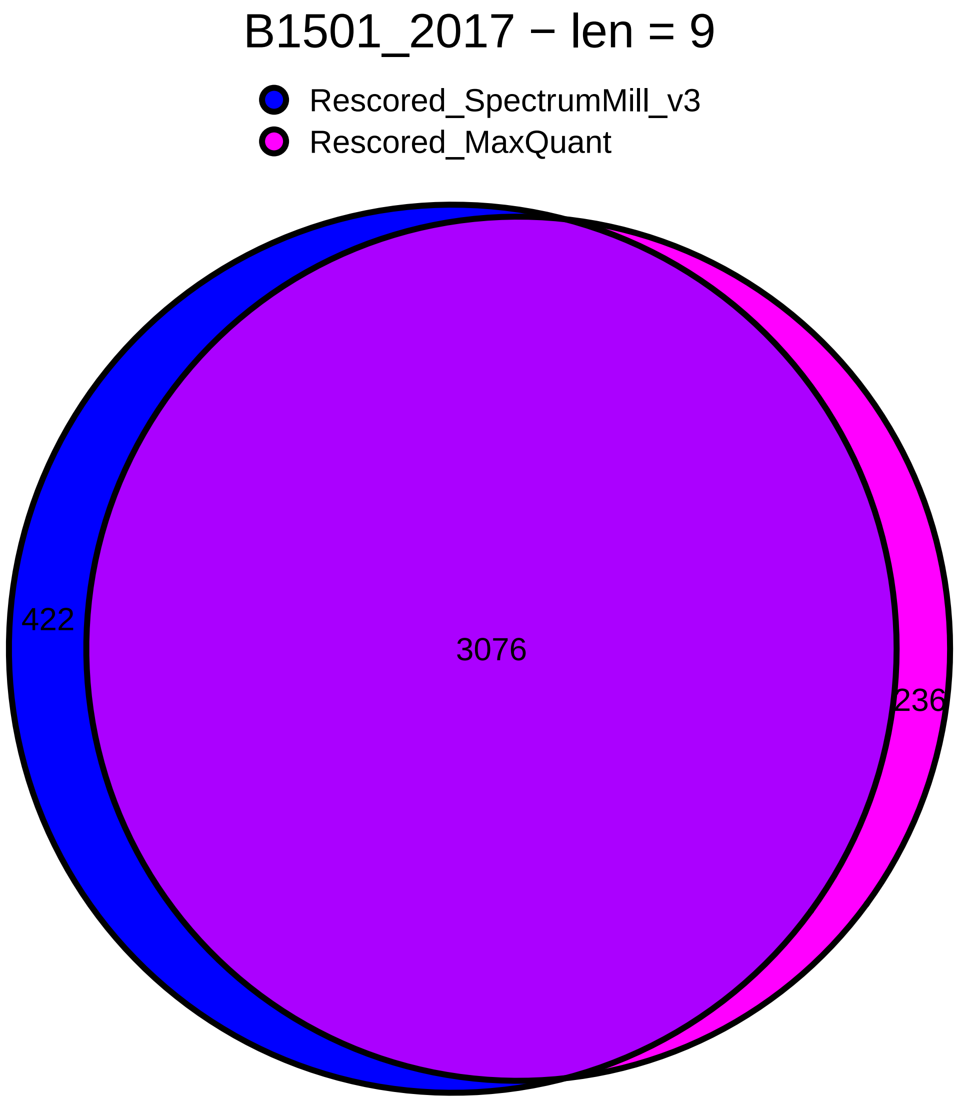
B1501_2017 − len = 9
Rescored_SpectrumMill_v3
Rescored_MaxQuant
422
3076
236

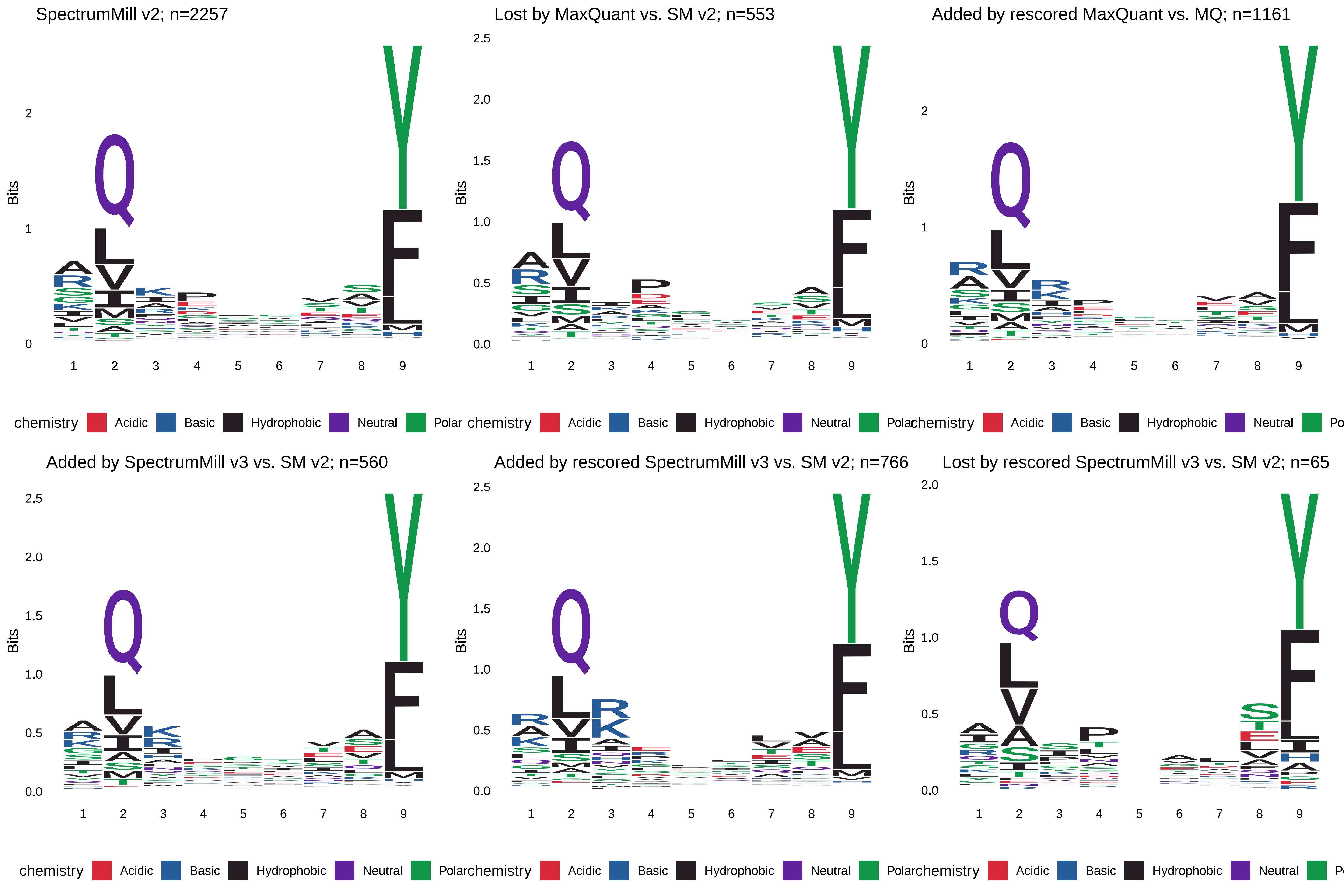
SpectrumMill v2; n=2257
Lost by MaxQuant vs. SM v2; n=553
Added by rescored MaxQuant vs. MQ; n=1161
Added by SpectrumMill v3 vs. SM v2; n=560
Added by rescored SpectrumMill v3 vs. SM v2; n=766
Lost by rescored SpectrumMill v3 vs. SM v2; n=65
Bits
1 2 3 4 5 6 7 8 9
chemistry
Acidic
Basic
Hydrophobic
Neutral
Polar

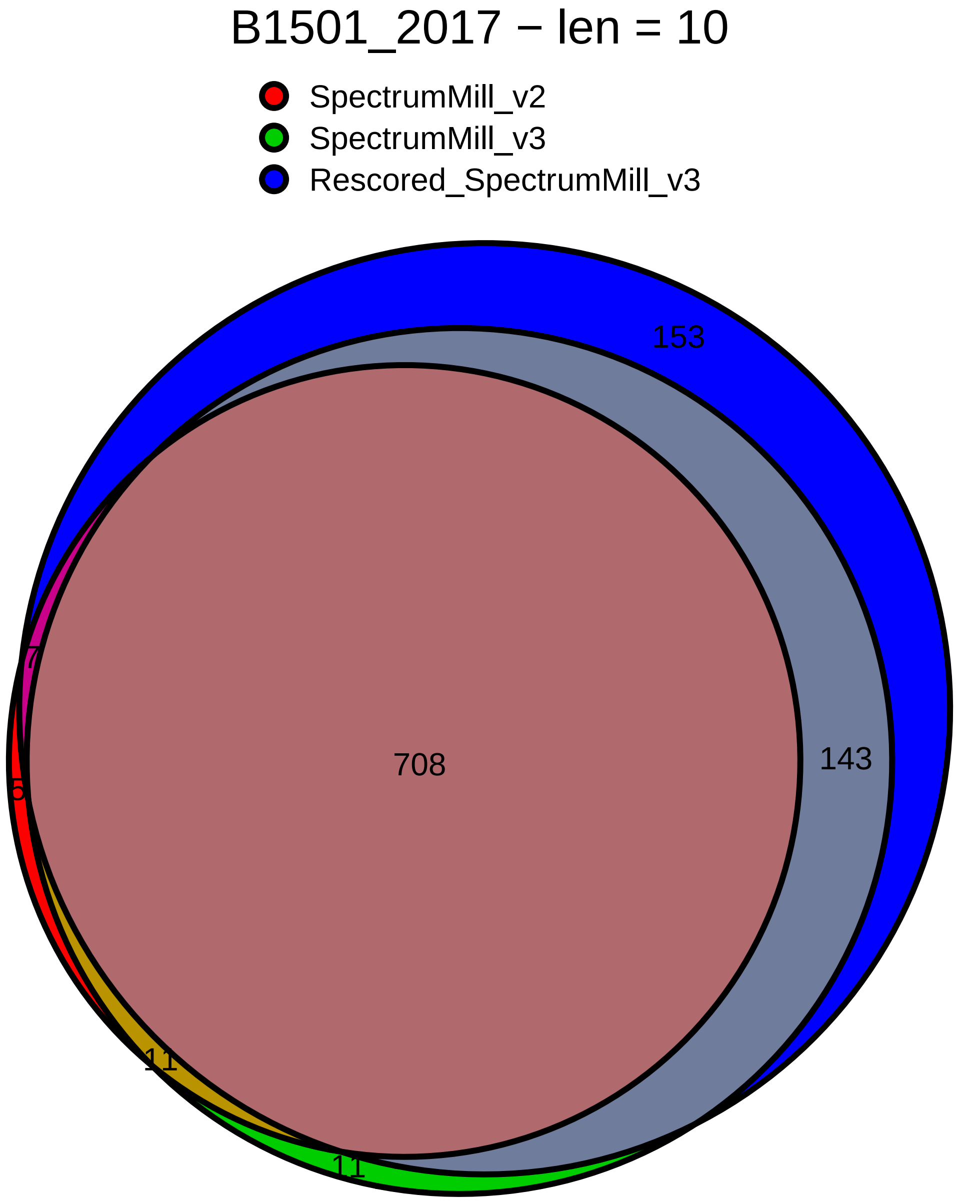
B1501_2017 − len = 10
SpectrumMill_v2
SpectrumMill_v3
Rescored_SpectrumMill_v3
153
7
708
143
5
11
11

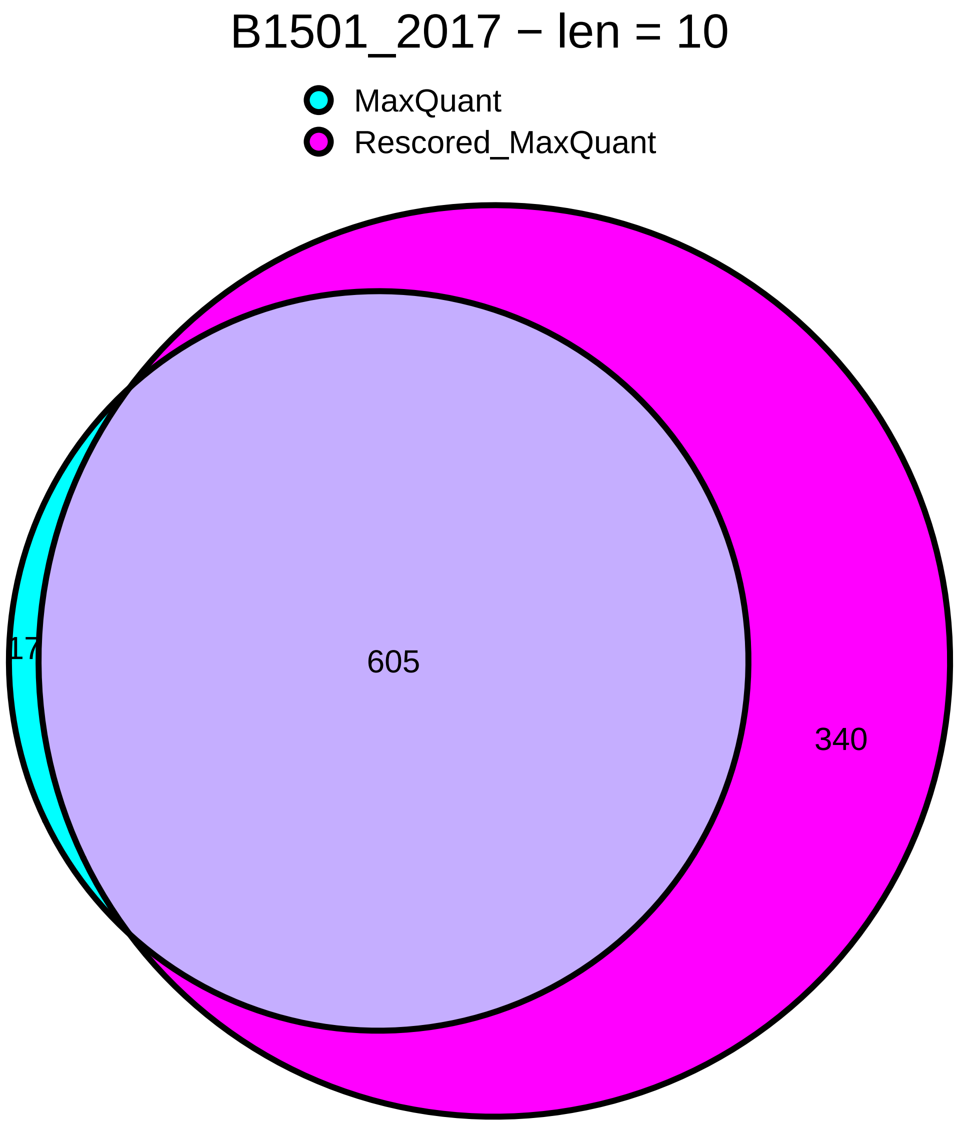
B1501_2017 − len = 10
MaxQuant
Rescored_MaxQuant
17
605
340

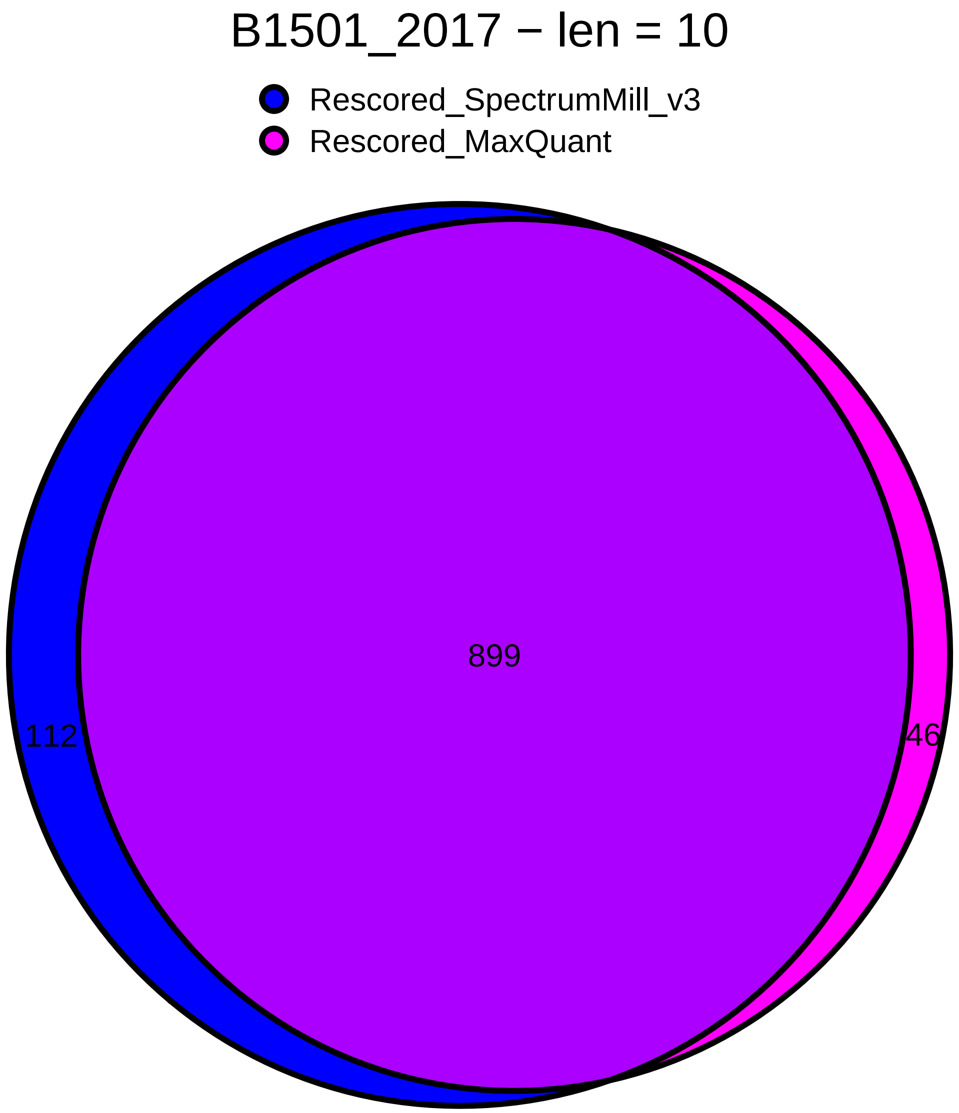
B1501_2017 − len = 10
Rescored_SpectrumMill_v3
Rescored_MaxQuant
899
112
46

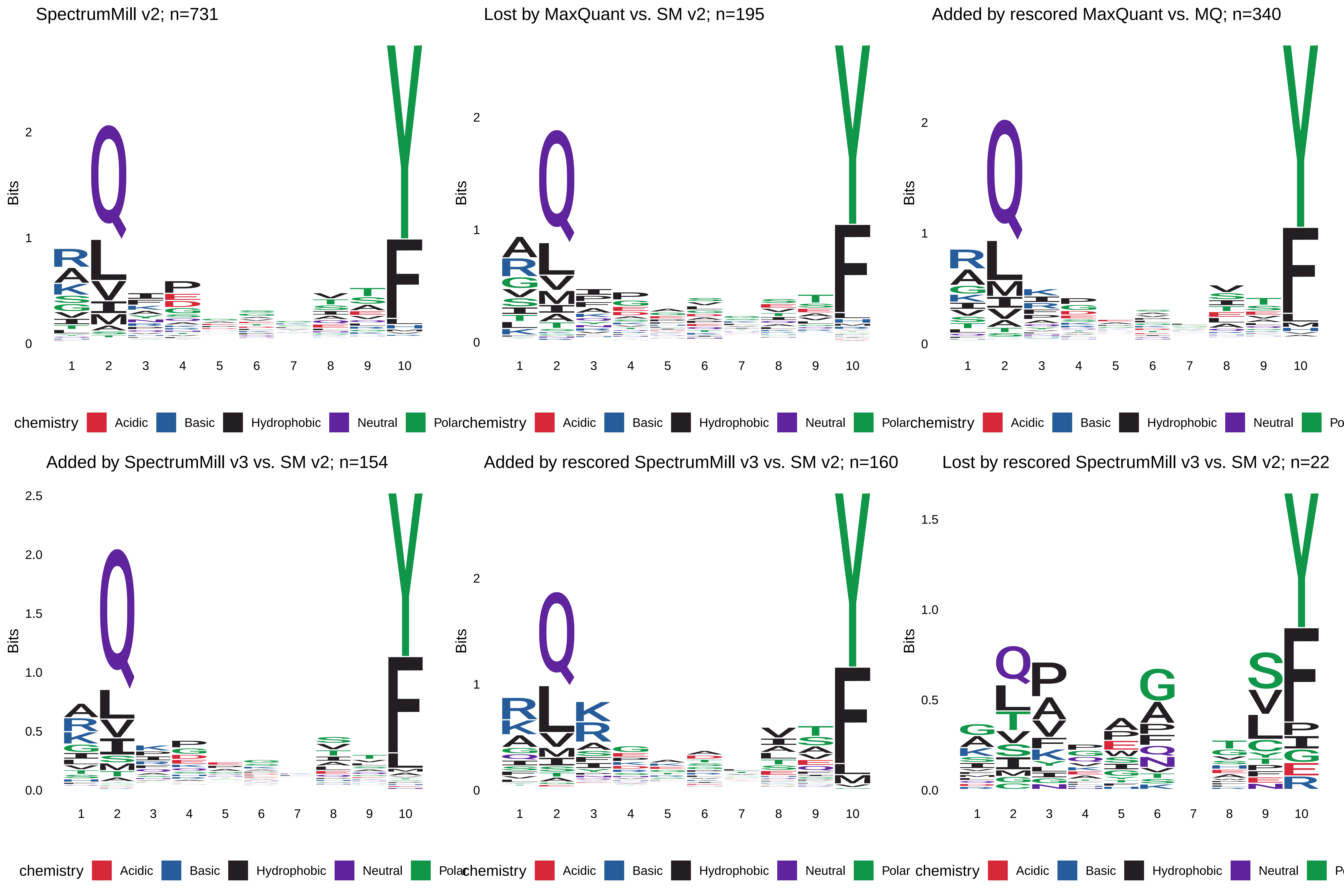
SpectrumMill v2; n=731
Lost by MaxQuant vs. SM v2; n=195
Added by rescored MaxQuant vs. MQ; n=340
Added by SpectrumMill v3 vs. SM v2; n=154
Added by rescored SpectrumMill v3 vs. SM v2; n=160
Lost by rescored SpectrumMill v3 vs. SM v2; n=22
Bits
1 2 3 4 5 6 7 8 9 10
chemistry
Acidic
Basic
Hydrophobic
Neutral
Polar

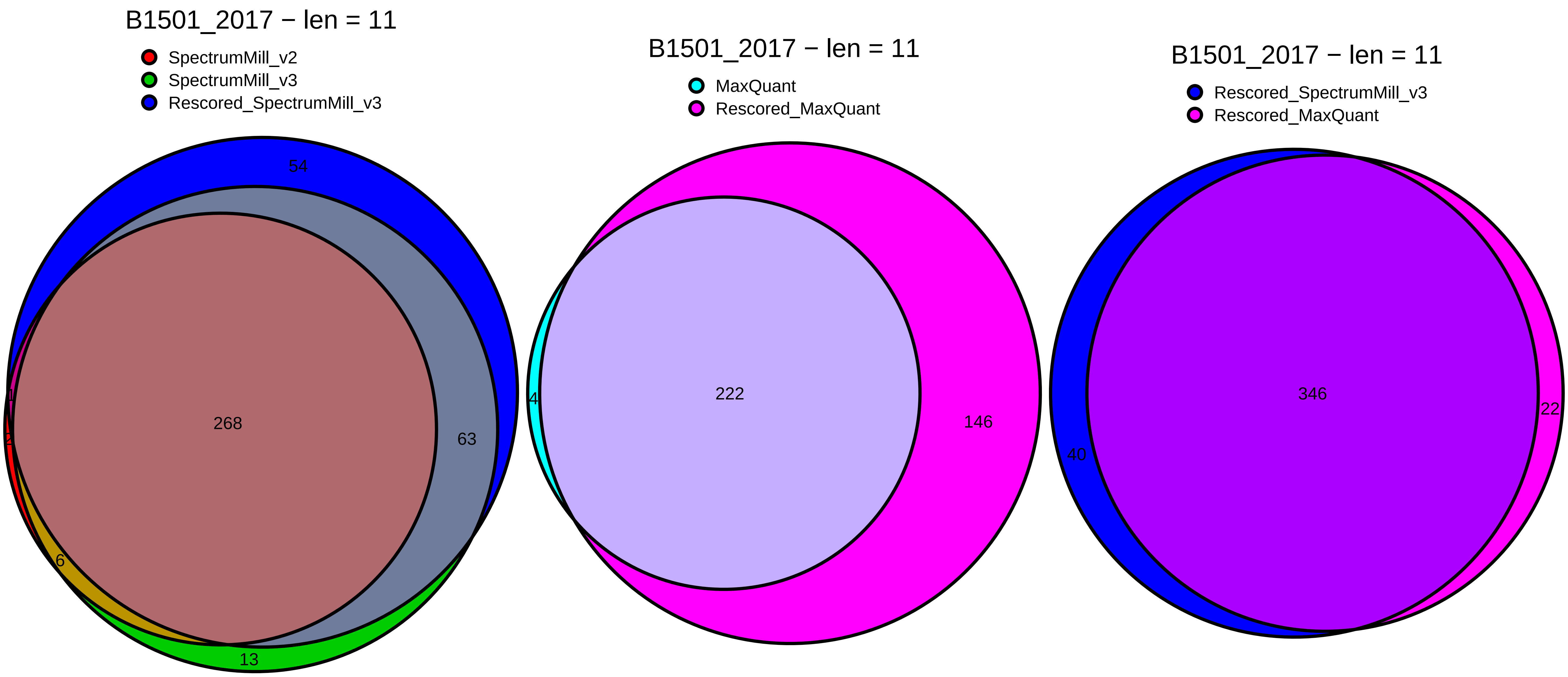

B1501_2017 − len = 11
SpectrumMill_v2
SpectrumMill_v3
Rescored_SpectrumMill_v3
54
268
63
6
13
B1501_2017 − len = 11
MaxQuant
Rescored_MaxQuant
222
146
B1501_2017 − len = 11
Rescored_SpectrumMill_v3
Rescored_MaxQuant
346
40
22

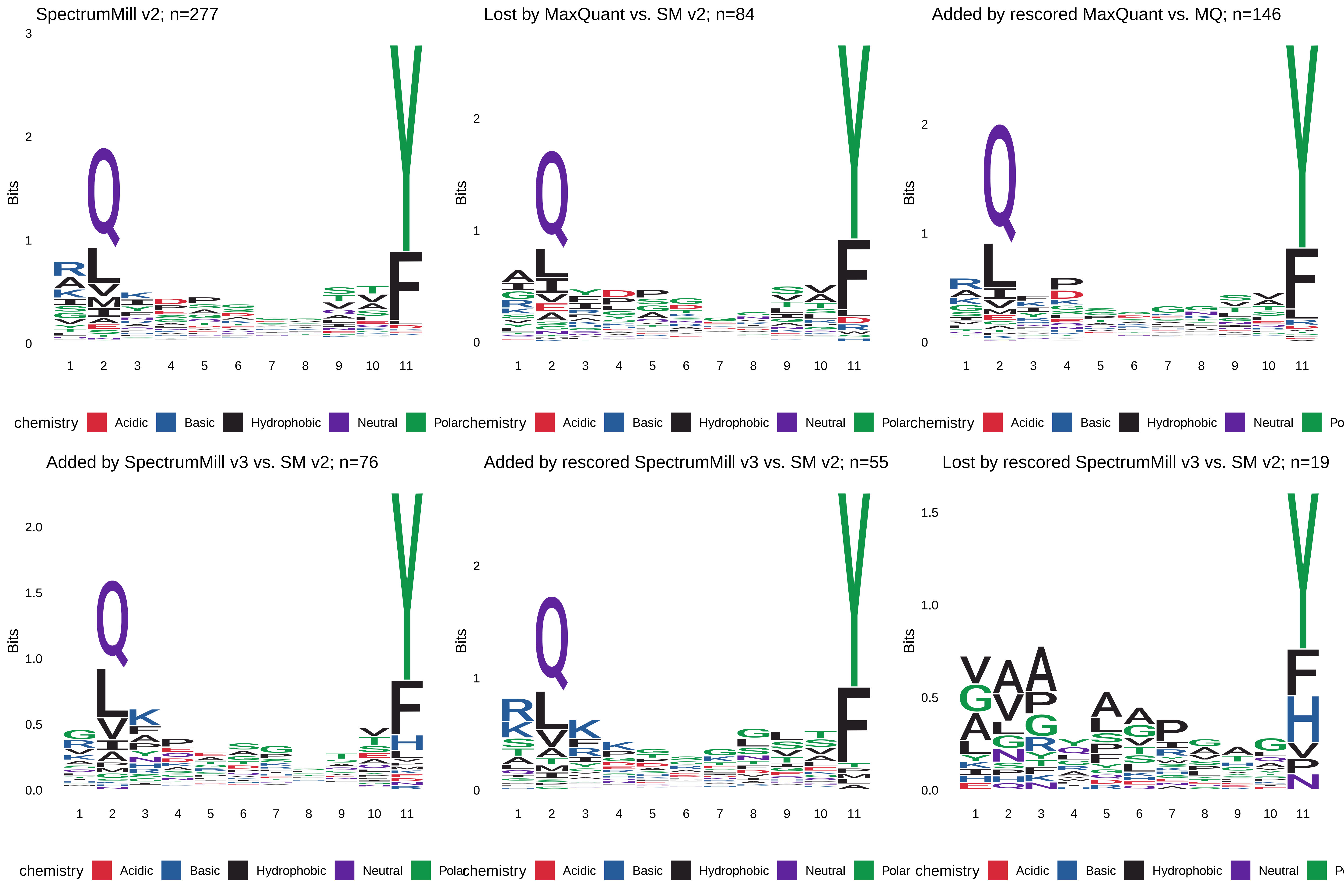
SpectrumMill v2; n=277
Lost by MaxQuant vs. SM v2; n=84
Added by rescored MaxQuant vs. MQ; n=146
Added by SpectrumMill v3 vs. SM v2; n=76
Added by rescored SpectrumMill v3 vs. SM v2; n=55
Lost by rescored SpectrumMill v3 vs. SM v2; n=19
Bits
1 2 3 4 5 6 7 8 9 10 11
chemistry
Acidic
Basic
Hydrophobic
Neutral
Polar

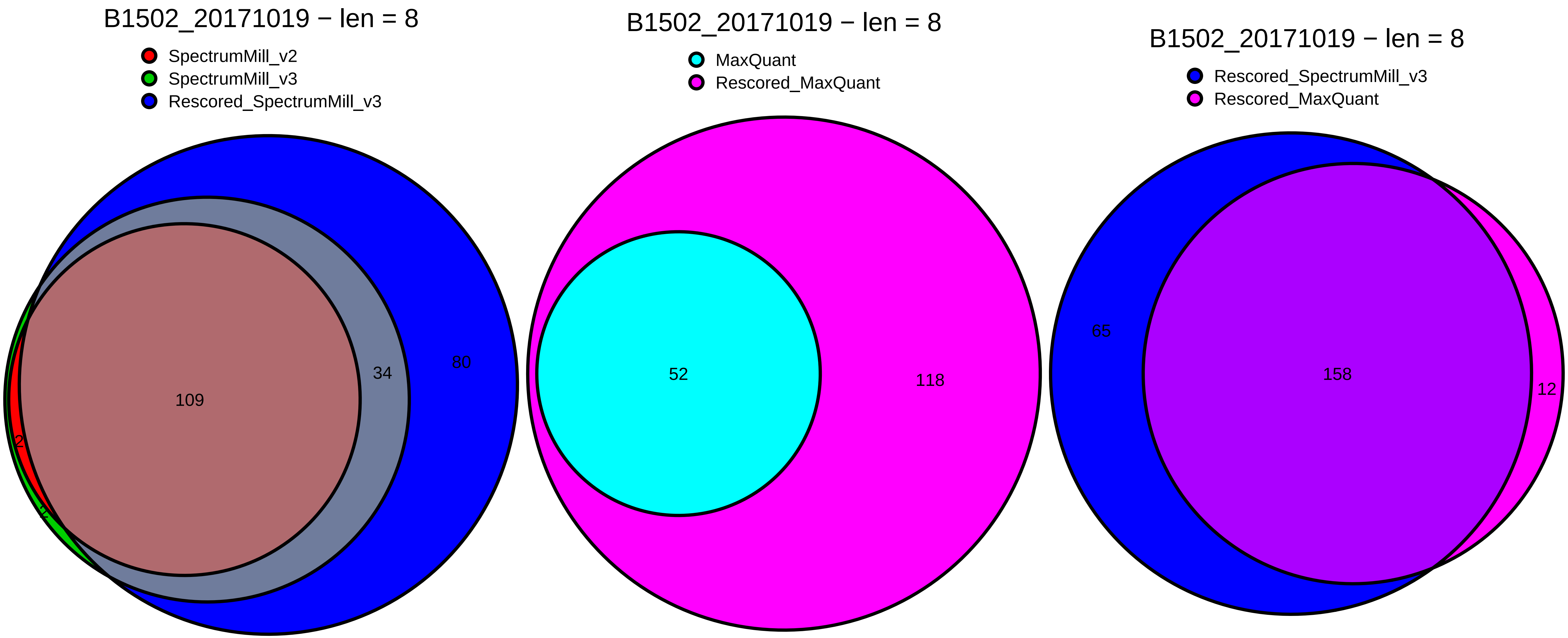

B1502_20171019 − len = 8
SpectrumMill_v2
SpectrumMill_v3
Rescored_SpectrumMill_v3
109
34
80
2
B1502_20171019 − len = 8
MaxQuant
Rescored_MaxQuant
52
118
B1502_20171019 − len = 8
Rescored_SpectrumMill_v3
Rescored_MaxQuant
65
158
12

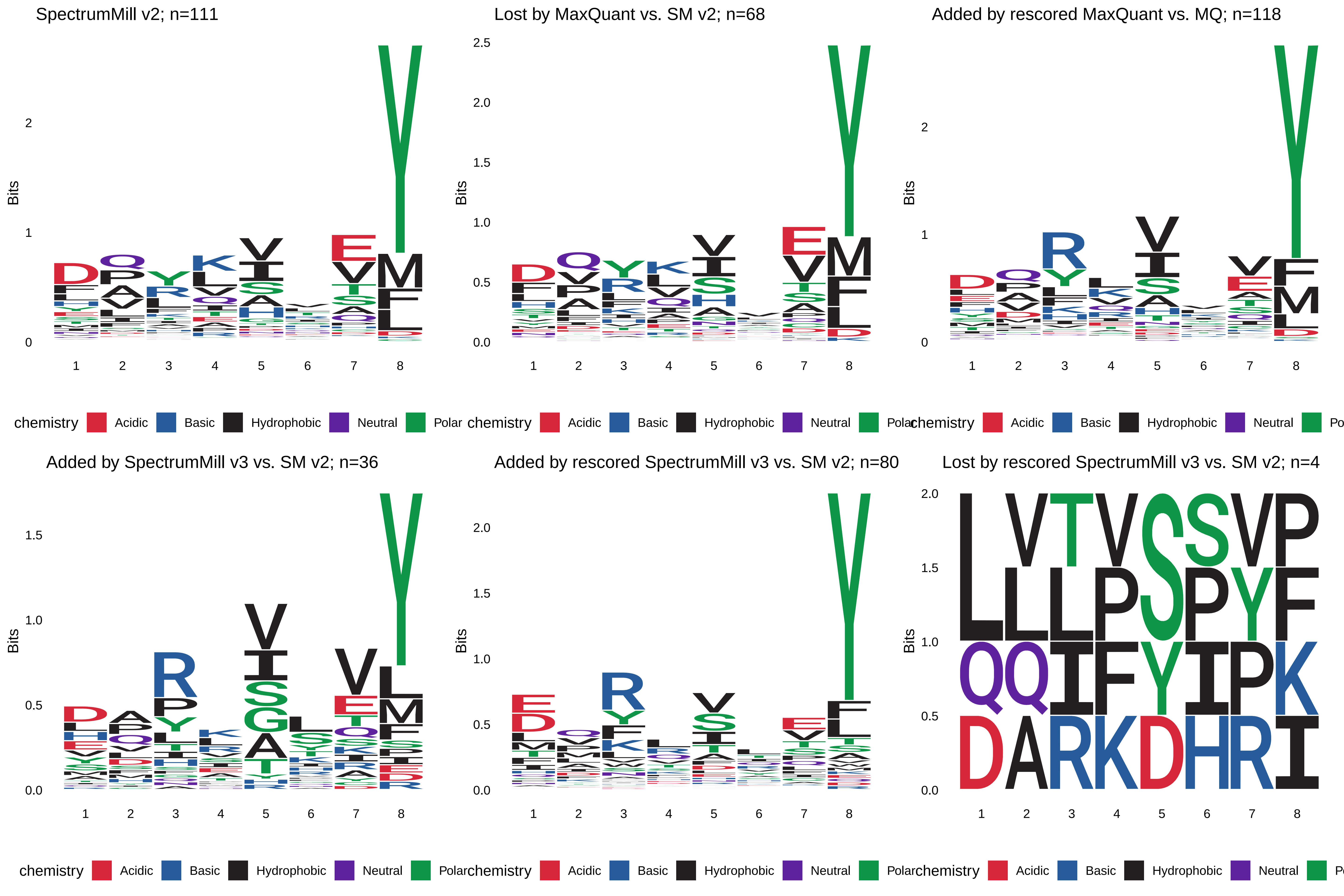

SpectrumMill v2; n=111
Lost by MaxQuant vs. SM v2; n=68
Added by rescored MaxQuant vs. MQ; n=118
Added by SpectrumMill v3 vs. SM v2; n=36
Added by rescored SpectrumMill v3 vs. SM v2; n=80
Lost by rescored SpectrumMill v3 vs. SM v2; n=4
Bits
1 2 3 4 5 6 7 8
chemistry
Acidic
Basic
Hydrophobic
Neutral
Polar

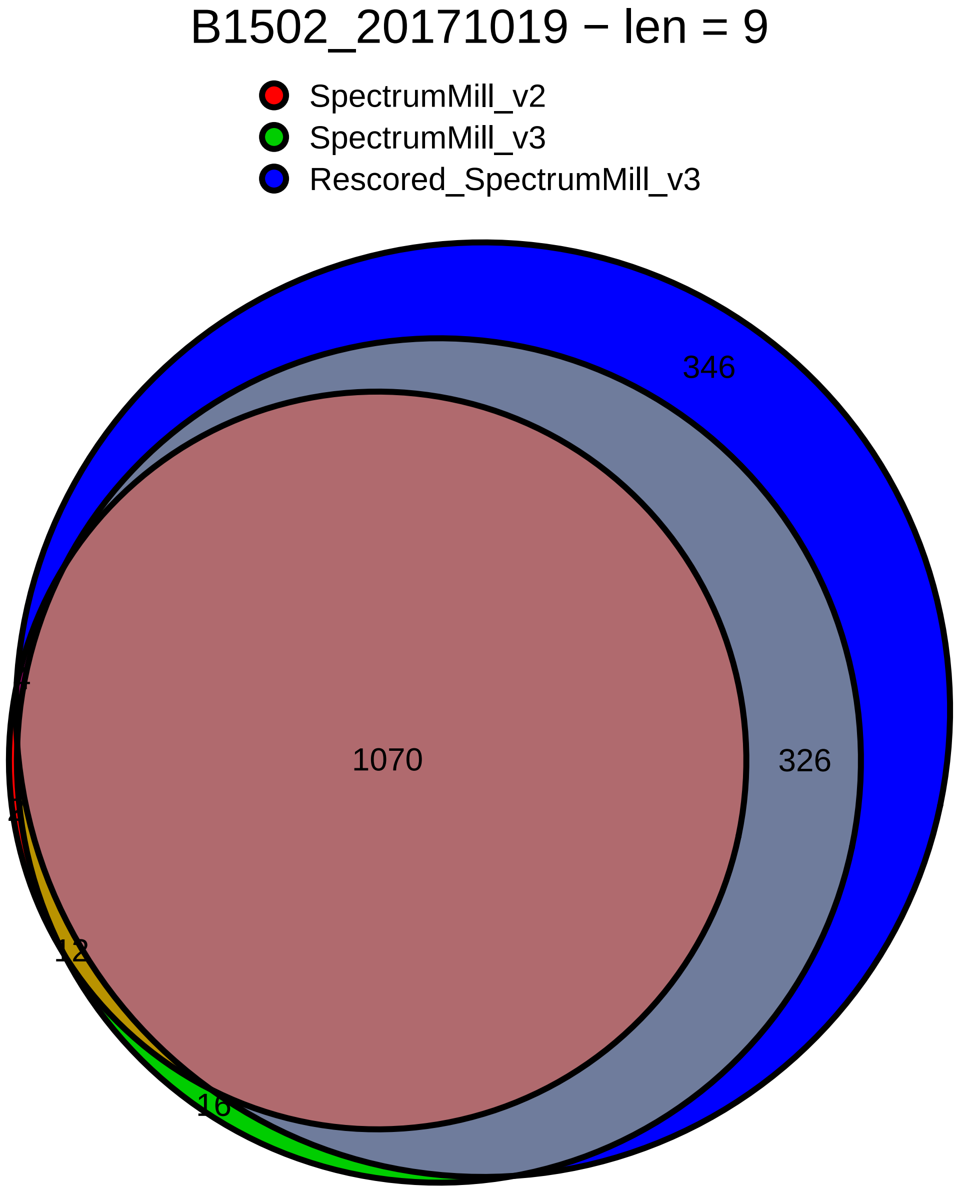
B1502_20171019 − len = 9
SpectrumMill_v2
SpectrumMill_v3
Rescored_SpectrumMill_v3
346
1070
326
12
16

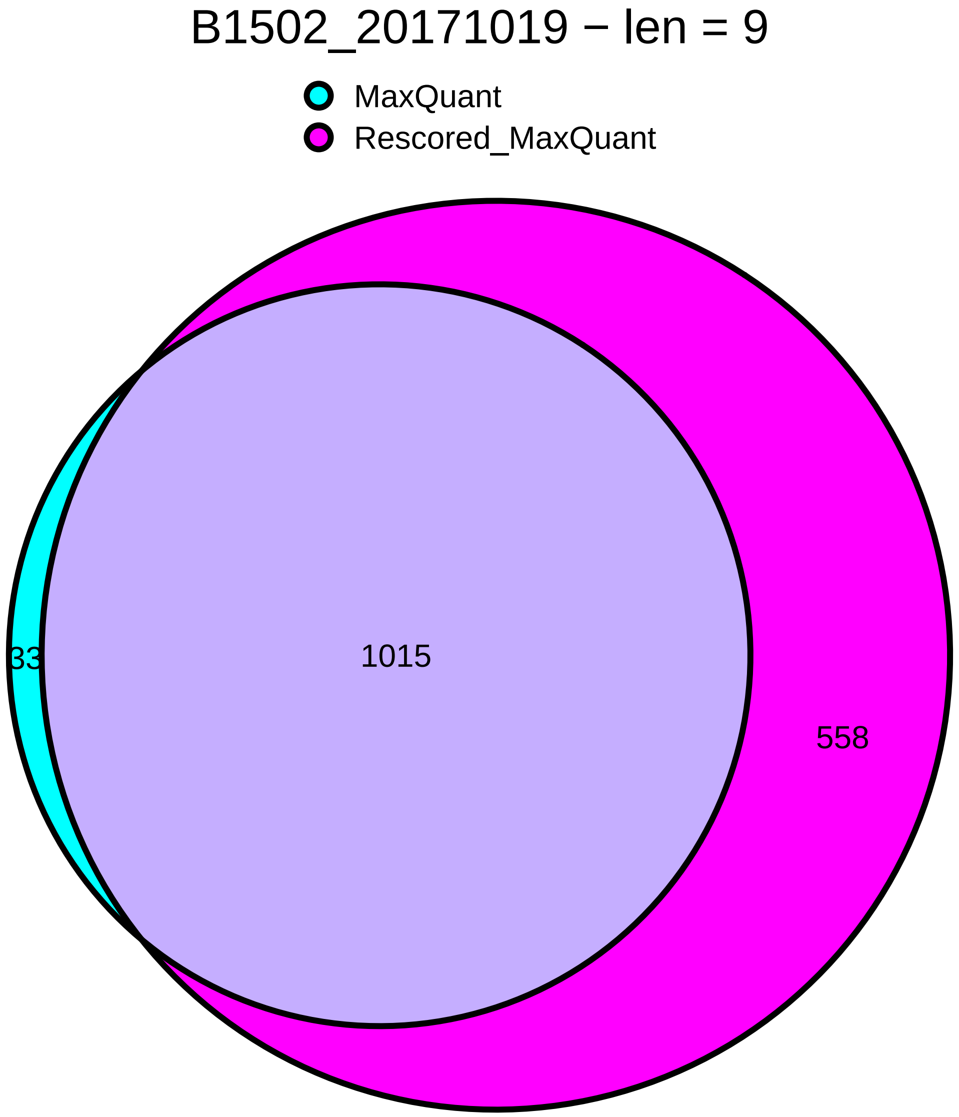
B1502_20171019 − len = 9
MaxQuant
Rescored_MaxQuant
33
1015
558

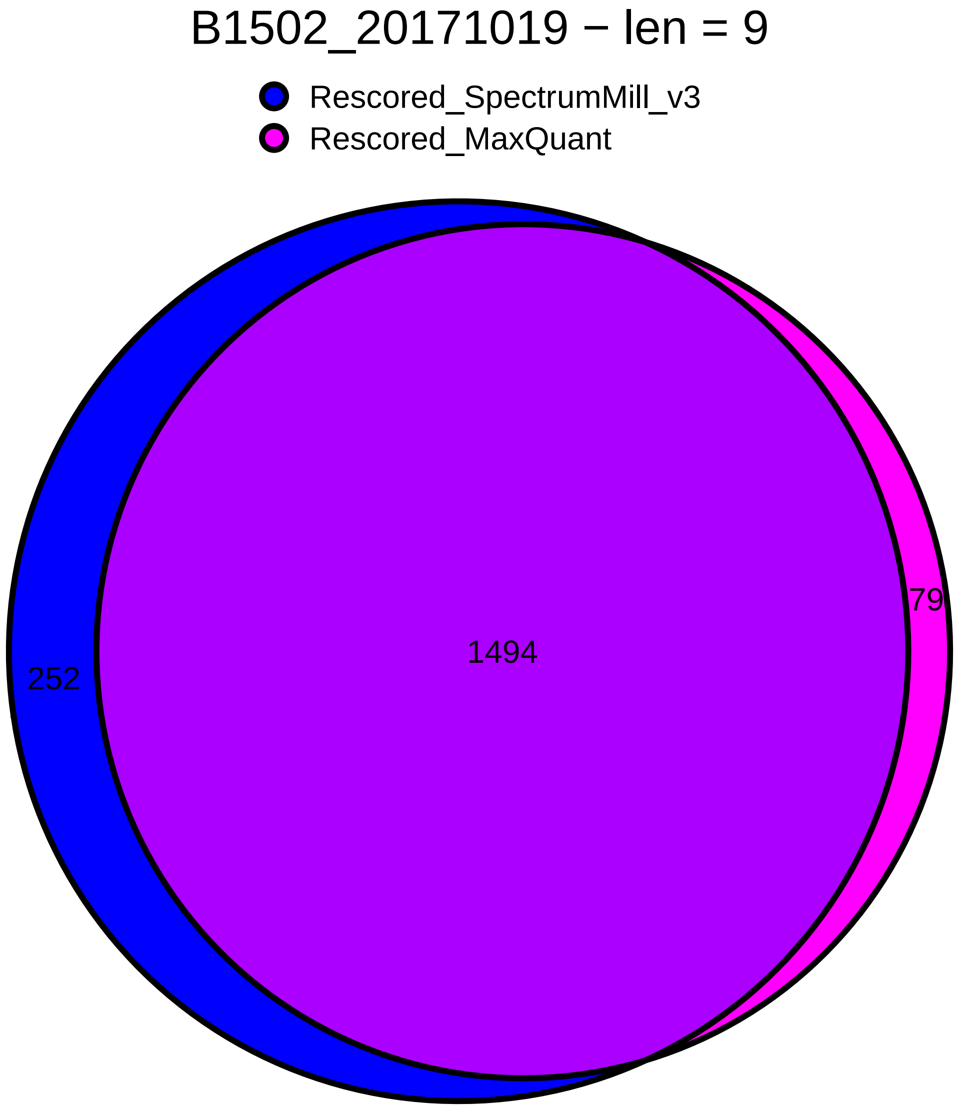
B1502_20171019 − len = 9
Rescored_SpectrumMill_v3
Rescored_MaxQuant
79
252
1494

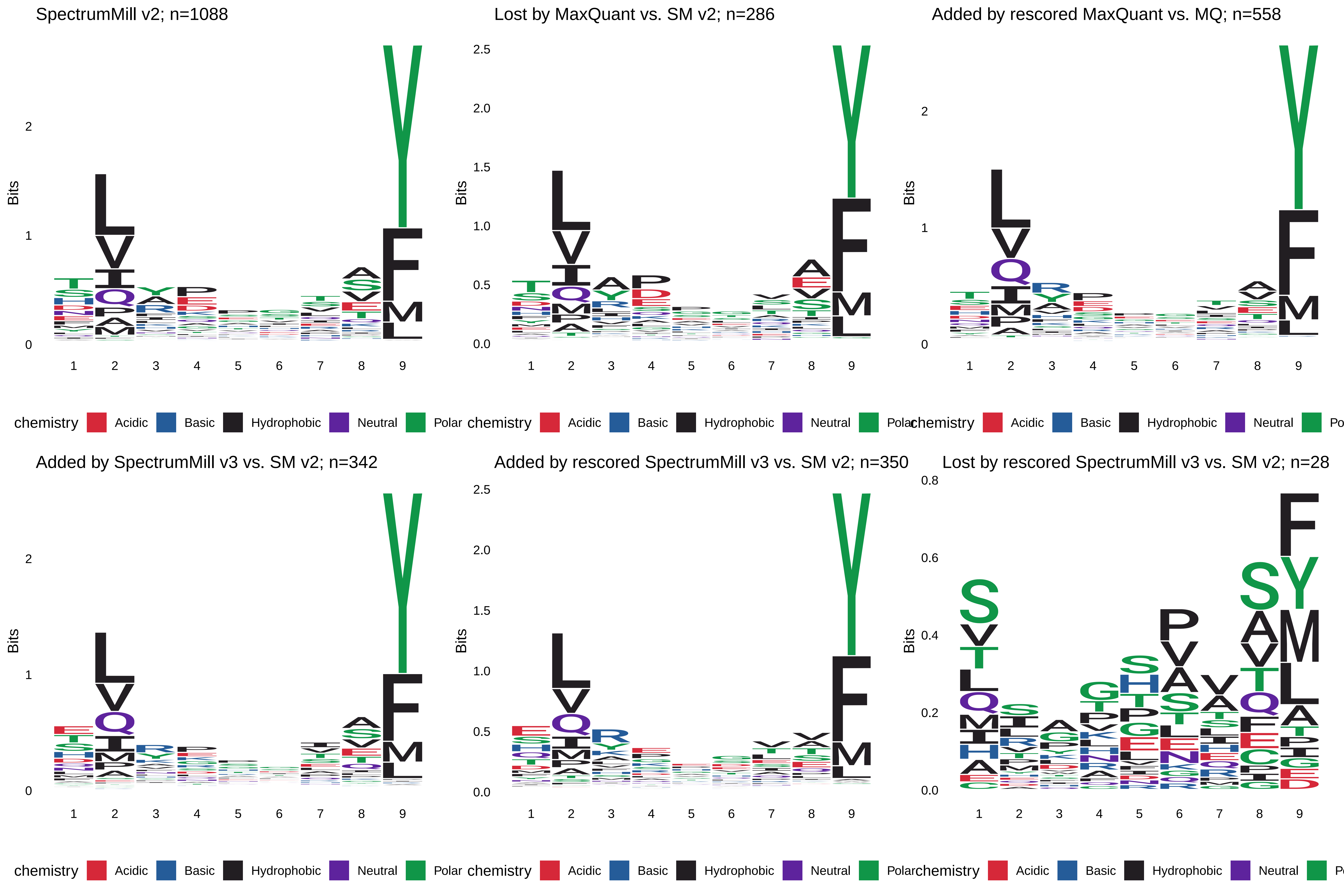
SpectrumMill v2; n=1088
Lost by MaxQuant vs. SM v2; n=286
Added by rescored MaxQuant vs. MQ; n=558
Added by SpectrumMill v3 vs. SM v2; n=342
Added by rescored SpectrumMill v3 vs. SM v2; n=350
Lost by rescored SpectrumMill v3 vs. SM v2; n=28
Bits
chemistry
Acidic
Basic
Hydrophobic
Neutral
Polar

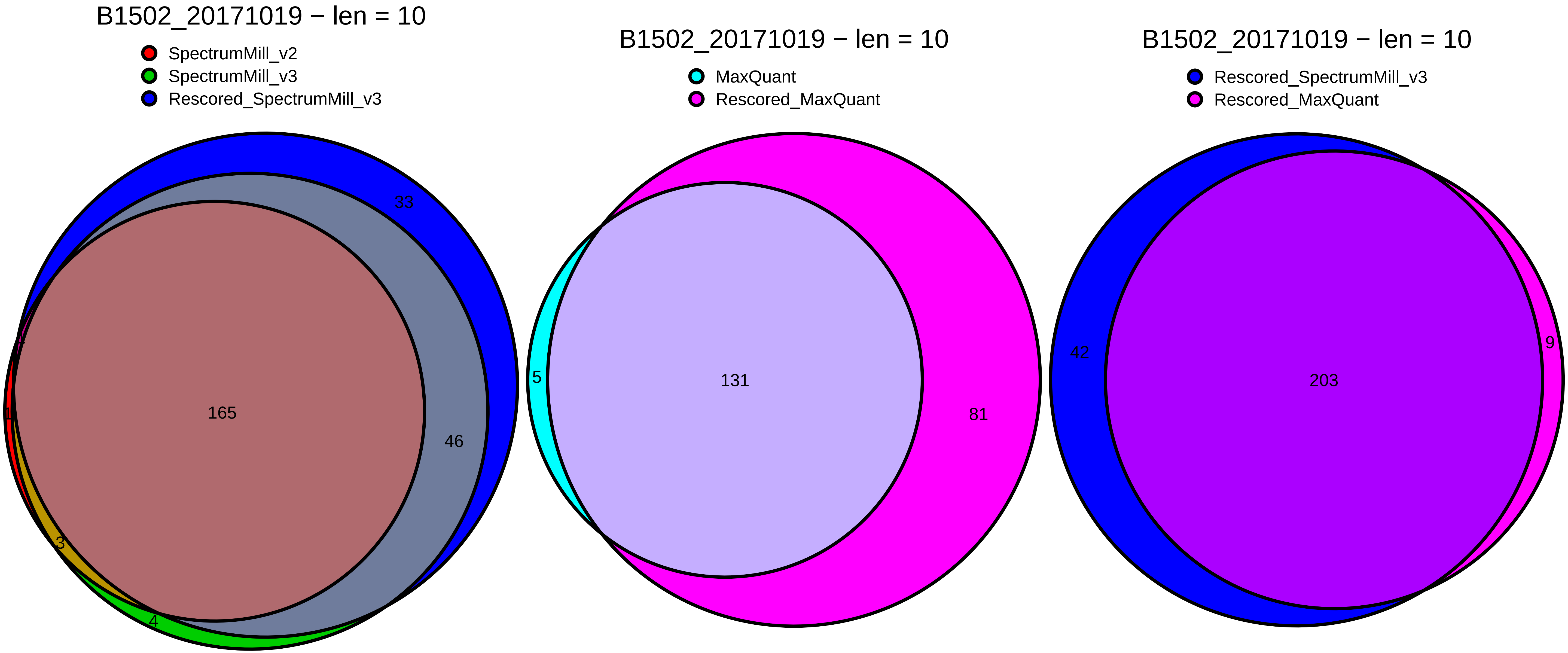
B1502_20171019 − len = 10
SpectrumMill_v2
SpectrumMill_v3
Rescored_SpectrumMill_v3
33
165
46
1
3
4
B1502_20171019 − len = 10
MaxQuant
Rescored_MaxQuant
5
131
81
B1502_20171019 − len = 10
Rescored_SpectrumMill_v3
Rescored_MaxQuant
42
203
9

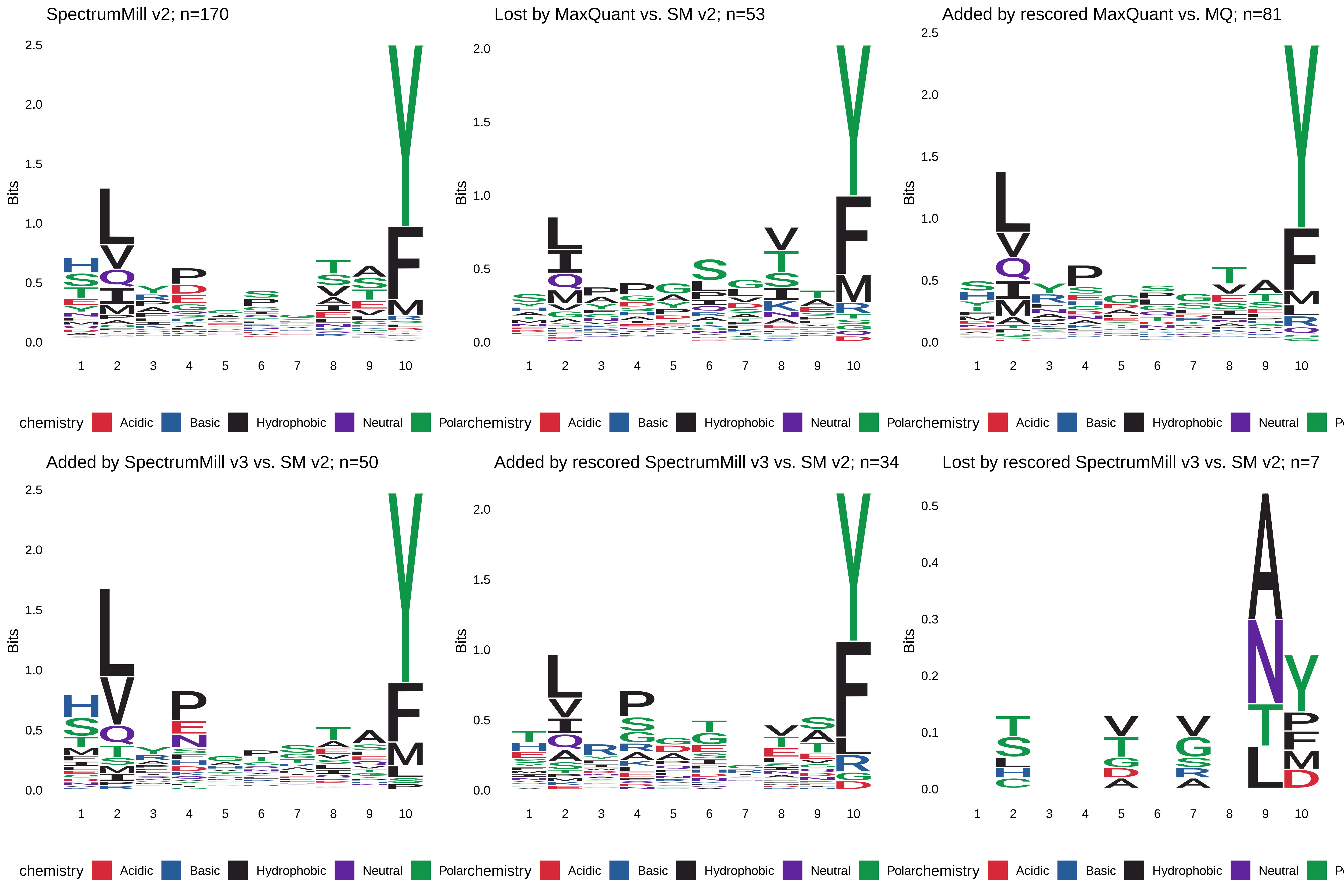
SpectrumMill v2; n=170
Lost by MaxQuant vs. SM v2; n=53
Added by rescored MaxQuant vs. MQ; n=81
Added by SpectrumMill v3 vs. SM v2; n=50
Added by rescored SpectrumMill v3 vs. SM v2; n=34
Lost by rescored SpectrumMill v3 vs. SM v2; n=7
Bits
chemistry
Acidic
Basic
Hydrophobic
Neutral
Polar

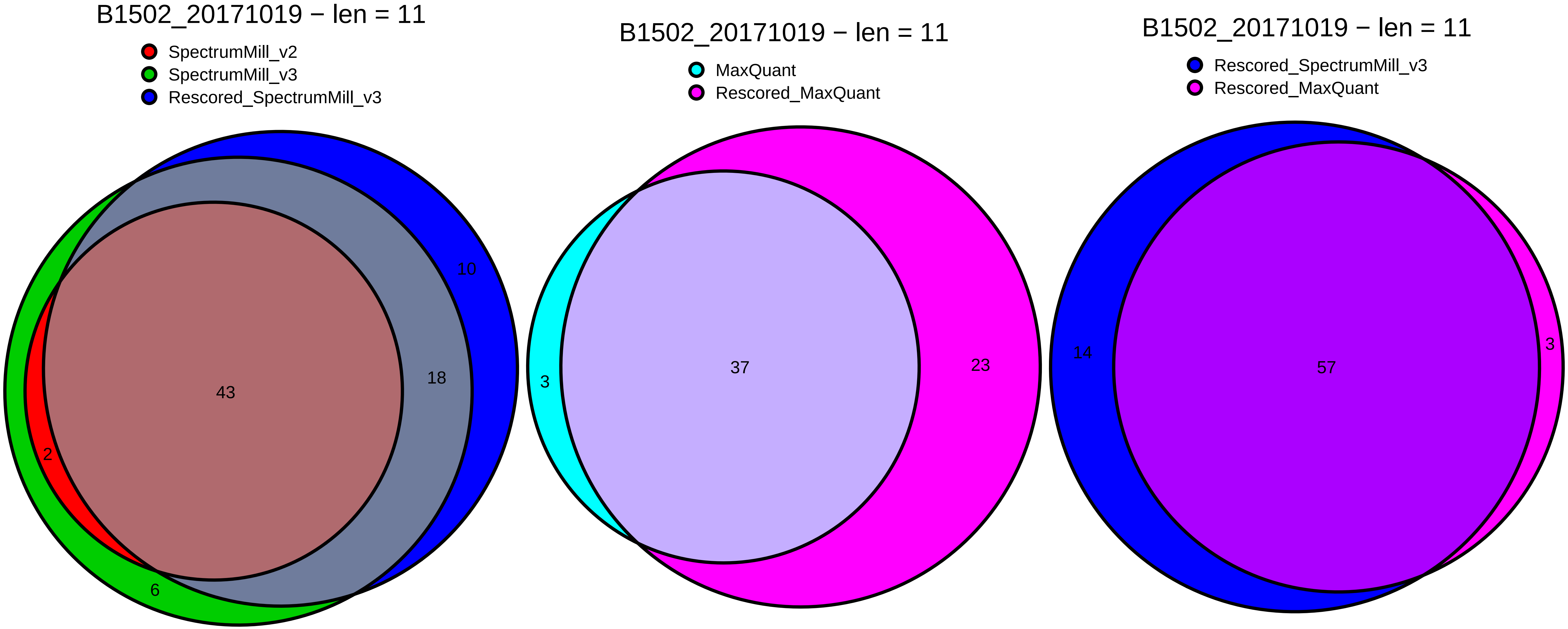
B1502_20171019 − len = 11
SpectrumMill_v2
SpectrumMill_v3
Rescored_SpectrumMill_v3
10
18
43
2
6
B1502_20171019 − len = 11
MaxQuant
Rescored_MaxQuant
3
37
23
B1502_20171019 − len = 11
Rescored_SpectrumMill_v3
Rescored_MaxQuant
14
57
3

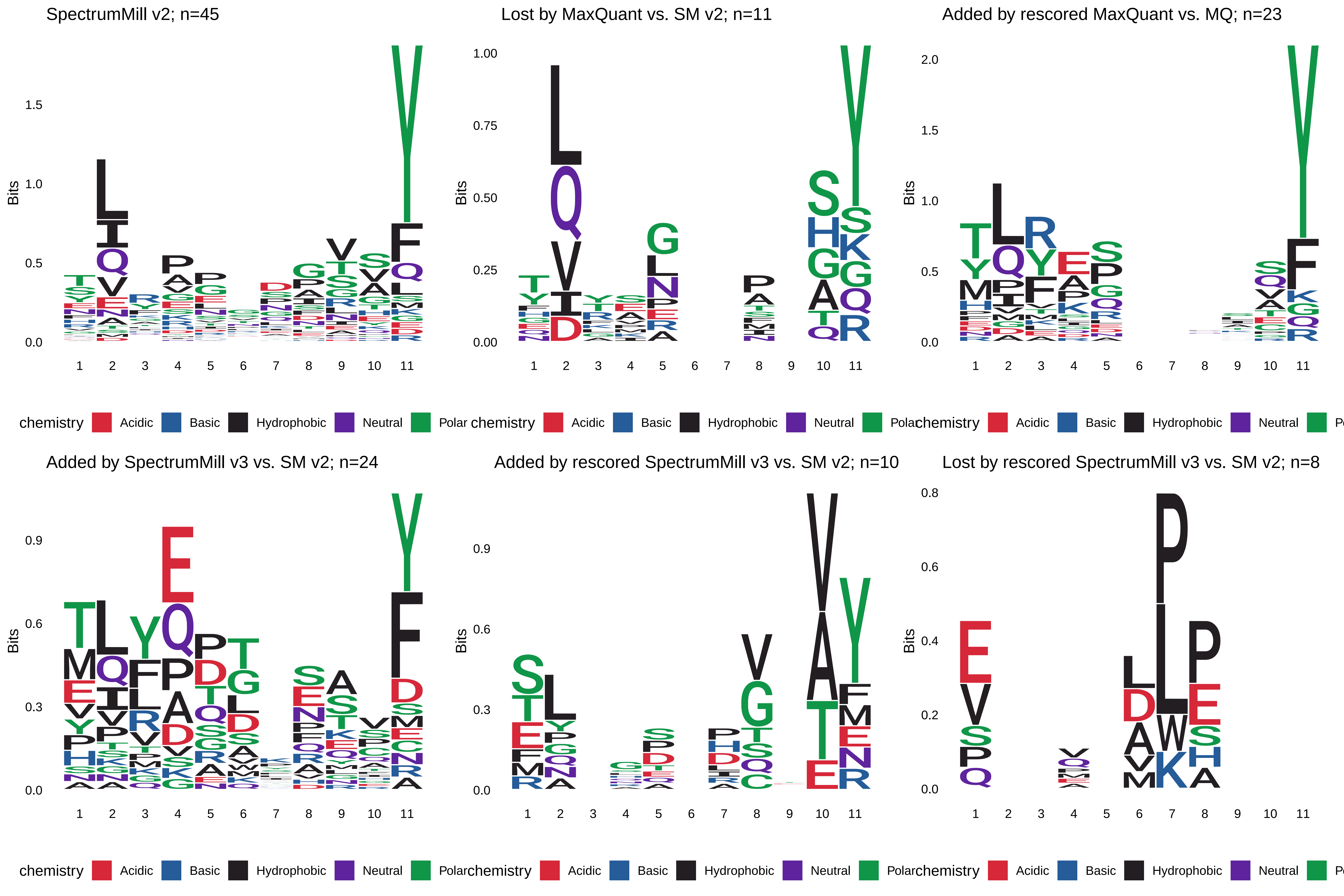
SpectrumMill v2; n=45
Lost by MaxQuant vs. SM v2; n=11
Added by rescored MaxQuant vs. MQ; n=23
Added by SpectrumMill v3 vs. SM v2; n=24
Added by rescored SpectrumMill v3 vs. SM v2; n=10
Lost by rescored SpectrumMill v3 vs. SM v2; n=8
Bits
1 2 3 4 5 6 7 8 9 10 11
chemistry Acidic Basic Hydrophobic Neutral Polar

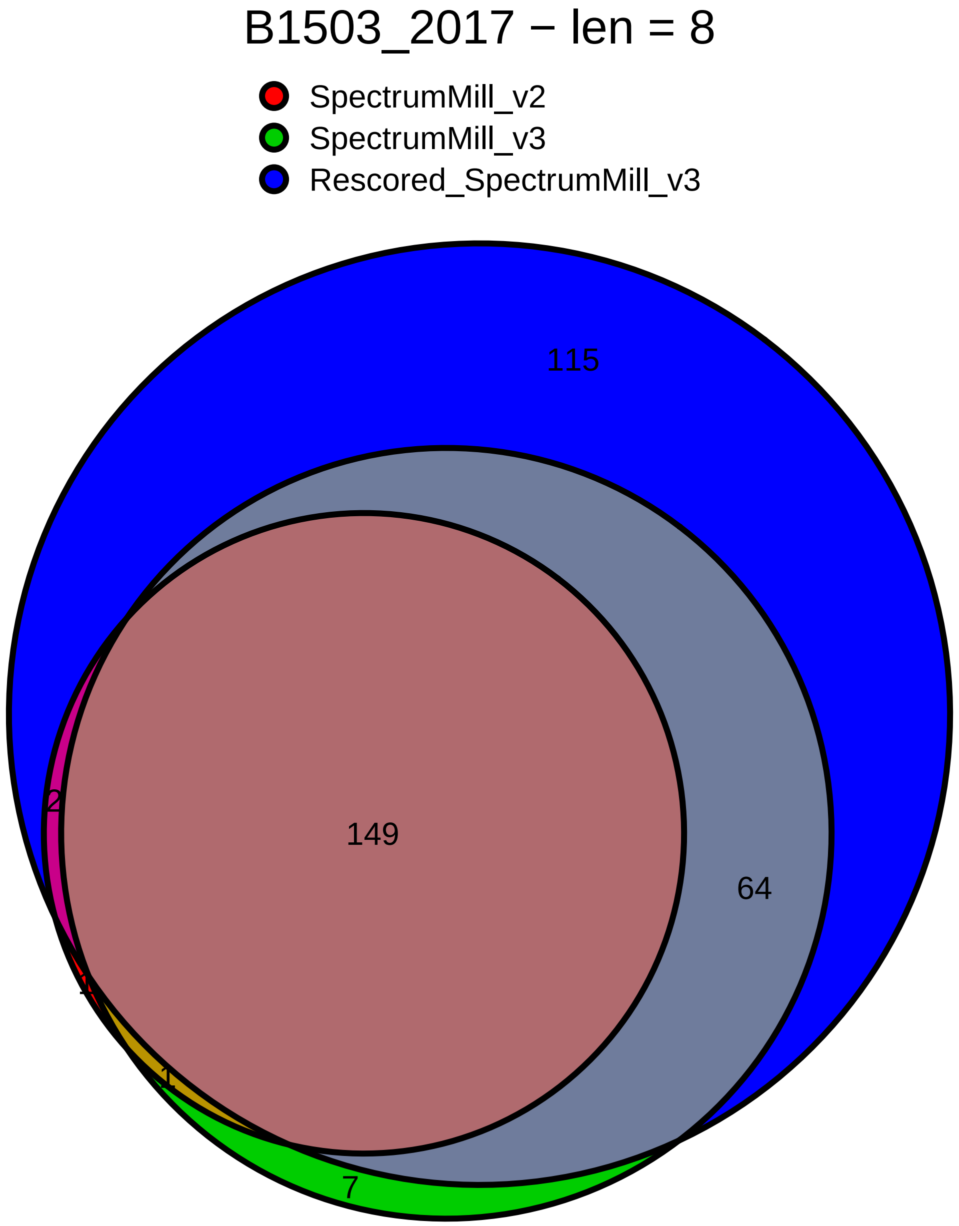
B1503_2017 − len = 8
SpectrumMill_v2
SpectrumMill_v3
Rescored_SpectrumMill_v3
115
149
64
7

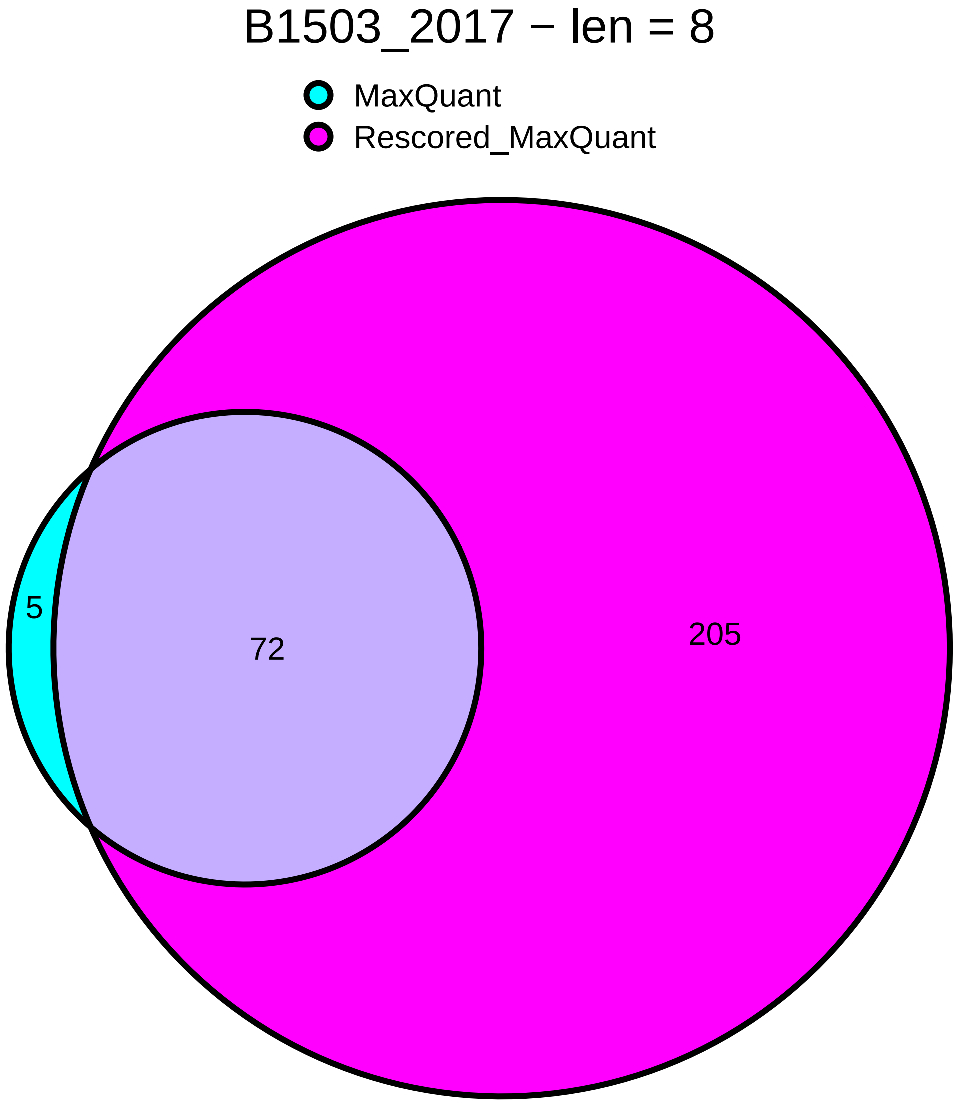
B1503_2017 − len = 8
MaxQuant
Rescored_MaxQuant
5
72
205

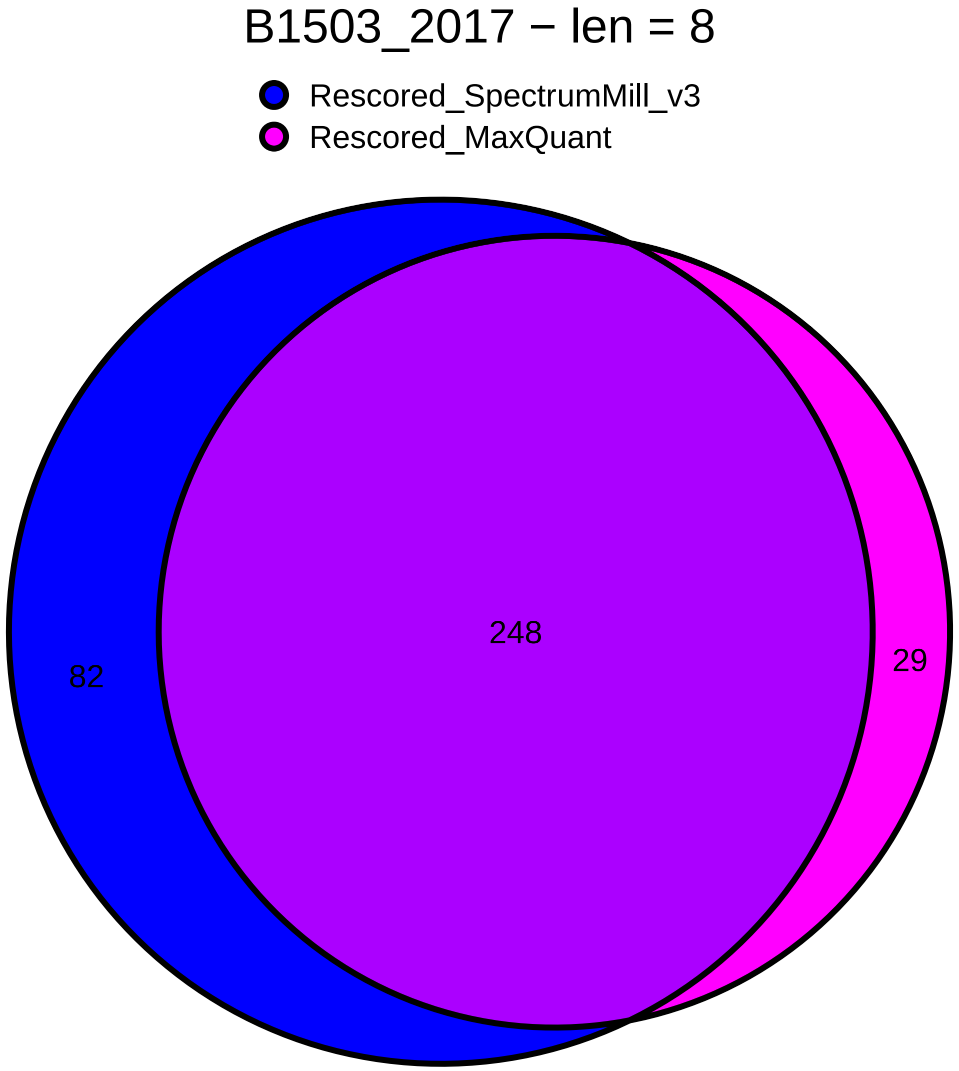
B1503_2017 − len = 8
Rescored_SpectrumMill_v3
Rescored_MaxQuant
82
248
29

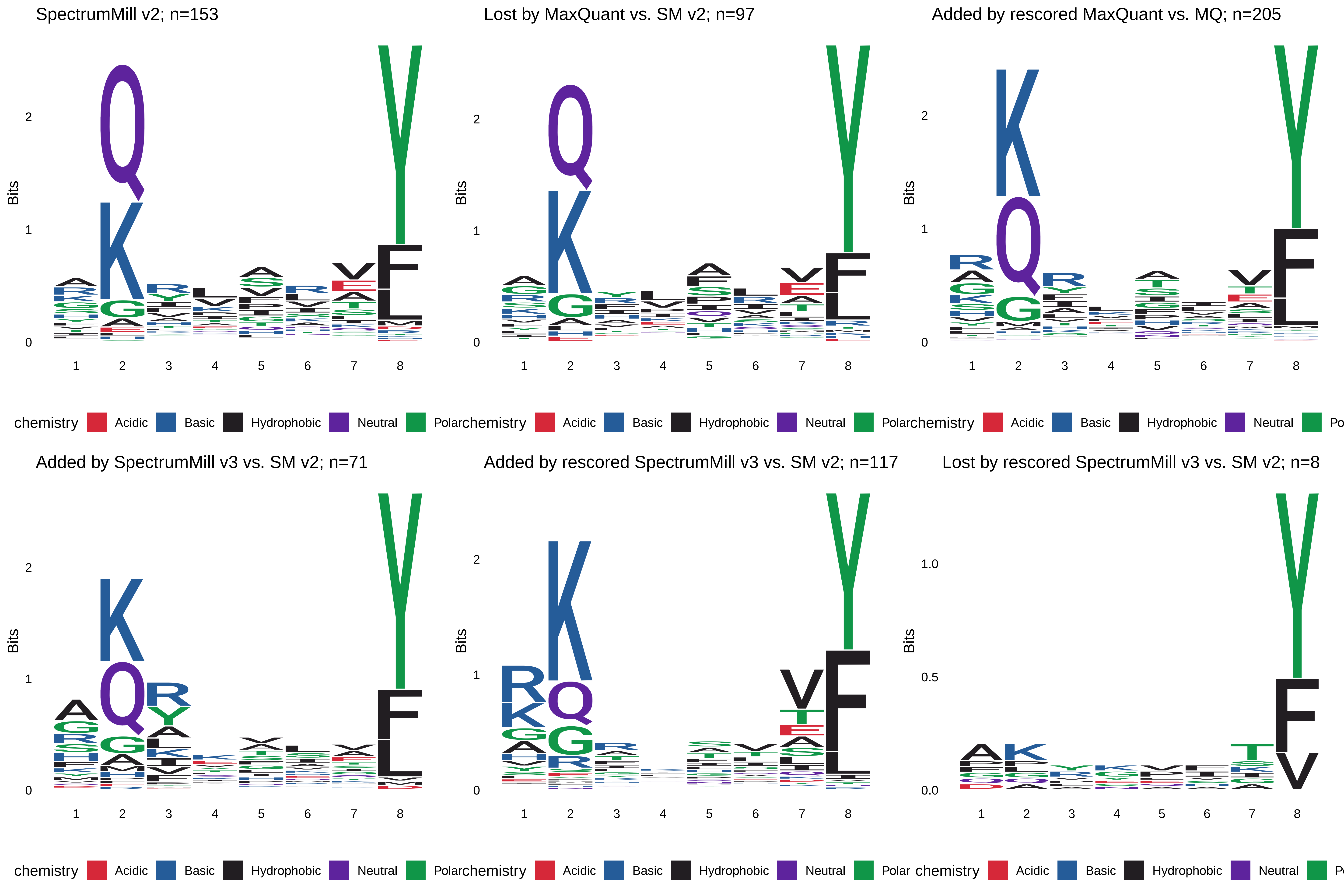
SpectrumMill v2; n=153
Lost by MaxQuant vs. SM v2; n=97
Added by rescored MaxQuant vs. MQ; n=205
Added by SpectrumMill v3 vs. SM v2; n=71
Added by rescored SpectrumMill v3 vs. SM v2; n=117
Lost by rescored SpectrumMill v3 vs. SM v2; n=8
Bits
1 2 3 4 5 6 7 8
chemistry
Acidic
Basic
Hydrophobic
Neutral
Polar

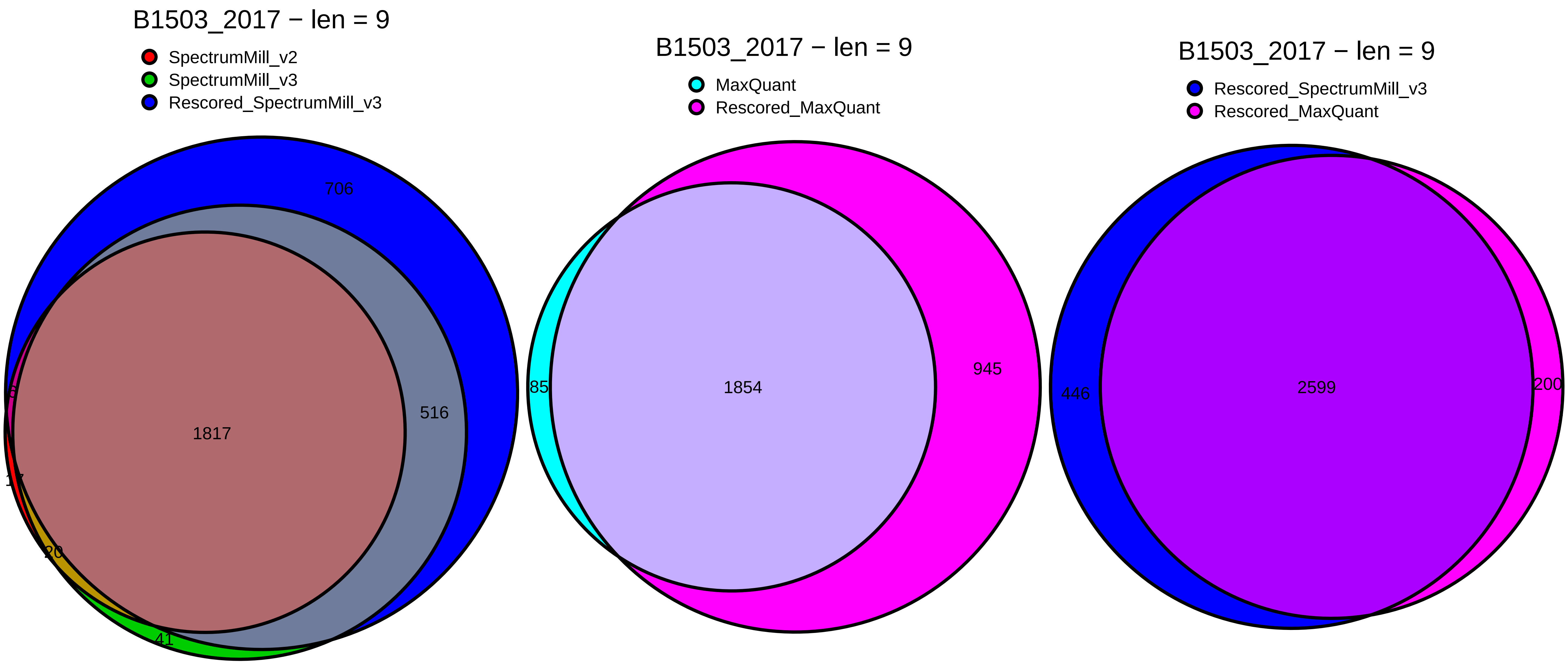
B1503_2017 − len = 9
SpectrumMill_v2
SpectrumMill_v3
Rescored_SpectrumMill_v3
706
516
1817
20
41
B1503_2017 − len = 9
MaxQuant
Rescored_MaxQuant
85
1854
945
B1503_2017 − len = 9
Rescored_SpectrumMill_v3
Rescored_MaxQuant
446
2599
200

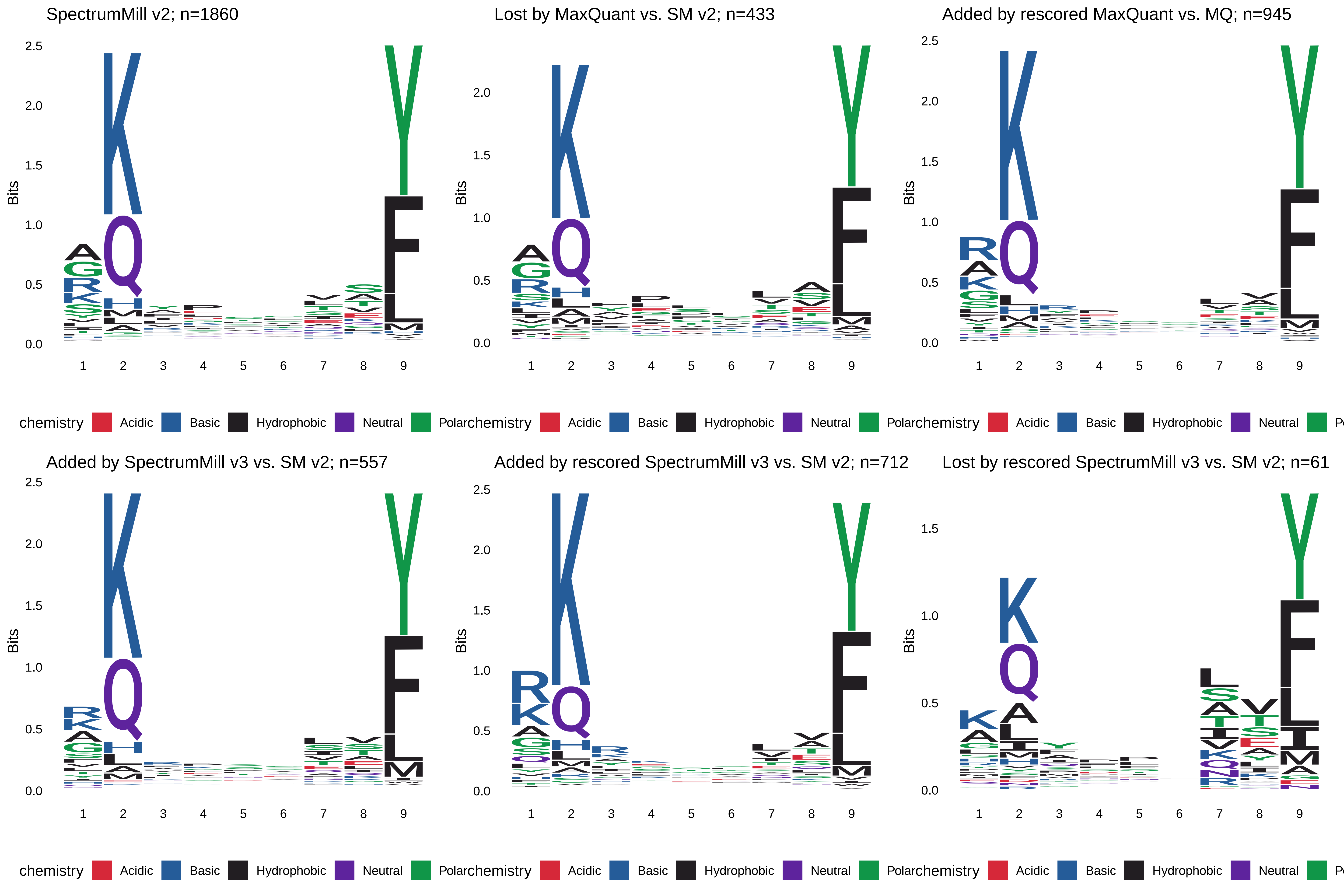
SpectrumMill v2; n=1860
Lost by MaxQuant vs. SM v2; n=433
Added by rescored MaxQuant vs. MQ; n=945
Added by SpectrumMill v3 vs. SM v2; n=557
Added by rescored SpectrumMill v3 vs. SM v2; n=712
Lost by rescored SpectrumMill v3 vs. SM v2; n=61
Bits
1 2 3 4 5 6 7 8 9
chemistry Acidic Basic Hydrophobic Neutral Polar

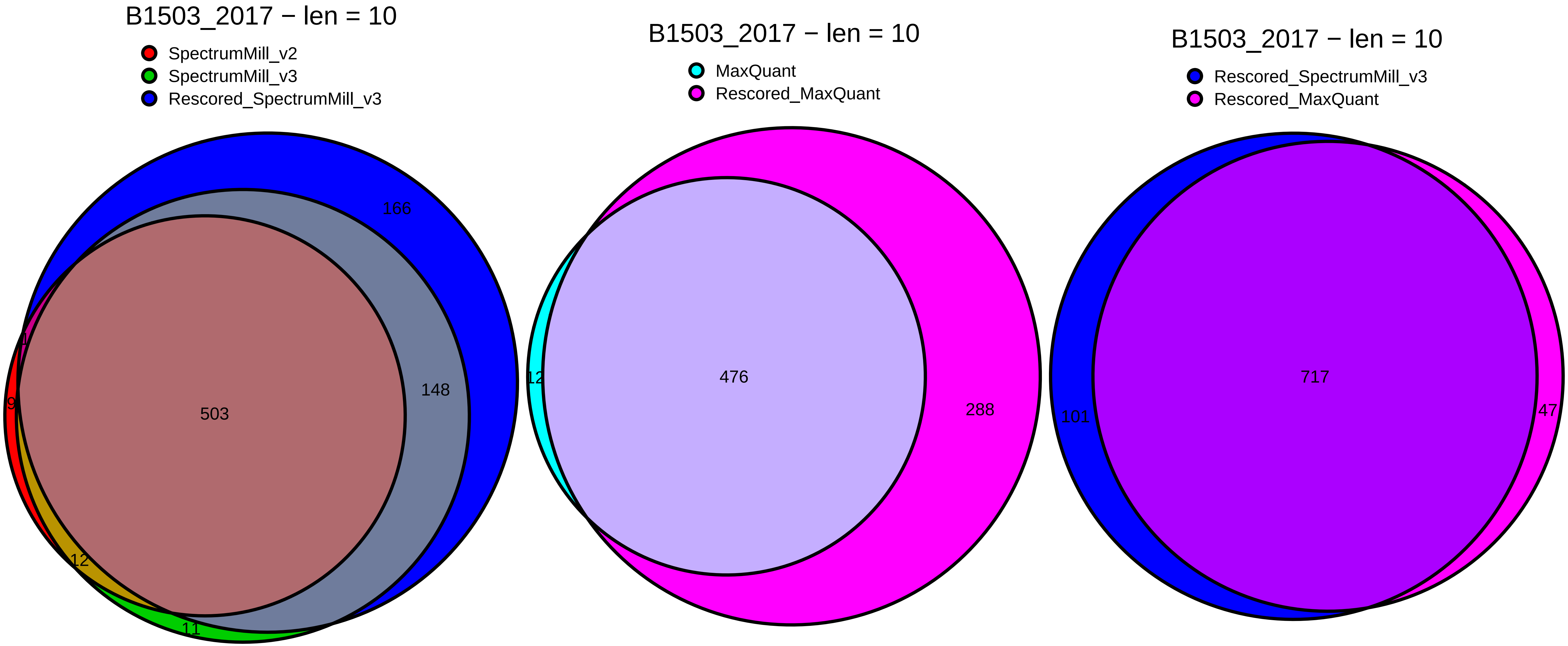
B1503_2017 − len = 10
SpectrumMill_v2
SpectrumMill_v3
Rescored_SpectrumMill_v3
166
148
503
12
11
B1503_2017 − len = 10
MaxQuant
Rescored_MaxQuant
476
288
B1503_2017 − len = 10
Rescored_SpectrumMill_v3
Rescored_MaxQuant
717
101
47

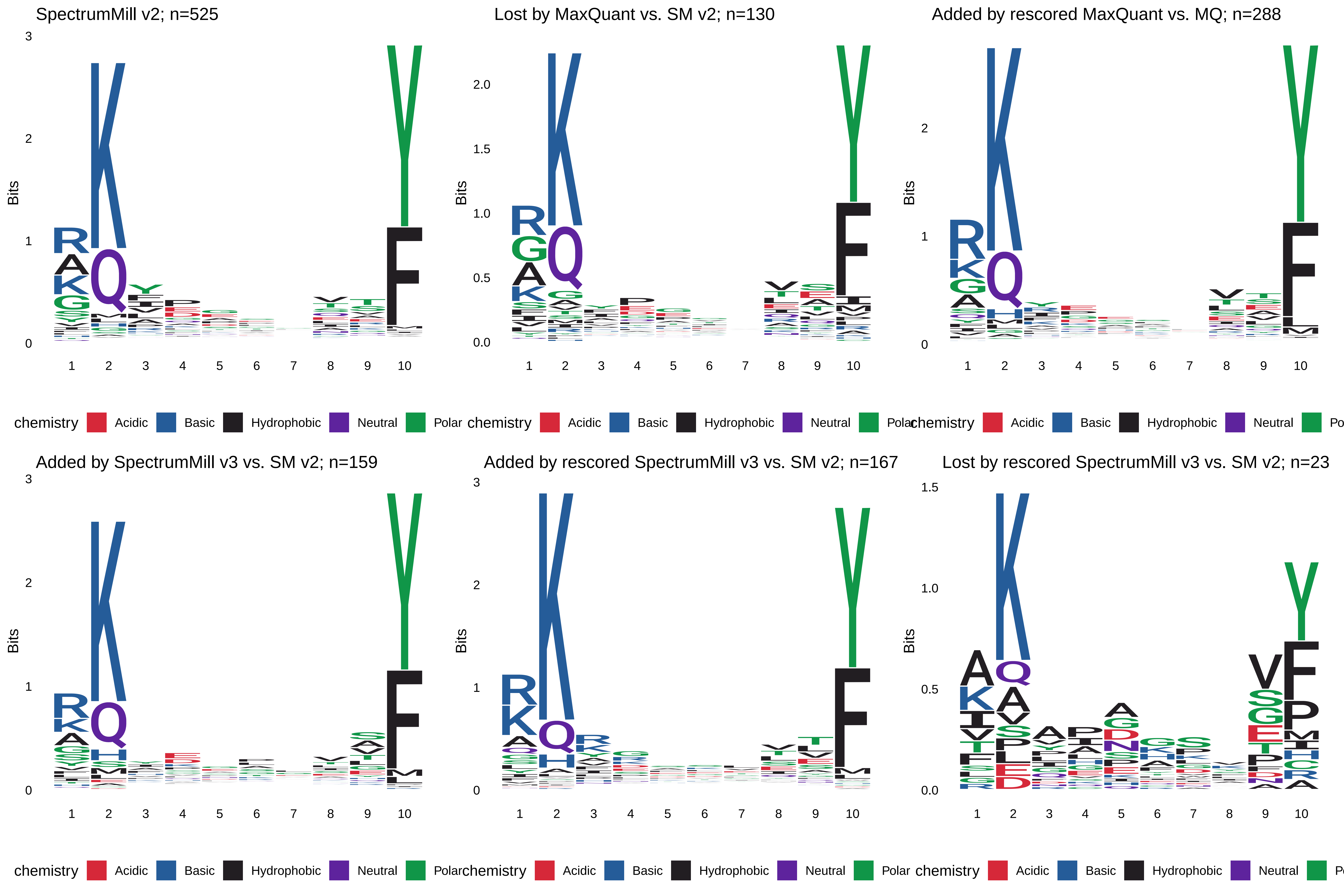
SpectrumMill v2; n=525
Lost by MaxQuant vs. SM v2; n=130
Added by rescored MaxQuant vs. MQ; n=288
Added by SpectrumMill v3 vs. SM v2; n=159
Added by rescored SpectrumMill v3 vs. SM v2; n=167
Lost by rescored SpectrumMill v3 vs. SM v2; n=23
Bits
1 2 3 4 5 6 7 8 9 10
chemistry
Acidic
Basic
Hydrophobic
Neutral
Polar

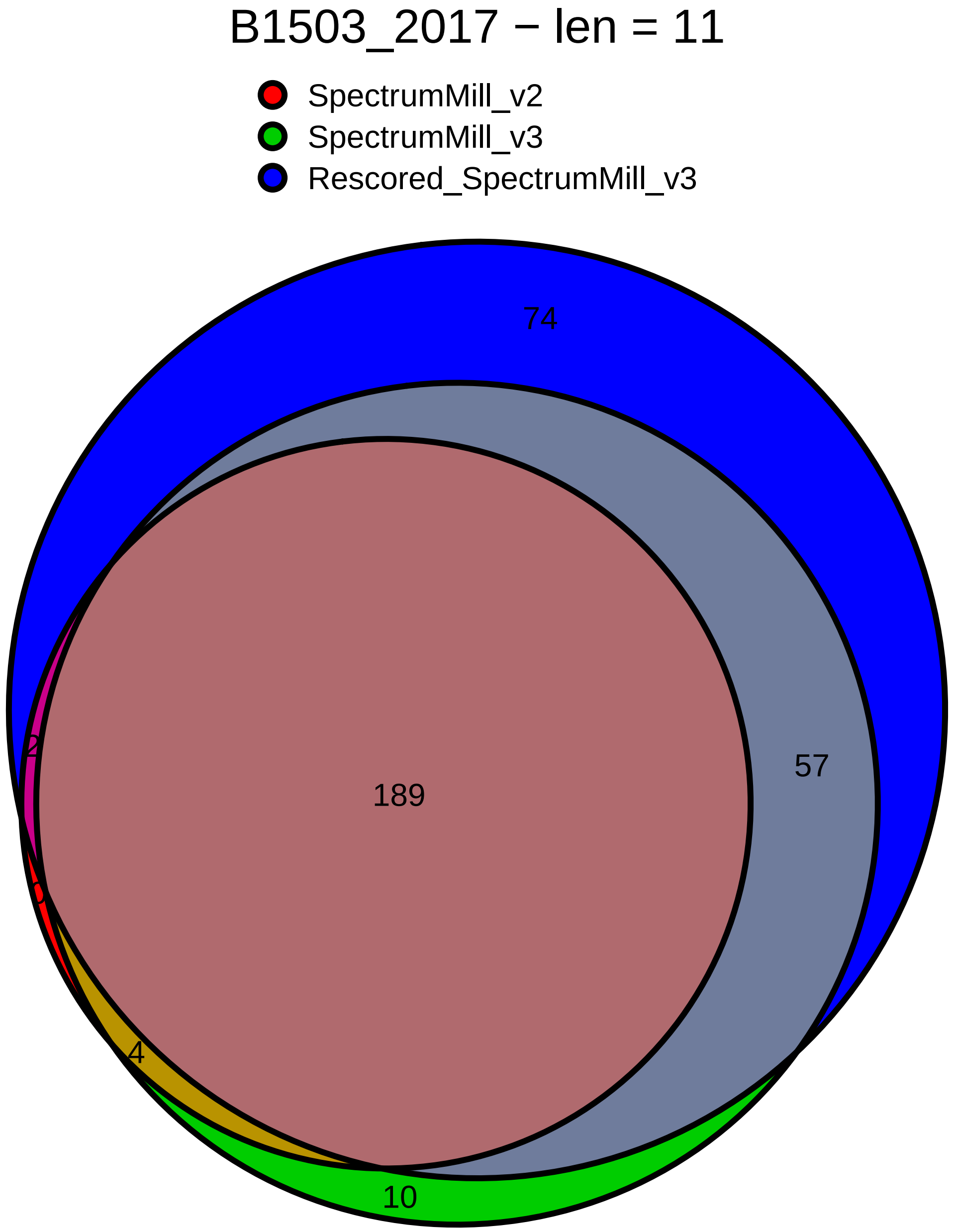
B1503_2017 − len = 11
SpectrumMill_v2
SpectrumMill_v3
Rescored_SpectrumMill_v3
74
2
189
57
4
10

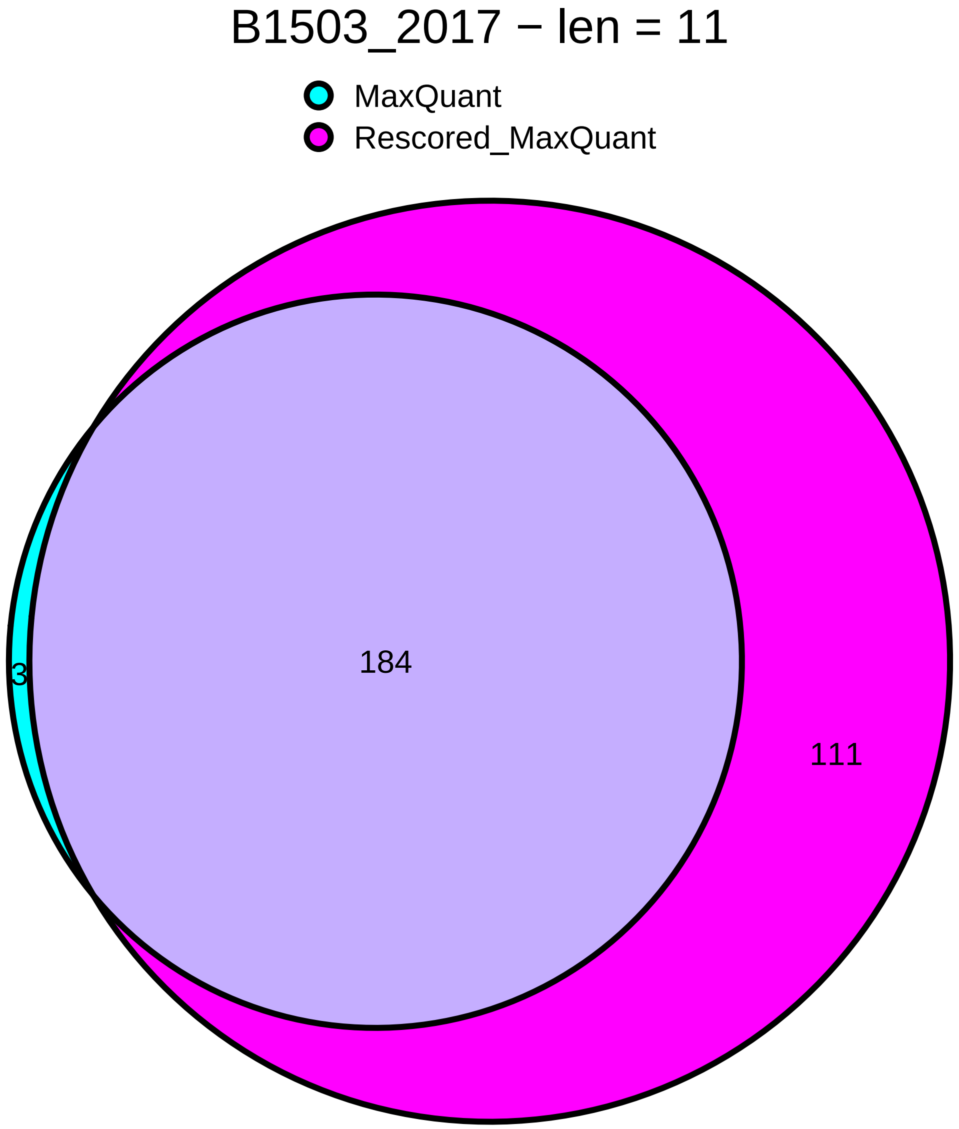
B1503_2017 − len = 11
MaxQuant
Rescored_MaxQuant
3
184
111

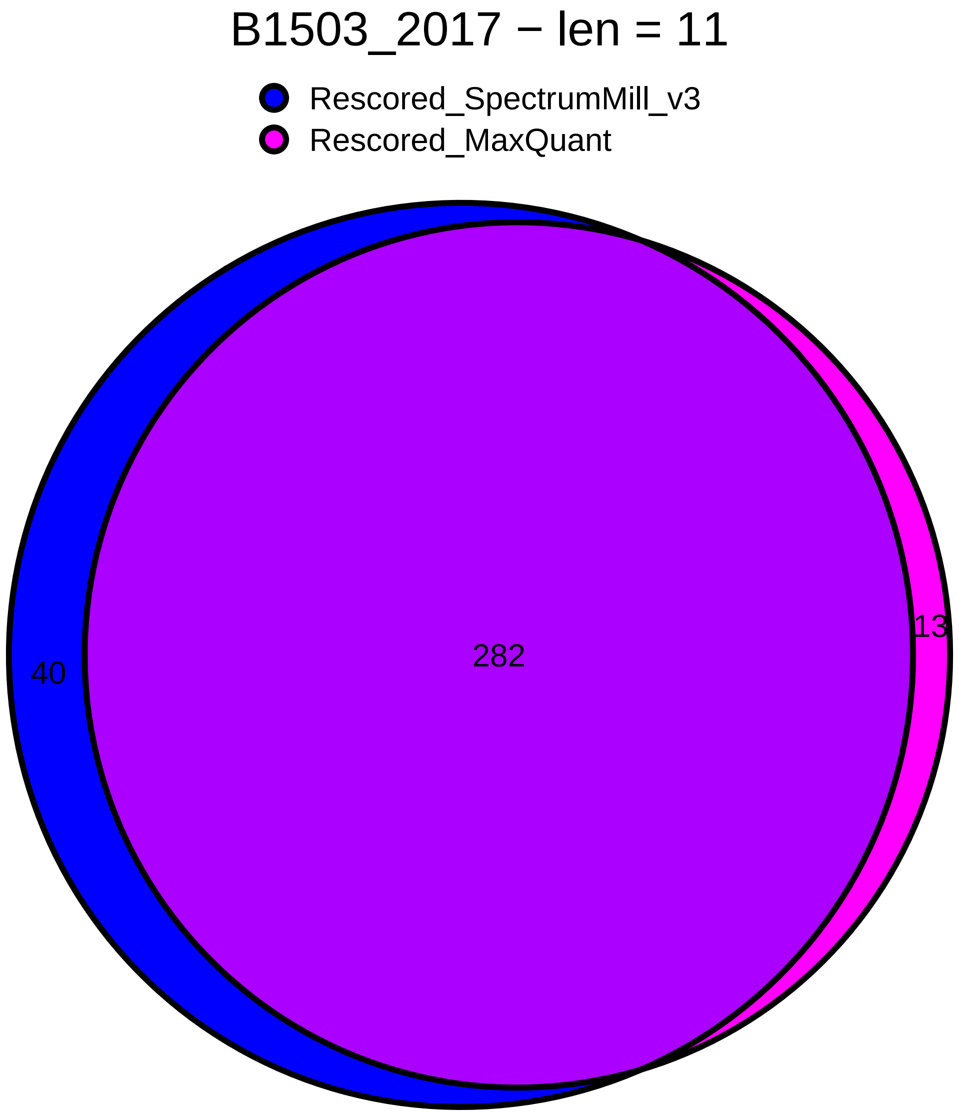
B1503_2017 − len = 11
Rescored_SpectrumMill_v3
Rescored_MaxQuant
40
282
13

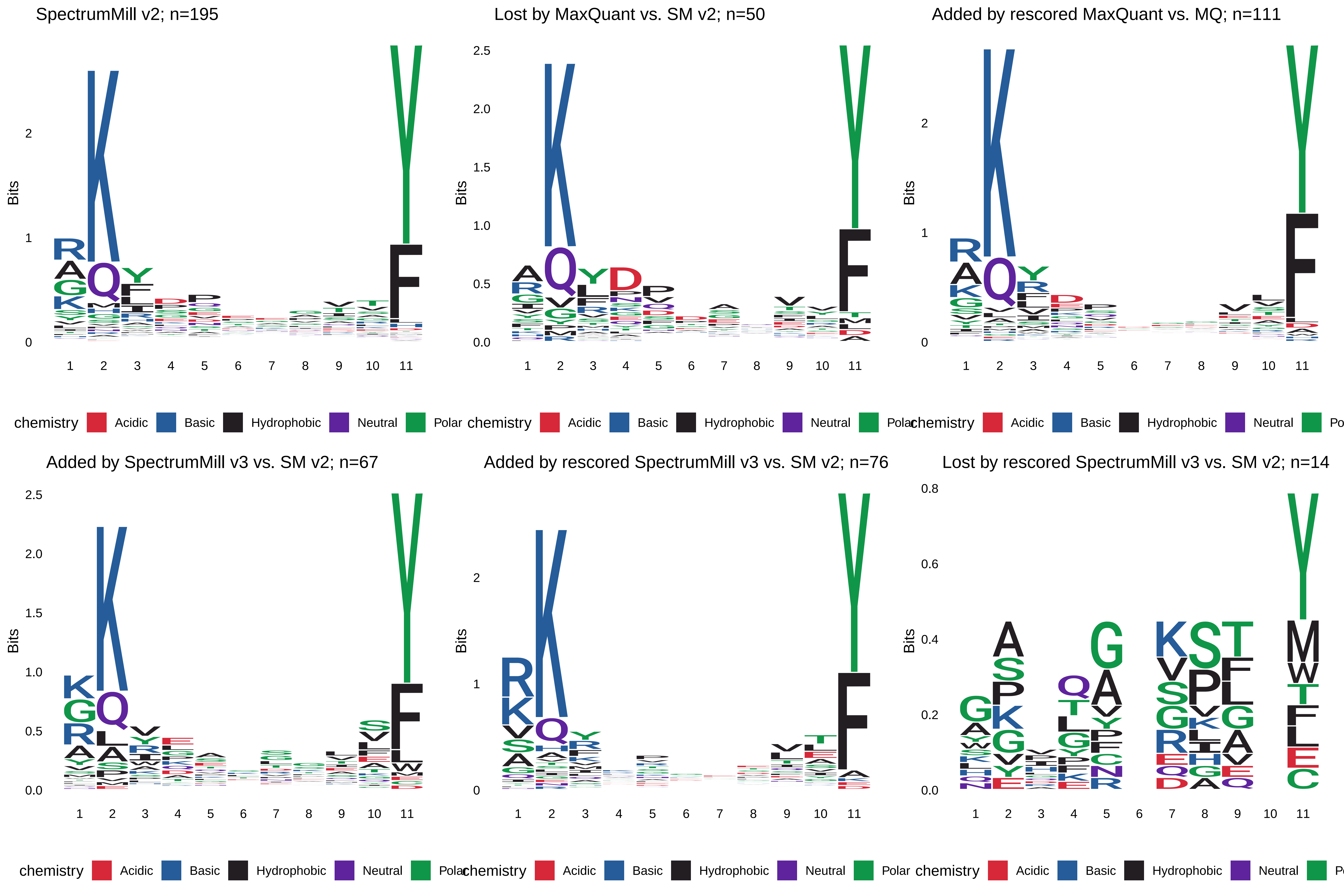
SpectrumMill v2; n=195
Lost by MaxQuant vs. SM v2; n=50
Added by rescored MaxQuant vs. MQ; n=111
Added by SpectrumMill v3 vs. SM v2; n=67
Added by rescored SpectrumMill v3 vs. SM v2; n=76
Lost by rescored SpectrumMill v3 vs. SM v2; n=14
Bits
chemistry
Acidic
Basic
Hydrophobic
Neutral
Polar

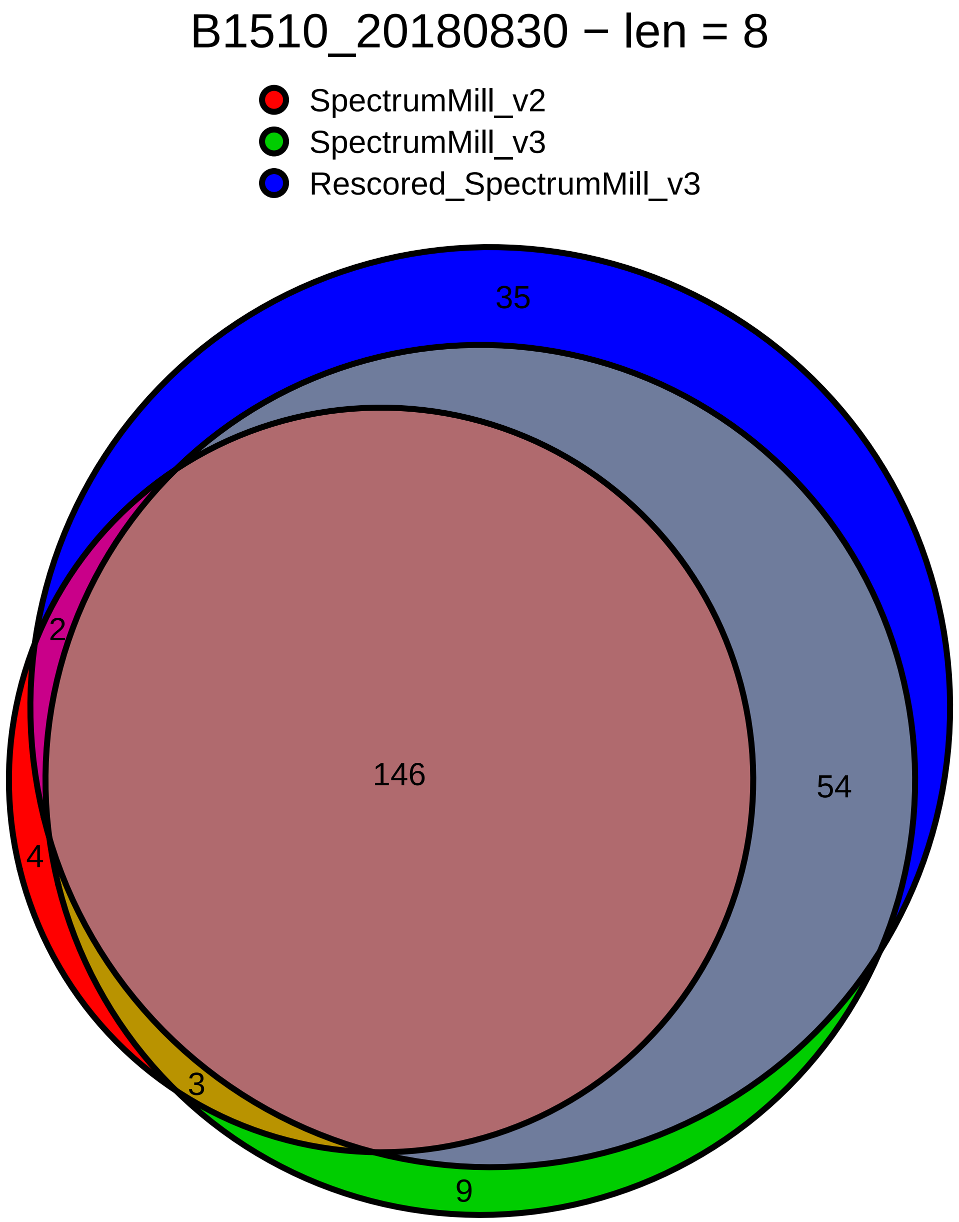
B1510_20180830 − len = 8
SpectrumMill_v2
SpectrumMill_v3
Rescored_SpectrumMill_v3
35
2
146
54
4
3
9

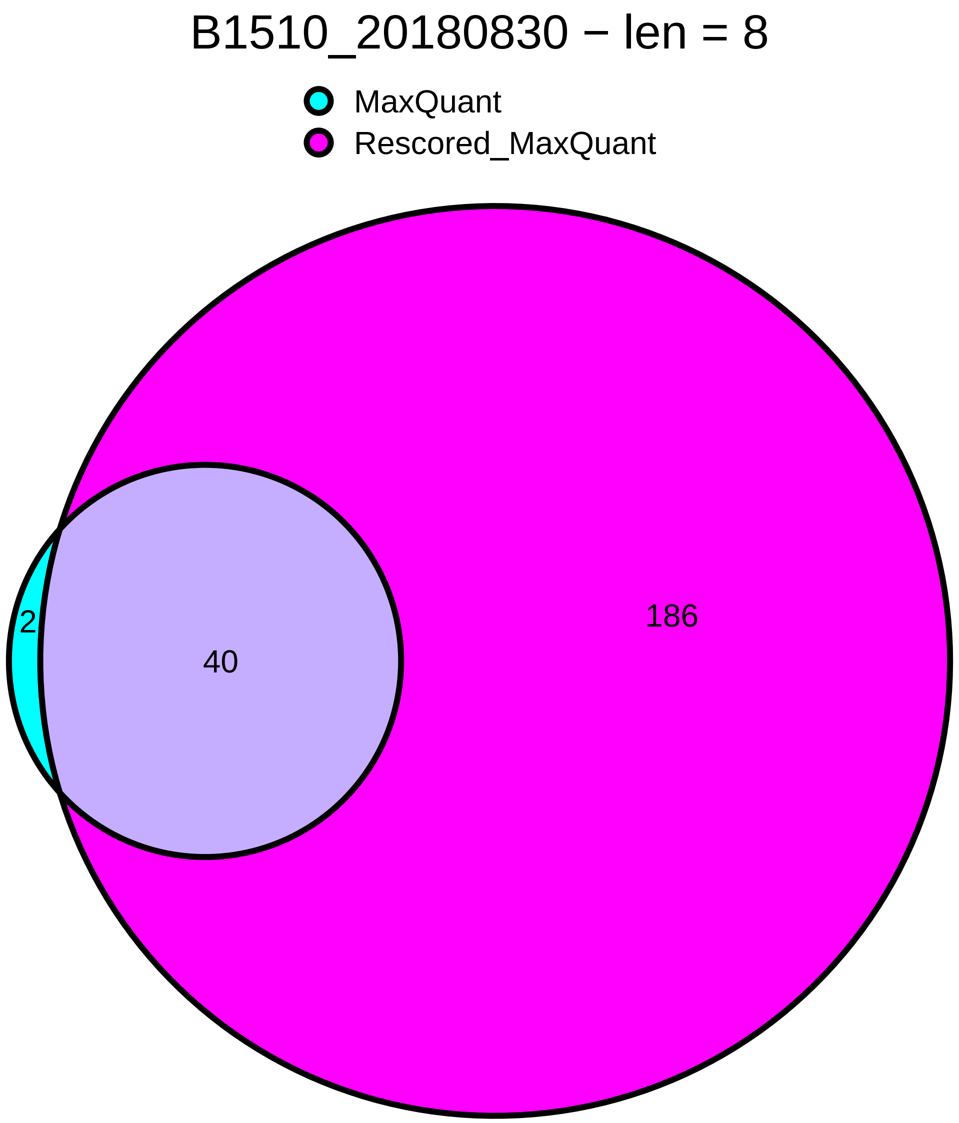
B1510_20180830 − len = 8
MaxQuant
Rescored_MaxQuant
2
40
186

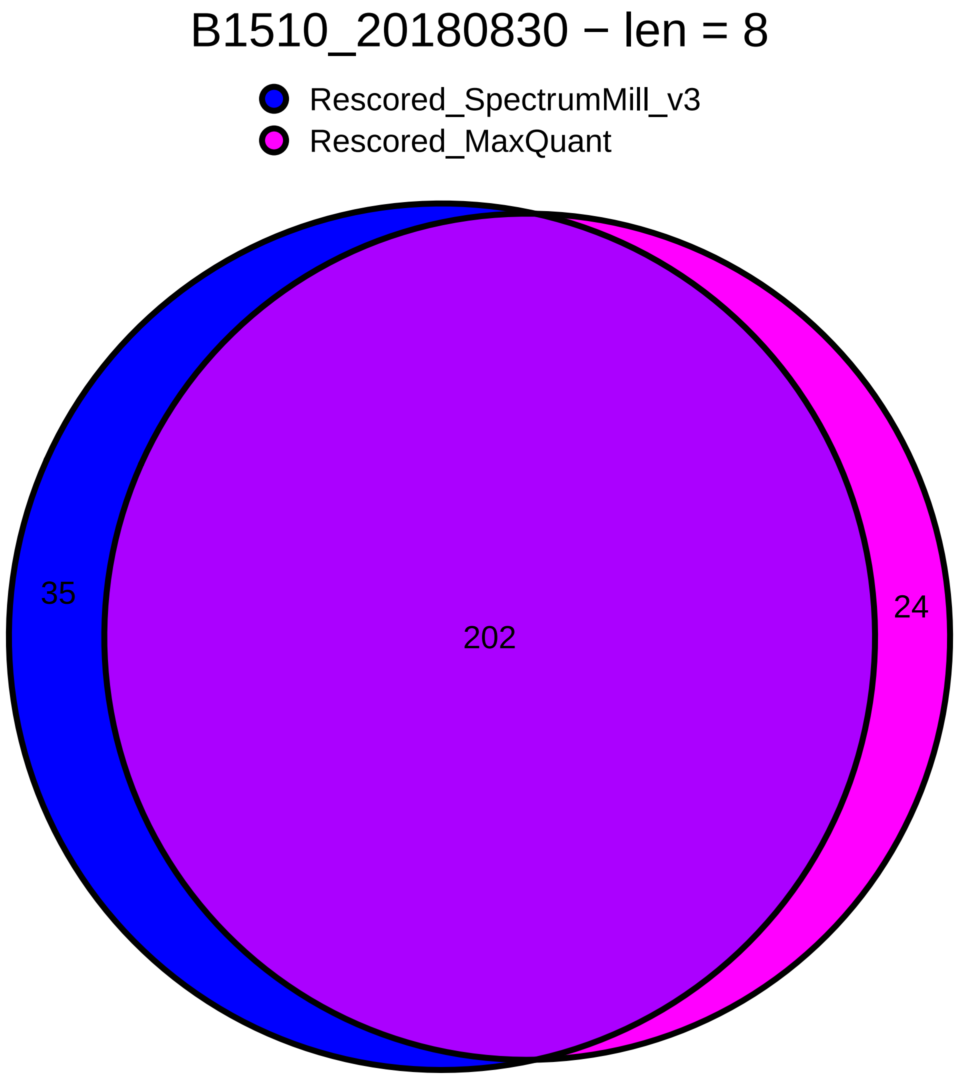
B1510_20180830 − len = 8
Rescored_SpectrumMill_v3
Rescored_MaxQuant
35
202
24

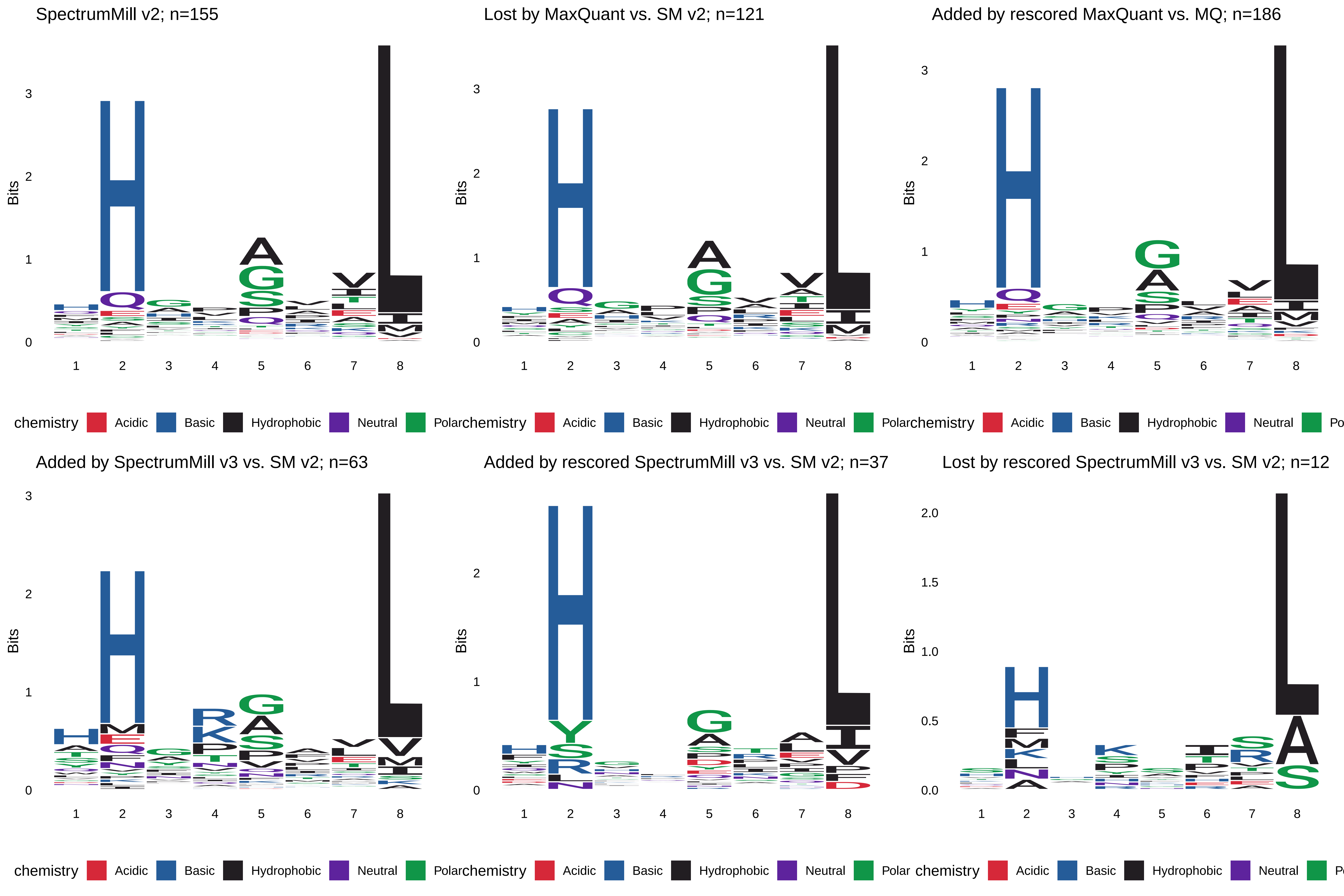
SpectrumMill v2; n=155
Lost by MaxQuant vs. SM v2; n=121
Added by rescored MaxQuant vs. MQ; n=186
Added by SpectrumMill v3 vs. SM v2; n=63
Added by rescored SpectrumMill v3 vs. SM v2; n=37
Lost by rescored SpectrumMill v3 vs. SM v2; n=12
Bits
1 2 3 4 5 6 7 8
chemistry Acidic Basic Hydrophobic Neutral Polar

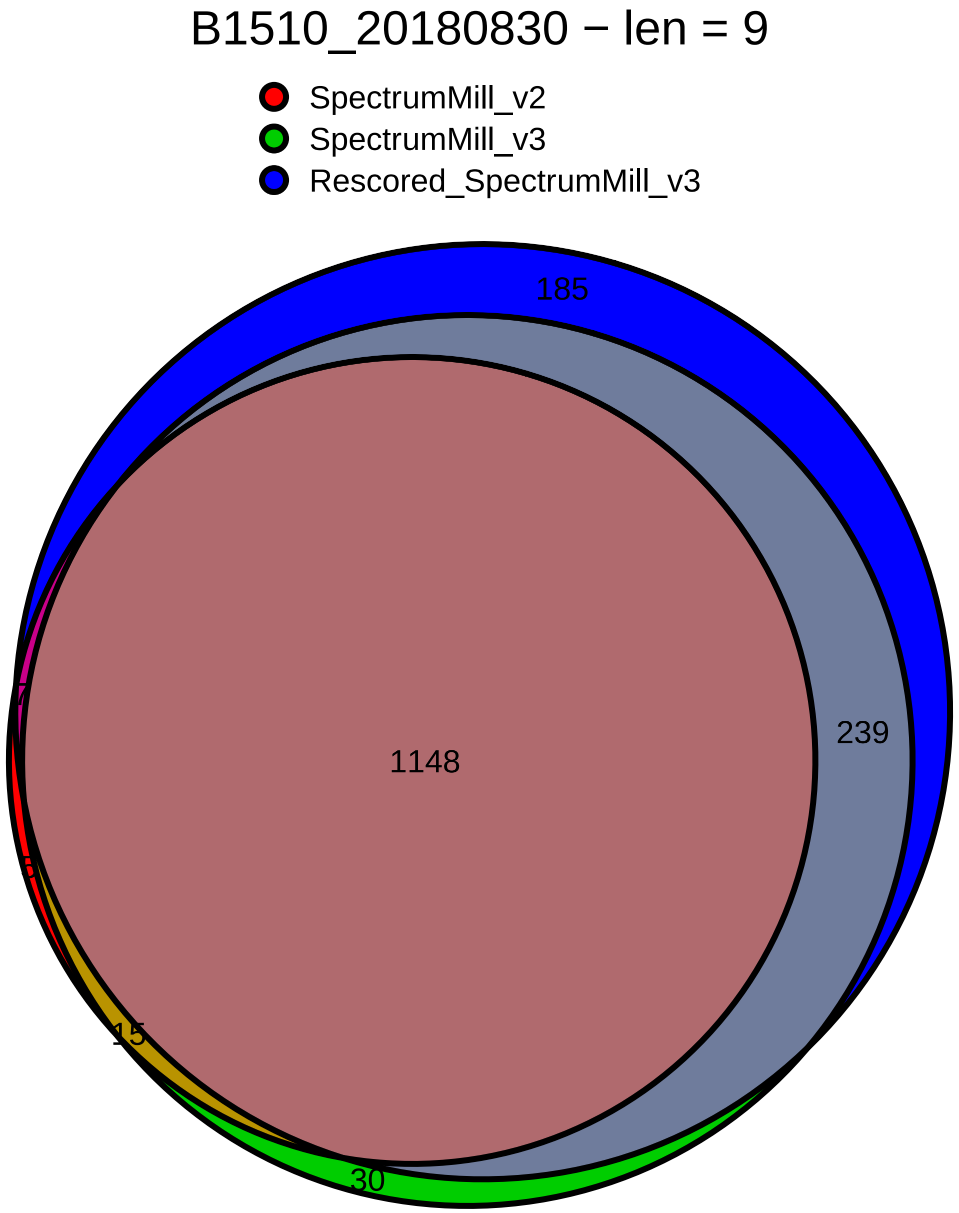
B1510_20180830 − len = 9
SpectrumMill_v2
SpectrumMill_v3
Rescored_SpectrumMill_v3
185
239
1148
15
30

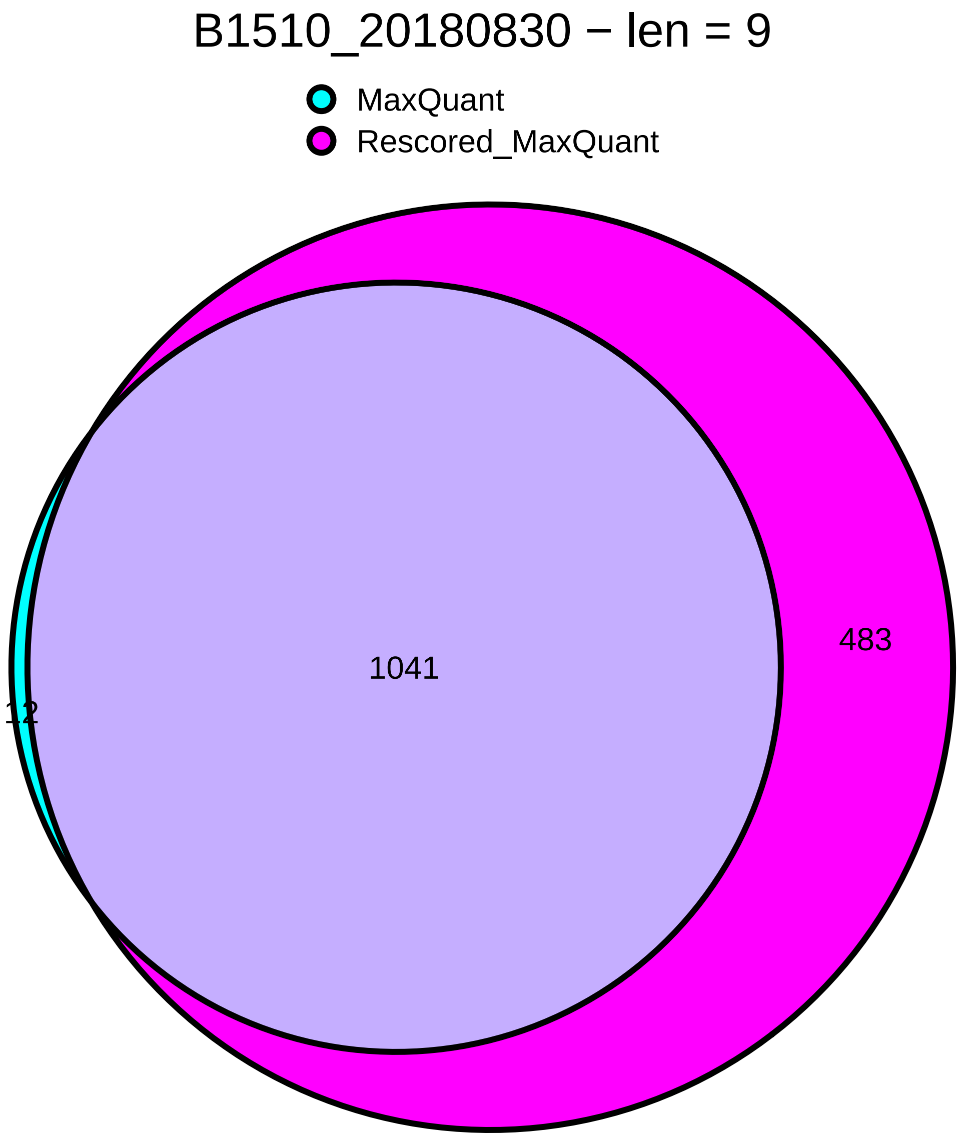
B1510_20180830 − len = 9
MaxQuant
Rescored_MaxQuant
1041
483
12

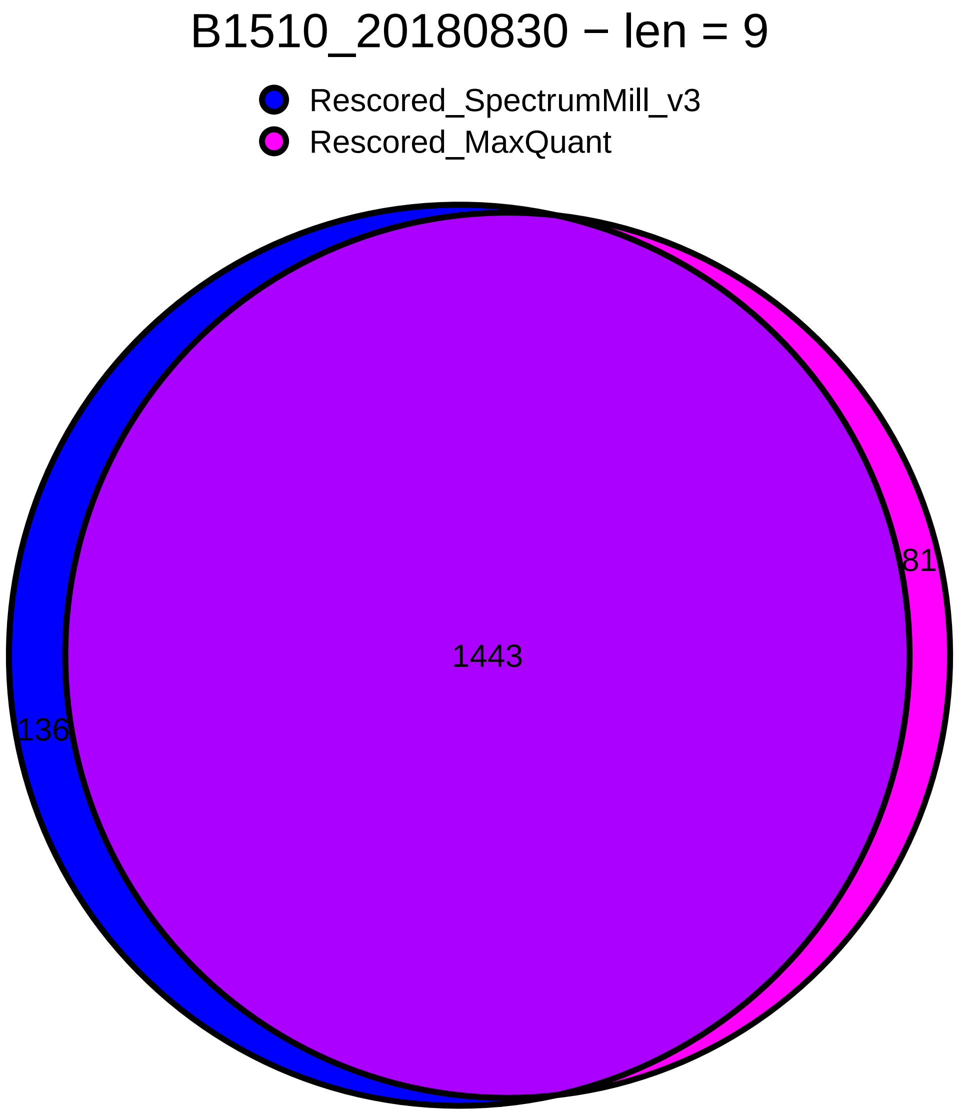
B1510_20180830 − len = 9
Rescored_SpectrumMill_v3
Rescored_MaxQuant
1443
136
81

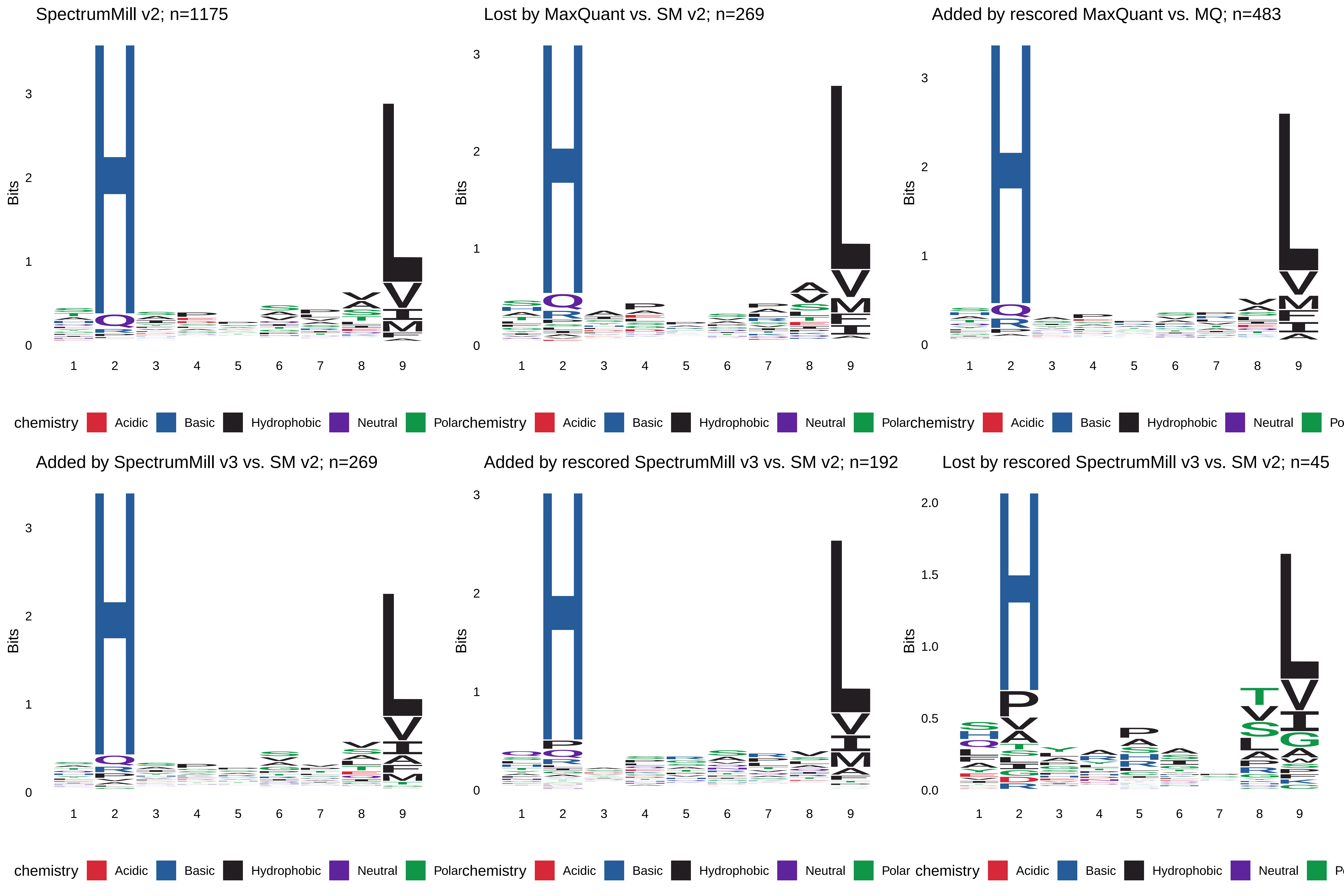
SpectrumMill v2; n=1175
Lost by MaxQuant vs. SM v2; n=269
Added by rescored MaxQuant vs. MQ; n=483
Added by SpectrumMill v3 vs. SM v2; n=269
Added by rescored SpectrumMill v3 vs. SM v2; n=192
Lost by rescored SpectrumMill v3 vs. SM v2; n=45
Bits
1 2 3 4 5 6 7 8 9
chemistry
Acidic
Basic
Hydrophobic
Neutral
Polar

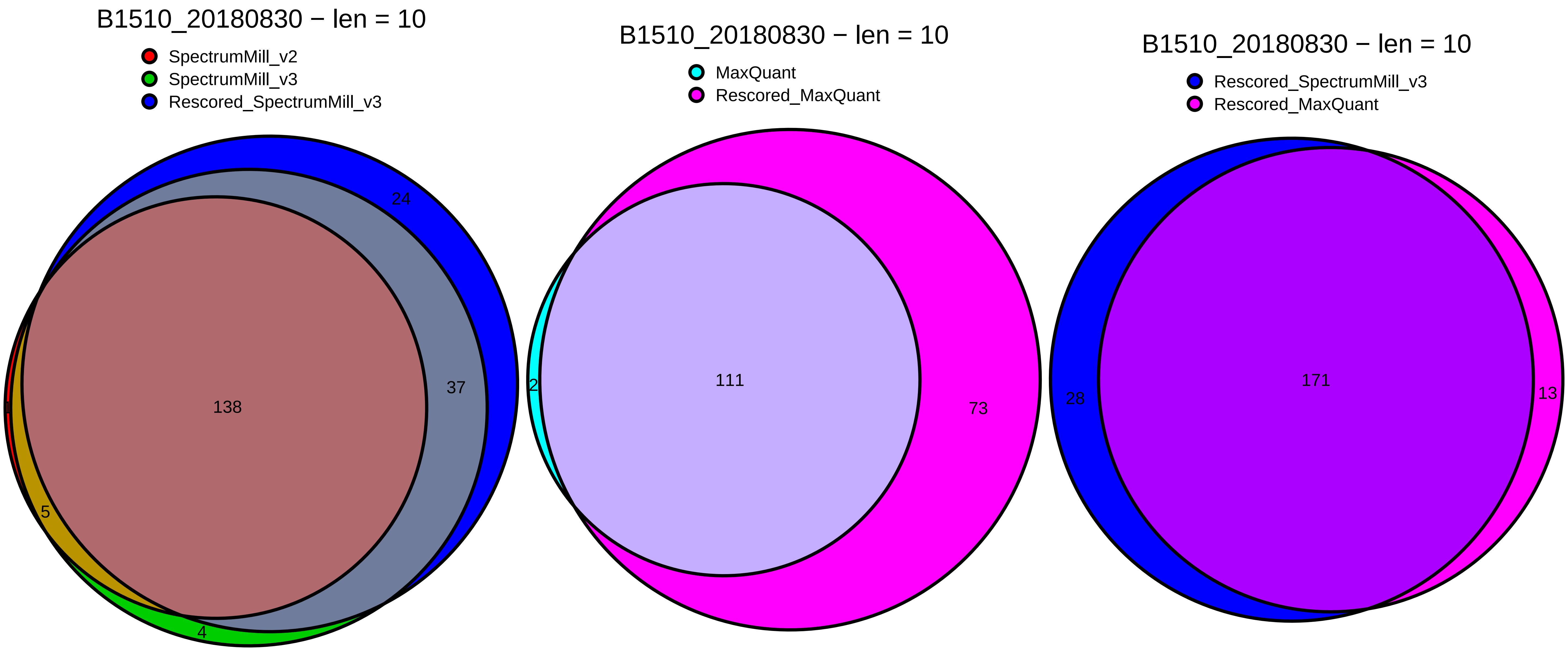

B1510_20180830 − len = 10
SpectrumMill_v2
SpectrumMill_v3
Rescored_SpectrumMill_v3
24
37
138
5
4
B1510_20180830 − len = 10
MaxQuant
Rescored_MaxQuant
2
111
73
B1510_20180830 − len = 10
Rescored_SpectrumMill_v3
Rescored_MaxQuant
28
171
13

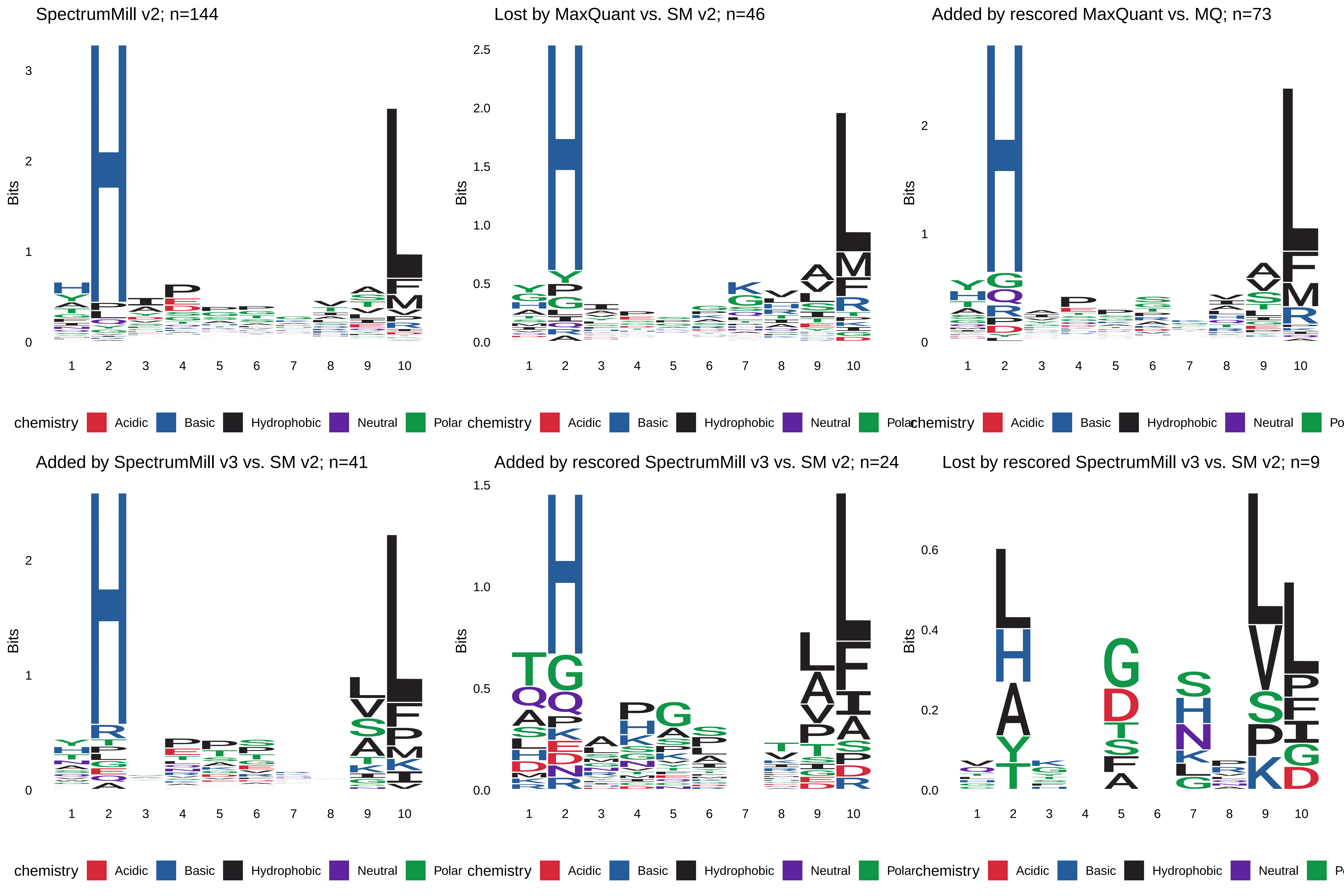
SpectrumMill v2; n=144
Lost by MaxQuant vs. SM v2; n=46
Added by rescored MaxQuant vs. MQ; n=73
Added by SpectrumMill v3 vs. SM v2; n=41
Added by rescored SpectrumMill v3 vs. SM v2; n=24
Lost by rescored SpectrumMill v3 vs. SM v2; n=9
Bits
1 2 3 4 5 6 7 8 9 10
chemistry
Acidic
Basic
Hydrophobic
Neutral
Polar

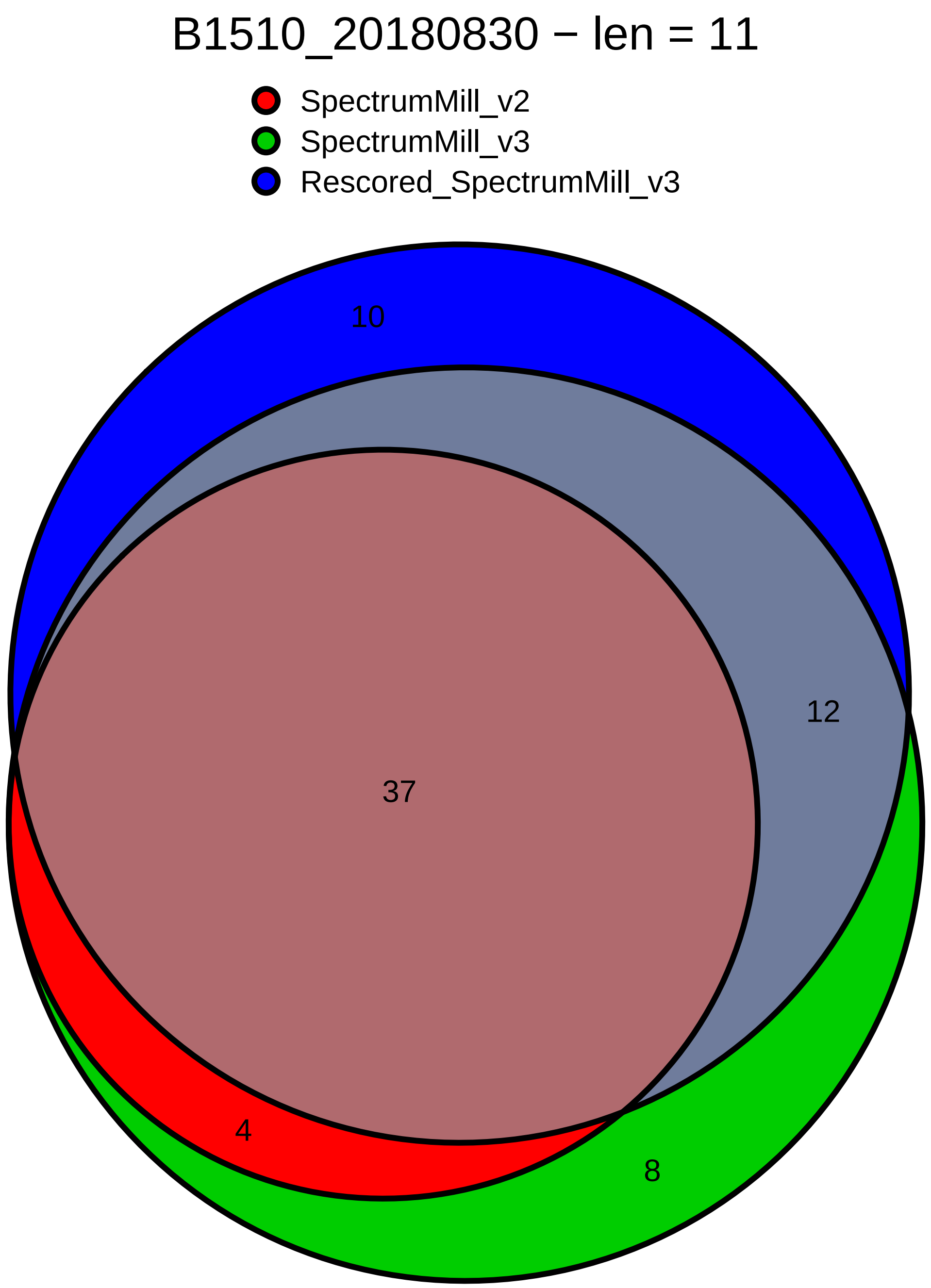
B1510_20180830 − len = 11
SpectrumMill_v2
SpectrumMill_v3
Rescored_SpectrumMill_v3
10
12
37
4
8

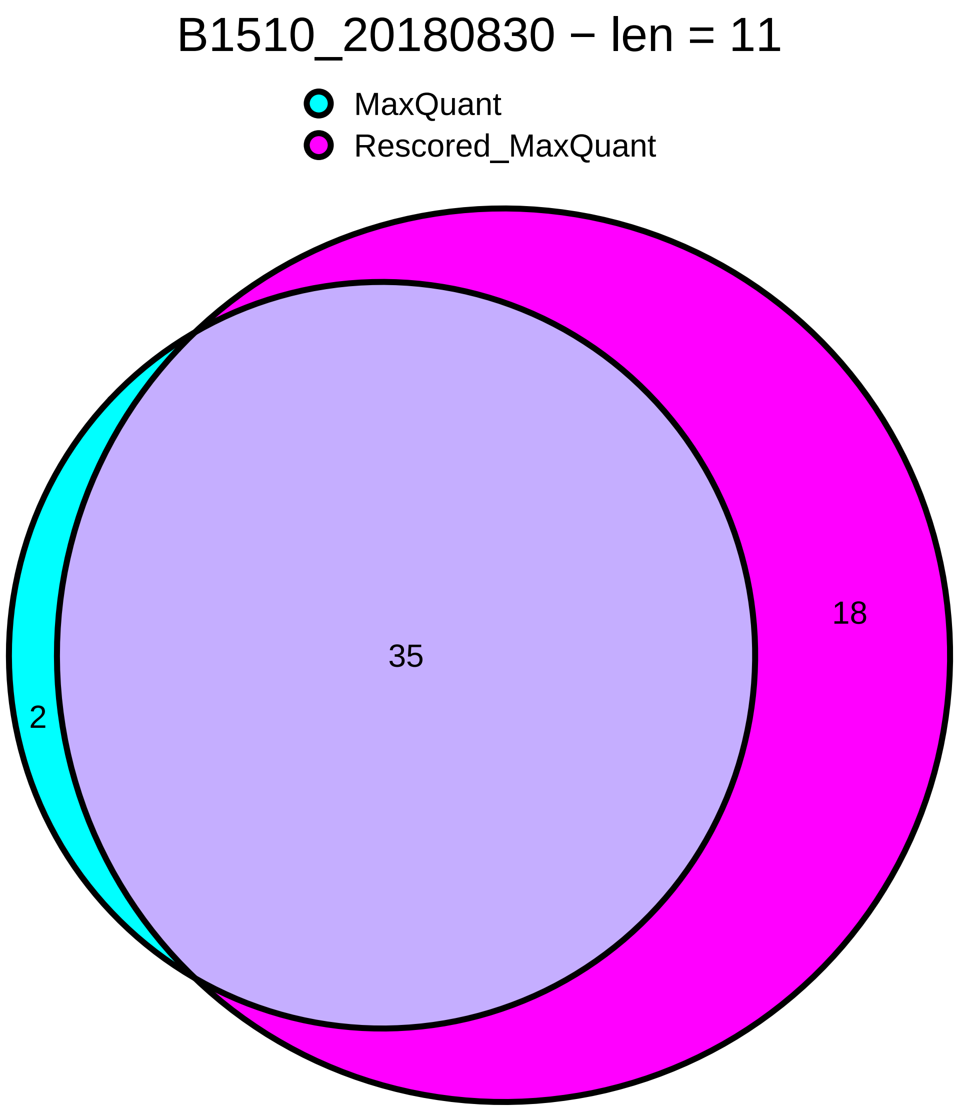
B1510_20180830 − len = 11
MaxQuant
Rescored_MaxQuant
35
18
2

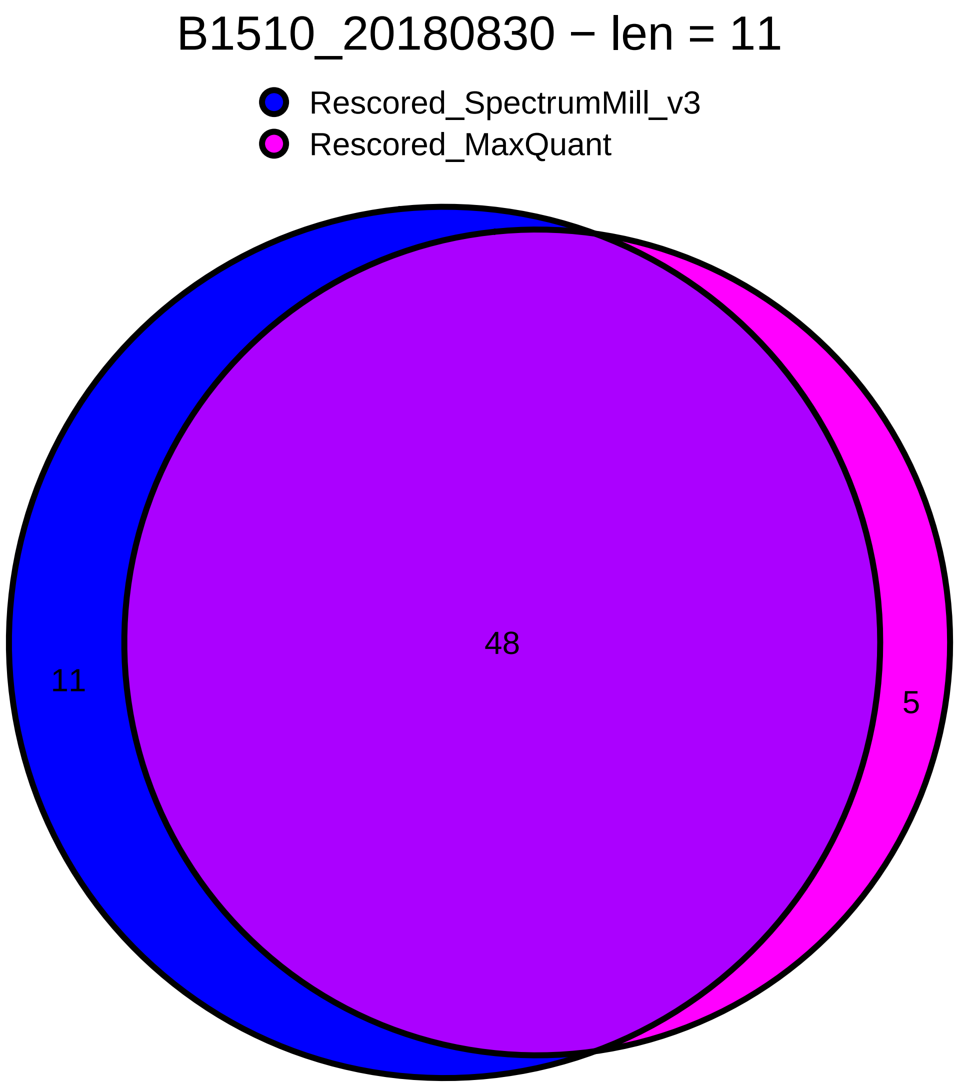
B1510_20180830 − len = 11
Rescored_SpectrumMill_v3
Rescored_MaxQuant
48
11
5

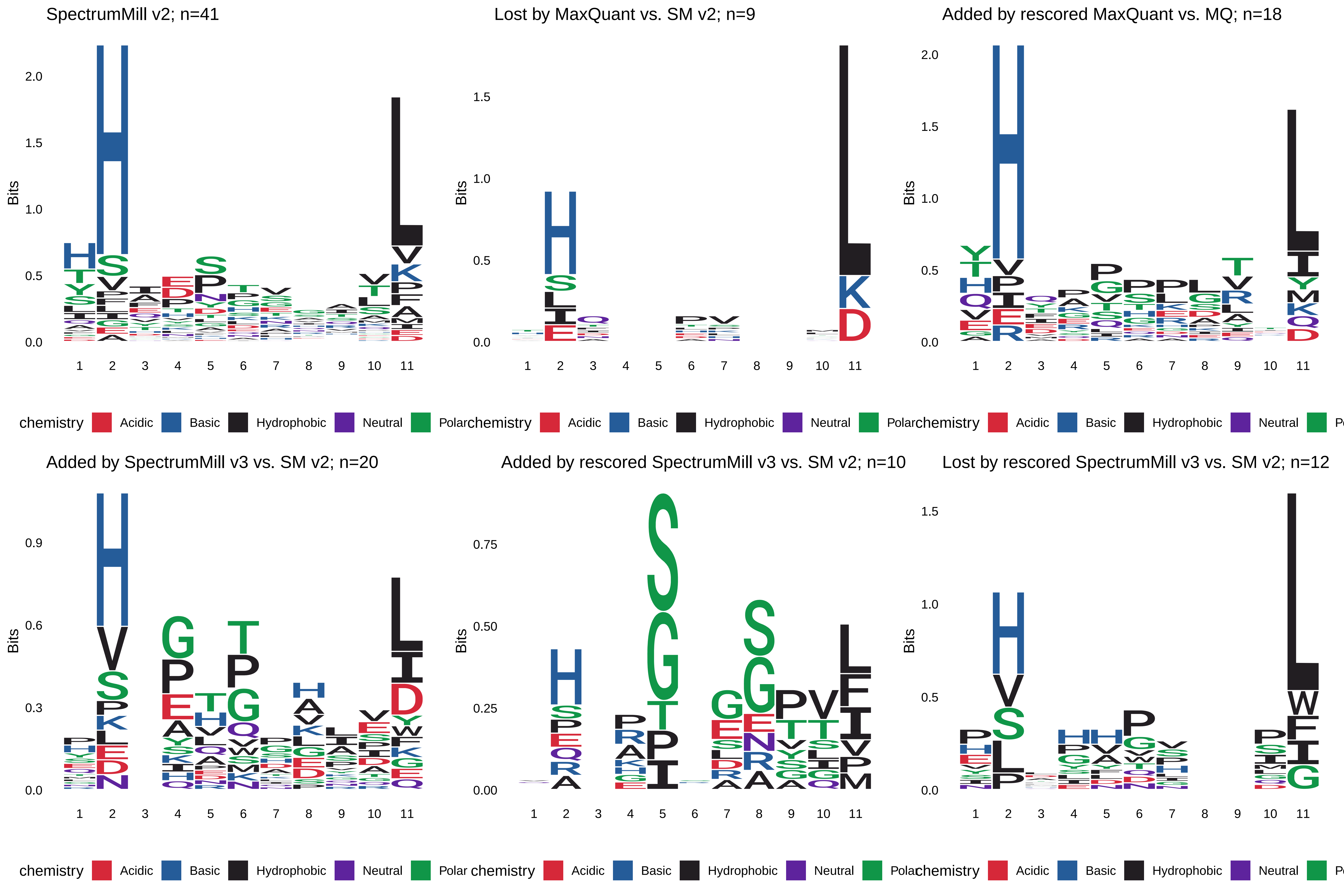
SpectrumMill v2; n=41
Lost by MaxQuant vs. SM v2; n=9
Added by rescored MaxQuant vs. MQ; n=18
Added by SpectrumMill v3 vs. SM v2; n=20
Added by rescored SpectrumMill v3 vs. SM v2; n=10
Lost by rescored SpectrumMill v3 vs. SM v2; n=12
Bits
1 2 3 4 5 6 7 8 9 10 11
chemistry Acidic Basic Hydrophobic Neutral Polar

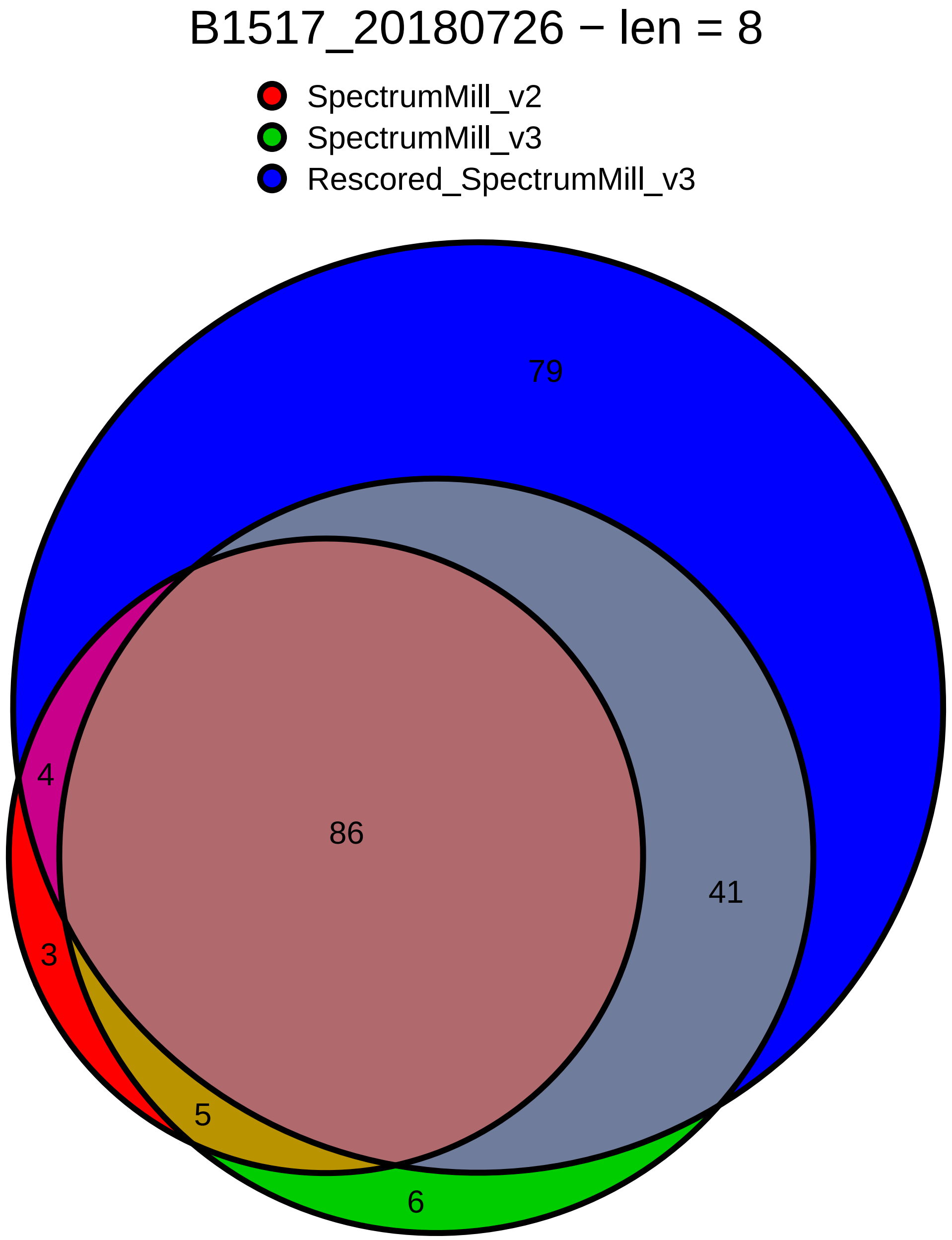

B1517_20180726 − len = 8
SpectrumMill_v2
SpectrumMill_v3
Rescored_SpectrumMill_v3
79
4
86
41
3
5
6

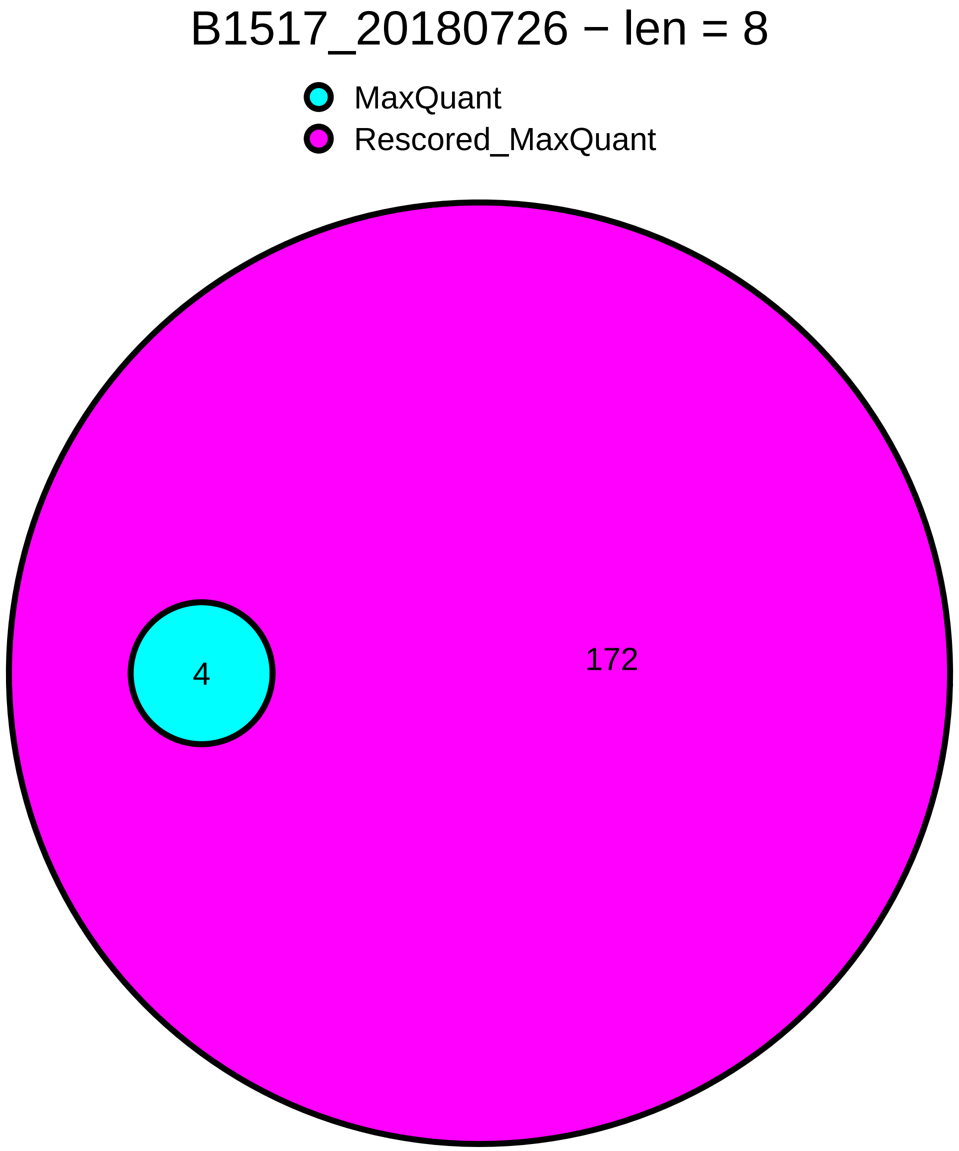

B1517_20180726 − len = 8
MaxQuant
Rescored_MaxQuant
4
172

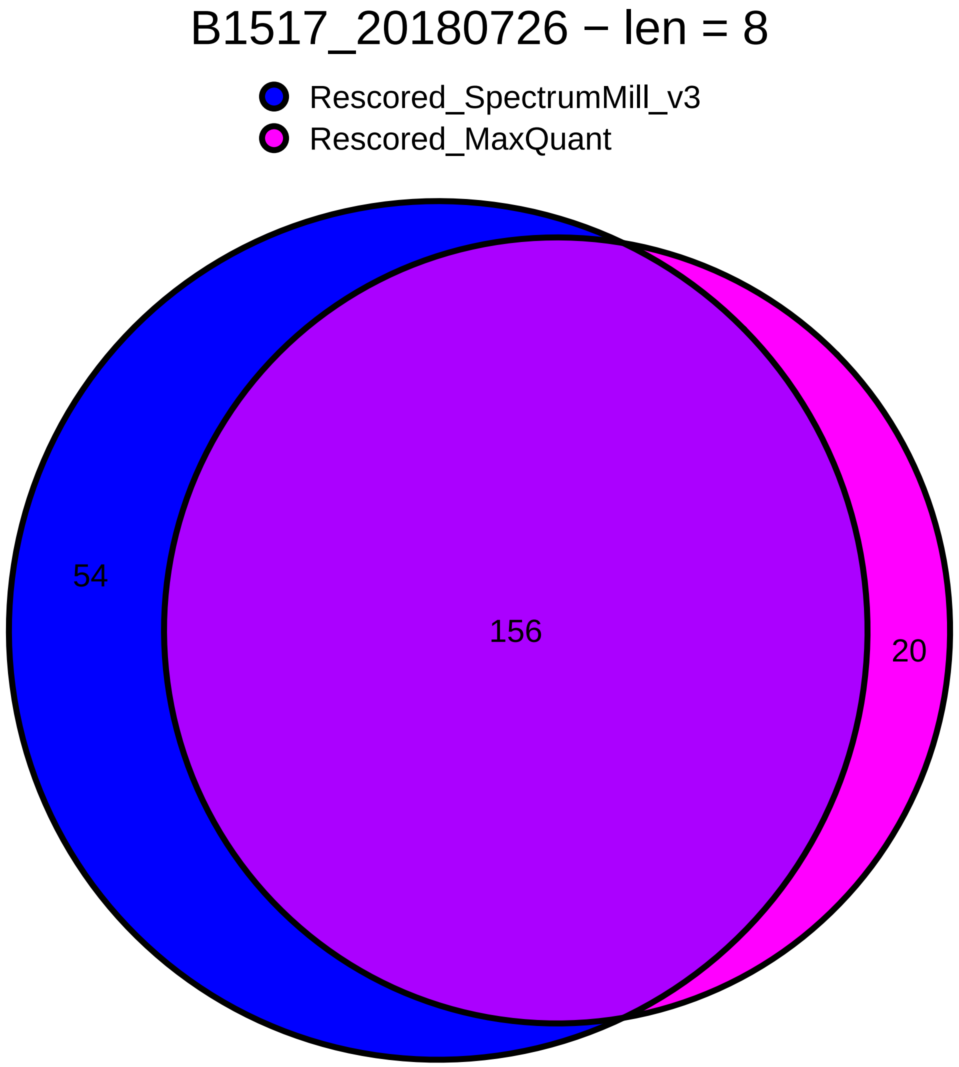

B1517_20180726 − len = 8
Rescored_SpectrumMill_v3
Rescored_MaxQuant
54
156
20

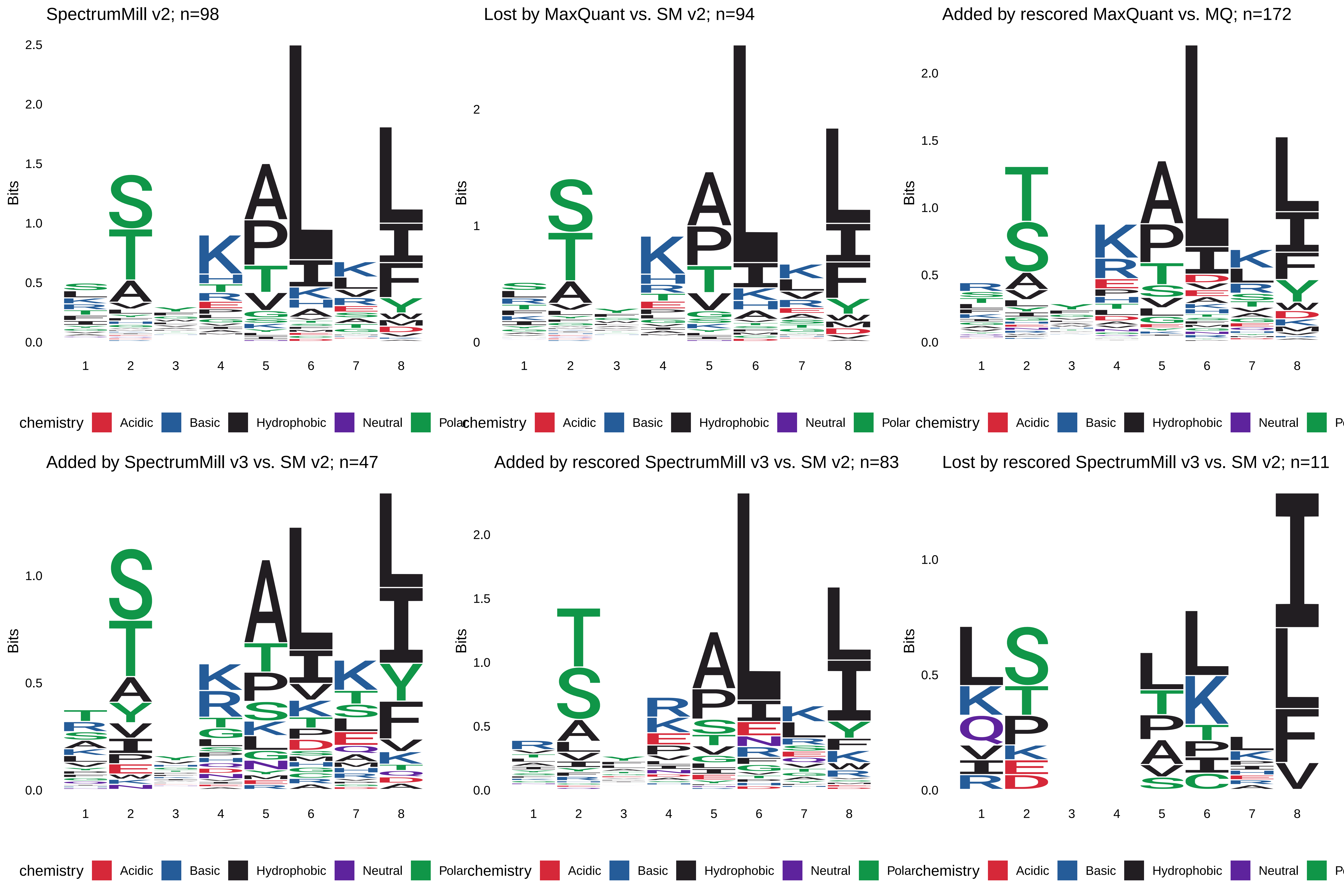

SpectrumMill v2; n=98
Lost by MaxQuant vs. SM v2; n=94
Added by rescored MaxQuant vs. MQ; n=172
Added by SpectrumMill v3 vs. SM v2; n=47
Added by rescored SpectrumMill v3 vs. SM v2; n=83
Lost by rescored SpectrumMill v3 vs. SM v2; n=11
Bits
1 2 3 4 5 6 7 8
chemistry
Acidic
Basic
Hydrophobic
Neutral
Polar

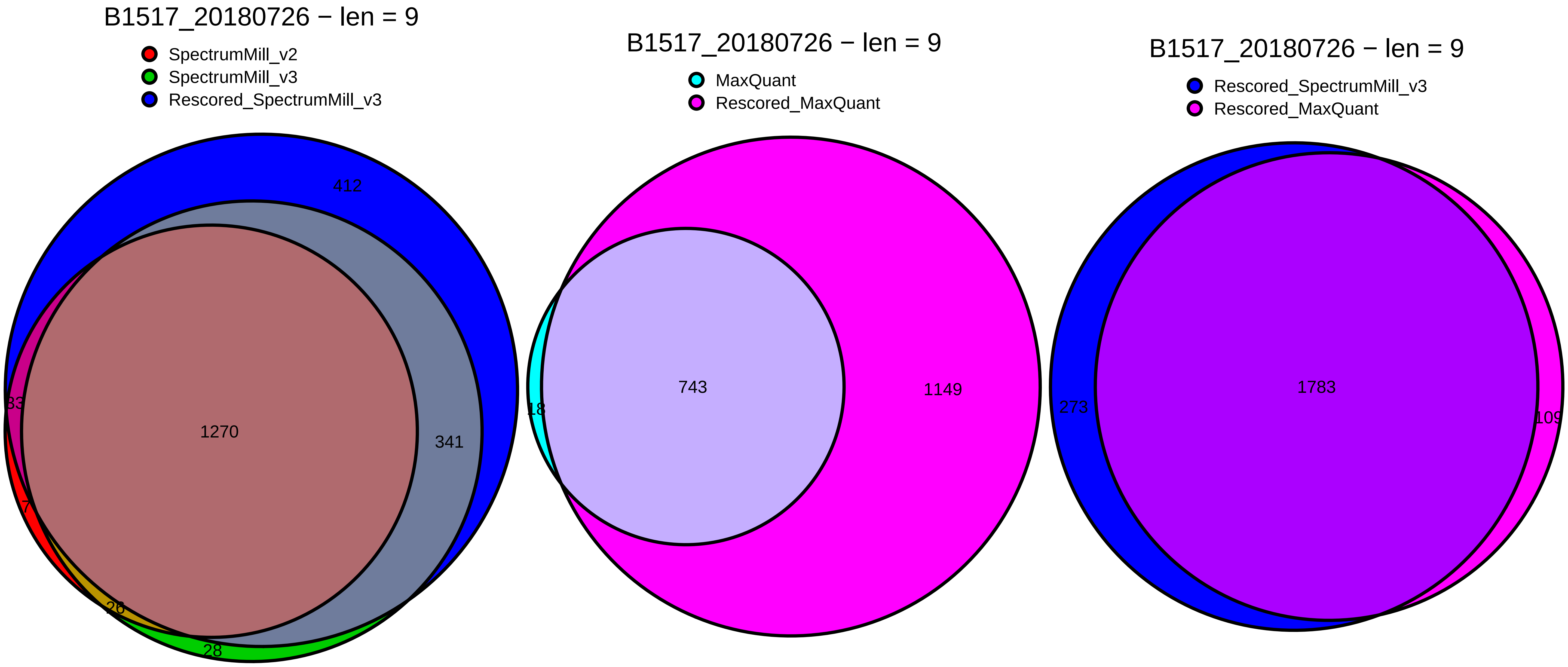
B1517_20180726 − len = 9
SpectrumMill_v2
SpectrumMill_v3
Rescored_SpectrumMill_v3
412
1270
341
7
28
28
B1517_20180726 − len = 9
MaxQuant
Rescored_MaxQuant
743
1149
B1517_20180726 − len = 9
Rescored_SpectrumMill_v3
Rescored_MaxQuant
1783
273

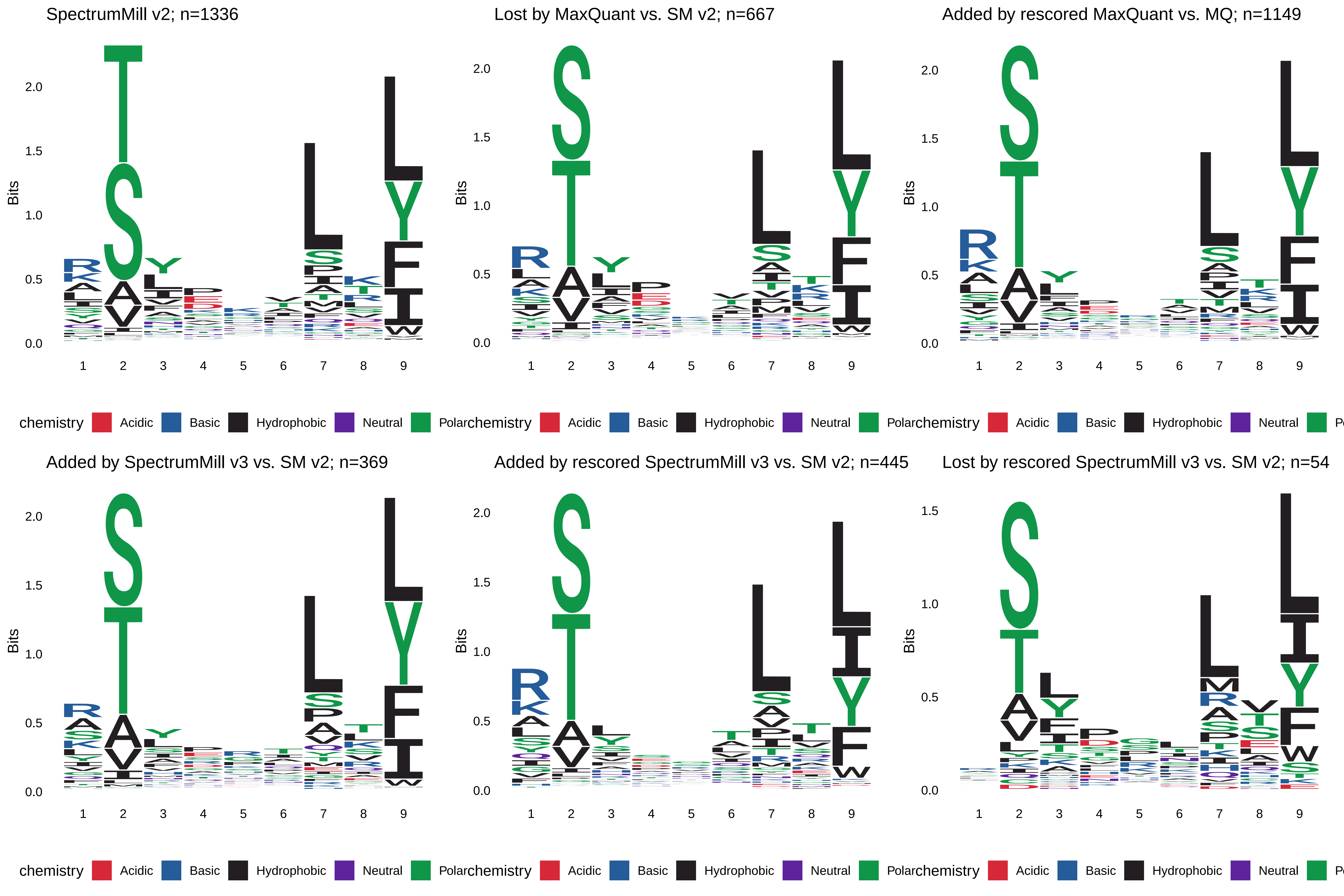
SpectrumMill v2; n=1336
Lost by MaxQuant vs. SM v2; n=667
Added by rescored MaxQuant vs. MQ; n=1149
Added by SpectrumMill v3 vs. SM v2; n=369
Added by rescored SpectrumMill v3 vs. SM v2; n=445
Lost by rescored SpectrumMill v3 vs. SM v2; n=54
Bits
1 2 3 4 5 6 7 8 9
chemistry Acidic Basic Hydrophobic Neutral Polarchemistry Acidic Basic Hydrophobic Neutral Polarchemistry Acidic Basic Hydrophobic Neutral

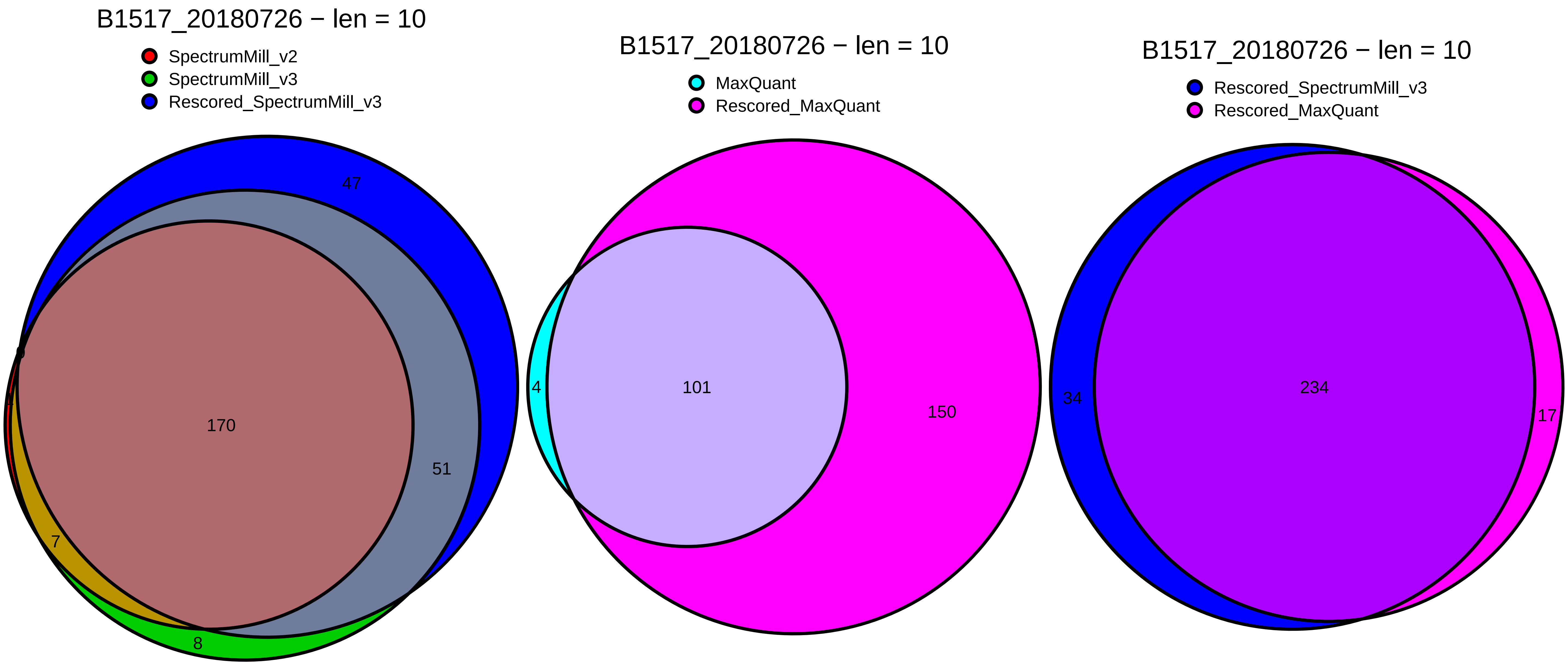
B1517_20180726 − len = 10
SpectrumMill_v2
SpectrumMill_v3
Rescored_SpectrumMill_v3
47
170
51
7
8
B1517_20180726 − len = 10
MaxQuant
Rescored_MaxQuant
4
101
150
B1517_20180726 − len = 10
Rescored_SpectrumMill_v3
Rescored_MaxQuant
34
234
17

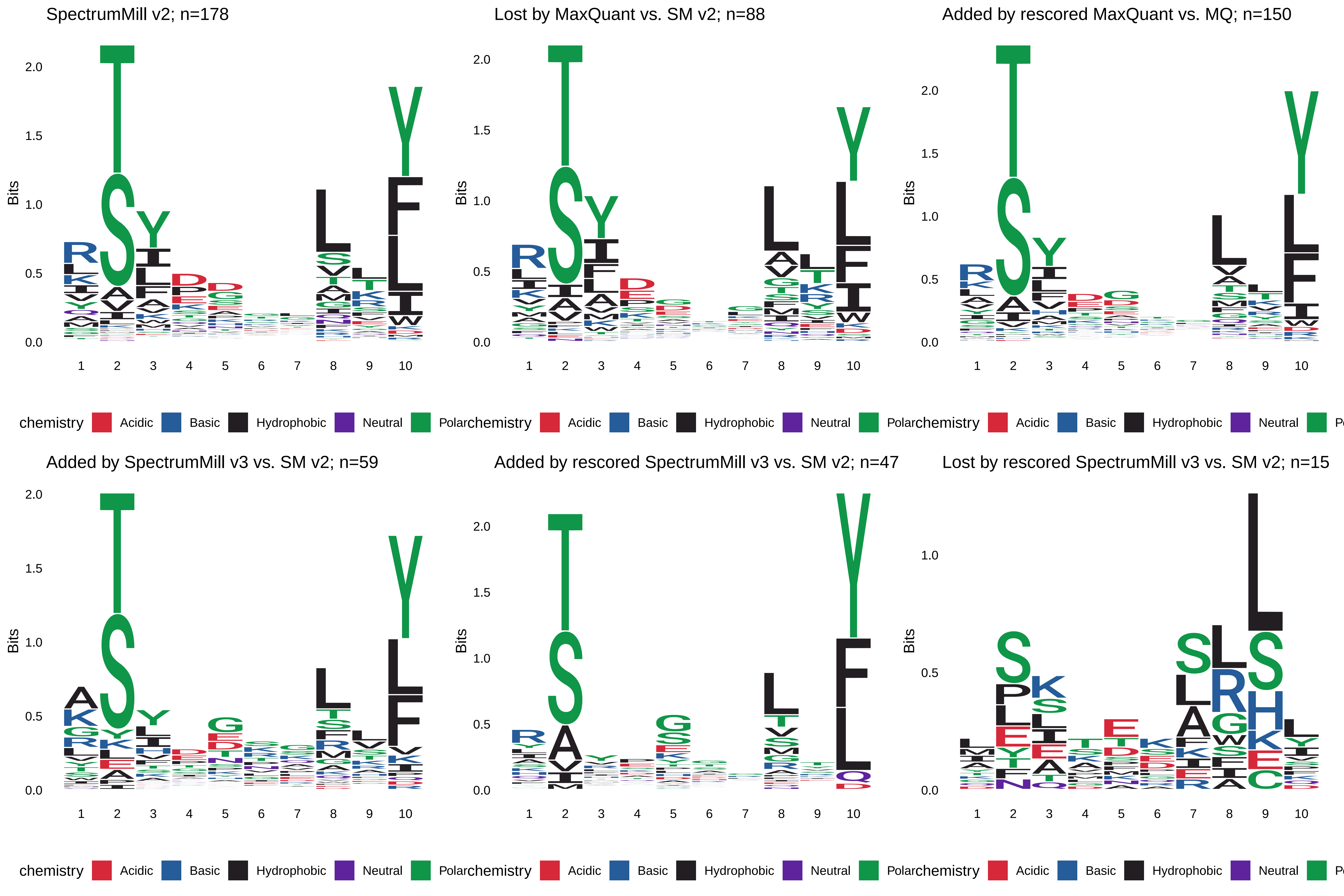
SpectrumMill v2; n=178
Lost by MaxQuant vs. SM v2; n=88
Added by rescored MaxQuant vs. MQ; n=150
Added by SpectrumMill v3 vs. SM v2; n=59
Added by rescored SpectrumMill v3 vs. SM v2; n=47
Lost by rescored SpectrumMill v3 vs. SM v2; n=15
Bits
0.0
0.5
1.0
1.5
2.0
1 2 3 4 5 6 7 8 9 10
chemistry
Acidic
Basic
Hydrophobic
Neutral
Polar

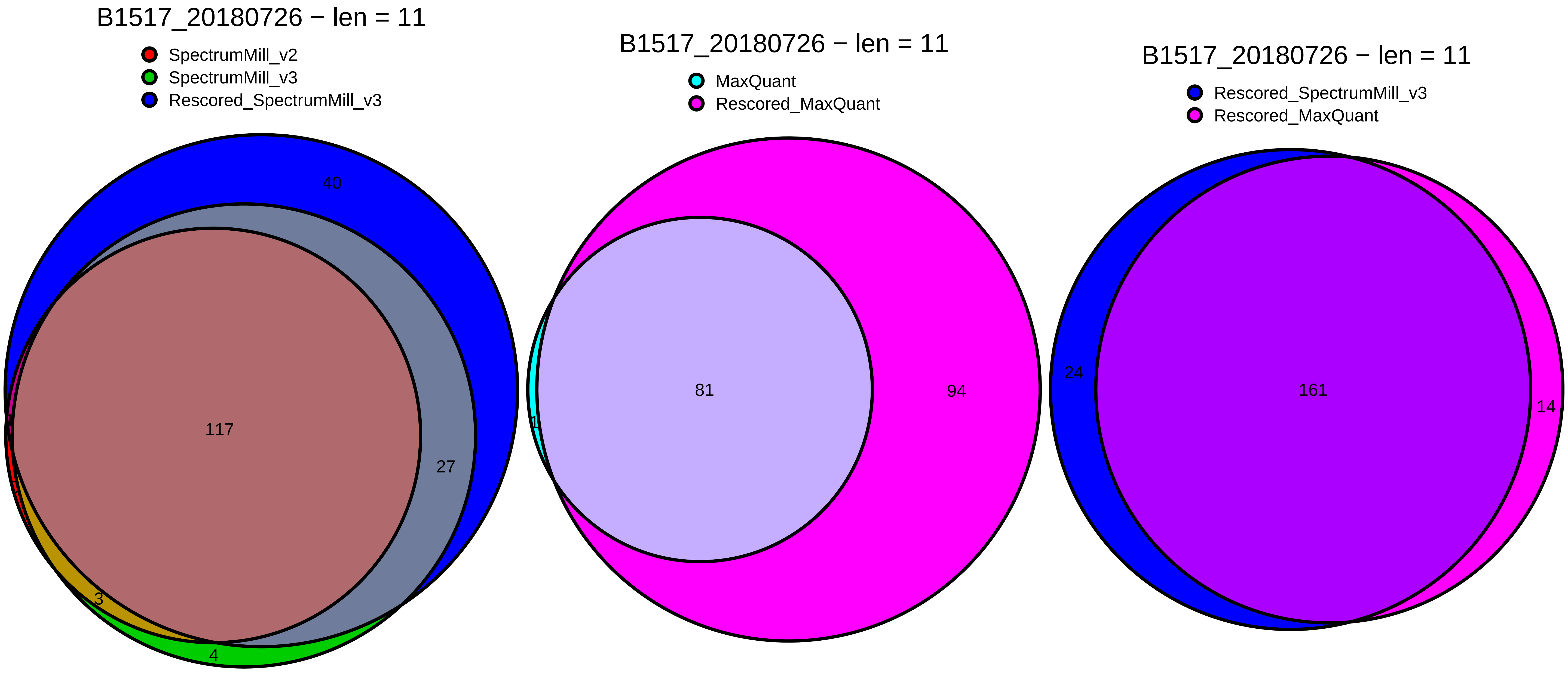

B1517_20180726 − len = 11
SpectrumMill_v2
SpectrumMill_v3
Rescored_SpectrumMill_v3
40
117
27
3
4
B1517_20180726 − len = 11
MaxQuant
Rescored_MaxQuant
81
94
B1517_20180726 − len = 11
Rescored_SpectrumMill_v3
Rescored_MaxQuant
24
161
14

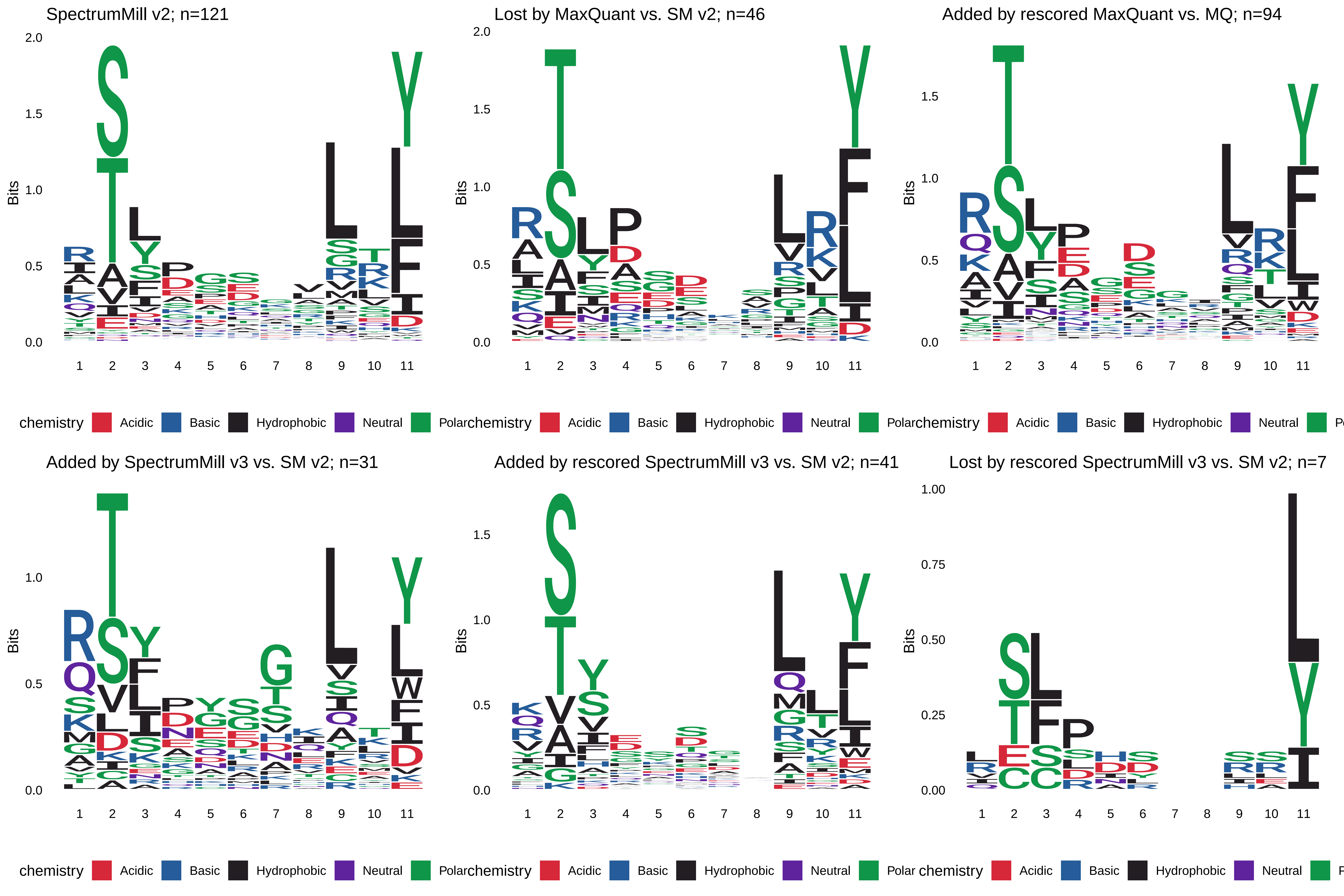
SpectrumMill v2; n=121
Lost by MaxQuant vs. SM v2; n=46
Added by rescored MaxQuant vs. MQ; n=94
Added by SpectrumMill v3 vs. SM v2; n=31
Added by rescored SpectrumMill v3 vs. SM v2; n=41
Lost by rescored SpectrumMill v3 vs. SM v2; n=7
Bits
1 2 3 4 5 6 7 8 9 10 11
chemistry Acidic Basic Hydrophobic Neutral Polar

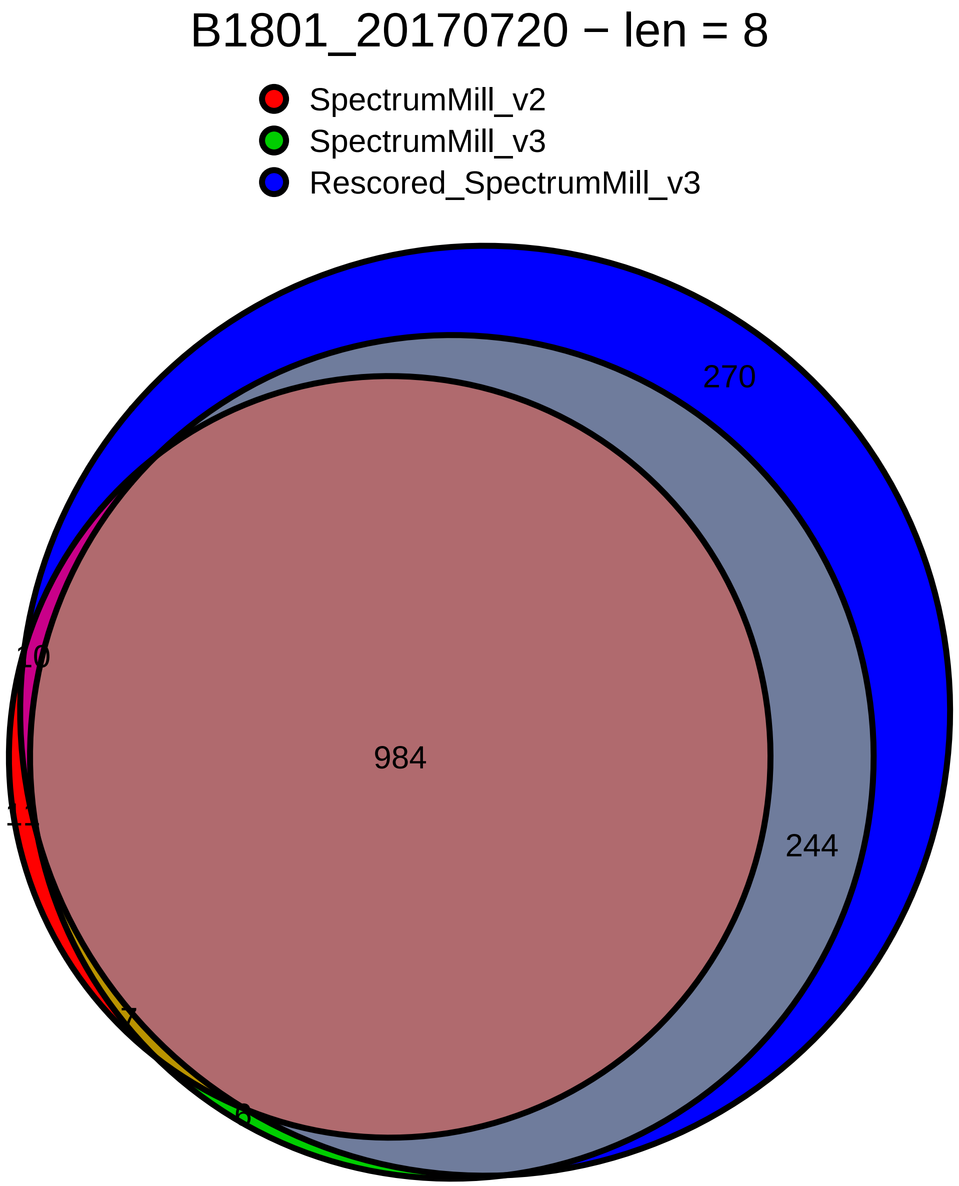
B1801_20170720 − len = 8
SpectrumMill_v2
SpectrumMill_v3
Rescored_SpectrumMill_v3
270
10
984
244
7

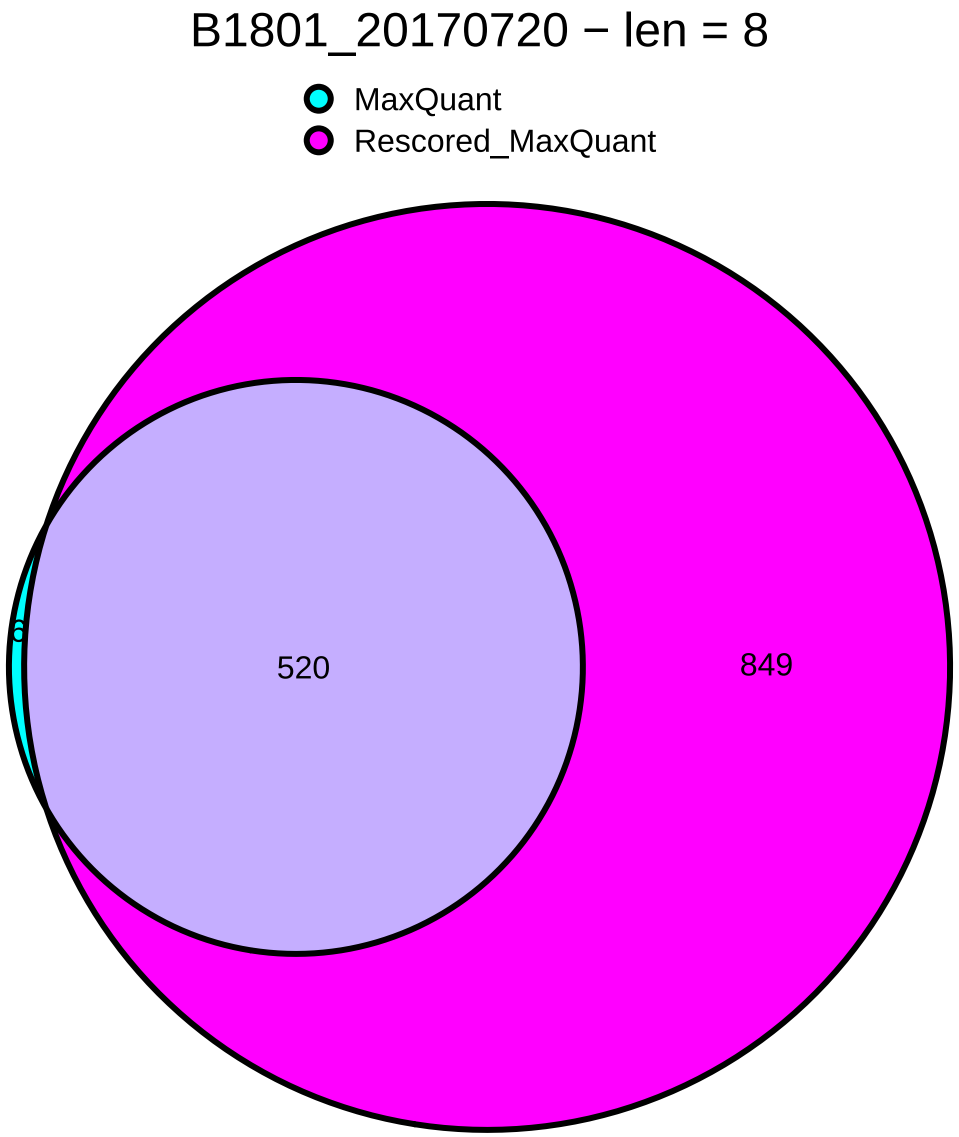
B1801_20170720 − len = 8
MaxQuant
Rescored_MaxQuant
520
849

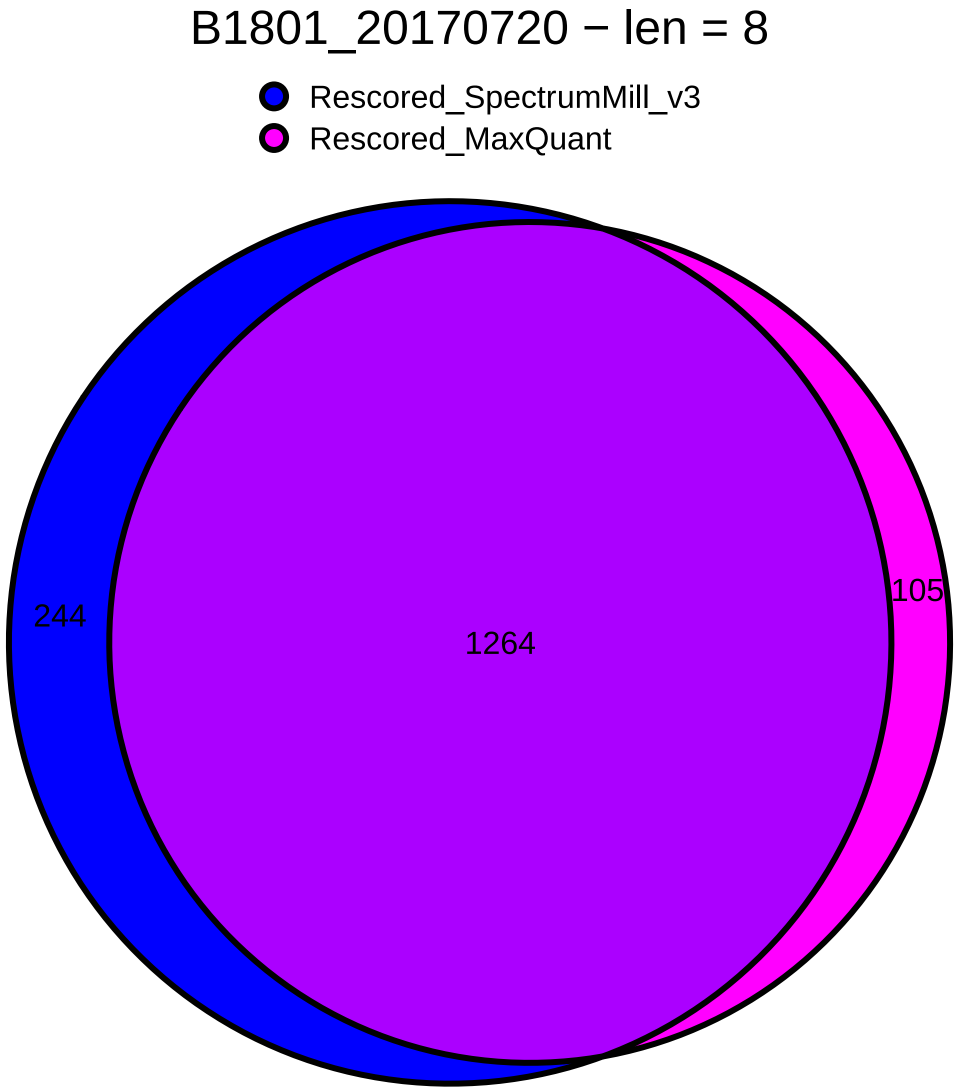
B1801_20170720 − len = 8
Rescored_SpectrumMill_v3
Rescored_MaxQuant
244
1264
105

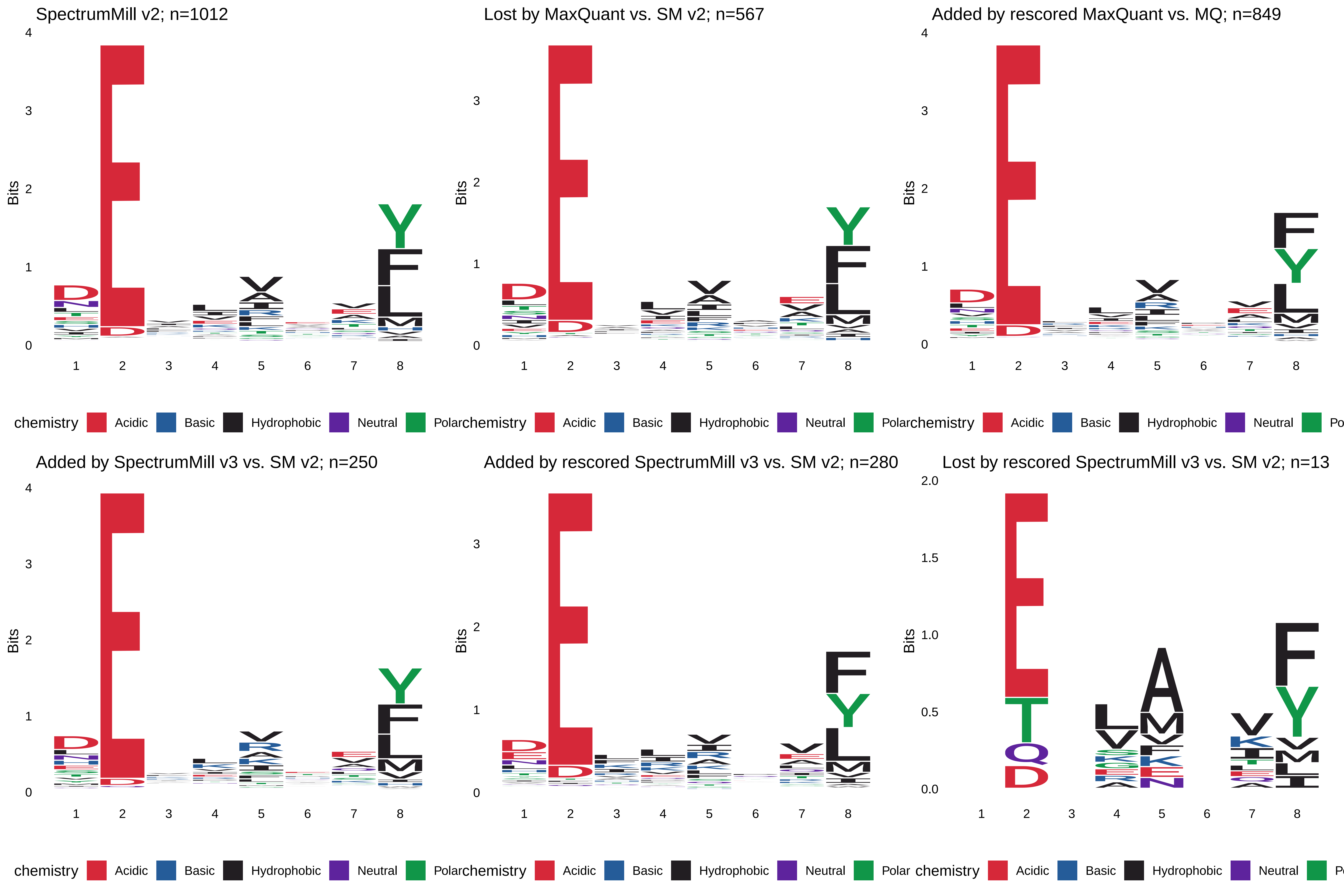
SpectrumMill v2; n=1012
Lost by MaxQuant vs. SM v2; n=567
Added by rescored MaxQuant vs. MQ; n=849
Added by SpectrumMill v3 vs. SM v2; n=250
Added by rescored SpectrumMill v3 vs. SM v2; n=280
Lost by rescored SpectrumMill v3 vs. SM v2; n=13
Bits
chemistry
Acidic
Basic
Hydrophobic
Neutral
Polar

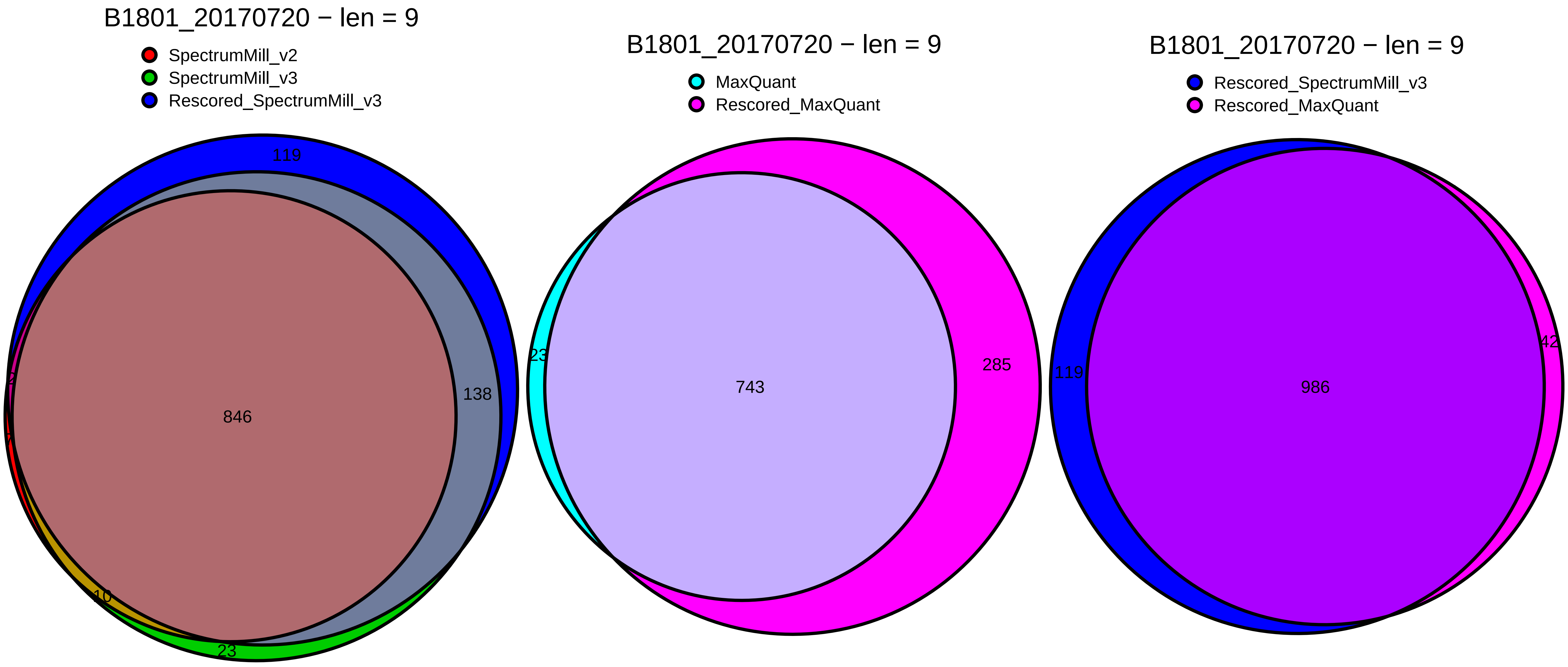

B1801_20170720 − len = 9
SpectrumMill_v2
SpectrumMill_v3
Rescored_SpectrumMill_v3
119
138
846
10
23
B1801_20170720 − len = 9
MaxQuant
Rescored_MaxQuant
23
743
285
B1801_20170720 − len = 9
Rescored_SpectrumMill_v3
Rescored_MaxQuant
119
986
42

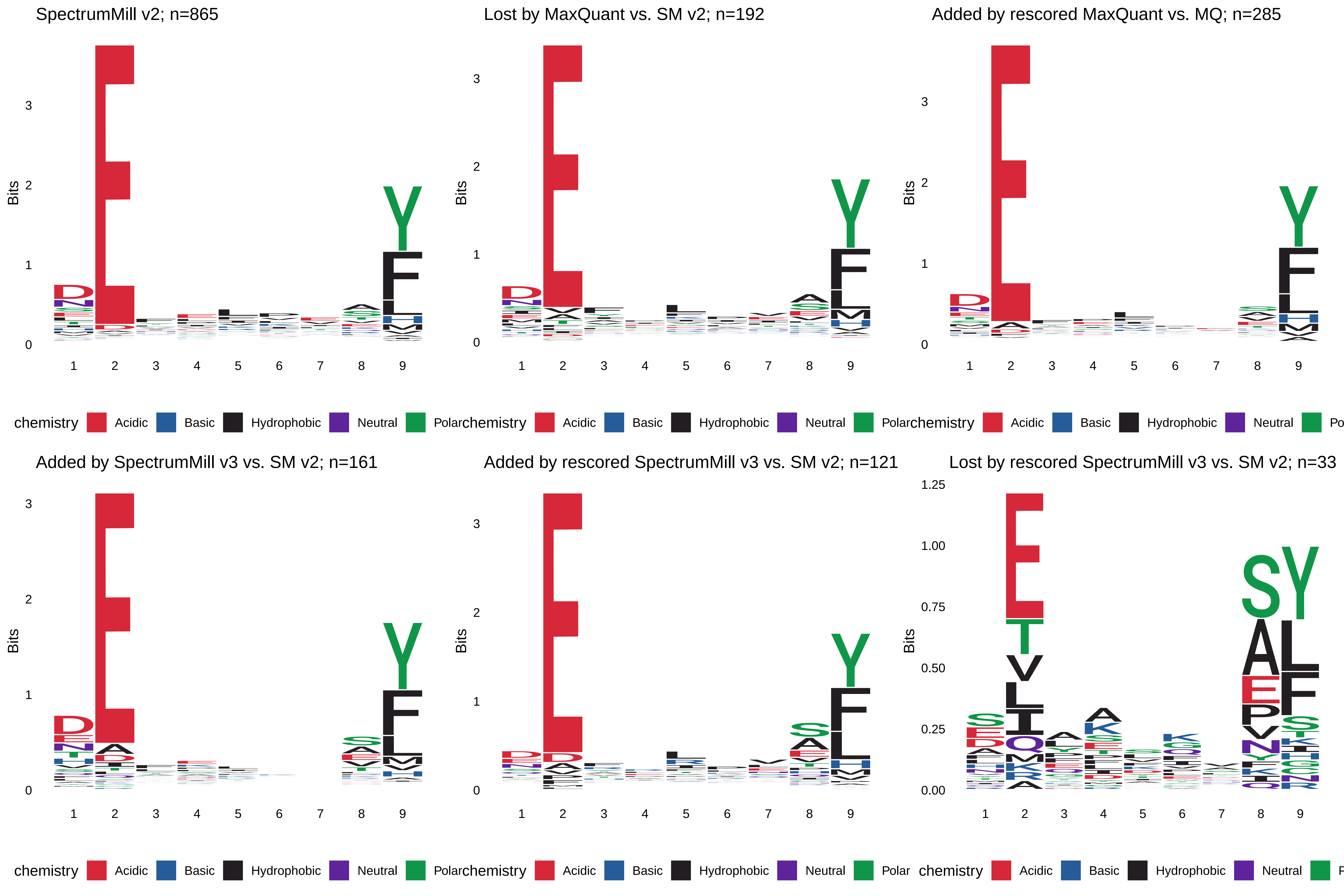
SpectrumMill v2; n=865
Lost by MaxQuant vs. SM v2; n=192
Added by rescored MaxQuant vs. MQ; n=285
Added by SpectrumMill v3 vs. SM v2; n=161
Added by rescored SpectrumMill v3 vs. SM v2; n=121
Lost by rescored SpectrumMill v3 vs. SM v2; n=33
Bits
0
1
2
3
0.00
0.25
0.50
0.75
1.00
1.25
1 2 3 4 5 6 7 8 9
chemistry
Acidic
Basic
Hydrophobic
Neutral
Polar

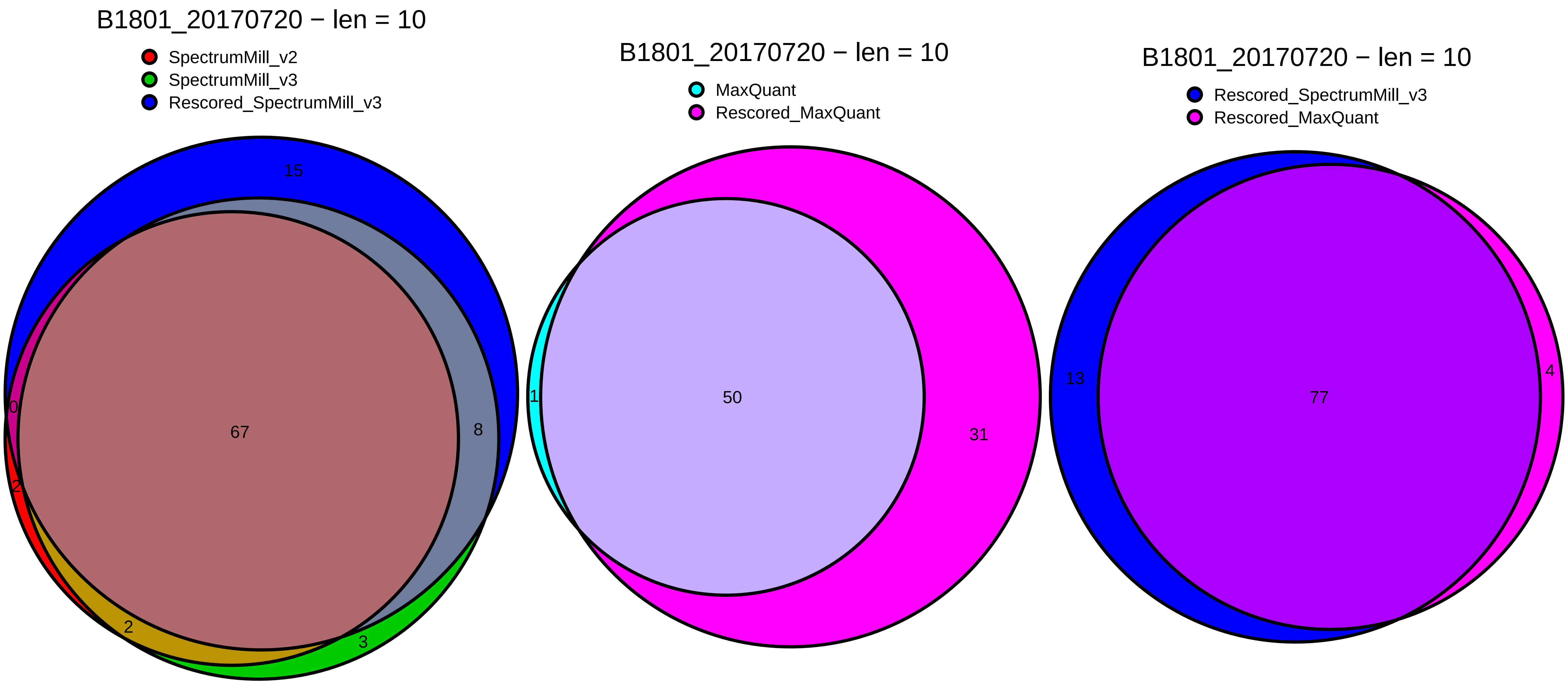
B1801_20170720 − len = 10
SpectrumMill_v2
SpectrumMill_v3
Rescored_SpectrumMill_v3
15
0
67
8
2
2
3
B1801_20170720 − len = 10
MaxQuant
Rescored_MaxQuant
1
50
31
B1801_20170720 − len = 10
Rescored_SpectrumMill_v3
Rescored_MaxQuant
13
77
4

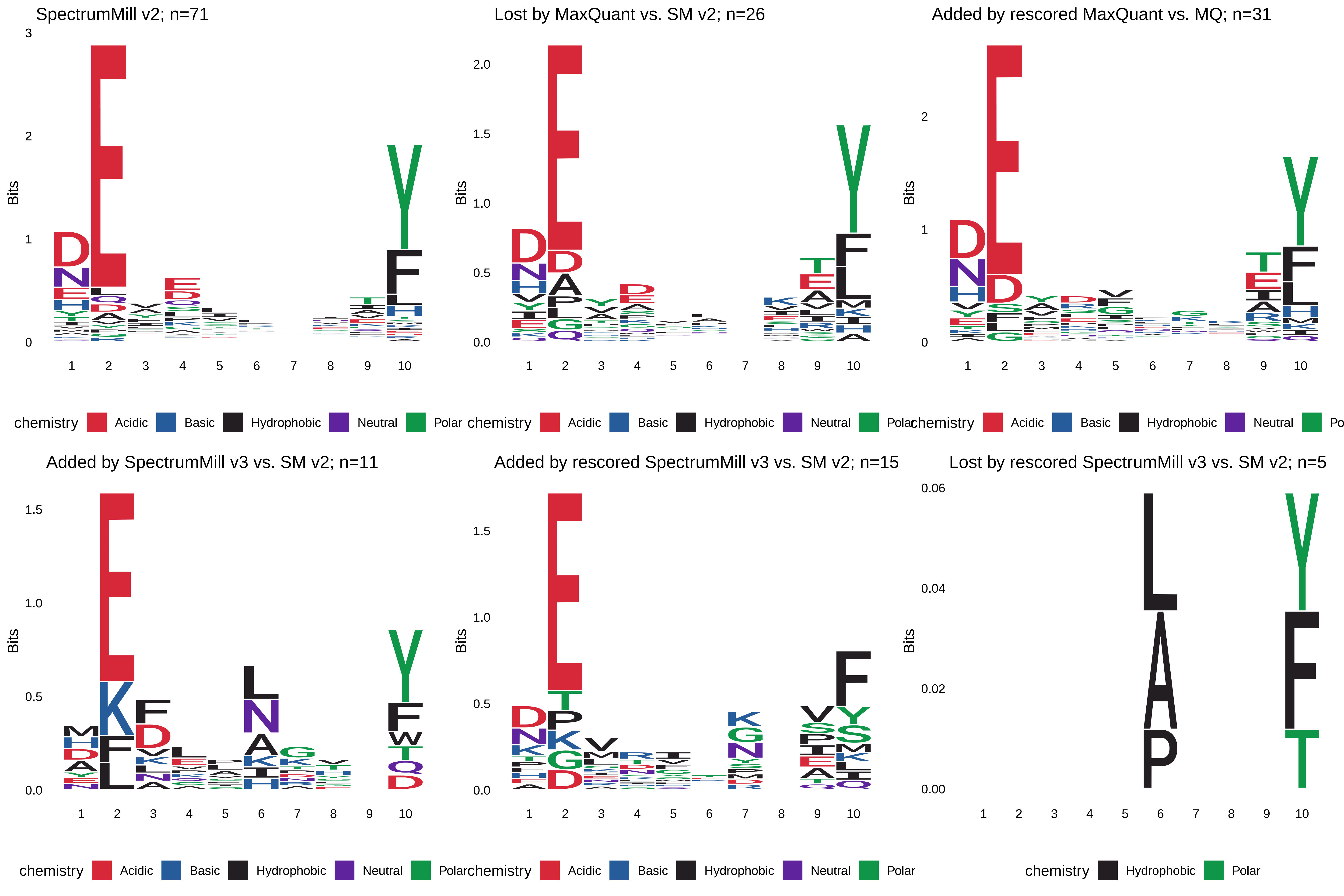
SpectrumMill v2; n=71
Lost by MaxQuant vs. SM v2; n=26
Added by rescored MaxQuant vs. MQ; n=31
Added by SpectrumMill v3 vs. SM v2; n=11
Added by rescored SpectrumMill v3 vs. SM v2; n=15
Lost by rescored SpectrumMill v3 vs. SM v2; n=5
Bits
1 2 3 4 5 6 7 8 9 10
chemistry
Acidic
Basic
Hydrophobic
Neutral
Polar

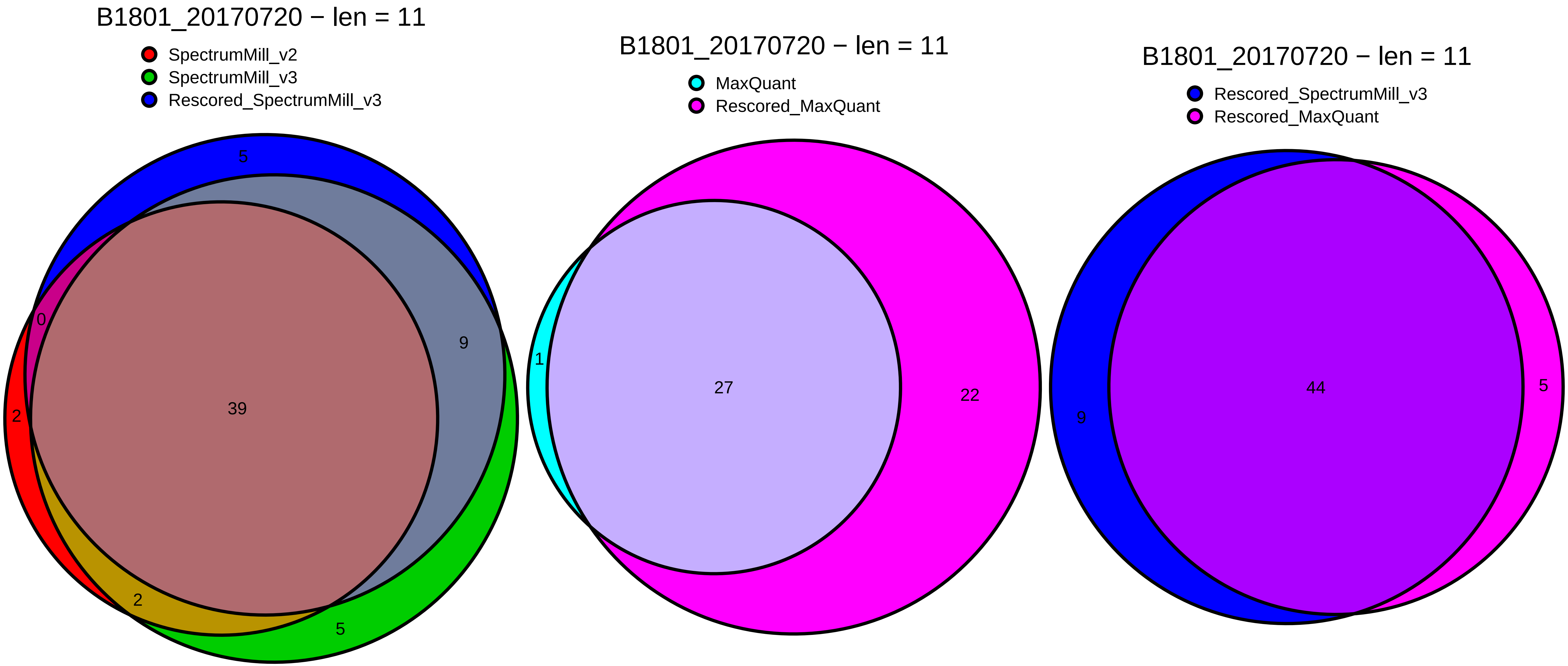
B1801_20170720 − len = 11
SpectrumMill_v2
SpectrumMill_v3
Rescored_SpectrumMill_v3
5
0
9
2
39
2
5
B1801_20170720 − len = 11
MaxQuant
Rescored_MaxQuant
1
27
22
B1801_20170720 − len = 11
Rescored_SpectrumMill_v3
Rescored_MaxQuant
9
44
5

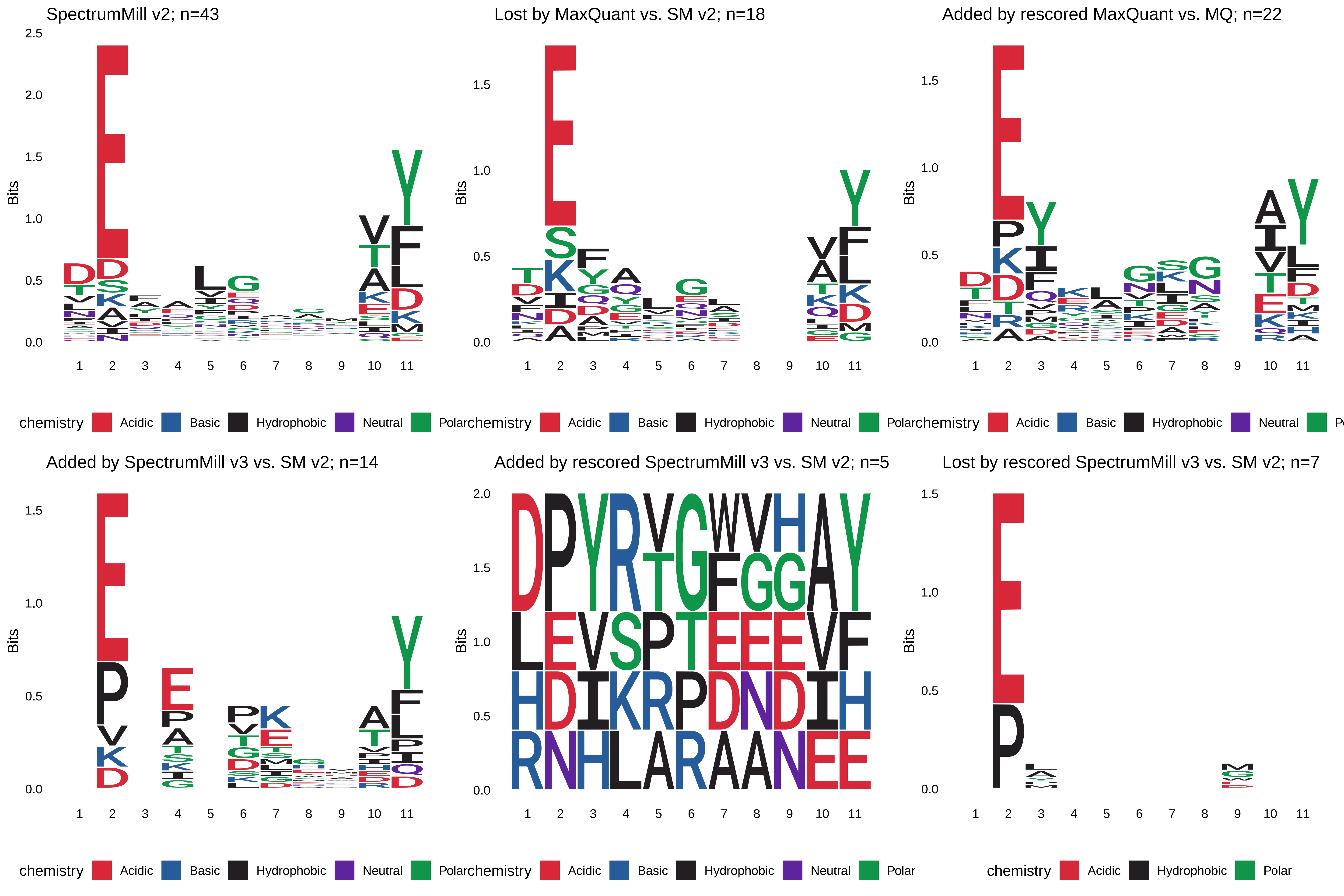
SpectrumMill v2; n=43
Lost by MaxQuant vs. SM v2; n=18
Added by rescored MaxQuant vs. MQ; n=22
Added by SpectrumMill v3 vs. SM v2; n=14
Added by rescored SpectrumMill v3 vs. SM v2; n=5
Lost by rescored SpectrumMill v3 vs. SM v2; n=7
Bits
1 2 3 4 5 6 7 8 9 10 11
chemistry Acidic Basic Hydrophobic Neutral Polar
chemistry Acidic Hydrophobic Polar

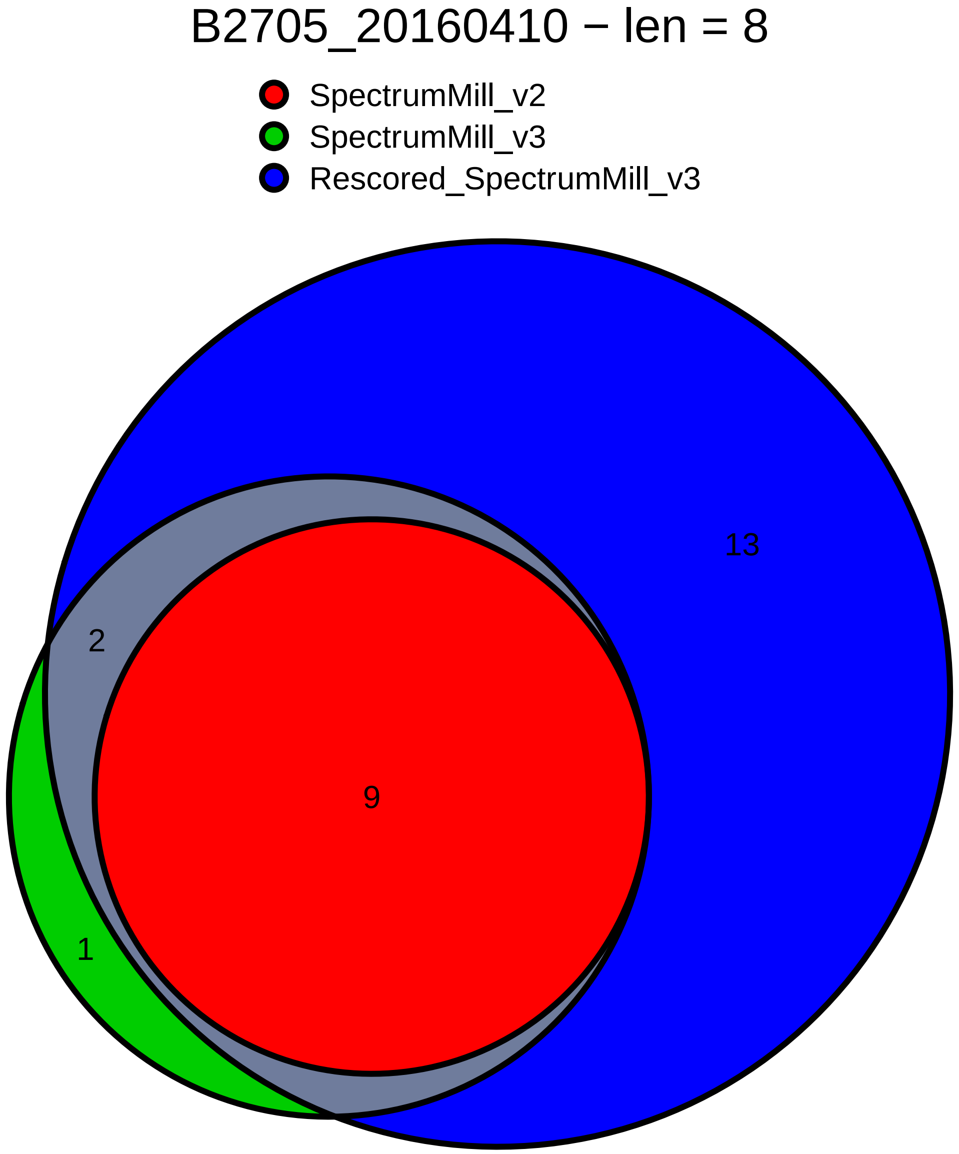

B2705_20160410 − len = 8
SpectrumMill_v2
SpectrumMill_v3
Rescored_SpectrumMill_v3
13
2
9
1

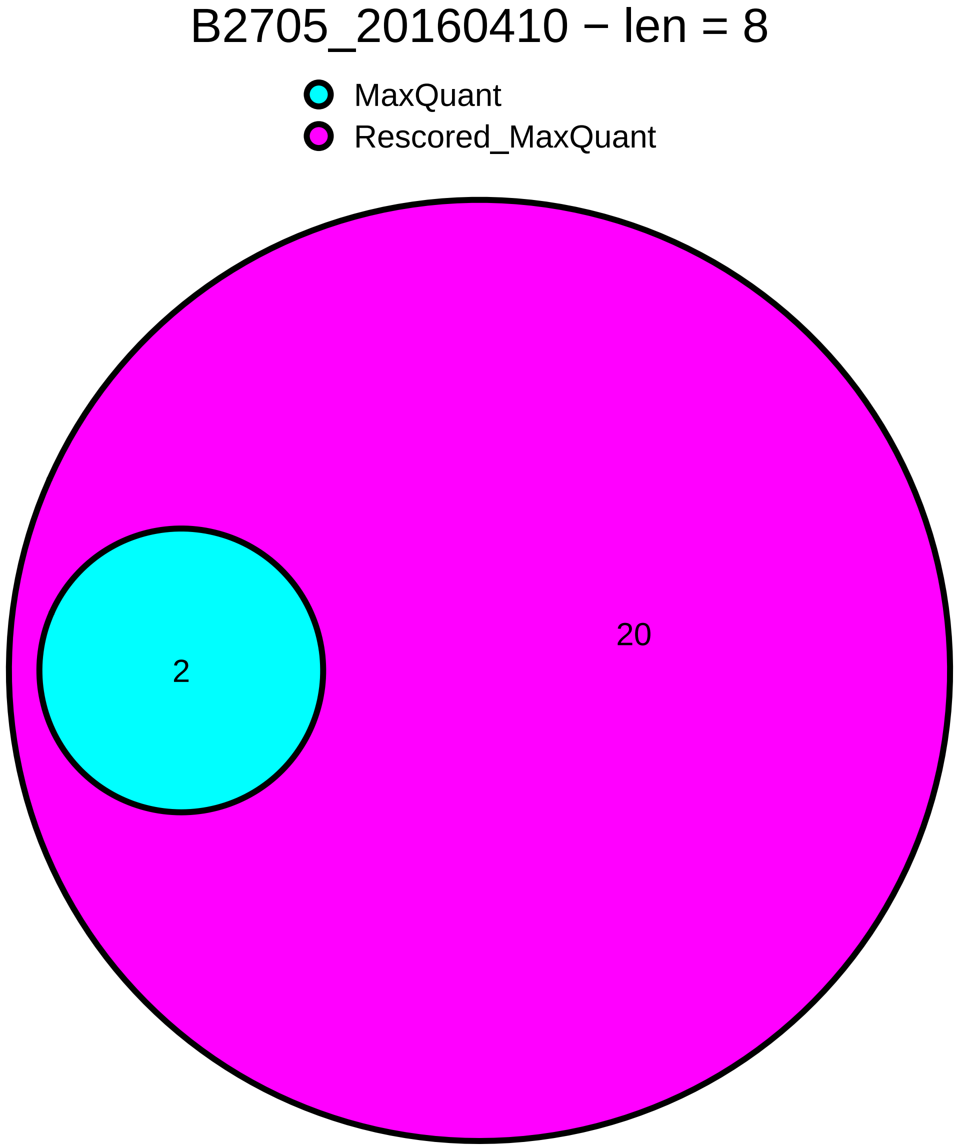

B2705_20160410 − len = 8
MaxQuant
Rescored_MaxQuant
2
20

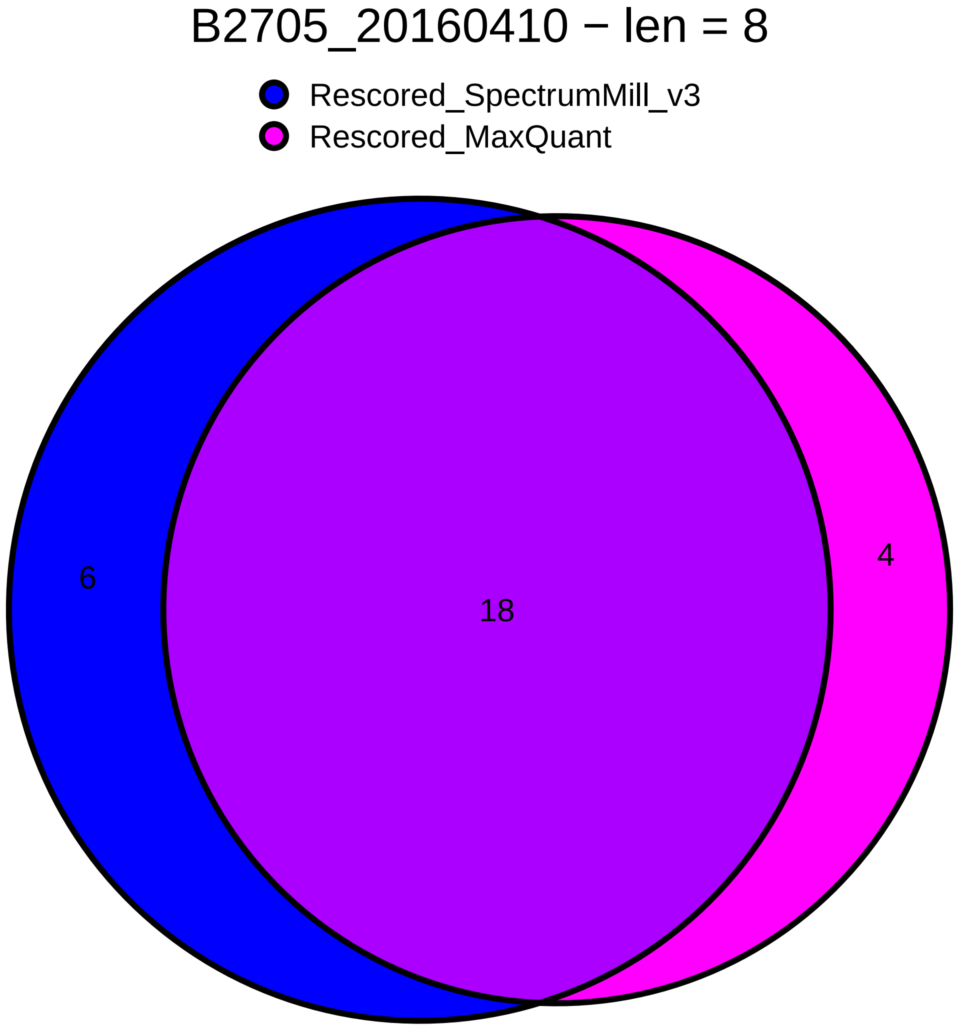

B2705_20160410 − len = 8
Rescored_SpectrumMill_v3
Rescored_MaxQuant
6
18
4

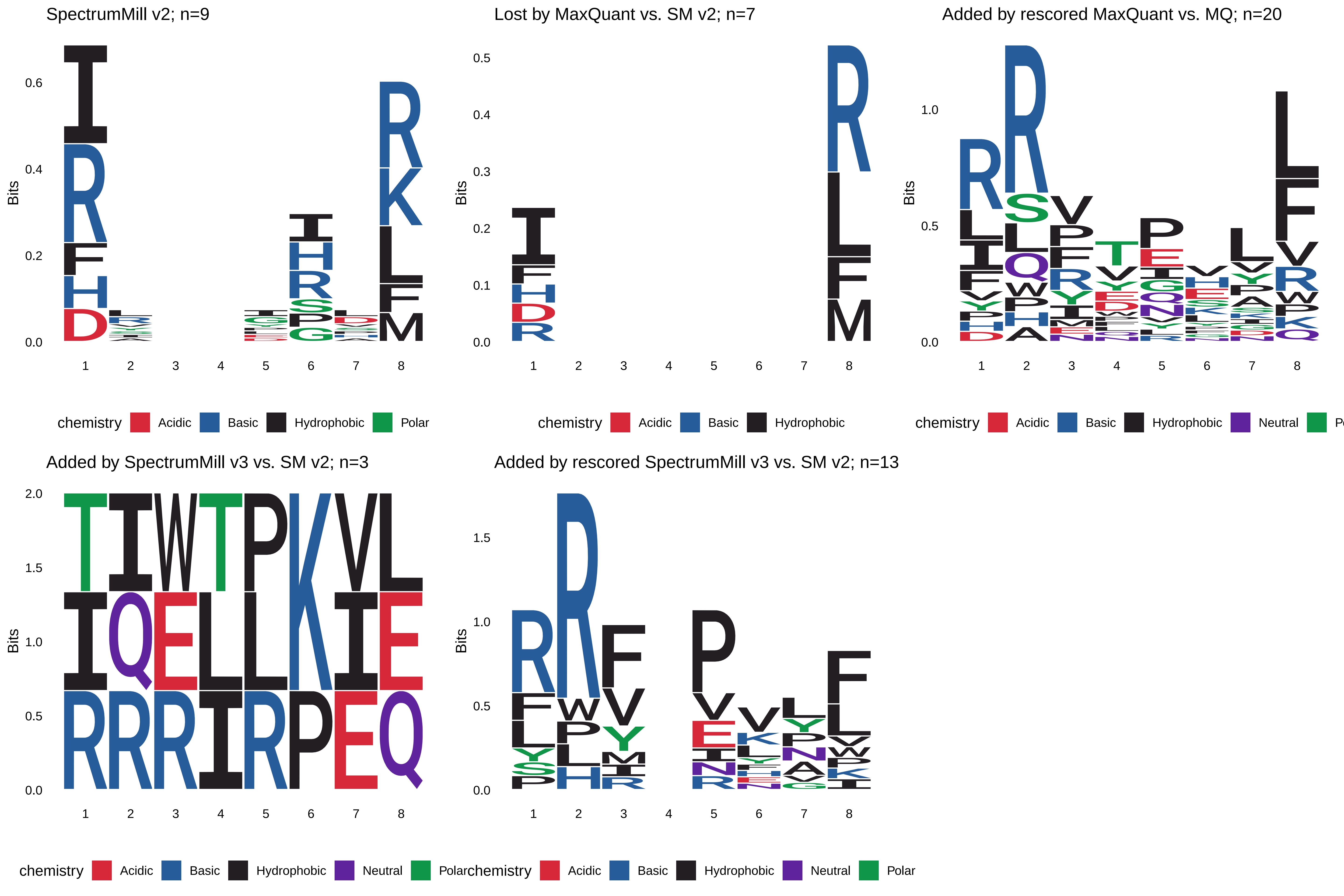

SpectrumMill v2; n=9
Lost by MaxQuant vs. SM v2; n=7
Added by rescored MaxQuant vs. MQ; n=20
Added by SpectrumMill v3 vs. SM v2; n=3
Added by rescored SpectrumMill v3 vs. SM v2; n=13
Bits
chemistry Acidic Basic Hydrophobic Polar
chemistry Acidic Basic Hydrophobic
chemistry Acidic Basic Hydrophobic Neutral
chemistry Acidic Basic Hydrophobic Neutral Polarchemistry Acidic Basic Hydrophobic Neutral Polar

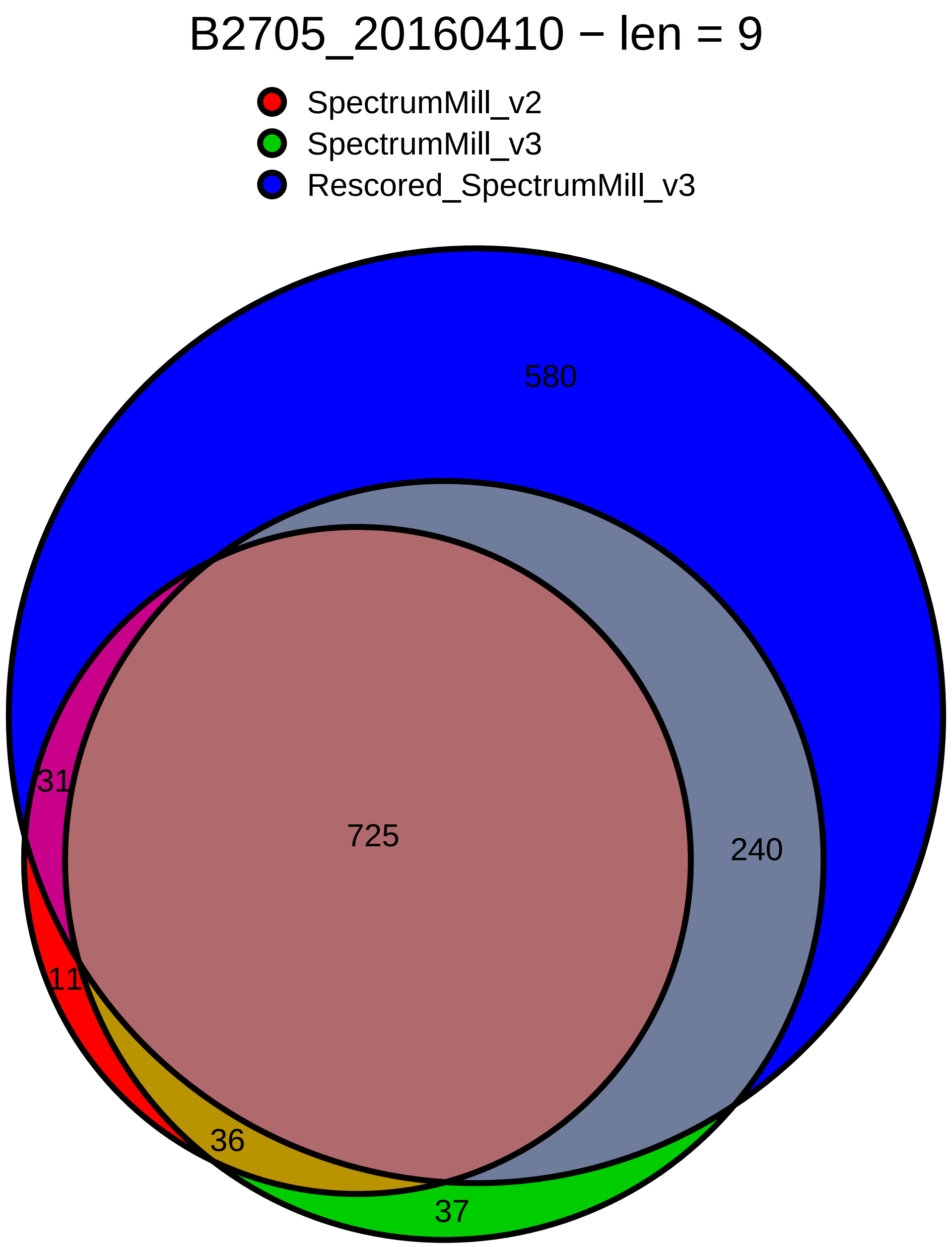
B2705_20160410 − len = 9
SpectrumMill_v2
SpectrumMill_v3
Rescored_SpectrumMill_v3
580
31
725
240
11
36
37

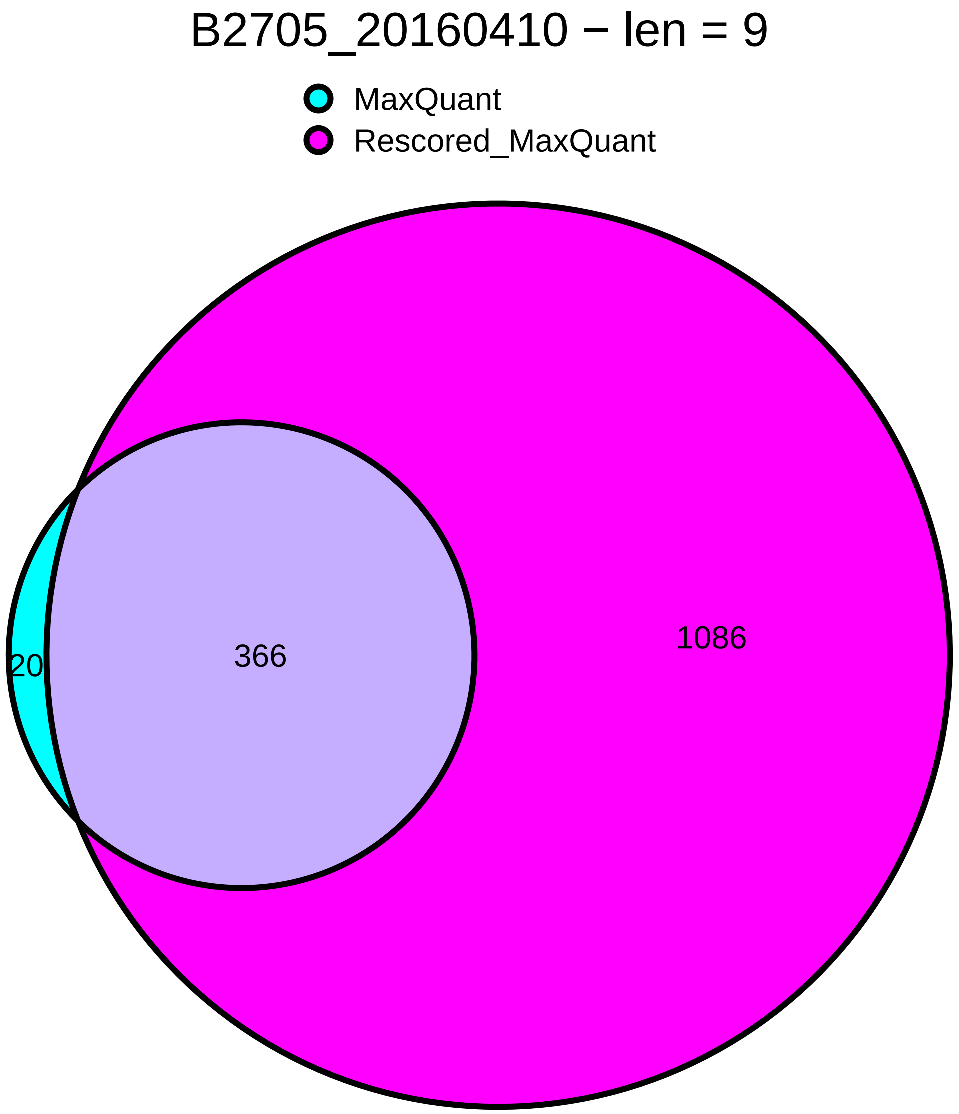
B2705_20160410 − len = 9
MaxQuant
Rescored_MaxQuant
20
366
1086

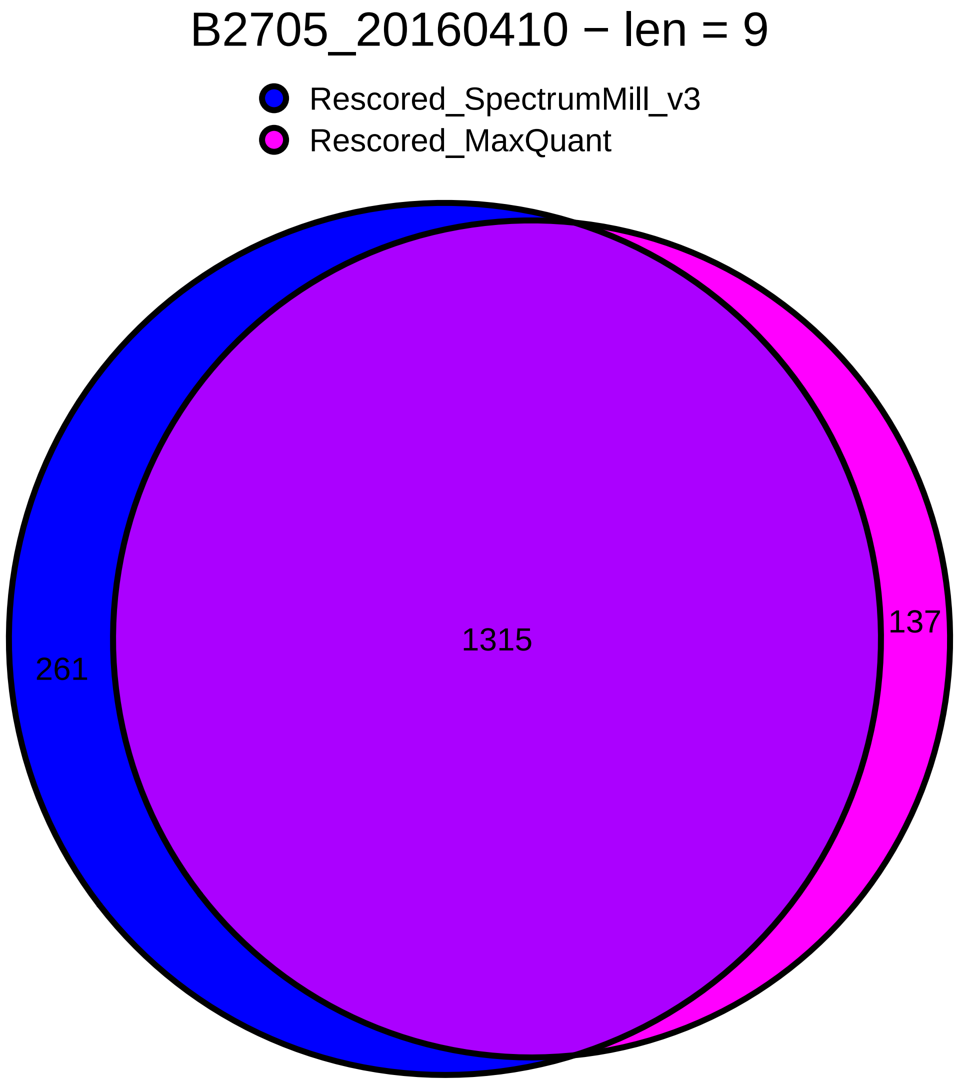
B2705_20160410 − len = 9
Rescored_SpectrumMill_v3
Rescored_MaxQuant
261
1315
137

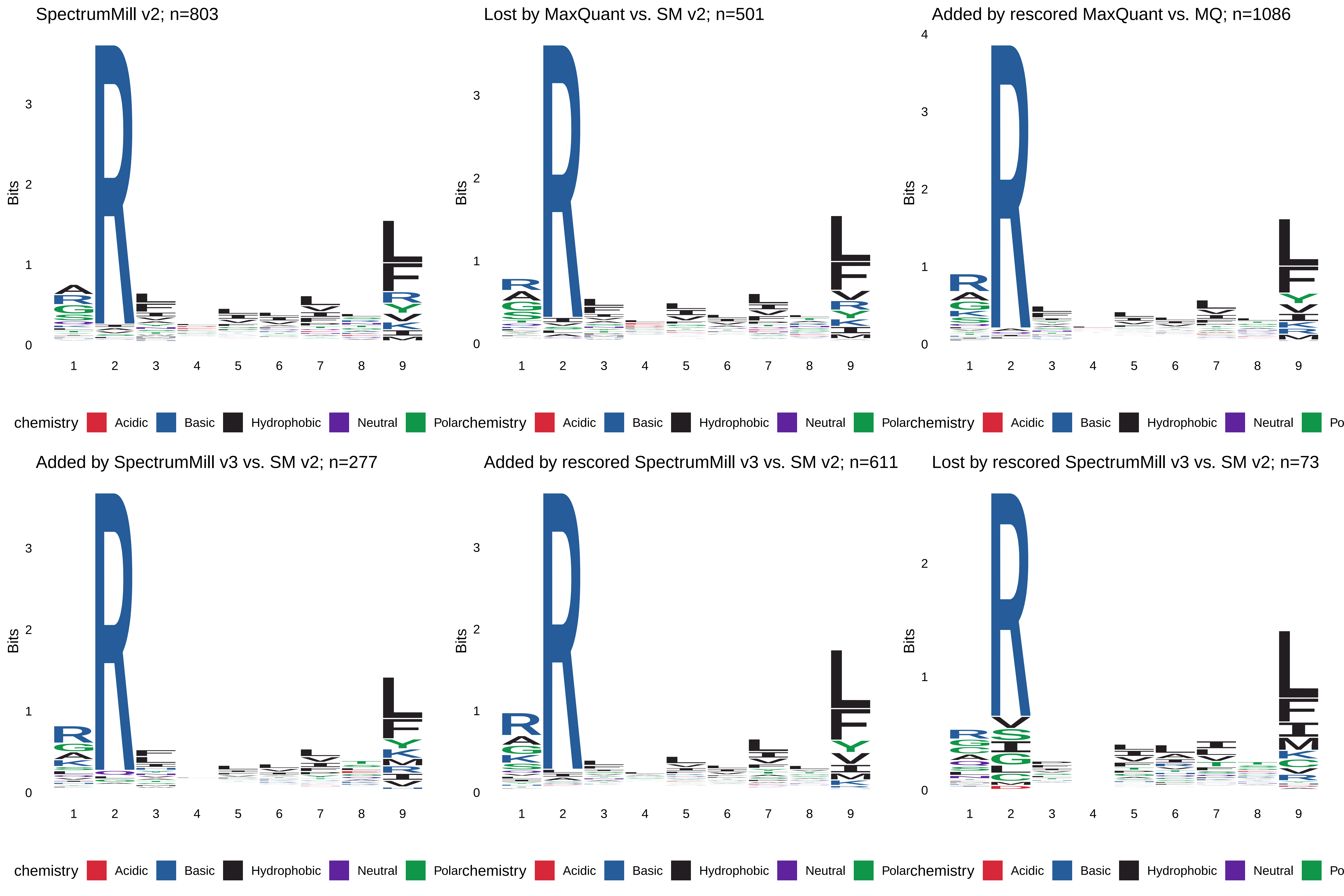
SpectrumMill v2; n=803
Lost by MaxQuant vs. SM v2; n=501
Added by rescored MaxQuant vs. MQ; n=1086
Added by SpectrumMill v3 vs. SM v2; n=277
Added by rescored SpectrumMill v3 vs. SM v2; n=611
Lost by rescored SpectrumMill v3 vs. SM v2; n=73
Bits
1 2 3 4 5 6 7 8 9
chemistry
Acidic
Basic
Hydrophobic
Neutral
Polar

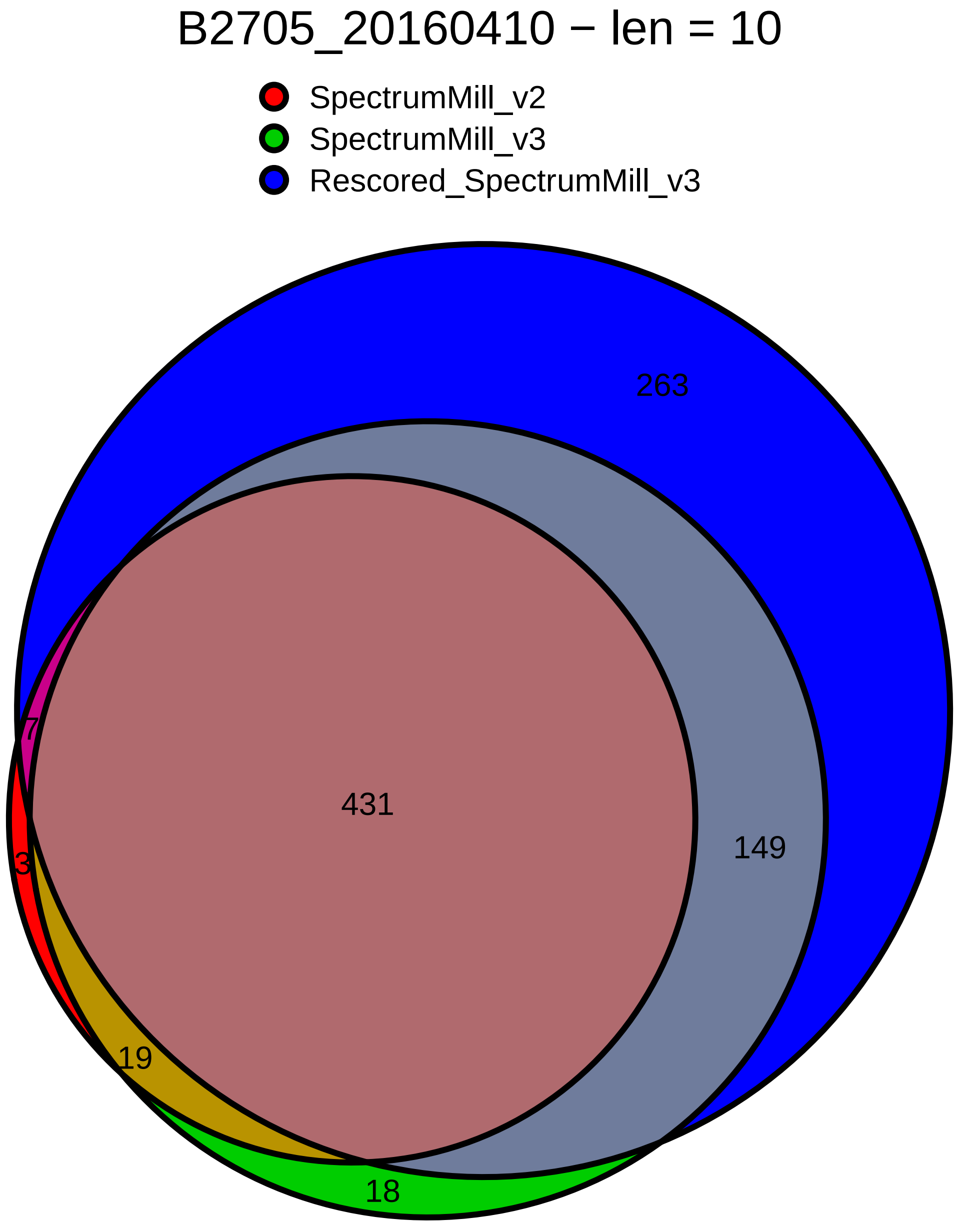
B2705_20160410 − len = 10
SpectrumMill_v2
SpectrumMill_v3
Rescored_SpectrumMill_v3
263
7
431
149
3
19
18

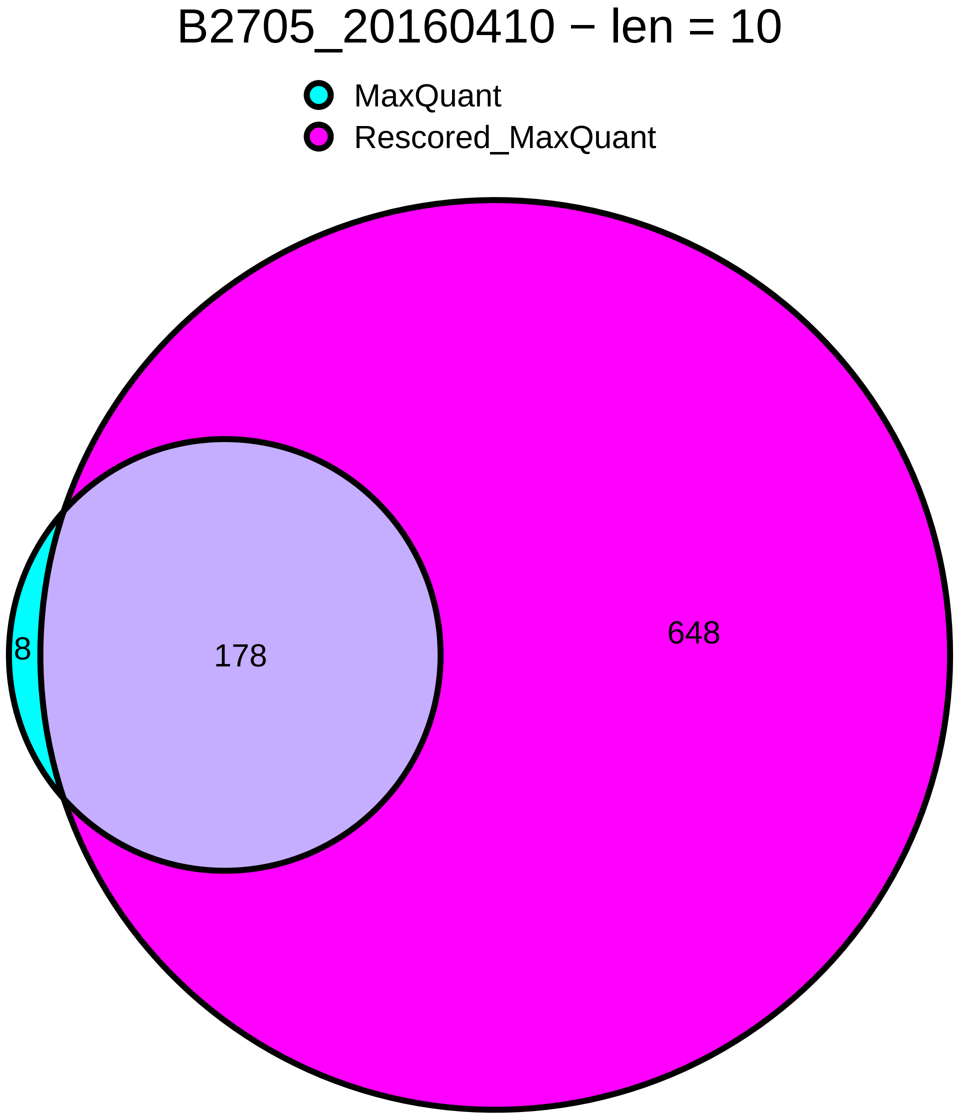
B2705_20160410 − len = 10
MaxQuant
Rescored_MaxQuant
8
178
648

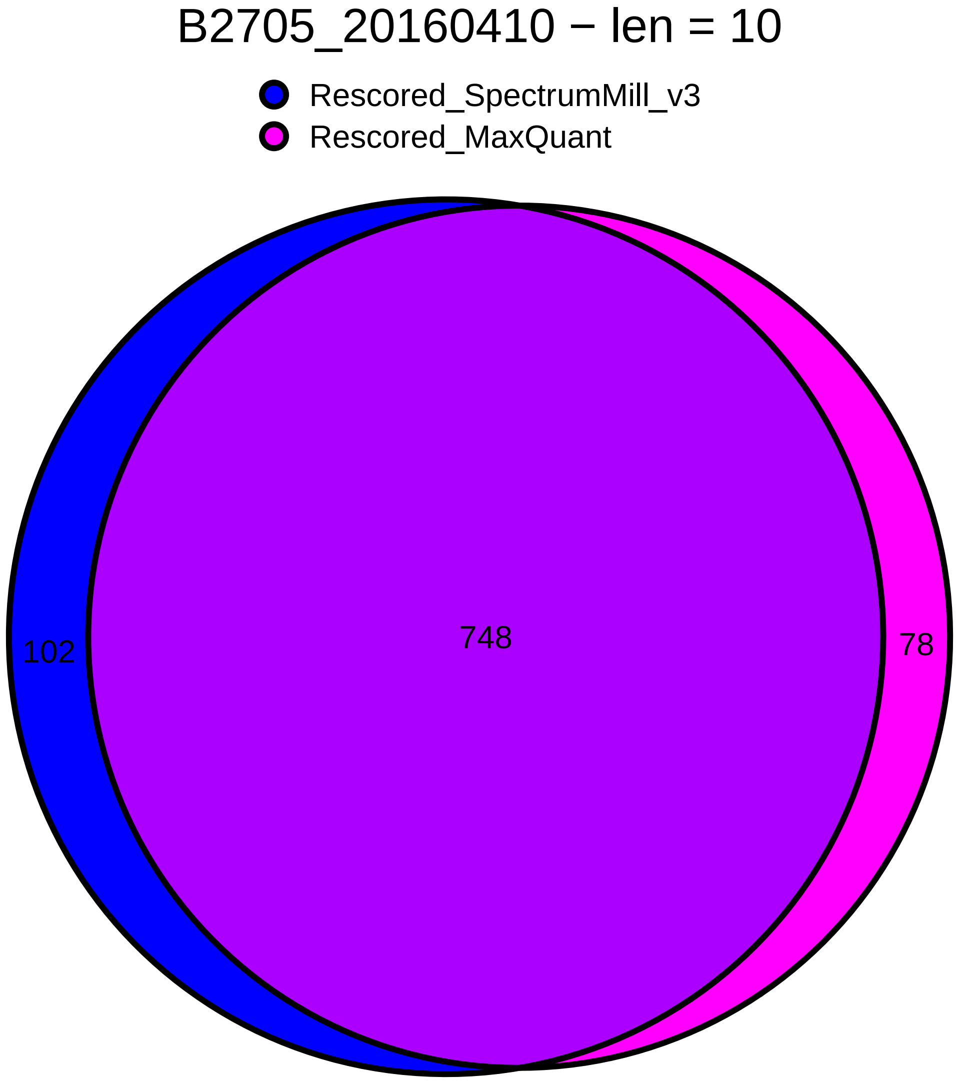
B2705_20160410 − len = 10
Rescored_SpectrumMill_v3
Rescored_MaxQuant
102
748
78

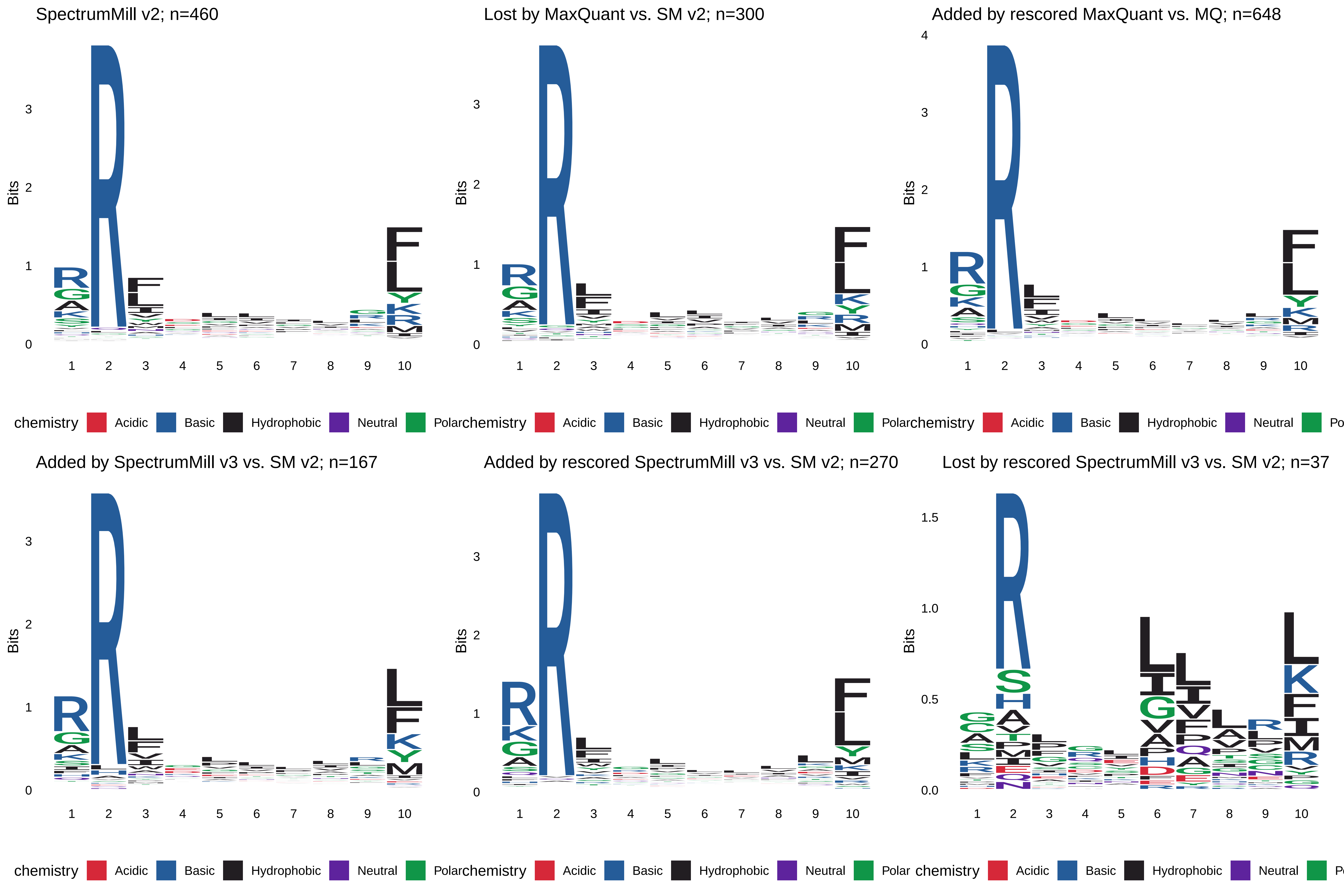

SpectrumMill v2; n=460
Lost by MaxQuant vs. SM v2; n=300
Added by rescored MaxQuant vs. MQ; n=648
Added by SpectrumMill v3 vs. SM v2; n=167
Added by rescored SpectrumMill v3 vs. SM v2; n=270
Lost by rescored SpectrumMill v3 vs. SM v2; n=37
Bits
chemistry
Acidic
Basic
Hydrophobic
Neutral
Polar

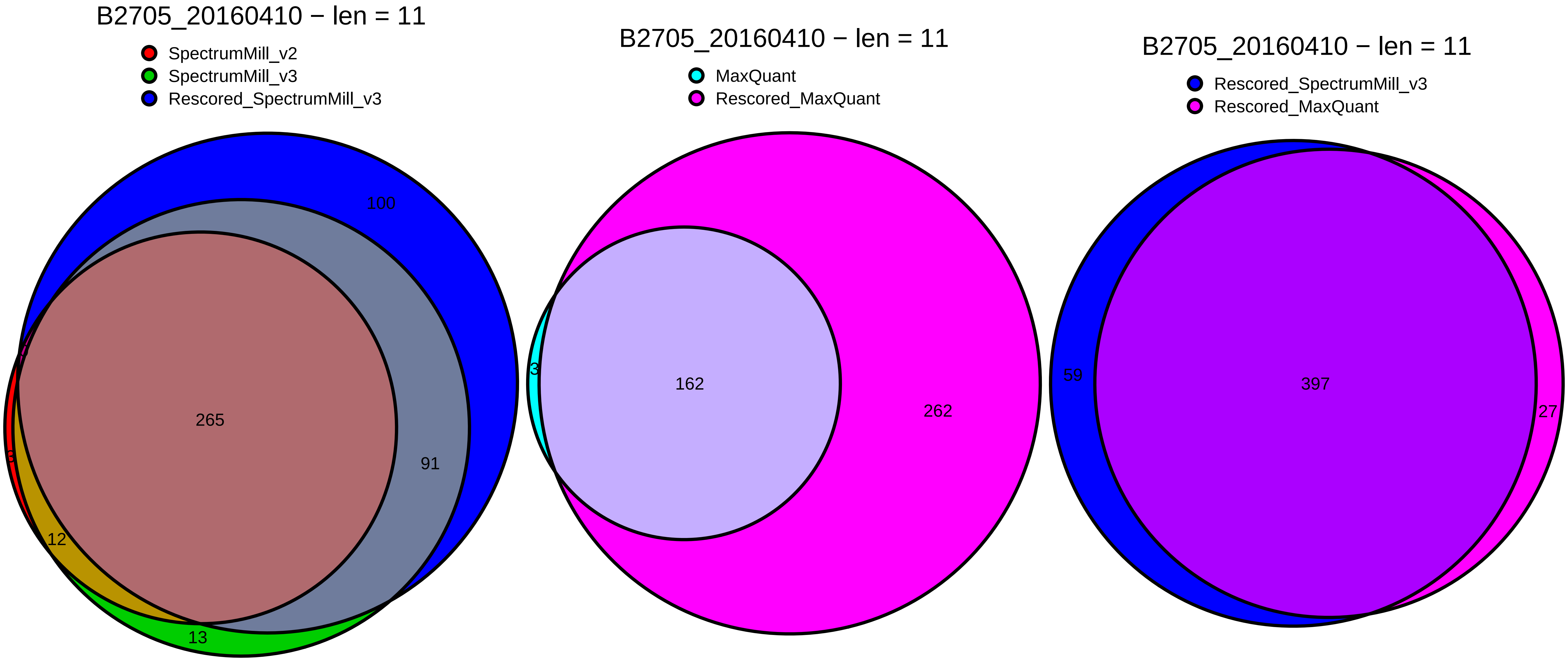

B2705_20160410 − len = 11
SpectrumMill_v2
SpectrumMill_v3
Rescored_SpectrumMill_v3
100
265
91
12
13
B2705_20160410 − len = 11
MaxQuant
Rescored_MaxQuant
162
262
B2705_20160410 − len = 11
Rescored_SpectrumMill_v3
Rescored_MaxQuant
59
397
27

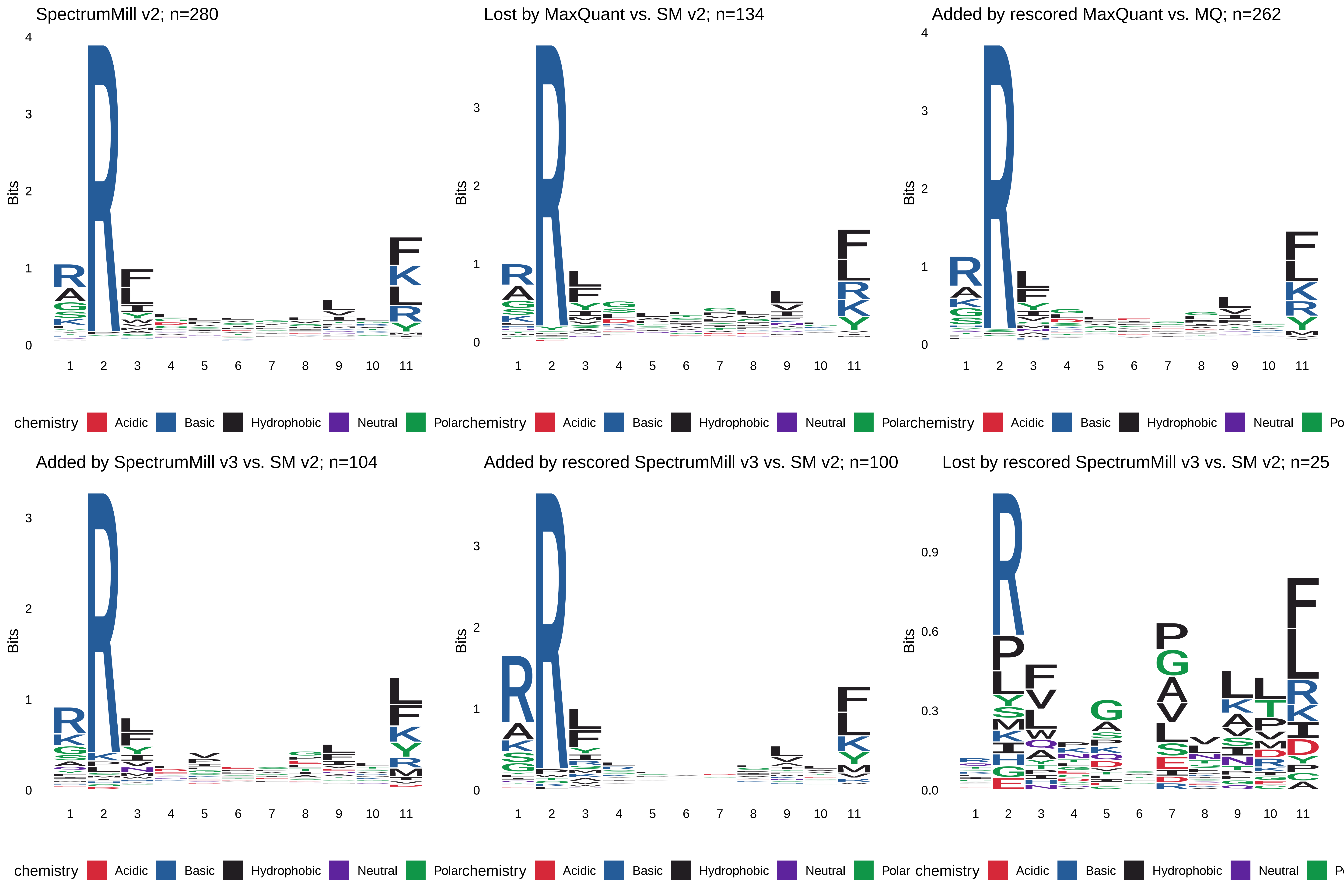
SpectrumMill v2; n=280
Lost by MaxQuant vs. SM v2; n=134
Added by rescored MaxQuant vs. MQ; n=262
Added by SpectrumMill v3 vs. SM v2; n=104
Added by rescored SpectrumMill v3 vs. SM v2; n=100
Lost by rescored SpectrumMill v3 vs. SM v2; n=25
Bits
1 2 3 4 5 6 7 8 9 10 11
chemistry Acidic Basic Hydrophobic Neutral Polar
chemistry Acidic Basic Hydrophobic Neutral Polar
chemistry Acidic Basic Hydrophobic Neutral Po

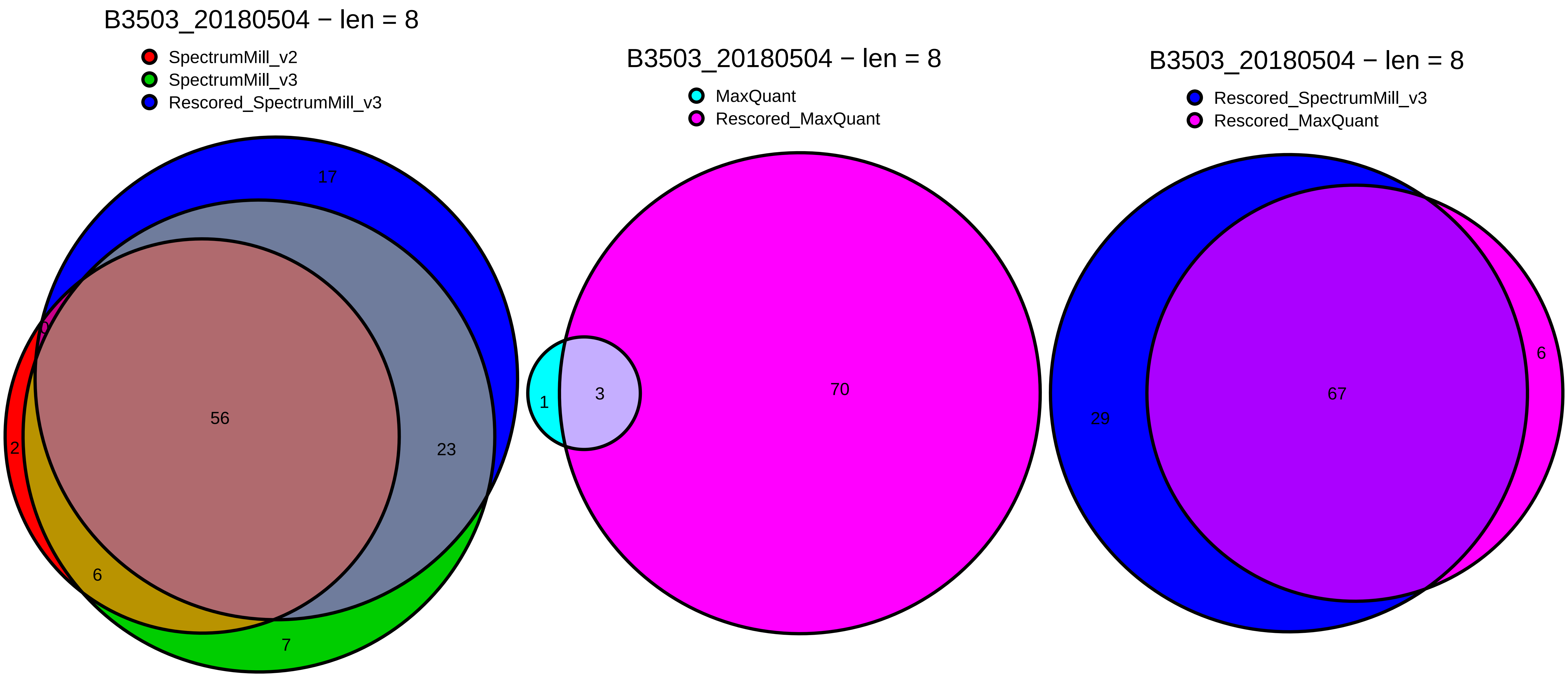

B3503_20180504 − len = 8
SpectrumMill_v2
SpectrumMill_v3
Rescored_SpectrumMill_v3
17
0
2
56
23
6
7
B3503_20180504 − len = 8
MaxQuant
Rescored_MaxQuant
1
3
70
B3503_20180504 − len = 8
Rescored_SpectrumMill_v3
Rescored_MaxQuant
29
67
6

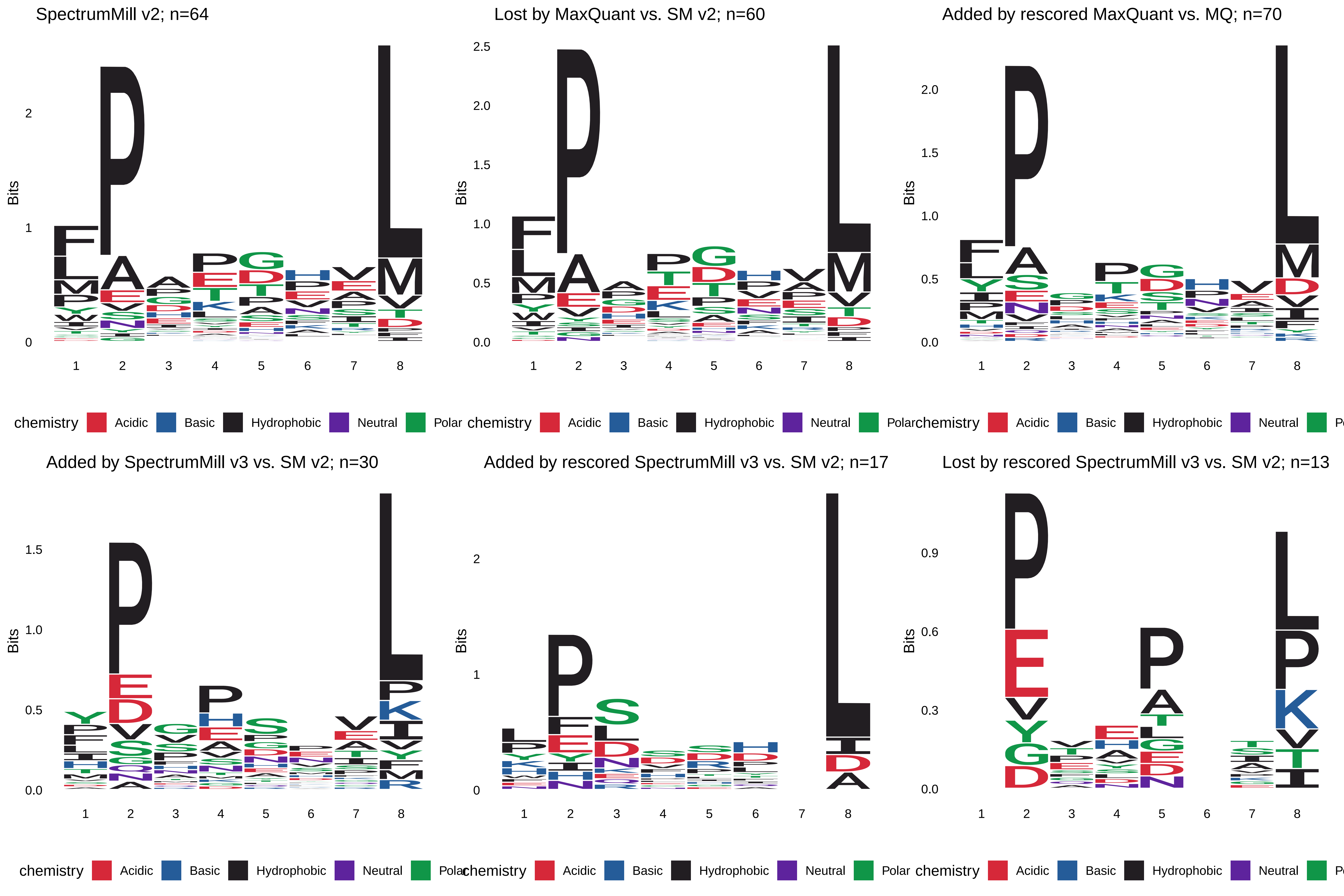
SpectrumMill v2; n=64
Lost by MaxQuant vs. SM v2; n=60
Added by rescored MaxQuant vs. MQ; n=70
Added by SpectrumMill v3 vs. SM v2; n=30
Added by rescored SpectrumMill v3 vs. SM v2; n=17
Lost by rescored SpectrumMill v3 vs. SM v2; n=13
Bits
1 2 3 4 5 6 7 8
chemistry
Acidic
Basic
Hydrophobic
Neutral
Polar

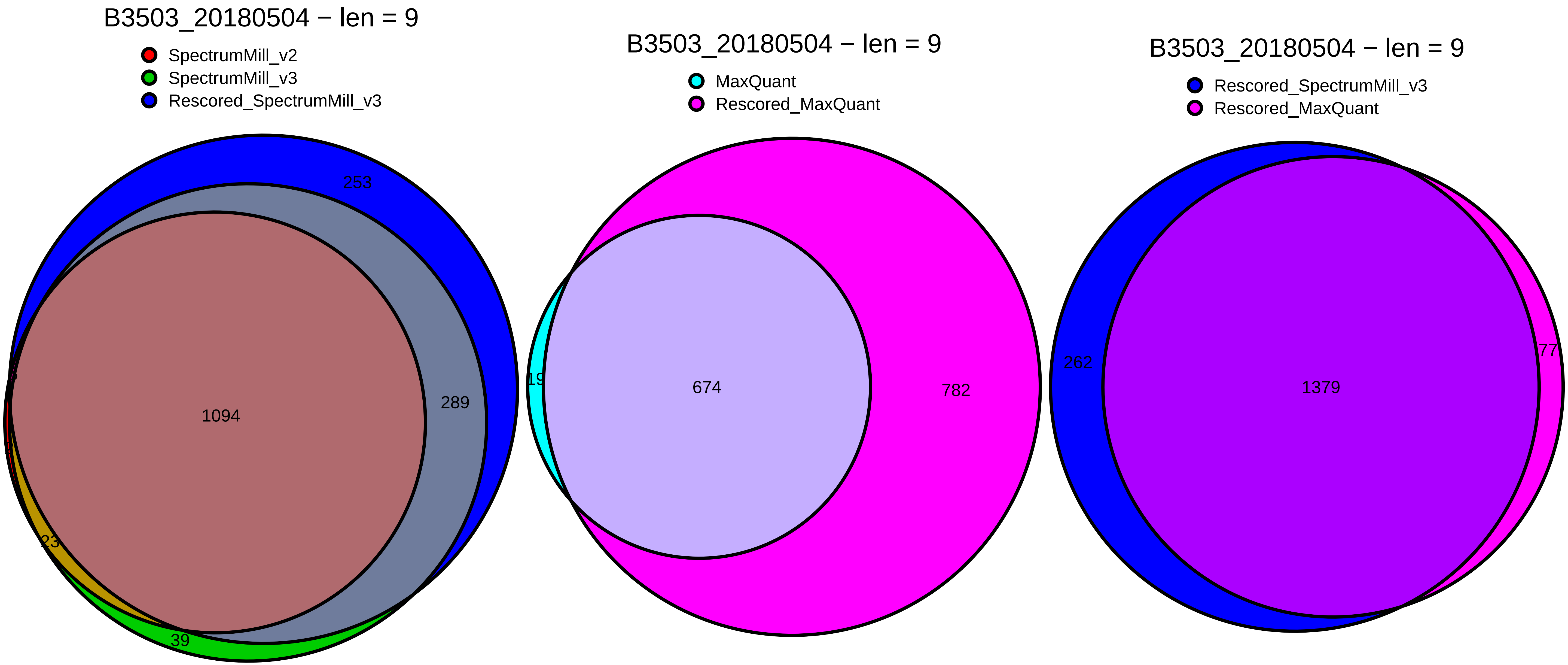

B3503_20180504 − len = 9
SpectrumMill_v2
SpectrumMill_v3
Rescored_SpectrumMill_v3
253
289
1094
23
39
B3503_20180504 − len = 9
MaxQuant
Rescored_MaxQuant
674
782
B3503_20180504 − len = 9
Rescored_SpectrumMill_v3
Rescored_MaxQuant
262
1379
77

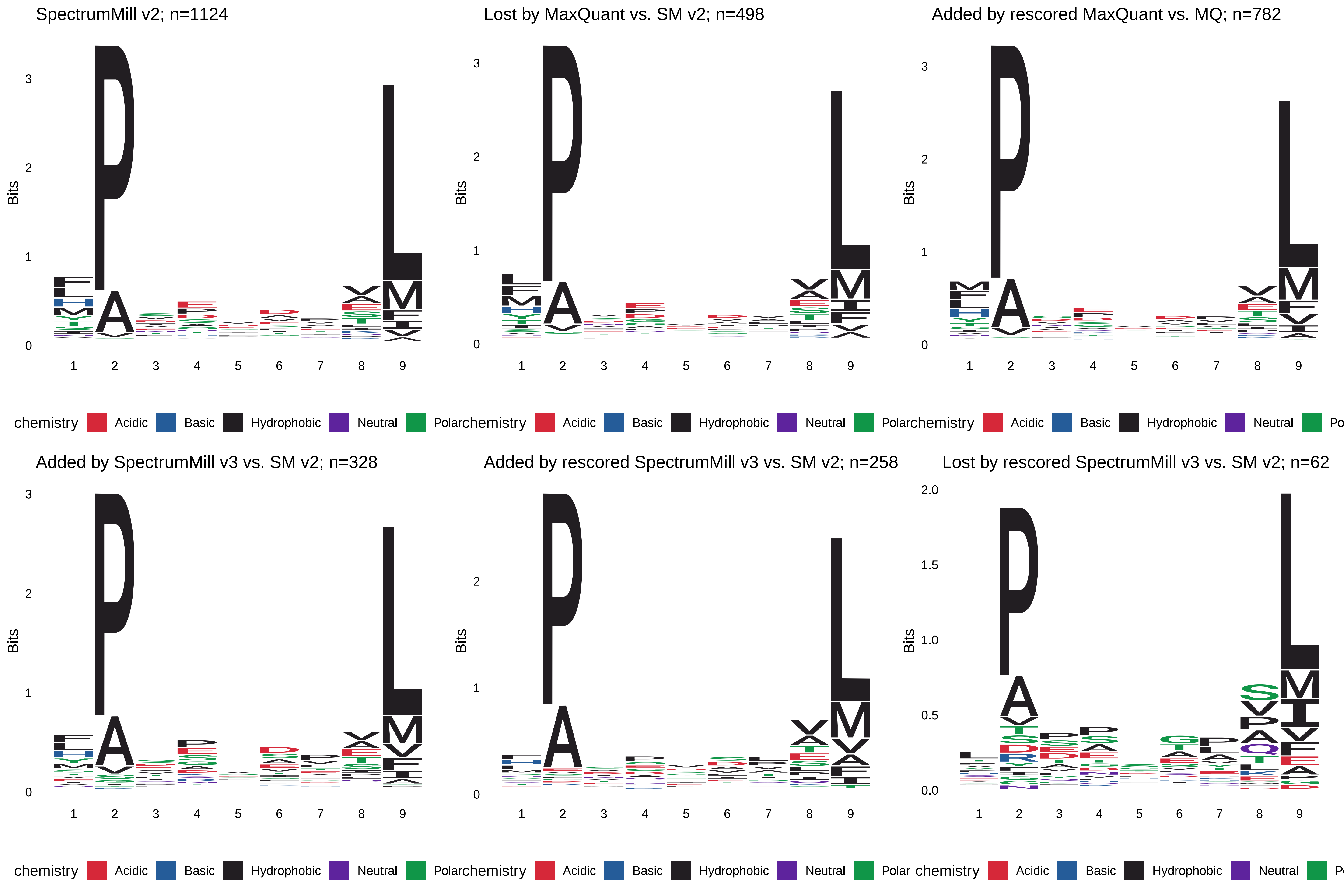
SpectrumMill v2; n=1124
Lost by MaxQuant vs. SM v2; n=498
Added by rescored MaxQuant vs. MQ; n=782
Added by SpectrumMill v3 vs. SM v2; n=328
Added by rescored SpectrumMill v3 vs. SM v2; n=258
Lost by rescored SpectrumMill v3 vs. SM v2; n=62
Bits
0
1
2
3
0.0
0.5
1.0
1.5
2.0
1 2 3 4 5 6 7 8 9
chemistry
Acidic
Basic
Hydrophobic
Neutral
Polar

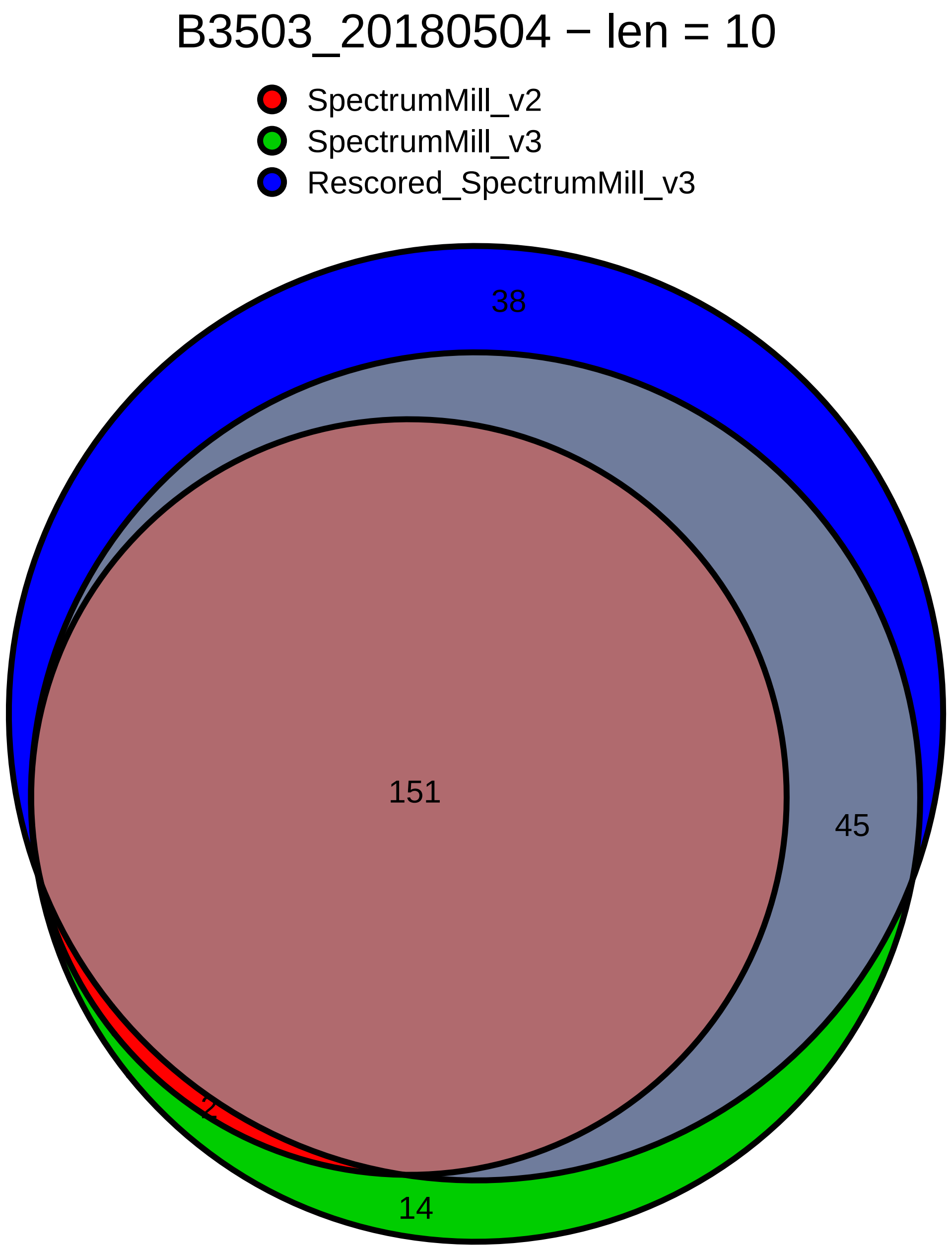
B3503_20180504 − len = 10
SpectrumMill_v2
SpectrumMill_v3
Rescored_SpectrumMill_v3
38
151
45
14

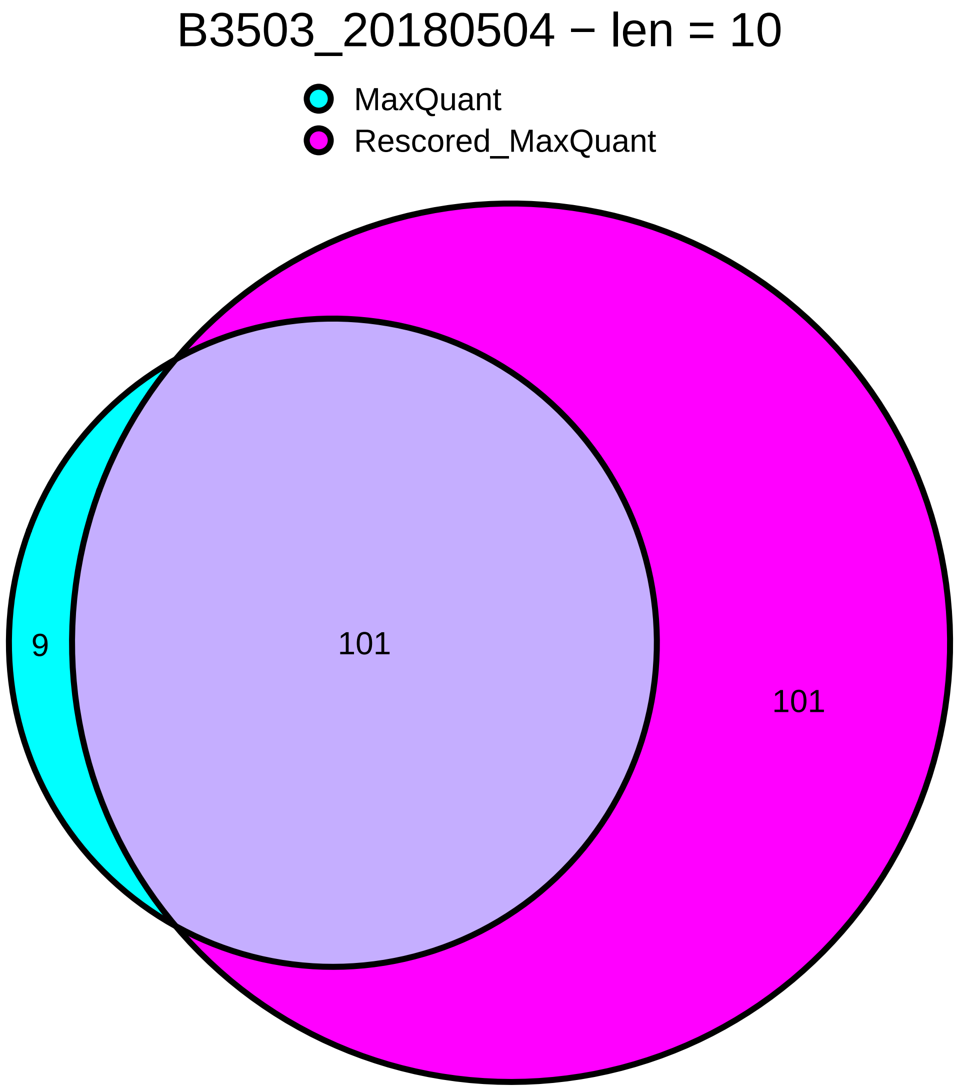
B3503_20180504 − len = 10
MaxQuant
Rescored_MaxQuant
9
101
101

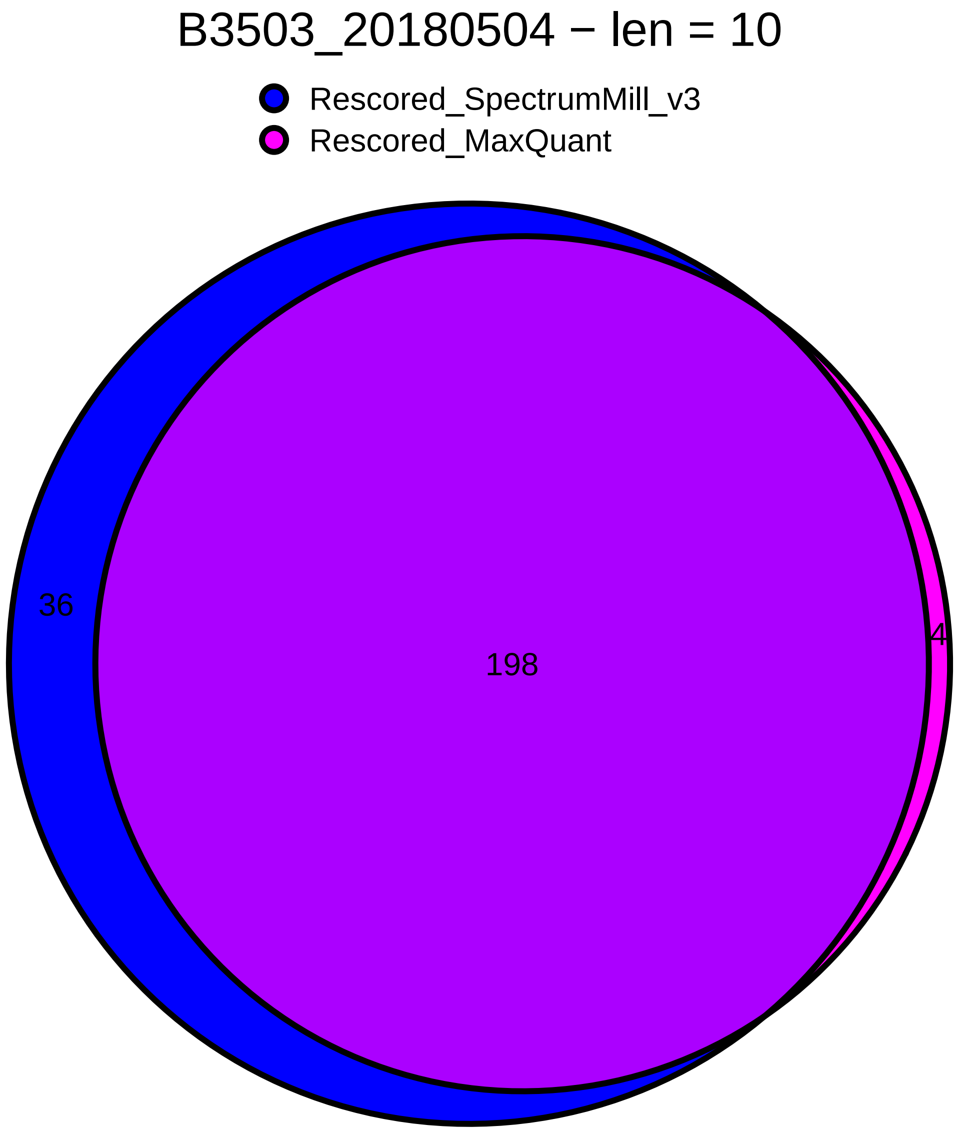
B3503_20180504 − len = 10
Rescored_SpectrumMill_v3
Rescored_MaxQuant
36
198
4

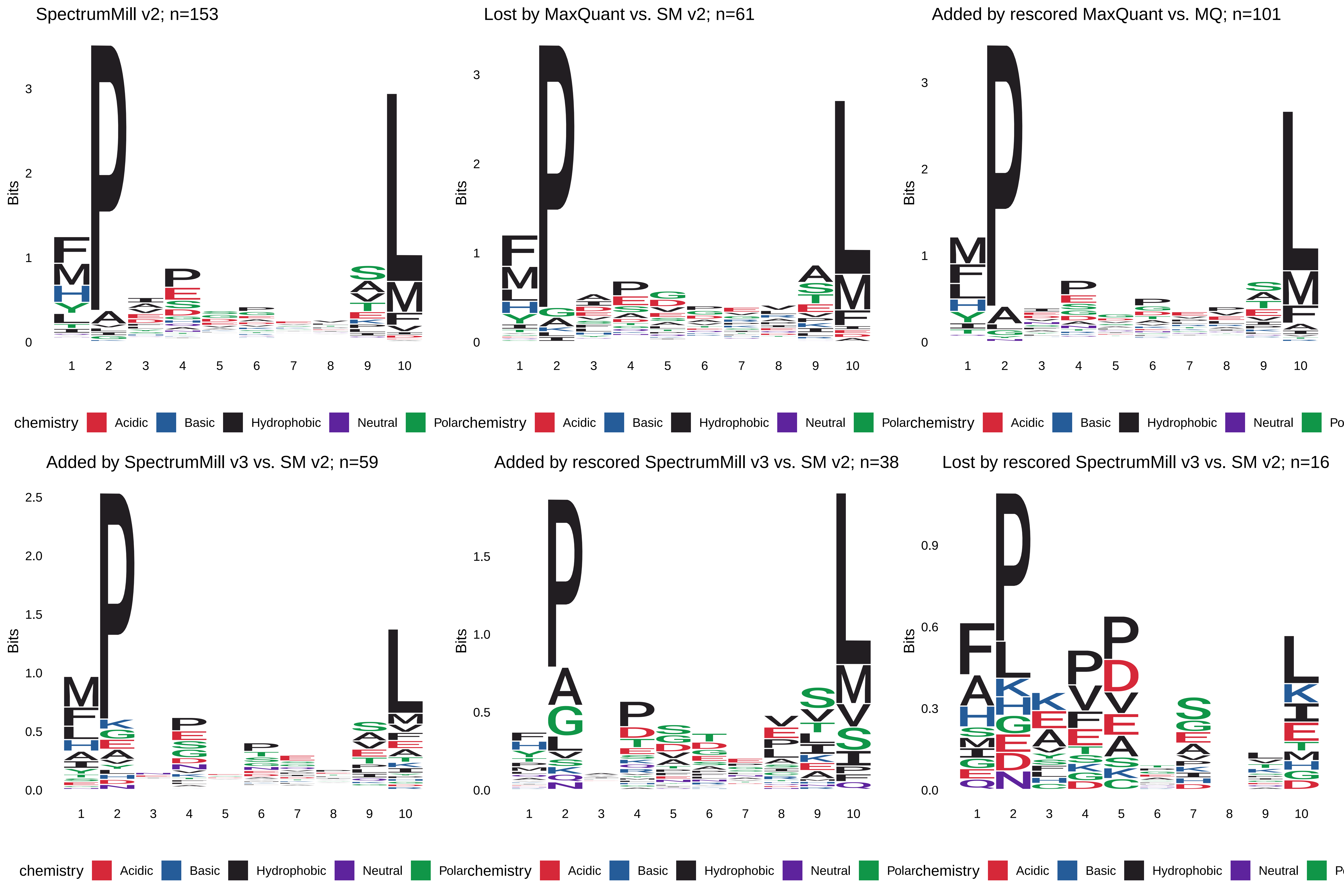
SpectrumMill v2; n=153
Lost by MaxQuant vs. SM v2; n=61
Added by rescored MaxQuant vs. MQ; n=101
Bits
chemistry Acidic Basic Hydrophobic Neutral Polar
Added by SpectrumMill v3 vs. SM v2; n=59
Added by rescored SpectrumMill v3 vs. SM v2; n=38
Lost by rescored SpectrumMill v3 vs. SM v2; n=16

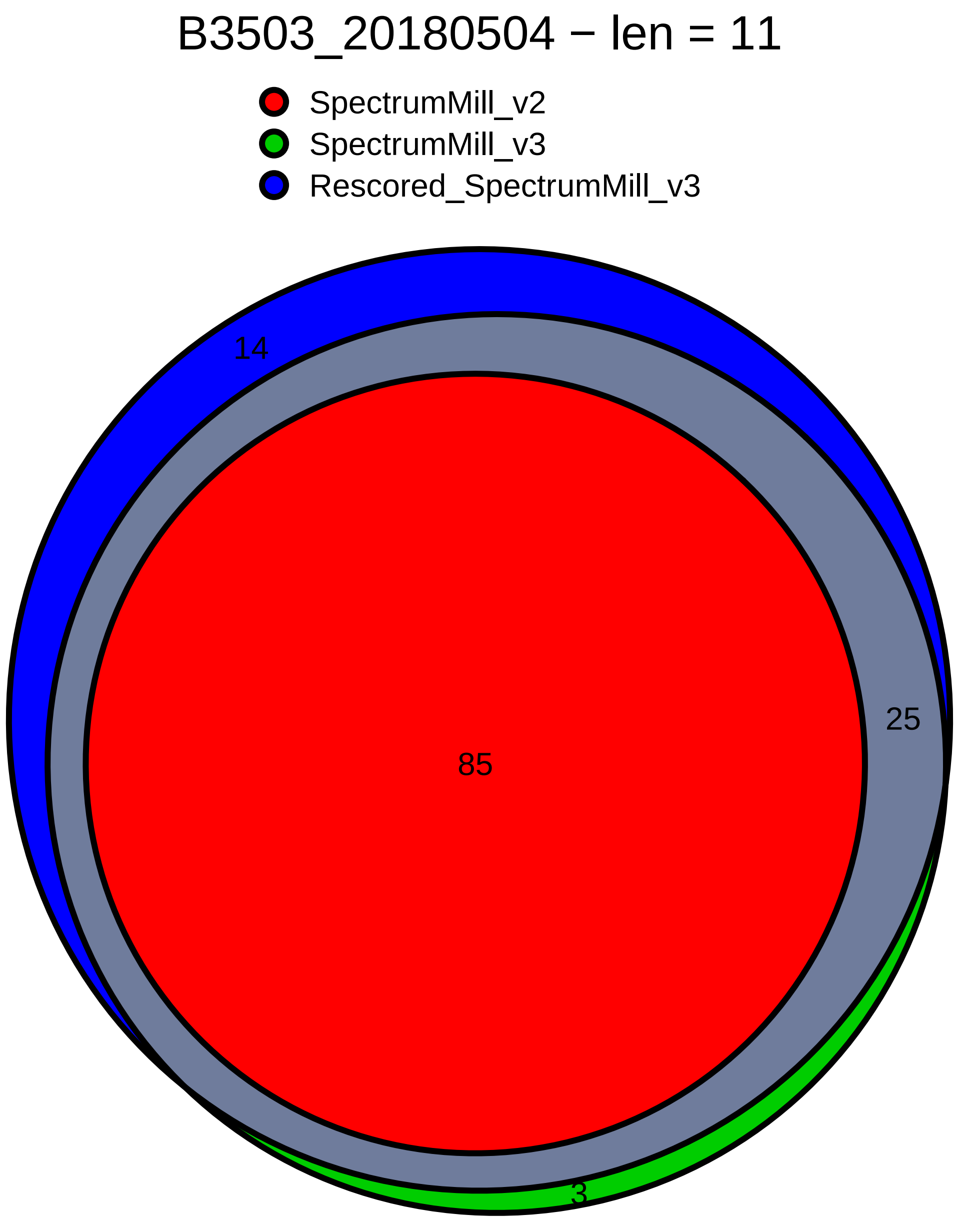
B3503_20180504 − len = 11
SpectrumMill_v2
SpectrumMill_v3
Rescored_SpectrumMill_v3
14
25
85
3
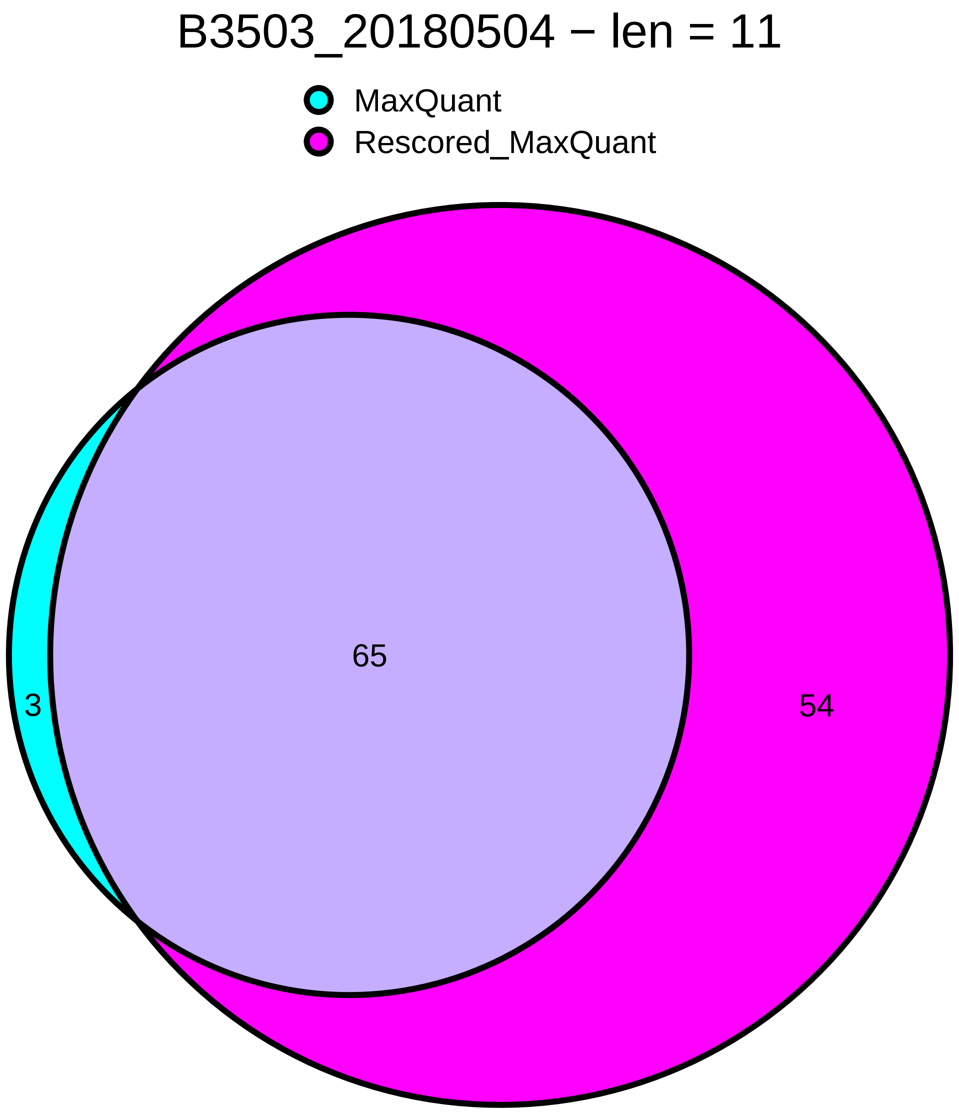
B3503_20180504 − len = 11
MaxQuant
Rescored_MaxQuant
3
65
54
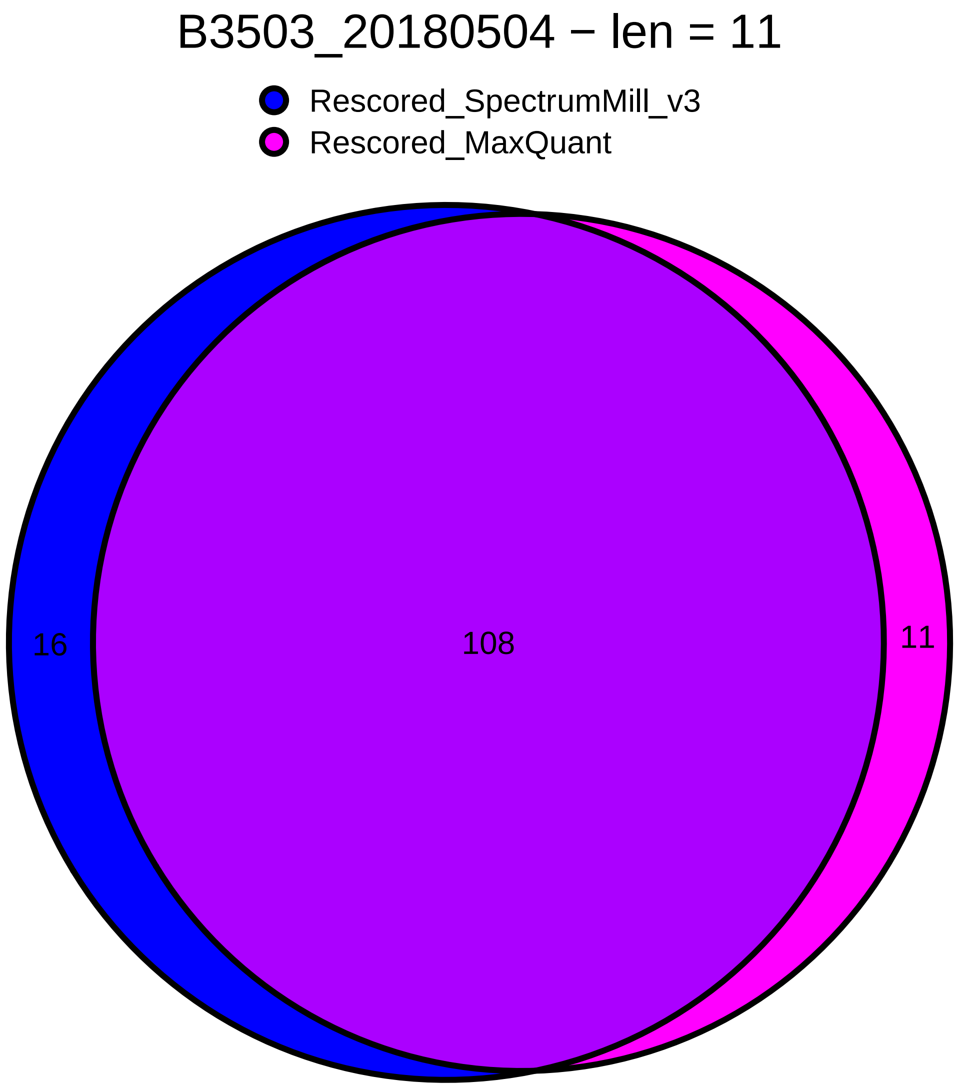
B3503_20180504 − len = 11
Rescored_SpectrumMill_v3
Rescored_MaxQuant
16
108
11

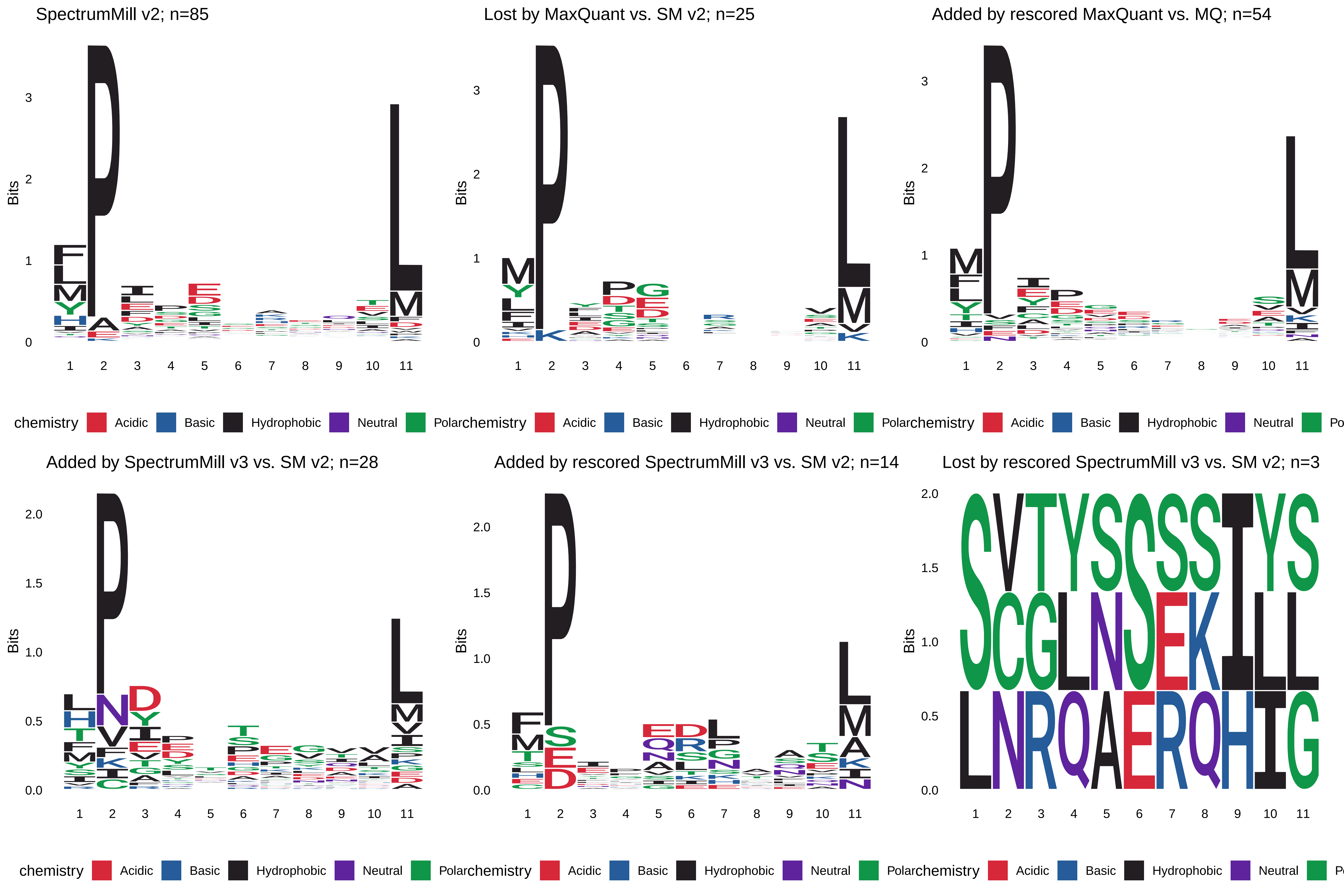

SpectrumMill v2; n=85
Lost by MaxQuant vs. SM v2; n=25
Added by rescored MaxQuant vs. MQ; n=54
Added by SpectrumMill v3 vs. SM v2; n=28
Added by rescored SpectrumMill v3 vs. SM v2; n=14
Lost by rescored SpectrumMill v3 vs. SM v2; n=3
Bits
chemistry
Acidic
Basic
Hydrophobic
Neutral
Polar

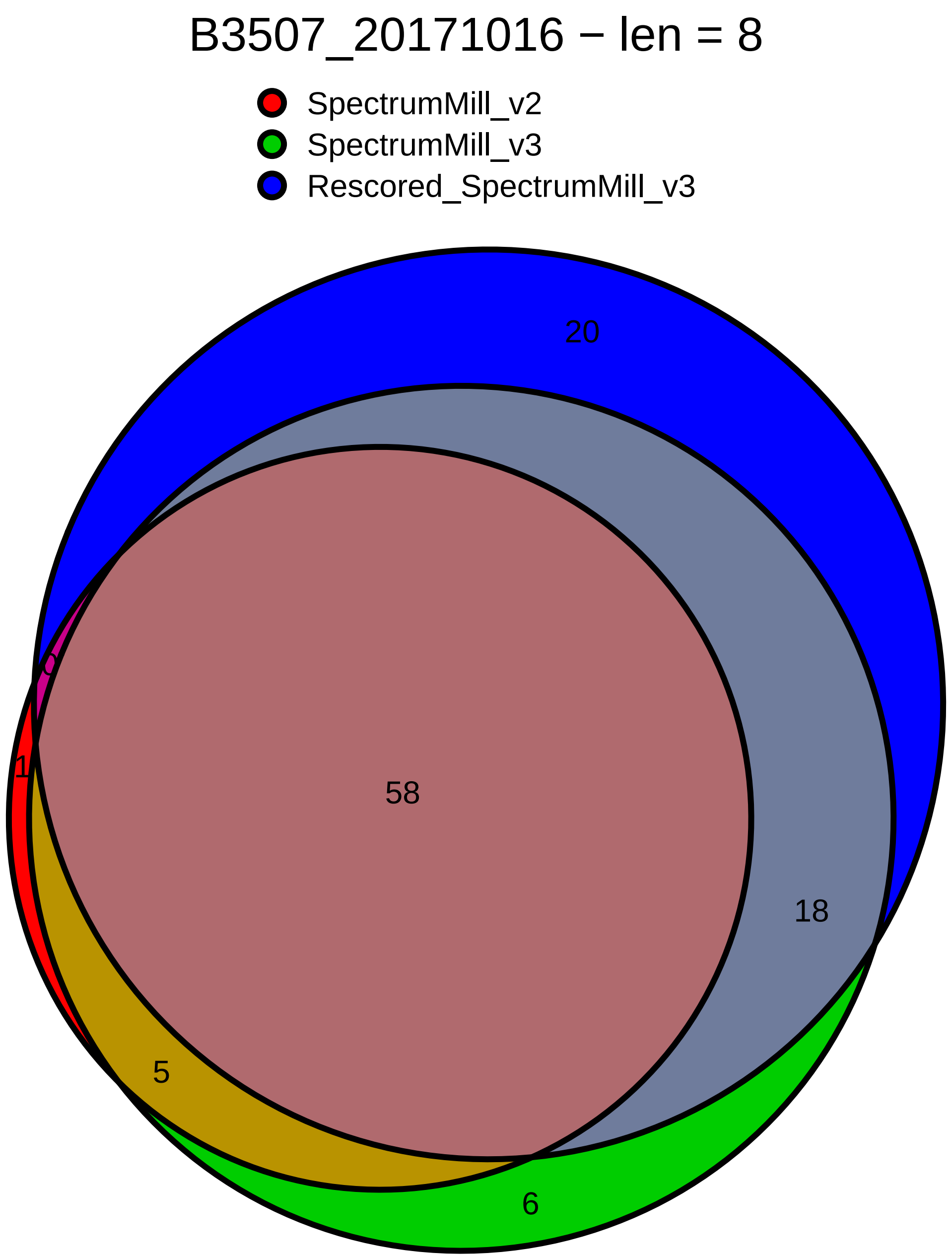
B3507_20171016 − len = 8
SpectrumMill_v2
SpectrumMill_v3
Rescored_SpectrumMill_v3
20
0
1
58
18
5
6

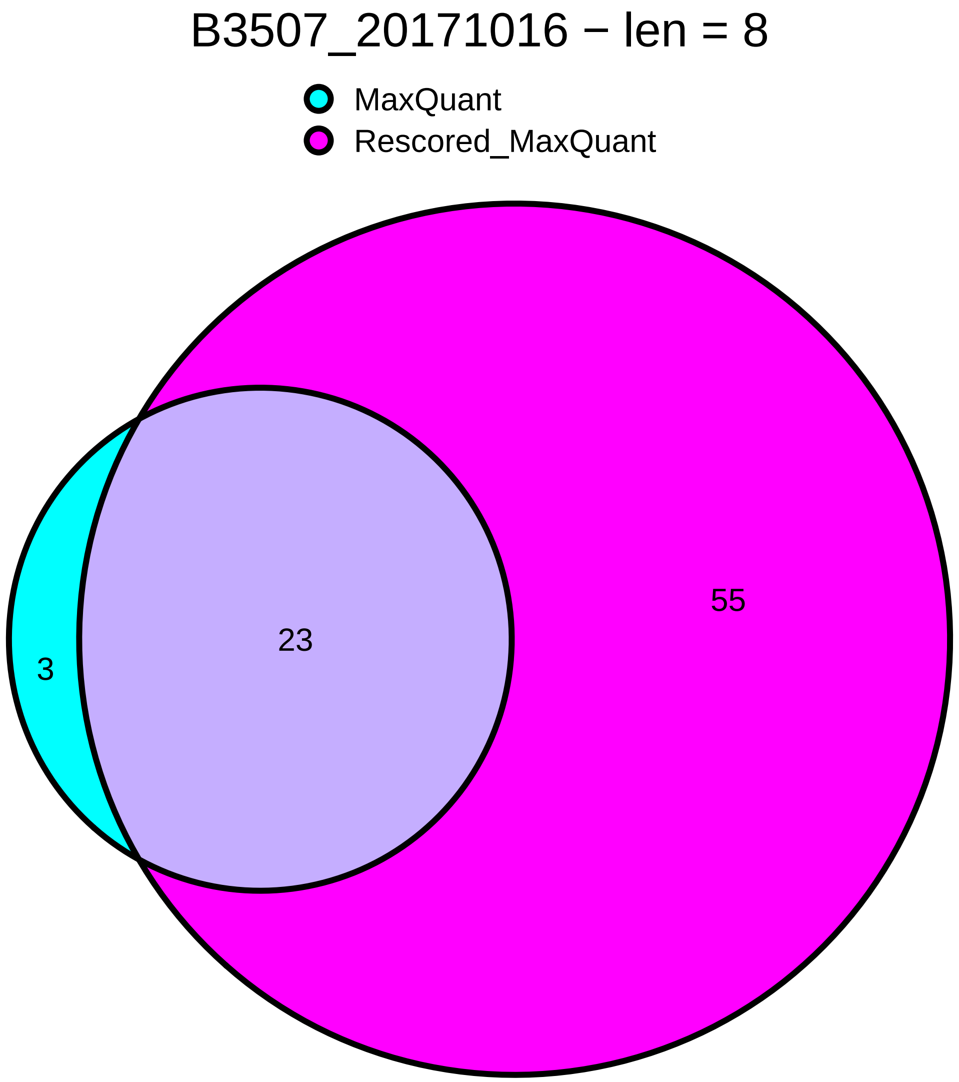
B3507_20171016 − len = 8
MaxQuant
Rescored_MaxQuant
3
23
55

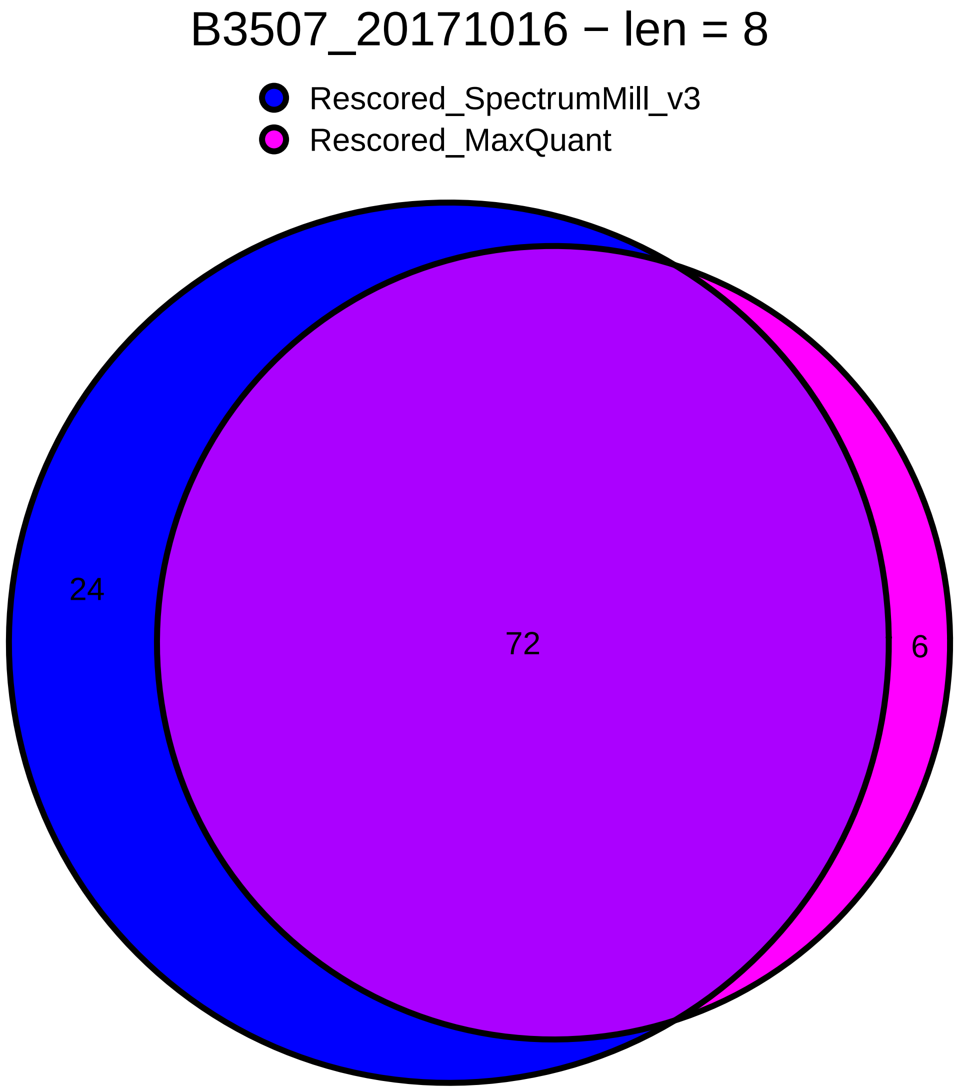
B3507_20171016 − len = 8
Rescored_SpectrumMill_v3
Rescored_MaxQuant
24
72
6

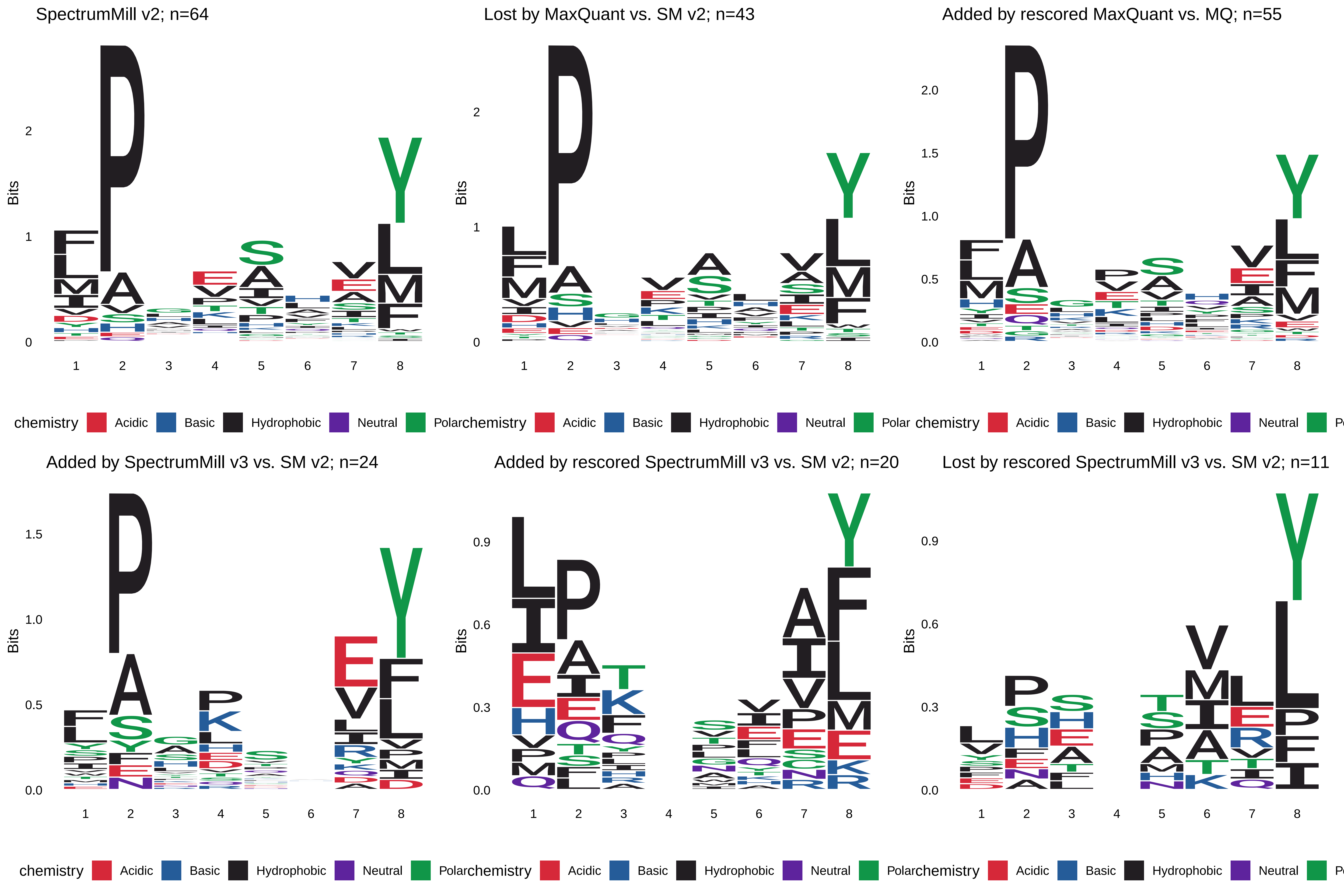
SpectrumMill v2; n=64
Lost by MaxQuant vs. SM v2; n=43
Added by rescored MaxQuant vs. MQ; n=55
Added by SpectrumMill v3 vs. SM v2; n=24
Added by rescored SpectrumMill v3 vs. SM v2; n=20
Lost by rescored SpectrumMill v3 vs. SM v2; n=11
Bits
1 2 3 4 5 6 7 8
chemistry
Acidic
Basic
Hydrophobic
Neutral
Polar

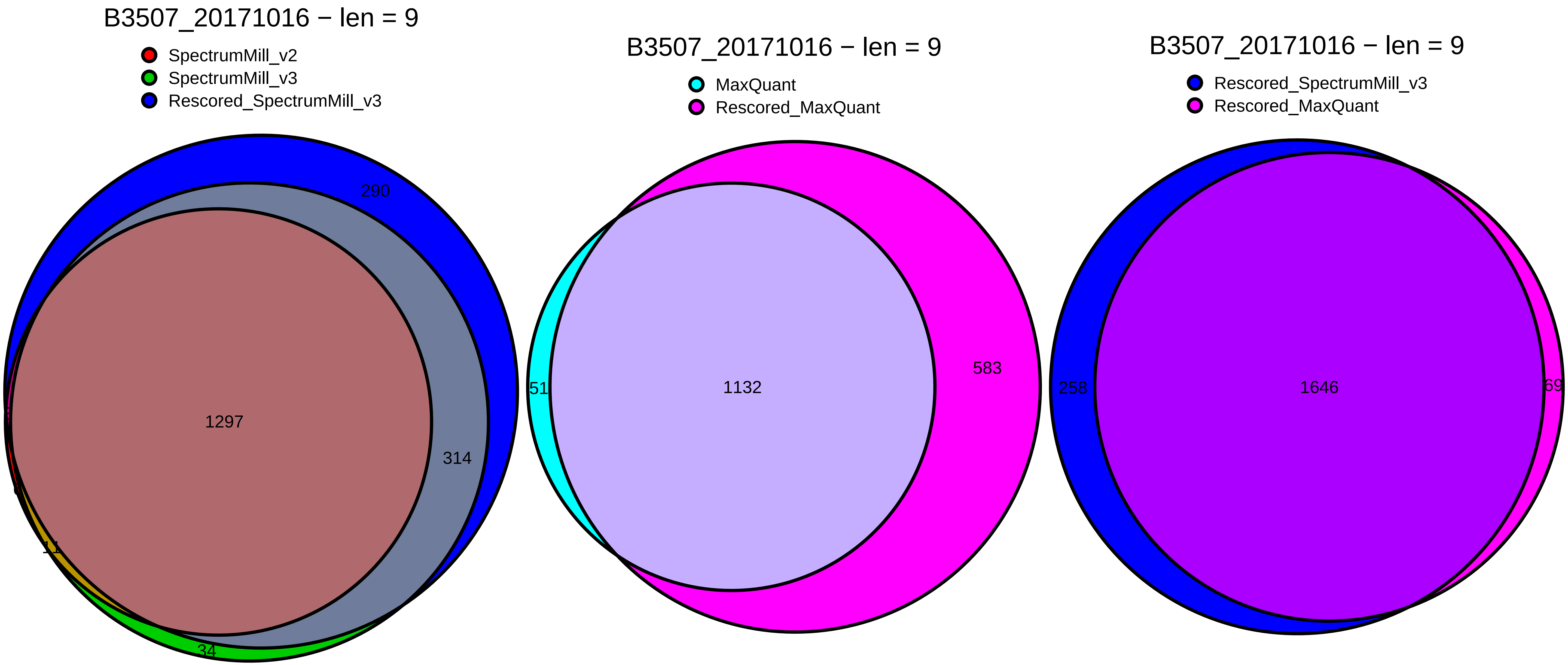

B3507_20171016 − len = 9
SpectrumMill_v2
SpectrumMill_v3
Rescored_SpectrumMill_v3
290
1297
314
34
B3507_20171016 − len = 9
MaxQuant
Rescored_MaxQuant
51
1132
583
B3507_20171016 − len = 9
Rescored_SpectrumMill_v3
Rescored_MaxQuant
258
1646
69

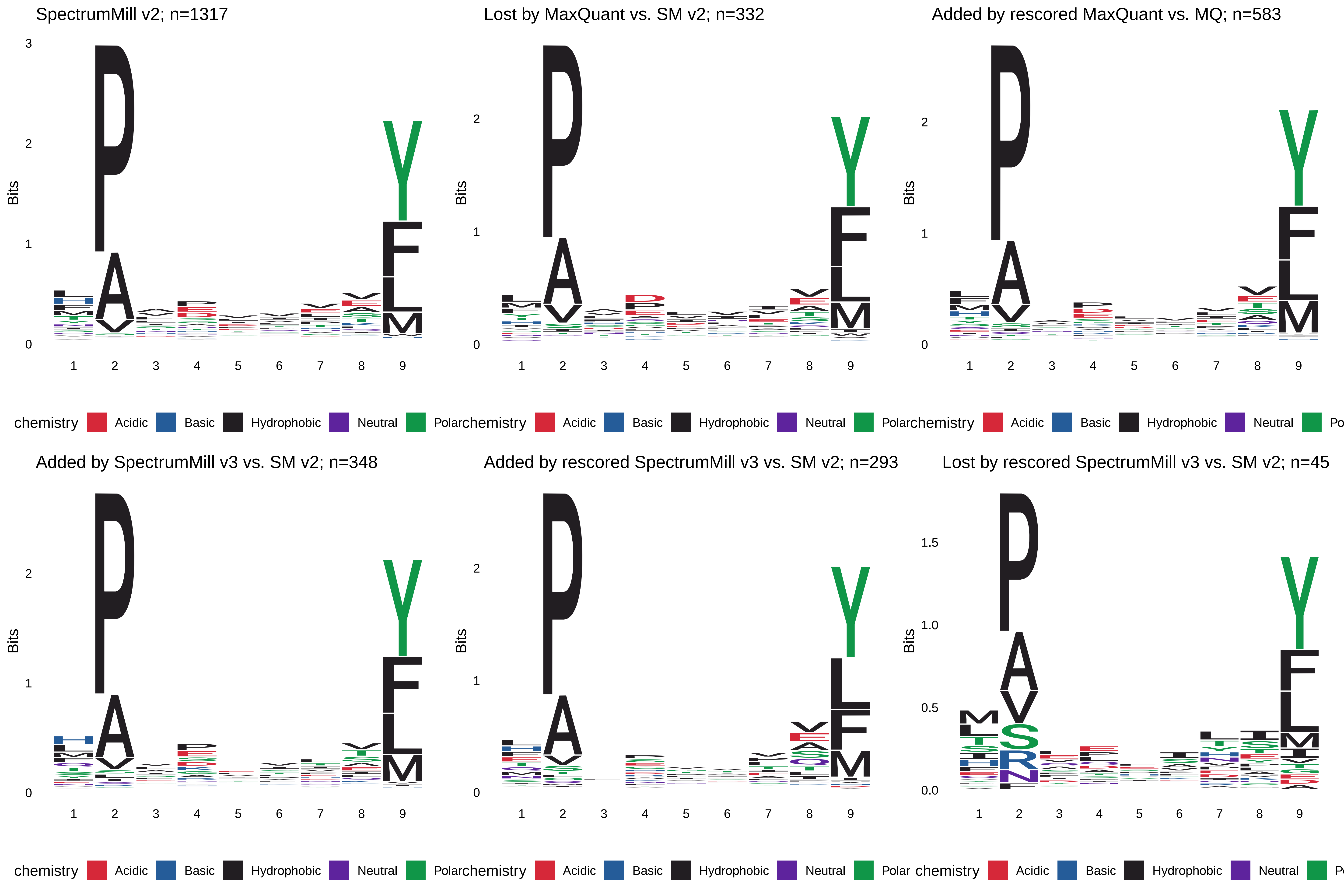
SpectrumMill v2; n=1317
Lost by MaxQuant vs. SM v2; n=332
Added by rescored MaxQuant vs. MQ; n=583
Added by SpectrumMill v3 vs. SM v2; n=348
Added by rescored SpectrumMill v3 vs. SM v2; n=293
Lost by rescored SpectrumMill v3 vs. SM v2; n=45
Bits
1 2 3 4 5 6 7 8 9
chemistry
Acidic
Basic
Hydrophobic
Neutral
Polar

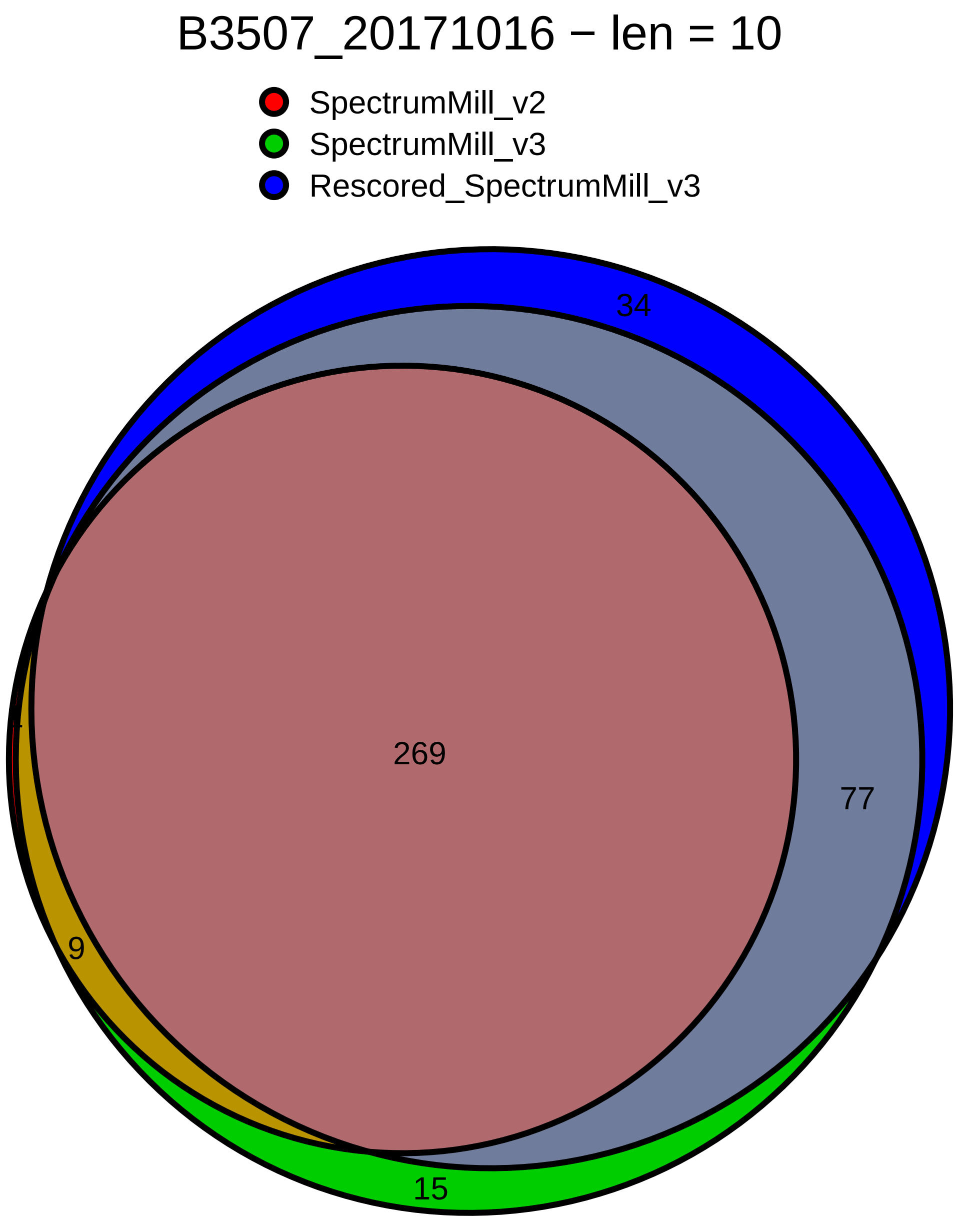
B3507_20171016 − len = 10
SpectrumMill_v2
SpectrumMill_v3
Rescored_SpectrumMill_v3
34
269
77
9
15

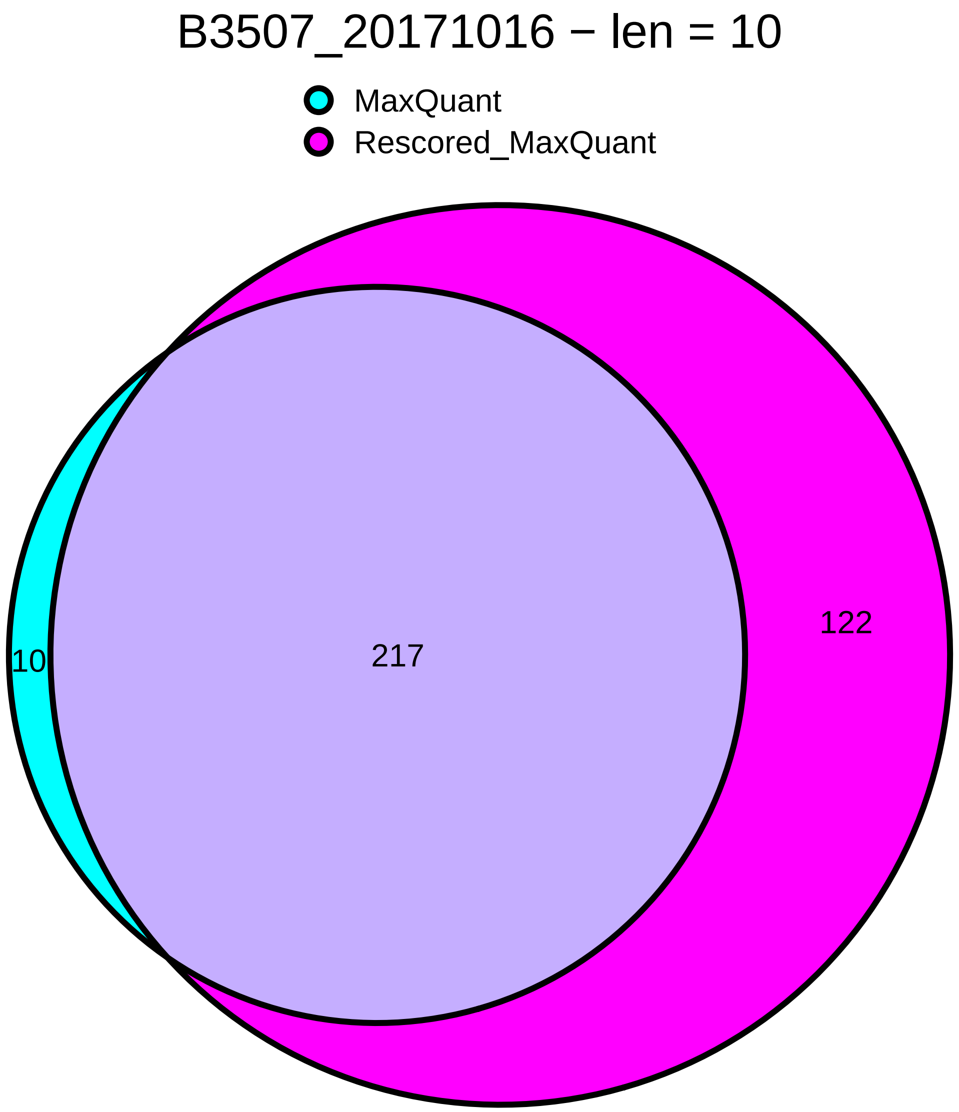
B3507_20171016 − len = 10
MaxQuant
Rescored_MaxQuant
10
217
122

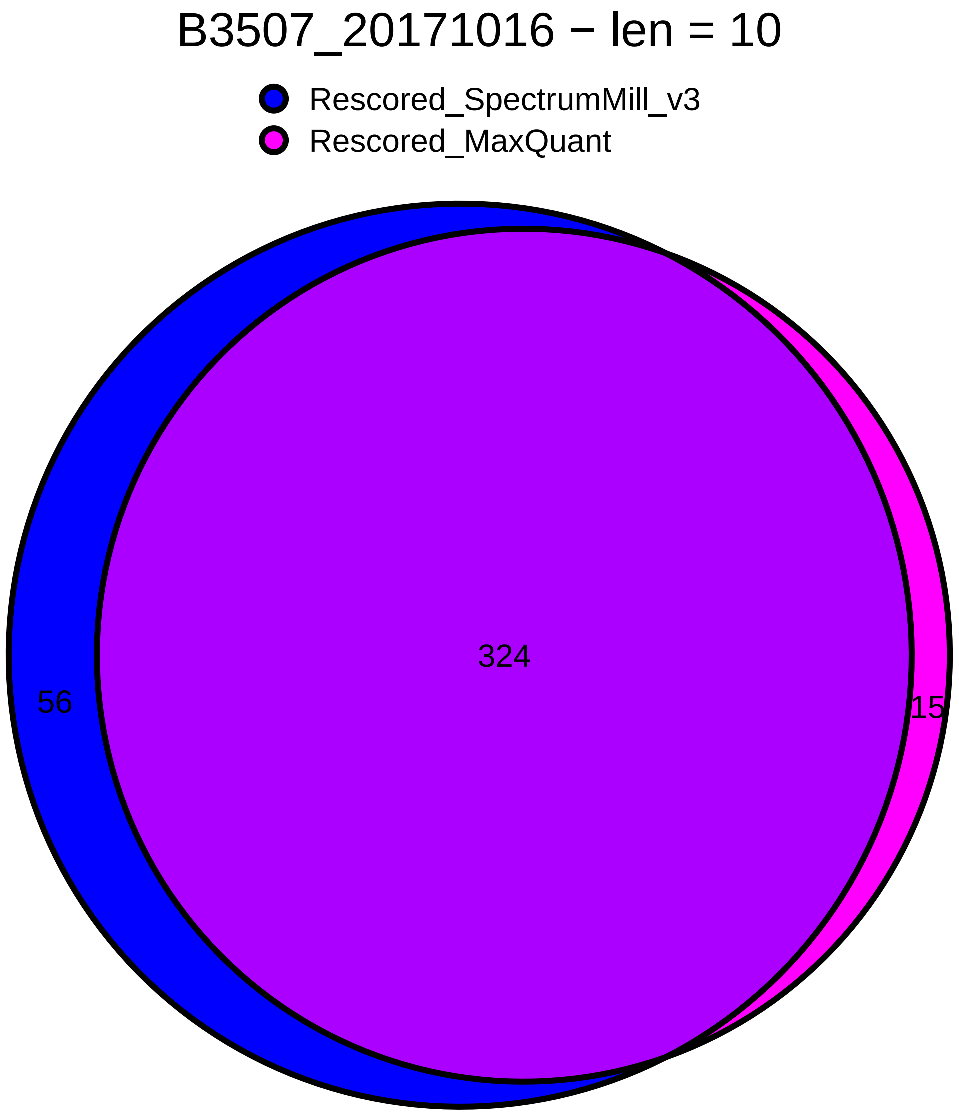
B3507_20171016 − len = 10
Rescored_SpectrumMill_v3
Rescored_MaxQuant
56
324
15

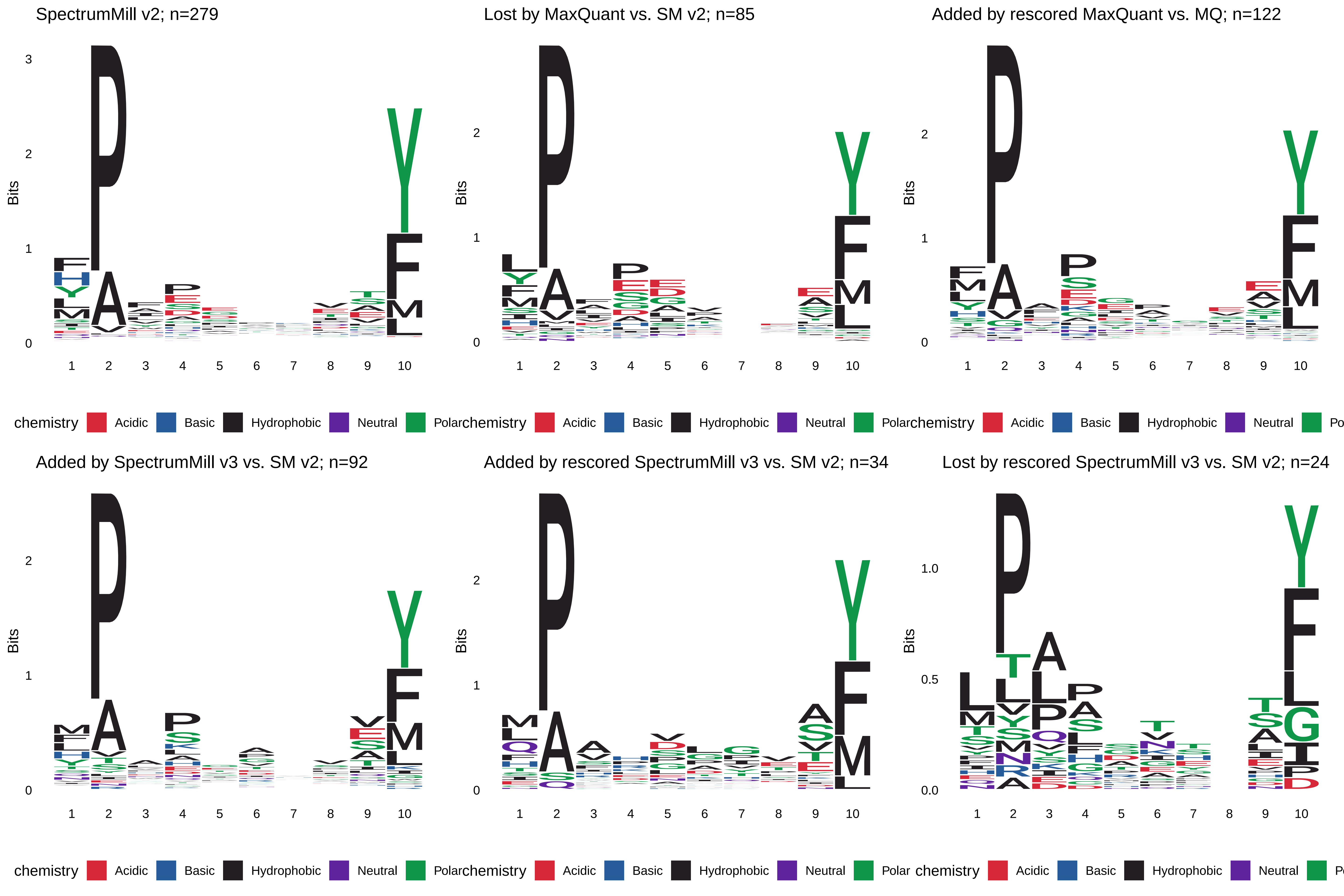

SpectrumMill v2; n=279
Lost by MaxQuant vs. SM v2; n=85
Added by rescored MaxQuant vs. MQ; n=122
Bits
0
1
2
3
1 2 3 4 5 6 7 8 9 10
chemistry Acidic Basic Hydrophobic Neutral Polar
Added by SpectrumMill v3 vs. SM v2; n=92
Added by rescored SpectrumMill v3 vs. SM v2; n=34
Lost by rescored SpectrumMill v3 vs. SM v2; n=24
0.0
0.5
1.0

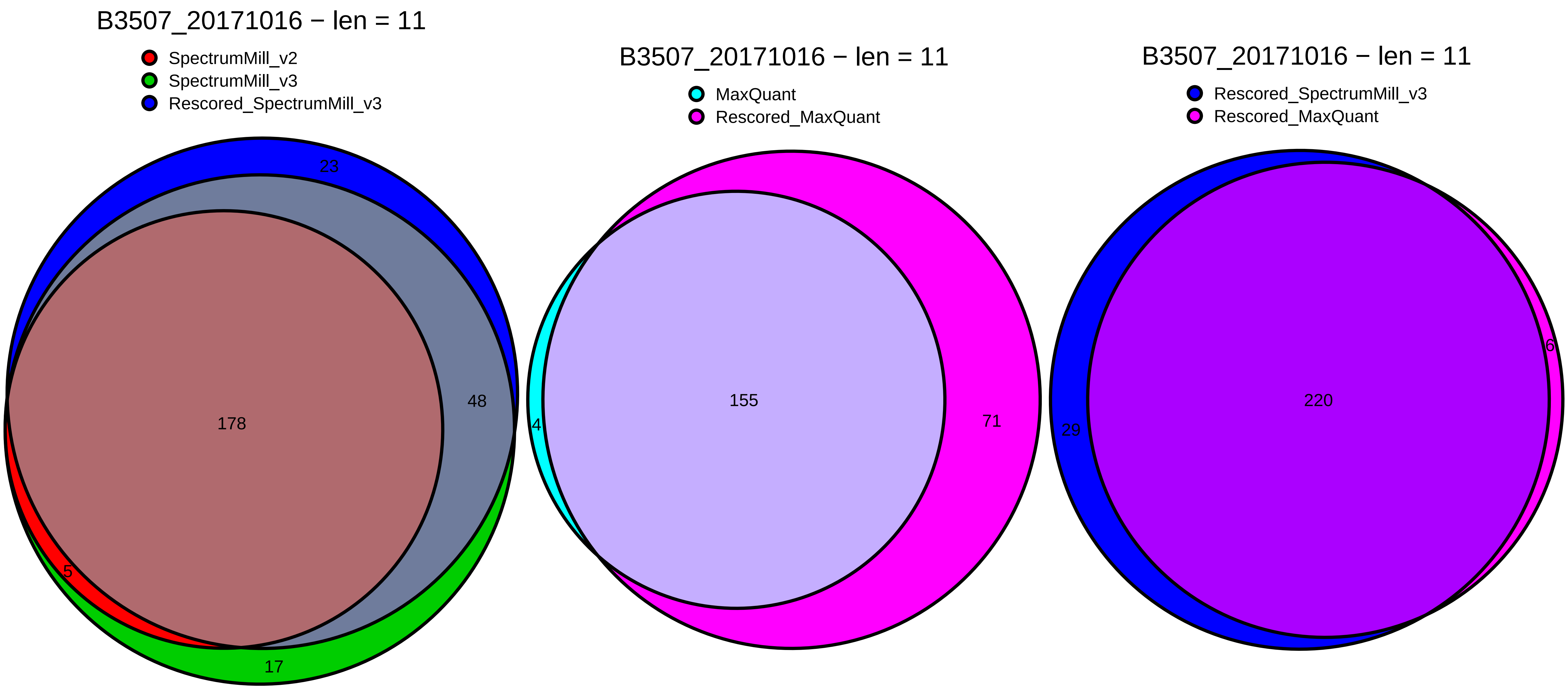

B3507_20171016 − len = 11
SpectrumMill_v2
SpectrumMill_v3
Rescored_SpectrumMill_v3
23
48
178
5
17
B3507_20171016 − len = 11
MaxQuant
Rescored_MaxQuant
4
155
71
B3507_20171016 − len = 11
Rescored_SpectrumMill_v3
Rescored_MaxQuant
6
220
29

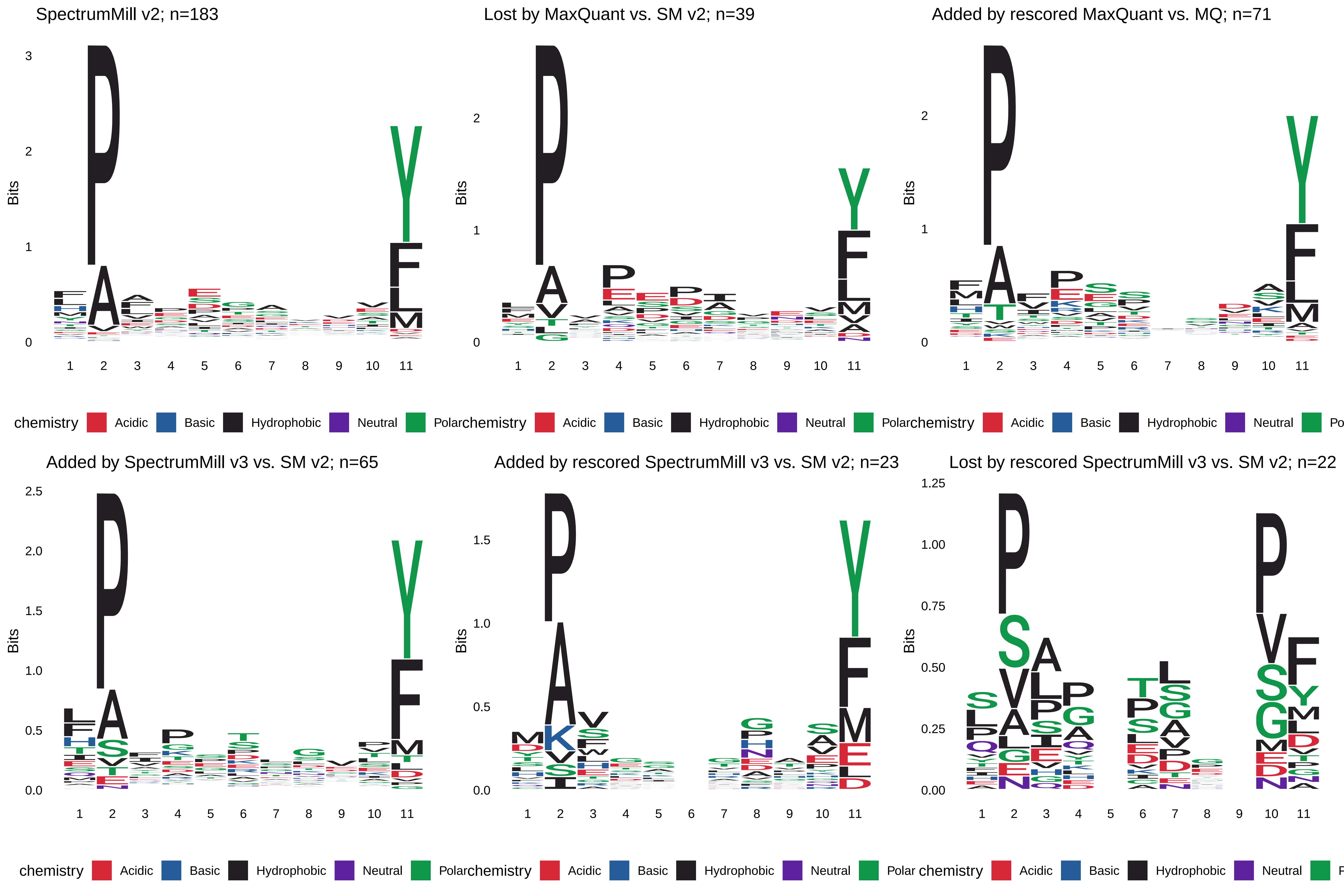
SpectrumMill v2; n=183
Lost by MaxQuant vs. SM v2; n=39
Added by rescored MaxQuant vs. MQ; n=71
Bits
1 2 3 4 5 6 7 8 9 10 11
chemistry Acidic Basic Hydrophobic Neutral Polarchemistry Acidic Basic Hydrophobic Neutral Polarchemistry Acidic Basic Hydrophobic Neutral Po
Added by SpectrumMill v3 vs. SM v2; n=65
Added by rescored SpectrumMill v3 vs. SM v2; n=23
Lost by rescored SpectrumMill v3 vs. SM v2; n=22
Bits
1 2 3 4 5 6 7 8 9 10 11
chemistry Acidic Basic Hydrophobic Neutral Polarchemistry Acidic Basic Hydrophobic Neutral Polar chemistry Acidic Basic Hydrophobic Neutral P

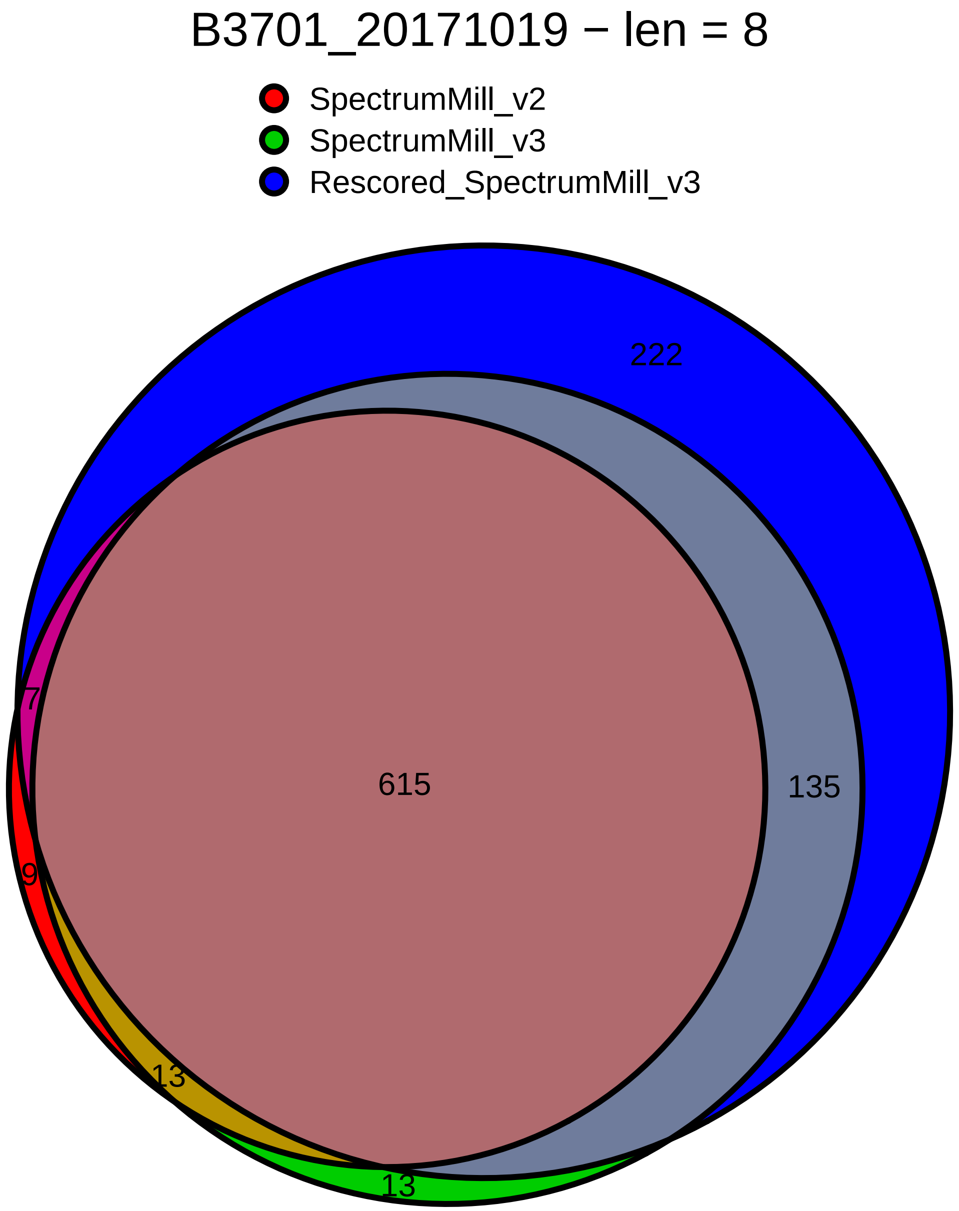
B3701_20171019 − len = 8
SpectrumMill_v2
SpectrumMill_v3
Rescored_SpectrumMill_v3
222
7
615
135
9
13
13

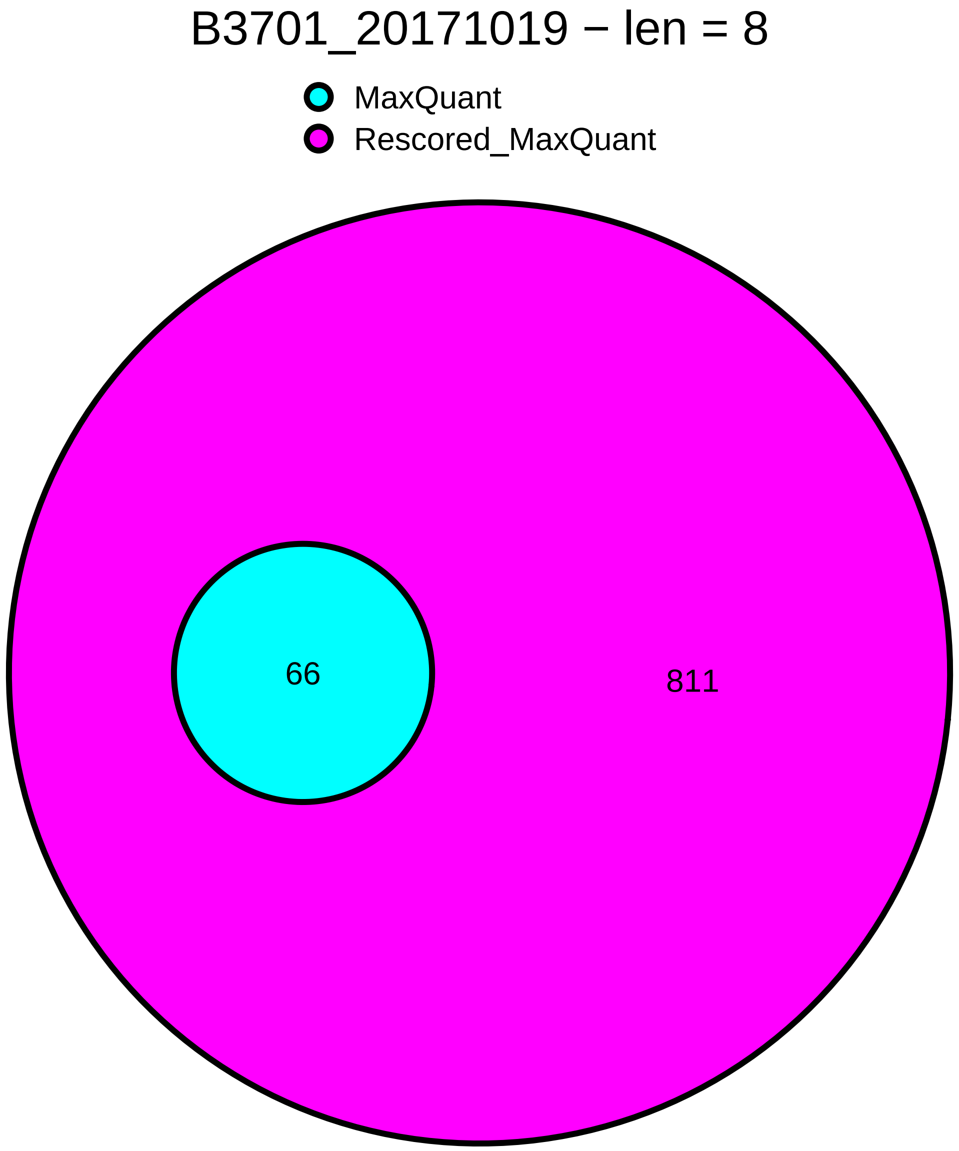
B3701_20171019 − len = 8
MaxQuant
Rescored_MaxQuant
66
811

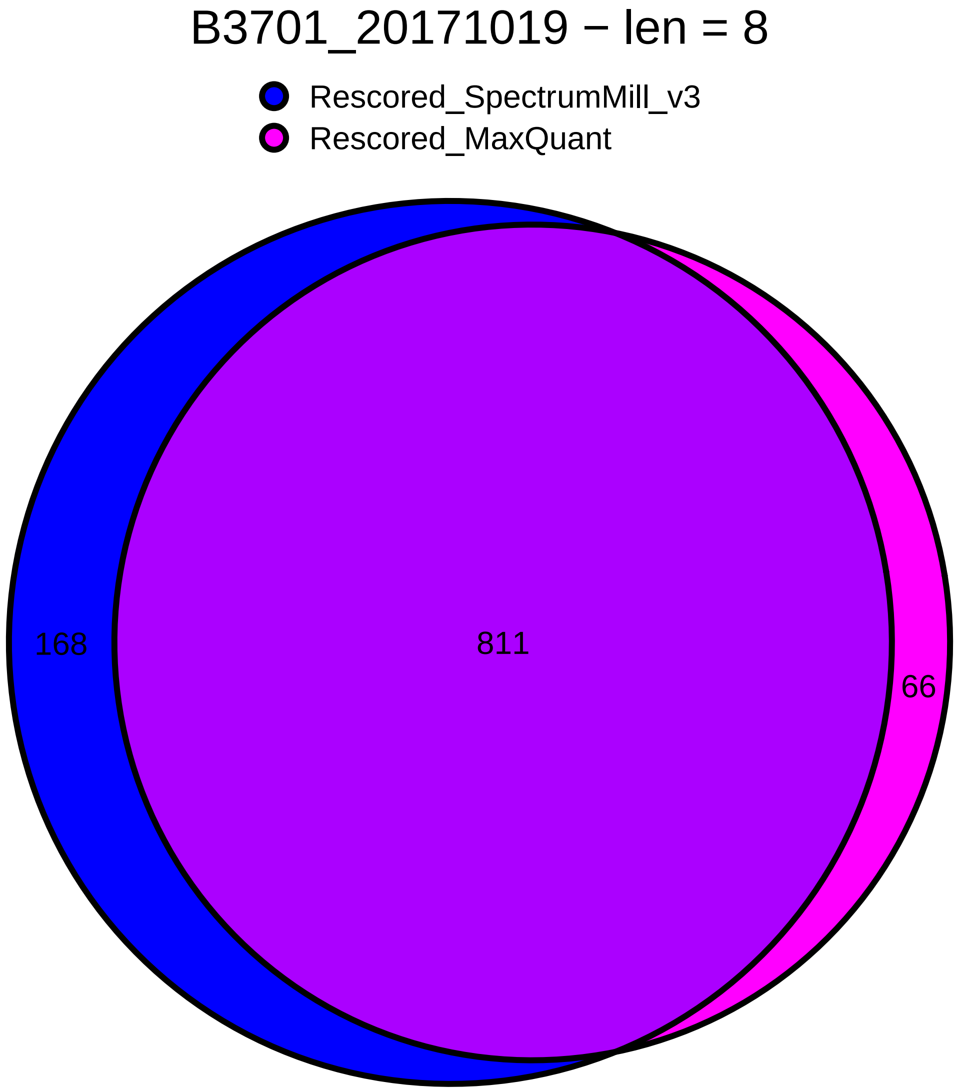
B3701_20171019 − len = 8
Rescored_SpectrumMill_v3
Rescored_MaxQuant
168
811
66

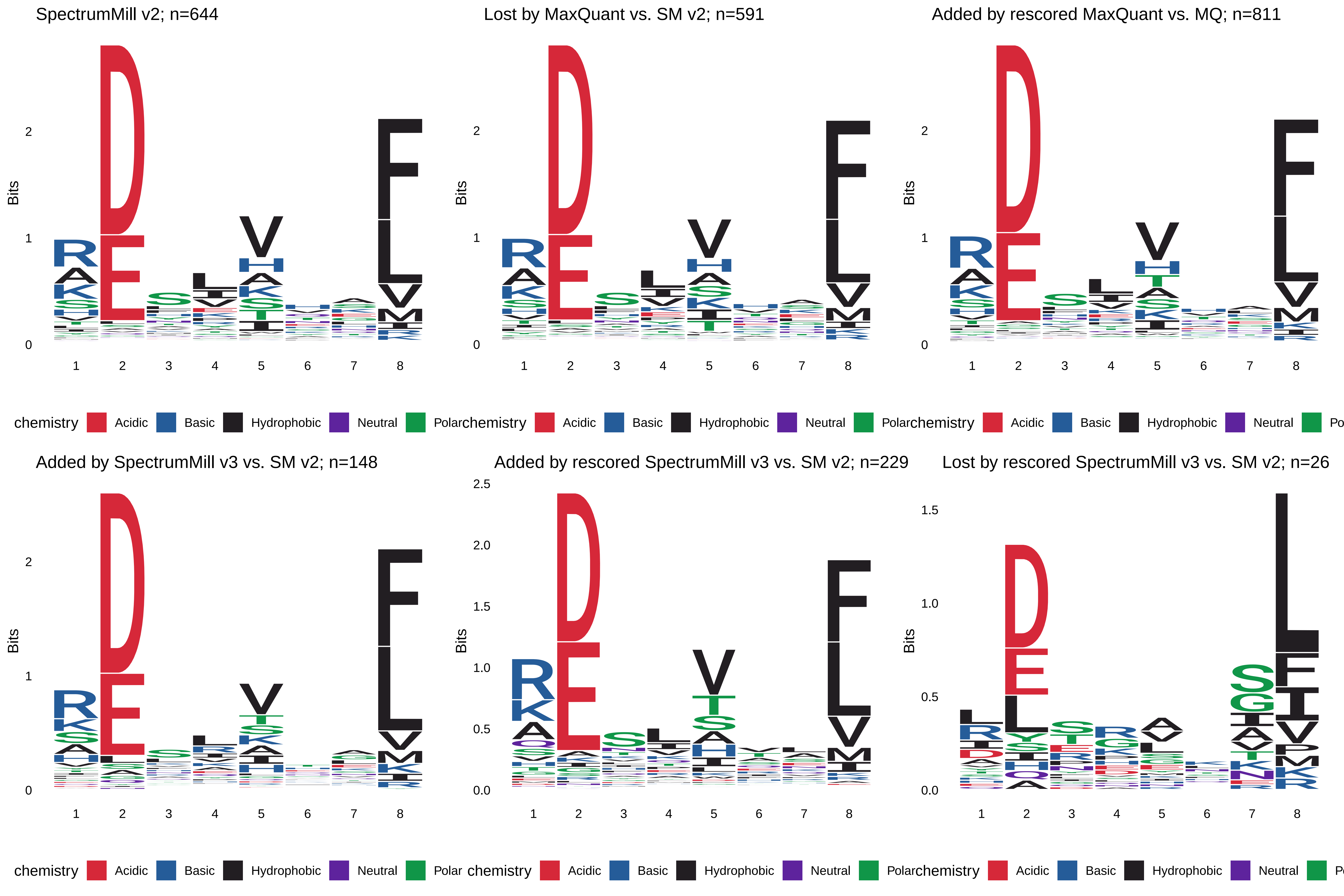
SpectrumMill v2; n=644
Lost by MaxQuant vs. SM v2; n=591
Added by rescored MaxQuant vs. MQ; n=811
Added by SpectrumMill v3 vs. SM v2; n=148
Added by rescored SpectrumMill v3 vs. SM v2; n=229
Lost by rescored SpectrumMill v3 vs. SM v2; n=26
Bits
0
0.0
0.5
1
1.0
1.5
2
2.0
2.5
1 2 3 4 5 6 7 8
chemistry
Acidic
Basic
Hydrophobic
Neutral
Polar

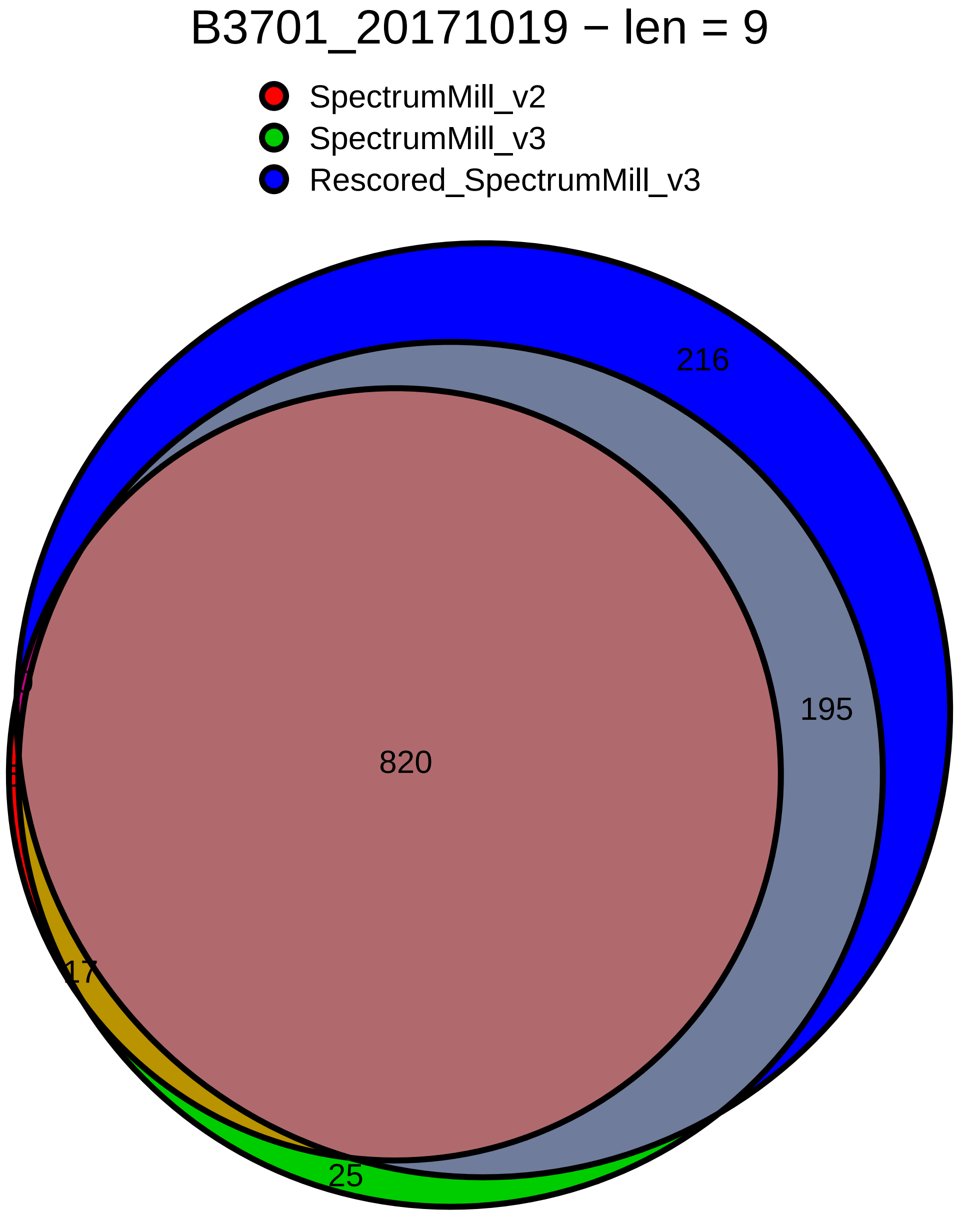
B3701_20171019 − len = 9
SpectrumMill_v2
SpectrumMill_v3
Rescored_SpectrumMill_v3
216
195
820
17
25

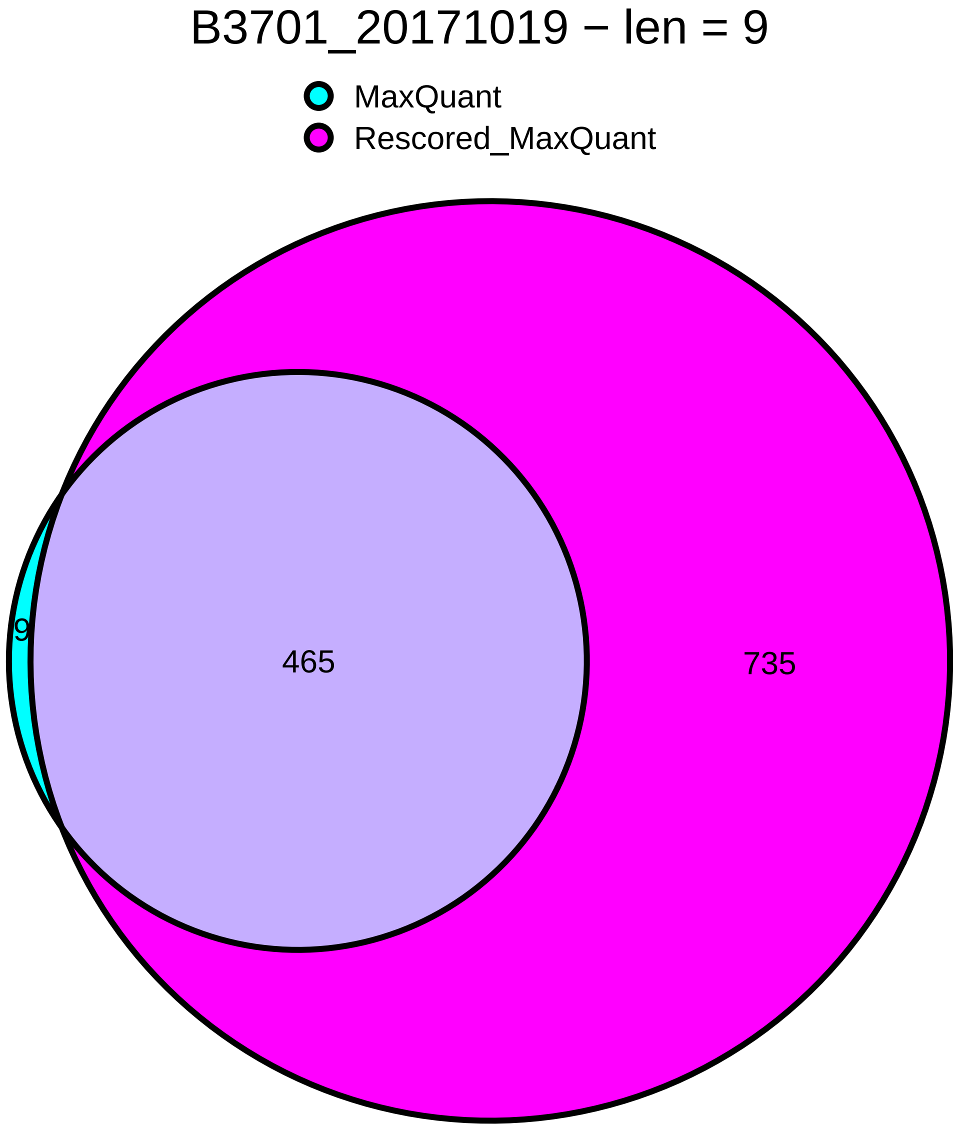
B3701_20171019 − len = 9
MaxQuant
Rescored_MaxQuant
9
465
735

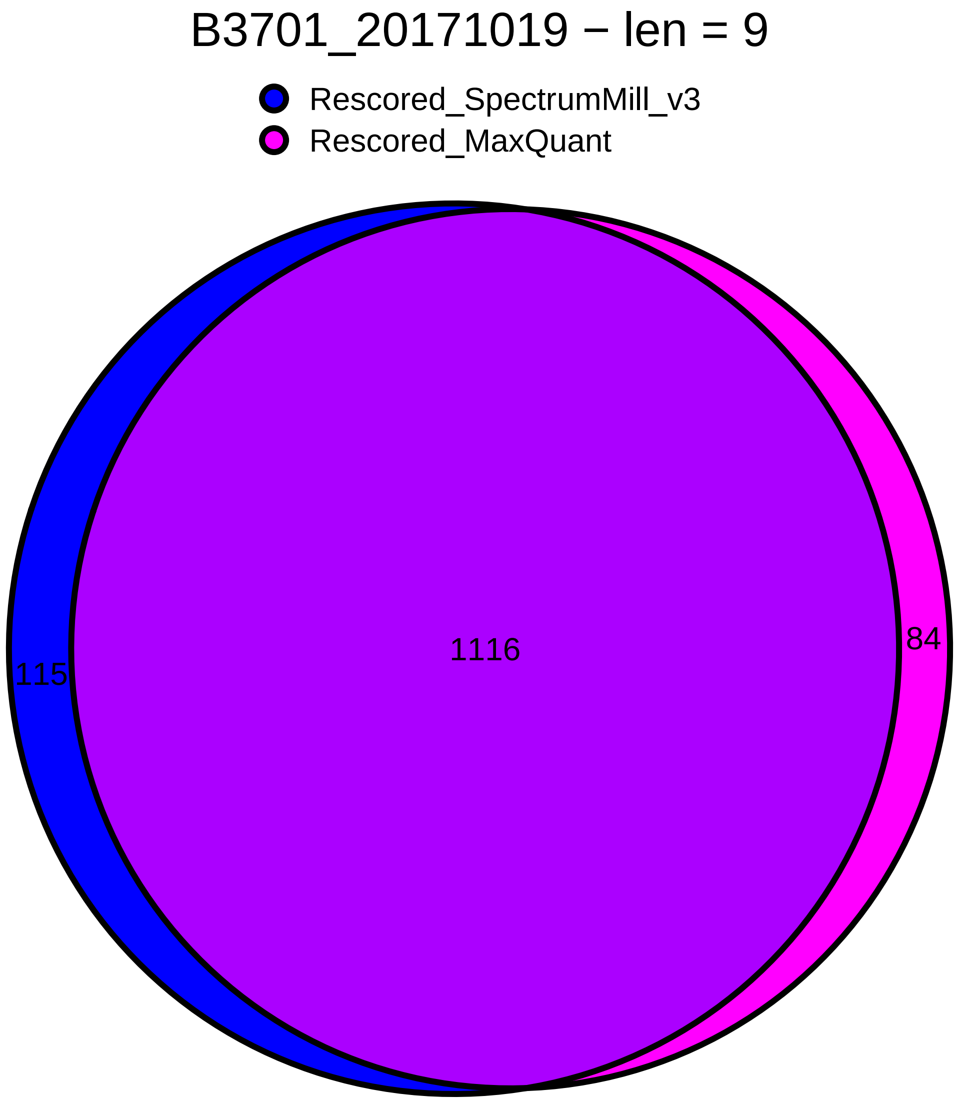
B3701_20171019 − len = 9
Rescored_SpectrumMill_v3
Rescored_MaxQuant
115
1116
84

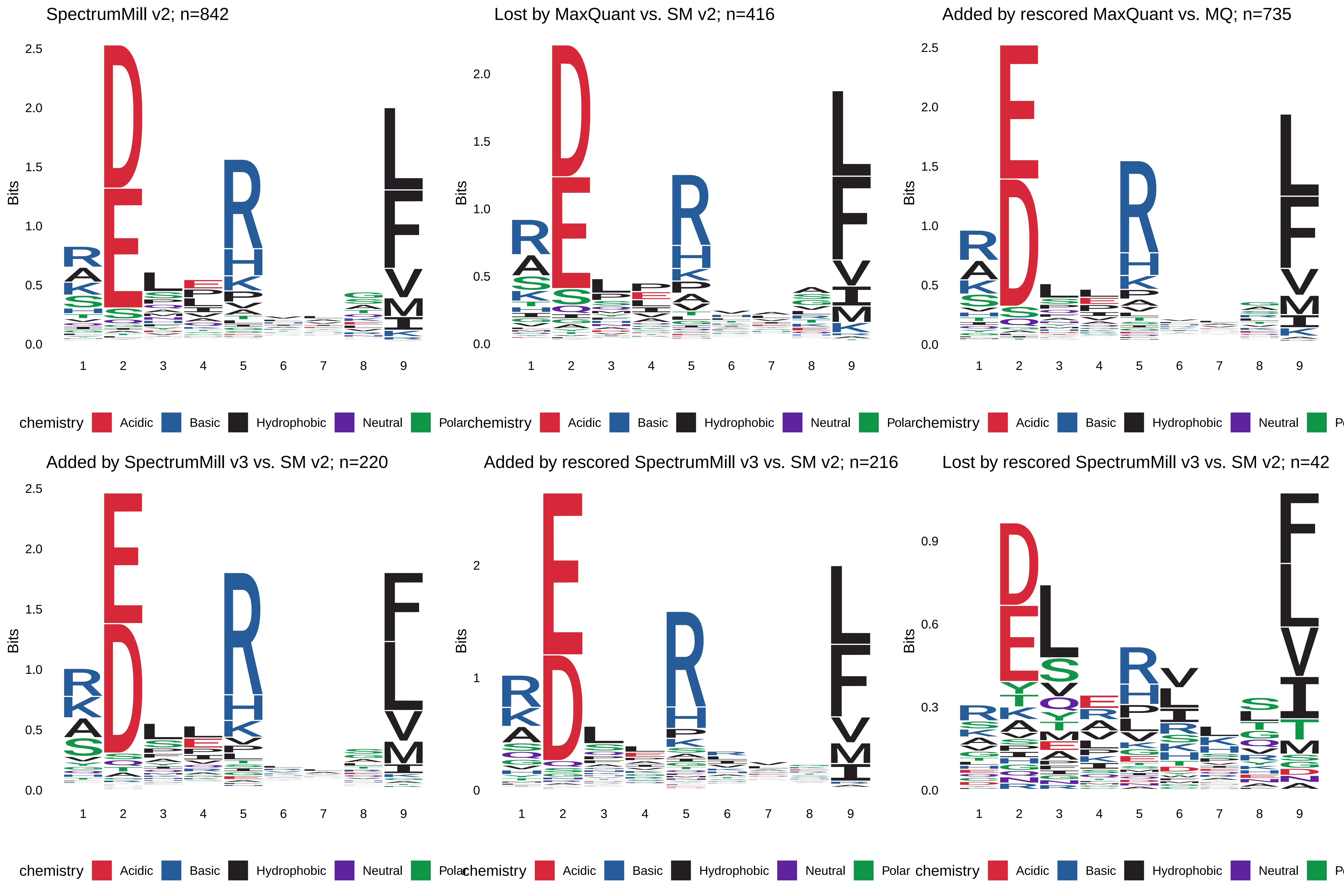
SpectrumMill v2; n=842
Lost by MaxQuant vs. SM v2; n=416
Added by rescored MaxQuant vs. MQ; n=735
Added by SpectrumMill v3 vs. SM v2; n=220
Added by rescored SpectrumMill v3 vs. SM v2; n=216
Lost by rescored SpectrumMill v3 vs. SM v2; n=42
Bits
1 2 3 4 5 6 7 8 9
chemistry Acidic Basic Hydrophobic Neutral Polar

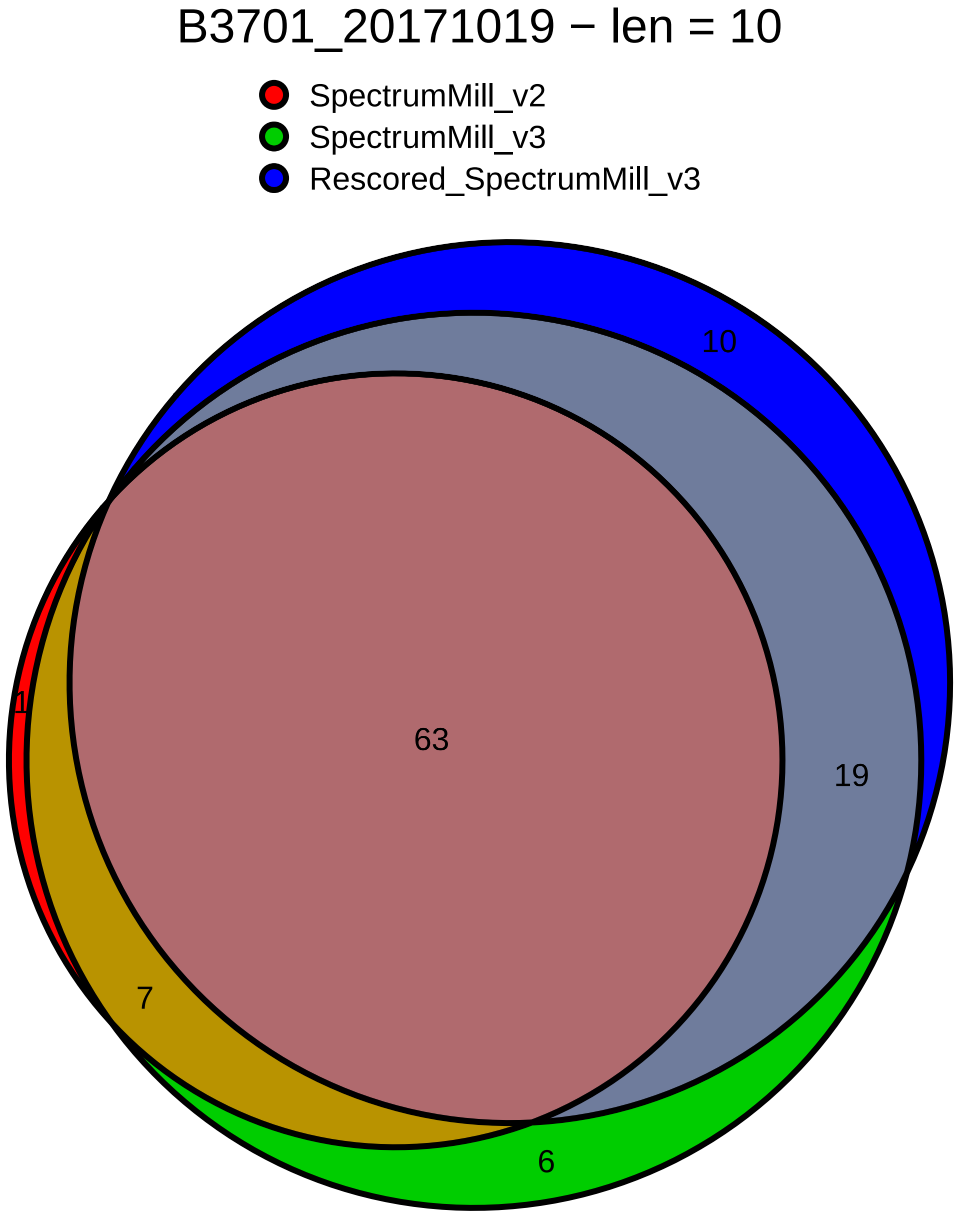
B3701_20171019 − len = 10
SpectrumMill_v2
SpectrumMill_v3
Rescored_SpectrumMill_v3
10
1
63
19
7
6

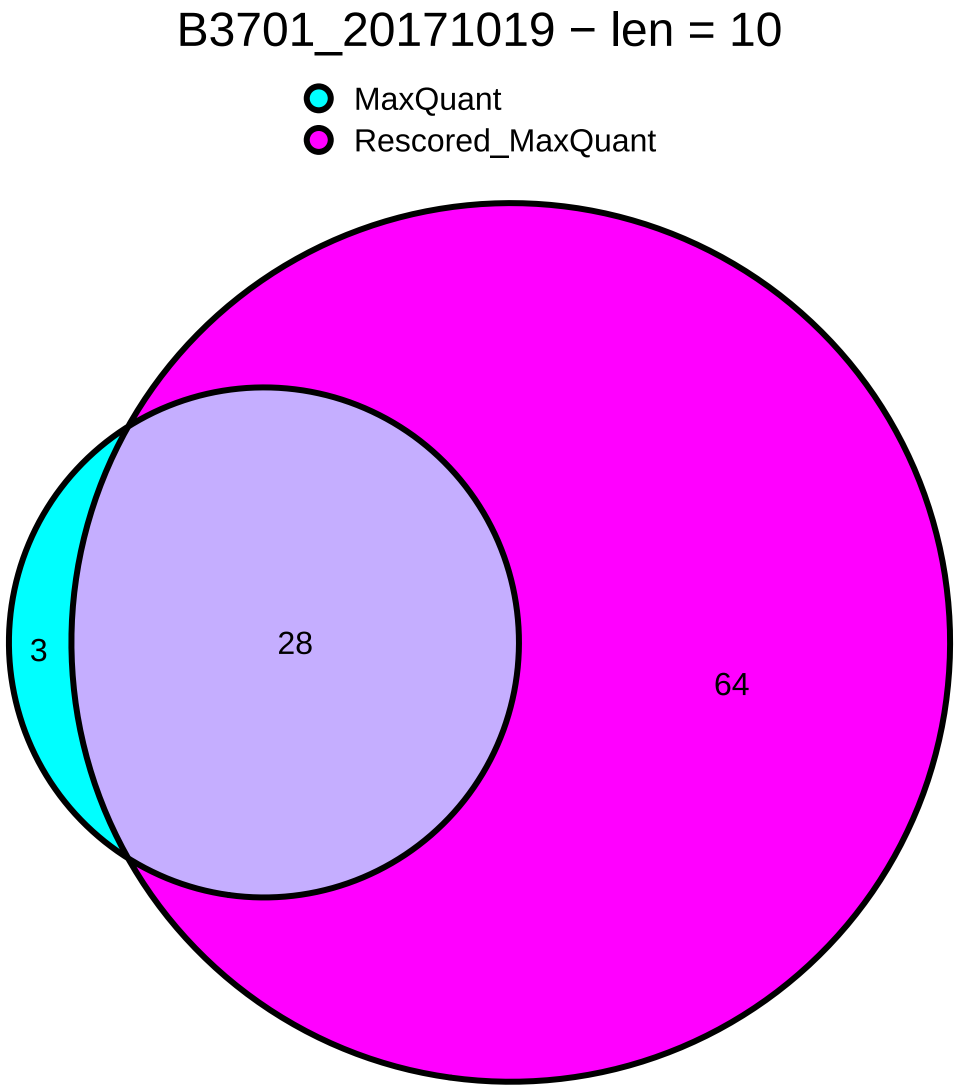
B3701_20171019 − len = 10
MaxQuant
Rescored_MaxQuant
3
28
64

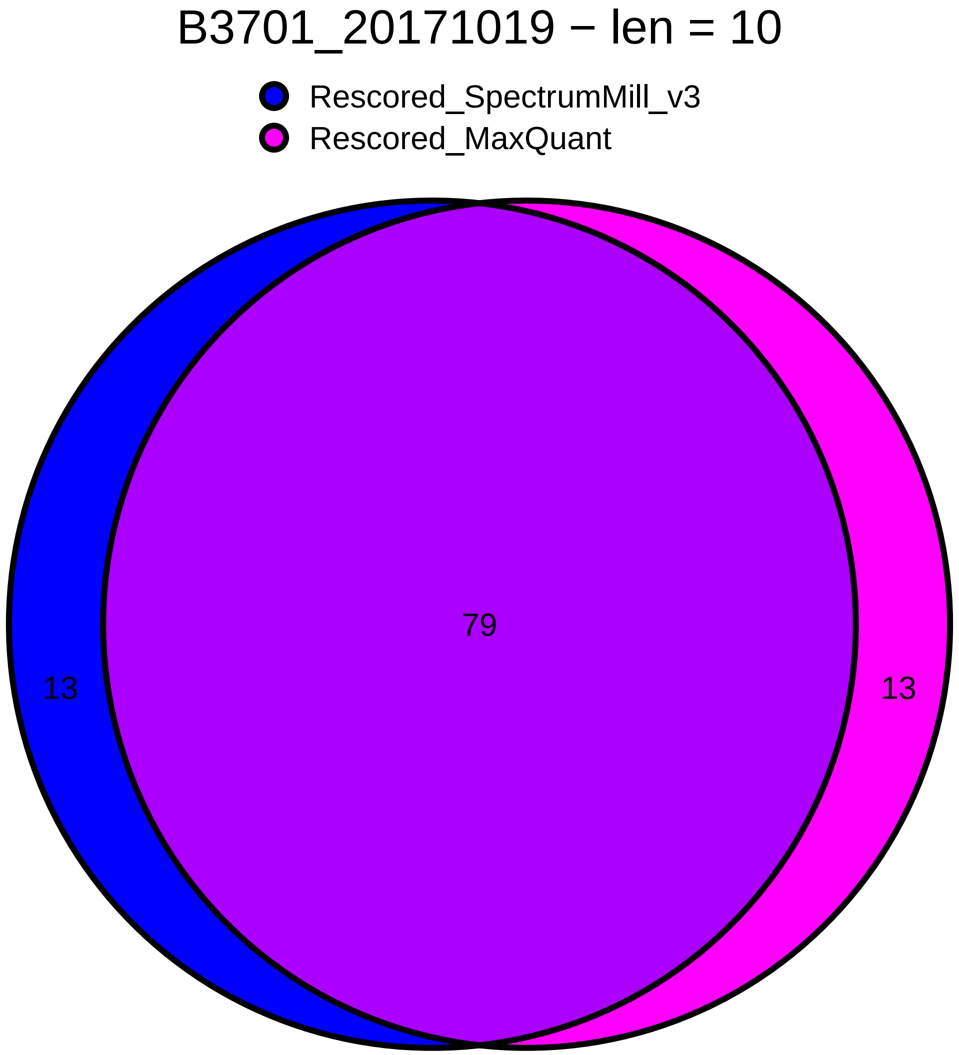
B3701_20171019 − len = 10
Rescored_SpectrumMill_v3
Rescored_MaxQuant
13
79
13

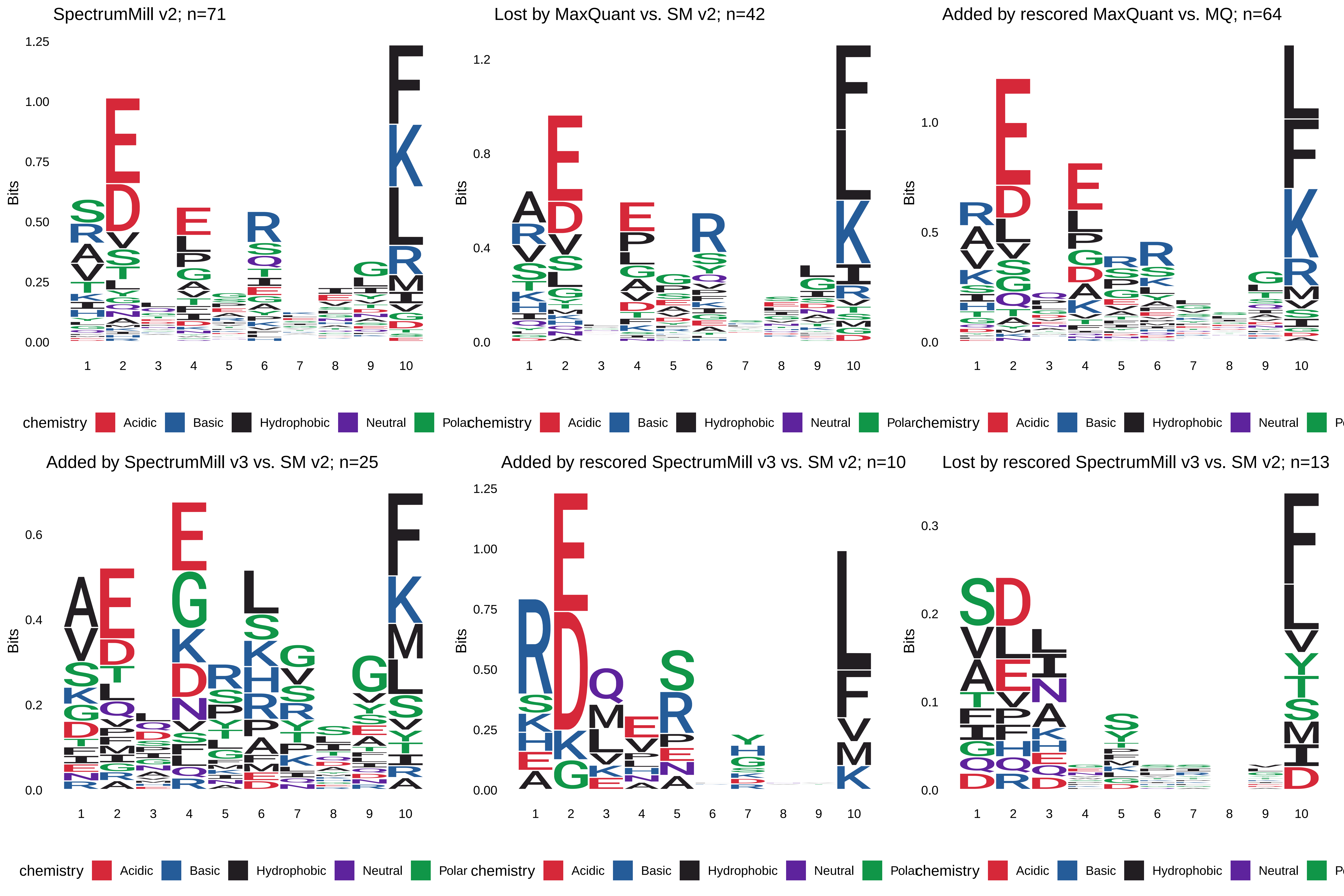
SpectrumMill v2; n=71
Lost by MaxQuant vs. SM v2; n=42
Added by rescored MaxQuant vs. MQ; n=64
Added by SpectrumMill v3 vs. SM v2; n=25
Added by rescored SpectrumMill v3 vs. SM v2; n=10
Lost by rescored SpectrumMill v3 vs. SM v2; n=13
Bits
chemistry
Acidic
Basic
Hydrophobic
Neutral
Polar

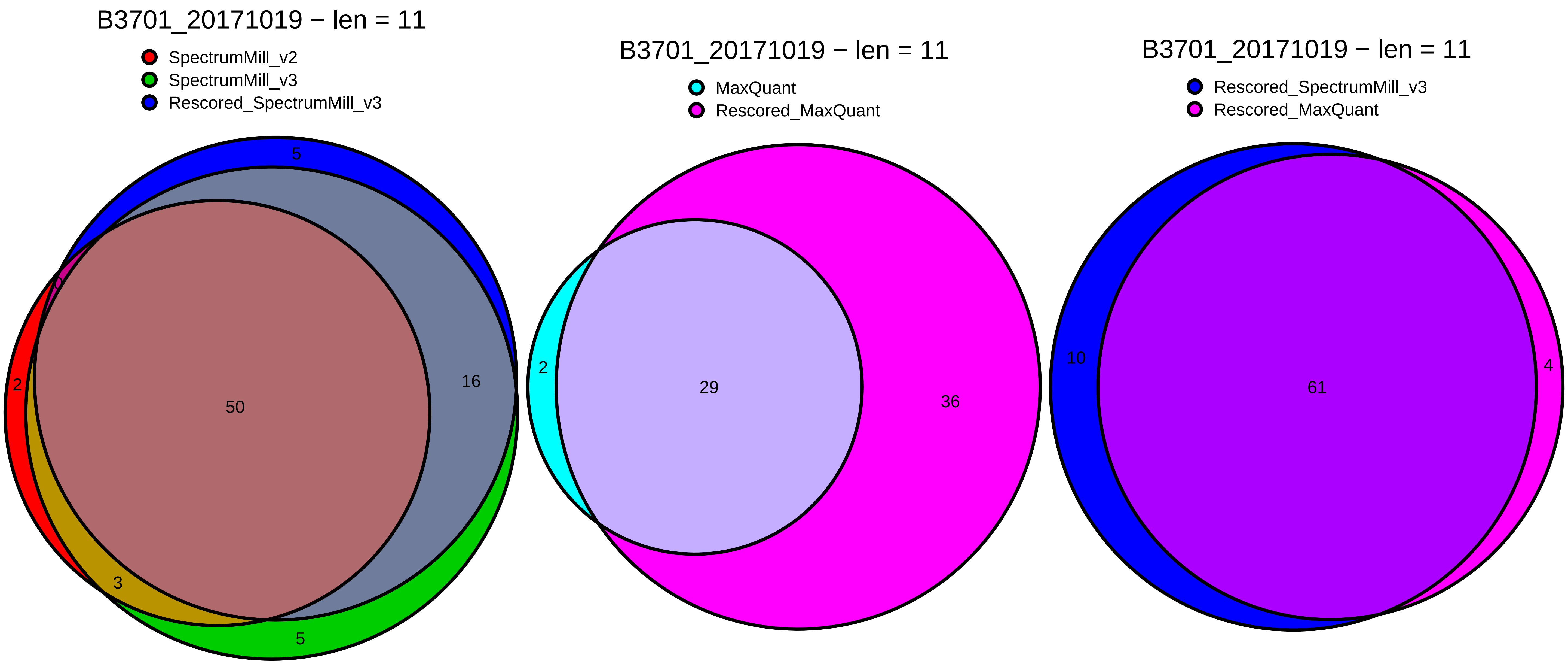

B3701_20171019 − len = 11
SpectrumMill_v2
SpectrumMill_v3
Rescored_SpectrumMill_v3
5
0
2
50
16
3
5
B3701_20171019 − len = 11
MaxQuant
Rescored_MaxQuant
2
29
36
B3701_20171019 − len = 11
Rescored_SpectrumMill_v3
Rescored_MaxQuant
10
61
4

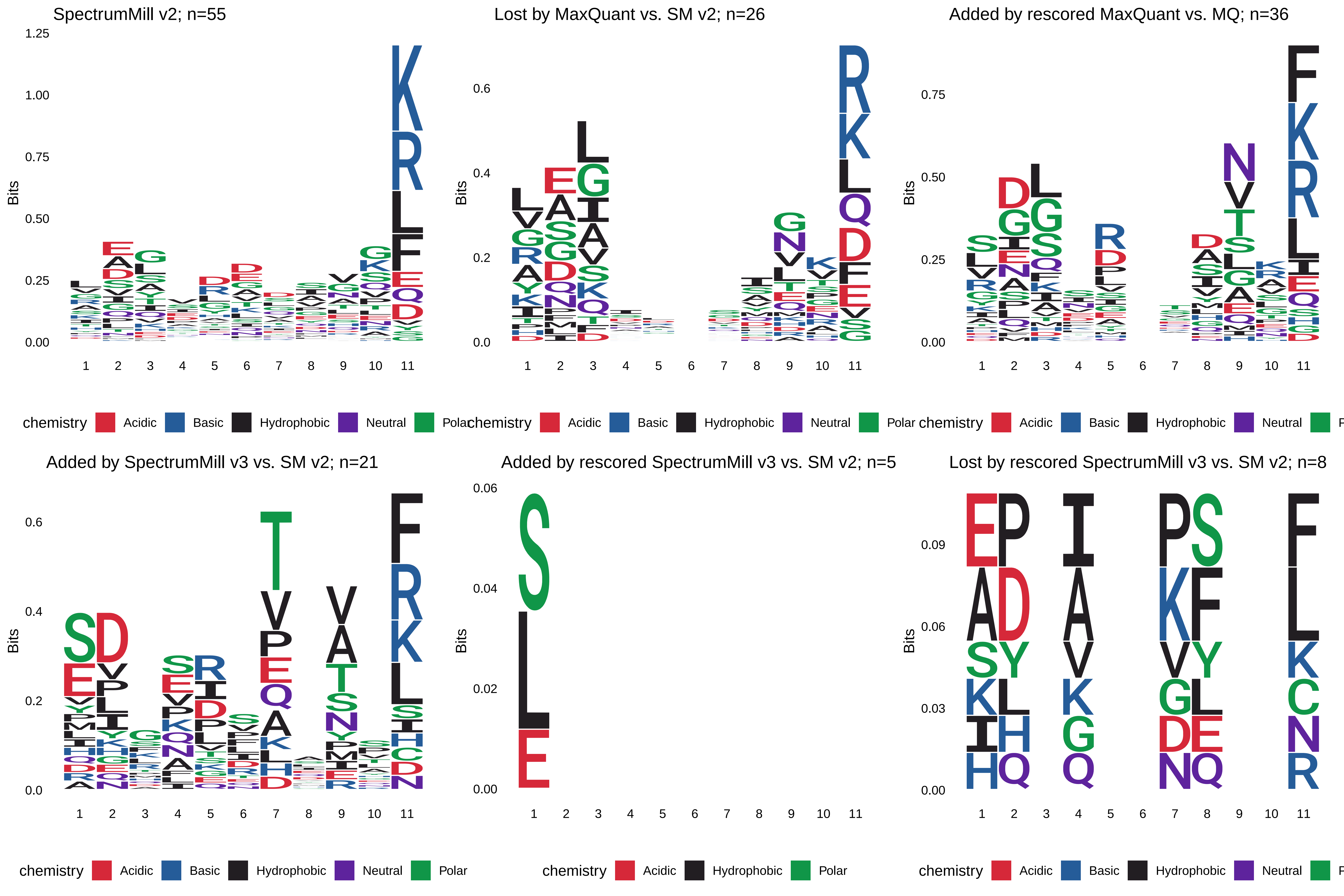

SpectrumMill v2; n=55
Lost by MaxQuant vs. SM v2; n=26
Added by rescored MaxQuant vs. MQ; n=36
Added by SpectrumMill v3 vs. SM v2; n=21
Added by rescored SpectrumMill v3 vs. SM v2; n=5
Lost by rescored SpectrumMill v3 vs. SM v2; n=8
Bits
1 2 3 4 5 6 7 8 9 10 11
chemistry Acidic Basic Hydrophobic Neutral Polar
chemistry Acidic Hydrophobic Polar

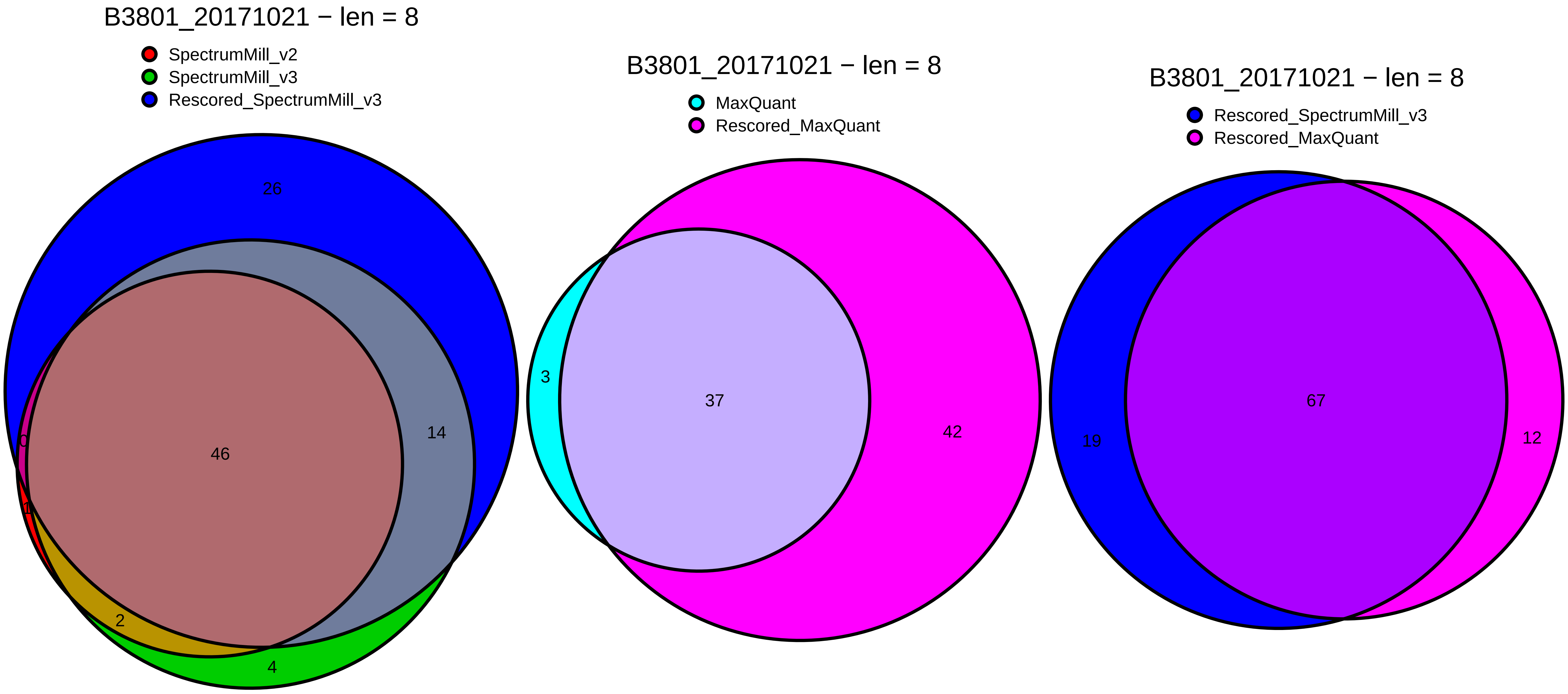

B3801_20171021 − len = 8
SpectrumMill_v2
SpectrumMill_v3
Rescored_SpectrumMill_v3
26
14
46
0
1
2
4
B3801_20171021 − len = 8
MaxQuant
Rescored_MaxQuant
3
37
42
B3801_20171021 − len = 8
Rescored_SpectrumMill_v3
Rescored_MaxQuant
67
19
12

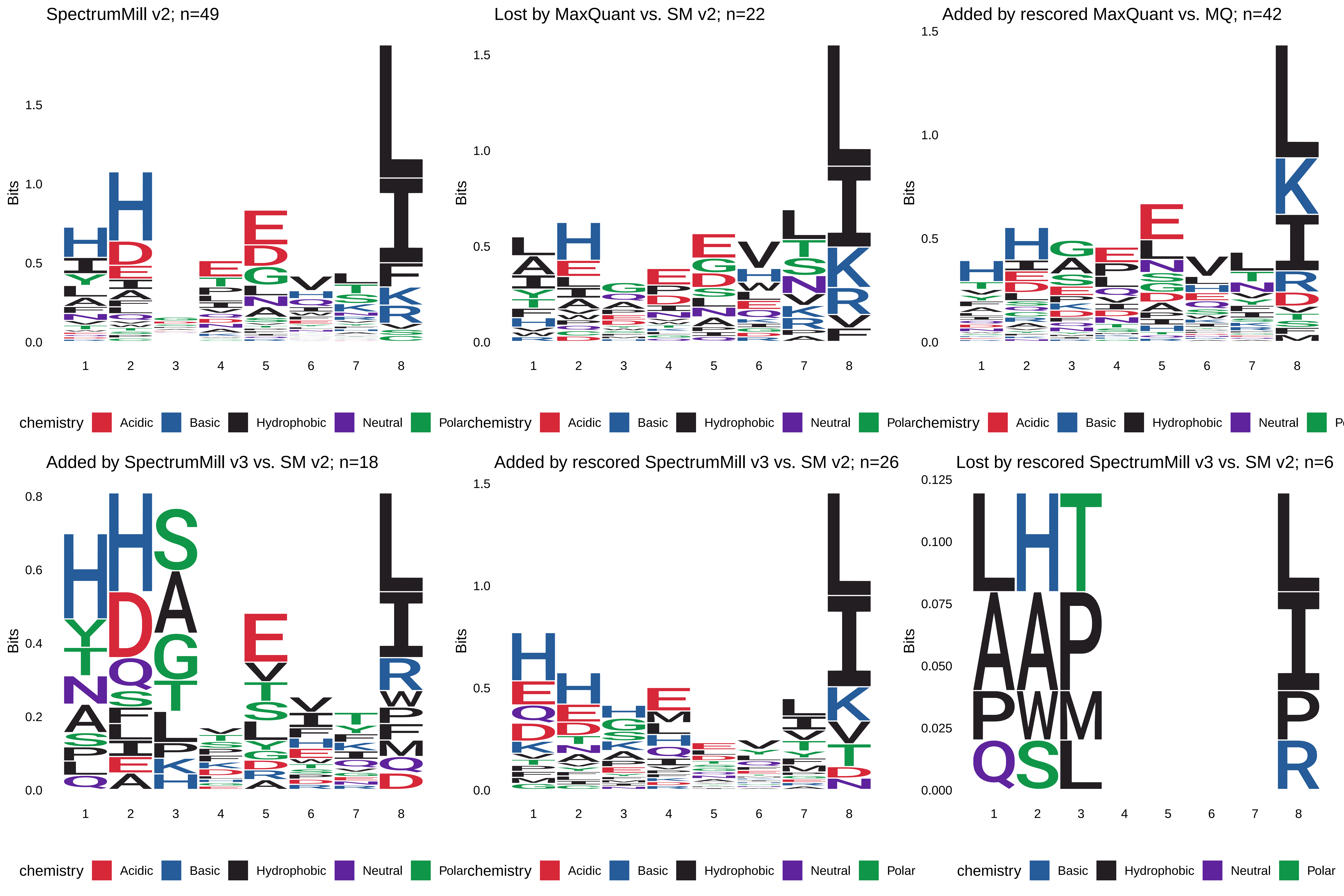

SpectrumMill v2; n=49
Lost by MaxQuant vs. SM v2; n=22
Added by rescored MaxQuant vs. MQ; n=42
Added by SpectrumMill v3 vs. SM v2; n=18
Added by rescored SpectrumMill v3 vs. SM v2; n=26
Lost by rescored SpectrumMill v3 vs. SM v2; n=6
Bits
1 2 3 4 5 6 7 8
chemistry Acidic Basic Hydrophobic Neutral Polar
chemistry Basic Hydrophobic Neutral Polar

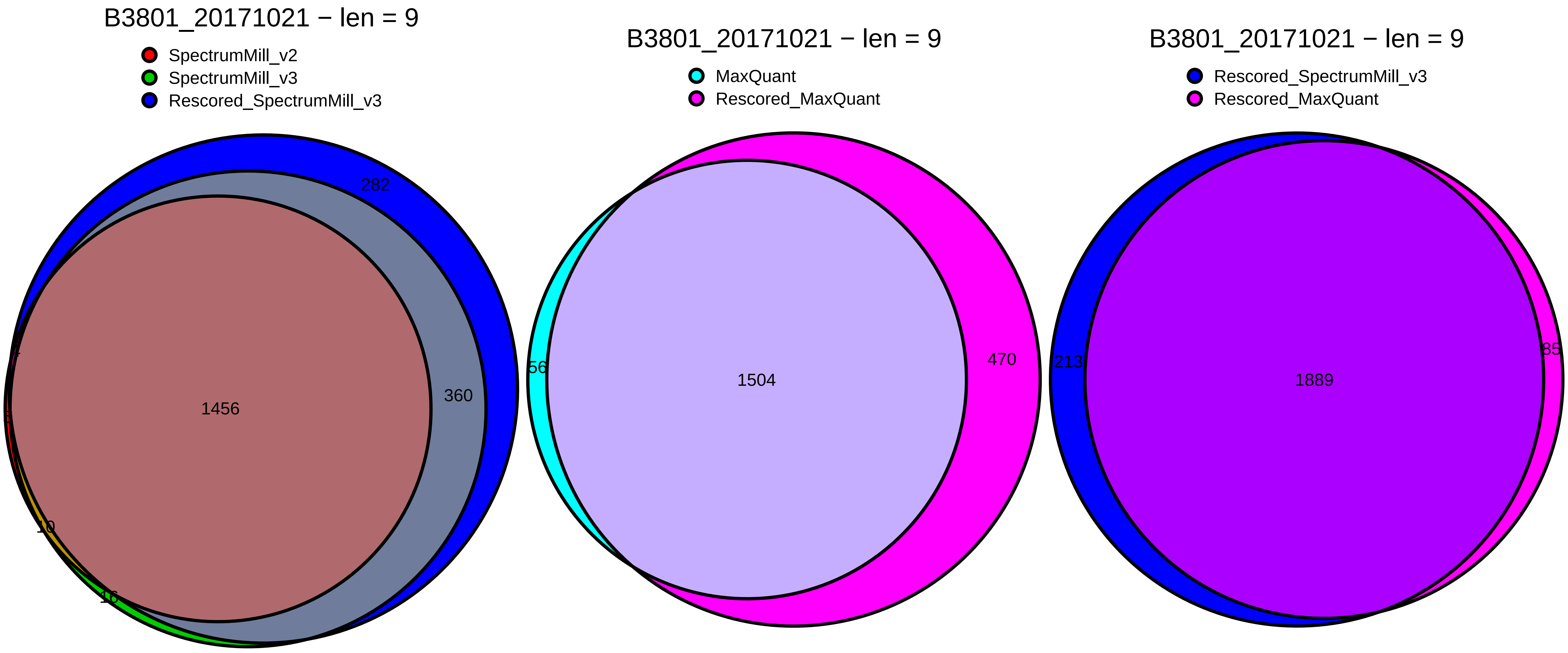

B3801_20171021 − len = 9
SpectrumMill_v2
SpectrumMill_v3
Rescored_SpectrumMill_v3
282
360
1456
10
16
B3801_20171021 − len = 9
MaxQuant
Rescored_MaxQuant
56
1504
470
B3801_20171021 − len = 9
Rescored_SpectrumMill_v3
Rescored_MaxQuant
213
1889
85

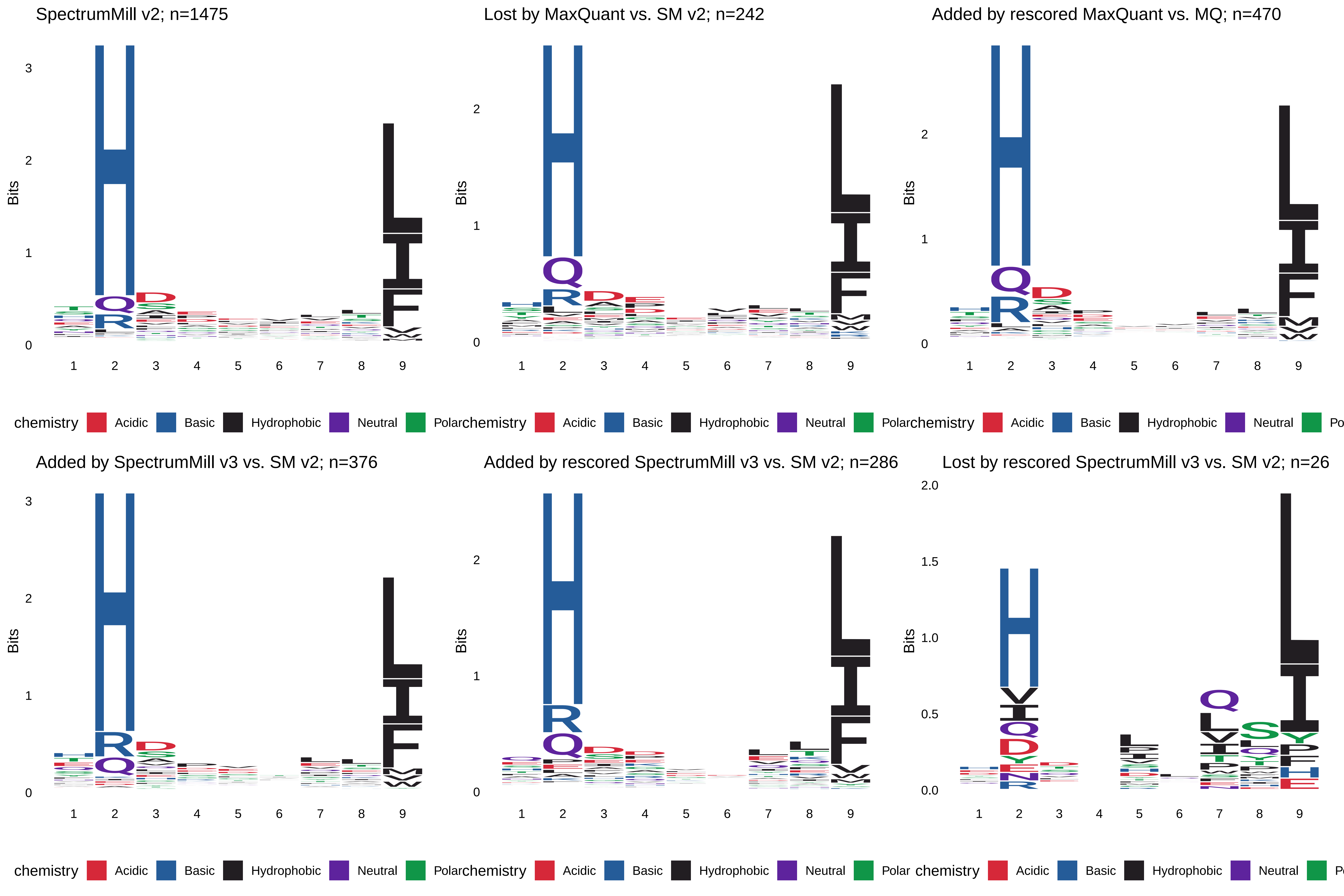
SpectrumMill v2; n=1475
Lost by MaxQuant vs. SM v2; n=242
Added by rescored MaxQuant vs. MQ; n=470
Added by SpectrumMill v3 vs. SM v2; n=376
Added by rescored SpectrumMill v3 vs. SM v2; n=286
Lost by rescored SpectrumMill v3 vs. SM v2; n=26
Bits
1 2 3 4 5 6 7 8 9
chemistry
Acidic
Basic
Hydrophobic
Neutral
Polar

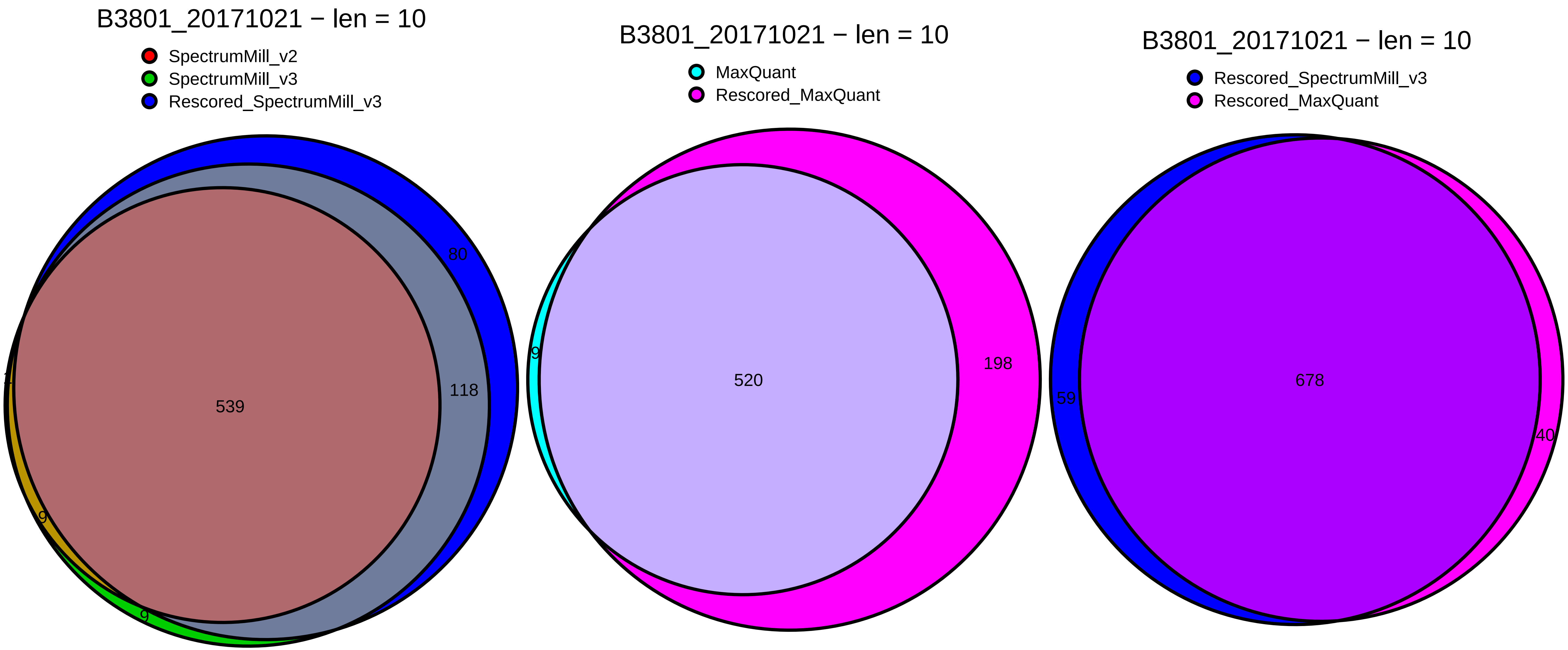
B3801_20171021 − len = 10
SpectrumMill_v2
SpectrumMill_v3
Rescored_SpectrumMill_v3
80
118
539
9
9
B3801_20171021 − len = 10
MaxQuant
Rescored_MaxQuant
9
520
198
B3801_20171021 − len = 10
Rescored_SpectrumMill_v3
Rescored_MaxQuant
59
678
40

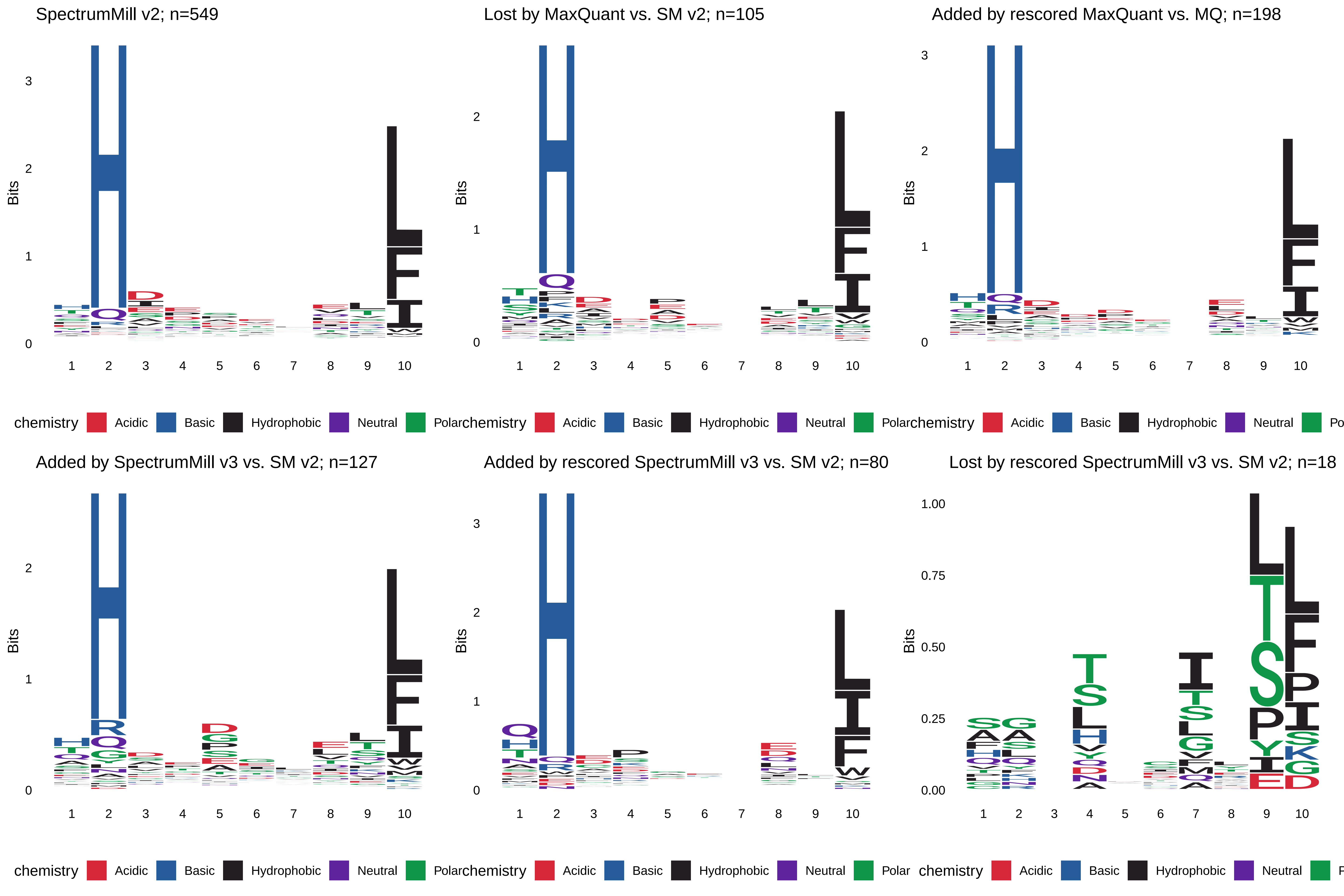
SpectrumMill v2; n=549
Lost by MaxQuant vs. SM v2; n=105
Added by rescored MaxQuant vs. MQ; n=198
Bits
chemistry
Acidic
Basic
Hydrophobic
Neutral
Polar
Added by SpectrumMill v3 vs. SM v2; n=127
Added by rescored SpectrumMill v3 vs. SM v2; n=80
Lost by rescored SpectrumMill v3 vs. SM v2; n=18

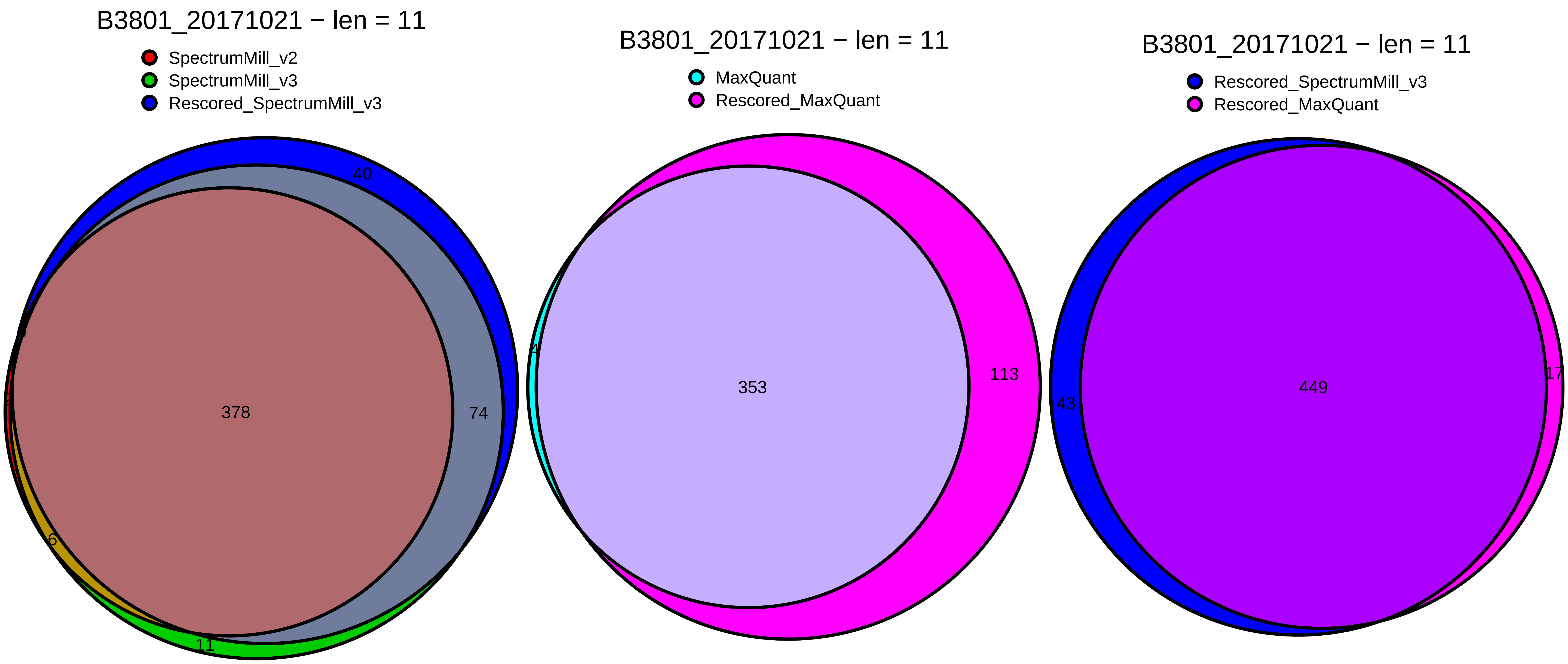
B3801_20171021 − len = 11
SpectrumMill_v2
SpectrumMill_v3
Rescored_SpectrumMill_v3
40
378
74
6
11
B3801_20171021 − len = 11
MaxQuant
Rescored_MaxQuant
4
353
113
B3801_20171021 − len = 11
Rescored_SpectrumMill_v3
Rescored_MaxQuant
43
449
17

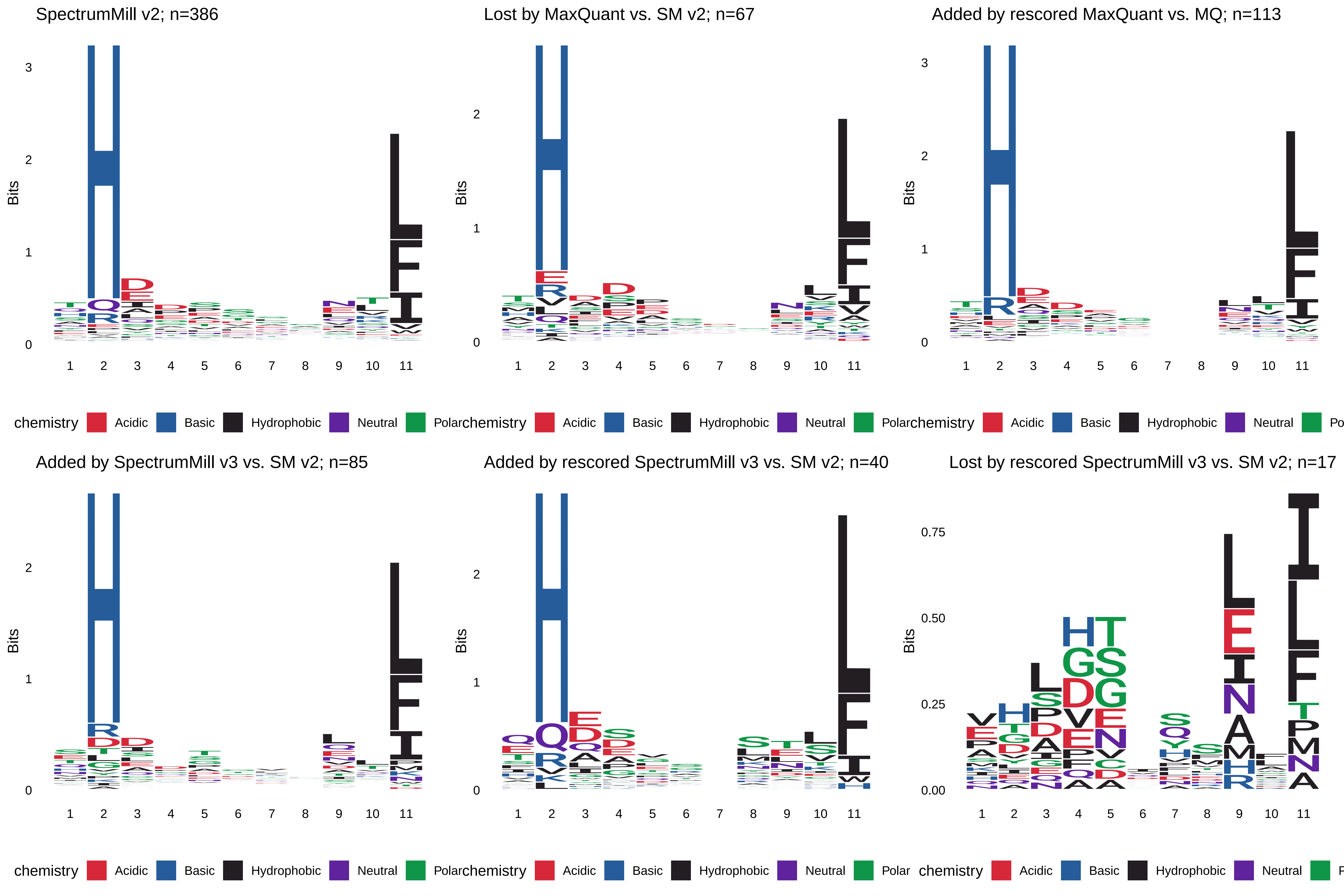
SpectrumMill v2; n=386
Lost by MaxQuant vs. SM v2; n=67
Added by rescored MaxQuant vs. MQ; n=113
Added by SpectrumMill v3 vs. SM v2; n=85
Added by rescored SpectrumMill v3 vs. SM v2; n=40
Lost by rescored SpectrumMill v3 vs. SM v2; n=17
Bits
1 2 3 4 5 6 7 8 9 10 11
chemistry
Acidic
Basic
Hydrophobic
Neutral
Polar

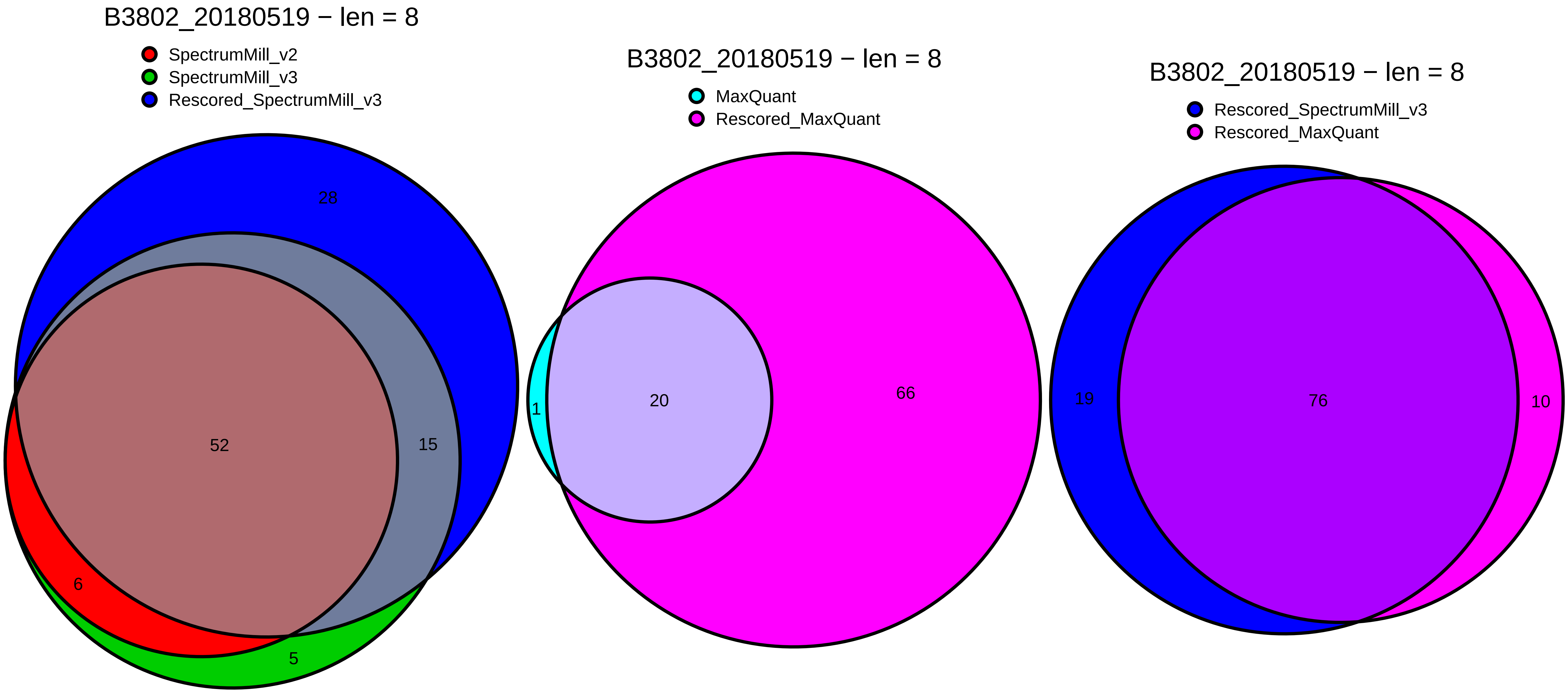
B3802_20180519 − len = 8
SpectrumMill_v2
SpectrumMill_v3
Rescored_SpectrumMill_v3
28
52
15
6
5
B3802_20180519 − len = 8
MaxQuant
Rescored_MaxQuant
1
20
66
B3802_20180519 − len = 8
Rescored_SpectrumMill_v3
Rescored_MaxQuant
19
76
10

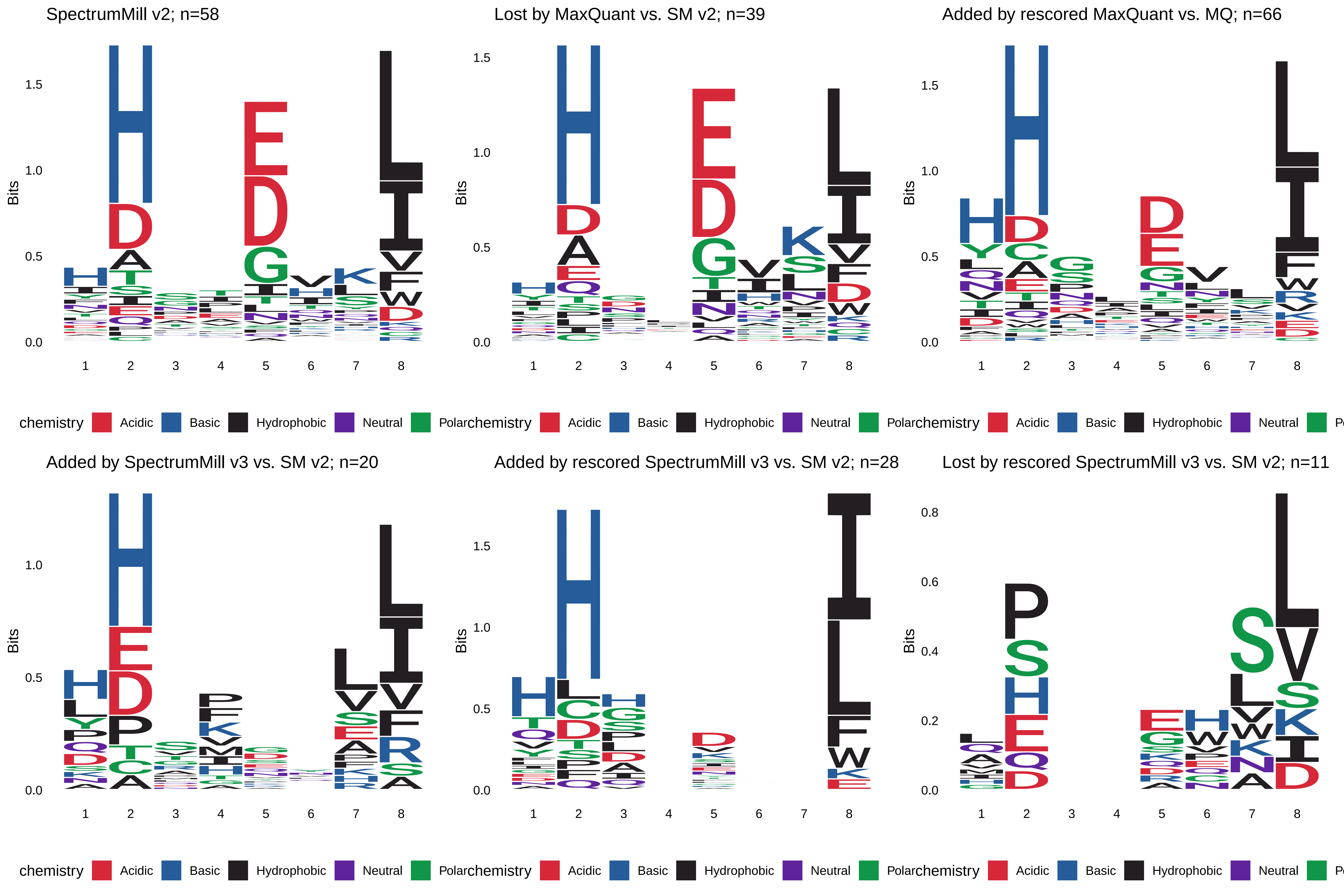
SpectrumMill v2; n=58
Lost by MaxQuant vs. SM v2; n=39
Added by rescored MaxQuant vs. MQ; n=66
Added by SpectrumMill v3 vs. SM v2; n=20
Added by rescored SpectrumMill v3 vs. SM v2; n=28
Lost by rescored SpectrumMill v3 vs. SM v2; n=11
Bits
1 2 3 4 5 6 7 8
chemistry Acidic Basic Hydrophobic Neutral Polarchemistry Acidic Basic Hydrophobic Neutral Polarchemistry Acidic Basic Hydrophobic Neutral
chemistry Acidic Basic Hydrophobic Neutral Polarchemistry Acidic Basic Hydrophobic Neutral Polarchemistry Acidic Basic Hydrophobic Neutral

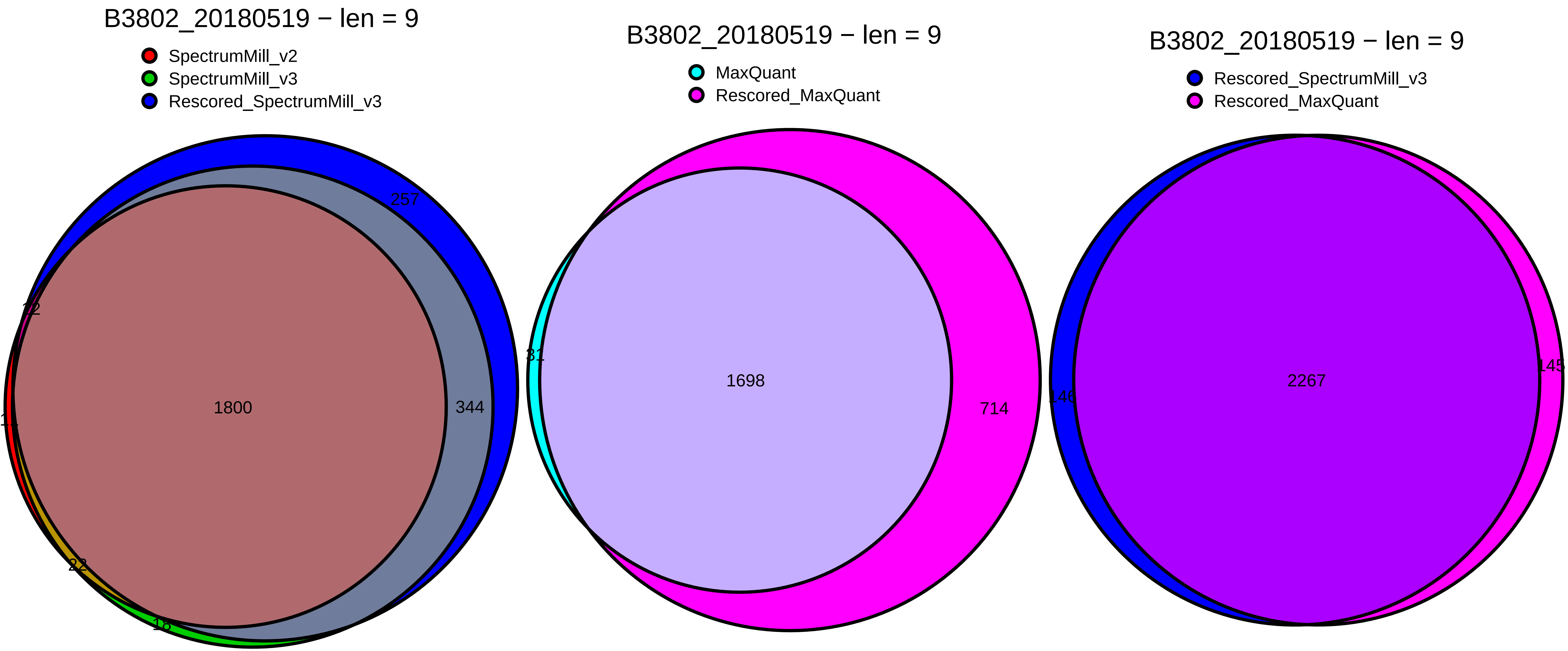
B3802_20180519 − len = 9
SpectrumMill_v2
SpectrumMill_v3
Rescored_SpectrumMill_v3
257
12
1800
344
22
18
B3802_20180519 − len = 9
MaxQuant
Rescored_MaxQuant
31
1698
714
B3802_20180519 − len = 9
Rescored_SpectrumMill_v3
Rescored_MaxQuant
146
2267
145

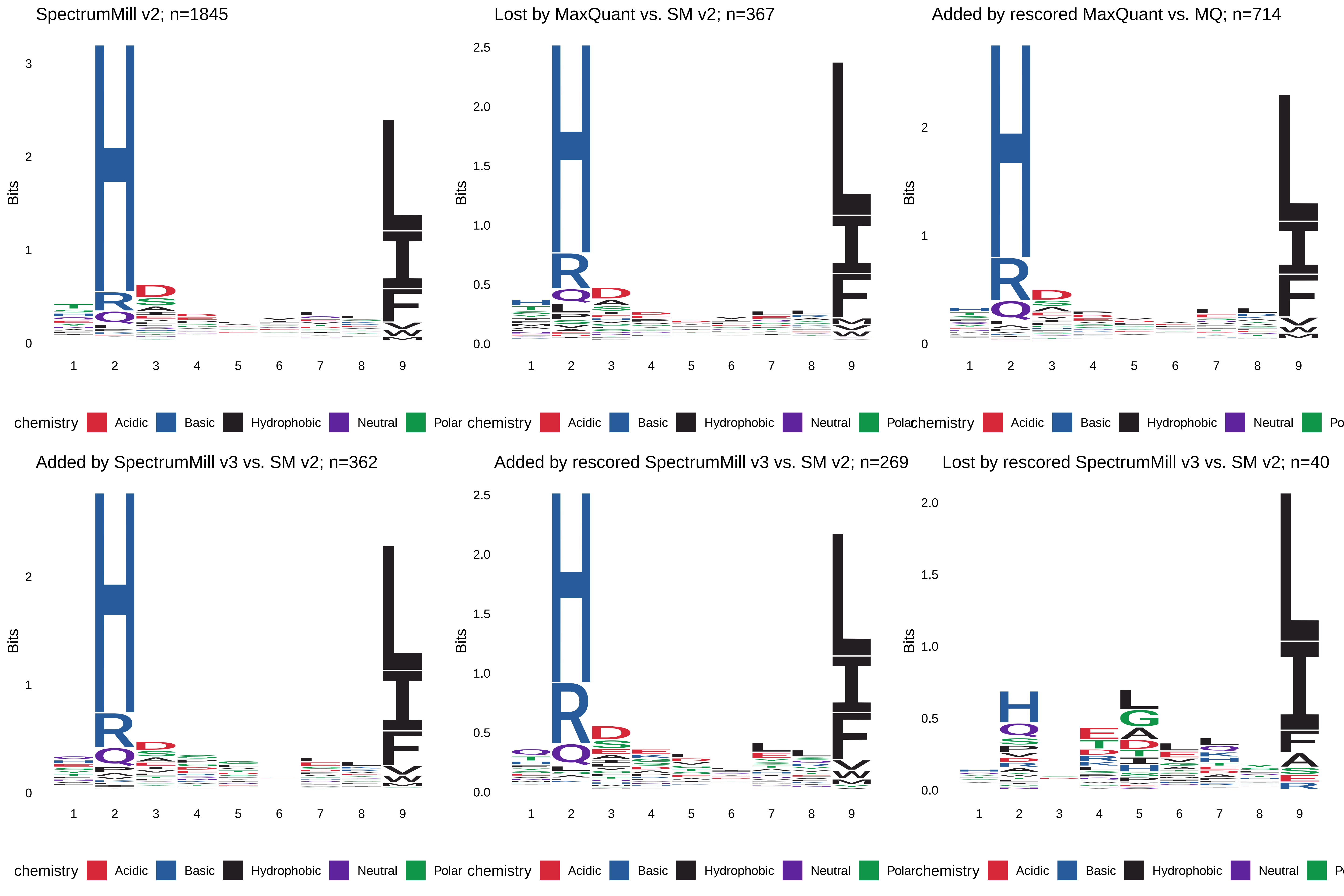
SpectrumMill v2; n=1845
Lost by MaxQuant vs. SM v2; n=367
Added by rescored MaxQuant vs. MQ; n=714
Added by SpectrumMill v3 vs. SM v2; n=362
Added by rescored SpectrumMill v3 vs. SM v2; n=269
Lost by rescored SpectrumMill v3 vs. SM v2; n=40
Bits
1 2 3 4 5 6 7 8 9
chemistry Acidic Basic Hydrophobic Neutral Polar

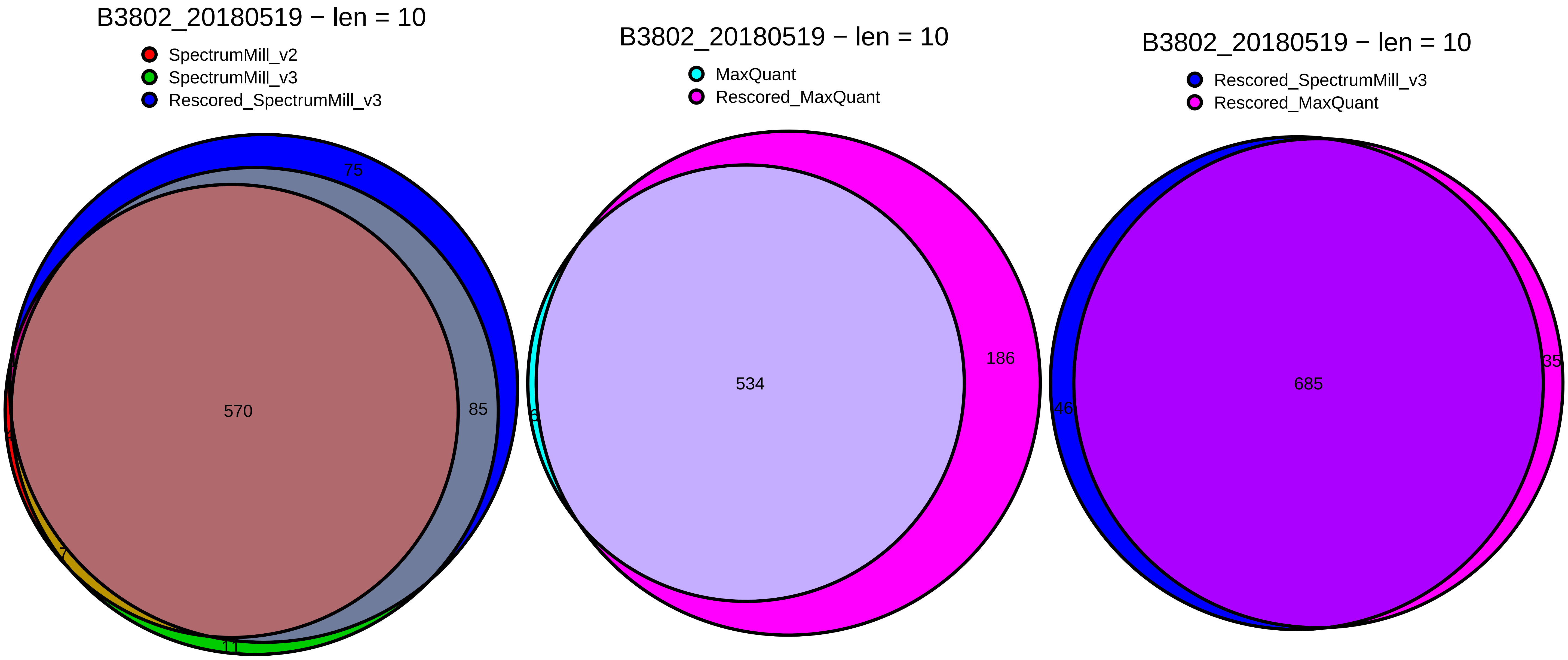

B3802_20180519 − len = 10
SpectrumMill_v2
SpectrumMill_v3
Rescored_SpectrumMill_v3
75
570
85
7
11
B3802_20180519 − len = 10
MaxQuant
Rescored_MaxQuant
534
186
B3802_20180519 − len = 10
Rescored_SpectrumMill_v3
Rescored_MaxQuant
685
46
35

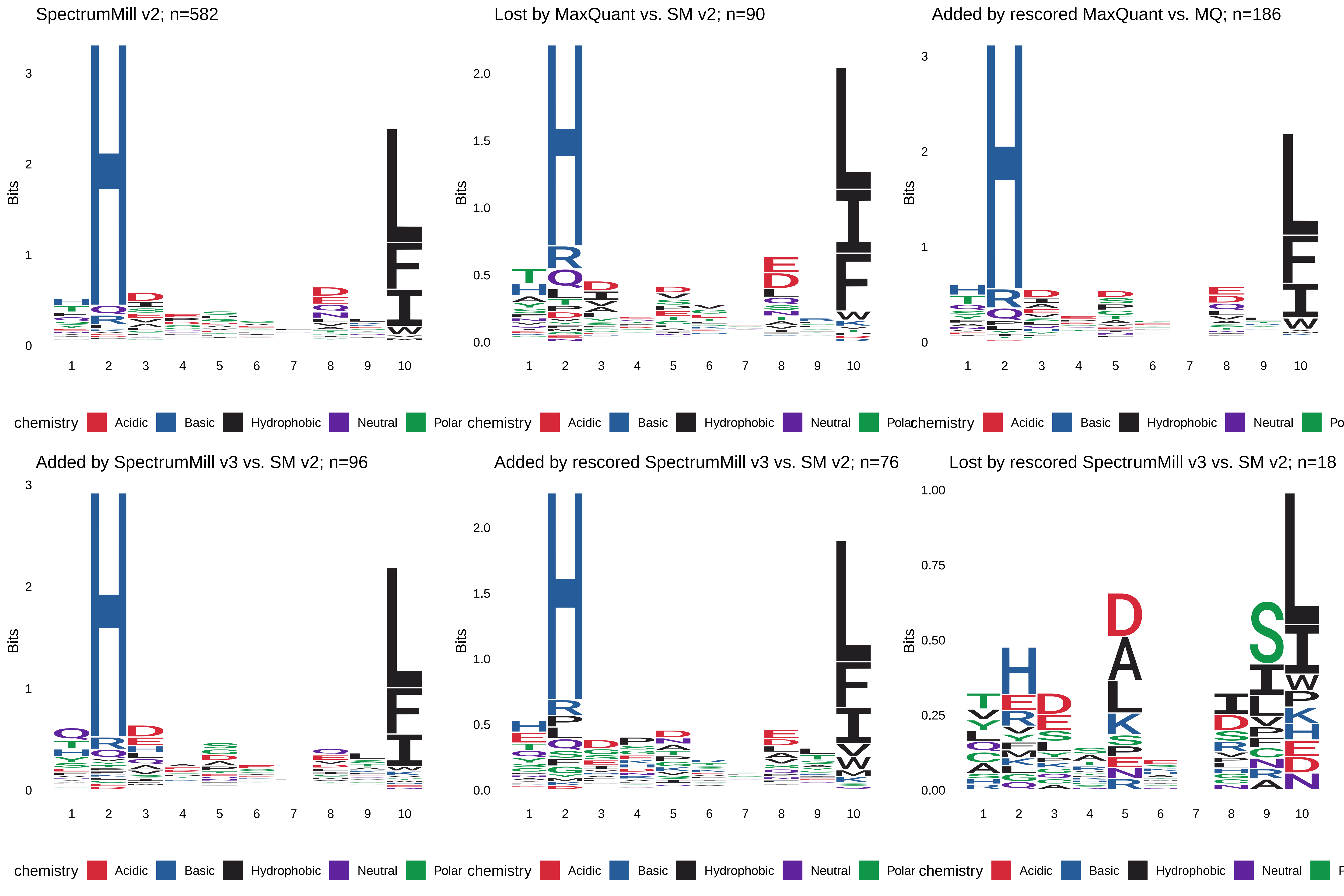

SpectrumMill v2; n=582
Lost by MaxQuant vs. SM v2; n=90
Added by rescored MaxQuant vs. MQ; n=186
Added by SpectrumMill v3 vs. SM v2; n=96
Added by rescored SpectrumMill v3 vs. SM v2; n=76
Lost by rescored SpectrumMill v3 vs. SM v2; n=18
Bits
1 2 3 4 5 6 7 8 9 10
chemistry Acidic Basic Hydrophobic Neutral Polar

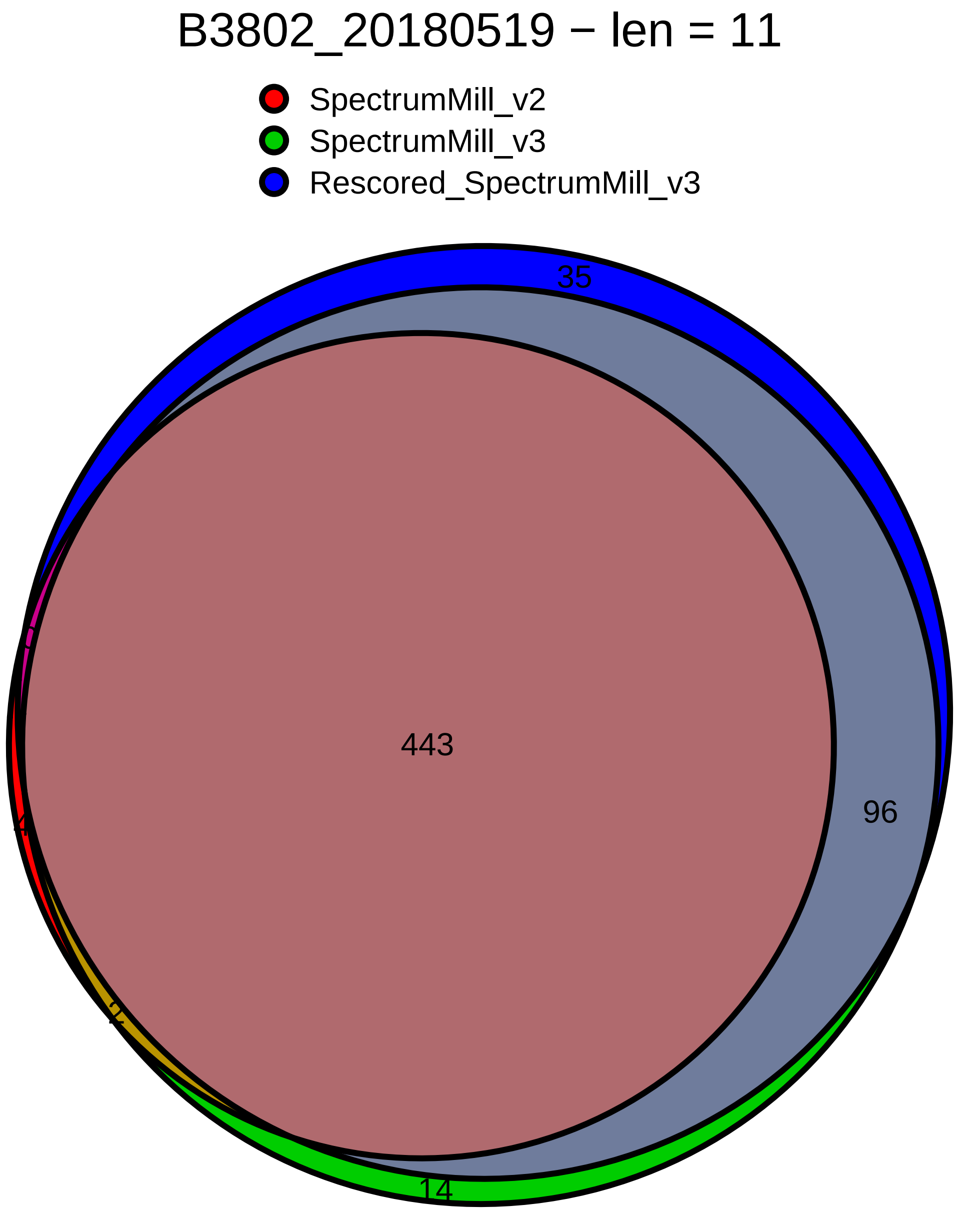
B3802_20180519 − len = 11
SpectrumMill_v2
SpectrumMill_v3
Rescored_SpectrumMill_v3
35
443
96
14

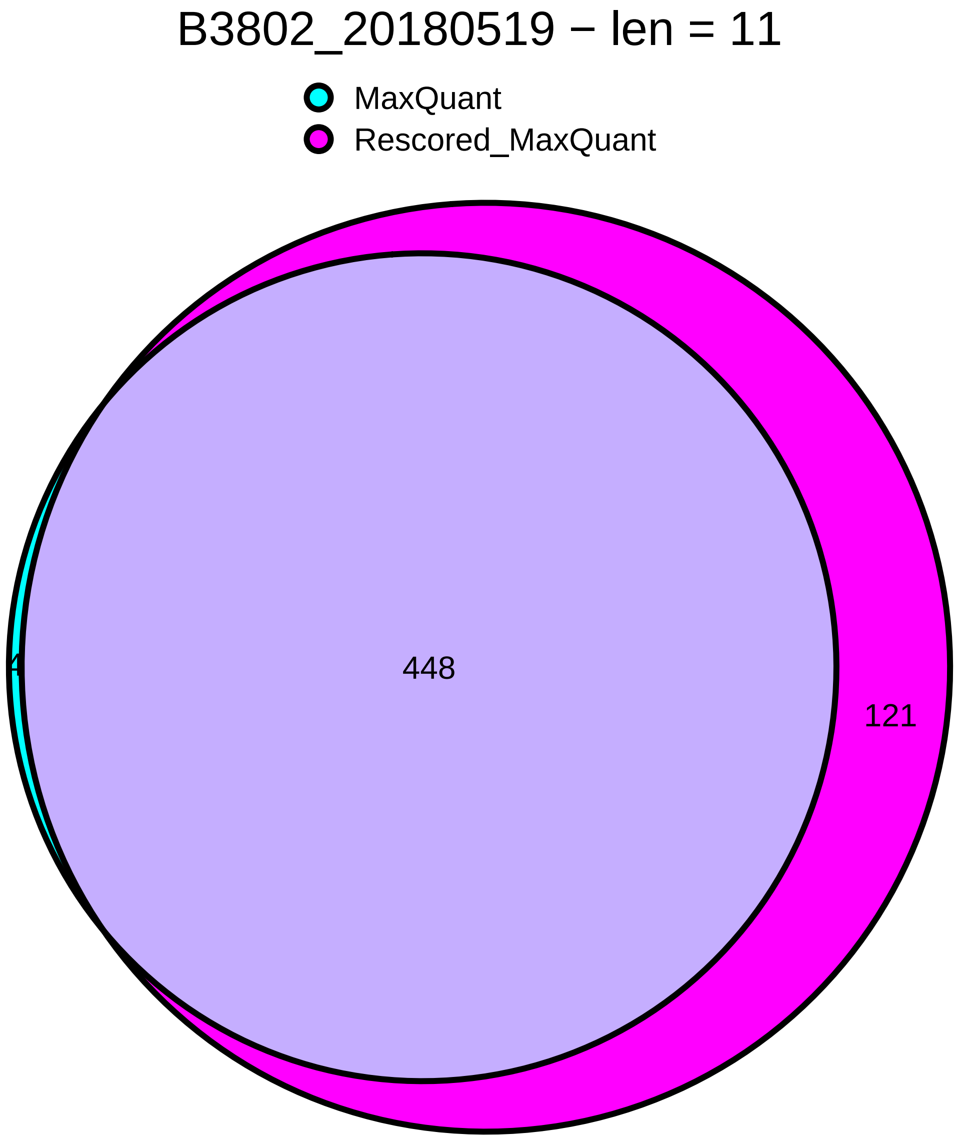
B3802_20180519 − len = 11
MaxQuant
Rescored_MaxQuant
448
121

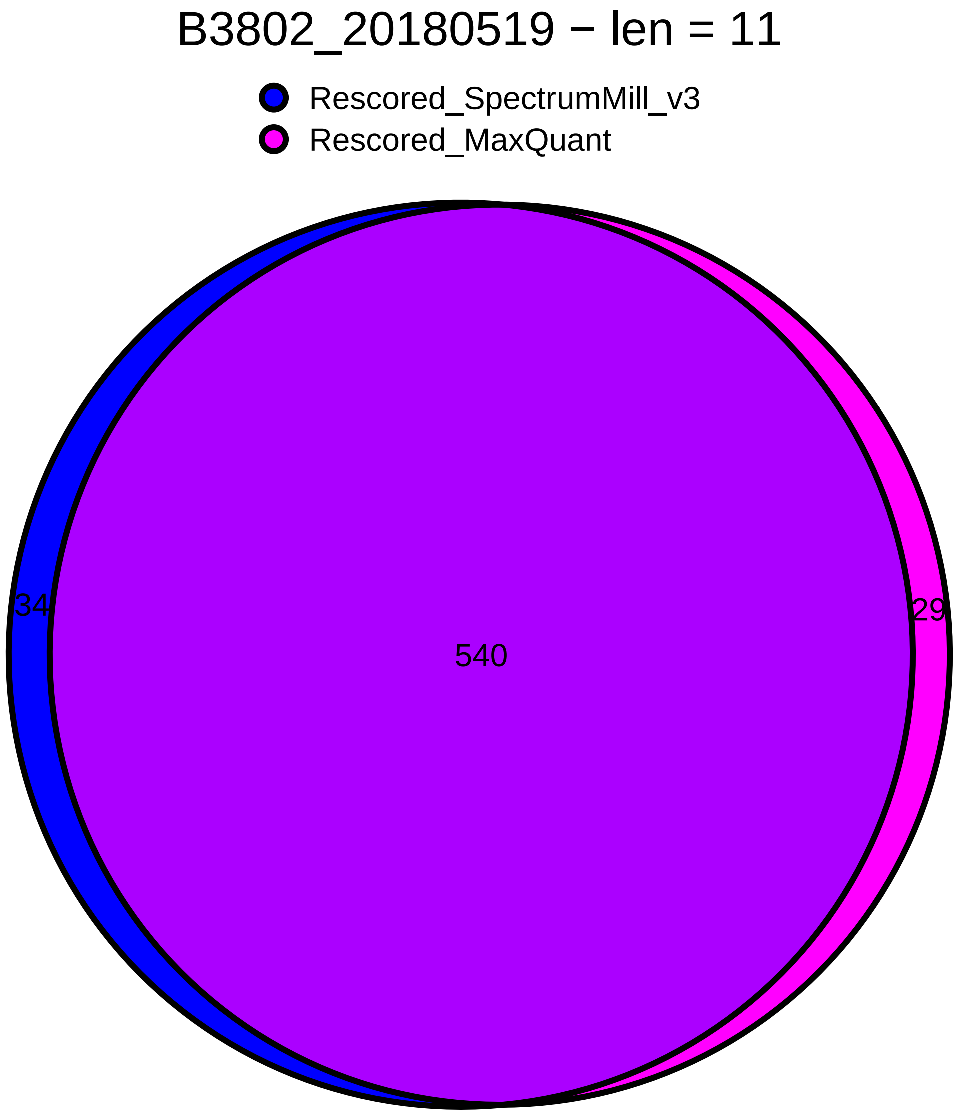
B3802_20180519 − len = 11
Rescored_SpectrumMill_v3
Rescored_MaxQuant
34
540
29

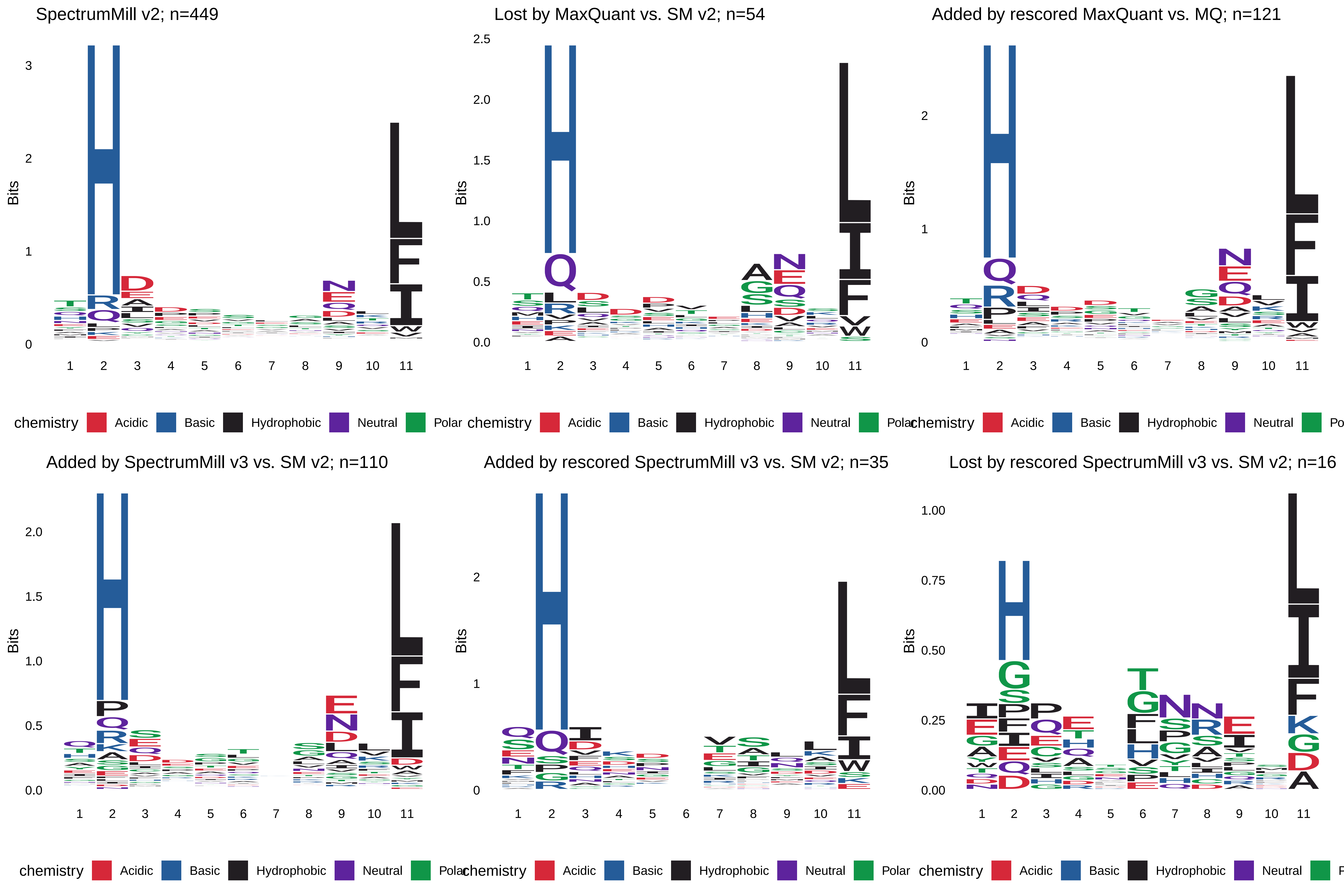
SpectrumMill v2; n=449
Lost by MaxQuant vs. SM v2; n=54
Added by rescored MaxQuant vs. MQ; n=121
Added by SpectrumMill v3 vs. SM v2; n=110
Added by rescored SpectrumMill v3 vs. SM v2; n=35
Lost by rescored SpectrumMill v3 vs. SM v2; n=16
Bits
1 2 3 4 5 6 7 8 9 10 11
chemistry
Acidic
Basic
Hydrophobic
Neutral
Polar

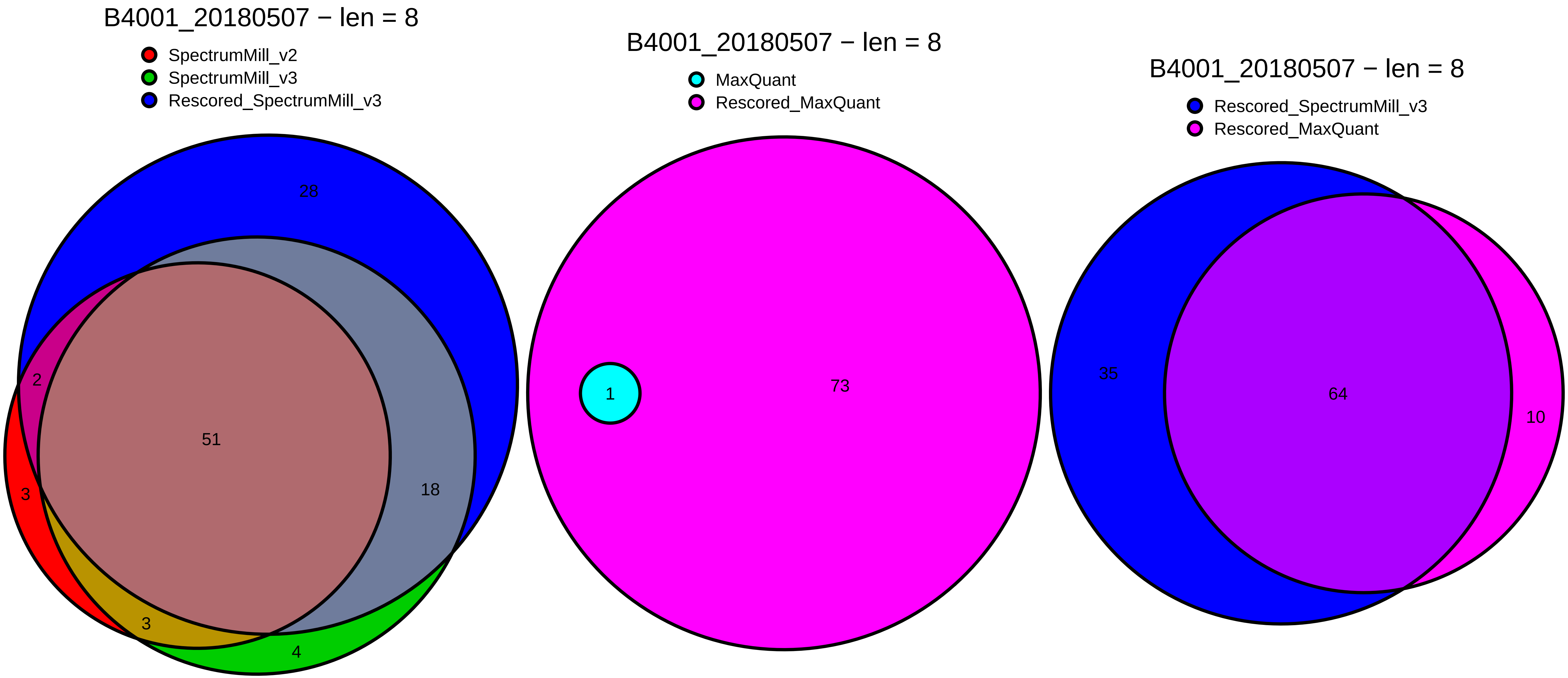

B4001_20180507 − len = 8
SpectrumMill_v2
SpectrumMill_v3
Rescored_SpectrumMill_v3
28
2
51
3
18
3
4
B4001_20180507 − len = 8
MaxQuant
Rescored_MaxQuant
1
73
B4001_20180507 − len = 8
Rescored_SpectrumMill_v3
Rescored_MaxQuant
35
64
10

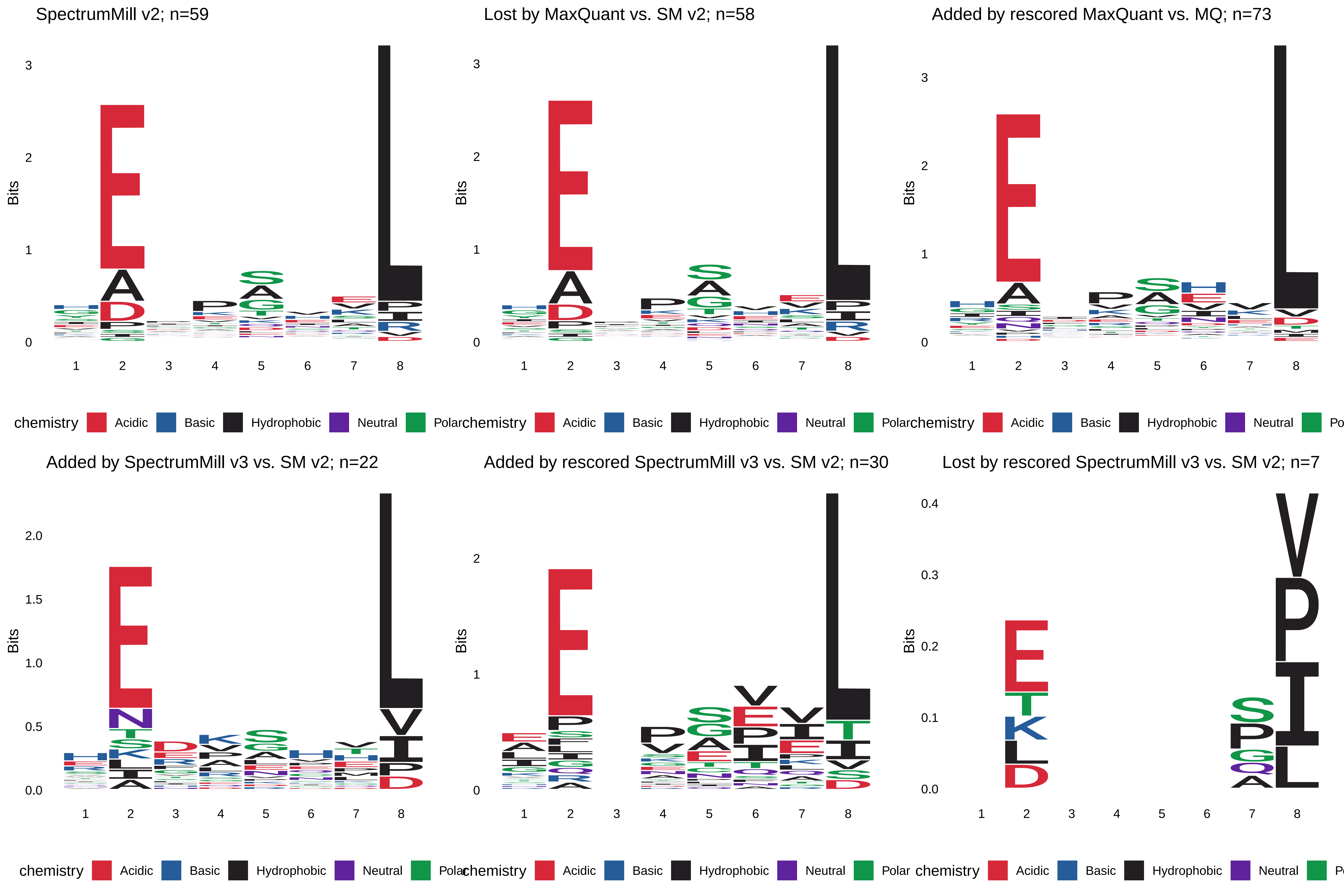
SpectrumMill v2; n=59
Lost by MaxQuant vs. SM v2; n=58
Added by rescored MaxQuant vs. MQ; n=73
Added by SpectrumMill v3 vs. SM v2; n=22
Added by rescored SpectrumMill v3 vs. SM v2; n=30
Lost by rescored SpectrumMill v3 vs. SM v2; n=7
Bits
1 2 3 4 5 6 7 8
chemistry
Acidic
Basic
Hydrophobic
Neutral
Polar

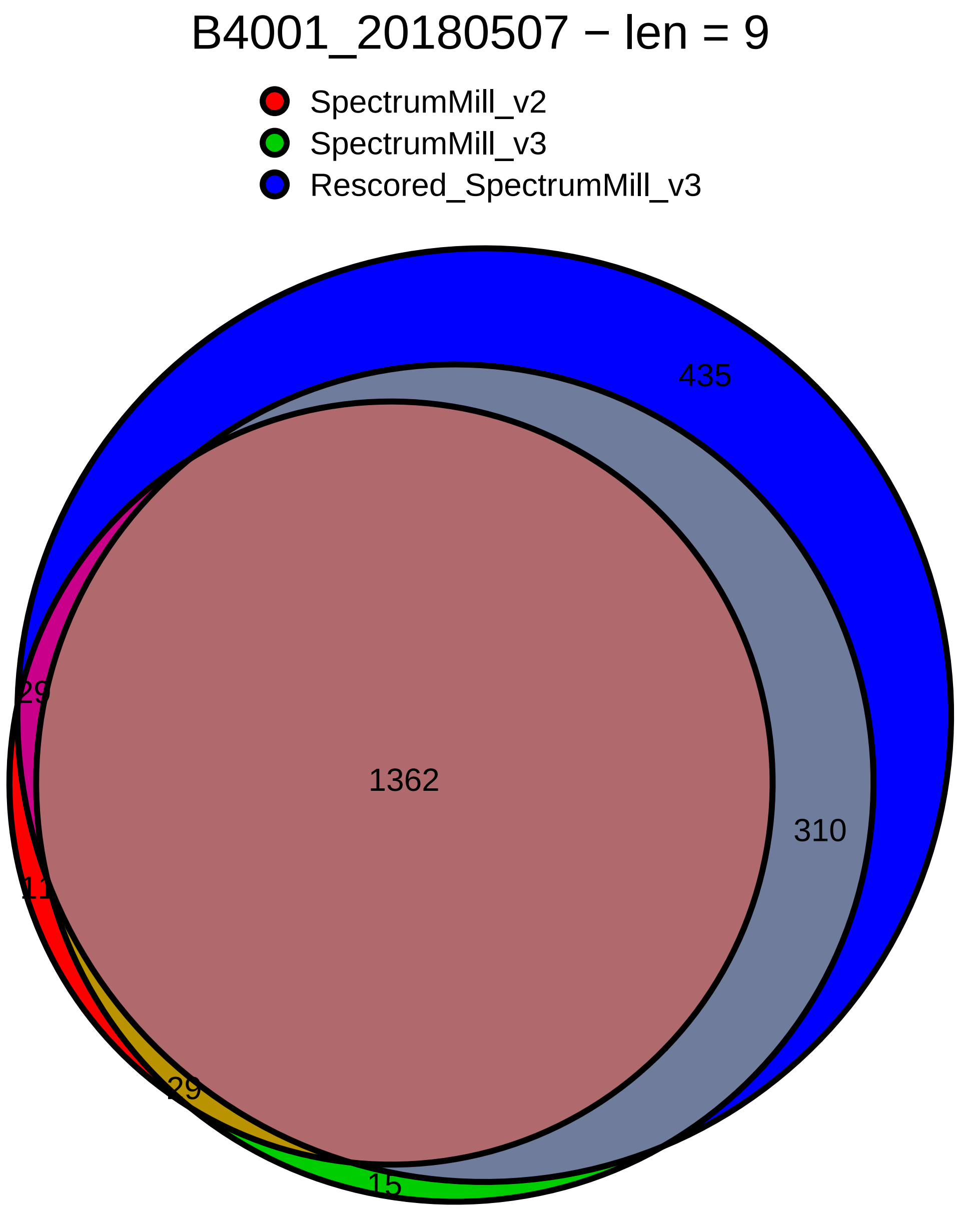
B4001_20180507 − len = 9
SpectrumMill_v2
SpectrumMill_v3
Rescored_SpectrumMill_v3
435
29
1362
310
11
29
15

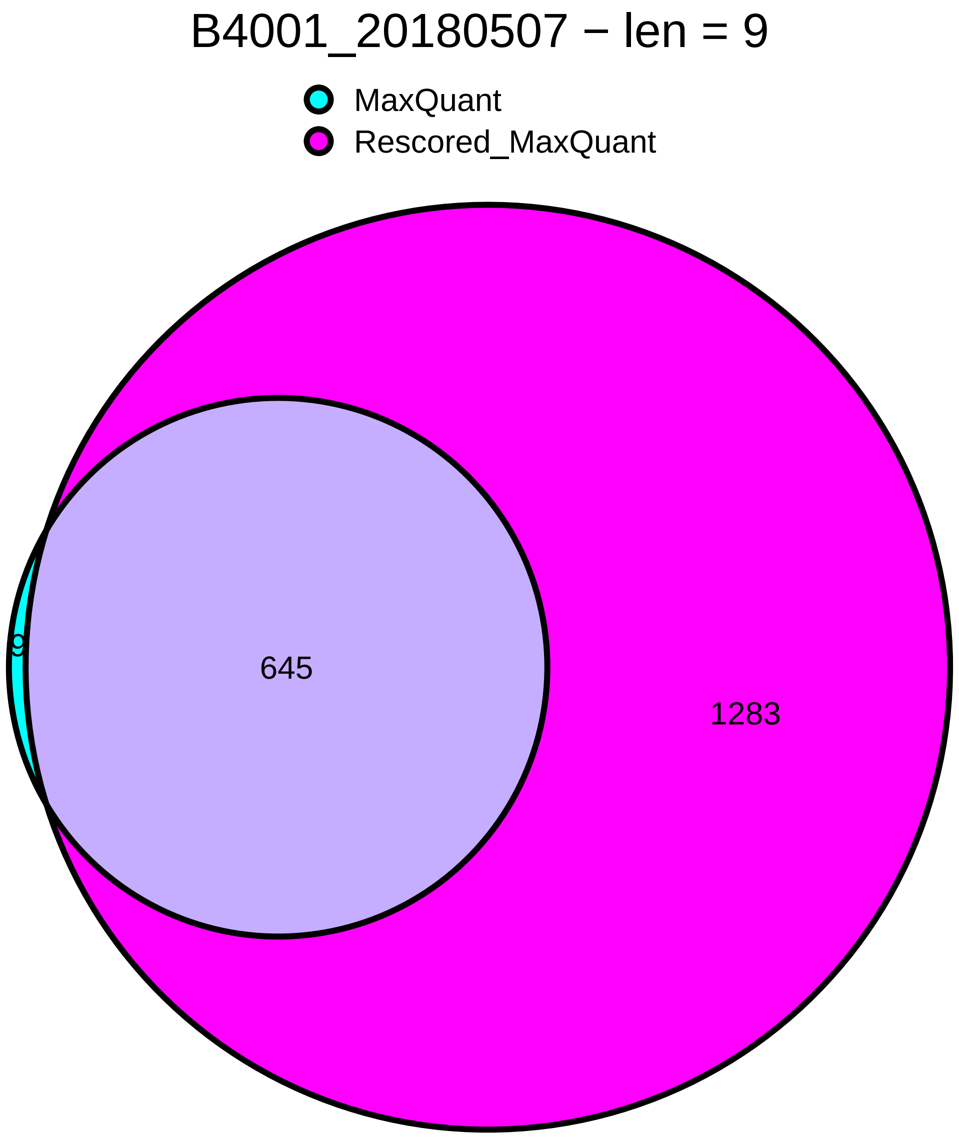
B4001_20180507 − len = 9
MaxQuant
Rescored_MaxQuant
645
1283

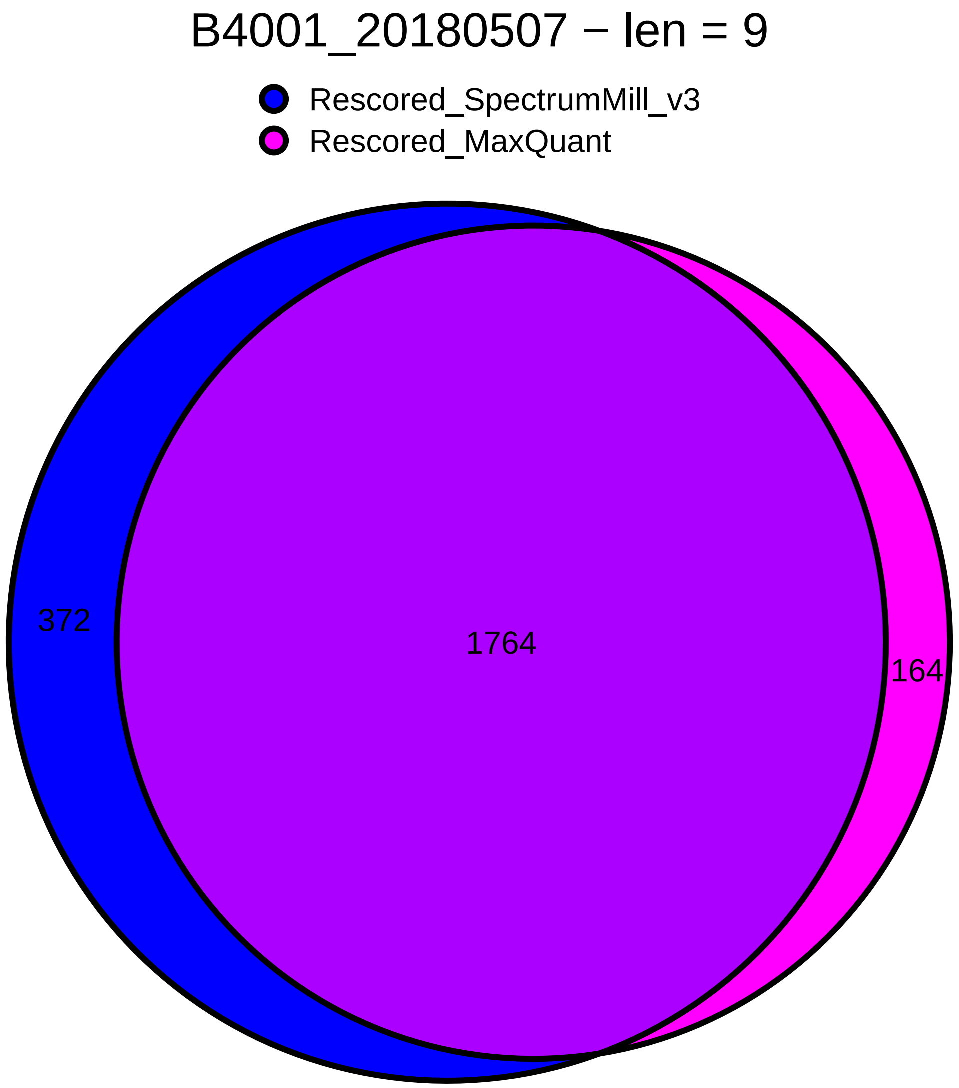
B4001_20180507 − len = 9
Rescored_SpectrumMill_v3
Rescored_MaxQuant
372
1764
164

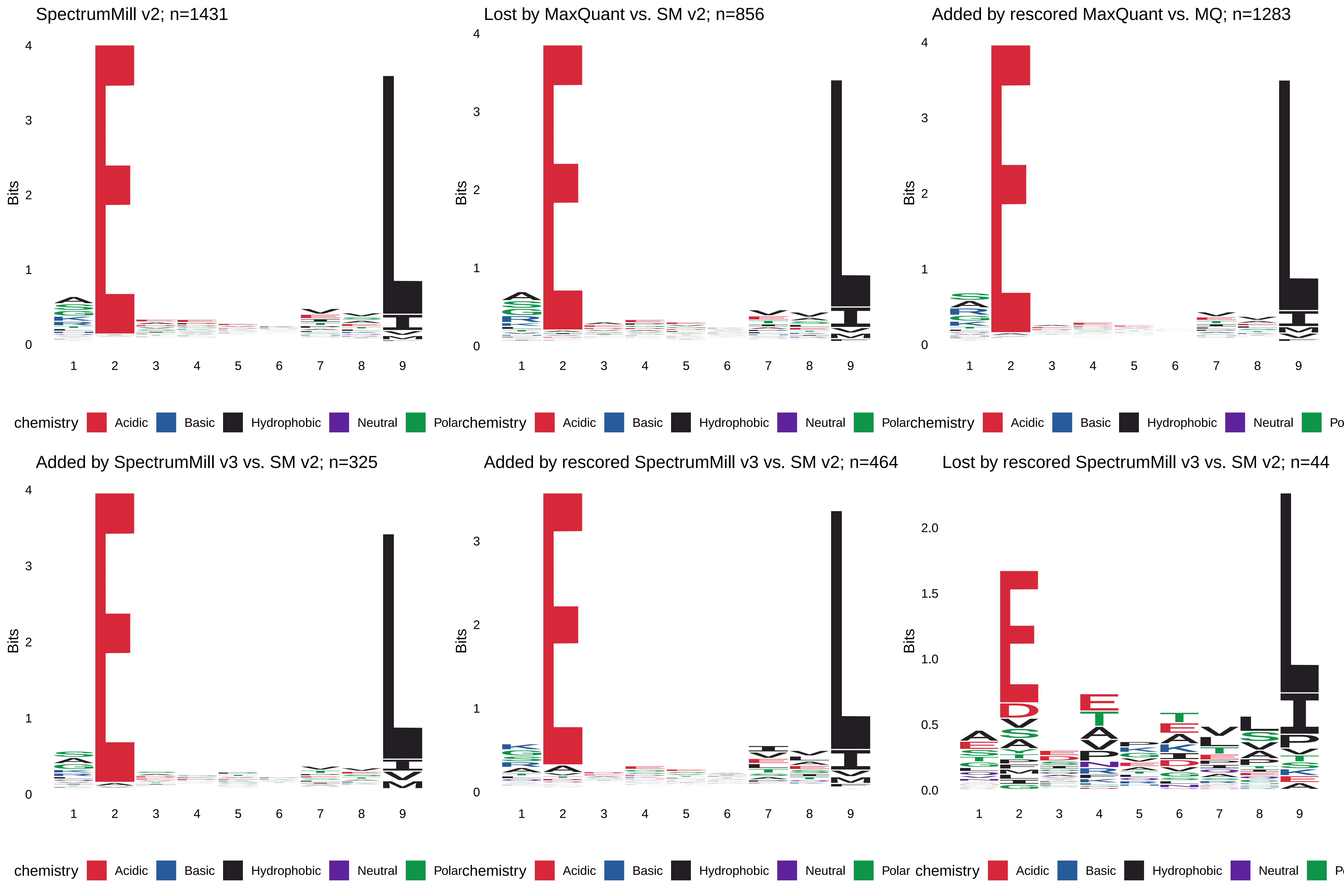
SpectrumMill v2; n=1431
Lost by MaxQuant vs. SM v2; n=856
Added by rescored MaxQuant vs. MQ; n=1283
Added by SpectrumMill v3 vs. SM v2; n=325
Added by rescored SpectrumMill v3 vs. SM v2; n=464
Lost by rescored SpectrumMill v3 vs. SM v2; n=44
Bits
chemistry
Acidic
Basic
Hydrophobic
Neutral
Polar

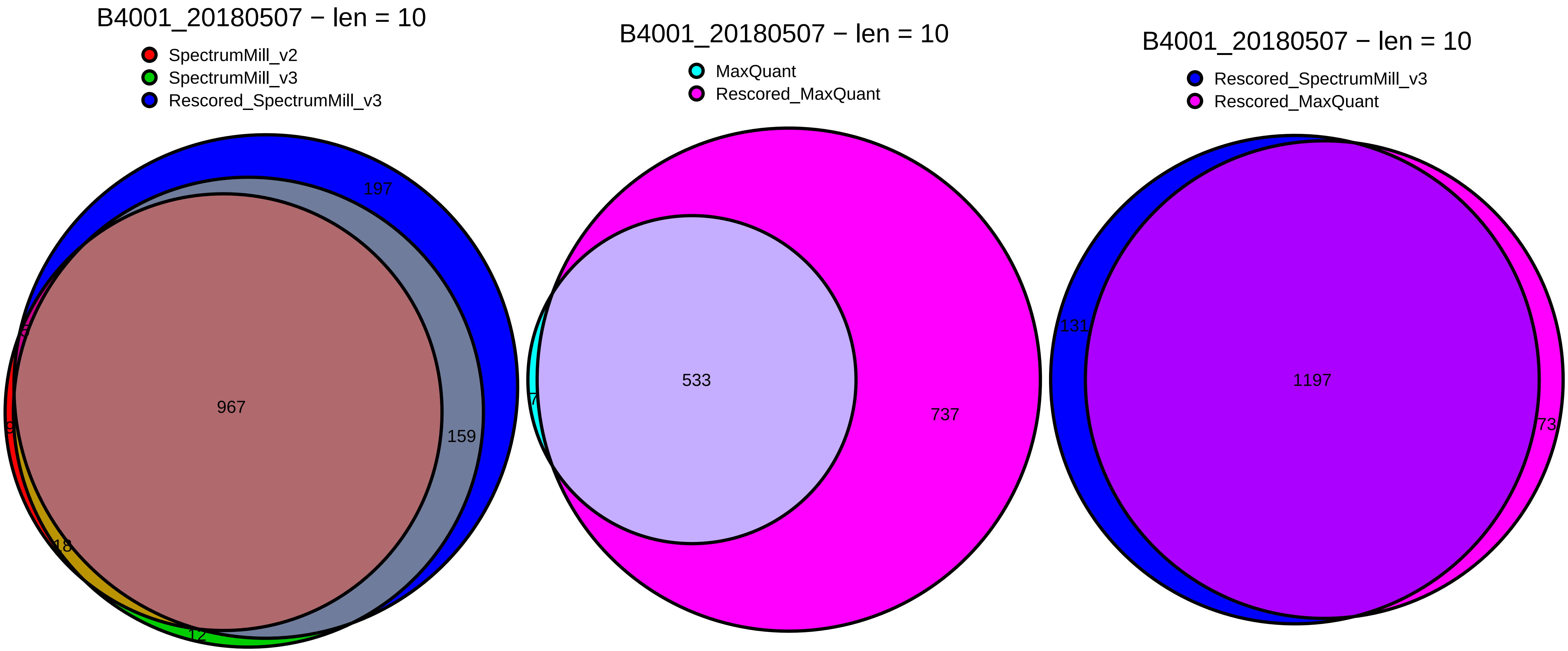
B4001_20180507 − len = 10
SpectrumMill_v2
SpectrumMill_v3
Rescored_SpectrumMill_v3
197
967
159
18
12
B4001_20180507 − len = 10
MaxQuant
Rescored_MaxQuant
533
737
B4001_20180507 − len = 10
Rescored_SpectrumMill_v3
Rescored_MaxQuant
131
1197

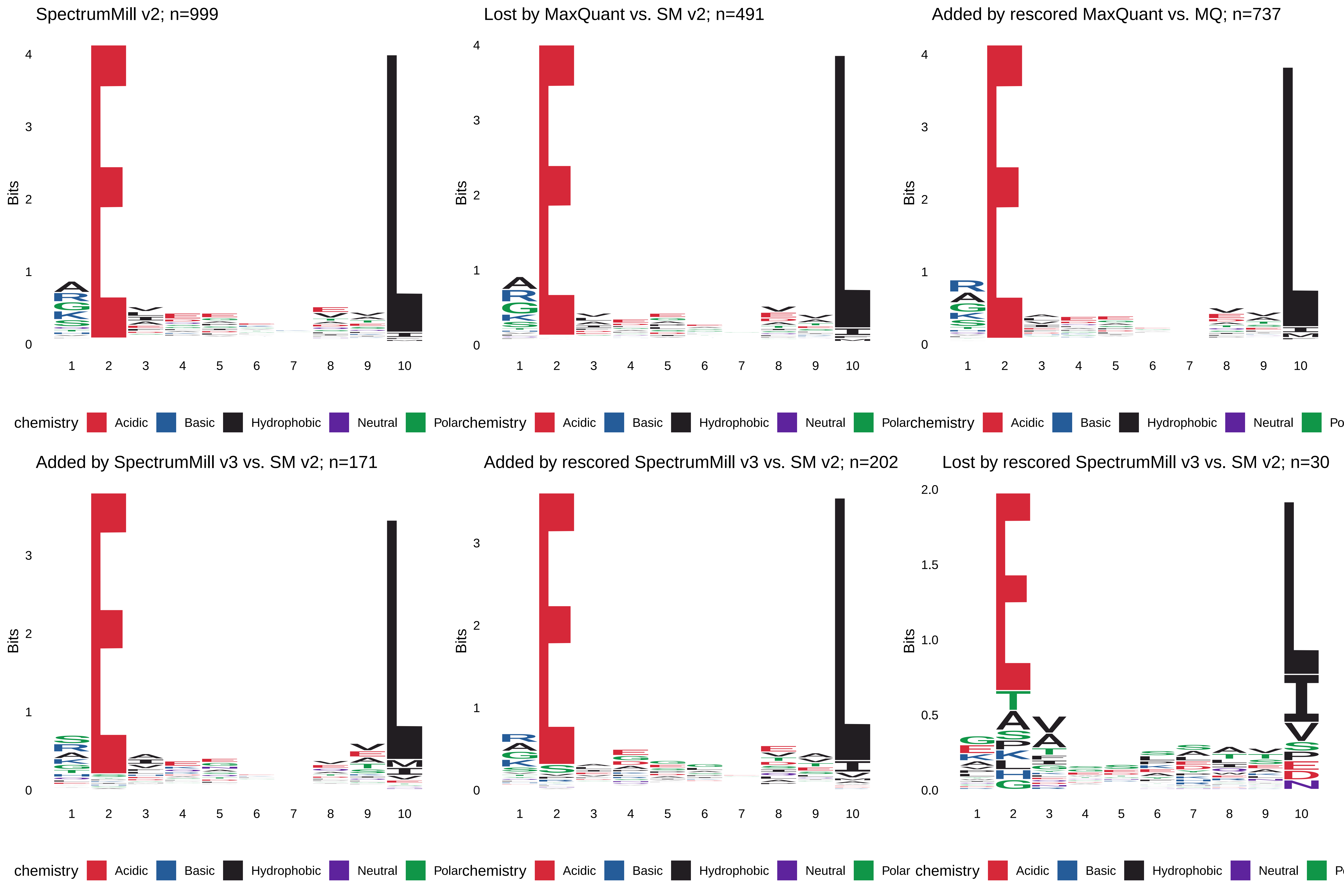
SpectrumMill v2; n=999
Lost by MaxQuant vs. SM v2; n=491
Added by rescored MaxQuant vs. MQ; n=737
Added by SpectrumMill v3 vs. SM v2; n=171
Added by rescored SpectrumMill v3 vs. SM v2; n=202
Lost by rescored SpectrumMill v3 vs. SM v2; n=30
Bits
chemistry
Acidic
Basic
Hydrophobic
Neutral
Polar

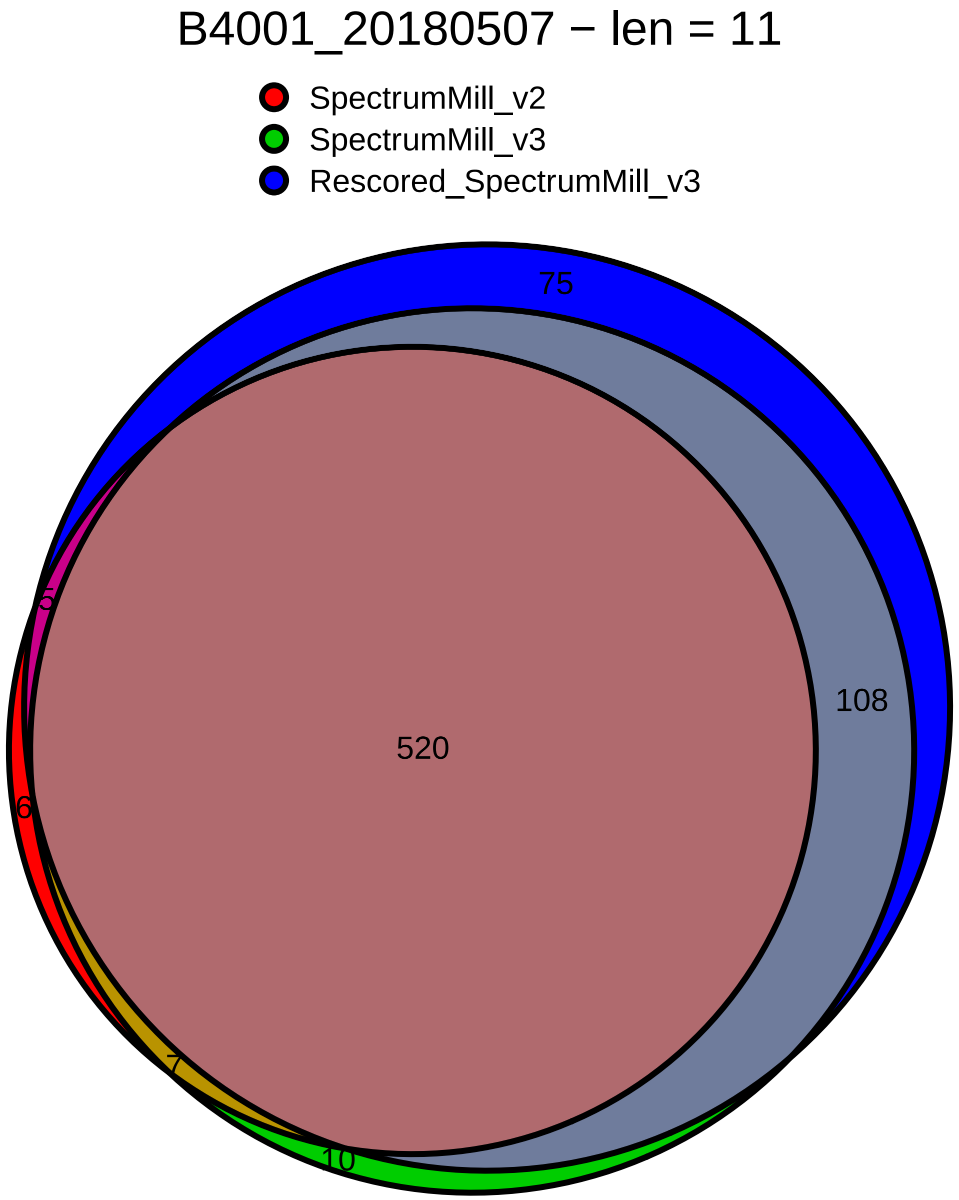
B4001_20180507 − len = 11
SpectrumMill_v2
SpectrumMill_v3
Rescored_SpectrumMill_v3
75
108
520
7
10

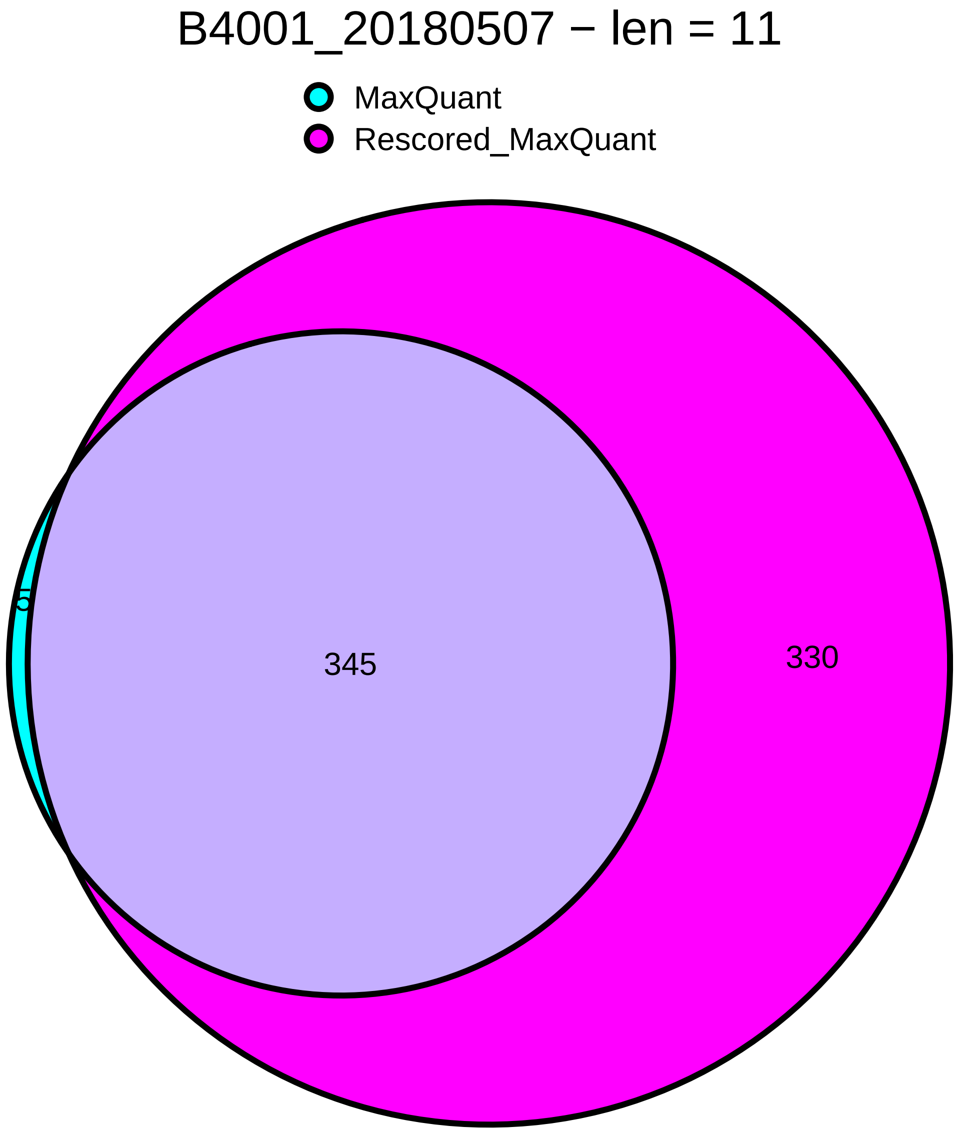
B4001_20180507 − len = 11
MaxQuant
Rescored_MaxQuant
345
330

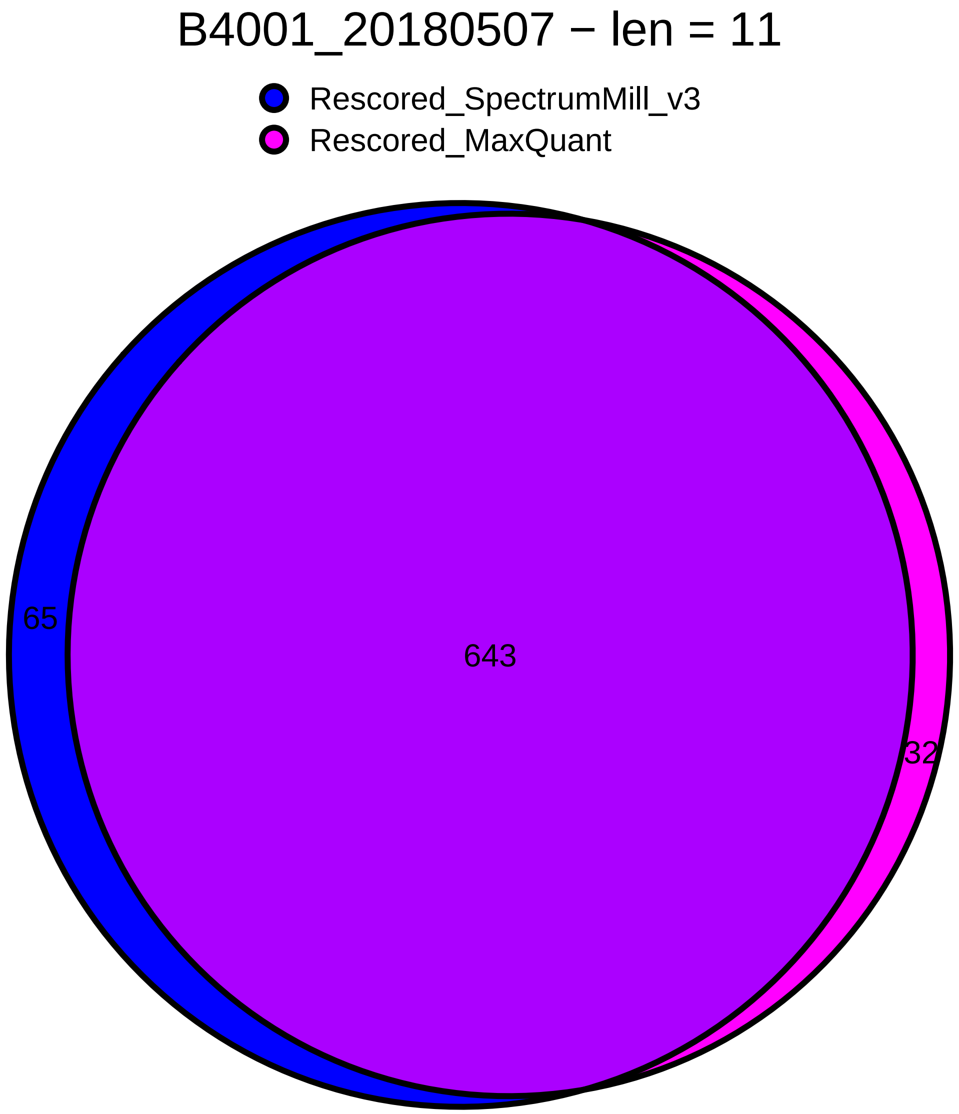
B4001_20180507 − len = 11
Rescored_SpectrumMill_v3
Rescored_MaxQuant
65
643
32

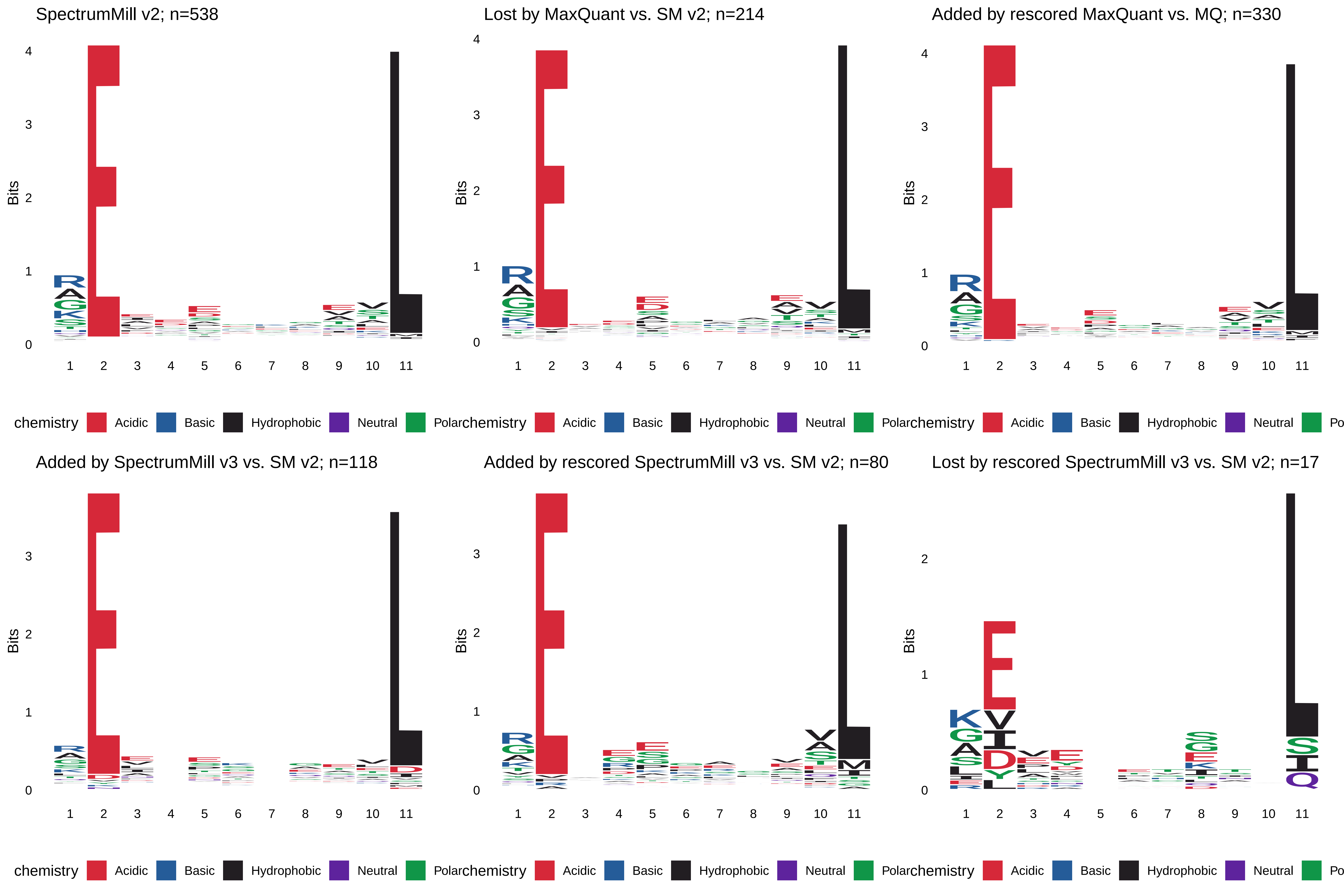
SpectrumMill v2; n=538
Lost by MaxQuant vs. SM v2; n=214
Added by rescored MaxQuant vs. MQ; n=330
Added by SpectrumMill v3 vs. SM v2; n=118
Added by rescored SpectrumMill v3 vs. SM v2; n=80
Lost by rescored SpectrumMill v3 vs. SM v2; n=17
Bits
chemistry
Acidic
Basic
Hydrophobic
Neutral
Polar

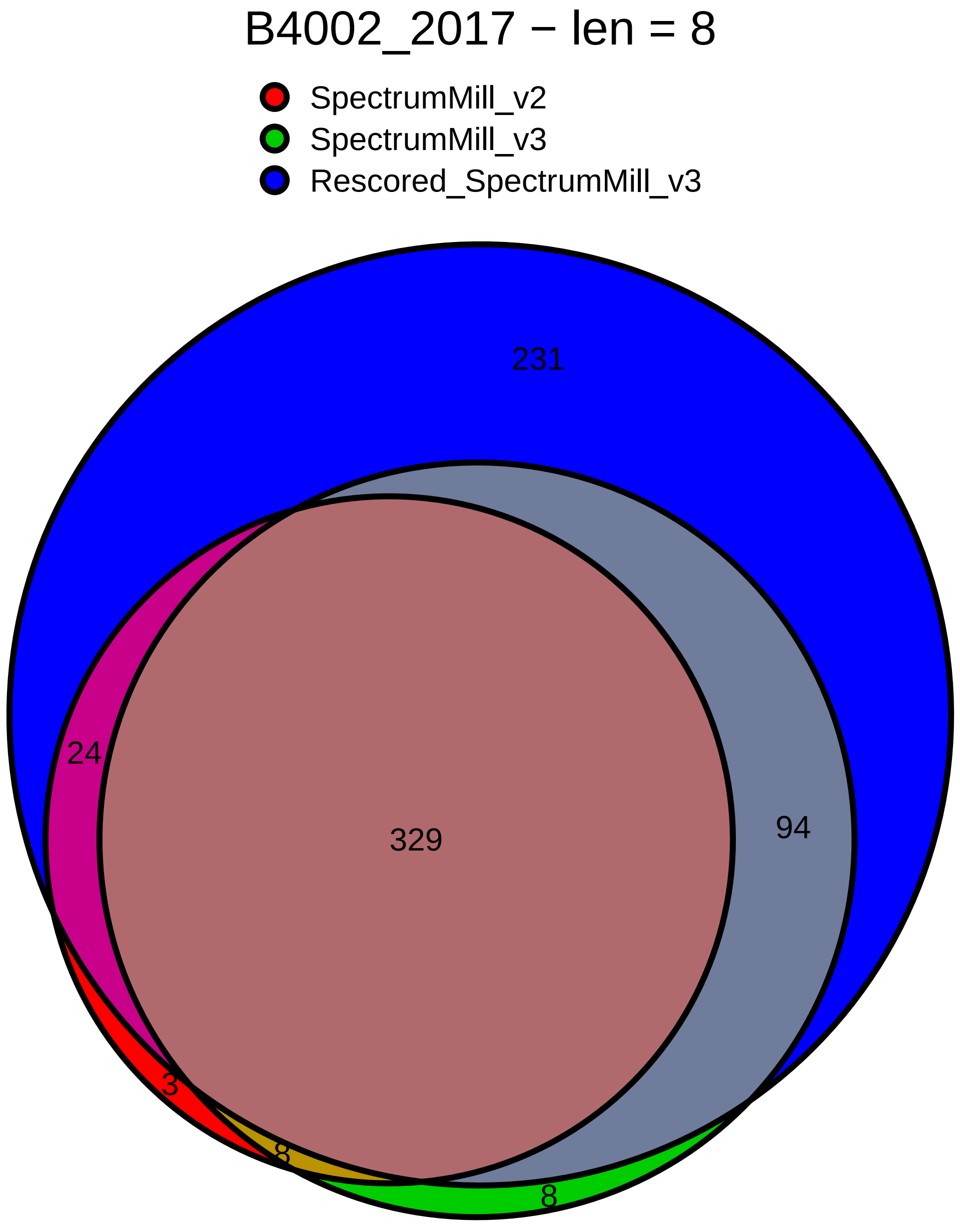
B4002_2017 − len = 8
SpectrumMill_v2
SpectrumMill_v3
Rescored_SpectrumMill_v3
231
24
329
94
3
8
8

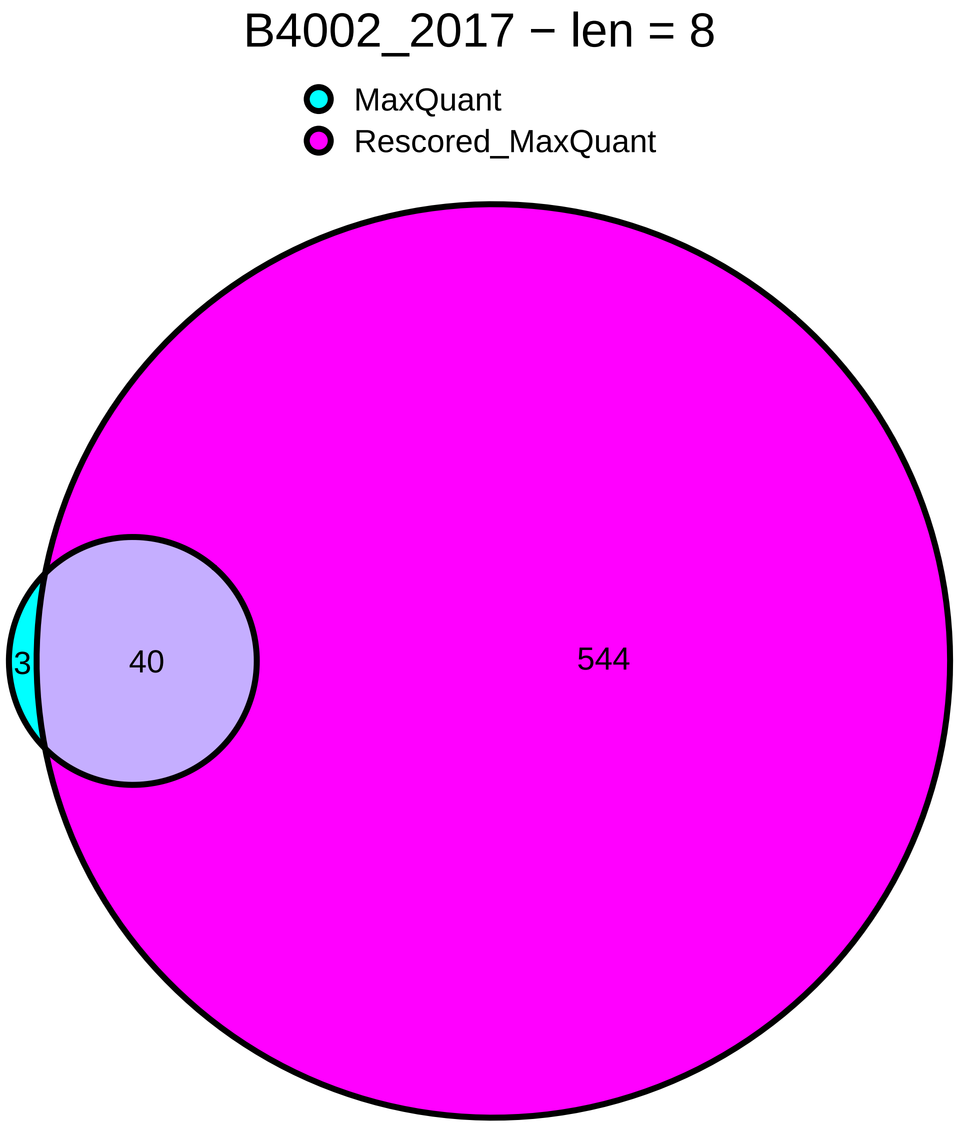
B4002_2017 − len = 8
MaxQuant
Rescored_MaxQuant
3
40
544

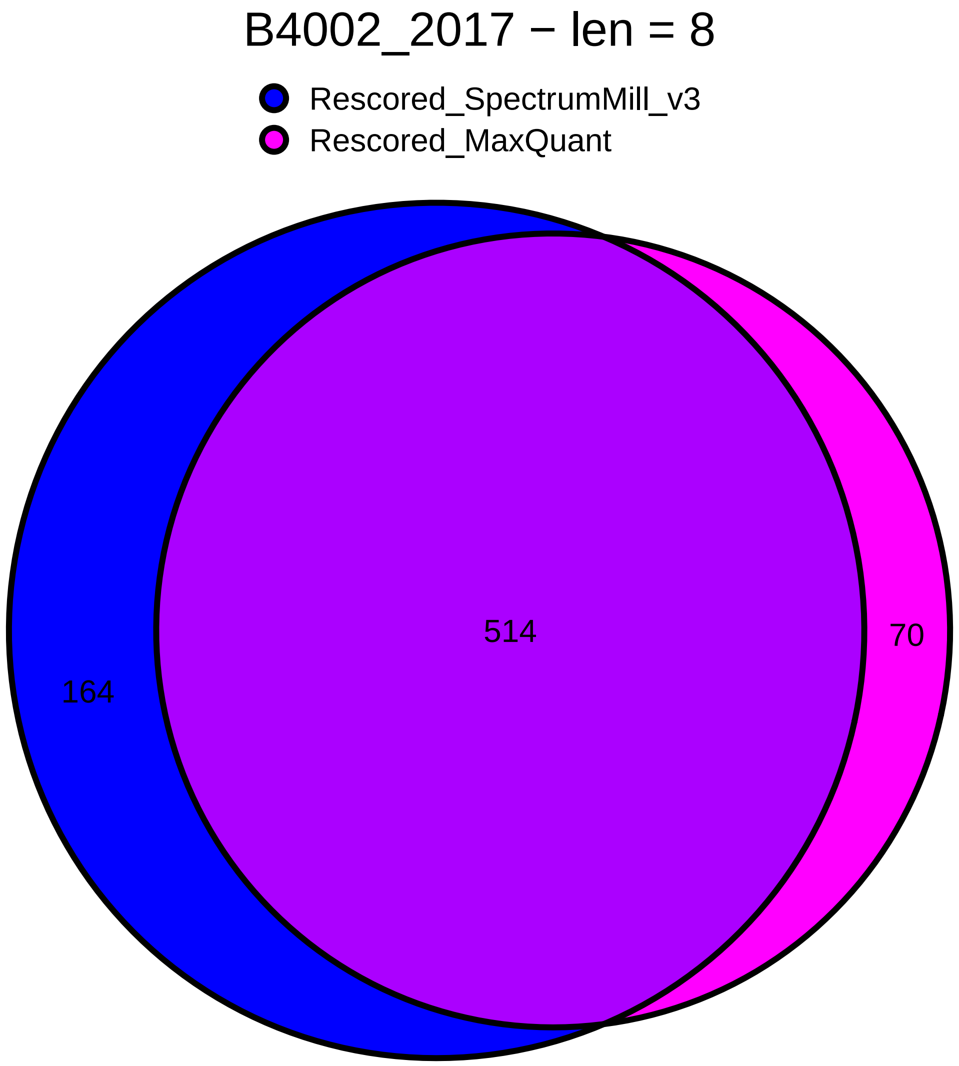
B4002_2017 − len = 8
Rescored_SpectrumMill_v3
Rescored_MaxQuant
164
514
70

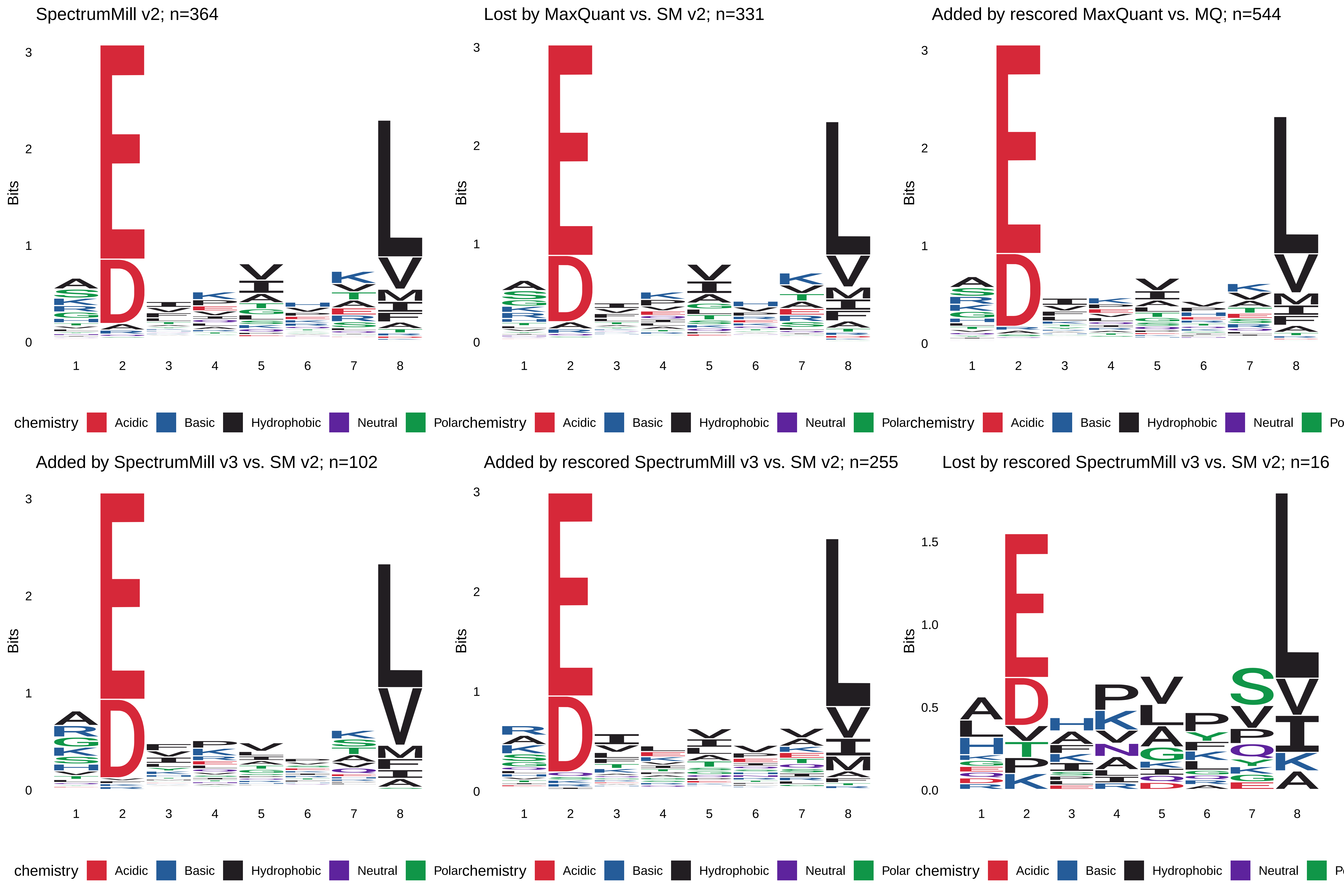
SpectrumMill v2; n=364
Lost by MaxQuant vs. SM v2; n=331
Added by rescored MaxQuant vs. MQ; n=544
Bits
0
1
2
3
1 2 3 4 5 6 7 8
chemistry
Acidic
Basic
Hydrophobic
Neutral
Polar
Added by SpectrumMill v3 vs. SM v2; n=102
Added by rescored SpectrumMill v3 vs. SM v2; n=255
Lost by rescored SpectrumMill v3 vs. SM v2; n=16
0.0
0.5
1.0
1.5

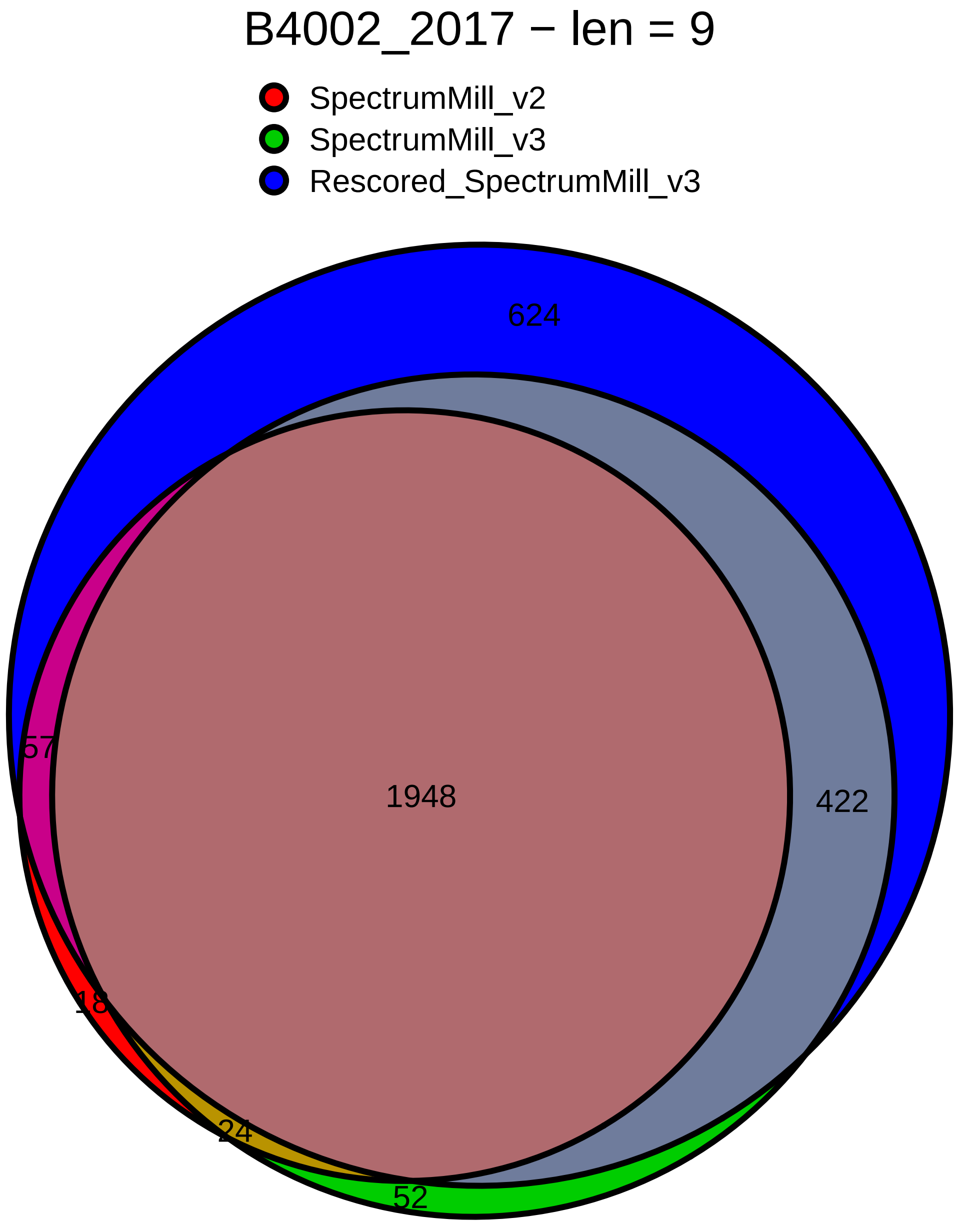

B4002_2017 − len = 9
SpectrumMill_v2
SpectrumMill_v3
Rescored_SpectrumMill_v3
624
57
1948
422
18
24
52

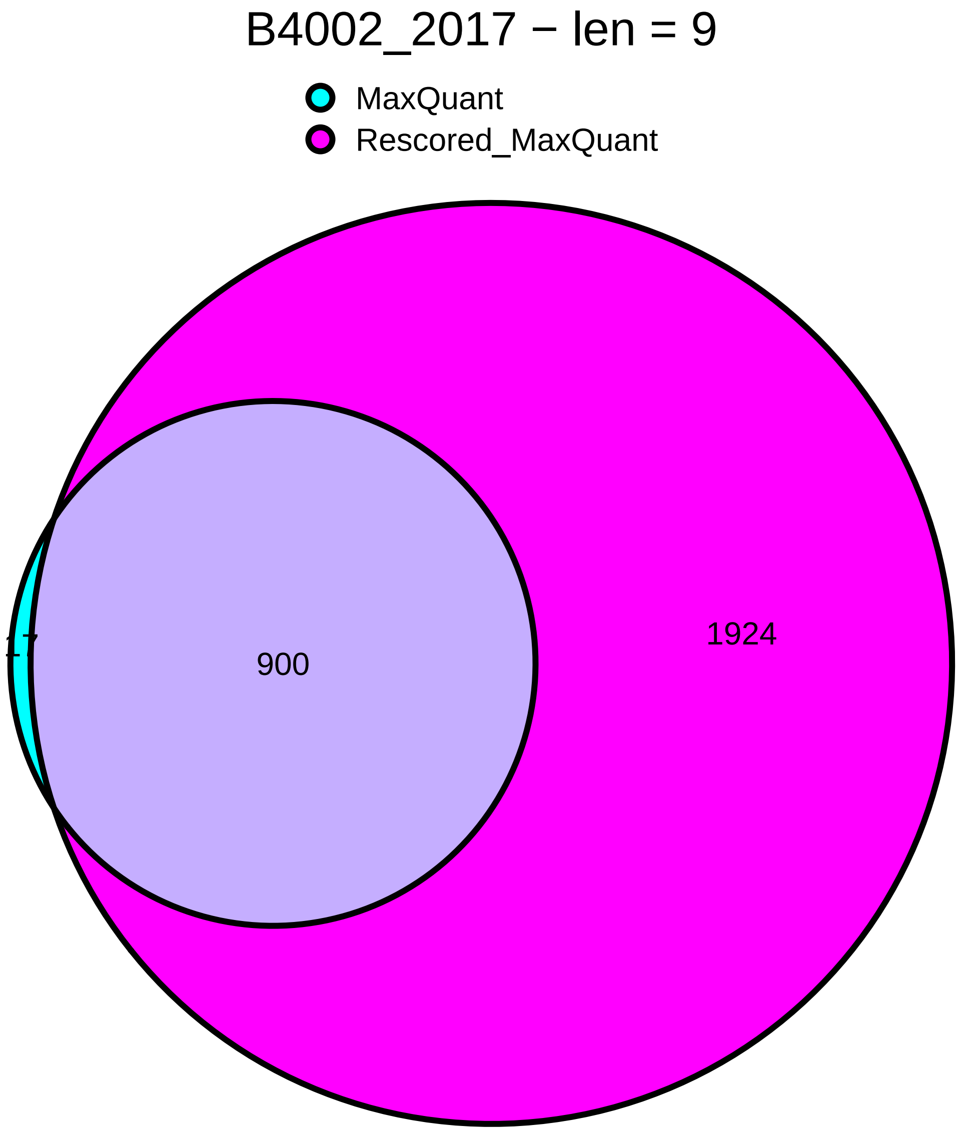

B4002_2017 − len = 9
MaxQuant
Rescored_MaxQuant
17
900
1924

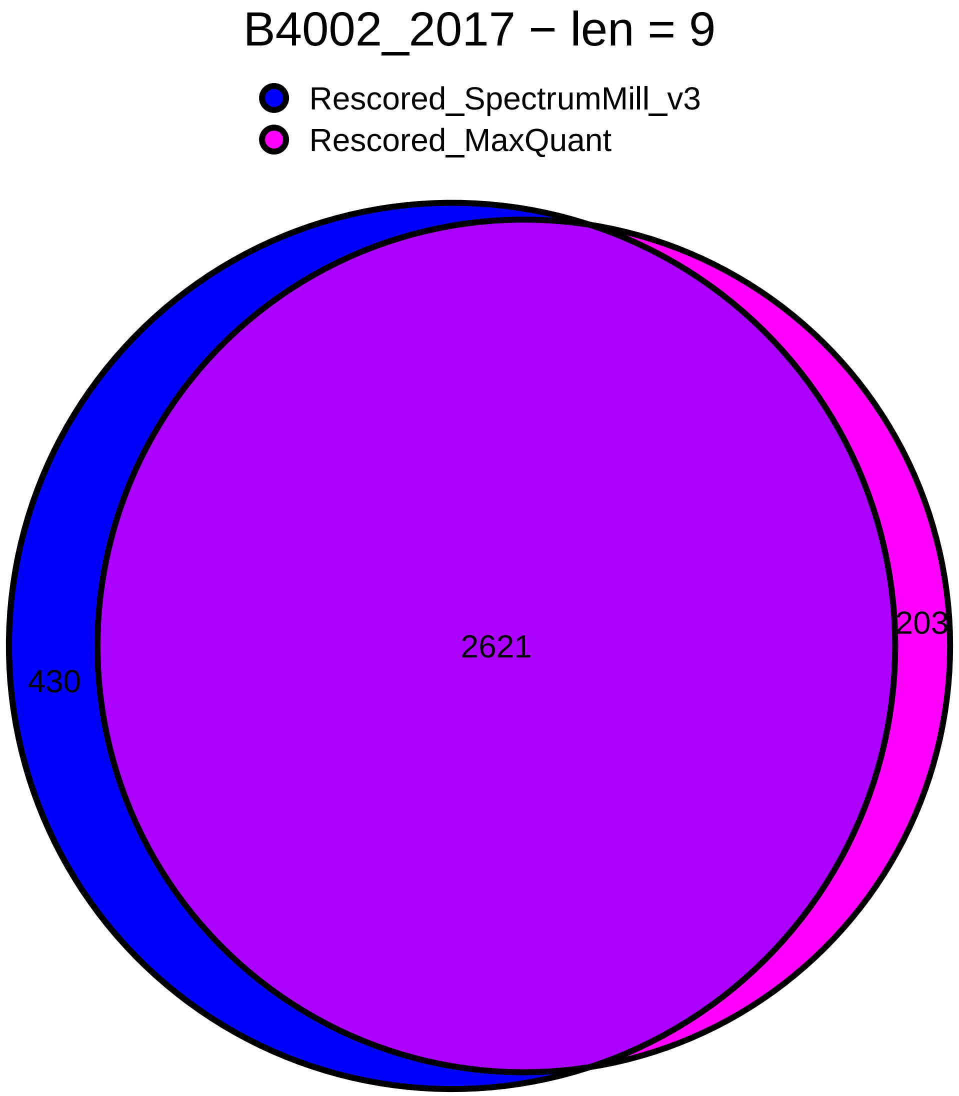

B4002_2017 − len = 9
Rescored_SpectrumMill_v3
Rescored_MaxQuant
430
2621
203

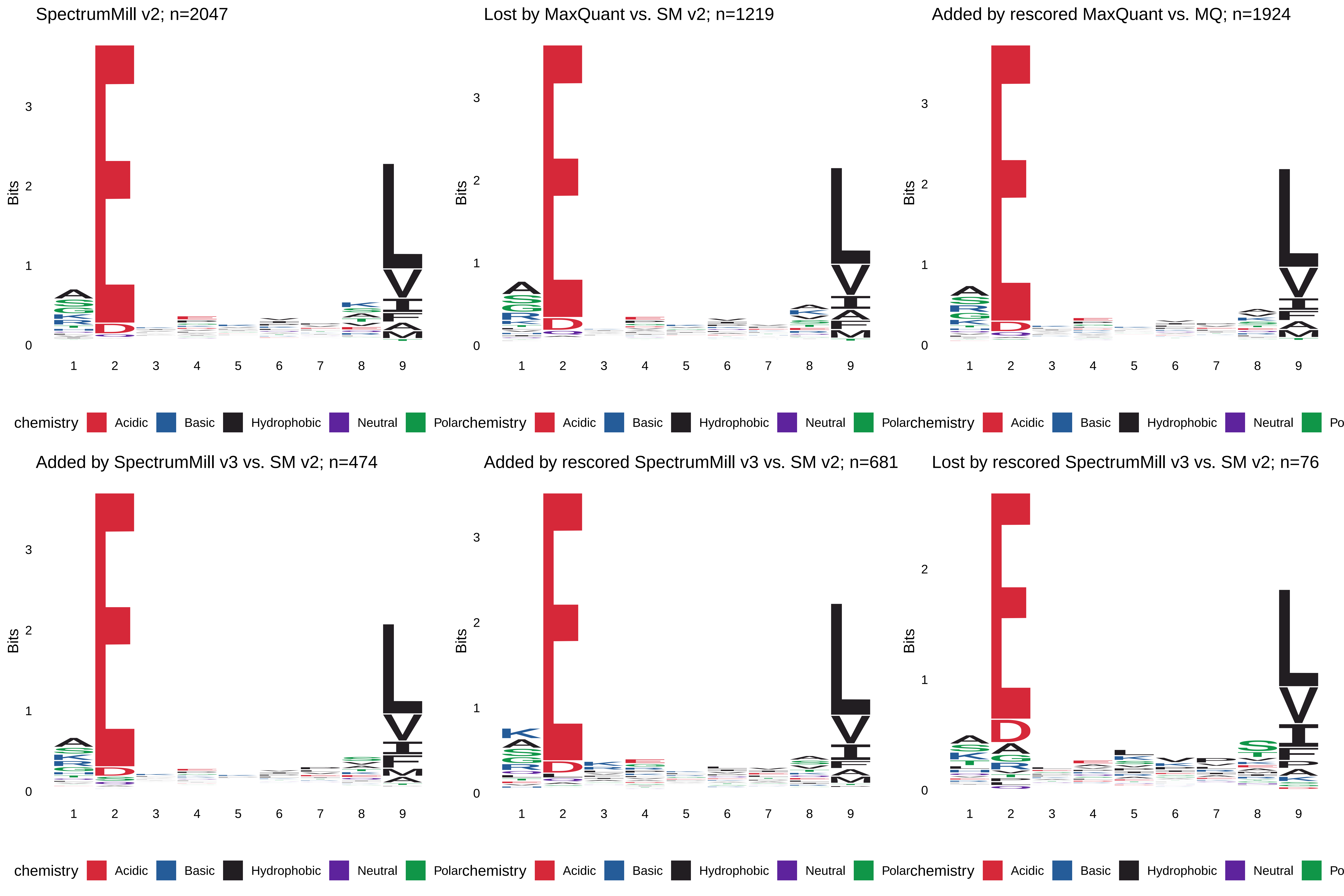
SpectrumMill v2; n=2047
Lost by MaxQuant vs. SM v2; n=1219
Added by rescored MaxQuant vs. MQ; n=1924
Added by SpectrumMill v3 vs. SM v2; n=474
Added by rescored SpectrumMill v3 vs. SM v2; n=681
Lost by rescored SpectrumMill v3 vs. SM v2; n=76
Bits
0
1
2
3
1 2 3 4 5 6 7 8 9
chemistry
Acidic
Basic
Hydrophobic
Neutral
Polar

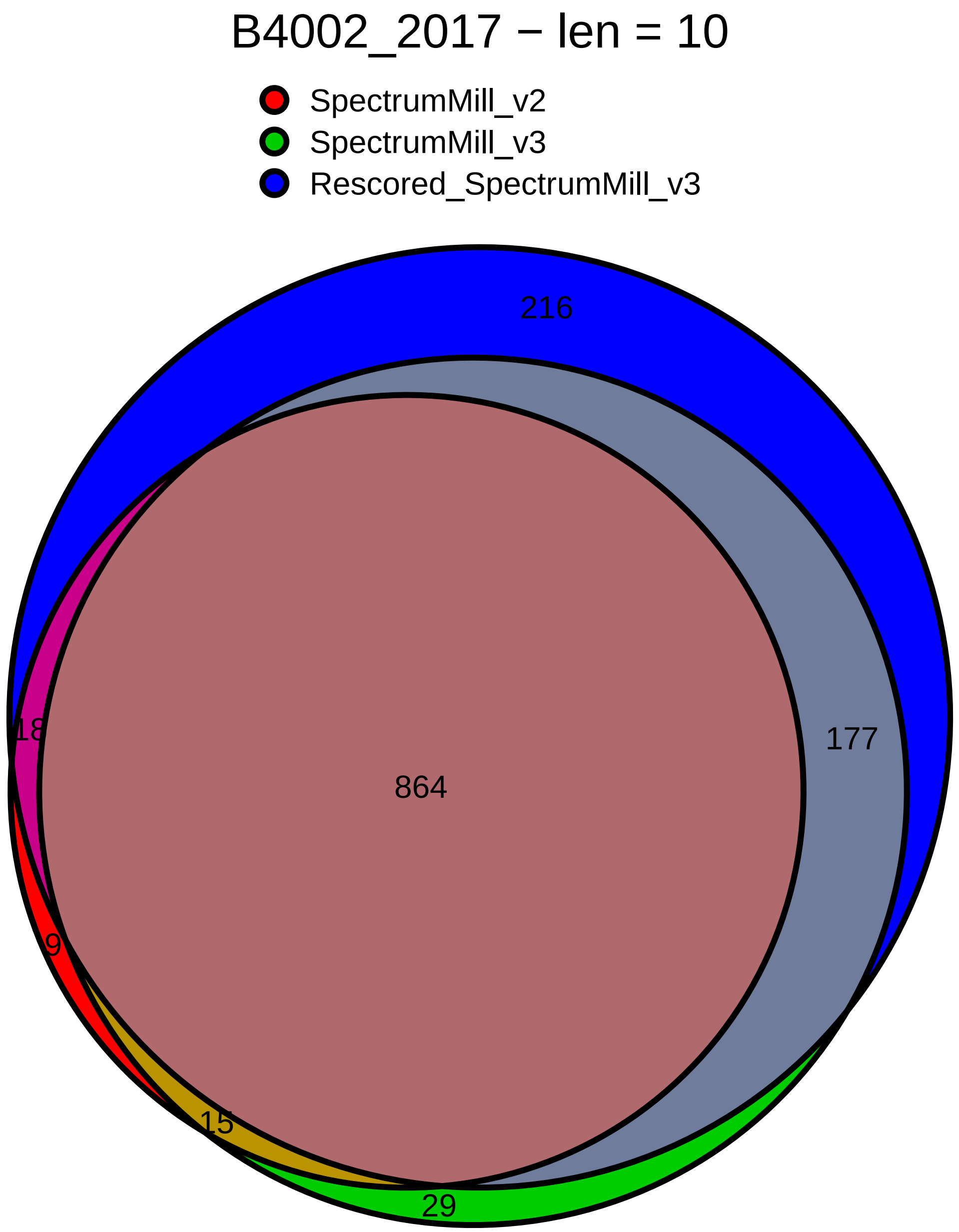
B4002_2017 − len = 10
SpectrumMill_v2
SpectrumMill_v3
Rescored_SpectrumMill_v3
216
18
864
177
9
15
29

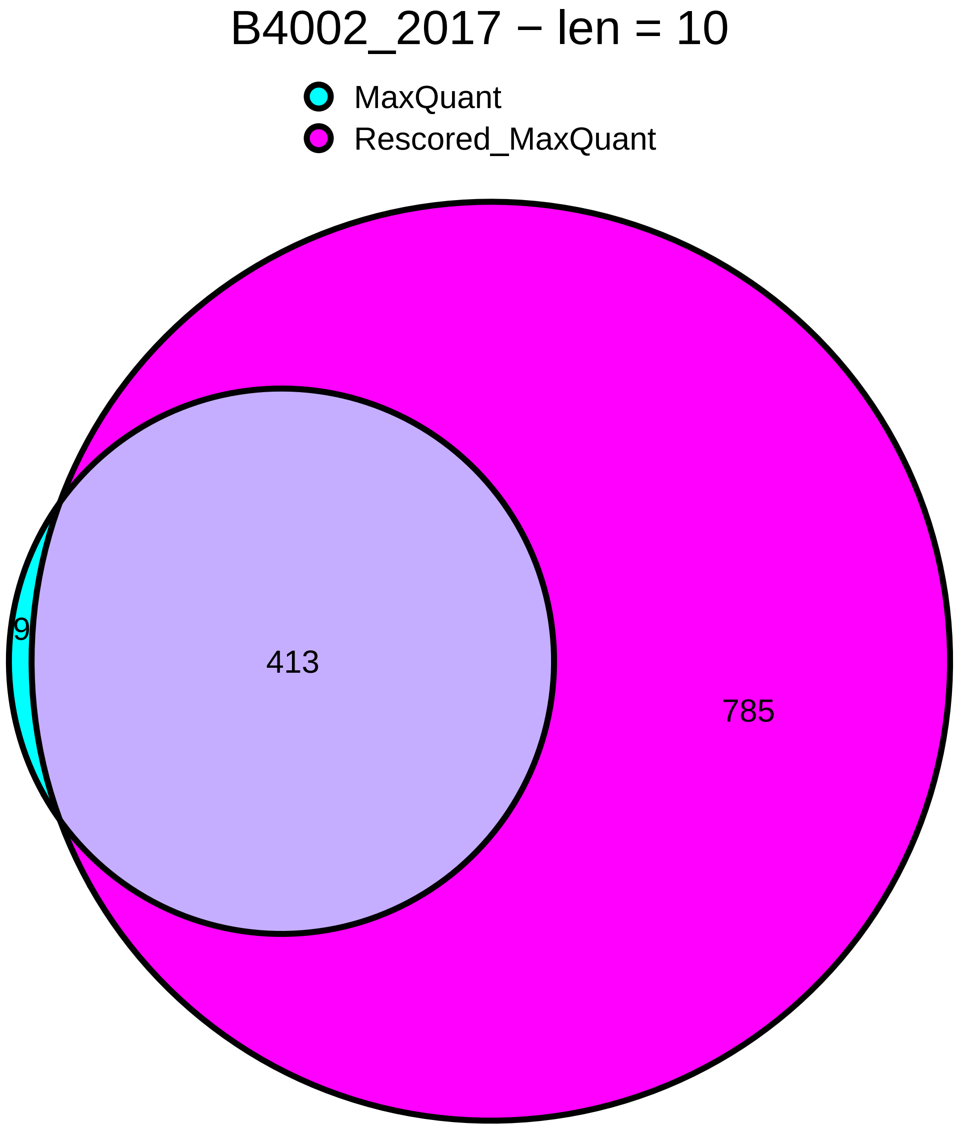
B4002_2017 − len = 10
MaxQuant
Rescored_MaxQuant
9
413
785

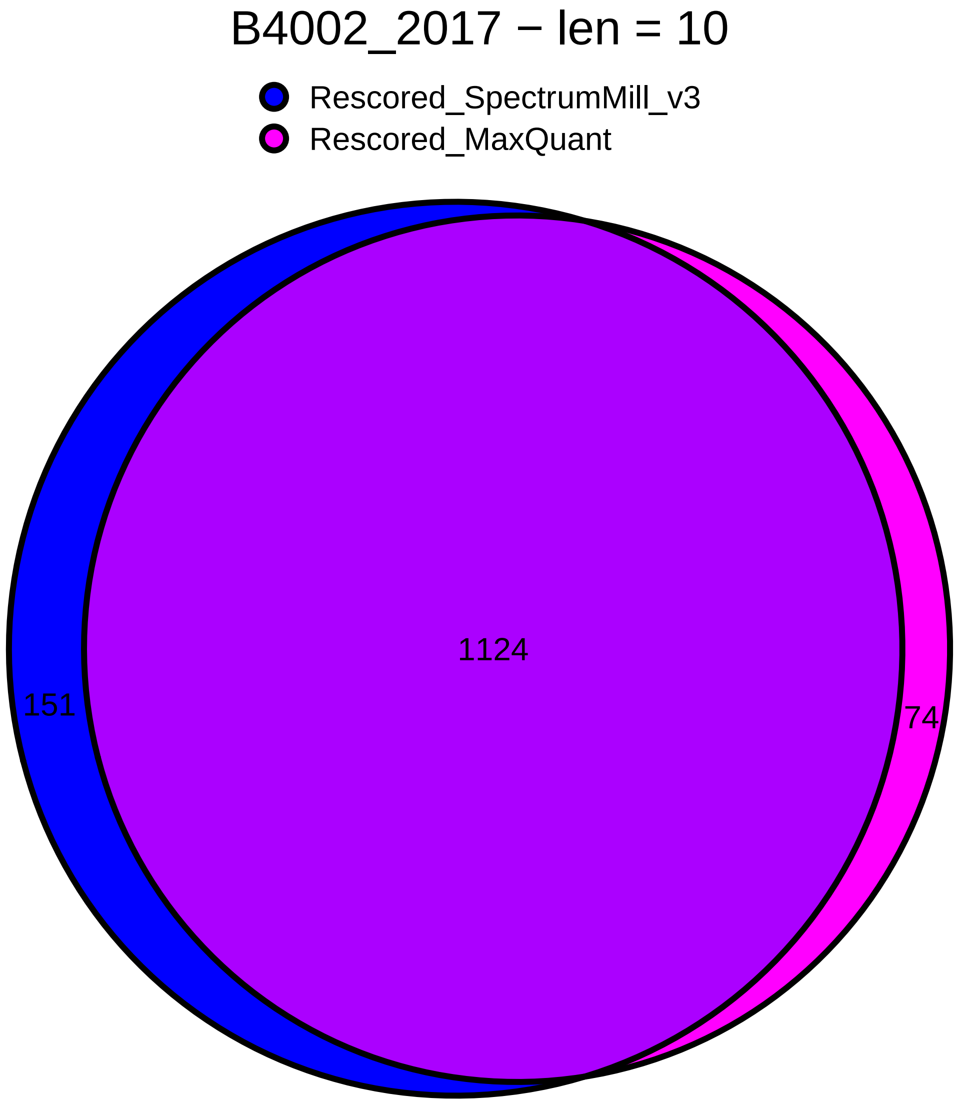
B4002_2017 − len = 10
Rescored_SpectrumMill_v3
Rescored_MaxQuant
151
1124
74

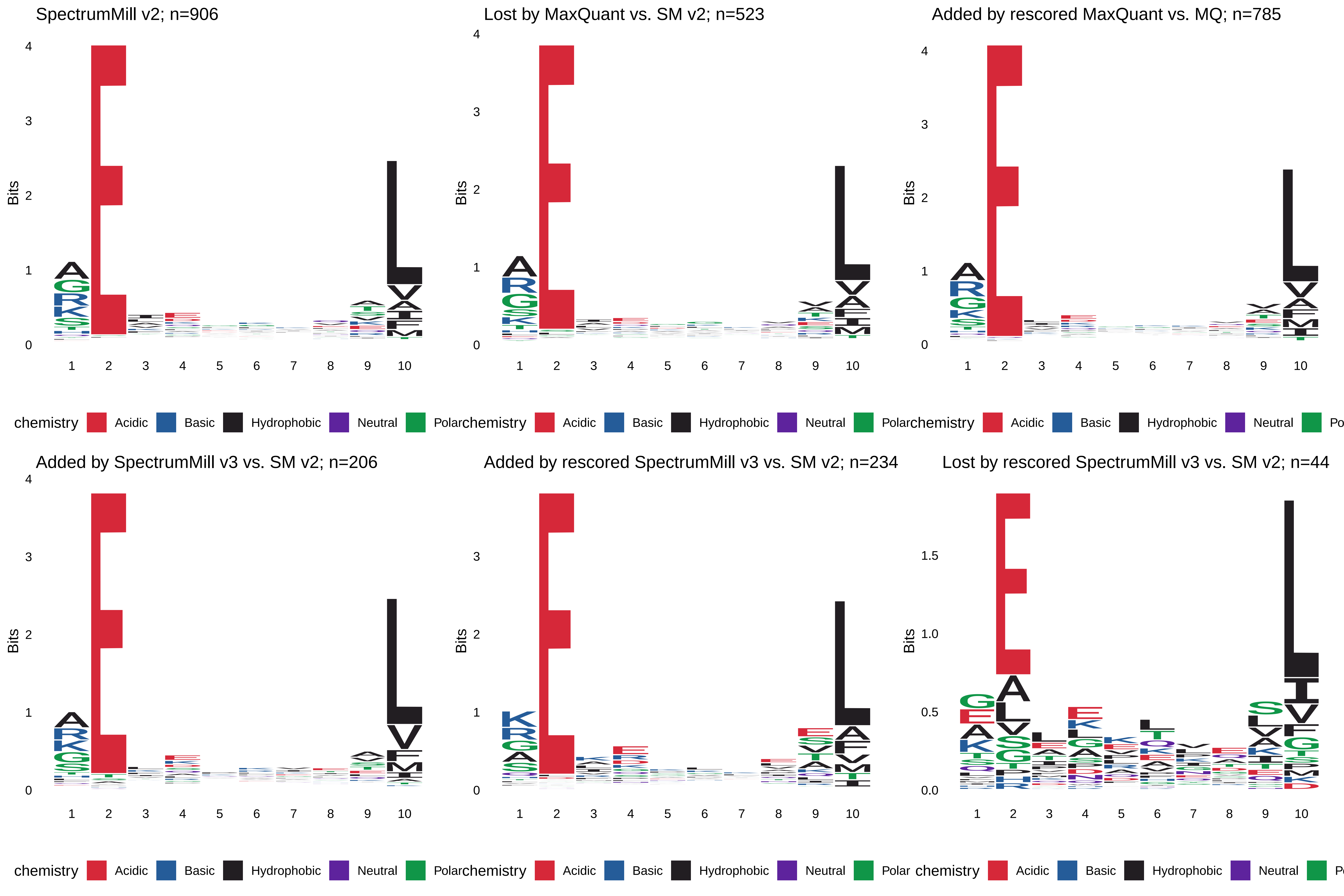
SpectrumMill v2; n=906
Lost by MaxQuant vs. SM v2; n=523
Added by rescored MaxQuant vs. MQ; n=785
Added by SpectrumMill v3 vs. SM v2; n=206
Added by rescored SpectrumMill v3 vs. SM v2; n=234
Lost by rescored SpectrumMill v3 vs. SM v2; n=44
Bits
0
1
2
3
4
0.0
0.5
1.0
1.5
1 2 3 4 5 6 7 8 9 10
chemistry
Acidic
Basic
Hydrophobic
Neutral
Polar

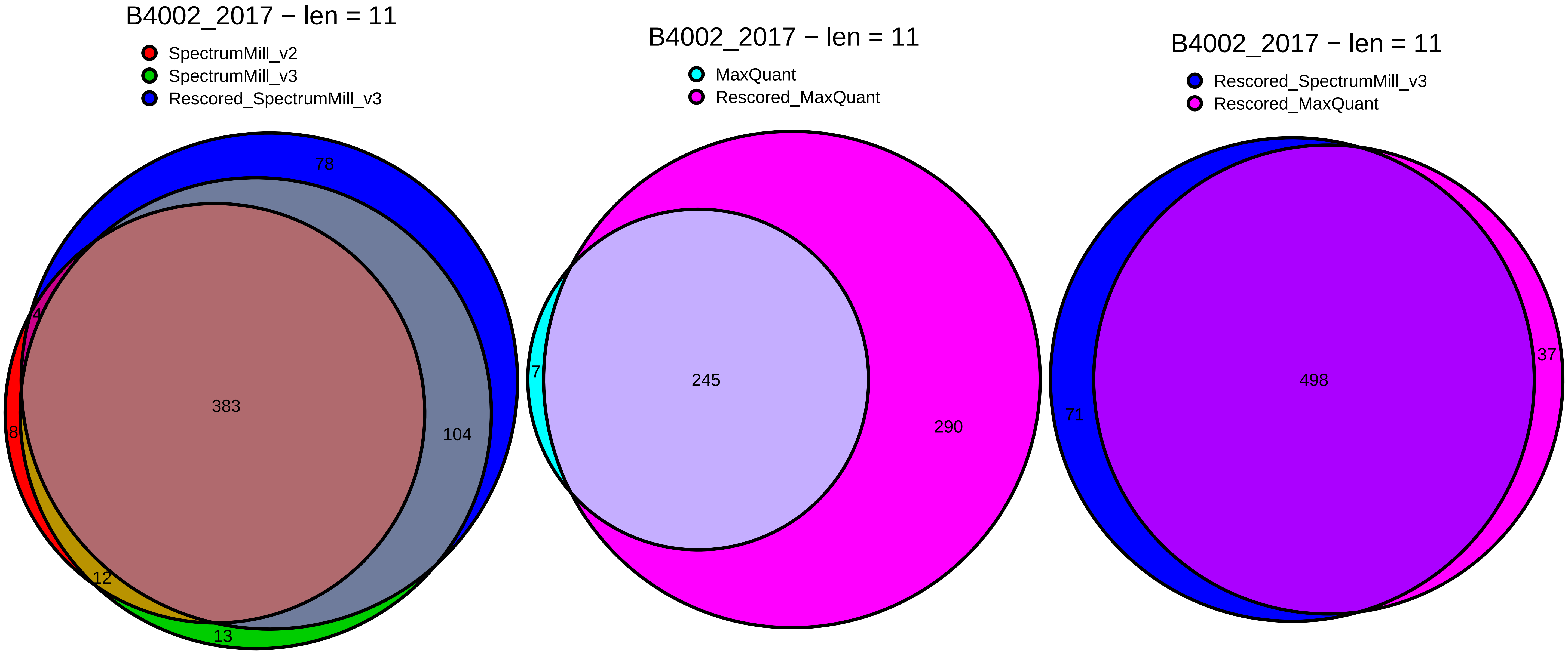

B4002_2017 − len = 11
SpectrumMill_v2
SpectrumMill_v3
Rescored_SpectrumMill_v3
78
4
383
104
8
12
13
B4002_2017 − len = 11
MaxQuant
Rescored_MaxQuant
7
245
290
B4002_2017 − len = 11
Rescored_SpectrumMill_v3
Rescored_MaxQuant
498
71
37

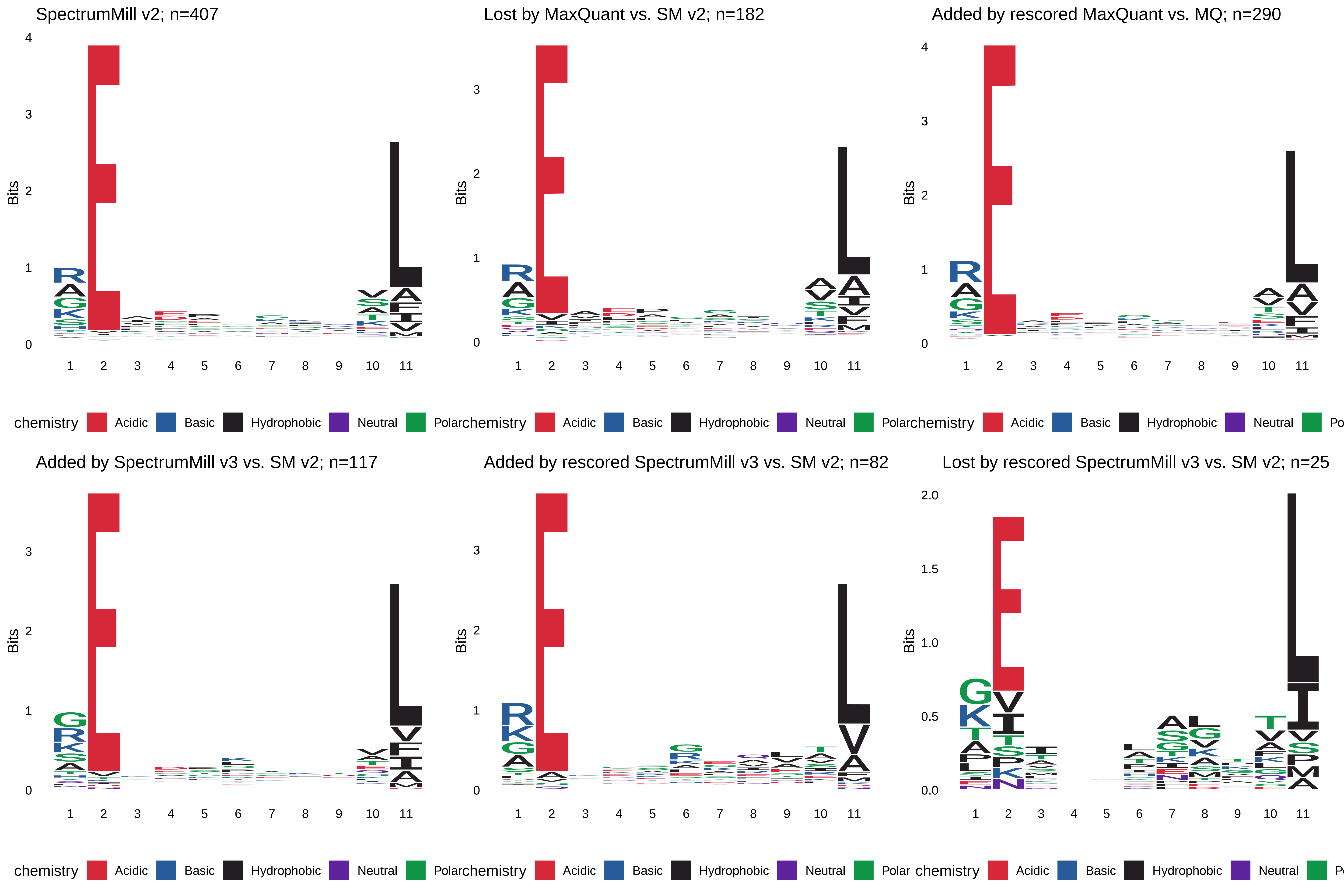

SpectrumMill v2; n=407
Lost by MaxQuant vs. SM v2; n=182
Added by rescored MaxQuant vs. MQ; n=290
Bits
1 2 3 4 5 6 7 8 9 10 11
chemistry Acidic Basic Hydrophobic Neutral Polar
Added by SpectrumMill v3 vs. SM v2; n=117
Added by rescored SpectrumMill v3 vs. SM v2; n=82
Lost by rescored SpectrumMill v3 vs. SM v2; n=25
Bits
1 2 3 4 5 6 7 8 9 10 11
chemistry Acidic Basic Hydrophobic Neutral Polar

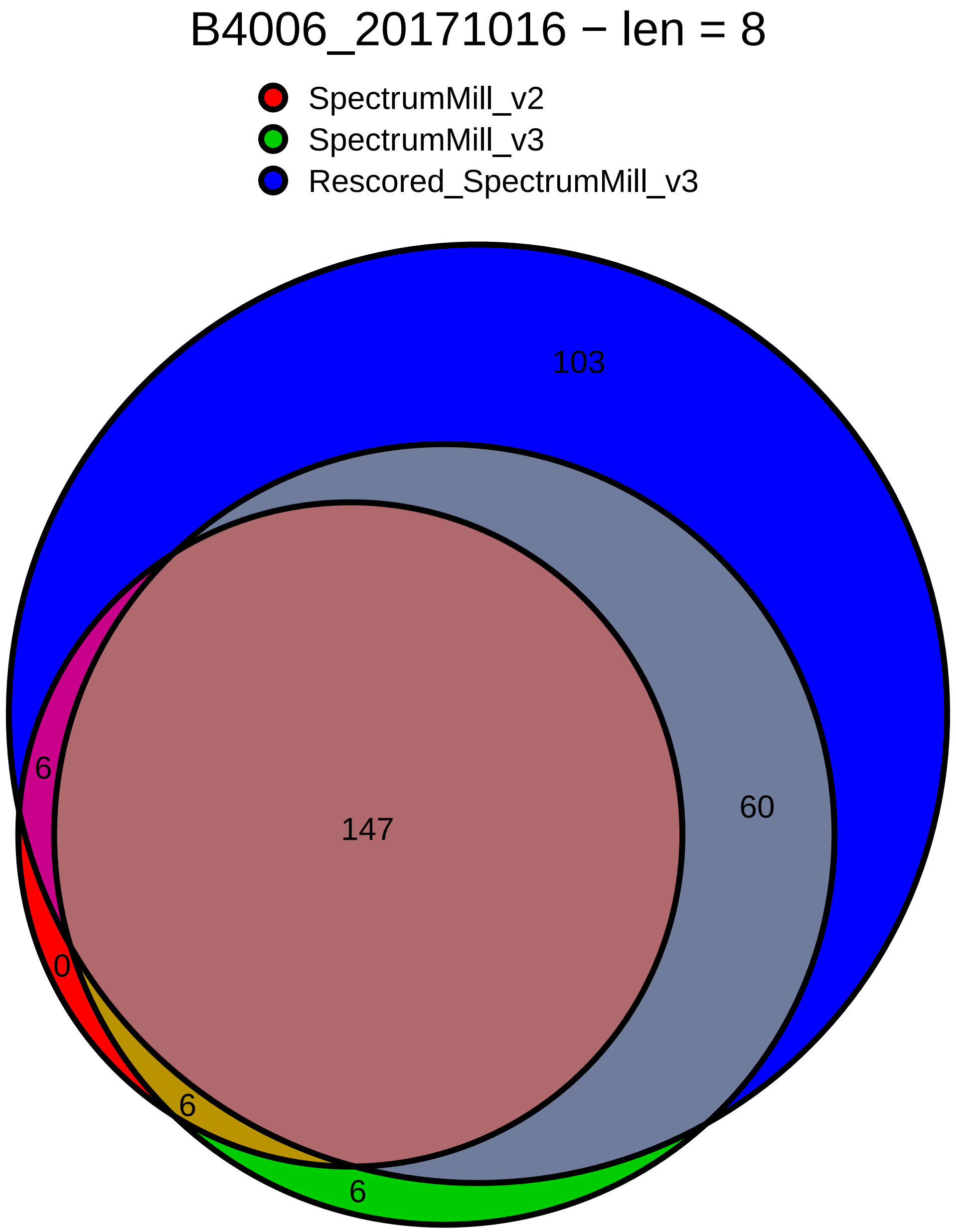
B4006_20171016 − len = 8
SpectrumMill_v2
SpectrumMill_v3
Rescored_SpectrumMill_v3
103
6
147
60
0
6
6
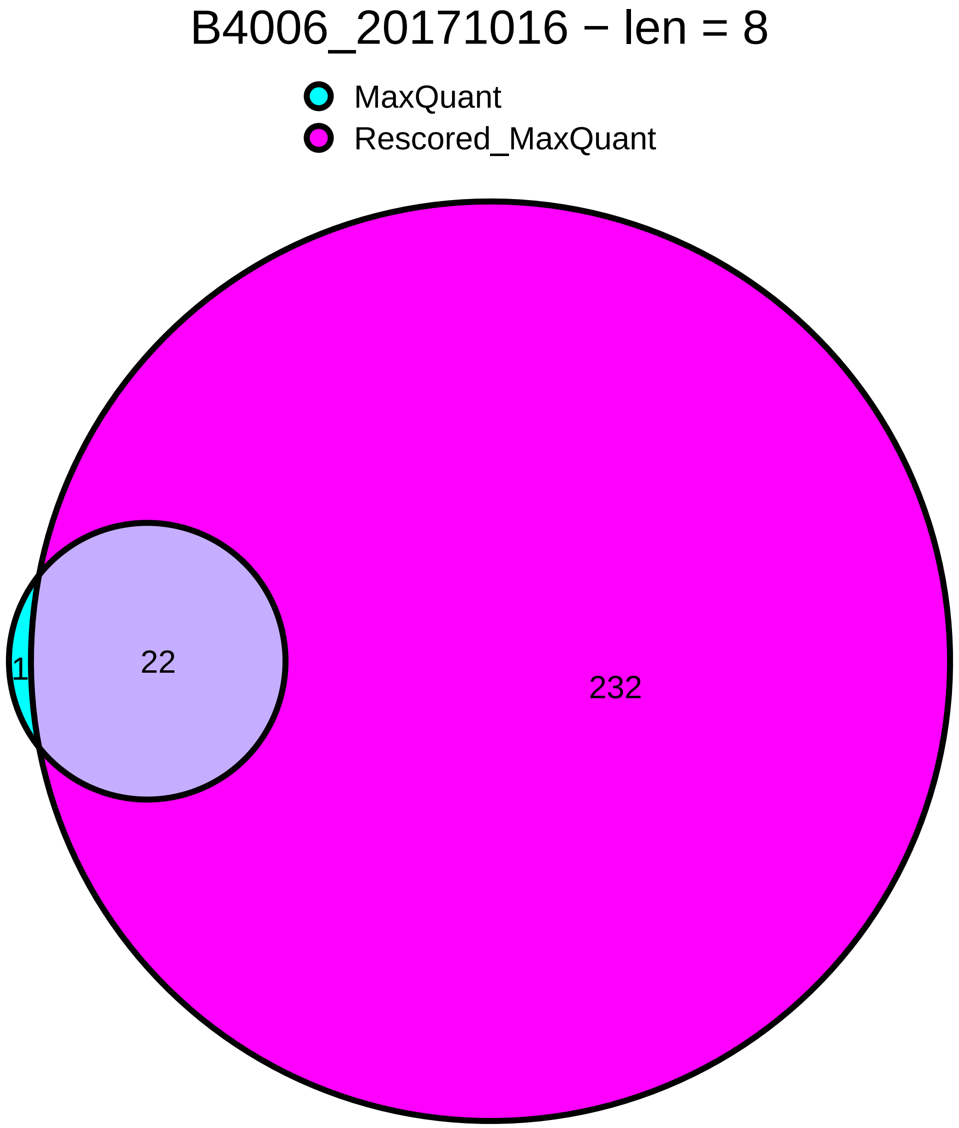
B4006_20171016 − len = 8
MaxQuant
Rescored_MaxQuant
1
22
232
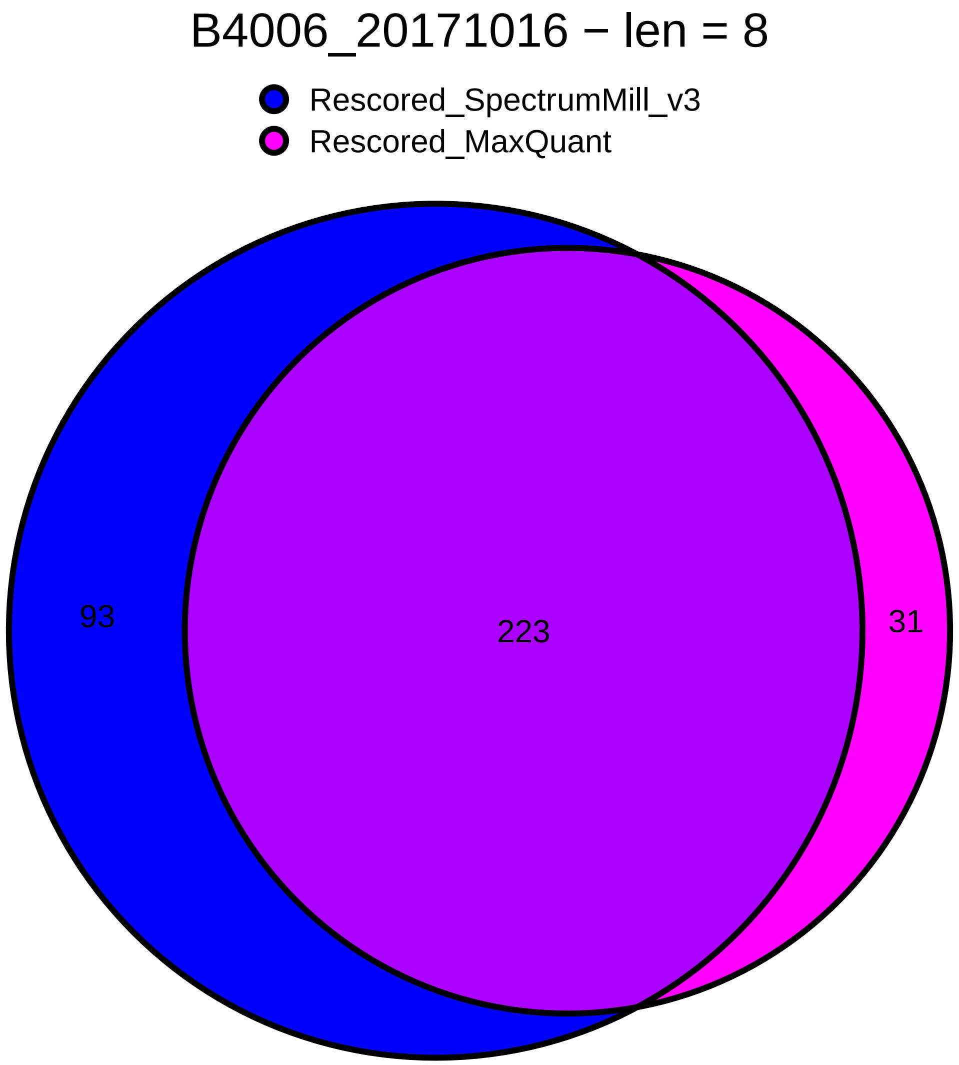
B4006_20171016 − len = 8
Rescored_SpectrumMill_v3
Rescored_MaxQuant
93
223
31

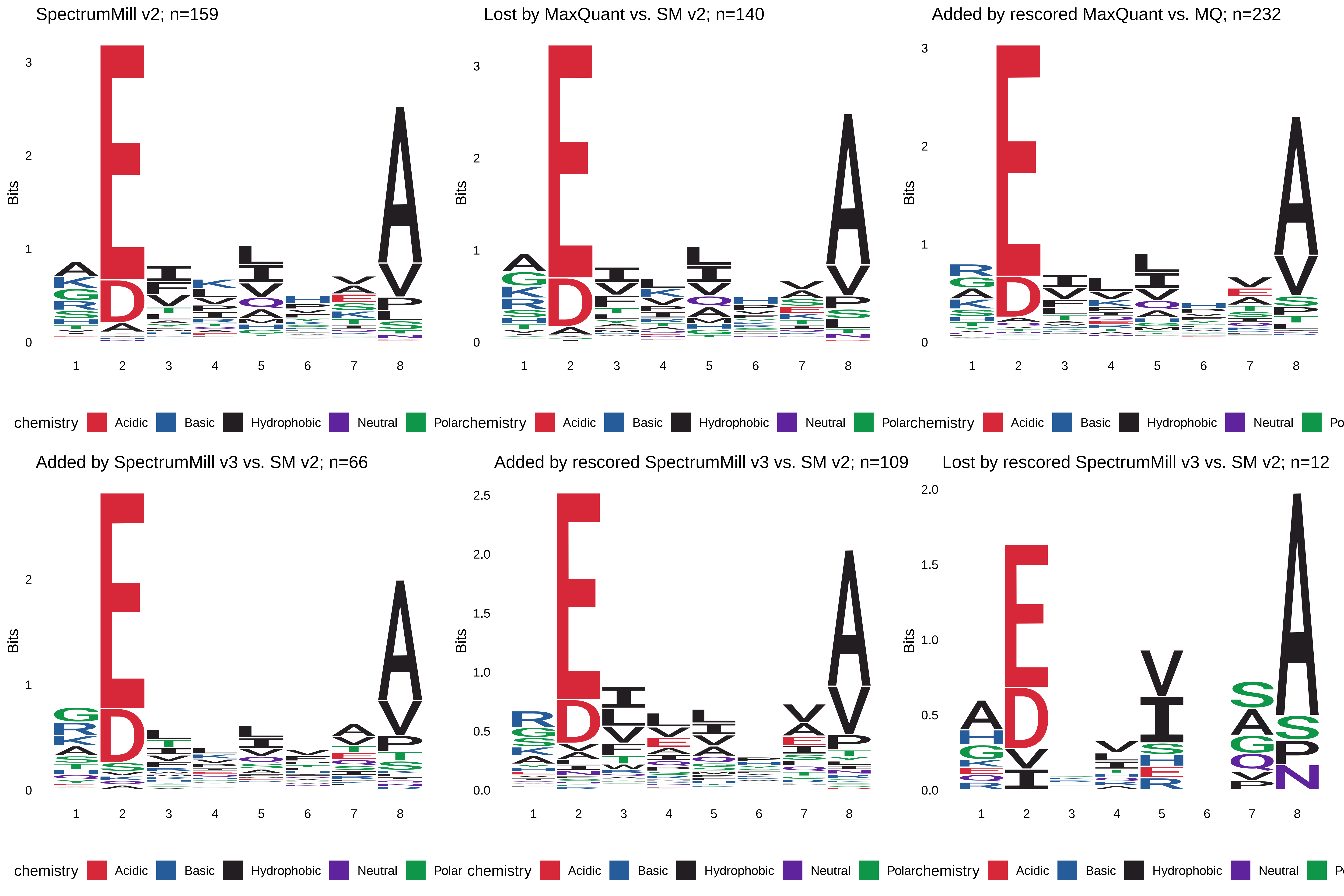
SpectrumMill v2; n=159
Lost by MaxQuant vs. SM v2; n=140
Added by rescored MaxQuant vs. MQ; n=232
Added by SpectrumMill v3 vs. SM v2; n=66
Added by rescored SpectrumMill v3 vs. SM v2; n=109
Lost by rescored SpectrumMill v3 vs. SM v2; n=12
Bits
1 2 3 4 5 6 7 8
chemistry
Acidic
Basic
Hydrophobic
Neutral
Polar

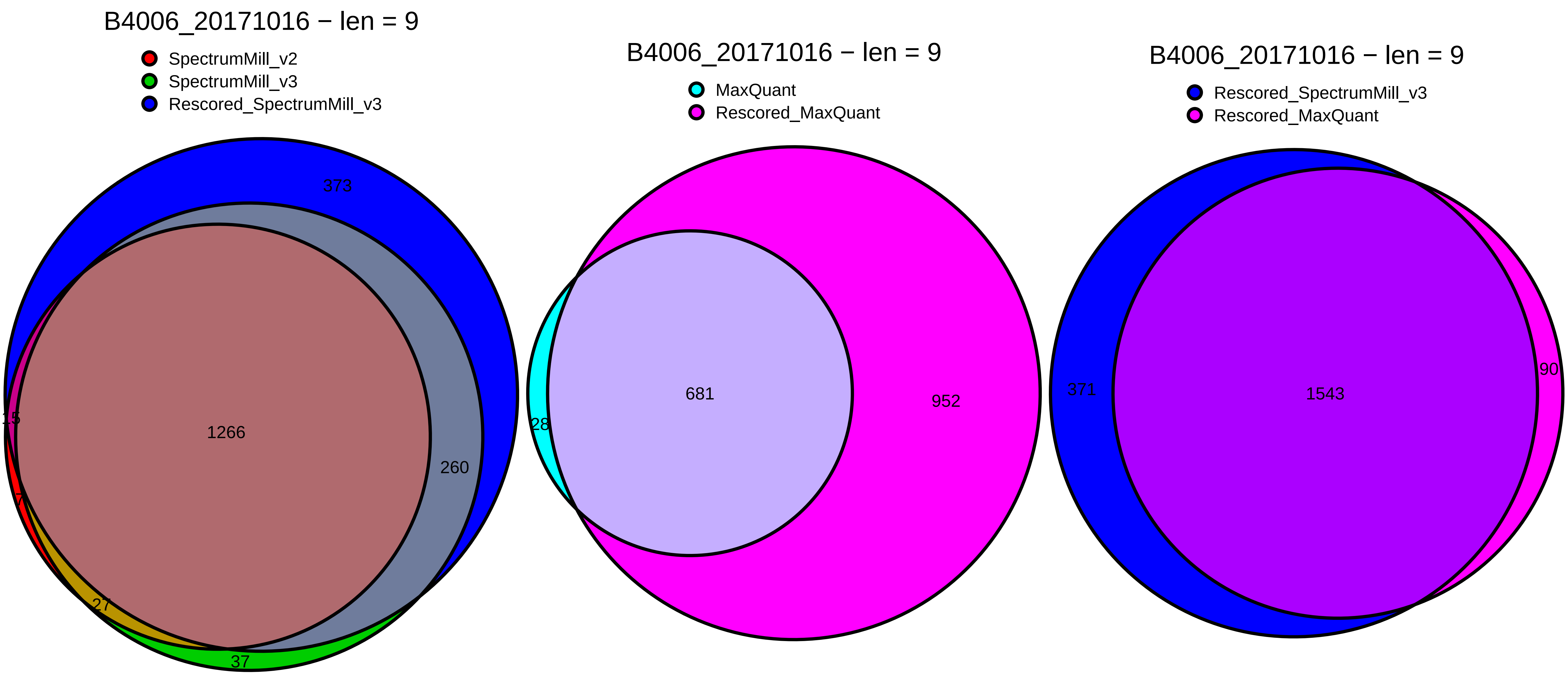
B4006_20171016 − len = 9
SpectrumMill_v2
SpectrumMill_v3
Rescored_SpectrumMill_v3
373
15
1266
260
7
27
37
B4006_20171016 − len = 9
MaxQuant
Rescored_MaxQuant
681
952
28
B4006_20171016 − len = 9
Rescored_SpectrumMill_v3
Rescored_MaxQuant
371
1543
90

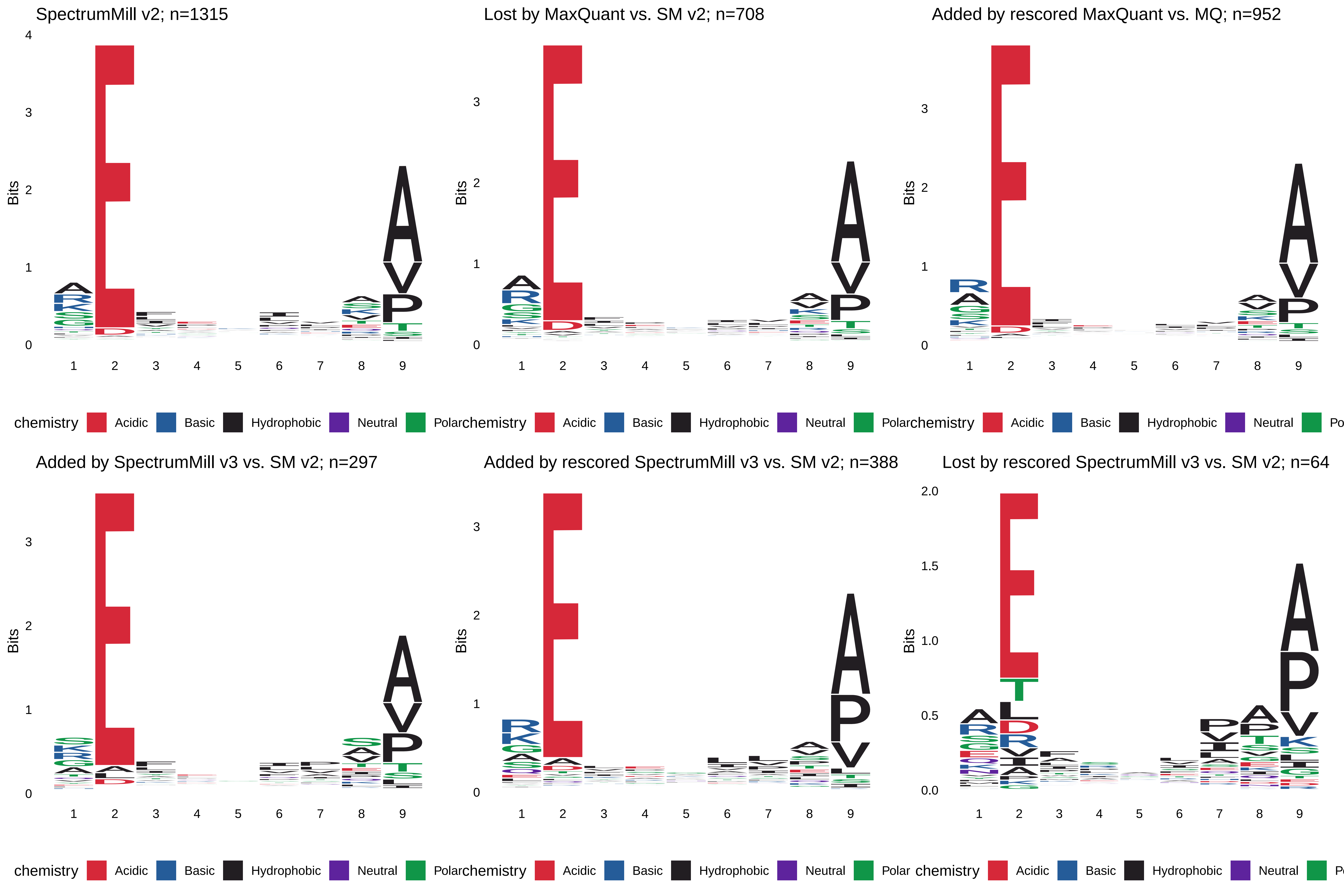

SpectrumMill v2; n=1315
Lost by MaxQuant vs. SM v2; n=708
Added by rescored MaxQuant vs. MQ; n=952
Added by SpectrumMill v3 vs. SM v2; n=297
Added by rescored SpectrumMill v3 vs. SM v2; n=388
Lost by rescored SpectrumMill v3 vs. SM v2; n=64
Bits
1 2 3 4 5 6 7 8 9
chemistry Acidic Basic Hydrophobic Neutral Polar

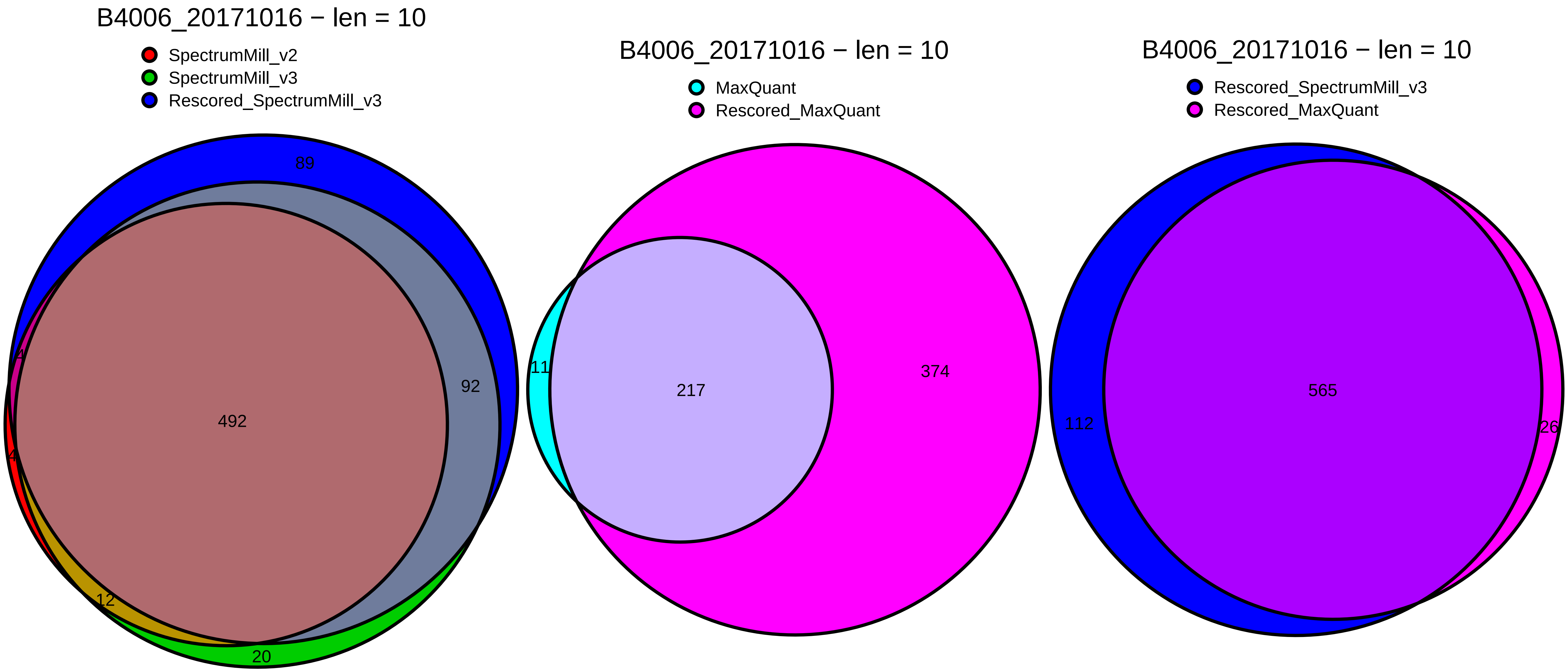

B4006_20171016 − len = 10
SpectrumMill_v2
SpectrumMill_v3
Rescored_SpectrumMill_v3
89
92
492
12
20
B4006_20171016 − len = 10
MaxQuant
Rescored_MaxQuant
11
217
374
B4006_20171016 − len = 10
Rescored_SpectrumMill_v3
Rescored_MaxQuant
112
565
26

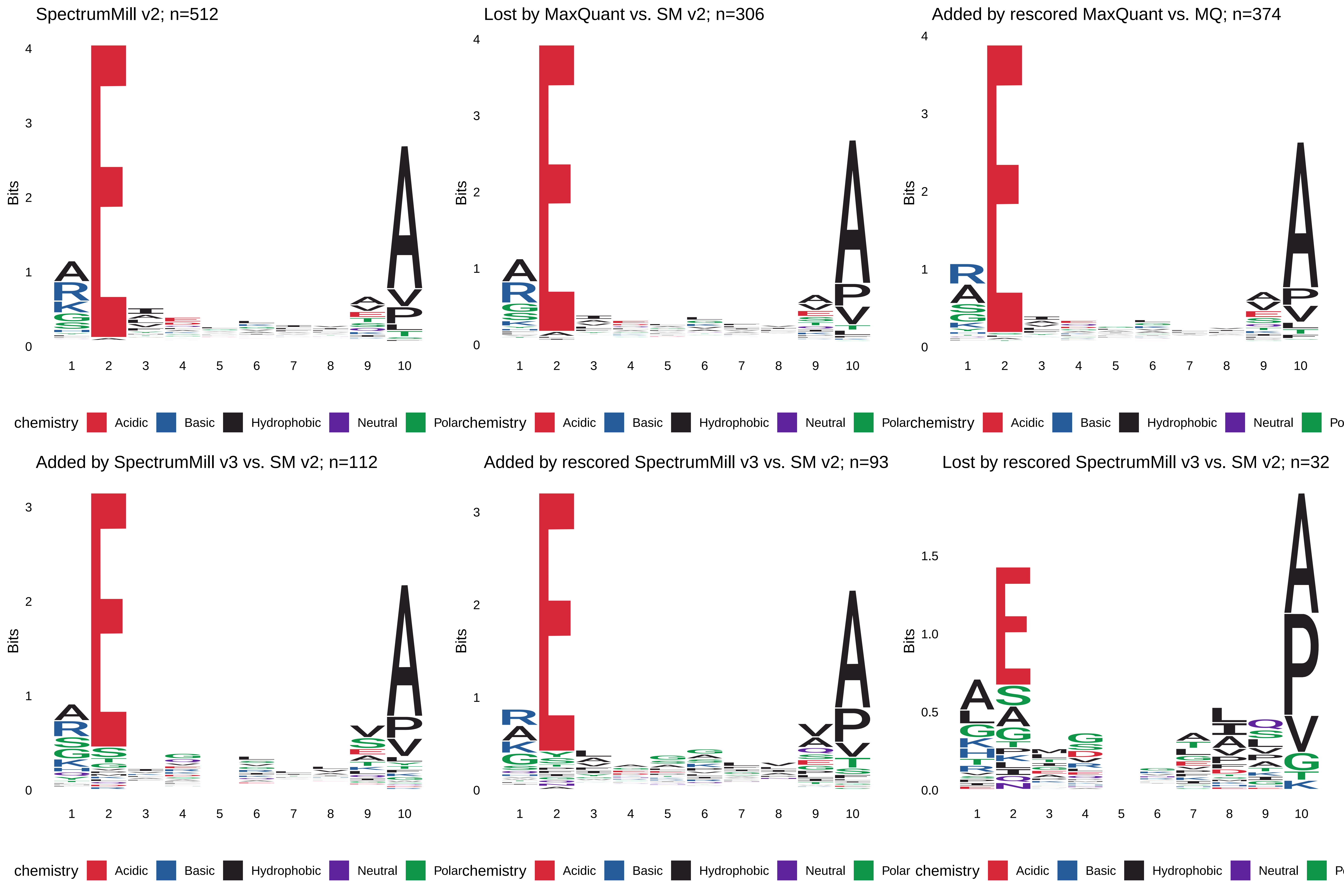
SpectrumMill v2; n=512
Lost by MaxQuant vs. SM v2; n=306
Added by rescored MaxQuant vs. MQ; n=374
Added by SpectrumMill v3 vs. SM v2; n=112
Added by rescored SpectrumMill v3 vs. SM v2; n=93
Lost by rescored SpectrumMill v3 vs. SM v2; n=32
Bits
1 2 3 4 5 6 7 8 9 10
chemistry Acidic Basic Hydrophobic Neutral Polar

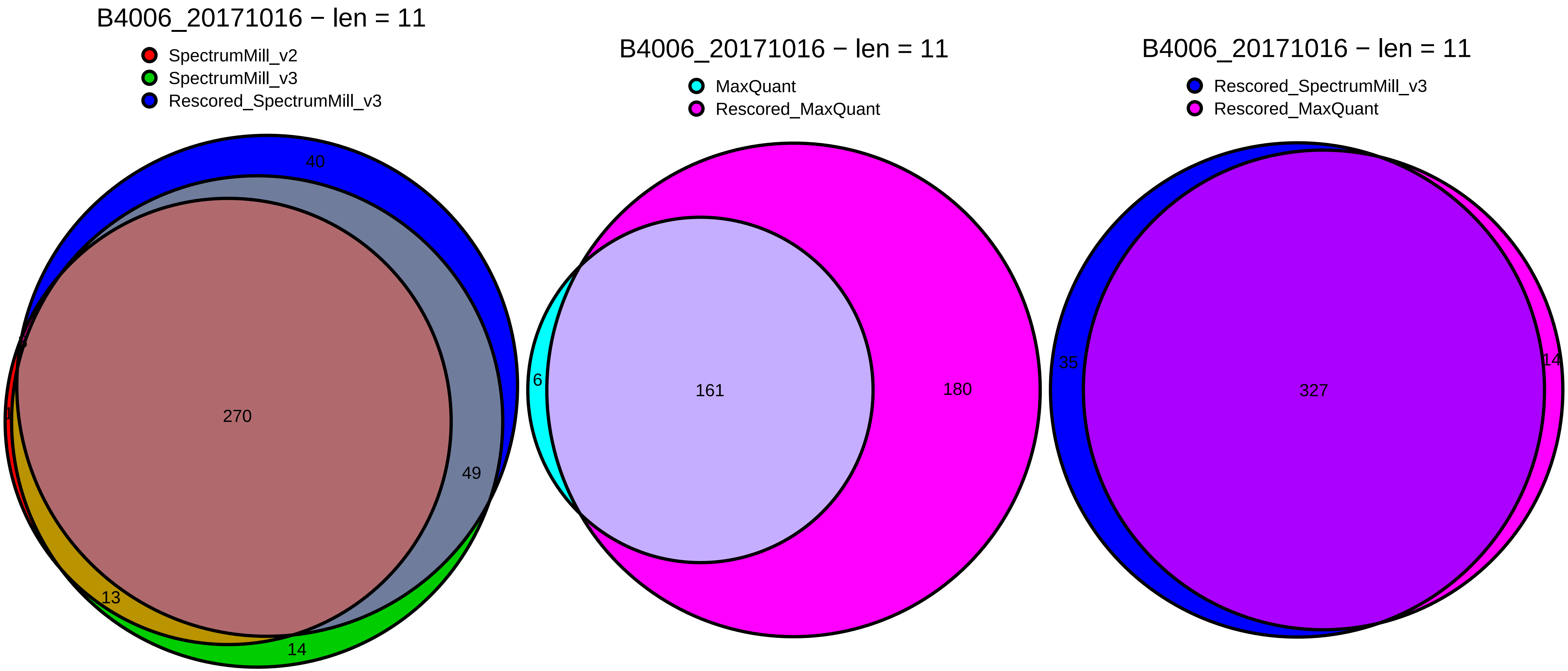
B4006_20171016 − len = 11
SpectrumMill_v2
SpectrumMill_v3
Rescored_SpectrumMill_v3
40
270
49
13
14
B4006_20171016 − len = 11
MaxQuant
Rescored_MaxQuant
6
161
180
B4006_20171016 − len = 11
Rescored_SpectrumMill_v3
Rescored_MaxQuant
35
327
14

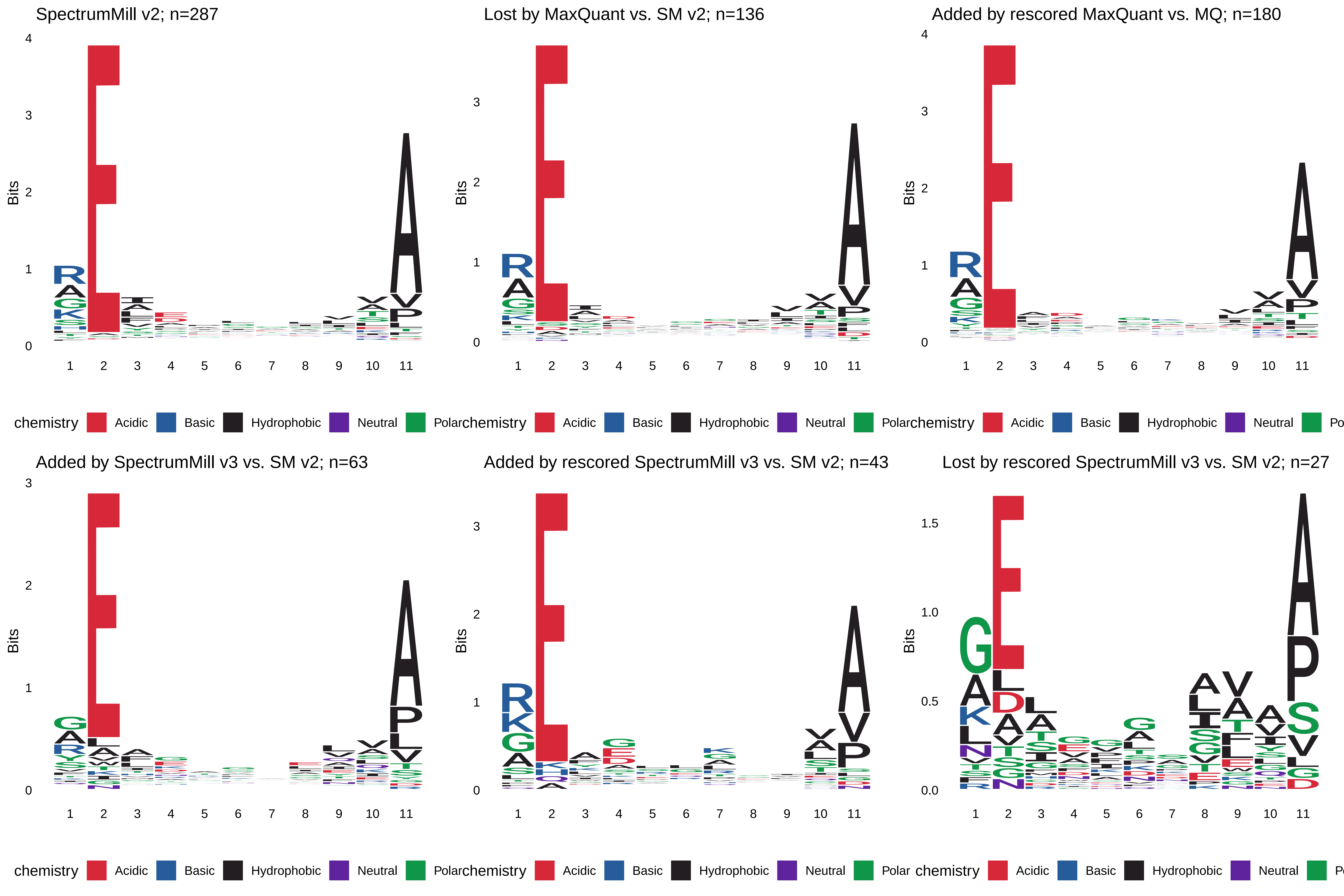
SpectrumMill v2; n=287
Lost by MaxQuant vs. SM v2; n=136
Added by rescored MaxQuant vs. MQ; n=180
Added by SpectrumMill v3 vs. SM v2; n=63
Added by rescored SpectrumMill v3 vs. SM v2; n=43
Lost by rescored SpectrumMill v3 vs. SM v2; n=27
Bits
chemistry
Acidic
Basic
Hydrophobic
Neutral
Polar

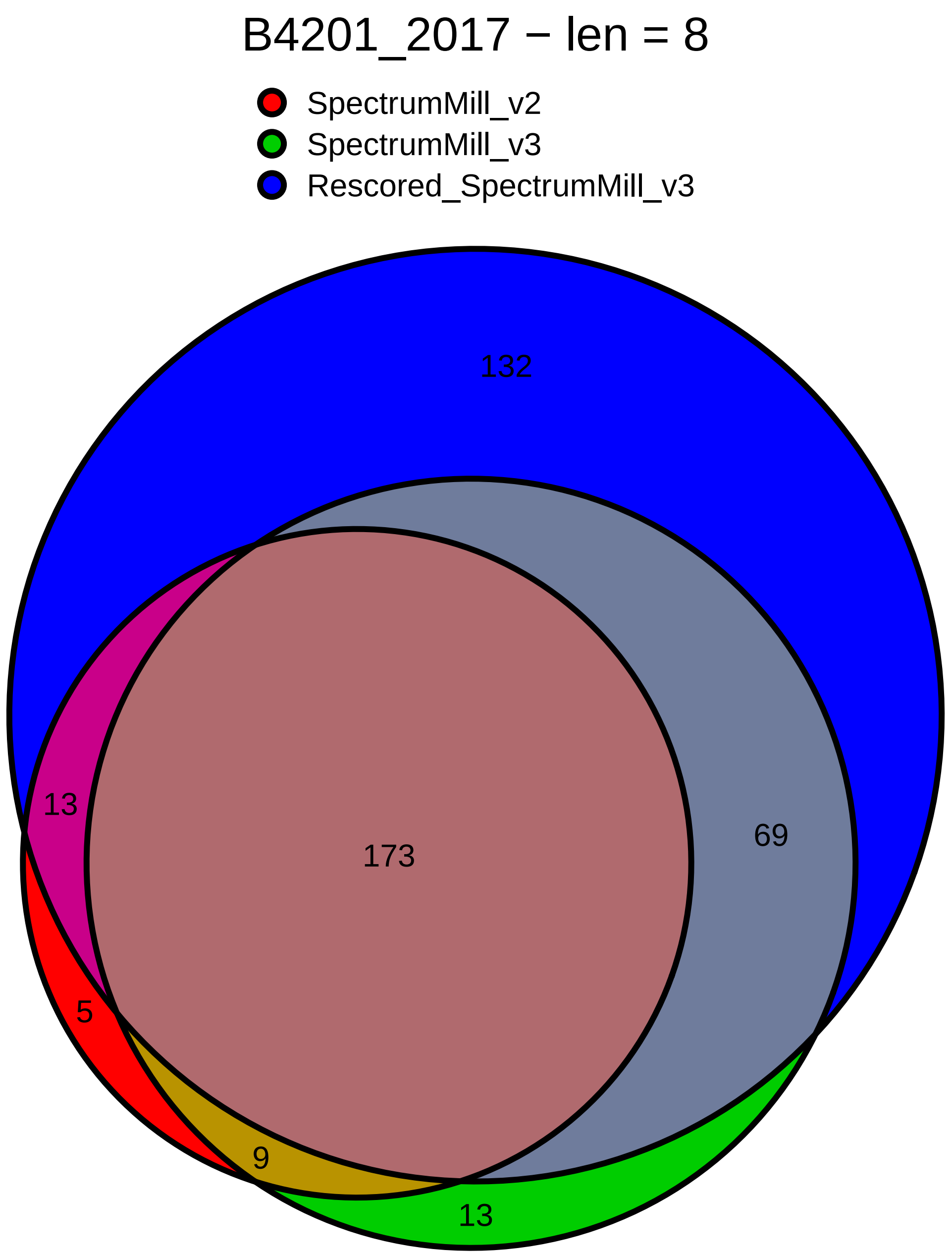
B4201_2017 − len = 8
SpectrumMill_v2
SpectrumMill_v3
Rescored_SpectrumMill_v3
132
13
173
69
5
9
13

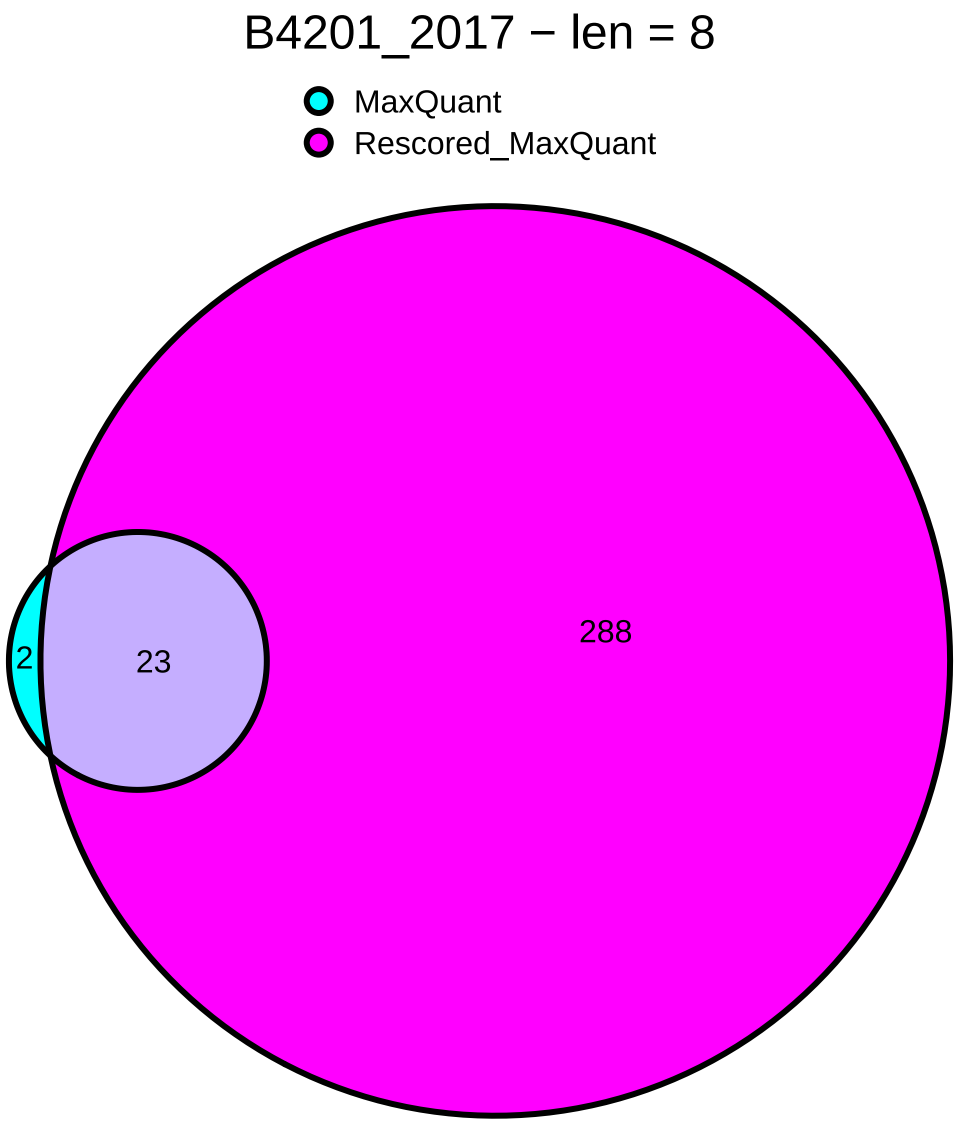
B4201_2017 − len = 8
MaxQuant
Rescored_MaxQuant
2
23
288

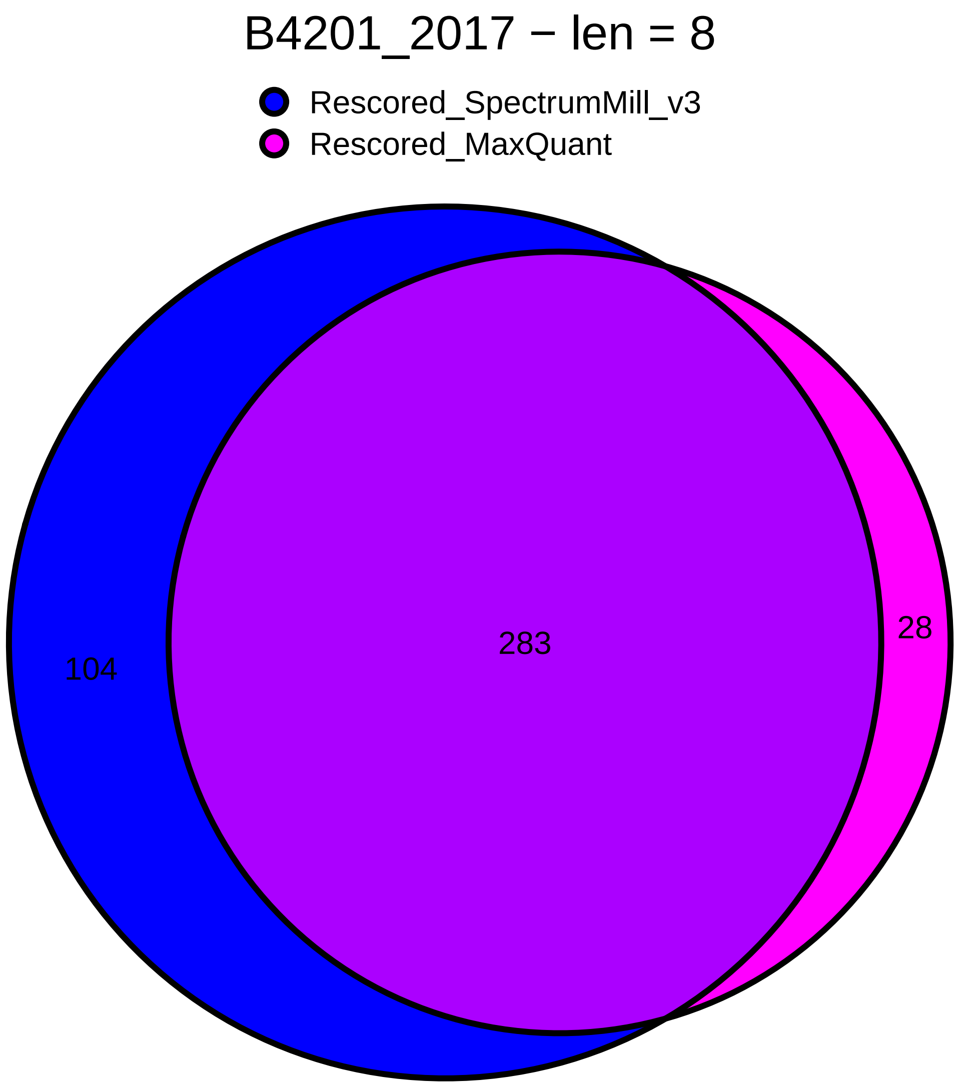
B4201_2017 − len = 8
Rescored_SpectrumMill_v3
Rescored_MaxQuant
104
283
28

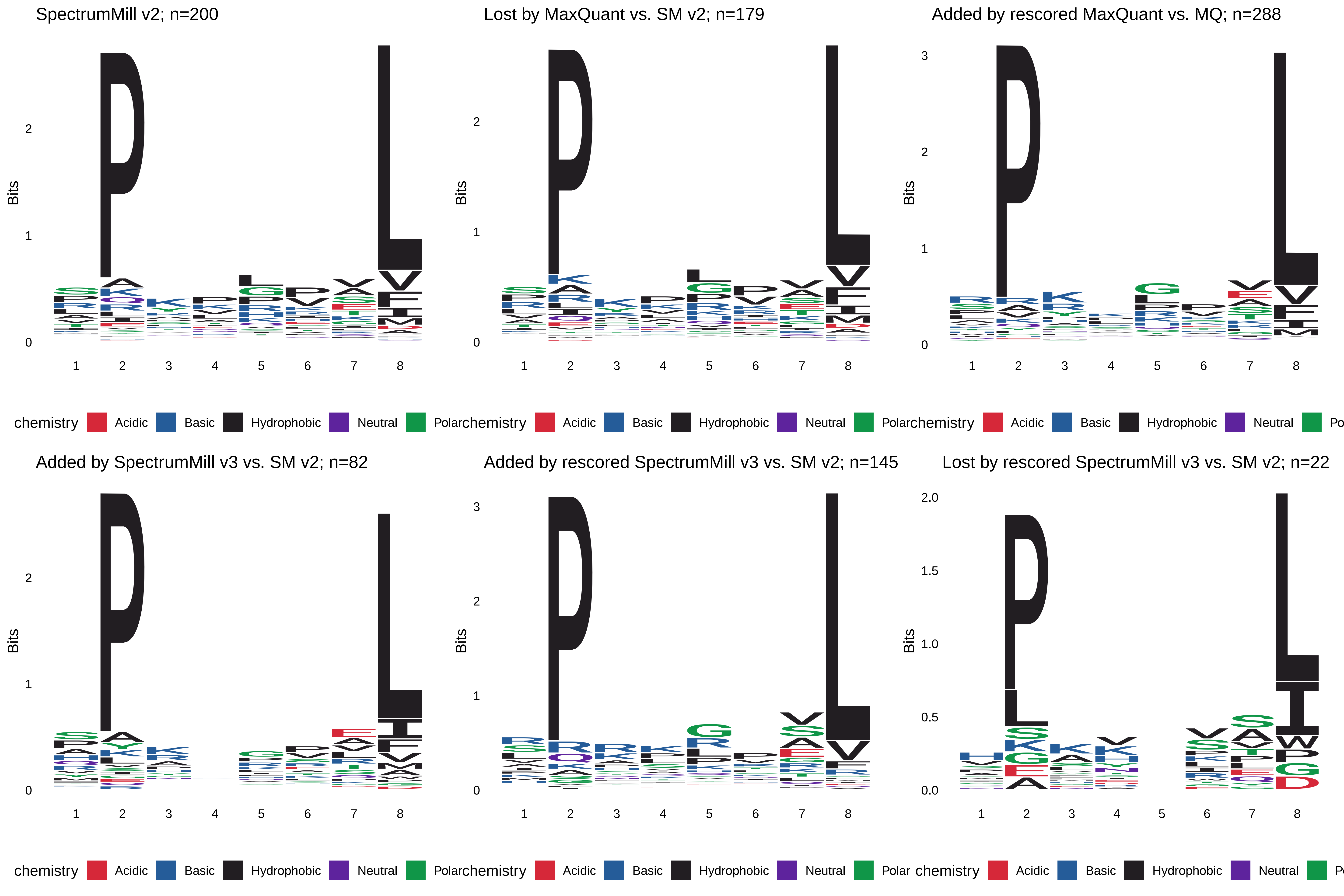
SpectrumMill v2; n=200
Lost by MaxQuant vs. SM v2; n=179
Added by rescored MaxQuant vs. MQ; n=288
Added by SpectrumMill v3 vs. SM v2; n=82
Added by rescored SpectrumMill v3 vs. SM v2; n=145
Lost by rescored SpectrumMill v3 vs. SM v2; n=22
Bits
chemistry
Acidic
Basic
Hydrophobic
Neutral
Polar

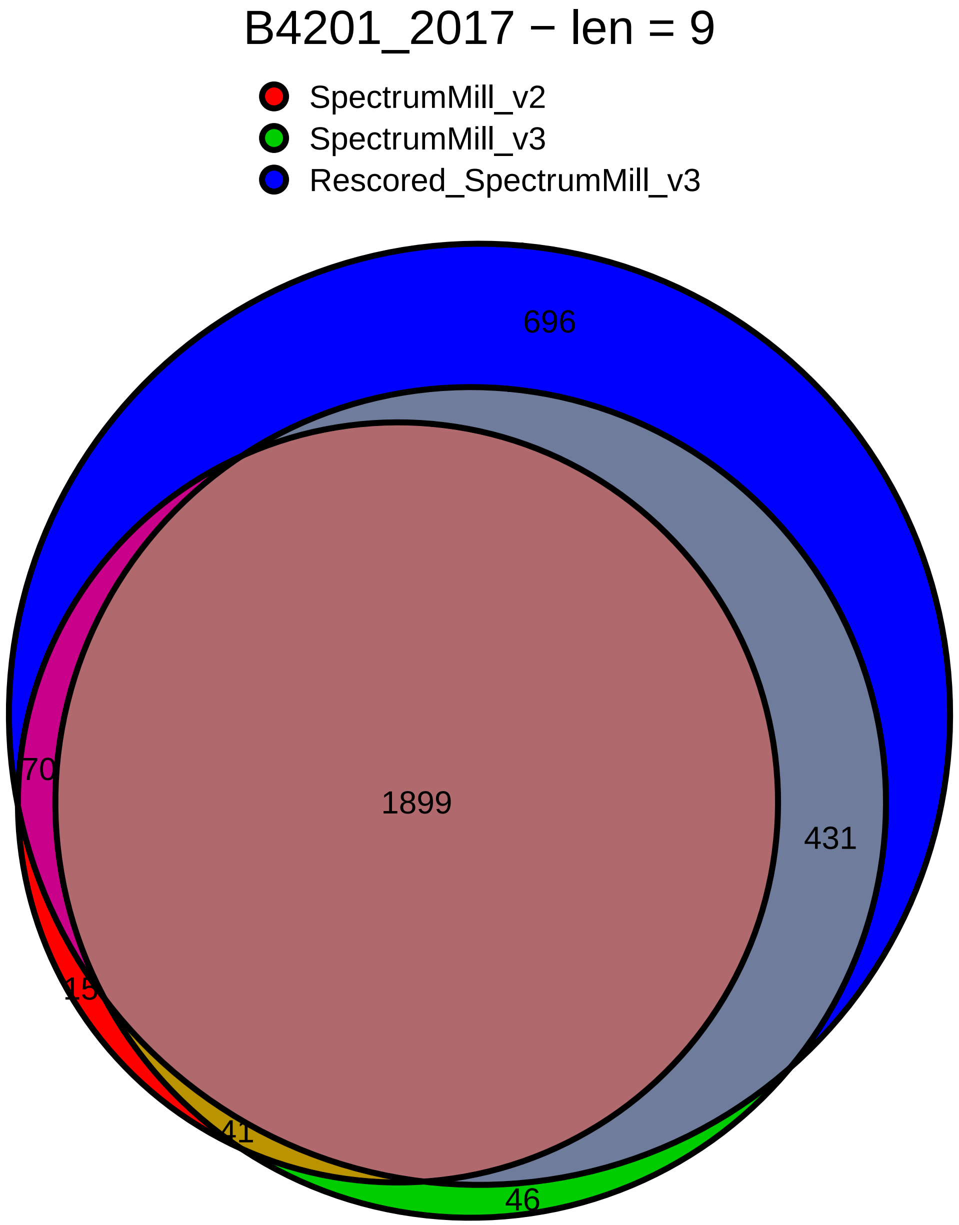
B4201_2017 − len = 9
SpectrumMill_v2
SpectrumMill_v3
Rescored_SpectrumMill_v3
696
70
1899
431
15
41
46

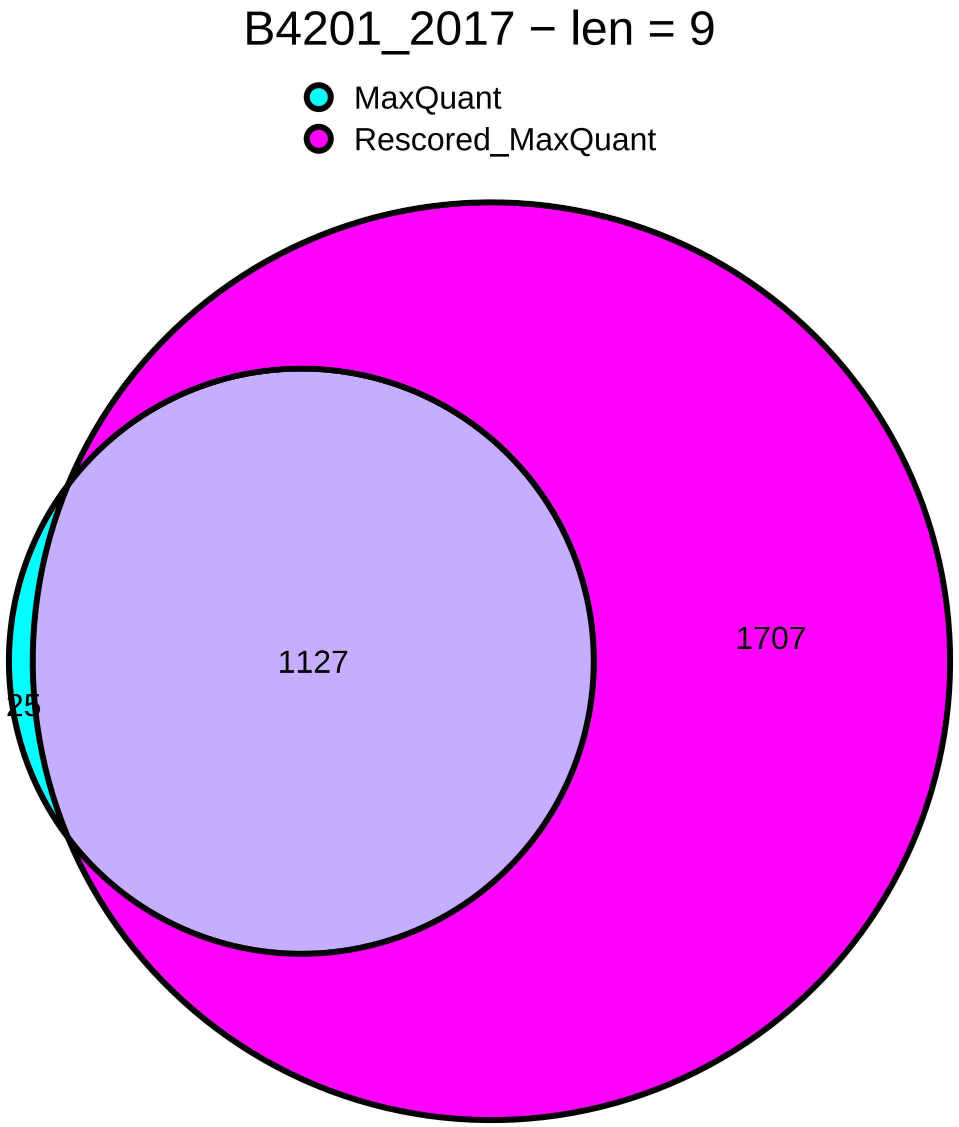
B4201_2017 − len = 9
MaxQuant
Rescored_MaxQuant
25
1127
1707

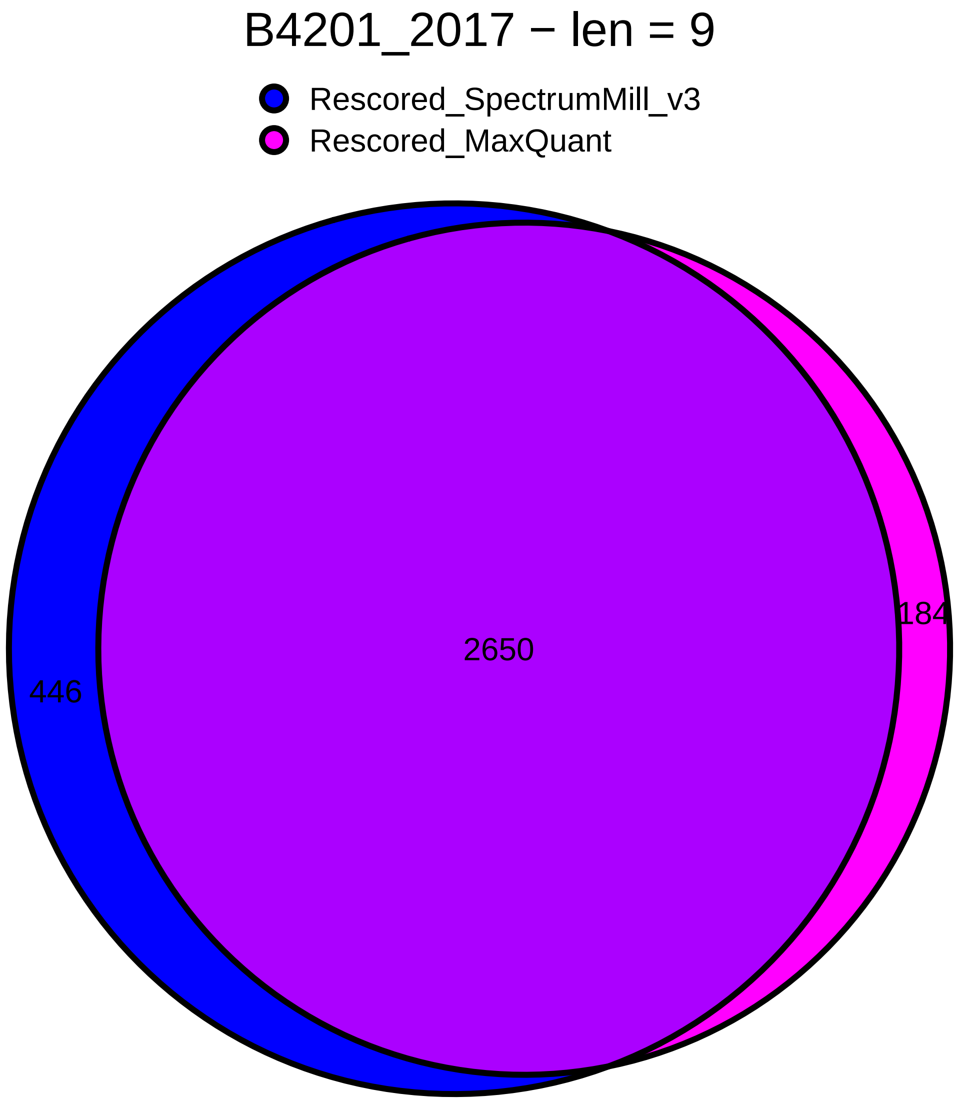
B4201_2017 − len = 9
Rescored_SpectrumMill_v3
Rescored_MaxQuant
446
2650
184

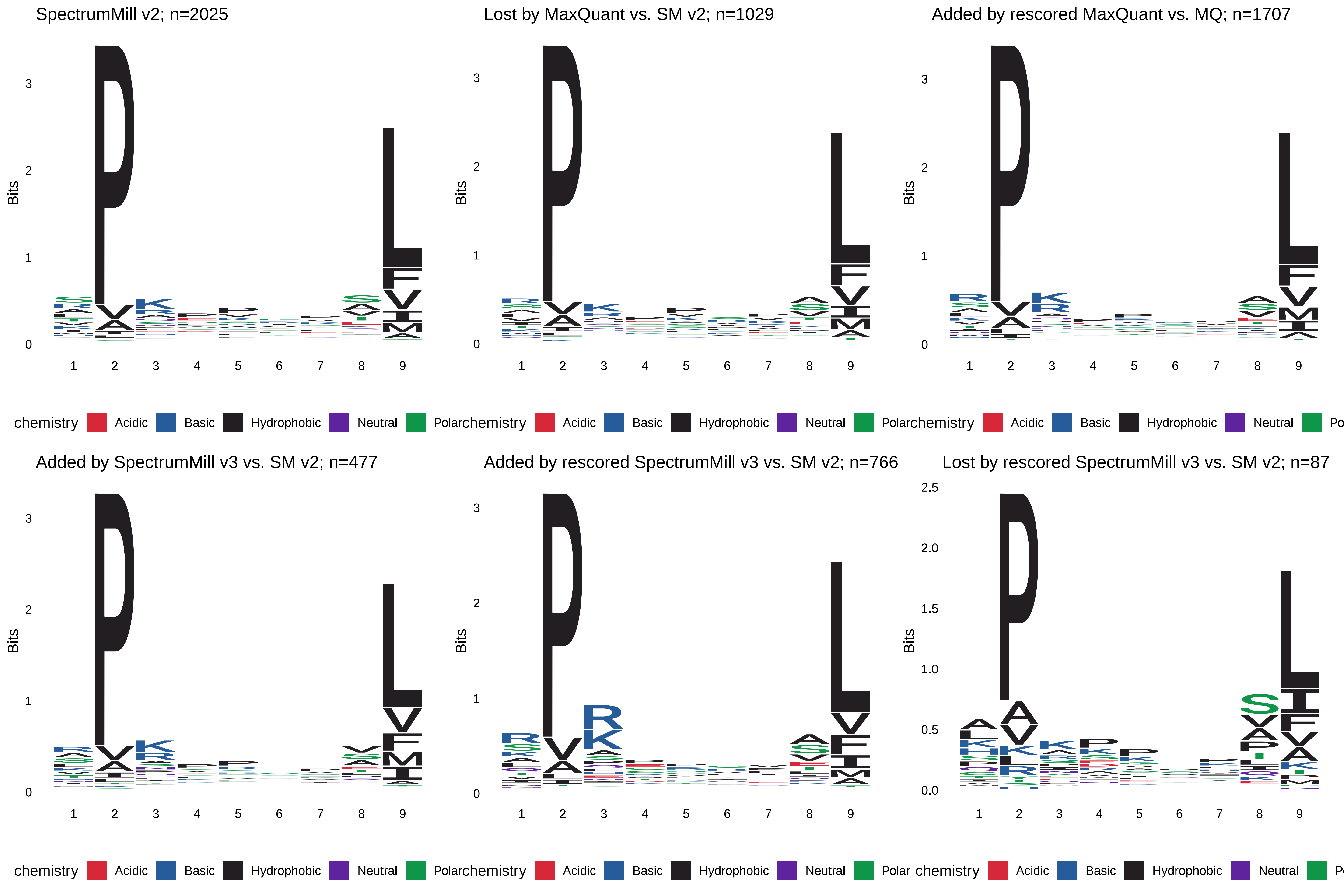
SpectrumMill v2; n=2025
Lost by MaxQuant vs. SM v2; n=1029
Added by rescored MaxQuant vs. MQ; n=1707
Added by SpectrumMill v3 vs. SM v2; n=477
Added by rescored SpectrumMill v3 vs. SM v2; n=766
Lost by rescored SpectrumMill v3 vs. SM v2; n=87
Bits
0
1
2
3
0.0
0.5
1.0
1.5
2.0
2.5
1 2 3 4 5 6 7 8 9
chemistry
Acidic
Basic
Hydrophobic
Neutral
Polar

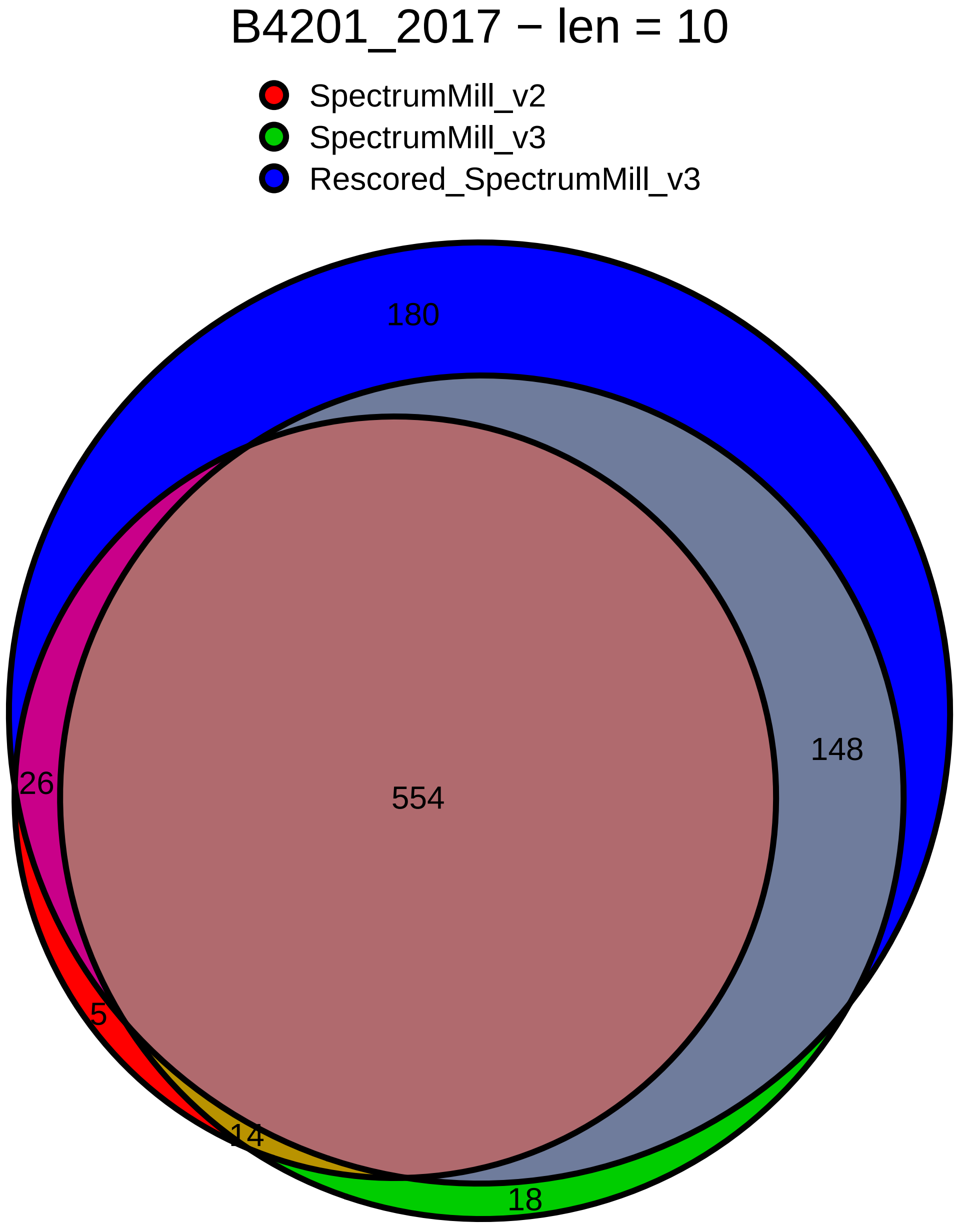
B4201_2017 − len = 10
SpectrumMill_v2
SpectrumMill_v3
Rescored_SpectrumMill_v3
180
148
26
554
5
14
18

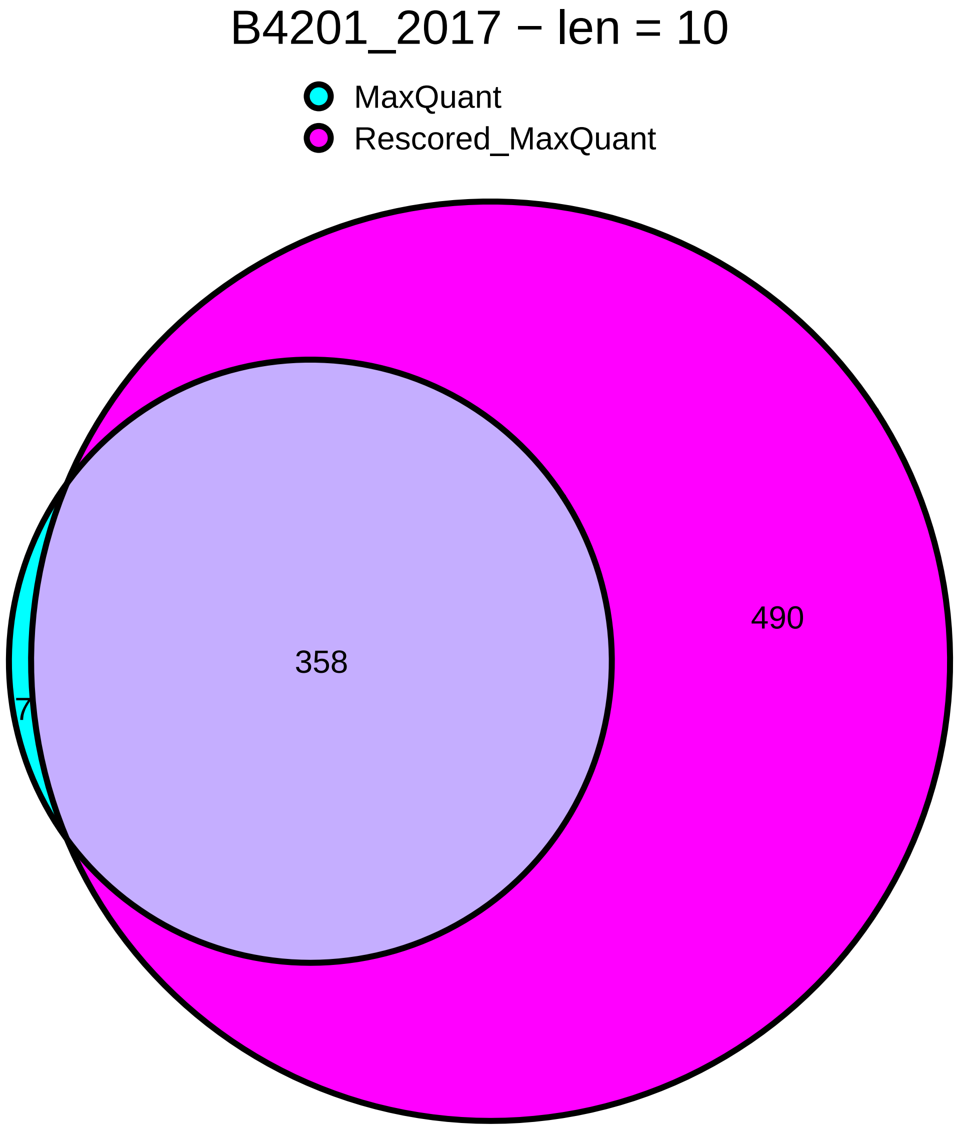
B4201_2017 − len = 10
MaxQuant
Rescored_MaxQuant
7
358
490

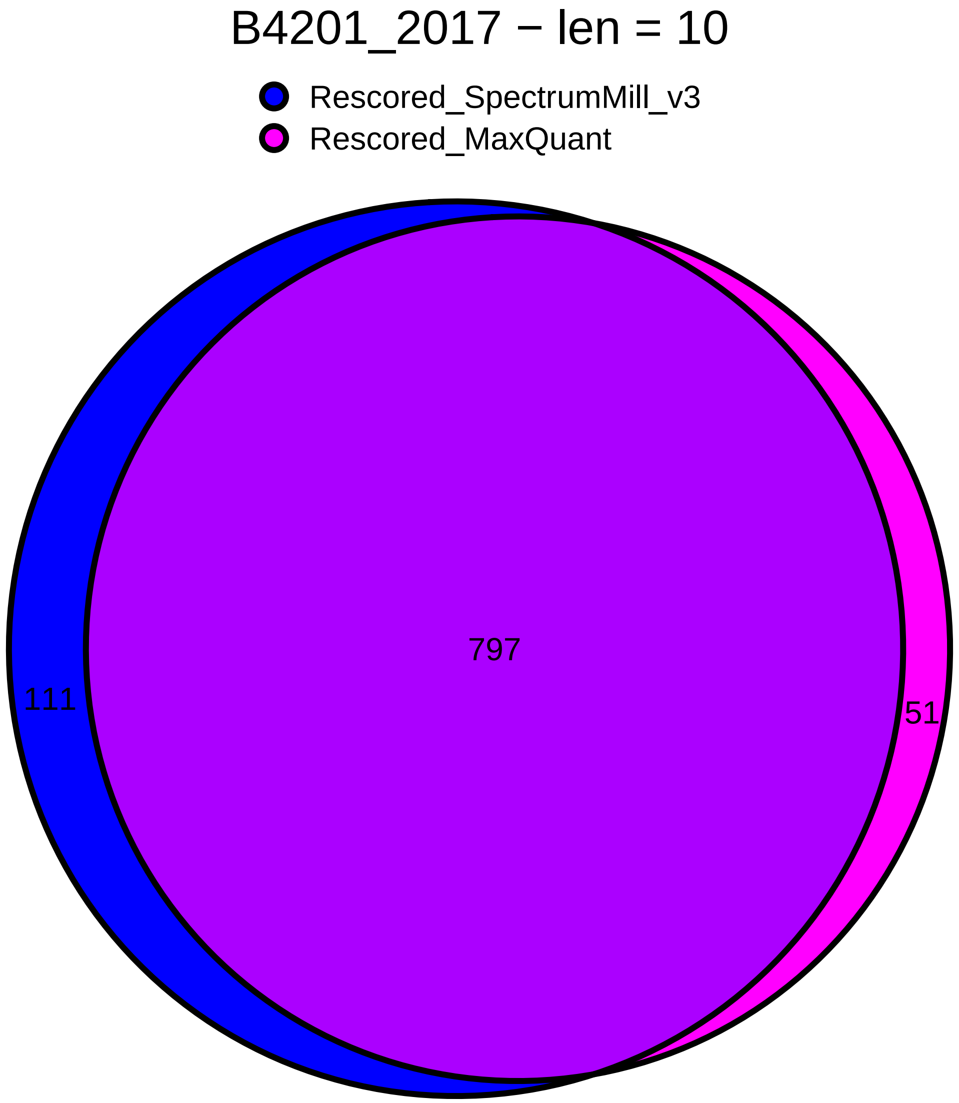
B4201_2017 − len = 10
Rescored_SpectrumMill_v3
Rescored_MaxQuant
111
797
51

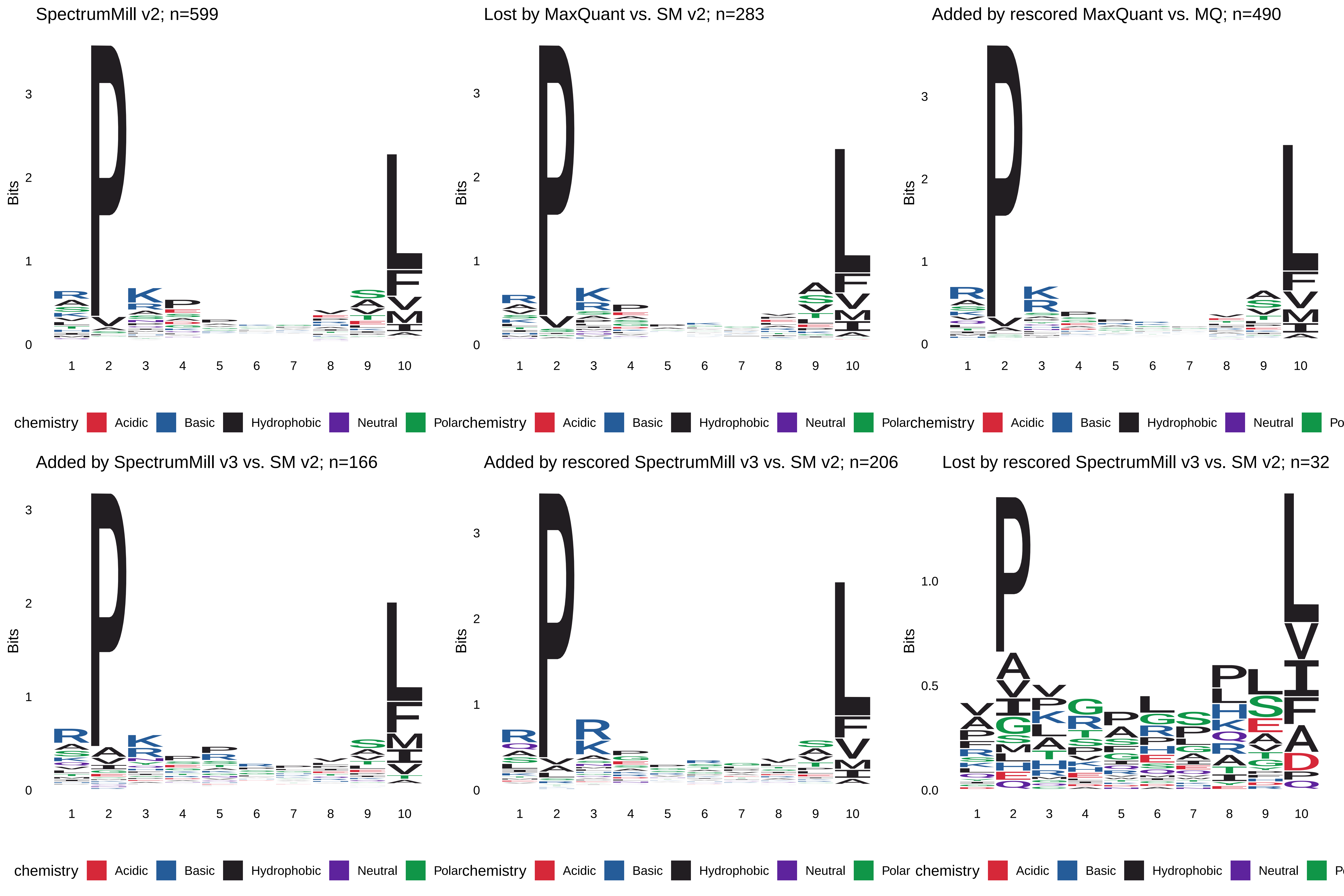
SpectrumMill v2; n=599
Lost by MaxQuant vs. SM v2; n=283
Added by rescored MaxQuant vs. MQ; n=490
Added by SpectrumMill v3 vs. SM v2; n=166
Added by rescored SpectrumMill v3 vs. SM v2; n=206
Lost by rescored SpectrumMill v3 vs. SM v2; n=32
Bits
0
1
2
3
0.0
0.5
1.0
1 2 3 4 5 6 7 8 9 10
chemistry
Acidic
Basic
Hydrophobic
Neutral
Polar

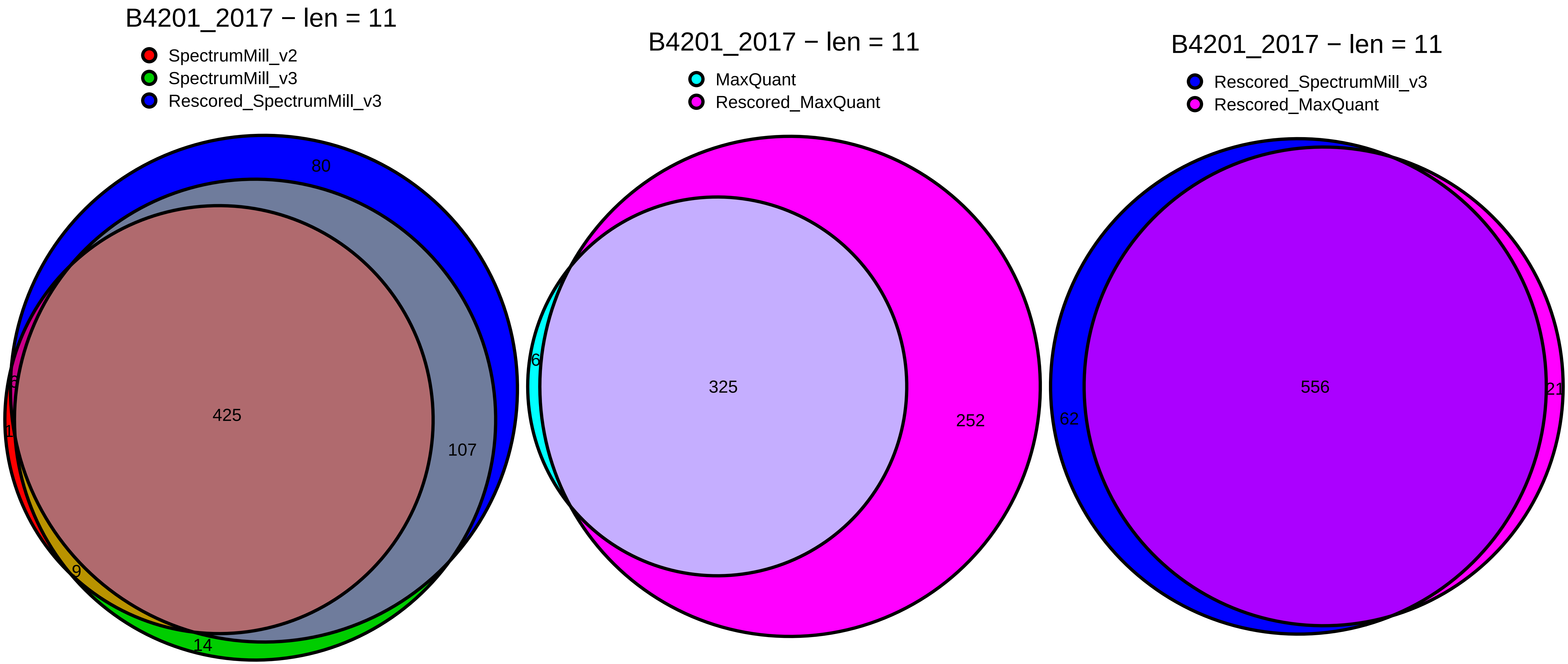

B4201_2017 − len = 11
SpectrumMill_v2
SpectrumMill_v3
Rescored_SpectrumMill_v3
80
425
107
9
14
B4201_2017 − len = 11
MaxQuant
Rescored_MaxQuant
6
325
252
B4201_2017 − len = 11
Rescored_SpectrumMill_v3
Rescored_MaxQuant
556
62
21

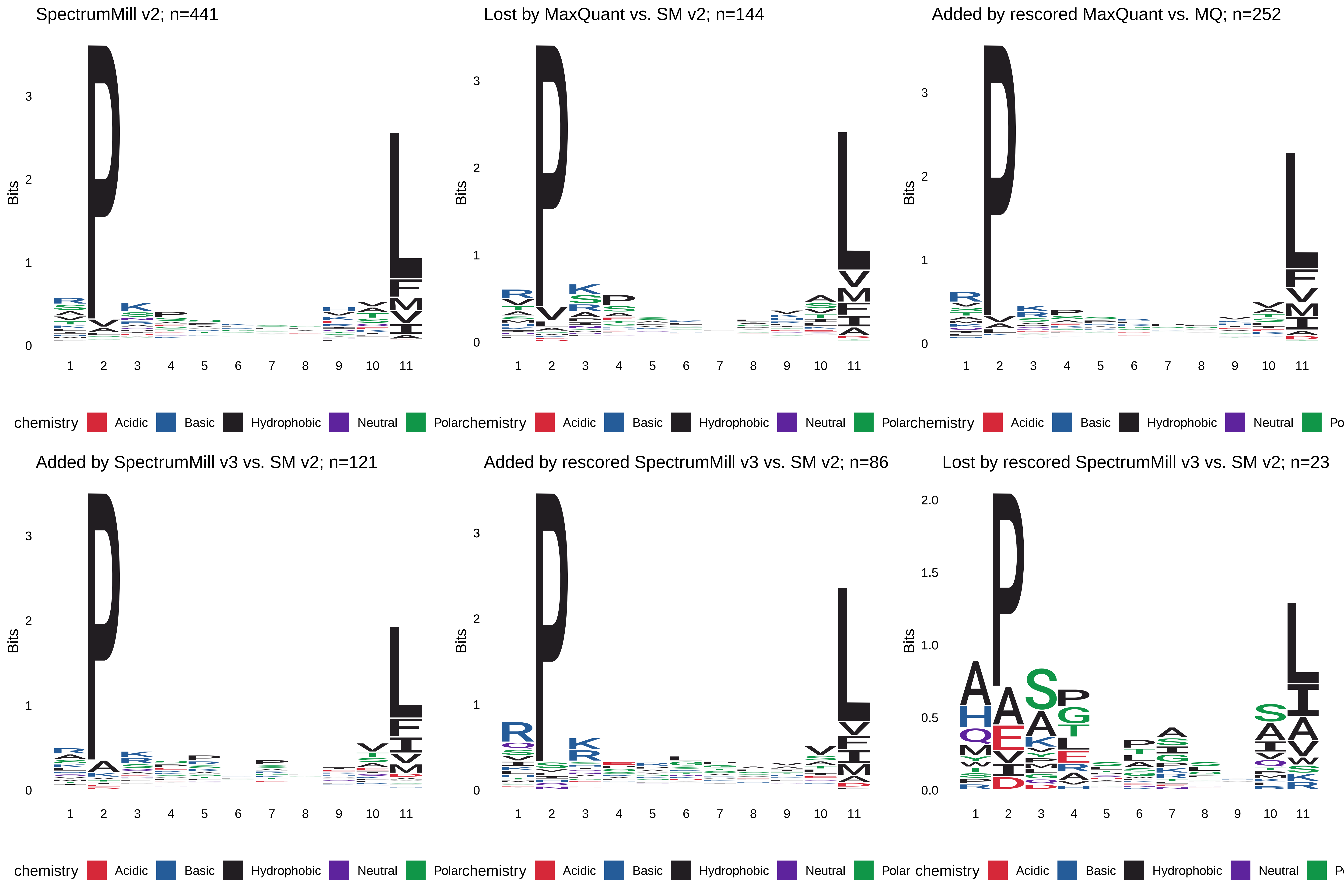
SpectrumMill v2; n=441
Lost by MaxQuant vs. SM v2; n=144
Added by rescored MaxQuant vs. MQ; n=252
Added by SpectrumMill v3 vs. SM v2; n=121
Added by rescored SpectrumMill v3 vs. SM v2; n=86
Lost by rescored SpectrumMill v3 vs. SM v2; n=23
Bits
0
1
2
3
0.0
0.5
1.0
1.5
2.0
1 2 3 4 5 6 7 8 9 10 11
chemistry
Acidic
Basic
Hydrophobic
Neutral
Polar

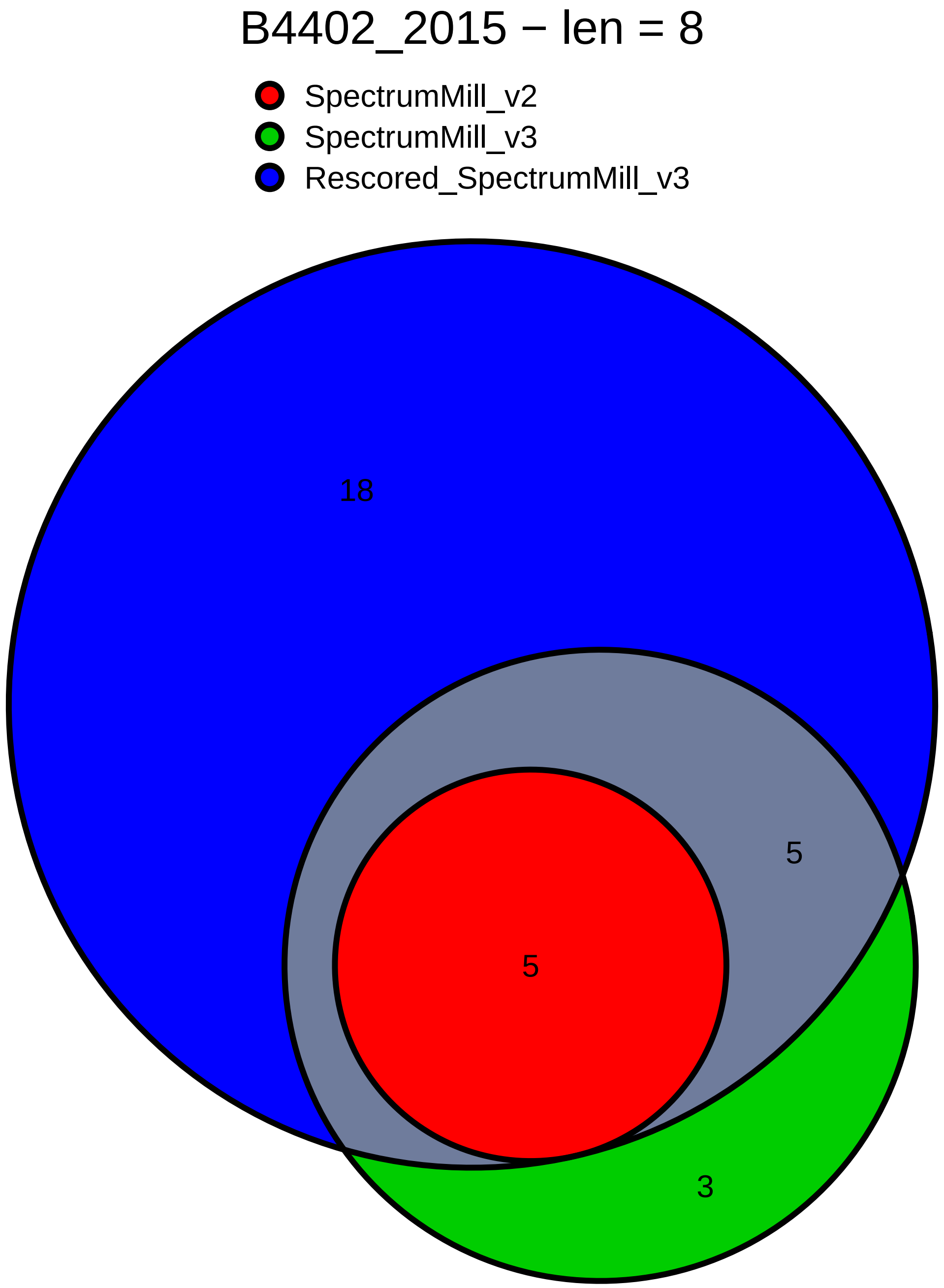
B4402_2015 − len = 8
SpectrumMill_v2
SpectrumMill_v3
Rescored_SpectrumMill_v3
18
5
5
3

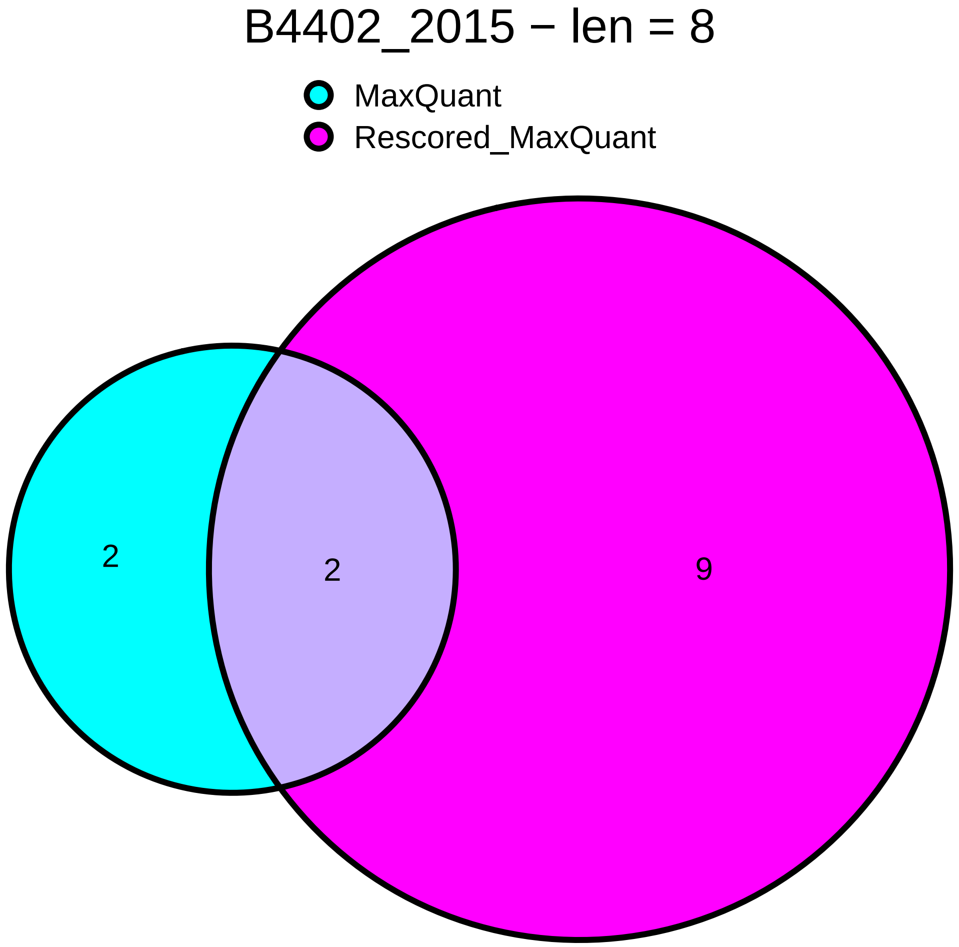
B4402_2015 − len = 8
MaxQuant
Rescored_MaxQuant
2
2
9

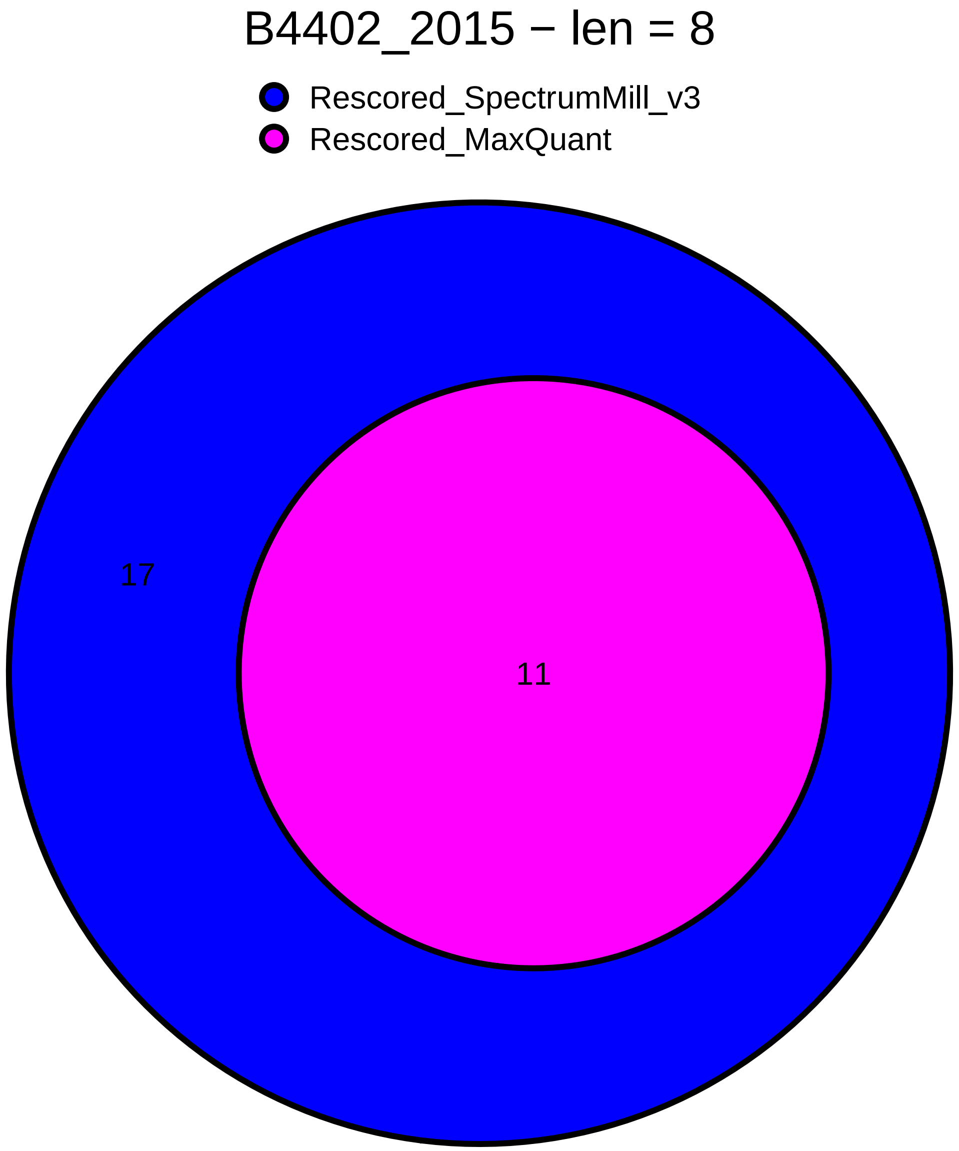
B4402_2015 − len = 8
Rescored_SpectrumMill_v3
Rescored_MaxQuant
17
11

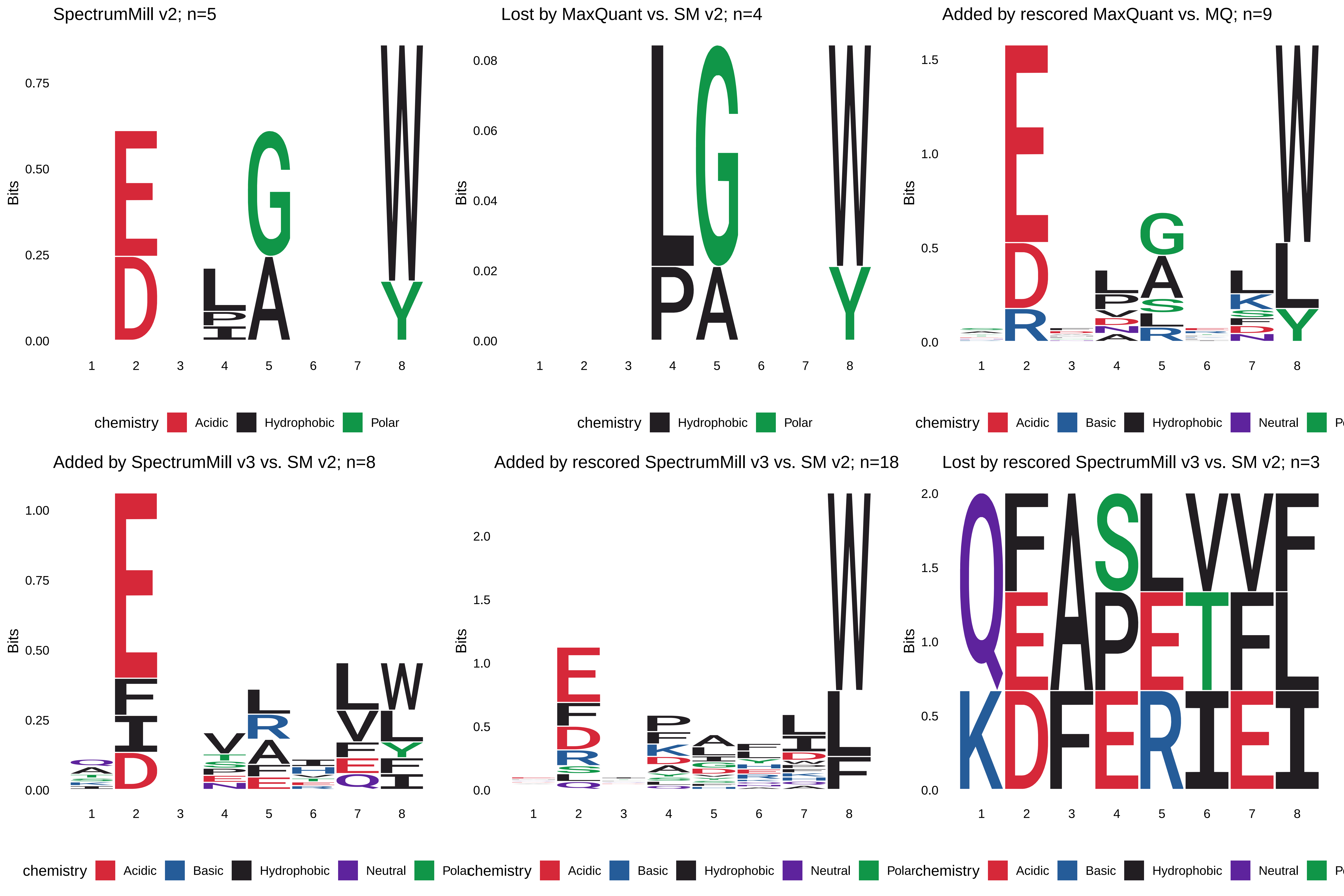
SpectrumMill v2; n=5
Lost by MaxQuant vs. SM v2; n=4
Added by rescored MaxQuant vs. MQ; n=9
Bits
0.00
0.25
0.50
0.75
0.02
0.04
0.06
0.08
0.0
0.5
1.0
1.5
1 2 3 4 5 6 7 8
chemistry
Acidic
Basic
Hydrophobic
Neutral
Polar
Added by SpectrumMill v3 vs. SM v2; n=8
Added by rescored SpectrumMill v3 vs. SM v2; n=18
Lost by rescored SpectrumMill v3 vs. SM v2; n=3
1.00
2.0

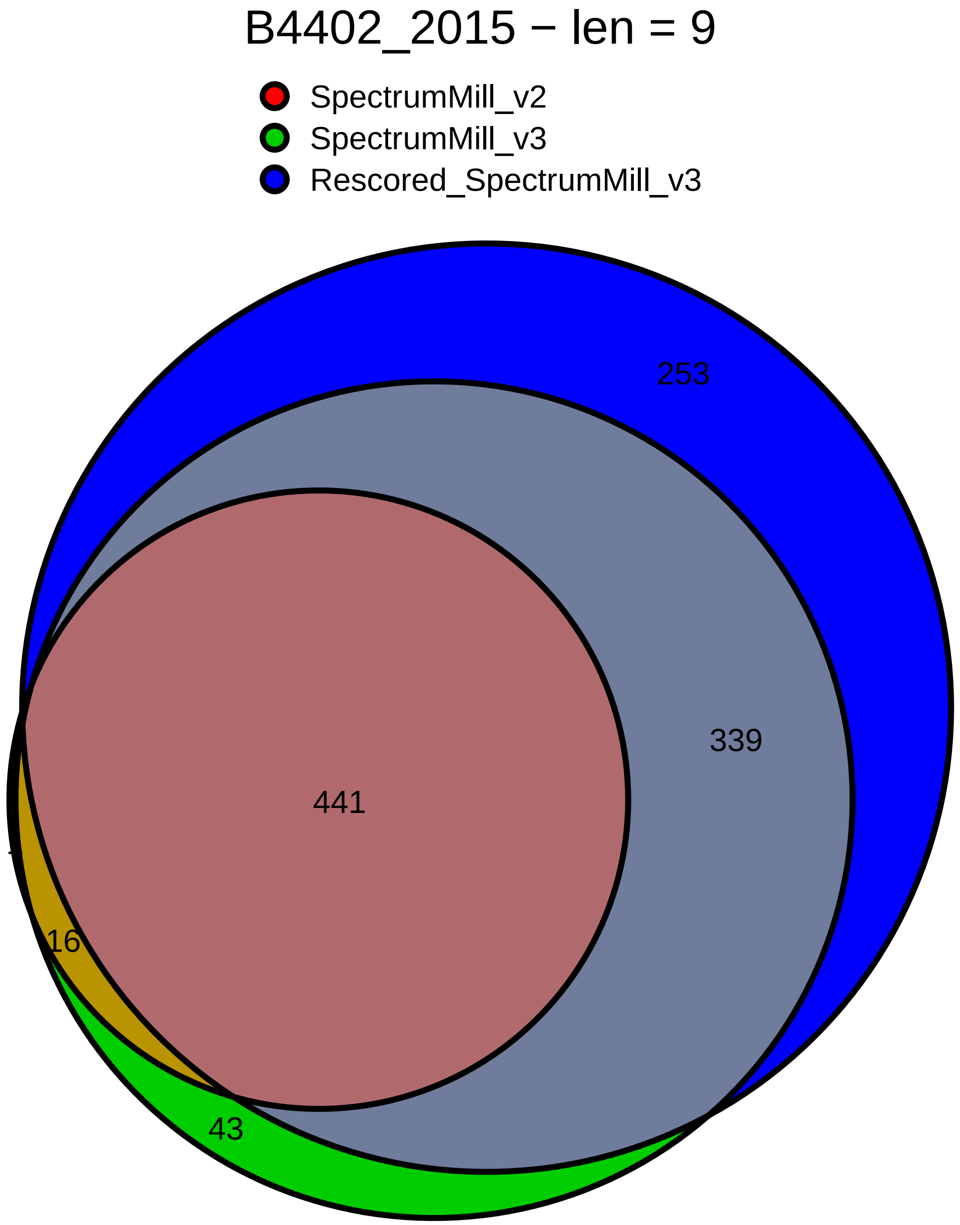
B4402_2015 − len = 9
SpectrumMill_v2
SpectrumMill_v3
Rescored_SpectrumMill_v3
253
339
441
16
43

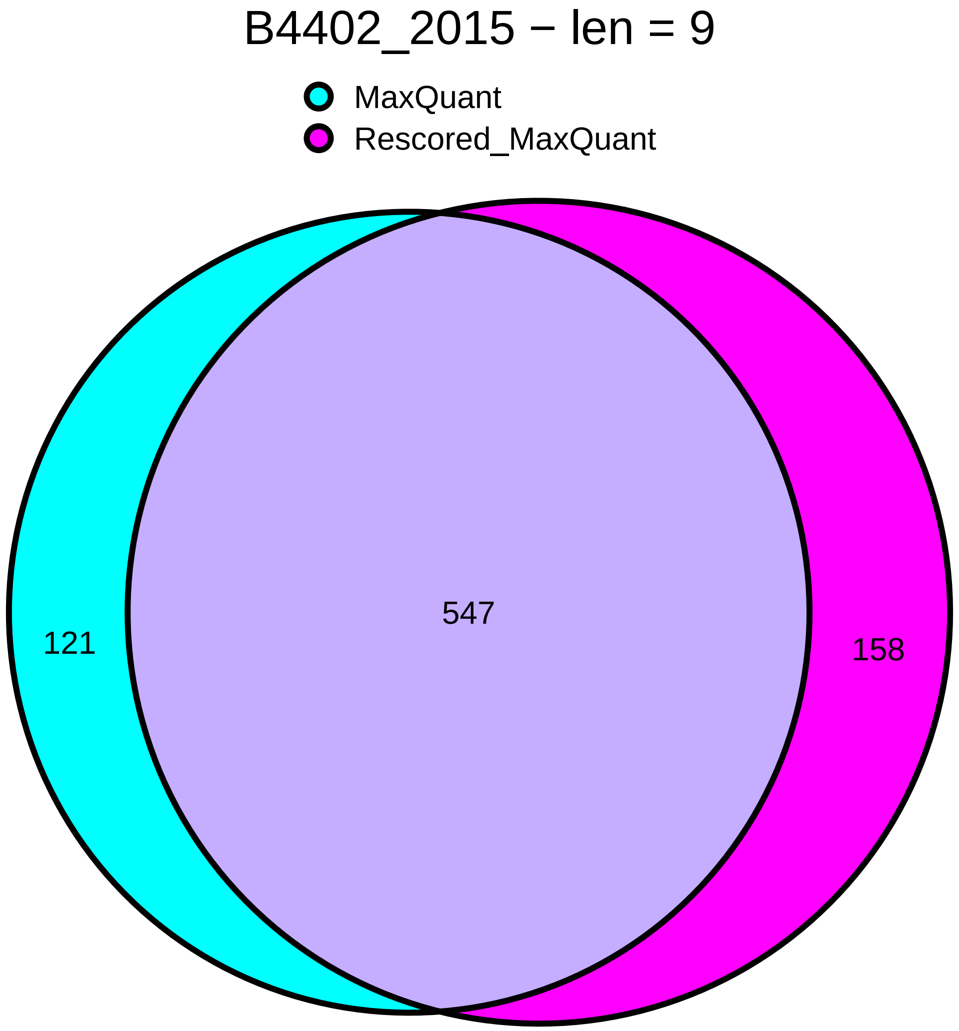
B4402_2015 − len = 9
MaxQuant
Rescored_MaxQuant
121
547
158

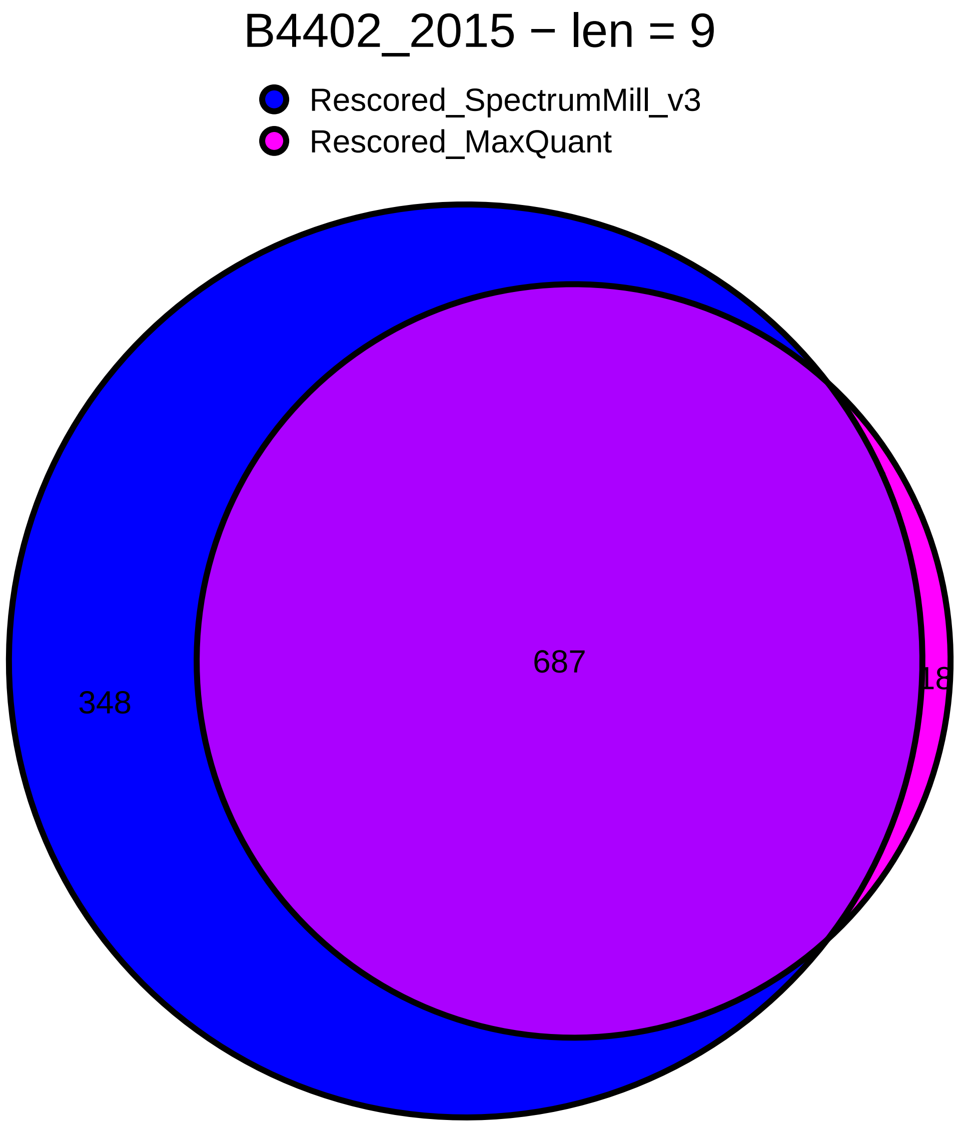
B4402_2015 − len = 9
Rescored_SpectrumMill_v3
Rescored_MaxQuant
348
687

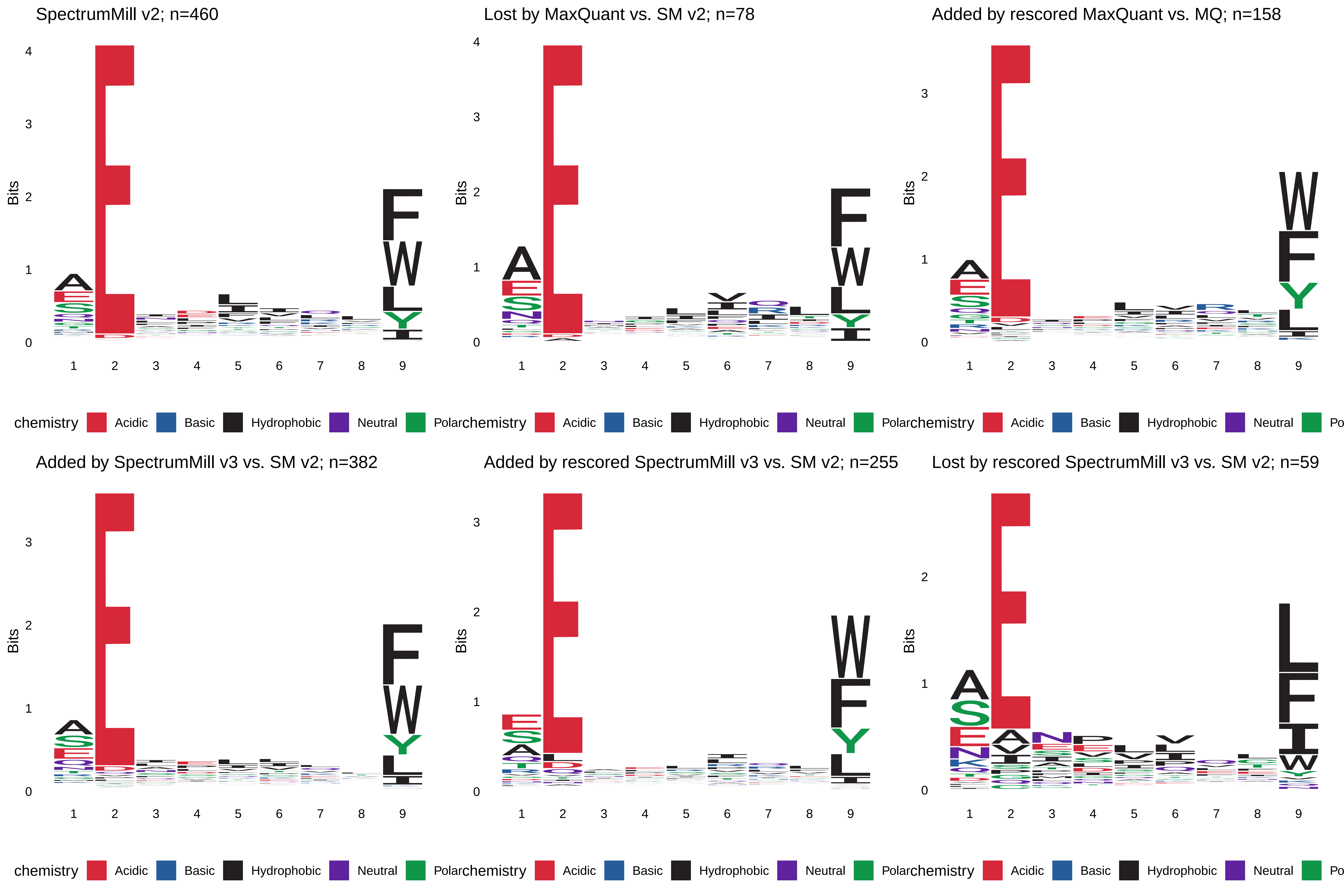

SpectrumMill v2; n=460
Lost by MaxQuant vs. SM v2; n=78
Added by rescored MaxQuant vs. MQ; n=158
Added by SpectrumMill v3 vs. SM v2; n=382
Added by rescored SpectrumMill v3 vs. SM v2; n=255
Lost by rescored SpectrumMill v3 vs. SM v2; n=59
Bits
1 2 3 4 5 6 7 8 9
chemistry
Acidic
Basic
Hydrophobic
Neutral
Polar

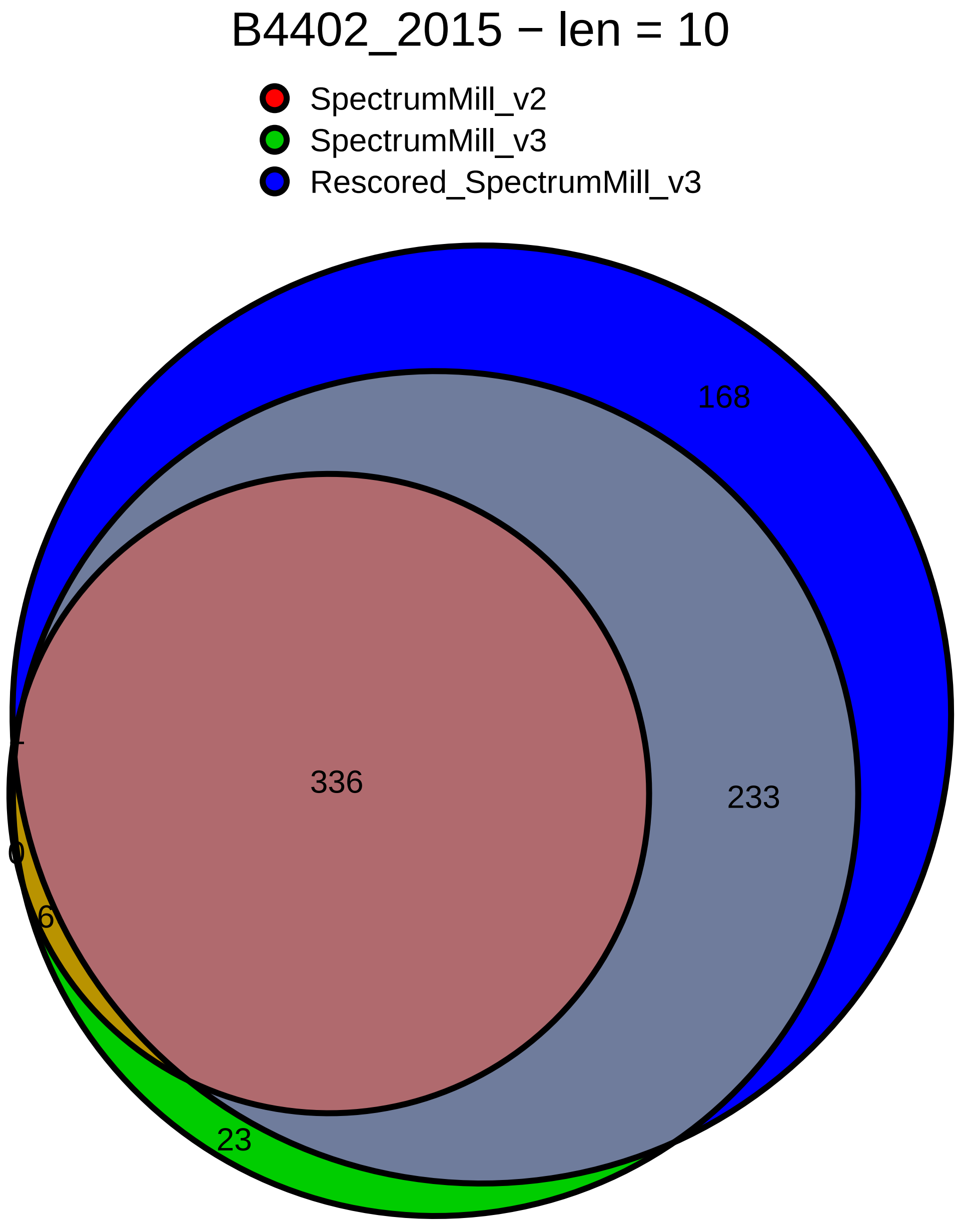
B4402_2015 − len = 10
SpectrumMill_v2
SpectrumMill_v3
Rescored_SpectrumMill_v3
168
336
233
0
6
23

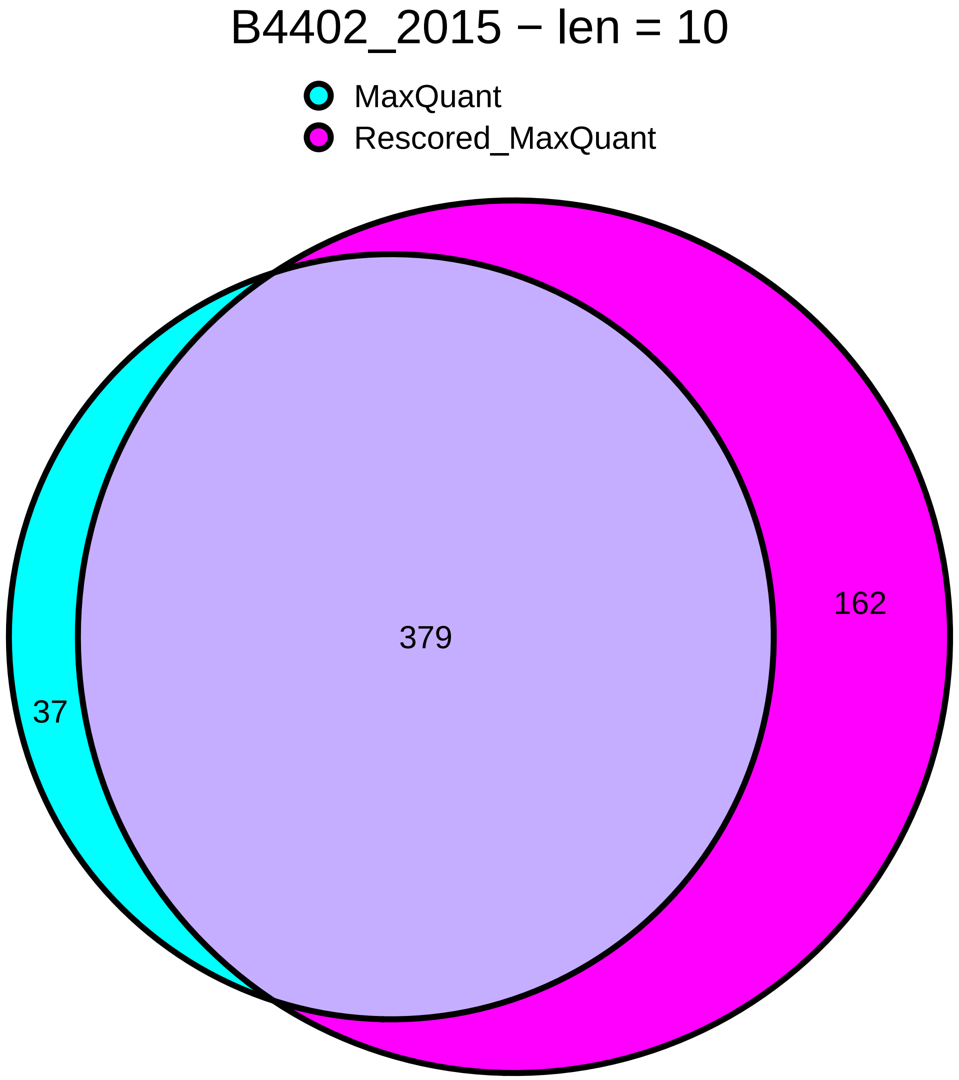
B4402_2015 − len = 10
MaxQuant
Rescored_MaxQuant
379
162
37

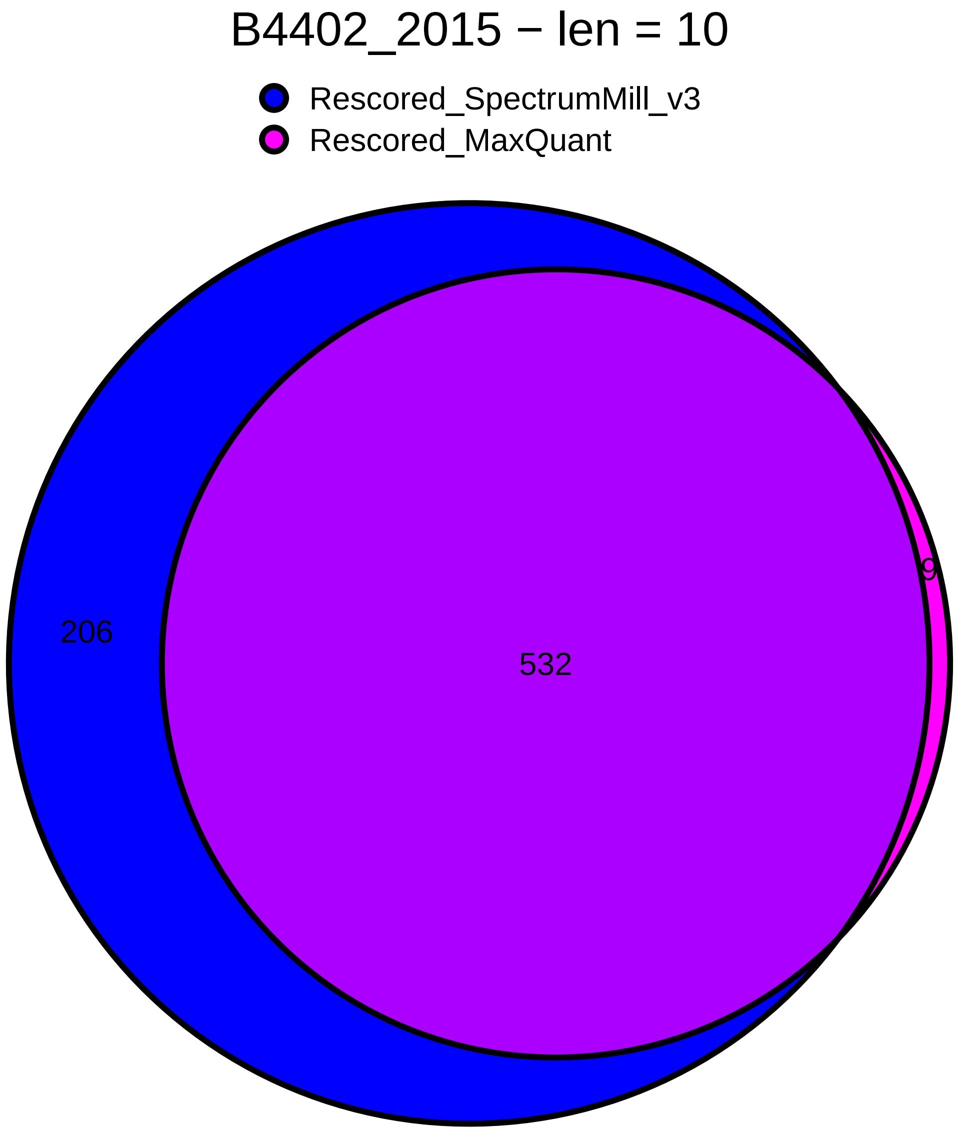
B4402_2015 − len = 10
Rescored_SpectrumMill_v3
Rescored_MaxQuant
206
532
9

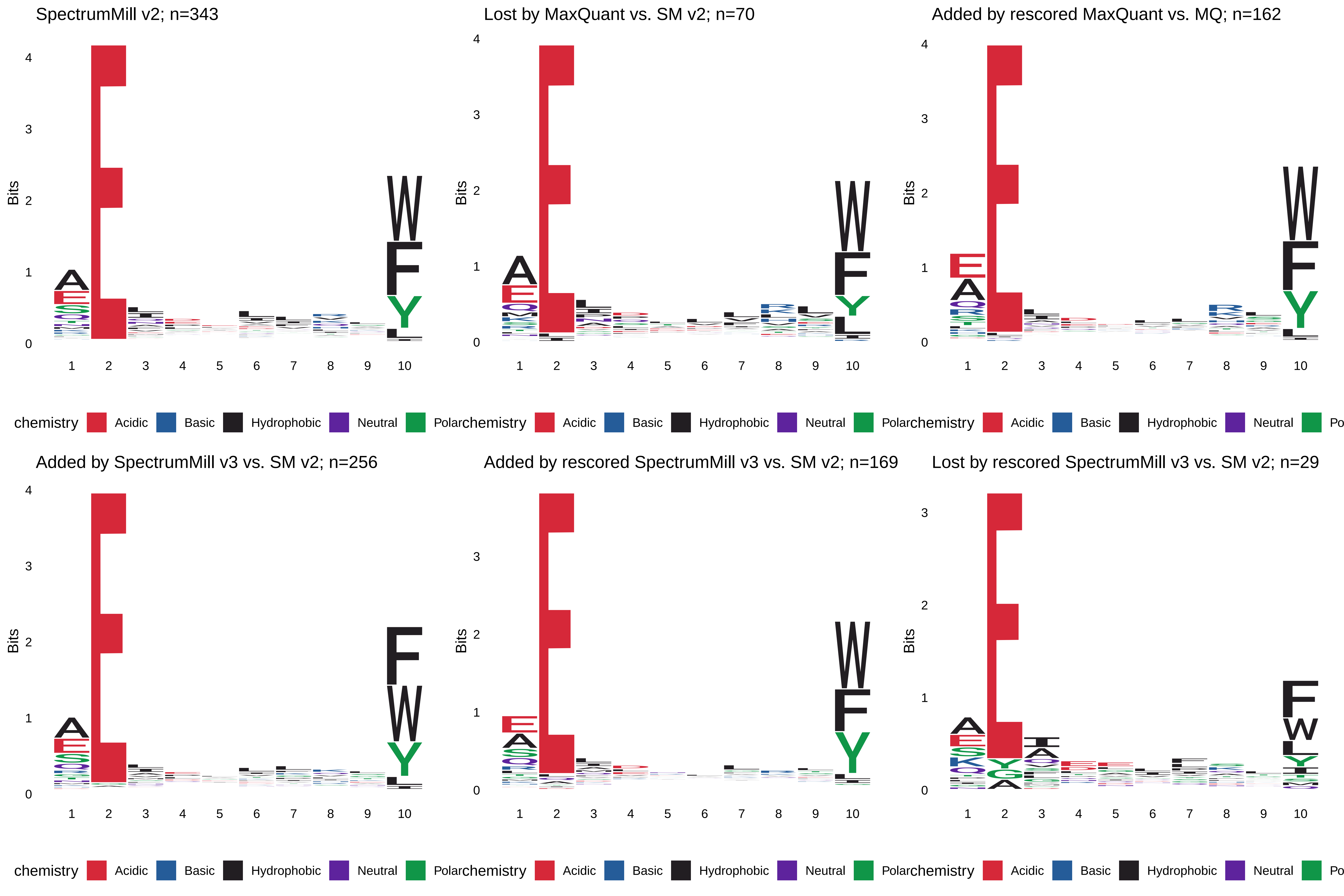
SpectrumMill v2; n=343
Lost by MaxQuant vs. SM v2; n=70
Added by rescored MaxQuant vs. MQ; n=162
Added by SpectrumMill v3 vs. SM v2; n=256
Added by rescored SpectrumMill v3 vs. SM v2; n=169
Lost by rescored SpectrumMill v3 vs. SM v2; n=29
Bits
1 2 3 4 5 6 7 8 9 10
chemistry
Acidic
Basic
Hydrophobic
Neutral
Polar

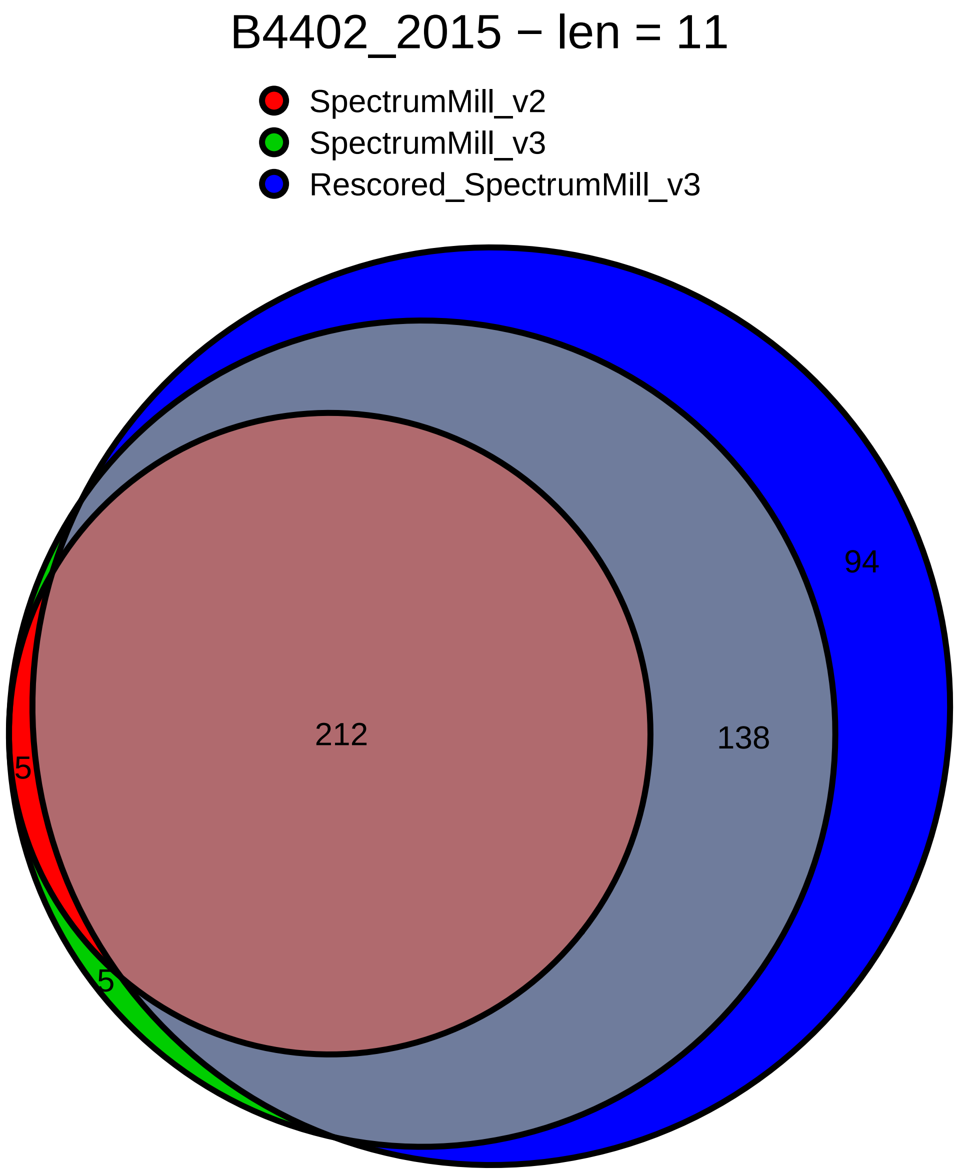
B4402_2015 − len = 11
SpectrumMill_v2
SpectrumMill_v3
Rescored_SpectrumMill_v3
94
5
212
138
5

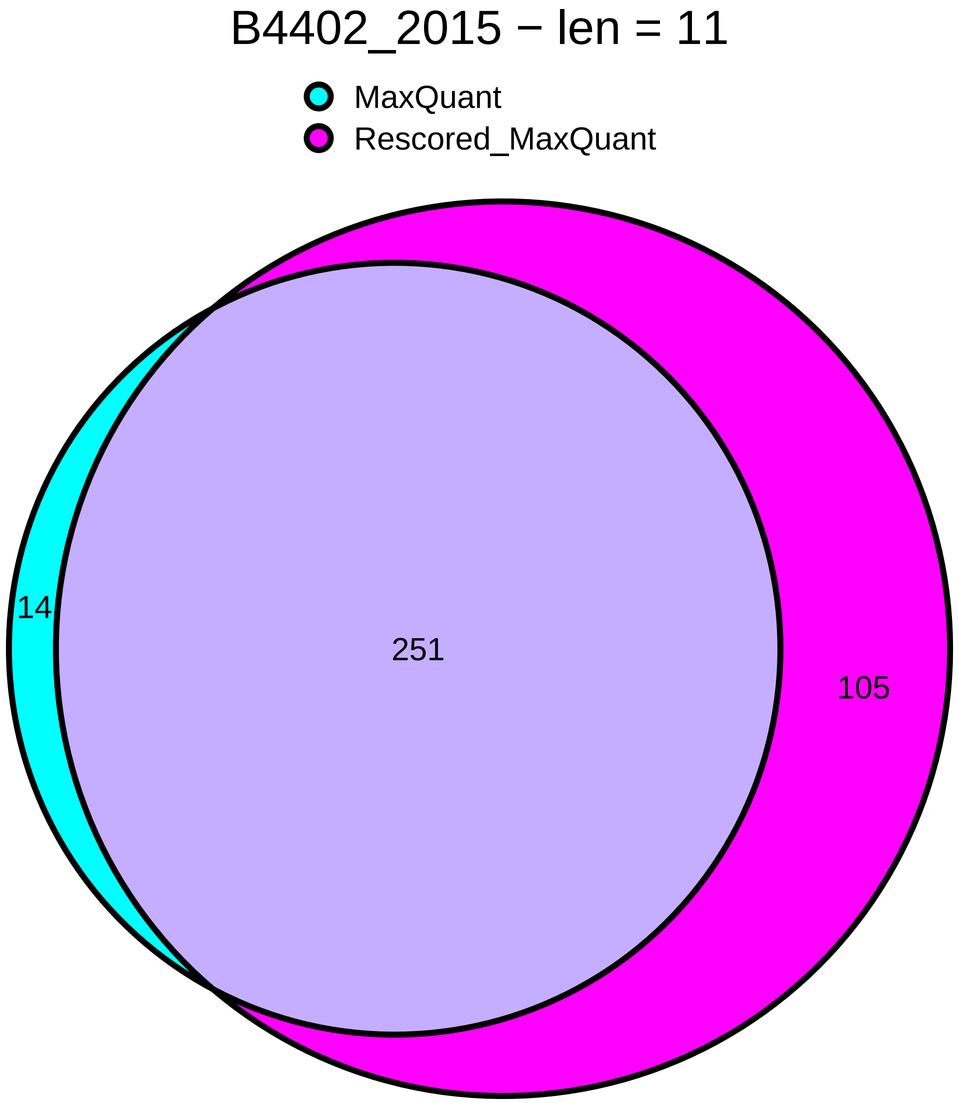
B4402_2015 − len = 11
MaxQuant
Rescored_MaxQuant
14
251
105

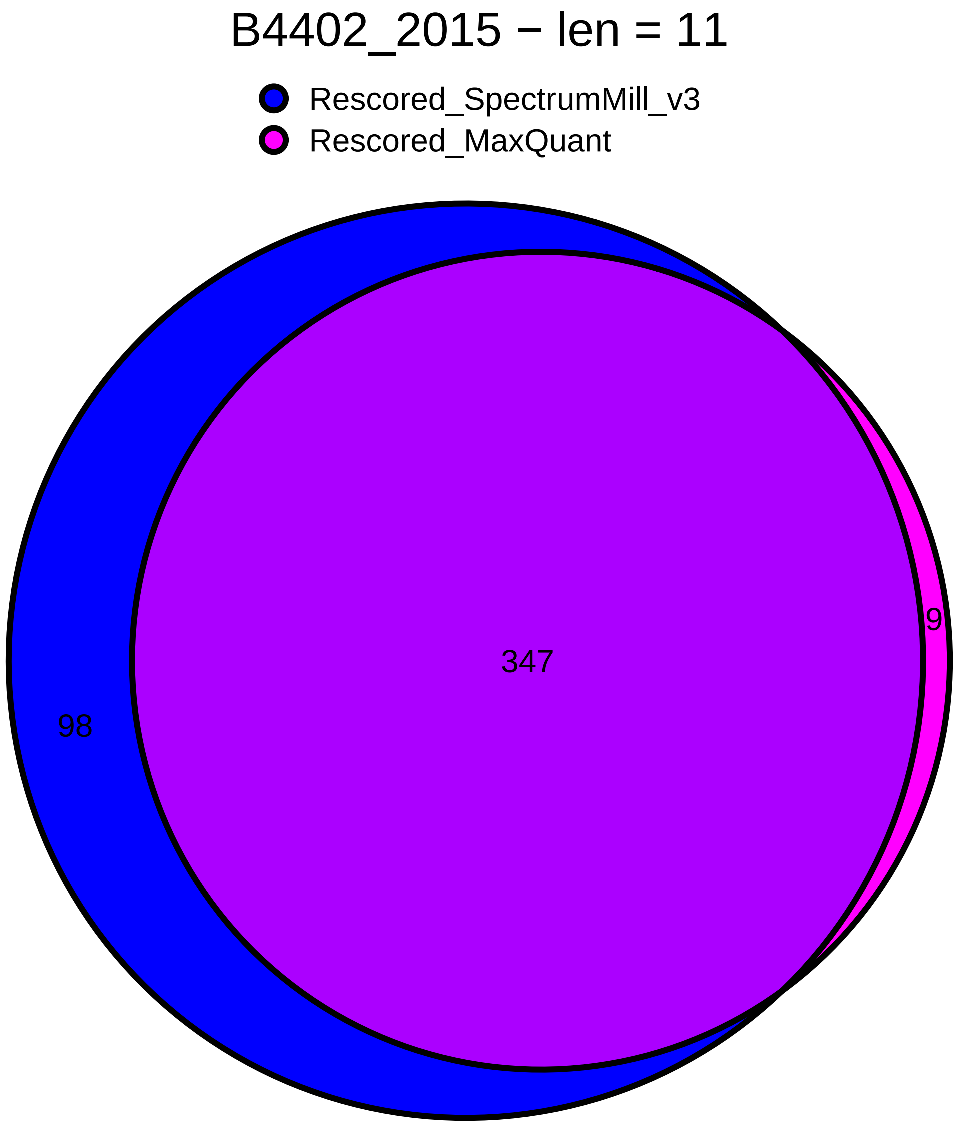
B4402_2015 − len = 11
Rescored_SpectrumMill_v3
Rescored_MaxQuant
9
347
98

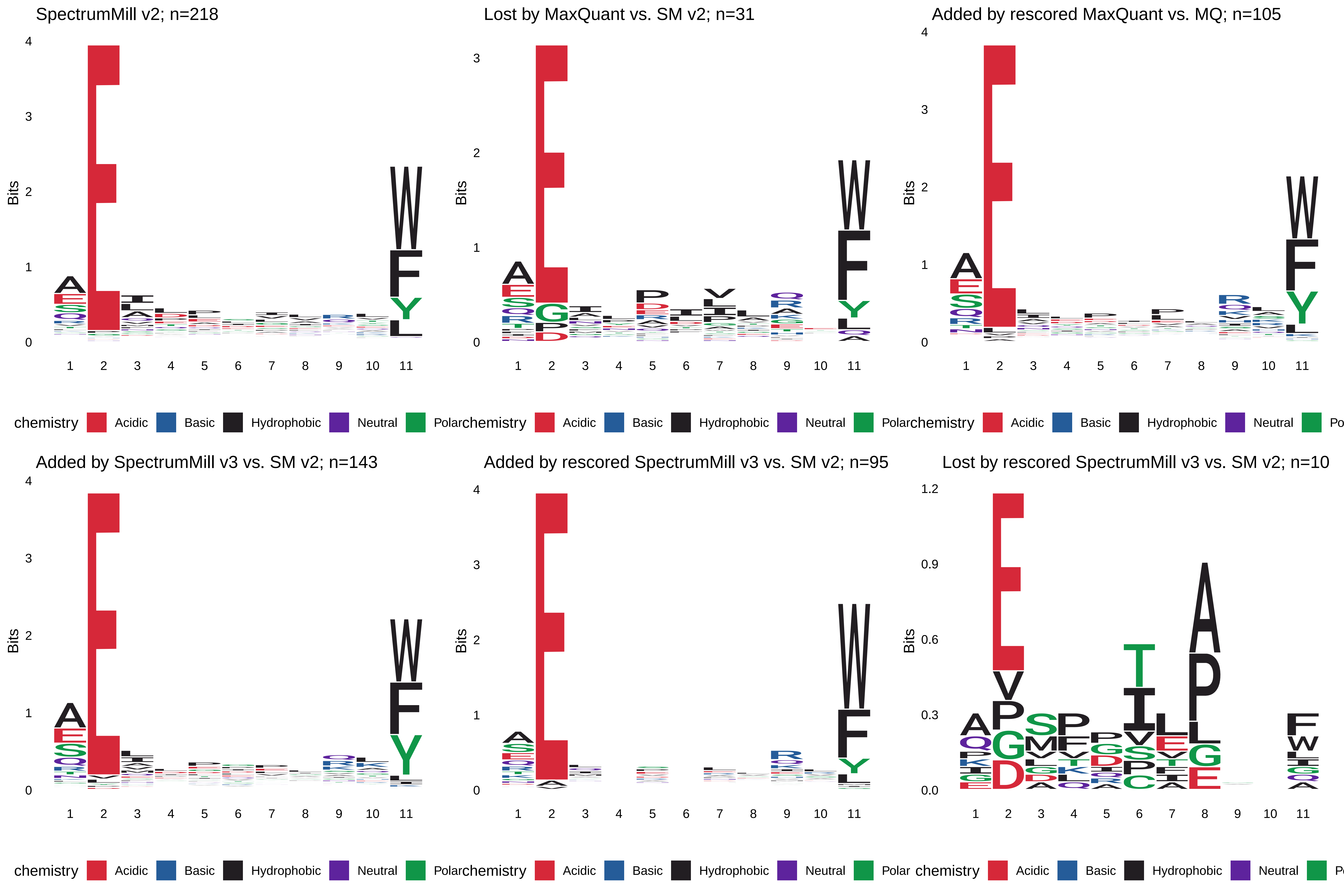
SpectrumMill v2; n=218
Lost by MaxQuant vs. SM v2; n=31
Added by rescored MaxQuant vs. MQ; n=105
Added by SpectrumMill v3 vs. SM v2; n=143
Added by rescored SpectrumMill v3 vs. SM v2; n=95
Lost by rescored SpectrumMill v3 vs. SM v2; n=10
Bits
0 1 2 3 4
0 1 2 3
0.0 0.3 0.6 0.9 1.2
1 2 3 4 5 6 7 8 9 10 11
chemistry Acidic Basic Hydrophobic Neutral Polarchemistry Acidic Basic Hydrophobic Neutral Polarchemistry Acidic Basic Hydrophobic Neutral
chemistry Acidic Basic Hydrophobic Neutral Polarchemistry Acidic Basic Hydrophobic Neutral Polar chemistry Acidic Basic Hydrophobic Neutral

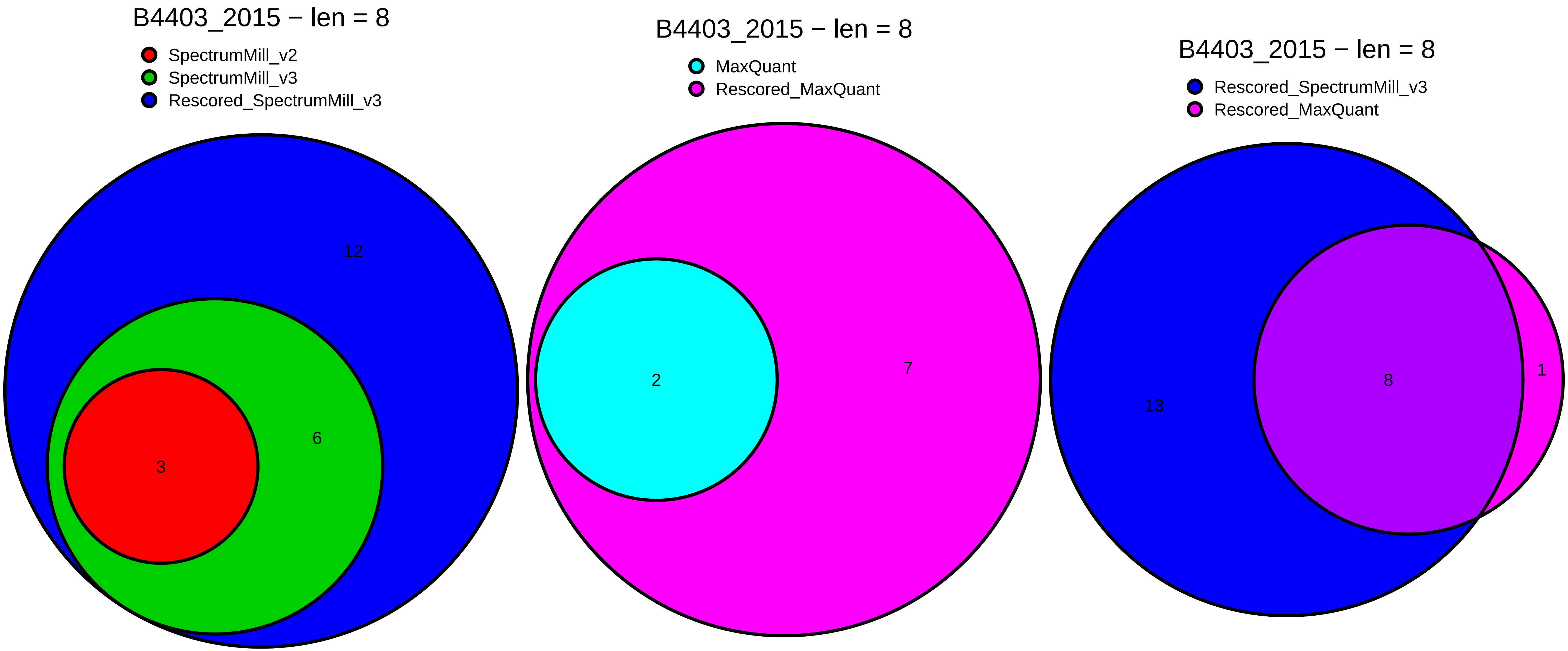

B4403_2015 − len = 8
SpectrumMill_v2
SpectrumMill_v3
Rescored_SpectrumMill_v3
12
6
3
B4403_2015 − len = 8
MaxQuant
Rescored_MaxQuant
2
7
B4403_2015 − len = 8
Rescored_SpectrumMill_v3
Rescored_MaxQuant
13
8
1

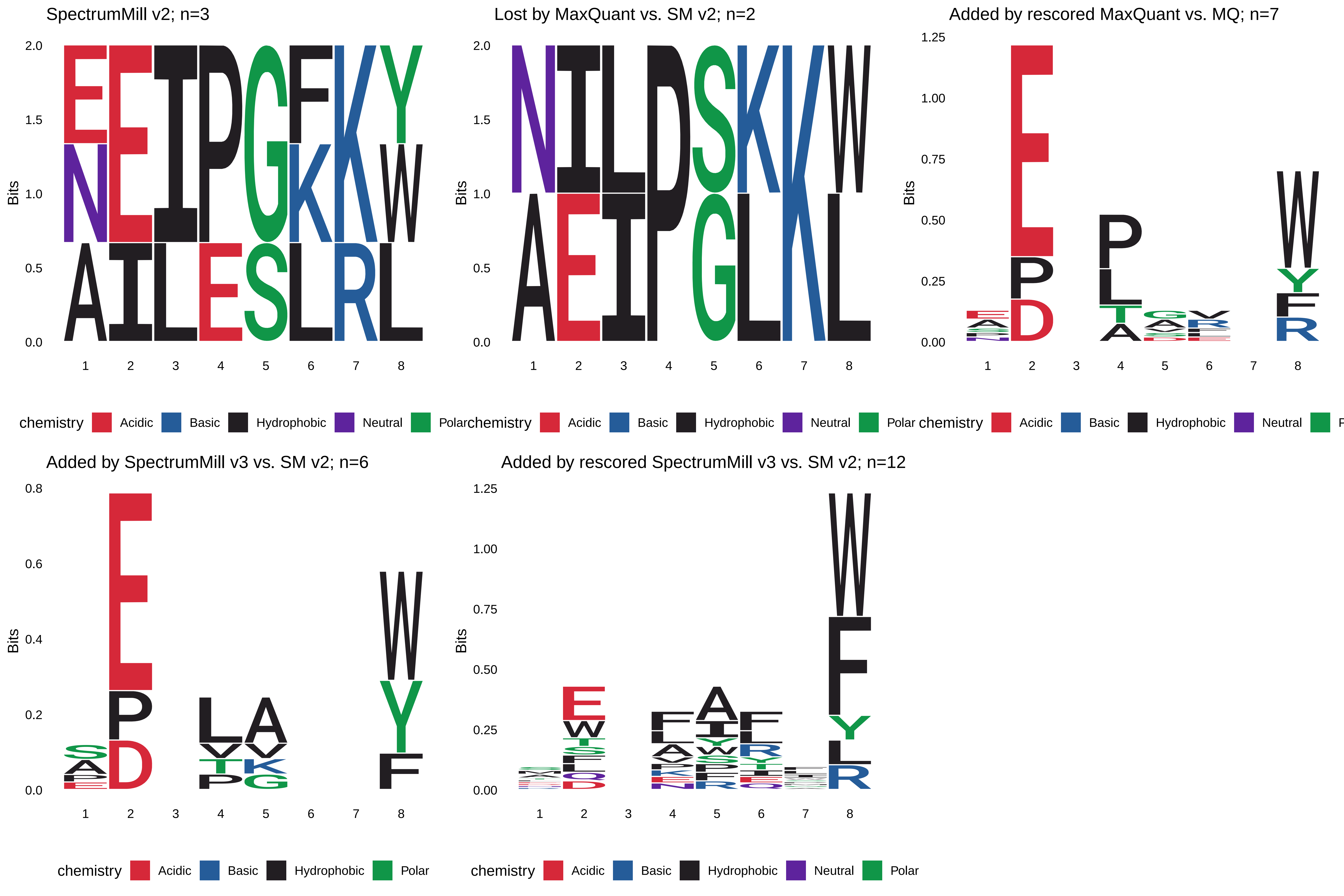

SpectrumMill v2; n=3
Lost by MaxQuant vs. SM v2; n=2
Added by rescored MaxQuant vs. MQ; n=7
Bits
1 2 3 4 5 6 7 8
chemistry
Acidic
Basic
Hydrophobic
Neutral
Polar
Added by SpectrumMill v3 vs. SM v2; n=6
Added by rescored SpectrumMill v3 vs. SM v2; n=12

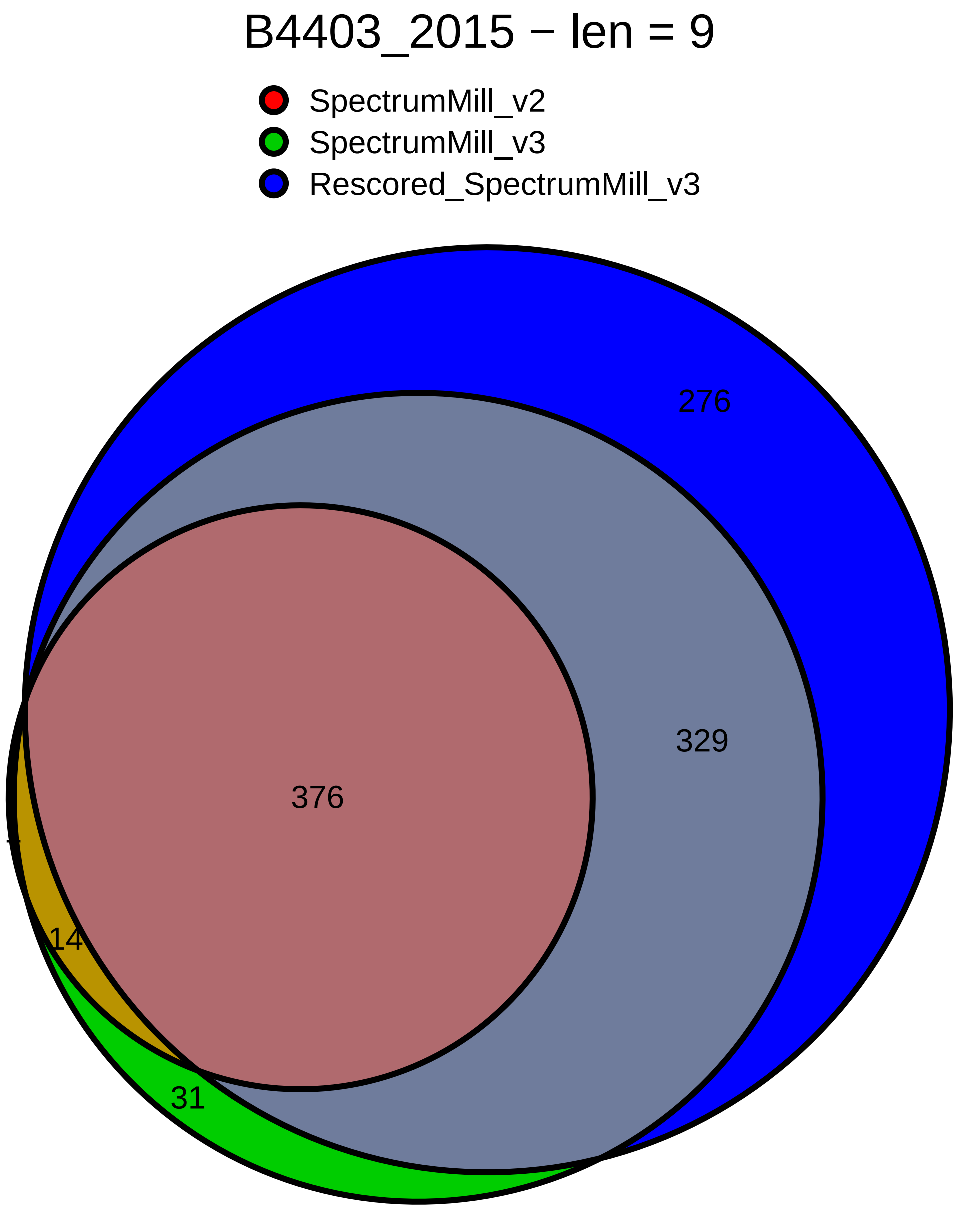
B4403_2015 − len = 9
SpectrumMill_v2
SpectrumMill_v3
Rescored_SpectrumMill_v3
276
329
376
14
31

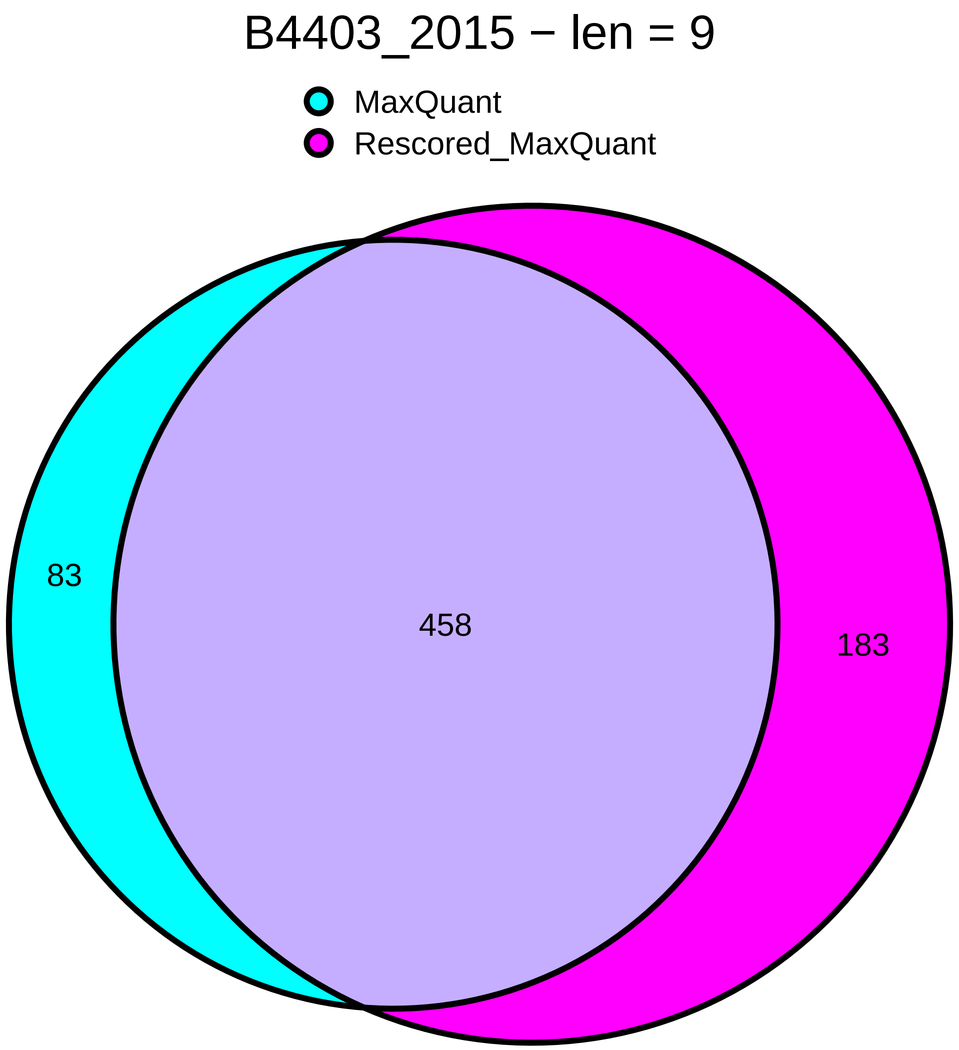
B4403_2015 − len = 9
MaxQuant
Rescored_MaxQuant
83
458
183

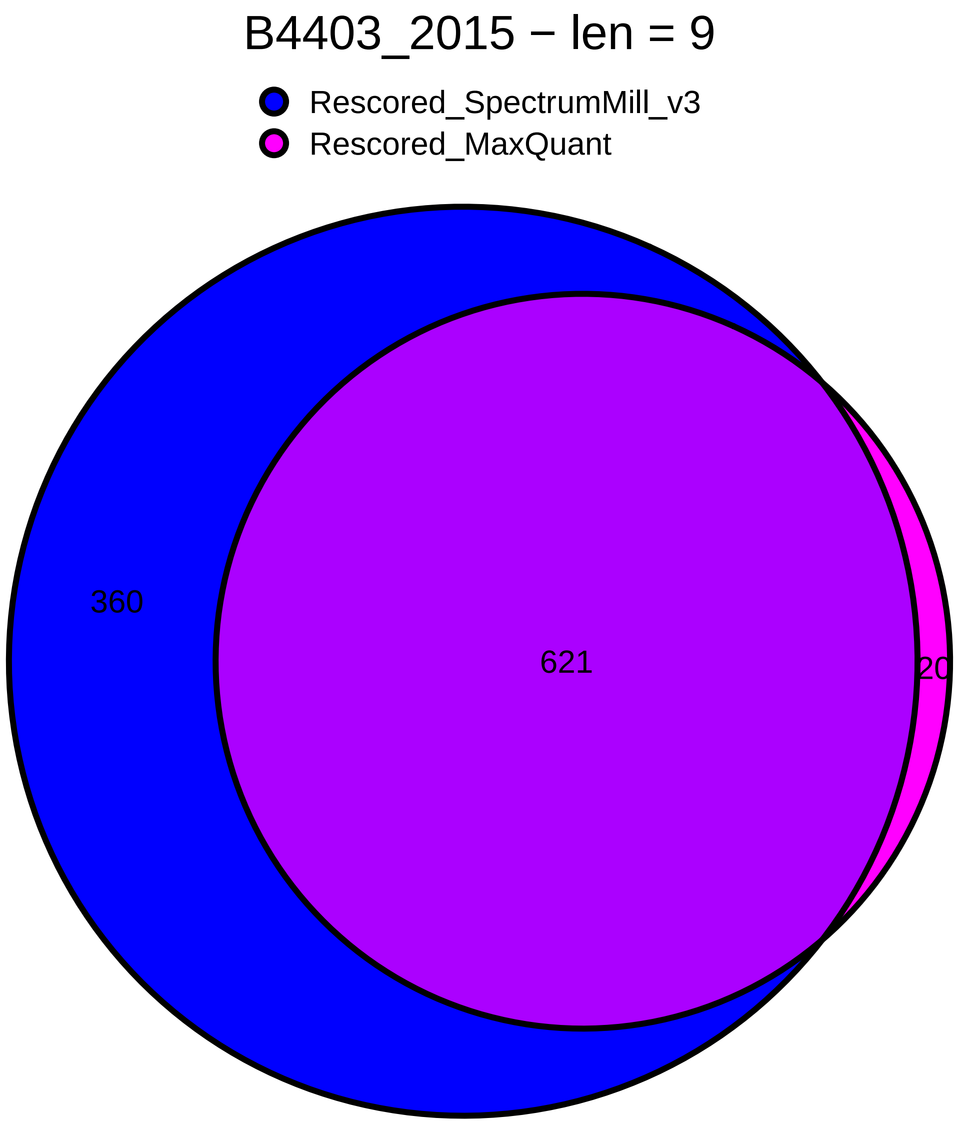
B4403_2015 − len = 9
Rescored_SpectrumMill_v3
Rescored_MaxQuant
360
621
20

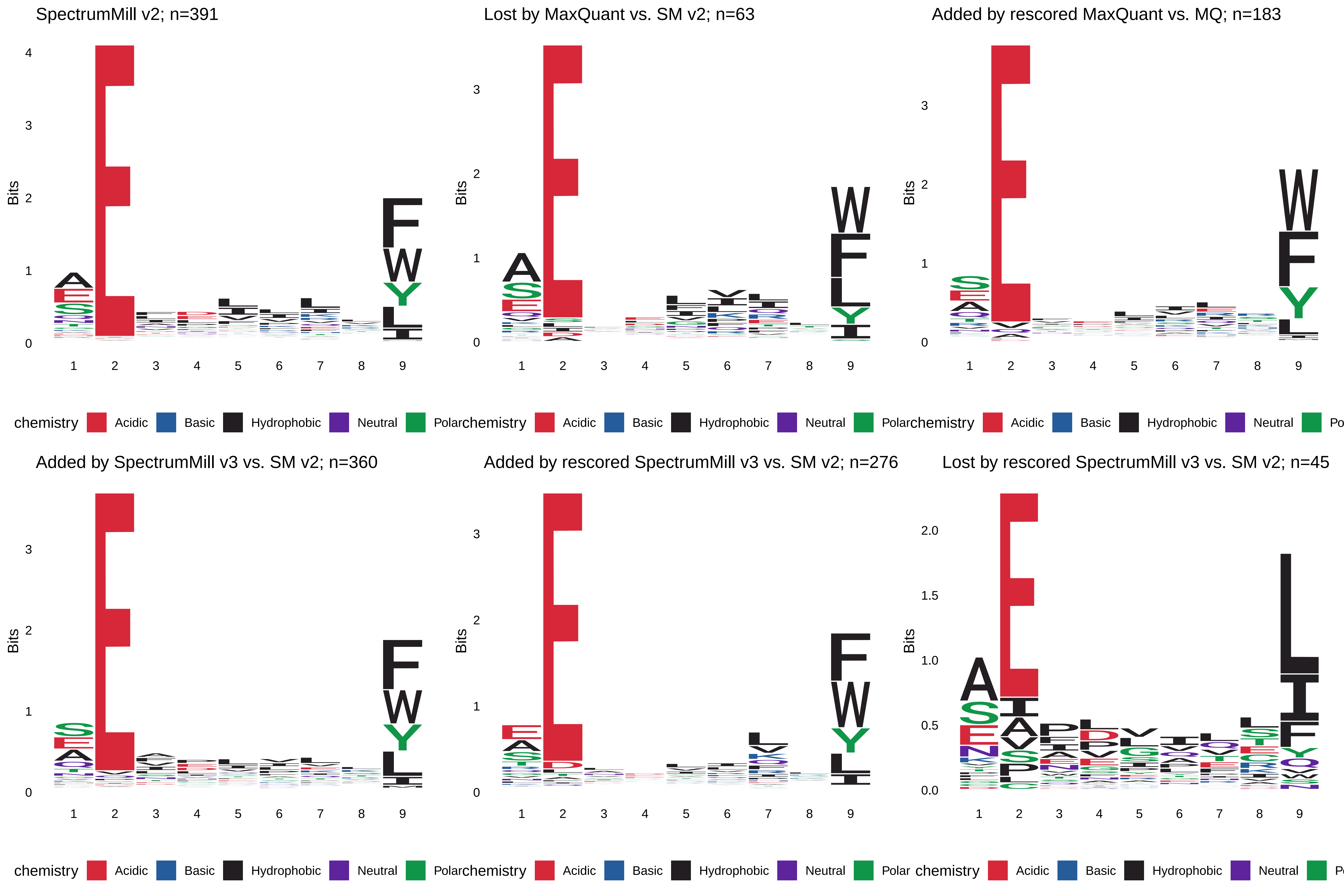
SpectrumMill v2; n=391
Lost by MaxQuant vs. SM v2; n=63
Added by rescored MaxQuant vs. MQ; n=183
Added by SpectrumMill v3 vs. SM v2; n=360
Added by rescored SpectrumMill v3 vs. SM v2; n=276
Lost by rescored SpectrumMill v3 vs. SM v2; n=45
Bits
chemistry
Acidic
Basic
Hydrophobic
Neutral
Polar

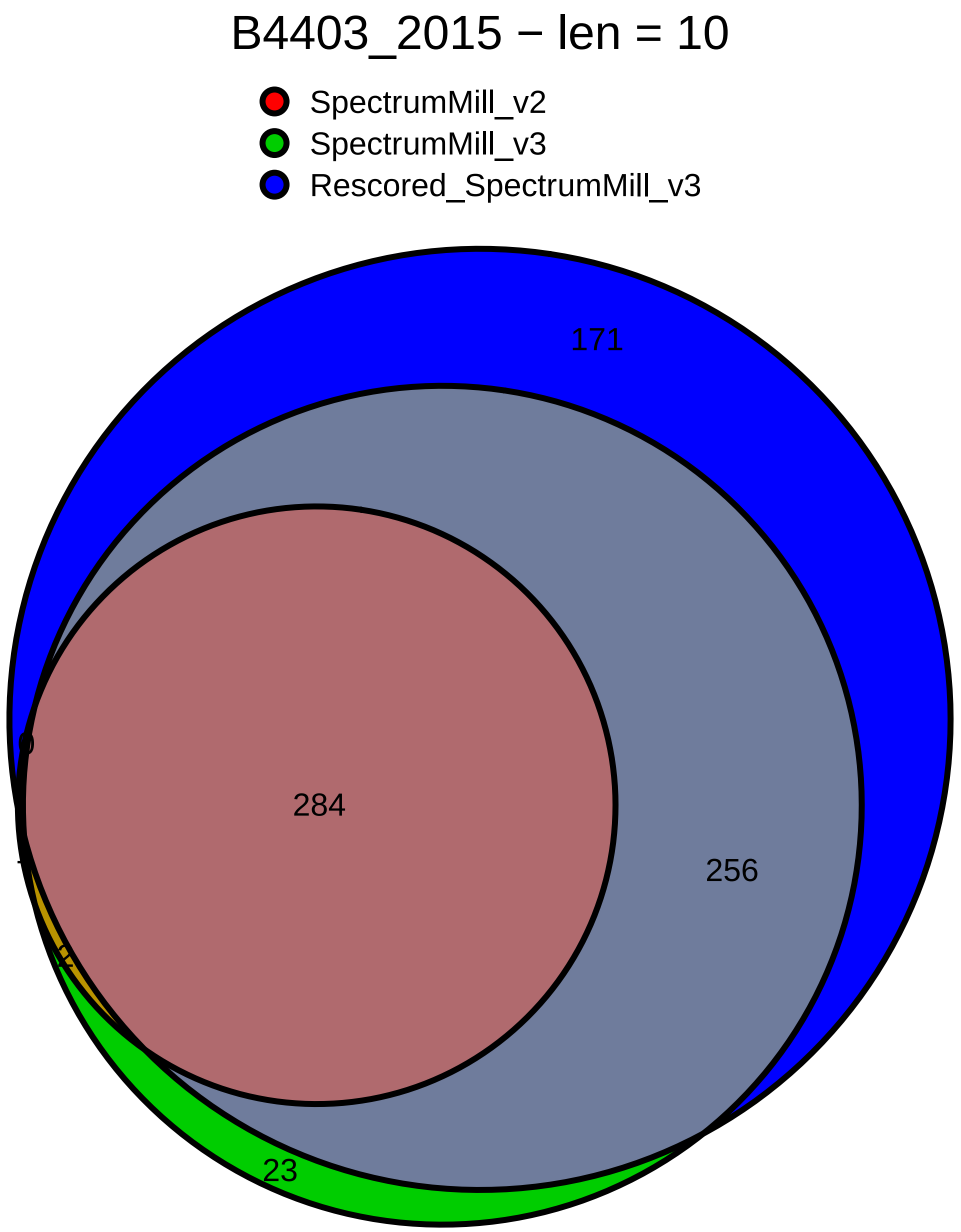
B4403_2015 − len = 10
SpectrumMill_v2
SpectrumMill_v3
Rescored_SpectrumMill_v3
171
284
256
23

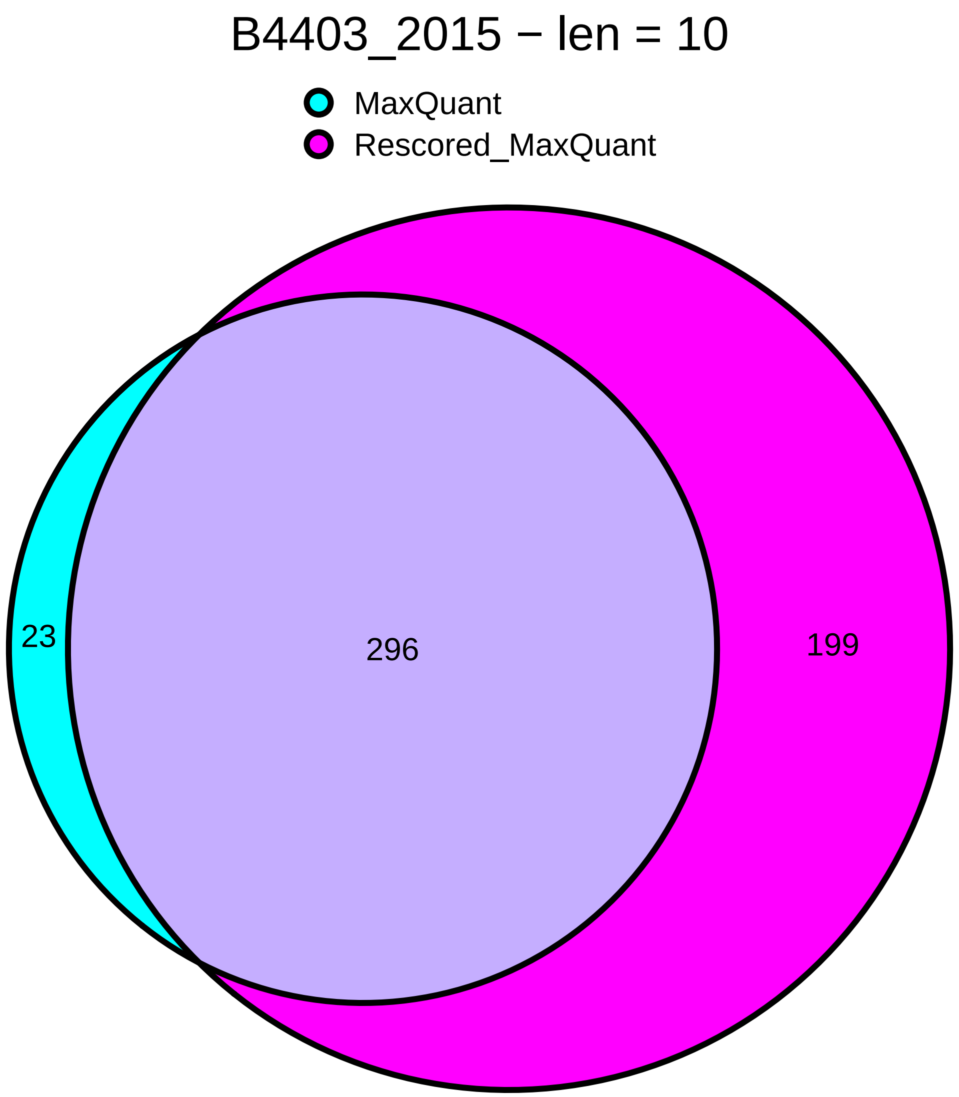
B4403_2015 − len = 10
MaxQuant
Rescored_MaxQuant
23
296
199

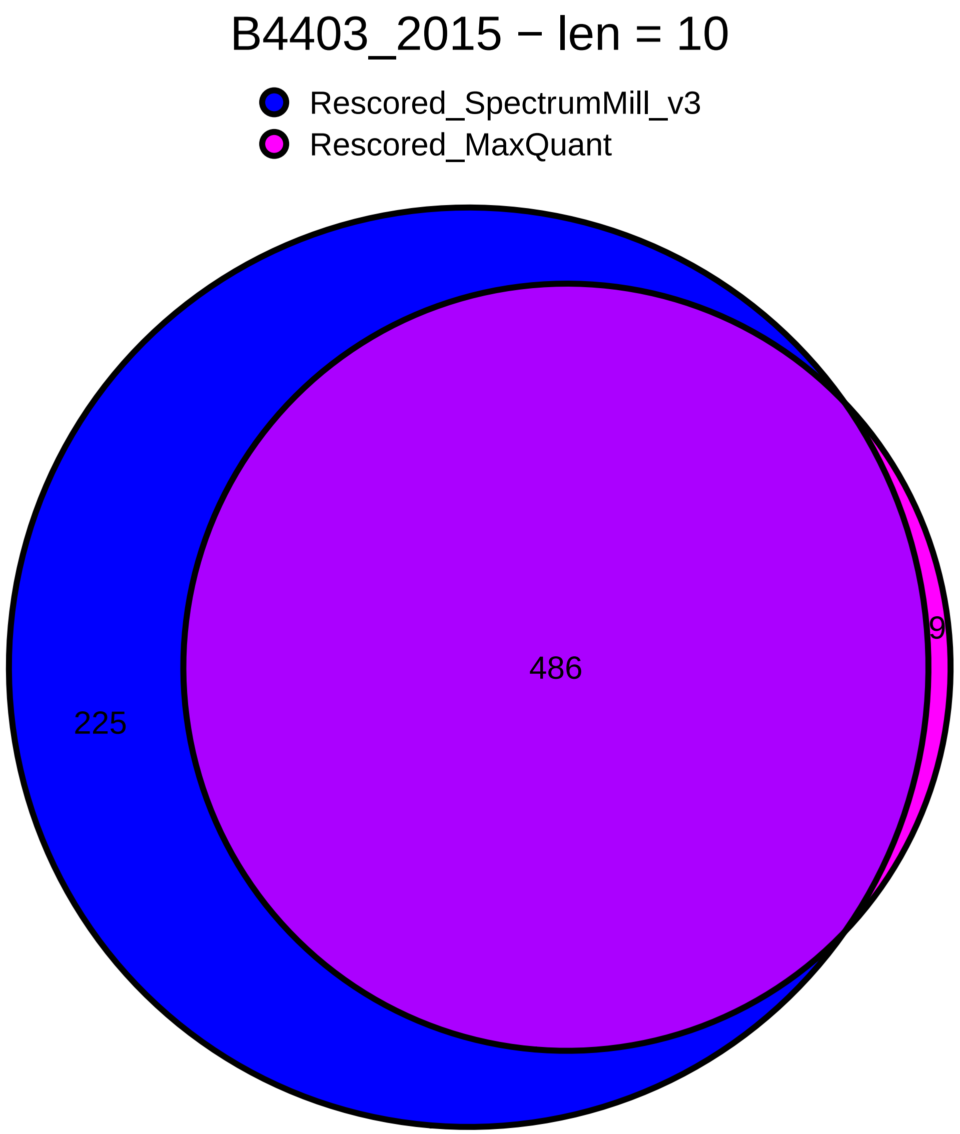
B4403_2015 − len = 10
Rescored_SpectrumMill_v3
Rescored_MaxQuant
225
486
9

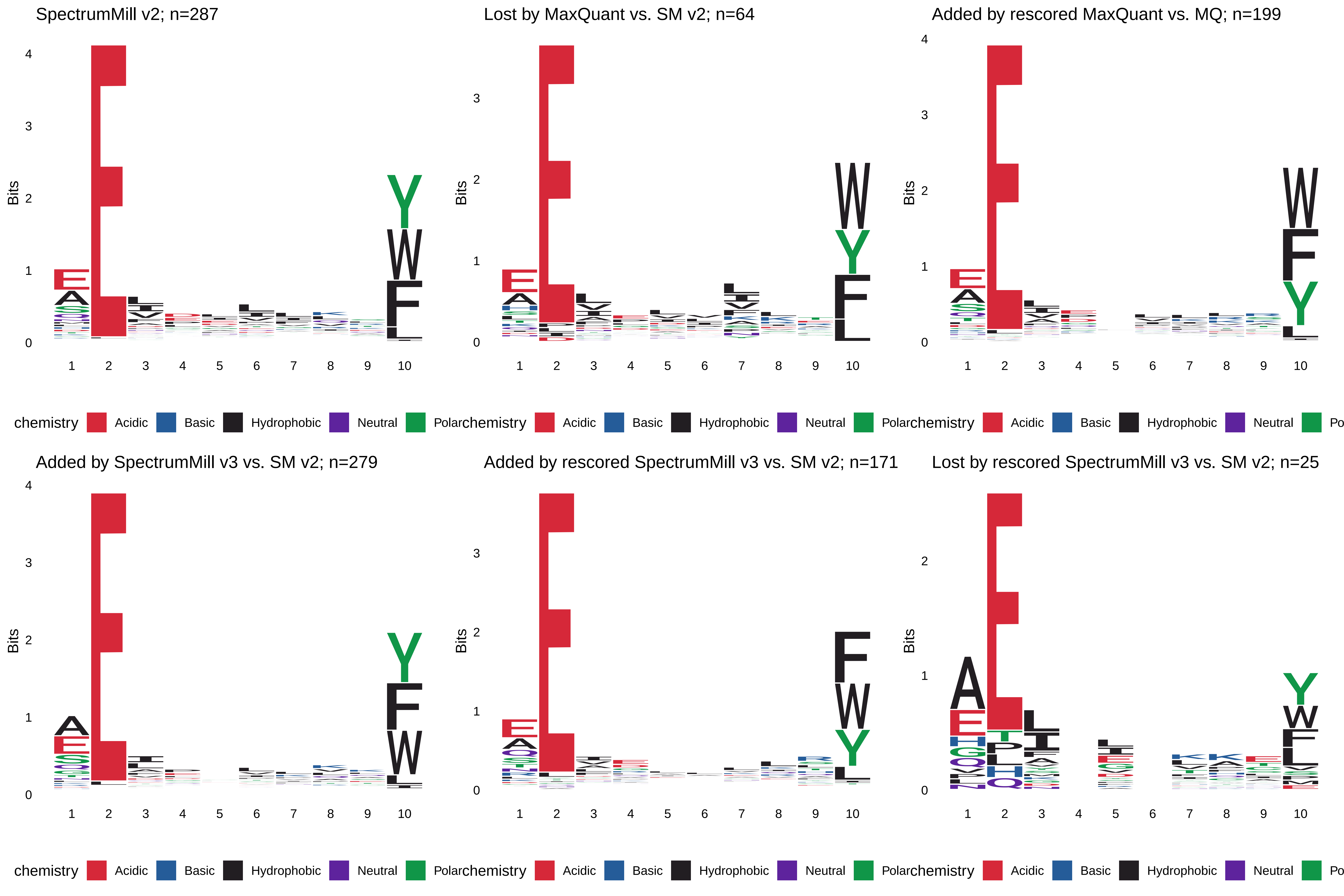
SpectrumMill v2; n=287
Lost by MaxQuant vs. SM v2; n=64
Added by rescored MaxQuant vs. MQ; n=199
Added by SpectrumMill v3 vs. SM v2; n=279
Added by rescored SpectrumMill v3 vs. SM v2; n=171
Lost by rescored SpectrumMill v3 vs. SM v2; n=25
Bits
1 2 3 4 5 6 7 8 9 10
chemistry
Acidic
Basic
Hydrophobic
Neutral
Polar

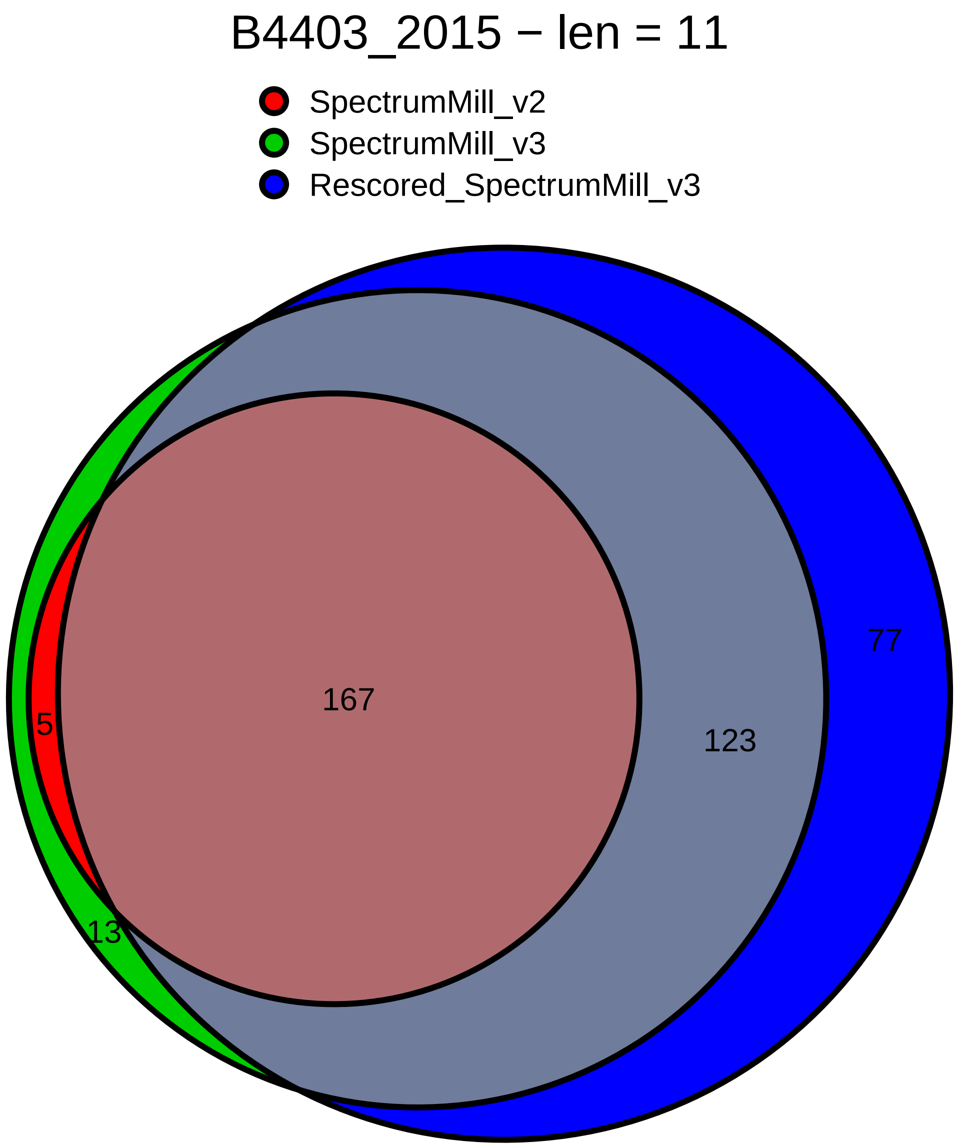

B4403_2015 − len = 11
SpectrumMill_v2
SpectrumMill_v3
Rescored_SpectrumMill_v3
77
5
167
123
13

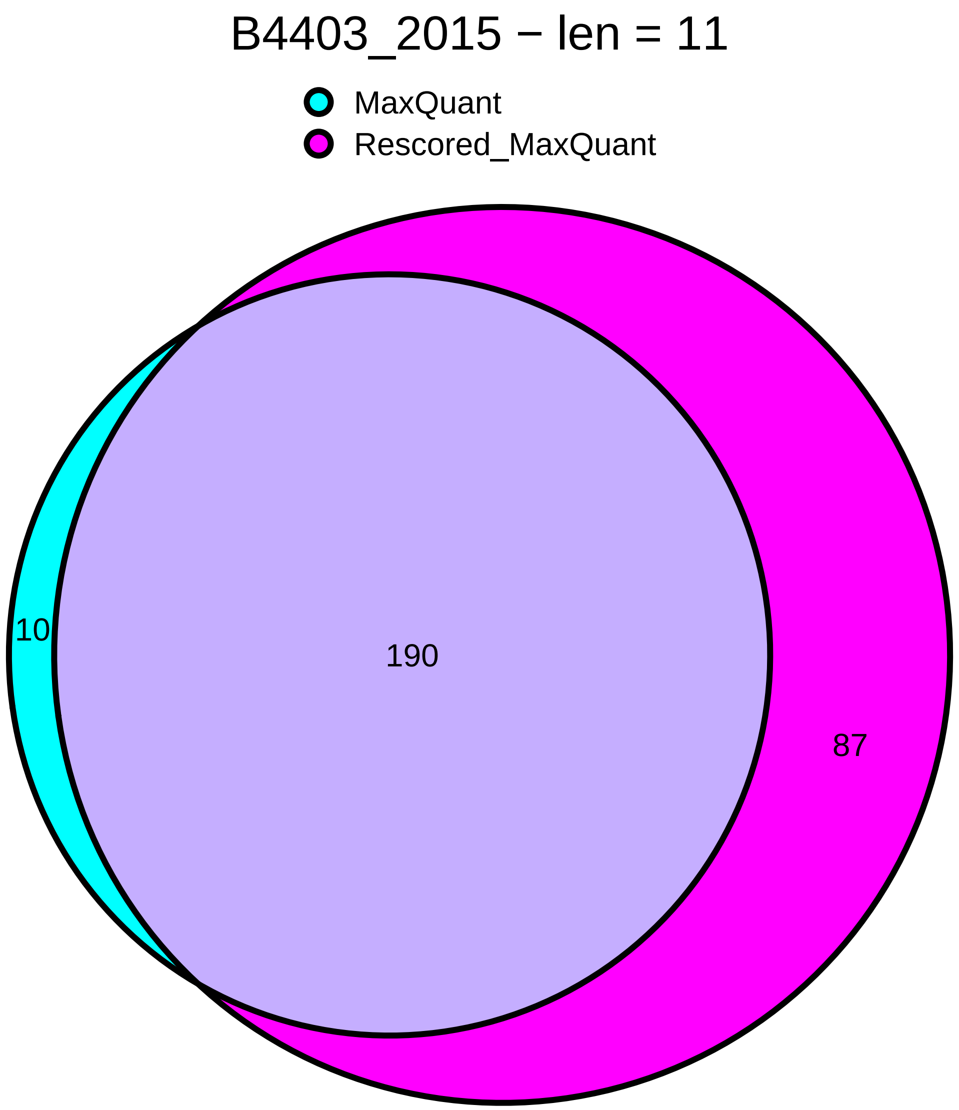

B4403_2015 − len = 11
MaxQuant
Rescored_MaxQuant
10
190
87

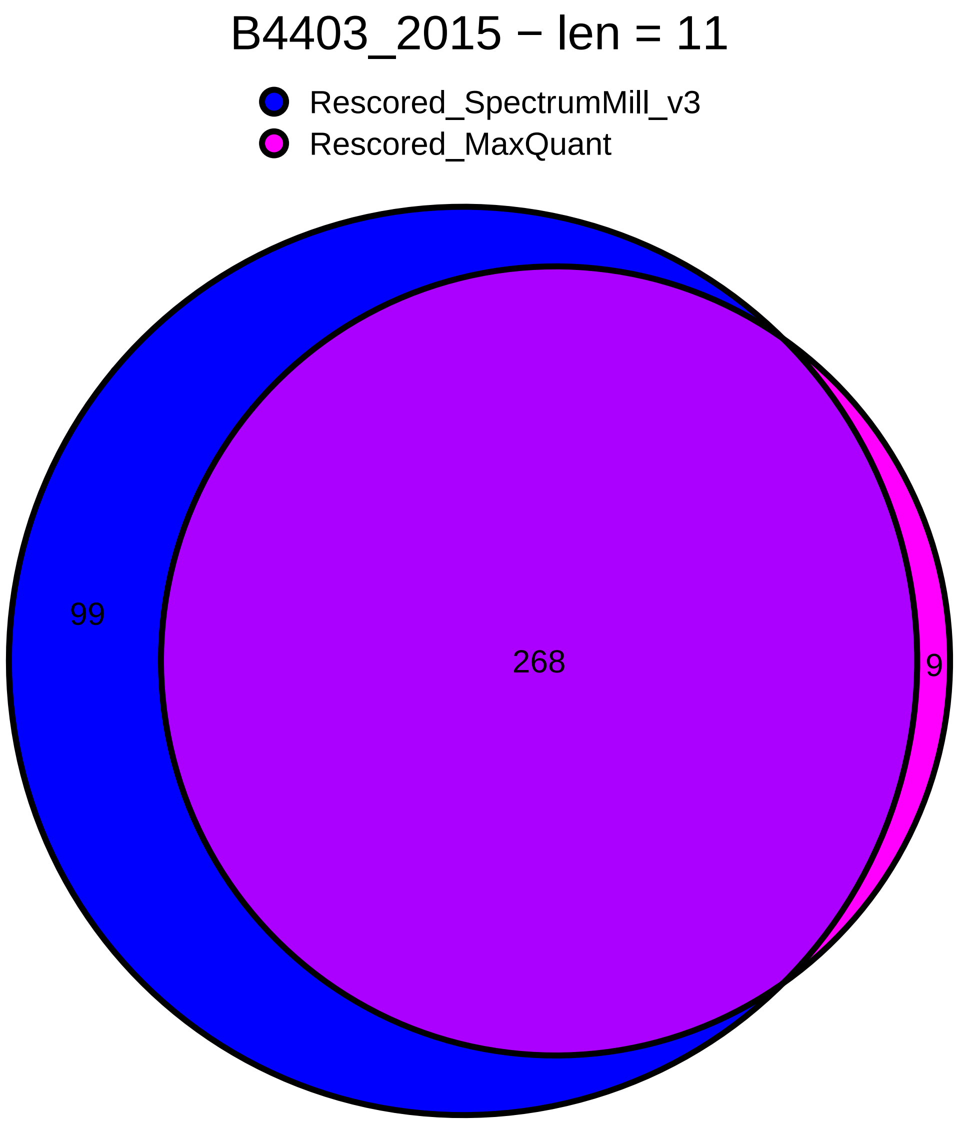

B4403_2015 − len = 11
Rescored_SpectrumMill_v3
Rescored_MaxQuant
99
268
9

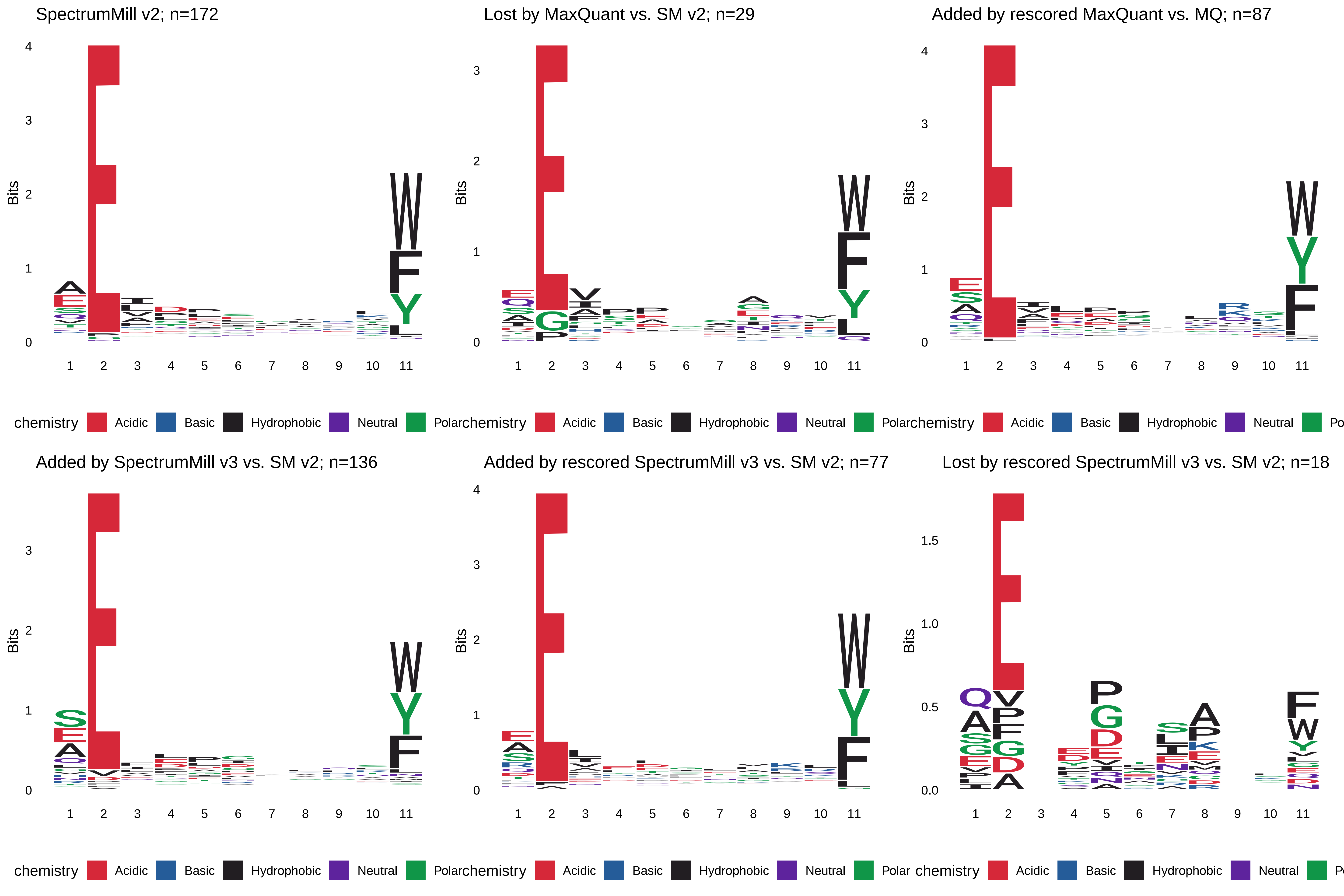

SpectrumMill v2; n=172
Lost by MaxQuant vs. SM v2; n=29
Added by rescored MaxQuant vs. MQ; n=87
Added by SpectrumMill v3 vs. SM v2; n=136
Added by rescored SpectrumMill v3 vs. SM v2; n=77
Lost by rescored SpectrumMill v3 vs. SM v2; n=18
Bits
1 2 3 4 5 6 7 8 9 10 11
chemistry Acidic Basic Hydrophobic Neutral Polar

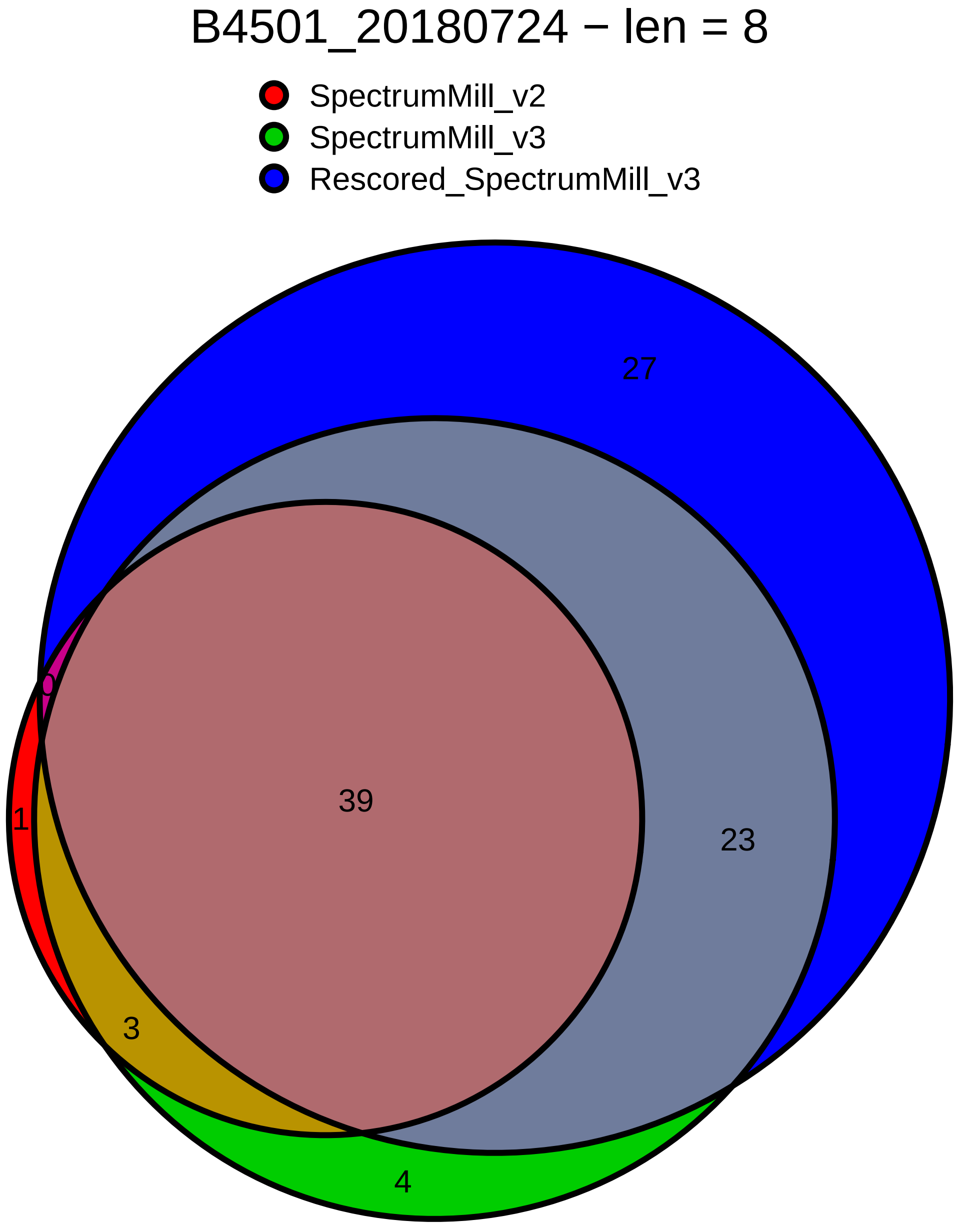
B4501_20180724 − len = 8
SpectrumMill_v2
SpectrumMill_v3
Rescored_SpectrumMill_v3
27
0
1
39
23
3
4

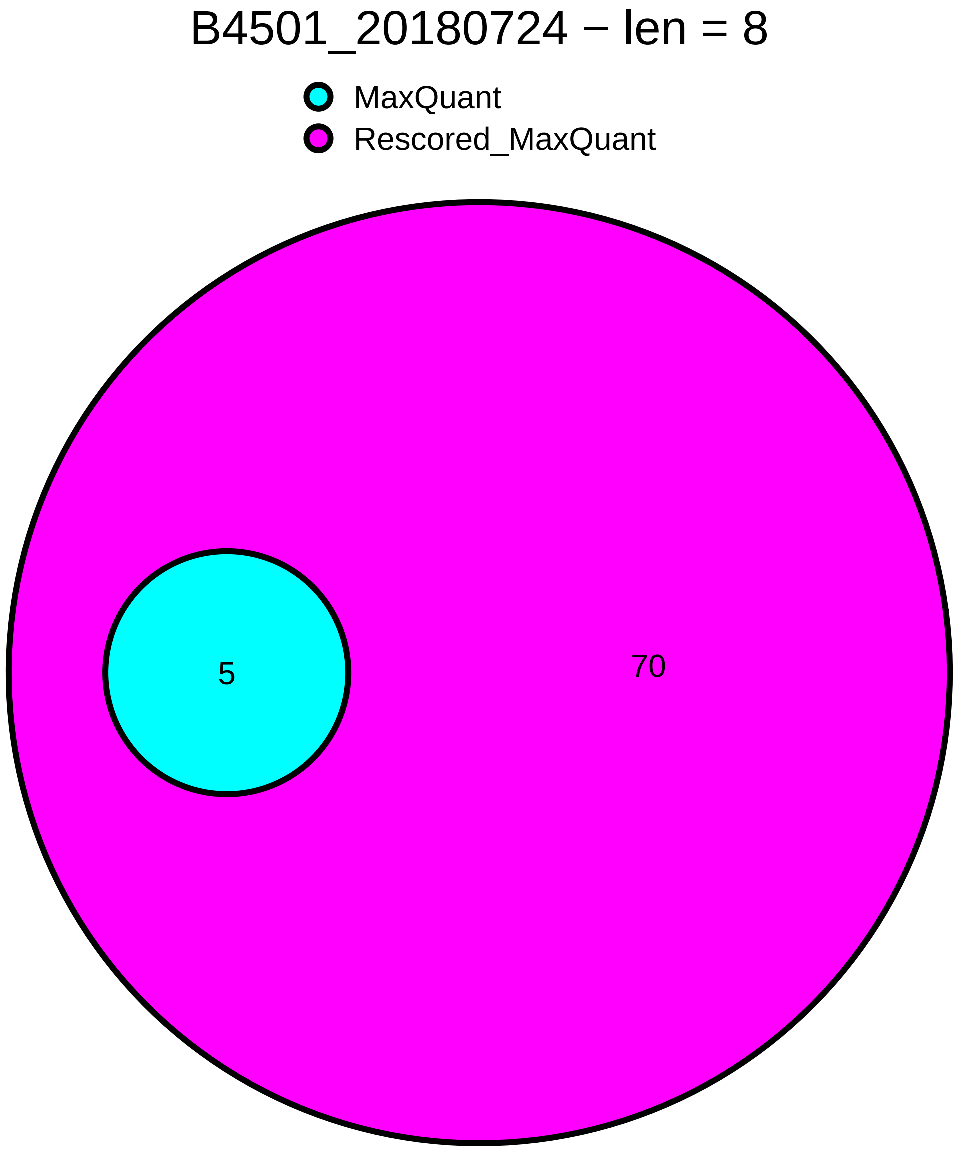
B4501_20180724 − len = 8
MaxQuant
Rescored_MaxQuant
5
70

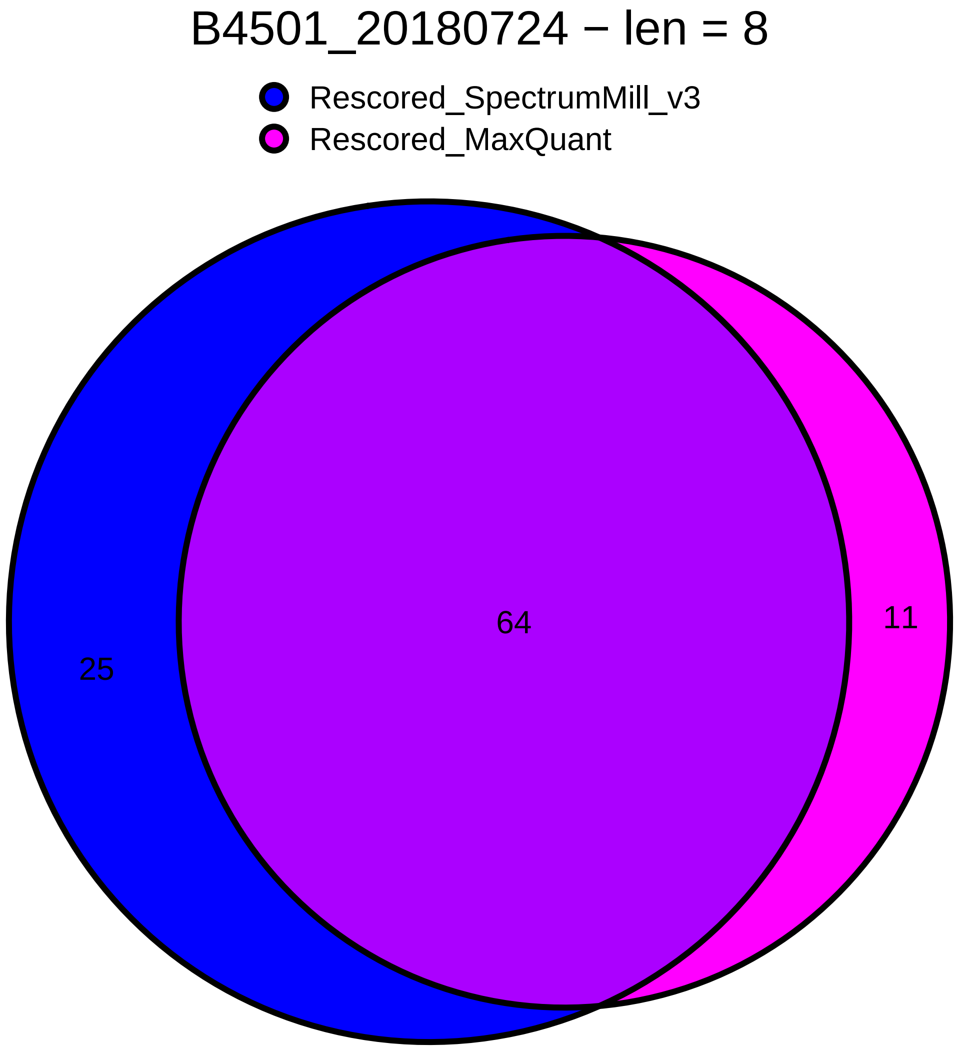
B4501_20180724 − len = 8
Rescored_SpectrumMill_v3
Rescored_MaxQuant
25
64
11

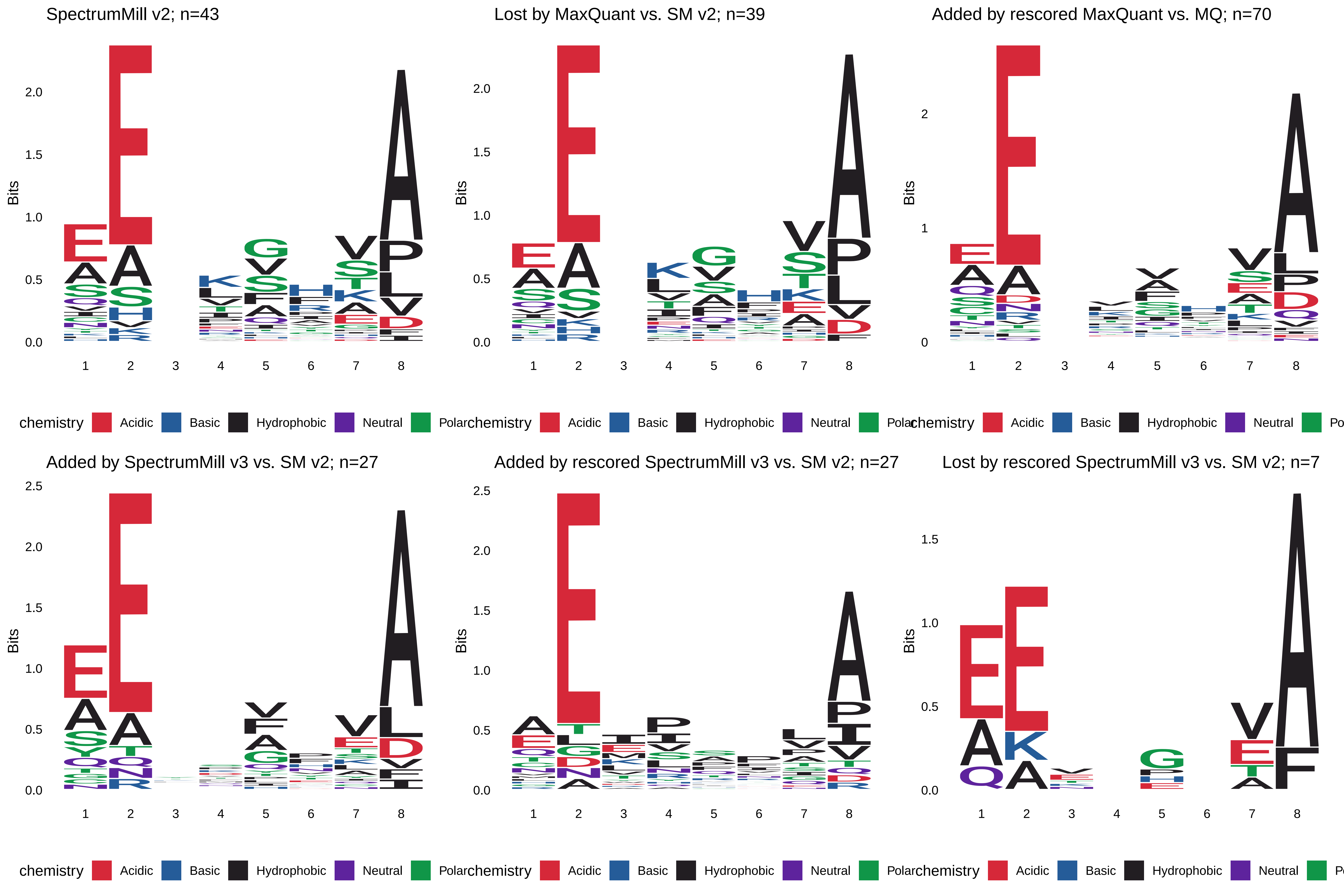
SpectrumMill v2; n=43
Lost by MaxQuant vs. SM v2; n=39
Added by rescored MaxQuant vs. MQ; n=70
Added by SpectrumMill v3 vs. SM v2; n=27
Added by rescored SpectrumMill v3 vs. SM v2; n=27
Lost by rescored SpectrumMill v3 vs. SM v2; n=7
Bits
1 2 3 4 5 6 7 8
chemistry Acidic Basic Hydrophobic Neutral Polar

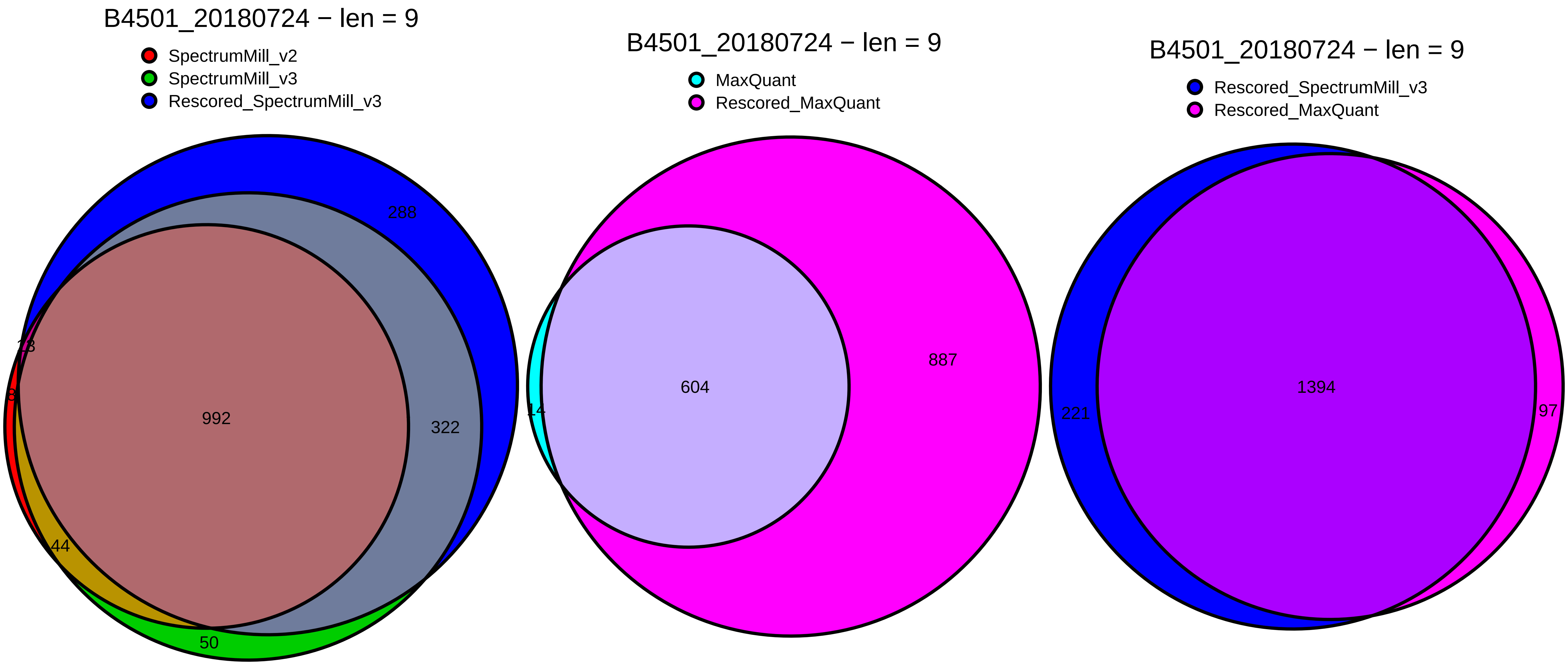

B4501_20180724 − len = 9
SpectrumMill_v2
SpectrumMill_v3
Rescored_SpectrumMill_v3
288
13
8
992
322
44
50
B4501_20180724 − len = 9
MaxQuant
Rescored_MaxQuant
604
887
14
B4501_20180724 − len = 9
Rescored_SpectrumMill_v3
Rescored_MaxQuant
1394
221
97

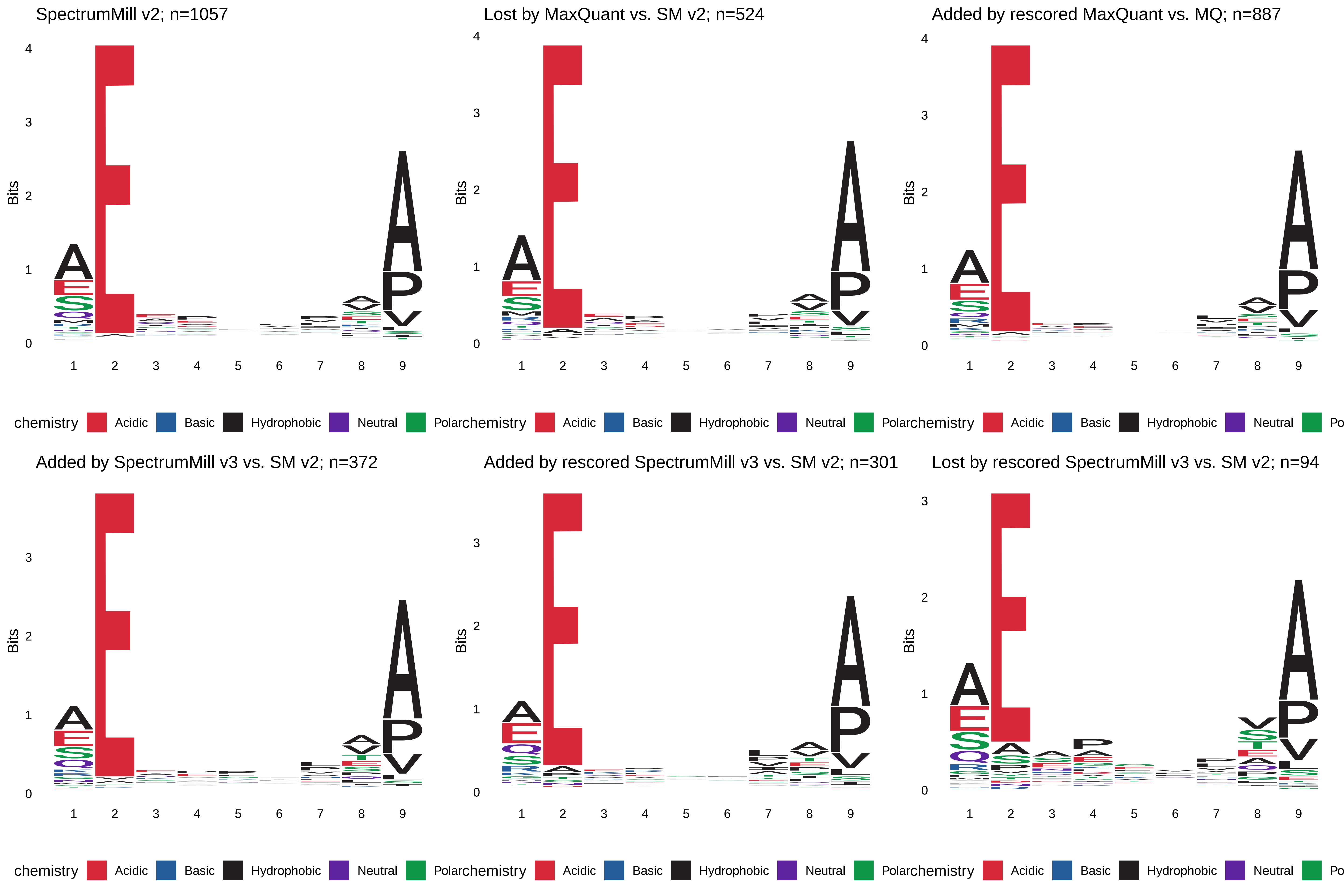
SpectrumMill v2; n=1057
Lost by MaxQuant vs. SM v2; n=524
Added by rescored MaxQuant vs. MQ; n=887
Added by SpectrumMill v3 vs. SM v2; n=372
Added by rescored SpectrumMill v3 vs. SM v2; n=301
Lost by rescored SpectrumMill v3 vs. SM v2; n=94
Bits
0
1
2
3
4
1 2 3 4 5 6 7 8 9
chemistry
Acidic
Basic
Hydrophobic
Neutral
Polar

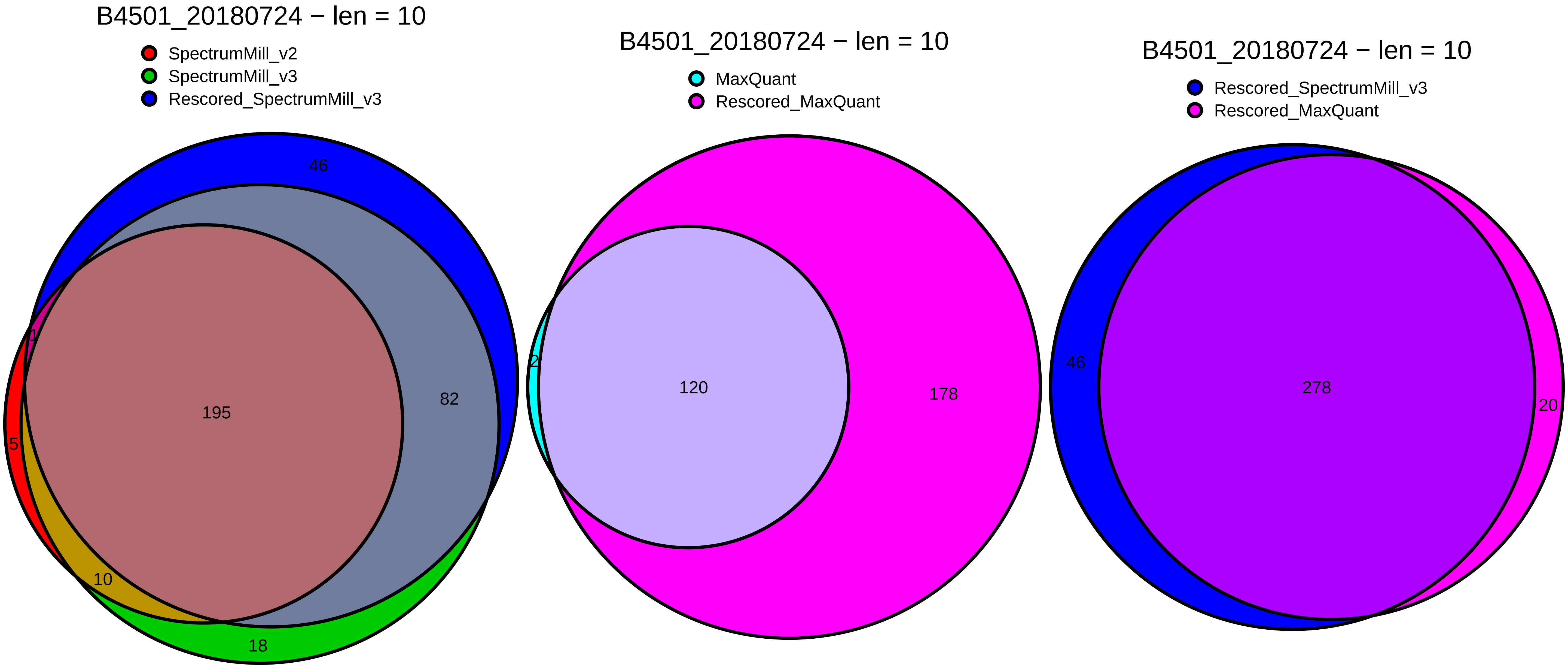
B4501_20180724 − len = 10
SpectrumMill_v2
SpectrumMill_v3
Rescored_SpectrumMill_v3
46
1
195
82
5
10
18
B4501_20180724 − len = 10
MaxQuant
Rescored_MaxQuant
2
120
178
B4501_20180724 − len = 10
Rescored_SpectrumMill_v3
Rescored_MaxQuant
46
278
20

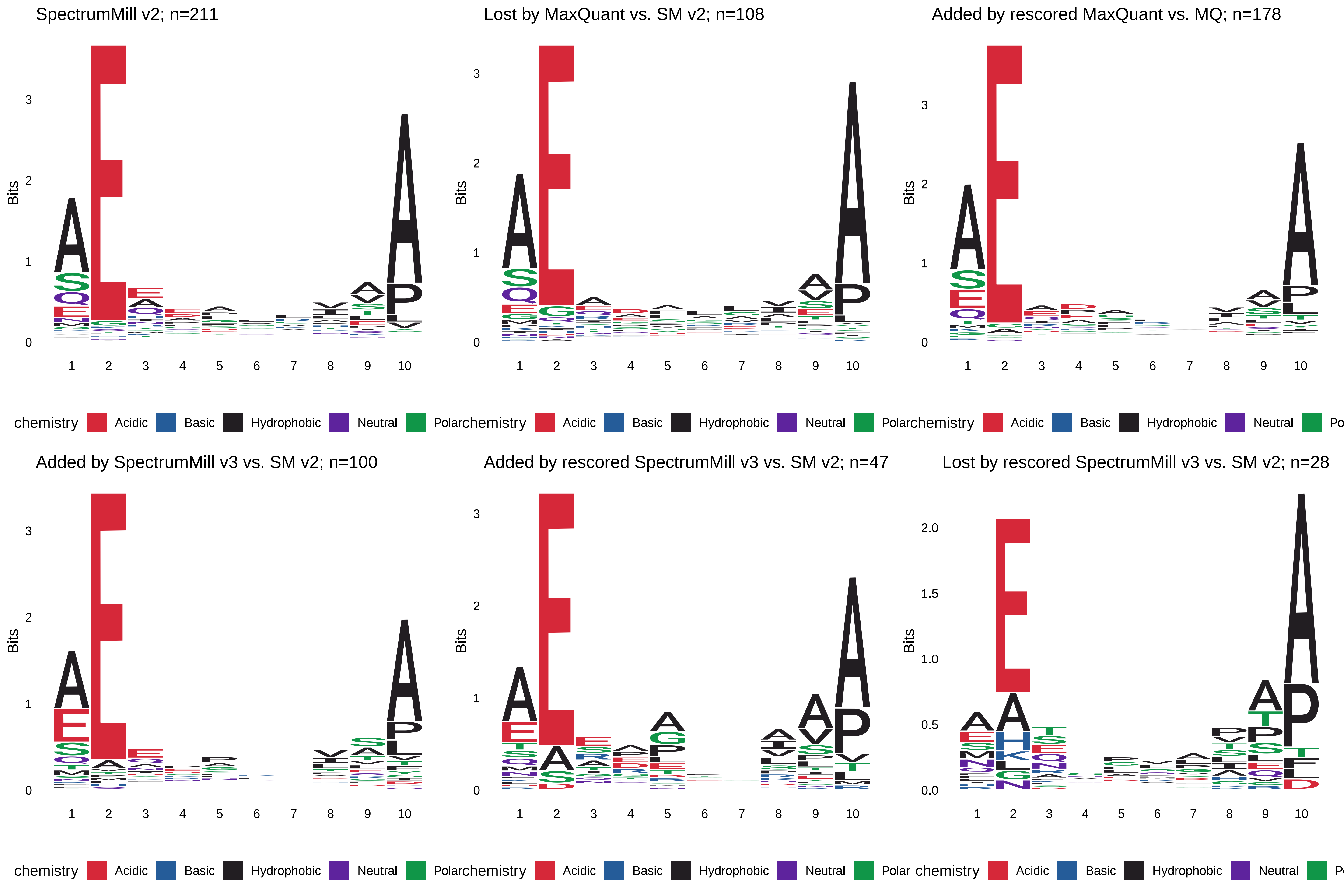

SpectrumMill v2; n=211
Lost by MaxQuant vs. SM v2; n=108
Added by rescored MaxQuant vs. MQ; n=178
Added by SpectrumMill v3 vs. SM v2; n=100
Added by rescored SpectrumMill v3 vs. SM v2; n=47
Lost by rescored SpectrumMill v3 vs. SM v2; n=28
Bits
0
0.5
1
1.0
1.5
2
2.0
3
1 2 3 4 5 6 7 8 9 10
chemistry
Acidic
Basic
Hydrophobic
Neutral
Polar

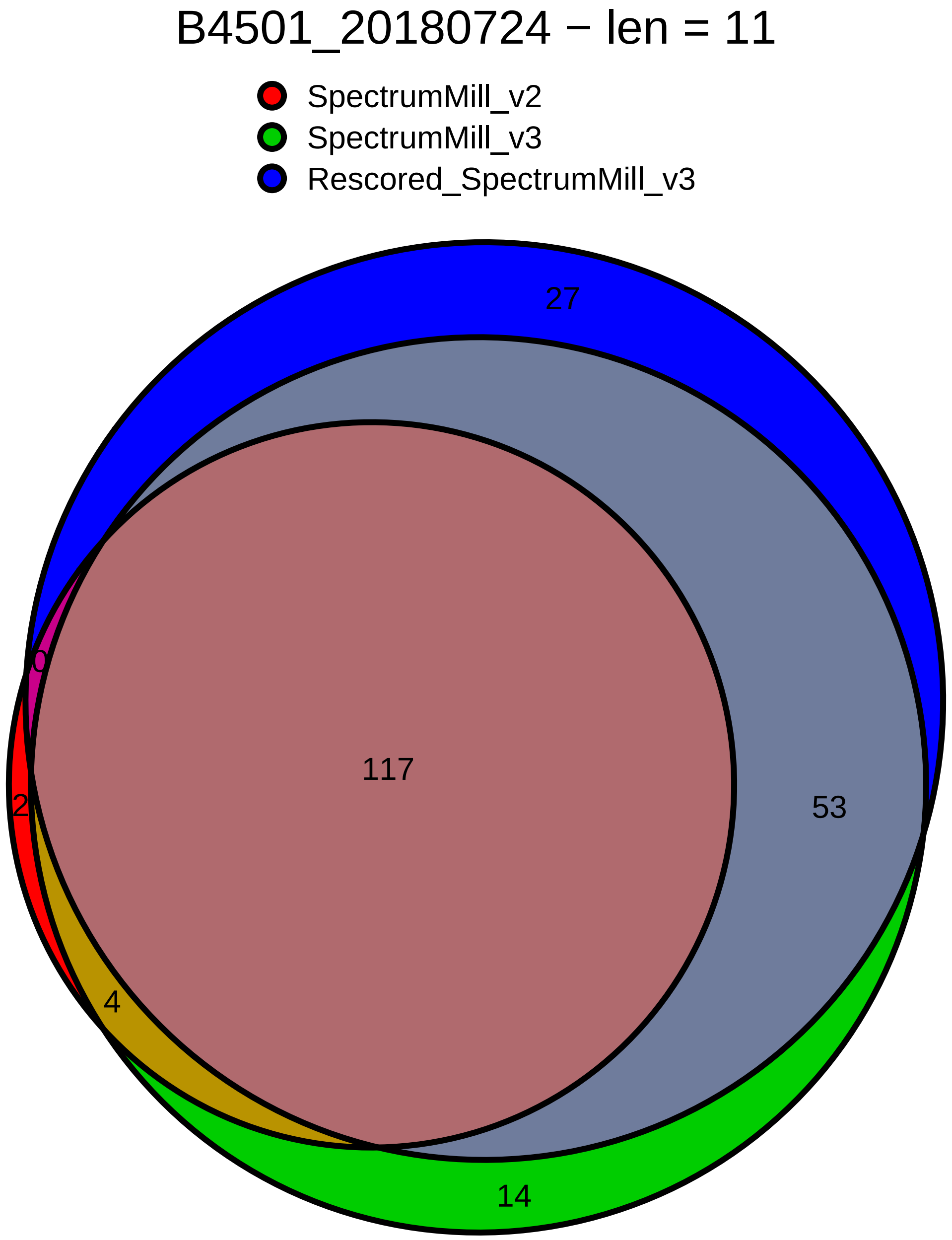
B4501_20180724 − len = 11
SpectrumMill_v2
SpectrumMill_v3
Rescored_SpectrumMill_v3
27
0
117
53
2
4
14

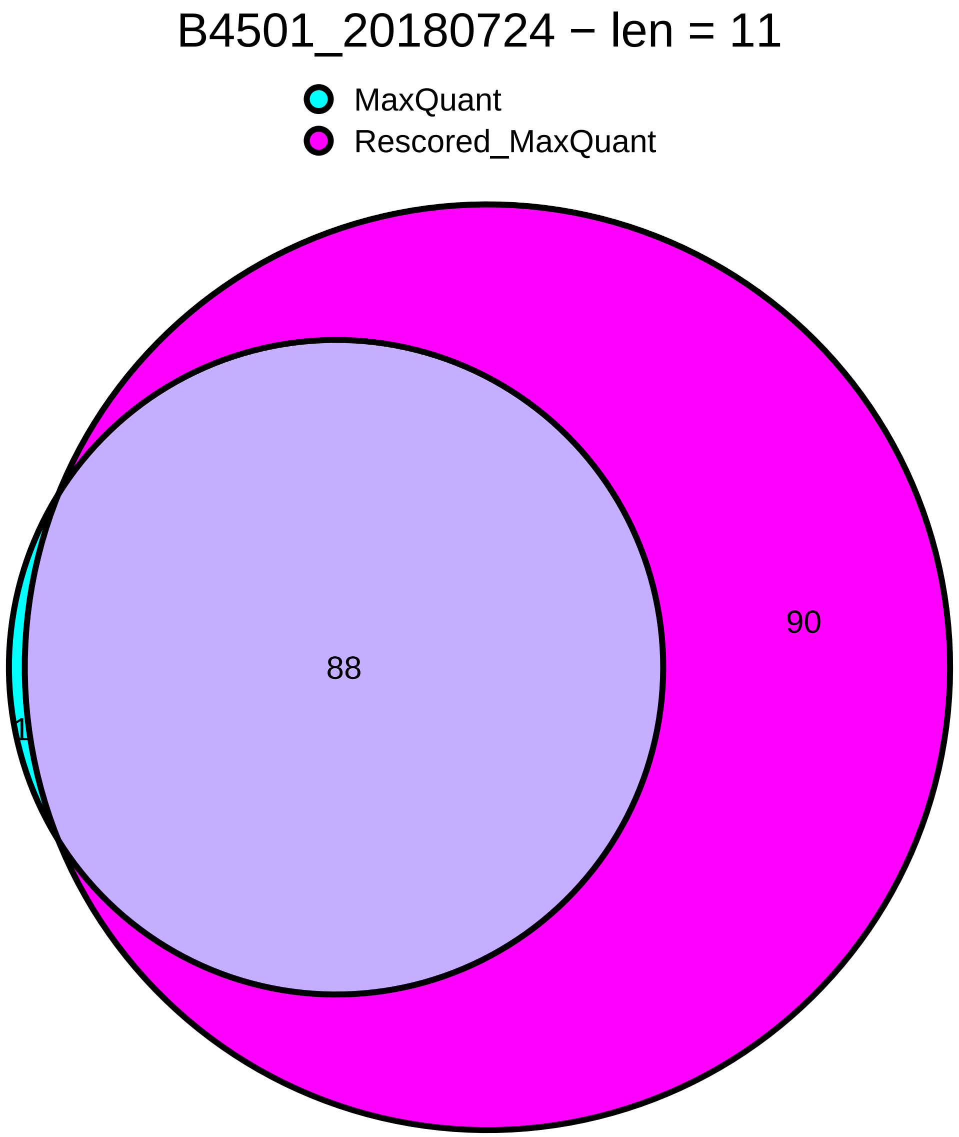
B4501_20180724 − len = 11
MaxQuant
Rescored_MaxQuant
88
90
1

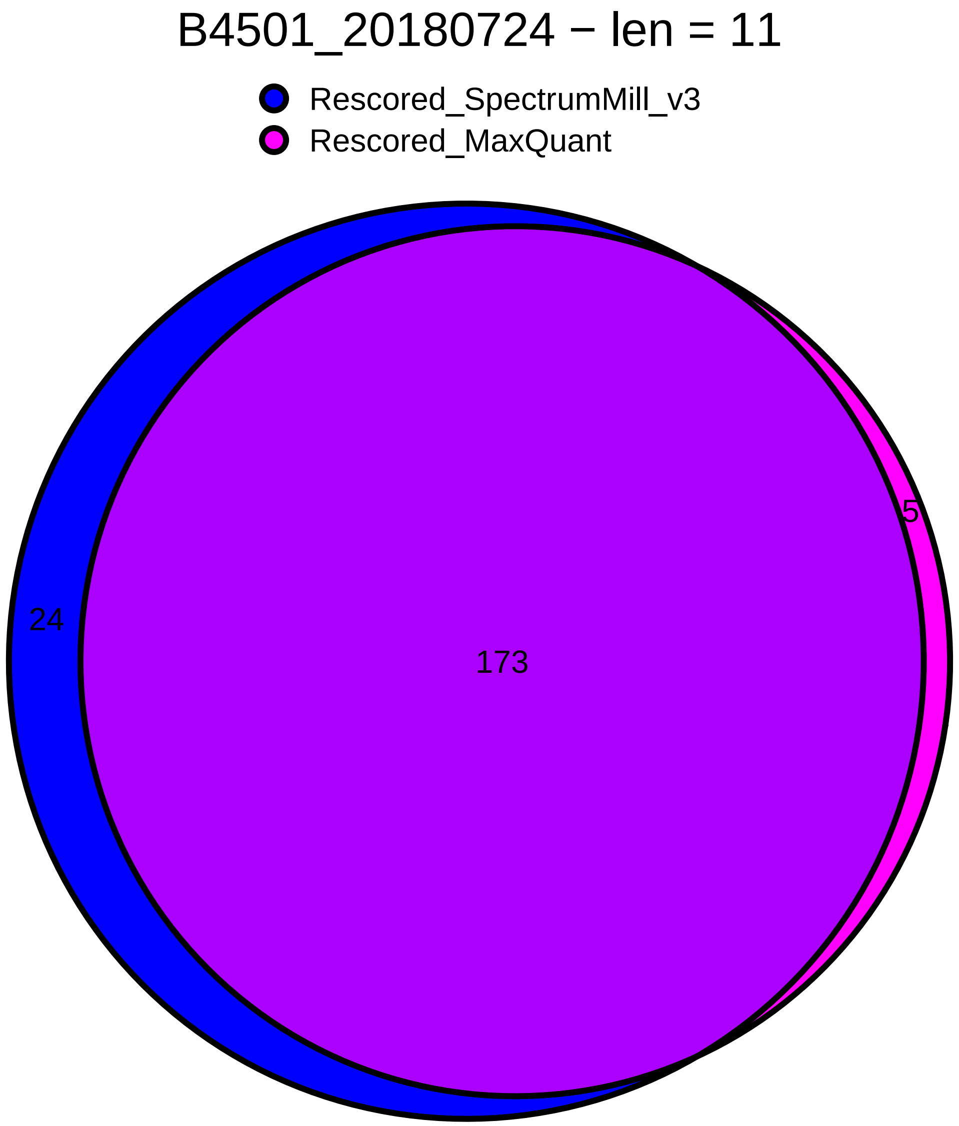
B4501_20180724 − len = 11
Rescored_SpectrumMill_v3
Rescored_MaxQuant
24
173
5

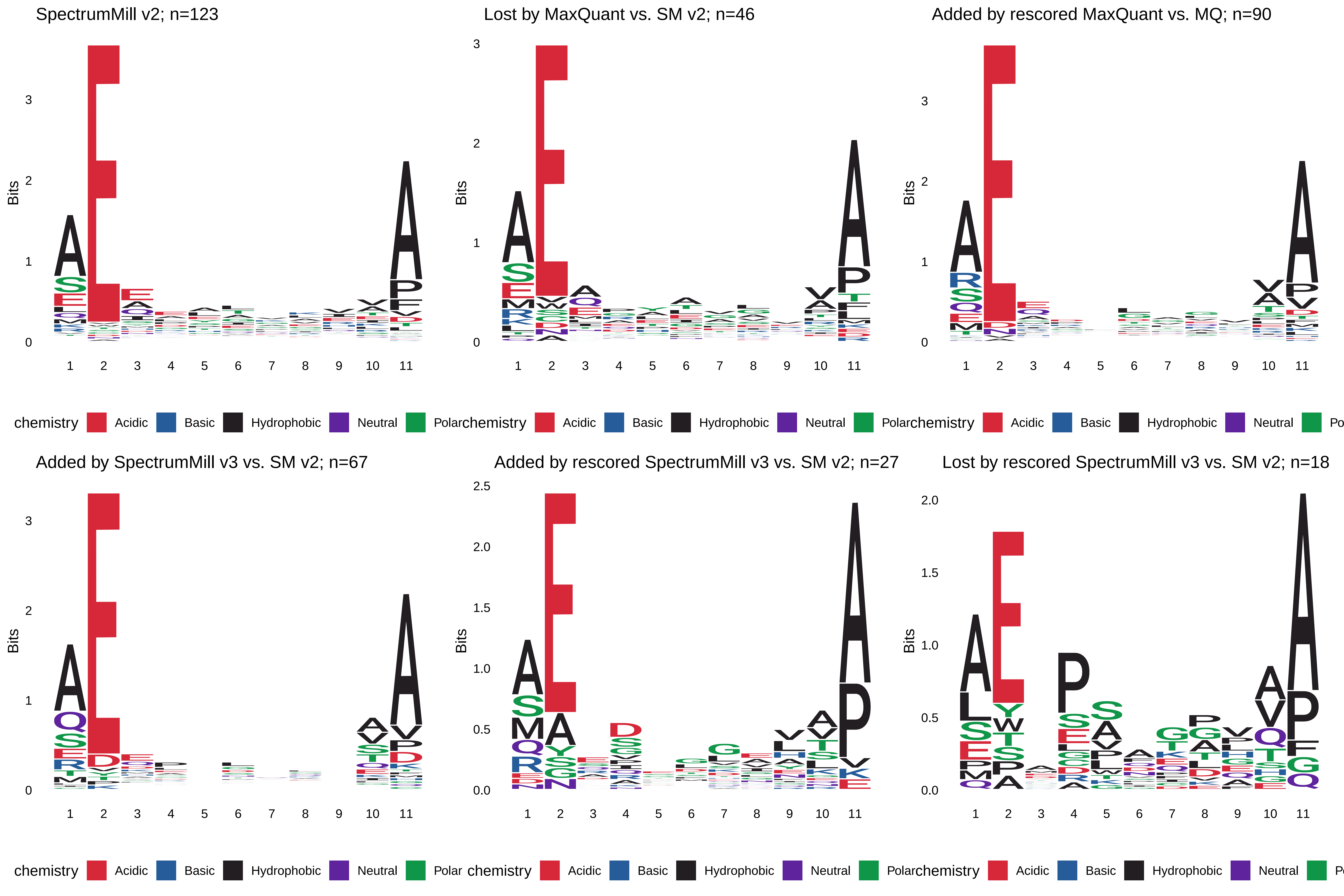

SpectrumMill v2; n=123
Lost by MaxQuant vs. SM v2; n=46
Added by rescored MaxQuant vs. MQ; n=90
Bits
chemistry Acidic Basic Hydrophobic Neutral Polar
Added by SpectrumMill v3 vs. SM v2; n=67
Added by rescored SpectrumMill v3 vs. SM v2; n=27
Lost by rescored SpectrumMill v3 vs. SM v2; n=18
Bits
chemistry Acidic Basic Hydrophobic Neutral Polar

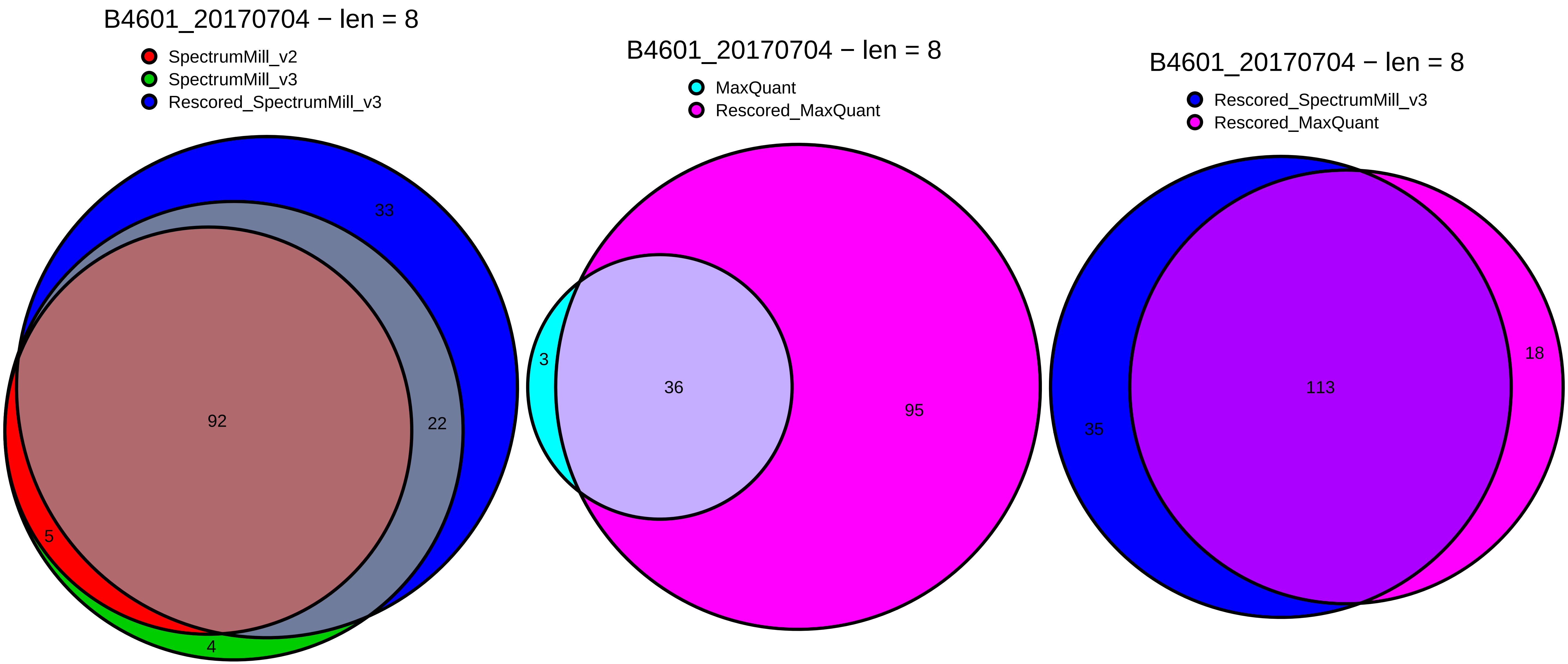
B4601_20170704 − len = 8
SpectrumMill_v2
SpectrumMill_v3
Rescored_SpectrumMill_v3
33
92
22
5
4
B4601_20170704 − len = 8
MaxQuant
Rescored_MaxQuant
3
36
95
B4601_20170704 − len = 8
Rescored_SpectrumMill_v3
Rescored_MaxQuant
18
113
35

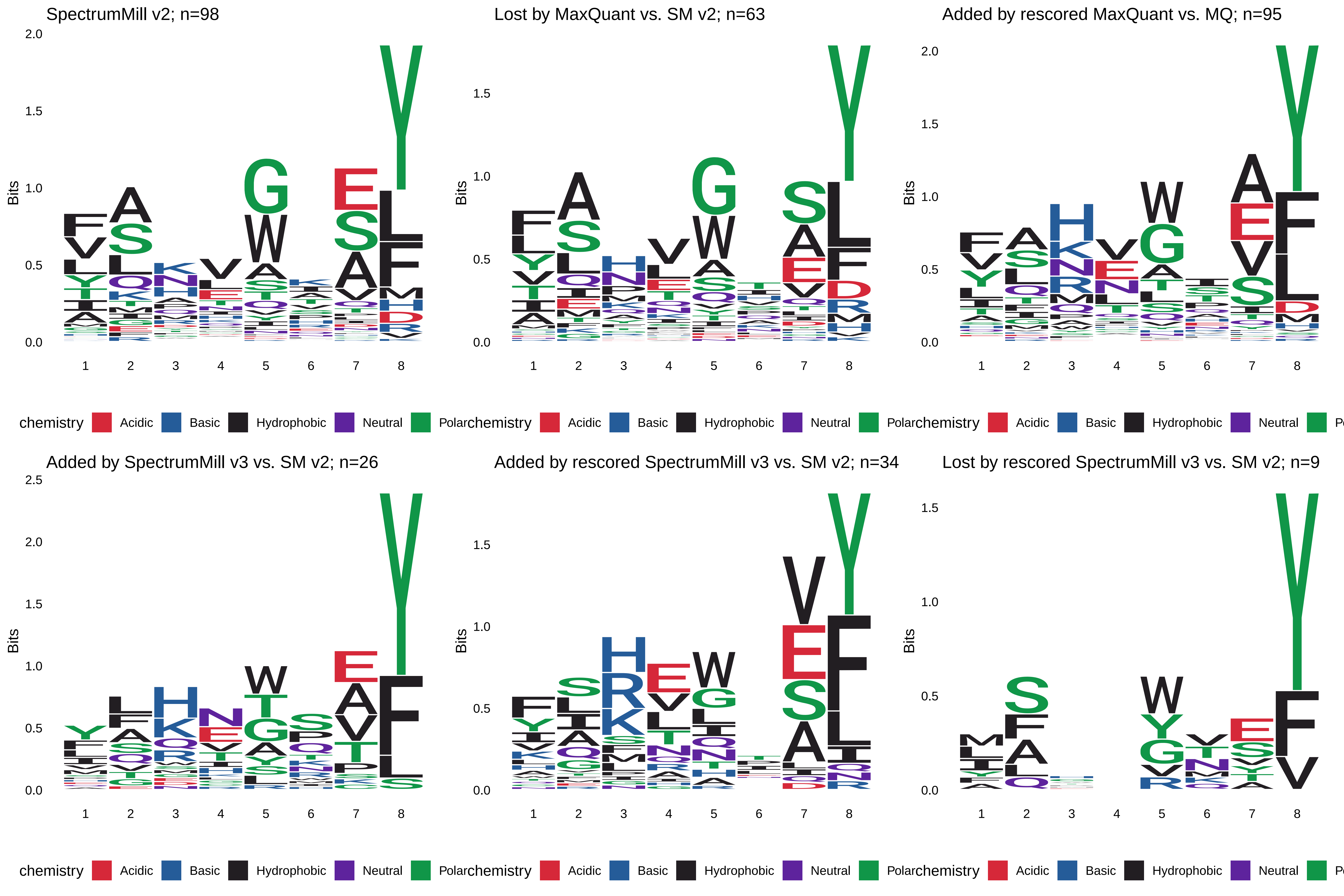
SpectrumMill v2; n=98
Lost by MaxQuant vs. SM v2; n=63
Added by rescored MaxQuant vs. MQ; n=95
Added by SpectrumMill v3 vs. SM v2; n=26
Added by rescored SpectrumMill v3 vs. SM v2; n=34
Lost by rescored SpectrumMill v3 vs. SM v2; n=9
Bits
1 2 3 4 5 6 7 8
chemistry Acidic Basic Hydrophobic Neutral Polar

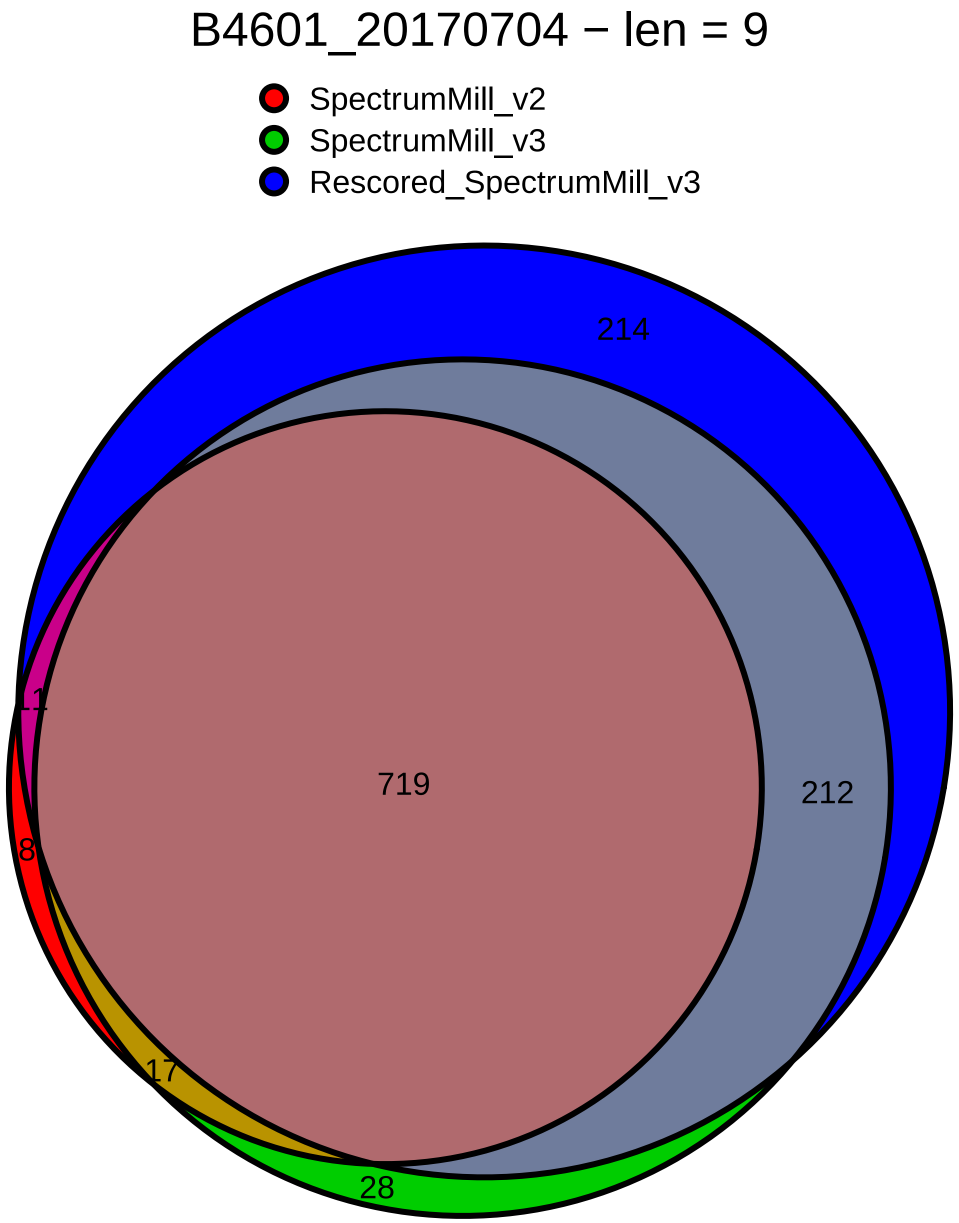
B4601_20170704 − len = 9
SpectrumMill_v2
SpectrumMill_v3
Rescored_SpectrumMill_v3
214
212
719
8
17
28

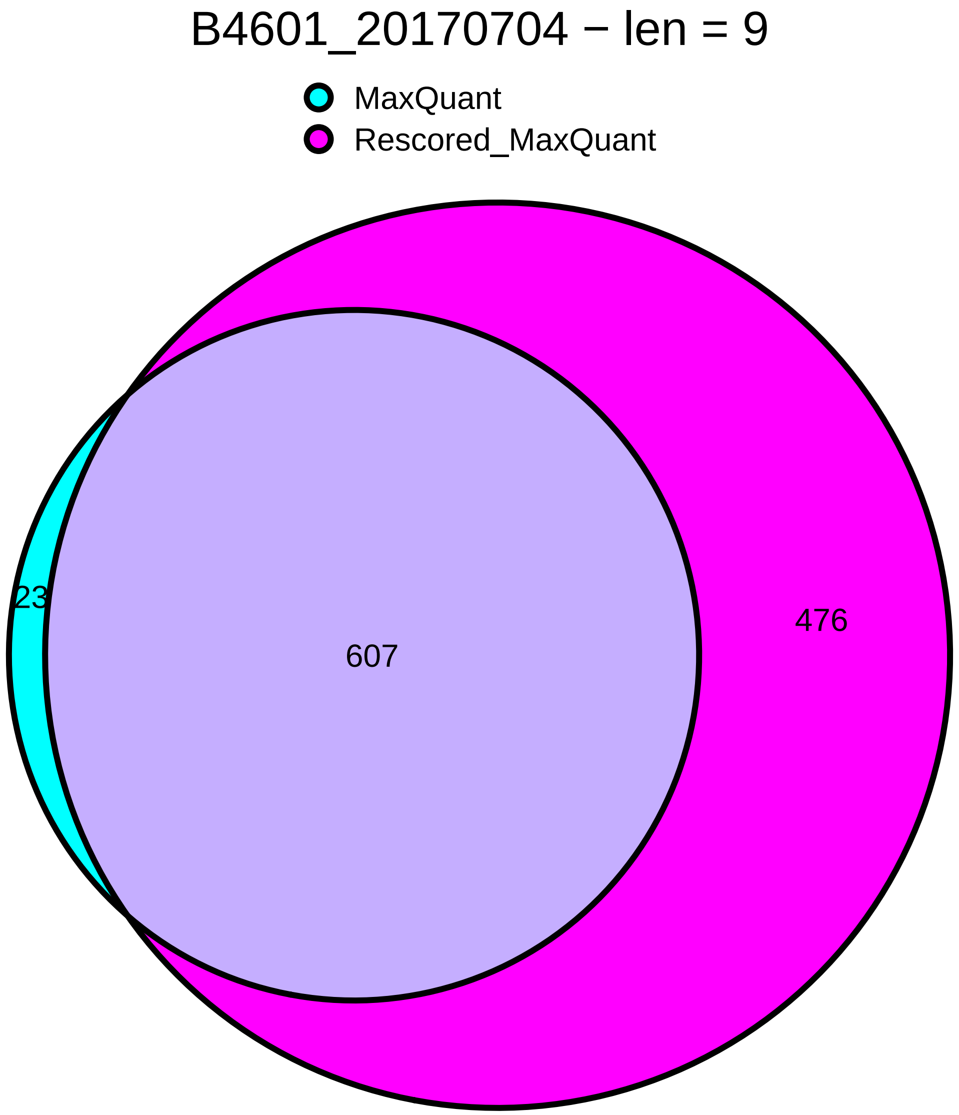
B4601_20170704 − len = 9
MaxQuant
Rescored_MaxQuant
23
607
476

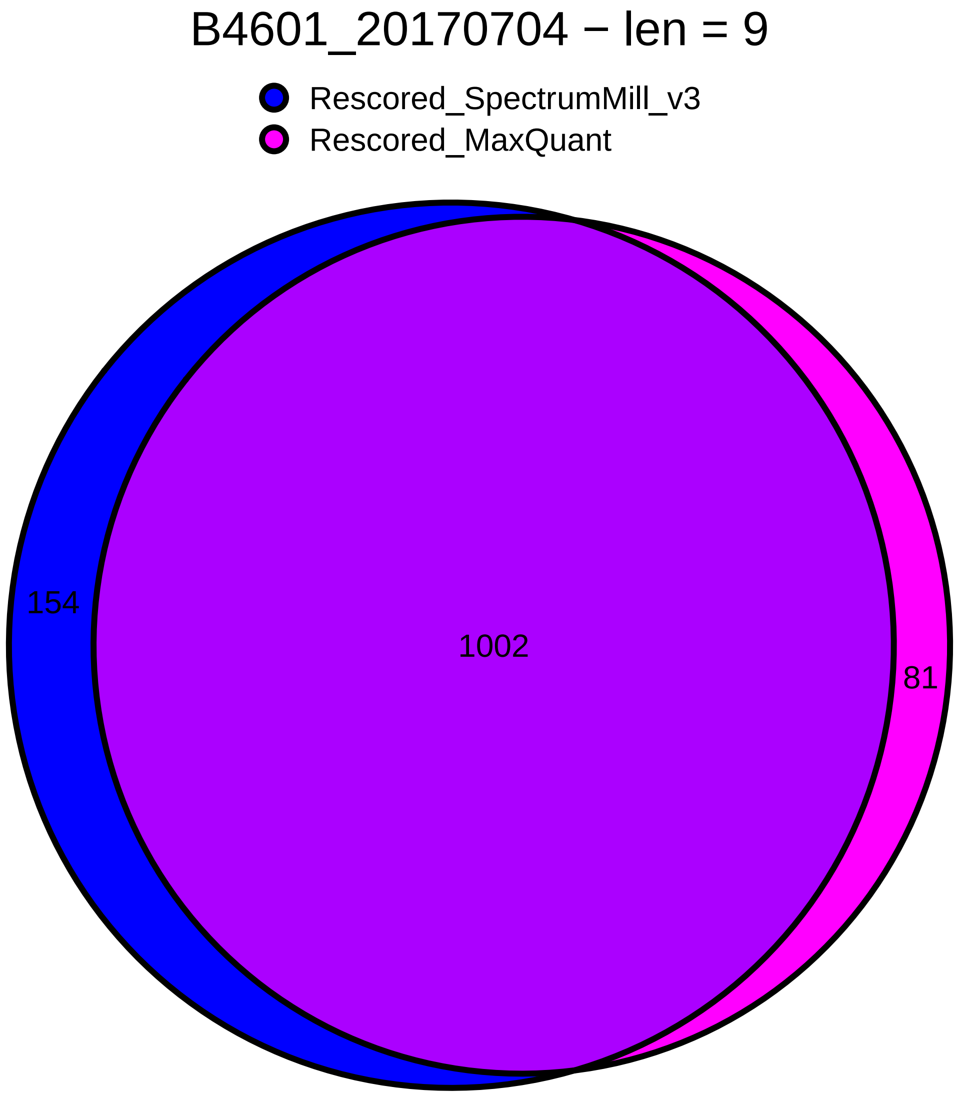
B4601_20170704 − len = 9
Rescored_SpectrumMill_v3
Rescored_MaxQuant
154
1002
81

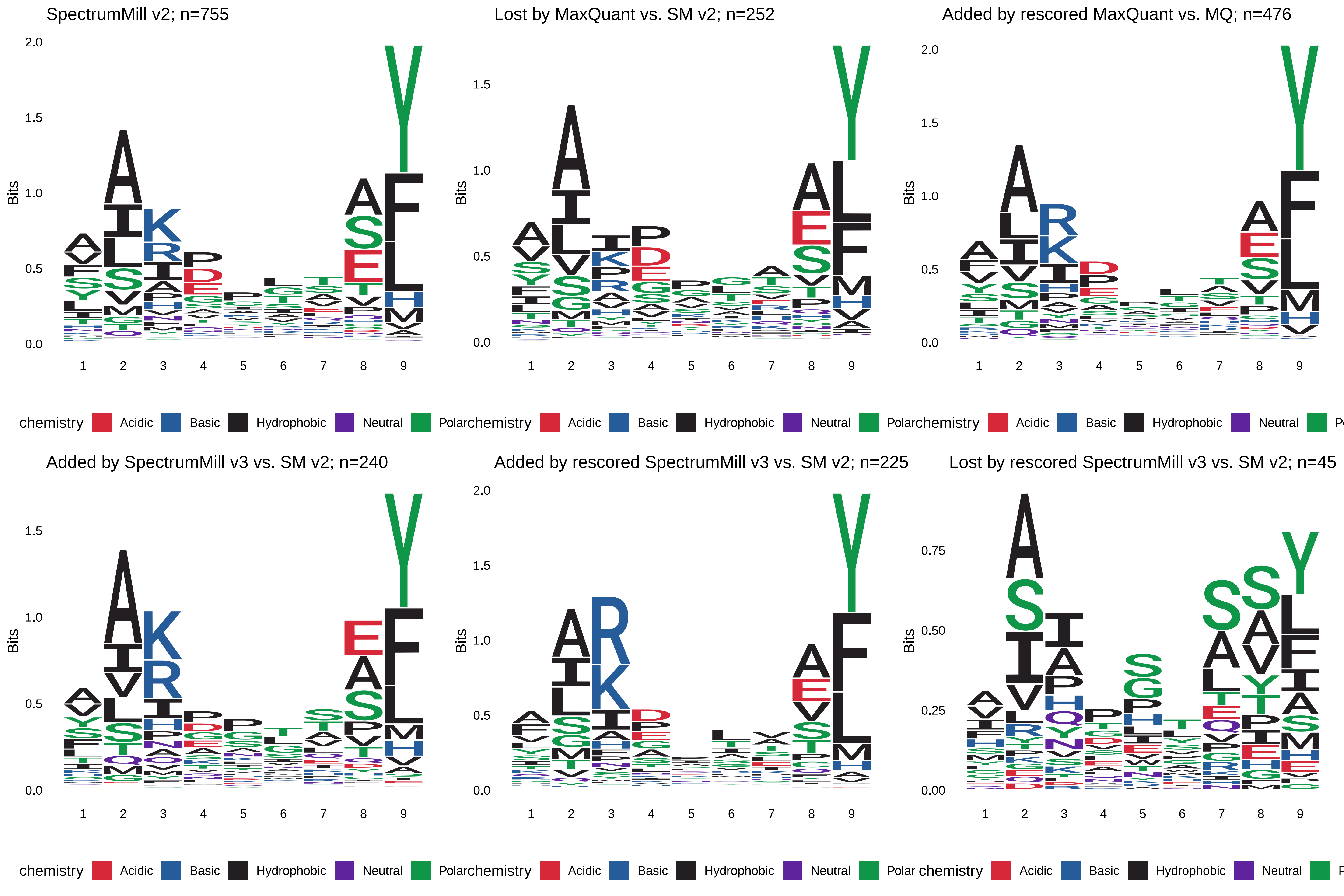
SpectrumMill v2; n=755
Lost by MaxQuant vs. SM v2; n=252
Added by rescored MaxQuant vs. MQ; n=476
Bits
0.0
0.5
1.0
1.5
2.0
1
2
3
4
5
6
7
8
9
chemistry
Acidic
Basic
Hydrophobic
Neutral
Polar
Added by SpectrumMill v3 vs. SM v2; n=240
Added by rescored SpectrumMill v3 vs. SM v2; n=225
Lost by rescored SpectrumMill v3 vs. SM v2; n=45
0.00
0.25
0.50
0.75

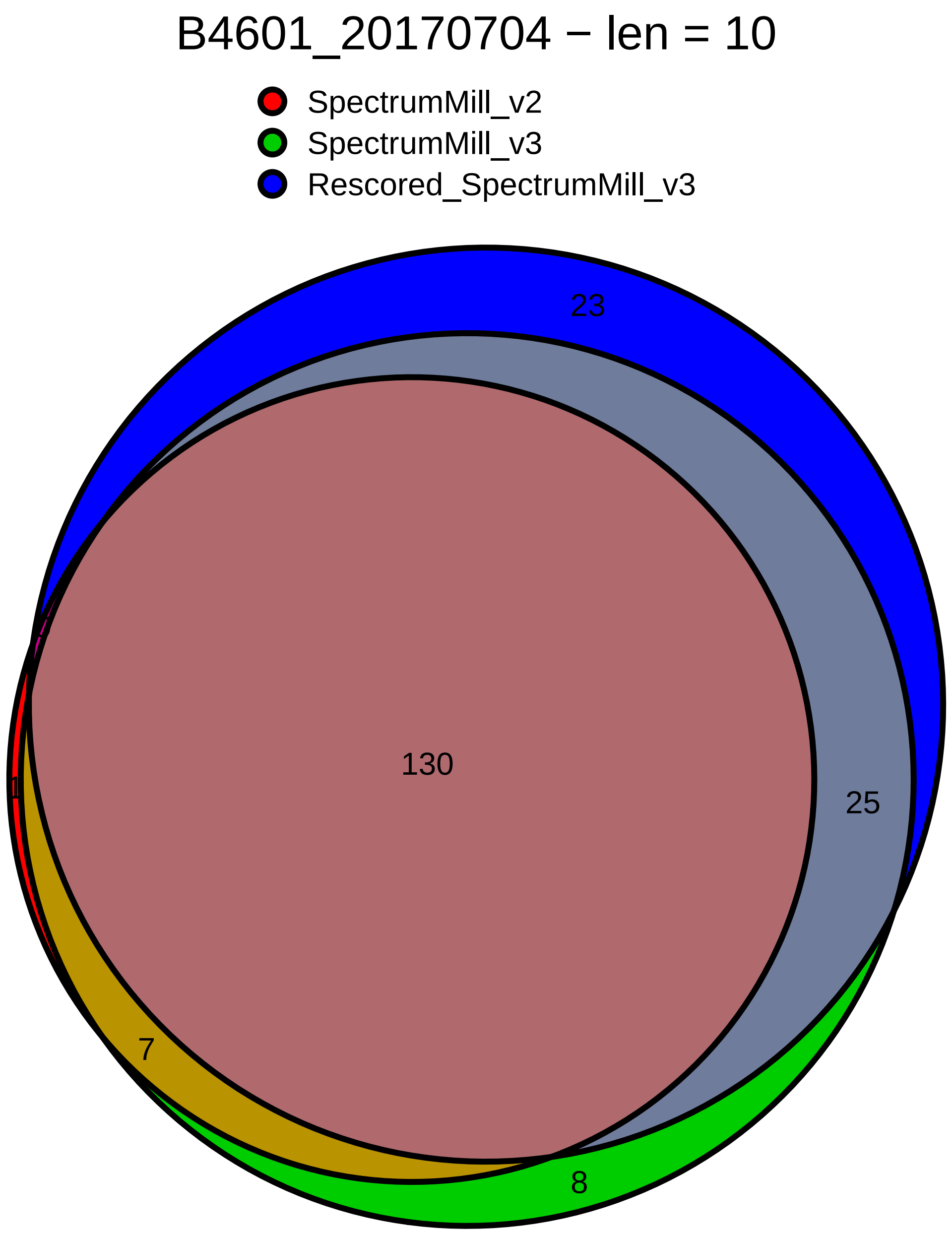
B4601_20170704 − len = 10
SpectrumMill_v2
SpectrumMill_v3
Rescored_SpectrumMill_v3
23
1
130
25
7
8

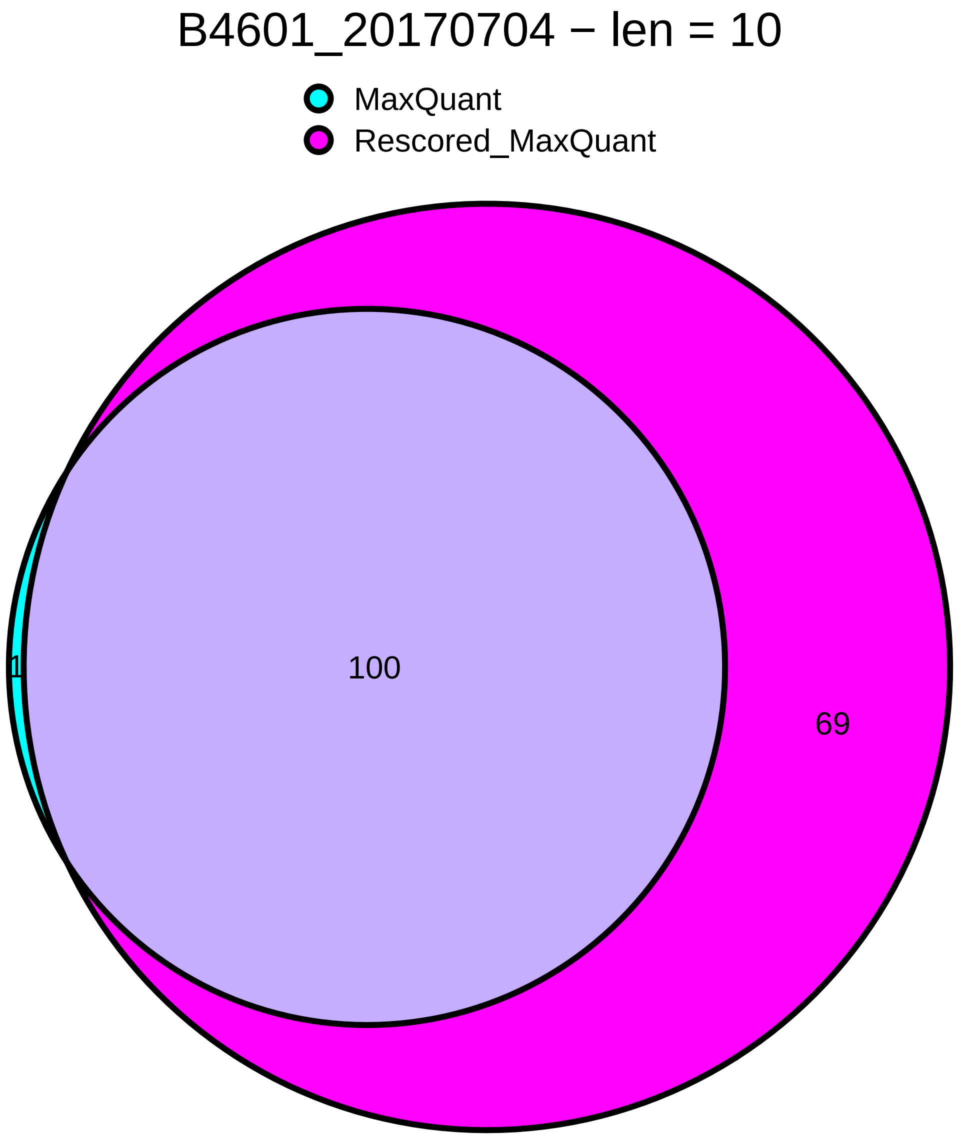
B4601_20170704 − len = 10
MaxQuant
Rescored_MaxQuant
1
100
69

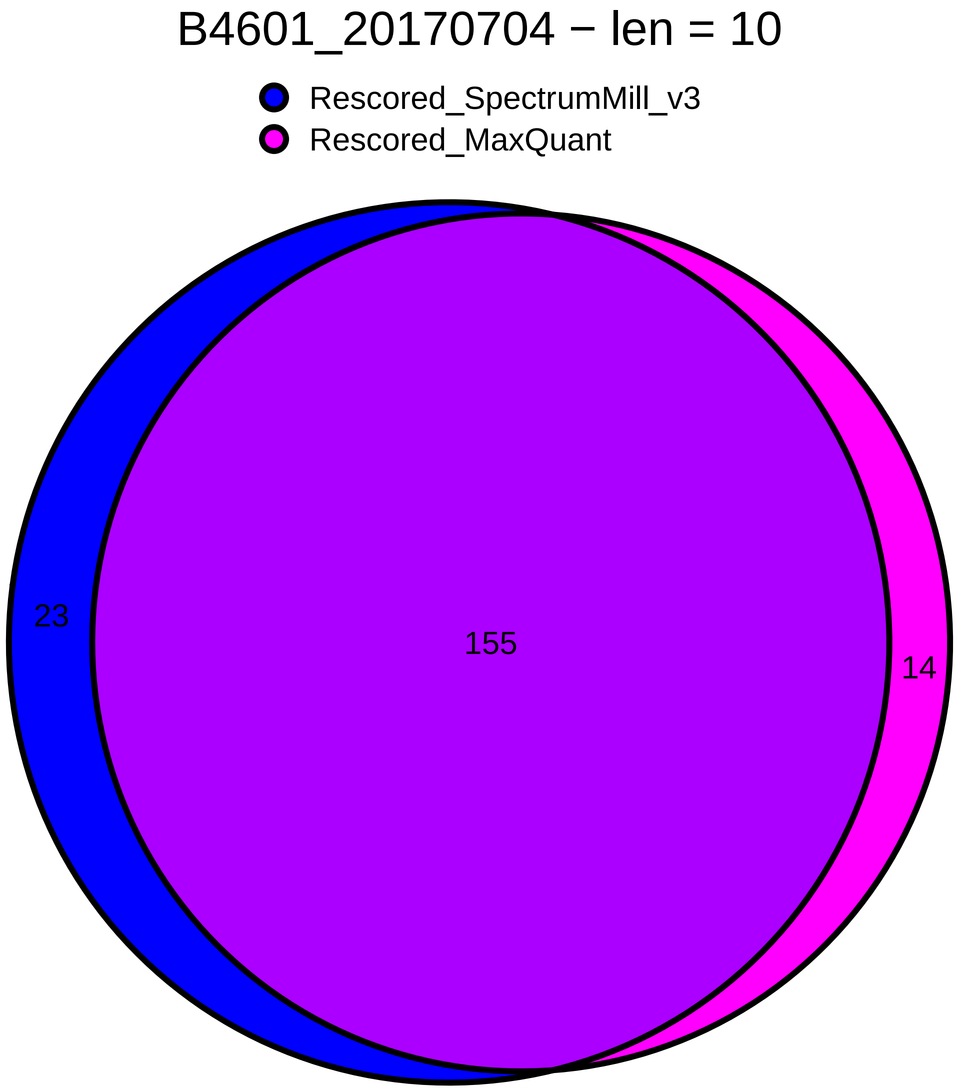
B4601_20170704 − len = 10
Rescored_SpectrumMill_v3
Rescored_MaxQuant
23
155
14

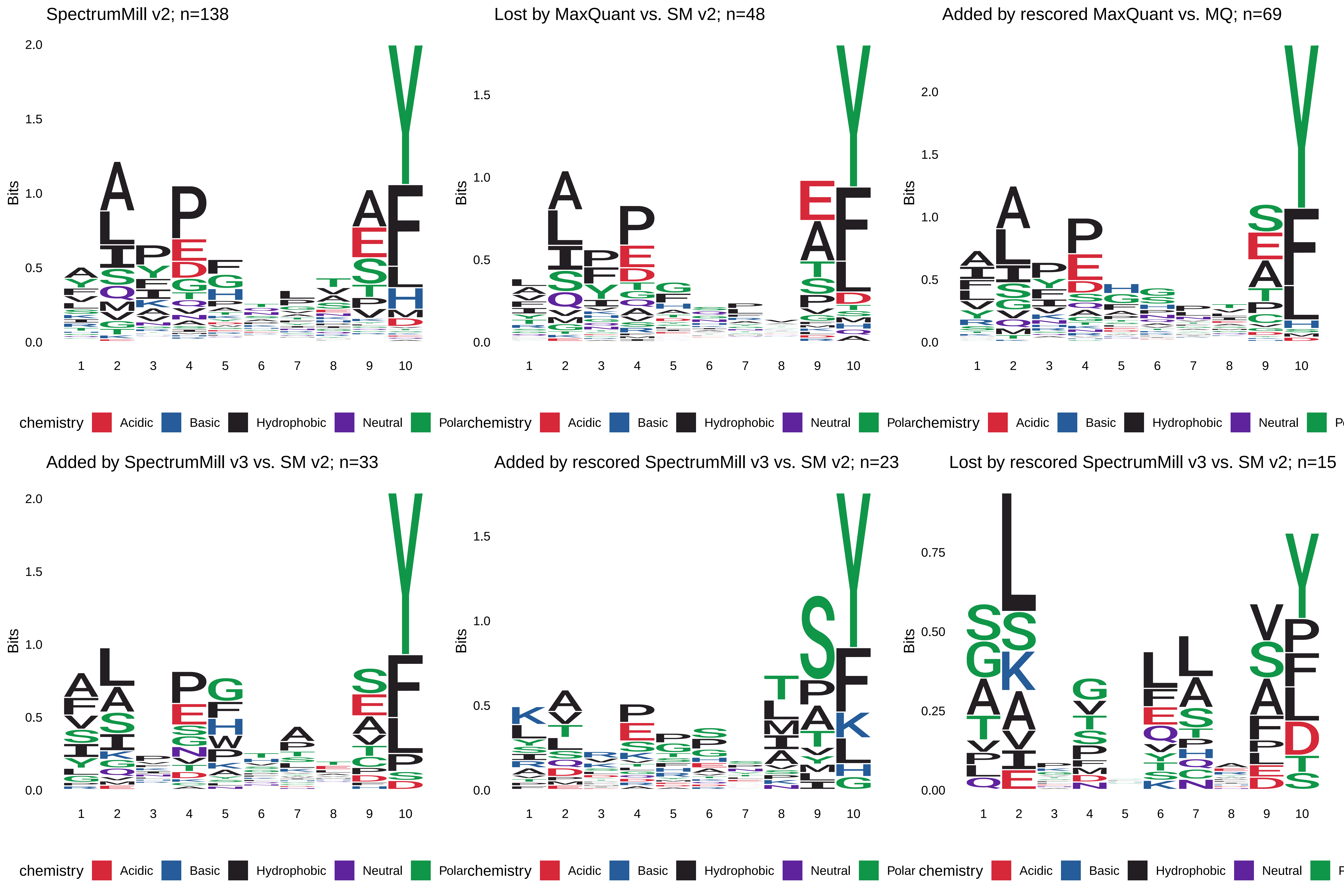

SpectrumMill v2; n=138
Lost by MaxQuant vs. SM v2; n=48
Added by rescored MaxQuant vs. MQ; n=69
Added by SpectrumMill v3 vs. SM v2; n=33
Added by rescored SpectrumMill v3 vs. SM v2; n=23
Lost by rescored SpectrumMill v3 vs. SM v2; n=15
Bits
chemistry
Acidic
Basic
Hydrophobic
Neutral
Polar

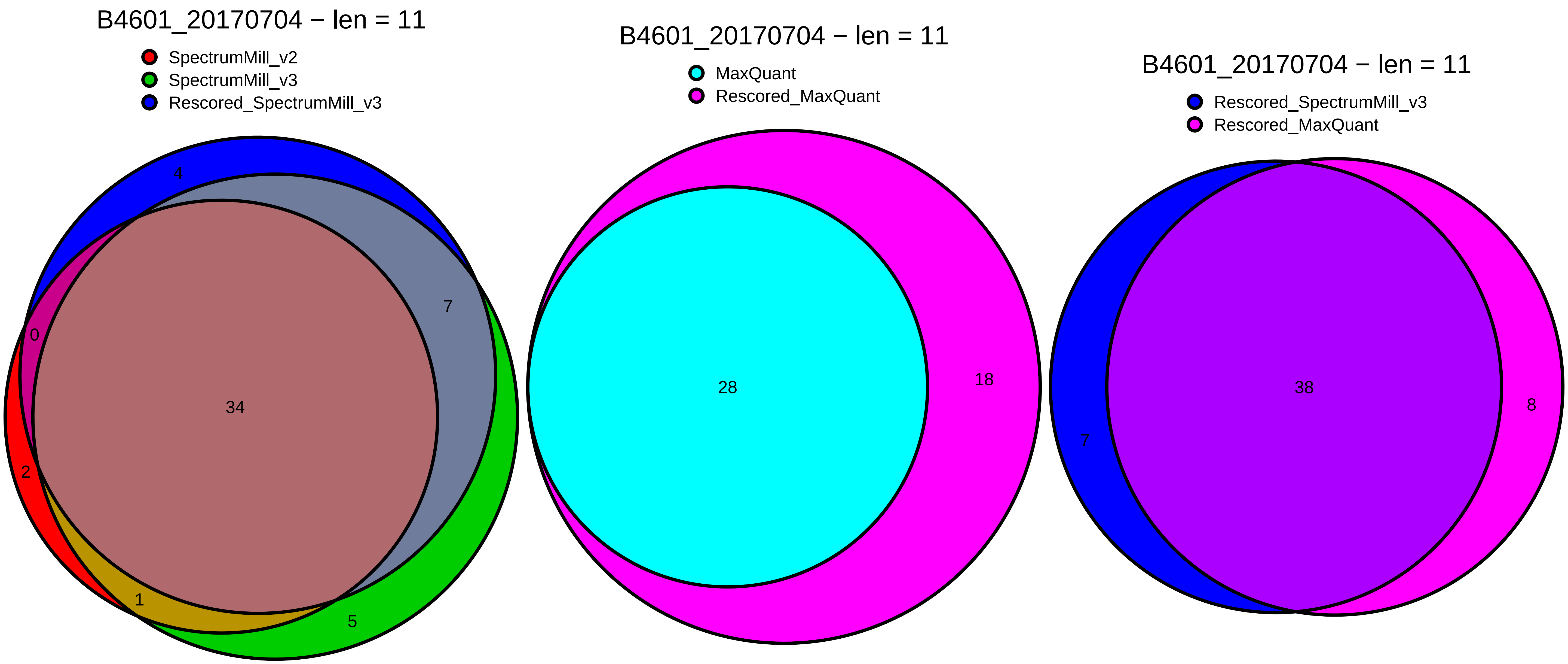
B4601_20170704 − len = 11
SpectrumMill_v2
SpectrumMill_v3
Rescored_SpectrumMill_v3
4
7
0
34
2
1
5
B4601_20170704 − len = 11
MaxQuant
Rescored_MaxQuant
28
18
B4601_20170704 − len = 11
Rescored_SpectrumMill_v3
Rescored_MaxQuant
38
7
8

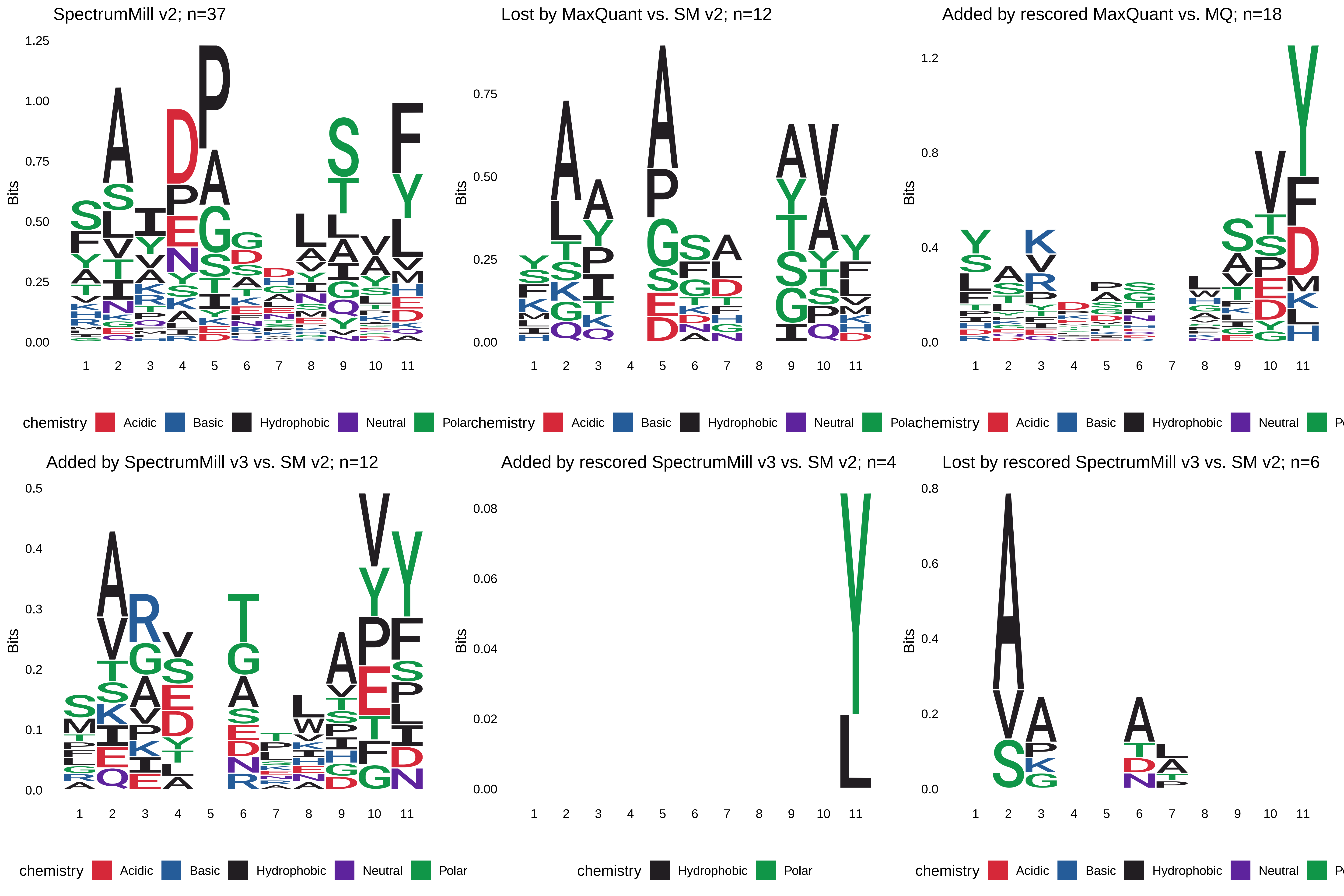
SpectrumMill v2; n=37
Lost by MaxQuant vs. SM v2; n=12
Added by rescored MaxQuant vs. MQ; n=18
Added by SpectrumMill v3 vs. SM v2; n=12
Added by rescored SpectrumMill v3 vs. SM v2; n=4
Lost by rescored SpectrumMill v3 vs. SM v2; n=6
Bits
chemistry
Acidic
Basic
Hydrophobic
Neutral
Polar

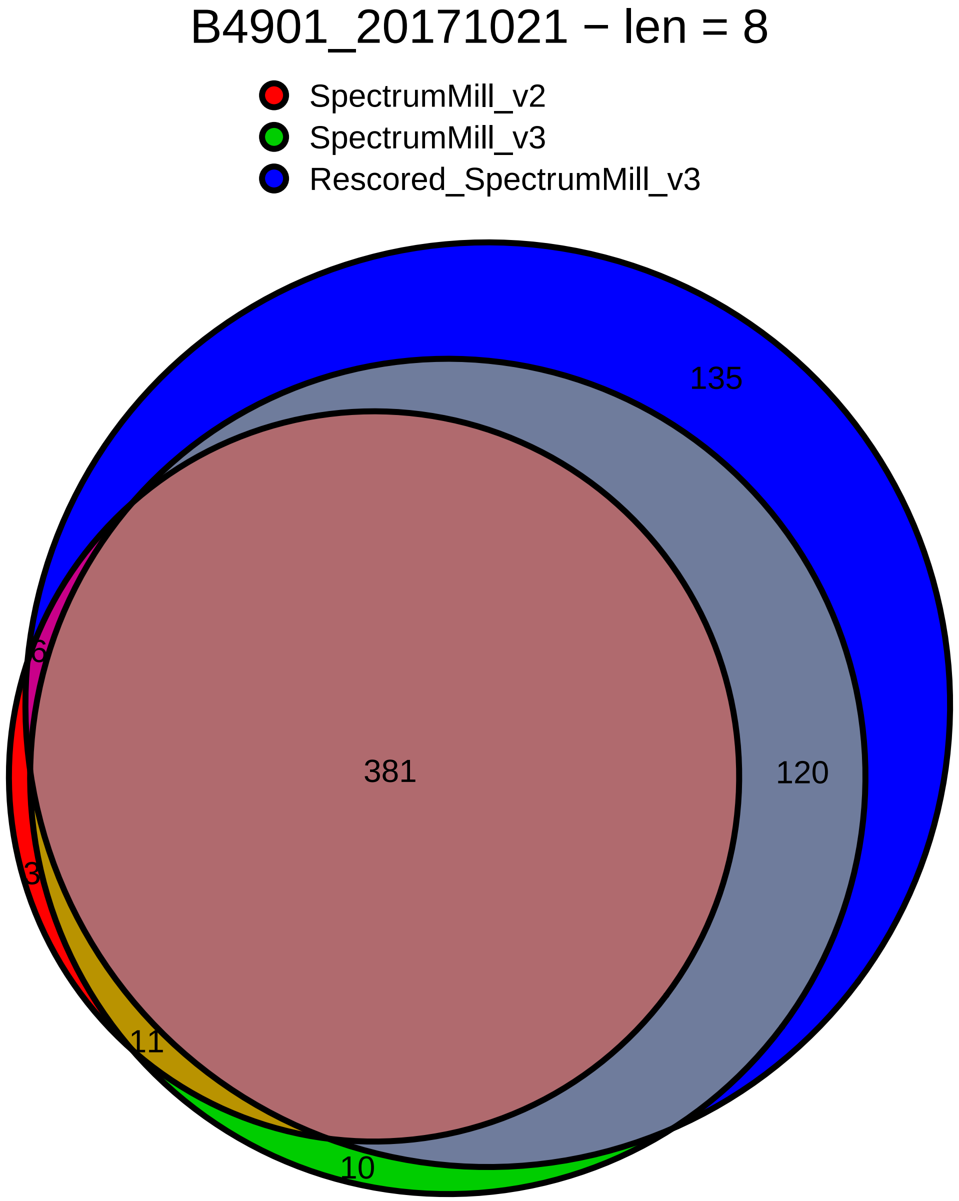
B4901_20171021 − len = 8
SpectrumMill_v2
SpectrumMill_v3
Rescored_SpectrumMill_v3
135
120
381
3
11
10

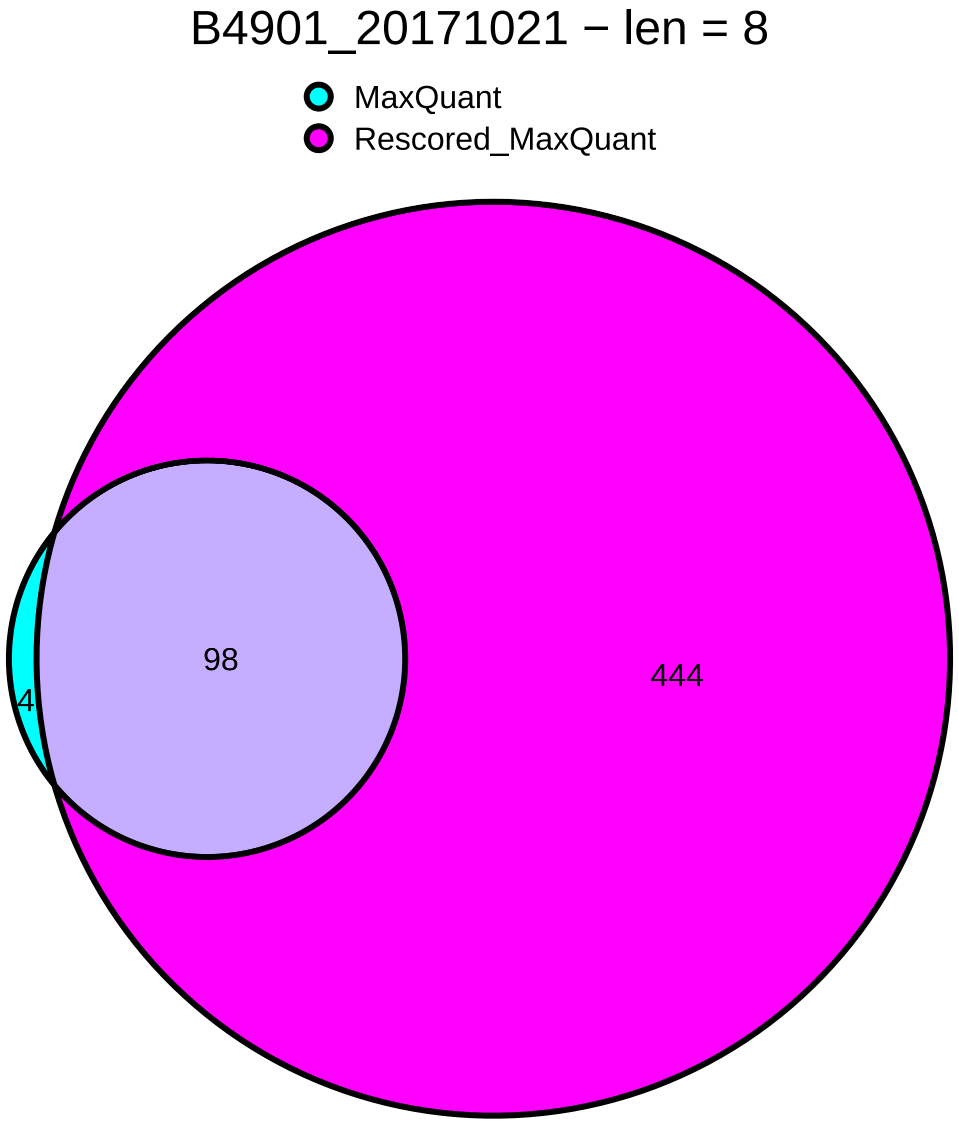
B4901_20171021 − len = 8
MaxQuant
Rescored_MaxQuant
4
98
444

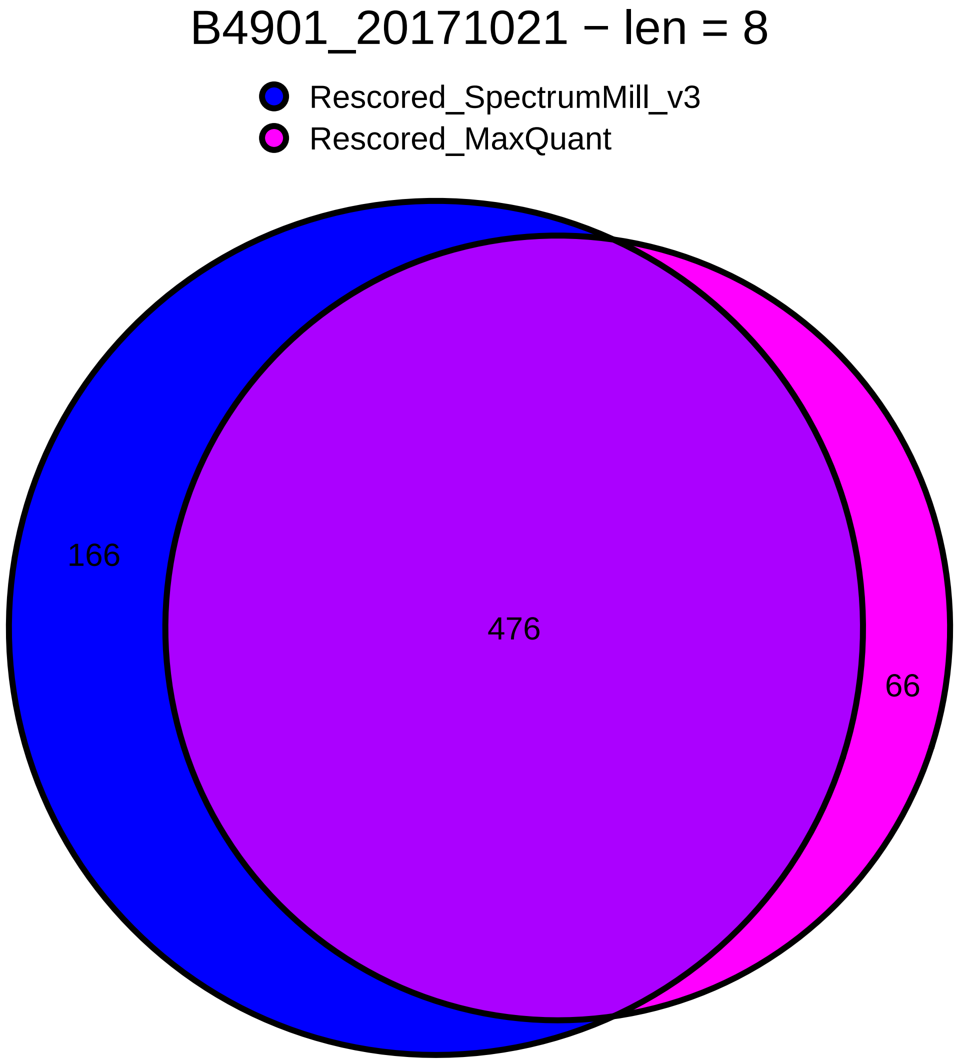
B4901_20171021 − len = 8
Rescored_SpectrumMill_v3
Rescored_MaxQuant
166
476
66

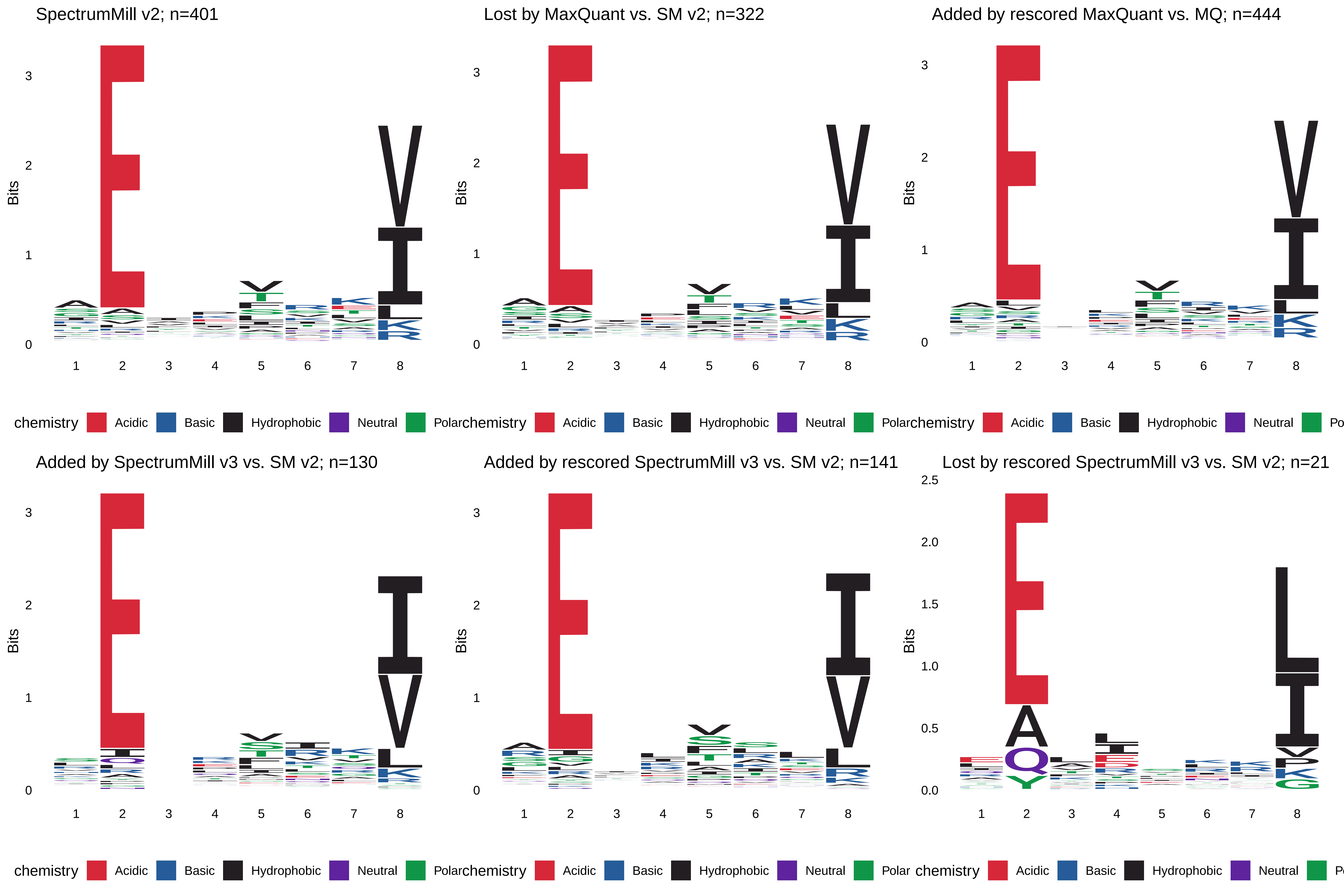
SpectrumMill v2; n=401
Lost by MaxQuant vs. SM v2; n=322
Added by rescored MaxQuant vs. MQ; n=444
Added by SpectrumMill v3 vs. SM v2; n=130
Added by rescored SpectrumMill v3 vs. SM v2; n=141
Lost by rescored SpectrumMill v3 vs. SM v2; n=21
Bits
chemistry
Acidic
Basic
Hydrophobic
Neutral
Polar

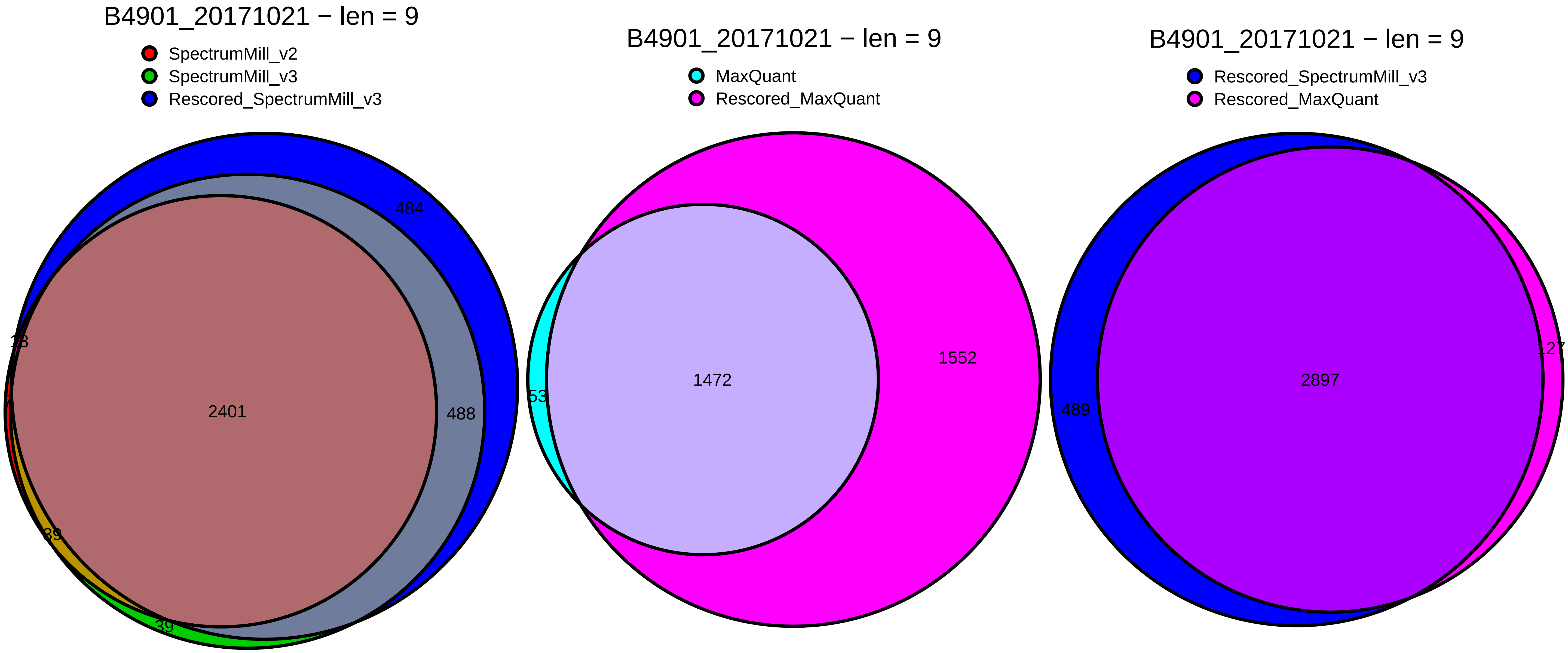
B4901_20171021 − len = 9
SpectrumMill_v2
SpectrumMill_v3
Rescored_SpectrumMill_v3
484
2401
488
39
39
B4901_20171021 − len = 9
MaxQuant
Rescored_MaxQuant
53
1472
1552
B4901_20171021 − len = 9
Rescored_SpectrumMill_v3
Rescored_MaxQuant
489
2897
127

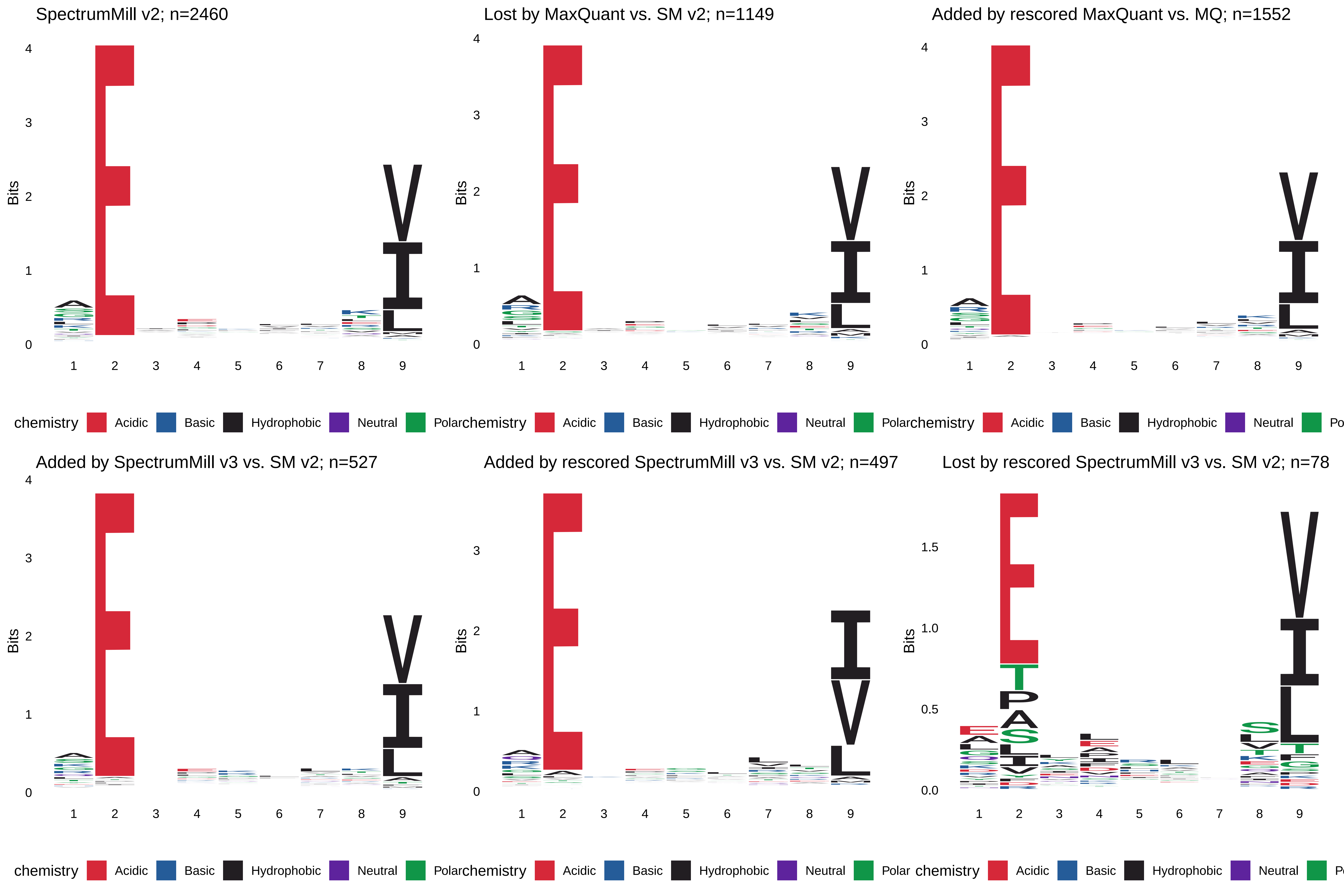

SpectrumMill v2; n=2460
Lost by MaxQuant vs. SM v2; n=1149
Added by rescored MaxQuant vs. MQ; n=1552
Added by SpectrumMill v3 vs. SM v2; n=527
Added by rescored SpectrumMill v3 vs. SM v2; n=497
Lost by rescored SpectrumMill v3 vs. SM v2; n=78
Bits
chemistry
Acidic
Basic
Hydrophobic
Neutral
Polar

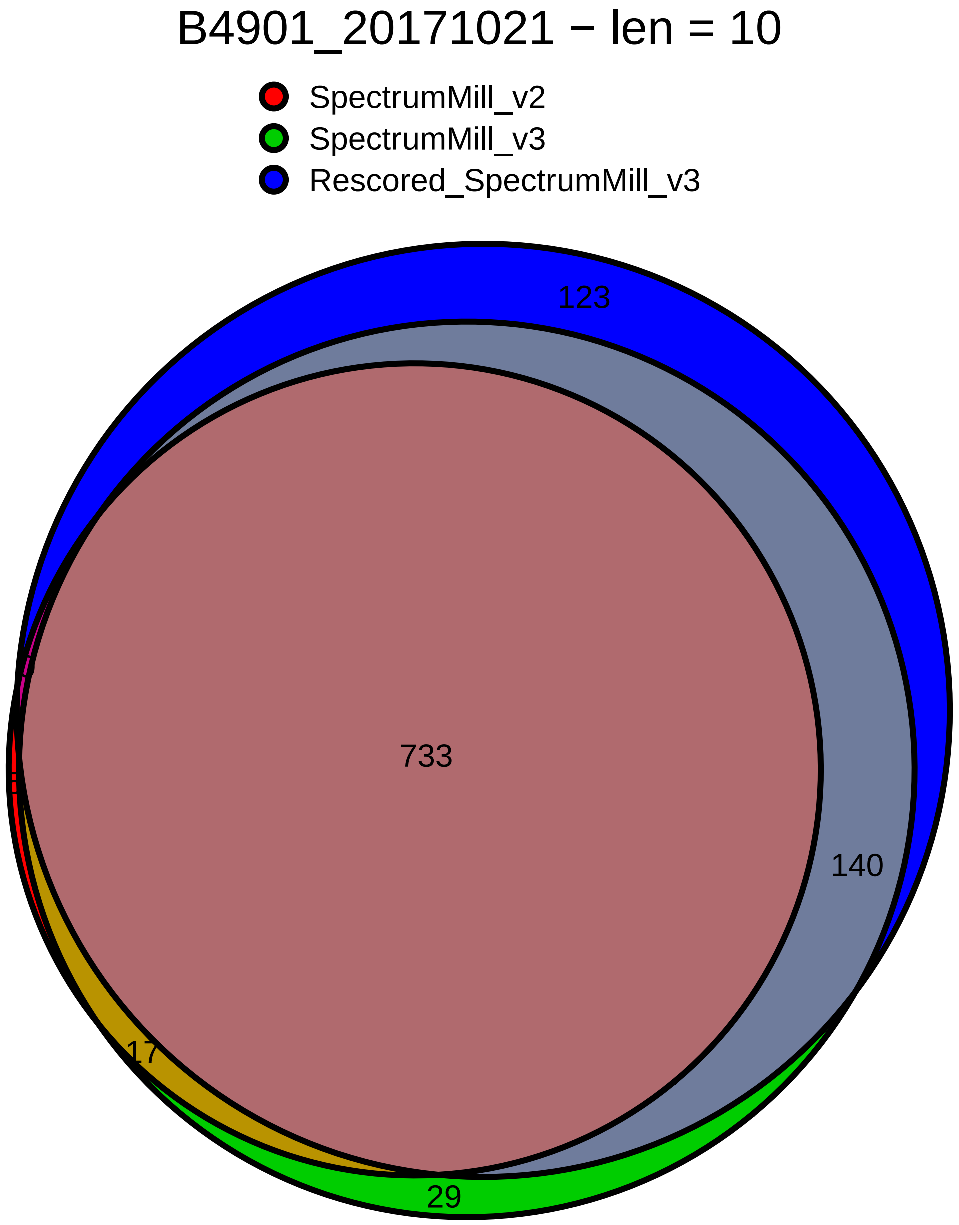
B4901_20171021 − len = 10
SpectrumMill_v2
SpectrumMill_v3
Rescored_SpectrumMill_v3
123
733
140
17
29

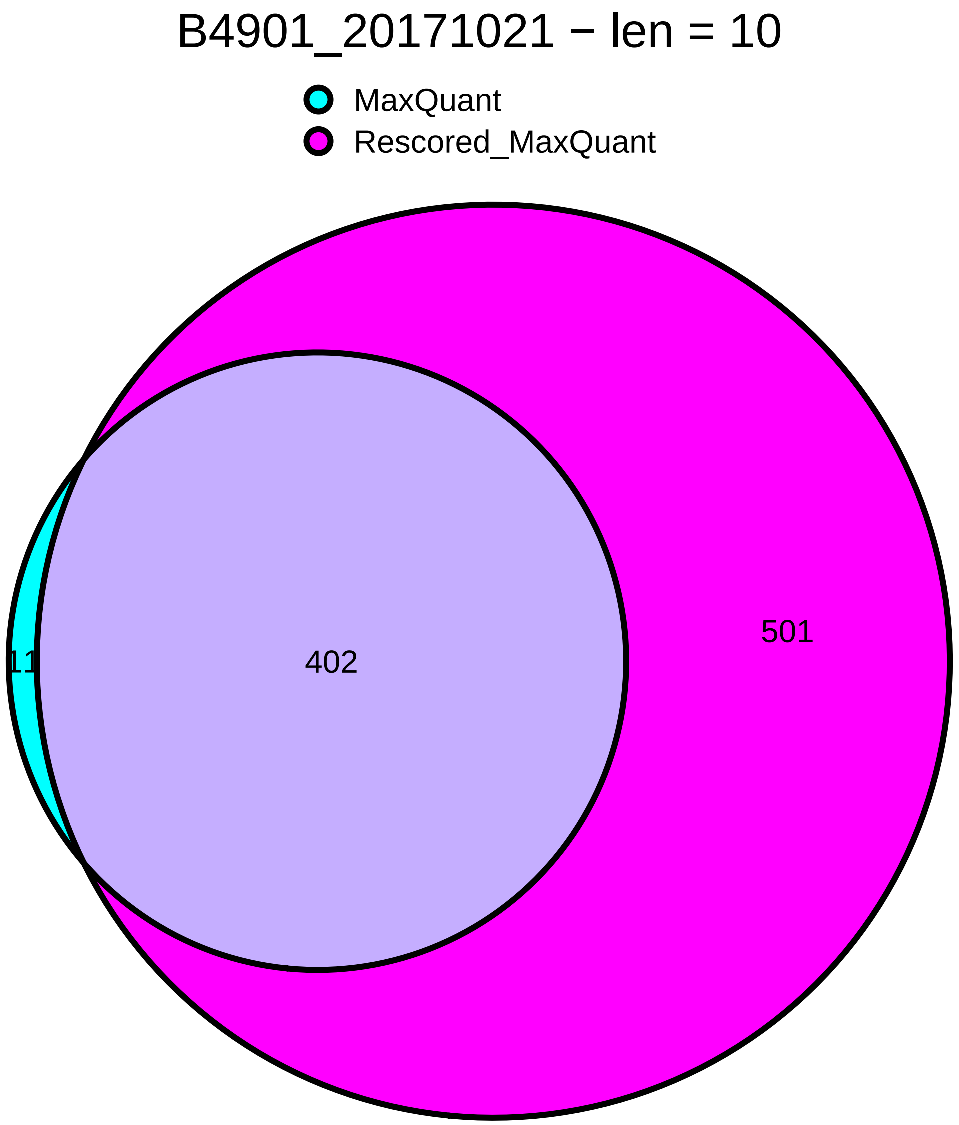
B4901_20171021 − len = 10
MaxQuant
Rescored_MaxQuant
402
501

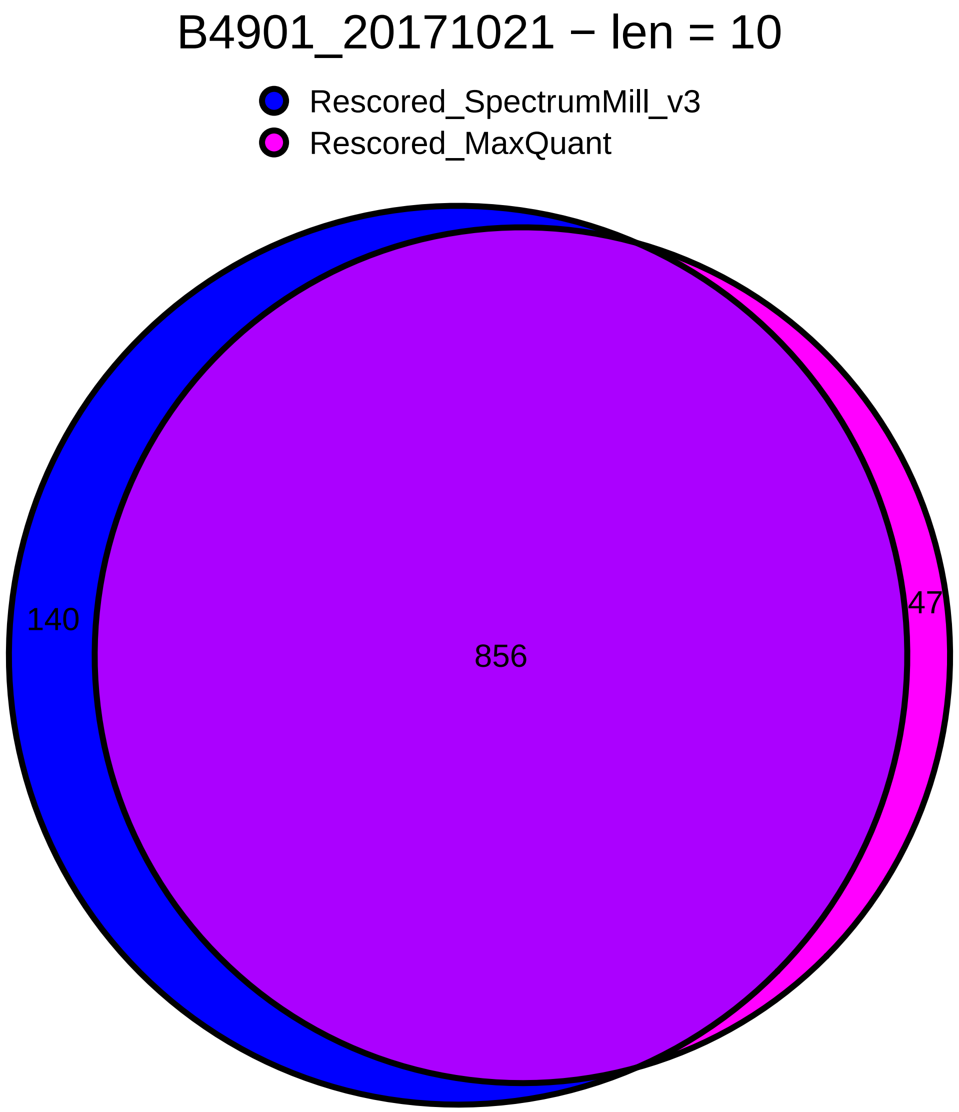
B4901_20171021 − len = 10
Rescored_SpectrumMill_v3
Rescored_MaxQuant
140
856
47

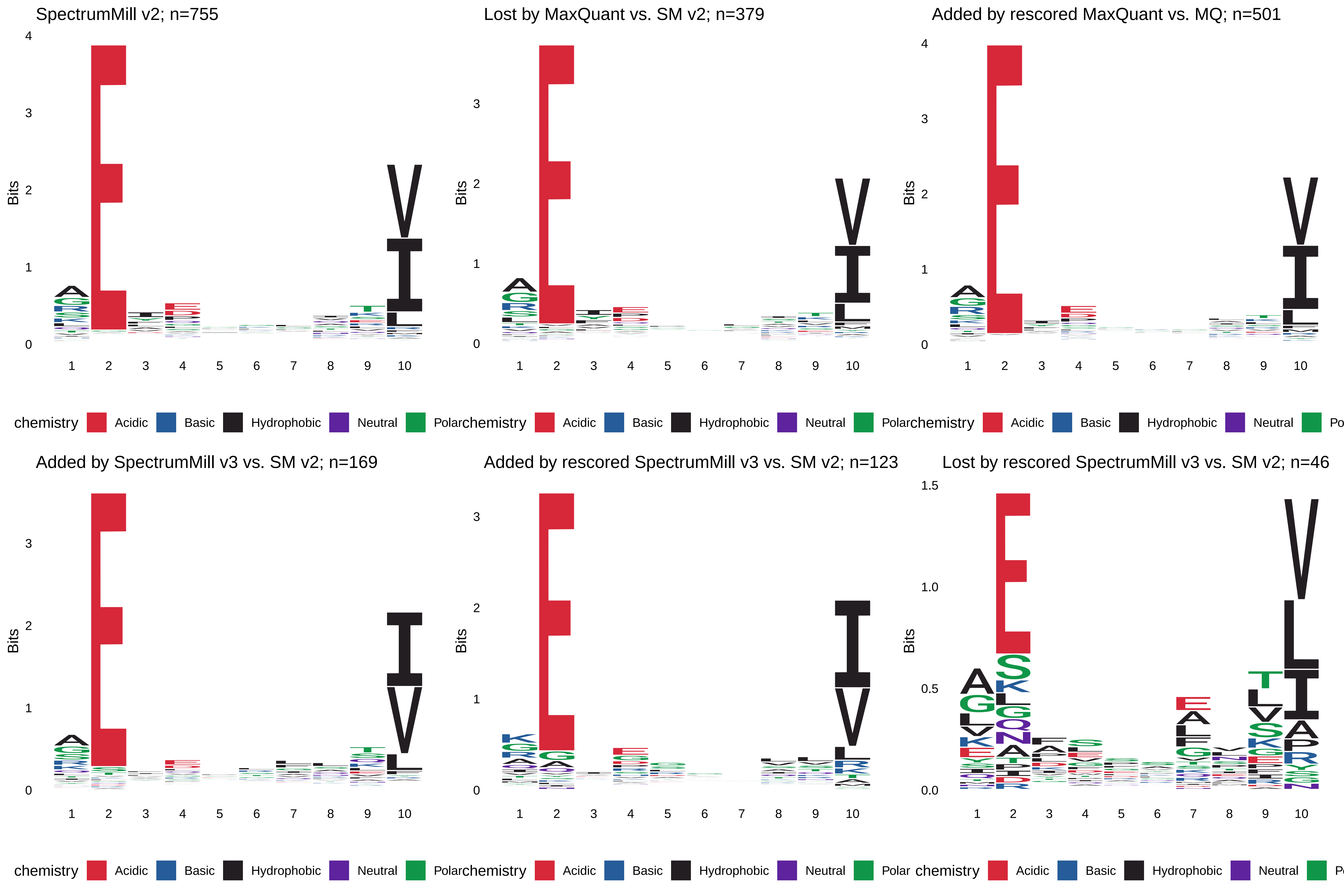
SpectrumMill v2; n=755
Lost by MaxQuant vs. SM v2; n=379
Added by rescored MaxQuant vs. MQ; n=501
Added by SpectrumMill v3 vs. SM v2; n=169
Added by rescored SpectrumMill v3 vs. SM v2; n=123
Lost by rescored SpectrumMill v3 vs. SM v2; n=46
Bits
1 2 3 4 5 6 7 8 9 10
chemistry Acidic Basic Hydrophobic Neutral Polar

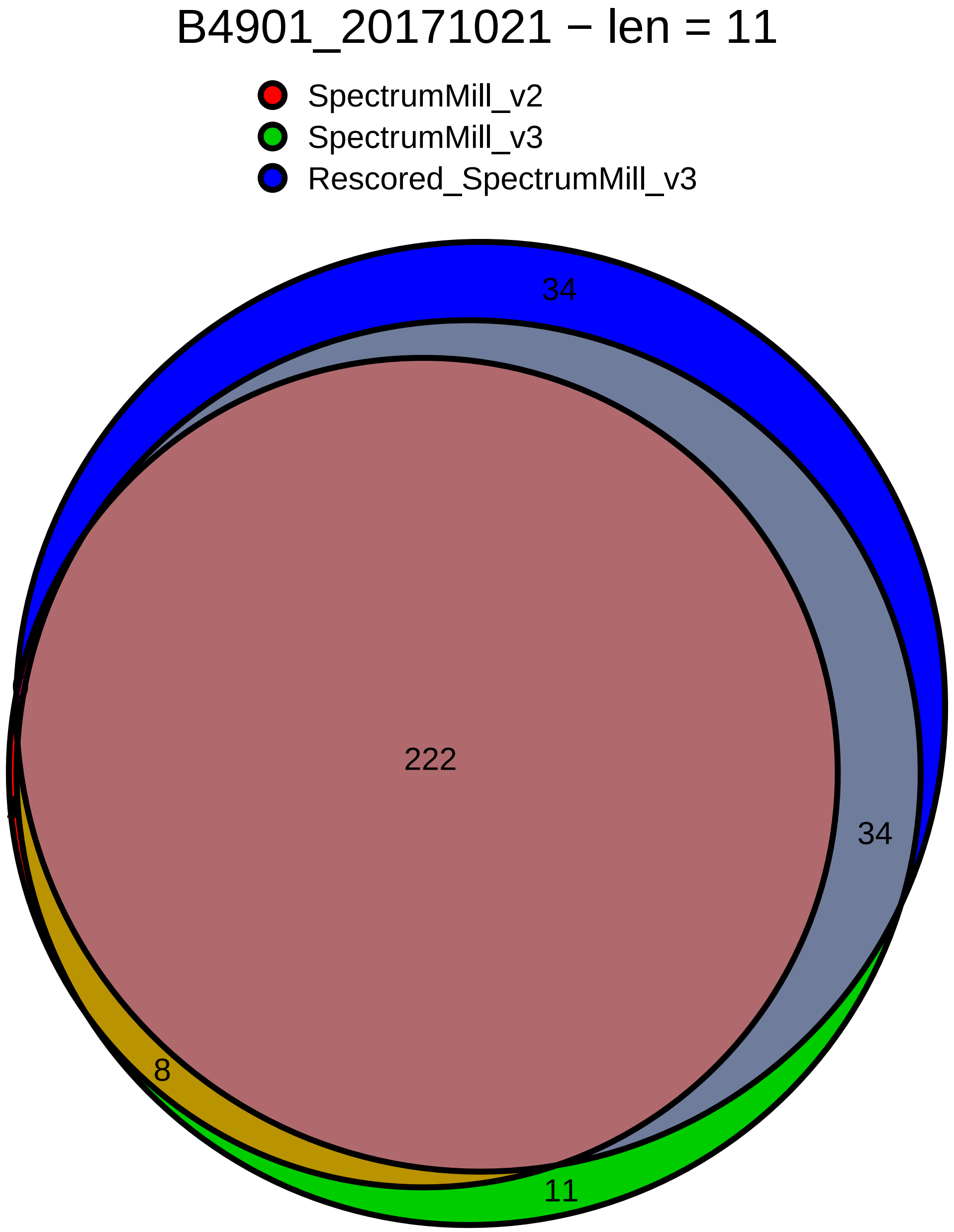
B4901_20171021 − len = 11
SpectrumMill_v2
SpectrumMill_v3
Rescored_SpectrumMill_v3
34
222
34
8
11

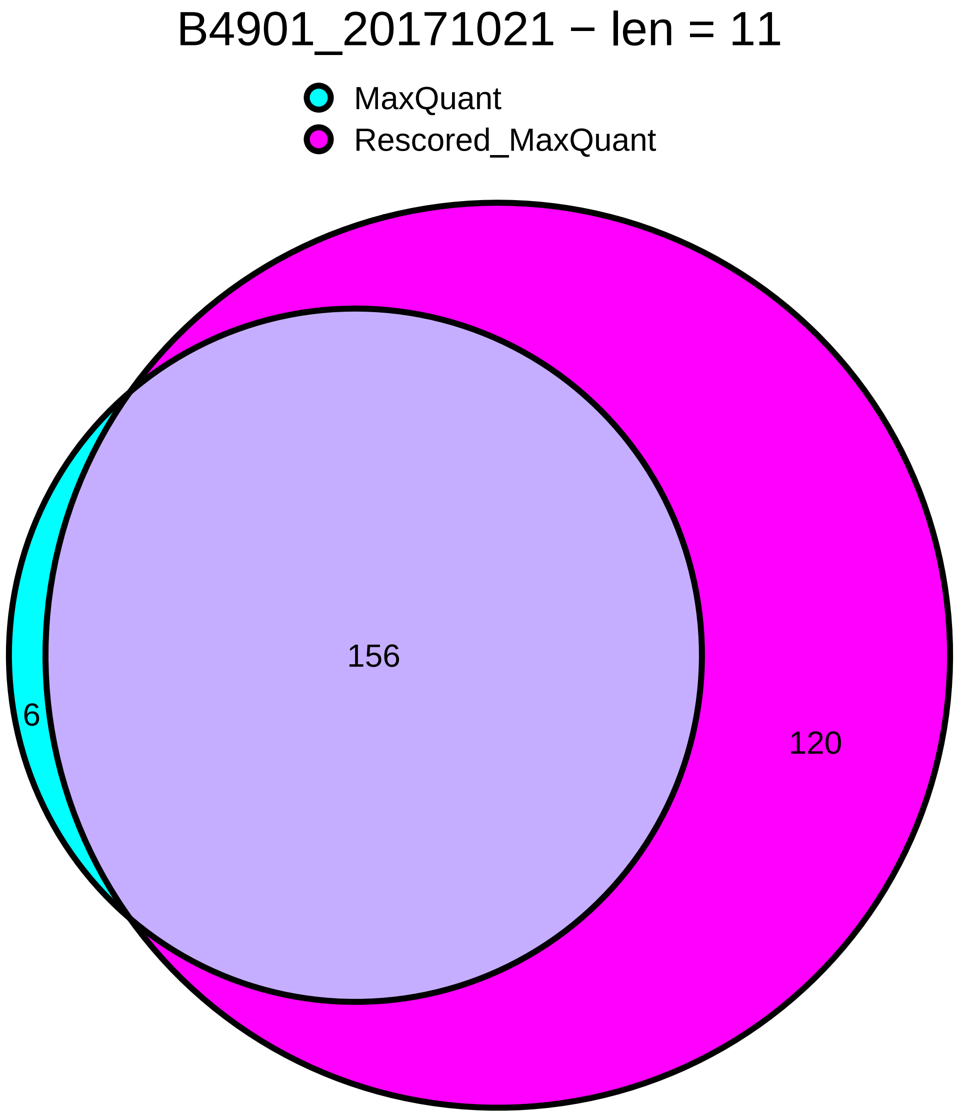
B4901_20171021 − len = 11
MaxQuant
Rescored_MaxQuant
156
6
120

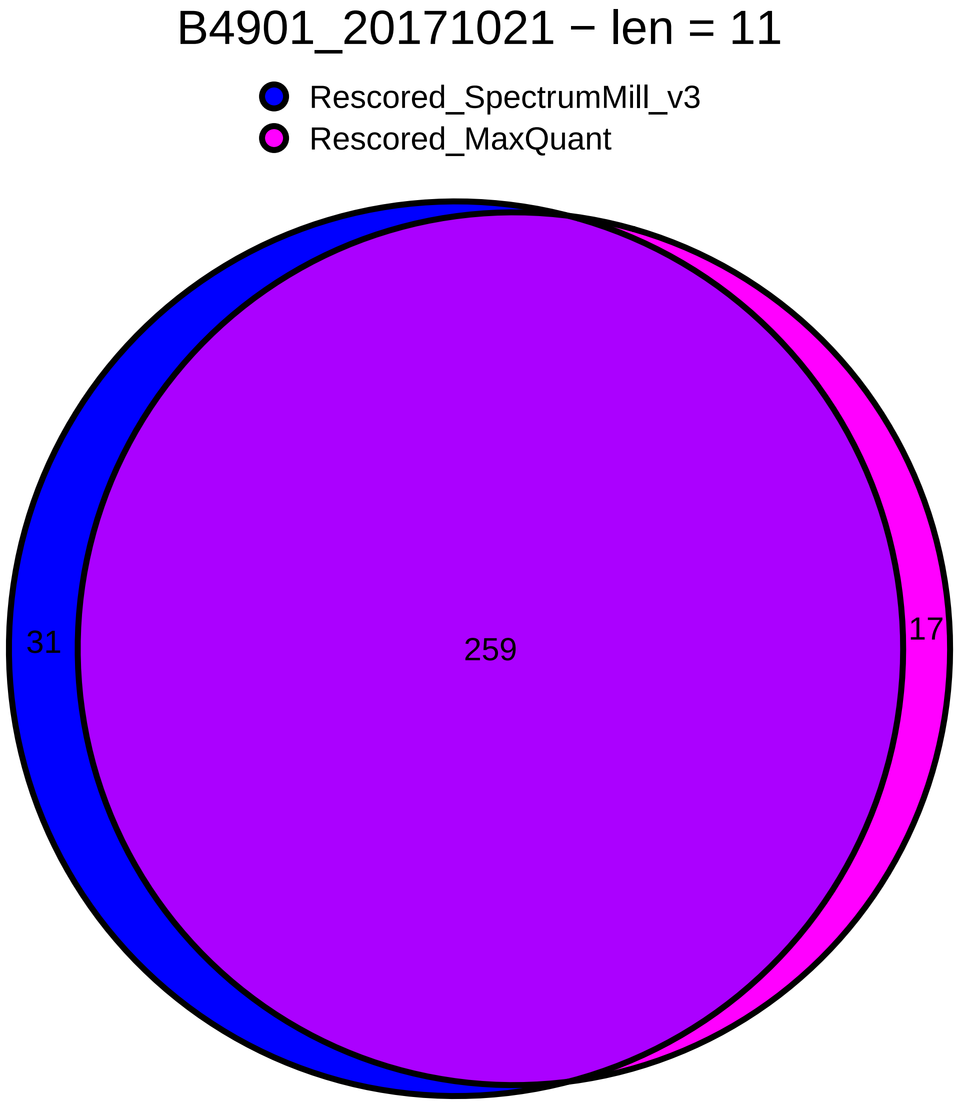
B4901_20171021 − len = 11
Rescored_SpectrumMill_v3
Rescored_MaxQuant
31
259
17

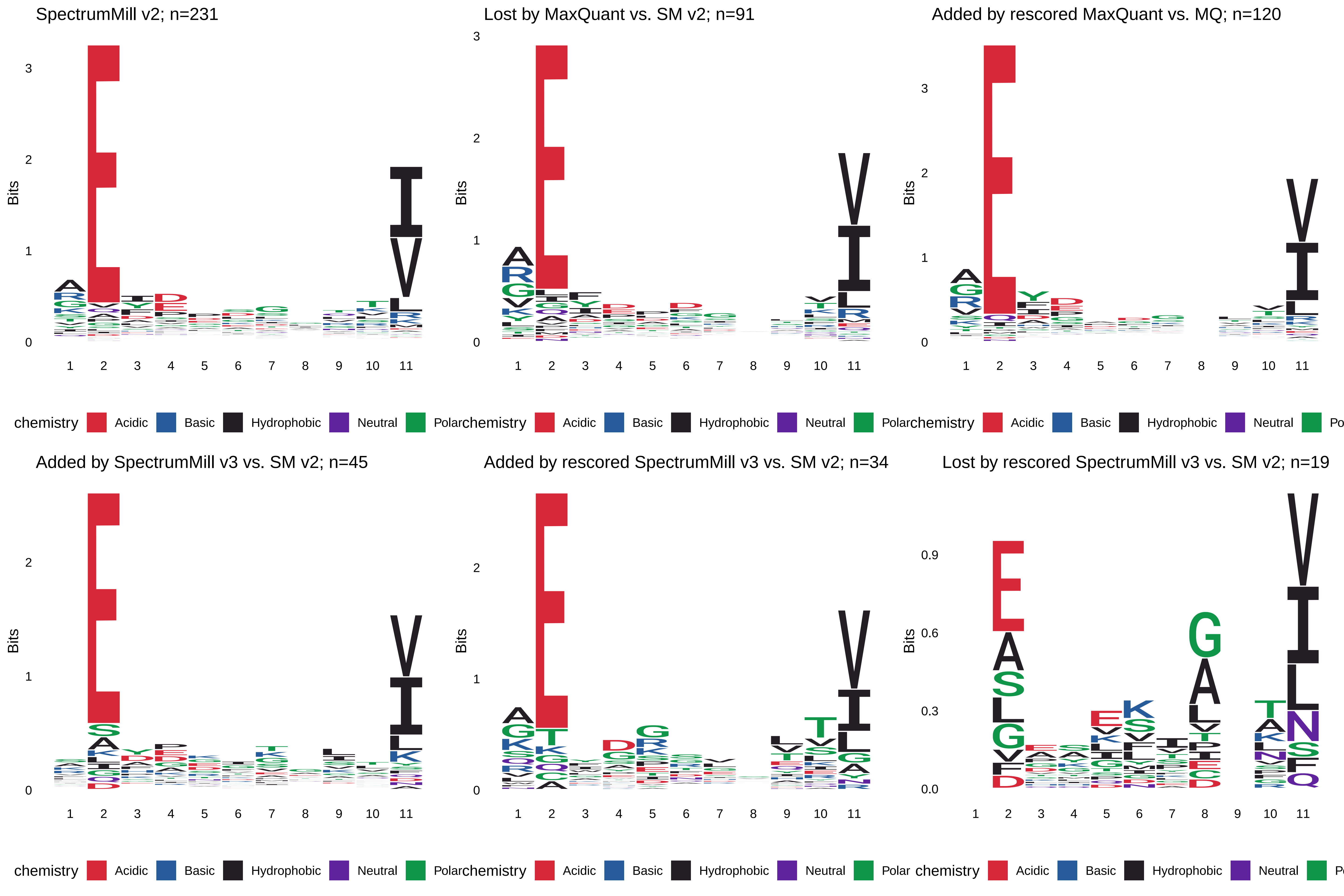

SpectrumMill v2; n=231
Lost by MaxQuant vs. SM v2; n=91
Added by rescored MaxQuant vs. MQ; n=120
Added by SpectrumMill v3 vs. SM v2; n=45
Added by rescored SpectrumMill v3 vs. SM v2; n=34
Lost by rescored SpectrumMill v3 vs. SM v2; n=19
Bits
1 2 3 4 5 6 7 8 9 10 11
chemistry
Acidic
Basic
Hydrophobic
Neutral
Polar

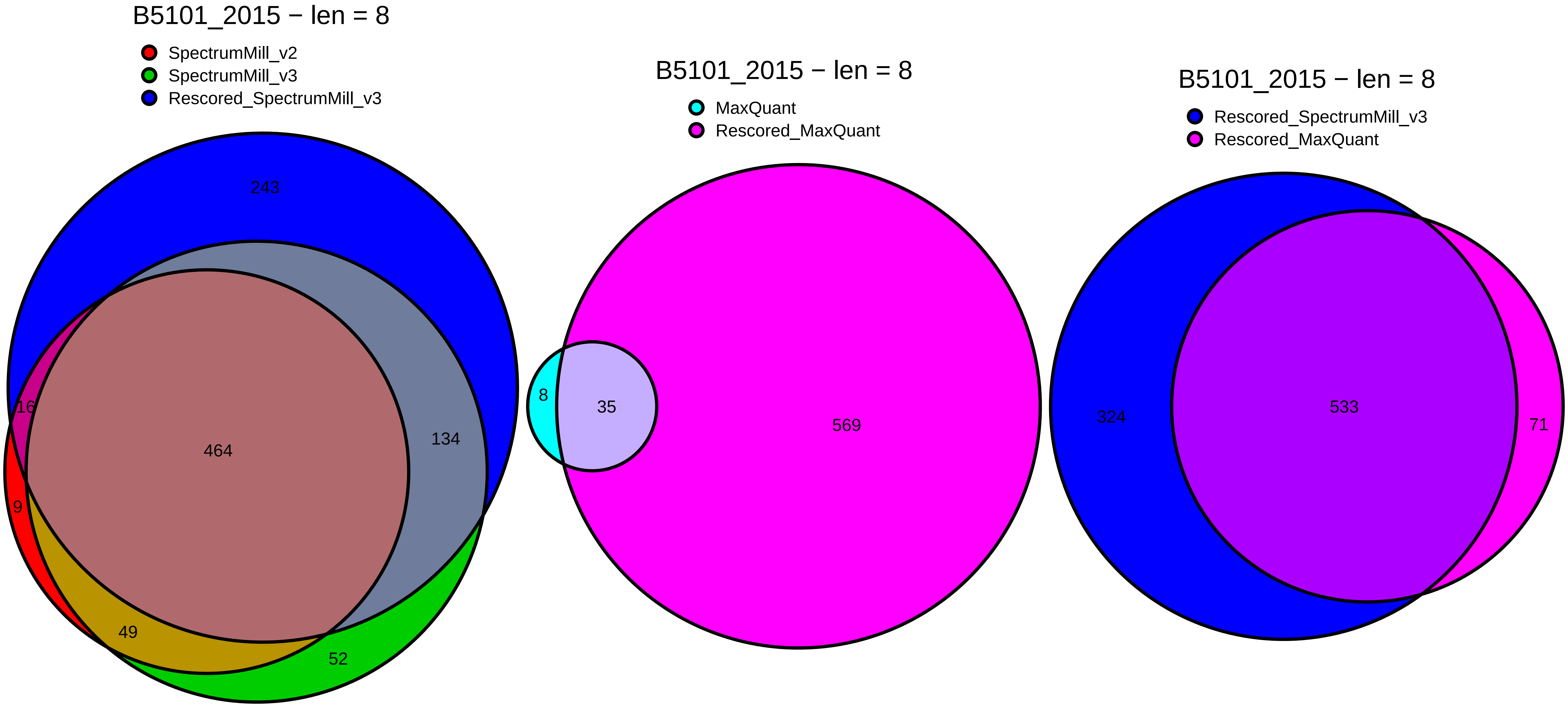

B5101_2015 − len = 8
SpectrumMill_v2
SpectrumMill_v3
Rescored_SpectrumMill_v3
243
16
464
134
9
49
52
B5101_2015 − len = 8
MaxQuant
Rescored_MaxQuant
8
35
569
B5101_2015 − len = 8
Rescored_SpectrumMill_v3
Rescored_MaxQuant
324
533
71

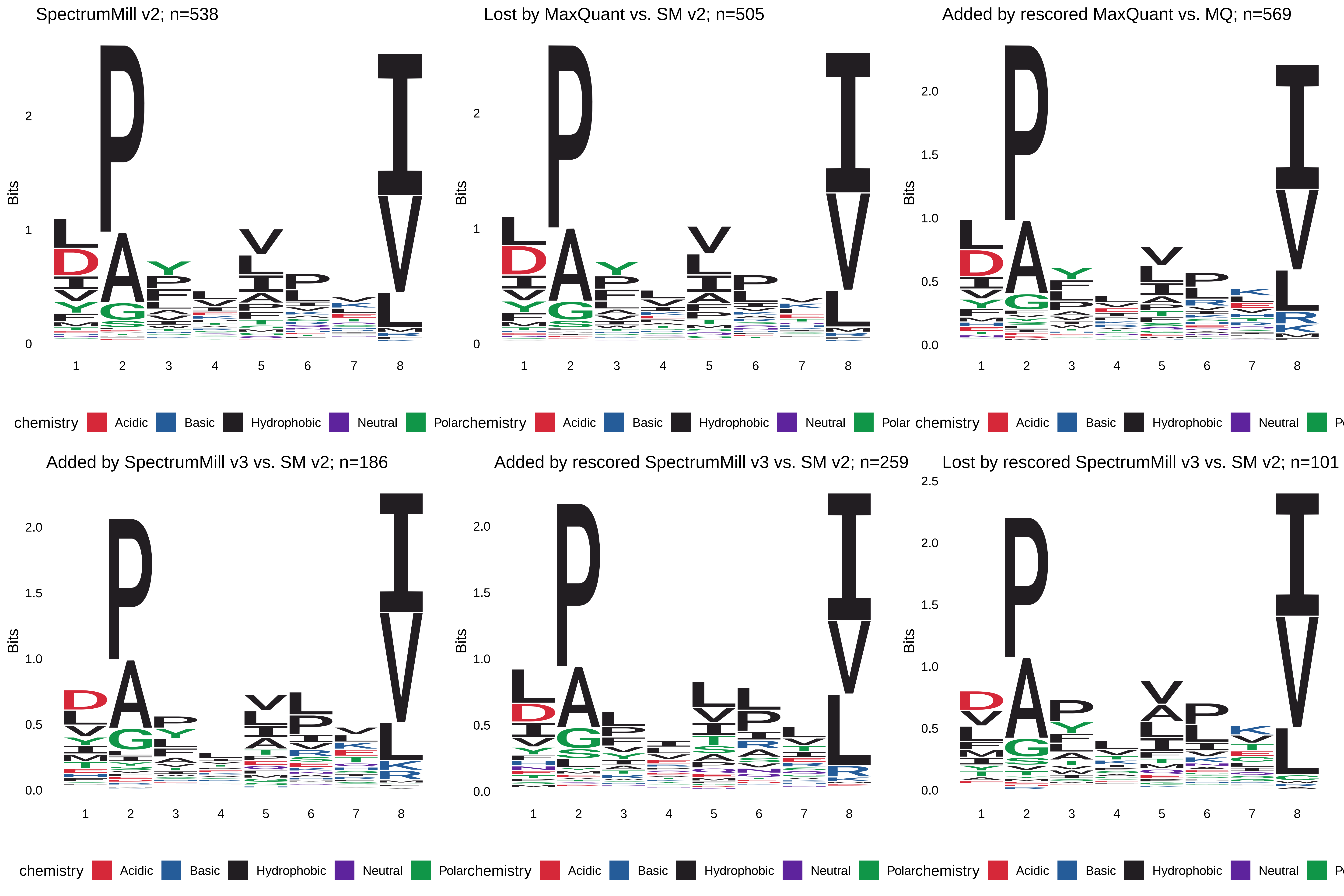
SpectrumMill v2; n=538
Lost by MaxQuant vs. SM v2; n=505
Added by rescored MaxQuant vs. MQ; n=569
Added by SpectrumMill v3 vs. SM v2; n=186
Added by rescored SpectrumMill v3 vs. SM v2; n=259
Lost by rescored SpectrumMill v3 vs. SM v2; n=101
Bits
1 2 3 4 5 6 7 8
chemistry Acidic Basic Hydrophobic Neutral Polar

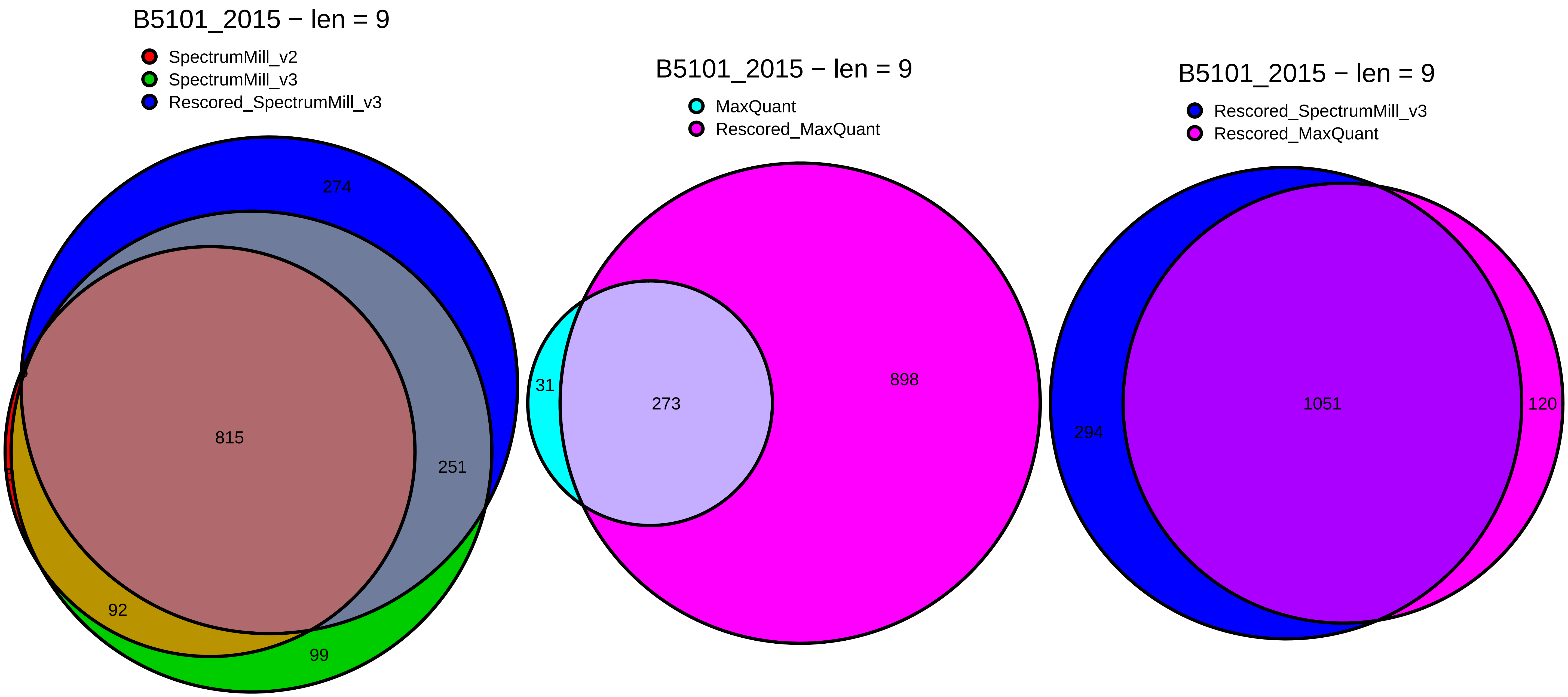
B5101_2015 − len = 9
SpectrumMill_v2
SpectrumMill_v3
Rescored_SpectrumMill_v3
274
815
251
92
99
B5101_2015 − len = 9
MaxQuant
Rescored_MaxQuant
31
273
898
B5101_2015 − len = 9
Rescored_SpectrumMill_v3
Rescored_MaxQuant
294
1051
120

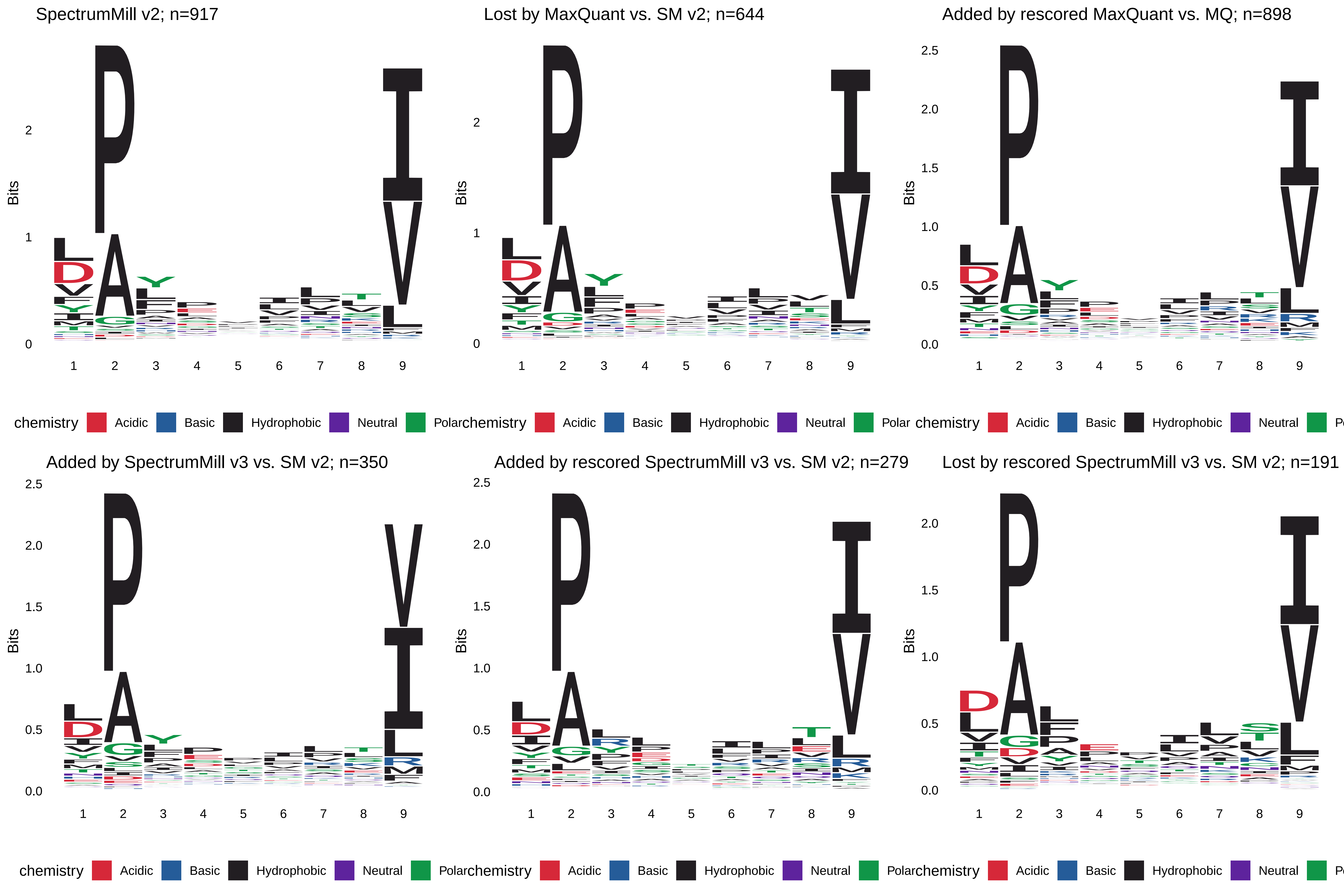

SpectrumMill v2; n=917
Lost by MaxQuant vs. SM v2; n=644
Added by rescored MaxQuant vs. MQ; n=898
Added by SpectrumMill v3 vs. SM v2; n=350
Added by rescored SpectrumMill v3 vs. SM v2; n=279
Lost by rescored SpectrumMill v3 vs. SM v2; n=191
Bits
1 2 3 4 5 6 7 8 9
chemistry
Acidic
Basic
Hydrophobic
Neutral
Polar

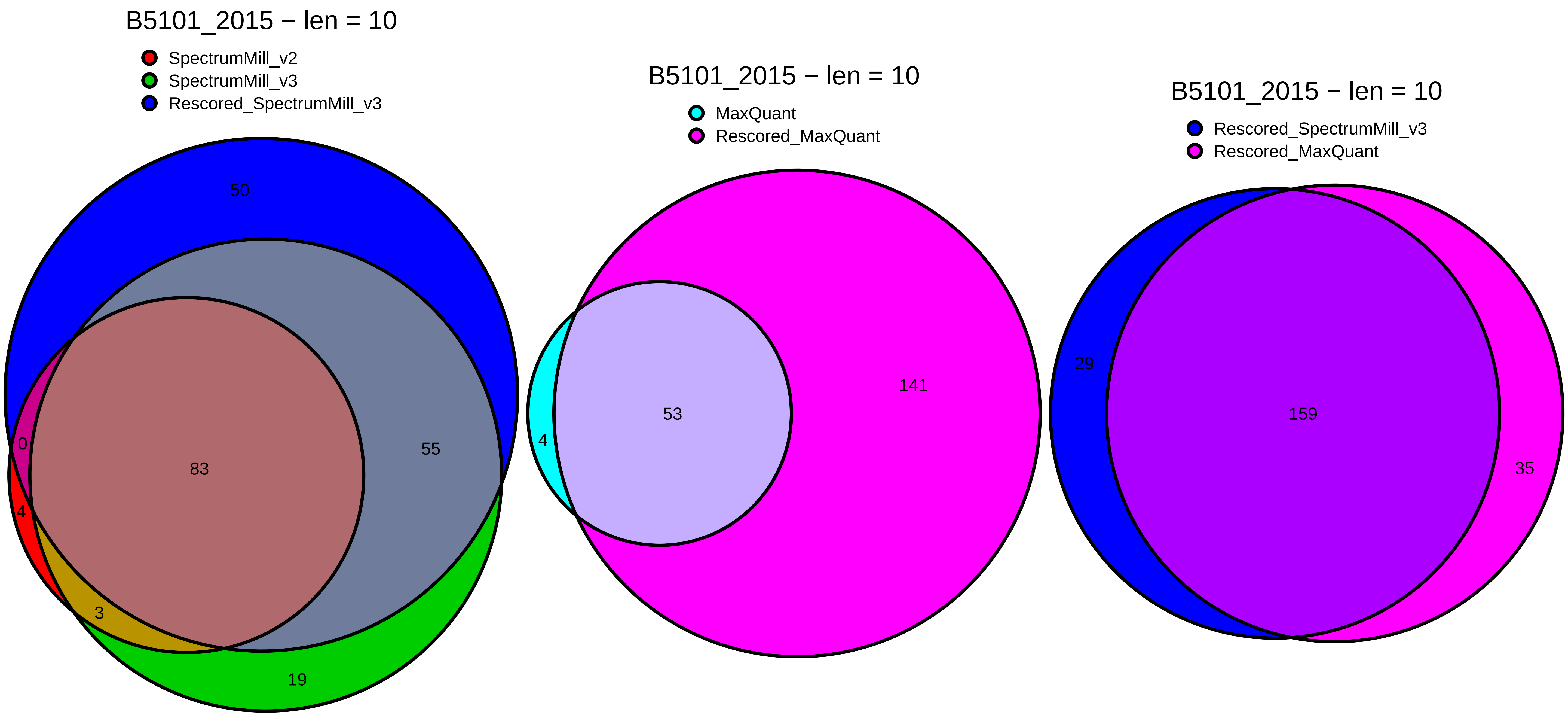
B5101_2015 − len = 10
SpectrumMill_v2
SpectrumMill_v3
Rescored_SpectrumMill_v3
50
0
83
55
4
3
19
B5101_2015 − len = 10
MaxQuant
Rescored_MaxQuant
4
53
141
B5101_2015 − len = 10
Rescored_SpectrumMill_v3
Rescored_MaxQuant
29
159
35

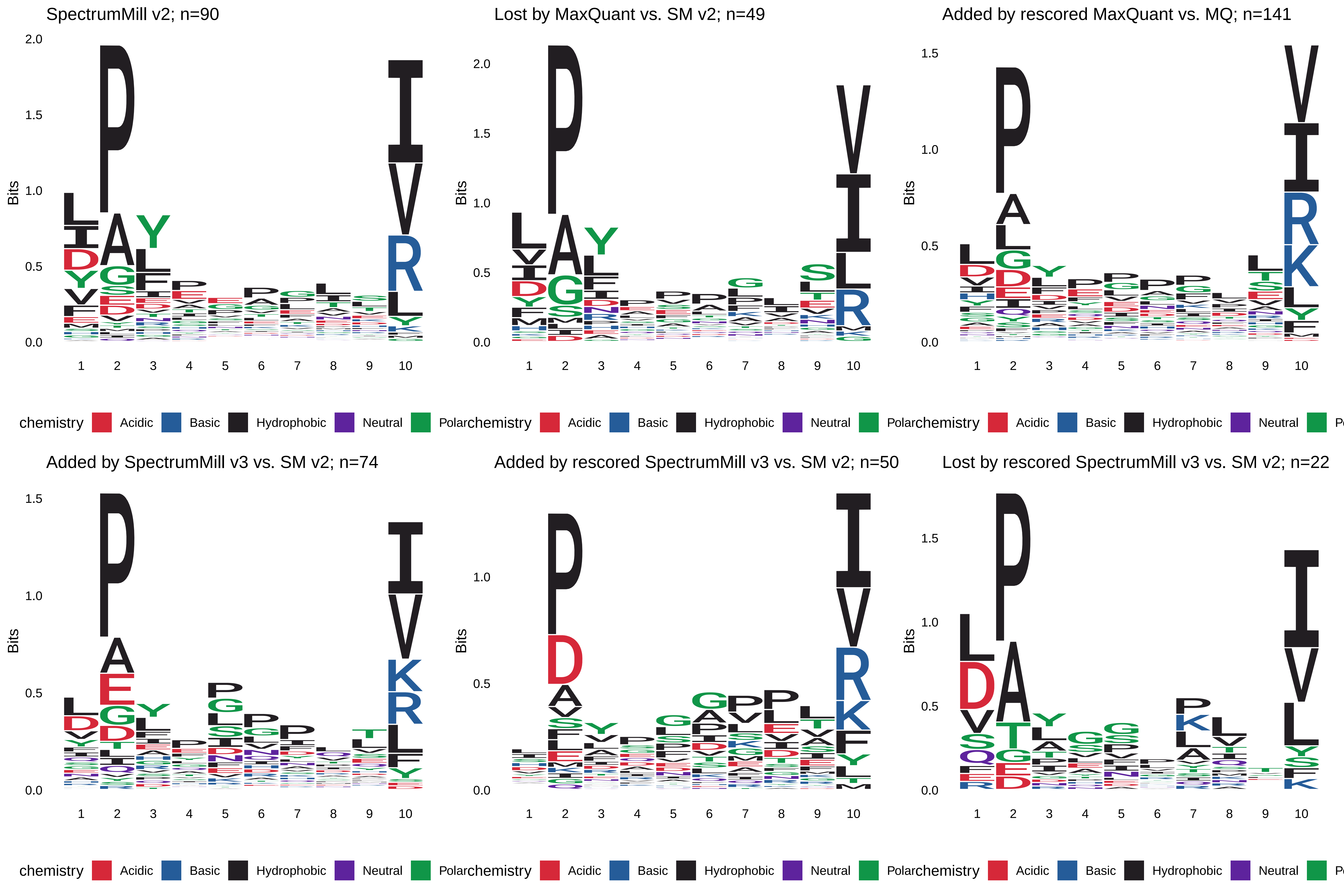

SpectrumMill v2; n=90
Lost by MaxQuant vs. SM v2; n=49
Added by rescored MaxQuant vs. MQ; n=141
Added by SpectrumMill v3 vs. SM v2; n=74
Added by rescored SpectrumMill v3 vs. SM v2; n=50
Lost by rescored SpectrumMill v3 vs. SM v2; n=22
Bits
1 2 3 4 5 6 7 8 9 10
chemistry
Acidic
Basic
Hydrophobic
Neutral
Polar

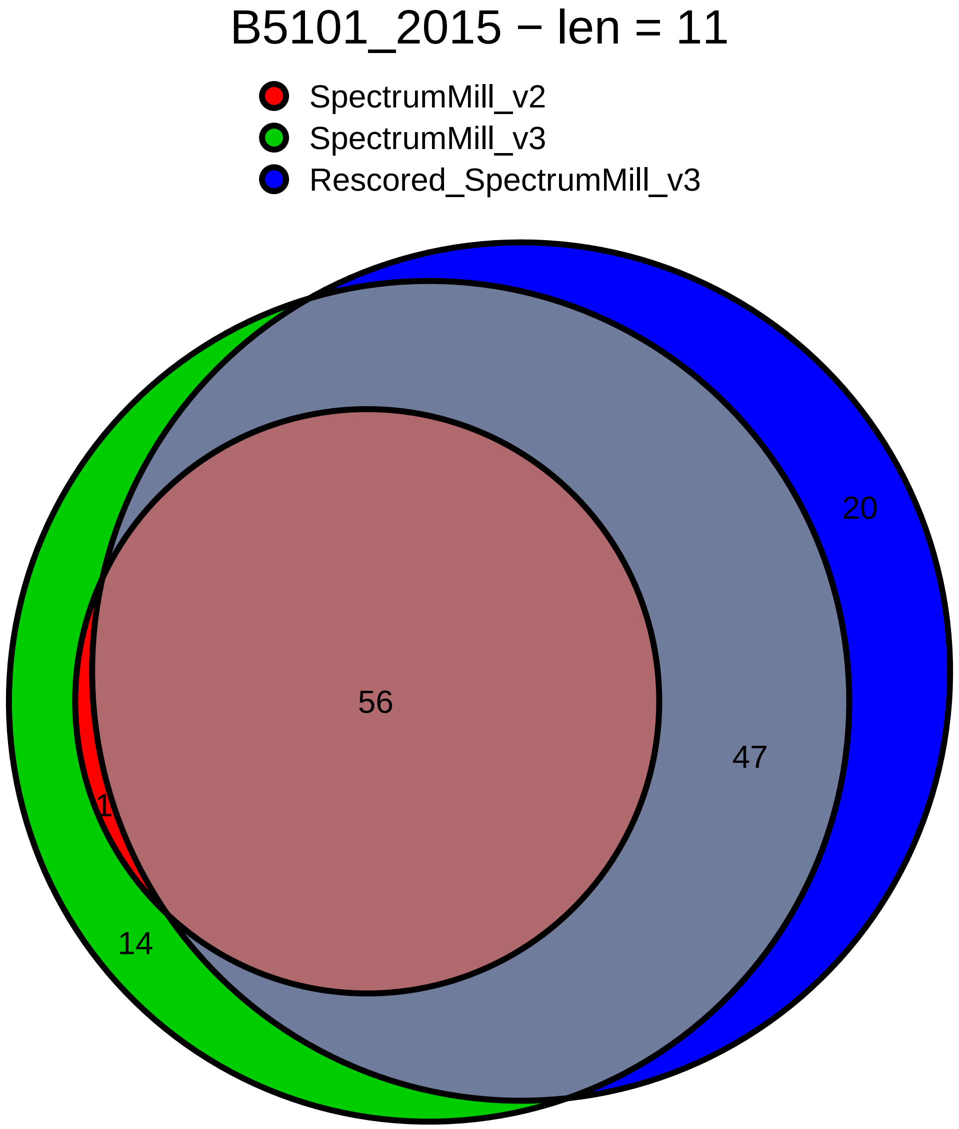
B5101_2015 − len = 11
SpectrumMill_v2
SpectrumMill_v3
Rescored_SpectrumMill_v3
20
56
47
1
14

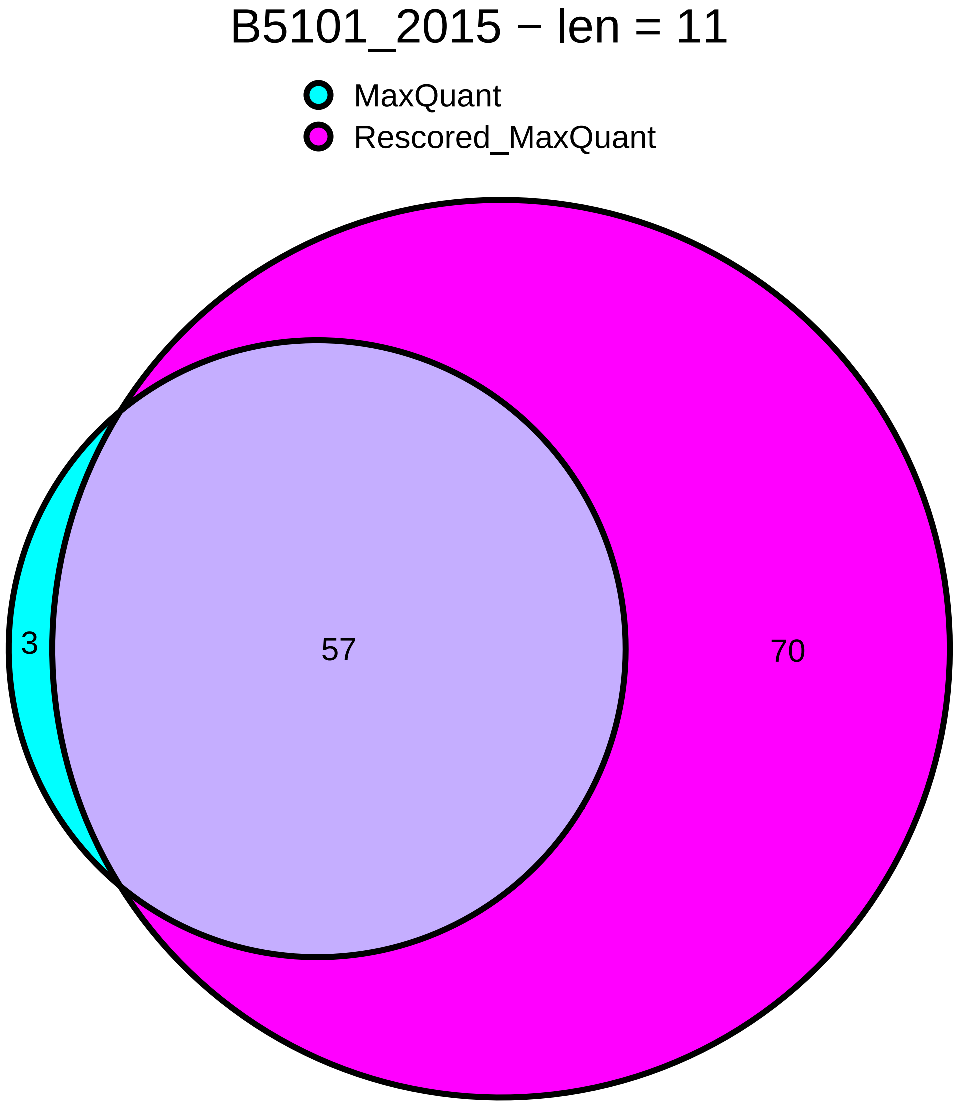
B5101_2015 − len = 11
MaxQuant
Rescored_MaxQuant
3
57
70

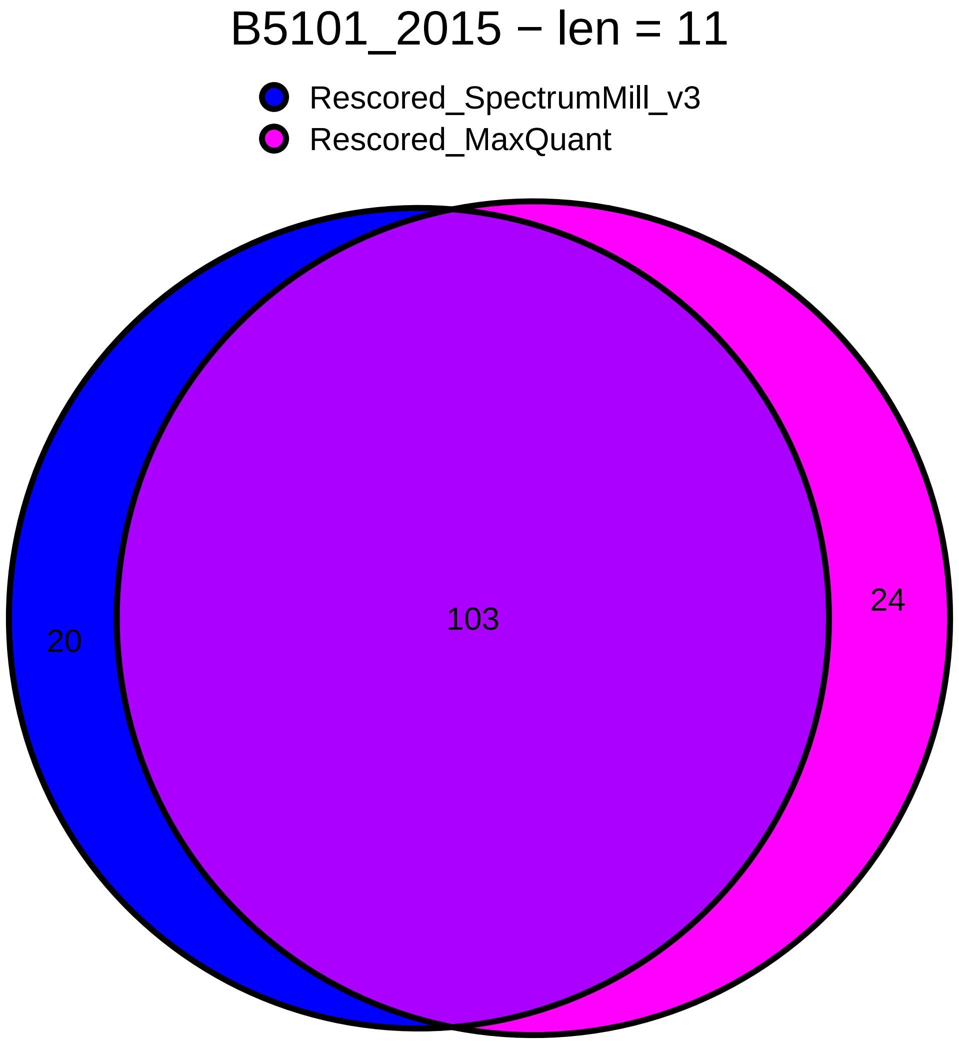
B5101_2015 − len = 11
Rescored_SpectrumMill_v3
Rescored_MaxQuant
20
103
24

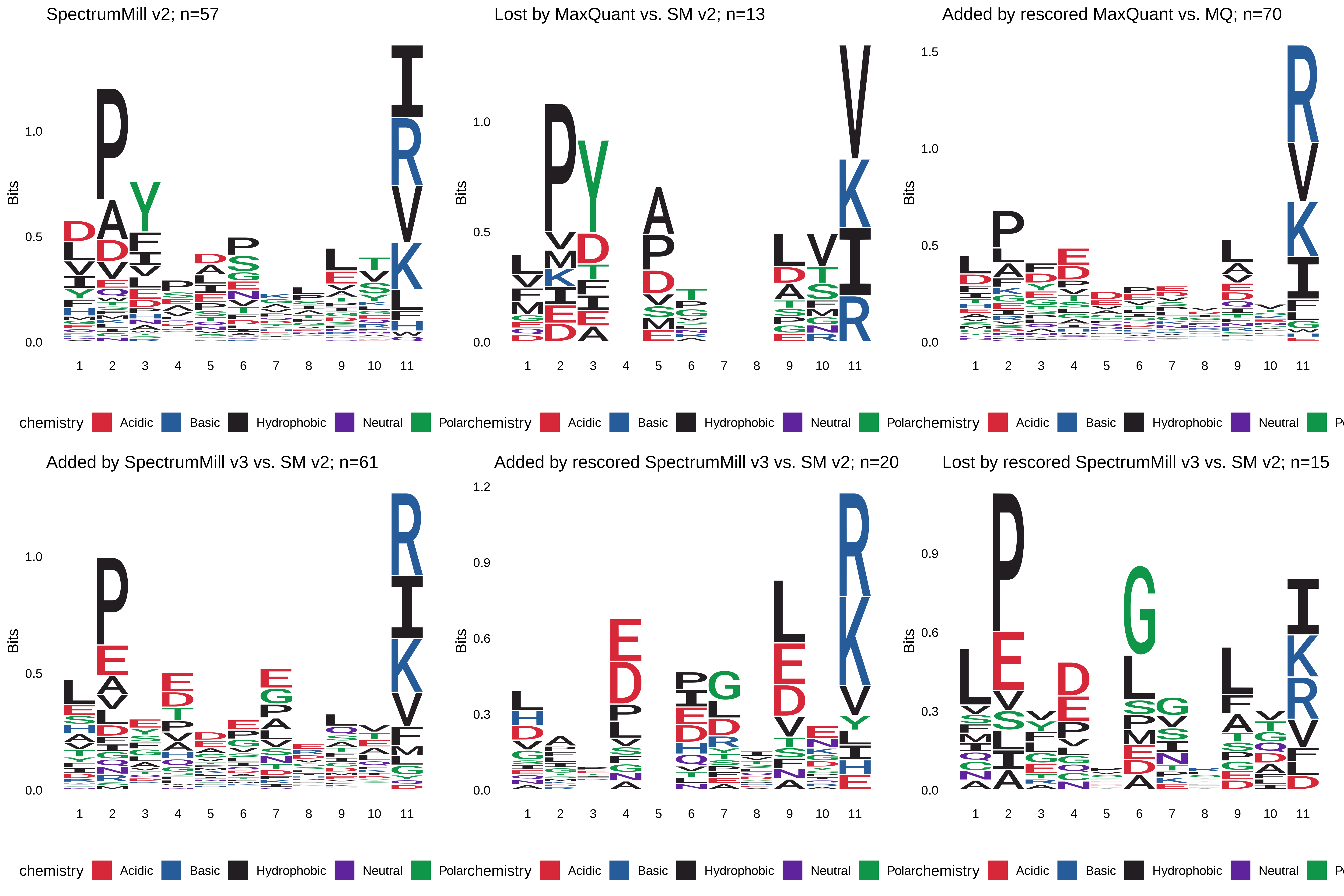

SpectrumMill v2; n=57
Lost by MaxQuant vs. SM v2; n=13
Added by rescored MaxQuant vs. MQ; n=70
Added by SpectrumMill v3 vs. SM v2; n=61
Added by rescored SpectrumMill v3 vs. SM v2; n=20
Lost by rescored SpectrumMill v3 vs. SM v2; n=15
Bits
0.0
0.3
0.5
0.6
0.9
1.0
1.2
1.5
1 2 3 4 5 6 7 8 9 10 11
chemistry
Acidic
Basic
Hydrophobic
Neutral
Polar

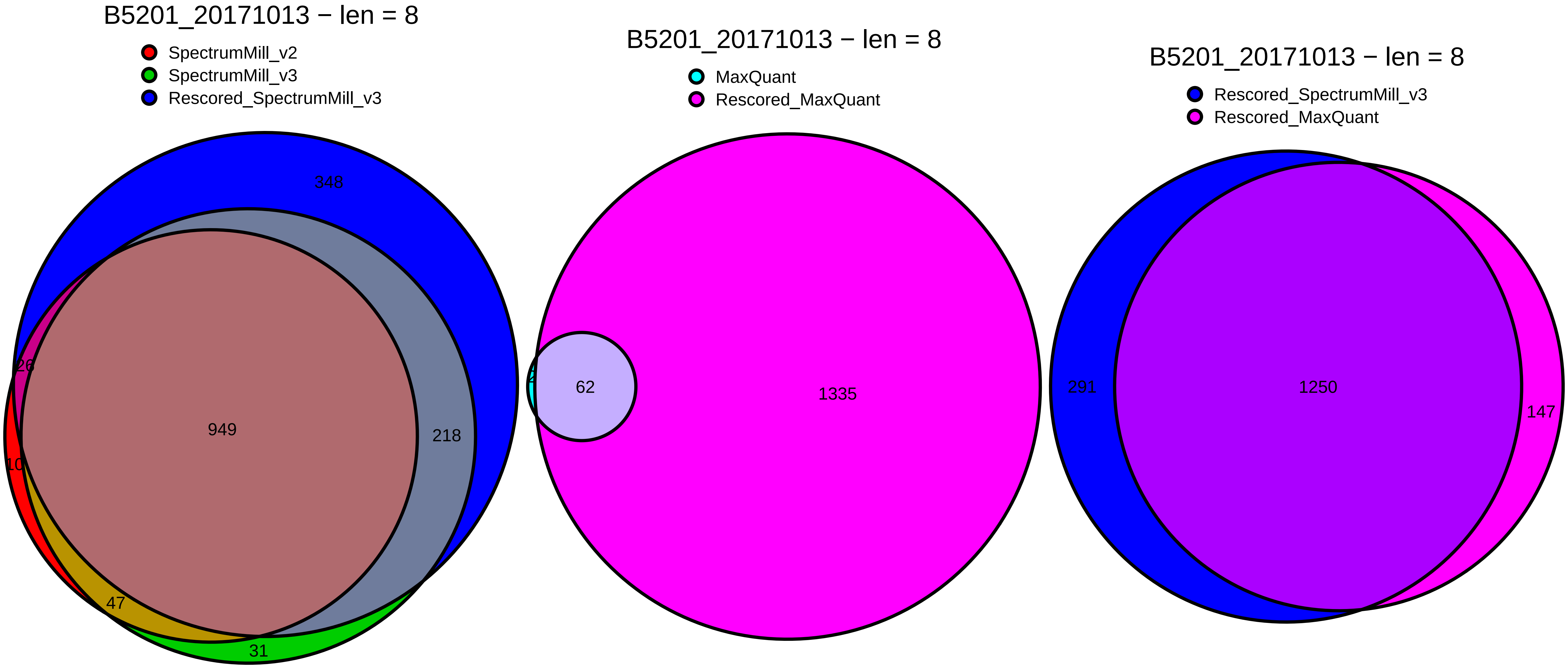
B5201_20171013 − len = 8
SpectrumMill_v2
SpectrumMill_v3
Rescored_SpectrumMill_v3
348
26
949
218
10
47
31
B5201_20171013 − len = 8
MaxQuant
Rescored_MaxQuant
62
1335
B5201_20171013 − len = 8
Rescored_SpectrumMill_v3
Rescored_MaxQuant
291
1250
147

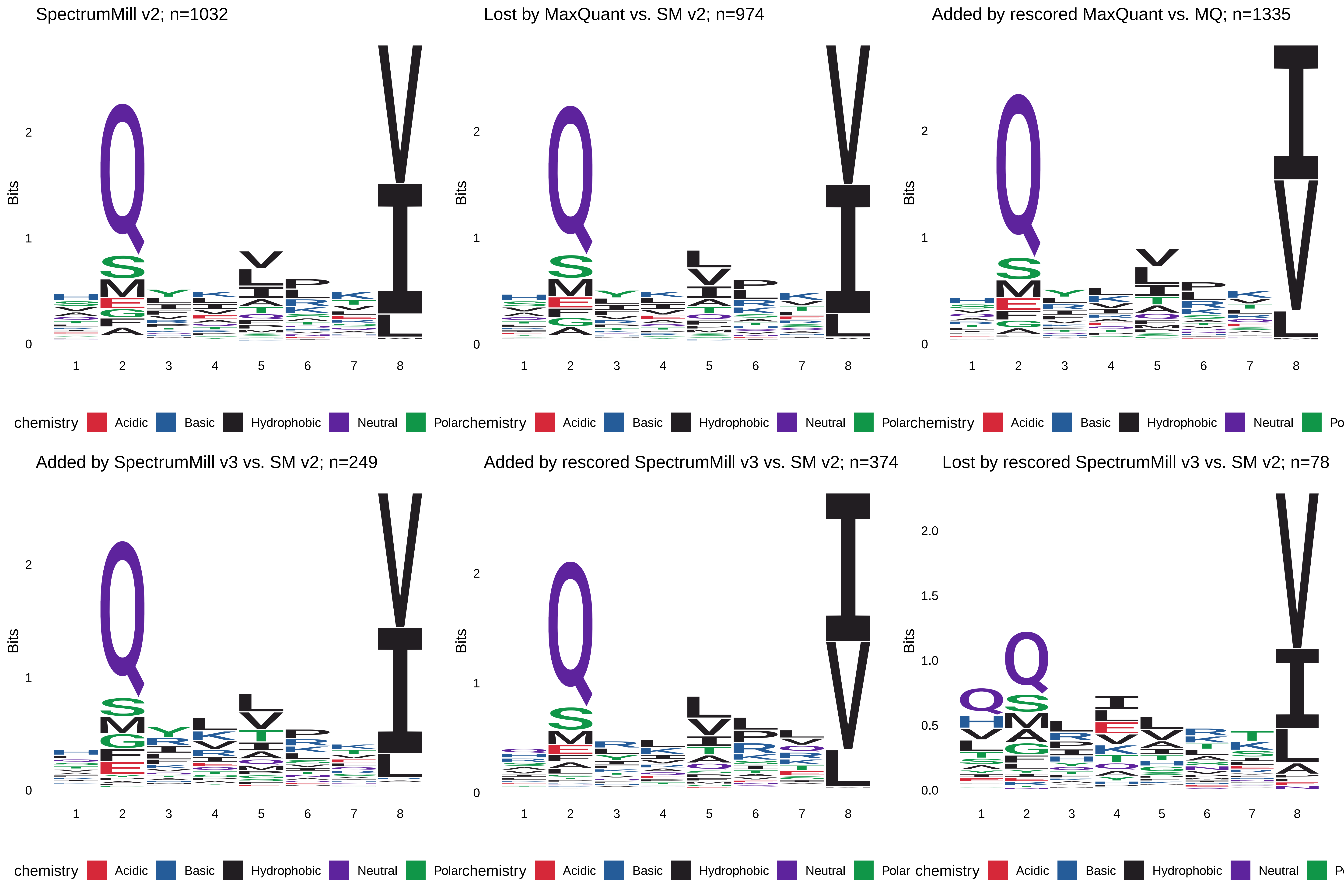

SpectrumMill v2; n=1032
Lost by MaxQuant vs. SM v2; n=974
Added by rescored MaxQuant vs. MQ; n=1335
Added by SpectrumMill v3 vs. SM v2; n=249
Added by rescored SpectrumMill v3 vs. SM v2; n=374
Lost by rescored SpectrumMill v3 vs. SM v2; n=78
Bits
0
1
2
0.0
0.5
1.0
1.5
2.0
1
2
3
4
5
6
7
8
chemistry
Acidic
Basic
Hydrophobic
Neutral
Polar

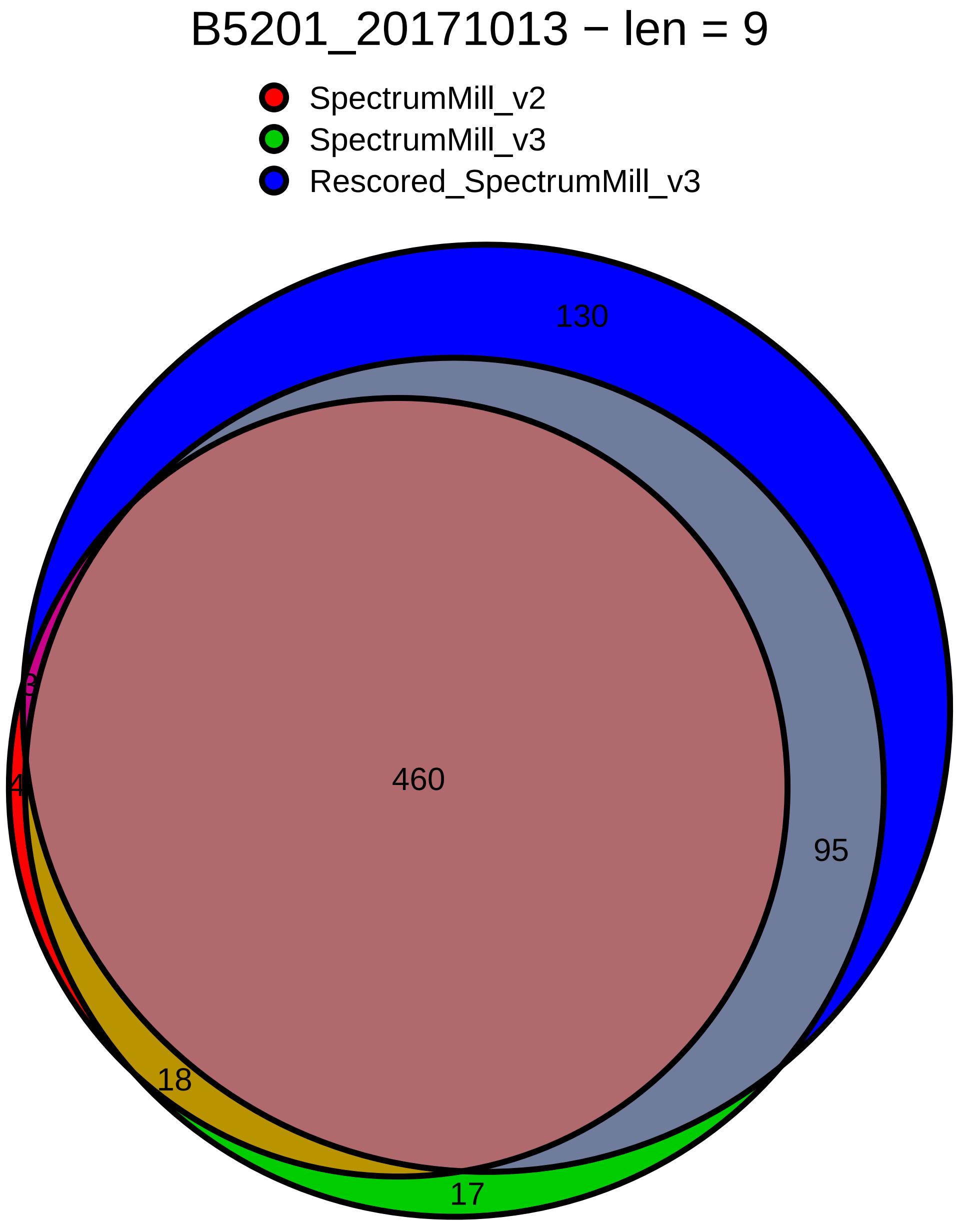
B5201_20171013 − len = 9
SpectrumMill_v2
SpectrumMill_v3
Rescored_SpectrumMill_v3
130
460
95
18
17

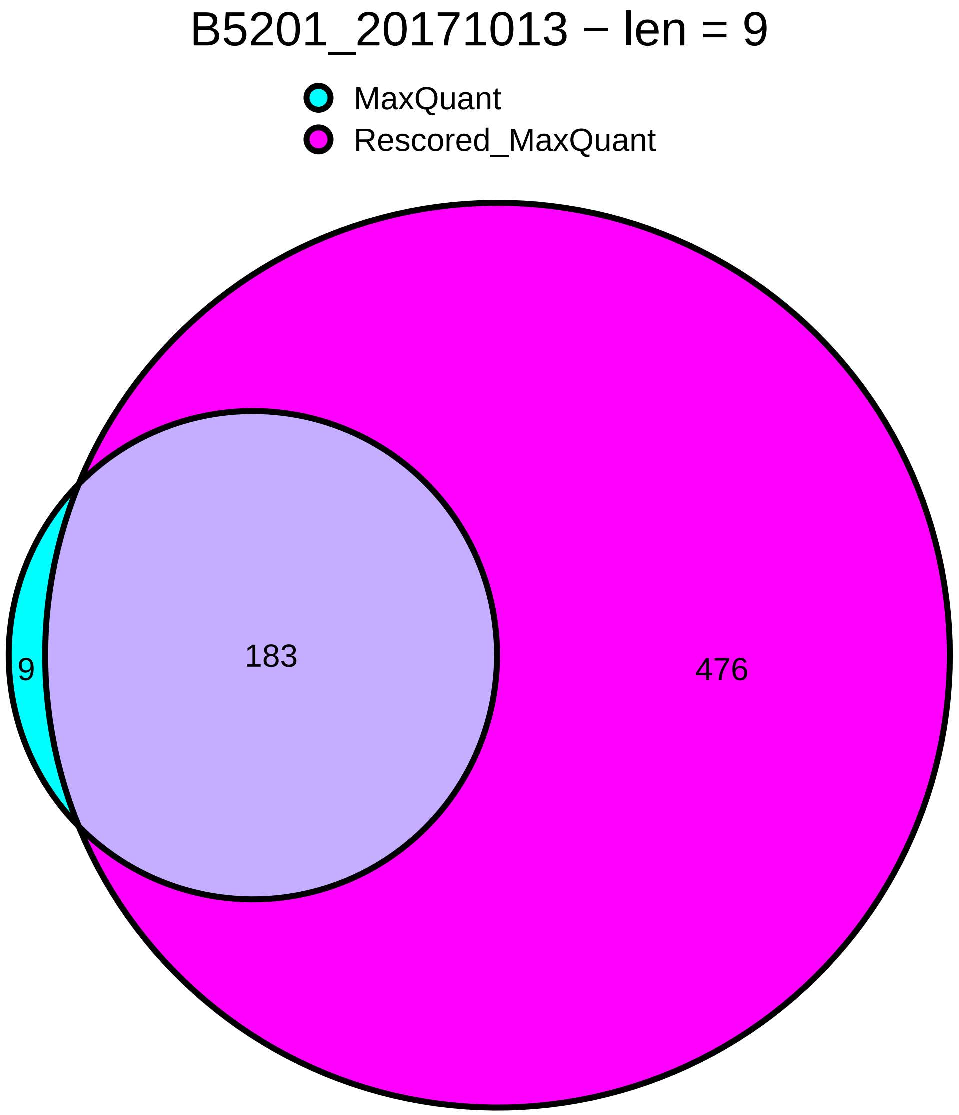
B5201_20171013 − len = 9
MaxQuant
Rescored_MaxQuant
9
183
476

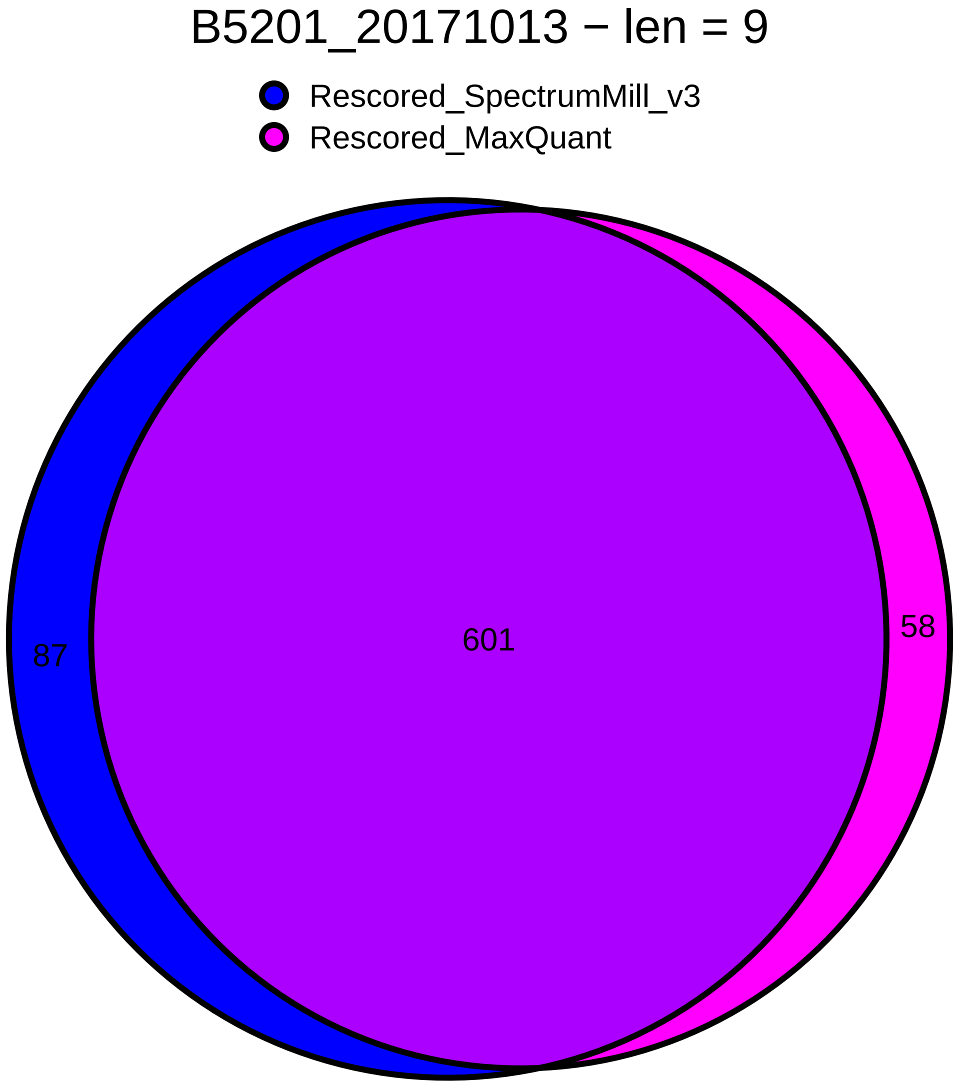
B5201_20171013 − len = 9
Rescored_SpectrumMill_v3
Rescored_MaxQuant
87
601
58

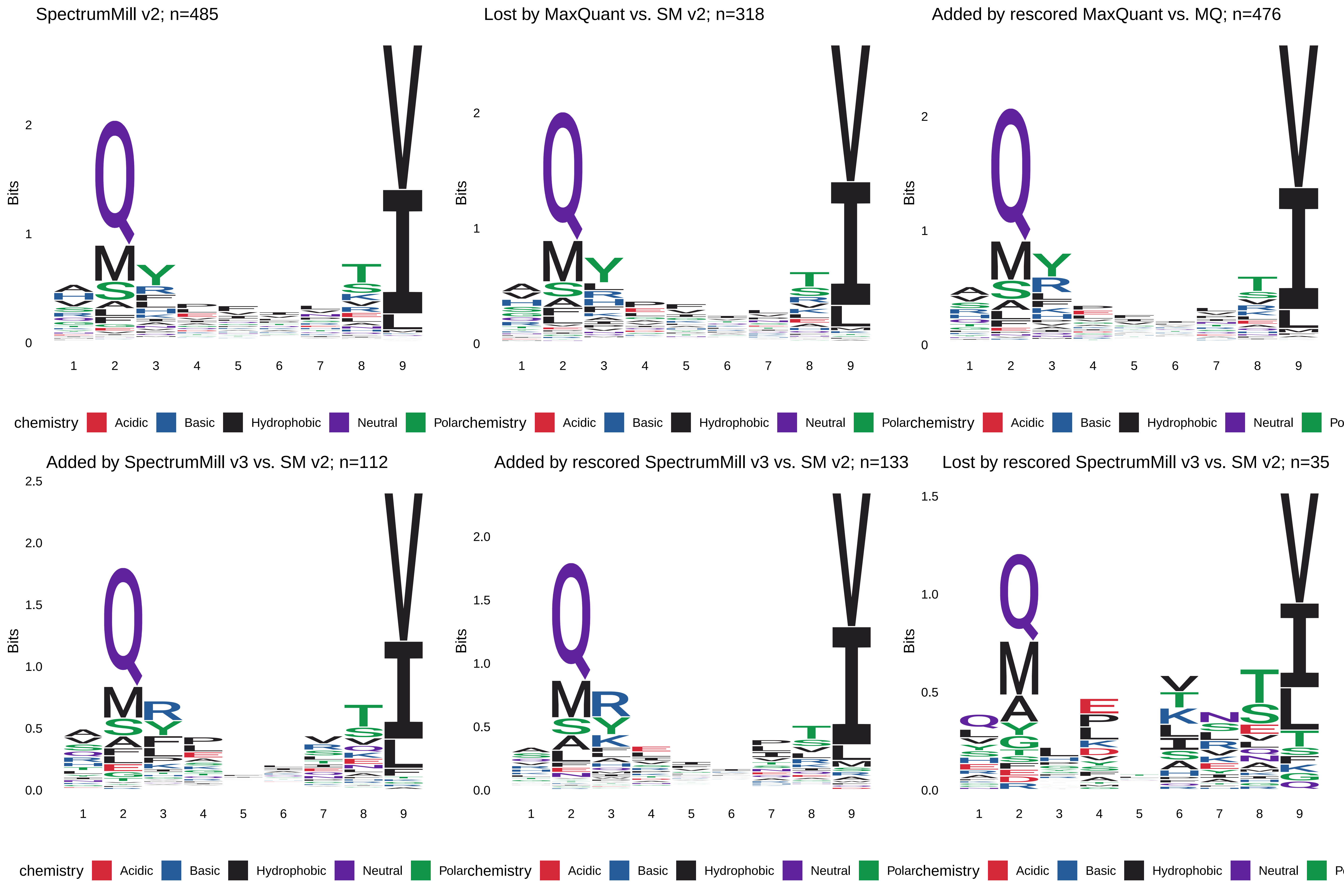
SpectrumMill v2; n=485
Lost by MaxQuant vs. SM v2; n=318
Added by rescored MaxQuant vs. MQ; n=476
Added by SpectrumMill v3 vs. SM v2; n=112
Added by rescored SpectrumMill v3 vs. SM v2; n=133
Lost by rescored SpectrumMill v3 vs. SM v2; n=35
Bits
1 2 3 4 5 6 7 8 9
chemistry
Acidic
Basic
Hydrophobic
Neutral
Polar

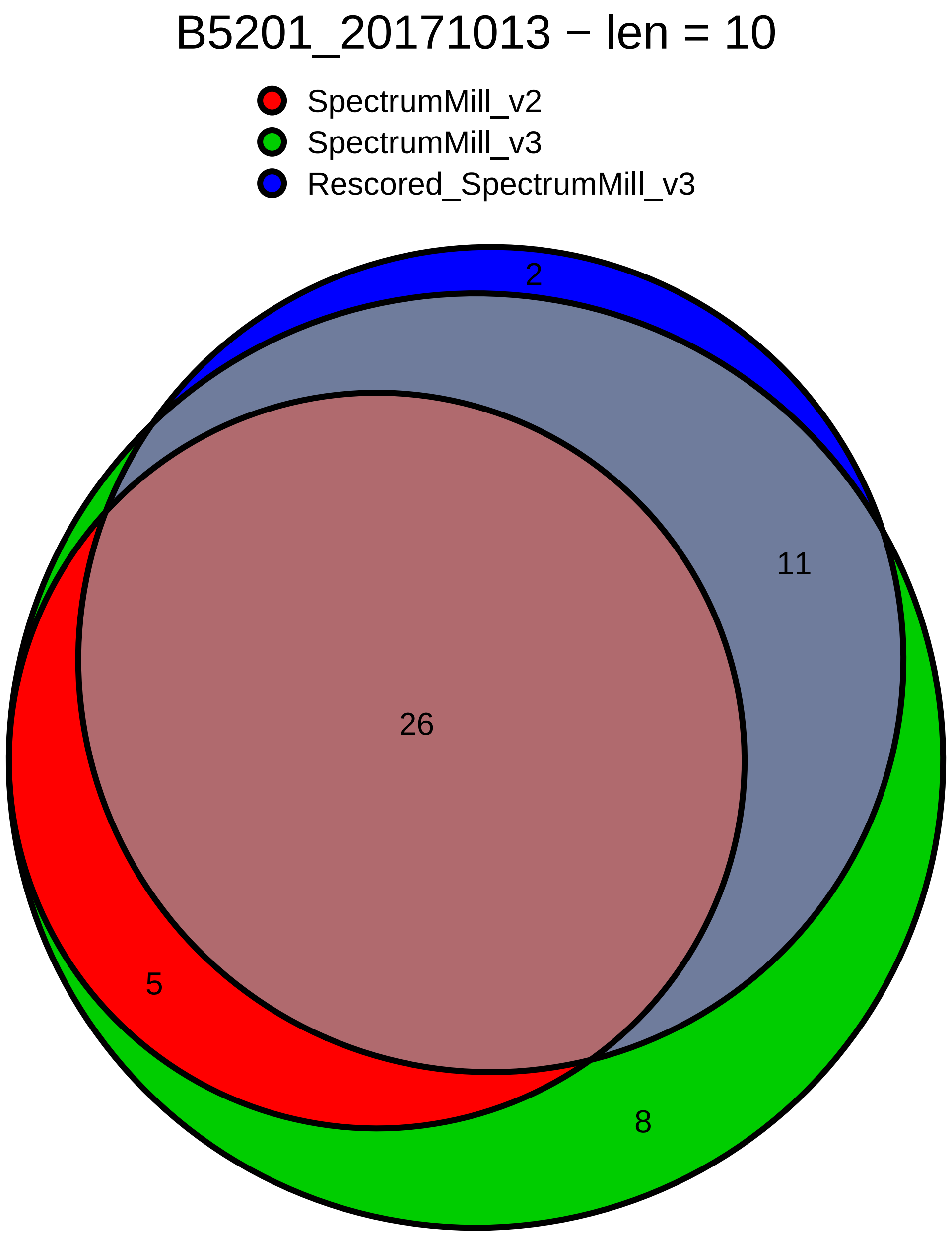
B5201_20171013 − len = 10
SpectrumMill_v2
SpectrumMill_v3
Rescored_SpectrumMill_v3
2
11
26
5
8

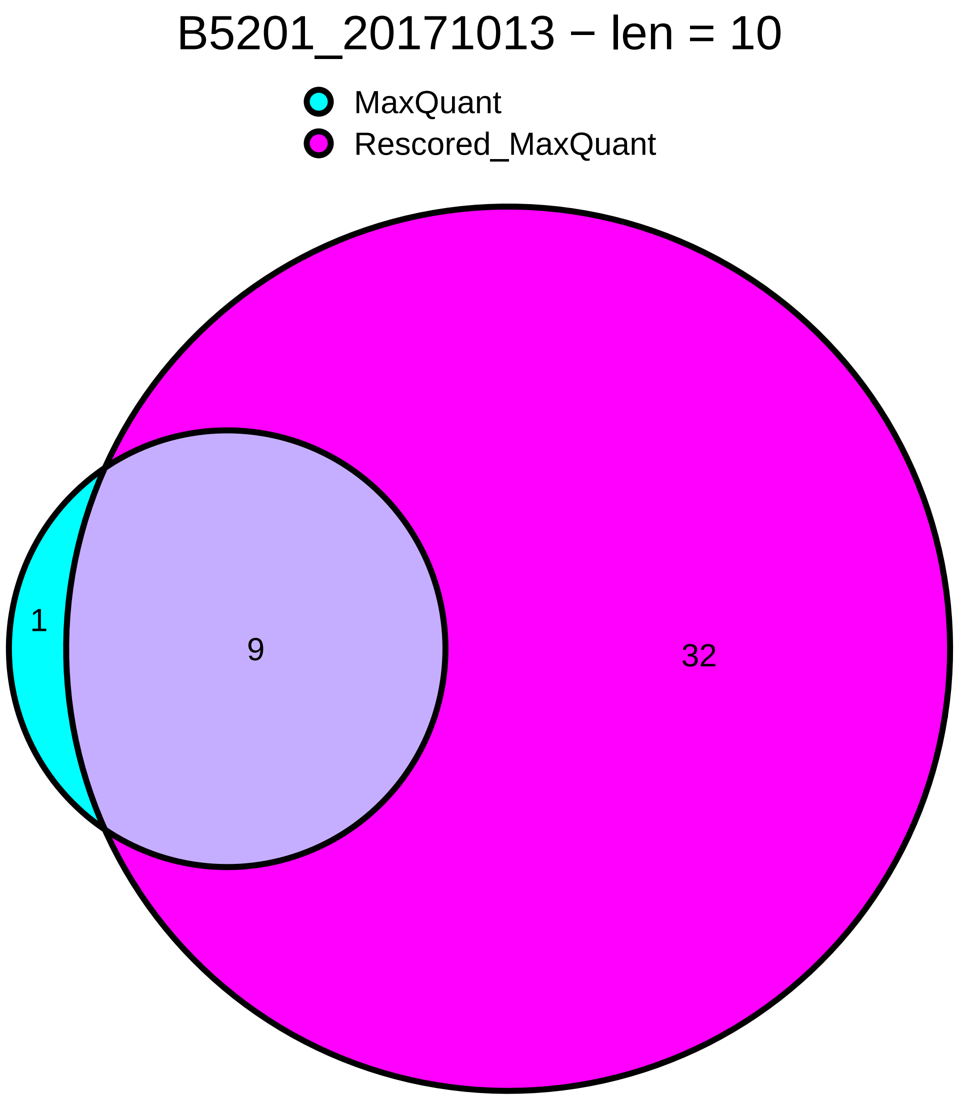
B5201_20171013 − len = 10
MaxQuant
Rescored_MaxQuant
1
9
32

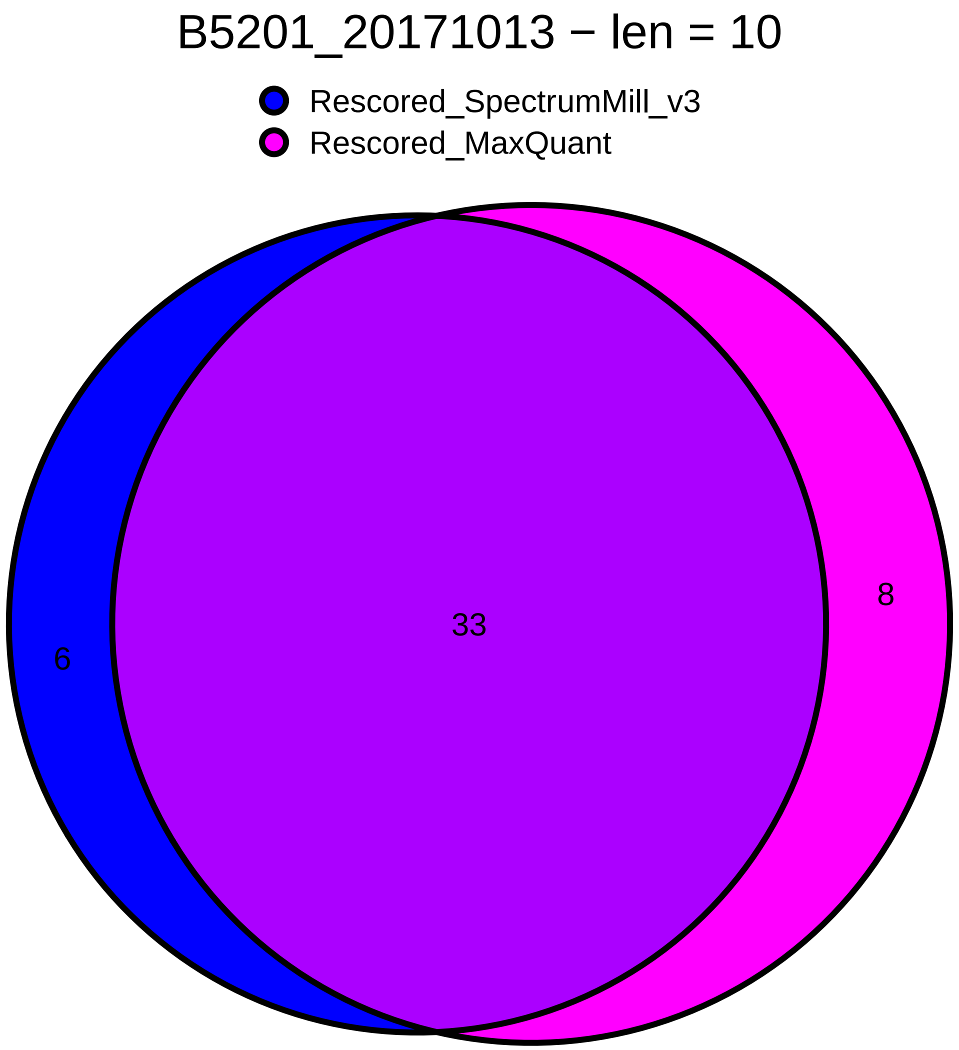
B5201_20171013 − len = 10
Rescored_SpectrumMill_v3
Rescored_MaxQuant
6
33
8

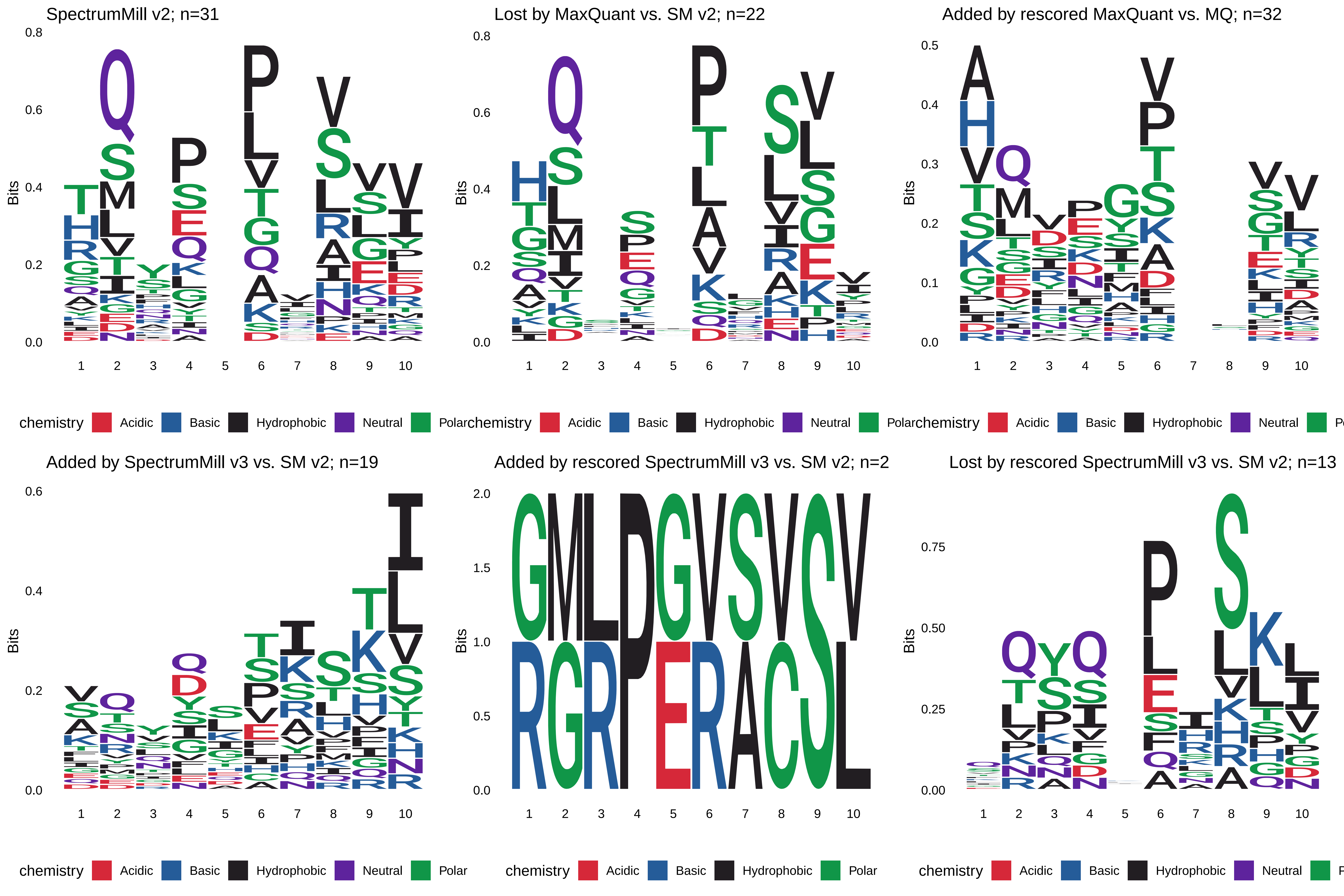
SpectrumMill v2; n=31
Lost by MaxQuant vs. SM v2; n=22
Added by rescored MaxQuant vs. MQ; n=32
Added by SpectrumMill v3 vs. SM v2; n=19
Added by rescored SpectrumMill v3 vs. SM v2; n=2
Lost by rescored SpectrumMill v3 vs. SM v2; n=13
Bits
chemistry
Acidic
Basic
Hydrophobic
Neutral
Polar

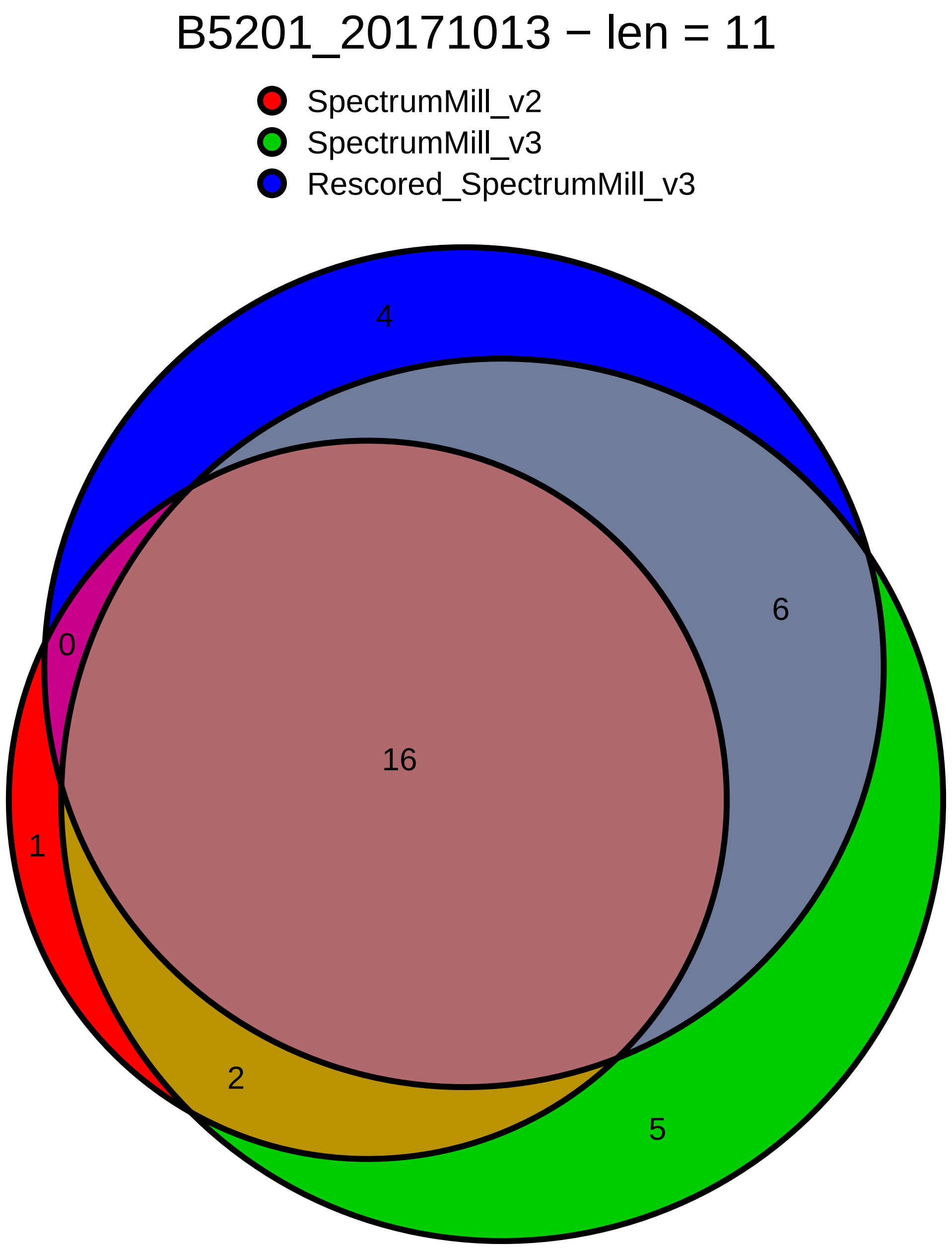
B5201_20171013 − len = 11
SpectrumMill_v2
SpectrumMill_v3
Rescored_SpectrumMill_v3
4
6
0
16
1
2
5

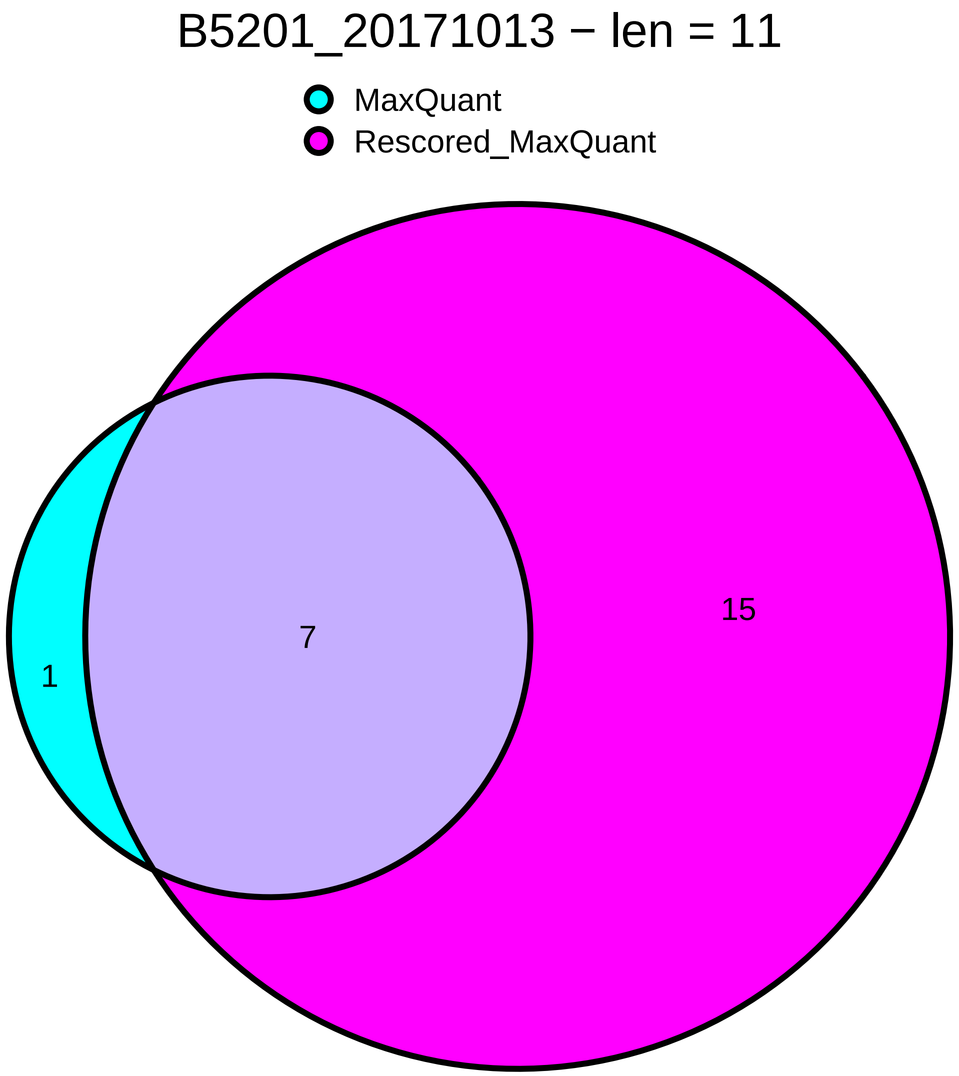
B5201_20171013 − len = 11
MaxQuant
Rescored_MaxQuant
1
7
15

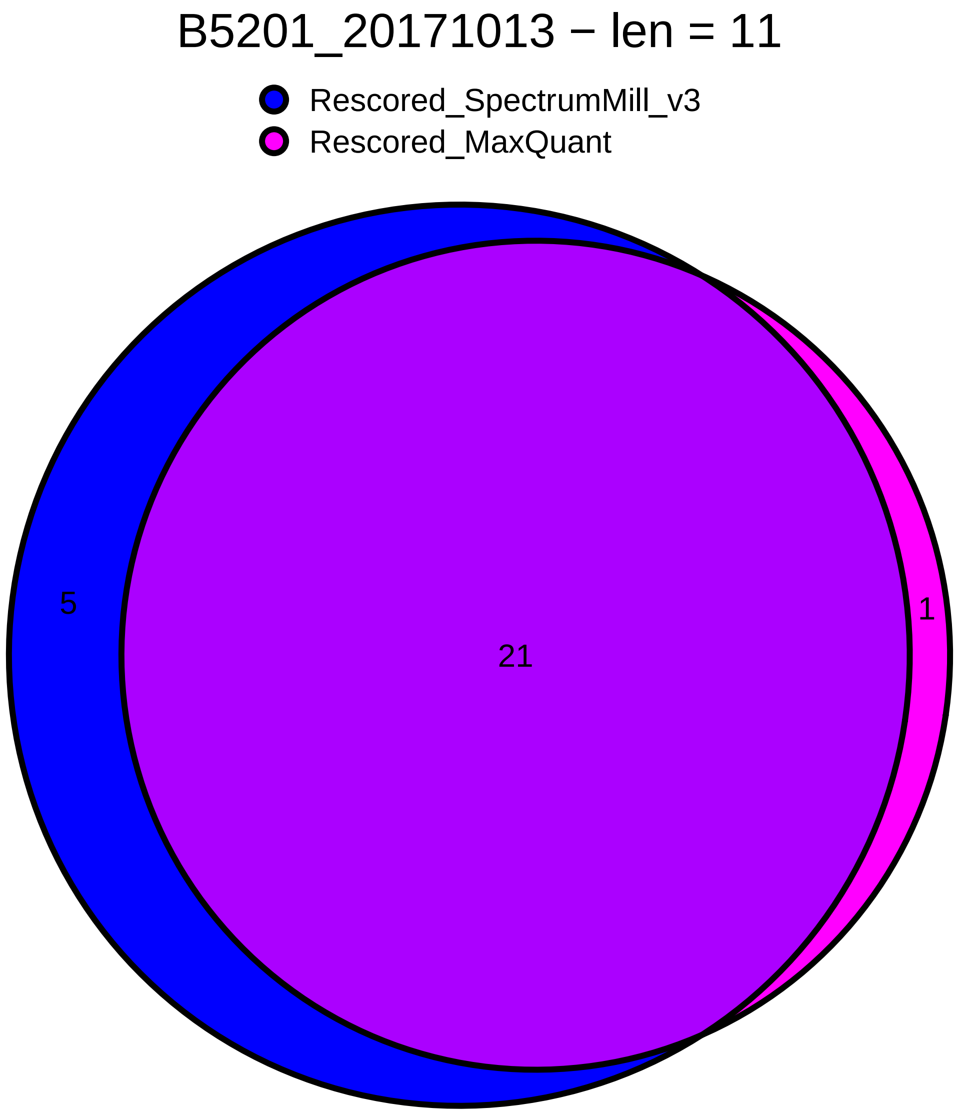
B5201_20171013 − len = 11
Rescored_SpectrumMill_v3
Rescored_MaxQuant
5
21
1

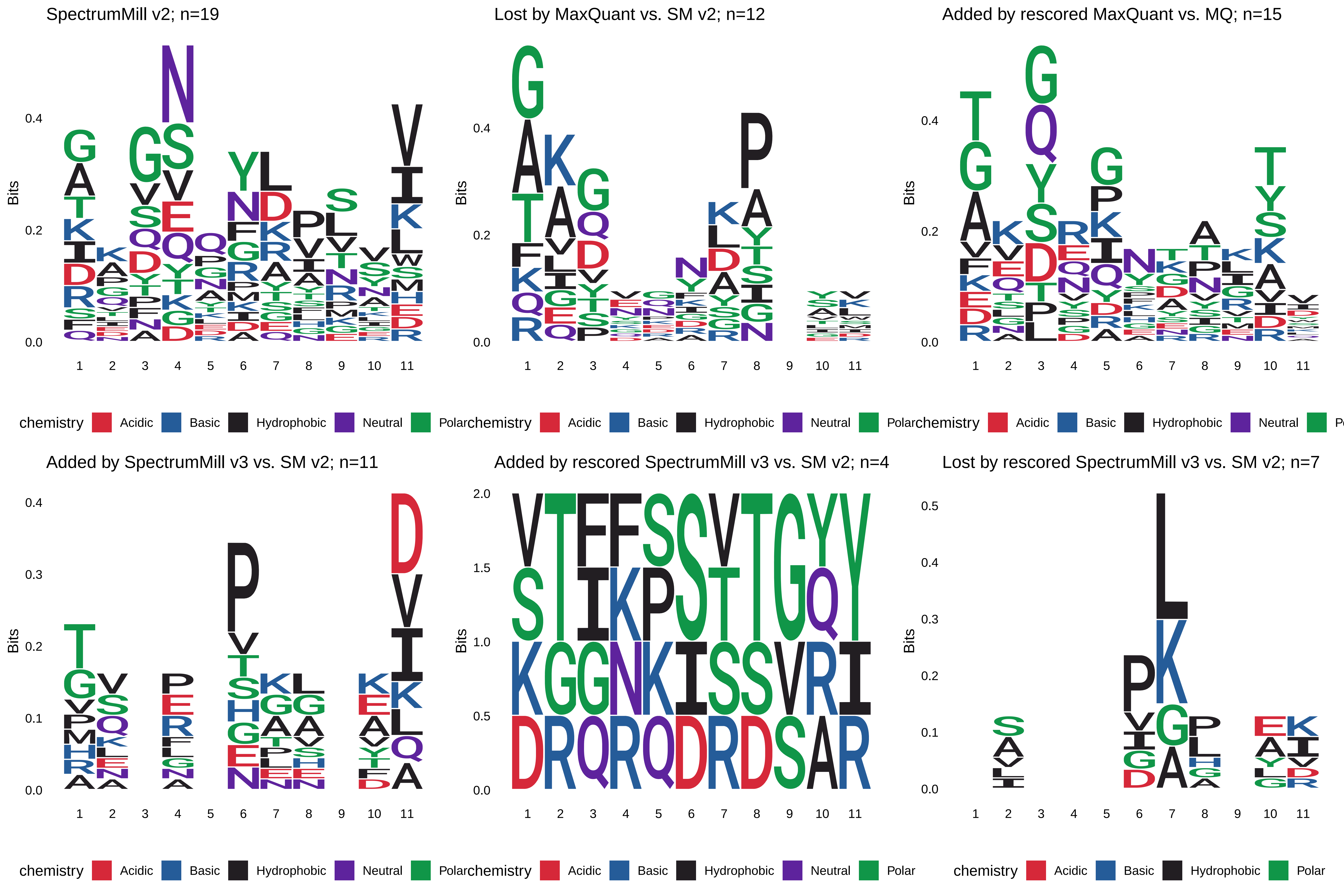
SpectrumMill v2; n=19
Lost by MaxQuant vs. SM v2; n=12
Added by rescored MaxQuant vs. MQ; n=15
Added by SpectrumMill v3 vs. SM v2; n=11
Added by rescored SpectrumMill v3 vs. SM v2; n=4
Lost by rescored SpectrumMill v3 vs. SM v2; n=7
Bits
chemistry Acidic Basic Hydrophobic Neutral Polarchemistry Acidic Basic Hydrophobic Neutral Polarchemistry Acidic Basic Hydrophobic Neutral
chemistry Acidic Basic Hydrophobic Neutral Polarchemistry Acidic Basic Hydrophobic Neutral Polar
chemistry Acidic Basic Hydrophobic Polar

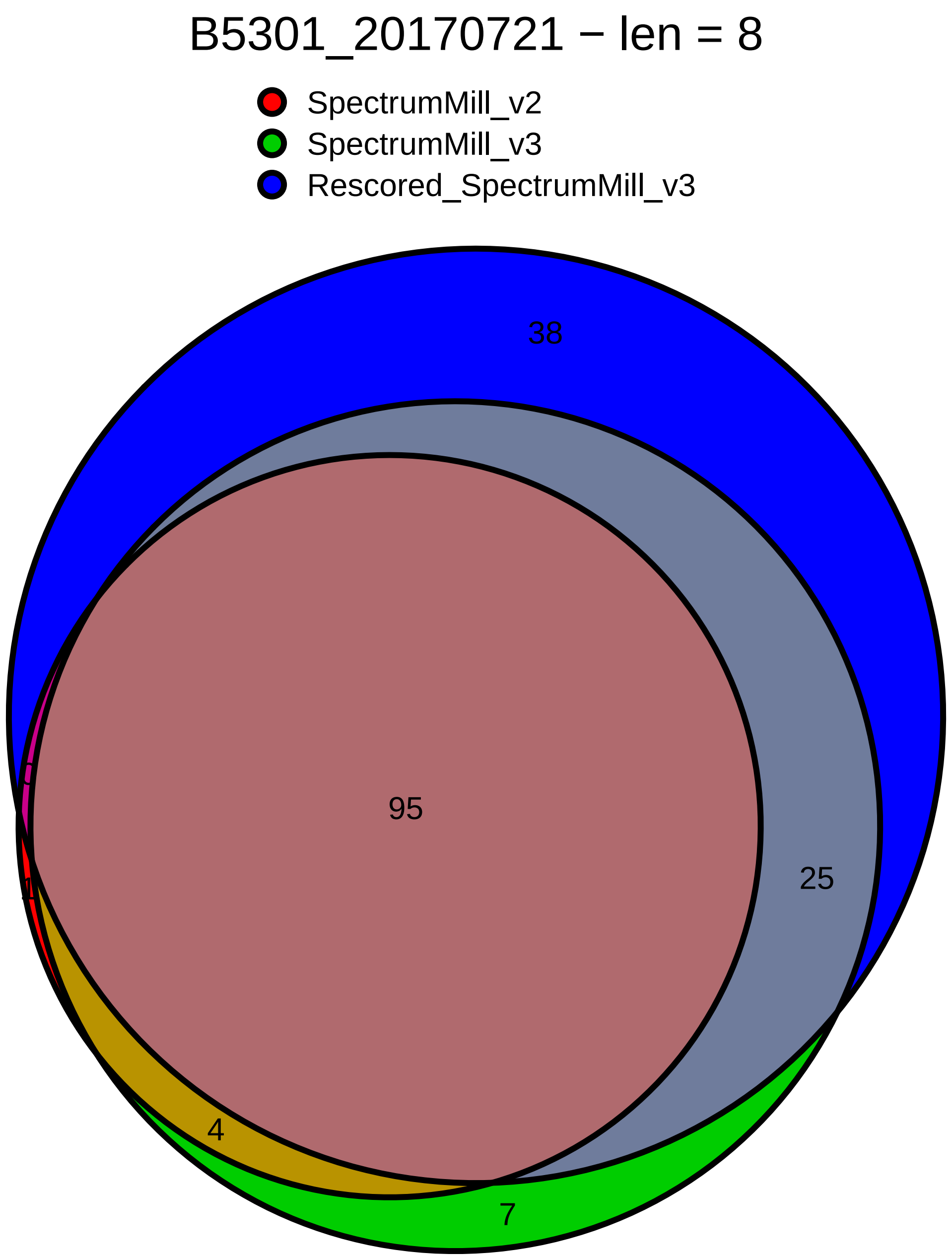
B5301_20170721 − len = 8
SpectrumMill_v2
SpectrumMill_v3
Rescored_SpectrumMill_v3
38
95
25
4
7

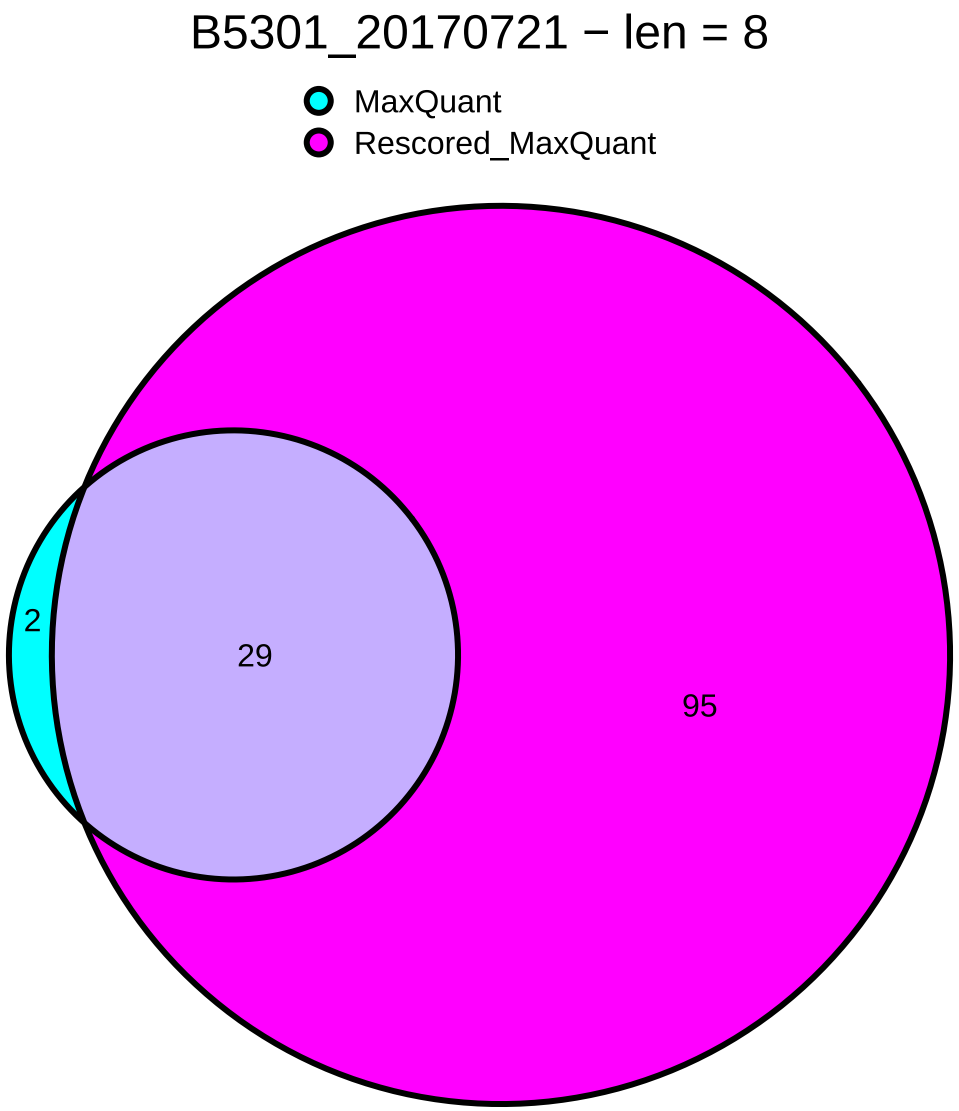
B5301_20170721 − len = 8
MaxQuant
Rescored_MaxQuant
2
29
95

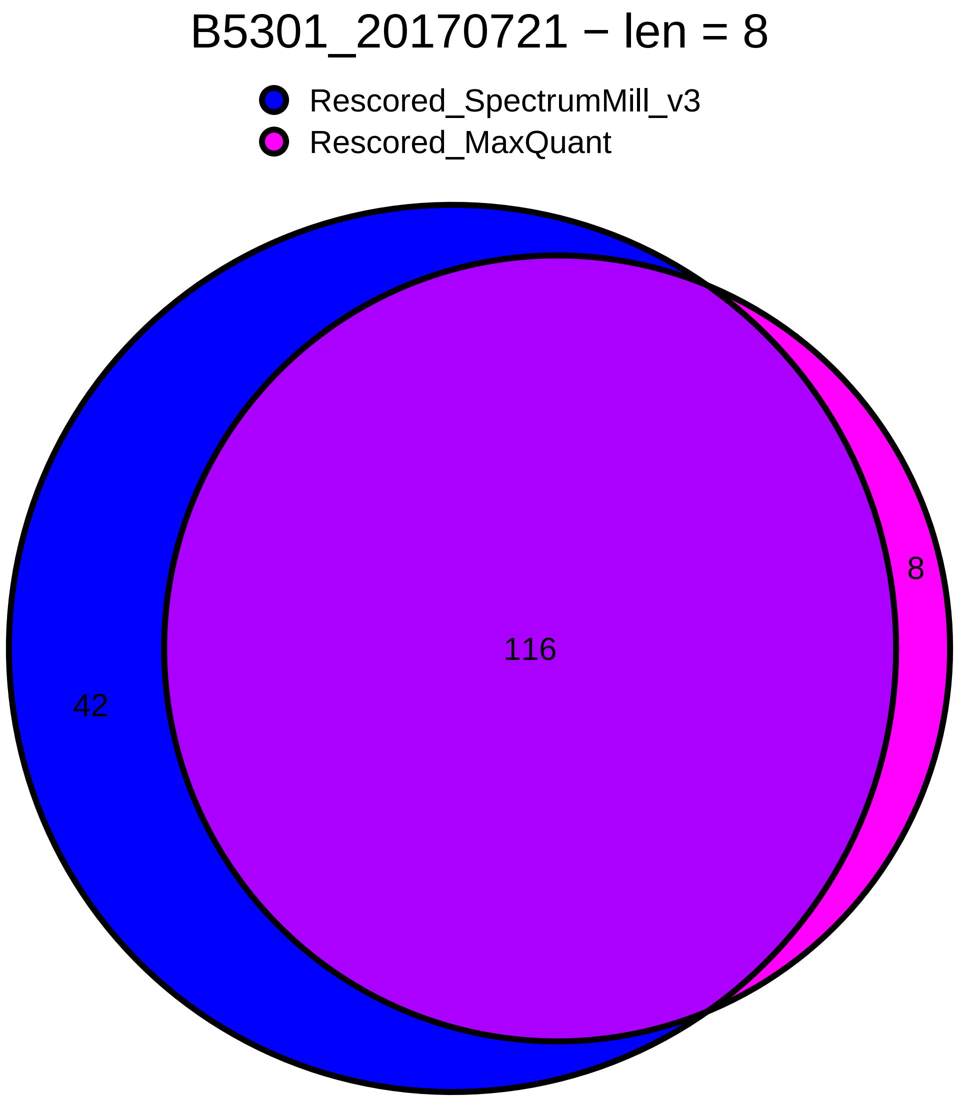
B5301_20170721 − len = 8
Rescored_SpectrumMill_v3
Rescored_MaxQuant
42
116
8

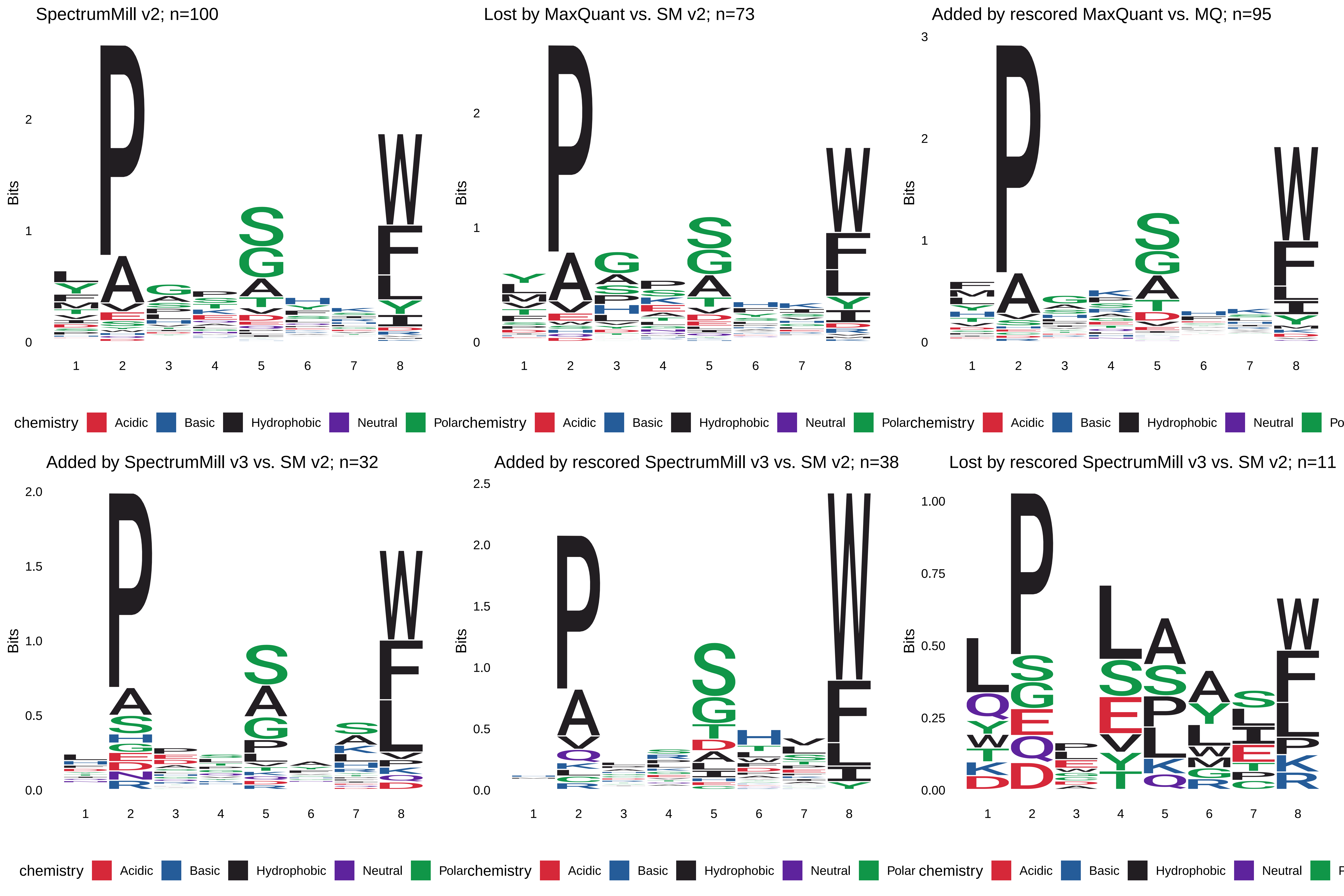
SpectrumMill v2; n=100
Lost by MaxQuant vs. SM v2; n=73
Added by rescored MaxQuant vs. MQ; n=95
Added by SpectrumMill v3 vs. SM v2; n=32
Added by rescored SpectrumMill v3 vs. SM v2; n=38
Lost by rescored SpectrumMill v3 vs. SM v2; n=11
Bits
1 2 3 4 5 6 7 8
chemistry
Acidic
Basic
Hydrophobic
Neutral
Polar

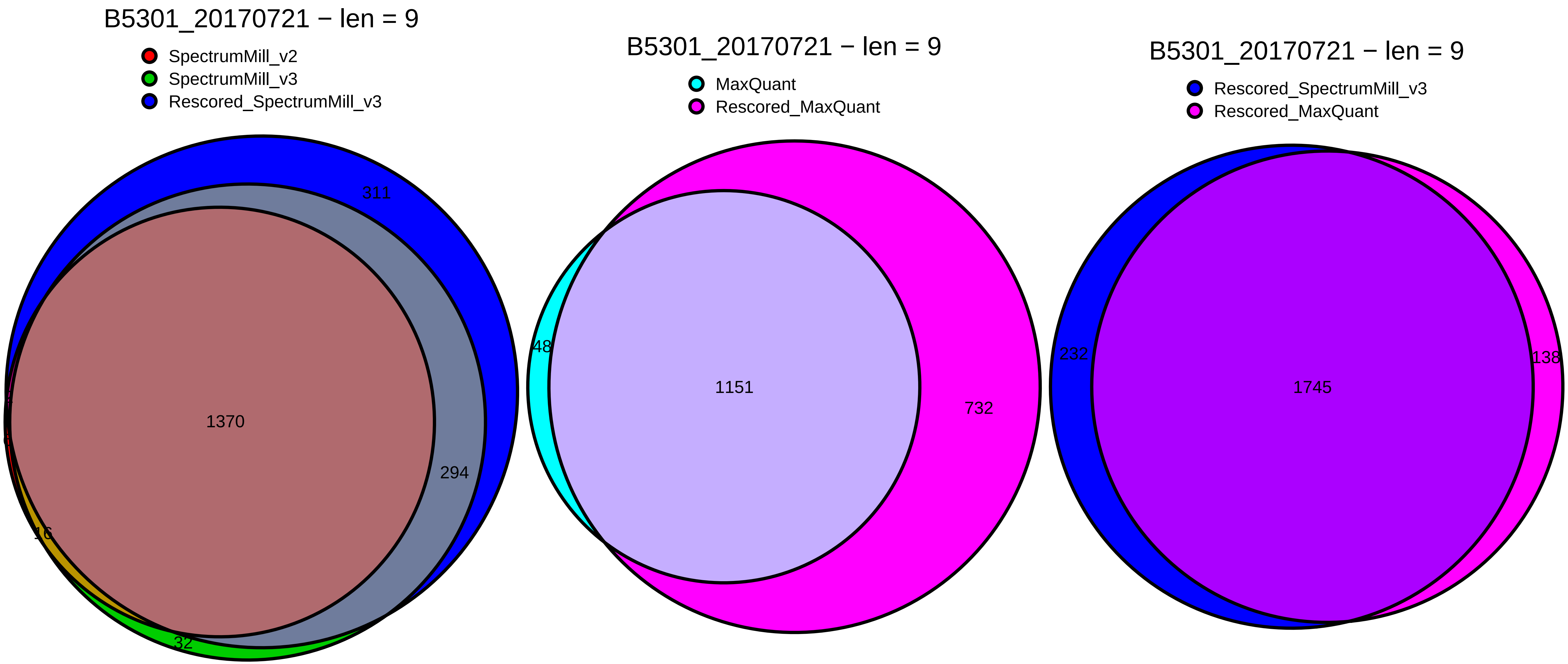

B5301_20170721 − len = 9
SpectrumMill_v2
SpectrumMill_v3
Rescored_SpectrumMill_v3
311
1370
294
16
32
B5301_20170721 − len = 9
MaxQuant
Rescored_MaxQuant
48
1151
732
B5301_20170721 − len = 9
Rescored_SpectrumMill_v3
Rescored_MaxQuant
232
1745
138

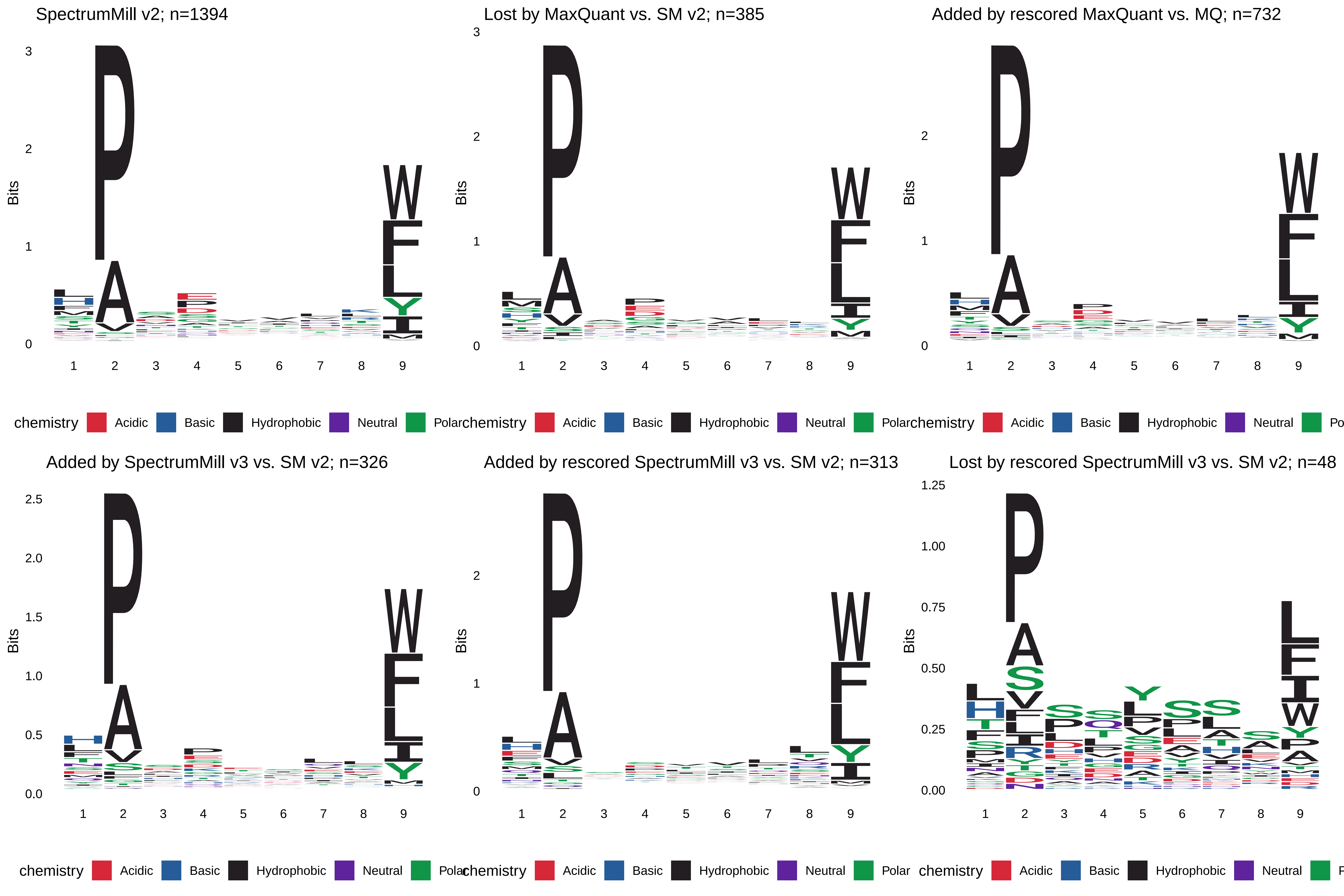
SpectrumMill v2; n=1394
Lost by MaxQuant vs. SM v2; n=385
Added by rescored MaxQuant vs. MQ; n=732
Added by SpectrumMill v3 vs. SM v2; n=326
Added by rescored SpectrumMill v3 vs. SM v2; n=313
Lost by rescored SpectrumMill v3 vs. SM v2; n=48
Bits
chemistry
Acidic
Basic
Hydrophobic
Neutral
Polar

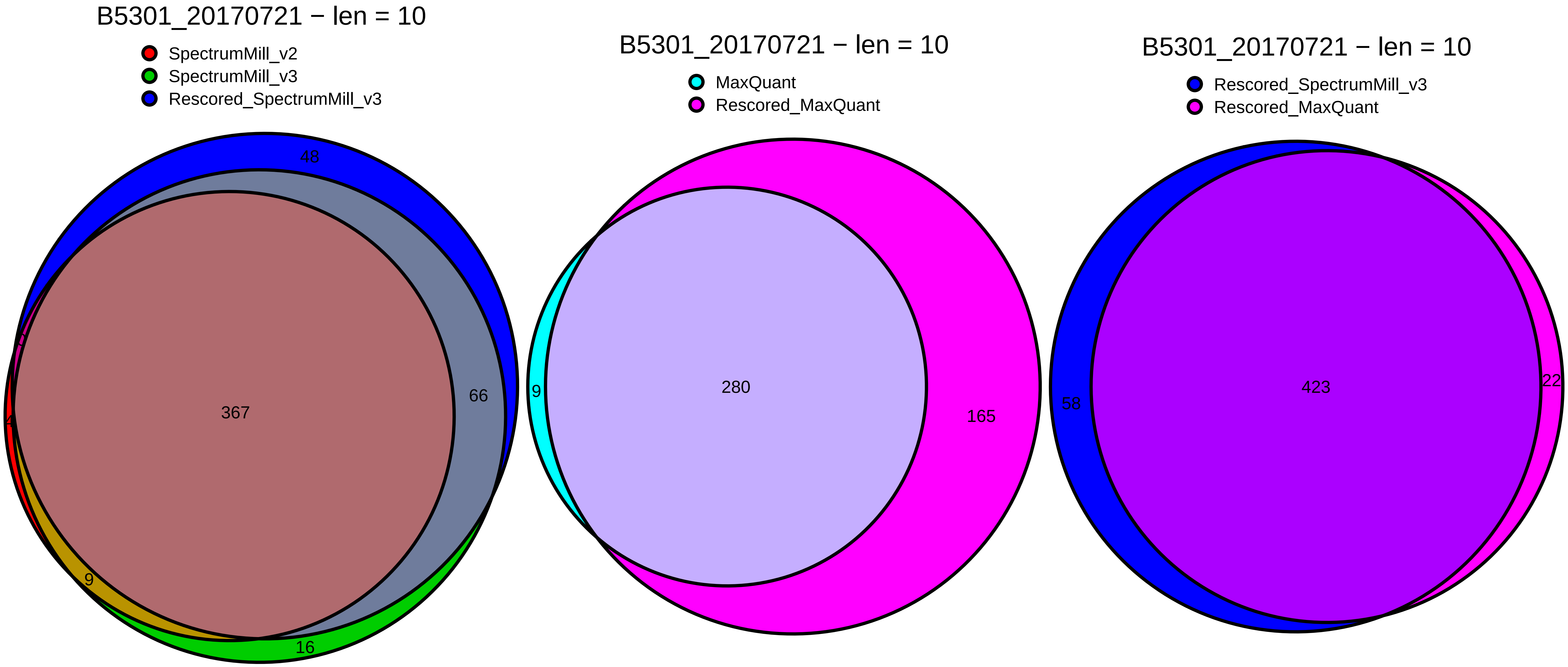
B5301_20170721 − len = 10
SpectrumMill_v2
SpectrumMill_v3
Rescored_SpectrumMill_v3
48
66
367
9
16
B5301_20170721 − len = 10
MaxQuant
Rescored_MaxQuant
9
280
165
B5301_20170721 − len = 10
Rescored_SpectrumMill_v3
Rescored_MaxQuant
58
423
22

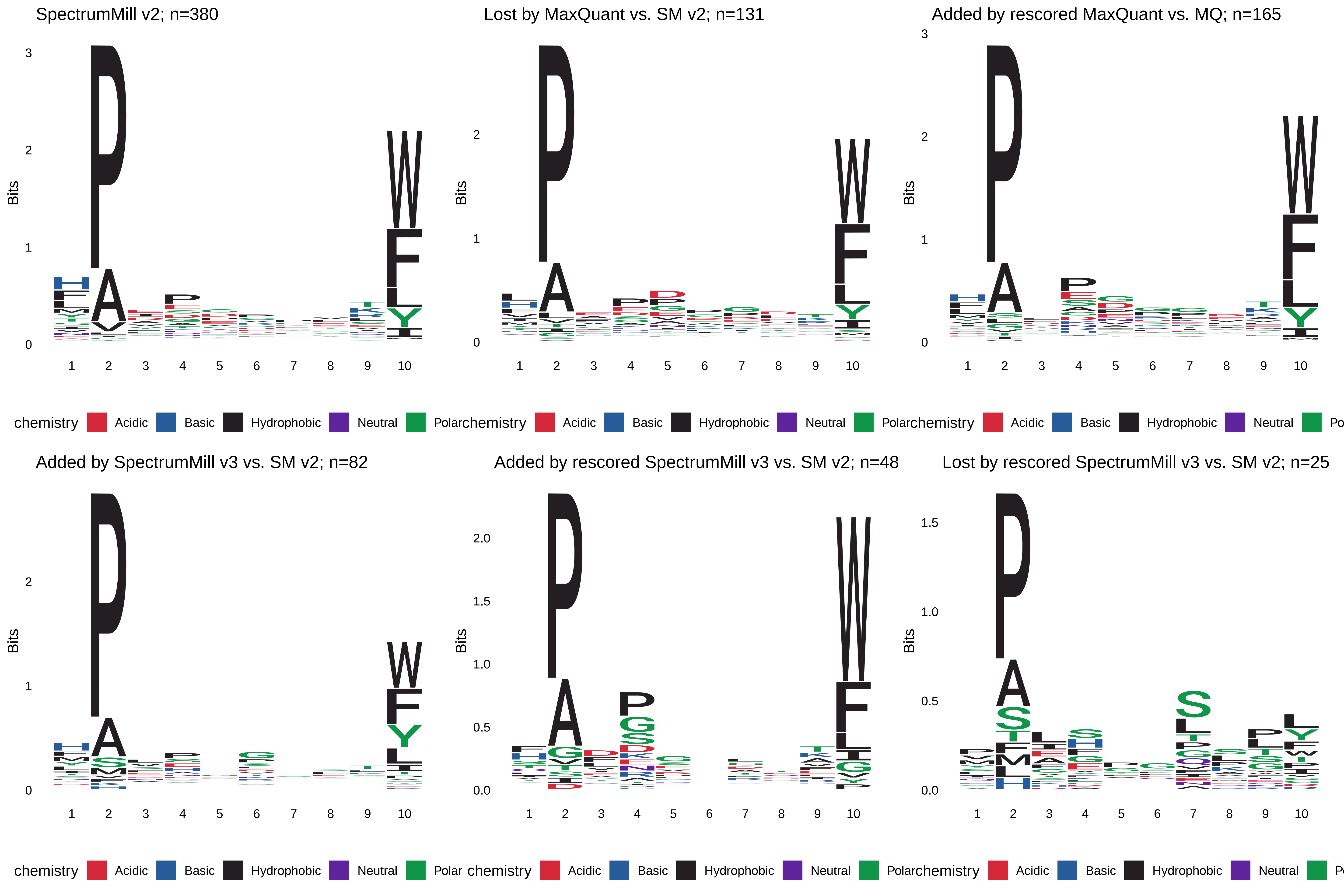
SpectrumMill v2; n=380
Lost by MaxQuant vs. SM v2; n=131
Added by rescored MaxQuant vs. MQ; n=165
Added by SpectrumMill v3 vs. SM v2; n=82
Added by rescored SpectrumMill v3 vs. SM v2; n=48
Lost by rescored SpectrumMill v3 vs. SM v2; n=25
Bits
chemistry
Acidic
Basic
Hydrophobic
Neutral
Polar

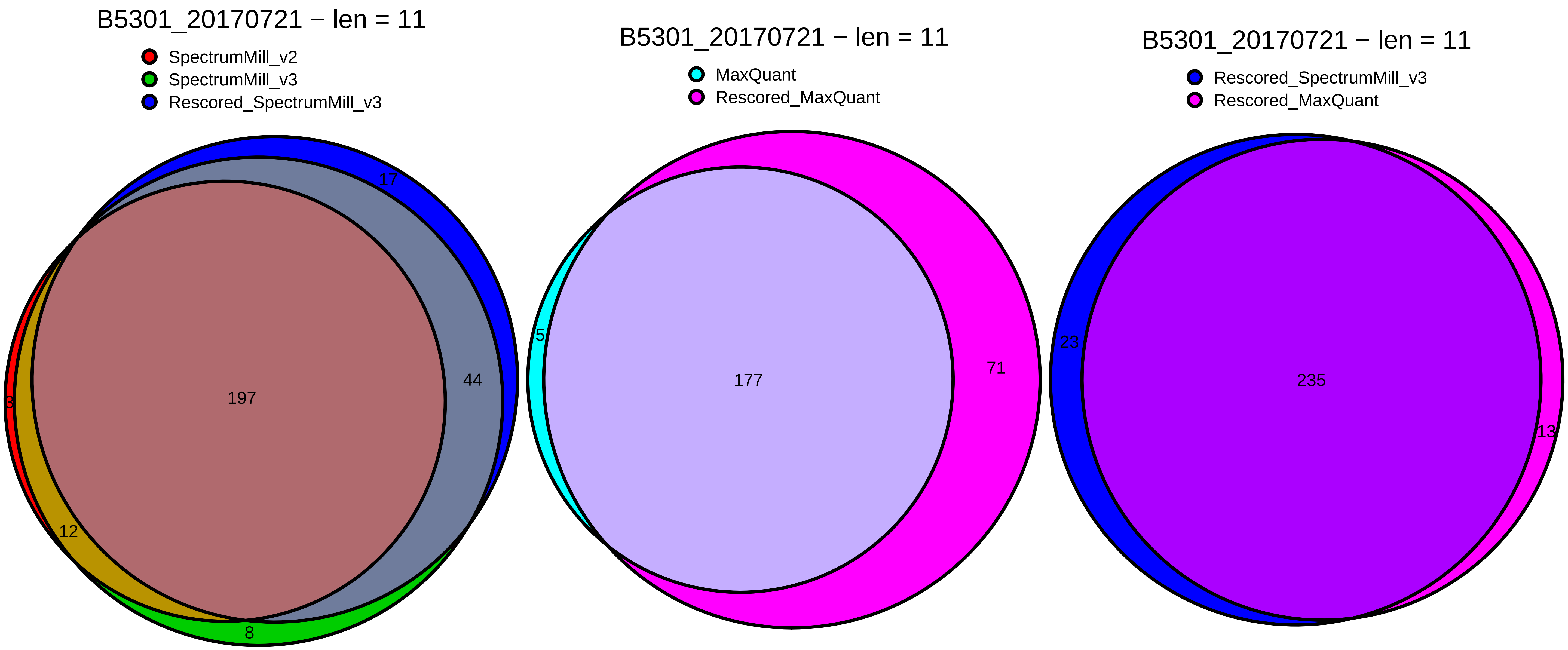
B5301_20170721 − len = 11
SpectrumMill_v2
SpectrumMill_v3
Rescored_SpectrumMill_v3
17
44
197
3
12
8
B5301_20170721 − len = 11
MaxQuant
Rescored_MaxQuant
5
177
71
B5301_20170721 − len = 11
Rescored_SpectrumMill_v3
Rescored_MaxQuant
23
235
13

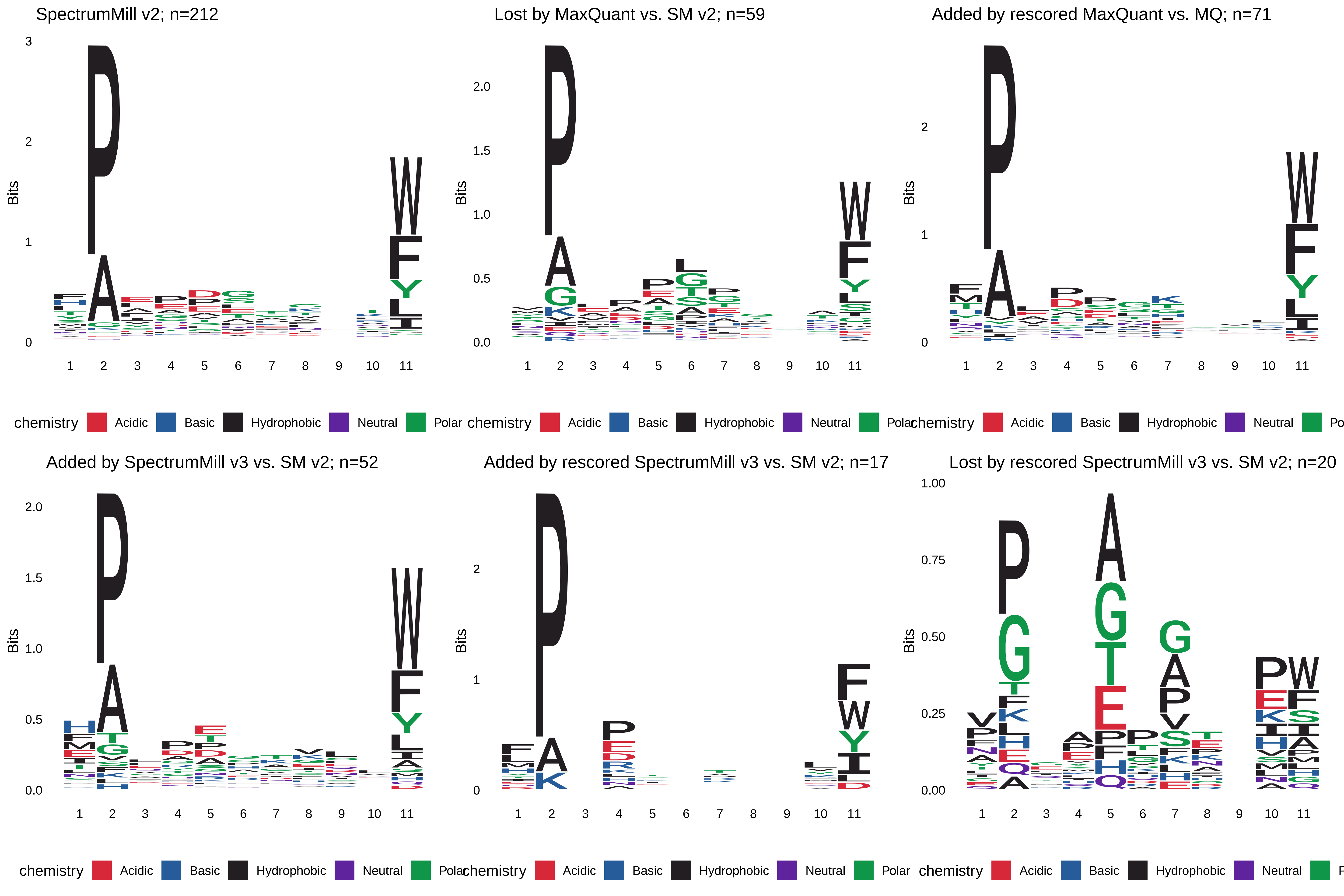
SpectrumMill v2; n=212
Lost by MaxQuant vs. SM v2; n=59
Added by rescored MaxQuant vs. MQ; n=71
Added by SpectrumMill v3 vs. SM v2; n=52
Added by rescored SpectrumMill v3 vs. SM v2; n=17
Lost by rescored SpectrumMill v3 vs. SM v2; n=20
Bits
1 2 3 4 5 6 7 8 9 10 11
chemistry Acidic Basic Hydrophobic Neutral Polar

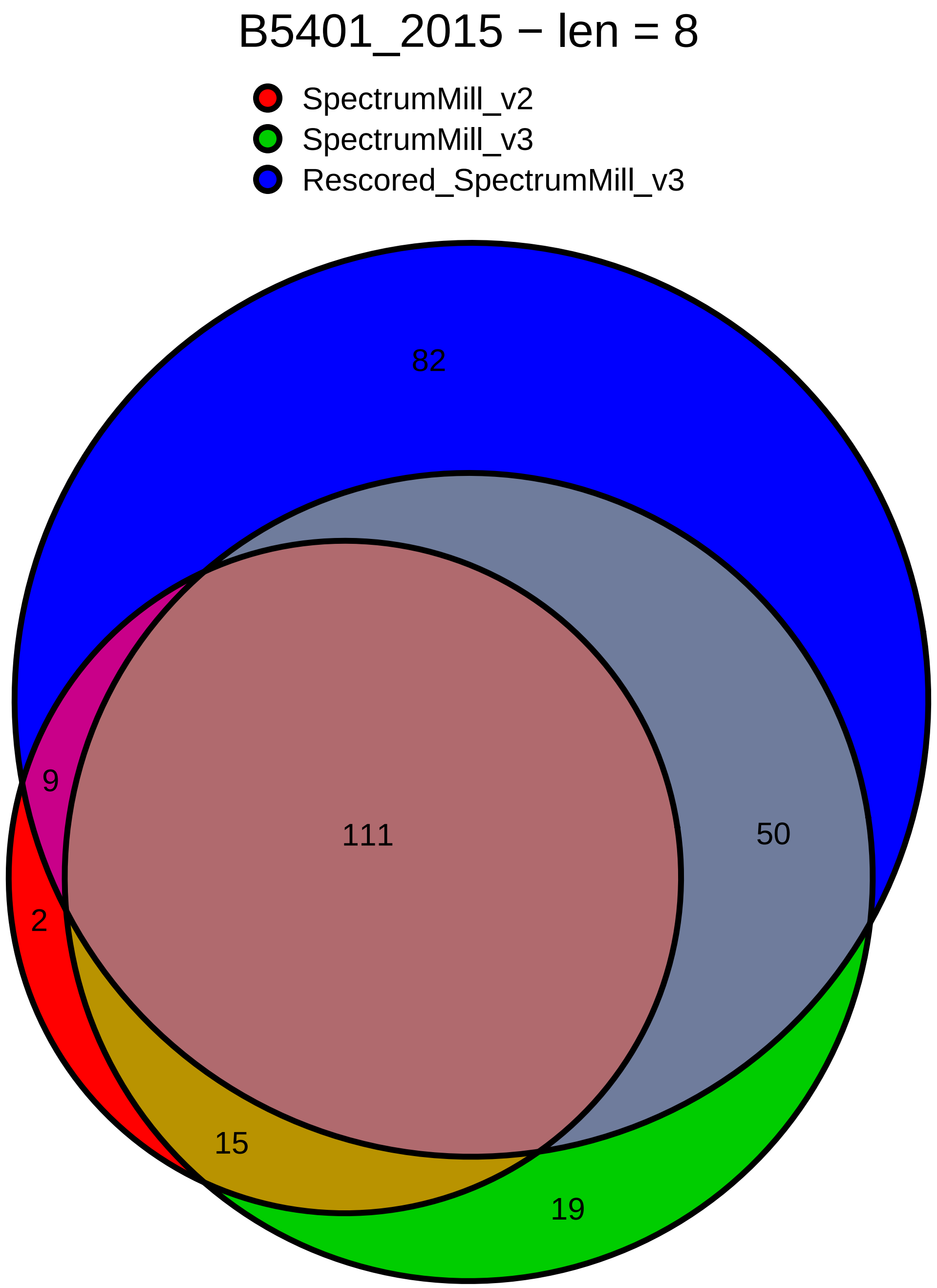
B5401_2015 − len = 8
SpectrumMill_v2
SpectrumMill_v3
Rescored_SpectrumMill_v3
82
9
111
50
2
15
19

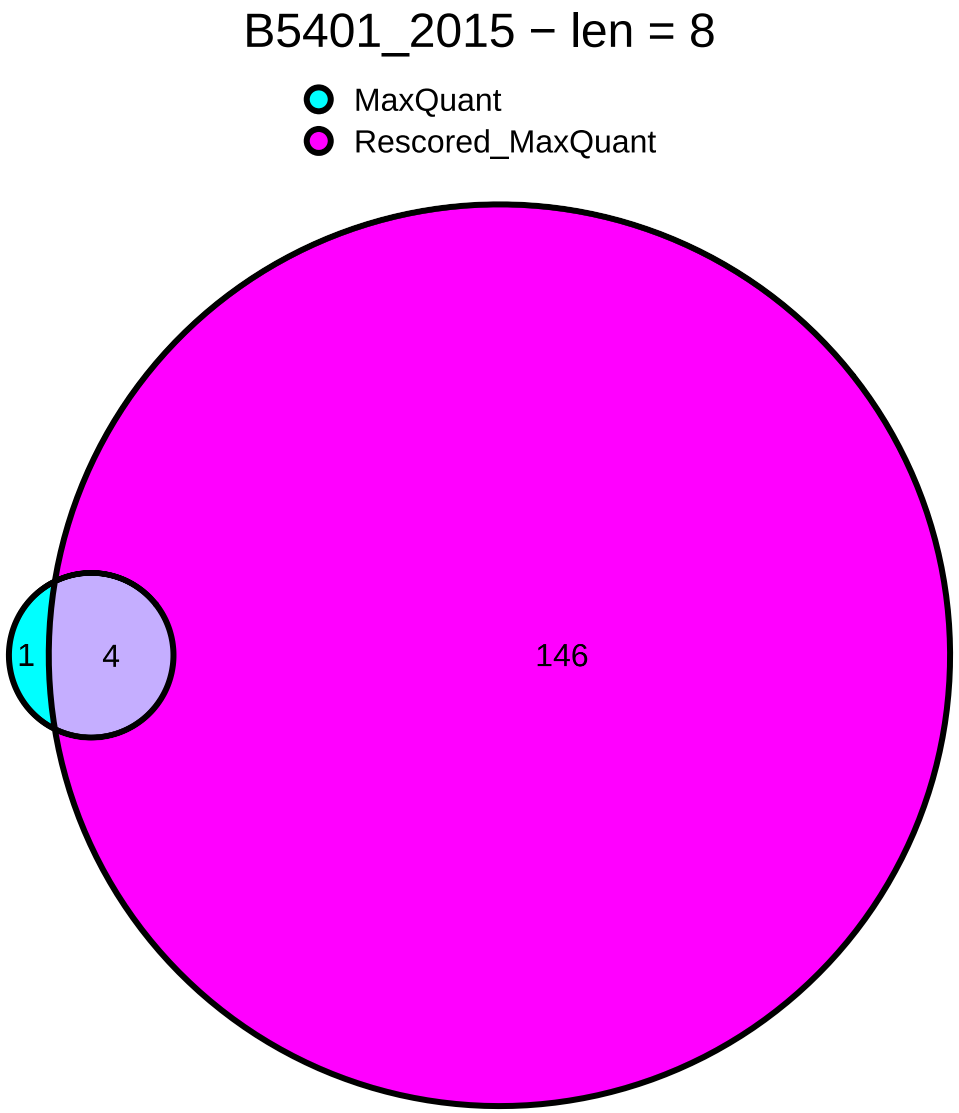
B5401_2015 − len = 8
MaxQuant
Rescored_MaxQuant
1
4
146

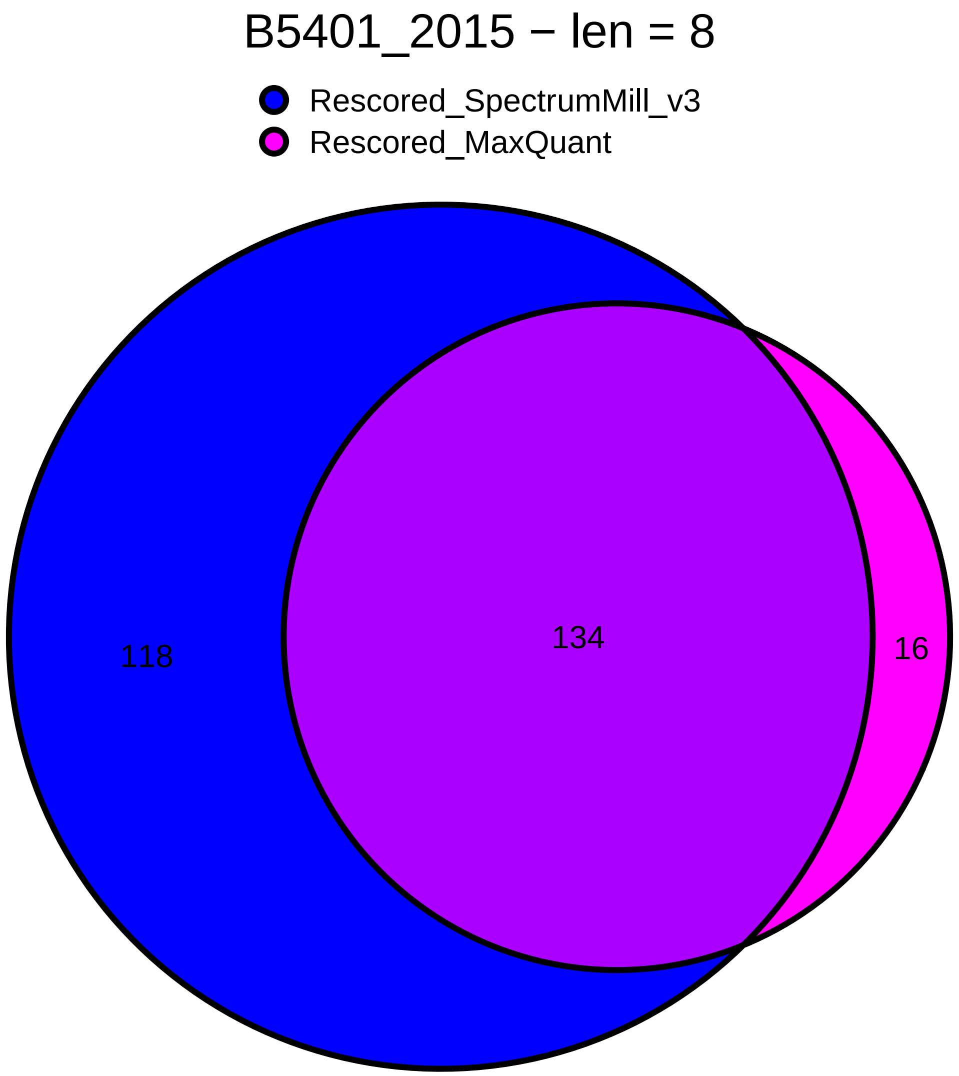
B5401_2015 − len = 8
Rescored_SpectrumMill_v3
Rescored_MaxQuant
118
134
16

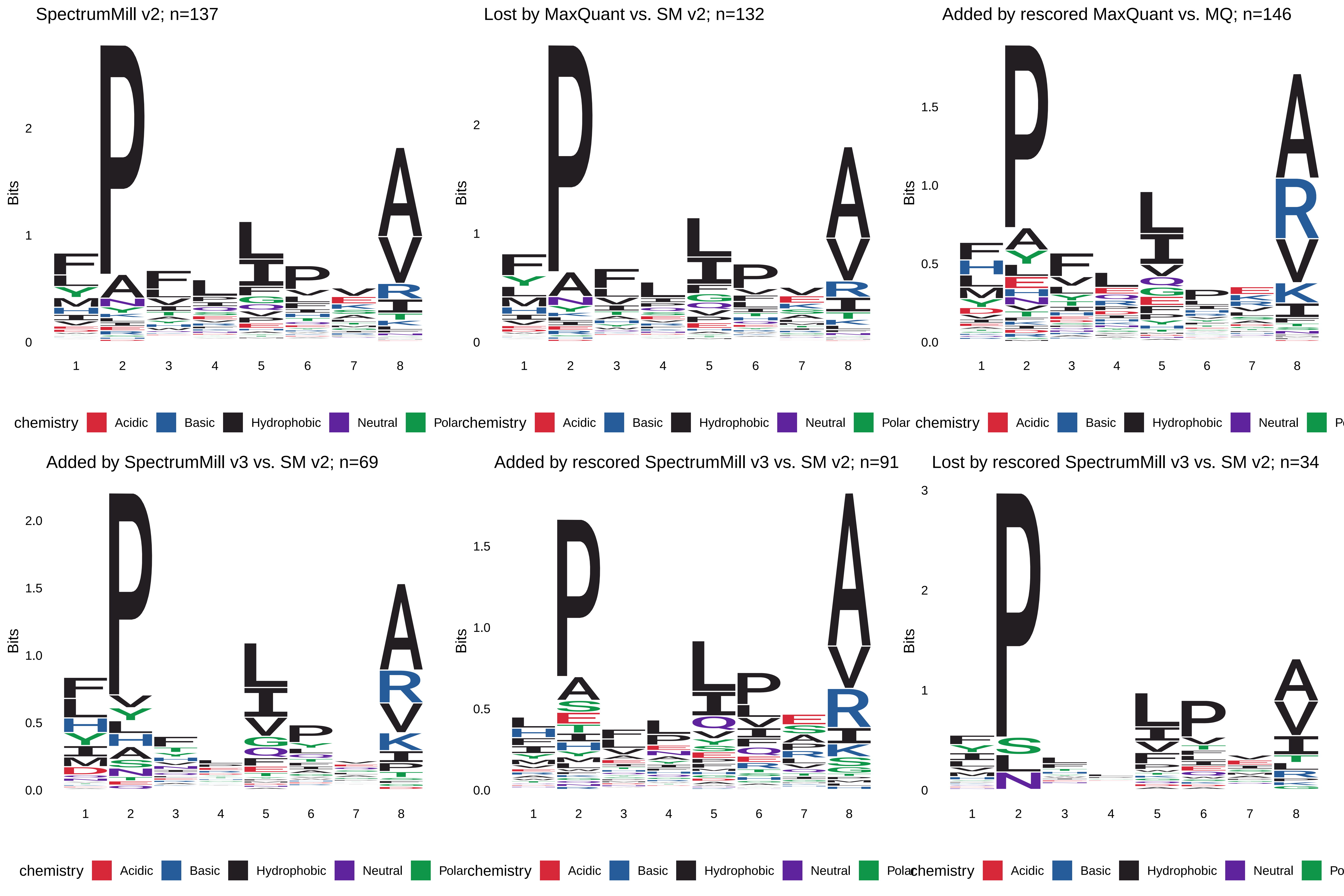
SpectrumMill v2; n=137
Lost by MaxQuant vs. SM v2; n=132
Added by rescored MaxQuant vs. MQ; n=146
Added by SpectrumMill v3 vs. SM v2; n=69
Added by rescored SpectrumMill v3 vs. SM v2; n=91
Lost by rescored SpectrumMill v3 vs. SM v2; n=34
Bits
1 2 3 4 5 6 7 8
chemistry Acidic Basic Hydrophobic Neutral Polar

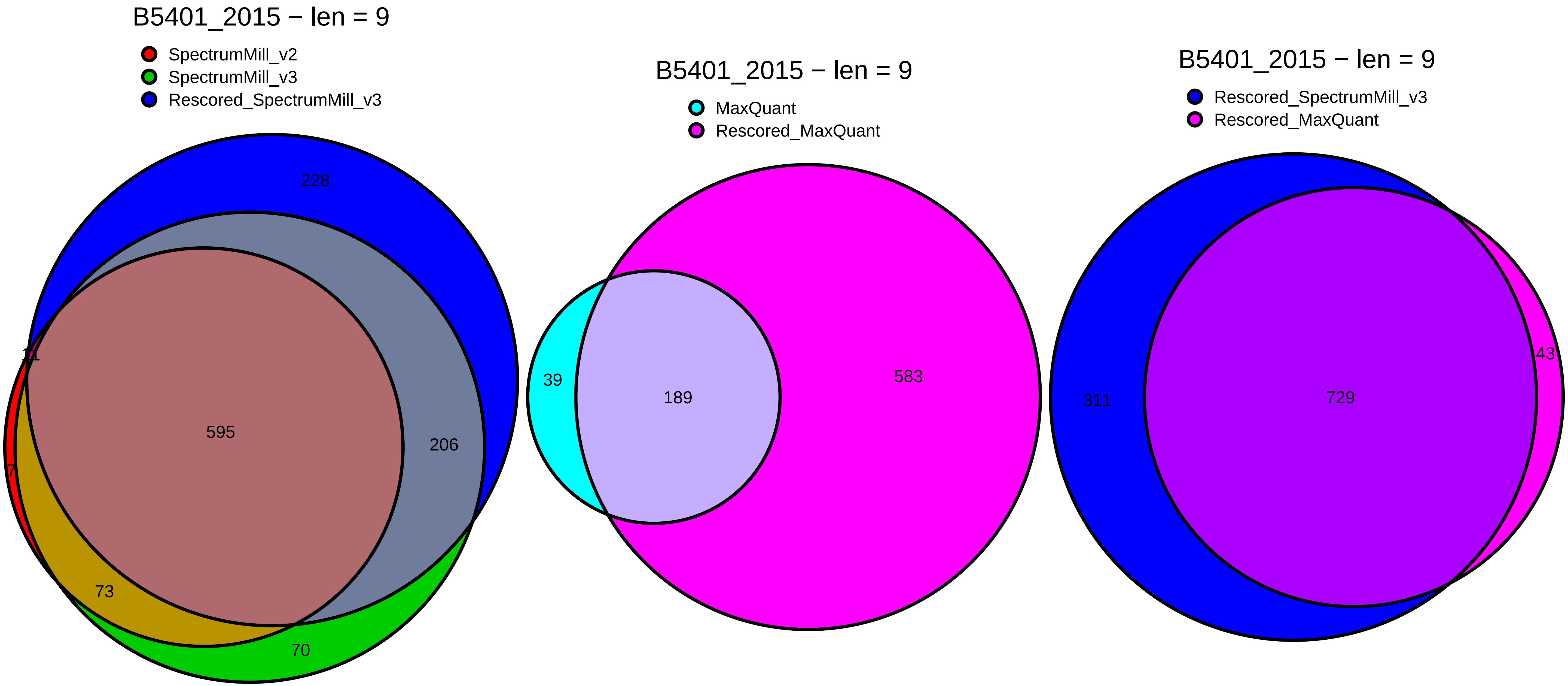
B5401_2015 − len = 9
SpectrumMill_v2
SpectrumMill_v3
Rescored_SpectrumMill_v3
228
11
595
206
7
73
70
B5401_2015 − len = 9
MaxQuant
Rescored_MaxQuant
39
189
583
B5401_2015 − len = 9
Rescored_SpectrumMill_v3
Rescored_MaxQuant
311
729
43

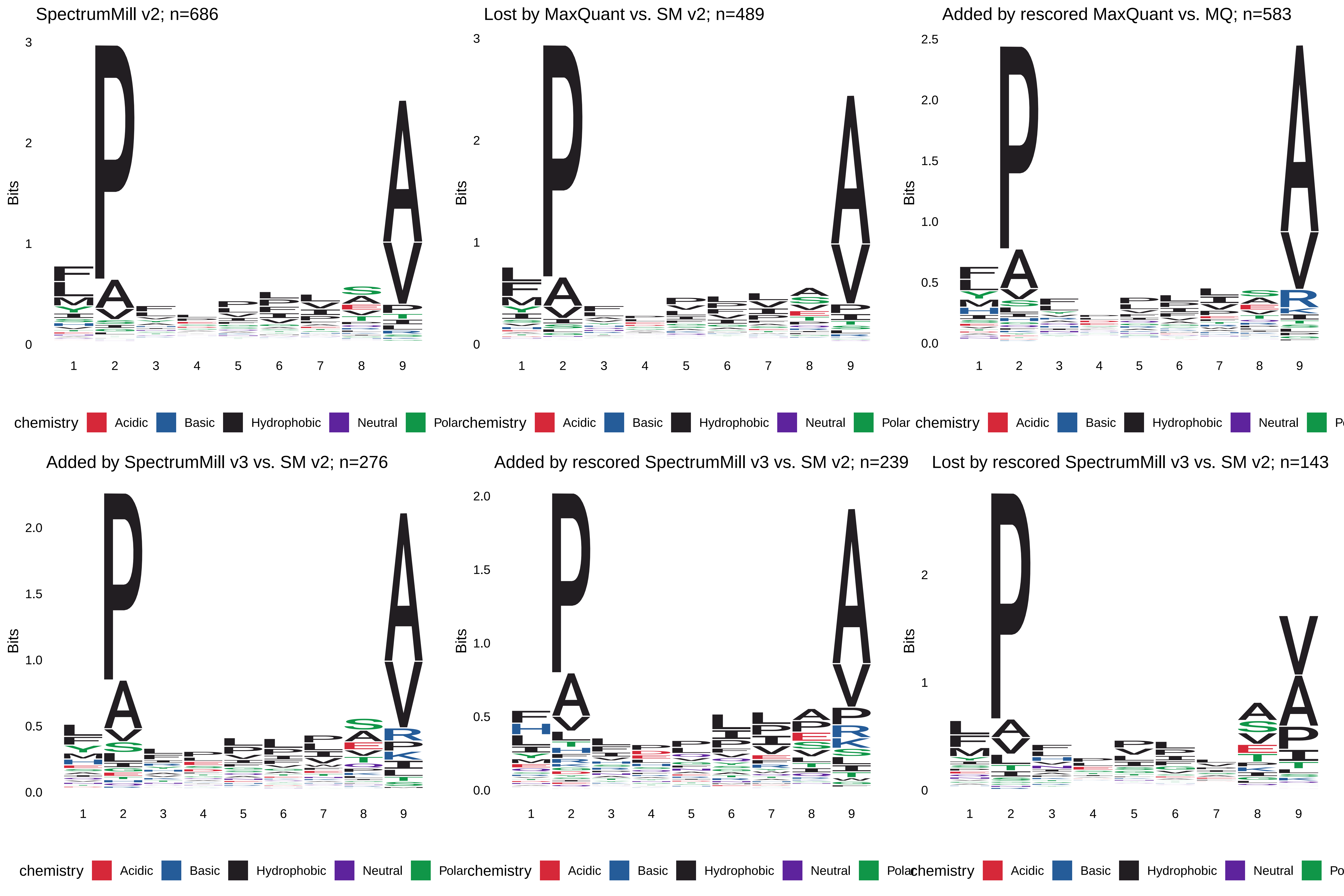
SpectrumMill v2; n=686
Lost by MaxQuant vs. SM v2; n=489
Added by rescored MaxQuant vs. MQ; n=583
Added by SpectrumMill v3 vs. SM v2; n=276
Added by rescored SpectrumMill v3 vs. SM v2; n=239
Lost by rescored SpectrumMill v3 vs. SM v2; n=143
Bits
1 2 3 4 5 6 7 8 9
chemistry
Acidic
Basic
Hydrophobic
Neutral
Polar

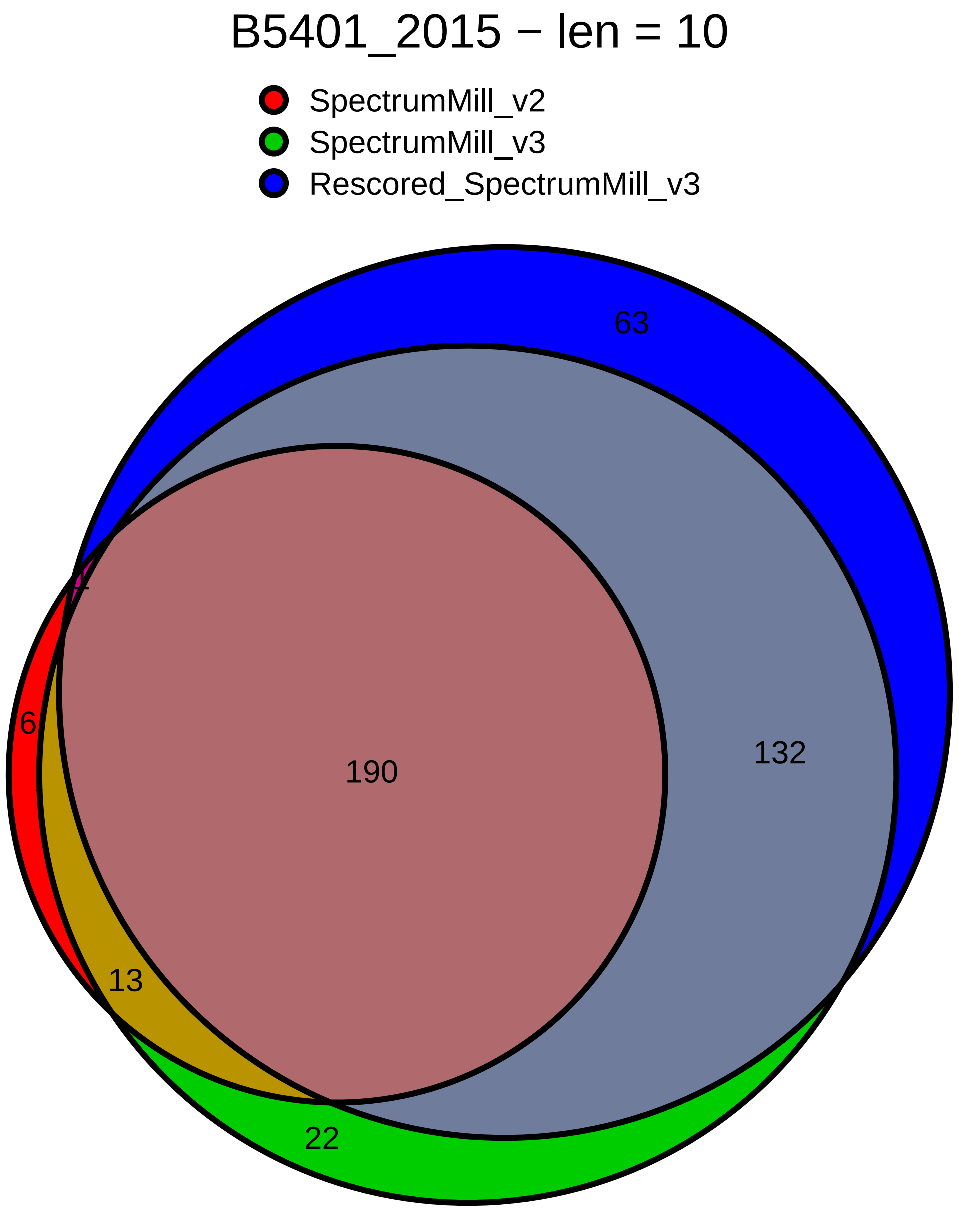
B5401_2015 − len = 10
SpectrumMill_v2
SpectrumMill_v3
Rescored_SpectrumMill_v3
63
6
190
132
13
22

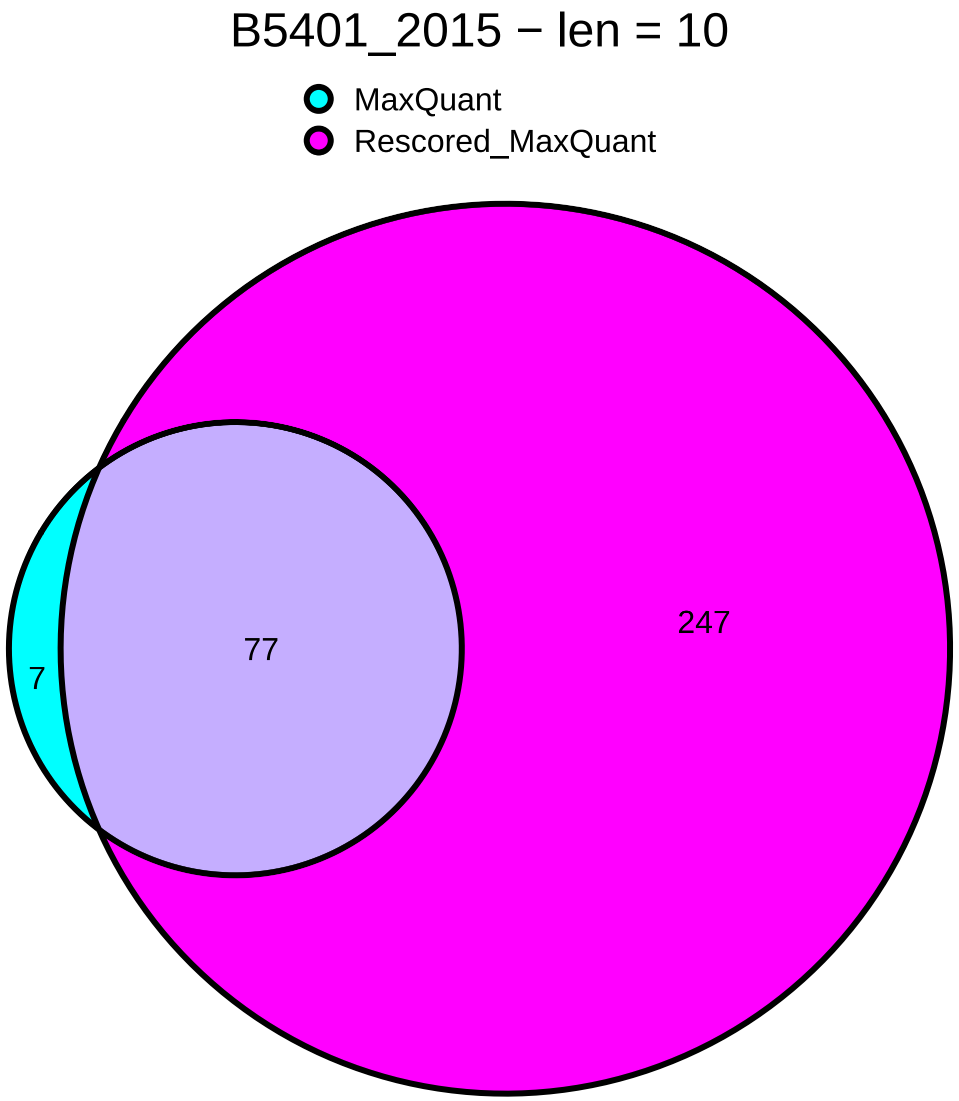
B5401_2015 − len = 10
MaxQuant
Rescored_MaxQuant
7
77
247

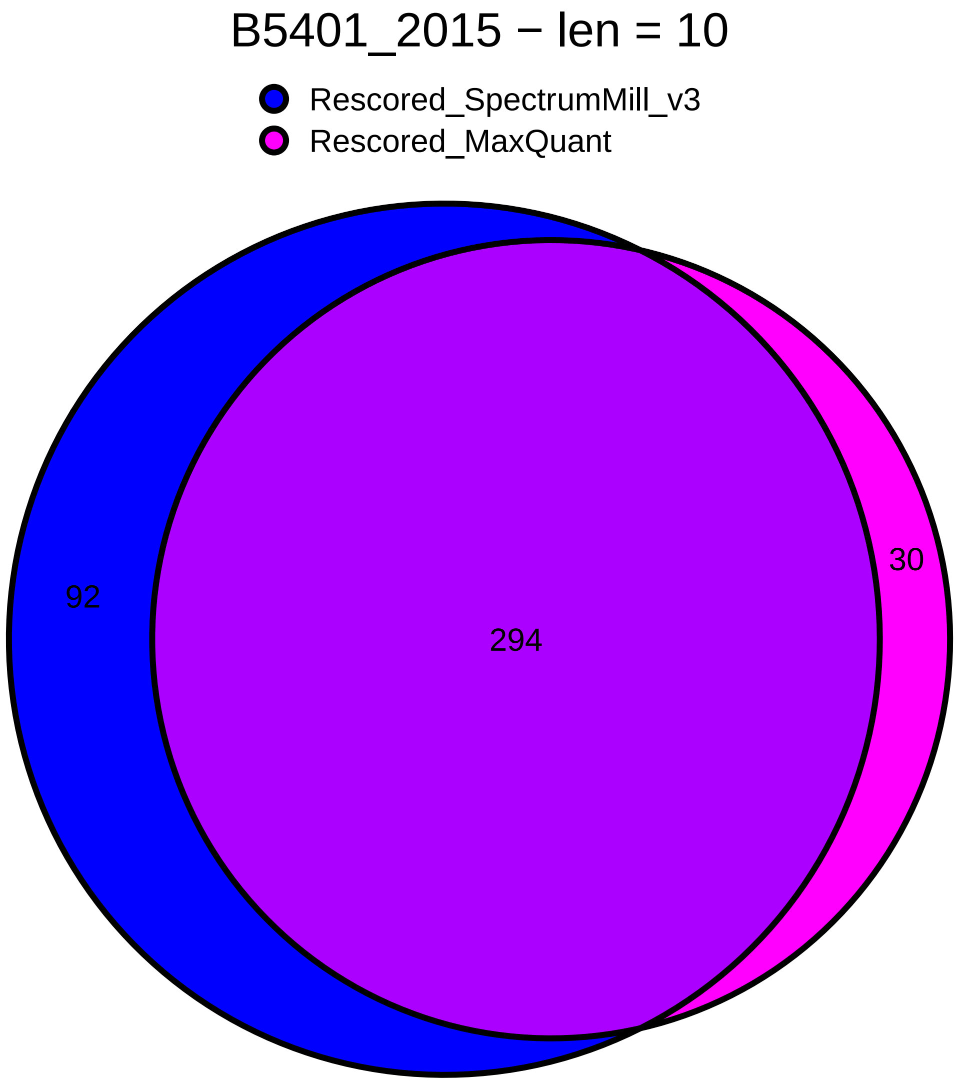
B5401_2015 − len = 10
Rescored_SpectrumMill_v3
Rescored_MaxQuant
92
294
30

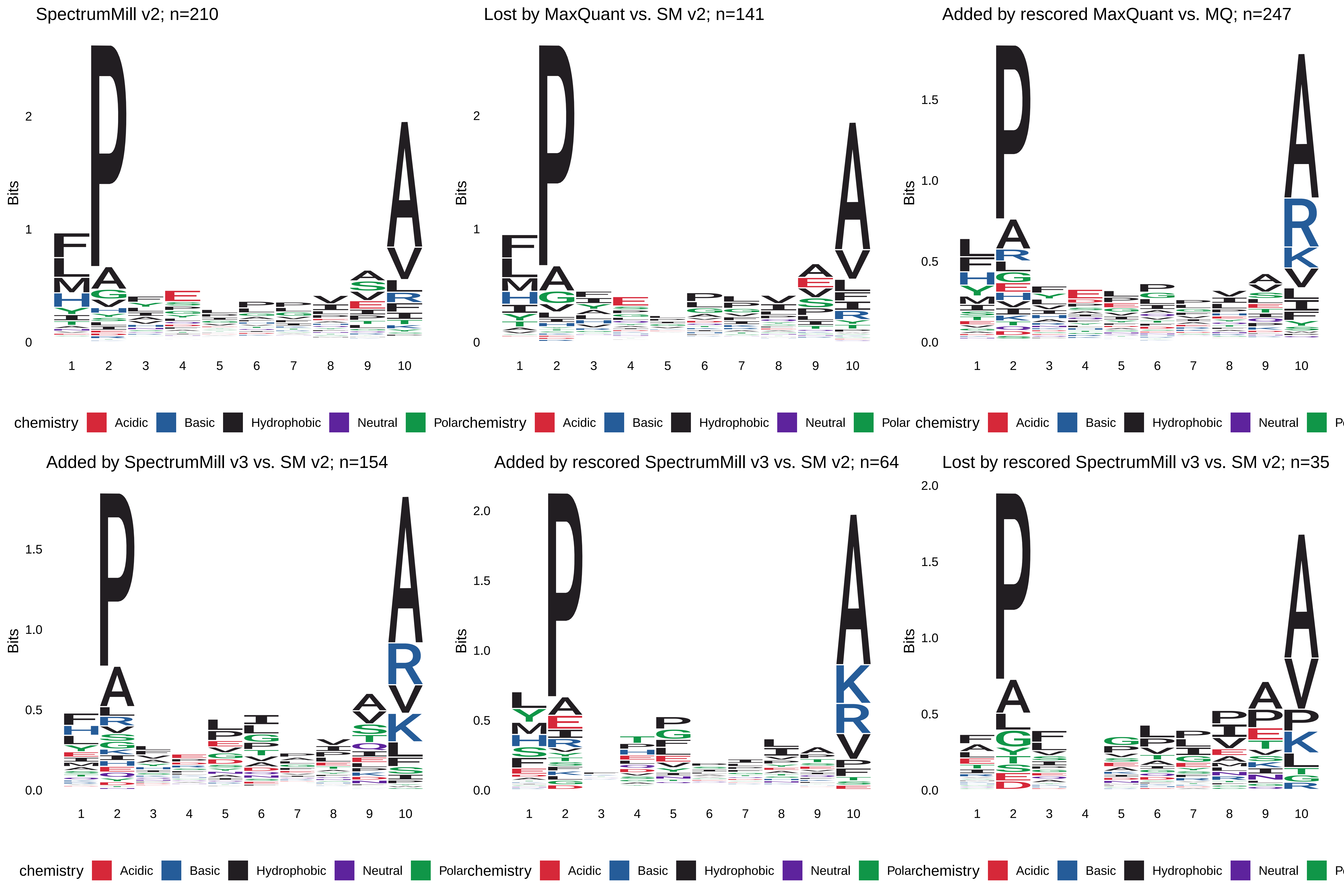
SpectrumMill v2; n=210
Lost by MaxQuant vs. SM v2; n=141
Added by rescored MaxQuant vs. MQ; n=247
Bits
1 2 3 4 5 6 7 8 9 10
chemistry Acidic Basic Hydrophobic Neutral Polar
Added by SpectrumMill v3 vs. SM v2; n=154
Added by rescored SpectrumMill v3 vs. SM v2; n=64
Lost by rescored SpectrumMill v3 vs. SM v2; n=35
Bits
1 2 3 4 5 6 7 8 9 10
chemistry Acidic Basic Hydrophobic Neutral Polar

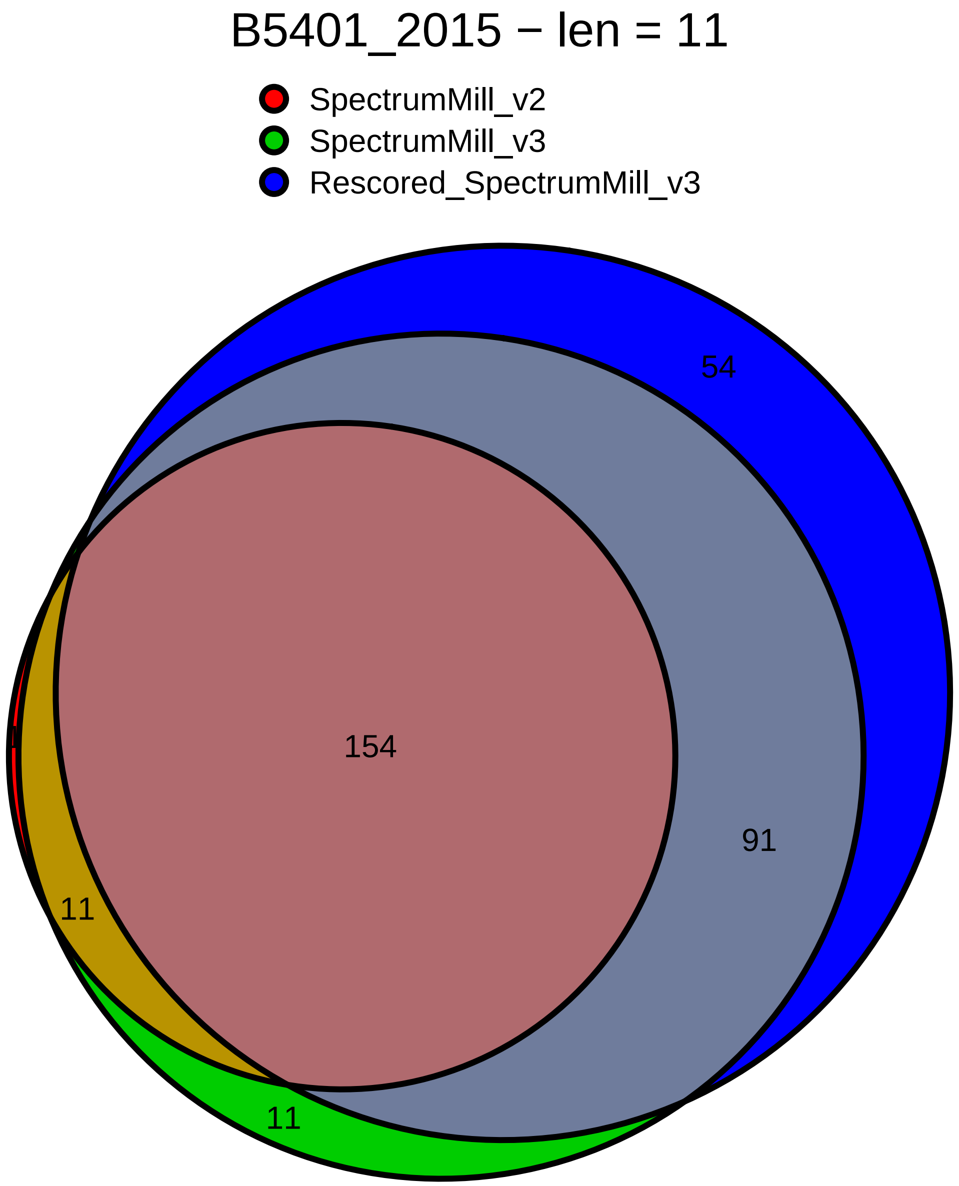
B5401_2015 − len = 11
SpectrumMill_v2
SpectrumMill_v3
Rescored_SpectrumMill_v3
54
154
91
11
11
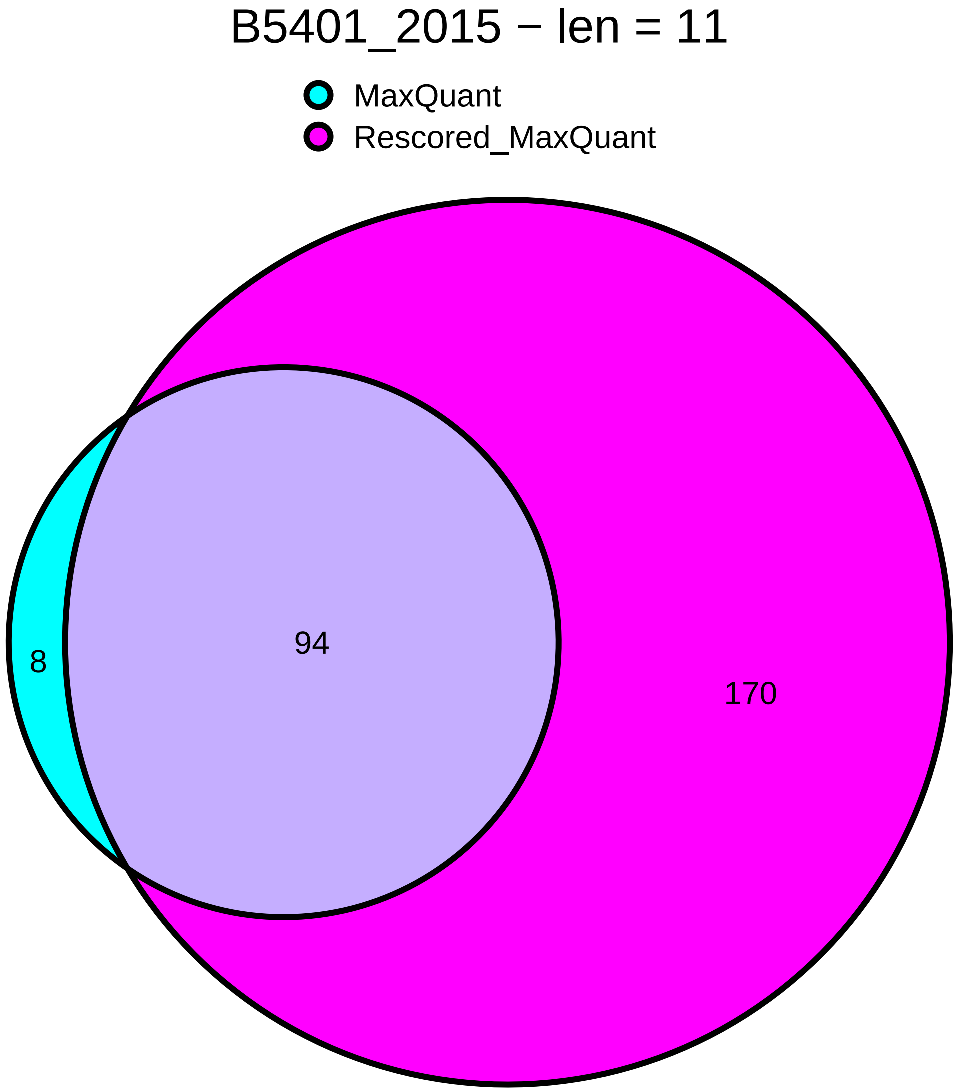
B5401_2015 − len = 11
MaxQuant
Rescored_MaxQuant
8
94
170
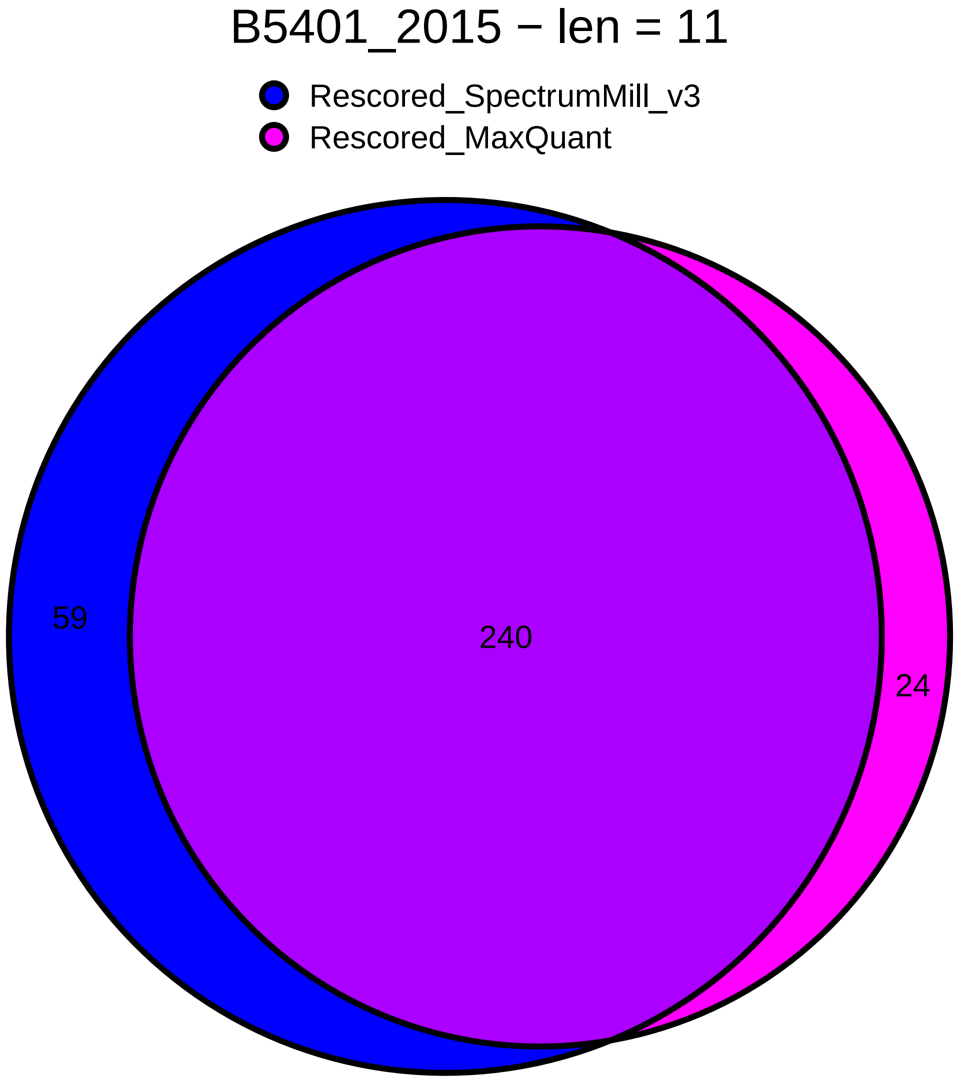
B5401_2015 − len = 11
Rescored_SpectrumMill_v3
Rescored_MaxQuant
59
240
24

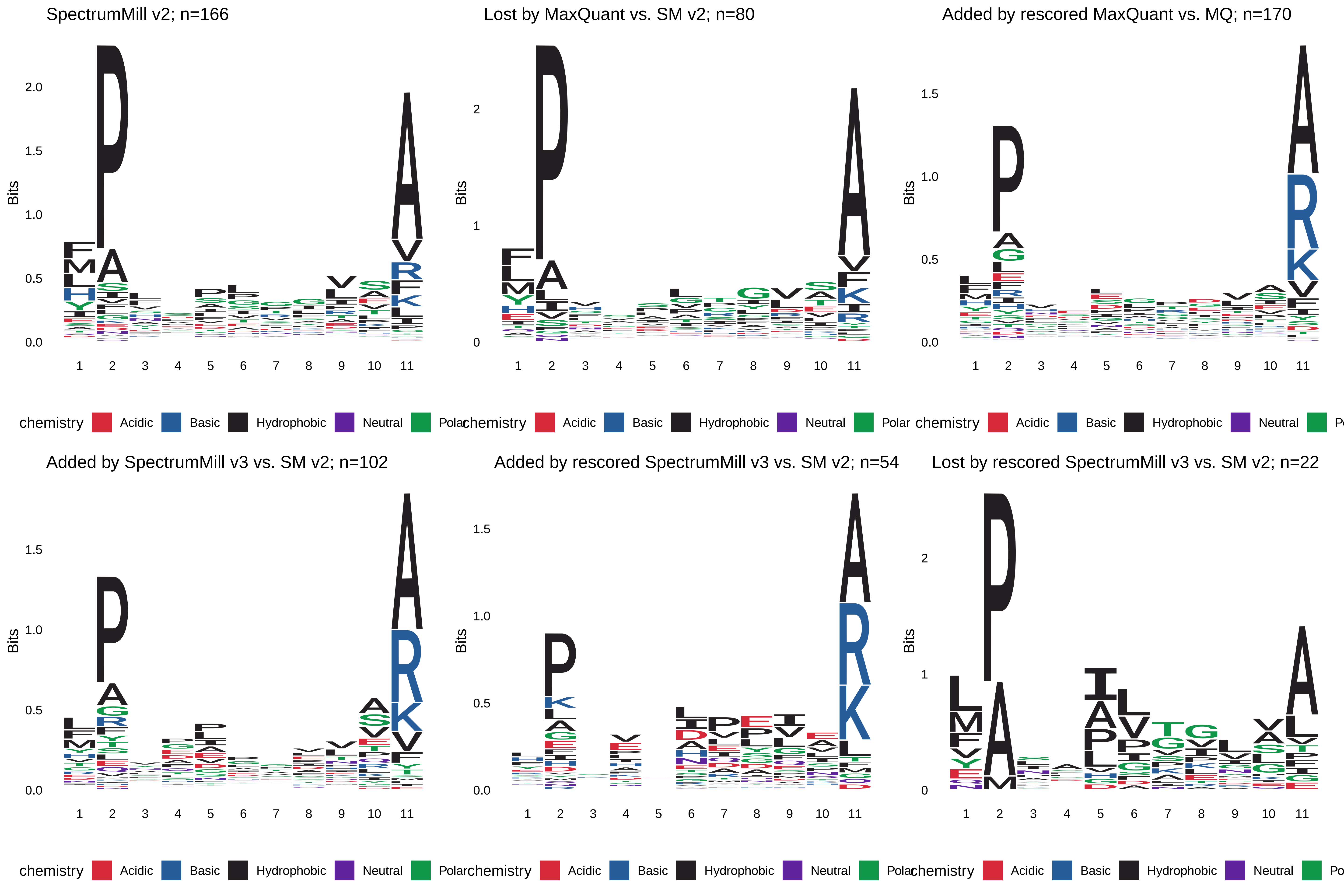

SpectrumMill v2; n=166
Lost by MaxQuant vs. SM v2; n=80
Added by rescored MaxQuant vs. MQ; n=170
Added by SpectrumMill v3 vs. SM v2; n=102
Added by rescored SpectrumMill v3 vs. SM v2; n=54
Lost by rescored SpectrumMill v3 vs. SM v2; n=22
Bits
chemistry
Acidic
Basic
Hydrophobic
Neutral
Polar

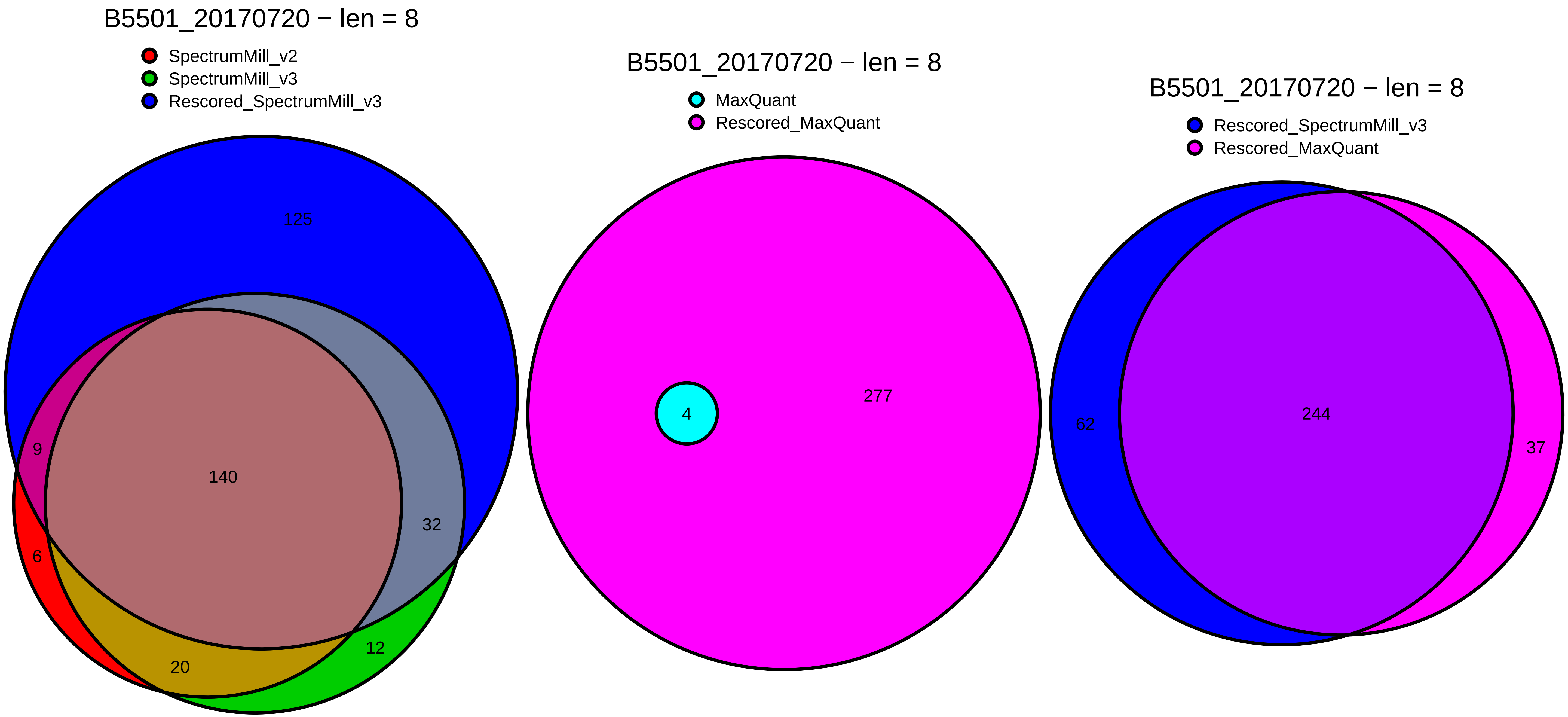
B5501_20170720 − len = 8
SpectrumMill_v2
SpectrumMill_v3
Rescored_SpectrumMill_v3
125
9
140
32
6
20
12
B5501_20170720 − len = 8
MaxQuant
Rescored_MaxQuant
4
277
B5501_20170720 − len = 8
Rescored_SpectrumMill_v3
Rescored_MaxQuant
62
244
37

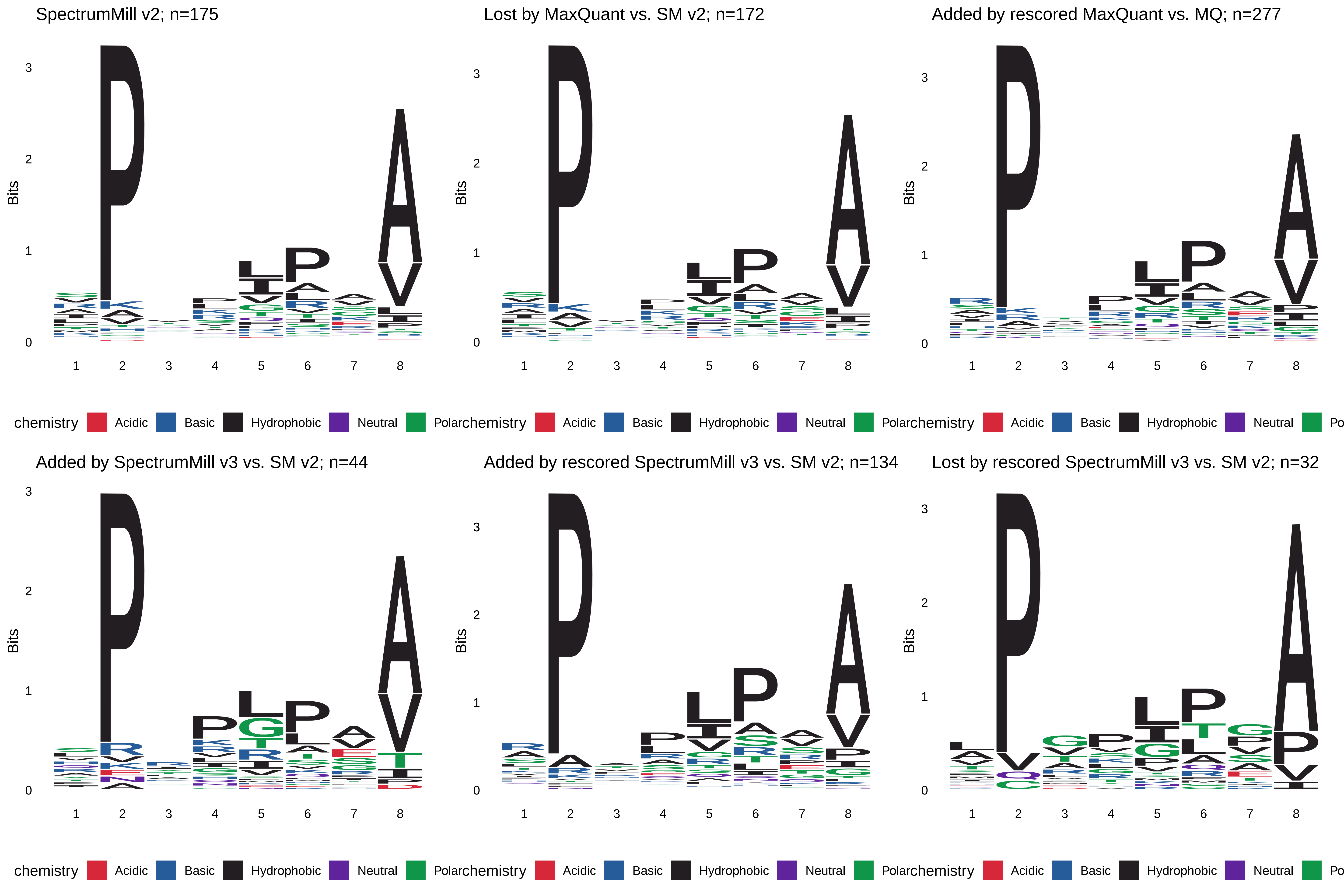

SpectrumMill v2; n=175
Lost by MaxQuant vs. SM v2; n=172
Added by rescored MaxQuant vs. MQ; n=277
Added by SpectrumMill v3 vs. SM v2; n=44
Added by rescored SpectrumMill v3 vs. SM v2; n=134
Lost by rescored SpectrumMill v3 vs. SM v2; n=32
Bits
0
1
2
3
1 2 3 4 5 6 7 8
chemistry
Acidic
Basic
Hydrophobic
Neutral
Polar

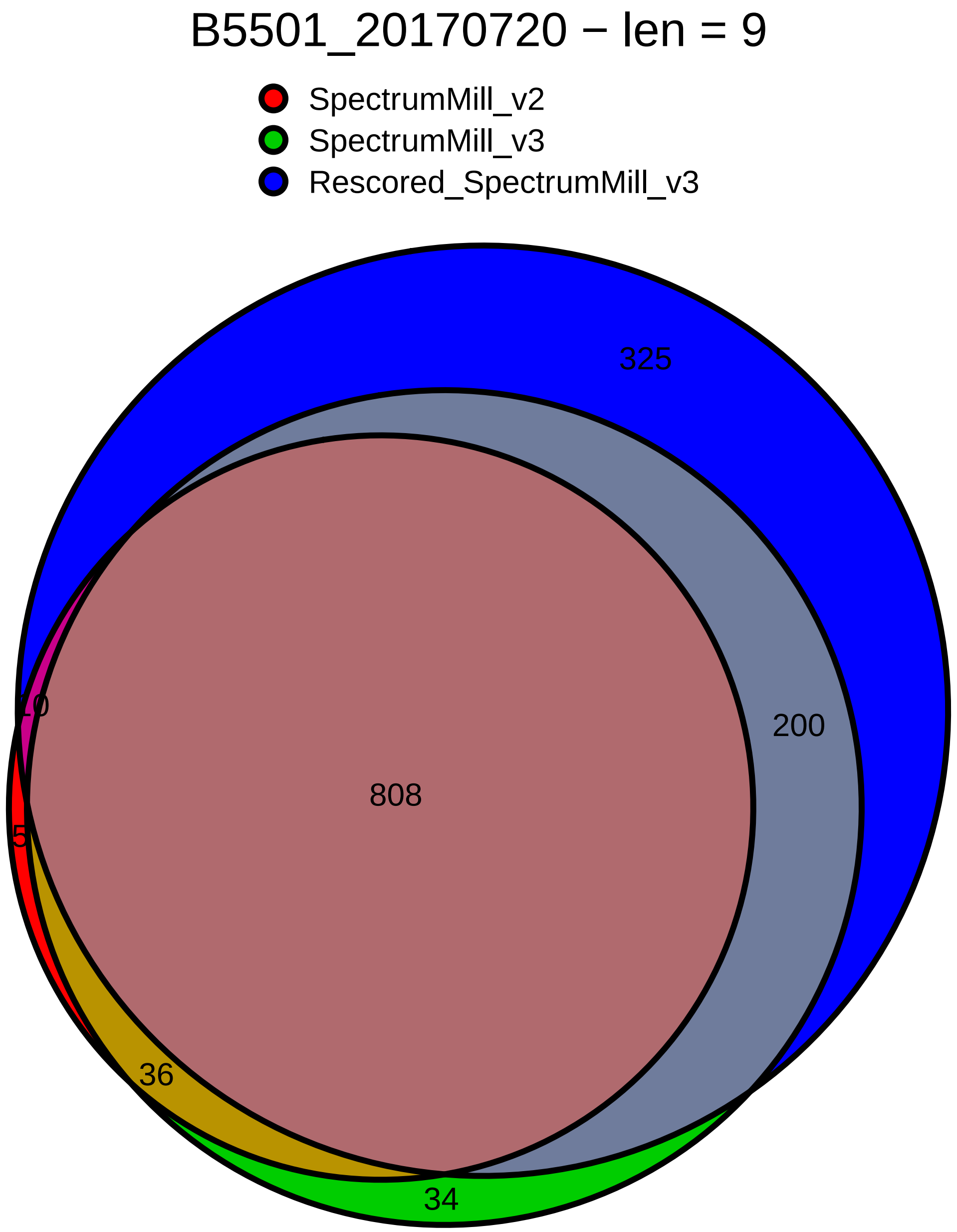
B5501_20170720 − len = 9
SpectrumMill_v2
SpectrumMill_v3
Rescored_SpectrumMill_v3
325
10
200
808
5
36
34

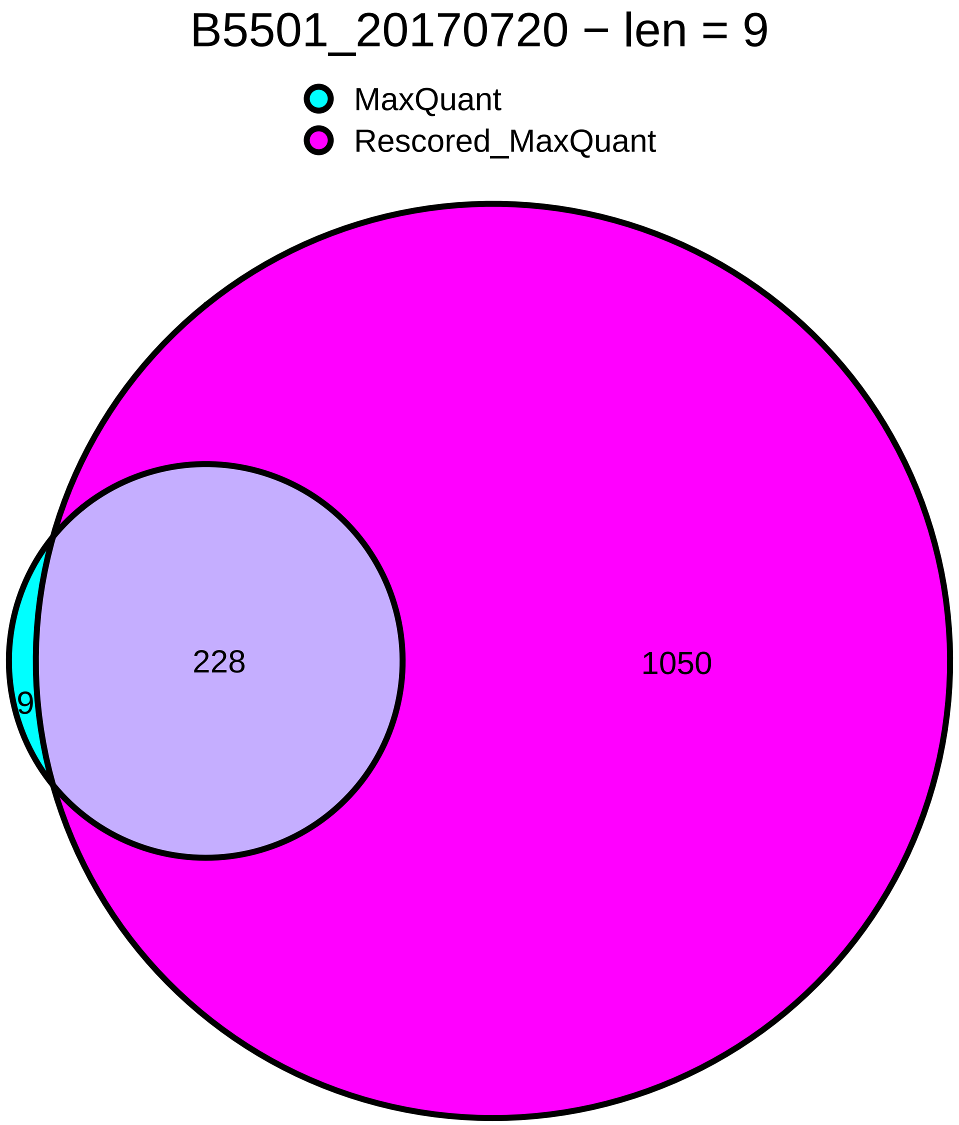
B5501_20170720 − len = 9
MaxQuant
Rescored_MaxQuant
9
228
1050

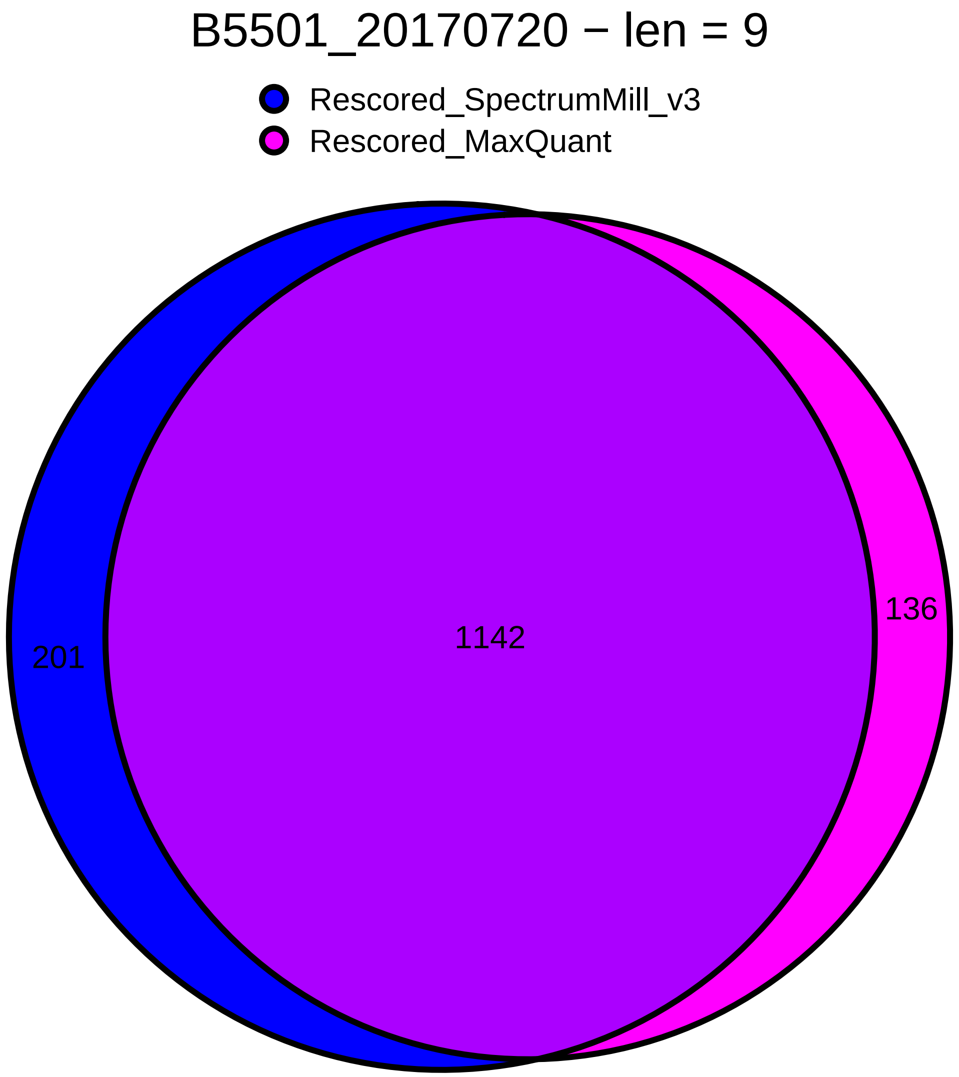
B5501_20170720 − len = 9
Rescored_SpectrumMill_v3
Rescored_MaxQuant
201
1142
136

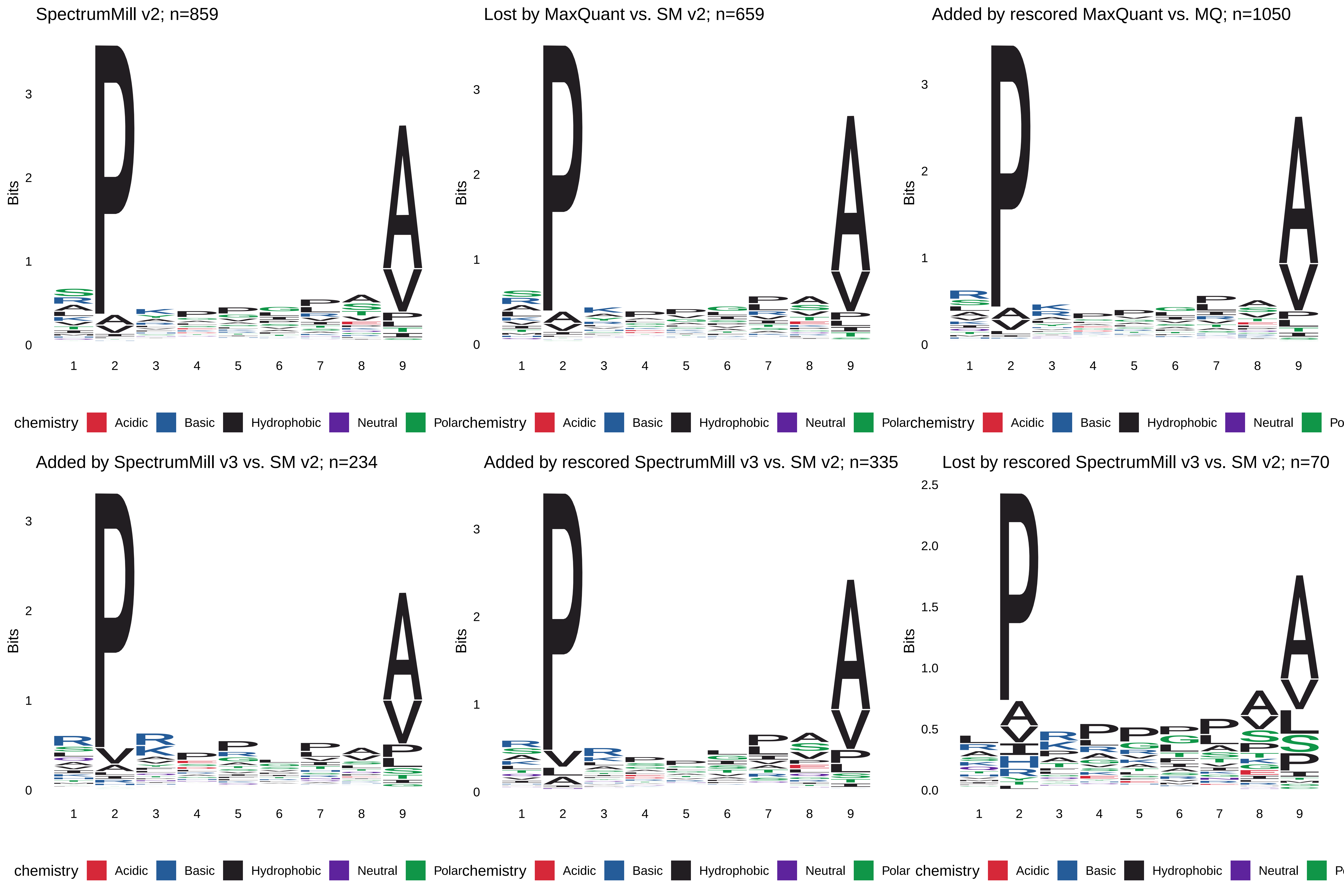
SpectrumMill v2; n=859
Lost by MaxQuant vs. SM v2; n=659
Added by rescored MaxQuant vs. MQ; n=1050
Added by SpectrumMill v3 vs. SM v2; n=234
Added by rescored SpectrumMill v3 vs. SM v2; n=335
Lost by rescored SpectrumMill v3 vs. SM v2; n=70
Bits
chemistry
Acidic
Basic
Hydrophobic
Neutral
Polar

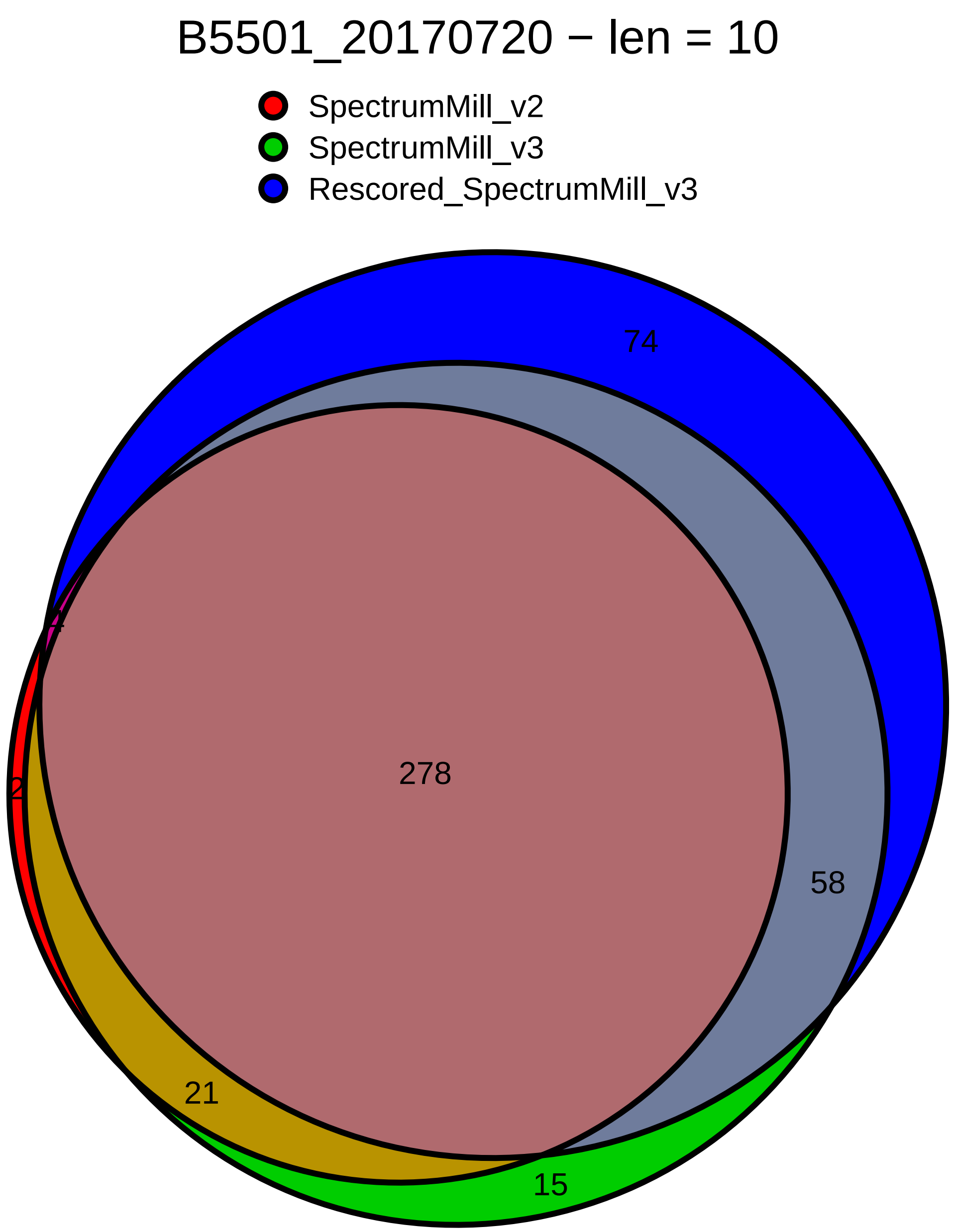
B5501_20170720 − len = 10
SpectrumMill_v2
SpectrumMill_v3
Rescored_SpectrumMill_v3
74
278
58
21
15

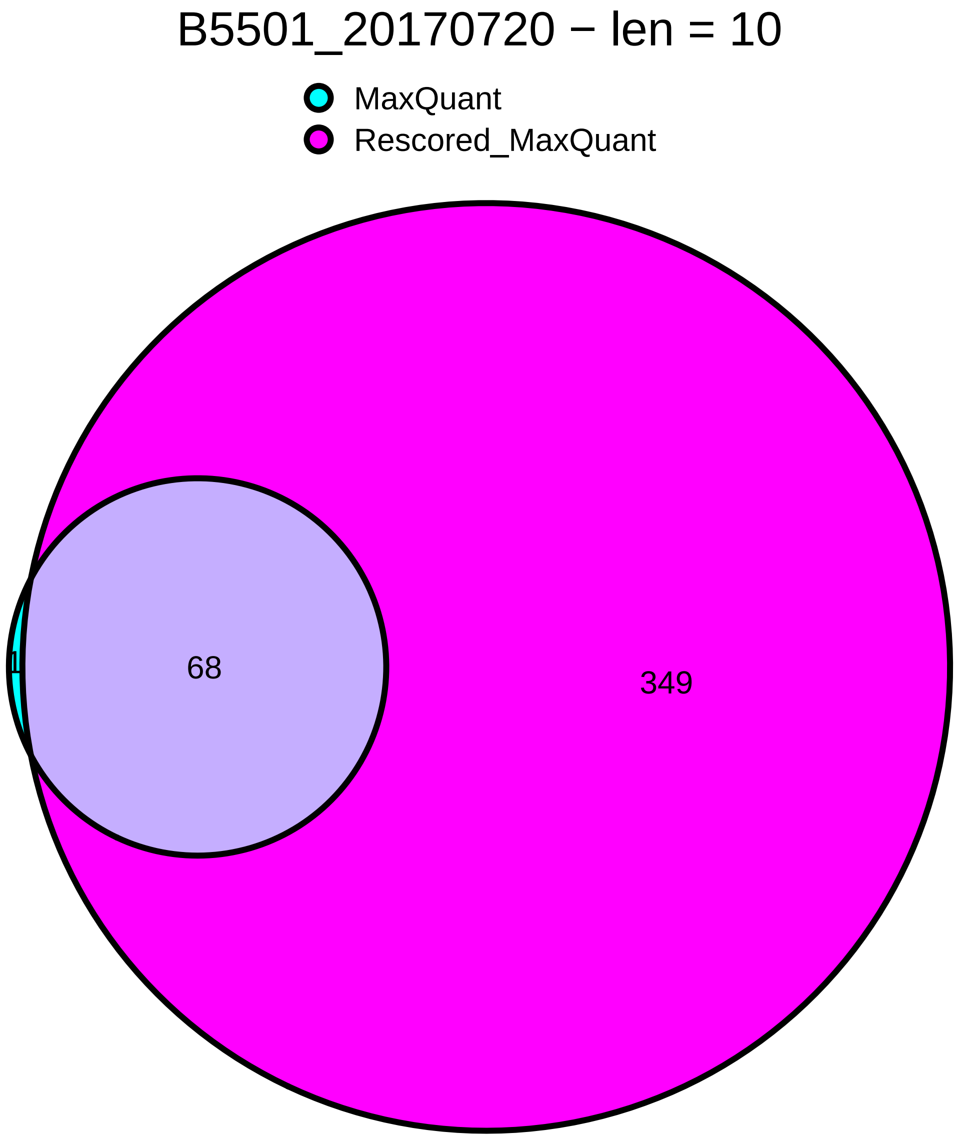
B5501_20170720 − len = 10
MaxQuant
Rescored_MaxQuant
68
349

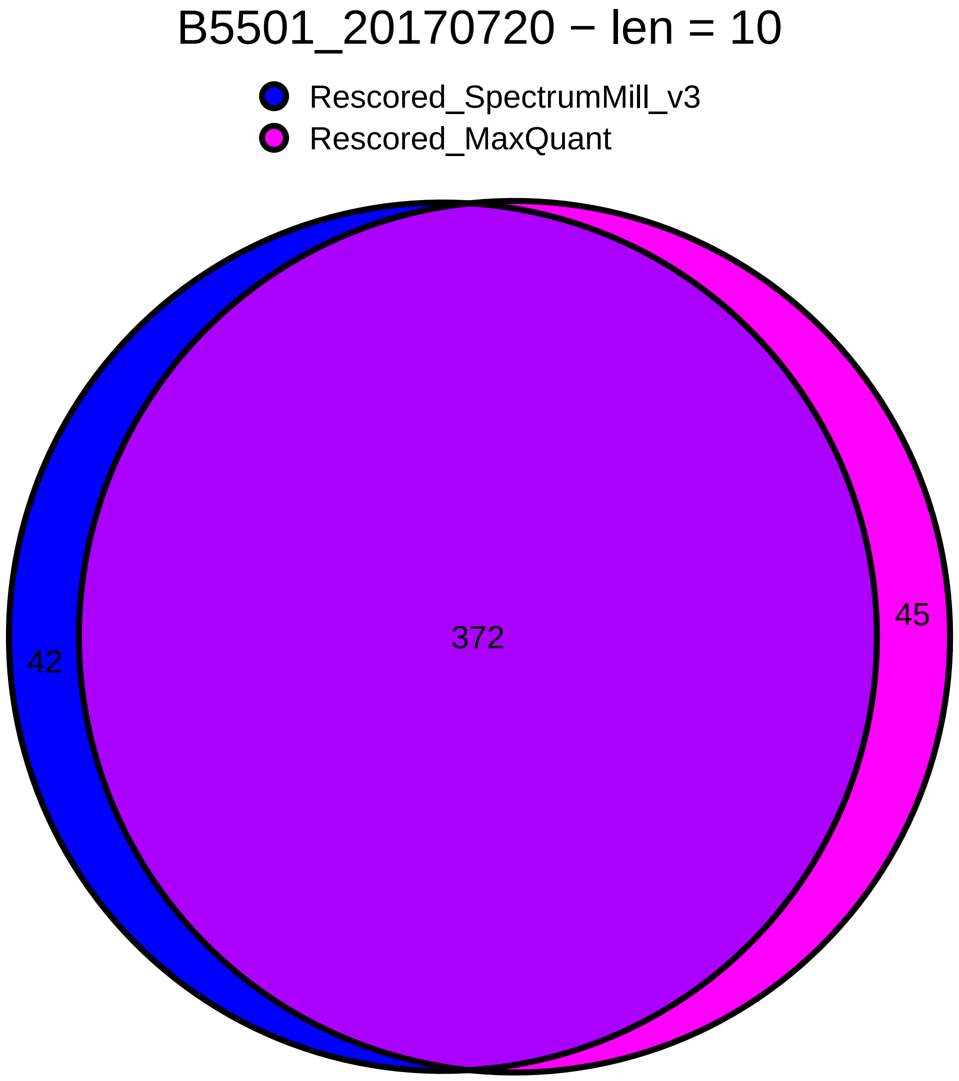
B5501_20170720 − len = 10
Rescored_SpectrumMill_v3
Rescored_MaxQuant
42
372
45

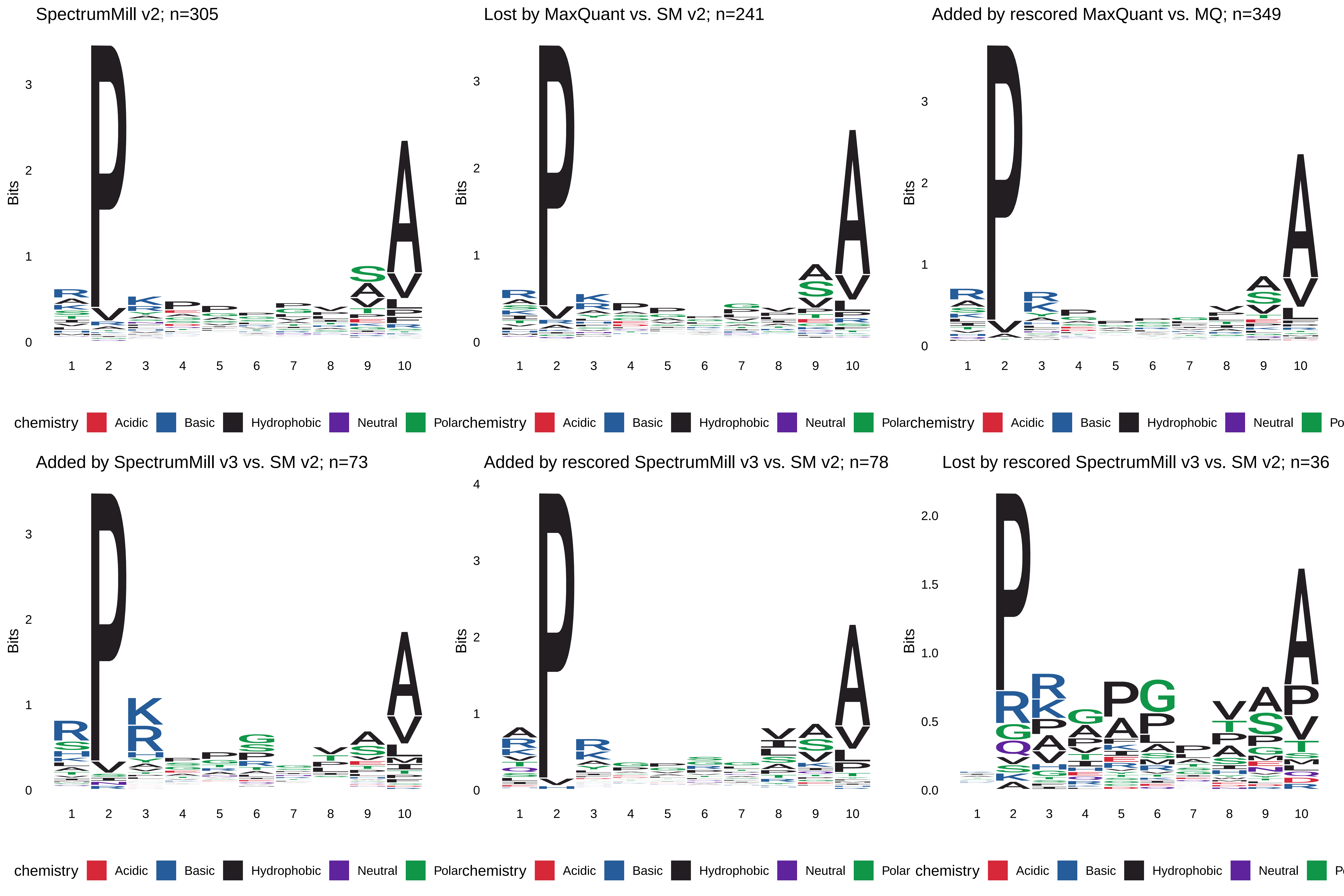
SpectrumMill v2; n=305
Lost by MaxQuant vs. SM v2; n=241
Added by rescored MaxQuant vs. MQ; n=349
Added by SpectrumMill v3 vs. SM v2; n=73
Added by rescored SpectrumMill v3 vs. SM v2; n=78
Lost by rescored SpectrumMill v3 vs. SM v2; n=36
Bits
1 2 3 4 5 6 7 8 9 10
chemistry
Acidic
Basic
Hydrophobic
Neutral
Polar

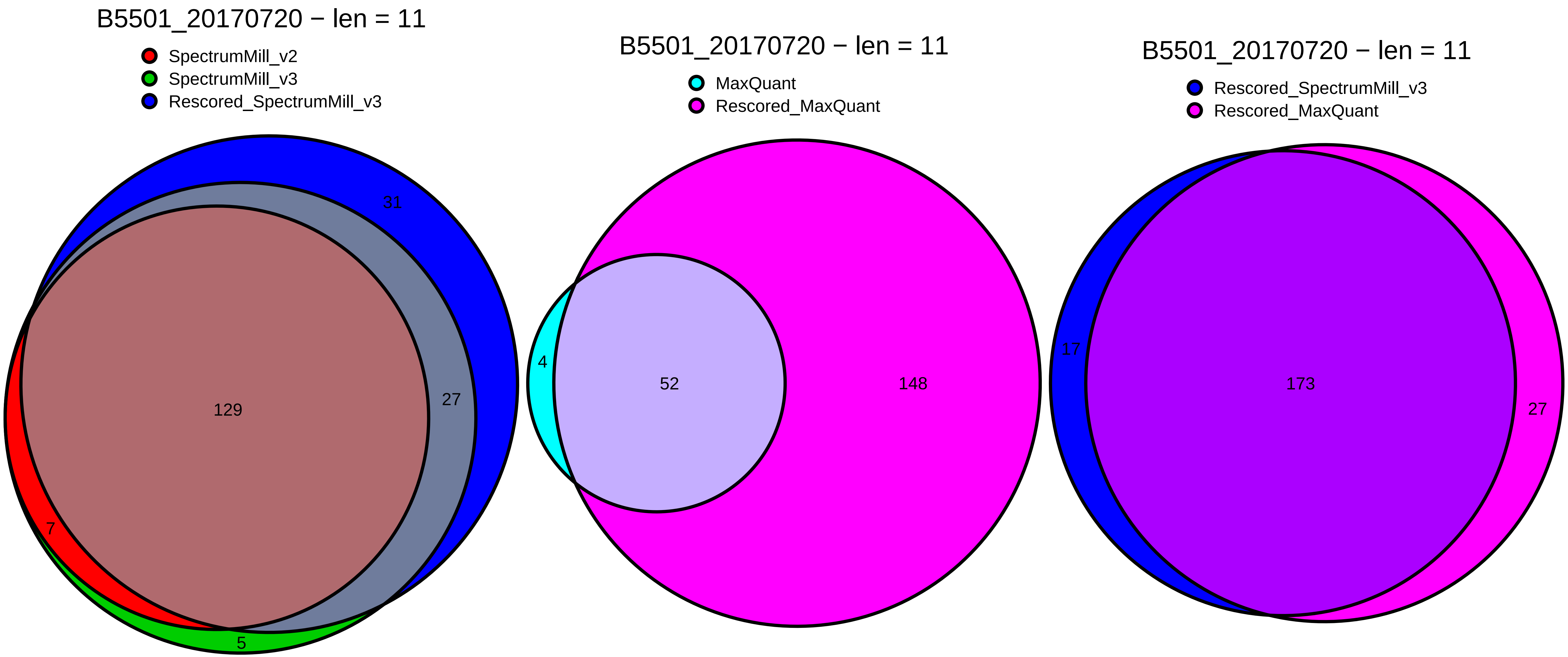
B5501_20170720 − len = 11
SpectrumMill_v2
SpectrumMill_v3
Rescored_SpectrumMill_v3
31
27
129
7
5
B5501_20170720 − len = 11
MaxQuant
Rescored_MaxQuant
4
52
148
B5501_20170720 − len = 11
Rescored_SpectrumMill_v3
Rescored_MaxQuant
17
173
27

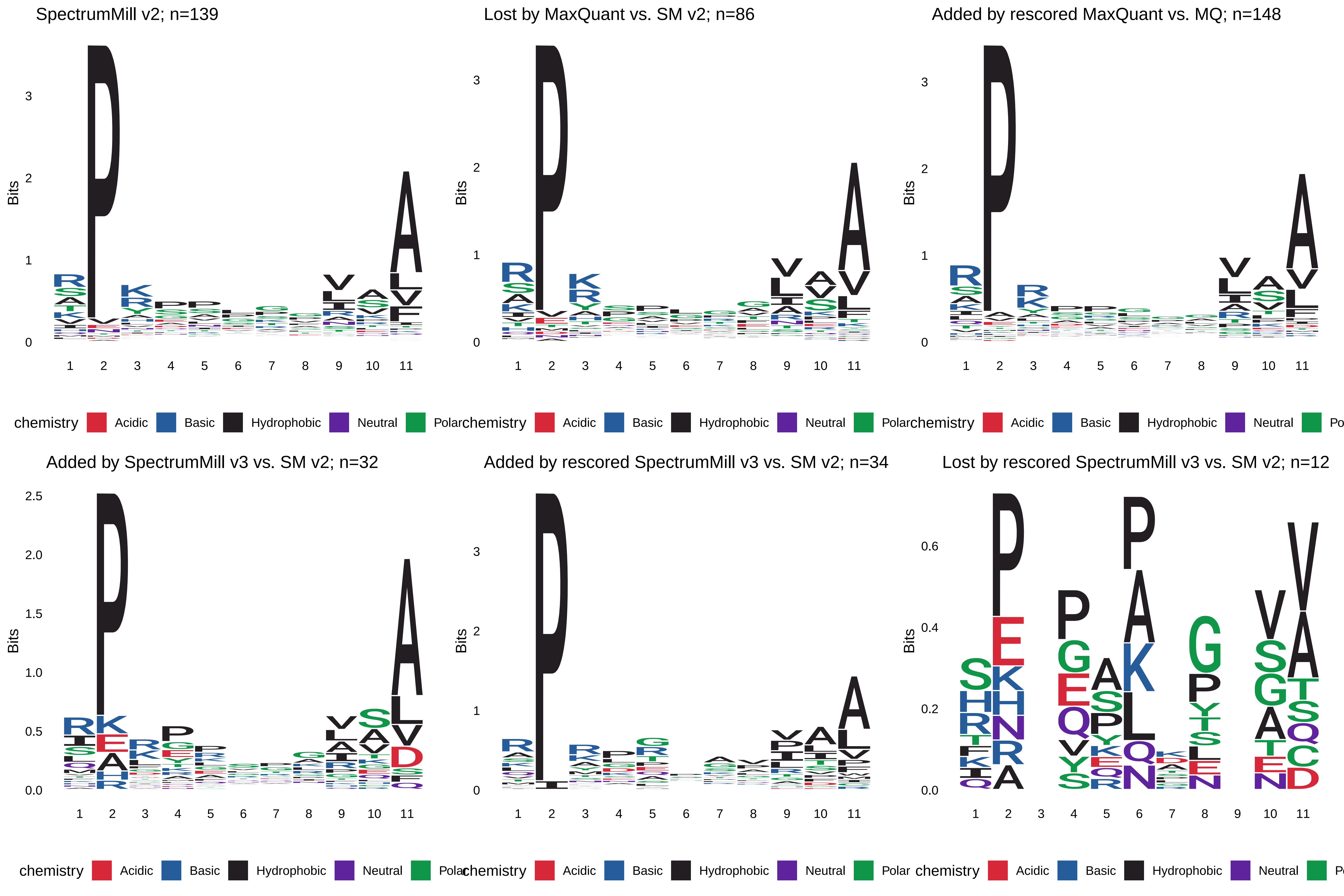
SpectrumMill v2; n=139
Lost by MaxQuant vs. SM v2; n=86
Added by rescored MaxQuant vs. MQ; n=148
Added by SpectrumMill v3 vs. SM v2; n=32
Added by rescored SpectrumMill v3 vs. SM v2; n=34
Lost by rescored SpectrumMill v3 vs. SM v2; n=12
Bits
1 2 3 4 5 6 7 8 9 10 11
chemistry
Acidic
Basic
Hydrophobic
Neutral
Polar

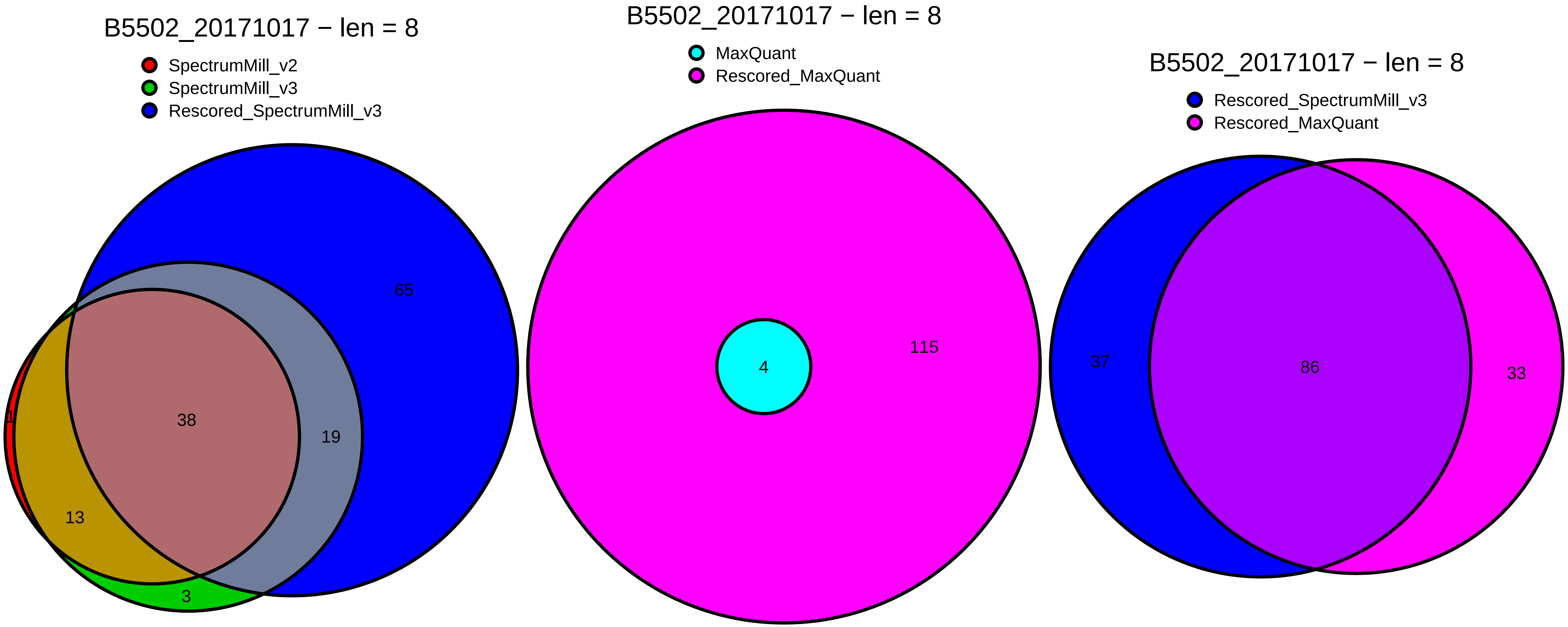
B5502_20171017 − len = 8
SpectrumMill_v2
SpectrumMill_v3
Rescored_SpectrumMill_v3
65
1
38
19
13
3
B5502_20171017 − len = 8
MaxQuant
Rescored_MaxQuant
4
115
B5502_20171017 − len = 8
Rescored_SpectrumMill_v3
Rescored_MaxQuant
37
86
33

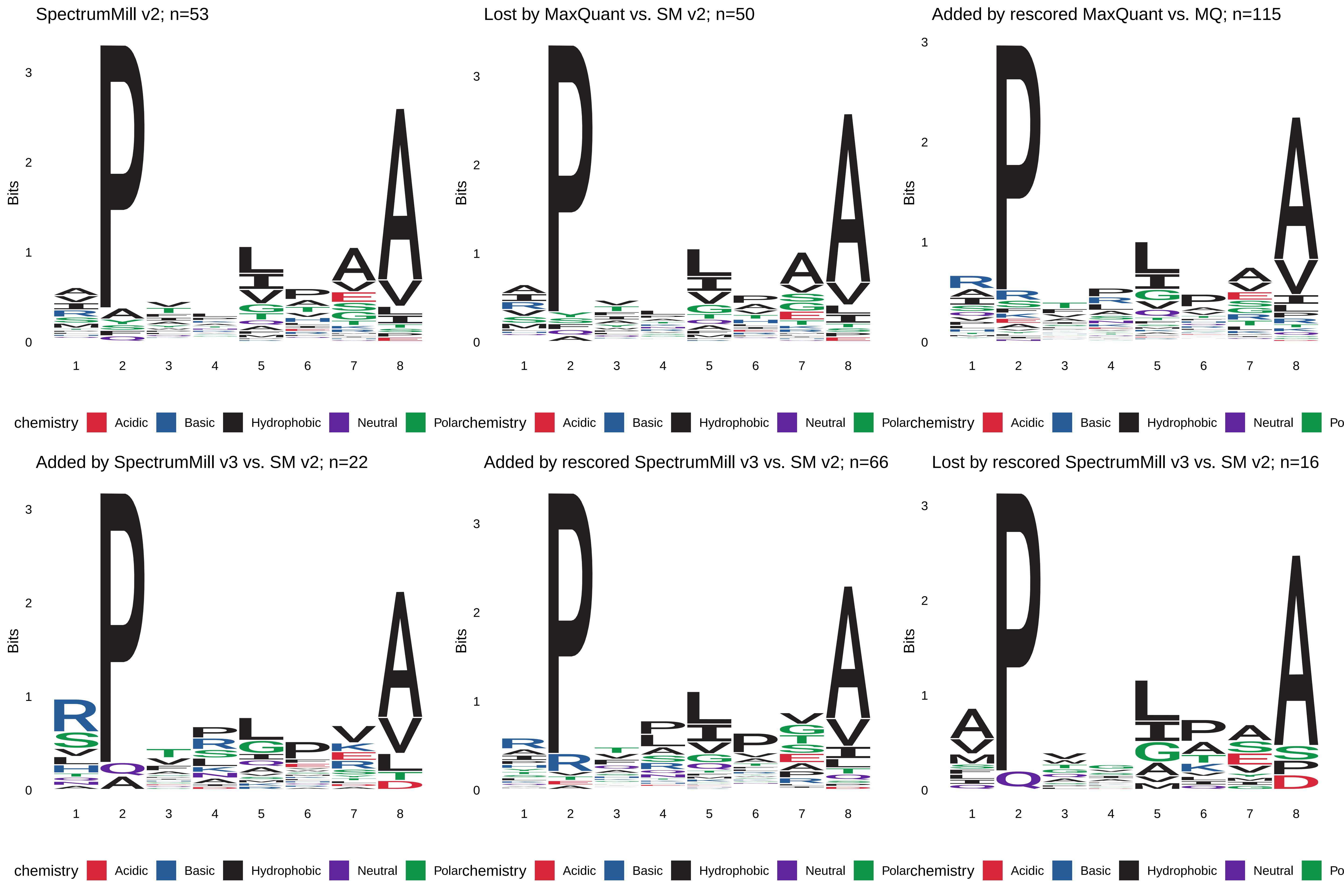

SpectrumMill v2; n=53
Lost by MaxQuant vs. SM v2; n=50
Added by rescored MaxQuant vs. MQ; n=115
Added by SpectrumMill v3 vs. SM v2; n=22
Added by rescored SpectrumMill v3 vs. SM v2; n=66
Lost by rescored SpectrumMill v3 vs. SM v2; n=16
Bits
0
1
2
3
1 2 3 4 5 6 7 8
chemistry Acidic Basic Hydrophobic Neutral Polarchemistry Acidic Basic Hydrophobic Neutral Polarchemistry Acidic Basic Hydrophobic Neutral Po
chemistry Acidic Basic Hydrophobic Neutral Polarchemistry Acidic Basic Hydrophobic Neutral Polarchemistry Acidic Basic Hydrophobic Neutral Po

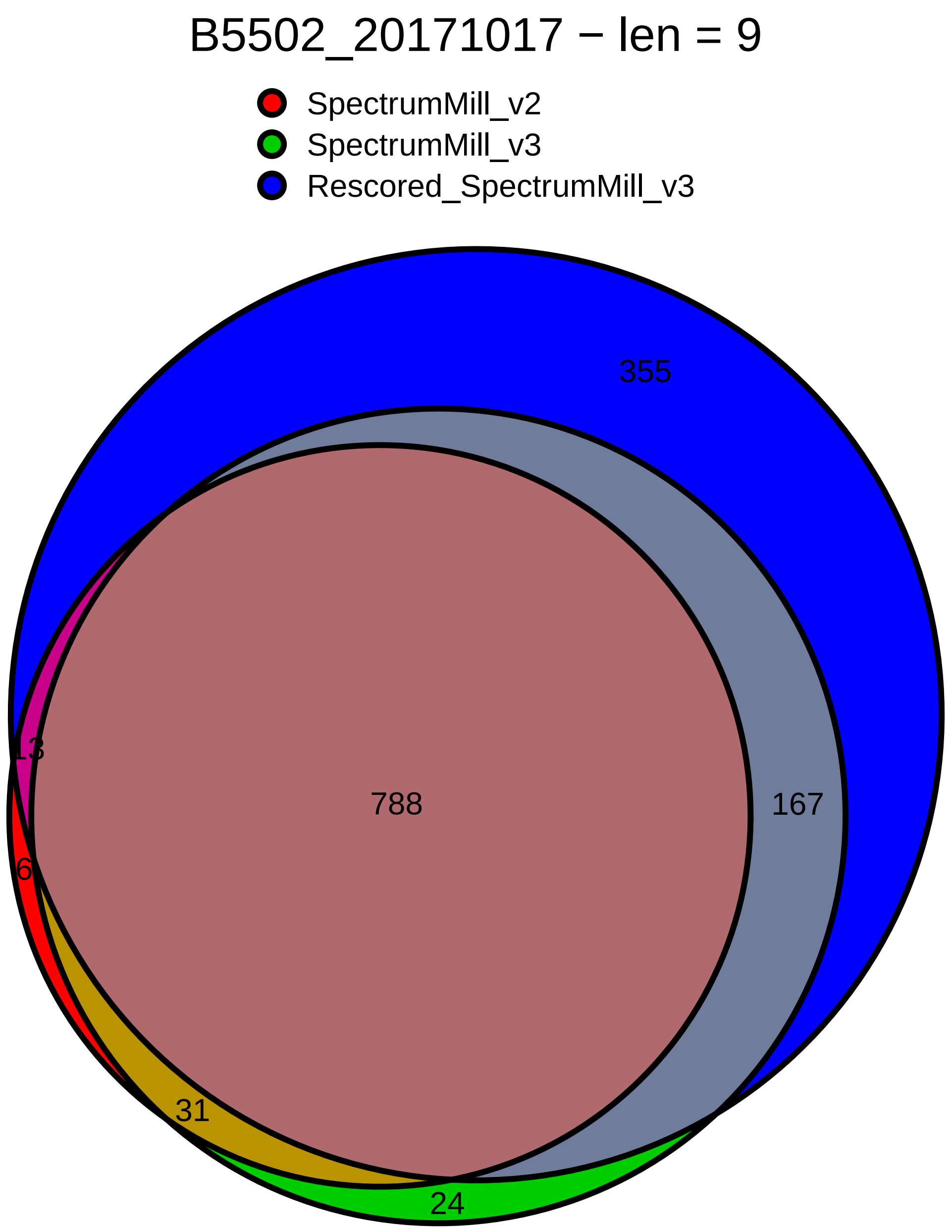

B5502_20171017 − len = 9
SpectrumMill_v2
SpectrumMill_v3
Rescored_SpectrumMill_v3
355
13
788
167
6
31
24

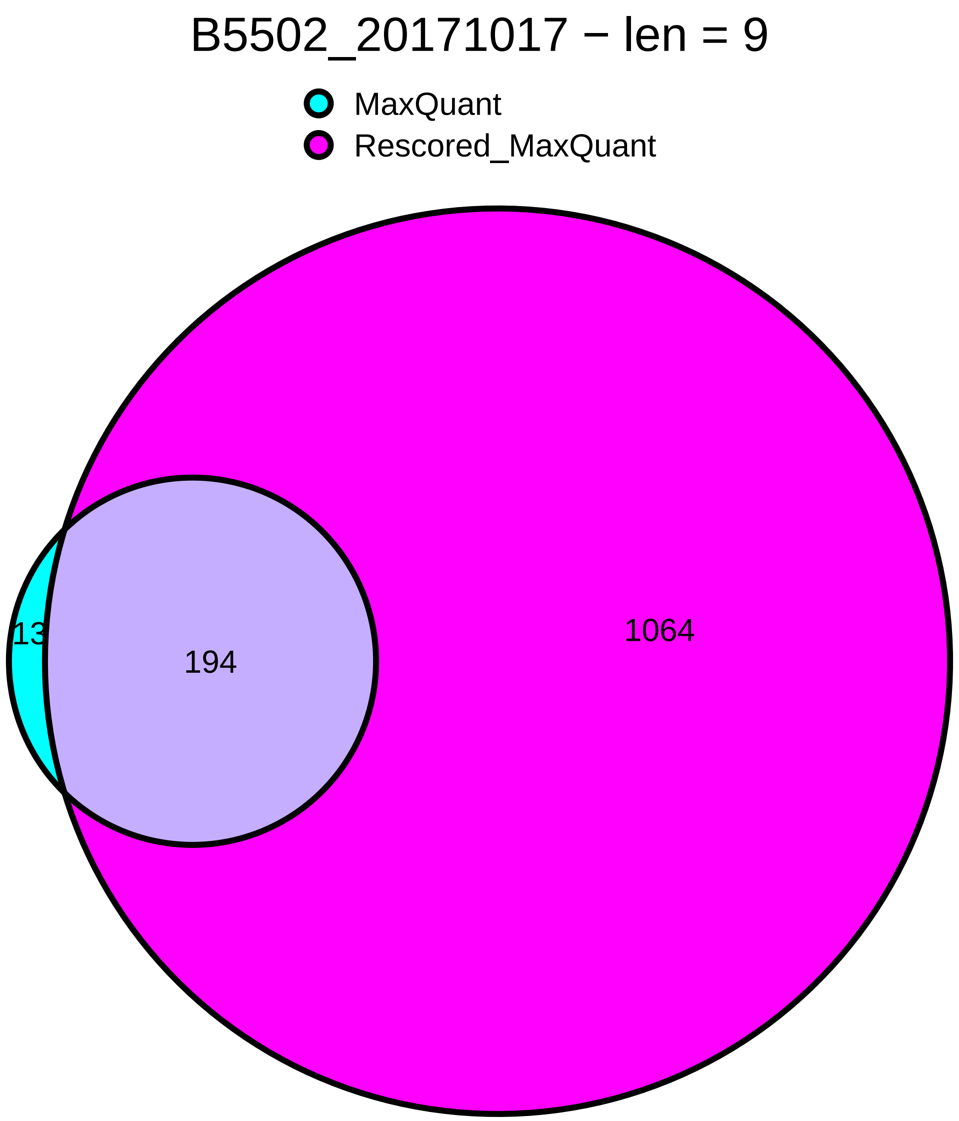

B5502_20171017 − len = 9
MaxQuant
Rescored_MaxQuant
13
194
1064

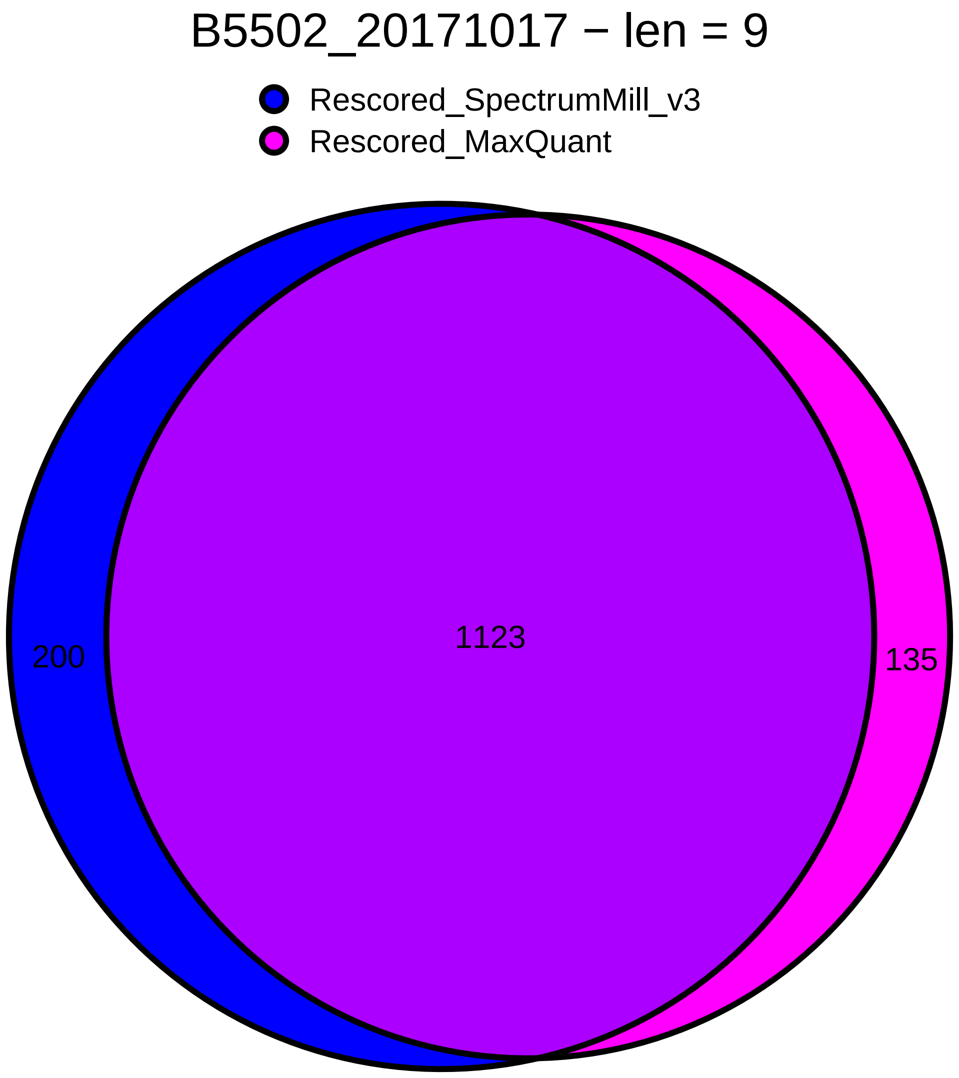

B5502_20171017 − len = 9
Rescored_SpectrumMill_v3
Rescored_MaxQuant
200
1123
135

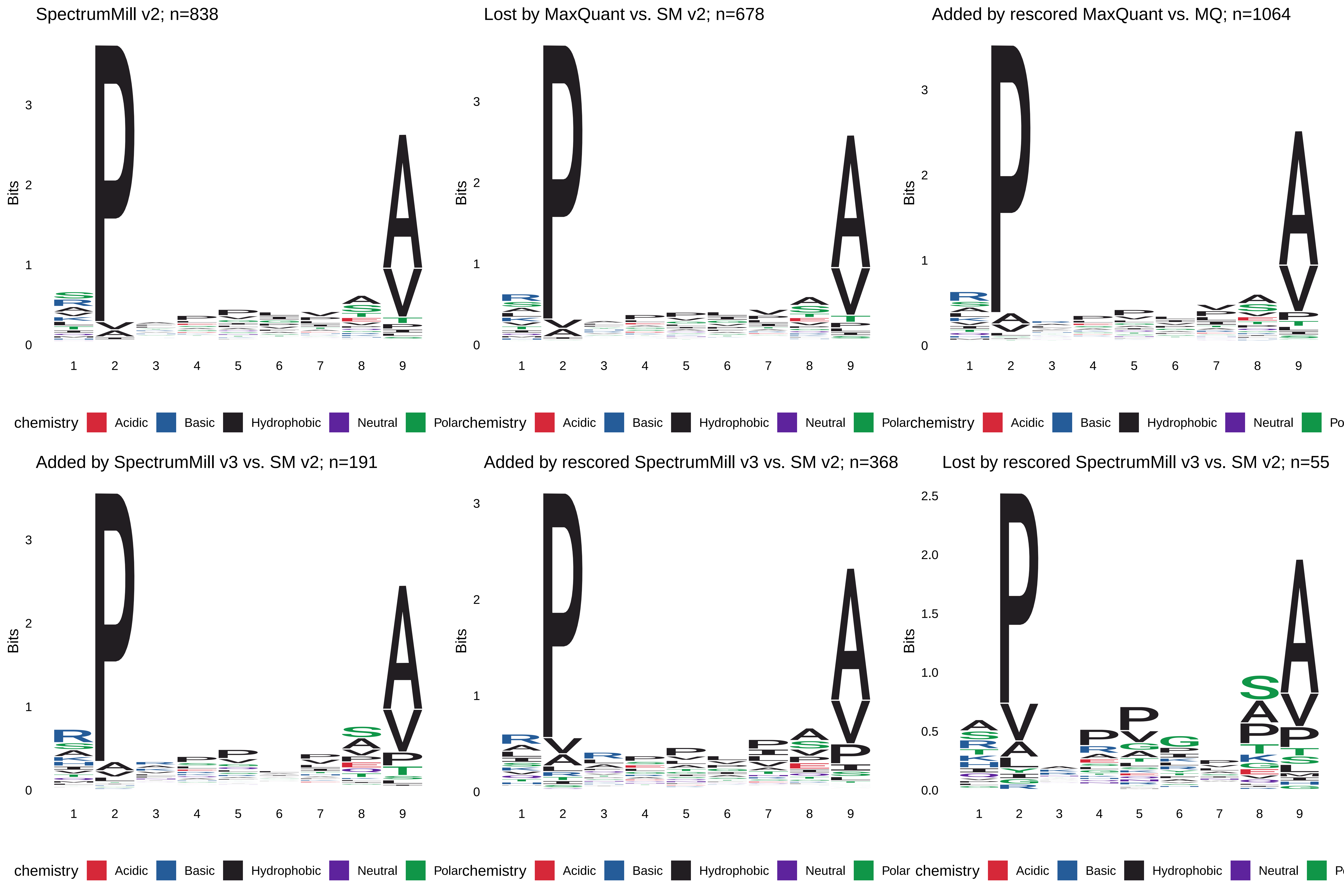
SpectrumMill v2; n=838
Lost by MaxQuant vs. SM v2; n=678
Added by rescored MaxQuant vs. MQ; n=1064
Added by SpectrumMill v3 vs. SM v2; n=191
Added by rescored SpectrumMill v3 vs. SM v2; n=368
Lost by rescored SpectrumMill v3 vs. SM v2; n=55
Bits
1 2 3 4 5 6 7 8 9
chemistry
Acidic
Basic
Hydrophobic
Neutral
Polar

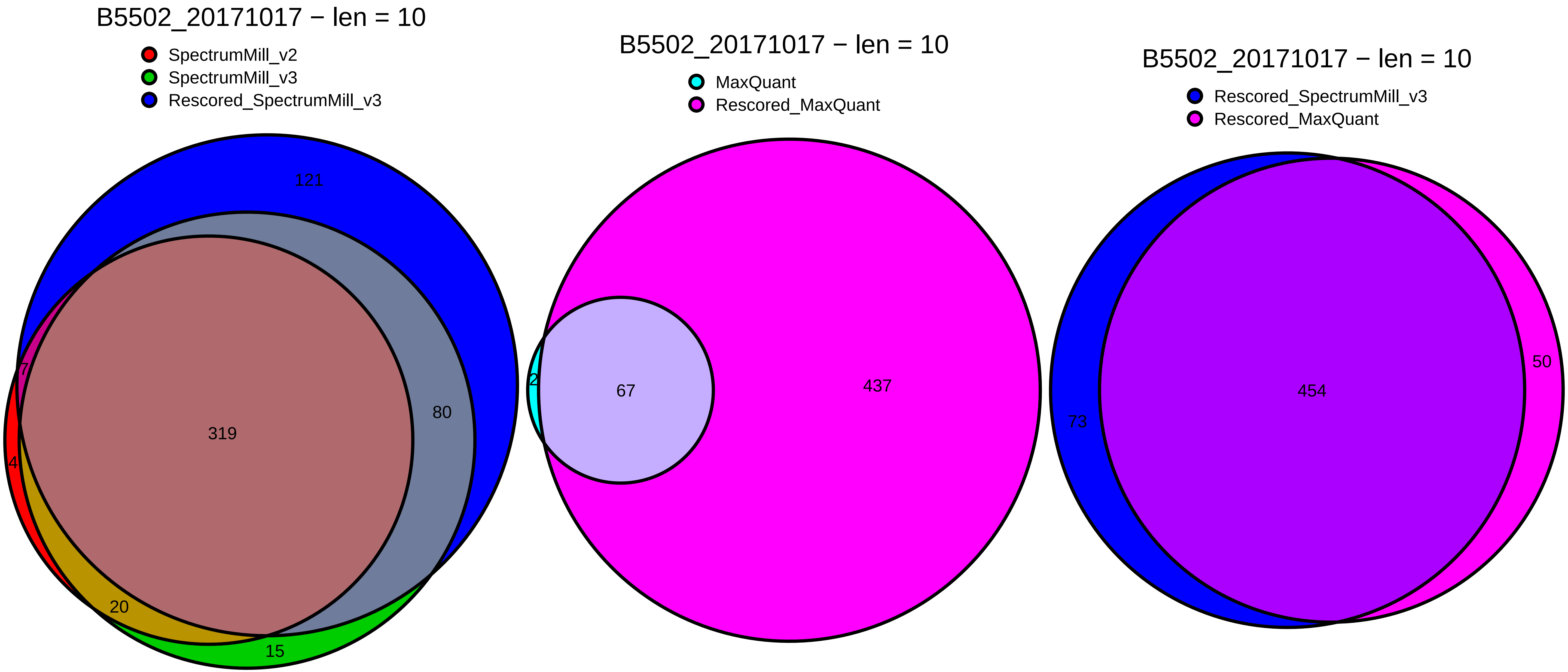
B5502_20171017 − len = 10
SpectrumMill_v2
SpectrumMill_v3
Rescored_SpectrumMill_v3
121
7
80
319
4
20
15
B5502_20171017 − len = 10
MaxQuant
Rescored_MaxQuant
2
67
437
B5502_20171017 − len = 10
Rescored_SpectrumMill_v3
Rescored_MaxQuant
50
454
73

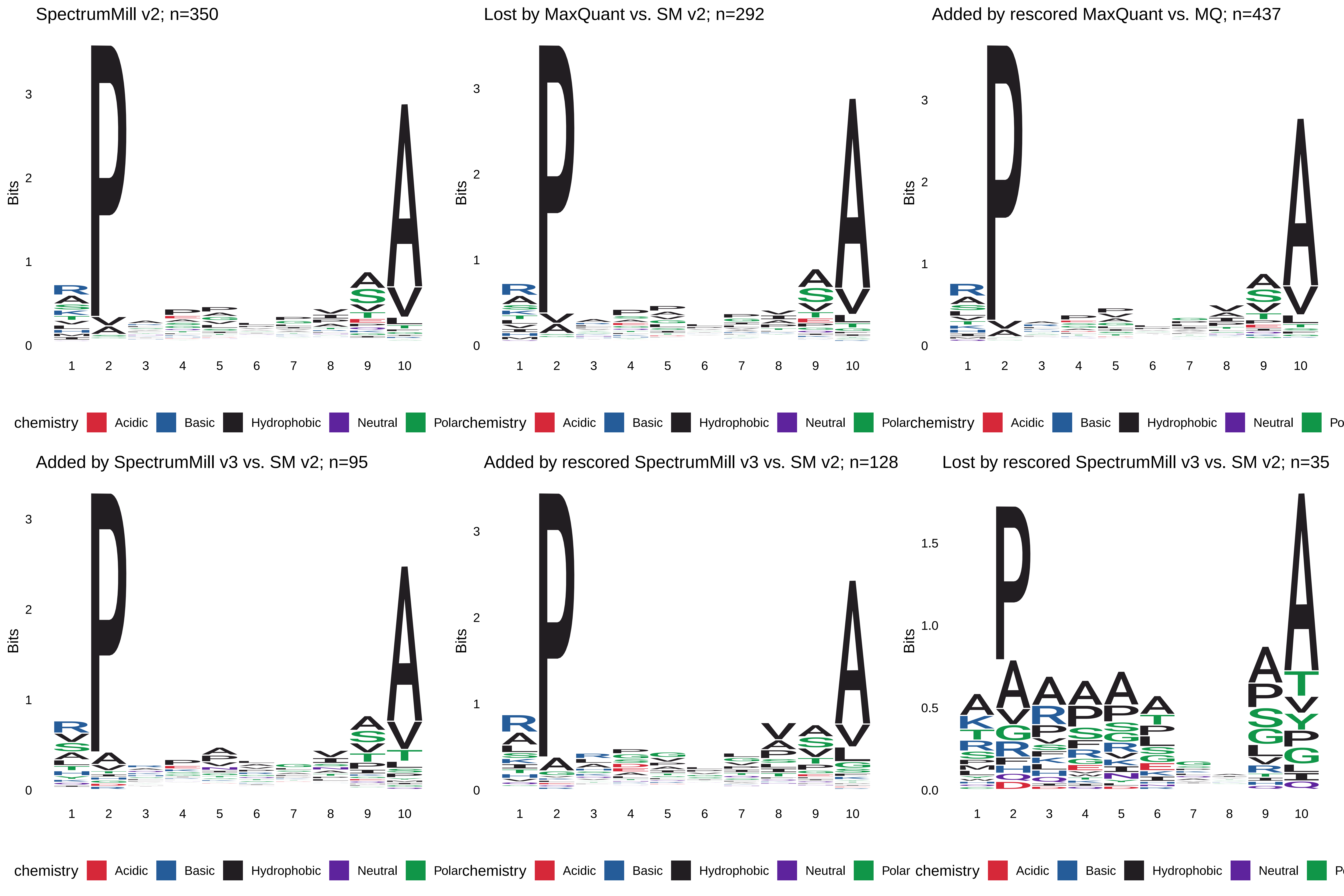

SpectrumMill v2; n=350
Lost by MaxQuant vs. SM v2; n=292
Added by rescored MaxQuant vs. MQ; n=437
Added by SpectrumMill v3 vs. SM v2; n=95
Added by rescored SpectrumMill v3 vs. SM v2; n=128
Lost by rescored SpectrumMill v3 vs. SM v2; n=35
Bits
0
1
2
3
0.0
0.5
1.0
1.5
1 2 3 4 5 6 7 8 9 10
chemistry
Acidic
Basic
Hydrophobic
Neutral
Polar

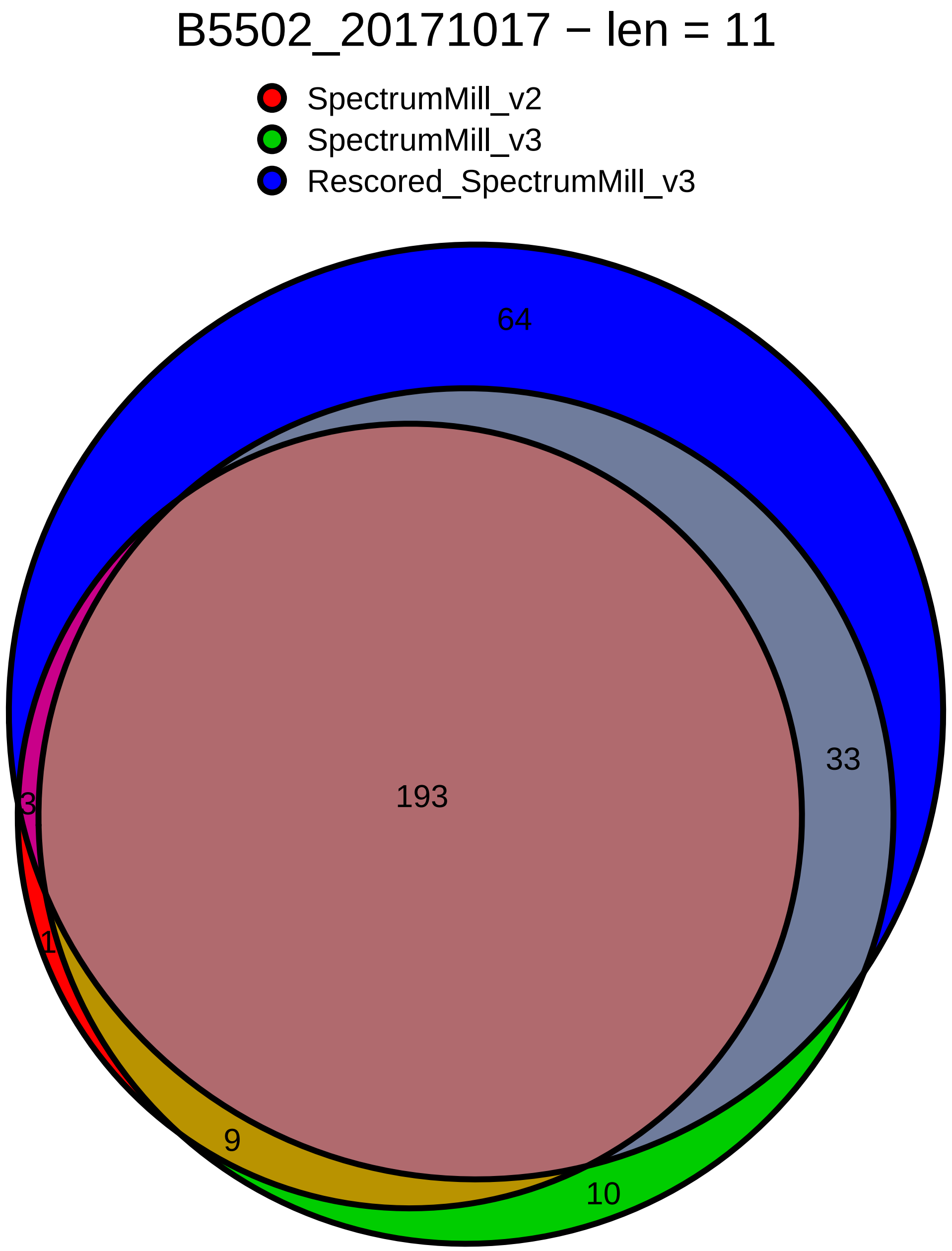
B5502_20171017 − len = 11
SpectrumMill_v2
SpectrumMill_v3
Rescored_SpectrumMill_v3
64
33
193
3
1
9
10

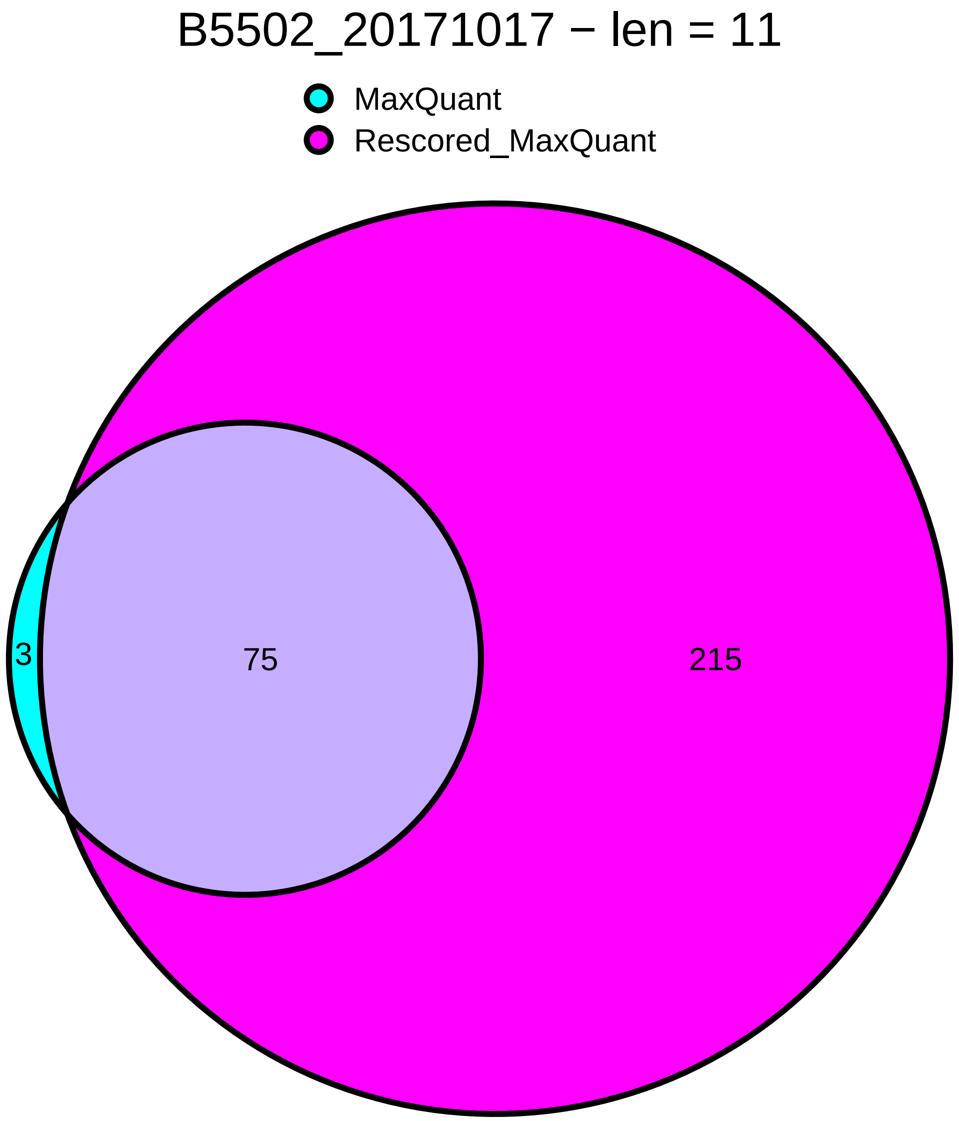
B5502_20171017 − len = 11
MaxQuant
Rescored_MaxQuant
3
75
215

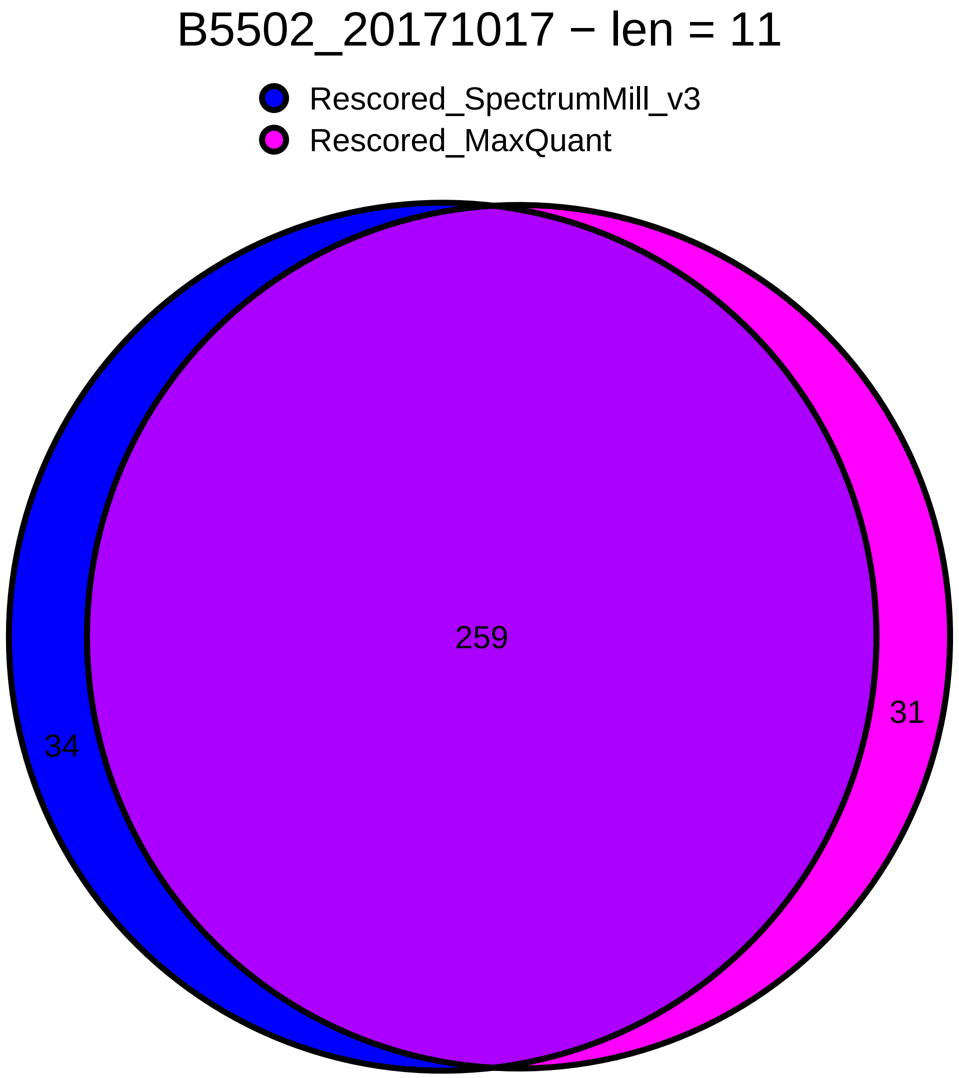
B5502_20171017 − len = 11
Rescored_SpectrumMill_v3
Rescored_MaxQuant
259
34
31

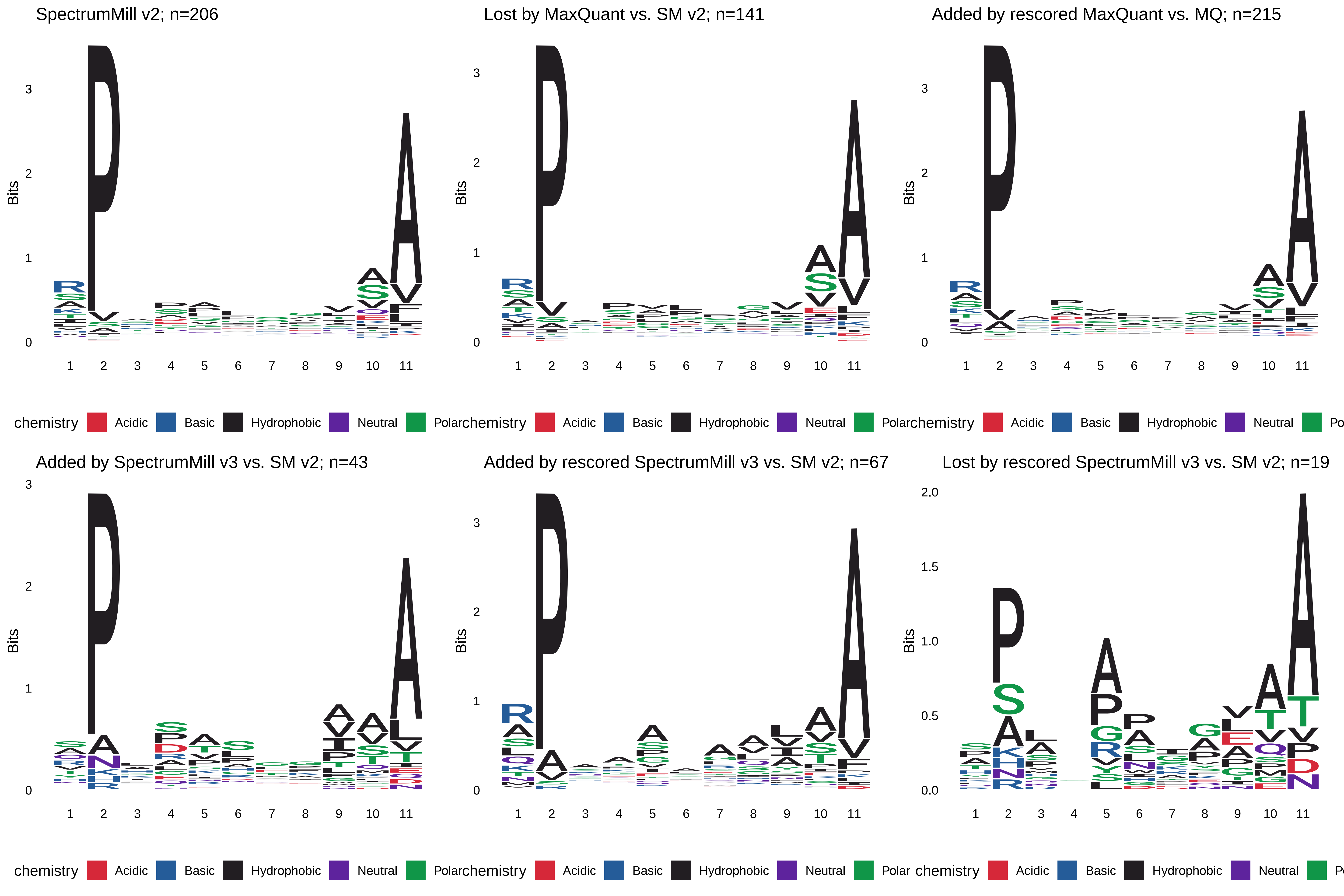
SpectrumMill v2; n=206
Lost by MaxQuant vs. SM v2; n=141
Added by rescored MaxQuant vs. MQ; n=215
Added by SpectrumMill v3 vs. SM v2; n=43
Added by rescored SpectrumMill v3 vs. SM v2; n=67
Lost by rescored SpectrumMill v3 vs. SM v2; n=19
Bits
chemistry
Acidic
Basic
Hydrophobic
Neutral
Polar

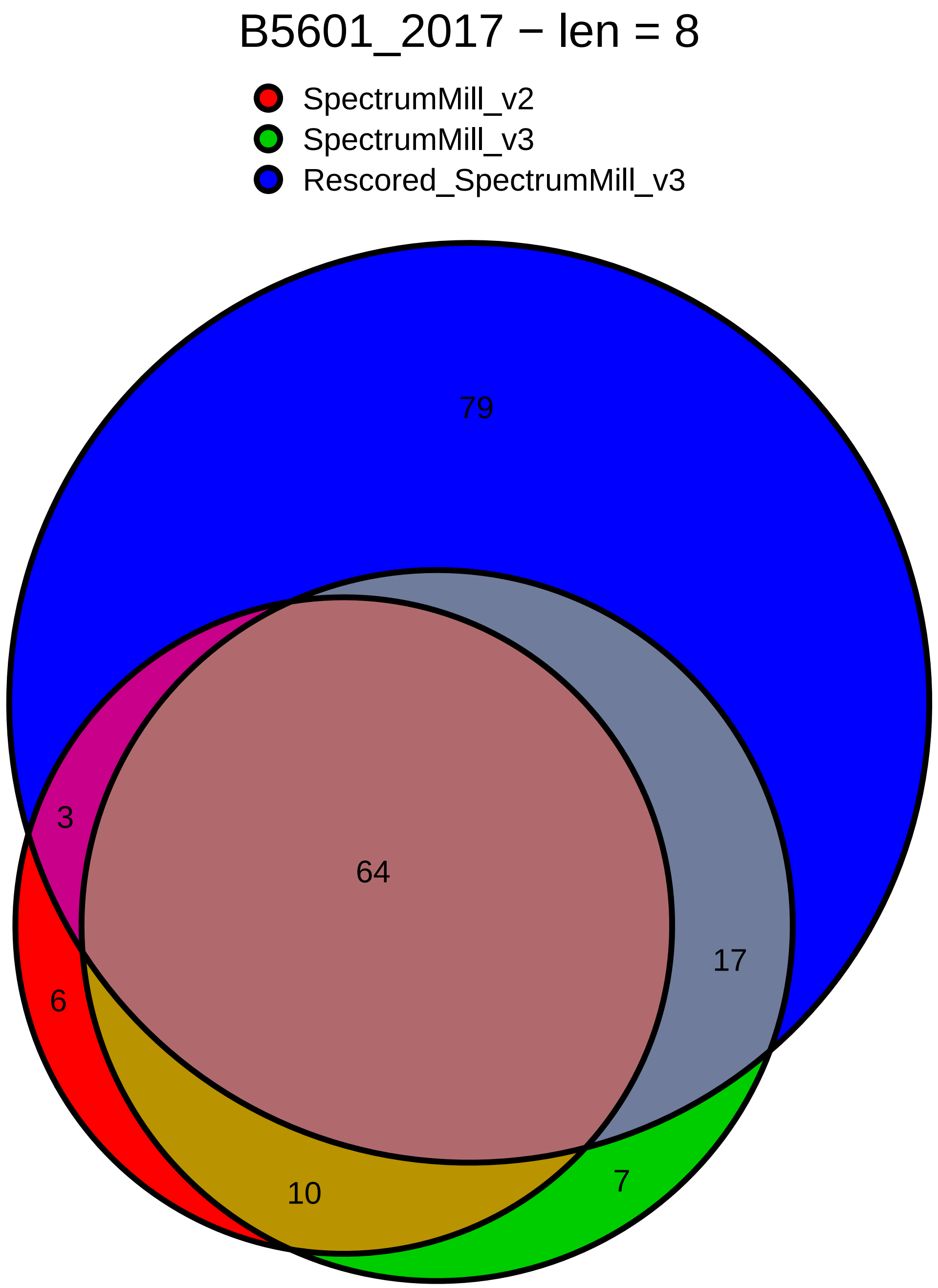

B5601_2017 − len = 8
SpectrumMill_v2
SpectrumMill_v3
Rescored_SpectrumMill_v3
79
3
64
17
6
10
7

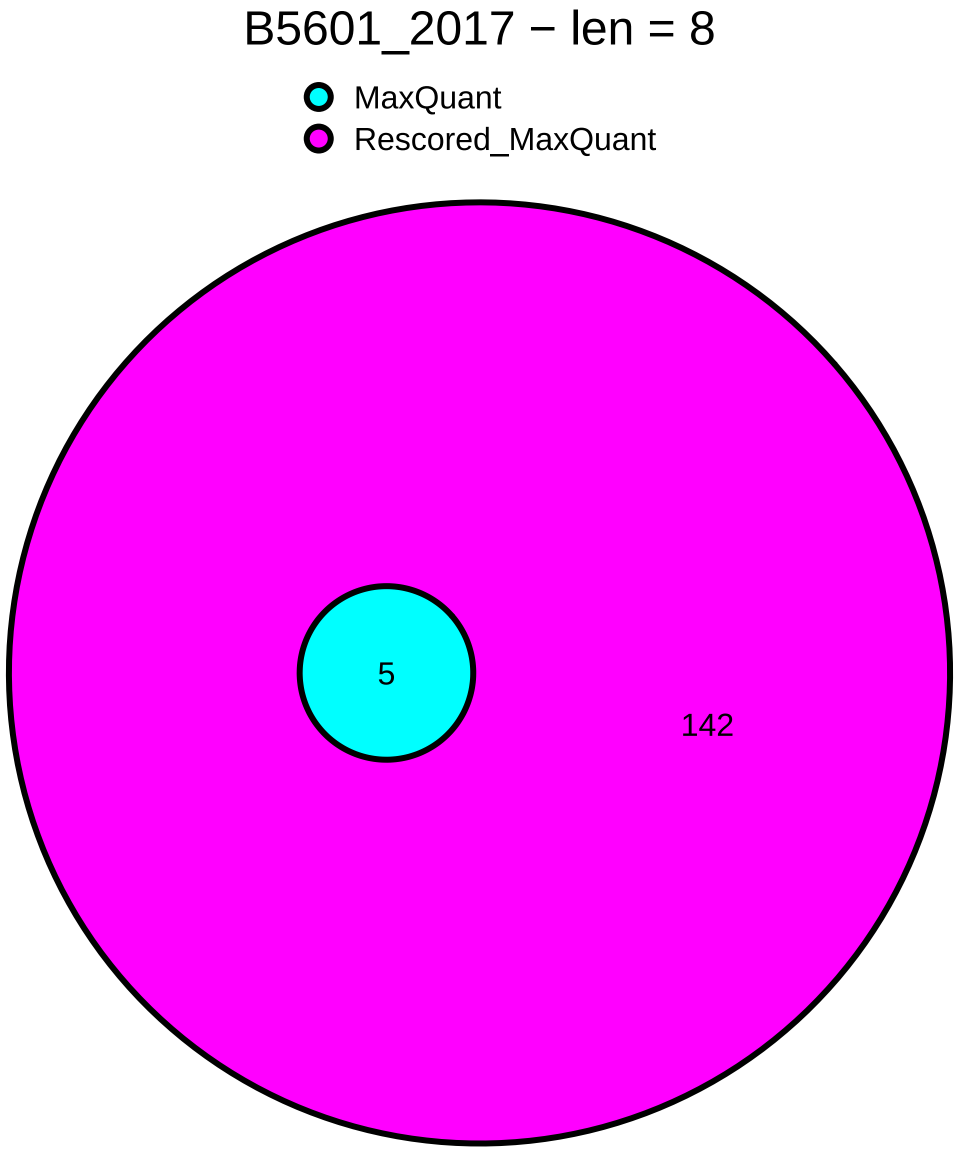

B5601_2017 − len = 8
MaxQuant
Rescored_MaxQuant
5
142

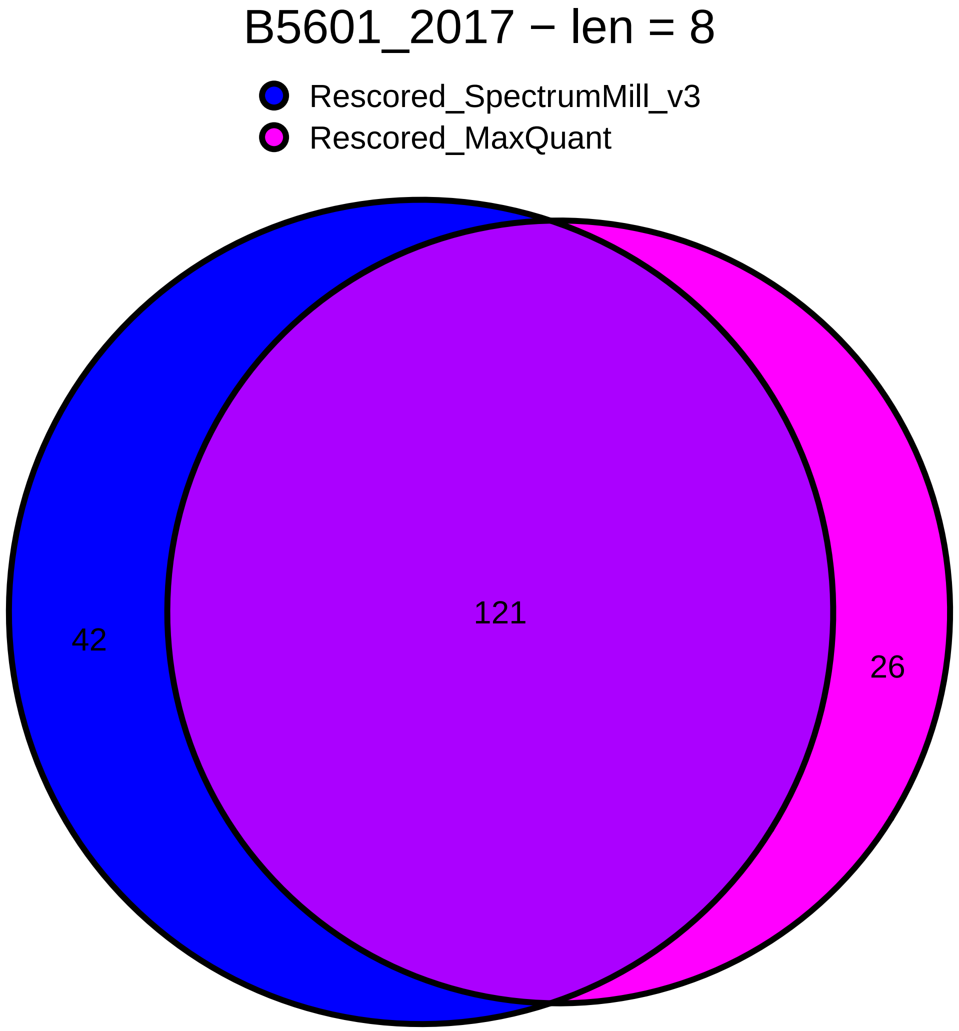

B5601_2017 − len = 8
Rescored_SpectrumMill_v3
Rescored_MaxQuant
42
121
26

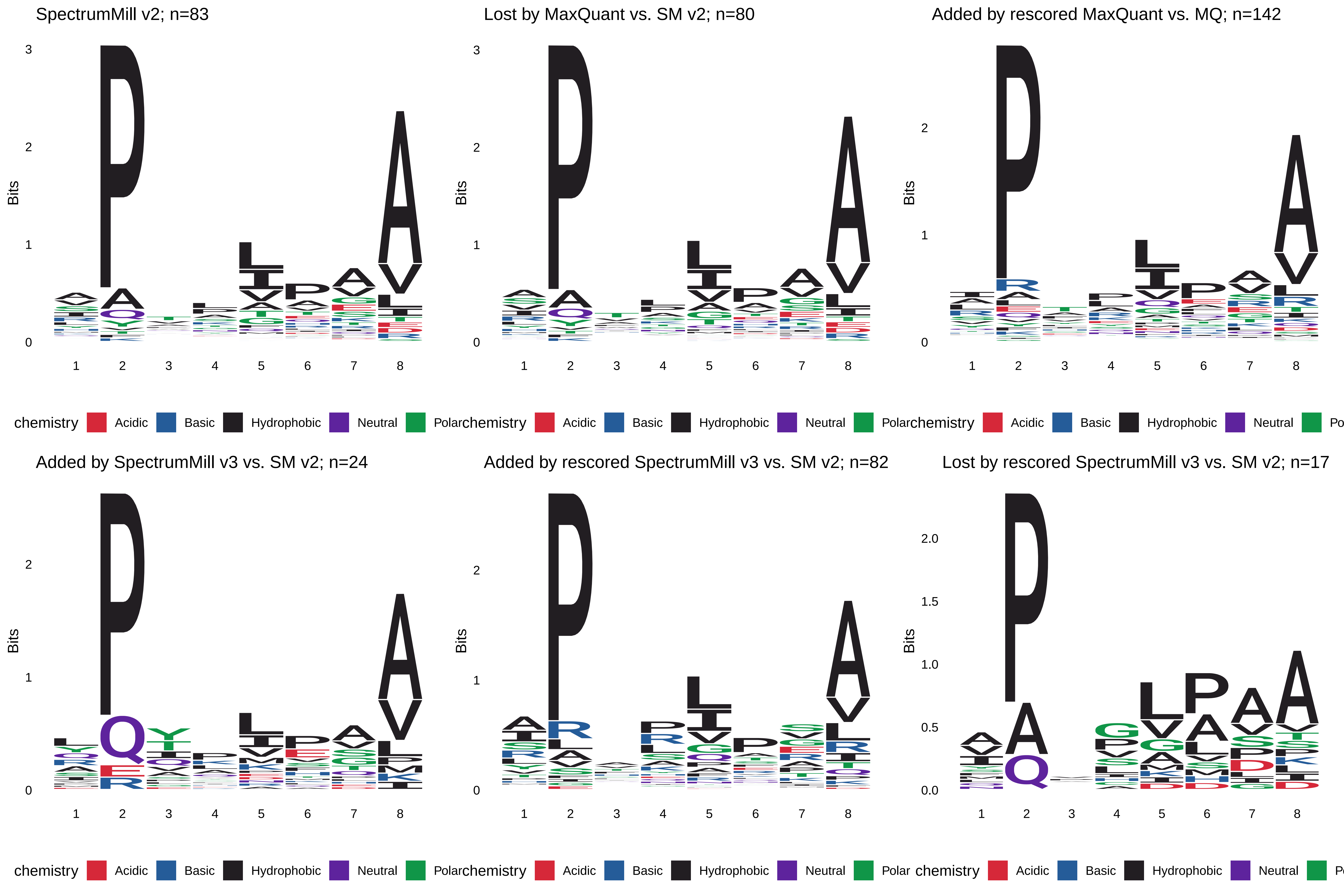
SpectrumMill v2; n=83
Lost by MaxQuant vs. SM v2; n=80
Added by rescored MaxQuant vs. MQ; n=142
Bits
0
1
2
3
1 2 3 4 5 6 7 8
chemistry Acidic Basic Hydrophobic Neutral Polar
Added by SpectrumMill v3 vs. SM v2; n=24
Added by rescored SpectrumMill v3 vs. SM v2; n=82
Lost by rescored SpectrumMill v3 vs. SM v2; n=17
0.0
0.5
1.0
1.5
2.0
chemistry Acidic Basic Hydrophobic Neutral Polar

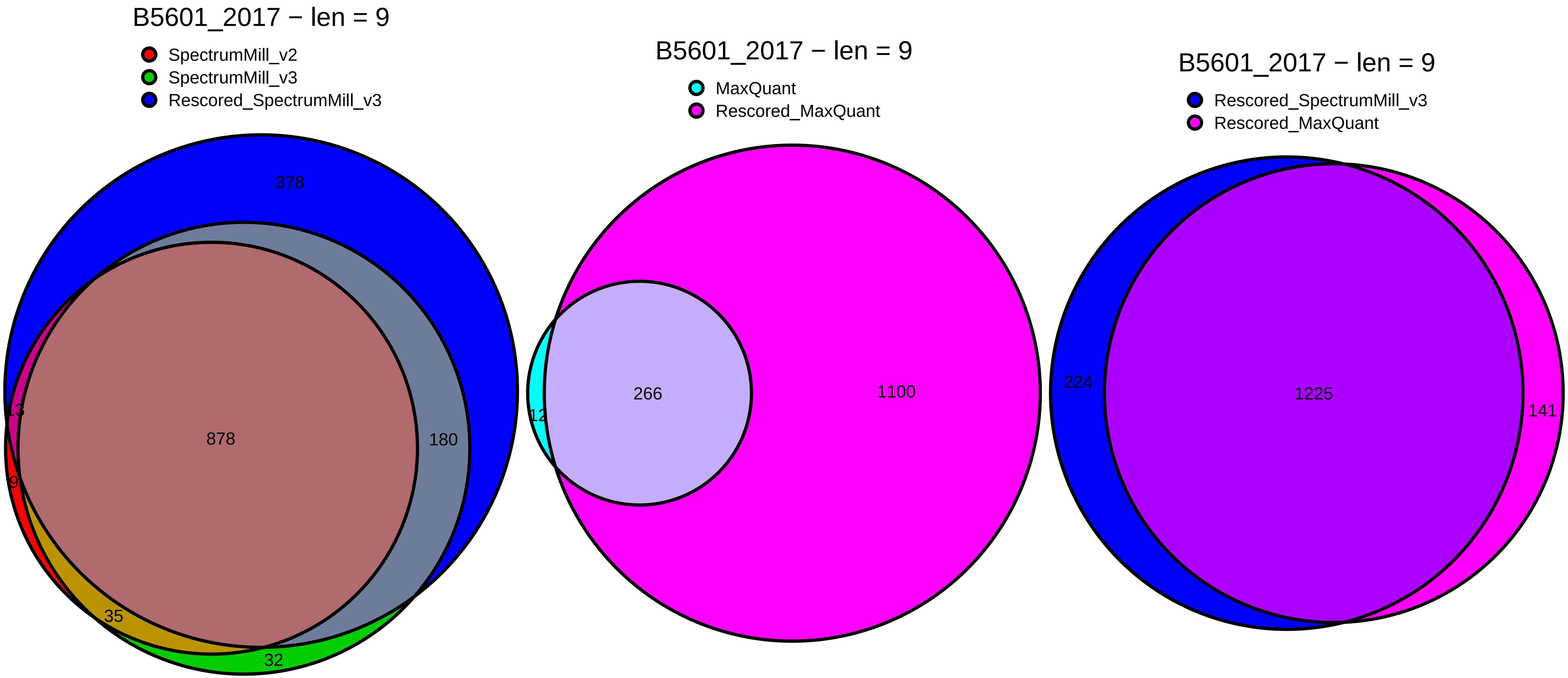
B5601_2017 − len = 9
SpectrumMill_v2
SpectrumMill_v3
Rescored_SpectrumMill_v3
378
878
180
9
35
32
B5601_2017 − len = 9
MaxQuant
Rescored_MaxQuant
266
1100
B5601_2017 − len = 9
Rescored_SpectrumMill_v3
Rescored_MaxQuant
224
1225
141

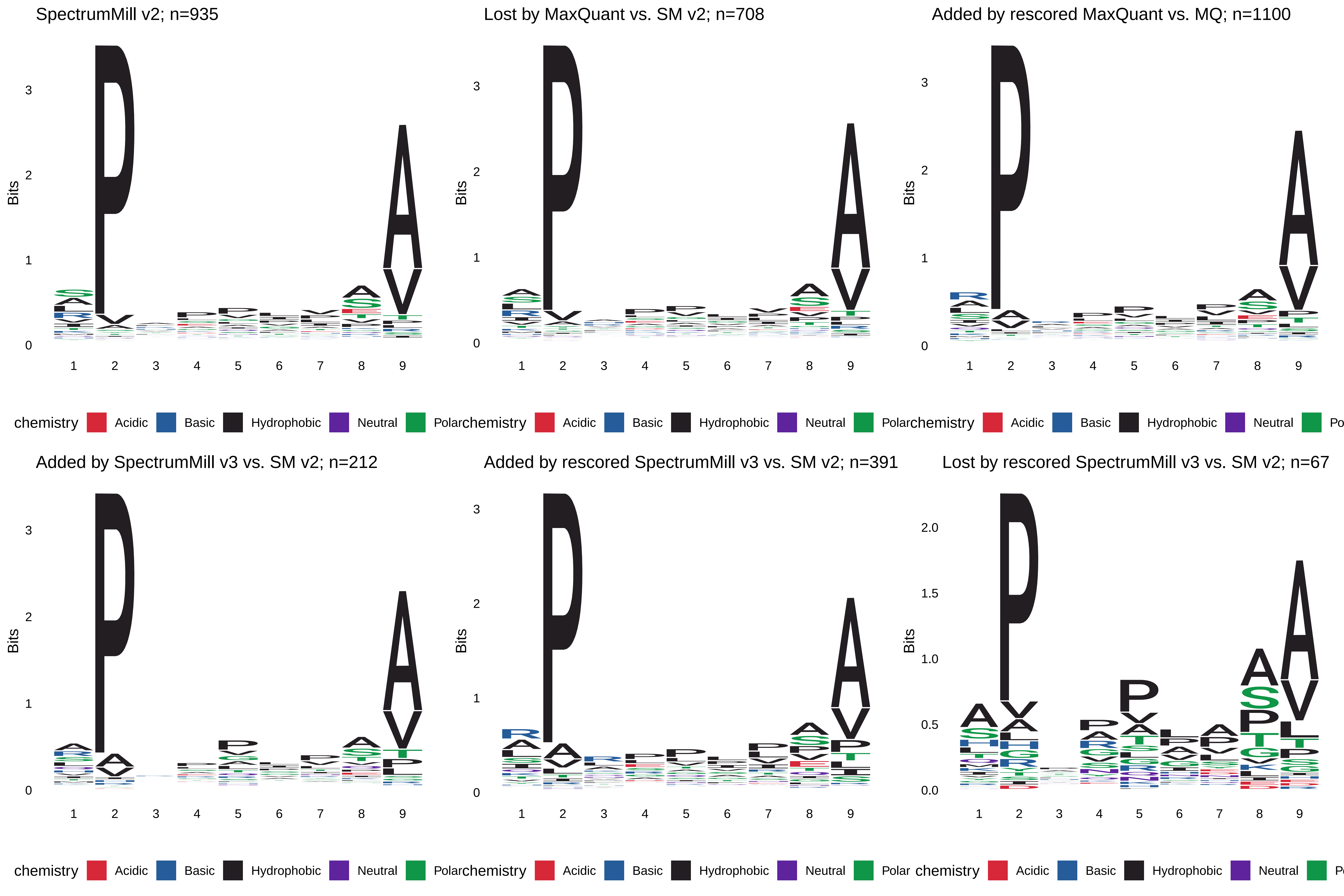
SpectrumMill v2; n=935
Lost by MaxQuant vs. SM v2; n=708
Added by rescored MaxQuant vs. MQ; n=1100
Added by SpectrumMill v3 vs. SM v2; n=212
Added by rescored SpectrumMill v3 vs. SM v2; n=391
Lost by rescored SpectrumMill v3 vs. SM v2; n=67
Bits
0
1
2
3
0.0
0.5
1.0
1.5
2.0
1 2 3 4 5 6 7 8 9
chemistry
Acidic
Basic
Hydrophobic
Neutral
Polar

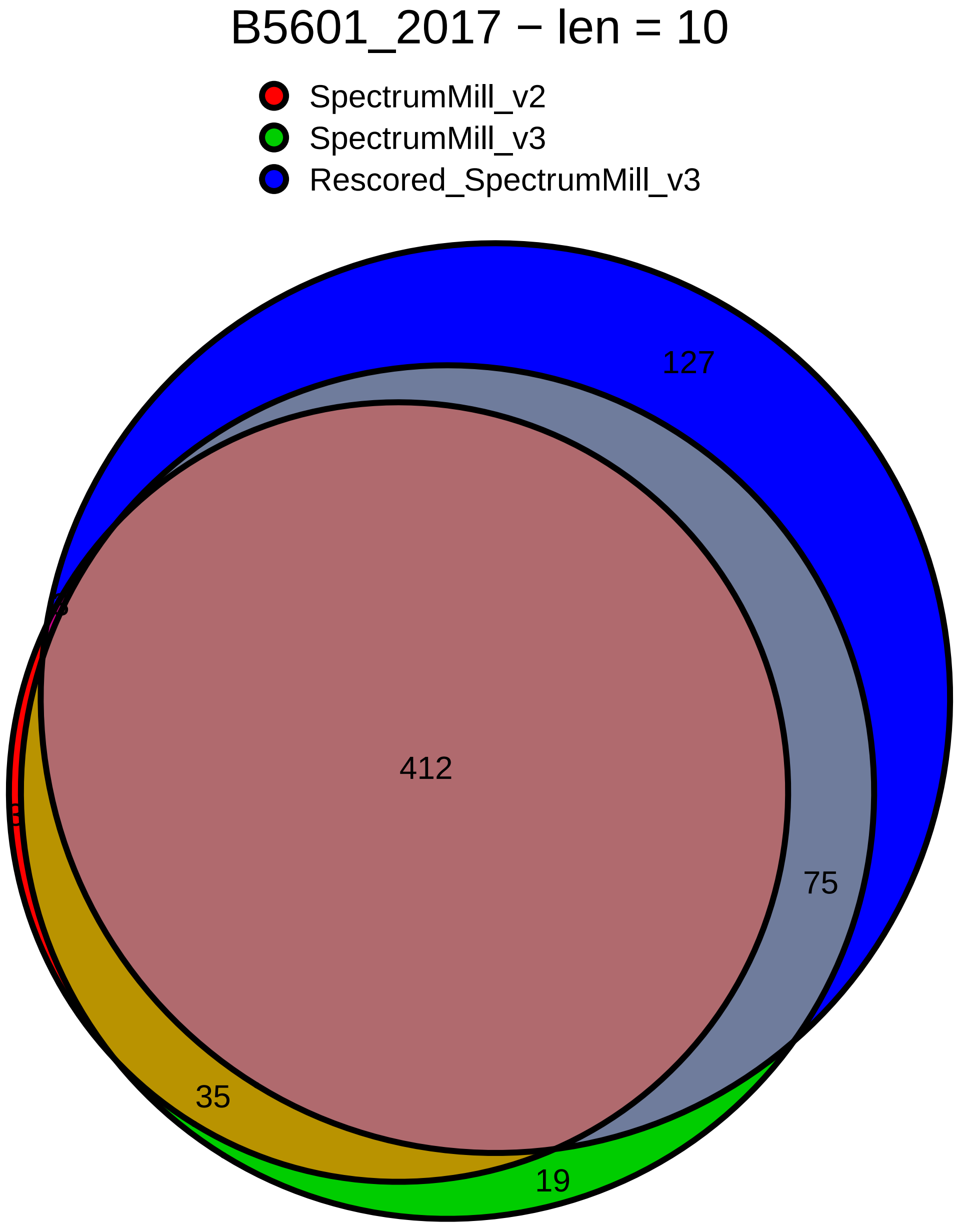
B5601_2017 − len = 10
SpectrumMill_v2
SpectrumMill_v3
Rescored_SpectrumMill_v3
127
412
75
35
19

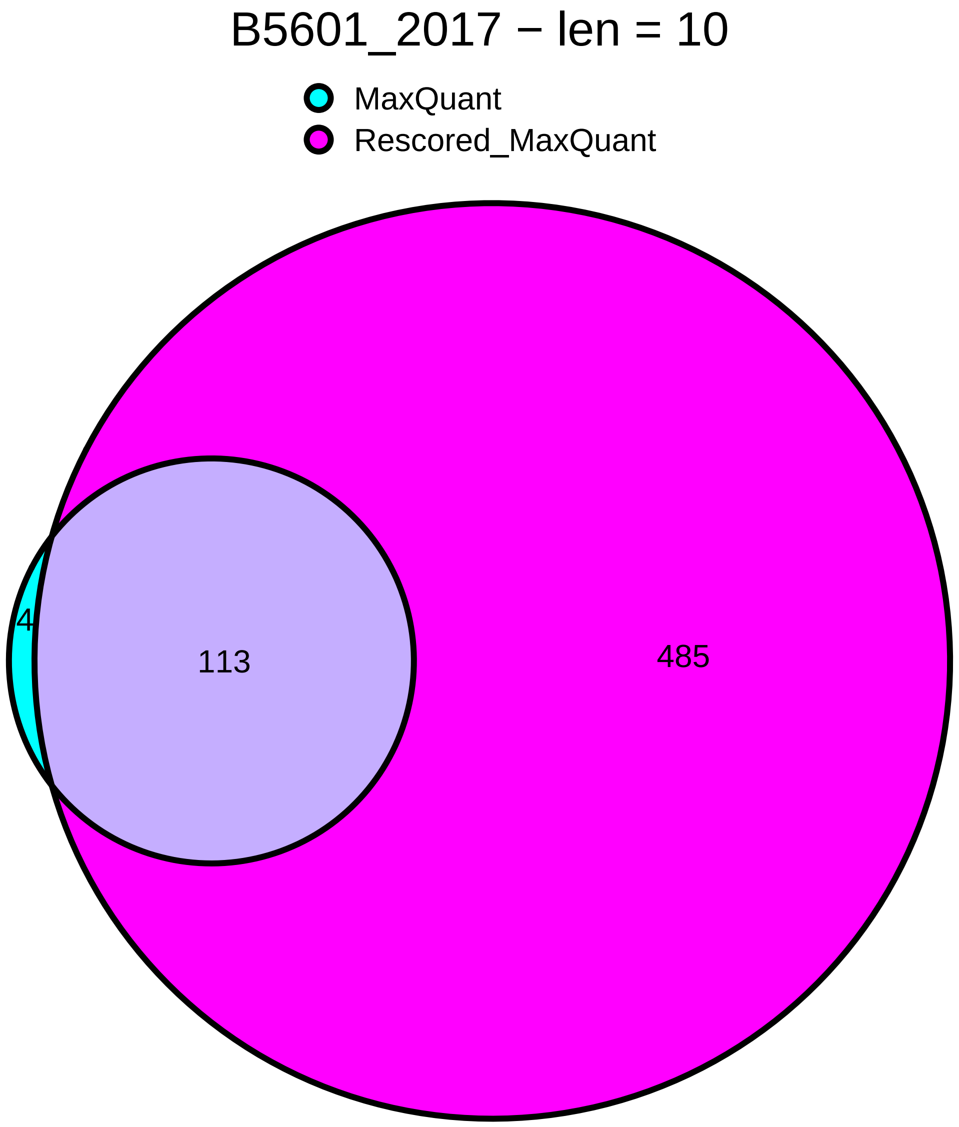
B5601_2017 − len = 10
MaxQuant
Rescored_MaxQuant
4
113
485

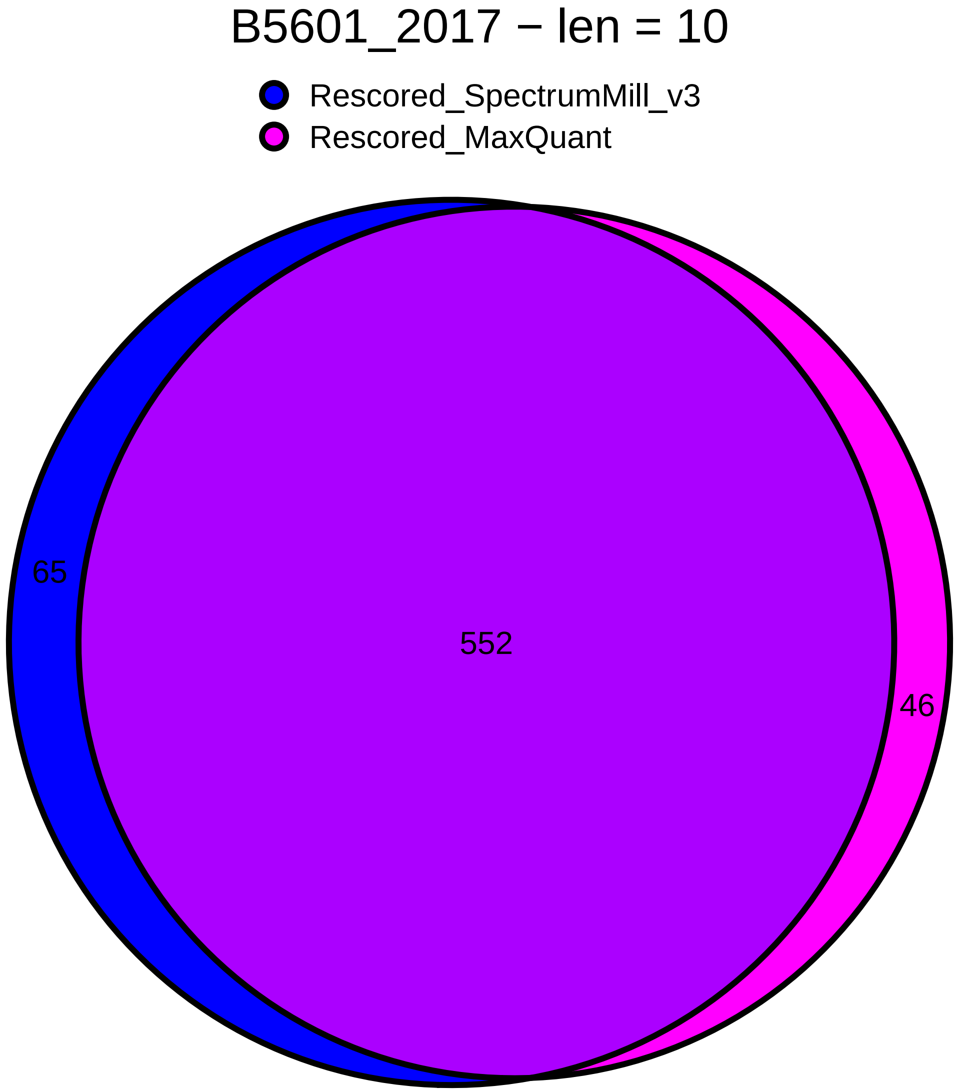
B5601_2017 − len = 10
Rescored_SpectrumMill_v3
Rescored_MaxQuant
65
552
46

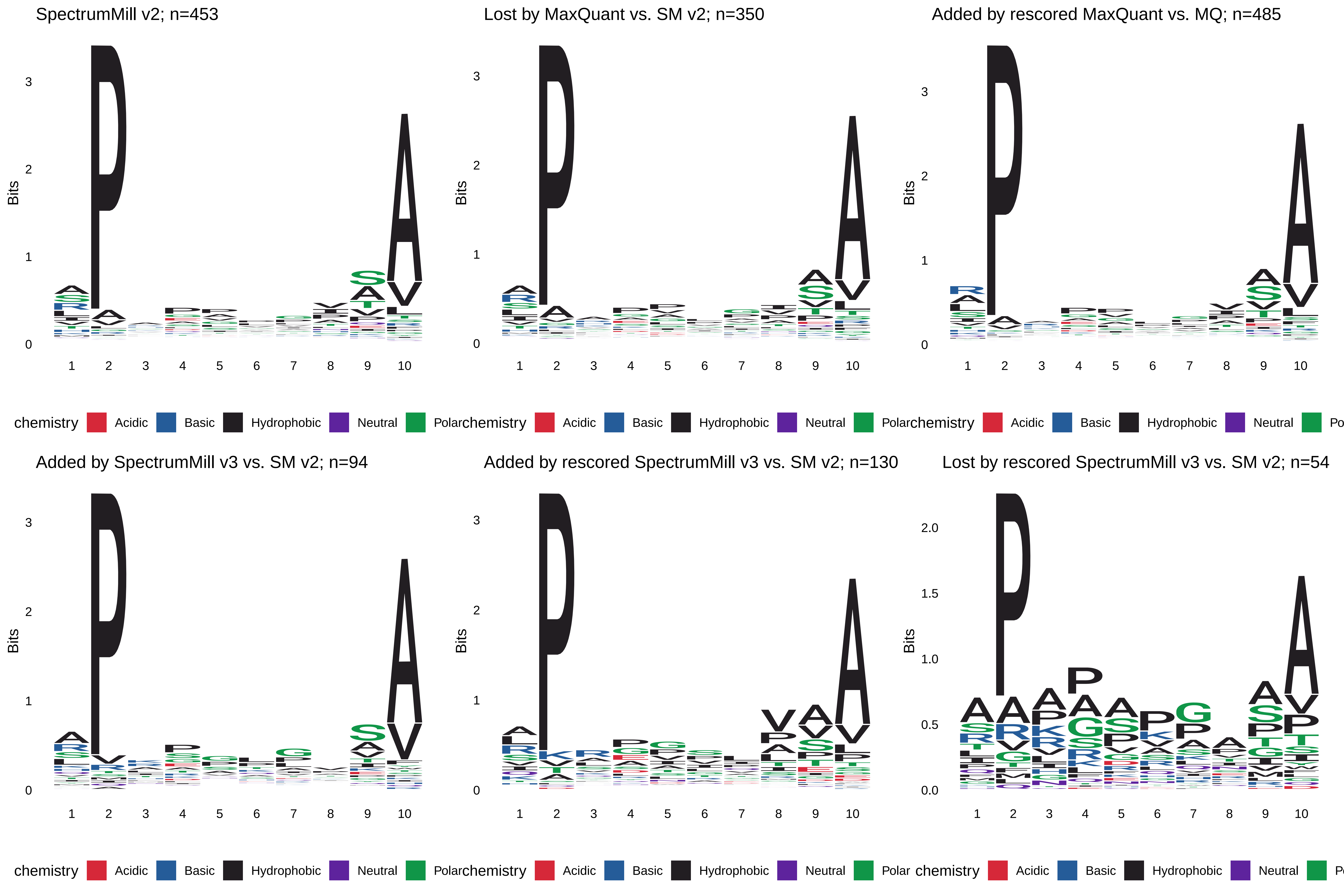

SpectrumMill v2; n=453
Lost by MaxQuant vs. SM v2; n=350
Added by rescored MaxQuant vs. MQ; n=485
Added by SpectrumMill v3 vs. SM v2; n=94
Added by rescored SpectrumMill v3 vs. SM v2; n=130
Lost by rescored SpectrumMill v3 vs. SM v2; n=54
Bits
1 2 3 4 5 6 7 8 9 10
chemistry
Acidic
Basic
Hydrophobic
Neutral
Polar

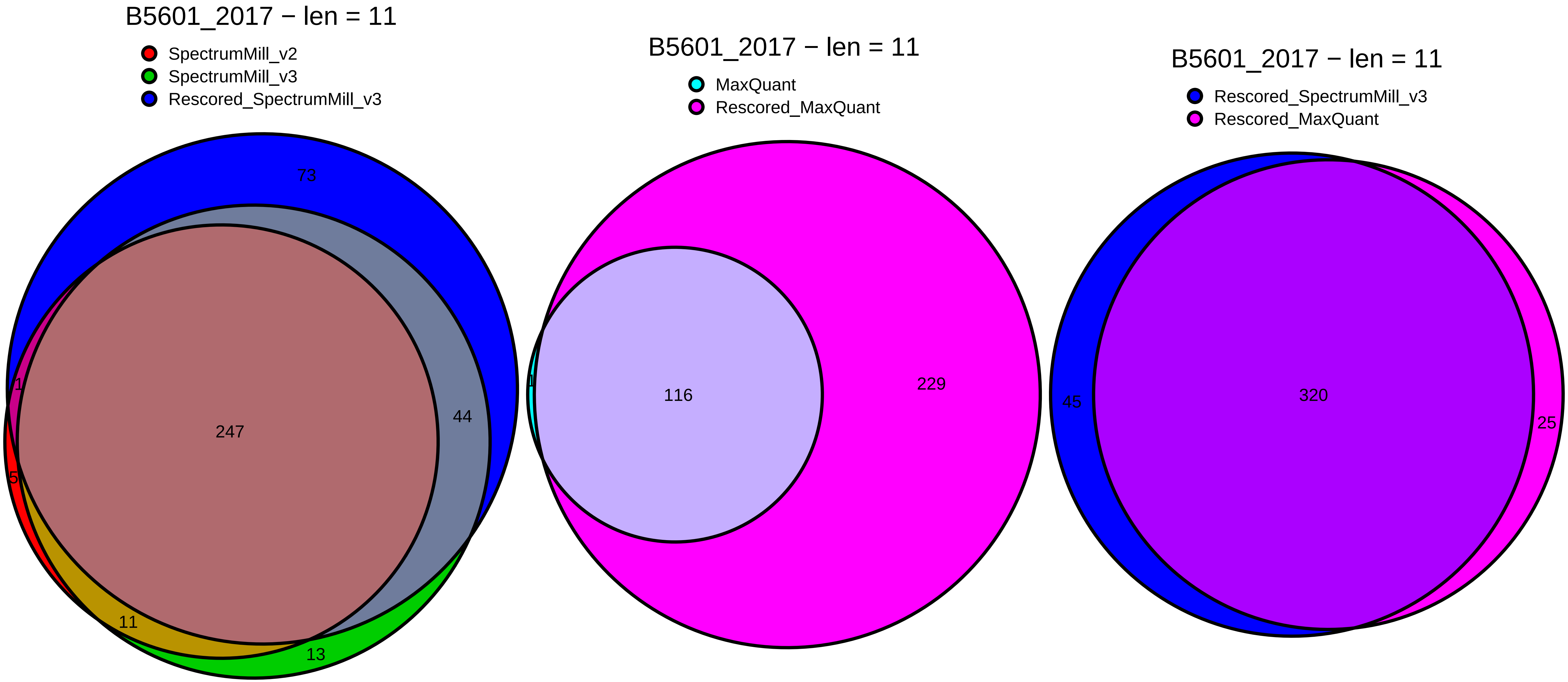

B5601_2017 − len = 11
SpectrumMill_v2
SpectrumMill_v3
Rescored_SpectrumMill_v3
73
44
247
11
13
B5601_2017 − len = 11
MaxQuant
Rescored_MaxQuant
116
229
B5601_2017 − len = 11
Rescored_SpectrumMill_v3
Rescored_MaxQuant
45
320
25

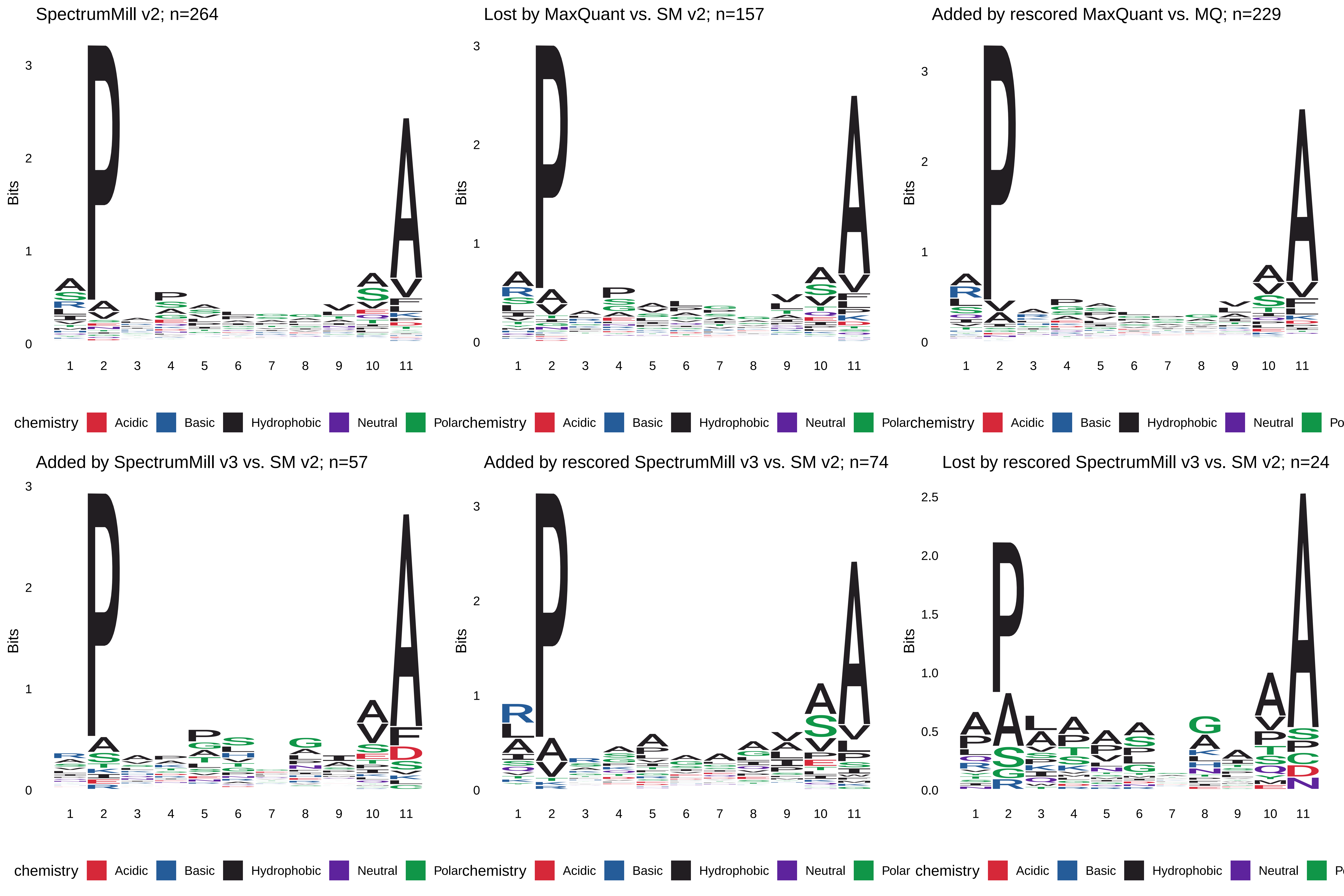

SpectrumMill v2; n=264
Lost by MaxQuant vs. SM v2; n=157
Added by rescored MaxQuant vs. MQ; n=229
Added by SpectrumMill v3 vs. SM v2; n=57
Added by rescored SpectrumMill v3 vs. SM v2; n=74
Lost by rescored SpectrumMill v3 vs. SM v2; n=24
Bits
1 2 3 4 5 6 7 8 9 10 11
chemistry
Acidic
Basic
Hydrophobic
Neutral
Polar

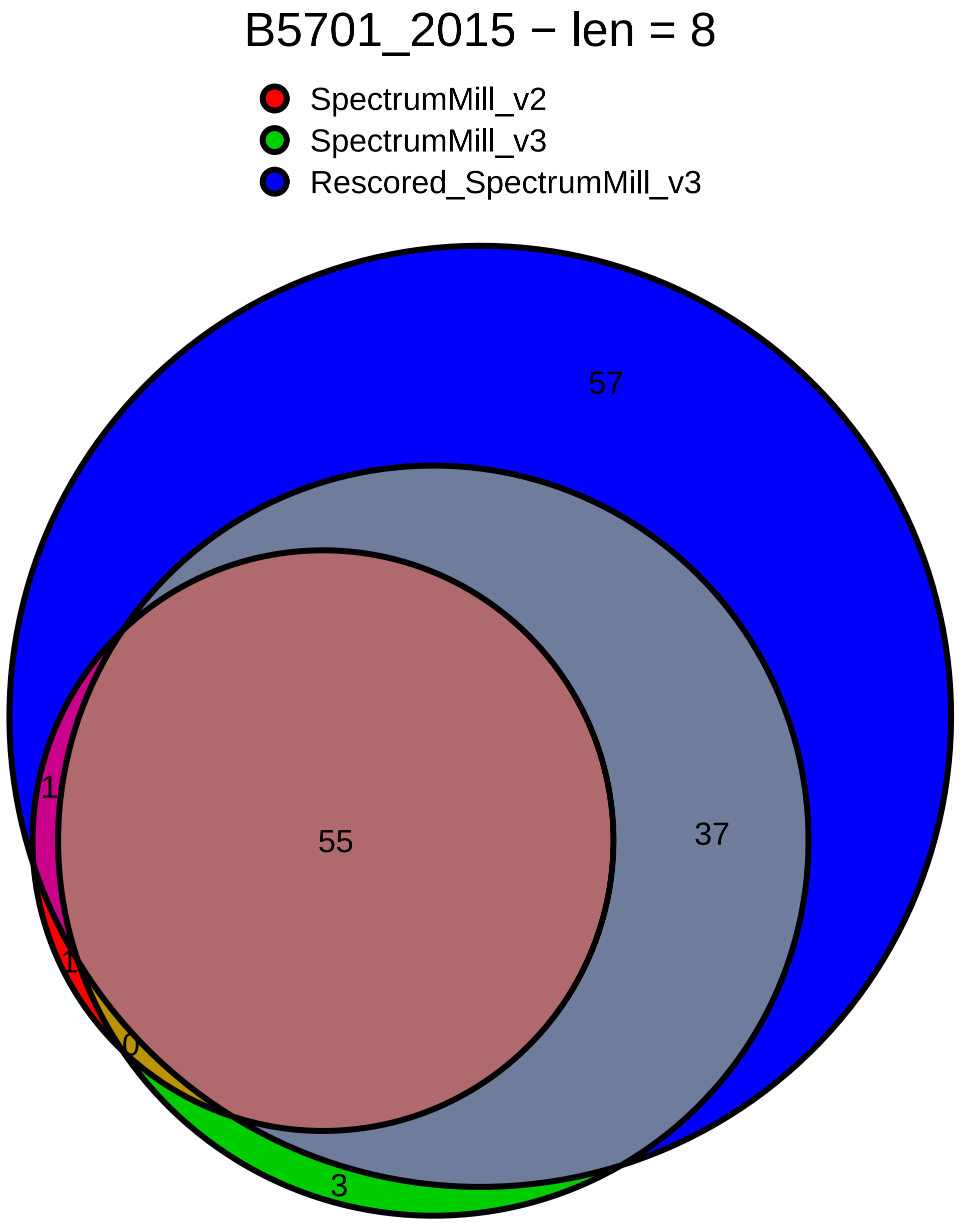
B5701_2015 − len = 8
SpectrumMill_v2
SpectrumMill_v3
Rescored_SpectrumMill_v3
57
1
55
37
1
0
3
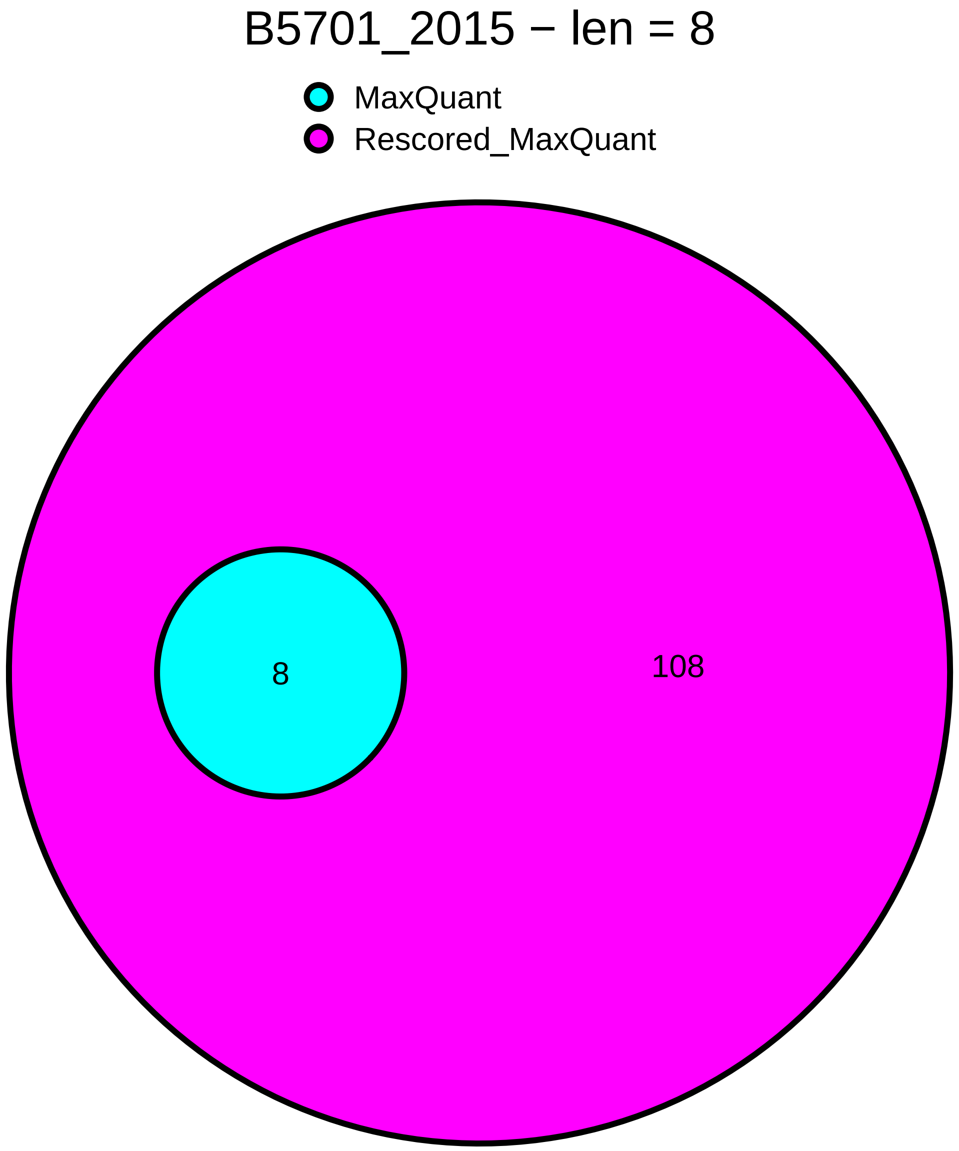
B5701_2015 − len = 8
MaxQuant
Rescored_MaxQuant
8
108
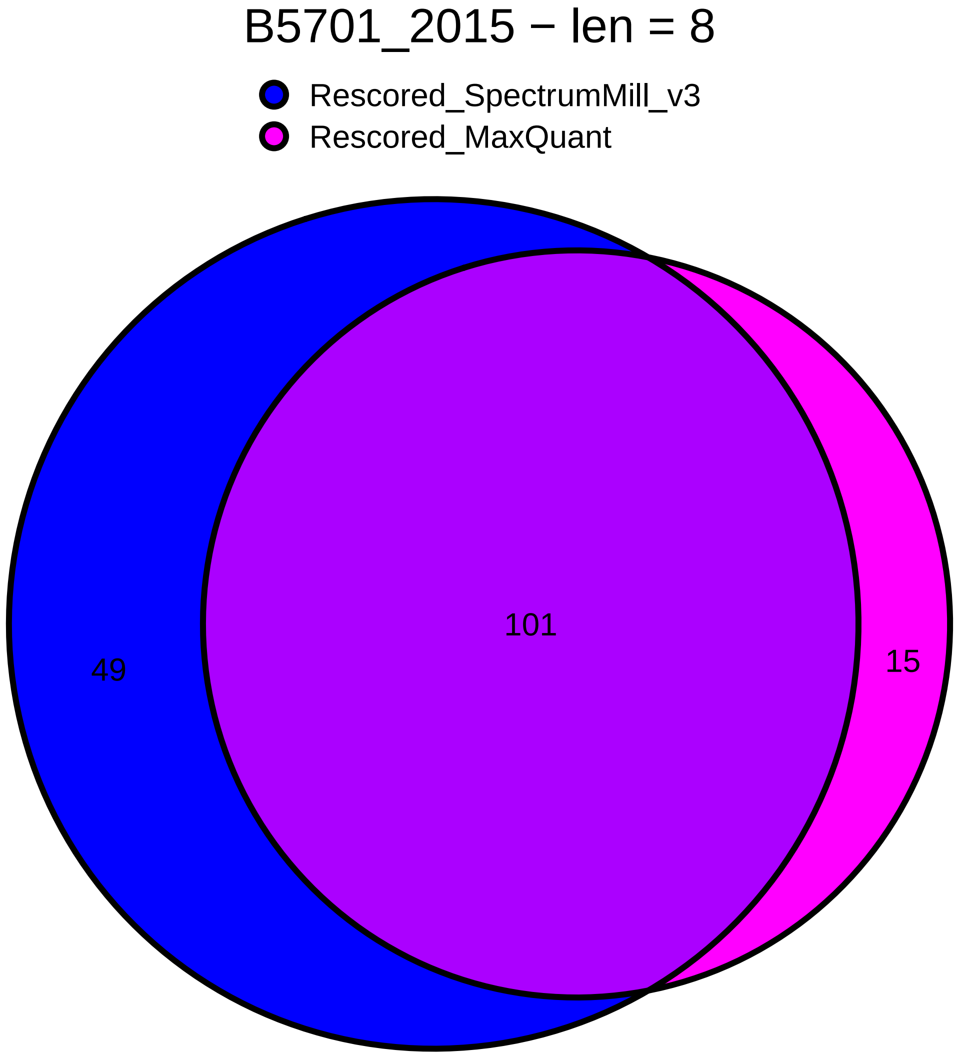
B5701_2015 − len = 8
Rescored_SpectrumMill_v3
Rescored_MaxQuant
49
101
15

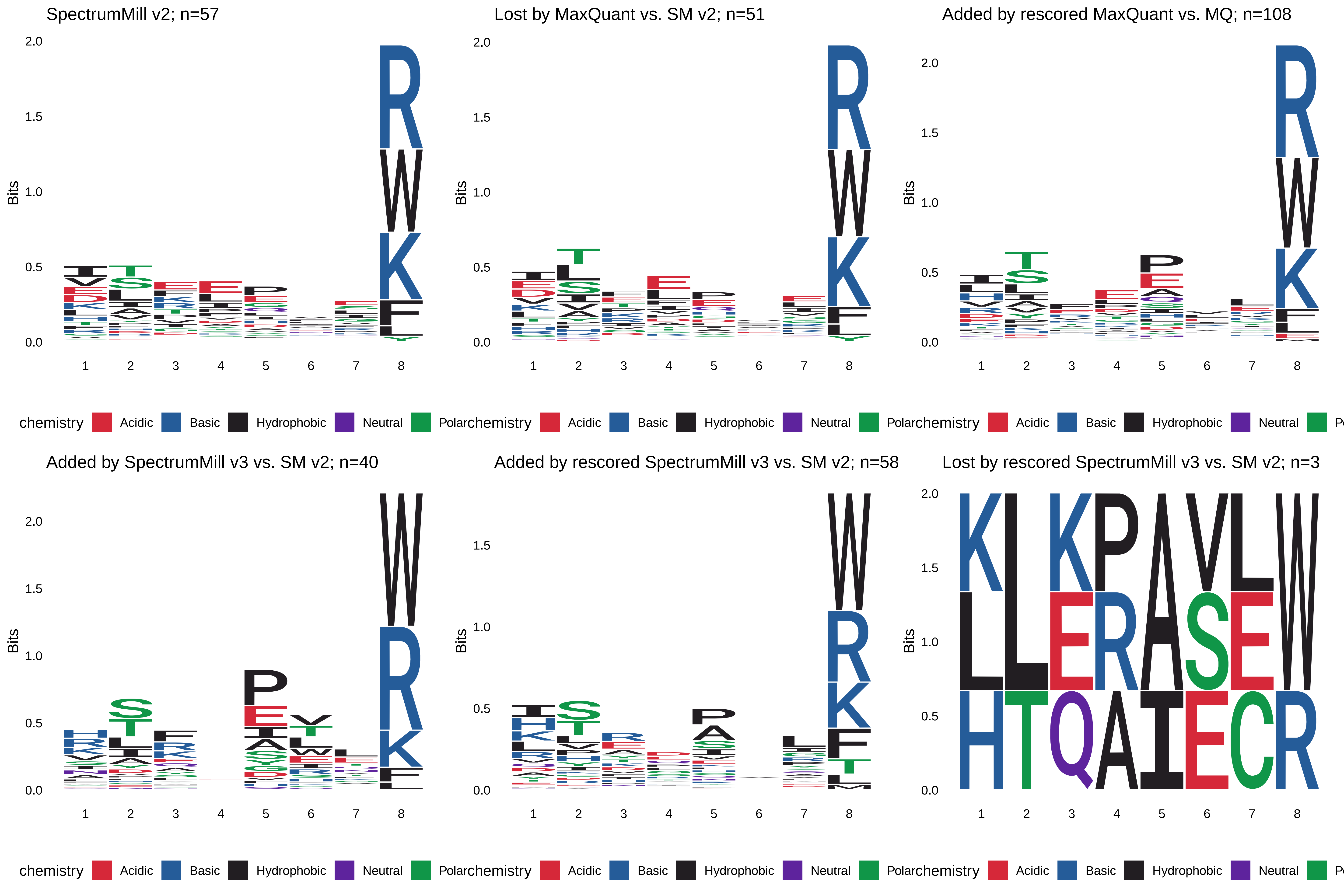
SpectrumMill v2; n=57
Lost by MaxQuant vs. SM v2; n=51
Added by rescored MaxQuant vs. MQ; n=108
Added by SpectrumMill v3 vs. SM v2; n=40
Added by rescored SpectrumMill v3 vs. SM v2; n=58
Lost by rescored SpectrumMill v3 vs. SM v2; n=3
Bits
chemistry
Acidic
Basic
Hydrophobic
Neutral
Polar

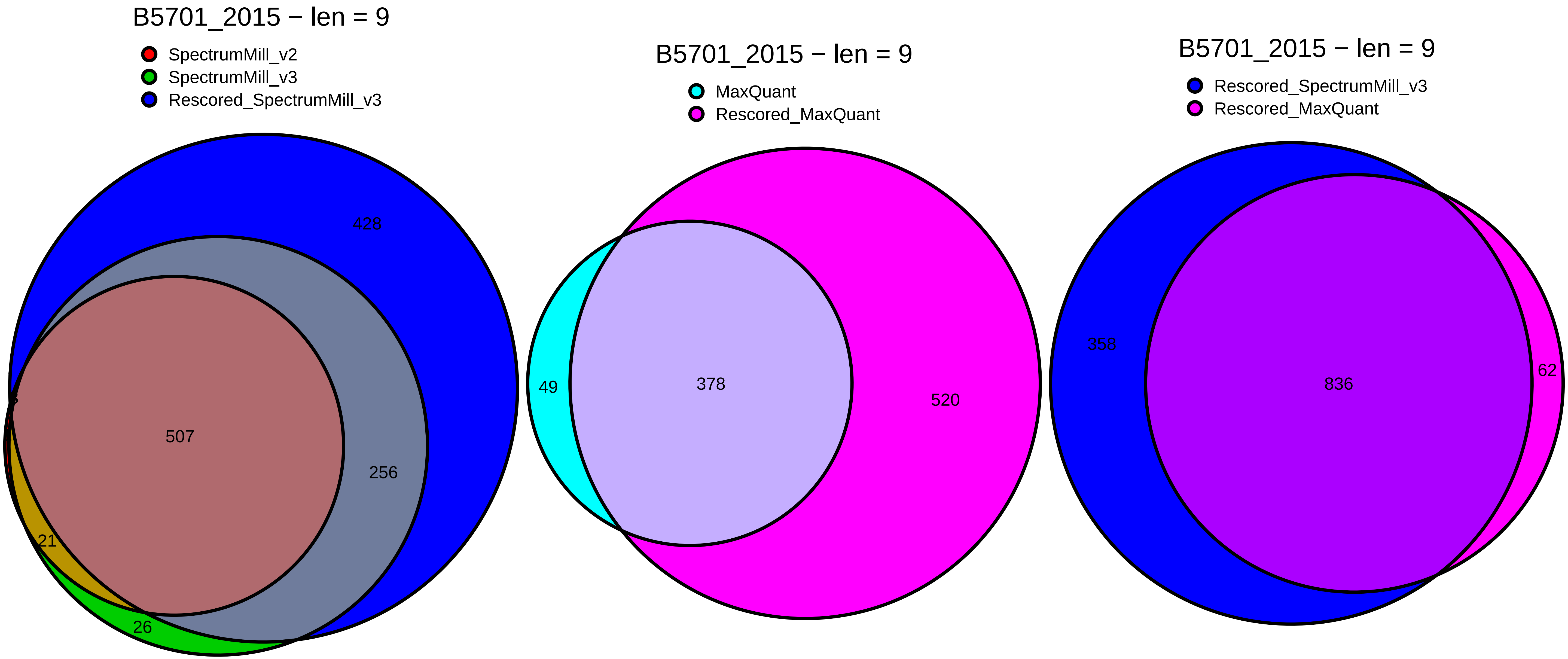
B5701_2015 − len = 9
SpectrumMill_v2
SpectrumMill_v3
Rescored_SpectrumMill_v3
428
507
256
21
26
B5701_2015 − len = 9
MaxQuant
Rescored_MaxQuant
49
378
520
B5701_2015 − len = 9
Rescored_SpectrumMill_v3
Rescored_MaxQuant
358
836
62

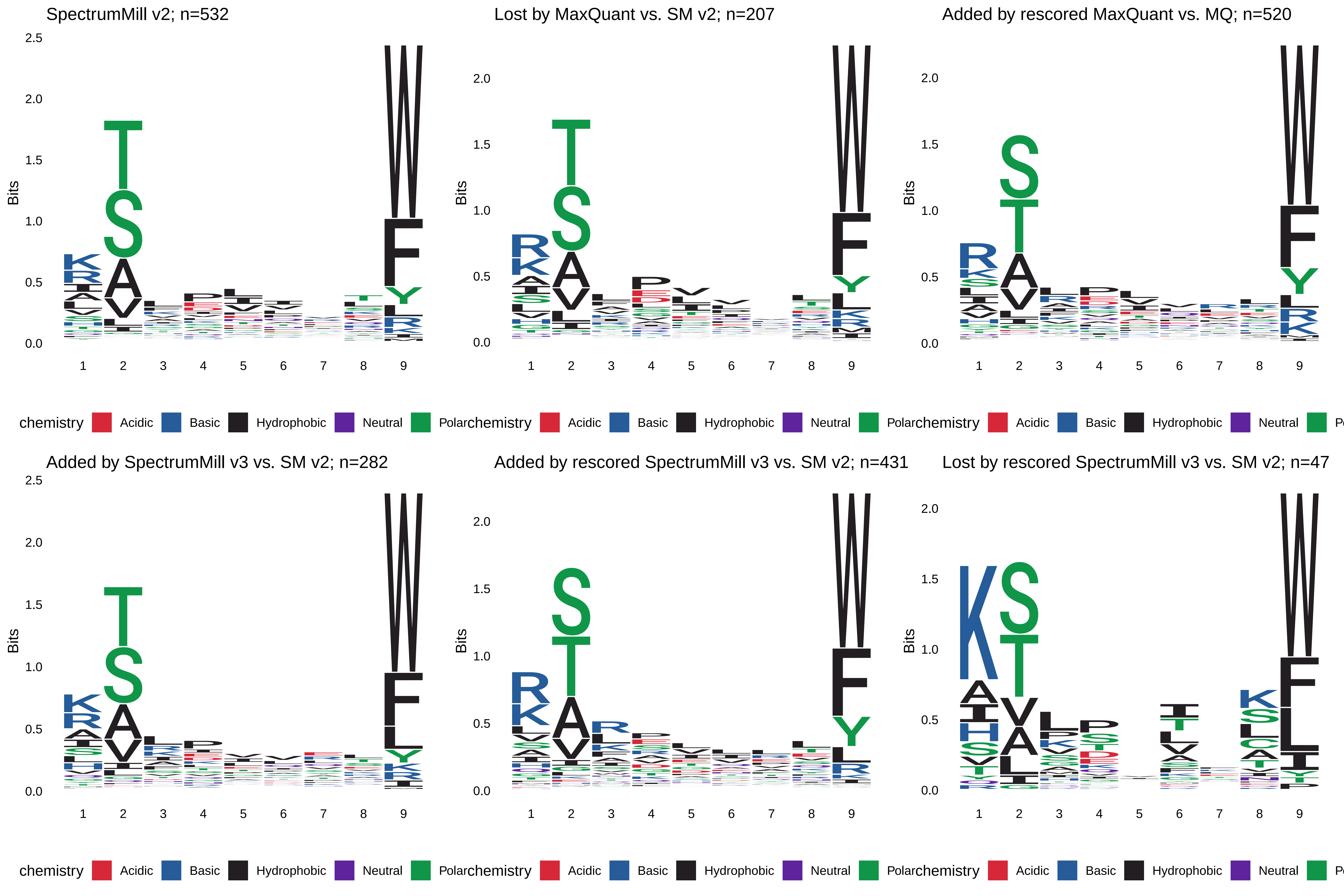
SpectrumMill v2; n=532
Lost by MaxQuant vs. SM v2; n=207
Added by rescored MaxQuant vs. MQ; n=520
Added by SpectrumMill v3 vs. SM v2; n=282
Added by rescored SpectrumMill v3 vs. SM v2; n=431
Lost by rescored SpectrumMill v3 vs. SM v2; n=47
Bits
0.0
0.5
1.0
1.5
2.0
2.5
1 2 3 4 5 6 7 8 9
chemistry
Acidic
Basic
Hydrophobic
Neutral
Polar

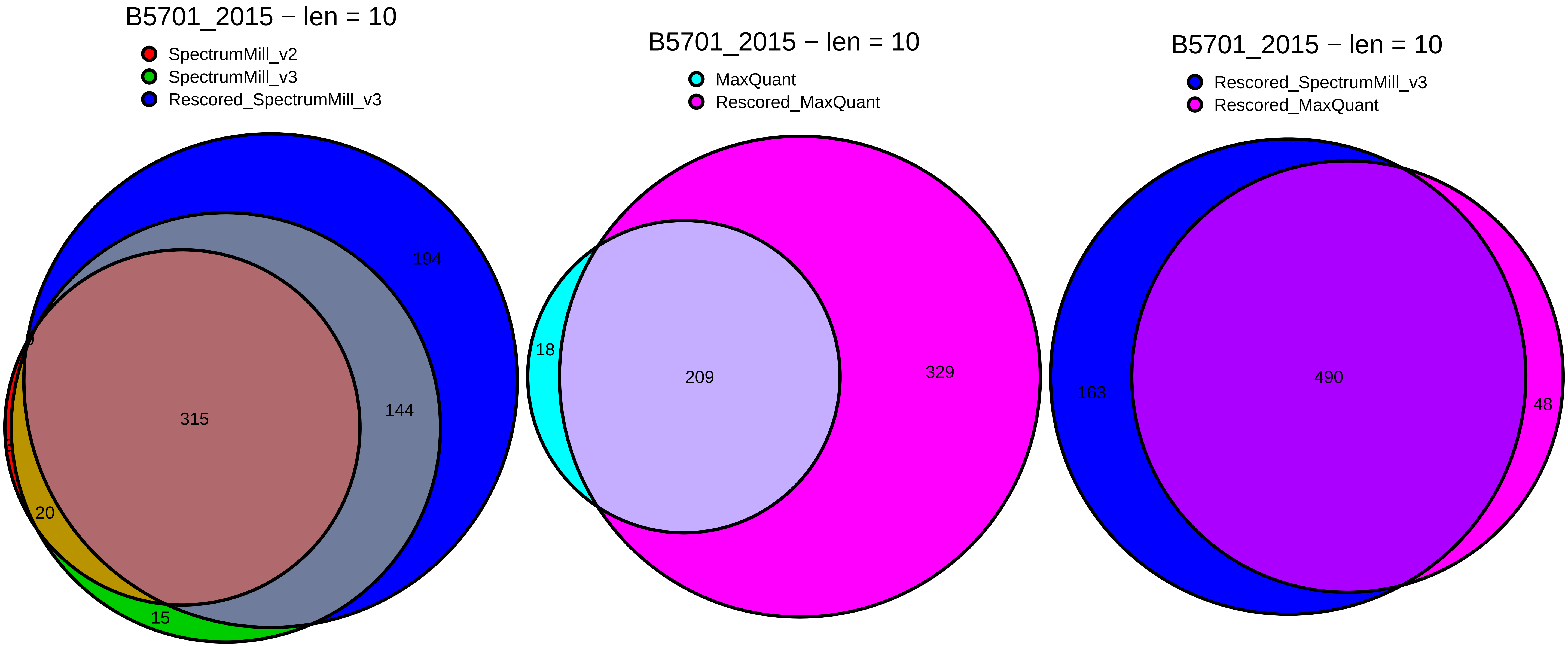
B5701_2015 − len = 10
SpectrumMill_v2
SpectrumMill_v3
Rescored_SpectrumMill_v3
194
0
315
144
20
15
B5701_2015 − len = 10
MaxQuant
Rescored_MaxQuant
18
209
329
B5701_2015 − len = 10
Rescored_SpectrumMill_v3
Rescored_MaxQuant
163
490
48

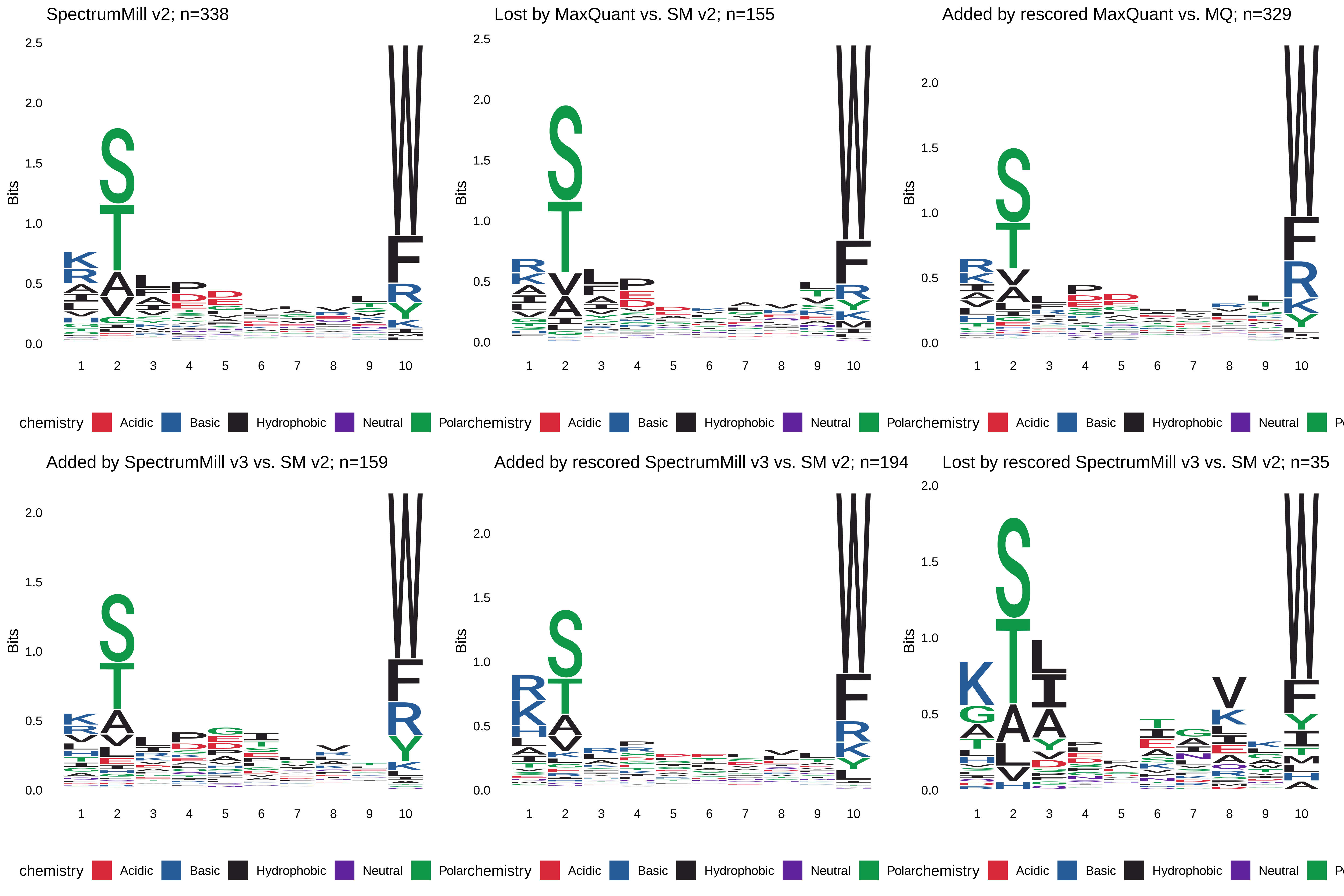
SpectrumMill v2; n=338
Lost by MaxQuant vs. SM v2; n=155
Added by rescored MaxQuant vs. MQ; n=329
Added by SpectrumMill v3 vs. SM v2; n=159
Added by rescored SpectrumMill v3 vs. SM v2; n=194
Lost by rescored SpectrumMill v3 vs. SM v2; n=35
Bits
0.0
0.5
1.0
1.5
2.0
2.5
1 2 3 4 5 6 7 8 9 10
chemistry
Acidic
Basic
Hydrophobic
Neutral
Polar

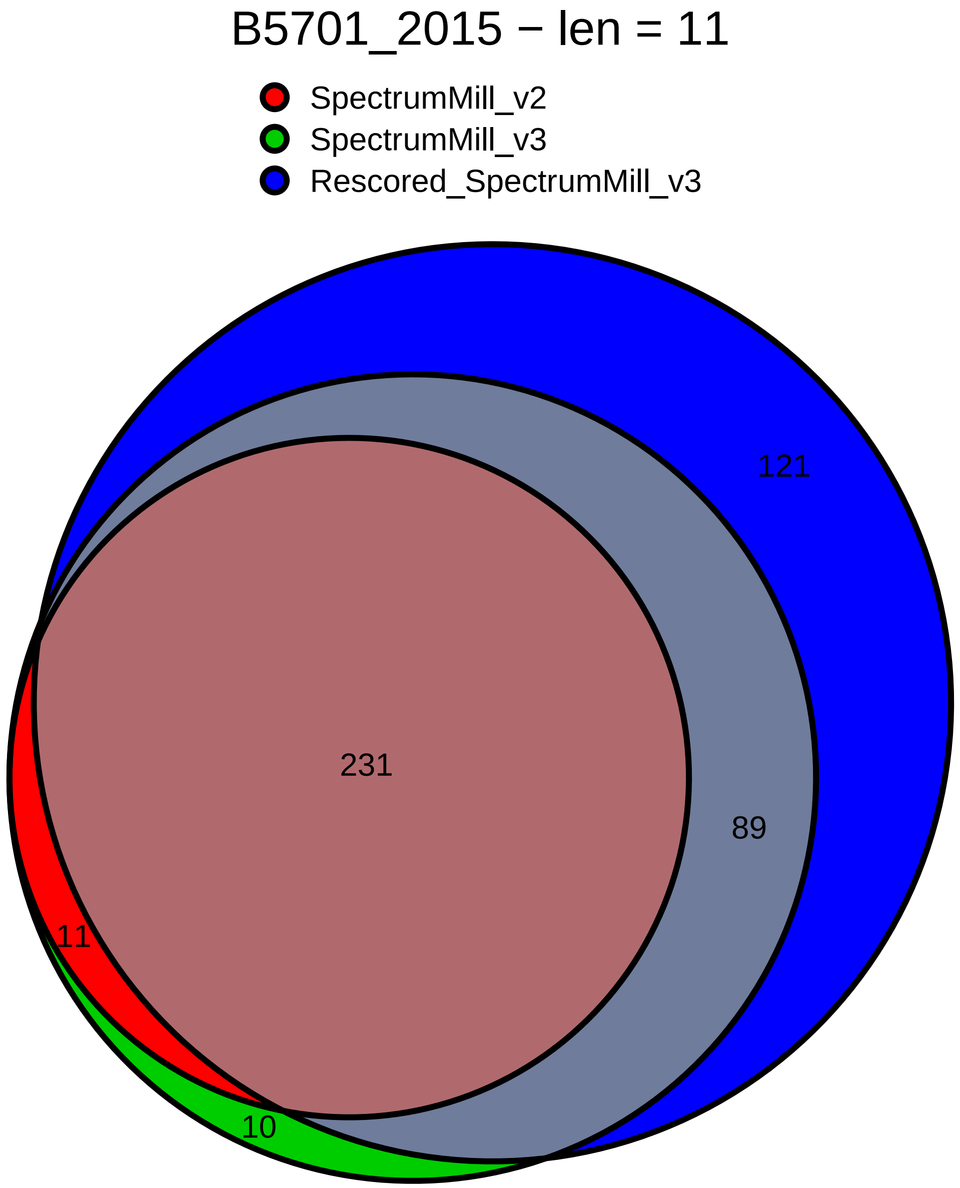
B5701_2015 − len = 11
SpectrumMill_v2
SpectrumMill_v3
Rescored_SpectrumMill_v3
121
231
89
11
10

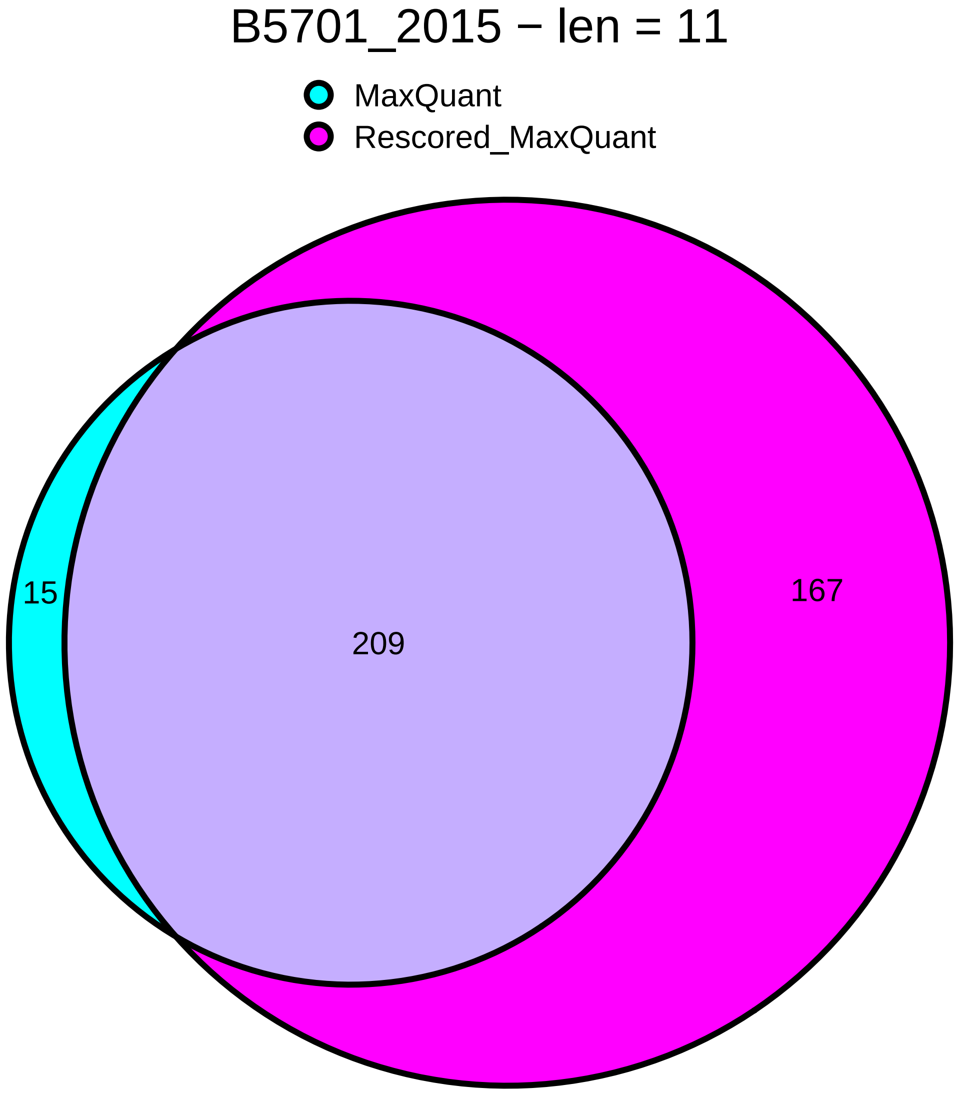
B5701_2015 − len = 11
MaxQuant
Rescored_MaxQuant
15
209
167

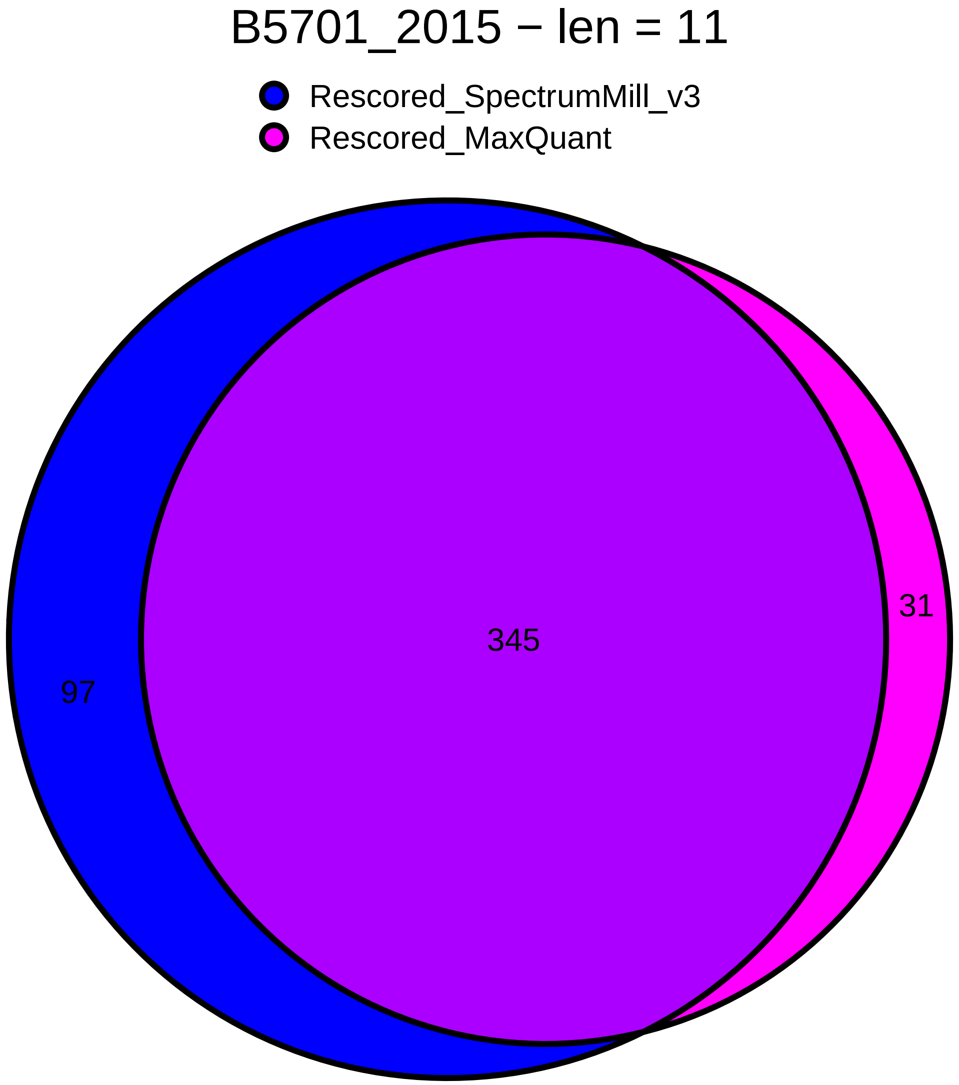
B5701_2015 − len = 11
Rescored_SpectrumMill_v3
Rescored_MaxQuant
97
345
31

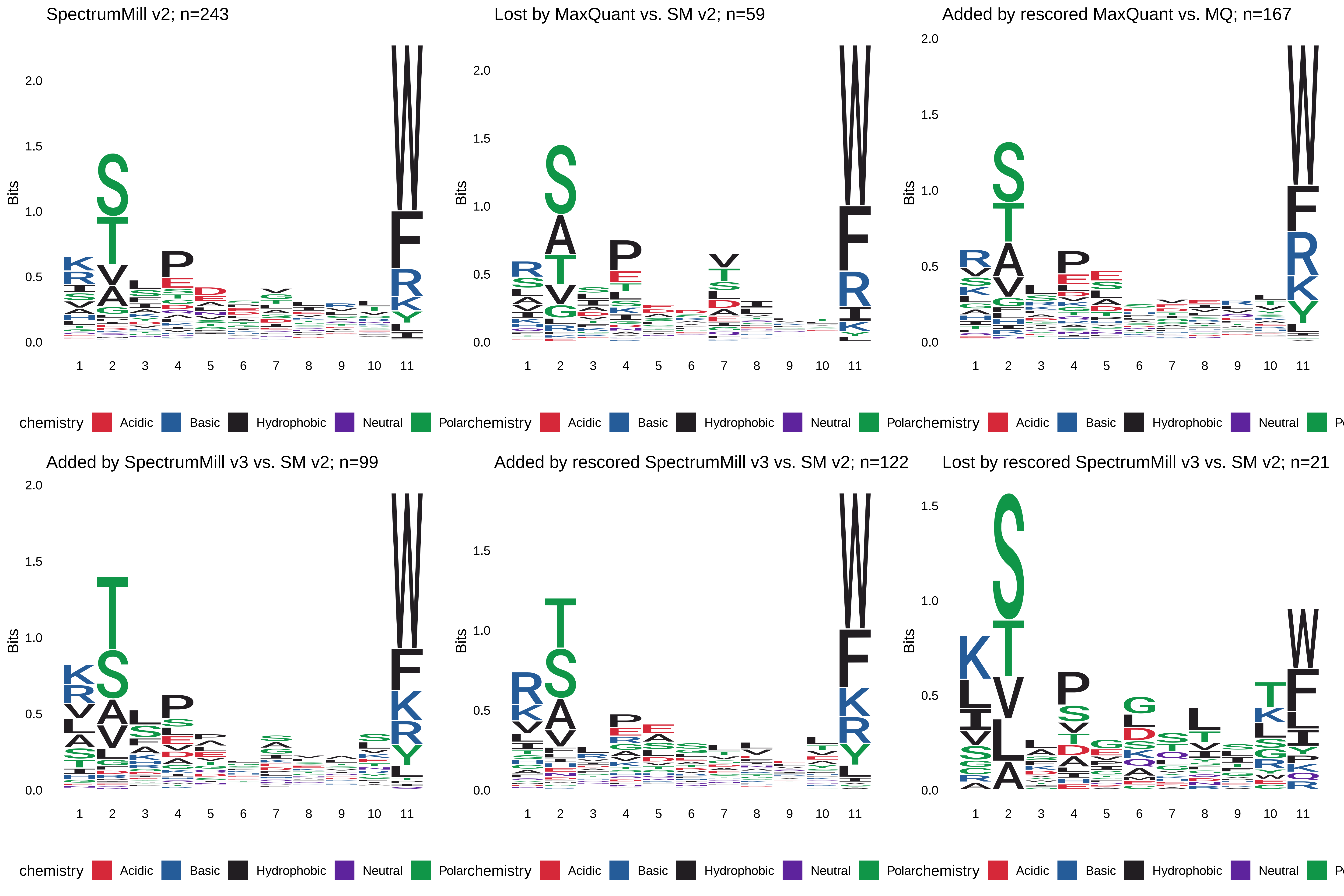
SpectrumMill v2; n=243
Lost by MaxQuant vs. SM v2; n=59
Added by rescored MaxQuant vs. MQ; n=167
Added by SpectrumMill v3 vs. SM v2; n=99
Added by rescored SpectrumMill v3 vs. SM v2; n=122
Lost by rescored SpectrumMill v3 vs. SM v2; n=21
Bits
chemistry Acidic Basic Hydrophobic Neutral Polar

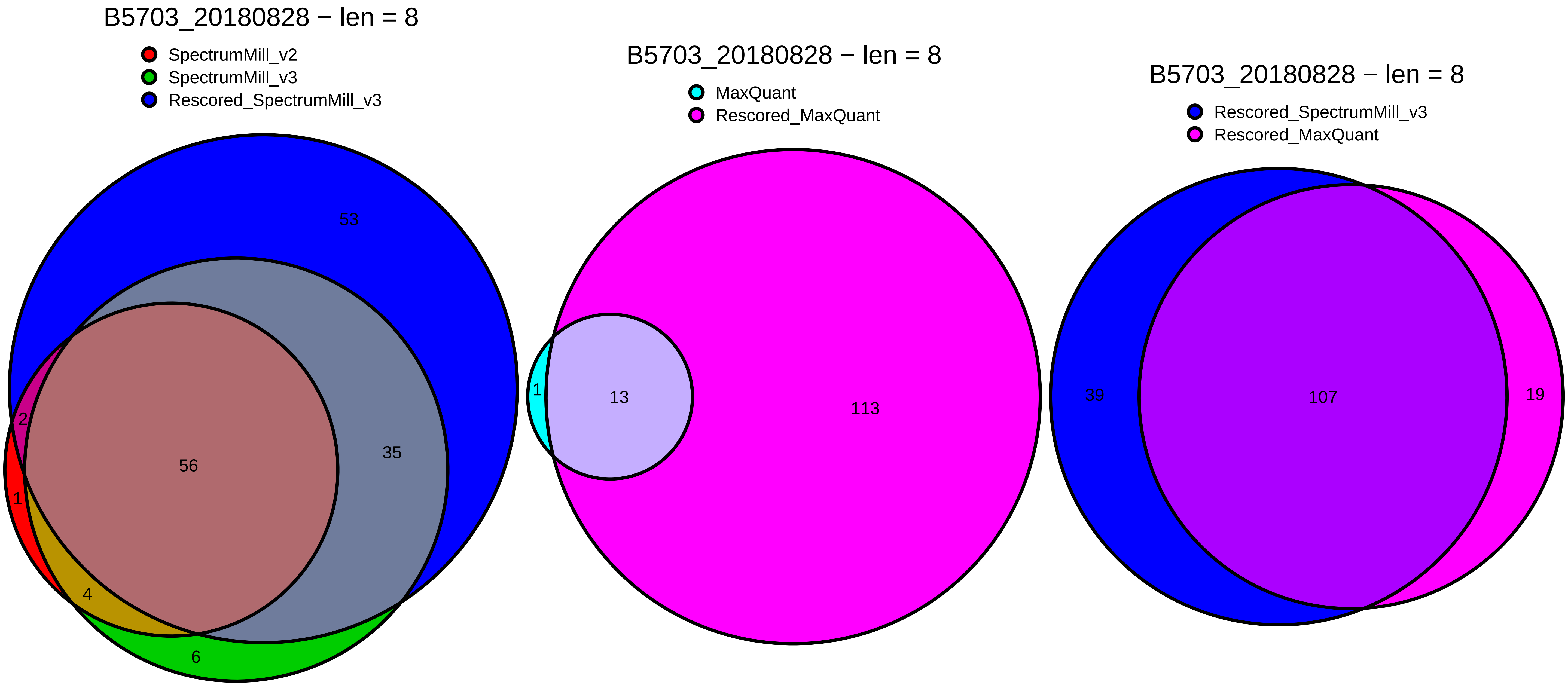
B5703_20180828 − len = 8
SpectrumMill_v2
SpectrumMill_v3
Rescored_SpectrumMill_v3
53
2
56
35
1
4
6
B5703_20180828 − len = 8
MaxQuant
Rescored_MaxQuant
1
13
113
B5703_20180828 − len = 8
Rescored_SpectrumMill_v3
Rescored_MaxQuant
39
107
19

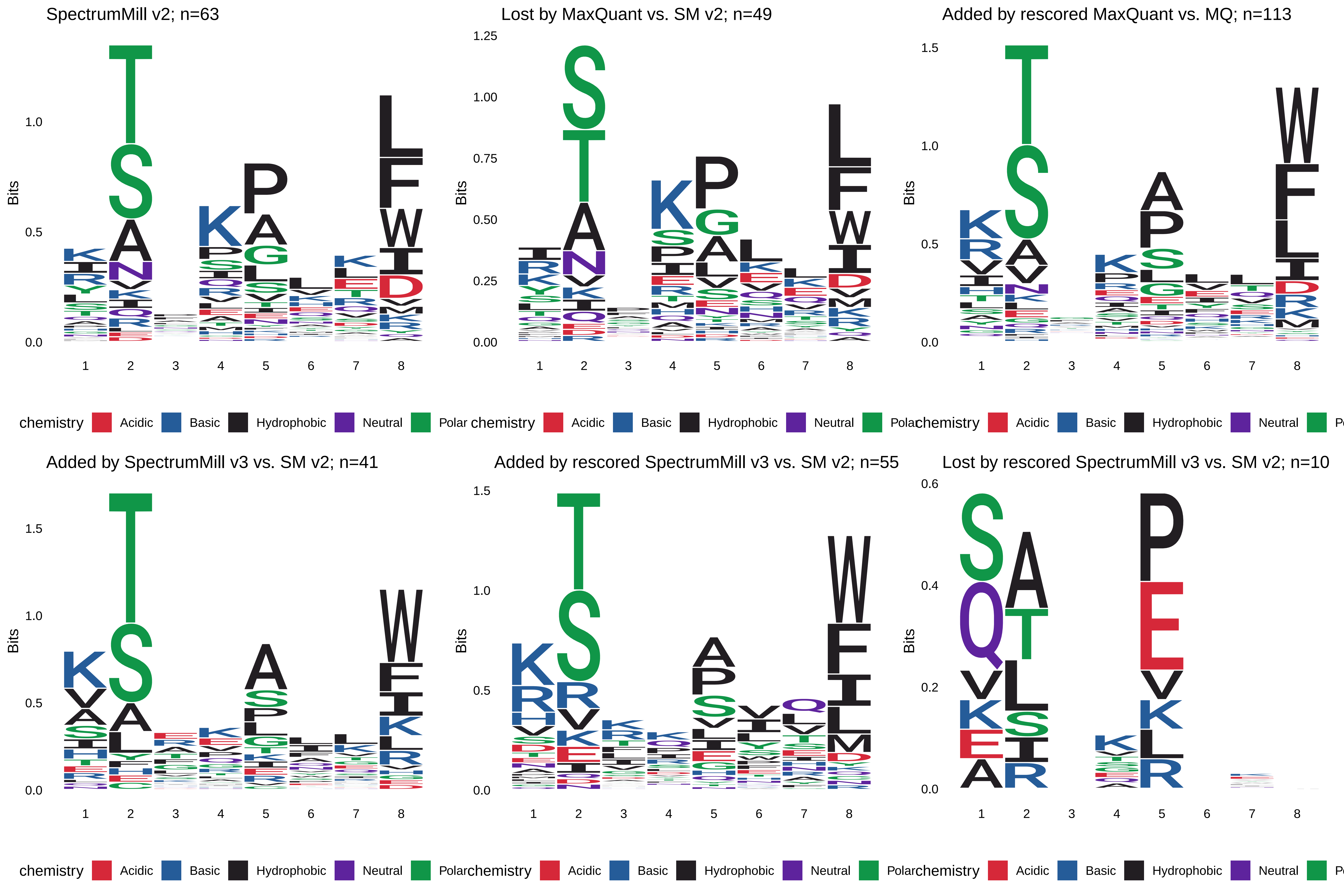
SpectrumMill v2; n=63
Lost by MaxQuant vs. SM v2; n=49
Added by rescored MaxQuant vs. MQ; n=113
Bits
1 2 3 4 5 6 7 8
chemistry Acidic Basic Hydrophobic Neutral Polar
Added by SpectrumMill v3 vs. SM v2; n=41
Added by rescored SpectrumMill v3 vs. SM v2; n=55
Lost by rescored SpectrumMill v3 vs. SM v2; n=10
Bits
1 2 3 4 5 6 7 8
chemistry Acidic Basic Hydrophobic Neutral Polar

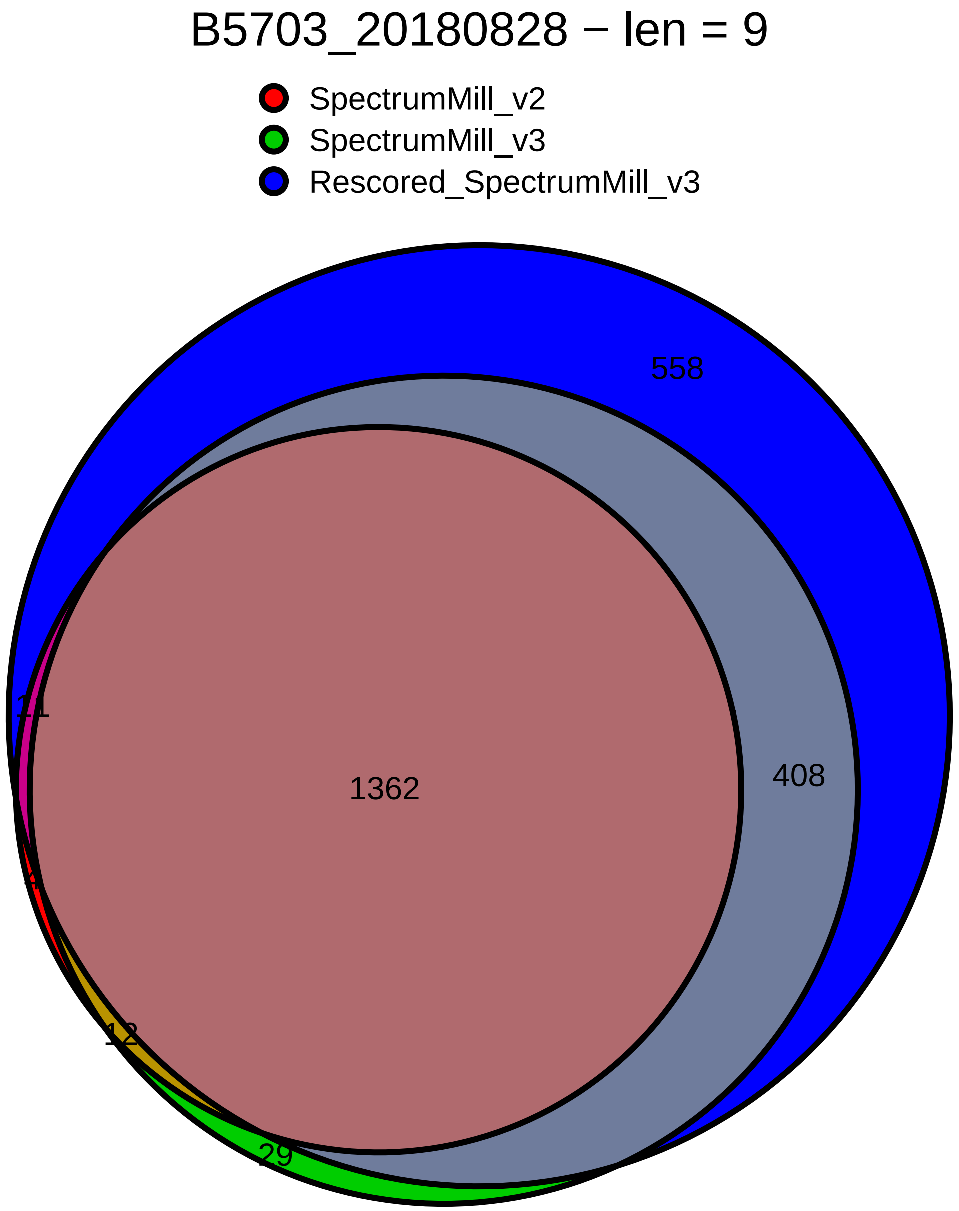
B5703_20180828 − len = 9
SpectrumMill_v2
SpectrumMill_v3
Rescored_SpectrumMill_v3
558
11
1362
408
12
29

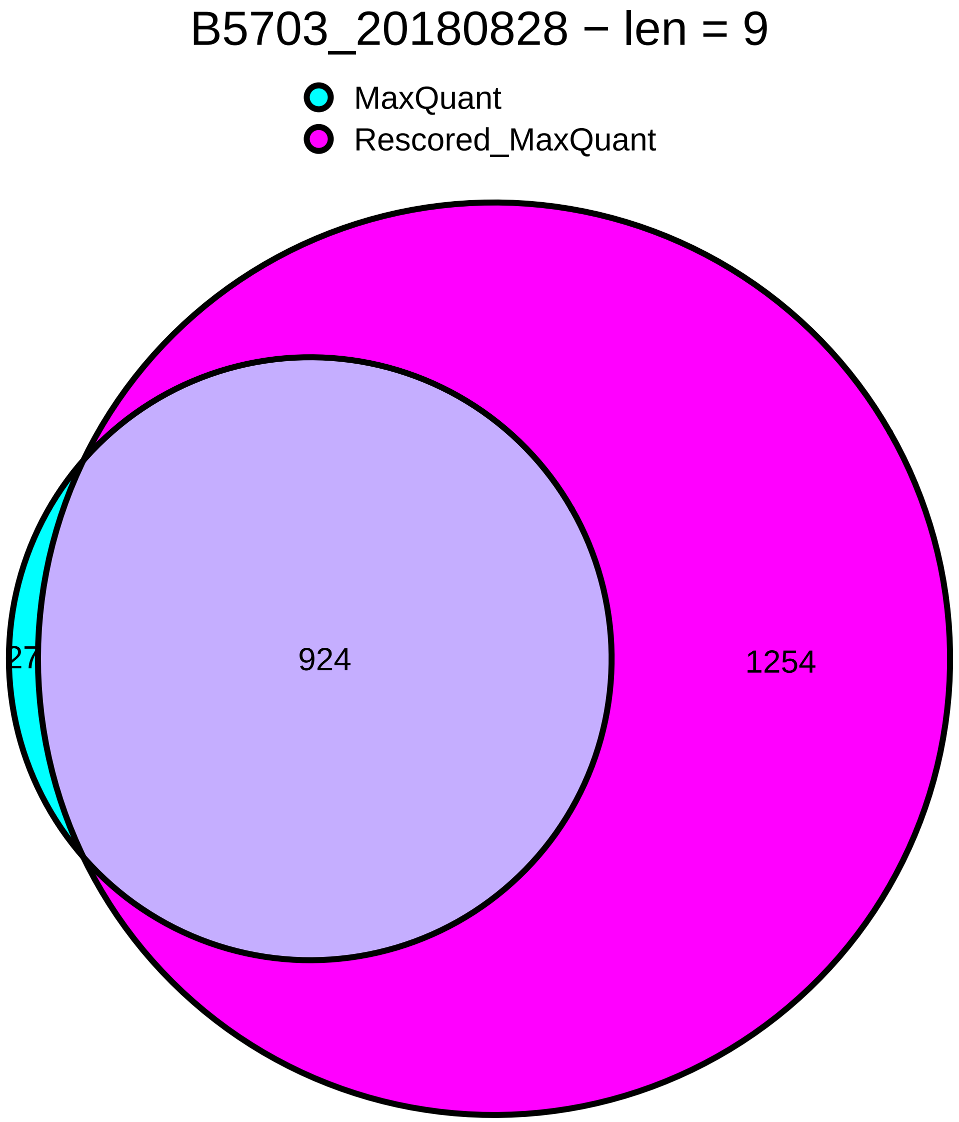
B5703_20180828 − len = 9
MaxQuant
Rescored_MaxQuant
27
924
1254

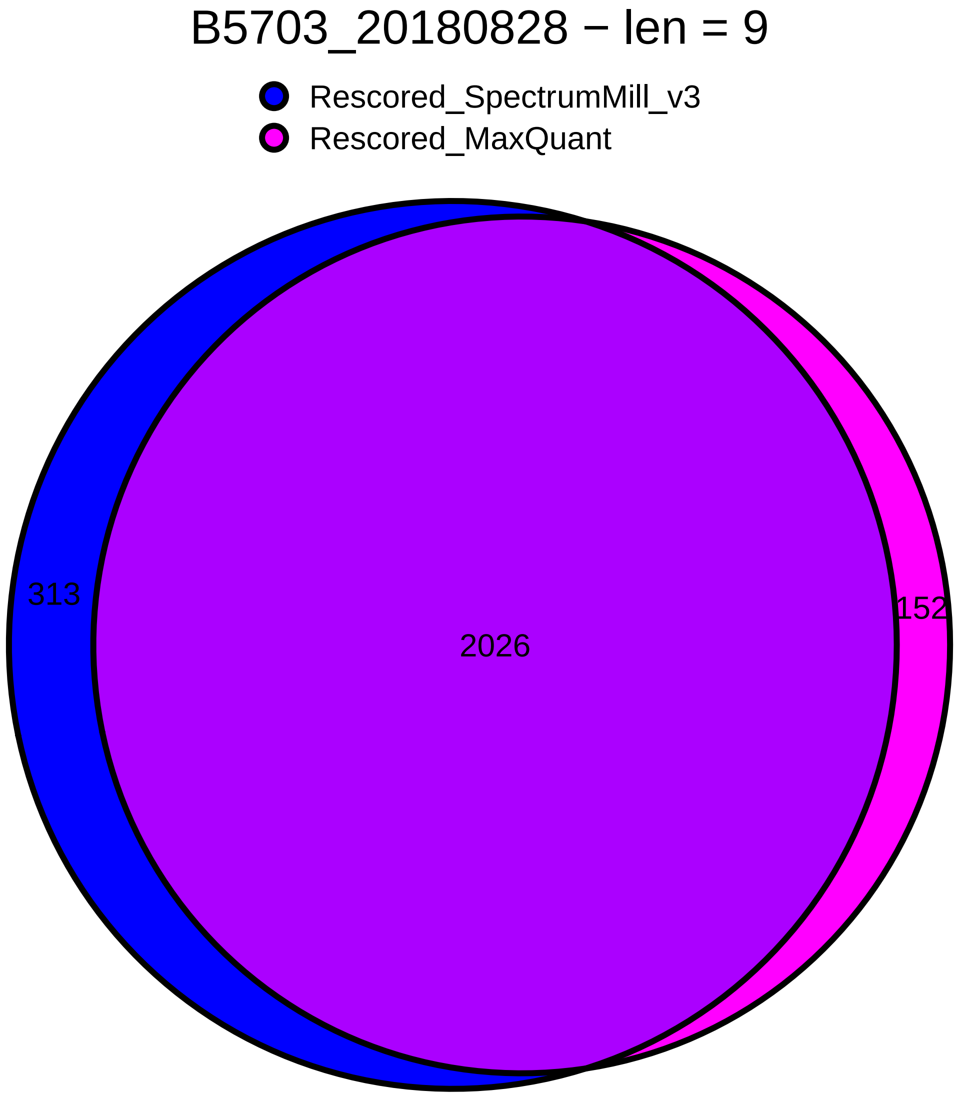
B5703_20180828 − len = 9
Rescored_SpectrumMill_v3
Rescored_MaxQuant
313
2026
152

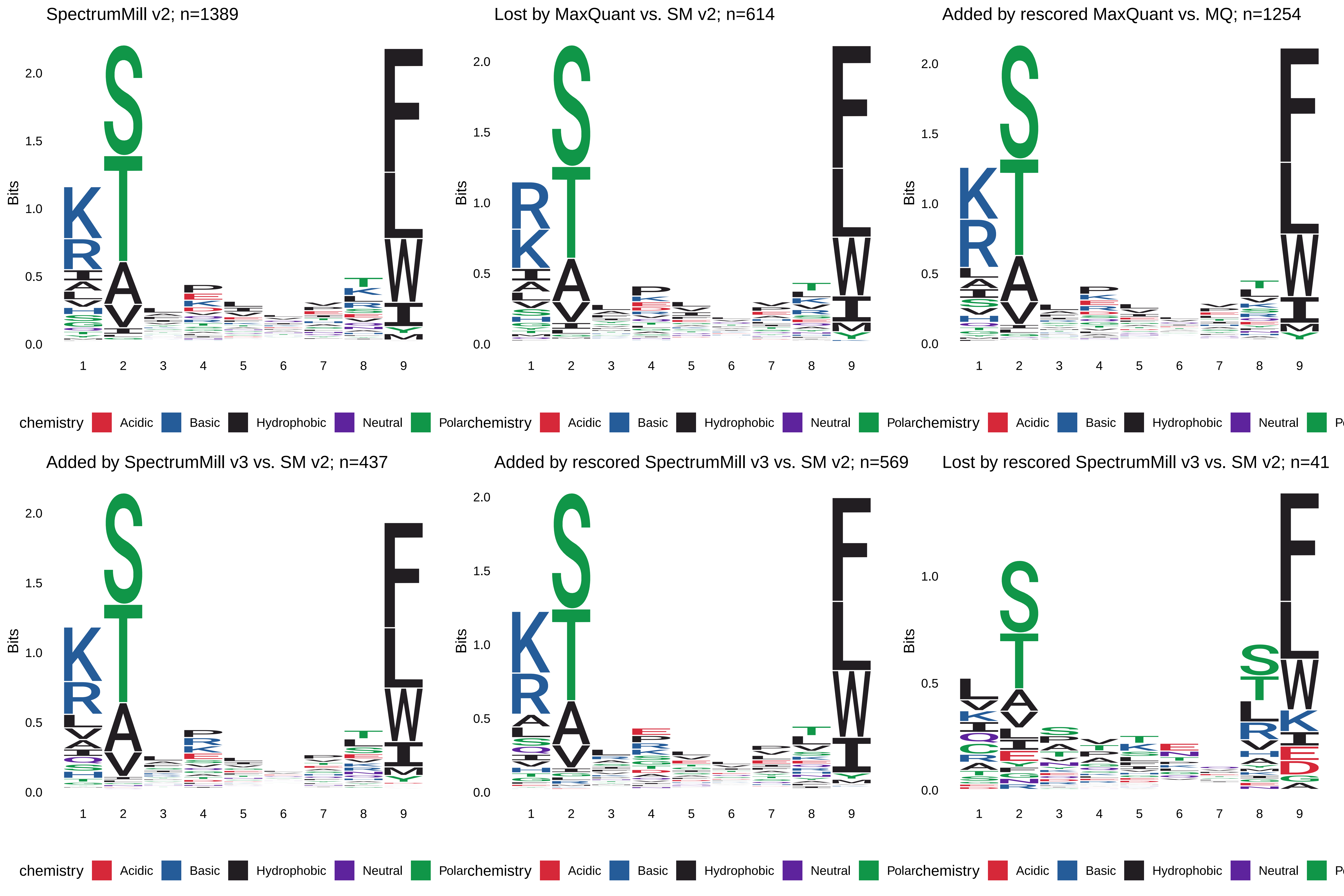

SpectrumMill v2; n=1389
Lost by MaxQuant vs. SM v2; n=614
Added by rescored MaxQuant vs. MQ; n=1254
Added by SpectrumMill v3 vs. SM v2; n=437
Added by rescored SpectrumMill v3 vs. SM v2; n=569
Lost by rescored SpectrumMill v3 vs. SM v2; n=41
Bits
0.0
0.5
1.0
1.5
2.0
1 2 3 4 5 6 7 8 9
chemistry Acidic Basic Hydrophobic Neutral Polarchemistry Acidic Basic Hydrophobic Neutral Polarchemistry Acidic Basic Hydrophobic Neutral

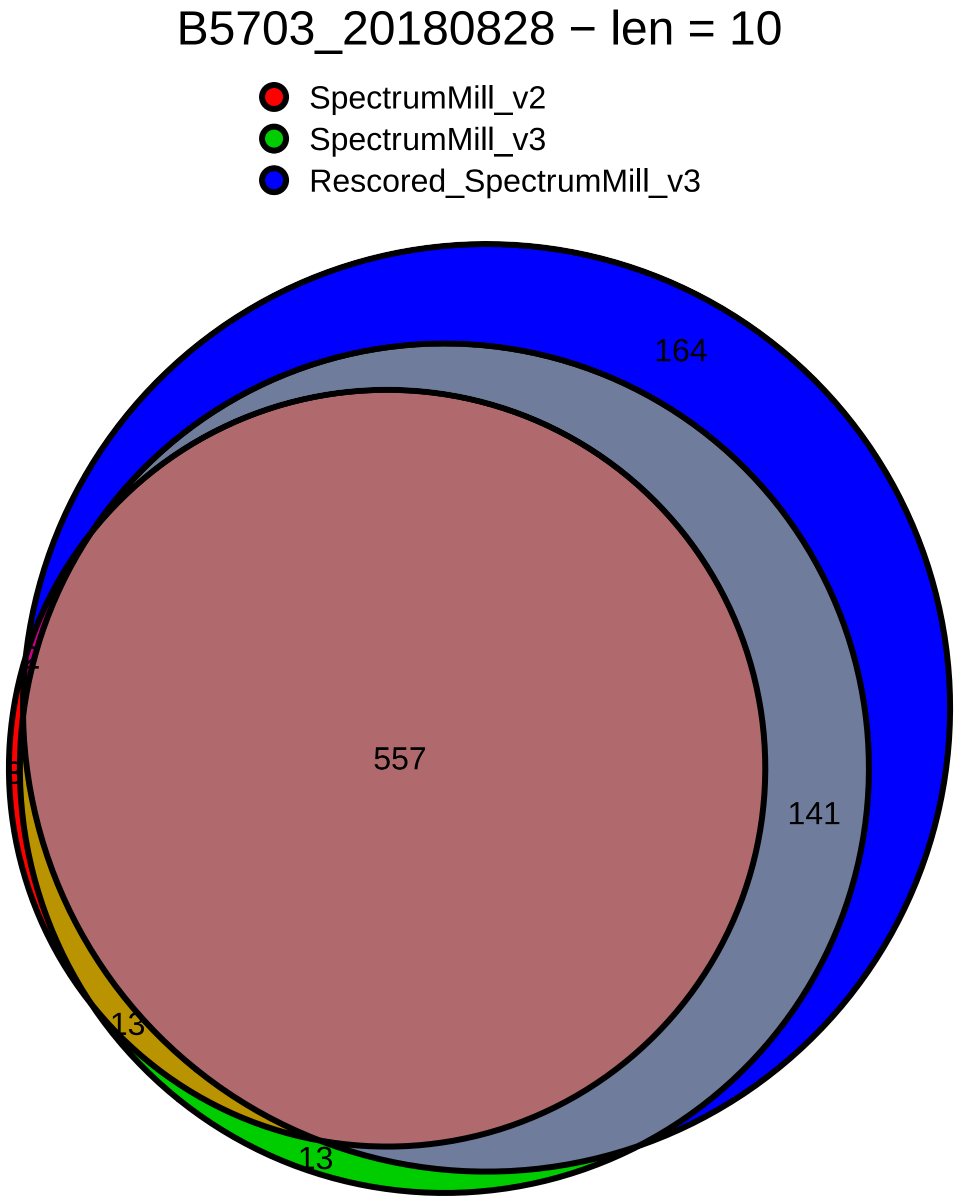
B5703_20180828 − len = 10
SpectrumMill_v2
SpectrumMill_v3
Rescored_SpectrumMill_v3
164
557
141
13
13

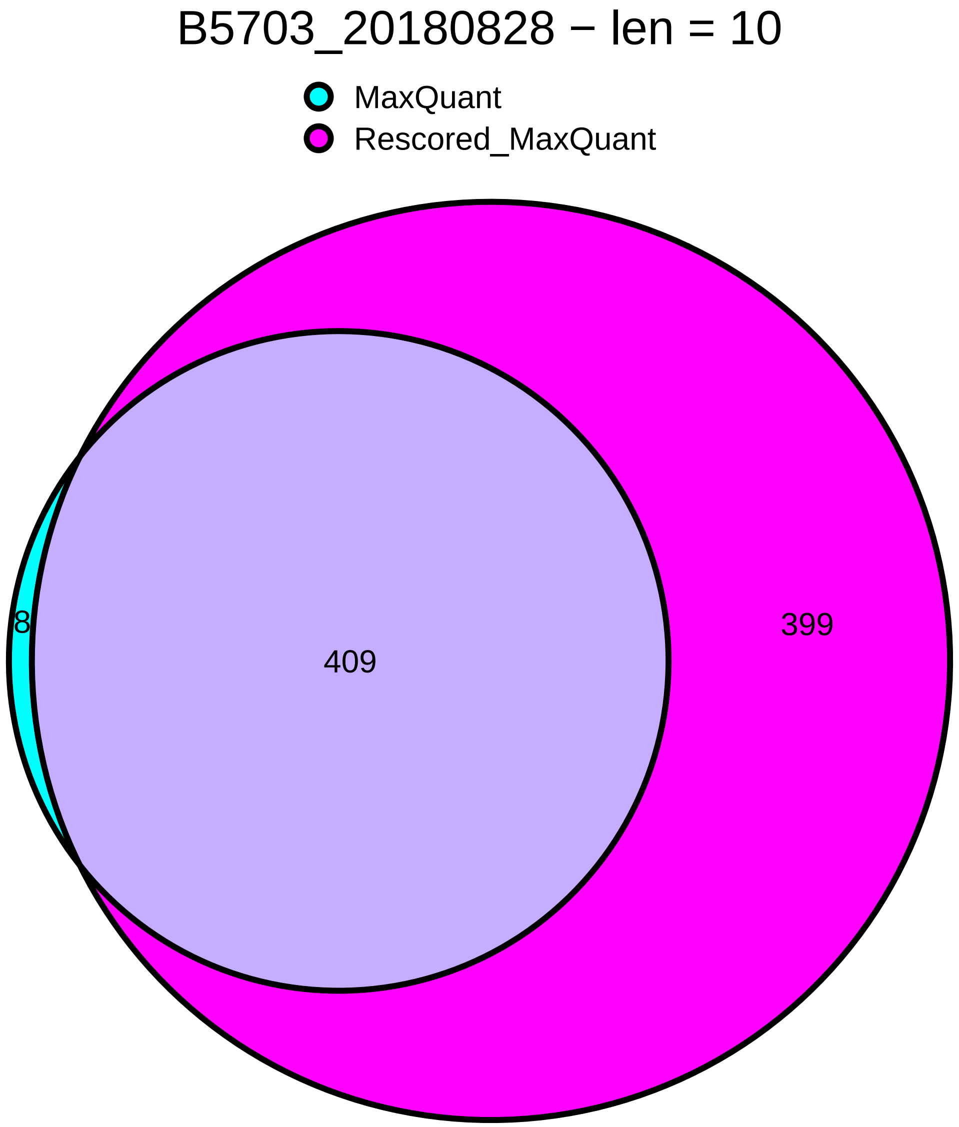
B5703_20180828 − len = 10
MaxQuant
Rescored_MaxQuant
8
409
399

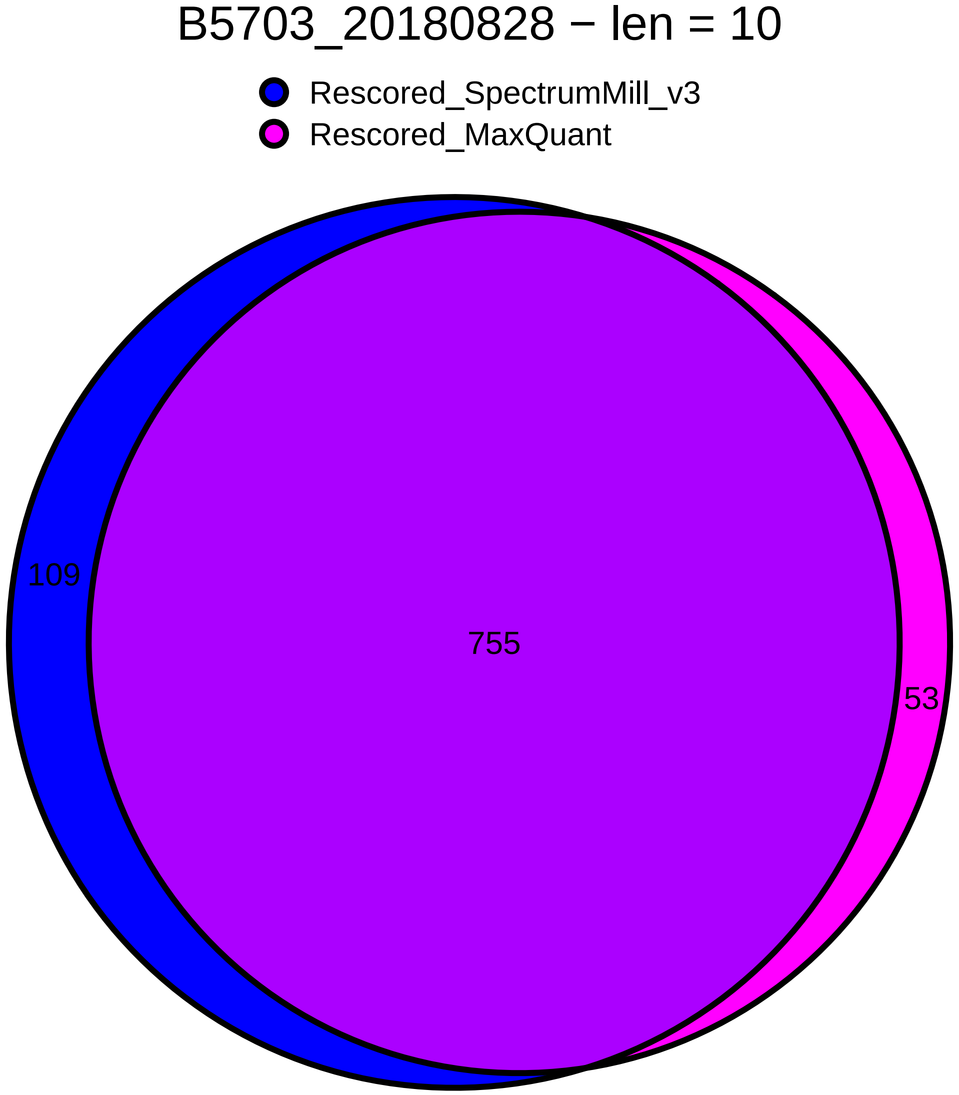
B5703_20180828 − len = 10
Rescored_SpectrumMill_v3
Rescored_MaxQuant
109
755
53

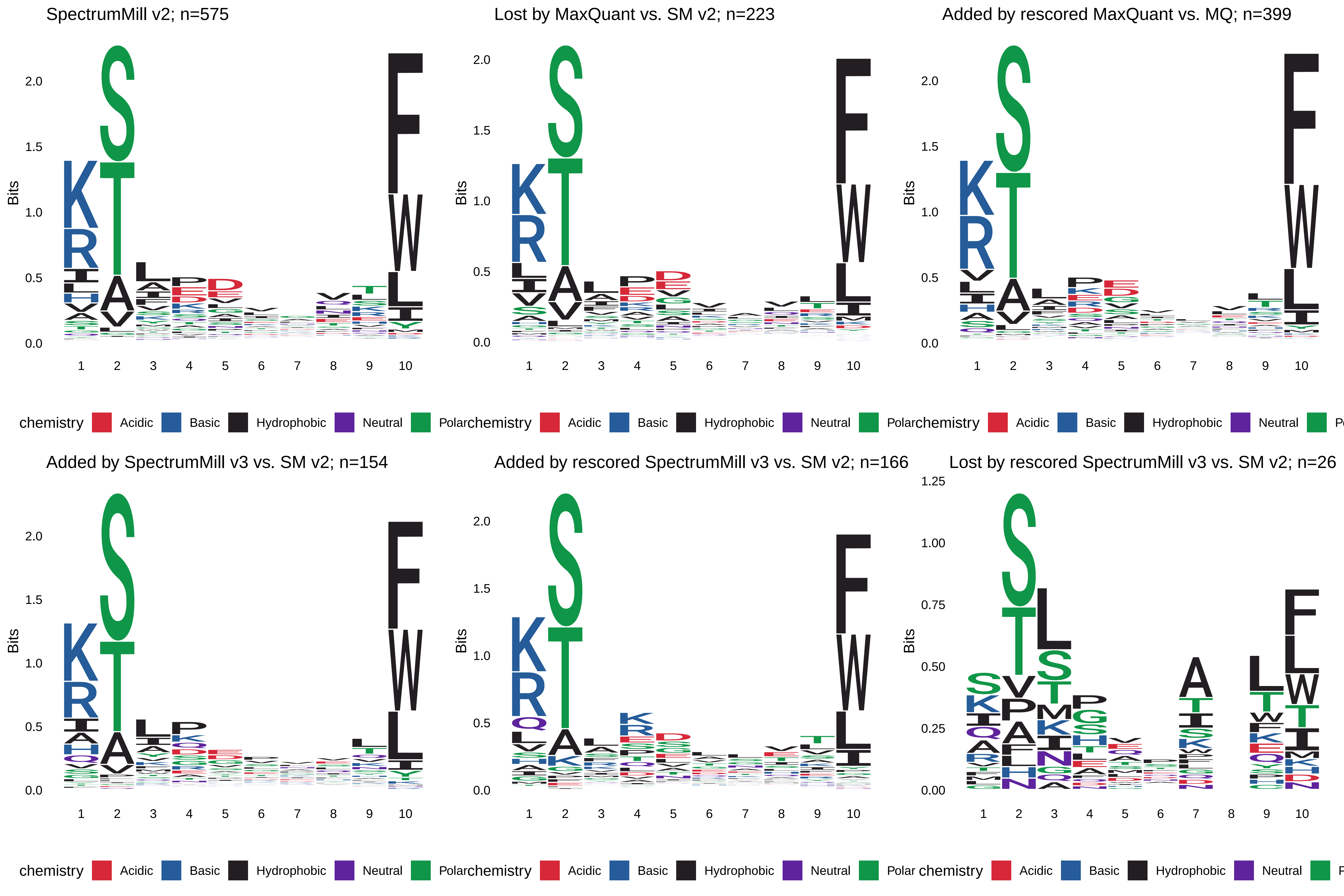
SpectrumMill v2; n=575
Lost by MaxQuant vs. SM v2; n=223
Added by rescored MaxQuant vs. MQ; n=399
Added by SpectrumMill v3 vs. SM v2; n=154
Added by rescored SpectrumMill v3 vs. SM v2; n=166
Lost by rescored SpectrumMill v3 vs. SM v2; n=26
Bits
chemistry
Acidic
Basic
Hydrophobic
Neutral
Polar

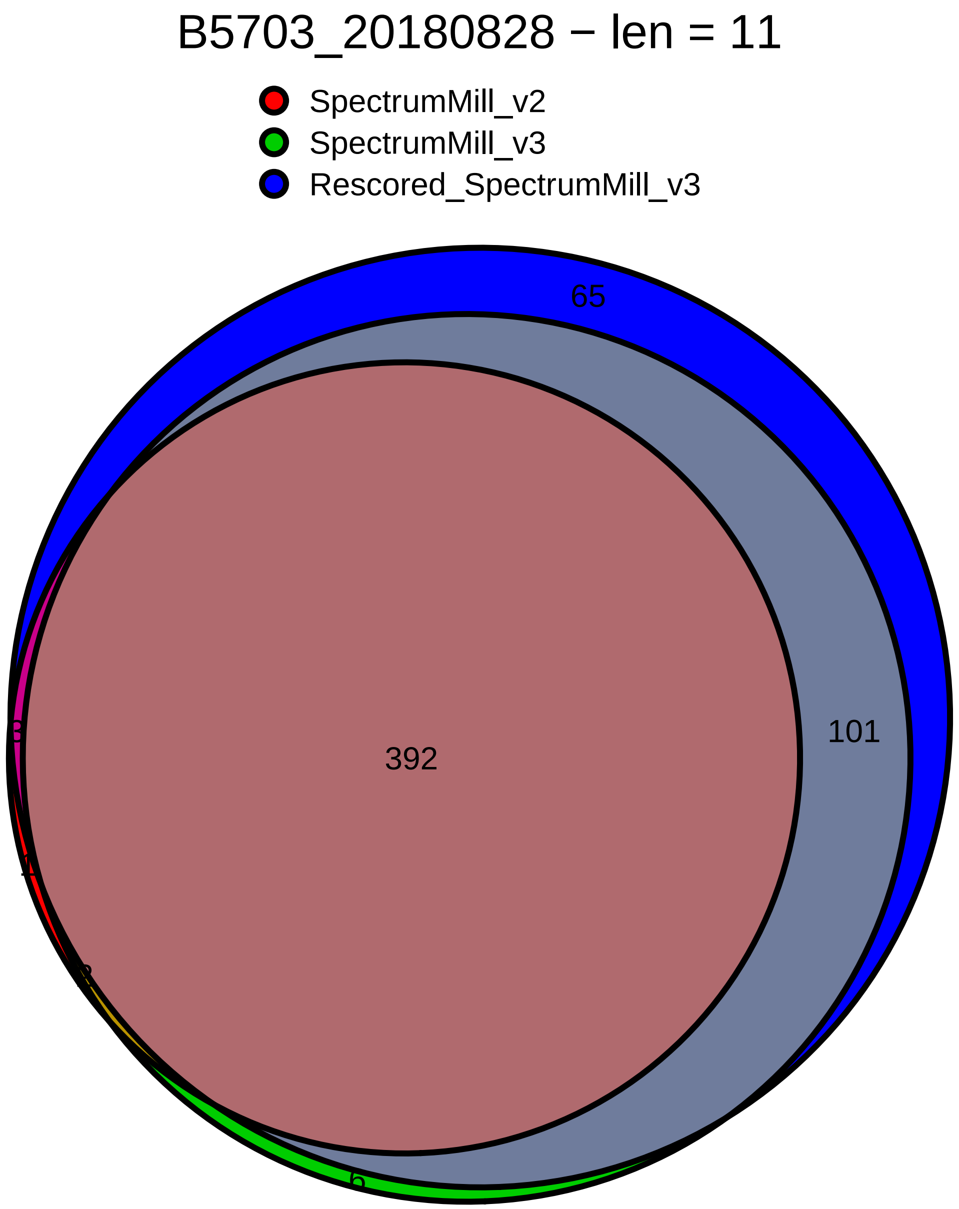

B5703_20180828 − len = 11
SpectrumMill_v2
SpectrumMill_v3
Rescored_SpectrumMill_v3
65
101
392

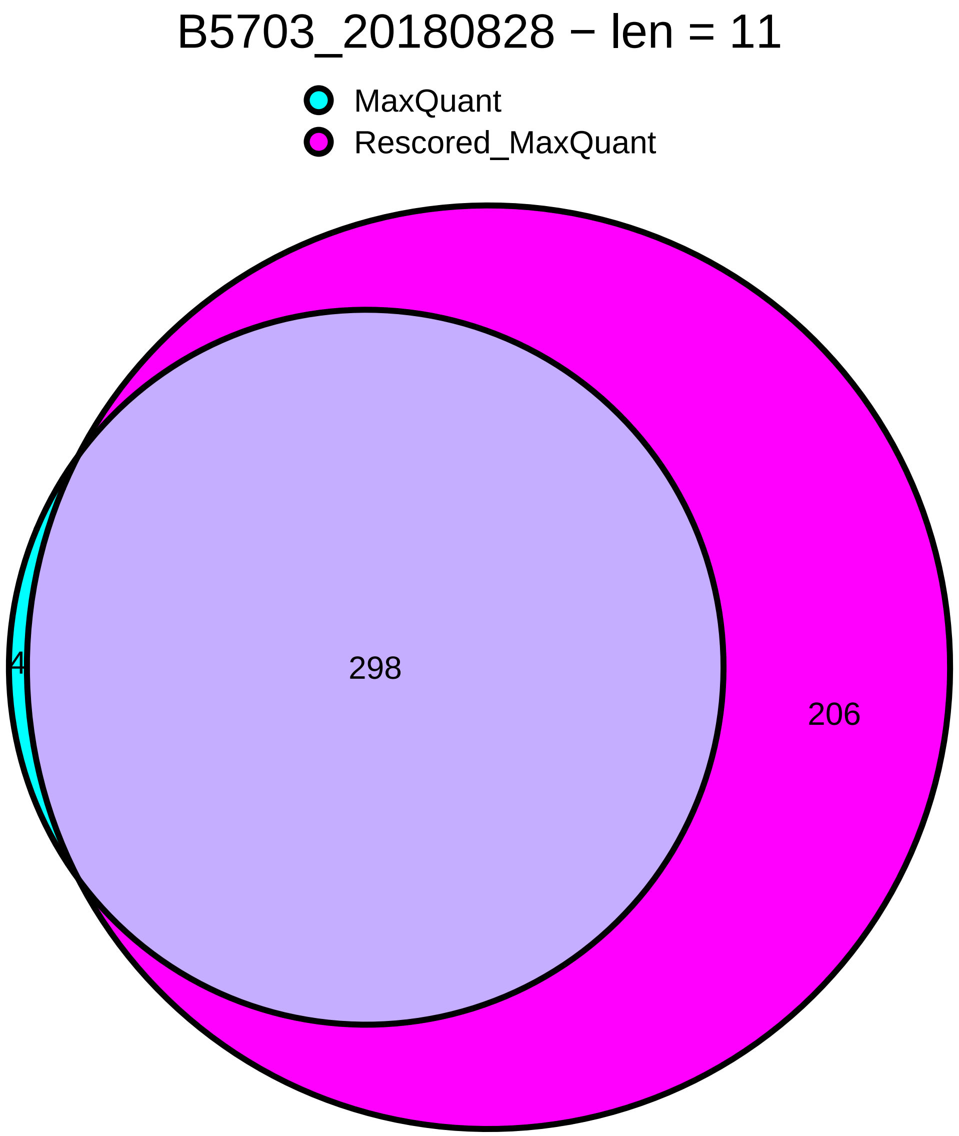

B5703_20180828 − len = 11
MaxQuant
Rescored_MaxQuant
4
298
206

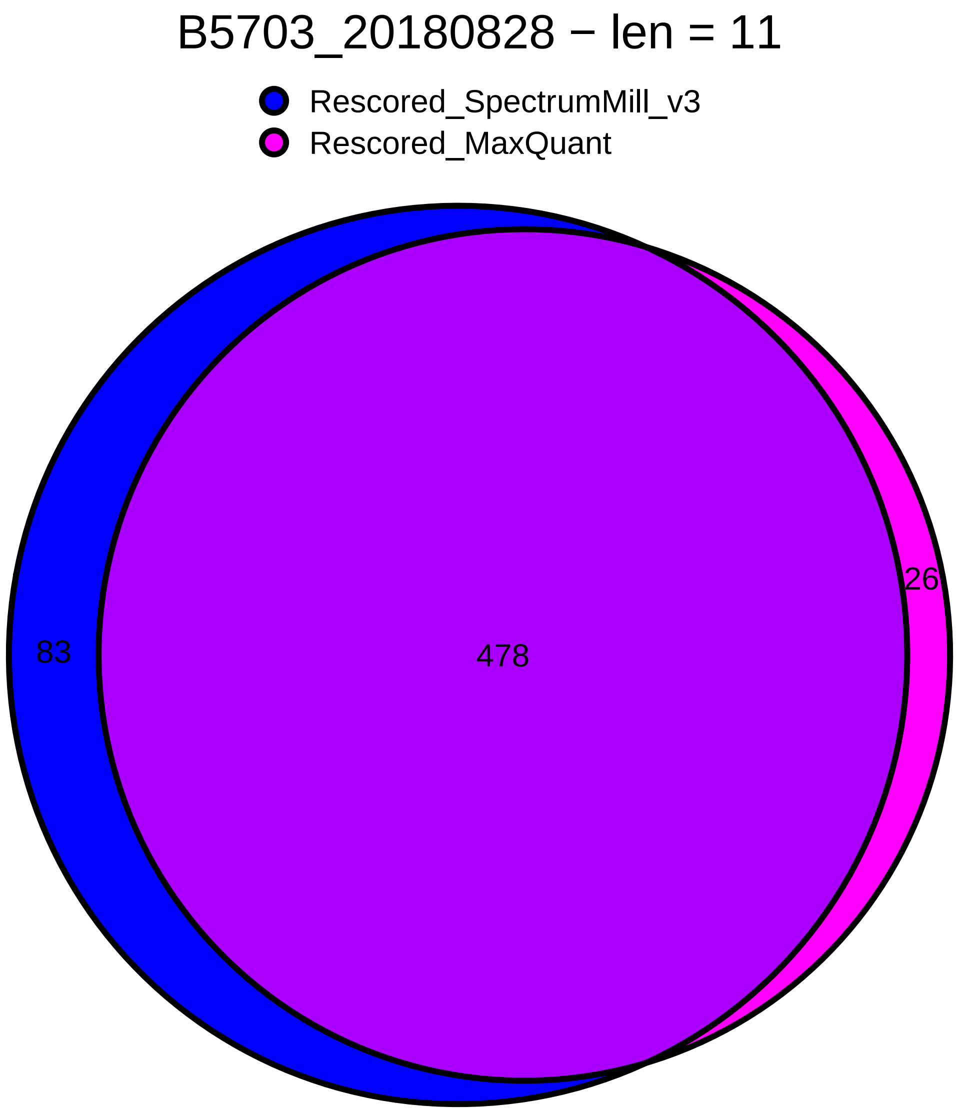

B5703_20180828 − len = 11
Rescored_SpectrumMill_v3
Rescored_MaxQuant
83
478
26

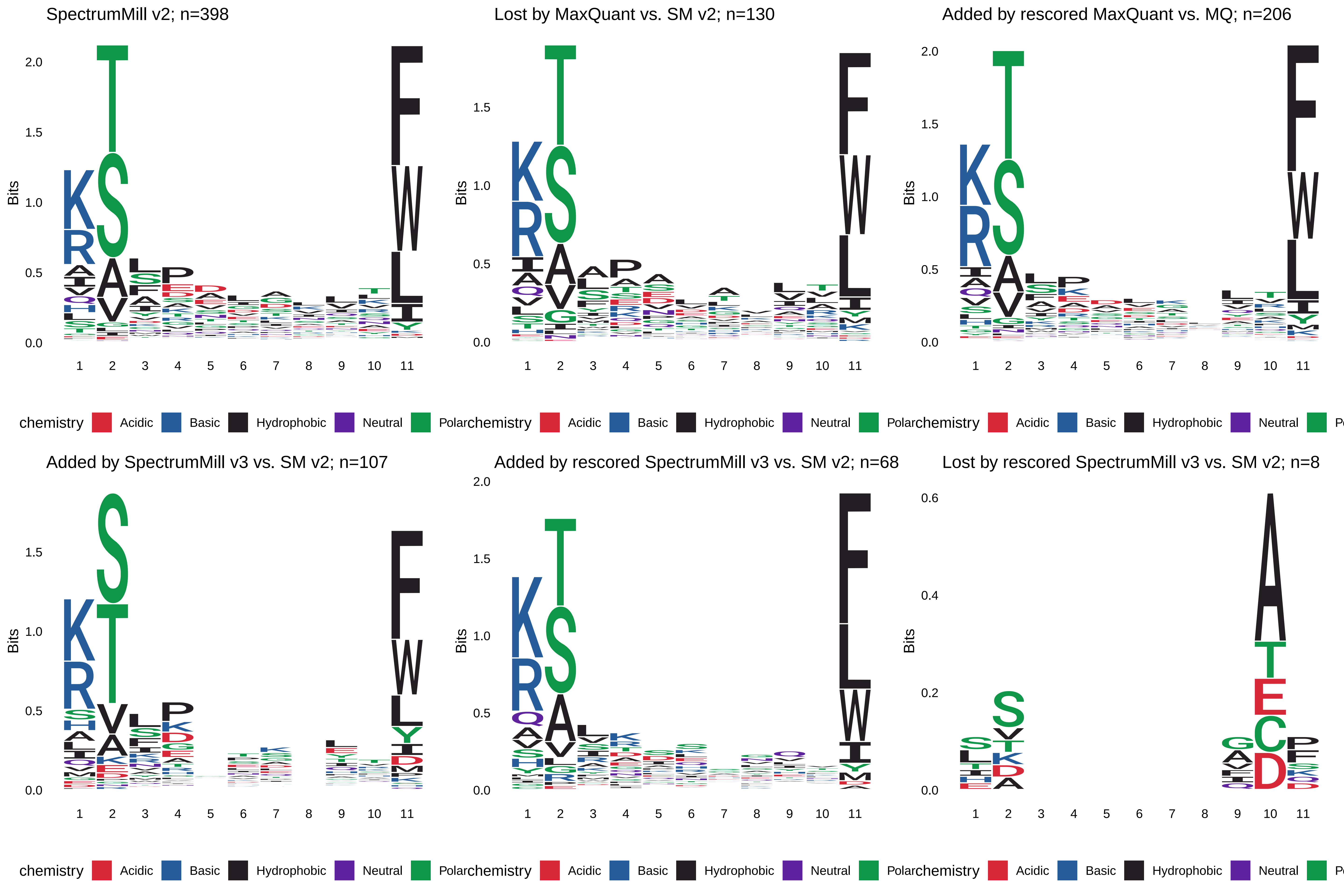
SpectrumMill v2; n=398
Lost by MaxQuant vs. SM v2; n=130
Added by rescored MaxQuant vs. MQ; n=206
Added by SpectrumMill v3 vs. SM v2; n=107
Added by rescored SpectrumMill v3 vs. SM v2; n=68
Lost by rescored SpectrumMill v3 vs. SM v2; n=8
Bits
1 2 3 4 5 6 7 8 9 10 11
chemistry
Acidic
Basic
Hydrophobic
Neutral
Polar

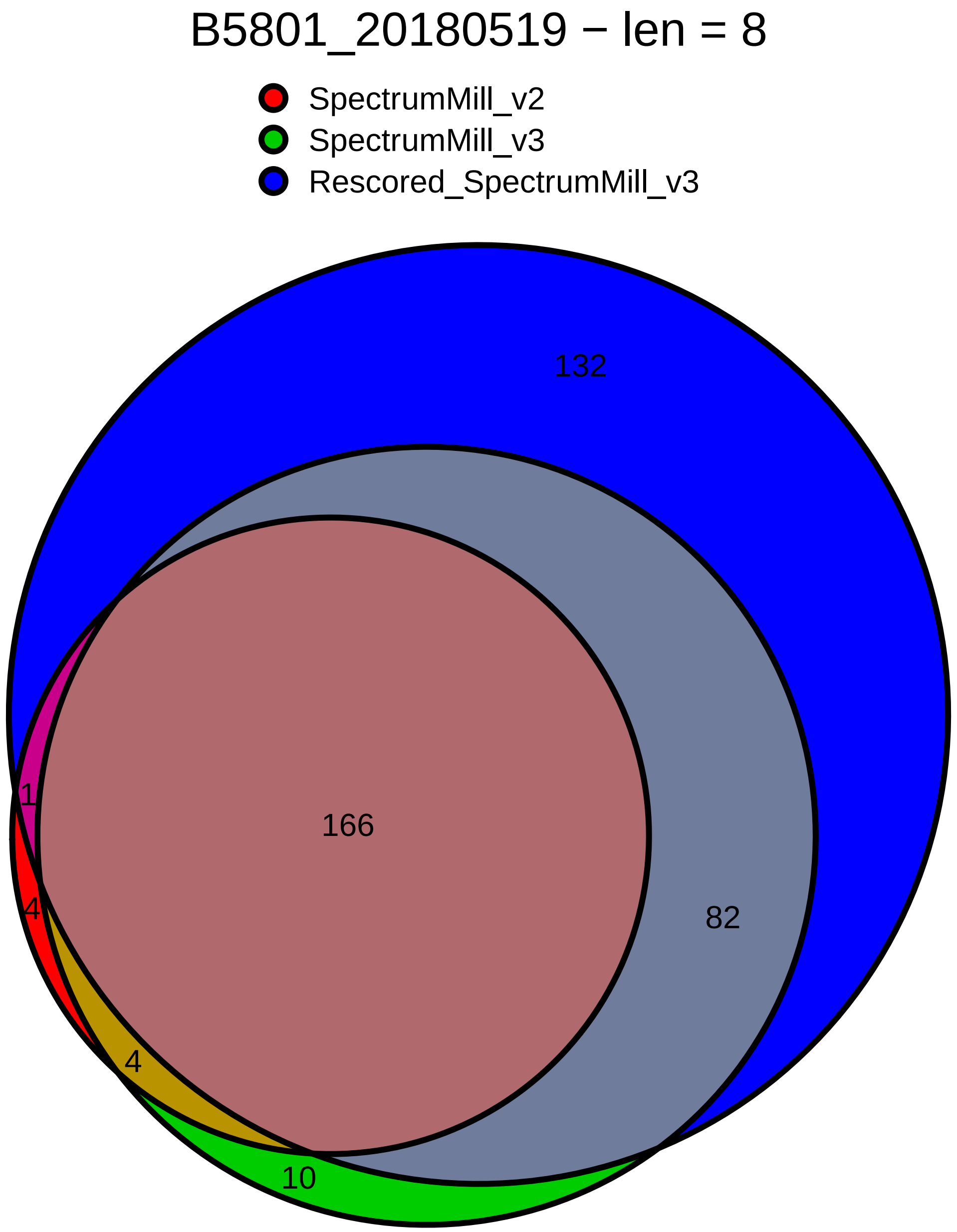
B5801_20180519 − len = 8
SpectrumMill_v2
SpectrumMill_v3
Rescored_SpectrumMill_v3
132
166
82
4
4
10

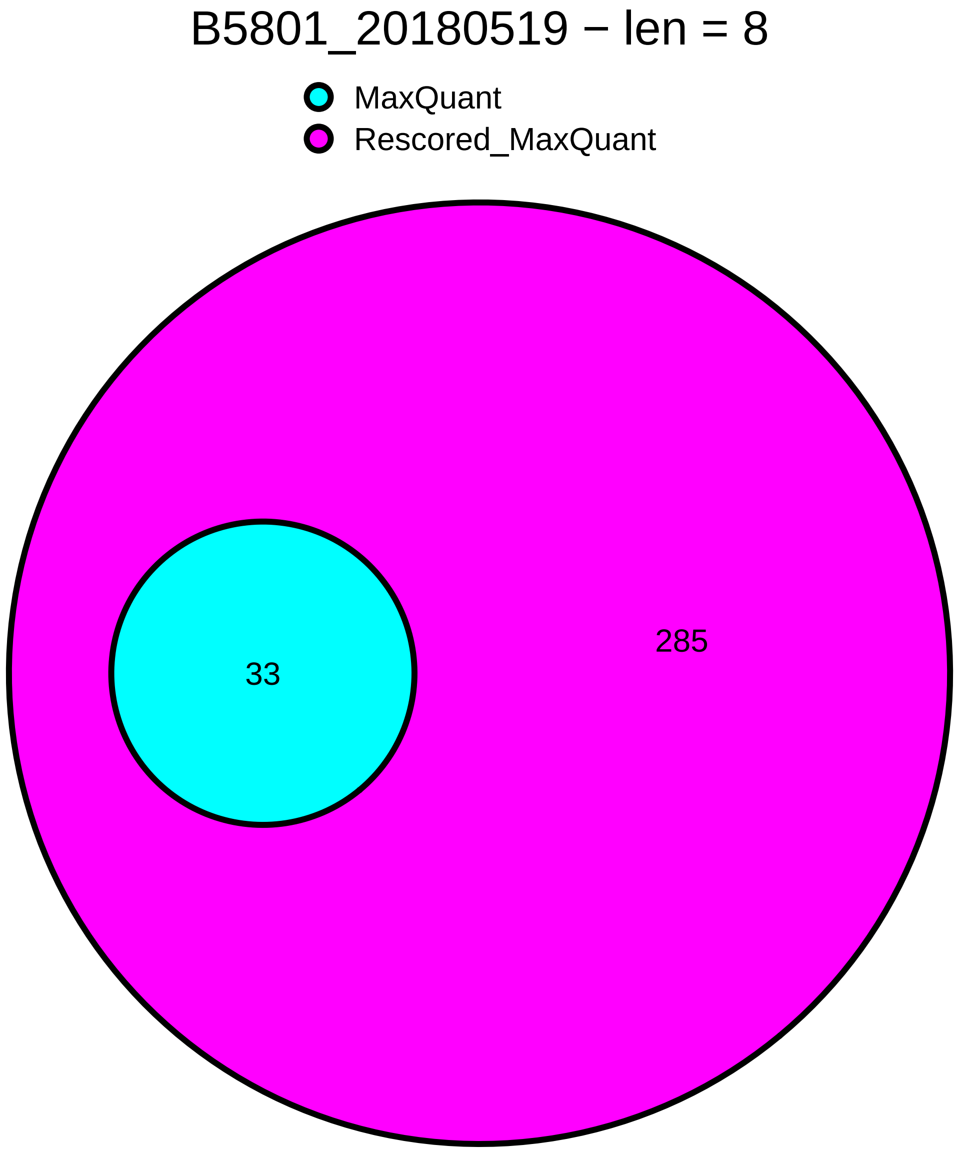
B5801_20180519 − len = 8
MaxQuant
Rescored_MaxQuant
33
285

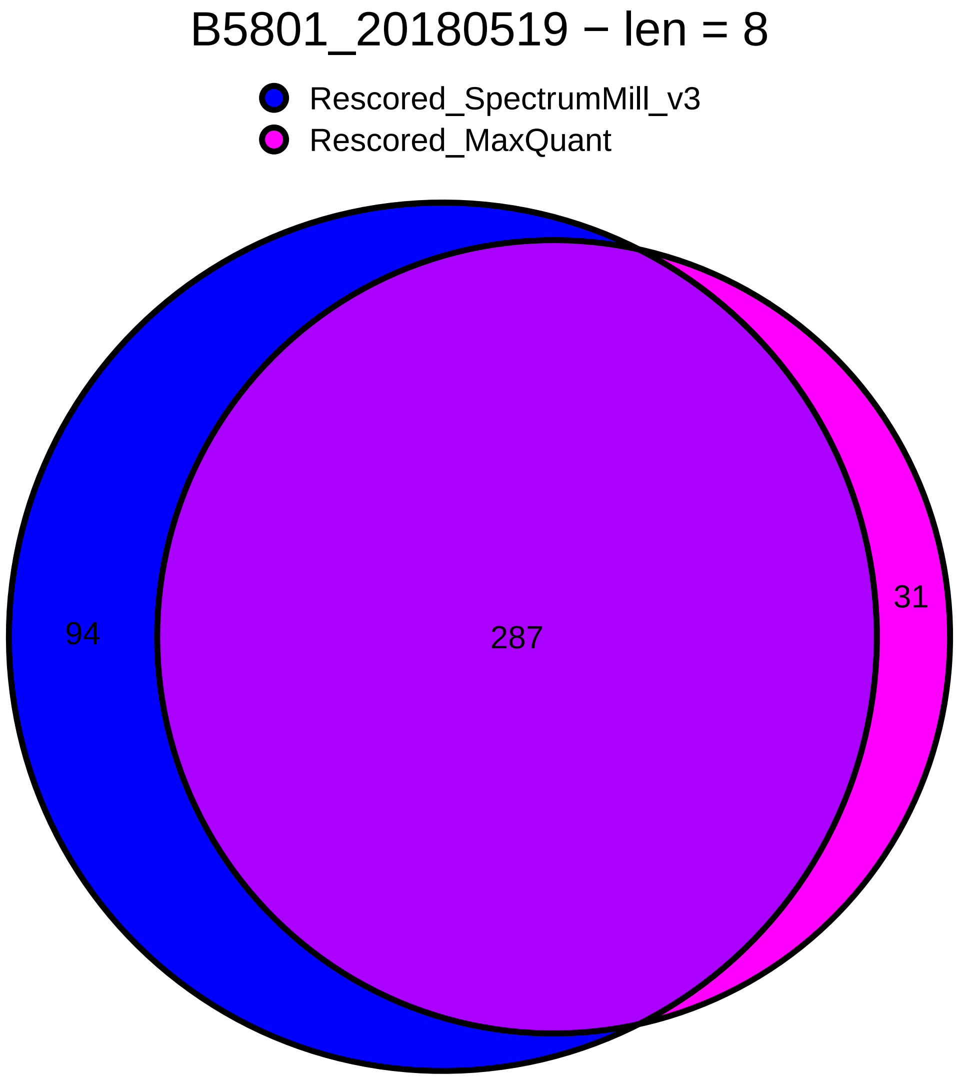
B5801_20180519 − len = 8
Rescored_SpectrumMill_v3
Rescored_MaxQuant
94
287
31

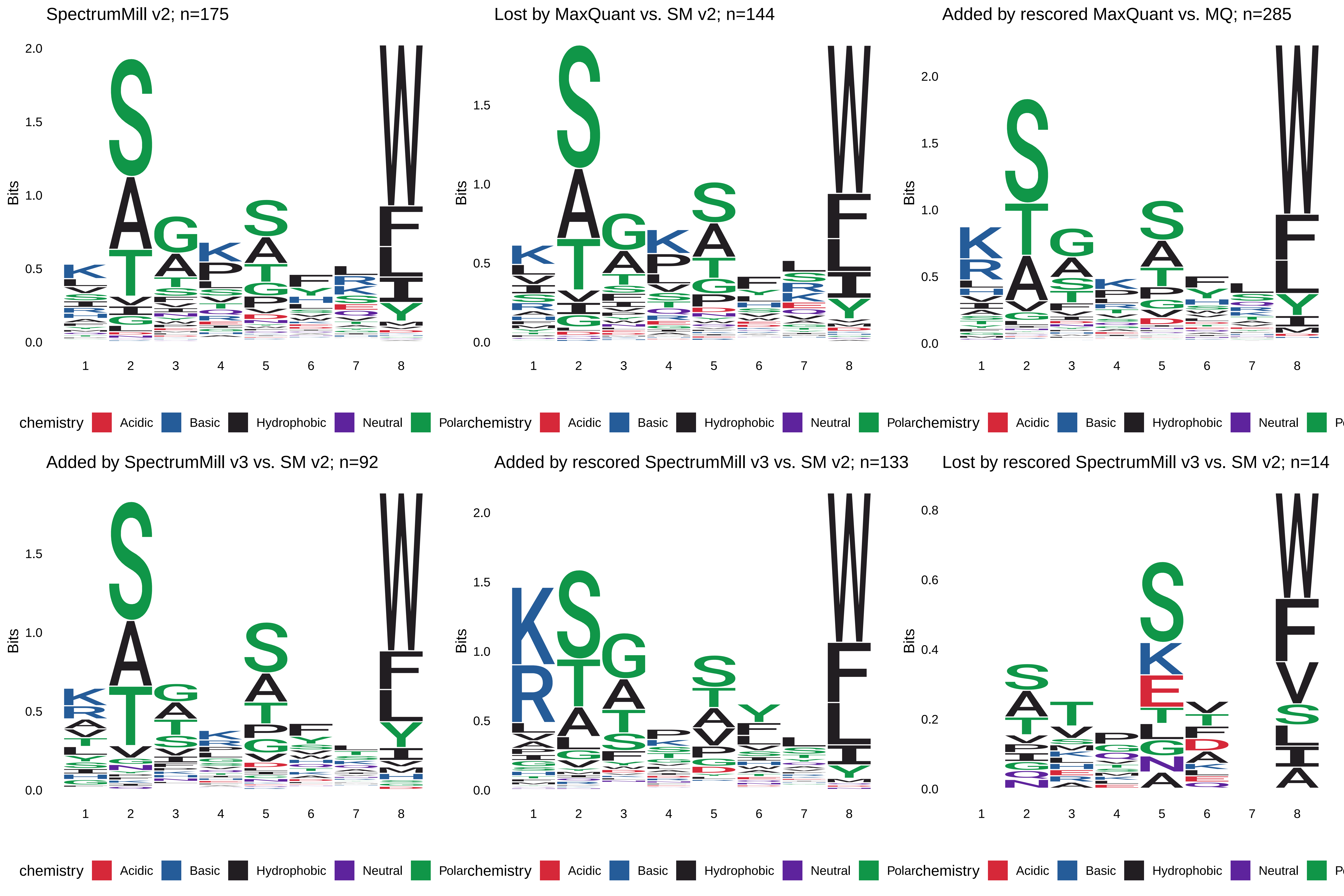
SpectrumMill v2; n=175
Lost by MaxQuant vs. SM v2; n=144
Added by rescored MaxQuant vs. MQ; n=285
Added by SpectrumMill v3 vs. SM v2; n=92
Added by rescored SpectrumMill v3 vs. SM v2; n=133
Lost by rescored SpectrumMill v3 vs. SM v2; n=14
Bits
1 2 3 4 5 6 7 8
chemistry Acidic Basic Hydrophobic Neutral Polarchemistry Acidic Basic Hydrophobic Neutral Polarchemistry Acidic Basic Hydrophobic Neutral Po
chemistry Acidic Basic Hydrophobic Neutral Polarchemistry Acidic Basic Hydrophobic Neutral Polarchemistry Acidic Basic Hydrophobic Neutral Po

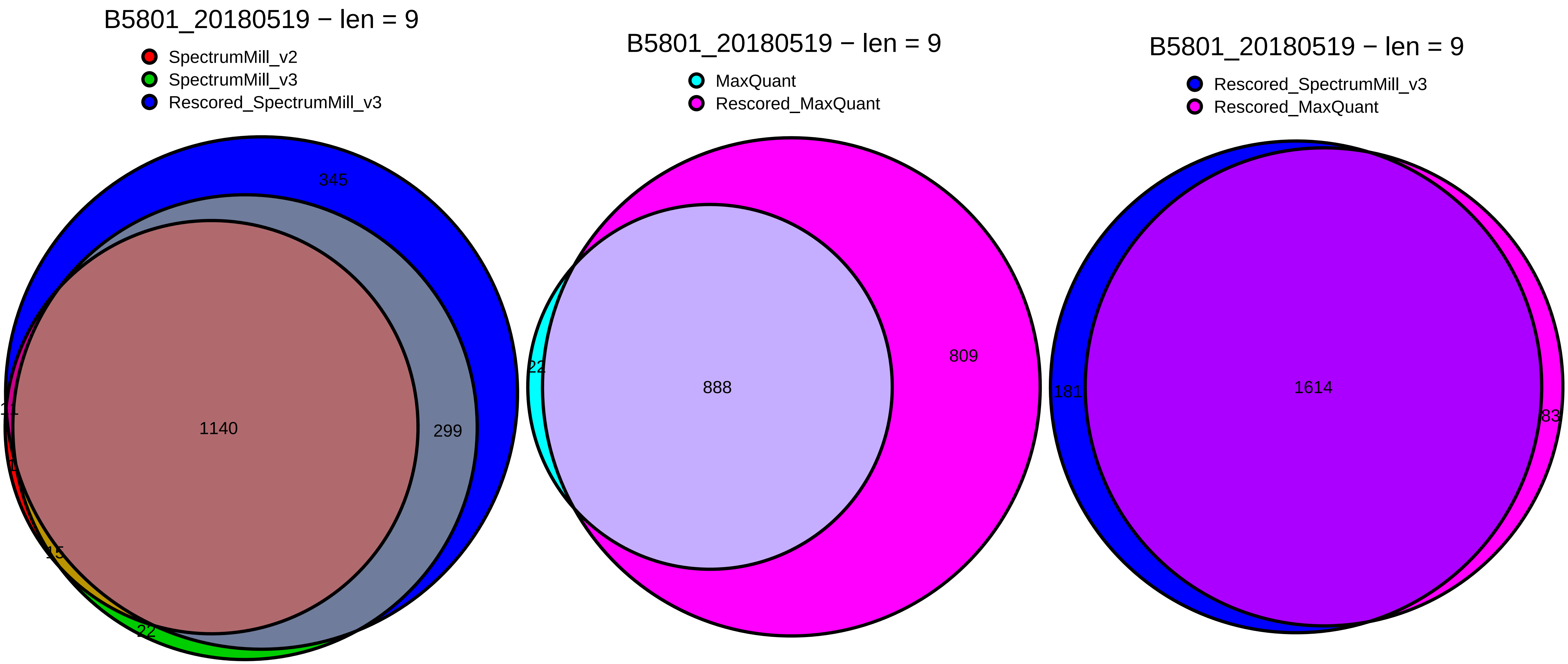
B5801_20180519 − len = 9
SpectrumMill_v2
SpectrumMill_v3
Rescored_SpectrumMill_v3
345
1140
299
15
22
B5801_20180519 − len = 9
MaxQuant
Rescored_MaxQuant
22
888
809
B5801_20180519 − len = 9
Rescored_SpectrumMill_v3
Rescored_MaxQuant
181
1614
83

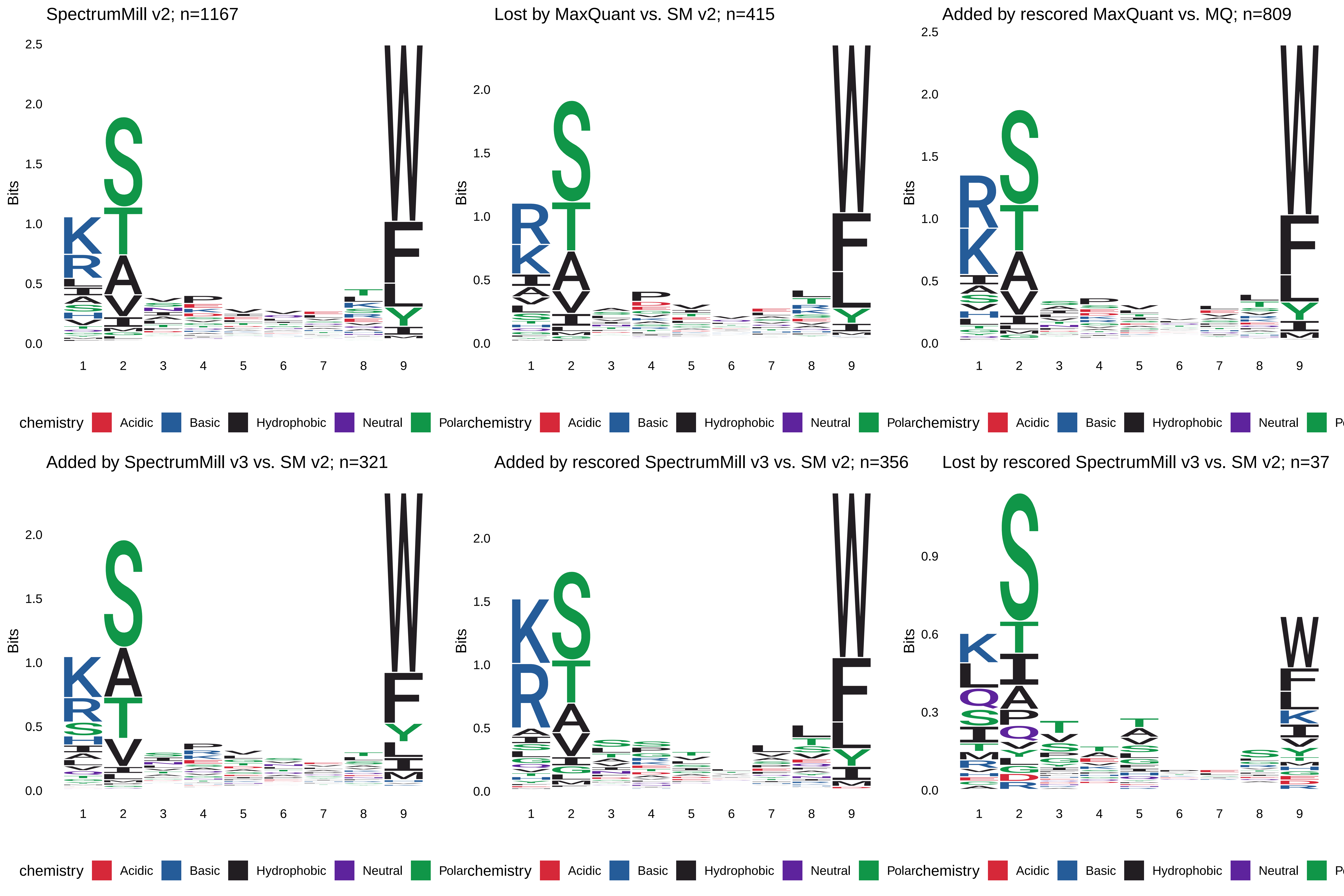
SpectrumMill v2; n=1167
Lost by MaxQuant vs. SM v2; n=415
Added by rescored MaxQuant vs. MQ; n=809
Added by SpectrumMill v3 vs. SM v2; n=321
Added by rescored SpectrumMill v3 vs. SM v2; n=356
Lost by rescored SpectrumMill v3 vs. SM v2; n=37
Bits
1 2 3 4 5 6 7 8 9
chemistry Acidic Basic Hydrophobic Neutral Polar
chemistry Acidic Basic Hydrophobic Neutral Polar
chemistry Acidic Basic Hydrophobic Neutral

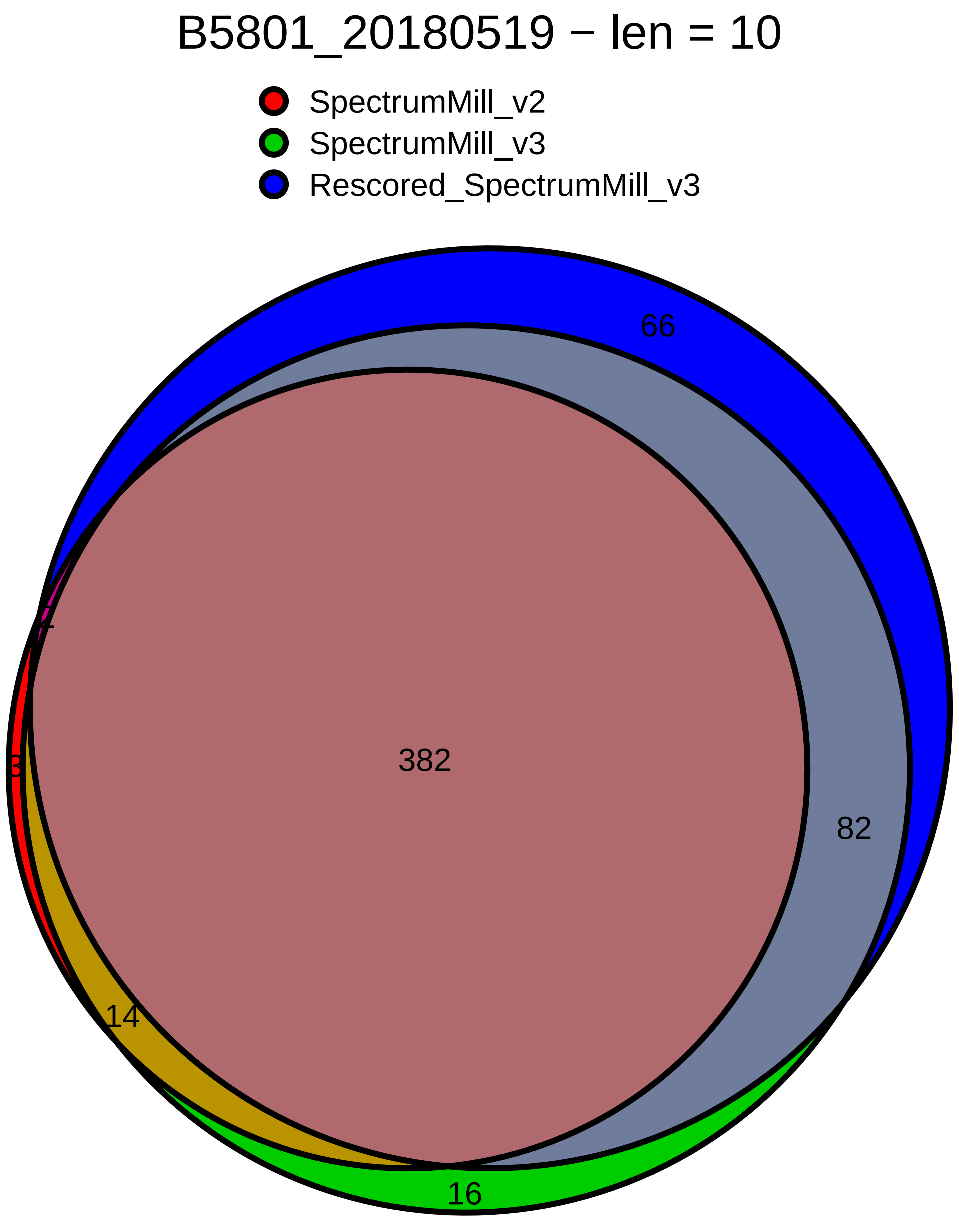
B5801_20180519 − len = 10
SpectrumMill_v2
SpectrumMill_v3
Rescored_SpectrumMill_v3
66
382
82
3
14
16

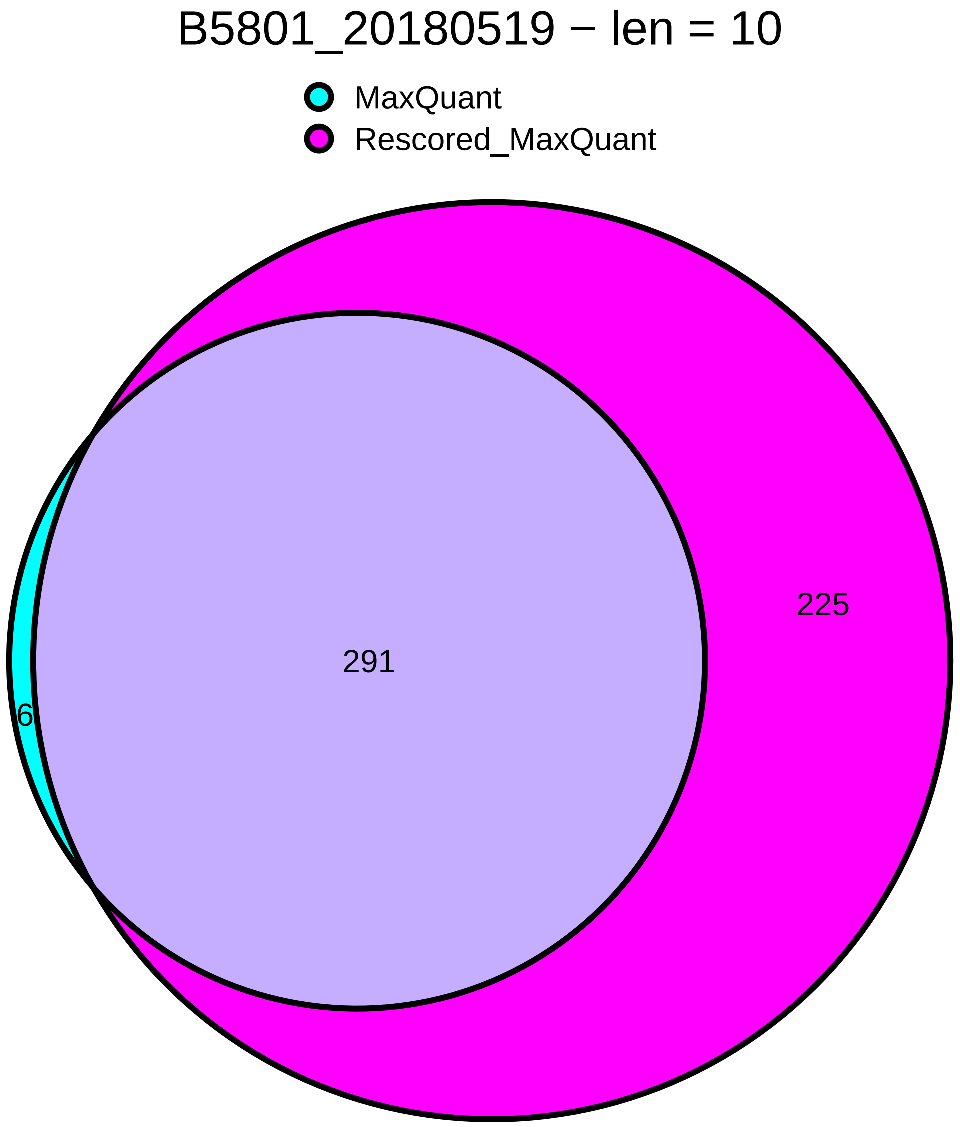
B5801_20180519 − len = 10
MaxQuant
Rescored_MaxQuant
291
225
6

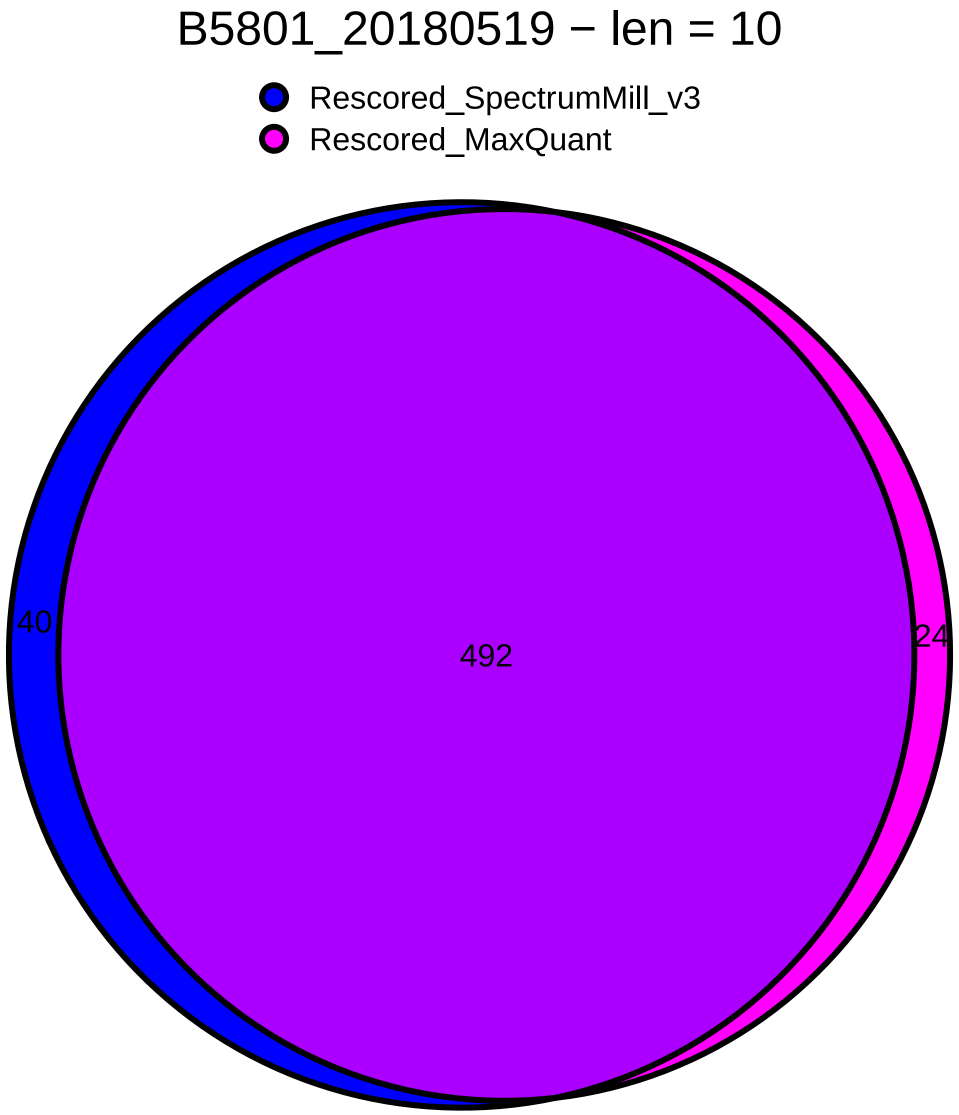
B5801_20180519 − len = 10
Rescored_SpectrumMill_v3
Rescored_MaxQuant
40
492
24

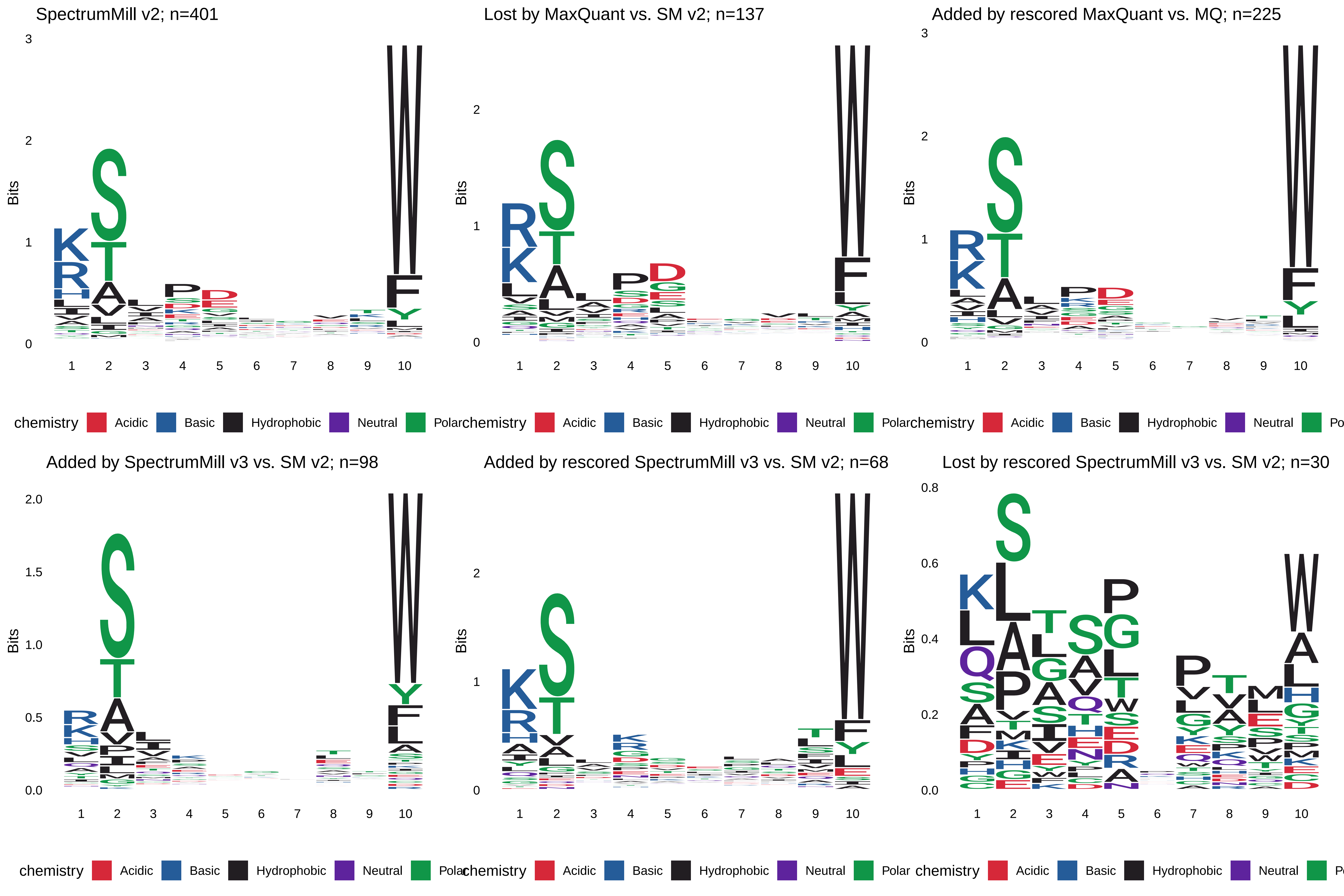
SpectrumMill v2; n=401
Lost by MaxQuant vs. SM v2; n=137
Added by rescored MaxQuant vs. MQ; n=225
Added by SpectrumMill v3 vs. SM v2; n=98
Added by rescored SpectrumMill v3 vs. SM v2; n=68
Lost by rescored SpectrumMill v3 vs. SM v2; n=30
Bits
0
1
2
3
0.0
0.5
1.0
1.5
2.0
0.2
0.4
0.6
0.8
1 2 3 4 5 6 7 8 9 10
chemistry
Acidic
Basic
Hydrophobic
Neutral
Polar

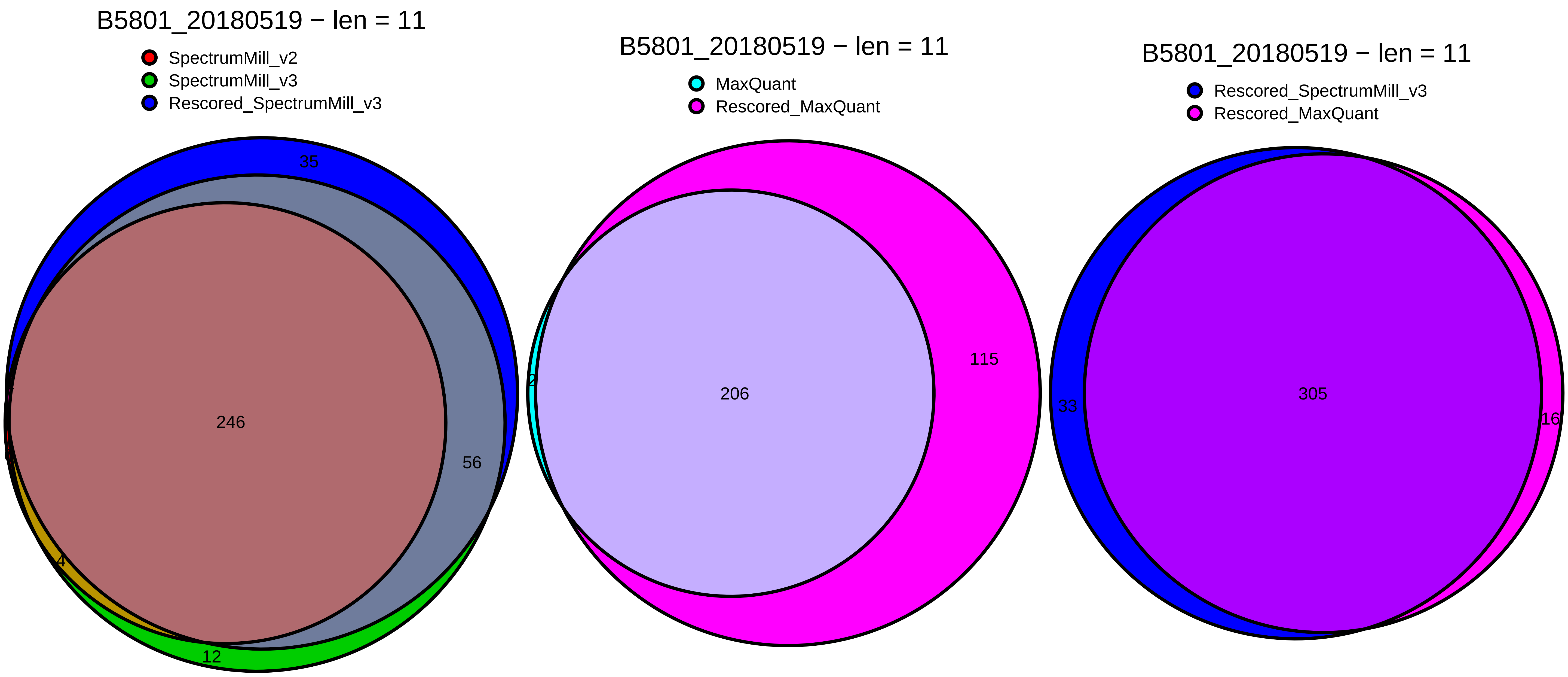
B5801_20180519 − len = 11
SpectrumMill_v2
SpectrumMill_v3
Rescored_SpectrumMill_v3
35
246
56
4
12
B5801_20180519 − len = 11
MaxQuant
Rescored_MaxQuant
2
206
115
B5801_20180519 − len = 11
Rescored_SpectrumMill_v3
Rescored_MaxQuant
33
305
16

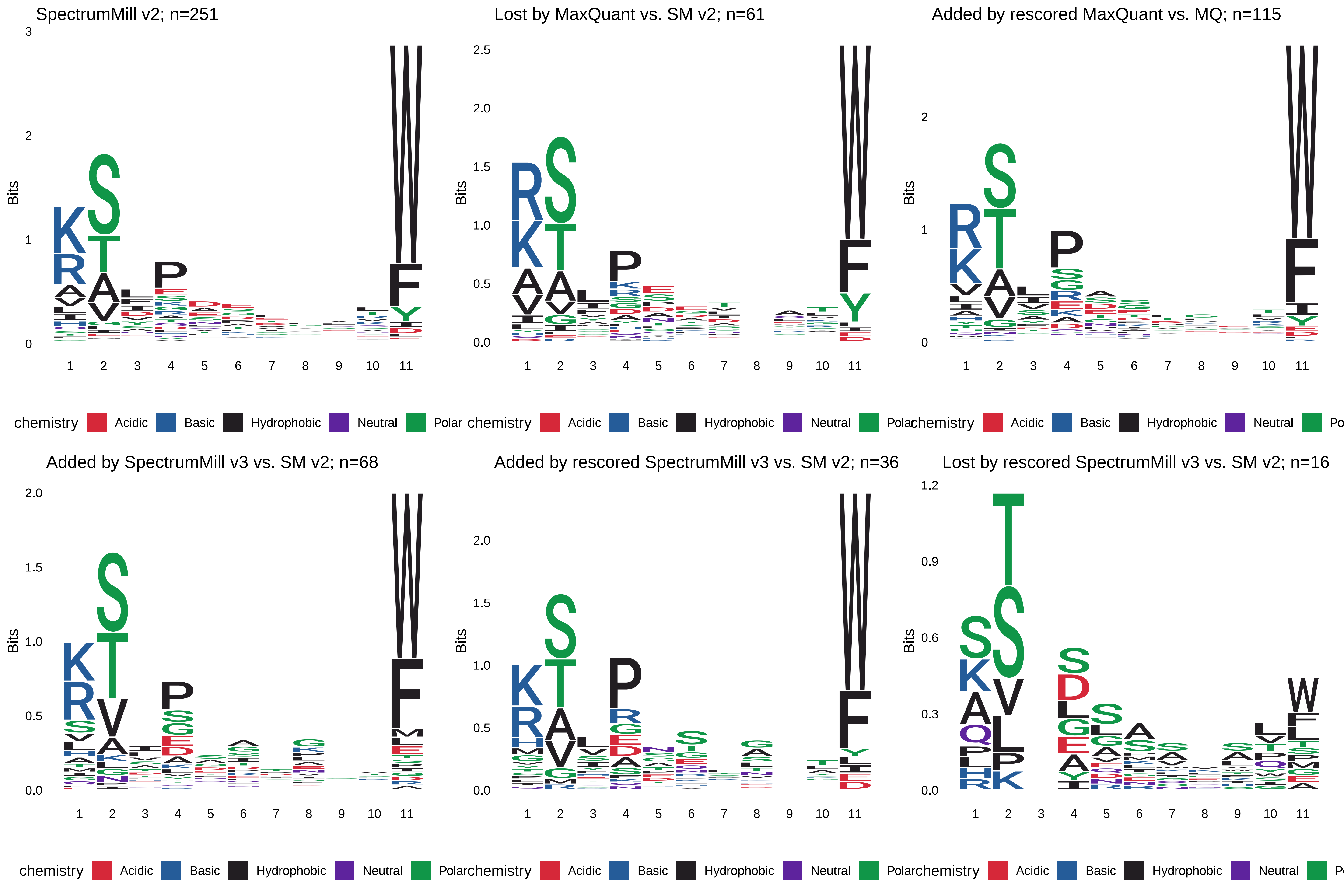
SpectrumMill v2; n=251
Lost by MaxQuant vs. SM v2; n=61
Added by rescored MaxQuant vs. MQ; n=115
Added by SpectrumMill v3 vs. SM v2; n=68
Added by rescored SpectrumMill v3 vs. SM v2; n=36
Lost by rescored SpectrumMill v3 vs. SM v2; n=16
Bits
1 2 3 4 5 6 7 8 9 10 11
chemistry Acidic Basic Hydrophobic Neutral Polar

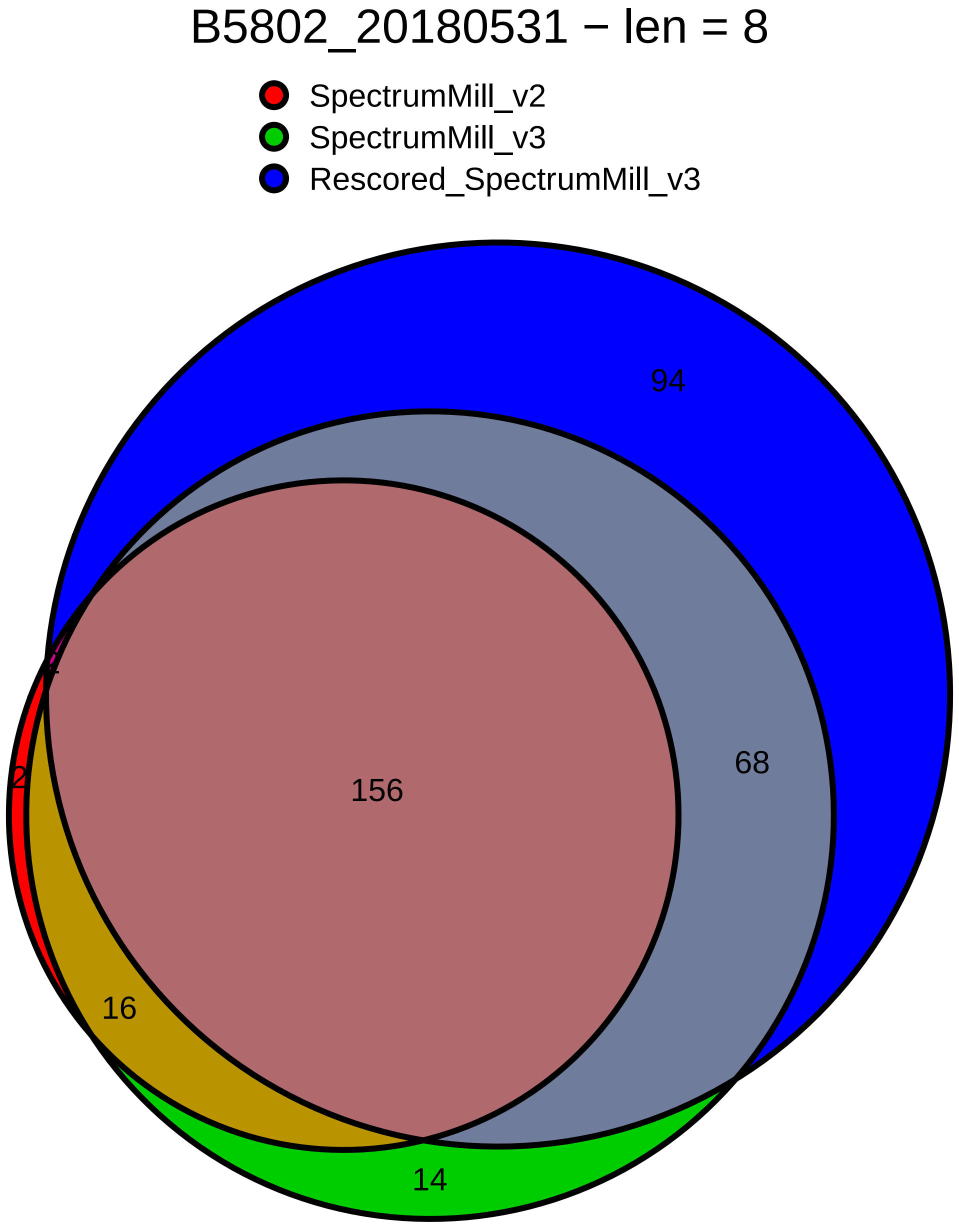
B5802_20180531 − len = 8
SpectrumMill_v2
SpectrumMill_v3
Rescored_SpectrumMill_v3
94
68
156
16
14

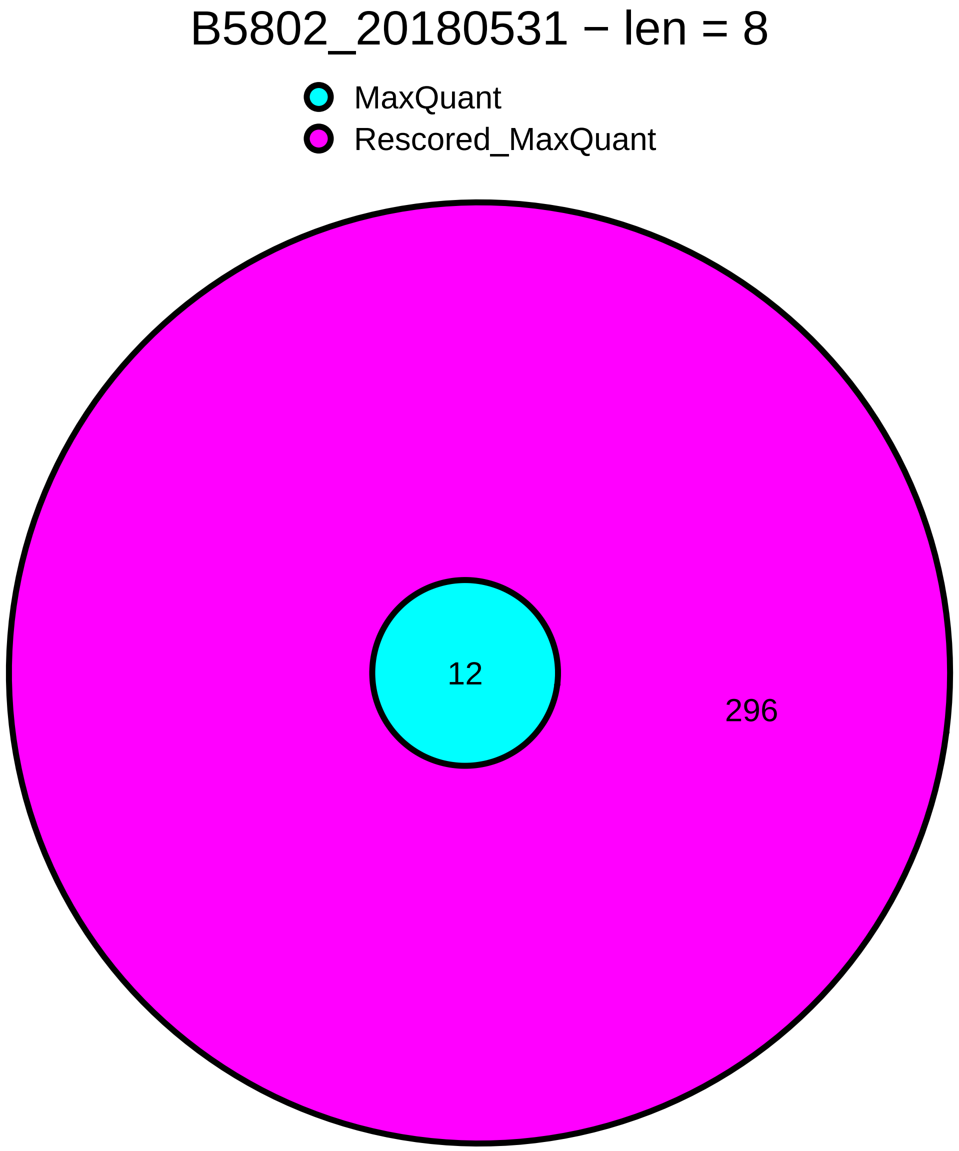
B5802_20180531 − len = 8
MaxQuant
Rescored_MaxQuant
12
296

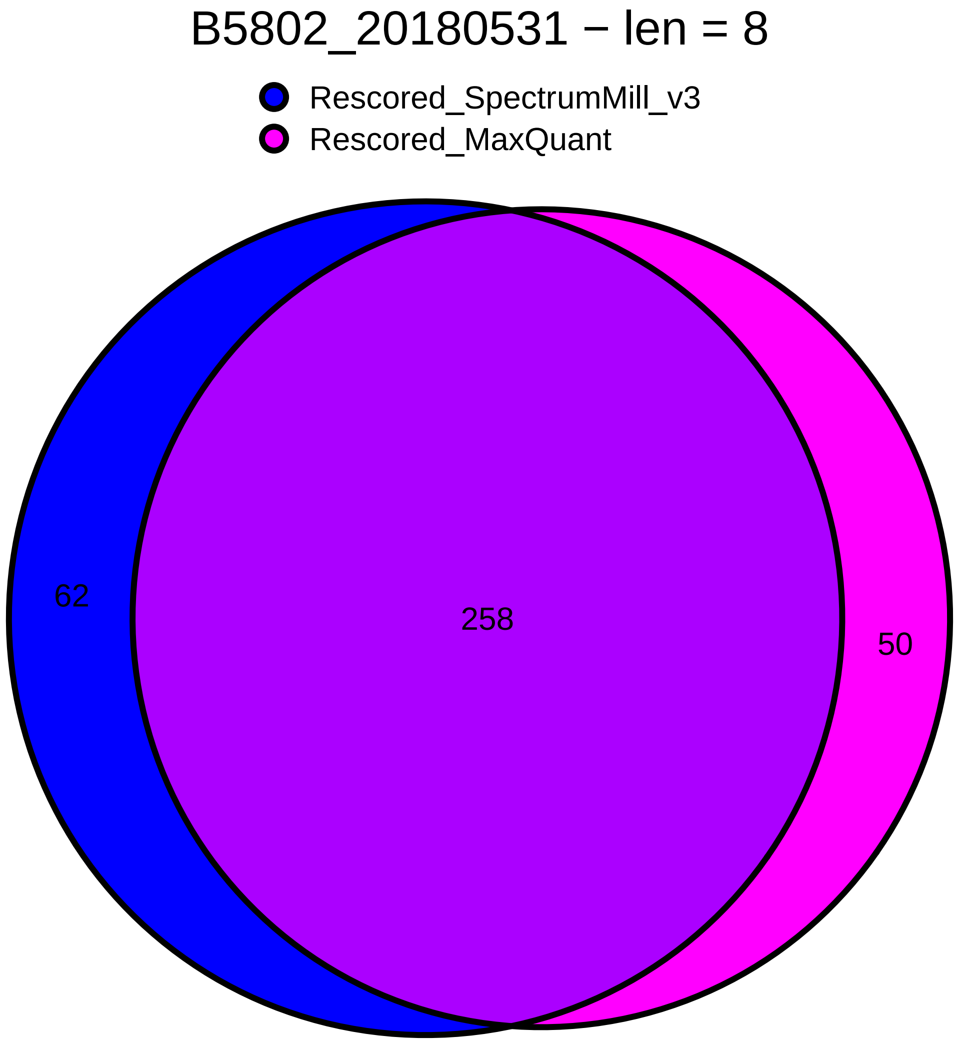
B5802_20180531 − len = 8
Rescored_SpectrumMill_v3
Rescored_MaxQuant
62
258
50

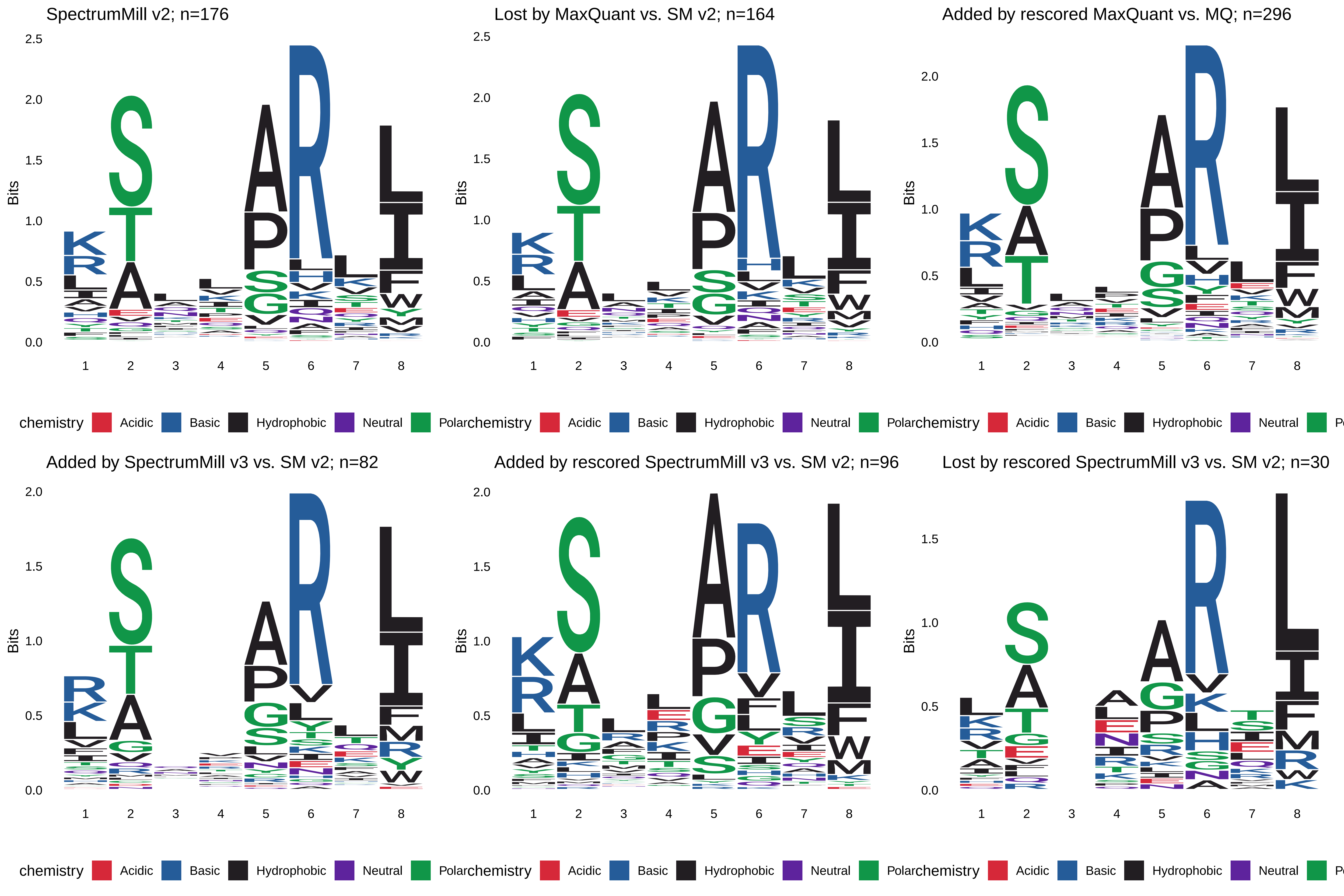
SpectrumMill v2; n=176
Lost by MaxQuant vs. SM v2; n=164
Added by rescored MaxQuant vs. MQ; n=296
Added by SpectrumMill v3 vs. SM v2; n=82
Added by rescored SpectrumMill v3 vs. SM v2; n=96
Lost by rescored SpectrumMill v3 vs. SM v2; n=30
Bits
1 2 3 4 5 6 7 8
chemistry Acidic Basic Hydrophobic Neutral Polar

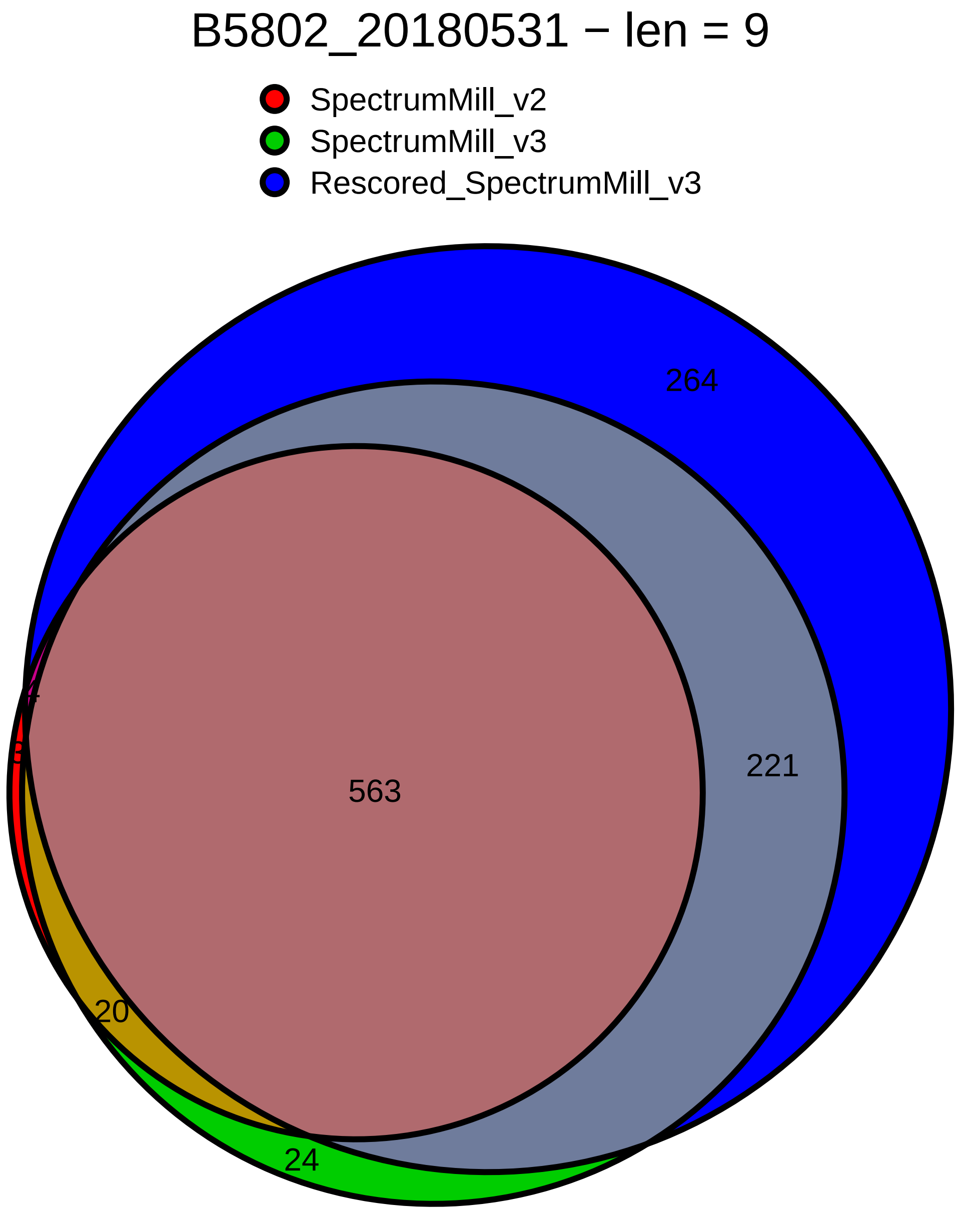

B5802_20180531 − len = 9
SpectrumMill_v2
SpectrumMill_v3
Rescored_SpectrumMill_v3
264
4
3
563
221
20
24

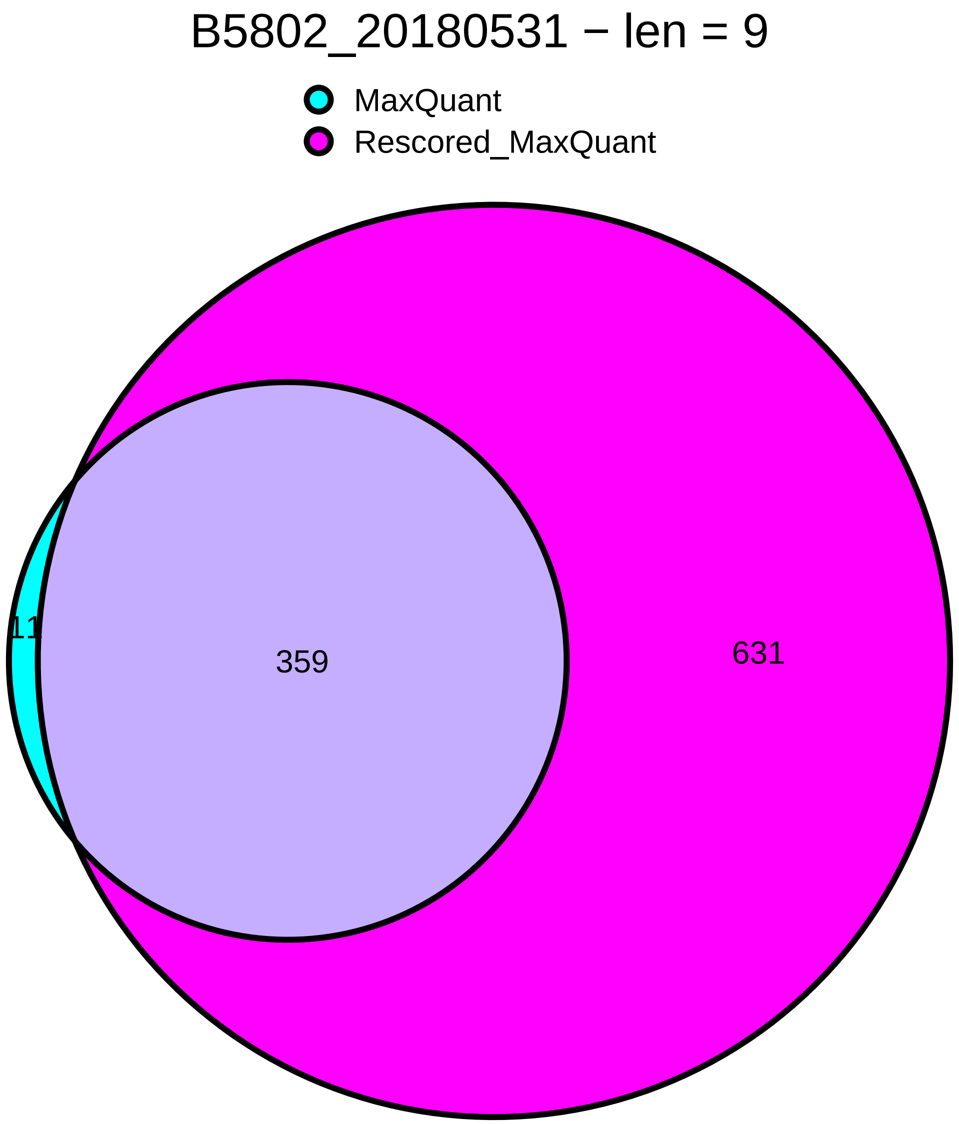

B5802_20180531 − len = 9
MaxQuant
Rescored_MaxQuant
11
359
631

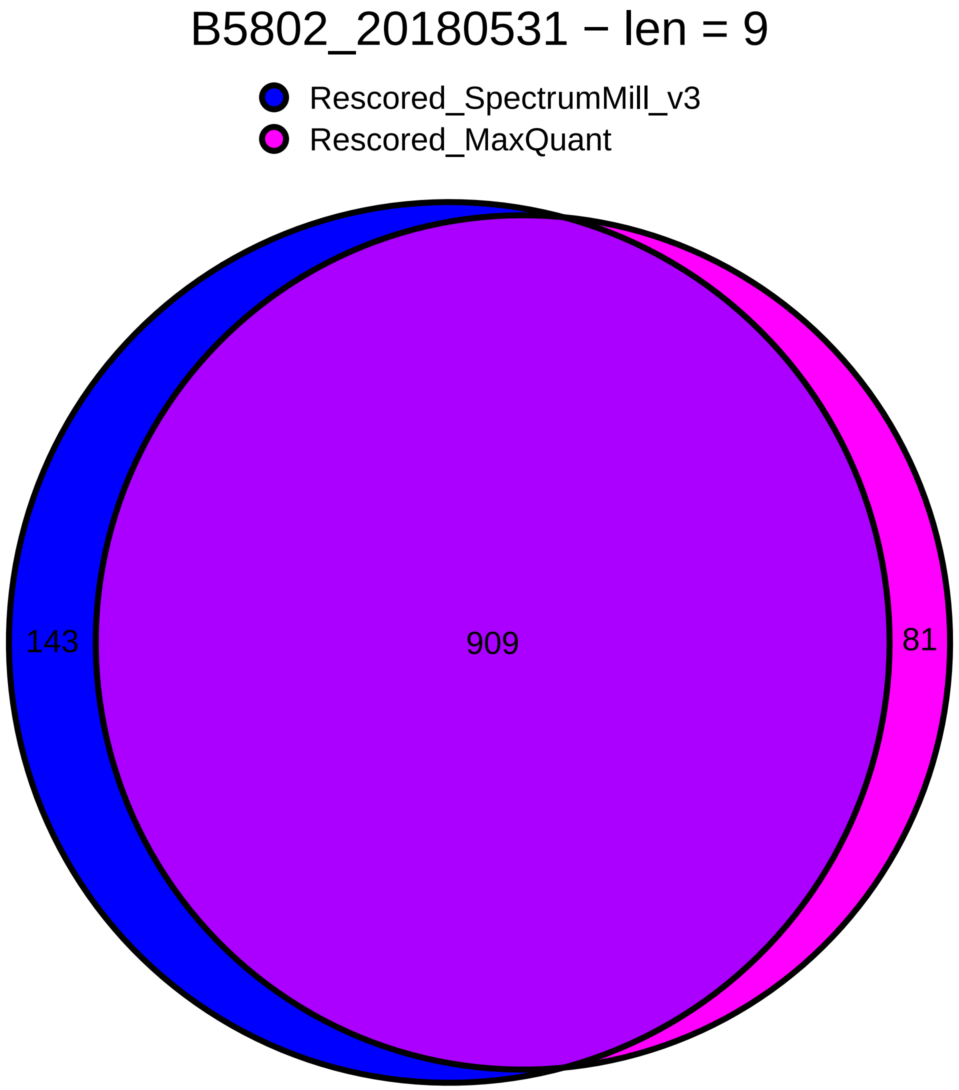

B5802_20180531 − len = 9
Rescored_SpectrumMill_v3
Rescored_MaxQuant
143
909
81

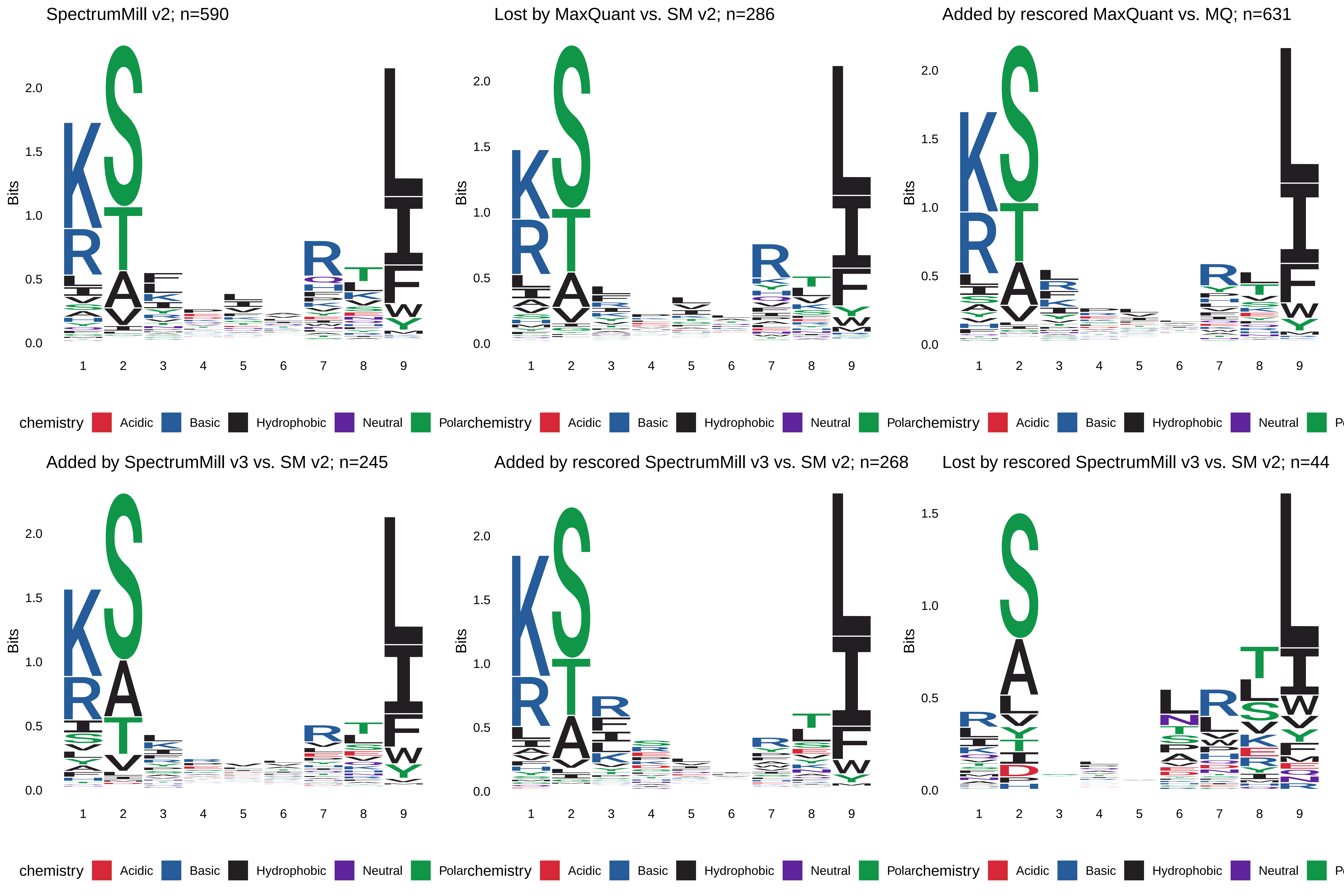
SpectrumMill v2; n=590
Lost by MaxQuant vs. SM v2; n=286
Added by rescored MaxQuant vs. MQ; n=631
Added by SpectrumMill v3 vs. SM v2; n=245
Added by rescored SpectrumMill v3 vs. SM v2; n=268
Lost by rescored SpectrumMill v3 vs. SM v2; n=44
Bits
chemistry
Acidic
Basic
Hydrophobic
Neutral
Polar

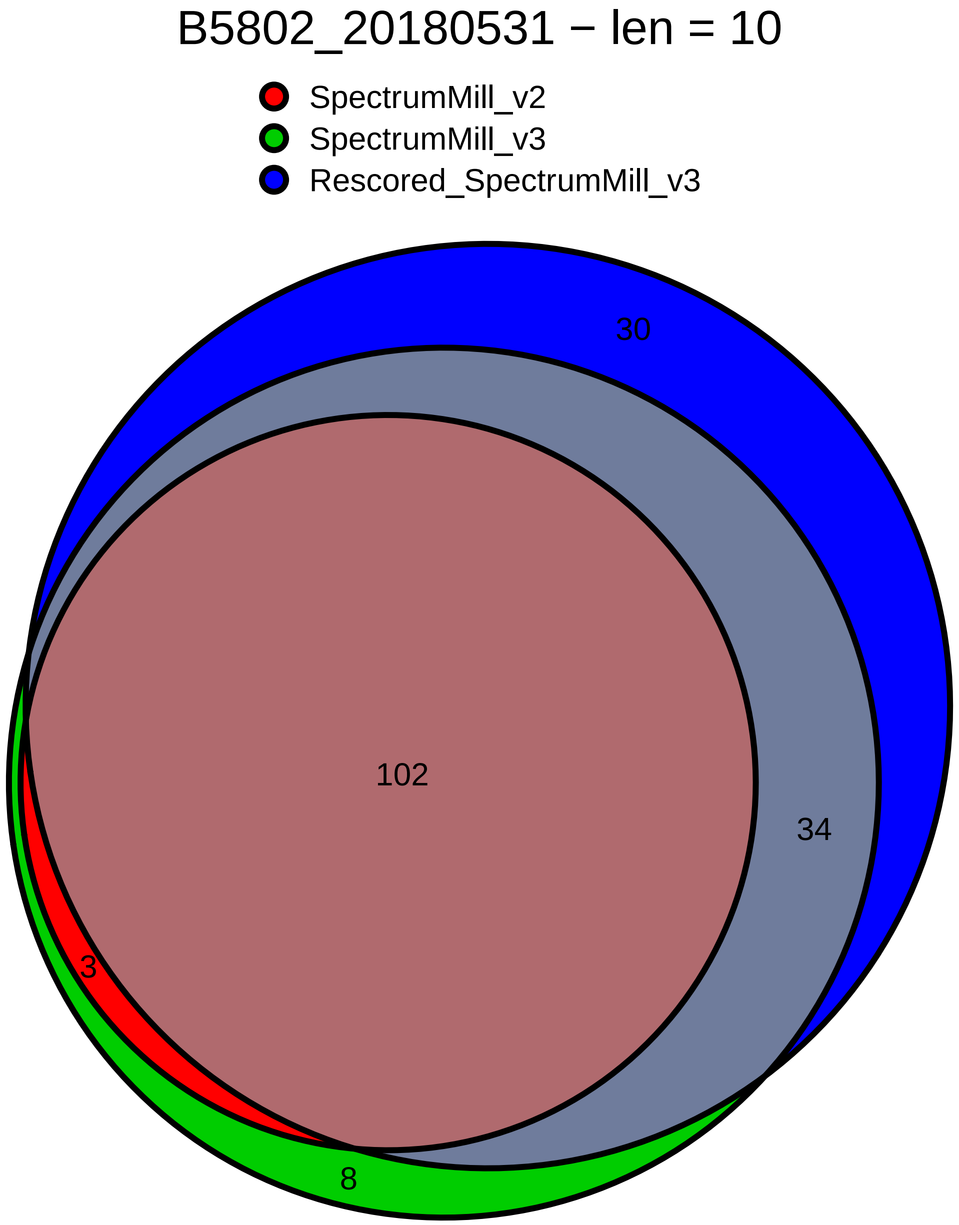
B5802_20180531 − len = 10
SpectrumMill_v2
SpectrumMill_v3
Rescored_SpectrumMill_v3
30
102
34
3
8

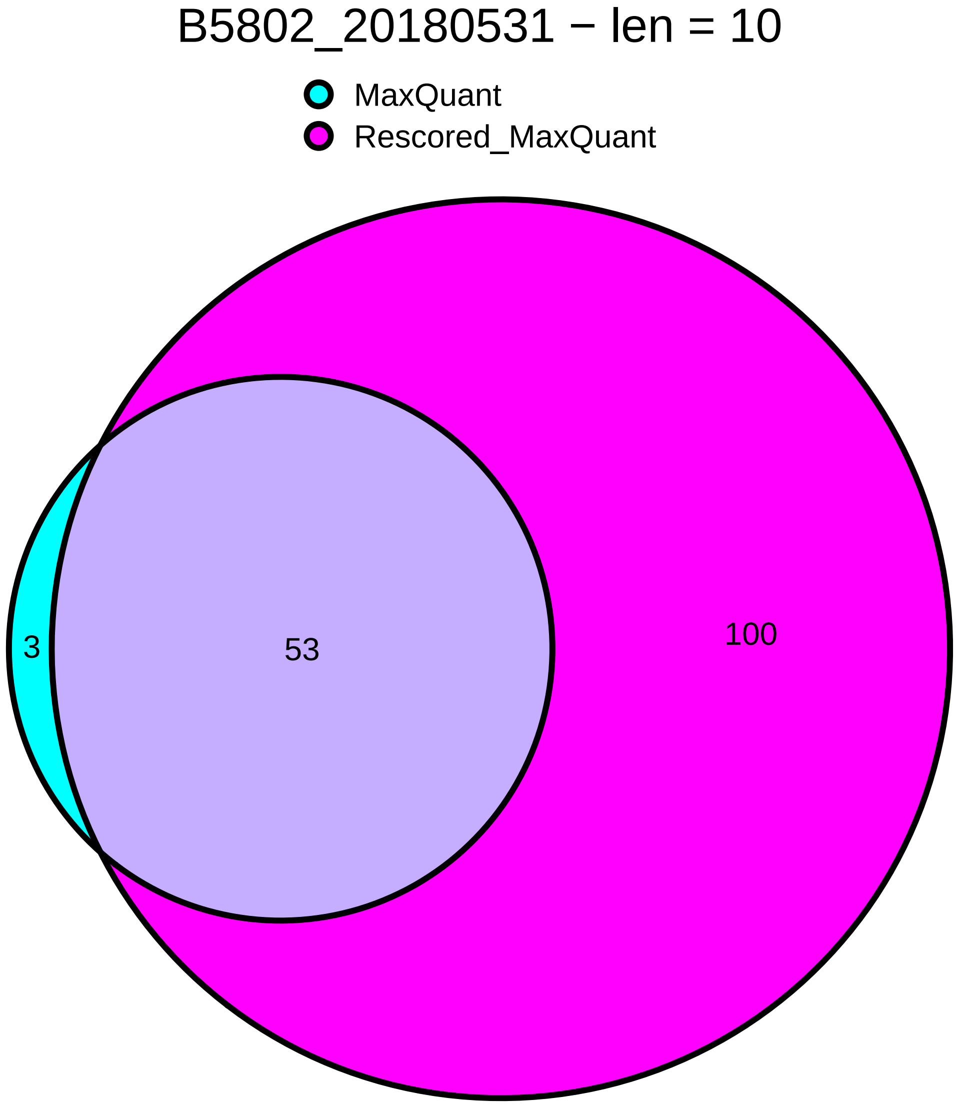
B5802_20180531 − len = 10
MaxQuant
Rescored_MaxQuant
3
53
100

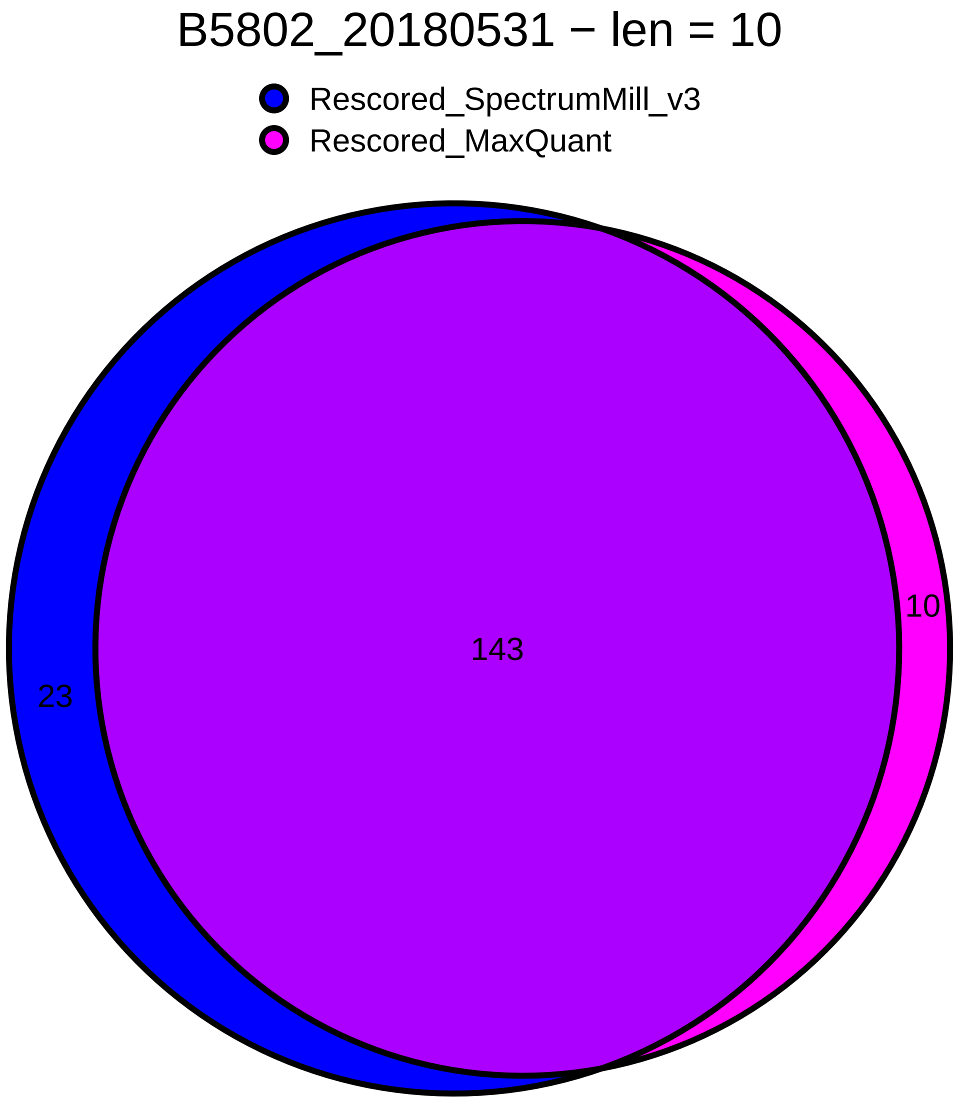
B5802_20180531 − len = 10
Rescored_SpectrumMill_v3
Rescored_MaxQuant
23
143
10

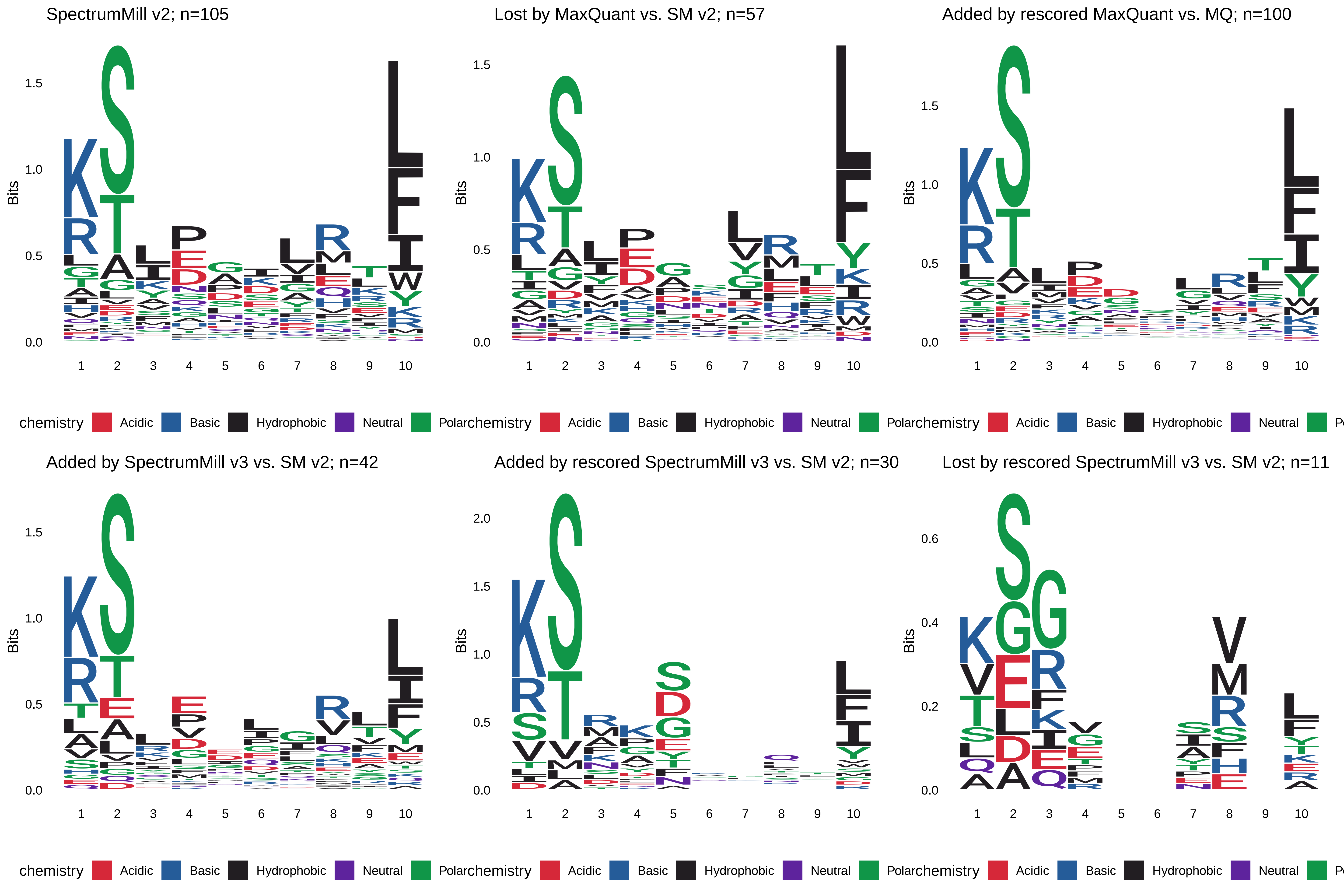

SpectrumMill v2; n=105
Lost by MaxQuant vs. SM v2; n=57
Added by rescored MaxQuant vs. MQ; n=100
Bits
1 2 3 4 5 6 7 8 9 10
chemistry Acidic Basic Hydrophobic Neutral Polarchemistry Acidic Basic Hydrophobic Neutral Polarchemistry Acidic Basic Hydrophobic Neutral
Added by SpectrumMill v3 vs. SM v2; n=42
Added by rescored SpectrumMill v3 vs. SM v2; n=30
Lost by rescored SpectrumMill v3 vs. SM v2; n=11
Bits
1 2 3 4 5 6 7 8 9 10
chemistry Acidic Basic Hydrophobic Neutral Polarchemistry Acidic Basic Hydrophobic Neutral Polarchemistry Acidic Basic Hydrophobic Neutral

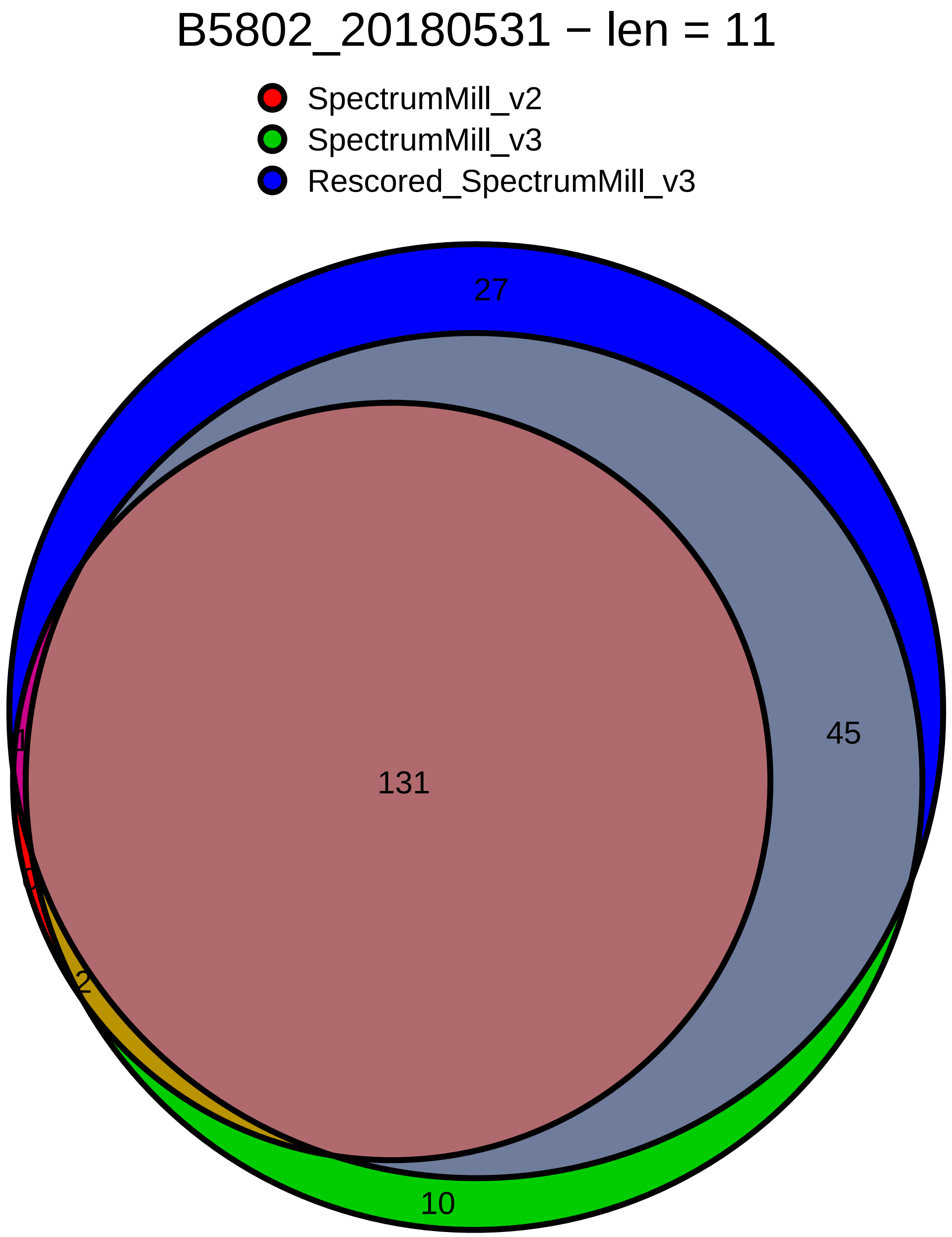
B5802_20180531 − len = 11
SpectrumMill_v2
SpectrumMill_v3
Rescored_SpectrumMill_v3
27
45
131
2
10

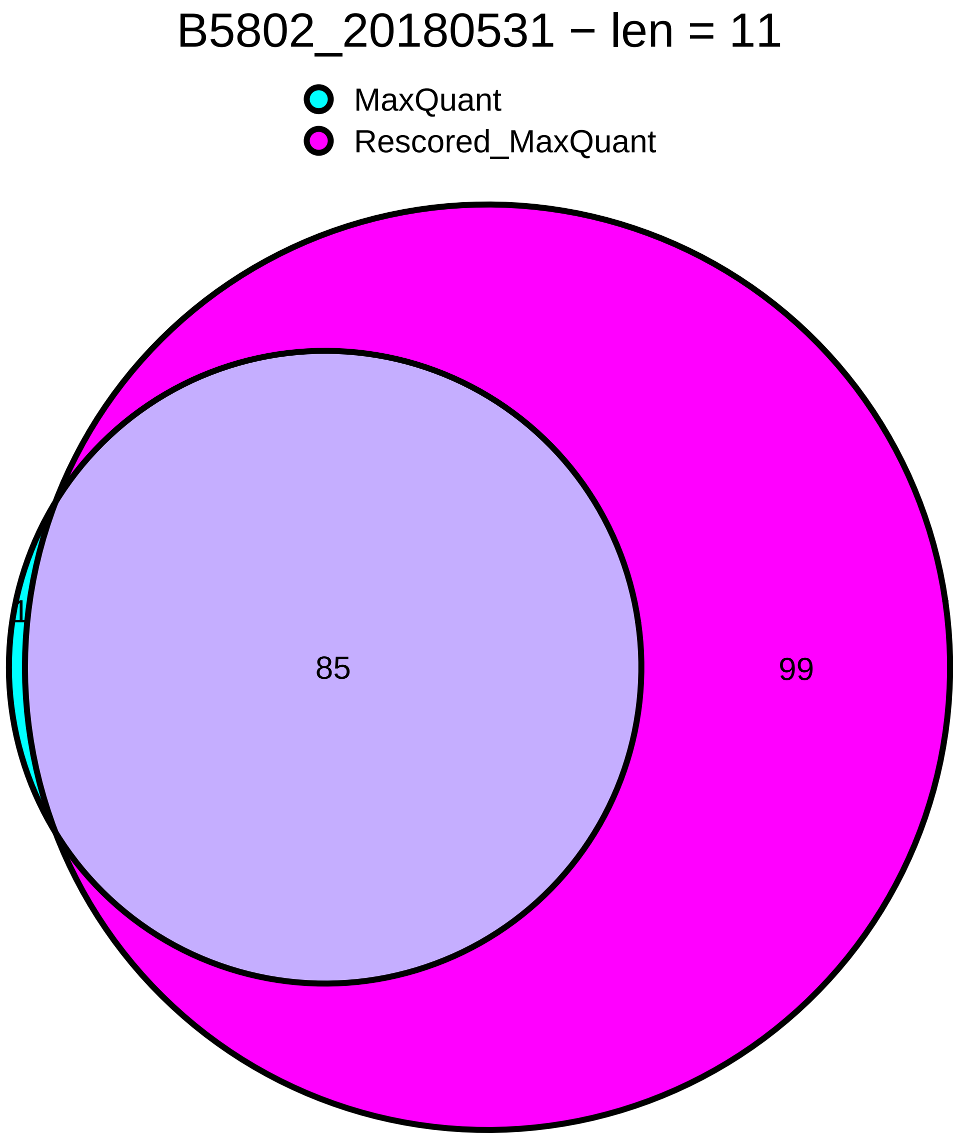
B5802_20180531 − len = 11
MaxQuant
Rescored_MaxQuant
85
99

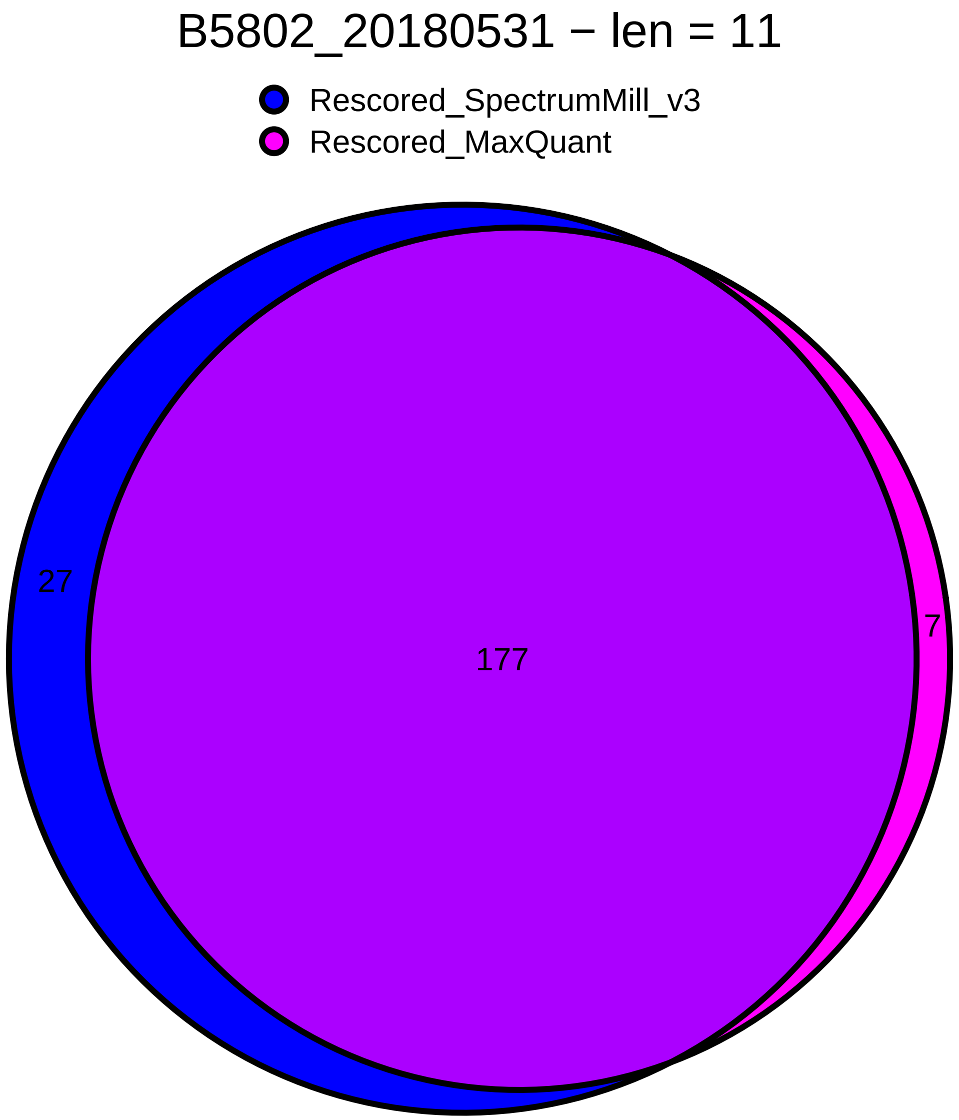
B5802_20180531 − len = 11
Rescored_SpectrumMill_v3
Rescored_MaxQuant
27
177
7

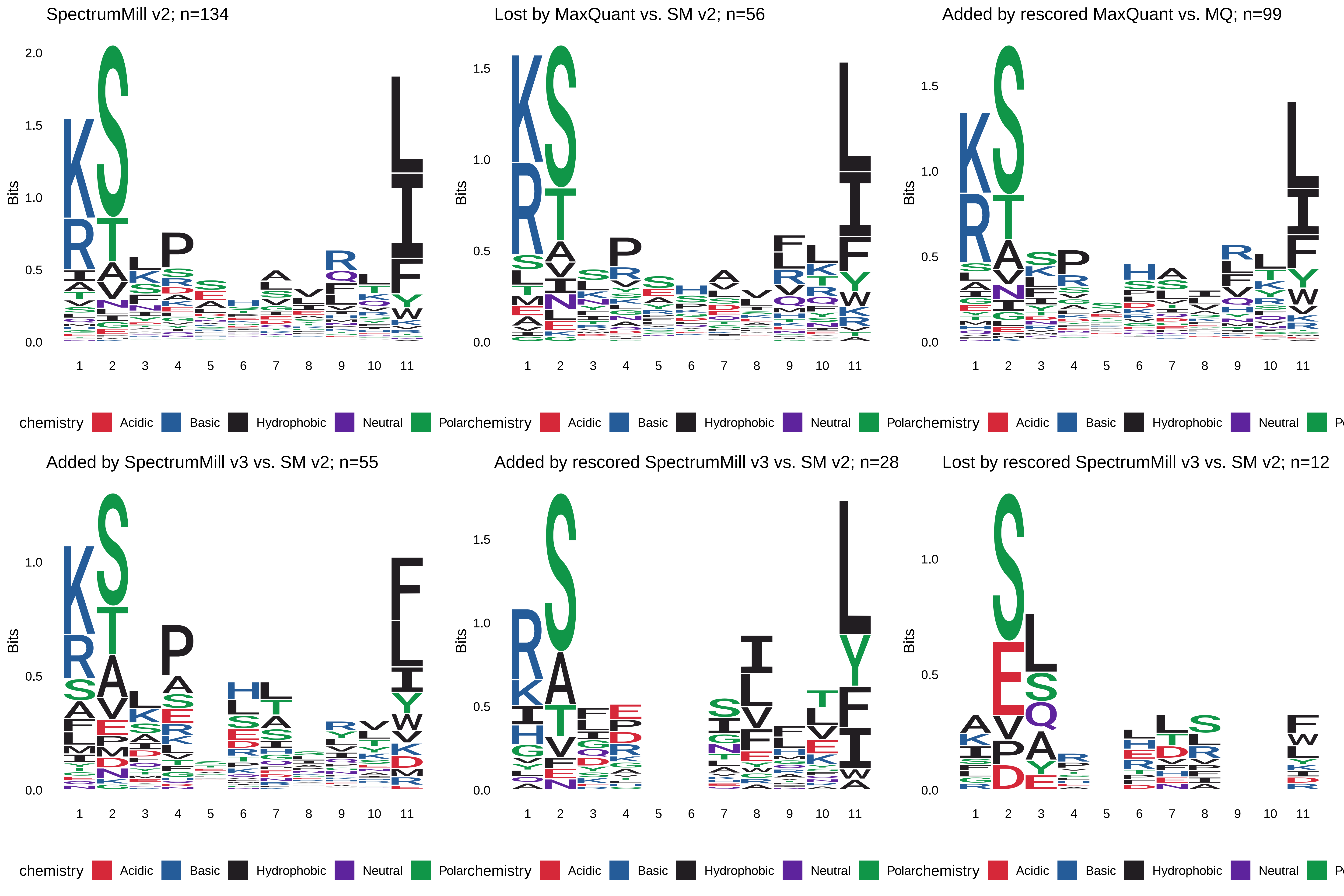

SpectrumMill v2; n=134
Lost by MaxQuant vs. SM v2; n=56
Added by rescored MaxQuant vs. MQ; n=99
Added by SpectrumMill v3 vs. SM v2; n=55
Added by rescored SpectrumMill v3 vs. SM v2; n=28
Lost by rescored SpectrumMill v3 vs. SM v2; n=12
Bits
0.0
0.5
1.0
1.5
2.0
1 2 3 4 5 6 7 8 9 10 11
chemistry Acidic Basic Hydrophobic Neutral Polarchemistry Acidic Basic Hydrophobic Neutral Polarchemistry Acidic Basic Hydrophobic Neutral
chemistry Acidic Basic Hydrophobic Neutral Polarchemistry Acidic Basic Hydrophobic Neutral Polarchemistry Acidic Basic Hydrophobic Neutral

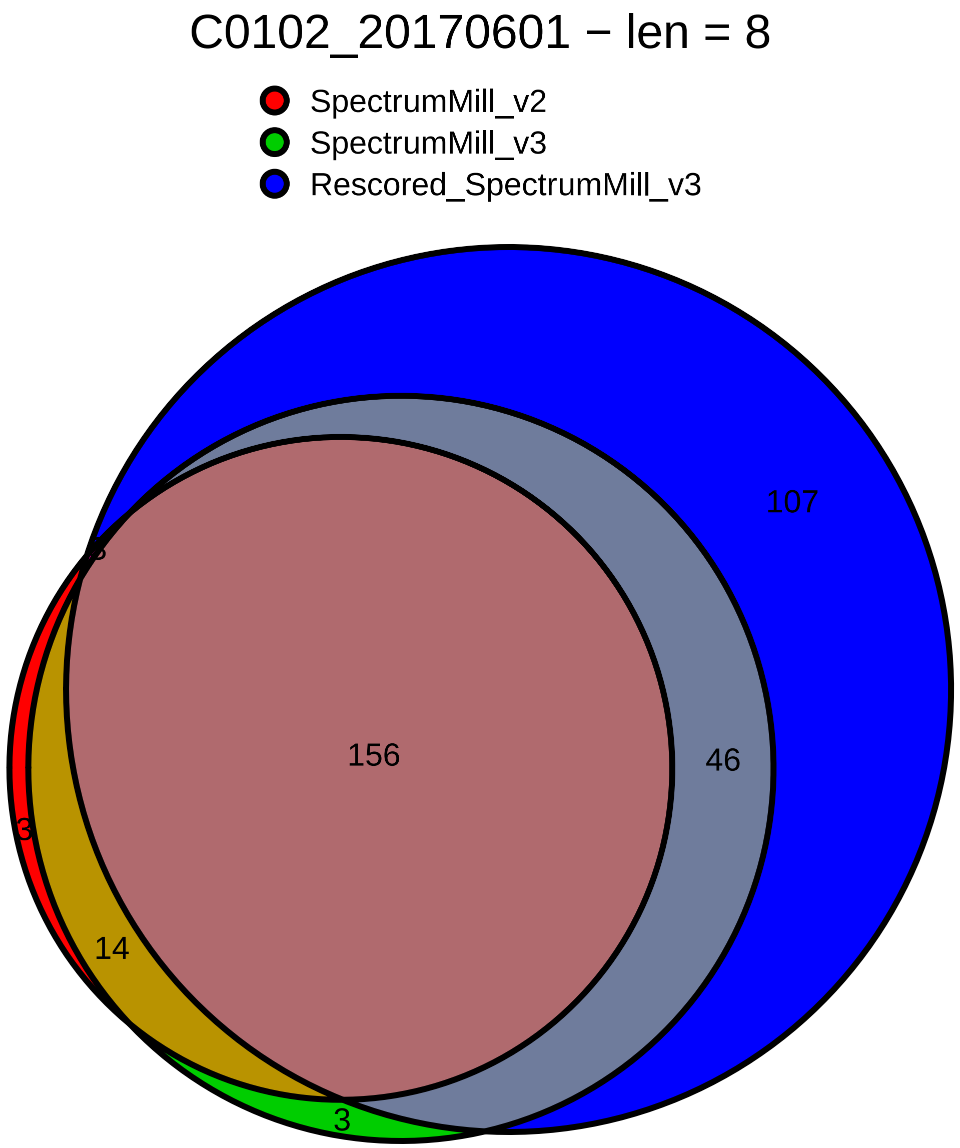
C0102_20170601 − len = 8
SpectrumMill_v2
SpectrumMill_v3
Rescored_SpectrumMill_v3
107
156
46
3
14
3

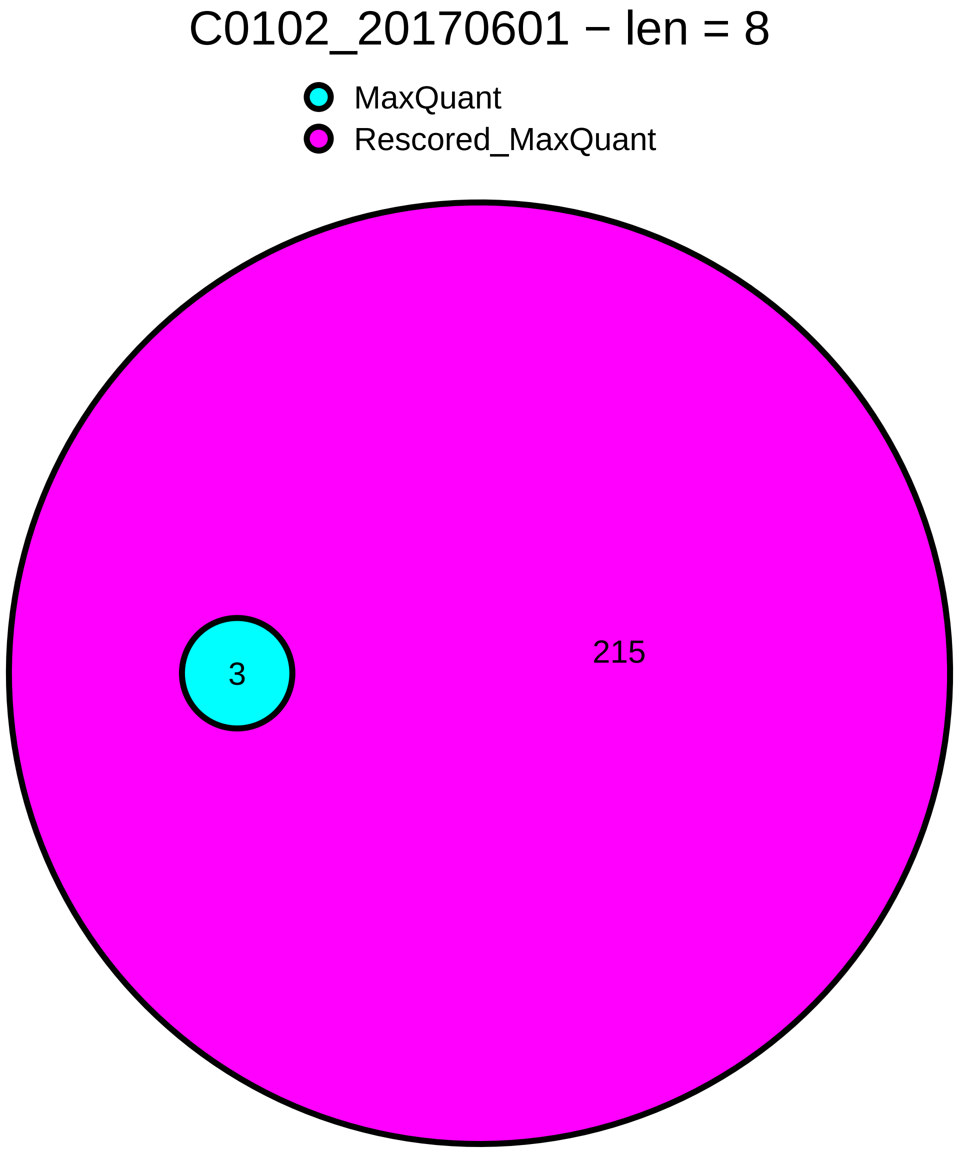
C0102_20170601 − len = 8
MaxQuant
Rescored_MaxQuant
3
215

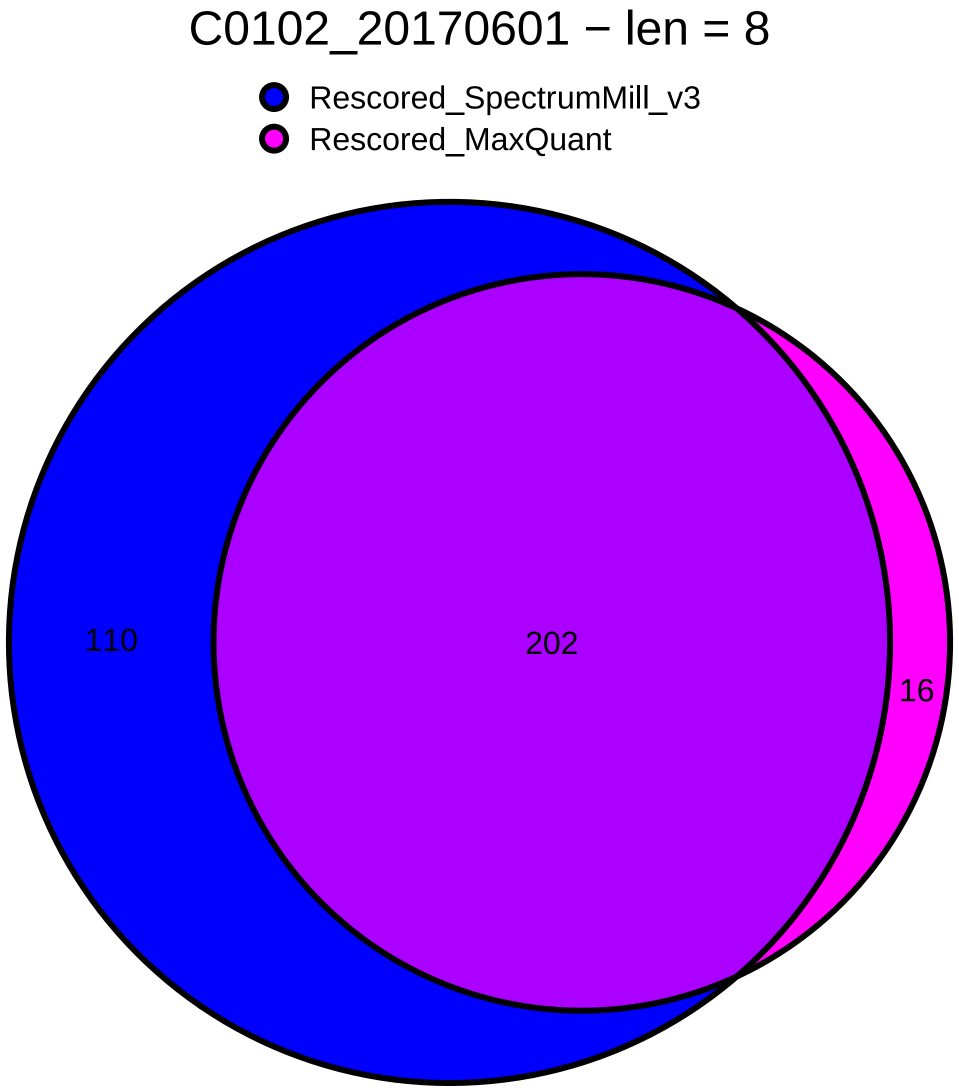
C0102_20170601 − len = 8
Rescored_SpectrumMill_v3
Rescored_MaxQuant
110
202
16

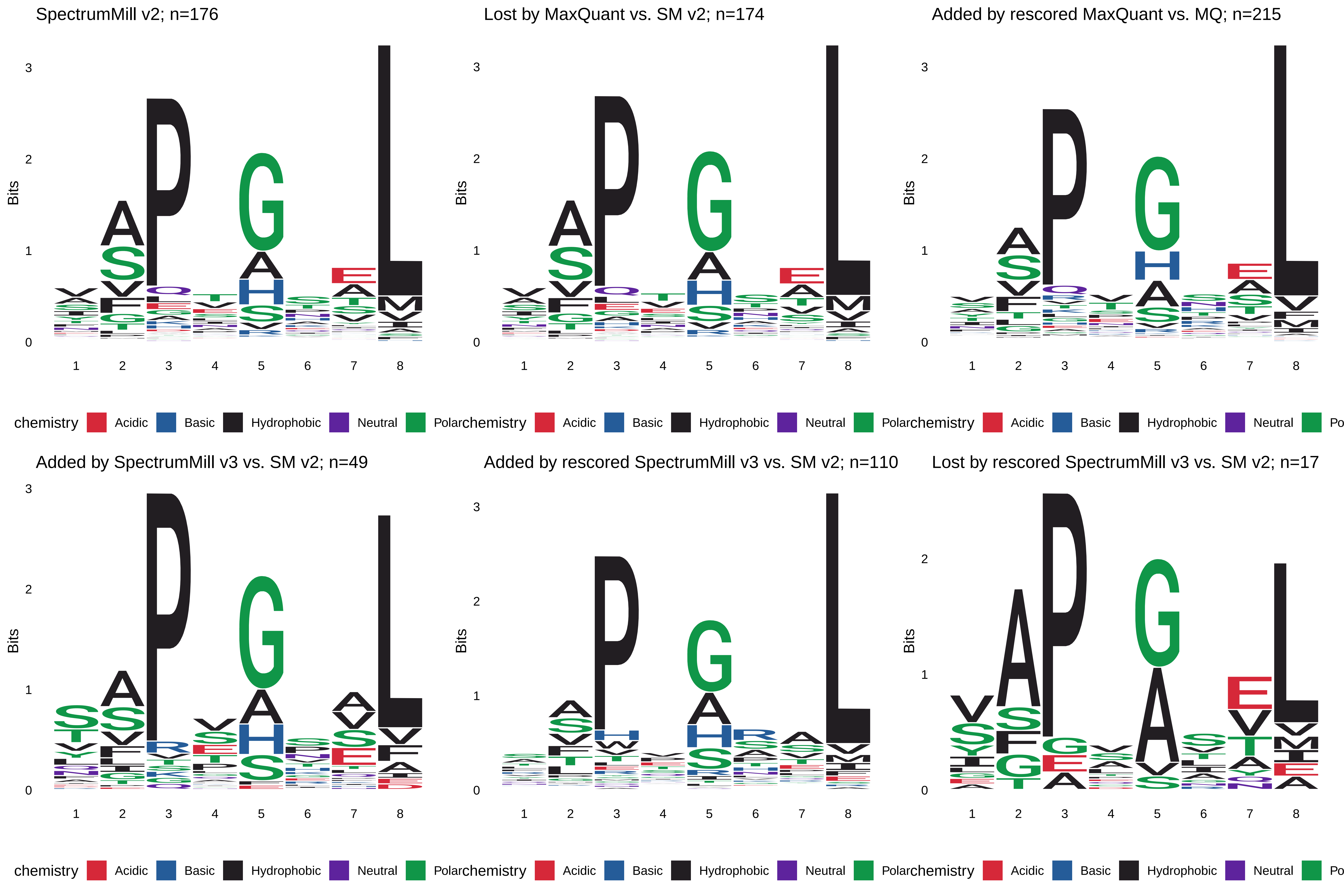
SpectrumMill v2; n=176
Lost by MaxQuant vs. SM v2; n=174
Added by rescored MaxQuant vs. MQ; n=215
Added by SpectrumMill v3 vs. SM v2; n=49
Added by rescored SpectrumMill v3 vs. SM v2; n=110
Lost by rescored SpectrumMill v3 vs. SM v2; n=17
Bits
1 2 3 4 5 6 7 8
chemistry Acidic Basic Hydrophobic Neutral Polar

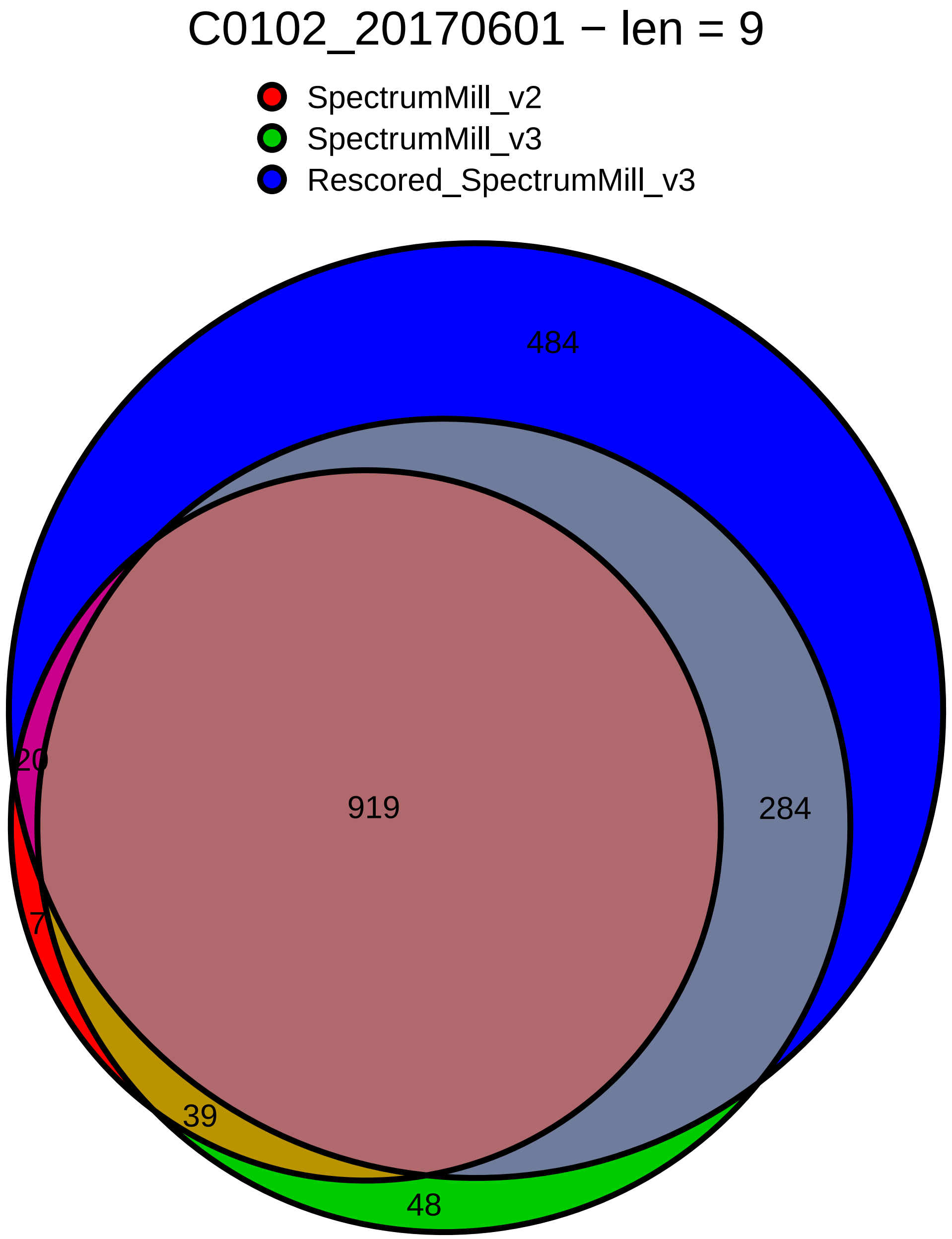
C0102_20170601 − len = 9
SpectrumMill_v2
SpectrumMill_v3
Rescored_SpectrumMill_v3
484
20
919
284
7
39
48

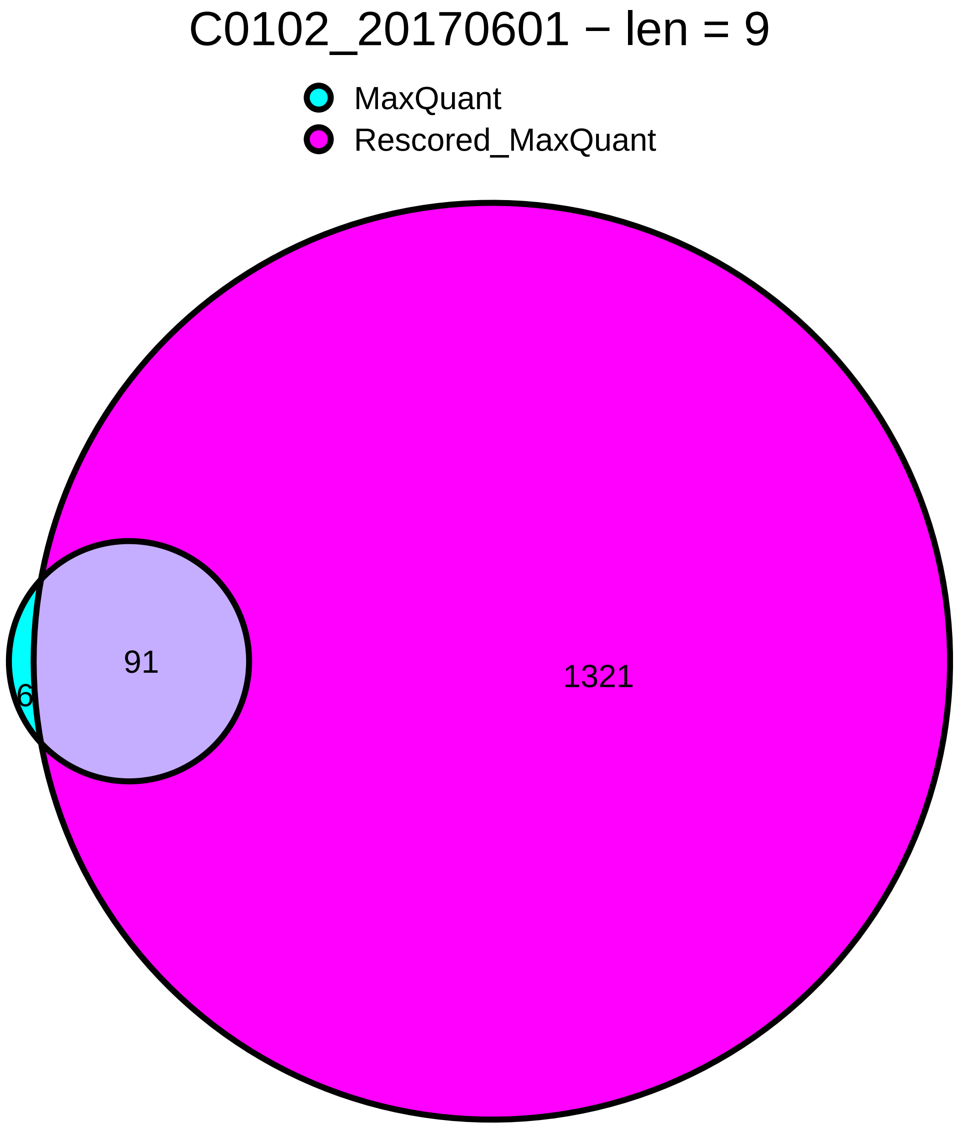
C0102_20170601 − len = 9
MaxQuant
Rescored_MaxQuant
6
91
1321

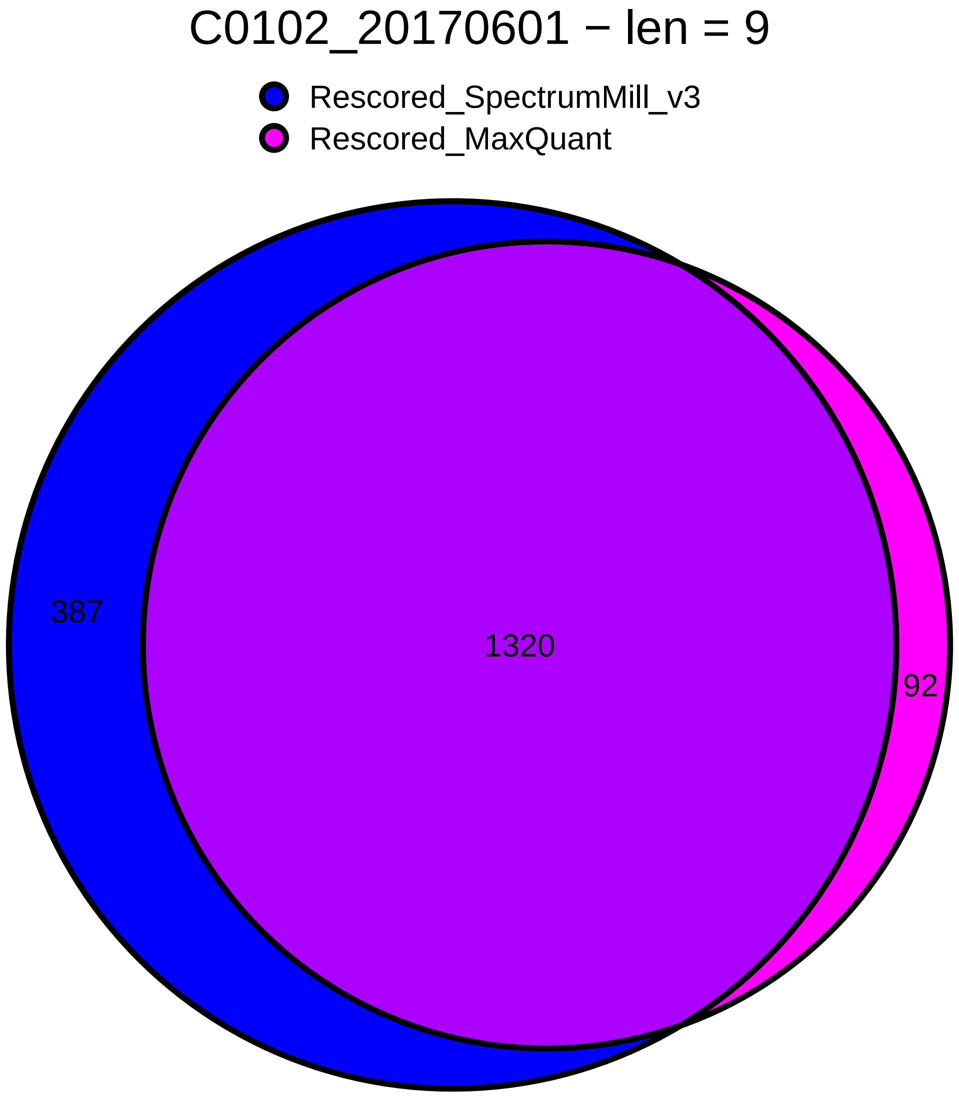
C0102_20170601 − len = 9
Rescored_SpectrumMill_v3
Rescored_MaxQuant
387
1320
92

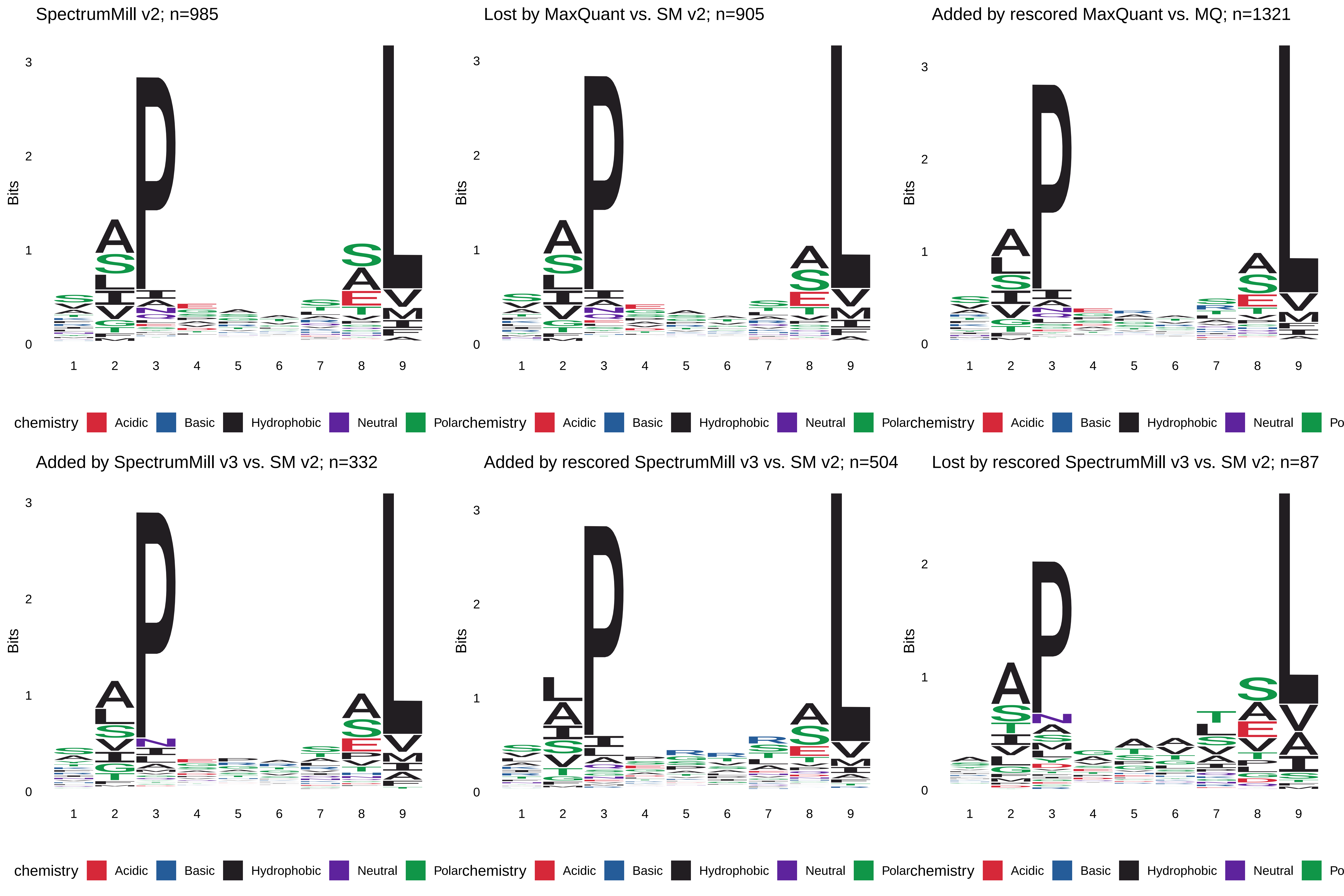
SpectrumMill v2; n=985
Lost by MaxQuant vs. SM v2; n=905
Added by rescored MaxQuant vs. MQ; n=1321
Added by SpectrumMill v3 vs. SM v2; n=332
Added by rescored SpectrumMill v3 vs. SM v2; n=504
Lost by rescored SpectrumMill v3 vs. SM v2; n=87
Bits
0
1
2
3
1 2 3 4 5 6 7 8 9
chemistry
Acidic
Basic
Hydrophobic
Neutral
Polar

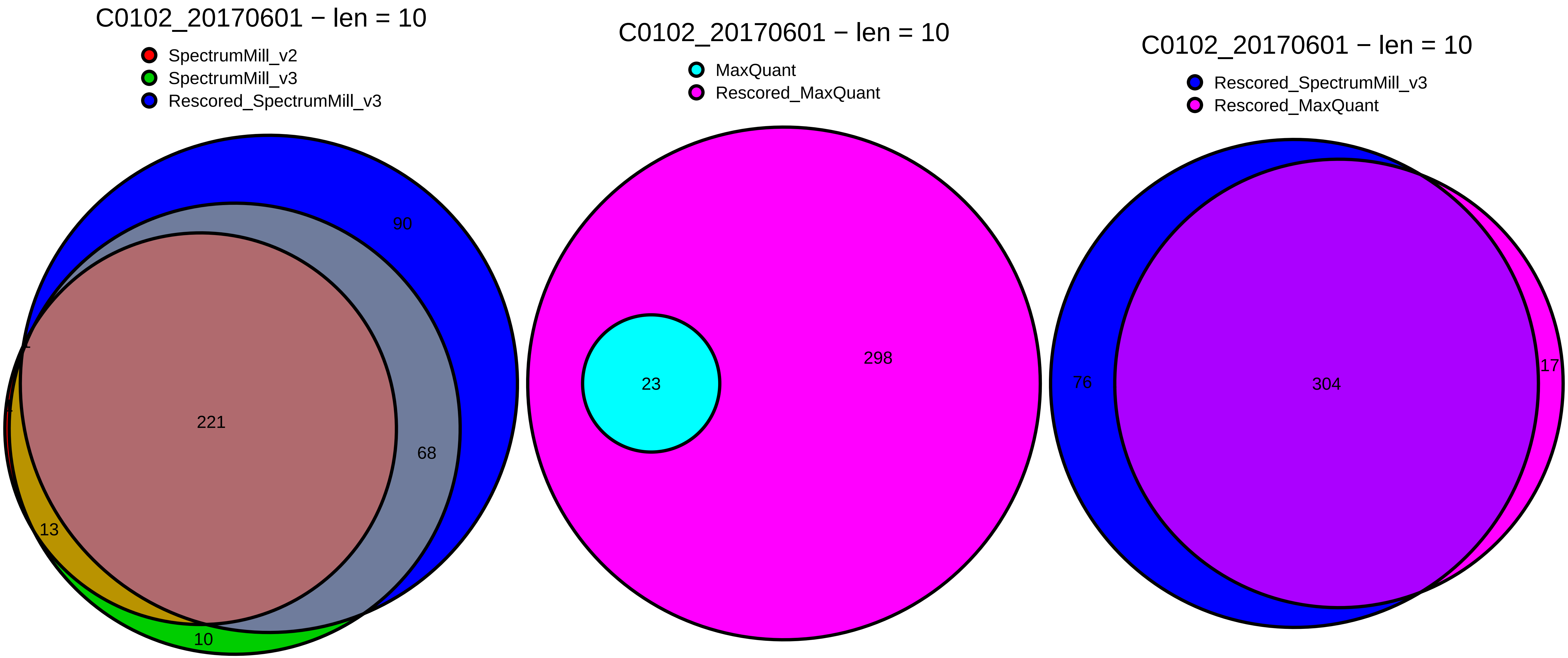
C0102_20170601 − len = 10
SpectrumMill_v2
SpectrumMill_v3
Rescored_SpectrumMill_v3
90
221
68
13
10
C0102_20170601 − len = 10
MaxQuant
Rescored_MaxQuant
23
298
C0102_20170601 − len = 10
Rescored_SpectrumMill_v3
Rescored_MaxQuant
76
304
17

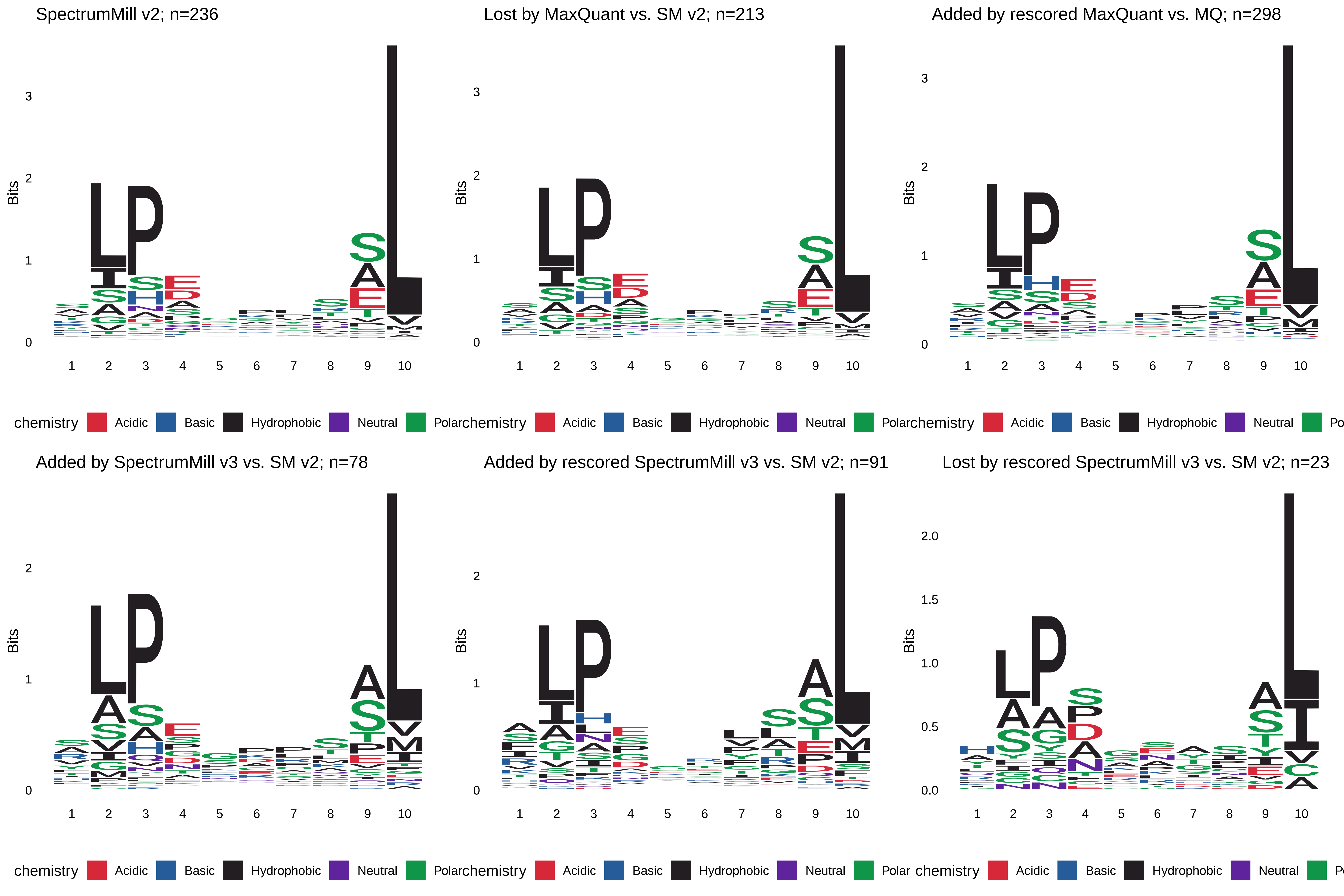
SpectrumMill v2; n=236
Lost by MaxQuant vs. SM v2; n=213
Added by rescored MaxQuant vs. MQ; n=298
Added by SpectrumMill v3 vs. SM v2; n=78
Added by rescored SpectrumMill v3 vs. SM v2; n=91
Lost by rescored SpectrumMill v3 vs. SM v2; n=23
Bits
1 2 3 4 5 6 7 8 9 10
chemistry
Acidic
Basic
Hydrophobic
Neutral
Polar

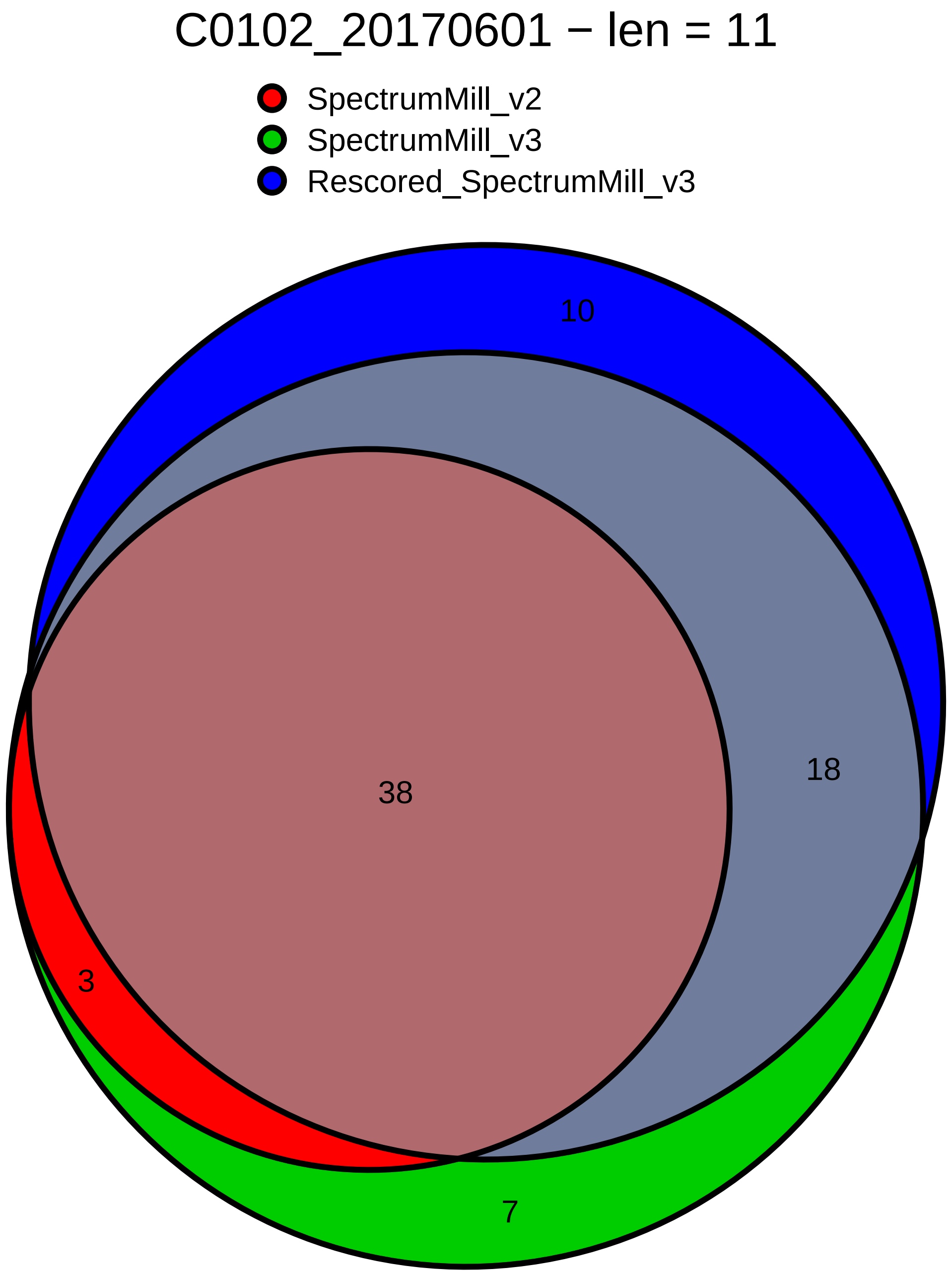
C0102_20170601 − len = 11
SpectrumMill_v2
SpectrumMill_v3
Rescored_SpectrumMill_v3
10
18
38
3
7

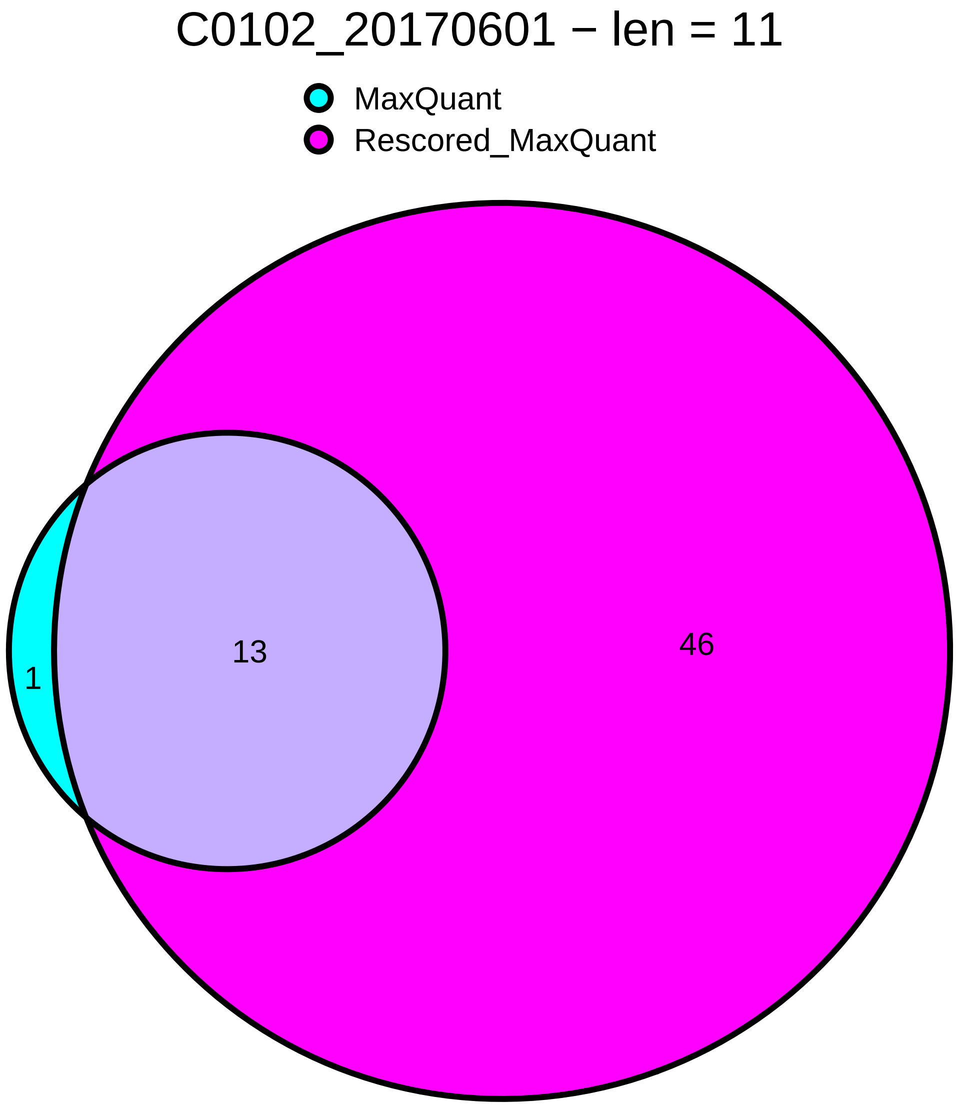
C0102_20170601 − len = 11
MaxQuant
Rescored_MaxQuant
1
13
46

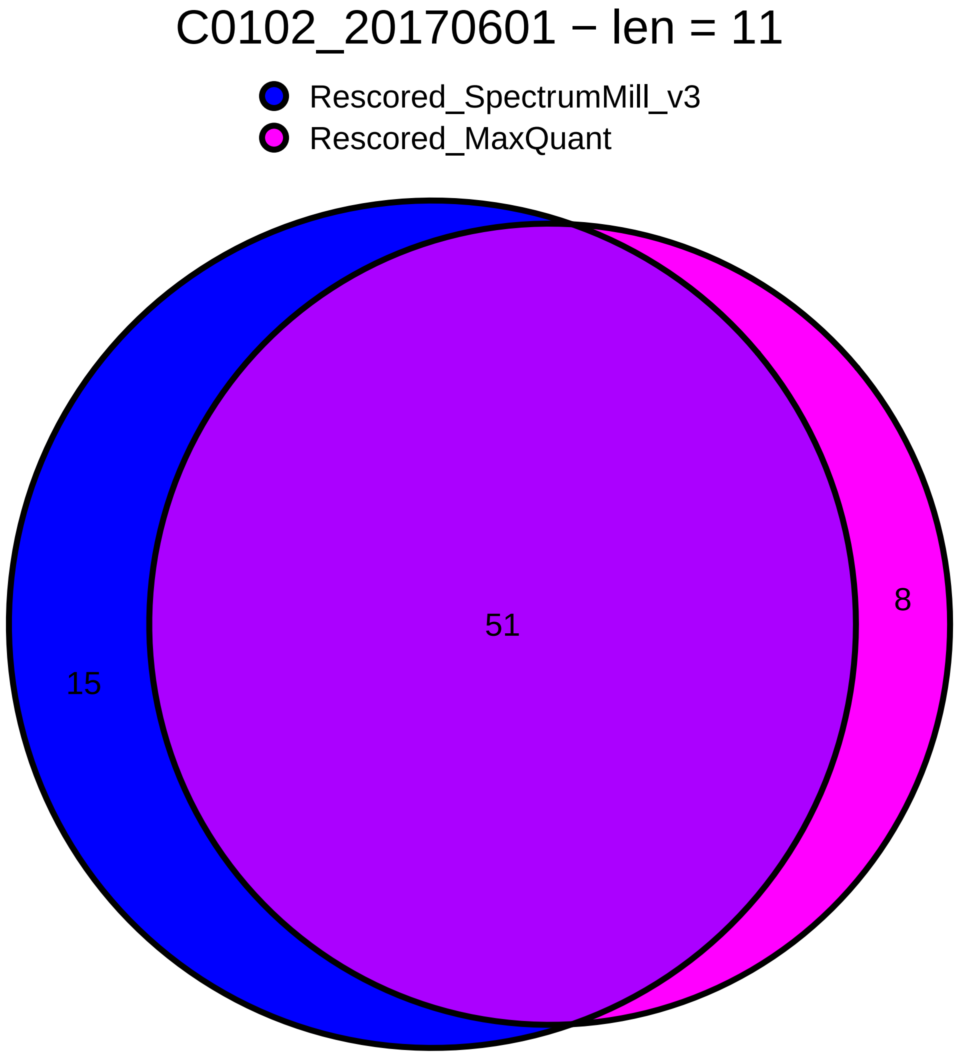
C0102_20170601 − len = 11
Rescored_SpectrumMill_v3
Rescored_MaxQuant
15
51
8

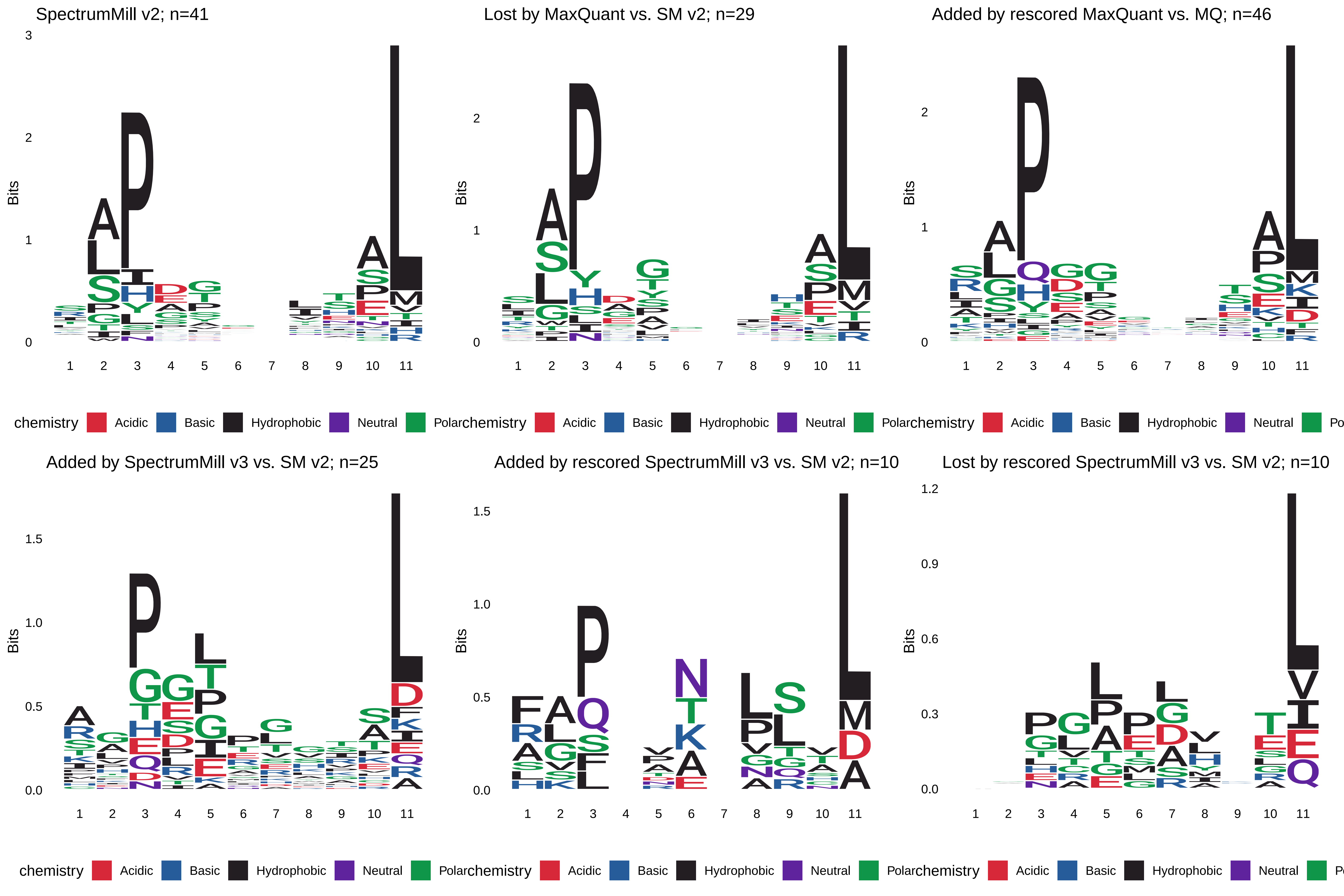
SpectrumMill v2; n=41
Lost by MaxQuant vs. SM v2; n=29
Added by rescored MaxQuant vs. MQ; n=46
Added by SpectrumMill v3 vs. SM v2; n=25
Added by rescored SpectrumMill v3 vs. SM v2; n=10
Lost by rescored SpectrumMill v3 vs. SM v2; n=10
Bits
chemistry
Acidic
Basic
Hydrophobic
Neutral
Polar

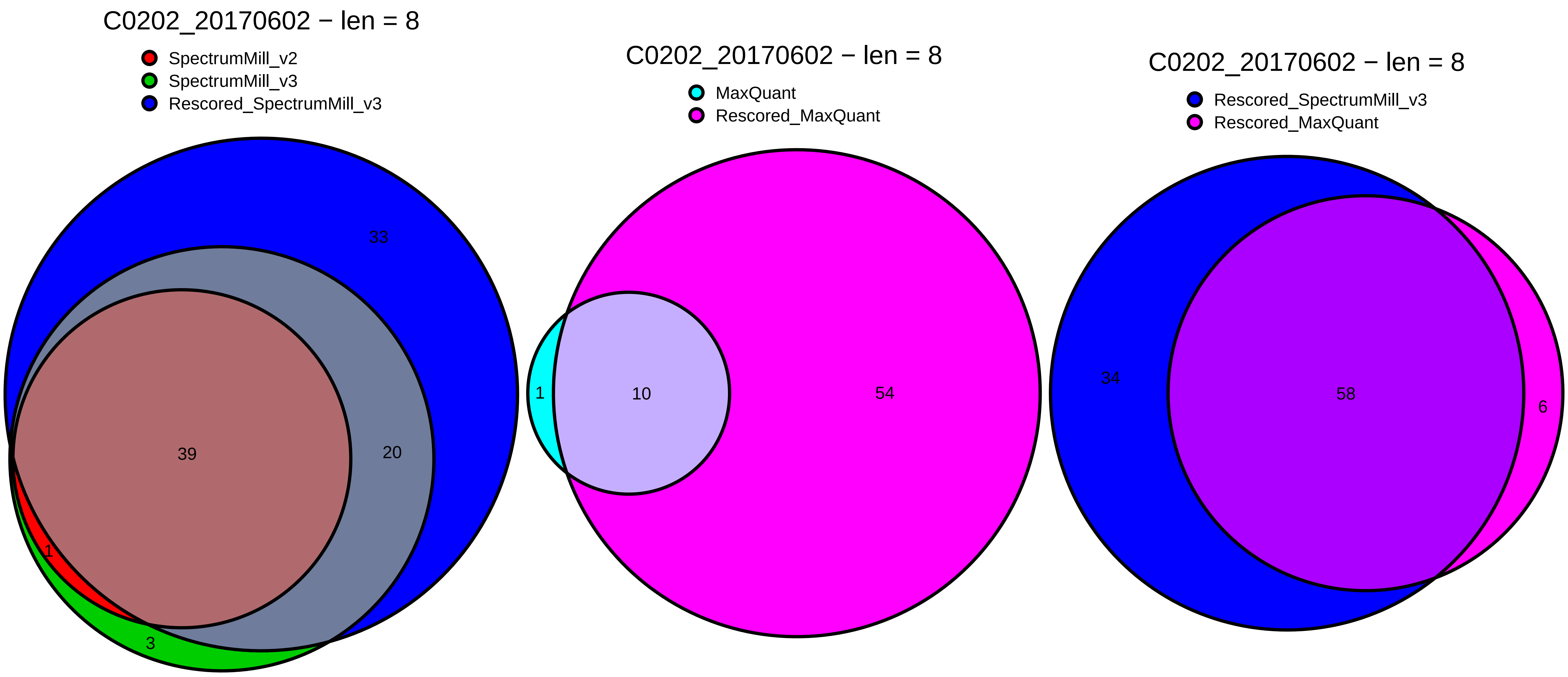

C0202_20170602 − len = 8
SpectrumMill_v2
SpectrumMill_v3
Rescored_SpectrumMill_v3
33
39
20
1
3
C0202_20170602 − len = 8
MaxQuant
Rescored_MaxQuant
1
10
54
C0202_20170602 − len = 8
Rescored_SpectrumMill_v3
Rescored_MaxQuant
34
58
6

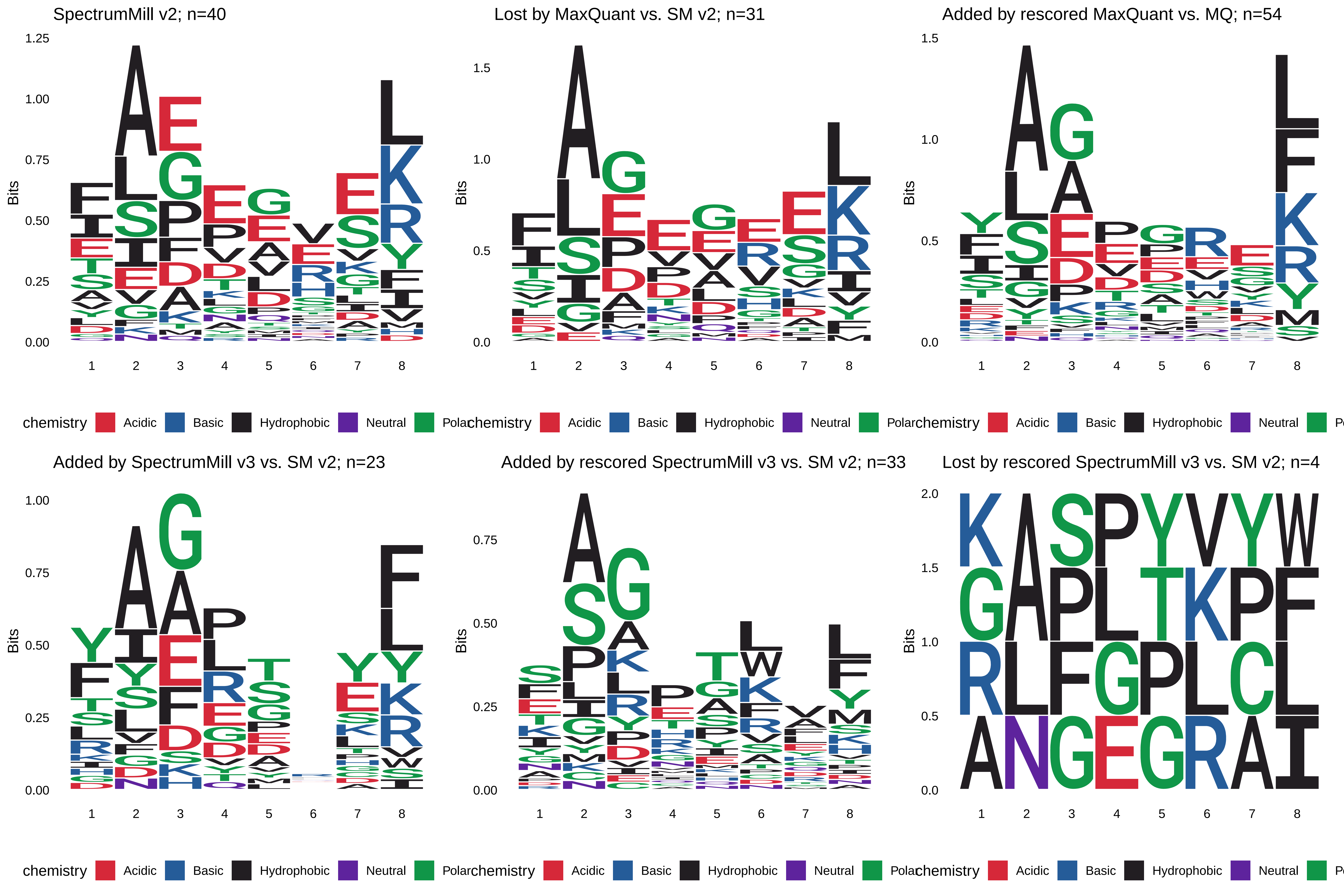
SpectrumMill v2; n=40
Lost by MaxQuant vs. SM v2; n=31
Added by rescored MaxQuant vs. MQ; n=54
Added by SpectrumMill v3 vs. SM v2; n=23
Added by rescored SpectrumMill v3 vs. SM v2; n=33
Lost by rescored SpectrumMill v3 vs. SM v2; n=4
Bits
chemistry
Acidic
Basic
Hydrophobic
Neutral
Polar

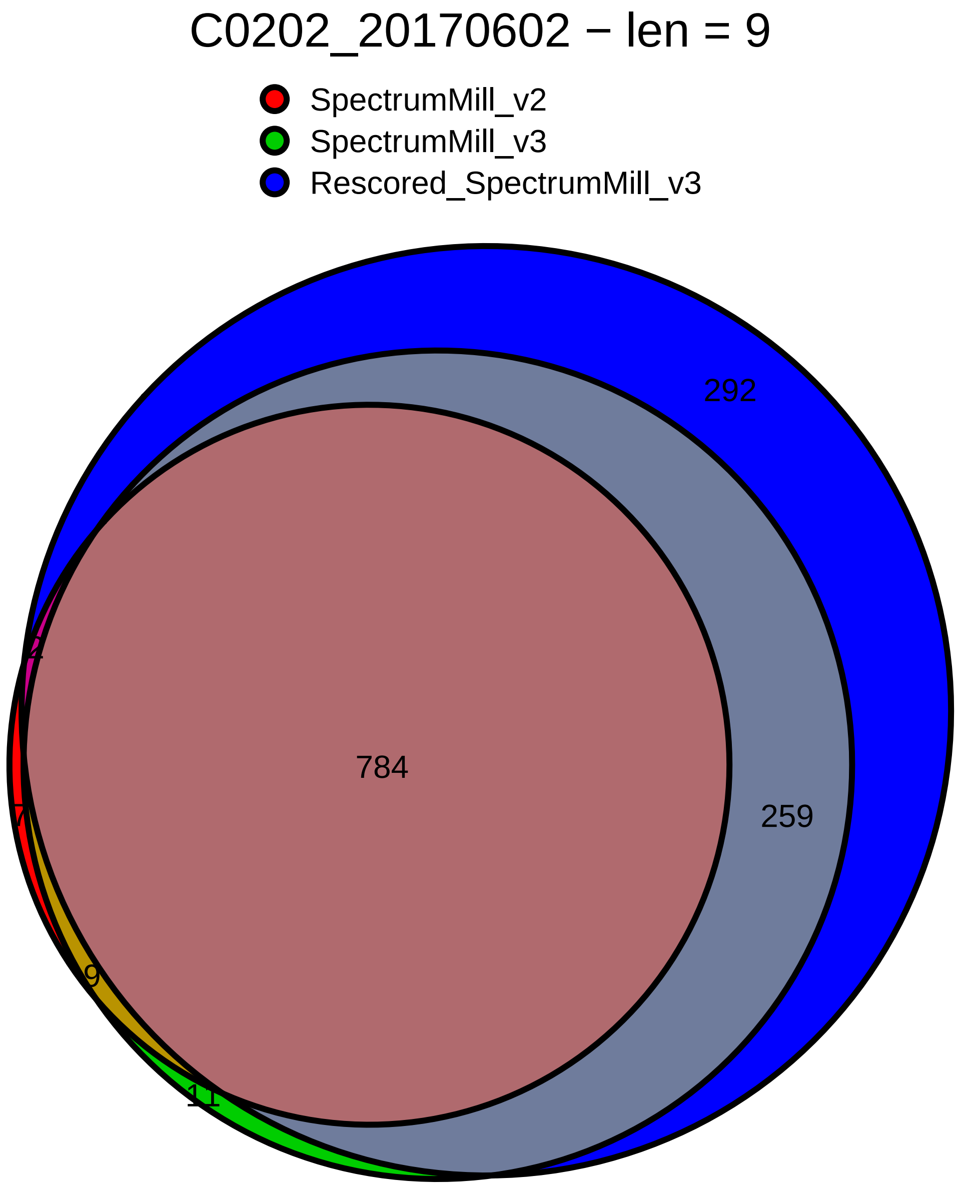
C0202_20170602 − len = 9
SpectrumMill_v2
SpectrumMill_v3
Rescored_SpectrumMill_v3
292
784
259
9
11

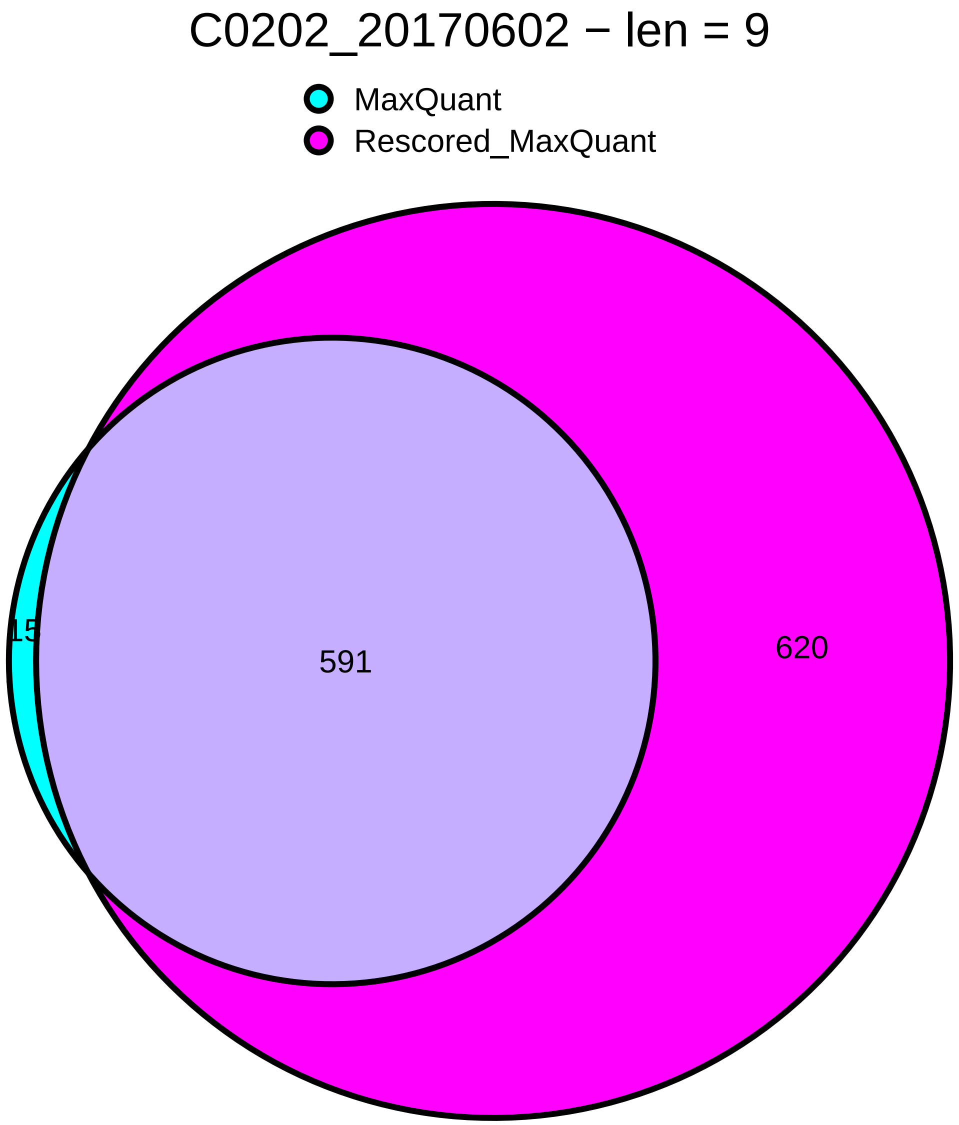
C0202_20170602 − len = 9
MaxQuant
Rescored_MaxQuant
591
620

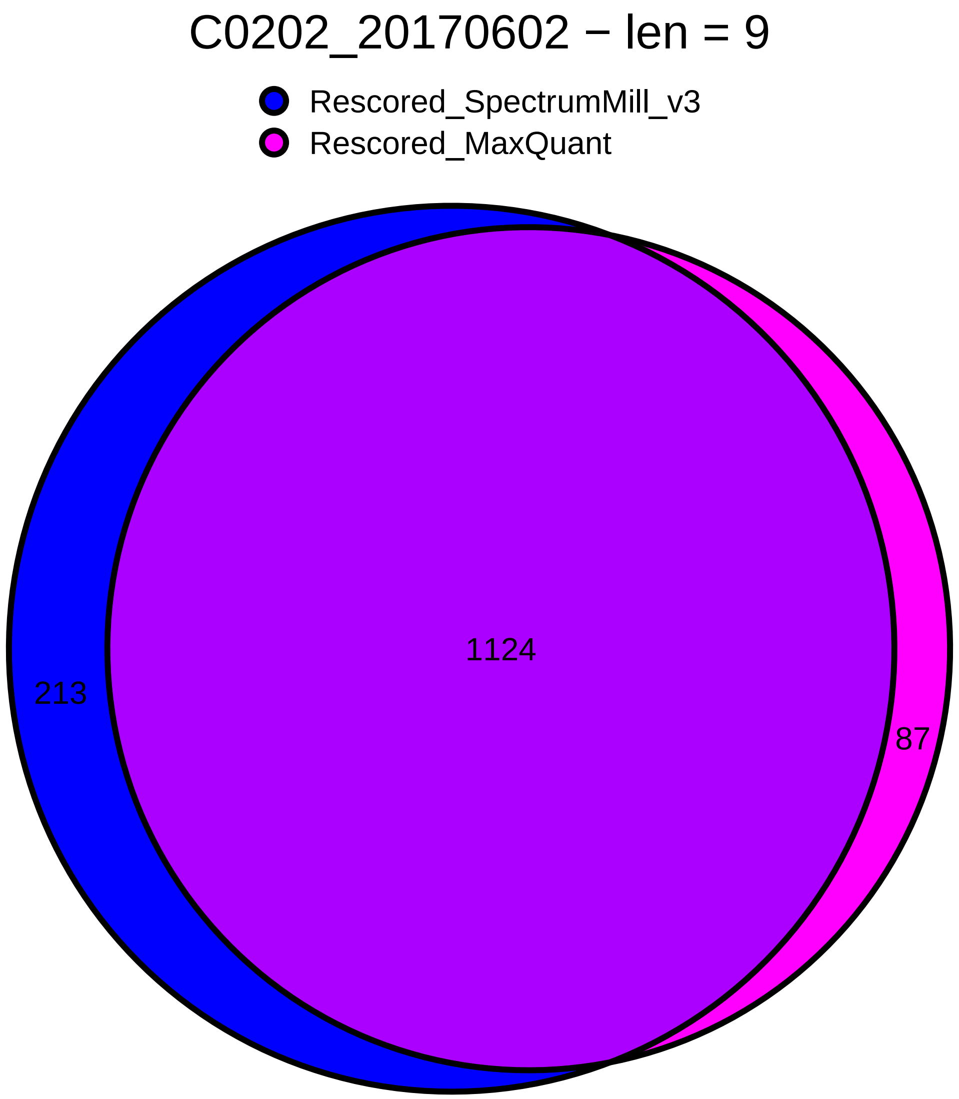
C0202_20170602 − len = 9
Rescored_SpectrumMill_v3
Rescored_MaxQuant
1124
213
87

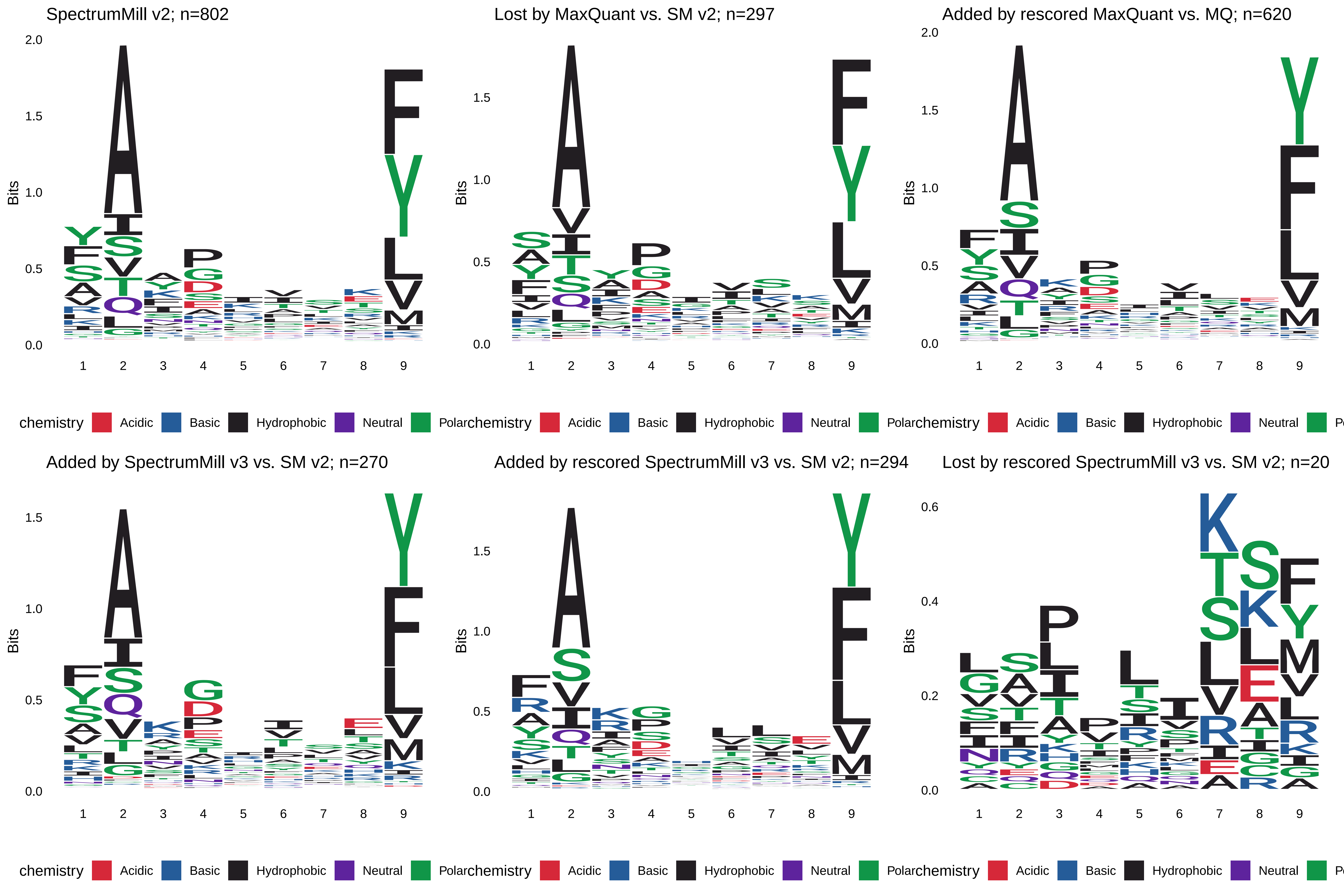
SpectrumMill v2; n=802
Lost by MaxQuant vs. SM v2; n=297
Added by rescored MaxQuant vs. MQ; n=620
Added by SpectrumMill v3 vs. SM v2; n=270
Added by rescored SpectrumMill v3 vs. SM v2; n=294
Lost by rescored SpectrumMill v3 vs. SM v2; n=20
Bits
1 2 3 4 5 6 7 8 9
chemistry
Acidic
Basic
Hydrophobic
Neutral
Polar

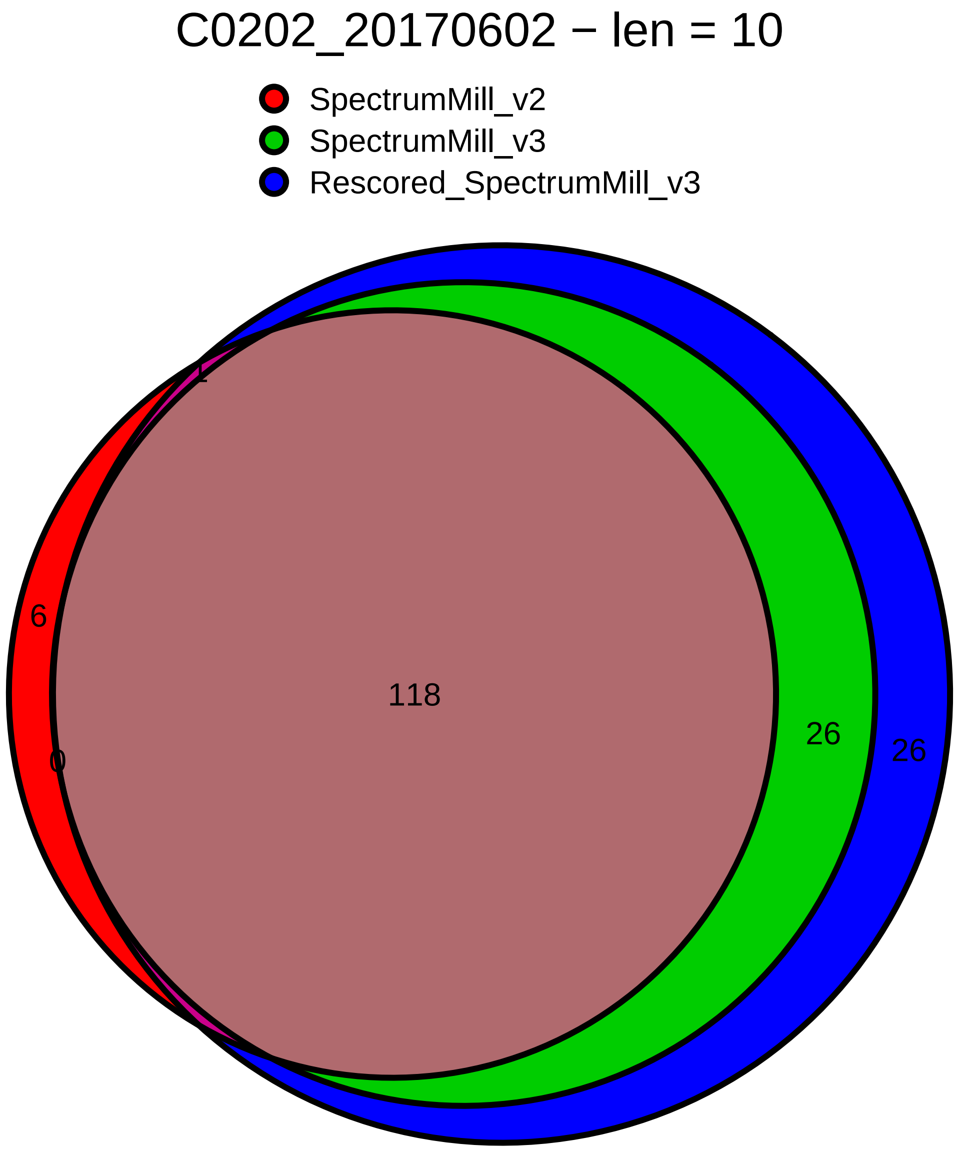
C0202_20170602 − len = 10
SpectrumMill_v2
SpectrumMill_v3
Rescored_SpectrumMill_v3
1
6
118
0
26
26

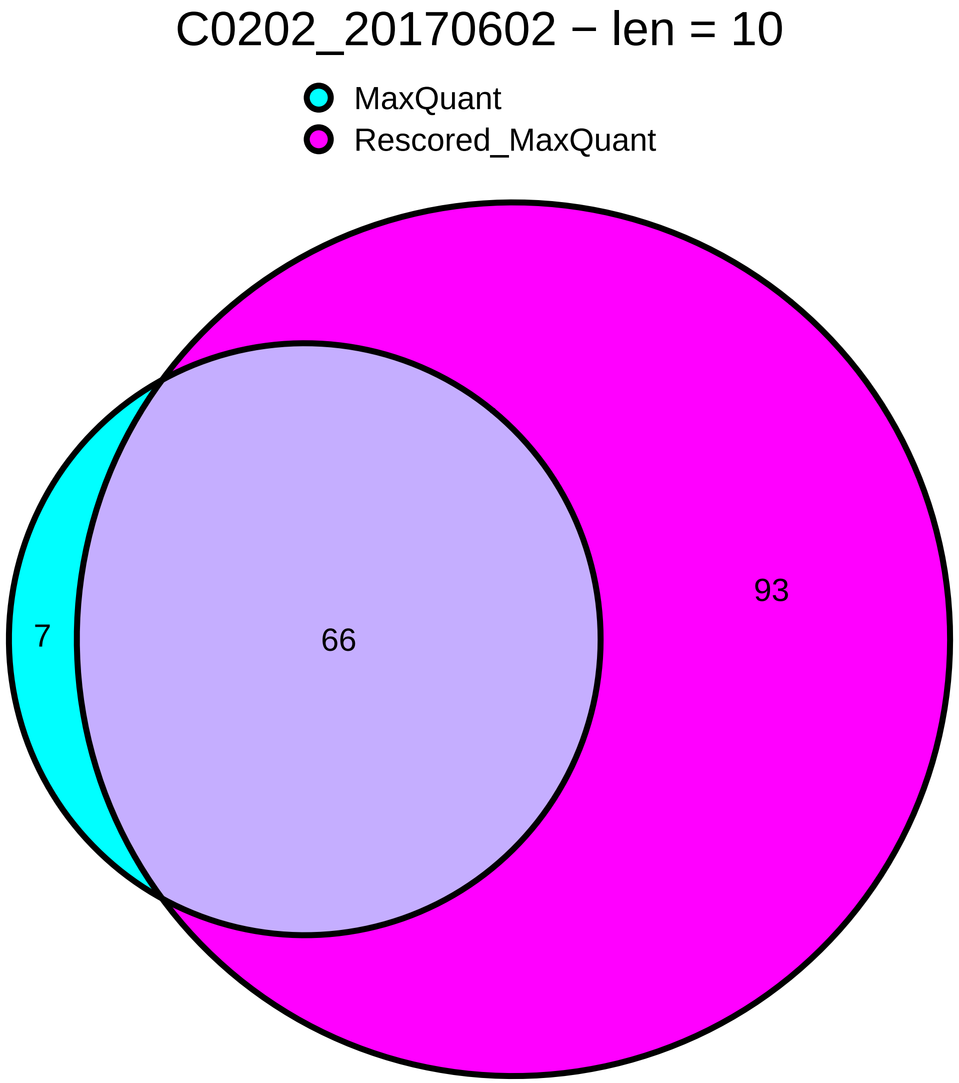
C0202_20170602 − len = 10
MaxQuant
Rescored_MaxQuant
7
66
93

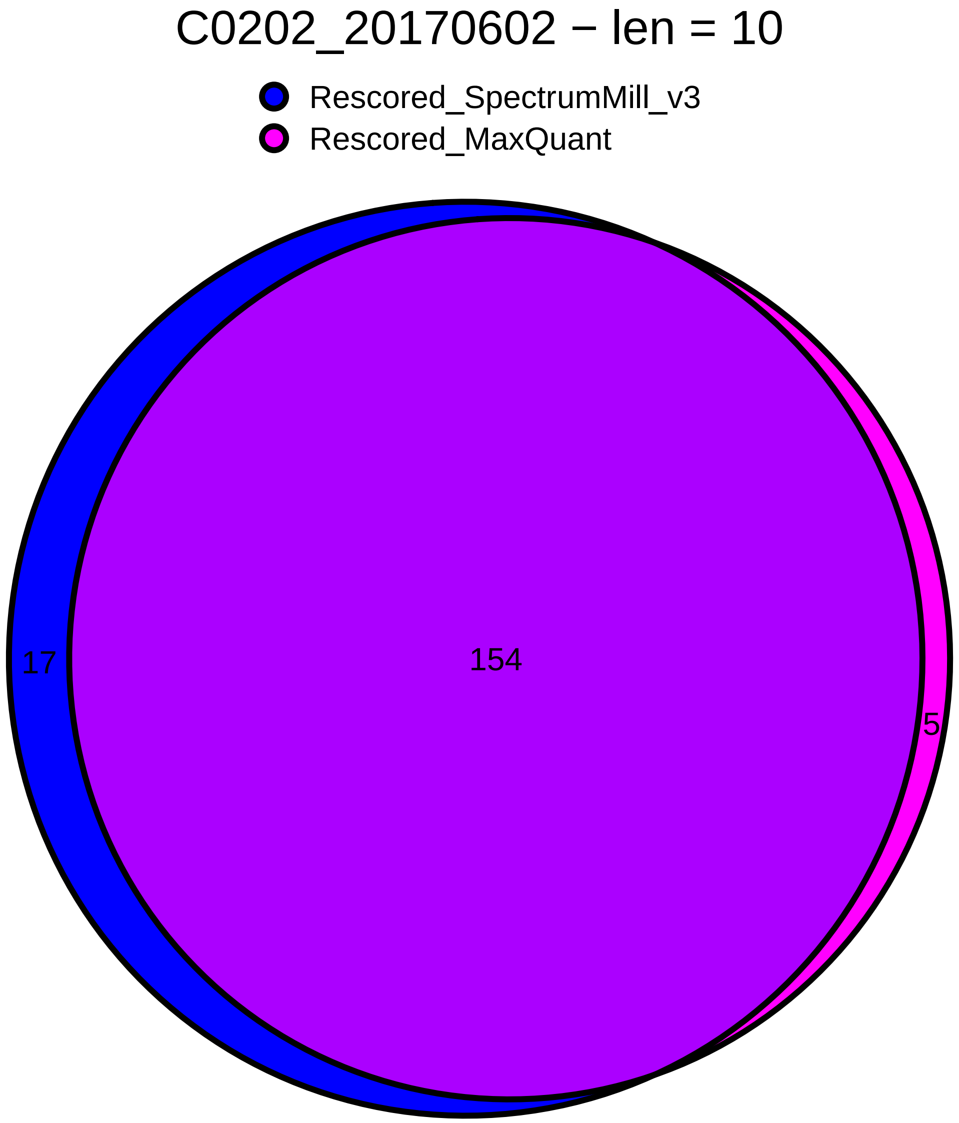
C0202_20170602 − len = 10
Rescored_SpectrumMill_v3
Rescored_MaxQuant
17
154
5

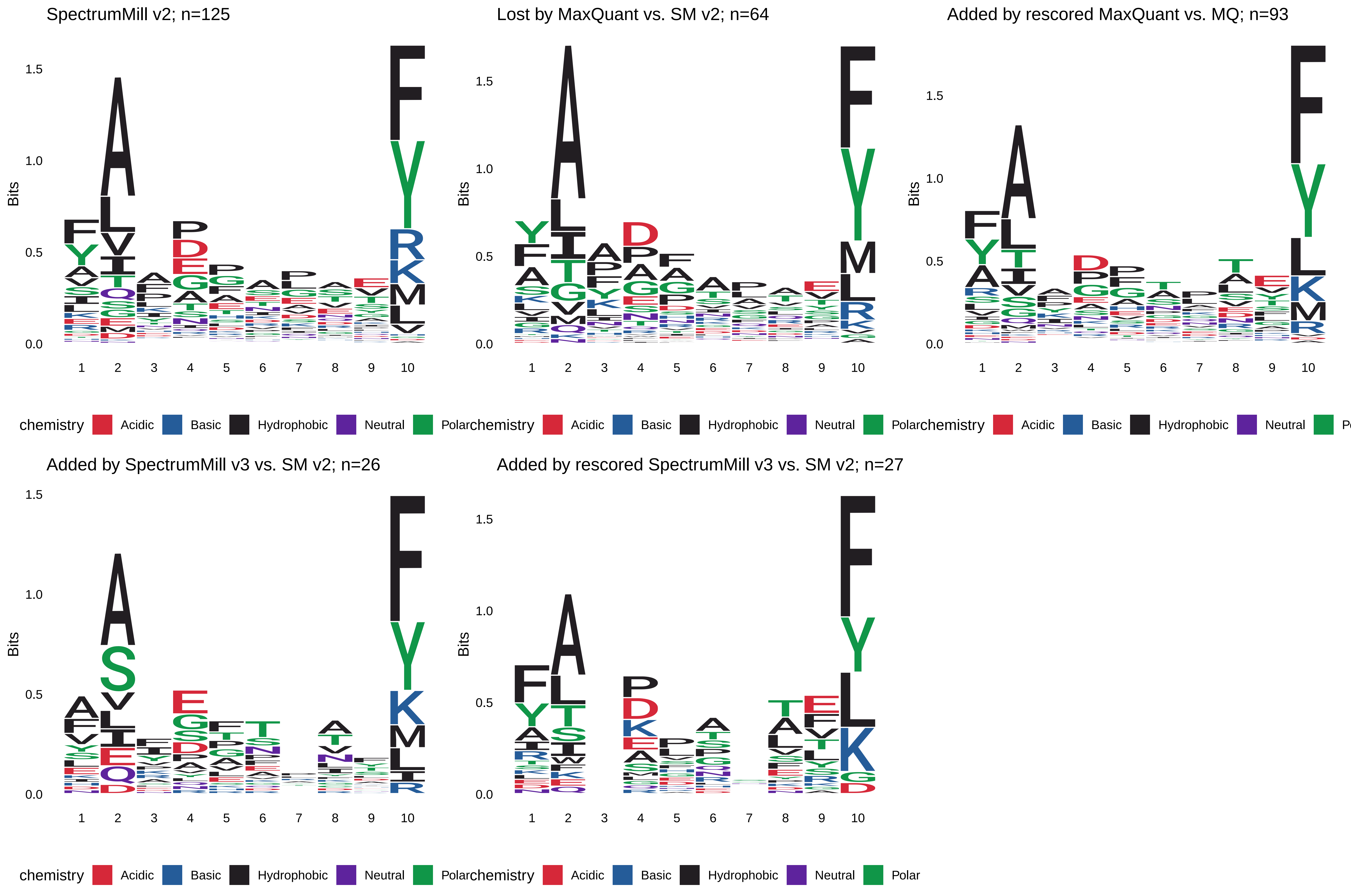
SpectrumMill v2; n=125
Lost by MaxQuant vs. SM v2; n=64
Added by rescored MaxQuant vs. MQ; n=93
Added by SpectrumMill v3 vs. SM v2; n=26
Added by rescored SpectrumMill v3 vs. SM v2; n=27
Bits
1 2 3 4 5 6 7 8 9 10
chemistry
Acidic
Basic
Hydrophobic
Neutral
Polar

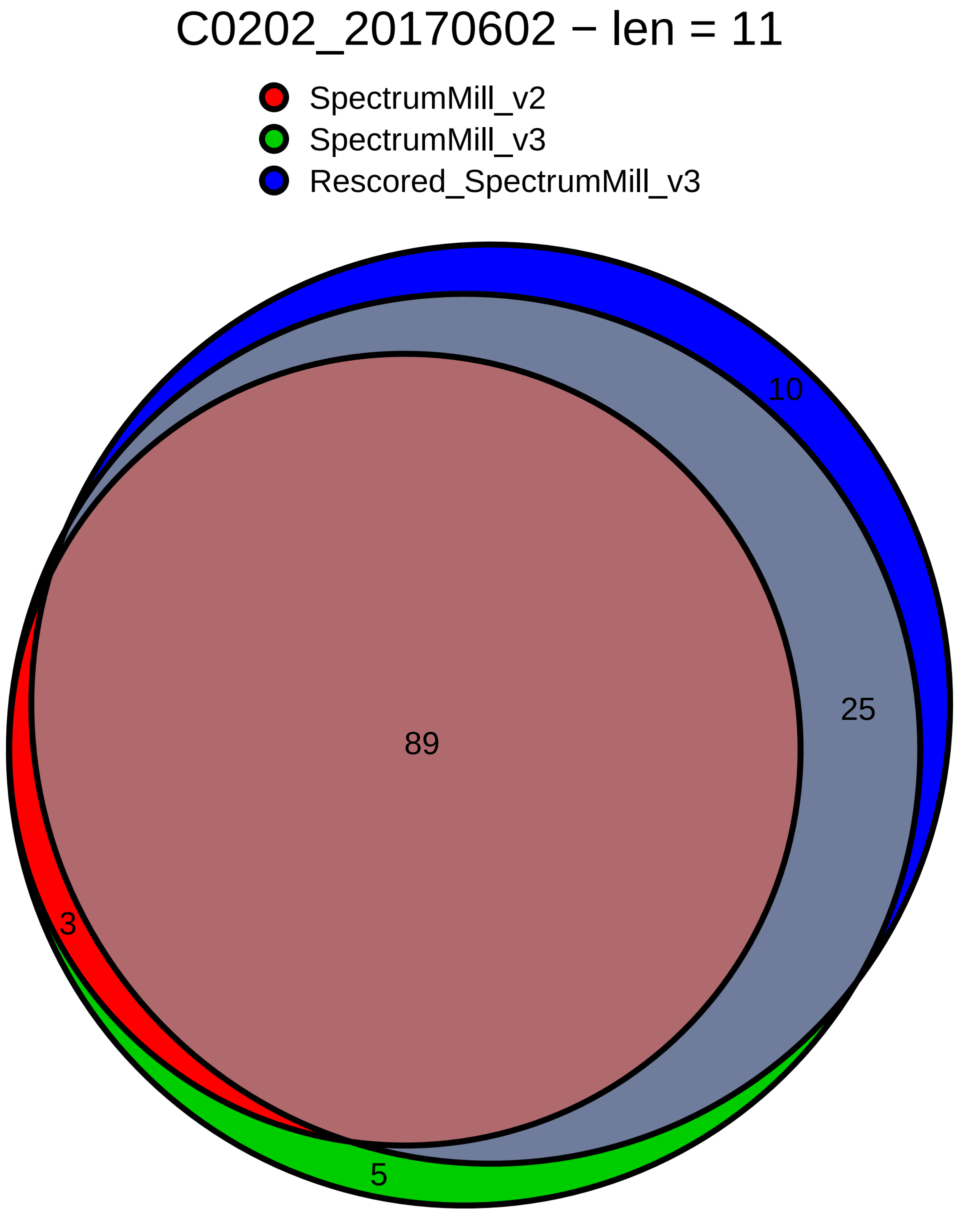
C0202_20170602 − len = 11
SpectrumMill_v2
SpectrumMill_v3
Rescored_SpectrumMill_v3
10
25
89
3
5

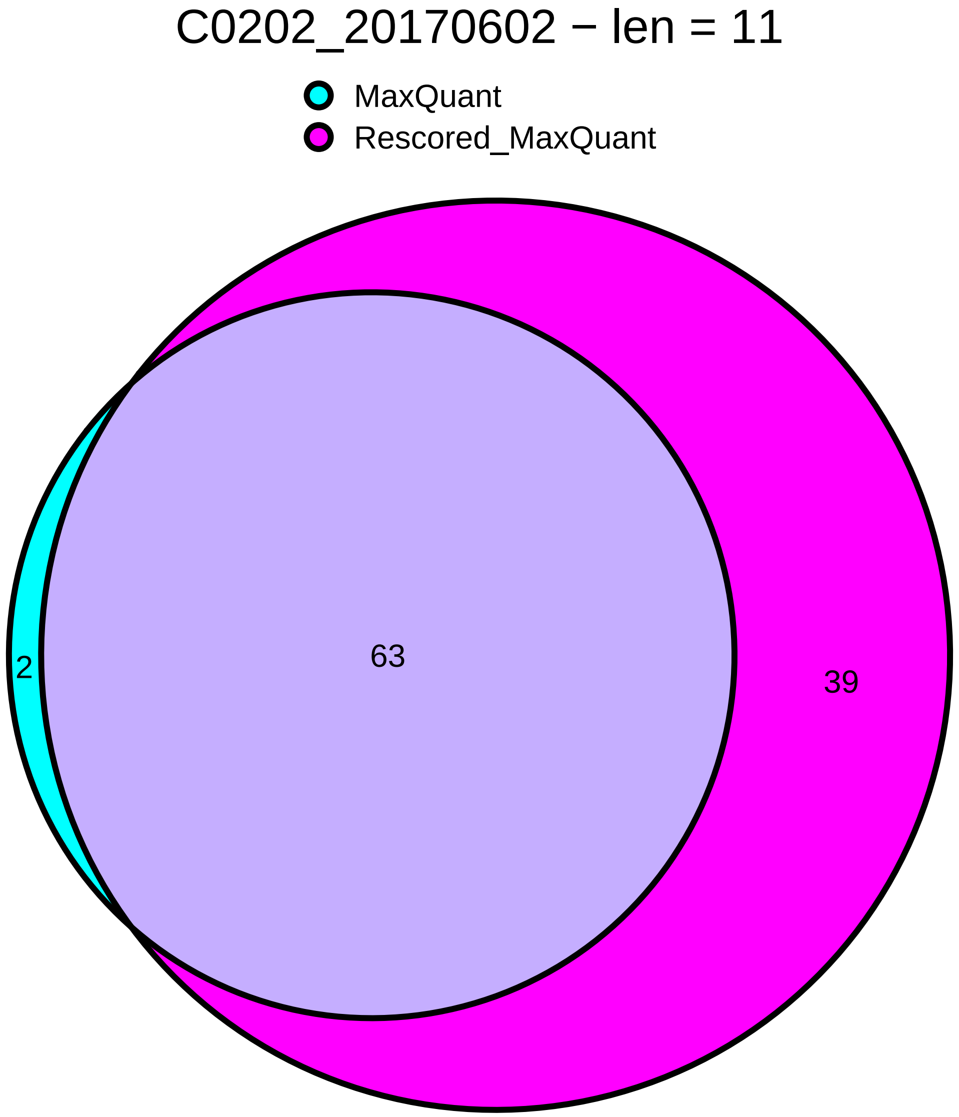
C0202_20170602 − len = 11
MaxQuant
Rescored_MaxQuant
2
63
39

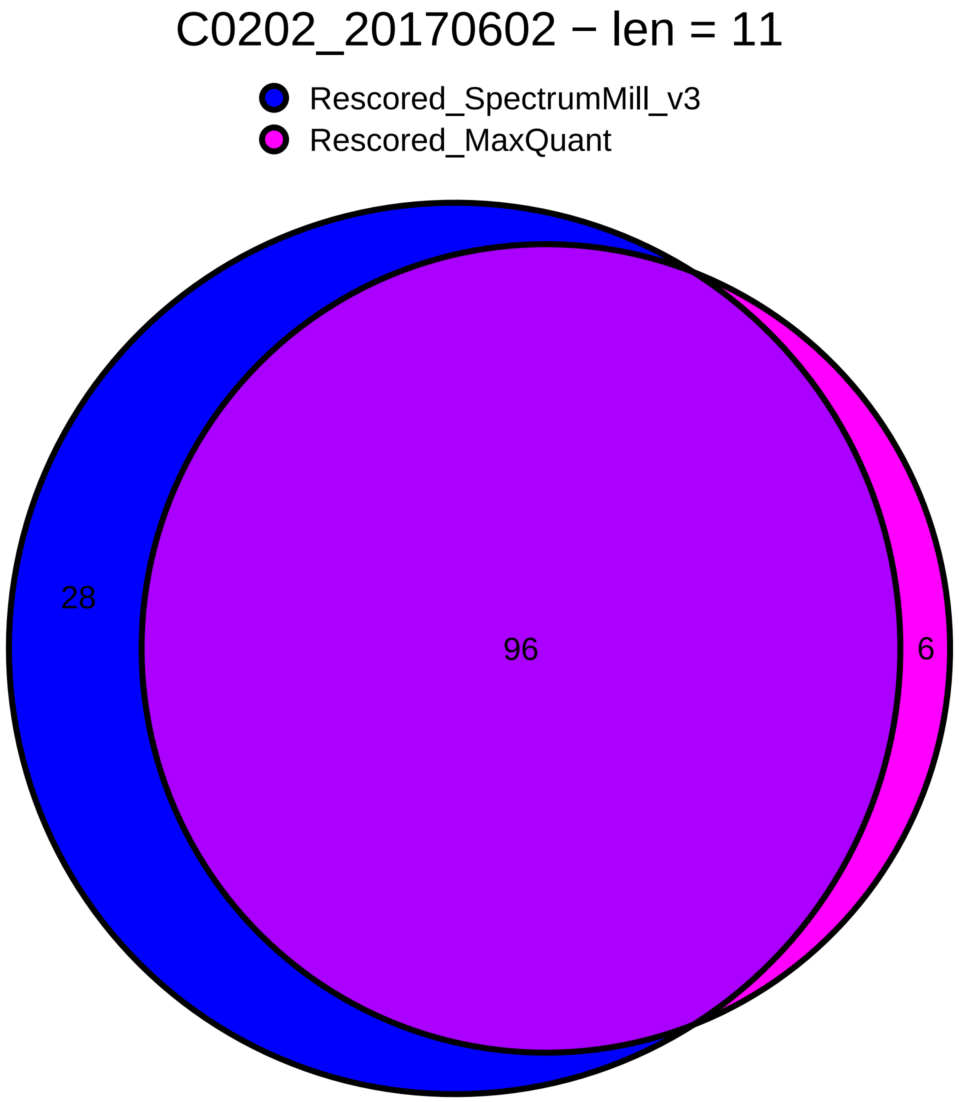
C0202_20170602 − len = 11
Rescored_SpectrumMill_v3
Rescored_MaxQuant
28
96
6

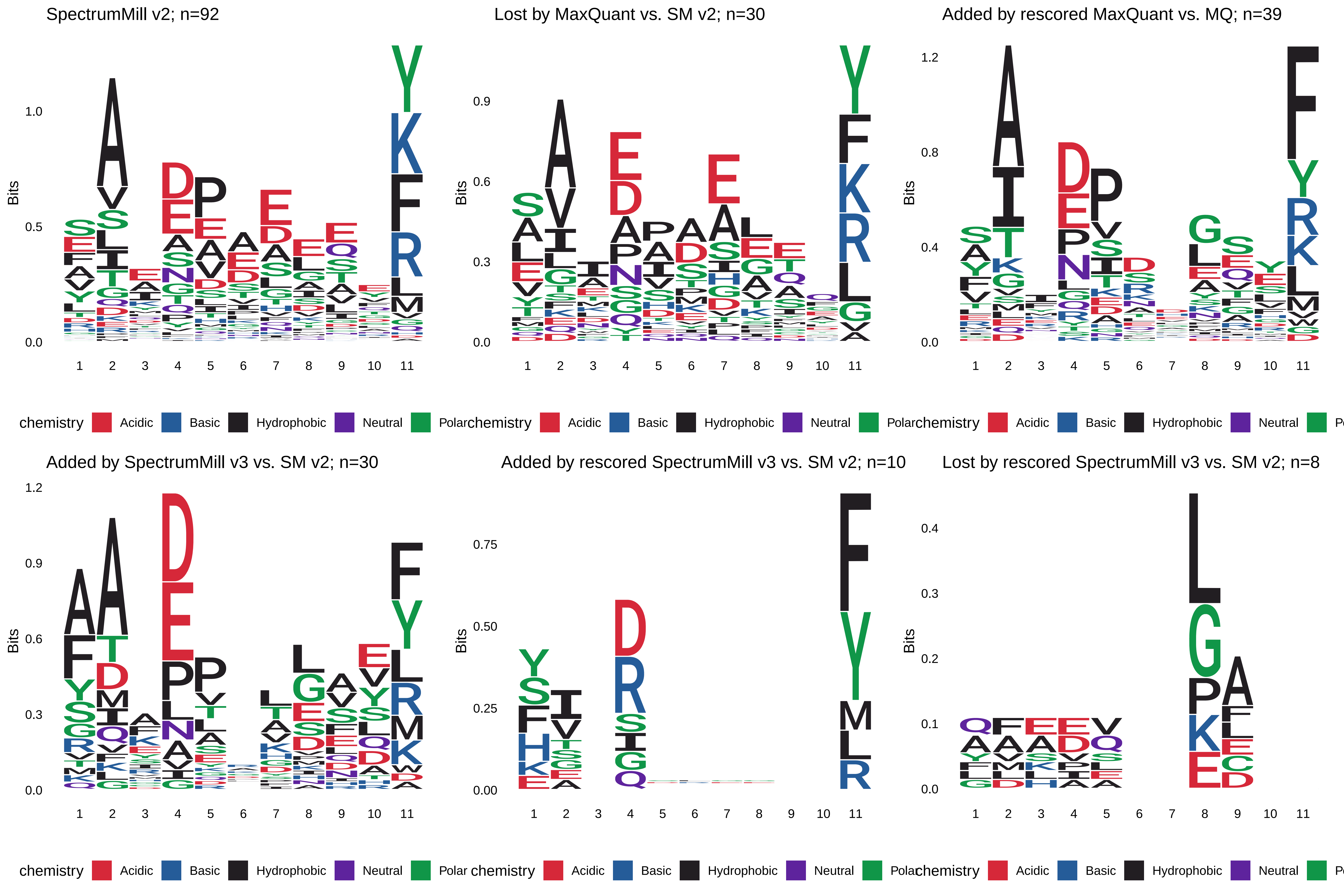
SpectrumMill v2; n=92
Lost by MaxQuant vs. SM v2; n=30
Added by rescored MaxQuant vs. MQ; n=39
Added by SpectrumMill v3 vs. SM v2; n=30
Added by rescored SpectrumMill v3 vs. SM v2; n=10
Lost by rescored SpectrumMill v3 vs. SM v2; n=8
Bits
chemistry
Acidic
Basic
Hydrophobic
Neutral
Polar

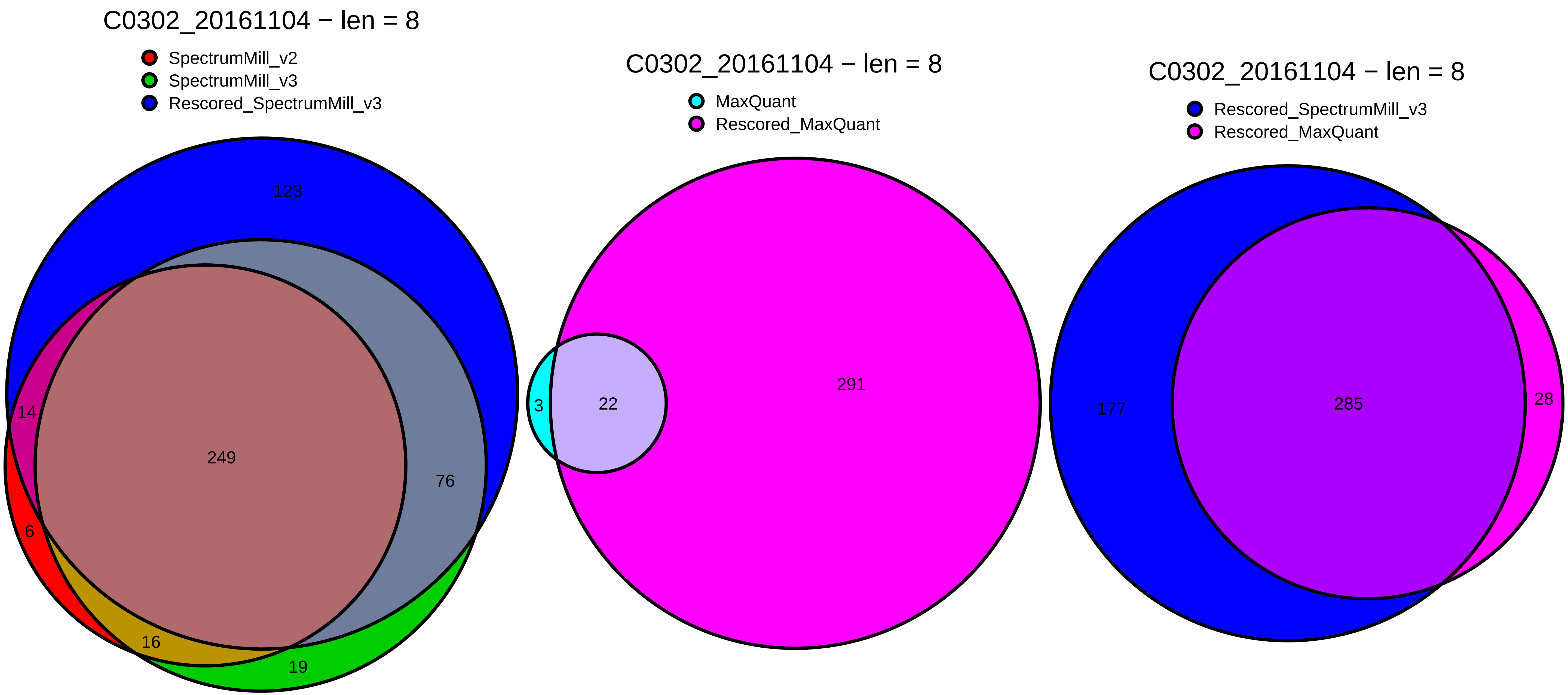
C0302_20161104 − len = 8
SpectrumMill_v2
SpectrumMill_v3
Rescored_SpectrumMill_v3
123
14
249
76
6
16
19
C0302_20161104 − len = 8
MaxQuant
Rescored_MaxQuant
3
22
291
C0302_20161104 − len = 8
Rescored_SpectrumMill_v3
Rescored_MaxQuant
177
285
28

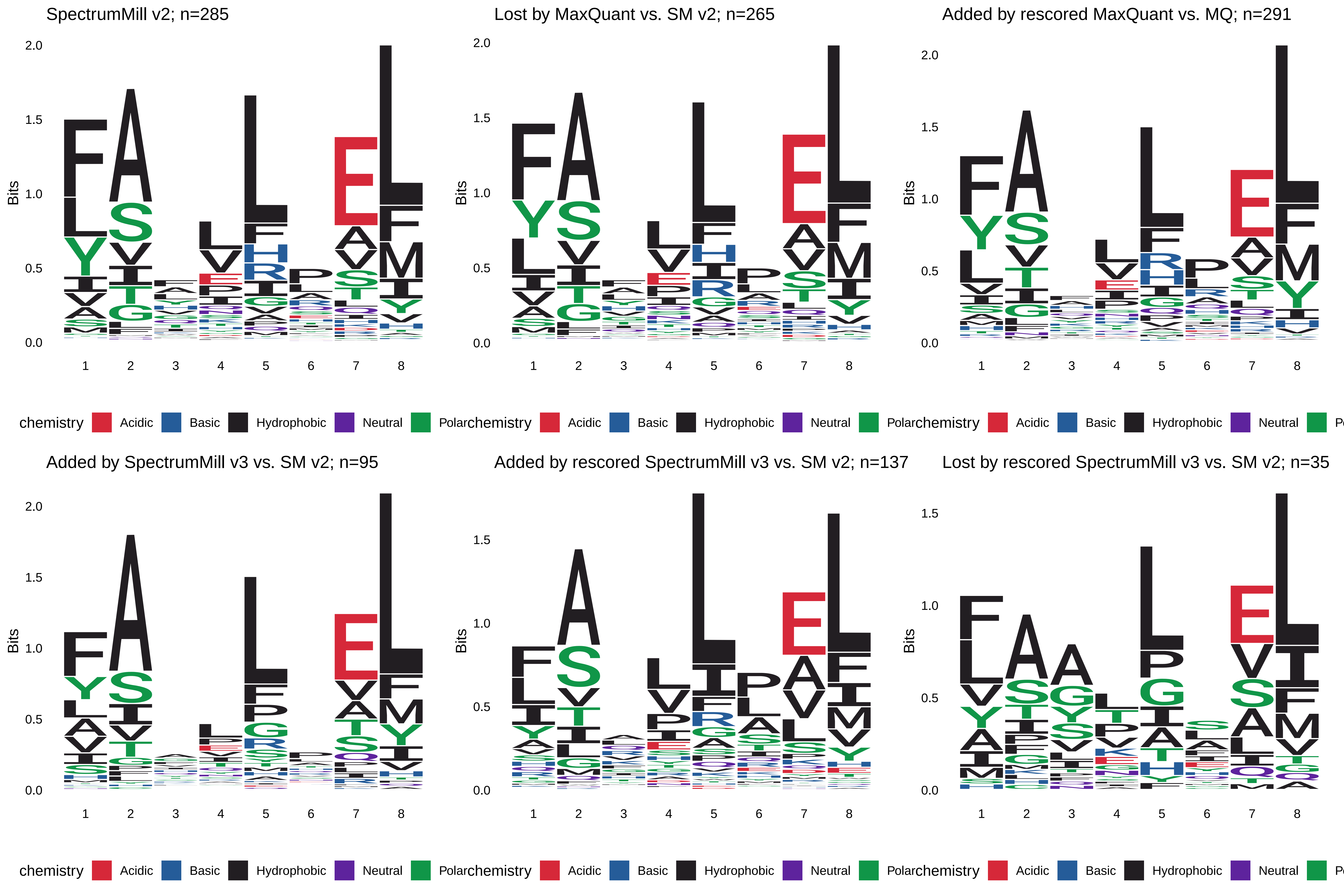
SpectrumMill v2; n=285
Lost by MaxQuant vs. SM v2; n=265
Added by rescored MaxQuant vs. MQ; n=291
Bits
chemistry Acidic Basic Hydrophobic Neutral Polar
Added by SpectrumMill v3 vs. SM v2; n=95
Added by rescored SpectrumMill v3 vs. SM v2; n=137
Lost by rescored SpectrumMill v3 vs. SM v2; n=35
Bits
chemistry Acidic Basic Hydrophobic Neutral Polar

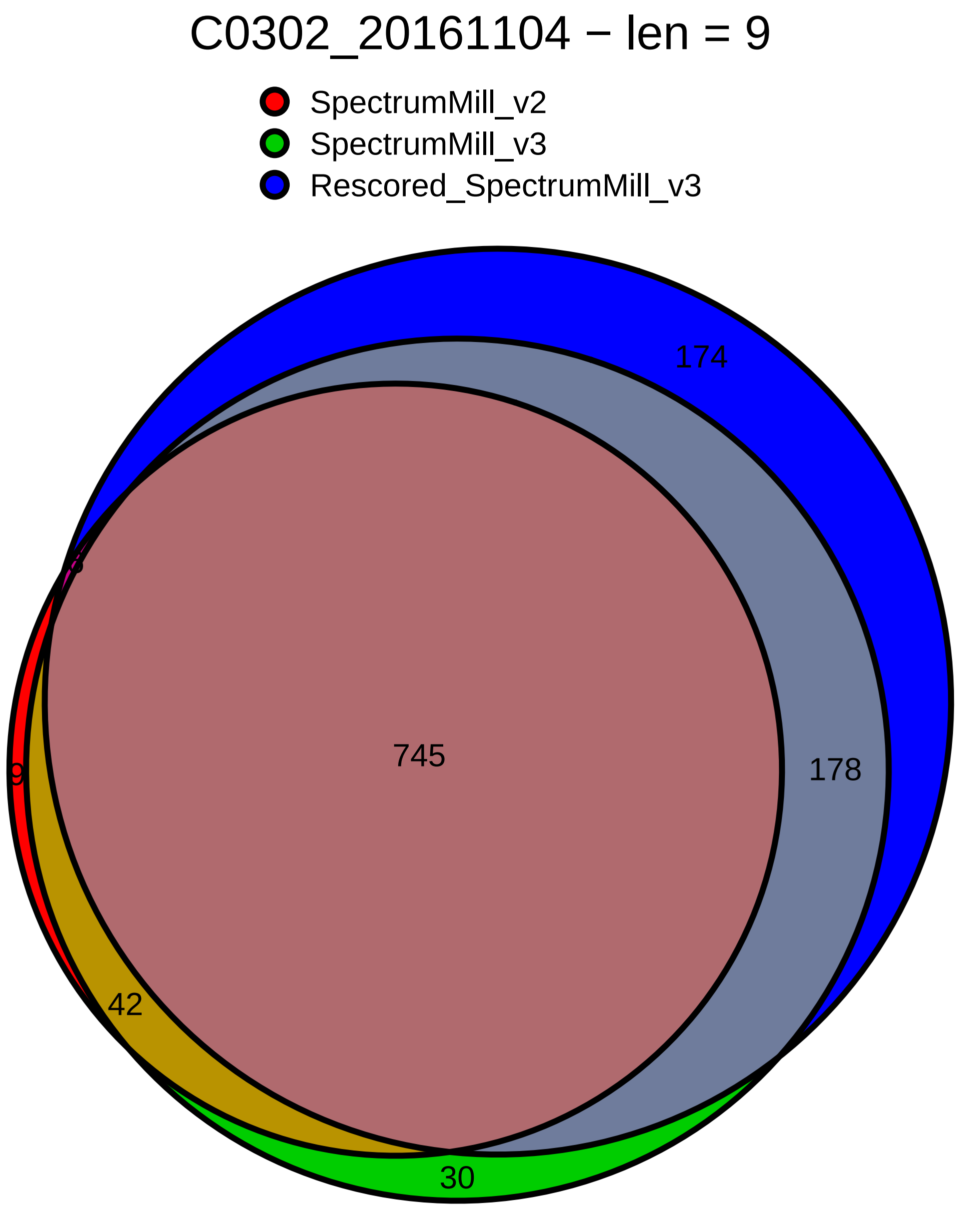
C0302_20161104 − len = 9
SpectrumMill_v2
SpectrumMill_v3
Rescored_SpectrumMill_v3
174
745
178
9
42
30

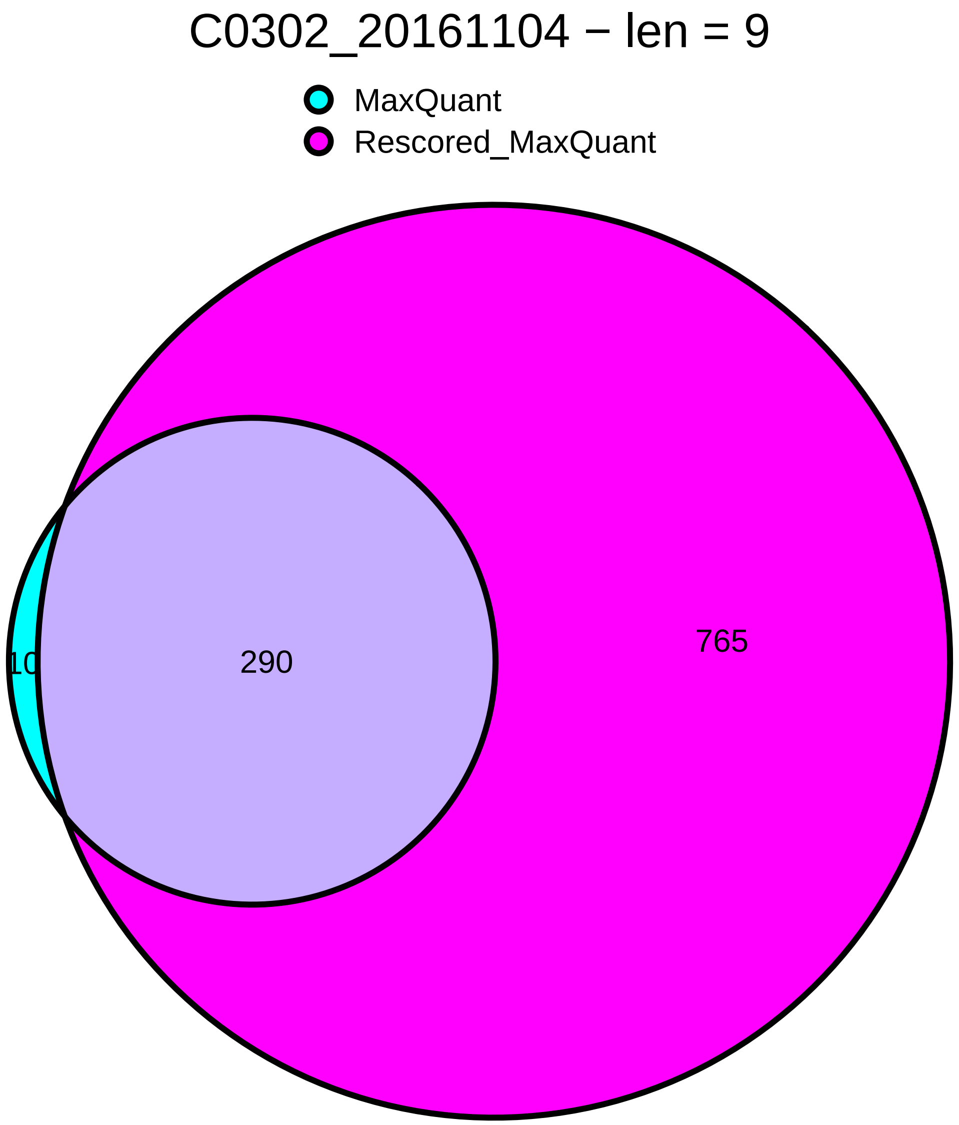
C0302_20161104 − len = 9
MaxQuant
Rescored_MaxQuant
10
290
765

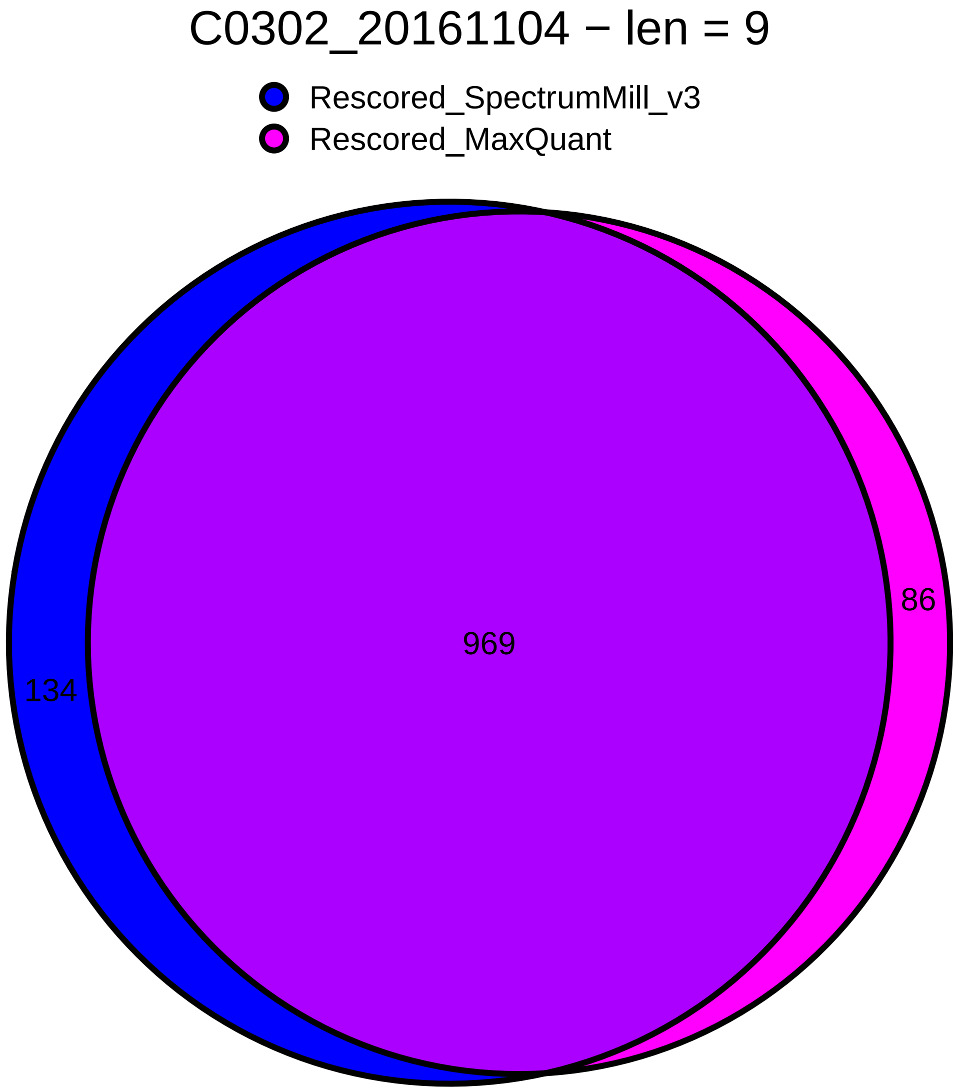
C0302_20161104 − len = 9
Rescored_SpectrumMill_v3
Rescored_MaxQuant
134
969
86

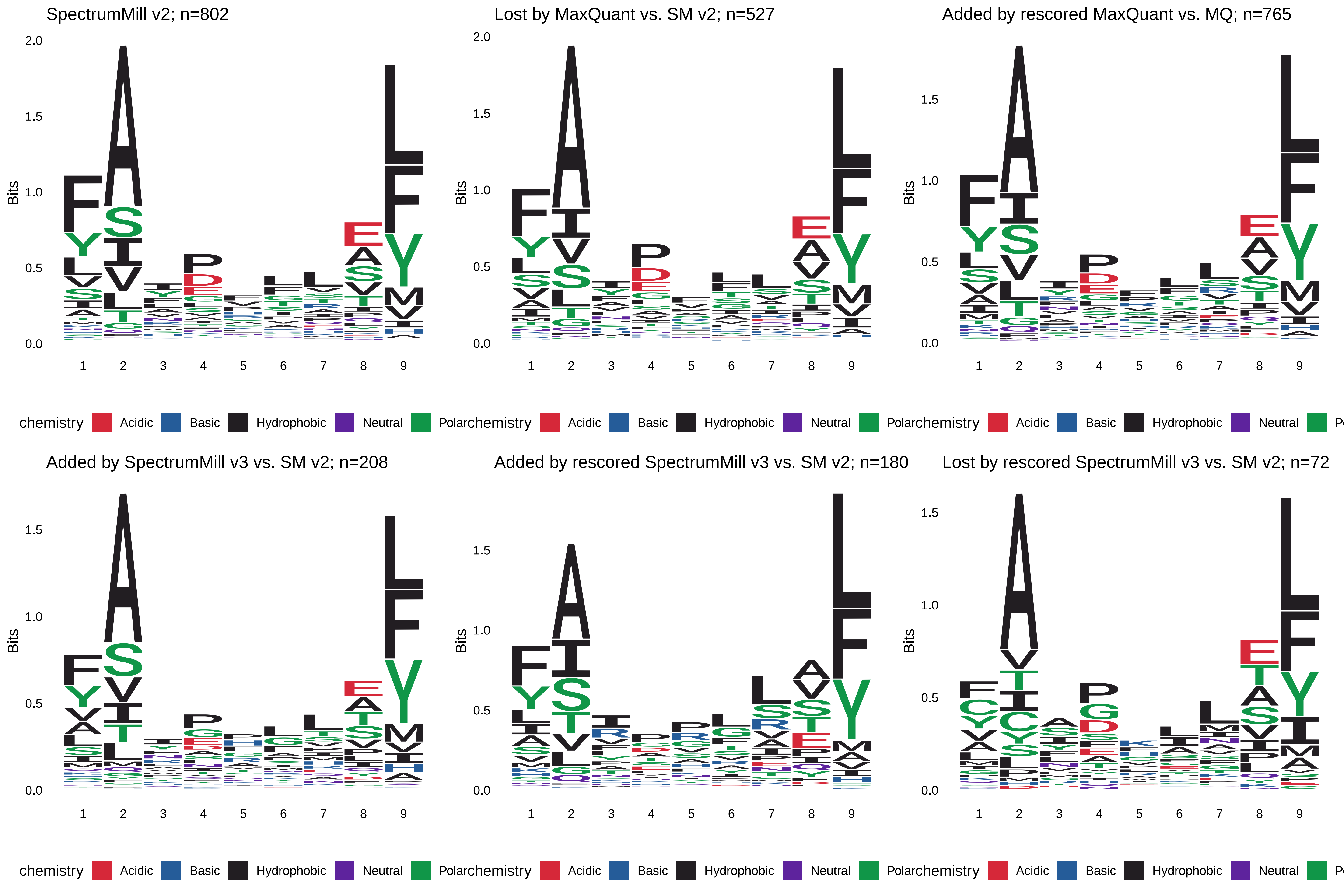
SpectrumMill v2; n=802
Lost by MaxQuant vs. SM v2; n=527
Added by rescored MaxQuant vs. MQ; n=765
Added by SpectrumMill v3 vs. SM v2; n=208
Added by rescored SpectrumMill v3 vs. SM v2; n=180
Lost by rescored SpectrumMill v3 vs. SM v2; n=72
Bits
1 2 3 4 5 6 7 8 9
chemistry
Acidic
Basic
Hydrophobic
Neutral
Polar

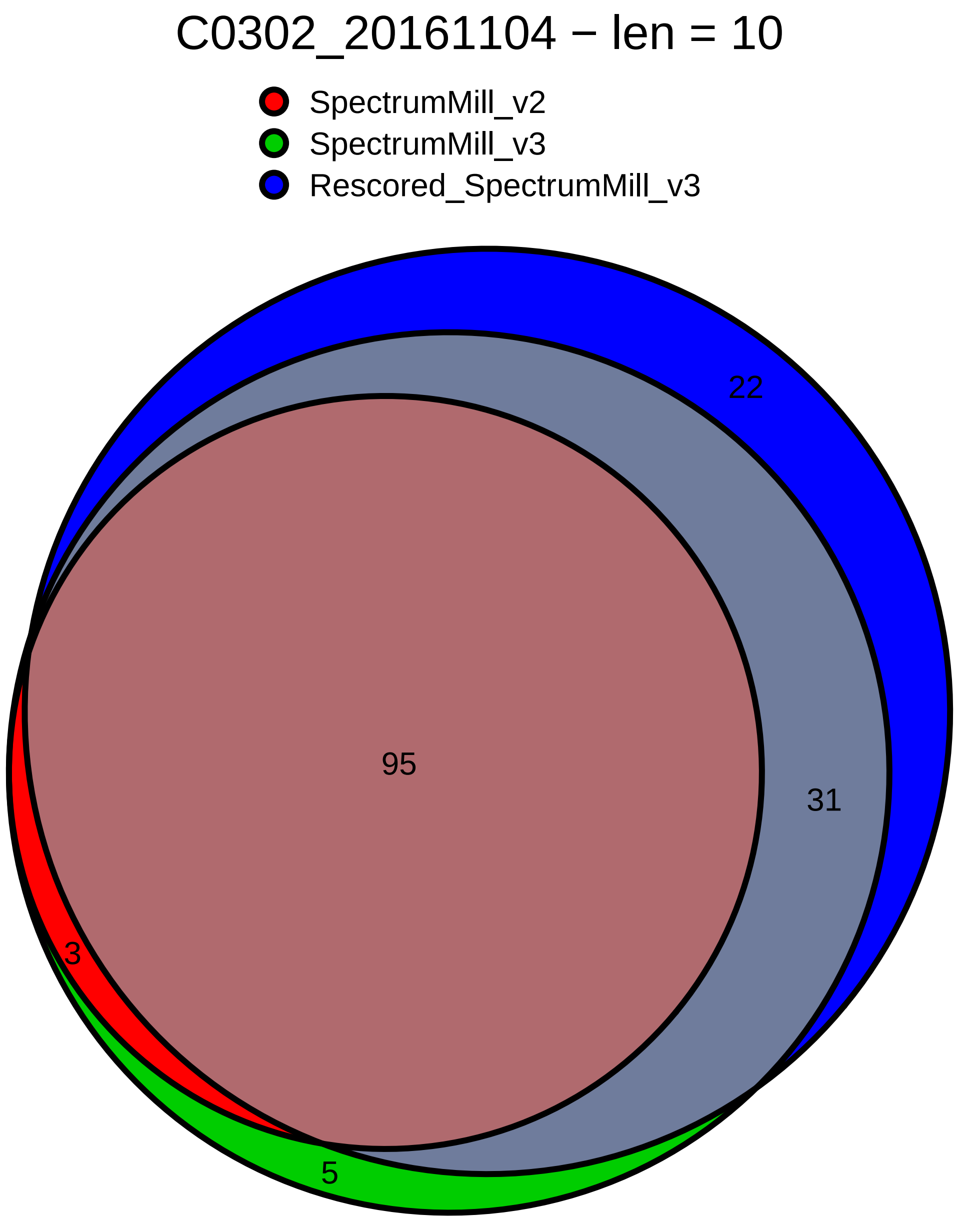

C0302_20161104 − len = 10
SpectrumMill_v2
SpectrumMill_v3
Rescored_SpectrumMill_v3
22
95
31
3
5

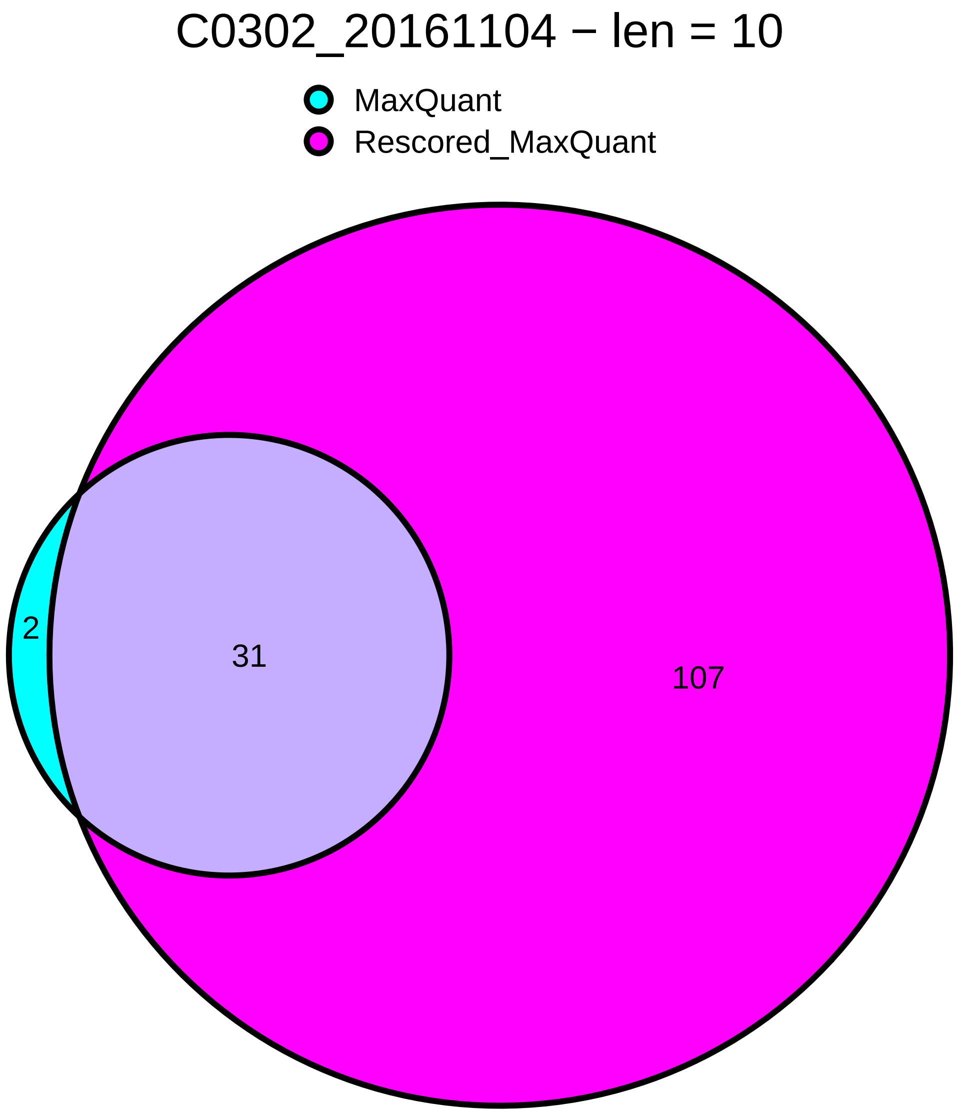

C0302_20161104 − len = 10
MaxQuant
Rescored_MaxQuant
2
31
107

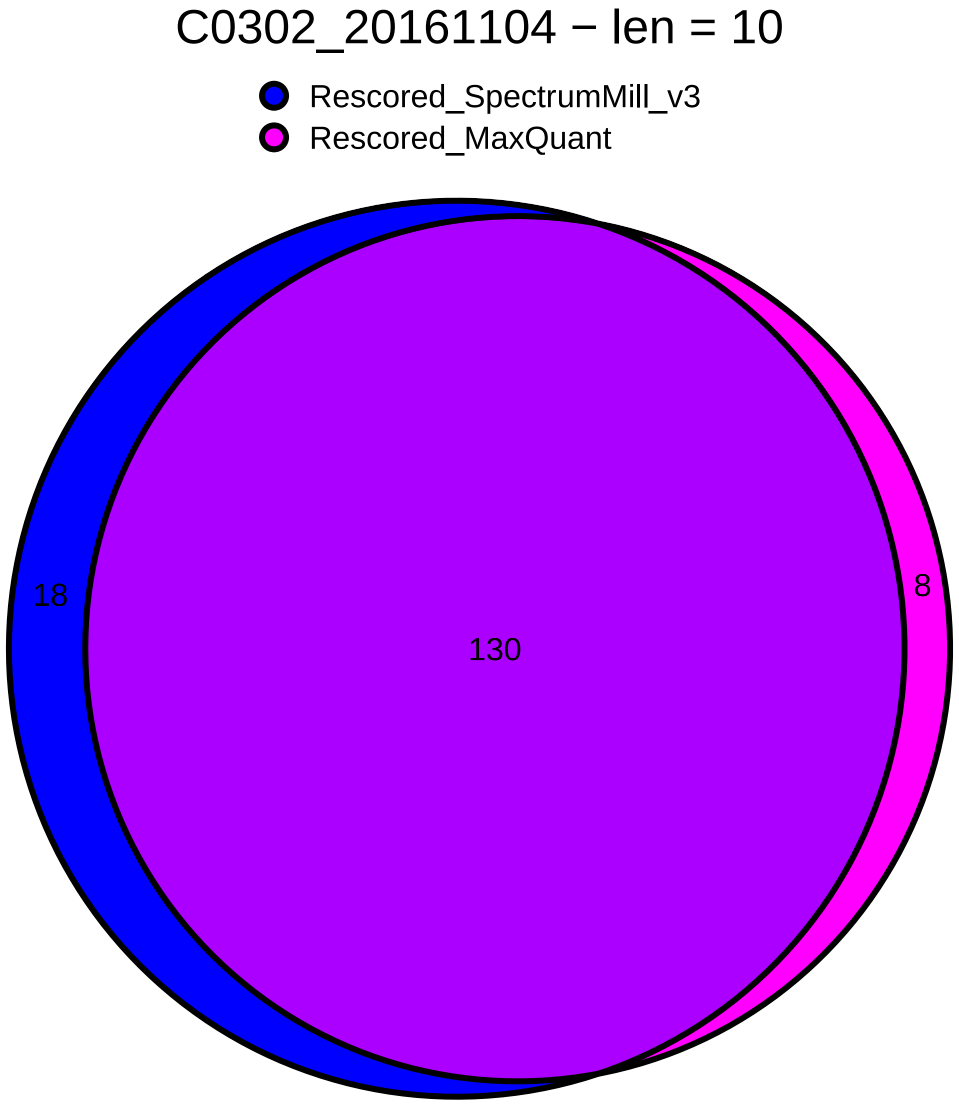

C0302_20161104 − len = 10
Rescored_SpectrumMill_v3
Rescored_MaxQuant
18
130
8

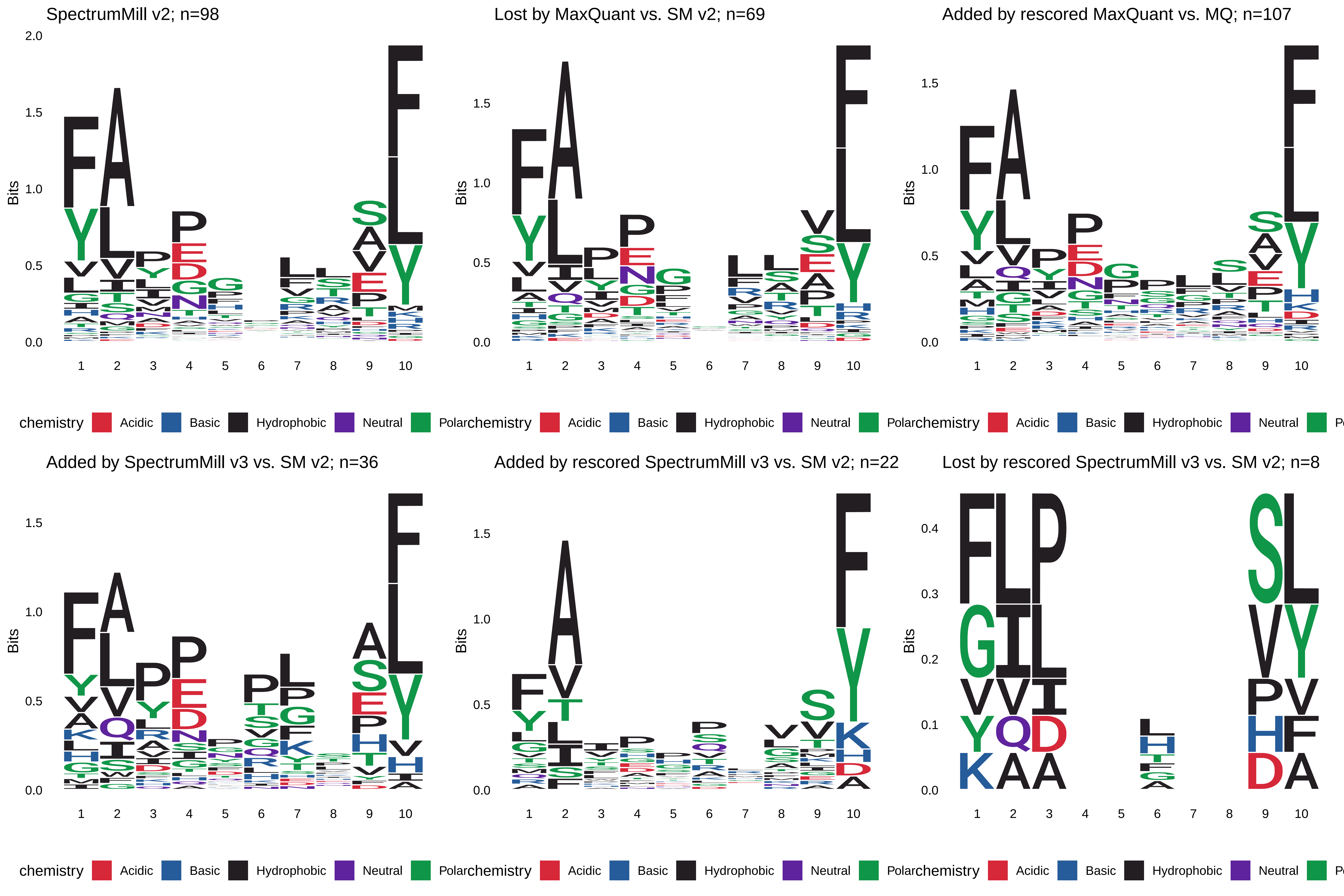
SpectrumMill v2; n=98
Lost by MaxQuant vs. SM v2; n=69
Added by rescored MaxQuant vs. MQ; n=107
Bits
1 2 3 4 5 6 7 8 9 10
chemistry Acidic Basic Hydrophobic Neutral Polarchemistry Acidic Basic Hydrophobic Neutral Polarchemistry Acidic Basic Hydrophobic Neutral
Added by SpectrumMill v3 vs. SM v2; n=36
Added by rescored SpectrumMill v3 vs. SM v2; n=22
Lost by rescored SpectrumMill v3 vs. SM v2; n=8
Bits
1 2 3 4 5 6 7 8 9 10
chemistry Acidic Basic Hydrophobic Neutral Polarchemistry Acidic Basic Hydrophobic Neutral Polarchemistry Acidic Basic Hydrophobic Neutral

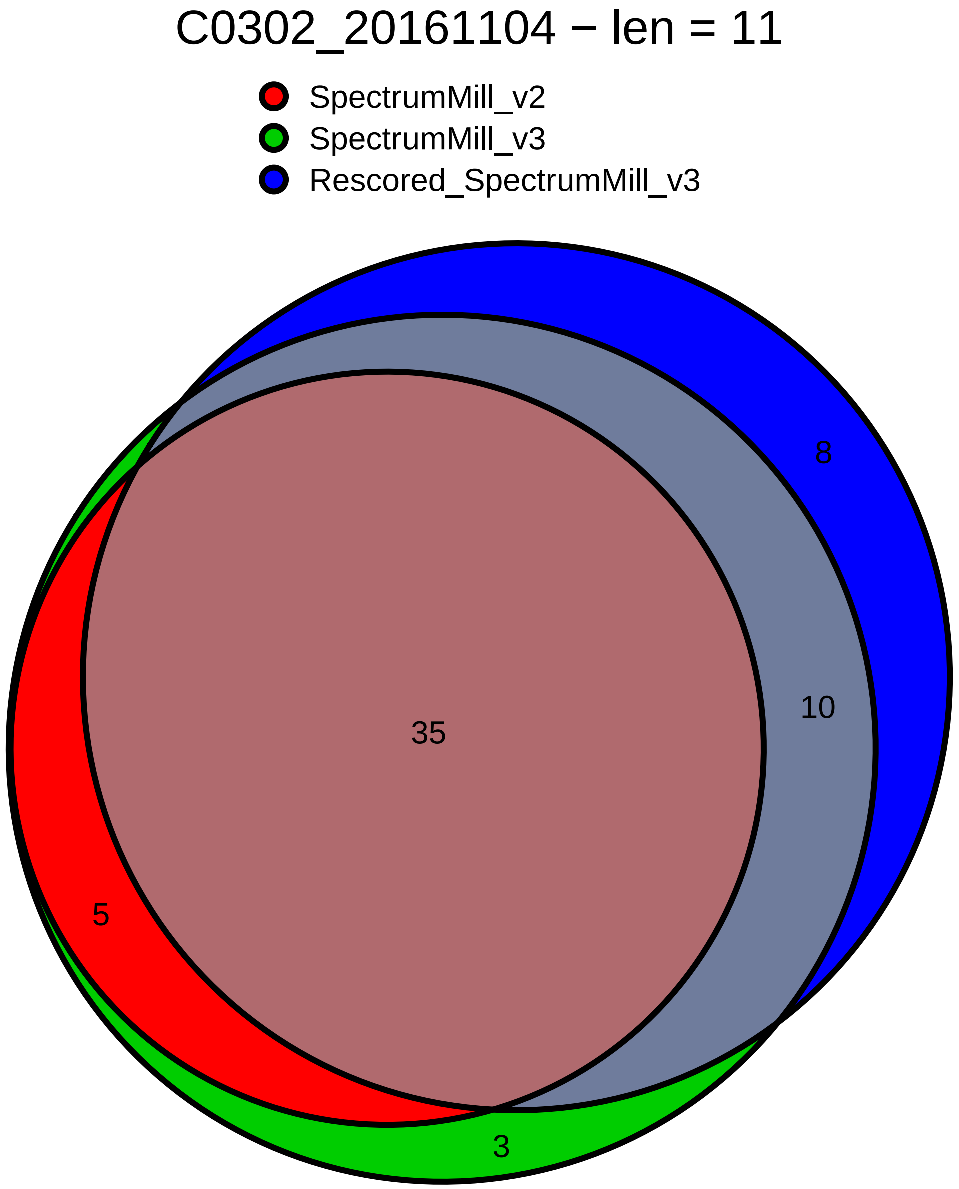

C0302_20161104 − len = 11
SpectrumMill_v2
SpectrumMill_v3
Rescored_SpectrumMill_v3
8
10
35
5
3

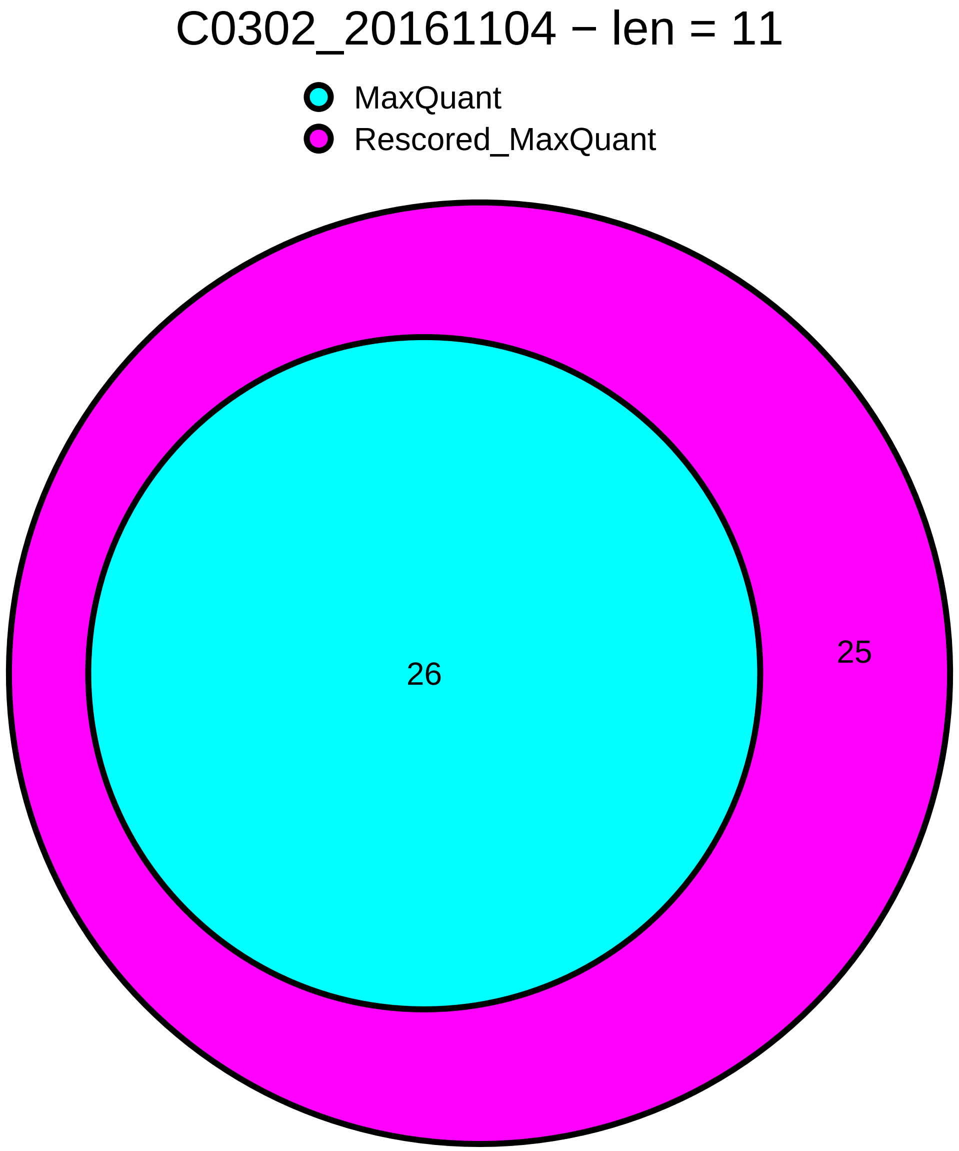

C0302_20161104 − len = 11
MaxQuant
Rescored_MaxQuant
26
25

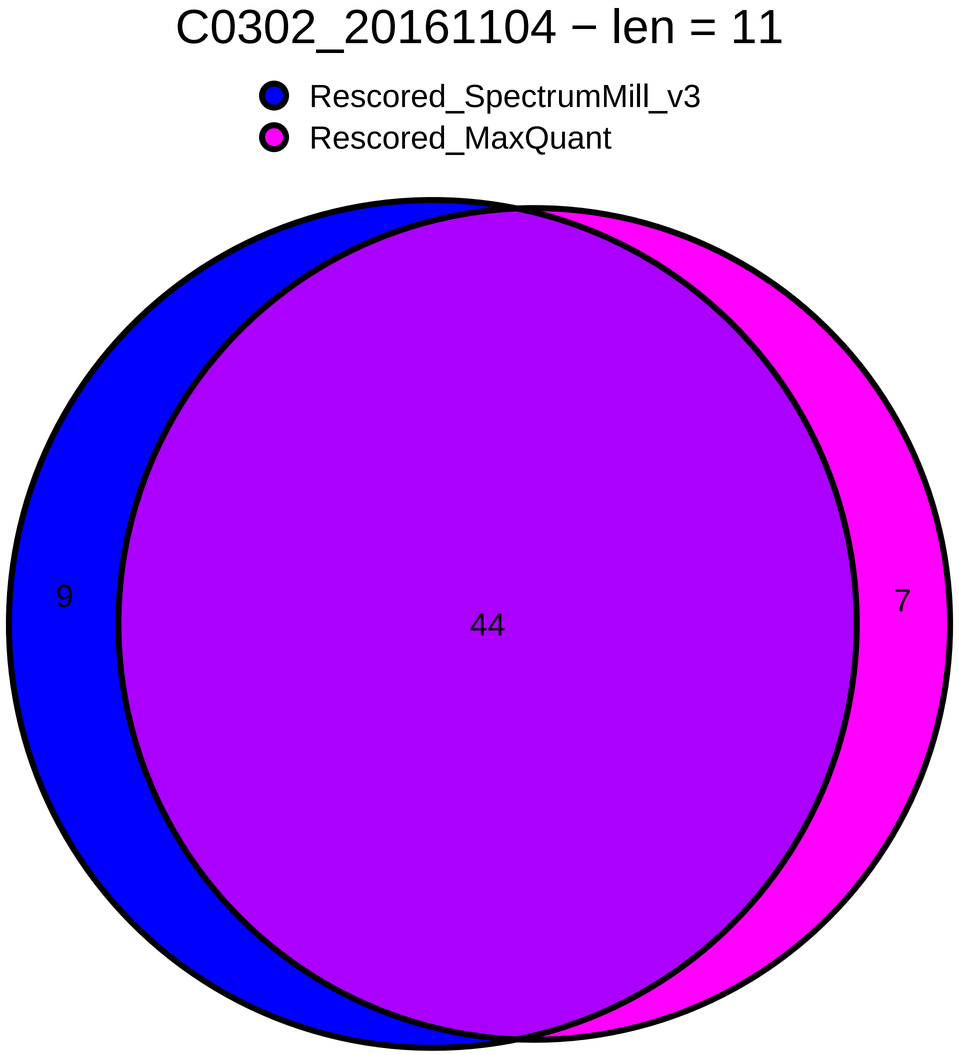

C0302_20161104 − len = 11
Rescored_SpectrumMill_v3
Rescored_MaxQuant
9
44
7

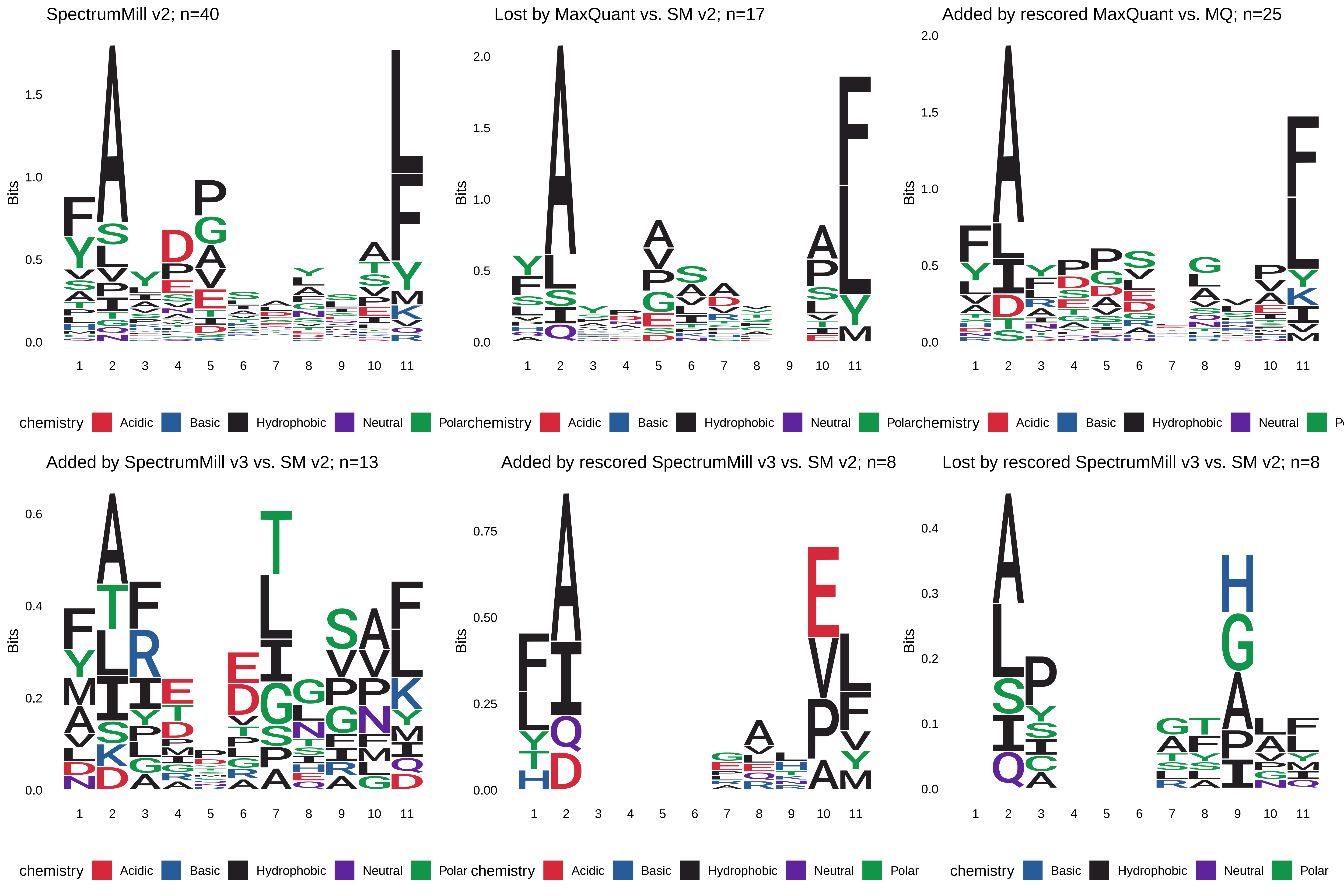
SpectrumMill v2; n=40
Lost by MaxQuant vs. SM v2; n=17
Added by rescored MaxQuant vs. MQ; n=25
Added by SpectrumMill v3 vs. SM v2; n=13
Added by rescored SpectrumMill v3 vs. SM v2; n=8
Lost by rescored SpectrumMill v3 vs. SM v2; n=8
Bits
1 2 3 4 5 6 7 8 9 10 11
chemistry Acidic Basic Hydrophobic Neutral Polar
chemistry Acidic Basic Hydrophobic Neutral Polar
chemistry Acidic Basic Hydrophobic Neutral Po
chemistry Acidic Basic Hydrophobic Neutral Polar
chemistry Acidic Basic Hydrophobic Neutral Polar
chemistry Basic Hydrophobic Neutral Polar

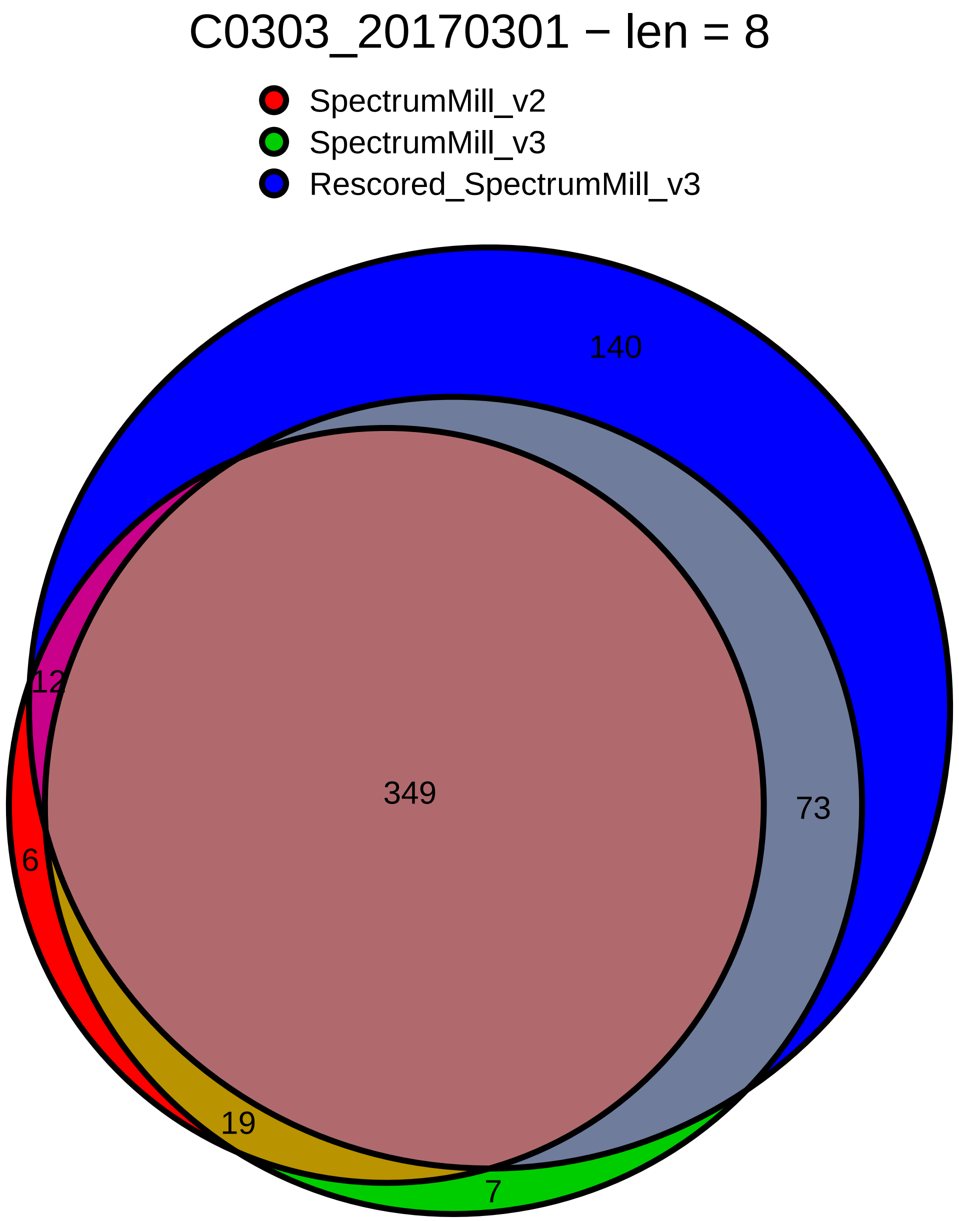
C0303_20170301 − len = 8
SpectrumMill_v2
SpectrumMill_v3
Rescored_SpectrumMill_v3
140
12
349
73
6
19
7

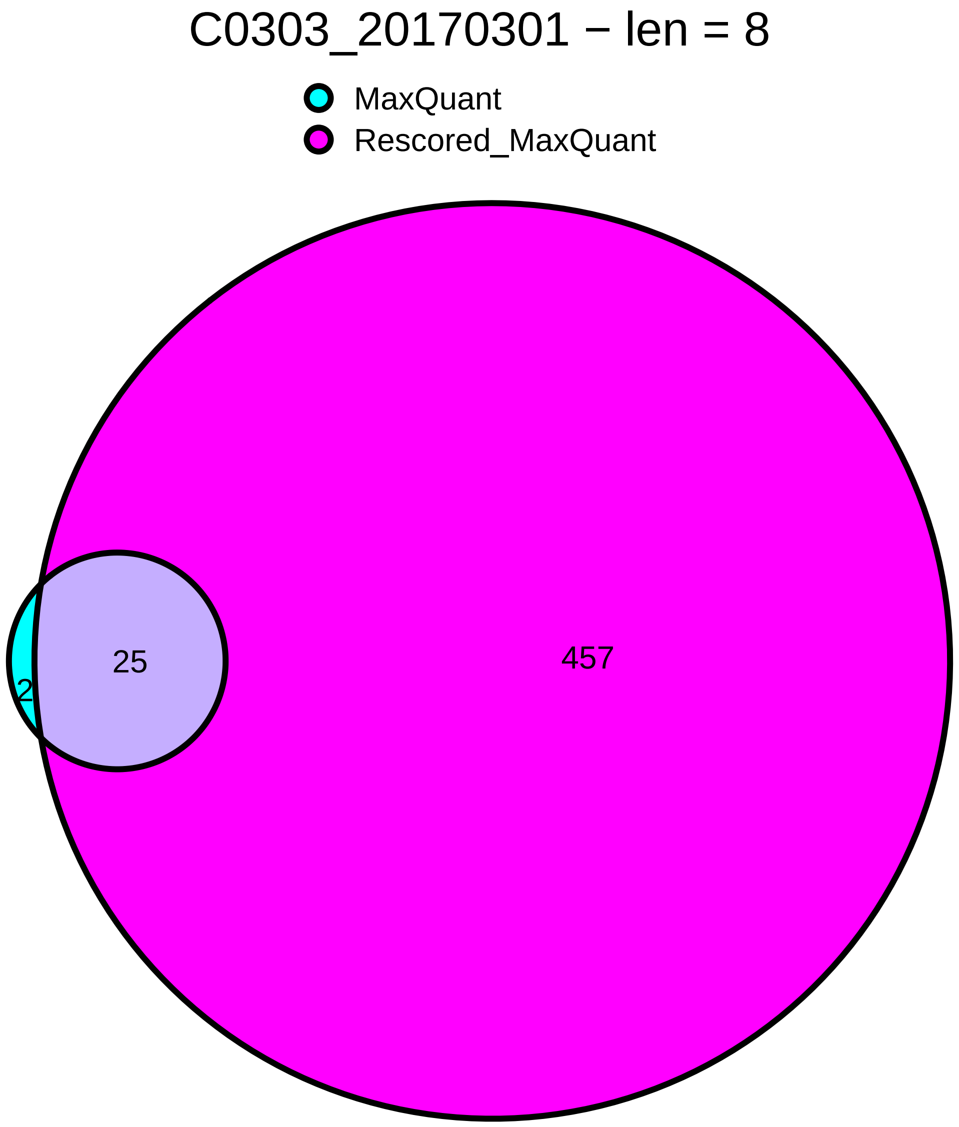
C0303_20170301 − len = 8
MaxQuant
Rescored_MaxQuant
2
25
457

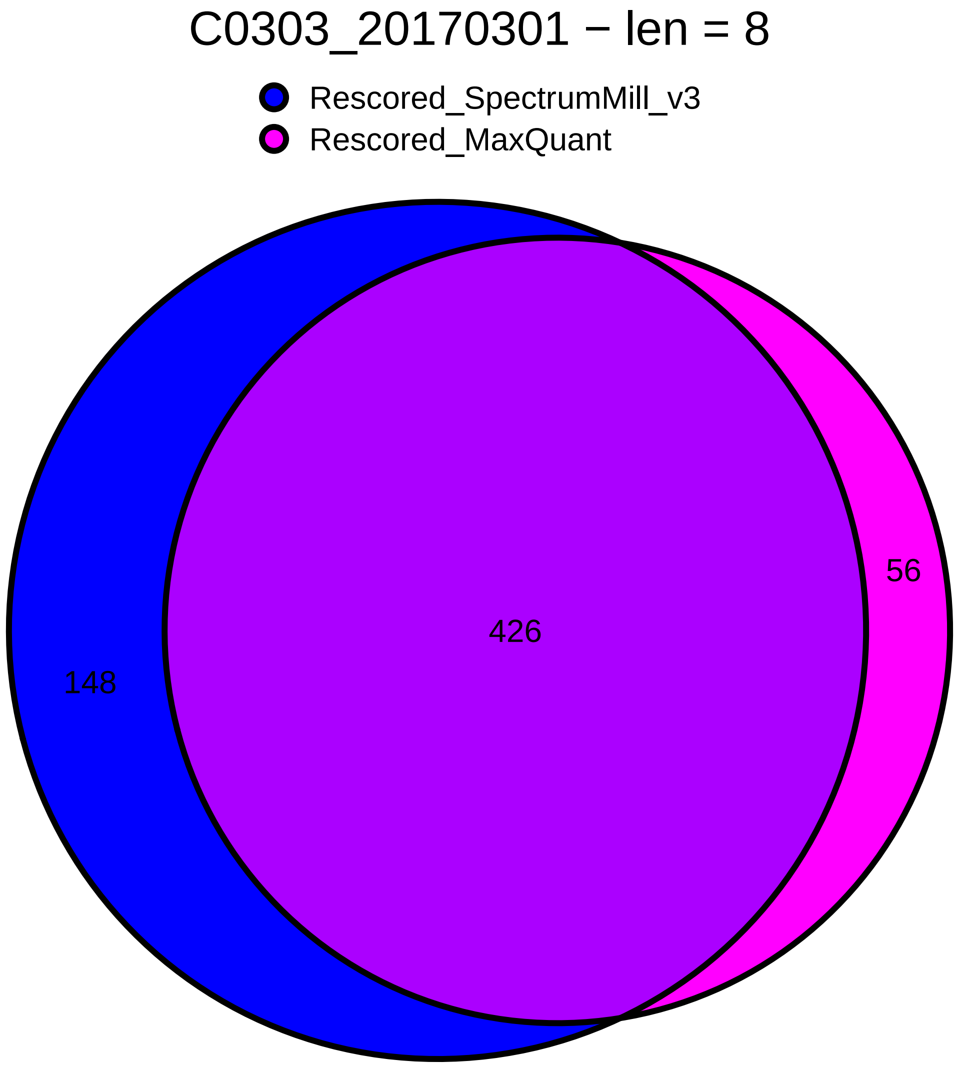
C0303_20170301 − len = 8
Rescored_SpectrumMill_v3
Rescored_MaxQuant
148
426
56

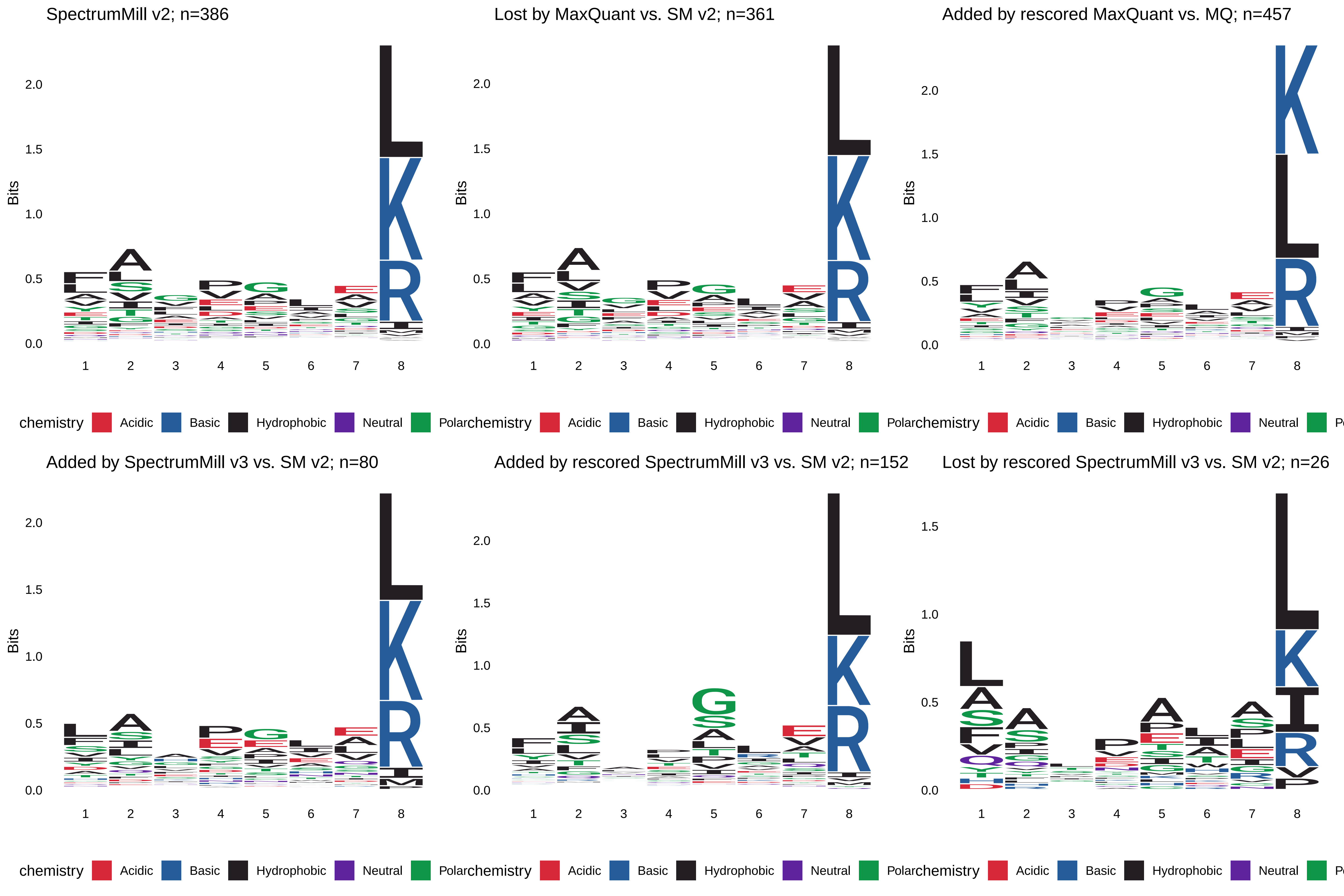
SpectrumMill v2; n=386
Lost by MaxQuant vs. SM v2; n=361
Added by rescored MaxQuant vs. MQ; n=457
Added by SpectrumMill v3 vs. SM v2; n=80
Added by rescored SpectrumMill v3 vs. SM v2; n=152
Lost by rescored SpectrumMill v3 vs. SM v2; n=26
Bits
0.0
0.5
1.0
1.5
2.0
1 2 3 4 5 6 7 8
chemistry
Acidic
Basic
Hydrophobic
Neutral
Polar

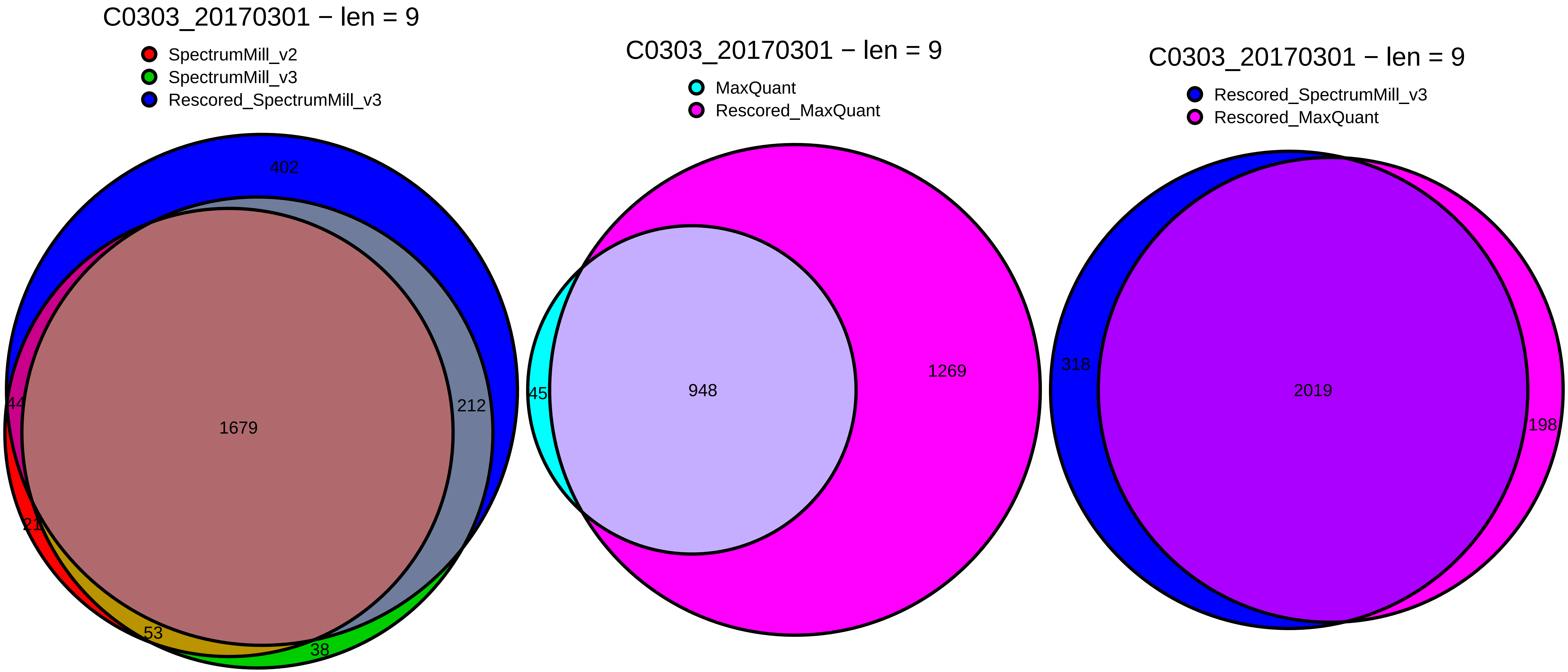

C0303_20170301 − len = 9
SpectrumMill_v2
SpectrumMill_v3
Rescored_SpectrumMill_v3
402
1679
212
53
38
C0303_20170301 − len = 9
MaxQuant
Rescored_MaxQuant
45
948
1269
C0303_20170301 − len = 9
Rescored_SpectrumMill_v3
Rescored_MaxQuant
318
2019
198

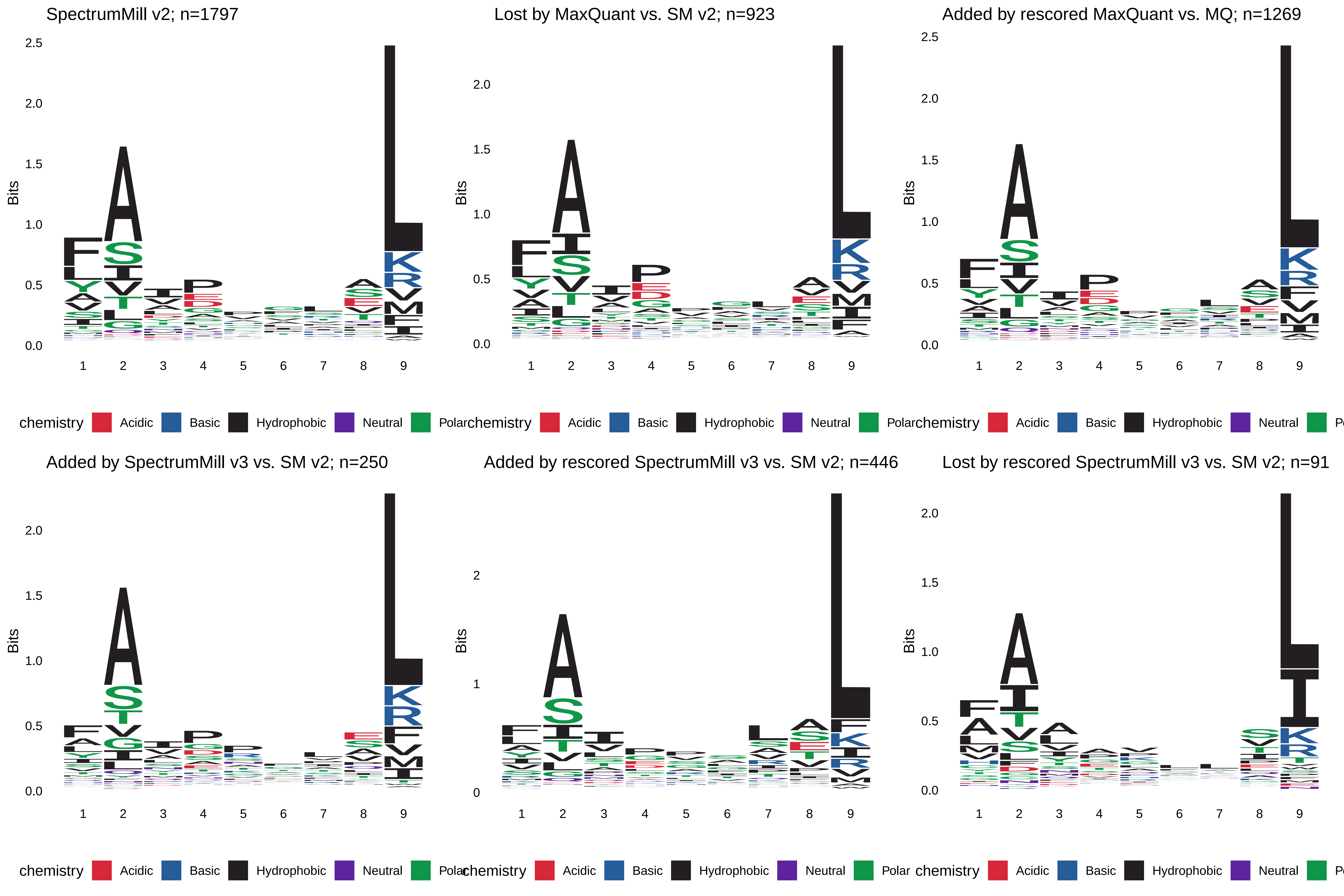
SpectrumMill v2; n=1797
Lost by MaxQuant vs. SM v2; n=923
Added by rescored MaxQuant vs. MQ; n=1269
Added by SpectrumMill v3 vs. SM v2; n=250
Added by rescored SpectrumMill v3 vs. SM v2; n=446
Lost by rescored SpectrumMill v3 vs. SM v2; n=91
Bits
1 2 3 4 5 6 7 8 9
chemistry Acidic Basic Hydrophobic Neutral Polar

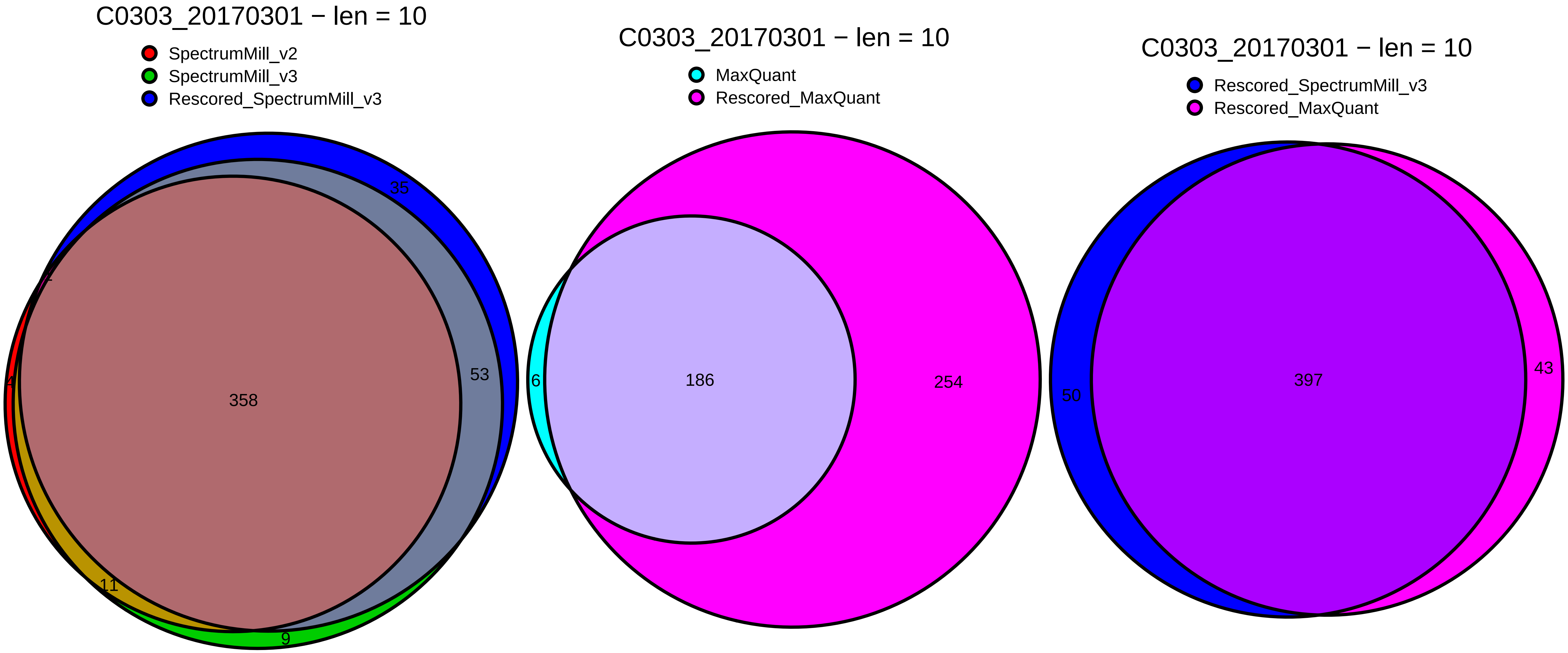
C0303_20170301 − len = 10
SpectrumMill_v2
SpectrumMill_v3
Rescored_SpectrumMill_v3
35
1
4
53
358
11
9
C0303_20170301 − len = 10
MaxQuant
Rescored_MaxQuant
6
186
254
C0303_20170301 − len = 10
Rescored_SpectrumMill_v3
Rescored_MaxQuant
50
397
43

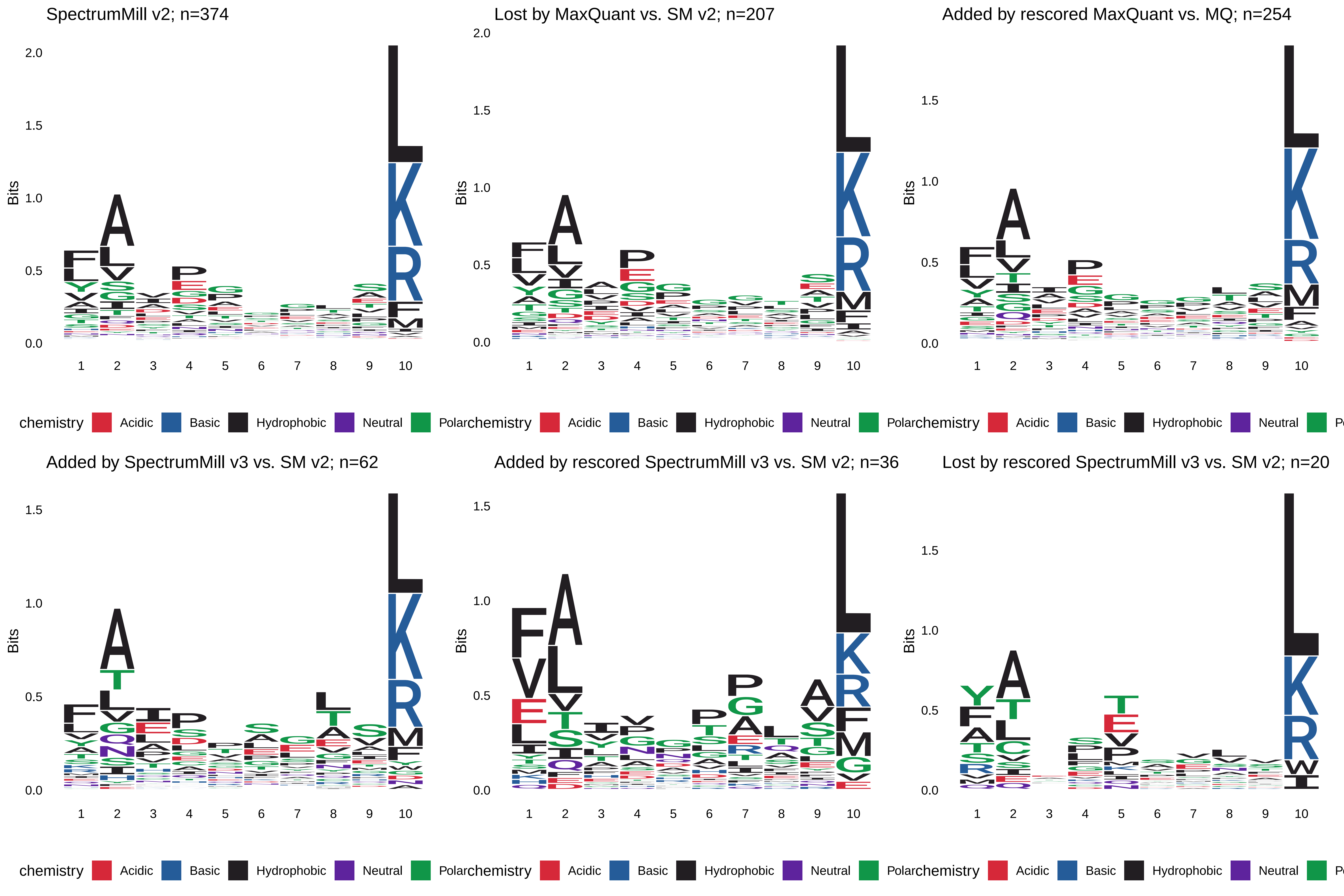
SpectrumMill v2; n=374
Lost by MaxQuant vs. SM v2; n=207
Added by rescored MaxQuant vs. MQ; n=254
Added by SpectrumMill v3 vs. SM v2; n=62
Added by rescored SpectrumMill v3 vs. SM v2; n=36
Lost by rescored SpectrumMill v3 vs. SM v2; n=20
Bits
1 2 3 4 5 6 7 8 9 10
chemistry Acidic Basic Hydrophobic Neutral Polar

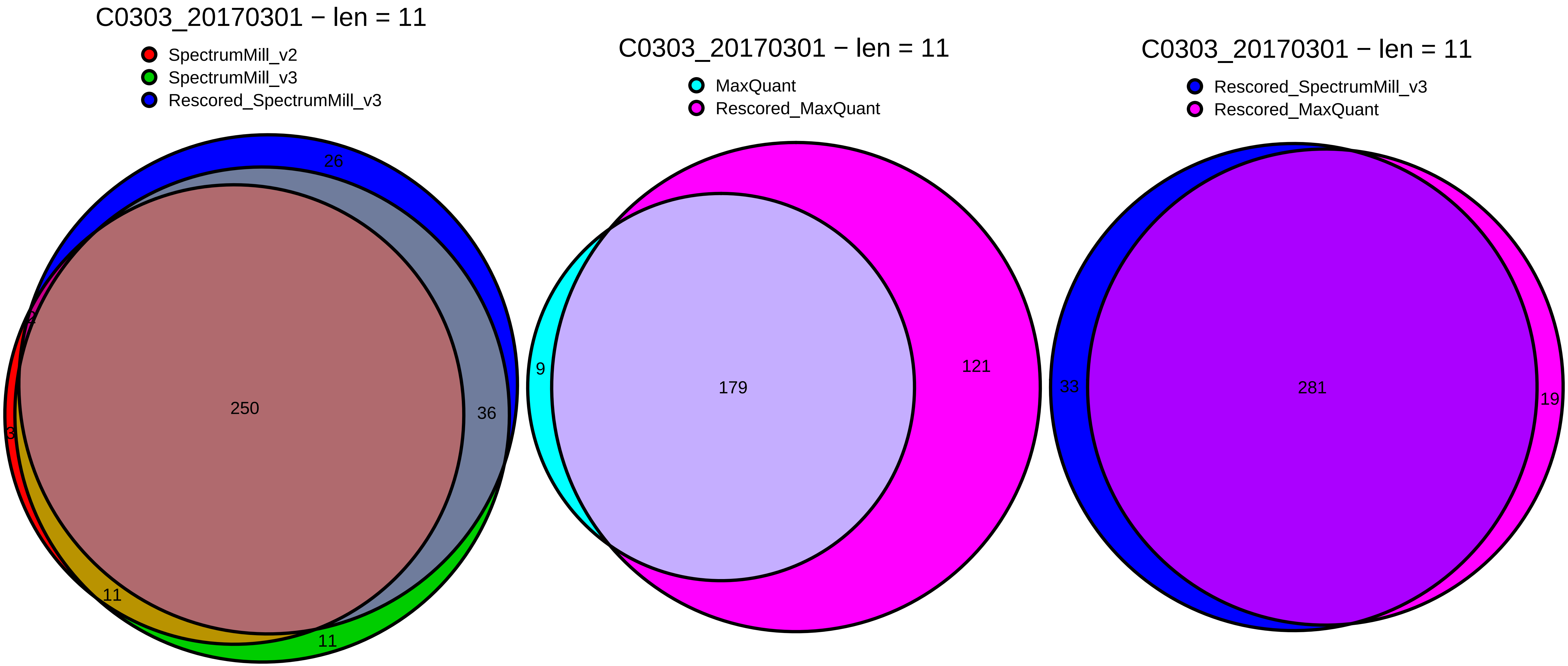
C0303_20170301 − len = 11
SpectrumMill_v2
SpectrumMill_v3
Rescored_SpectrumMill_v3
26
2
36
250
3
11
11
C0303_20170301 − len = 11
MaxQuant
Rescored_MaxQuant
9
179
121
C0303_20170301 − len = 11
Rescored_SpectrumMill_v3
Rescored_MaxQuant
33
281
19

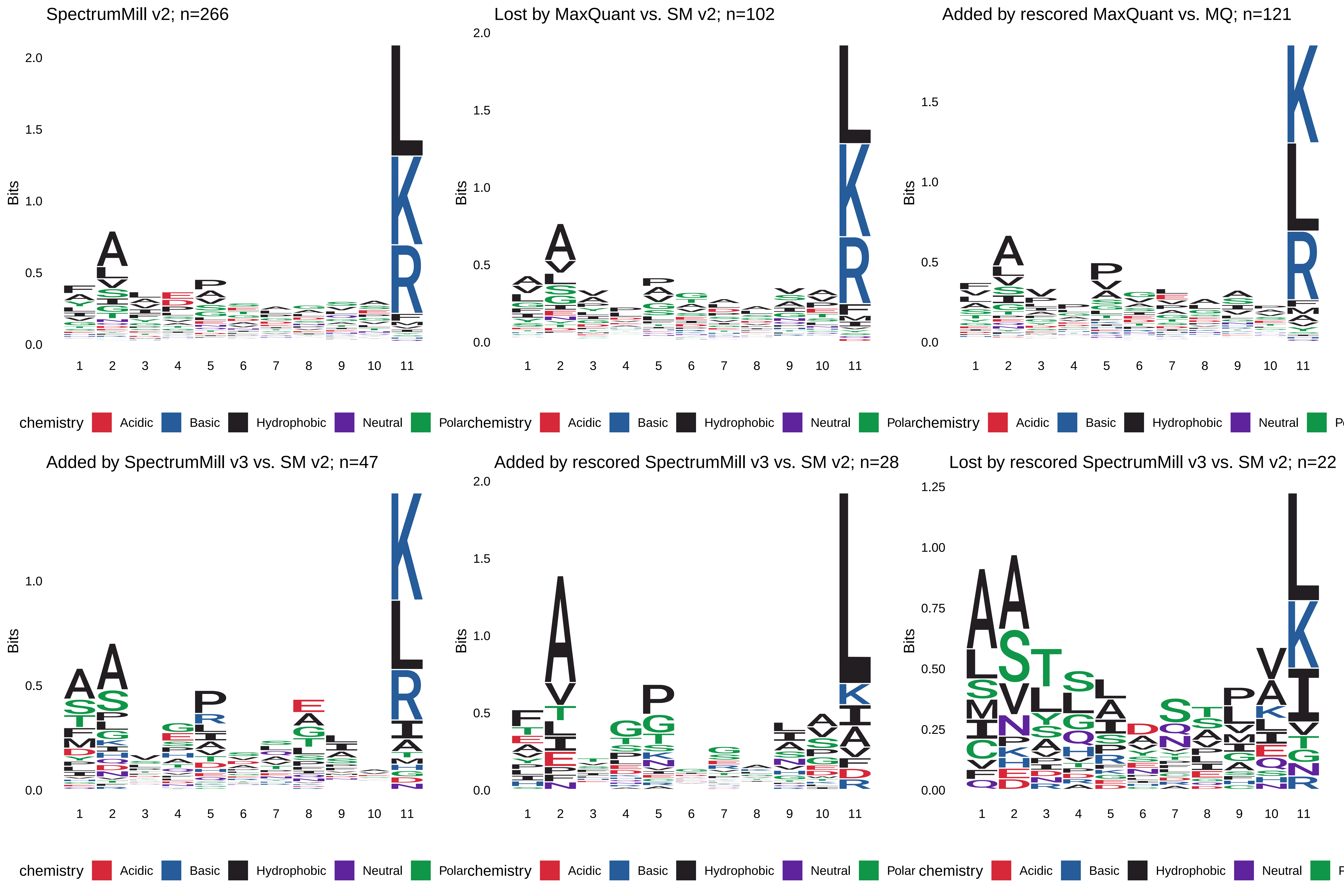
SpectrumMill v2; n=266
Lost by MaxQuant vs. SM v2; n=102
Added by rescored MaxQuant vs. MQ; n=121
Added by SpectrumMill v3 vs. SM v2; n=47
Added by rescored SpectrumMill v3 vs. SM v2; n=28
Lost by rescored SpectrumMill v3 vs. SM v2; n=22
Bits
1 2 3 4 5 6 7 8 9 10 11
chemistry Acidic Basic Hydrophobic Neutral Polar

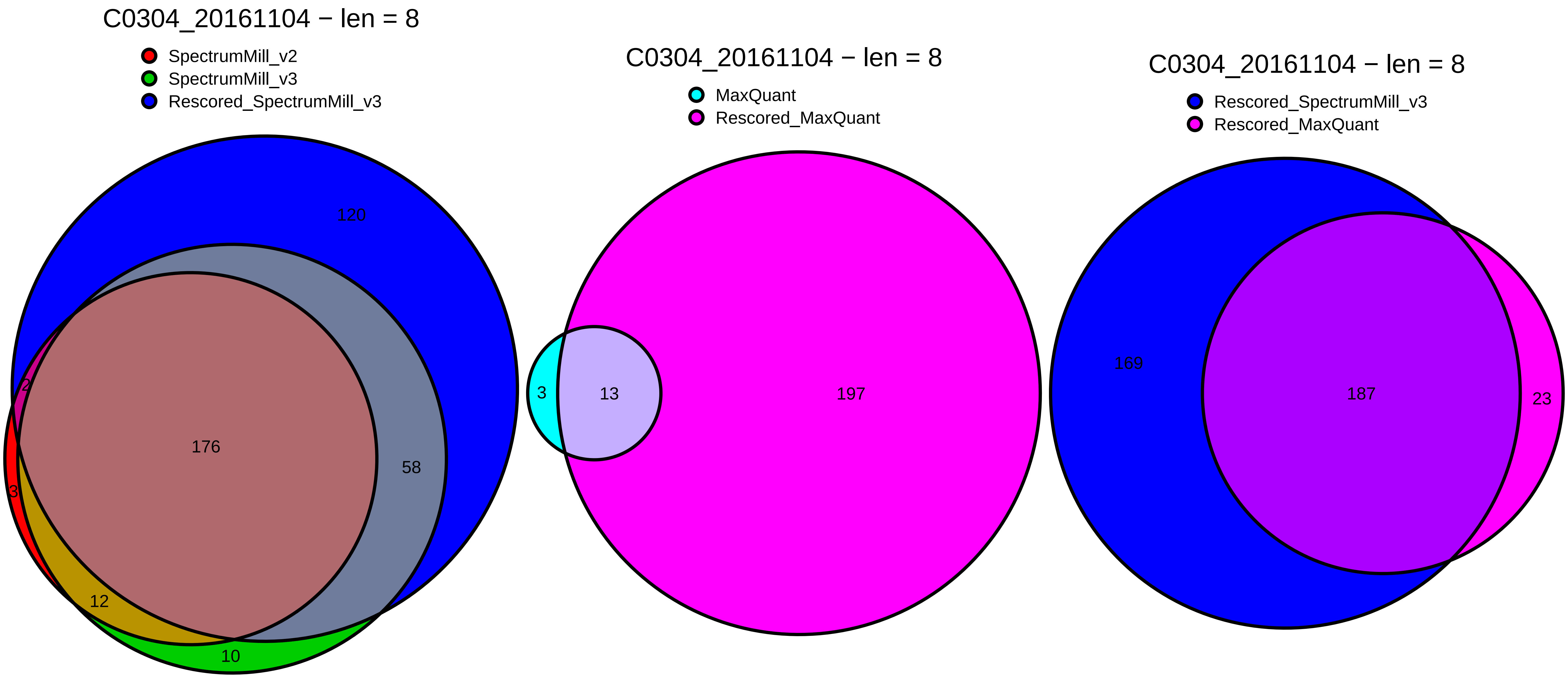
C0304_20161104 − len = 8
SpectrumMill_v2
SpectrumMill_v3
Rescored_SpectrumMill_v3
120
2
176
58
3
12
10
C0304_20161104 − len = 8
MaxQuant
Rescored_MaxQuant
3
13
197
C0304_20161104 − len = 8
Rescored_SpectrumMill_v3
Rescored_MaxQuant
169
187
23

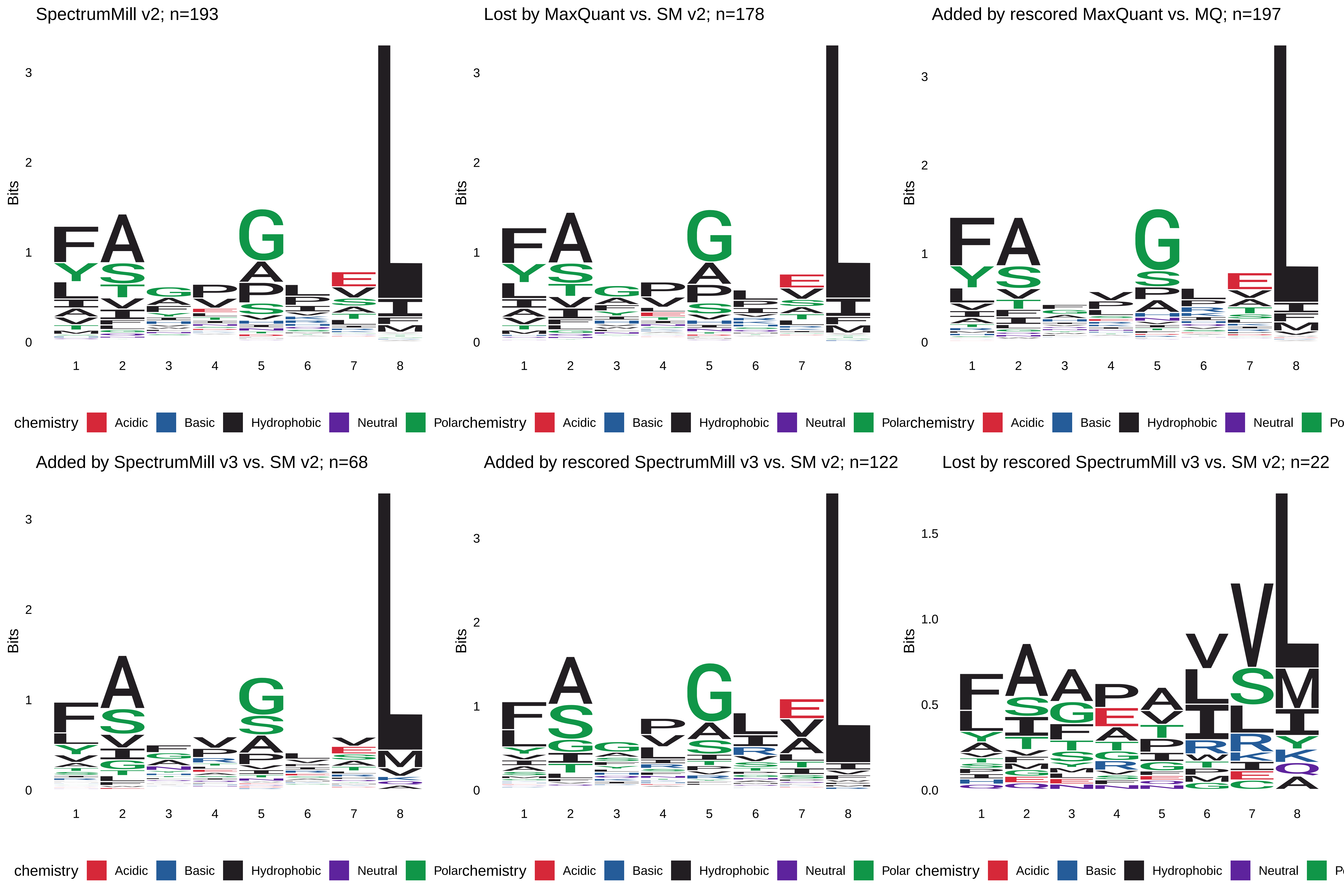
SpectrumMill v2; n=193
Lost by MaxQuant vs. SM v2; n=178
Added by rescored MaxQuant vs. MQ; n=197
Added by SpectrumMill v3 vs. SM v2; n=68
Added by rescored SpectrumMill v3 vs. SM v2; n=122
Lost by rescored SpectrumMill v3 vs. SM v2; n=22
Bits
1 2 3 4 5 6 7 8
chemistry Acidic Basic Hydrophobic Neutral Polar

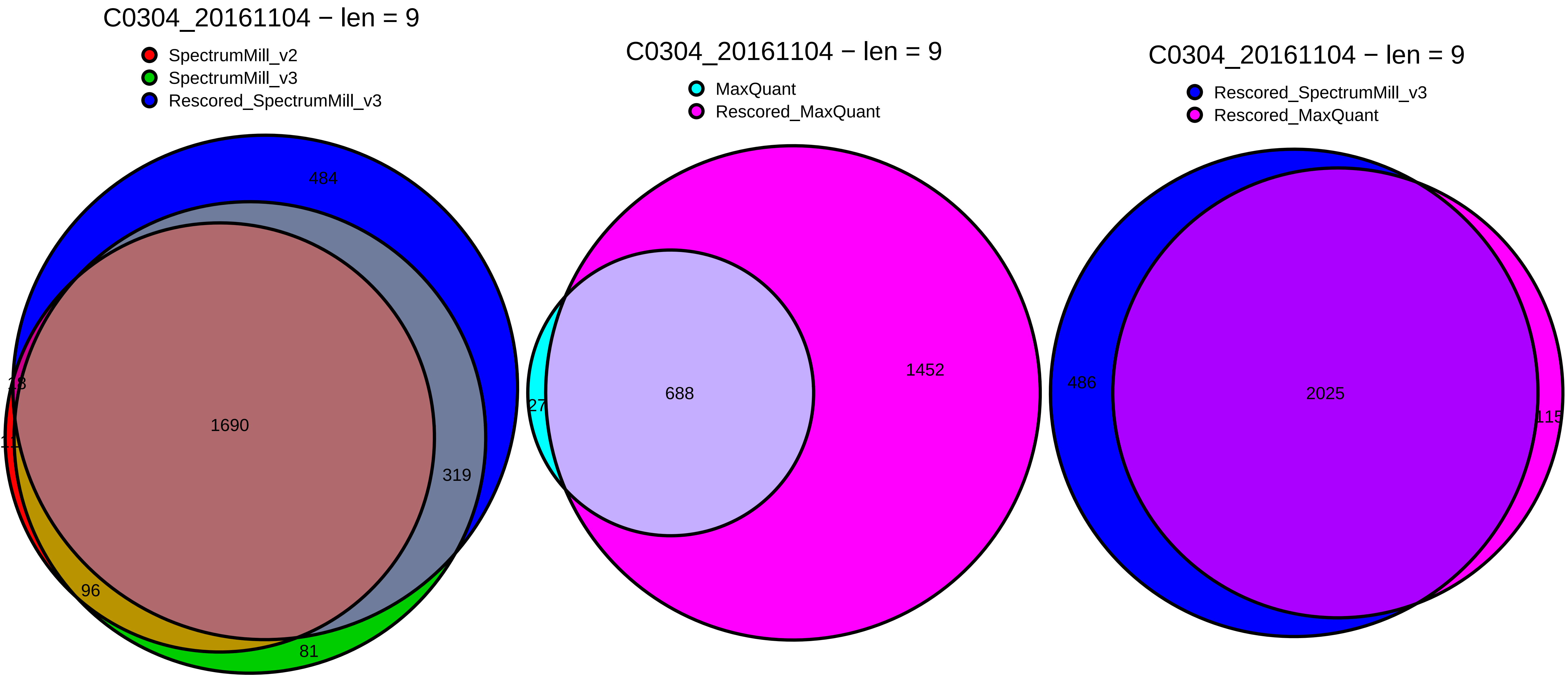
C0304_20161104 − len = 9
SpectrumMill_v2
SpectrumMill_v3
Rescored_SpectrumMill_v3
484
1690
319
96
81
C0304_20161104 − len = 9
MaxQuant
Rescored_MaxQuant
27
688
1452
C0304_20161104 − len = 9
Rescored_SpectrumMill_v3
Rescored_MaxQuant
486
2025

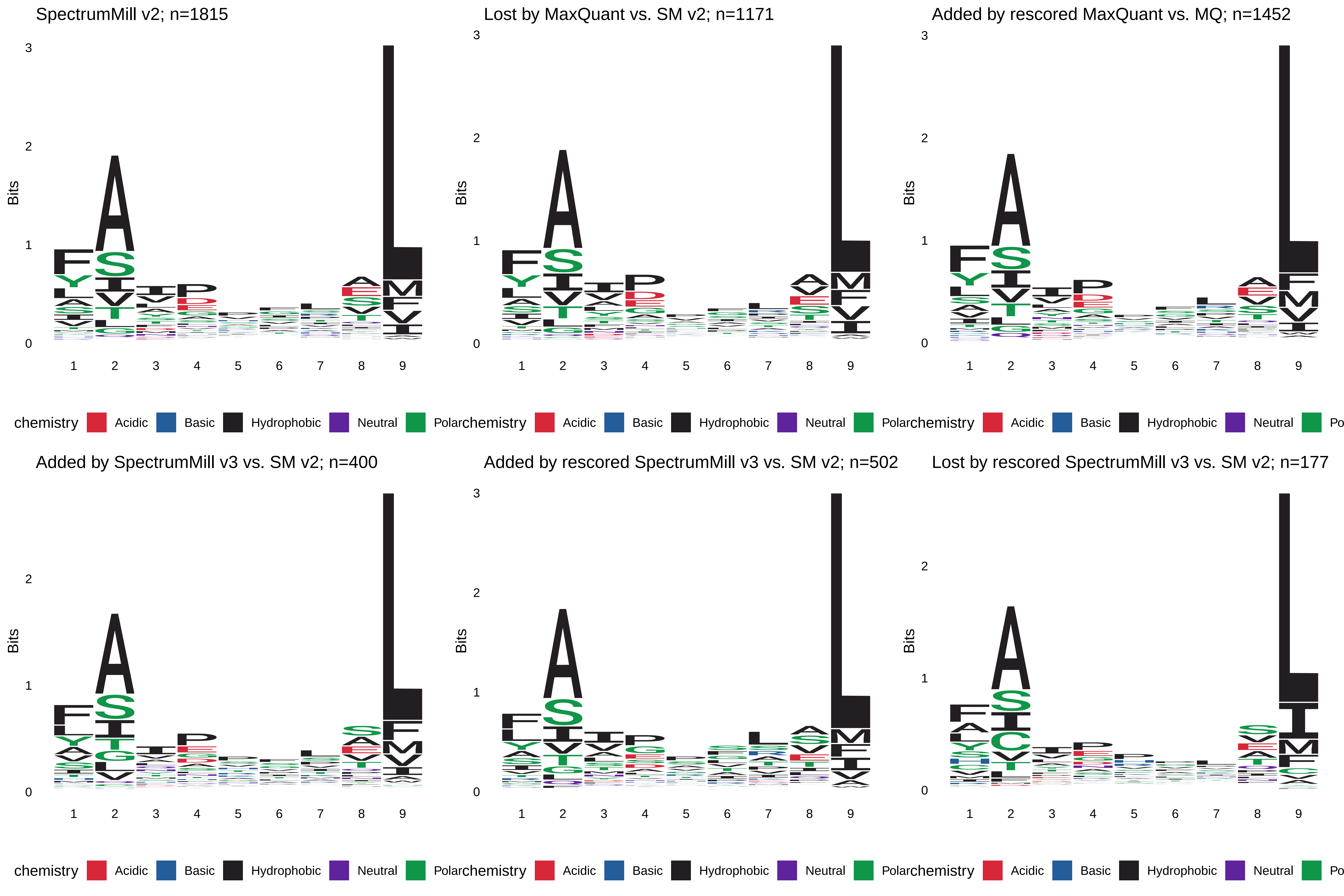
SpectrumMill v2; n=1815
Lost by MaxQuant vs. SM v2; n=1171
Added by rescored MaxQuant vs. MQ; n=1452
Added by SpectrumMill v3 vs. SM v2; n=400
Added by rescored SpectrumMill v3 vs. SM v2; n=502
Lost by rescored SpectrumMill v3 vs. SM v2; n=177
Bits
0
1
2
3
1
2
3
4
5
6
7
8
9
chemistry
Acidic
Basic
Hydrophobic
Neutral
Polar

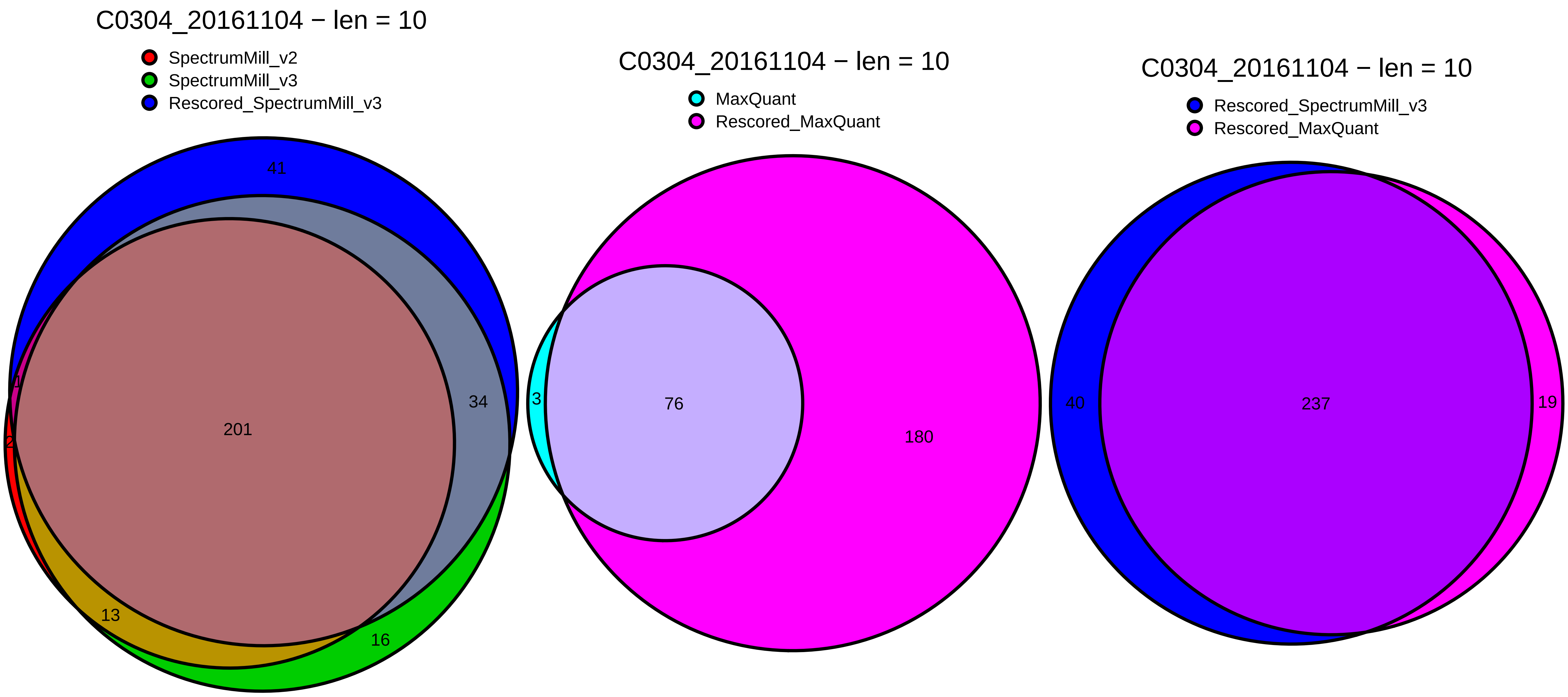
C0304_20161104 − len = 10
SpectrumMill_v2
SpectrumMill_v3
Rescored_SpectrumMill_v3
41
1
34
201
2
13
16
C0304_20161104 − len = 10
MaxQuant
Rescored_MaxQuant
3
76
180
C0304_20161104 − len = 10
Rescored_SpectrumMill_v3
Rescored_MaxQuant
40
237
19

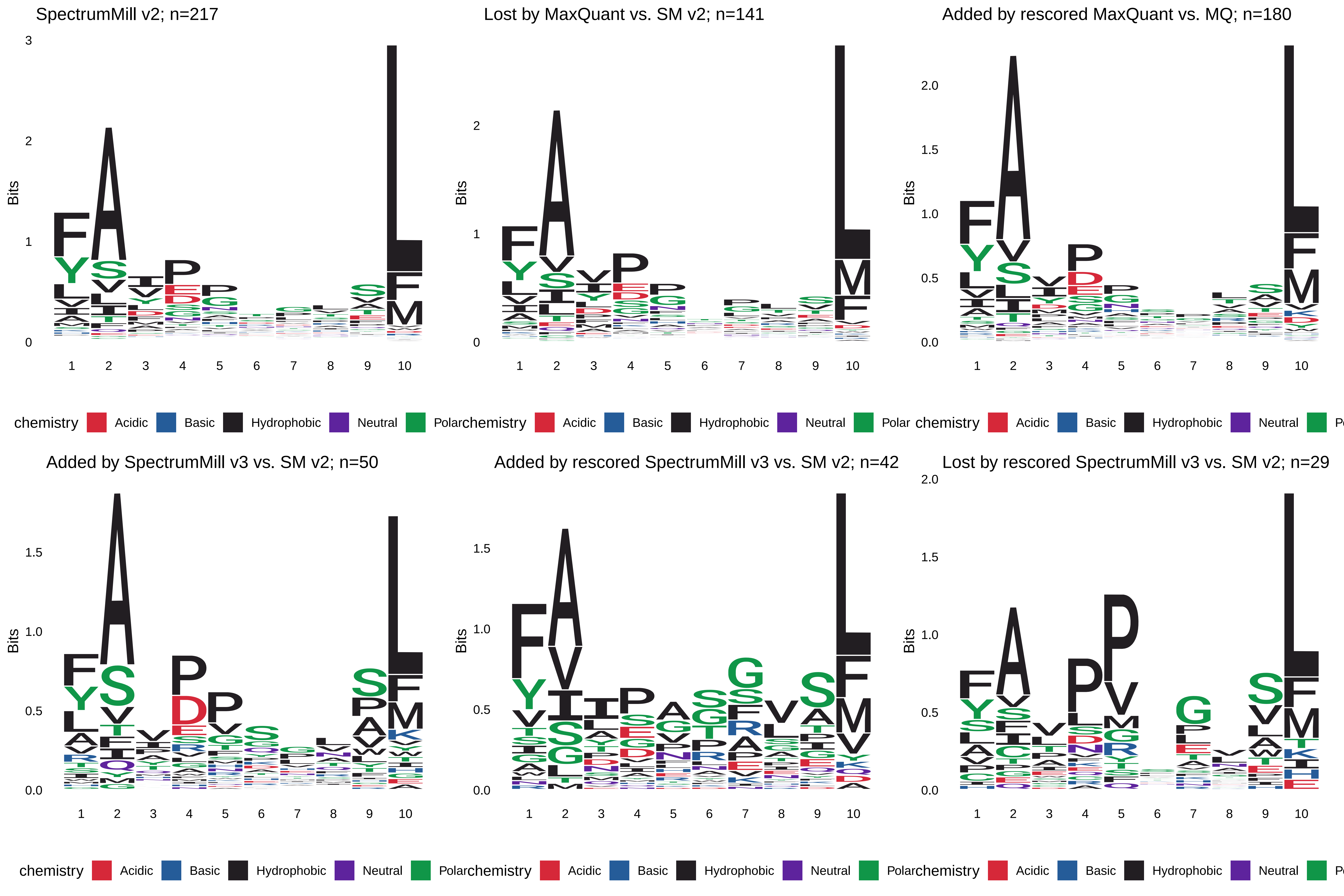
SpectrumMill v2; n=217
Lost by MaxQuant vs. SM v2; n=141
Added by rescored MaxQuant vs. MQ; n=180
Added by SpectrumMill v3 vs. SM v2; n=50
Added by rescored SpectrumMill v3 vs. SM v2; n=42
Lost by rescored SpectrumMill v3 vs. SM v2; n=29
Bits
1 2 3 4 5 6 7 8 9 10
chemistry
Acidic
Basic
Hydrophobic
Neutral
Polar

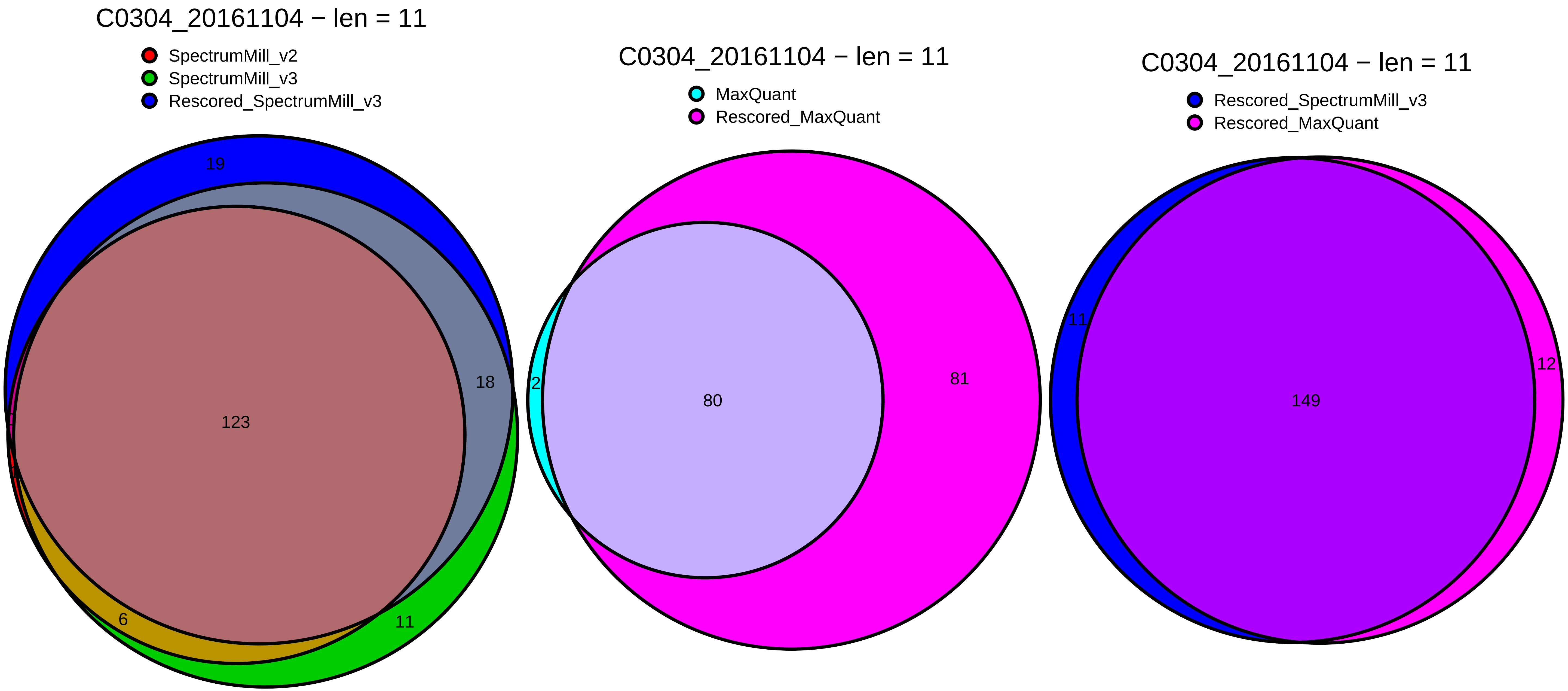
C0304_20161104 − len = 11
SpectrumMill_v2
SpectrumMill_v3
Rescored_SpectrumMill_v3
19
18
123
6
11
C0304_20161104 − len = 11
MaxQuant
Rescored_MaxQuant
2
80
81
C0304_20161104 − len = 11
Rescored_SpectrumMill_v3
Rescored_MaxQuant
11
149
12

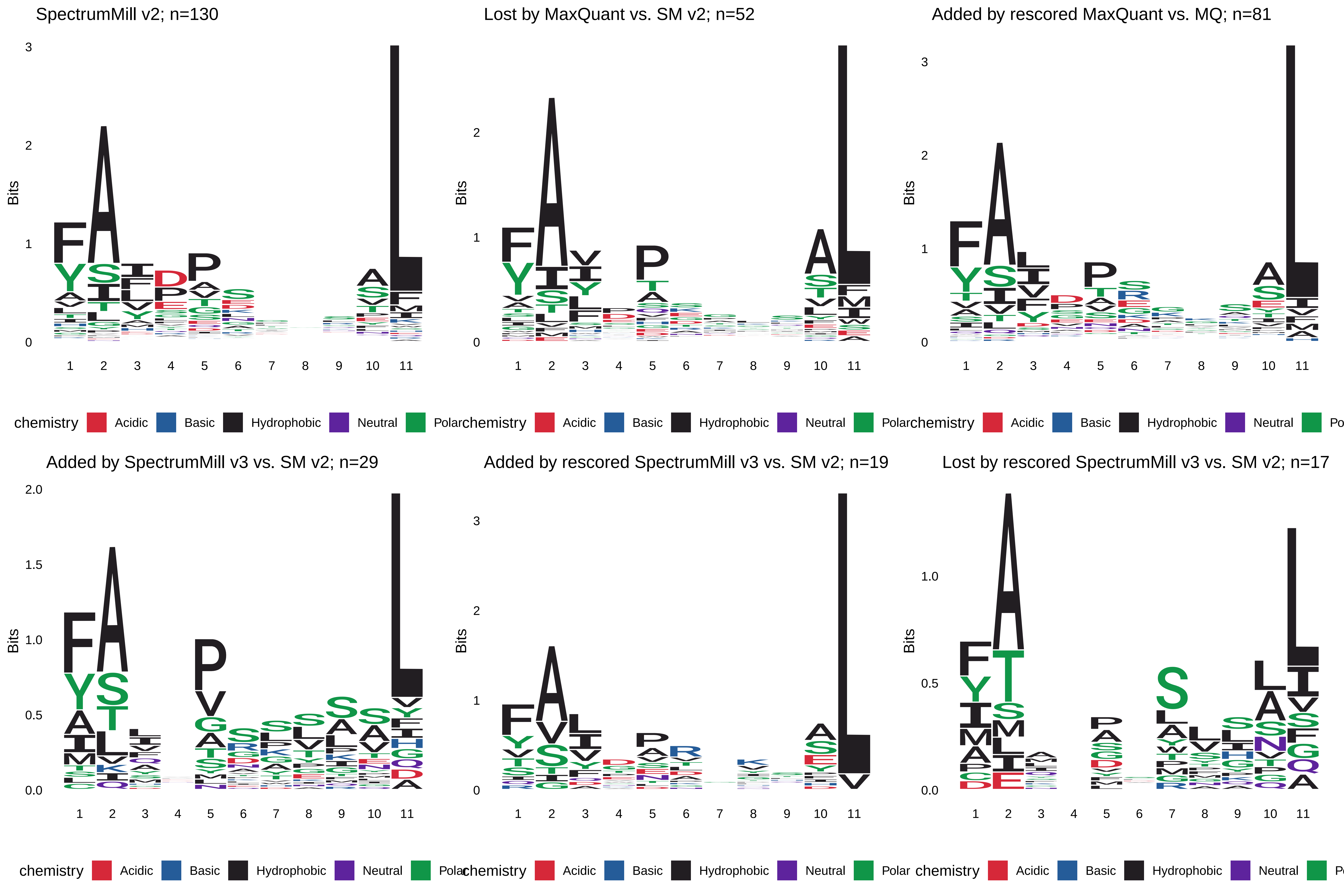

SpectrumMill v2; n=130
Lost by MaxQuant vs. SM v2; n=52
Added by rescored MaxQuant vs. MQ; n=81
Added by SpectrumMill v3 vs. SM v2; n=29
Added by rescored SpectrumMill v3 vs. SM v2; n=19
Lost by rescored SpectrumMill v3 vs. SM v2; n=17
Bits
1 2 3 4 5 6 7 8 9 10 11
chemistry
Acidic
Basic
Hydrophobic
Neutral
Polar

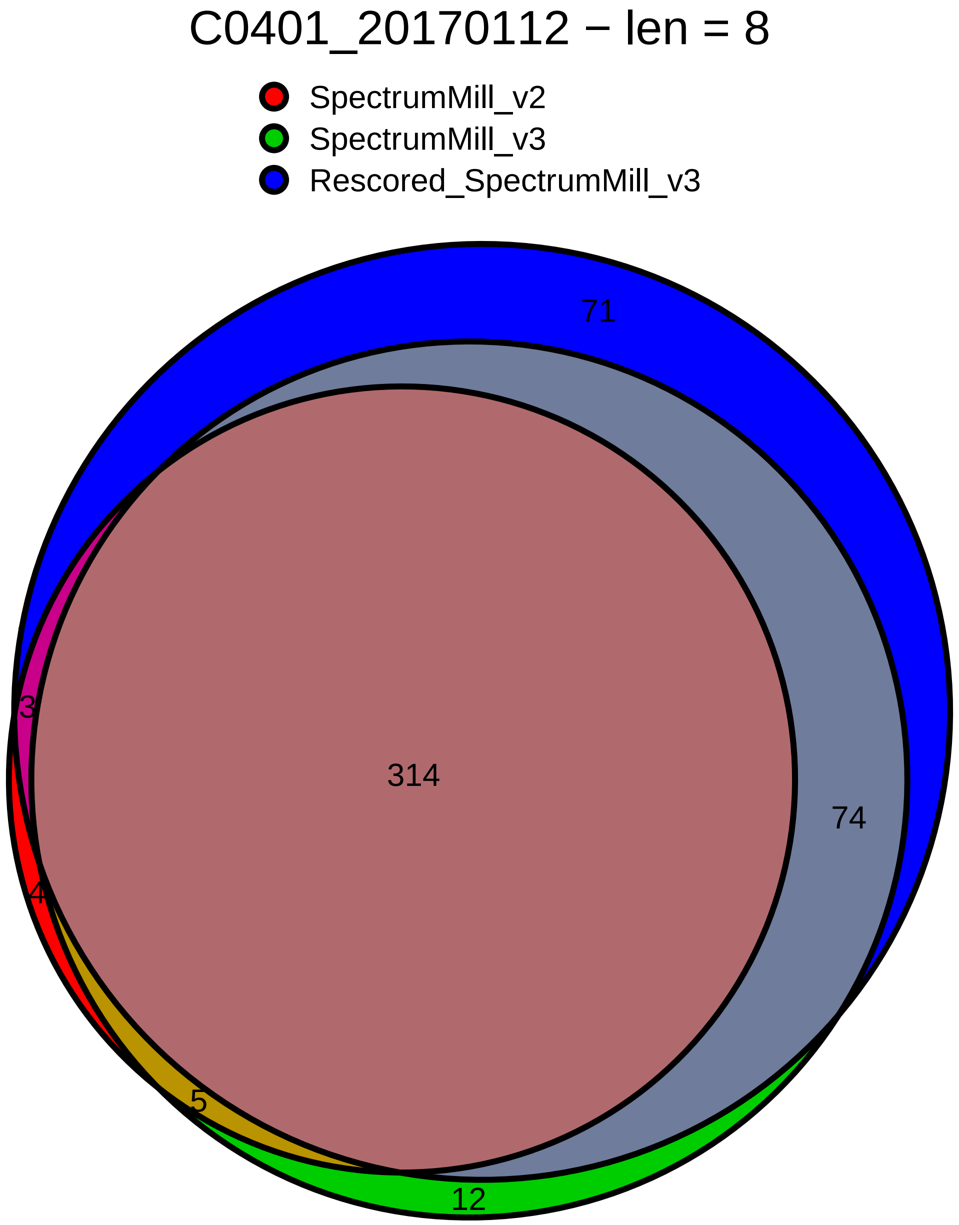
C0401_20170112 − len = 8
SpectrumMill_v2
SpectrumMill_v3
Rescored_SpectrumMill_v3
71
3
314
74
4
5
12

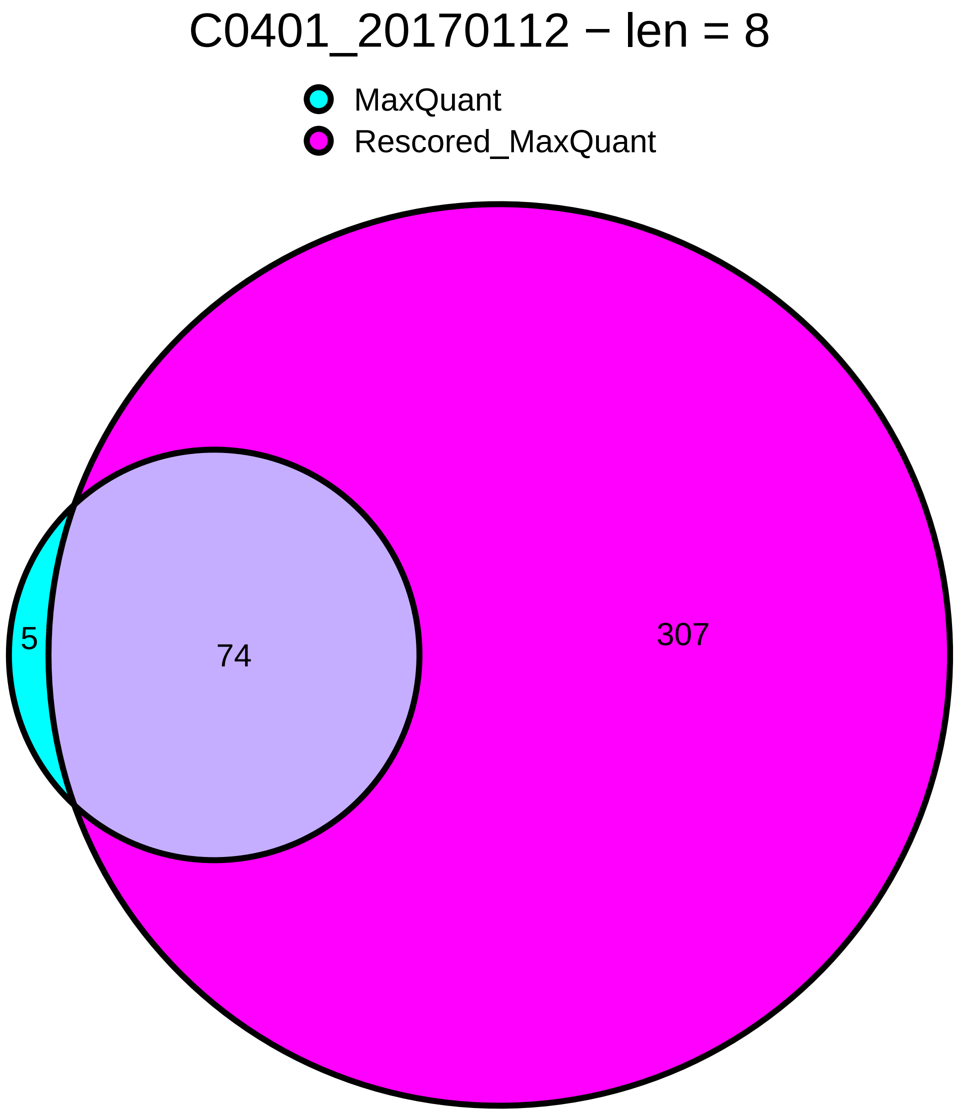
C0401_20170112 − len = 8
MaxQuant
Rescored_MaxQuant
5
74
307

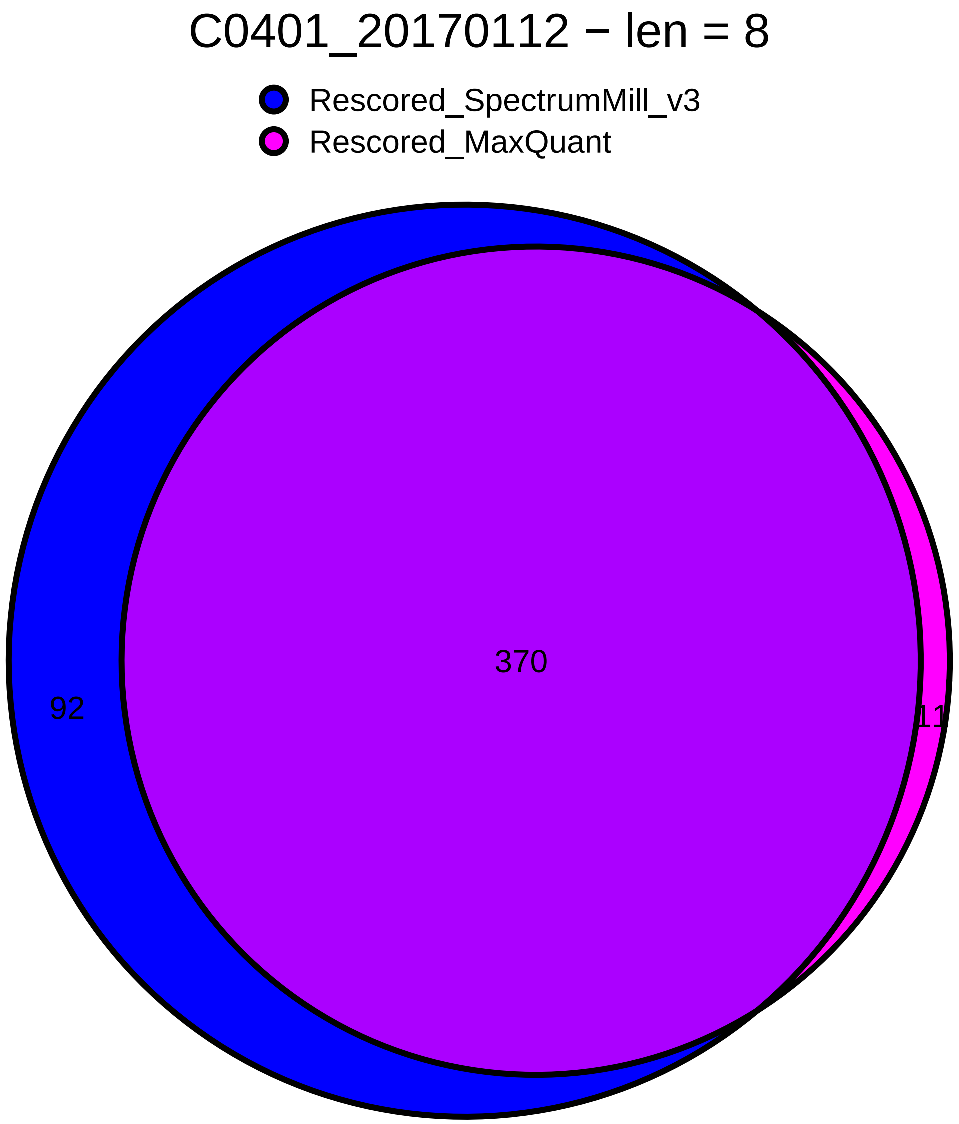
C0401_20170112 − len = 8
Rescored_SpectrumMill_v3
Rescored_MaxQuant
92
370
11

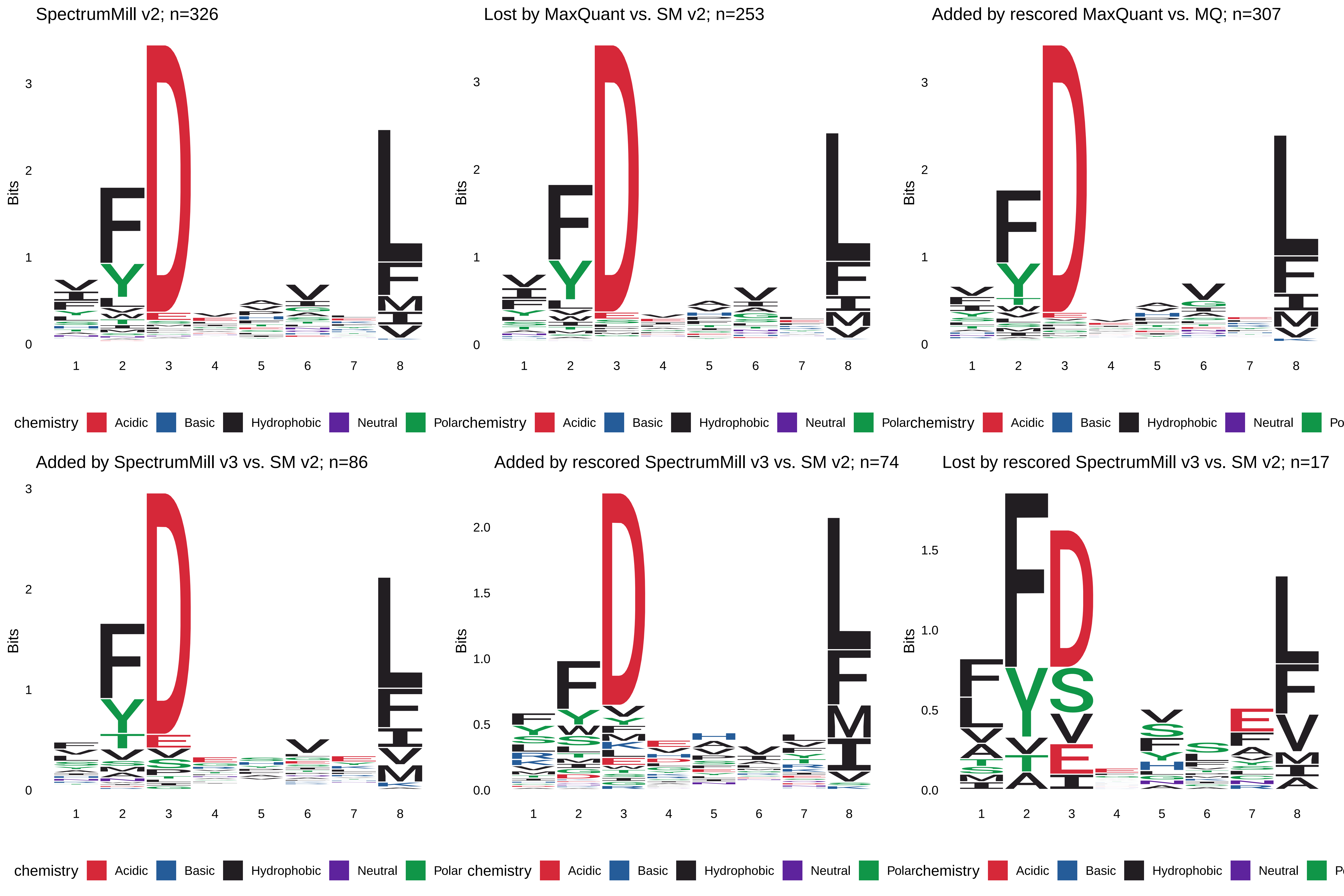
SpectrumMill v2; n=326
Lost by MaxQuant vs. SM v2; n=253
Added by rescored MaxQuant vs. MQ; n=307
Added by SpectrumMill v3 vs. SM v2; n=86
Added by rescored SpectrumMill v3 vs. SM v2; n=74
Lost by rescored SpectrumMill v3 vs. SM v2; n=17
Bits
1 2 3 4 5 6 7 8
chemistry Acidic Basic Hydrophobic Neutral Polar

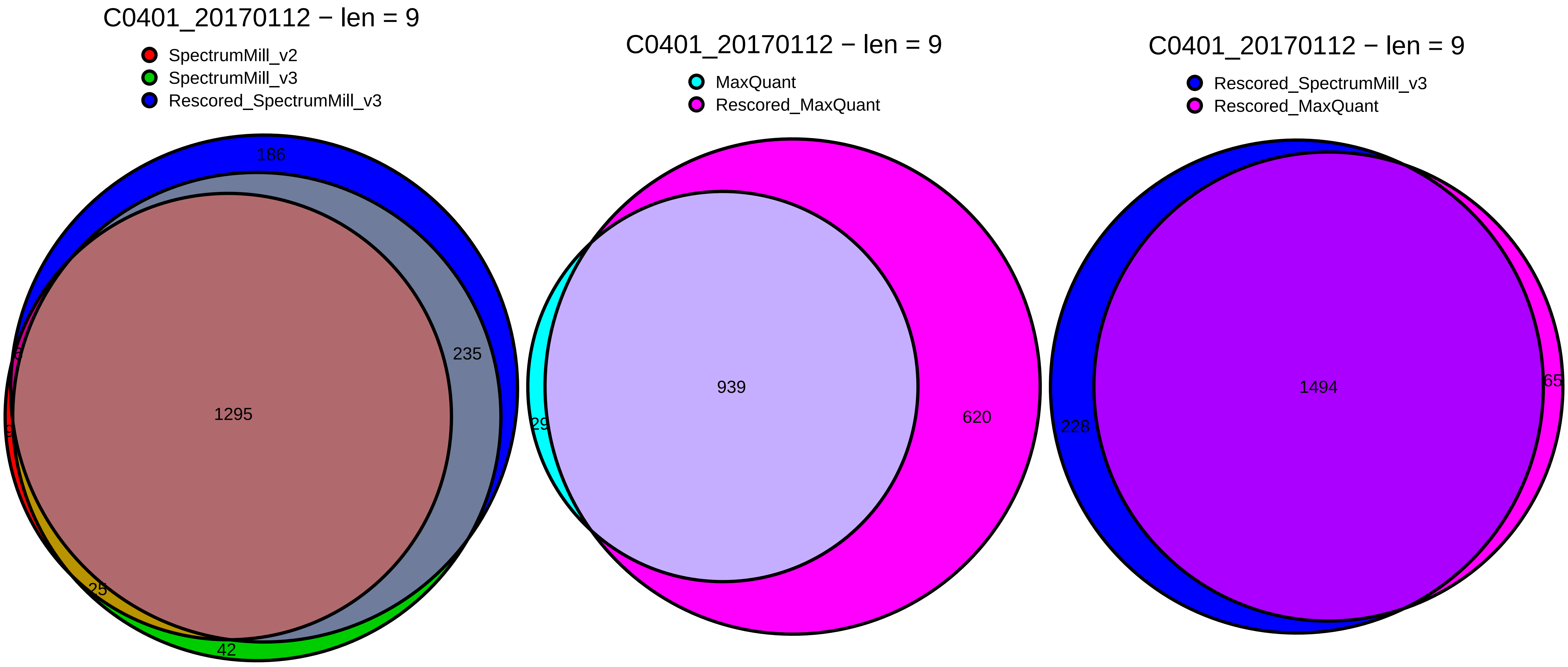
C0401_20170112 − len = 9
SpectrumMill_v2
SpectrumMill_v3
Rescored_SpectrumMill_v3
186
235
1295
25
42
C0401_20170112 − len = 9
MaxQuant
Rescored_MaxQuant
29
939
620
C0401_20170112 − len = 9
Rescored_SpectrumMill_v3
Rescored_MaxQuant
1494
228
65

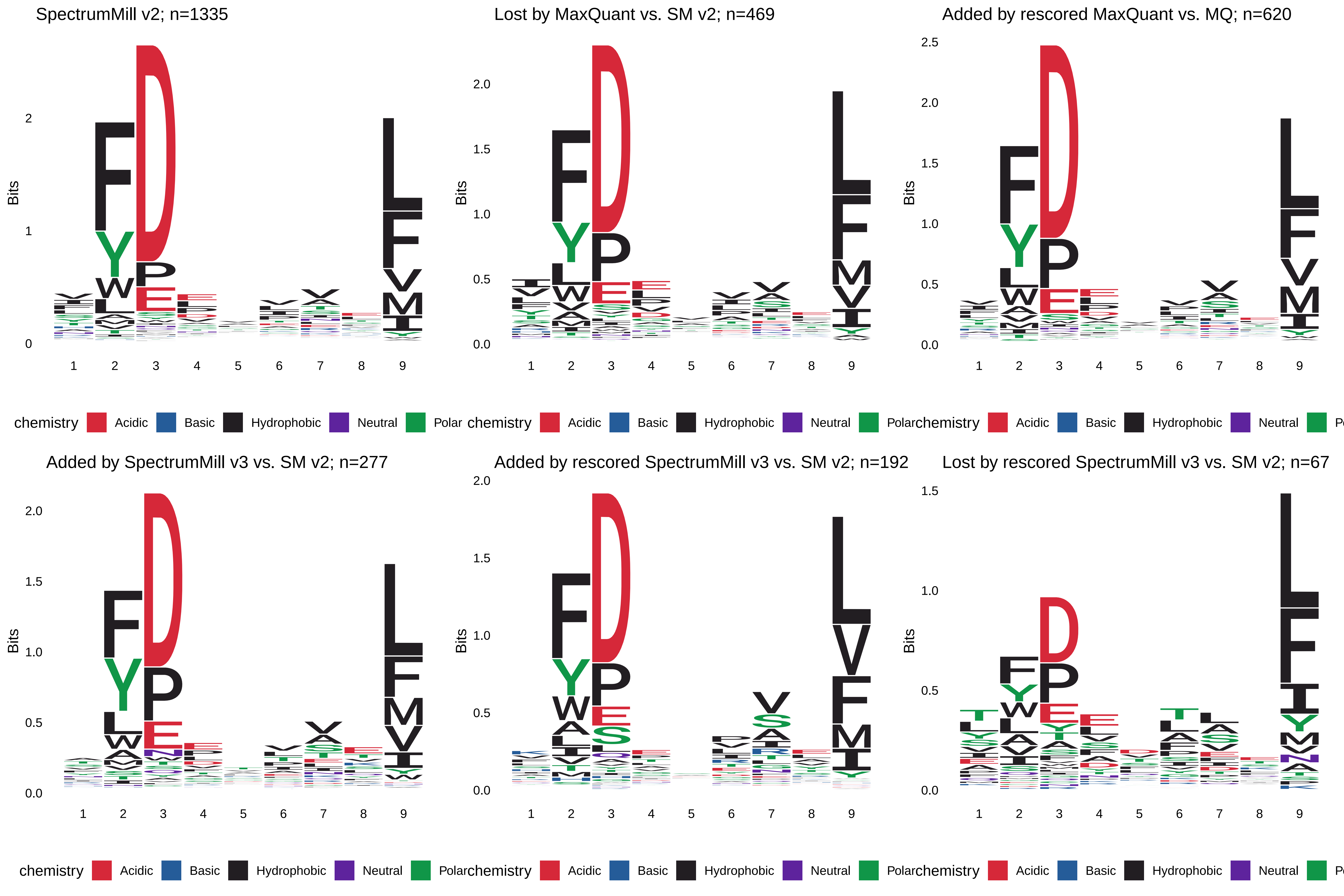

SpectrumMill v2; n=1335
Lost by MaxQuant vs. SM v2; n=469
Added by rescored MaxQuant vs. MQ; n=620
Added by SpectrumMill v3 vs. SM v2; n=277
Added by rescored SpectrumMill v3 vs. SM v2; n=192
Lost by rescored SpectrumMill v3 vs. SM v2; n=67
Bits
1 2 3 4 5 6 7 8 9
chemistry Acidic Basic Hydrophobic Neutral Polar

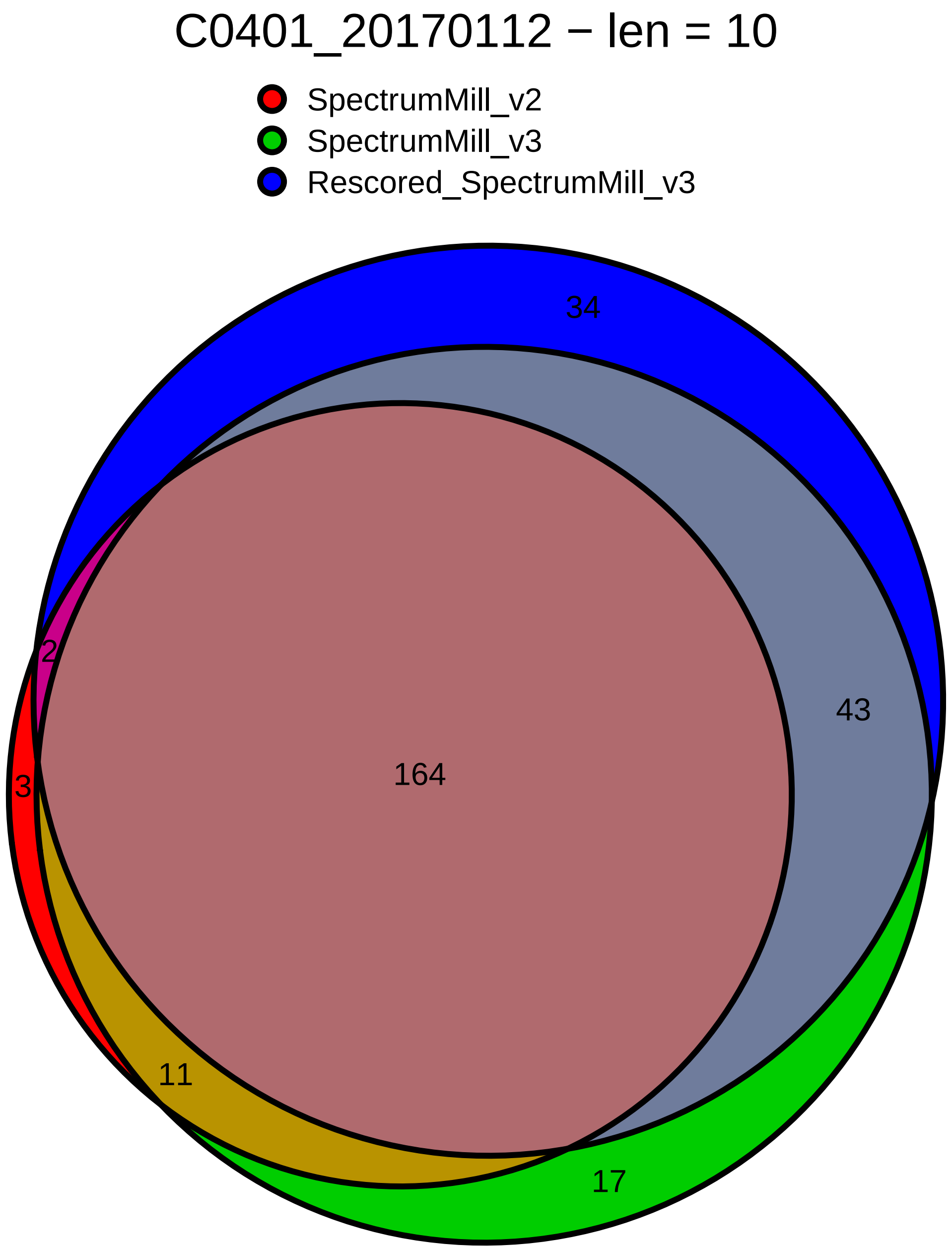
C0401_20170112 − len = 10
SpectrumMill_v2
SpectrumMill_v3
Rescored_SpectrumMill_v3
34
2
43
164
3
11
17

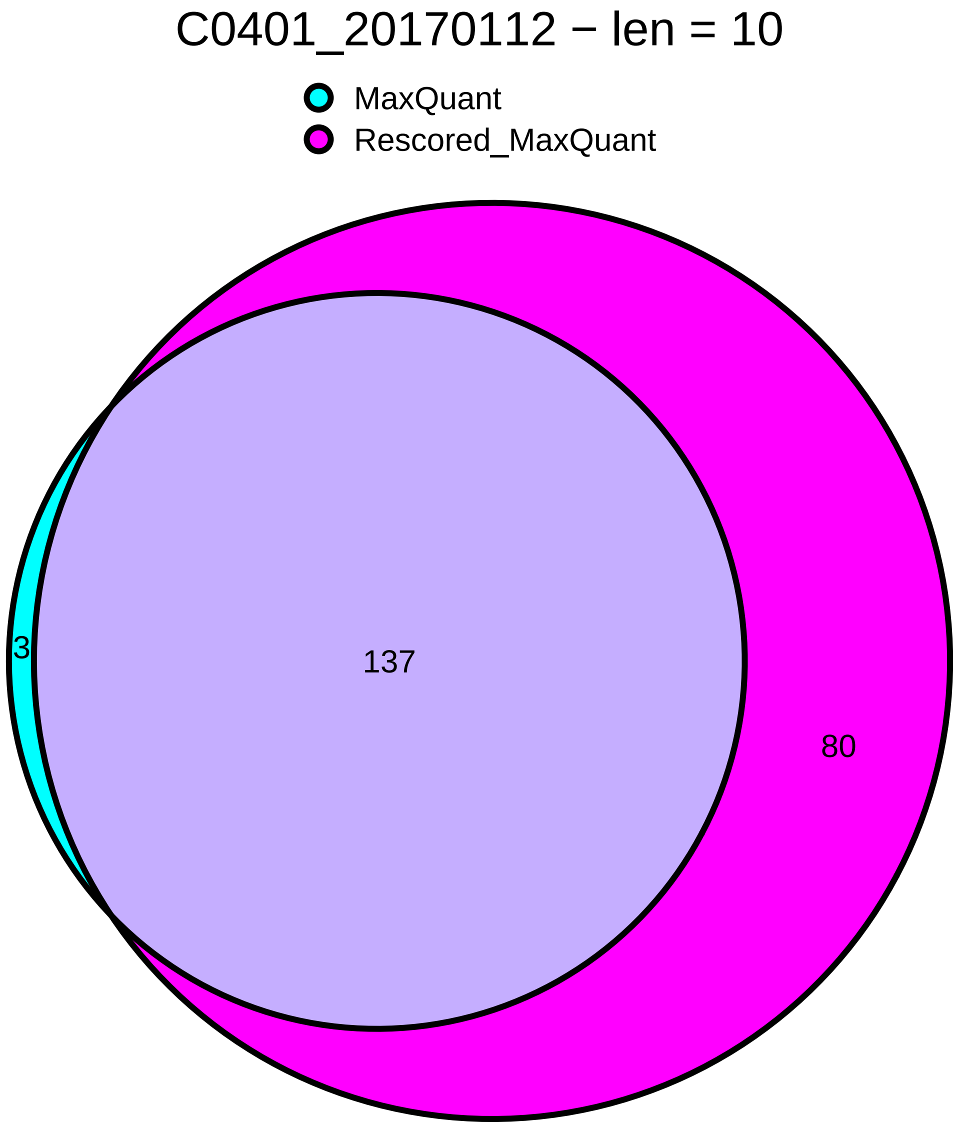
C0401_20170112 − len = 10
MaxQuant
Rescored_MaxQuant
3
137
80

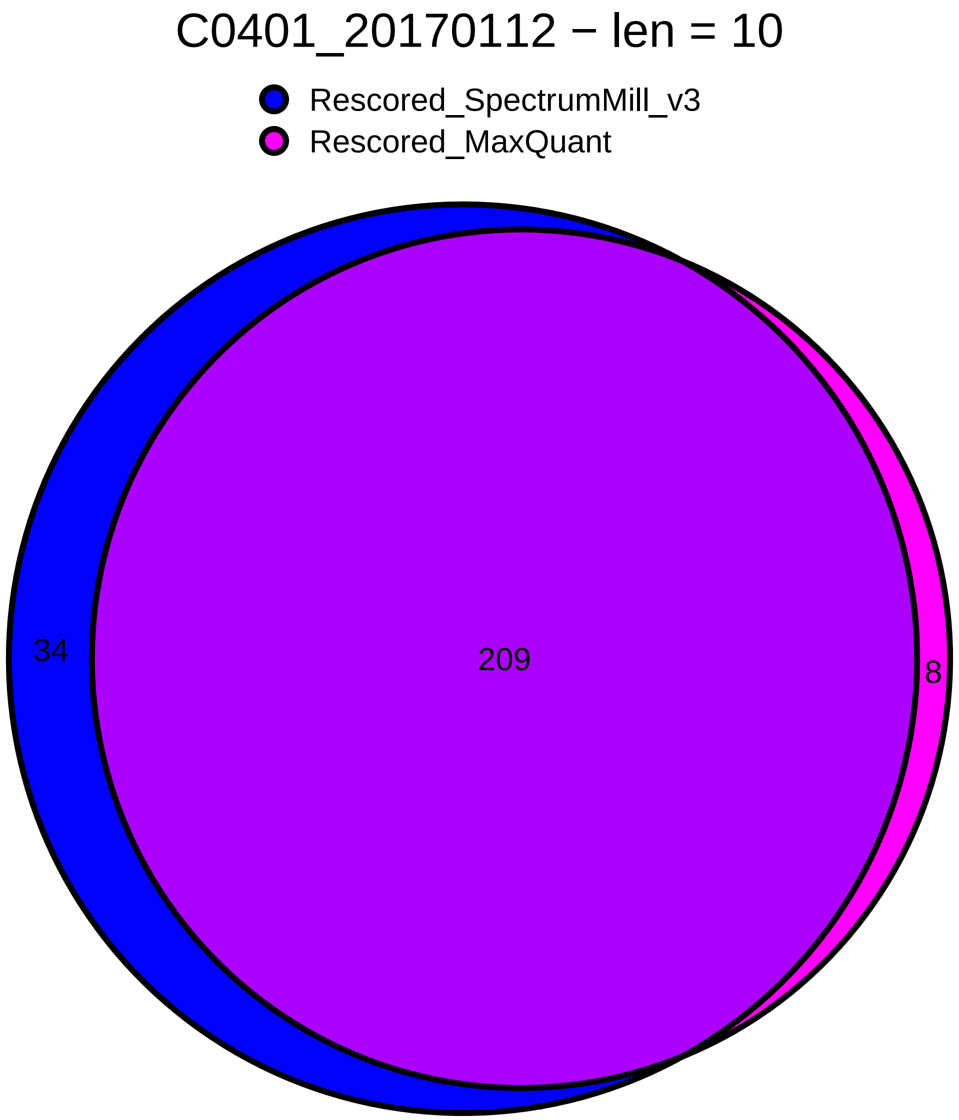
C0401_20170112 − len = 10
Rescored_SpectrumMill_v3
Rescored_MaxQuant
34
209
8

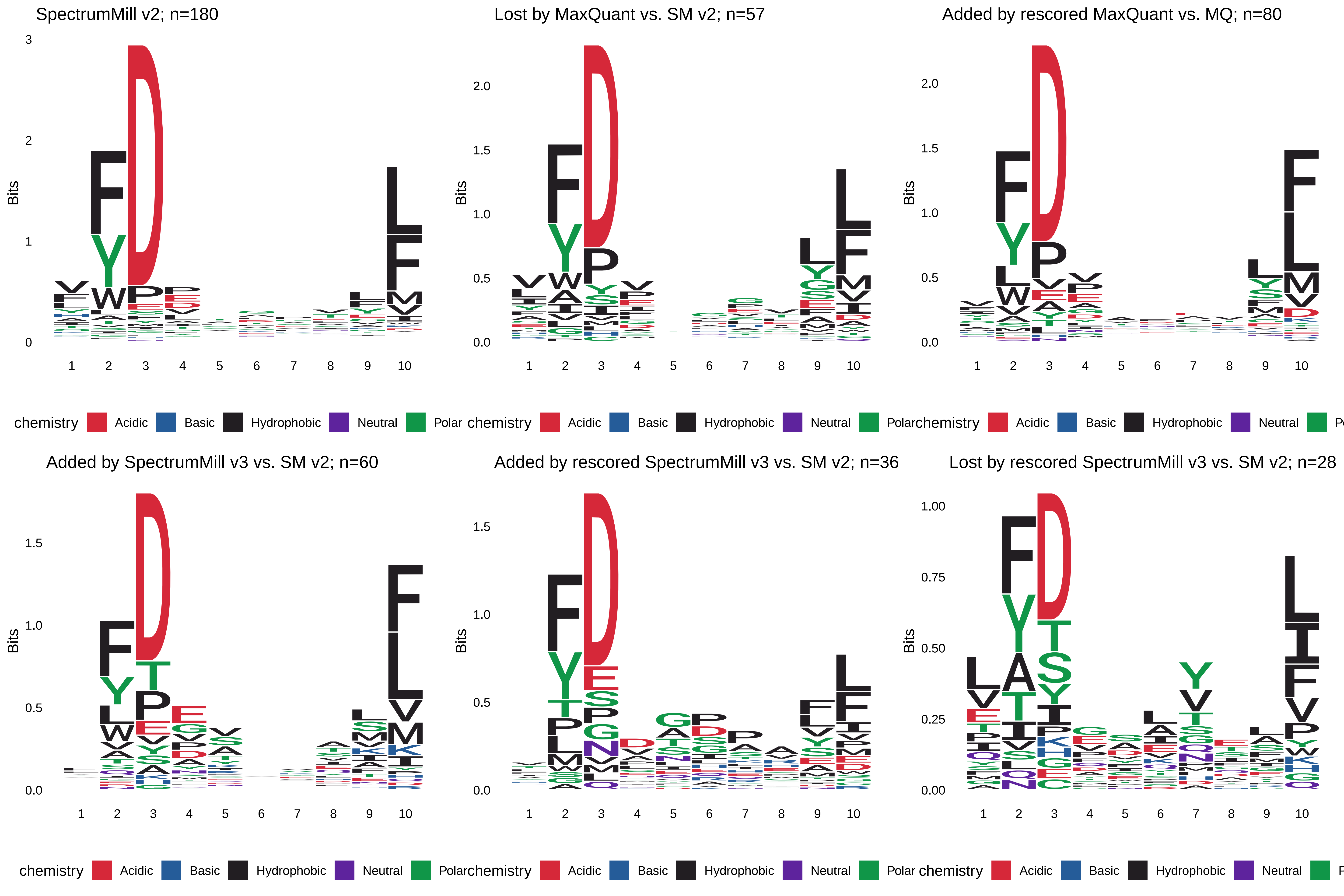
SpectrumMill v2; n=180
Lost by MaxQuant vs. SM v2; n=57
Added by rescored MaxQuant vs. MQ; n=80
Added by SpectrumMill v3 vs. SM v2; n=60
Added by rescored SpectrumMill v3 vs. SM v2; n=36
Lost by rescored SpectrumMill v3 vs. SM v2; n=28
Bits
1 2 3 4 5 6 7 8 9 10
chemistry
Acidic
Basic
Hydrophobic
Neutral
Polar

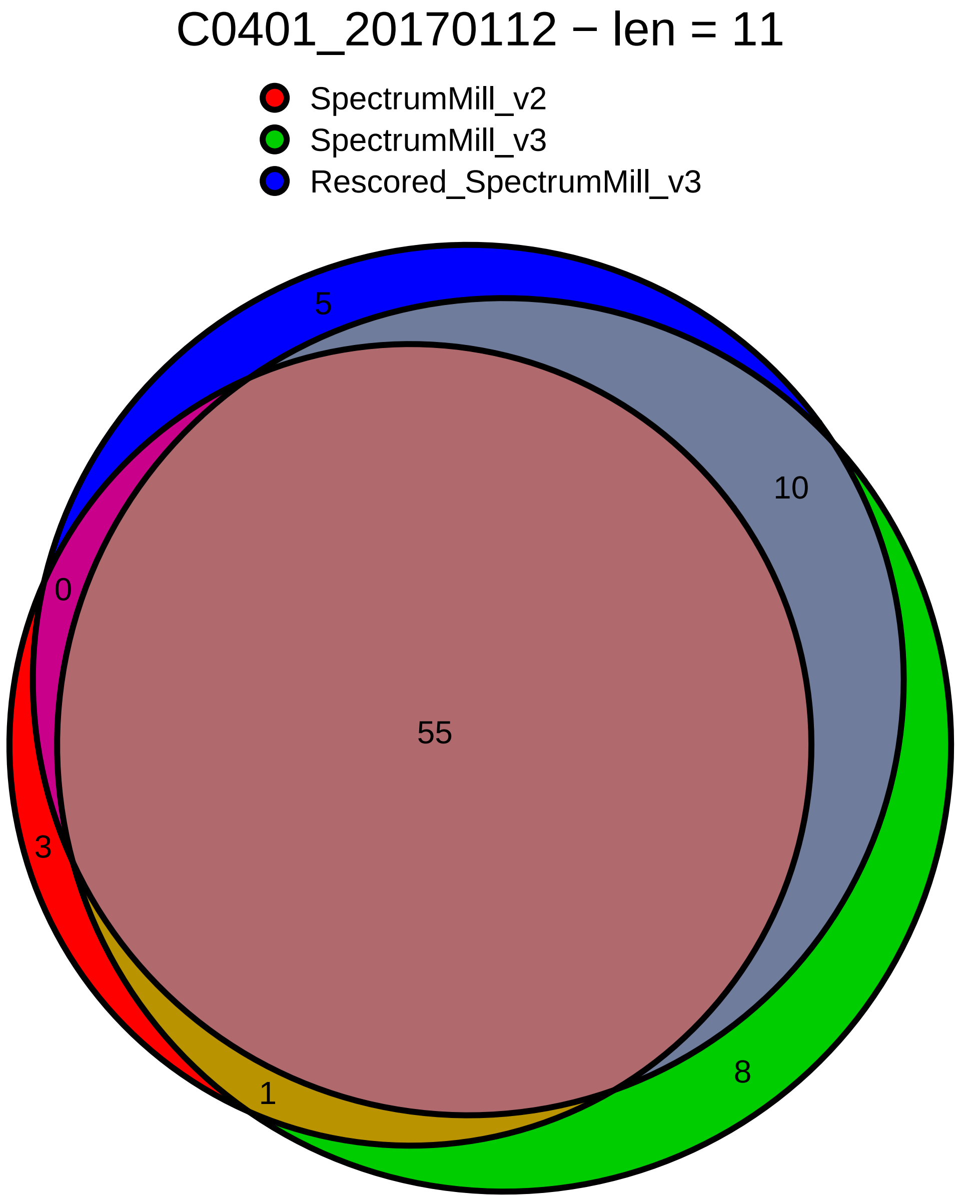
C0401_20170112 − len = 11
SpectrumMill_v2
SpectrumMill_v3
Rescored_SpectrumMill_v3
5
10
0
55
3
1
8

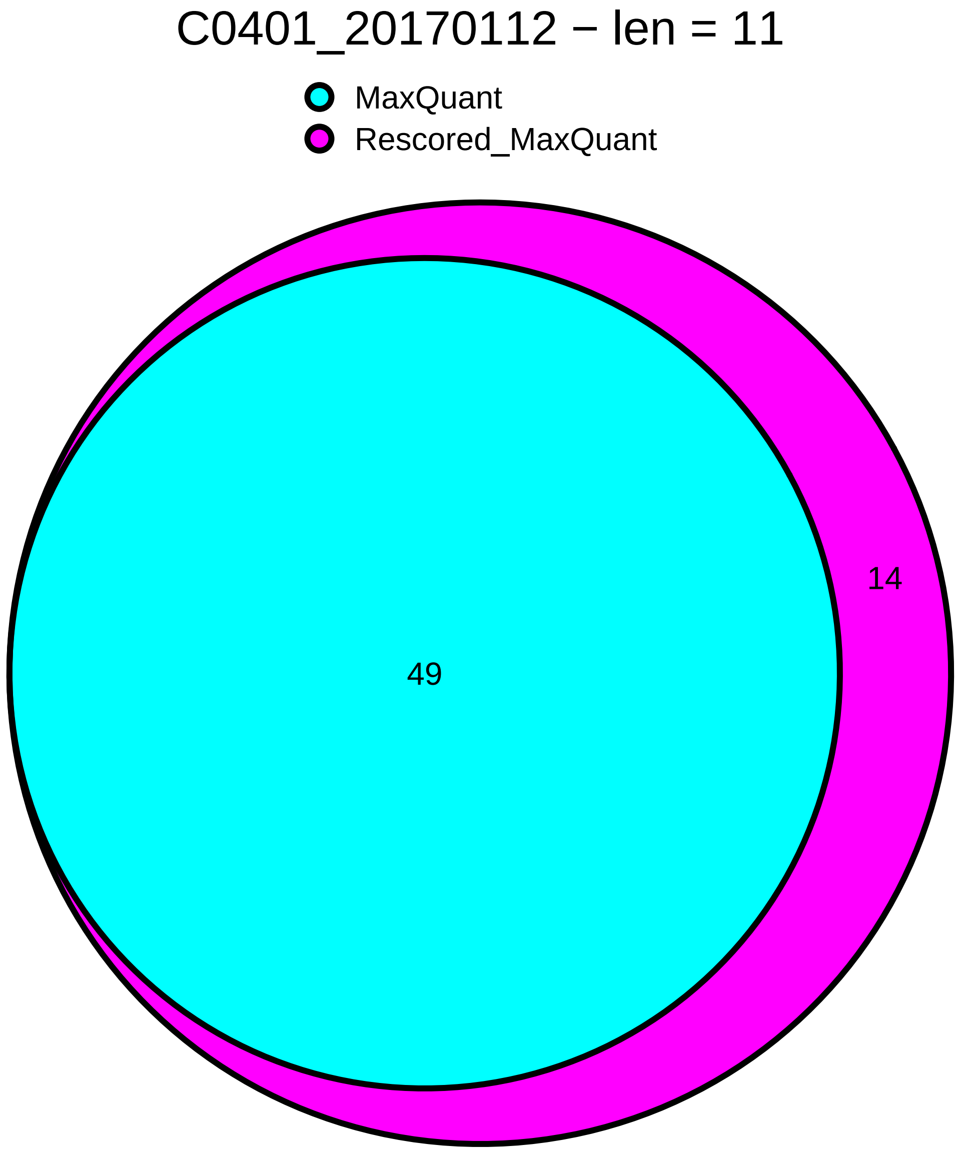
C0401_20170112 − len = 11
MaxQuant
Rescored_MaxQuant
49
14

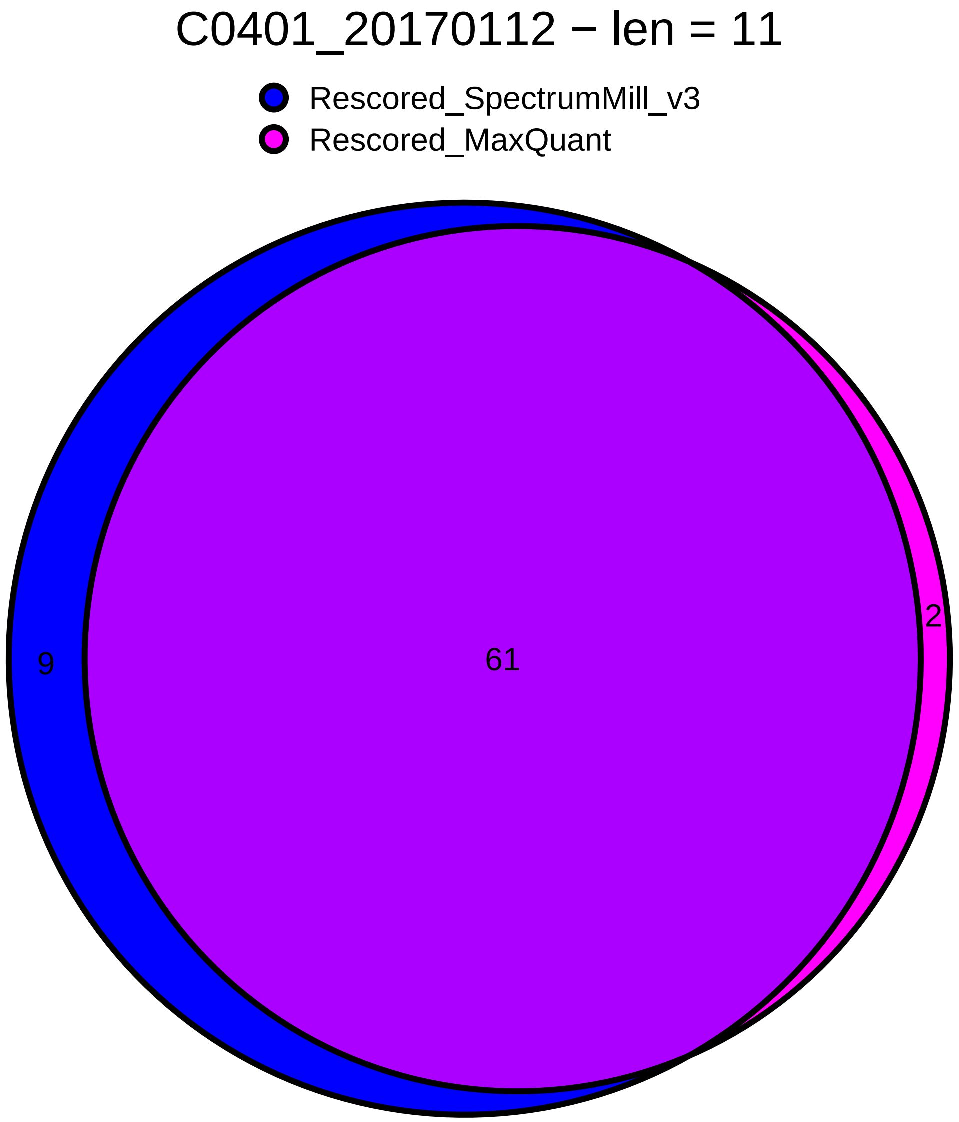
C0401_20170112 − len = 11
Rescored_SpectrumMill_v3
Rescored_MaxQuant
9
61
2

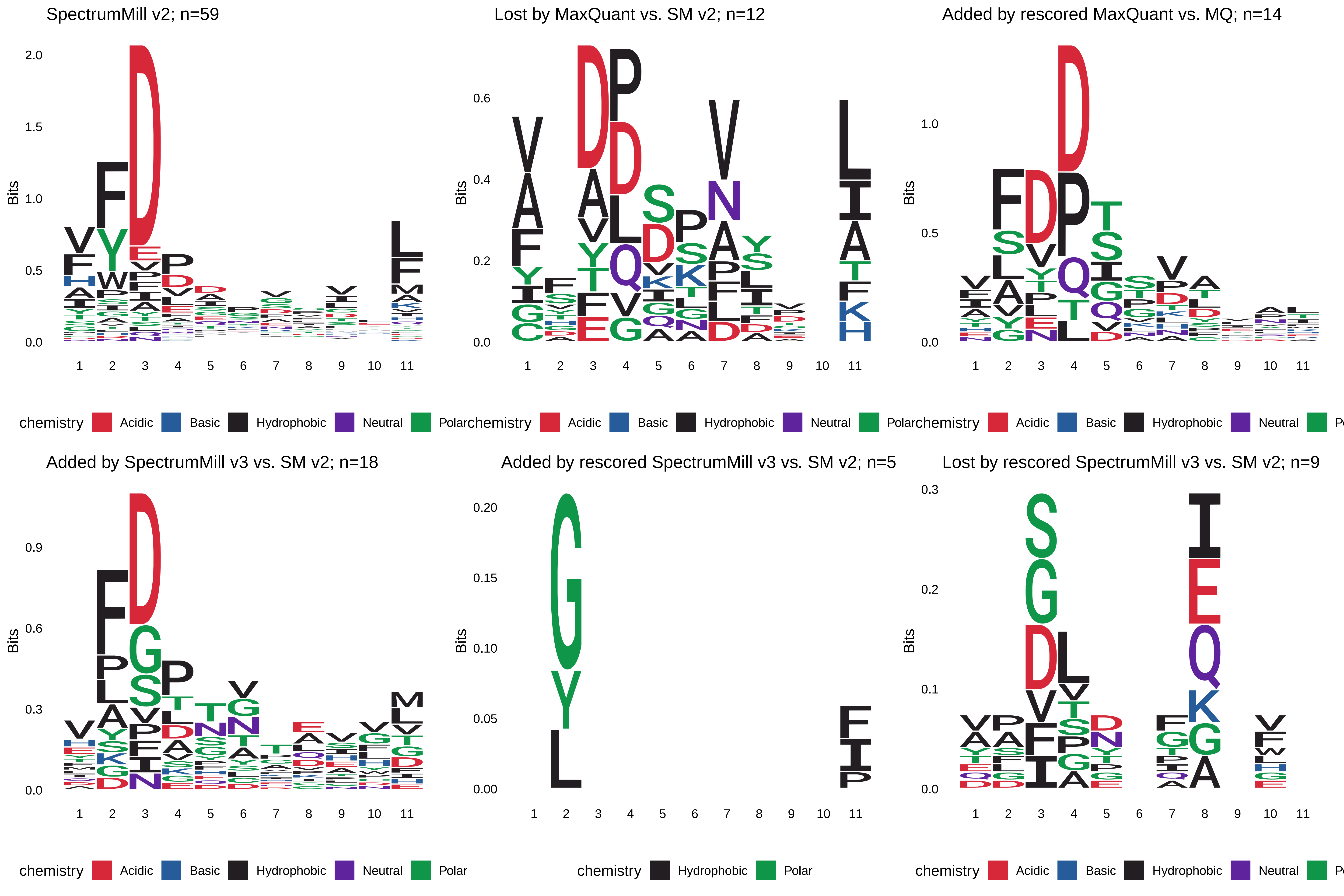
SpectrumMill v2; n=59
Lost by MaxQuant vs. SM v2; n=12
Added by rescored MaxQuant vs. MQ; n=14
Added by SpectrumMill v3 vs. SM v2; n=18
Added by rescored SpectrumMill v3 vs. SM v2; n=5
Lost by rescored SpectrumMill v3 vs. SM v2; n=9
Bits
1 2 3 4 5 6 7 8 9 10 11
chemistry Acidic Basic Hydrophobic Neutral Polar
chemistry Hydrophobic Polar

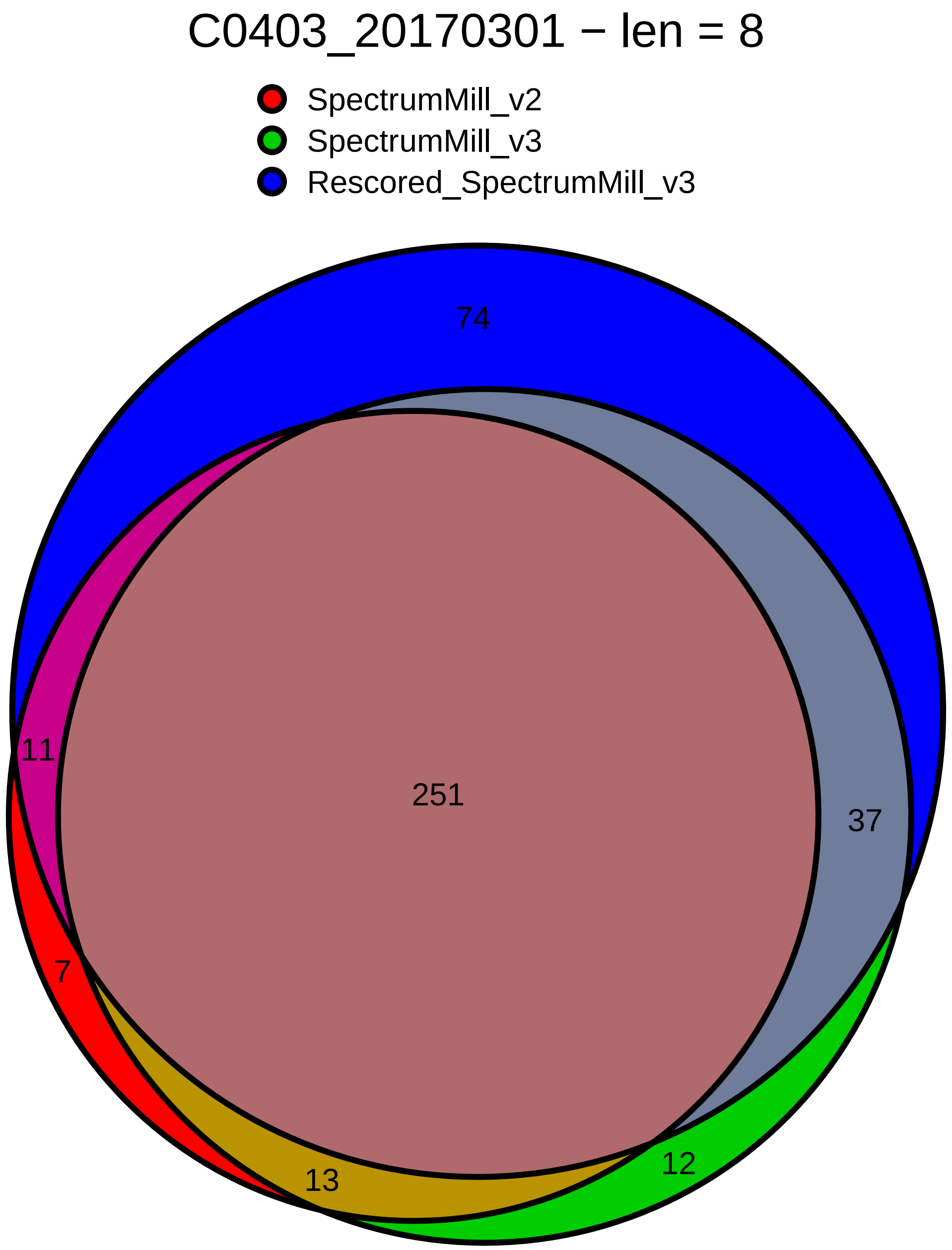
C0403_20170301 − len = 8
SpectrumMill_v2
SpectrumMill_v3
Rescored_SpectrumMill_v3
74
11
251
37
7
13
12

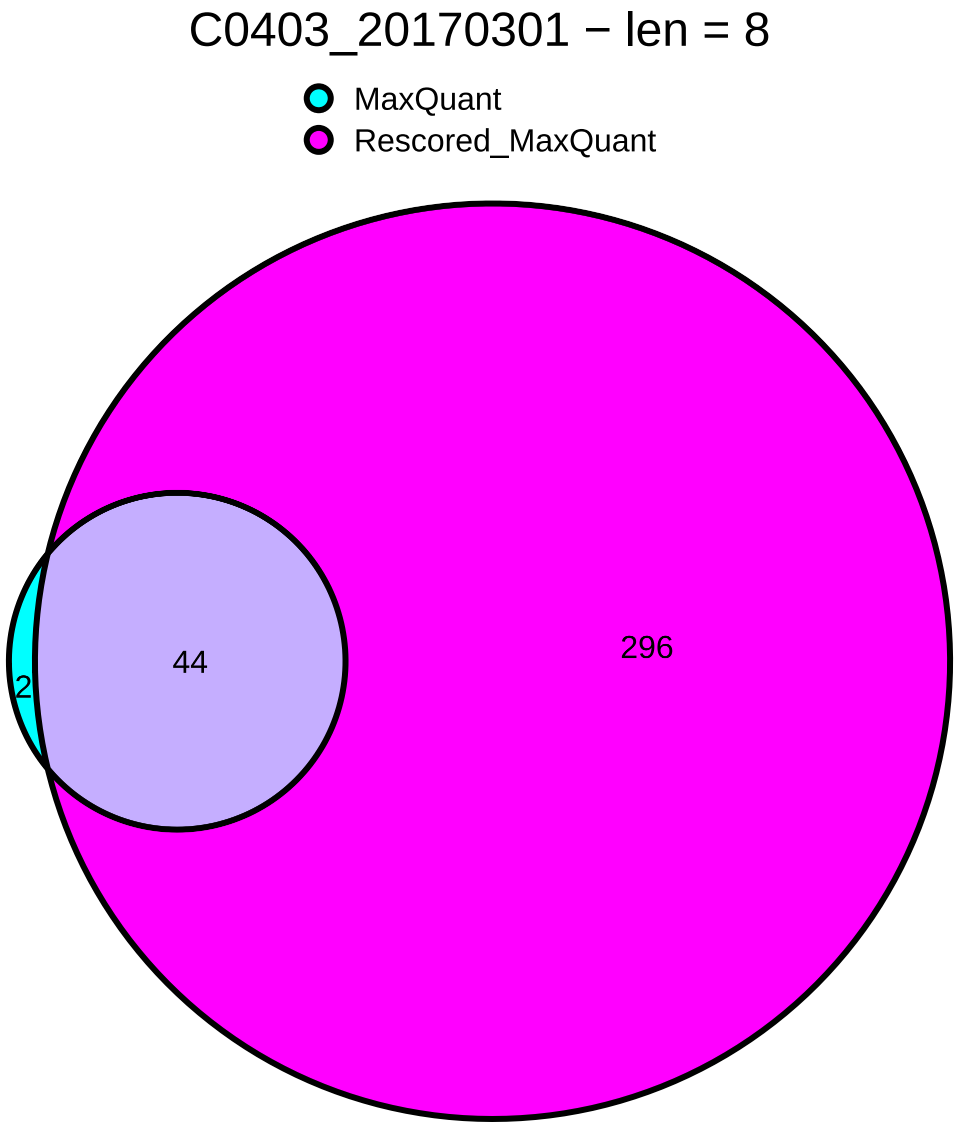
C0403_20170301 − len = 8
MaxQuant
Rescored_MaxQuant
2
44
296

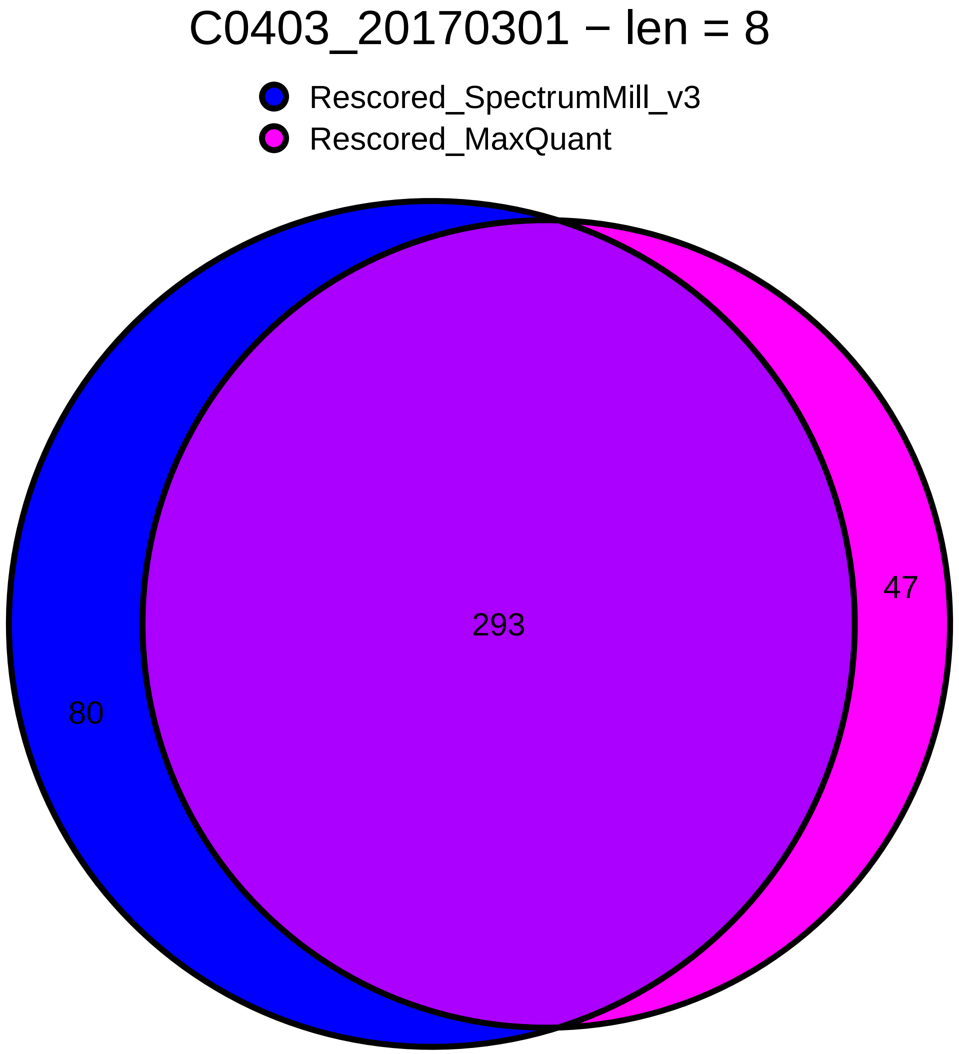
C0403_20170301 − len = 8
Rescored_SpectrumMill_v3
Rescored_MaxQuant
80
293
47

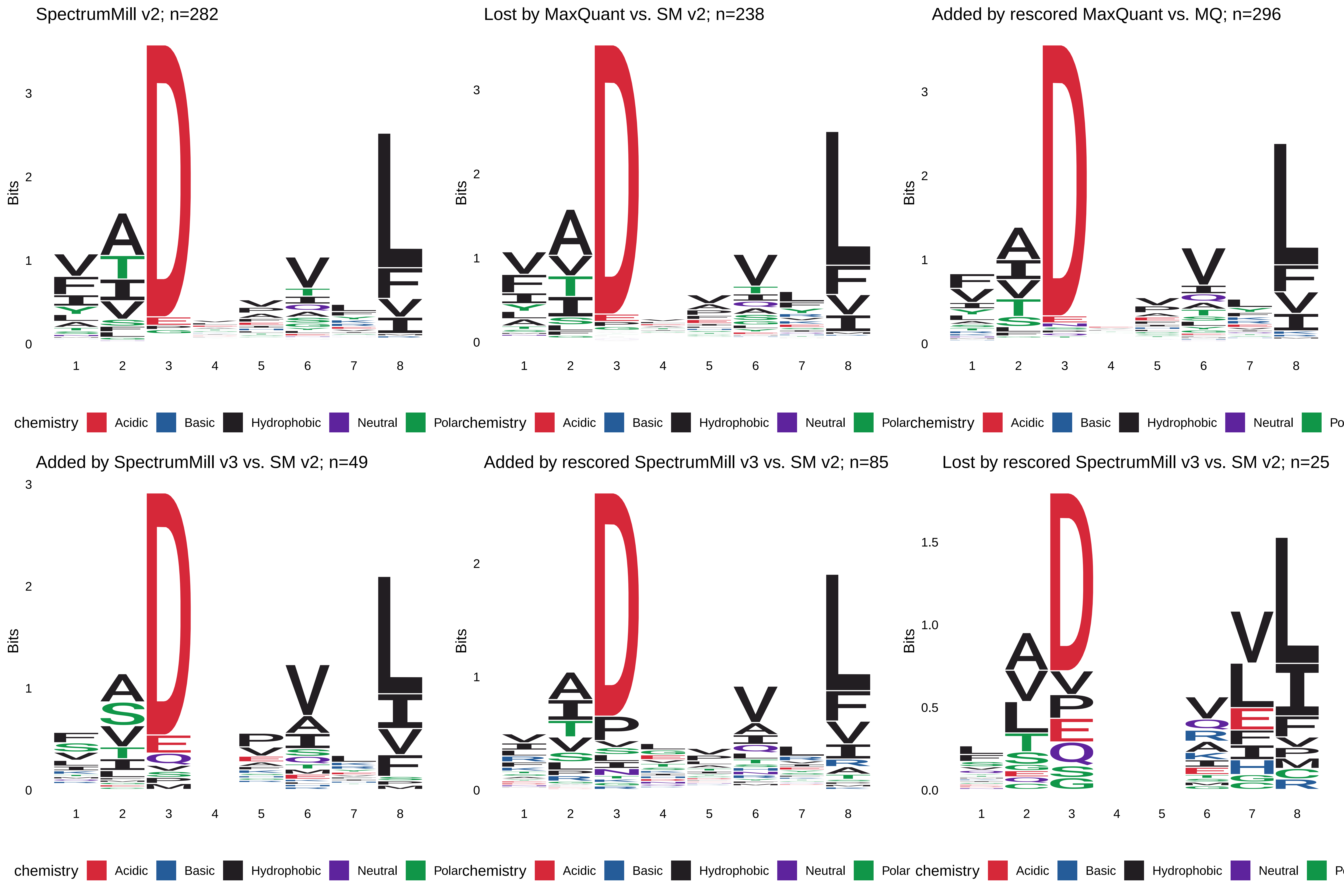
SpectrumMill v2; n=282
Lost by MaxQuant vs. SM v2; n=238
Added by rescored MaxQuant vs. MQ; n=296
Added by SpectrumMill v3 vs. SM v2; n=49
Added by rescored SpectrumMill v3 vs. SM v2; n=85
Lost by rescored SpectrumMill v3 vs. SM v2; n=25
Bits
1
2
3
4
5
6
7
8
chemistry
Acidic
Basic
Hydrophobic
Neutral
Polar

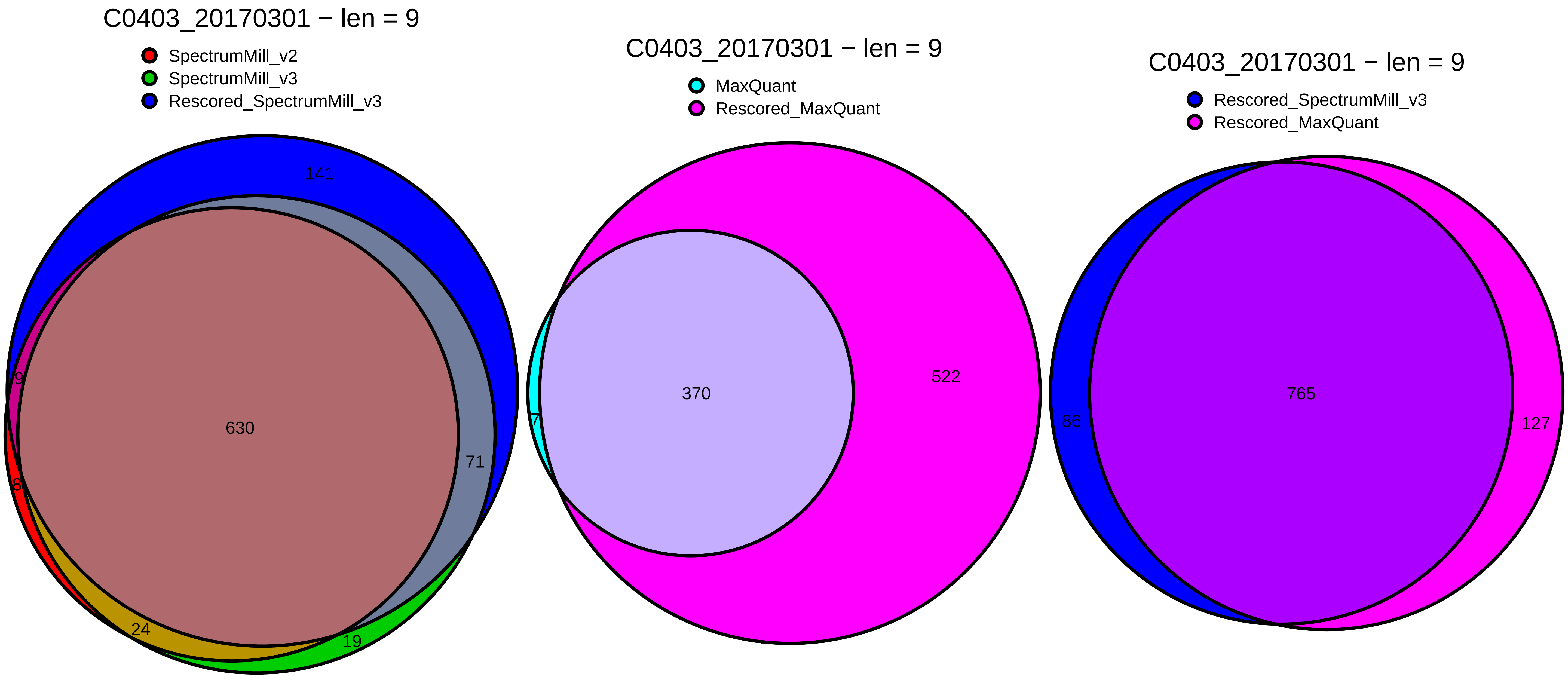
C0403_20170301 − len = 9
SpectrumMill_v2
SpectrumMill_v3
Rescored_SpectrumMill_v3
141
9
630
71
8
24
19
C0403_20170301 − len = 9
MaxQuant
Rescored_MaxQuant
7
370
522
C0403_20170301 − len = 9
Rescored_SpectrumMill_v3
Rescored_MaxQuant
86
765
127

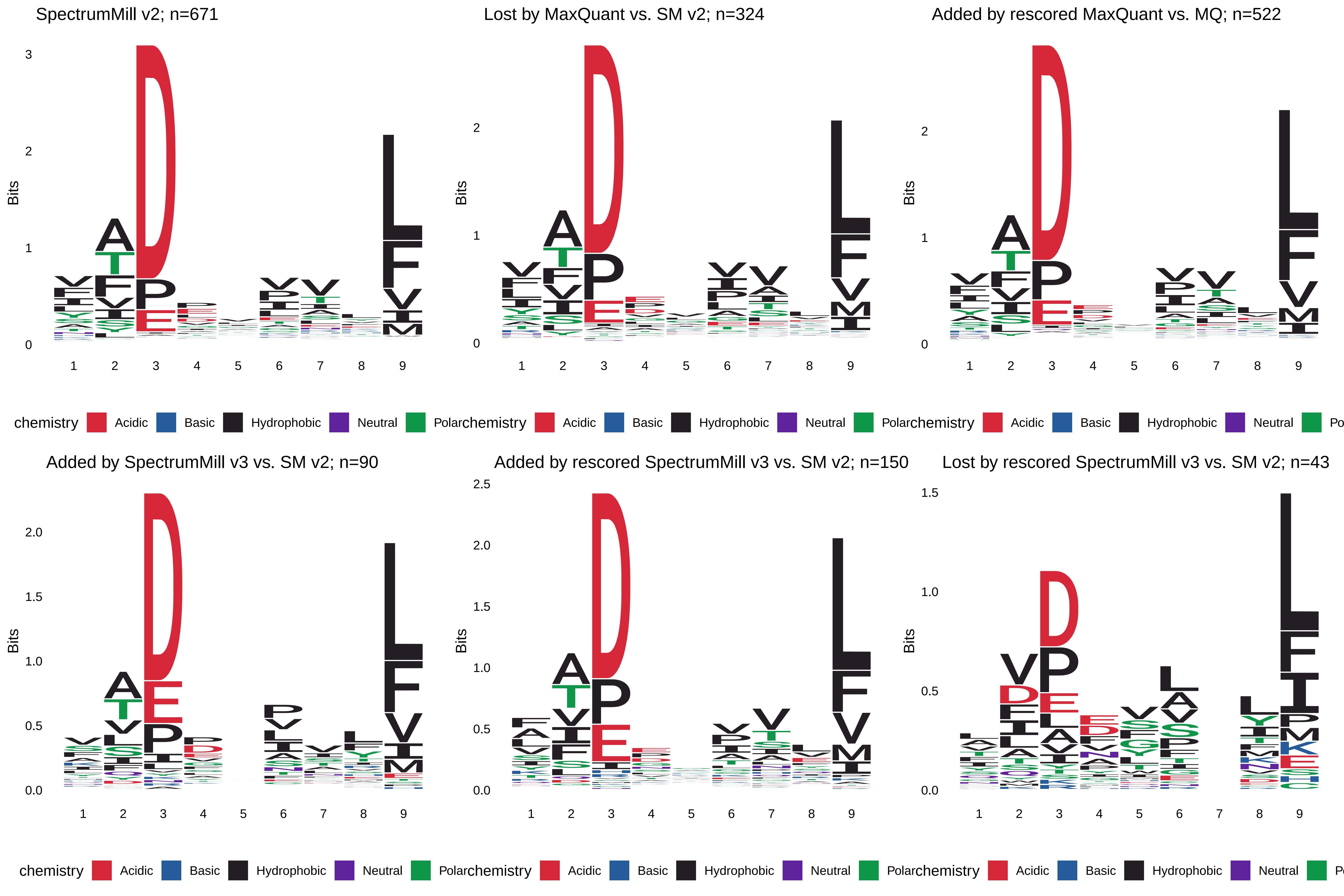
SpectrumMill v2; n=671
Lost by MaxQuant vs. SM v2; n=324
Added by rescored MaxQuant vs. MQ; n=522
Added by SpectrumMill v3 vs. SM v2; n=90
Added by rescored SpectrumMill v3 vs. SM v2; n=150
Lost by rescored SpectrumMill v3 vs. SM v2; n=43
Bits
1 2 3 4 5 6 7 8 9
chemistry Acidic Basic Hydrophobic Neutral Polar

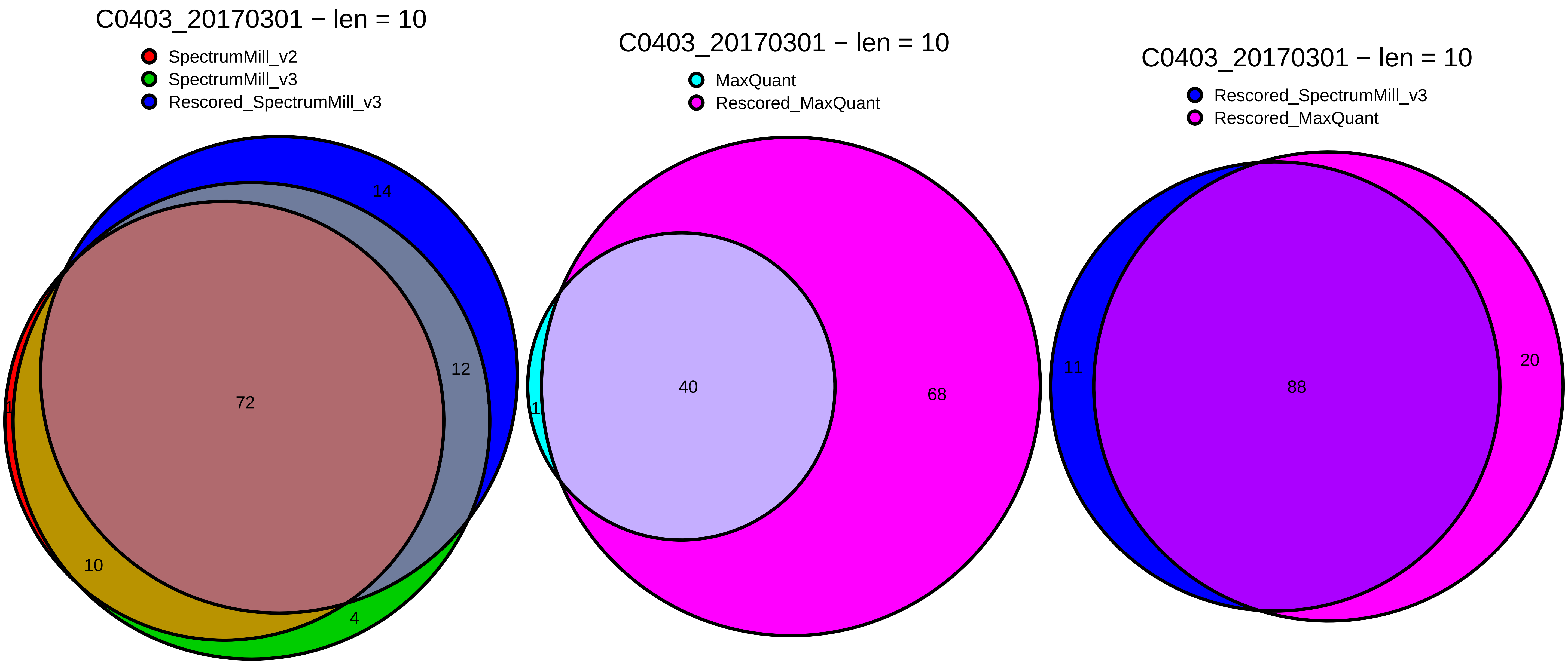

C0403_20170301 − len = 10
SpectrumMill_v2
SpectrumMill_v3
Rescored_SpectrumMill_v3
14
12
1
72
10
4
C0403_20170301 − len = 10
MaxQuant
Rescored_MaxQuant
1
40
68
C0403_20170301 − len = 10
Rescored_SpectrumMill_v3
Rescored_MaxQuant
11
88
20

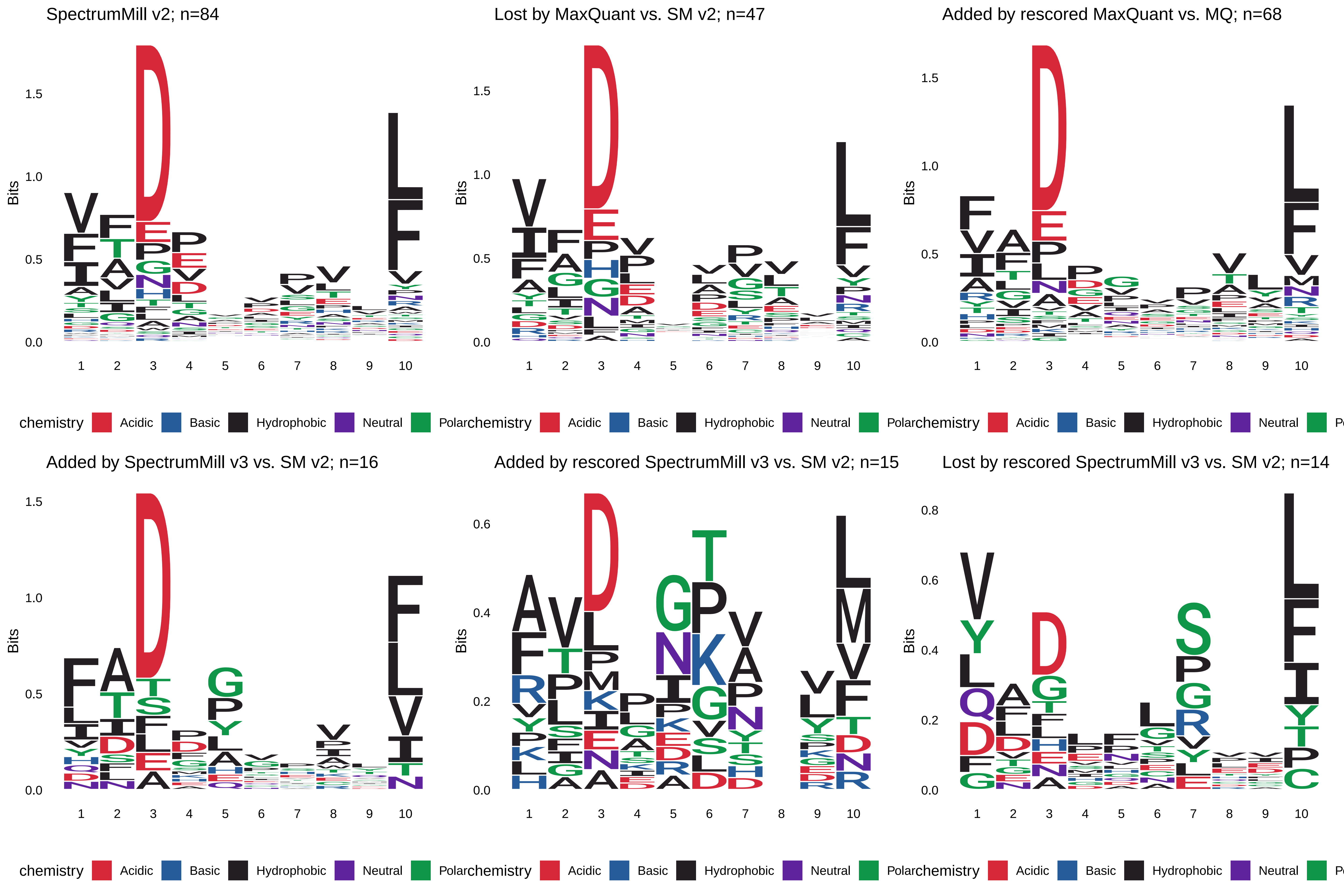
SpectrumMill v2; n=84
Lost by MaxQuant vs. SM v2; n=47
Added by rescored MaxQuant vs. MQ; n=68
Bits
1.5
1.0
0.5
0.0
1 2 3 4 5 6 7 8 9 10
chemistry Acidic Basic Hydrophobic Neutral Polarchemistry Acidic Basic Hydrophobic Neutral Polarchemistry Acidic Basic Hydrophobic Neutral Po
Added by SpectrumMill v3 vs. SM v2; n=16
Added by rescored SpectrumMill v3 vs. SM v2; n=15
Lost by rescored SpectrumMill v3 vs. SM v2; n=14
0.8
0.6
0.4
0.2
chemistry Acidic Basic Hydrophobic Neutral Polarchemistry Acidic Basic Hydrophobic Neutral Polarchemistry Acidic Basic Hydrophobic Neutral Po

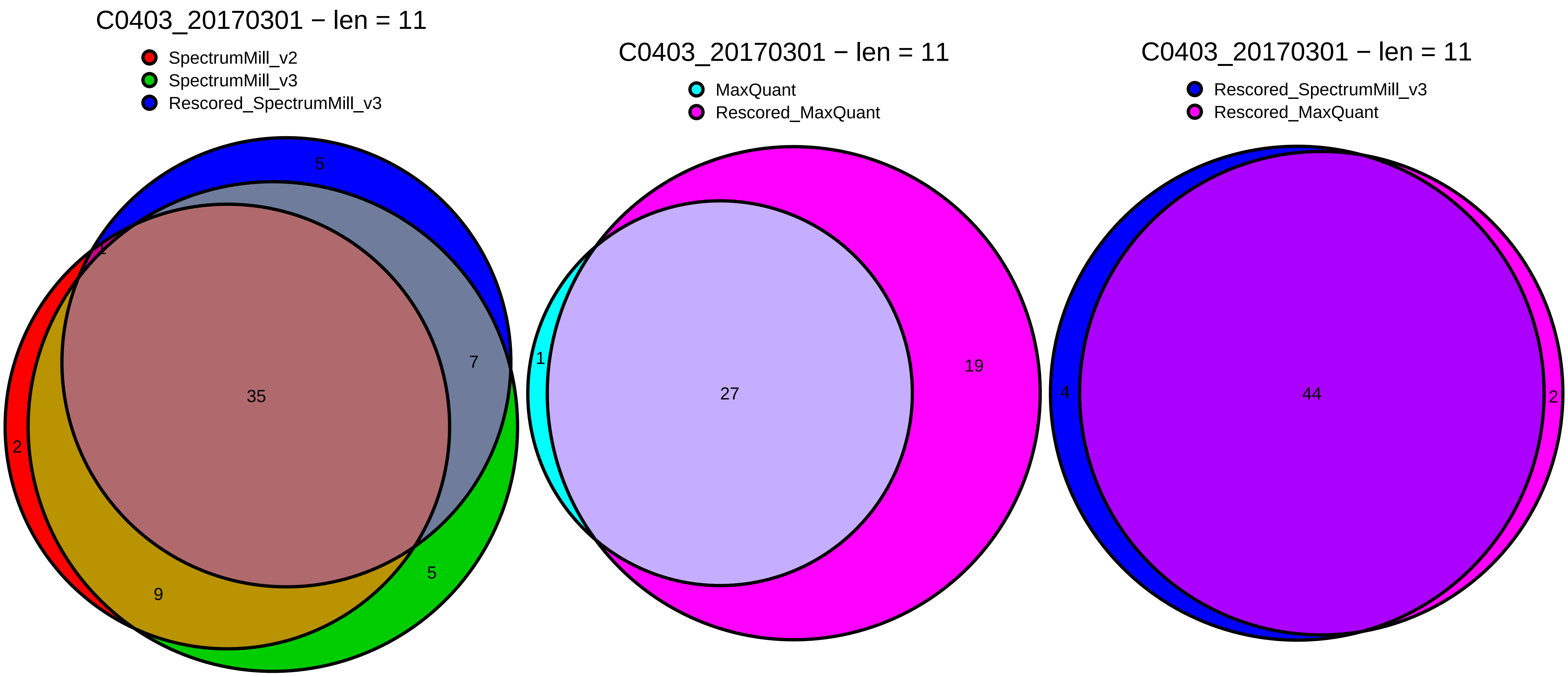

C0403_20170301 − len = 11
SpectrumMill_v2
SpectrumMill_v3
Rescored_SpectrumMill_v3
5
1
7
35
2
9
5
C0403_20170301 − len = 11
MaxQuant
Rescored_MaxQuant
1
27
19
C0403_20170301 − len = 11
Rescored_SpectrumMill_v3
Rescored_MaxQuant
4
44
2

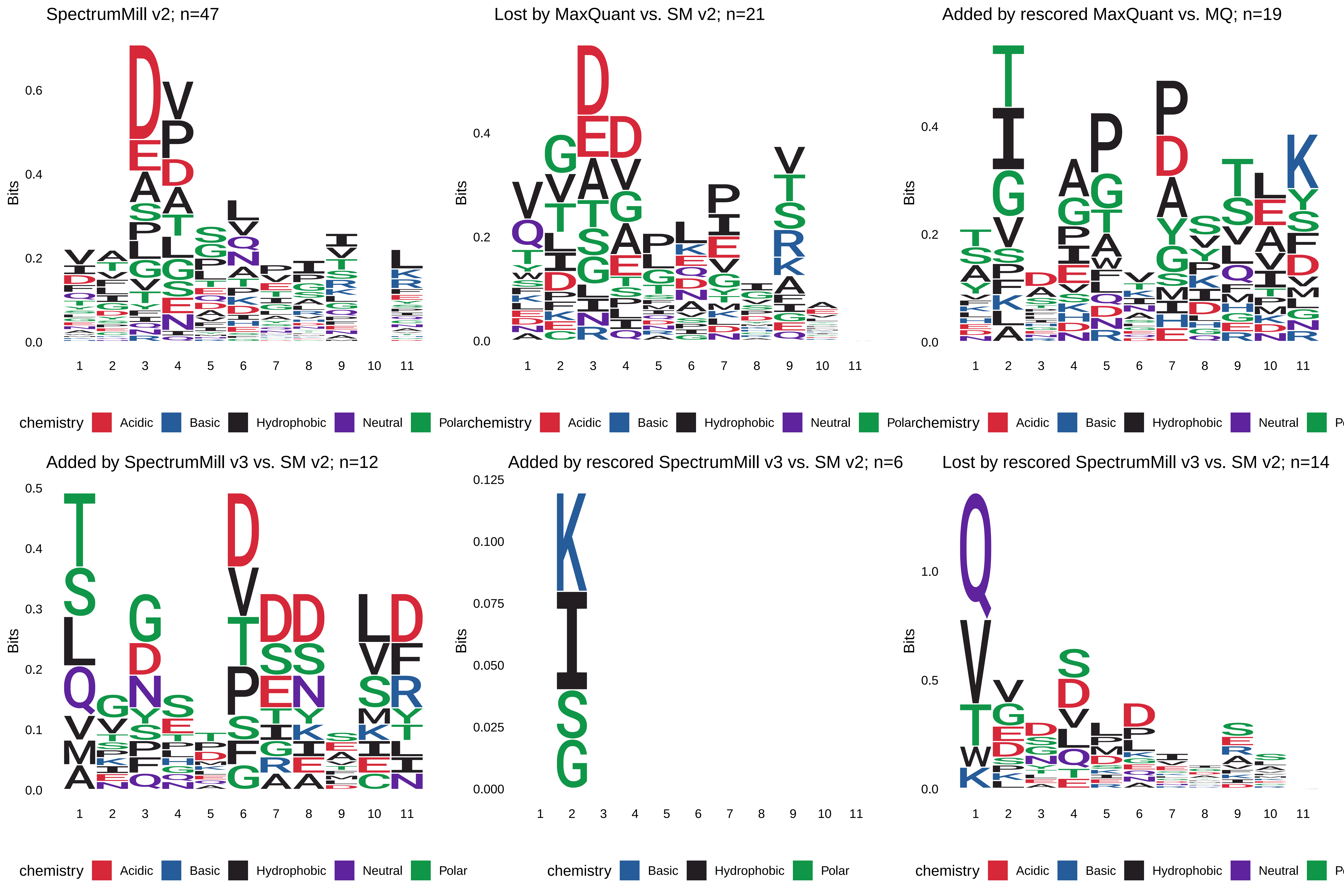
SpectrumMill v2; n=47
Lost by MaxQuant vs. SM v2; n=21
Added by rescored MaxQuant vs. MQ; n=19
Added by SpectrumMill v3 vs. SM v2; n=12
Added by rescored SpectrumMill v3 vs. SM v2; n=6
Lost by rescored SpectrumMill v3 vs. SM v2; n=14
Bits
1 2 3 4 5 6 7 8 9 10 11
chemistry Acidic Basic Hydrophobic Neutral Polar
chemistry Basic Hydrophobic Polar

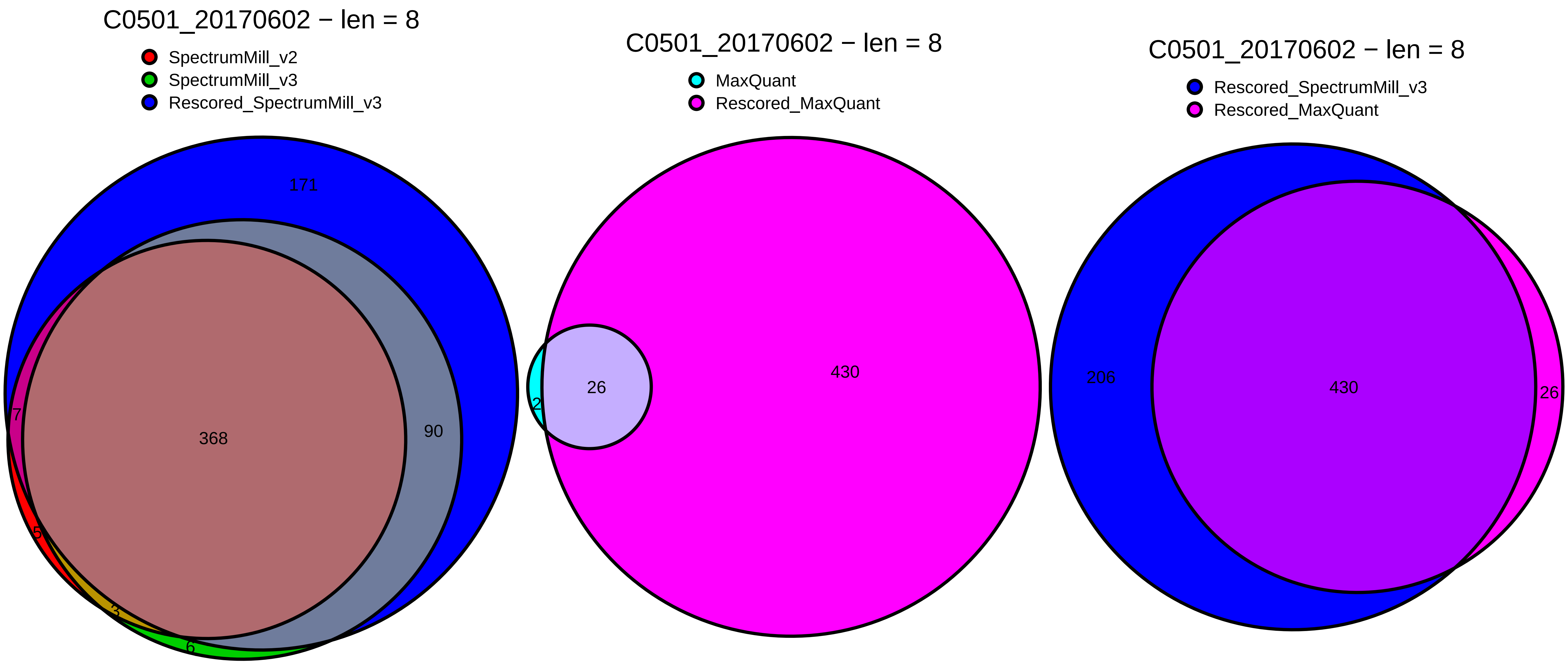
C0501_20170602 − len = 8
SpectrumMill_v2
SpectrumMill_v3
Rescored_SpectrumMill_v3
171
7
368
90
5
3
6
C0501_20170602 − len = 8
MaxQuant
Rescored_MaxQuant
2
26
430
C0501_20170602 − len = 8
Rescored_SpectrumMill_v3
Rescored_MaxQuant
206
430
26

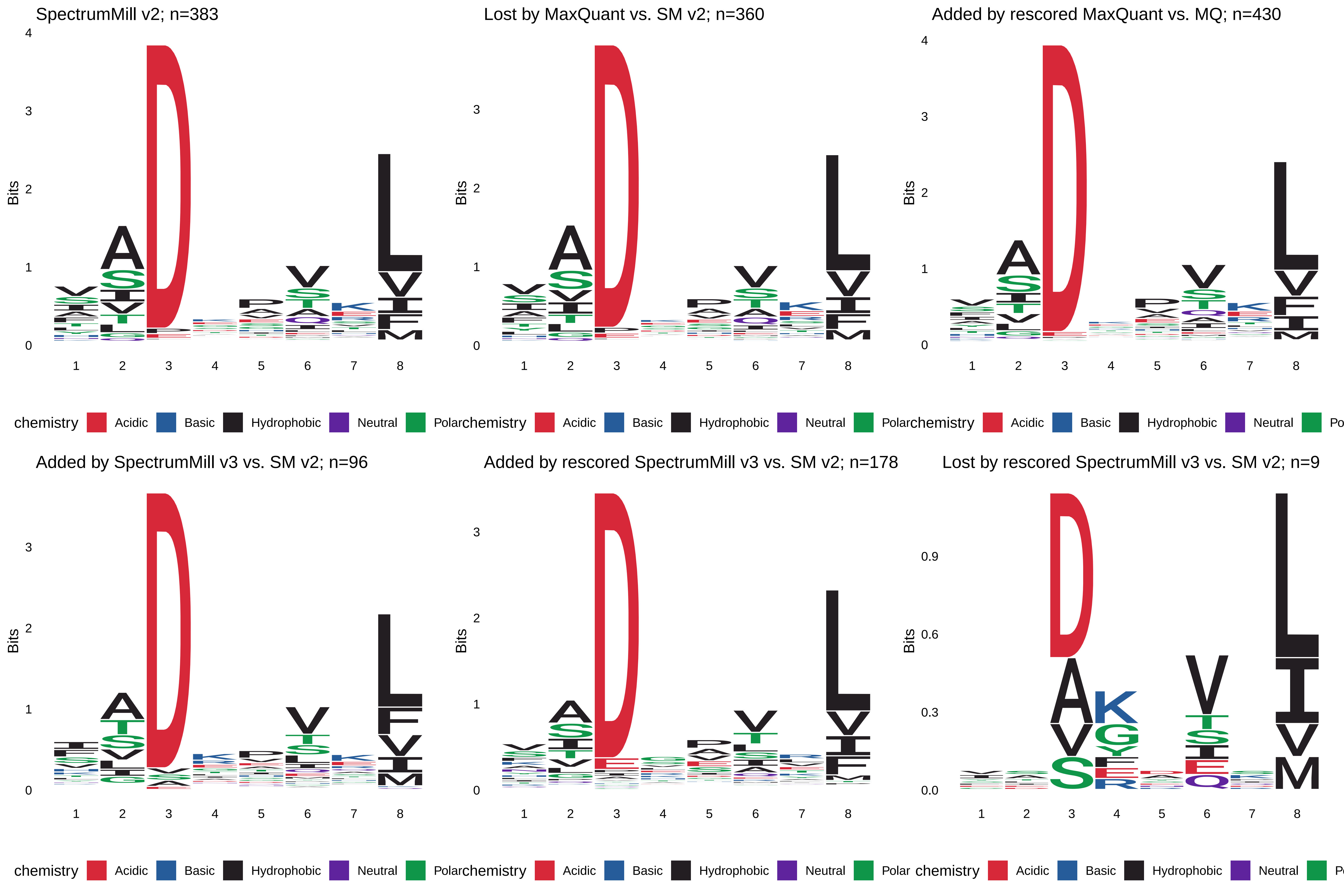
SpectrumMill v2; n=383
Lost by MaxQuant vs. SM v2; n=360
Added by rescored MaxQuant vs. MQ; n=430
Added by SpectrumMill v3 vs. SM v2; n=96
Added by rescored SpectrumMill v3 vs. SM v2; n=178
Lost by rescored SpectrumMill v3 vs. SM v2; n=9
Bits
chemistry
Acidic
Basic
Hydrophobic
Neutral
Polar

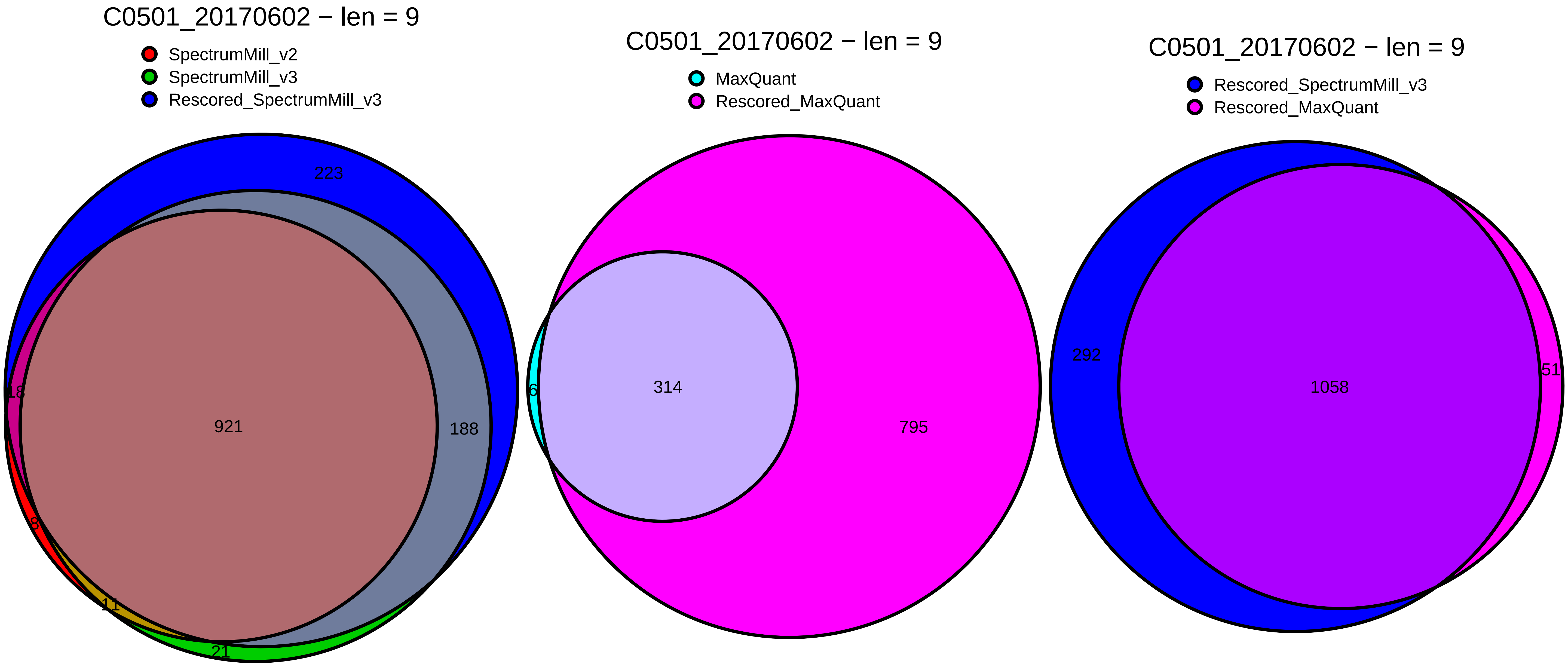
C0501_20170602 − len = 9
SpectrumMill_v2
SpectrumMill_v3
Rescored_SpectrumMill_v3
223
18
921
188
8
11
21
C0501_20170602 − len = 9
MaxQuant
Rescored_MaxQuant
6
314
795
C0501_20170602 − len = 9
Rescored_SpectrumMill_v3
Rescored_MaxQuant
292
1058
51

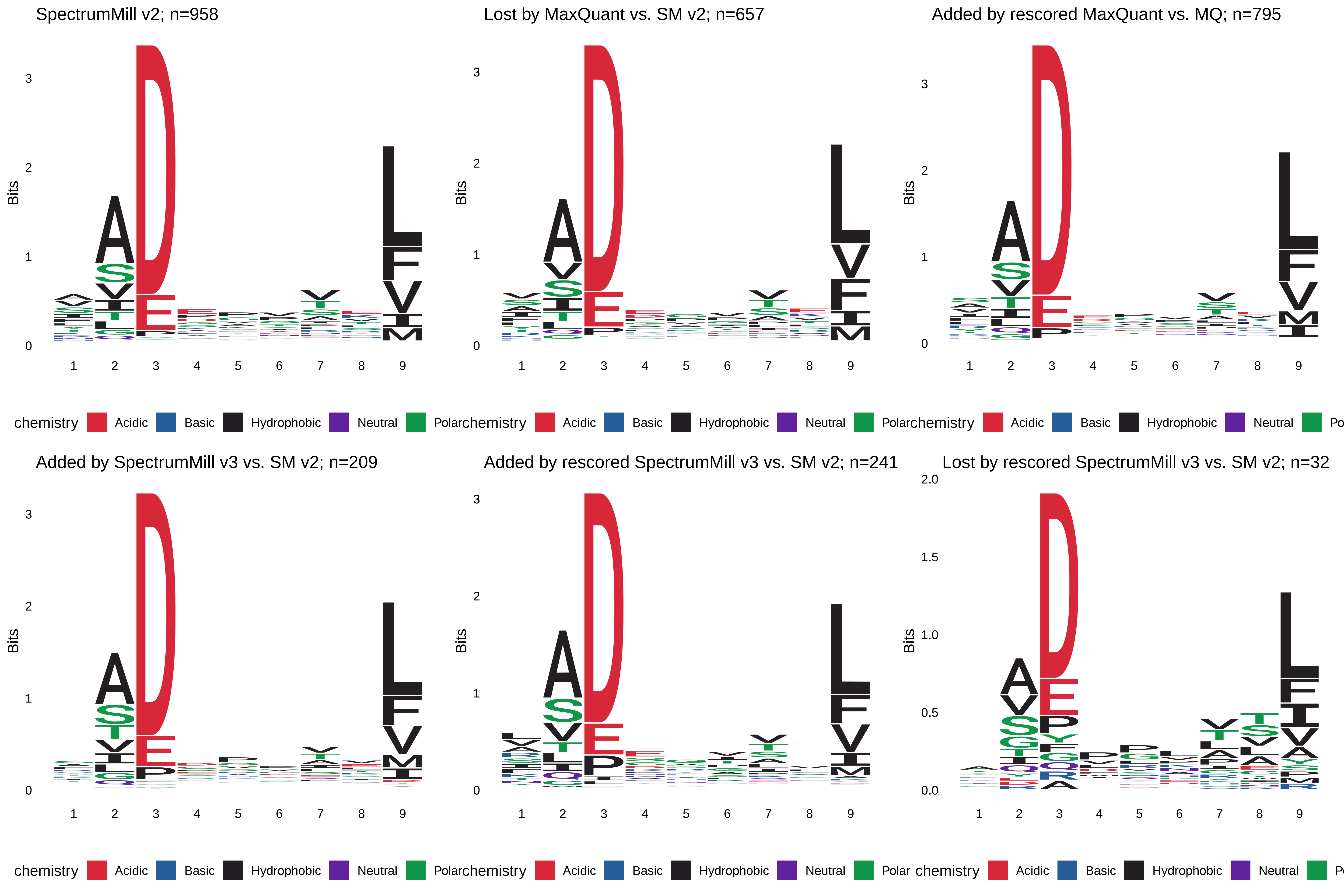
SpectrumMill v2; n=958
Lost by MaxQuant vs. SM v2; n=657
Added by rescored MaxQuant vs. MQ; n=795
Added by SpectrumMill v3 vs. SM v2; n=209
Added by rescored SpectrumMill v3 vs. SM v2; n=241
Lost by rescored SpectrumMill v3 vs. SM v2; n=32
Bits
0
1
2
3
0.0
0.5
1.0
1.5
2.0
1 2 3 4 5 6 7 8 9
chemistry
Acidic
Basic
Hydrophobic
Neutral
Polar

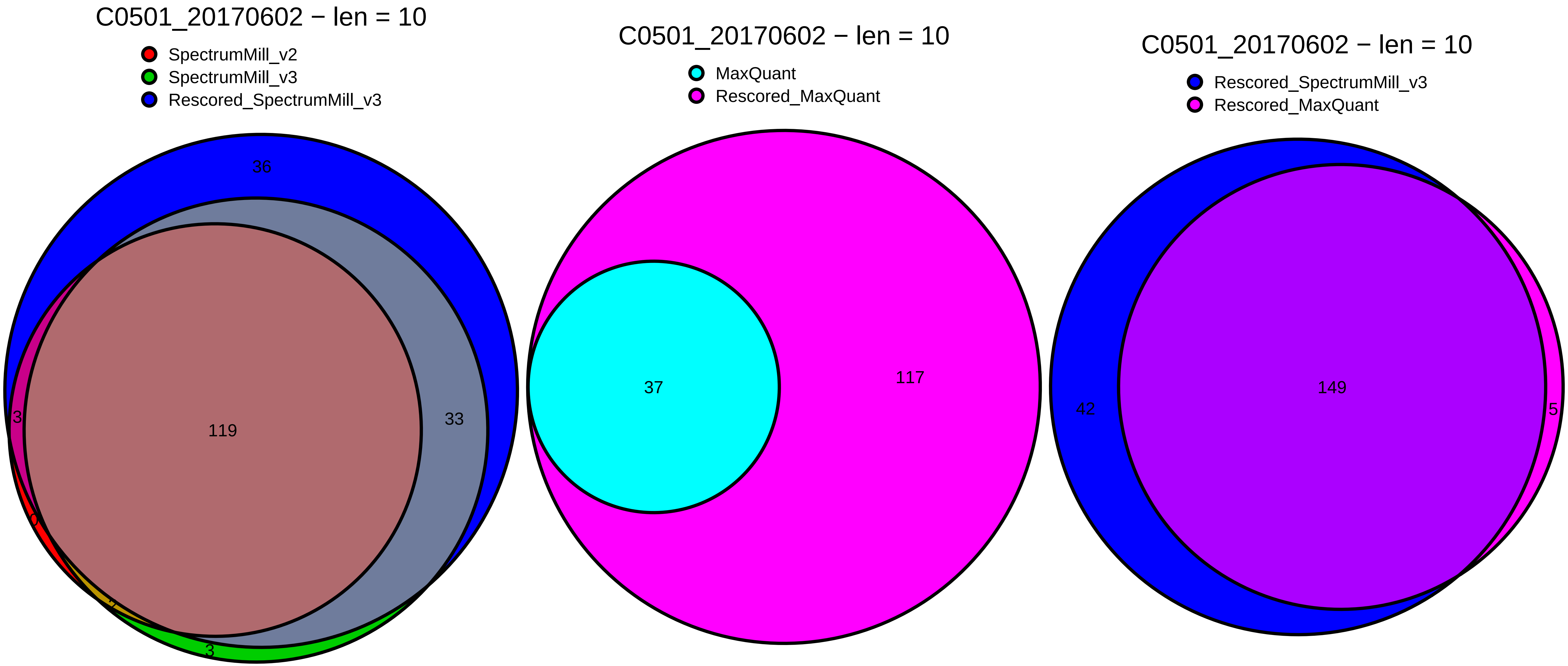
C0501_20170602 − len = 10
SpectrumMill_v2
SpectrumMill_v3
Rescored_SpectrumMill_v3
36
3
119
33
0
3
C0501_20170602 − len = 10
MaxQuant
Rescored_MaxQuant
37
117
C0501_20170602 − len = 10
Rescored_SpectrumMill_v3
Rescored_MaxQuant
42
149
5

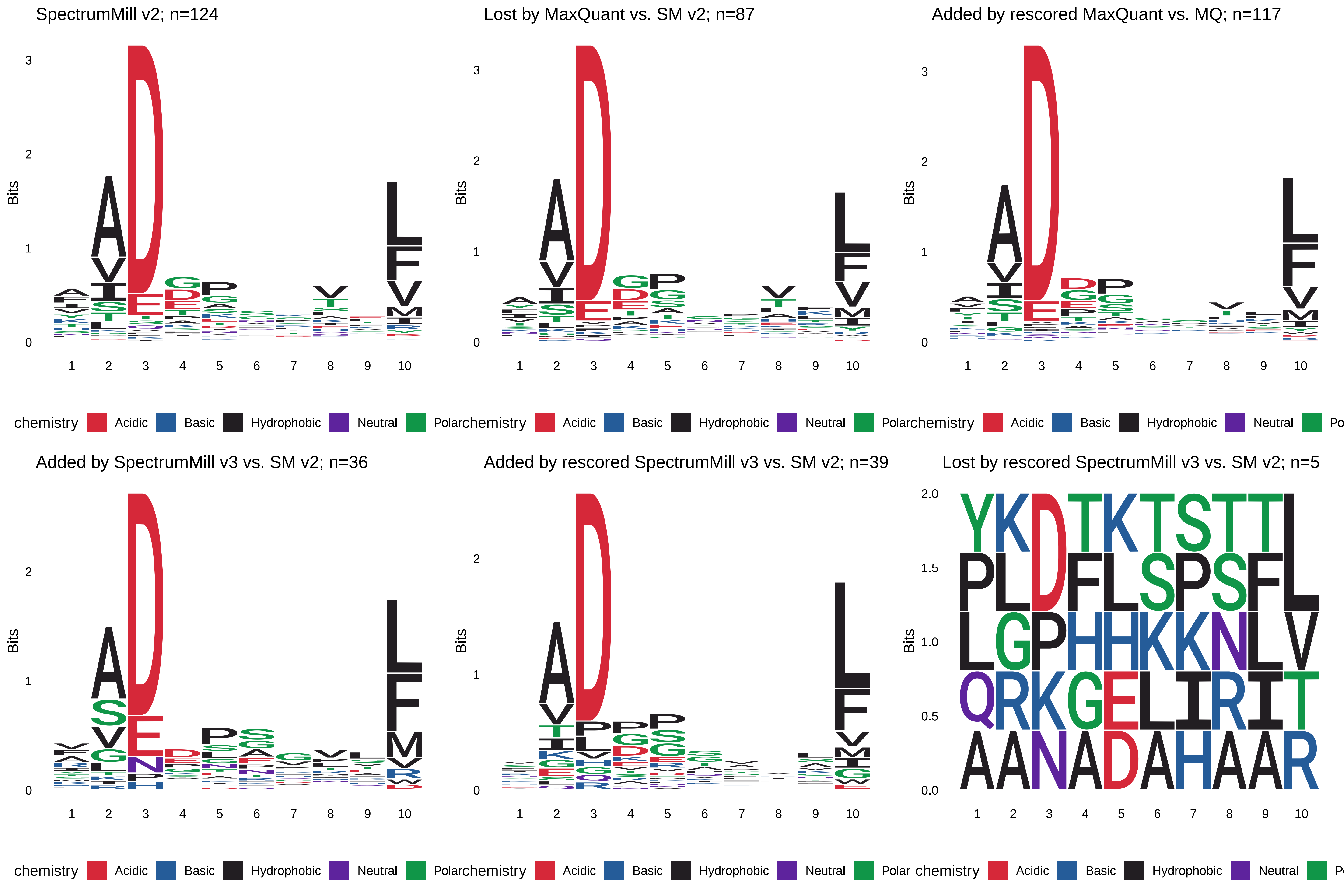
SpectrumMill v2; n=124
Lost by MaxQuant vs. SM v2; n=87
Added by rescored MaxQuant vs. MQ; n=117
Added by SpectrumMill v3 vs. SM v2; n=36
Added by rescored SpectrumMill v3 vs. SM v2; n=39
Lost by rescored SpectrumMill v3 vs. SM v2; n=5
Bits
chemistry Acidic Basic Hydrophobic Neutral Polarchemistry Acidic Basic Hydrophobic Neutral Polarchemistry Acidic Basic Hydrophobic Neutral Po
chemistry Acidic Basic Hydrophobic Neutral Polarchemistry Acidic Basic Hydrophobic Neutral Polar chemistry Acidic Basic Hydrophobic Neutral P

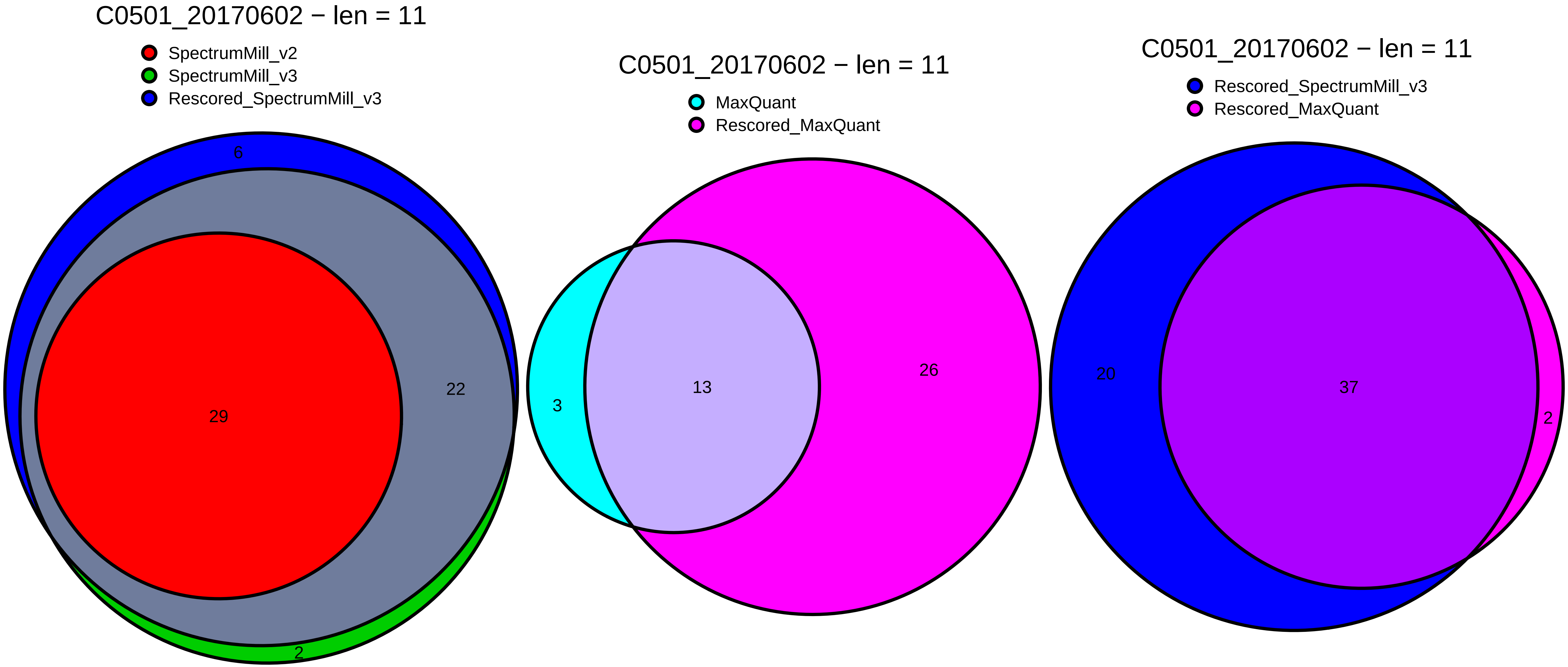
C0501_20170602 − len = 11
SpectrumMill_v2
SpectrumMill_v3
Rescored_SpectrumMill_v3
6
22
29
2
C0501_20170602 − len = 11
MaxQuant
Rescored_MaxQuant
3
13
26
C0501_20170602 − len = 11
Rescored_SpectrumMill_v3
Rescored_MaxQuant
20
37
2

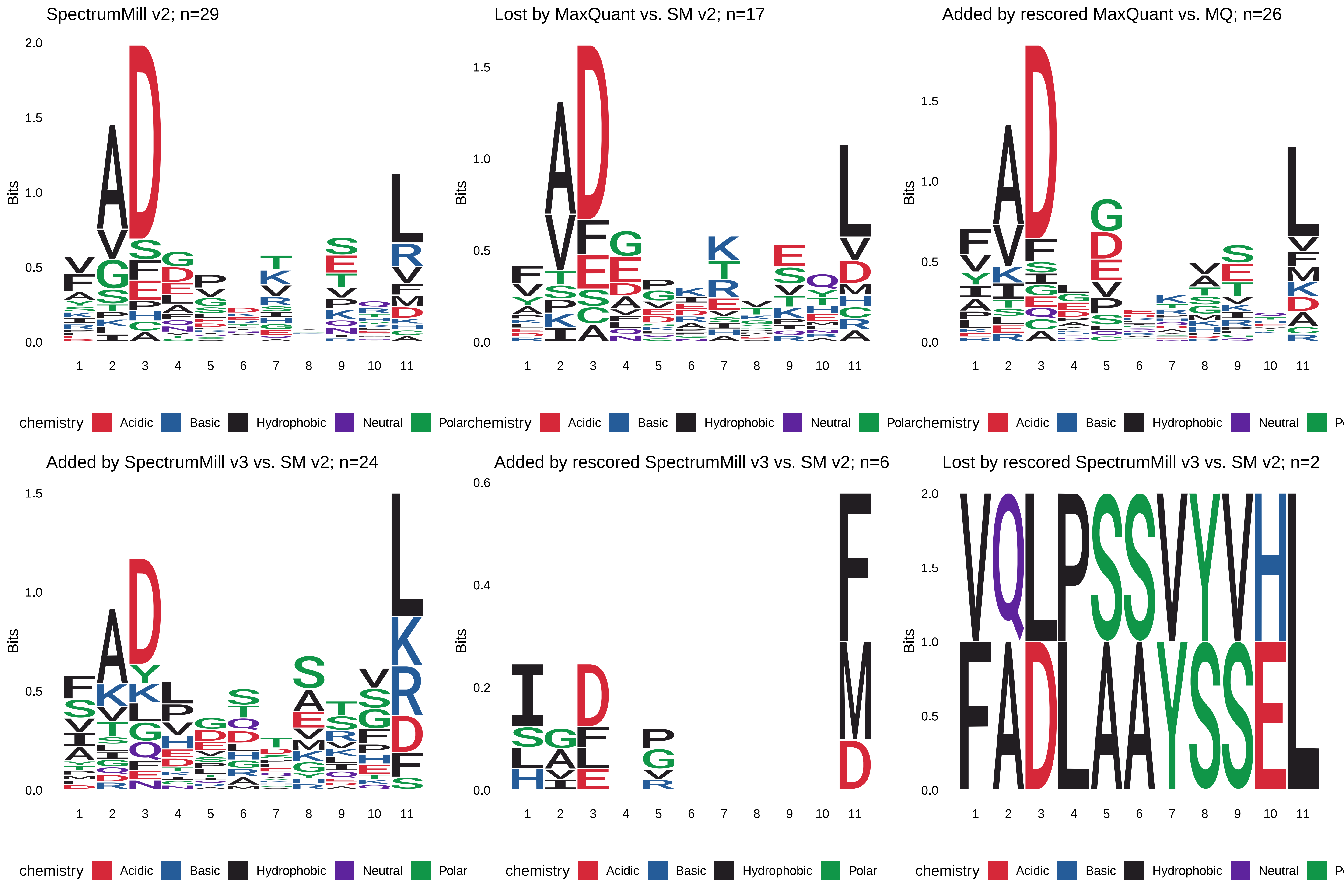

SpectrumMill v2; n=29
Lost by MaxQuant vs. SM v2; n=17
Added by rescored MaxQuant vs. MQ; n=26
Added by SpectrumMill v3 vs. SM v2; n=24
Added by rescored SpectrumMill v3 vs. SM v2; n=6
Lost by rescored SpectrumMill v3 vs. SM v2; n=2
Bits
chemistry Acidic Basic Hydrophobic Neutral Polar

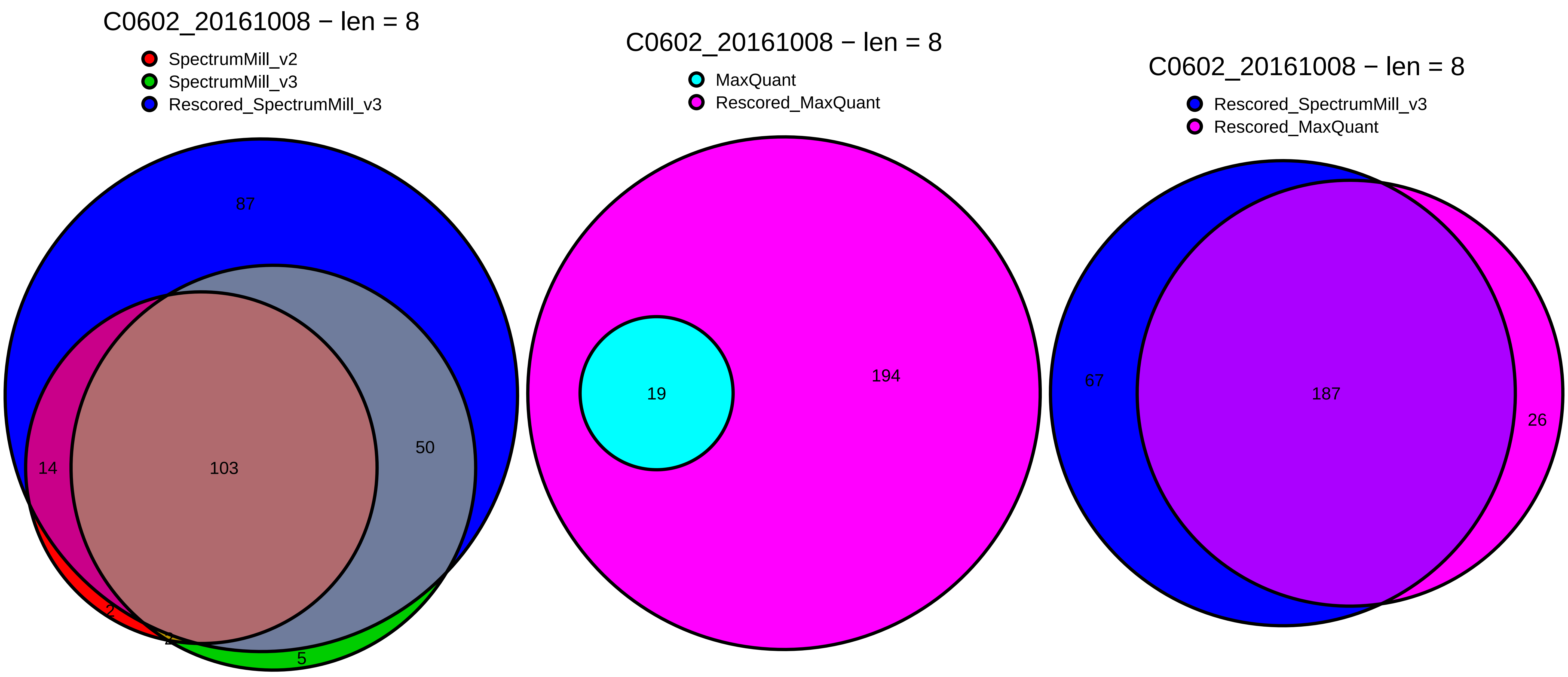
C0602_20161008 − len = 8
SpectrumMill_v2
SpectrumMill_v3
Rescored_SpectrumMill_v3
87
14
103
50
2
2
5
C0602_20161008 − len = 8
MaxQuant
Rescored_MaxQuant
19
194
C0602_20161008 − len = 8
Rescored_SpectrumMill_v3
Rescored_MaxQuant
67
187
26

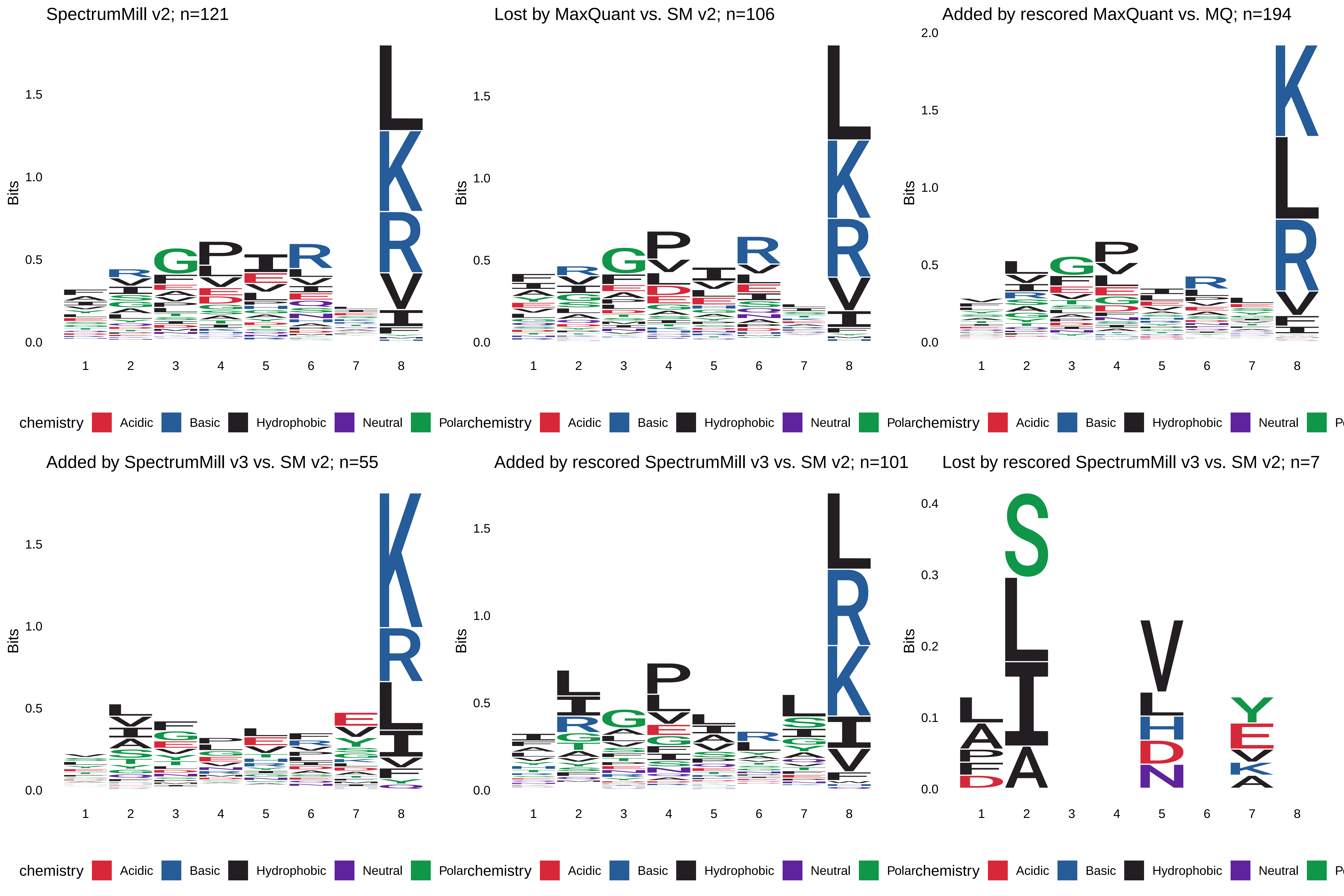

SpectrumMill v2; n=121
Lost by MaxQuant vs. SM v2; n=106
Added by rescored MaxQuant vs. MQ; n=194
Added by SpectrumMill v3 vs. SM v2; n=55
Added by rescored SpectrumMill v3 vs. SM v2; n=101
Lost by rescored SpectrumMill v3 vs. SM v2; n=7
Bits
1 2 3 4 5 6 7 8
chemistry Acidic Basic Hydrophobic Neutral Polar

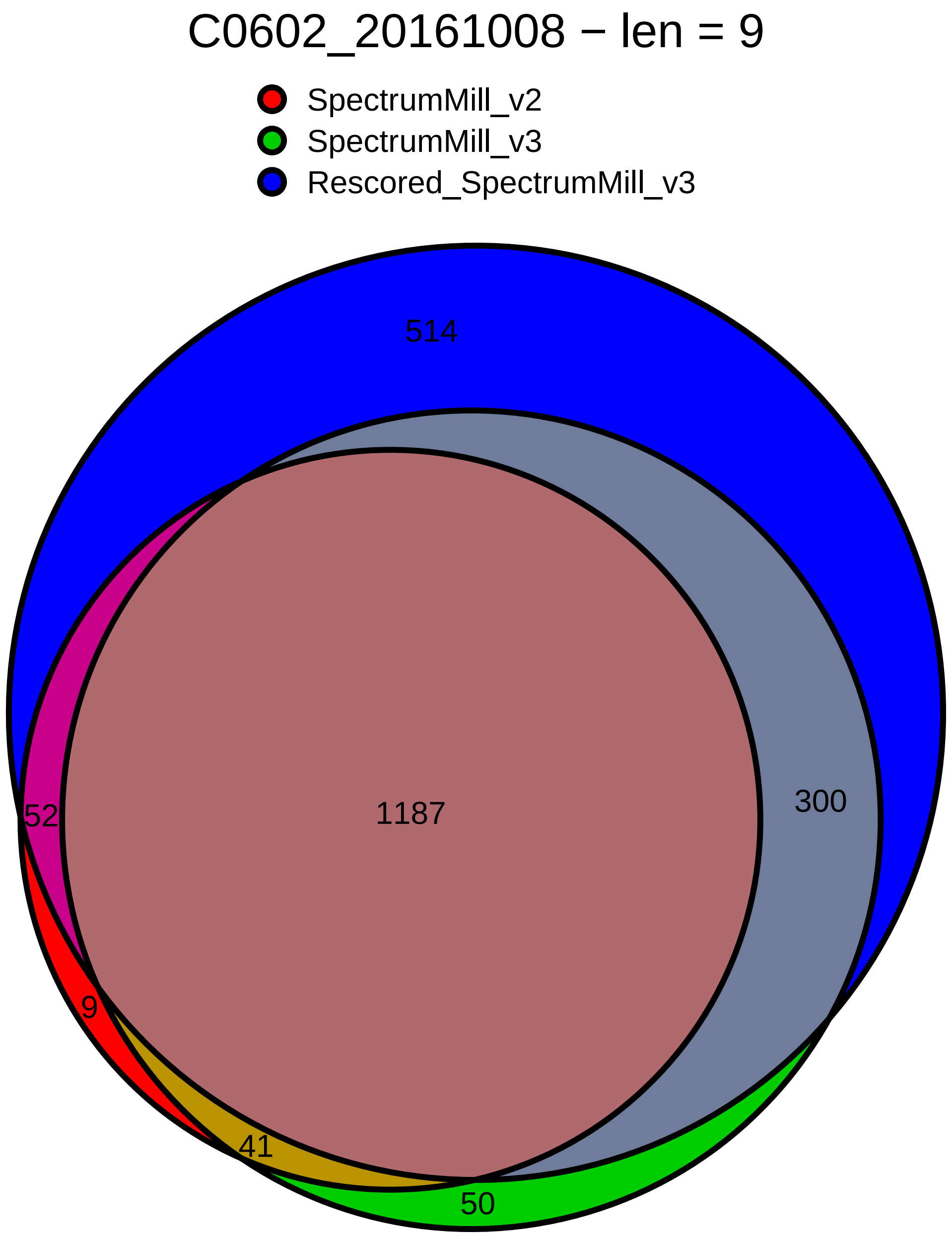

C0602_20161008 − len = 9
SpectrumMill_v2
SpectrumMill_v3
Rescored_SpectrumMill_v3
514
52
1187
300
9
41
50

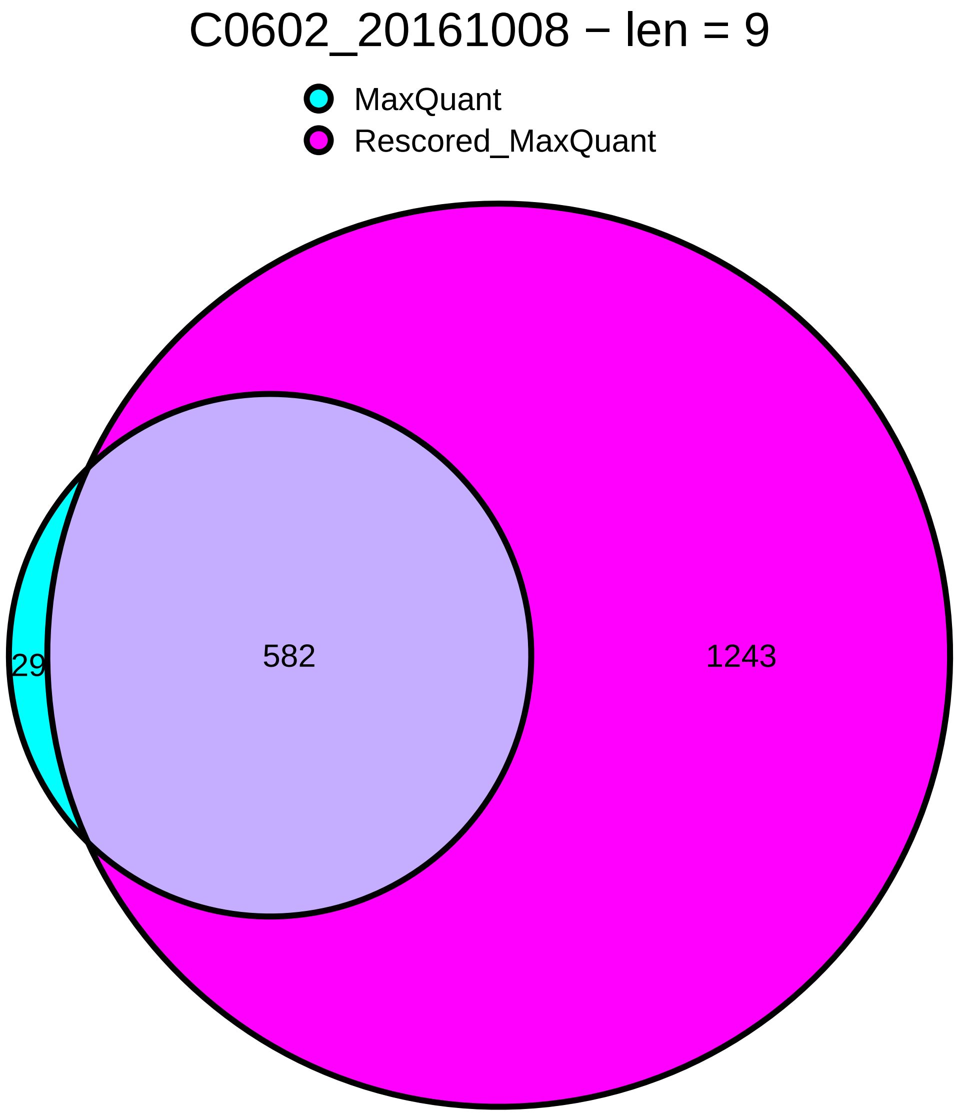

C0602_20161008 − len = 9
MaxQuant
Rescored_MaxQuant
29
582
1243

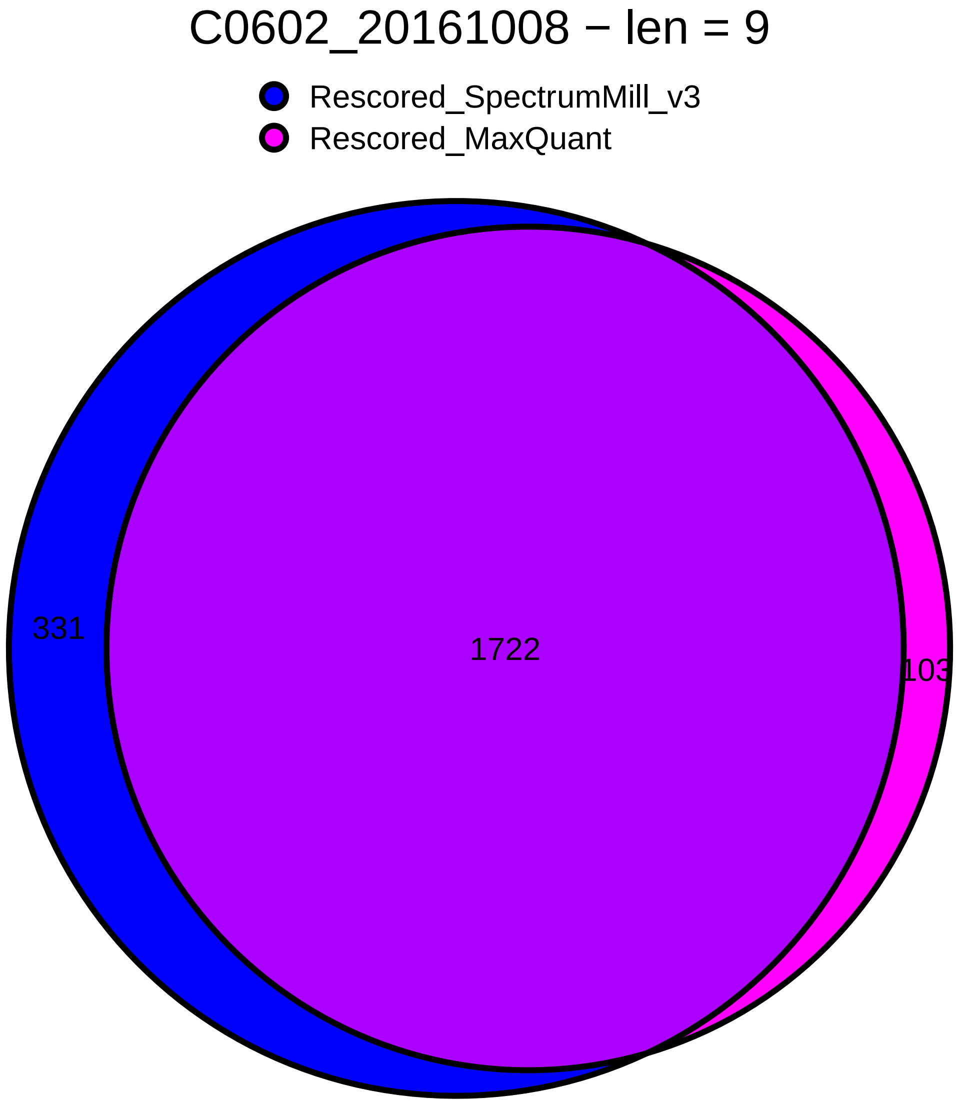

C0602_20161008 − len = 9
Rescored_SpectrumMill_v3
Rescored_MaxQuant
331
1722
103

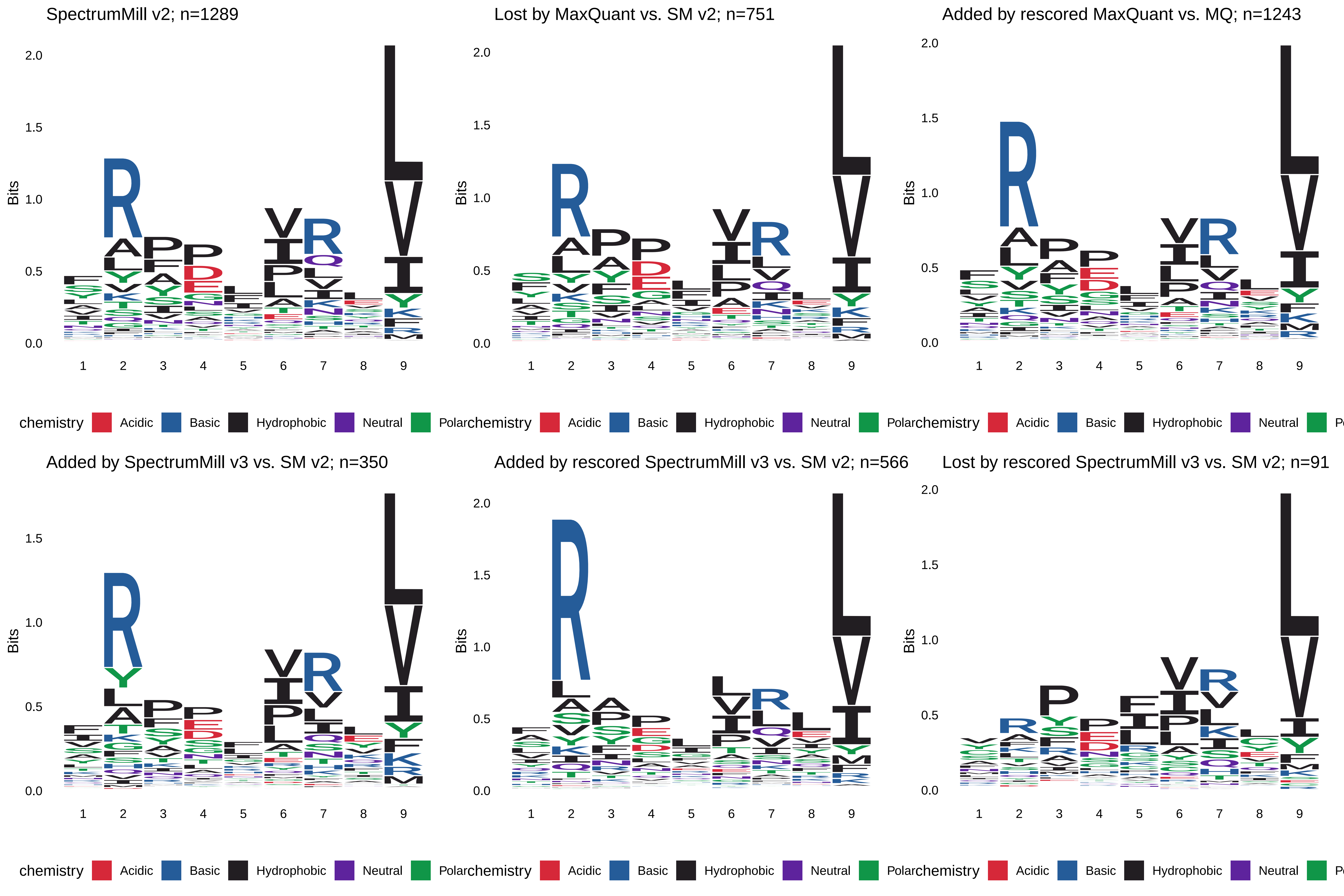
SpectrumMill v2; n=1289
Lost by MaxQuant vs. SM v2; n=751
Added by rescored MaxQuant vs. MQ; n=1243
Added by SpectrumMill v3 vs. SM v2; n=350
Added by rescored SpectrumMill v3 vs. SM v2; n=566
Lost by rescored SpectrumMill v3 vs. SM v2; n=91
Bits
0.0
0.5
1.0
1.5
2.0
1
2
3
4
5
6
7
8
9
chemistry
Acidic
Basic
Hydrophobic
Neutral
Polar

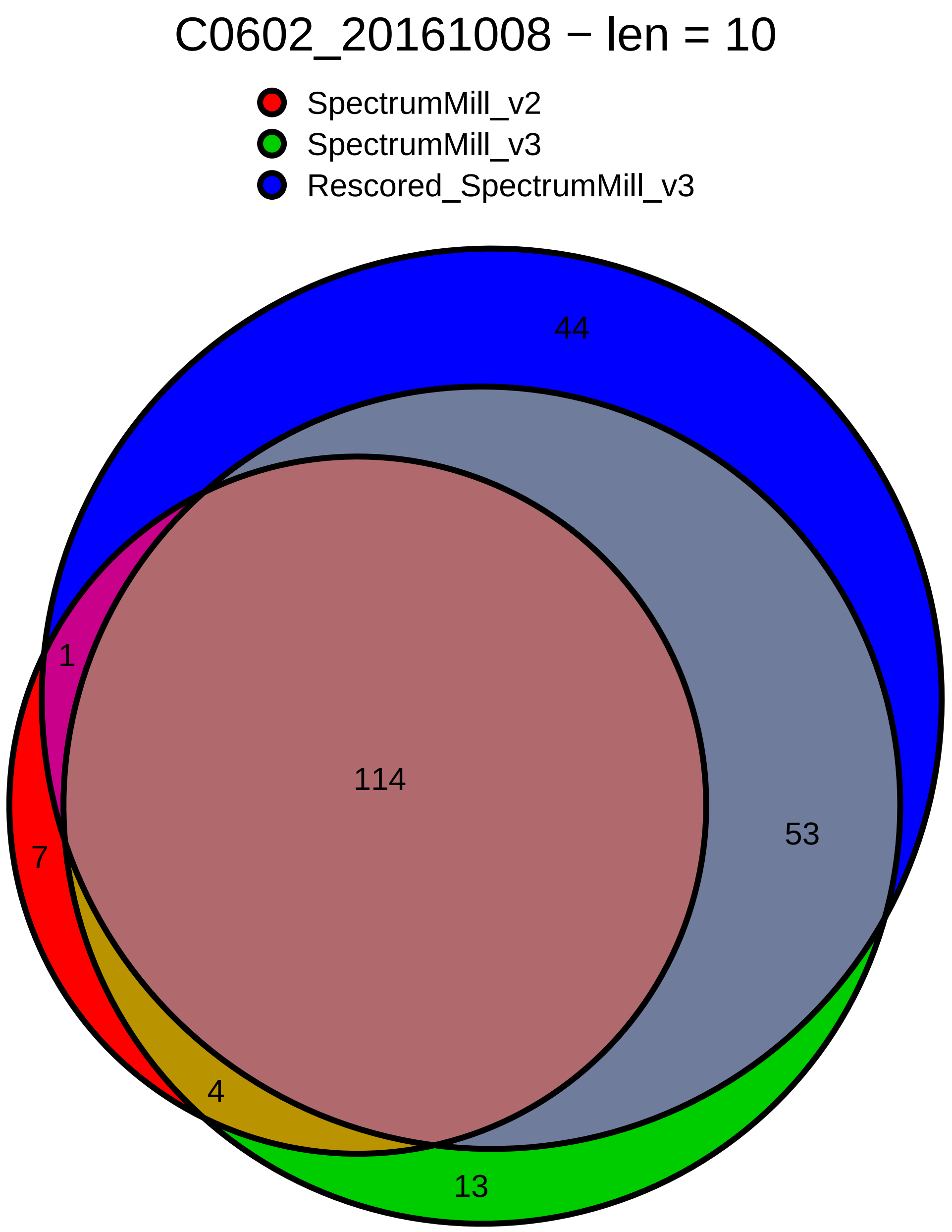
C0602_20161008 − len = 10
SpectrumMill_v2
SpectrumMill_v3
Rescored_SpectrumMill_v3
44
1
114
53
7
4
13

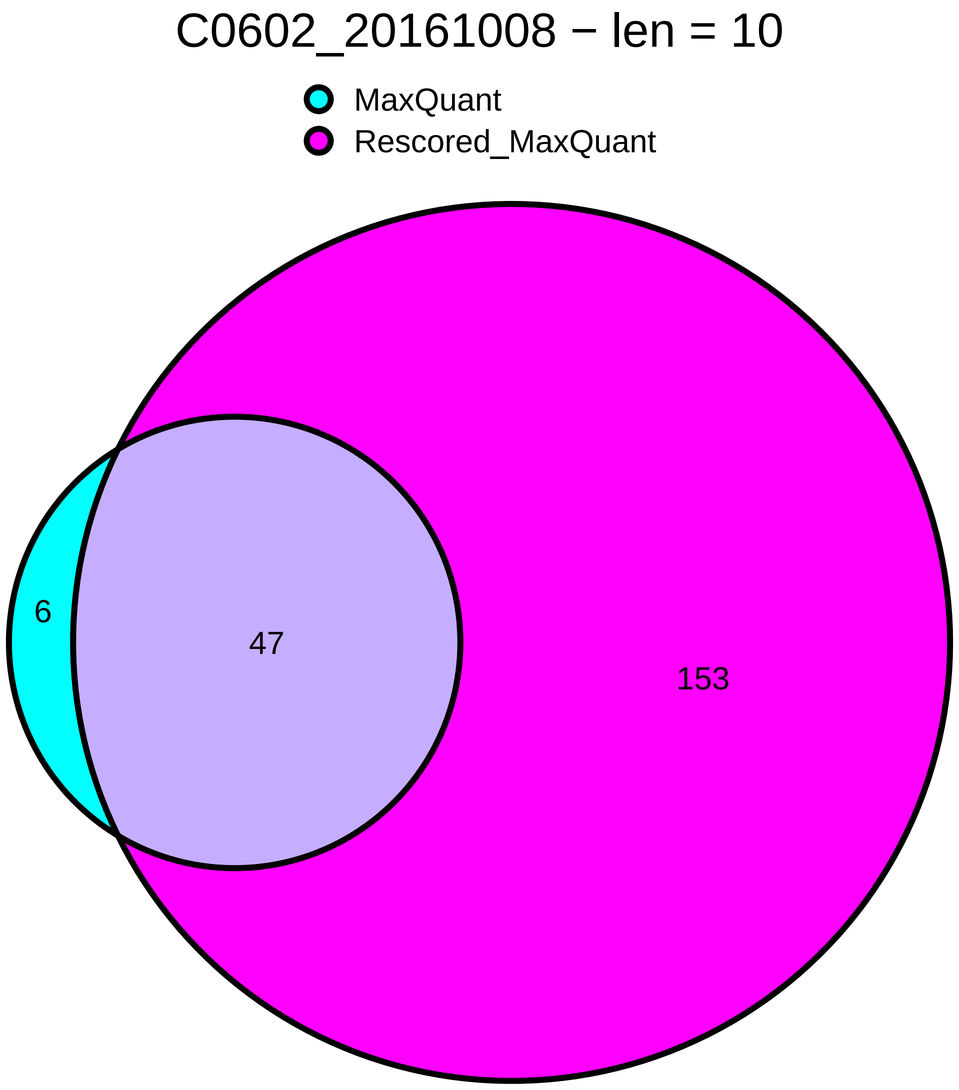
C0602_20161008 − len = 10
MaxQuant
Rescored_MaxQuant
6
47
153

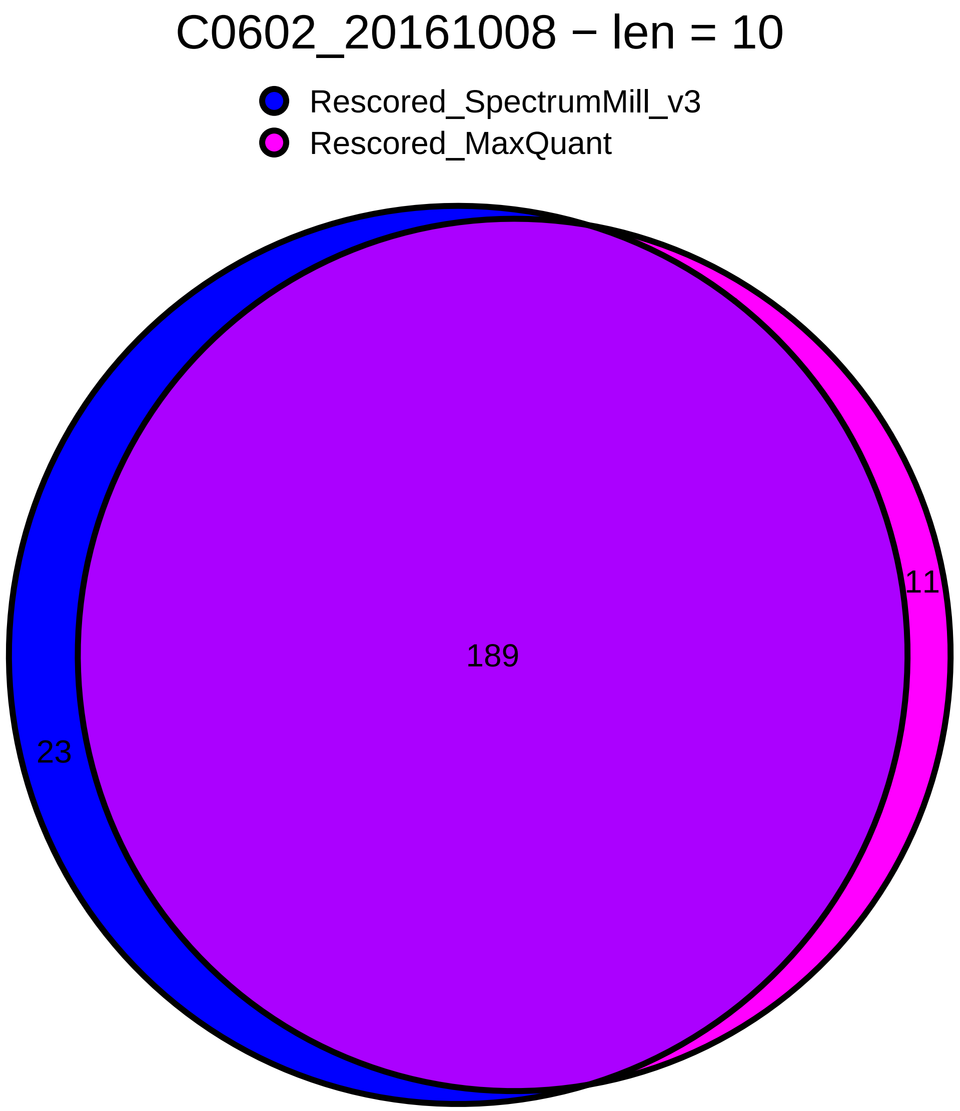
C0602_20161008 − len = 10
Rescored_SpectrumMill_v3
Rescored_MaxQuant
189
23
11

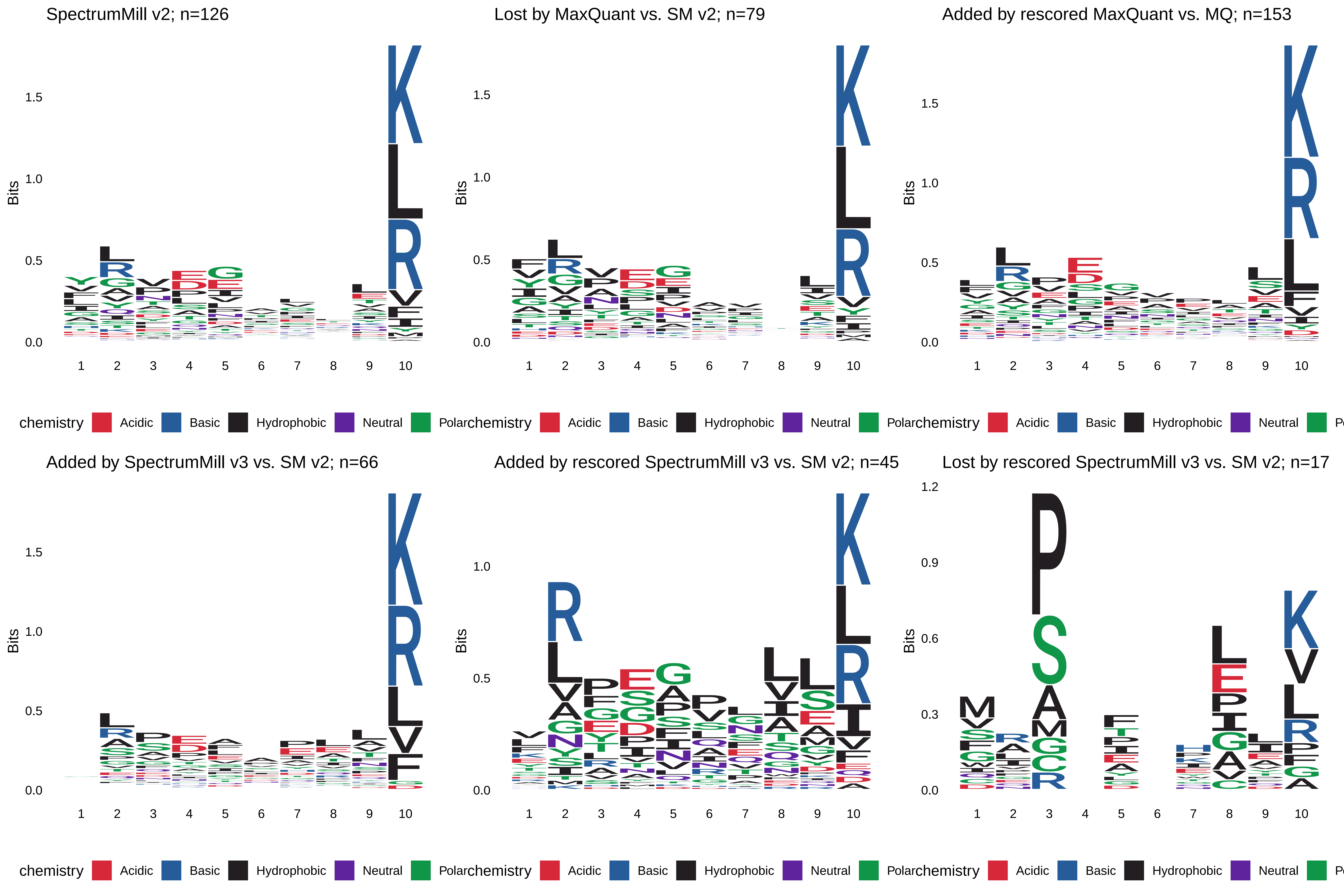
SpectrumMill v2; n=126
Lost by MaxQuant vs. SM v2; n=79
Added by rescored MaxQuant vs. MQ; n=153
Bits
1.5
1.0
0.5
0.0
1 2 3 4 5 6 7 8 9 10
chemistry
Acidic
Basic
Hydrophobic
Neutral
Polar
Added by SpectrumMill v3 vs. SM v2; n=66
Added by rescored SpectrumMill v3 vs. SM v2; n=45
Lost by rescored SpectrumMill v3 vs. SM v2; n=17
1.2
0.9
0.6
0.3

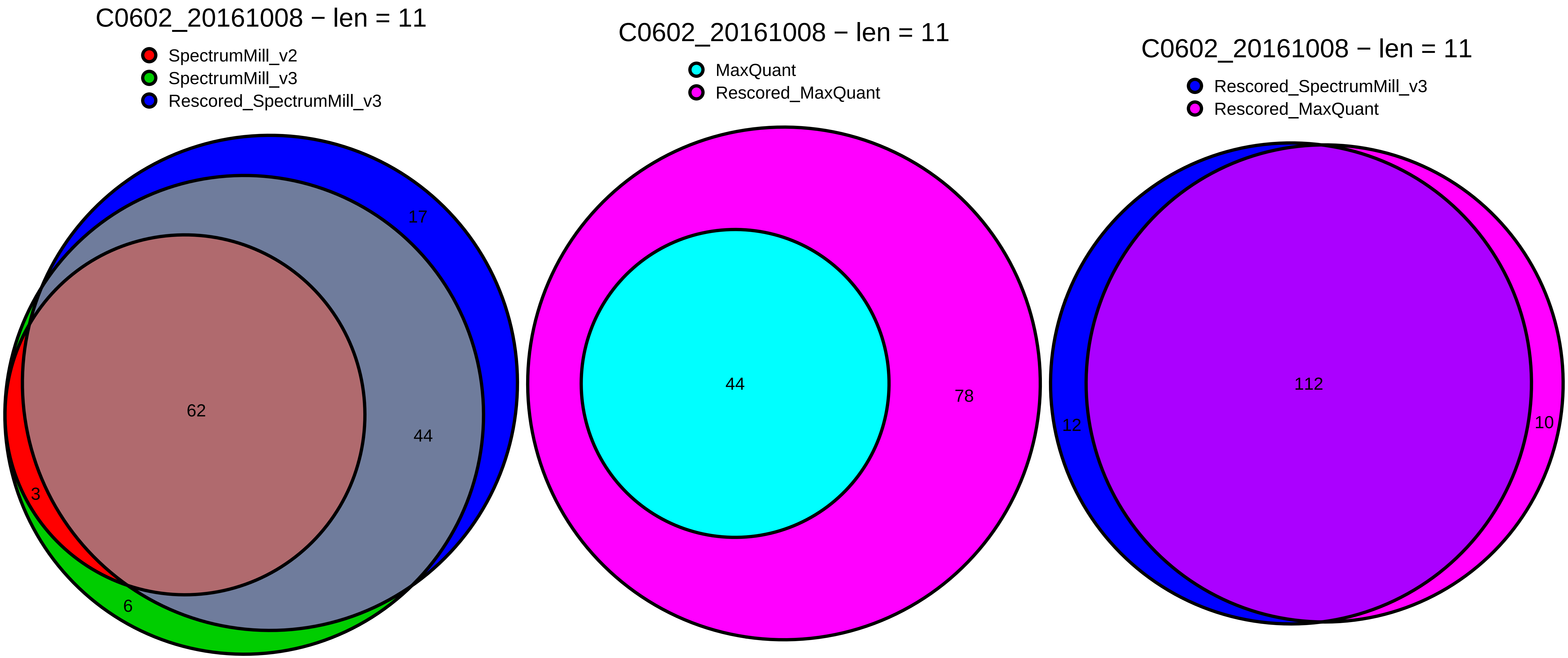
C0602_20161008 − len = 11
SpectrumMill_v2
SpectrumMill_v3
Rescored_SpectrumMill_v3
17
62
44
3
6
C0602_20161008 − len = 11
MaxQuant
Rescored_MaxQuant
44
78
C0602_20161008 − len = 11
Rescored_SpectrumMill_v3
Rescored_MaxQuant
112
12
10

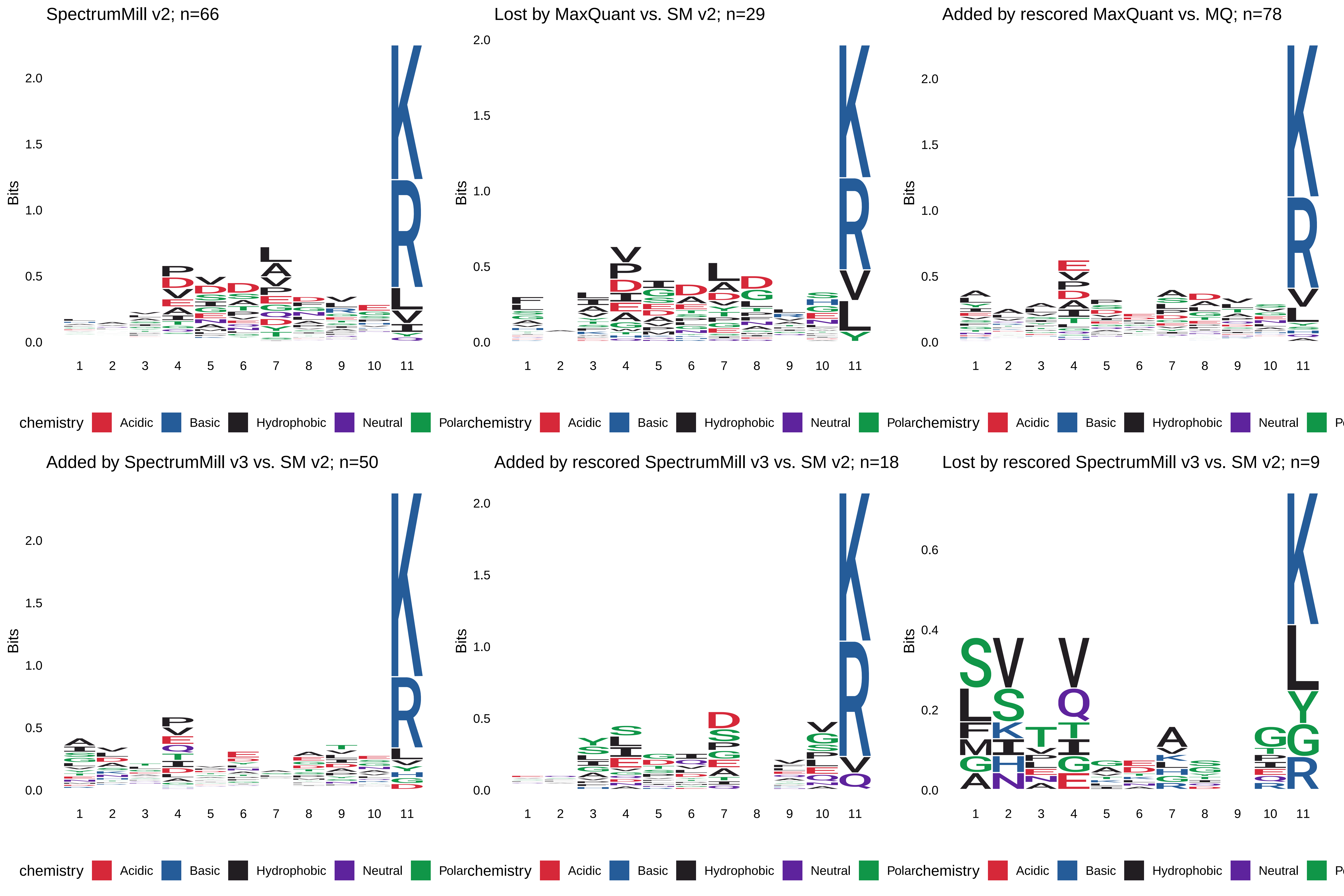
SpectrumMill v2; n=66
Lost by MaxQuant vs. SM v2; n=29
Added by rescored MaxQuant vs. MQ; n=78
Added by SpectrumMill v3 vs. SM v2; n=50
Added by rescored SpectrumMill v3 vs. SM v2; n=18
Lost by rescored SpectrumMill v3 vs. SM v2; n=9
Bits
chemistry
Acidic
Basic
Hydrophobic
Neutral
Polar

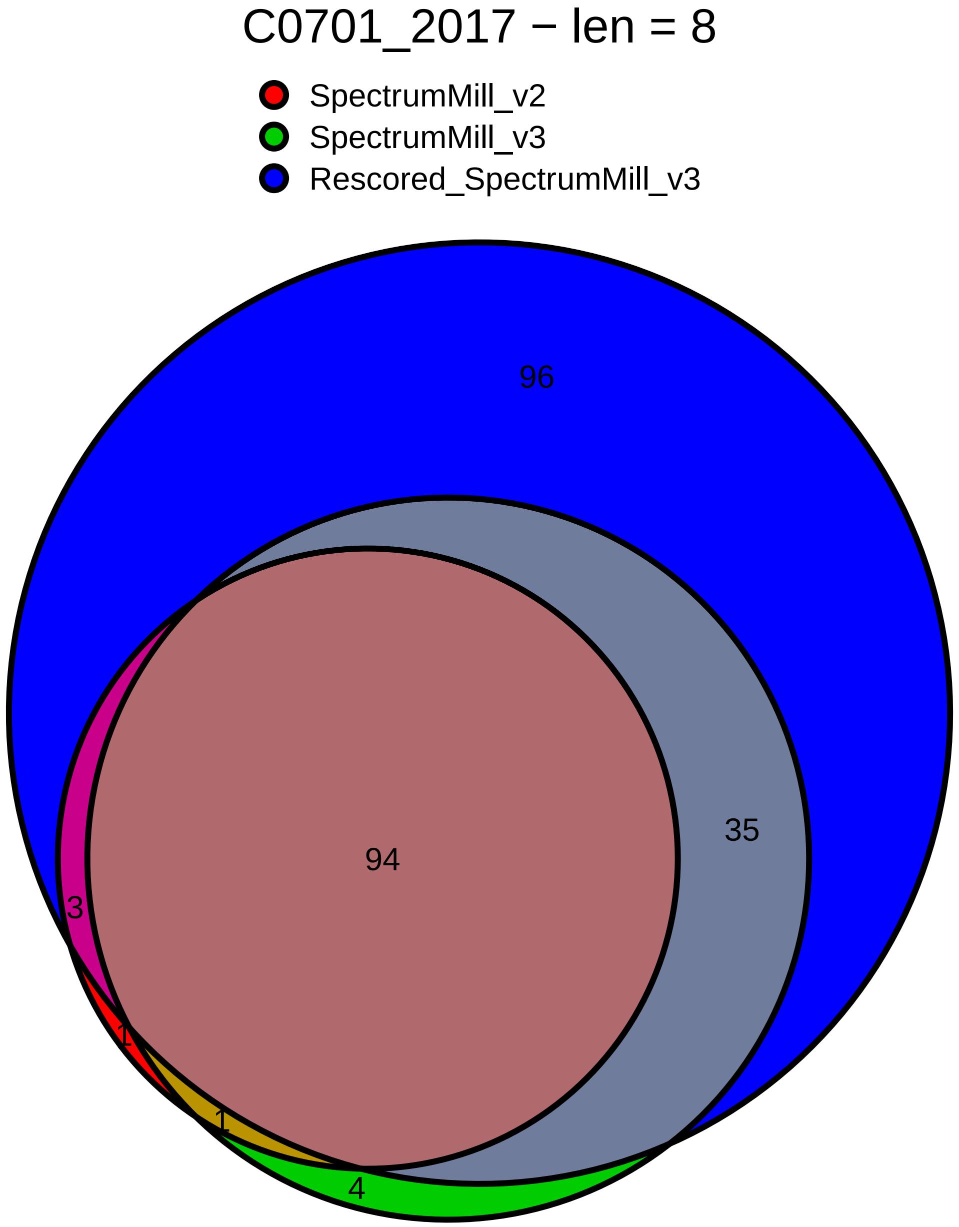
C0701_2017 − len = 8
SpectrumMill_v2
SpectrumMill_v3
Rescored_SpectrumMill_v3
96
35
94
3
1
1
4

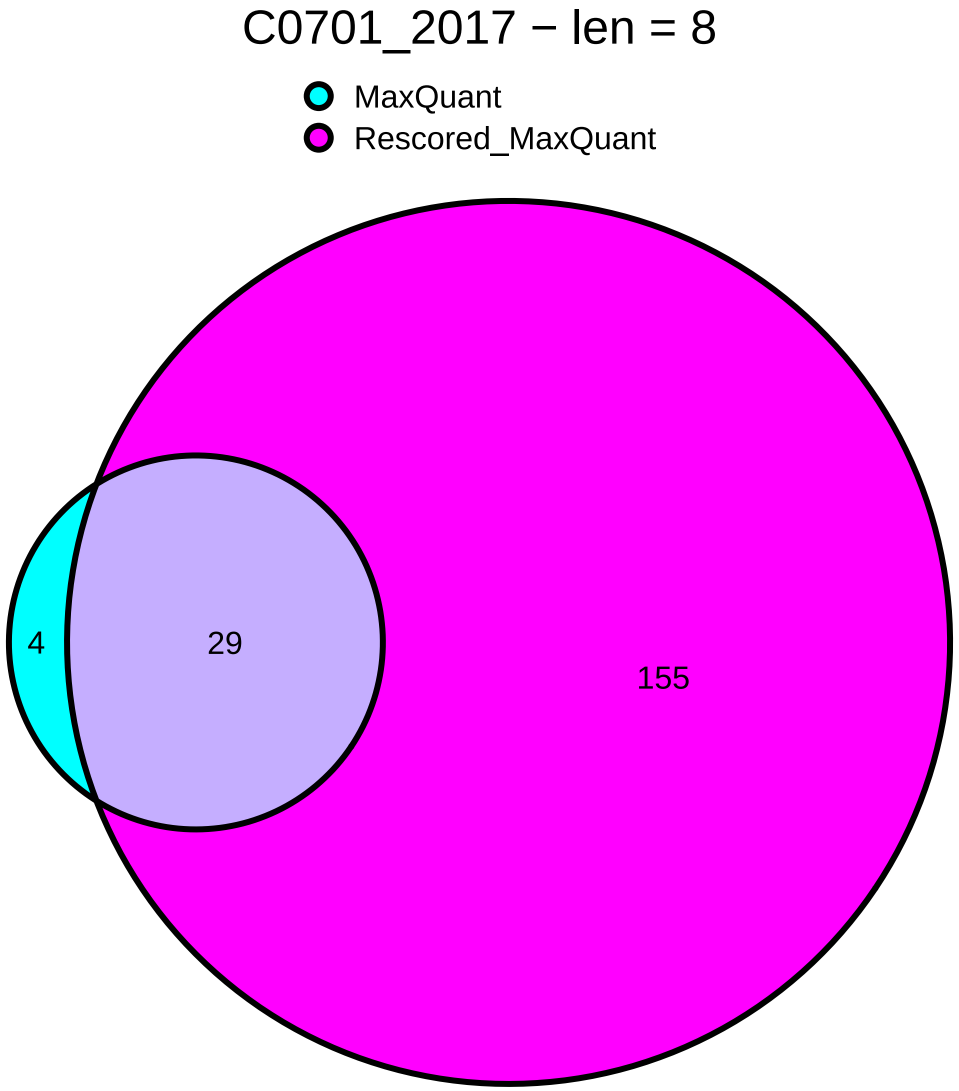
C0701_2017 − len = 8
MaxQuant
Rescored_MaxQuant
4
29
155

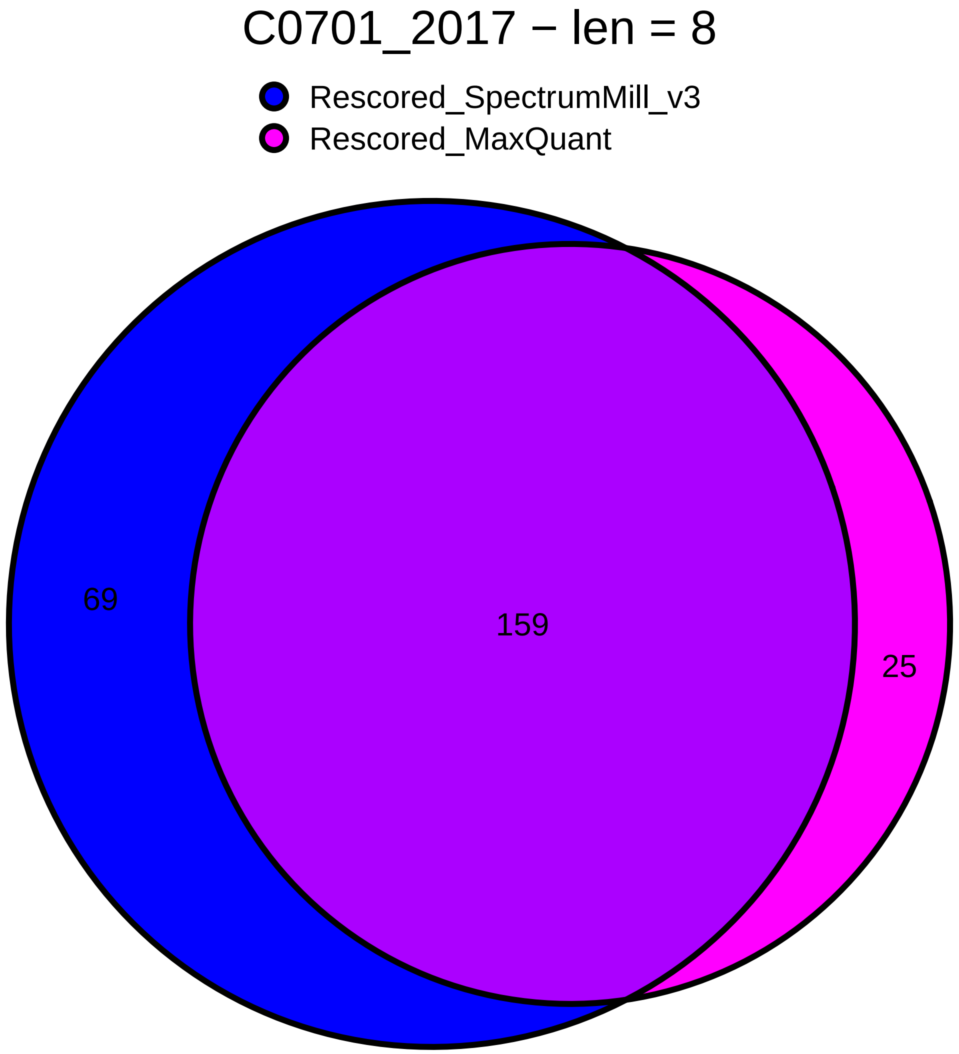
C0701_2017 − len = 8
Rescored_SpectrumMill_v3
Rescored_MaxQuant
69
159
25

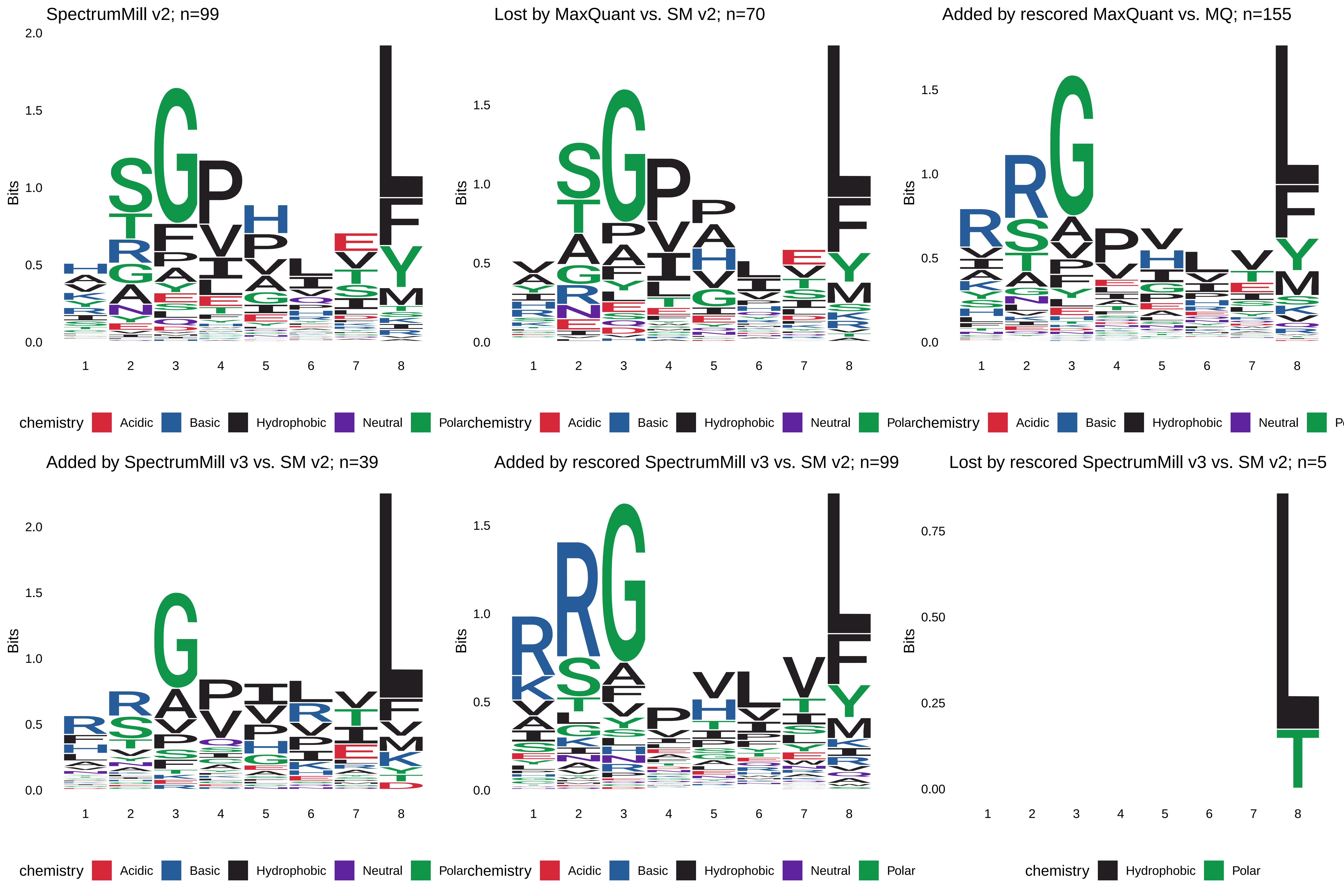

SpectrumMill v2; n=99
Lost by MaxQuant vs. SM v2; n=70
Added by rescored MaxQuant vs. MQ; n=155
Added by SpectrumMill v3 vs. SM v2; n=39
Added by rescored SpectrumMill v3 vs. SM v2; n=99
Lost by rescored SpectrumMill v3 vs. SM v2; n=5
Bits
1 2 3 4 5 6 7 8
chemistry Acidic Basic Hydrophobic Neutral Polarchemistry Acidic Basic Hydrophobic Neutral Polarchemistry Acidic Basic Hydrophobic Neutral Po
chemistry Acidic Basic Hydrophobic Neutral Polarchemistry Acidic Basic Hydrophobic Neutral Polar
chemistry Hydrophobic Polar

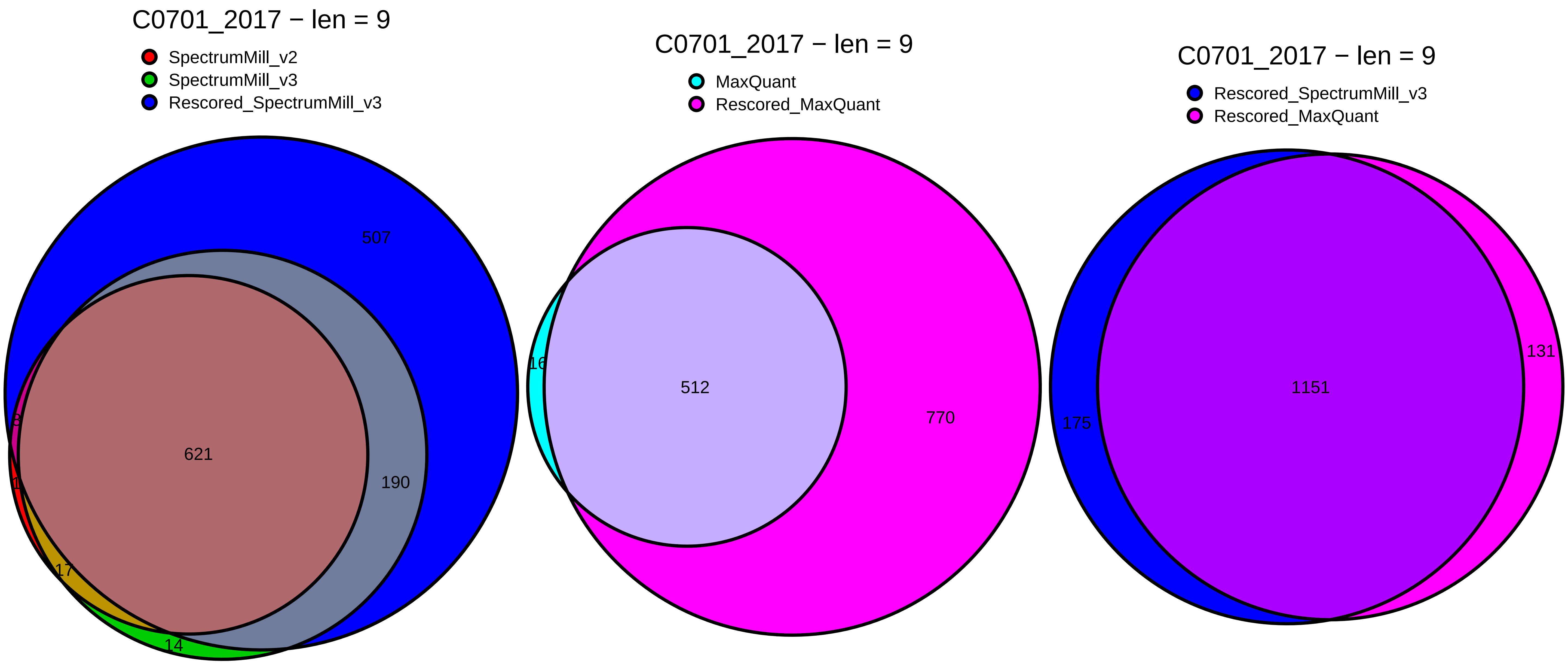
C0701_2017 − len = 9
SpectrumMill_v2
SpectrumMill_v3
Rescored_SpectrumMill_v3
507
621
190
17
14
C0701_2017 − len = 9
MaxQuant
Rescored_MaxQuant
16
512
770
C0701_2017 − len = 9
Rescored_SpectrumMill_v3
Rescored_MaxQuant
131
1151
175

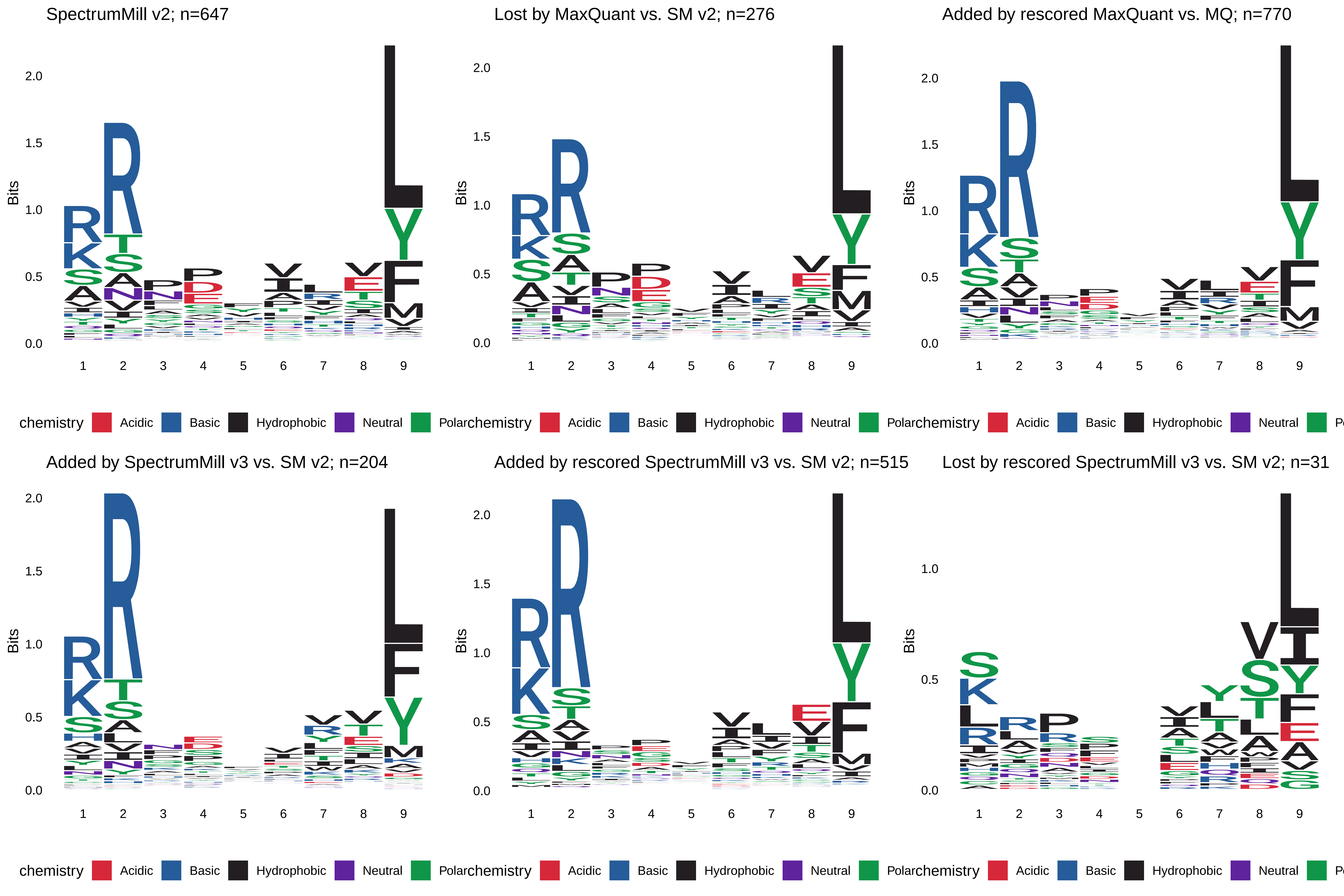

SpectrumMill v2; n=647
Lost by MaxQuant vs. SM v2; n=276
Added by rescored MaxQuant vs. MQ; n=770
Added by SpectrumMill v3 vs. SM v2; n=204
Added by rescored SpectrumMill v3 vs. SM v2; n=515
Lost by rescored SpectrumMill v3 vs. SM v2; n=31
Bits
1 2 3 4 5 6 7 8 9
chemistry Acidic Basic Hydrophobic Neutral Polar

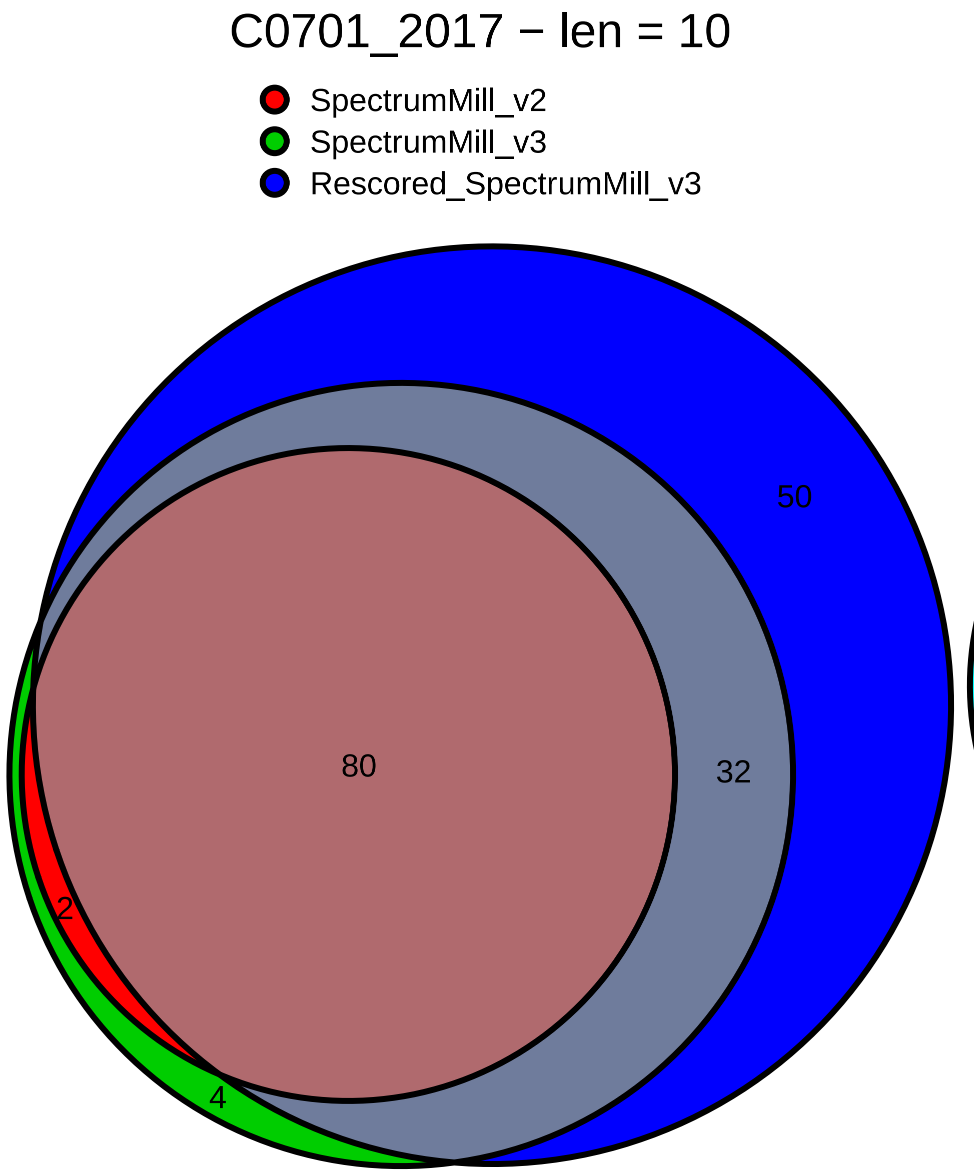
C0701_2017 − len = 10
SpectrumMill_v2
SpectrumMill_v3
Rescored_SpectrumMill_v3
50
80
32
2
4

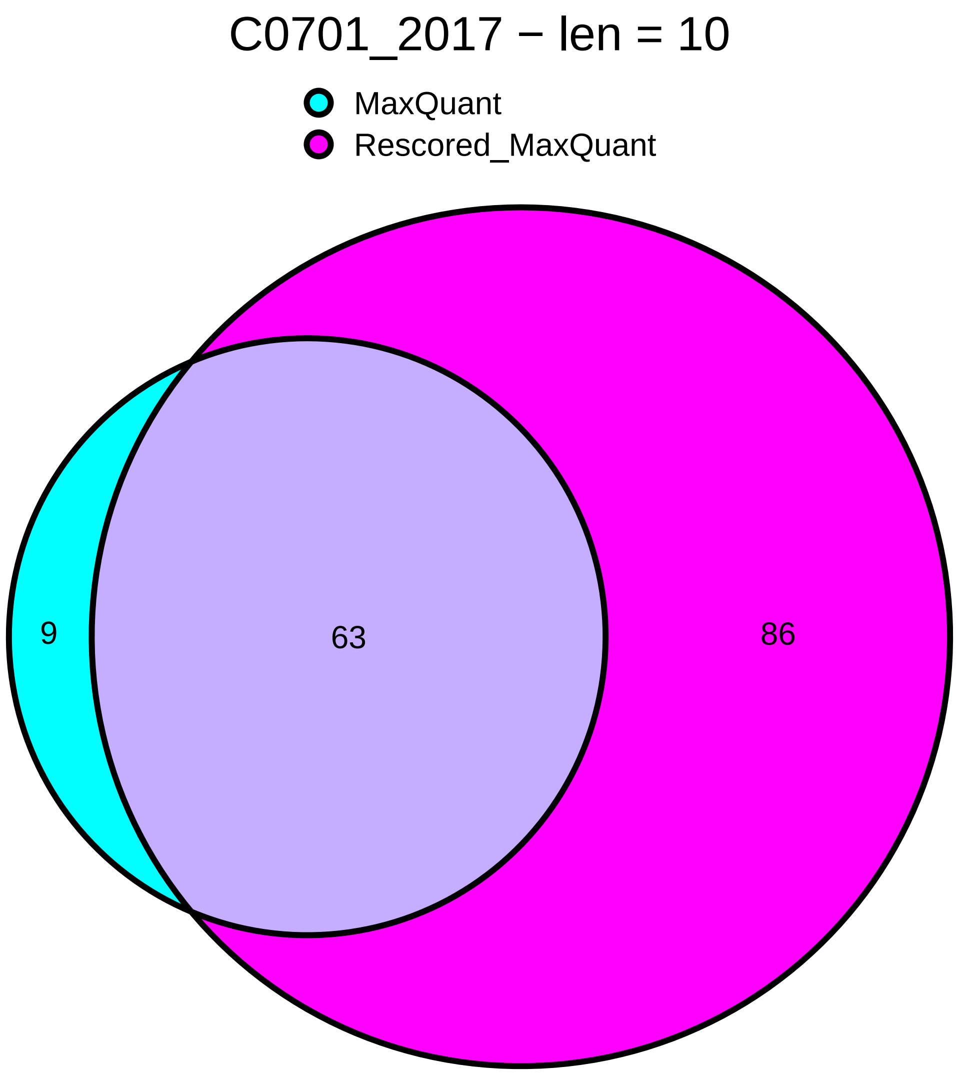
C0701_2017 − len = 10
MaxQuant
Rescored_MaxQuant
9
63
86

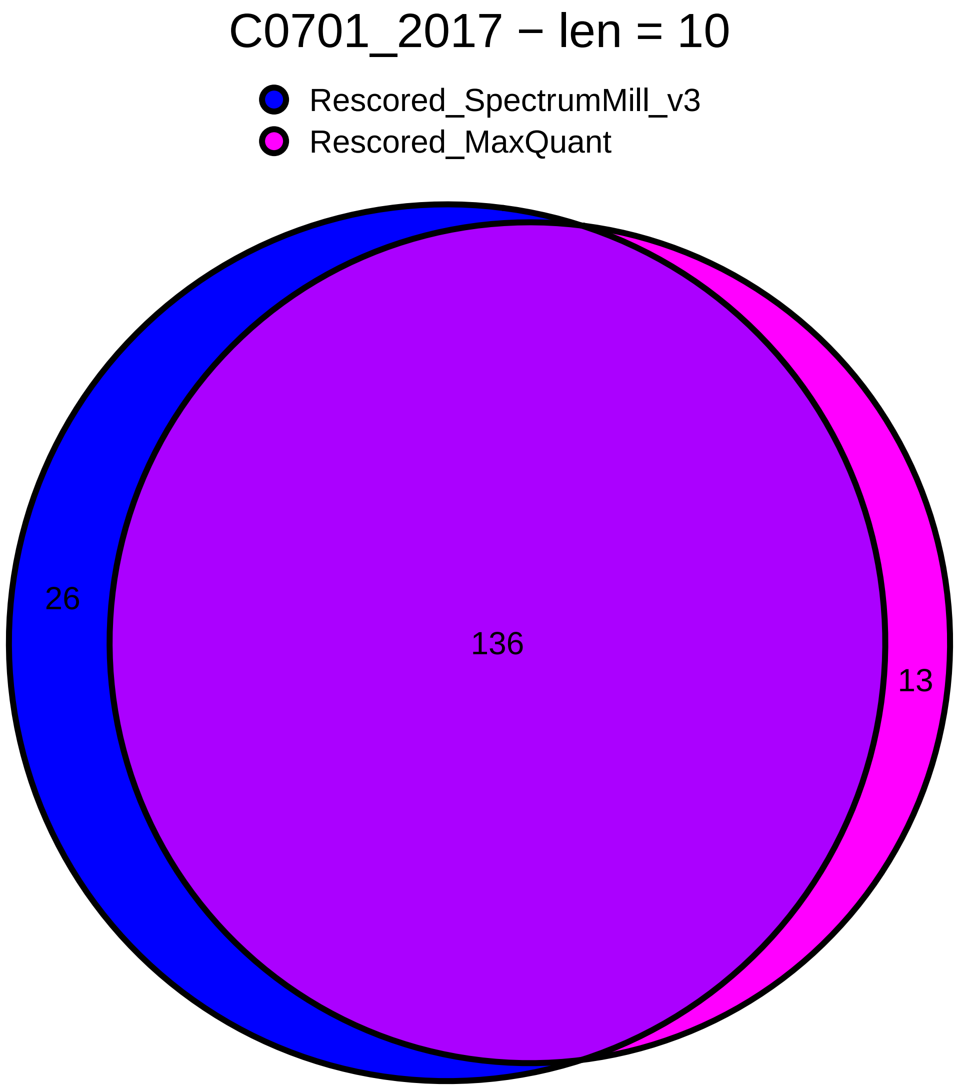
C0701_2017 − len = 10
Rescored_SpectrumMill_v3
Rescored_MaxQuant
26
136
13

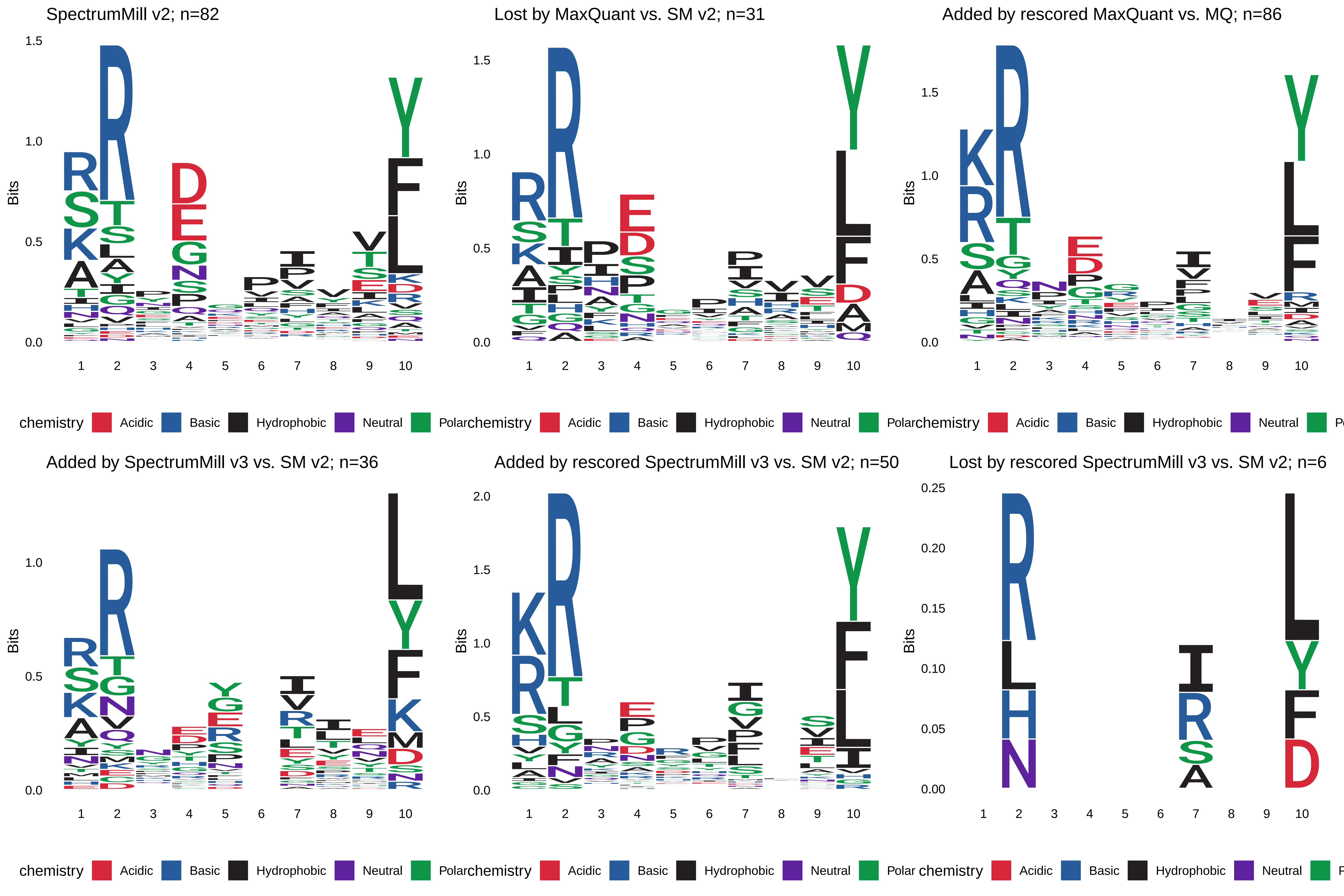
SpectrumMill v2; n=82
Lost by MaxQuant vs. SM v2; n=31
Added by rescored MaxQuant vs. MQ; n=86
Added by SpectrumMill v3 vs. SM v2; n=36
Added by rescored SpectrumMill v3 vs. SM v2; n=50
Lost by rescored SpectrumMill v3 vs. SM v2; n=6
Bits
1 2 3 4 5 6 7 8 9 10
chemistry
Acidic
Basic
Hydrophobic
Neutral
Polar

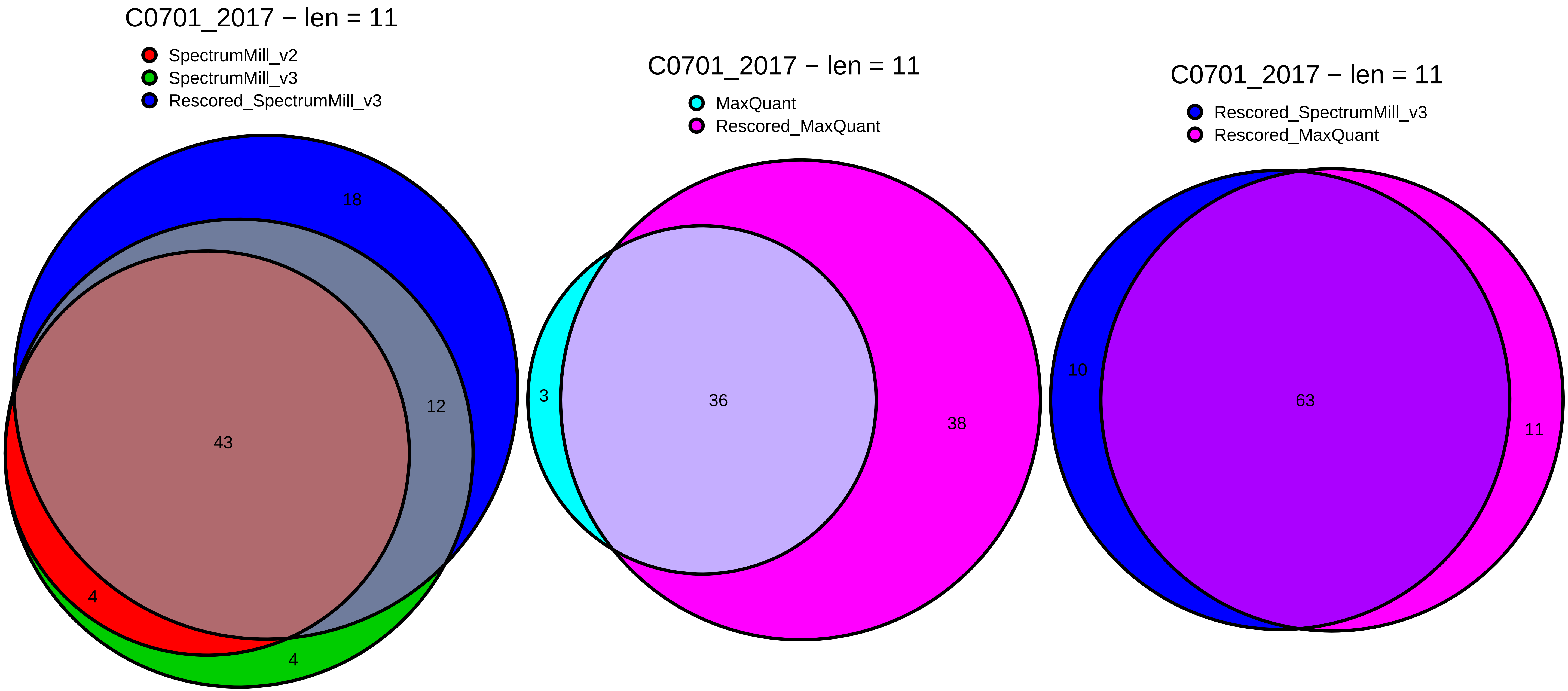
C0701_2017 − len = 11
SpectrumMill_v2
SpectrumMill_v3
Rescored_SpectrumMill_v3
18
12
43
4
4
C0701_2017 − len = 11
MaxQuant
Rescored_MaxQuant
3
36
38
C0701_2017 − len = 11
Rescored_SpectrumMill_v3
Rescored_MaxQuant
10
63
11

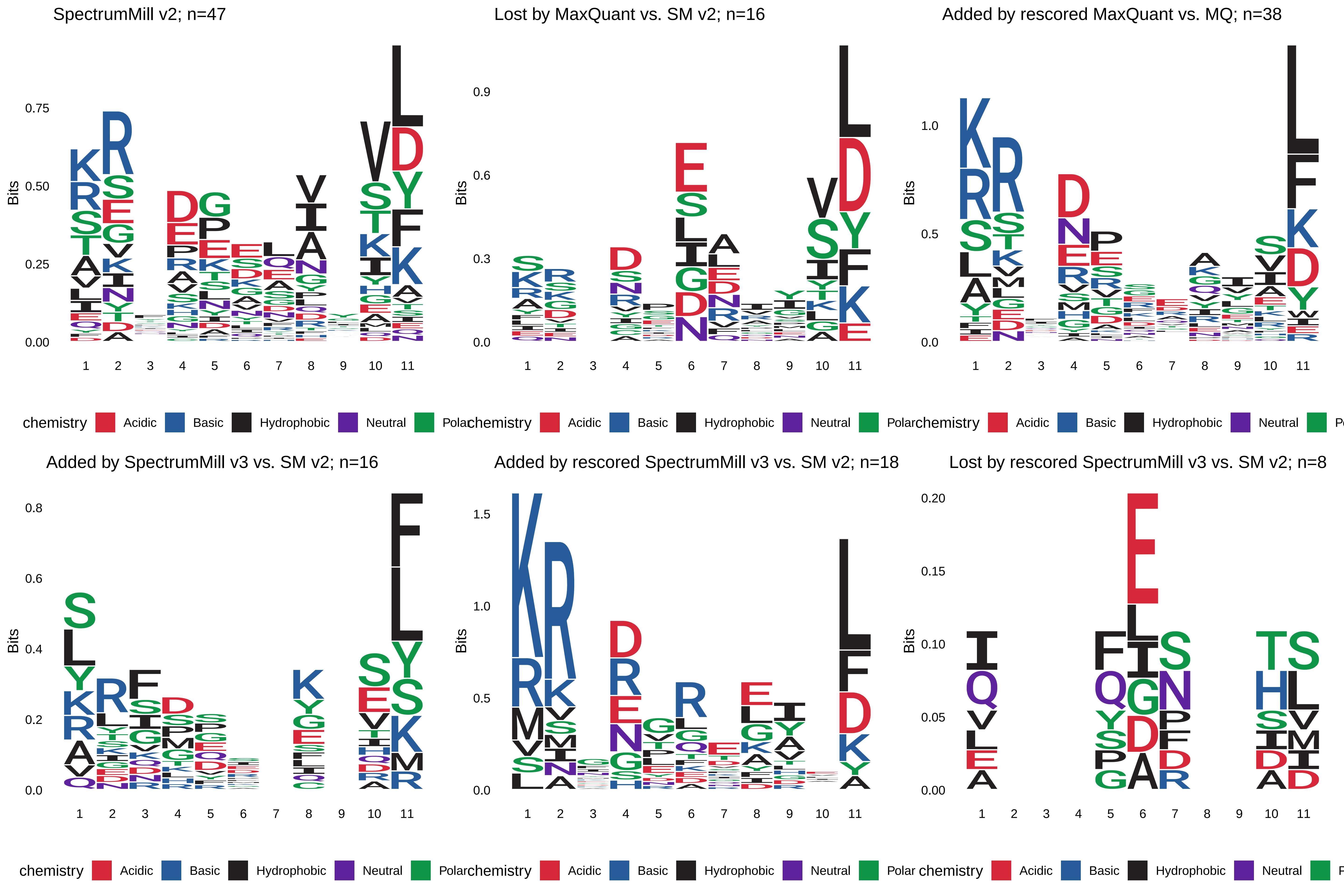

SpectrumMill v2; n=47
Lost by MaxQuant vs. SM v2; n=16
Added by rescored MaxQuant vs. MQ; n=38
Bits
0.00
0.25
0.50
0.75
0.0
0.3
0.6
0.9
0.5
1.0
1 2 3 4 5 6 7 8 9 10 11
chemistry Acidic Basic Hydrophobic Neutral Polar
Added by SpectrumMill v3 vs. SM v2; n=16
Added by rescored SpectrumMill v3 vs. SM v2; n=18
Lost by rescored SpectrumMill v3 vs. SM v2; n=8
0.2
0.4
0.6
0.8
1.5
0.05
0.10
0.15
0.20

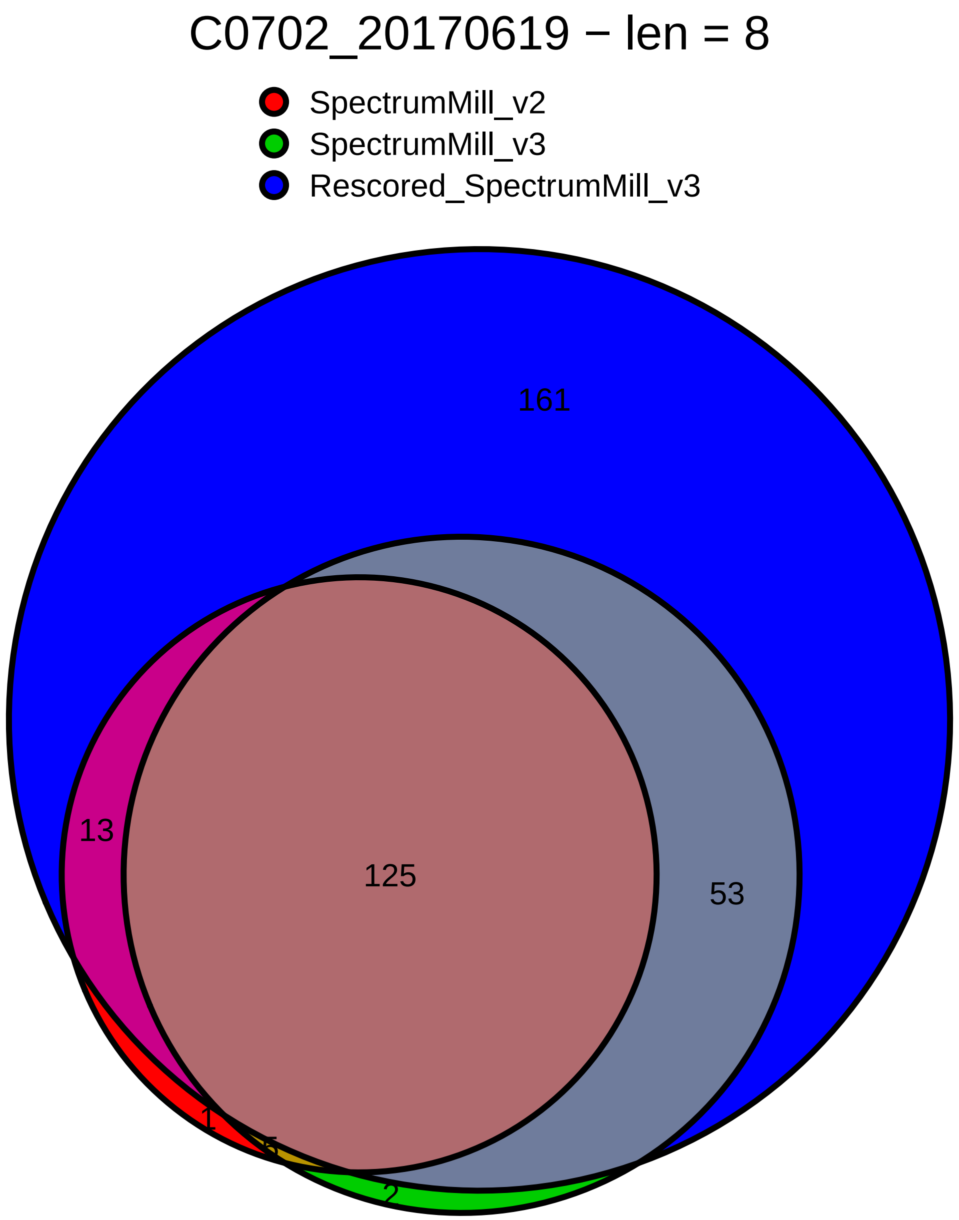
C0702_20170619 − len = 8
SpectrumMill_v2
SpectrumMill_v3
Rescored_SpectrumMill_v3
161
13
125
53
1
5
2

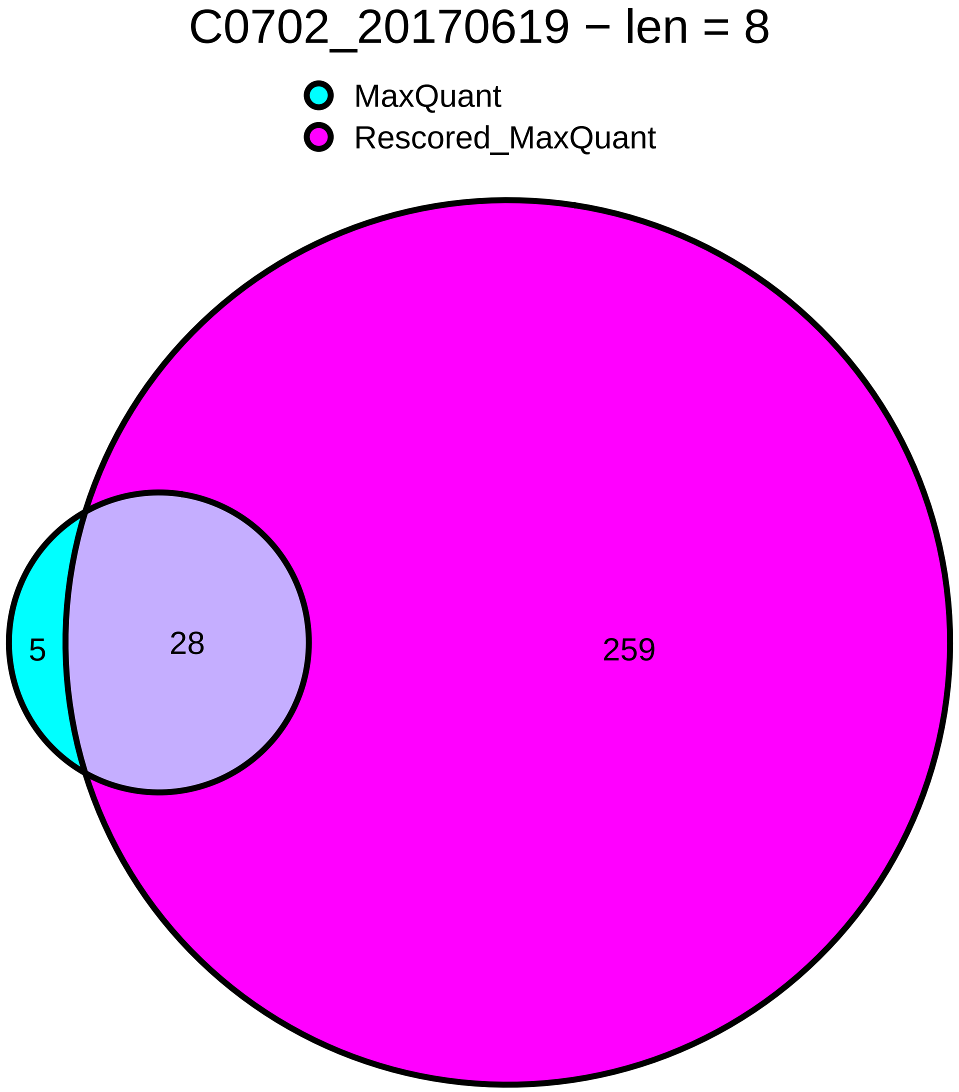
C0702_20170619 − len = 8
MaxQuant
Rescored_MaxQuant
5
28
259

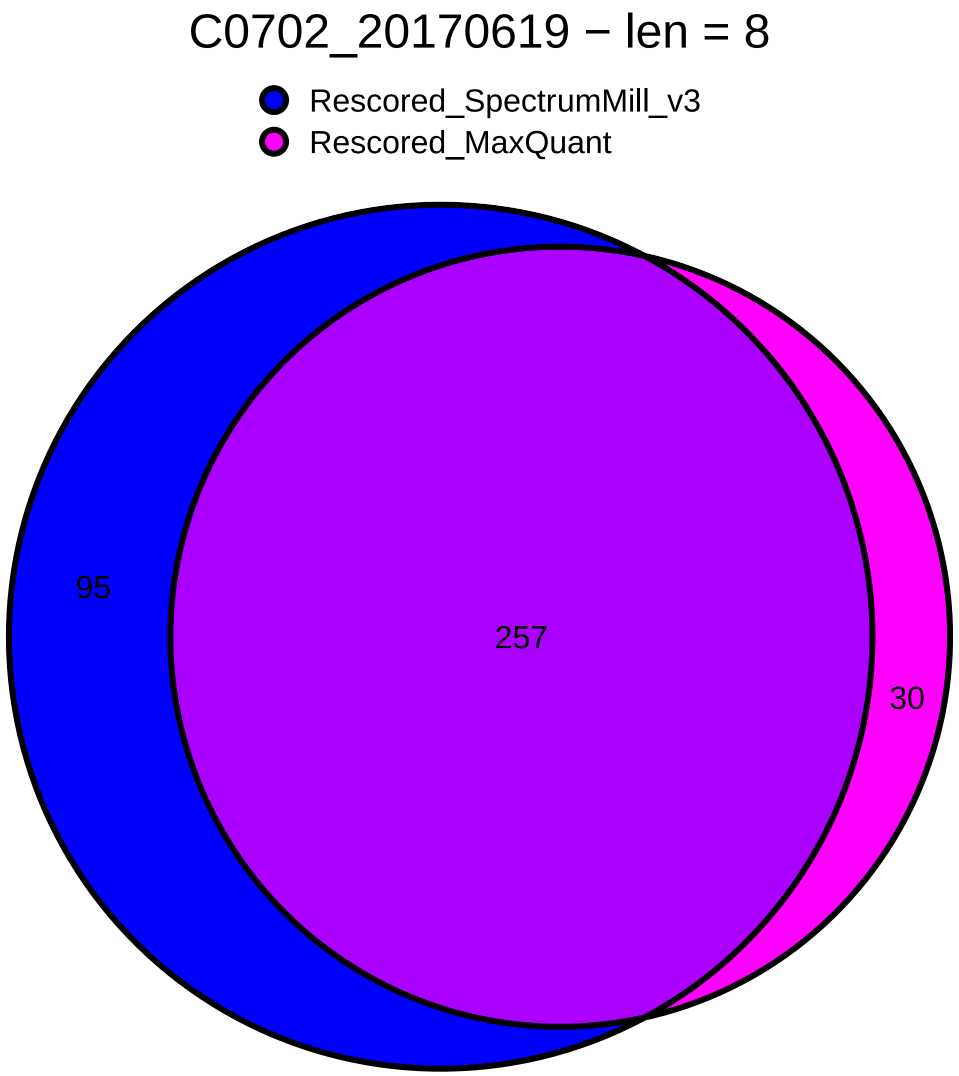
C0702_20170619 − len = 8
Rescored_SpectrumMill_v3
Rescored_MaxQuant
95
257
30

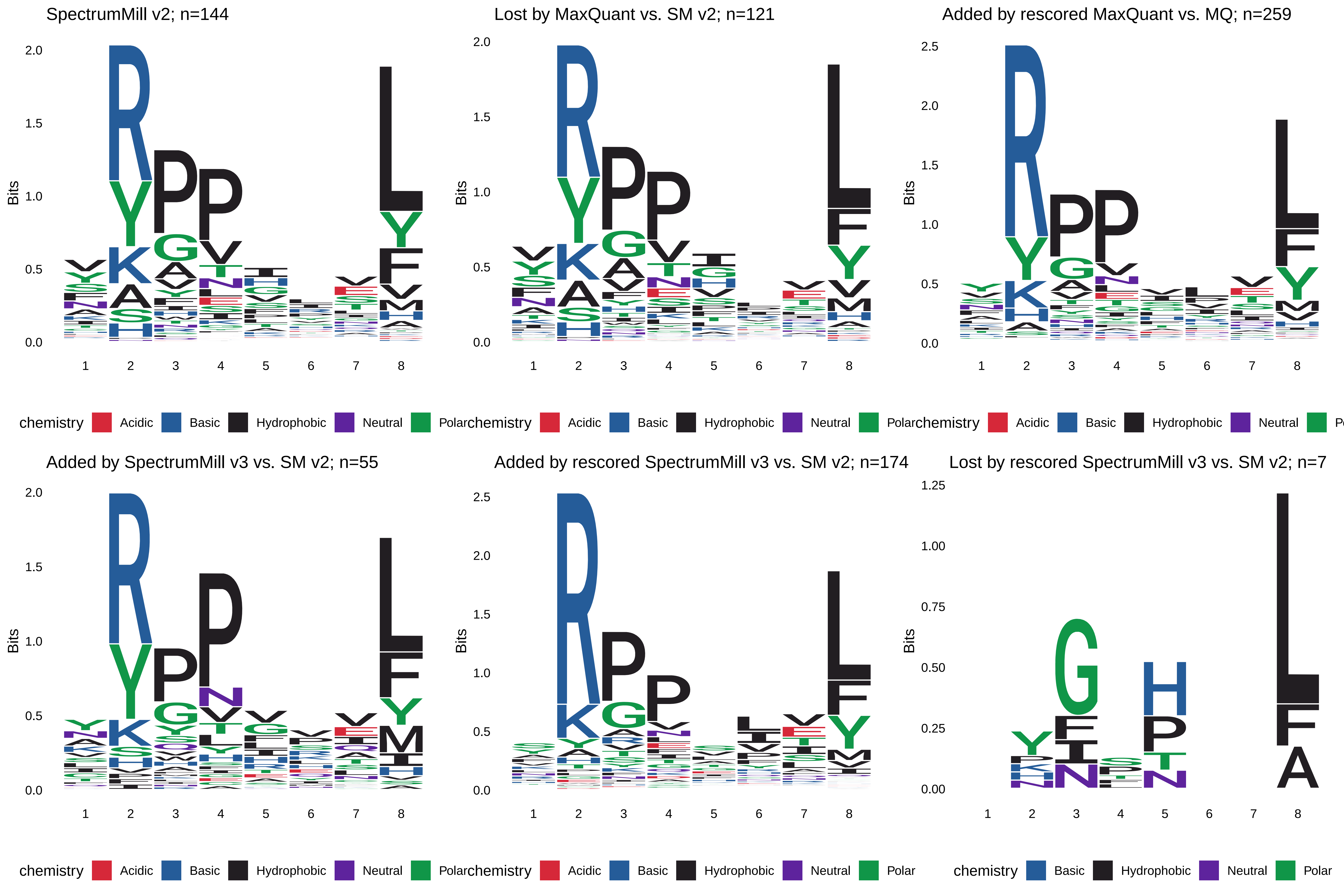

SpectrumMill v2; n=144
Lost by MaxQuant vs. SM v2; n=121
Added by rescored MaxQuant vs. MQ; n=259
Bits
chemistry Acidic Basic Hydrophobic Neutral Polar
Added by SpectrumMill v3 vs. SM v2; n=55
Added by rescored SpectrumMill v3 vs. SM v2; n=174
Lost by rescored SpectrumMill v3 vs. SM v2; n=7
Bits
chemistry Acidic Basic Hydrophobic Neutral Polar
chemistry Basic Hydrophobic Neutral Polar

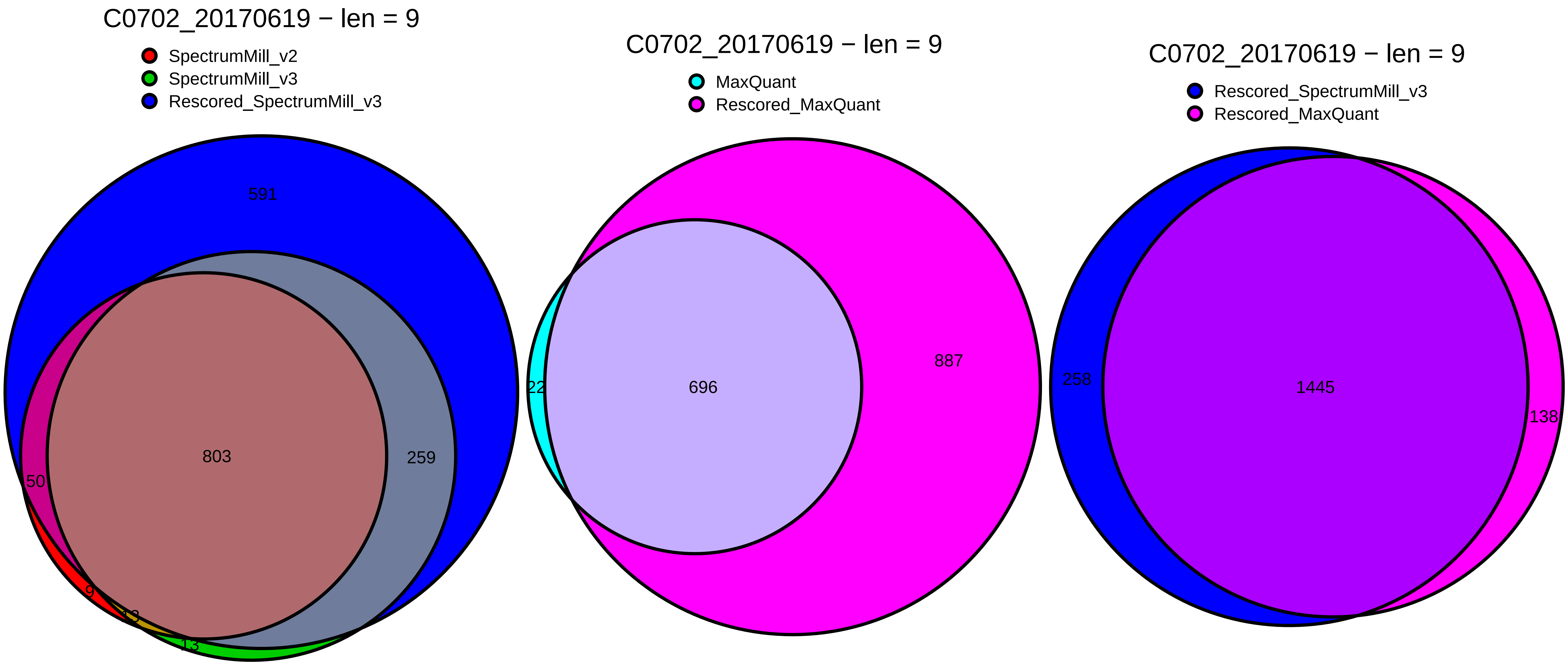
C0702_20170619 − len = 9
SpectrumMill_v2
SpectrumMill_v3
Rescored_SpectrumMill_v3
591
803
259
50
9
13
13
C0702_20170619 − len = 9
MaxQuant
Rescored_MaxQuant
22
696
887
C0702_20170619 − len = 9
Rescored_SpectrumMill_v3
Rescored_MaxQuant
258
1445
138

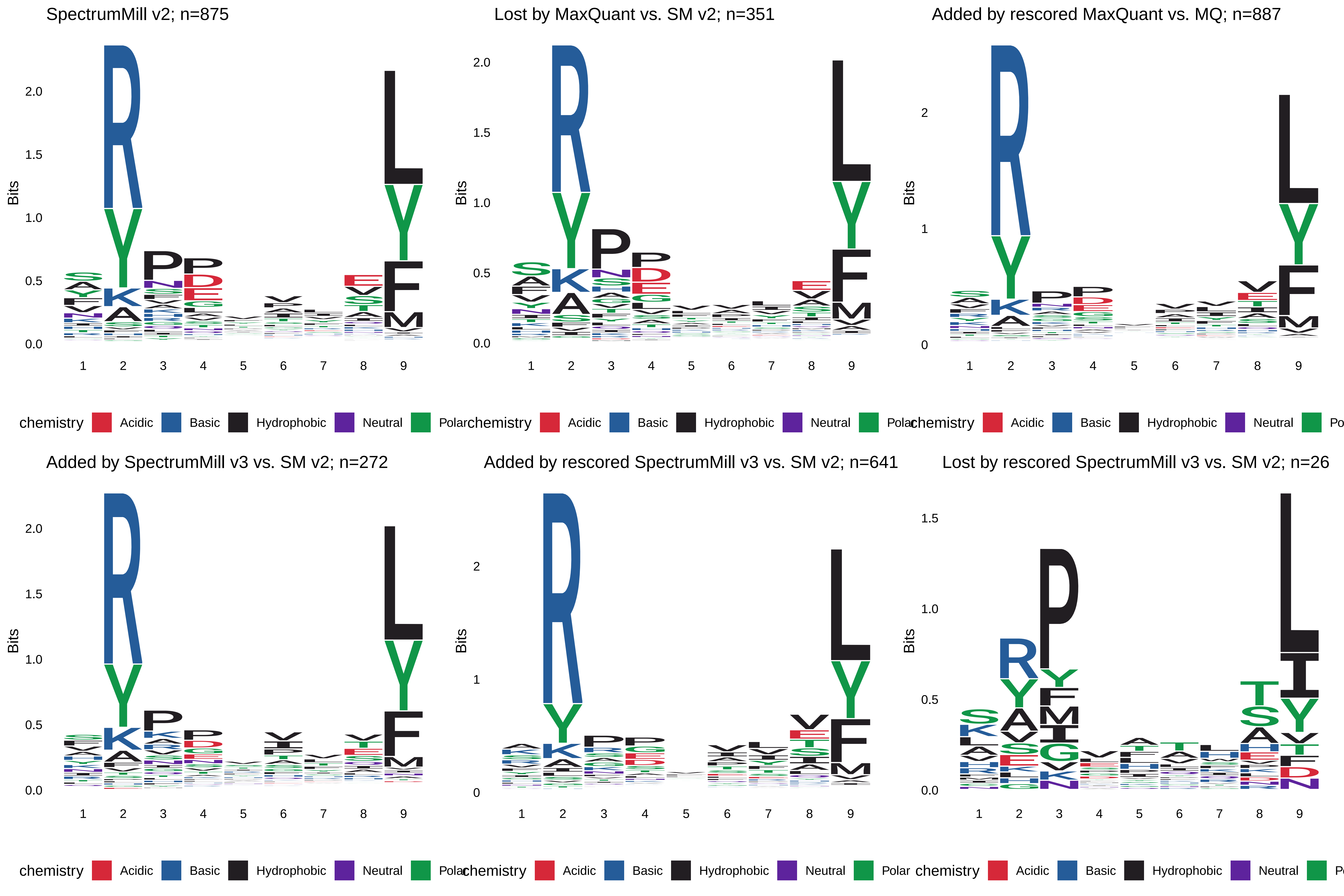
SpectrumMill v2; n=875
Lost by MaxQuant vs. SM v2; n=351
Added by rescored MaxQuant vs. MQ; n=887
Added by SpectrumMill v3 vs. SM v2; n=272
Added by rescored SpectrumMill v3 vs. SM v2; n=641
Lost by rescored SpectrumMill v3 vs. SM v2; n=26
Bits
1 2 3 4 5 6 7 8 9
chemistry
Acidic
Basic
Hydrophobic
Neutral
Polar

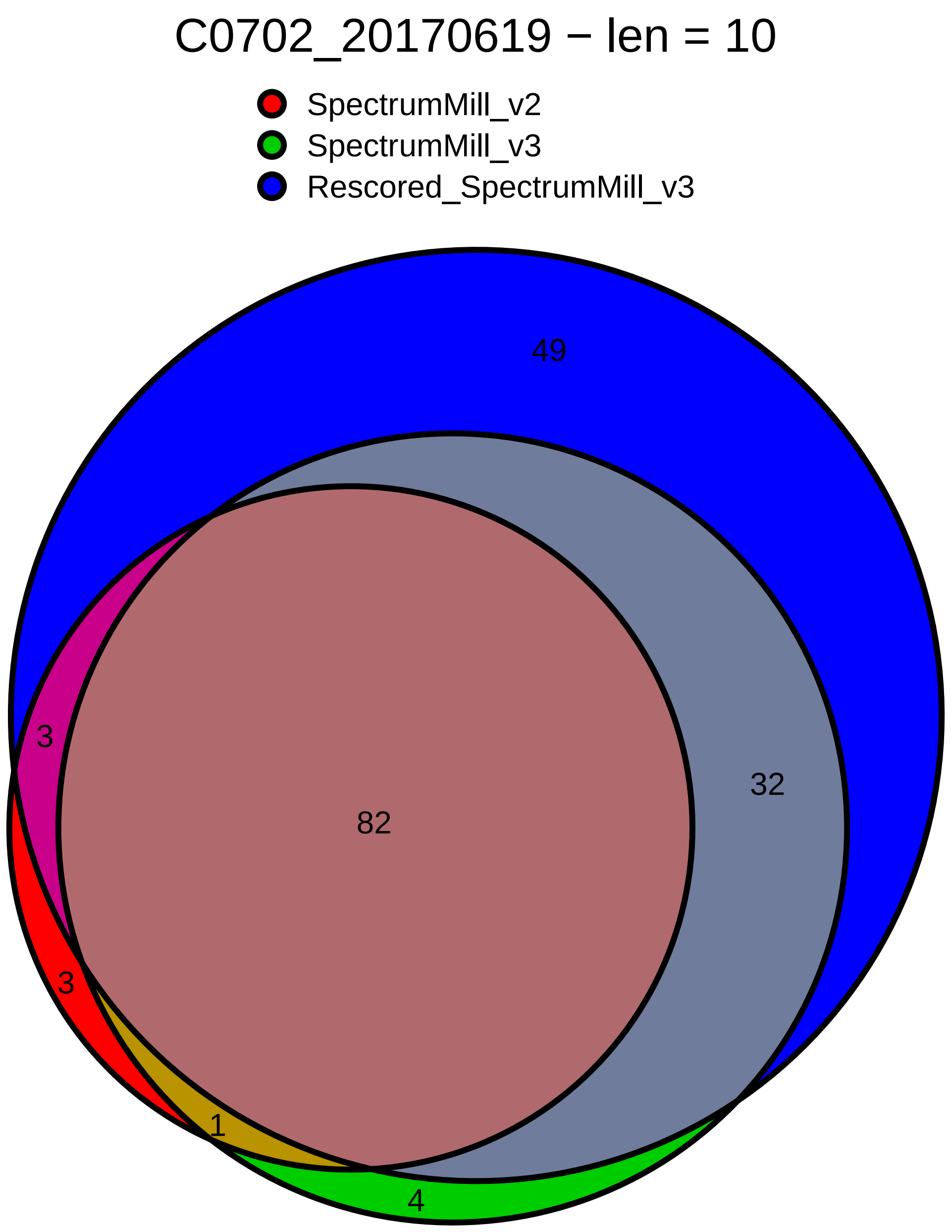
C0702_20170619 − len = 10
SpectrumMill_v2
SpectrumMill_v3
Rescored_SpectrumMill_v3
49
3
82
32
3
1
4
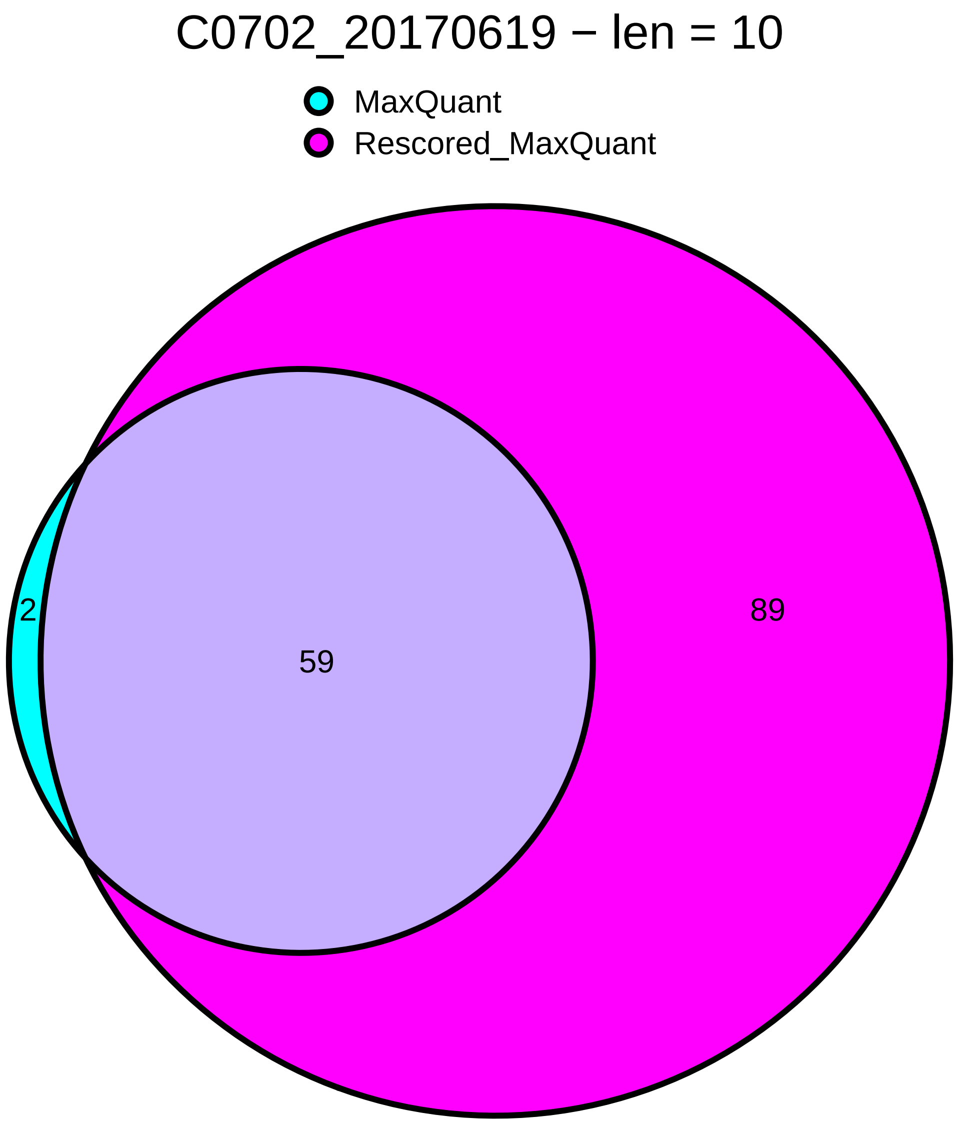
C0702_20170619 − len = 10
MaxQuant
Rescored_MaxQuant
2
59
89
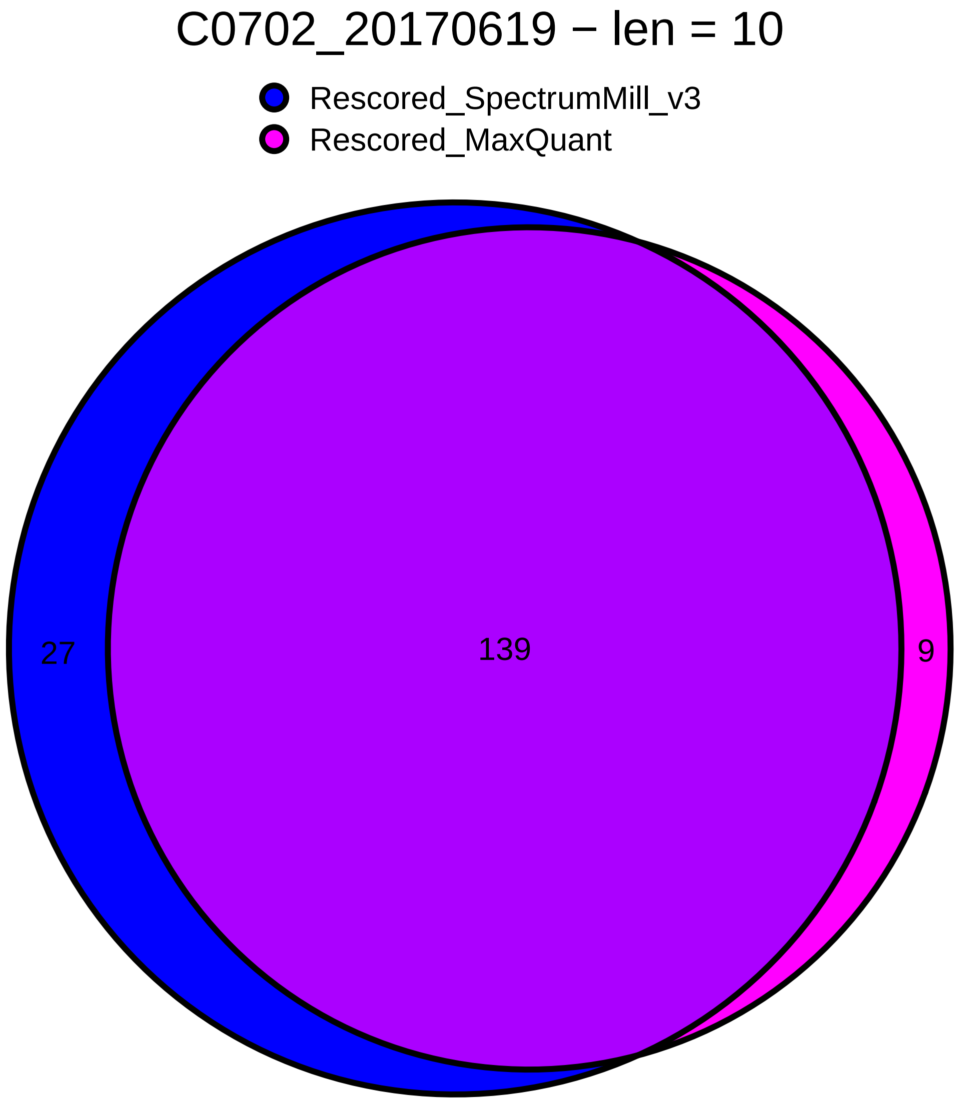
C0702_20170619 − len = 10
Rescored_SpectrumMill_v3
Rescored_MaxQuant
27
139
9

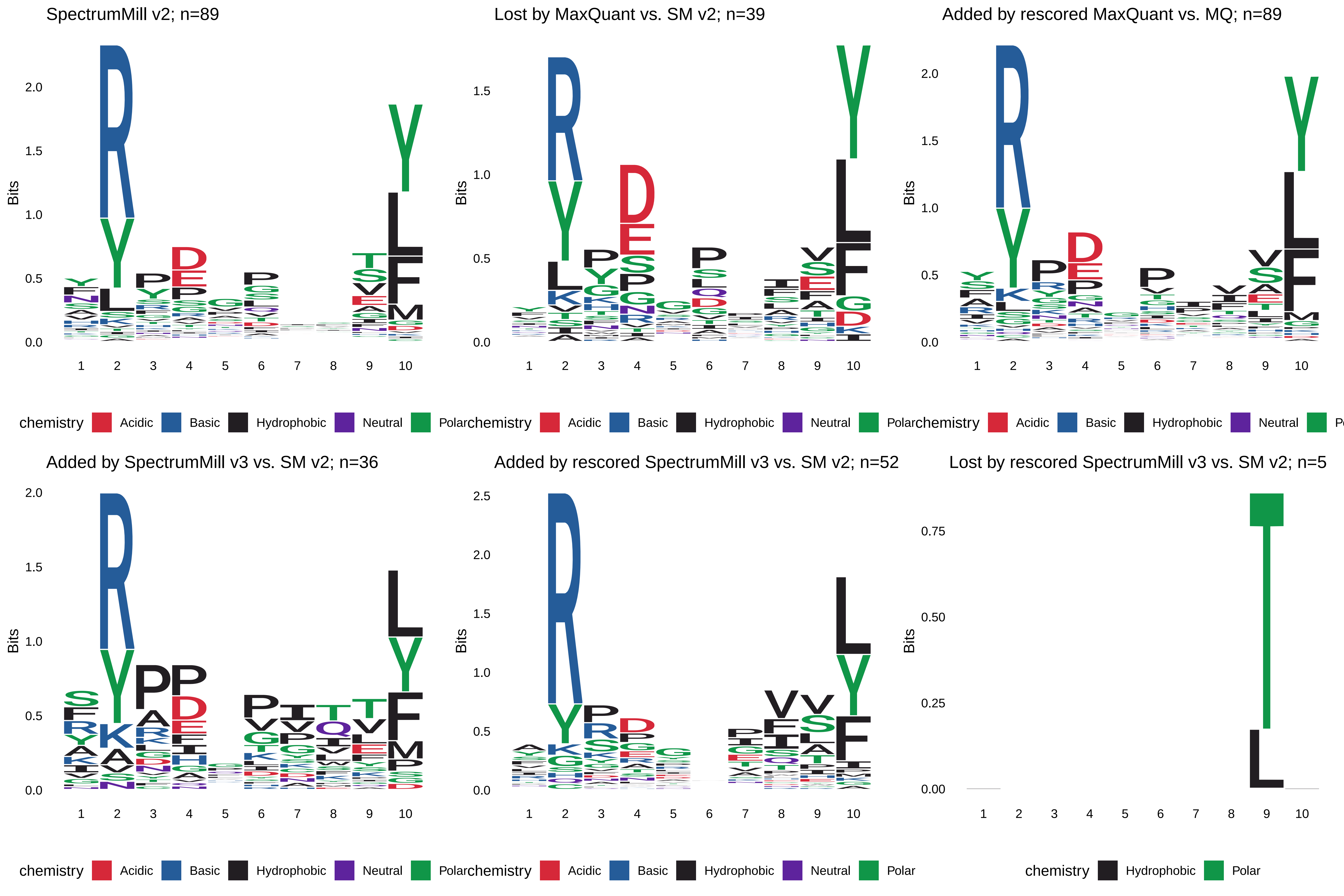
SpectrumMill v2; n=89
Lost by MaxQuant vs. SM v2; n=39
Added by rescored MaxQuant vs. MQ; n=89
Added by SpectrumMill v3 vs. SM v2; n=36
Added by rescored SpectrumMill v3 vs. SM v2; n=52
Lost by rescored SpectrumMill v3 vs. SM v2; n=5
Bits
1 2 3 4 5 6 7 8 9 10
chemistry Acidic Basic Hydrophobic Neutral Polar
chemistry Hydrophobic Polar

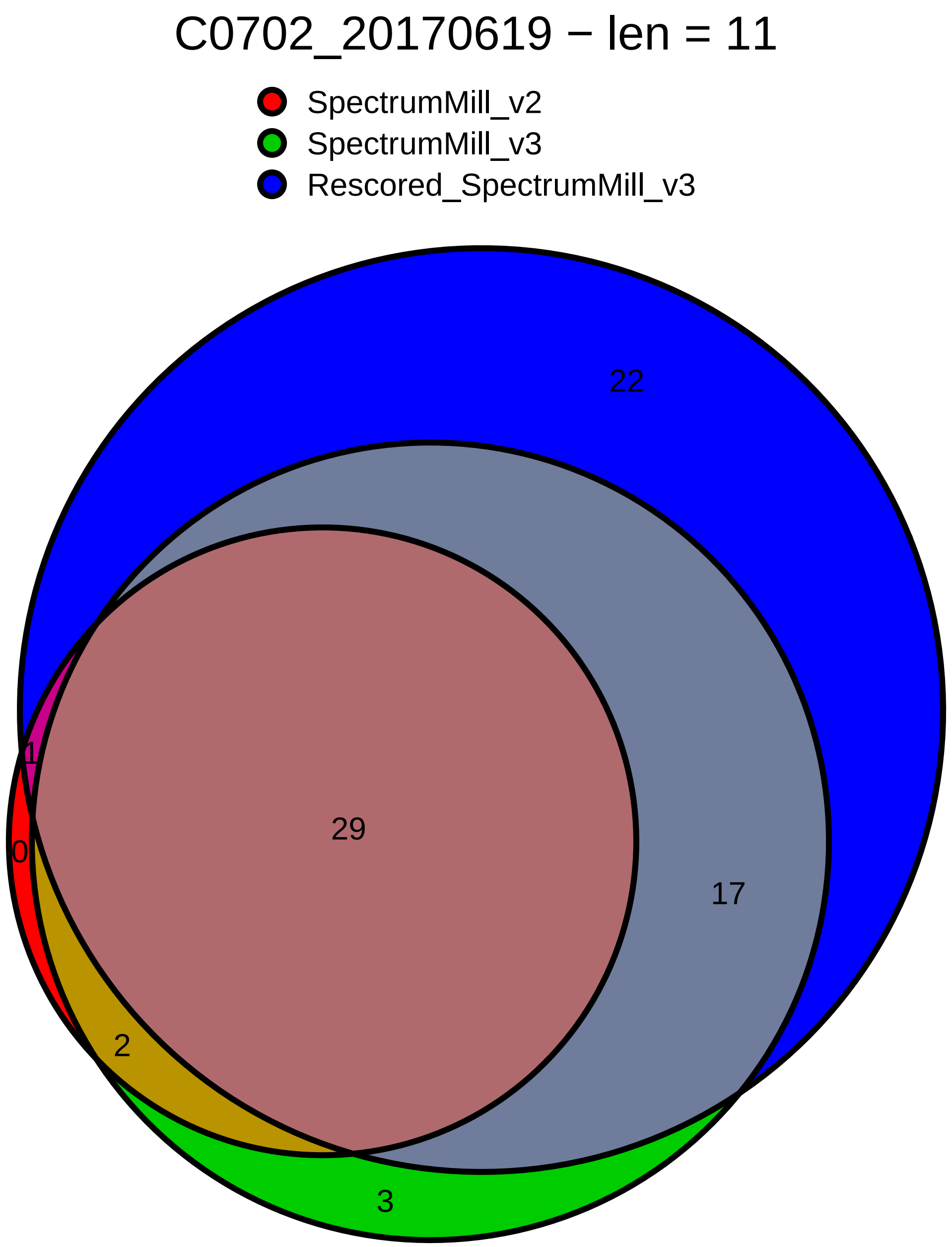
C0702_20170619 − len = 11
SpectrumMill_v2
SpectrumMill_v3
Rescored_SpectrumMill_v3
22
1
0
29
17
2
3

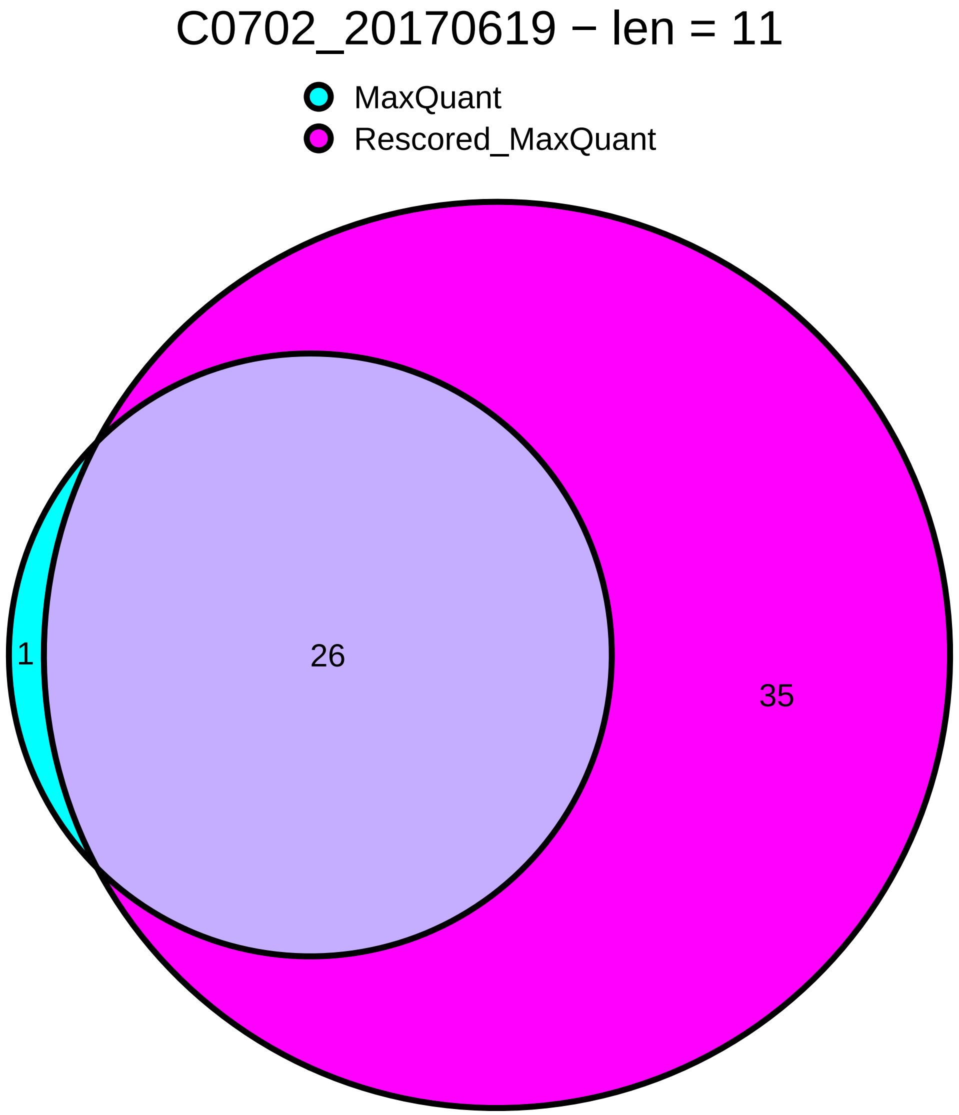
C0702_20170619 − len = 11
MaxQuant
Rescored_MaxQuant
1
26
35

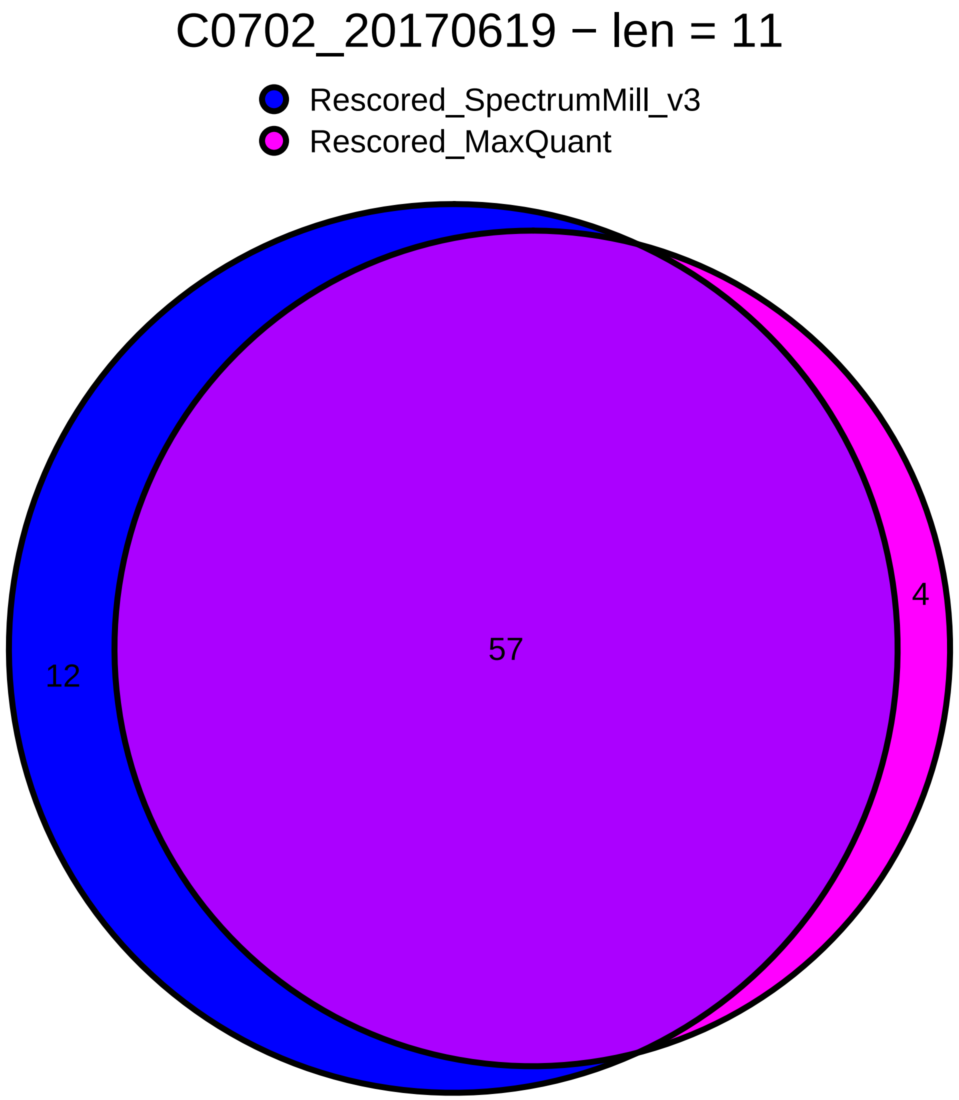
C0702_20170619 − len = 11
Rescored_SpectrumMill_v3
Rescored_MaxQuant
12
57
4

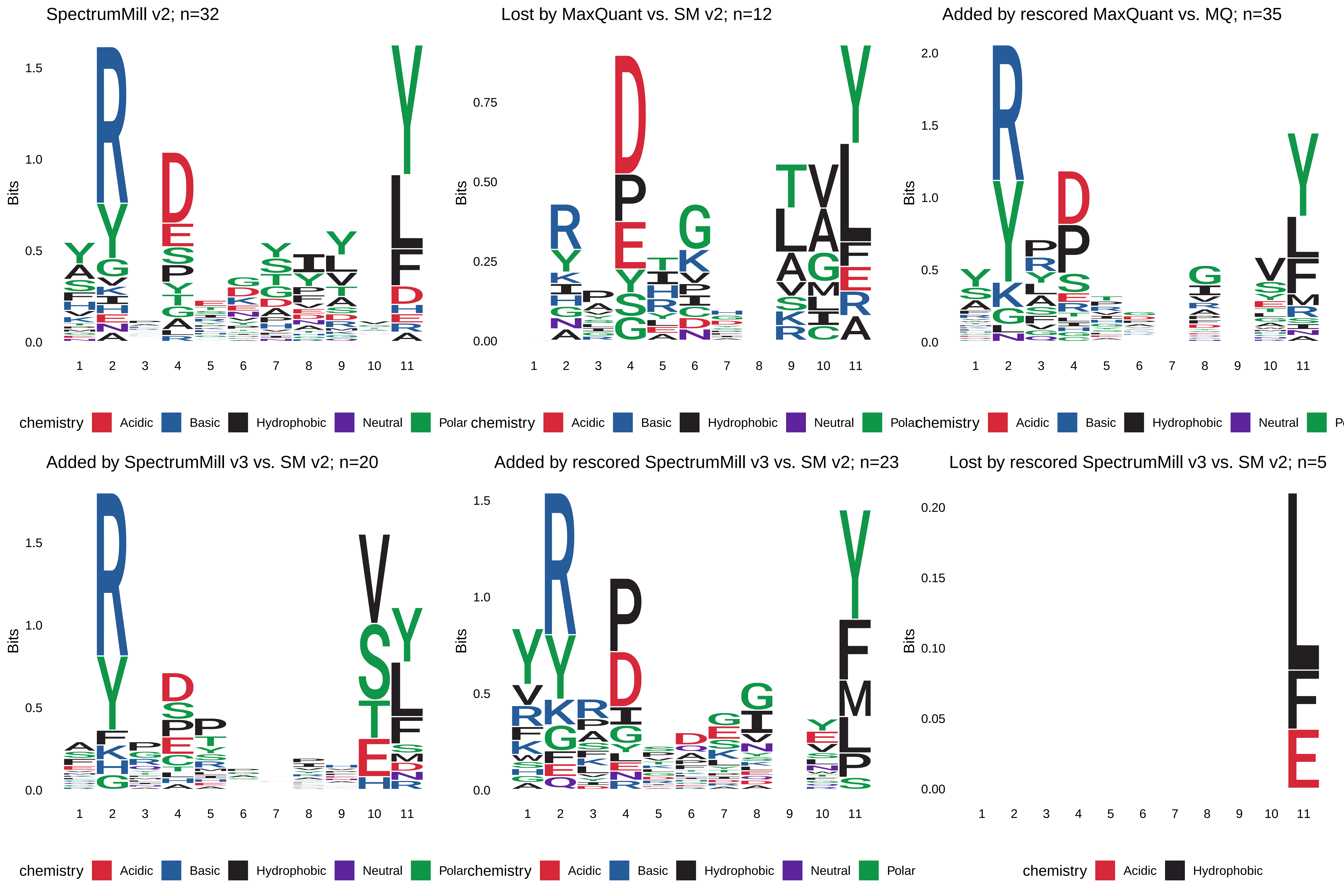

SpectrumMill v2; n=32
Lost by MaxQuant vs. SM v2; n=12
Added by rescored MaxQuant vs. MQ; n=35
Added by SpectrumMill v3 vs. SM v2; n=20
Added by rescored SpectrumMill v3 vs. SM v2; n=23
Lost by rescored SpectrumMill v3 vs. SM v2; n=5
Bits
1 2 3 4 5 6 7 8 9 10 11
chemistry
Acidic
Basic
Hydrophobic
Neutral
Polar

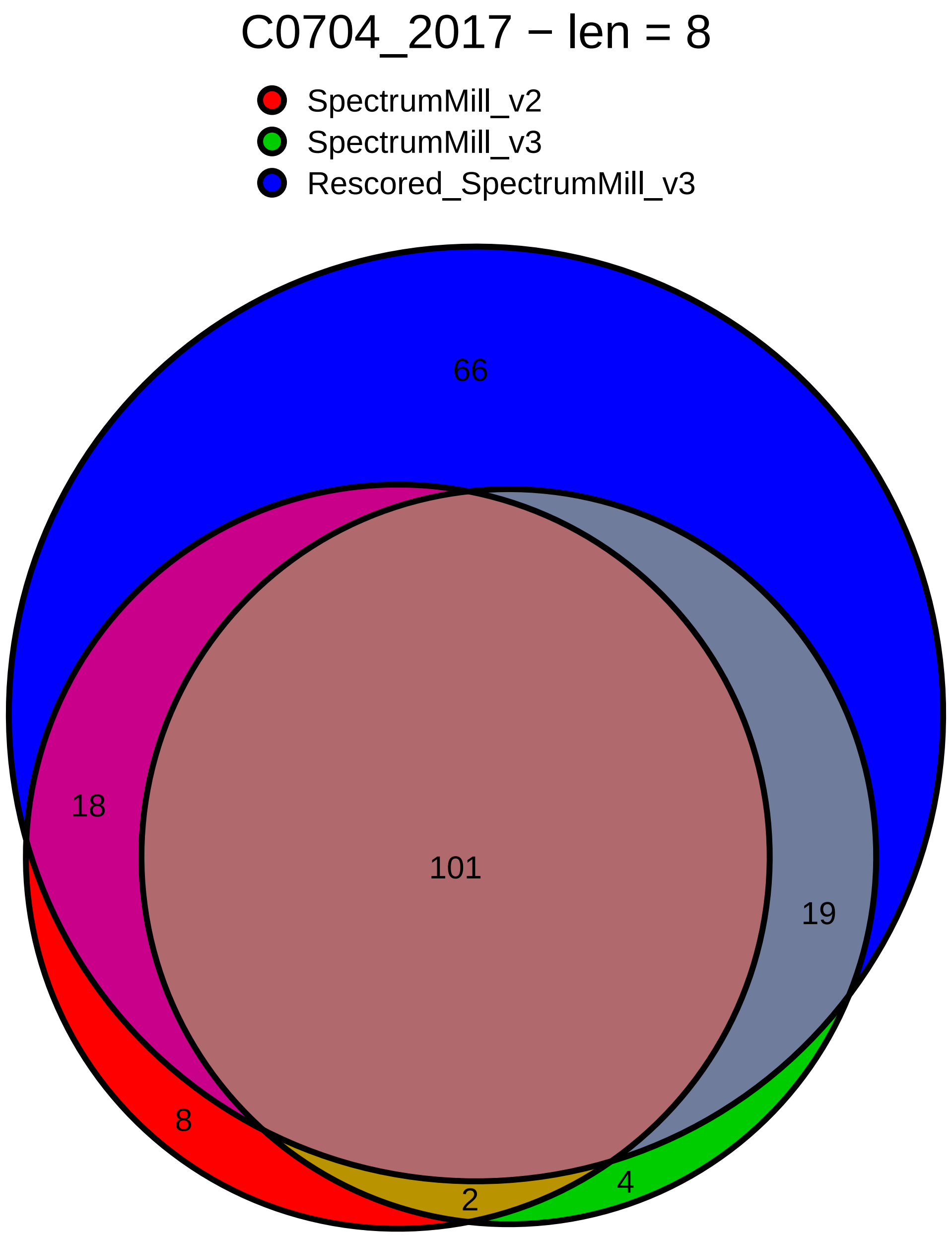
C0704_2017 − len = 8
SpectrumMill_v2
SpectrumMill_v3
Rescored_SpectrumMill_v3
66
18
101
19
8
2
4

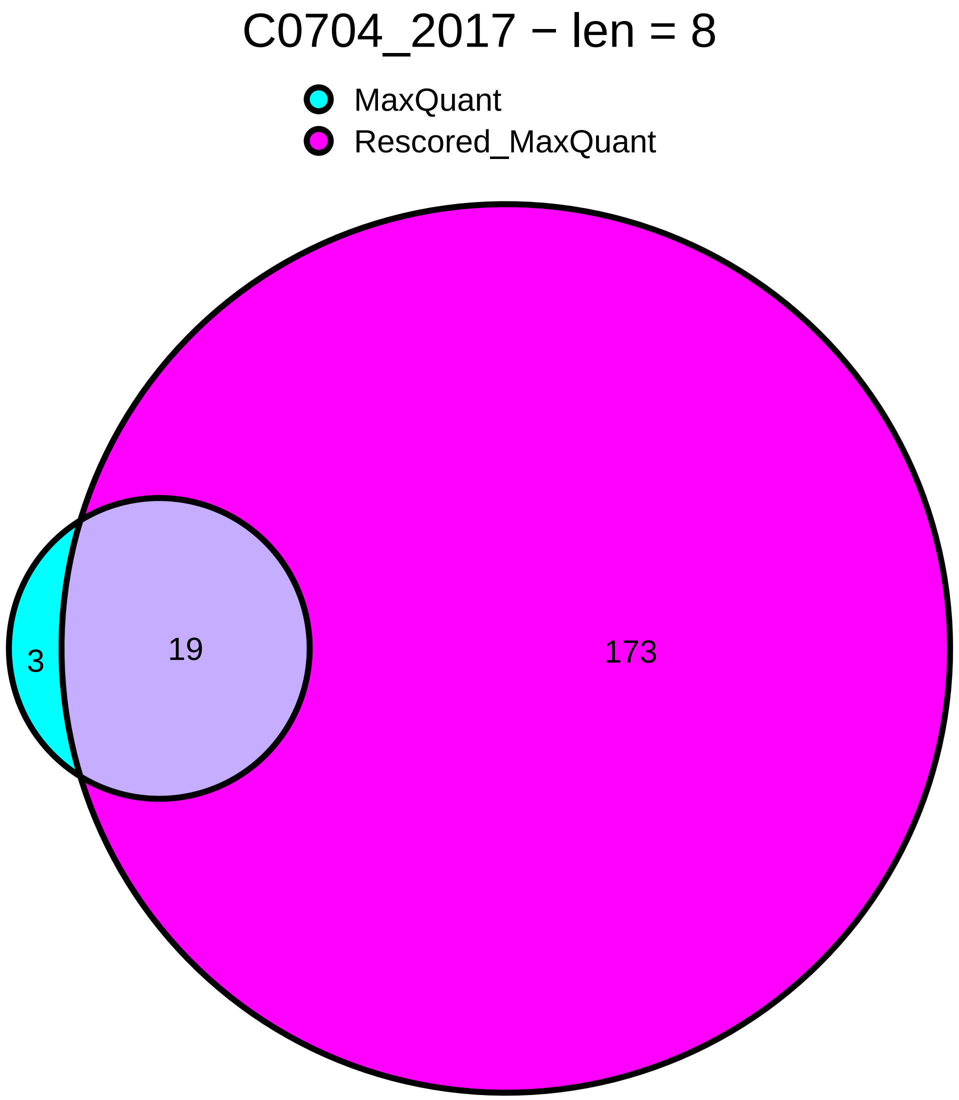
C0704_2017 − len = 8
MaxQuant
Rescored_MaxQuant
3
19
173

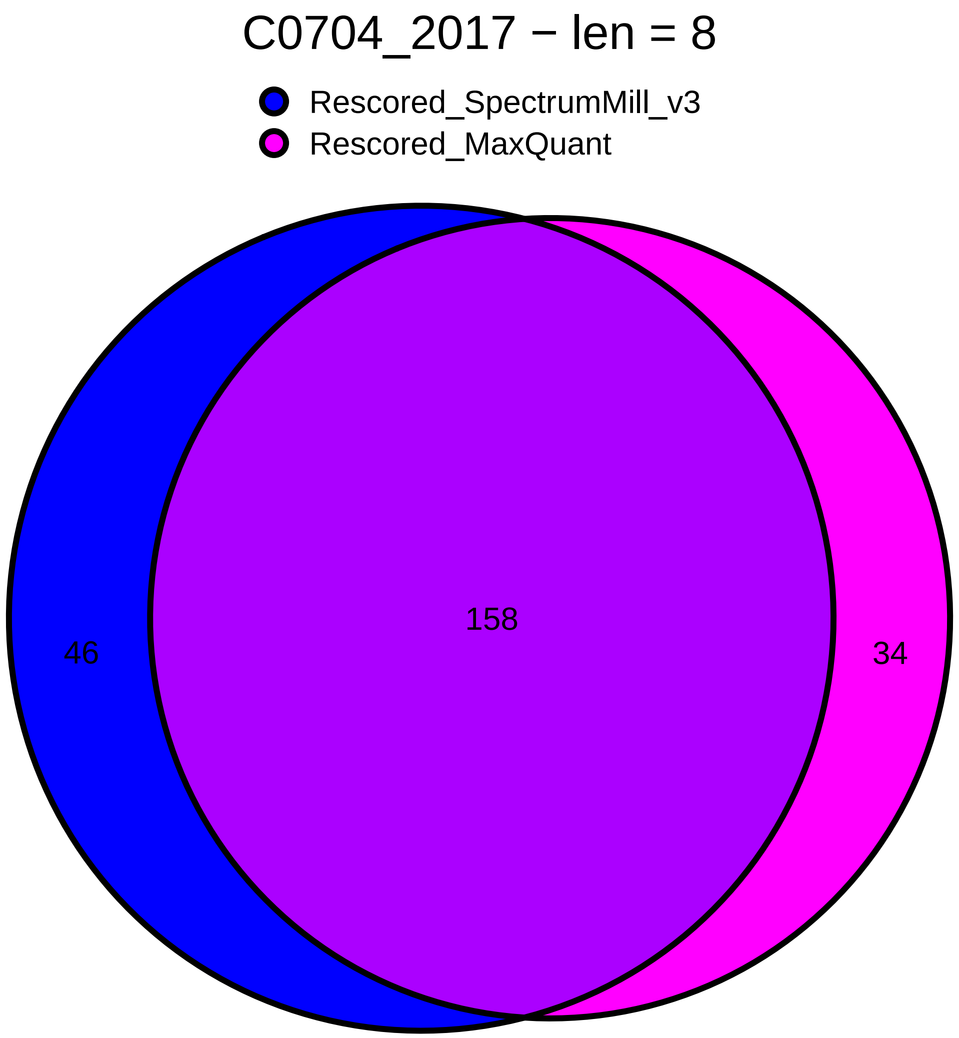
C0704_2017 − len = 8
Rescored_SpectrumMill_v3
Rescored_MaxQuant
46
158
34

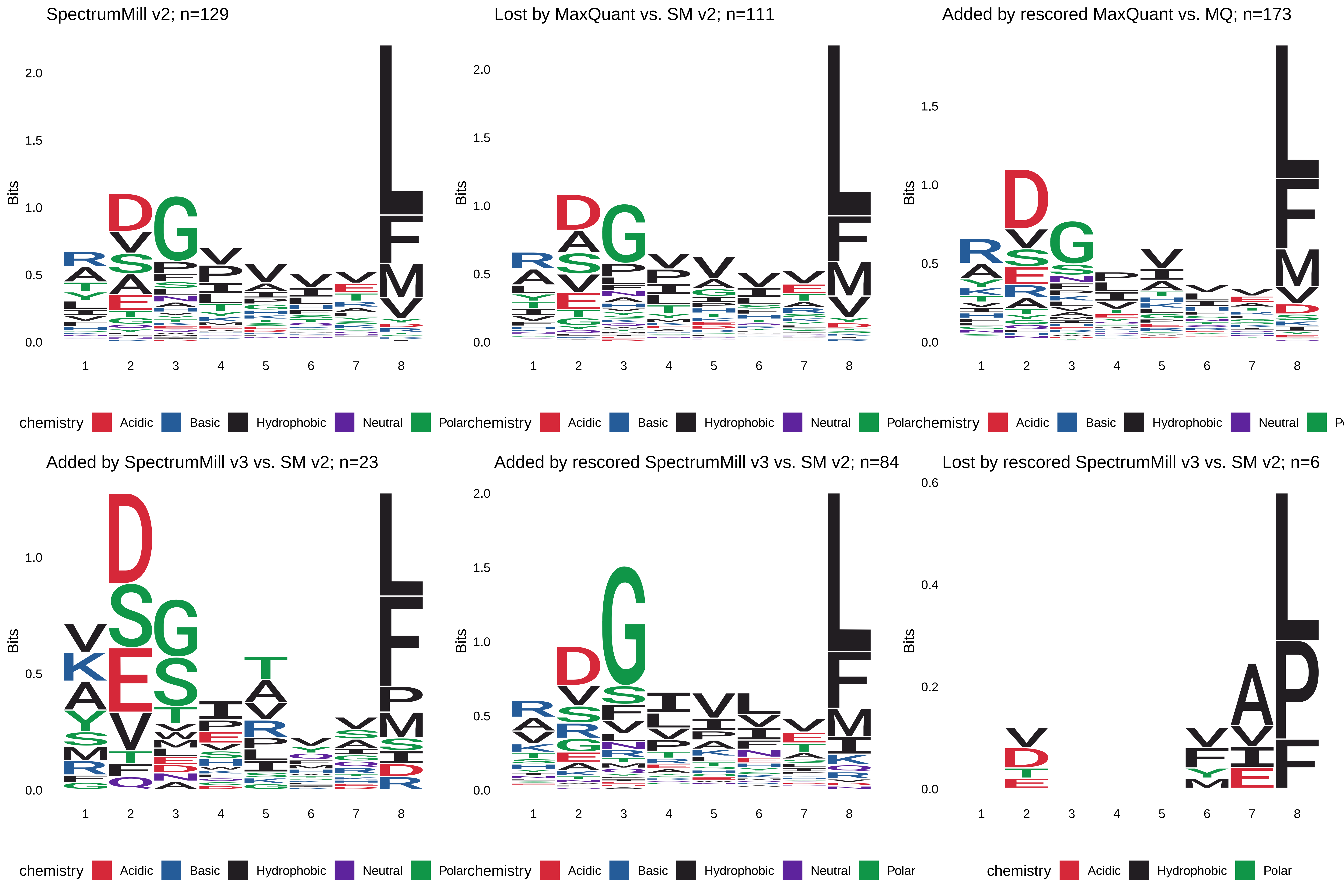

SpectrumMill v2; n=129
Lost by MaxQuant vs. SM v2; n=111
Added by rescored MaxQuant vs. MQ; n=173
Added by SpectrumMill v3 vs. SM v2; n=23
Added by rescored SpectrumMill v3 vs. SM v2; n=84
Lost by rescored SpectrumMill v3 vs. SM v2; n=6
Bits
chemistry
Acidic
Basic
Hydrophobic
Neutral
Polar

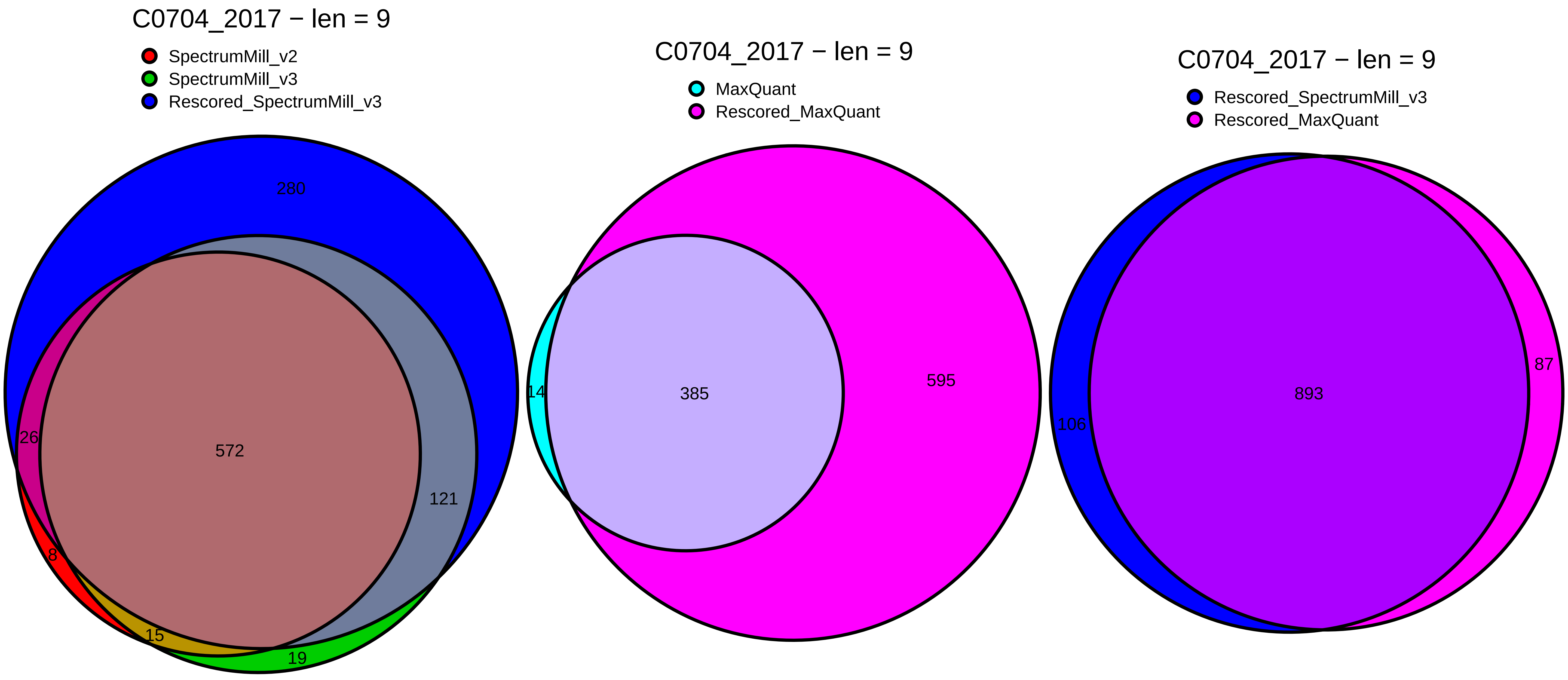

C0704_2017 − len = 9
SpectrumMill_v2
SpectrumMill_v3
Rescored_SpectrumMill_v3
280
26
572
121
8
15
19
C0704_2017 − len = 9
MaxQuant
Rescored_MaxQuant
14
385
595
C0704_2017 − len = 9
Rescored_SpectrumMill_v3
Rescored_MaxQuant
106
893
87

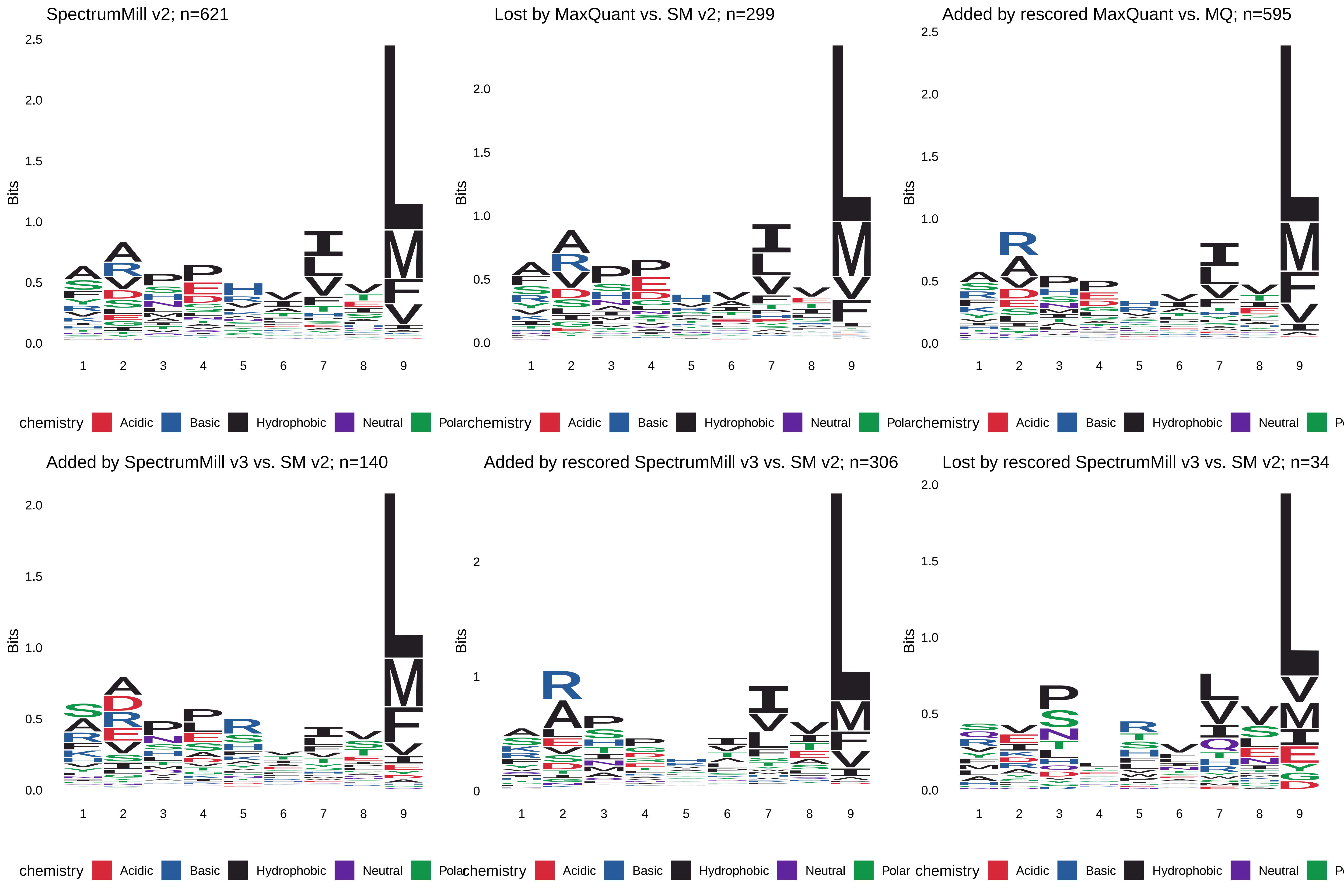
SpectrumMill v2; n=621
Lost by MaxQuant vs. SM v2; n=299
Added by rescored MaxQuant vs. MQ; n=595
Added by SpectrumMill v3 vs. SM v2; n=140
Added by rescored SpectrumMill v3 vs. SM v2; n=306
Lost by rescored SpectrumMill v3 vs. SM v2; n=34
Bits
1 2 3 4 5 6 7 8 9
chemistry Acidic Basic Hydrophobic Neutral Polar

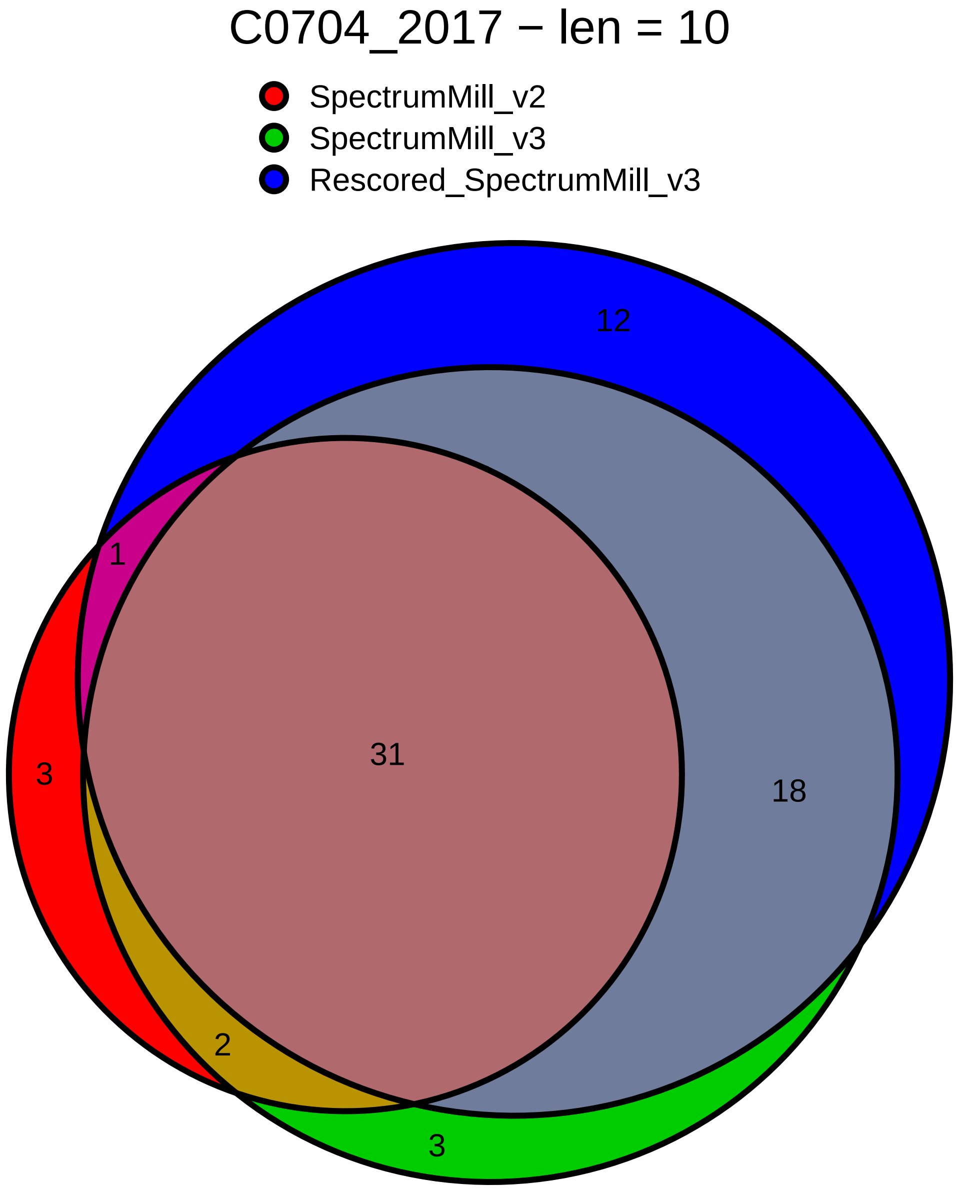

C0704_2017 − len = 10
SpectrumMill_v2
SpectrumMill_v3
Rescored_SpectrumMill_v3
12
1
3
31
18
2
3

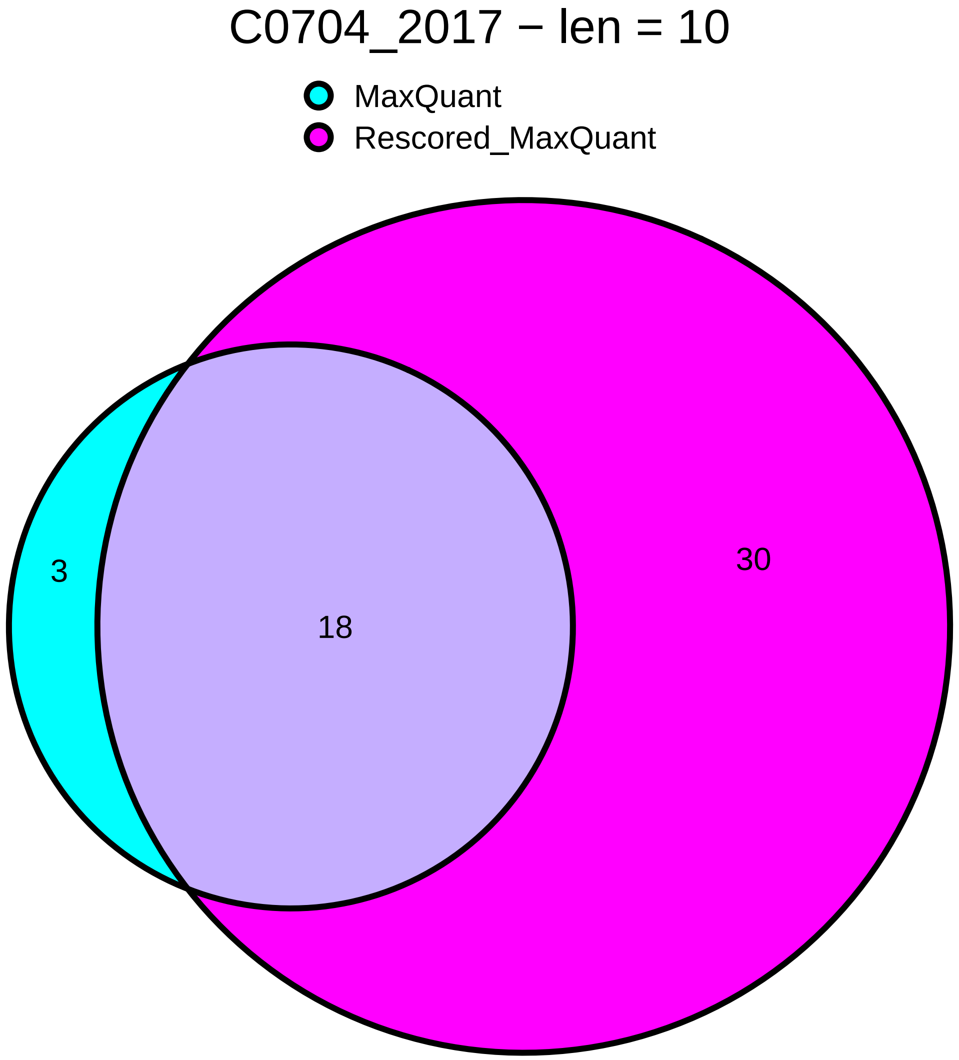

C0704_2017 − len = 10
MaxQuant
Rescored_MaxQuant
3
18
30

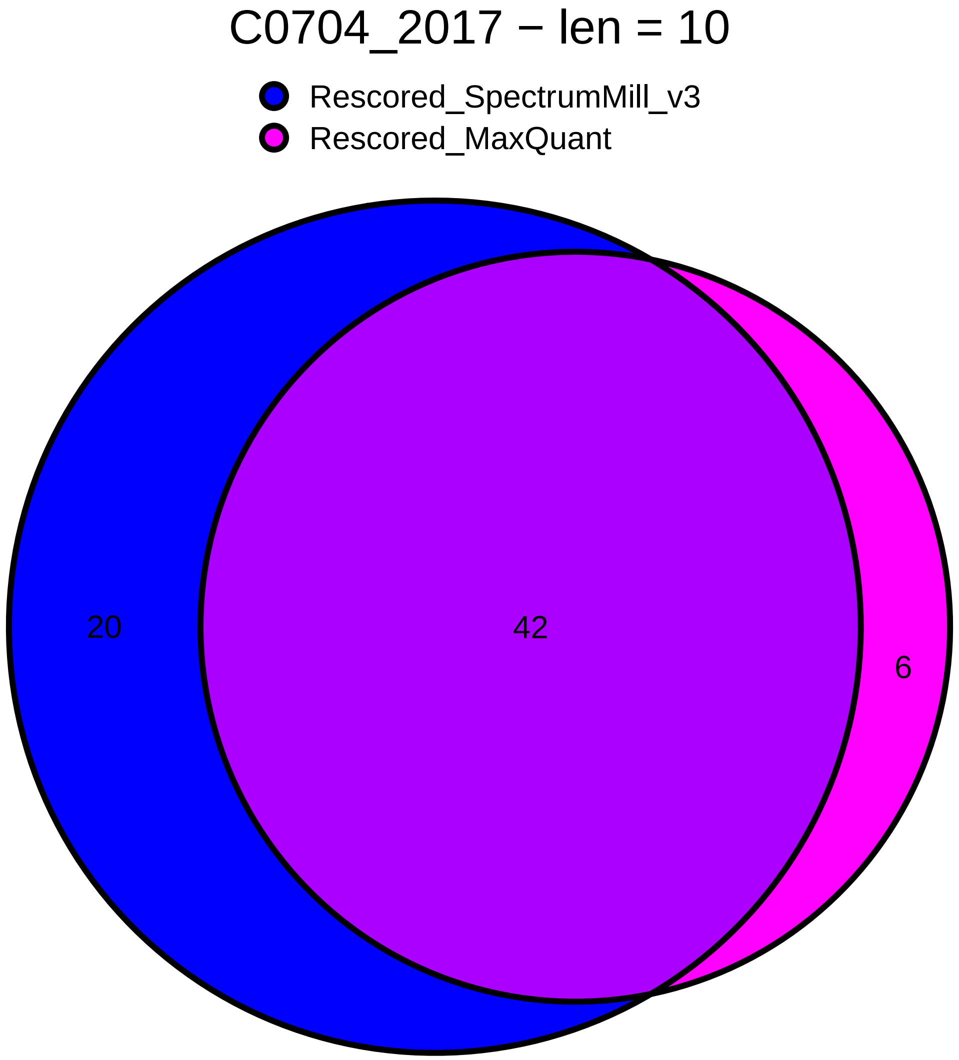

C0704_2017 − len = 10
Rescored_SpectrumMill_v3
Rescored_MaxQuant
20
42
6

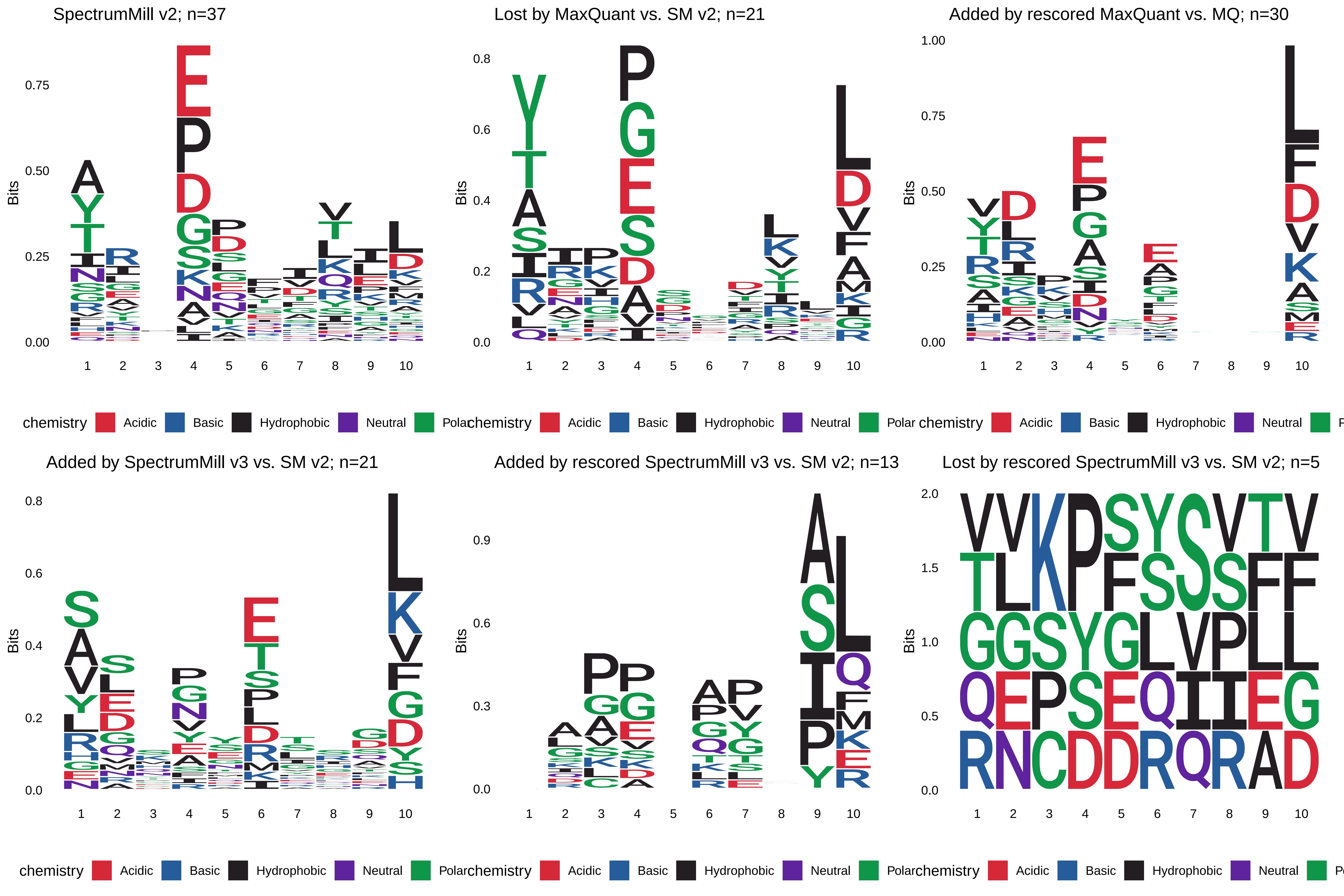
SpectrumMill v2; n=37
Lost by MaxQuant vs. SM v2; n=21
Added by rescored MaxQuant vs. MQ; n=30
Added by SpectrumMill v3 vs. SM v2; n=21
Added by rescored SpectrumMill v3 vs. SM v2; n=13
Lost by rescored SpectrumMill v3 vs. SM v2; n=5
Bits
1 2 3 4 5 6 7 8 9 10
chemistry
Acidic
Basic
Hydrophobic
Neutral
Polar

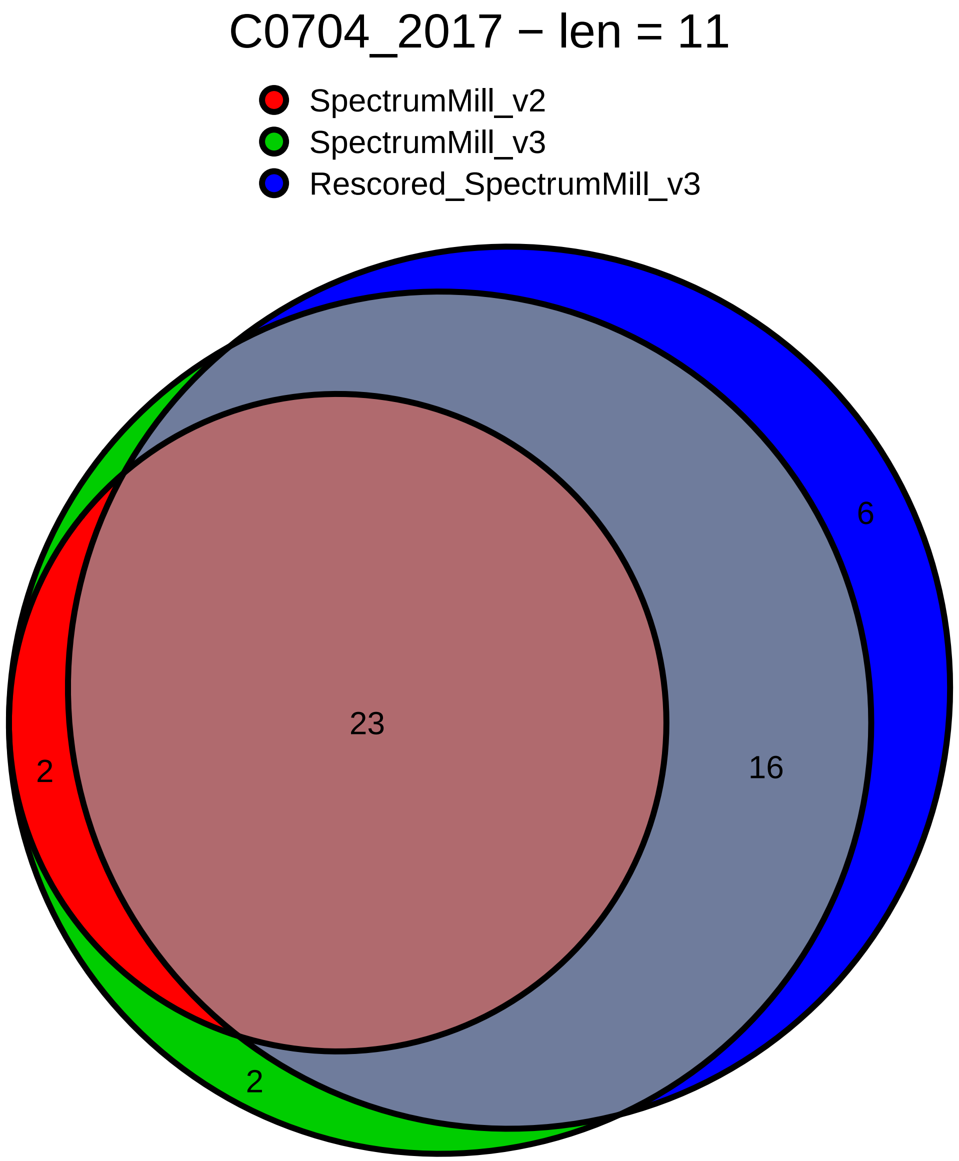
C0704_2017 − len = 11
SpectrumMill_v2
SpectrumMill_v3
Rescored_SpectrumMill_v3
6
23
16
2
2

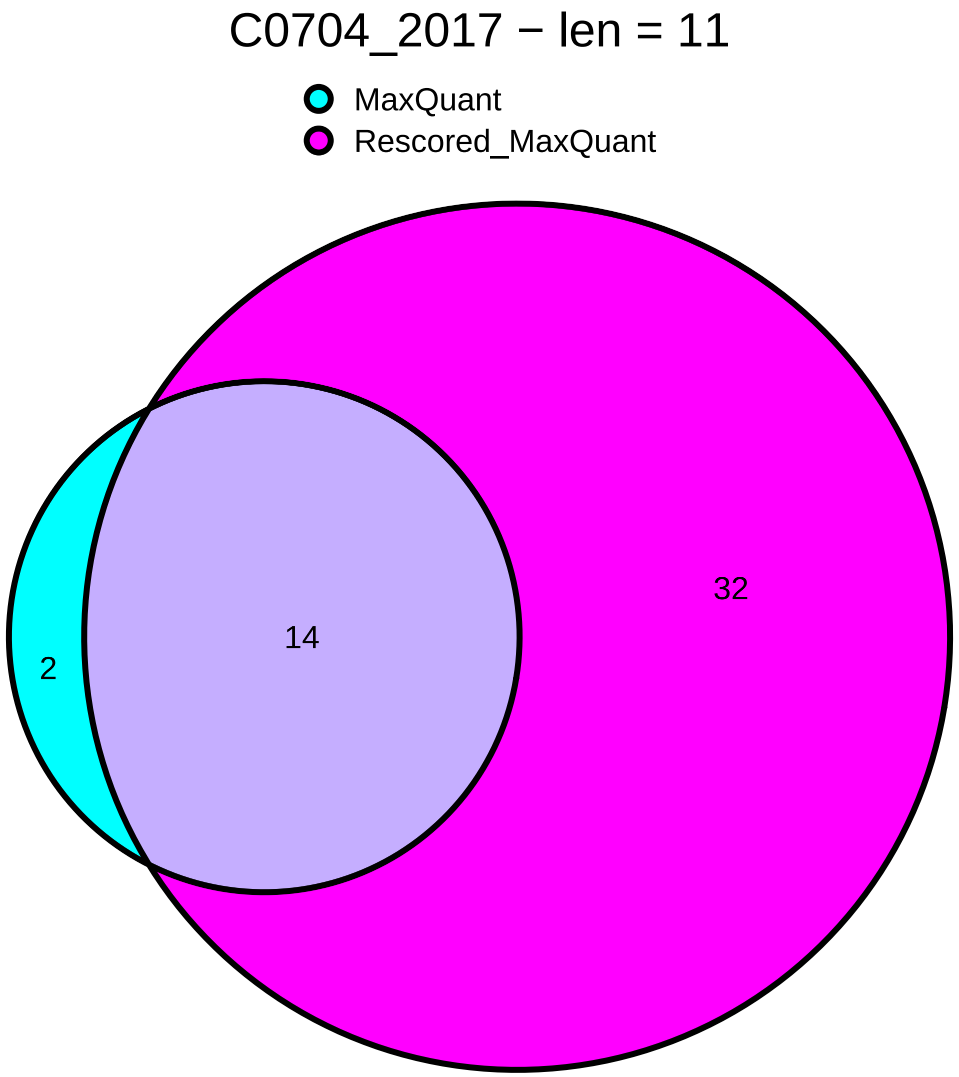
C0704_2017 − len = 11
MaxQuant
Rescored_MaxQuant
2
14
32

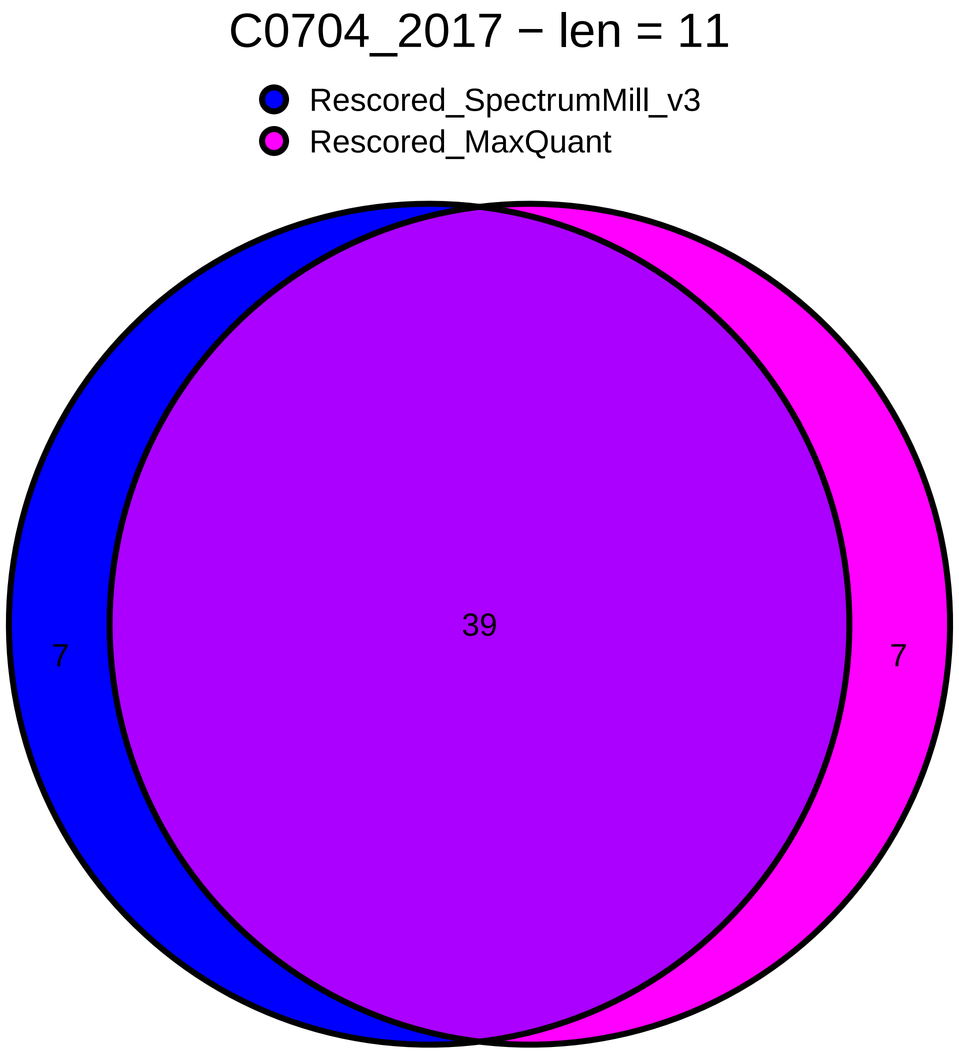
C0704_2017 − len = 11
Rescored_SpectrumMill_v3
Rescored_MaxQuant
7
39
7

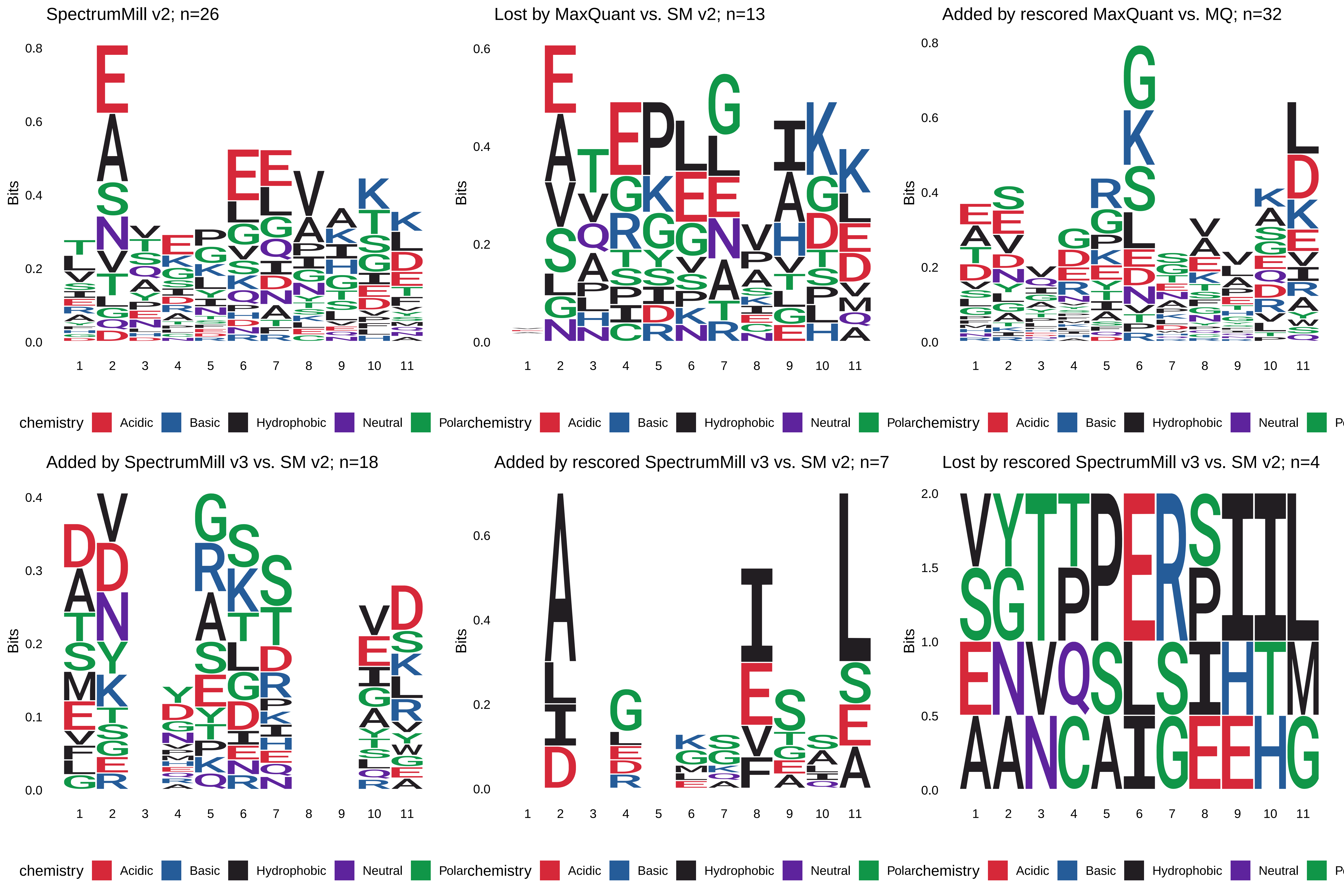

SpectrumMill v2; n=26
Lost by MaxQuant vs. SM v2; n=13
Added by rescored MaxQuant vs. MQ; n=32
Added by SpectrumMill v3 vs. SM v2; n=18
Added by rescored SpectrumMill v3 vs. SM v2; n=7
Lost by rescored SpectrumMill v3 vs. SM v2; n=4
Bits
1 2 3 4 5 6 7 8 9 10 11
chemistry Acidic Basic Hydrophobic Neutral Polar

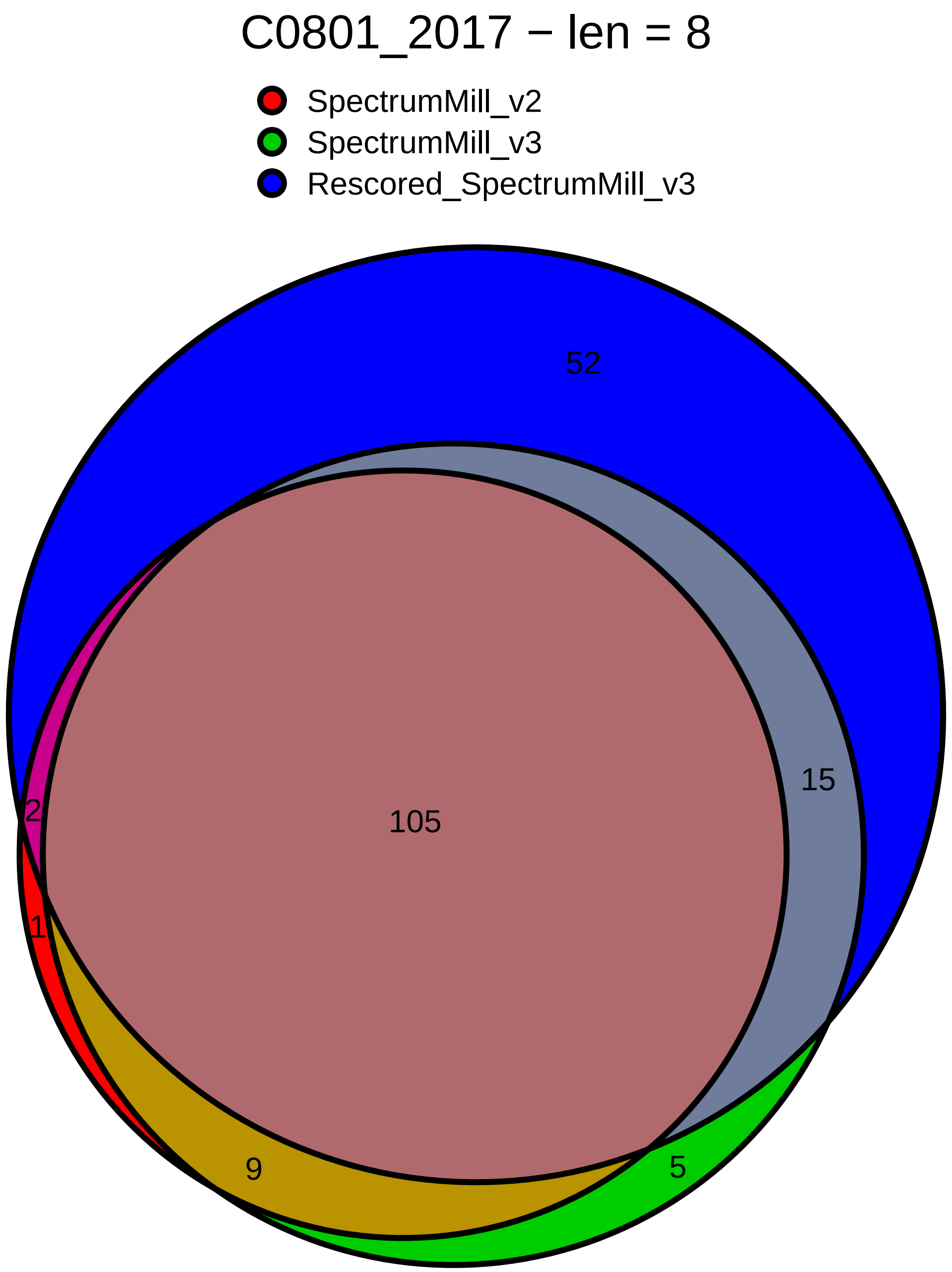
C0801_2017 − len = 8
SpectrumMill_v2
SpectrumMill_v3
Rescored_SpectrumMill_v3
52
15
2
105
1
9
5

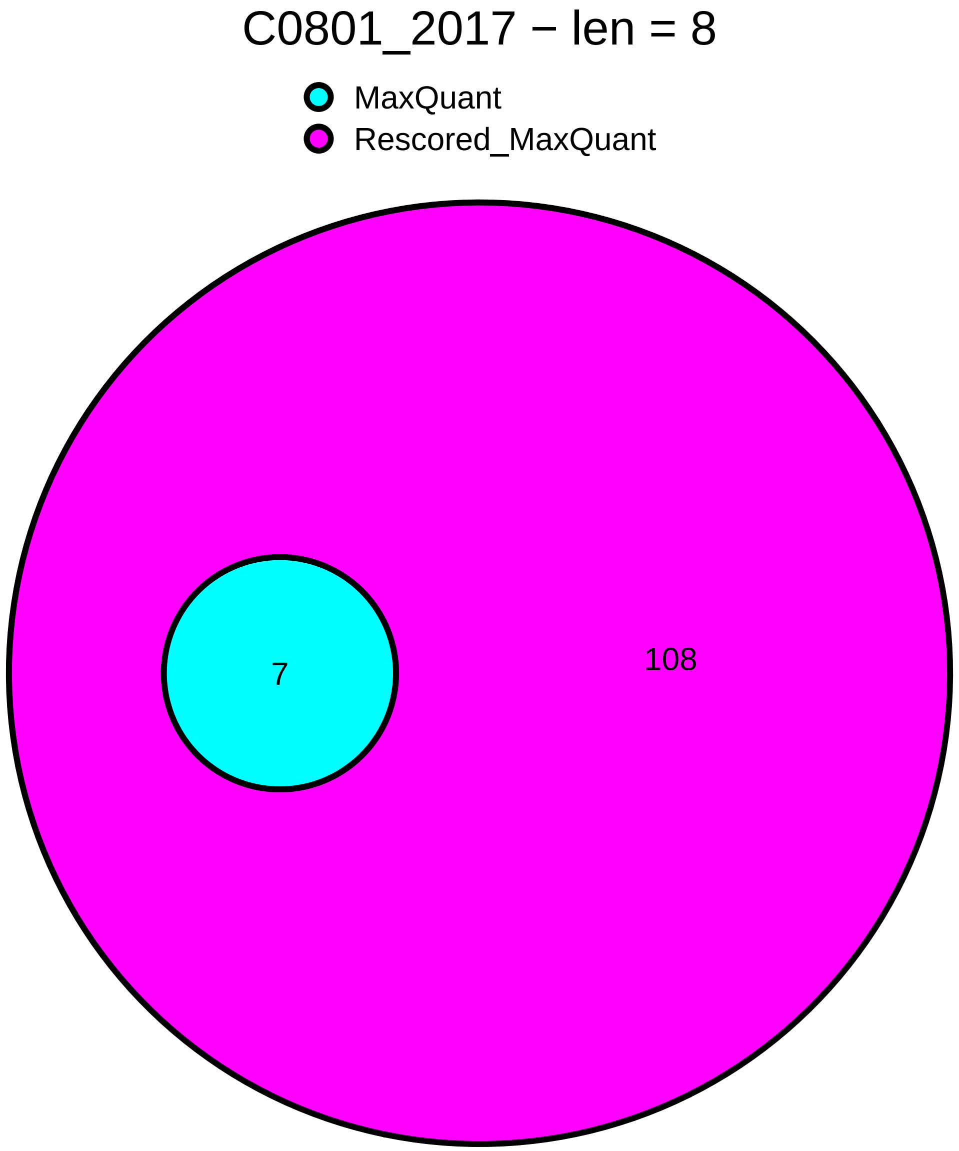
C0801_2017 − len = 8
MaxQuant
Rescored_MaxQuant
7
108

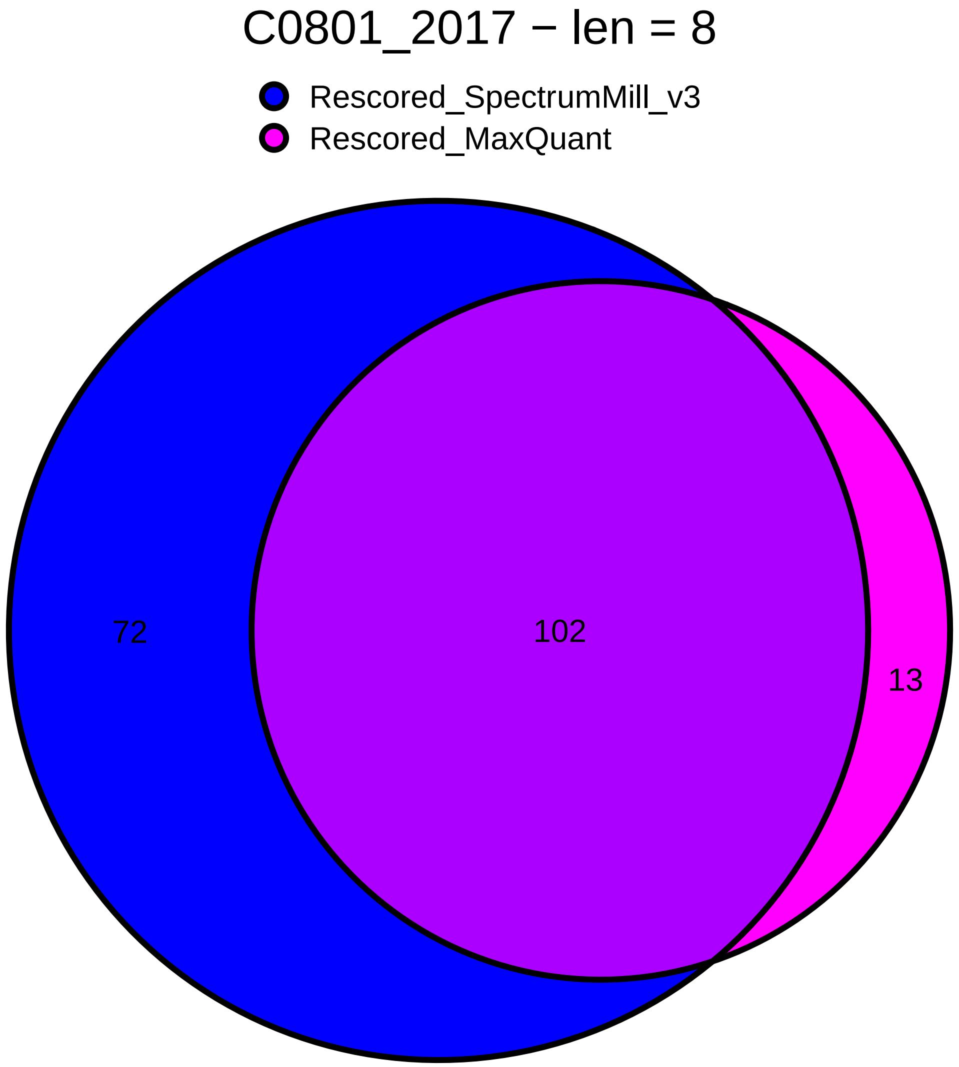
C0801_2017 − len = 8
Rescored_SpectrumMill_v3
Rescored_MaxQuant
72
102
13

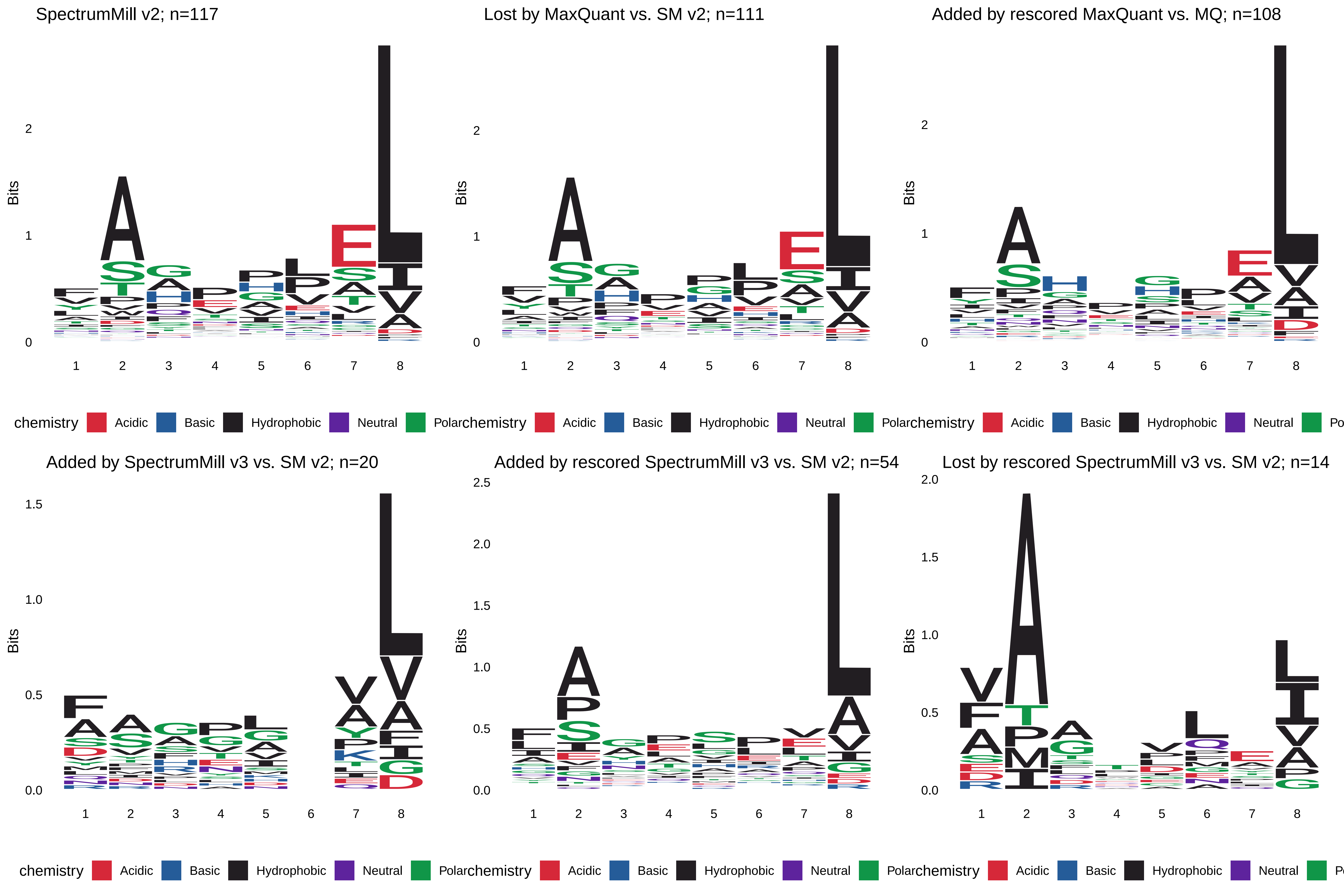
SpectrumMill v2; n=117
Lost by MaxQuant vs. SM v2; n=111
Added by rescored MaxQuant vs. MQ; n=108
Added by SpectrumMill v3 vs. SM v2; n=20
Added by rescored SpectrumMill v3 vs. SM v2; n=54
Lost by rescored SpectrumMill v3 vs. SM v2; n=14
Bits
1 2 3 4 5 6 7 8
chemistry
Acidic
Basic
Hydrophobic
Neutral
Polar

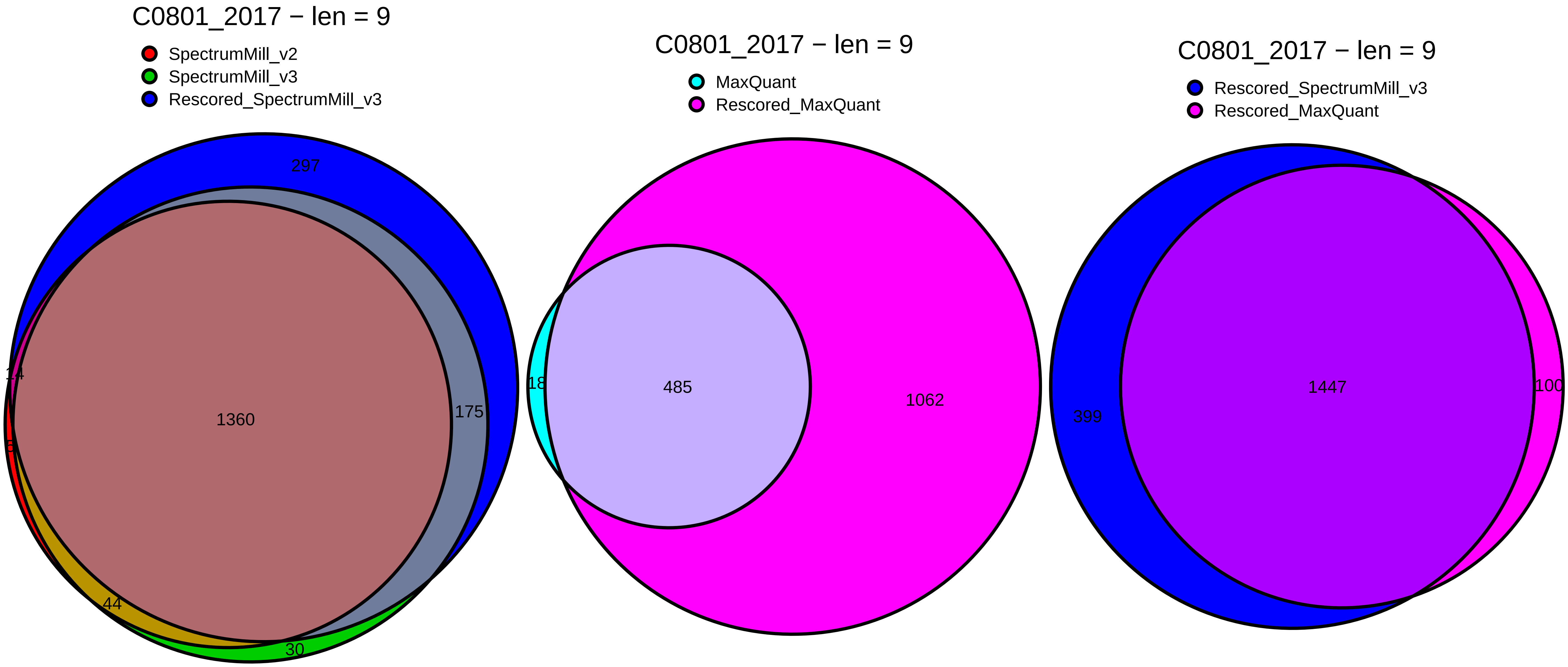
C0801_2017 − len = 9
SpectrumMill_v2
SpectrumMill_v3
Rescored_SpectrumMill_v3
297
14
1360
175
44
30
C0801_2017 − len = 9
MaxQuant
Rescored_MaxQuant
18
485
1062
C0801_2017 − len = 9
Rescored_SpectrumMill_v3
Rescored_MaxQuant
399
1447
100

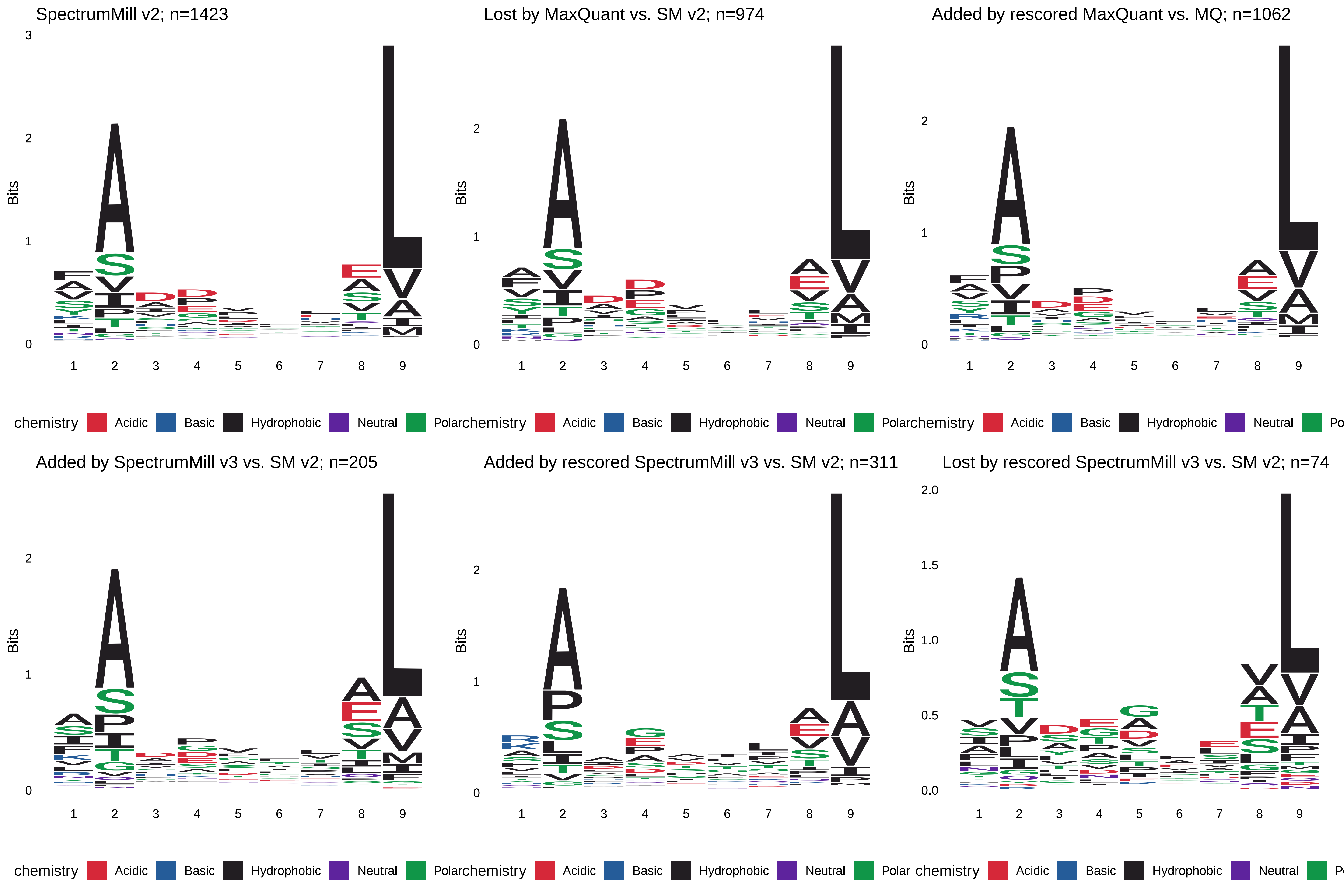
SpectrumMill v2; n=1423
Lost by MaxQuant vs. SM v2; n=974
Added by rescored MaxQuant vs. MQ; n=1062
Added by SpectrumMill v3 vs. SM v2; n=205
Added by rescored SpectrumMill v3 vs. SM v2; n=311
Lost by rescored SpectrumMill v3 vs. SM v2; n=74
Bits
1 2 3 4 5 6 7 8 9
chemistry
Acidic
Basic
Hydrophobic
Neutral
Polar

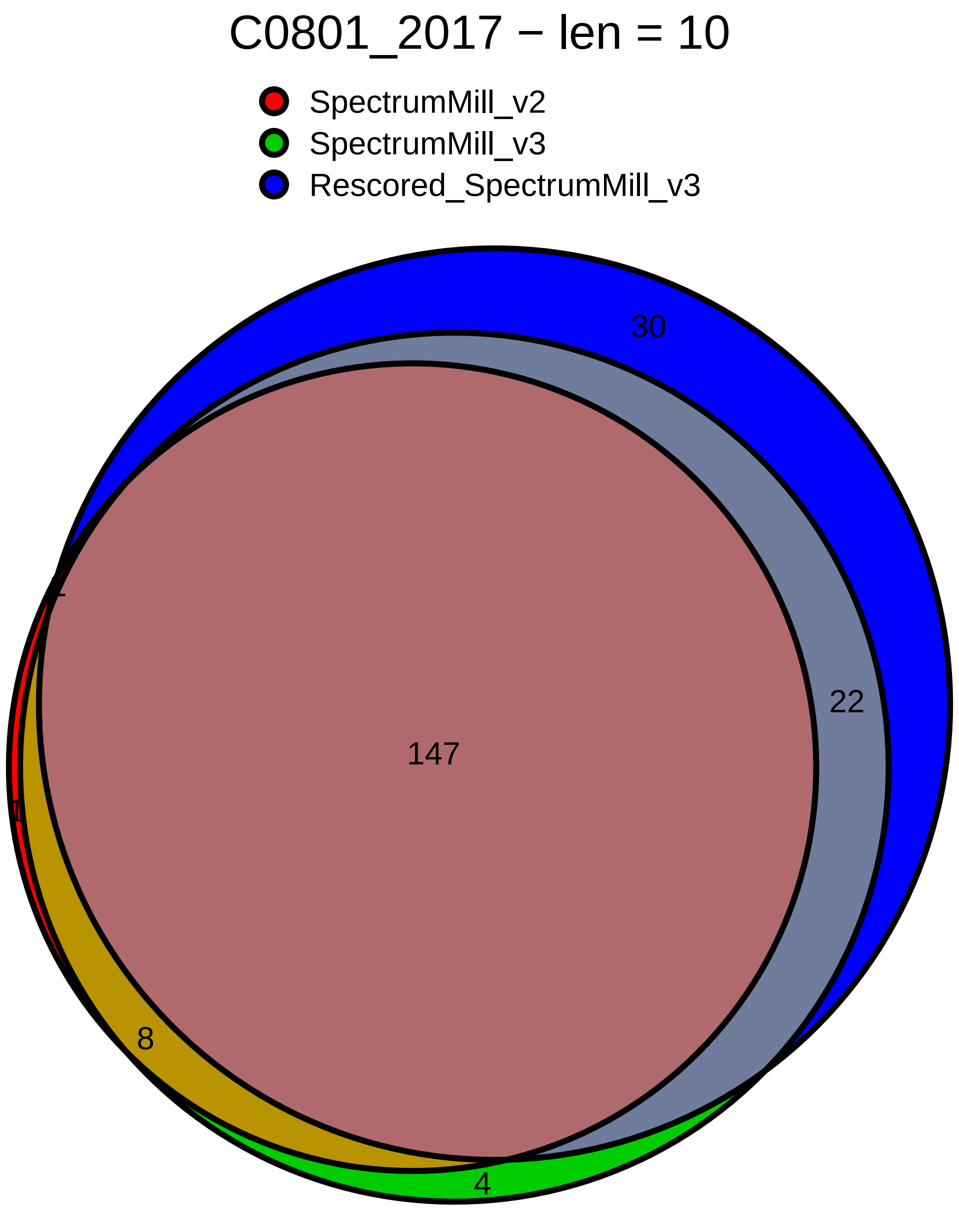
C0801_2017 − len = 10
SpectrumMill_v2
SpectrumMill_v3
Rescored_SpectrumMill_v3
30
22
147
8
4

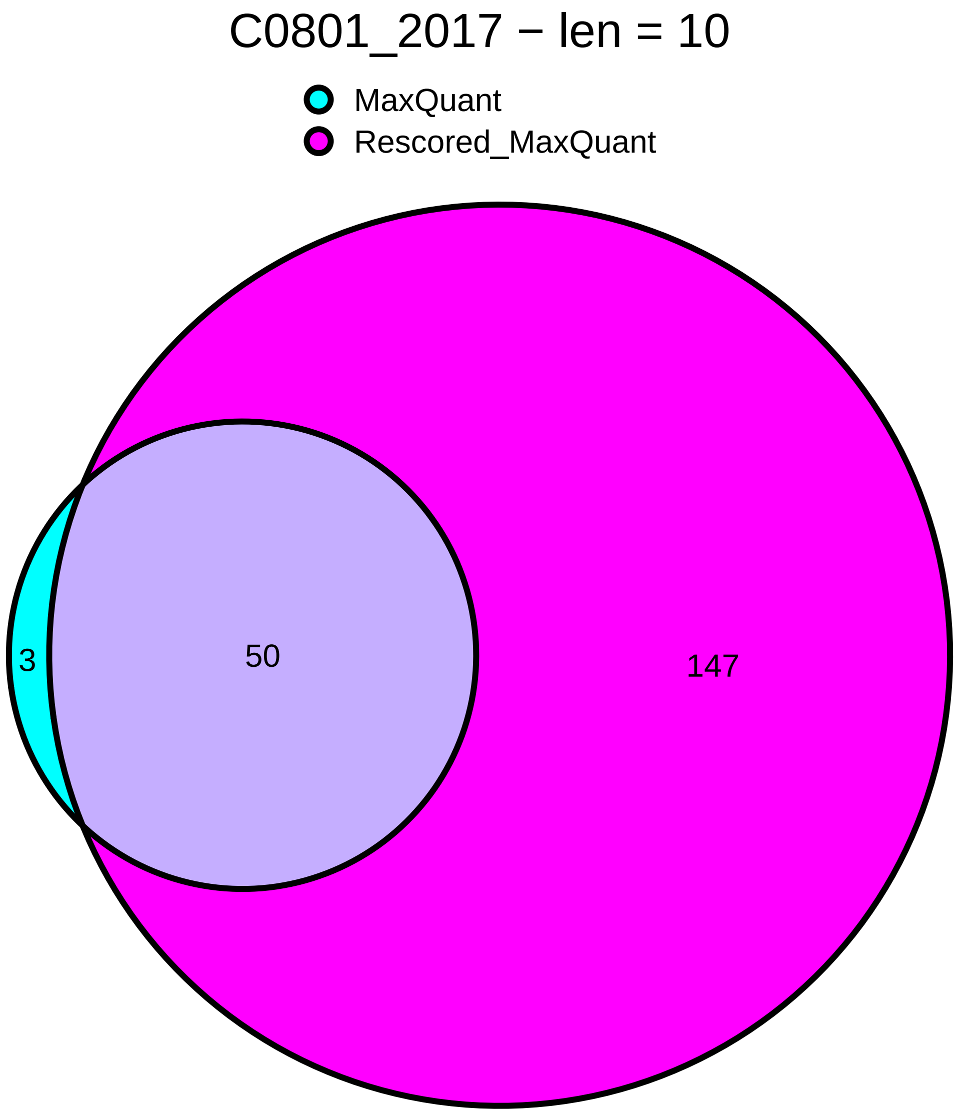
C0801_2017 − len = 10
MaxQuant
Rescored_MaxQuant
3
50
147

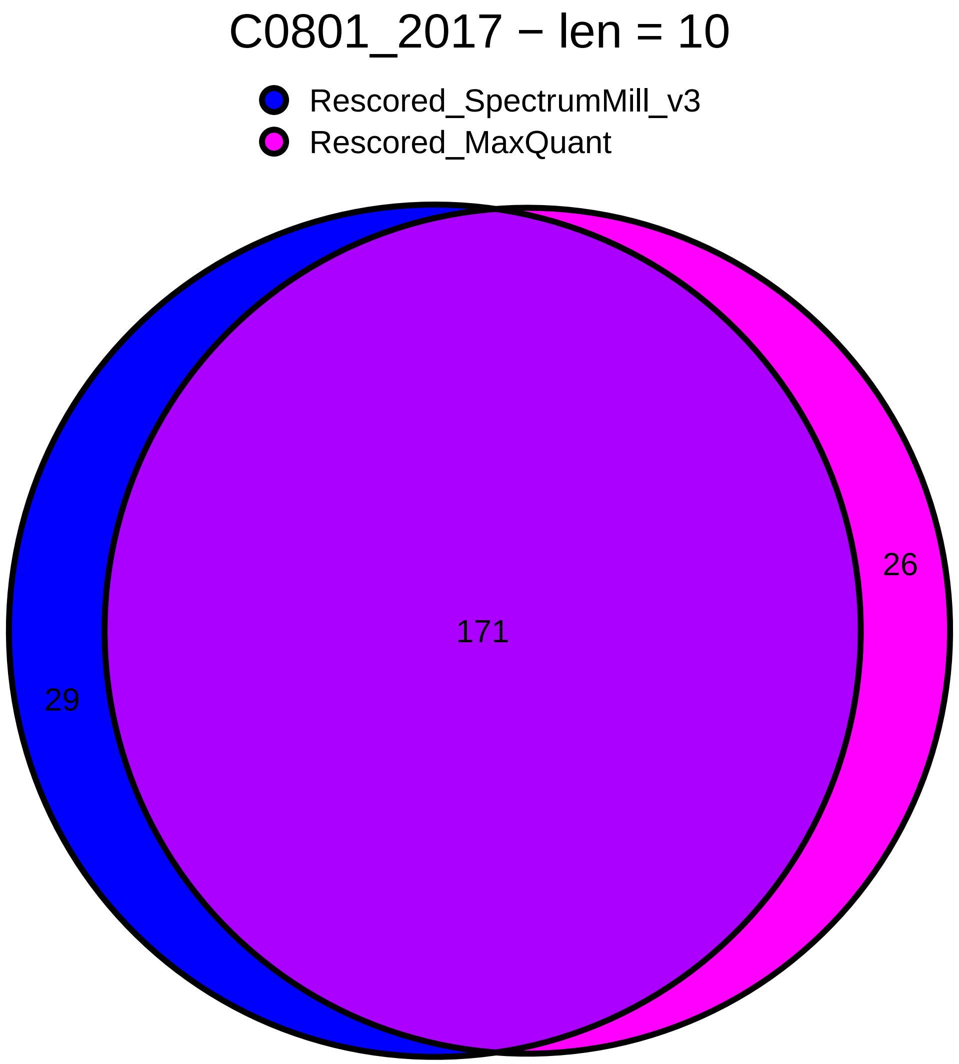
C0801_2017 − len = 10
Rescored_SpectrumMill_v3
Rescored_MaxQuant
26
171
29

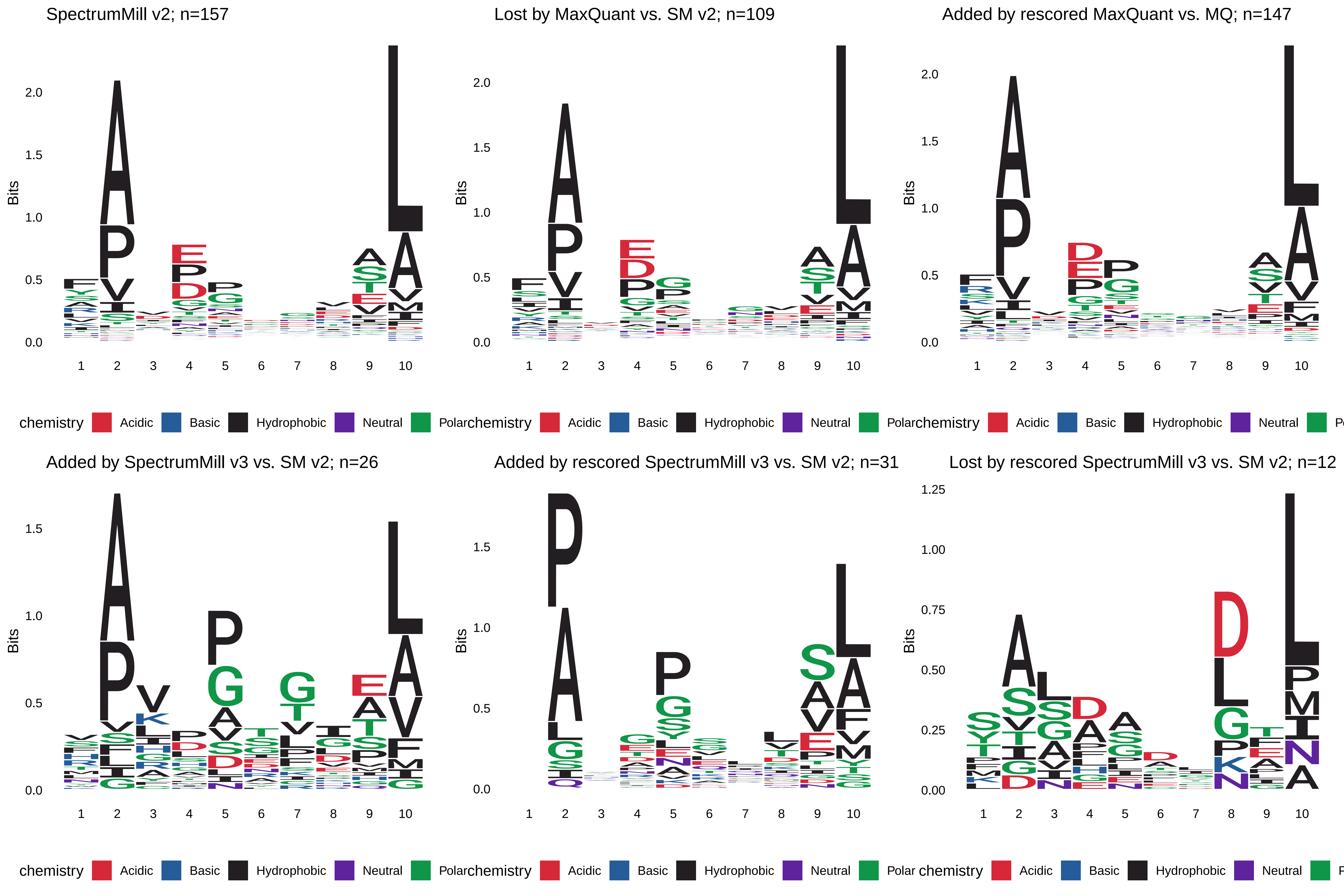
SpectrumMill v2; n=157
Lost by MaxQuant vs. SM v2; n=109
Added by rescored MaxQuant vs. MQ; n=147
Added by SpectrumMill v3 vs. SM v2; n=26
Added by rescored SpectrumMill v3 vs. SM v2; n=31
Lost by rescored SpectrumMill v3 vs. SM v2; n=12
Bits
1 2 3 4 5 6 7 8 9 10
chemistry Acidic Basic Hydrophobic Neutral Polar

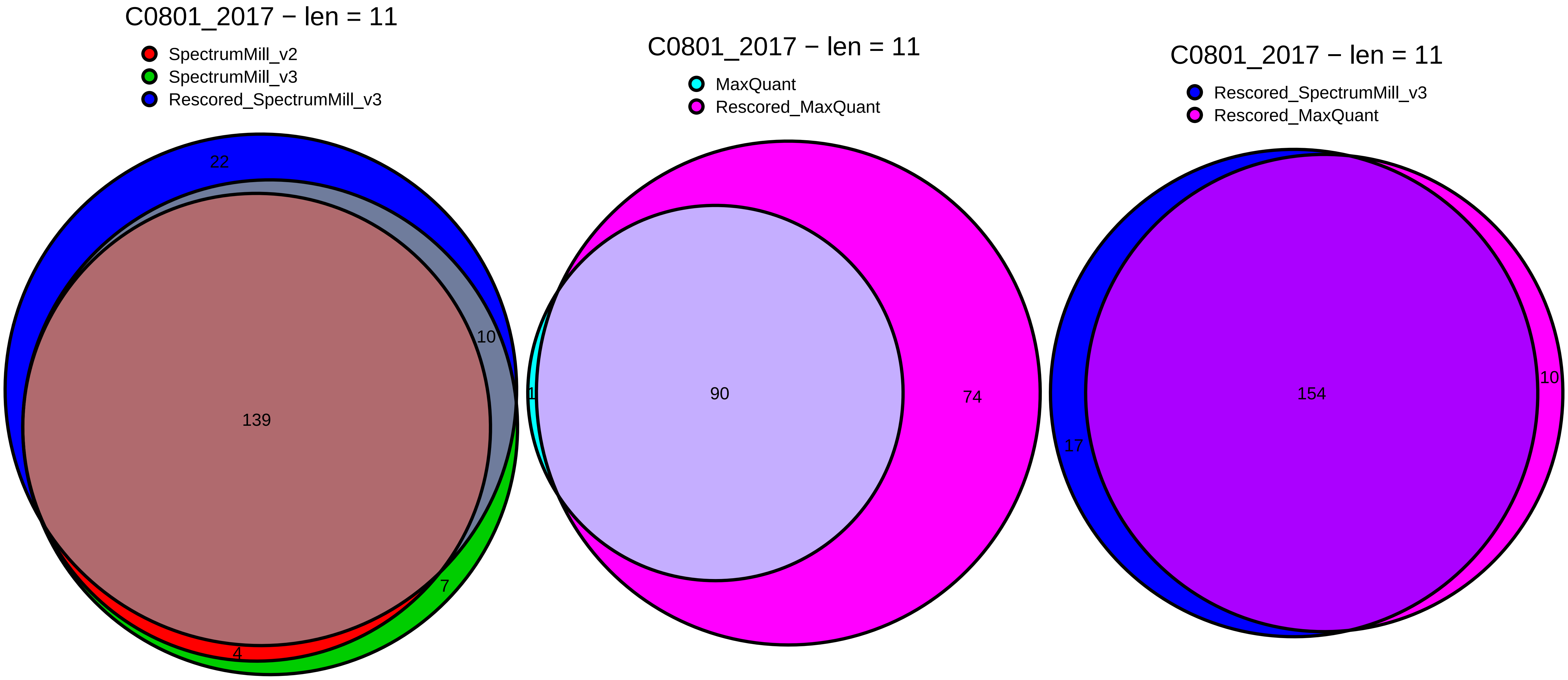
C0801_2017 − len = 11
SpectrumMill_v2
SpectrumMill_v3
Rescored_SpectrumMill_v3
22
10
139
7
4
C0801_2017 − len = 11
MaxQuant
Rescored_MaxQuant
1
90
74
C0801_2017 − len = 11
Rescored_SpectrumMill_v3
Rescored_MaxQuant
154
10
17

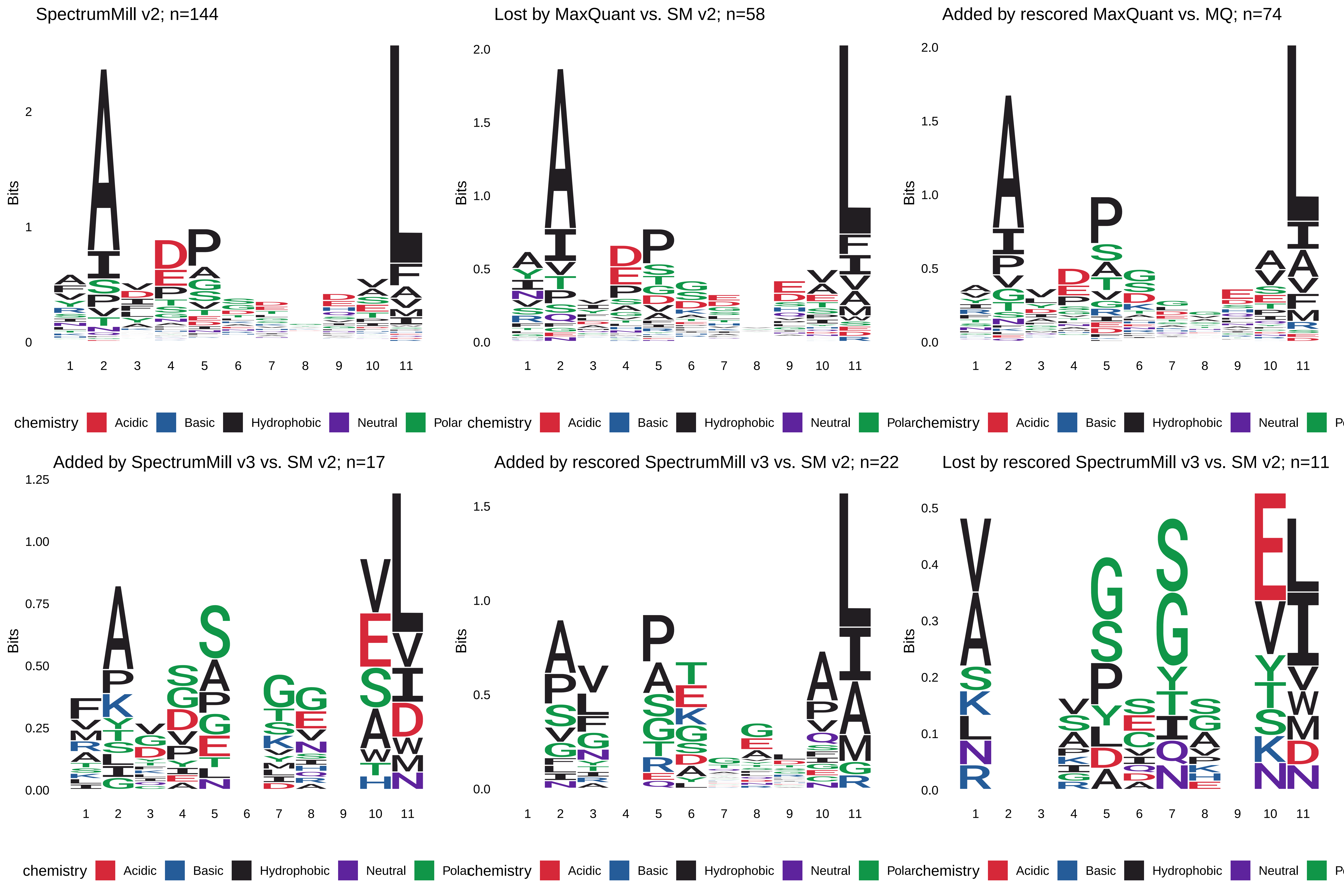

SpectrumMill v2; n=144
Lost by MaxQuant vs. SM v2; n=58
Added by rescored MaxQuant vs. MQ; n=74
Added by SpectrumMill v3 vs. SM v2; n=17
Added by rescored SpectrumMill v3 vs. SM v2; n=22
Lost by rescored SpectrumMill v3 vs. SM v2; n=11
Bits
chemistry
Acidic
Basic
Hydrophobic
Neutral
Polar

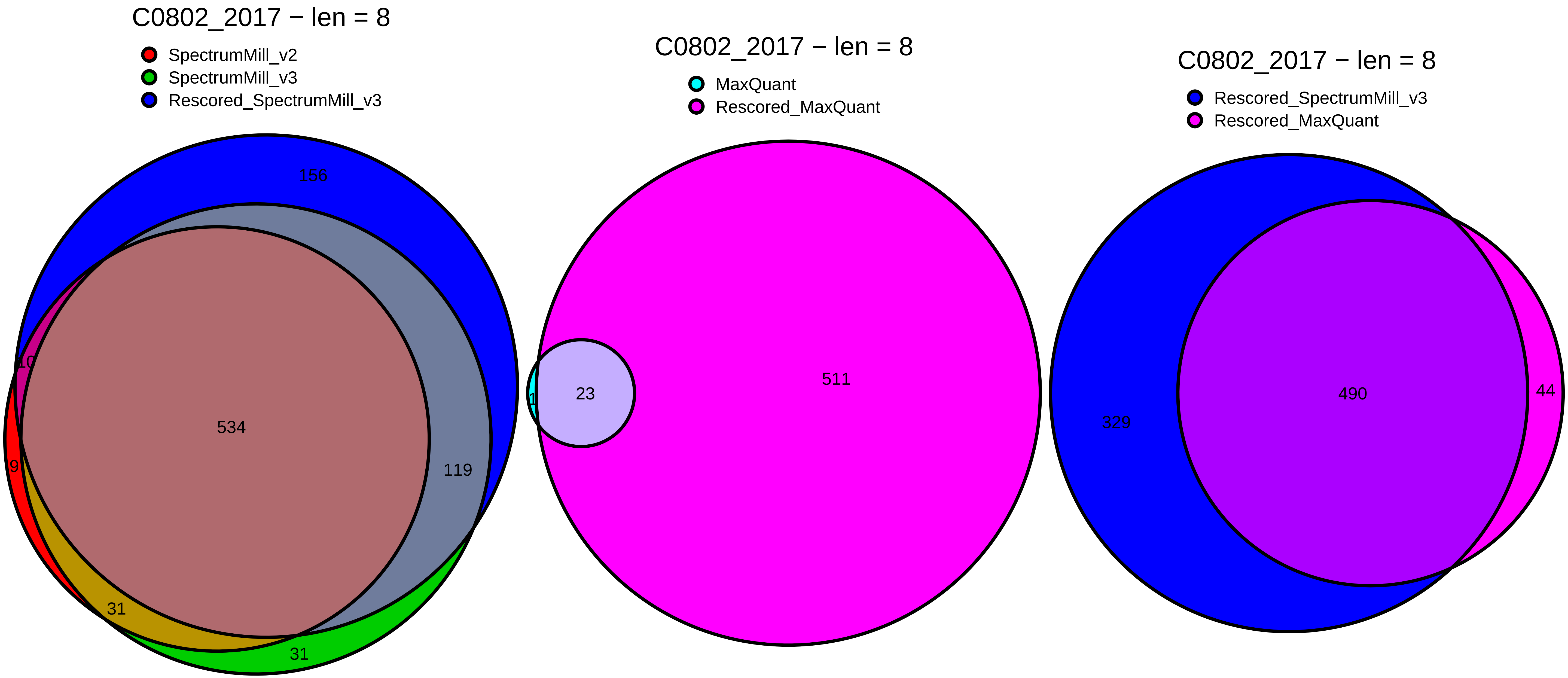
C0802_2017 − len = 8
SpectrumMill_v2
SpectrumMill_v3
Rescored_SpectrumMill_v3
156
10
534
9
119
31
31
C0802_2017 − len = 8
MaxQuant
Rescored_MaxQuant
23
511
C0802_2017 − len = 8
Rescored_SpectrumMill_v3
Rescored_MaxQuant
329
490
44

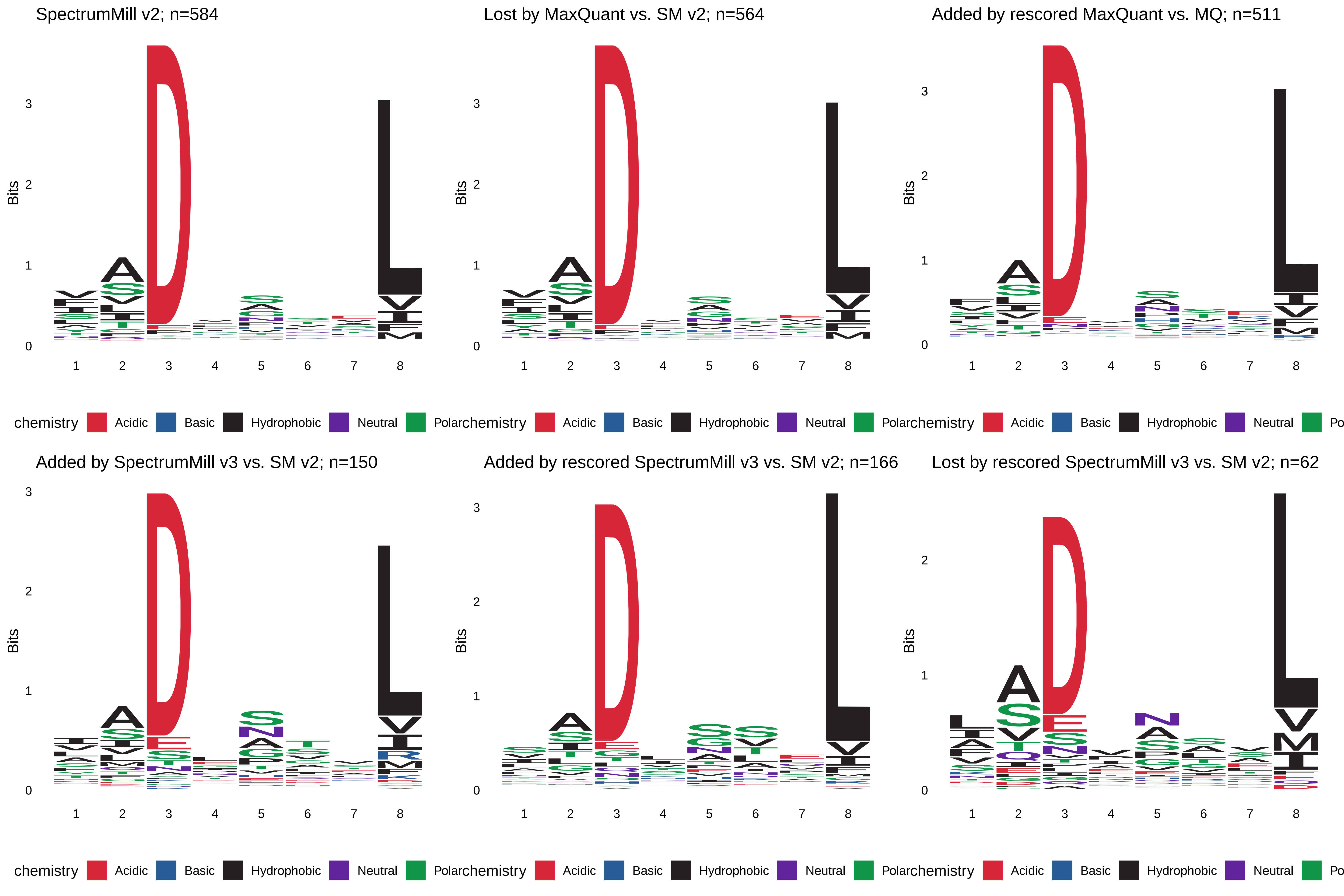
SpectrumMill v2; n=584
Lost by MaxQuant vs. SM v2; n=564
Added by rescored MaxQuant vs. MQ; n=511
Added by SpectrumMill v3 vs. SM v2; n=150
Added by rescored SpectrumMill v3 vs. SM v2; n=166
Lost by rescored SpectrumMill v3 vs. SM v2; n=62
Bits
0
1
2
3
1 2 3 4 5 6 7 8
chemistry
Acidic
Basic
Hydrophobic
Neutral
Polar

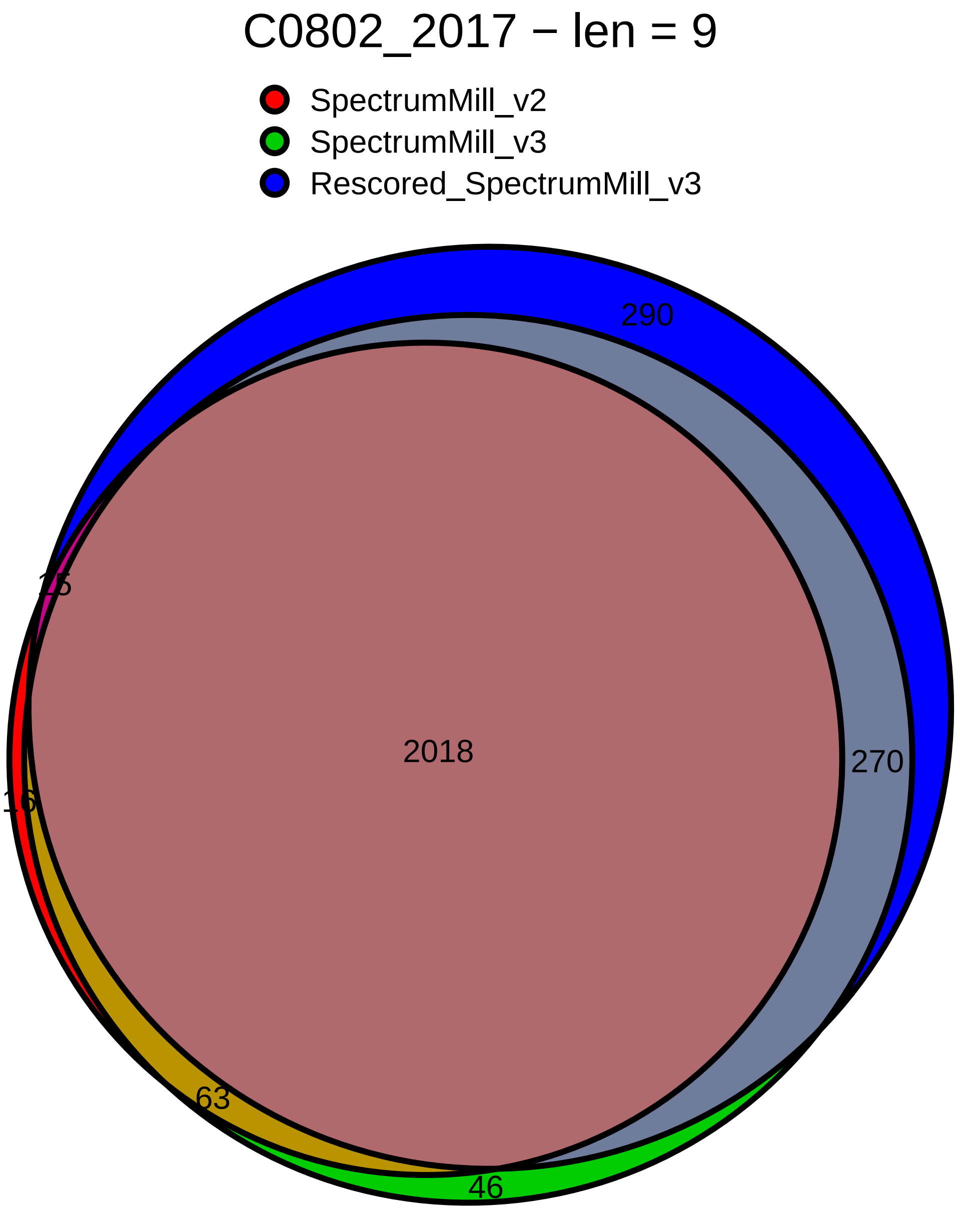
C0802_2017 − len = 9
SpectrumMill_v2
SpectrumMill_v3
Rescored_SpectrumMill_v3
290
15
2018
270
16
63
46

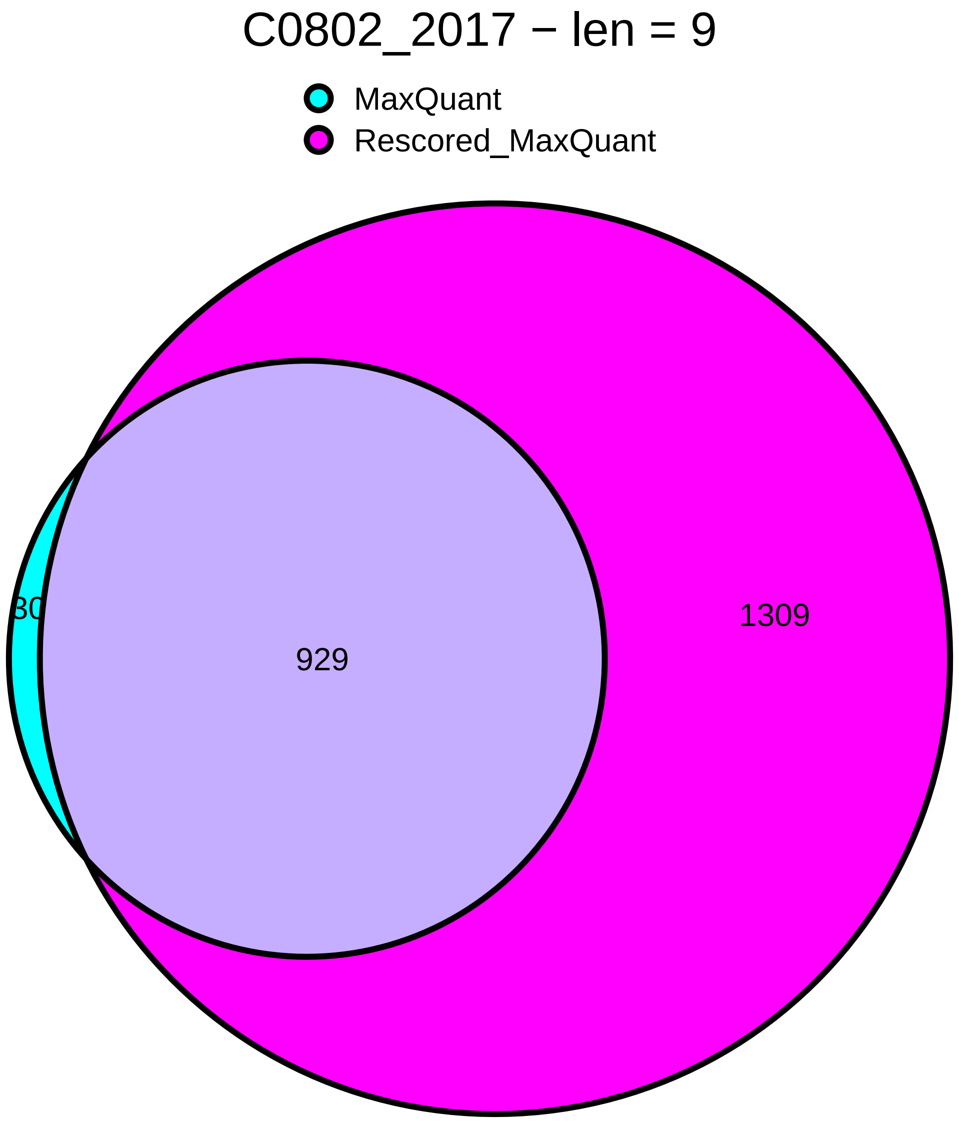
C0802_2017 − len = 9
MaxQuant
Rescored_MaxQuant
30
929
1309

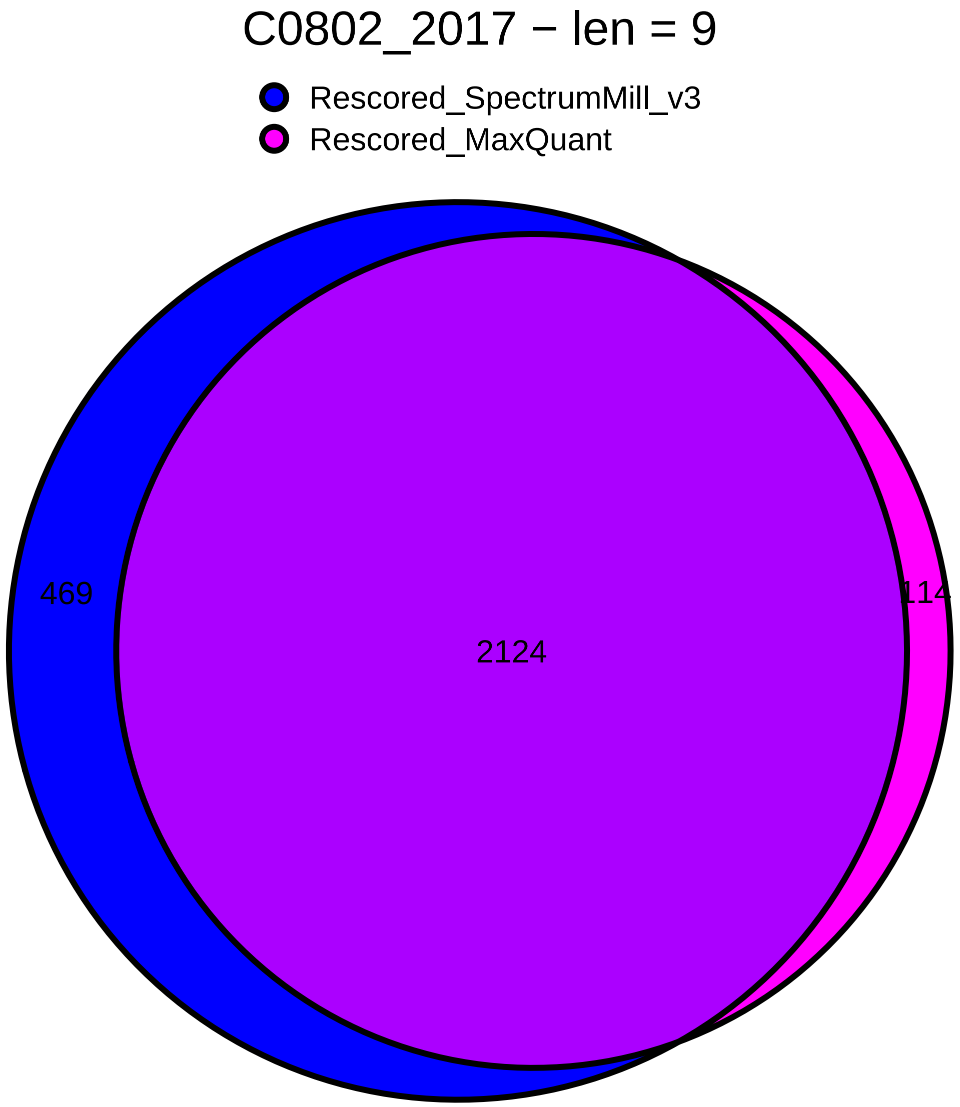
C0802_2017 − len = 9
Rescored_SpectrumMill_v3
Rescored_MaxQuant
469
2124
114

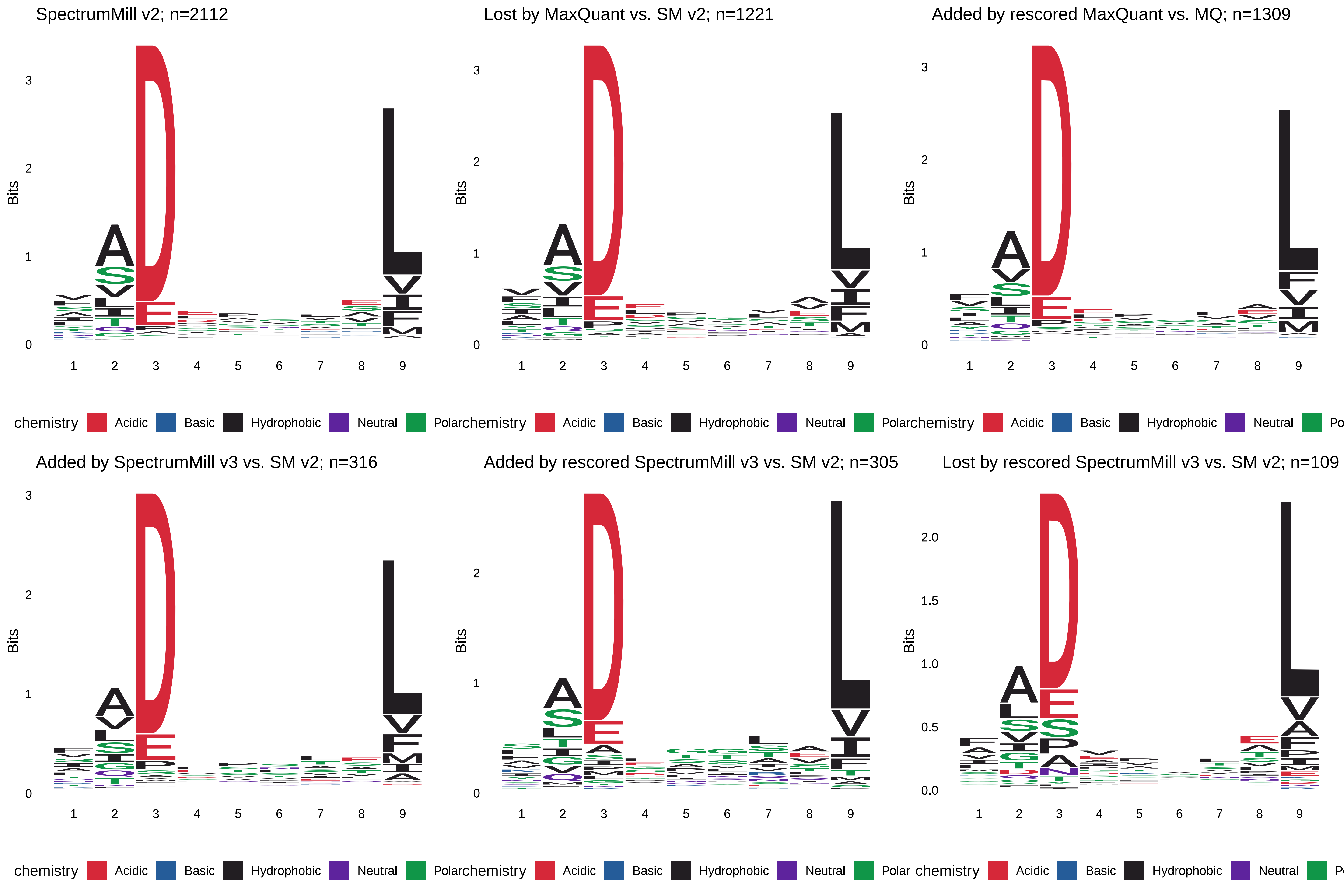
SpectrumMill v2; n=2112
Lost by MaxQuant vs. SM v2; n=1221
Added by rescored MaxQuant vs. MQ; n=1309
Added by SpectrumMill v3 vs. SM v2; n=316
Added by rescored SpectrumMill v3 vs. SM v2; n=305
Lost by rescored SpectrumMill v3 vs. SM v2; n=109
Bits
1 2 3 4 5 6 7 8 9
chemistry Acidic Basic Hydrophobic Neutral Polar

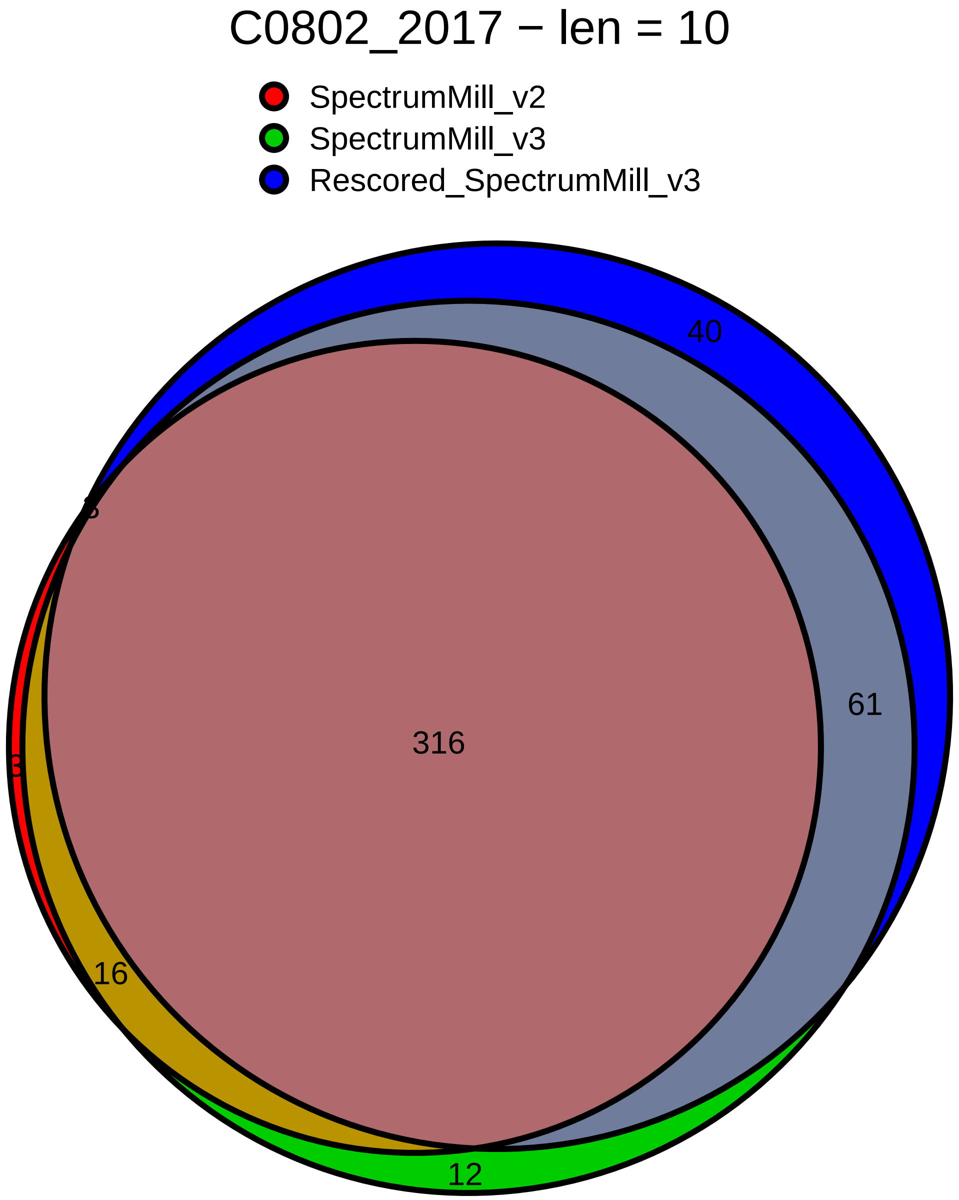
C0802_2017 − len = 10
SpectrumMill_v2
SpectrumMill_v3
Rescored_SpectrumMill_v3
40
61
316
16
12

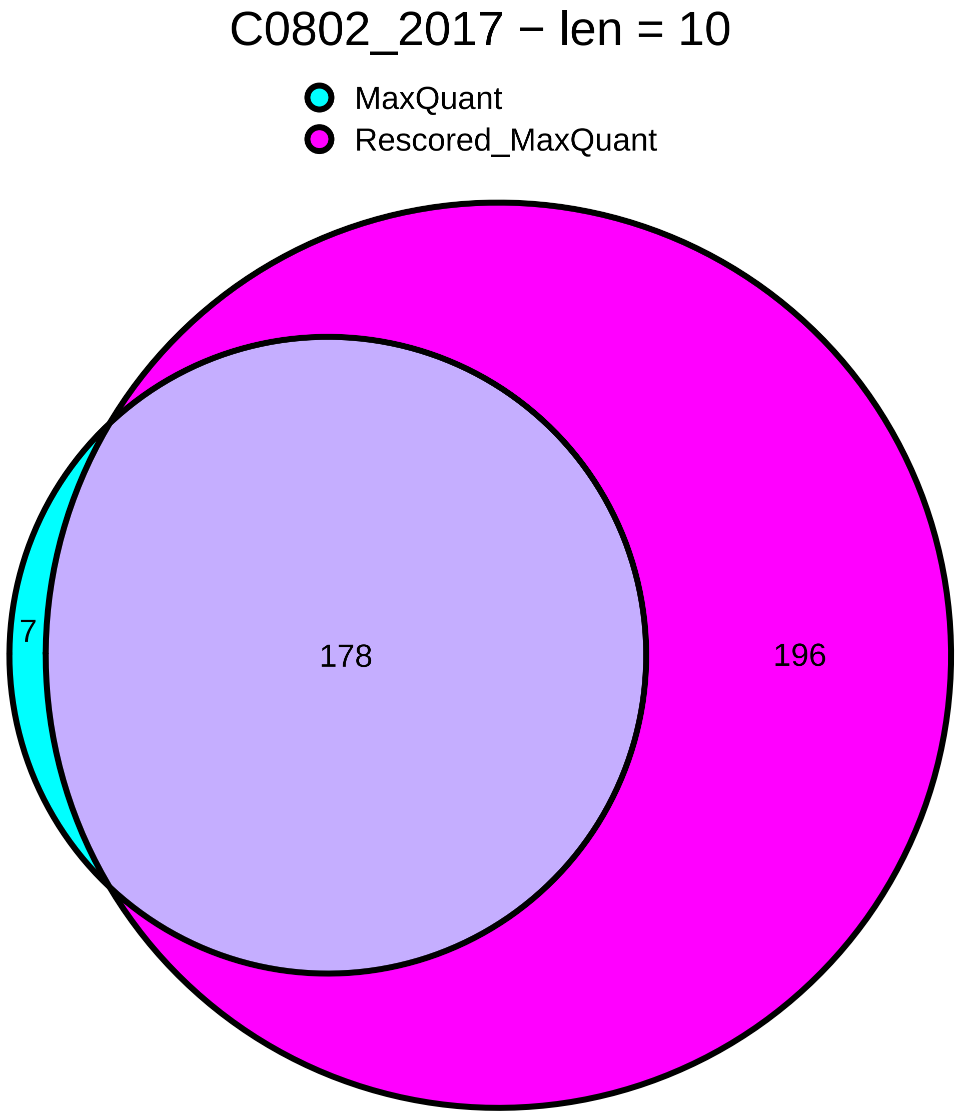
C0802_2017 − len = 10
MaxQuant
Rescored_MaxQuant
7
178
196

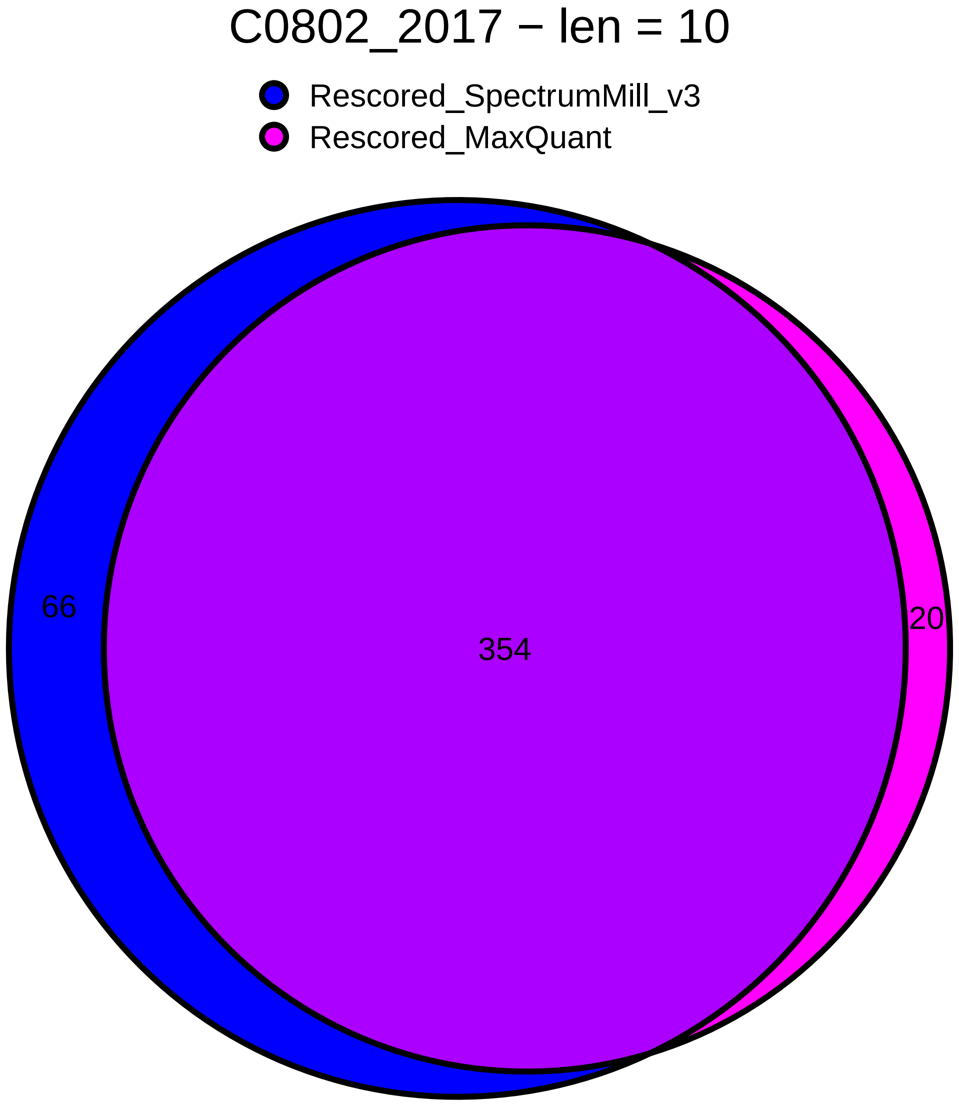
C0802_2017 − len = 10
Rescored_SpectrumMill_v3
Rescored_MaxQuant
66
354
20

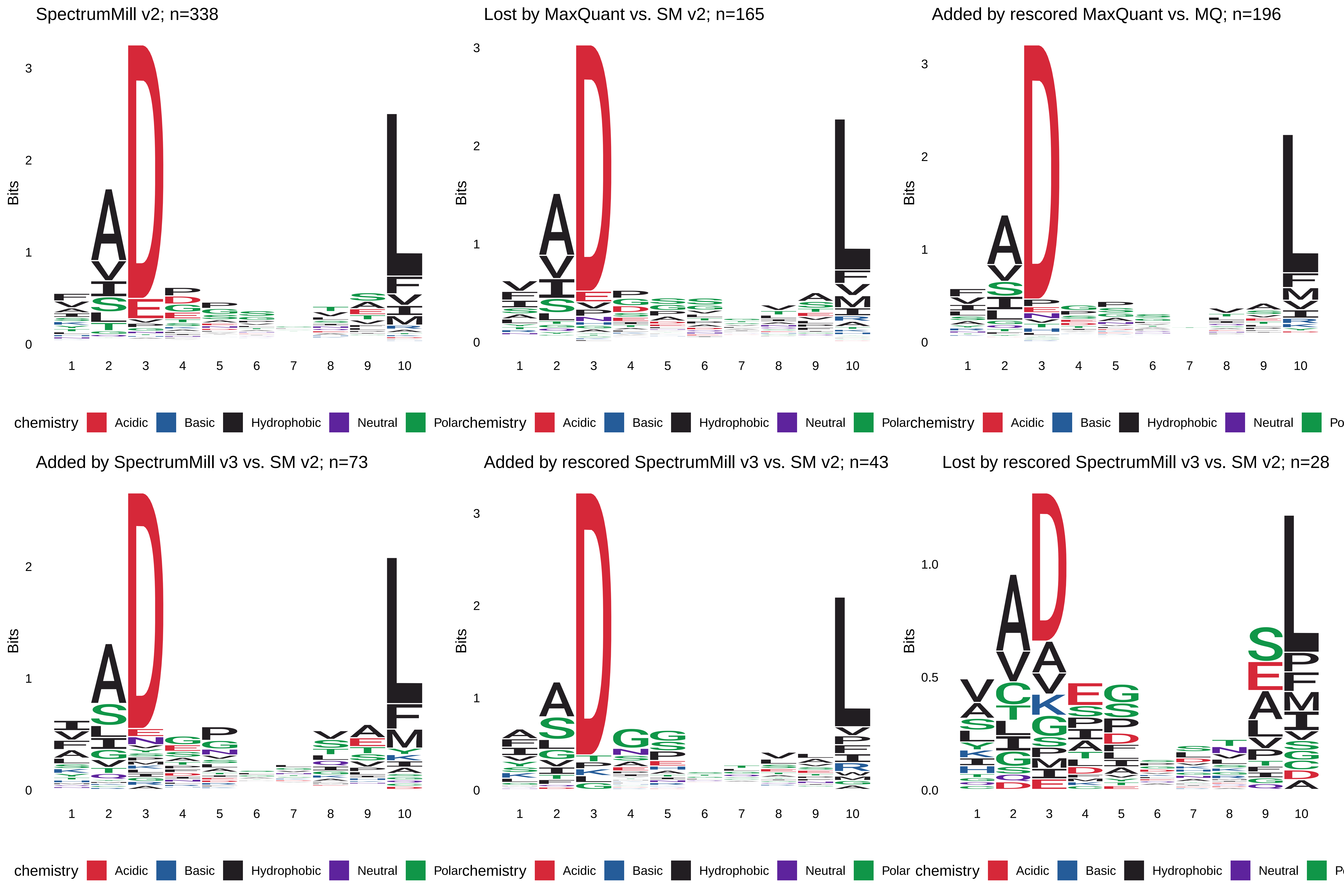

SpectrumMill v2; n=338
Lost by MaxQuant vs. SM v2; n=165
Added by rescored MaxQuant vs. MQ; n=196
Added by SpectrumMill v3 vs. SM v2; n=73
Added by rescored SpectrumMill v3 vs. SM v2; n=43
Lost by rescored SpectrumMill v3 vs. SM v2; n=28
Bits
chemistry
Acidic
Basic
Hydrophobic
Neutral
Polar

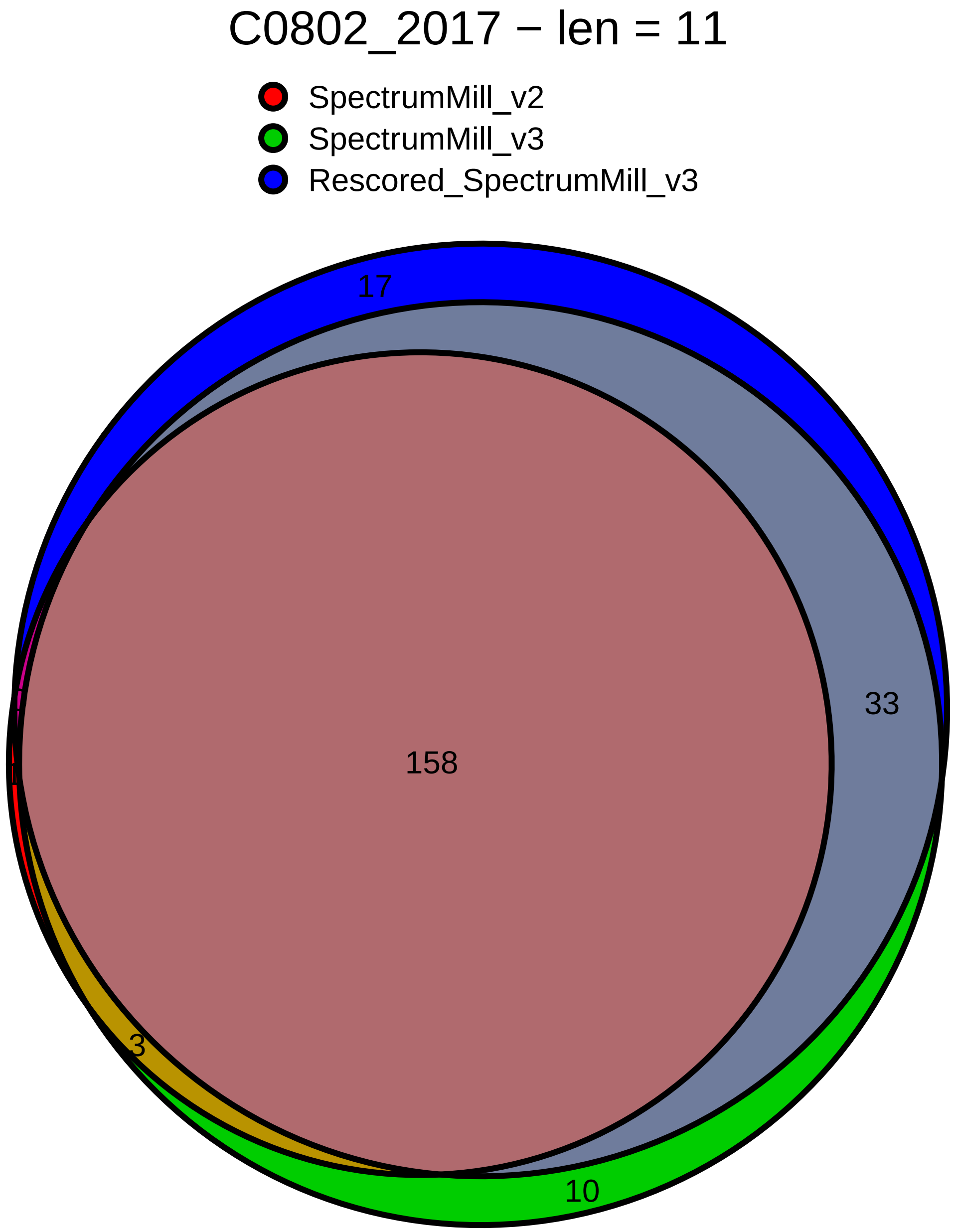
C0802_2017 − len = 11
SpectrumMill_v2
SpectrumMill_v3
Rescored_SpectrumMill_v3
17
33
158
3
10

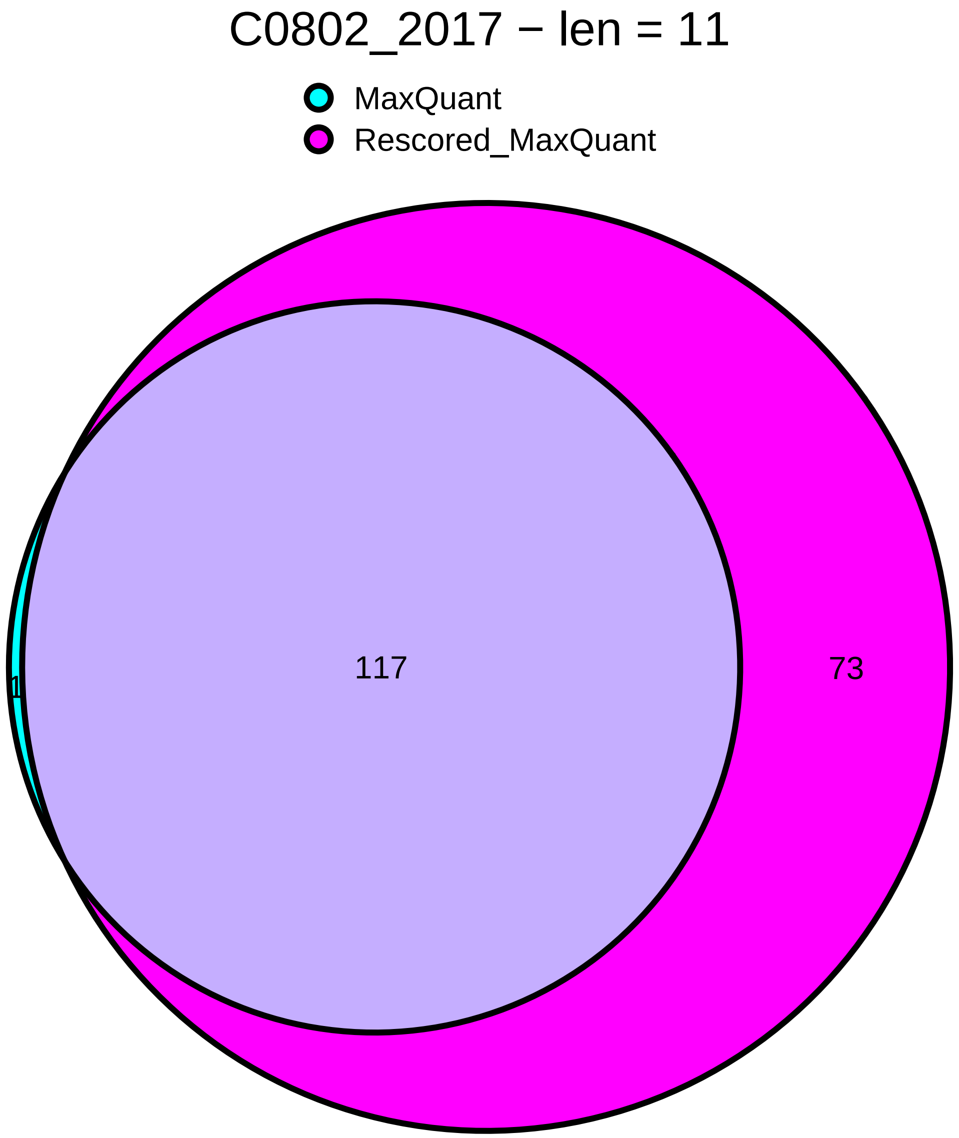
C0802_2017 − len = 11
MaxQuant
Rescored_MaxQuant
117
73

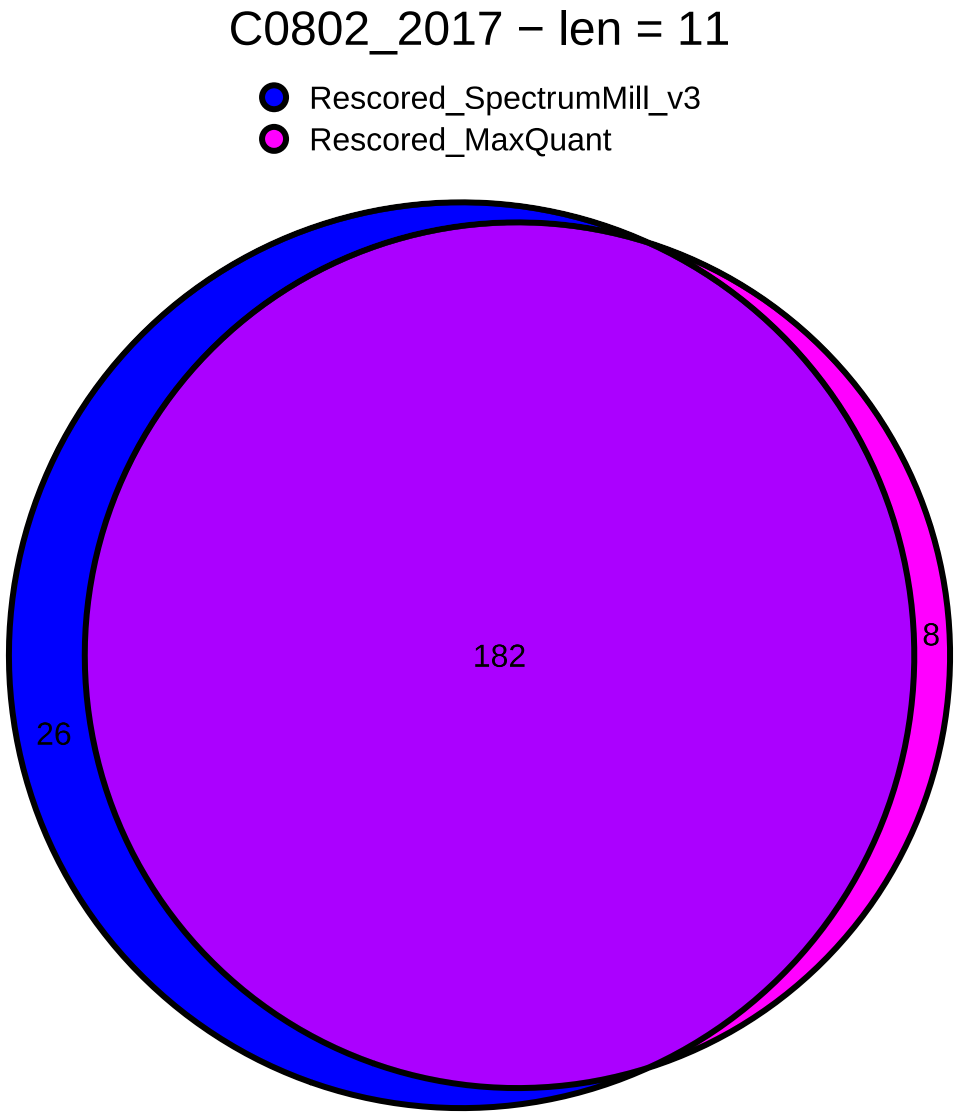
C0802_2017 − len = 11
Rescored_SpectrumMill_v3
Rescored_MaxQuant
182
26
8

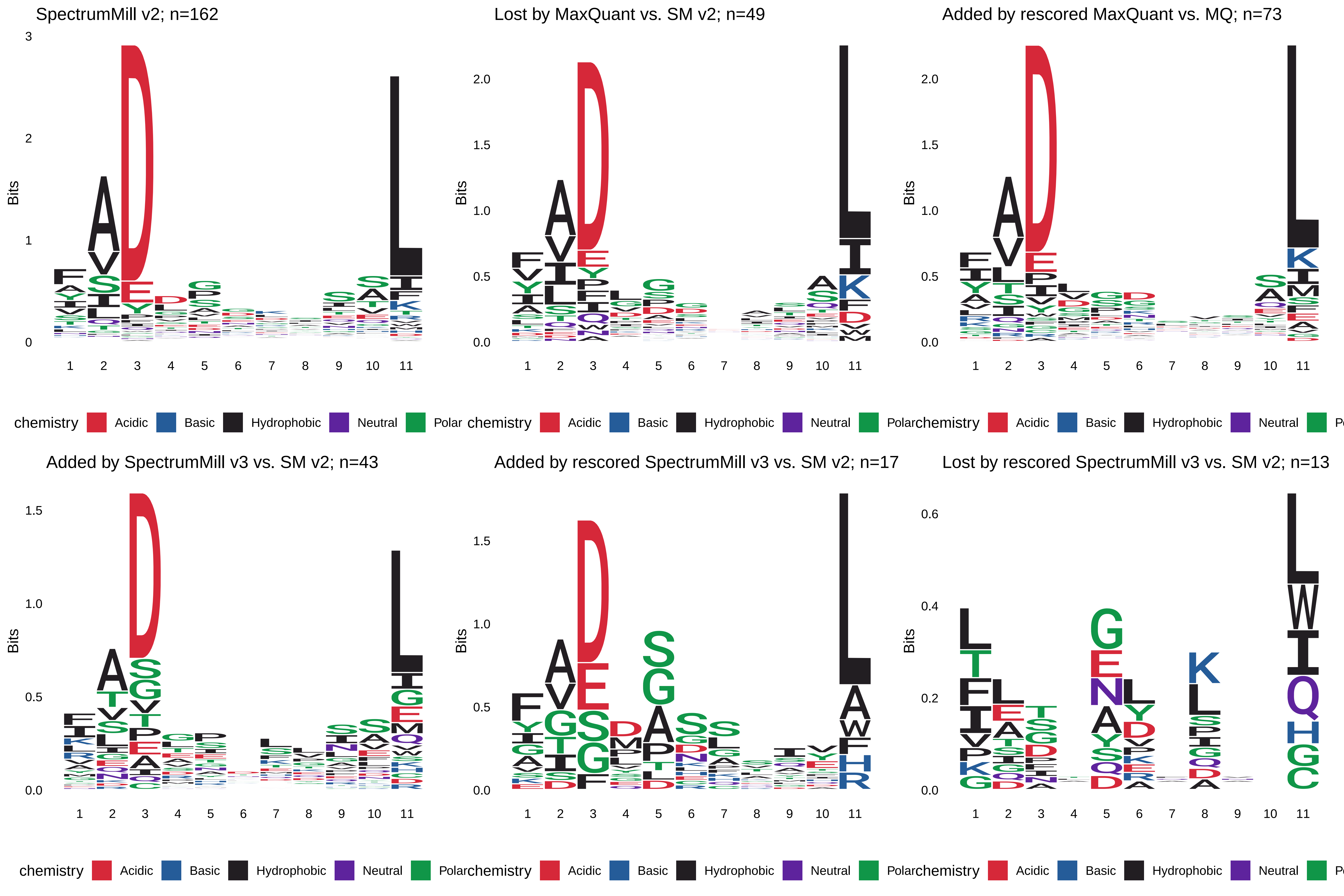
SpectrumMill v2; n=162
Lost by MaxQuant vs. SM v2; n=49
Added by rescored MaxQuant vs. MQ; n=73
Added by SpectrumMill v3 vs. SM v2; n=43
Added by rescored SpectrumMill v3 vs. SM v2; n=17
Lost by rescored SpectrumMill v3 vs. SM v2; n=13
Bits
chemistry
Acidic
Basic
Hydrophobic
Neutral
Polar

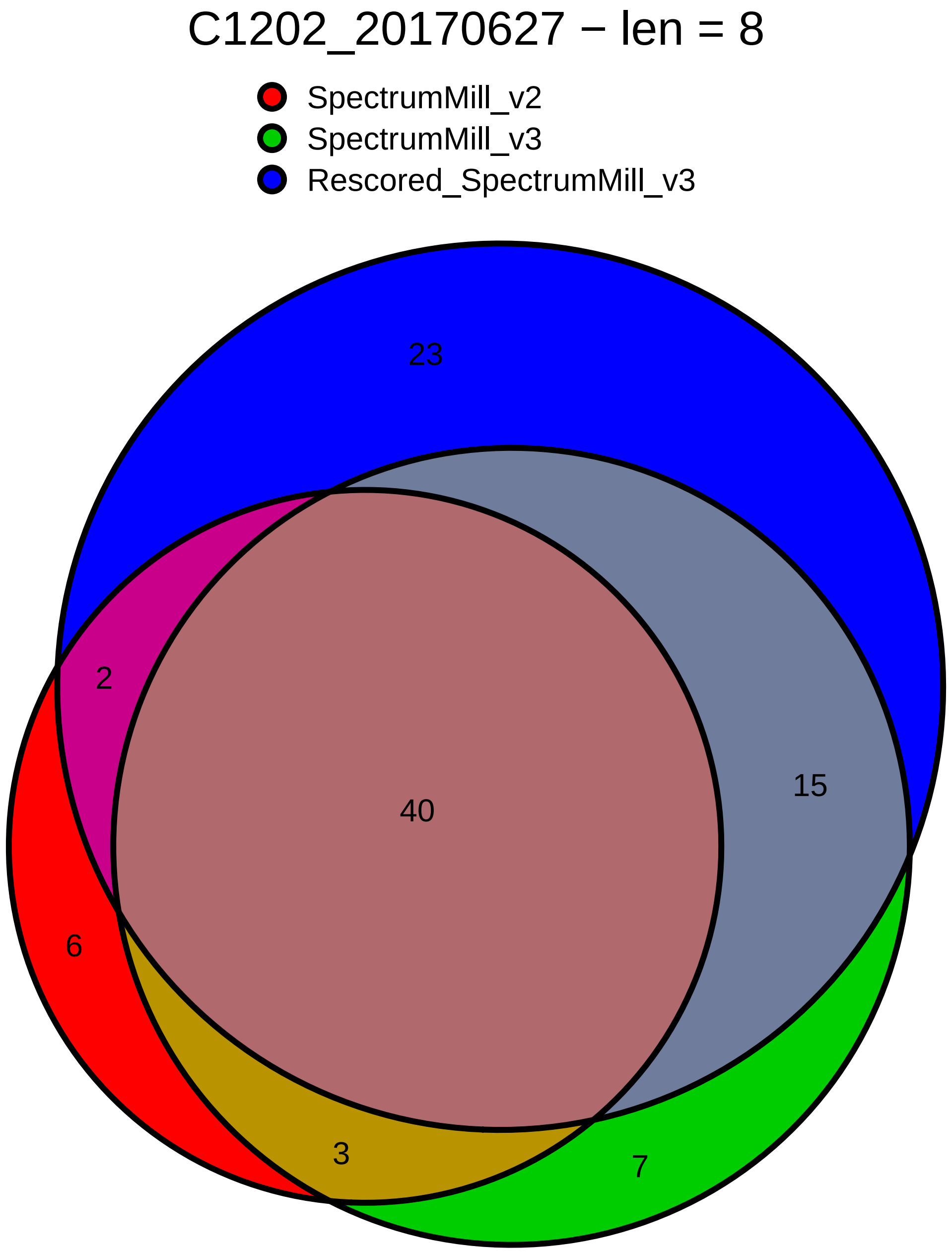
C1202_20170627 − len = 8
SpectrumMill_v2
SpectrumMill_v3
Rescored_SpectrumMill_v3
23
2
40
15
6
3
7

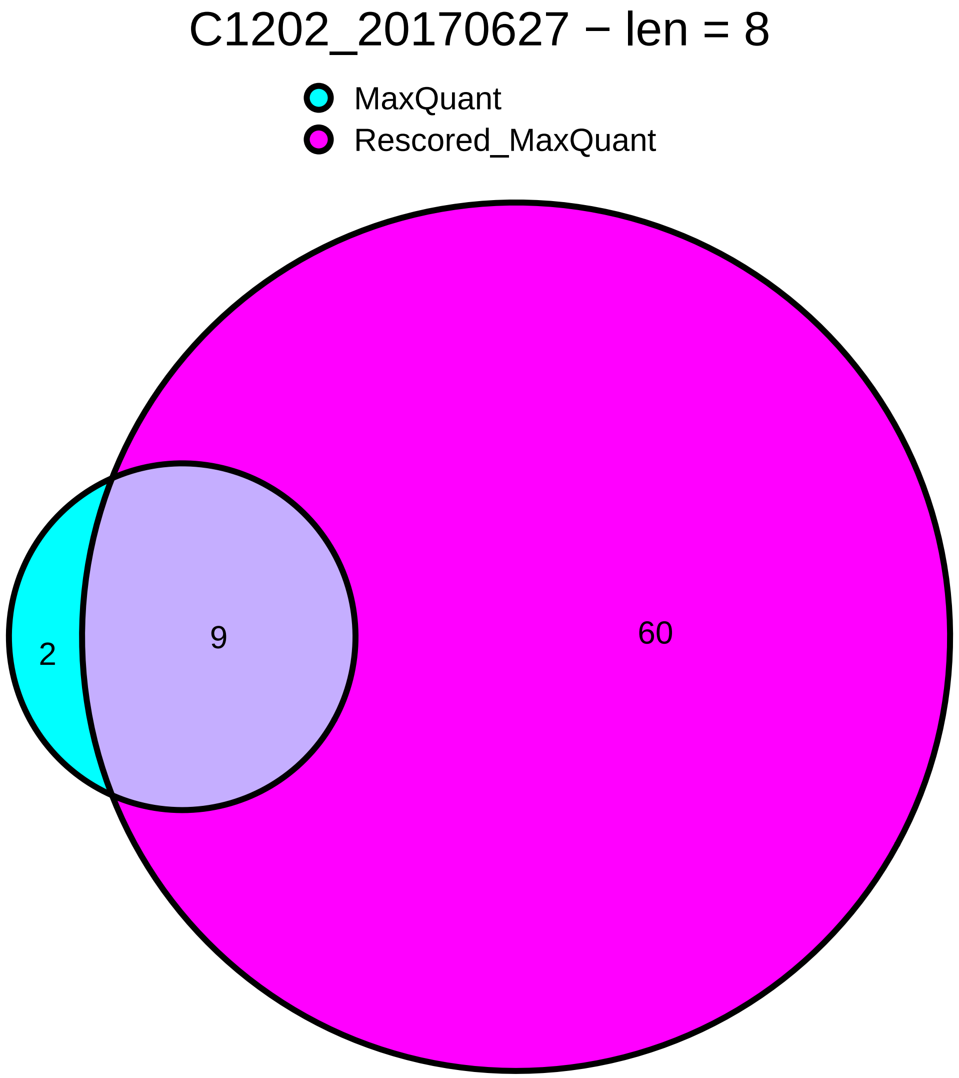
C1202_20170627 − len = 8
MaxQuant
Rescored_MaxQuant
2
9
60

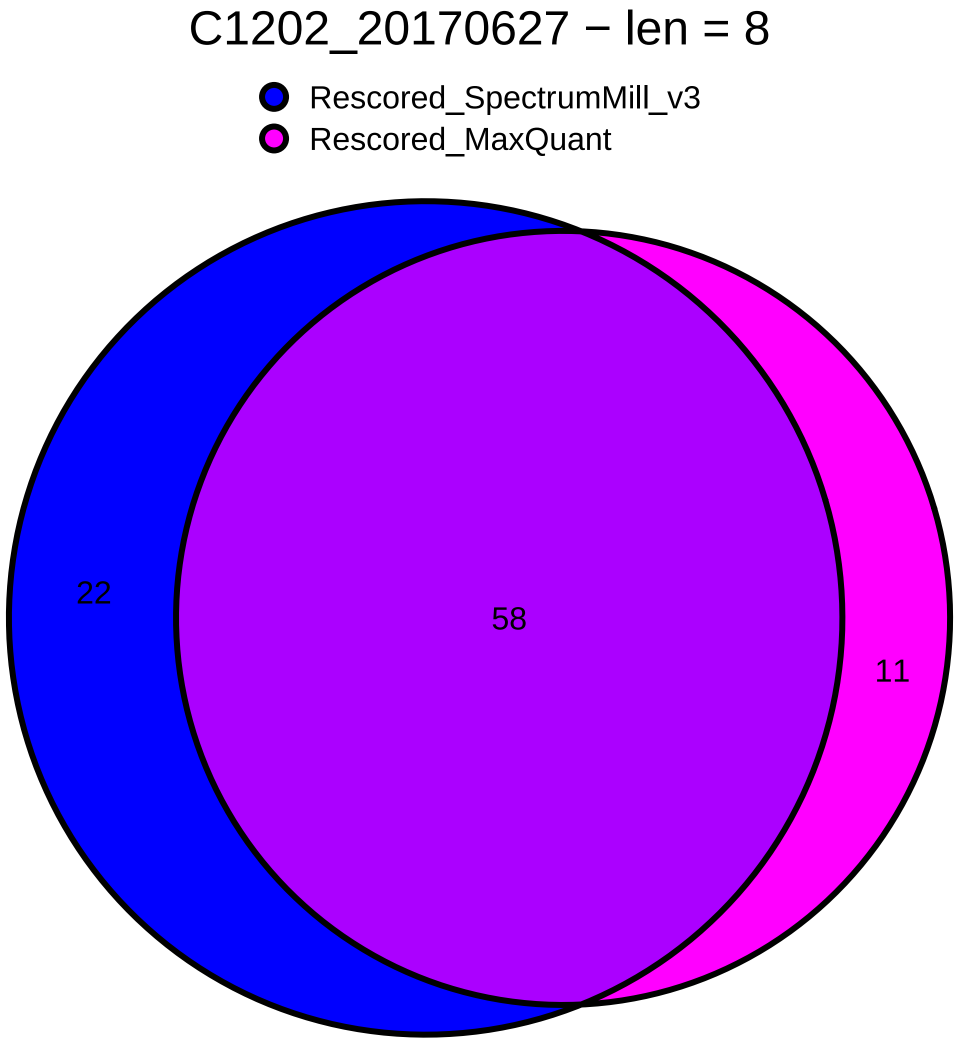
C1202_20170627 − len = 8
Rescored_SpectrumMill_v3
Rescored_MaxQuant
22
58
11

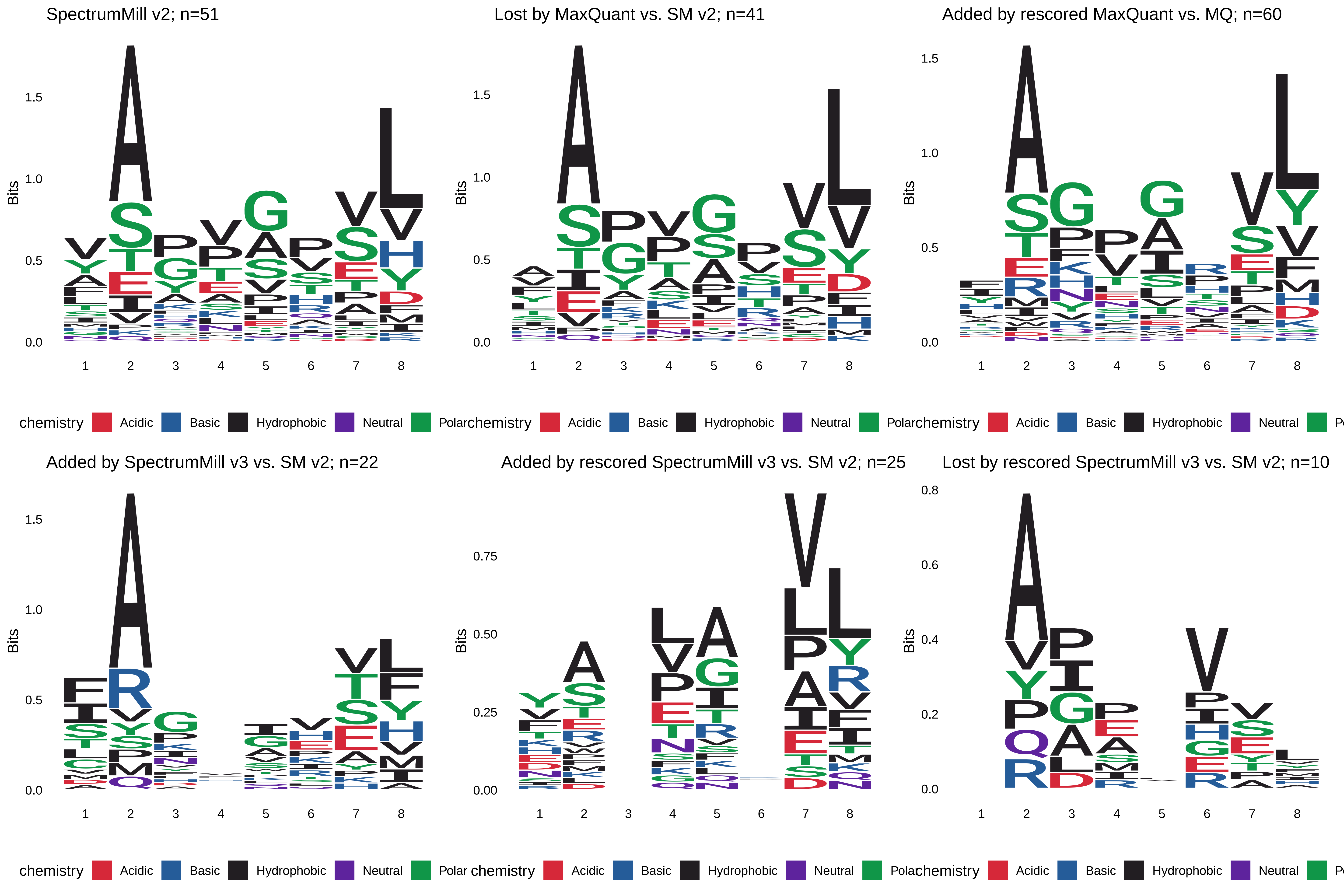
SpectrumMill v2; n=51
Lost by MaxQuant vs. SM v2; n=41
Added by rescored MaxQuant vs. MQ; n=60
Added by SpectrumMill v3 vs. SM v2; n=22
Added by rescored SpectrumMill v3 vs. SM v2; n=25
Lost by rescored SpectrumMill v3 vs. SM v2; n=10
Bits
1 2 3 4 5 6 7 8
chemistry Acidic Basic Hydrophobic Neutral Polar

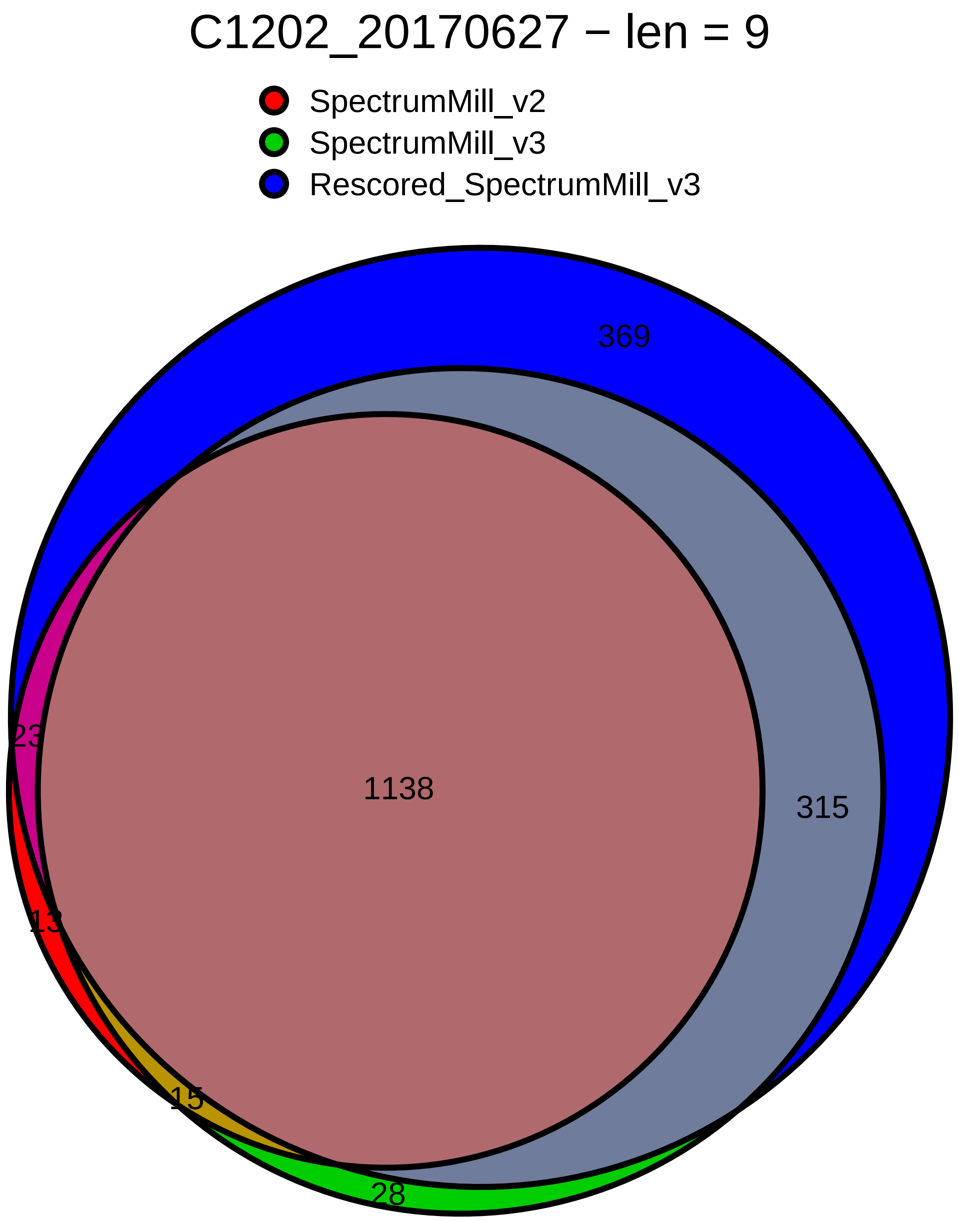
C1202_20170627 − len = 9
SpectrumMill_v2
SpectrumMill_v3
Rescored_SpectrumMill_v3
369
23
1138
315
13
15
28

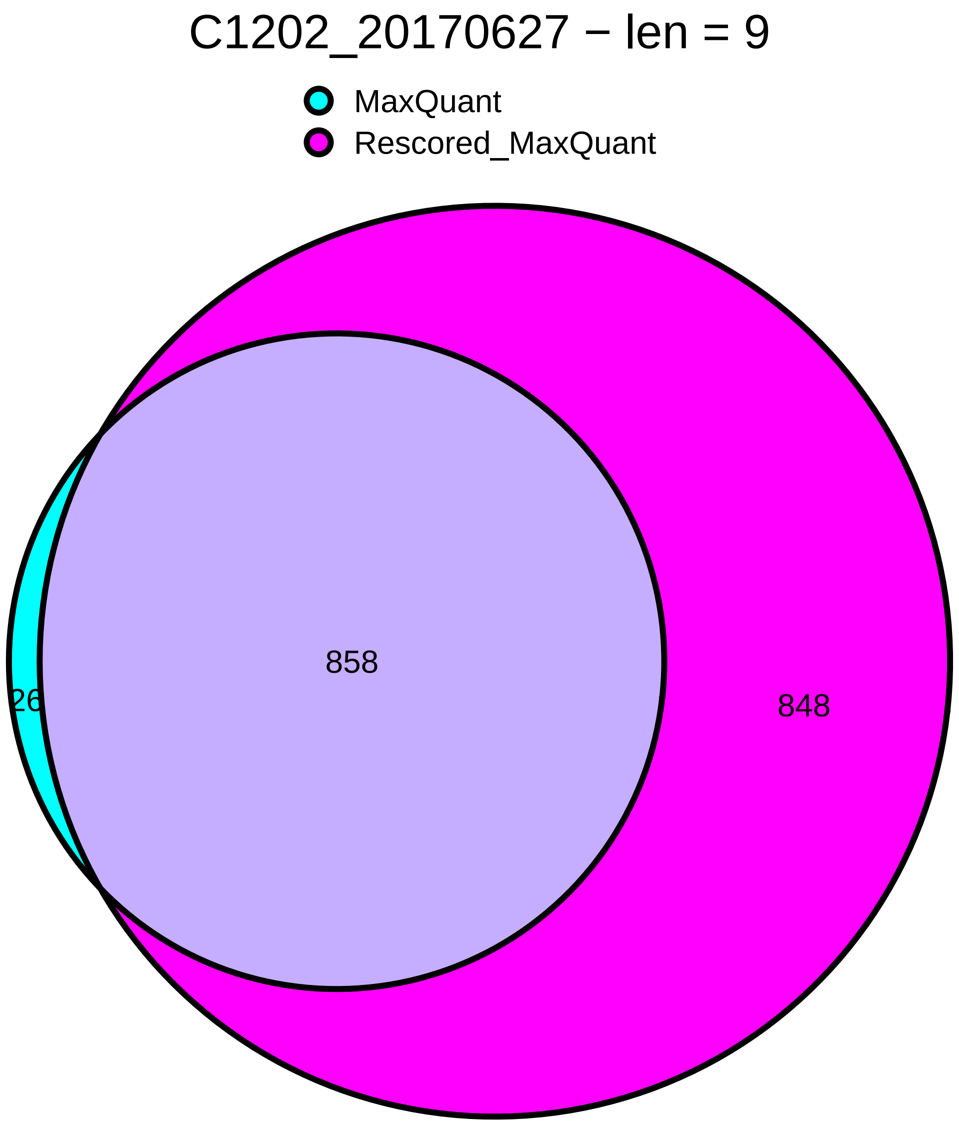
C1202_20170627 − len = 9
MaxQuant
Rescored_MaxQuant
26
858
848

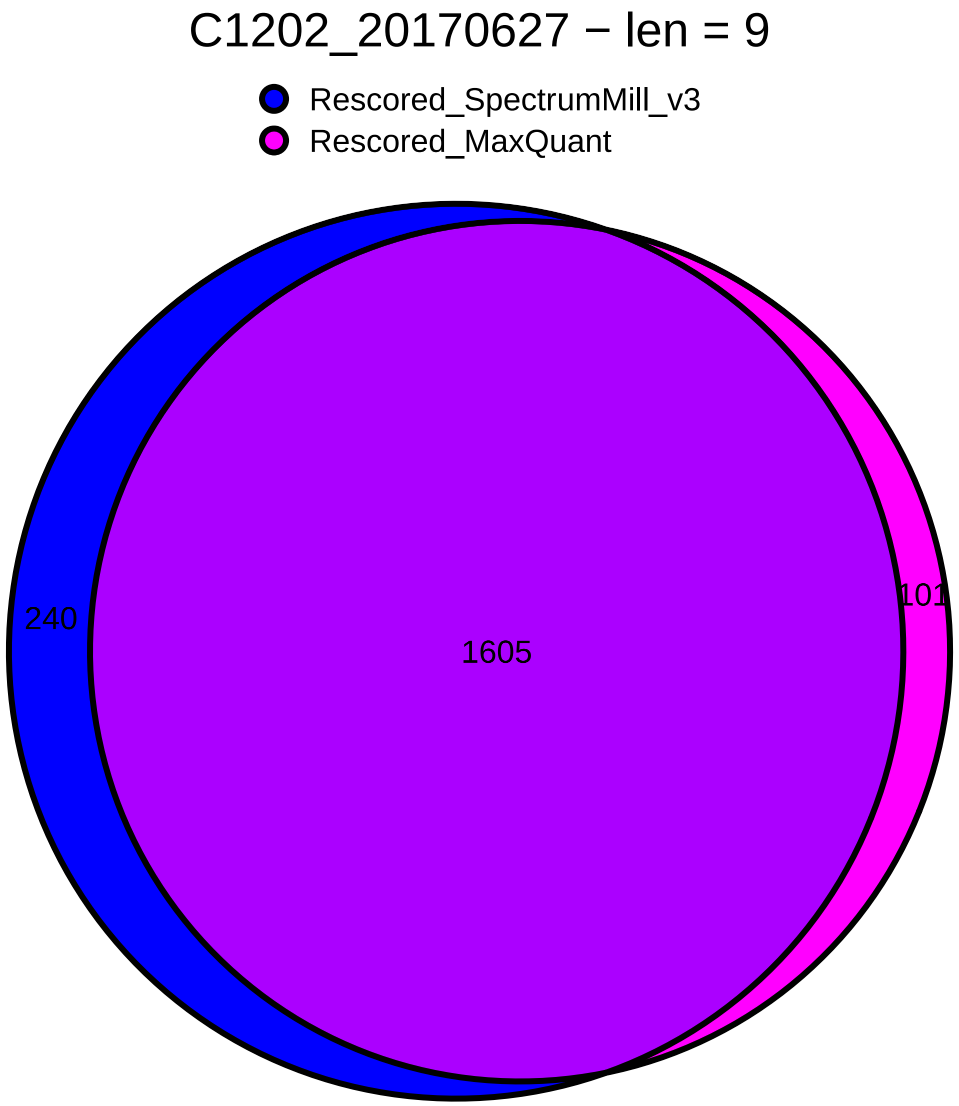
C1202_20170627 − len = 9
Rescored_SpectrumMill_v3
Rescored_MaxQuant
240
1605
101

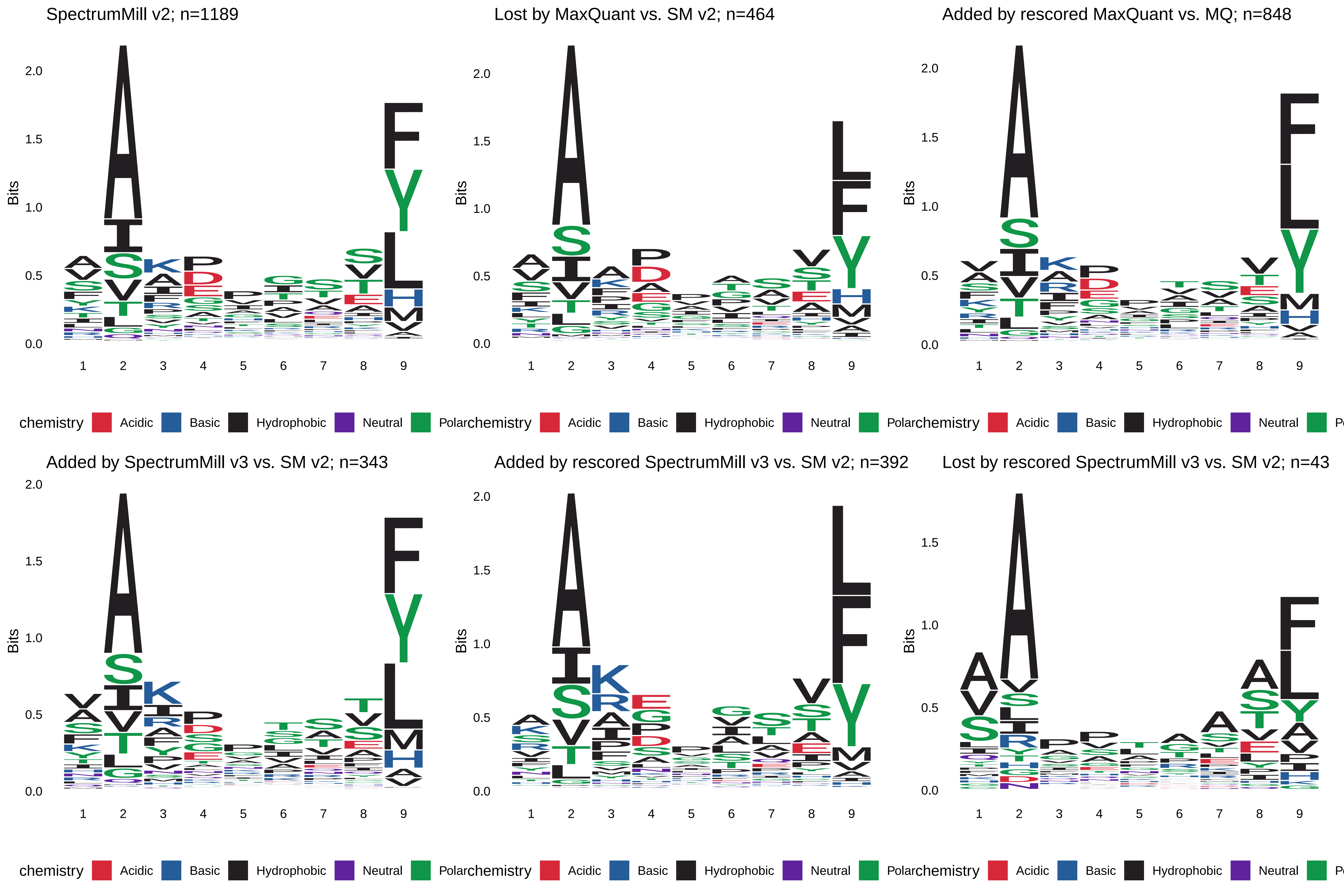
SpectrumMill v2; n=1189
Lost by MaxQuant vs. SM v2; n=464
Added by rescored MaxQuant vs. MQ; n=848
Bits
0.0
0.5
1.0
1.5
2.0
1 2 3 4 5 6 7 8 9
chemistry Acidic Basic Hydrophobic Neutral Polarchemistry Acidic Basic Hydrophobic Neutral Polarchemistry Acidic Basic Hydrophobic Neutral P
Added by SpectrumMill v3 vs. SM v2; n=343
Added by rescored SpectrumMill v3 vs. SM v2; n=392
Lost by rescored SpectrumMill v3 vs. SM v2; n=43
Bits
0.0
0.5
1.0
1.5
2.0
1 2 3 4 5 6 7 8 9
chemistry Acidic Basic Hydrophobic Neutral Polarchemistry Acidic Basic Hydrophobic Neutral Polarchemistry Acidic Basic Hydrophobic Neutral P

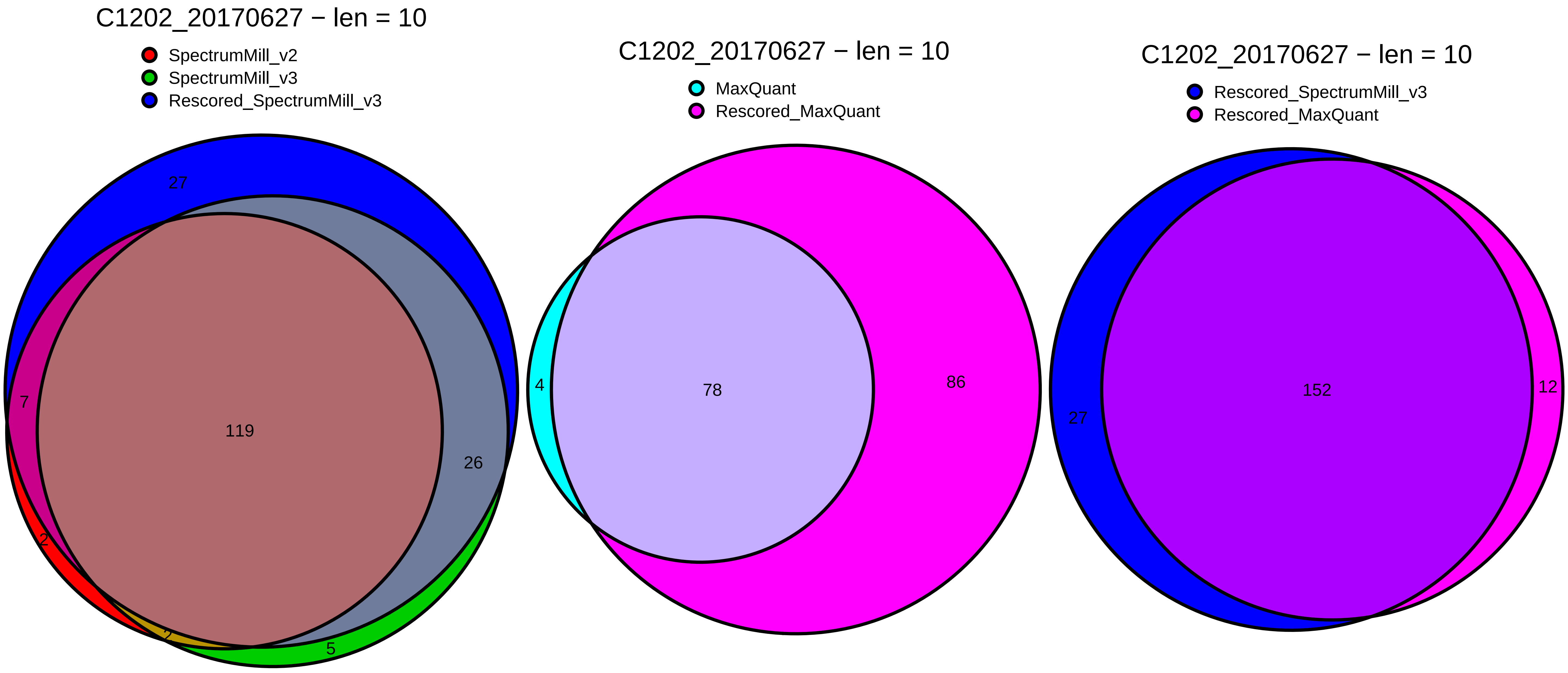
C1202_20170627 − len = 10
SpectrumMill_v2
SpectrumMill_v3
Rescored_SpectrumMill_v3
27
7
119
26
2
2
5
C1202_20170627 − len = 10
MaxQuant
Rescored_MaxQuant
4
78
86
C1202_20170627 − len = 10
Rescored_SpectrumMill_v3
Rescored_MaxQuant
27
152
12

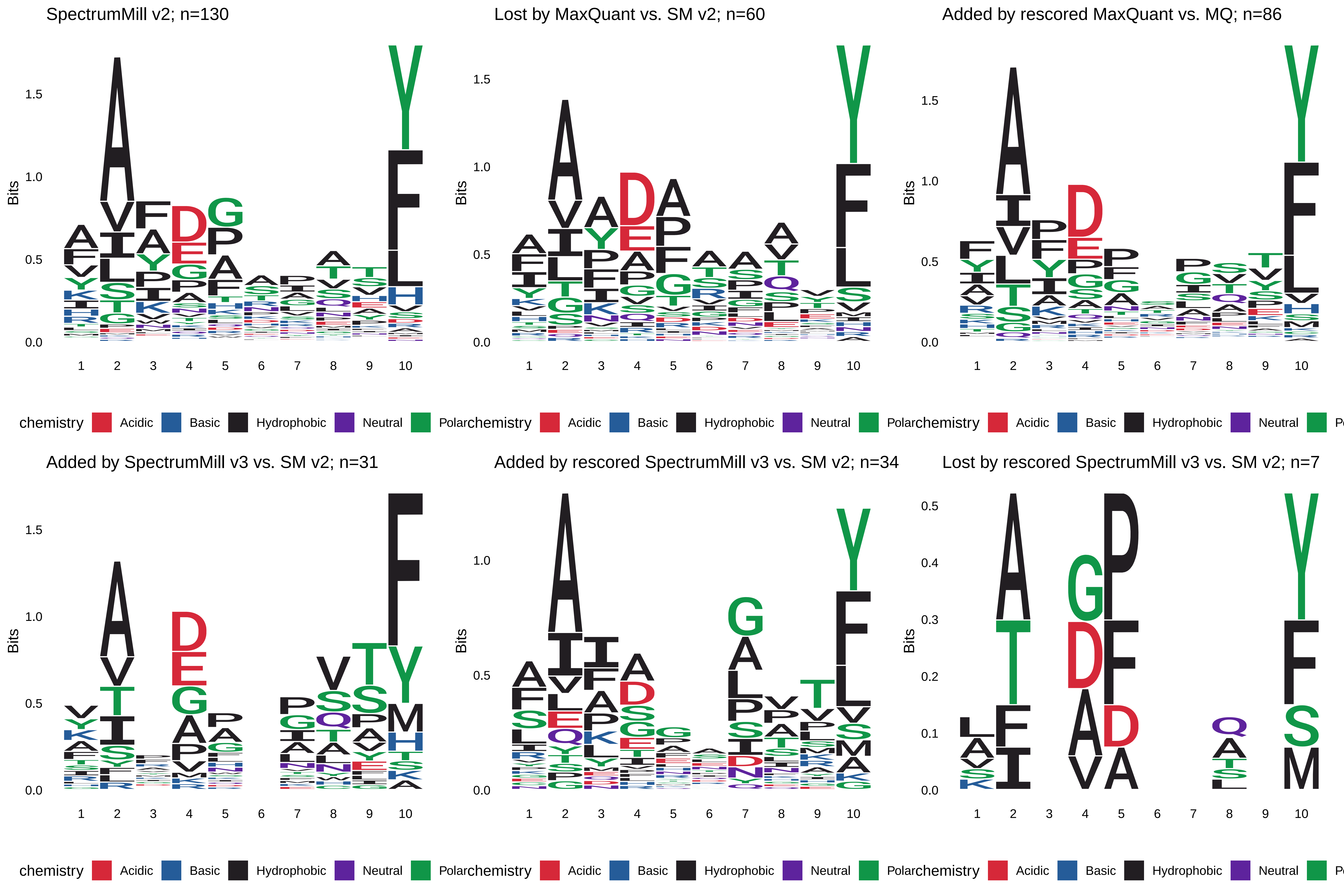
SpectrumMill v2; n=130
Lost by MaxQuant vs. SM v2; n=60
Added by rescored MaxQuant vs. MQ; n=86
Bits
chemistry Acidic Basic Hydrophobic Neutral Polarchemistry Acidic Basic Hydrophobic Neutral Polarchemistry Acidic Basic Hydrophobic Neutral Po
Added by SpectrumMill v3 vs. SM v2; n=31
Added by rescored SpectrumMill v3 vs. SM v2; n=34
Lost by rescored SpectrumMill v3 vs. SM v2; n=7
chemistry Acidic Basic Hydrophobic Neutral Polarchemistry Acidic Basic Hydrophobic Neutral Polarchemistry Acidic Basic Hydrophobic Neutral Po

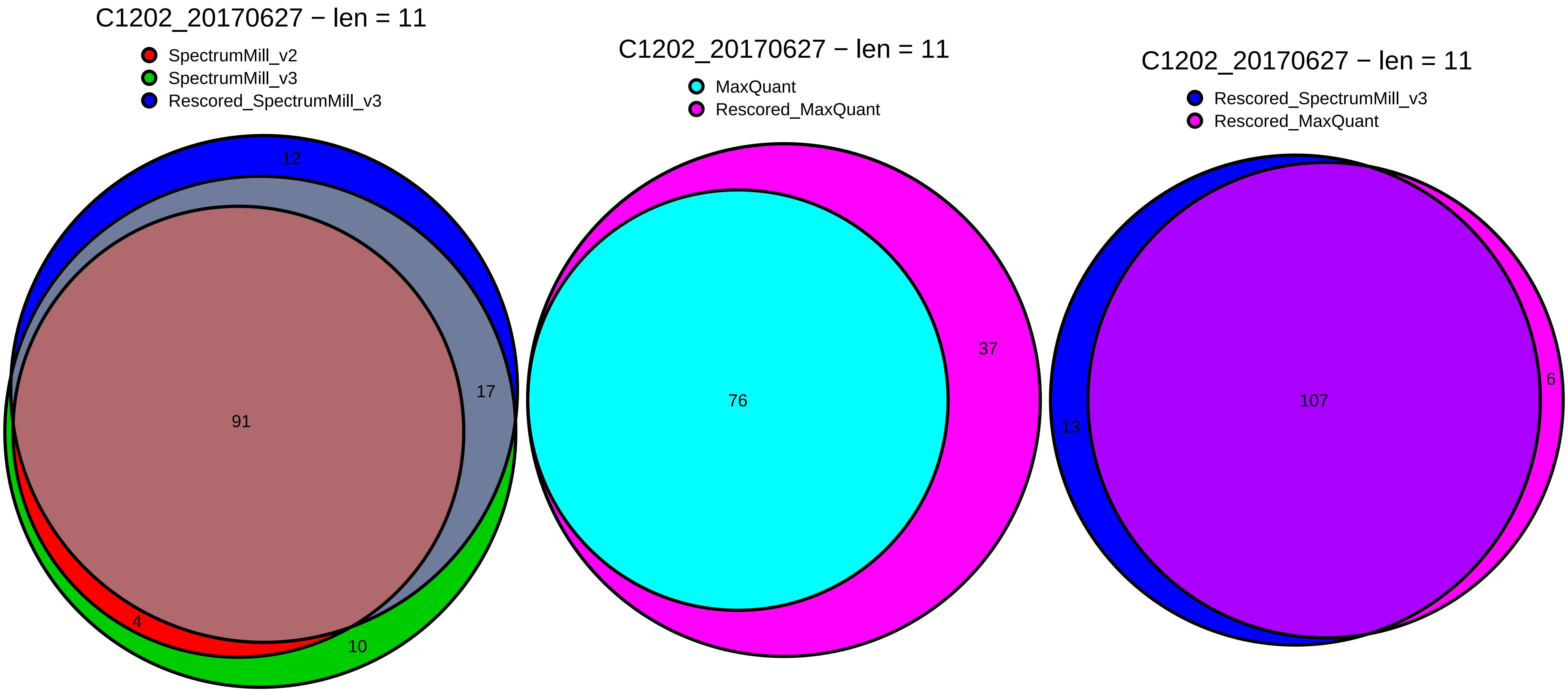
C1202_20170627 − len = 11
SpectrumMill_v2
SpectrumMill_v3
Rescored_SpectrumMill_v3
12
17
91
4
10
C1202_20170627 − len = 11
MaxQuant
Rescored_MaxQuant
37
76
C1202_20170627 − len = 11
Rescored_SpectrumMill_v3
Rescored_MaxQuant
6
107
13

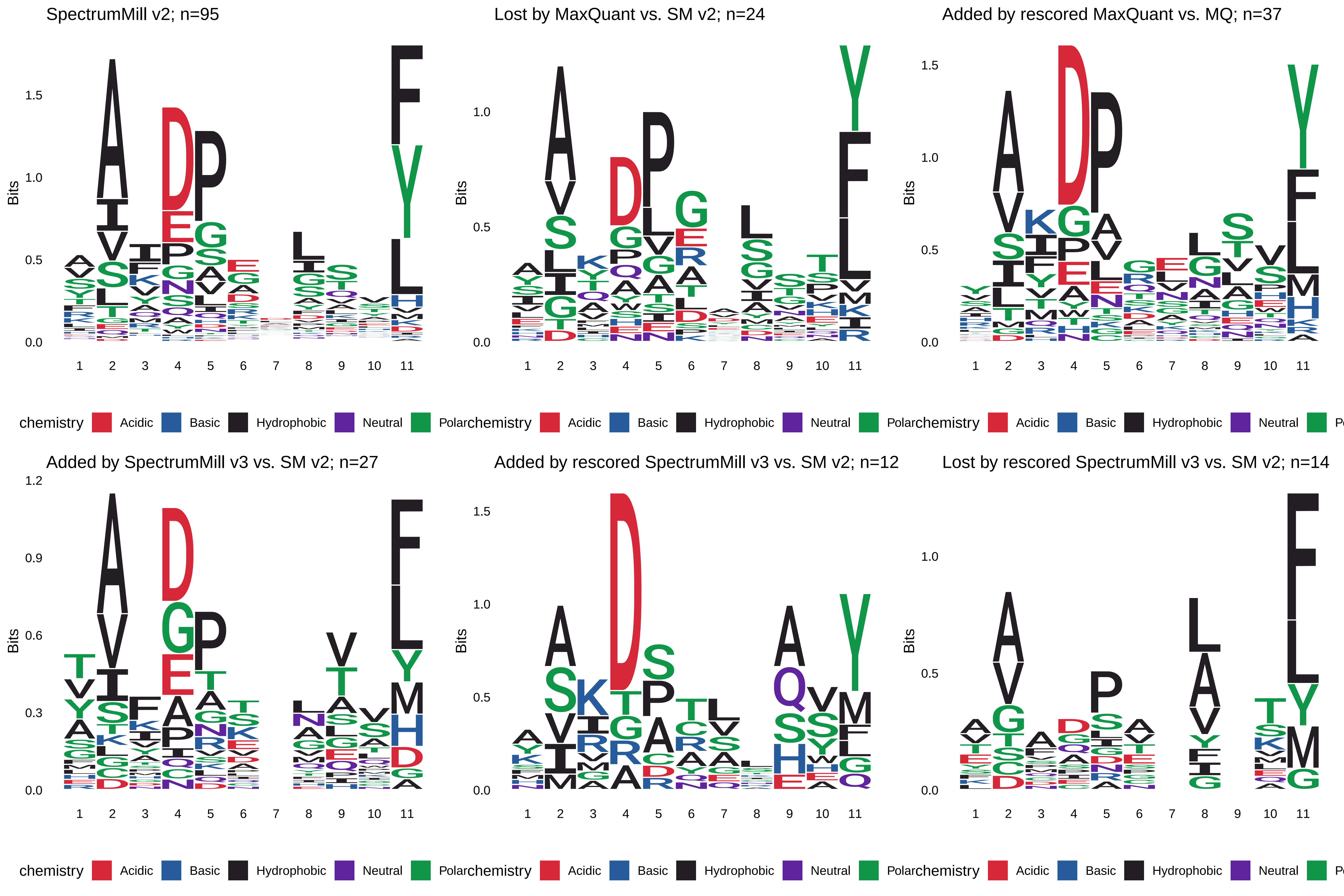
SpectrumMill v2; n=95
Lost by MaxQuant vs. SM v2; n=24
Added by rescored MaxQuant vs. MQ; n=37
Added by SpectrumMill v3 vs. SM v2; n=27
Added by rescored SpectrumMill v3 vs. SM v2; n=12
Lost by rescored SpectrumMill v3 vs. SM v2; n=14
Bits
1 2 3 4 5 6 7 8 9 10 11
chemistry Acidic Basic Hydrophobic Neutral Polar

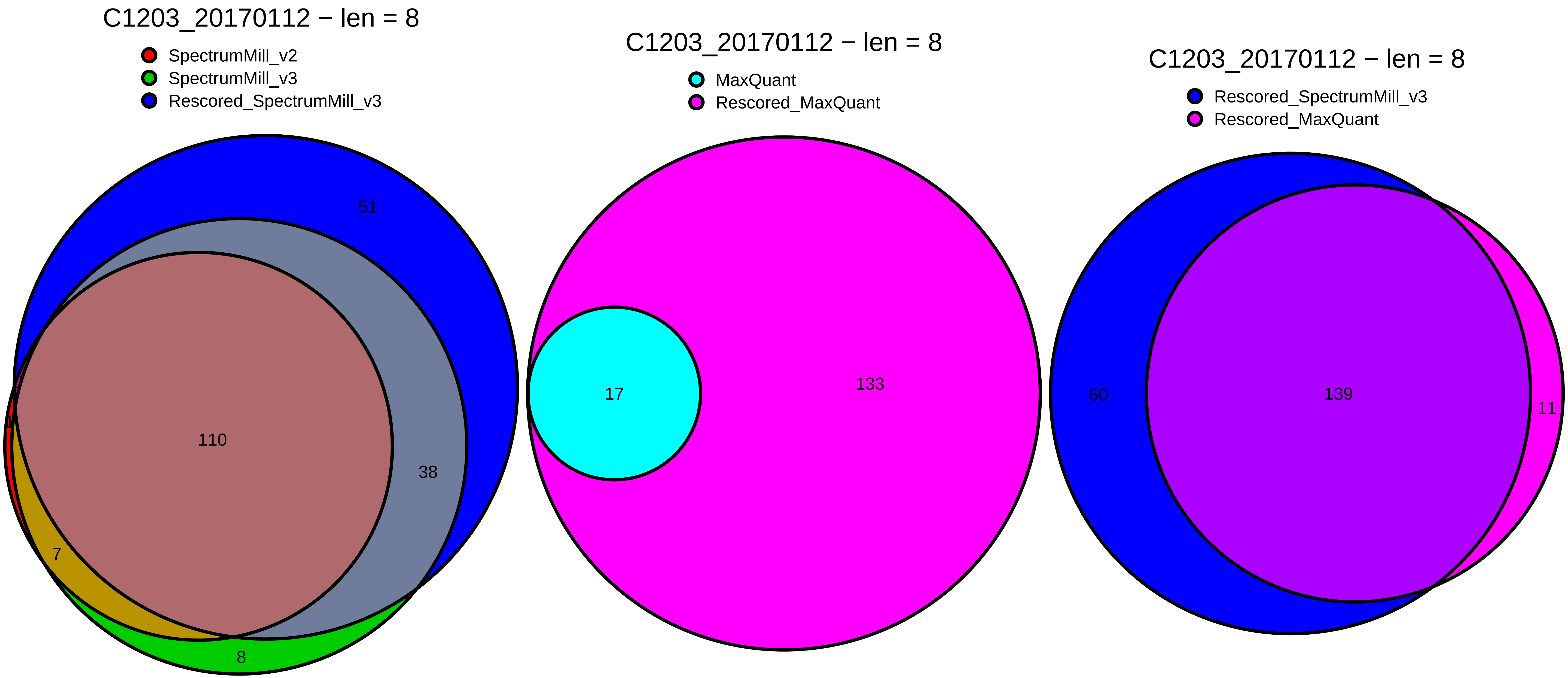
C1203_20170112 − len = 8
SpectrumMill_v2
SpectrumMill_v3
Rescored_SpectrumMill_v3
51
110
38
7
8
C1203_20170112 − len = 8
MaxQuant
Rescored_MaxQuant
17
133
C1203_20170112 − len = 8
Rescored_SpectrumMill_v3
Rescored_MaxQuant
60
139
11

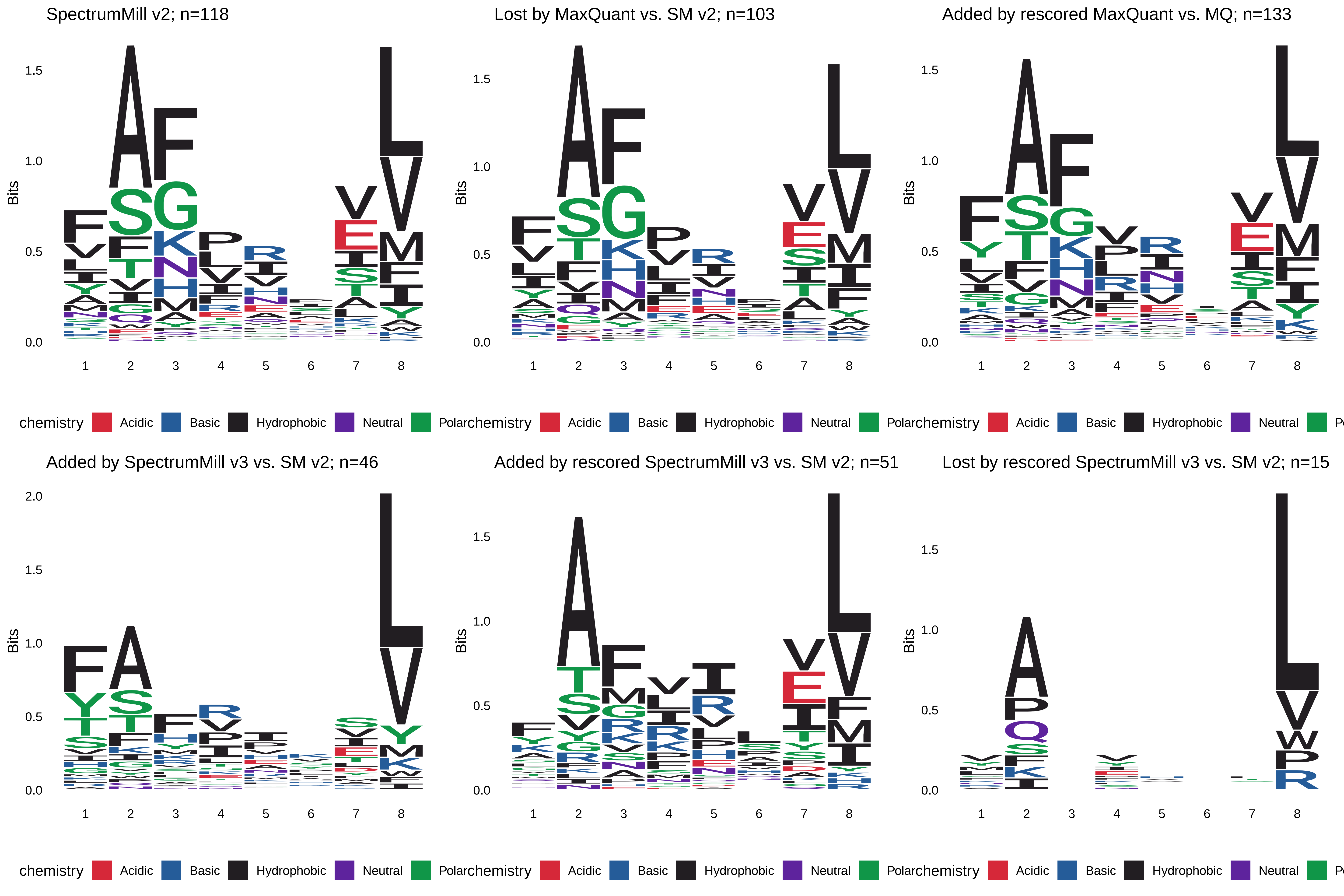
SpectrumMill v2; n=118
Lost by MaxQuant vs. SM v2; n=103
Added by rescored MaxQuant vs. MQ; n=133
Added by SpectrumMill v3 vs. SM v2; n=46
Added by rescored SpectrumMill v3 vs. SM v2; n=51
Lost by rescored SpectrumMill v3 vs. SM v2; n=15
Bits
0.0
0.5
1.0
1.5
2.0
1 2 3 4 5 6 7 8
chemistry
Acidic
Basic
Hydrophobic
Neutral
Polar

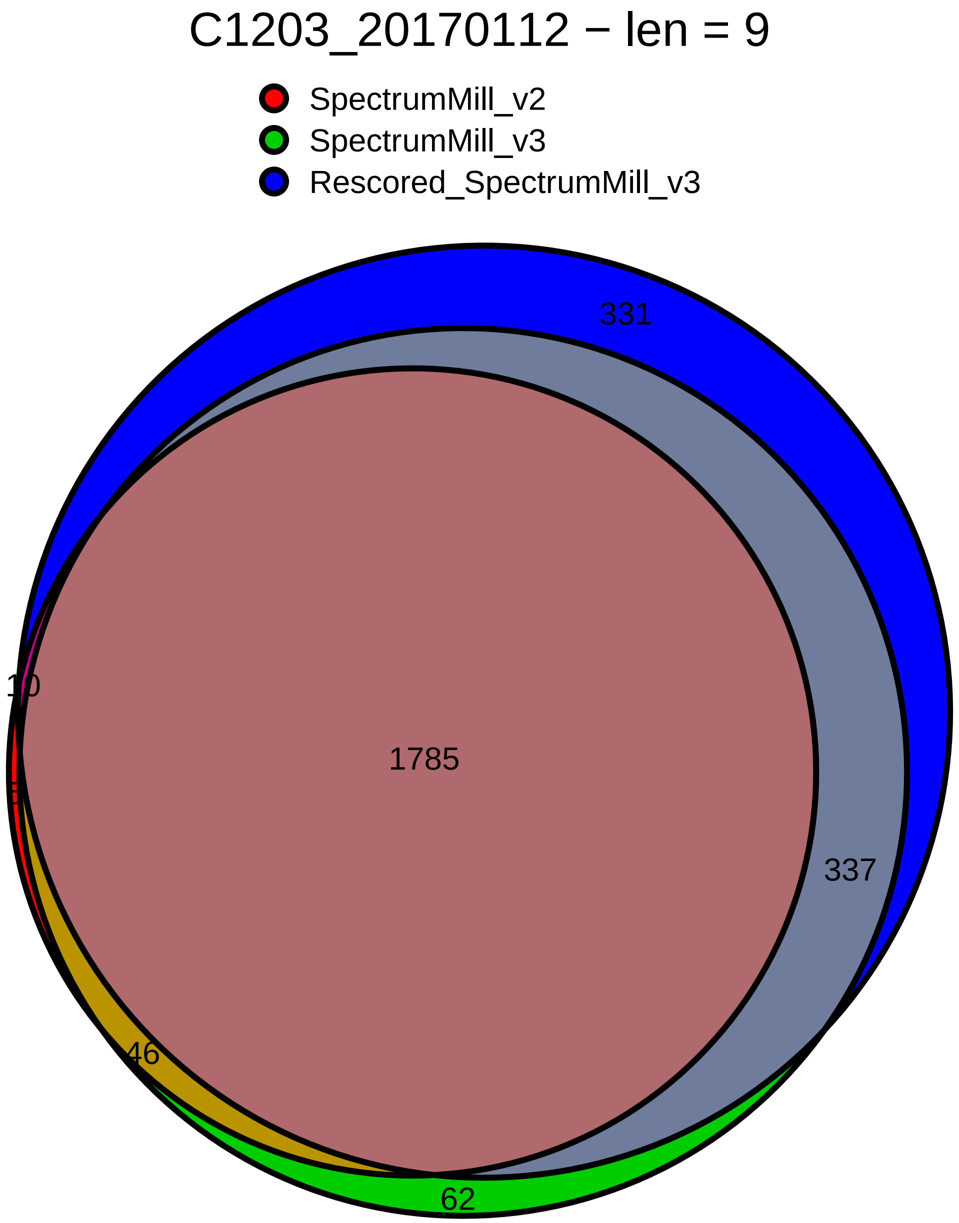
C1203_20170112 − len = 9
SpectrumMill_v2
SpectrumMill_v3
Rescored_SpectrumMill_v3
331
1785
337
46
62

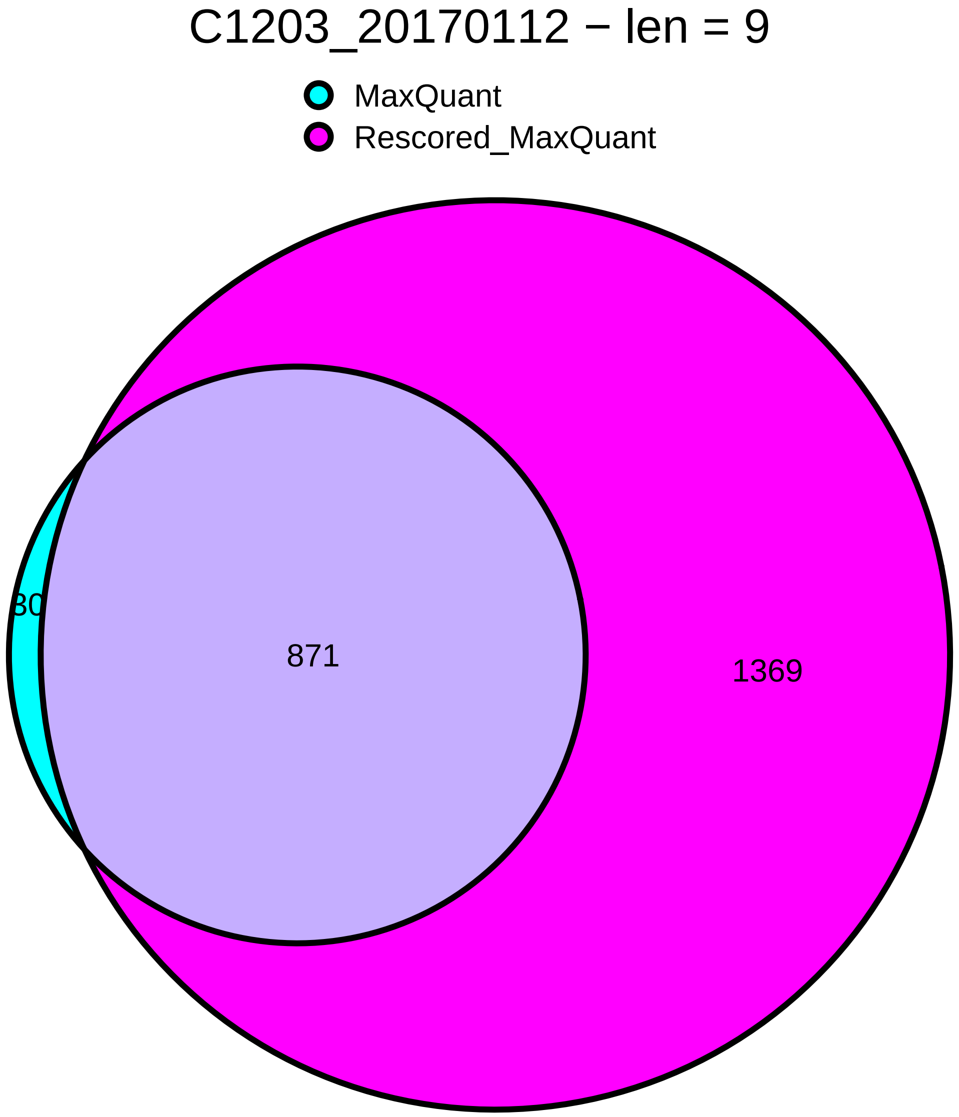
C1203_20170112 − len = 9
MaxQuant
Rescored_MaxQuant
871
1369

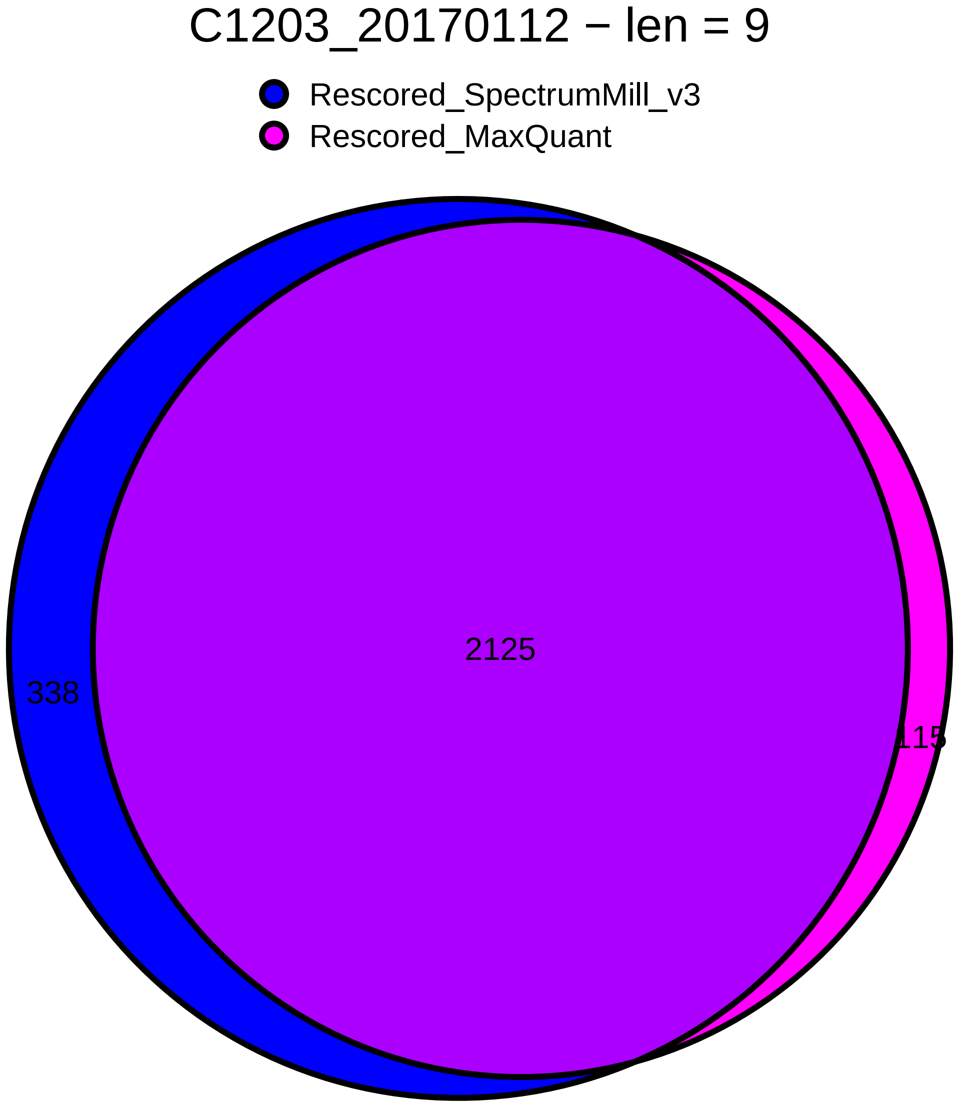
C1203_20170112 − len = 9
Rescored_SpectrumMill_v3
Rescored_MaxQuant
2125
338
115

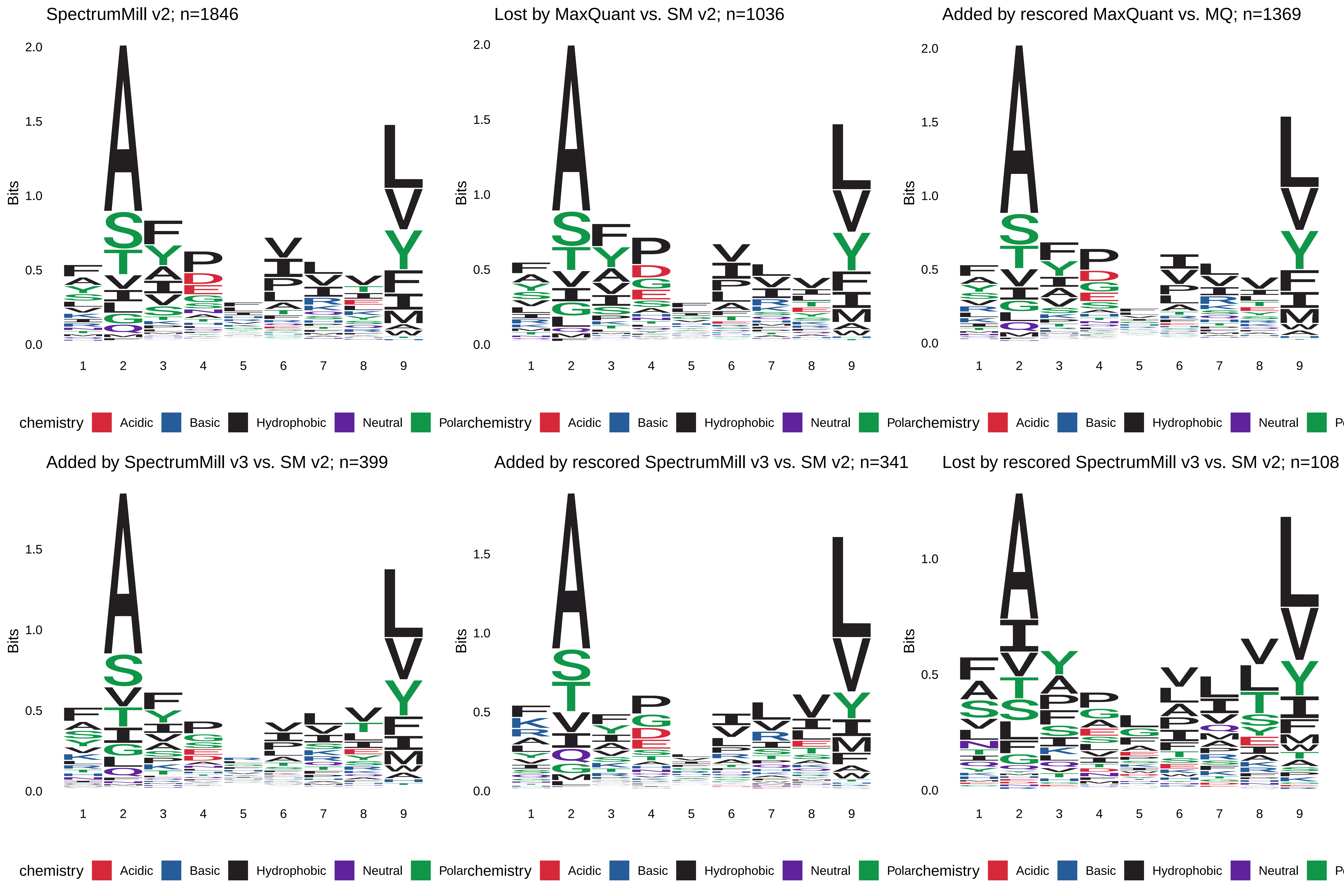
SpectrumMill v2; n=1846
Lost by MaxQuant vs. SM v2; n=1036
Added by rescored MaxQuant vs. MQ; n=1369
Added by SpectrumMill v3 vs. SM v2; n=399
Added by rescored SpectrumMill v3 vs. SM v2; n=341
Lost by rescored SpectrumMill v3 vs. SM v2; n=108
Bits
0.0
0.5
1.0
1.5
2.0
1 2 3 4 5 6 7 8 9
chemistry
Acidic
Basic
Hydrophobic
Neutral
Polar

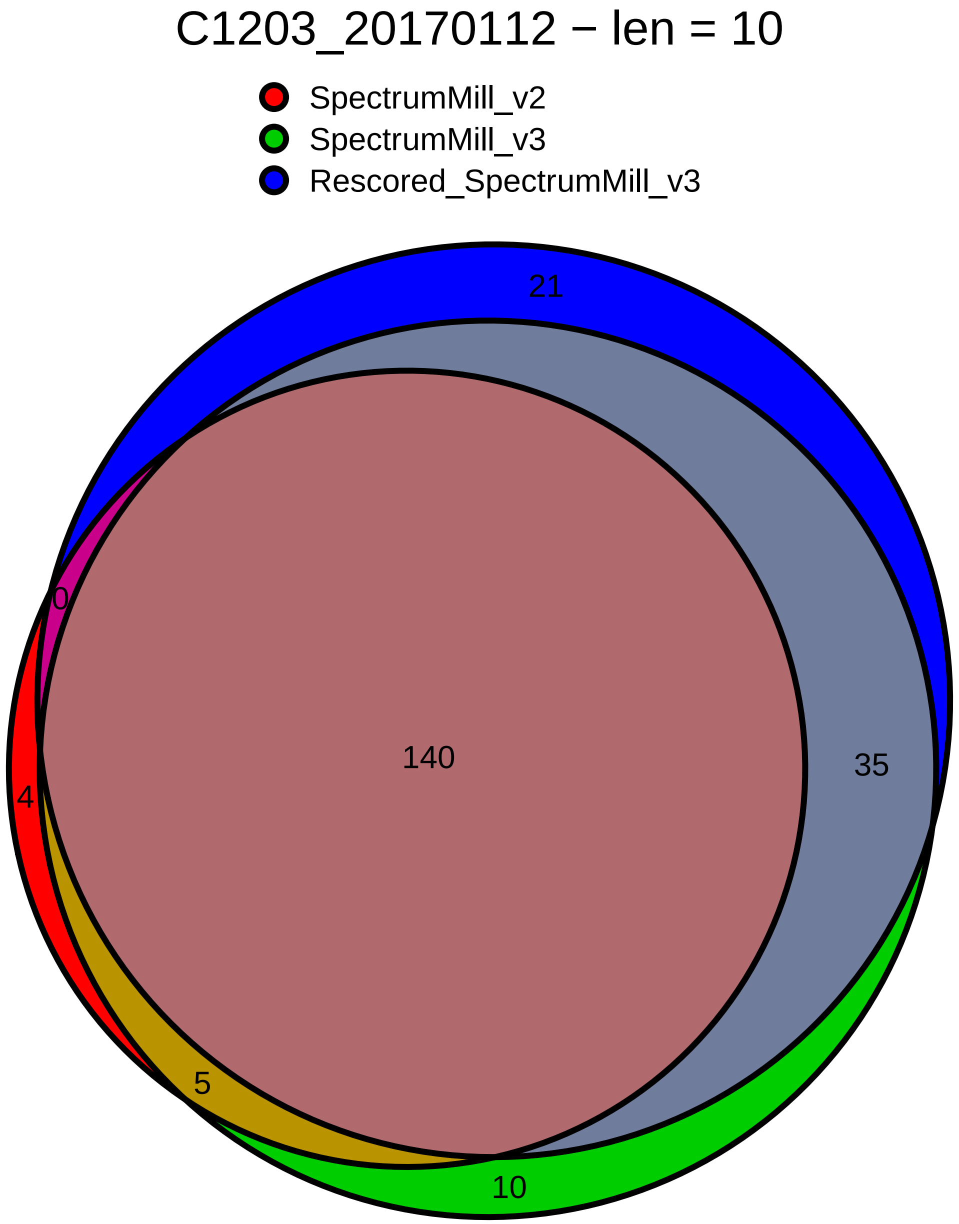
C1203_20170112 − len = 10
SpectrumMill_v2
SpectrumMill_v3
Rescored_SpectrumMill_v3
21
0
140
35
4
5
10

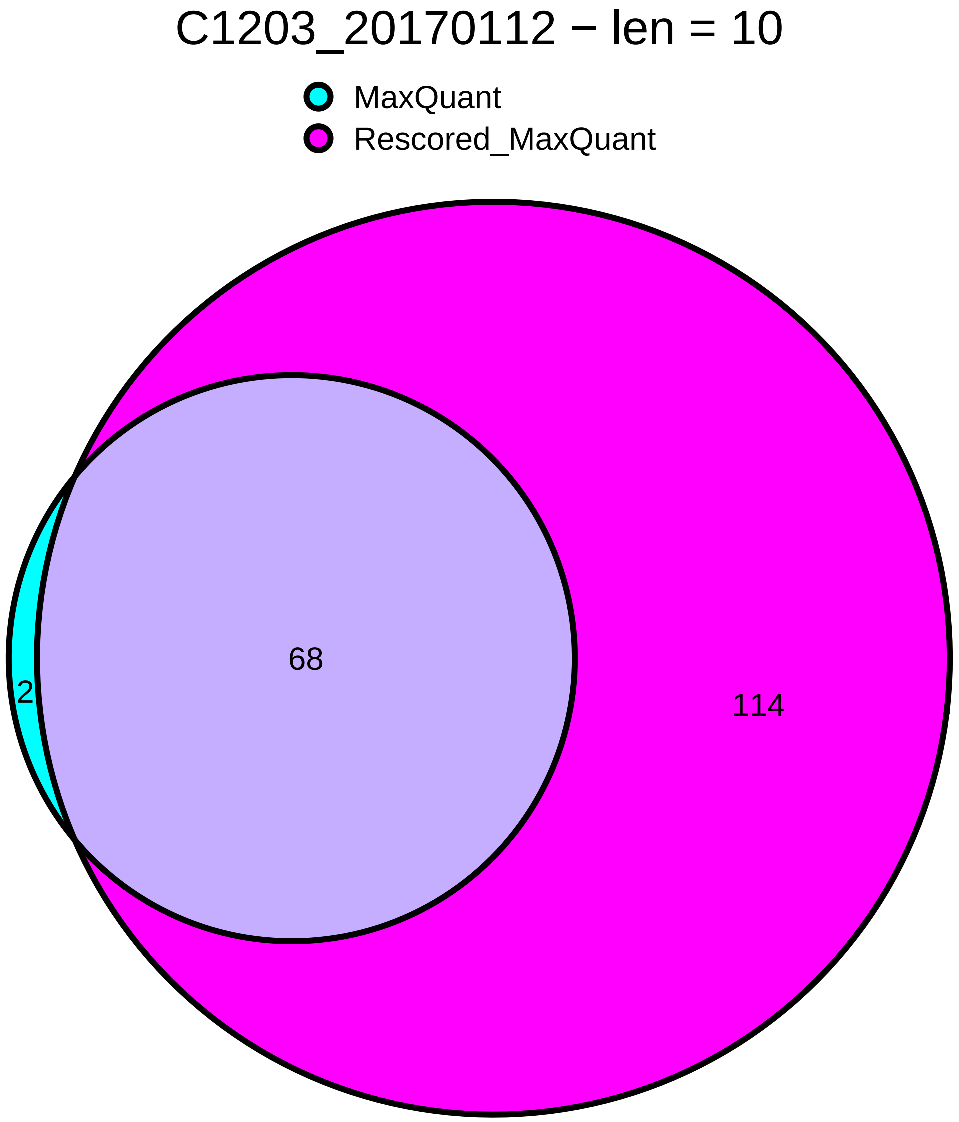
C1203_20170112 − len = 10
MaxQuant
Rescored_MaxQuant
2
68
114

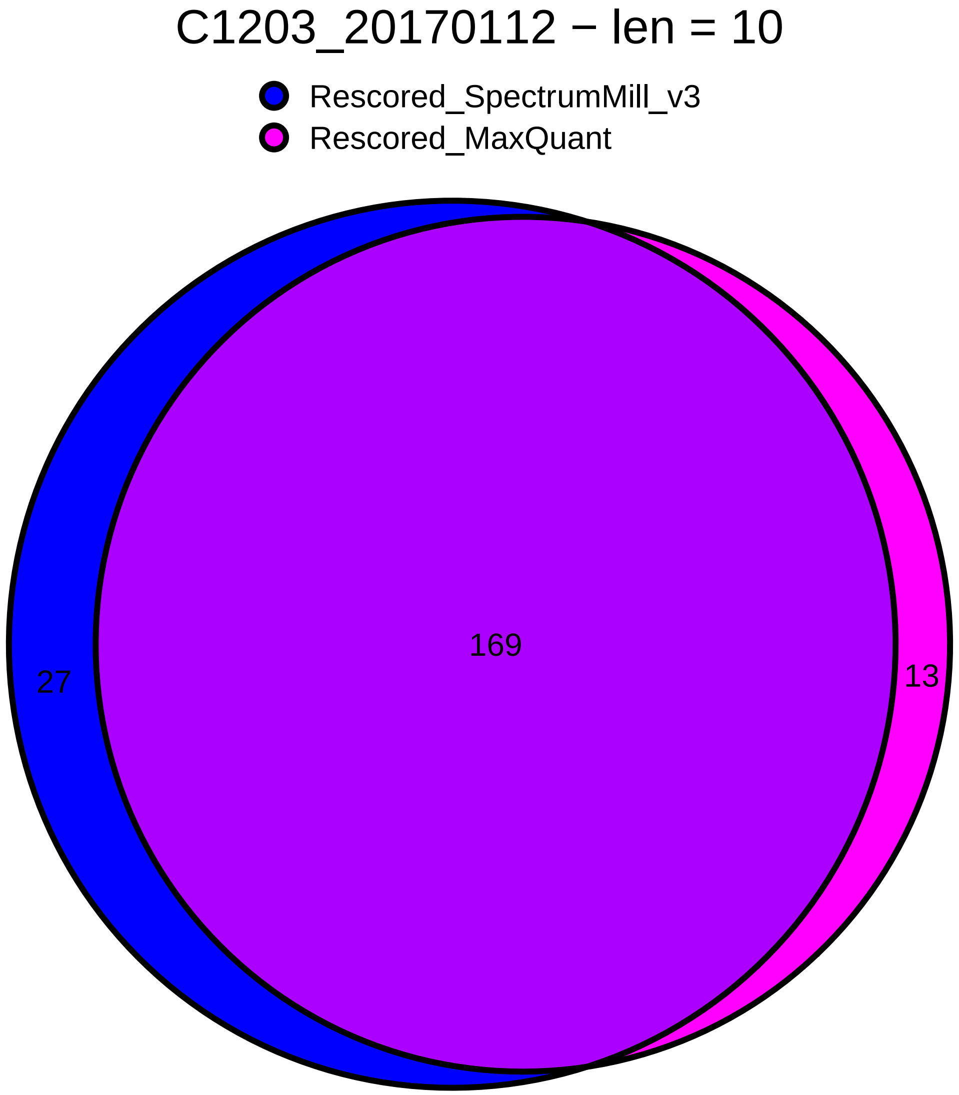
C1203_20170112 − len = 10
Rescored_SpectrumMill_v3
Rescored_MaxQuant
27
169
13

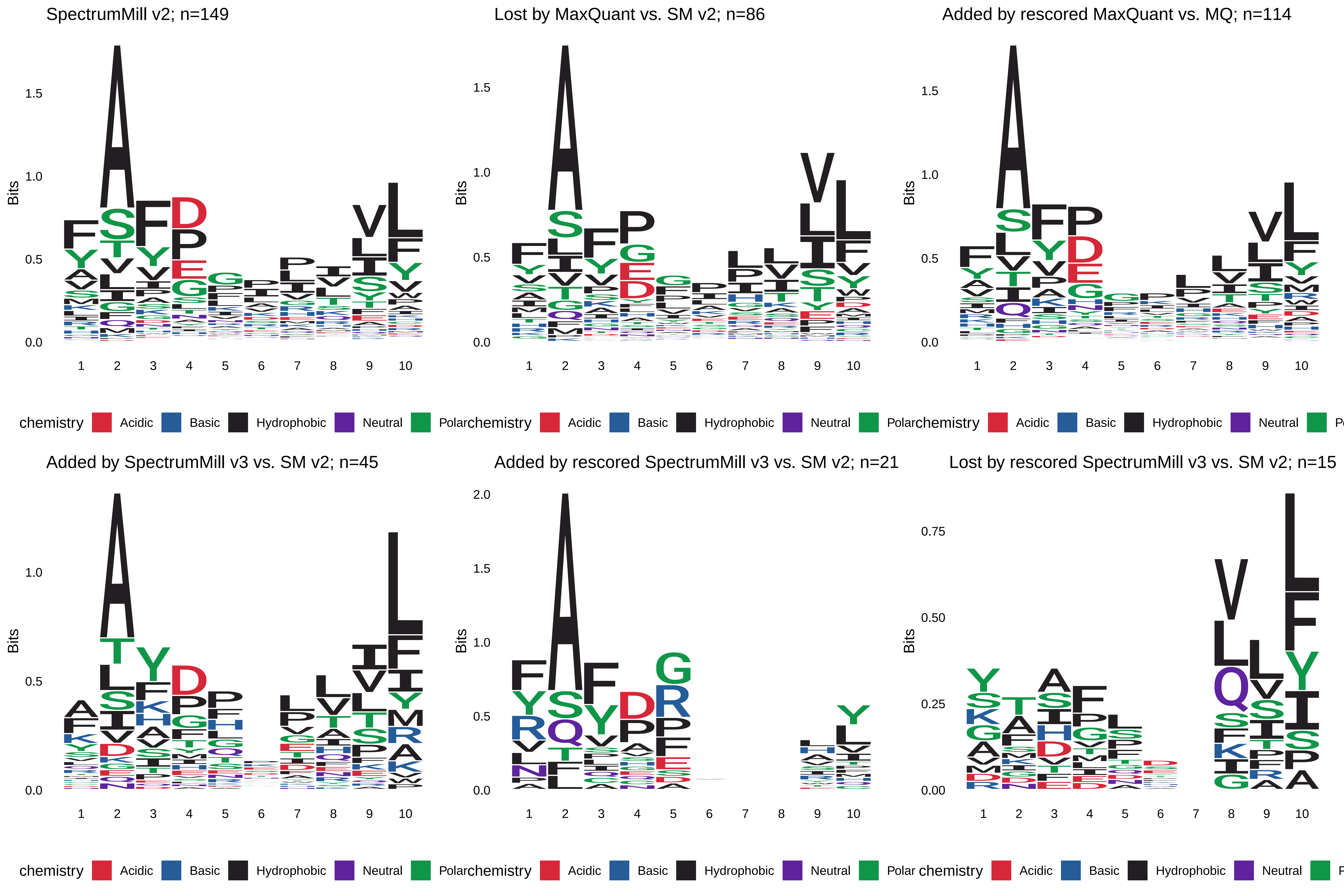
SpectrumMill v2; n=149
Lost by MaxQuant vs. SM v2; n=86
Added by rescored MaxQuant vs. MQ; n=114
Added by SpectrumMill v3 vs. SM v2; n=45
Added by rescored SpectrumMill v3 vs. SM v2; n=21
Lost by rescored SpectrumMill v3 vs. SM v2; n=15
Bits
1 2 3 4 5 6 7 8 9 10
chemistry Acidic Basic Hydrophobic Neutral Polar

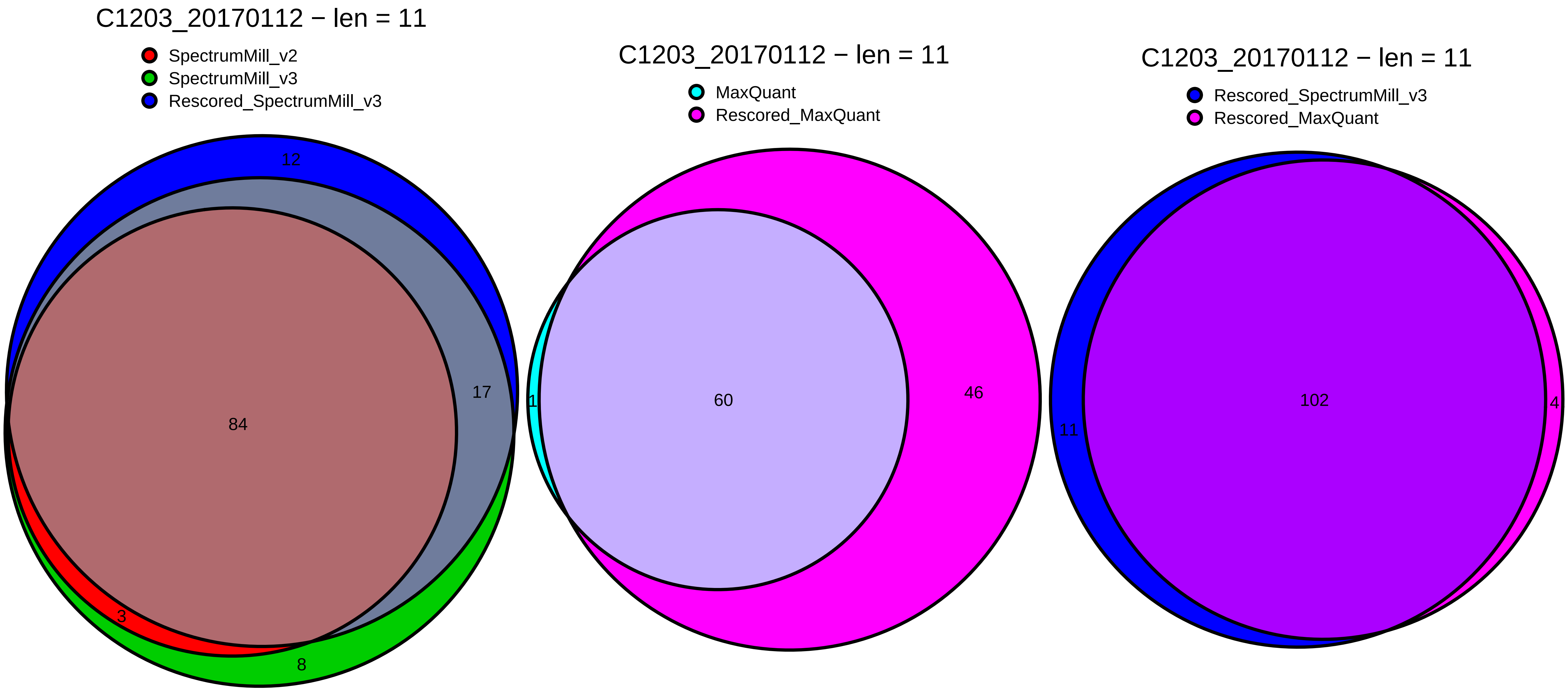
C1203_20170112 − len = 11
SpectrumMill_v2
SpectrumMill_v3
Rescored_SpectrumMill_v3
12
17
84
3
8
C1203_20170112 − len = 11
MaxQuant
Rescored_MaxQuant
1
60
46
C1203_20170112 − len = 11
Rescored_SpectrumMill_v3
Rescored_MaxQuant
11
102
4

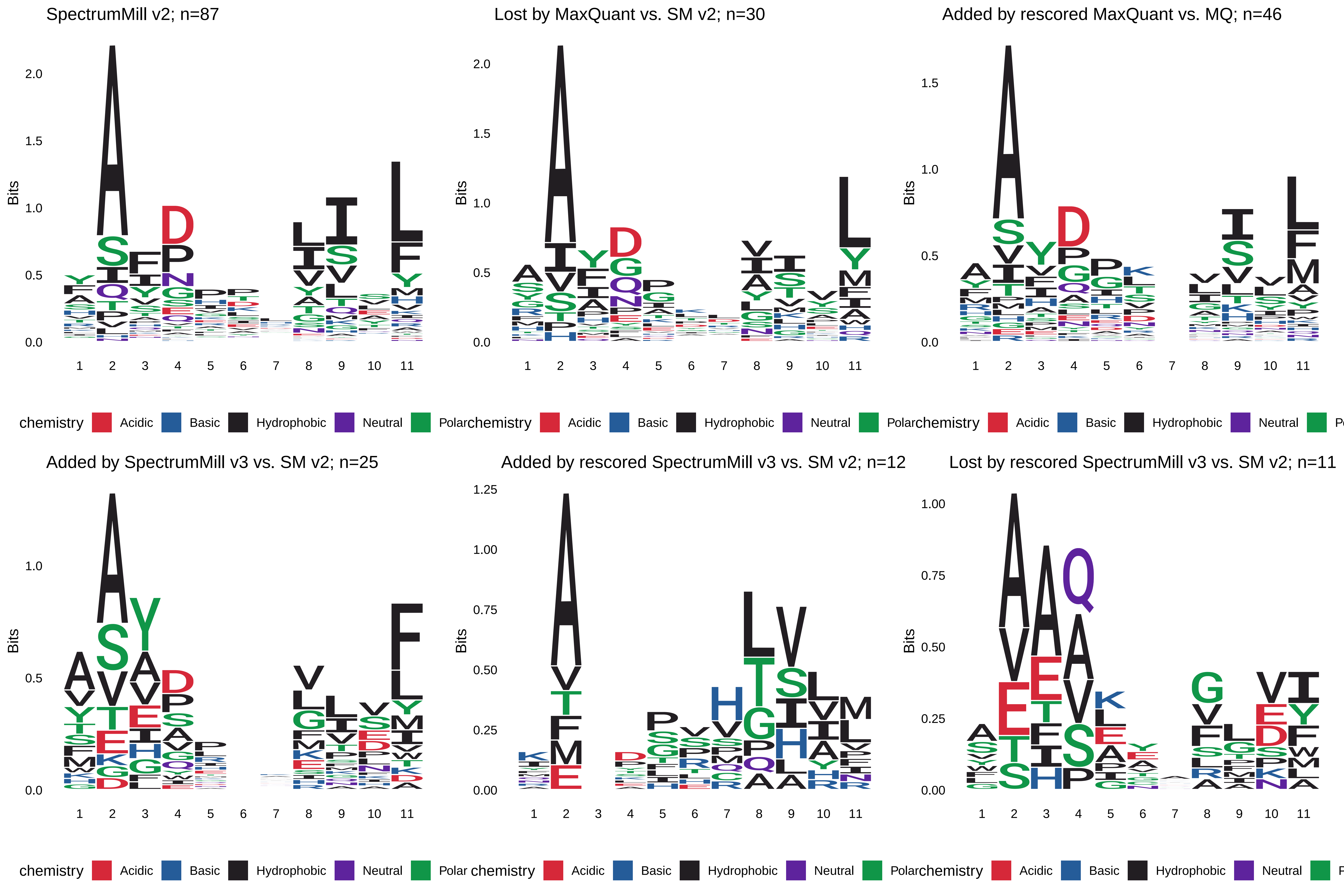
SpectrumMill v2; n=87
Lost by MaxQuant vs. SM v2; n=30
Added by rescored MaxQuant vs. MQ; n=46
Bits
chemistry Acidic Basic Hydrophobic Neutral Polar
Added by SpectrumMill v3 vs. SM v2; n=25
Added by rescored SpectrumMill v3 vs. SM v2; n=12
Lost by rescored SpectrumMill v3 vs. SM v2; n=11
Bits
chemistry Acidic Basic Hydrophobic Neutral Polar

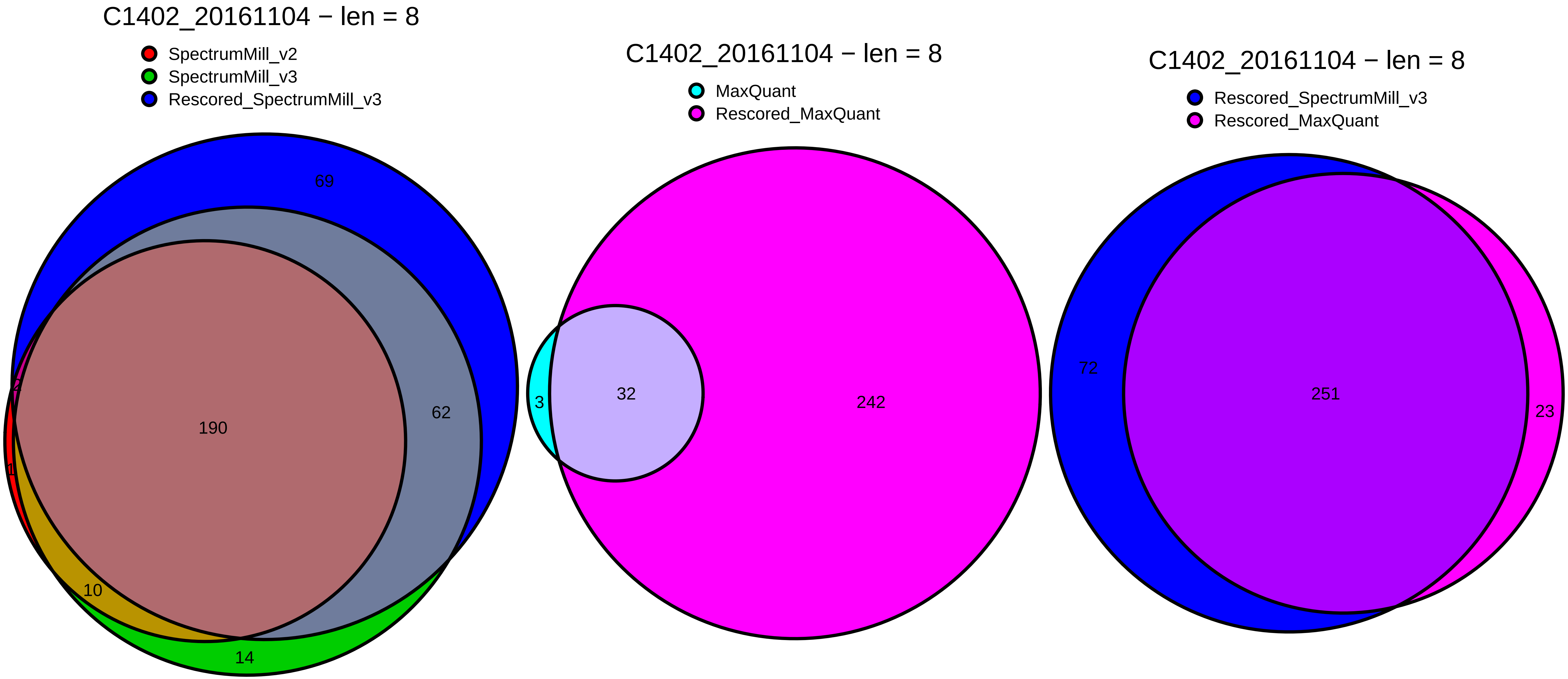
C1402_20161104 − len = 8
SpectrumMill_v2
SpectrumMill_v3
Rescored_SpectrumMill_v3
69
62
190
10
14
C1402_20161104 − len = 8
MaxQuant
Rescored_MaxQuant
3
32
242
C1402_20161104 − len = 8
Rescored_SpectrumMill_v3
Rescored_MaxQuant
72
251
23

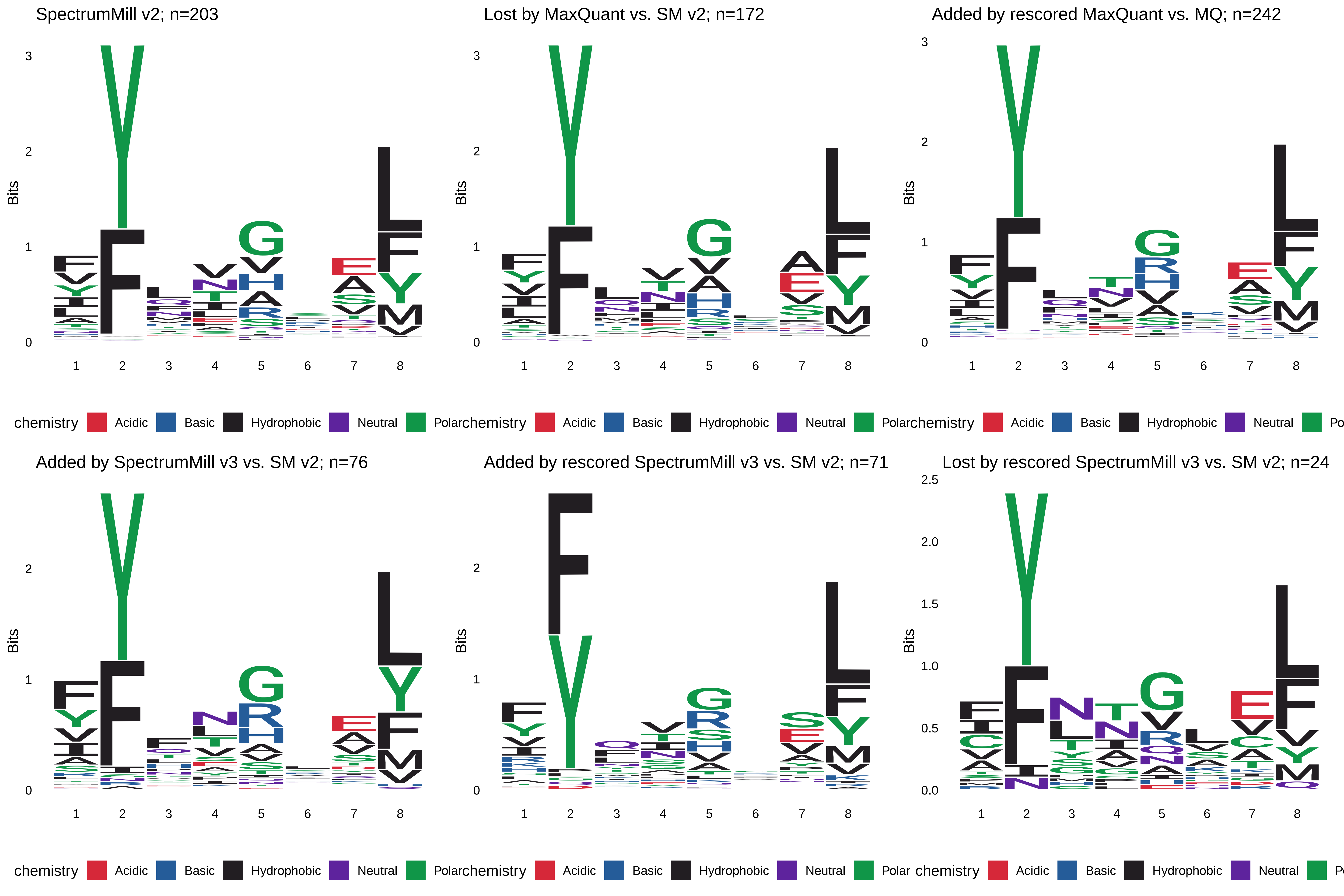

SpectrumMill v2; n=203
Lost by MaxQuant vs. SM v2; n=172
Added by rescored MaxQuant vs. MQ; n=242
Added by SpectrumMill v3 vs. SM v2; n=76
Added by rescored SpectrumMill v3 vs. SM v2; n=71
Lost by rescored SpectrumMill v3 vs. SM v2; n=24
Bits
chemistry
Acidic
Basic
Hydrophobic
Neutral
Polar

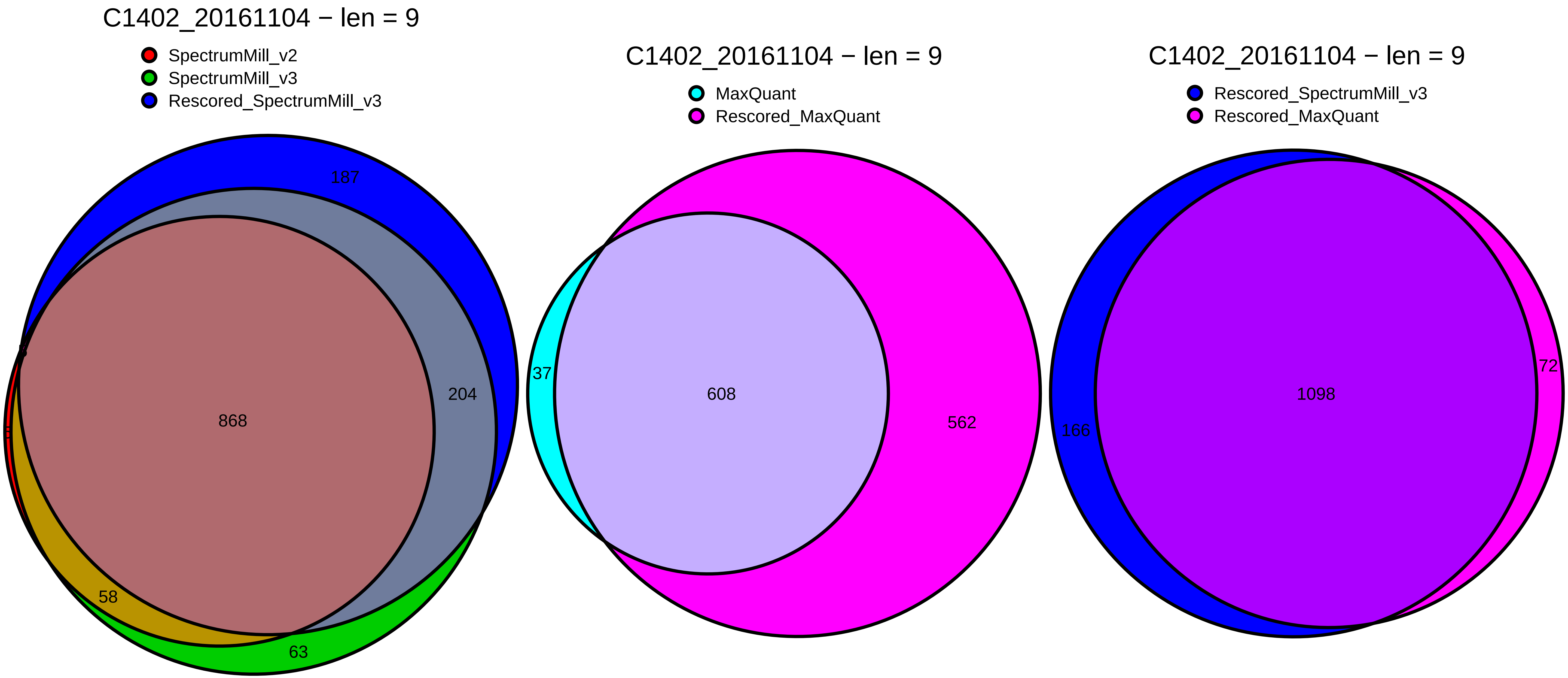
C1402_20161104 − len = 9
SpectrumMill_v2
SpectrumMill_v3
Rescored_SpectrumMill_v3
187
204
868
58
63
C1402_20161104 − len = 9
MaxQuant
Rescored_MaxQuant
37
608
562
C1402_20161104 − len = 9
Rescored_SpectrumMill_v3
Rescored_MaxQuant
1098
166
72

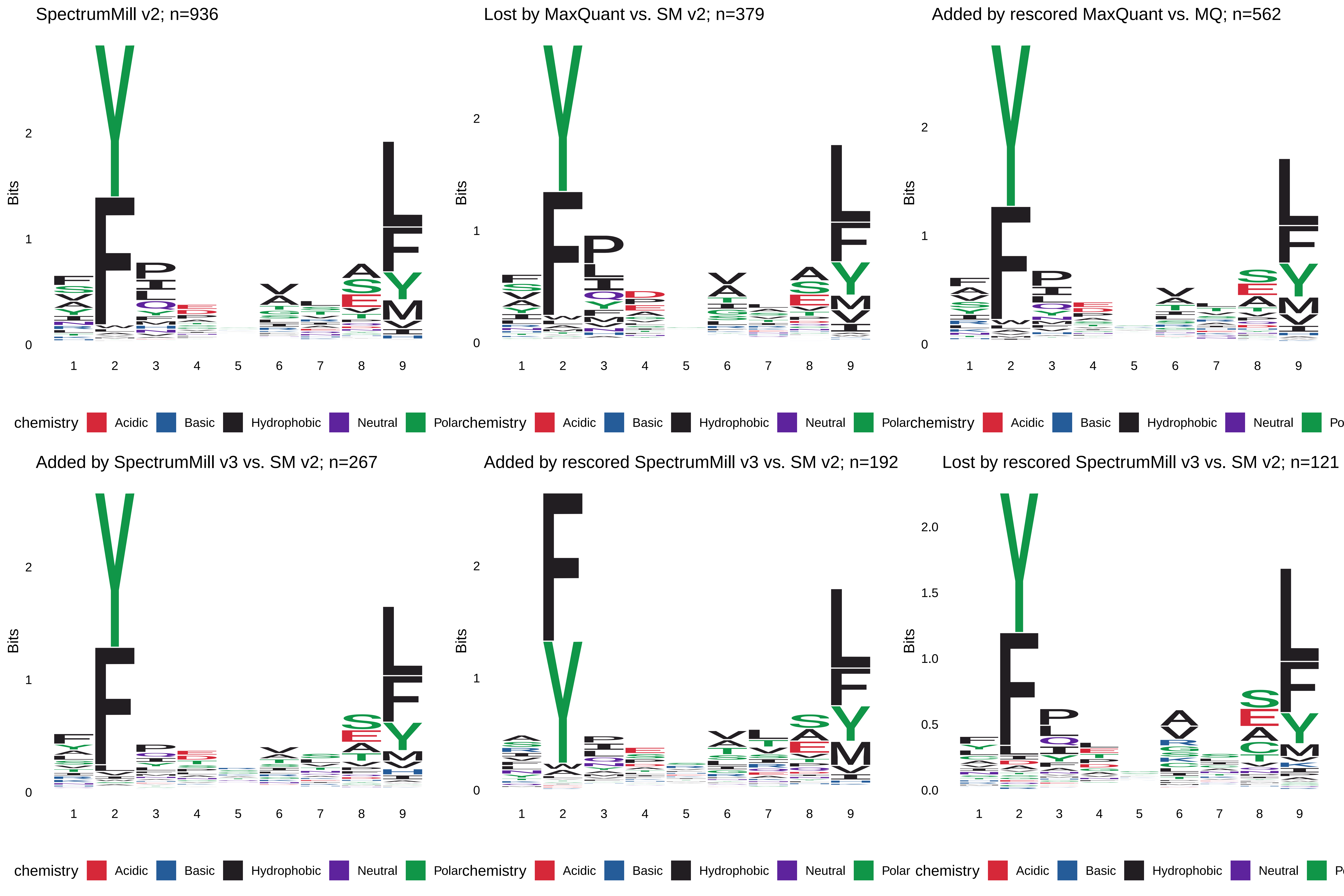
SpectrumMill v2; n=936
Lost by MaxQuant vs. SM v2; n=379
Added by rescored MaxQuant vs. MQ; n=562
Bits
0
1
2
1 2 3 4 5 6 7 8 9
chemistry Acidic Basic Hydrophobic Neutral Polar
Added by SpectrumMill v3 vs. SM v2; n=267
Added by rescored SpectrumMill v3 vs. SM v2; n=192
Lost by rescored SpectrumMill v3 vs. SM v2; n=121
0.0
0.5
1.0
1.5
2.0

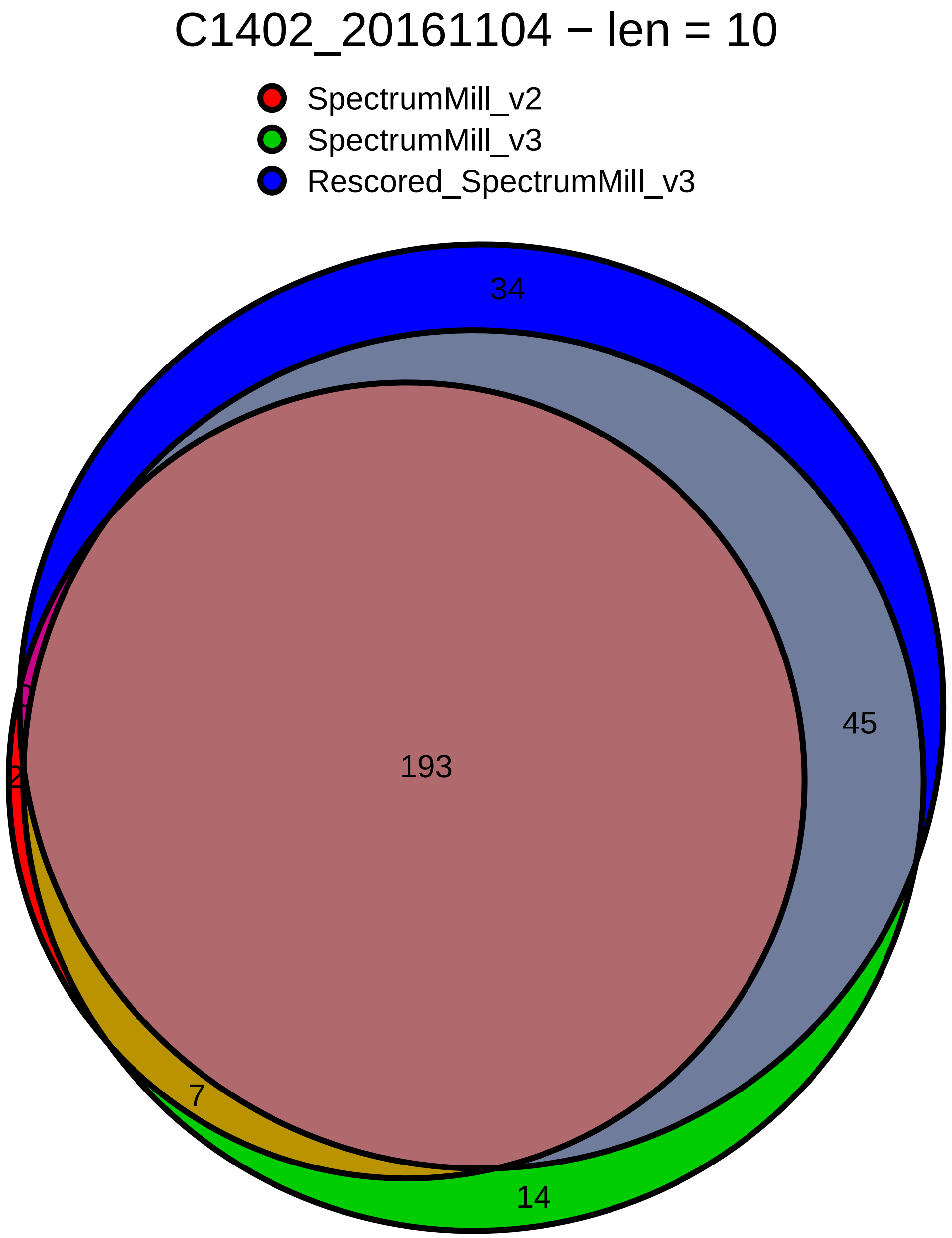
C1402_20161104 − len = 10
SpectrumMill_v2
SpectrumMill_v3
Rescored_SpectrumMill_v3
34
45
193
2
7
14

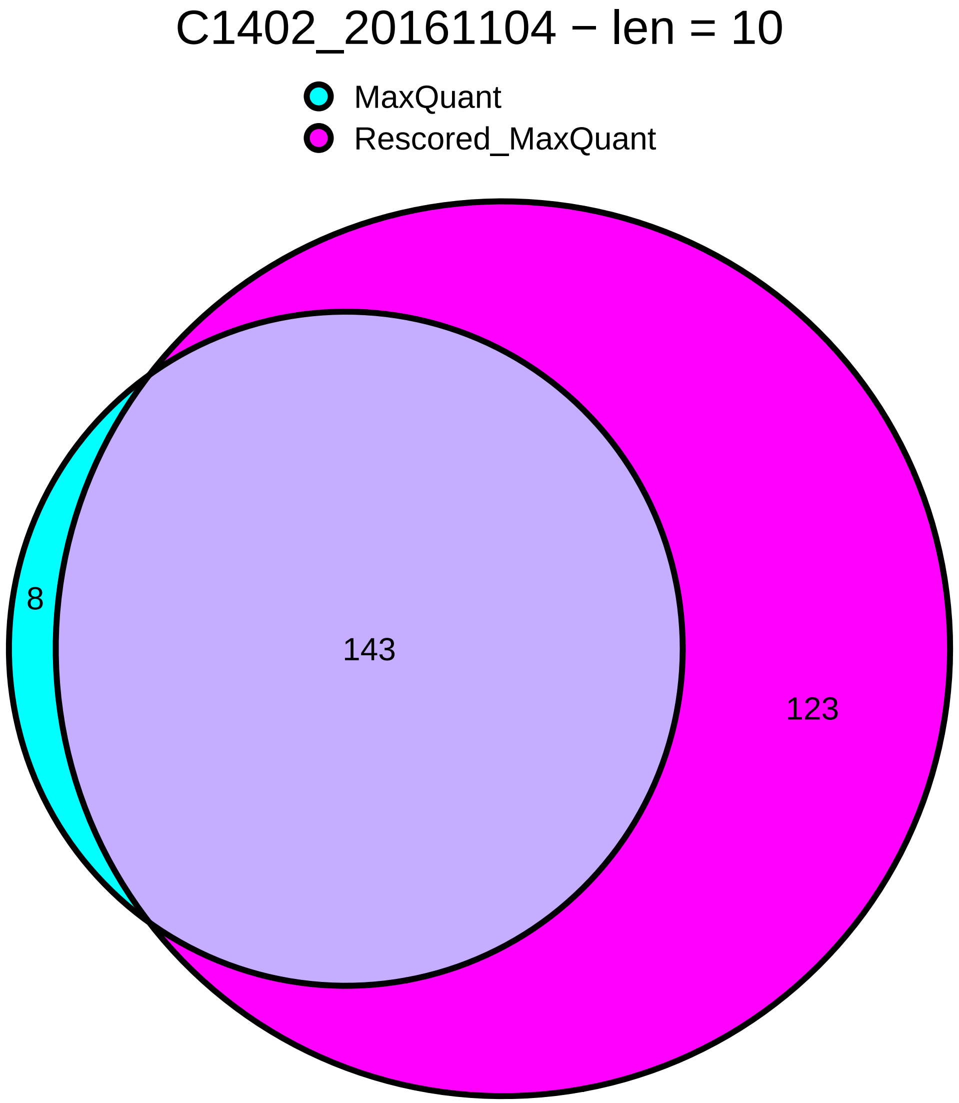
C1402_20161104 − len = 10
MaxQuant
Rescored_MaxQuant
8
143
123

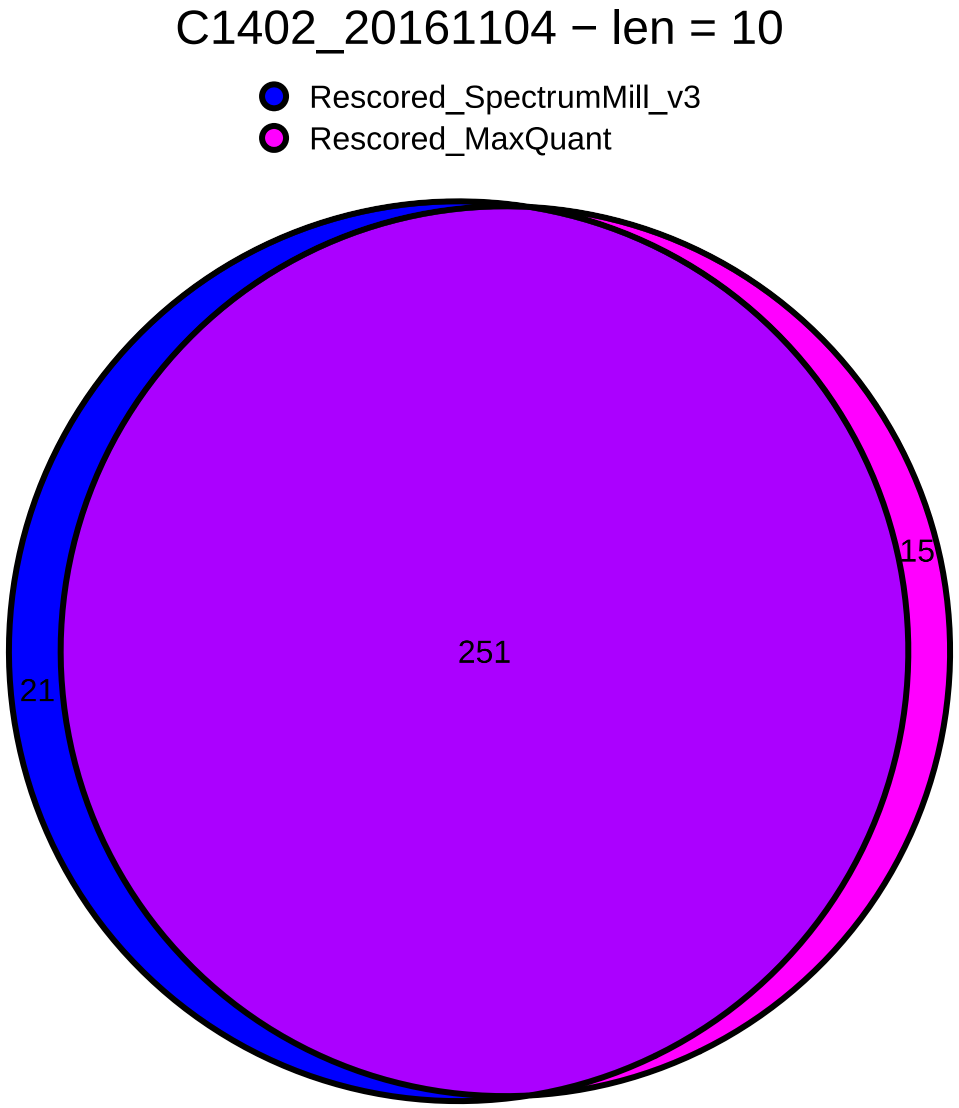
C1402_20161104 − len = 10
Rescored_SpectrumMill_v3
Rescored_MaxQuant
21
251
15

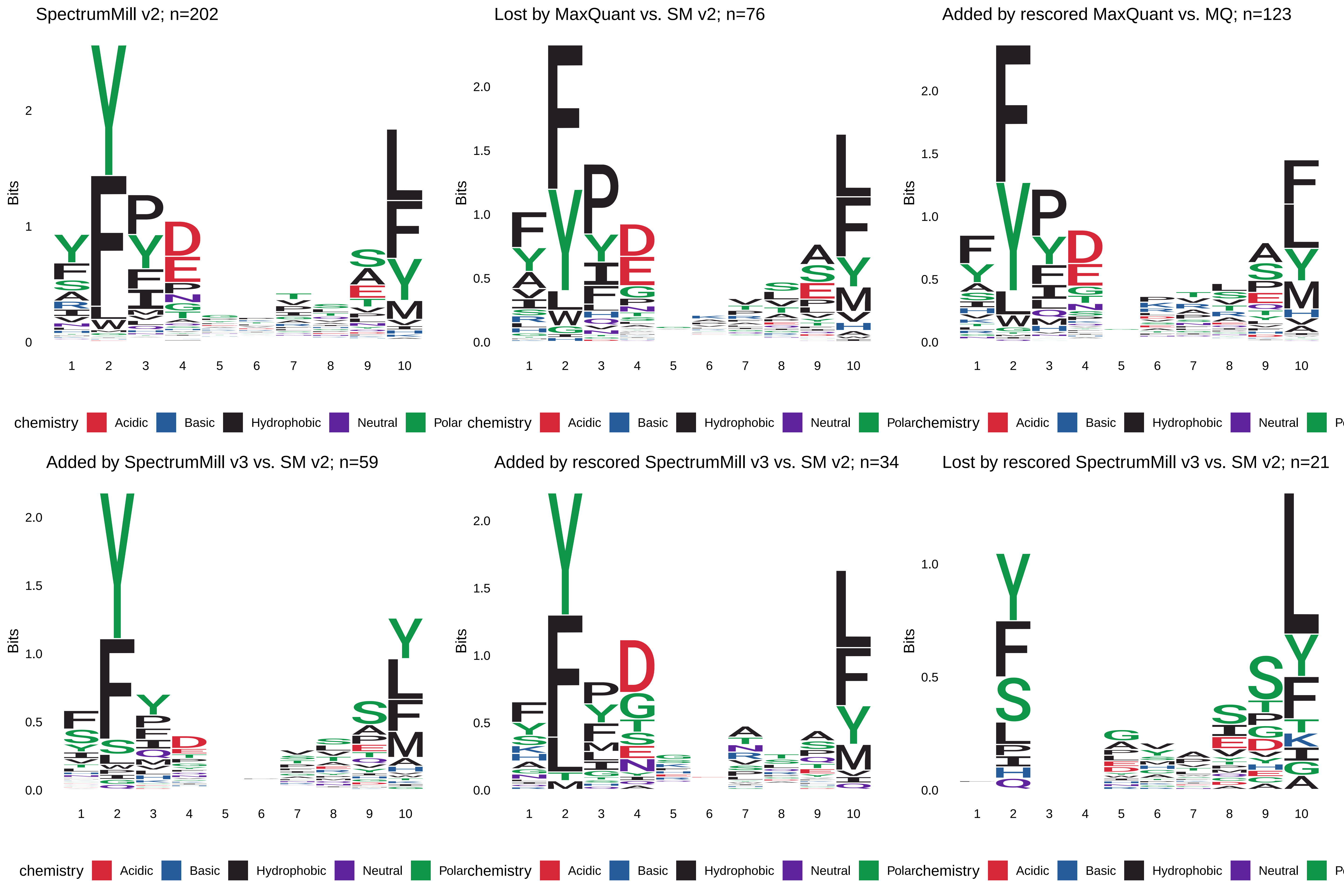
SpectrumMill v2; n=202
Lost by MaxQuant vs. SM v2; n=76
Added by rescored MaxQuant vs. MQ; n=123
Added by SpectrumMill v3 vs. SM v2; n=59
Added by rescored SpectrumMill v3 vs. SM v2; n=34
Lost by rescored SpectrumMill v3 vs. SM v2; n=21
Bits
chemistry
Acidic
Basic
Hydrophobic
Neutral
Polar

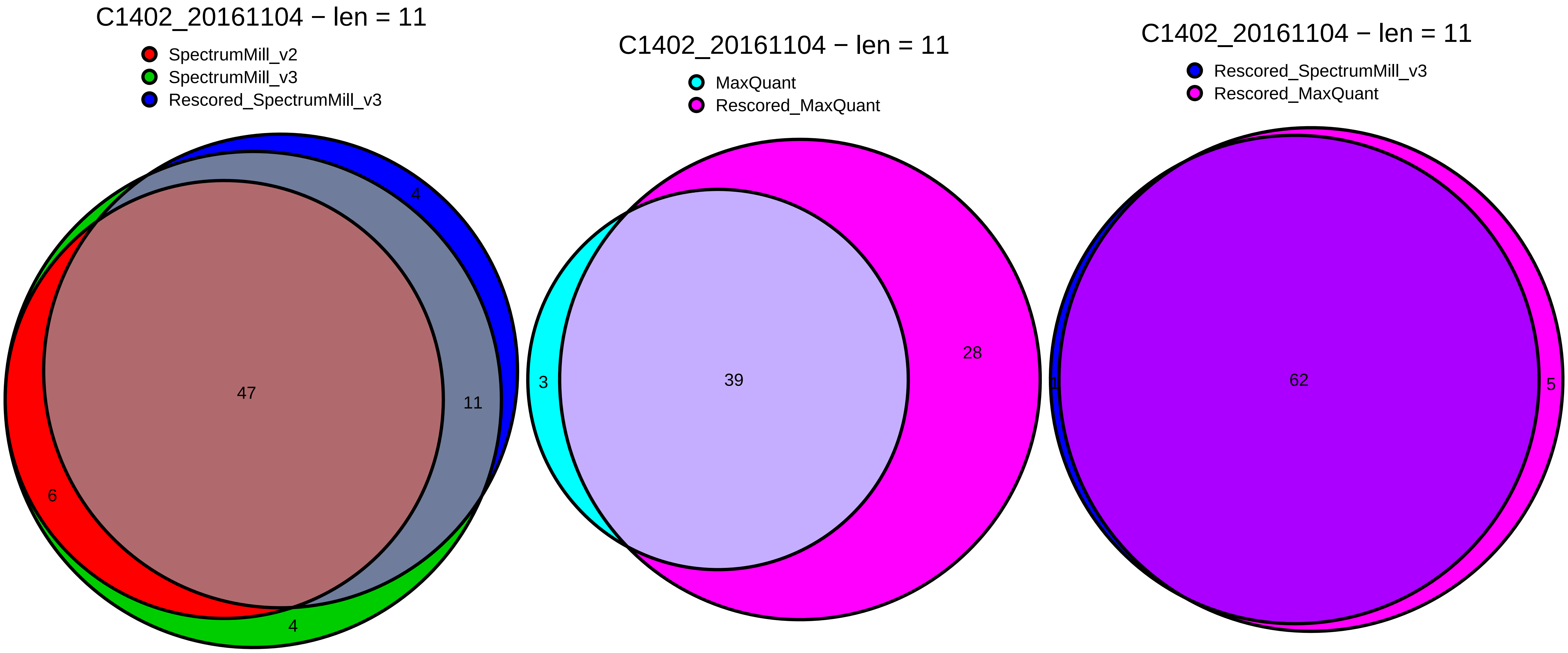
C1402_20161104 − len = 11
SpectrumMill_v2
SpectrumMill_v3
Rescored_SpectrumMill_v3
4
47
11
6
4
C1402_20161104 − len = 11
MaxQuant
Rescored_MaxQuant
3
39
28
C1402_20161104 − len = 11
Rescored_SpectrumMill_v3
Rescored_MaxQuant
1
62
5

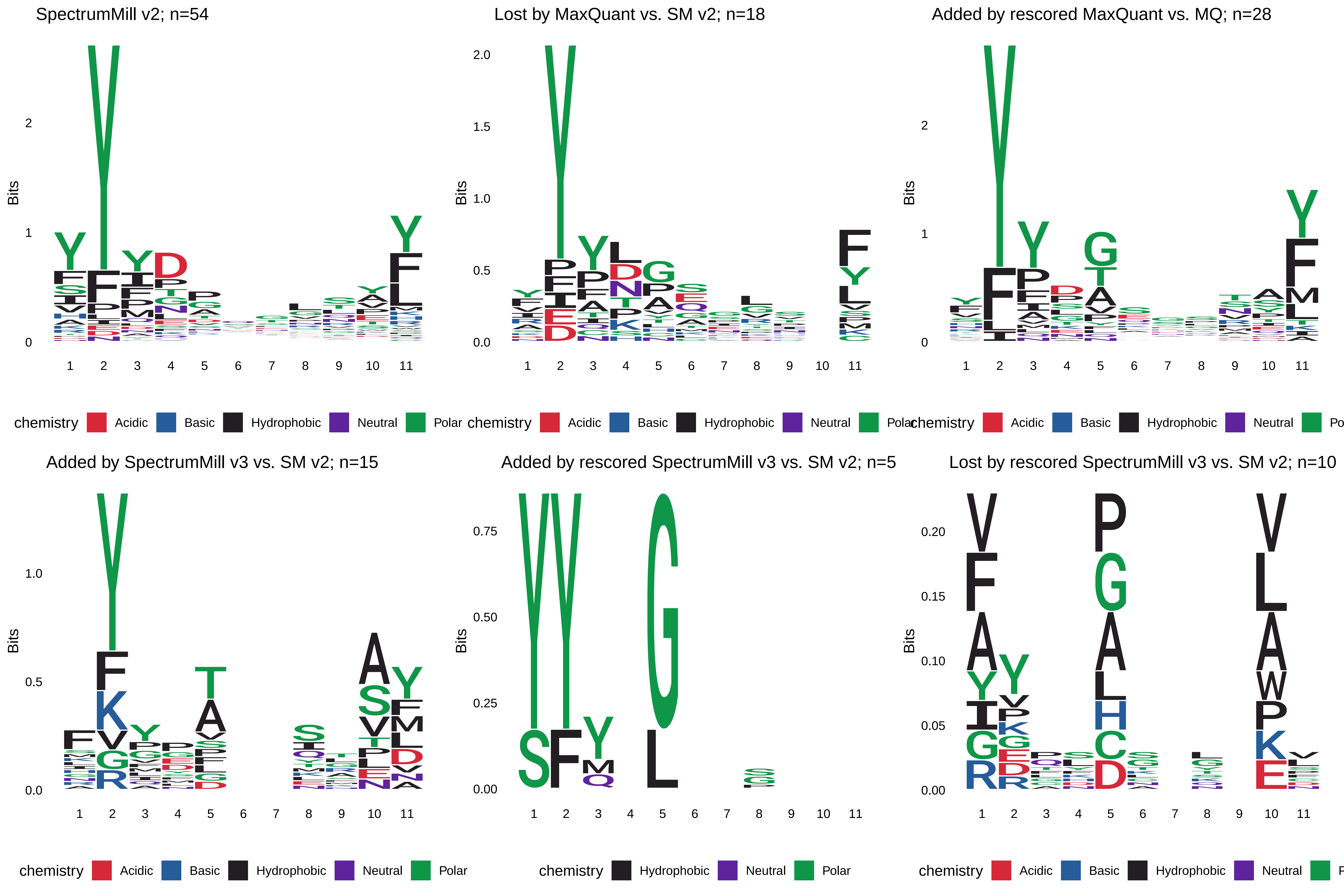

SpectrumMill v2; n=54
Lost by MaxQuant vs. SM v2; n=18
Added by rescored MaxQuant vs. MQ; n=28
Added by SpectrumMill v3 vs. SM v2; n=15
Added by rescored SpectrumMill v3 vs. SM v2; n=5
Lost by rescored SpectrumMill v3 vs. SM v2; n=10
Bits
1 2 3 4 5 6 7 8 9 10 11
chemistry
Acidic
Basic
Hydrophobic
Neutral
Polar

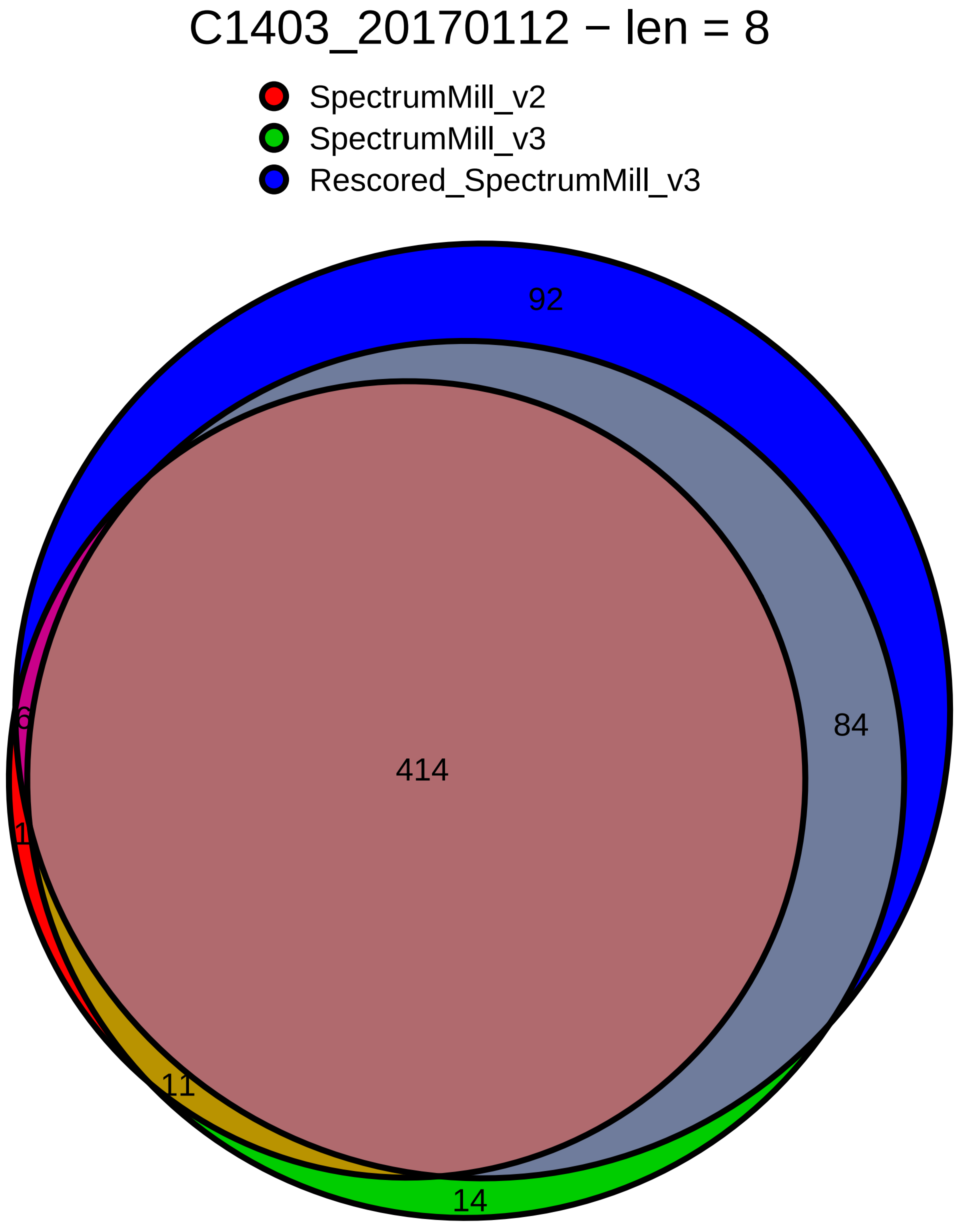
C1403_20170112 − len = 8
SpectrumMill_v2
SpectrumMill_v3
Rescored_SpectrumMill_v3
92
84
414
11
14

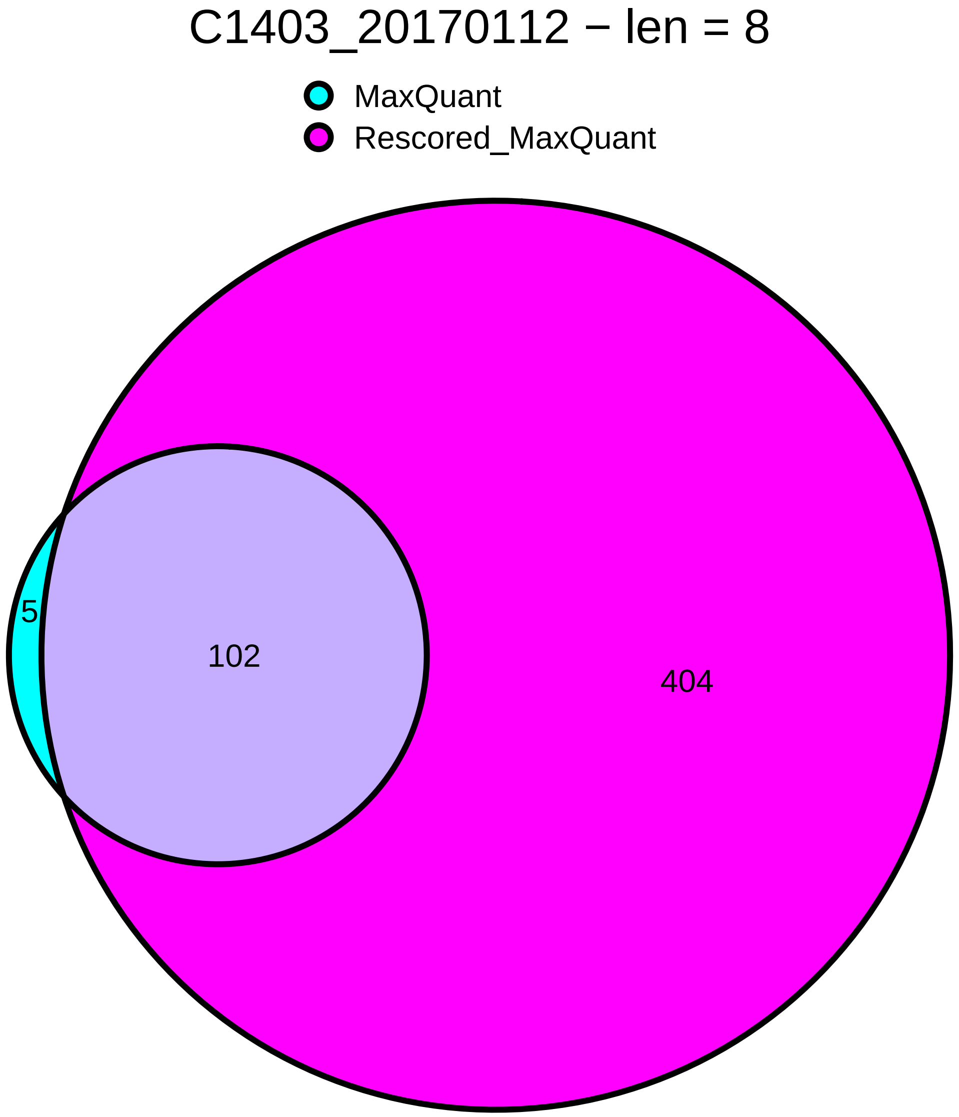
C1403_20170112 − len = 8
MaxQuant
Rescored_MaxQuant
5
102
404

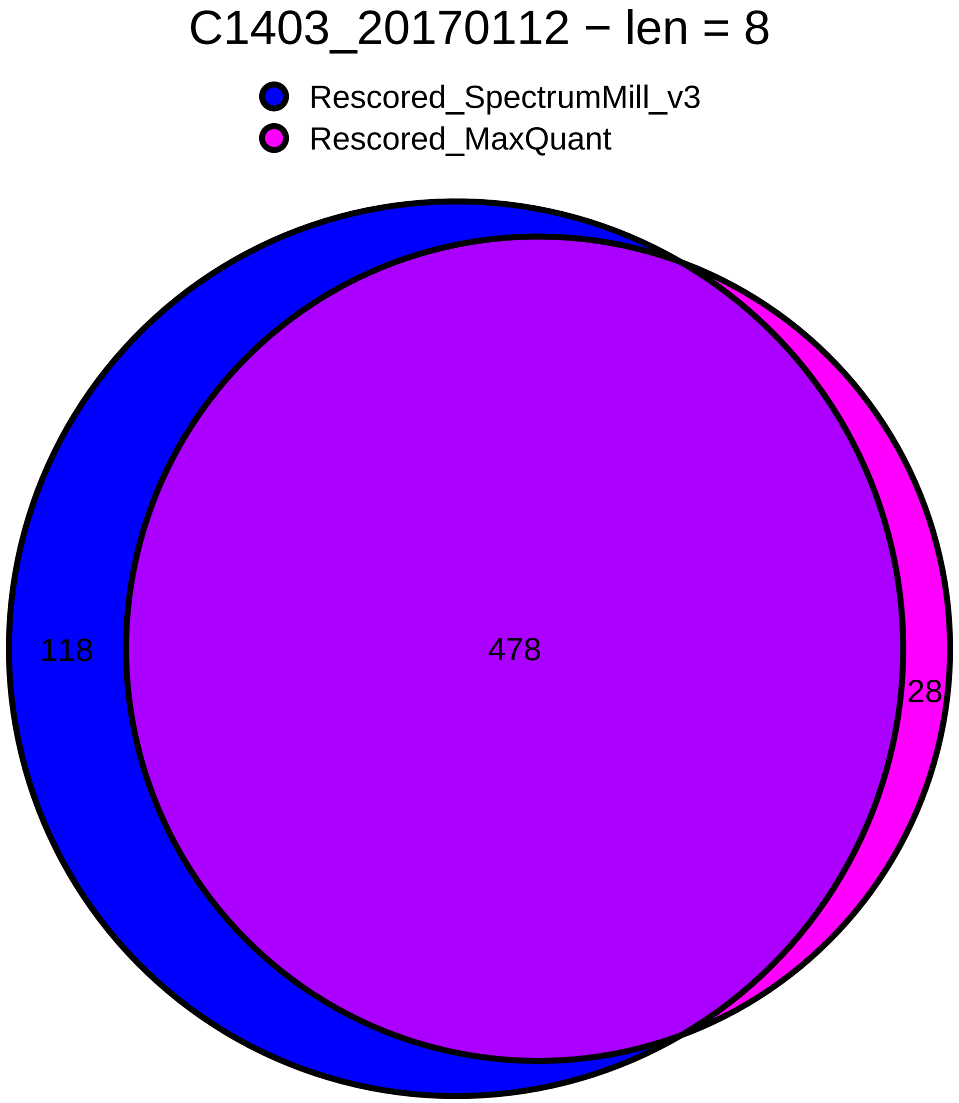
C1403_20170112 − len = 8
Rescored_SpectrumMill_v3
Rescored_MaxQuant
118
478
28

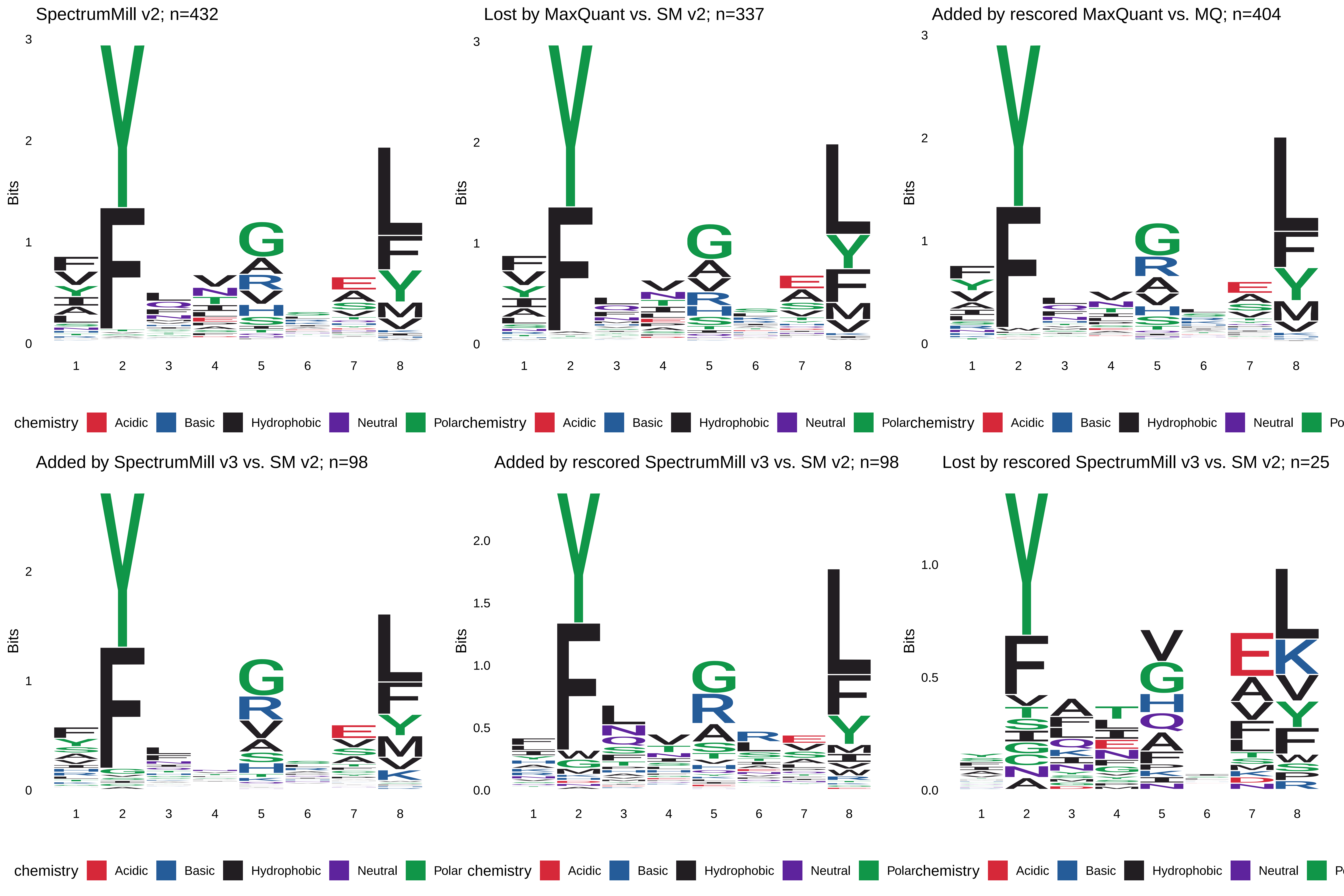
SpectrumMill v2; n=432
Lost by MaxQuant vs. SM v2; n=337
Added by rescored MaxQuant vs. MQ; n=404
Bits
0
1
2
3
1 2 3 4 5 6 7 8
chemistry Acidic Basic Hydrophobic Neutral Polar
Added by SpectrumMill v3 vs. SM v2; n=98
Added by rescored SpectrumMill v3 vs. SM v2; n=98
Lost by rescored SpectrumMill v3 vs. SM v2; n=25
0.0
0.5
1.0
1.5
2.0
chemistry Acidic Basic Hydrophobic Neutral Polar

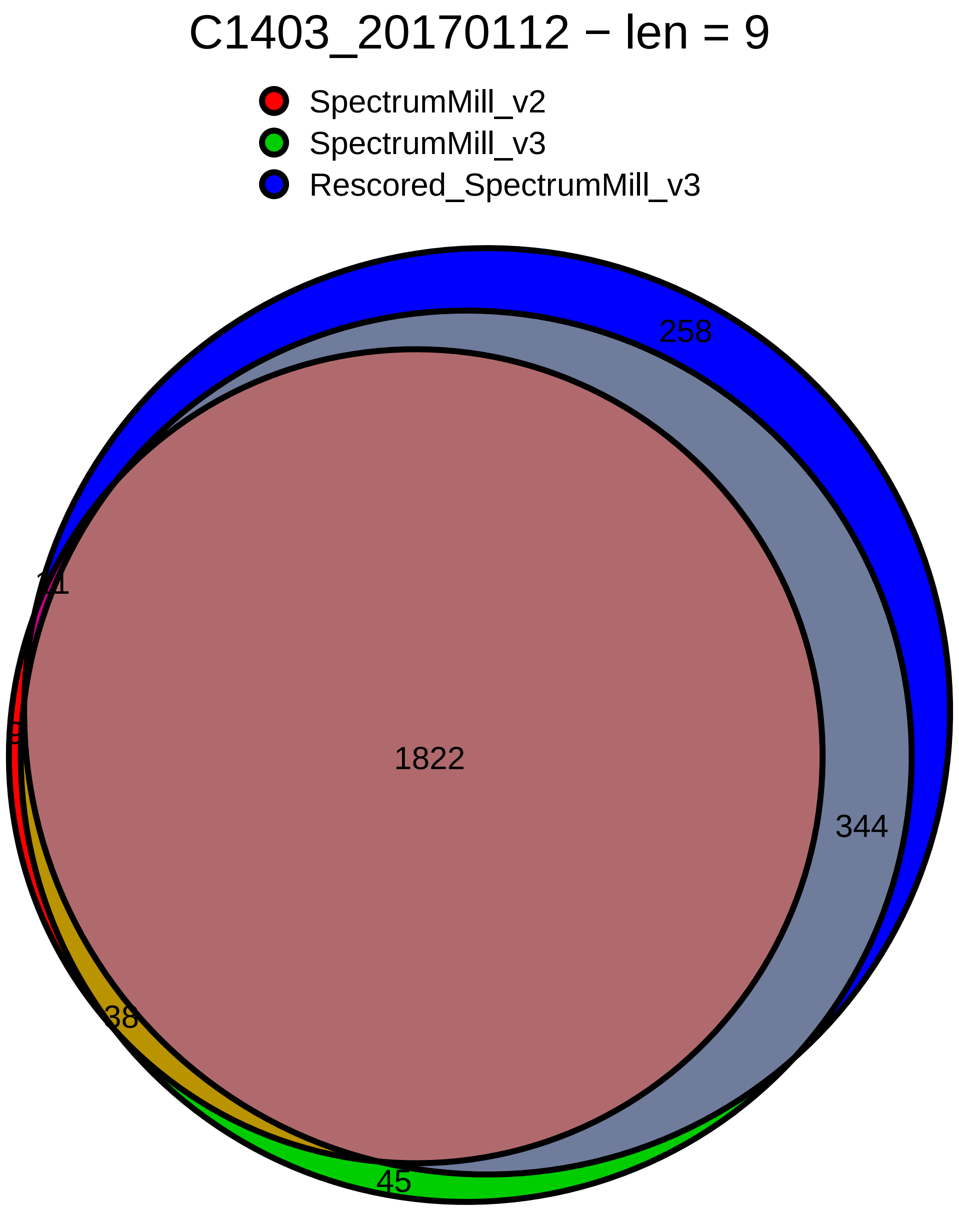
C1403_20170112 − len = 9
SpectrumMill_v2
SpectrumMill_v3
Rescored_SpectrumMill_v3
258
1822
344
38
45

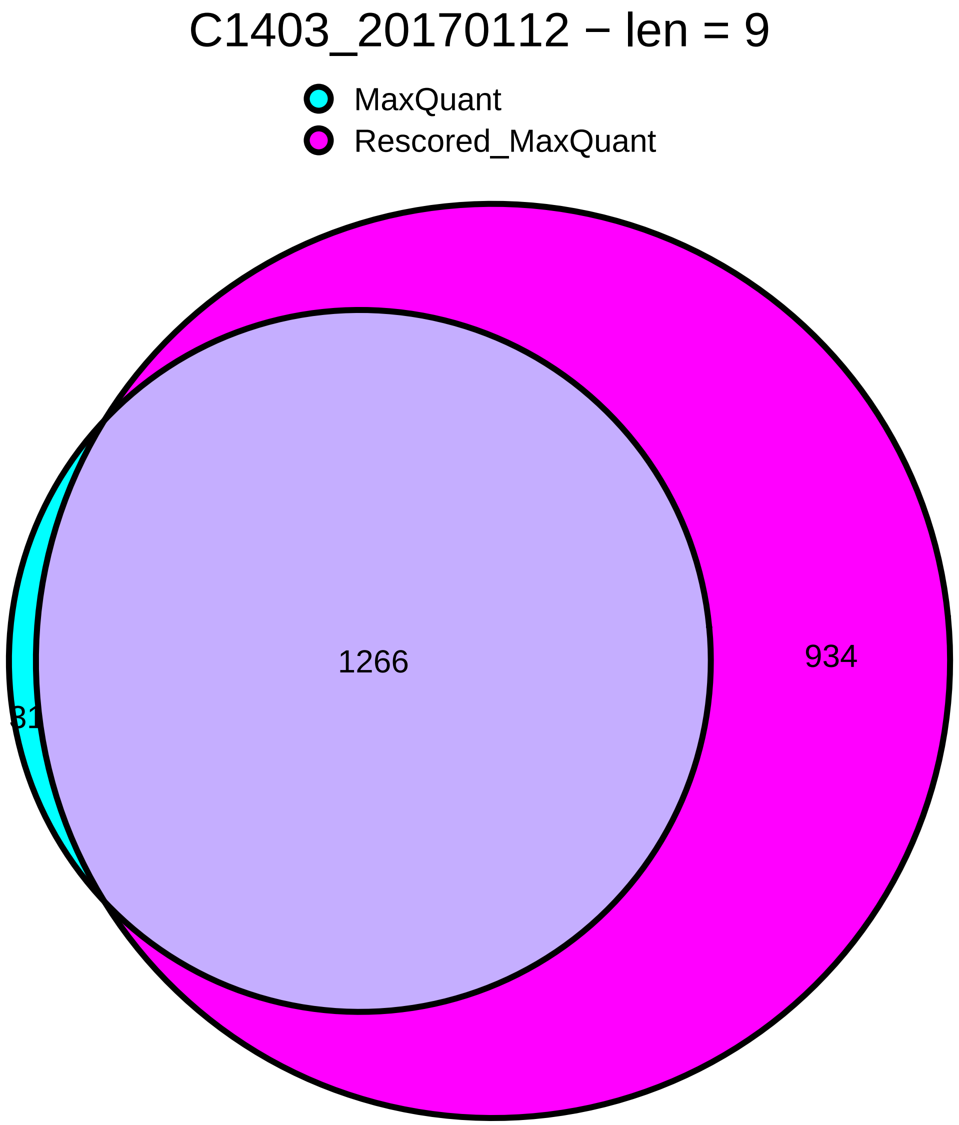
C1403_20170112 − len = 9
MaxQuant
Rescored_MaxQuant
1266
934

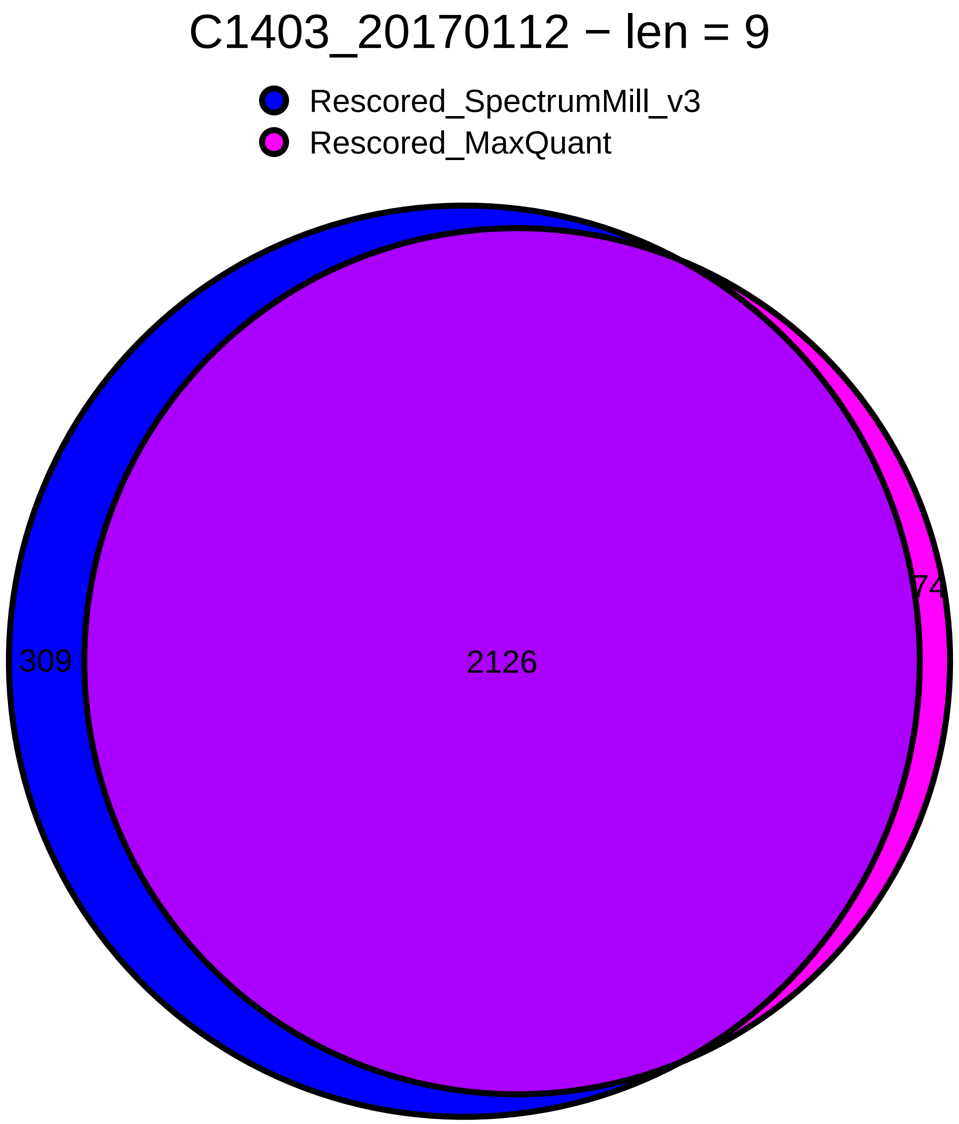
C1403_20170112 − len = 9
Rescored_SpectrumMill_v3
Rescored_MaxQuant
309
2126

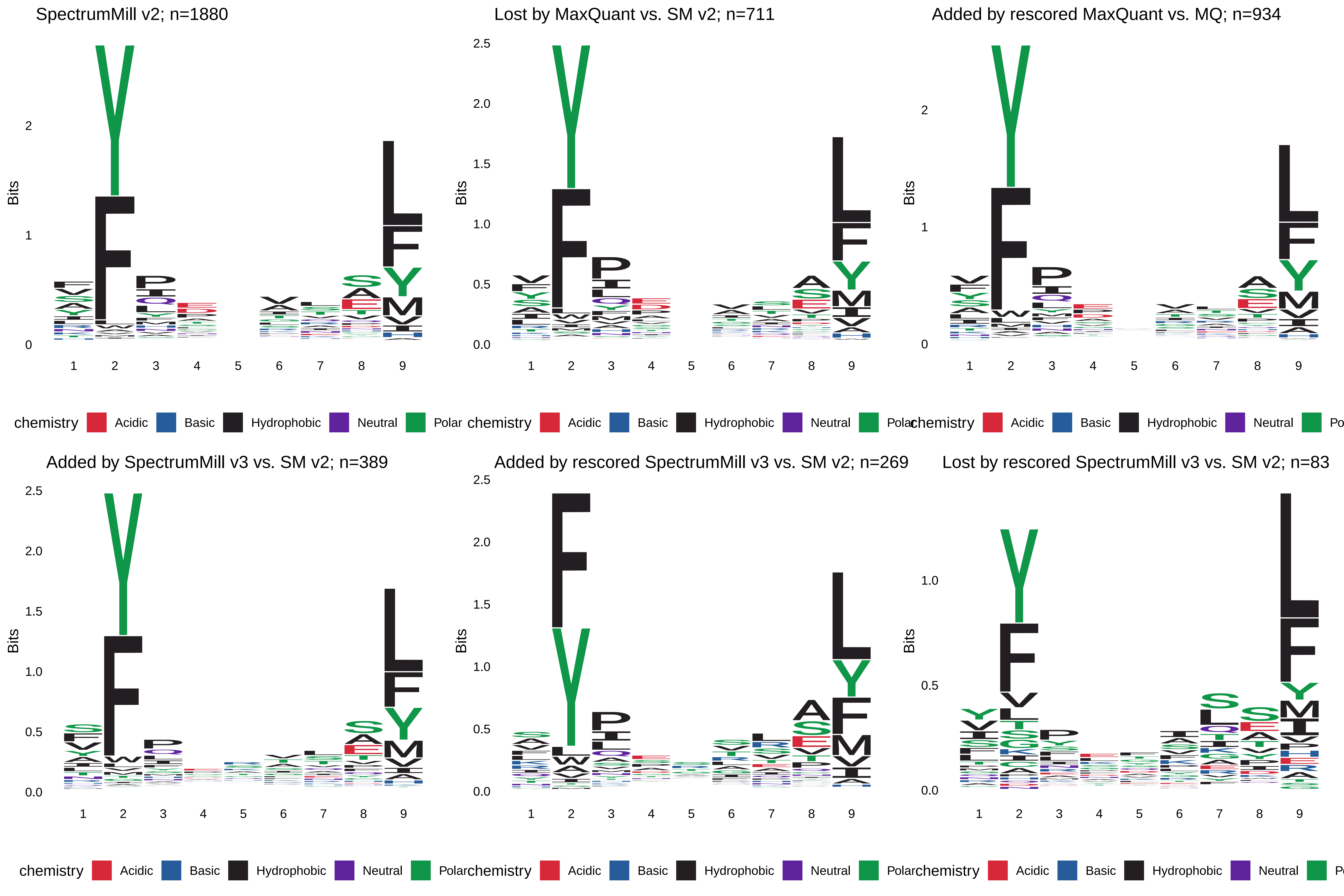
SpectrumMill v2; n=1880
Lost by MaxQuant vs. SM v2; n=711
Added by rescored MaxQuant vs. MQ; n=934
Added by SpectrumMill v3 vs. SM v2; n=389
Added by rescored SpectrumMill v3 vs. SM v2; n=269
Lost by rescored SpectrumMill v3 vs. SM v2; n=83
Bits
1 2 3 4 5 6 7 8 9
chemistry
Acidic
Basic
Hydrophobic
Neutral
Polar

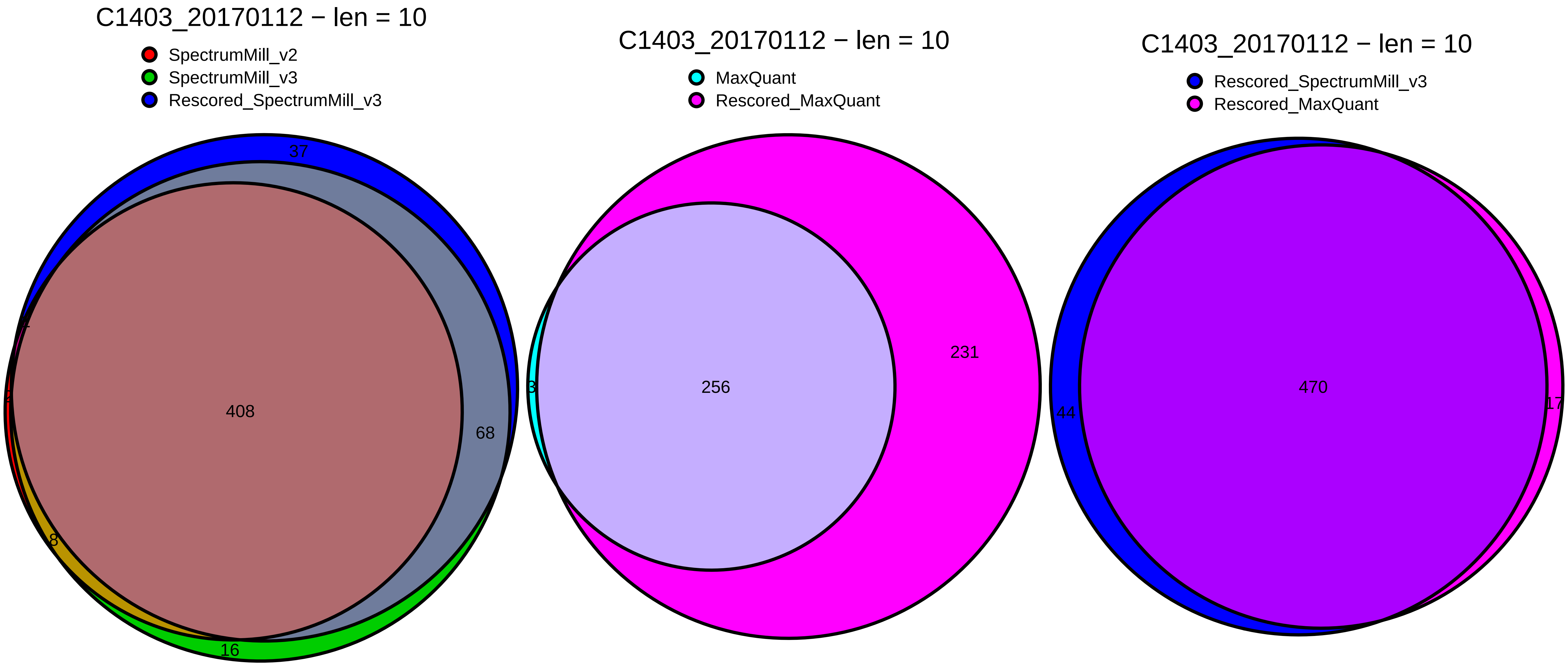
C1403_20170112 − len = 10
SpectrumMill_v2
SpectrumMill_v3
Rescored_SpectrumMill_v3
37
408
68
8
16
C1403_20170112 − len = 10
MaxQuant
Rescored_MaxQuant
3
256
231
C1403_20170112 − len = 10
Rescored_SpectrumMill_v3
Rescored_MaxQuant
470
44

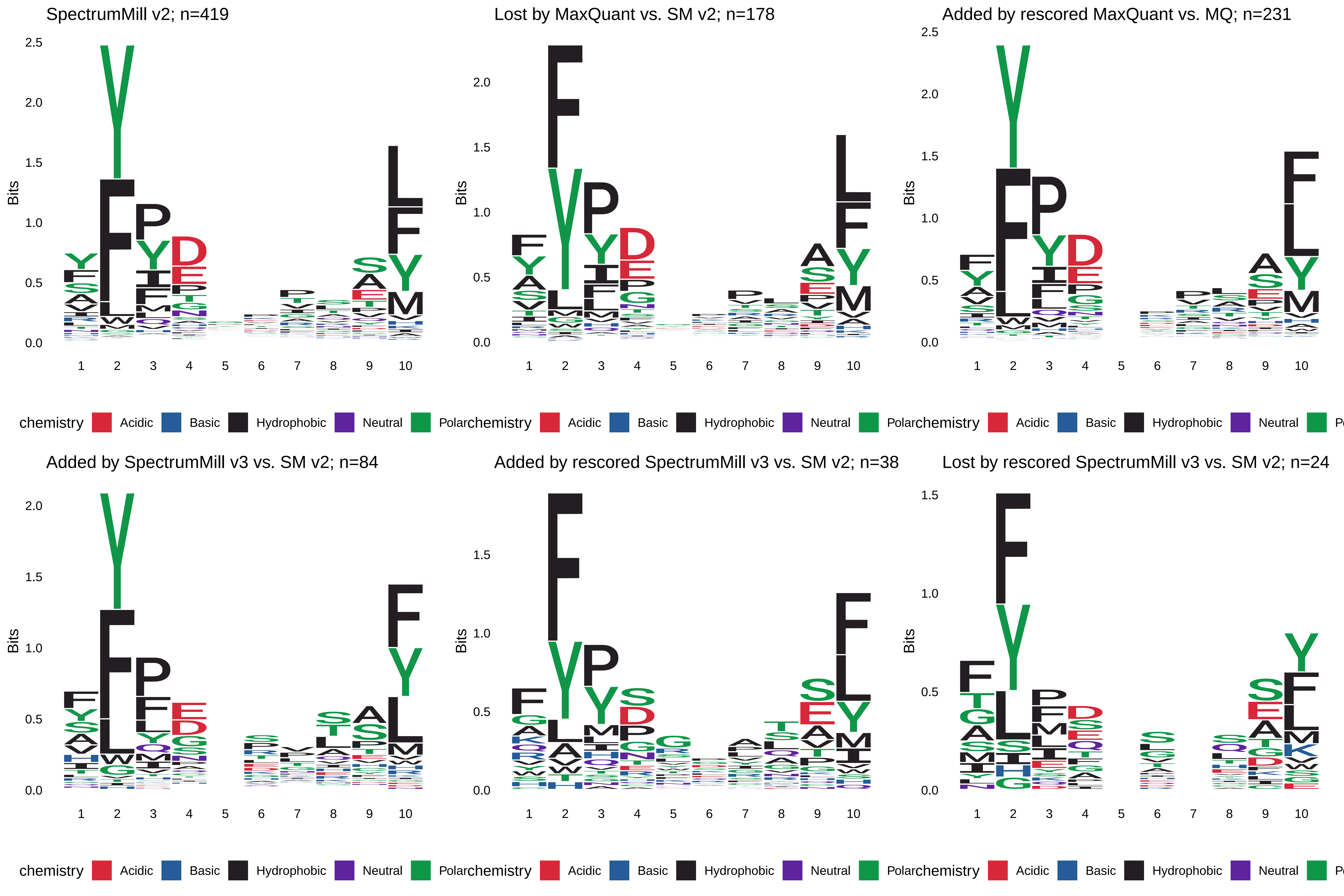
SpectrumMill v2; n=419
Lost by MaxQuant vs. SM v2; n=178
Added by rescored MaxQuant vs. MQ; n=231
Added by SpectrumMill v3 vs. SM v2; n=84
Added by rescored SpectrumMill v3 vs. SM v2; n=38
Lost by rescored SpectrumMill v3 vs. SM v2; n=24
Bits
chemistry
Acidic
Basic
Hydrophobic
Neutral
Polar

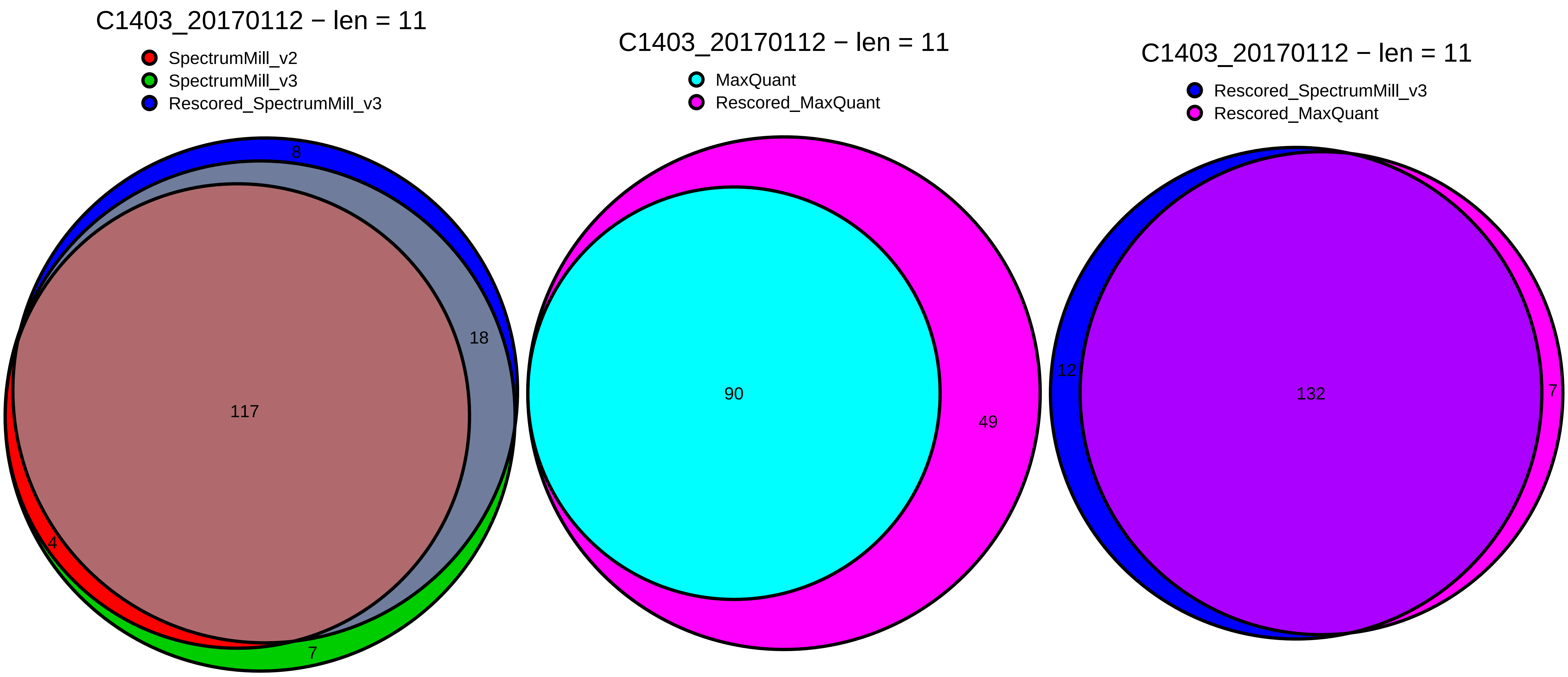
C1403_20170112 − len = 11
SpectrumMill_v2
SpectrumMill_v3
Rescored_SpectrumMill_v3
8
18
117
4
7
C1403_20170112 − len = 11
MaxQuant
Rescored_MaxQuant
90
49
C1403_20170112 − len = 11
Rescored_SpectrumMill_v3
Rescored_MaxQuant
12
132
7

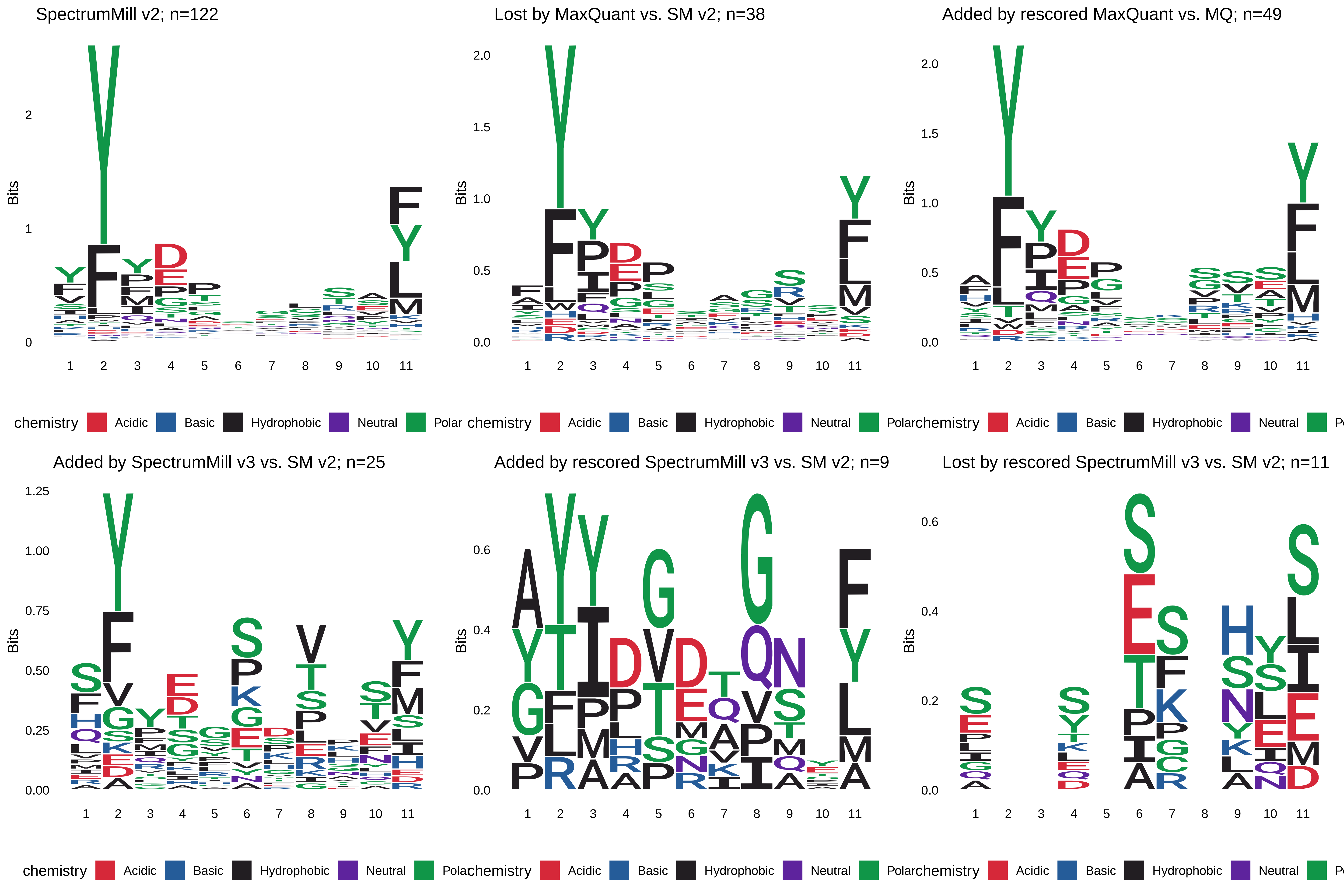
SpectrumMill v2; n=122
Lost by MaxQuant vs. SM v2; n=38
Added by rescored MaxQuant vs. MQ; n=49
Added by SpectrumMill v3 vs. SM v2; n=25
Added by rescored SpectrumMill v3 vs. SM v2; n=9
Lost by rescored SpectrumMill v3 vs. SM v2; n=11
Bits
1 2 3 4 5 6 7 8 9 10 11
chemistry
Acidic
Basic
Hydrophobic
Neutral
Polar

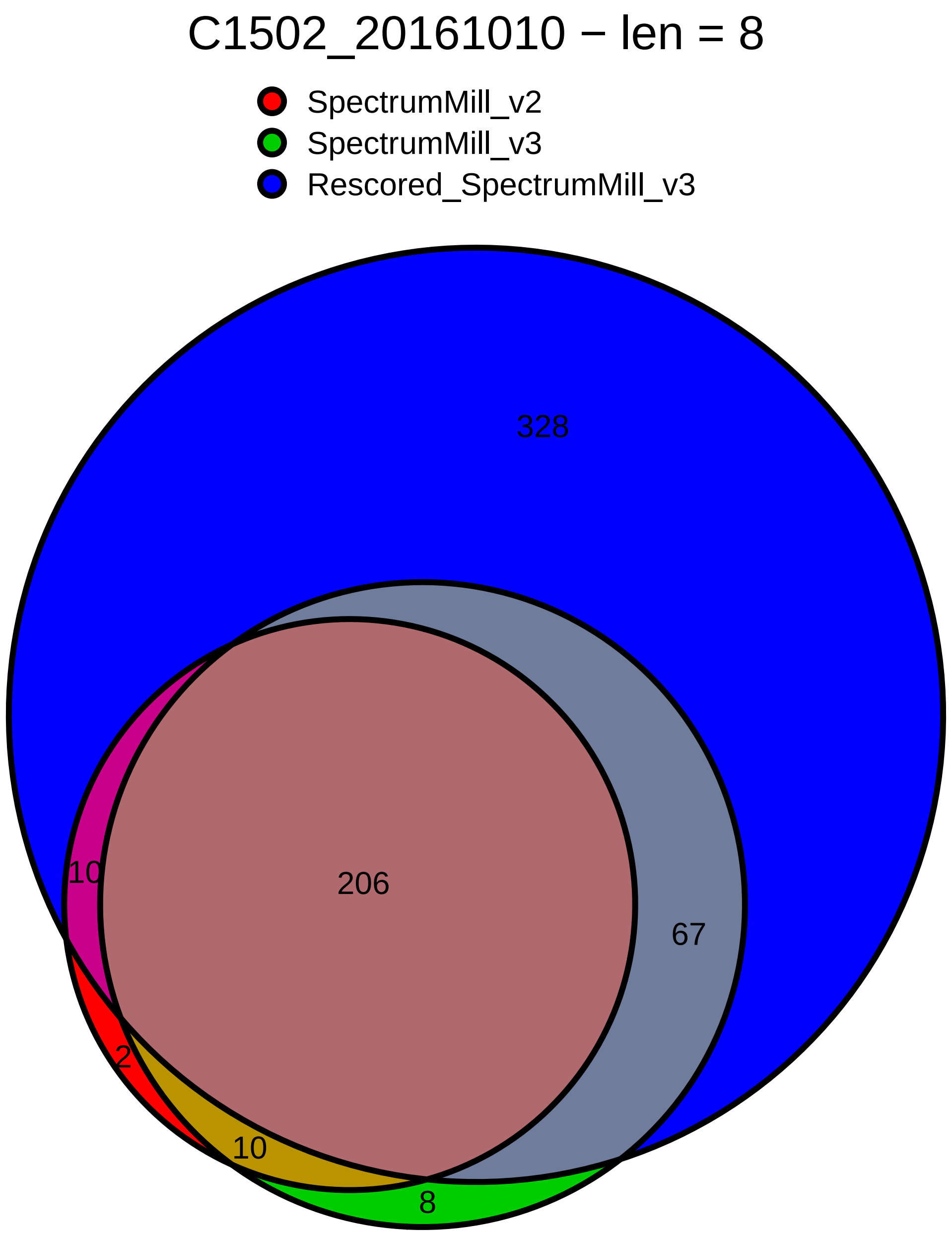
C1502_20161010 − len = 8
SpectrumMill_v2
SpectrumMill_v3
Rescored_SpectrumMill_v3
328
10
206
67
2
10
8

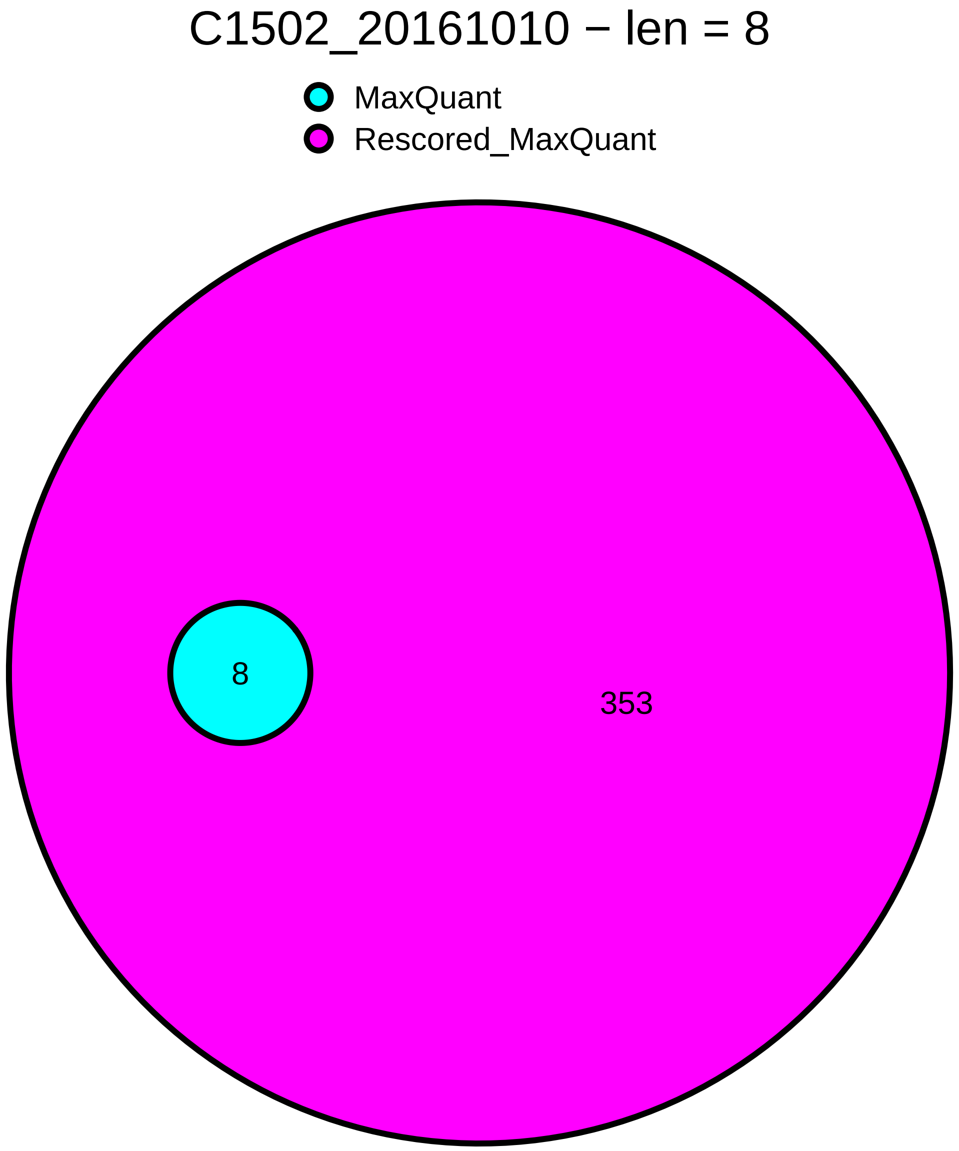
C1502_20161010 − len = 8
MaxQuant
Rescored_MaxQuant
8
353

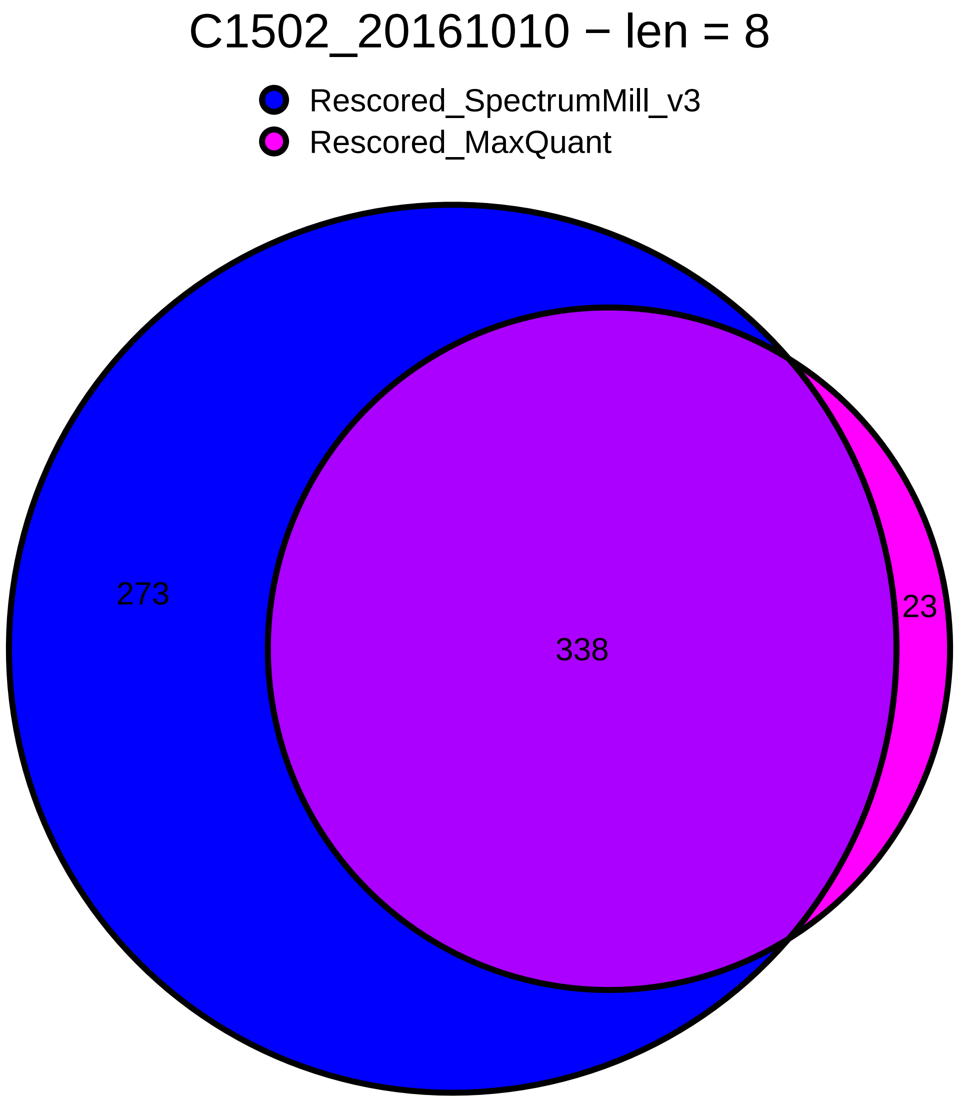
C1502_20161010 − len = 8
Rescored_SpectrumMill_v3
Rescored_MaxQuant
273
338
23

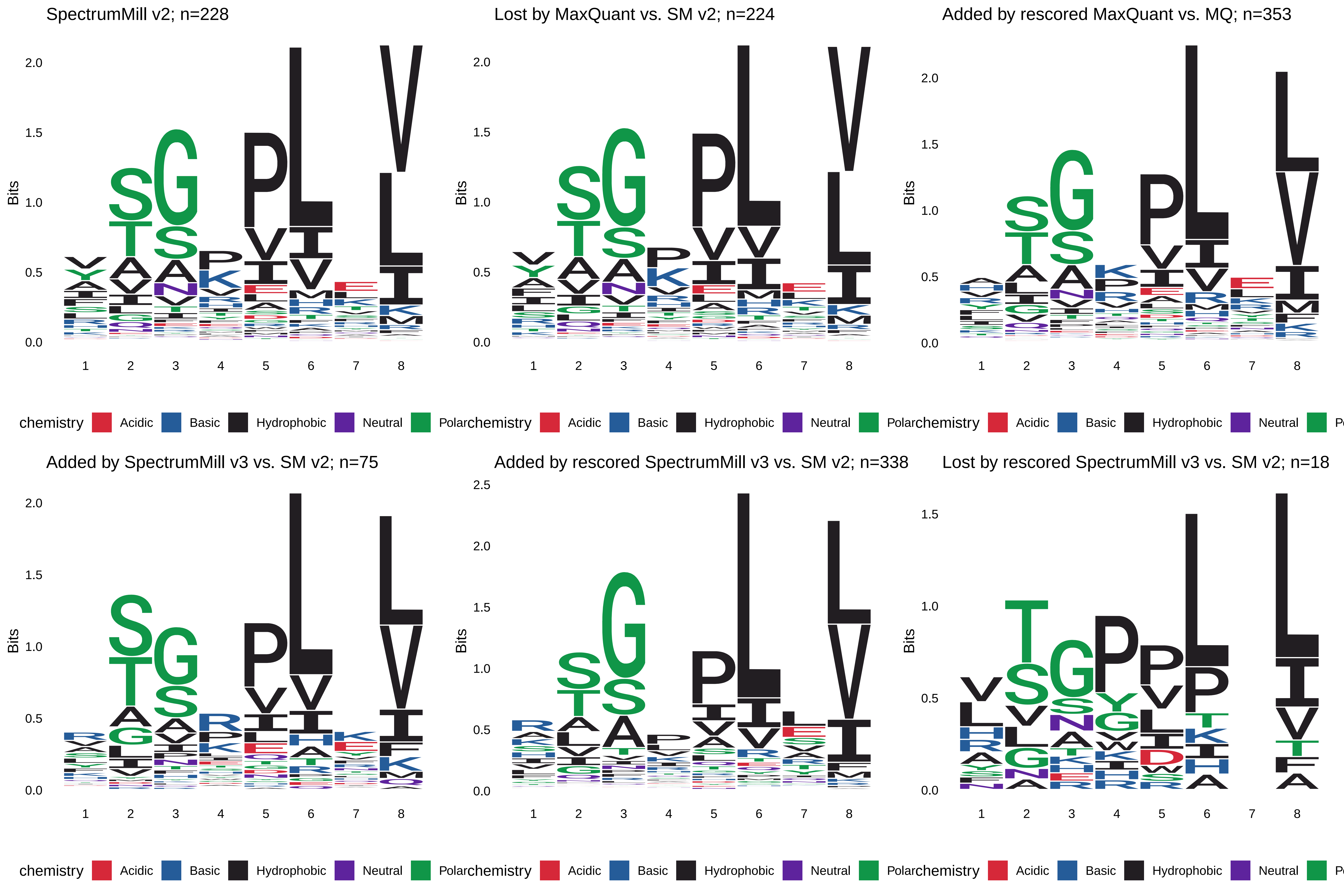
SpectrumMill v2; n=228
Lost by MaxQuant vs. SM v2; n=224
Added by rescored MaxQuant vs. MQ; n=353
Added by SpectrumMill v3 vs. SM v2; n=75
Added by rescored SpectrumMill v3 vs. SM v2; n=338
Lost by rescored SpectrumMill v3 vs. SM v2; n=18
Bits
chemistry
Acidic
Basic
Hydrophobic
Neutral
Polar

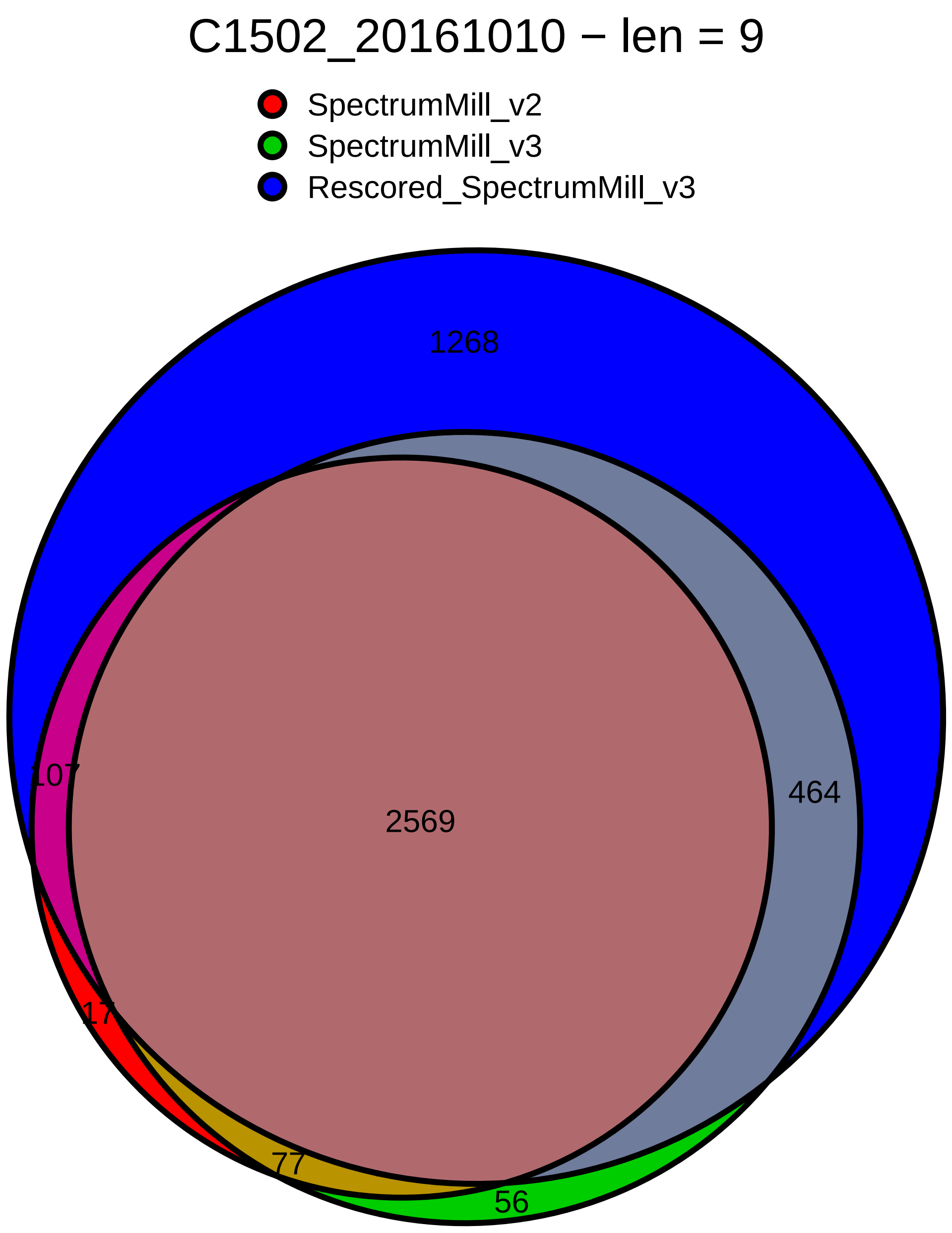
C1502_20161010 − len = 9
SpectrumMill_v2
SpectrumMill_v3
Rescored_SpectrumMill_v3
1268
107
2569
464
17
77
56

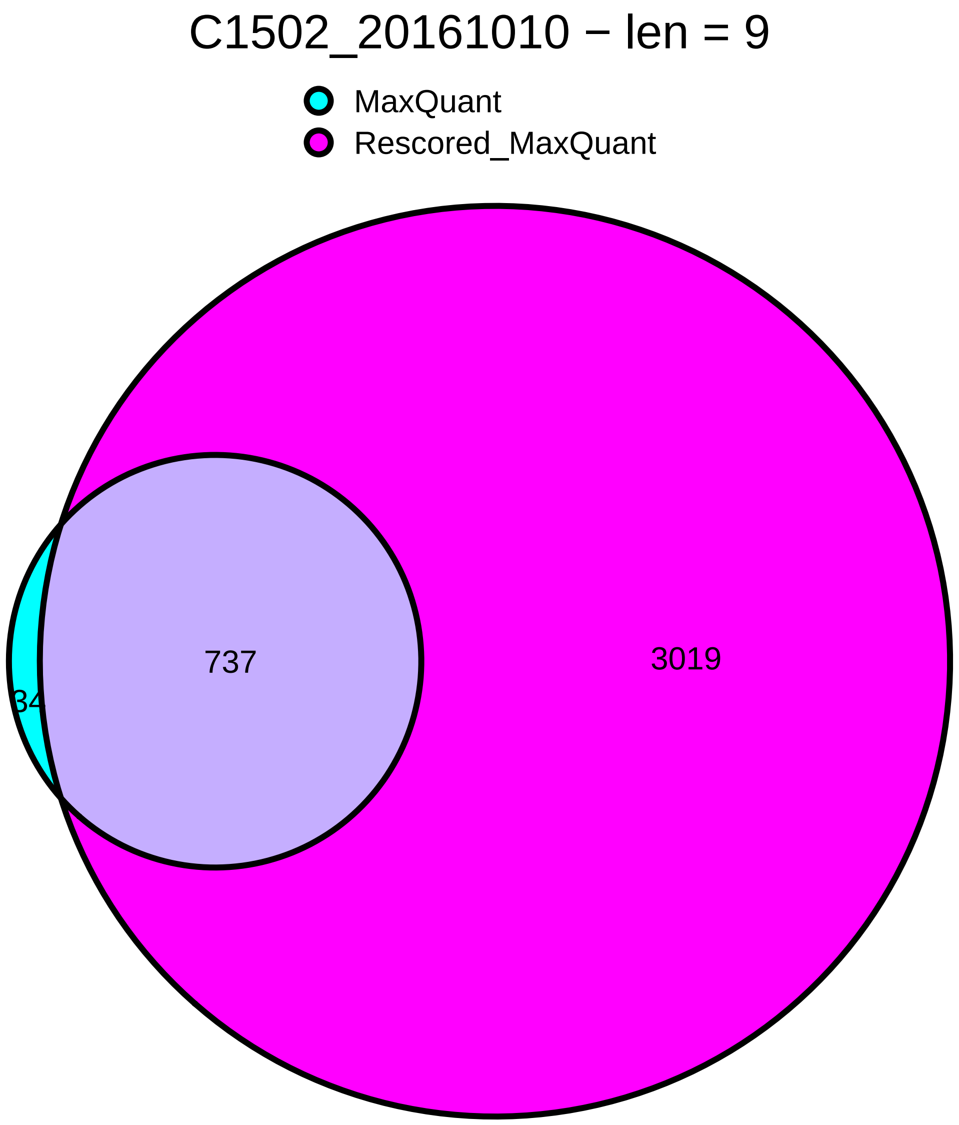
C1502_20161010 − len = 9
MaxQuant
Rescored_MaxQuant
34
737
3019

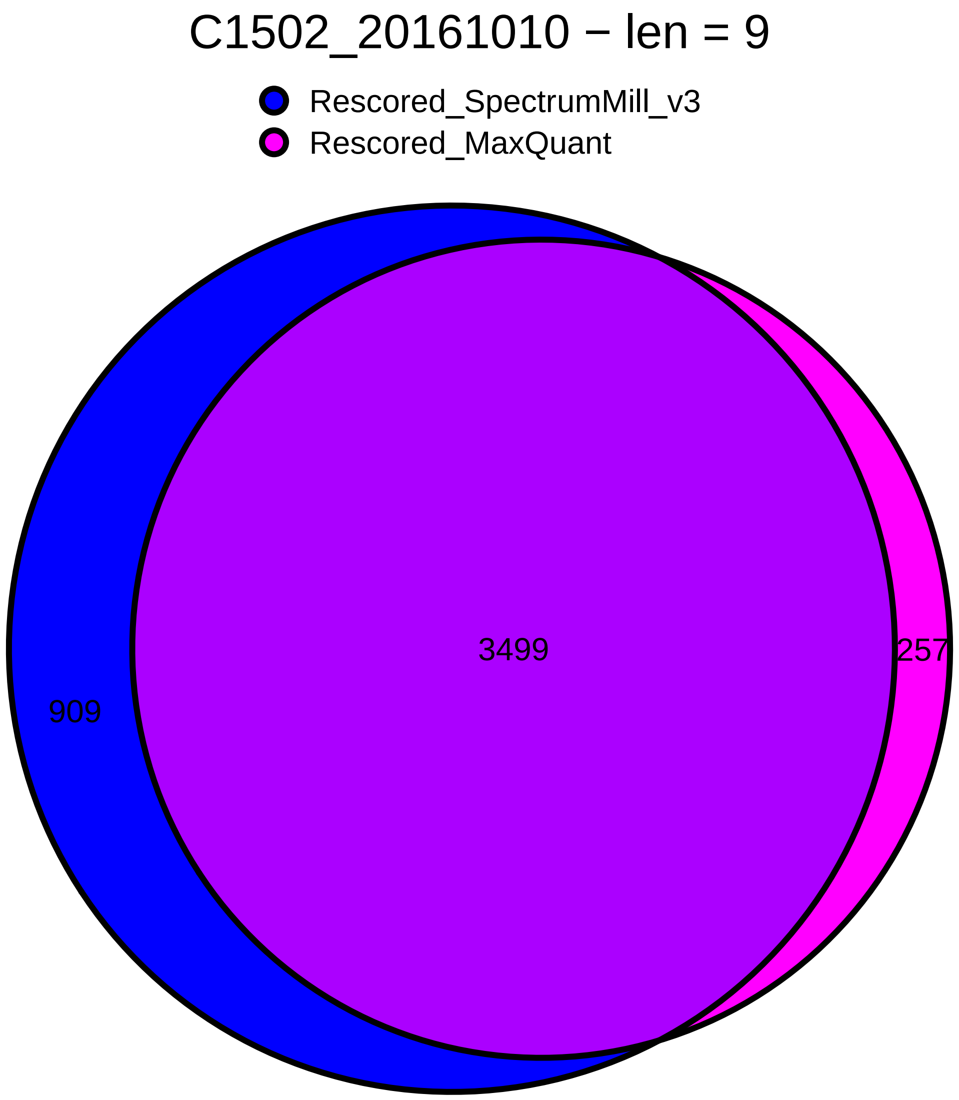
C1502_20161010 − len = 9
Rescored_SpectrumMill_v3
Rescored_MaxQuant
3499
909
257

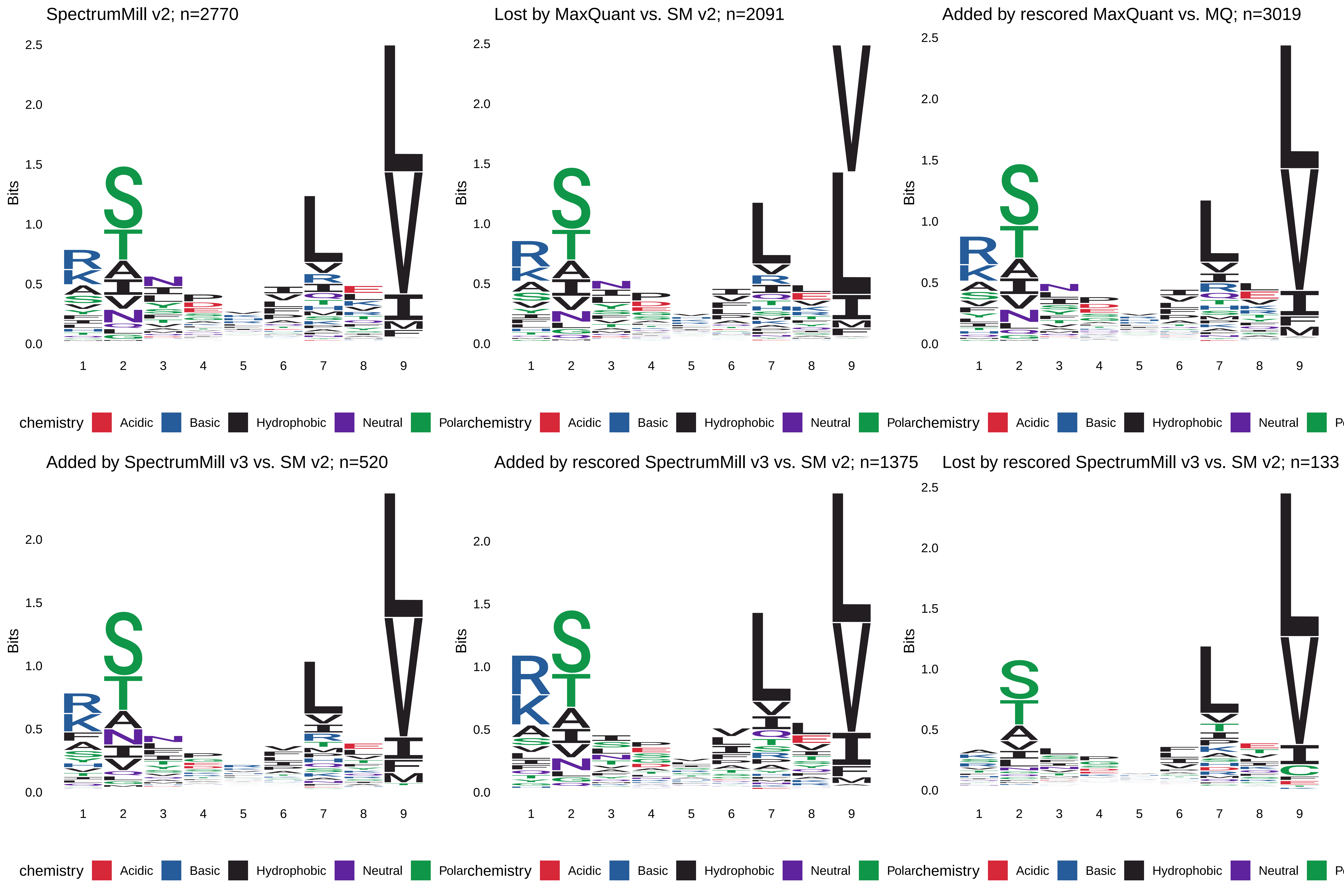
SpectrumMill v2; n=2770
Lost by MaxQuant vs. SM v2; n=2091
Added by rescored MaxQuant vs. MQ; n=3019
Added by SpectrumMill v3 vs. SM v2; n=520
Added by rescored SpectrumMill v3 vs. SM v2; n=1375
Lost by rescored SpectrumMill v3 vs. SM v2; n=133
Bits
0.0
0.5
1.0
1.5
2.0
2.5
1 2 3 4 5 6 7 8 9
chemistry
Acidic
Basic
Hydrophobic
Neutral
Polar

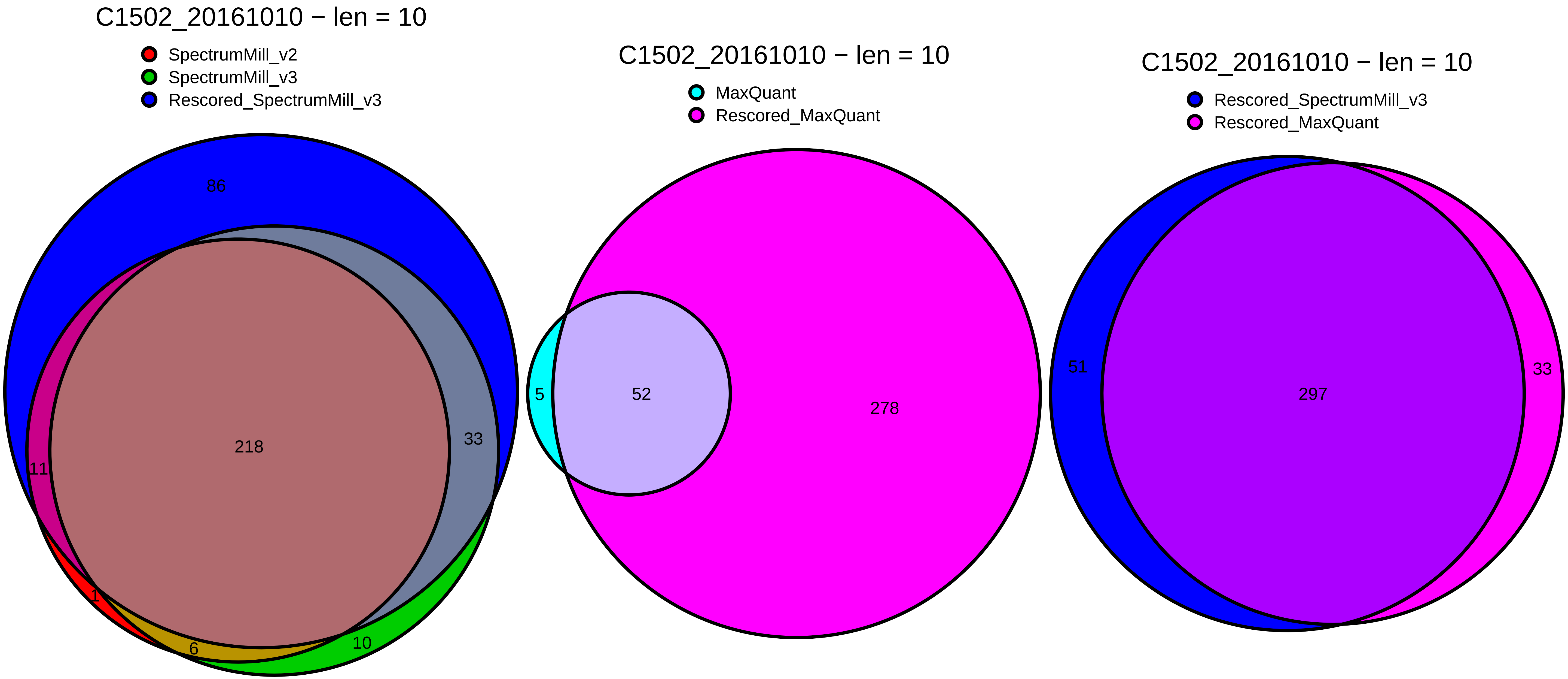

C1502_20161010 − len = 10
SpectrumMill_v2
SpectrumMill_v3
Rescored_SpectrumMill_v3
86
218
33
11
1
6
10
C1502_20161010 − len = 10
MaxQuant
Rescored_MaxQuant
5
52
278
C1502_20161010 − len = 10
Rescored_SpectrumMill_v3
Rescored_MaxQuant
51
297
33

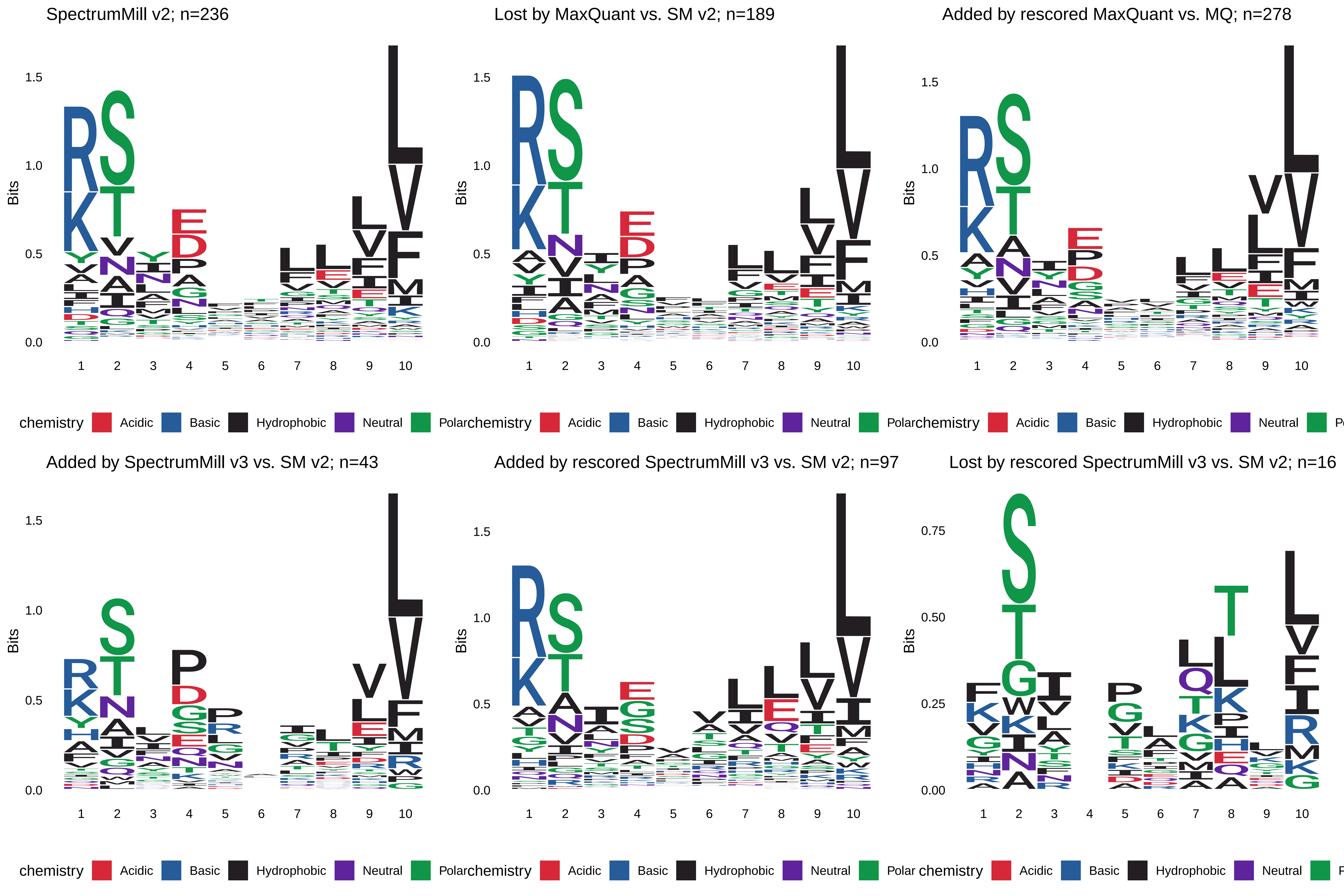
SpectrumMill v2; n=236
Lost by MaxQuant vs. SM v2; n=189
Added by rescored MaxQuant vs. MQ; n=278
Added by SpectrumMill v3 vs. SM v2; n=43
Added by rescored SpectrumMill v3 vs. SM v2; n=97
Lost by rescored SpectrumMill v3 vs. SM v2; n=16
Bits
0.0
0.5
1.0
1.5
0.00
0.25
0.50
0.75
1
2
3
4
5
6
7
8
9
10
chemistry
Acidic
Basic
Hydrophobic
Neutral
Polar

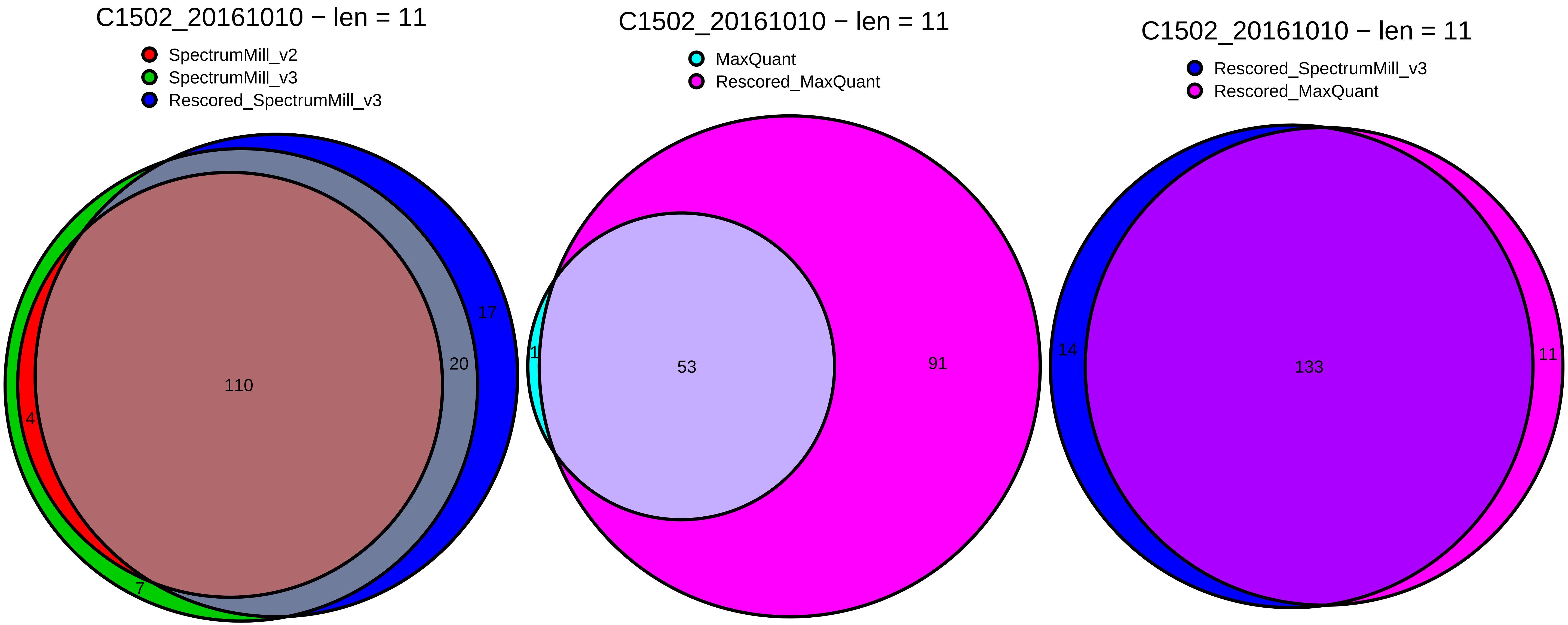

C1502_20161010 − len = 11
SpectrumMill_v2
SpectrumMill_v3
Rescored_SpectrumMill_v3
17
20
110
4
7
C1502_20161010 − len = 11
MaxQuant
Rescored_MaxQuant
1
53
91
C1502_20161010 − len = 11
Rescored_SpectrumMill_v3
Rescored_MaxQuant
14
133
11

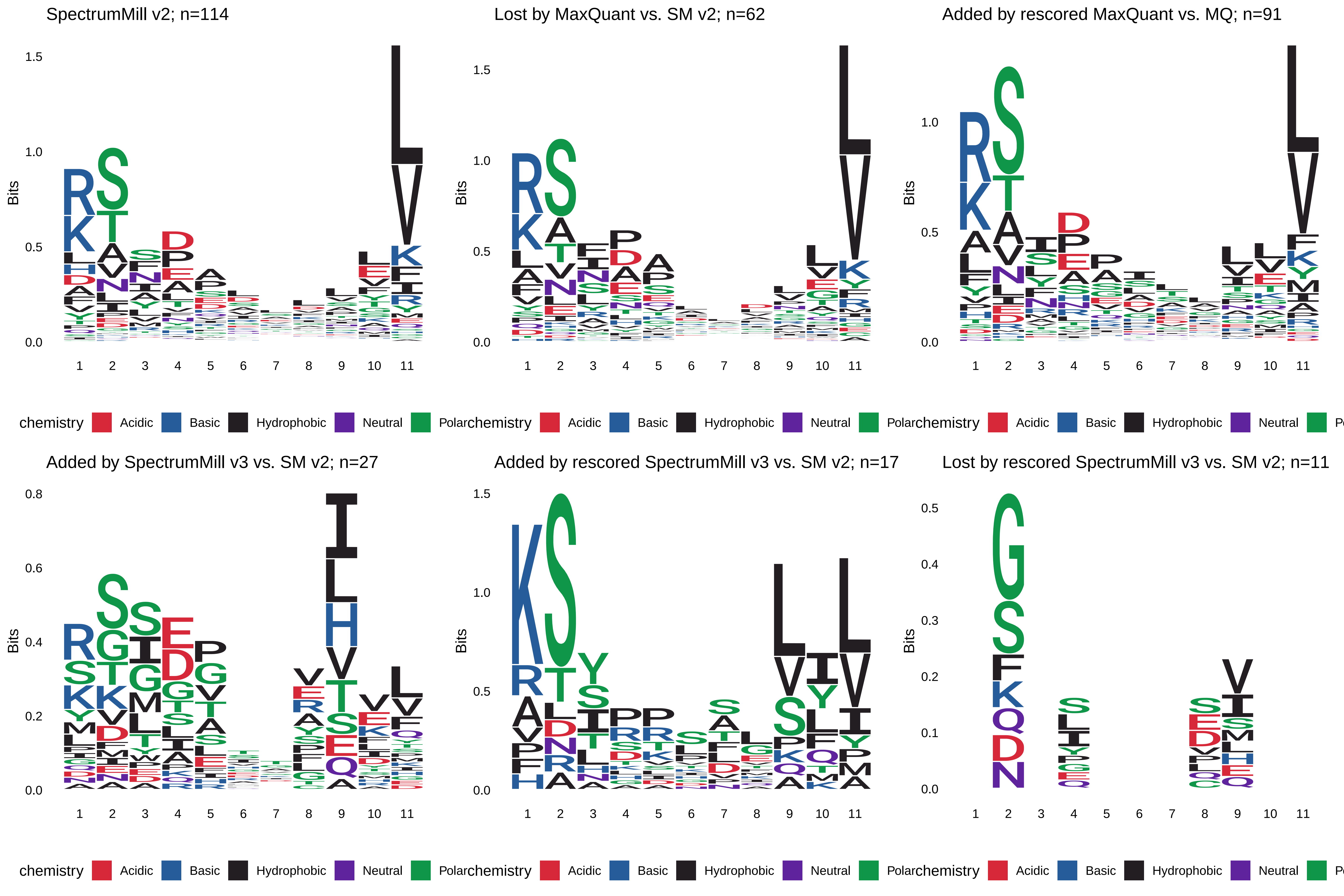
SpectrumMill v2; n=114
Lost by MaxQuant vs. SM v2; n=62
Added by rescored MaxQuant vs. MQ; n=91
Added by SpectrumMill v3 vs. SM v2; n=27
Added by rescored SpectrumMill v3 vs. SM v2; n=17
Lost by rescored SpectrumMill v3 vs. SM v2; n=11
Bits
1 2 3 4 5 6 7 8 9 10 11
chemistry
Acidic
Basic
Hydrophobic
Neutral
Polar

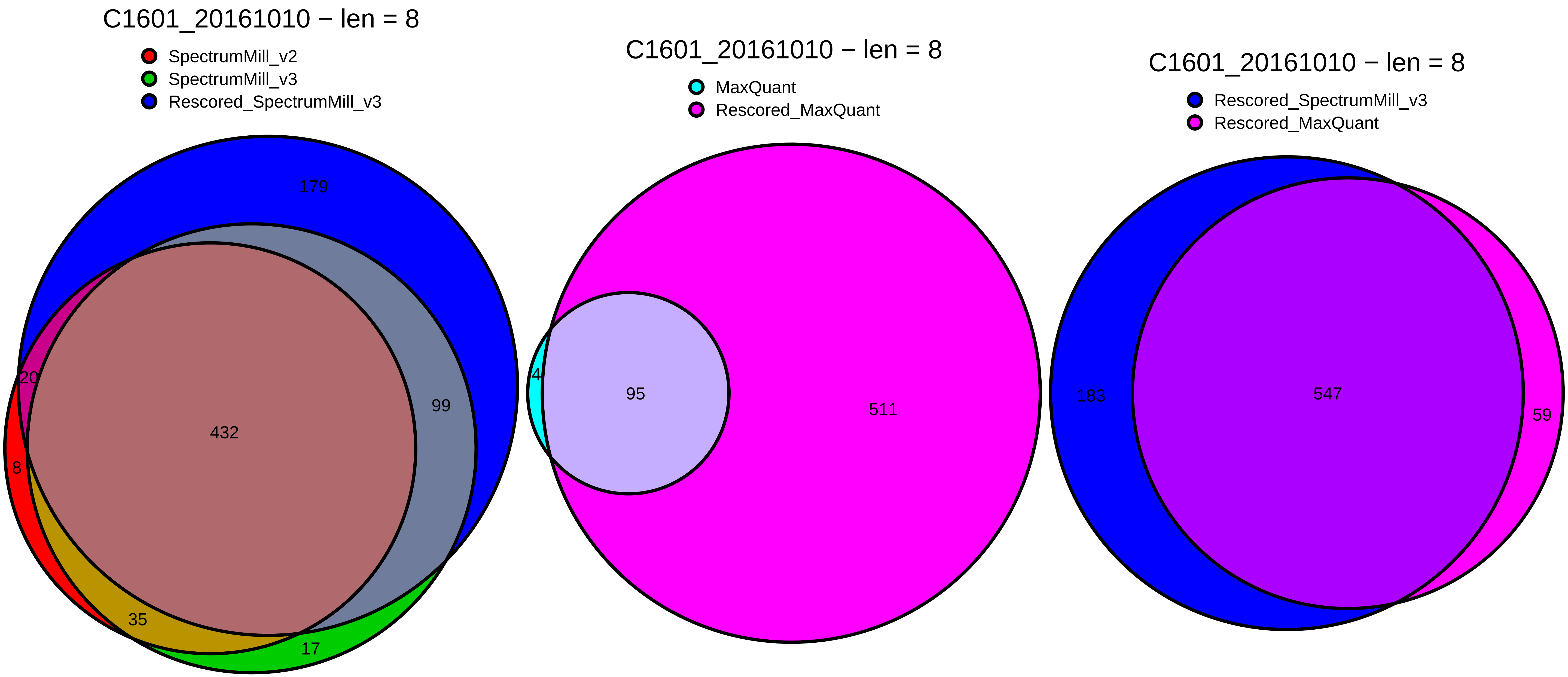
C1601_20161010 − len = 8
SpectrumMill_v2
SpectrumMill_v3
Rescored_SpectrumMill_v3
179
20
99
432
8
35
17
C1601_20161010 − len = 8
MaxQuant
Rescored_MaxQuant
4
95
511
C1601_20161010 − len = 8
Rescored_SpectrumMill_v3
Rescored_MaxQuant
183
547
59

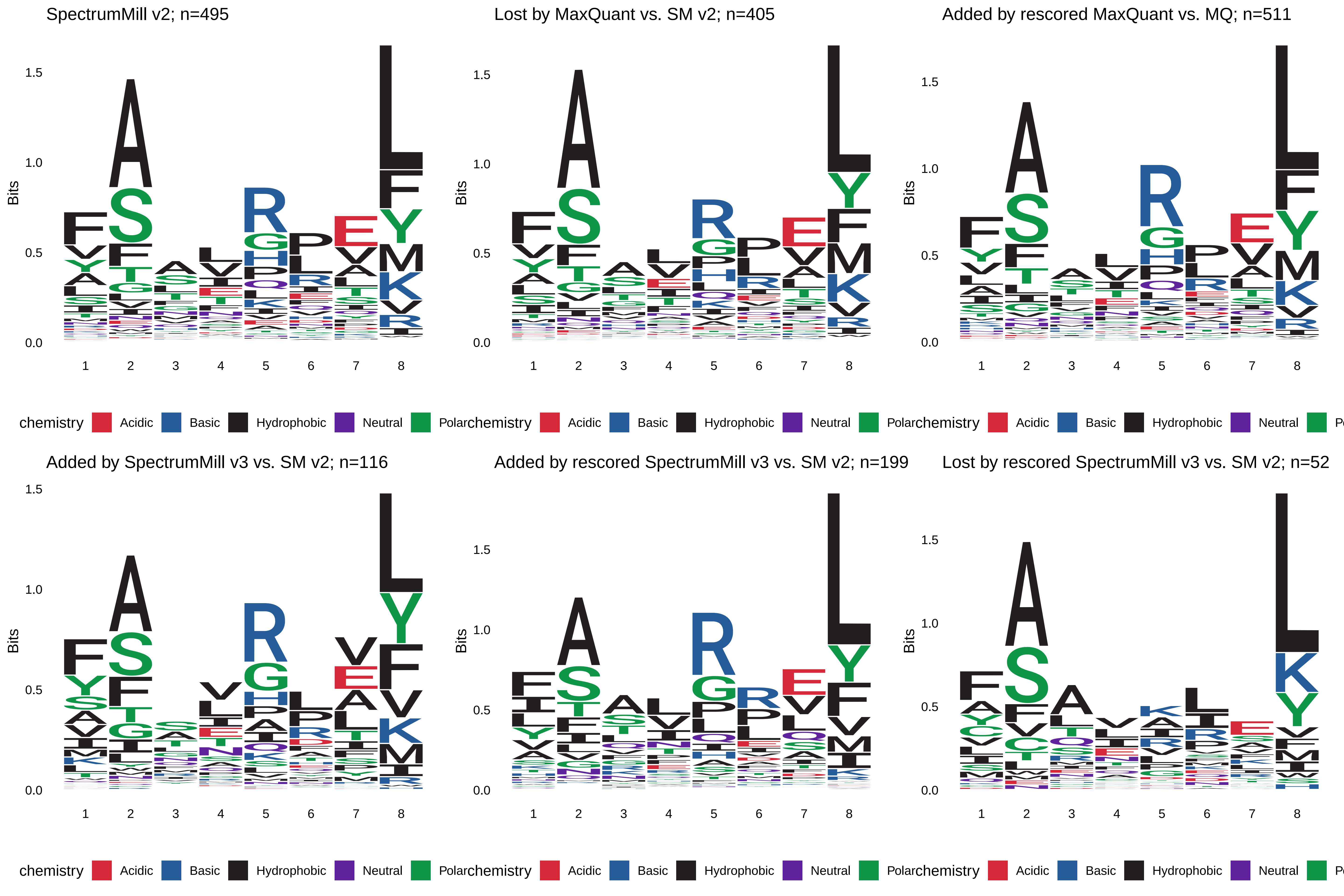
SpectrumMill v2; n=495
Lost by MaxQuant vs. SM v2; n=405
Added by rescored MaxQuant vs. MQ; n=511
Bits
0.0
0.5
1.0
1.5
1
2
3
4
5
6
7
8
chemistry Acidic Basic Hydrophobic Neutral Polarchemistry Acidic Basic Hydrophobic Neutral Polarchemistry Acidic Basic Hydrophobic Neutral
Added by SpectrumMill v3 vs. SM v2; n=116
Added by rescored SpectrumMill v3 vs. SM v2; n=199
Lost by rescored SpectrumMill v3 vs. SM v2; n=52
chemistry Acidic Basic Hydrophobic Neutral Polarchemistry Acidic Basic Hydrophobic Neutral Polarchemistry Acidic Basic Hydrophobic Neutral

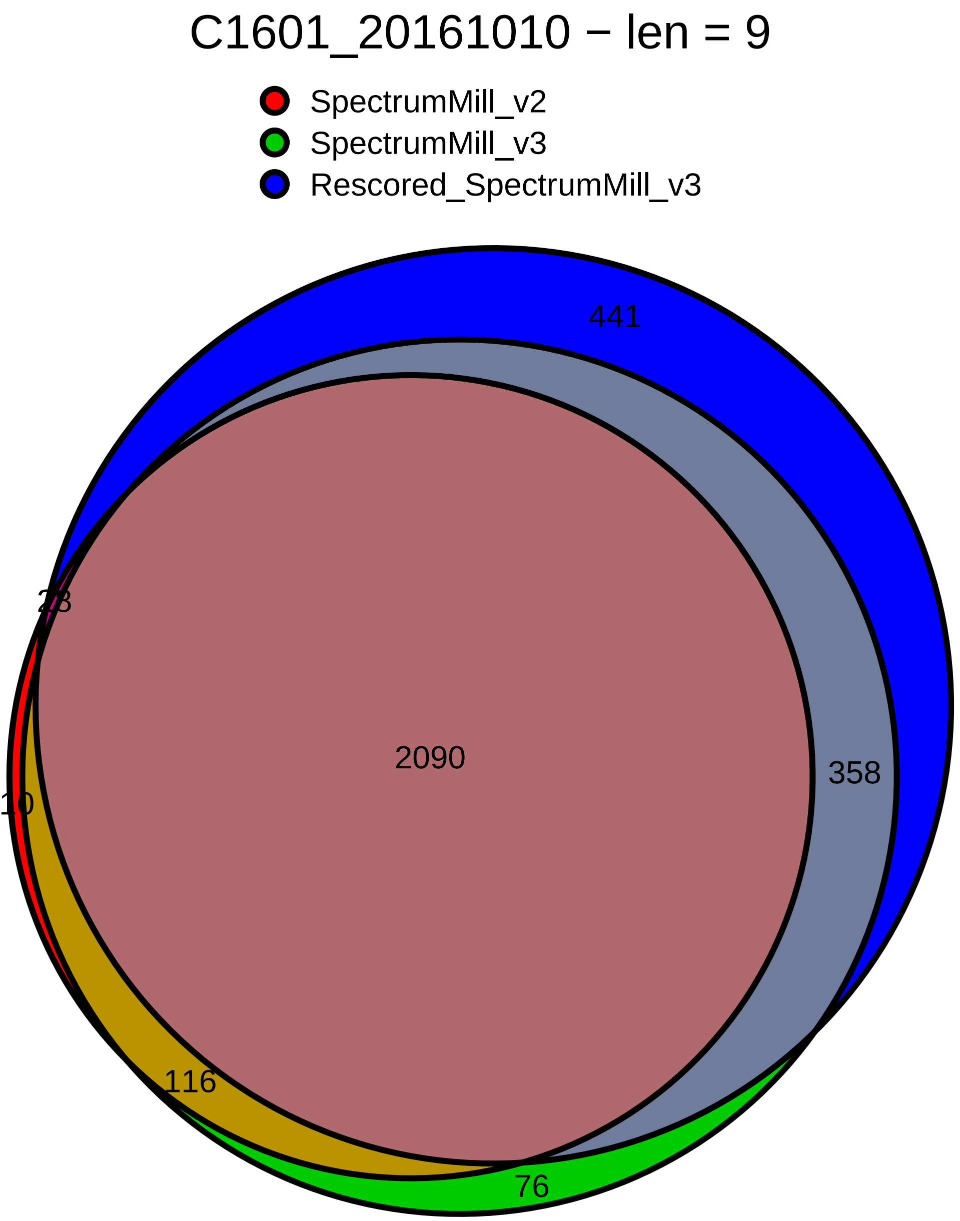
C1601_20161010 − len = 9
SpectrumMill_v2
SpectrumMill_v3
Rescored_SpectrumMill_v3
441
23
10
2090
358
116
76

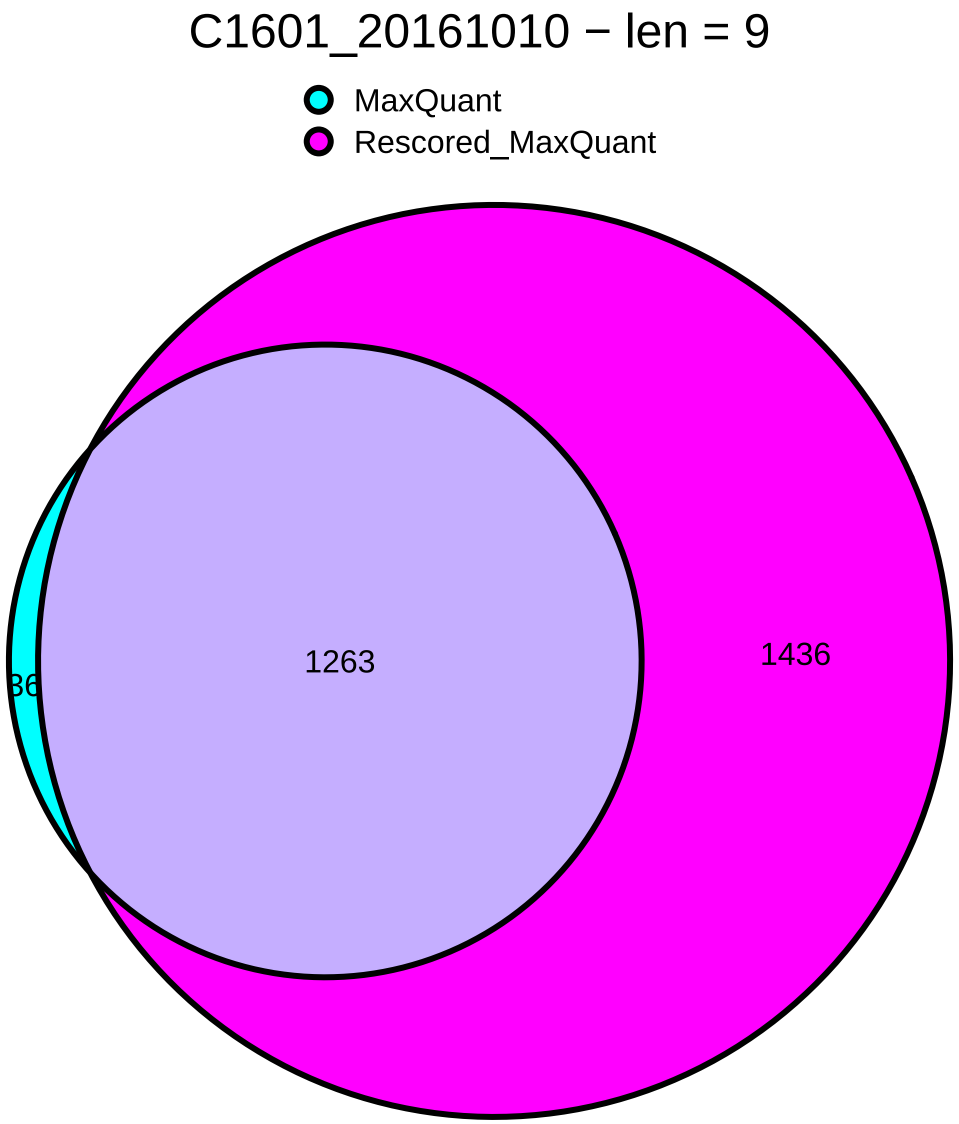
C1601_20161010 − len = 9
MaxQuant
Rescored_MaxQuant
36
1263
1436

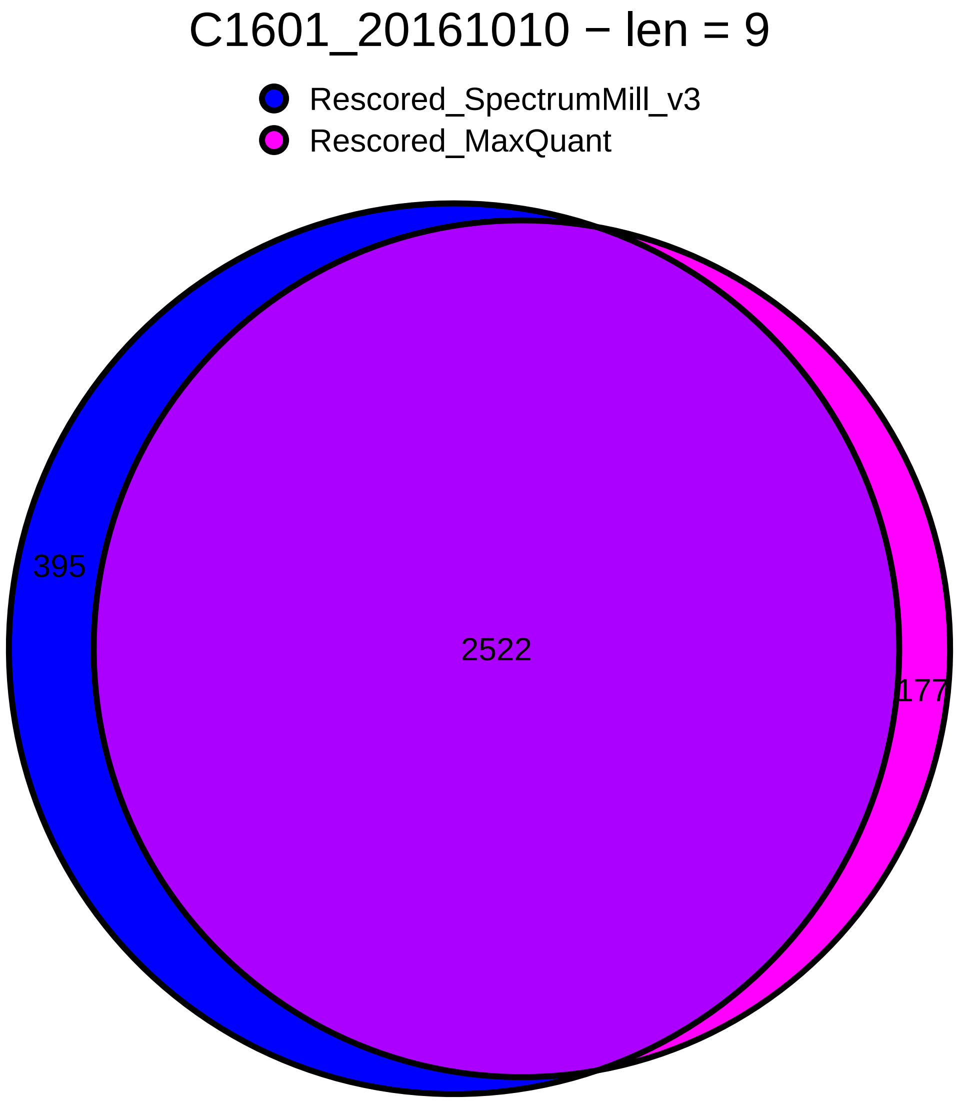
C1601_20161010 − len = 9
Rescored_SpectrumMill_v3
Rescored_MaxQuant
395
2522
177

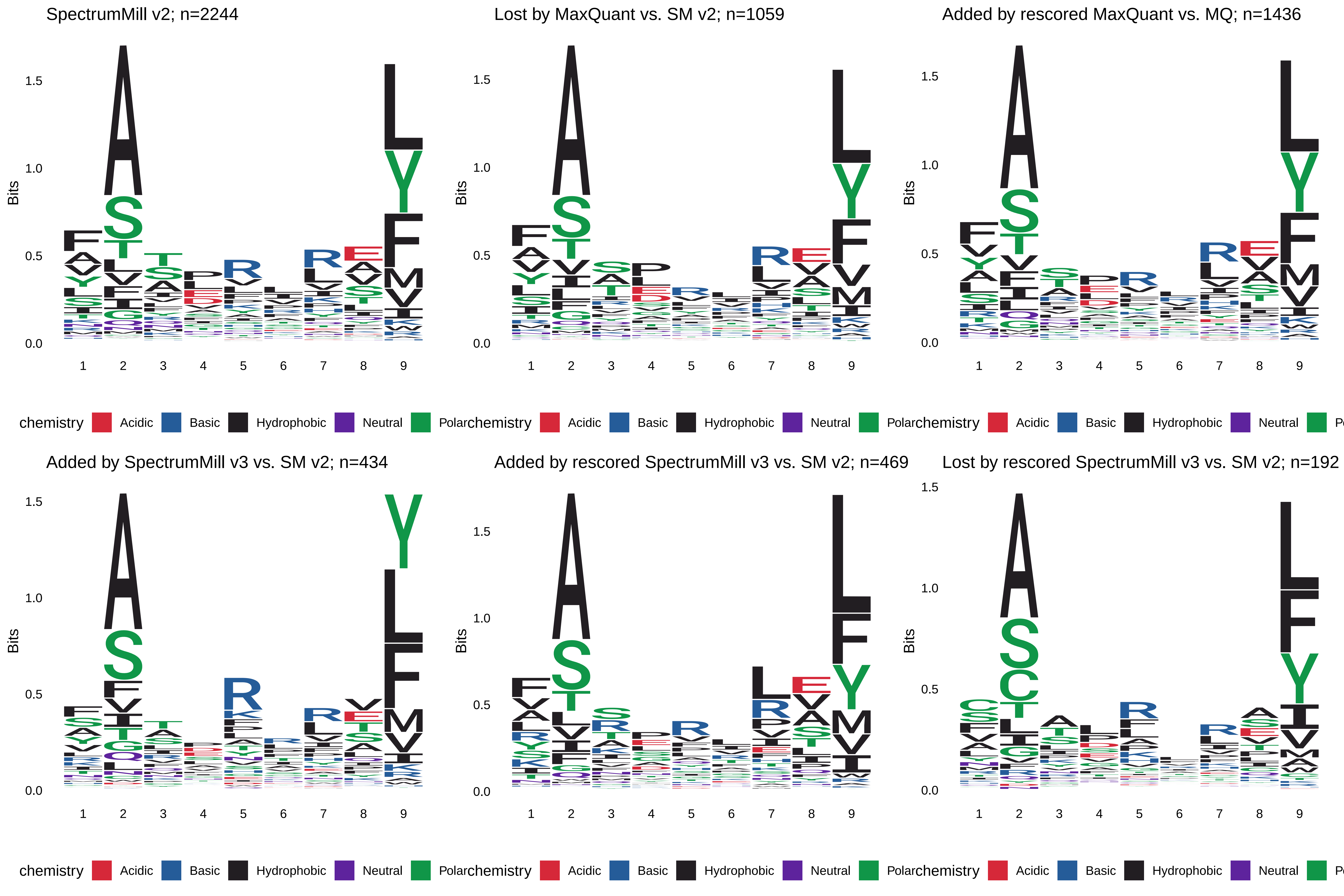

SpectrumMill v2; n=2244
Lost by MaxQuant vs. SM v2; n=1059
Added by rescored MaxQuant vs. MQ; n=1436
Added by SpectrumMill v3 vs. SM v2; n=434
Added by rescored SpectrumMill v3 vs. SM v2; n=469
Lost by rescored SpectrumMill v3 vs. SM v2; n=192
Bits
0.0
0.5
1.0
1.5
1
2
3
4
5
6
7
8
9
chemistry
Acidic
Basic
Hydrophobic
Neutral
Polar

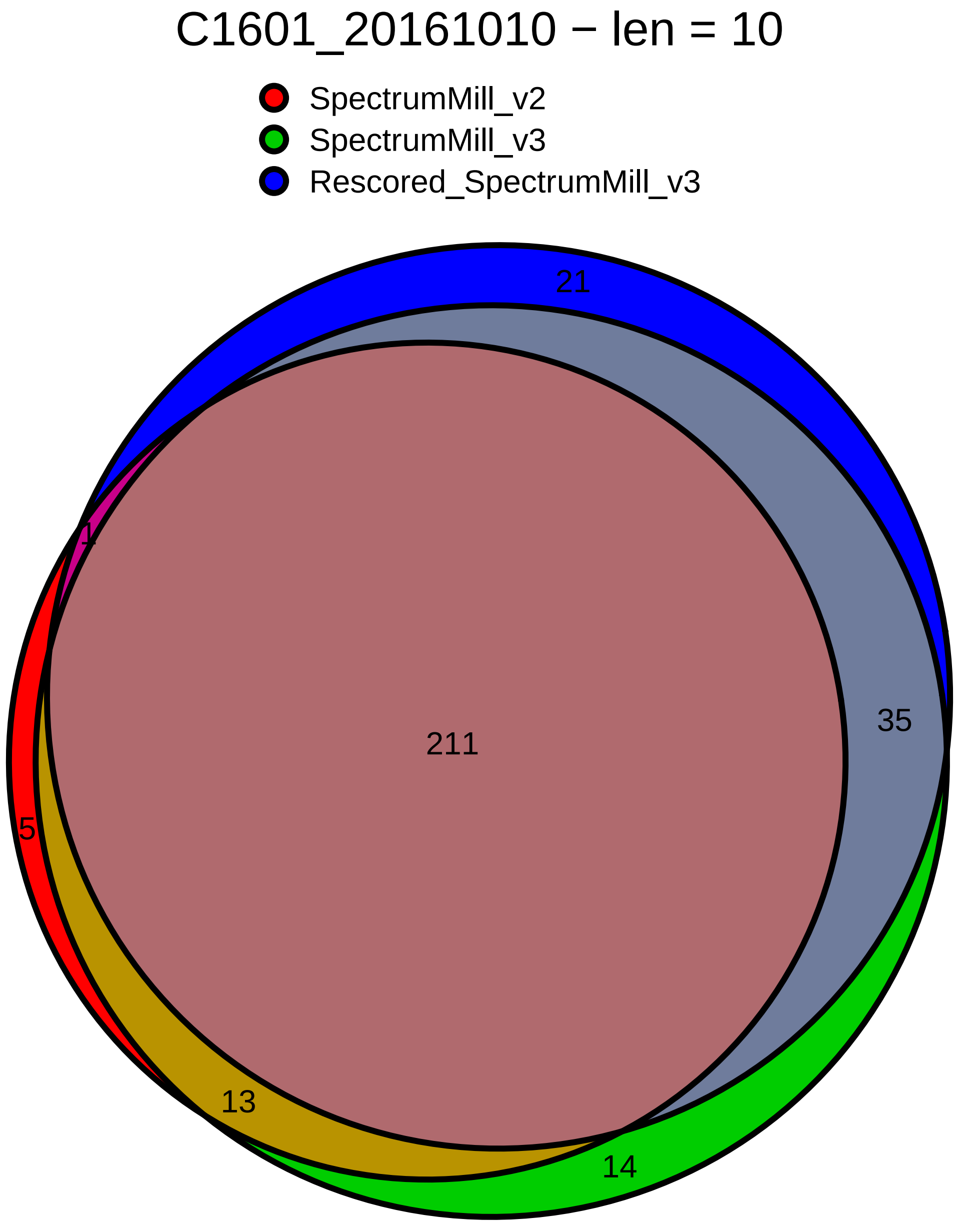
C1601_20161010 − len = 10
SpectrumMill_v2
SpectrumMill_v3
Rescored_SpectrumMill_v3
21
1
35
211
5
13
14

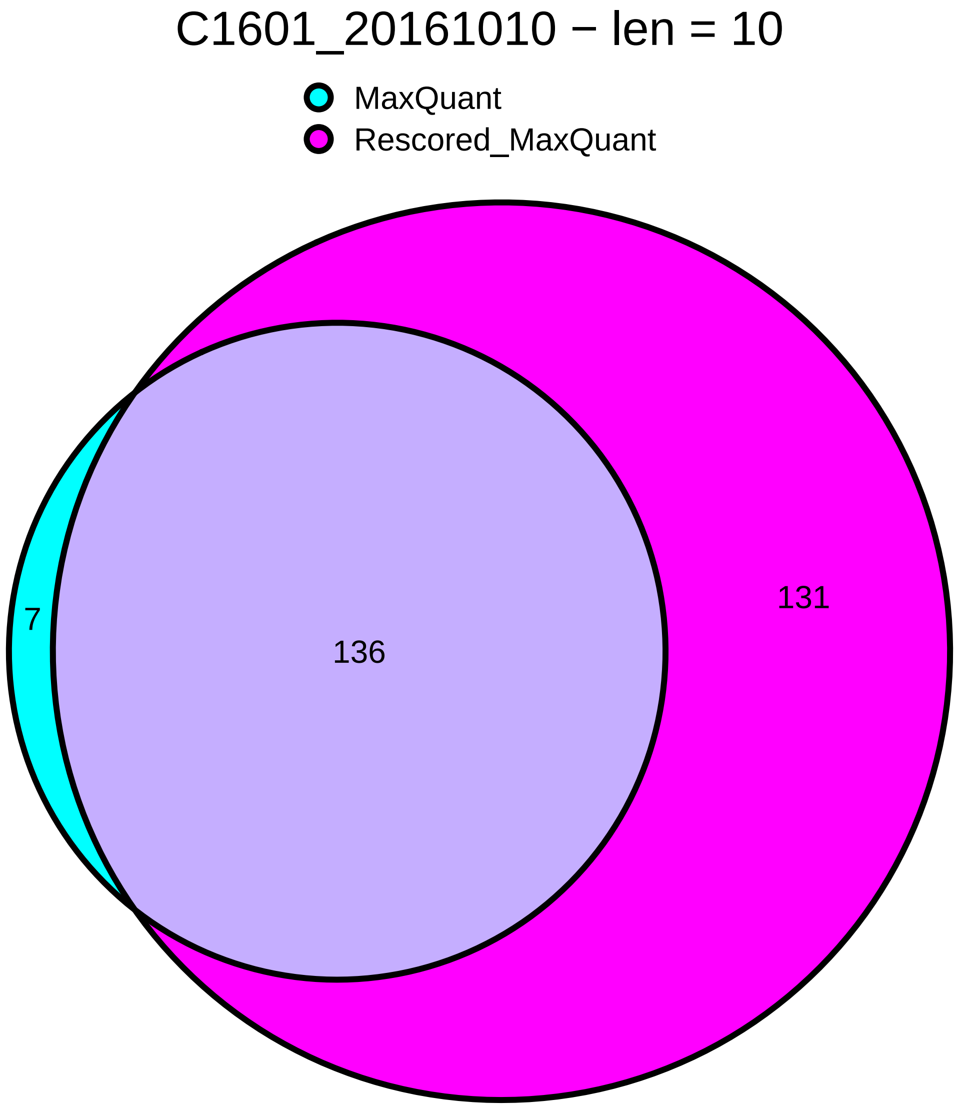
C1601_20161010 − len = 10
MaxQuant
Rescored_MaxQuant
7
136
131

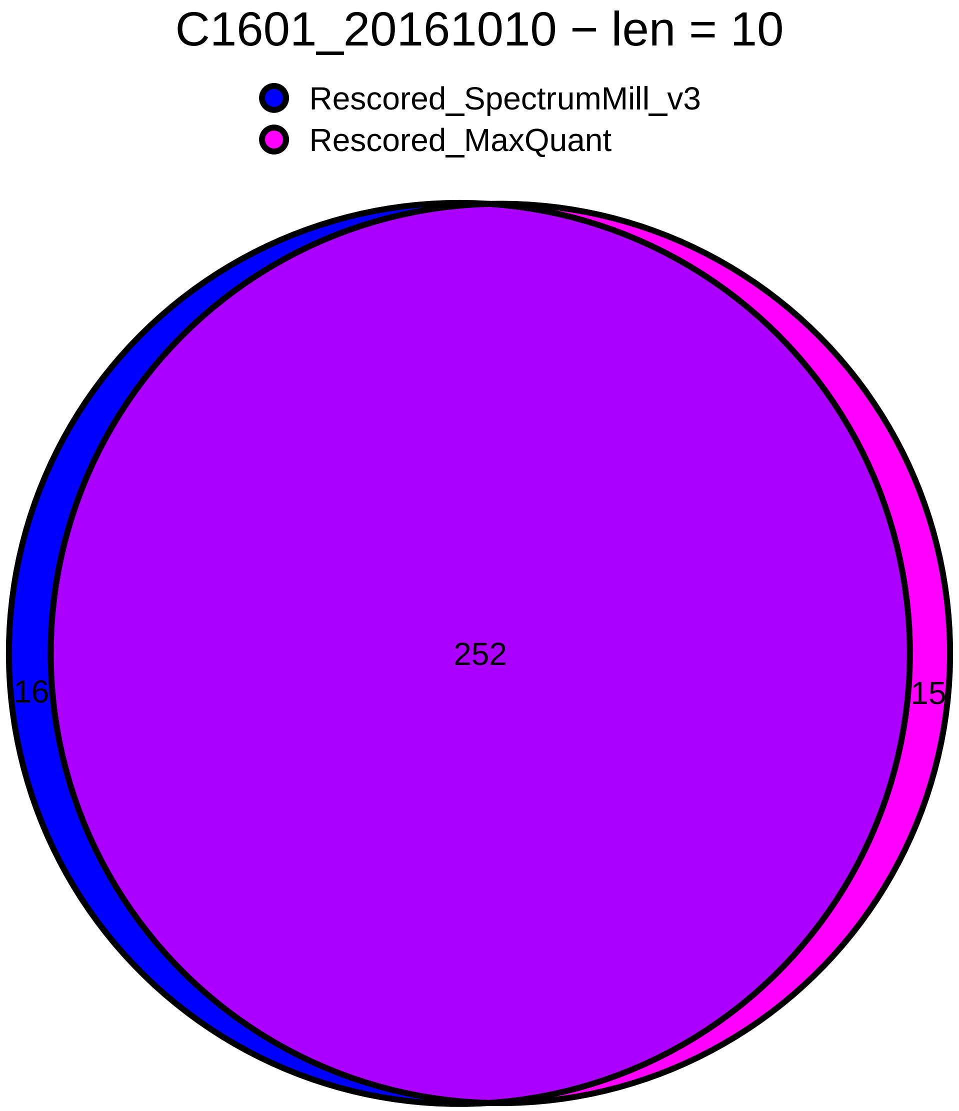
C1601_20161010 − len = 10
Rescored_SpectrumMill_v3
Rescored_MaxQuant
16
252
15

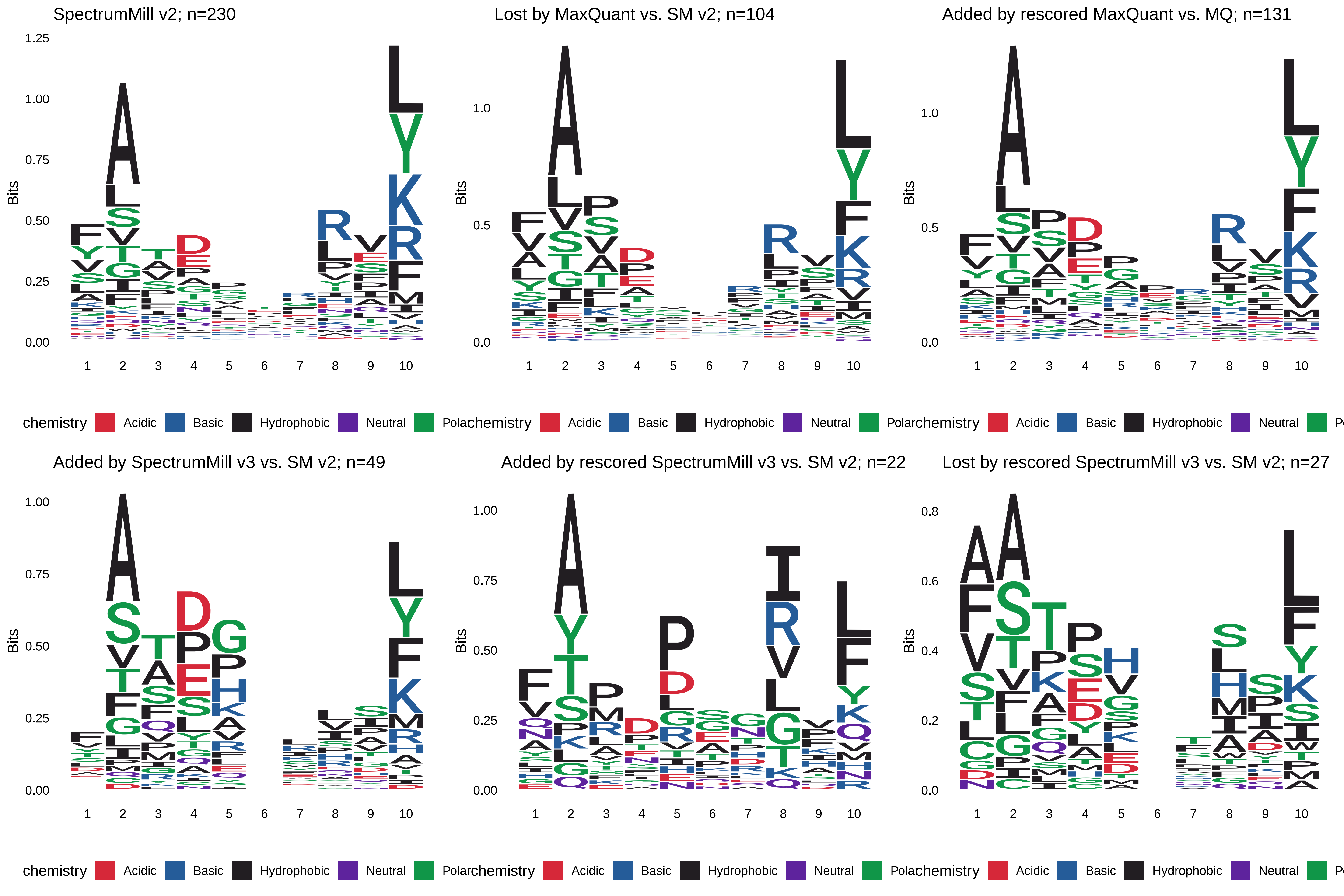
SpectrumMill v2; n=230
Lost by MaxQuant vs. SM v2; n=104
Added by rescored MaxQuant vs. MQ; n=131
Added by SpectrumMill v3 vs. SM v2; n=49
Added by rescored SpectrumMill v3 vs. SM v2; n=22
Lost by rescored SpectrumMill v3 vs. SM v2; n=27
Bits
1 2 3 4 5 6 7 8 9 10
chemistry
Acidic
Basic
Hydrophobic
Neutral
Polar

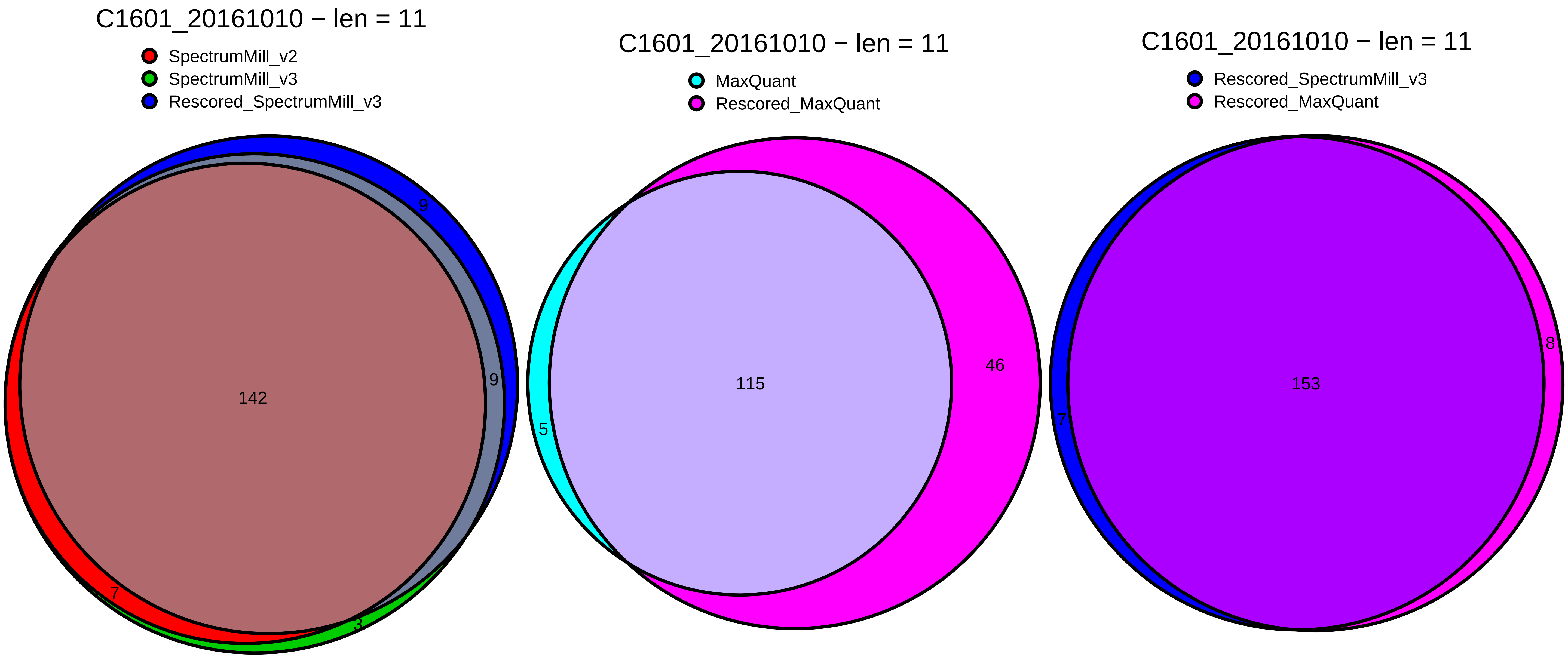

C1601_20161010 − len = 11
SpectrumMill_v2
SpectrumMill_v3
Rescored_SpectrumMill_v3
9
9
142
7
3
C1601_20161010 − len = 11
MaxQuant
Rescored_MaxQuant
115
46
5
C1601_20161010 − len = 11
Rescored_SpectrumMill_v3
Rescored_MaxQuant
153
8
7

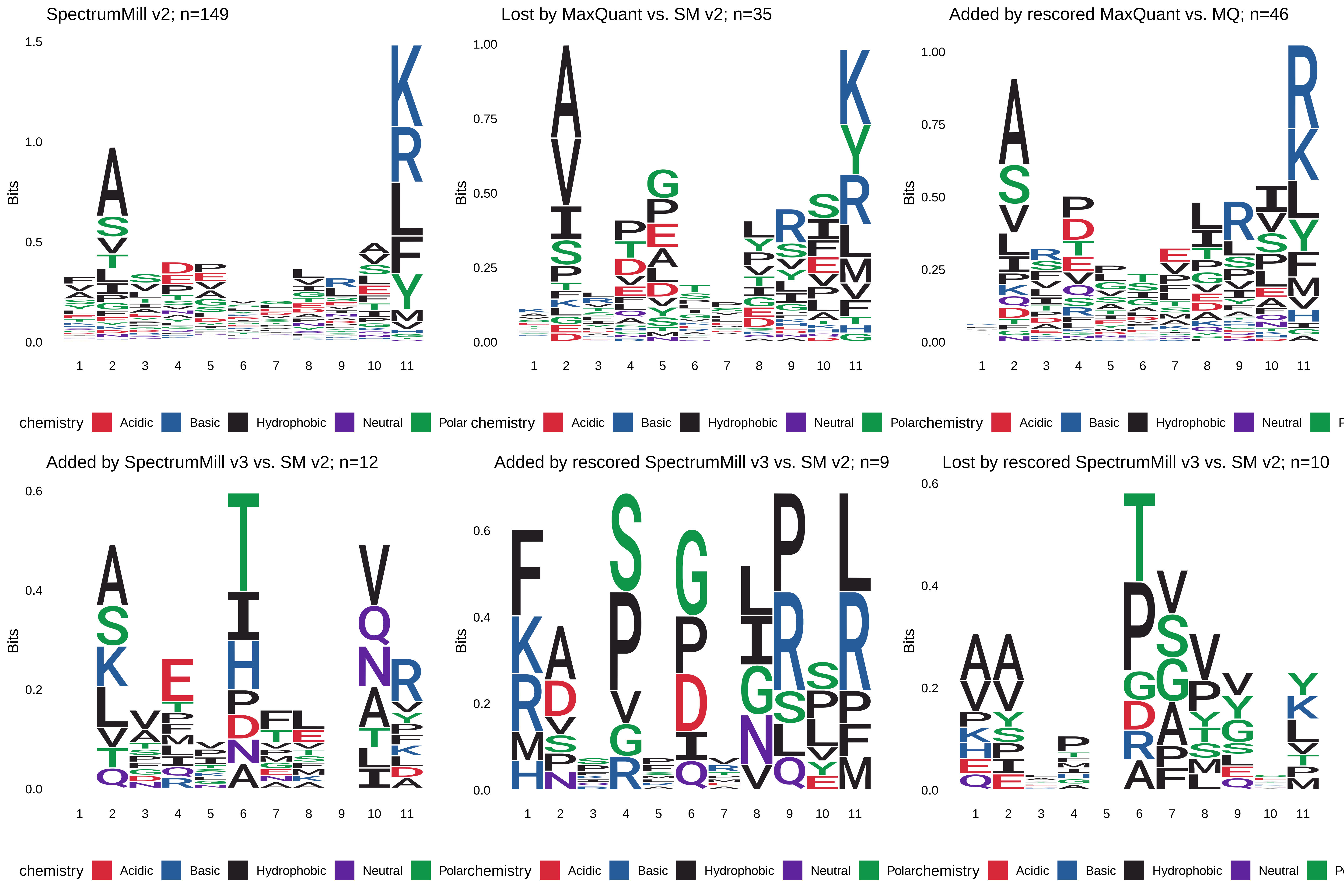

SpectrumMill v2; n=149
Lost by MaxQuant vs. SM v2; n=35
Added by rescored MaxQuant vs. MQ; n=46
Added by SpectrumMill v3 vs. SM v2; n=12
Added by rescored SpectrumMill v3 vs. SM v2; n=9
Lost by rescored SpectrumMill v3 vs. SM v2; n=10
Bits
1 2 3 4 5 6 7 8 9 10 11
chemistry Acidic Basic Hydrophobic Neutral Polar

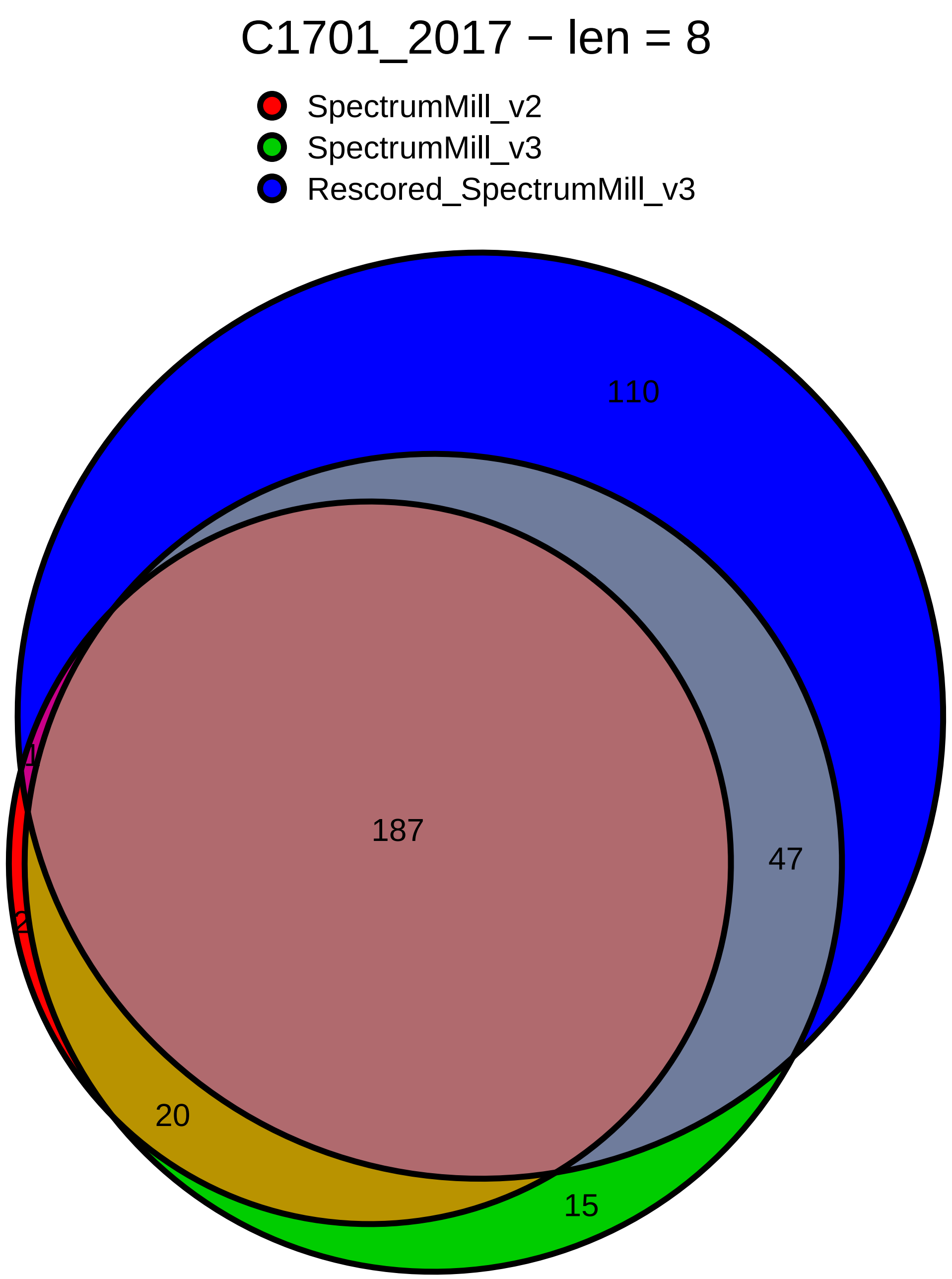
C1701_2017 − len = 8
SpectrumMill_v2
SpectrumMill_v3
Rescored_SpectrumMill_v3
110
187
47
20
15

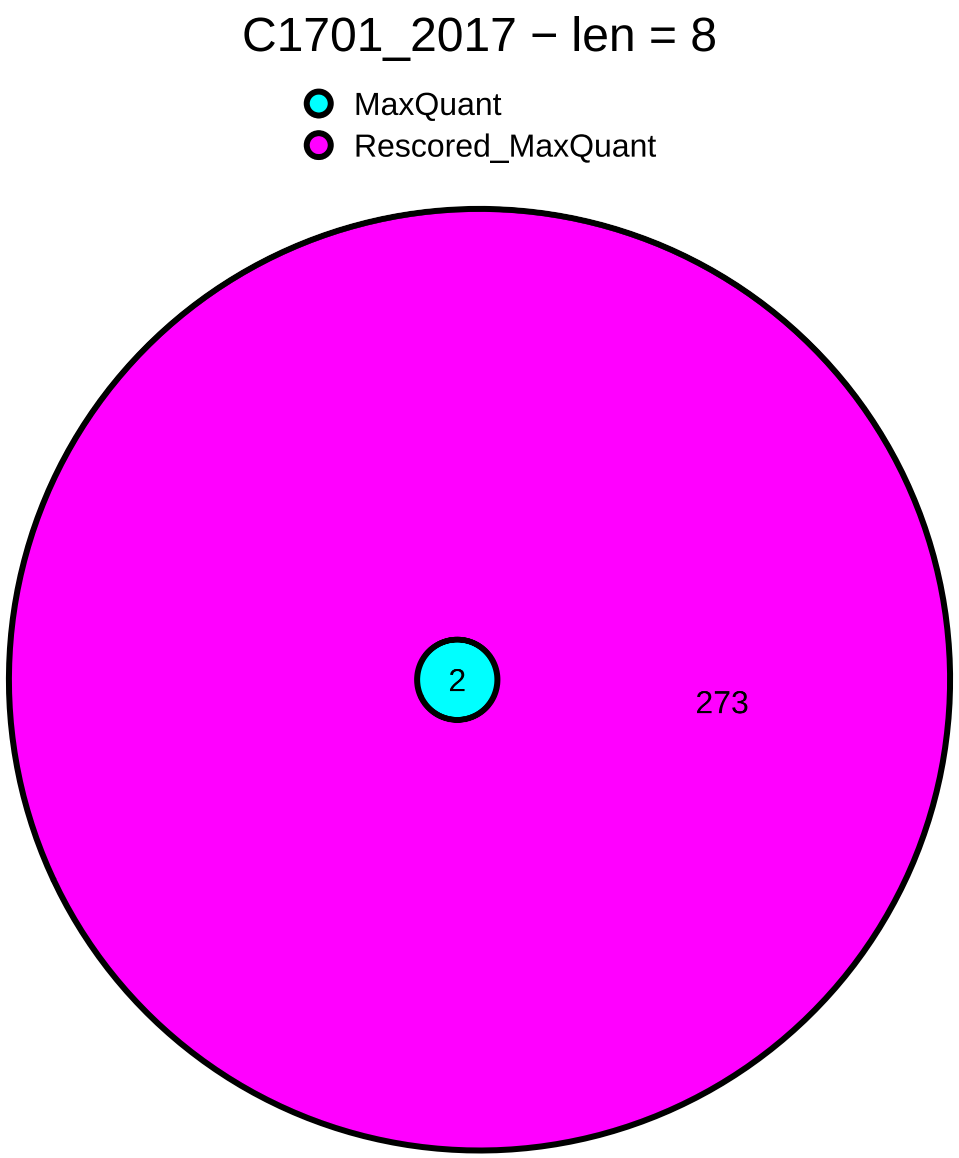
C1701_2017 − len = 8
MaxQuant
Rescored_MaxQuant
2
273

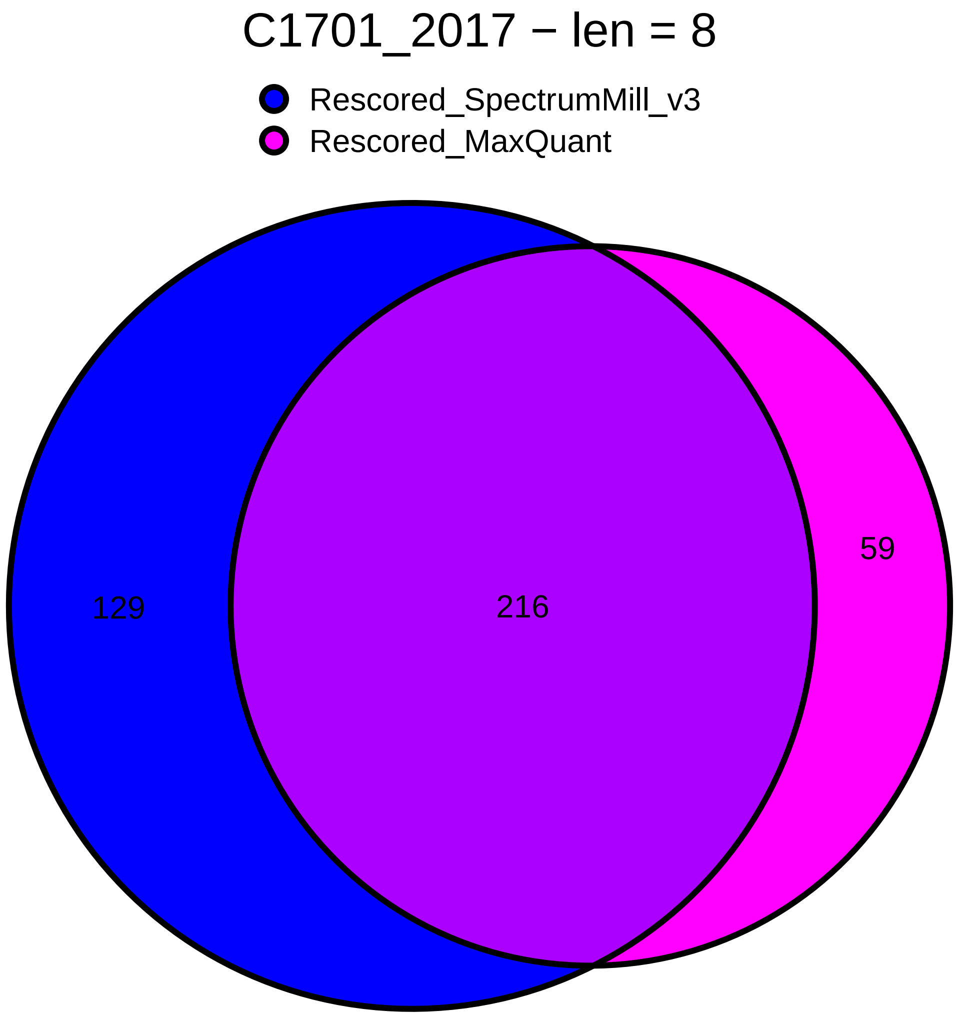
C1701_2017 − len = 8
Rescored_SpectrumMill_v3
Rescored_MaxQuant
129
216
59

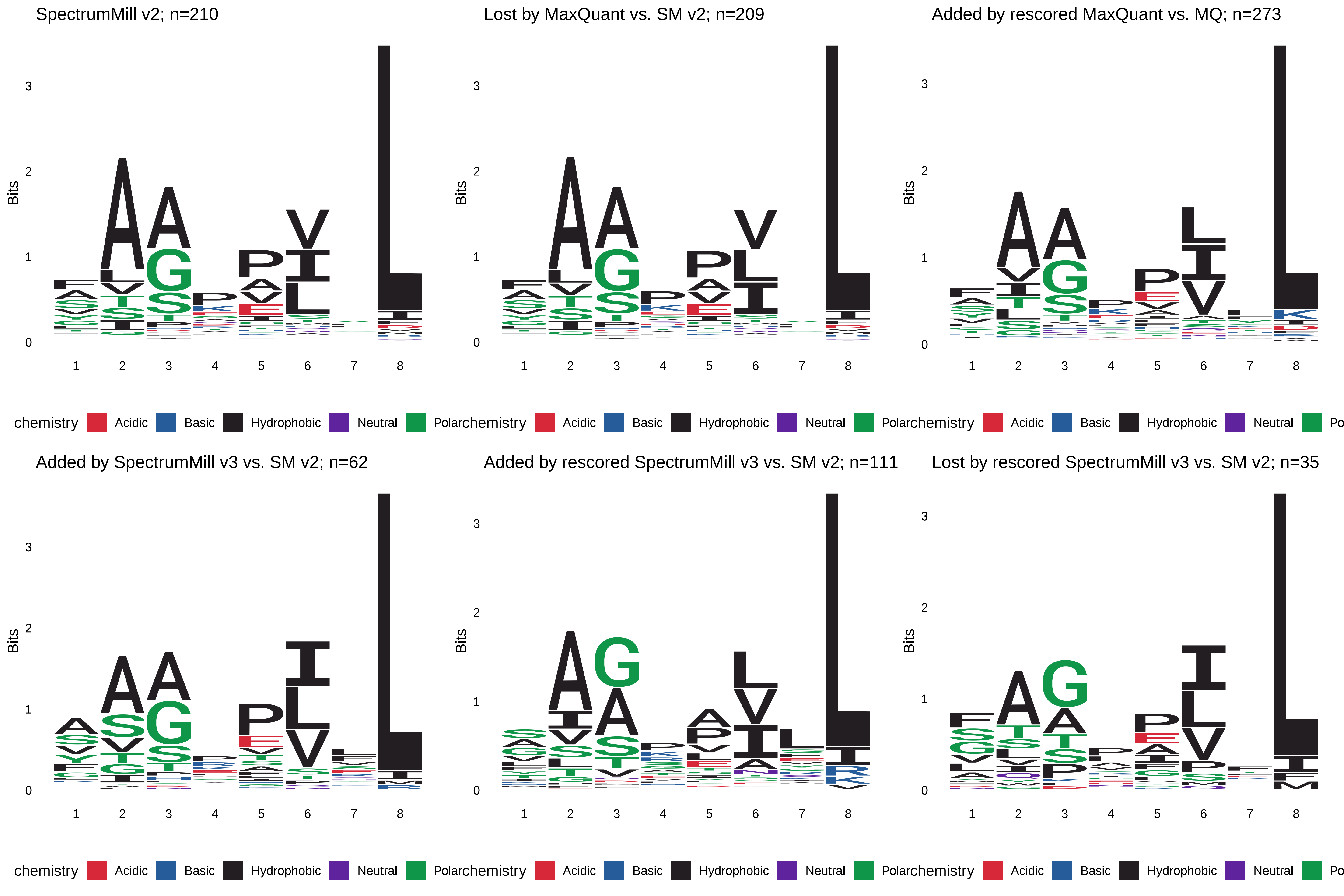
SpectrumMill v2; n=210
Lost by MaxQuant vs. SM v2; n=209
Added by rescored MaxQuant vs. MQ; n=273
Added by SpectrumMill v3 vs. SM v2; n=62
Added by rescored SpectrumMill v3 vs. SM v2; n=111
Lost by rescored SpectrumMill v3 vs. SM v2; n=35
Bits
0
1
2
3
1 2 3 4 5 6 7 8
chemistry
Acidic
Basic
Hydrophobic
Neutral
Polar

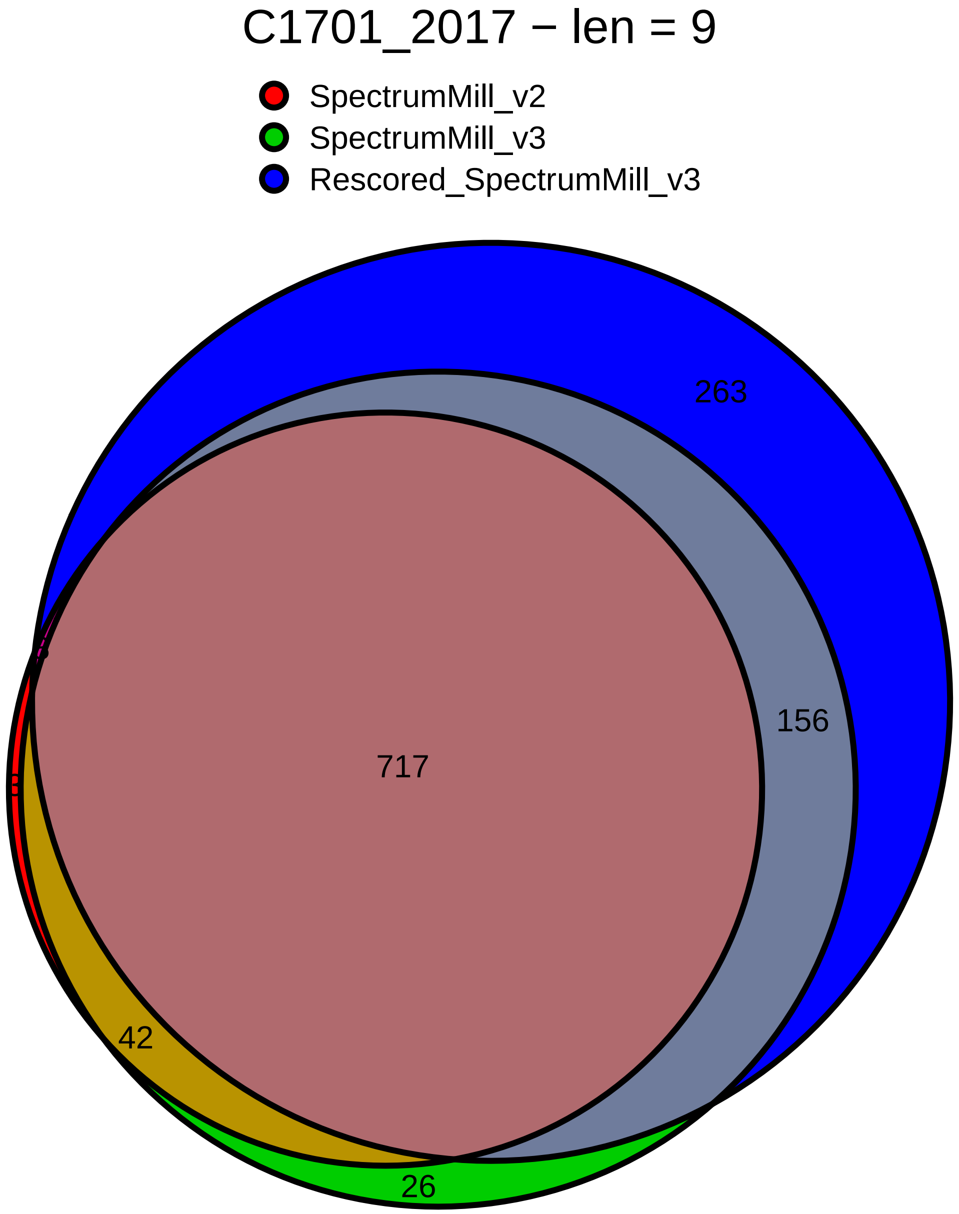
C1701_2017 − len = 9
SpectrumMill_v2
SpectrumMill_v3
Rescored_SpectrumMill_v3
263
156
717
42
26

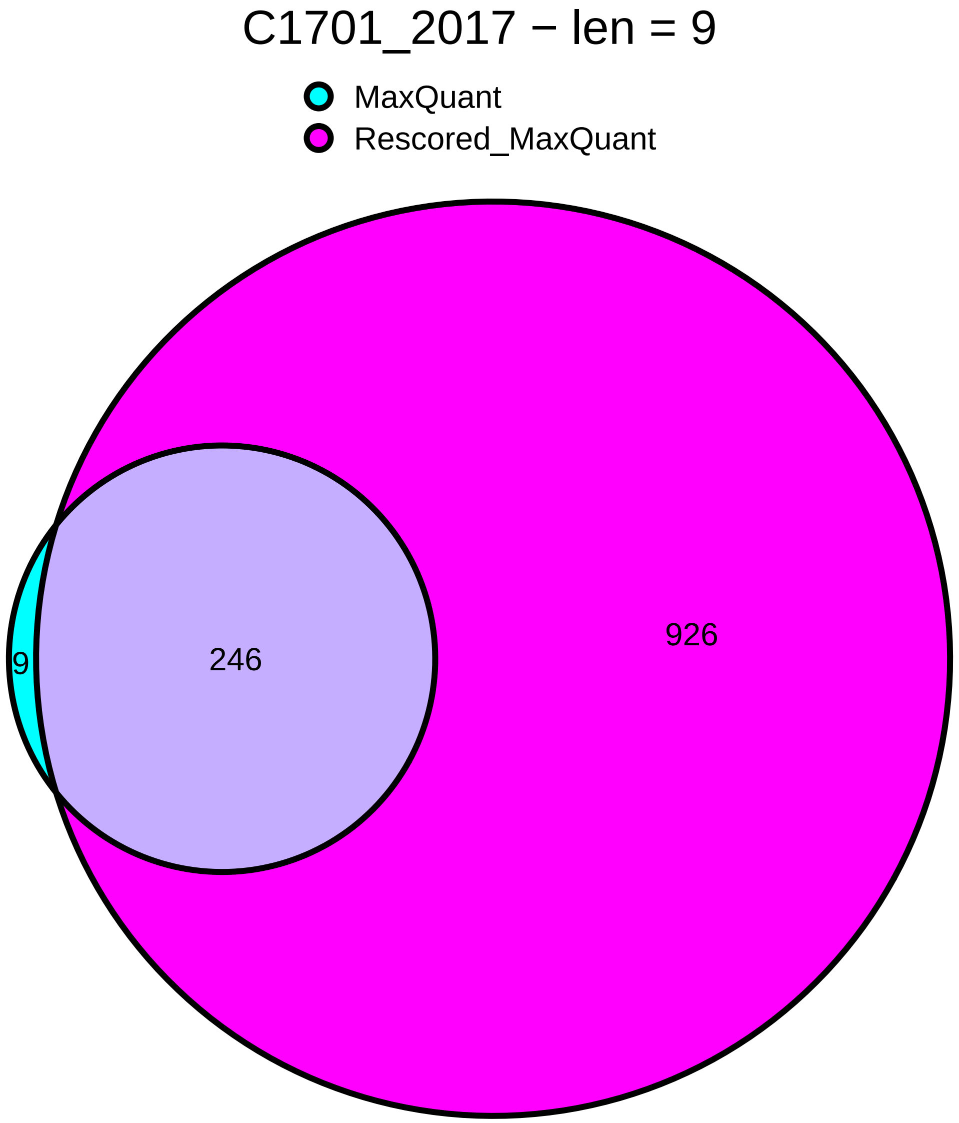
C1701_2017 − len = 9
MaxQuant
Rescored_MaxQuant
9
246
926

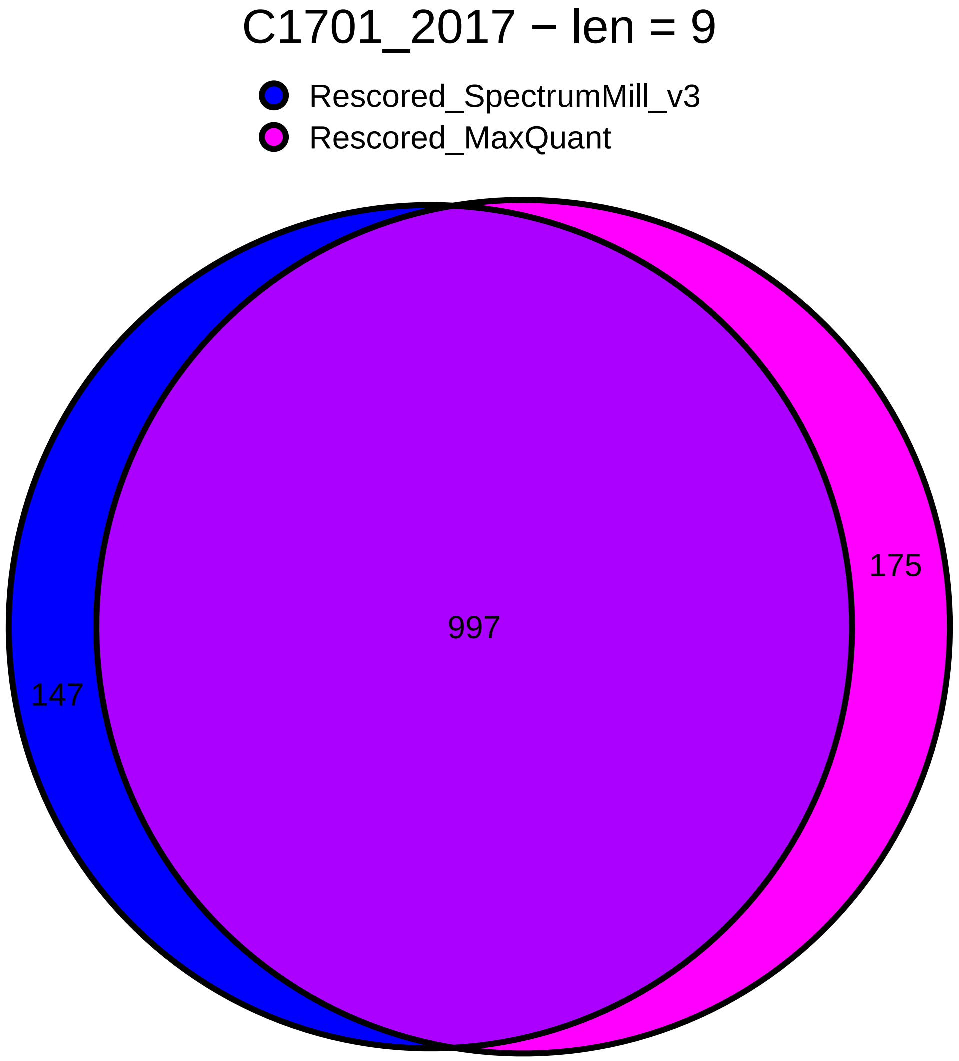
C1701_2017 − len = 9
Rescored_SpectrumMill_v3
Rescored_MaxQuant
147
997
175

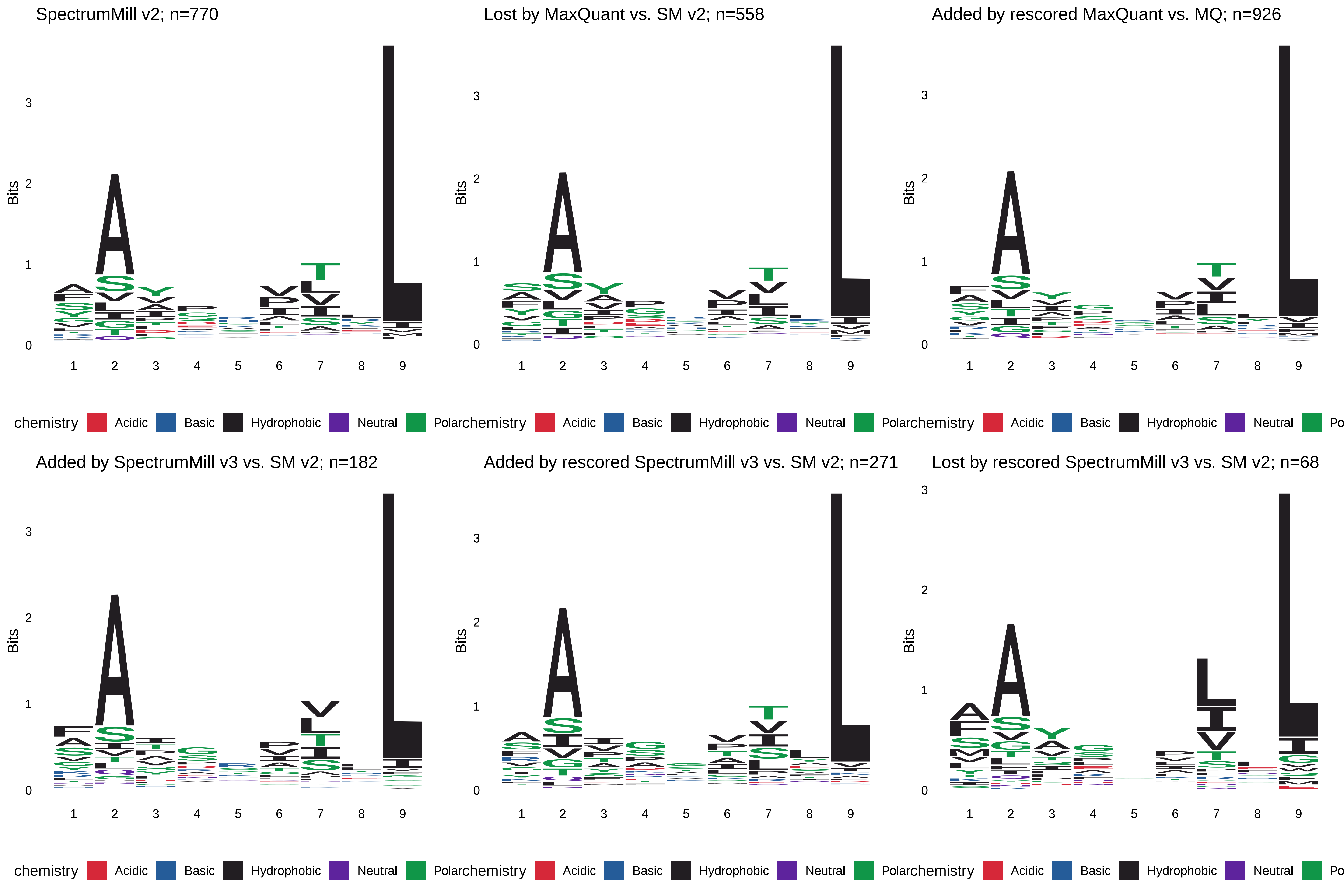
SpectrumMill v2; n=770
Lost by MaxQuant vs. SM v2; n=558
Added by rescored MaxQuant vs. MQ; n=926
Added by SpectrumMill v3 vs. SM v2; n=182
Added by rescored SpectrumMill v3 vs. SM v2; n=271
Lost by rescored SpectrumMill v3 vs. SM v2; n=68
Bits
1 2 3 4 5 6 7 8 9
chemistry Acidic Basic Hydrophobic Neutral Polar

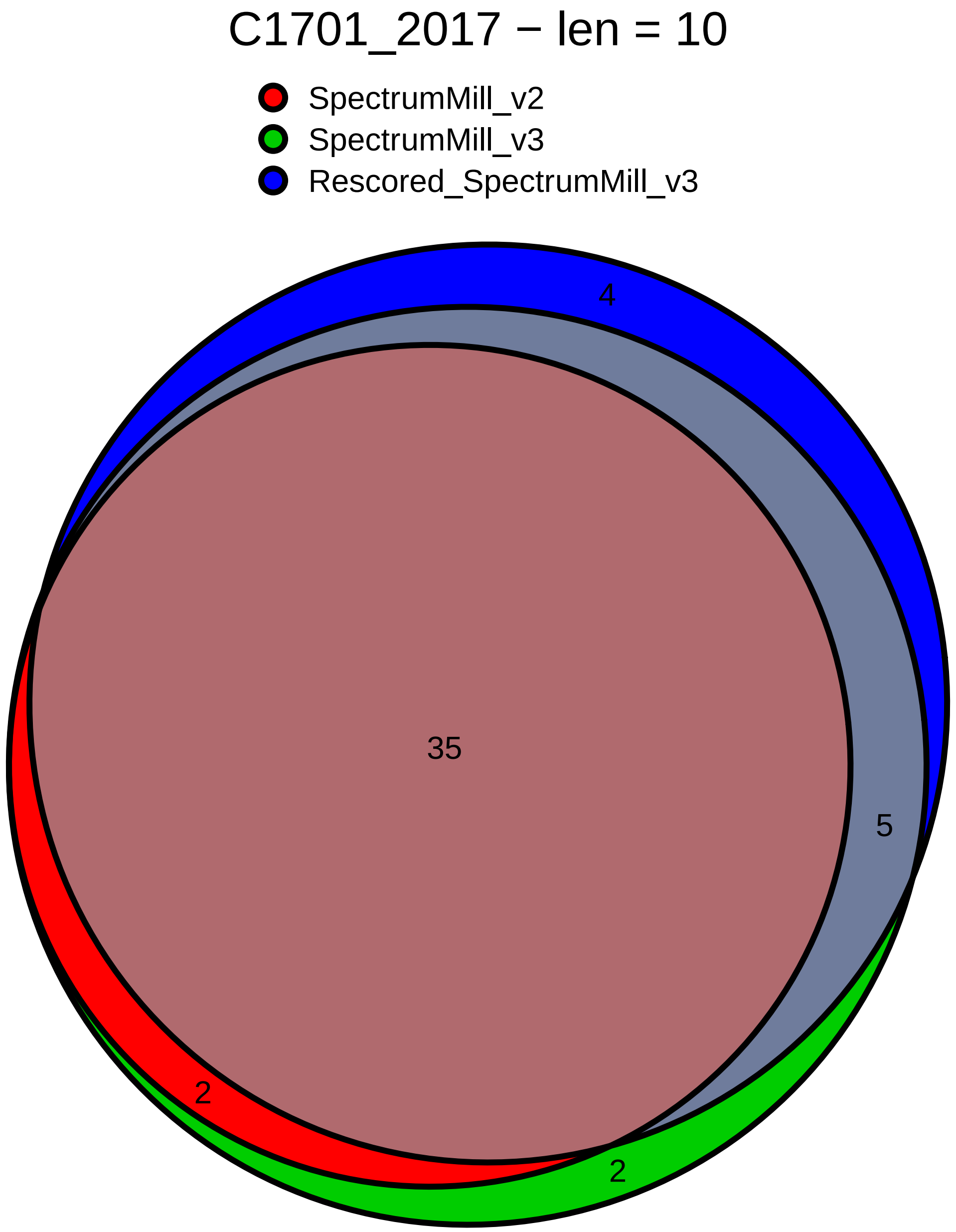
C1701_2017 − len = 10
SpectrumMill_v2
SpectrumMill_v3
Rescored_SpectrumMill_v3
4
35
5
2
2

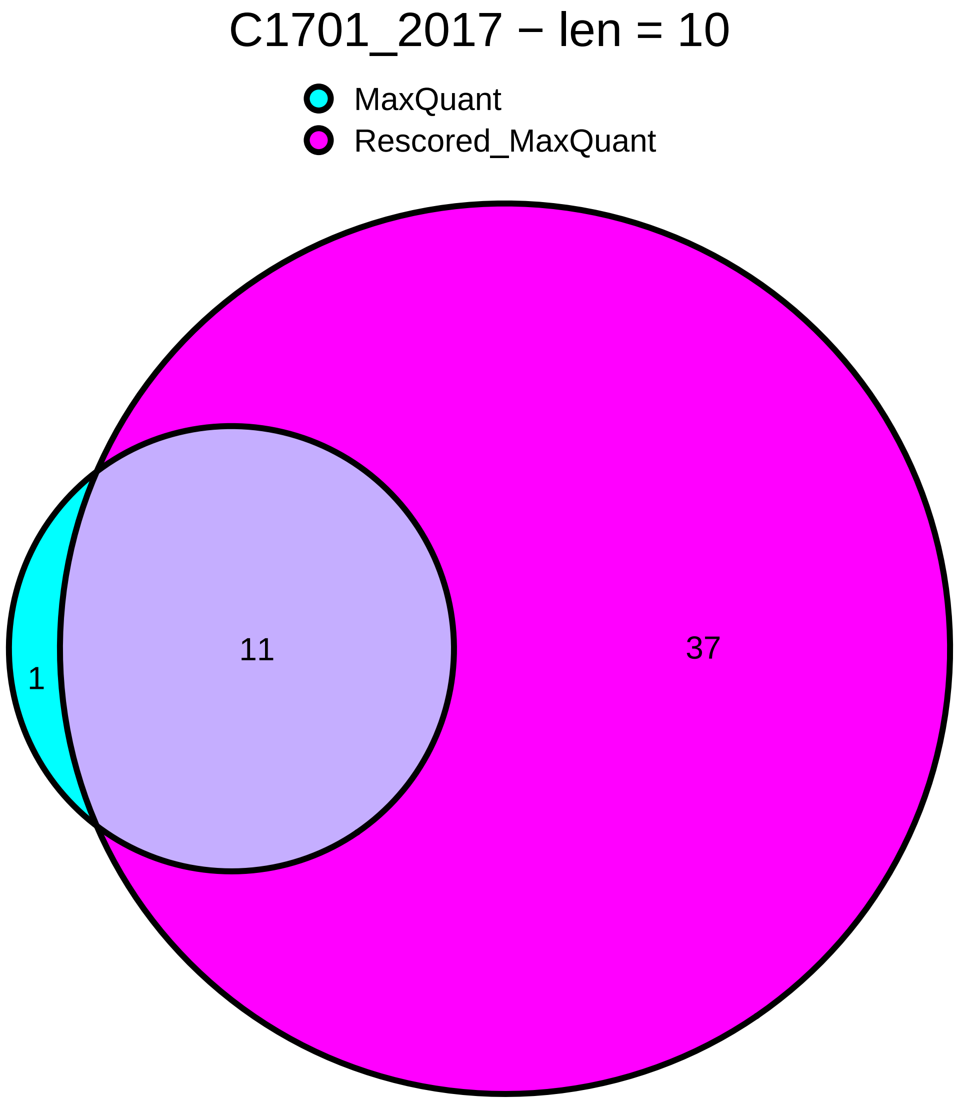
C1701_2017 − len = 10
MaxQuant
Rescored_MaxQuant
1
11
37

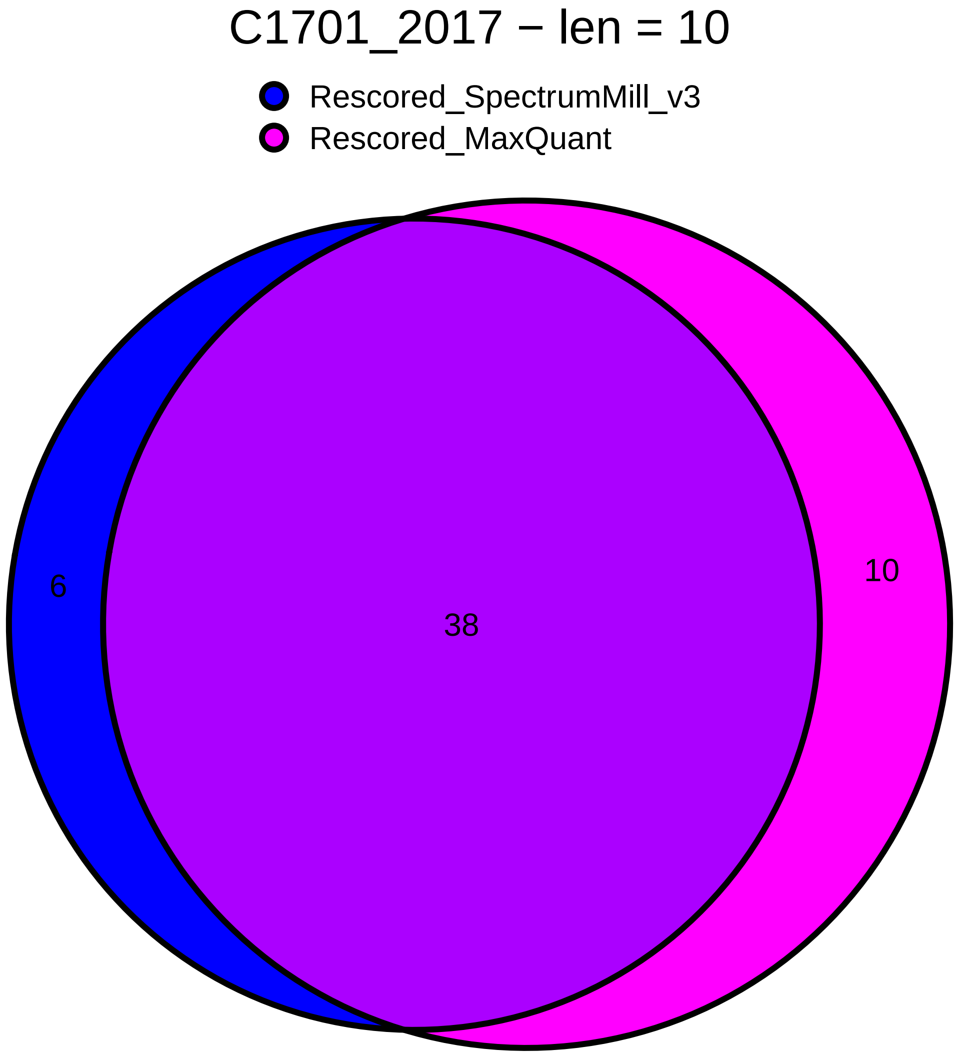
C1701_2017 − len = 10
Rescored_SpectrumMill_v3
Rescored_MaxQuant
6
38
10

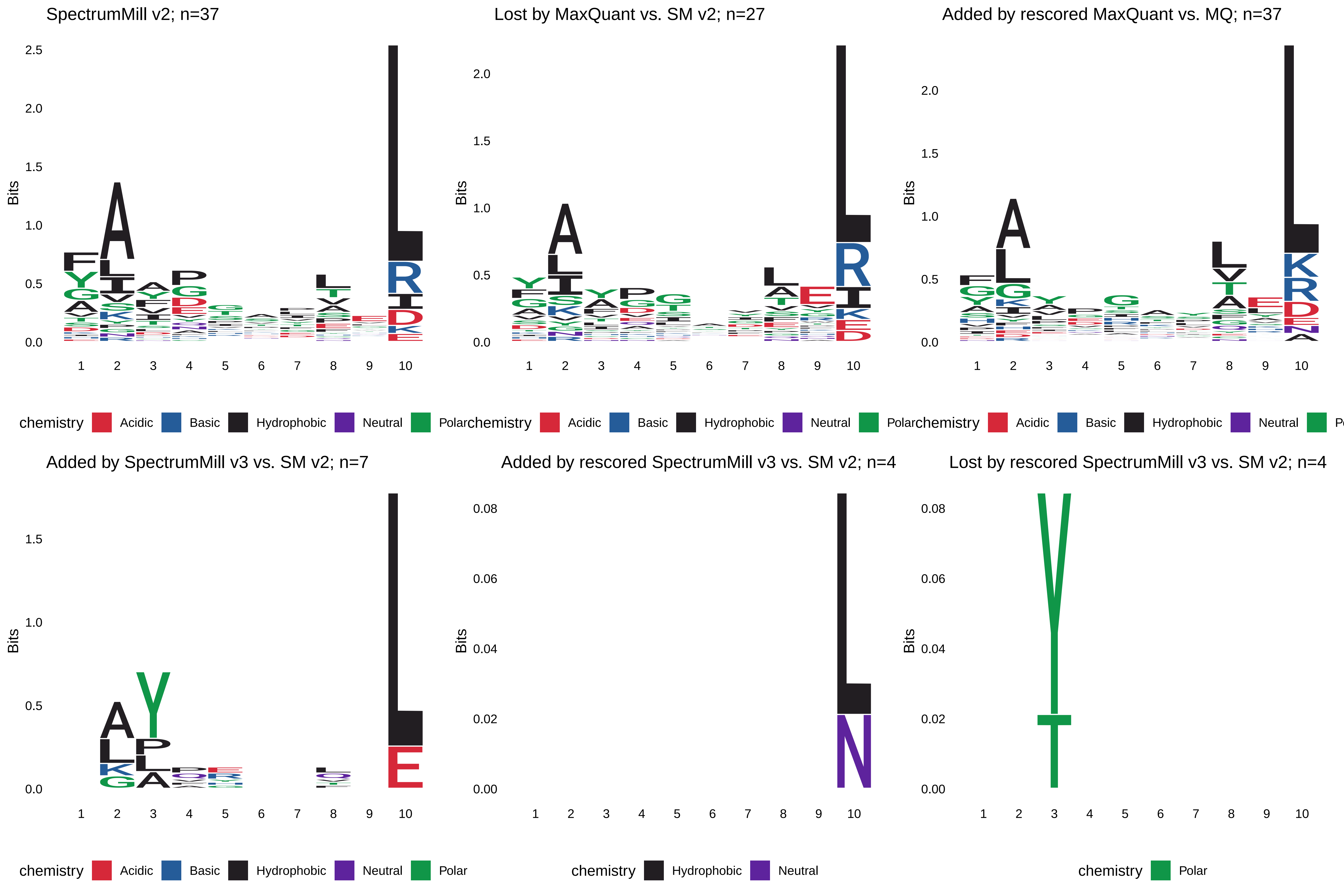
SpectrumMill v2; n=37
Lost by MaxQuant vs. SM v2; n=27
Added by rescored MaxQuant vs. MQ; n=37
Added by SpectrumMill v3 vs. SM v2; n=7
Added by rescored SpectrumMill v3 vs. SM v2; n=4
Lost by rescored SpectrumMill v3 vs. SM v2; n=4
Bits
1 2 3 4 5 6 7 8 9 10
chemistry Acidic Basic Hydrophobic Neutral Polar

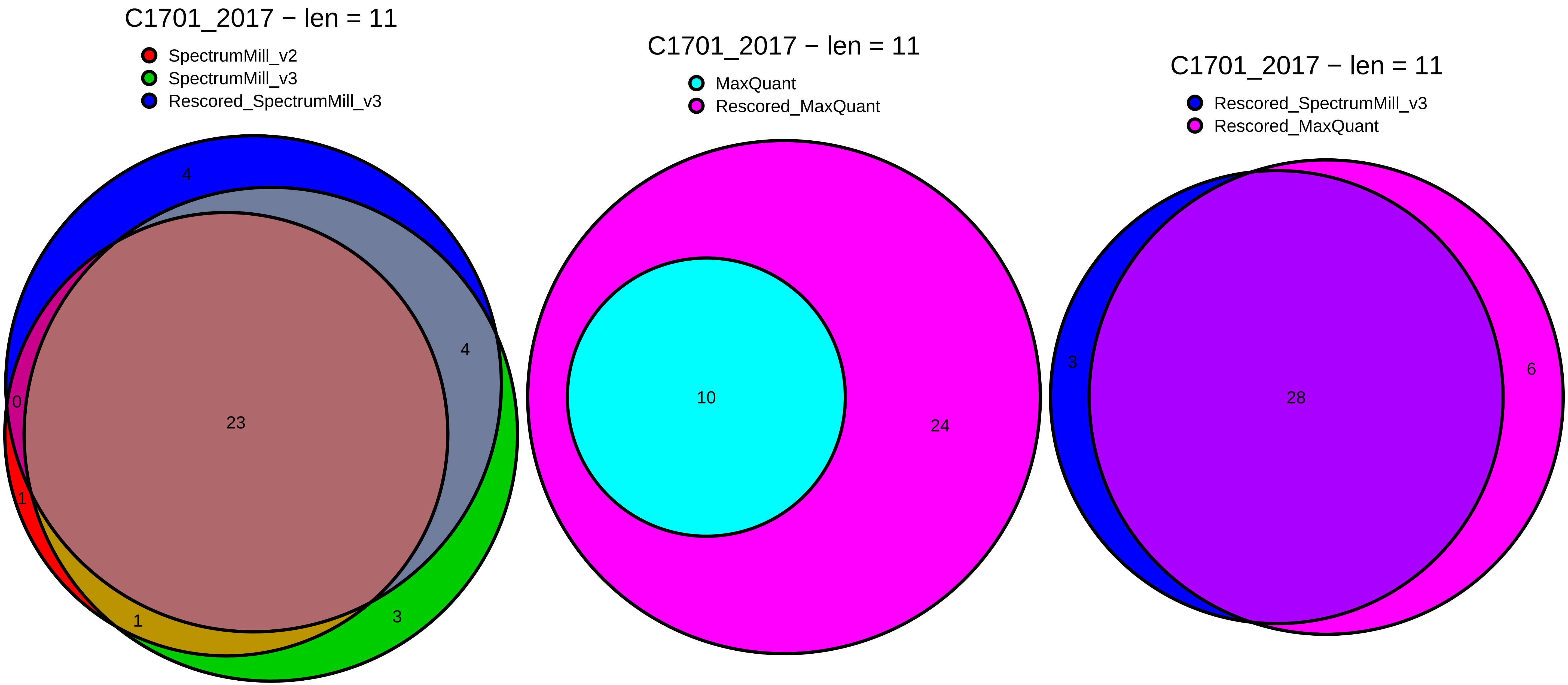

C1701_2017 − len = 11
SpectrumMill_v2
SpectrumMill_v3
Rescored_SpectrumMill_v3
4
4
0
23
1
1
3
C1701_2017 − len = 11
MaxQuant
Rescored_MaxQuant
10
24
C1701_2017 − len = 11
Rescored_SpectrumMill_v3
Rescored_MaxQuant
3
28
6

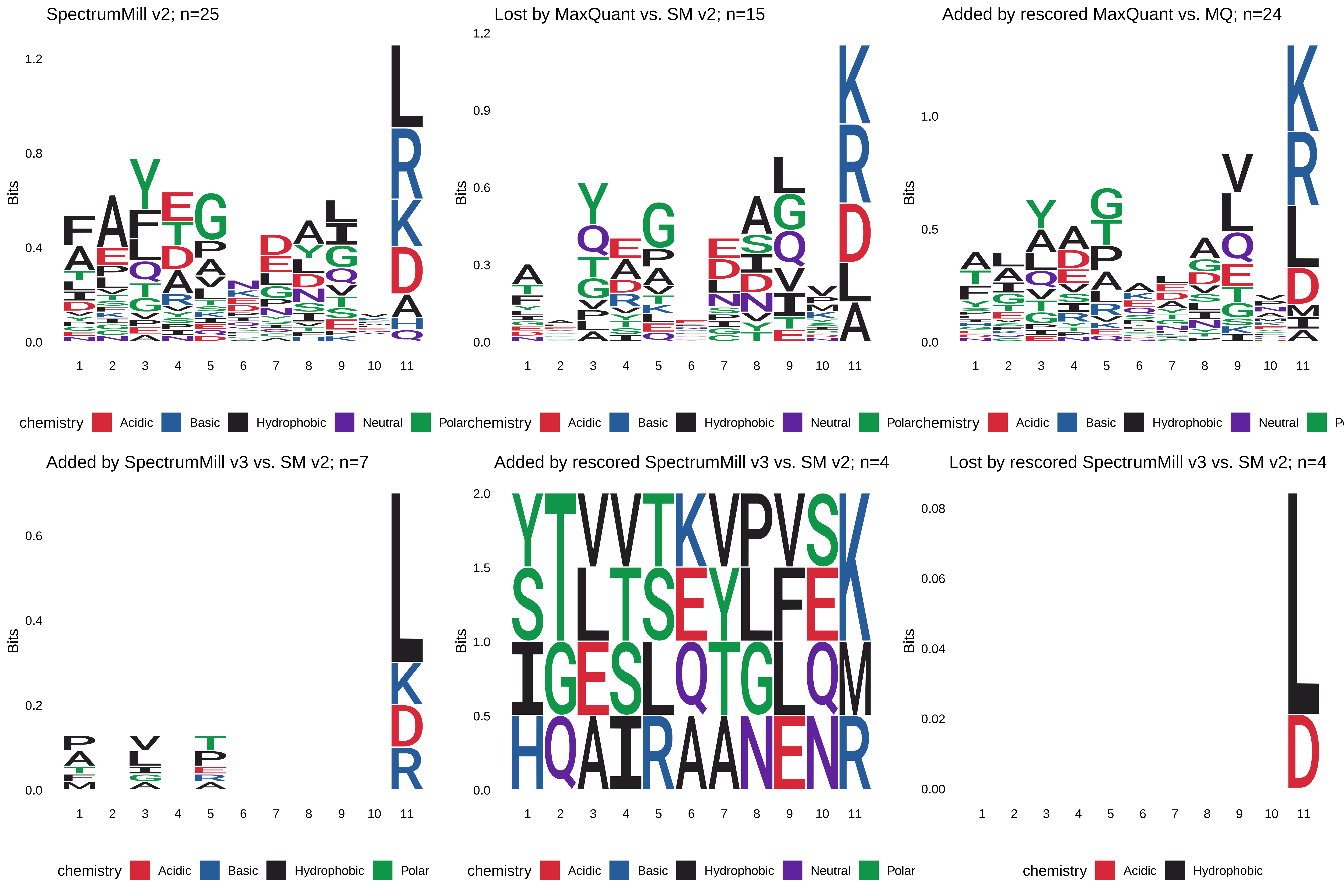
SpectrumMill v2; n=25
Lost by MaxQuant vs. SM v2; n=15
Added by rescored MaxQuant vs. MQ; n=24
Added by SpectrumMill v3 vs. SM v2; n=7
Added by rescored SpectrumMill v3 vs. SM v2; n=4
Lost by rescored SpectrumMill v3 vs. SM v2; n=4
Bits
1 2 3 4 5 6 7 8 9 10 11
chemistry Acidic Basic Hydrophobic Neutral Polar
chemistry Acidic Basic Hydrophobic Polar
chemistry Acidic Hydrophobic

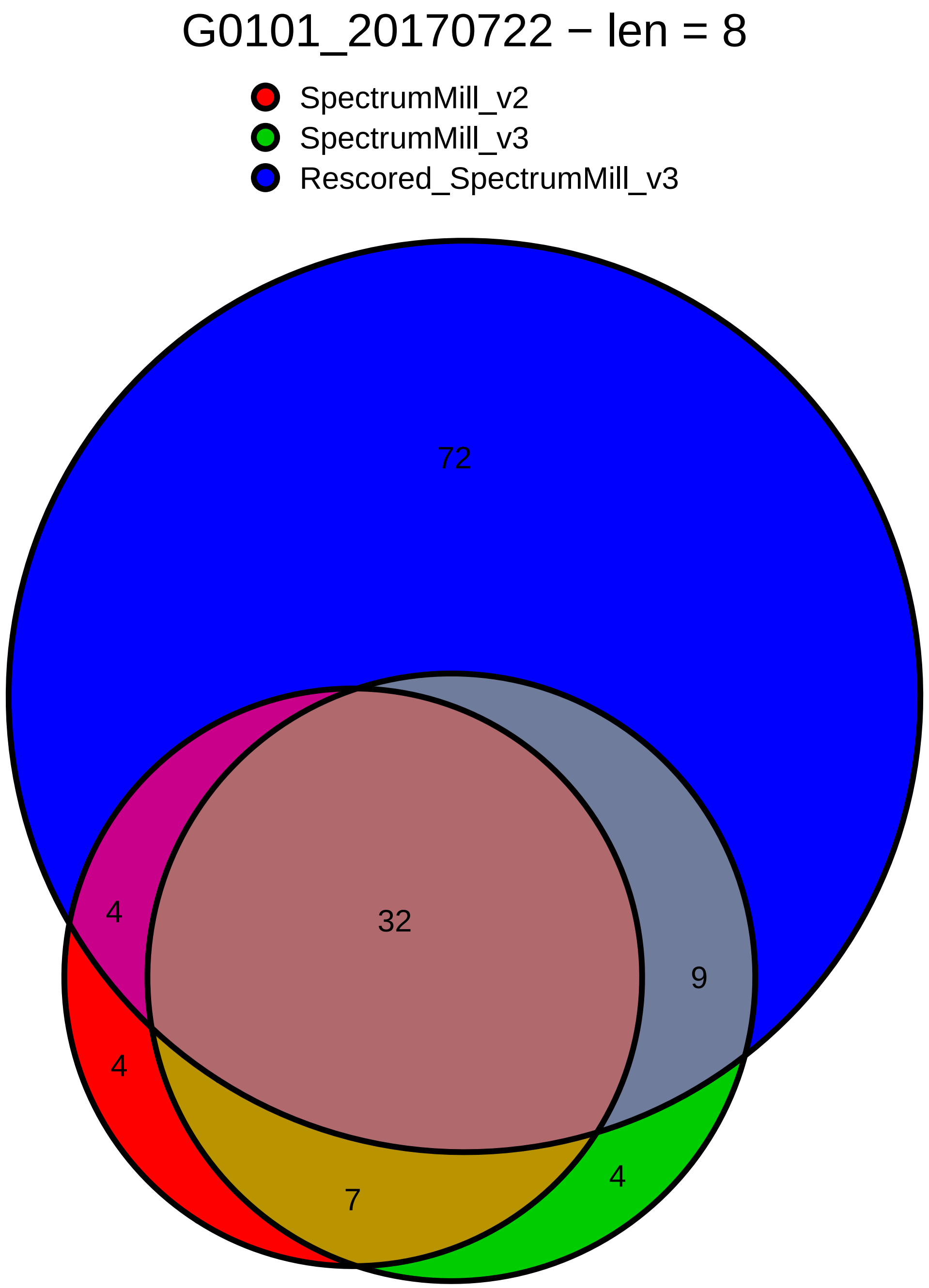
G0101_20170722 − len = 8
SpectrumMill_v2
SpectrumMill_v3
Rescored_SpectrumMill_v3
72
4
32
9
4
7
4

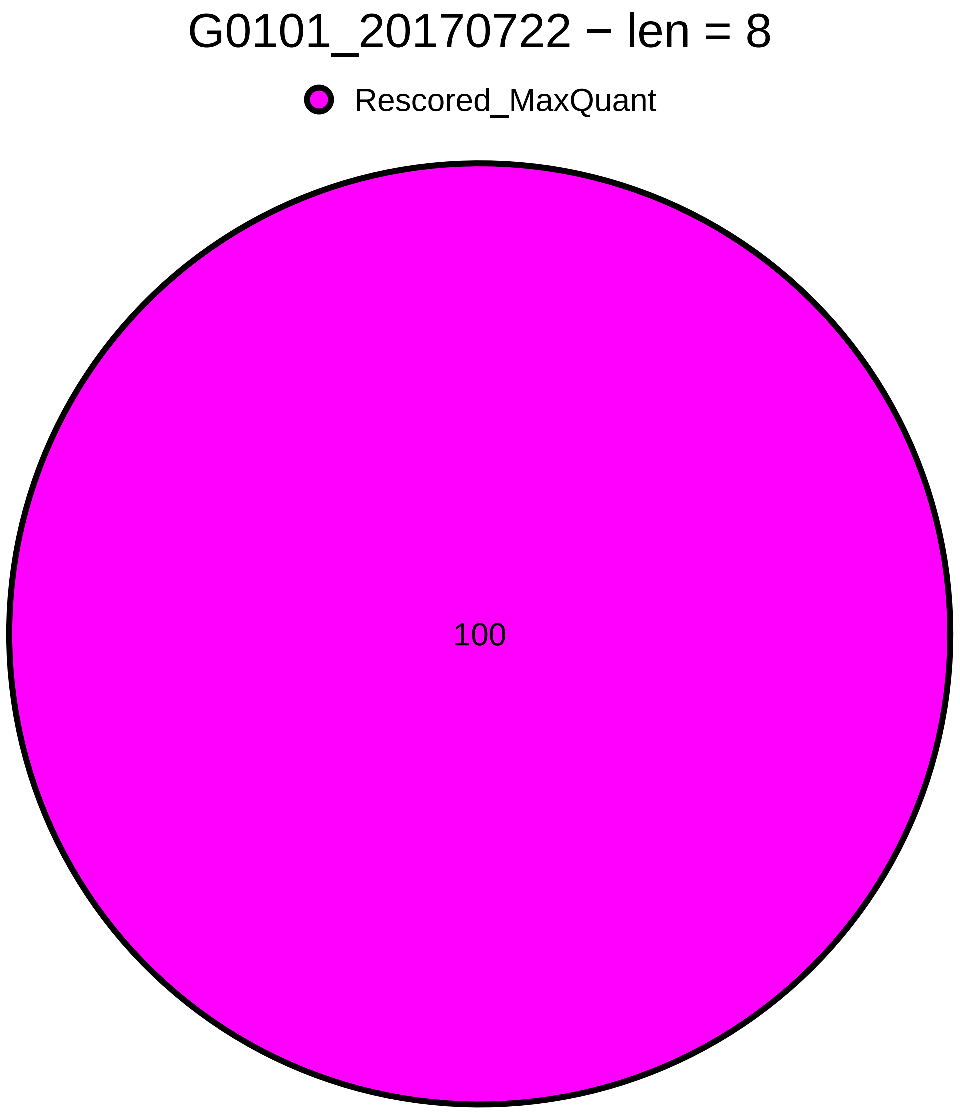
G0101_20170722 − len = 8
Rescored_MaxQuant
100

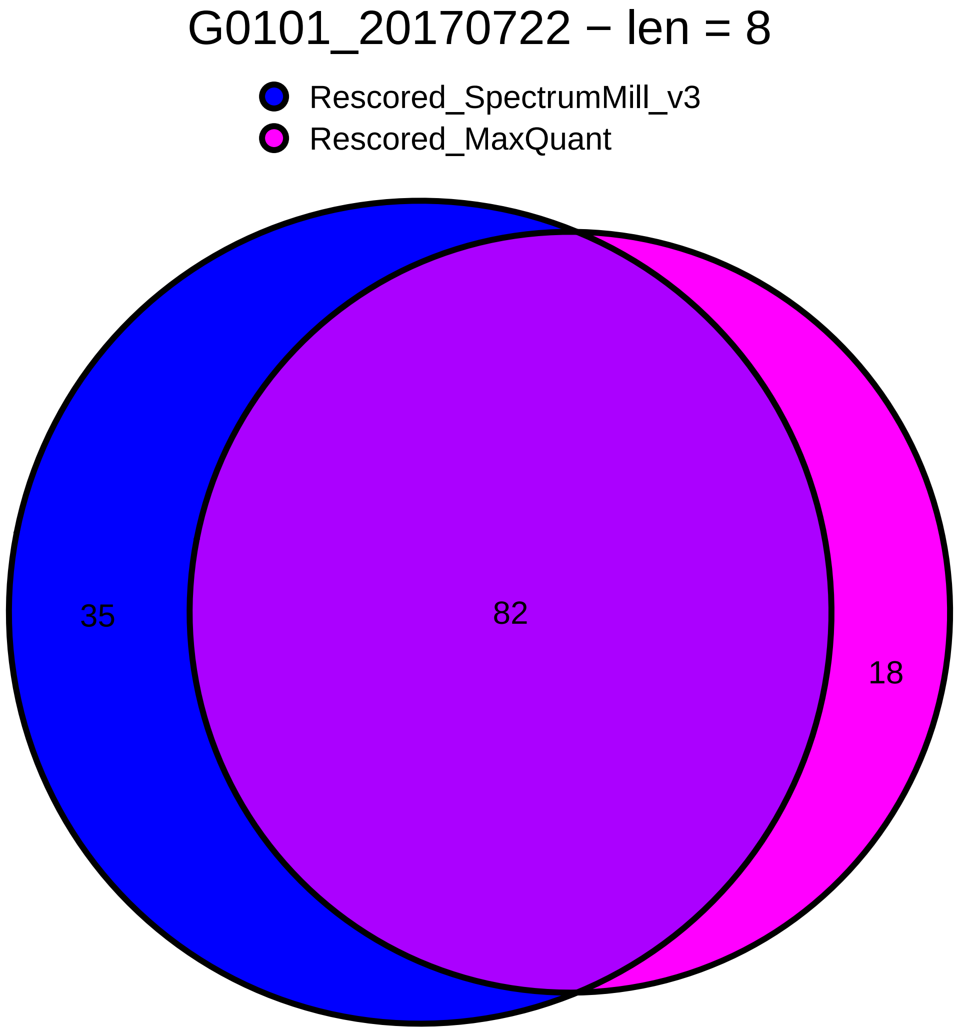
G0101_20170722 − len = 8
Rescored_SpectrumMill_v3
Rescored_MaxQuant
35
82
18

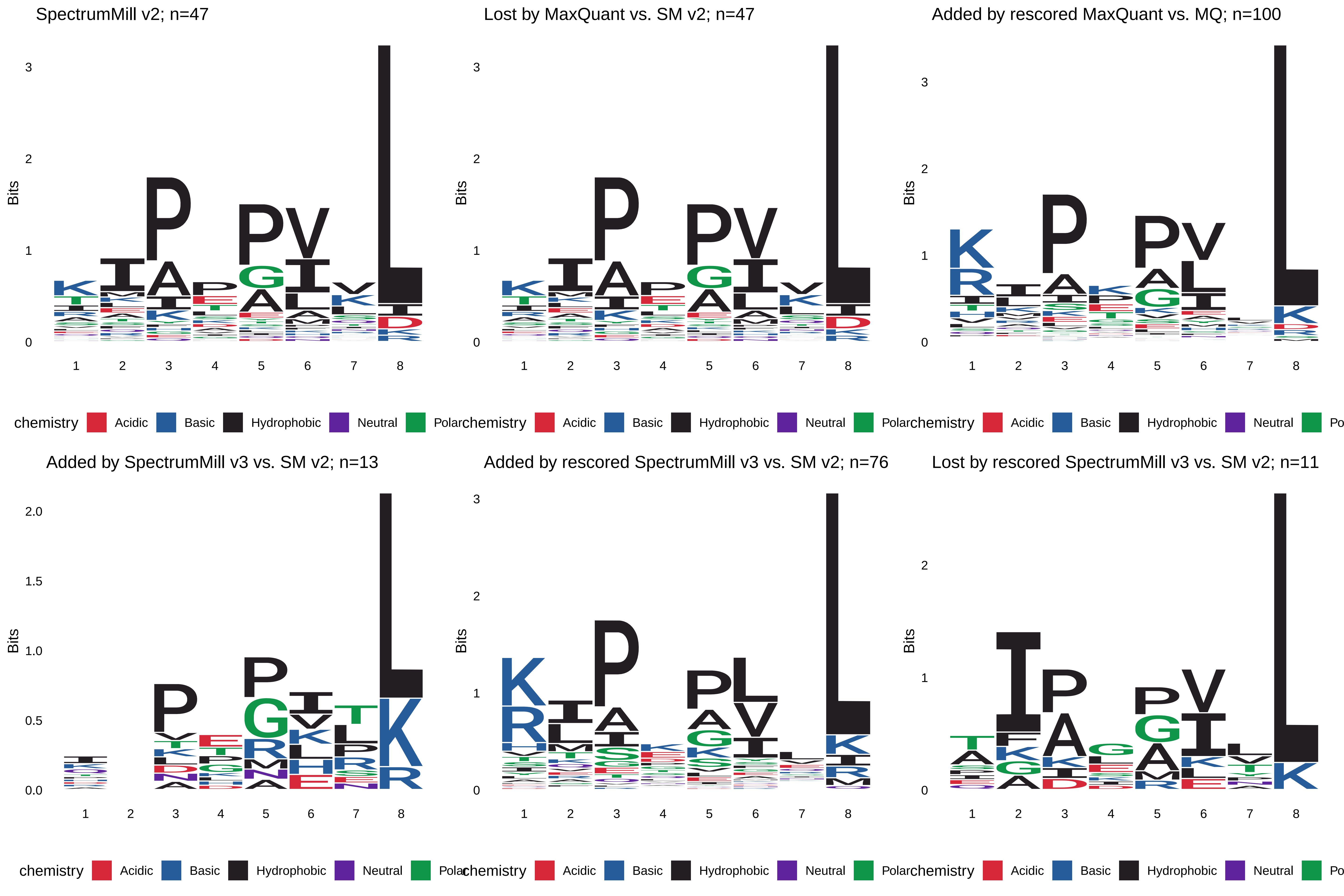
SpectrumMill v2; n=47
Lost by MaxQuant vs. SM v2; n=47
Added by rescored MaxQuant vs. MQ; n=100
Added by SpectrumMill v3 vs. SM v2; n=13
Added by rescored SpectrumMill v3 vs. SM v2; n=76
Lost by rescored SpectrumMill v3 vs. SM v2; n=11
Bits
chemistry
Acidic
Basic
Hydrophobic
Neutral
Polar

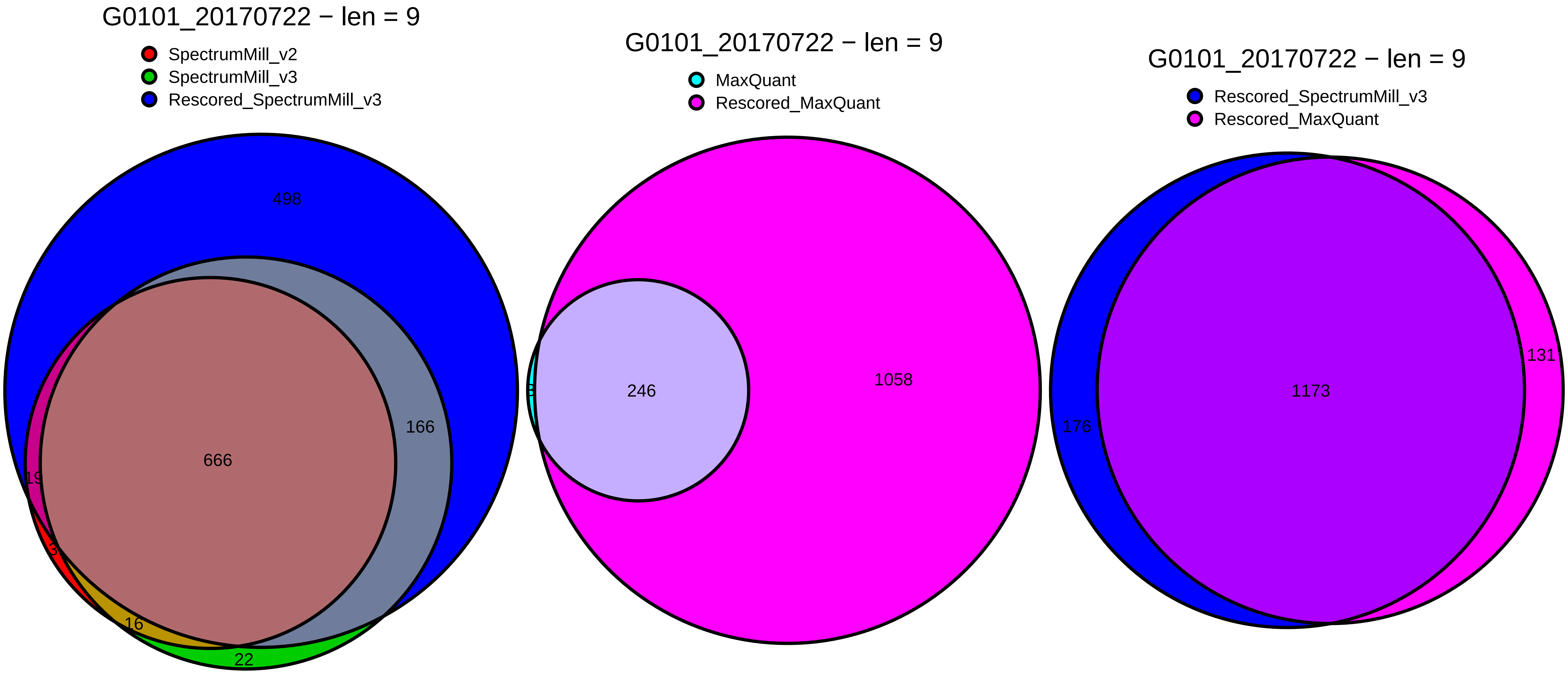

G0101_20170722 − len = 9
SpectrumMill_v2
SpectrumMill_v3
Rescored_SpectrumMill_v3
498
166
666
16
22
G0101_20170722 − len = 9
MaxQuant
Rescored_MaxQuant
246
1058
G0101_20170722 − len = 9
Rescored_SpectrumMill_v3
Rescored_MaxQuant
1173
176
131

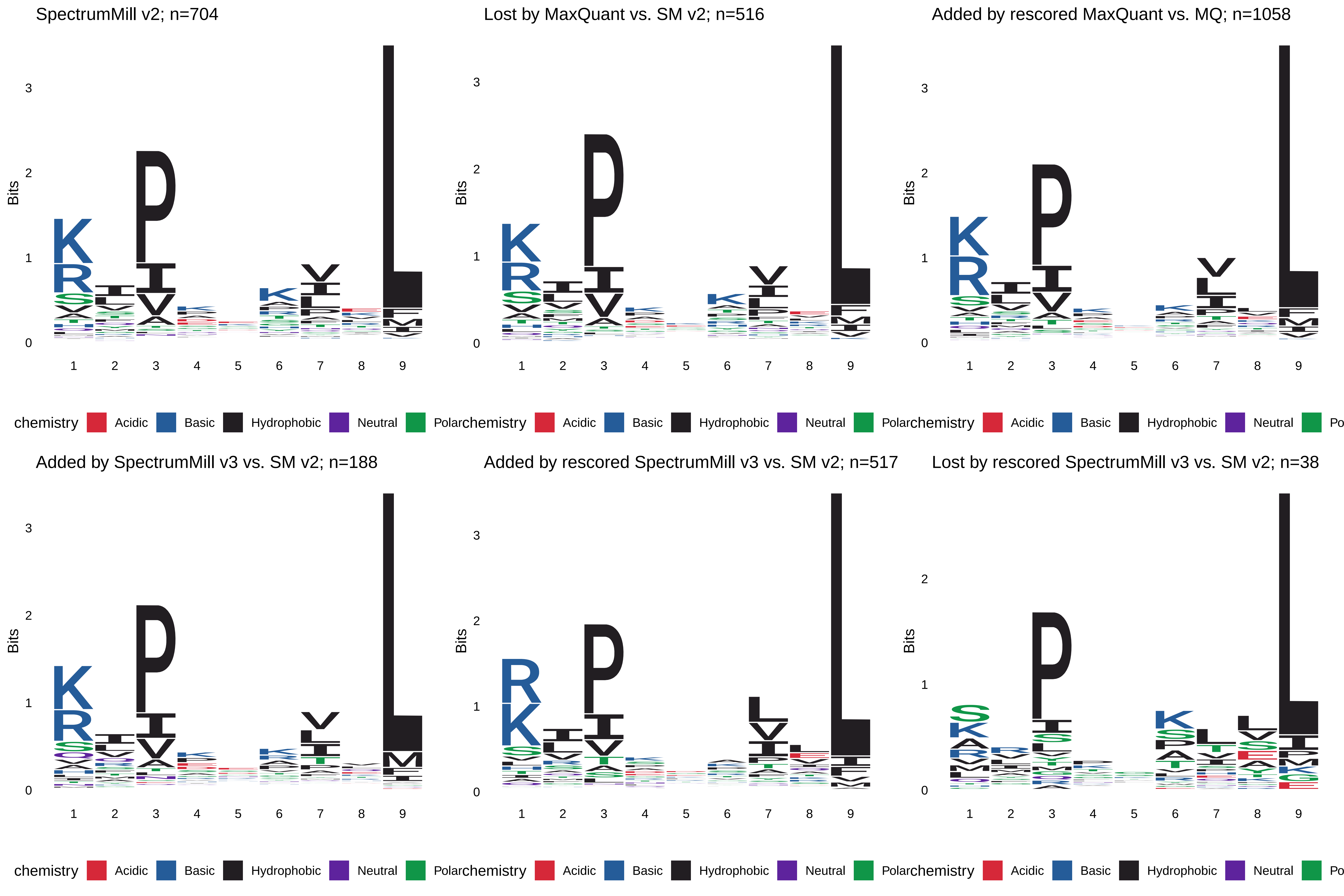

SpectrumMill v2; n=704
Lost by MaxQuant vs. SM v2; n=516
Added by rescored MaxQuant vs. MQ; n=1058
Added by SpectrumMill v3 vs. SM v2; n=188
Added by rescored SpectrumMill v3 vs. SM v2; n=517
Lost by rescored SpectrumMill v3 vs. SM v2; n=38
Bits
0
1
2
3
1
2
3
4
5
6
7
8
9
chemistry
Acidic
Basic
Hydrophobic
Neutral
Polar

G0101_20170722 − len = 10

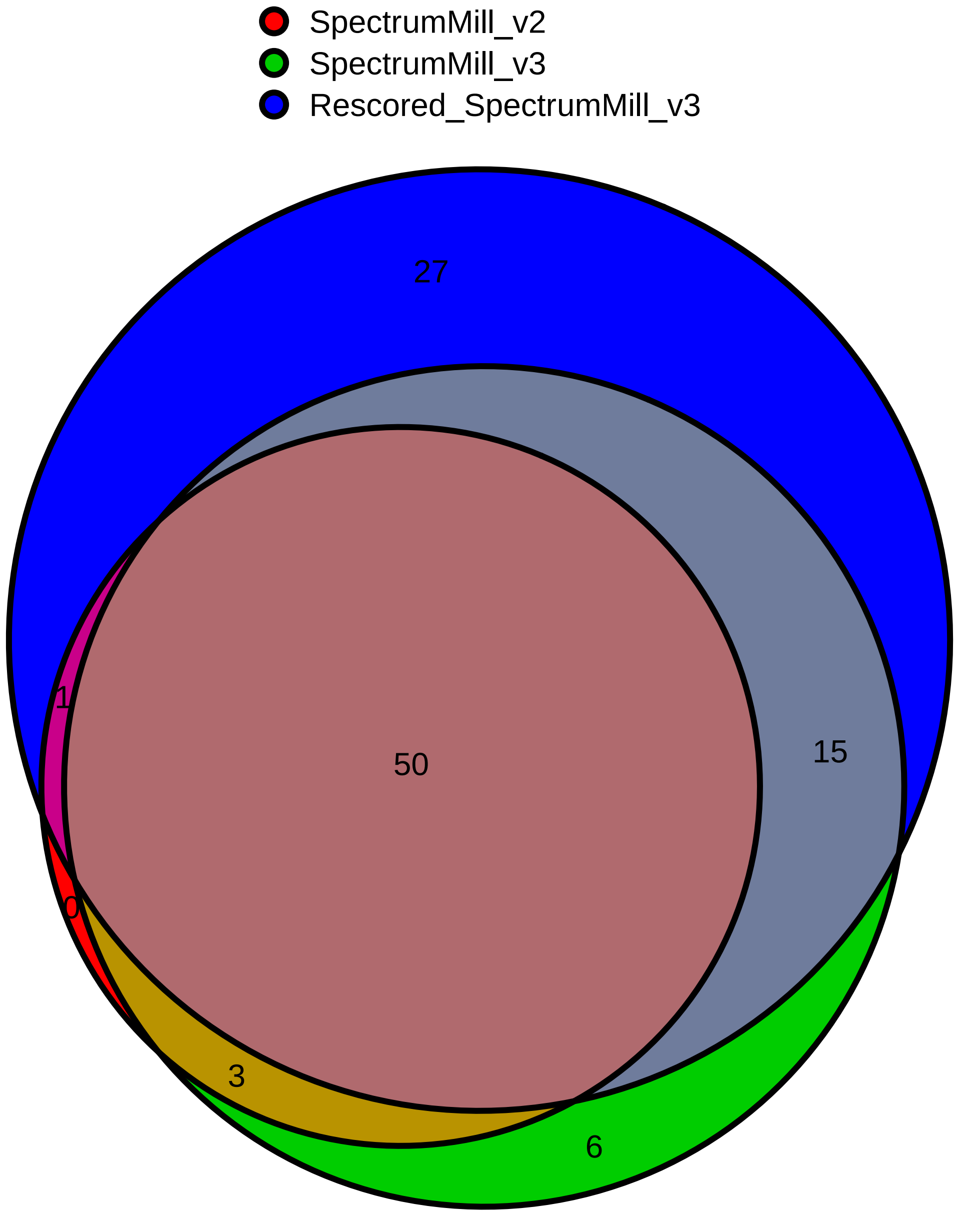

G0101_20170722 − len = 10

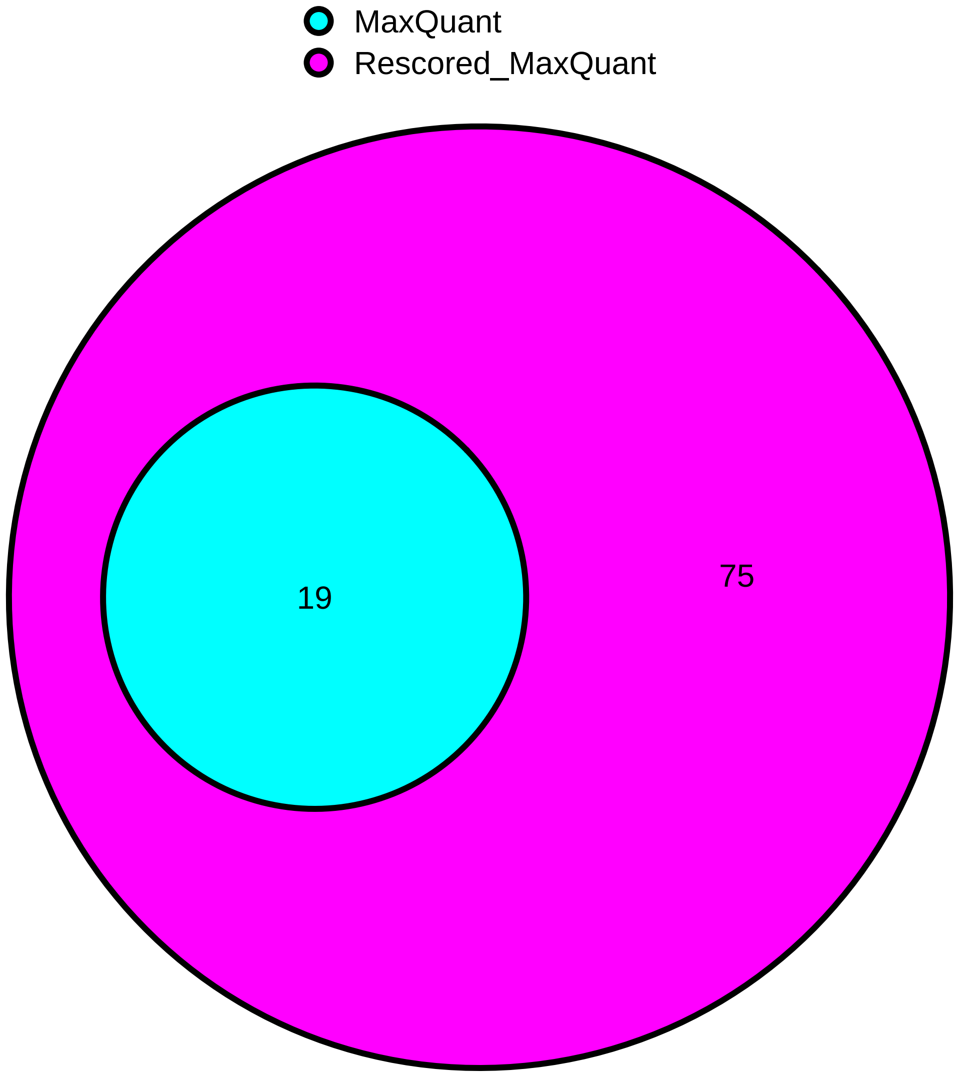

G0101_20170722 − len = 10

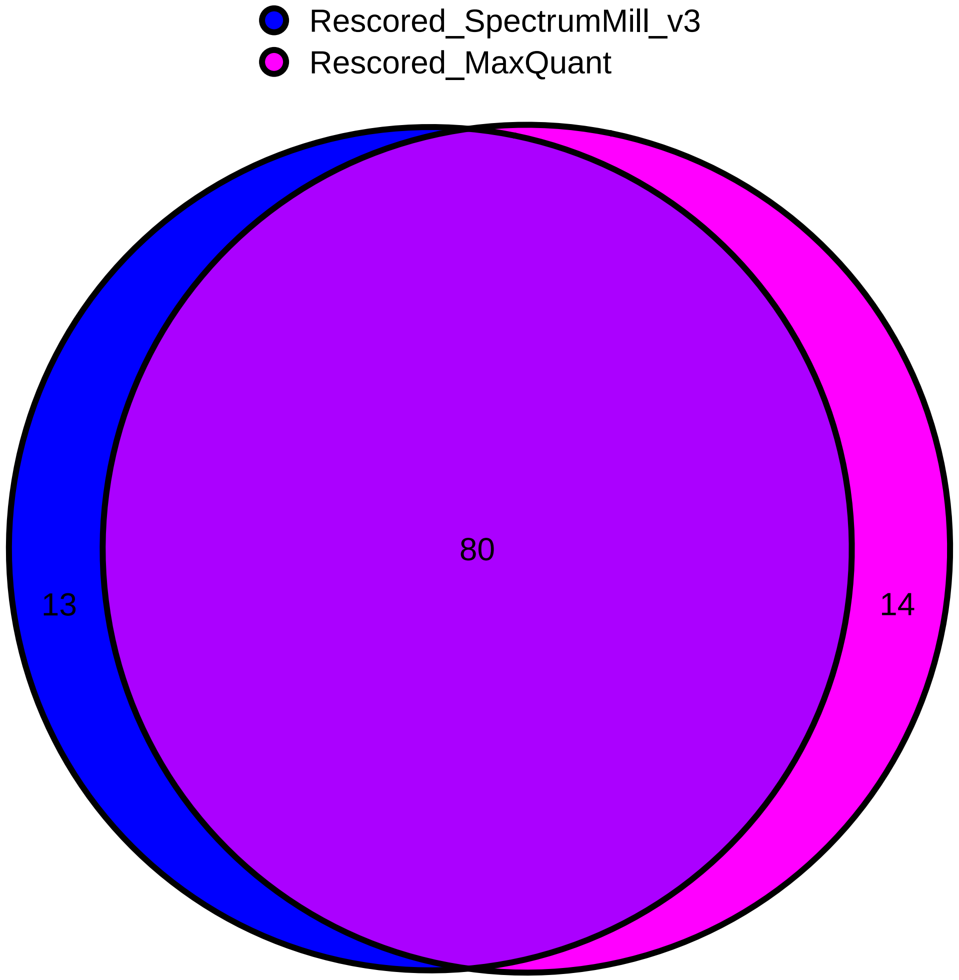

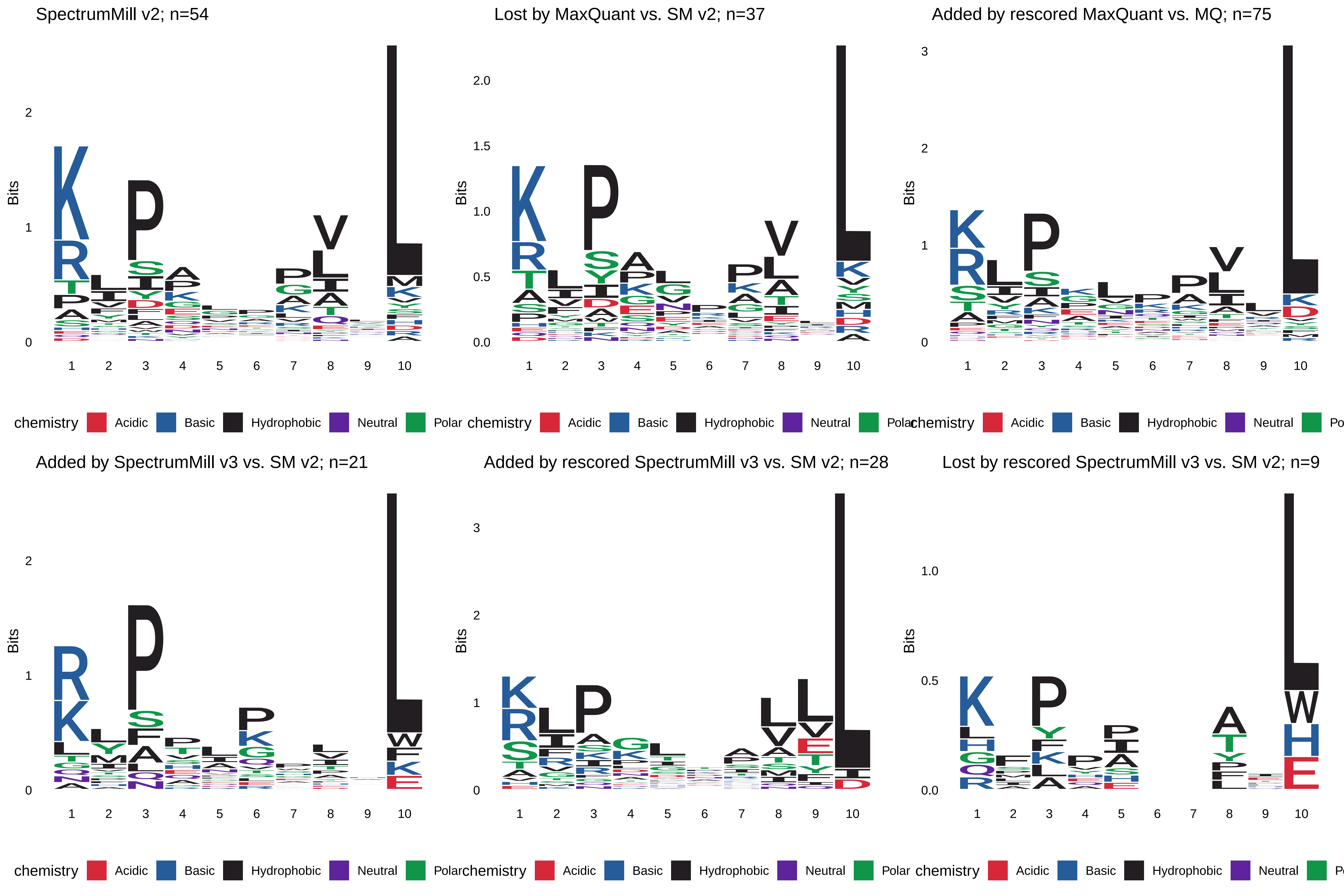
SpectrumMill v2; n=54
Lost by MaxQuant vs. SM v2; n=37
Added by rescored MaxQuant vs. MQ; n=75
Added by SpectrumMill v3 vs. SM v2; n=21
Added by rescored SpectrumMill v3 vs. SM v2; n=28
Lost by rescored SpectrumMill v3 vs. SM v2; n=9
Bits
1 2 3 4 5 6 7 8 9 10
chemistry
Acidic
Basic
Hydrophobic
Neutral
Polar

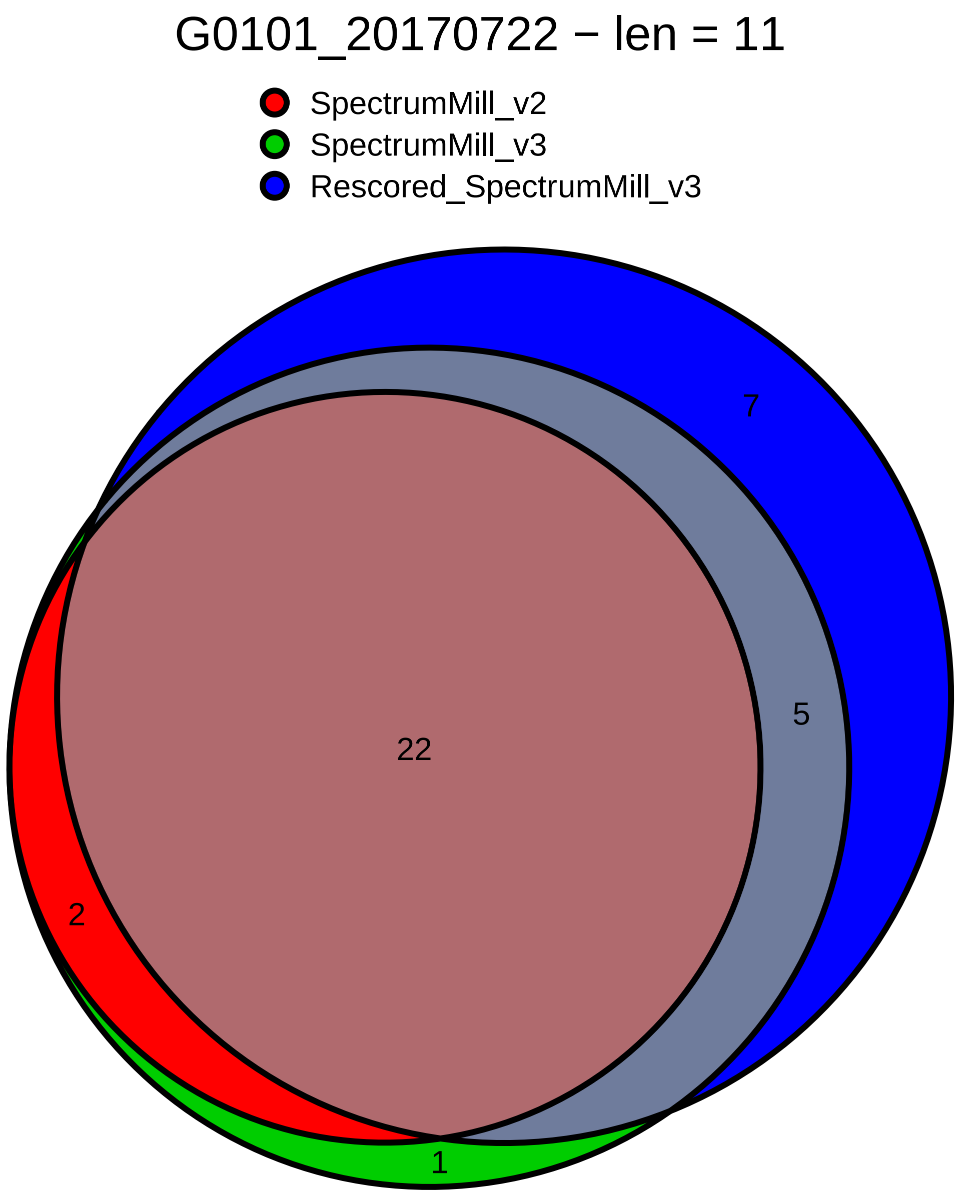
G0101_20170722 − len = 11
SpectrumMill_v2
SpectrumMill_v3
Rescored_SpectrumMill_v3
7
5
22
2
1

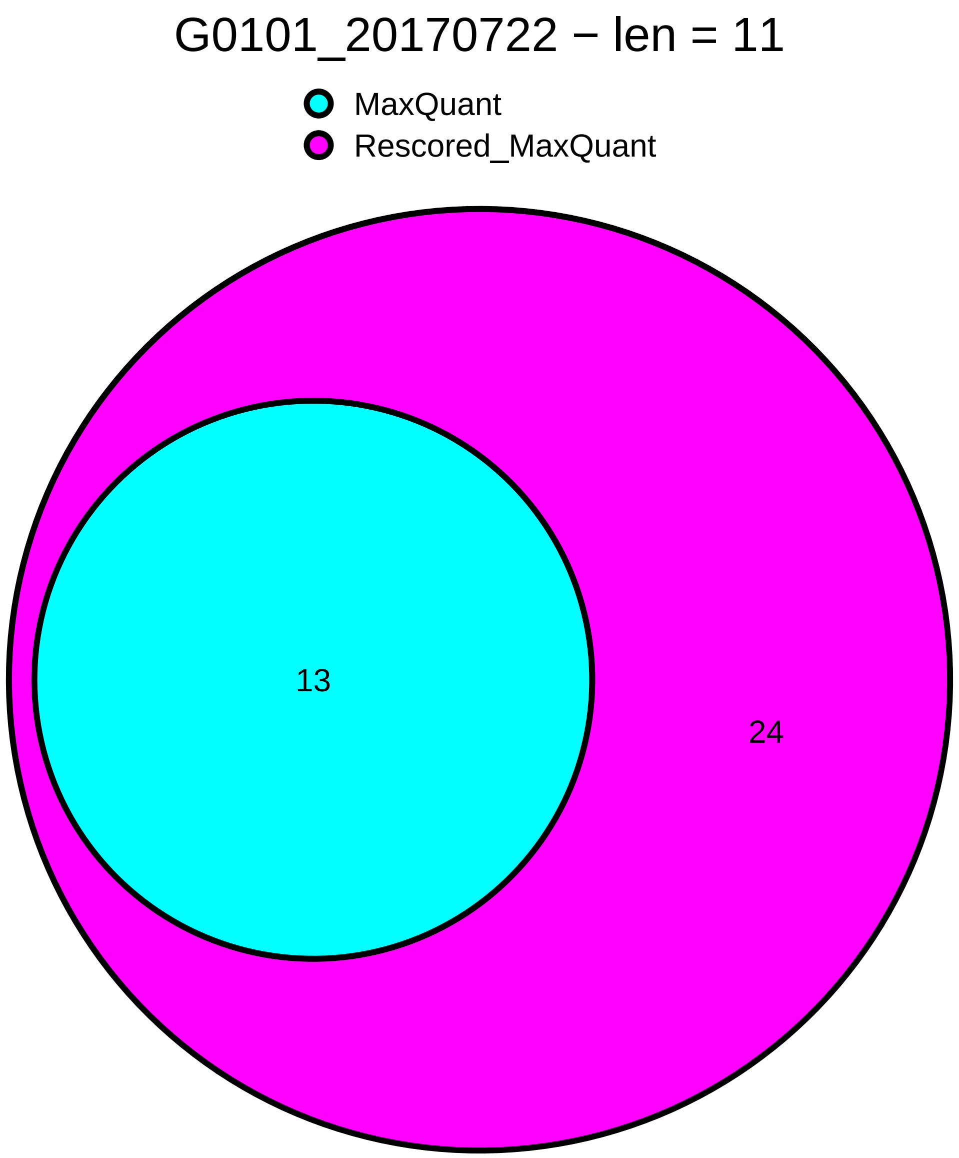
G0101_20170722 − len = 11
MaxQuant
Rescored_MaxQuant
13
24

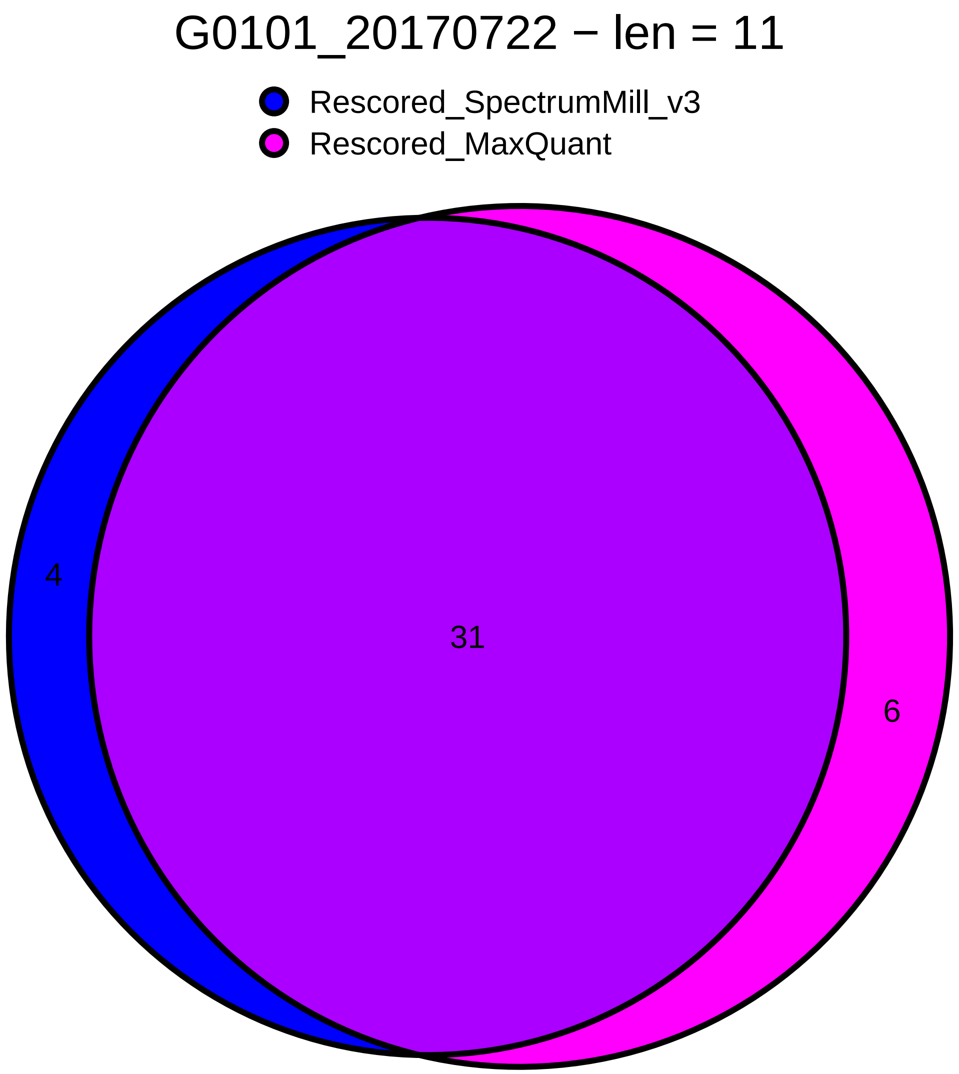
G0101_20170722 − len = 11
Rescored_SpectrumMill_v3
Rescored_MaxQuant
4
31
6

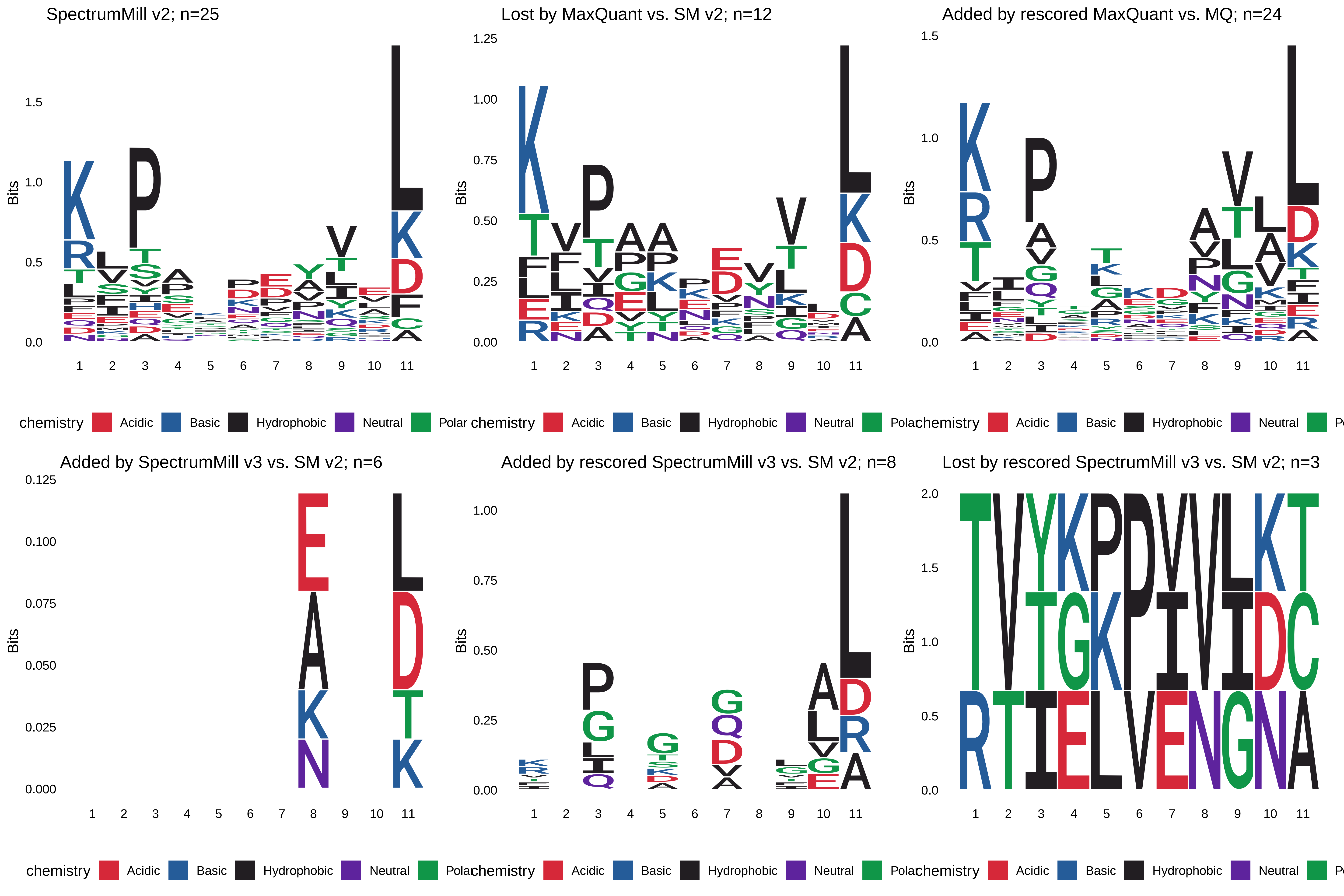

SpectrumMill v2; n=25
Lost by MaxQuant vs. SM v2; n=12
Added by rescored MaxQuant vs. MQ; n=24
Added by SpectrumMill v3 vs. SM v2; n=6
Added by rescored SpectrumMill v3 vs. SM v2; n=8
Lost by rescored SpectrumMill v3 vs. SM v2; n=3
Bits
1 2 3 4 5 6 7 8 9 10 11
chemistry
Acidic
Basic
Hydrophobic
Neutral
Polar

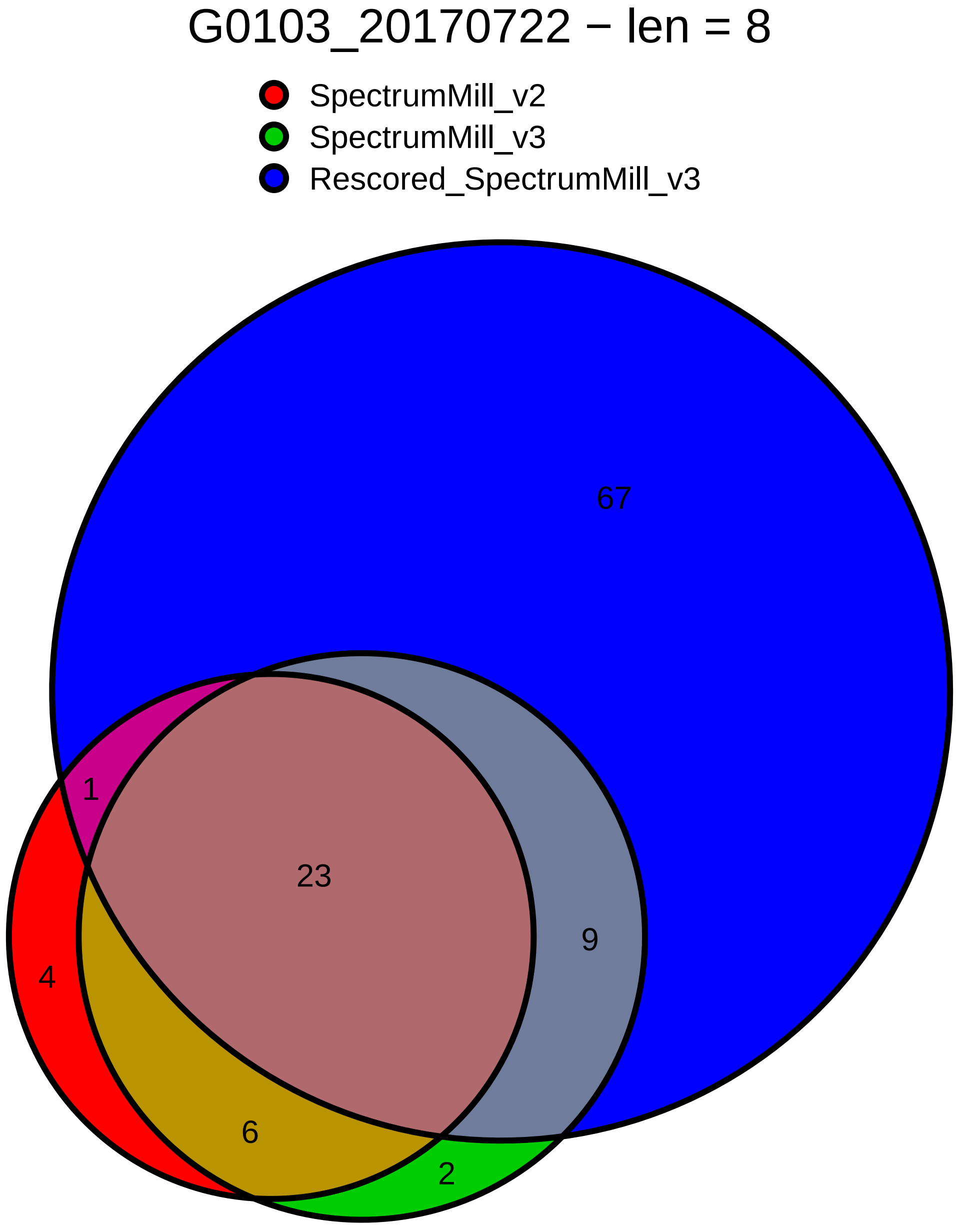
G0103_20170722 − len = 8
SpectrumMill_v2
SpectrumMill_v3
Rescored_SpectrumMill_v3
67
1
23
9
4
6
2

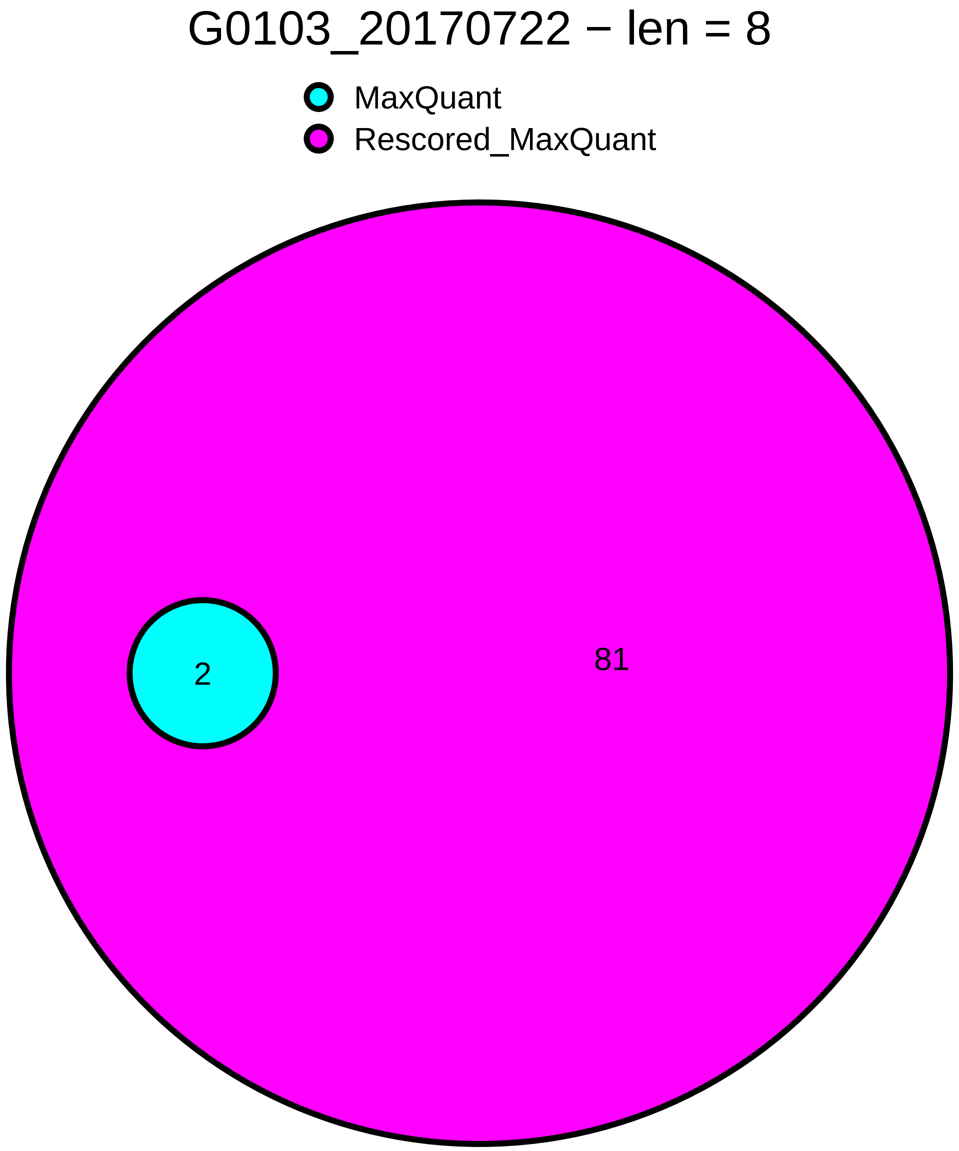
G0103_20170722 − len = 8
MaxQuant
Rescored_MaxQuant
2
81

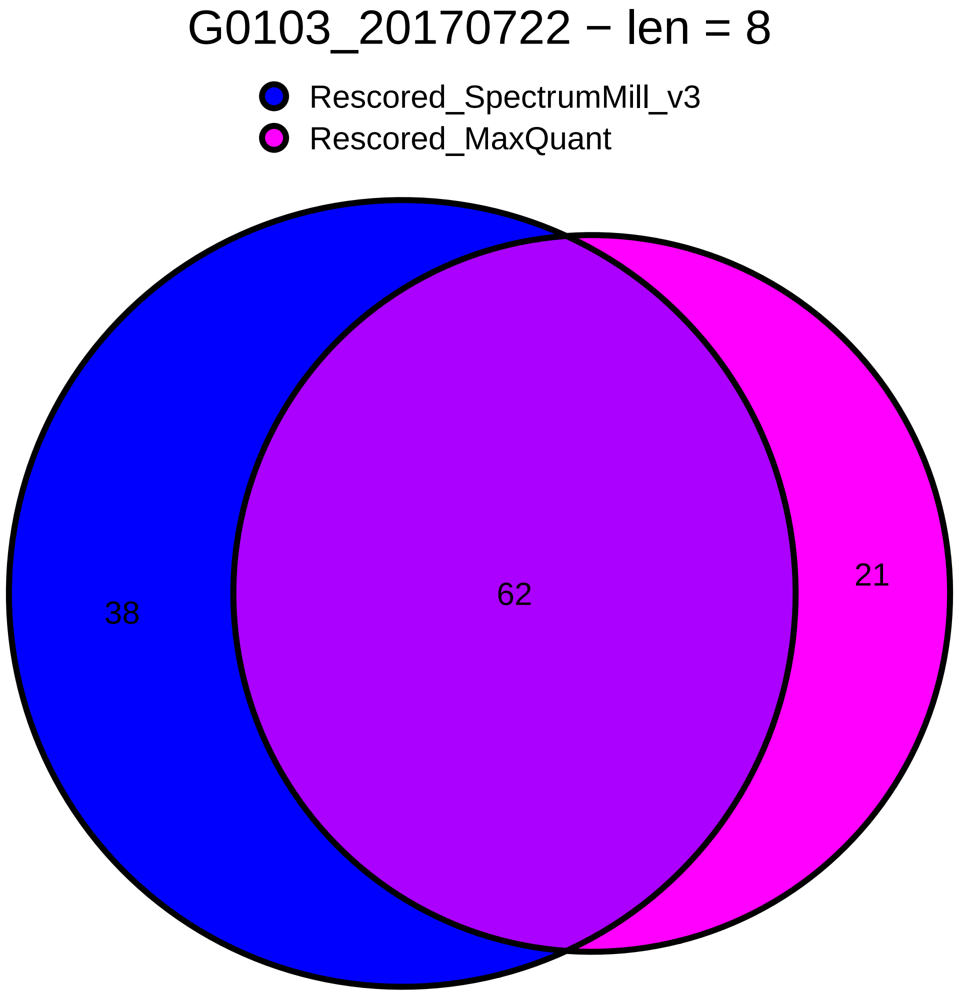
G0103_20170722 − len = 8
Rescored_SpectrumMill_v3
Rescored_MaxQuant
38
62
21

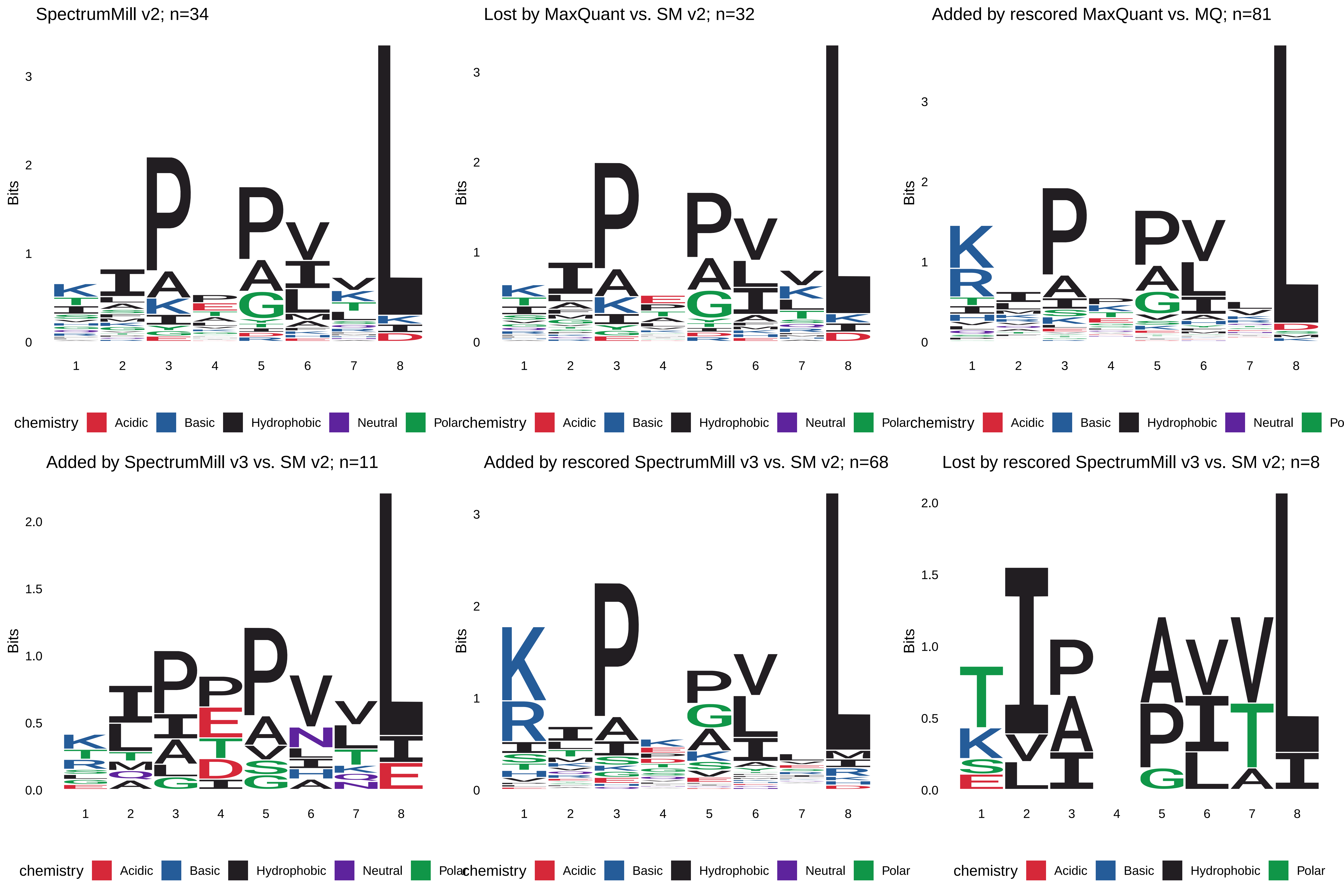

SpectrumMill v2; n=34
Lost by MaxQuant vs. SM v2; n=32
Added by rescored MaxQuant vs. MQ; n=81
Added by SpectrumMill v3 vs. SM v2; n=11
Added by rescored SpectrumMill v3 vs. SM v2; n=68
Lost by rescored SpectrumMill v3 vs. SM v2; n=8
Bits
0
0.0
0.5
1
1.0
1.5
2
2.0
3
1
2
3
4
5
6
7
8
chemistry
Acidic
Basic
Hydrophobic
Neutral
Polar

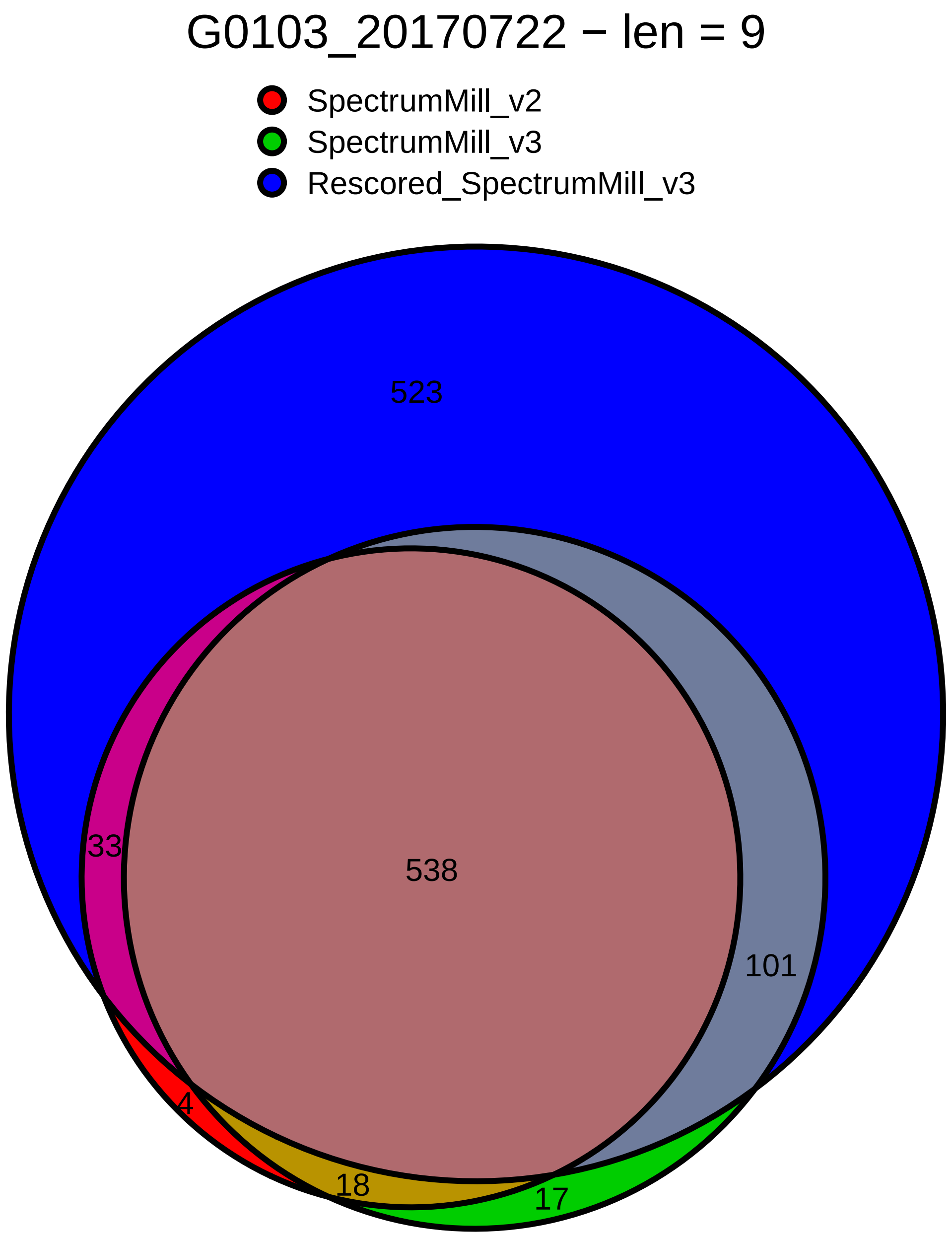
G0103_20170722 − len = 9
SpectrumMill_v2
SpectrumMill_v3
Rescored_SpectrumMill_v3
523
33
538
101
4
18
17

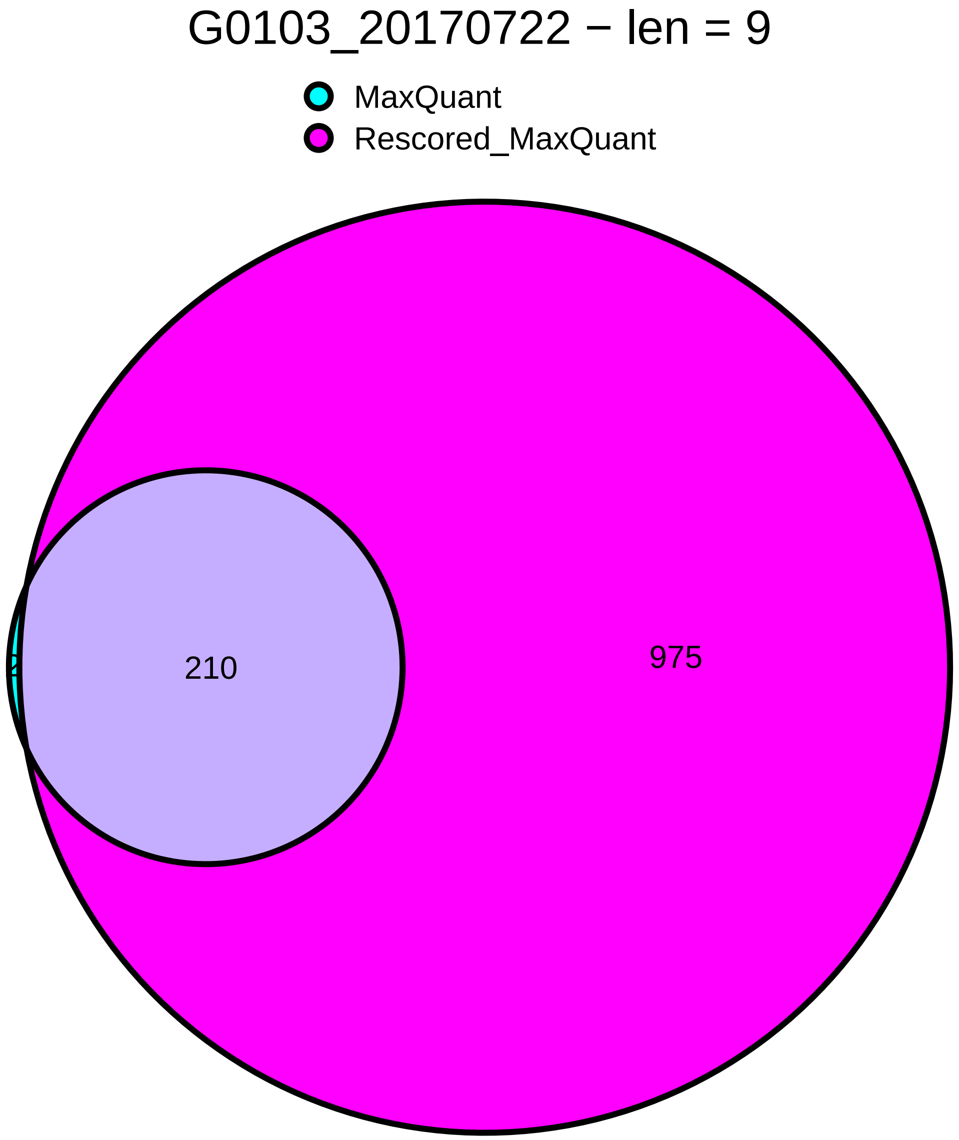
G0103_20170722 − len = 9
MaxQuant
Rescored_MaxQuant
210
975

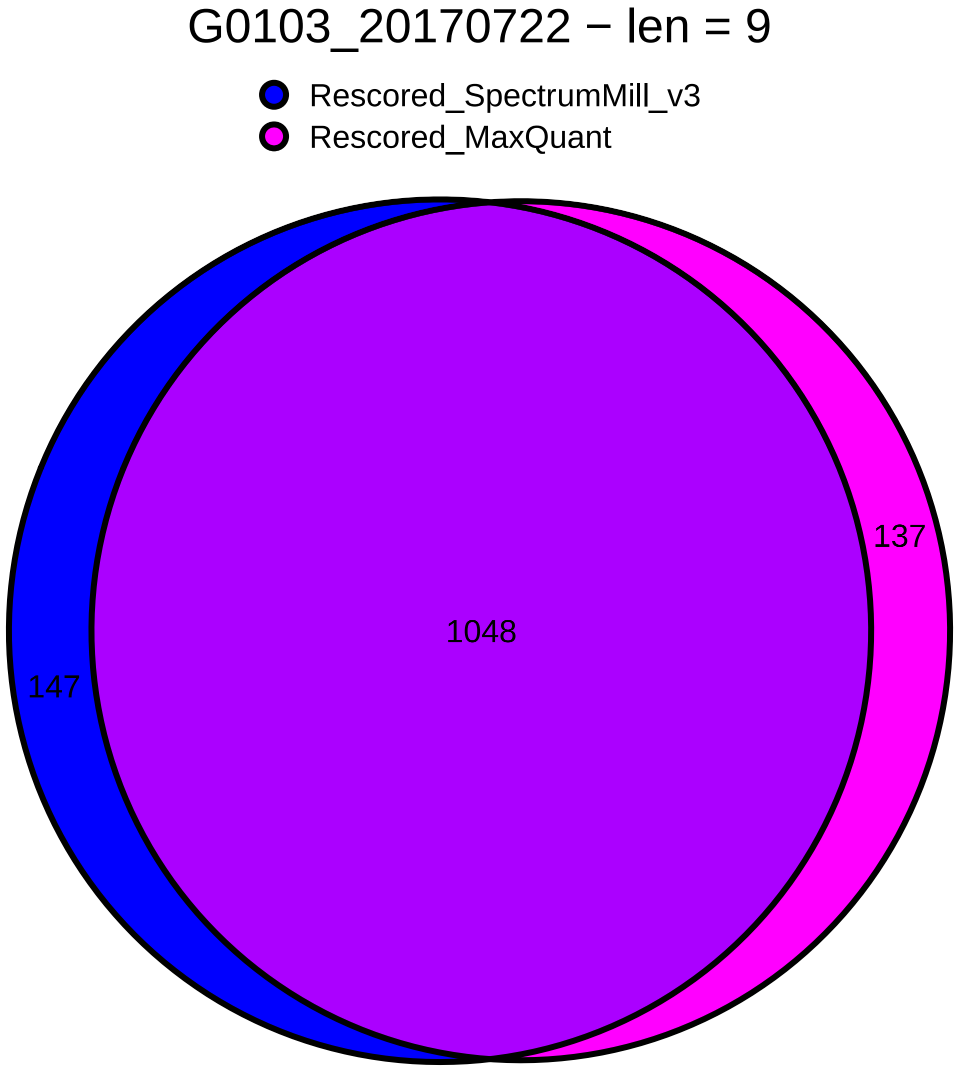
G0103_20170722 − len = 9
Rescored_SpectrumMill_v3
Rescored_MaxQuant
137
1048
147

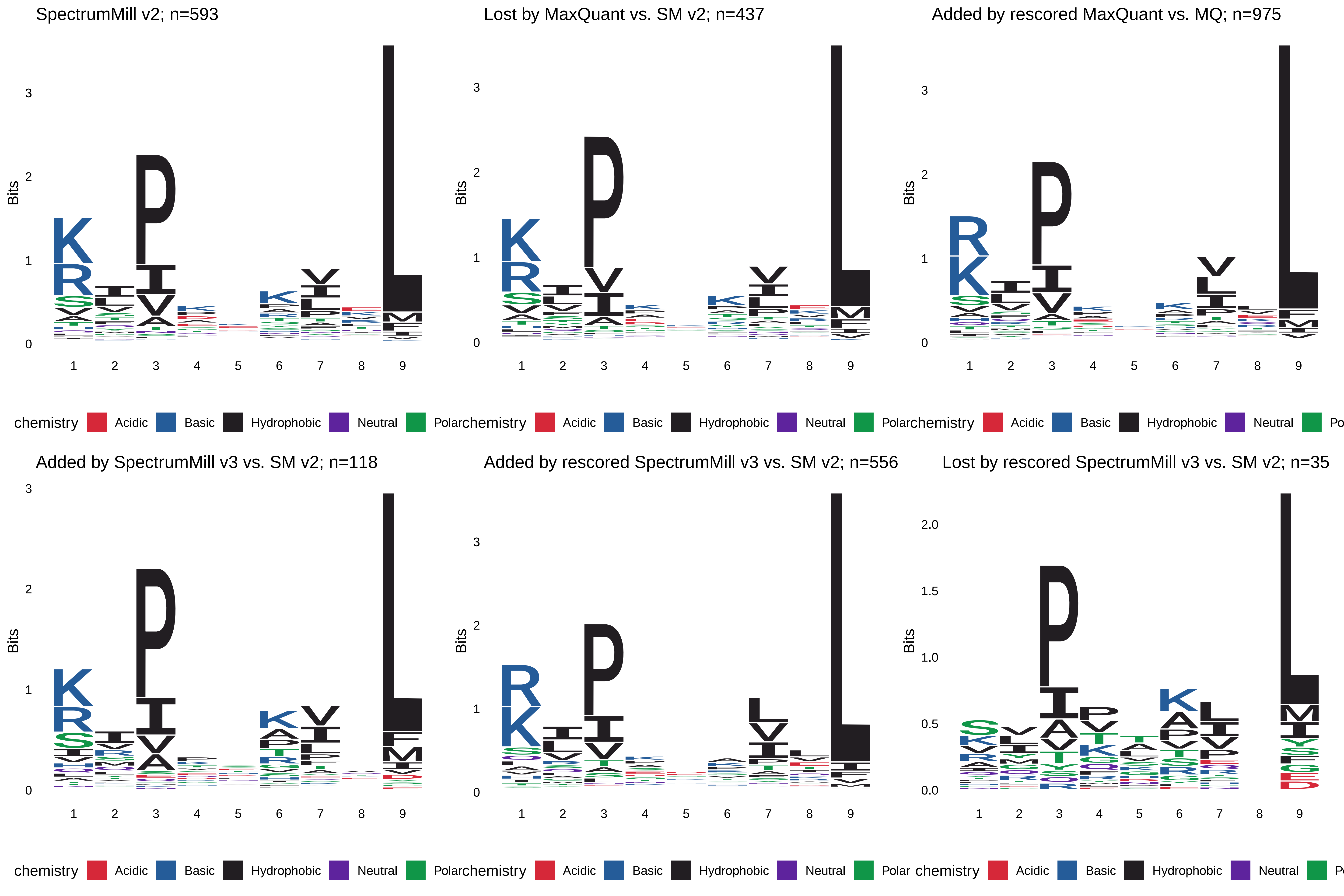
SpectrumMill v2; n=593
Lost by MaxQuant vs. SM v2; n=437
Added by rescored MaxQuant vs. MQ; n=975
Added by SpectrumMill v3 vs. SM v2; n=118
Added by rescored SpectrumMill v3 vs. SM v2; n=556
Lost by rescored SpectrumMill v3 vs. SM v2; n=35
Bits
1 2 3 4 5 6 7 8 9
chemistry
Acidic
Basic
Hydrophobic
Neutral
Polar

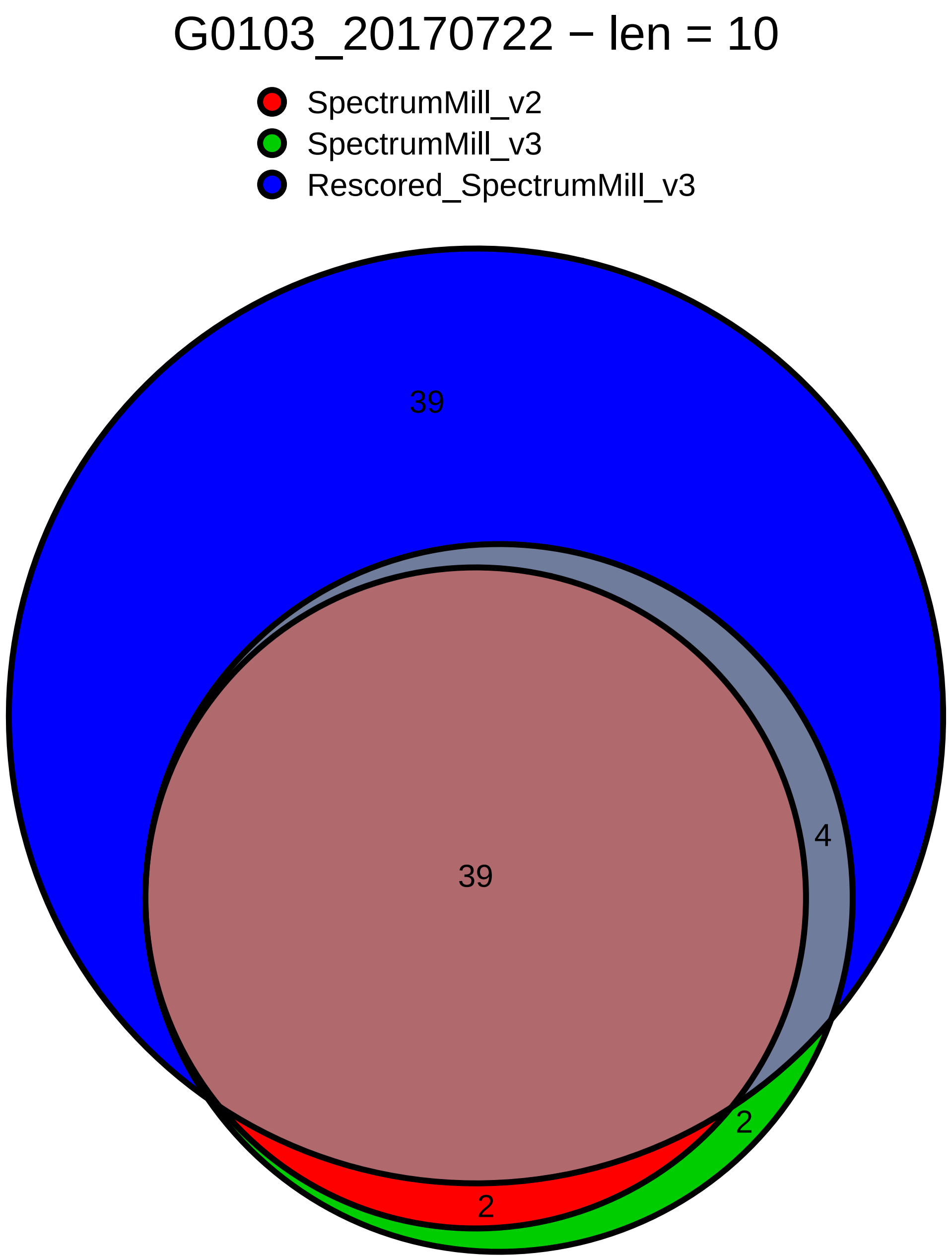
G0103_20170722 − len = 10
SpectrumMill_v2
SpectrumMill_v3
Rescored_SpectrumMill_v3
39
4
39
2
2

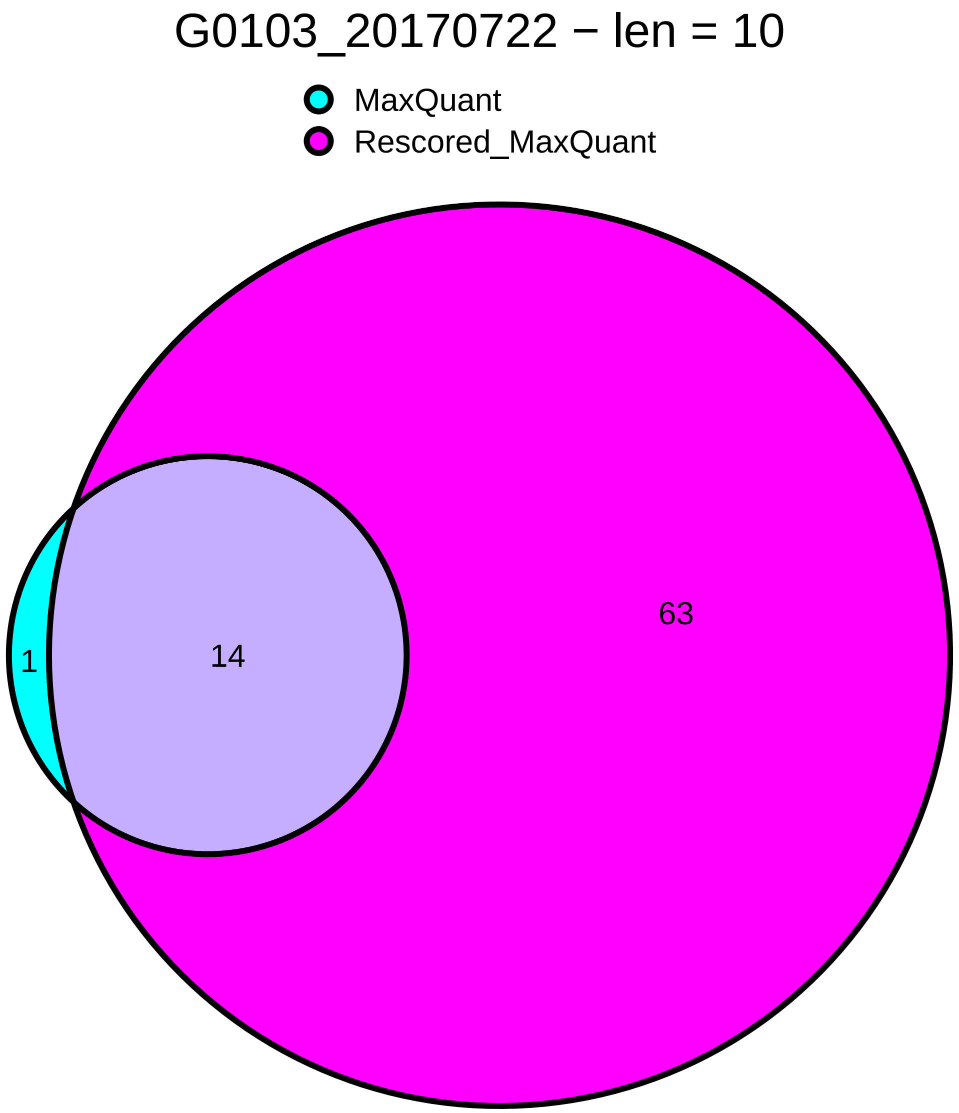
G0103_20170722 − len = 10
MaxQuant
Rescored_MaxQuant
1
14
63

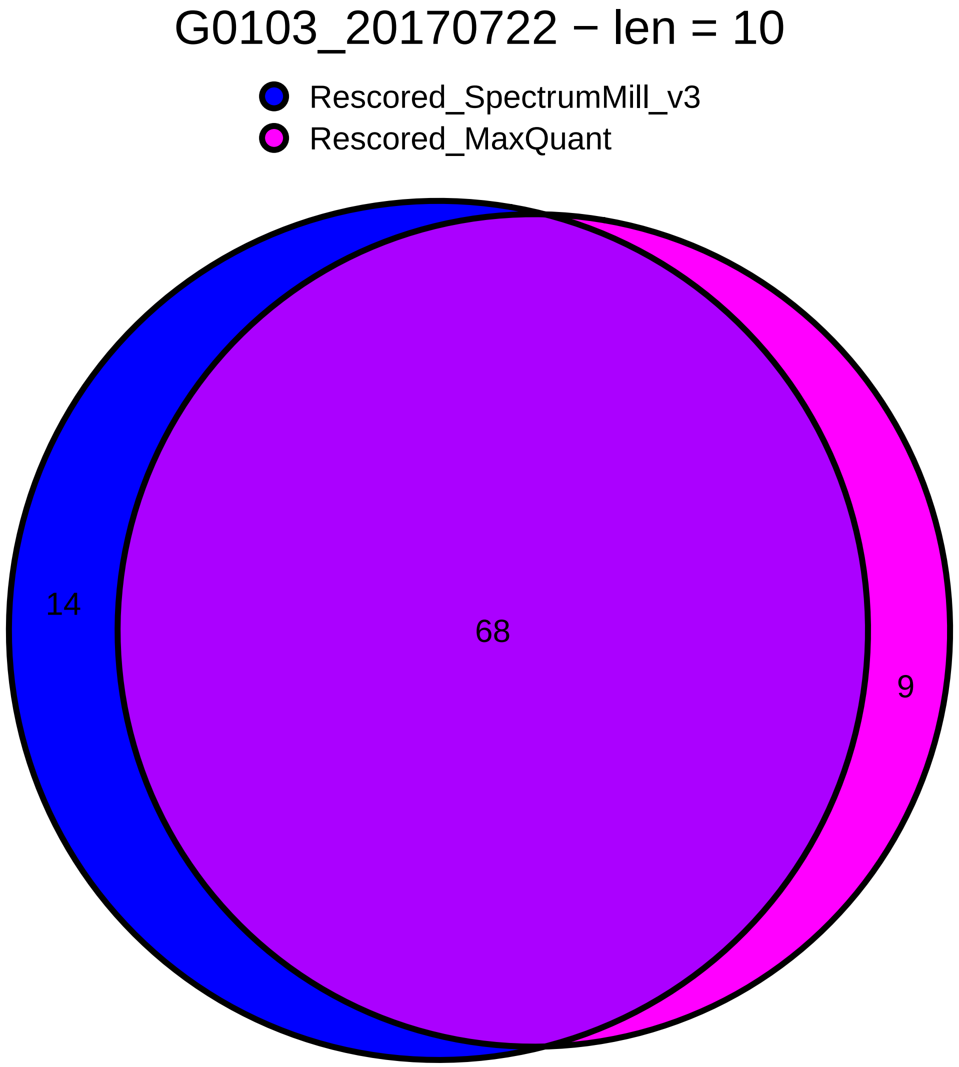
G0103_20170722 − len = 10
Rescored_SpectrumMill_v3
Rescored_MaxQuant
14
68
9

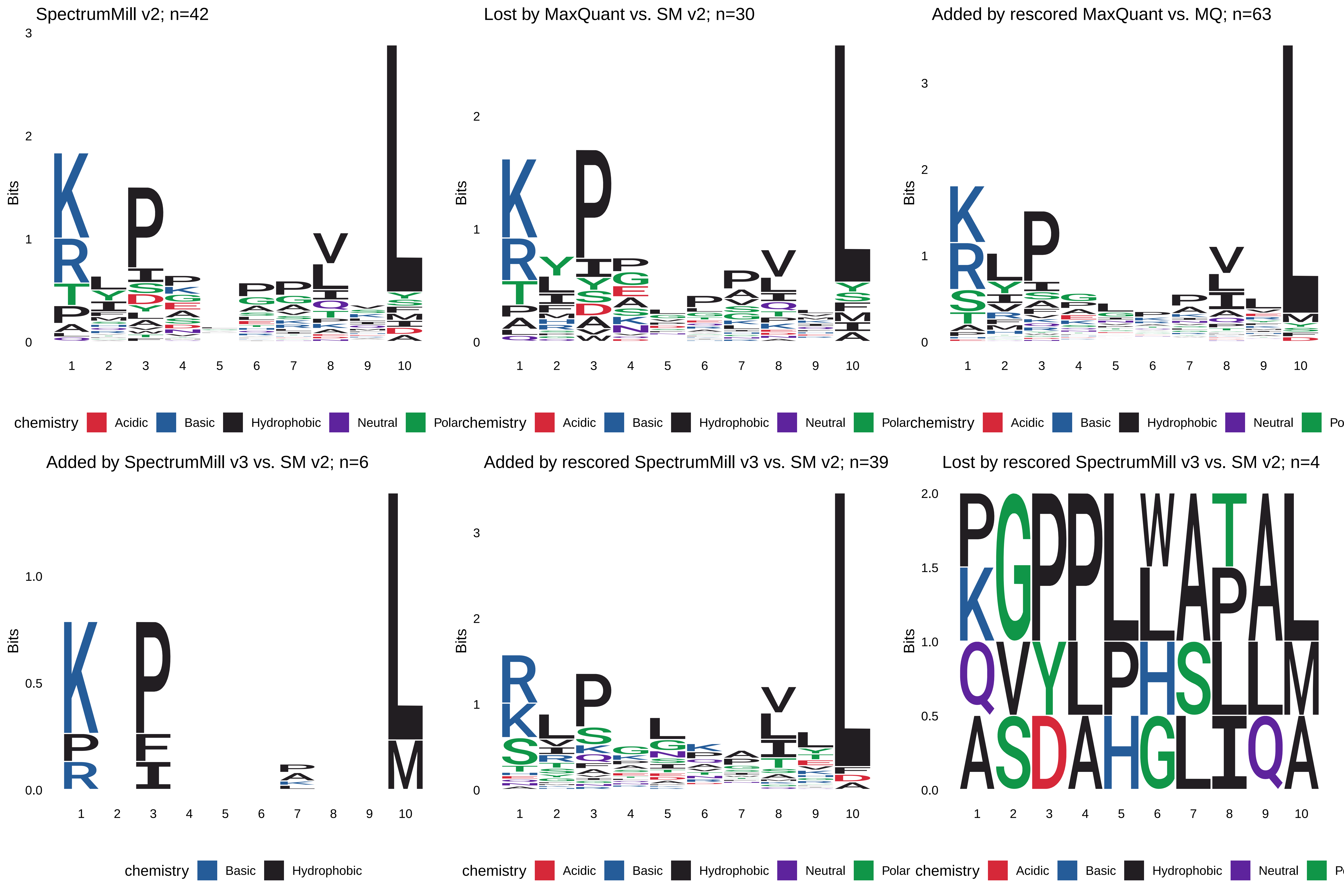
SpectrumMill v2; n=42
Lost by MaxQuant vs. SM v2; n=30
Added by rescored MaxQuant vs. MQ; n=63
Bits
1 2 3 4 5 6 7 8 9 10
chemistry Acidic Basic Hydrophobic Neutral Polarchemistry Acidic Basic Hydrophobic Neutral Polarchemistry Acidic Basic Hydrophobic Neutral Po
Added by SpectrumMill v3 vs. SM v2; n=6
Added by rescored SpectrumMill v3 vs. SM v2; n=39
Lost by rescored SpectrumMill v3 vs. SM v2; n=4
chemistry Basic Hydrophobic
chemistry Acidic Basic Hydrophobic Neutral Polar chemistry Acidic Basic Hydrophobic Neutral P

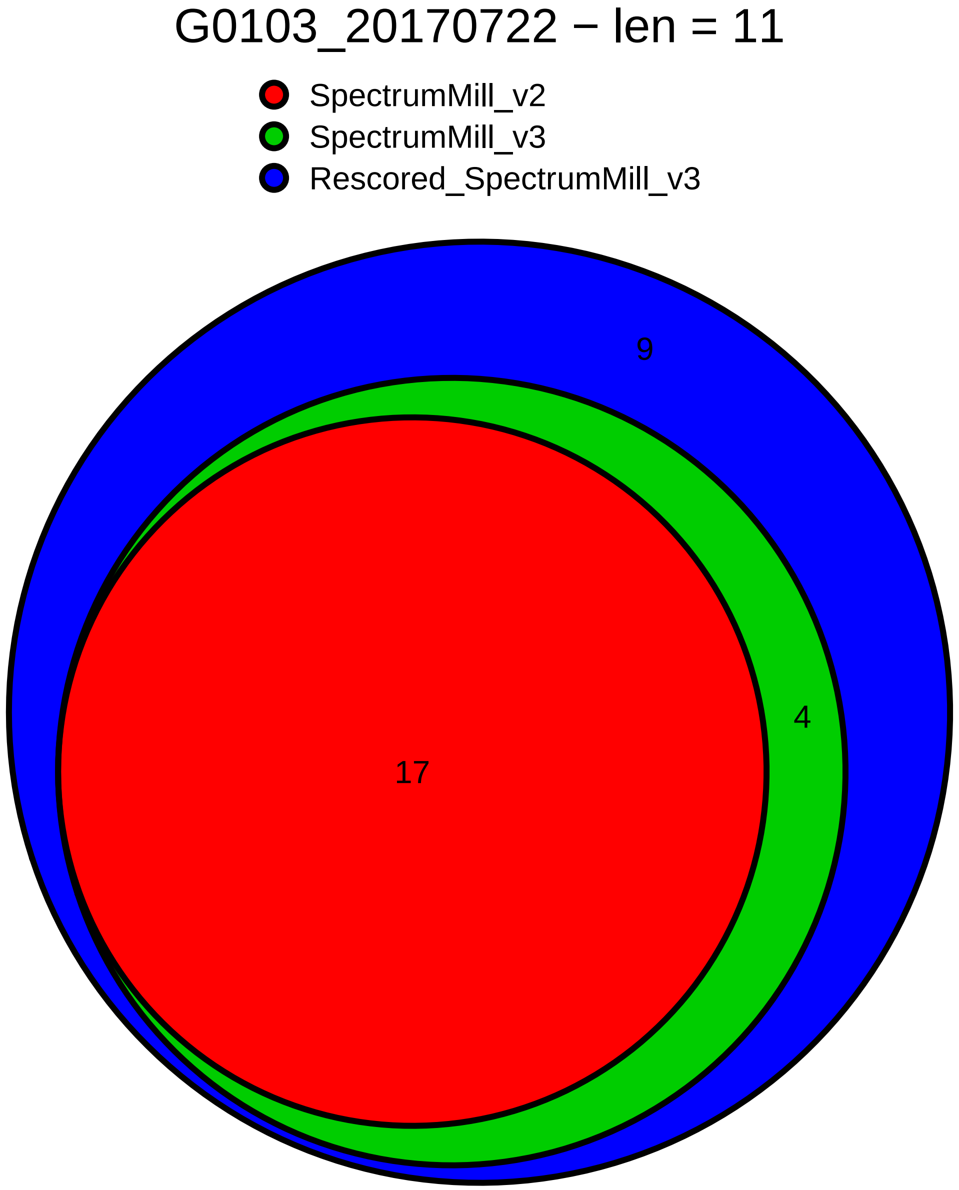
G0103_20170722 − len = 11
SpectrumMill_v2
SpectrumMill_v3
Rescored_SpectrumMill_v3
9
4
17

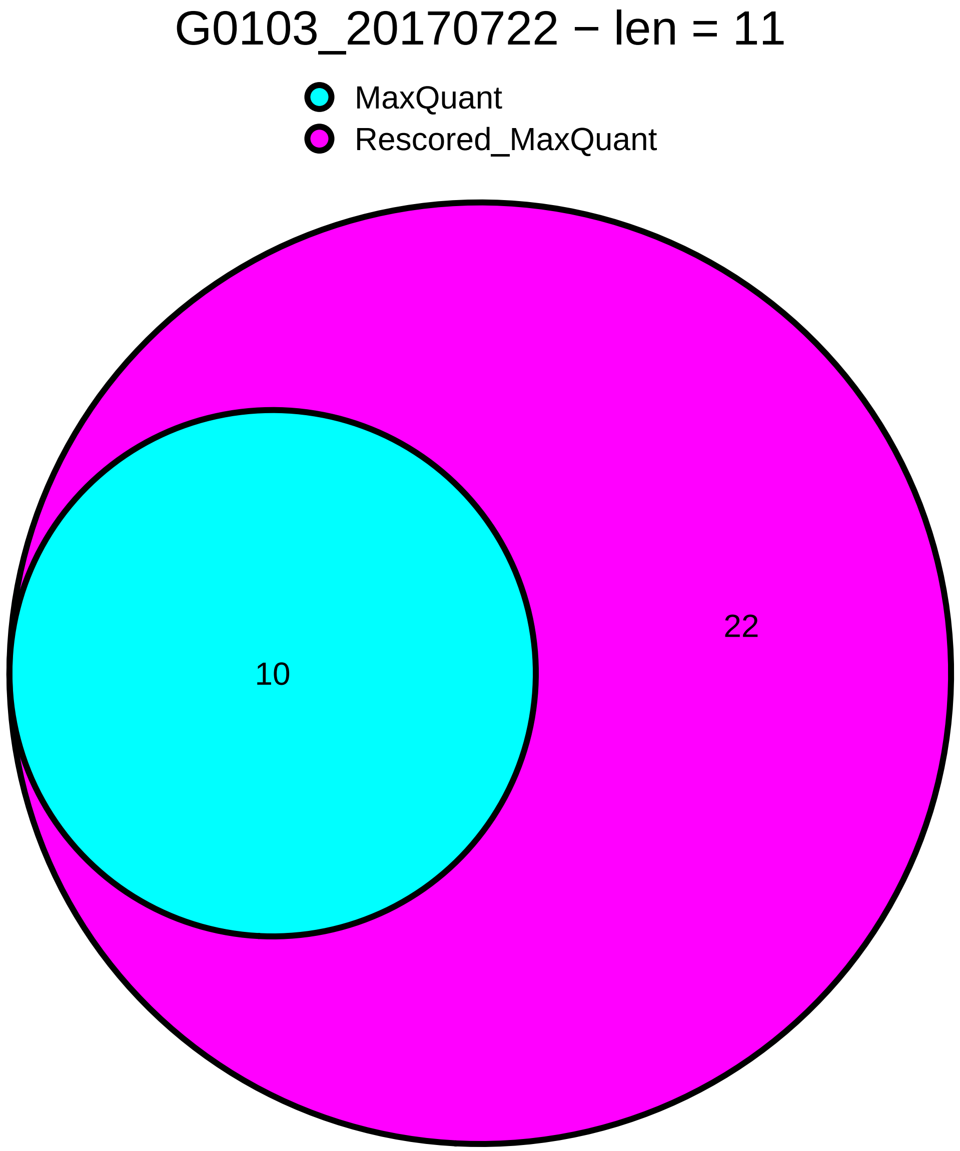
G0103_20170722 − len = 11
MaxQuant
Rescored_MaxQuant
10
22

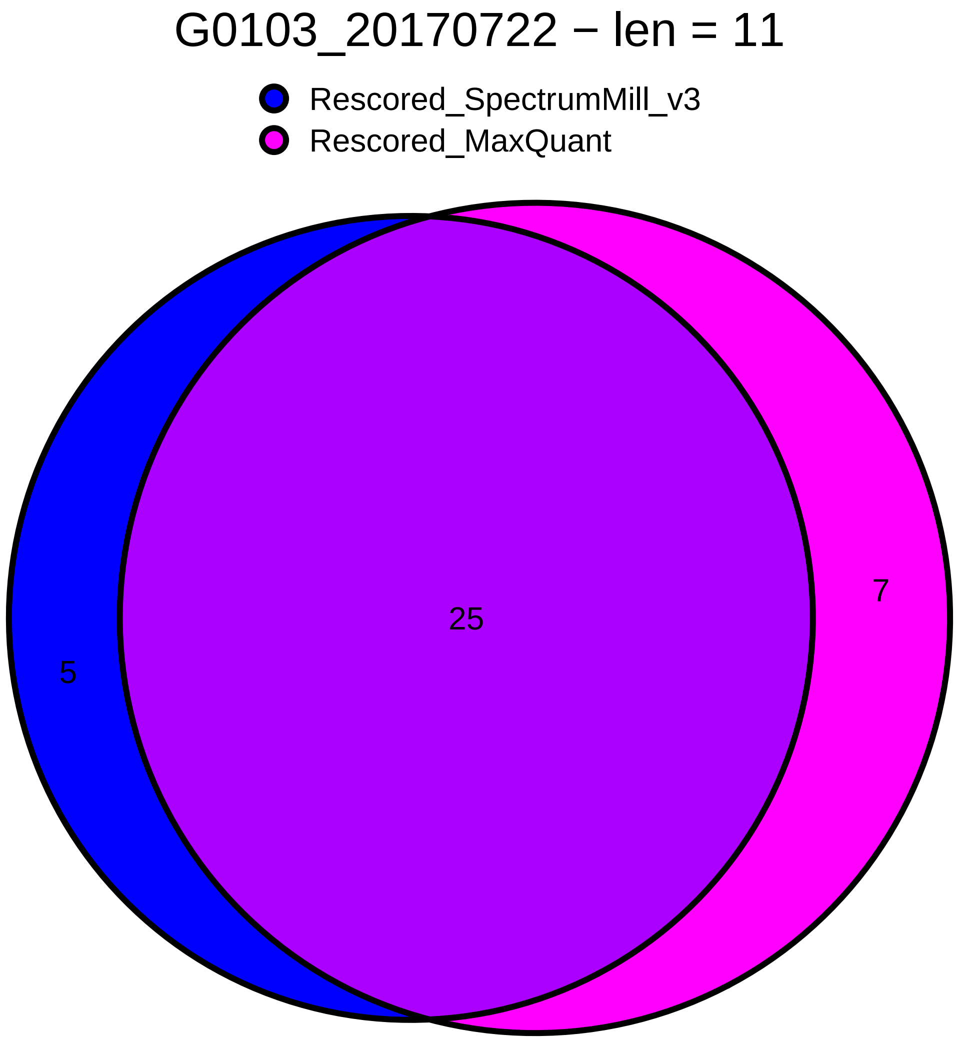
G0103_20170722 − len = 11
Rescored_SpectrumMill_v3
Rescored_MaxQuant
5
25
7

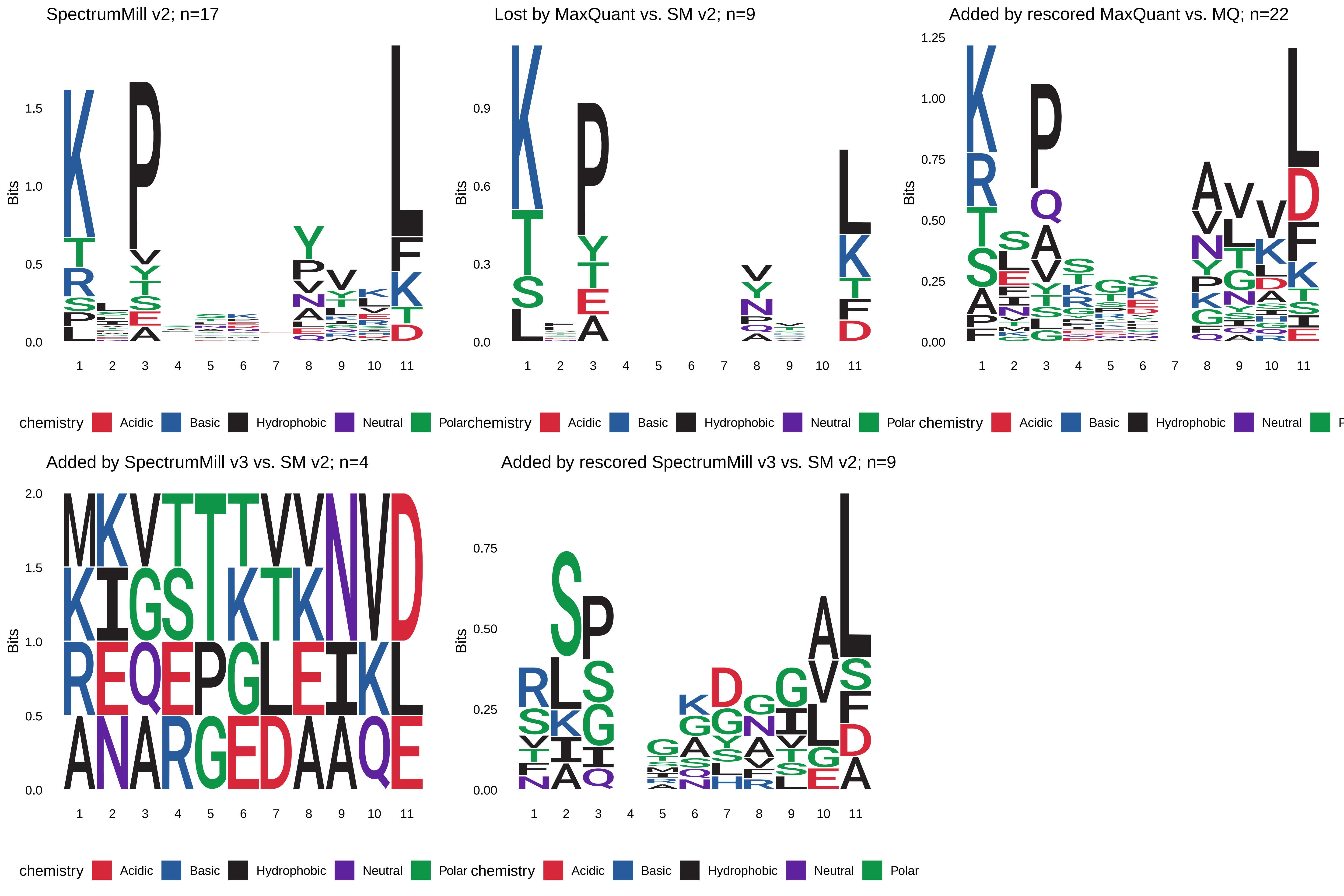
SpectrumMill v2; n=17
Lost by MaxQuant vs. SM v2; n=9
Added by rescored MaxQuant vs. MQ; n=22
Added by SpectrumMill v3 vs. SM v2; n=4
Added by rescored SpectrumMill v3 vs. SM v2; n=9
Bits
1 2 3 4 5 6 7 8 9 10 11
chemistry
Acidic
Basic
Hydrophobic
Neutral
Polar

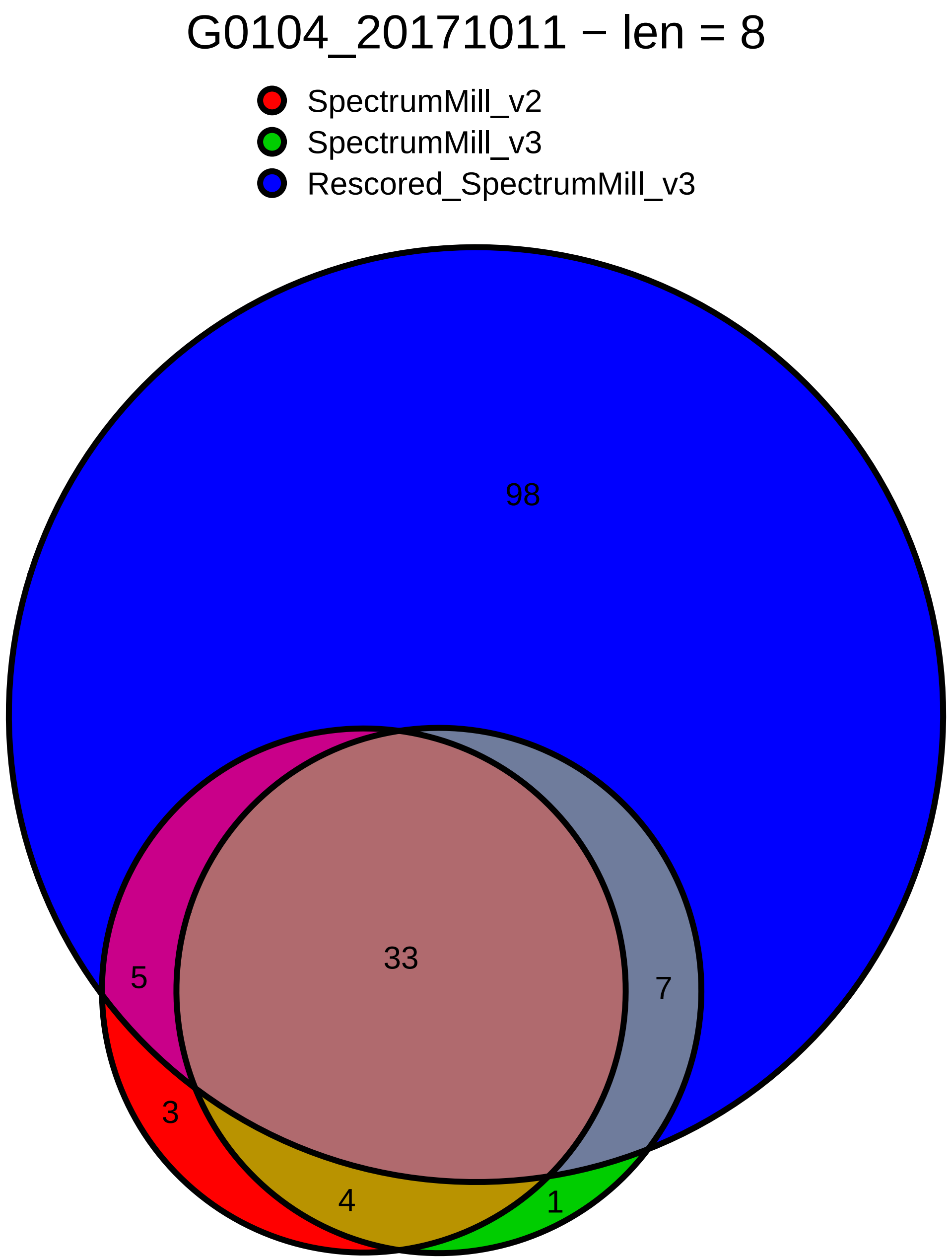

G0104_20171011 − len = 8
SpectrumMill_v2
SpectrumMill_v3
Rescored_SpectrumMill_v3
98
5
33
7
3
4
1

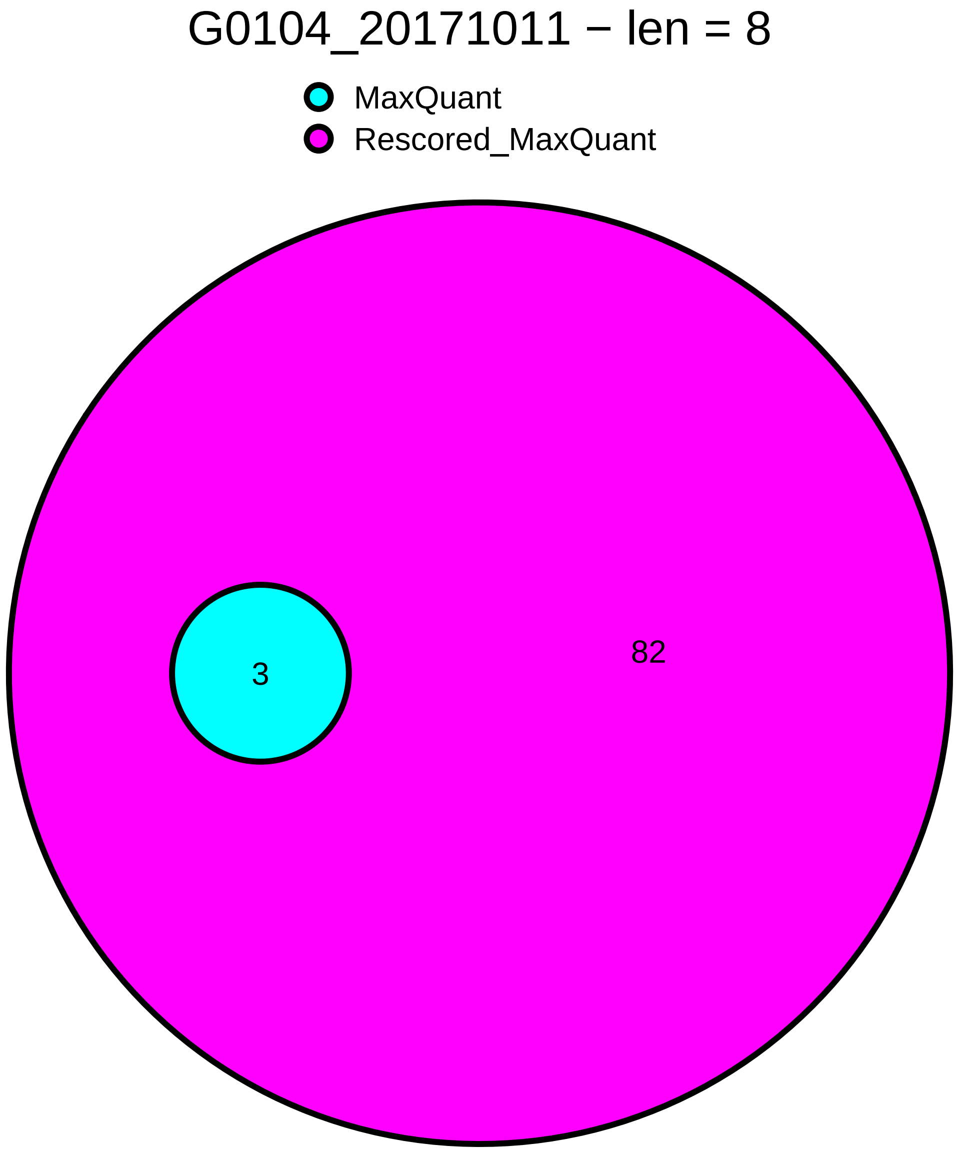

G0104_20171011 − len = 8
MaxQuant
Rescored_MaxQuant
3
82

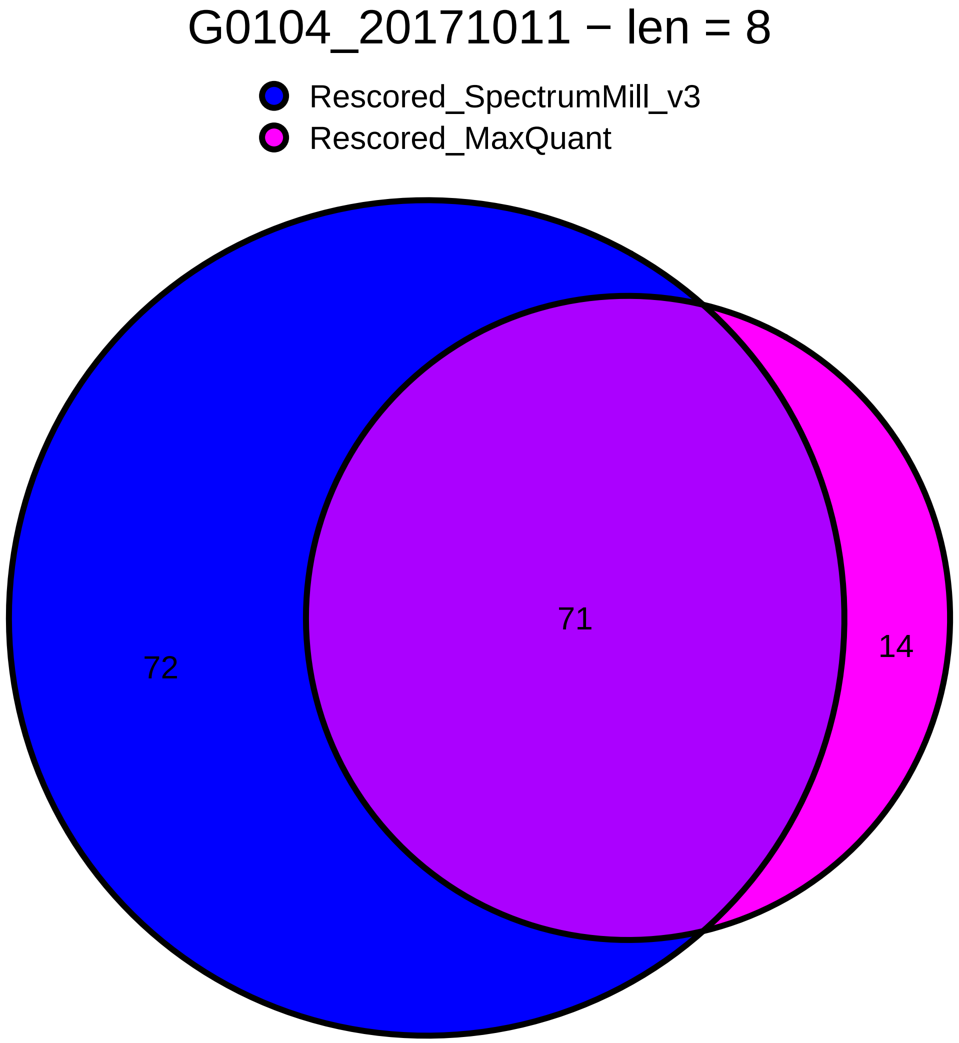

G0104_20171011 − len = 8
Rescored_SpectrumMill_v3
Rescored_MaxQuant
72
71
14

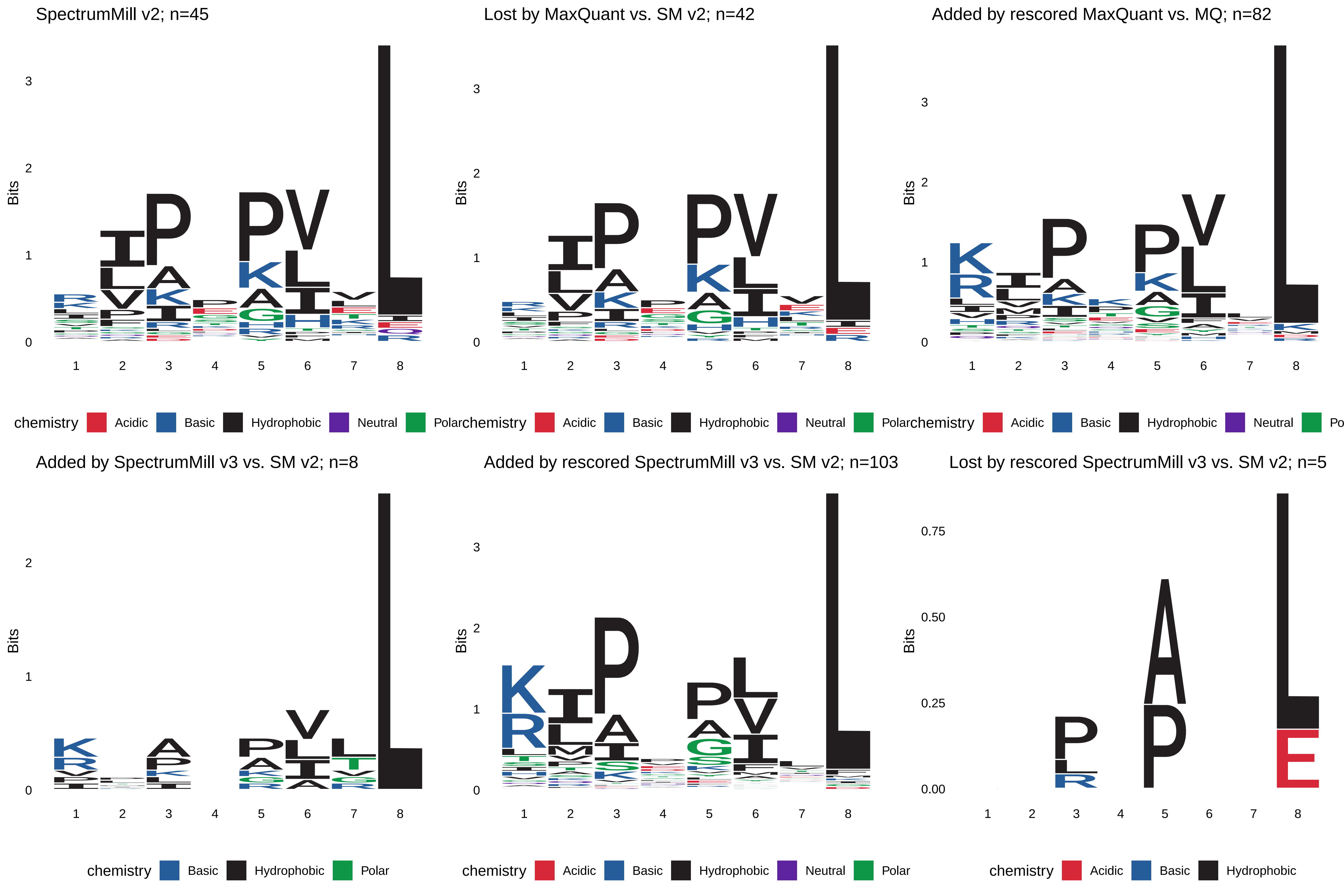
SpectrumMill v2; n=45
Lost by MaxQuant vs. SM v2; n=42
Added by rescored MaxQuant vs. MQ; n=82
Bits
chemistry Acidic Basic Hydrophobic Neutral Polar
Added by SpectrumMill v3 vs. SM v2; n=8
Added by rescored SpectrumMill v3 vs. SM v2; n=103
Lost by rescored SpectrumMill v3 vs. SM v2; n=5
chemistry Basic Hydrophobic Polar
chemistry Acidic Basic Hydrophobic Neutral Polar
chemistry Acidic Basic Hydrophobic

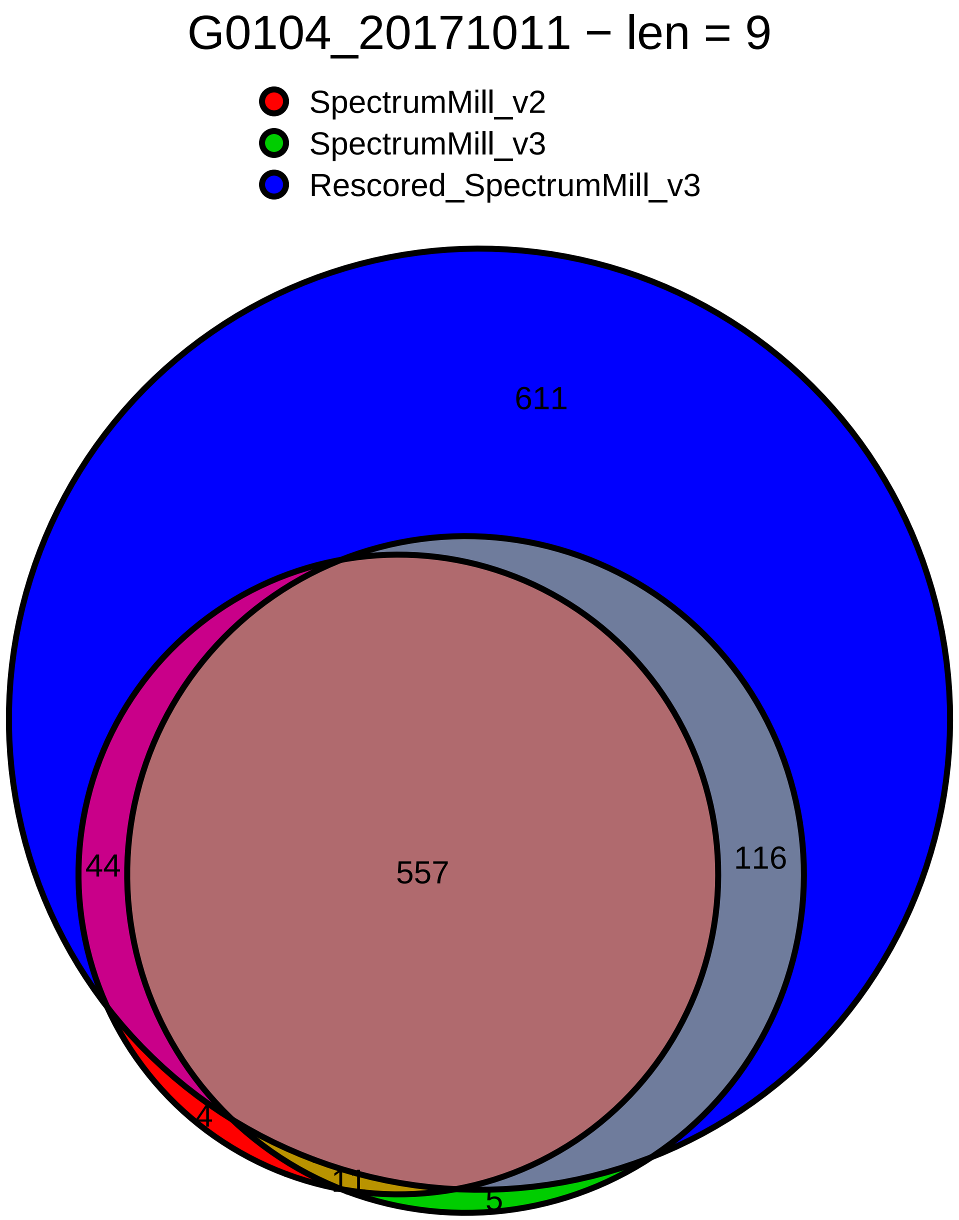
G0104_20171011 − len = 9
SpectrumMill_v2
SpectrumMill_v3
Rescored_SpectrumMill_v3
611
44
557
116
4
11
5

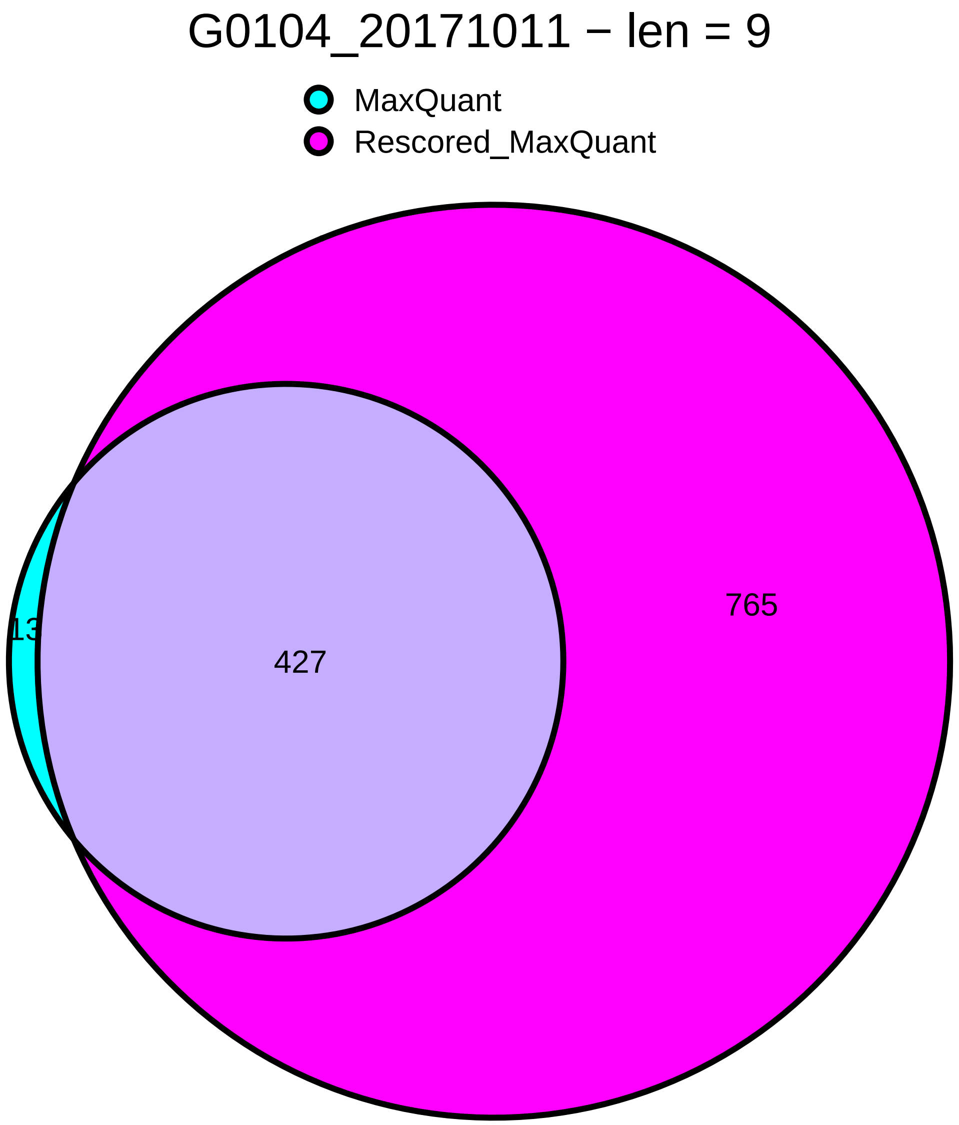
G0104_20171011 − len = 9
MaxQuant
Rescored_MaxQuant
13
427
765

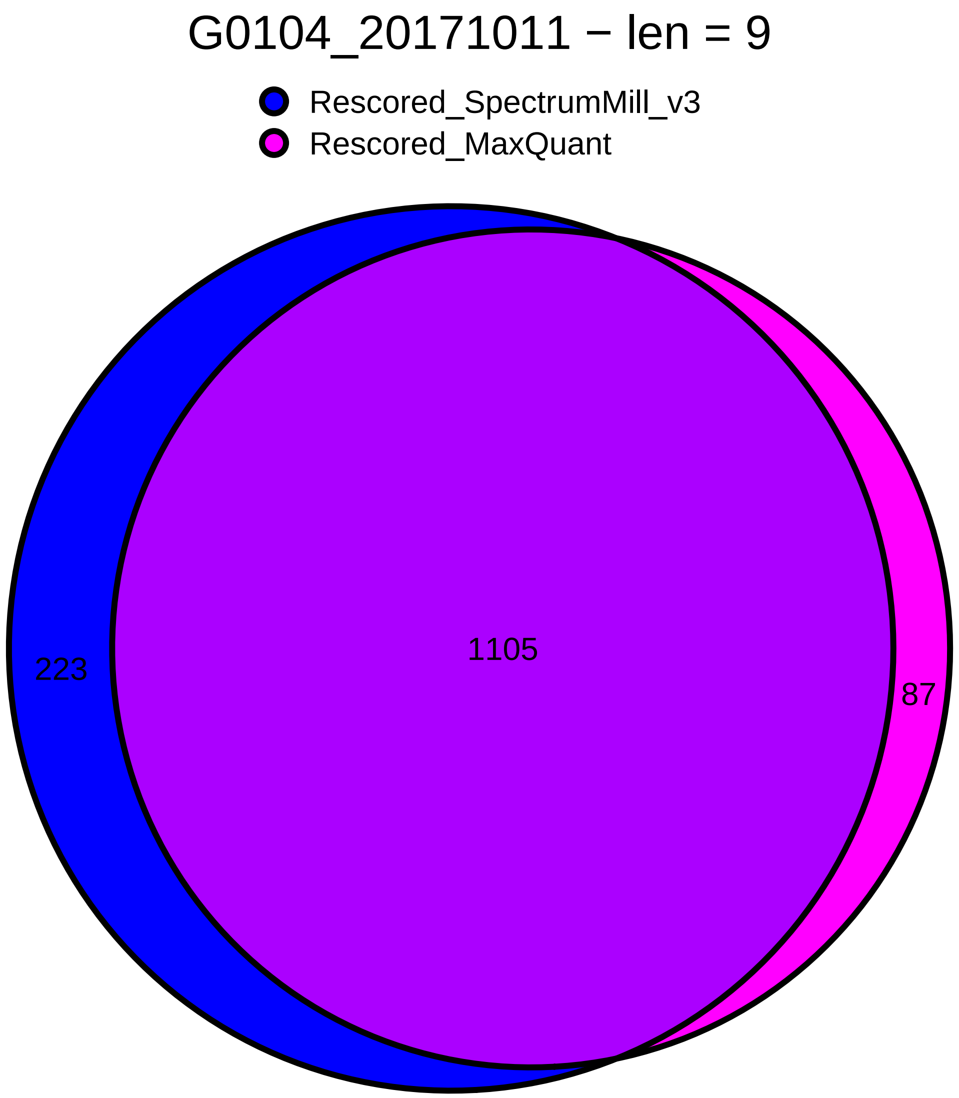
G0104_20171011 − len = 9
Rescored_SpectrumMill_v3
Rescored_MaxQuant
223
1105
87

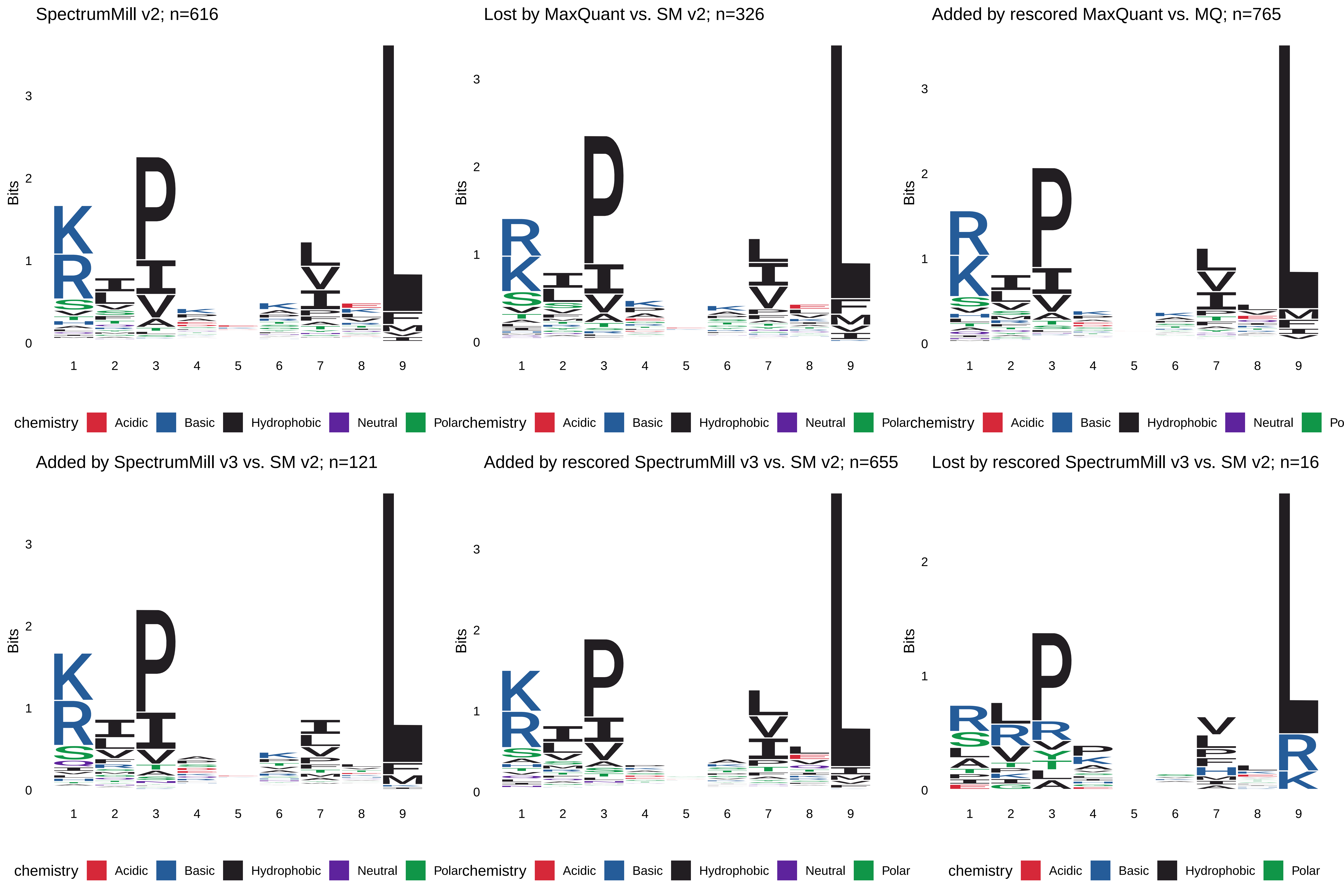
SpectrumMill v2; n=616
Lost by MaxQuant vs. SM v2; n=326
Added by rescored MaxQuant vs. MQ; n=765
Added by SpectrumMill v3 vs. SM v2; n=121
Added by rescored SpectrumMill v3 vs. SM v2; n=655
Lost by rescored SpectrumMill v3 vs. SM v2; n=16
Bits
1 2 3 4 5 6 7 8 9
chemistry Acidic Basic Hydrophobic Neutral Polar
chemistry Acidic Basic Hydrophobic Polar

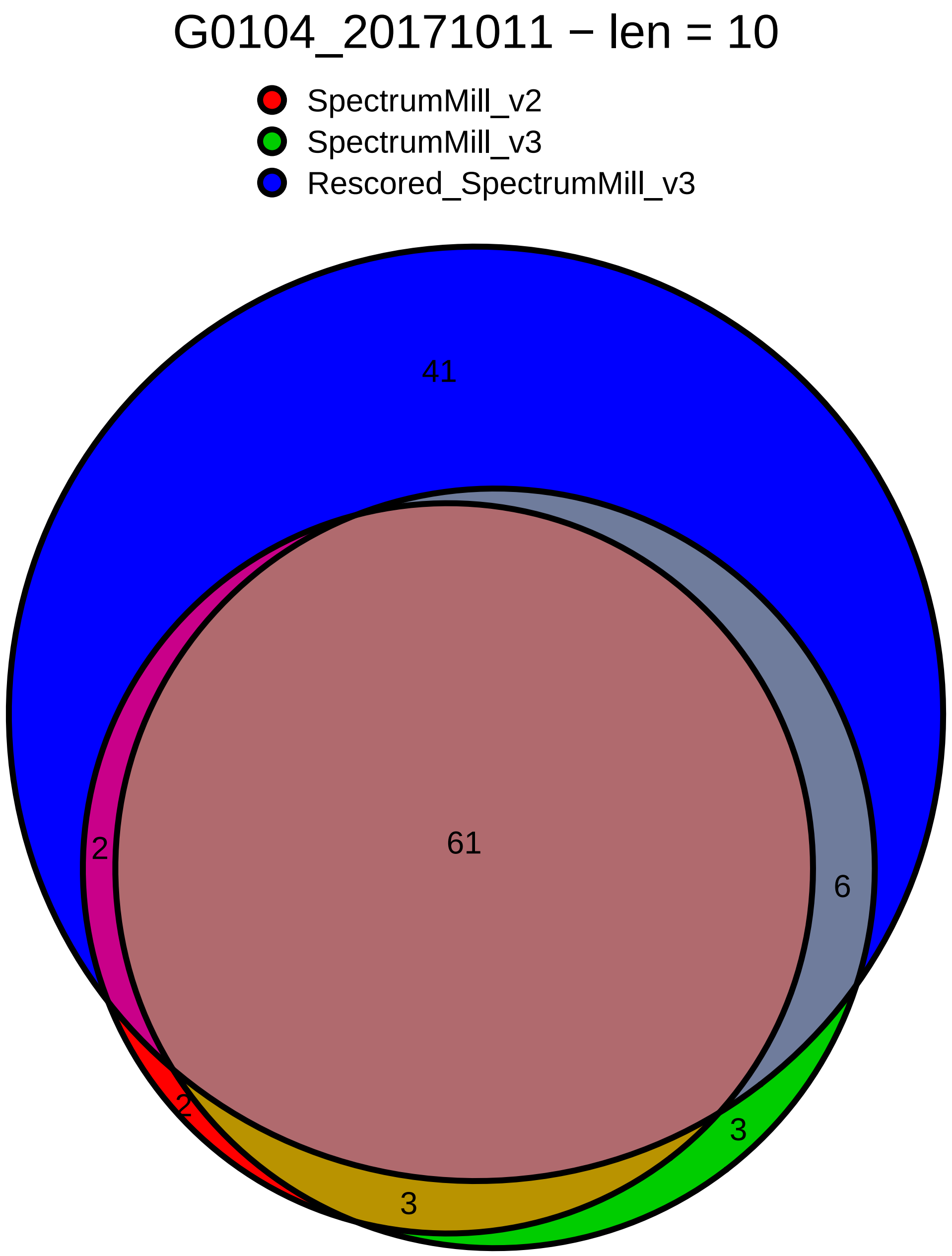
G0104_20171011 − len = 10
SpectrumMill_v2
SpectrumMill_v3
Rescored_SpectrumMill_v3
41
2
61
6
2
3
3

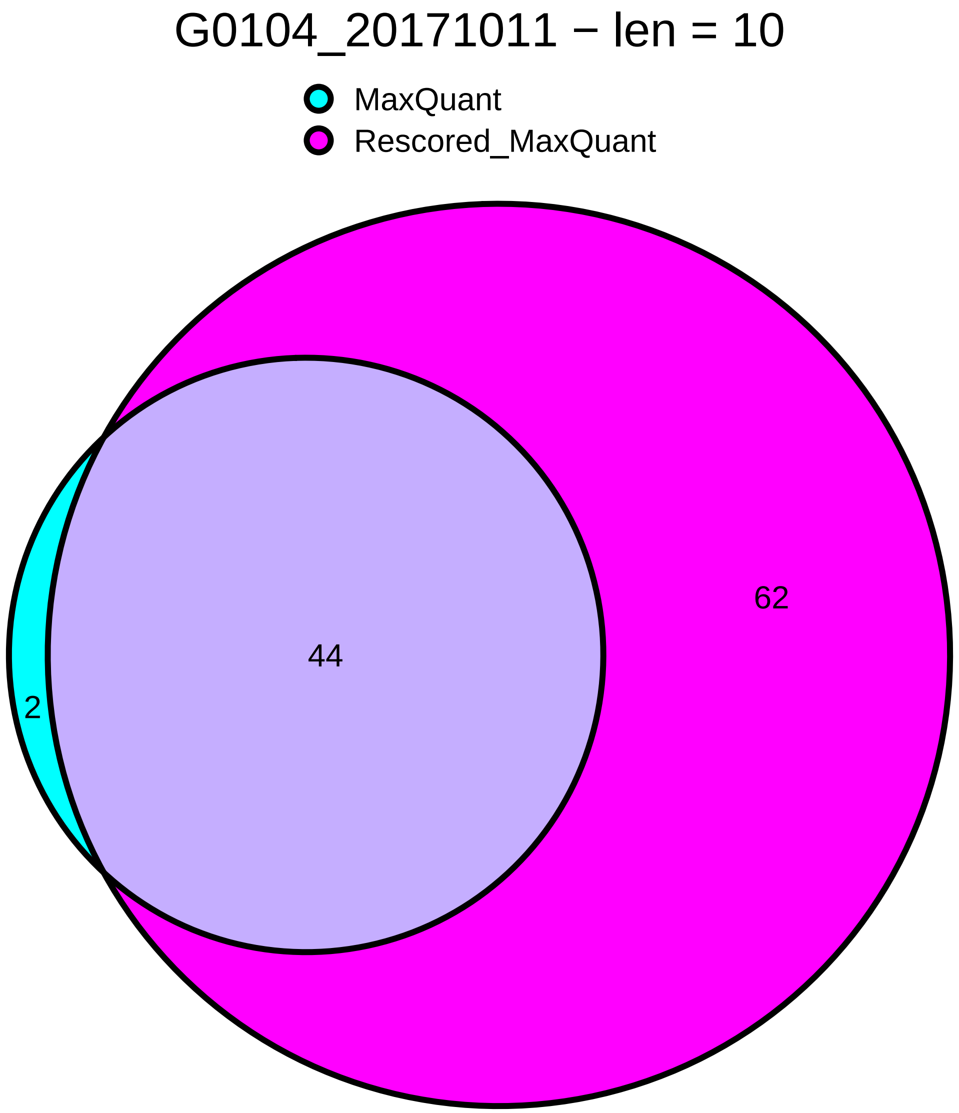
G0104_20171011 − len = 10
MaxQuant
Rescored_MaxQuant
2
44
62

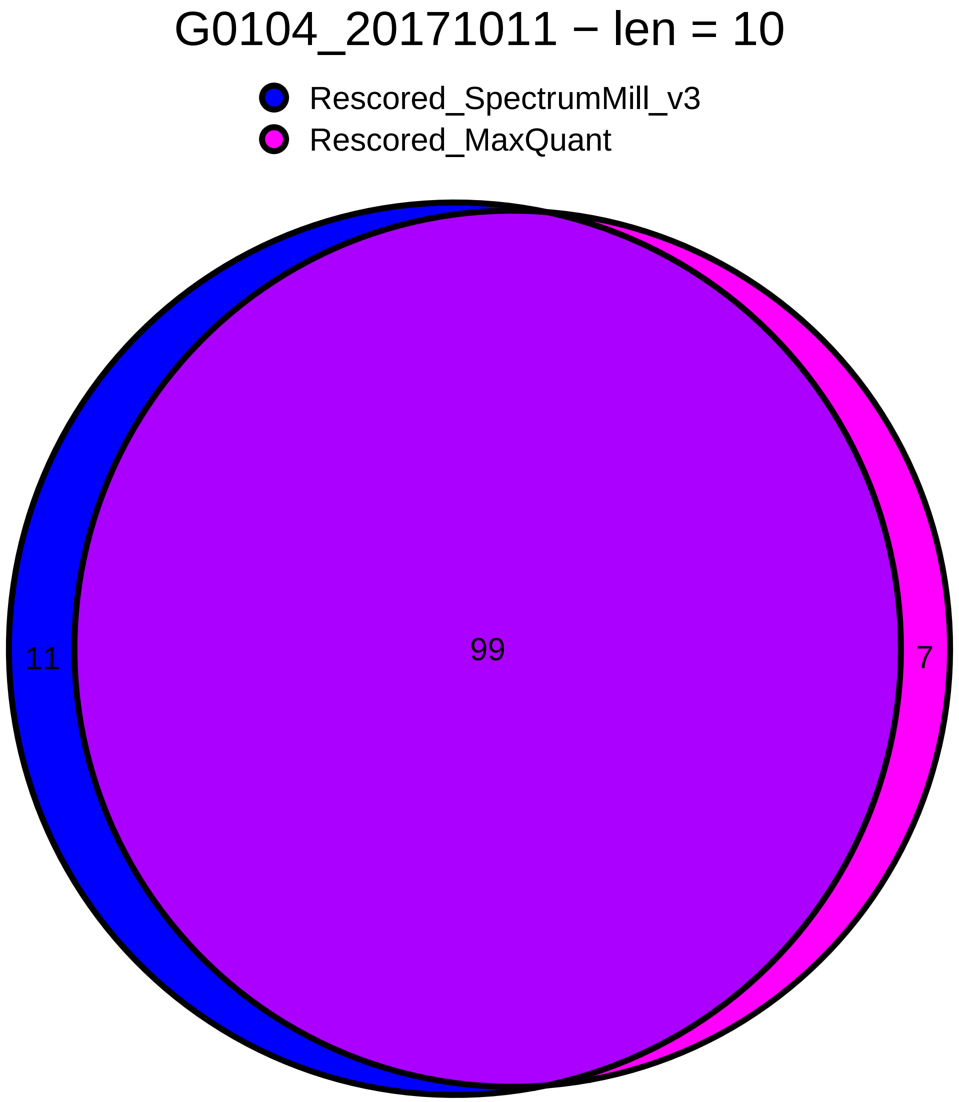
G0104_20171011 − len = 10
Rescored_SpectrumMill_v3
Rescored_MaxQuant
11
99
7

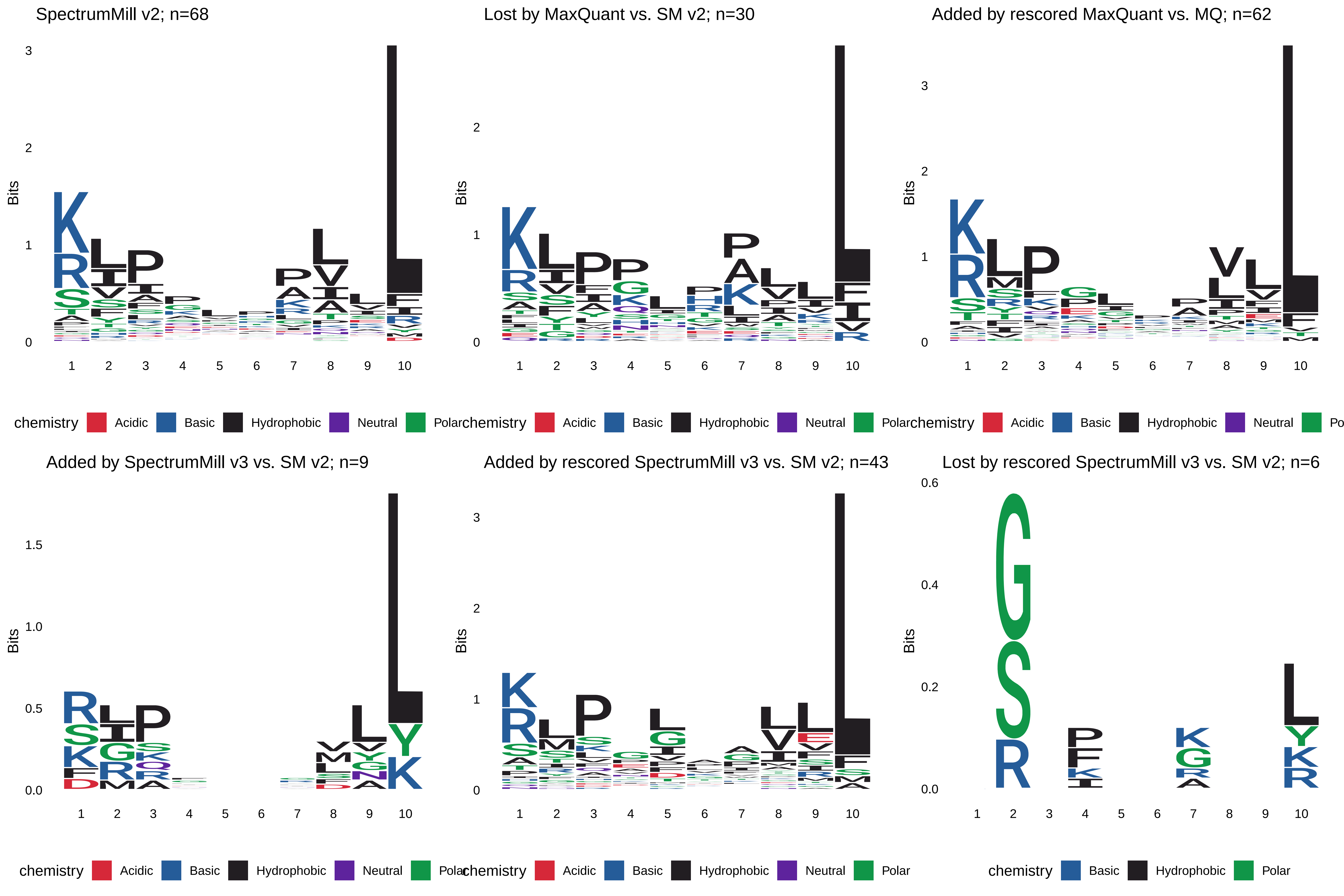
SpectrumMill v2; n=68
Lost by MaxQuant vs. SM v2; n=30
Added by rescored MaxQuant vs. MQ; n=62
Added by SpectrumMill v3 vs. SM v2; n=9
Added by rescored SpectrumMill v3 vs. SM v2; n=43
Lost by rescored SpectrumMill v3 vs. SM v2; n=6
Bits
chemistry
Acidic
Basic
Hydrophobic
Neutral
Polar

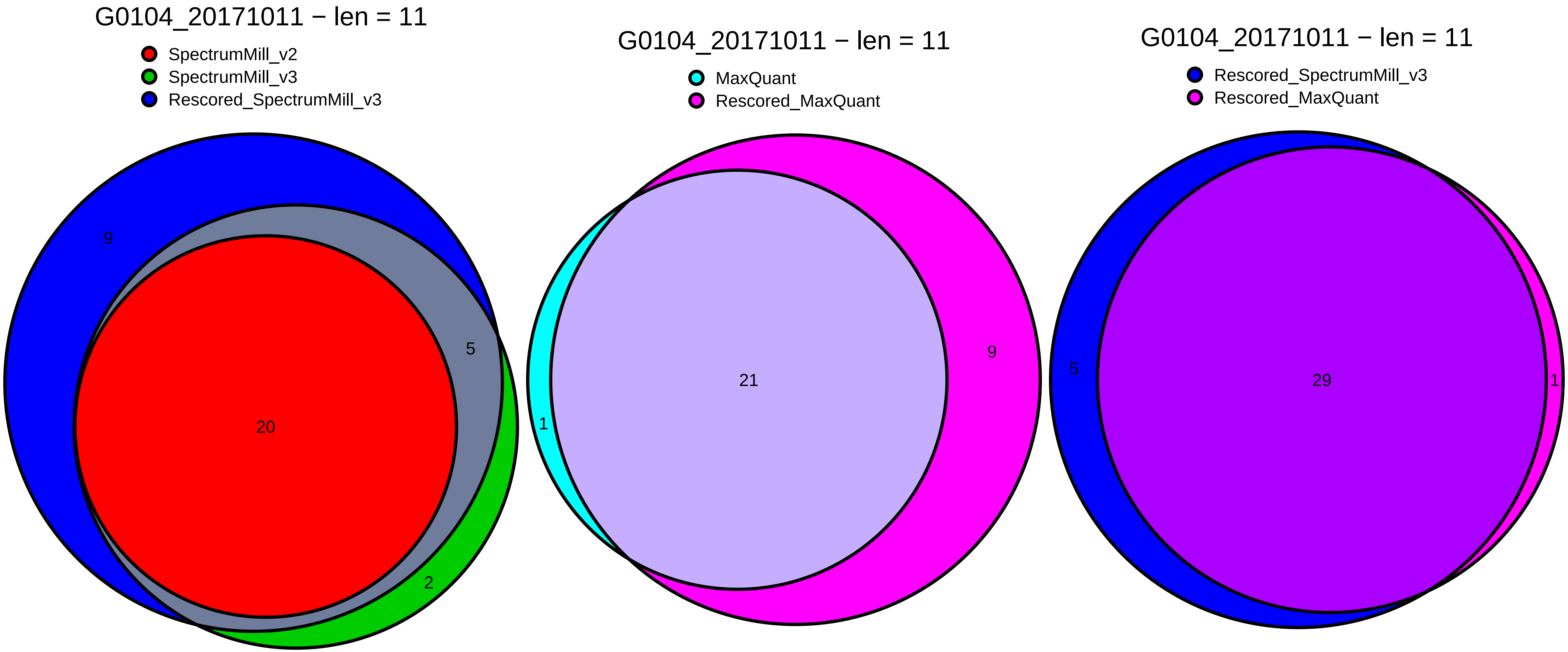
G0104_20171011 − len = 11
SpectrumMill_v2
SpectrumMill_v3
Rescored_SpectrumMill_v3
9
5
20
2
G0104_20171011 − len = 11
MaxQuant
Rescored_MaxQuant
1
21
9
G0104_20171011 − len = 11
Rescored_SpectrumMill_v3
Rescored_MaxQuant
5
29
1

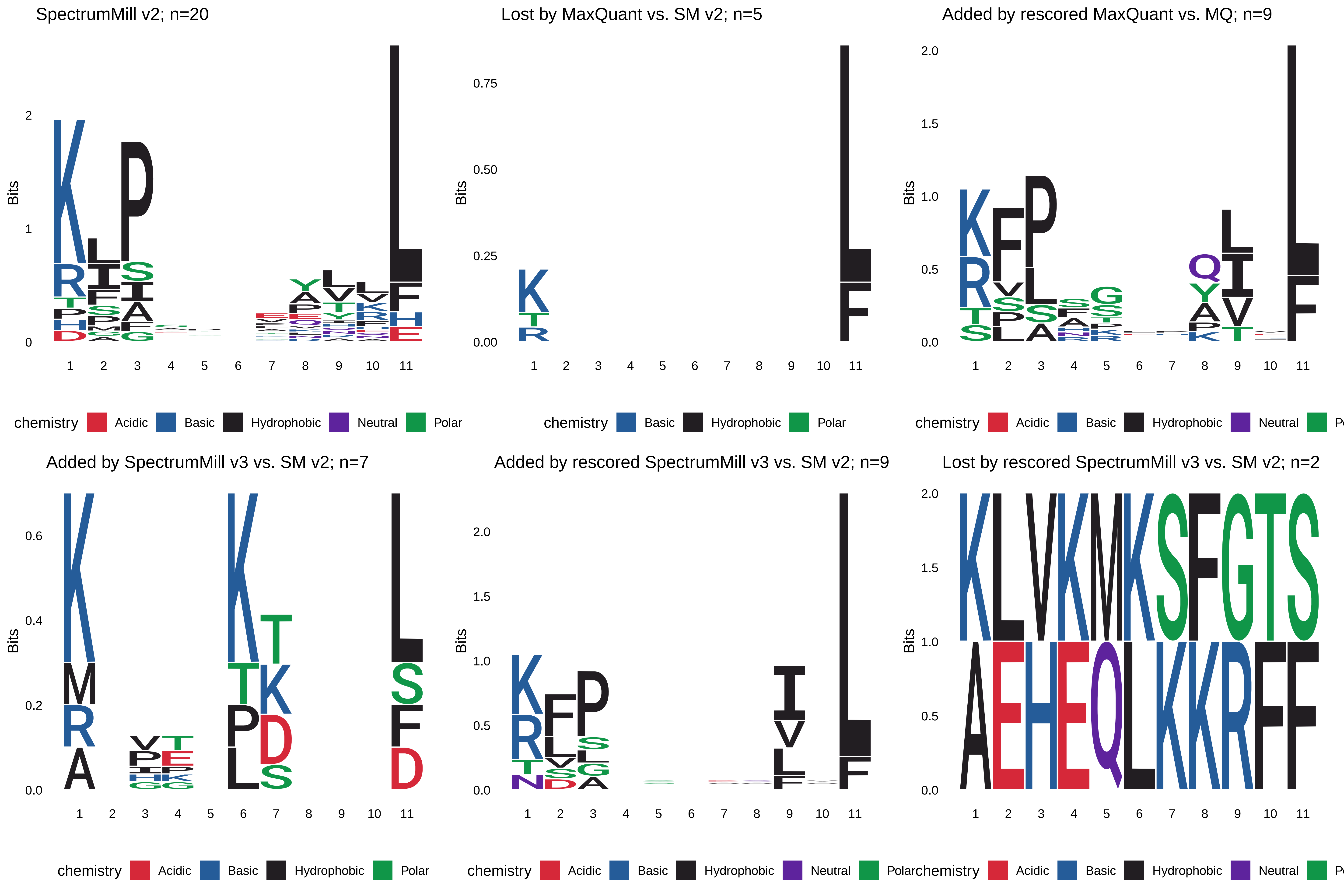

SpectrumMill v2; n=20
Lost by MaxQuant vs. SM v2; n=5
Added by rescored MaxQuant vs. MQ; n=9
Added by SpectrumMill v3 vs. SM v2; n=7
Added by rescored SpectrumMill v3 vs. SM v2; n=9
Lost by rescored SpectrumMill v3 vs. SM v2; n=2
Bits
1 2 3 4 5 6 7 8 9 10 11
chemistry Acidic Basic Hydrophobic Neutral Polar
chemistry Basic Hydrophobic Polar
chemistry Acidic Basic Hydrophobic Polar

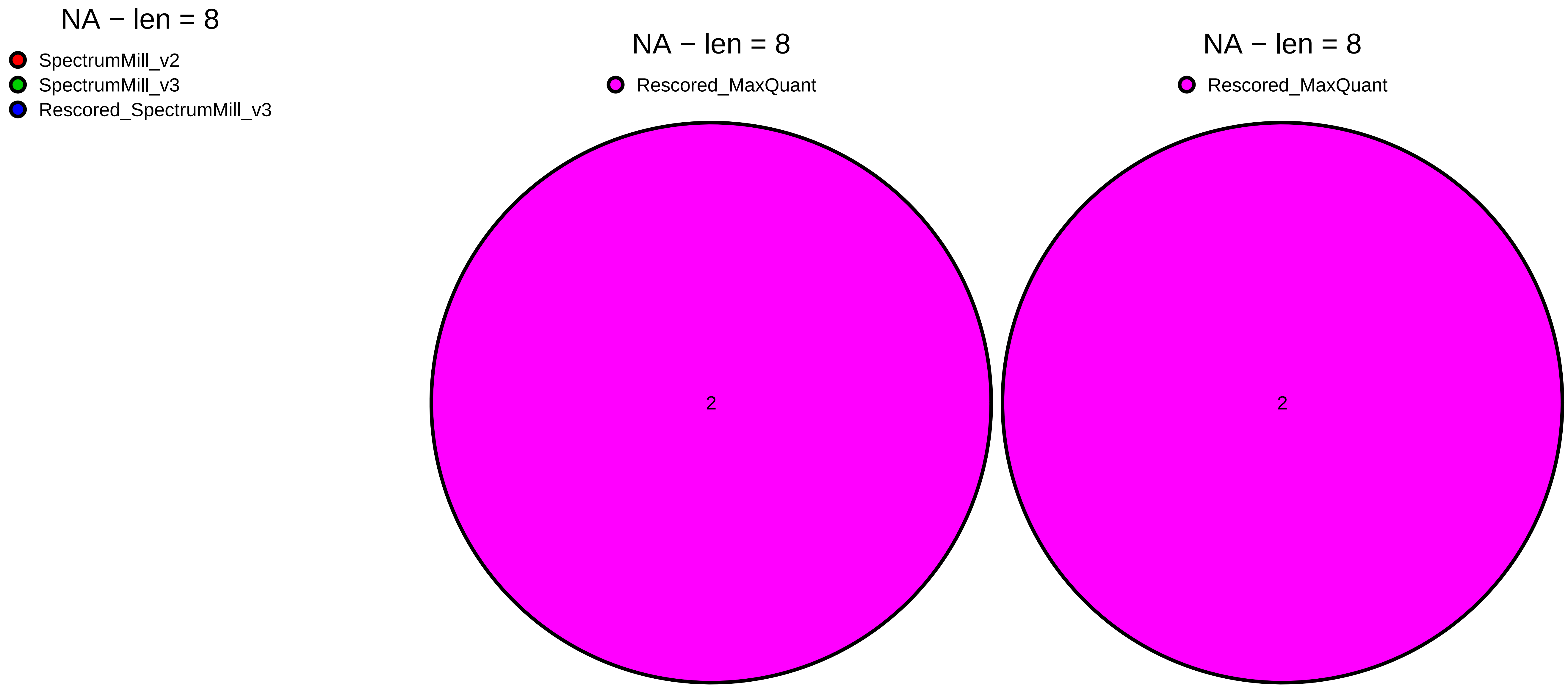
NA − len = 8
SpectrumMill_v2
SpectrumMill_v3
Rescored_SpectrumMill_v3
NA − len = 8
Rescored_MaxQuant
2
NA − len = 8
Rescored_MaxQuant
2

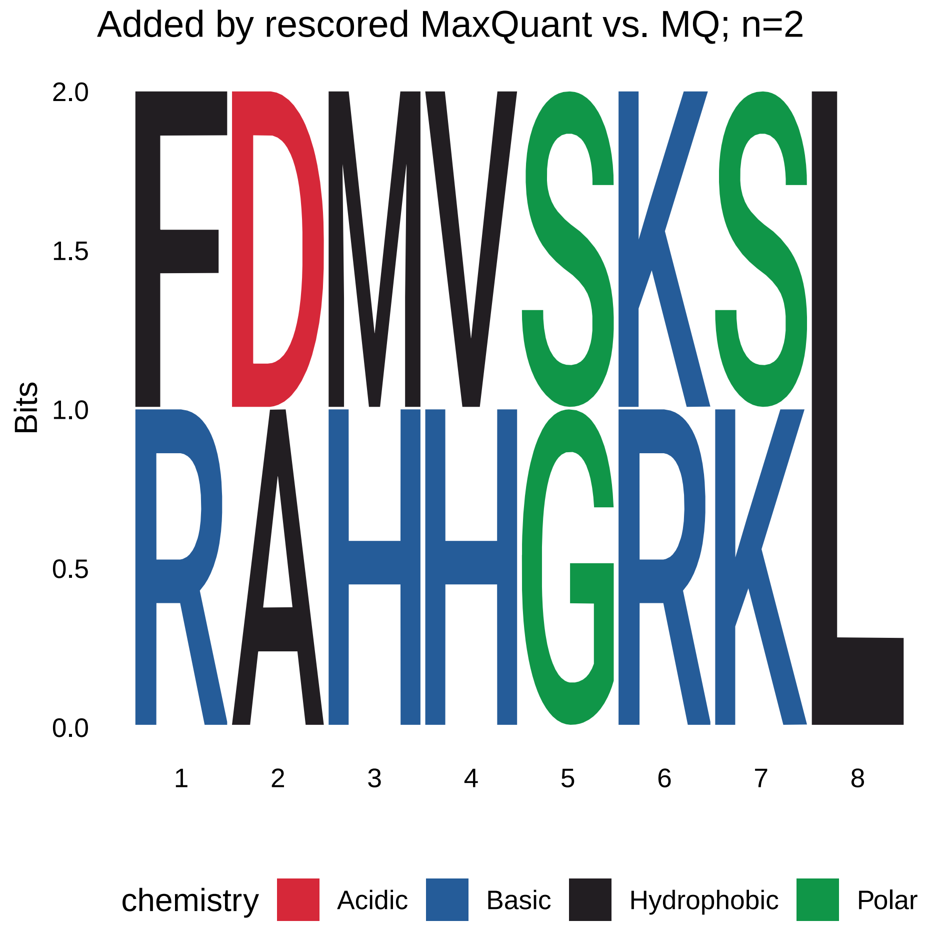

Added by rescored MaxQuant vs. MQ; n=2
Bits
2.0
1.5
1.0
0.5
0.0
1
2
3
4
5
6
7
8
chemistry
Acidic
Basic
Hydrophobic
Polar

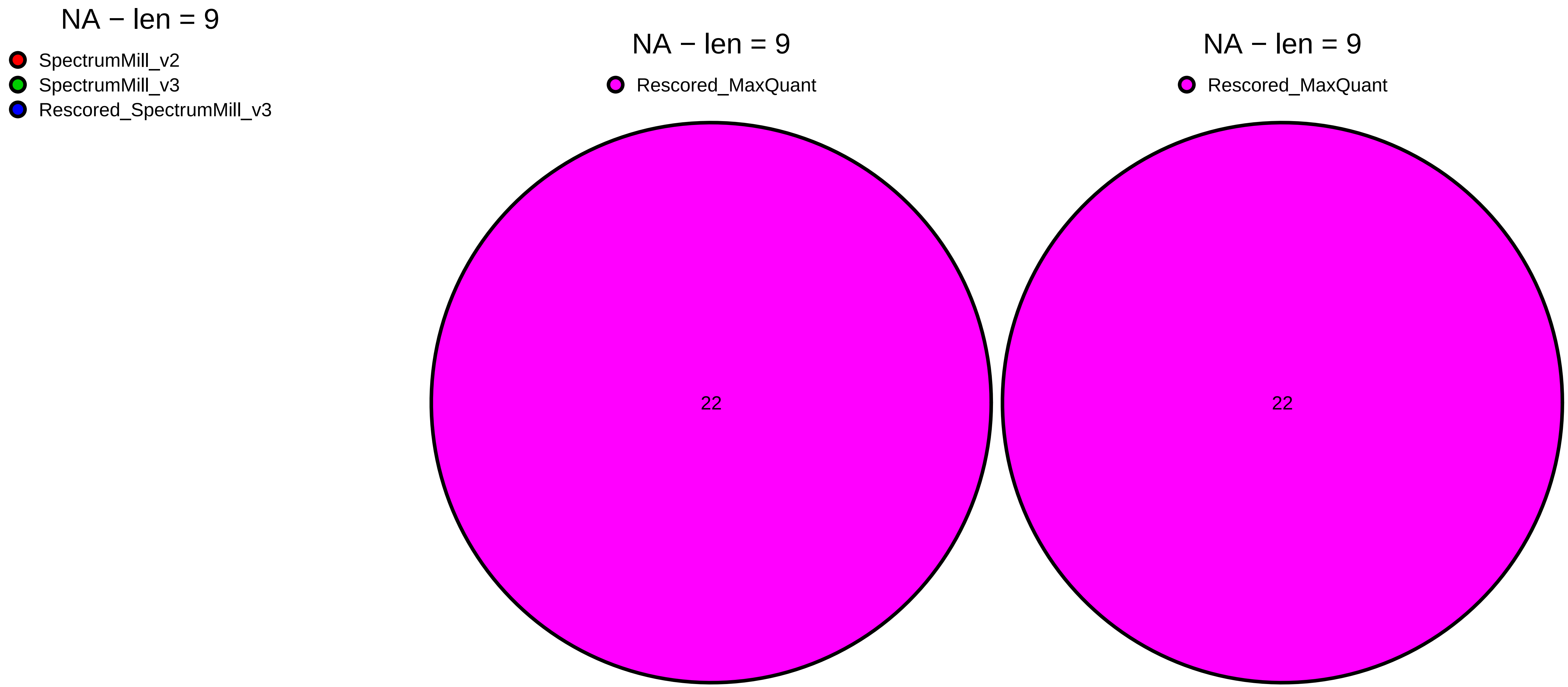
NA − len = 9
SpectrumMill_v2
SpectrumMill_v3
Rescored_SpectrumMill_v3
NA − len = 9
Rescored_MaxQuant
22
NA − len = 9
Rescored_MaxQuant
22

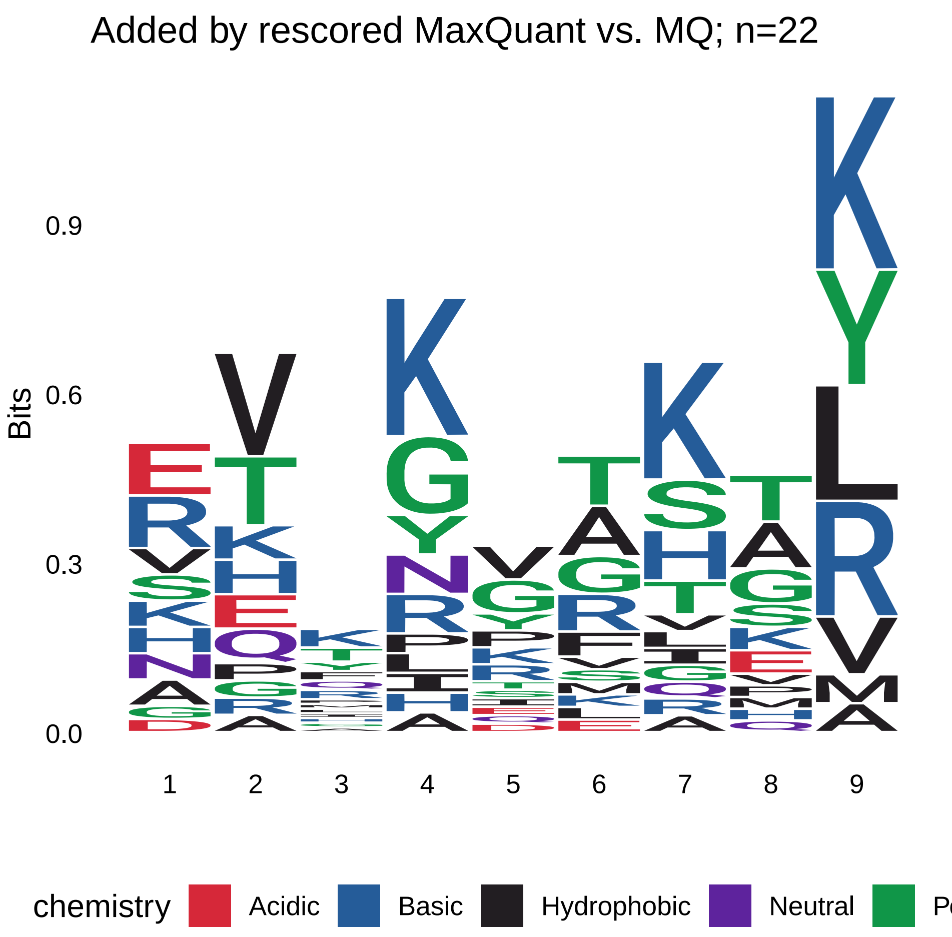
Added by rescored MaxQuant vs. MQ; n=22
Bits
0.9
0.6
0.3
0.0
1
2
3
4
5
6
7
8
9
chemistry
Acidic
Basic
Hydrophobic
Neutral

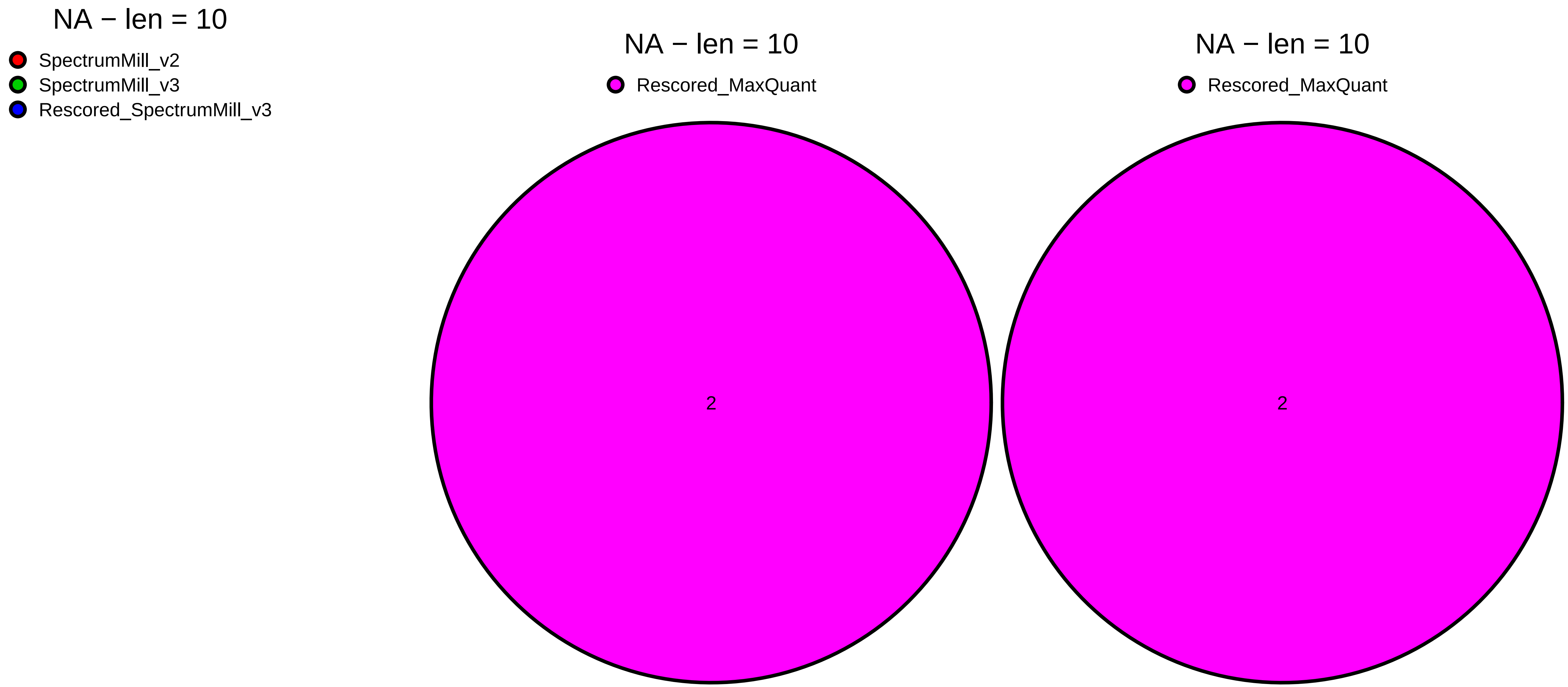
NA − len = 10
SpectrumMill_v2
SpectrumMill_v3
Rescored_SpectrumMill_v3
NA − len = 10
Rescored_MaxQuant
2
NA − len = 10
Rescored_MaxQuant
2

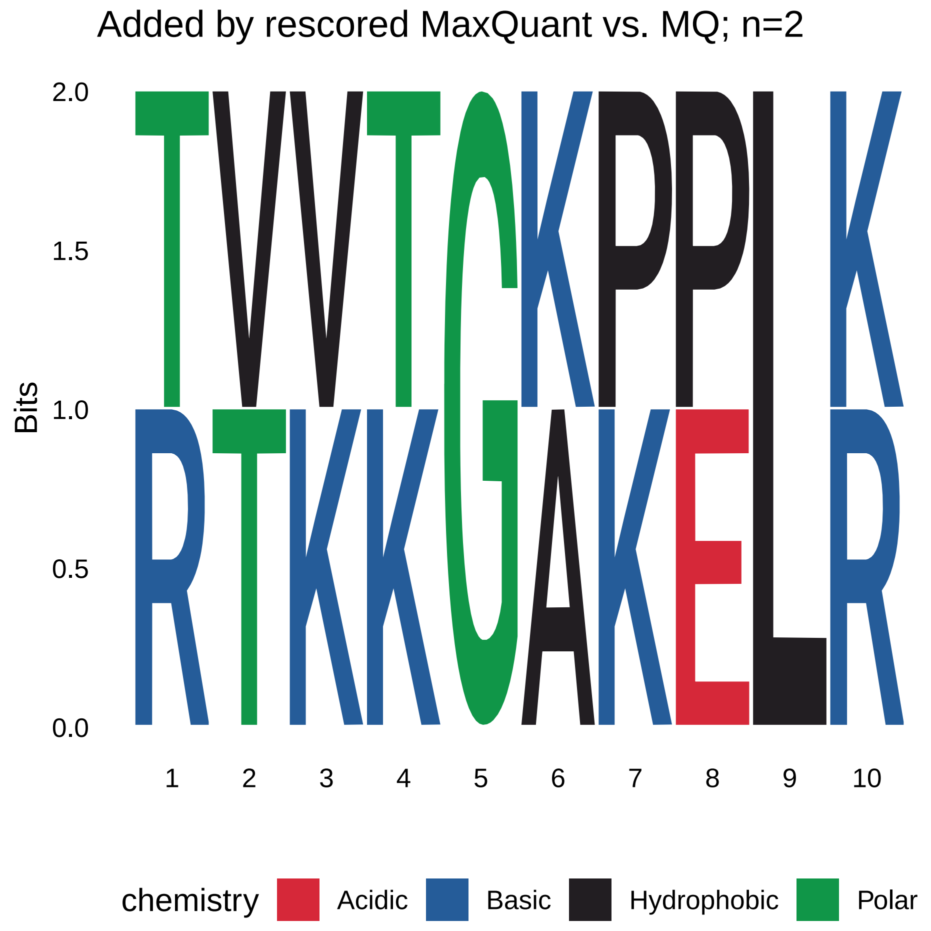

Added by rescored MaxQuant vs. MQ; n=2
Bits
2.0
1.5
1.0
0.5
0.0
1
2
3
4
5
6
7
8
9
10
chemistry
Acidic
Basic
Hydrophobic
Polar

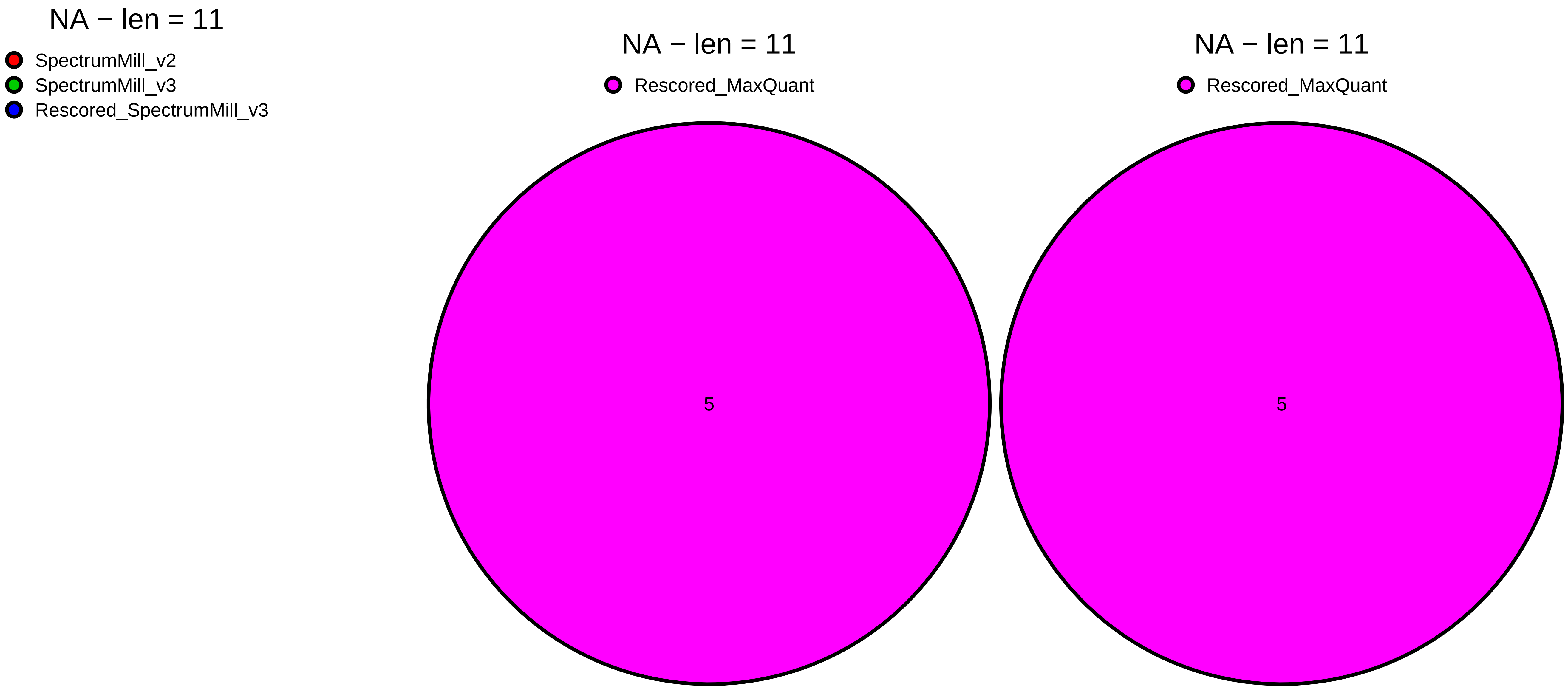
NA − len = 11
SpectrumMill_v2
SpectrumMill_v3
Rescored_SpectrumMill_v3
NA − len = 11
Rescored_MaxQuant
5
NA − len = 11
Rescored_MaxQuant
5

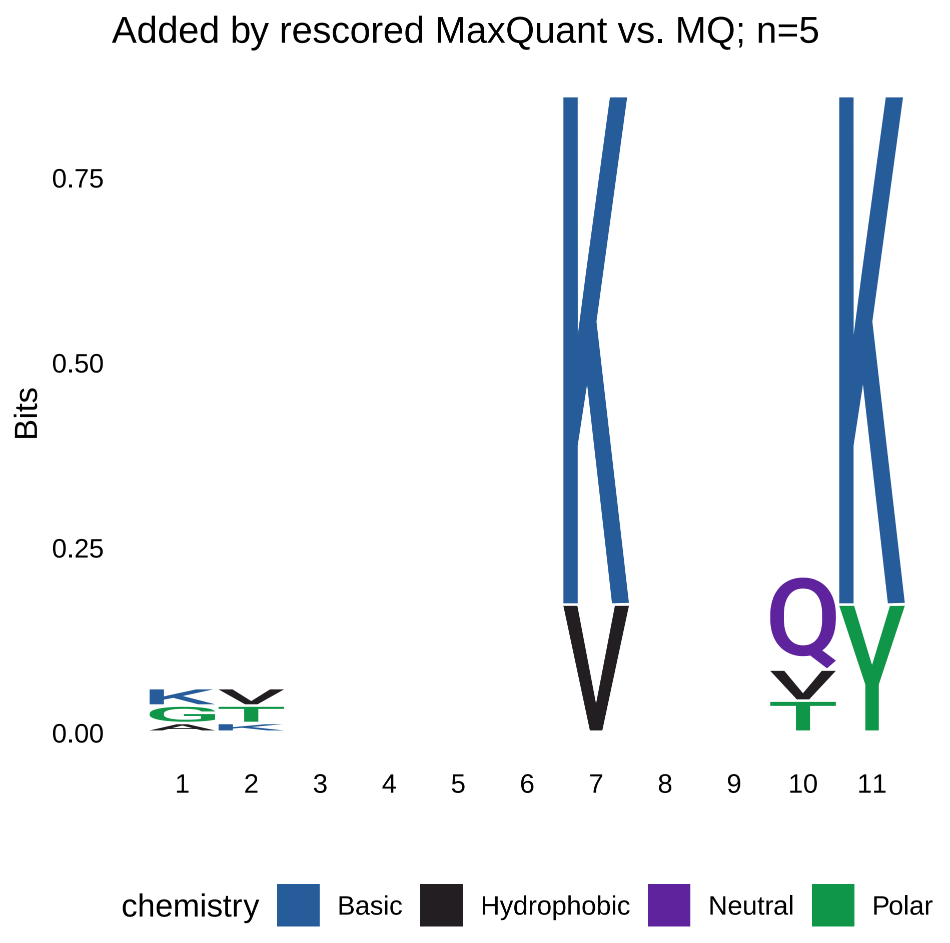

Added by rescored MaxQuant vs. MQ; n=5
Bits
0.75
0.50
0.25
0.00
1
2
3
4
5
6
7
8
9
10
11
chemistry
Basic
Hydrophobic
Neutral
Polar
