# Supplementary material for: Deep learning boosts sensitivity of mass spectrometry-based immunopeptidomics
Source: Nat Commun. 2021 Jun 7;12:3346. doi: 10.1038/s41467-021-23713-9 (PMC8184761; doi:10.1038/s41467-021-23713-9)
Supplement: Supplementary file 9 — Supplementary Data 6 [file 41467_2021_23713_MOESM9_ESM.pdf]

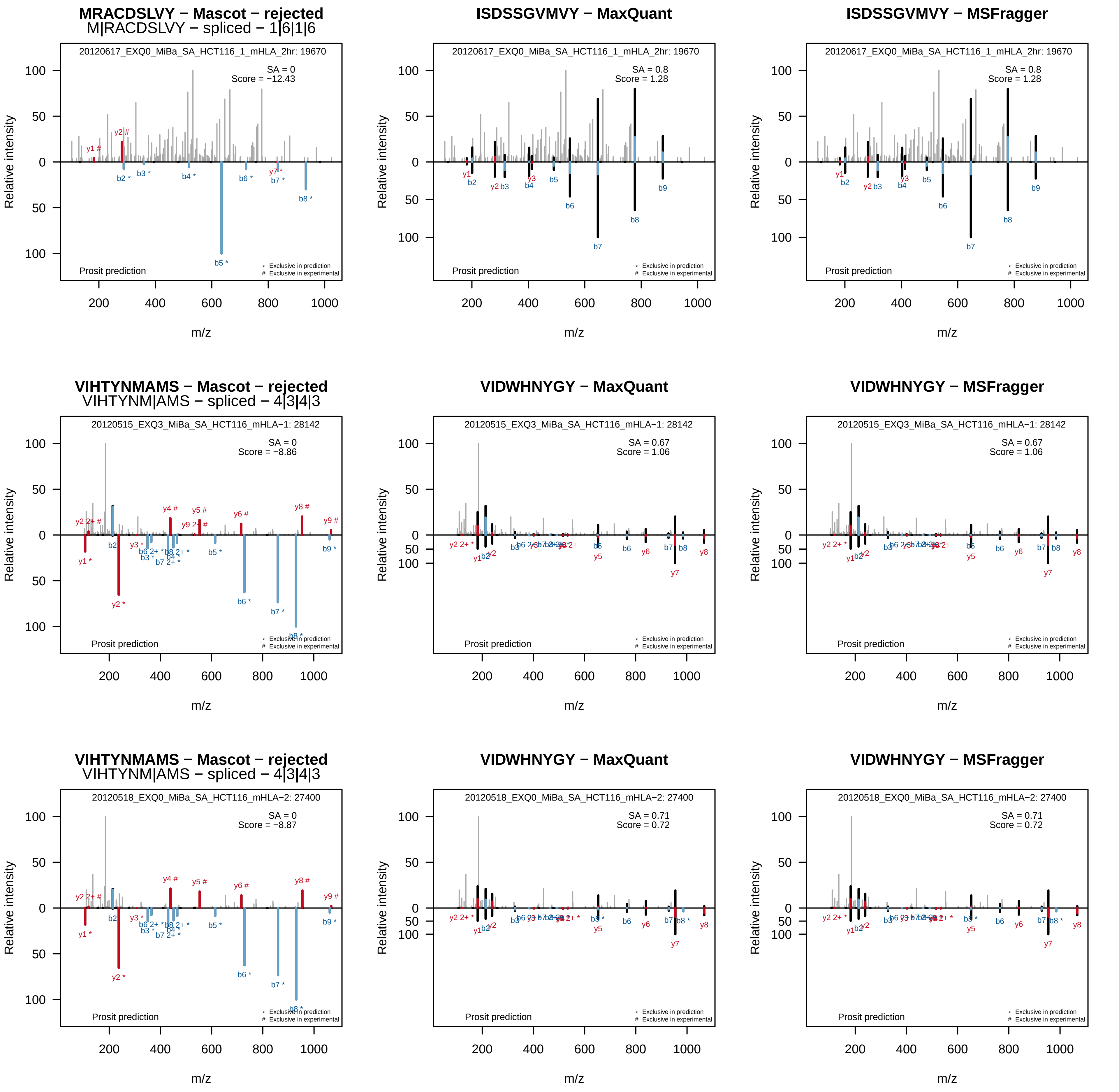
MRACDSLVY − Mascot − rejected
M|RACDSLVY − spliced − 1|6|1|6
20120617_EXQ0_MiBa_SA_HCT116_1_mHLA_2hr: 19670
SA = 0
Score = −12.43
ISDSSGVMVY − MaxQuant
20120617_EXQ0_MiBa_SA_HCT116_1_mHLA_2hr: 19670
SA = 0.8
Score = 1.28
ISDSSGVMVY − MSFragger
20120617_EXQ0_MiBa_SA_HCT116_1_mHLA_2hr: 19670
SA = 0.8
Score = 1.28
VIHTYNMAMS − Mascot − rejected
VIHTYNM|AMS − spliced − 4|3|4|3
20120515_EXQ3_MiBa_SA_HCT116_mHLA−1: 28142
SA = 0
Score = −8.86
VIDWHNYGY − MaxQuant
20120515_EXQ3_MiBa_SA_HCT116_mHLA−1: 28142
SA = 0.67
Score = 1.06
VIDWHNYGY − MSFragger
20120515_EXQ3_MiBa_SA_HCT116_mHLA−1: 28142
SA = 0.67
Score = 1.06
VIHTYNMAMS − Mascot − rejected
VIHTYNM|AMS − spliced − 4|3|4|3
20120518_EXQ0_MiBa_SA_HCT116_mHLA−2: 27400
SA = 0
Score = −8.87
VIDWHNYGY − MaxQuant
20120518_EXQ0_MiBa_SA_HCT116_mHLA−2: 27400
SA = 0.71
Score = 0.72
VIDWHNYGY − MSFragger
20120518_EXQ0_MiBa_SA_HCT116_mHLA−2: 27400
SA = 0.71
Score = 0.72
Relative intensity
m/z
Prosit prediction
⋆ Exclusive in prediction
Exclusive in experimental

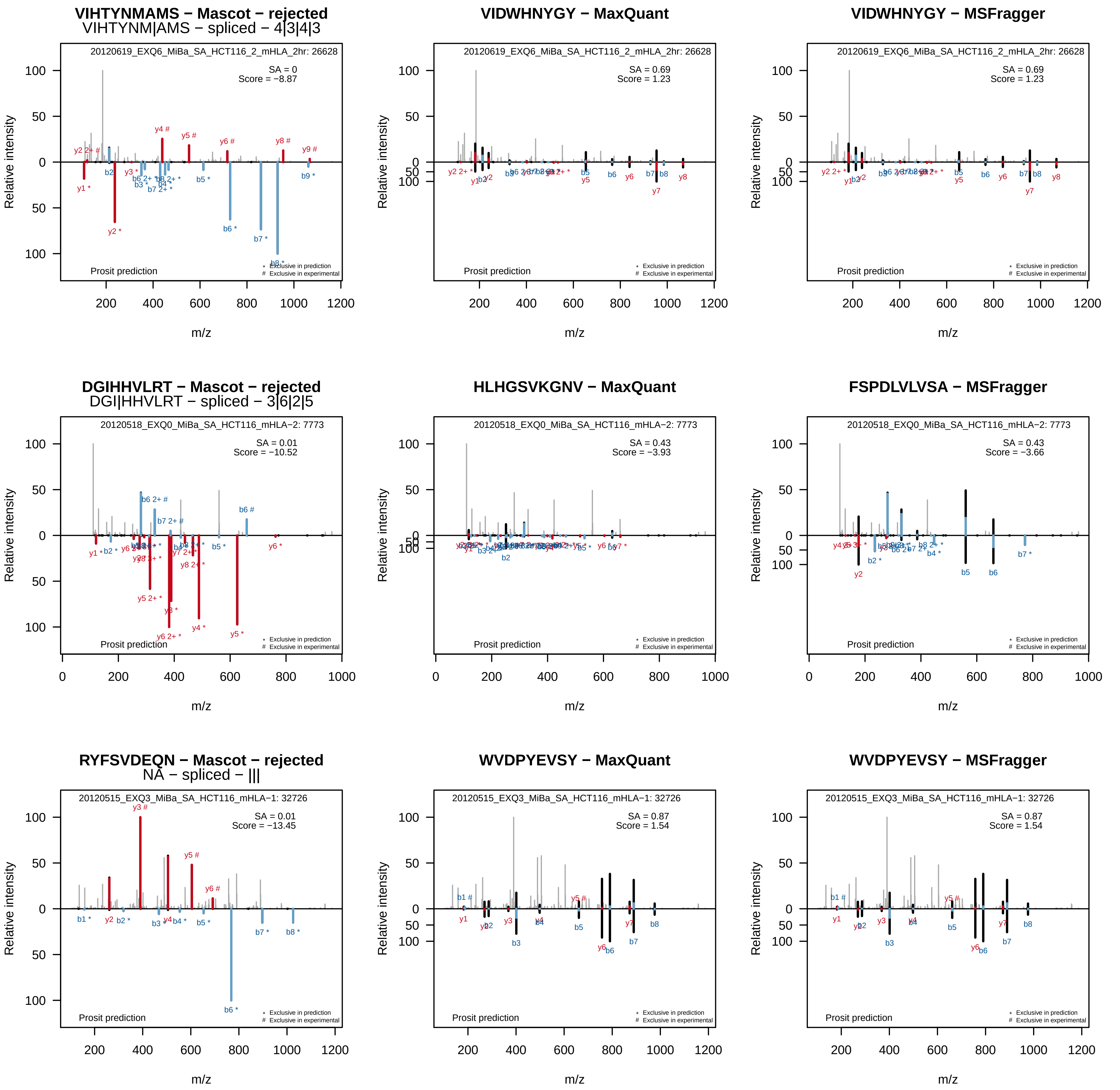

VIHTYNMAMS − Mascot − rejected
VIHTYNM|AMS − spliced − 4|3|4|3
20120619_EXQ6_MiBa_SA_HCT116_2_mHLA_2hr: 26628
SA = 0
Score = −8.87
VIDWHNYGY − MaxQuant
20120619_EXQ6_MiBa_SA_HCT116_2_mHLA_2hr: 26628
SA = 0.69
Score = 1.23
VIDWHNYGY − MSFragger
20120619_EXQ6_MiBa_SA_HCT116_2_mHLA_2hr: 26628
SA = 0.69
Score = 1.23
DGIHHVLRT − Mascot − rejected
DGI|HHVLRT − spliced − 3|6|2|5
20120518_EXQ0_MiBa_SA_HCT116_mHLA−2: 7773
SA = 0.01
Score = −10.52
HLHGSVKGNV − MaxQuant
20120518_EXQ0_MiBa_SA_HCT116_mHLA−2: 7773
SA = 0.43
Score = −3.93
FSPDLVLVSA − MSFragger
20120518_EXQ0_MiBa_SA_HCT116_mHLA−2: 7773
SA = 0.43
Score = −3.66
RYFSVDEQN − Mascot − rejected
NA − spliced − |||
20120515_EXQ3_MiBa_SA_HCT116_mHLA−1: 32726
SA = 0.01
Score = −13.45
WVDPYEVSY − MaxQuant
20120515_EXQ3_MiBa_SA_HCT116_mHLA−1: 32726
SA = 0.87
Score = 1.54
WVDPYEVSY − MSFragger
20120515_EXQ3_MiBa_SA_HCT116_mHLA−1: 32726
SA = 0.87
Score = 1.54
Relative intensity
m/z
Prosit prediction
⋆ Exclusive in prediction
Exclusive in experimental

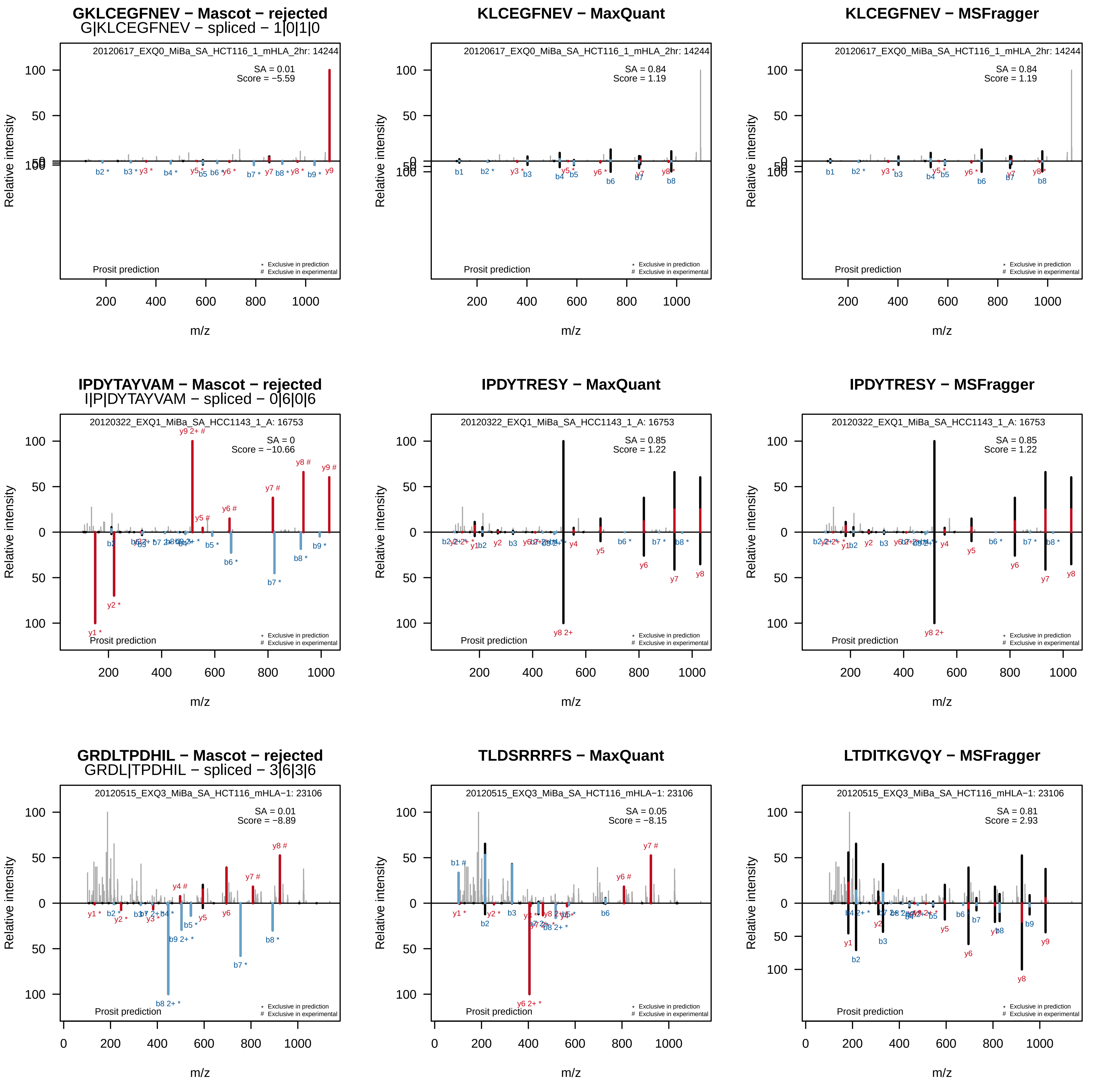
GKLCEGFNEV − Mascot − rejected
G|KLCEGFNEV − spliced − 1|0|1|0
20120617_EXQ0_MiBa_SA_HCT116_1_mHLA_2hr: 14244
SA = 0.01
Score = −5.59
KLCEGFNEV − MaxQuant
20120617_EXQ0_MiBa_SA_HCT116_1_mHLA_2hr: 14244
SA = 0.84
Score = 1.19
KLCEGFNEV − MSFragger
20120617_EXQ0_MiBa_SA_HCT116_1_mHLA_2hr: 14244
SA = 0.84
Score = 1.19
IPDYTAYVAM − Mascot − rejected
I|P|DYTAYVAM − spliced − 0|6|0|6
20120322_EXQ1_MiBa_SA_HCC1143_1_A: 16753
SA = 0
Score = −10.66
IPDYTRESY − MaxQuant
20120322_EXQ1_MiBa_SA_HCC1143_1_A: 16753
SA = 0.85
Score = 1.22
IPDYTRESY − MSFragger
20120322_EXQ1_MiBa_SA_HCC1143_1_A: 16753
SA = 0.85
Score = 1.22
GRDLTPDHIL − Mascot − rejected
GRDL|TPDHIL − spliced − 3|6|3|6
20120515_EXQ3_MiBa_SA_HCT116_mHLA−1: 23106
SA = 0.01
Score = −8.89
TLDSRRRFS − MaxQuant
20120515_EXQ3_MiBa_SA_HCT116_mHLA−1: 23106
SA = 0.05
Score = −8.15
LTDITKGVQY − MSFragger
20120515_EXQ3_MiBa_SA_HCT116_mHLA−1: 23106
SA = 0.81
Score = 2.93
Relative intensity
m/z
Prosit prediction
⋆ Exclusive in prediction
Exclusive in experimental

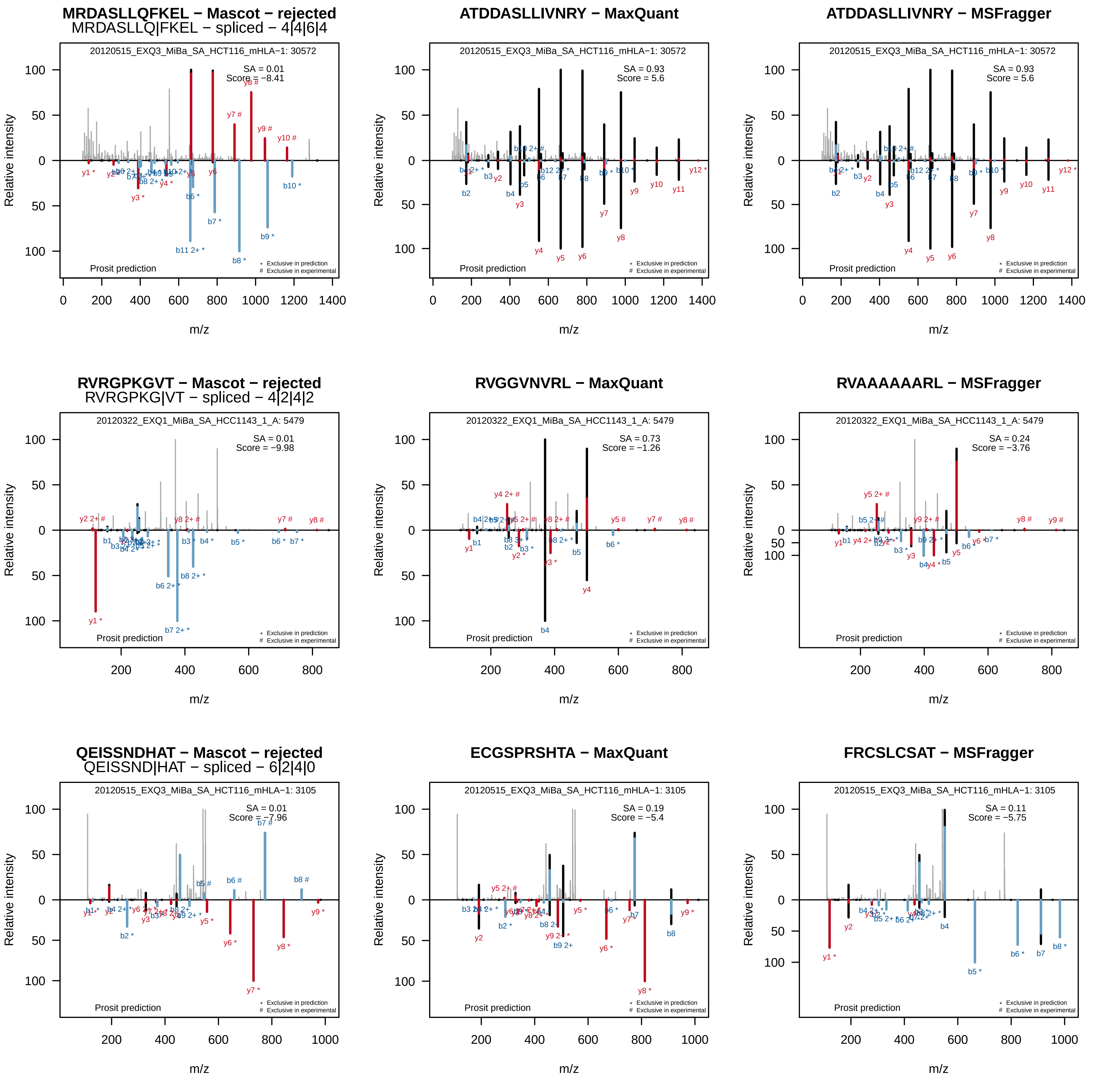

MRDASLLQFKEL − Mascot − rejected
MRDASLLQ|FKEL − spliced − 4|4|6|4
20120515_EXQ3_MiBa_SA_HCT116_mHLA−1: 30572
SA = 0.01
Score = −8.41
ATDDASLLIVNRY − MaxQuant
20120515_EXQ3_MiBa_SA_HCT116_mHLA−1: 30572
SA = 0.93
Score = 5.6
ATDDASLLIVNRY − MSFragger
20120515_EXQ3_MiBa_SA_HCT116_mHLA−1: 30572
SA = 0.93
Score = 5.6
RVRGPKGVT − Mascot − rejected
RVRGPKG|VT − spliced − 4|2|4|2
20120322_EXQ1_MiBa_SA_HCC1143_1_A: 5479
SA = 0.01
Score = −9.98
RVGGVNVRL − MaxQuant
20120322_EXQ1_MiBa_SA_HCC1143_1_A: 5479
SA = 0.73
Score = −1.26
RVAAAAAARL − MSFragger
20120322_EXQ1_MiBa_SA_HCC1143_1_A: 5479
SA = 0.24
Score = −3.76
QEISSNDHAT − Mascot − rejected
QEISSND|HAT − spliced − 6|2|4|0
20120515_EXQ3_MiBa_SA_HCT116_mHLA−1: 3105
SA = 0.01
Score = −7.96
ECGSPRSHTA − MaxQuant
20120515_EXQ3_MiBa_SA_HCT116_mHLA−1: 3105
SA = 0.19
Score = −5.4
FRCSLCSAT − MSFragger
20120515_EXQ3_MiBa_SA_HCT116_mHLA−1: 3105
SA = 0.11
Score = −5.75
Prosit prediction
* Exclusive in prediction
# Exclusive in experimental
Relative intensity
m/z

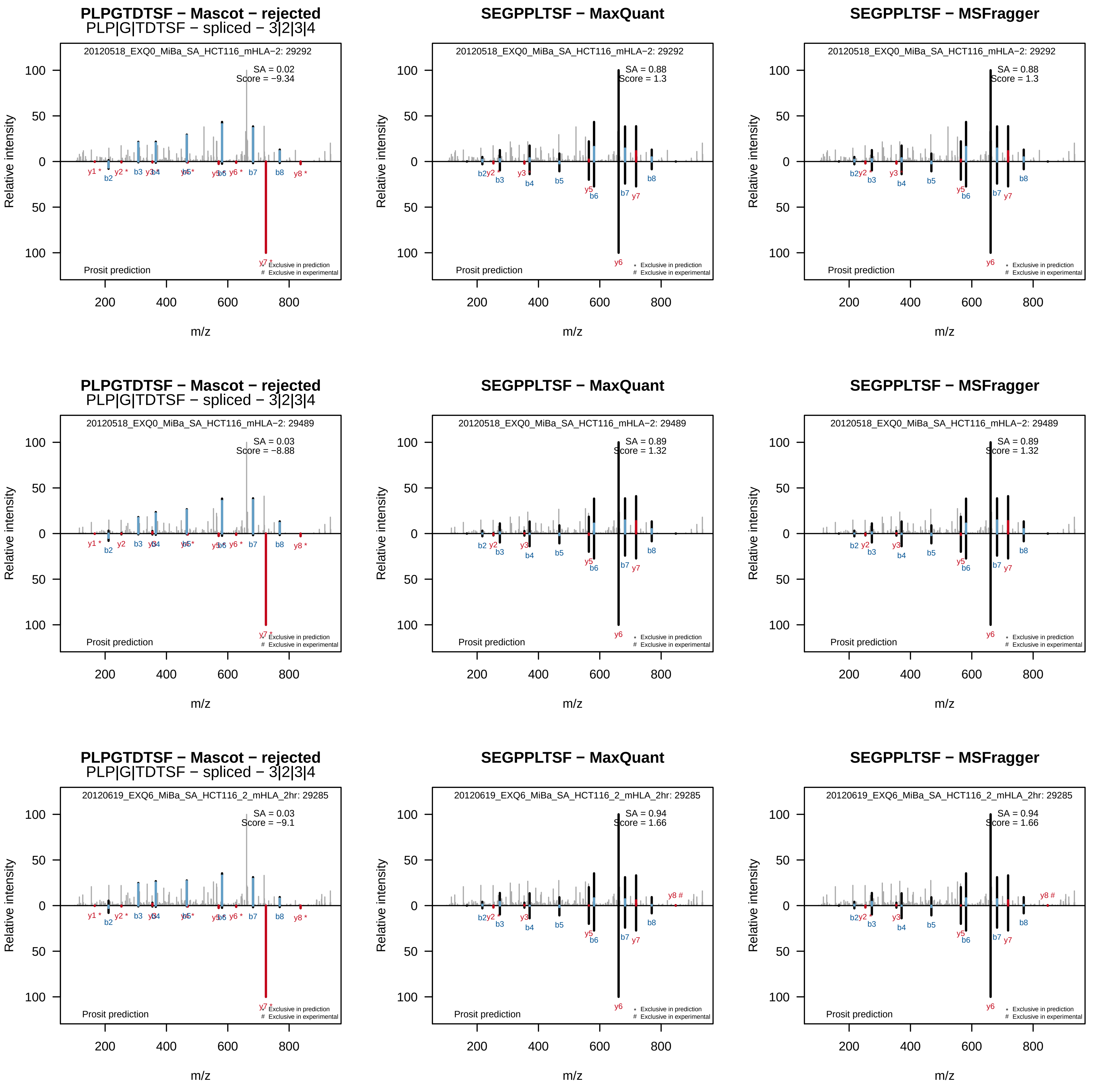

PLPGTDTSF − Mascot − rejected
PLP|G|TDTSF − spliced − 3|2|3|4
20120518_EXQ0_MiBa_SA_HCT116_mHLA−2: 29292
SA = 0.02
Score = −9.34
Prosit prediction
* Exclusive in prediction
# Exclusive in experimental
SEGPPLTSF − MaxQuant
20120518_EXQ0_MiBa_SA_HCT116_mHLA−2: 29292
SA = 0.88
Score = 1.3
Prosit prediction
* Exclusive in prediction
# Exclusive in experimental
SEGPPLTSF − MSFragger
20120518_EXQ0_MiBa_SA_HCT116_mHLA−2: 29292
SA = 0.88
Score = 1.3
Prosit prediction
* Exclusive in prediction
# Exclusive in experimental
PLPGTDTSF − Mascot − rejected
PLP|G|TDTSF − spliced − 3|2|3|4
20120518_EXQ0_MiBa_SA_HCT116_mHLA−2: 29489
SA = 0.03
Score = −8.88
Prosit prediction
* Exclusive in prediction
# Exclusive in experimental
SEGPPLTSF − MaxQuant
20120518_EXQ0_MiBa_SA_HCT116_mHLA−2: 29489
SA = 0.89
Score = 1.32
Prosit prediction
* Exclusive in prediction
# Exclusive in experimental
SEGPPLTSF − MSFragger
20120518_EXQ0_MiBa_SA_HCT116_mHLA−2: 29489
SA = 0.89
Score = 1.32
Prosit prediction
* Exclusive in prediction
# Exclusive in experimental
PLPGTDTSF − Mascot − rejected
PLP|G|TDTSF − spliced − 3|2|3|4
20120619_EXQ6_MiBa_SA_HCT116_2_mHLA_2hr: 29285
SA = 0.03
Score = −9.1
Prosit prediction
* Exclusive in prediction
# Exclusive in experimental
SEGPPLTSF − MaxQuant
20120619_EXQ6_MiBa_SA_HCT116_2_mHLA_2hr: 29285
SA = 0.94
Score = 1.66
Prosit prediction
* Exclusive in prediction
# Exclusive in experimental
SEGPPLTSF − MSFragger
20120619_EXQ6_MiBa_SA_HCT116_2_mHLA_2hr: 29285
SA = 0.94
Score = 1.66
Prosit prediction
* Exclusive in prediction
# Exclusive in experimental
Relative intensity
m/z

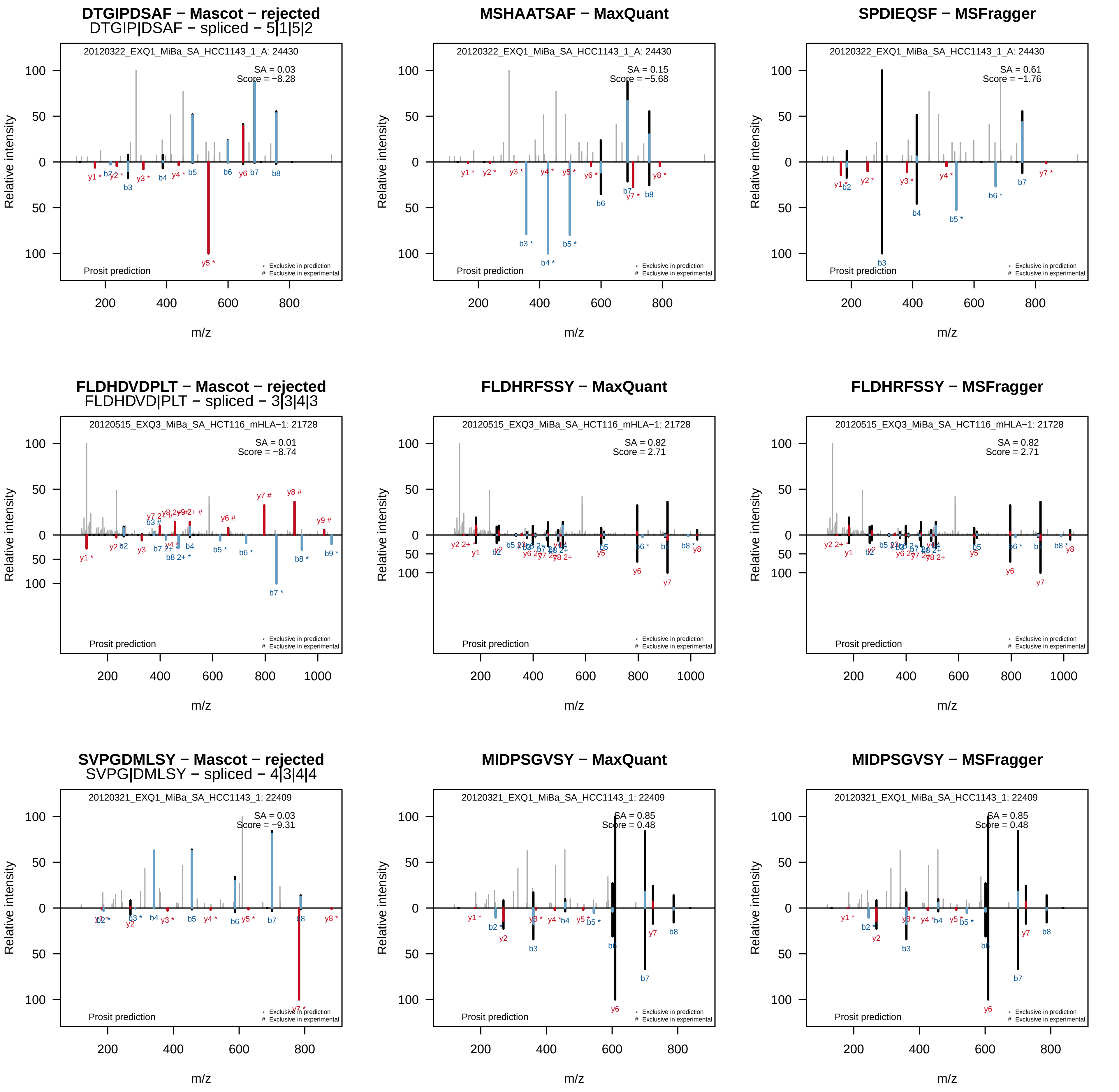
DTGIPDSAF − Mascot − rejected
DTGIP|DSAF − spliced − 5|1|5|2
20120322_EXQ1_MiBa_SA_HCC1143_1_A: 24430
SA = 0.03
Score = −8.28
MSHAATSAF − MaxQuant
20120322_EXQ1_MiBa_SA_HCC1143_1_A: 24430
SA = 0.15
Score = −5.68
SPDIEQSF − MSFragger
20120322_EXQ1_MiBa_SA_HCC1143_1_A: 24430
SA = 0.61
Score = −1.76
FLDHDVDPLT − Mascot − rejected
FLDHDVD|PLT − spliced − 3|3|4|3
20120515_EXQ3_MiBa_SA_HCT116_mHLA−1: 21728
SA = 0.01
Score = −8.74
FLDHRFSSY − MaxQuant
20120515_EXQ3_MiBa_SA_HCT116_mHLA−1: 21728
SA = 0.82
Score = 2.71
FLDHRFSSY − MSFragger
20120515_EXQ3_MiBa_SA_HCT116_mHLA−1: 21728
SA = 0.82
Score = 2.71
SVPGDMLSY − Mascot − rejected
SVPG|DMLSY − spliced − 4|3|4|4
20120321_EXQ1_MiBa_SA_HCC1143_1: 22409
SA = 0.03
Score = −9.31
MIDPSGVSY − MaxQuant
20120321_EXQ1_MiBa_SA_HCC1143_1: 22409
SA = 0.85
Score = 0.48
MIDPSGVSY − MSFragger
20120321_EXQ1_MiBa_SA_HCC1143_1: 22409
SA = 0.85
Score = 0.48
Relative intensity
m/z
Prosit prediction
* Exclusive in prediction
# Exclusive in experimental

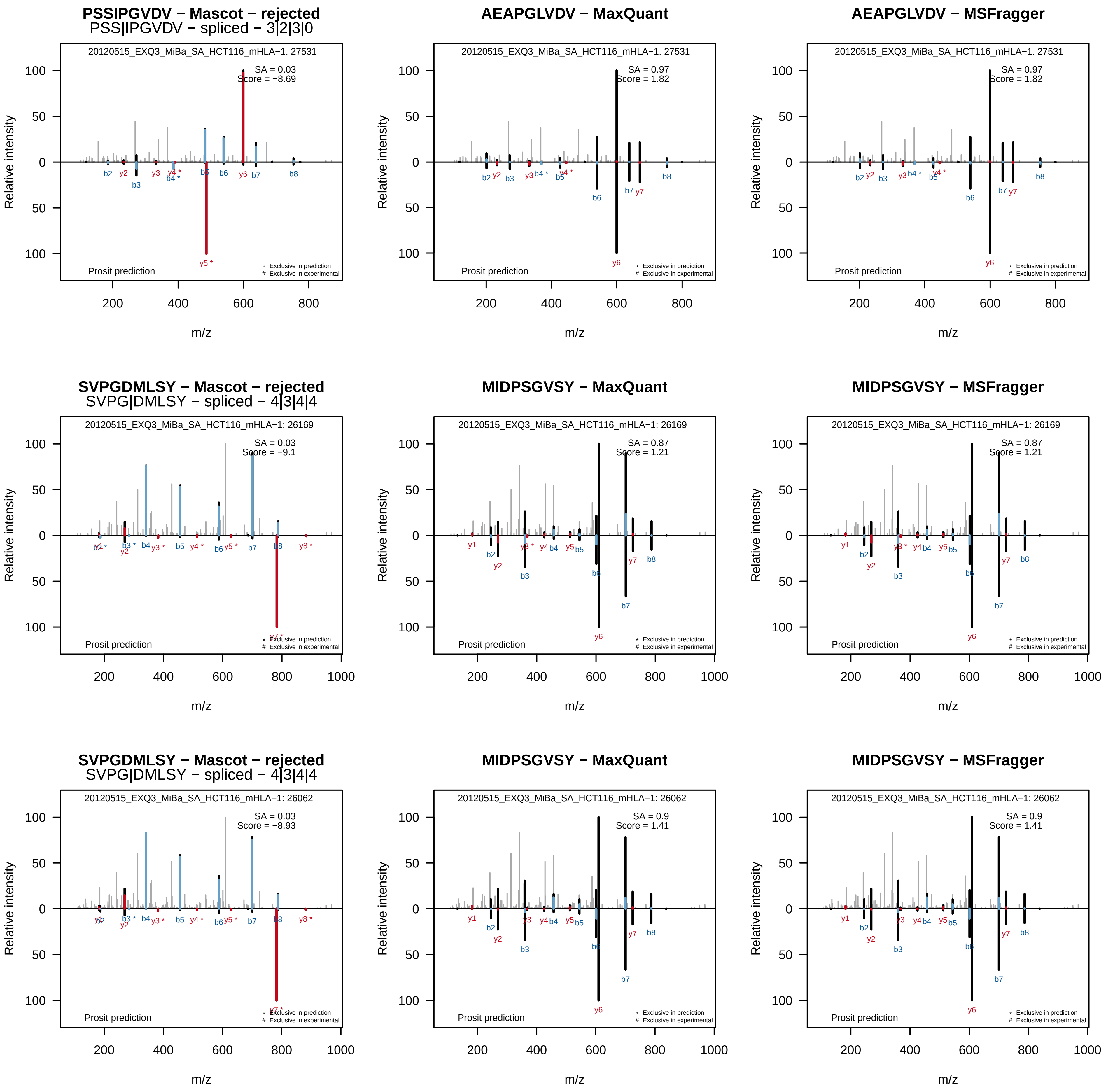
PSSIPGVDV − Mascot − rejected
PSS|IPGVDV − spliced − 3|2|3|0
20120515_EXQ3_MiBa_SA_HCT116_mHLA−1: 27531
SA = 0.03
Score = −8.69
Prosit prediction
AEAPGLVDV − MaxQuant
20120515_EXQ3_MiBa_SA_HCT116_mHLA−1: 27531
SA = 0.97
Score = 1.82
Prosit prediction
AEAPGLVDV − MSFragger
20120515_EXQ3_MiBa_SA_HCT116_mHLA−1: 27531
SA = 0.97
Score = 1.82
Prosit prediction
SVPGDMLSY − Mascot − rejected
SVPG|DMLSY − spliced − 4|3|4|4
20120515_EXQ3_MiBa_SA_HCT116_mHLA−1: 26169
SA = 0.03
Score = −9.1
Prosit prediction
MIDPSGVSY − MaxQuant
20120515_EXQ3_MiBa_SA_HCT116_mHLA−1: 26169
SA = 0.87
Score = 1.21
Prosit prediction
MIDPSGVSY − MSFragger
20120515_EXQ3_MiBa_SA_HCT116_mHLA−1: 26169
SA = 0.87
Score = 1.21
Prosit prediction
SVPGDMLSY − Mascot − rejected
SVPG|DMLSY − spliced − 4|3|4|4
20120515_EXQ3_MiBa_SA_HCT116_mHLA−1: 26062
SA = 0.03
Score = −8.93
Prosit prediction
MIDPSGVSY − MaxQuant
20120515_EXQ3_MiBa_SA_HCT116_mHLA−1: 26062
SA = 0.9
Score = 1.41
Prosit prediction
MIDPSGVSY − MSFragger
20120515_EXQ3_MiBa_SA_HCT116_mHLA−1: 26062
SA = 0.9
Score = 1.41
Prosit prediction
⋆ Exclusive in prediction
Exclusive in experimental
Relative intensity
m/z

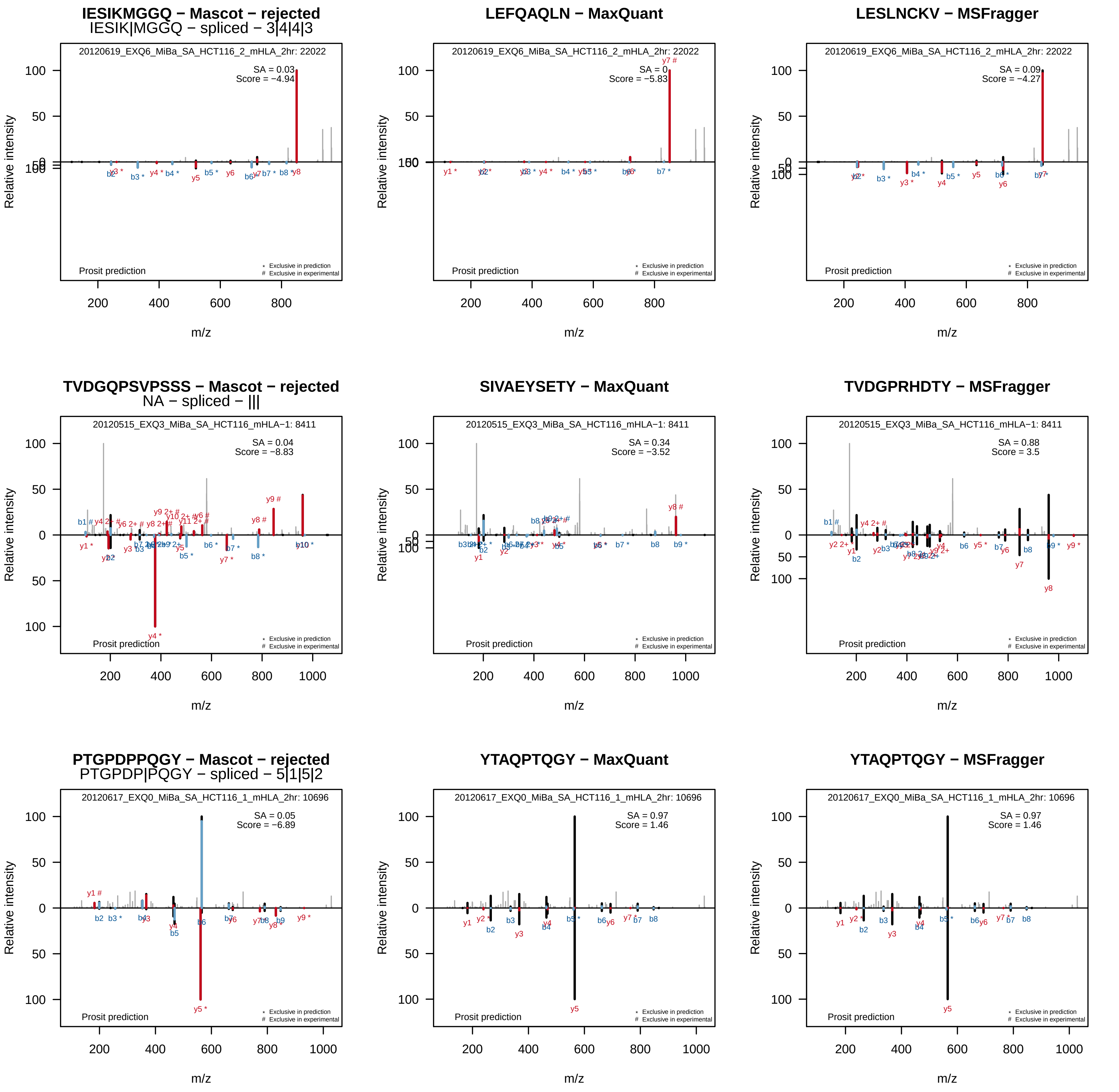
IESIKMGGQ − Mascot − rejected
IESIK|MGGQ − spliced − 3|4|4|3
20120619_EXQ6_MiBa_SA_HCT116_2_mHLA_2hr: 22022
SA = 0.03
Score = −4.94
LEFQAQLN − MaxQuant
20120619_EXQ6_MiBa_SA_HCT116_2_mHLA_2hr: 22022
SA = 0
Score = −5.83
LESLNCKV − MSFragger
20120619_EXQ6_MiBa_SA_HCT116_2_mHLA_2hr: 22022
SA = 0.09
Score = −4.27
TVDGQPSVPSSS − Mascot − rejected
NA − spliced − |||
20120515_EXQ3_MiBa_SA_HCT116_mHLA−1: 8411
SA = 0.04
Score = −8.83
SIVAEYSETY − MaxQuant
20120515_EXQ3_MiBa_SA_HCT116_mHLA−1: 8411
SA = 0.34
Score = −3.52
TVDGPRHDTY − MSFragger
20120515_EXQ3_MiBa_SA_HCT116_mHLA−1: 8411
SA = 0.88
Score = 3.5
PTGPDPPQGY − Mascot − rejected
PTGPDP|PQGY − spliced − 5|1|5|2
20120617_EXQ0_MiBa_SA_HCT116_1_mHLA_2hr: 10696
SA = 0.05
Score = −6.89
YTAQPTQGY − MaxQuant
20120617_EXQ0_MiBa_SA_HCT116_1_mHLA_2hr: 10696
SA = 0.97
Score = 1.46
YTAQPTQGY − MSFragger
20120617_EXQ0_MiBa_SA_HCT116_1_mHLA_2hr: 10696
SA = 0.97
Score = 1.46
Relative intensity
m/z
Prosit prediction
* Exclusive in prediction
# Exclusive in experimental

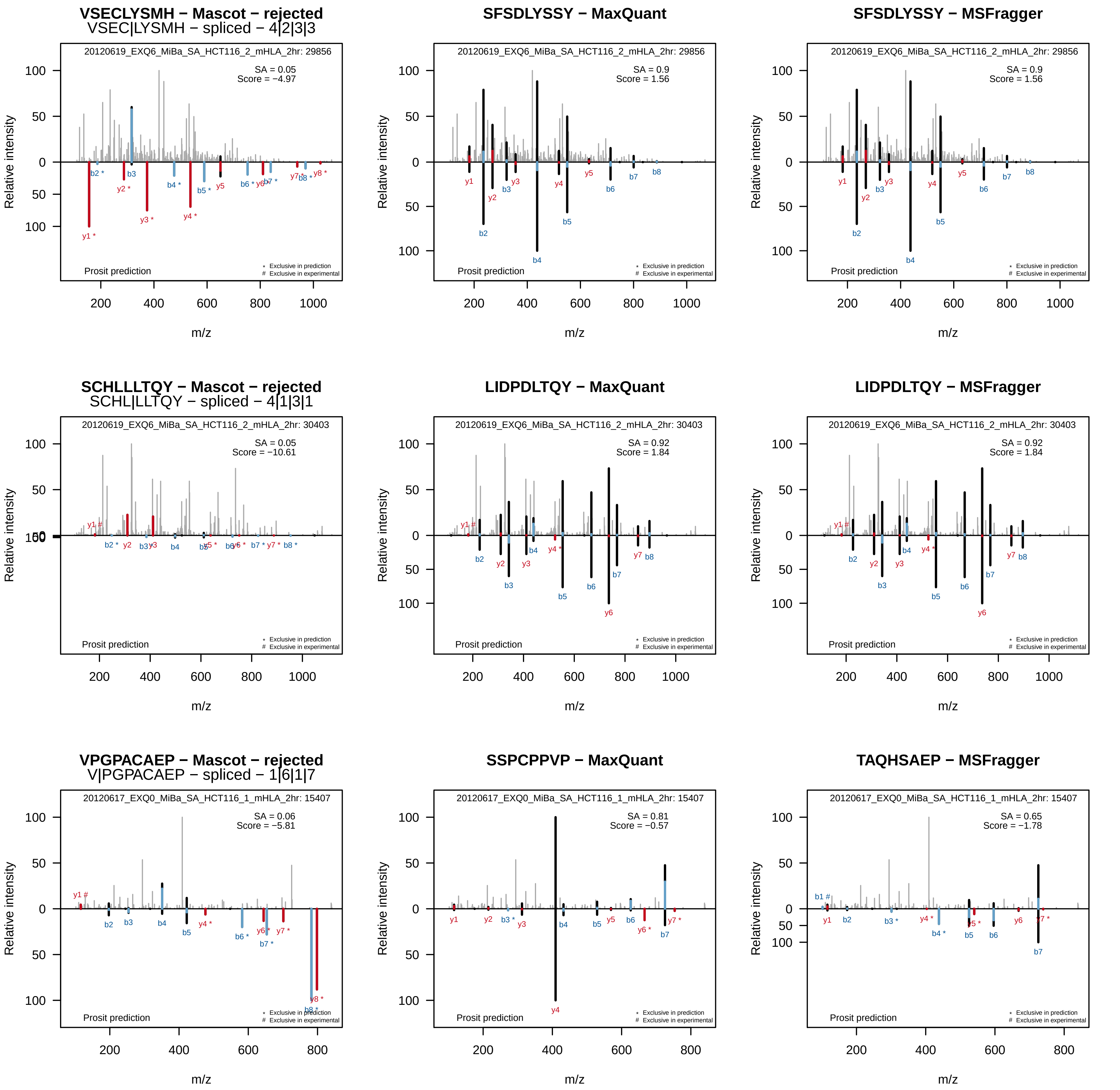

VSECLYSMH − Mascot − rejected
VSEC|LYSMH − spliced − 4|2|3|3
20120619_EXQ6_MiBa_SA_HCT116_2_mHLA_2hr: 29856
SA = 0.05
Score = −4.97
SFSDLYSSY − MaxQuant
20120619_EXQ6_MiBa_SA_HCT116_2_mHLA_2hr: 29856
SA = 0.9
Score = 1.56
SFSDLYSSY − MSFragger
20120619_EXQ6_MiBa_SA_HCT116_2_mHLA_2hr: 29856
SA = 0.9
Score = 1.56
SCHLLLTQY − Mascot − rejected
SCHL|LLTQY − spliced − 4|1|3|1
20120619_EXQ6_MiBa_SA_HCT116_2_mHLA_2hr: 30403
SA = 0.05
Score = −10.61
LIDPDLTQY − MaxQuant
20120619_EXQ6_MiBa_SA_HCT116_2_mHLA_2hr: 30403
SA = 0.92
Score = 1.84
LIDPDLTQY − MSFragger
20120619_EXQ6_MiBa_SA_HCT116_2_mHLA_2hr: 30403
SA = 0.92
Score = 1.84
VPGPACAEP − Mascot − rejected
V|PGPACAEP − spliced − 1|6|1|7
20120617_EXQ0_MiBa_SA_HCT116_1_mHLA_2hr: 15407
SA = 0.06
Score = −5.81
SSPCPPVP − MaxQuant
20120617_EXQ0_MiBa_SA_HCT116_1_mHLA_2hr: 15407
SA = 0.81
Score = −0.57
TAQHSAEP − MSFragger
20120617_EXQ0_MiBa_SA_HCT116_1_mHLA_2hr: 15407
SA = 0.65
Score = −1.78
Prosit prediction
⋆ Exclusive in prediction
Exclusive in experimental
Relative intensity
m/z

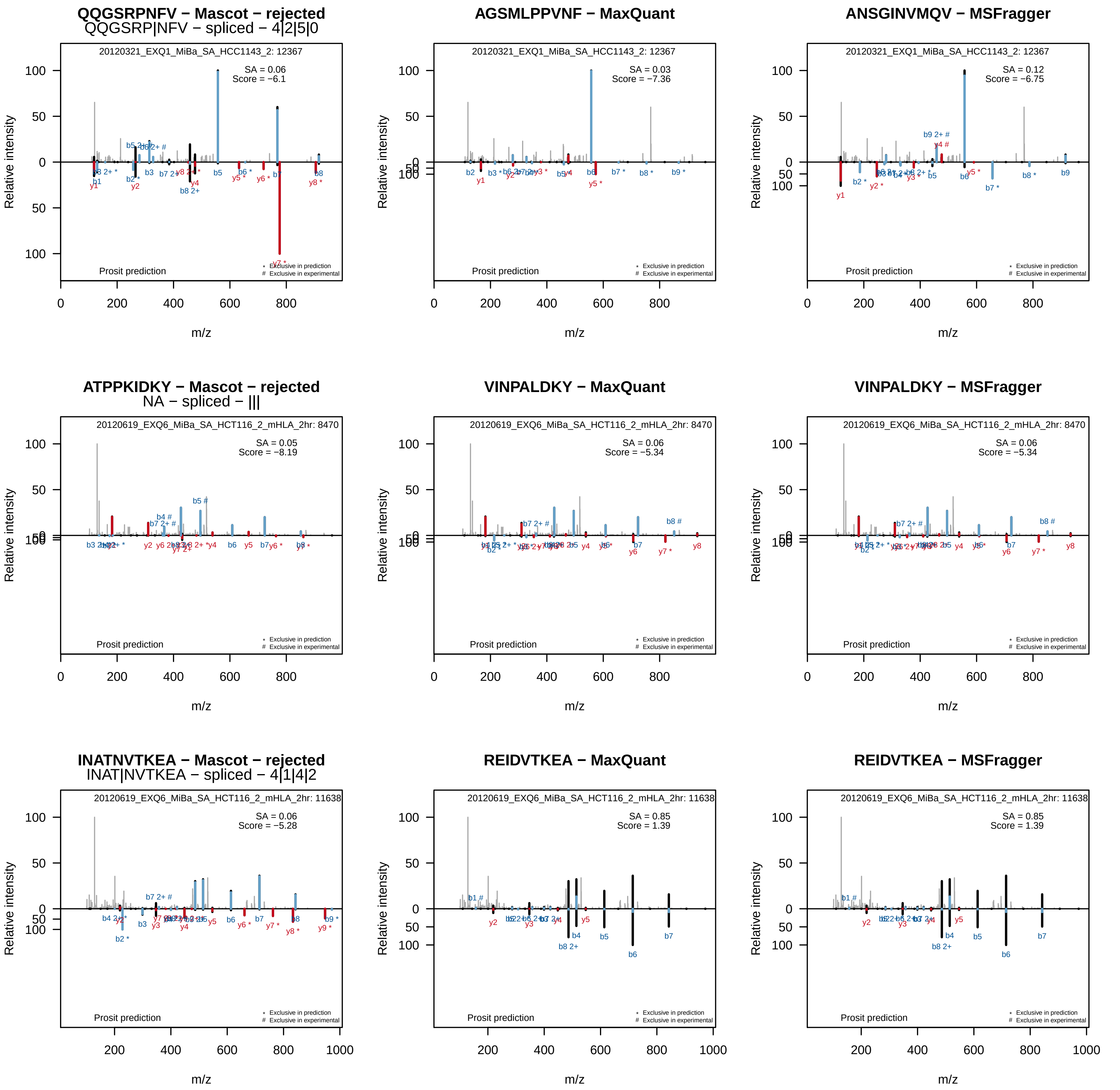
QQGSRPNFV − Mascot − rejected
QQGSRP|NFV − spliced − 4|2|5|0
20120321_EXQ1_MiBa_SA_HCC1143_2: 12367
SA = 0.06
Score = −6.1
AGSMLPPVNF − MaxQuant
20120321_EXQ1_MiBa_SA_HCC1143_2: 12367
SA = 0.03
Score = −7.36
ANSGINVMQV − MSFragger
20120321_EXQ1_MiBa_SA_HCC1143_2: 12367
SA = 0.12
Score = −6.75
ATPPKIDKY − Mascot − rejected
NA − spliced − |||
20120619_EXQ6_MiBa_SA_HCT116_2_mHLA_2hr: 8470
SA = 0.05
Score = −8.19
VINPALDKY − MaxQuant
20120619_EXQ6_MiBa_SA_HCT116_2_mHLA_2hr: 8470
SA = 0.06
Score = −5.34
VINPALDKY − MSFragger
20120619_EXQ6_MiBa_SA_HCT116_2_mHLA_2hr: 8470
SA = 0.06
Score = −5.34
INATNVTKEA − Mascot − rejected
INAT|NVTKEA − spliced − 4|1|4|2
20120619_EXQ6_MiBa_SA_HCT116_2_mHLA_2hr: 11638
SA = 0.06
Score = −5.28
REIDVTKEA − MaxQuant
20120619_EXQ6_MiBa_SA_HCT116_2_mHLA_2hr: 11638
SA = 0.85
Score = 1.39
REIDVTKEA − MSFragger
20120619_EXQ6_MiBa_SA_HCT116_2_mHLA_2hr: 11638
SA = 0.85
Score = 1.39
Relative intensity
m/z
Prosit prediction
⋆ Exclusive in prediction
Exclusive in experimental

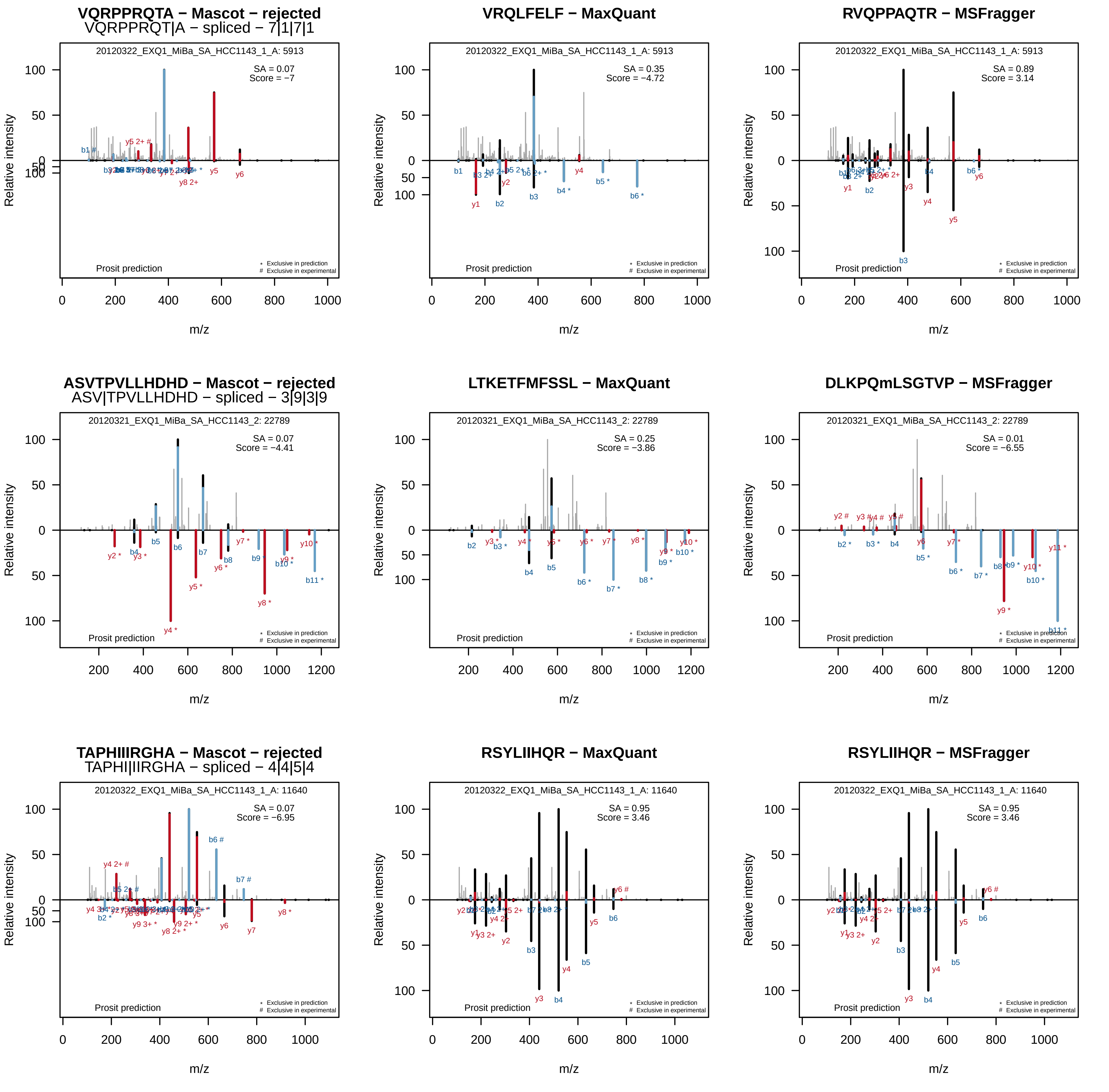
VQRPPRQTA − Mascot − rejected
VQRPPRQT|A − spliced − 7|1|7|1
20120322_EXQ1_MiBa_SA_HCC1143_1_A: 5913
SA = 0.07
Score = −7
VRQLFELF − MaxQuant
20120322_EXQ1_MiBa_SA_HCC1143_1_A: 5913
SA = 0.35
Score = −4.72
RVQPPAQTR − MSFragger
20120322_EXQ1_MiBa_SA_HCC1143_1_A: 5913
SA = 0.89
Score = 3.14
ASVTPVLLHDHD − Mascot − rejected
ASV|TPVLLHDHD − spliced − 3|9|3|9
20120321_EXQ1_MiBa_SA_HCC1143_2: 22789
SA = 0.07
Score = −4.41
LTKETFMFSSL − MaxQuant
20120321_EXQ1_MiBa_SA_HCC1143_2: 22789
SA = 0.25
Score = −3.86
DLKPQmLSGTVP − MSFragger
20120321_EXQ1_MiBa_SA_HCC1143_2: 22789
SA = 0.01
Score = −6.55
TAPHIIIRGHA − Mascot − rejected
TAPHI|IIRGHA − spliced − 4|4|5|4
20120322_EXQ1_MiBa_SA_HCC1143_1_A: 11640
SA = 0.07
Score = −6.95
RSYLIIHQR − MaxQuant
20120322_EXQ1_MiBa_SA_HCC1143_1_A: 11640
SA = 0.95
Score = 3.46
RSYLIIHQR − MSFragger
20120322_EXQ1_MiBa_SA_HCC1143_1_A: 11640
SA = 0.95
Score = 3.46
Relative intensity
m/z
Prosit prediction
* Exclusive in prediction
# Exclusive in experimental

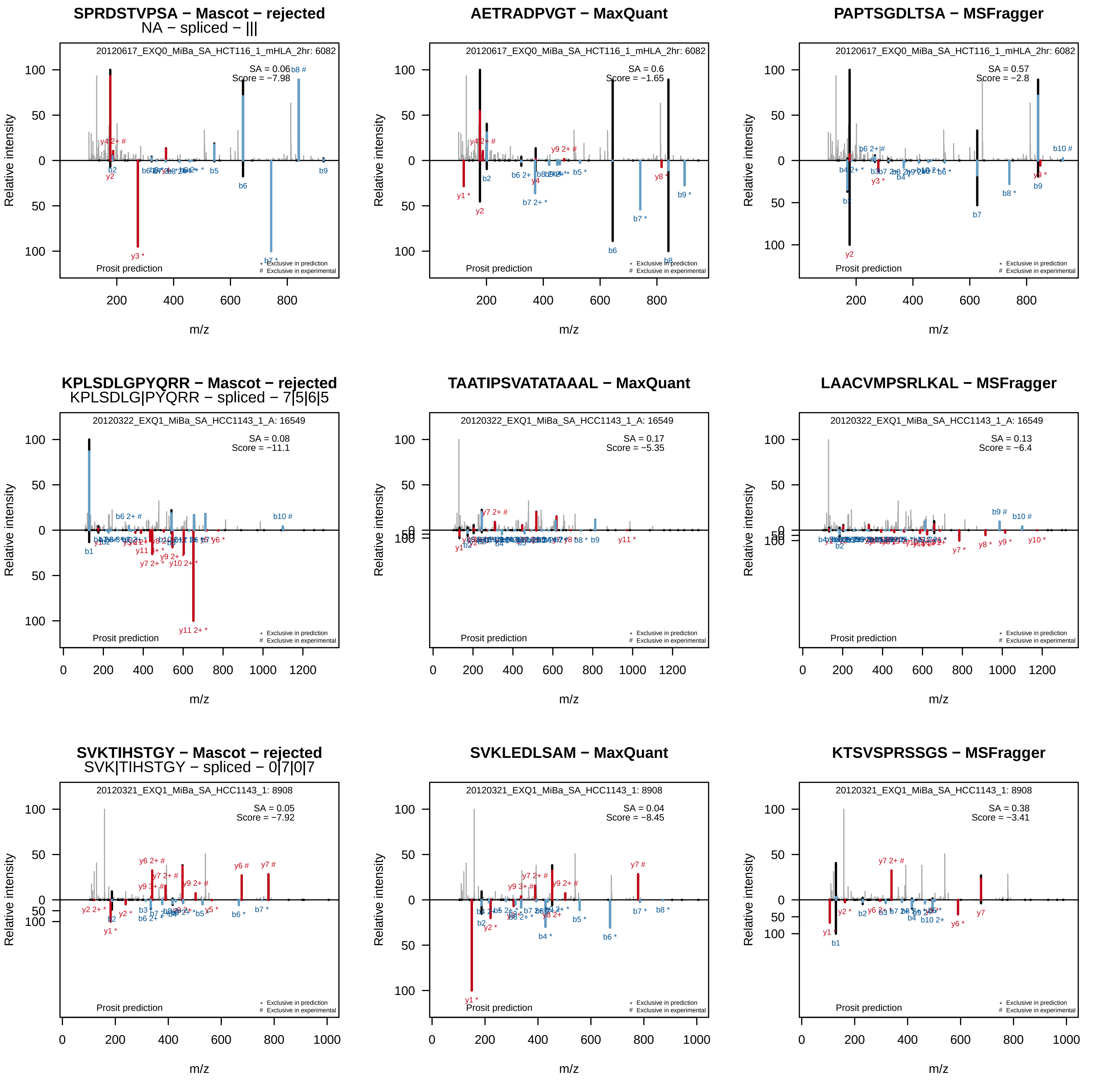
SPRDSTVPSA − Mascot − rejected
NA − spliced − |||
20120617_EXQ0_MiBa_SA_HCT116_1_mHLA_2hr: 6082
SA = 0.06
Score = −7.98
AETRADPVGT − MaxQuant
20120617_EXQ0_MiBa_SA_HCT116_1_mHLA_2hr: 6082
SA = 0.6
Score = −1.65
PAPTSGDLTSA − MSFragger
20120617_EXQ0_MiBa_SA_HCT116_1_mHLA_2hr: 6082
SA = 0.57
Score = −2.8
KPLSDLGPYQRR − Mascot − rejected
KPLSDLG|PYQRR − spliced − 7|5|6|5
20120322_EXQ1_MiBa_SA_HCC1143_1_A: 16549
SA = 0.08
Score = −11.1
TAATIPSVATATAAAL − MaxQuant
20120322_EXQ1_MiBa_SA_HCC1143_1_A: 16549
SA = 0.17
Score = −5.35
LAACVMPSRLKAL − MSFragger
20120322_EXQ1_MiBa_SA_HCC1143_1_A: 16549
SA = 0.13
Score = −6.4
SVKTIHSTGY − Mascot − rejected
SVK|TIHSTGY − spliced − 0|7|0|7
20120321_EXQ1_MiBa_SA_HCC1143_1: 8908
SA = 0.05
Score = −7.92
SVKLEDLSAM − MaxQuant
20120321_EXQ1_MiBa_SA_HCC1143_1: 8908
SA = 0.04
Score = −8.45
KTSVSPRSSGS − MSFragger
20120321_EXQ1_MiBa_SA_HCC1143_1: 8908
SA = 0.38
Score = −3.41
Relative intensity
m/z
Prosit prediction
⋆ Exclusive in prediction
Exclusive in experimental

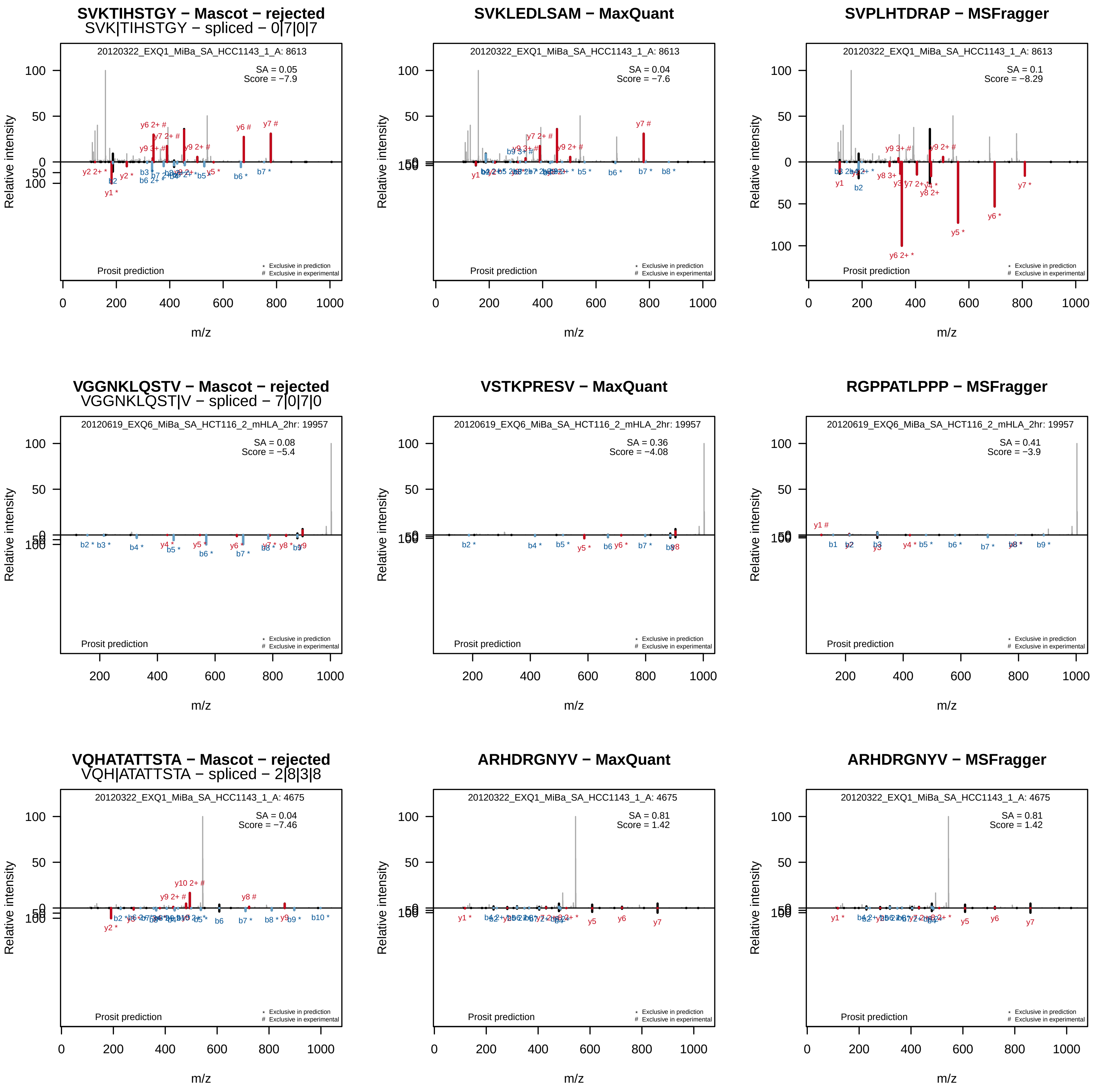
SVKTIHSTGY − Mascot − rejected
SVK|TIHSTGY − spliced − 0|7|0|7
20120322_EXQ1_MiBa_SA_HCC1143_1_A: 8613
SA = 0.05
Score = −7.9
SVKLEDLSAM − MaxQuant
20120322_EXQ1_MiBa_SA_HCC1143_1_A: 8613
SA = 0.04
Score = −7.6
SVPLHTDRAP − MSFragger
20120322_EXQ1_MiBa_SA_HCC1143_1_A: 8613
SA = 0.1
Score = −8.29
VGGNKLQSTV − Mascot − rejected
VGGNKLQST|V − spliced − 7|0|7|0
20120619_EXQ6_MiBa_SA_HCT116_2_mHLA_2hr: 19957
SA = 0.08
Score = −5.4
VSTKPRESV − MaxQuant
20120619_EXQ6_MiBa_SA_HCT116_2_mHLA_2hr: 19957
SA = 0.36
Score = −4.08
RGPPATLPPP − MSFragger
20120619_EXQ6_MiBa_SA_HCT116_2_mHLA_2hr: 19957
SA = 0.41
Score = −3.9
VQHATATTSTA − Mascot − rejected
VQH|ATATTSTA − spliced − 2|8|3|8
20120322_EXQ1_MiBa_SA_HCC1143_1_A: 4675
SA = 0.04
Score = −7.46
ARHDRGNYV − MaxQuant
20120322_EXQ1_MiBa_SA_HCC1143_1_A: 4675
SA = 0.81
Score = 1.42
ARHDRGNYV − MSFragger
20120322_EXQ1_MiBa_SA_HCC1143_1_A: 4675
SA = 0.81
Score = 1.42
Relative intensity
m/z
Prosit prediction
* Exclusive in prediction
# Exclusive in experimental

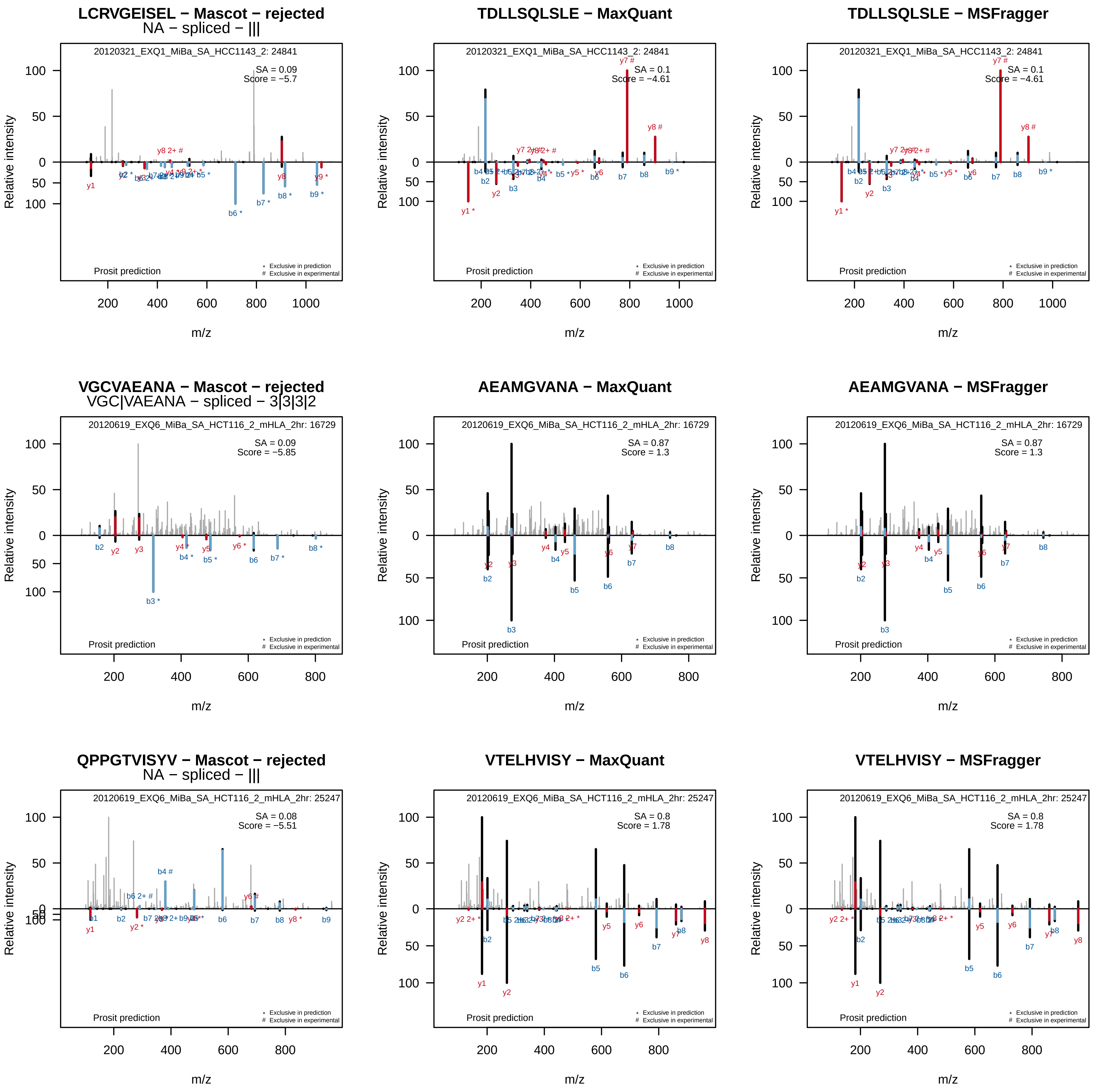
LCRVGEISEL − Mascot − rejected
NA − spliced − |||
20120321_EXQ1_MiBa_SA_HCC1143_2: 24841
SA = 0.09
Score = −5.7
TDLLSQLSLE − MaxQuant
20120321_EXQ1_MiBa_SA_HCC1143_2: 24841
SA = 0.1
Score = −4.61
TDLLSQLSLE − MSFragger
20120321_EXQ1_MiBa_SA_HCC1143_2: 24841
SA = 0.1
Score = −4.61
VGCVAEANA − Mascot − rejected
VGC|VAEANA − spliced − 3|3|3|2
20120619_EXQ6_MiBa_SA_HCT116_2_mHLA_2hr: 16729
SA = 0.09
Score = −5.85
AEAMGVANA − MaxQuant
20120619_EXQ6_MiBa_SA_HCT116_2_mHLA_2hr: 16729
SA = 0.87
Score = 1.3
AEAMGVANA − MSFragger
20120619_EXQ6_MiBa_SA_HCT116_2_mHLA_2hr: 16729
SA = 0.87
Score = 1.3
QPPGTVISYV − Mascot − rejected
NA − spliced − |||
20120619_EXQ6_MiBa_SA_HCT116_2_mHLA_2hr: 25247
SA = 0.08
Score = −5.51
VTELHVISY − MaxQuant
20120619_EXQ6_MiBa_SA_HCT116_2_mHLA_2hr: 25247
SA = 0.8
Score = 1.78
VTELHVISY − MSFragger
20120619_EXQ6_MiBa_SA_HCT116_2_mHLA_2hr: 25247
SA = 0.8
Score = 1.78
Relative intensity
m/z
Prosit prediction
* Exclusive in prediction
# Exclusive in experimental

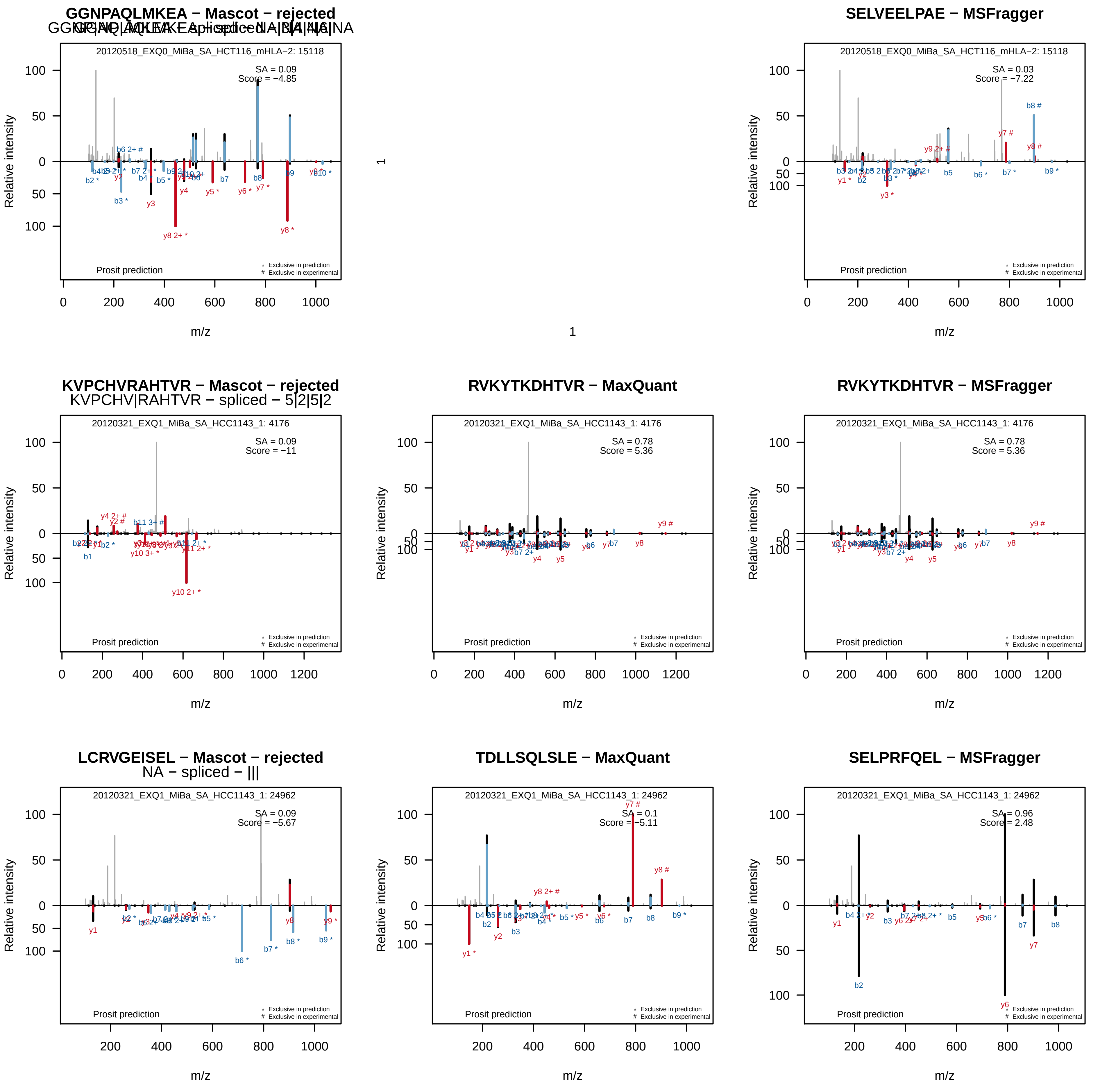

GGNPAQLMKEA − Mascot − rejected
20120518_EXQ0_MiBa_SA_HCT116_mHLA−2: 15118
SA = 0.09
Score = −4.85
SELVEELPAE − MSFragger
20120518_EXQ0_MiBa_SA_HCT116_mHLA−2: 15118
SA = 0.03
Score = −7.22
KVPCHVRAHTVR − Mascot − rejected
KVPCHV|RAHTVR − spliced − 5|2|5|2
20120321_EXQ1_MiBa_SA_HCC1143_1: 4176
SA = 0.09
Score = −11
RVKYTKDHTVR − MaxQuant
20120321_EXQ1_MiBa_SA_HCC1143_1: 4176
SA = 0.78
Score = 5.36
RVKYTKDHTVR − MSFragger
20120321_EXQ1_MiBa_SA_HCC1143_1: 4176
SA = 0.78
Score = 5.36
LCRVGEISEL − Mascot − rejected
NA − spliced − |||
20120321_EXQ1_MiBa_SA_HCC1143_1: 24962
SA = 0.09
Score = −5.67
TDLLSQLSLE − MaxQuant
20120321_EXQ1_MiBa_SA_HCC1143_1: 24962
SA = 0.1
Score = −5.11
SELPRFQEL − MSFragger
20120321_EXQ1_MiBa_SA_HCC1143_1: 24962
SA = 0.96
Score = 2.48
Relative intensity
m/z
Prosit prediction
* Exclusive in prediction
# Exclusive in experimental

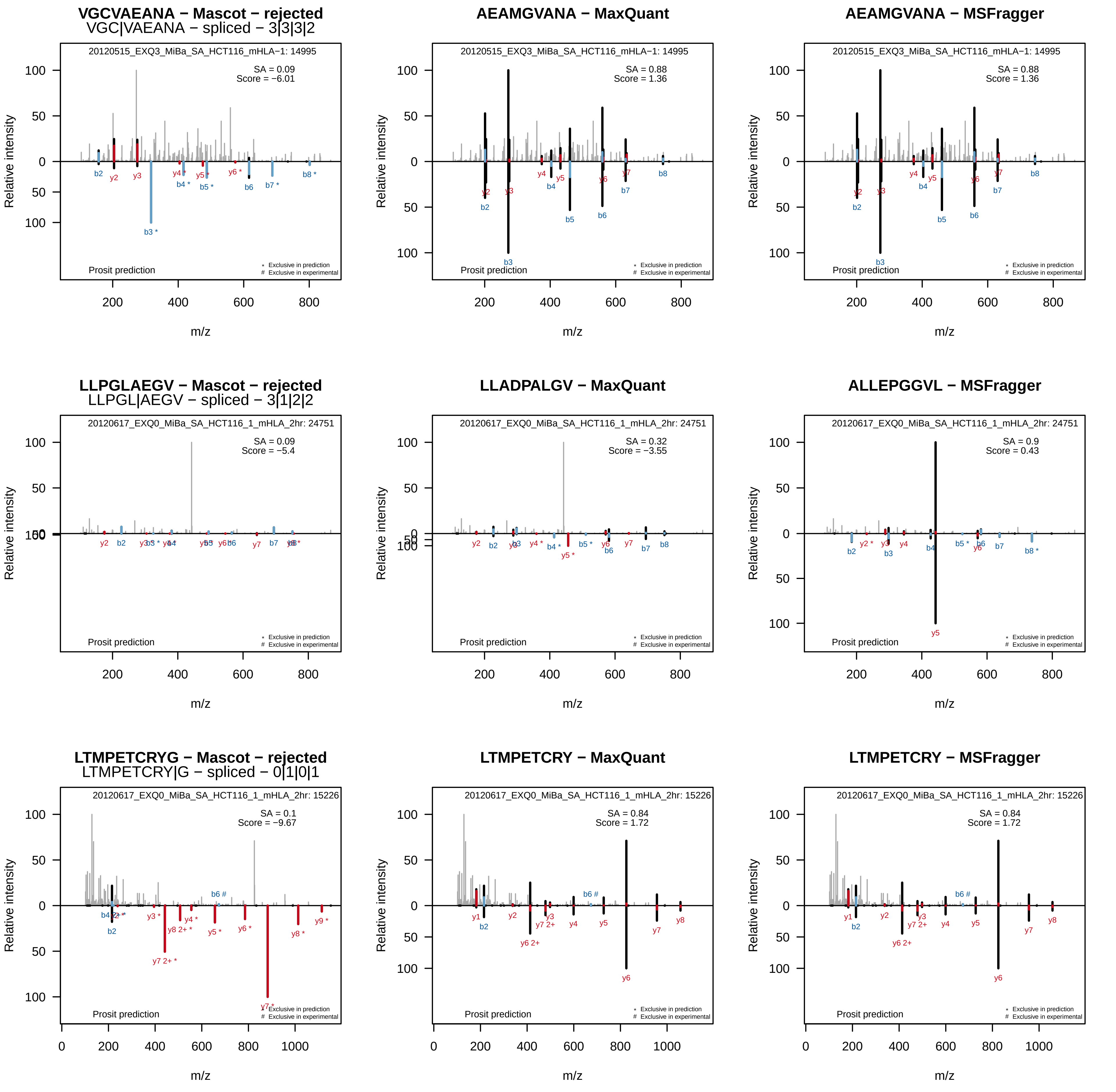

VGCVAEANA − Mascot − rejected
VGC|VAEANA − spliced − 3|3|3|2
20120515_EXQ3_MiBa_SA_HCT116_mHLA−1: 14995
SA = 0.09
Score = −6.01
AEAMGVANA − MaxQuant
20120515_EXQ3_MiBa_SA_HCT116_mHLA−1: 14995
SA = 0.88
Score = 1.36
AEAMGVANA − MSFragger
20120515_EXQ3_MiBa_SA_HCT116_mHLA−1: 14995
SA = 0.88
Score = 1.36
LLPGLAEGV − Mascot − rejected
LLPGL|AEGV − spliced − 3|1|2|2
20120617_EXQ0_MiBa_SA_HCT116_1_mHLA_2hr: 24751
SA = 0.09
Score = −5.4
LLADPALGV − MaxQuant
20120617_EXQ0_MiBa_SA_HCT116_1_mHLA_2hr: 24751
SA = 0.32
Score = −3.55
ALLEPGGVL − MSFragger
20120617_EXQ0_MiBa_SA_HCT116_1_mHLA_2hr: 24751
SA = 0.9
Score = 0.43
LTMPETCRYG − Mascot − rejected
LTMPETCRY|G − spliced − 0|1|0|1
20120617_EXQ0_MiBa_SA_HCT116_1_mHLA_2hr: 15226
SA = 0.1
Score = −9.67
LTMPETCRY − MaxQuant
20120617_EXQ0_MiBa_SA_HCT116_1_mHLA_2hr: 15226
SA = 0.84
Score = 1.72
LTMPETCRY − MSFragger
20120617_EXQ0_MiBa_SA_HCT116_1_mHLA_2hr: 15226
SA = 0.84
Score = 1.72
Relative intensity
m/z
Prosit prediction
⋆ Exclusive in prediction
Exclusive in experimental

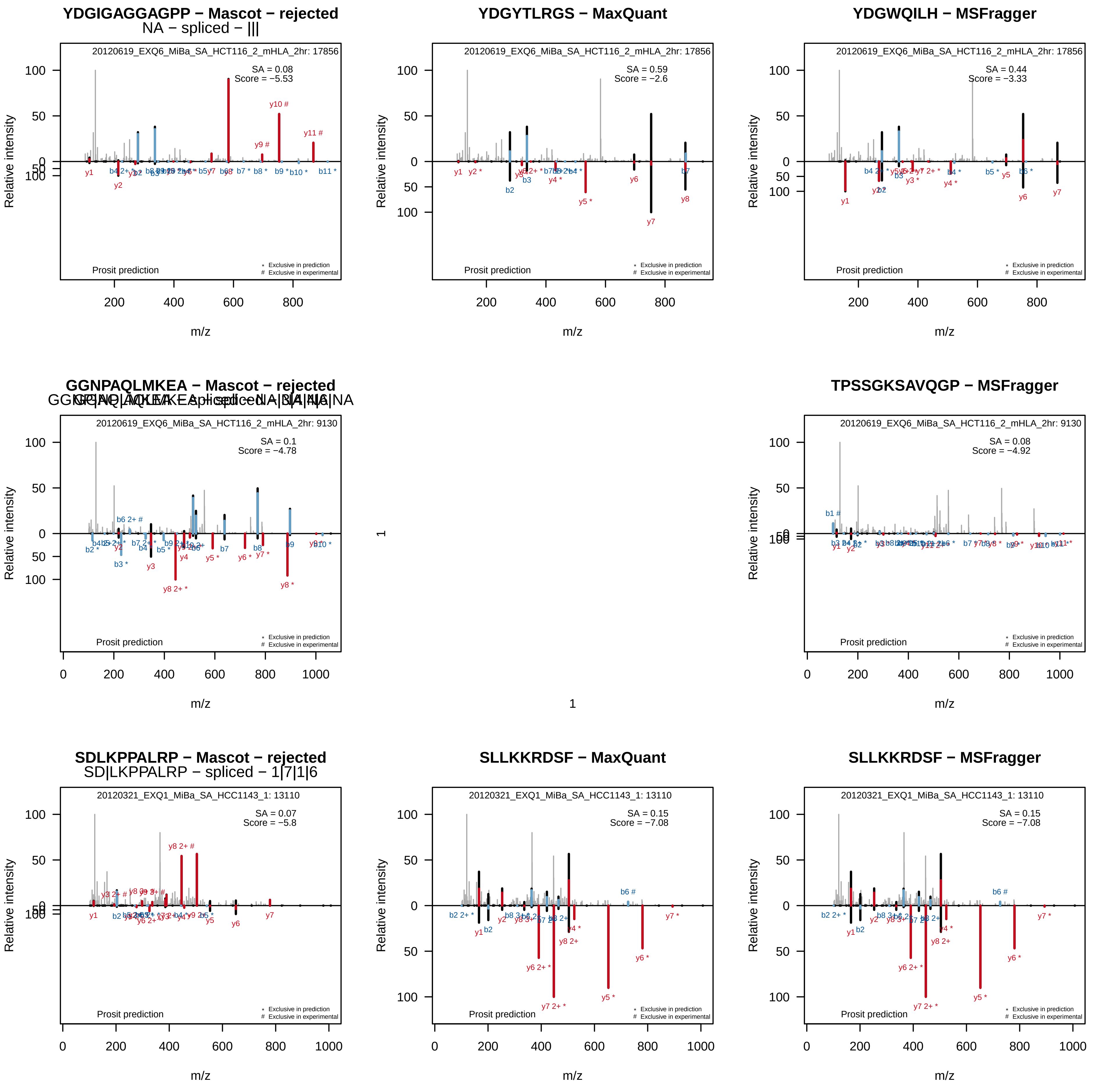
YDGIGAGGAGPP − Mascot − rejected
NA − spliced − |||
20120619_EXQ6_MiBa_SA_HCT116_2_mHLA_2hr: 17856
SA = 0.08
Score = −5.53
YDGYTLRGS − MaxQuant
20120619_EXQ6_MiBa_SA_HCT116_2_mHLA_2hr: 17856
SA = 0.59
Score = −2.6
YDGWQILH − MSFragger
20120619_EXQ6_MiBa_SA_HCT116_2_mHLA_2hr: 17856
SA = 0.44
Score = −3.33
GGNPAQLMKEA − Mascot − rejected
20120619_EXQ6_MiBa_SA_HCT116_2_mHLA_2hr: 9130
SA = 0.1
Score = −4.78
TPSSGKSAVQGP − MSFragger
20120619_EXQ6_MiBa_SA_HCT116_2_mHLA_2hr: 9130
SA = 0.08
Score = −4.92
SDLKPPALRP − Mascot − rejected
SD|LKPPALRP − spliced − 1|7|1|6
20120321_EXQ1_MiBa_SA_HCC1143_1: 13110
SA = 0.07
Score = −5.8
SLLKKRDSF − MaxQuant
20120321_EXQ1_MiBa_SA_HCC1143_1: 13110
SA = 0.15
Score = −7.08
SLLKKRDSF − MSFragger
20120321_EXQ1_MiBa_SA_HCC1143_1: 13110
SA = 0.15
Score = −7.08
Relative intensity
m/z
Prosit prediction
⋆ Exclusive in prediction
Exclusive in experimental

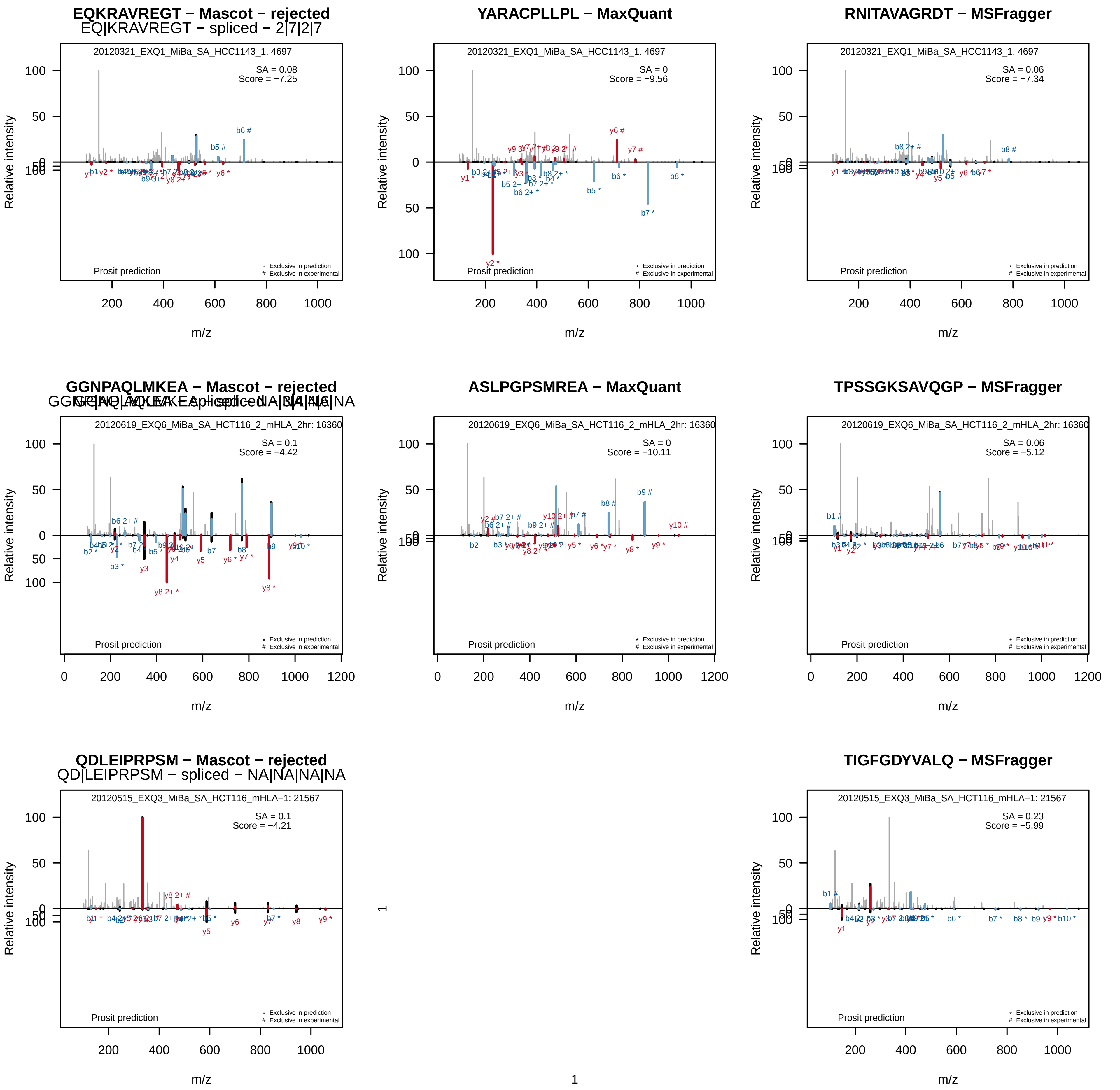

EQKRAVREGT − Mascot − rejected
EQ|KRAVREGT − spliced − 2|7|2|7
20120321_EXQ1_MiBa_SA_HCC1143_1: 4697
SA = 0.08
Score = −7.25
YARACPLLPL − MaxQuant
20120321_EXQ1_MiBa_SA_HCC1143_1: 4697
SA = 0
Score = −9.56
RNITAVAGRDT − MSFragger
20120321_EXQ1_MiBa_SA_HCC1143_1: 4697
SA = 0.06
Score = −7.34
GGNPAQLMKEA − Mascot − rejected
20120619_EXQ6_MiBa_SA_HCT116_2_mHLA_2hr: 16360
SA = 0.1
Score = −4.42
ASLPGPSMREA − MaxQuant
20120619_EXQ6_MiBa_SA_HCT116_2_mHLA_2hr: 16360
SA = 0
Score = −10.11
TPSSGKSAVQGP − MSFragger
20120619_EXQ6_MiBa_SA_HCT116_2_mHLA_2hr: 16360
SA = 0.06
Score = −5.12
QDLEIPRPSM − Mascot − rejected
QD|LEIPRPSM − spliced − NA|NA|NA|NA
20120515_EXQ3_MiBa_SA_HCT116_mHLA−1: 21567
SA = 0.1
Score = −4.21
TIGFGDYVALQ − MSFragger
20120515_EXQ3_MiBa_SA_HCT116_mHLA−1: 21567
SA = 0.23
Score = −5.99
Relative intensity
m/z
Prosit prediction
Exclusive in prediction
Exclusive in experimental

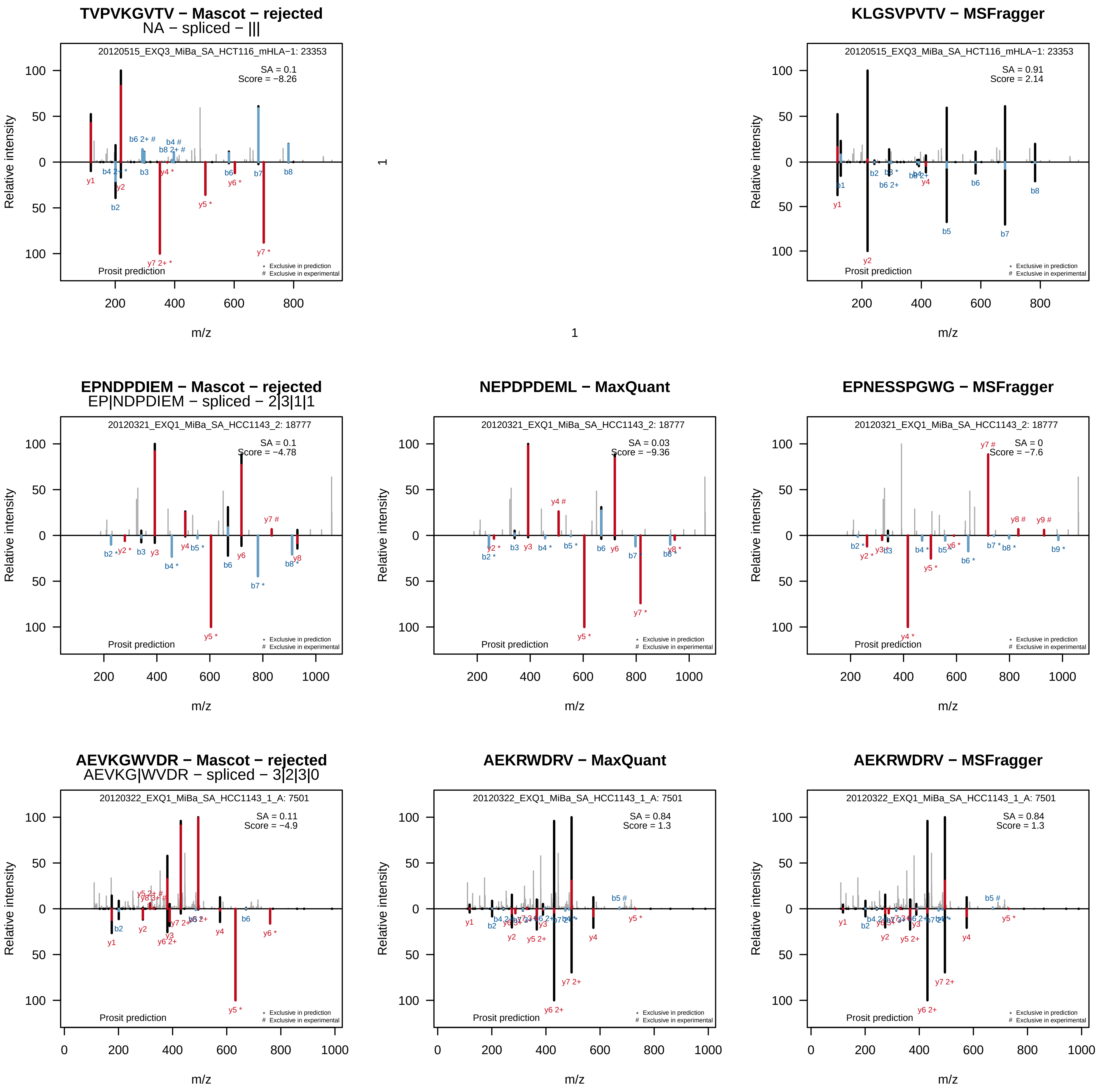

TVPVKGVTV − Mascot − rejected
NA − spliced − |||
20120515_EXQ3_MiBa_SA_HCT116_mHLA−1: 23353
SA = 0.1
Score = −8.26
KLGSVPVTV − MSFragger
20120515_EXQ3_MiBa_SA_HCT116_mHLA−1: 23353
SA = 0.91
Score = 2.14
EPNDPDIEM − Mascot − rejected
EP|NDPDIEM − spliced − 2|3|1|1
20120321_EXQ1_MiBa_SA_HCC1143_2: 18777
SA = 0.1
Score = −4.78
NEPDPDEML − MaxQuant
20120321_EXQ1_MiBa_SA_HCC1143_2: 18777
SA = 0.03
Score = −9.36
EPNESSPGWG − MSFragger
20120321_EXQ1_MiBa_SA_HCC1143_2: 18777
SA = 0
Score = −7.6
AEVKGWVDR − Mascot − rejected
AEVKG|WVDR − spliced − 3|2|3|0
20120322_EXQ1_MiBa_SA_HCC1143_1_A: 7501
SA = 0.11
Score = −4.9
AEKRWDRV − MaxQuant
20120322_EXQ1_MiBa_SA_HCC1143_1_A: 7501
SA = 0.84
Score = 1.3
AEKRWDRV − MSFragger
20120322_EXQ1_MiBa_SA_HCC1143_1_A: 7501
SA = 0.84
Score = 1.3
Relative intensity
m/z
Prosit prediction
* Exclusive in prediction
# Exclusive in experimental

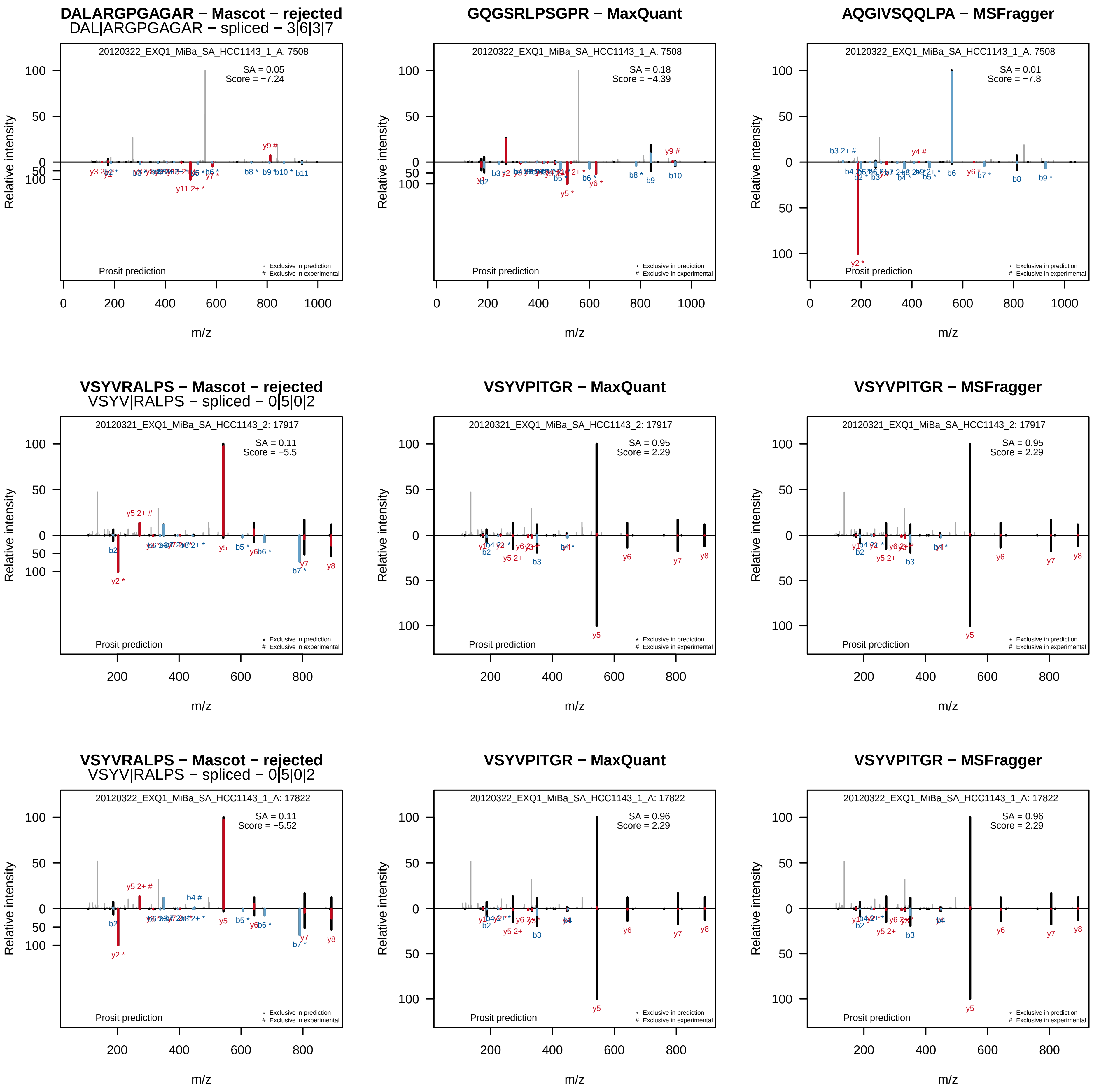
DALARGPGAGAR − Mascot − rejected
DAL|ARGPGAGAR − spliced − 3|6|3|7
20120322_EXQ1_MiBa_SA_HCC1143_1_A: 7508
SA = 0.05
Score = −7.24
GQGSRLPSGPR − MaxQuant
20120322_EXQ1_MiBa_SA_HCC1143_1_A: 7508
SA = 0.18
Score = −4.39
AQGIVSQQLPA − MSFragger
20120322_EXQ1_MiBa_SA_HCC1143_1_A: 7508
SA = 0.01
Score = −7.8
VSYVRALPS − Mascot − rejected
VSYV|RALPS − spliced − 0|5|0|2
20120321_EXQ1_MiBa_SA_HCC1143_2: 17917
SA = 0.11
Score = −5.5
VSYVPITGR − MaxQuant
20120321_EXQ1_MiBa_SA_HCC1143_2: 17917
SA = 0.95
Score = 2.29
VSYVPITGR − MSFragger
20120321_EXQ1_MiBa_SA_HCC1143_2: 17917
SA = 0.95
Score = 2.29
VSYVRALPS − Mascot − rejected
VSYV|RALPS − spliced − 0|5|0|2
20120322_EXQ1_MiBa_SA_HCC1143_1_A: 17822
SA = 0.11
Score = −5.52
VSYVPITGR − MaxQuant
20120322_EXQ1_MiBa_SA_HCC1143_1_A: 17822
SA = 0.96
Score = 2.29
VSYVPITGR − MSFragger
20120322_EXQ1_MiBa_SA_HCC1143_1_A: 17822
SA = 0.96
Score = 2.29
Relative intensity
m/z
Prosit prediction
* Exclusive in prediction
# Exclusive in experimental

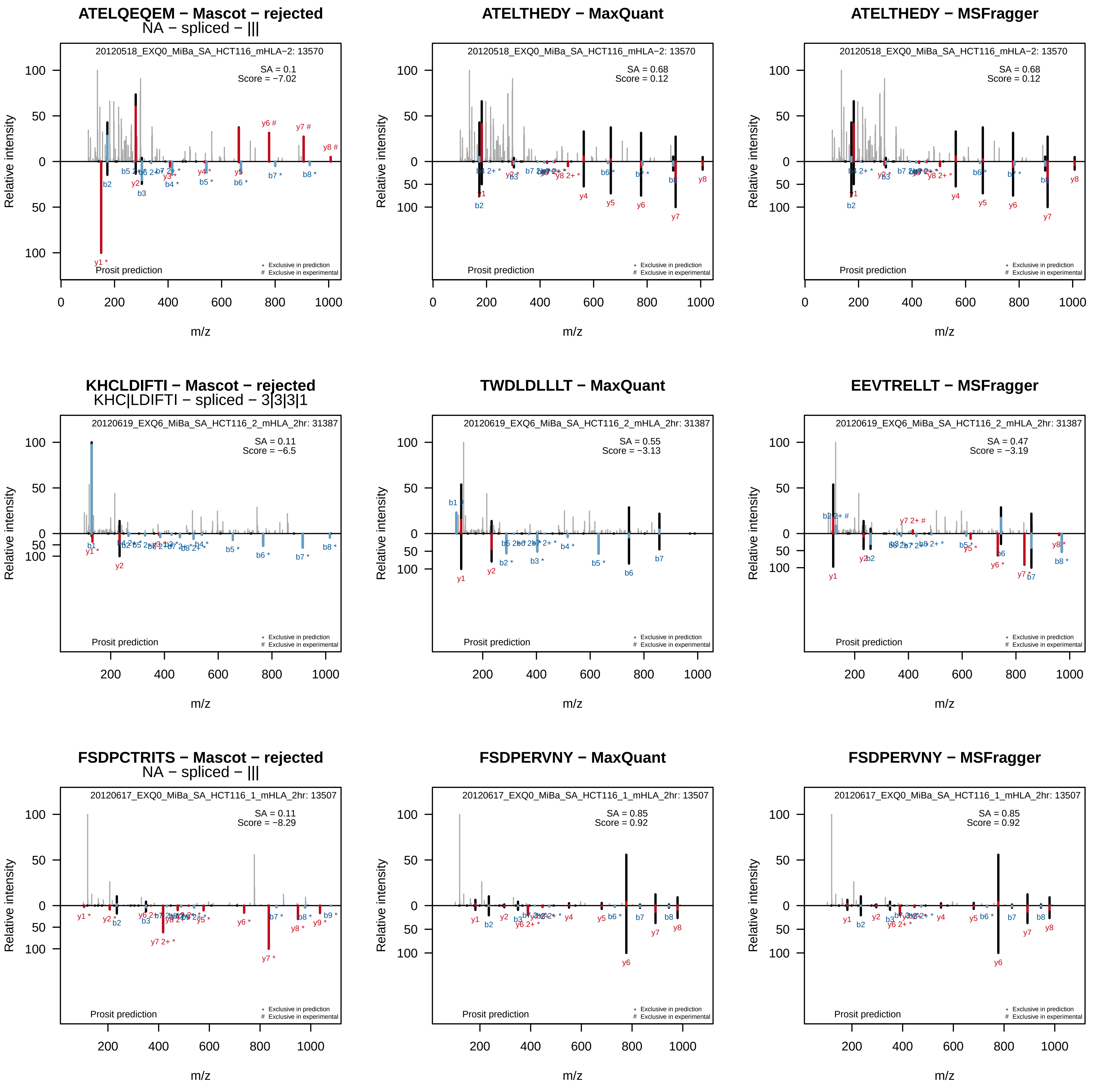

ATELQEQEM − Mascot − rejected
NA − spliced − |||
20120518_EXQ0_MiBa_SA_HCT116_mHLA−2: 13570
SA = 0.1
Score = −7.02
ATELTHEDY − MaxQuant
20120518_EXQ0_MiBa_SA_HCT116_mHLA−2: 13570
SA = 0.68
Score = 0.12
ATELTHEDY − MSFragger
20120518_EXQ0_MiBa_SA_HCT116_mHLA−2: 13570
SA = 0.68
Score = 0.12
KHCLDIFTI − Mascot − rejected
KHC|LDIFTI − spliced − 3|3|3|1
20120619_EXQ6_MiBa_SA_HCT116_2_mHLA_2hr: 31387
SA = 0.11
Score = −6.5
TWDLDLLLT − MaxQuant
20120619_EXQ6_MiBa_SA_HCT116_2_mHLA_2hr: 31387
SA = 0.55
Score = −3.13
EEVTRELLT − MSFragger
20120619_EXQ6_MiBa_SA_HCT116_2_mHLA_2hr: 31387
SA = 0.47
Score = −3.19
FSDPCTRITS − Mascot − rejected
NA − spliced − |||
20120617_EXQ0_MiBa_SA_HCT116_1_mHLA_2hr: 13507
SA = 0.11
Score = −8.29
FSDPERVNY − MaxQuant
20120617_EXQ0_MiBa_SA_HCT116_1_mHLA_2hr: 13507
SA = 0.85
Score = 0.92
FSDPERVNY − MSFragger
20120617_EXQ0_MiBa_SA_HCT116_1_mHLA_2hr: 13507
SA = 0.85
Score = 0.92
Relative intensity
m/z
Prosit prediction
* Exclusive in prediction
# Exclusive in experimental

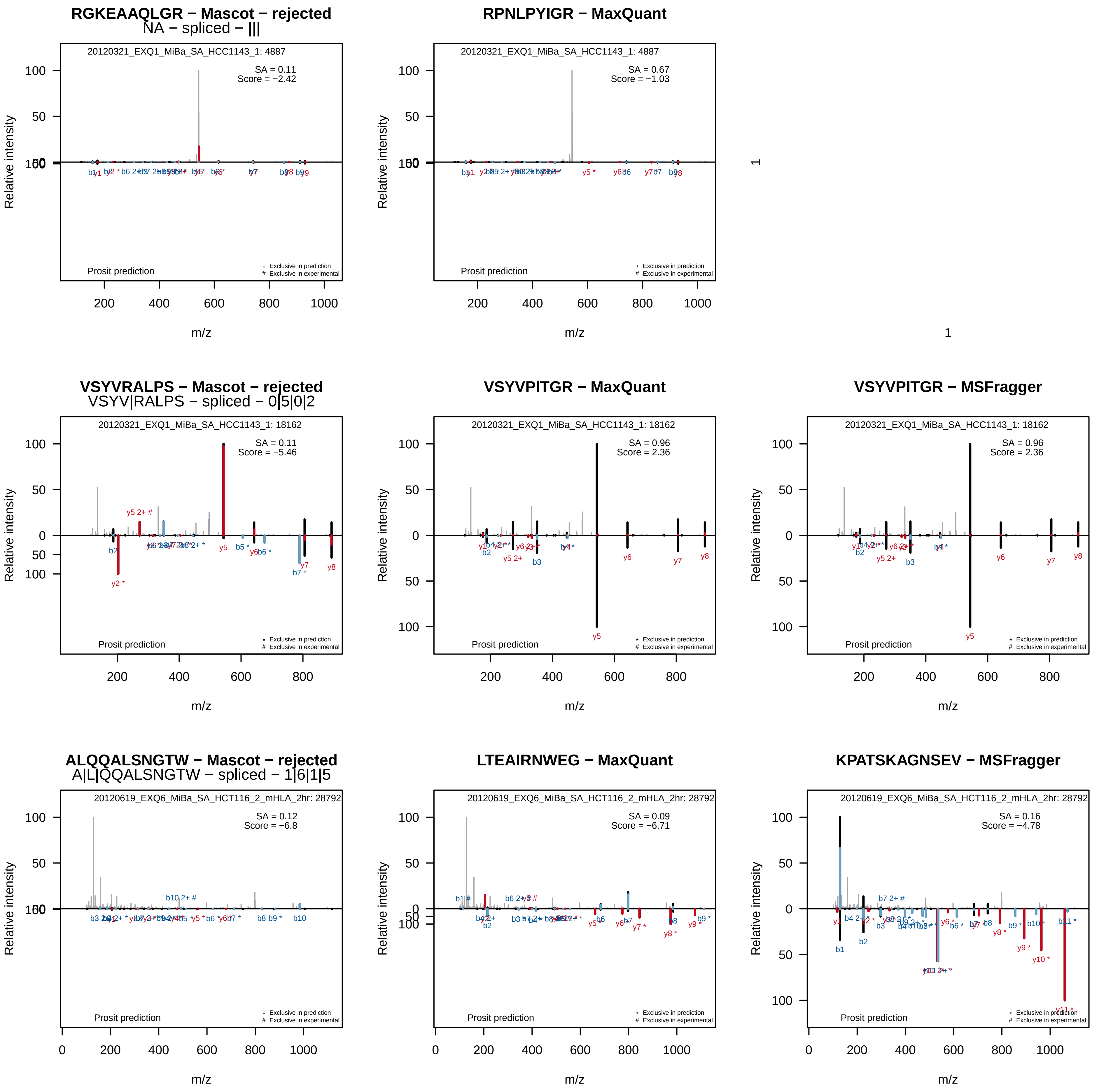

RGKEAAQLGR − Mascot − rejected
NA − spliced − |||
20120321_EXQ1_MiBa_SA_HCC1143_1: 4887
SA = 0.11
Score = −2.42
RPNLPYIGR − MaxQuant
20120321_EXQ1_MiBa_SA_HCC1143_1: 4887
SA = 0.67
Score = −1.03
VSYVRALPS − Mascot − rejected
VSYV|RALPS − spliced − 0|5|0|2
20120321_EXQ1_MiBa_SA_HCC1143_1: 18162
SA = 0.11
Score = −5.46
VSYVPITGR − MaxQuant
20120321_EXQ1_MiBa_SA_HCC1143_1: 18162
SA = 0.96
Score = 2.36
VSYVPITGR − MSFragger
20120321_EXQ1_MiBa_SA_HCC1143_1: 18162
SA = 0.96
Score = 2.36
ALQQALSNGTW − Mascot − rejected
A|L|QQALSNGTW − spliced − 1|6|1|5
20120619_EXQ6_MiBa_SA_HCT116_2_mHLA_2hr: 28792
SA = 0.12
Score = −6.8
LTEAIRNWEG − MaxQuant
20120619_EXQ6_MiBa_SA_HCT116_2_mHLA_2hr: 28792
SA = 0.09
Score = −6.71
KPATSKAGNSEV − MSFragger
20120619_EXQ6_MiBa_SA_HCT116_2_mHLA_2hr: 28792
SA = 0.16
Score = −4.78
Relative intensity
m/z
Prosit prediction
⋆ Exclusive in prediction
Exclusive in experimental

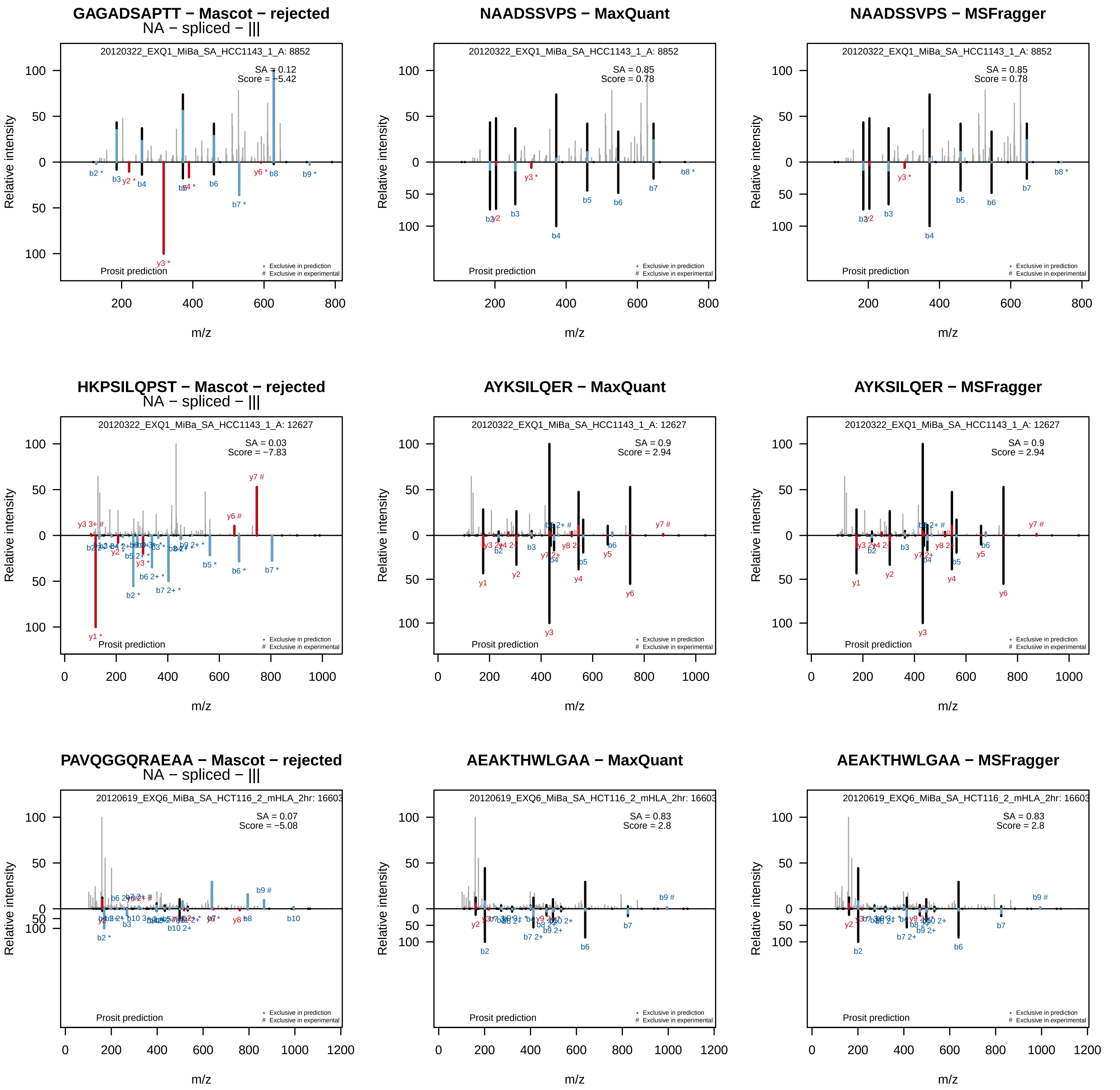
GAGADSAPTT − Mascot − rejected
NA − spliced − |||
20120322_EXQ1_MiBa_SA_HCC1143_1_A: 8852
SA = 0.12
Score = −5.42
Prosit prediction
NAADSSVPS − MaxQuant
20120322_EXQ1_MiBa_SA_HCC1143_1_A: 8852
SA = 0.85
Score = 0.78
Prosit prediction
NAADSSVPS − MSFragger
20120322_EXQ1_MiBa_SA_HCC1143_1_A: 8852
SA = 0.85
Score = 0.78
Prosit prediction
HKPSILQPST − Mascot − rejected
NA − spliced − |||
20120322_EXQ1_MiBa_SA_HCC1143_1_A: 12627
SA = 0.03
Score = −7.83
Prosit prediction
AYKSILQER − MaxQuant
20120322_EXQ1_MiBa_SA_HCC1143_1_A: 12627
SA = 0.9
Score = 2.94
Prosit prediction
AYKSILQER − MSFragger
20120322_EXQ1_MiBa_SA_HCC1143_1_A: 12627
SA = 0.9
Score = 2.94
Prosit prediction
PAVQGGQRAEAA − Mascot − rejected
NA − spliced − |||
20120619_EXQ6_MiBa_SA_HCT116_2_mHLA_2hr: 16603
SA = 0.07
Score = −5.08
Prosit prediction
AEAKTHWLGAA − MaxQuant
20120619_EXQ6_MiBa_SA_HCT116_2_mHLA_2hr: 16603
SA = 0.83
Score = 2.8
Prosit prediction
AEAKTHWLGAA − MSFragger
20120619_EXQ6_MiBa_SA_HCT116_2_mHLA_2hr: 16603
SA = 0.83
Score = 2.8
Prosit prediction
Relative intensity
m/z
* Exclusive in prediction
# Exclusive in experimental

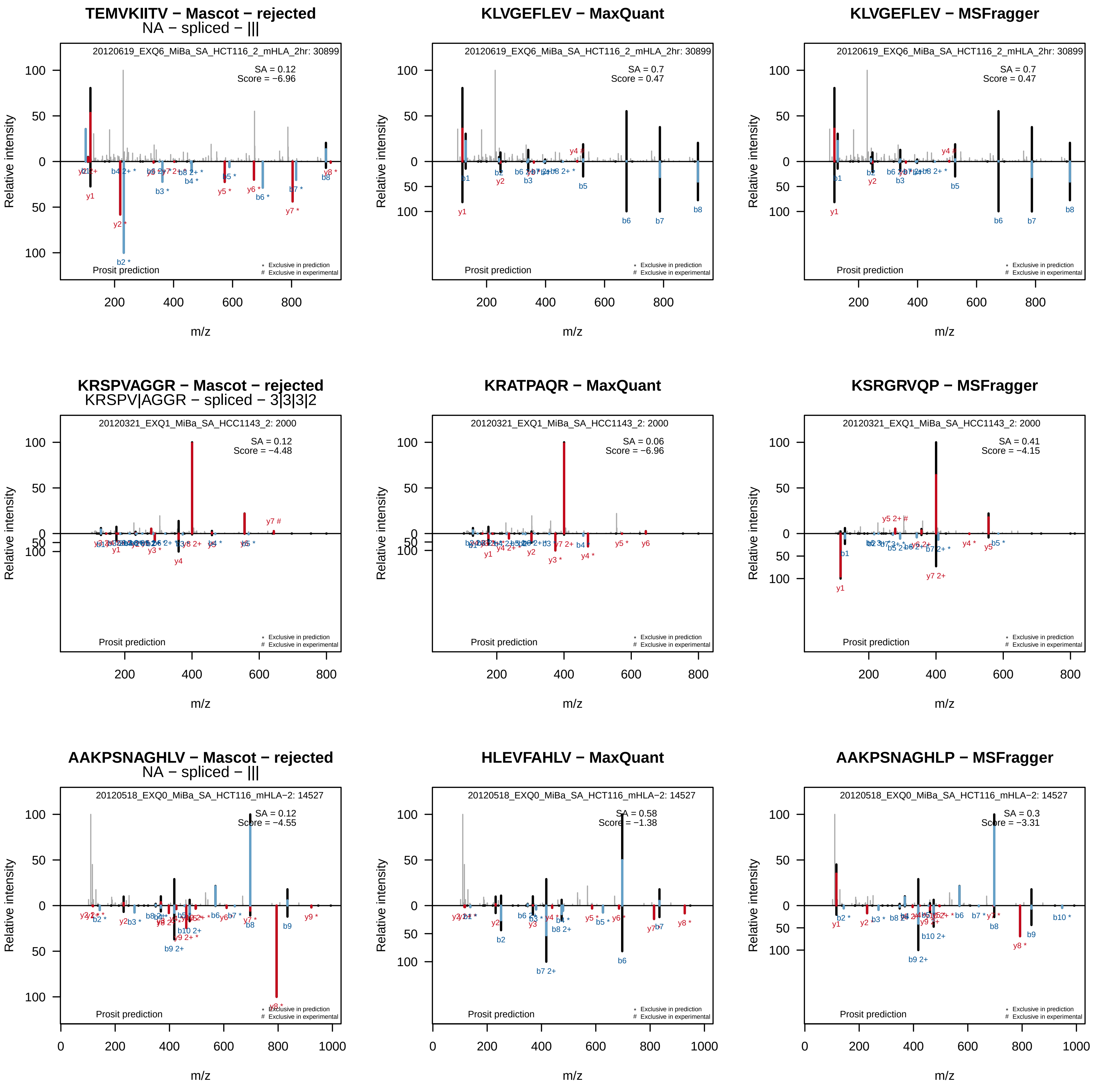
TEMVKIITV − Mascot − rejected
NA − spliced − |||
20120619_EXQ6_MiBa_SA_HCT116_2_mHLA_2hr: 30899
SA = 0.12
Score = −6.96
KLVGEFLEV − MaxQuant
20120619_EXQ6_MiBa_SA_HCT116_2_mHLA_2hr: 30899
SA = 0.7
Score = 0.47
KLVGEFLEV − MSFragger
20120619_EXQ6_MiBa_SA_HCT116_2_mHLA_2hr: 30899
SA = 0.7
Score = 0.47
KRSPVAGGR − Mascot − rejected
KRSPV|AGGR − spliced − 3|3|3|2
20120321_EXQ1_MiBa_SA_HCC1143_2: 2000
SA = 0.12
Score = −4.48
KRATPAQR − MaxQuant
20120321_EXQ1_MiBa_SA_HCC1143_2: 2000
SA = 0.06
Score = −6.96
KSRGRVQP − MSFragger
20120321_EXQ1_MiBa_SA_HCC1143_2: 2000
SA = 0.41
Score = −4.15
AAKPSNAGHLV − Mascot − rejected
NA − spliced − |||
20120518_EXQ0_MiBa_SA_HCT116_mHLA−2: 14527
SA = 0.12
Score = −4.55
HLEVFAHLV − MaxQuant
20120518_EXQ0_MiBa_SA_HCT116_mHLA−2: 14527
SA = 0.58
Score = −1.38
AAKPSNAGHLP − MSFragger
20120518_EXQ0_MiBa_SA_HCT116_mHLA−2: 14527
SA = 0.3
Score = −3.31
Relative intensity
m/z
Prosit prediction
⋆ Exclusive in prediction
Exclusive in experimental

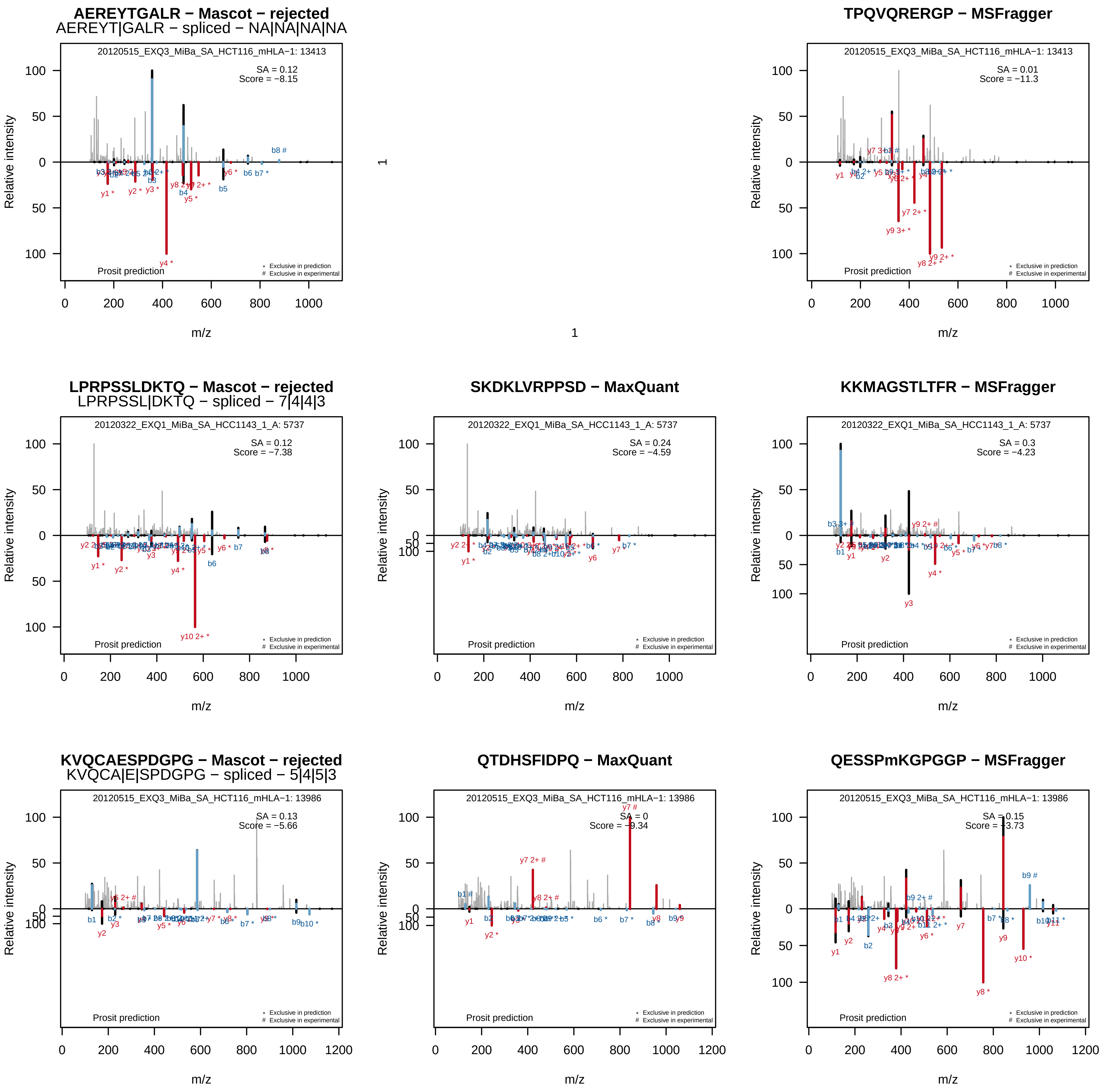
AEREYTGALR − Mascot − rejected
AEREYT|GALR − spliced − NA|NA|NA|NA
20120515_EXQ3_MiBa_SA_HCT116_mHLA−1: 13413
SA = 0.12
Score = −8.15
TPQVQRERGP − MSFragger
20120515_EXQ3_MiBa_SA_HCT116_mHLA−1: 13413
SA = 0.01
Score = −11.3
LPRPSSLDKTQ − Mascot − rejected
LPRPSSL|DKTQ − spliced − 7|4|4|3
20120322_EXQ1_MiBa_SA_HCC1143_1_A: 5737
SA = 0.12
Score = −7.38
SKDKLVRPPSD − MaxQuant
20120322_EXQ1_MiBa_SA_HCC1143_1_A: 5737
SA = 0.24
Score = −4.59
KKMAGSTLTFR − MSFragger
20120322_EXQ1_MiBa_SA_HCC1143_1_A: 5737
SA = 0.3
Score = −4.23
KVQCAESPDGPG − Mascot − rejected
KVQCA|E|SPDGPG − spliced − 5|4|5|3
20120515_EXQ3_MiBa_SA_HCT116_mHLA−1: 13986
SA = 0.13
Score = −5.66
QTDHSFIDPQ − MaxQuant
20120515_EXQ3_MiBa_SA_HCT116_mHLA−1: 13986
SA = 0
Score = −9.34
QESSPmKGPGGP − MSFragger
20120515_EXQ3_MiBa_SA_HCT116_mHLA−1: 13986
SA = 0.15
Score = −3.73
Relative intensity
m/z
Prosit prediction
* Exclusive in prediction
# Exclusive in experimental

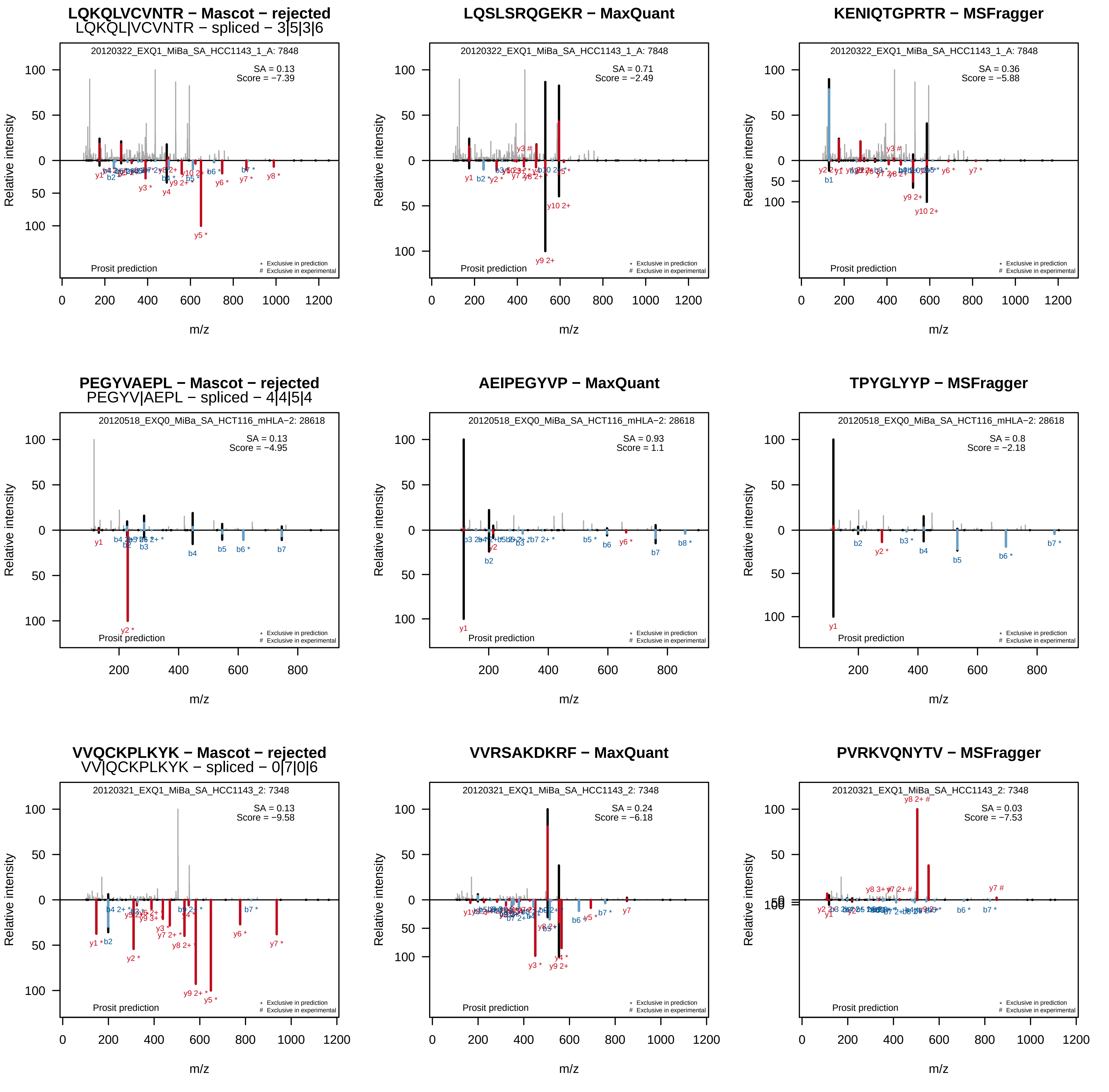
LQKQLVCVNTR − Mascot − rejected
LQKQL|VCVNTR − spliced − 3|5|3|6
20120322_EXQ1_MiBa_SA_HCC1143_1_A: 7848
SA = 0.13
Score = −7.39
LQSLSRQGEKR − MaxQuant
20120322_EXQ1_MiBa_SA_HCC1143_1_A: 7848
SA = 0.71
Score = −2.49
KENIQTGPRTR − MSFragger
20120322_EXQ1_MiBa_SA_HCC1143_1_A: 7848
SA = 0.36
Score = −5.88
PEGYVAEPL − Mascot − rejected
PEGYV|AEPL − spliced − 4|4|5|4
20120518_EXQ0_MiBa_SA_HCT116_mHLA−2: 28618
SA = 0.13
Score = −4.95
AEIPEGYVP − MaxQuant
20120518_EXQ0_MiBa_SA_HCT116_mHLA−2: 28618
SA = 0.93
Score = 1.1
TPYGLYYP − MSFragger
20120518_EXQ0_MiBa_SA_HCT116_mHLA−2: 28618
SA = 0.8
Score = −2.18
VVQCKPLKYK − Mascot − rejected
VV|QCKPLKYK − spliced − 0|7|0|6
20120321_EXQ1_MiBa_SA_HCC1143_2: 7348
SA = 0.13
Score = −9.58
VVRSAKDKRF − MaxQuant
20120321_EXQ1_MiBa_SA_HCC1143_2: 7348
SA = 0.24
Score = −6.18
PVRKVQNYTV − MSFragger
20120321_EXQ1_MiBa_SA_HCC1143_2: 7348
SA = 0.03
Score = −7.53
Relative intensity
m/z
Prosit prediction
* Exclusive in prediction
# Exclusive in experimental

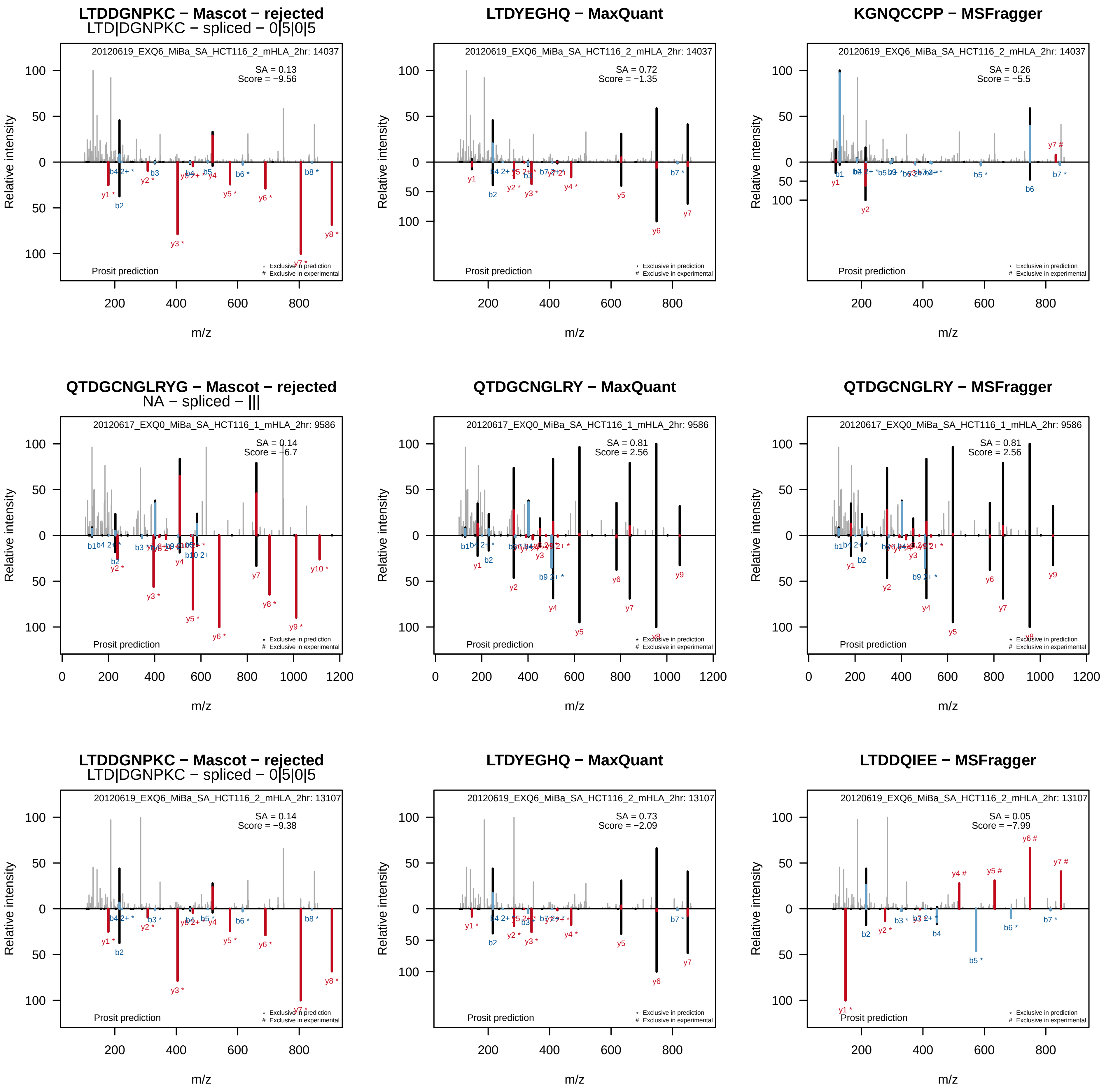
LTDDGNPKC − Mascot − rejected
LTD|DGNPKC − spliced − 0|5|0|5
20120619_EXQ6_MiBa_SA_HCT116_2_mHLA_2hr: 14037
SA = 0.13
Score = −9.56
Prosit prediction
⋆ Exclusive in prediction
Exclusive in experimental
Relative intensity
m/z
LTDYEGHQ − MaxQuant
20120619_EXQ6_MiBa_SA_HCT116_2_mHLA_2hr: 14037
SA = 0.72
Score = −1.35
KGNQCCPP − MSFragger
20120619_EXQ6_MiBa_SA_HCT116_2_mHLA_2hr: 14037
SA = 0.26
Score = −5.5
QTDGCNGLRYG − Mascot − rejected
NA − spliced − |||
20120617_EXQ0_MiBa_SA_HCT116_1_mHLA_2hr: 9586
SA = 0.14
Score = −6.7
QTDGCNGLRY − MaxQuant
20120617_EXQ0_MiBa_SA_HCT116_1_mHLA_2hr: 9586
SA = 0.81
Score = 2.56
QTDGCNGLRY − MSFragger
20120617_EXQ0_MiBa_SA_HCT116_1_mHLA_2hr: 9586
SA = 0.81
Score = 2.56
LTDDGNPKC − Mascot − rejected
LTD|DGNPKC − spliced − 0|5|0|5
20120619_EXQ6_MiBa_SA_HCT116_2_mHLA_2hr: 13107
SA = 0.14
Score = −9.38
LTDYEGHQ − MaxQuant
20120619_EXQ6_MiBa_SA_HCT116_2_mHLA_2hr: 13107
SA = 0.73
Score = −2.09
LTDDQIEE − MSFragger
20120619_EXQ6_MiBa_SA_HCT116_2_mHLA_2hr: 13107
SA = 0.05
Score = −7.99

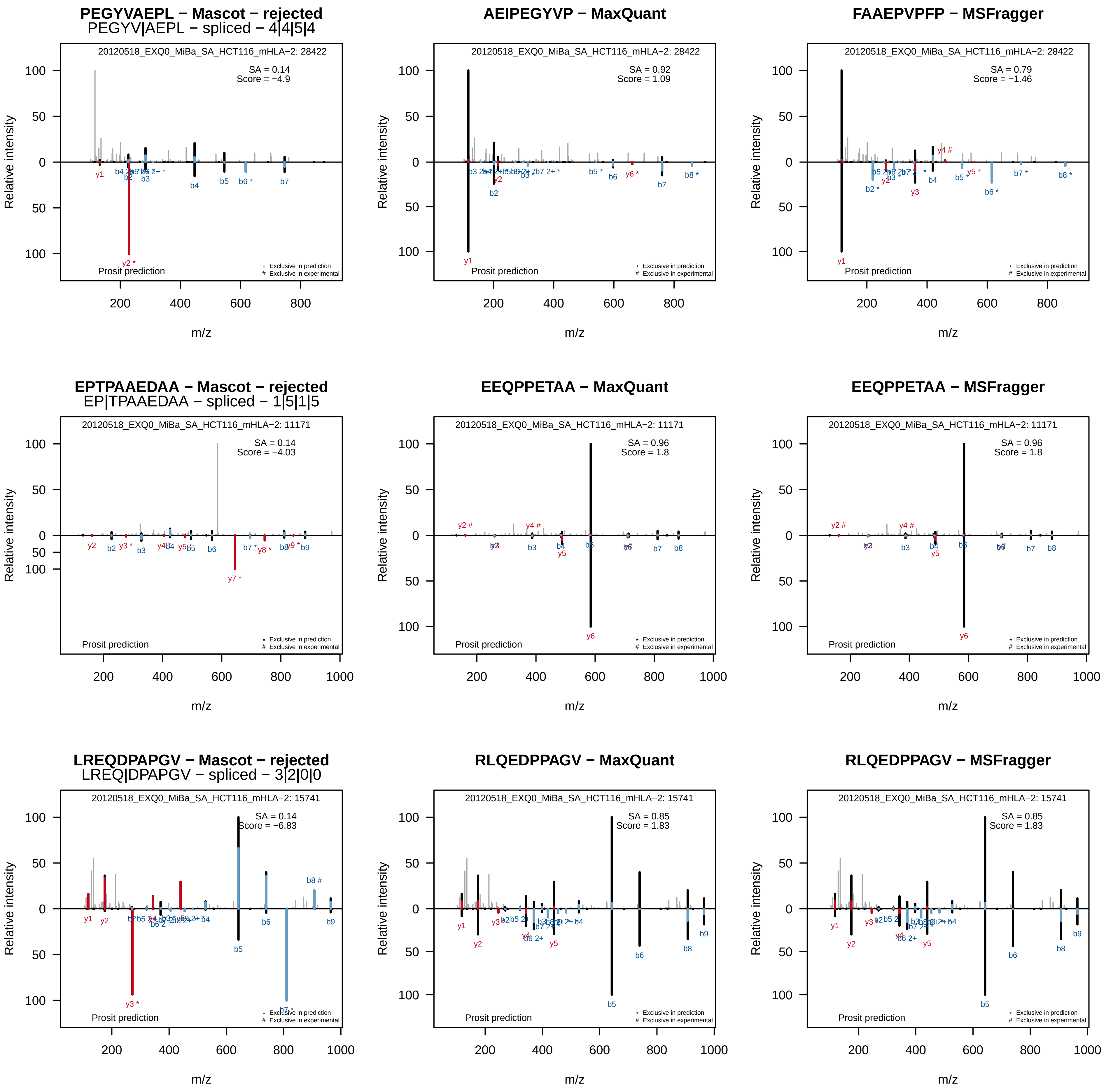
PEGYVAEPL − Mascot − rejected
PEGYV|AEPL − spliced − 4|4|5|4
20120518_EXQ0_MiBa_SA_HCT116_mHLA−2: 28422
SA = 0.14
Score = −4.9
AEIPEGYVP − MaxQuant
20120518_EXQ0_MiBa_SA_HCT116_mHLA−2: 28422
SA = 0.92
Score = 1.09
FAAEPVPFP − MSFragger
20120518_EXQ0_MiBa_SA_HCT116_mHLA−2: 28422
SA = 0.79
Score = −1.46
EPTPAAEDAA − Mascot − rejected
EP|TPAAEDAA − spliced − 1|5|1|5
20120518_EXQ0_MiBa_SA_HCT116_mHLA−2: 11171
SA = 0.14
Score = −4.03
EEQPPETAA − MaxQuant
20120518_EXQ0_MiBa_SA_HCT116_mHLA−2: 11171
SA = 0.96
Score = 1.8
EEQPPETAA − MSFragger
20120518_EXQ0_MiBa_SA_HCT116_mHLA−2: 11171
SA = 0.96
Score = 1.8
LREQDPAPGV − Mascot − rejected
LREQ|DPAPGV − spliced − 3|2|0|0
20120518_EXQ0_MiBa_SA_HCT116_mHLA−2: 15741
SA = 0.14
Score = −6.83
RLQEDPPAGV − MaxQuant
20120518_EXQ0_MiBa_SA_HCT116_mHLA−2: 15741
SA = 0.85
Score = 1.83
RLQEDPPAGV − MSFragger
20120518_EXQ0_MiBa_SA_HCT116_mHLA−2: 15741
SA = 0.85
Score = 1.83
Relative intensity
m/z
Prosit prediction
* Exclusive in prediction
# Exclusive in experimental

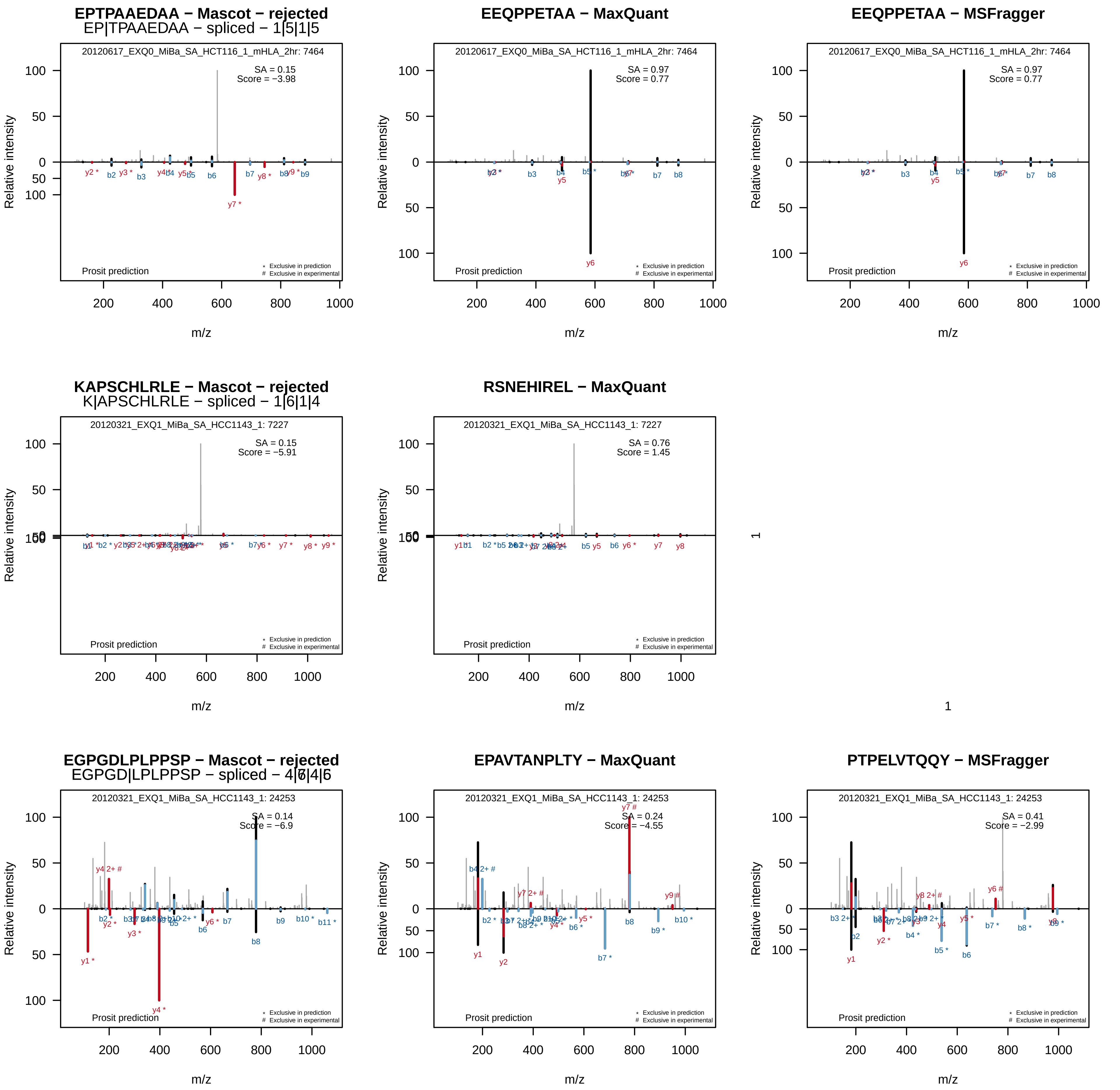

EPTPAAEDAA − Mascot − rejected
EP|TPAAEDAA − spliced − 1|5|1|5
20120617_EXQ0_MiBa_SA_HCT116_1_mHLA_2hr: 7464
SA = 0.15
Score = −3.98
EEQPPETAA − MaxQuant
20120617_EXQ0_MiBa_SA_HCT116_1_mHLA_2hr: 7464
SA = 0.97
Score = 0.77
EEQPPETAA − MSFragger
20120617_EXQ0_MiBa_SA_HCT116_1_mHLA_2hr: 7464
SA = 0.97
Score = 0.77
KAPSCHLRLE − Mascot − rejected
K|APSCHLRLE − spliced − 1|6|1|4
20120321_EXQ1_MiBa_SA_HCC1143_1: 7227
SA = 0.15
Score = −5.91
RSNEHIREL − MaxQuant
20120321_EXQ1_MiBa_SA_HCC1143_1: 7227
SA = 0.76
Score = 1.45
EGPGDLPLPPSP − Mascot − rejected
EGPGD|LPLPPSP − spliced − 4|6|4|6
20120321_EXQ1_MiBa_SA_HCC1143_1: 24253
SA = 0.14
Score = −6.9
EPAVTANPLTY − MaxQuant
20120321_EXQ1_MiBa_SA_HCC1143_1: 24253
SA = 0.24
Score = −4.55
PTPELVTQQY − MSFragger
20120321_EXQ1_MiBa_SA_HCC1143_1: 24253
SA = 0.41
Score = −2.99
Relative intensity
m/z
Prosit prediction
⋆ Exclusive in prediction
Exclusive in experimental

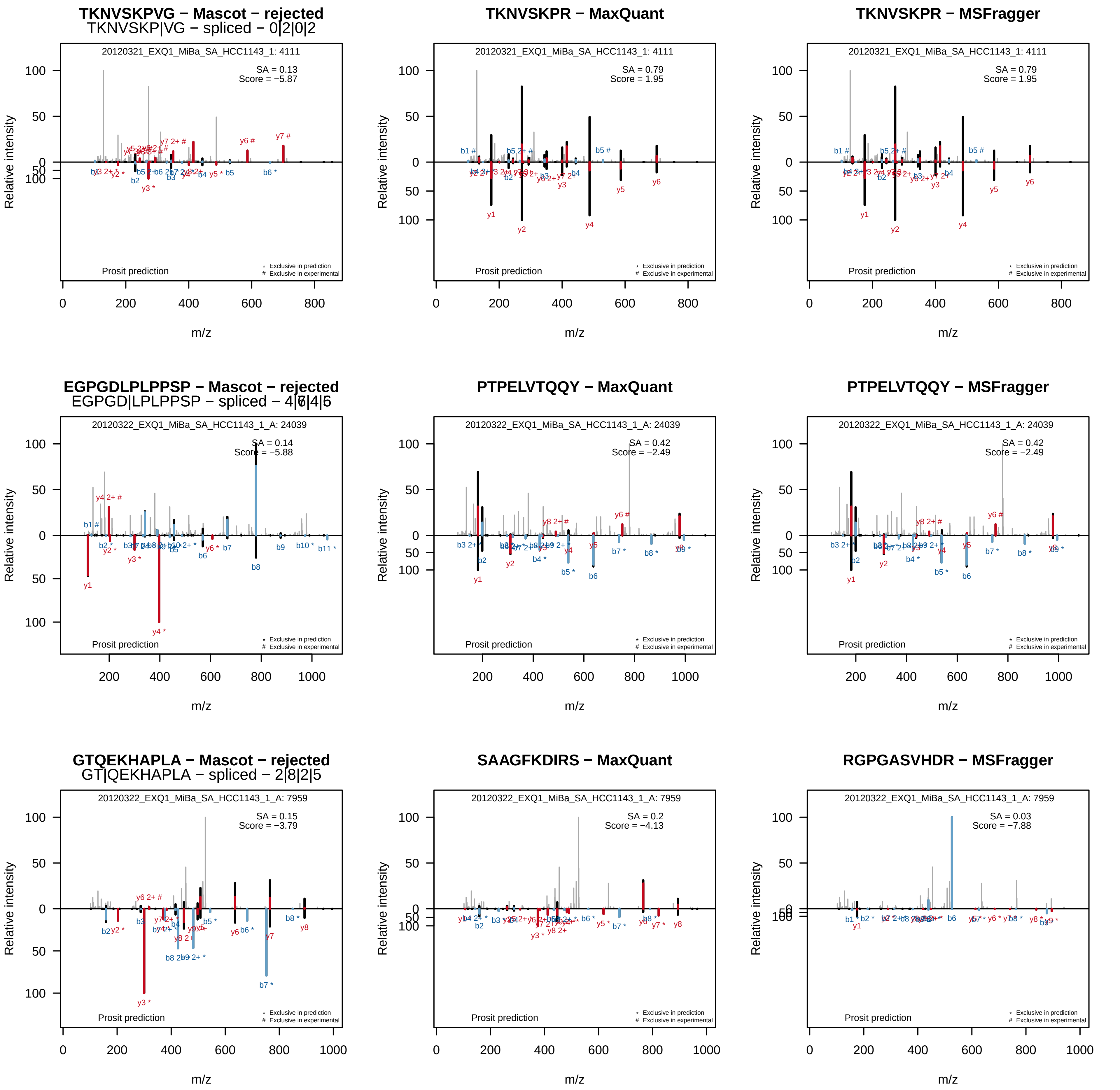

TKNVSKPVG − Mascot − rejected
TKNVSKP|VG − spliced − 0|2|0|2
20120321_EXQ1_MiBa_SA_HCC1143_1: 4111
SA = 0.13
Score = −5.87
TKNVSKPR − MaxQuant
20120321_EXQ1_MiBa_SA_HCC1143_1: 4111
SA = 0.79
Score = 1.95
TKNVSKPR − MSFragger
20120321_EXQ1_MiBa_SA_HCC1143_1: 4111
SA = 0.79
Score = 1.95
EGPGDLPLPPSP − Mascot − rejected
EGPGD|LPLPPSP − spliced − 4|7|4|6
20120322_EXQ1_MiBa_SA_HCC1143_1_A: 24039
SA = 0.14
Score = −5.88
PTPELVTQQY − MaxQuant
20120322_EXQ1_MiBa_SA_HCC1143_1_A: 24039
SA = 0.42
Score = −2.49
PTPELVTQQY − MSFragger
20120322_EXQ1_MiBa_SA_HCC1143_1_A: 24039
SA = 0.42
Score = −2.49
GTQEKHAPLA − Mascot − rejected
GT|QEKHAPLA − spliced − 2|8|2|5
20120322_EXQ1_MiBa_SA_HCC1143_1_A: 7959
SA = 0.15
Score = −3.79
SAAGFKDIRS − MaxQuant
20120322_EXQ1_MiBa_SA_HCC1143_1_A: 7959
SA = 0.2
Score = −4.13
RGPGASVHDR − MSFragger
20120322_EXQ1_MiBa_SA_HCC1143_1_A: 7959
SA = 0.03
Score = −7.88
Relative intensity
m/z
Prosit prediction
* Exclusive in prediction
# Exclusive in experimental

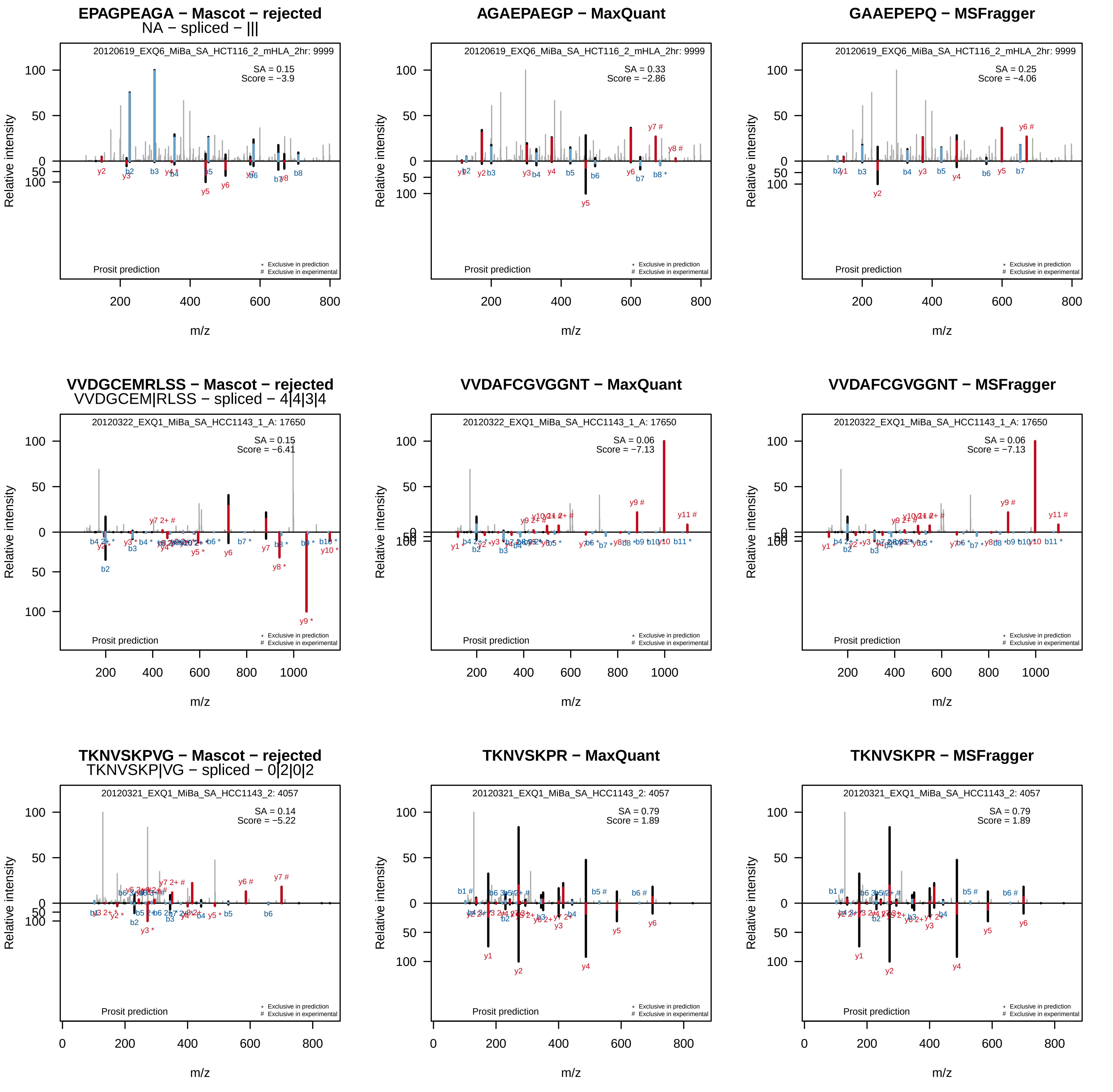
EPAGPEAGA − Mascot − rejected
NA − spliced − |||
20120619_EXQ6_MiBa_SA_HCT116_2_mHLA_2hr: 9999
SA = 0.15
Score = −3.9
AGAEPAEGP − MaxQuant
20120619_EXQ6_MiBa_SA_HCT116_2_mHLA_2hr: 9999
SA = 0.33
Score = −2.86
GAAEPEPQ − MSFragger
20120619_EXQ6_MiBa_SA_HCT116_2_mHLA_2hr: 9999
SA = 0.25
Score = −4.06
VVDGCEMRLSS − Mascot − rejected
VVDGCEM|RLSS − spliced − 4|4|3|4
20120322_EXQ1_MiBa_SA_HCC1143_1_A: 17650
SA = 0.15
Score = −6.41
VVDAFCGVGGNT − MaxQuant
20120322_EXQ1_MiBa_SA_HCC1143_1_A: 17650
SA = 0.06
Score = −7.13
VVDAFCGVGGNT − MSFragger
20120322_EXQ1_MiBa_SA_HCC1143_1_A: 17650
SA = 0.06
Score = −7.13
TKNVSKPVG − Mascot − rejected
TKNVSKP|VG − spliced − 0|2|0|2
20120321_EXQ1_MiBa_SA_HCC1143_2: 4057
SA = 0.14
Score = −5.22
TKNVSKPR − MaxQuant
20120321_EXQ1_MiBa_SA_HCC1143_2: 4057
SA = 0.79
Score = 1.89
TKNVSKPR − MSFragger
20120321_EXQ1_MiBa_SA_HCC1143_2: 4057
SA = 0.79
Score = 1.89
Relative intensity
m/z
Prosit prediction
* Exclusive in prediction
# Exclusive in experimental

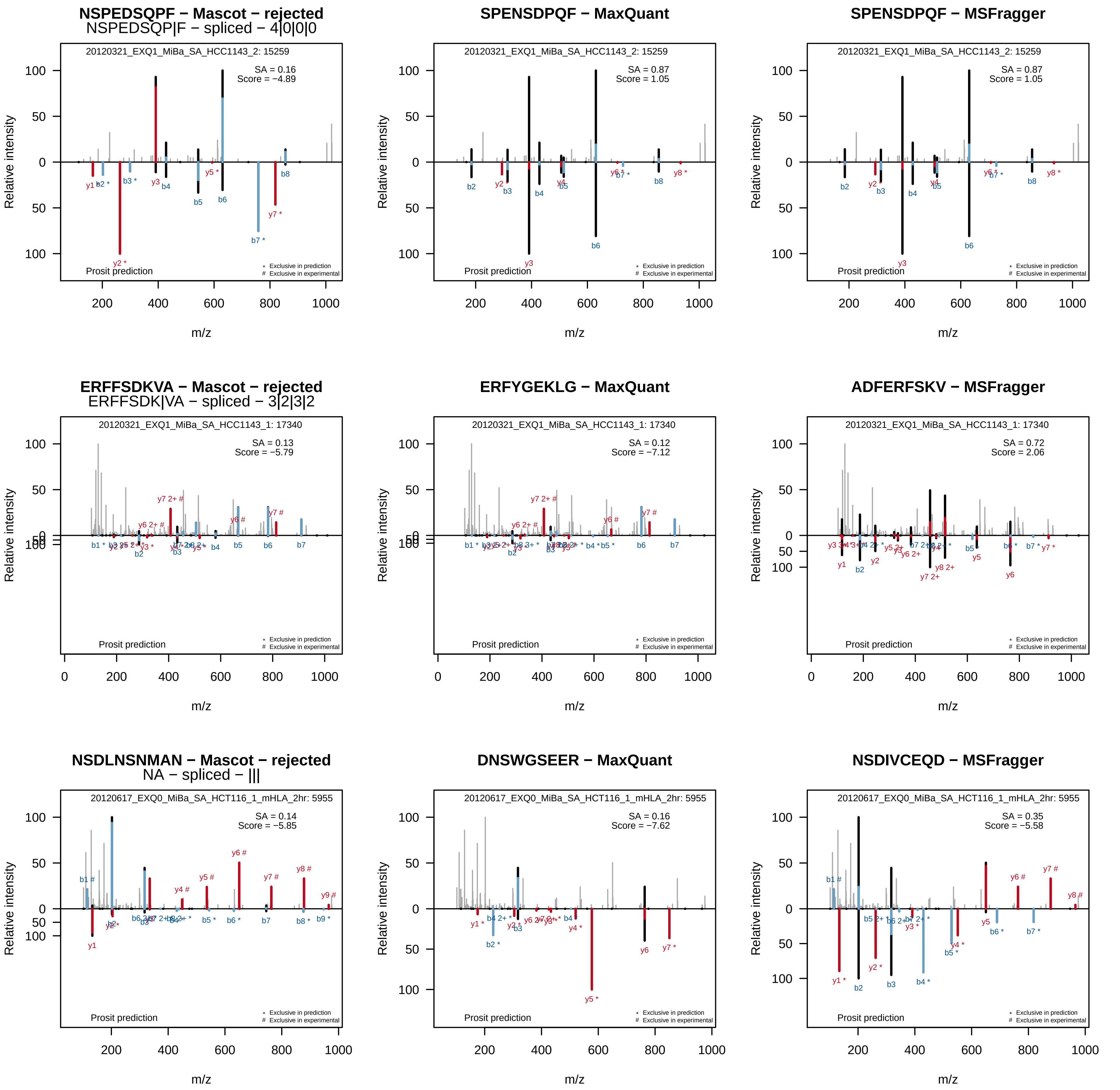
NSPEDSQPF − Mascot − rejected
NSPEDSQP|F − spliced − 4|0|0|0
20120321_EXQ1_MiBa_SA_HCC1143_2: 15259
SA = 0.16
Score = −4.89
SPENSDPQF − MaxQuant
20120321_EXQ1_MiBa_SA_HCC1143_2: 15259
SA = 0.87
Score = 1.05
SPENSDPQF − MSFragger
20120321_EXQ1_MiBa_SA_HCC1143_2: 15259
SA = 0.87
Score = 1.05
ERFFSDKVA − Mascot − rejected
ERFFSDK|VA − spliced − 3|2|3|2
20120321_EXQ1_MiBa_SA_HCC1143_1: 17340
SA = 0.13
Score = −5.79
ERFYGEKLG − MaxQuant
20120321_EXQ1_MiBa_SA_HCC1143_1: 17340
SA = 0.12
Score = −7.12
ADFERFSKV − MSFragger
20120321_EXQ1_MiBa_SA_HCC1143_1: 17340
SA = 0.72
Score = 2.06
NSDLNSNMAN − Mascot − rejected
NA − spliced − |||
20120617_EXQ0_MiBa_SA_HCT116_1_mHLA_2hr: 5955
SA = 0.14
Score = −5.85
DNSWGSEER − MaxQuant
20120617_EXQ0_MiBa_SA_HCT116_1_mHLA_2hr: 5955
SA = 0.16
Score = −7.62
NSDIVCEQD − MSFragger
20120617_EXQ0_MiBa_SA_HCT116_1_mHLA_2hr: 5955
SA = 0.35
Score = −5.58
Relative intensity
m/z
Prosit prediction
* Exclusive in prediction
# Exclusive in experimental

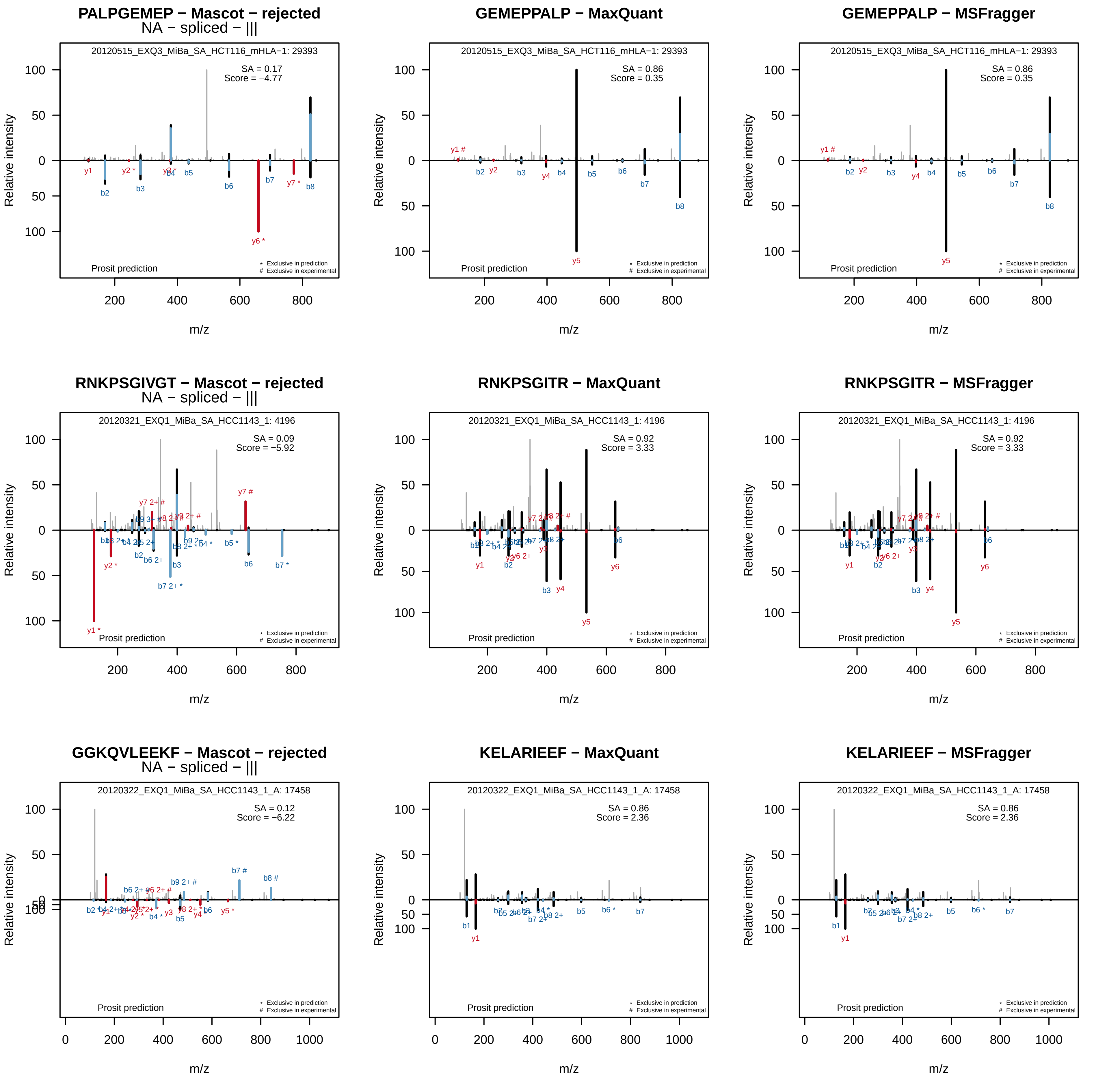

PALPGEMEP − Mascot − rejected
NA − spliced − |||
20120515_EXQ3_MiBa_SA_HCT116_mHLA−1: 29393
SA = 0.17
Score = −4.77
GEMEPPALP − MaxQuant
20120515_EXQ3_MiBa_SA_HCT116_mHLA−1: 29393
SA = 0.86
Score = 0.35
GEMEPPALP − MSFragger
20120515_EXQ3_MiBa_SA_HCT116_mHLA−1: 29393
SA = 0.86
Score = 0.35
RNKPSGIVGT − Mascot − rejected
NA − spliced − |||
20120321_EXQ1_MiBa_SA_HCC1143_1: 4196
SA = 0.09
Score = −5.92
RNKPSGITR − MaxQuant
20120321_EXQ1_MiBa_SA_HCC1143_1: 4196
SA = 0.92
Score = 3.33
RNKPSGITR − MSFragger
20120321_EXQ1_MiBa_SA_HCC1143_1: 4196
SA = 0.92
Score = 3.33
GGKQVLEEKF − Mascot − rejected
NA − spliced − |||
20120322_EXQ1_MiBa_SA_HCC1143_1_A: 17458
SA = 0.12
Score = −6.22
KELARIEEF − MaxQuant
20120322_EXQ1_MiBa_SA_HCC1143_1_A: 17458
SA = 0.86
Score = 2.36
KELARIEEF − MSFragger
20120322_EXQ1_MiBa_SA_HCC1143_1_A: 17458
SA = 0.86
Score = 2.36
Relative intensity
m/z
Prosit prediction
⋆ Exclusive in prediction
Exclusive in experimental

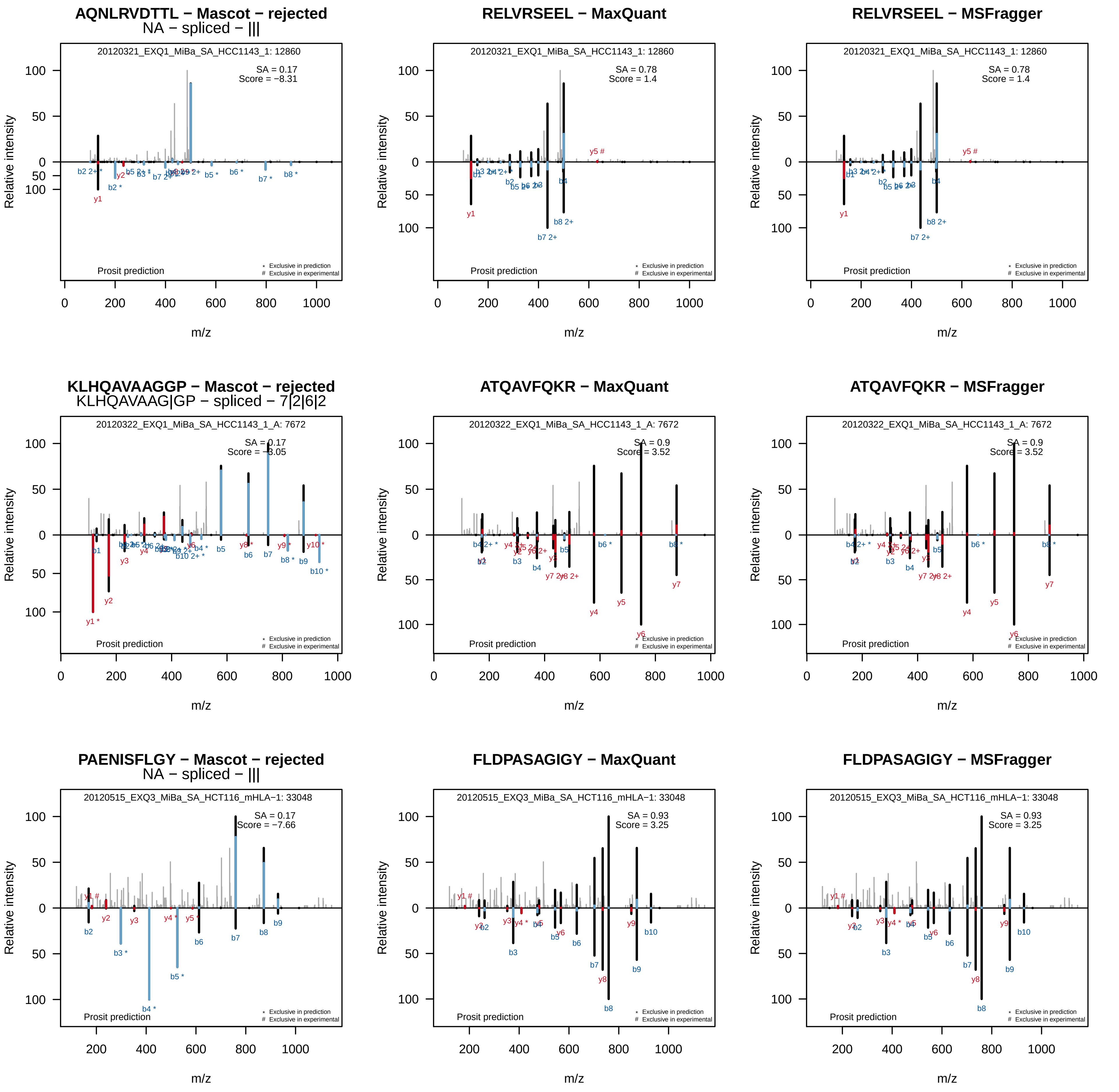

AQNLRVDTTL − Mascot − rejected
NA − spliced − |||
20120321_EXQ1_MiBa_SA_HCC1143_1: 12860
SA = 0.17
Score = −8.31
RELVRSEEL − MaxQuant
20120321_EXQ1_MiBa_SA_HCC1143_1: 12860
SA = 0.78
Score = 1.4
RELVRSEEL − MSFragger
20120321_EXQ1_MiBa_SA_HCC1143_1: 12860
SA = 0.78
Score = 1.4
KLHQAVAAGGP − Mascot − rejected
KLHQAVAAG|GP − spliced − 7|2|6|2
20120322_EXQ1_MiBa_SA_HCC1143_1_A: 7672
SA = 0.17
Score = −3.05
ATQAVFQKR − MaxQuant
20120322_EXQ1_MiBa_SA_HCC1143_1_A: 7672
SA = 0.9
Score = 3.52
ATQAVFQKR − MSFragger
20120322_EXQ1_MiBa_SA_HCC1143_1_A: 7672
SA = 0.9
Score = 3.52
PAENISFLGY − Mascot − rejected
NA − spliced − |||
20120515_EXQ3_MiBa_SA_HCT116_mHLA−1: 33048
SA = 0.17
Score = −7.66
FLDPASAGIGY − MaxQuant
20120515_EXQ3_MiBa_SA_HCT116_mHLA−1: 33048
SA = 0.93
Score = 3.25
FLDPASAGIGY − MSFragger
20120515_EXQ3_MiBa_SA_HCT116_mHLA−1: 33048
SA = 0.93
Score = 3.25
Relative intensity
m/z
Prosit prediction
* Exclusive in prediction
# Exclusive in experimental

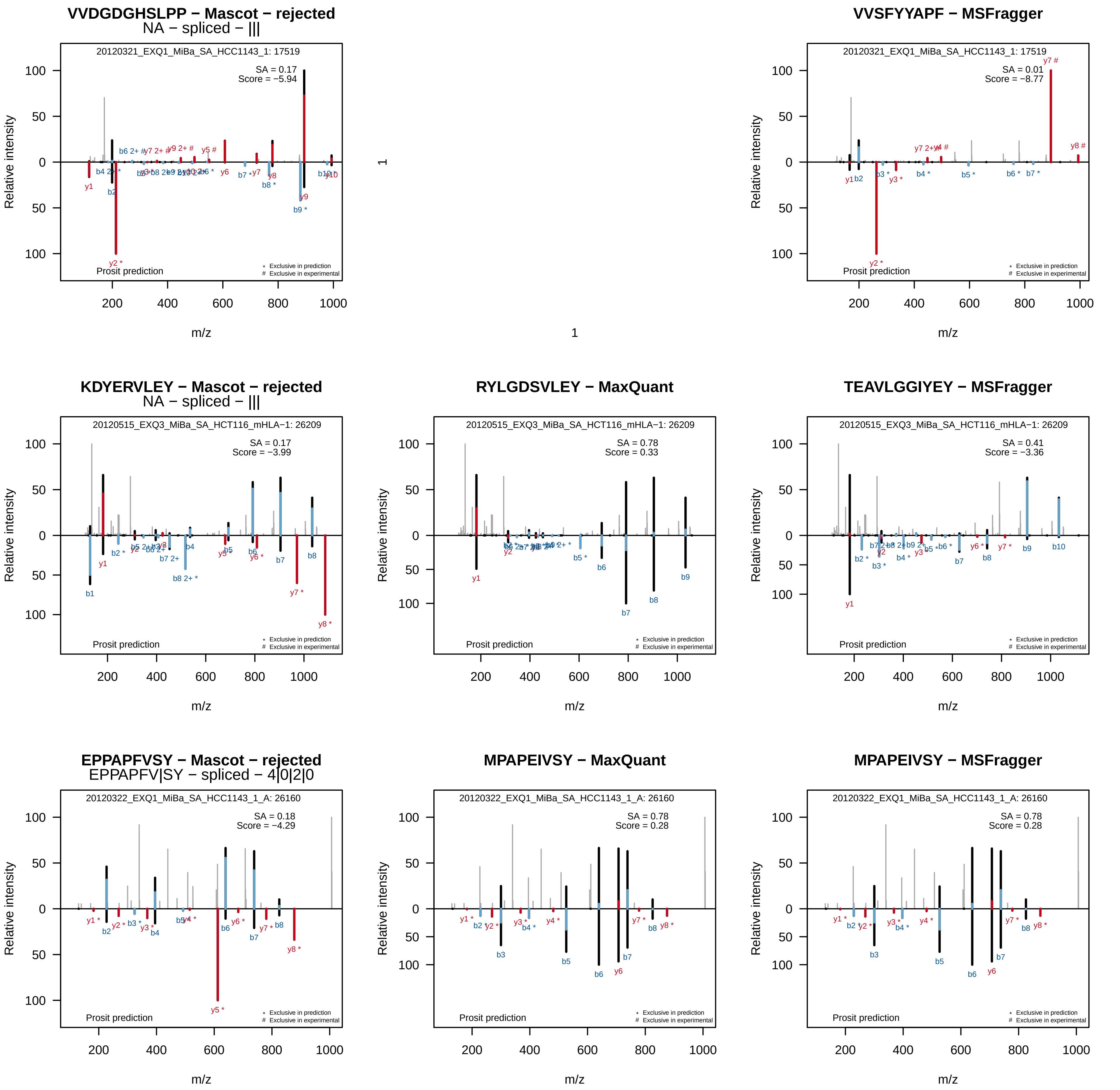
VVDGDGHSLPP − Mascot − rejected
NA − spliced − |||
20120321_EXQ1_MiBa_SA_HCC1143_1: 17519
SA = 0.17
Score = −5.94
VVSFYYAPF − MSFragger
20120321_EXQ1_MiBa_SA_HCC1143_1: 17519
SA = 0.01
Score = −8.77
KDYERVLEY − Mascot − rejected
NA − spliced − |||
20120515_EXQ3_MiBa_SA_HCT116_mHLA−1: 26209
SA = 0.17
Score = −3.99
RYLGDSVLEY − MaxQuant
20120515_EXQ3_MiBa_SA_HCT116_mHLA−1: 26209
SA = 0.78
Score = 0.33
TEAVLGGIYEY − MSFragger
20120515_EXQ3_MiBa_SA_HCT116_mHLA−1: 26209
SA = 0.41
Score = −3.36
EPPAPFVSY − Mascot − rejected
EPPAPFV|SY − spliced − 4|0|2|0
20120322_EXQ1_MiBa_SA_HCC1143_1_A: 26160
SA = 0.18
Score = −4.29
MPAPEIVSY − MaxQuant
20120322_EXQ1_MiBa_SA_HCC1143_1_A: 26160
SA = 0.78
Score = 0.28
MPAPEIVSY − MSFragger
20120322_EXQ1_MiBa_SA_HCC1143_1_A: 26160
SA = 0.78
Score = 0.28
Relative intensity
m/z
Prosit prediction
* Exclusive in prediction
# Exclusive in experimental

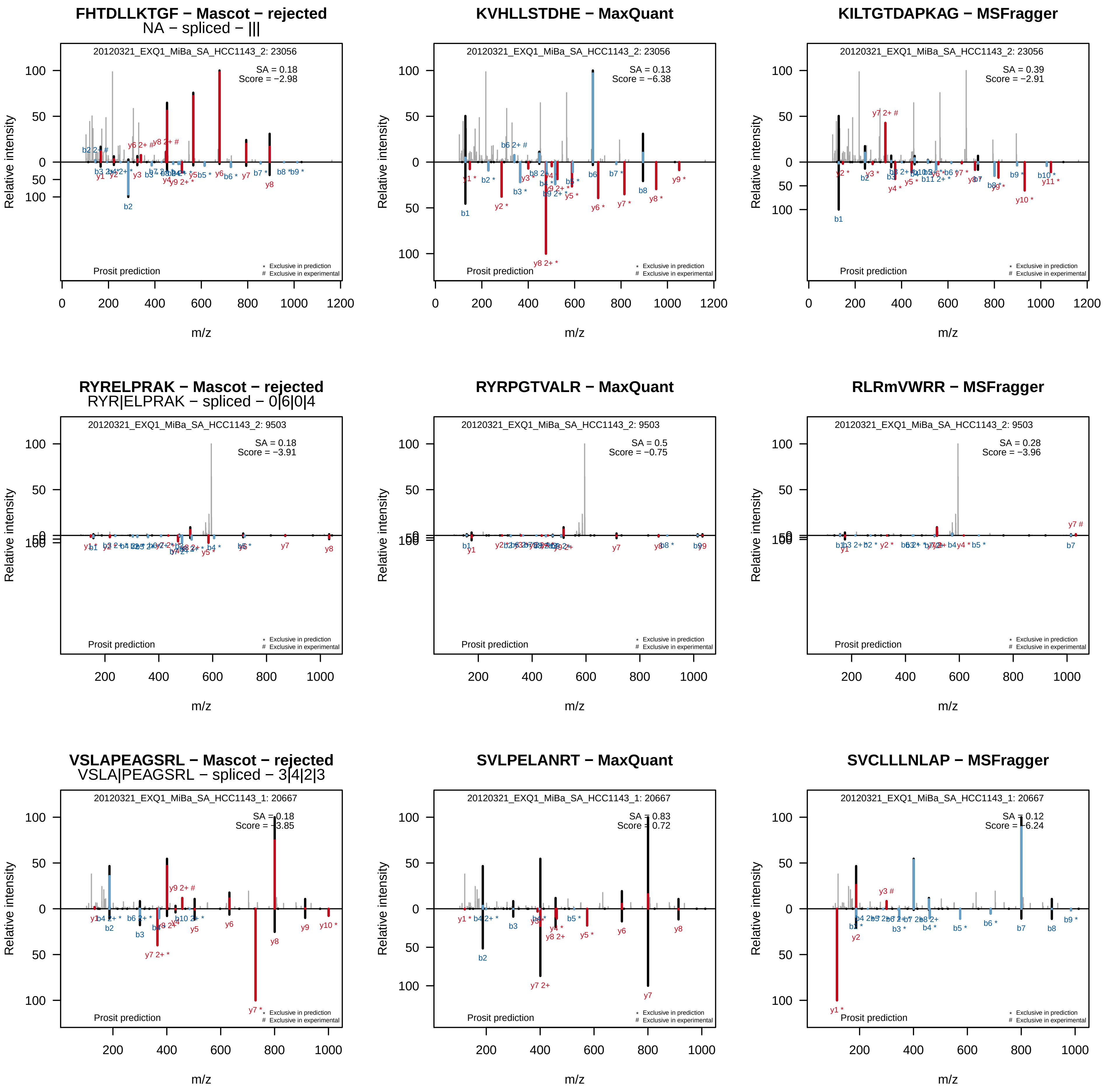
FHTDLLKTGF − Mascot − rejected
NA − spliced − |||
20120321_EXQ1_MiBa_SA_HCC1143_2: 23056
SA = 0.18
Score = −2.98
KVHLLSTDHE − MaxQuant
20120321_EXQ1_MiBa_SA_HCC1143_2: 23056
SA = 0.13
Score = −6.38
KILTGTDAPKAG − MSFragger
20120321_EXQ1_MiBa_SA_HCC1143_2: 23056
SA = 0.39
Score = −2.91
RYRELPRAK − Mascot − rejected
RYR|ELPRAK − spliced − 0|6|0|4
20120321_EXQ1_MiBa_SA_HCC1143_2: 9503
SA = 0.18
Score = −3.91
RYRPGTVALR − MaxQuant
20120321_EXQ1_MiBa_SA_HCC1143_2: 9503
SA = 0.5
Score = −0.75
RLRmVWRR − MSFragger
20120321_EXQ1_MiBa_SA_HCC1143_2: 9503
SA = 0.28
Score = −3.96
VSLAPEAGSRL − Mascot − rejected
VSLA|PEAGSRL − spliced − 3|4|2|3
20120321_EXQ1_MiBa_SA_HCC1143_1: 20667
SA = 0.18
Score = −3.85
SVLPELANRT − MaxQuant
20120321_EXQ1_MiBa_SA_HCC1143_1: 20667
SA = 0.83
Score = 0.72
SVCLLLNLAP − MSFragger
20120321_EXQ1_MiBa_SA_HCC1143_1: 20667
SA = 0.12
Score = −6.24
Relative intensity
m/z
Prosit prediction
* Exclusive in prediction
# Exclusive in experimental

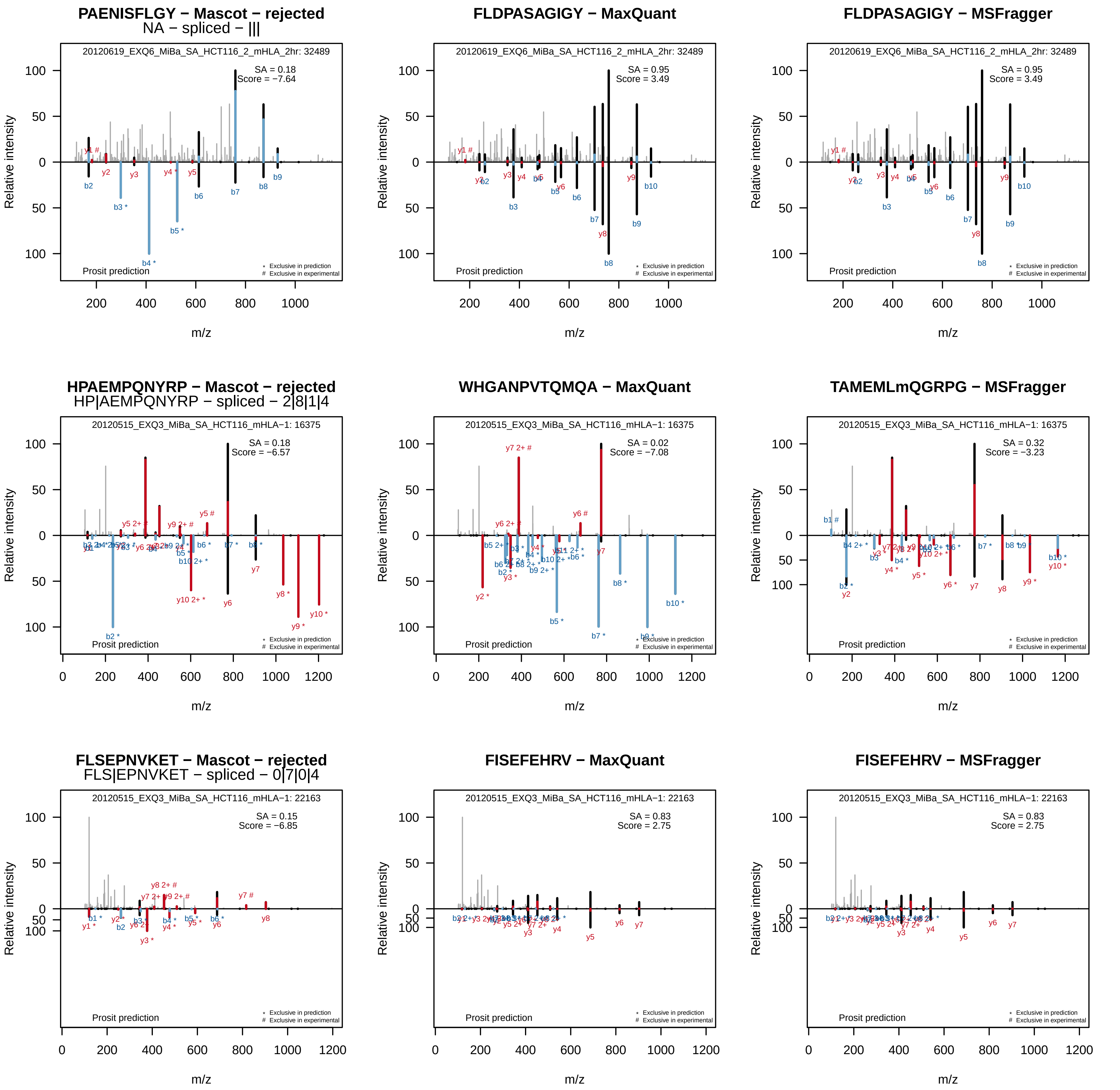
PAENISFLGY − Mascot − rejected
NA − spliced − |||
20120619_EXQ6_MiBa_SA_HCT116_2_mHLA_2hr: 32489
SA = 0.18
Score = −7.64
FLDPASAGIGY − MaxQuant
20120619_EXQ6_MiBa_SA_HCT116_2_mHLA_2hr: 32489
SA = 0.95
Score = 3.49
FLDPASAGIGY − MSFragger
20120619_EXQ6_MiBa_SA_HCT116_2_mHLA_2hr: 32489
SA = 0.95
Score = 3.49
HPAEMPQNYRP − Mascot − rejected
HP|AEMPQNYRP − spliced − 2|8|1|4
20120515_EXQ3_MiBa_SA_HCT116_mHLA−1: 16375
SA = 0.18
Score = −6.57
WHGANPVTQMQA − MaxQuant
20120515_EXQ3_MiBa_SA_HCT116_mHLA−1: 16375
SA = 0.02
Score = −7.08
TAMEMLmQGRPG − MSFragger
20120515_EXQ3_MiBa_SA_HCT116_mHLA−1: 16375
SA = 0.32
Score = −3.23
FLSEPNVKET − Mascot − rejected
FLS|EPNVKET − spliced − 0|7|0|4
20120515_EXQ3_MiBa_SA_HCT116_mHLA−1: 22163
SA = 0.15
Score = −6.85
FISEFEHRV − MaxQuant
20120515_EXQ3_MiBa_SA_HCT116_mHLA−1: 22163
SA = 0.83
Score = 2.75
FISEFEHRV − MSFragger
20120515_EXQ3_MiBa_SA_HCT116_mHLA−1: 22163
SA = 0.83
Score = 2.75
Relative intensity
m/z
Prosit prediction
⋆ Exclusive in prediction
Exclusive in experimental

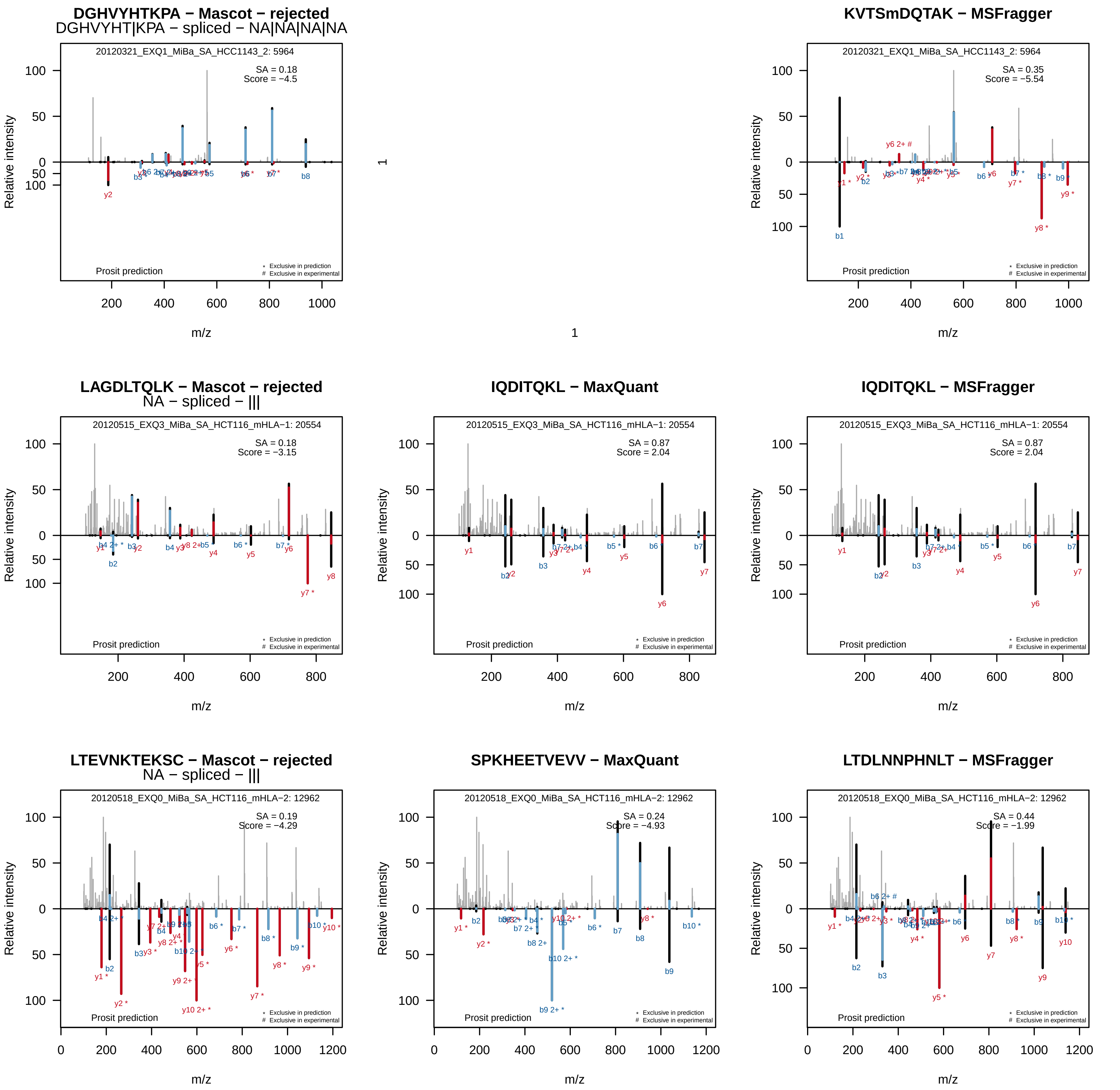
DGHVYHTKPA − Mascot − rejected
DGHVYHT|KPA − spliced − NA|NA|NA|NA
20120321_EXQ1_MiBa_SA_HCC1143_2: 5964
SA = 0.18
Score = −4.5
KVTSmDQTAK − MSFragger
20120321_EXQ1_MiBa_SA_HCC1143_2: 5964
SA = 0.35
Score = −5.54
LAGDLTQLK − Mascot − rejected
NA − spliced − |||
20120515_EXQ3_MiBa_SA_HCT116_mHLA−1: 20554
SA = 0.18
Score = −3.15
IQDITQKL − MaxQuant
20120515_EXQ3_MiBa_SA_HCT116_mHLA−1: 20554
SA = 0.87
Score = 2.04
IQDITQKL − MSFragger
20120515_EXQ3_MiBa_SA_HCT116_mHLA−1: 20554
SA = 0.87
Score = 2.04
LTEVNKTEKSC − Mascot − rejected
NA − spliced − |||
20120518_EXQ0_MiBa_SA_HCT116_mHLA−2: 12962
SA = 0.19
Score = −4.29
SPKHEETVEVV − MaxQuant
20120518_EXQ0_MiBa_SA_HCT116_mHLA−2: 12962
SA = 0.24
Score = −4.93
LTDLNNPHNLT − MSFragger
20120518_EXQ0_MiBa_SA_HCT116_mHLA−2: 12962
SA = 0.44
Score = −1.99
Relative intensity
m/z
Prosit prediction
* Exclusive in prediction
# Exclusive in experimental
1
1

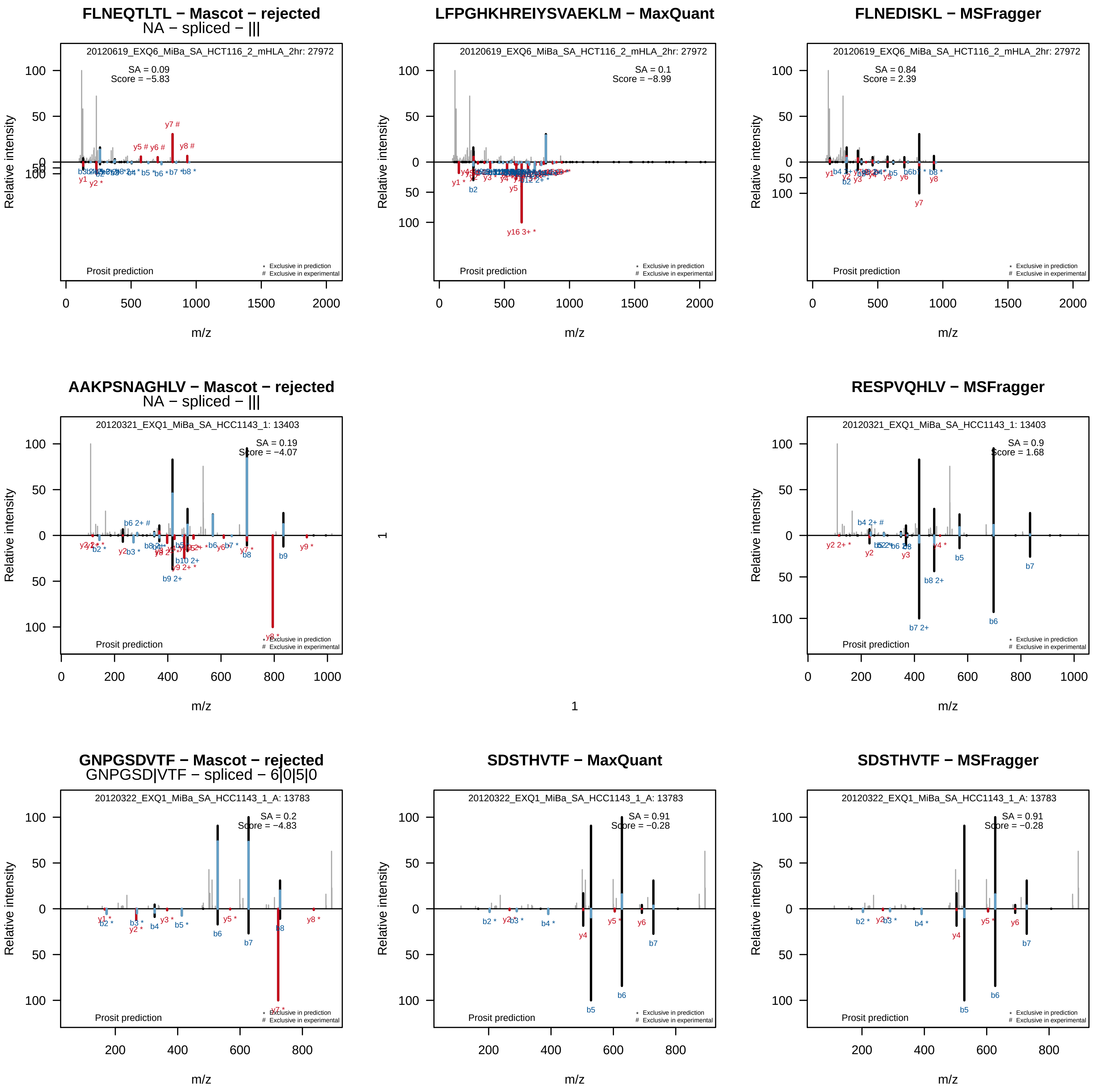
FLNEQTLTL − Mascot − rejected
NA − spliced − |||
20120619_EXQ6_MiBa_SA_HCT116_2_mHLA_2hr: 27972
SA = 0.09
Score = −5.83
LFPGHKHREIYSVAEKLM − MaxQuant
20120619_EXQ6_MiBa_SA_HCT116_2_mHLA_2hr: 27972
SA = 0.1
Score = −8.99
FLNEDISKL − MSFragger
20120619_EXQ6_MiBa_SA_HCT116_2_mHLA_2hr: 27972
SA = 0.84
Score = 2.39
AAKPSNAGHLV − Mascot − rejected
NA − spliced − |||
20120321_EXQ1_MiBa_SA_HCC1143_1: 13403
SA = 0.19
Score = −4.07
RESPVQHLV − MSFragger
20120321_EXQ1_MiBa_SA_HCC1143_1: 13403
SA = 0.9
Score = 1.68
GNPGSDVTF − Mascot − rejected
GNPGSD|VTF − spliced − 6|0|5|0
20120322_EXQ1_MiBa_SA_HCC1143_1_A: 13783
SA = 0.2
Score = −4.83
SDSTHVTF − MaxQuant
20120322_EXQ1_MiBa_SA_HCC1143_1_A: 13783
SA = 0.91
Score = −0.28
SDSTHVTF − MSFragger
20120322_EXQ1_MiBa_SA_HCC1143_1_A: 13783
SA = 0.91
Score = −0.28
Relative intensity
m/z
Prosit prediction
* Exclusive in prediction
# Exclusive in experimental

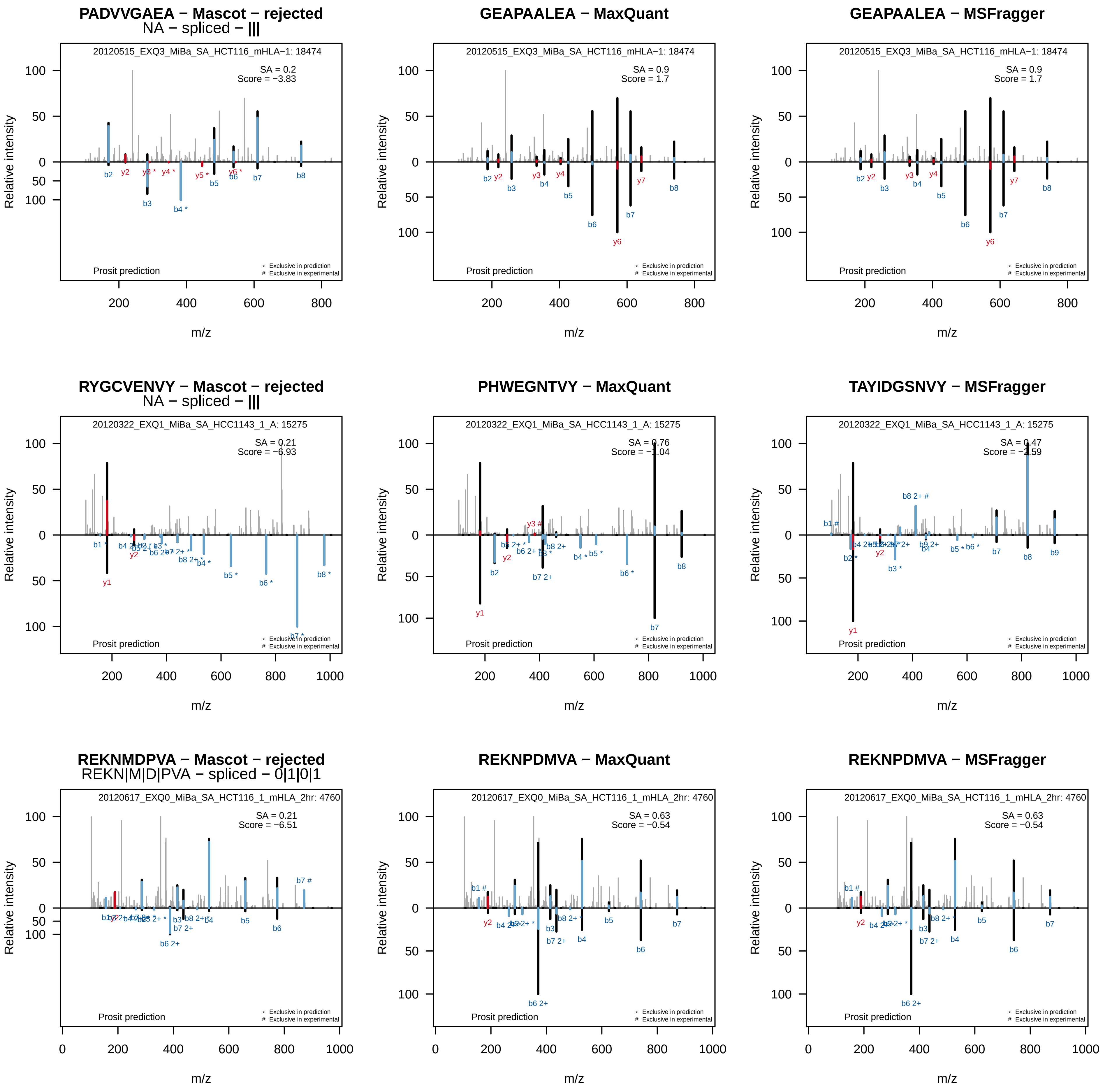

PADVVGAEA − Mascot − rejected
NA − spliced − |||
20120515_EXQ3_MiBa_SA_HCT116_mHLA−1: 18474
SA = 0.2
Score = −3.83
GEAPAALEA − MaxQuant
20120515_EXQ3_MiBa_SA_HCT116_mHLA−1: 18474
SA = 0.9
Score = 1.7
GEAPAALEA − MSFragger
20120515_EXQ3_MiBa_SA_HCT116_mHLA−1: 18474
SA = 0.9
Score = 1.7
RYGCVENVY − Mascot − rejected
NA − spliced − |||
20120322_EXQ1_MiBa_SA_HCC1143_1_A: 15275
SA = 0.21
Score = −6.93
PHWEGNTVY − MaxQuant
20120322_EXQ1_MiBa_SA_HCC1143_1_A: 15275
SA = 0.76
Score = −1.04
TAYIDGSNVY − MSFragger
20120322_EXQ1_MiBa_SA_HCC1143_1_A: 15275
SA = 0.47
Score = −2.59
REKNMDPVA − Mascot − rejected
REKN|M|D|PVA − spliced − 0|1|0|1
20120617_EXQ0_MiBa_SA_HCT116_1_mHLA_2hr: 4760
SA = 0.21
Score = −6.51
REKNPDMVA − MaxQuant
20120617_EXQ0_MiBa_SA_HCT116_1_mHLA_2hr: 4760
SA = 0.63
Score = −0.54
REKNPDMVA − MSFragger
20120617_EXQ0_MiBa_SA_HCT116_1_mHLA_2hr: 4760
SA = 0.63
Score = −0.54
Relative intensity
m/z
Prosit prediction
⋆ Exclusive in prediction
Exclusive in experimental

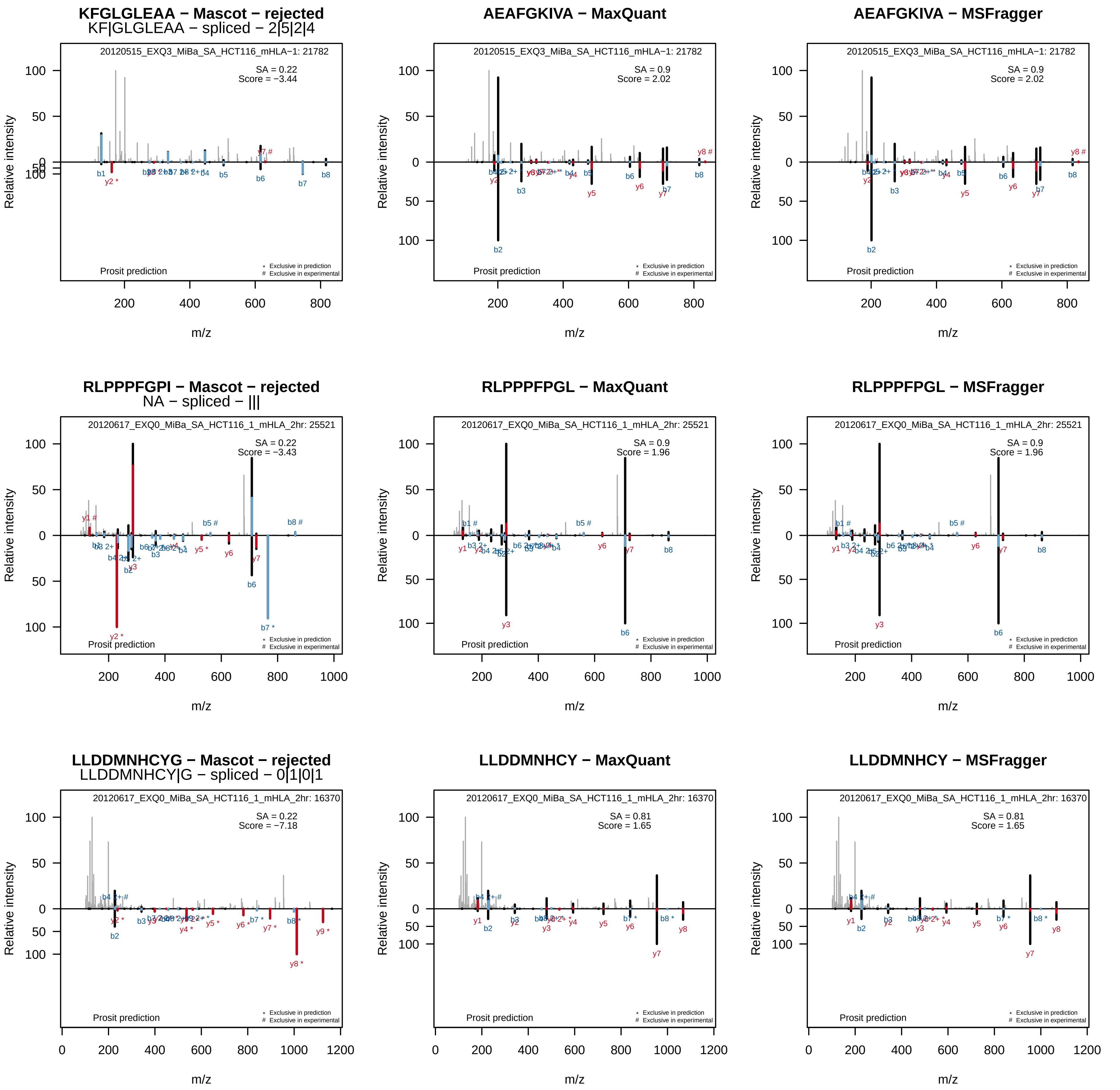

KFGLGLEAA − Mascot − rejected
KF|GLGLEAA − spliced − 2|5|2|4
20120515_EXQ3_MiBa_SA_HCT116_mHLA−1: 21782
SA = 0.22
Score = −3.44
AEAFGKIVA − MaxQuant
20120515_EXQ3_MiBa_SA_HCT116_mHLA−1: 21782
SA = 0.9
Score = 2.02
AEAFGKIVA − MSFragger
20120515_EXQ3_MiBa_SA_HCT116_mHLA−1: 21782
SA = 0.9
Score = 2.02
RLPPPFGPI − Mascot − rejected
NA − spliced − |||
20120617_EXQ0_MiBa_SA_HCT116_1_mHLA_2hr: 25521
SA = 0.22
Score = −3.43
RLPPPFPGL − MaxQuant
20120617_EXQ0_MiBa_SA_HCT116_1_mHLA_2hr: 25521
SA = 0.9
Score = 1.96
RLPPPFPGL − MSFragger
20120617_EXQ0_MiBa_SA_HCT116_1_mHLA_2hr: 25521
SA = 0.9
Score = 1.96
LLDDMNHCYG − Mascot − rejected
LLDDMNHCY|G − spliced − 0|1|0|1
20120617_EXQ0_MiBa_SA_HCT116_1_mHLA_2hr: 16370
SA = 0.22
Score = −7.18
LLDDMNHCY − MaxQuant
20120617_EXQ0_MiBa_SA_HCT116_1_mHLA_2hr: 16370
SA = 0.81
Score = 1.65
LLDDMNHCY − MSFragger
20120617_EXQ0_MiBa_SA_HCT116_1_mHLA_2hr: 16370
SA = 0.81
Score = 1.65
Relative intensity
m/z
Prosit prediction
* Exclusive in prediction
# Exclusive in experimental

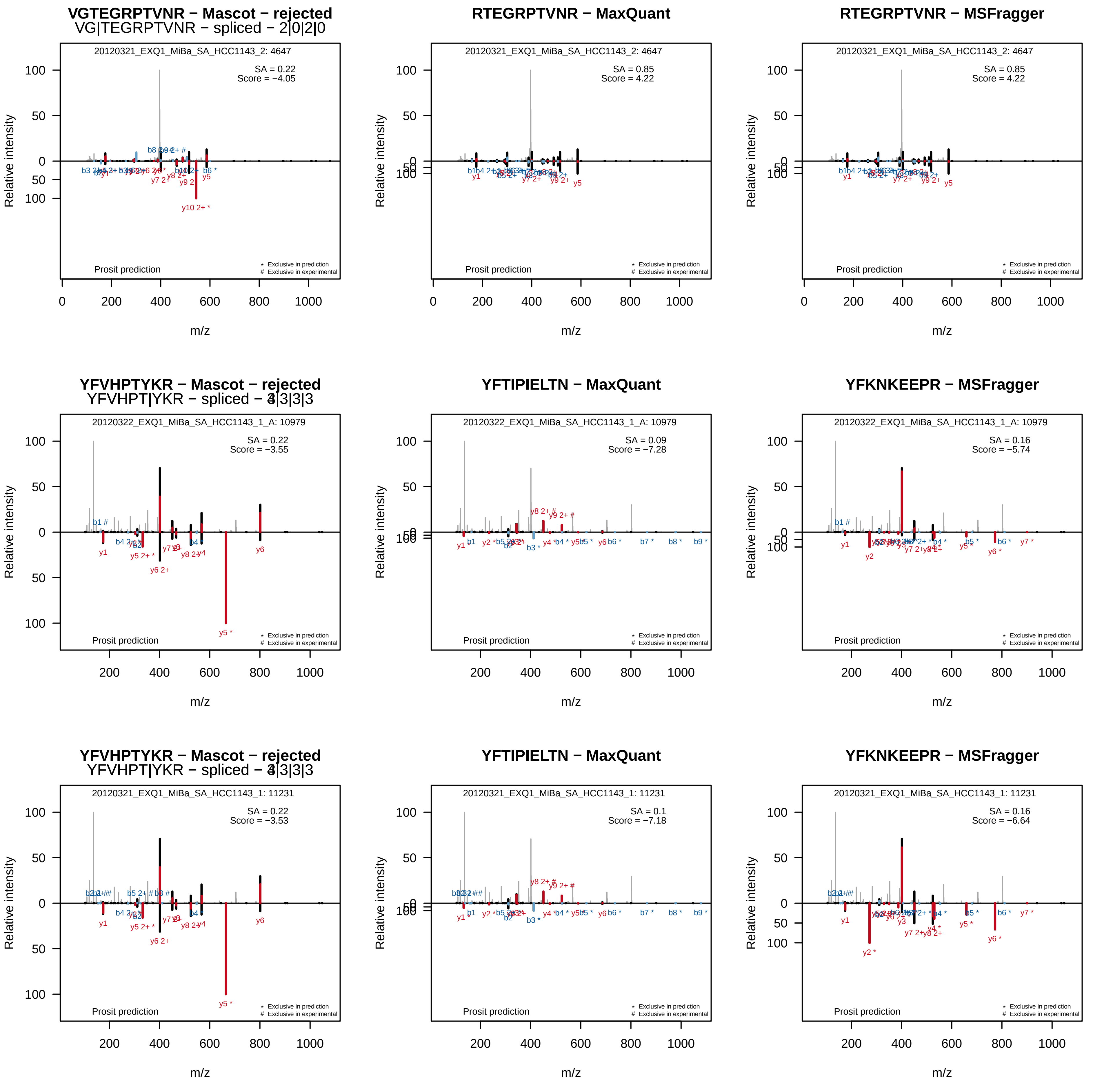
VGTEGRPTVNR − Mascot − rejected
VG|TEGRPTVNR − spliced − 2|0|2|0
20120321_EXQ1_MiBa_SA_HCC1143_2: 4647
SA = 0.22
Score = −4.05
RTEGRPTVNR − MaxQuant
20120321_EXQ1_MiBa_SA_HCC1143_2: 4647
SA = 0.85
Score = 4.22
RTEGRPTVNR − MSFragger
20120321_EXQ1_MiBa_SA_HCC1143_2: 4647
SA = 0.85
Score = 4.22
YFVHPTYKR − Mascot − rejected
YFVHPT|YKR − spliced
20120322_EXQ1_MiBa_SA_HCC1143_1_A: 10979
SA = 0.22
Score = −3.55
YFTIPIELTN − MaxQuant
20120322_EXQ1_MiBa_SA_HCC1143_1_A: 10979
SA = 0.09
Score = −7.28
YFKNKEEPR − MSFragger
20120322_EXQ1_MiBa_SA_HCC1143_1_A: 10979
SA = 0.16
Score = −5.74
YFVHPTYKR − Mascot − rejected
YFVHPT|YKR − spliced
20120321_EXQ1_MiBa_SA_HCC1143_1: 11231
SA = 0.22
Score = −3.53
YFTIPIELTN − MaxQuant
20120321_EXQ1_MiBa_SA_HCC1143_1: 11231
SA = 0.1
Score = −7.18
YFKNKEEPR − MSFragger
20120321_EXQ1_MiBa_SA_HCC1143_1: 11231
SA = 0.16
Score = −6.64
Prosit prediction
⋆ Exclusive in prediction
Exclusive in experimental
Relative intensity
m/z

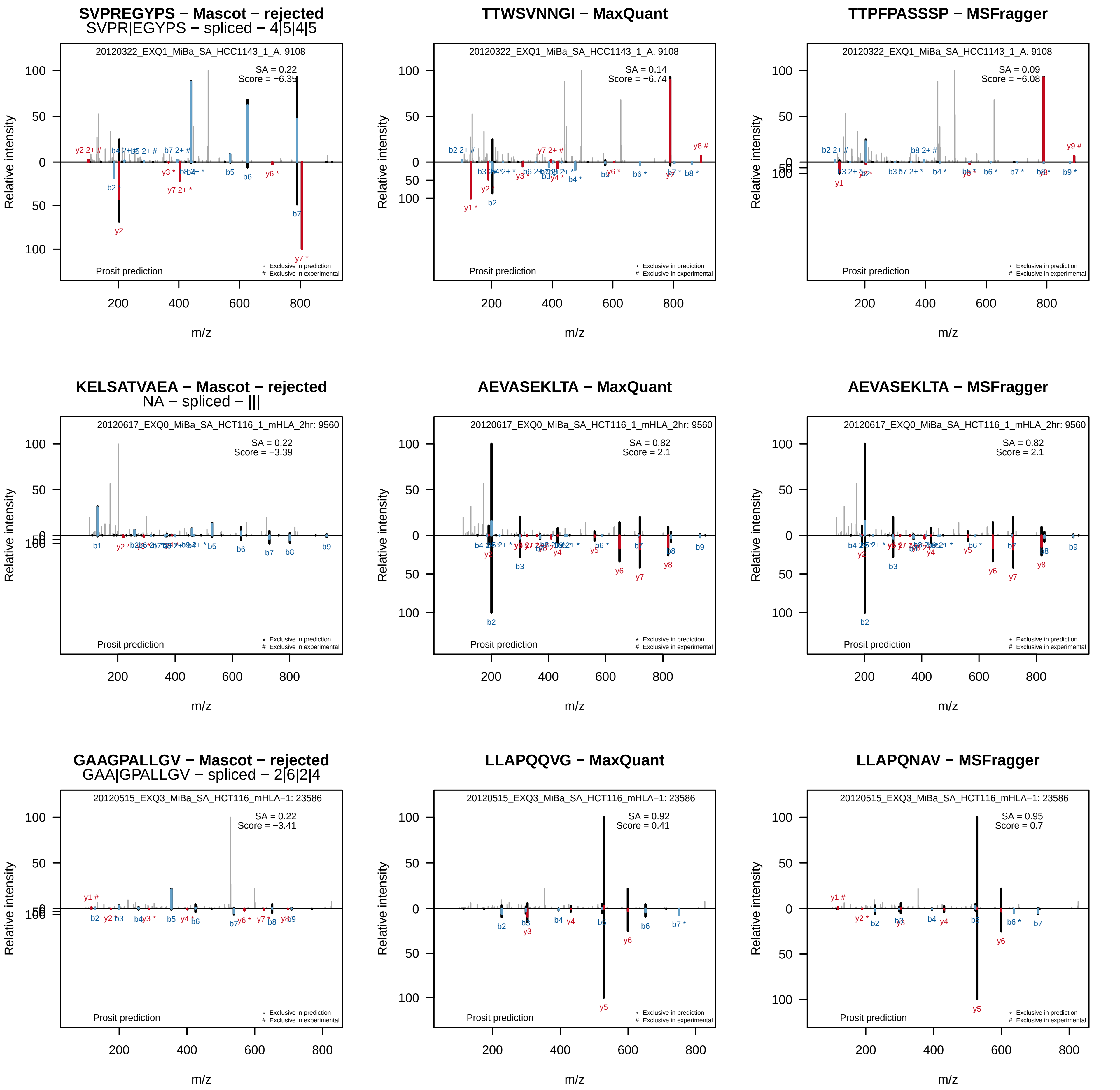
SVPREGYPS − Mascot − rejected
SVPR|EGYPS − spliced − 4|5|4|5
20120322_EXQ1_MiBa_SA_HCC1143_1_A: 9108
SA = 0.22
Score = −6.35
TTWSVNNGI − MaxQuant
20120322_EXQ1_MiBa_SA_HCC1143_1_A: 9108
SA = 0.14
Score = −6.74
TTPFPASSSP − MSFragger
20120322_EXQ1_MiBa_SA_HCC1143_1_A: 9108
SA = 0.09
Score = −6.08
KELSATVAEA − Mascot − rejected
NA − spliced − |||
20120617_EXQ0_MiBa_SA_HCT116_1_mHLA_2hr: 9560
SA = 0.22
Score = −3.39
AEVASEKLTA − MaxQuant
20120617_EXQ0_MiBa_SA_HCT116_1_mHLA_2hr: 9560
SA = 0.82
Score = 2.1
AEVASEKLTA − MSFragger
20120617_EXQ0_MiBa_SA_HCT116_1_mHLA_2hr: 9560
SA = 0.82
Score = 2.1
GAAGPALLGV − Mascot − rejected
GAA|GPALLGV − spliced − 2|6|2|4
20120515_EXQ3_MiBa_SA_HCT116_mHLA−1: 23586
SA = 0.22
Score = −3.41
LLAPQQVG − MaxQuant
20120515_EXQ3_MiBa_SA_HCT116_mHLA−1: 23586
SA = 0.92
Score = 0.41
LLAPQNAV − MSFragger
20120515_EXQ3_MiBa_SA_HCT116_mHLA−1: 23586
SA = 0.95
Score = 0.7
Relative intensity
m/z
Prosit prediction
⋆ Exclusive in prediction
Exclusive in experimental

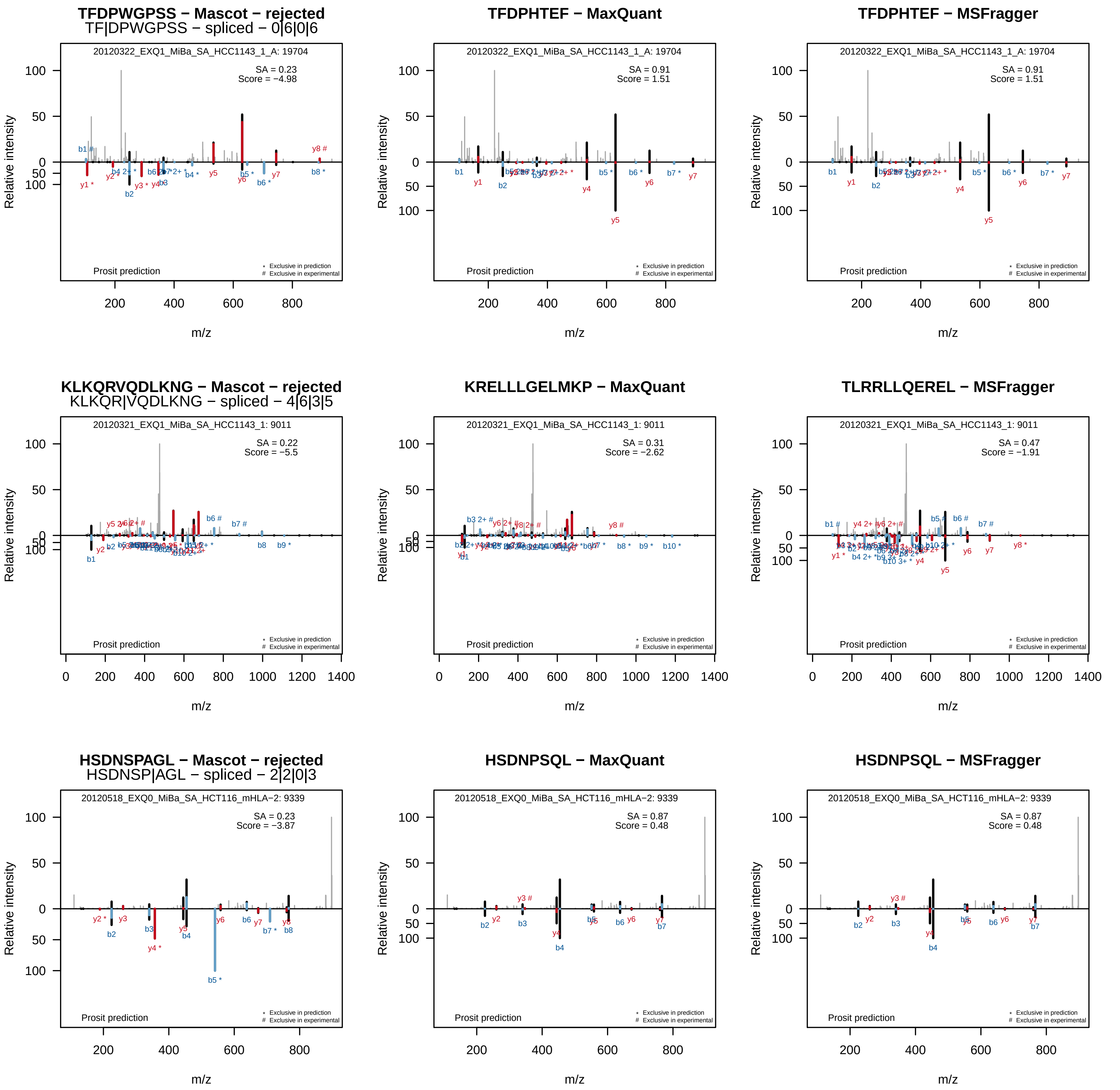

TFDPWGPSS − Mascot − rejected
TF|DPWGPSS − spliced − 0|6|0|6
TFDPHTEF − MaxQuant
TFDPHTEF − MSFragger
20120322_EXQ1_MiBa_SA_HCC1143_1_A: 19704
SA = 0.23
Score = −4.98
SA = 0.91
Score = 1.51
KLKQRVQDLKNG − Mascot − rejected
KLKQR|VQDLKNG − spliced − 4|6|3|5
KRELLLGELMKP − MaxQuant
TLRRLLQEREL − MSFragger
20120321_EXQ1_MiBa_SA_HCC1143_1: 9011
SA = 0.22
Score = −5.5
SA = 0.31
Score = −2.62
SA = 0.47
Score = −1.91
HSDNSPAGL − Mascot − rejected
HSDNSP|AGL − spliced − 2|2|0|3
HSDNPSQL − MaxQuant
HSDNPSQL − MSFragger
20120518_EXQ0_MiBa_SA_HCT116_mHLA−2: 9339
SA = 0.23
Score = −3.87
SA = 0.87
Score = 0.48
Relative intensity
m/z
Prosit prediction
* Exclusive in prediction
# Exclusive in experimental

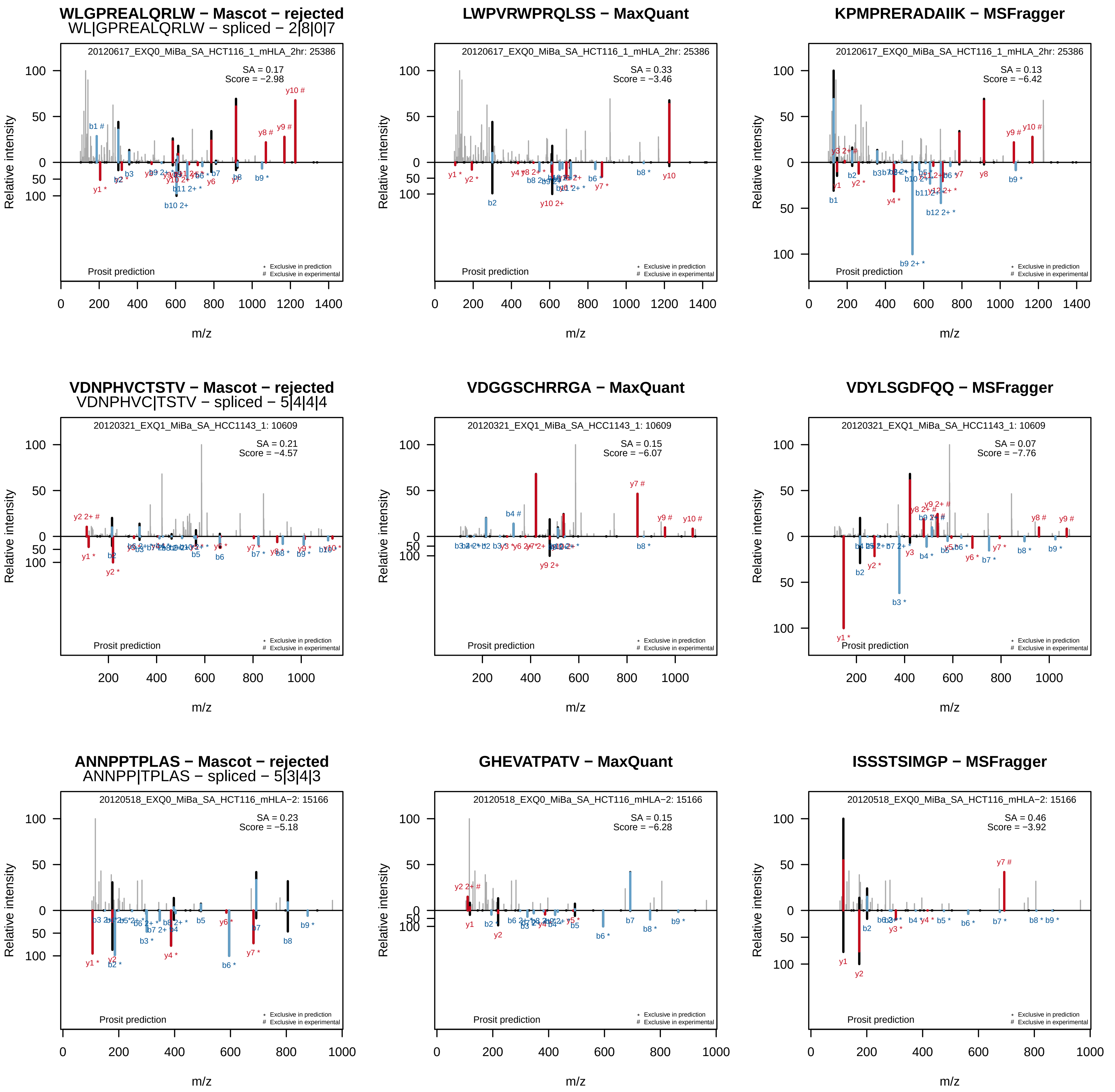
WLGPREALQRLW − Mascot − rejected
WL|GPREALQRLW − spliced − 2|8|0|7
20120617_EXQ0_MiBa_SA_HCT116_1_mHLA_2hr: 25386
SA = 0.17
Score = −2.98
LWPVRWPRQLSS − MaxQuant
20120617_EXQ0_MiBa_SA_HCT116_1_mHLA_2hr: 25386
SA = 0.33
Score = −3.46
KPMPRERADAIIK − MSFragger
20120617_EXQ0_MiBa_SA_HCT116_1_mHLA_2hr: 25386
SA = 0.13
Score = −6.42
VDNPHVCTSTV − Mascot − rejected
VDNPHVC|TSTV − spliced − 5|4|4|4
20120321_EXQ1_MiBa_SA_HCC1143_1: 10609
SA = 0.21
Score = −4.57
VDGGSCHRRGA − MaxQuant
20120321_EXQ1_MiBa_SA_HCC1143_1: 10609
SA = 0.15
Score = −6.07
VDYLSGDFQQ − MSFragger
20120321_EXQ1_MiBa_SA_HCC1143_1: 10609
SA = 0.07
Score = −7.76
ANNPPTPLAS − Mascot − rejected
ANNPP|TPLAS − spliced − 5|3|4|3
20120518_EXQ0_MiBa_SA_HCT116_mHLA−2: 15166
SA = 0.23
Score = −5.18
GHEVATPATV − MaxQuant
20120518_EXQ0_MiBa_SA_HCT116_mHLA−2: 15166
SA = 0.15
Score = −6.28
ISSSTSIMGP − MSFragger
20120518_EXQ0_MiBa_SA_HCT116_mHLA−2: 15166
SA = 0.46
Score = −3.92
Relative intensity
m/z
Prosit prediction
⋆ Exclusive in prediction
Exclusive in experimental

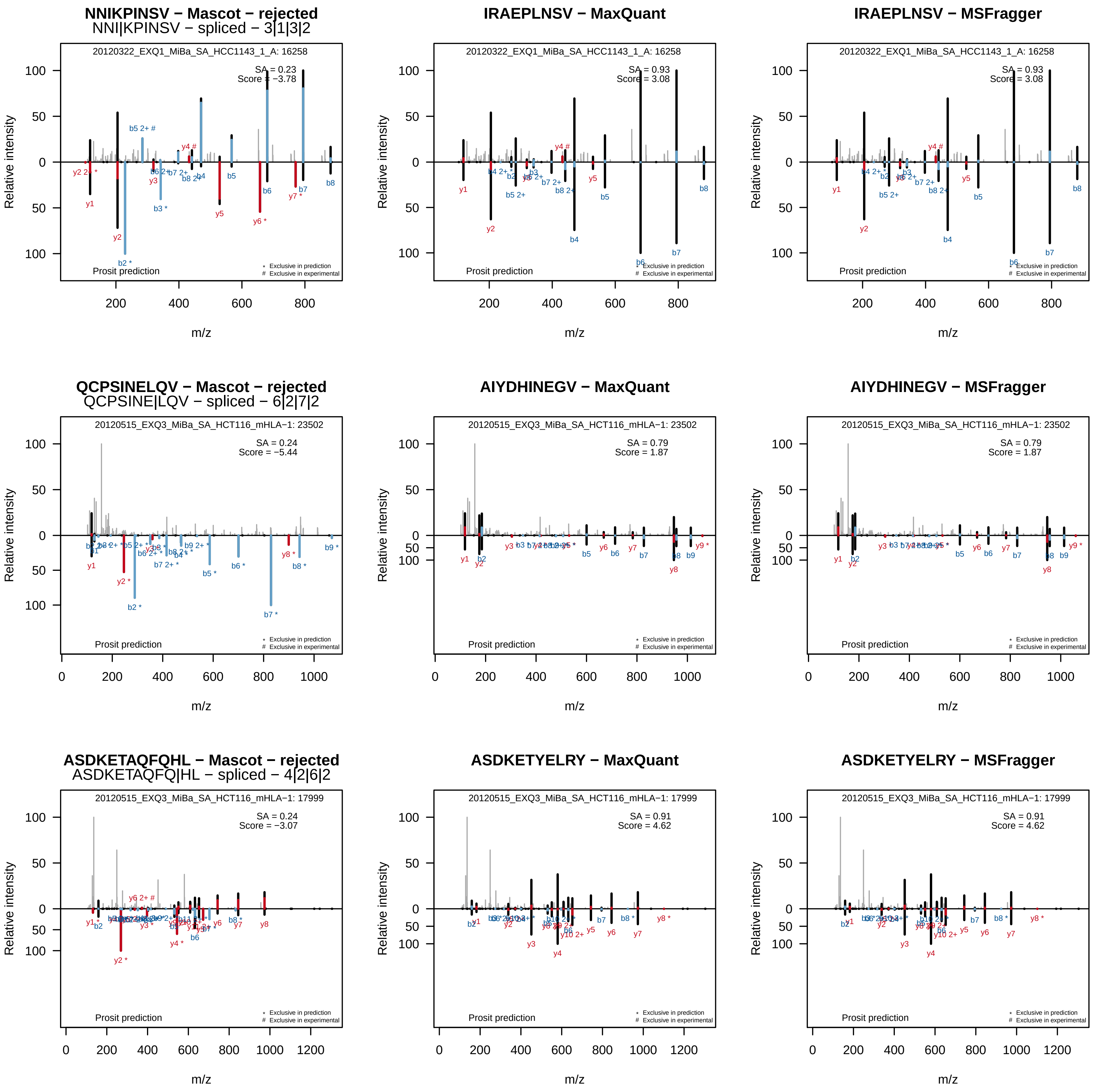
NNIKPINSV − Mascot − rejected
NNI|KPINSV − spliced − 3|1|3|2
IRAEPLNSV − MaxQuant
IRAEPLNSV − MSFragger
20120322_EXQ1_MiBa_SA_HCC1143_1_A: 16258
SA = 0.23
Score = −3.78
SA = 0.93
Score = 3.08
QCPSINELQV − Mascot − rejected
QCPSINE|LQV − spliced − 6|2|7|2
AIYDHINEGV − MaxQuant
AIYDHINEGV − MSFragger
20120515_EXQ3_MiBa_SA_HCT116_mHLA−1: 23502
SA = 0.24
Score = −5.44
SA = 0.79
Score = 1.87
ASDKETAQFQHL − Mascot − rejected
ASDKETAQFQ|HL − spliced − 4|2|6|2
ASDKETYELRY − MaxQuant
ASDKETYELRY − MSFragger
20120515_EXQ3_MiBa_SA_HCT116_mHLA−1: 17999
SA = 0.24
Score = −3.07
SA = 0.91
Score = 4.62
Relative intensity
m/z
Prosit prediction
* Exclusive in prediction
# Exclusive in experimental

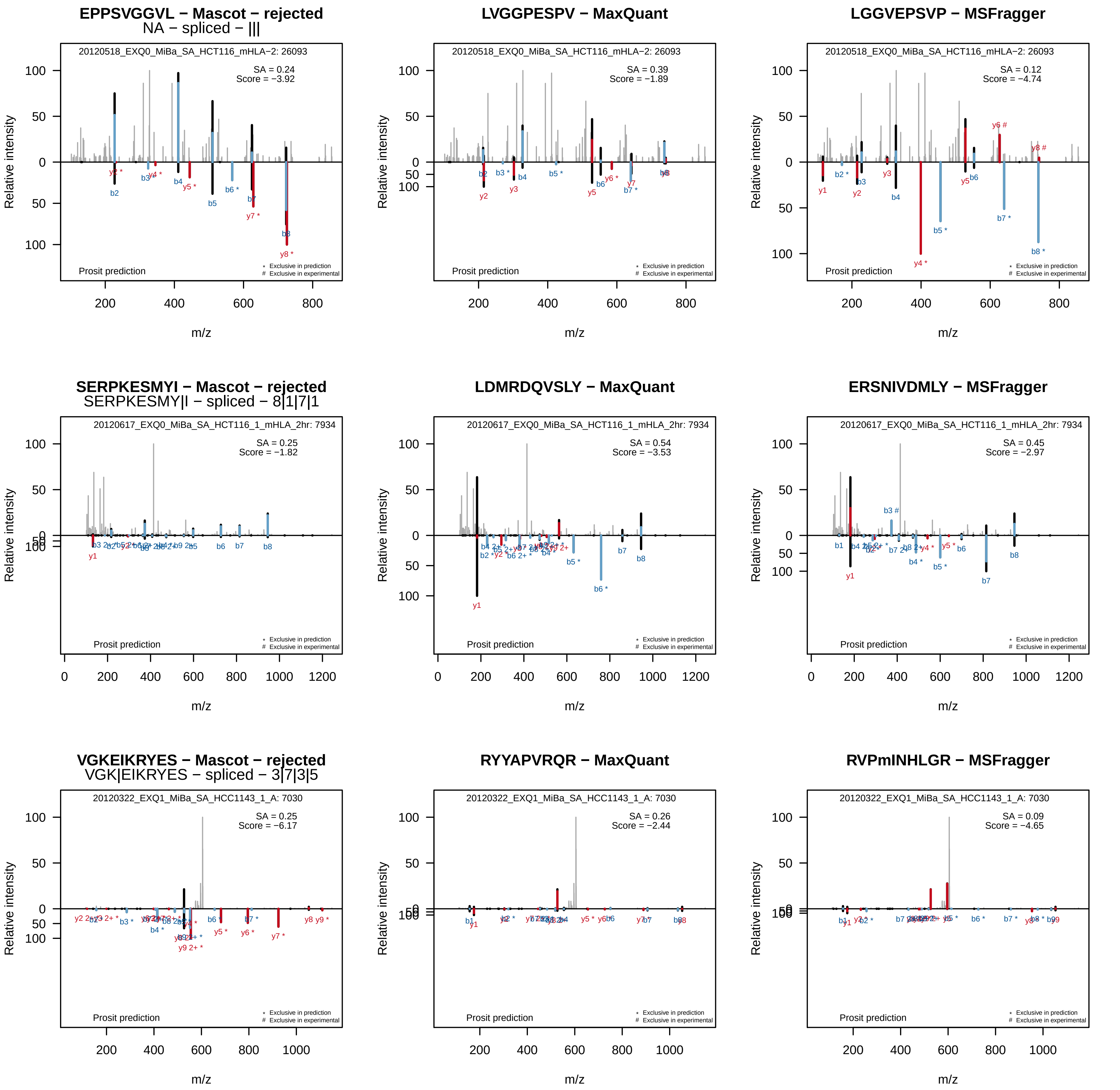
EPPSVGGVL − Mascot − rejected
NA − spliced − |||
20120518_EXQ0_MiBa_SA_HCT116_mHLA−2: 26093
SA = 0.24
Score = −3.92
LVGGPESPV − MaxQuant
20120518_EXQ0_MiBa_SA_HCT116_mHLA−2: 26093
SA = 0.39
Score = −1.89
LGGVEPSVP − MSFragger
20120518_EXQ0_MiBa_SA_HCT116_mHLA−2: 26093
SA = 0.12
Score = −4.74
SERPKESMYI − Mascot − rejected
SERPKESMY|I − spliced − 8|1|7|1
20120617_EXQ0_MiBa_SA_HCT116_1_mHLA_2hr: 7934
SA = 0.25
Score = −1.82
LDMRDQVSLY − MaxQuant
20120617_EXQ0_MiBa_SA_HCT116_1_mHLA_2hr: 7934
SA = 0.54
Score = −3.53
ERSNIVDMLY − MSFragger
20120617_EXQ0_MiBa_SA_HCT116_1_mHLA_2hr: 7934
SA = 0.45
Score = −2.97
VGKEIKRYES − Mascot − rejected
VGK|EIKRYES − spliced − 3|7|3|5
20120322_EXQ1_MiBa_SA_HCC1143_1_A: 7030
SA = 0.25
Score = −6.17
RYYAPVRQR − MaxQuant
20120322_EXQ1_MiBa_SA_HCC1143_1_A: 7030
SA = 0.26
Score = −2.44
RVPmINHLGR − MSFragger
20120322_EXQ1_MiBa_SA_HCC1143_1_A: 7030
SA = 0.09
Score = −4.65
Relative intensity
m/z
Prosit prediction
⋆ Exclusive in prediction
Exclusive in experimental

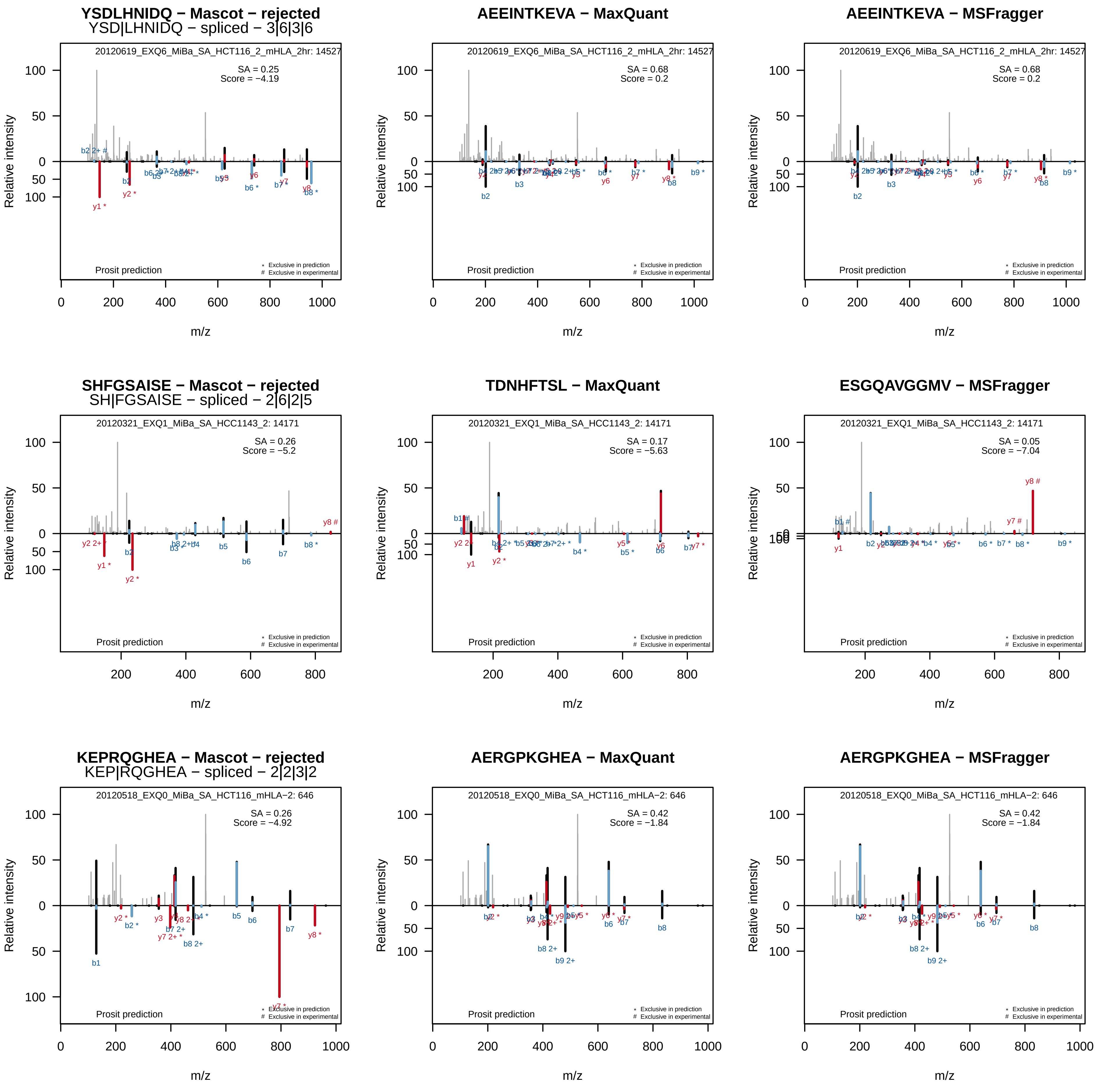
YSDLHNIDQ − Mascot − rejected
YSD|LHNIDQ − spliced − 3|6|3|6
20120619_EXQ6_MiBa_SA_HCT116_2_mHLA_2hr: 14527
SA = 0.25
Score = −4.19
AEEINTKEVA − MaxQuant
20120619_EXQ6_MiBa_SA_HCT116_2_mHLA_2hr: 14527
SA = 0.68
Score = 0.2
AEEINTKEVA − MSFragger
20120619_EXQ6_MiBa_SA_HCT116_2_mHLA_2hr: 14527
SA = 0.68
Score = 0.2
SHFGSAISE − Mascot − rejected
SH|FGSAISE − spliced − 2|6|2|5
20120321_EXQ1_MiBa_SA_HCC1143_2: 14171
SA = 0.26
Score = −5.2
TDNHFTSL − MaxQuant
20120321_EXQ1_MiBa_SA_HCC1143_2: 14171
SA = 0.17
Score = −5.63
ESGQAVGGMV − MSFragger
20120321_EXQ1_MiBa_SA_HCC1143_2: 14171
SA = 0.05
Score = −7.04
KEPRQGHEA − Mascot − rejected
KEP|RQGHEA − spliced − 2|2|3|2
20120518_EXQ0_MiBa_SA_HCT116_mHLA−2: 646
SA = 0.26
Score = −4.92
AERGPKGHEA − MaxQuant
20120518_EXQ0_MiBa_SA_HCT116_mHLA−2: 646
SA = 0.42
Score = −1.84
AERGPKGHEA − MSFragger
20120518_EXQ0_MiBa_SA_HCT116_mHLA−2: 646
SA = 0.42
Score = −1.84
Relative intensity
m/z
Prosit prediction
* Exclusive in prediction
# Exclusive in experimental

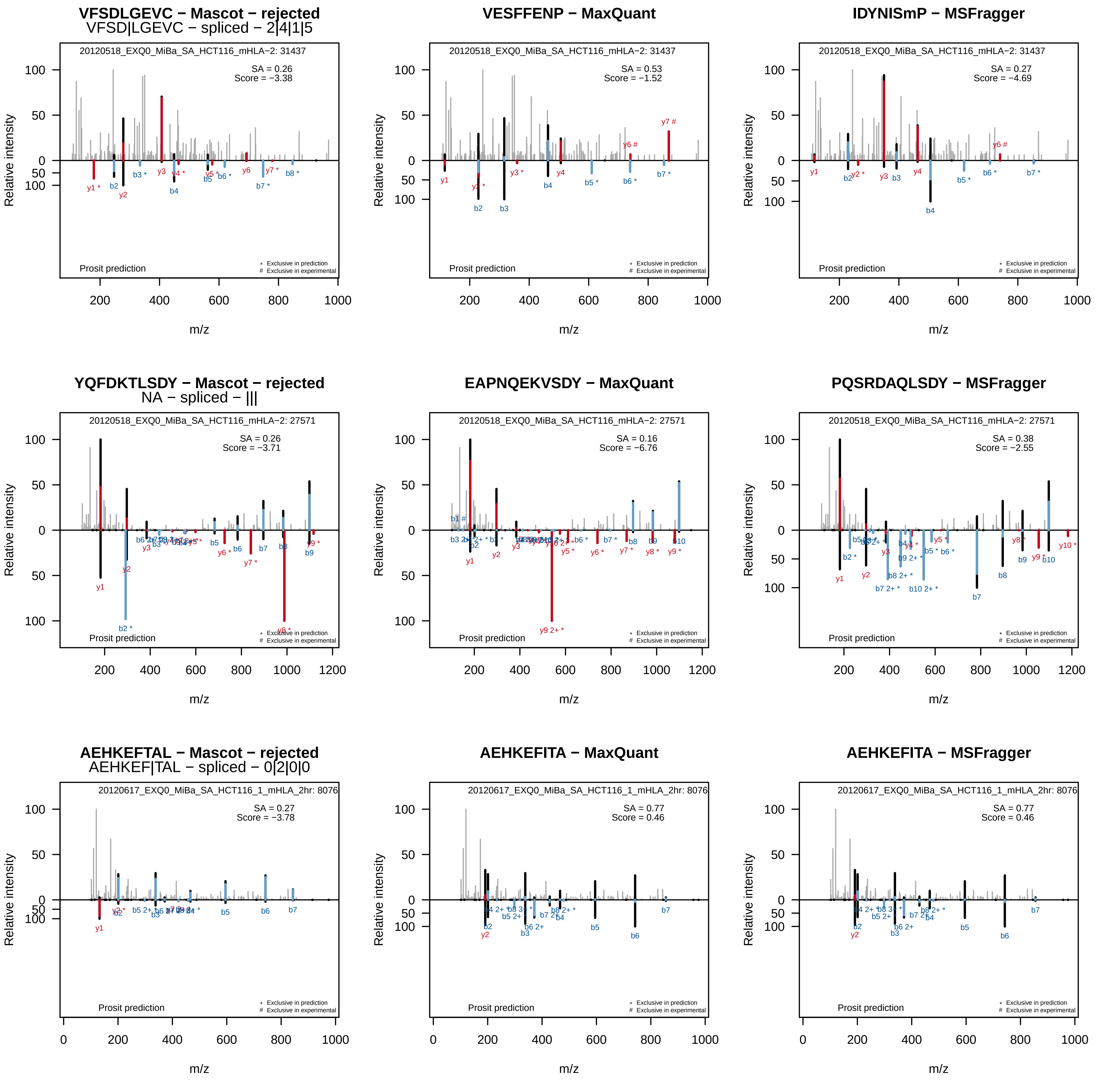

VFSDLGEVC − Mascot − rejected
VFSD|LGEVC − spliced − 2|4|1|5
20120518_EXQ0_MiBa_SA_HCT116_mHLA−2: 31437
SA = 0.26
Score = −3.38
VESFFENP − MaxQuant
20120518_EXQ0_MiBa_SA_HCT116_mHLA−2: 31437
SA = 0.53
Score = −1.52
IDYNISmP − MSFragger
20120518_EXQ0_MiBa_SA_HCT116_mHLA−2: 31437
SA = 0.27
Score = −4.69
YQFDKTLSDY − Mascot − rejected
NA − spliced − |||
20120518_EXQ0_MiBa_SA_HCT116_mHLA−2: 27571
SA = 0.26
Score = −3.71
EAPNQEKVSDY − MaxQuant
20120518_EXQ0_MiBa_SA_HCT116_mHLA−2: 27571
SA = 0.16
Score = −6.76
PQSRDAQLSDY − MSFragger
20120518_EXQ0_MiBa_SA_HCT116_mHLA−2: 27571
SA = 0.38
Score = −2.55
AEHKEFTAL − Mascot − rejected
AEHKEF|TAL − spliced − 0|2|0|0
20120617_EXQ0_MiBa_SA_HCT116_1_mHLA_2hr: 8076
SA = 0.27
Score = −3.78
AEHKEFITA − MaxQuant
20120617_EXQ0_MiBa_SA_HCT116_1_mHLA_2hr: 8076
SA = 0.77
Score = 0.46
AEHKEFITA − MSFragger
20120617_EXQ0_MiBa_SA_HCT116_1_mHLA_2hr: 8076
SA = 0.77
Score = 0.46
Relative intensity
m/z
Prosit prediction
* Exclusive in prediction
# Exclusive in experimental

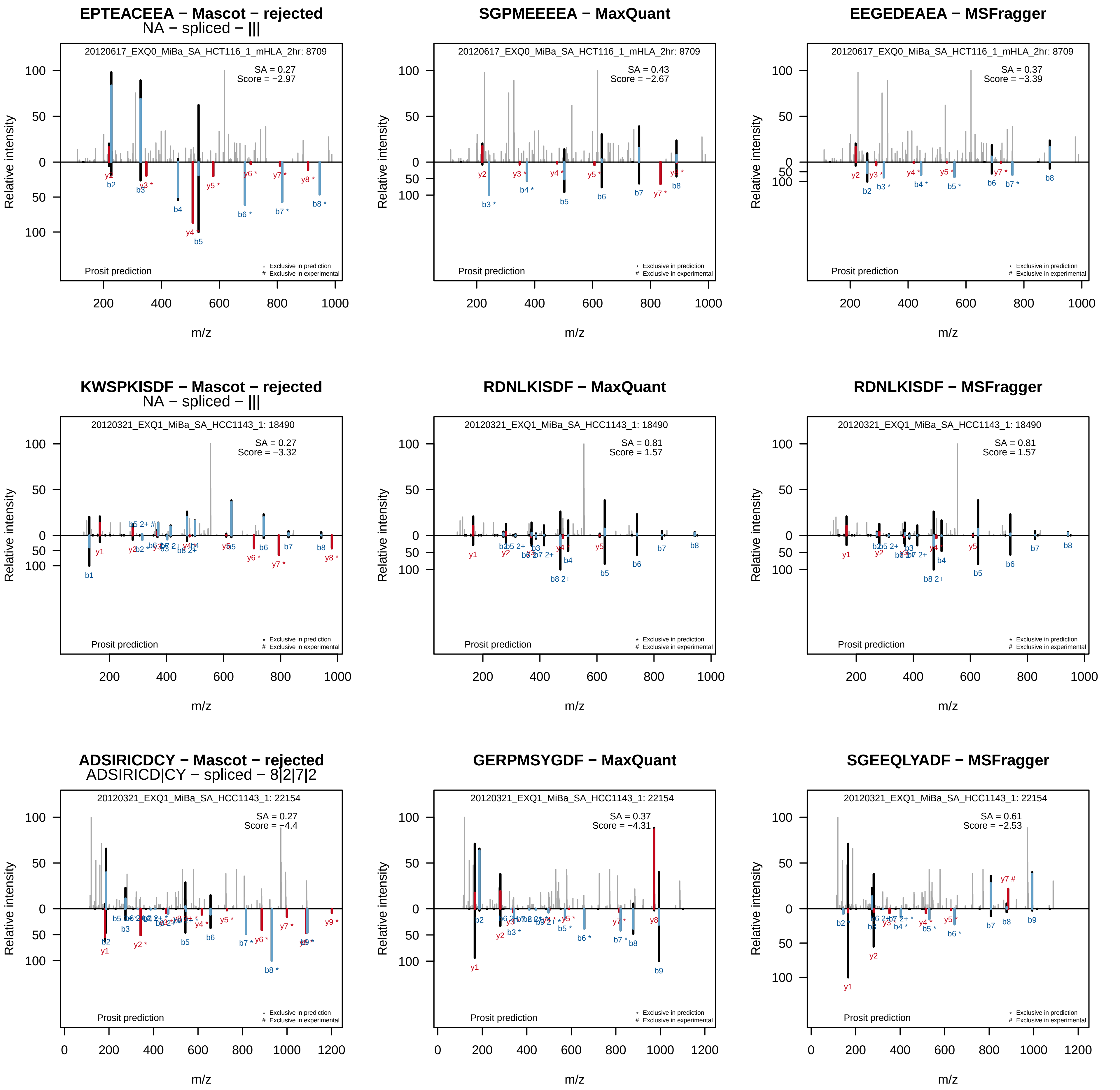
EPTEACEEA − Mascot − rejected
NA − spliced − |||
20120617_EXQ0_MiBa_SA_HCT116_1_mHLA_2hr: 8709
SA = 0.27
Score = −2.97
SGPMEEEEA − MaxQuant
20120617_EXQ0_MiBa_SA_HCT116_1_mHLA_2hr: 8709
SA = 0.43
Score = −2.67
EEGEDEAEA − MSFragger
20120617_EXQ0_MiBa_SA_HCT116_1_mHLA_2hr: 8709
SA = 0.37
Score = −3.39
KWSPKISDF − Mascot − rejected
NA − spliced − |||
20120321_EXQ1_MiBa_SA_HCC1143_1: 18490
SA = 0.27
Score = −3.32
RDNLKISDF − MaxQuant
20120321_EXQ1_MiBa_SA_HCC1143_1: 18490
SA = 0.81
Score = 1.57
RDNLKISDF − MSFragger
20120321_EXQ1_MiBa_SA_HCC1143_1: 18490
SA = 0.81
Score = 1.57
ADSIRICDCY − Mascot − rejected
ADSIRICD|CY − spliced − 8|2|7|2
20120321_EXQ1_MiBa_SA_HCC1143_1: 22154
SA = 0.27
Score = −4.4
GERPMSYGDF − MaxQuant
20120321_EXQ1_MiBa_SA_HCC1143_1: 22154
SA = 0.37
Score = −4.31
SGEEQLYADF − MSFragger
20120321_EXQ1_MiBa_SA_HCC1143_1: 22154
SA = 0.61
Score = −2.53
Relative intensity
m/z
Prosit prediction
⋆ Exclusive in prediction
Exclusive in experimental

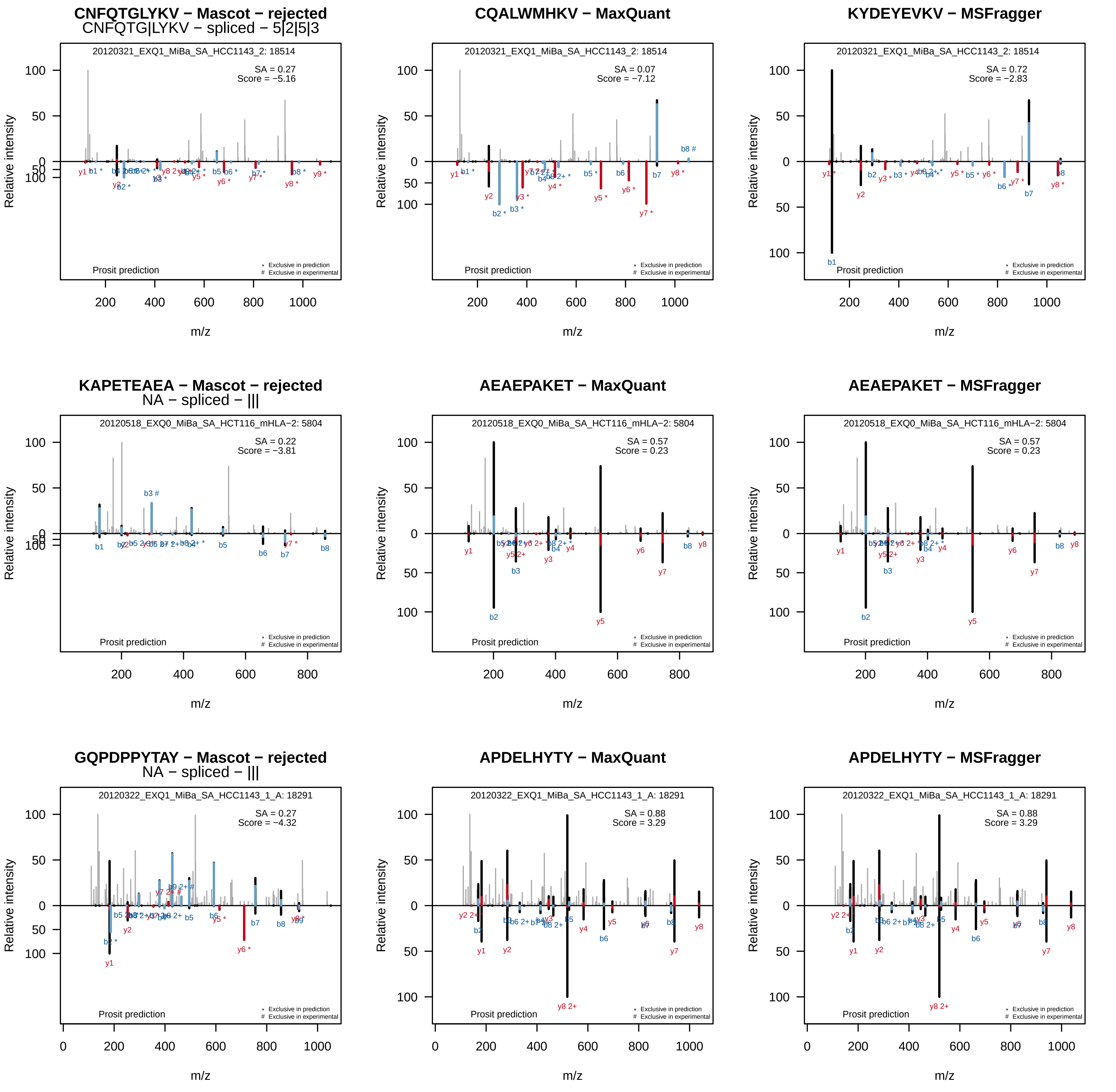

CNFQTGLYKV − Mascot − rejected
CNFQTG|LYKV − spliced − 5|2|5|3
20120321_EXQ1_MiBa_SA_HCC1143_2: 18514
SA = 0.27
Score = −5.16
Prosit prediction
CQALWMHKV − MaxQuant
20120321_EXQ1_MiBa_SA_HCC1143_2: 18514
SA = 0.07
Score = −7.12
Prosit prediction
KYDEYEVKV − MSFragger
20120321_EXQ1_MiBa_SA_HCC1143_2: 18514
SA = 0.72
Score = −2.83
Prosit prediction
KAPETEAEA − Mascot − rejected
NA − spliced − |||
20120518_EXQ0_MiBa_SA_HCT116_mHLA−2: 5804
SA = 0.22
Score = −3.81
Prosit prediction
AEAEPAKET − MaxQuant
20120518_EXQ0_MiBa_SA_HCT116_mHLA−2: 5804
SA = 0.57
Score = 0.23
Prosit prediction
AEAEPAKET − MSFragger
20120518_EXQ0_MiBa_SA_HCT116_mHLA−2: 5804
SA = 0.57
Score = 0.23
Prosit prediction
GQPDPPYTAY − Mascot − rejected
NA − spliced − |||
20120322_EXQ1_MiBa_SA_HCC1143_1_A: 18291
SA = 0.27
Score = −4.32
Prosit prediction
APDELHYTY − MaxQuant
20120322_EXQ1_MiBa_SA_HCC1143_1_A: 18291
SA = 0.88
Score = 3.29
Prosit prediction
APDELHYTY − MSFragger
20120322_EXQ1_MiBa_SA_HCC1143_1_A: 18291
SA = 0.88
Score = 3.29
Prosit prediction
* Exclusive in prediction
# Exclusive in experimental
Relative intensity
m/z

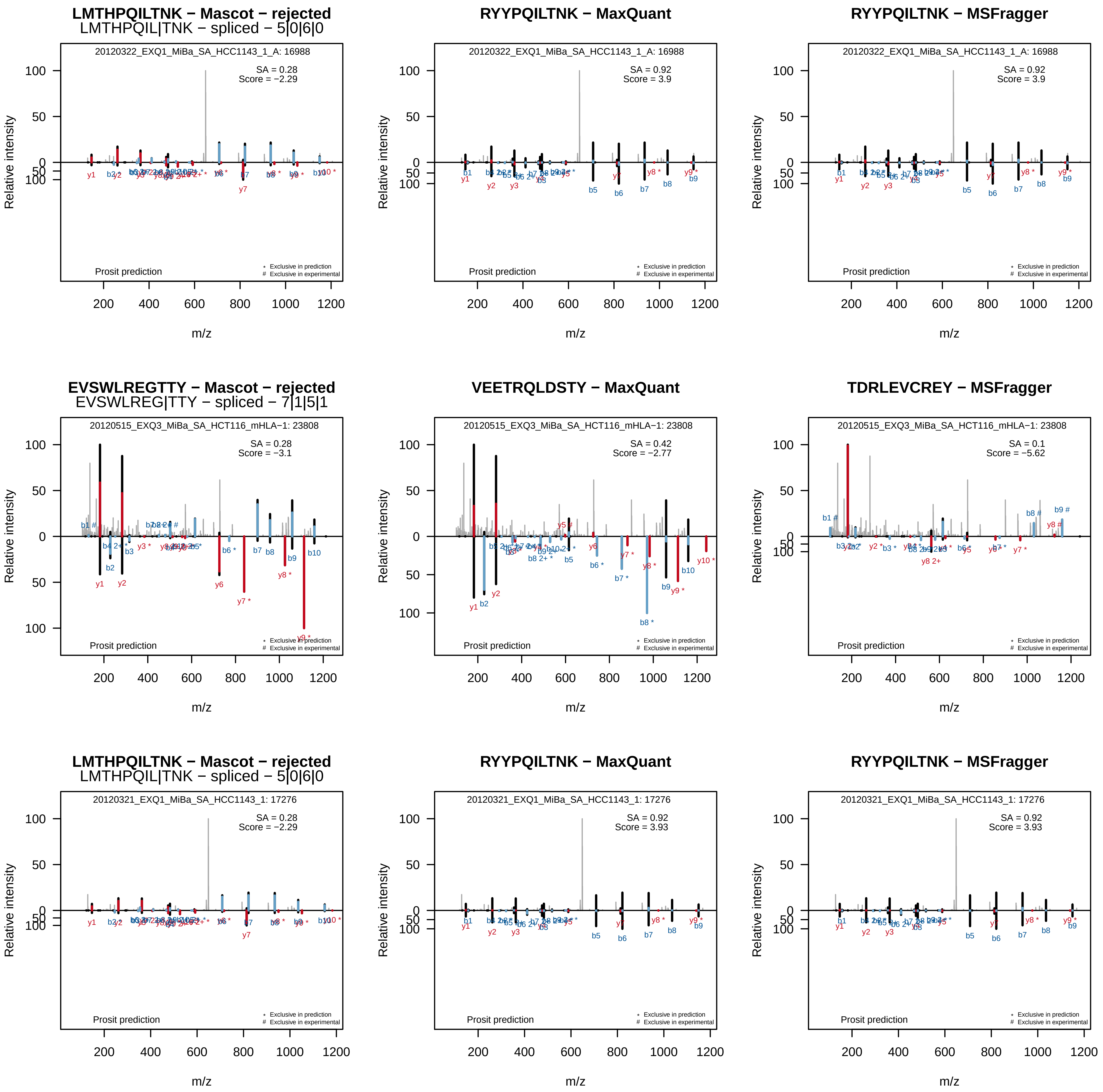

LMTHPQILTNK − Mascot − rejected
LMTHPQIL|TNK − spliced − 5|0|6|0
20120322_EXQ1_MiBa_SA_HCC1143_1_A: 16988
SA = 0.28
Score = −2.29
RYYPQILTNK − MaxQuant
20120322_EXQ1_MiBa_SA_HCC1143_1_A: 16988
SA = 0.92
Score = 3.9
RYYPQILTNK − MSFragger
20120322_EXQ1_MiBa_SA_HCC1143_1_A: 16988
SA = 0.92
Score = 3.9
EVSWLREGTTY − Mascot − rejected
EVSWLREG|TTY − spliced − 7|1|5|1
20120515_EXQ3_MiBa_SA_HCT116_mHLA−1: 23808
SA = 0.28
Score = −3.1
VEETRQLDSTY − MaxQuant
20120515_EXQ3_MiBa_SA_HCT116_mHLA−1: 23808
SA = 0.42
Score = −2.77
TDRLEVCREY − MSFragger
20120515_EXQ3_MiBa_SA_HCT116_mHLA−1: 23808
SA = 0.1
Score = −5.62
LMTHPQILTNK − Mascot − rejected
LMTHPQIL|TNK − spliced − 5|0|6|0
20120321_EXQ1_MiBa_SA_HCC1143_1: 17276
SA = 0.28
Score = −2.29
RYYPQILTNK − MaxQuant
20120321_EXQ1_MiBa_SA_HCC1143_1: 17276
SA = 0.92
Score = 3.93
RYYPQILTNK − MSFragger
20120321_EXQ1_MiBa_SA_HCC1143_1: 17276
SA = 0.92
Score = 3.93
Relative intensity
m/z
Prosit prediction
* Exclusive in prediction
# Exclusive in experimental

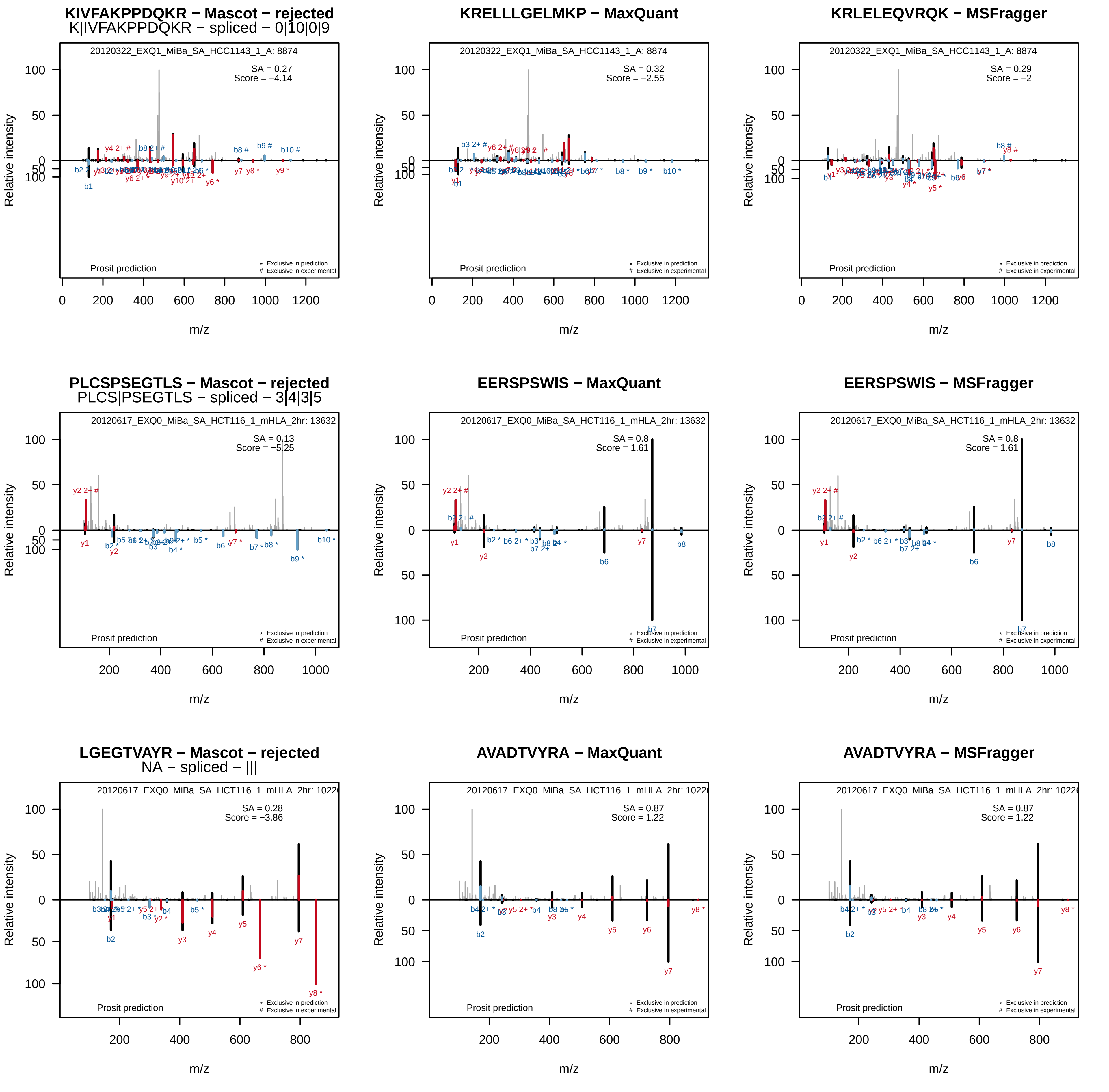
KIVFAKPPDQKR − Mascot − rejected
K|IVFAKPPDQKR − spliced − 0|10|0|9
20120322_EXQ1_MiBa_SA_HCC1143_1_A: 8874
SA = 0.27
Score = −4.14
KRELLLGELMKP − MaxQuant
20120322_EXQ1_MiBa_SA_HCC1143_1_A: 8874
SA = 0.32
Score = −2.55
KRLELEQVRQK − MSFragger
20120322_EXQ1_MiBa_SA_HCC1143_1_A: 8874
SA = 0.29
Score = −2
PLCSPSEGTLS − Mascot − rejected
PLCS|PSEGTLS − spliced − 3|4|3|5
20120617_EXQ0_MiBa_SA_HCT116_1_mHLA_2hr: 13632
SA = 0.13
Score = −5.25
EERSPSWIS − MaxQuant
20120617_EXQ0_MiBa_SA_HCT116_1_mHLA_2hr: 13632
SA = 0.8
Score = 1.61
EERSPSWIS − MSFragger
20120617_EXQ0_MiBa_SA_HCT116_1_mHLA_2hr: 13632
SA = 0.8
Score = 1.61
LGEGTVAYR − Mascot − rejected
NA − spliced − |||
20120617_EXQ0_MiBa_SA_HCT116_1_mHLA_2hr: 10226
SA = 0.28
Score = −3.86
AVADTVYRA − MaxQuant
20120617_EXQ0_MiBa_SA_HCT116_1_mHLA_2hr: 10226
SA = 0.87
Score = 1.22
AVADTVYRA − MSFragger
20120617_EXQ0_MiBa_SA_HCT116_1_mHLA_2hr: 10226
SA = 0.87
Score = 1.22
Relative intensity
m/z
Prosit prediction
* Exclusive in prediction
# Exclusive in experimental

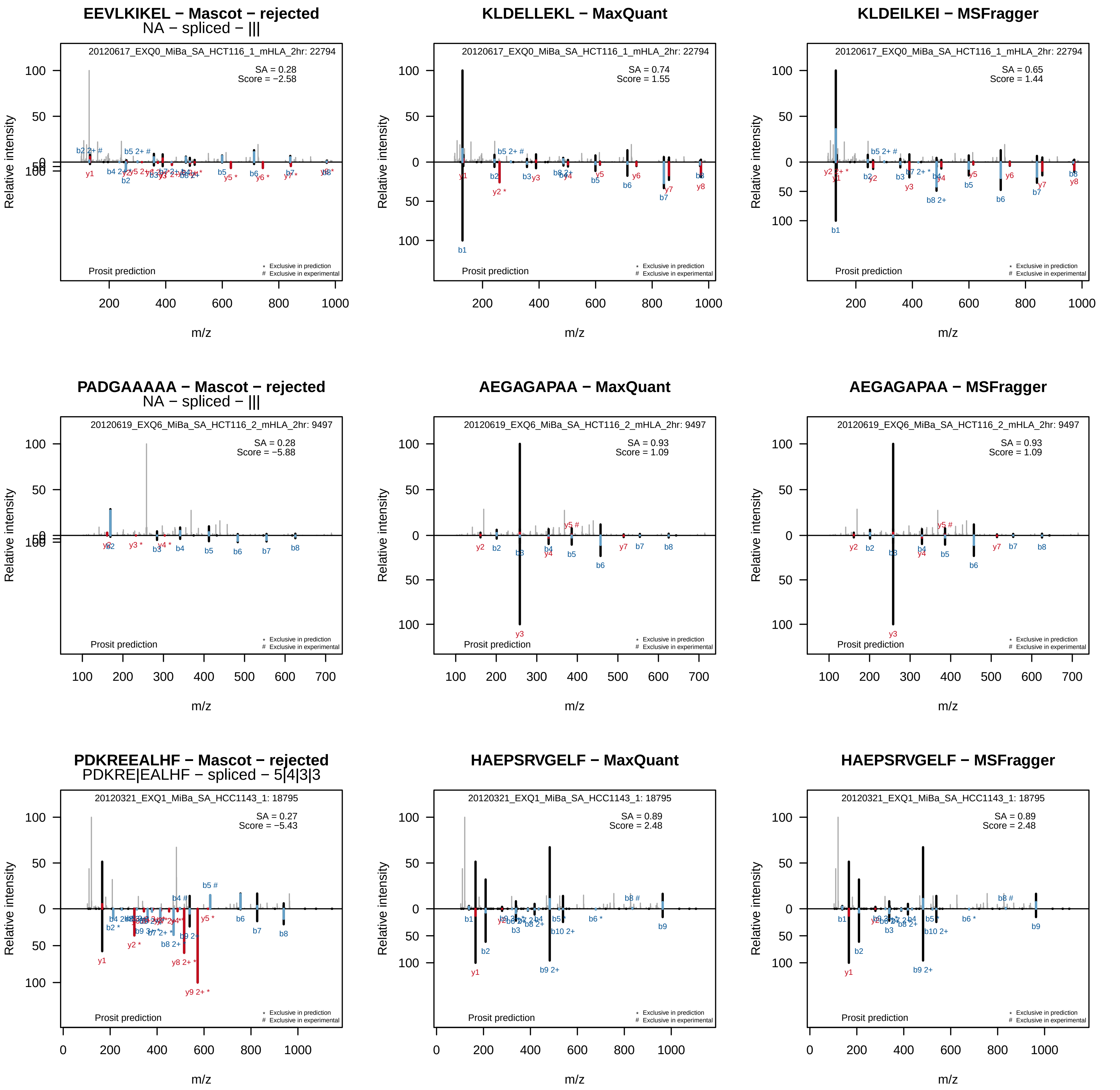

EEVLKIKEL − Mascot − rejected
NA − spliced − |||
20120617_EXQ0_MiBa_SA_HCT116_1_mHLA_2hr: 22794
SA = 0.28
Score = −2.58
Prosit prediction
Exclusive in prediction
Exclusive in experimental
Relative intensity
m/z
KLDELLEKL − MaxQuant
20120617_EXQ0_MiBa_SA_HCT116_1_mHLA_2hr: 22794
SA = 0.74
Score = 1.55
Prosit prediction
KLDEILKEI − MSFragger
20120617_EXQ0_MiBa_SA_HCT116_1_mHLA_2hr: 22794
SA = 0.65
Score = 1.44
Prosit prediction
PADGAAAAA − Mascot − rejected
NA − spliced − |||
20120619_EXQ6_MiBa_SA_HCT116_2_mHLA_2hr: 9497
SA = 0.28
Score = −5.88
Prosit prediction
AEGAGAPAA − MaxQuant
20120619_EXQ6_MiBa_SA_HCT116_2_mHLA_2hr: 9497
SA = 0.93
Score = 1.09
Prosit prediction
AEGAGAPAA − MSFragger
20120619_EXQ6_MiBa_SA_HCT116_2_mHLA_2hr: 9497
SA = 0.93
Score = 1.09
Prosit prediction
PDKREEALHF − Mascot − rejected
PDKRE|EALHF − spliced − 5|4|3|3
20120321_EXQ1_MiBa_SA_HCC1143_1: 18795
SA = 0.27
Score = −5.43
Prosit prediction
HAEPSRVGELF − MaxQuant
20120321_EXQ1_MiBa_SA_HCC1143_1: 18795
SA = 0.89
Score = 2.48
Prosit prediction
HAEPSRVGELF − MSFragger
20120321_EXQ1_MiBa_SA_HCC1143_1: 18795
SA = 0.89
Score = 2.48
Prosit prediction

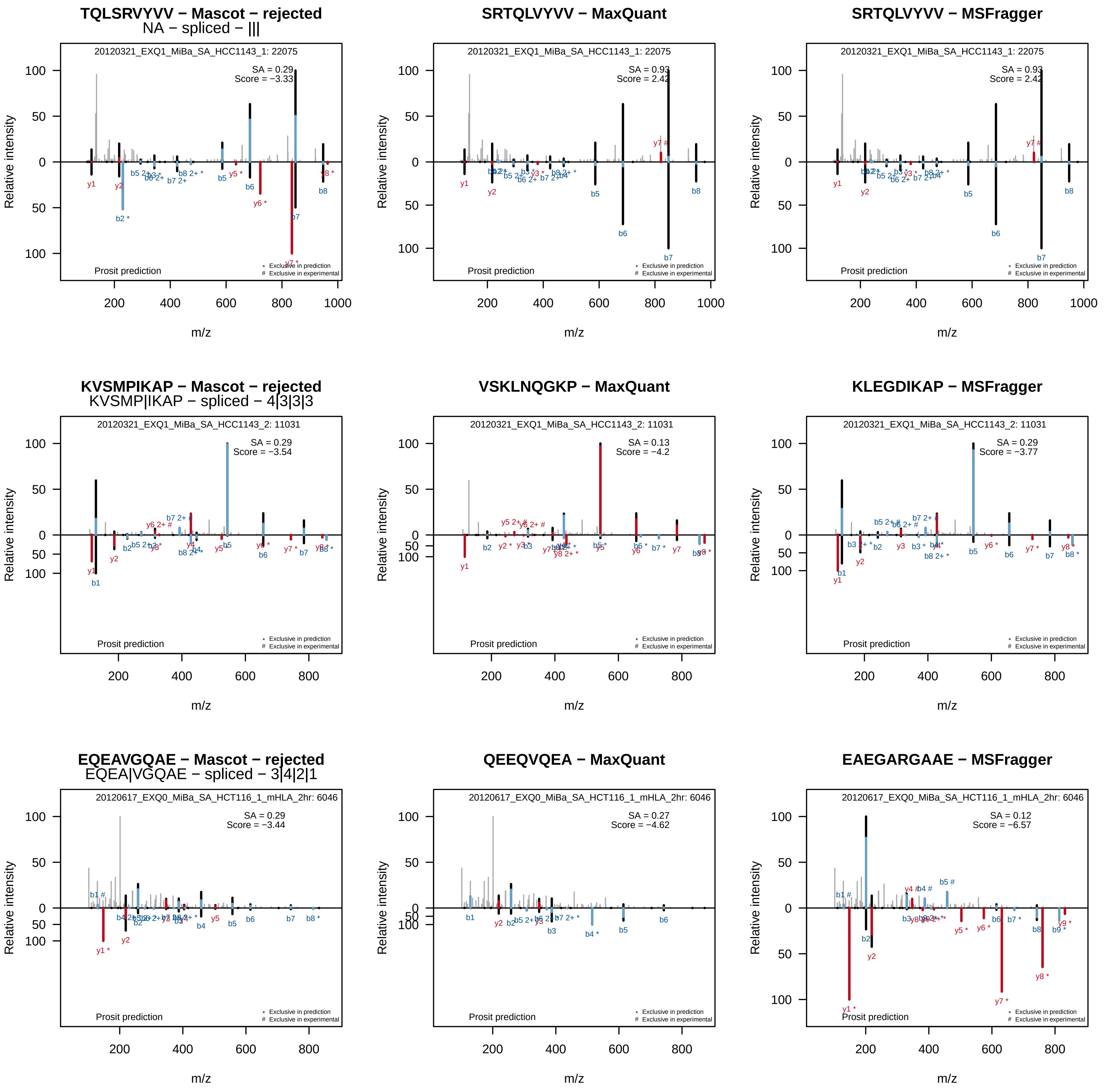

TQLSRVYVV − Mascot − rejected
NA − spliced − |||
20120321_EXQ1_MiBa_SA_HCC1143_1: 22075
SA = 0.29
Score = −3.33
SRTQLVYVV − MaxQuant
20120321_EXQ1_MiBa_SA_HCC1143_1: 22075
SA = 0.93
Score = 2.42
SRTQLVYVV − MSFragger
20120321_EXQ1_MiBa_SA_HCC1143_1: 22075
SA = 0.93
Score = 2.42
KVSMPIKAP − Mascot − rejected
KVSMP|IKAP − spliced − 4|3|3|3
20120321_EXQ1_MiBa_SA_HCC1143_2: 11031
SA = 0.29
Score = −3.54
VSKLNQGKP − MaxQuant
20120321_EXQ1_MiBa_SA_HCC1143_2: 11031
SA = 0.13
Score = −4.2
KLEGDIKAP − MSFragger
20120321_EXQ1_MiBa_SA_HCC1143_2: 11031
SA = 0.29
Score = −3.77
EQEAVGQAE − Mascot − rejected
EQEA|VGQAE − spliced − 3|4|2|1
20120617_EXQ0_MiBa_SA_HCT116_1_mHLA_2hr: 6046
SA = 0.29
Score = −3.44
QEEQVQEA − MaxQuant
20120617_EXQ0_MiBa_SA_HCT116_1_mHLA_2hr: 6046
SA = 0.27
Score = −4.62
EAEGARGAAE − MSFragger
20120617_EXQ0_MiBa_SA_HCT116_1_mHLA_2hr: 6046
SA = 0.12
Score = −6.57
Relative intensity
m/z
Prosit prediction
⋆ Exclusive in prediction
Exclusive in experimental

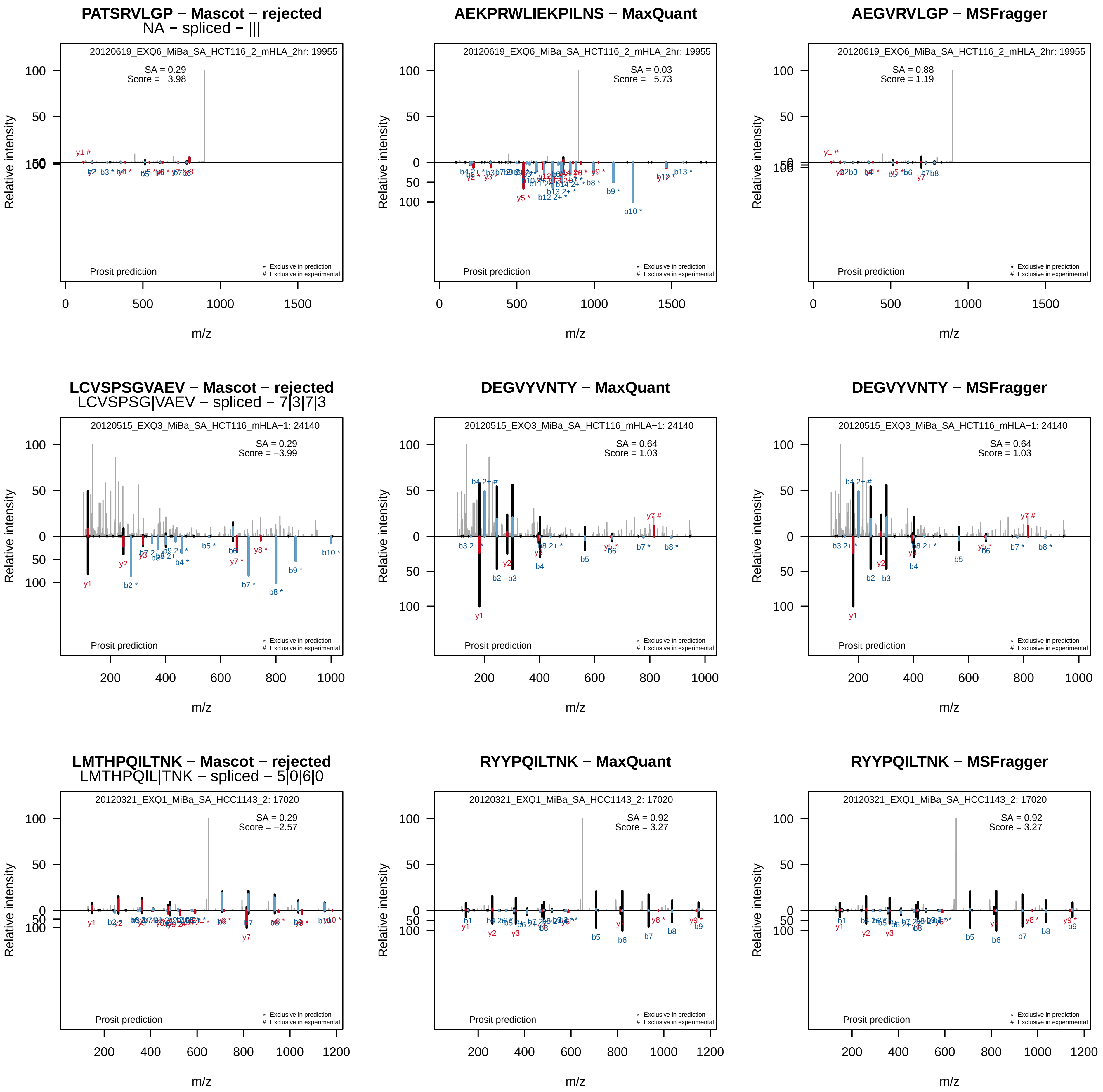
PATSRVLGP − Mascot − rejected
NA − spliced − |||
20120619_EXQ6_MiBa_SA_HCT116_2_mHLA_2hr: 19955
SA = 0.29
Score = −3.98
AEKPRWLIEKPILNS − MaxQuant
20120619_EXQ6_MiBa_SA_HCT116_2_mHLA_2hr: 19955
SA = 0.03
Score = −5.73
AEGVRVLGP − MSFragger
20120619_EXQ6_MiBa_SA_HCT116_2_mHLA_2hr: 19955
SA = 0.88
Score = 1.19
LCVSPSGVAEV − Mascot − rejected
LCVSPSG|VAEV − spliced − 7|3|7|3
20120515_EXQ3_MiBa_SA_HCT116_mHLA−1: 24140
SA = 0.29
Score = −3.99
DEGVYVVNTY − MaxQuant
20120515_EXQ3_MiBa_SA_HCT116_mHLA−1: 24140
SA = 0.64
Score = 1.03
DEGVYVVNTY − MSFragger
20120515_EXQ3_MiBa_SA_HCT116_mHLA−1: 24140
SA = 0.64
Score = 1.03
LMTHPQILTNK − Mascot − rejected
LMTHPQIL|TNK − spliced − 5|0|6|0
20120321_EXQ1_MiBa_SA_HCC1143_2: 17020
SA = 0.29
Score = −2.57
RYYPQILTNK − MaxQuant
20120321_EXQ1_MiBa_SA_HCC1143_2: 17020
SA = 0.92
Score = 3.27
RYYPQILTNK − MSFragger
20120321_EXQ1_MiBa_SA_HCC1143_2: 17020
SA = 0.92
Score = 3.27
Relative intensity
m/z
Prosit prediction
* Exclusive in prediction
# Exclusive in experimental

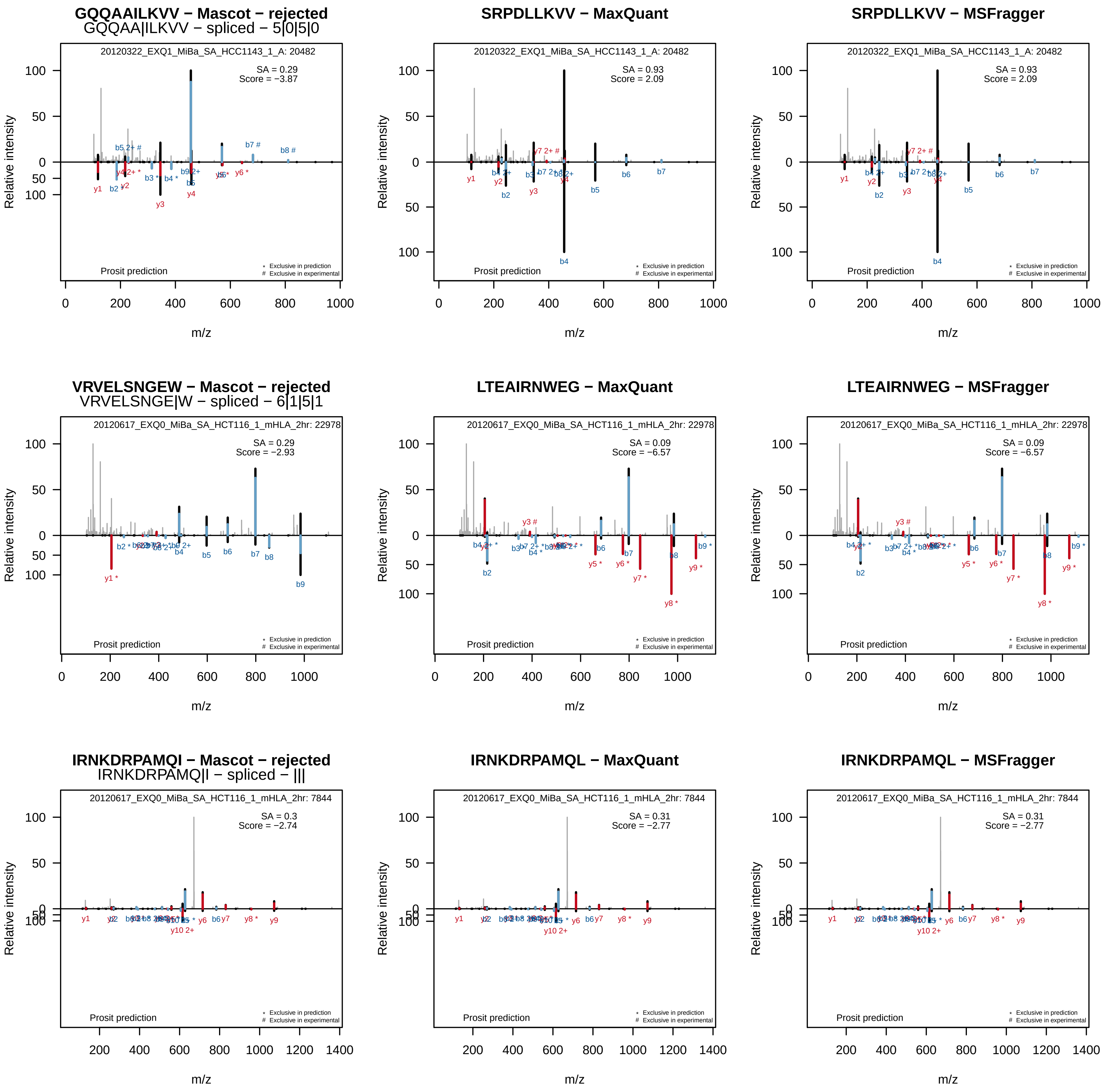
GQQAAILKVV − Mascot − rejected
GQQAA|ILKVV − spliced − 5|0|5|0
20120322_EXQ1_MiBa_SA_HCC1143_1_A: 20482
SA = 0.29
Score = −3.87
SRPDLLKVV − MaxQuant
20120322_EXQ1_MiBa_SA_HCC1143_1_A: 20482
SA = 0.93
Score = 2.09
SRPDLLKVV − MSFragger
20120322_EXQ1_MiBa_SA_HCC1143_1_A: 20482
SA = 0.93
Score = 2.09
VRVELSNGEW − Mascot − rejected
VRVELSNGE|W − spliced − 6|1|5|1
20120617_EXQ0_MiBa_SA_HCT116_1_mHLA_2hr: 22978
SA = 0.29
Score = −2.93
LTEAIRNWEG − MaxQuant
20120617_EXQ0_MiBa_SA_HCT116_1_mHLA_2hr: 22978
SA = 0.09
Score = −6.57
LTEAIRNWEG − MSFragger
20120617_EXQ0_MiBa_SA_HCT116_1_mHLA_2hr: 22978
SA = 0.09
Score = −6.57
IRNKDRPAMQI − Mascot − rejected
IRNKDRPAMQ|I − spliced − |||
20120617_EXQ0_MiBa_SA_HCT116_1_mHLA_2hr: 7844
SA = 0.3
Score = −2.74
IRNKDRPAMQL − MaxQuant
20120617_EXQ0_MiBa_SA_HCT116_1_mHLA_2hr: 7844
SA = 0.31
Score = −2.77
IRNKDRPAMQL − MSFragger
20120617_EXQ0_MiBa_SA_HCT116_1_mHLA_2hr: 7844
SA = 0.31
Score = −2.77
Relative intensity
m/z
Prosit prediction
* Exclusive in prediction
# Exclusive in experimental

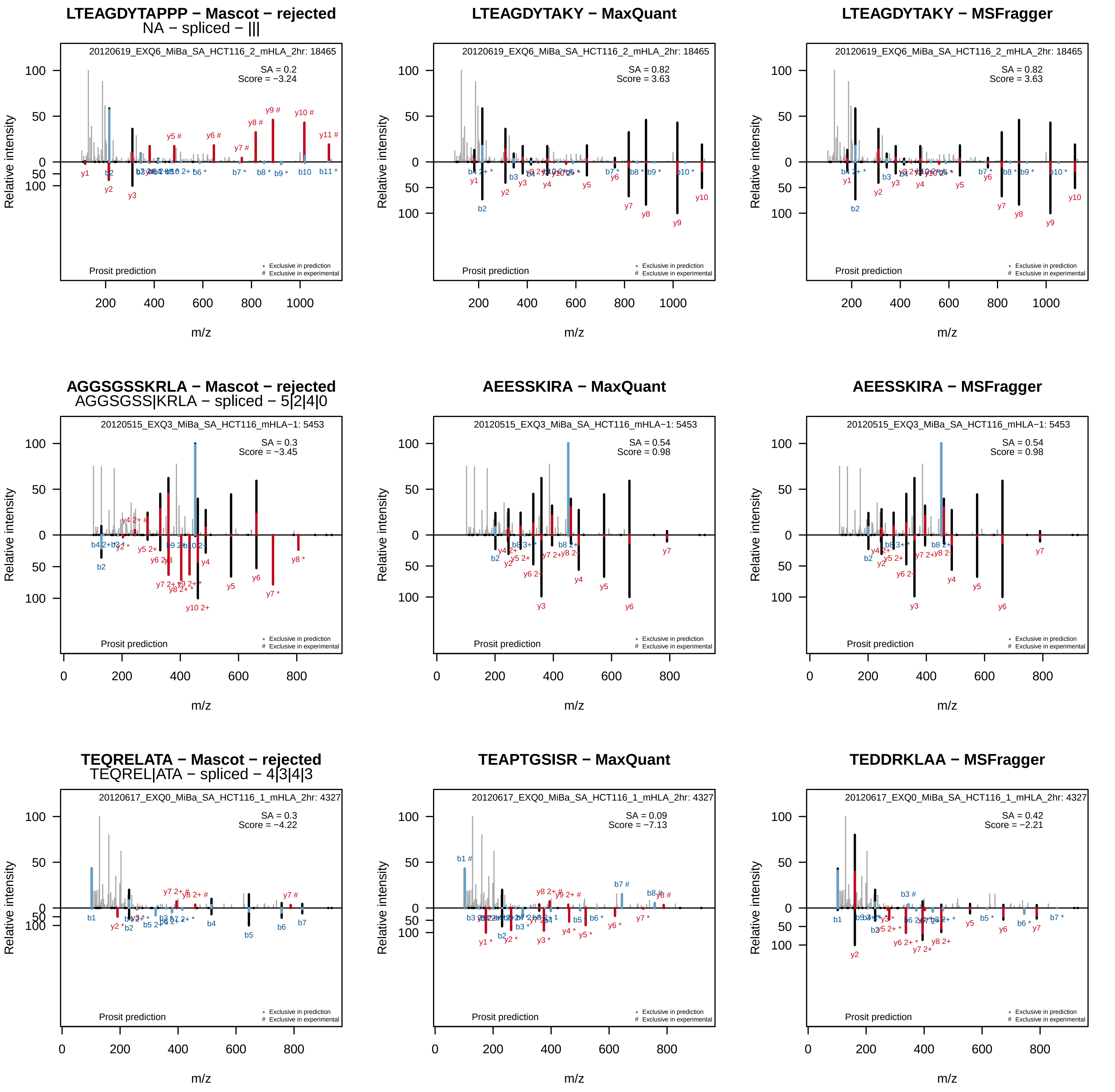
LTEAGDYTAPPP − Mascot − rejected
NA − spliced − |||
20120619_EXQ6_MiBa_SA_HCT116_2_mHLA_2hr: 18465
SA = 0.2
Score = −3.24
LTEAGDYTAKY − MaxQuant
20120619_EXQ6_MiBa_SA_HCT116_2_mHLA_2hr: 18465
SA = 0.82
Score = 3.63
LTEAGDYTAKY − MSFragger
20120619_EXQ6_MiBa_SA_HCT116_2_mHLA_2hr: 18465
SA = 0.82
Score = 3.63
AGGSGSSKRLA − Mascot − rejected
AGGSGSS|KRLA − spliced − 5|2|4|0
20120515_EXQ3_MiBa_SA_HCT116_mHLA−1: 5453
SA = 0.3
Score = −3.45
AEESSKIRA − MaxQuant
20120515_EXQ3_MiBa_SA_HCT116_mHLA−1: 5453
SA = 0.54
Score = 0.98
AEESSKIRA − MSFragger
20120515_EXQ3_MiBa_SA_HCT116_mHLA−1: 5453
SA = 0.54
Score = 0.98
TEQRELATA − Mascot − rejected
TEQREL|ATA − spliced − 4|3|4|3
20120617_EXQ0_MiBa_SA_HCT116_1_mHLA_2hr: 4327
SA = 0.3
Score = −4.22
TEAPTGSISR − MaxQuant
20120617_EXQ0_MiBa_SA_HCT116_1_mHLA_2hr: 4327
SA = 0.09
Score = −7.13
TEDDRKLAA − MSFragger
20120617_EXQ0_MiBa_SA_HCT116_1_mHLA_2hr: 4327
SA = 0.42
Score = −2.21
Relative intensity
m/z
Prosit prediction
⋆ Exclusive in prediction
Exclusive in experimental

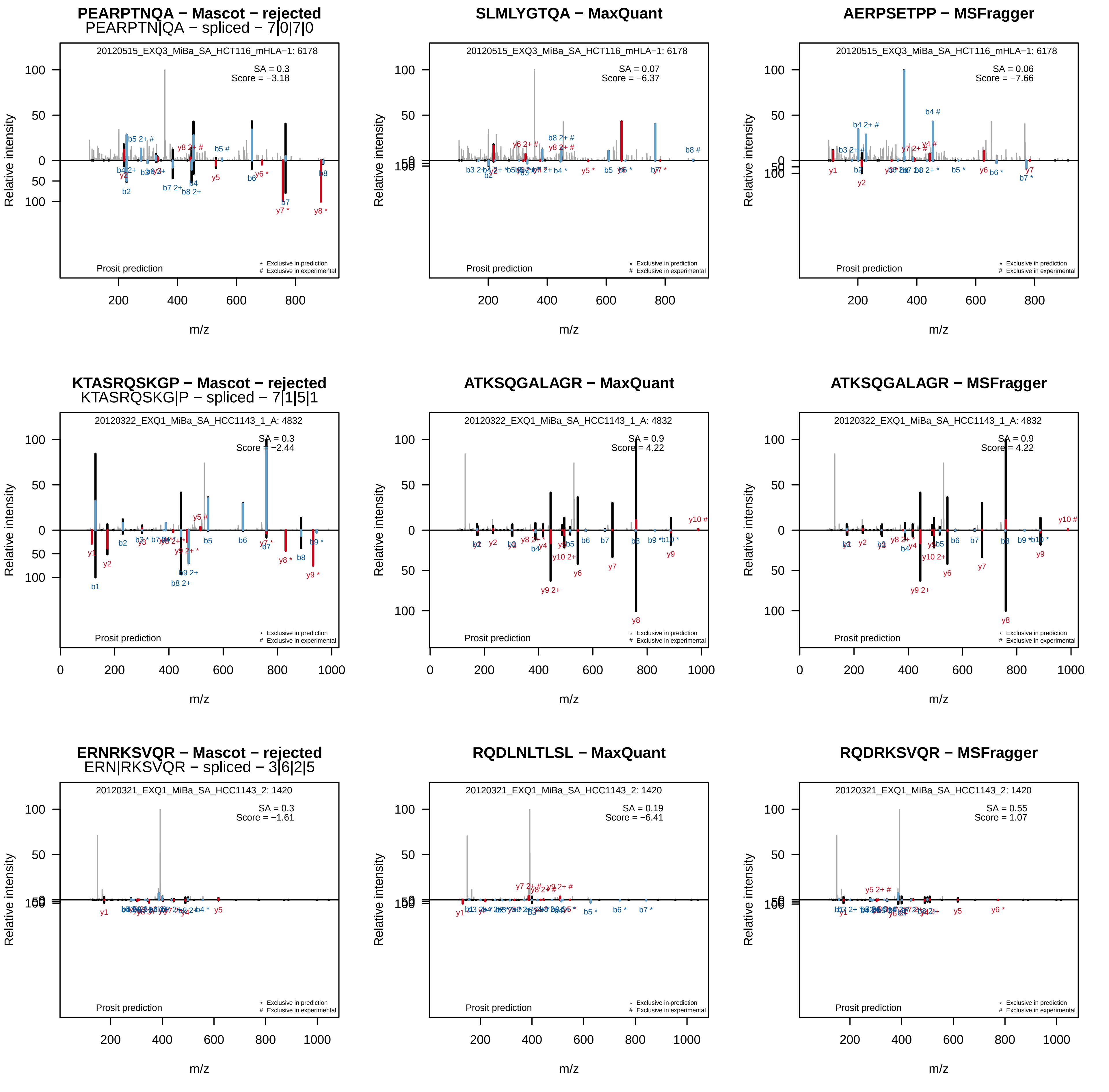
PEARPTNQA − Mascot − rejected
PEARPTN|QA − spliced − 7|0|7|0
20120515_EXQ3_MiBa_SA_HCT116_mHLA−1: 6178
SA = 0.3
Score = −3.18
SLMLYGTQA − MaxQuant
20120515_EXQ3_MiBa_SA_HCT116_mHLA−1: 6178
SA = 0.07
Score = −6.37
AERPSETPP − MSFragger
20120515_EXQ3_MiBa_SA_HCT116_mHLA−1: 6178
SA = 0.06
Score = −7.66
KTASRQSKGP − Mascot − rejected
KTASRQSKG|P − spliced − 7|1|5|1
20120322_EXQ1_MiBa_SA_HCC1143_1_A: 4832
SA = 0.3
Score = −2.44
ATKSQGALAGR − MaxQuant
20120322_EXQ1_MiBa_SA_HCC1143_1_A: 4832
SA = 0.9
Score = 4.22
ATKSQGALAGR − MSFragger
20120322_EXQ1_MiBa_SA_HCC1143_1_A: 4832
SA = 0.9
Score = 4.22
ERNRKSVQR − Mascot − rejected
ERN|RKSVQR − spliced − 3|6|2|5
20120321_EXQ1_MiBa_SA_HCC1143_2: 1420
SA = 0.3
Score = −1.61
RQDLNLTLSL − MaxQuant
20120321_EXQ1_MiBa_SA_HCC1143_2: 1420
SA = 0.19
Score = −6.41
RQDRKSVQR − MSFragger
20120321_EXQ1_MiBa_SA_HCC1143_2: 1420
SA = 0.55
Score = 1.07
Relative intensity
m/z
Prosit prediction
* Exclusive in prediction
# Exclusive in experimental

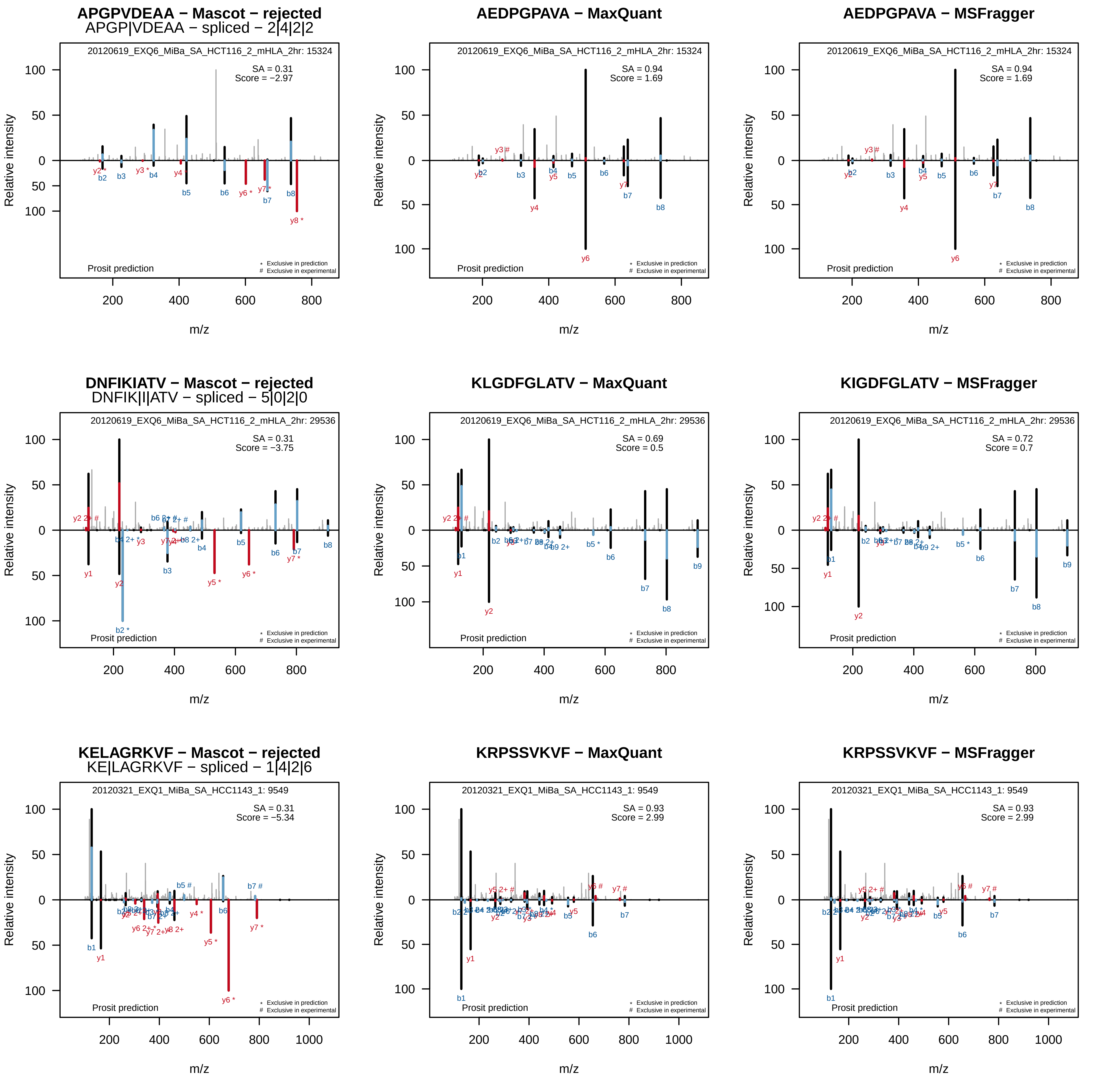

APGPVDEAA − Mascot − rejected
APGP|VDEAA − spliced − 2|4|2|2
20120619_EXQ6_MiBa_SA_HCT116_2_mHLA_2hr: 15324
SA = 0.31
Score = −2.97
AEDPGPAVA − MaxQuant
20120619_EXQ6_MiBa_SA_HCT116_2_mHLA_2hr: 15324
SA = 0.94
Score = 1.69
AEDPGPAVA − MSFragger
20120619_EXQ6_MiBa_SA_HCT116_2_mHLA_2hr: 15324
SA = 0.94
Score = 1.69
DNFIKIATV − Mascot − rejected
DNFIK|I|ATV − spliced − 5|0|2|0
20120619_EXQ6_MiBa_SA_HCT116_2_mHLA_2hr: 29536
SA = 0.31
Score = −3.75
KLGDFGLATV − MaxQuant
20120619_EXQ6_MiBa_SA_HCT116_2_mHLA_2hr: 29536
SA = 0.69
Score = 0.5
KIGDFGLATV − MSFragger
20120619_EXQ6_MiBa_SA_HCT116_2_mHLA_2hr: 29536
SA = 0.72
Score = 0.7
KELAGRKVF − Mascot − rejected
KE|LAGRKVF − spliced − 1|4|2|6
20120321_EXQ1_MiBa_SA_HCC1143_1: 9549
SA = 0.31
Score = −5.34
KRPSSVKVF − MaxQuant
20120321_EXQ1_MiBa_SA_HCC1143_1: 9549
SA = 0.93
Score = 2.99
KRPSSVKVF − MSFragger
20120321_EXQ1_MiBa_SA_HCC1143_1: 9549
SA = 0.93
Score = 2.99
Relative intensity
m/z
Prosit prediction
* Exclusive in prediction
# Exclusive in experimental

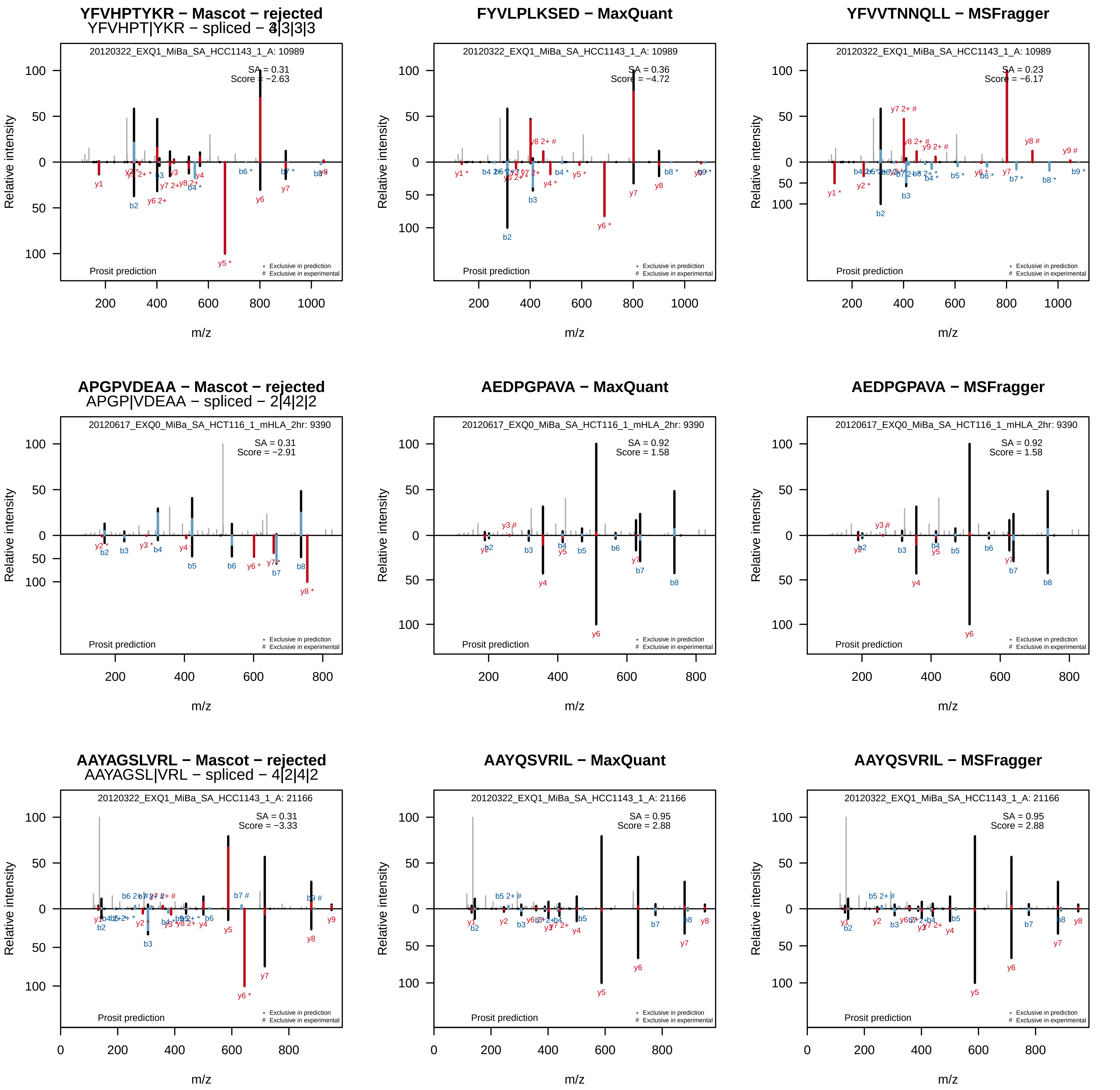
YFVHPTYKR − Mascot − rejected
YFVHPT|YKR − spliced − 3|3|3|3
20120322_EXQ1_MiBa_SA_HCC1143_1_A: 10989
SA = 0.31
Score = −2.63
FYVLPLKSED − MaxQuant
20120322_EXQ1_MiBa_SA_HCC1143_1_A: 10989
SA = 0.36
Score = −4.72
YFVVTNNQLL − MSFragger
20120322_EXQ1_MiBa_SA_HCC1143_1_A: 10989
SA = 0.23
Score = −6.17
APGPVDEAA − Mascot − rejected
APGP|VDEAA − spliced − 2|4|2|2
20120617_EXQ0_MiBa_SA_HCT116_1_mHLA_2hr: 9390
SA = 0.31
Score = −2.91
AEDPGPAVA − MaxQuant
20120617_EXQ0_MiBa_SA_HCT116_1_mHLA_2hr: 9390
SA = 0.92
Score = 1.58
AEDPGPAVA − MSFragger
20120617_EXQ0_MiBa_SA_HCT116_1_mHLA_2hr: 9390
SA = 0.92
Score = 1.58
AAYAGSLVRL − Mascot − rejected
AAYAGSL|VRL − spliced − 4|2|4|2
20120322_EXQ1_MiBa_SA_HCC1143_1_A: 21166
SA = 0.31
Score = −3.33
AAYQSVRIL − MaxQuant
20120322_EXQ1_MiBa_SA_HCC1143_1_A: 21166
SA = 0.95
Score = 2.88
AAYQSVRIL − MSFragger
20120322_EXQ1_MiBa_SA_HCC1143_1_A: 21166
SA = 0.95
Score = 2.88
Relative intensity
m/z
Prosit prediction
* Exclusive in prediction
# Exclusive in experimental

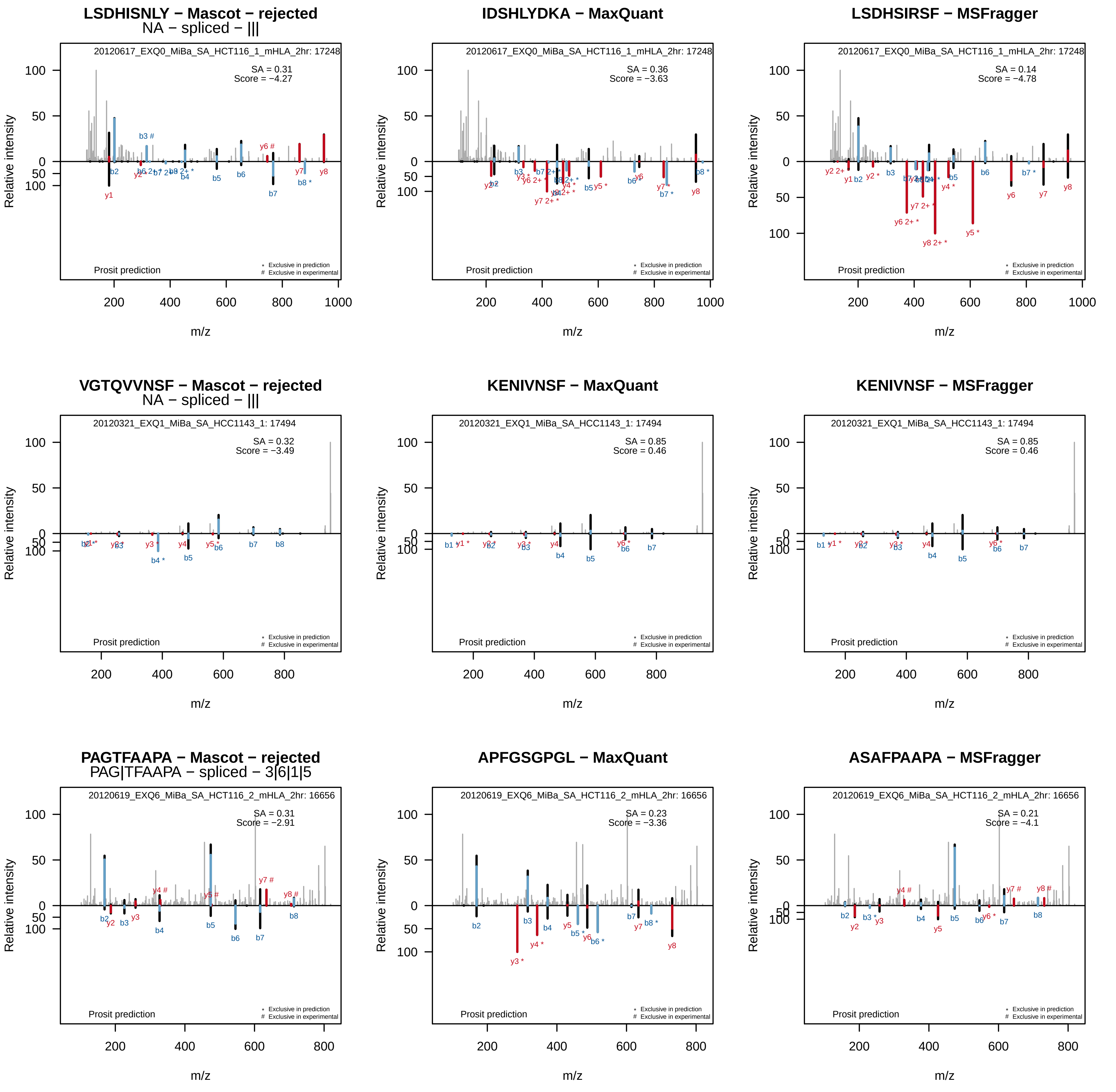

LSDHISNLY − Mascot − rejected
NA − spliced − |||
20120617_EXQ0_MiBa_SA_HCT116_1_mHLA_2hr: 17248
SA = 0.31
Score = −4.27
IDSHLYDKA − MaxQuant
20120617_EXQ0_MiBa_SA_HCT116_1_mHLA_2hr: 17248
SA = 0.36
Score = −3.63
LSDHSIRSF − MSFragger
20120617_EXQ0_MiBa_SA_HCT116_1_mHLA_2hr: 17248
SA = 0.14
Score = −4.78
VGTQVVNSF − Mascot − rejected
NA − spliced − |||
20120321_EXQ1_MiBa_SA_HCC1143_1: 17494
SA = 0.32
Score = −3.49
KENIVNSF − MaxQuant
20120321_EXQ1_MiBa_SA_HCC1143_1: 17494
SA = 0.85
Score = 0.46
KENIVNSF − MSFragger
20120321_EXQ1_MiBa_SA_HCC1143_1: 17494
SA = 0.85
Score = 0.46
PAGTFAAPA − Mascot − rejected
PAG|TFAAPA − spliced − 3|6|1|5
20120619_EXQ6_MiBa_SA_HCT116_2_mHLA_2hr: 16656
SA = 0.31
Score = −2.91
APFGSGPGL − MaxQuant
20120619_EXQ6_MiBa_SA_HCT116_2_mHLA_2hr: 16656
SA = 0.23
Score = −3.36
ASAFPAAPA − MSFragger
20120619_EXQ6_MiBa_SA_HCT116_2_mHLA_2hr: 16656
SA = 0.21
Score = −4.1
Relative intensity
m/z
Prosit prediction
* Exclusive in prediction
# Exclusive in experimental

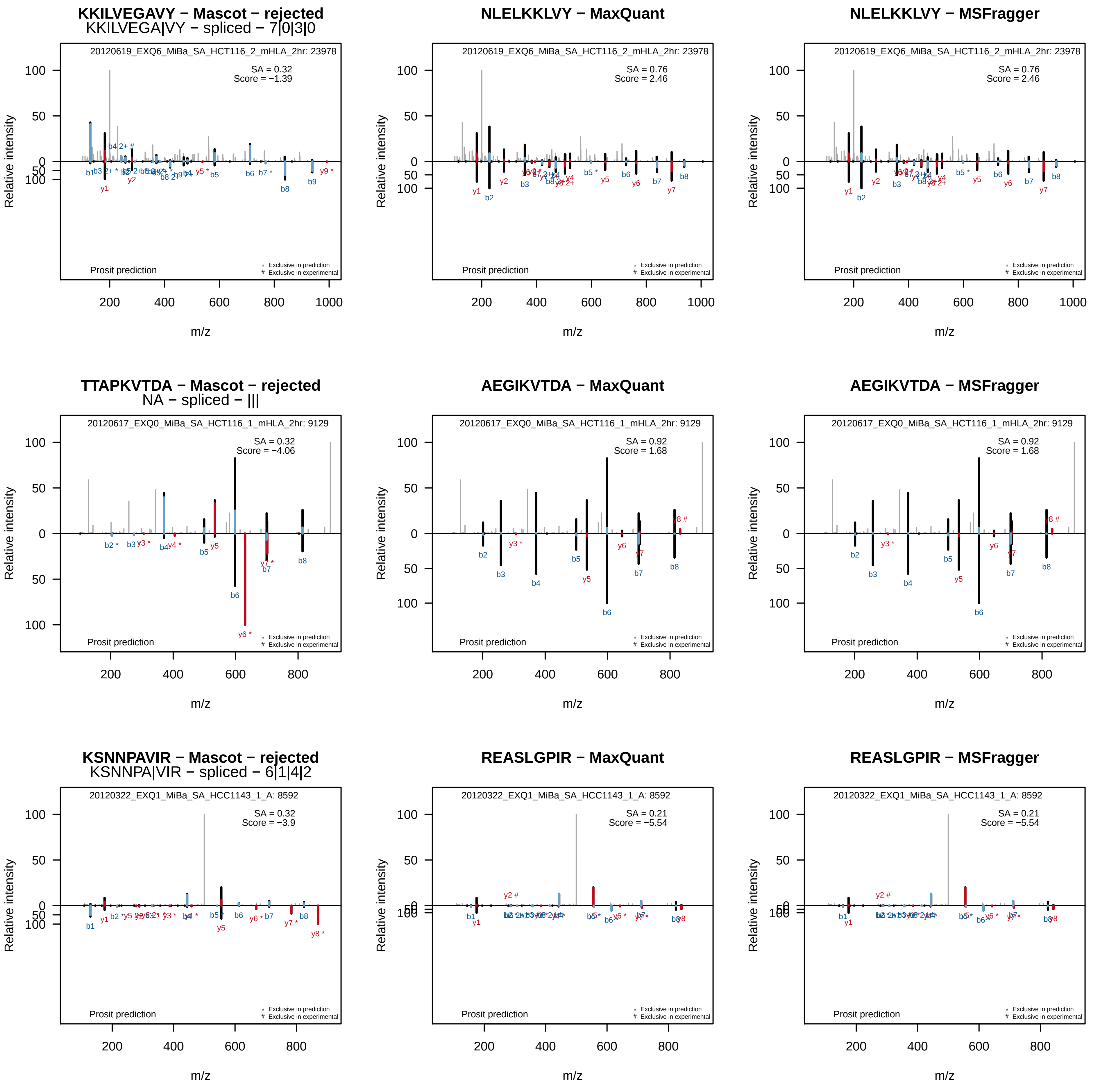

KKILVEGAVY − Mascot − rejected
KKILVEGA|VY − spliced − 7|0|3|0
20120619_EXQ6_MiBa_SA_HCT116_2_mHLA_2hr: 23978
SA = 0.32
Score = −1.39
NLELKKLVY − MaxQuant
20120619_EXQ6_MiBa_SA_HCT116_2_mHLA_2hr: 23978
SA = 0.76
Score = 2.46
NLELKKLVY − MSFragger
20120619_EXQ6_MiBa_SA_HCT116_2_mHLA_2hr: 23978
SA = 0.76
Score = 2.46
TTAPKVTDA − Mascot − rejected
NA − spliced − |||
20120617_EXQ0_MiBa_SA_HCT116_1_mHLA_2hr: 9129
SA = 0.32
Score = −4.06
AEGIKVTDA − MaxQuant
20120617_EXQ0_MiBa_SA_HCT116_1_mHLA_2hr: 9129
SA = 0.92
Score = 1.68
AEGIKVTDA − MSFragger
20120617_EXQ0_MiBa_SA_HCT116_1_mHLA_2hr: 9129
SA = 0.92
Score = 1.68
KSNNPAVIR − Mascot − rejected
KSNNPA|VIR − spliced − 6|1|4|2
20120322_EXQ1_MiBa_SA_HCC1143_1_A: 8592
SA = 0.32
Score = −3.9
REASLGPIR − MaxQuant
20120322_EXQ1_MiBa_SA_HCC1143_1_A: 8592
SA = 0.21
Score = −5.54
REASLGPIR − MSFragger
20120322_EXQ1_MiBa_SA_HCC1143_1_A: 8592
SA = 0.21
Score = −5.54
Relative intensity
m/z
Prosit prediction
⋆ Exclusive in prediction
Exclusive in experimental

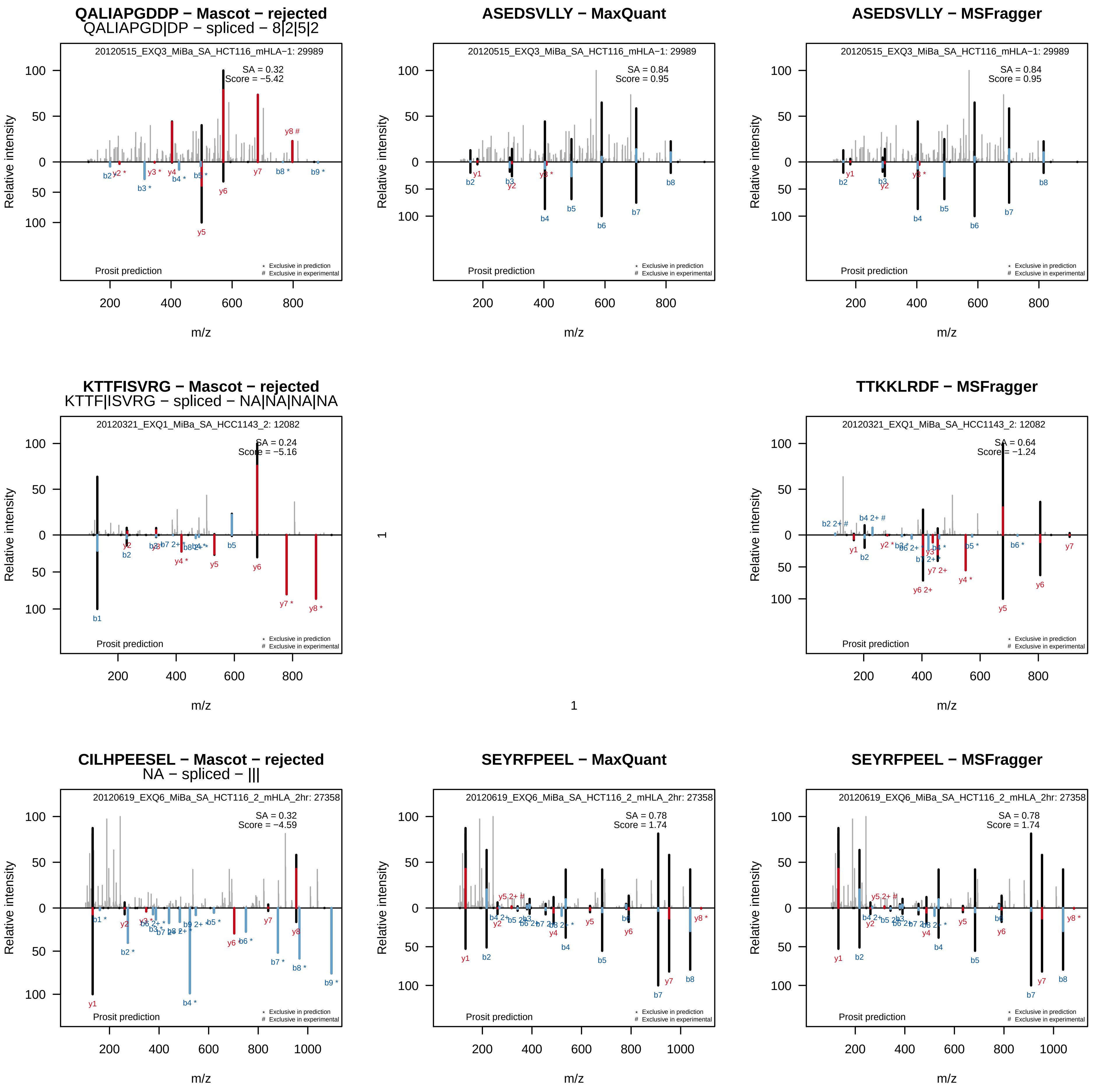

QALIAPGDDP − Mascot − rejected
QALIAPGD|DP − spliced − 8|2|5|2
20120515_EXQ3_MiBa_SA_HCT116_mHLA−1: 29989
SA = 0.32
Score = −5.42
ASEDSVLLY − MaxQuant
20120515_EXQ3_MiBa_SA_HCT116_mHLA−1: 29989
SA = 0.84
Score = 0.95
ASEDSVLLY − MSFragger
20120515_EXQ3_MiBa_SA_HCT116_mHLA−1: 29989
SA = 0.84
Score = 0.95
KTTFISVRG − Mascot − rejected
KTTF|ISVRG − spliced − NA|NA|NA|NA
20120321_EXQ1_MiBa_SA_HCC1143_2: 12082
SA = 0.24
Score = −5.16
TTKKLRDF − MSFragger
20120321_EXQ1_MiBa_SA_HCC1143_2: 12082
SA = 0.64
Score = −1.24
CILHPEESEL − Mascot − rejected
NA − spliced − |||
20120619_EXQ6_MiBa_SA_HCT116_2_mHLA_2hr: 27358
SA = 0.32
Score = −4.59
SEYRFPEEL − MaxQuant
20120619_EXQ6_MiBa_SA_HCT116_2_mHLA_2hr: 27358
SA = 0.78
Score = 1.74
SEYRFPEEL − MSFragger
20120619_EXQ6_MiBa_SA_HCT116_2_mHLA_2hr: 27358
SA = 0.78
Score = 1.74
Relative intensity
m/z
Prosit prediction
* Exclusive in prediction
# Exclusive in experimental

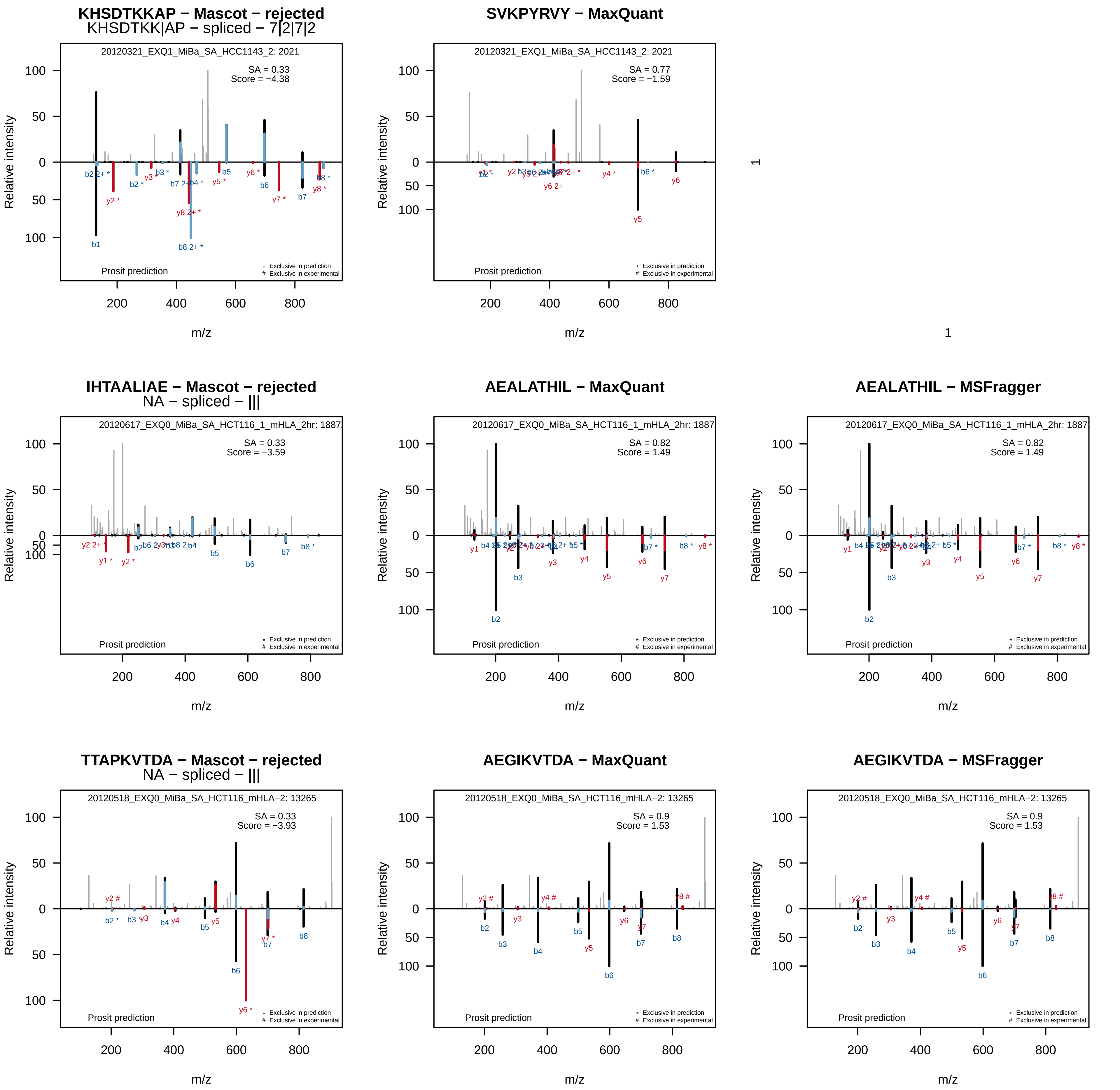
KHSDTKKAP − Mascot − rejected
KHSDTKK|AP − spliced − 7|2|7|2
20120321_EXQ1_MiBa_SA_HCC1143_2: 2021
SA = 0.33
Score = −4.38
SVKPYRVY − MaxQuant
20120321_EXQ1_MiBa_SA_HCC1143_2: 2021
SA = 0.77
Score = −1.59
IHTAALIAE − Mascot − rejected
NA − spliced − |||
20120617_EXQ0_MiBa_SA_HCT116_1_mHLA_2hr: 1887
SA = 0.33
Score = −3.59
AEALATHIL − MaxQuant
20120617_EXQ0_MiBa_SA_HCT116_1_mHLA_2hr: 1887
SA = 0.82
Score = 1.49
AEALATHIL − MSFragger
20120617_EXQ0_MiBa_SA_HCT116_1_mHLA_2hr: 1887
SA = 0.82
Score = 1.49
TTAPKVTDA − Mascot − rejected
NA − spliced − |||
20120518_EXQ0_MiBa_SA_HCT116_mHLA−2: 13265
SA = 0.33
Score = −3.93
AEGIKVTDA − MaxQuant
20120518_EXQ0_MiBa_SA_HCT116_mHLA−2: 13265
SA = 0.9
Score = 1.53
AEGIKVTDA − MSFragger
20120518_EXQ0_MiBa_SA_HCT116_mHLA−2: 13265
SA = 0.9
Score = 1.53
Relative intensity
m/z
Prosit prediction
* Exclusive in prediction
# Exclusive in experimental

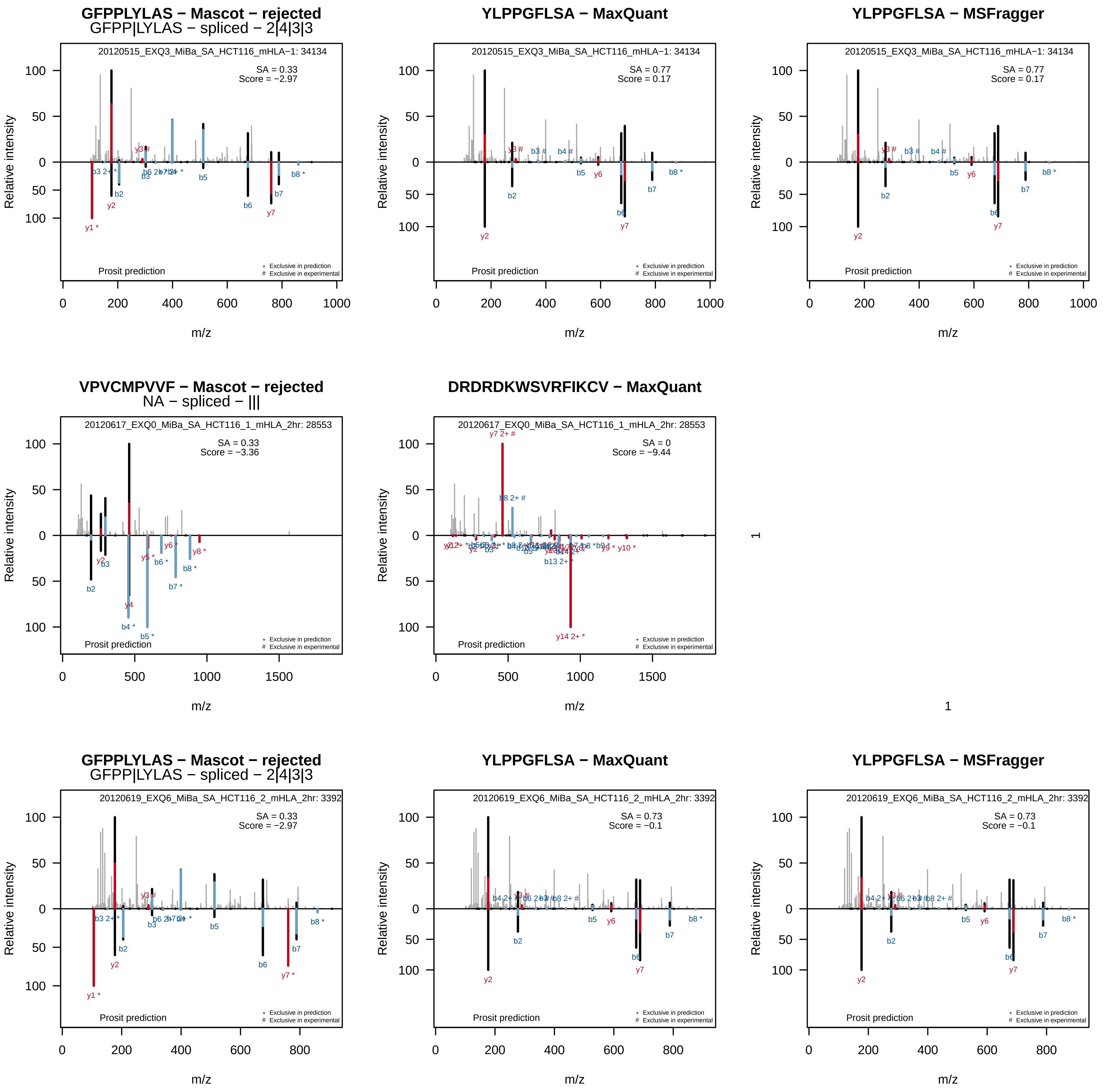
GFPPLYLAS − Mascot − rejected
GFPP|LYLAS − spliced − 2|4|3|3
20120515_EXQ3_MiBa_SA_HCT116_mHLA−1: 34134
SA = 0.33
Score = −2.97
YLPPGFLSA − MaxQuant
20120515_EXQ3_MiBa_SA_HCT116_mHLA−1: 34134
SA = 0.77
Score = 0.17
YLPPGFLSA − MSFragger
20120515_EXQ3_MiBa_SA_HCT116_mHLA−1: 34134
SA = 0.77
Score = 0.17
VPVCMPVVF − Mascot − rejected
NA − spliced − |||
20120617_EXQ0_MiBa_SA_HCT116_1_mHLA_2hr: 28553
SA = 0.33
Score = −3.36
DRDRDKWSVRFIKCV − MaxQuant
20120617_EXQ0_MiBa_SA_HCT116_1_mHLA_2hr: 28553
SA = 0
Score = −9.44
GFPPLYLAS − Mascot − rejected
GFPP|LYLAS − spliced − 2|4|3|3
20120619_EXQ6_MiBa_SA_HCT116_2_mHLA_2hr: 3392
SA = 0.33
Score = −2.97
YLPPGFLSA − MaxQuant
20120619_EXQ6_MiBa_SA_HCT116_2_mHLA_2hr: 3392
SA = 0.73
Score = −0.1
YLPPGFLSA − MSFragger
20120619_EXQ6_MiBa_SA_HCT116_2_mHLA_2hr: 3392
SA = 0.73
Score = −0.1
Relative intensity
m/z
Prosit prediction
* Exclusive in prediction
# Exclusive in experimental

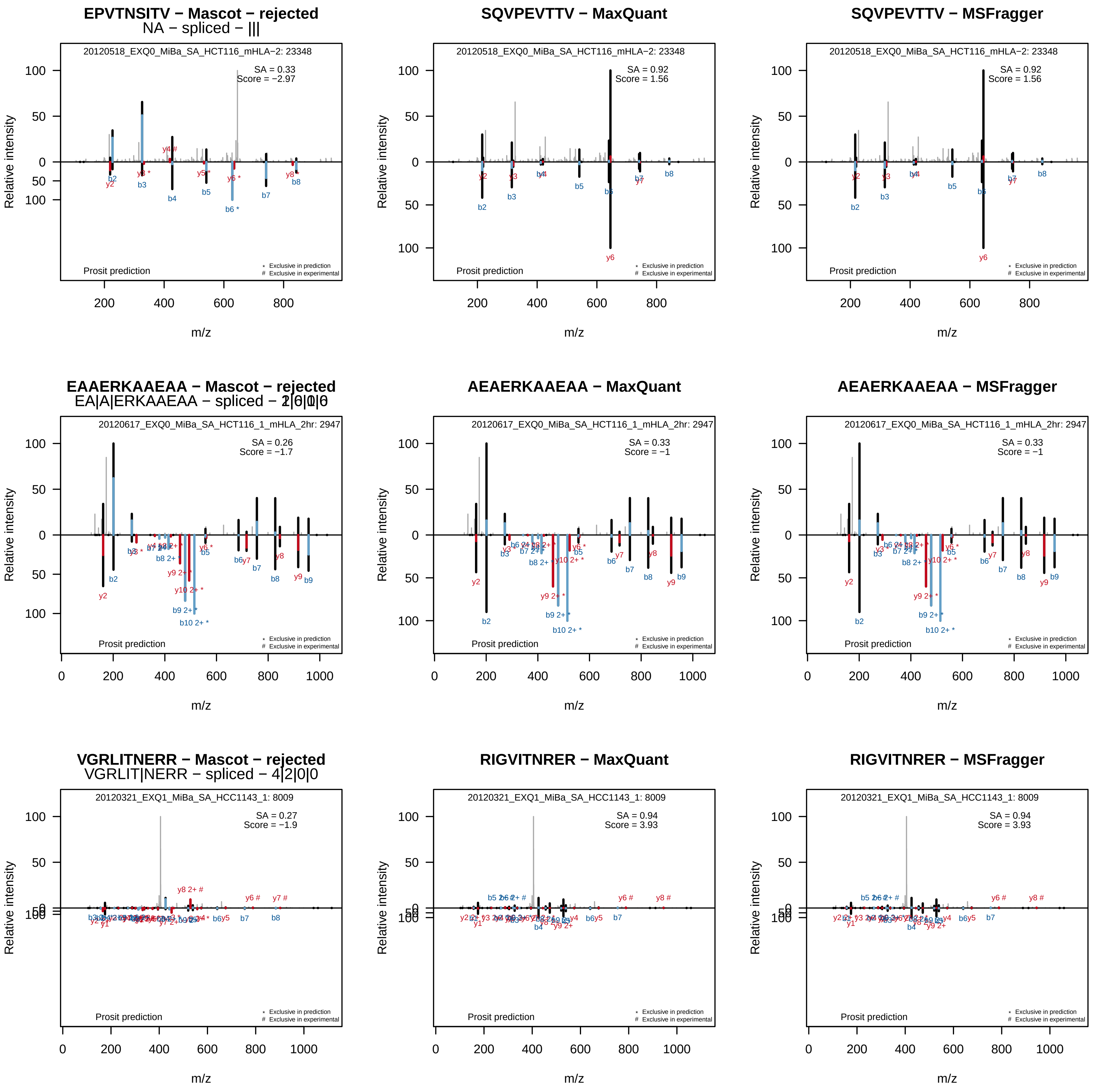
EPVTNSITV − Mascot − rejected
NA − spliced − |||
20120518_EXQ0_MiBa_SA_HCT116_mHLA−2: 23348
SA = 0.33
Score = −2.97
SQVPEVTTV − MaxQuant
20120518_EXQ0_MiBa_SA_HCT116_mHLA−2: 23348
SA = 0.92
Score = 1.56
SQVPEVTTV − MSFragger
20120518_EXQ0_MiBa_SA_HCT116_mHLA−2: 23348
SA = 0.92
Score = 1.56
EAAERKAAEAA − Mascot − rejected
20120617_EXQ0_MiBa_SA_HCT116_1_mHLA_2hr: 2947
SA = 0.26
Score = −1.7
AEAERKAAEAA − MaxQuant
20120617_EXQ0_MiBa_SA_HCT116_1_mHLA_2hr: 2947
SA = 0.33
Score = −1
AEAERKAAEAA − MSFragger
20120617_EXQ0_MiBa_SA_HCT116_1_mHLA_2hr: 2947
SA = 0.33
Score = −1
VGRLITNERR − Mascot − rejected
VGRLIT|NERR − spliced − 4|2|0|0
20120321_EXQ1_MiBa_SA_HCC1143_1: 8009
SA = 0.27
Score = −1.9
RIGVITNRER − MaxQuant
20120321_EXQ1_MiBa_SA_HCC1143_1: 8009
SA = 0.94
Score = 3.93
RIGVITNRER − MSFragger
20120321_EXQ1_MiBa_SA_HCC1143_1: 8009
SA = 0.94
Score = 3.93
Prosit prediction
* Exclusive in prediction
# Exclusive in experimental
Relative intensity
m/z

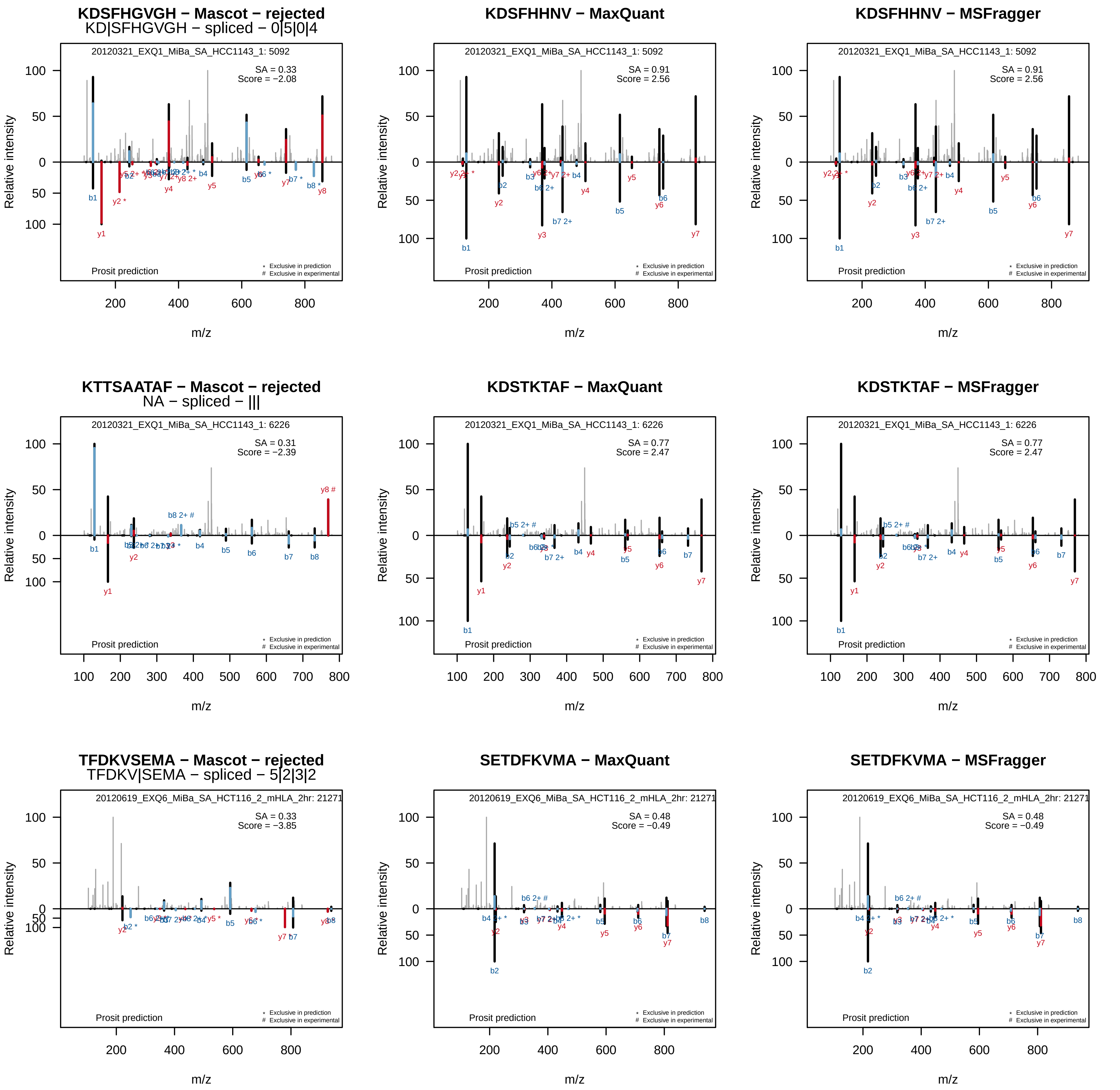

KDSFHGVGH − Mascot − rejected
KD|SFHGVGH − spliced − 0|5|0|4
20120321_EXQ1_MiBa_SA_HCC1143_1: 5092
SA = 0.33
Score = −2.08
KDSFHHNV − MaxQuant
20120321_EXQ1_MiBa_SA_HCC1143_1: 5092
SA = 0.91
Score = 2.56
KDSFHHNV − MSFragger
20120321_EXQ1_MiBa_SA_HCC1143_1: 5092
SA = 0.91
Score = 2.56
KTTSAATAF − Mascot − rejected
NA − spliced − |||
20120321_EXQ1_MiBa_SA_HCC1143_1: 6226
SA = 0.31
Score = −2.39
KDSTKTAF − MaxQuant
20120321_EXQ1_MiBa_SA_HCC1143_1: 6226
SA = 0.77
Score = 2.47
KDSTKTAF − MSFragger
20120321_EXQ1_MiBa_SA_HCC1143_1: 6226
SA = 0.77
Score = 2.47
TFDKVSEMA − Mascot − rejected
TFDKV|SEMA − spliced − 5|2|3|2
20120619_EXQ6_MiBa_SA_HCT116_2_mHLA_2hr: 21271
SA = 0.33
Score = −3.85
SETDFKVMA − MaxQuant
20120619_EXQ6_MiBa_SA_HCT116_2_mHLA_2hr: 21271
SA = 0.48
Score = −0.49
SETDFKVMA − MSFragger
20120619_EXQ6_MiBa_SA_HCT116_2_mHLA_2hr: 21271
SA = 0.48
Score = −0.49
Relative intensity
m/z
Prosit prediction
⋆ Exclusive in prediction
Exclusive in experimental

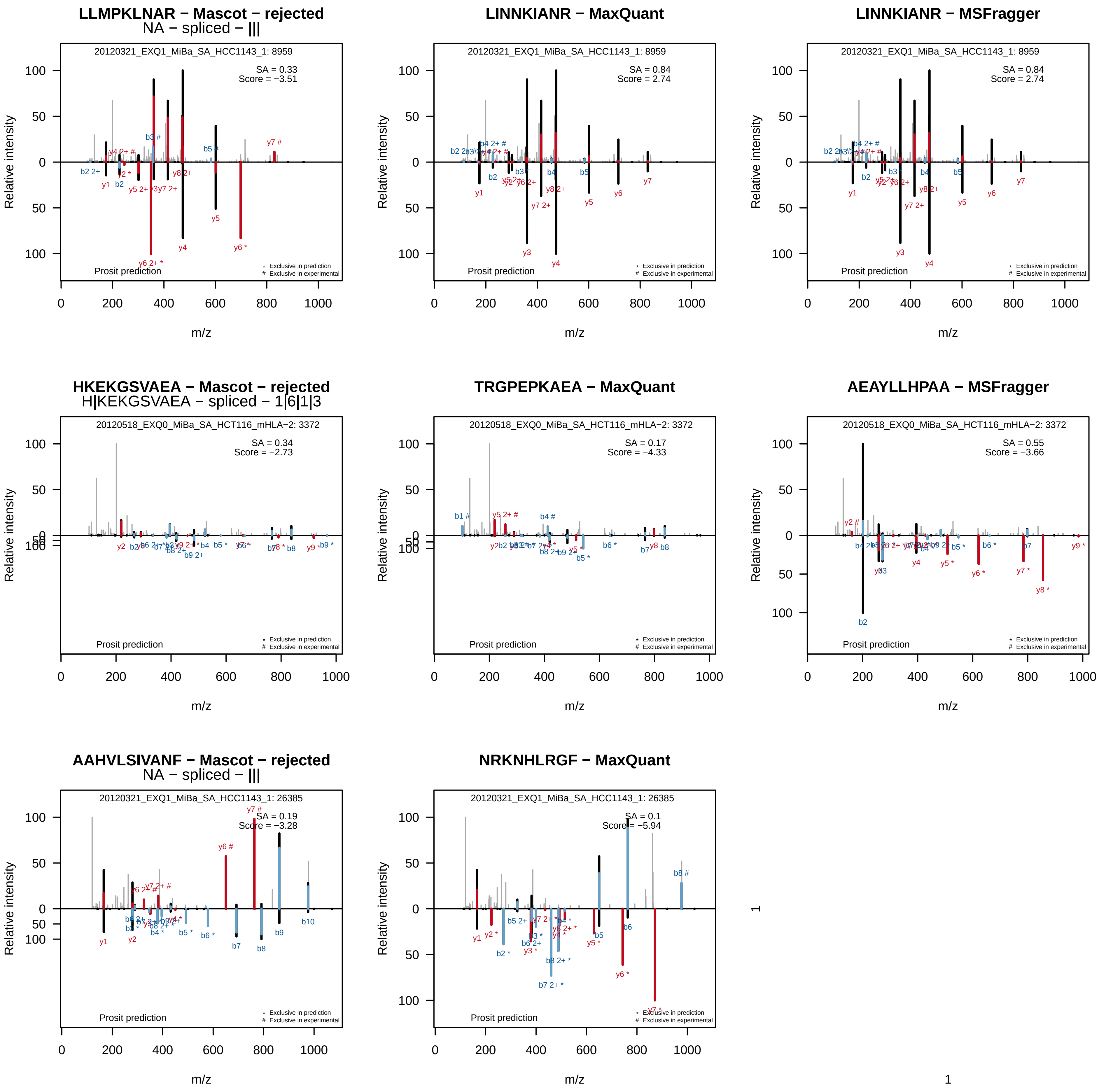
LLMPKLNAR − Mascot − rejected
NA − spliced − |||
20120321_EXQ1_MiBa_SA_HCC1143_1: 8959
SA = 0.33
Score = −3.51
LINNKIANR − MaxQuant
20120321_EXQ1_MiBa_SA_HCC1143_1: 8959
SA = 0.84
Score = 2.74
LINNKIANR − MSFragger
20120321_EXQ1_MiBa_SA_HCC1143_1: 8959
SA = 0.84
Score = 2.74
HKEKGSVAEA − Mascot − rejected
H|KEKGSVAEA − spliced − 1|6|1|3
20120518_EXQ0_MiBa_SA_HCT116_mHLA−2: 3372
SA = 0.34
Score = −2.73
TRGPEPKAEA − MaxQuant
20120518_EXQ0_MiBa_SA_HCT116_mHLA−2: 3372
SA = 0.17
Score = −4.33
AEAYLLHPAA − MSFragger
20120518_EXQ0_MiBa_SA_HCT116_mHLA−2: 3372
SA = 0.55
Score = −3.66
AAHVLSIVANF − Mascot − rejected
NA − spliced − |||
20120321_EXQ1_MiBa_SA_HCC1143_1: 26385
SA = 0.19
Score = −3.28
NRKNHLRGF − MaxQuant
20120321_EXQ1_MiBa_SA_HCC1143_1: 26385
SA = 0.1
Score = −5.94
Relative intensity
m/z
Prosit prediction
⋆ Exclusive in prediction
Exclusive in experimental
1
1

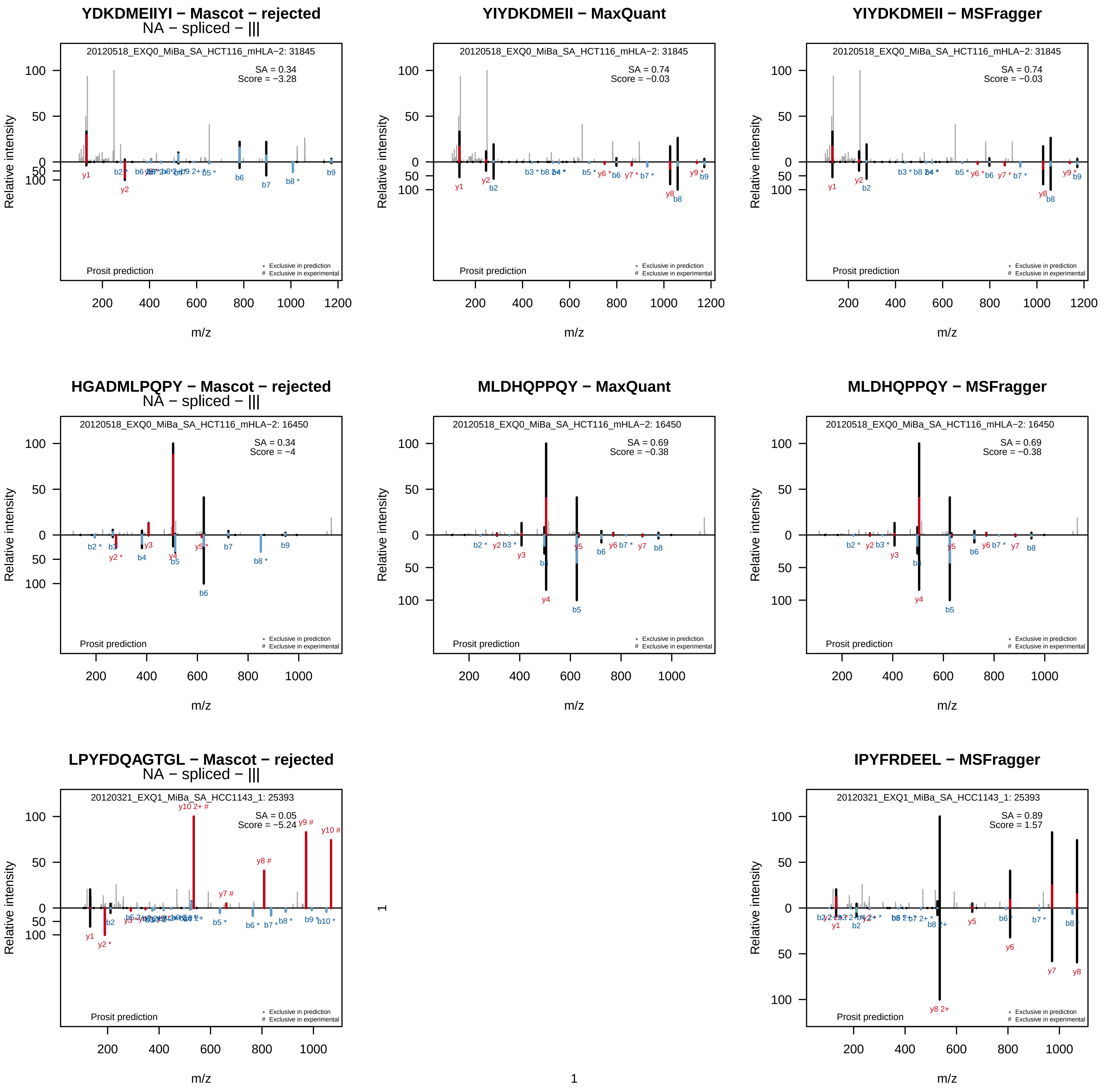
YDKDMEIIYI − Mascot − rejected
NA − spliced − |||
20120518_EXQ0_MiBa_SA_HCT116_mHLA−2: 31845
SA = 0.34
Score = −3.28
YIYDKDMEII − MaxQuant
20120518_EXQ0_MiBa_SA_HCT116_mHLA−2: 31845
SA = 0.74
Score = −0.03
YIYDKDMEII − MSFragger
20120518_EXQ0_MiBa_SA_HCT116_mHLA−2: 31845
SA = 0.74
Score = −0.03
HGADMLPQPY − Mascot − rejected
NA − spliced − |||
20120518_EXQ0_MiBa_SA_HCT116_mHLA−2: 16450
SA = 0.34
Score = −4
MLDHQPPQY − MaxQuant
20120518_EXQ0_MiBa_SA_HCT116_mHLA−2: 16450
SA = 0.69
Score = −0.38
MLDHQPPQY − MSFragger
20120518_EXQ0_MiBa_SA_HCT116_mHLA−2: 16450
SA = 0.69
Score = −0.38
LPYFDQAGTGL − Mascot − rejected
NA − spliced − |||
20120321_EXQ1_MiBa_SA_HCC1143_1: 25393
SA = 0.05
Score = −5.24
IPYFRDEEL − MSFragger
20120321_EXQ1_MiBa_SA_HCC1143_1: 25393
SA = 0.89
Score = 1.57
Relative intensity
m/z
Prosit prediction
⋆ Exclusive in prediction
Exclusive in experimental

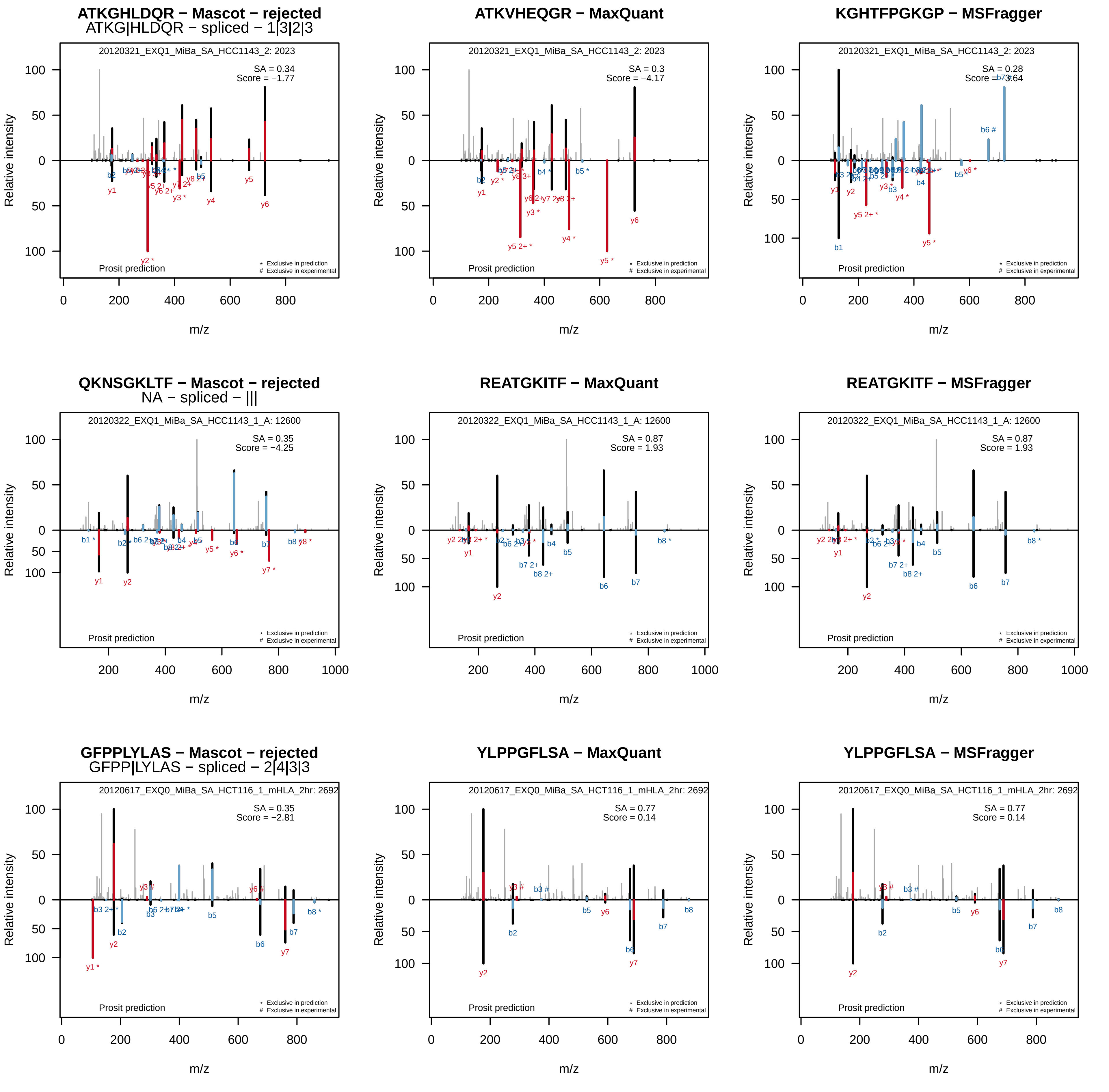
ATKGHLDQR − Mascot − rejected
ATKG|HLDQR − spliced − 1|3|2|3
20120321_EXQ1_MiBa_SA_HCC1143_2: 2023
SA = 0.34
Score = −1.77
ATKVHEQGR − MaxQuant
20120321_EXQ1_MiBa_SA_HCC1143_2: 2023
SA = 0.3
Score = −4.17
KGHTFPGKGP − MSFragger
20120321_EXQ1_MiBa_SA_HCC1143_2: 2023
SA = 0.28
QKNSGKLTF − Mascot − rejected
NA − spliced − |||
20120322_EXQ1_MiBa_SA_HCC1143_1_A: 12600
SA = 0.35
Score = −4.25
REATGKITF − MaxQuant
20120322_EXQ1_MiBa_SA_HCC1143_1_A: 12600
SA = 0.87
Score = 1.93
REATGKITF − MSFragger
20120322_EXQ1_MiBa_SA_HCC1143_1_A: 12600
SA = 0.87
Score = 1.93
GFPPLYLAS − Mascot − rejected
GFPP|LYLAS − spliced − 2|4|3|3
20120617_EXQ0_MiBa_SA_HCT116_1_mHLA_2hr: 2692
SA = 0.35
Score = −2.81
YLPPGFLSA − MaxQuant
20120617_EXQ0_MiBa_SA_HCT116_1_mHLA_2hr: 2692
SA = 0.77
Score = 0.14
YLPPGFLSA − MSFragger
20120617_EXQ0_MiBa_SA_HCT116_1_mHLA_2hr: 2692
SA = 0.77
Score = 0.14
Relative intensity
m/z
Prosit prediction
* Exclusive in prediction
# Exclusive in experimental

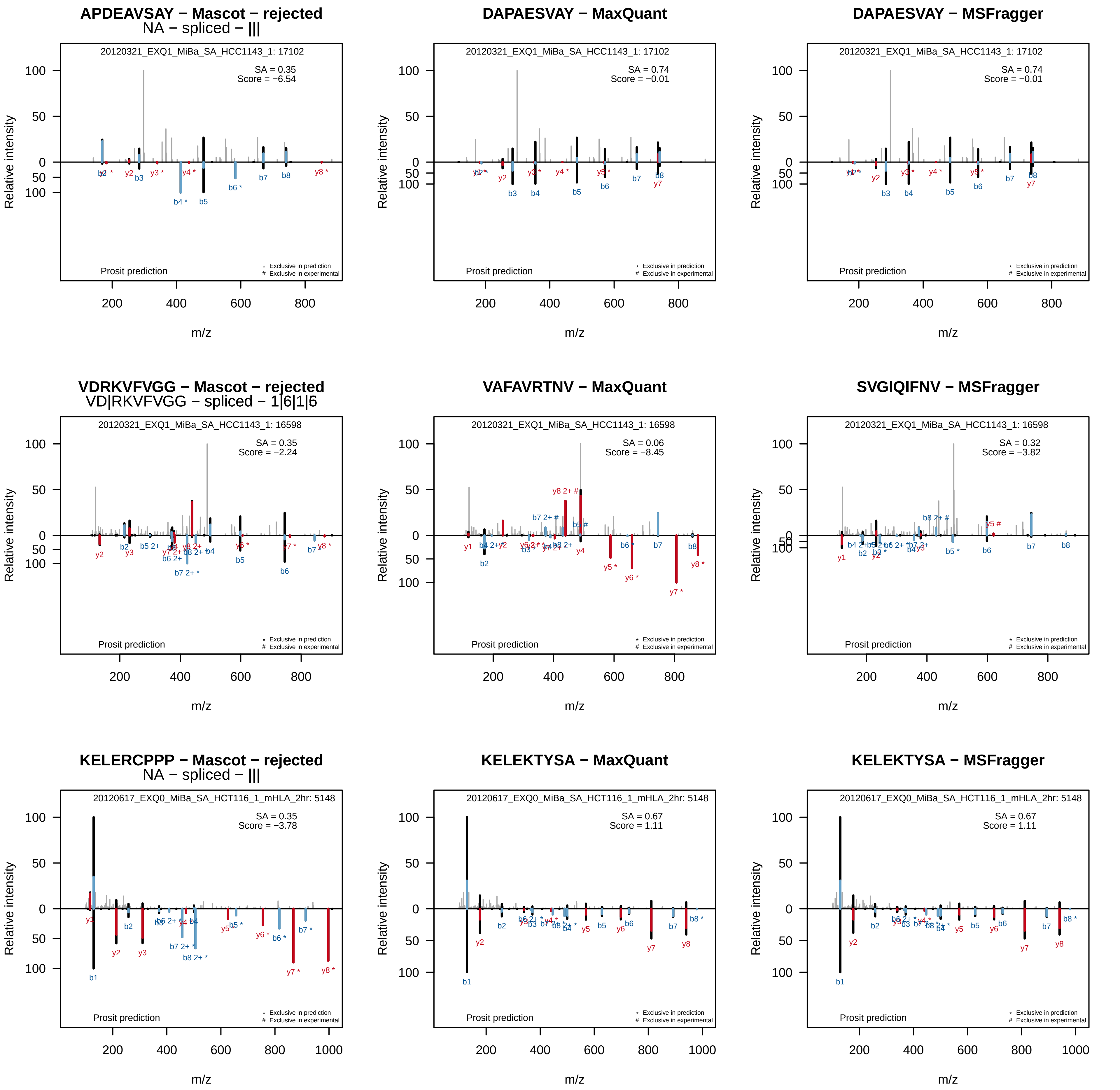

APDEAVSAY − Mascot − rejected
NA − spliced − |||
20120321_EXQ1_MiBa_SA_HCC1143_1: 17102
SA = 0.35
Score = −6.54
DAPAESVAY − MaxQuant
20120321_EXQ1_MiBa_SA_HCC1143_1: 17102
SA = 0.74
Score = −0.01
DAPAESVAY − MSFragger
20120321_EXQ1_MiBa_SA_HCC1143_1: 17102
SA = 0.74
Score = −0.01
VDRKVFVGG − Mascot − rejected
VD|RKVFVGG − spliced − 1|6|1|6
20120321_EXQ1_MiBa_SA_HCC1143_1: 16598
SA = 0.35
Score = −2.24
VAFAVRTNV − MaxQuant
20120321_EXQ1_MiBa_SA_HCC1143_1: 16598
SA = 0.06
Score = −8.45
SVGIQIFNV − MSFragger
20120321_EXQ1_MiBa_SA_HCC1143_1: 16598
SA = 0.32
Score = −3.82
KELERCPPP − Mascot − rejected
NA − spliced − |||
20120617_EXQ0_MiBa_SA_HCT116_1_mHLA_2hr: 5148
SA = 0.35
Score = −3.78
KELEKTYSA − MaxQuant
20120617_EXQ0_MiBa_SA_HCT116_1_mHLA_2hr: 5148
SA = 0.67
Score = 1.11
KELEKTYSA − MSFragger
20120617_EXQ0_MiBa_SA_HCT116_1_mHLA_2hr: 5148
SA = 0.67
Score = 1.11
Relative intensity
m/z
Prosit prediction
* Exclusive in prediction
# Exclusive in experimental

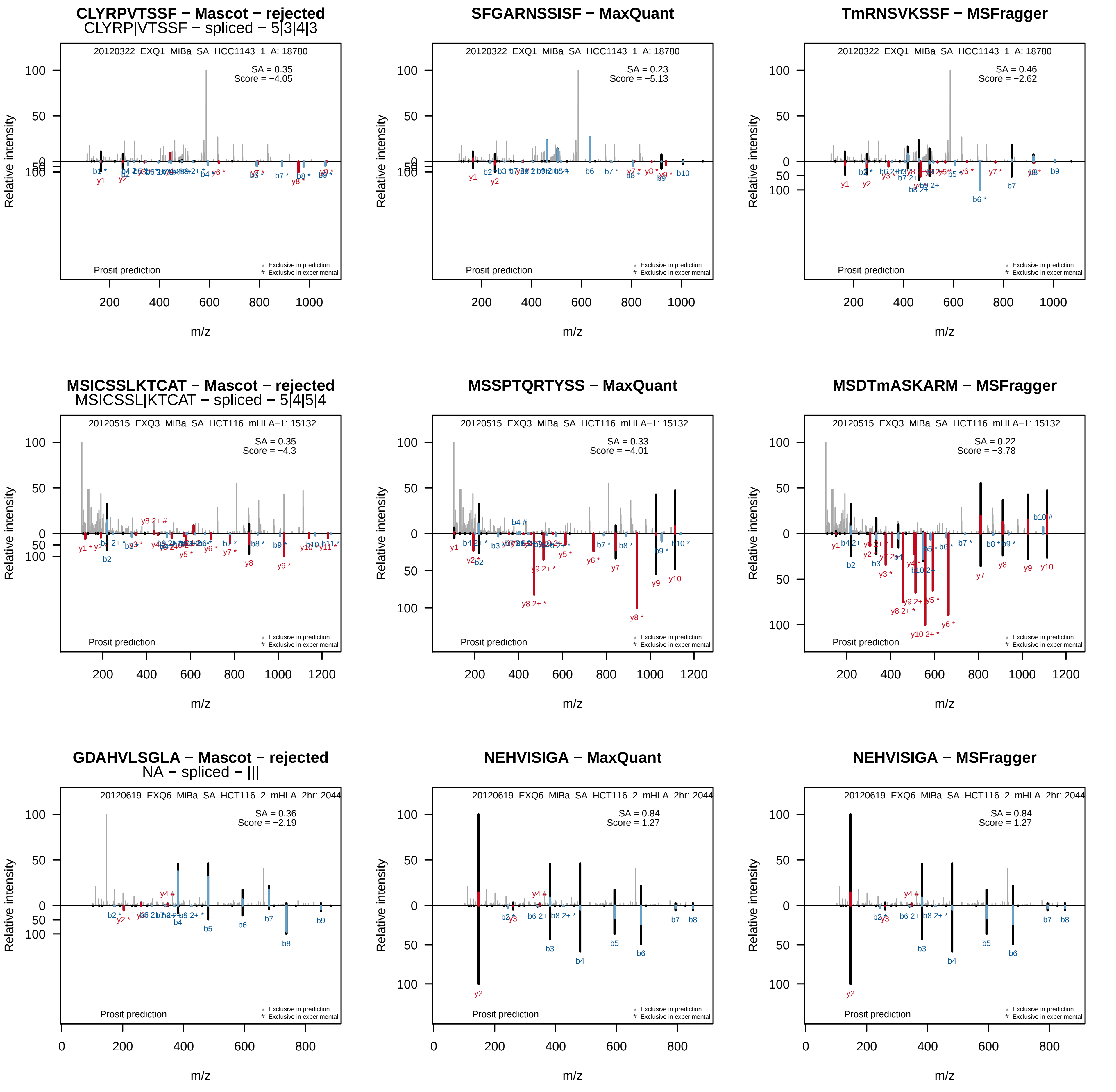
CLYRPVTSSF − Mascot − rejected
CLYRP|VTSSF − spliced − 5|3|4|3
20120322_EXQ1_MiBa_SA_HCC1143_1_A: 18780
SA = 0.35
Score = −4.05
SFGARNSSISF − MaxQuant
20120322_EXQ1_MiBa_SA_HCC1143_1_A: 18780
SA = 0.23
Score = −5.13
TmRNSVKSSF − MSFragger
20120322_EXQ1_MiBa_SA_HCC1143_1_A: 18780
SA = 0.46
Score = −2.62
MSICSSLKTCAT − Mascot − rejected
MSICSSL|KTCAT − spliced − 5|4|5|4
20120515_EXQ3_MiBa_SA_HCT116_mHLA−1: 15132
SA = 0.35
Score = −4.3
MSSPTQRTYSS − MaxQuant
20120515_EXQ3_MiBa_SA_HCT116_mHLA−1: 15132
SA = 0.33
Score = −4.01
MSDTmASKARM − MSFragger
20120515_EXQ3_MiBa_SA_HCT116_mHLA−1: 15132
SA = 0.22
Score = −3.78
GDAHVLSGLA − Mascot − rejected
NA − spliced − |||
20120619_EXQ6_MiBa_SA_HCT116_2_mHLA_2hr: 2044
SA = 0.36
Score = −2.19
NEHVISIGA − MaxQuant
20120619_EXQ6_MiBa_SA_HCT116_2_mHLA_2hr: 2044
SA = 0.84
Score = 1.27
NEHVISIGA − MSFragger
20120619_EXQ6_MiBa_SA_HCT116_2_mHLA_2hr: 2044
SA = 0.84
Score = 1.27
Relative intensity
m/z
Prosit prediction
⋆ Exclusive in prediction
Exclusive in experimental

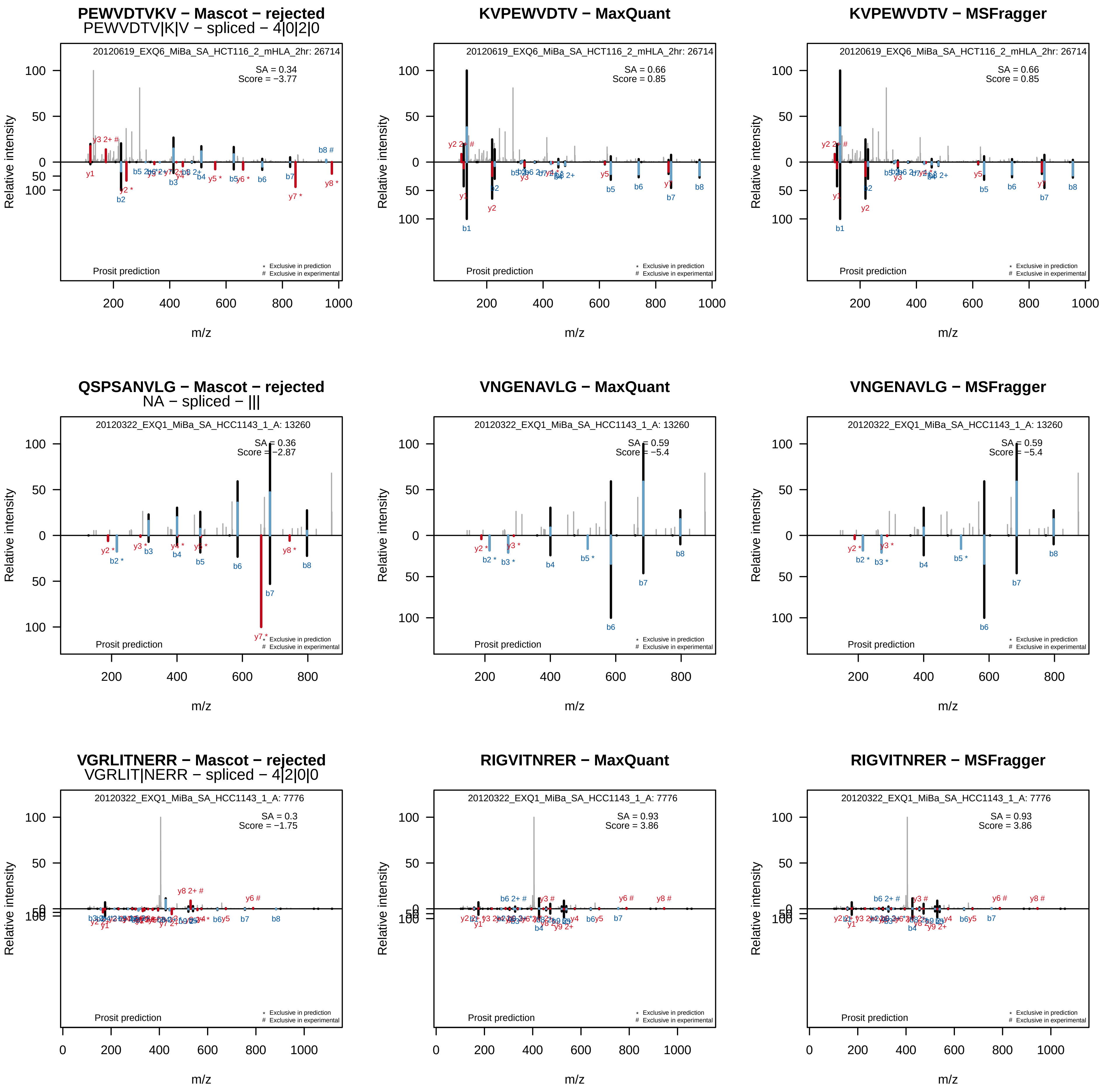

PEWVDTVKV − Mascot − rejected
PEWVDTV|K|V − spliced − 4|0|2|0
20120619_EXQ6_MiBa_SA_HCT116_2_mHLA_2hr: 26714
SA = 0.34
Score = −3.77
KVPEWVDTV − MaxQuant
20120619_EXQ6_MiBa_SA_HCT116_2_mHLA_2hr: 26714
SA = 0.66
Score = 0.85
KVPEWVDTV − MSFragger
20120619_EXQ6_MiBa_SA_HCT116_2_mHLA_2hr: 26714
SA = 0.66
Score = 0.85
QSPSANVLG − Mascot − rejected
NA − spliced − |||
20120322_EXQ1_MiBa_SA_HCC1143_1_A: 13260
SA = 0.36
Score = −2.87
VNGENAVLG − MaxQuant
20120322_EXQ1_MiBa_SA_HCC1143_1_A: 13260
SA = 0.59
Score = −5.4
VNGENAVLG − MSFragger
20120322_EXQ1_MiBa_SA_HCC1143_1_A: 13260
SA = 0.59
Score = −5.4
VGRLITNERR − Mascot − rejected
VGRLIT|NERR − spliced − 4|2|0|0
20120322_EXQ1_MiBa_SA_HCC1143_1_A: 7776
SA = 0.3
Score = −1.75
RIGVITNRER − MaxQuant
20120322_EXQ1_MiBa_SA_HCC1143_1_A: 7776
SA = 0.93
Score = 3.86
RIGVITNRER − MSFragger
20120322_EXQ1_MiBa_SA_HCC1143_1_A: 7776
SA = 0.93
Score = 3.86
Relative intensity
m/z
Prosit prediction
* Exclusive in prediction
# Exclusive in experimental

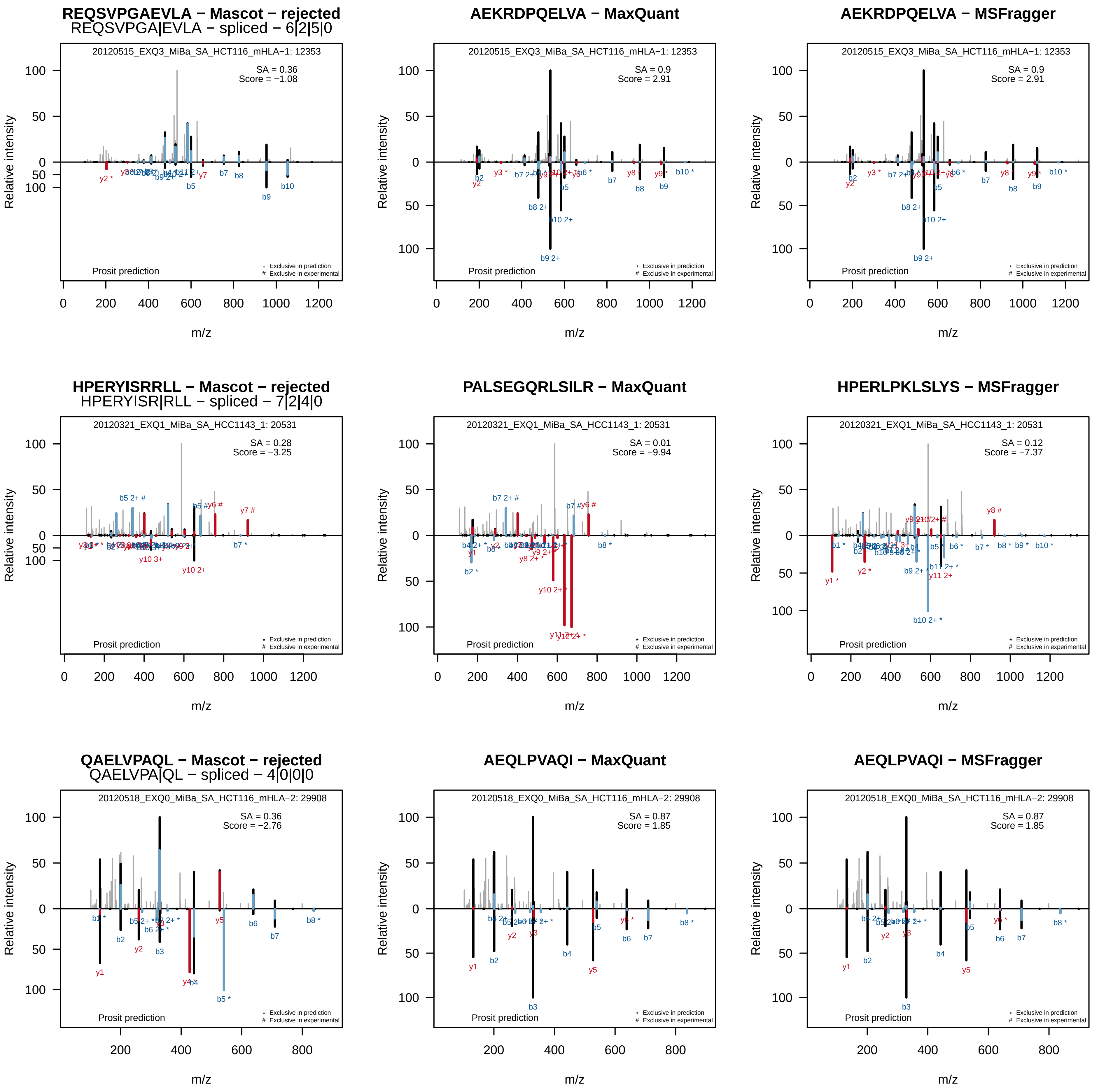

REQSVPGAEVLA − Mascot − rejected
REQSVPGA|EVLA − spliced − 6|2|5|0
20120515_EXQ3_MiBa_SA_HCT116_mHLA−1: 12353
SA = 0.36
Score = −1.08
AEKRDPQELVA − MaxQuant
20120515_EXQ3_MiBa_SA_HCT116_mHLA−1: 12353
SA = 0.9
Score = 2.91
AEKRDPQELVA − MSFragger
20120515_EXQ3_MiBa_SA_HCT116_mHLA−1: 12353
SA = 0.9
Score = 2.91
HPERYISRRLL − Mascot − rejected
HPERYISR|RLL − spliced − 7|2|4|0
20120321_EXQ1_MiBa_SA_HCC1143_1: 20531
SA = 0.28
Score = −3.25
PALSEGQRLSILR − MaxQuant
20120321_EXQ1_MiBa_SA_HCC1143_1: 20531
SA = 0.01
Score = −9.94
HPERLPKLSLYS − MSFragger
20120321_EXQ1_MiBa_SA_HCC1143_1: 20531
SA = 0.12
Score = −7.37
QAELVPAQL − Mascot − rejected
QAELVPA|QL − spliced − 4|0|0|0
20120518_EXQ0_MiBa_SA_HCT116_mHLA−2: 29908
SA = 0.36
Score = −2.76
AEQLPVAQI − MaxQuant
20120518_EXQ0_MiBa_SA_HCT116_mHLA−2: 29908
SA = 0.87
Score = 1.85
AEQLPVAQI − MSFragger
20120518_EXQ0_MiBa_SA_HCT116_mHLA−2: 29908
SA = 0.87
Score = 1.85
Relative intensity
m/z
Prosit prediction
⋆ Exclusive in prediction
Exclusive in experimental

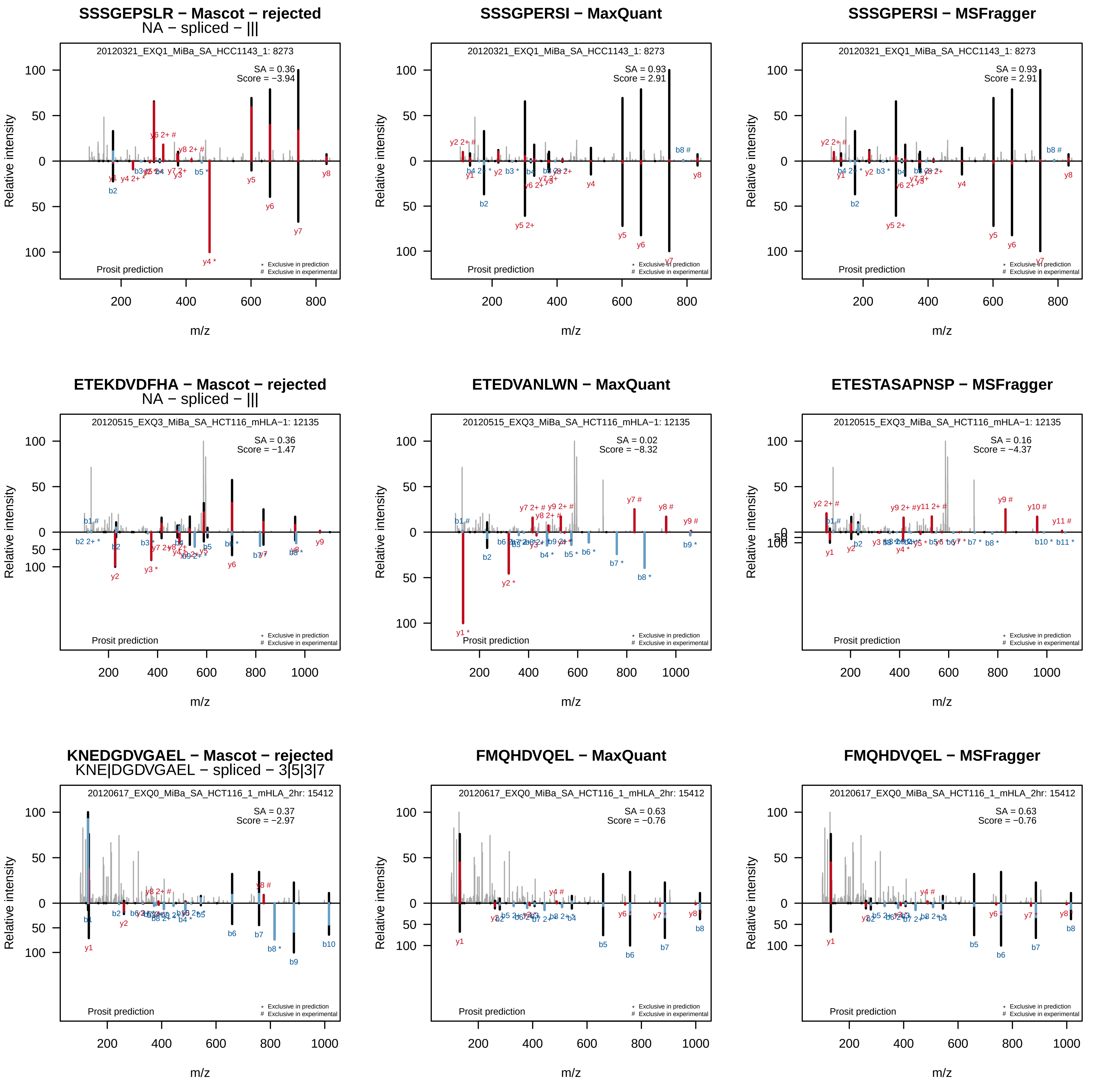

SSSGEPSLR − Mascot − rejected
NA − spliced − |||
20120321_EXQ1_MiBa_SA_HCC1143_1: 8273
SA = 0.36
Score = −3.94
SSSGPERSI − MaxQuant
20120321_EXQ1_MiBa_SA_HCC1143_1: 8273
SA = 0.93
Score = 2.91
SSSGPERSI − MSFragger
20120321_EXQ1_MiBa_SA_HCC1143_1: 8273
SA = 0.93
Score = 2.91
ETEKDVDFHA − Mascot − rejected
NA − spliced − |||
20120515_EXQ3_MiBa_SA_HCT116_mHLA−1: 12135
SA = 0.36
Score = −1.47
ETEDVANLWN − MaxQuant
20120515_EXQ3_MiBa_SA_HCT116_mHLA−1: 12135
SA = 0.02
Score = −8.32
ETESTASAPNSP − MSFragger
20120515_EXQ3_MiBa_SA_HCT116_mHLA−1: 12135
SA = 0.16
Score = −4.37
KNEDGDVGAEL − Mascot − rejected
KNE|DGDVGAEL − spliced − 3|5|3|7
20120617_EXQ0_MiBa_SA_HCT116_1_mHLA_2hr: 15412
SA = 0.37
Score = −2.97
FMQHDVQEL − MaxQuant
20120617_EXQ0_MiBa_SA_HCT116_1_mHLA_2hr: 15412
SA = 0.63
Score = −0.76
FMQHDVQEL − MSFragger
20120617_EXQ0_MiBa_SA_HCT116_1_mHLA_2hr: 15412
SA = 0.63
Score = −0.76
Relative intensity
m/z
Prosit prediction
* Exclusive in prediction
# Exclusive in experimental

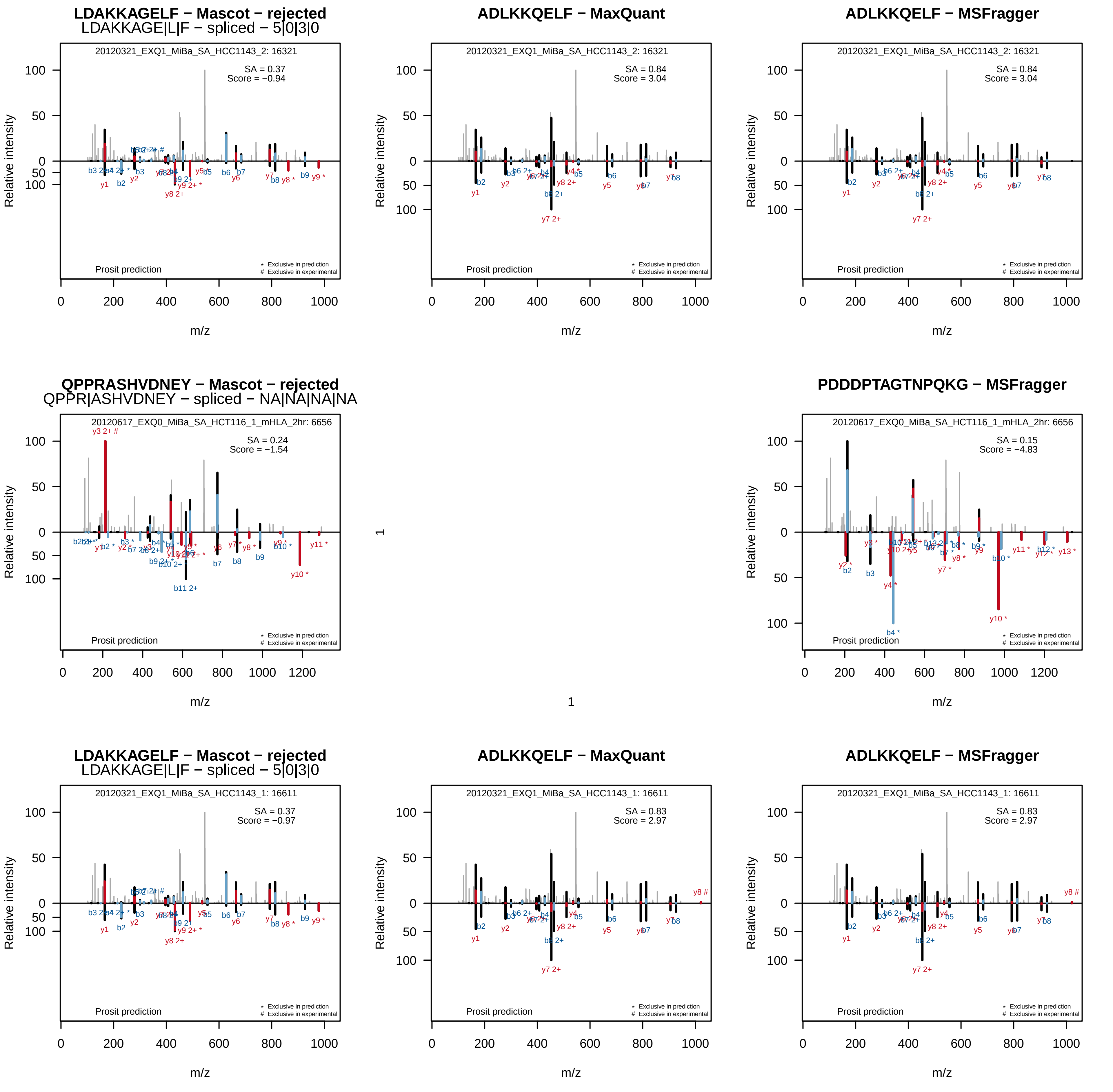
LDAKKAGELF − Mascot − rejected
LDAKKAGE|L|F − spliced − 5|0|3|0
20120321_EXQ1_MiBa_SA_HCC1143_2: 16321
SA = 0.37
Score = −0.94
ADLKKQELF − MaxQuant
20120321_EXQ1_MiBa_SA_HCC1143_2: 16321
SA = 0.84
Score = 3.04
ADLKKQELF − MSFragger
20120321_EXQ1_MiBa_SA_HCC1143_2: 16321
SA = 0.84
Score = 3.04
QPPRASHVDNEY − Mascot − rejected
QPPR|ASHVDNEY − spliced − NA|NA|NA|NA
20120617_EXQ0_MiBa_SA_HCT116_1_mHLA_2hr: 6656
SA = 0.24
Score = −1.54
PDDDPTAGTNPQKG − MSFragger
20120617_EXQ0_MiBa_SA_HCT116_1_mHLA_2hr: 6656
SA = 0.15
Score = −4.83
LDAKKAGELF − Mascot − rejected
LDAKKAGE|L|F − spliced − 5|0|3|0
20120321_EXQ1_MiBa_SA_HCC1143_1: 16611
SA = 0.37
Score = −0.97
ADLKKQELF − MaxQuant
20120321_EXQ1_MiBa_SA_HCC1143_1: 16611
SA = 0.83
Score = 2.97
ADLKKQELF − MSFragger
20120321_EXQ1_MiBa_SA_HCC1143_1: 16611
SA = 0.83
Score = 2.97
Relative intensity
m/z
Prosit prediction
* Exclusive in prediction
# Exclusive in experimental

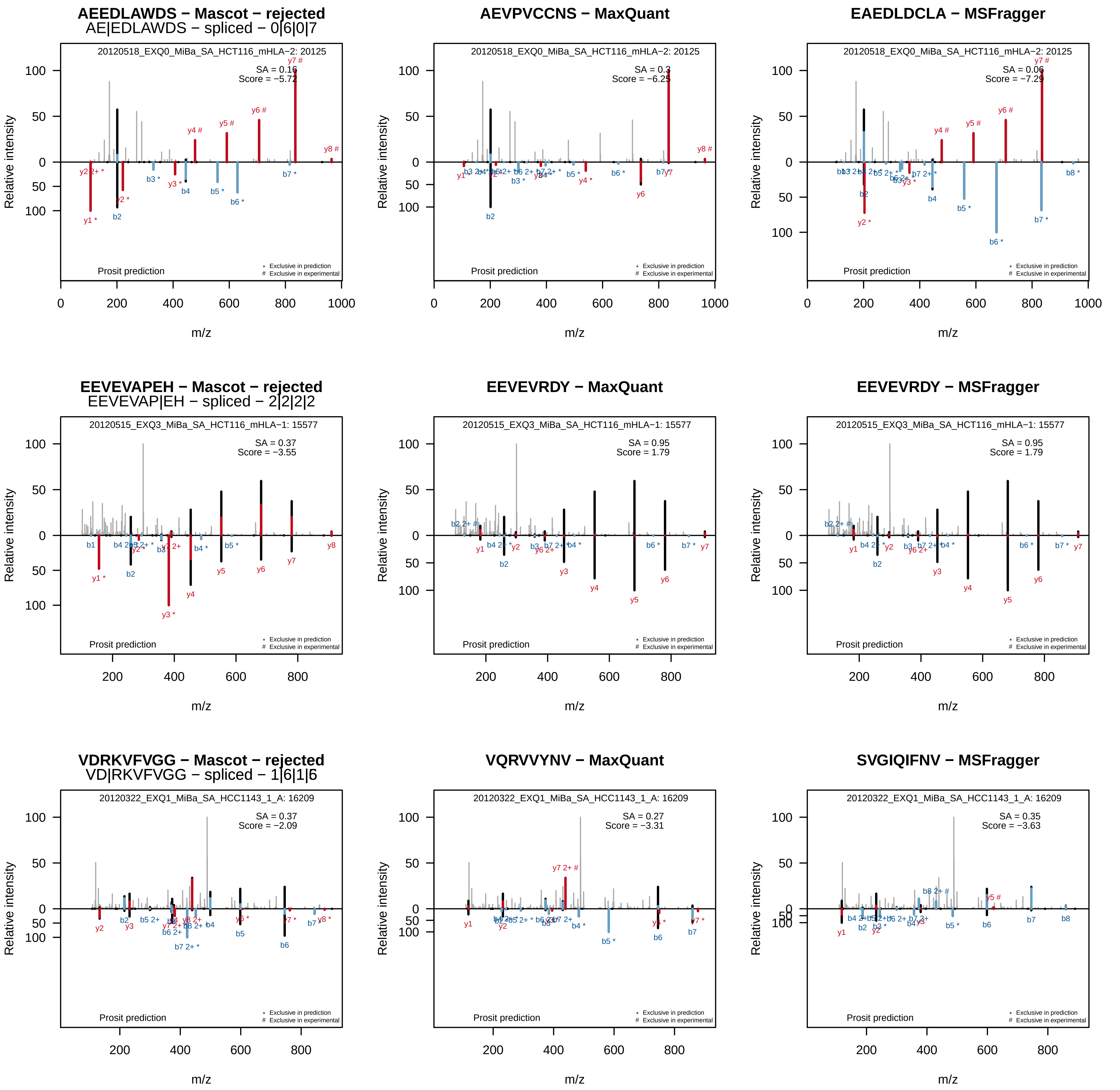
AEEDLAWDS − Mascot − rejected
AE|EDLAWDS − spliced − 0|6|0|7
20120518_EXQ0_MiBa_SA_HCT116_mHLA−2: 20125
SA = 0.16
Score = −5.72
AEVPVCCNS − MaxQuant
20120518_EXQ0_MiBa_SA_HCT116_mHLA−2: 20125
SA = 0.3
Score = −6.25
EAEDLDCLA − MSFragger
20120518_EXQ0_MiBa_SA_HCT116_mHLA−2: 20125
SA = 0.06
Score = −7.29
EEVEVAPEH − Mascot − rejected
EEVEVAP|EH − spliced − 2|2|2|2
20120515_EXQ3_MiBa_SA_HCT116_mHLA−1: 15577
SA = 0.37
Score = −3.55
EEVEVRDY − MaxQuant
20120515_EXQ3_MiBa_SA_HCT116_mHLA−1: 15577
SA = 0.95
Score = 1.79
EEVEVRDY − MSFragger
20120515_EXQ3_MiBa_SA_HCT116_mHLA−1: 15577
SA = 0.95
Score = 1.79
VDRKVFVGG − Mascot − rejected
VD|RKVFVGG − spliced − 1|6|1|6
20120322_EXQ1_MiBa_SA_HCC1143_1_A: 16209
SA = 0.37
Score = −2.09
VQRVVYNV − MaxQuant
20120322_EXQ1_MiBa_SA_HCC1143_1_A: 16209
SA = 0.27
Score = −3.31
SVGIQIFNV − MSFragger
20120322_EXQ1_MiBa_SA_HCC1143_1_A: 16209
SA = 0.35
Score = −3.63
Relative intensity
m/z
Prosit prediction
* Exclusive in prediction
# Exclusive in experimental

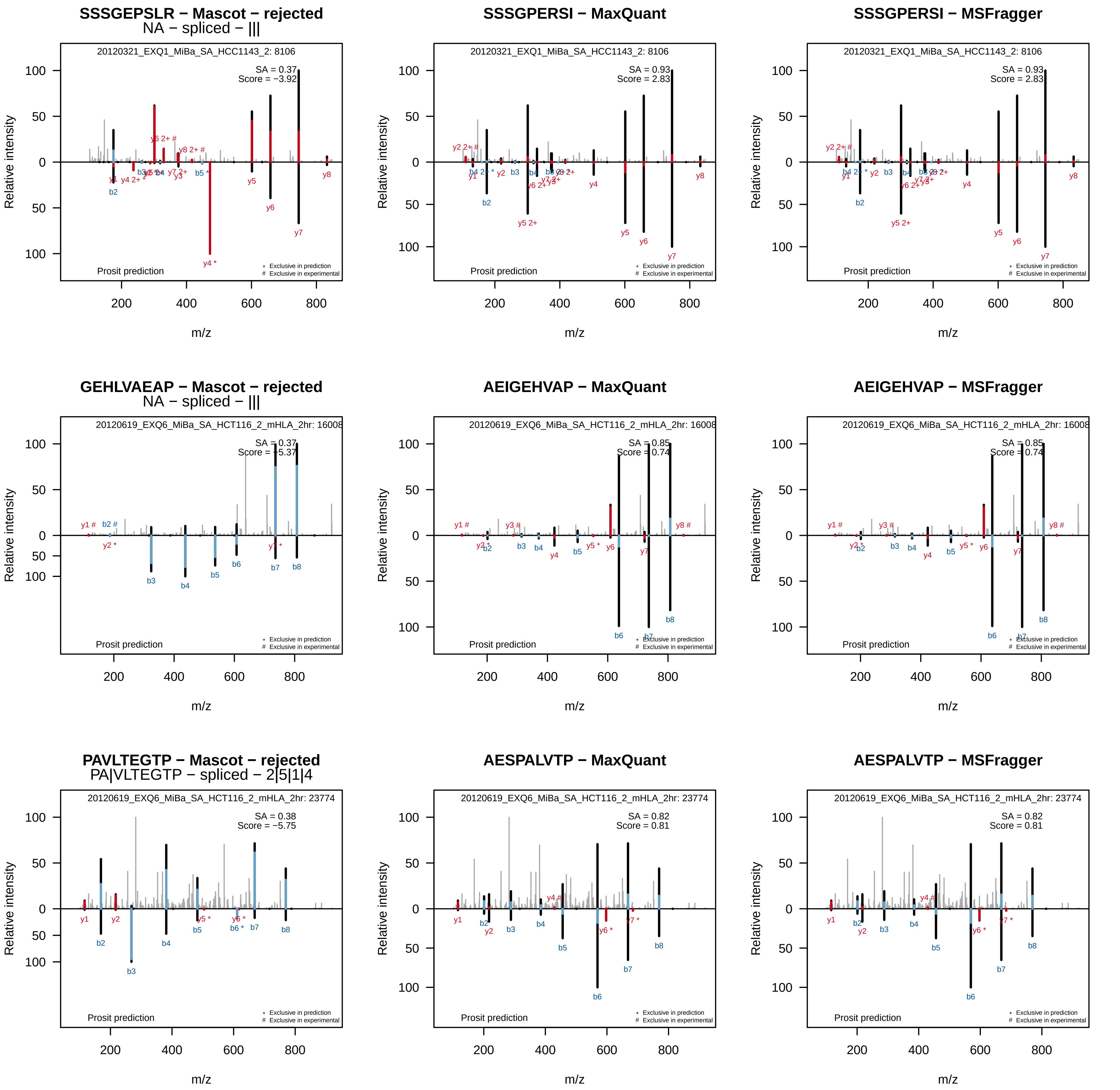
SSSGEPSLR − Mascot − rejected
NA − spliced − |||
20120321_EXQ1_MiBa_SA_HCC1143_2: 8106
SA = 0.37
Score = −3.92
SSSGPERSI − MaxQuant
20120321_EXQ1_MiBa_SA_HCC1143_2: 8106
SA = 0.93
Score = 2.83
SSSGPERSI − MSFragger
20120321_EXQ1_MiBa_SA_HCC1143_2: 8106
SA = 0.93
Score = 2.83
GEHLVAEAP − Mascot − rejected
NA − spliced − |||
20120619_EXQ6_MiBa_SA_HCT116_2_mHLA_2hr: 16008
SA = 0.37
Score = −5.37
AEIGEHVAP − MaxQuant
20120619_EXQ6_MiBa_SA_HCT116_2_mHLA_2hr: 16008
SA = 0.85
Score = 0.74
AEIGEHVAP − MSFragger
20120619_EXQ6_MiBa_SA_HCT116_2_mHLA_2hr: 16008
SA = 0.85
Score = 0.74
PAVLTEGTP − Mascot − rejected
PA|VLTEGTP − spliced − 2|5|1|4
20120619_EXQ6_MiBa_SA_HCT116_2_mHLA_2hr: 23774
SA = 0.38
Score = −5.75
AESPALVTP − MaxQuant
20120619_EXQ6_MiBa_SA_HCT116_2_mHLA_2hr: 23774
SA = 0.82
Score = 0.81
AESPALVTP − MSFragger
20120619_EXQ6_MiBa_SA_HCT116_2_mHLA_2hr: 23774
SA = 0.82
Score = 0.81
Relative intensity
m/z
Prosit prediction
⋆ Exclusive in prediction
Exclusive in experimental

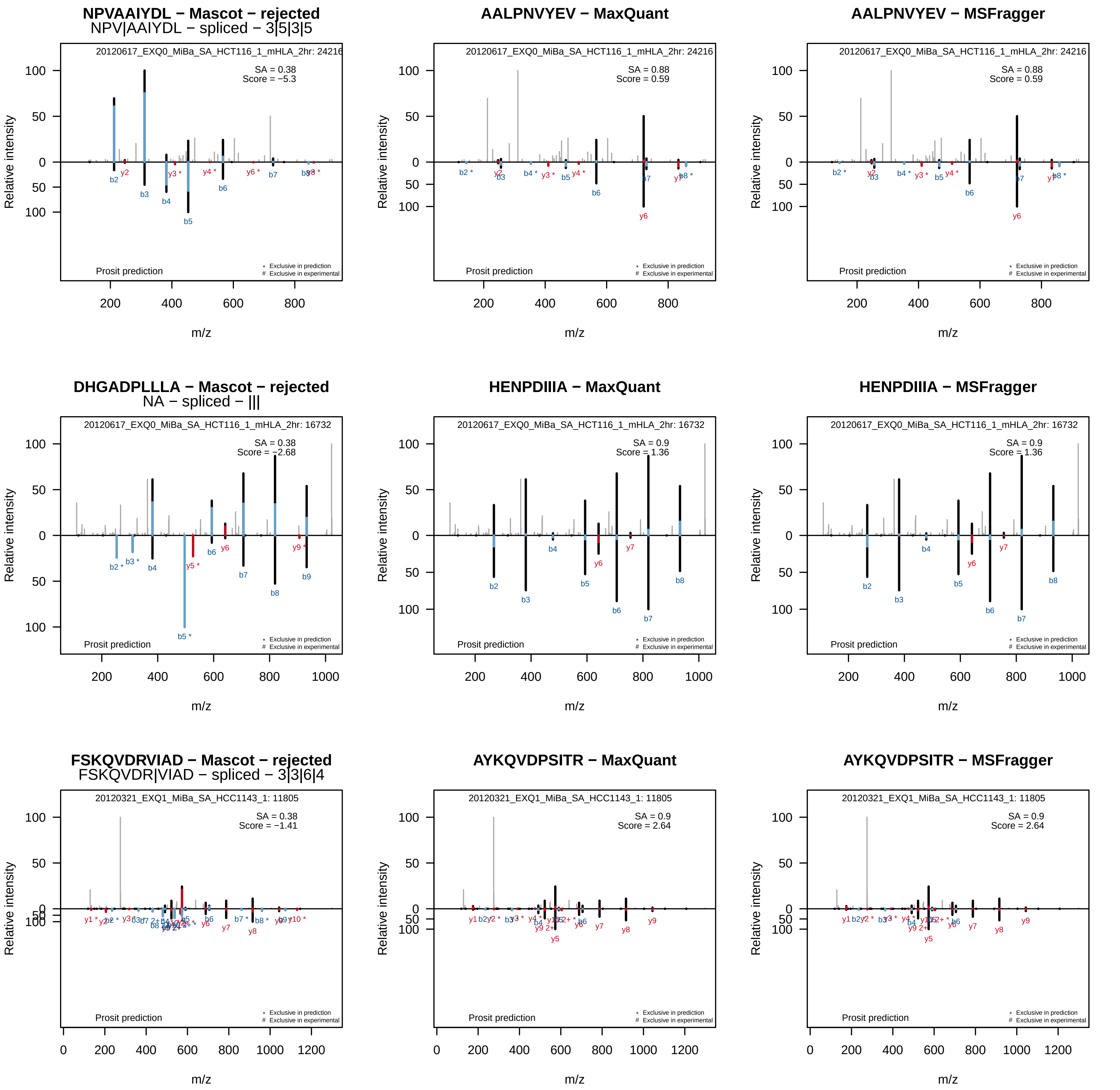

NPVAAIYDL − Mascot − rejected
NPV|AAIYDL − spliced − 3|5|3|5
20120617_EXQ0_MiBa_SA_HCT116_1_mHLA_2hr: 24216
SA = 0.38
Score = −5.3
AALPNVYEV − MaxQuant
20120617_EXQ0_MiBa_SA_HCT116_1_mHLA_2hr: 24216
SA = 0.88
Score = 0.59
AALPNVYEV − MSFragger
20120617_EXQ0_MiBa_SA_HCT116_1_mHLA_2hr: 24216
SA = 0.88
Score = 0.59
DHGADPLLLA − Mascot − rejected
NA − spliced − |||
20120617_EXQ0_MiBa_SA_HCT116_1_mHLA_2hr: 16732
SA = 0.38
Score = −2.68
HENPDIIIA − MaxQuant
20120617_EXQ0_MiBa_SA_HCT116_1_mHLA_2hr: 16732
SA = 0.9
Score = 1.36
HENPDIIIA − MSFragger
20120617_EXQ0_MiBa_SA_HCT116_1_mHLA_2hr: 16732
SA = 0.9
Score = 1.36
FSKQVDRVIAD − Mascot − rejected
FSKQVDR|VIAD − spliced − 3|3|6|4
20120321_EXQ1_MiBa_SA_HCC1143_1: 11805
SA = 0.38
Score = −1.41
AYKQVDPSITR − MaxQuant
20120321_EXQ1_MiBa_SA_HCC1143_1: 11805
SA = 0.9
Score = 2.64
AYKQVDPSITR − MSFragger
20120321_EXQ1_MiBa_SA_HCC1143_1: 11805
SA = 0.9
Score = 2.64
Relative intensity
m/z
Prosit prediction
⋆ Exclusive in prediction
Exclusive in experimental

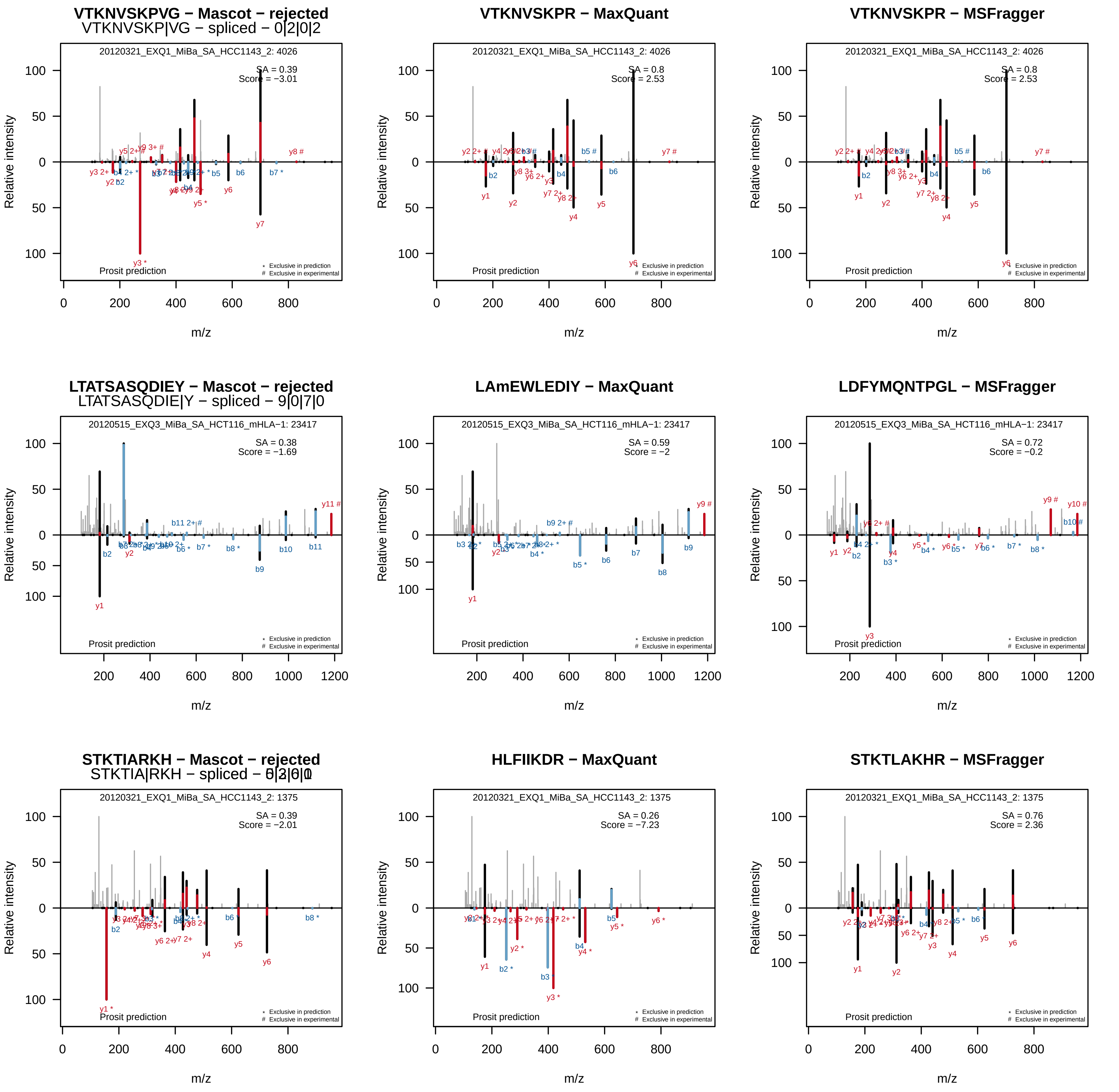
VTKNVSKPVG − Mascot − rejected
VTKNVSKP|VG − spliced − 0|2|0|2
20120321_EXQ1_MiBa_SA_HCC1143_2: 4026
SA = 0.39
Score = −3.01
VTKNVSKPR − MaxQuant
20120321_EXQ1_MiBa_SA_HCC1143_2: 4026
SA = 0.8
Score = 2.53
VTKNVSKPR − MSFragger
20120321_EXQ1_MiBa_SA_HCC1143_2: 4026
SA = 0.8
Score = 2.53
LTATSASQDIEY − Mascot − rejected
LTATSASQDIE|Y − spliced − 9|0|7|0
20120515_EXQ3_MiBa_SA_HCT116_mHLA−1: 23417
SA = 0.38
Score = −1.69
LAmEWLEDIY − MaxQuant
20120515_EXQ3_MiBa_SA_HCT116_mHLA−1: 23417
SA = 0.59
Score = −2
LDFYMQNTPGL − MSFragger
20120515_EXQ3_MiBa_SA_HCT116_mHLA−1: 23417
SA = 0.72
Score = −0.2
STKTIARKH − Mascot − rejected
STKTIA|RKH − spliced
20120321_EXQ1_MiBa_SA_HCC1143_2: 1375
SA = 0.39
Score = −2.01
HLFIIKDR − MaxQuant
20120321_EXQ1_MiBa_SA_HCC1143_2: 1375
SA = 0.26
Score = −7.23
STKTLAKHR − MSFragger
20120321_EXQ1_MiBa_SA_HCC1143_2: 1375
SA = 0.76
Score = 2.36
Relative intensity
m/z
Prosit prediction
* Exclusive in prediction
# Exclusive in experimental

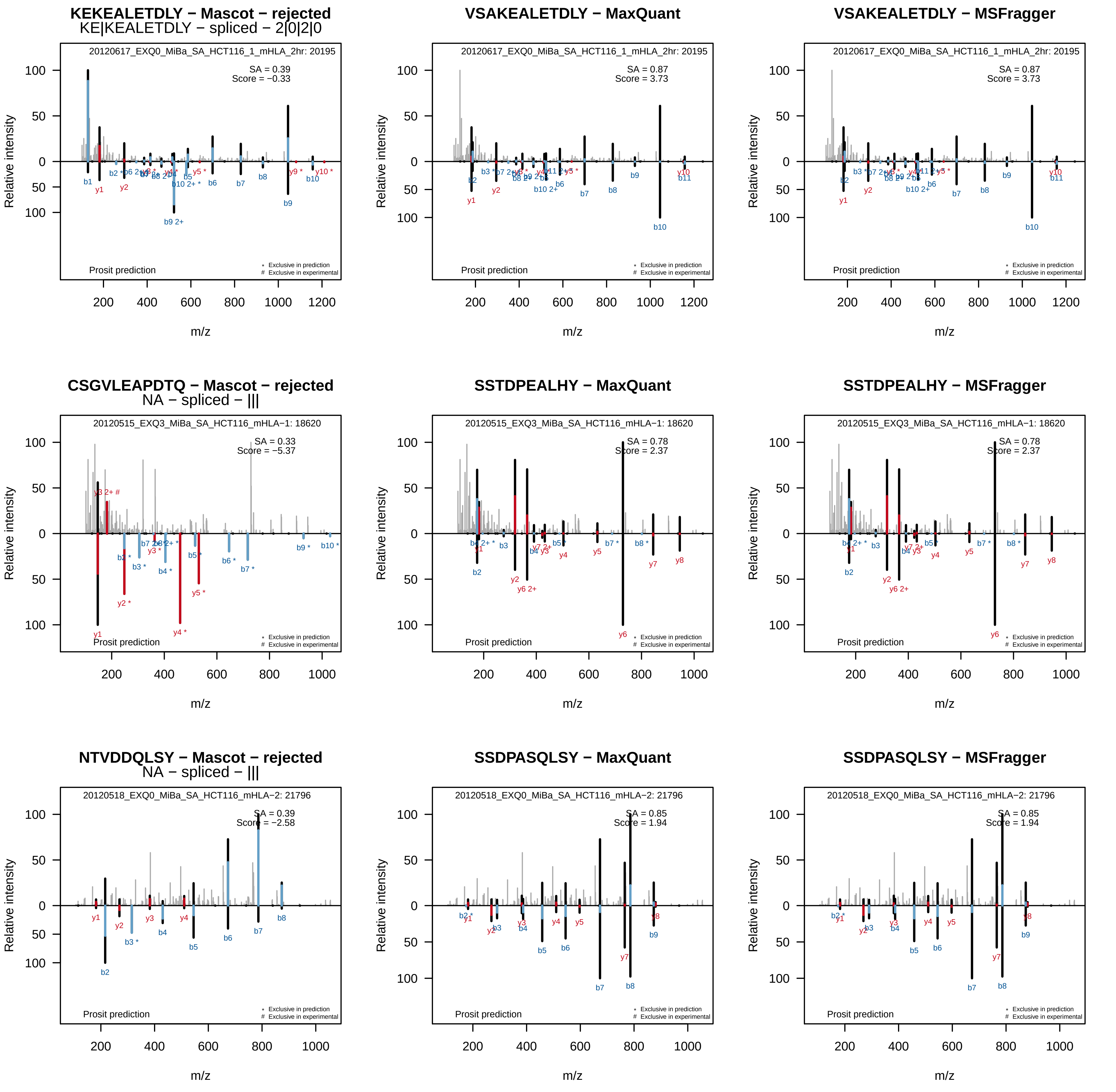
KEKEALETDLY − Mascot − rejected
KE|KEALETDLY − spliced − 2|0|2|0
20120617_EXQ0_MiBa_SA_HCT116_1_mHLA_2hr: 20195
SA = 0.39
Score = −0.33
VSAKEALETDLY − MaxQuant
20120617_EXQ0_MiBa_SA_HCT116_1_mHLA_2hr: 20195
SA = 0.87
Score = 3.73
VSAKEALETDLY − MSFragger
20120617_EXQ0_MiBa_SA_HCT116_1_mHLA_2hr: 20195
SA = 0.87
Score = 3.73
CSGVLEAPDTQ − Mascot − rejected
NA − spliced − |||
20120515_EXQ3_MiBa_SA_HCT116_mHLA−1: 18620
SA = 0.33
Score = −5.37
SSTDPEALHY − MaxQuant
20120515_EXQ3_MiBa_SA_HCT116_mHLA−1: 18620
SA = 0.78
Score = 2.37
SSTDPEALHY − MSFragger
20120515_EXQ3_MiBa_SA_HCT116_mHLA−1: 18620
SA = 0.78
Score = 2.37
NTVDDQLSY − Mascot − rejected
NA − spliced − |||
20120518_EXQ0_MiBa_SA_HCT116_mHLA−2: 21796
SA = 0.39
Score = −2.58
SSDPASQLSY − MaxQuant
20120518_EXQ0_MiBa_SA_HCT116_mHLA−2: 21796
SA = 0.85
Score = 1.94
SSDPASQLSY − MSFragger
20120518_EXQ0_MiBa_SA_HCT116_mHLA−2: 21796
SA = 0.85
Score = 1.94
Relative intensity
m/z
Prosit prediction
* Exclusive in prediction
# Exclusive in experimental

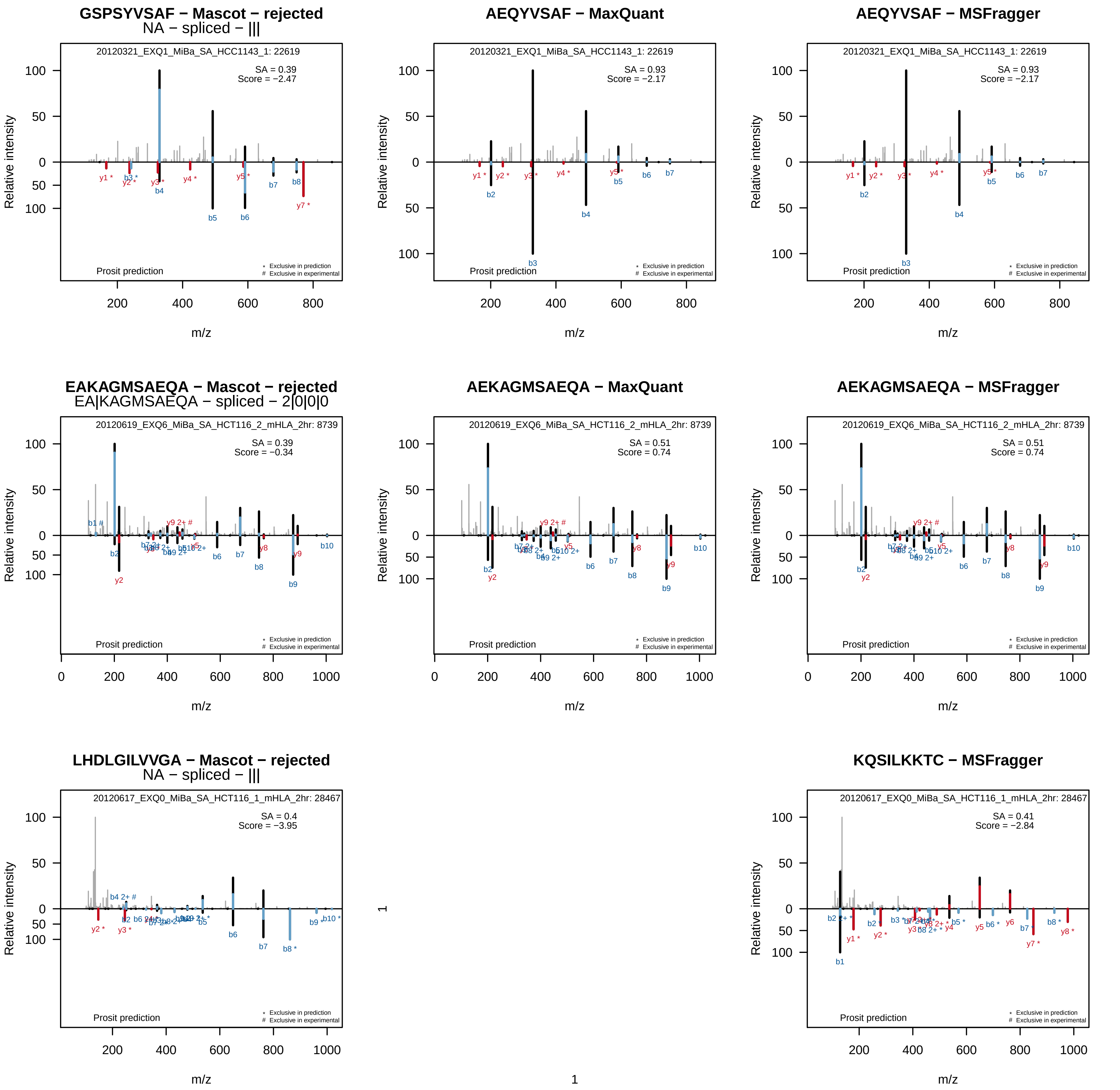

GSPSYVSAF − Mascot − rejected
NA − spliced − |||
20120321_EXQ1_MiBa_SA_HCC1143_1: 22619
SA = 0.39
Score = −2.47
AEQYVSAF − MaxQuant
20120321_EXQ1_MiBa_SA_HCC1143_1: 22619
SA = 0.93
Score = −2.17
AEQYVSAF − MSFragger
20120321_EXQ1_MiBa_SA_HCC1143_1: 22619
SA = 0.93
Score = −2.17
EAKAGMSAEQA − Mascot − rejected
EA|KAGMSAEQA − spliced − 2|0|0|0
20120619_EXQ6_MiBa_SA_HCT116_2_mHLA_2hr: 8739
SA = 0.39
Score = −0.34
AEKAGMSAEQA − MaxQuant
20120619_EXQ6_MiBa_SA_HCT116_2_mHLA_2hr: 8739
SA = 0.51
Score = 0.74
AEKAGMSAEQA − MSFragger
20120619_EXQ6_MiBa_SA_HCT116_2_mHLA_2hr: 8739
SA = 0.51
Score = 0.74
LHDLGILVVGA − Mascot − rejected
NA − spliced − |||
20120617_EXQ0_MiBa_SA_HCT116_1_mHLA_2hr: 28467
SA = 0.4
Score = −3.95
KQSILKKTC − MSFragger
20120617_EXQ0_MiBa_SA_HCT116_1_mHLA_2hr: 28467
SA = 0.41
Score = −2.84
Prosit prediction
* Exclusive in prediction
# Exclusive in experimental
Relative intensity
m/z
1

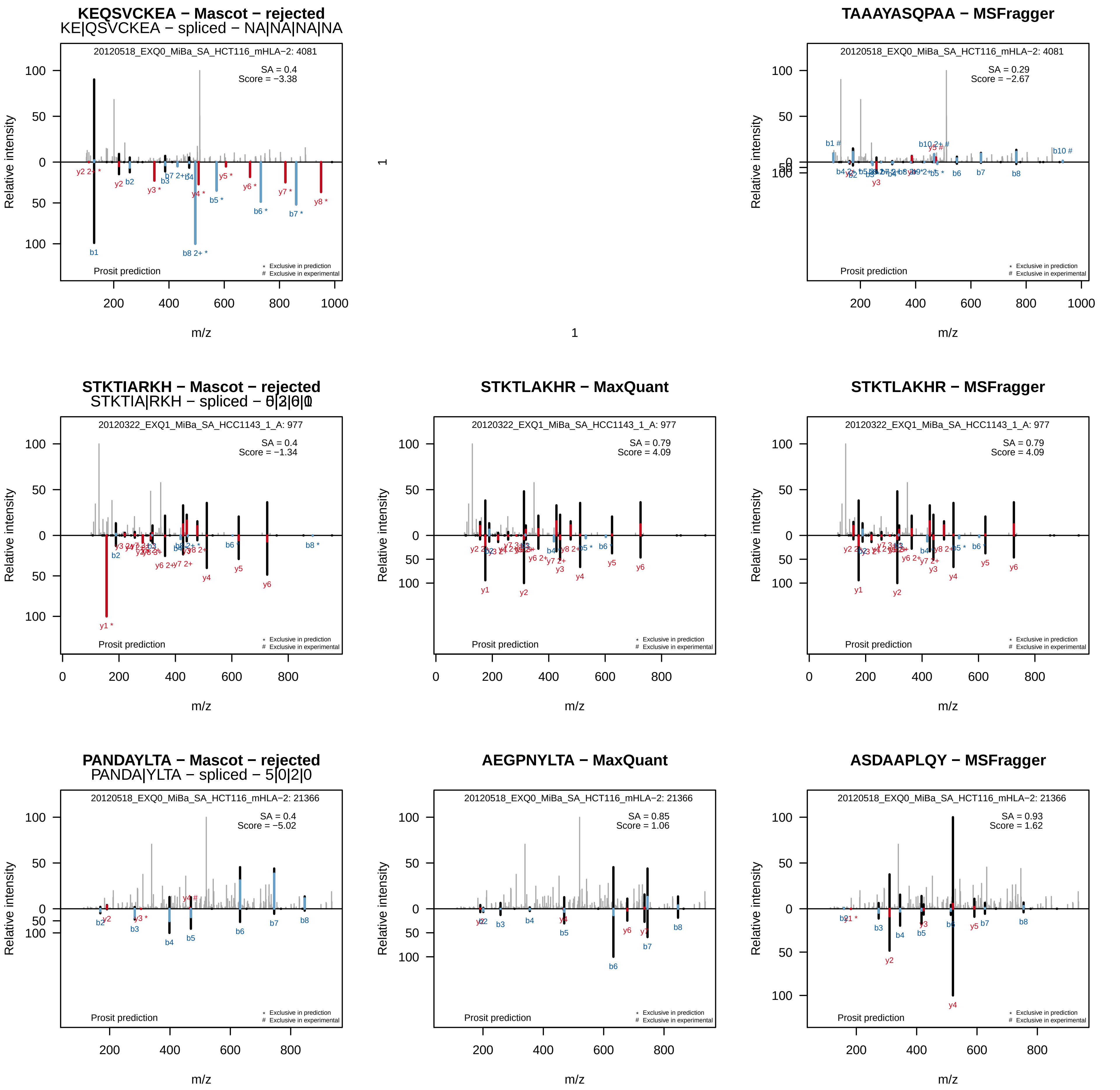
KEQSVCKEA − Mascot − rejected
KE|QSVCKEA − spliced − NA|NA|NA|NA
20120518_EXQ0_MiBa_SA_HCT116_mHLA−2: 4081
SA = 0.4
Score = −3.38
TAAAYASQPAA − MSFragger
20120518_EXQ0_MiBa_SA_HCT116_mHLA−2: 4081
SA = 0.29
Score = −2.67
STKTIARKH − Mascot − rejected
20120322_EXQ1_MiBa_SA_HCC1143_1_A: 977
SA = 0.4
Score = −1.34
STKTLAKHR − MaxQuant
20120322_EXQ1_MiBa_SA_HCC1143_1_A: 977
SA = 0.79
Score = 4.09
STKTLAKHR − MSFragger
20120322_EXQ1_MiBa_SA_HCC1143_1_A: 977
SA = 0.79
Score = 4.09
PANDAYLTA − Mascot − rejected
PANDA|YLTA − spliced − 5|0|2|0
20120518_EXQ0_MiBa_SA_HCT116_mHLA−2: 21366
SA = 0.4
Score = −5.02
AEGPNYLTA − MaxQuant
20120518_EXQ0_MiBa_SA_HCT116_mHLA−2: 21366
SA = 0.85
Score = 1.06
ASDAAPLQY − MSFragger
20120518_EXQ0_MiBa_SA_HCT116_mHLA−2: 21366
SA = 0.93
Score = 1.62
Relative intensity
m/z
Prosit prediction
* Exclusive in prediction
# Exclusive in experimental

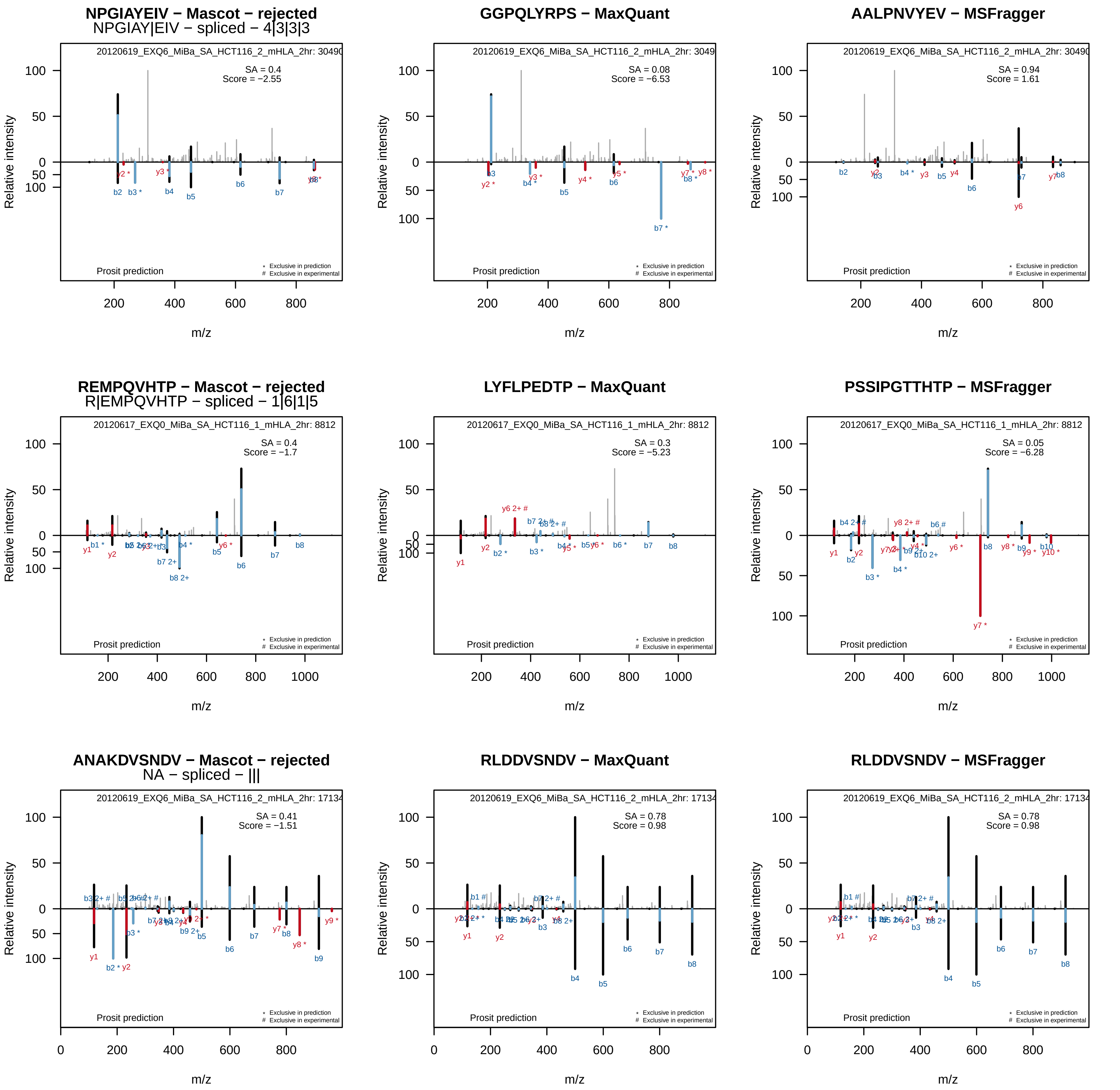

NPGIAYEIV − Mascot − rejected
NPGIAY|EIV − spliced − 4|3|3|3
20120619_EXQ6_MiBa_SA_HCT116_2_mHLA_2hr: 30490
SA = 0.4
Score = −2.55
GGPQLYRPS − MaxQuant
20120619_EXQ6_MiBa_SA_HCT116_2_mHLA_2hr: 3049
SA = 0.08
Score = −6.53
AALPNVYEV − MSFragger
20120619_EXQ6_MiBa_SA_HCT116_2_mHLA_2hr: 30490
SA = 0.94
Score = 1.61
REMPQVHTP − Mascot − rejected
R|EMPQVHTP − spliced − 1|6|1|5
20120617_EXQ0_MiBa_SA_HCT116_1_mHLA_2hr: 8812
SA = 0.4
Score = −1.7
LYFLPEDTP − MaxQuant
20120617_EXQ0_MiBa_SA_HCT116_1_mHLA_2hr: 8812
SA = 0.3
Score = −5.23
PSSIPGTTHTP − MSFragger
20120617_EXQ0_MiBa_SA_HCT116_1_mHLA_2hr: 8812
SA = 0.05
Score = −6.28
ANAKDVSNDV − Mascot − rejected
NA − spliced − |||
20120619_EXQ6_MiBa_SA_HCT116_2_mHLA_2hr: 17134
SA = 0.41
Score = −1.51
RLDDVSNDV − MaxQuant
20120619_EXQ6_MiBa_SA_HCT116_2_mHLA_2hr: 17134
SA = 0.78
Score = 0.98
RLDDVSNDV − MSFragger
20120619_EXQ6_MiBa_SA_HCT116_2_mHLA_2hr: 17134
SA = 0.78
Score = 0.98
Relative intensity
m/z
Prosit prediction
* Exclusive in prediction
# Exclusive in experimental

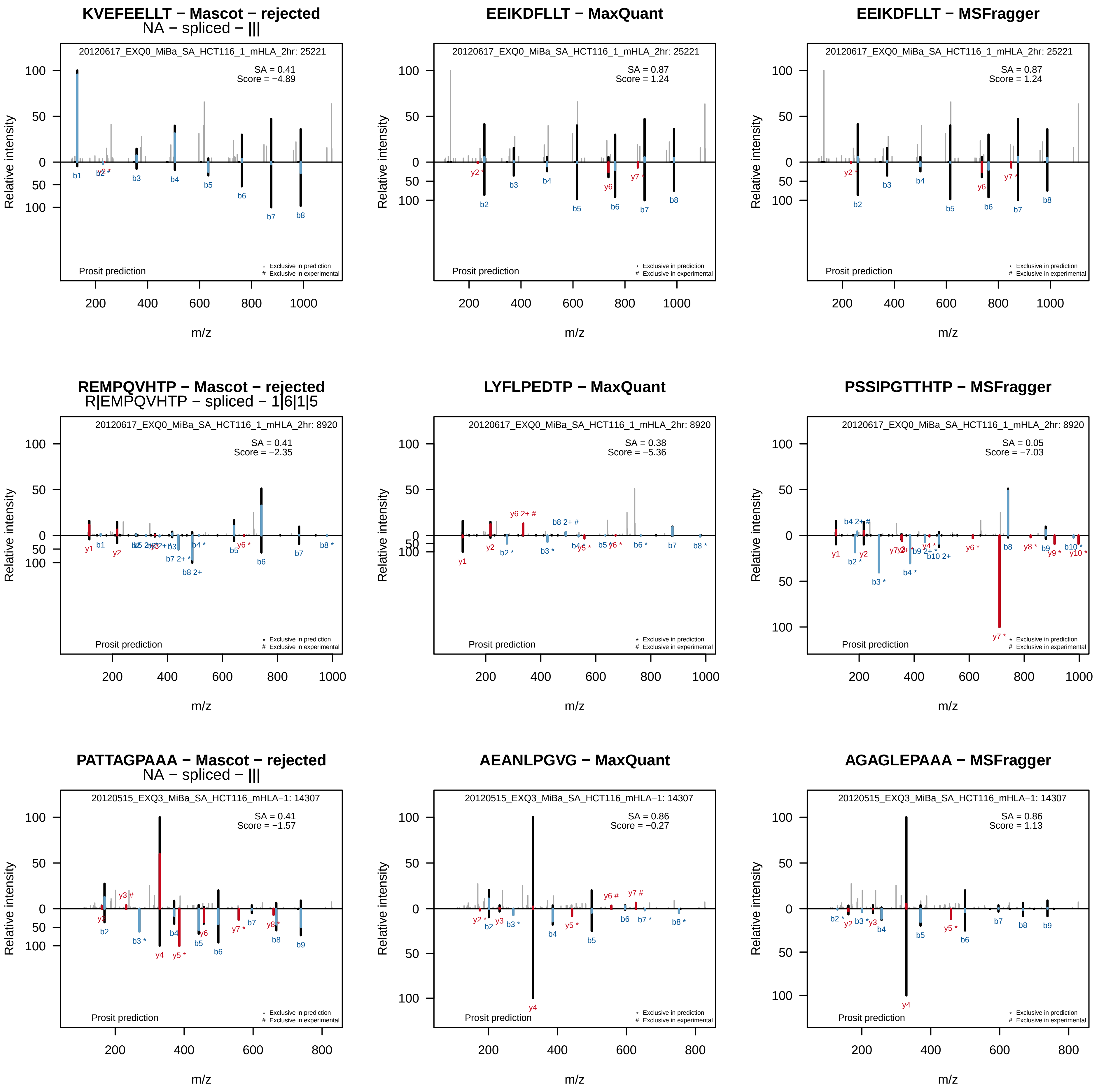
KVEFEELLT − Mascot − rejected
NA − spliced − |||
20120617_EXQ0_MiBa_SA_HCT116_1_mHLA_2hr: 25221
SA = 0.41
Score = −4.89
Prosit prediction
Relative intensity
m/z
EEIKDFLLT − MaxQuant
20120617_EXQ0_MiBa_SA_HCT116_1_mHLA_2hr: 25221
SA = 0.87
Score = 1.24
EEIKDFLLT − MSFragger
20120617_EXQ0_MiBa_SA_HCT116_1_mHLA_2hr: 25221
SA = 0.87
Score = 1.24
REMPQVHTP − Mascot − rejected
R|EMPQVHTP − spliced − 1|6|1|5
20120617_EXQ0_MiBa_SA_HCT116_1_mHLA_2hr: 8920
SA = 0.41
Score = −2.35
LYFLPEDTP − MaxQuant
20120617_EXQ0_MiBa_SA_HCT116_1_mHLA_2hr: 8920
SA = 0.38
Score = −5.36
PSSIPGTTHTP − MSFragger
20120617_EXQ0_MiBa_SA_HCT116_1_mHLA_2hr: 8920
SA = 0.05
Score = −7.03
PATTAGPAAA − Mascot − rejected
NA − spliced − |||
20120515_EXQ3_MiBa_SA_HCT116_mHLA−1: 14307
SA = 0.41
Score = −1.57
AEANLPGVG − MaxQuant
20120515_EXQ3_MiBa_SA_HCT116_mHLA−1: 14307
SA = 0.86
Score = −0.27
AGAGLEPAAA − MSFragger
20120515_EXQ3_MiBa_SA_HCT116_mHLA−1: 14307
SA = 0.86
Score = 1.13
* Exclusive in prediction
# Exclusive in experimental

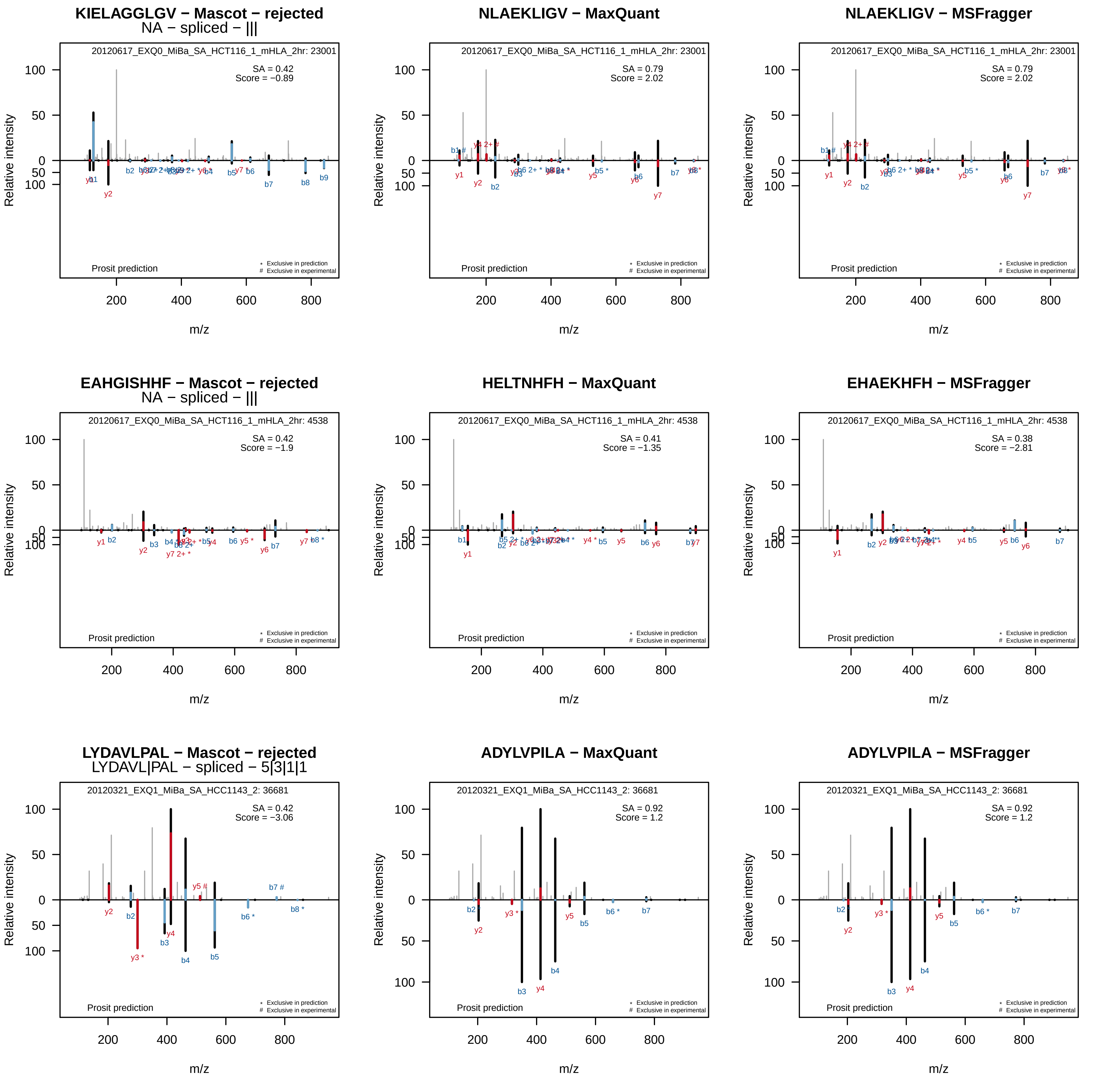
KIELAGGLGV − Mascot − rejected
NA − spliced − |||
20120617_EXQ0_MiBa_SA_HCT116_1_mHLA_2hr: 23001
SA = 0.42
Score = −0.89
NLAEKLIGV − MaxQuant
20120617_EXQ0_MiBa_SA_HCT116_1_mHLA_2hr: 23001
SA = 0.79
Score = 2.02
NLAEKLIGV − MSFragger
20120617_EXQ0_MiBa_SA_HCT116_1_mHLA_2hr: 23001
SA = 0.79
Score = 2.02
EAHGISHHF − Mascot − rejected
NA − spliced − |||
20120617_EXQ0_MiBa_SA_HCT116_1_mHLA_2hr: 4538
SA = 0.42
Score = −1.9
HELTNHFH − MaxQuant
20120617_EXQ0_MiBa_SA_HCT116_1_mHLA_2hr: 4538
SA = 0.41
Score = −1.35
EHAEKHFH − MSFragger
20120617_EXQ0_MiBa_SA_HCT116_1_mHLA_2hr: 4538
SA = 0.38
Score = −2.81
LYDAVLPAL − Mascot − rejected
LYDAVL|PAL − spliced − 5|3|1|1
20120321_EXQ1_MiBa_SA_HCC1143_2: 36681
SA = 0.42
Score = −3.06
ADYLVPILA − MaxQuant
20120321_EXQ1_MiBa_SA_HCC1143_2: 36681
SA = 0.92
Score = 1.2
ADYLVPILA − MSFragger
20120321_EXQ1_MiBa_SA_HCC1143_2: 36681
SA = 0.92
Score = 1.2
Relative intensity
m/z
Prosit prediction
* Exclusive in prediction
# Exclusive in experimental

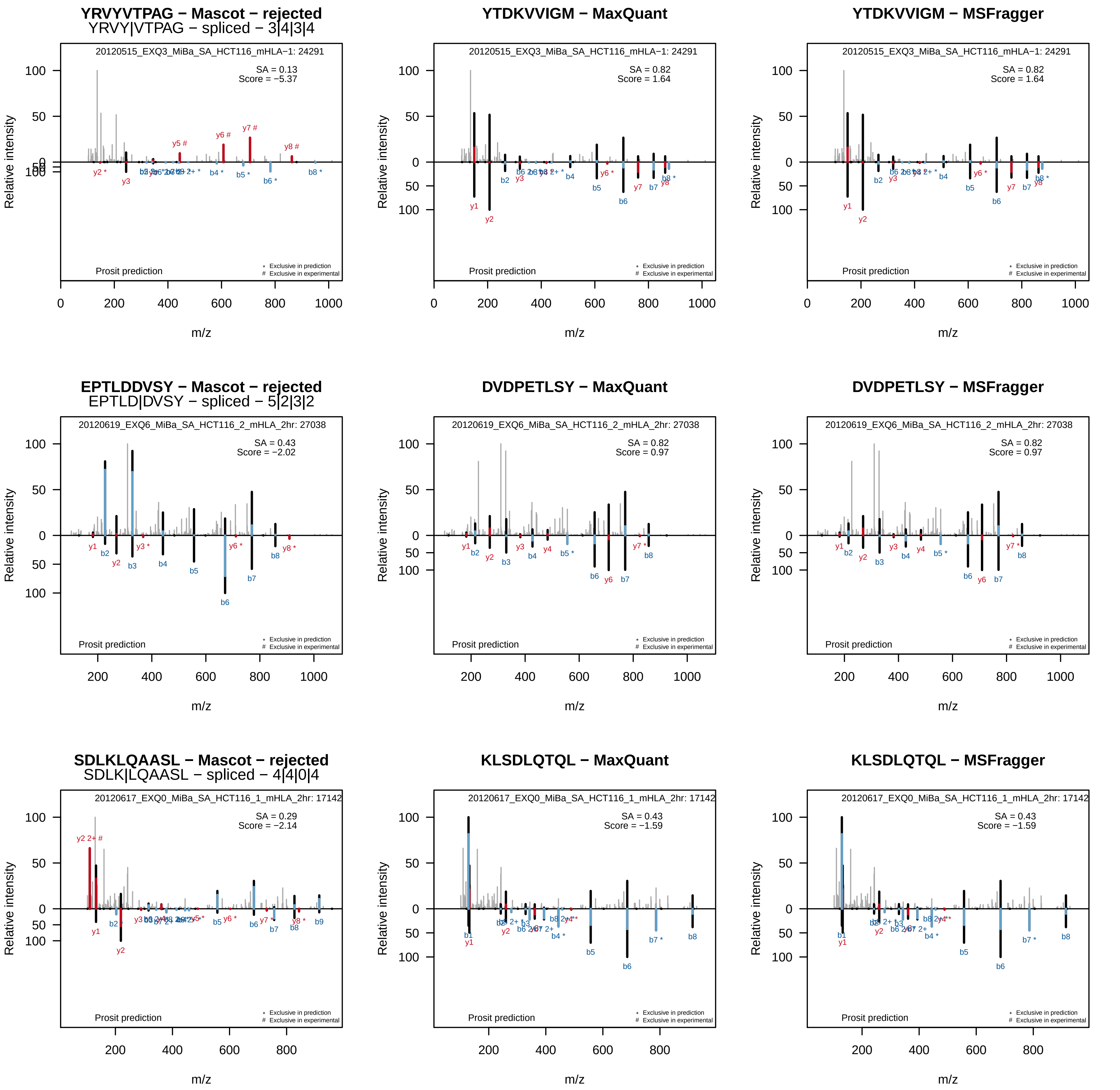

YRVYVTPAG − Mascot − rejected
YRVY|VTPAG − spliced − 3|4|3|4
20120515_EXQ3_MiBa_SA_HCT116_mHLA−1: 24291
SA = 0.13
Score = −5.37
YTDKVVIGM − MaxQuant
20120515_EXQ3_MiBa_SA_HCT116_mHLA−1: 24291
SA = 0.82
Score = 1.64
YTDKVVIGM − MSFragger
20120515_EXQ3_MiBa_SA_HCT116_mHLA−1: 24291
SA = 0.82
Score = 1.64
EPTLDDVSY − Mascot − rejected
EPTLD|DVSY − spliced − 5|2|3|2
20120619_EXQ6_MiBa_SA_HCT116_2_mHLA_2hr: 27038
SA = 0.43
Score = −2.02
DVDPETLSY − MaxQuant
20120619_EXQ6_MiBa_SA_HCT116_2_mHLA_2hr: 27038
SA = 0.82
Score = 0.97
DVDPETLSY − MSFragger
20120619_EXQ6_MiBa_SA_HCT116_2_mHLA_2hr: 27038
SA = 0.82
Score = 0.97
SDLKLQAASL − Mascot − rejected
SDLK|LQAASL − spliced − 4|4|0|4
20120617_EXQ0_MiBa_SA_HCT116_1_mHLA_2hr: 17142
SA = 0.29
Score = −2.14
KLSDLQTQL − MaxQuant
20120617_EXQ0_MiBa_SA_HCT116_1_mHLA_2hr: 17142
SA = 0.43
Score = −1.59
KLSDLQTQL − MSFragger
20120617_EXQ0_MiBa_SA_HCT116_1_mHLA_2hr: 17142
SA = 0.43
Score = −1.59
Relative intensity
m/z
Prosit prediction
⋆ Exclusive in prediction
Exclusive in experimental

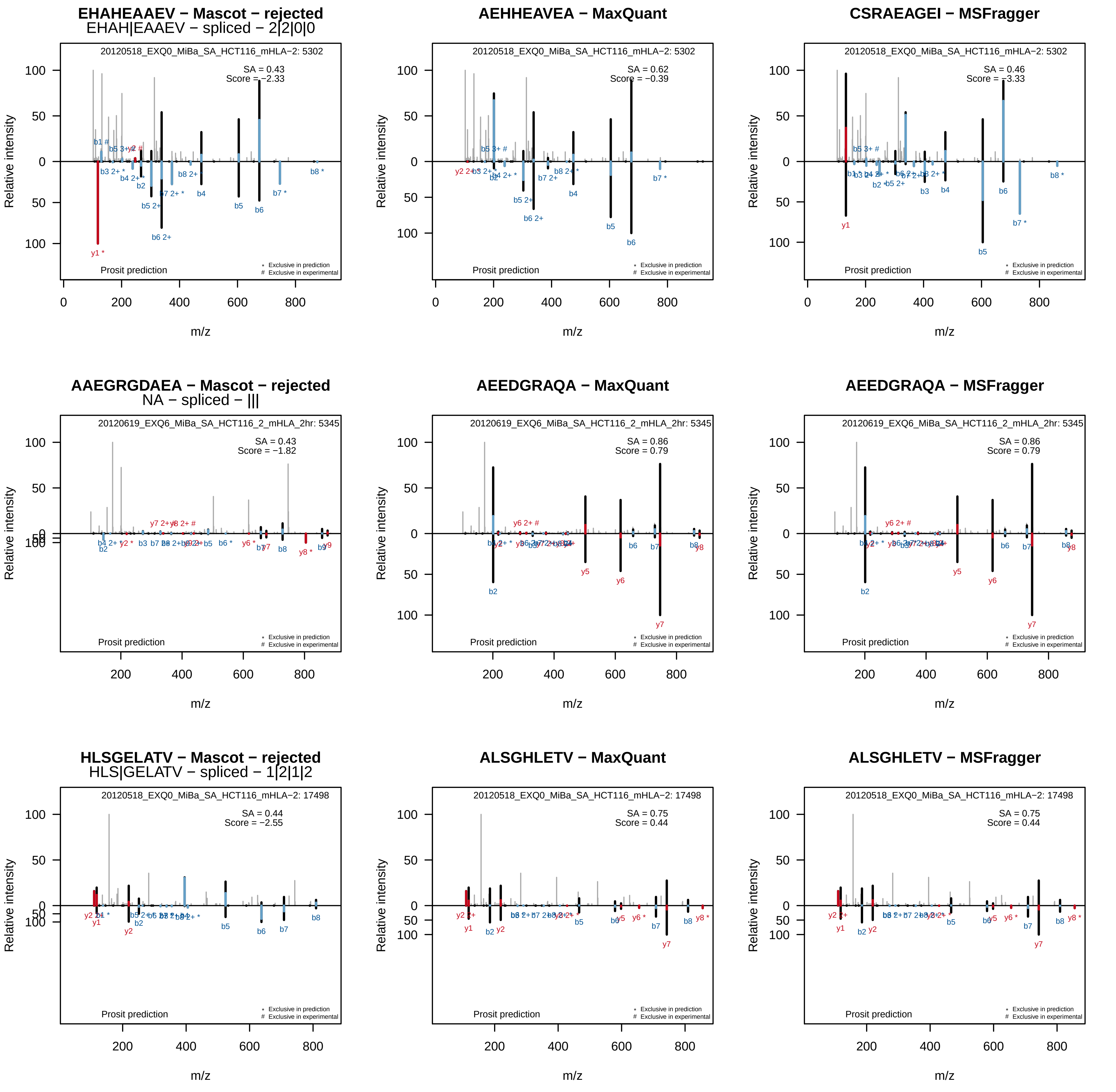
EHAHEAAEV − Mascot − rejected
EHAH|EAAEV − spliced − 2|2|0|0
20120518_EXQ0_MiBa_SA_HCT116_mHLA−2: 5302
SA = 0.43
Score = −2.33
AEHHEAVEA − MaxQuant
20120518_EXQ0_MiBa_SA_HCT116_mHLA−2: 5302
SA = 0.62
Score = −0.39
CSRAEAGEI − MSFragger
20120518_EXQ0_MiBa_SA_HCT116_mHLA−2: 5302
SA = 0.46
Score = −3.33
AAEGRGDAEA − Mascot − rejected
NA − spliced − |||
20120619_EXQ6_MiBa_SA_HCT116_2_mHLA_2hr: 5345
SA = 0.43
Score = −1.82
AEEDGRAQA − MaxQuant
20120619_EXQ6_MiBa_SA_HCT116_2_mHLA_2hr: 5345
SA = 0.86
Score = 0.79
AEEDGRAQA − MSFragger
20120619_EXQ6_MiBa_SA_HCT116_2_mHLA_2hr: 5345
SA = 0.86
Score = 0.79
HLSGELATV − Mascot − rejected
HLS|GELATV − spliced − 1|2|1|2
20120518_EXQ0_MiBa_SA_HCT116_mHLA−2: 17498
SA = 0.44
Score = −2.55
ALSGHLETV − MaxQuant
20120518_EXQ0_MiBa_SA_HCT116_mHLA−2: 17498
SA = 0.75
Score = 0.44
ALSGHLETV − MSFragger
20120518_EXQ0_MiBa_SA_HCT116_mHLA−2: 17498
SA = 0.75
Score = 0.44
Relative intensity
m/z
Prosit prediction
⋆ Exclusive in prediction
Exclusive in experimental

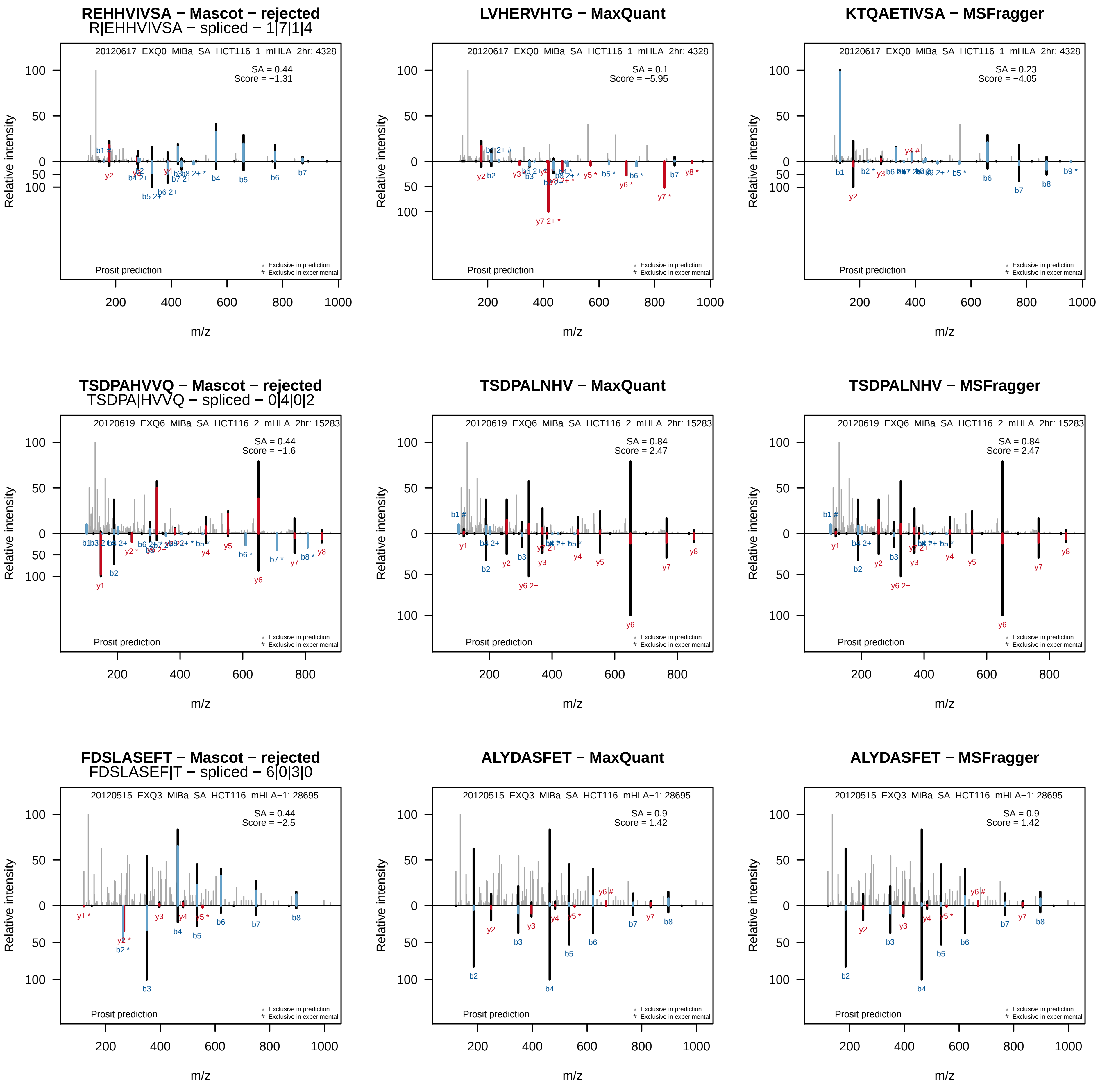

REHHVIVSA − Mascot − rejected
R|EHHVIVSA − spliced − 1|7|1|4
20120617_EXQ0_MiBa_SA_HCT116_1_mHLA_2hr: 4328
SA = 0.44
Score = −1.31
LVHERVHTG − MaxQuant
20120617_EXQ0_MiBa_SA_HCT116_1_mHLA_2hr: 4328
SA = 0.1
Score = −5.95
KTQAETIVSA − MSFragger
20120617_EXQ0_MiBa_SA_HCT116_1_mHLA_2hr: 4328
SA = 0.23
Score = −4.05
TSDPAHVVQ − Mascot − rejected
TSDPA|HVVQ − spliced − 0|4|0|2
20120619_EXQ6_MiBa_SA_HCT116_2_mHLA_2hr: 15283
SA = 0.44
Score = −1.6
TSDPALNHV − MaxQuant
20120619_EXQ6_MiBa_SA_HCT116_2_mHLA_2hr: 15283
SA = 0.84
Score = 2.47
TSDPALNHV − MSFragger
20120619_EXQ6_MiBa_SA_HCT116_2_mHLA_2hr: 15283
SA = 0.84
Score = 2.47
FDSLASEFT − Mascot − rejected
FDSLASEF|T − spliced − 6|0|3|0
20120515_EXQ3_MiBa_SA_HCT116_mHLA−1: 28695
SA = 0.44
Score = −2.5
ALYDASFET − MaxQuant
20120515_EXQ3_MiBa_SA_HCT116_mHLA−1: 28695
SA = 0.9
Score = 1.42
ALYDASFET − MSFragger
20120515_EXQ3_MiBa_SA_HCT116_mHLA−1: 28695
SA = 0.9
Score = 1.42
Relative intensity
m/z
Prosit prediction
⋆ Exclusive in prediction
Exclusive in experimental

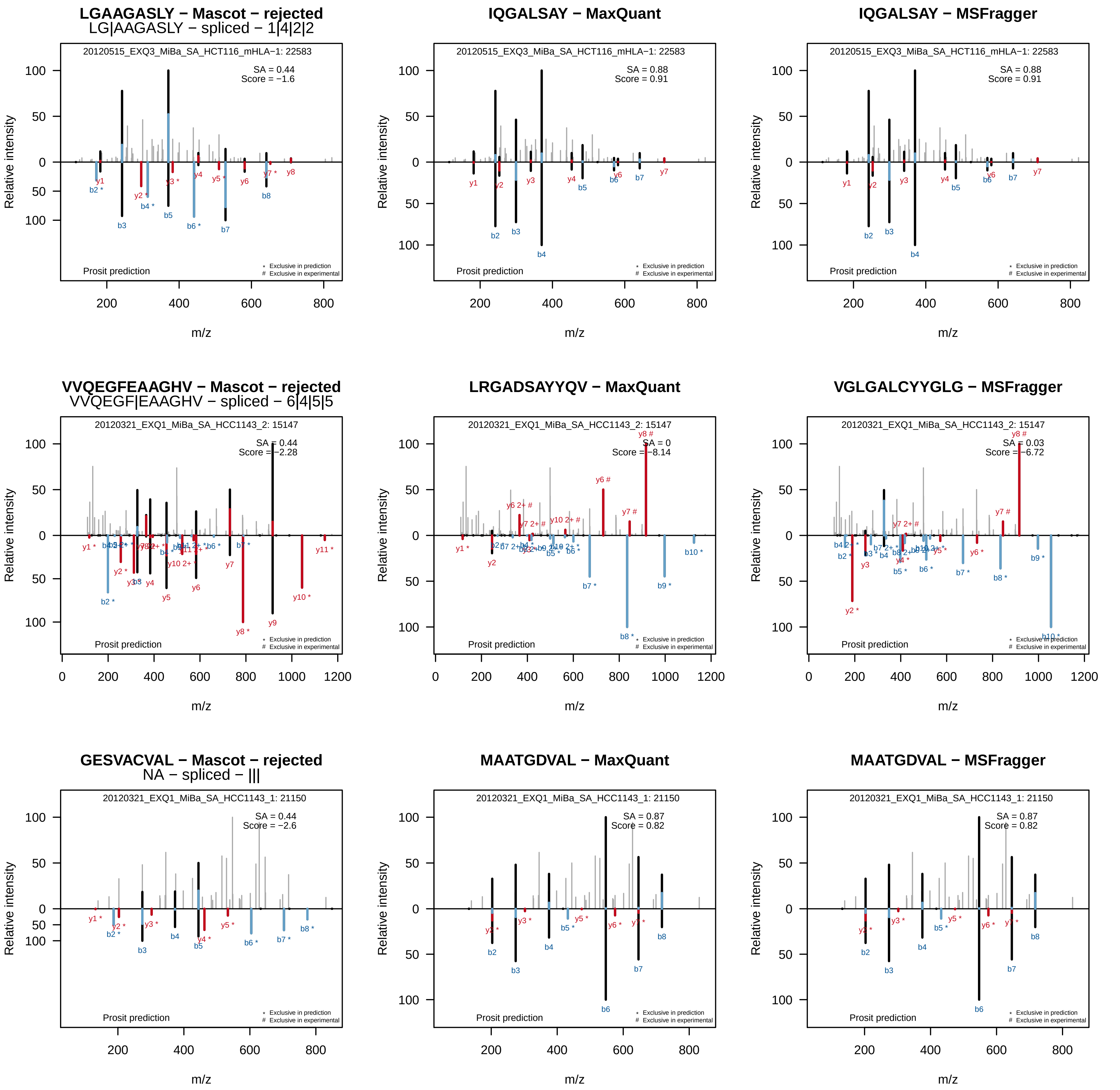
LGAAGASLY − Mascot − rejected
LG|AAGASLY − spliced − 1|4|2|2
20120515_EXQ3_MiBa_SA_HCT116_mHLA−1: 22583
SA = 0.44
Score = −1.6
IQGALSAY − MaxQuant
20120515_EXQ3_MiBa_SA_HCT116_mHLA−1: 22583
SA = 0.88
Score = 0.91
IQGALSAY − MSFragger
20120515_EXQ3_MiBa_SA_HCT116_mHLA−1: 22583
SA = 0.88
Score = 0.91
VVQEGFEAAGHV − Mascot − rejected
VVQEGF|EAAGHV − spliced − 6|4|5|5
20120321_EXQ1_MiBa_SA_HCC1143_2: 15147
SA = 0.44
Score = −2.28
LRGADSAYYQV − MaxQuant
20120321_EXQ1_MiBa_SA_HCC1143_2: 15147
SA = 0
Score = −8.14
VGLGALCYYGLG − MSFragger
20120321_EXQ1_MiBa_SA_HCC1143_2: 15147
SA = 0.03
Score = −6.72
GESVACVAL − Mascot − rejected
NA − spliced − |||
20120321_EXQ1_MiBa_SA_HCC1143_1: 21150
SA = 0.44
Score = −2.6
MAATGDVAL − MaxQuant
20120321_EXQ1_MiBa_SA_HCC1143_1: 21150
SA = 0.87
Score = 0.82
MAATGDVAL − MSFragger
20120321_EXQ1_MiBa_SA_HCC1143_1: 21150
SA = 0.87
Score = 0.82
Relative intensity
m/z
Prosit prediction
* Exclusive in prediction
# Exclusive in experimental

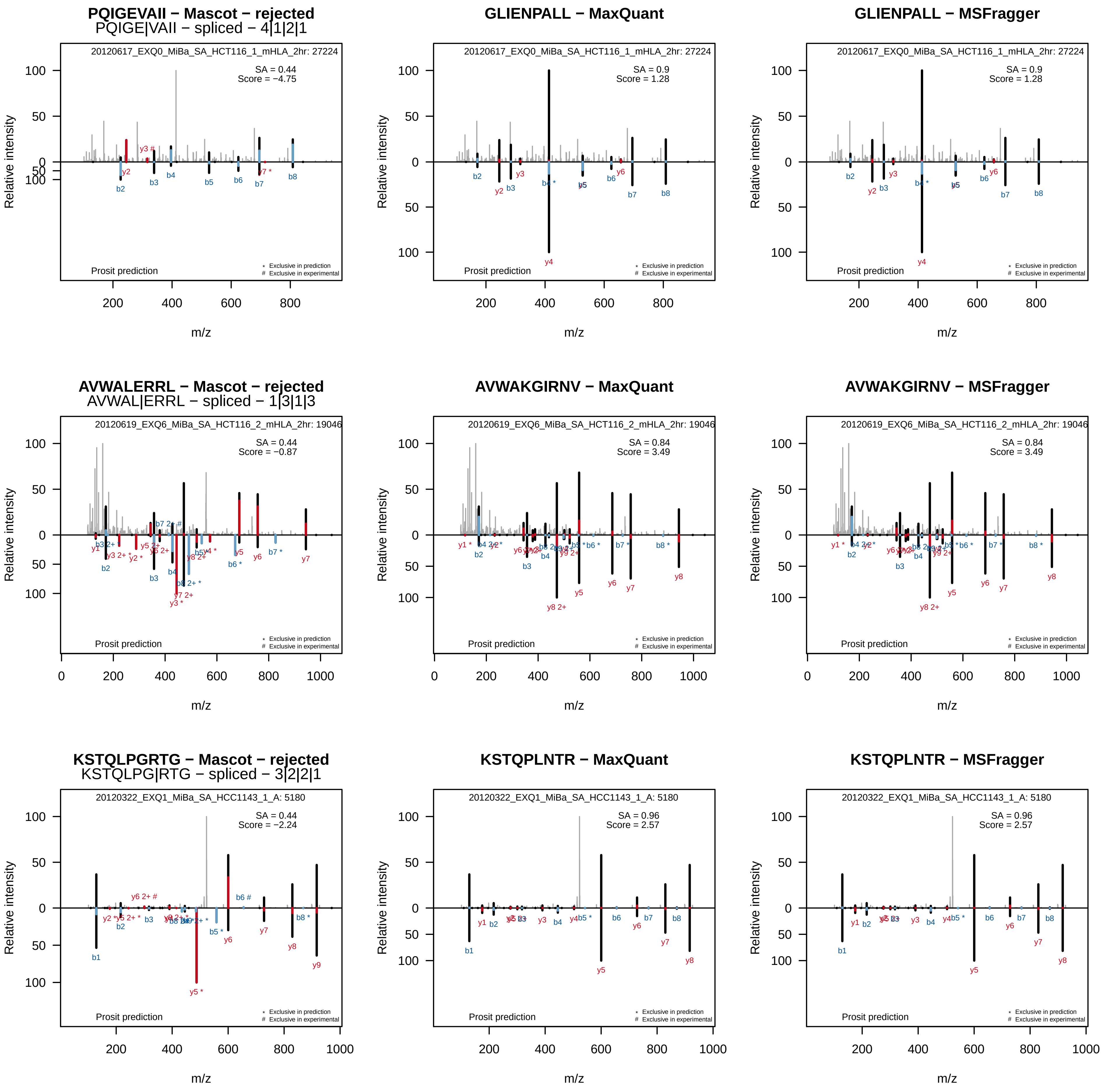
PQIGEVAII − Mascot − rejected
PQIGE|VAII − spliced − 4|1|2|1
20120617_EXQ0_MiBa_SA_HCT116_1_mHLA_2hr: 27224
SA = 0.44
Score = −4.75
GLIENPALL − MaxQuant
20120617_EXQ0_MiBa_SA_HCT116_1_mHLA_2hr: 27224
SA = 0.9
Score = 1.28
GLIENPALL − MSFragger
20120617_EXQ0_MiBa_SA_HCT116_1_mHLA_2hr: 27224
SA = 0.9
Score = 1.28
AVWALERRL − Mascot − rejected
AVWAL|ERRL − spliced − 1|3|1|3
20120619_EXQ6_MiBa_SA_HCT116_2_mHLA_2hr: 19046
SA = 0.44
Score = −0.87
AVWAKGIRNV − MaxQuant
20120619_EXQ6_MiBa_SA_HCT116_2_mHLA_2hr: 19046
SA = 0.84
Score = 3.49
AVWAKGIRNV − MSFragger
20120619_EXQ6_MiBa_SA_HCT116_2_mHLA_2hr: 19046
SA = 0.84
Score = 3.49
KSTQLPGRTG − Mascot − rejected
KSTQLPG|RTG − spliced − 3|2|2|1
20120322_EXQ1_MiBa_SA_HCC1143_1_A: 5180
SA = 0.44
Score = −2.24
KSTQPLNTR − MaxQuant
20120322_EXQ1_MiBa_SA_HCC1143_1_A: 5180
SA = 0.96
Score = 2.57
KSTQPLNTR − MSFragger
20120322_EXQ1_MiBa_SA_HCC1143_1_A: 5180
SA = 0.96
Score = 2.57
Relative intensity
m/z
Prosit prediction
* Exclusive in prediction
# Exclusive in experimental

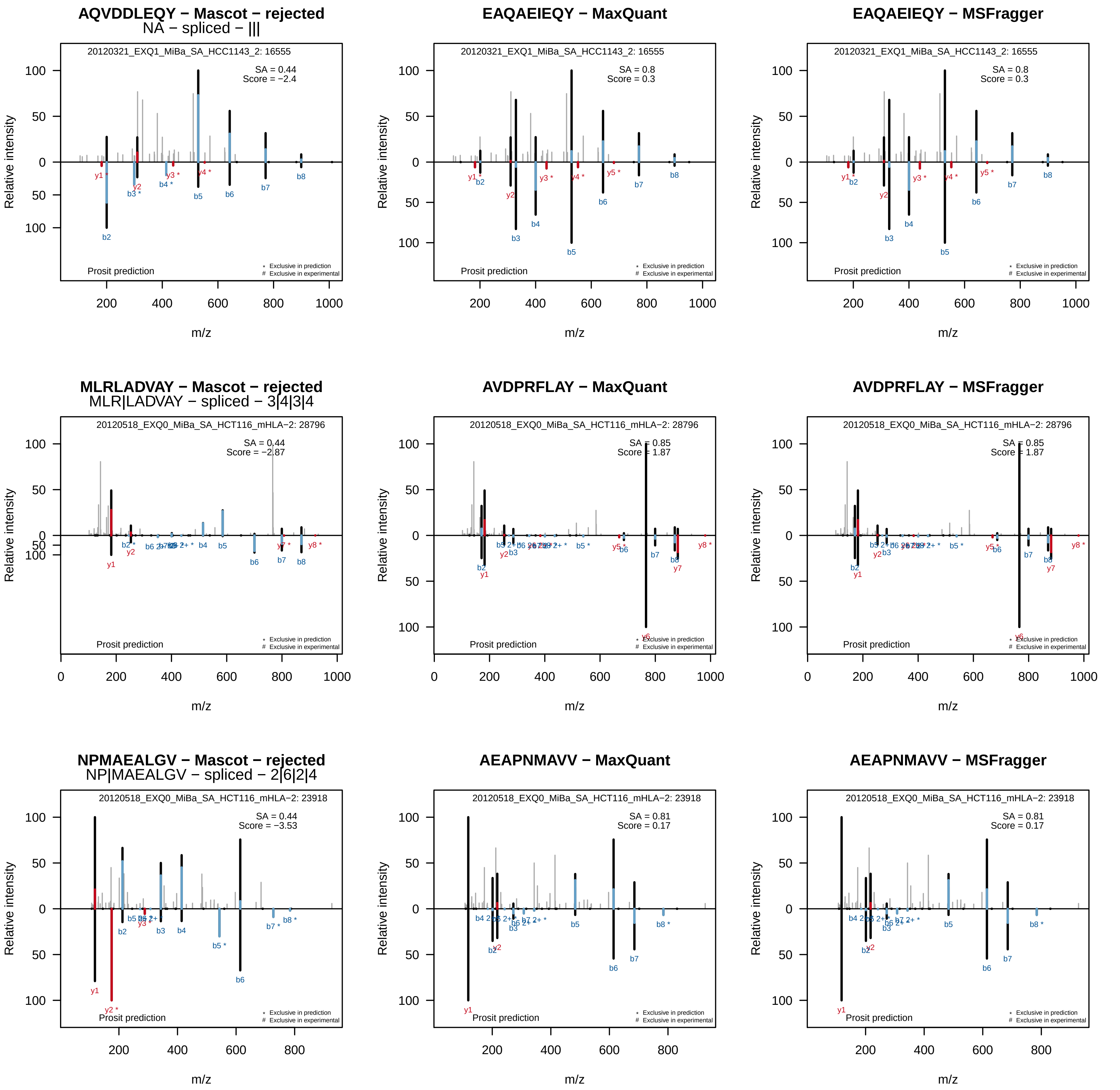
AQVDDLEQY − Mascot − rejected
NA − spliced − |||
20120321_EXQ1_MiBa_SA_HCC1143_2: 16555
SA = 0.44
Score = −2.4
Prosit prediction
EAQAEIEQY − MaxQuant
20120321_EXQ1_MiBa_SA_HCC1143_2: 16555
SA = 0.8
Score = 0.3
EAQAEIEQY − MSFragger
MLRLADVAY − Mascot − rejected
MLR|LADVAY − spliced − 3|4|3|4
20120518_EXQ0_MiBa_SA_HCT116_mHLA−2: 28796
SA = 0.44
Score = −2.87
AVDPRFLAY − MaxQuant
SA = 0.85
Score = 1.87
AVDPRFLAY − MSFragger
NPMAEALGV − Mascot − rejected
NP|MAEALGV − spliced − 2|6|2|4
20120518_EXQ0_MiBa_SA_HCT116_mHLA−2: 23918
SA = 0.44
Score = −3.53
AEAPNMAVV − MaxQuant
SA = 0.81
Score = 0.17
AEAPNMAVV − MSFragger
Relative intensity
m/z
⋆ Exclusive in prediction
Exclusive in experimental

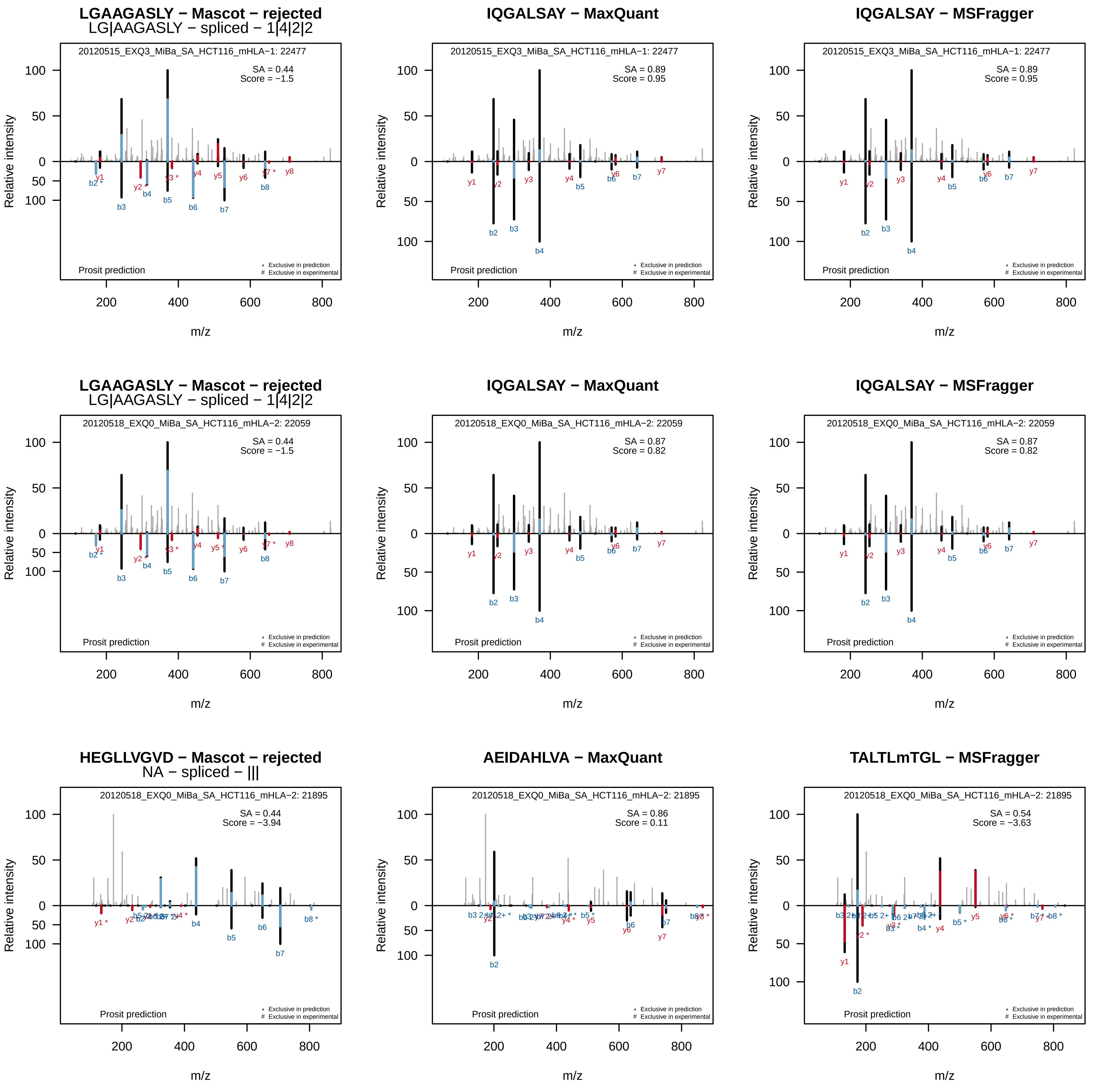

LGAAGASLY − Mascot − rejected
LG|AAGASLY − spliced − 1|4|2|2
20120515_EXQ3_MiBa_SA_HCT116_mHLA−1: 22477
SA = 0.44
Score = −1.5
IQGALSAY − MaxQuant
20120515_EXQ3_MiBa_SA_HCT116_mHLA−1: 22477
SA = 0.89
Score = 0.95
IQGALSAY − MSFragger
20120515_EXQ3_MiBa_SA_HCT116_mHLA−1: 22477
SA = 0.89
Score = 0.95
LGAAGASLY − Mascot − rejected
LG|AAGASLY − spliced − 1|4|2|2
20120518_EXQ0_MiBa_SA_HCT116_mHLA−2: 22059
SA = 0.44
Score = −1.5
IQGALSAY − MaxQuant
20120518_EXQ0_MiBa_SA_HCT116_mHLA−2: 22059
SA = 0.87
Score = 0.82
IQGALSAY − MSFragger
20120518_EXQ0_MiBa_SA_HCT116_mHLA−2: 22059
SA = 0.87
Score = 0.82
HEGLLVGVD − Mascot − rejected
NA − spliced − |||
20120518_EXQ0_MiBa_SA_HCT116_mHLA−2: 21895
SA = 0.44
Score = −3.94
AEIDAHLVA − MaxQuant
20120518_EXQ0_MiBa_SA_HCT116_mHLA−2: 21895
SA = 0.86
Score = 0.11
TALTLmTGL − MSFragger
20120518_EXQ0_MiBa_SA_HCT116_mHLA−2: 21895
SA = 0.54
Score = −3.63
Relative intensity
m/z
Prosit prediction
Exclusive in prediction
Exclusive in experimental

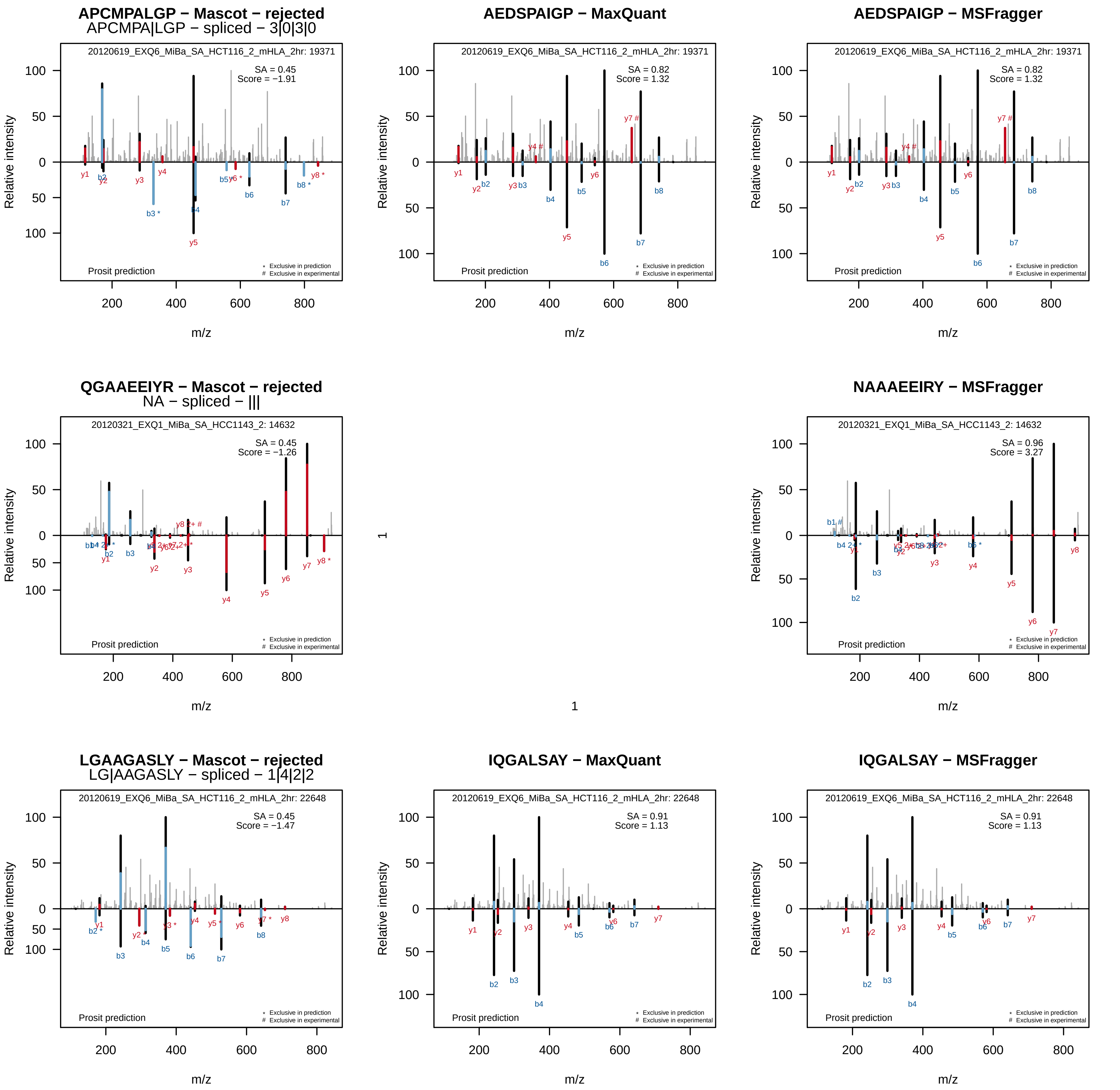
APCMPALGP − Mascot − rejected
APCMPA|LGP − spliced − 3|0|3|0
20120619_EXQ6_MiBa_SA_HCT116_2_mHLA_2hr: 19371
SA = 0.45
Score = −1.91
AEDSPAIGP − MaxQuant
20120619_EXQ6_MiBa_SA_HCT116_2_mHLA_2hr: 19371
SA = 0.82
Score = 1.32
AEDSPAIGP − MSFragger
20120619_EXQ6_MiBa_SA_HCT116_2_mHLA_2hr: 19371
SA = 0.82
Score = 1.32
QGAAEEIYR − Mascot − rejected
NA − spliced − |||
20120321_EXQ1_MiBa_SA_HCC1143_2: 14632
SA = 0.45
Score = −1.26
NAAAEEIRY − MSFragger
20120321_EXQ1_MiBa_SA_HCC1143_2: 14632
SA = 0.96
Score = 3.27
LGAAGASLY − Mascot − rejected
LG|AAGASLY − spliced − 1|4|2|2
20120619_EXQ6_MiBa_SA_HCT116_2_mHLA_2hr: 22648
SA = 0.45
Score = −1.47
IQGALSAY − MaxQuant
20120619_EXQ6_MiBa_SA_HCT116_2_mHLA_2hr: 22648
SA = 0.91
Score = 1.13
IQGALSAY − MSFragger
20120619_EXQ6_MiBa_SA_HCT116_2_mHLA_2hr: 22648
SA = 0.91
Score = 1.13
Relative intensity
m/z
Prosit prediction
* Exclusive in prediction
# Exclusive in experimental
1

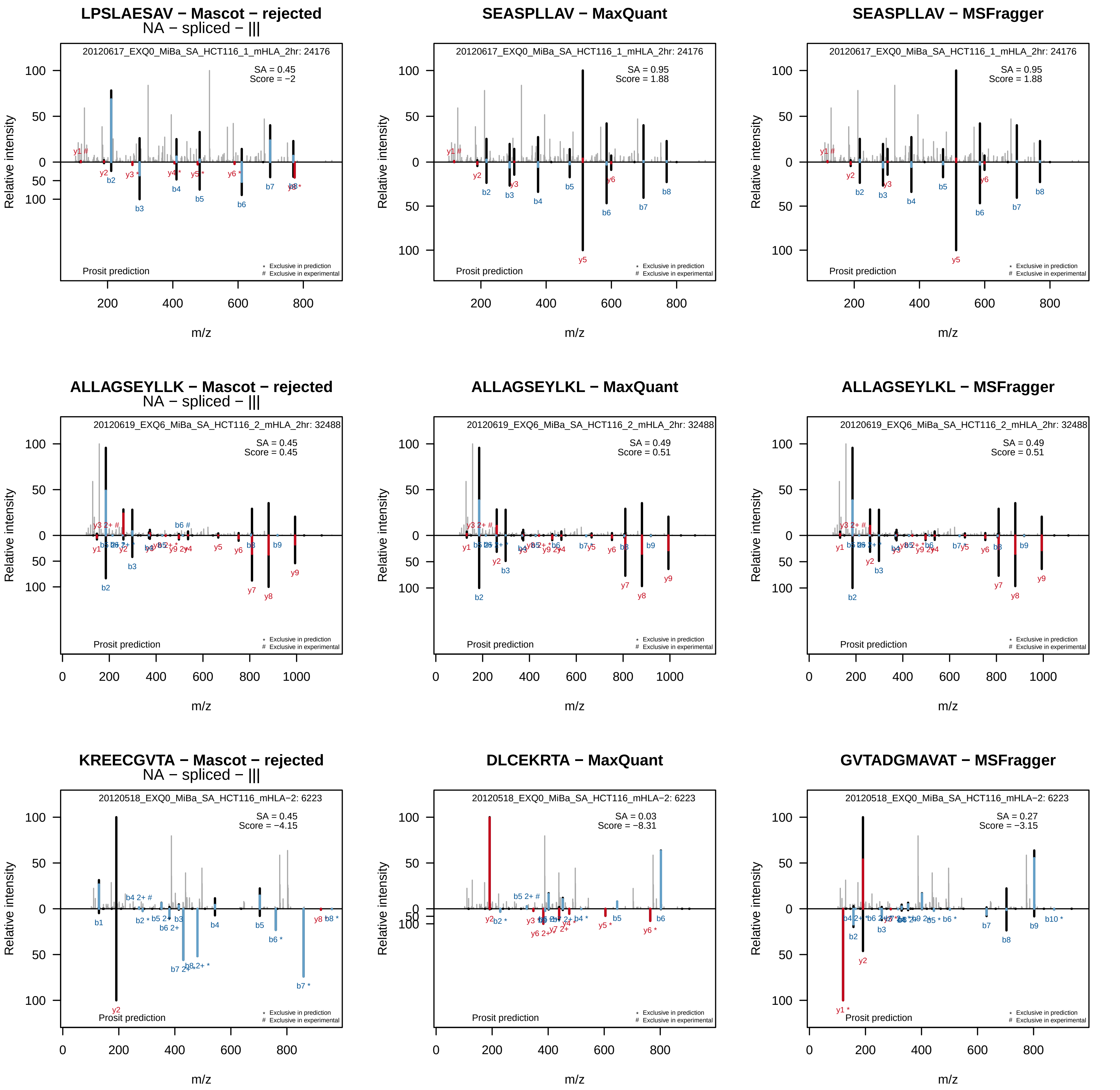

LPSLAESAV − Mascot − rejected
NA − spliced − |||
20120617_EXQ0_MiBa_SA_HCT116_1_mHLA_2hr: 24176
SA = 0.45
Score = −2
SEASPLLAV − MaxQuant
20120617_EXQ0_MiBa_SA_HCT116_1_mHLA_2hr: 24176
SA = 0.95
Score = 1.88
SEASPLLAV − MSFragger
20120617_EXQ0_MiBa_SA_HCT116_1_mHLA_2hr: 24176
SA = 0.95
Score = 1.88
ALLAGSEYLLK − Mascot − rejected
NA − spliced − |||
20120619_EXQ6_MiBa_SA_HCT116_2_mHLA_2hr: 32488
SA = 0.45
Score = 0.45
ALLAGSEYLKL − MaxQuant
20120619_EXQ6_MiBa_SA_HCT116_2_mHLA_2hr: 32488
SA = 0.49
Score = 0.51
ALLAGSEYLKL − MSFragger
20120619_EXQ6_MiBa_SA_HCT116_2_mHLA_2hr: 32488
SA = 0.49
Score = 0.51
KREECGVTA − Mascot − rejected
NA − spliced − |||
20120518_EXQ0_MiBa_SA_HCT116_mHLA−2: 6223
SA = 0.45
Score = −4.15
DLCEKRTA − MaxQuant
20120518_EXQ0_MiBa_SA_HCT116_mHLA−2: 6223
SA = 0.03
Score = −8.31
GVTADGMAVAT − MSFragger
20120518_EXQ0_MiBa_SA_HCT116_mHLA−2: 6223
SA = 0.27
Score = −3.15
Relative intensity
m/z
Prosit prediction
* Exclusive in prediction
# Exclusive in experimental

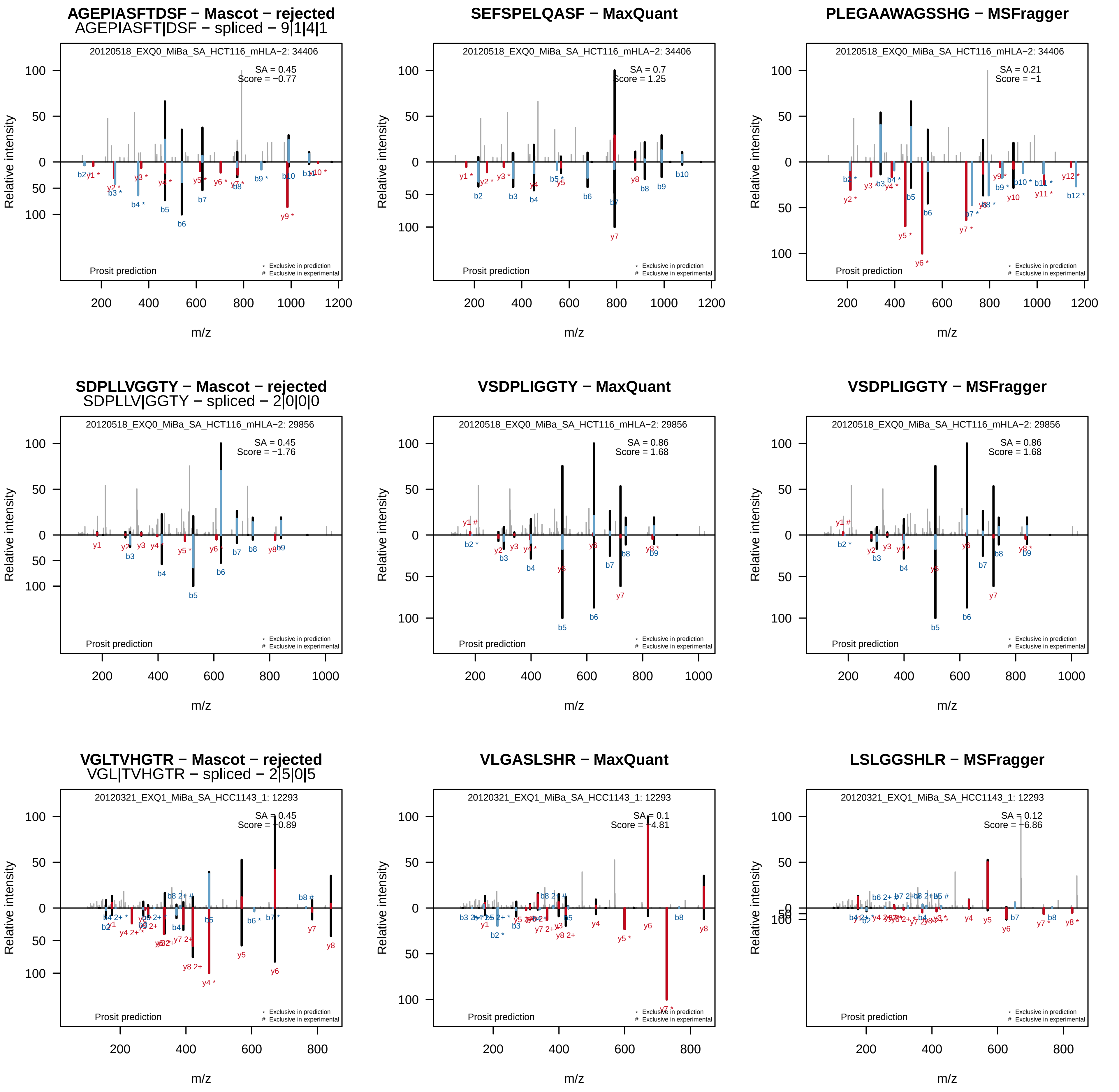
AGEPIASFTDSF − Mascot − rejected
AGEPIASFT|DSF − spliced − 9|1|4|1
20120518_EXQ0_MiBa_SA_HCT116_mHLA−2: 34406
SA = 0.45
Score = −0.77
SEFSPELQASF − MaxQuant
20120518_EXQ0_MiBa_SA_HCT116_mHLA−2: 34406
SA = 0.7
Score = 1.25
PLEGAAWAGSSHG − MSFragger
20120518_EXQ0_MiBa_SA_HCT116_mHLA−2: 34406
SA = 0.21
Score = −1
SDPLLVGGTY − Mascot − rejected
SDPLLV|GGTY − spliced − 2|0|0|0
20120518_EXQ0_MiBa_SA_HCT116_mHLA−2: 29856
SA = 0.45
Score = −1.76
VSDPLIGGTY − MaxQuant
20120518_EXQ0_MiBa_SA_HCT116_mHLA−2: 29856
SA = 0.86
Score = 1.68
VSDPLIGGTY − MSFragger
20120518_EXQ0_MiBa_SA_HCT116_mHLA−2: 29856
SA = 0.86
Score = 1.68
VGLTVHGTR − Mascot − rejected
VGL|TVHGTR − spliced − 2|5|0|5
20120321_EXQ1_MiBa_SA_HCC1143_1: 12293
SA = 0.45
Score = −0.89
VLGASLSHR − MaxQuant
20120321_EXQ1_MiBa_SA_HCC1143_1: 12293
SA = 0.1
Score = −4.81
LSLGGSHLR − MSFragger
20120321_EXQ1_MiBa_SA_HCC1143_1: 12293
SA = 0.12
Score = −6.86
Relative intensity
m/z
Prosit prediction
* Exclusive in prediction
# Exclusive in experimental

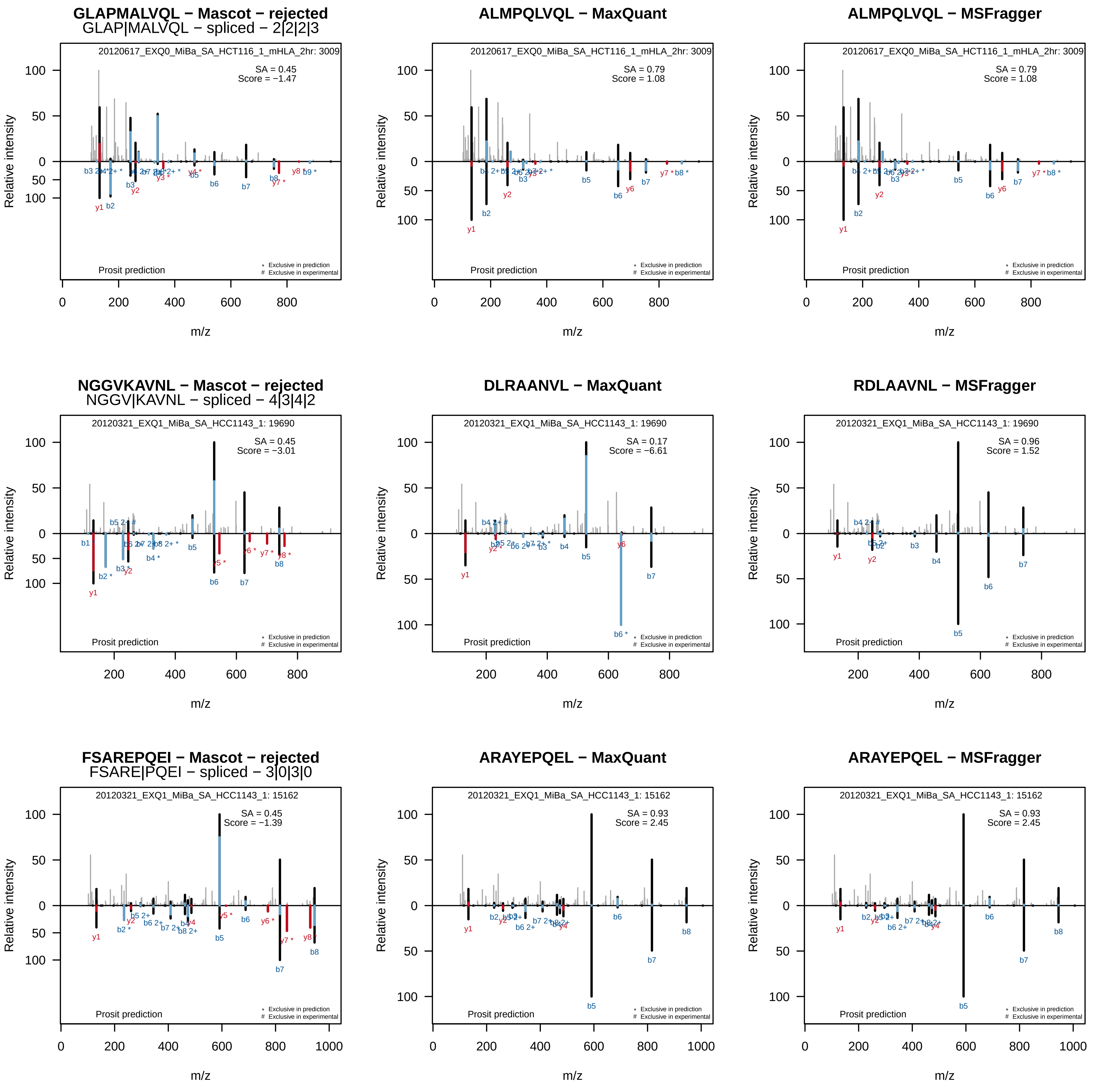
GLAPMALVQL − Mascot − rejected
GLAP|MALVQL − spliced − 2|2|2|3
20120617_EXQ0_MiBa_SA_HCT116_1_mHLA_2hr: 3009
SA = 0.45
Score = −1.47
ALMPQLVQL − MaxQuant
20120617_EXQ0_MiBa_SA_HCT116_1_mHLA_2hr: 3009
SA = 0.79
Score = 1.08
ALMPQLVQL − MSFragger
20120617_EXQ0_MiBa_SA_HCT116_1_mHLA_2hr: 3009
SA = 0.79
Score = 1.08
NGGVKAVNL − Mascot − rejected
NGGV|KAVNL − spliced − 4|3|4|2
20120321_EXQ1_MiBa_SA_HCC1143_1: 19690
SA = 0.45
Score = −3.01
DLRAANVL − MaxQuant
20120321_EXQ1_MiBa_SA_HCC1143_1: 19690
SA = 0.17
Score = −6.61
RDLAAVNL − MSFragger
20120321_EXQ1_MiBa_SA_HCC1143_1: 19690
SA = 0.96
Score = 1.52
FSAREPQEI − Mascot − rejected
FSARE|PQEI − spliced − 3|0|3|0
20120321_EXQ1_MiBa_SA_HCC1143_1: 15162
SA = 0.45
Score = −1.39
ARAYEPQEL − MaxQuant
20120321_EXQ1_MiBa_SA_HCC1143_1: 15162
SA = 0.93
Score = 2.45
ARAYEPQEL − MSFragger
20120321_EXQ1_MiBa_SA_HCC1143_1: 15162
SA = 0.93
Score = 2.45
Relative intensity
m/z
Prosit prediction
⋆ Exclusive in prediction
Exclusive in experimental

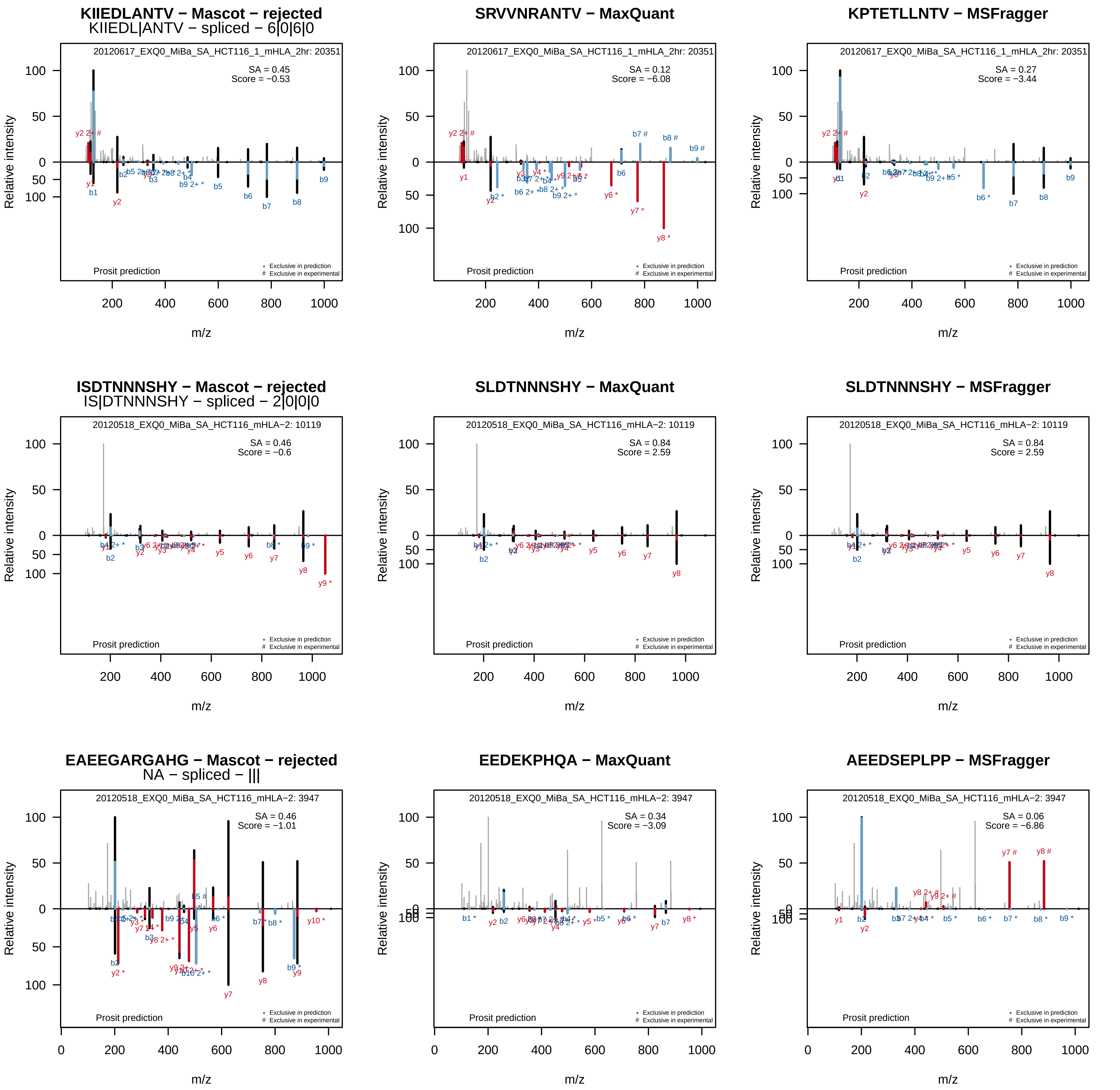
KIIEDLANTV − Mascot − rejected
KIIEDL|ANTV − spliced − 6|0|6|0
20120617_EXQ0_MiBa_SA_HCT116_1_mHLA_2hr: 20351
SA = 0.45
Score = −0.53
SRVVNRANTV − MaxQuant
20120617_EXQ0_MiBa_SA_HCT116_1_mHLA_2hr: 20351
SA = 0.12
Score = −6.08
KPTETLLNTV − MSFragger
20120617_EXQ0_MiBa_SA_HCT116_1_mHLA_2hr: 20351
SA = 0.27
Score = −3.44
ISDTNNNSHY − Mascot − rejected
IS|DTNNNSHY − spliced − 2|0|0|0
20120518_EXQ0_MiBa_SA_HCT116_mHLA−2: 10119
SA = 0.46
Score = −0.6
SLDTNNNSHY − MaxQuant
20120518_EXQ0_MiBa_SA_HCT116_mHLA−2: 10119
SA = 0.84
Score = 2.59
SLDTNNNSHY − MSFragger
20120518_EXQ0_MiBa_SA_HCT116_mHLA−2: 10119
SA = 0.84
Score = 2.59
EAEEGARGAHG − Mascot − rejected
NA − spliced − |||
20120518_EXQ0_MiBa_SA_HCT116_mHLA−2: 3947
SA = 0.46
Score = −1.01
EEDEKPHQA − MaxQuant
20120518_EXQ0_MiBa_SA_HCT116_mHLA−2: 3947
SA = 0.34
Score = −3.09
AEEDSEPLPP − MSFragger
20120518_EXQ0_MiBa_SA_HCT116_mHLA−2: 3947
SA = 0.06
Score = −6.86
Prosit prediction
* Exclusive in prediction
# Exclusive in experimental
Relative intensity
m/z

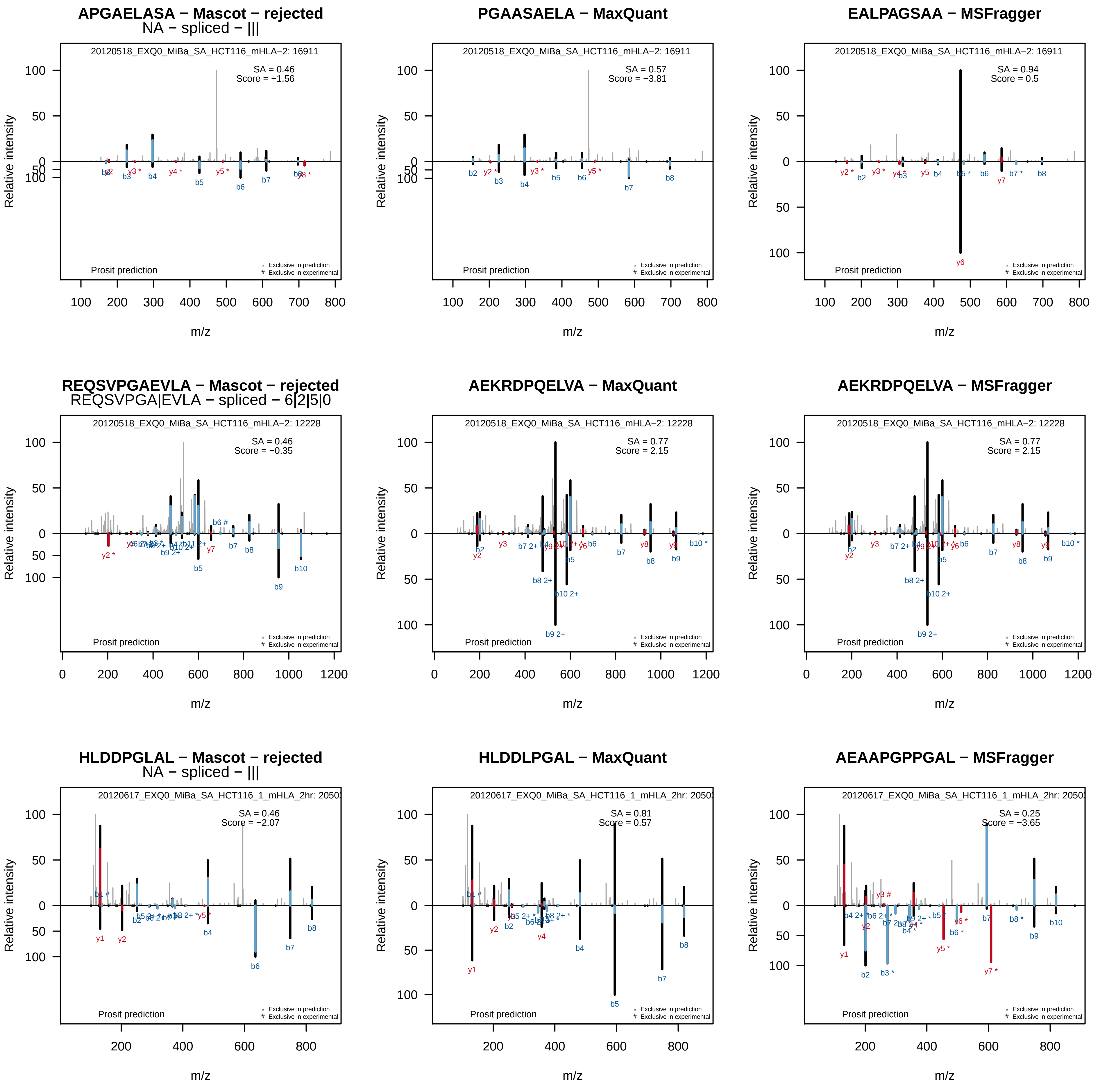
APGAELASA − Mascot − rejected
NA − spliced − |||
20120518_EXQ0_MiBa_SA_HCT116_mHLA−2: 16911
SA = 0.46
Score = −1.56
PGAASAELA − MaxQuant
20120518_EXQ0_MiBa_SA_HCT116_mHLA−2: 16911
SA = 0.57
Score = −3.81
EALPAGSAA − MSFragger
20120518_EXQ0_MiBa_SA_HCT116_mHLA−2: 16911
SA = 0.94
Score = 0.5
REQSVPGAEVLA − Mascot − rejected
REQSVPGA|EVLA − spliced − 6|2|5|0
20120518_EXQ0_MiBa_SA_HCT116_mHLA−2: 12228
SA = 0.46
Score = −0.35
AEKRDPQELVA − MaxQuant
20120518_EXQ0_MiBa_SA_HCT116_mHLA−2: 12228
SA = 0.77
Score = 2.15
AEKRDPQELVA − MSFragger
20120518_EXQ0_MiBa_SA_HCT116_mHLA−2: 12228
SA = 0.77
Score = 2.15
HLDDPGLAL − Mascot − rejected
NA − spliced − |||
20120617_EXQ0_MiBa_SA_HCT116_1_mHLA_2hr: 2050
SA = 0.46
Score = −2.07
HLDDLPGAL − MaxQuant
20120617_EXQ0_MiBa_SA_HCT116_1_mHLA_2hr: 2050
SA = 0.81
Score = 0.57
AEAAPGPPGAL − MSFragger
20120617_EXQ0_MiBa_SA_HCT116_1_mHLA_2hr: 2050
SA = 0.25
Score = −3.65
Relative intensity
m/z
Prosit prediction
* Exclusive in prediction
# Exclusive in experimental

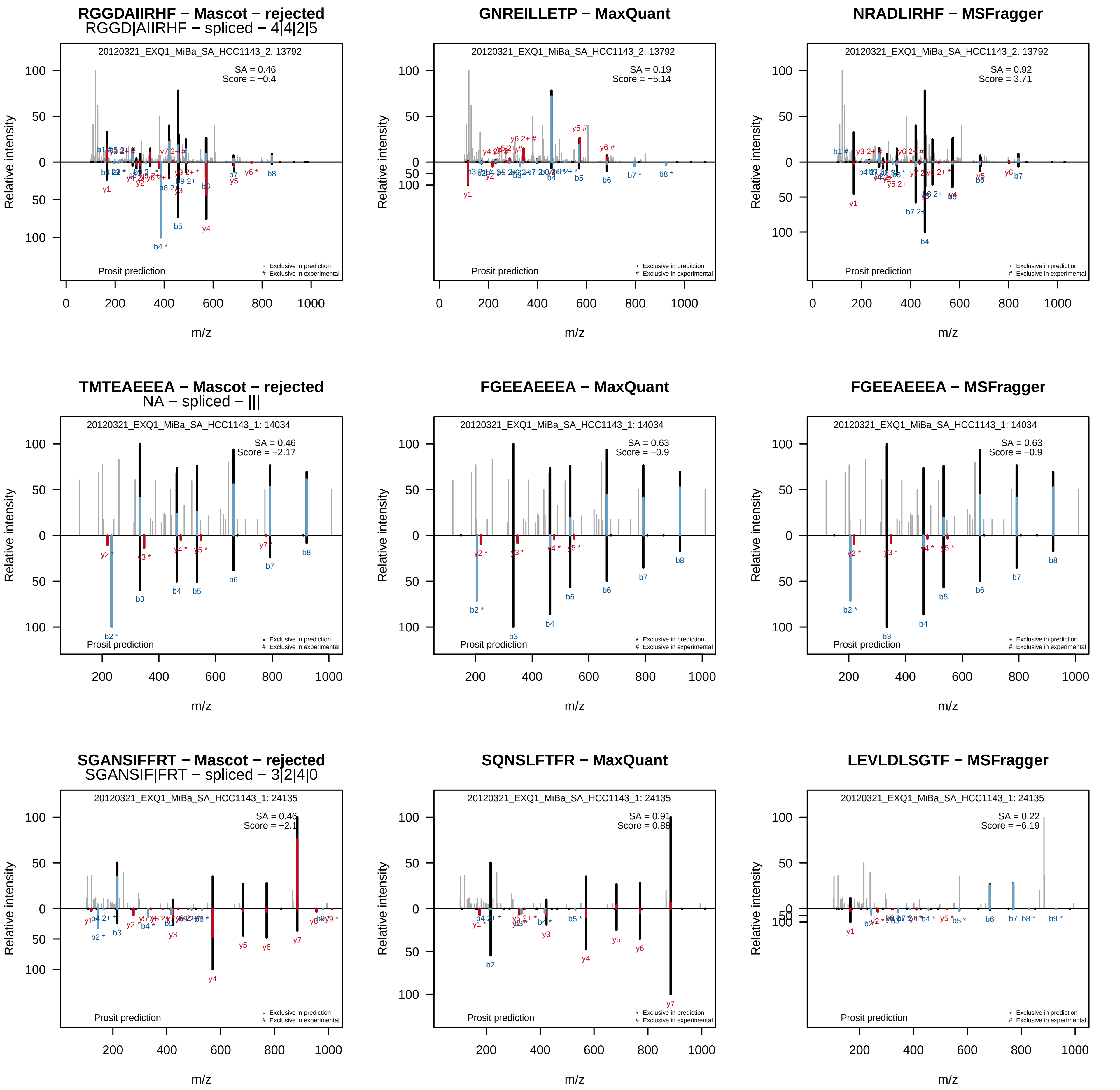

RGGDAIIRHF − Mascot − rejected
RGGD|AIIRHF − spliced − 4|4|2|5
20120321_EXQ1_MiBa_SA_HCC1143_2: 13792
SA = 0.46
Score = −0.4
GNREILLETP − MaxQuant
20120321_EXQ1_MiBa_SA_HCC1143_2: 13792
SA = 0.19
Score = −5.14
NRADLIRHF − MSFragger
20120321_EXQ1_MiBa_SA_HCC1143_2: 13792
SA = 0.92
Score = 3.71
TMTEAEEEA − Mascot − rejected
NA − spliced − |||
20120321_EXQ1_MiBa_SA_HCC1143_1: 14034
SA = 0.46
Score = −2.17
FGEEAEEEA − MaxQuant
20120321_EXQ1_MiBa_SA_HCC1143_1: 14034
SA = 0.63
Score = −0.9
FGEEAEEEA − MSFragger
20120321_EXQ1_MiBa_SA_HCC1143_1: 14034
SA = 0.63
Score = −0.9
SGANSIFFRT − Mascot − rejected
SGANSIF|FRT − spliced − 3|2|4|0
20120321_EXQ1_MiBa_SA_HCC1143_1: 24135
SA = 0.46
Score = −2.1
SQNSLFTFR − MaxQuant
20120321_EXQ1_MiBa_SA_HCC1143_1: 24135
SA = 0.91
Score = 0.88
LEVLDLSGTF − MSFragger
20120321_EXQ1_MiBa_SA_HCC1143_1: 24135
SA = 0.22
Score = −6.19
Relative intensity
m/z
Prosit prediction
⋆ Exclusive in prediction
Exclusive in experimental

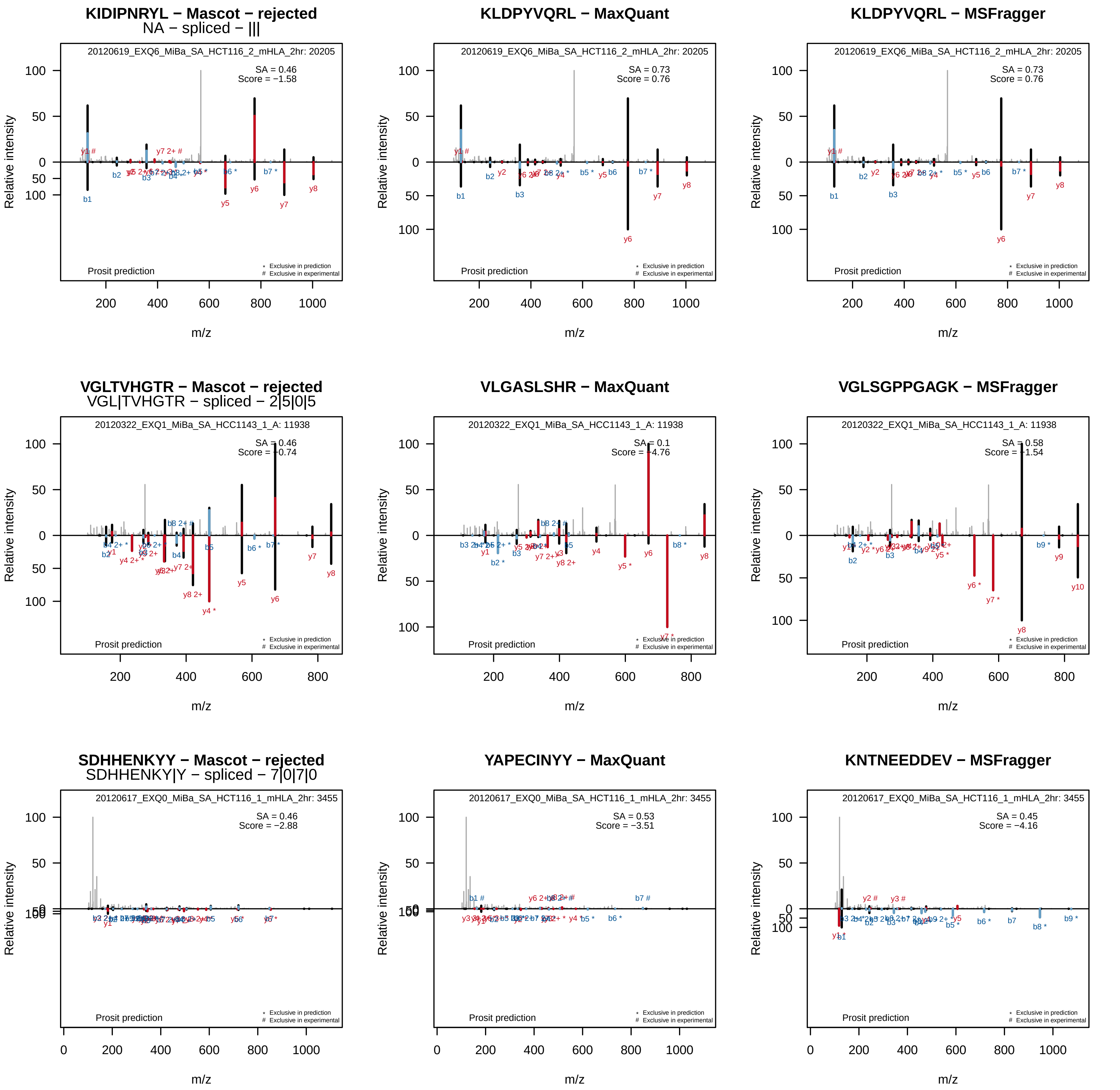
KIDIPNRYL − Mascot − rejected
NA − spliced − |||
20120619_EXQ6_MiBa_SA_HCT116_2_mHLA_2hr: 20205
SA = 0.46
Score = −1.58
KLDPYVQRL − MaxQuant
20120619_EXQ6_MiBa_SA_HCT116_2_mHLA_2hr: 20205
SA = 0.73
Score = 0.76
KLDPYVQRL − MSFragger
20120619_EXQ6_MiBa_SA_HCT116_2_mHLA_2hr: 20205
SA = 0.73
Score = 0.76
VGLTVHGTR − Mascot − rejected
VGL|TVHGTR − spliced − 2|5|0|5
20120322_EXQ1_MiBa_SA_HCC1143_1_A: 11938
SA = 0.46
Score = −0.74
VLGASLSHR − MaxQuant
20120322_EXQ1_MiBa_SA_HCC1143_1_A: 11938
SA = 0.1
Score = −4.76
VGLSGPPGAGK − MSFragger
20120322_EXQ1_MiBa_SA_HCC1143_1_A: 11938
SA = 0.58
Score = −1.54
SDHHENKYY − Mascot − rejected
SDHHENKY|Y − spliced − 7|0|7|0
20120617_EXQ0_MiBa_SA_HCT116_1_mHLA_2hr: 3455
SA = 0.46
Score = −2.88
YAPECINYY − MaxQuant
20120617_EXQ0_MiBa_SA_HCT116_1_mHLA_2hr: 3455
SA = 0.53
Score = −3.51
KNTNEEDDEV − MSFragger
20120617_EXQ0_MiBa_SA_HCT116_1_mHLA_2hr: 3455
SA = 0.45
Score = −4.16
Relative intensity
m/z
Prosit prediction
* Exclusive in prediction
# Exclusive in experimental

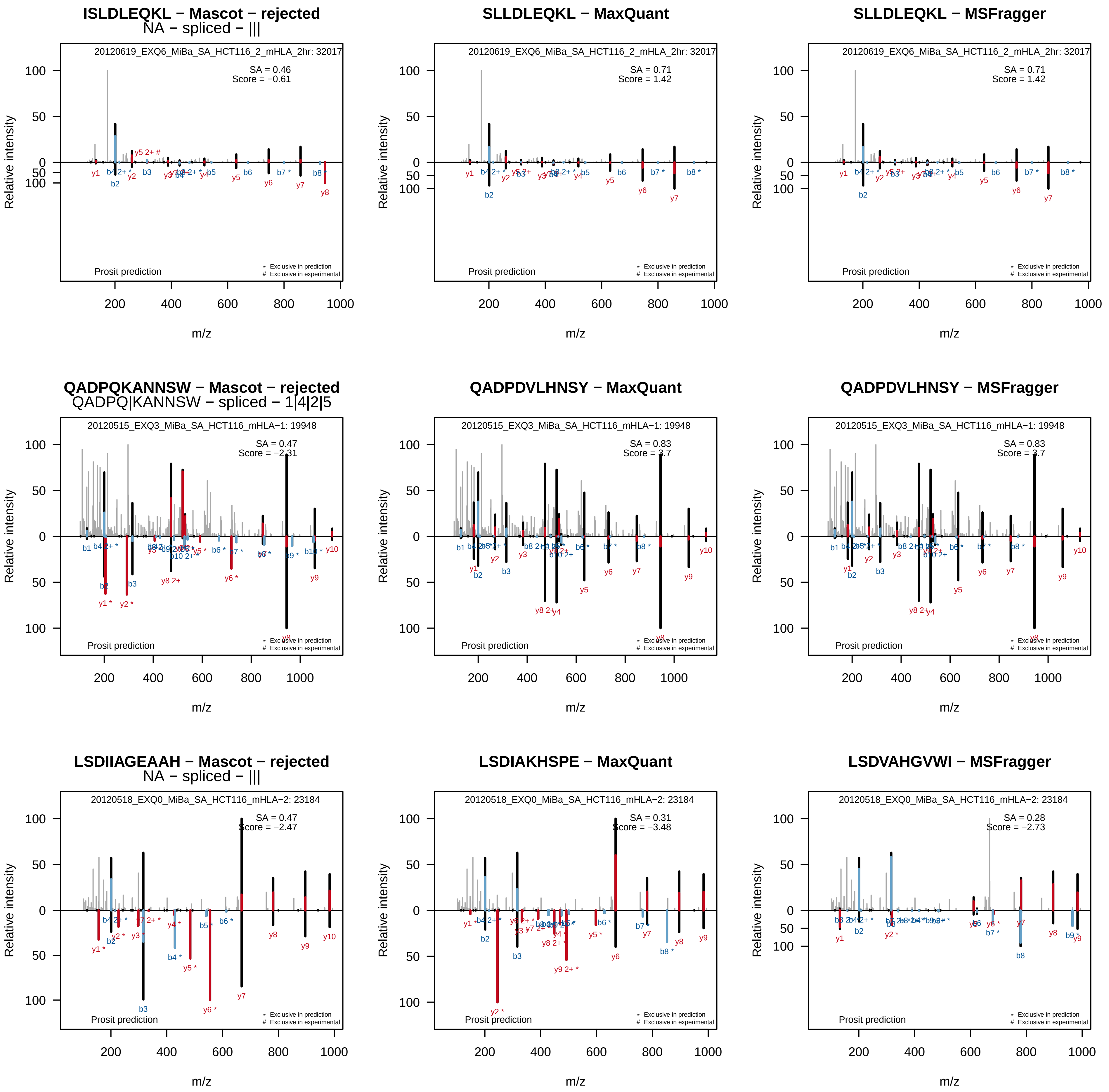
ISLDLEQKL − Mascot − rejected
NA − spliced − |||
20120619_EXQ6_MiBa_SA_HCT116_2_mHLA_2hr: 32017
SA = 0.46
Score = −0.61
SLLDLEQKL − MaxQuant
20120619_EXQ6_MiBa_SA_HCT116_2_mHLA_2hr: 32017
SA = 0.71
Score = 1.42
SLLDLEQKL − MSFragger
20120619_EXQ6_MiBa_SA_HCT116_2_mHLA_2hr: 32017
SA = 0.71
Score = 1.42
QADPQKANNSW − Mascot − rejected
QADPQ|KANNSW − spliced − 1|4|2|5
20120515_EXQ3_MiBa_SA_HCT116_mHLA−1: 19948
SA = 0.47
Score = −2.31
QADPDVLHNSY − MaxQuant
20120515_EXQ3_MiBa_SA_HCT116_mHLA−1: 19948
SA = 0.83
Score = 3.7
QADPDVLHNSY − MSFragger
20120515_EXQ3_MiBa_SA_HCT116_mHLA−1: 19948
SA = 0.83
Score = 3.7
LSDIIAGEAAH − Mascot − rejected
NA − spliced − |||
20120518_EXQ0_MiBa_SA_HCT116_mHLA−2: 23184
SA = 0.47
Score = −2.47
LSDIAKHSPE − MaxQuant
20120518_EXQ0_MiBa_SA_HCT116_mHLA−2: 23184
SA = 0.31
Score = −3.48
LSDVAHGVWI − MSFragger
20120518_EXQ0_MiBa_SA_HCT116_mHLA−2: 23184
SA = 0.28
Score = −2.73
Relative intensity
m/z
Prosit prediction
* Exclusive in prediction
# Exclusive in experimental

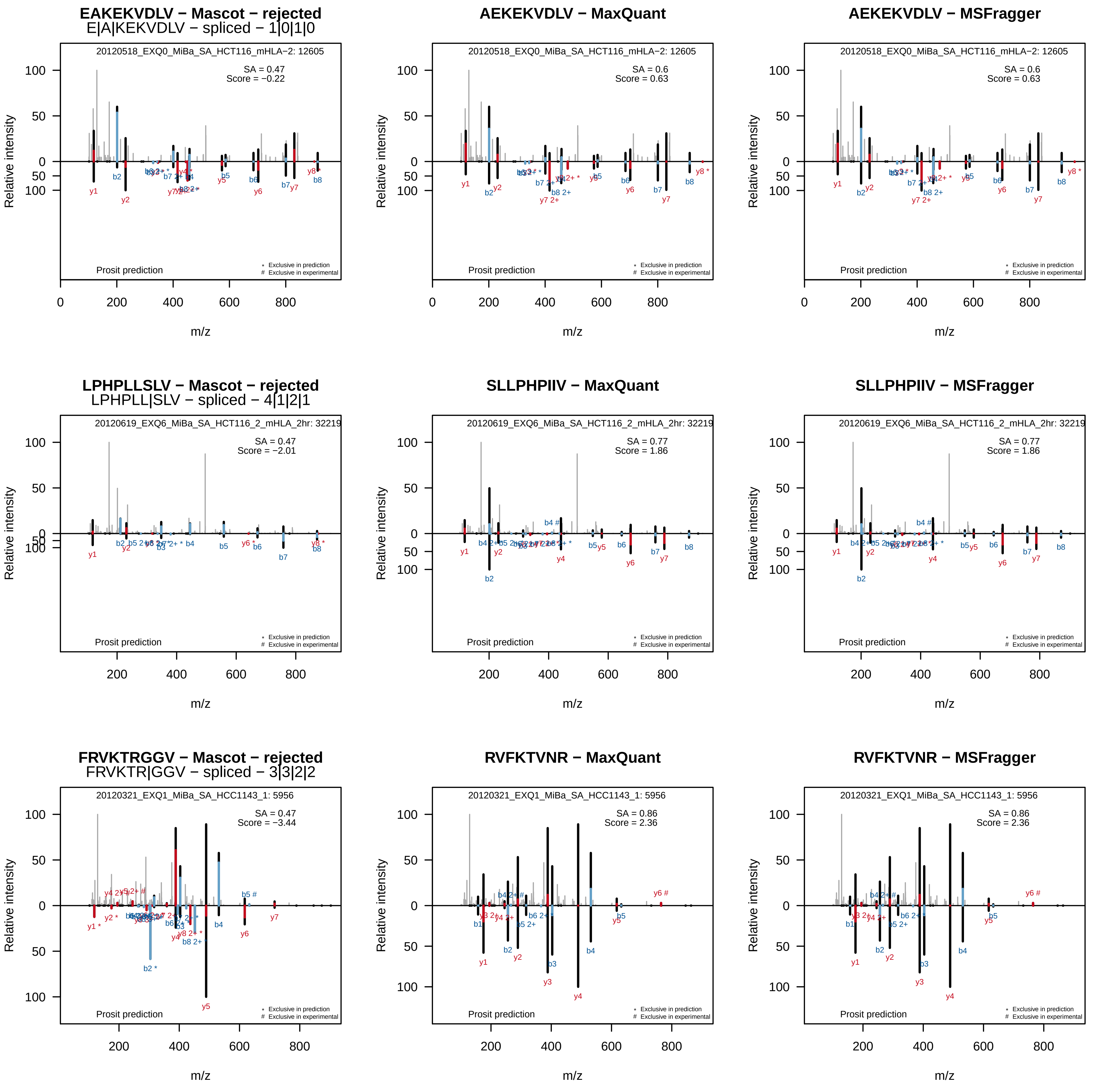

EAKEKVDLV − Mascot − rejected
E|A|KEKVDLV − spliced − 1|0|1|0
20120518_EXQ0_MiBa_SA_HCT116_mHLA−2: 12605
SA = 0.47
Score = −0.22
AEKEKVDLV − MaxQuant
20120518_EXQ0_MiBa_SA_HCT116_mHLA−2: 12605
SA = 0.6
Score = 0.63
AEKEKVDLV − MSFragger
20120518_EXQ0_MiBa_SA_HCT116_mHLA−2: 12605
SA = 0.6
Score = 0.63
LPHPLLSLV − Mascot − rejected
LPHPLL|SLV − spliced − 4|1|2|1
20120619_EXQ6_MiBa_SA_HCT116_2_mHLA_2hr: 32219
SA = 0.47
Score = −2.01
SLLPHPIIV − MaxQuant
20120619_EXQ6_MiBa_SA_HCT116_2_mHLA_2hr: 32219
SA = 0.77
Score = 1.86
SLLPHPIIV − MSFragger
20120619_EXQ6_MiBa_SA_HCT116_2_mHLA_2hr: 32219
SA = 0.77
Score = 1.86
FRVKTRGGV − Mascot − rejected
FRVKTR|GGV − spliced − 3|3|2|2
20120321_EXQ1_MiBa_SA_HCC1143_1: 5956
SA = 0.47
Score = −3.44
RVFKTVNR − MaxQuant
20120321_EXQ1_MiBa_SA_HCC1143_1: 5956
SA = 0.86
Score = 2.36
RVFKTVNR − MSFragger
20120321_EXQ1_MiBa_SA_HCC1143_1: 5956
SA = 0.86
Score = 2.36
Relative intensity
m/z
Prosit prediction
⋆ Exclusive in prediction
Exclusive in experimental

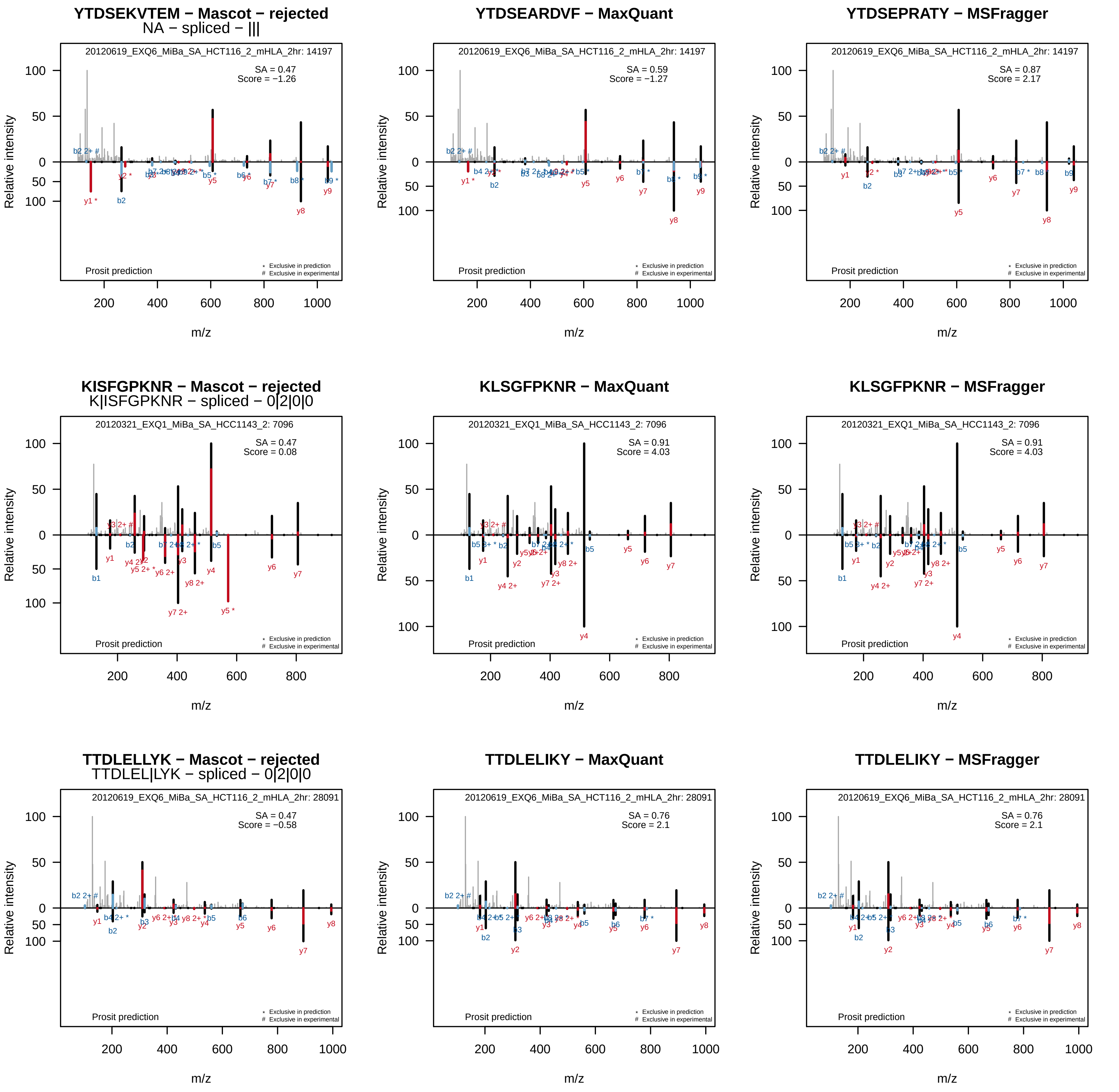

YTDSEKVTEM − Mascot − rejected
NA − spliced − |||
20120619_EXQ6_MiBa_SA_HCT116_2_mHLA_2hr: 14197
SA = 0.47
Score = −1.26
YTDSEARDVF − MaxQuant
20120619_EXQ6_MiBa_SA_HCT116_2_mHLA_2hr: 14197
SA = 0.59
Score = −1.27
YTDSEPRATY − MSFragger
20120619_EXQ6_MiBa_SA_HCT116_2_mHLA_2hr: 14197
SA = 0.87
Score = 2.17
KISFGPKNR − Mascot − rejected
K|ISFGPKNR − spliced − 0|2|0|0
20120321_EXQ1_MiBa_SA_HCC1143_2: 7096
SA = 0.47
Score = 0.08
KLSGFPKNR − MaxQuant
20120321_EXQ1_MiBa_SA_HCC1143_2: 7096
SA = 0.91
Score = 4.03
KLSGFPKNR − MSFragger
20120321_EXQ1_MiBa_SA_HCC1143_2: 7096
SA = 0.91
Score = 4.03
TTDLELLYK − Mascot − rejected
TTDLEL|LYK − spliced − 0|2|0|0
20120619_EXQ6_MiBa_SA_HCT116_2_mHLA_2hr: 28091
SA = 0.47
Score = −0.58
TTDLELIKY − MaxQuant
20120619_EXQ6_MiBa_SA_HCT116_2_mHLA_2hr: 28091
SA = 0.76
Score = 2.1
TTDLELIKY − MSFragger
20120619_EXQ6_MiBa_SA_HCT116_2_mHLA_2hr: 28091
SA = 0.76
Score = 2.1
Relative intensity
m/z
Prosit prediction
* Exclusive in prediction
# Exclusive in experimental

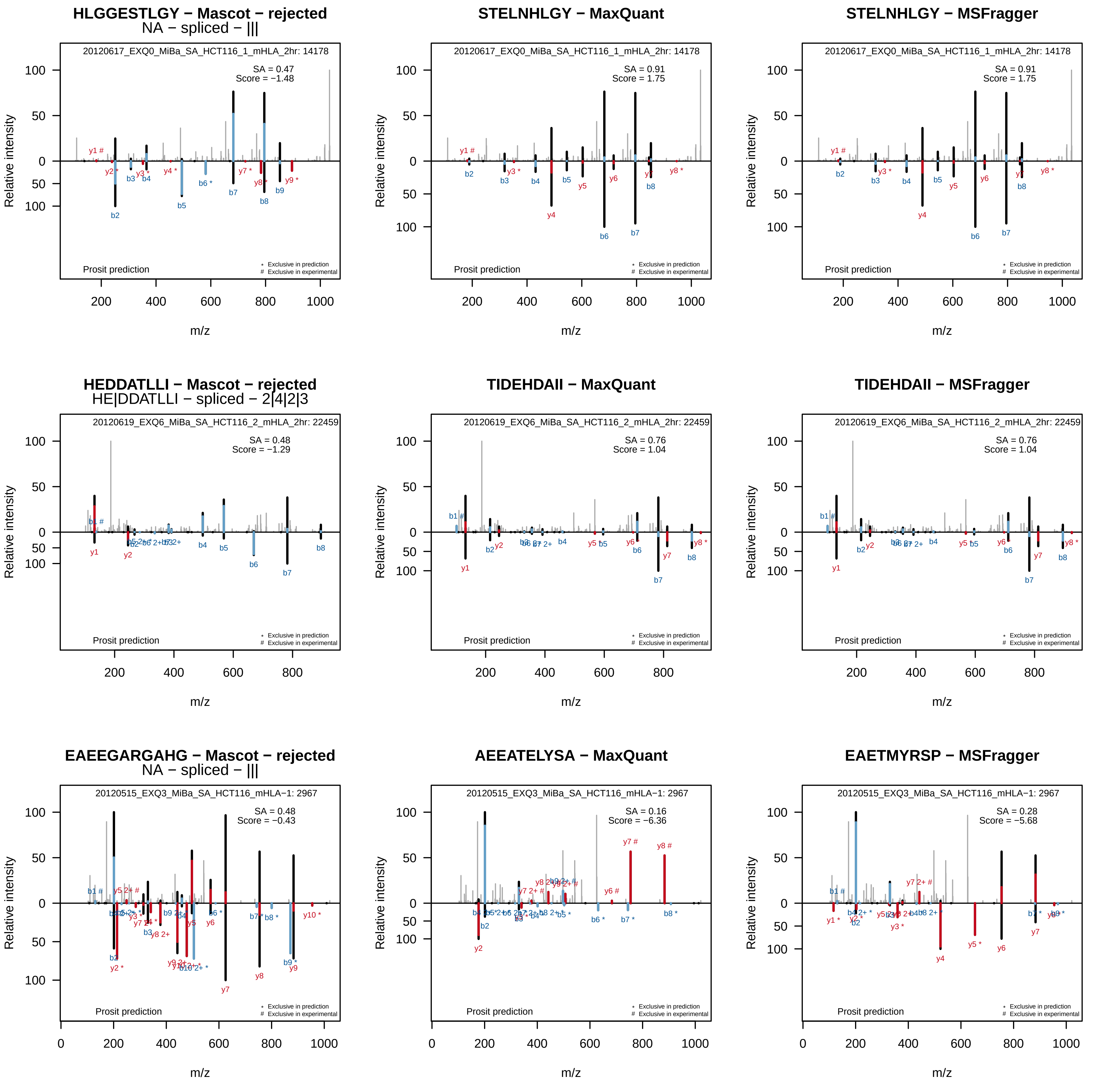

HLGGESTLGY − Mascot − rejected
NA − spliced − |||
20120617_EXQ0_MiBa_SA_HCT116_1_mHLA_2hr: 14178
SA = 0.47
Score = −1.48
STELNHLGY − MaxQuant
20120617_EXQ0_MiBa_SA_HCT116_1_mHLA_2hr: 14178
SA = 0.91
Score = 1.75
STELNHLGY − MSFragger
20120617_EXQ0_MiBa_SA_HCT116_1_mHLA_2hr: 14178
SA = 0.91
Score = 1.75
HEDDATLLI − Mascot − rejected
HE|DDATLLI − spliced − 2|4|2|3
20120619_EXQ6_MiBa_SA_HCT116_2_mHLA_2hr: 22459
SA = 0.48
Score = −1.29
TIDEHDAII − MaxQuant
20120619_EXQ6_MiBa_SA_HCT116_2_mHLA_2hr: 22459
SA = 0.76
Score = 1.04
TIDEHDAII − MSFragger
20120619_EXQ6_MiBa_SA_HCT116_2_mHLA_2hr: 22459
SA = 0.76
Score = 1.04
EAEEGARGAHG − Mascot − rejected
NA − spliced − |||
20120515_EXQ3_MiBa_SA_HCT116_mHLA−1: 2967
SA = 0.48
Score = −0.43
AEEATELYSA − MaxQuant
20120515_EXQ3_MiBa_SA_HCT116_mHLA−1: 2967
SA = 0.16
Score = −6.36
EAETMYRSP − MSFragger
20120515_EXQ3_MiBa_SA_HCT116_mHLA−1: 2967
SA = 0.28
Score = −5.68
Relative intensity
m/z
Prosit prediction
⋆ Exclusive in prediction
Exclusive in experimental

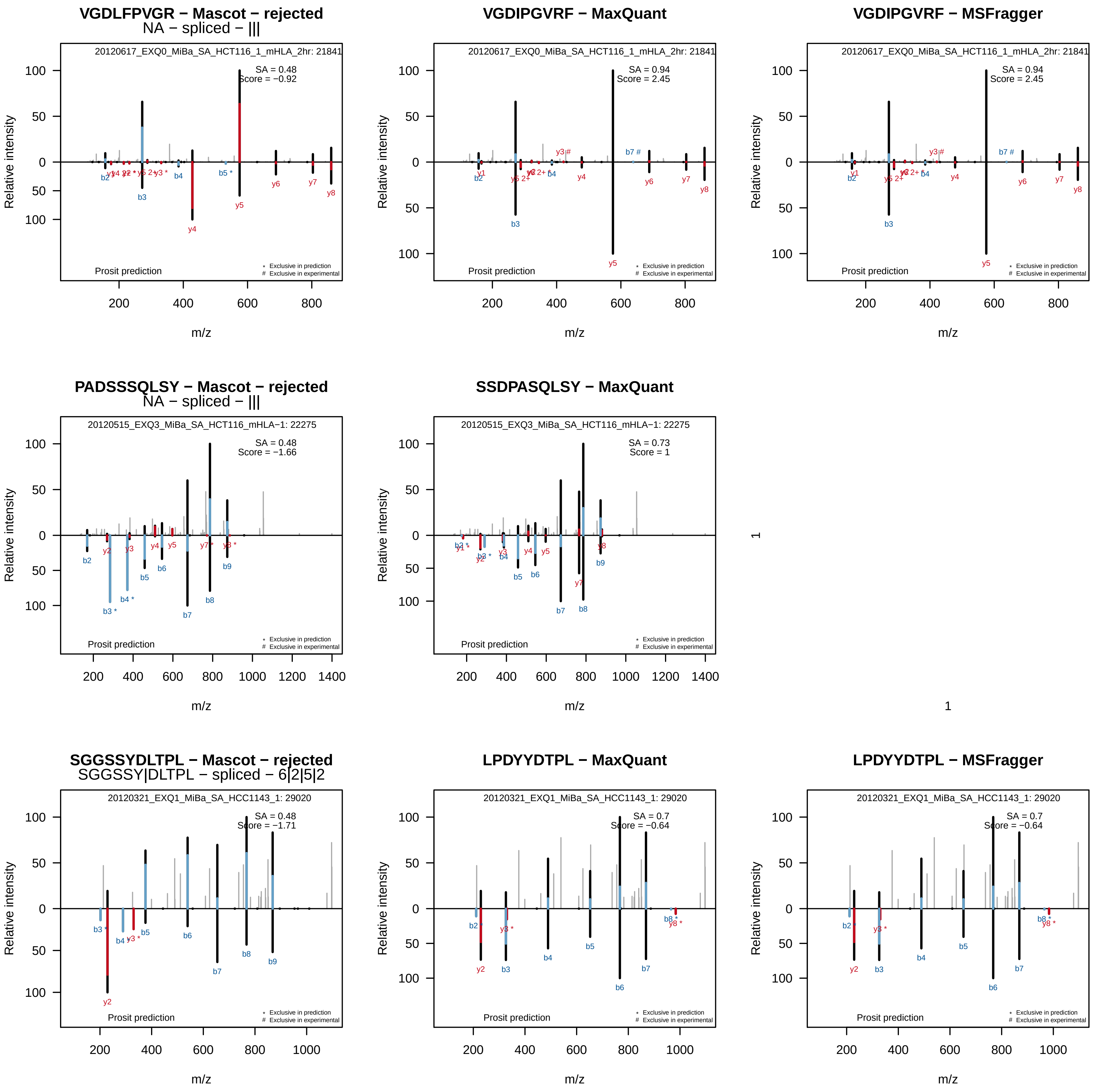
VGDLFPVGR − Mascot − rejected
NA − spliced − |||
20120617_EXQ0_MiBa_SA_HCT116_1_mHLA_2hr: 21841
SA = 0.48
Score = −0.92
VGDIPGVRF − MaxQuant
20120617_EXQ0_MiBa_SA_HCT116_1_mHLA_2hr: 21841
SA = 0.94
Score = 2.45
VGDIPGVRF − MSFragger
20120617_EXQ0_MiBa_SA_HCT116_1_mHLA_2hr: 21841
SA = 0.94
Score = 2.45
PADSSSQLSY − Mascot − rejected
NA − spliced − |||
20120515_EXQ3_MiBa_SA_HCT116_mHLA−1: 22275
SA = 0.48
Score = −1.66
SSDPASQLSY − MaxQuant
20120515_EXQ3_MiBa_SA_HCT116_mHLA−1: 22275
SA = 0.73
Score = 1
SGGSSYDLTPL − Mascot − rejected
SGGSSY|DLTPL − spliced − 6|2|5|2
20120321_EXQ1_MiBa_SA_HCC1143_1: 29020
SA = 0.48
Score = −1.71
LPDYYDTPL − MaxQuant
20120321_EXQ1_MiBa_SA_HCC1143_1: 29020
SA = 0.7
Score = −0.64
LPDYYDTPL − MSFragger
20120321_EXQ1_MiBa_SA_HCC1143_1: 29020
SA = 0.7
Score = −0.64
Relative intensity
m/z
Prosit prediction
* Exclusive in prediction
# Exclusive in experimental
1

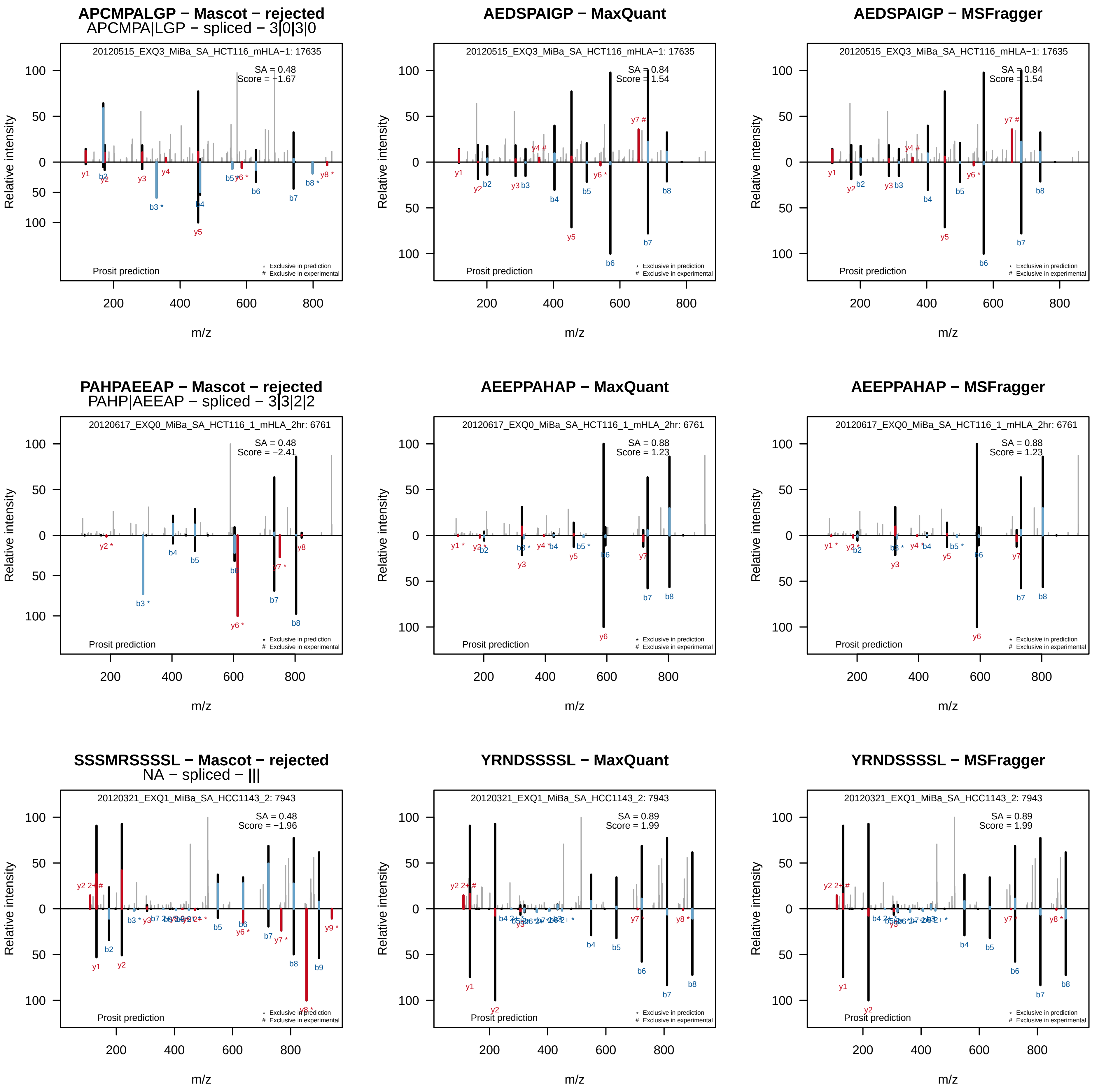

APCMPALGP − Mascot − rejected
APCMPA|LGP − spliced − 3|0|3|0
20120515_EXQ3_MiBa_SA_HCT116_mHLA−1: 17635
SA = 0.48
Score = −1.67
AEDSPAIGP − MaxQuant
20120515_EXQ3_MiBa_SA_HCT116_mHLA−1: 17635
SA = 0.84
Score = 1.54
AEDSPAIGP − MSFragger
20120515_EXQ3_MiBa_SA_HCT116_mHLA−1: 17635
SA = 0.84
Score = 1.54
PAHPAEEAP − Mascot − rejected
PAHP|AEEAP − spliced − 3|3|2|2
20120617_EXQ0_MiBa_SA_HCT116_1_mHLA_2hr: 6761
SA = 0.48
Score = −2.41
AEEPPAHAP − MaxQuant
20120617_EXQ0_MiBa_SA_HCT116_1_mHLA_2hr: 6761
SA = 0.88
Score = 1.23
AEEPPAHAP − MSFragger
20120617_EXQ0_MiBa_SA_HCT116_1_mHLA_2hr: 6761
SA = 0.88
Score = 1.23
SSSMRSSSSL − Mascot − rejected
NA − spliced − |||
20120321_EXQ1_MiBa_SA_HCC1143_2: 7943
SA = 0.48
Score = −1.96
YRNDSSSSL − MaxQuant
20120321_EXQ1_MiBa_SA_HCC1143_2: 7943
SA = 0.89
Score = 1.99
YRNDSSSSL − MSFragger
20120321_EXQ1_MiBa_SA_HCC1143_2: 7943
SA = 0.89
Score = 1.99
Relative intensity
m/z
Prosit prediction
* Exclusive in prediction
# Exclusive in experimental

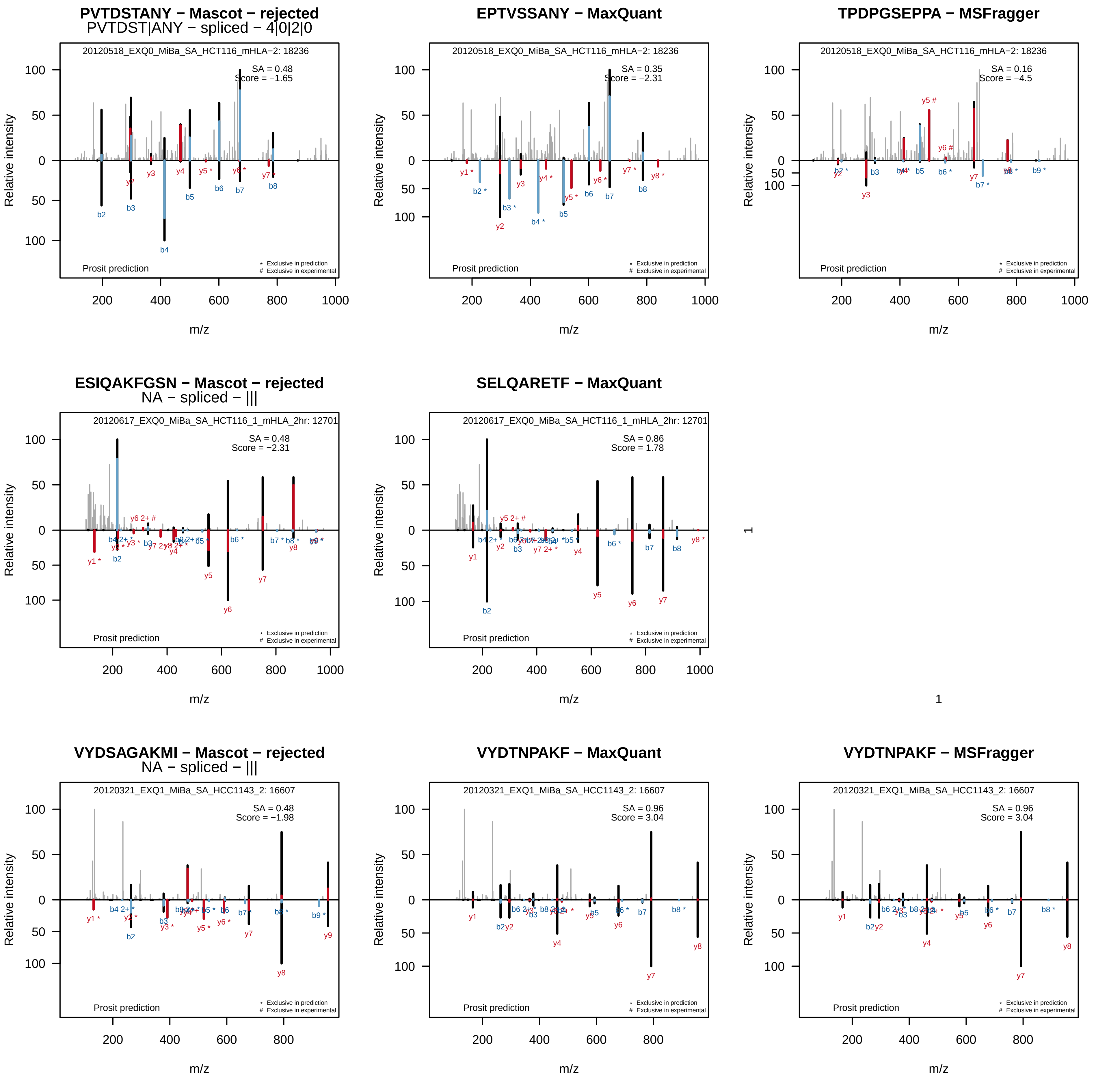
PVTDSTANY − Mascot − rejected
PVTDST|ANY − spliced − 4|0|2|0
20120518_EXQ0_MiBa_SA_HCT116_mHLA−2: 18236
SA = 0.48
Score = −1.65
EPTVSSANY − MaxQuant
20120518_EXQ0_MiBa_SA_HCT116_mHLA−2: 18236
SA = 0.35
Score = −2.31
TPDPGSEPPA − MSFragger
20120518_EXQ0_MiBa_SA_HCT116_mHLA−2: 18236
SA = 0.16
Score = −4.5
ESIQAKFGSN − Mascot − rejected
NA − spliced − |||
20120617_EXQ0_MiBa_SA_HCT116_1_mHLA_2hr: 12701
SA = 0.48
Score = −2.31
SELQARETF − MaxQuant
20120617_EXQ0_MiBa_SA_HCT116_1_mHLA_2hr: 12701
SA = 0.86
Score = 1.78
VYDSAGAKMI − Mascot − rejected
NA − spliced − |||
20120321_EXQ1_MiBa_SA_HCC1143_2: 16607
SA = 0.48
Score = −1.98
VYDTNPAKF − MaxQuant
20120321_EXQ1_MiBa_SA_HCC1143_2: 16607
SA = 0.96
Score = 3.04
VYDTNPAKF − MSFragger
20120321_EXQ1_MiBa_SA_HCC1143_2: 16607
SA = 0.96
Score = 3.04
Relative intensity
m/z
Prosit prediction
⋆ Exclusive in prediction
Exclusive in experimental

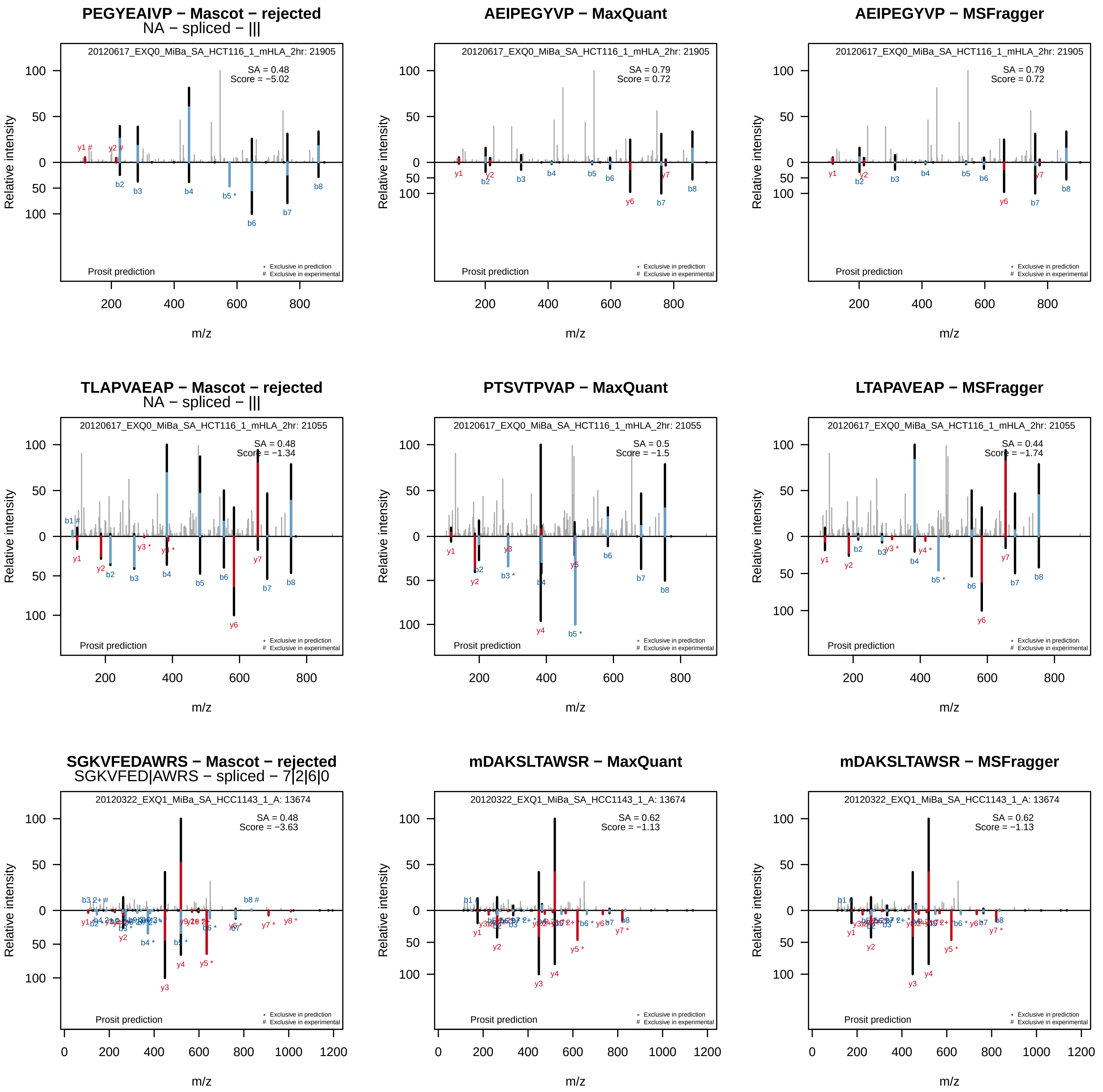
PEGYEAIVP − Mascot − rejected
NA − spliced − |||
20120617_EXQ0_MiBa_SA_HCT116_1_mHLA_2hr: 21905
SA = 0.48
Score = −5.02
AEIPEGYVP − MaxQuant
20120617_EXQ0_MiBa_SA_HCT116_1_mHLA_2hr: 21905
SA = 0.79
Score = 0.72
AEIPEGYVP − MSFragger
20120617_EXQ0_MiBa_SA_HCT116_1_mHLA_2hr: 21905
SA = 0.79
Score = 0.72
TLAPVAEAP − Mascot − rejected
NA − spliced − |||
20120617_EXQ0_MiBa_SA_HCT116_1_mHLA_2hr: 21055
SA = 0.48
Score = −1.34
PTSVTPVAP − MaxQuant
20120617_EXQ0_MiBa_SA_HCT116_1_mHLA_2hr: 21055
SA = 0.5
Score = −1.5
LTAPAVEAP − MSFragger
20120617_EXQ0_MiBa_SA_HCT116_1_mHLA_2hr: 21055
SA = 0.44
Score = −1.74
SGKVFEDAWRS − Mascot − rejected
SGKVFED|AWRS − spliced − 7|2|6|0
20120322_EXQ1_MiBa_SA_HCC1143_1_A: 13674
SA = 0.48
Score = −3.63
mDAKSLTAWSR − MaxQuant
20120322_EXQ1_MiBa_SA_HCC1143_1_A: 13674
SA = 0.62
Score = −1.13
mDAKSLTAWSR − MSFragger
20120322_EXQ1_MiBa_SA_HCC1143_1_A: 13674
SA = 0.62
Score = −1.13
Relative intensity
m/z
Prosit prediction
⋆ Exclusive in prediction
Exclusive in experimental

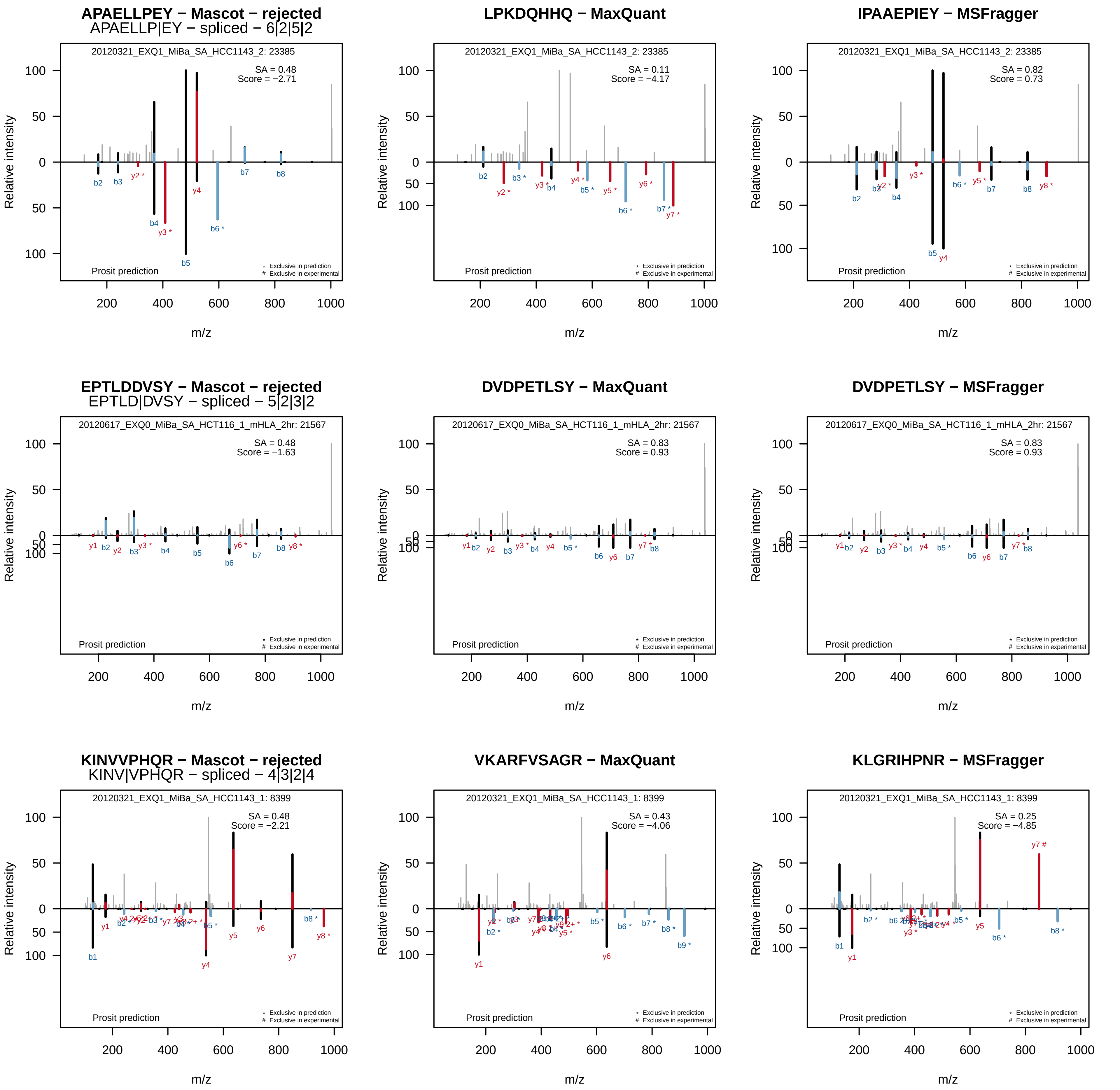
APAELLPEY − Mascot − rejected
APAELLP|EY − spliced − 6|2|5|2
20120321_EXQ1_MiBa_SA_HCC1143_2: 23385
SA = 0.48
Score = −2.71
Prosit prediction
LPKDQHHQ − MaxQuant
20120321_EXQ1_MiBa_SA_HCC1143_2: 23385
SA = 0.11
Score = −4.17
Prosit prediction
IPAAEPIEY − MSFragger
20120321_EXQ1_MiBa_SA_HCC1143_2: 23385
SA = 0.82
Score = 0.73
Prosit prediction
EPTLDDVSY − Mascot − rejected
EPTLD|DVSY − spliced − 5|2|3|2
20120617_EXQ0_MiBa_SA_HCT116_1_mHLA_2hr: 21567
SA = 0.48
Score = −1.63
Prosit prediction
DVDPETLSY − MaxQuant
20120617_EXQ0_MiBa_SA_HCT116_1_mHLA_2hr: 21567
SA = 0.83
Score = 0.93
Prosit prediction
DVDPETLSY − MSFragger
20120617_EXQ0_MiBa_SA_HCT116_1_mHLA_2hr: 21567
SA = 0.83
Score = 0.93
Prosit prediction
KINVVPHQR − Mascot − rejected
KINV|VPHQR − spliced − 4|3|2|4
20120321_EXQ1_MiBa_SA_HCC1143_1: 8399
SA = 0.48
Score = −2.21
Prosit prediction
VKARFVSAGR − MaxQuant
20120321_EXQ1_MiBa_SA_HCC1143_1: 8399
SA = 0.43
Score = −4.06
Prosit prediction
KLGRIHPNR − MSFragger
20120321_EXQ1_MiBa_SA_HCC1143_1: 8399
SA = 0.25
Score = −4.85
Prosit prediction
* Exclusive in prediction
# Exclusive in experimental
Relative intensity
m/z

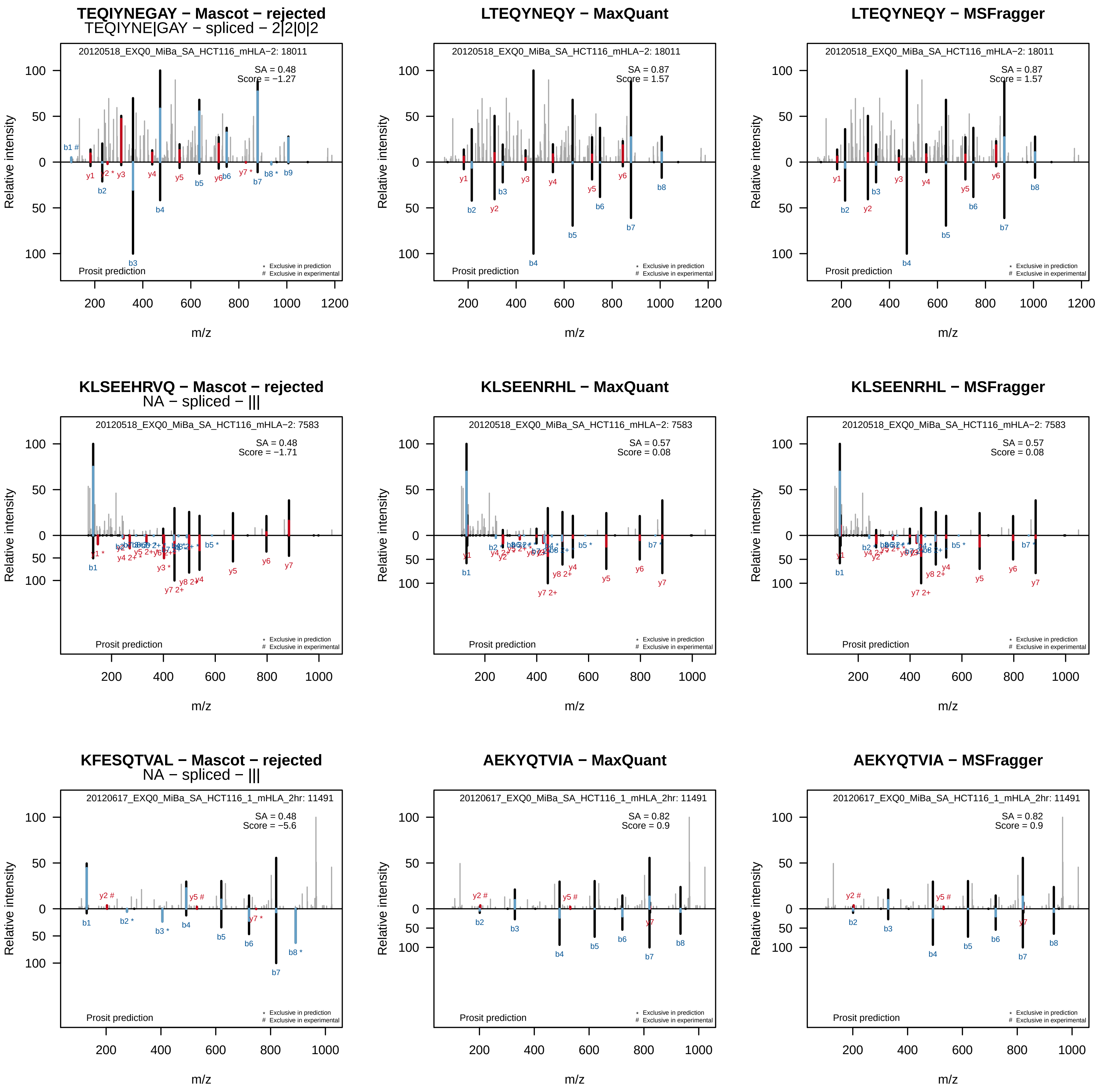

TEQIYNEGAY − Mascot − rejected
TEQIYNE|GAY − spliced − 2|2|0|2
20120518_EXQ0_MiBa_SA_HCT116_mHLA−2: 18011
SA = 0.48
Score = −1.27
LTEQYNEQY − MaxQuant
20120518_EXQ0_MiBa_SA_HCT116_mHLA−2: 18011
SA = 0.87
Score = 1.57
LTEQYNEQY − MSFragger
20120518_EXQ0_MiBa_SA_HCT116_mHLA−2: 18011
SA = 0.87
Score = 1.57
KLSEEHRVQ − Mascot − rejected
NA − spliced − |||
20120518_EXQ0_MiBa_SA_HCT116_mHLA−2: 7583
SA = 0.48
Score = −1.71
KLSEENRHL − MaxQuant
20120518_EXQ0_MiBa_SA_HCT116_mHLA−2: 7583
SA = 0.57
Score = 0.08
KLSEENRHL − MSFragger
20120518_EXQ0_MiBa_SA_HCT116_mHLA−2: 7583
SA = 0.57
Score = 0.08
KFESQTVAL − Mascot − rejected
NA − spliced − |||
20120617_EXQ0_MiBa_SA_HCT116_1_mHLA_2hr: 11491
SA = 0.48
Score = −5.6
AEKYQTVIA − MaxQuant
20120617_EXQ0_MiBa_SA_HCT116_1_mHLA_2hr: 11491
SA = 0.82
Score = 0.9
AEKYQTVIA − MSFragger
20120617_EXQ0_MiBa_SA_HCT116_1_mHLA_2hr: 11491
SA = 0.82
Score = 0.9
Relative intensity
m/z
Prosit prediction
* Exclusive in prediction
# Exclusive in experimental

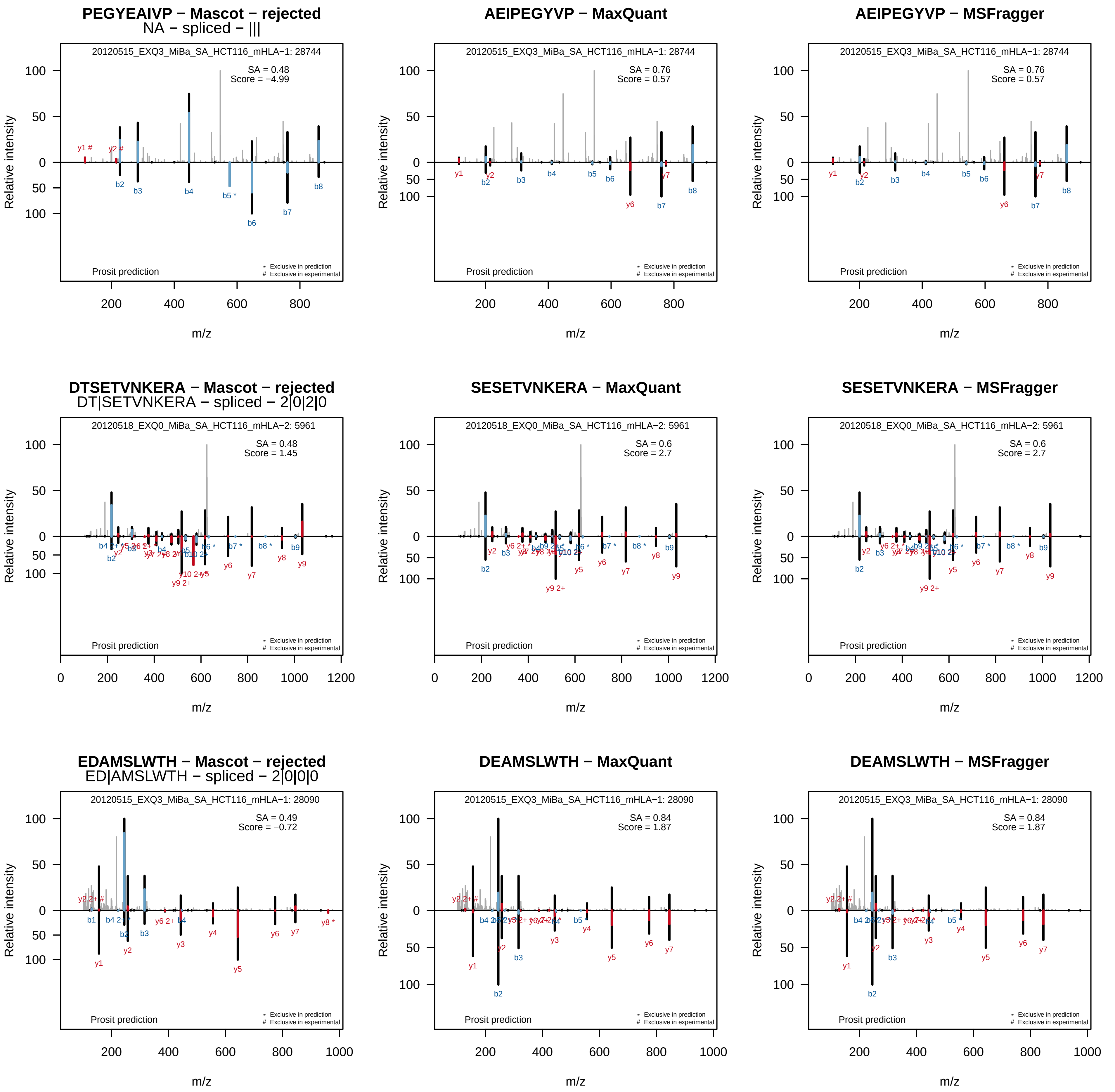
PEGYEAIVP − Mascot − rejected
NA − spliced − |||
20120515_EXQ3_MiBa_SA_HCT116_mHLA−1: 28744
SA = 0.48
Score = −4.99
AEIPEGYVP − MaxQuant
20120515_EXQ3_MiBa_SA_HCT116_mHLA−1: 28744
SA = 0.76
Score = 0.57
AEIPEGYVP − MSFragger
20120515_EXQ3_MiBa_SA_HCT116_mHLA−1: 28744
SA = 0.76
Score = 0.57
DTSETVNKERA − Mascot − rejected
DT|SETVNKERA − spliced − 2|0|2|0
20120518_EXQ0_MiBa_SA_HCT116_mHLA−2: 5961
SA = 0.48
Score = 1.45
SESETVNKERA − MaxQuant
20120518_EXQ0_MiBa_SA_HCT116_mHLA−2: 5961
SA = 0.6
Score = 2.7
SESETVNKERA − MSFragger
20120518_EXQ0_MiBa_SA_HCT116_mHLA−2: 5961
SA = 0.6
Score = 2.7
EDAMSLWTH − Mascot − rejected
ED|AMSLWTH − spliced − 2|0|0|0
20120515_EXQ3_MiBa_SA_HCT116_mHLA−1: 28090
SA = 0.49
Score = −0.72
DEAMSLWTH − MaxQuant
20120515_EXQ3_MiBa_SA_HCT116_mHLA−1: 28090
SA = 0.84
Score = 1.87
DEAMSLWTH − MSFragger
20120515_EXQ3_MiBa_SA_HCT116_mHLA−1: 28090
SA = 0.84
Score = 1.87
Relative intensity
m/z
Prosit prediction
⋆ Exclusive in prediction
Exclusive in experimental

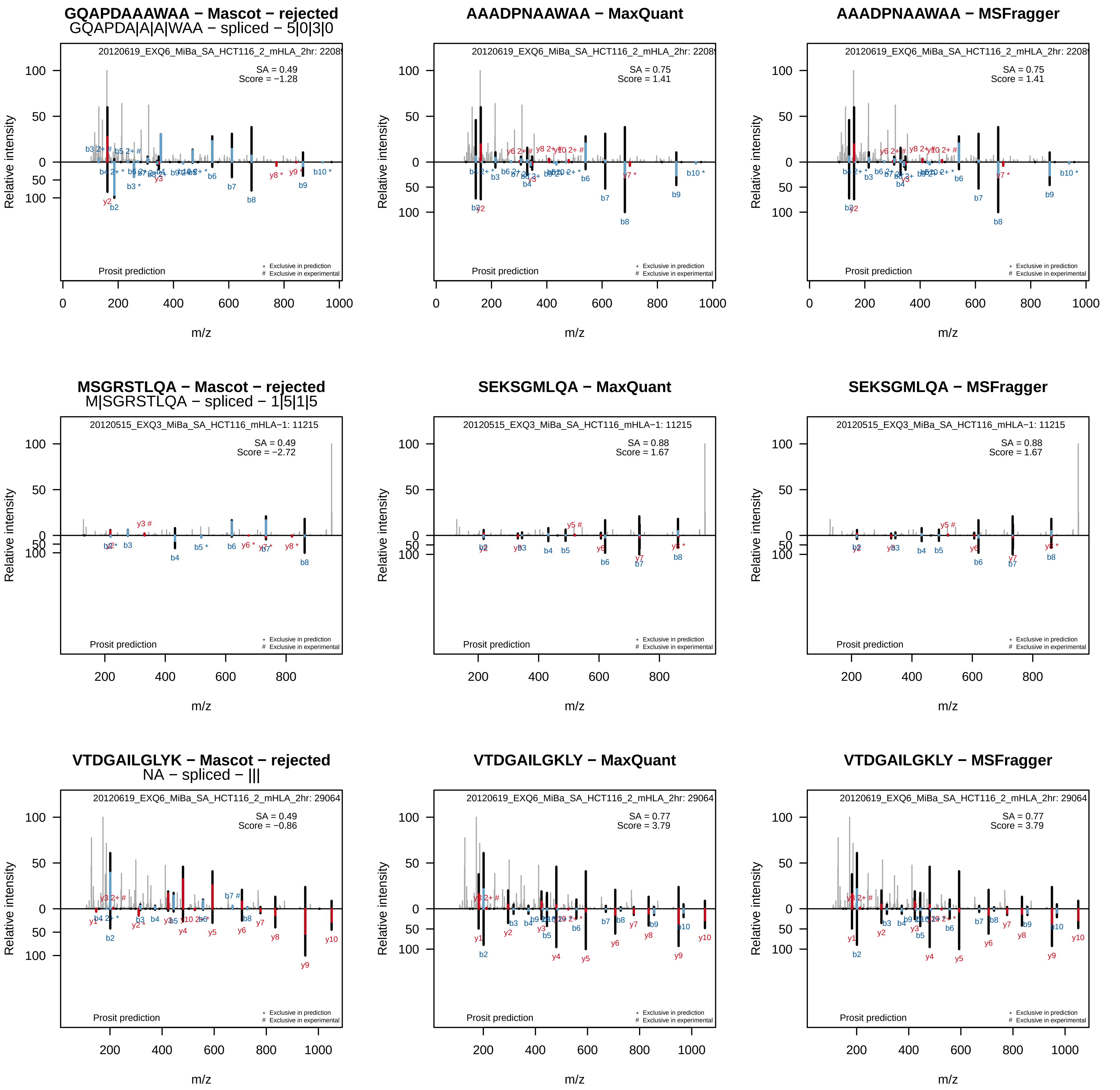
GQAPDAAAWAA − Mascot − rejected
GQAPDA|A|A|WAA − spliced − 5|0|3|0
20120619_EXQ6_MiBa_SA_HCT116_2_mHLA_2hr: 2208
SA = 0.49
Score = −1.28
AAADPNAAWAA − MaxQuant
20120619_EXQ6_MiBa_SA_HCT116_2_mHLA_2hr: 2208
SA = 0.75
Score = 1.41
AAADPNAAWAA − MSFragger
20120619_EXQ6_MiBa_SA_HCT116_2_mHLA_2hr: 2208
SA = 0.75
Score = 1.41
MSGRSTLQA − Mascot − rejected
M|SGRSTLQA − spliced − 1|5|1|5
20120515_EXQ3_MiBa_SA_HCT116_mHLA−1: 11215
SA = 0.49
Score = −2.72
SEKSGMLQA − MaxQuant
20120515_EXQ3_MiBa_SA_HCT116_mHLA−1: 11215
SA = 0.88
Score = 1.67
SEKSGMLQA − MSFragger
20120515_EXQ3_MiBa_SA_HCT116_mHLA−1: 11215
SA = 0.88
Score = 1.67
VTDGAILGLYK − Mascot − rejected
NA − spliced − |||
20120619_EXQ6_MiBa_SA_HCT116_2_mHLA_2hr: 29064
SA = 0.49
Score = −0.86
VTDGAILGKLY − MaxQuant
20120619_EXQ6_MiBa_SA_HCT116_2_mHLA_2hr: 29064
SA = 0.77
Score = 3.79
VTDGAILGKLY − MSFragger
20120619_EXQ6_MiBa_SA_HCT116_2_mHLA_2hr: 29064
SA = 0.77
Score = 3.79
Relative intensity
m/z
Prosit prediction
* Exclusive in prediction
# Exclusive in experimental

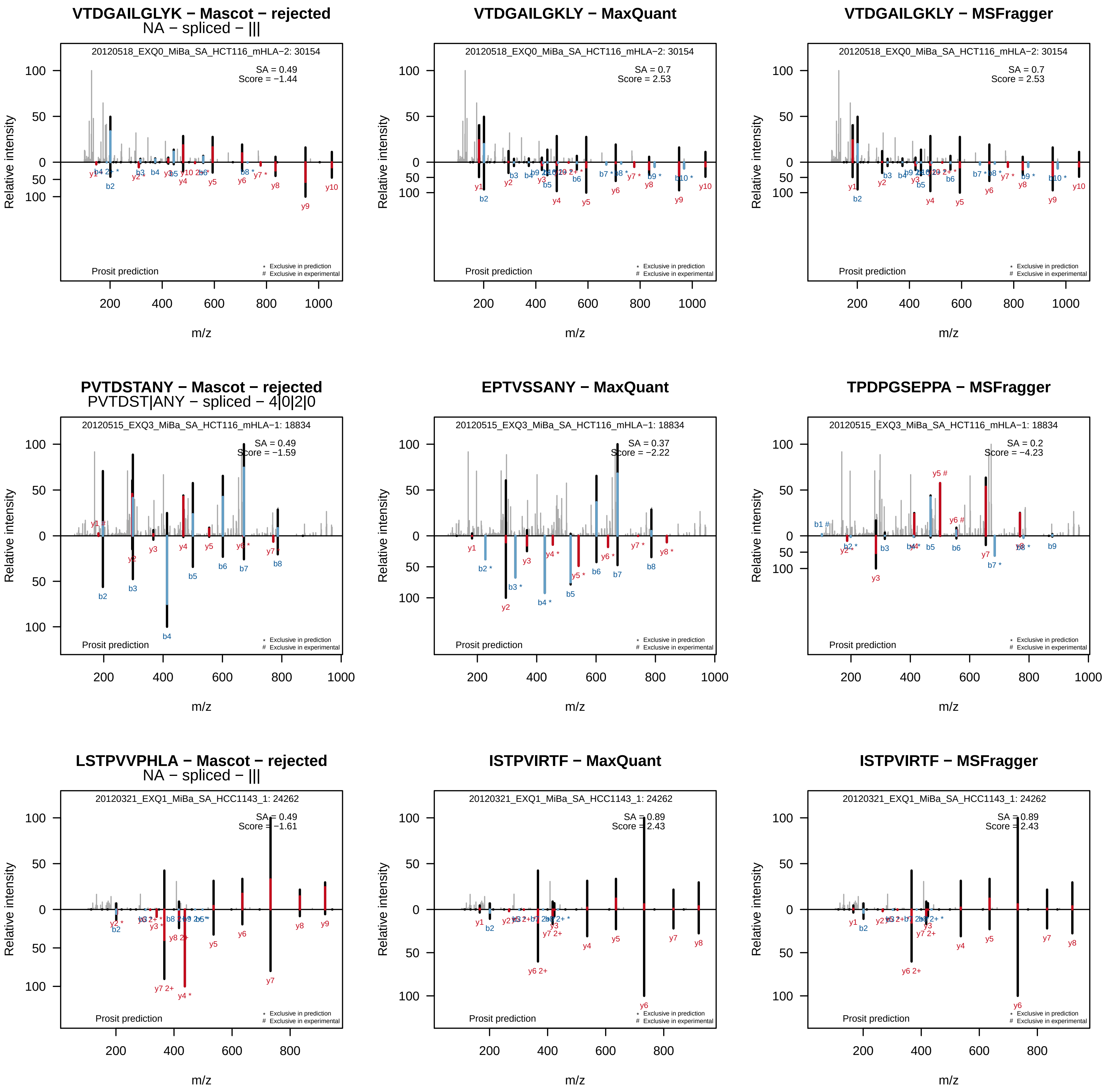
VTDGAILGLYK − Mascot − rejected
NA − spliced − |||
20120518_EXQ0_MiBa_SA_HCT116_mHLA−2: 30154
SA = 0.49
Score = −1.44
VTDGAILGKLY − MaxQuant
20120518_EXQ0_MiBa_SA_HCT116_mHLA−2: 30154
SA = 0.7
Score = 2.53
VTDGAILGKLY − MSFragger
20120518_EXQ0_MiBa_SA_HCT116_mHLA−2: 30154
SA = 0.7
Score = 2.53
PVTDSTANY − Mascot − rejected
PVTDST|ANY − spliced − 4|0|2|0
20120515_EXQ3_MiBa_SA_HCT116_mHLA−1: 18834
SA = 0.49
Score = −1.59
EPTVSSANY − MaxQuant
20120515_EXQ3_MiBa_SA_HCT116_mHLA−1: 18834
SA = 0.37
Score = −2.22
TPDPGSEPPA − MSFragger
20120515_EXQ3_MiBa_SA_HCT116_mHLA−1: 18834
SA = 0.2
Score = −4.23
LSTPVVPHLA − Mascot − rejected
NA − spliced − |||
20120321_EXQ1_MiBa_SA_HCC1143_1: 24262
SA = 0.49
Score = −1.61
ISTPVIRTF − MaxQuant
20120321_EXQ1_MiBa_SA_HCC1143_1: 24262
SA = 0.89
Score = 2.43
ISTPVIRTF − MSFragger
20120321_EXQ1_MiBa_SA_HCC1143_1: 24262
SA = 0.89
Score = 2.43
Relative intensity
m/z
Prosit prediction
* Exclusive in prediction
# Exclusive in experimental

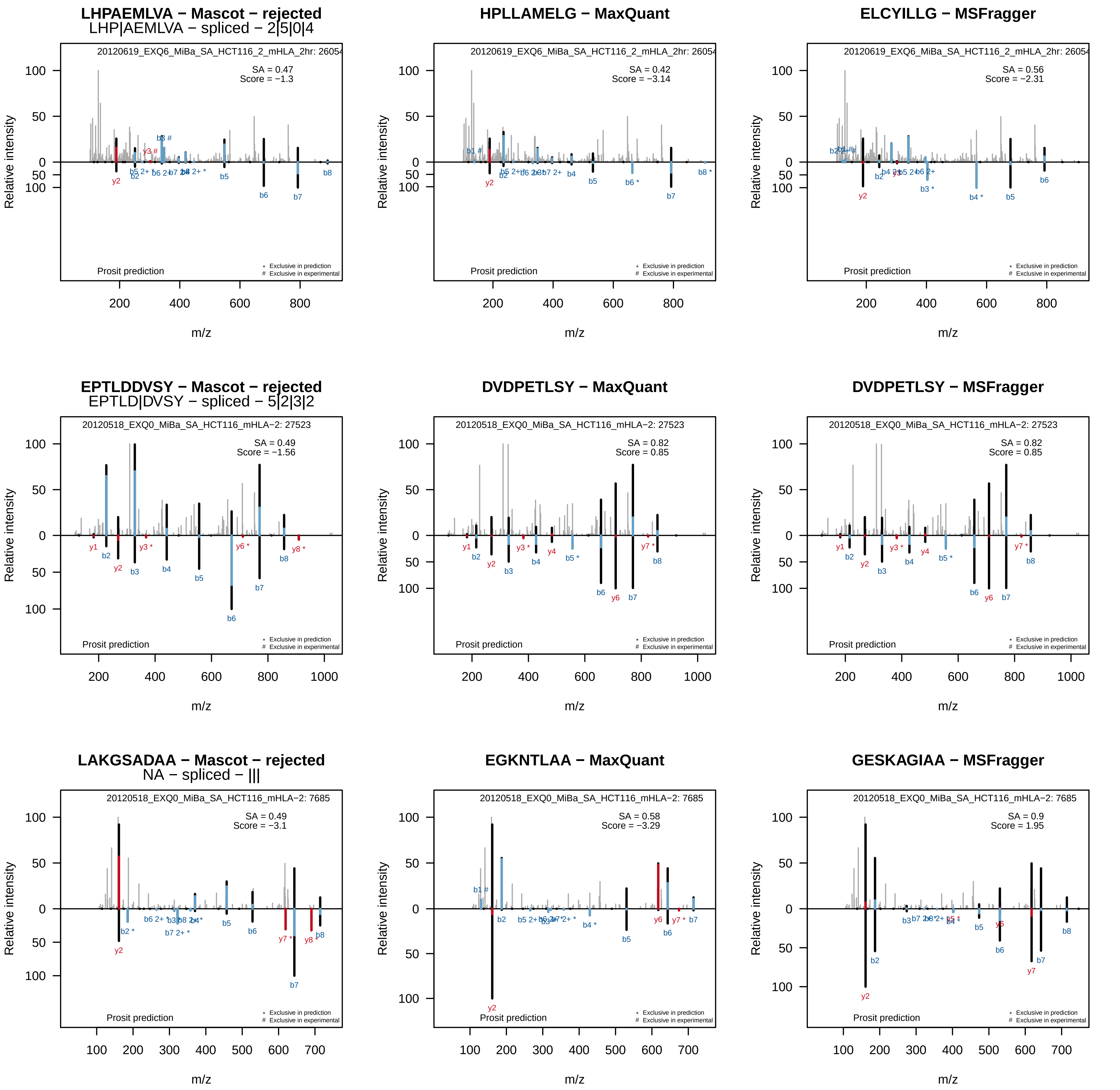

LHPAEMLVA − Mascot − rejected
LHP|AEMLVA − spliced − 2|5|0|4
20120619_EXQ6_MiBa_SA_HCT116_2_mHLA_2hr: 26054
SA = 0.47
Score = −1.3
HPLLAMELG − MaxQuant
20120619_EXQ6_MiBa_SA_HCT116_2_mHLA_2hr: 26054
SA = 0.42
Score = −3.14
ELCYILLG − MSFragger
20120619_EXQ6_MiBa_SA_HCT116_2_mHLA_2hr: 26054
SA = 0.56
Score = −2.31
EPTLDDVSY − Mascot − rejected
EPTLD|DVSY − spliced − 5|2|3|2
20120518_EXQ0_MiBa_SA_HCT116_mHLA−2: 27523
SA = 0.49
Score = −1.56
DVDPETLSY − MaxQuant
20120518_EXQ0_MiBa_SA_HCT116_mHLA−2: 27523
SA = 0.82
Score = 0.85
DVDPETLSY − MSFragger
20120518_EXQ0_MiBa_SA_HCT116_mHLA−2: 27523
SA = 0.82
Score = 0.85
LAKGSADAA − Mascot − rejected
NA − spliced − |||
20120518_EXQ0_MiBa_SA_HCT116_mHLA−2: 7685
SA = 0.49
Score = −3.1
EGKNTLAA − MaxQuant
20120518_EXQ0_MiBa_SA_HCT116_mHLA−2: 7685
SA = 0.58
Score = −3.29
GESKAGIAA − MSFragger
20120518_EXQ0_MiBa_SA_HCT116_mHLA−2: 7685
SA = 0.9
Score = 1.95
Relative intensity
m/z
Prosit prediction
* Exclusive in prediction
# Exclusive in experimental

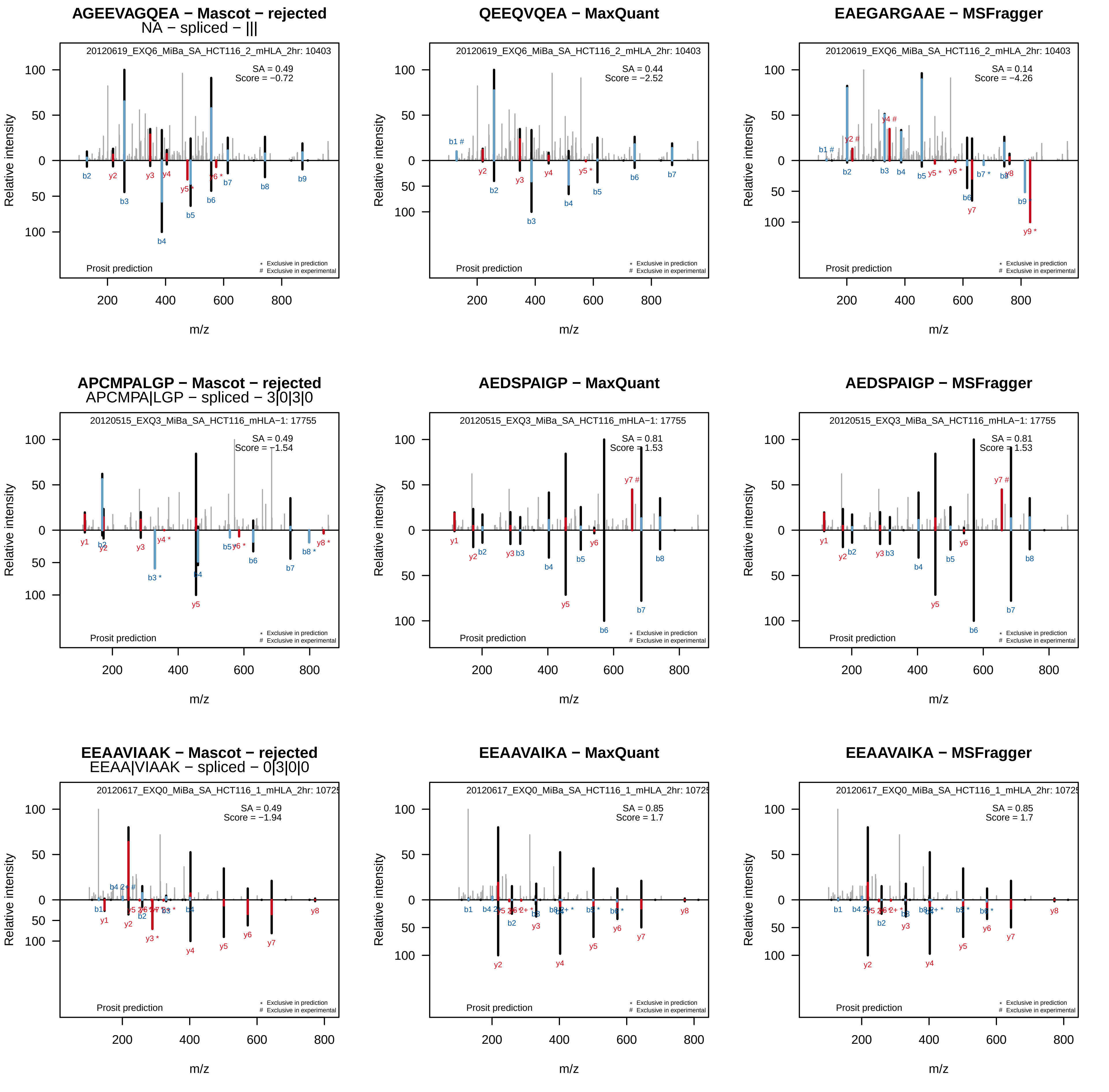

AGEEVAGQEA − Mascot − rejected
NA − spliced − |||
20120619_EXQ6_MiBa_SA_HCT116_2_mHLA_2hr: 10403
SA = 0.49
Score = −0.72
QEEQVQEA − MaxQuant
20120619_EXQ6_MiBa_SA_HCT116_2_mHLA_2hr: 10403
SA = 0.44
Score = −2.52
EAEGARGAAE − MSFragger
20120619_EXQ6_MiBa_SA_HCT116_2_mHLA_2hr: 10403
SA = 0.14
Score = −4.26
APCMPALGP − Mascot − rejected
APCMPA|LGP − spliced − 3|0|3|0
20120515_EXQ3_MiBa_SA_HCT116_mHLA−1: 17755
SA = 0.49
Score = −1.54
AEDSPAIGP − MaxQuant
20120515_EXQ3_MiBa_SA_HCT116_mHLA−1: 17755
SA = 0.81
Score = 1.53
AEDSPAIGP − MSFragger
20120515_EXQ3_MiBa_SA_HCT116_mHLA−1: 17755
SA = 0.81
Score = 1.53
EEAAVIAAK − Mascot − rejected
EEAA|VIAAK − spliced − 0|3|0|0
20120617_EXQ0_MiBa_SA_HCT116_1_mHLA_2hr: 10725
SA = 0.49
Score = −1.94
EEAAVAIKA − MaxQuant
20120617_EXQ0_MiBa_SA_HCT116_1_mHLA_2hr: 10725
SA = 0.85
Score = 1.7
EEAAVAIKA − MSFragger
20120617_EXQ0_MiBa_SA_HCT116_1_mHLA_2hr: 10725
SA = 0.85
Score = 1.7
Relative intensity
m/z
Prosit prediction
* Exclusive in prediction
# Exclusive in experimental

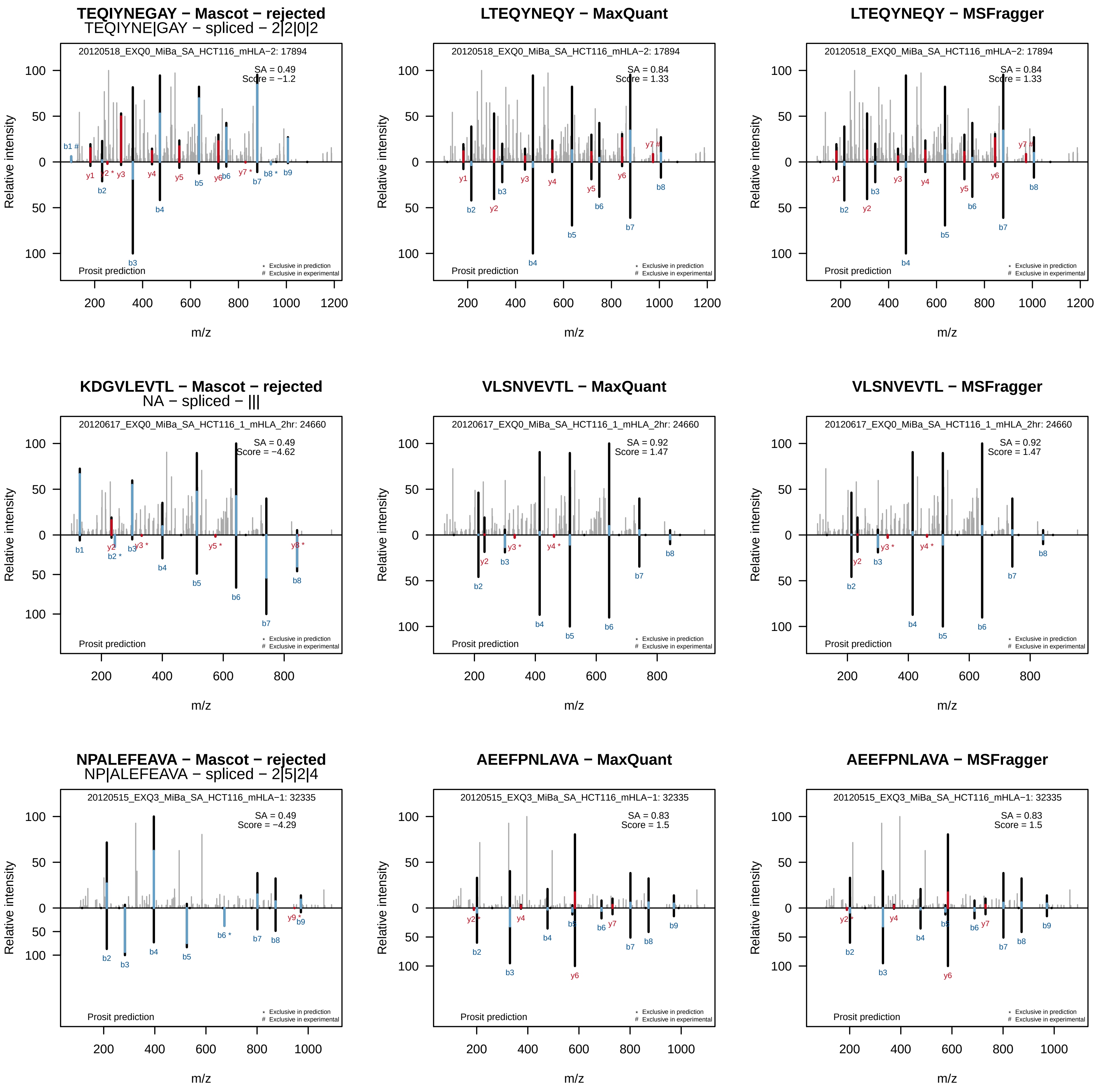
TEQIYNEGAY − Mascot − rejected
TEQIYNE|GAY − spliced − 2|2|0|2
20120518_EXQ0_MiBa_SA_HCT116_mHLA−2: 17894
SA = 0.49
Score = −1.2
LTEQYNEQY − MaxQuant
20120518_EXQ0_MiBa_SA_HCT116_mHLA−2: 17894
SA = 0.84
Score = 1.33
LTEQYNEQY − MSFragger
20120518_EXQ0_MiBa_SA_HCT116_mHLA−2: 17894
SA = 0.84
Score = 1.33
KDGVLEVTL − Mascot − rejected
NA − spliced − |||
20120617_EXQ0_MiBa_SA_HCT116_1_mHLA_2hr: 24660
SA = 0.49
Score = −4.62
VLSNVEVTL − MaxQuant
20120617_EXQ0_MiBa_SA_HCT116_1_mHLA_2hr: 24660
SA = 0.92
Score = 1.47
VLSNVEVTL − MSFragger
20120617_EXQ0_MiBa_SA_HCT116_1_mHLA_2hr: 24660
SA = 0.92
Score = 1.47
NPALEFEAVA − Mascot − rejected
NP|ALEFEAVA − spliced − 2|5|2|4
20120515_EXQ3_MiBa_SA_HCT116_mHLA−1: 32335
SA = 0.49
Score = −4.29
AEEFPNLAVA − MaxQuant
20120515_EXQ3_MiBa_SA_HCT116_mHLA−1: 32335
SA = 0.83
Score = 1.5
AEEFPNLAVA − MSFragger
20120515_EXQ3_MiBa_SA_HCT116_mHLA−1: 32335
SA = 0.83
Score = 1.5
Relative intensity
m/z
Prosit prediction
⋆ Exclusive in prediction
Exclusive in experimental

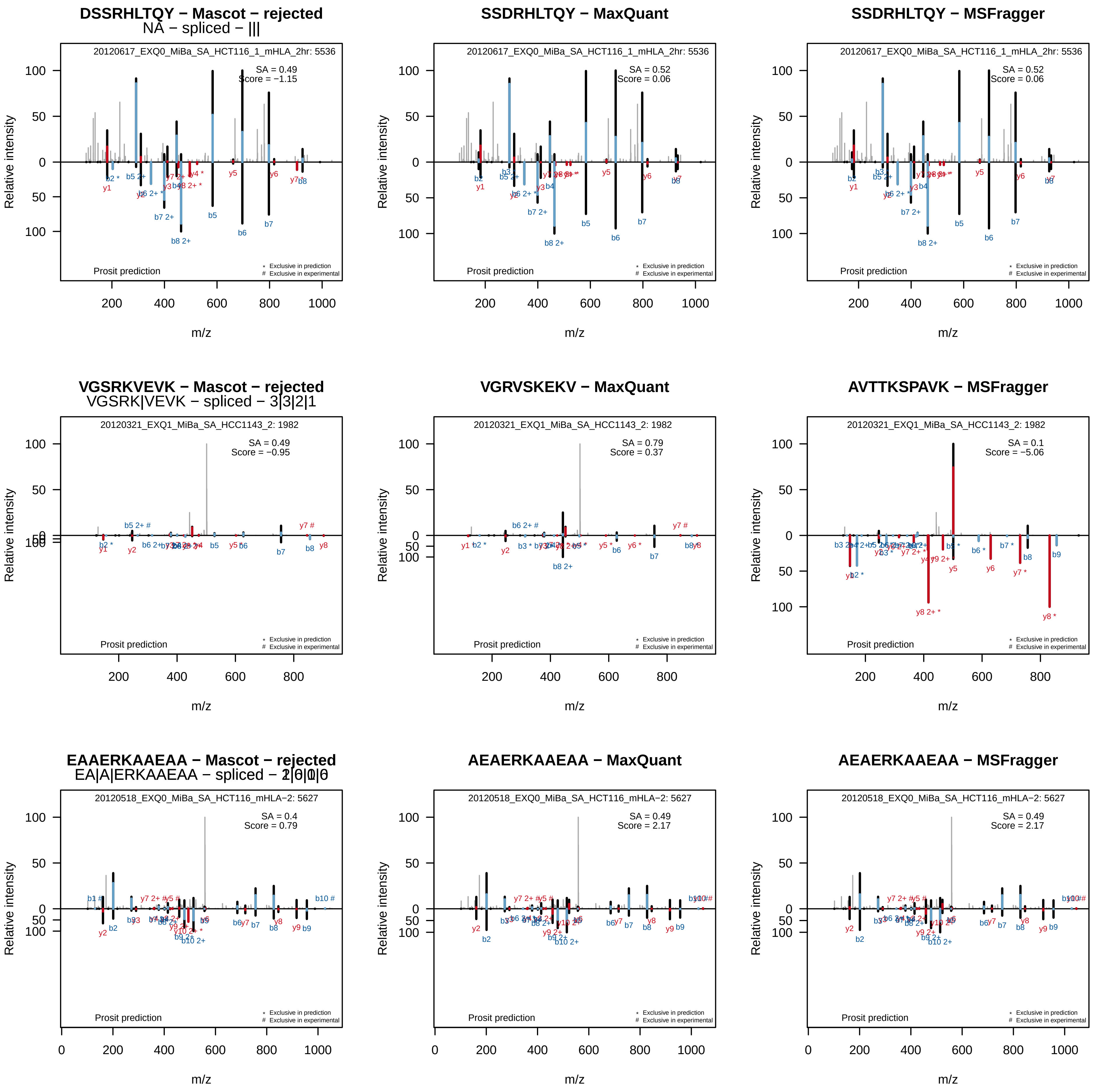
DSSRHLTQY − Mascot − rejected
NA − spliced − |||
20120617_EXQ0_MiBa_SA_HCT116_1_mHLA_2hr: 5536
SA = 0.49
Score = −1.15
SSDRHLTQY − MaxQuant
20120617_EXQ0_MiBa_SA_HCT116_1_mHLA_2hr: 5536
SA = 0.52
Score = 0.06
SSDRHLTQY − MSFragger
20120617_EXQ0_MiBa_SA_HCT116_1_mHLA_2hr: 5536
SA = 0.52
Score = 0.06
VGSRKVEVK − Mascot − rejected
VGSRK|VEVK − spliced − 3|3|2|1
20120321_EXQ1_MiBa_SA_HCC1143_2: 1982
SA = 0.49
Score = −0.95
VGRVSKEKV − MaxQuant
20120321_EXQ1_MiBa_SA_HCC1143_2: 1982
SA = 0.79
Score = 0.37
AVTTKSPAVK − MSFragger
20120321_EXQ1_MiBa_SA_HCC1143_2: 1982
SA = 0.1
Score = −5.06
EAAERKAAEAA − Mascot − rejected
EA|A|ERKAAEAA − spliced
20120518_EXQ0_MiBa_SA_HCT116_mHLA−2: 5627
SA = 0.4
Score = 0.79
AEAERKAAEAA − MaxQuant
20120518_EXQ0_MiBa_SA_HCT116_mHLA−2: 5627
SA = 0.49
Score = 2.17
AEAERKAAEAA − MSFragger
20120518_EXQ0_MiBa_SA_HCT116_mHLA−2: 5627
SA = 0.49
Score = 2.17
Relative intensity
m/z
Prosit prediction
⋆ Exclusive in prediction
Exclusive in experimental

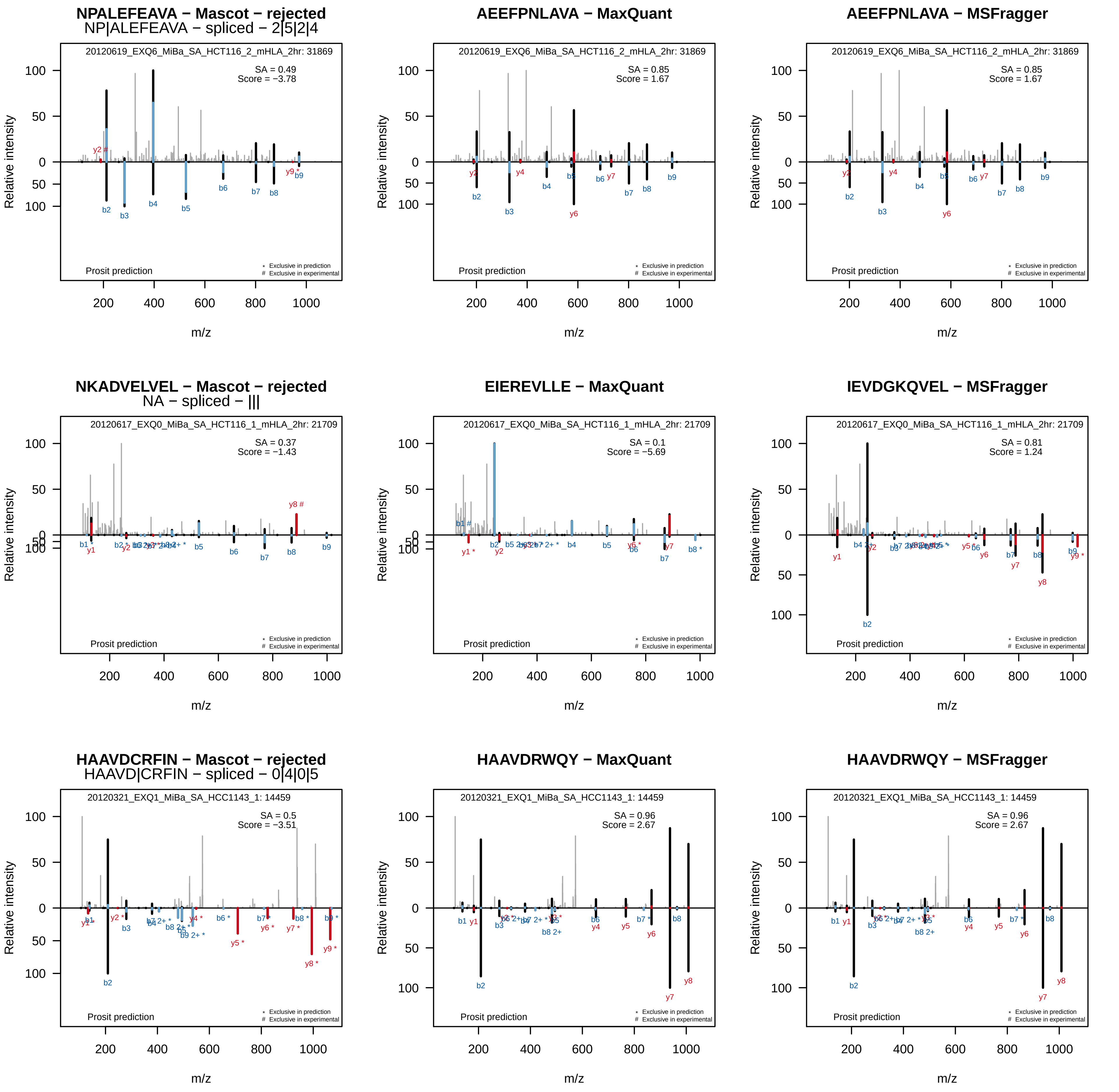

NPALEFEAVA − Mascot − rejected
NP|ALEFEAVA − spliced − 2|5|2|4
20120619_EXQ6_MiBa_SA_HCT116_2_mHLA_2hr: 31869
SA = 0.49
Score = −3.78
AEEFPNLAVA − MaxQuant
20120619_EXQ6_MiBa_SA_HCT116_2_mHLA_2hr: 31869
SA = 0.85
Score = 1.67
AEEFPNLAVA − MSFragger
20120619_EXQ6_MiBa_SA_HCT116_2_mHLA_2hr: 31869
SA = 0.85
Score = 1.67
NKADVELVEL − Mascot − rejected
NA − spliced − |||
20120617_EXQ0_MiBa_SA_HCT116_1_mHLA_2hr: 21709
SA = 0.37
Score = −1.43
EIEREVLLE − MaxQuant
20120617_EXQ0_MiBa_SA_HCT116_1_mHLA_2hr: 21709
SA = 0.1
Score = −5.69
IEVDGKQVEL − MSFragger
20120617_EXQ0_MiBa_SA_HCT116_1_mHLA_2hr: 21709
SA = 0.81
Score = 1.24
HAAVDCRFIN − Mascot − rejected
HAAVD|CRFIN − spliced − 0|4|0|5
20120321_EXQ1_MiBa_SA_HCC1143_1: 14459
SA = 0.5
Score = −3.51
HAAVDRWQY − MaxQuant
20120321_EXQ1_MiBa_SA_HCC1143_1: 14459
SA = 0.96
Score = 2.67
HAAVDRWQY − MSFragger
20120321_EXQ1_MiBa_SA_HCC1143_1: 14459
SA = 0.96
Score = 2.67
Relative intensity
m/z
Prosit prediction
* Exclusive in prediction
# Exclusive in experimental

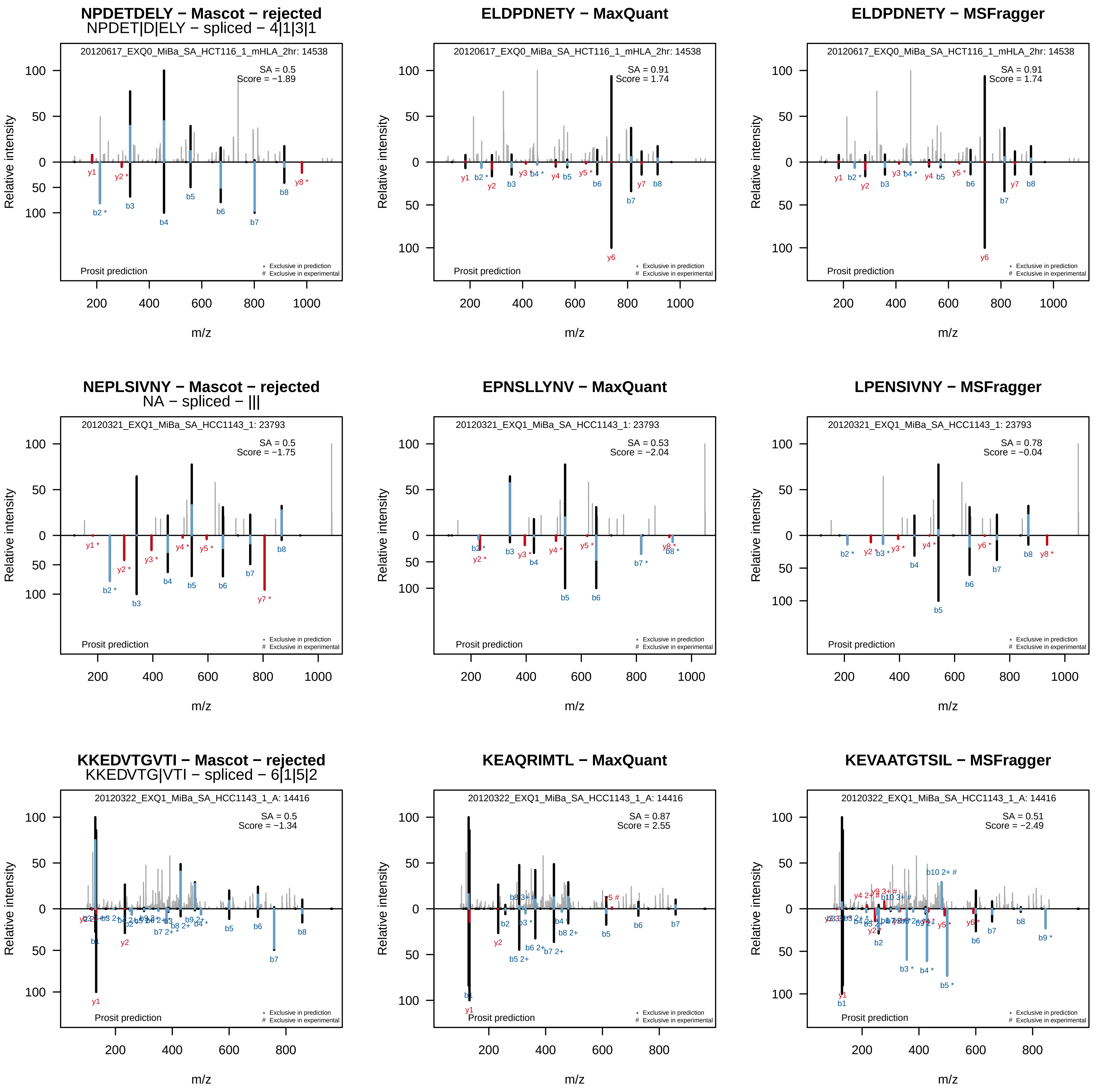
NPDETDELY − Mascot − rejected
NPDET|D|ELY − spliced − 4|1|3|1
20120617_EXQ0_MiBa_SA_HCT116_1_mHLA_2hr: 14538
SA = 0.5
Score = −1.89
ELDPDNETY − MaxQuant
20120617_EXQ0_MiBa_SA_HCT116_1_mHLA_2hr: 14538
SA = 0.91
Score = 1.74
ELDPDNETY − MSFragger
20120617_EXQ0_MiBa_SA_HCT116_1_mHLA_2hr: 14538
SA = 0.91
Score = 1.74
NEPLSIVNY − Mascot − rejected
NA − spliced − |||
20120321_EXQ1_MiBa_SA_HCC1143_1: 23793
SA = 0.5
Score = −1.75
EPNSLLYNV − MaxQuant
20120321_EXQ1_MiBa_SA_HCC1143_1: 23793
SA = 0.53
Score = −2.04
LPENSIVNY − MSFragger
20120321_EXQ1_MiBa_SA_HCC1143_1: 23793
SA = 0.78
Score = −0.04
KKEDVTGVTI − Mascot − rejected
KKEDVTG|VTI − spliced − 6|1|5|2
20120322_EXQ1_MiBa_SA_HCC1143_1_A: 14416
SA = 0.5
Score = −1.34
KEAQRIMTL − MaxQuant
20120322_EXQ1_MiBa_SA_HCC1143_1_A: 14416
SA = 0.87
Score = 2.55
KEVAATGTSIL − MSFragger
20120322_EXQ1_MiBa_SA_HCC1143_1_A: 14416
SA = 0.51
Score = −2.49
Relative intensity
m/z
Prosit prediction
⋆ Exclusive in prediction
Exclusive in experimental

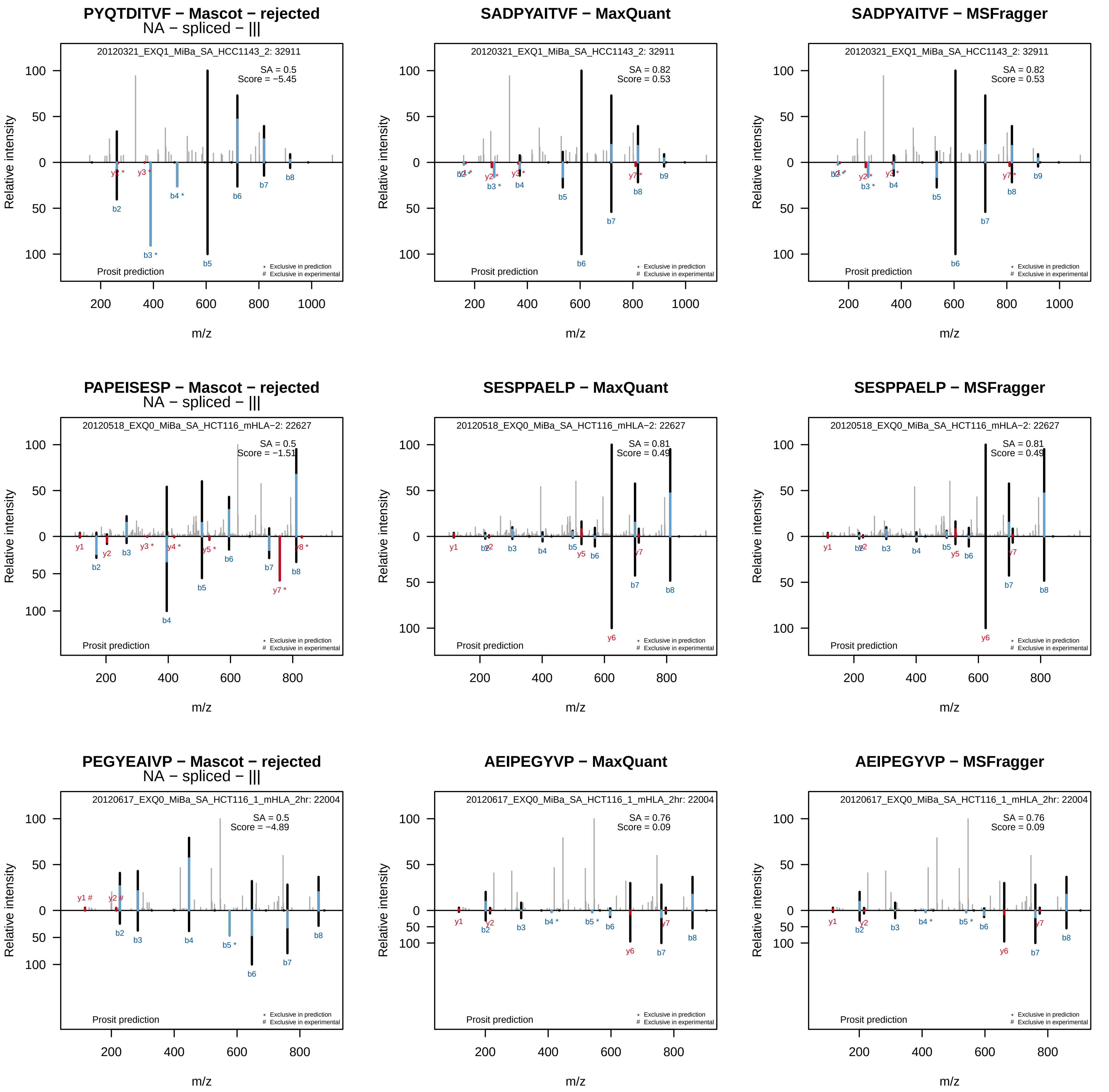

PYQTDITVF − Mascot − rejected
NA − spliced − |||
20120321_EXQ1_MiBa_SA_HCC1143_2: 32911
SA = 0.5
Score = −5.45
SADPYAITVF − MaxQuant
20120321_EXQ1_MiBa_SA_HCC1143_2: 32911
SA = 0.82
Score = 0.53
SADPYAITVF − MSFragger
20120321_EXQ1_MiBa_SA_HCC1143_2: 32911
SA = 0.82
Score = 0.53
PAPEISESP − Mascot − rejected
NA − spliced − |||
20120518_EXQ0_MiBa_SA_HCT116_mHLA−2: 22627
SA = 0.5
Score = −1.51
SESPPAELP − MaxQuant
20120518_EXQ0_MiBa_SA_HCT116_mHLA−2: 22627
SA = 0.81
Score = 0.49
SESPPAELP − MSFragger
20120518_EXQ0_MiBa_SA_HCT116_mHLA−2: 22627
SA = 0.81
Score = 0.49
PEGYEAIVP − Mascot − rejected
NA − spliced − |||
20120617_EXQ0_MiBa_SA_HCT116_1_mHLA_2hr: 22004
SA = 0.5
Score = −4.89
AEIPEGYVP − MaxQuant
20120617_EXQ0_MiBa_SA_HCT116_1_mHLA_2hr: 22004
SA = 0.76
Score = 0.09
AEIPEGYVP − MSFragger
20120617_EXQ0_MiBa_SA_HCT116_1_mHLA_2hr: 22004
SA = 0.76
Score = 0.09
Relative intensity
m/z
Prosit prediction
* Exclusive in prediction
# Exclusive in experimental

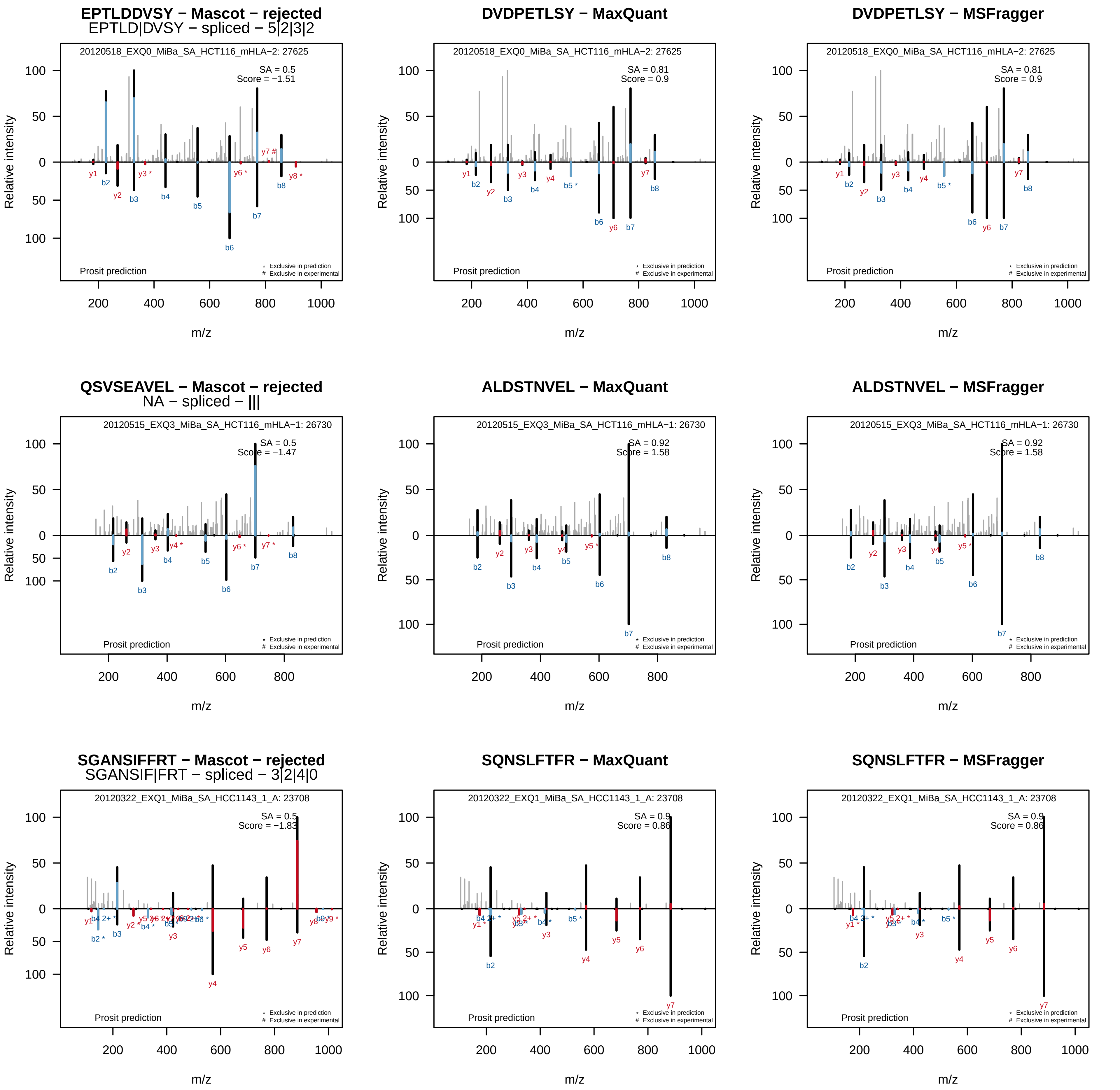

EPTLDDVSY − Mascot − rejected
EPTLD|DVSY − spliced − 5|2|3|2
20120518_EXQ0_MiBa_SA_HCT116_mHLA−2: 27625
SA = 0.5
Score = −1.51
DVDPETLSY − MaxQuant
20120518_EXQ0_MiBa_SA_HCT116_mHLA−2: 27625
SA = 0.81
Score = 0.9
DVDPETLSY − MSFragger
20120518_EXQ0_MiBa_SA_HCT116_mHLA−2: 27625
SA = 0.81
Score = 0.9
QSVSEAVEL − Mascot − rejected
NA − spliced − |||
20120515_EXQ3_MiBa_SA_HCT116_mHLA−1: 26730
SA = 0.5
Score = −1.47
ALDSTNVEL − MaxQuant
20120515_EXQ3_MiBa_SA_HCT116_mHLA−1: 26730
SA = 0.92
Score = 1.58
ALDSTNVEL − MSFragger
20120515_EXQ3_MiBa_SA_HCT116_mHLA−1: 26730
SA = 0.92
Score = 1.58
SGANSIFFRT − Mascot − rejected
SGANSIF|FRT − spliced − 3|2|4|0
20120322_EXQ1_MiBa_SA_HCC1143_1_A: 23708
SA = 0.5
Score = −1.83
SQNSLFTFR − MaxQuant
20120322_EXQ1_MiBa_SA_HCC1143_1_A: 23708
SA = 0.9
Score = 0.86
SQNSLFTFR − MSFragger
20120322_EXQ1_MiBa_SA_HCC1143_1_A: 23708
SA = 0.9
Score = 0.86
Relative intensity
m/z
Prosit prediction
* Exclusive in prediction
# Exclusive in experimental

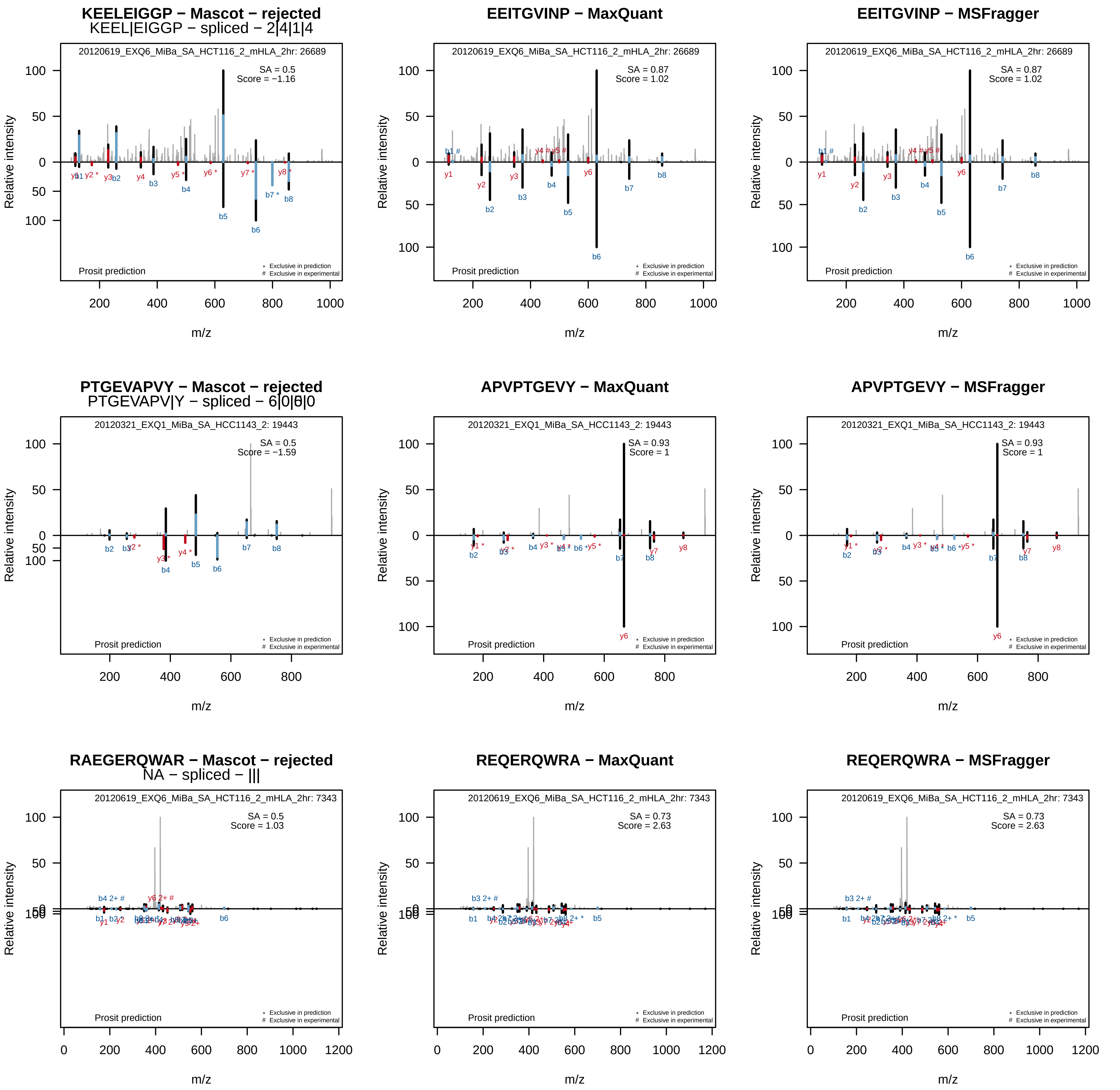
KEELEIGGP − Mascot − rejected
KEEL|EIGGP − spliced − 2|4|1|4
20120619_EXQ6_MiBa_SA_HCT116_2_mHLA_2hr: 26689
SA = 0.5
Score = −1.16
Prosit prediction
Exclusive in prediction
Exclusive in experimental
EEITGVINP − MaxQuant
20120619_EXQ6_MiBa_SA_HCT116_2_mHLA_2hr: 26689
SA = 0.87
Score = 1.02
Prosit prediction
EEITGVINP − MSFragger
20120619_EXQ6_MiBa_SA_HCT116_2_mHLA_2hr: 26689
SA = 0.87
Score = 1.02
Prosit prediction
PTGEVAPVY − Mascot − rejected
PTGEVAPV|Y − spliced − 6|0|6|0
20120321_EXQ1_MiBa_SA_HCC1143_2: 19443
SA = 0.5
Score = −1.59
Prosit prediction
APVPTGEVY − MaxQuant
20120321_EXQ1_MiBa_SA_HCC1143_2: 19443
SA = 0.93
Score = 1
Prosit prediction
APVPTGEVY − MSFragger
20120321_EXQ1_MiBa_SA_HCC1143_2: 19443
SA = 0.93
Score = 1
Prosit prediction
RAEGERQWAR − Mascot − rejected
NA − spliced − |||
20120619_EXQ6_MiBa_SA_HCT116_2_mHLA_2hr: 7343
SA = 0.5
Score = 1.03
Prosit prediction
REQERQWRA − MaxQuant
20120619_EXQ6_MiBa_SA_HCT116_2_mHLA_2hr: 7343
SA = 0.73
Score = 2.63
Prosit prediction
REQERQWRA − MSFragger
20120619_EXQ6_MiBa_SA_HCT116_2_mHLA_2hr: 7343
SA = 0.73
Score = 2.63
Prosit prediction
Relative intensity
m/z

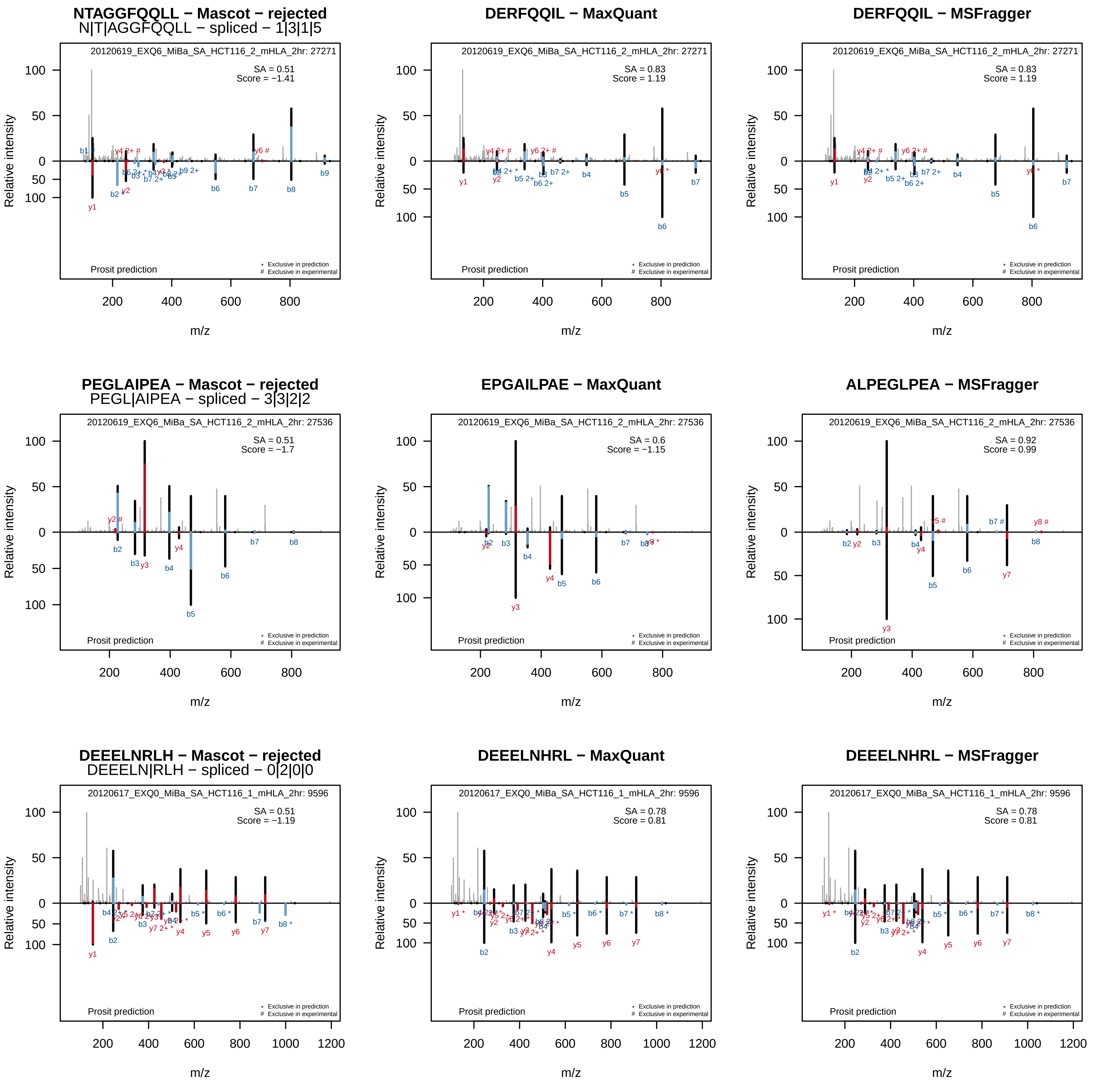
NTAGGFQQLL − Mascot − rejected
N|T|AGGFQQLL − spliced − 1|3|1|5
20120619_EXQ6_MiBa_SA_HCT116_2_mHLA_2hr: 27271
SA = 0.51
Score = −1.41
DERFQQIL − MaxQuant
20120619_EXQ6_MiBa_SA_HCT116_2_mHLA_2hr: 27271
SA = 0.83
Score = 1.19
DERFQQIL − MSFragger
20120619_EXQ6_MiBa_SA_HCT116_2_mHLA_2hr: 27271
SA = 0.83
Score = 1.19
PEGLAIPEA − Mascot − rejected
PEGL|AIPEA − spliced − 3|3|2|2
20120619_EXQ6_MiBa_SA_HCT116_2_mHLA_2hr: 27536
SA = 0.51
Score = −1.7
EPGAILPAE − MaxQuant
20120619_EXQ6_MiBa_SA_HCT116_2_mHLA_2hr: 27536
SA = 0.6
Score = −1.15
ALPEGLPEA − MSFragger
20120619_EXQ6_MiBa_SA_HCT116_2_mHLA_2hr: 27536
SA = 0.92
Score = 0.99
DEEELNRLH − Mascot − rejected
DEEELN|RLH − spliced − 0|2|0|0
20120617_EXQ0_MiBa_SA_HCT116_1_mHLA_2hr: 9596
SA = 0.51
Score = −1.19
DEEELNHRL − MaxQuant
20120617_EXQ0_MiBa_SA_HCT116_1_mHLA_2hr: 9596
SA = 0.78
Score = 0.81
DEEELNHRL − MSFragger
20120617_EXQ0_MiBa_SA_HCT116_1_mHLA_2hr: 9596
SA = 0.78
Score = 0.81
Relative intensity
m/z
Prosit prediction
⋆ Exclusive in prediction
Exclusive in experimental

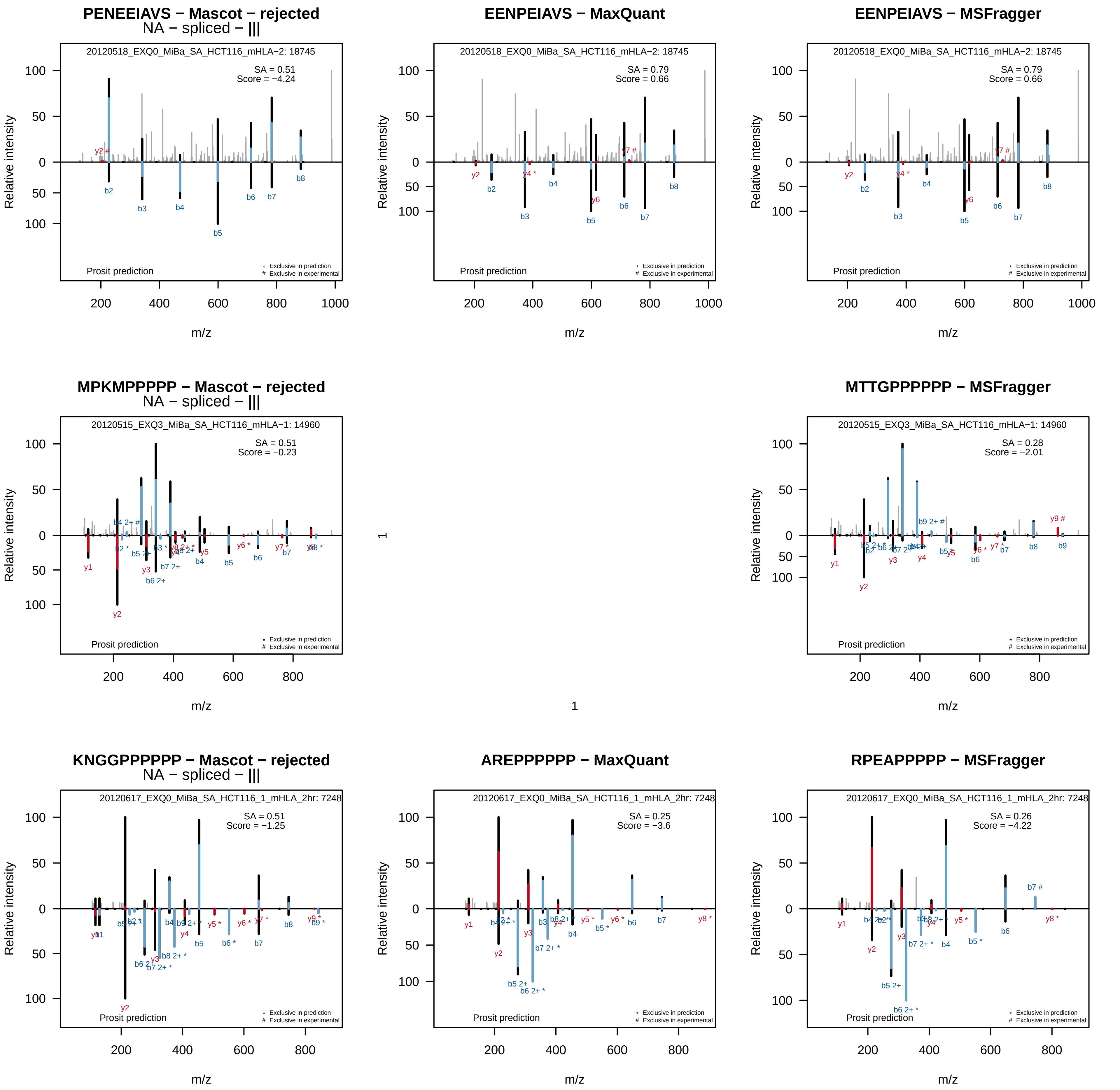

PENEEIAVS − Mascot − rejected
NA − spliced − |||
20120518_EXQ0_MiBa_SA_HCT116_mHLA−2: 18745
SA = 0.51
Score = −4.24
EENPEIAVS − MaxQuant
20120518_EXQ0_MiBa_SA_HCT116_mHLA−2: 18745
SA = 0.79
Score = 0.66
EENPEIAVS − MSFragger
20120518_EXQ0_MiBa_SA_HCT116_mHLA−2: 18745
SA = 0.79
Score = 0.66
MPKMPPPPP − Mascot − rejected
NA − spliced − |||
20120515_EXQ3_MiBa_SA_HCT116_mHLA−1: 14960
SA = 0.51
Score = −0.23
MTTGPPPPPP − MSFragger
20120515_EXQ3_MiBa_SA_HCT116_mHLA−1: 14960
SA = 0.28
Score = −2.01
KNGGPPPPPP − Mascot − rejected
NA − spliced − |||
20120617_EXQ0_MiBa_SA_HCT116_1_mHLA_2hr: 7248
SA = 0.51
Score = −1.25
AREPPPPPP − MaxQuant
20120617_EXQ0_MiBa_SA_HCT116_1_mHLA_2hr: 7248
SA = 0.25
Score = −3.6
RPEAPPPPP − MSFragger
20120617_EXQ0_MiBa_SA_HCT116_1_mHLA_2hr: 7248
SA = 0.26
Score = −4.22
Relative intensity
m/z
Prosit prediction
⋆ Exclusive in prediction
Exclusive in experimental

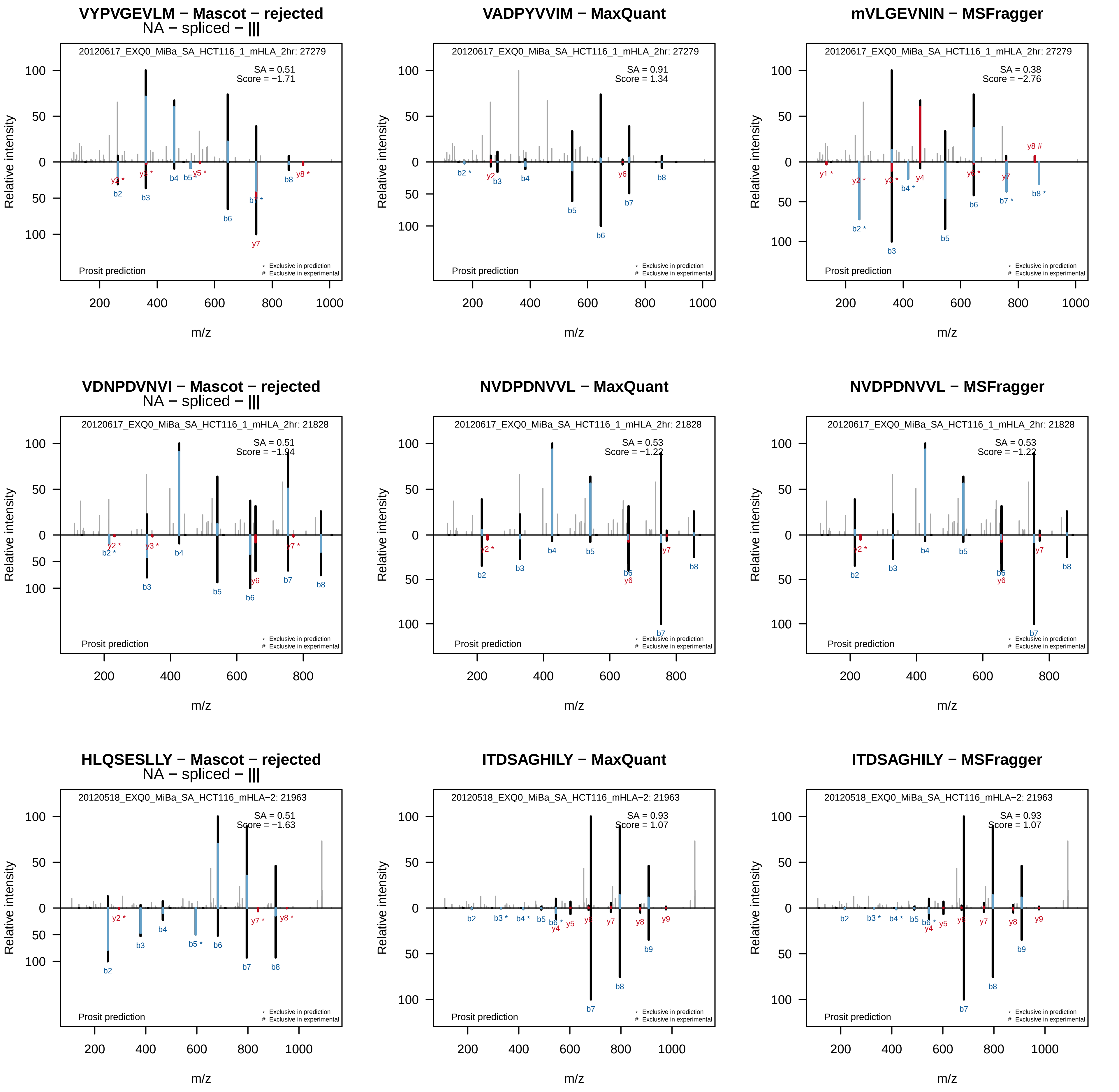
VYPVGEVLM − Mascot − rejected
NA − spliced − |||
20120617_EXQ0_MiBa_SA_HCT116_1_mHLA_2hr: 27279
SA = 0.51
Score = −1.71
VADPYVVIM − MaxQuant
20120617_EXQ0_MiBa_SA_HCT116_1_mHLA_2hr: 27279
SA = 0.91
Score = 1.34
mVLGEVNIN − MSFragger
20120617_EXQ0_MiBa_SA_HCT116_1_mHLA_2hr: 27279
SA = 0.38
Score = −2.76
VDNPDVNVI − Mascot − rejected
NA − spliced − |||
20120617_EXQ0_MiBa_SA_HCT116_1_mHLA_2hr: 21828
SA = 0.51
Score = −1.94
NVDPDNVVL − MaxQuant
20120617_EXQ0_MiBa_SA_HCT116_1_mHLA_2hr: 21828
SA = 0.53
Score = −1.22
NVDPDNVVL − MSFragger
20120617_EXQ0_MiBa_SA_HCT116_1_mHLA_2hr: 21828
SA = 0.53
Score = −1.22
HLQSESLLY − Mascot − rejected
NA − spliced − |||
20120518_EXQ0_MiBa_SA_HCT116_mHLA−2: 21963
SA = 0.51
Score = −1.63
ITDSAGHILY − MaxQuant
20120518_EXQ0_MiBa_SA_HCT116_mHLA−2: 21963
SA = 0.93
Score = 1.07
ITDSAGHILY − MSFragger
20120518_EXQ0_MiBa_SA_HCT116_mHLA−2: 21963
SA = 0.93
Score = 1.07
Relative intensity
m/z
Prosit prediction
⋆ Exclusive in prediction
Exclusive in experimental

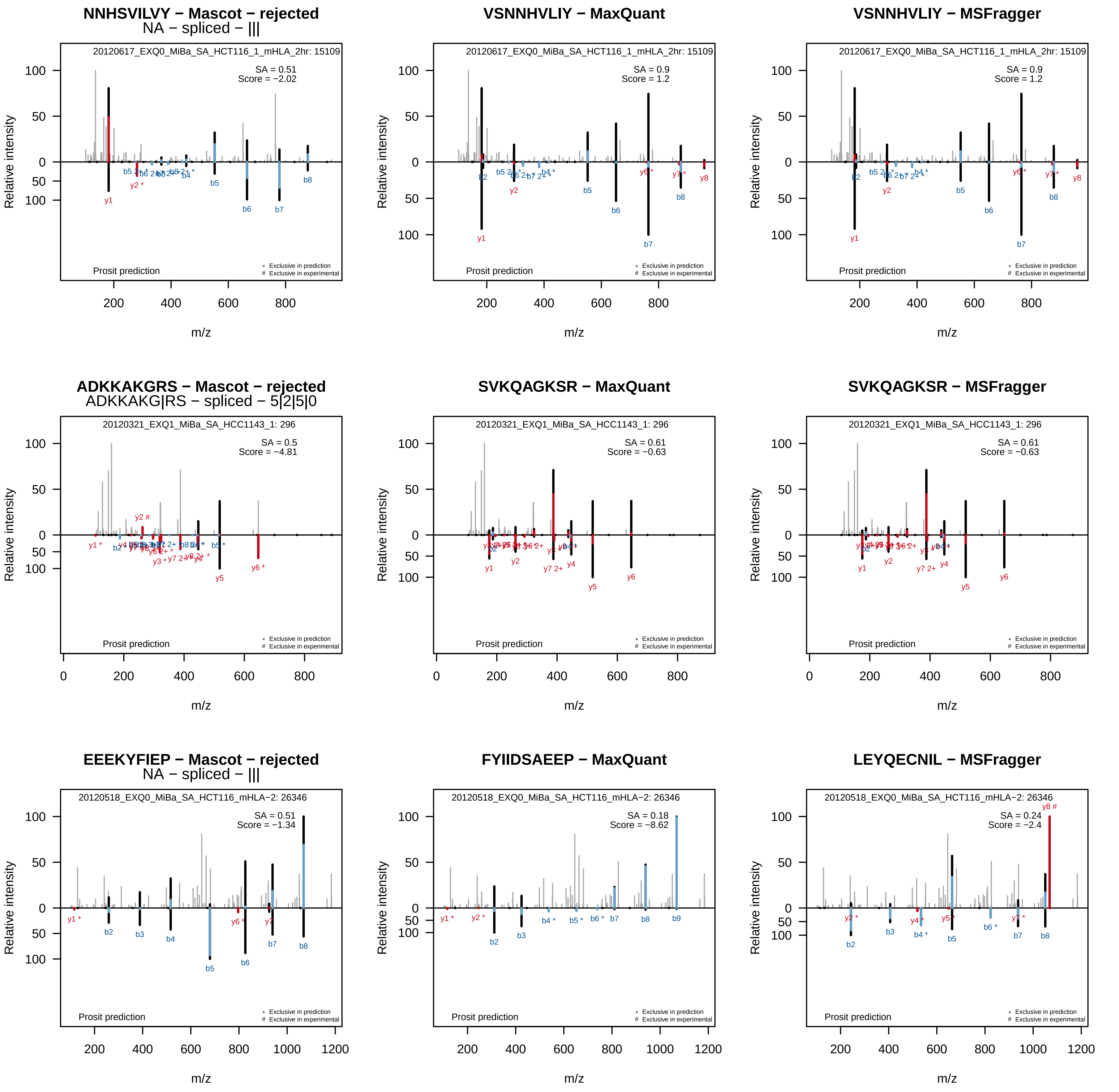

NNHSVILVY − Mascot − rejected
NA − spliced − |||
20120617_EXQ0_MiBa_SA_HCT116_1_mHLA_2hr: 15109
SA = 0.51
Score = −2.02
VSNNHVLIY − MaxQuant
20120617_EXQ0_MiBa_SA_HCT116_1_mHLA_2hr: 15109
SA = 0.9
Score = 1.2
VSNNHVLIY − MSFragger
20120617_EXQ0_MiBa_SA_HCT116_1_mHLA_2hr: 15109
SA = 0.9
Score = 1.2
ADKKAKGRS − Mascot − rejected
ADKKAKG|RS − spliced − 5|2|5|0
20120321_EXQ1_MiBa_SA_HCC1143_1: 296
SA = 0.5
Score = −4.81
SVKQAGKSR − MaxQuant
20120321_EXQ1_MiBa_SA_HCC1143_1: 296
SA = 0.61
Score = −0.63
SVKQAGKSR − MSFragger
20120321_EXQ1_MiBa_SA_HCC1143_1: 296
SA = 0.61
Score = −0.63
EEEKYFIEP − Mascot − rejected
NA − spliced − |||
20120518_EXQ0_MiBa_SA_HCT116_mHLA−2: 26346
SA = 0.51
Score = −1.34
FYIIDSAEEP − MaxQuant
20120518_EXQ0_MiBa_SA_HCT116_mHLA−2: 26346
SA = 0.18
Score = −8.62
LEYQECNIL − MSFragger
20120518_EXQ0_MiBa_SA_HCT116_mHLA−2: 26346
SA = 0.24
Score = −2.4
Relative intensity
m/z
Prosit prediction
⋆ Exclusive in prediction
Exclusive in experimental

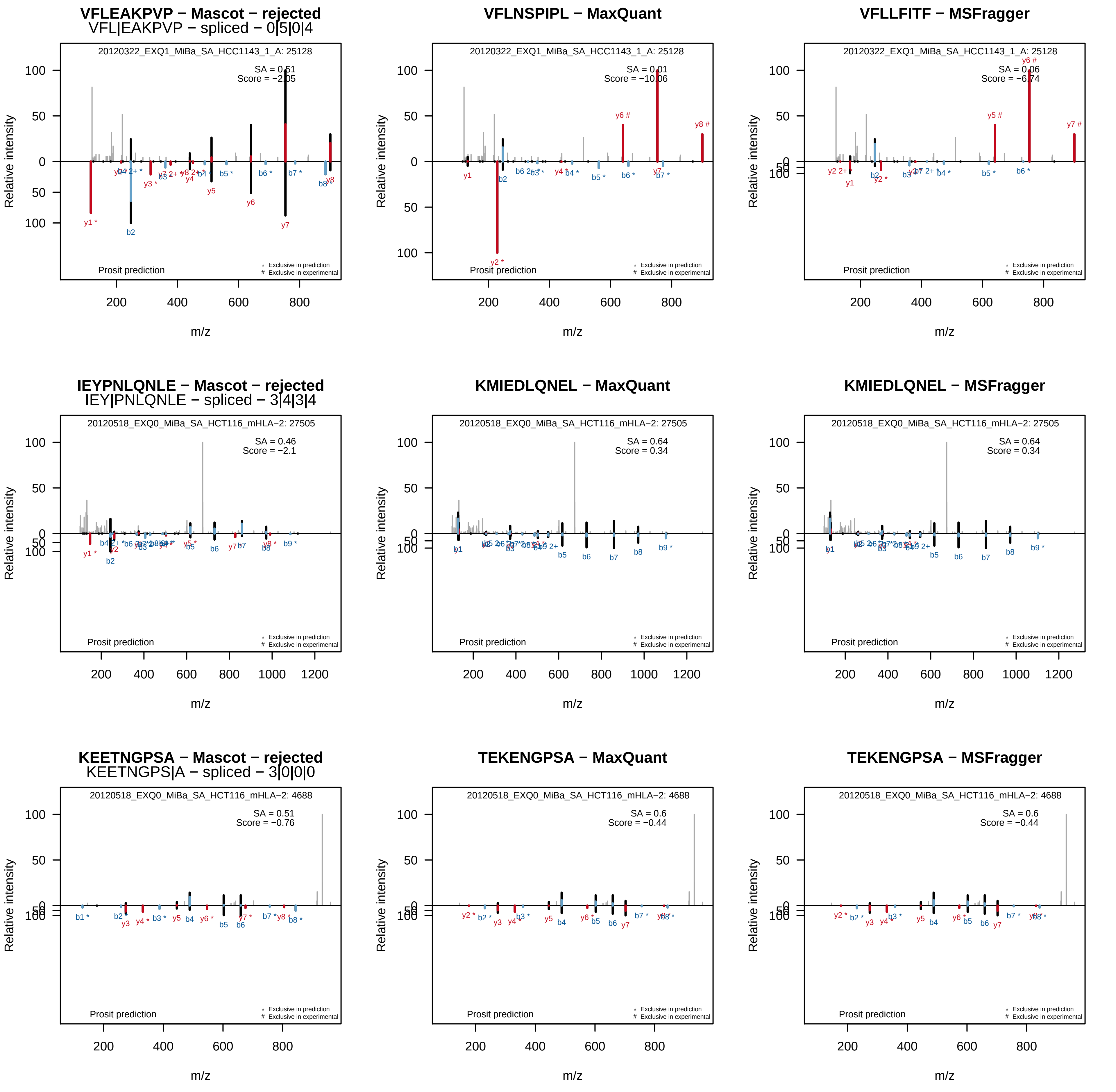

VFLEAKPVP − Mascot − rejected
VFL|EAKPVP − spliced − 0|5|0|4
20120322_EXQ1_MiBa_SA_HCC1143_1_A: 25128
SA = 0.51
Score = −2.05
VFLNSPIPL − MaxQuant
20120322_EXQ1_MiBa_SA_HCC1143_1_A: 25128
SA = 0.01
Score = −10.06
VFLLFITF − MSFragger
20120322_EXQ1_MiBa_SA_HCC1143_1_A: 25128
SA = 0.06
Score = −6.74
IEYPNLQNLE − Mascot − rejected
IEY|PNLQNLE − spliced − 3|4|3|4
20120518_EXQ0_MiBa_SA_HCT116_mHLA−2: 27505
SA = 0.46
Score = −2.1
KMIEDLQNEL − MaxQuant
20120518_EXQ0_MiBa_SA_HCT116_mHLA−2: 27505
SA = 0.64
Score = 0.34
KMIEDLQNEL − MSFragger
20120518_EXQ0_MiBa_SA_HCT116_mHLA−2: 27505
SA = 0.64
Score = 0.34
KEETNGPSA − Mascot − rejected
KEETNGPS|A − spliced − 3|0|0|0
20120518_EXQ0_MiBa_SA_HCT116_mHLA−2: 4688
SA = 0.51
Score = −0.76
TEKENGPSA − MaxQuant
20120518_EXQ0_MiBa_SA_HCT116_mHLA−2: 4688
SA = 0.6
Score = −0.44
TEKENGPSA − MSFragger
20120518_EXQ0_MiBa_SA_HCT116_mHLA−2: 4688
SA = 0.6
Score = −0.44
Relative intensity
m/z
Prosit prediction
⋆ Exclusive in prediction
Exclusive in experimental

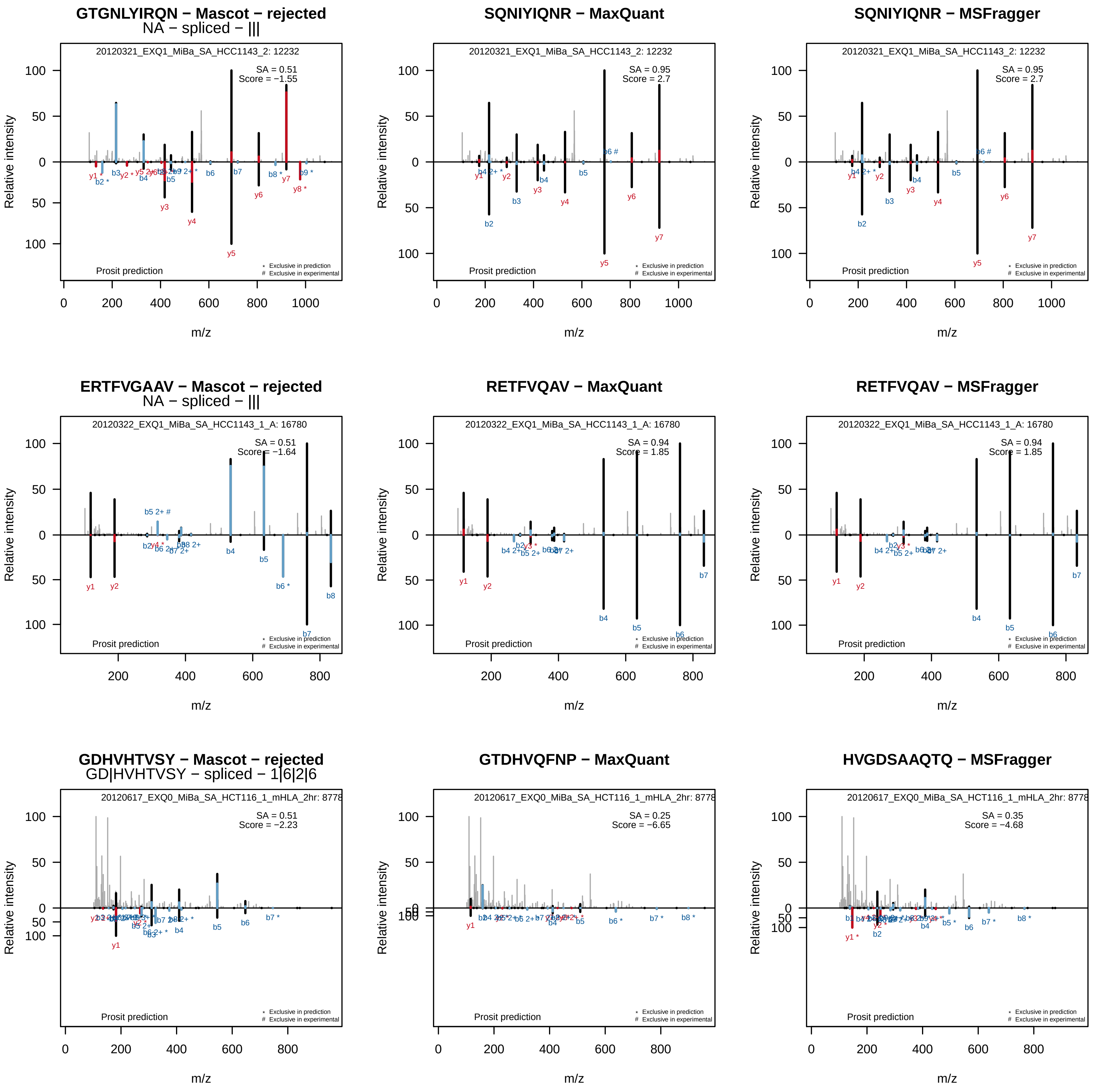
GTGNLYIRQN − Mascot − rejected
NA − spliced − |||
20120321_EXQ1_MiBa_SA_HCC1143_2: 12232
SA = 0.51
Score = −1.55
SQNIYIQNR − MaxQuant
20120321_EXQ1_MiBa_SA_HCC1143_2: 12232
SA = 0.95
Score = 2.7
SQNIYIQNR − MSFragger
20120321_EXQ1_MiBa_SA_HCC1143_2: 12232
SA = 0.95
Score = 2.7
ERTFVGAAV − Mascot − rejected
NA − spliced − |||
20120322_EXQ1_MiBa_SA_HCC1143_1_A: 16780
SA = 0.51
Score = −1.64
RETFVQAV − MaxQuant
20120322_EXQ1_MiBa_SA_HCC1143_1_A: 16780
SA = 0.94
Score = 1.85
RETFVQAV − MSFragger
20120322_EXQ1_MiBa_SA_HCC1143_1_A: 16780
SA = 0.94
Score = 1.85
GDHVHTVSY − Mascot − rejected
GD|HVHTVSY − spliced − 1|6|2|6
20120617_EXQ0_MiBa_SA_HCT116_1_mHLA_2hr: 8778
SA = 0.51
Score = −2.23
GTDHVQFNP − MaxQuant
20120617_EXQ0_MiBa_SA_HCT116_1_mHLA_2hr: 8778
SA = 0.25
Score = −6.65
HVGDSAAQTQ − MSFragger
20120617_EXQ0_MiBa_SA_HCT116_1_mHLA_2hr: 8778
SA = 0.35
Score = −4.68
Relative intensity
m/z
Prosit prediction
⋆ Exclusive in prediction
Exclusive in experimental

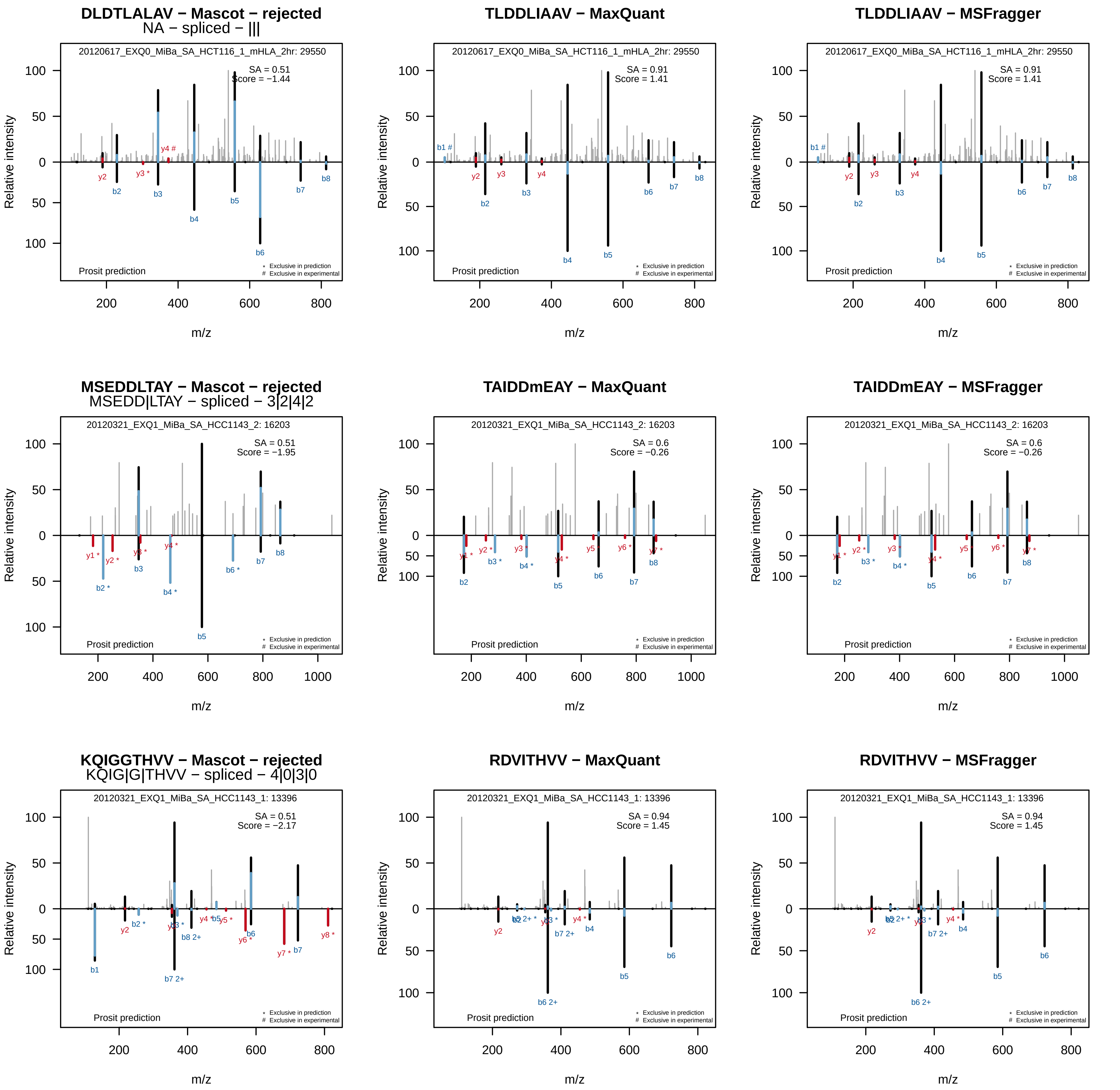
DLDTLALAV − Mascot − rejected
NA − spliced − |||
20120617_EXQ0_MiBa_SA_HCT116_1_mHLA_2hr: 29550
SA = 0.51
Score = −1.44
TLDDLIAAV − MaxQuant
20120617_EXQ0_MiBa_SA_HCT116_1_mHLA_2hr: 29550
SA = 0.91
Score = 1.41
TLDDLIAAV − MSFragger
20120617_EXQ0_MiBa_SA_HCT116_1_mHLA_2hr: 29550
SA = 0.91
Score = 1.41
MSEDDLTAY − Mascot − rejected
MSEDD|LTAY − spliced − 3|2|4|2
20120321_EXQ1_MiBa_SA_HCC1143_2: 16203
SA = 0.51
Score = −1.95
TAIDDmEAY − MaxQuant
20120321_EXQ1_MiBa_SA_HCC1143_2: 16203
SA = 0.6
Score = −0.26
TAIDDmEAY − MSFragger
20120321_EXQ1_MiBa_SA_HCC1143_2: 16203
SA = 0.6
Score = −0.26
KQIGGTHVV − Mascot − rejected
KQIG|G|THVV − spliced − 4|0|3|0
20120321_EXQ1_MiBa_SA_HCC1143_1: 13396
SA = 0.51
Score = −2.17
RDVITHVV − MaxQuant
20120321_EXQ1_MiBa_SA_HCC1143_1: 13396
SA = 0.94
Score = 1.45
RDVITHVV − MSFragger
20120321_EXQ1_MiBa_SA_HCC1143_1: 13396
SA = 0.94
Score = 1.45
Relative intensity
m/z
Prosit prediction
⋆ Exclusive in prediction
Exclusive in experimental

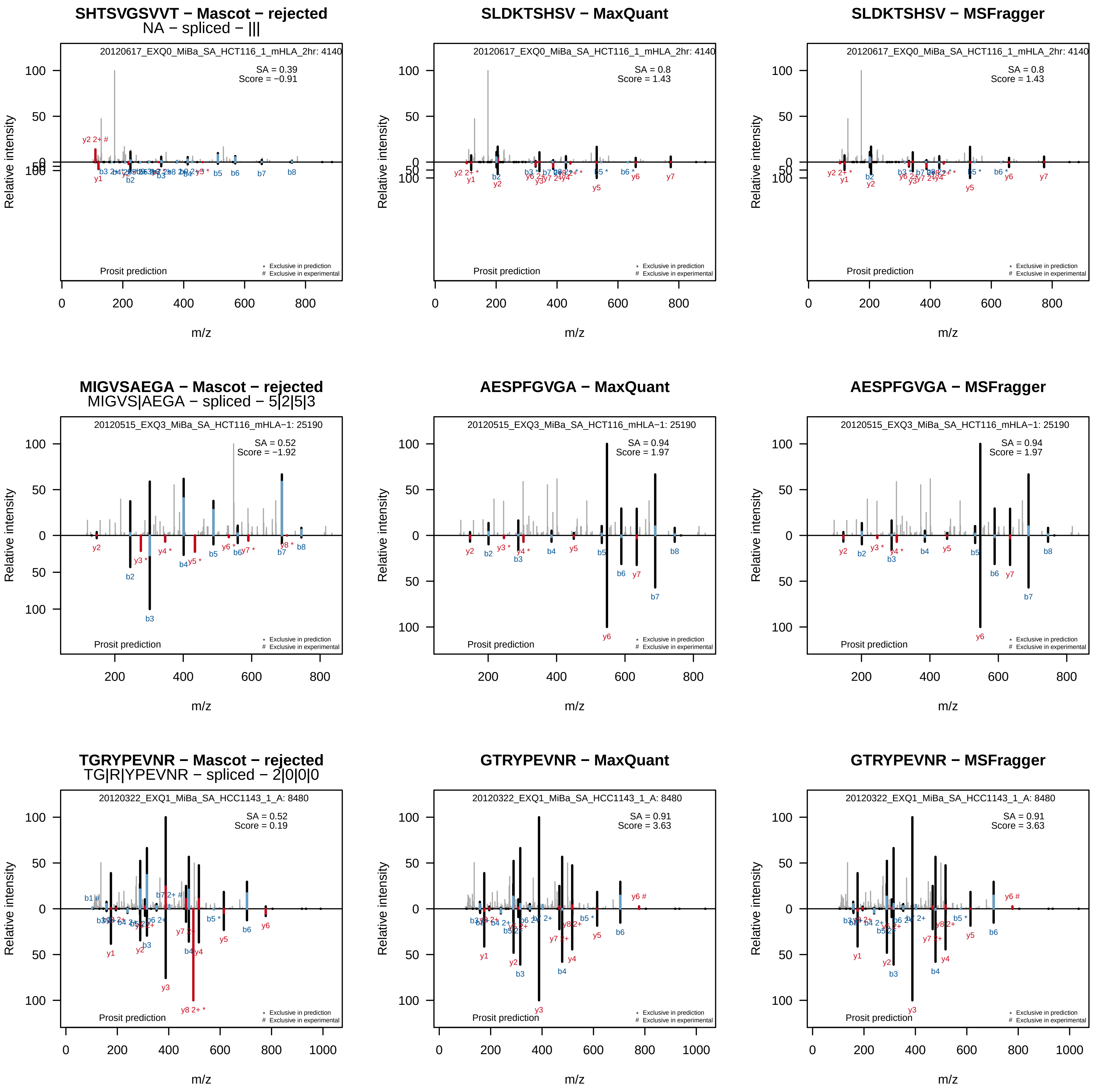
SHTSVGSVVT − Mascot − rejected
NA − spliced − |||
20120617_EXQ0_MiBa_SA_HCT116_1_mHLA_2hr: 4140
SA = 0.39
Score = −0.91
SLDKTSHSV − MaxQuant
20120617_EXQ0_MiBa_SA_HCT116_1_mHLA_2hr: 4140
SA = 0.8
Score = 1.43
SLDKTSHSV − MSFragger
20120617_EXQ0_MiBa_SA_HCT116_1_mHLA_2hr: 4140
SA = 0.8
Score = 1.43
MIGVSAEGA − Mascot − rejected
MIGVS|AEGA − spliced − 5|2|5|3
20120515_EXQ3_MiBa_SA_HCT116_mHLA−1: 25190
SA = 0.52
Score = −1.92
AESPFGVGA − MaxQuant
20120515_EXQ3_MiBa_SA_HCT116_mHLA−1: 25190
SA = 0.94
Score = 1.97
AESPFGVGA − MSFragger
20120515_EXQ3_MiBa_SA_HCT116_mHLA−1: 25190
SA = 0.94
Score = 1.97
TGRYPEVNR − Mascot − rejected
TG|R|YPEVNR − spliced − 2|0|0|0
20120322_EXQ1_MiBa_SA_HCC1143_1_A: 8480
SA = 0.52
Score = 0.19
GTRYPEVNR − MaxQuant
20120322_EXQ1_MiBa_SA_HCC1143_1_A: 8480
SA = 0.91
Score = 3.63
GTRYPEVNR − MSFragger
20120322_EXQ1_MiBa_SA_HCC1143_1_A: 8480
SA = 0.91
Score = 3.63
Relative intensity
m/z
Prosit prediction
⋆ Exclusive in prediction
Exclusive in experimental

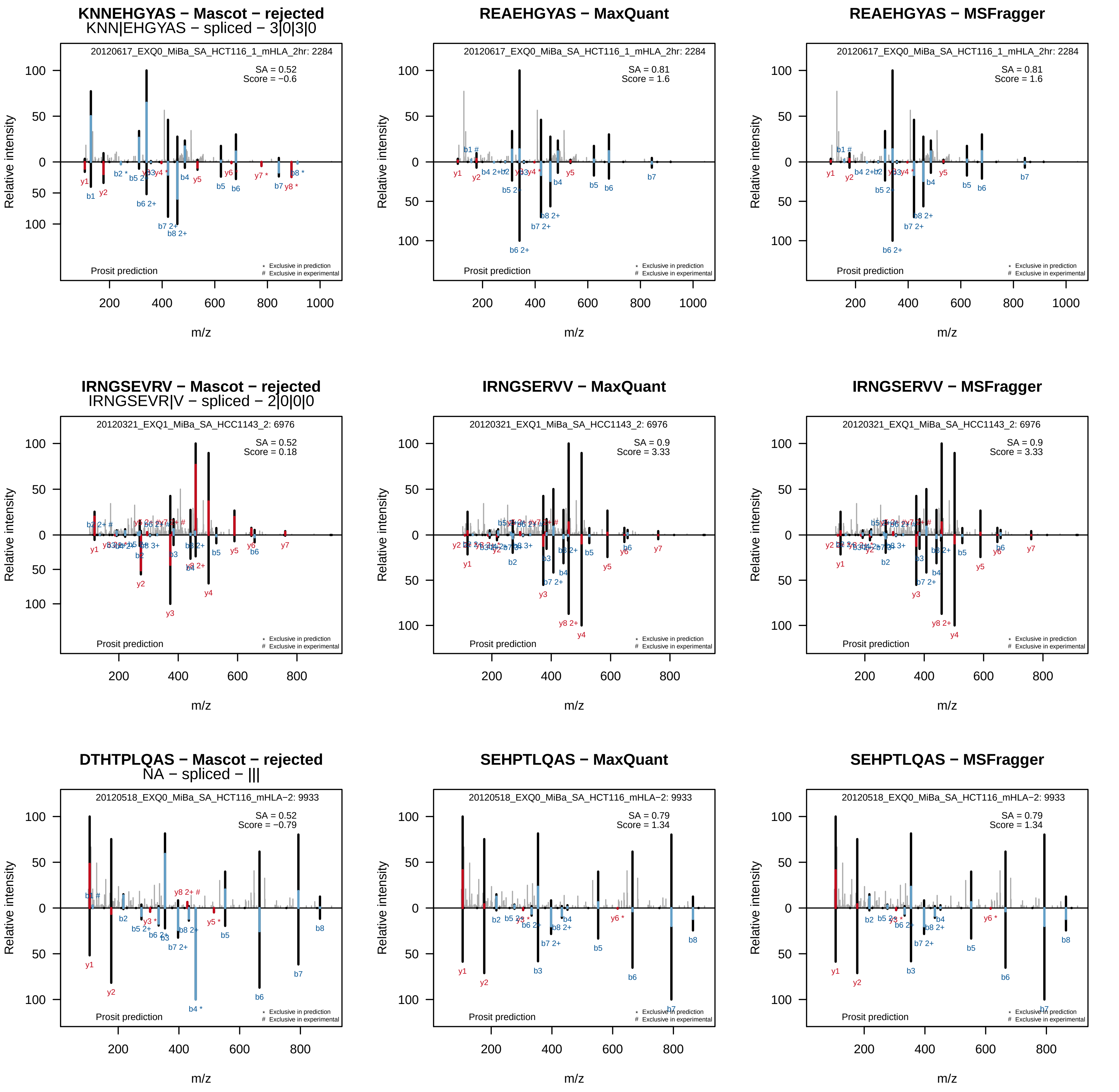

KNNEHGYAS − Mascot − rejected
KNN|EHGYAS − spliced − 3|0|3|0
20120617_EXQ0_MiBa_SA_HCT116_1_mHLA_2hr: 2284
SA = 0.52
Score = −0.6
REAEHGYAS − MaxQuant
20120617_EXQ0_MiBa_SA_HCT116_1_mHLA_2hr: 2284
SA = 0.81
Score = 1.6
REAEHGYAS − MSFragger
20120617_EXQ0_MiBa_SA_HCT116_1_mHLA_2hr: 2284
SA = 0.81
Score = 1.6
IRNGSEVRV − Mascot − rejected
IRNGSEVR|V − spliced − 2|0|0|0
20120321_EXQ1_MiBa_SA_HCC1143_2: 6976
SA = 0.52
Score = 0.18
IRNGSERVV − MaxQuant
20120321_EXQ1_MiBa_SA_HCC1143_2: 6976
SA = 0.9
Score = 3.33
IRNGSERVV − MSFragger
20120321_EXQ1_MiBa_SA_HCC1143_2: 6976
SA = 0.9
Score = 3.33
DTHTPLQAS − Mascot − rejected
NA − spliced − |||
20120518_EXQ0_MiBa_SA_HCT116_mHLA−2: 9933
SA = 0.52
Score = −0.79
SEHPTLQAS − MaxQuant
20120518_EXQ0_MiBa_SA_HCT116_mHLA−2: 9933
SA = 0.79
Score = 1.34
SEHPTLQAS − MSFragger
20120518_EXQ0_MiBa_SA_HCT116_mHLA−2: 9933
SA = 0.79
Score = 1.34
Relative intensity
m/z
Prosit prediction
⋆ Exclusive in prediction
Exclusive in experimental

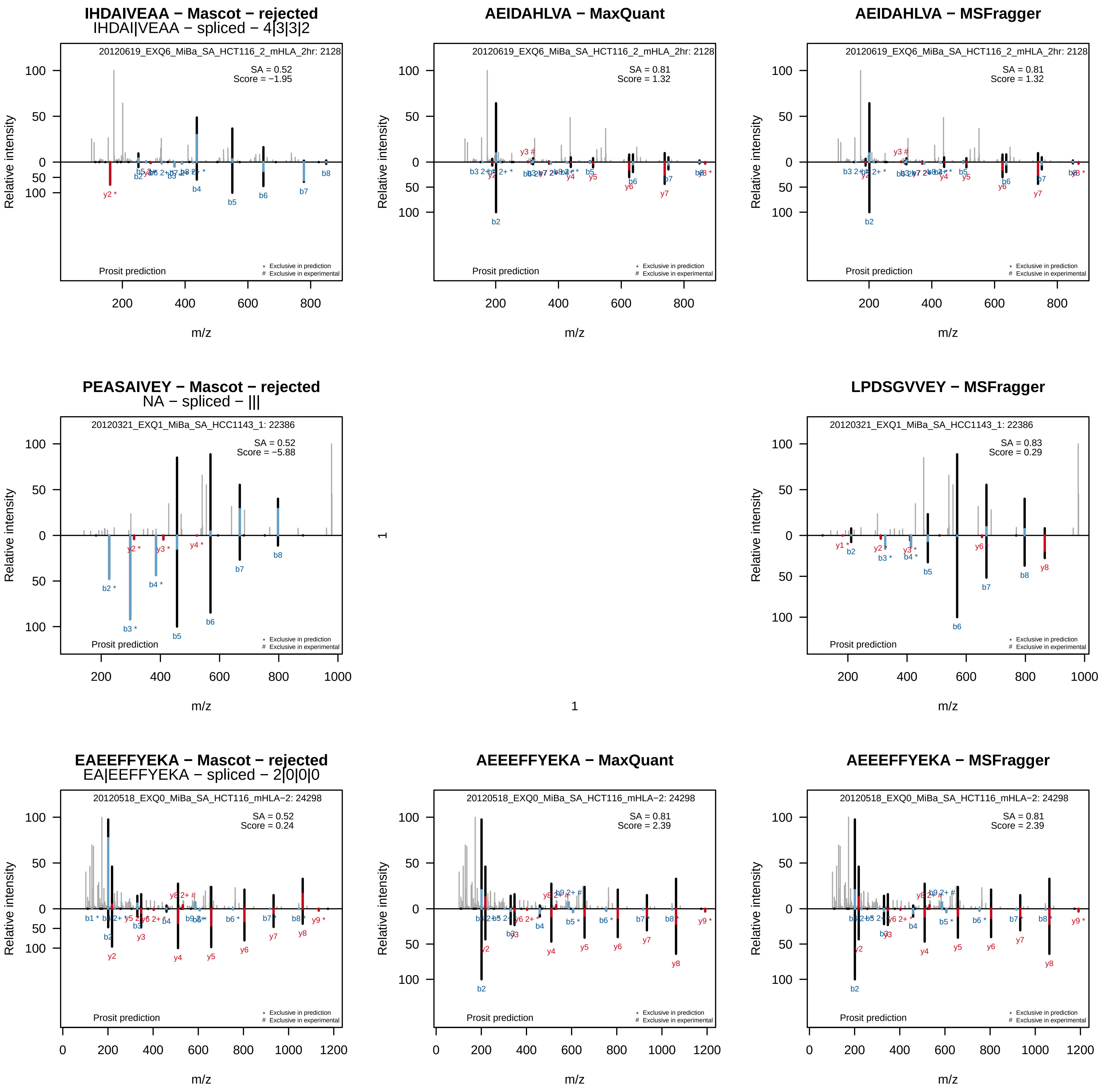
IHDAIVEAA − Mascot − rejected
IHDAI|VEAA − spliced − 4|3|3|2
20120619_EXQ6_MiBa_SA_HCT116_2_mHLA_2hr: 2128
SA = 0.52
Score = −1.95
AEIDAHLVA − MaxQuant
20120619_EXQ6_MiBa_SA_HCT116_2_mHLA_2hr: 2128
SA = 0.81
Score = 1.32
AEIDAHLVA − MSFragger
20120619_EXQ6_MiBa_SA_HCT116_2_mHLA_2hr: 2128
SA = 0.81
Score = 1.32
PEASAIVEY − Mascot − rejected
NA − spliced − |||
20120321_EXQ1_MiBa_SA_HCC1143_1: 22386
SA = 0.52
Score = −5.88
LPDSGVVEY − MSFragger
20120321_EXQ1_MiBa_SA_HCC1143_1: 22386
SA = 0.83
Score = 0.29
EAEEFFYEKA − Mascot − rejected
EA|EEFFYEKA − spliced − 2|0|0|0
20120518_EXQ0_MiBa_SA_HCT116_mHLA−2: 24298
SA = 0.52
Score = 0.24
AEEEFFYEKA − MaxQuant
20120518_EXQ0_MiBa_SA_HCT116_mHLA−2: 24298
SA = 0.81
Score = 2.39
AEEEFFYEKA − MSFragger
20120518_EXQ0_MiBa_SA_HCT116_mHLA−2: 24298
SA = 0.81
Score = 2.39
Relative intensity
m/z
Prosit prediction
⋆ Exclusive in prediction
Exclusive in experimental
1

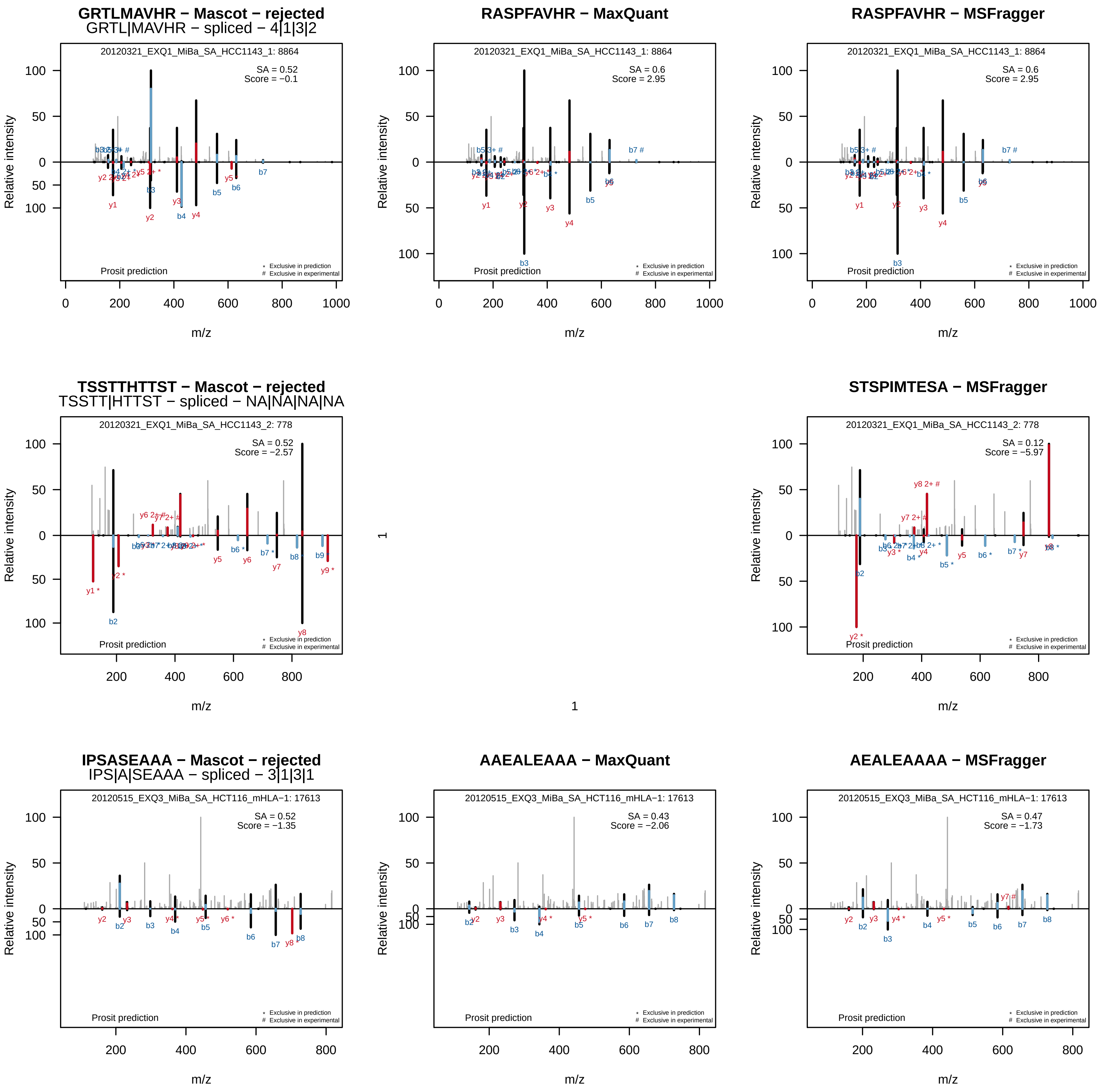
GRTLMAVHR − Mascot − rejected
GRTL|MAVHR − spliced − 4|1|3|2
20120321_EXQ1_MiBa_SA_HCC1143_1: 8864
SA = 0.52
Score = −0.1
RASPFAVHR − MaxQuant
20120321_EXQ1_MiBa_SA_HCC1143_1: 8864
SA = 0.6
Score = 2.95
RASPFAVHR − MSFragger
20120321_EXQ1_MiBa_SA_HCC1143_1: 8864
SA = 0.6
Score = 2.95
TSSTTHTTST − Mascot − rejected
TSSTT|HTTST − spliced − NA|NA|NA|NA
20120321_EXQ1_MiBa_SA_HCC1143_2: 778
SA = 0.52
Score = −2.57
STSPIMTESA − MSFragger
20120321_EXQ1_MiBa_SA_HCC1143_2: 778
SA = 0.12
Score = −5.97
IPSASEAAA − Mascot − rejected
IPS|A|SEAAA − spliced − 3|1|3|1
20120515_EXQ3_MiBa_SA_HCT116_mHLA−1: 17613
SA = 0.52
Score = −1.35
AAEALEAAA − MaxQuant
20120515_EXQ3_MiBa_SA_HCT116_mHLA−1: 17613
SA = 0.43
Score = −2.06
AEALEAAAA − MSFragger
20120515_EXQ3_MiBa_SA_HCT116_mHLA−1: 17613
SA = 0.47
Score = −1.73
Relative intensity
m/z
Prosit prediction
⋆ Exclusive in prediction
Exclusive in experimental

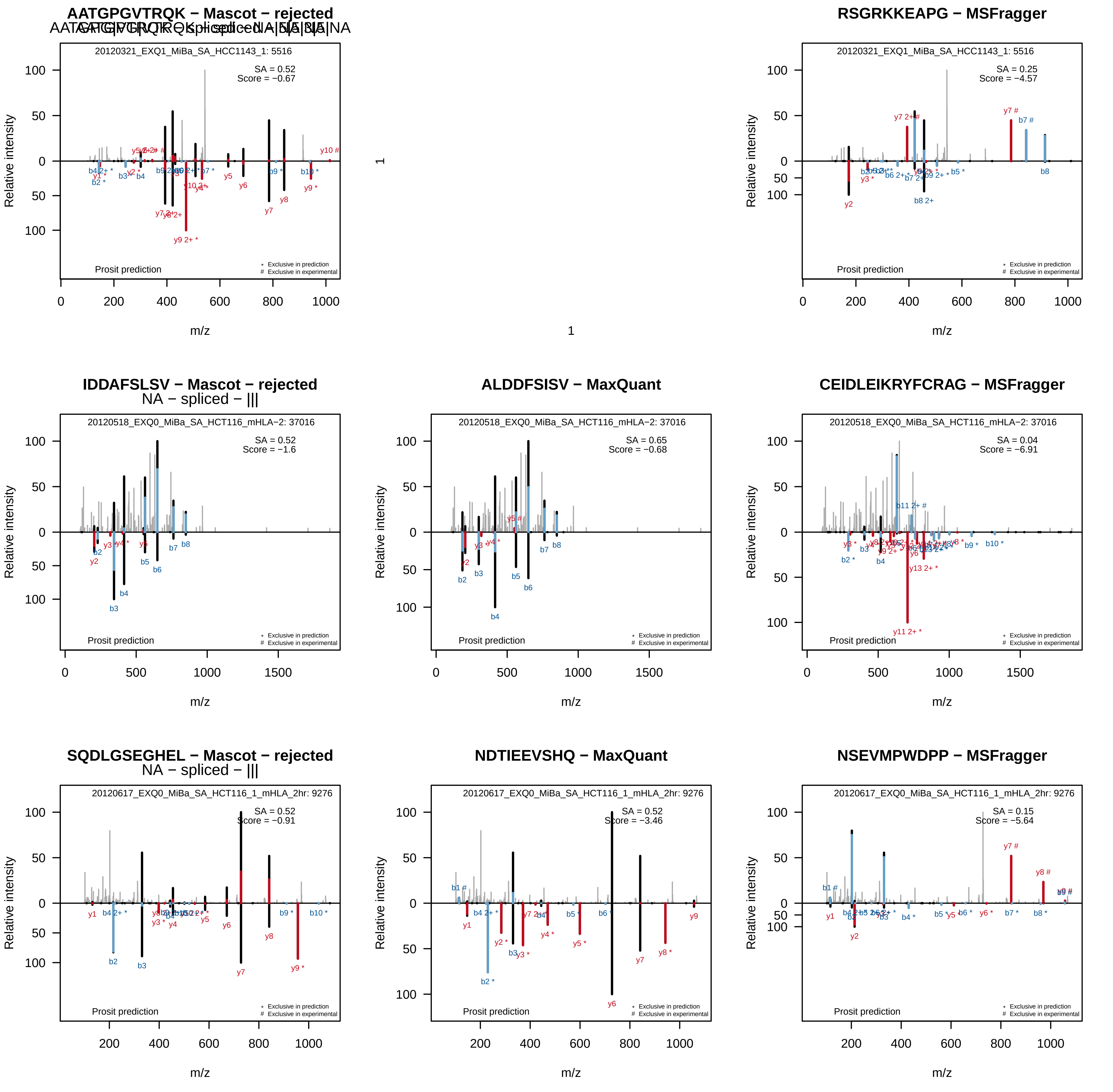
AATGPGVTRQK − Mascot − rejected
20120321_EXQ1_MiBa_SA_HCC1143_1: 5516
SA = 0.52
Score = −0.67
Prosit prediction
⋆ Exclusive in prediction
Exclusive in experimental
RSGRKKEAPG − MSFragger
20120321_EXQ1_MiBa_SA_HCC1143_1: 5516
SA = 0.25
Score = −4.57
IDDAFSLSV − Mascot − rejected
NA − spliced − |||
20120518_EXQ0_MiBa_SA_HCT116_mHLA−2: 37016
SA = 0.52
Score = −1.6
ALDDFSISV − MaxQuant
20120518_EXQ0_MiBa_SA_HCT116_mHLA−2: 37016
SA = 0.65
Score = −0.68
CEIDLEIKRYFCRAG − MSFragger
20120518_EXQ0_MiBa_SA_HCT116_mHLA−2: 37016
SA = 0.04
Score = −6.91
SQDLGSEGHEL − Mascot − rejected
NA − spliced − |||
20120617_EXQ0_MiBa_SA_HCT116_1_mHLA_2hr: 9276
SA = 0.52
Score = −0.91
NDTIEEVSHQ − MaxQuant
20120617_EXQ0_MiBa_SA_HCT116_1_mHLA_2hr: 9276
SA = 0.52
Score = −3.46
NSEVMPWDPP − MSFragger
20120617_EXQ0_MiBa_SA_HCT116_1_mHLA_2hr: 9276
SA = 0.15
Score = −5.64
Relative intensity
m/z

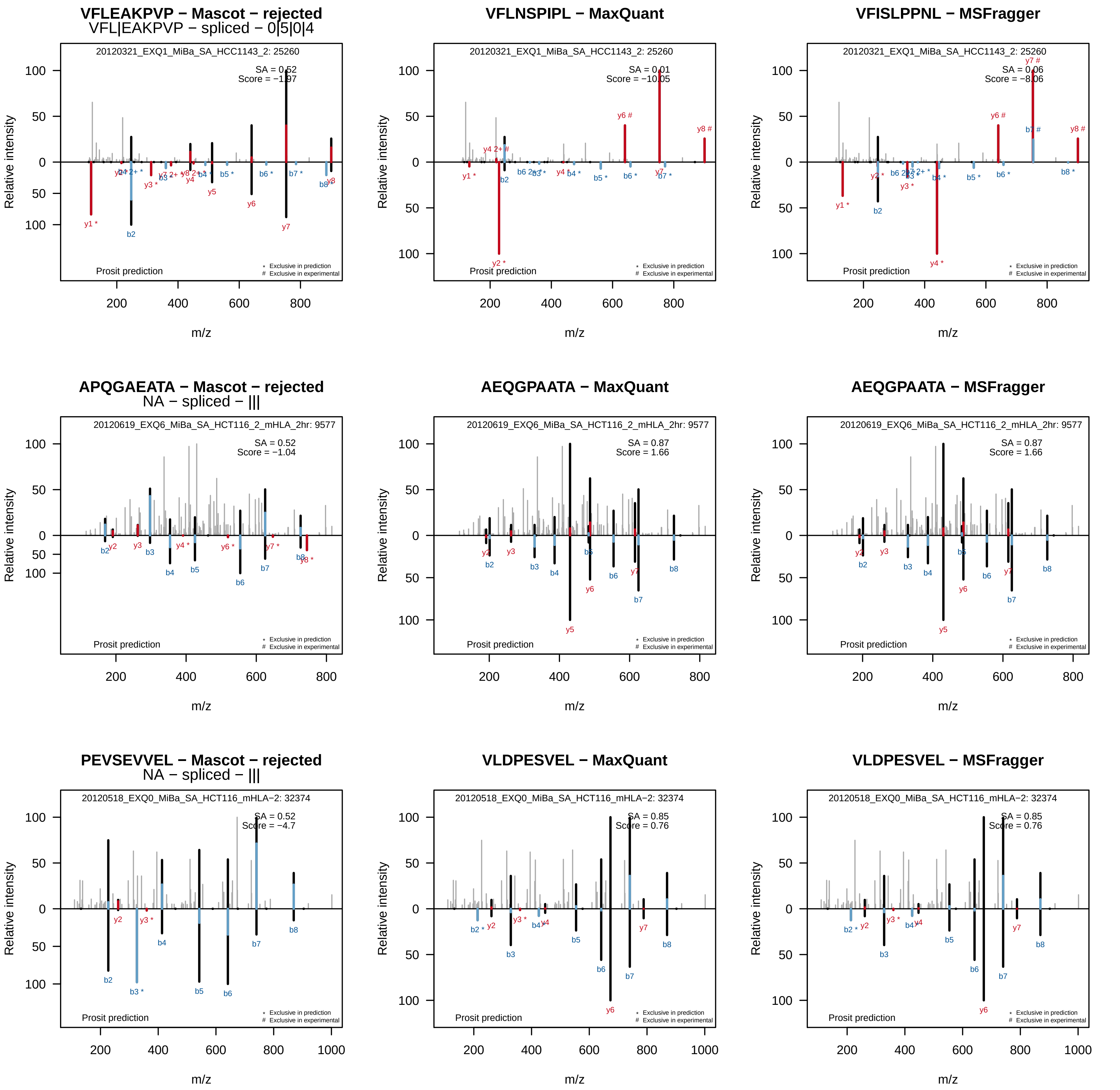
VFLEAKPVP − Mascot − rejected
VFL|EAKPVP − spliced − 0|5|0|4
20120321_EXQ1_MiBa_SA_HCC1143_2: 25260
SA = 0.52
Score = −1.97
VFLNSPIPL − MaxQuant
20120321_EXQ1_MiBa_SA_HCC1143_2: 25260
SA = 0.01
Score = −10.05
VFISLPPNL − MSFragger
20120321_EXQ1_MiBa_SA_HCC1143_2: 25260
SA = 0.06
Score = −8.06
APQGAEATA − Mascot − rejected
NA − spliced − |||
20120619_EXQ6_MiBa_SA_HCT116_2_mHLA_2hr: 9577
SA = 0.52
Score = −1.04
AEQGPAATA − MaxQuant
20120619_EXQ6_MiBa_SA_HCT116_2_mHLA_2hr: 9577
SA = 0.87
Score = 1.66
AEQGPAATA − MSFragger
20120619_EXQ6_MiBa_SA_HCT116_2_mHLA_2hr: 9577
SA = 0.87
Score = 1.66
PEVSEVVEL − Mascot − rejected
NA − spliced − |||
20120518_EXQ0_MiBa_SA_HCT116_mHLA−2: 32374
SA = 0.52
Score = −4.7
VLDPESVEL − MaxQuant
20120518_EXQ0_MiBa_SA_HCT116_mHLA−2: 32374
SA = 0.85
Score = 0.76
VLDPESVEL − MSFragger
20120518_EXQ0_MiBa_SA_HCT116_mHLA−2: 32374
SA = 0.85
Score = 0.76
Relative intensity
m/z
Prosit prediction
⋆ Exclusive in prediction
Exclusive in experimental

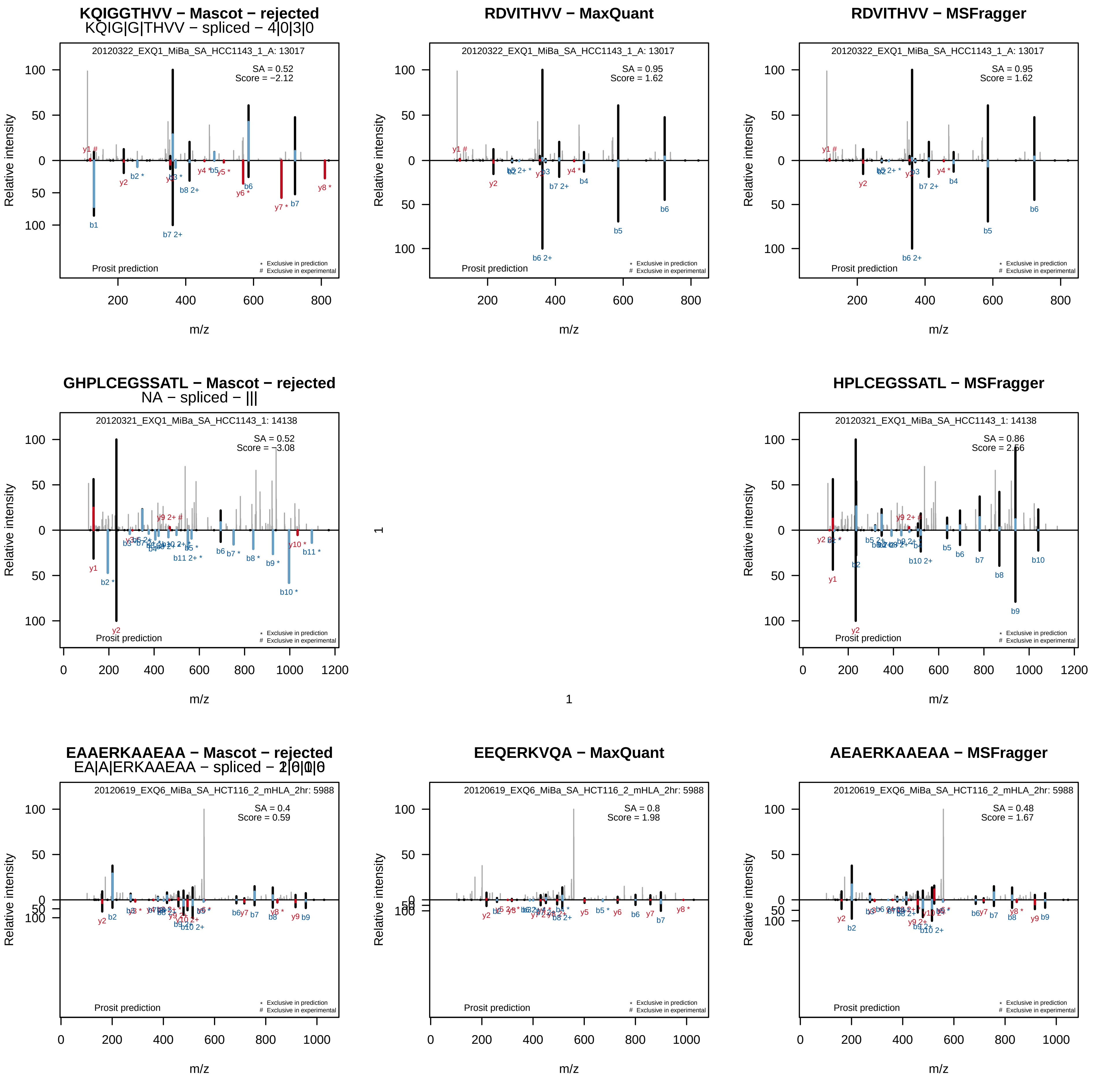
KQIGGTHVV − Mascot − rejected
KQIG|G|THVV − spliced − 4|0|3|0
20120322_EXQ1_MiBa_SA_HCC1143_1_A: 13017
SA = 0.52
Score = −2.12
RDVITHVV − MaxQuant
20120322_EXQ1_MiBa_SA_HCC1143_1_A: 13017
SA = 0.95
Score = 1.62
RDVITHVV − MSFragger
20120322_EXQ1_MiBa_SA_HCC1143_1_A: 13017
SA = 0.95
Score = 1.62
GHPLCEGSSATL − Mascot − rejected
NA − spliced − |||
20120321_EXQ1_MiBa_SA_HCC1143_1: 14138
SA = 0.52
Score = −3.08
HPLCEGSSATL − MSFragger
20120321_EXQ1_MiBa_SA_HCC1143_1: 14138
SA = 0.86
Score = 2.56
EAAERKAAEAA − Mascot − rejected
EA|A|ERKAAEAA − spliced − 2|0|1|0
20120619_EXQ6_MiBa_SA_HCT116_2_mHLA_2hr: 5988
SA = 0.4
Score = 0.59
EEQERKVQA − MaxQuant
20120619_EXQ6_MiBa_SA_HCT116_2_mHLA_2hr: 5988
SA = 0.8
Score = 1.98
AEAERKAAEAA − MSFragger
20120619_EXQ6_MiBa_SA_HCT116_2_mHLA_2hr: 5988
SA = 0.48
Score = 1.67
Relative intensity
m/z
Prosit prediction
⋆ Exclusive in prediction
Exclusive in experimental

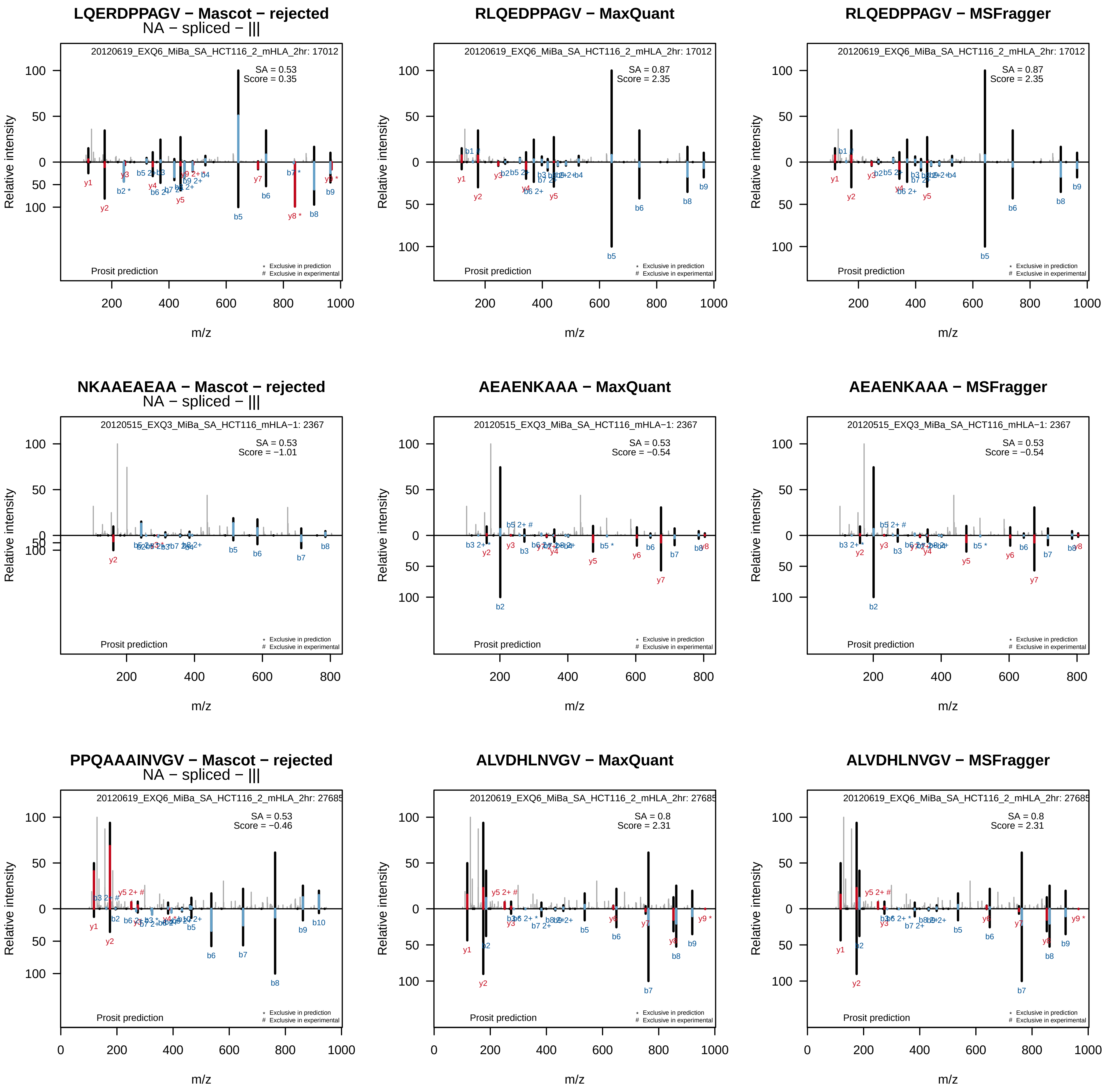
LQERDPPAGV − Mascot − rejected
NA − spliced − |||
20120619_EXQ6_MiBa_SA_HCT116_2_mHLA_2hr: 17012
SA = 0.53
Score = 0.35
RLQEDPPAGV − MaxQuant
20120619_EXQ6_MiBa_SA_HCT116_2_mHLA_2hr: 17012
SA = 0.87
Score = 2.35
RLQEDPPAGV − MSFragger
20120619_EXQ6_MiBa_SA_HCT116_2_mHLA_2hr: 17012
SA = 0.87
Score = 2.35
NKAAEAEAA − Mascot − rejected
NA − spliced − |||
20120515_EXQ3_MiBa_SA_HCT116_mHLA−1: 2367
SA = 0.53
Score = −1.01
AEAENKAAA − MaxQuant
20120515_EXQ3_MiBa_SA_HCT116_mHLA−1: 2367
SA = 0.53
Score = −0.54
AEAENKAAA − MSFragger
20120515_EXQ3_MiBa_SA_HCT116_mHLA−1: 2367
SA = 0.53
Score = −0.54
PPQAAAINVGV − Mascot − rejected
NA − spliced − |||
20120619_EXQ6_MiBa_SA_HCT116_2_mHLA_2hr: 27685
SA = 0.53
Score = −0.46
ALVDHLNVGV − MaxQuant
20120619_EXQ6_MiBa_SA_HCT116_2_mHLA_2hr: 27685
SA = 0.8
Score = 2.31
ALVDHLNVGV − MSFragger
20120619_EXQ6_MiBa_SA_HCT116_2_mHLA_2hr: 27685
SA = 0.8
Score = 2.31
Relative intensity
m/z
Prosit prediction
* Exclusive in prediction
# Exclusive in experimental

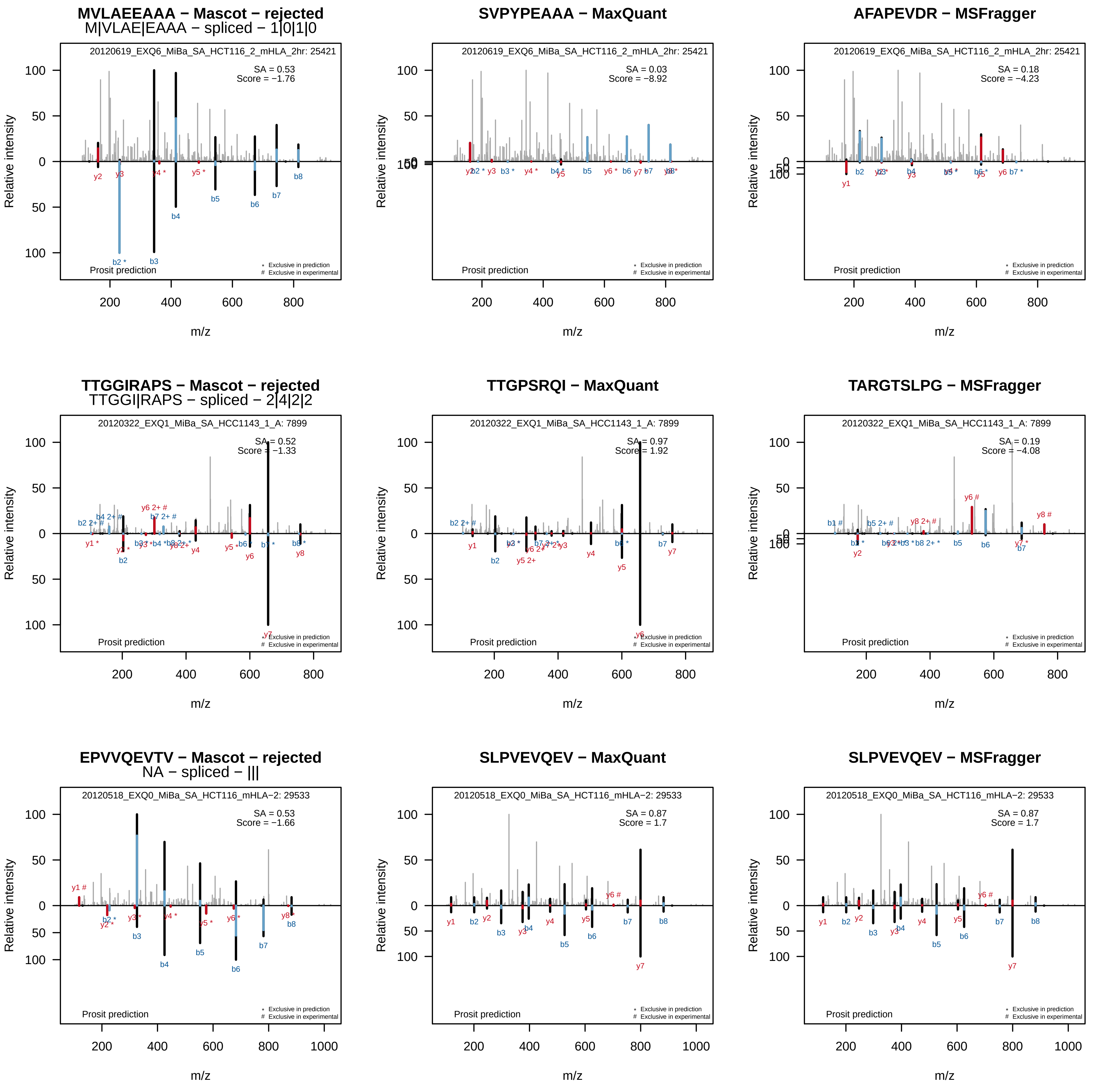

MVLAEEAAA − Mascot − rejected
M|VLAE|EAAA − spliced − 1|0|1|0
20120619_EXQ6_MiBa_SA_HCT116_2_mHLA_2hr: 25421
SA = 0.53
Score = −1.76
SVPYPEAAA − MaxQuant
20120619_EXQ6_MiBa_SA_HCT116_2_mHLA_2hr: 25421
SA = 0.03
Score = −8.92
AFAPEVDR − MSFragger
20120619_EXQ6_MiBa_SA_HCT116_2_mHLA_2hr: 25421
SA = 0.18
Score = −4.23
TTGGIRAPS − Mascot − rejected
TTGGI|RAPS − spliced − 2|4|2|2
20120322_EXQ1_MiBa_SA_HCC1143_1_A: 7899
SA = 0.52
Score = −1.33
TTGPSRQI − MaxQuant
20120322_EXQ1_MiBa_SA_HCC1143_1_A: 7899
SA = 0.97
Score = 1.92
TARGTSLPG − MSFragger
20120322_EXQ1_MiBa_SA_HCC1143_1_A: 7899
SA = 0.19
Score = −4.08
EPVVQEVTV − Mascot − rejected
NA − spliced − |||
20120518_EXQ0_MiBa_SA_HCT116_mHLA−2: 29533
SA = 0.53
Score = −1.66
SLPVEVQEV − MaxQuant
20120518_EXQ0_MiBa_SA_HCT116_mHLA−2: 29533
SA = 0.87
Score = 1.7
SLPVEVQEV − MSFragger
20120518_EXQ0_MiBa_SA_HCT116_mHLA−2: 29533
SA = 0.87
Score = 1.7
Relative intensity
m/z
Prosit prediction
* Exclusive in prediction
# Exclusive in experimental

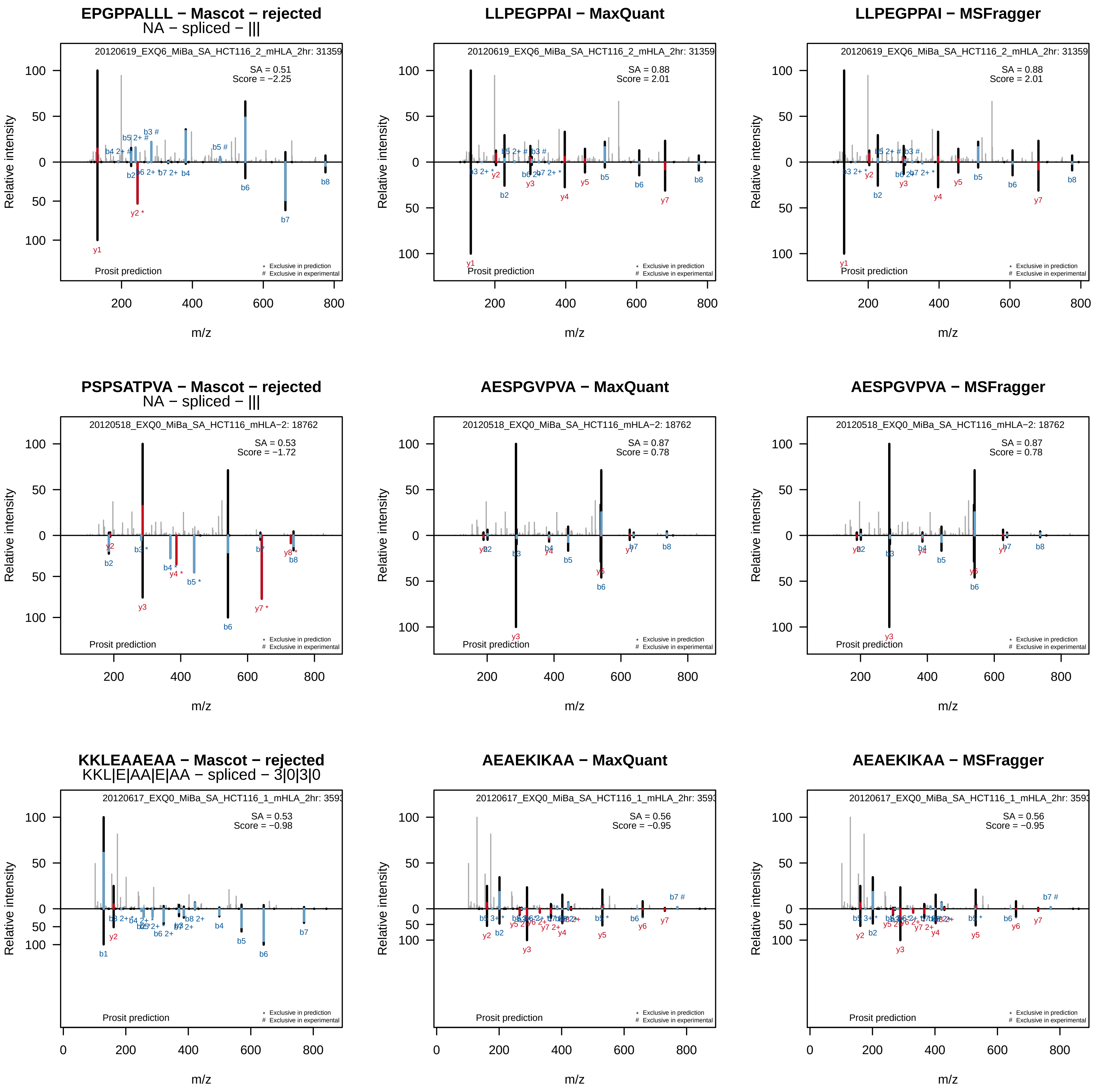

EPGPPALLL − Mascot − rejected
NA − spliced − |||
20120619_EXQ6_MiBa_SA_HCT116_2_mHLA_2hr: 31359
SA = 0.51
Score = −2.25
Prosit prediction
⋆ Exclusive in prediction
Exclusive in experimental
LLPEGPPAI − MaxQuant
20120619_EXQ6_MiBa_SA_HCT116_2_mHLA_2hr: 31359
SA = 0.88
Score = 2.01
LLPEGPPAI − MSFragger
20120619_EXQ6_MiBa_SA_HCT116_2_mHLA_2hr: 31359
SA = 0.88
Score = 2.01
PSPSATPVA − Mascot − rejected
NA − spliced − |||
20120518_EXQ0_MiBa_SA_HCT116_mHLA−2: 18762
SA = 0.53
Score = −1.72
AESPGVPVA − MaxQuant
20120518_EXQ0_MiBa_SA_HCT116_mHLA−2: 18762
SA = 0.87
Score = 0.78
AESPGVPVA − MSFragger
20120518_EXQ0_MiBa_SA_HCT116_mHLA−2: 18762
SA = 0.87
Score = 0.78
KKLEAAEAA − Mascot − rejected
KKL|E|AA|E|AA − spliced − 3|0|3|0
20120617_EXQ0_MiBa_SA_HCT116_1_mHLA_2hr: 3593
SA = 0.53
Score = −0.98
AEAEKIKAA − MaxQuant
20120617_EXQ0_MiBa_SA_HCT116_1_mHLA_2hr: 3593
SA = 0.56
Score = −0.95
AEAEKIKAA − MSFragger
20120617_EXQ0_MiBa_SA_HCT116_1_mHLA_2hr: 3593
SA = 0.56
Score = −0.95
Relative intensity
m/z

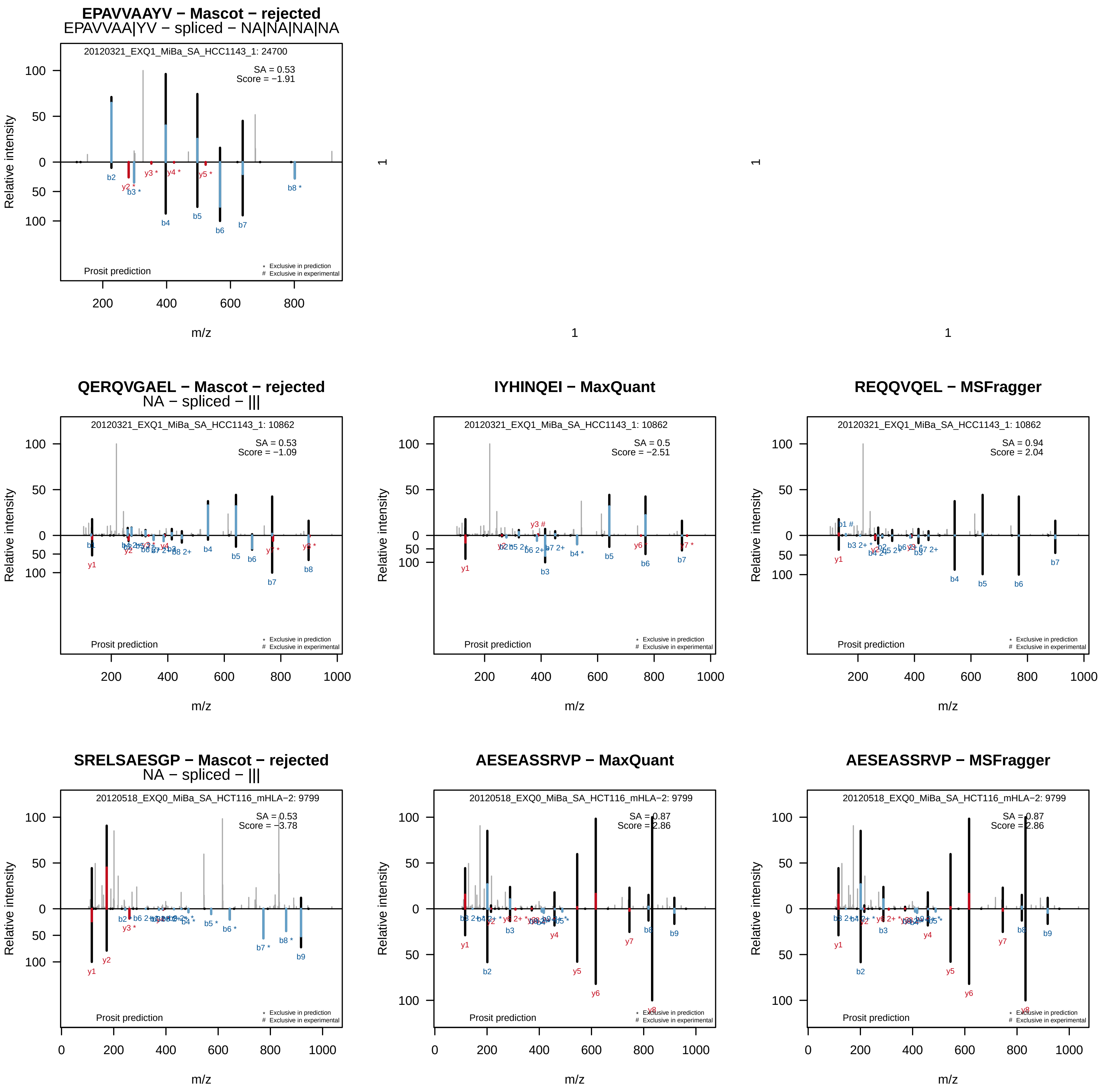
EPAVVAAYV − Mascot − rejected
EPAVVAA|YV − spliced − NA|NA|NA|NA
20120321_EXQ1_MiBa_SA_HCC1143_1: 24700
SA = 0.53
Score = −1.91
Relative intensity
m/z
Prosit prediction
⋆ Exclusive in prediction
Exclusive in experimental
QERQVGAEL − Mascot − rejected
NA − spliced − |||
20120321_EXQ1_MiBa_SA_HCC1143_1: 10862
SA = 0.53
Score = −1.09
IYHINQEI − MaxQuant
20120321_EXQ1_MiBa_SA_HCC1143_1: 10862
SA = 0.5
Score = −2.51
REQQVQEL − MSFragger
20120321_EXQ1_MiBa_SA_HCC1143_1: 10862
SA = 0.94
Score = 2.04
SRELSAESGP − Mascot − rejected
NA − spliced − |||
20120518_EXQ0_MiBa_SA_HCT116_mHLA−2: 9799
SA = 0.53
Score = −3.78
AESEASSRVP − MaxQuant
20120518_EXQ0_MiBa_SA_HCT116_mHLA−2: 9799
SA = 0.87
Score = 2.86
AESEASSRVP − MSFragger
20120518_EXQ0_MiBa_SA_HCT116_mHLA−2: 9799
SA = 0.87
Score = 2.86

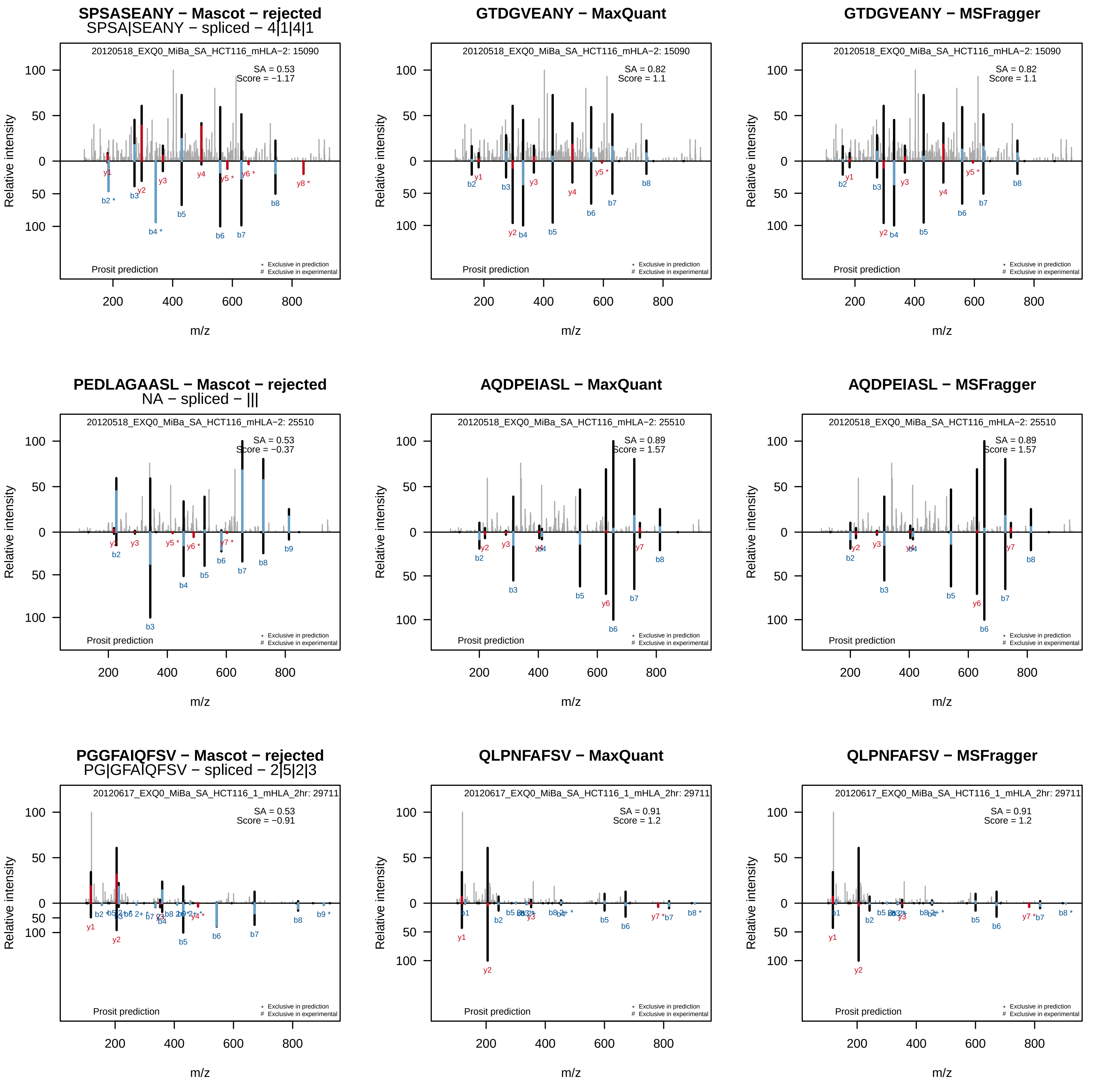

SPSASEANY − Mascot − rejected
SPSA|SEANY − spliced − 4|1|4|1
20120518_EXQ0_MiBa_SA_HCT116_mHLA−2: 15090
SA = 0.53
Score = −1.17
GTDGVEANY − MaxQuant
20120518_EXQ0_MiBa_SA_HCT116_mHLA−2: 15090
SA = 0.82
Score = 1.1
GTDGVEANY − MSFragger
20120518_EXQ0_MiBa_SA_HCT116_mHLA−2: 15090
SA = 0.82
Score = 1.1
PEDLAGAASL − Mascot − rejected
NA − spliced − |||
20120518_EXQ0_MiBa_SA_HCT116_mHLA−2: 25510
SA = 0.53
Score = −0.37
AQDPEIASL − MaxQuant
20120518_EXQ0_MiBa_SA_HCT116_mHLA−2: 25510
SA = 0.89
Score = 1.57
AQDPEIASL − MSFragger
20120518_EXQ0_MiBa_SA_HCT116_mHLA−2: 25510
SA = 0.89
Score = 1.57
PGGFAIQFSV − Mascot − rejected
PG|GFAIQFSV − spliced − 2|5|2|3
20120617_EXQ0_MiBa_SA_HCT116_1_mHLA_2hr: 29711
SA = 0.53
Score = −0.91
QLPNFAFSV − MaxQuant
20120617_EXQ0_MiBa_SA_HCT116_1_mHLA_2hr: 29711
SA = 0.91
Score = 1.2
QLPNFAFSV − MSFragger
20120617_EXQ0_MiBa_SA_HCT116_1_mHLA_2hr: 29711
SA = 0.91
Score = 1.2
Relative intensity
m/z
Prosit prediction
⋆ Exclusive in prediction
Exclusive in experimental

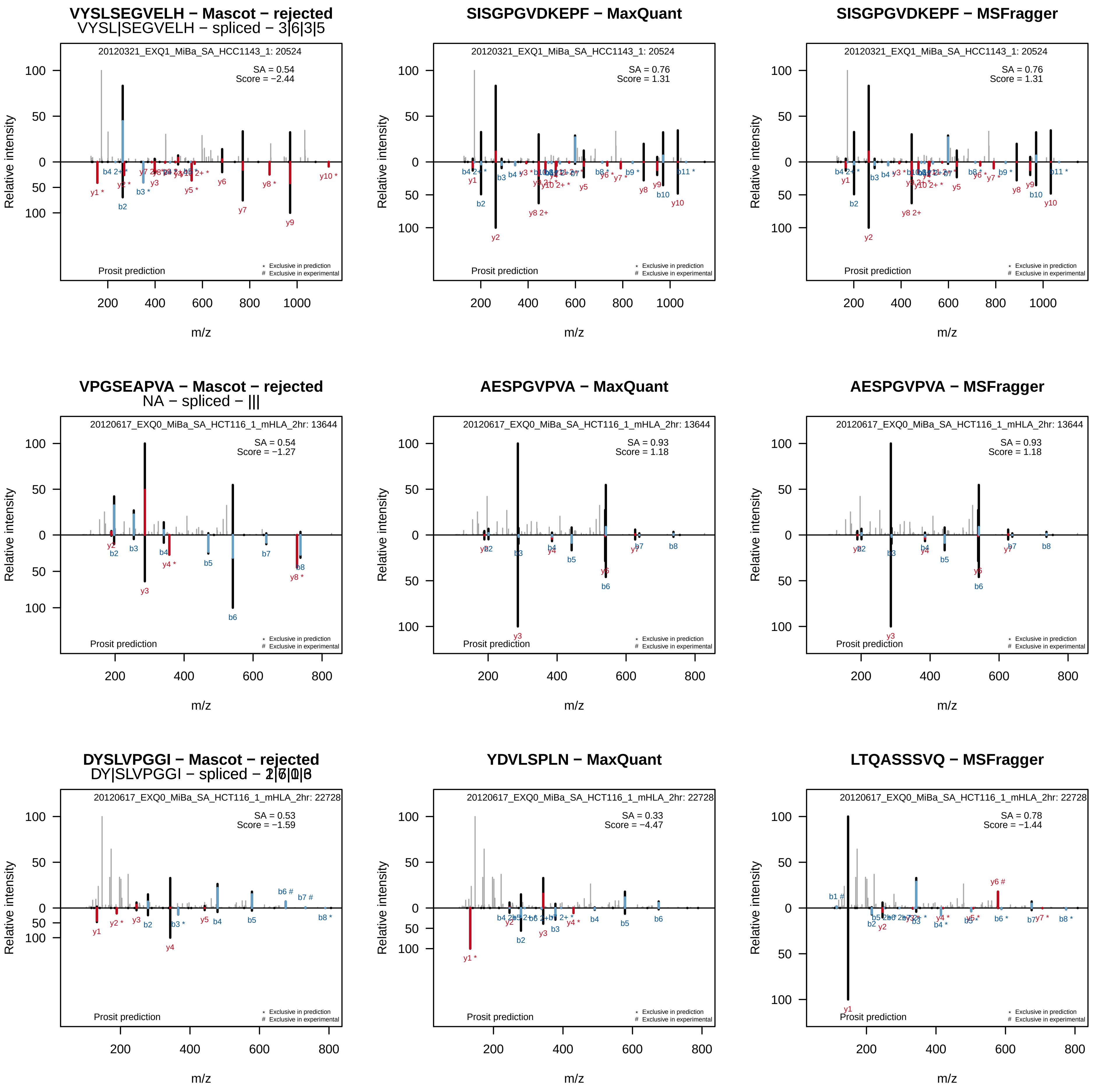
VYSLSEGVELH − Mascot − rejected
VYSL|SEGVELH − spliced − 3|6|3|5
20120321_EXQ1_MiBa_SA_HCC1143_1: 20524
SA = 0.54
Score = −2.44
SISGPGVDKEPF − MaxQuant
20120321_EXQ1_MiBa_SA_HCC1143_1: 20524
SA = 0.76
Score = 1.31
SISGPGVDKEPF − MSFragger
20120321_EXQ1_MiBa_SA_HCC1143_1: 20524
SA = 0.76
Score = 1.31
VPGSEAPVA − Mascot − rejected
NA − spliced − |||
20120617_EXQ0_MiBa_SA_HCT116_1_mHLA_2hr: 13644
SA = 0.54
Score = −1.27
AESPGVPVA − MaxQuant
20120617_EXQ0_MiBa_SA_HCT116_1_mHLA_2hr: 13644
SA = 0.93
Score = 1.18
AESPGVPVA − MSFragger
20120617_EXQ0_MiBa_SA_HCT116_1_mHLA_2hr: 13644
SA = 0.93
Score = 1.18
DYSLVPGGI − Mascot − rejected
DY|SLVPGGI − spliced − 2|7|0|3
20120617_EXQ0_MiBa_SA_HCT116_1_mHLA_2hr: 22728
SA = 0.53
Score = −1.59
YDVLSPLN − MaxQuant
20120617_EXQ0_MiBa_SA_HCT116_1_mHLA_2hr: 22728
SA = 0.33
Score = −4.47
LTQASSSVQ − MSFragger
20120617_EXQ0_MiBa_SA_HCT116_1_mHLA_2hr: 22728
SA = 0.78
Score = −1.44
Relative intensity
m/z
Prosit prediction
* Exclusive in prediction
# Exclusive in experimental

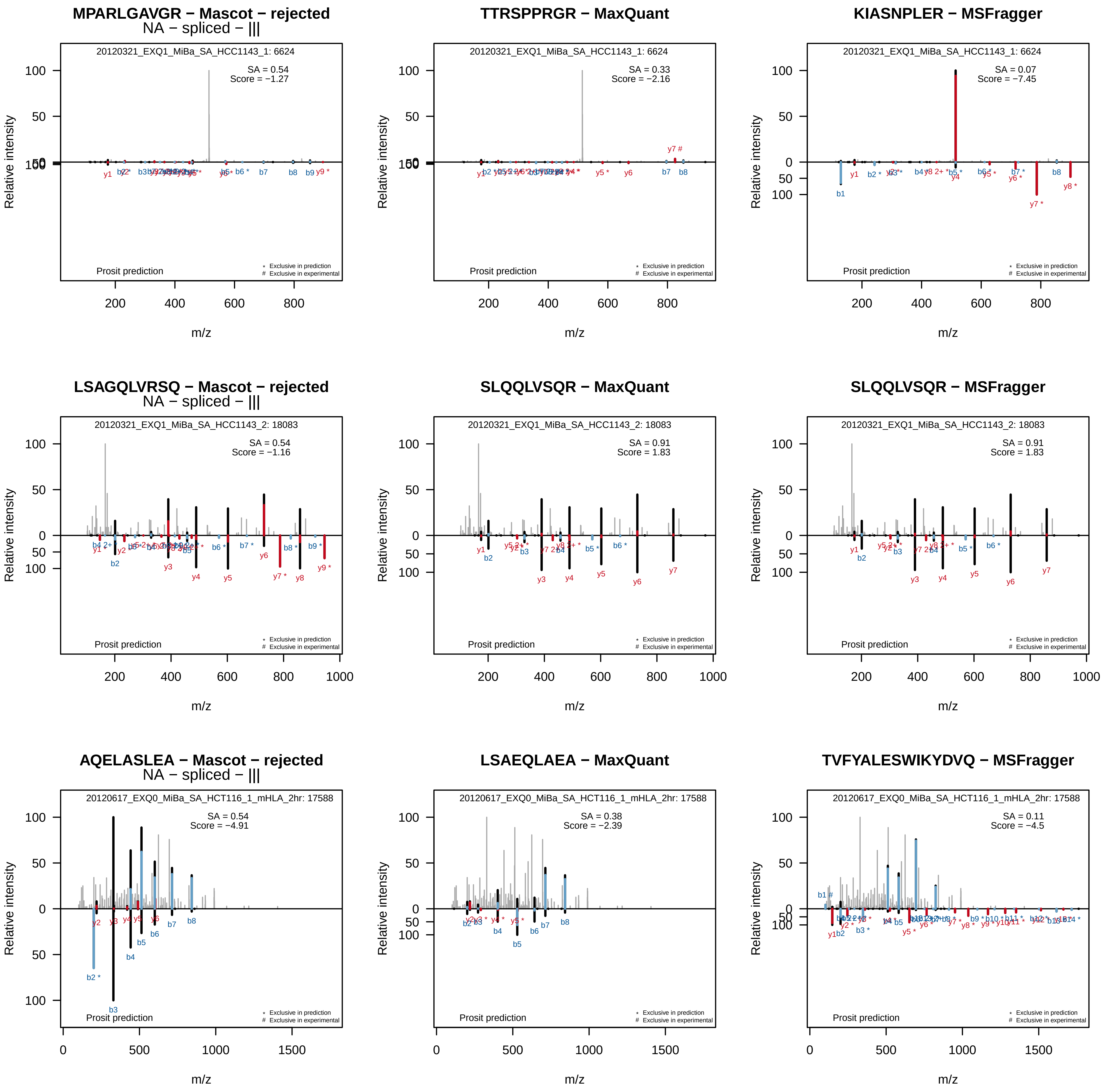
MPARLGAVGR − Mascot − rejected
NA − spliced − |||
20120321_EXQ1_MiBa_SA_HCC1143_1: 6624
SA = 0.54
Score = −1.27
TTRSPPRGR − MaxQuant
20120321_EXQ1_MiBa_SA_HCC1143_1: 6624
SA = 0.33
Score = −2.16
KIASNPLER − MSFragger
20120321_EXQ1_MiBa_SA_HCC1143_1: 6624
SA = 0.07
Score = −7.45
LSAGQLVRSQ − Mascot − rejected
NA − spliced − |||
20120321_EXQ1_MiBa_SA_HCC1143_2: 18083
SA = 0.54
Score = −1.16
SLQQLVSQR − MaxQuant
20120321_EXQ1_MiBa_SA_HCC1143_2: 18083
SA = 0.91
Score = 1.83
SLQQLVSQR − MSFragger
20120321_EXQ1_MiBa_SA_HCC1143_2: 18083
SA = 0.91
Score = 1.83
AQELASLEA − Mascot − rejected
NA − spliced − |||
20120617_EXQ0_MiBa_SA_HCT116_1_mHLA_2hr: 17588
SA = 0.54
Score = −4.91
LSAEQLAEA − MaxQuant
20120617_EXQ0_MiBa_SA_HCT116_1_mHLA_2hr: 17588
SA = 0.38
Score = −2.39
TVFYALESWIKYDVQ − MSFragger
20120617_EXQ0_MiBa_SA_HCT116_1_mHLA_2hr: 17588
SA = 0.11
Score = −4.5
Relative intensity
m/z
Prosit prediction
⋆ Exclusive in prediction
Exclusive in experimental

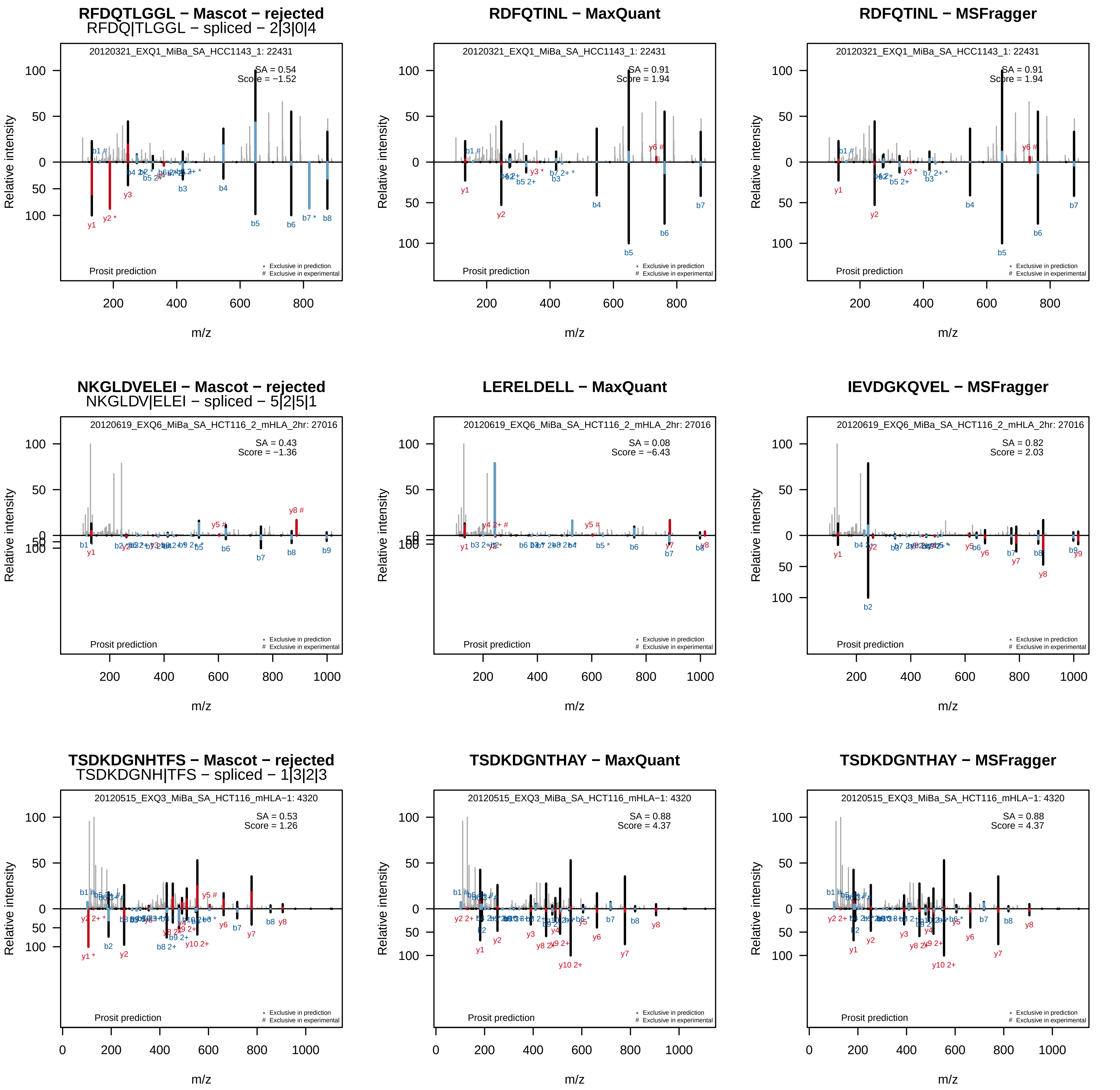
RFDQTLGGL − Mascot − rejected
RFDQ|TLGGL − spliced − 2|3|0|4
20120321_EXQ1_MiBa_SA_HCC1143_1: 22431
SA = 0.54
Score = −1.52
RDFQTINL − MaxQuant
20120321_EXQ1_MiBa_SA_HCC1143_1: 22431
SA = 0.91
Score = 1.94
RDFQTINL − MSFragger
20120321_EXQ1_MiBa_SA_HCC1143_1: 22431
SA = 0.91
Score = 1.94
NKGLDVELEI − Mascot − rejected
NKGLDV|ELEI − spliced − 5|2|5|1
20120619_EXQ6_MiBa_SA_HCT116_2_mHLA_2hr: 27016
SA = 0.43
Score = −1.36
LERELDELL − MaxQuant
20120619_EXQ6_MiBa_SA_HCT116_2_mHLA_2hr: 27016
SA = 0.08
Score = −6.43
IEVDGKQVEL − MSFragger
20120619_EXQ6_MiBa_SA_HCT116_2_mHLA_2hr: 27016
SA = 0.82
Score = 2.03
TSDKDGNHTFS − Mascot − rejected
TSDKDGNH|TFS − spliced − 1|3|2|3
20120515_EXQ3_MiBa_SA_HCT116_mHLA−1: 4320
SA = 0.53
Score = 1.26
TSDKDGNTHAY − MaxQuant
20120515_EXQ3_MiBa_SA_HCT116_mHLA−1: 4320
SA = 0.88
Score = 4.37
TSDKDGNTHAY − MSFragger
20120515_EXQ3_MiBa_SA_HCT116_mHLA−1: 4320
SA = 0.88
Score = 4.37
Relative intensity
m/z
Prosit prediction
* Exclusive in prediction
# Exclusive in experimental

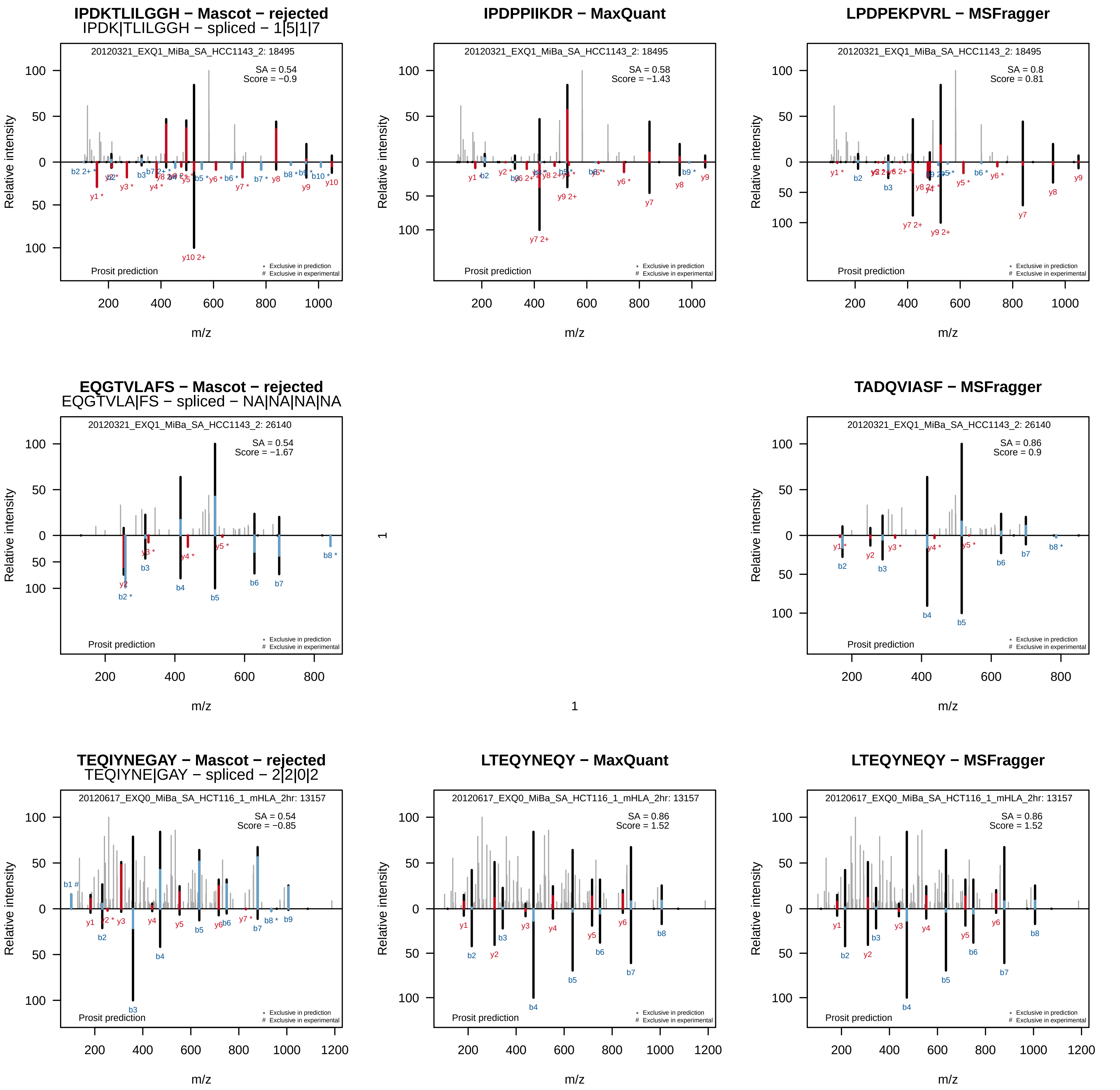

IPDKTLILGGH − Mascot − rejected
IPDK|TLILGGH − spliced − 1|5|1|7
20120321_EXQ1_MiBa_SA_HCC1143_2: 18495
SA = 0.54
Score = −0.9
IPDPPIIKDR − MaxQuant
20120321_EXQ1_MiBa_SA_HCC1143_2: 18495
SA = 0.58
Score = −1.43
LPDPEKPVRL − MSFragger
20120321_EXQ1_MiBa_SA_HCC1143_2: 18495
SA = 0.8
Score = 0.81
EQGTVLAFS − Mascot − rejected
EQGTVLA|FS − spliced − NA|NA|NA|NA
20120321_EXQ1_MiBa_SA_HCC1143_2: 26140
SA = 0.54
Score = −1.67
TADQVIASF − MSFragger
20120321_EXQ1_MiBa_SA_HCC1143_2: 26140
SA = 0.86
Score = 0.9
TEQIYNEGAY − Mascot − rejected
TEQIYNE|GAY − spliced − 2|2|0|2
20120617_EXQ0_MiBa_SA_HCT116_1_mHLA_2hr: 13157
SA = 0.54
Score = −0.85
LTEQYNEQY − MaxQuant
20120617_EXQ0_MiBa_SA_HCT116_1_mHLA_2hr: 13157
SA = 0.86
Score = 1.52
LTEQYNEQY − MSFragger
20120617_EXQ0_MiBa_SA_HCT116_1_mHLA_2hr: 13157
SA = 0.86
Score = 1.52
Relative intensity
m/z
Prosit prediction
⋆ Exclusive in prediction
Exclusive in experimental

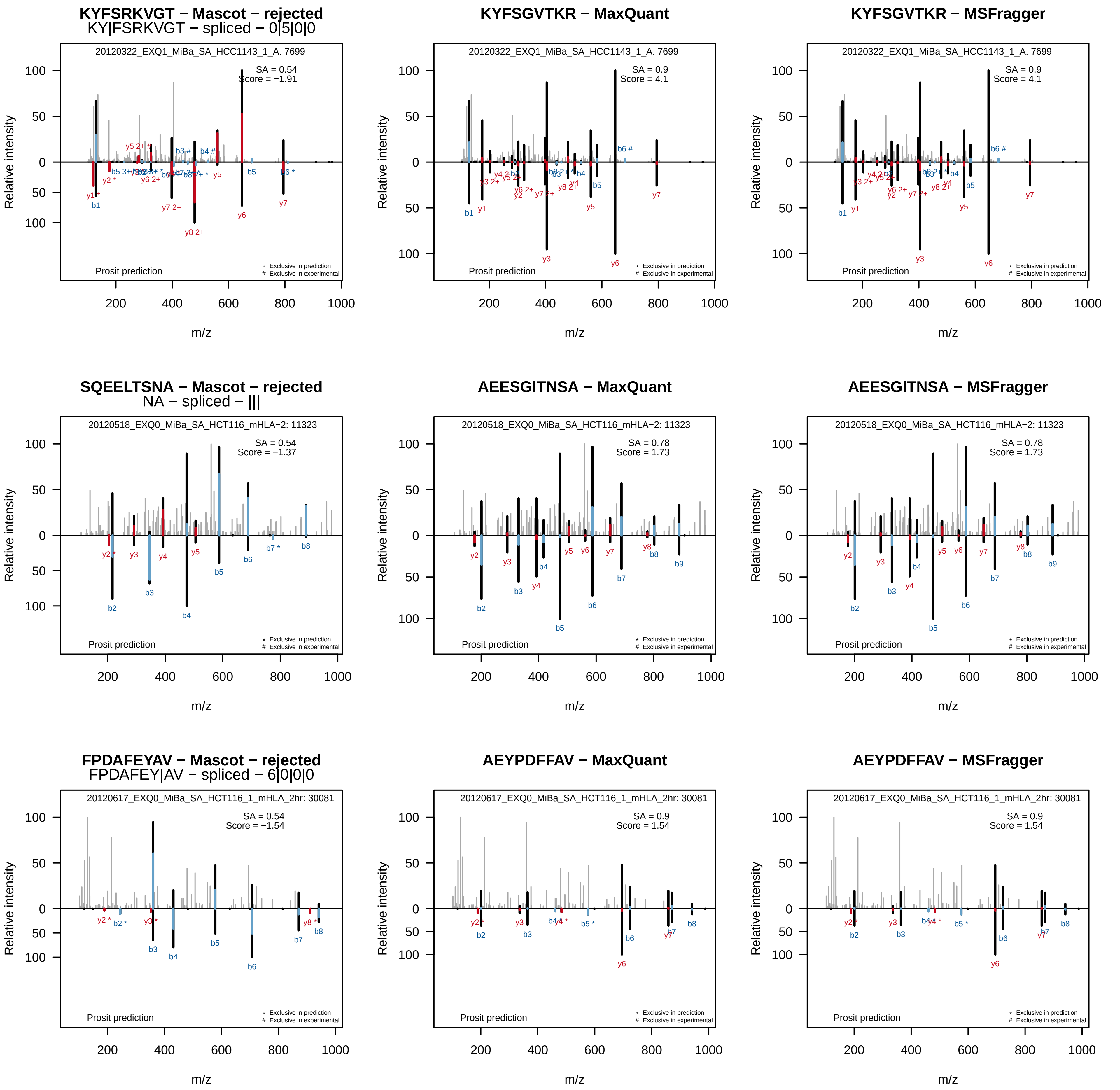

KYFSRKVGT − Mascot − rejected
KY|FSRKVGT − spliced − 0|5|0|0
KYFSGVTKR − MaxQuant
KYFSGVTKR − MSFragger
20120322_EXQ1_MiBa_SA_HCC1143_1_A: 7699
SA = 0.54
Score = −1.91
SA = 0.9
Score = 4.1
SQEELTSNA − Mascot − rejected
NA − spliced − |||
AEESGITNSA − MaxQuant
AEESGITNSA − MSFragger
20120518_EXQ0_MiBa_SA_HCT116_mHLA−2: 11323
SA = 0.54
Score = −1.37
SA = 0.78
Score = 1.73
FPDAFEYAV − Mascot − rejected
FPDAFEY|AV − spliced − 6|0|0|0
AEYPDFFAV − MaxQuant
AEYPDFFAV − MSFragger
20120617_EXQ0_MiBa_SA_HCT116_1_mHLA_2hr: 30081
SA = 0.54
Score = −1.54
SA = 0.9
Score = 1.54
Prosit prediction
⋆ Exclusive in prediction
Exclusive in experimental
Relative intensity
m/z

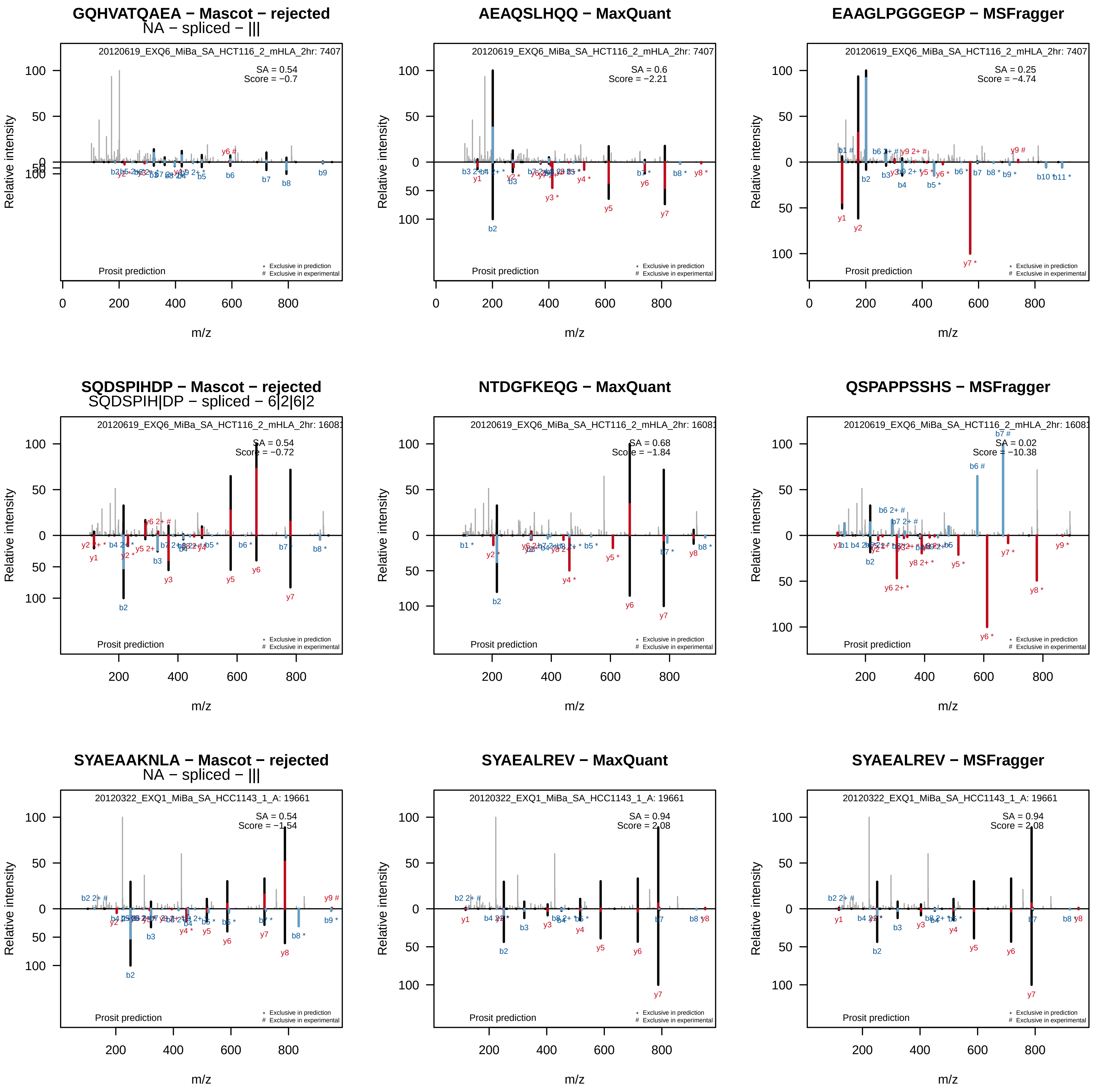

GQHVATQAEA − Mascot − rejected
NA − spliced − |||
20120619_EXQ6_MiBa_SA_HCT116_2_mHLA_2hr: 7407
SA = 0.54
Score = −0.7
AEAQSLHQQ − MaxQuant
20120619_EXQ6_MiBa_SA_HCT116_2_mHLA_2hr: 7407
SA = 0.6
Score = −2.21
EAAGLPGGGEGP − MSFragger
20120619_EXQ6_MiBa_SA_HCT116_2_mHLA_2hr: 7407
SA = 0.25
Score = −4.74
SQDSPIHDP − Mascot − rejected
SQDSPIH|DP − spliced − 6|2|6|2
20120619_EXQ6_MiBa_SA_HCT116_2_mHLA_2hr: 16081
SA = 0.54
Score = −0.72
NTDGFKEQG − MaxQuant
20120619_EXQ6_MiBa_SA_HCT116_2_mHLA_2hr: 16081
SA = 0.68
Score = −1.84
QSPAPPSSHS − MSFragger
20120619_EXQ6_MiBa_SA_HCT116_2_mHLA_2hr: 16081
SA = 0.02
Score = −10.38
SYAEAAKNLA − Mascot − rejected
NA − spliced − |||
20120322_EXQ1_MiBa_SA_HCC1143_1_A: 19661
SA = 0.54
Score = −1.54
SYAEALREV − MaxQuant
20120322_EXQ1_MiBa_SA_HCC1143_1_A: 19661
SA = 0.94
Score = 2.08
SYAEALREV − MSFragger
20120322_EXQ1_MiBa_SA_HCC1143_1_A: 19661
SA = 0.94
Score = 2.08
Relative intensity
m/z
Prosit prediction
* Exclusive in prediction
# Exclusive in experimental

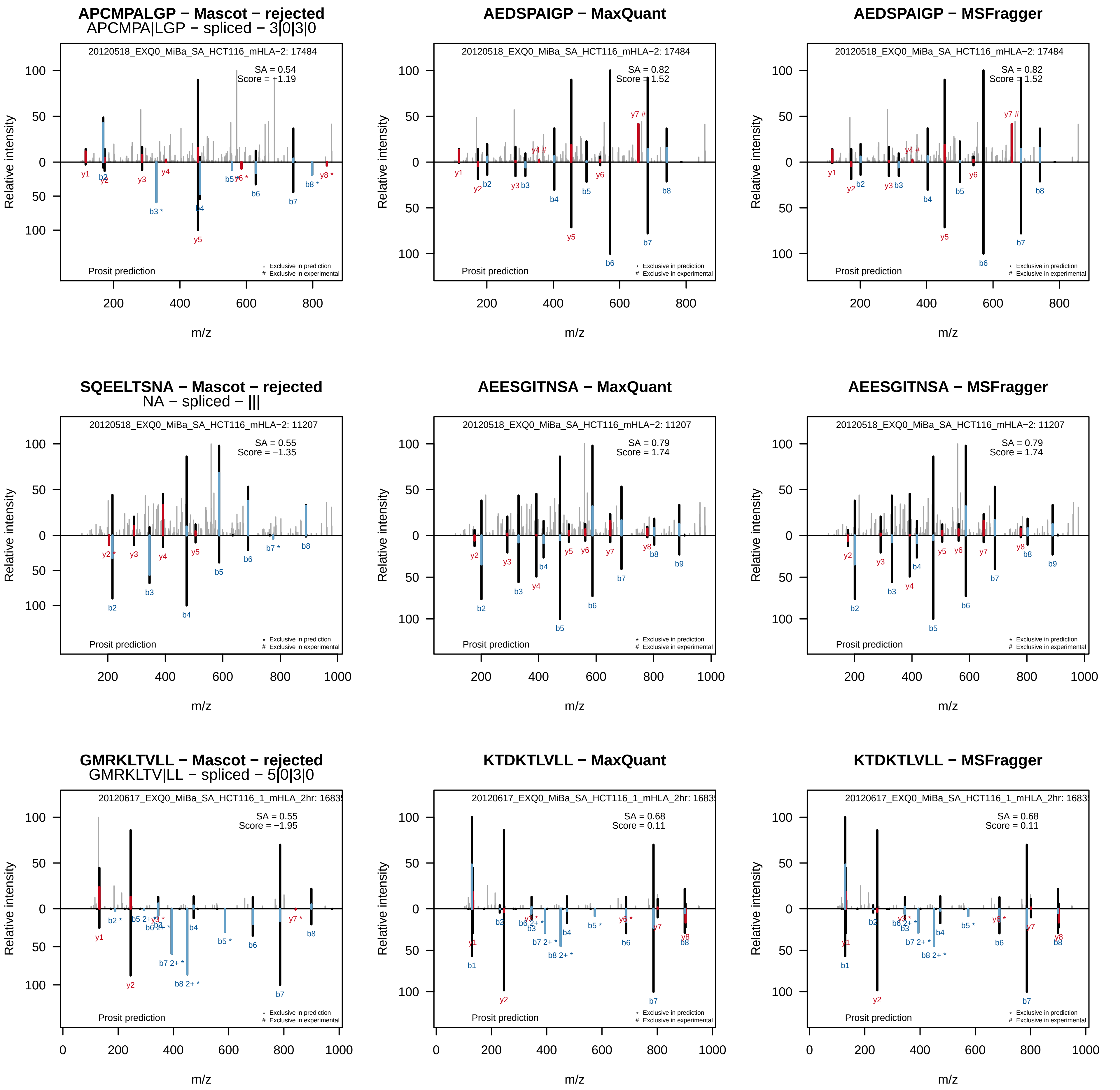

APCMPALGP − Mascot − rejected
APCMPA|LGP − spliced − 3|0|3|0
AEDSPAIGP − MaxQuant
AEDSPAIGP − MSFragger
20120518_EXQ0_MiBa_SA_HCT116_mHLA-2: 17484
SA = 0.54
Score = −1.19
SA = 0.82
Score = 1.52
SQEELTSNA − Mascot − rejected
NA − spliced − |||
AEESGITNSA − MaxQuant
AEESGITNSA − MSFragger
20120518_EXQ0_MiBa_SA_HCT116_mHLA-2: 11207
SA = 0.55
Score = −1.35
SA = 0.79
Score = 1.74
GMRKLTVLL − Mascot − rejected
GMRKLTV|LL − spliced − 5|0|3|0
KTDKTLVLL − MaxQuant
KTDKTLVLL − MSFragger
20120617_EXQ0_MiBa_SA_HCT116_1_mHLA_2hr: 1683
SA = 0.55
Score = −1.95
SA = 0.68
Score = 0.11
Relative intensity
m/z
Prosit prediction
* Exclusive in prediction
# Exclusive in experimental

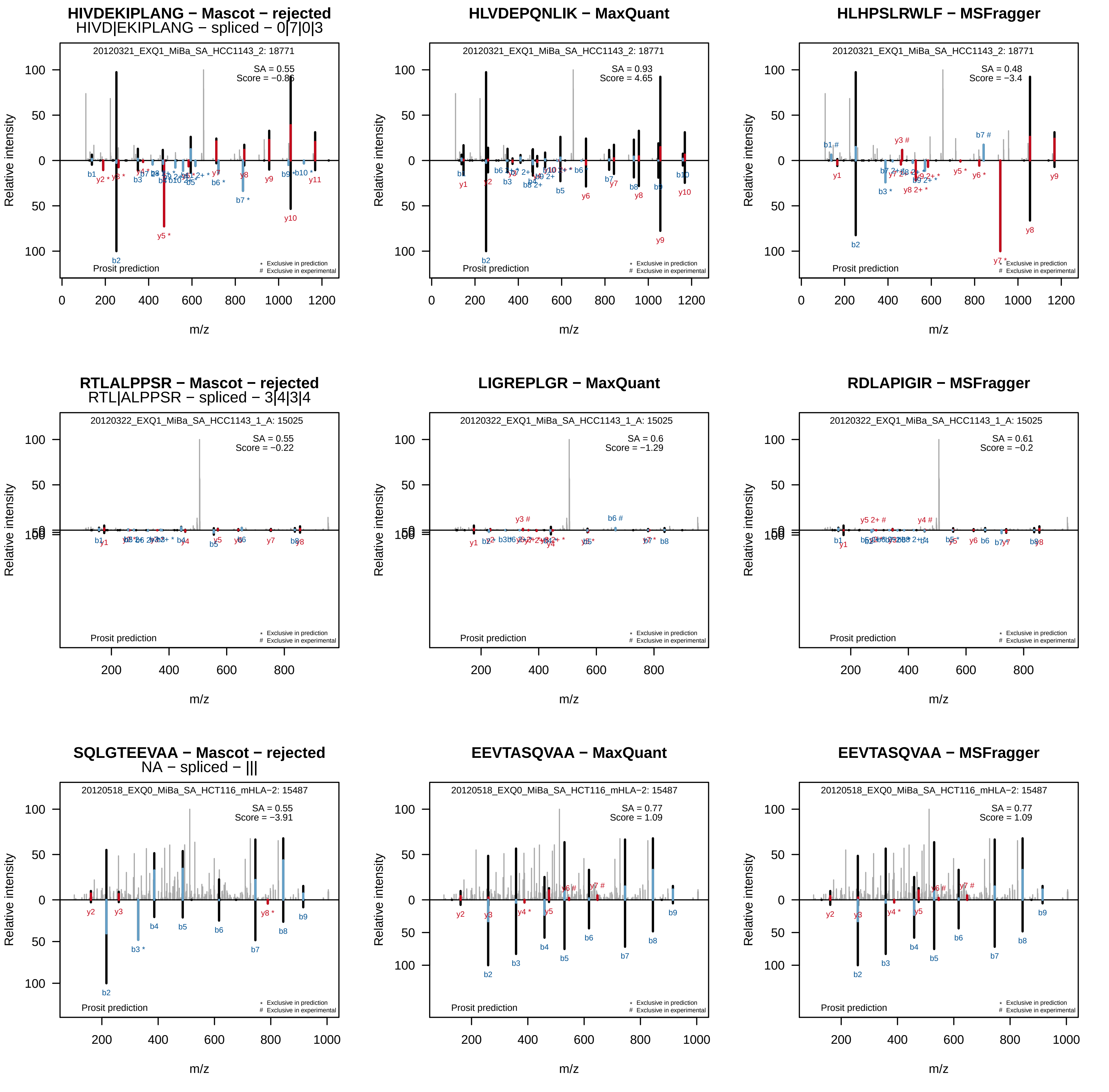

HIVDEKIPLANG − Mascot − rejected
HIVD|EKIPLANG − spliced − 0|7|0|3
20120321_EXQ1_MiBa_SA_HCC1143_2: 18771
SA = 0.55
Score = −0.86
HLVDEPQNLIK − MaxQuant
20120321_EXQ1_MiBa_SA_HCC1143_2: 18771
SA = 0.93
Score = 4.65
HLHPSLRWLF − MSFragger
20120321_EXQ1_MiBa_SA_HCC1143_2: 18771
SA = 0.48
Score = −3.4
RTLALPPSR − Mascot − rejected
RTL|ALPPSR − spliced − 3|4|3|4
20120322_EXQ1_MiBa_SA_HCC1143_1_A: 15025
SA = 0.55
Score = −0.22
LIGREPLGR − MaxQuant
20120322_EXQ1_MiBa_SA_HCC1143_1_A: 15025
SA = 0.6
Score = −1.29
RDLAPIGIR − MSFragger
20120322_EXQ1_MiBa_SA_HCC1143_1_A: 15025
SA = 0.61
Score = −0.2
SQLGTEEVAA − Mascot − rejected
NA − spliced − |||
20120518_EXQ0_MiBa_SA_HCT116_mHLA−2: 15487
SA = 0.55
Score = −3.91
EEVTASQVAA − MaxQuant
20120518_EXQ0_MiBa_SA_HCT116_mHLA−2: 15487
SA = 0.77
Score = 1.09
EEVTASQVAA − MSFragger
20120518_EXQ0_MiBa_SA_HCT116_mHLA−2: 15487
SA = 0.77
Score = 1.09
Relative intensity
m/z
Prosit prediction
⋆ Exclusive in prediction
Exclusive in experimental

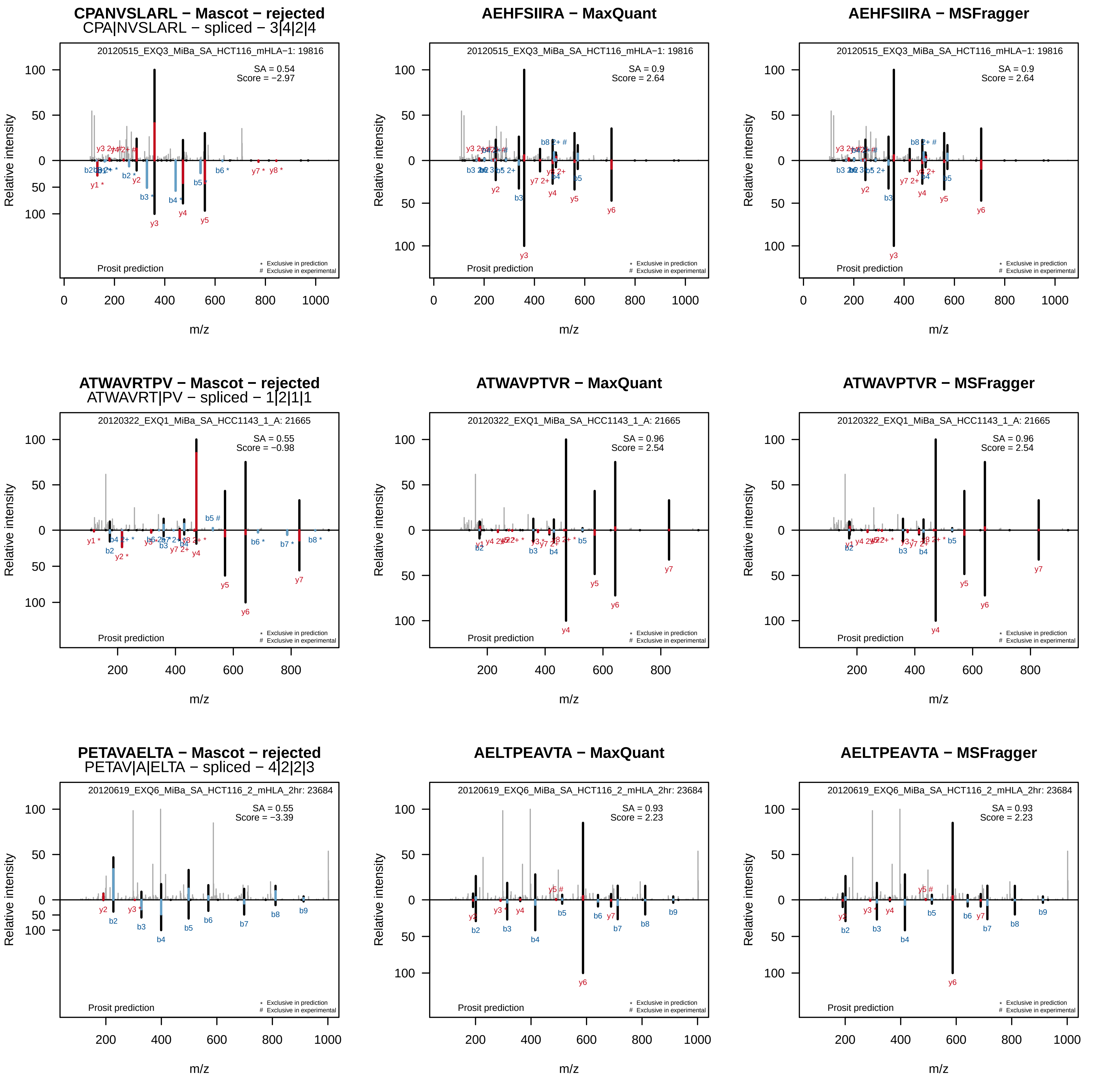

CPANVSLARL − Mascot − rejected
CPA|NVSLARL − spliced − 3|4|2|4
20120515_EXQ3_MiBa_SA_HCT116_mHLA−1: 19816
SA = 0.54
Score = −2.97
AEHFSIIRA − MaxQuant
20120515_EXQ3_MiBa_SA_HCT116_mHLA−1: 19816
SA = 0.9
Score = 2.64
AEHFSIIRA − MSFragger
20120515_EXQ3_MiBa_SA_HCT116_mHLA−1: 19816
SA = 0.9
Score = 2.64
ATWAVRTPV − Mascot − rejected
ATWAVRT|PV − spliced − 1|2|1|1
20120322_EXQ1_MiBa_SA_HCC1143_1_A: 21665
SA = 0.55
Score = −0.98
ATWAVPTVR − MaxQuant
20120322_EXQ1_MiBa_SA_HCC1143_1_A: 21665
SA = 0.96
Score = 2.54
ATWAVPTVR − MSFragger
20120322_EXQ1_MiBa_SA_HCC1143_1_A: 21665
SA = 0.96
Score = 2.54
PETAVAELTA − Mascot − rejected
PETAV|A|ELTA − spliced − 4|2|2|3
20120619_EXQ6_MiBa_SA_HCT116_2_mHLA_2hr: 23684
SA = 0.55
Score = −3.39
AELTPEAVTA − MaxQuant
20120619_EXQ6_MiBa_SA_HCT116_2_mHLA_2hr: 23684
SA = 0.93
Score = 2.23
AELTPEAVTA − MSFragger
20120619_EXQ6_MiBa_SA_HCT116_2_mHLA_2hr: 23684
SA = 0.93
Score = 2.23
Relative intensity
m/z
Prosit prediction
* Exclusive in prediction
# Exclusive in experimental

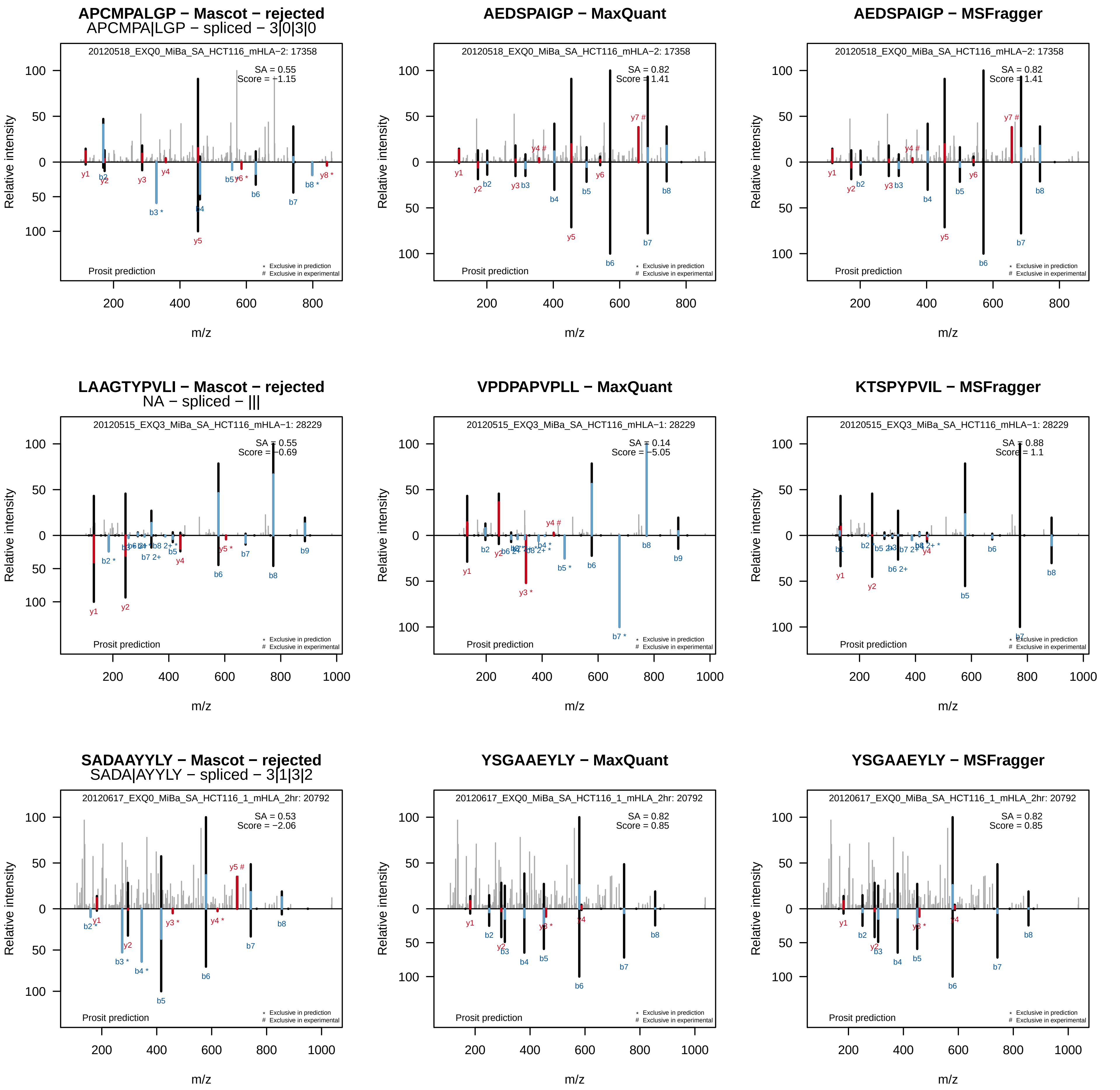
APCMPALGP − Mascot − rejected
APCMPA|LGP − spliced − 3|0|3|0
20120518_EXQ0_MiBa_SA_HCT116_mHLA−2: 17358
SA = 0.55
Score = −1.15
AEDSPAIGP − MaxQuant
20120518_EXQ0_MiBa_SA_HCT116_mHLA−2: 17358
SA = 0.82
Score = 1.41
AEDSPAIGP − MSFragger
20120518_EXQ0_MiBa_SA_HCT116_mHLA−2: 17358
SA = 0.82
Score = 1.41
LAAGTYPVLI − Mascot − rejected
NA − spliced − |||
20120515_EXQ3_MiBa_SA_HCT116_mHLA−1: 28229
SA = 0.55
Score = −0.69
VPDPAPVPLL − MaxQuant
20120515_EXQ3_MiBa_SA_HCT116_mHLA−1: 28229
SA = 0.14
Score = −5.05
KTSPYPVIL − MSFragger
20120515_EXQ3_MiBa_SA_HCT116_mHLA−1: 28229
SA = 0.88
Score = 1.1
SADAAYYLY − Mascot − rejected
SADA|AYYLY − spliced − 3|1|3|2
20120617_EXQ0_MiBa_SA_HCT116_1_mHLA_2hr: 20792
SA = 0.53
Score = −2.06
YSGAAEYLY − MaxQuant
20120617_EXQ0_MiBa_SA_HCT116_1_mHLA_2hr: 20792
SA = 0.82
Score = 0.85
YSGAAEYLY − MSFragger
20120617_EXQ0_MiBa_SA_HCT116_1_mHLA_2hr: 20792
SA = 0.82
Score = 0.85
Relative intensity
m/z
Prosit prediction
* Exclusive in prediction
# Exclusive in experimental

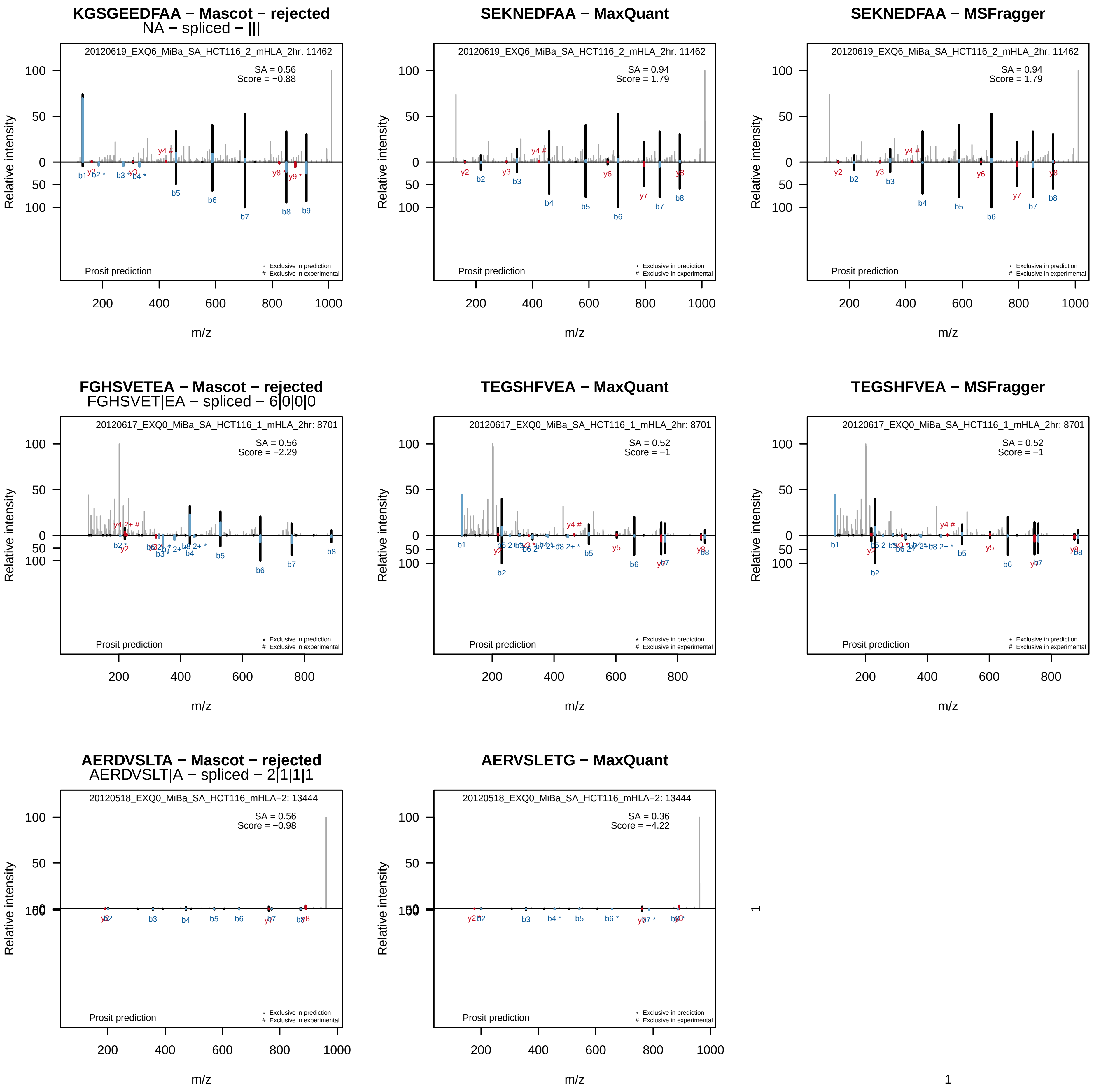

KGSGEEDFAA − Mascot − rejected
NA − spliced − |||
20120619_EXQ6_MiBa_SA_HCT116_2_mHLA_2hr: 11462
SA = 0.56
Score = −0.88
SEKNEDFAA − MaxQuant
20120619_EXQ6_MiBa_SA_HCT116_2_mHLA_2hr: 11462
SA = 0.94
Score = 1.79
SEKNEDFAA − MSFragger
20120619_EXQ6_MiBa_SA_HCT116_2_mHLA_2hr: 11462
SA = 0.94
Score = 1.79
FGHSVETEA − Mascot − rejected
FGHSVET|EA − spliced − 6|0|0|0
20120617_EXQ0_MiBa_SA_HCT116_1_mHLA_2hr: 8701
SA = 0.56
Score = −2.29
TEGSHFVEA − MaxQuant
20120617_EXQ0_MiBa_SA_HCT116_1_mHLA_2hr: 8701
SA = 0.52
Score = −1
TEGSHFVEA − MSFragger
20120617_EXQ0_MiBa_SA_HCT116_1_mHLA_2hr: 8701
SA = 0.52
Score = −1
AERDVSLTA − Mascot − rejected
AERDVSLT|A − spliced − 2|1|1|1
20120518_EXQ0_MiBa_SA_HCT116_mHLA−2: 13444
SA = 0.56
Score = −0.98
AERVSLETG − MaxQuant
20120518_EXQ0_MiBa_SA_HCT116_mHLA−2: 13444
SA = 0.36
Score = −4.22
Relative intensity
m/z
Prosit prediction
⋆ Exclusive in prediction
Exclusive in experimental

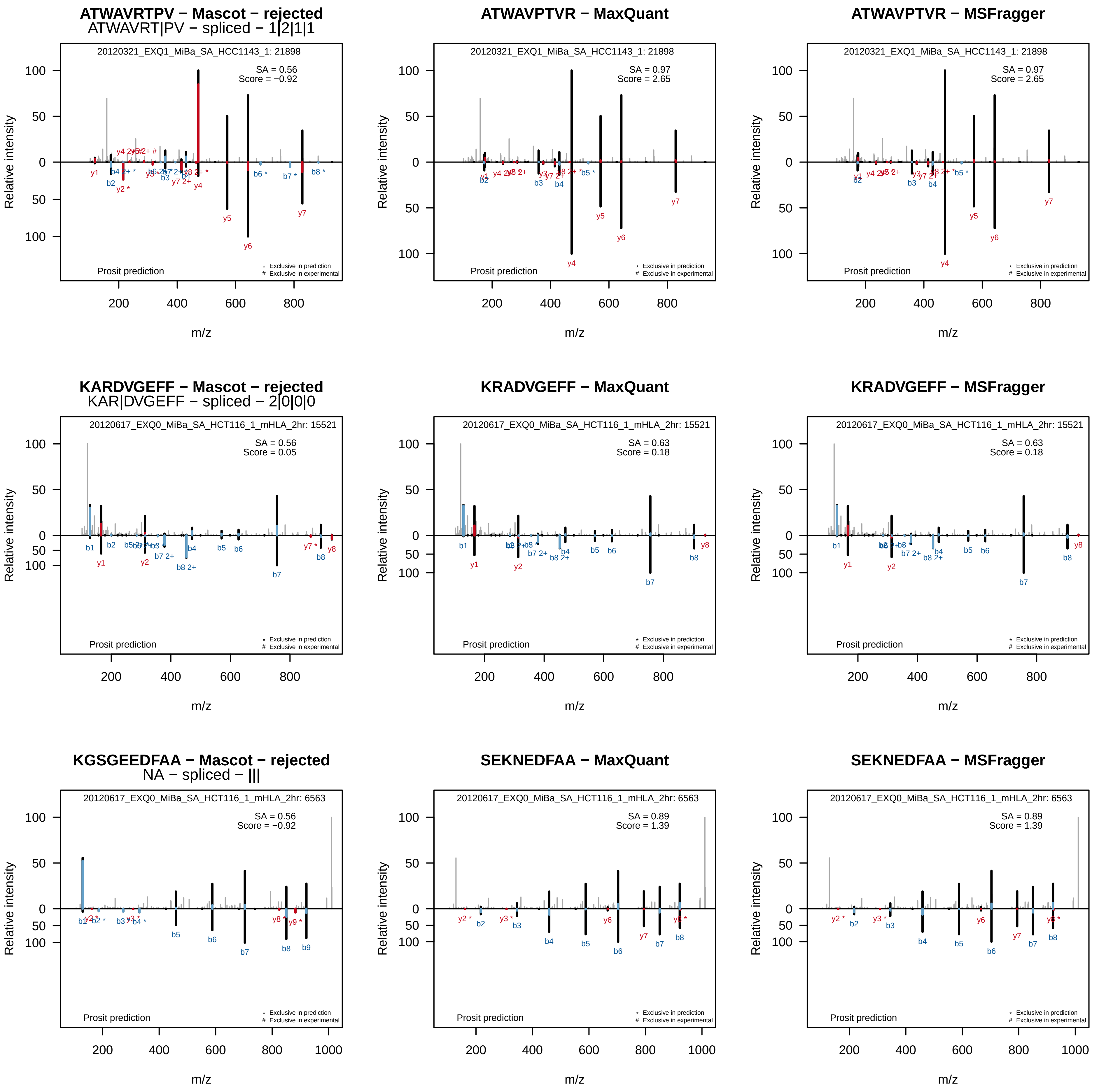

ATWAVRTPV − Mascot − rejected
ATWAVRT|PV − spliced − 1|2|1|1
20120321_EXQ1_MiBa_SA_HCC1143_1: 21898
SA = 0.56
Score = −0.92
ATWAVPTVR − MaxQuant
20120321_EXQ1_MiBa_SA_HCC1143_1: 21898
SA = 0.97
Score = 2.65
ATWAVPTVR − MSFragger
20120321_EXQ1_MiBa_SA_HCC1143_1: 21898
SA = 0.97
Score = 2.65
KARDVGEFF − Mascot − rejected
KAR|DVGEFF − spliced − 2|0|0|0
20120617_EXQ0_MiBa_SA_HCT116_1_mHLA_2hr: 15521
SA = 0.56
Score = 0.05
KRADVGEFF − MaxQuant
20120617_EXQ0_MiBa_SA_HCT116_1_mHLA_2hr: 15521
SA = 0.63
Score = 0.18
KRADVGEFF − MSFragger
20120617_EXQ0_MiBa_SA_HCT116_1_mHLA_2hr: 15521
SA = 0.63
Score = 0.18
KGSGEEDFAA − Mascot − rejected
NA − spliced − |||
20120617_EXQ0_MiBa_SA_HCT116_1_mHLA_2hr: 6563
SA = 0.56
Score = −0.92
SEKNEDFAA − MaxQuant
20120617_EXQ0_MiBa_SA_HCT116_1_mHLA_2hr: 6563
SA = 0.89
Score = 1.39
SEKNEDFAA − MSFragger
20120617_EXQ0_MiBa_SA_HCT116_1_mHLA_2hr: 6563
SA = 0.89
Score = 1.39
Relative intensity
m/z
Prosit prediction
* Exclusive in prediction
# Exclusive in experimental

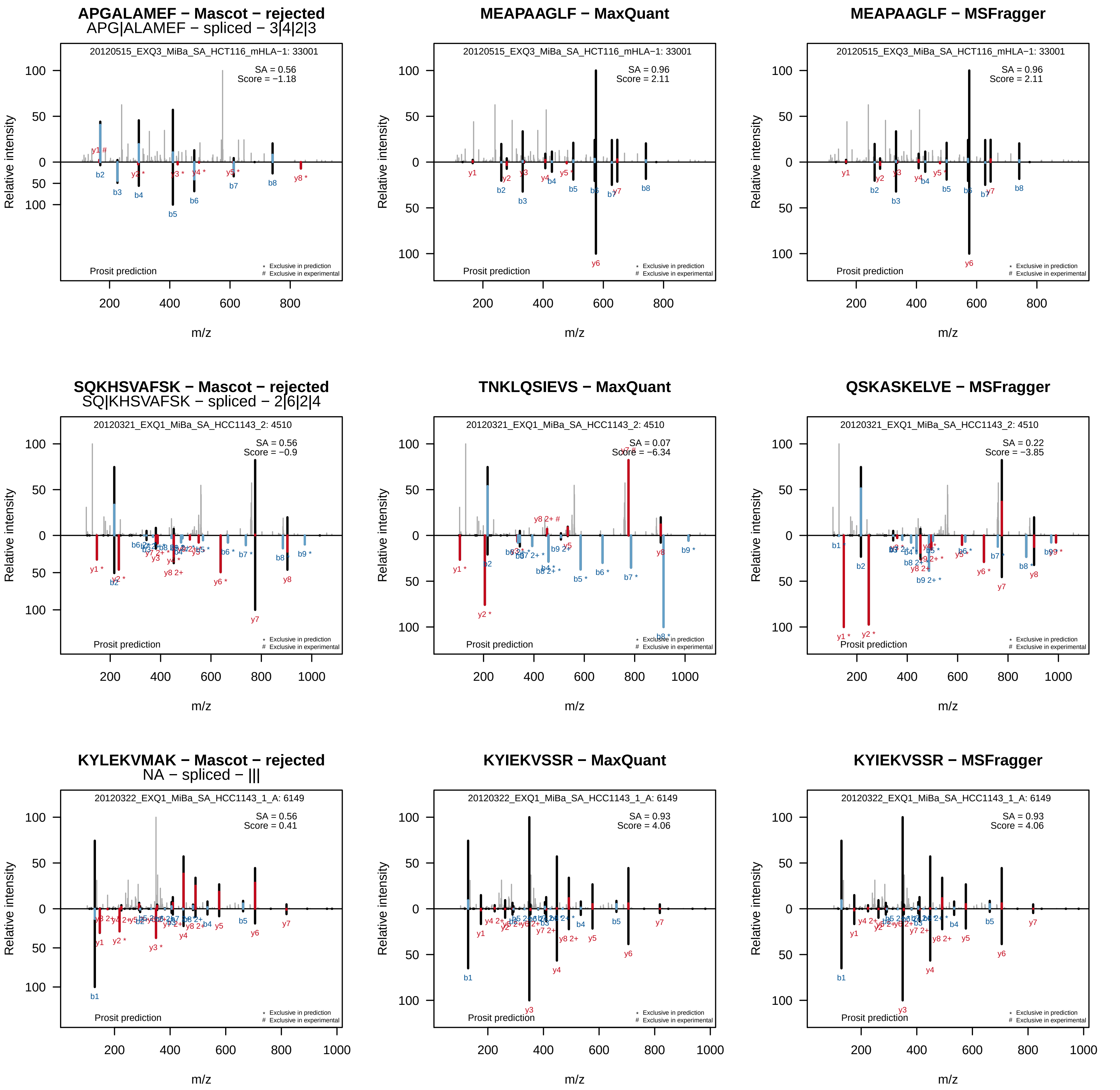

APGALAMEF − Mascot − rejected
APG|ALAMEF − spliced − 3|4|2|3
20120515_EXQ3_MiBa_SA_HCT116_mHLA−1: 33001
SA = 0.56
Score = −1.18
MEAPAAGLF − MaxQuant
20120515_EXQ3_MiBa_SA_HCT116_mHLA−1: 33001
SA = 0.96
Score = 2.11
MEAPAAGLF − MSFragger
20120515_EXQ3_MiBa_SA_HCT116_mHLA−1: 33001
SA = 0.96
Score = 2.11
SQKHSVAFSK − Mascot − rejected
SQ|KHSVAFSK − spliced − 2|6|2|4
20120321_EXQ1_MiBa_SA_HCC1143_2: 4510
SA = 0.56
Score = −0.9
TNKLQSIEVS − MaxQuant
20120321_EXQ1_MiBa_SA_HCC1143_2: 4510
SA = 0.07
Score = −6.34
QSKASKELVE − MSFragger
20120321_EXQ1_MiBa_SA_HCC1143_2: 4510
SA = 0.22
Score = −3.85
KYLEKVMAK − Mascot − rejected
NA − spliced − |||
20120322_EXQ1_MiBa_SA_HCC1143_1_A: 6149
SA = 0.56
Score = 0.41
KYIEKVSSR − MaxQuant
20120322_EXQ1_MiBa_SA_HCC1143_1_A: 6149
SA = 0.93
Score = 4.06
KYIEKVSSR − MSFragger
20120322_EXQ1_MiBa_SA_HCC1143_1_A: 6149
SA = 0.93
Score = 4.06
Relative intensity
m/z
Prosit prediction
* Exclusive in prediction
# Exclusive in experimental

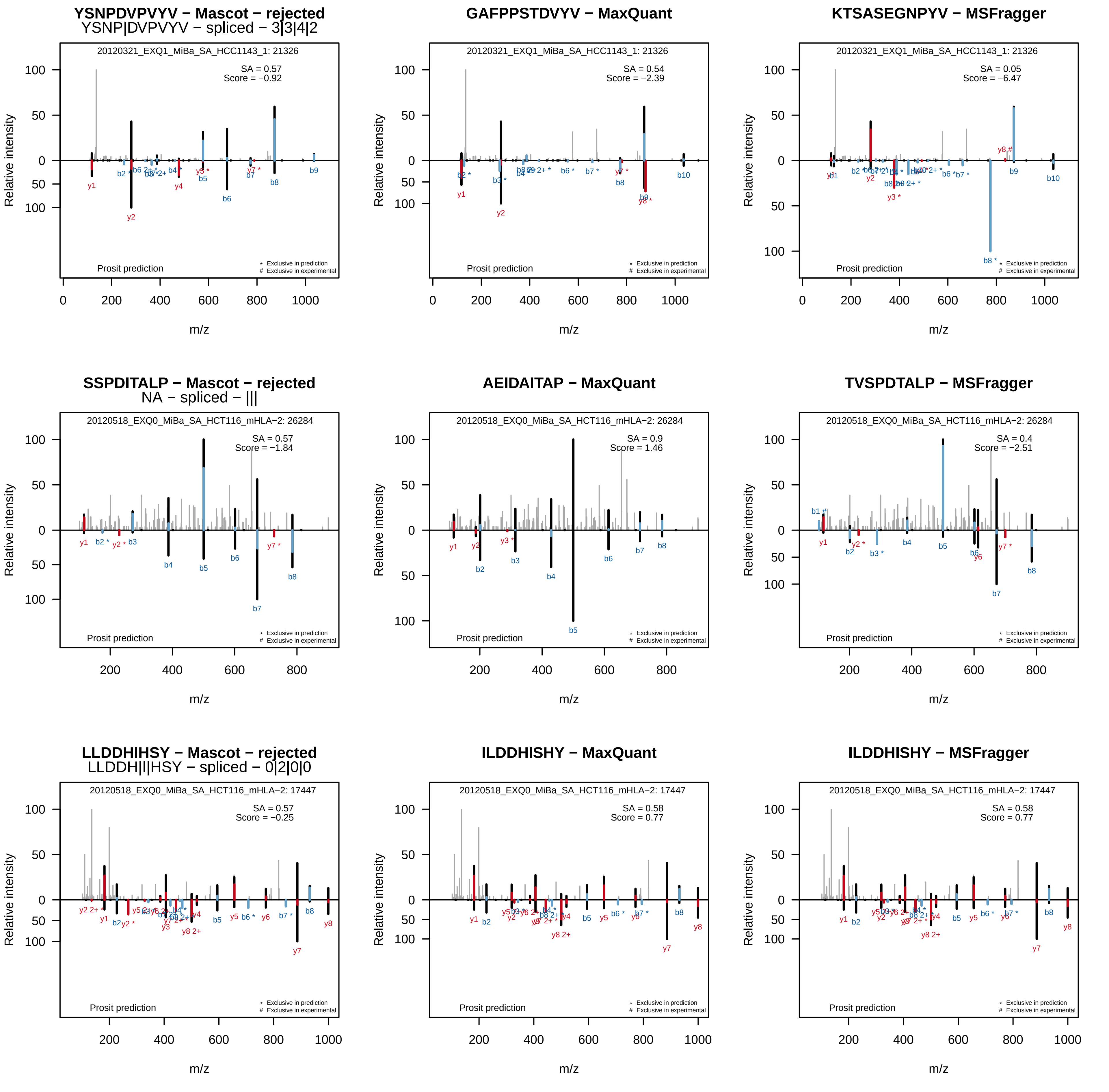

YSNPDVPVYV − Mascot − rejected
YSNP|DVPVYV − spliced − 3|3|4|2
20120321_EXQ1_MiBa_SA_HCC1143_1: 21326
SA = 0.57
Score = −0.92
GAFPPSTDVYV − MaxQuant
20120321_EXQ1_MiBa_SA_HCC1143_1: 21326
SA = 0.54
Score = −2.39
KTSASEGNPYV − MSFragger
20120321_EXQ1_MiBa_SA_HCC1143_1: 21326
SA = 0.05
Score = −6.47
SSPDITALP − Mascot − rejected
NA − spliced − |||
20120518_EXQ0_MiBa_SA_HCT116_mHLA−2: 26284
SA = 0.57
Score = −1.84
AEIDAITAP − MaxQuant
20120518_EXQ0_MiBa_SA_HCT116_mHLA−2: 26284
SA = 0.9
Score = 1.46
TVSPDTALP − MSFragger
20120518_EXQ0_MiBa_SA_HCT116_mHLA−2: 26284
SA = 0.4
Score = −2.51
LLDDHIHSY − Mascot − rejected
LLDDH|I|HSY − spliced − 0|2|0|0
20120518_EXQ0_MiBa_SA_HCT116_mHLA−2: 17447
SA = 0.57
Score = −0.25
ILDDHISHY − MaxQuant
20120518_EXQ0_MiBa_SA_HCT116_mHLA−2: 17447
SA = 0.58
Score = 0.77
ILDDHISHY − MSFragger
20120518_EXQ0_MiBa_SA_HCT116_mHLA−2: 17447
SA = 0.58
Score = 0.77
Relative intensity
m/z
Prosit prediction
* Exclusive in prediction
# Exclusive in experimental

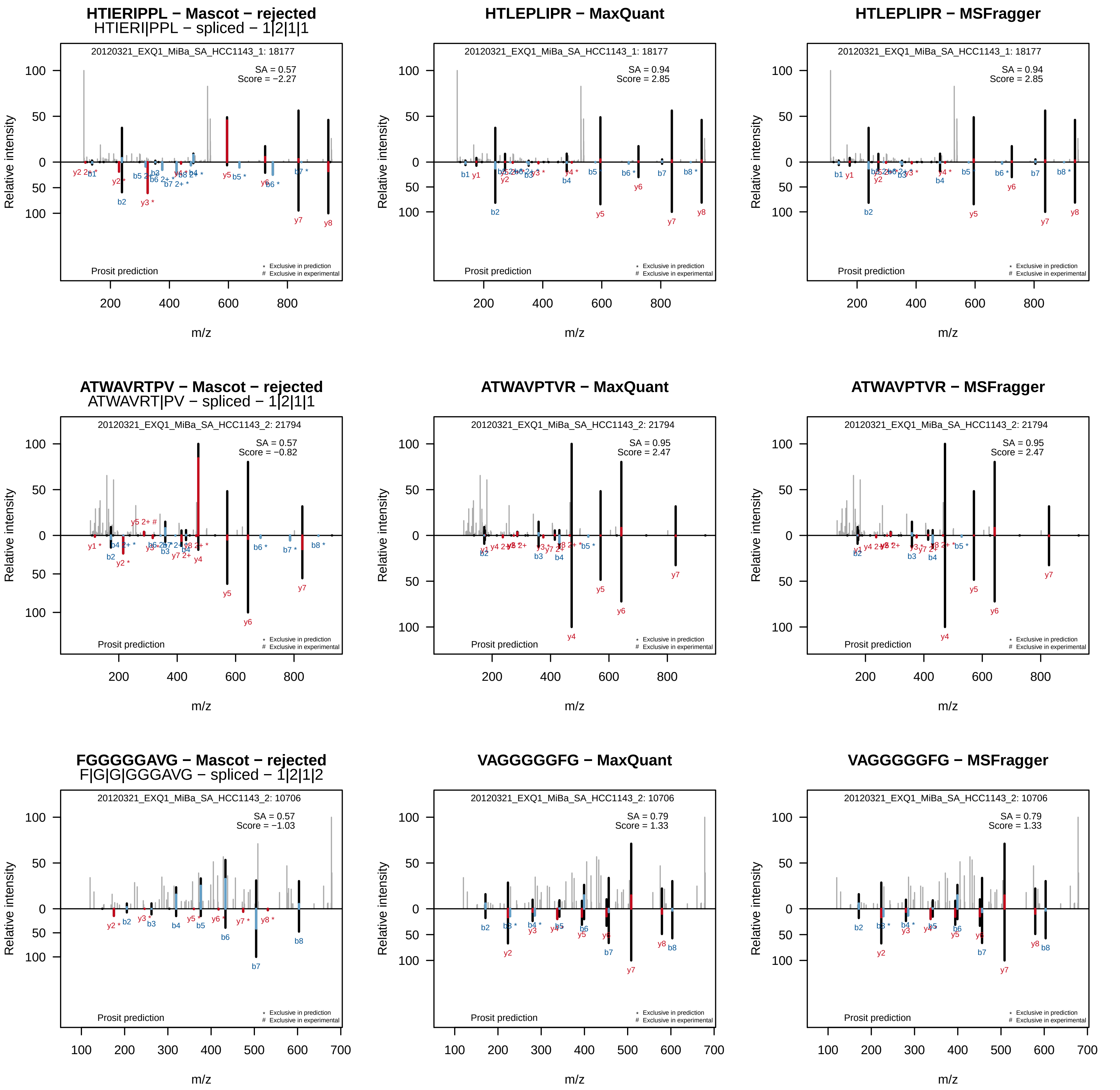
HTIERIPPL − Mascot − rejected
HTIERI|PPL − spliced − 1|2|1|1
20120321_EXQ1_MiBa_SA_HCC1143_1: 18177
SA = 0.57
Score = −2.27
HTLEPLIPR − MaxQuant
20120321_EXQ1_MiBa_SA_HCC1143_1: 18177
SA = 0.94
Score = 2.85
HTLEPLIPR − MSFragger
20120321_EXQ1_MiBa_SA_HCC1143_1: 18177
SA = 0.94
Score = 2.85
ATWAVRTPV − Mascot − rejected
ATWAVRT|PV − spliced − 1|2|1|1
20120321_EXQ1_MiBa_SA_HCC1143_2: 21794
SA = 0.57
Score = −0.82
ATWAVPTVR − MaxQuant
20120321_EXQ1_MiBa_SA_HCC1143_2: 21794
SA = 0.95
Score = 2.47
ATWAVPTVR − MSFragger
20120321_EXQ1_MiBa_SA_HCC1143_2: 21794
SA = 0.95
Score = 2.47
FGGGGGAVG − Mascot − rejected
F|G|G|GGGAVG − spliced − 1|2|1|2
20120321_EXQ1_MiBa_SA_HCC1143_2: 10706
SA = 0.57
Score = −1.03
VAGGGGGFG − MaxQuant
20120321_EXQ1_MiBa_SA_HCC1143_2: 10706
SA = 0.79
Score = 1.33
VAGGGGGFG − MSFragger
20120321_EXQ1_MiBa_SA_HCC1143_2: 10706
SA = 0.79
Score = 1.33
Relative intensity
m/z
Prosit prediction
⋆ Exclusive in prediction
Exclusive in experimental

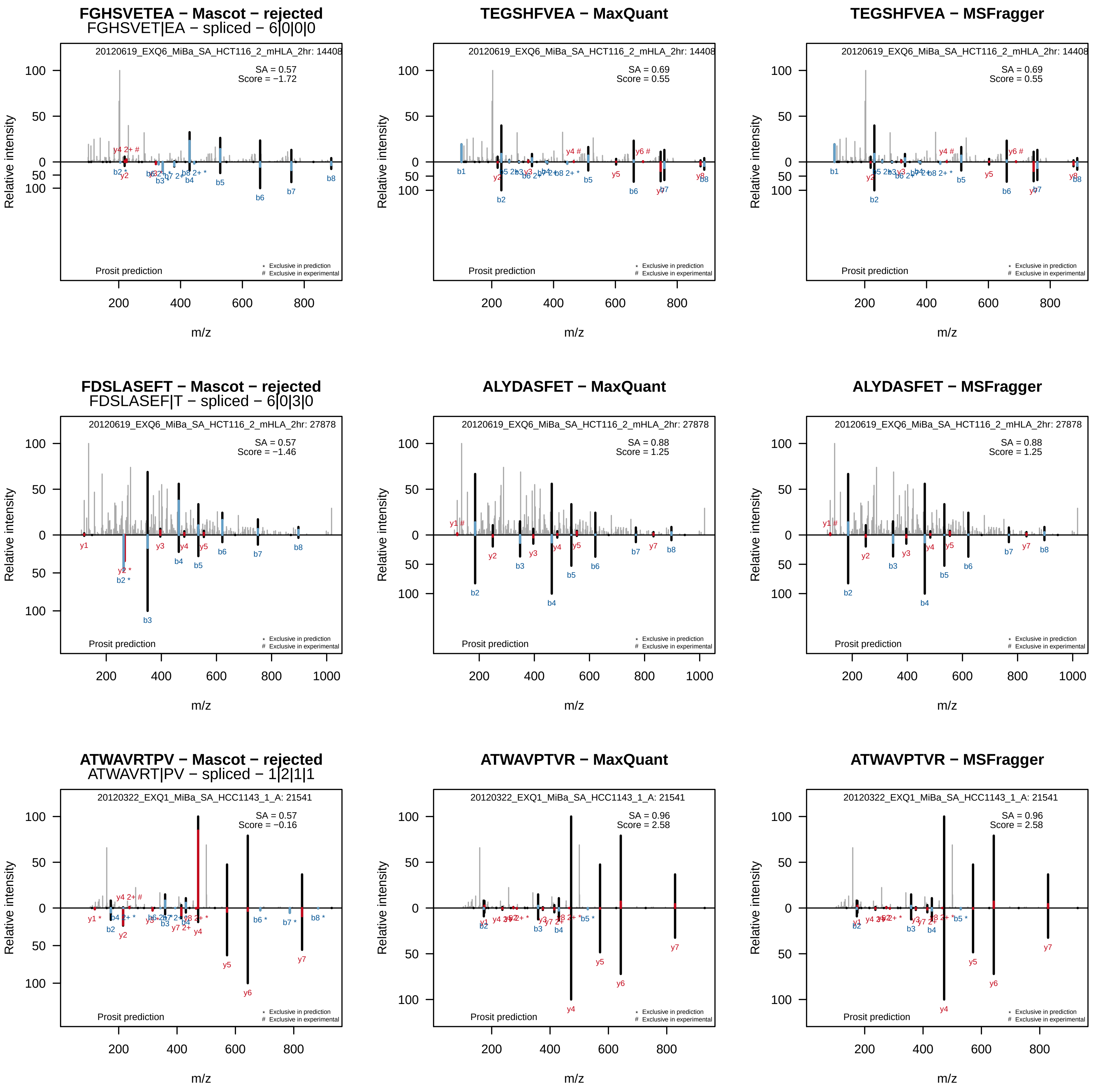
FGHSVETEA − Mascot − rejected
FGHSVET|EA − spliced − 6|0|0|0
20120619_EXQ6_MiBa_SA_HCT116_2_mHLA_2hr: 14408
SA = 0.57
Score = −1.72
TEGSHFVEA − MaxQuant
20120619_EXQ6_MiBa_SA_HCT116_2_mHLA_2hr: 14408
SA = 0.69
Score = 0.55
TEGSHFVEA − MSFragger
20120619_EXQ6_MiBa_SA_HCT116_2_mHLA_2hr: 14408
SA = 0.69
Score = 0.55
FDSLASEFT − Mascot − rejected
FDSLASEF|T − spliced − 6|0|3|0
20120619_EXQ6_MiBa_SA_HCT116_2_mHLA_2hr: 27878
SA = 0.57
Score = −1.46
ALYDASFET − MaxQuant
20120619_EXQ6_MiBa_SA_HCT116_2_mHLA_2hr: 27878
SA = 0.88
Score = 1.25
ALYDASFET − MSFragger
20120619_EXQ6_MiBa_SA_HCT116_2_mHLA_2hr: 27878
SA = 0.88
Score = 1.25
ATWAVRTPV − Mascot − rejected
ATWAVRT|PV − spliced − 1|2|1|1
20120322_EXQ1_MiBa_SA_HCC1143_1_A: 21541
SA = 0.57
Score = −0.16
ATWAVPTVR − MaxQuant
20120322_EXQ1_MiBa_SA_HCC1143_1_A: 21541
SA = 0.96
Score = 2.58
ATWAVPTVR − MSFragger
20120322_EXQ1_MiBa_SA_HCC1143_1_A: 21541
SA = 0.96
Score = 2.58
Relative intensity
m/z
Prosit prediction
* Exclusive in prediction
# Exclusive in experimental

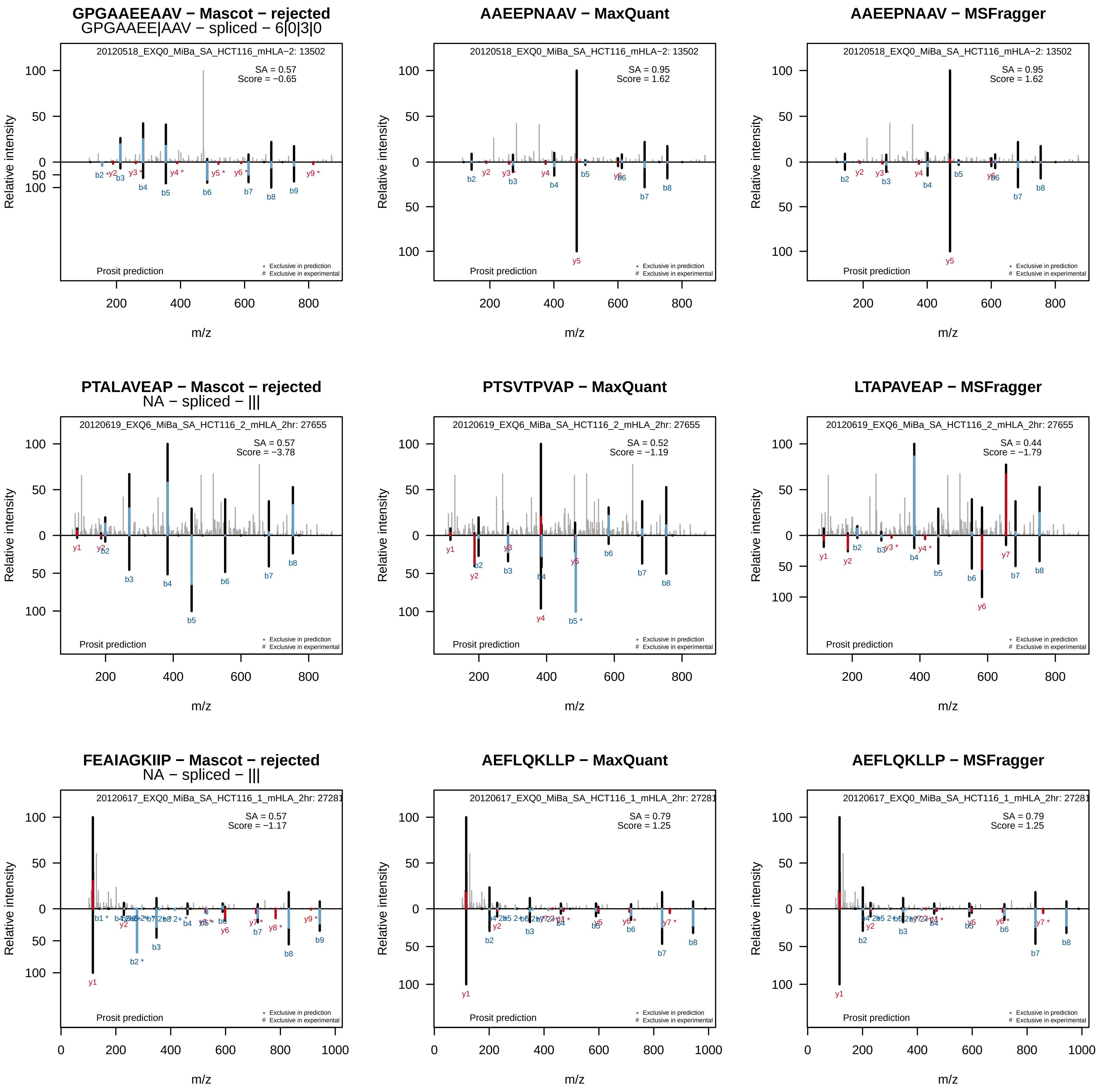

GPGAAEEAAV − Mascot − rejected
GPGAAEE|AAV − spliced − 6|0|3|0
20120518_EXQ0_MiBa_SA_HCT116_mHLA−2: 13502
SA = 0.57
Score = −0.65
AAEEPNAAV − MaxQuant
20120518_EXQ0_MiBa_SA_HCT116_mHLA−2: 13502
SA = 0.95
Score = 1.62
AAEEPNAAV − MSFragger
20120518_EXQ0_MiBa_SA_HCT116_mHLA−2: 13502
SA = 0.95
Score = 1.62
PTALAVEAP − Mascot − rejected
NA − spliced − |||
20120619_EXQ6_MiBa_SA_HCT116_2_mHLA_2hr: 27655
SA = 0.57
Score = −3.78
PTSVTPVAP − MaxQuant
20120619_EXQ6_MiBa_SA_HCT116_2_mHLA_2hr: 27655
SA = 0.52
Score = −1.19
LTAPAVEAP − MSFragger
20120619_EXQ6_MiBa_SA_HCT116_2_mHLA_2hr: 27655
SA = 0.44
Score = −1.79
FEAIAGKIIP − Mascot − rejected
NA − spliced − |||
20120617_EXQ0_MiBa_SA_HCT116_1_mHLA_2hr: 27281
SA = 0.57
Score = −1.17
AEFLQKLLP − MaxQuant
20120617_EXQ0_MiBa_SA_HCT116_1_mHLA_2hr: 27281
SA = 0.79
Score = 1.25
AEFLQKLLP − MSFragger
20120617_EXQ0_MiBa_SA_HCT116_1_mHLA_2hr: 27281
SA = 0.79
Score = 1.25
Relative intensity
m/z
Prosit prediction
⋆ Exclusive in prediction
Exclusive in experimental

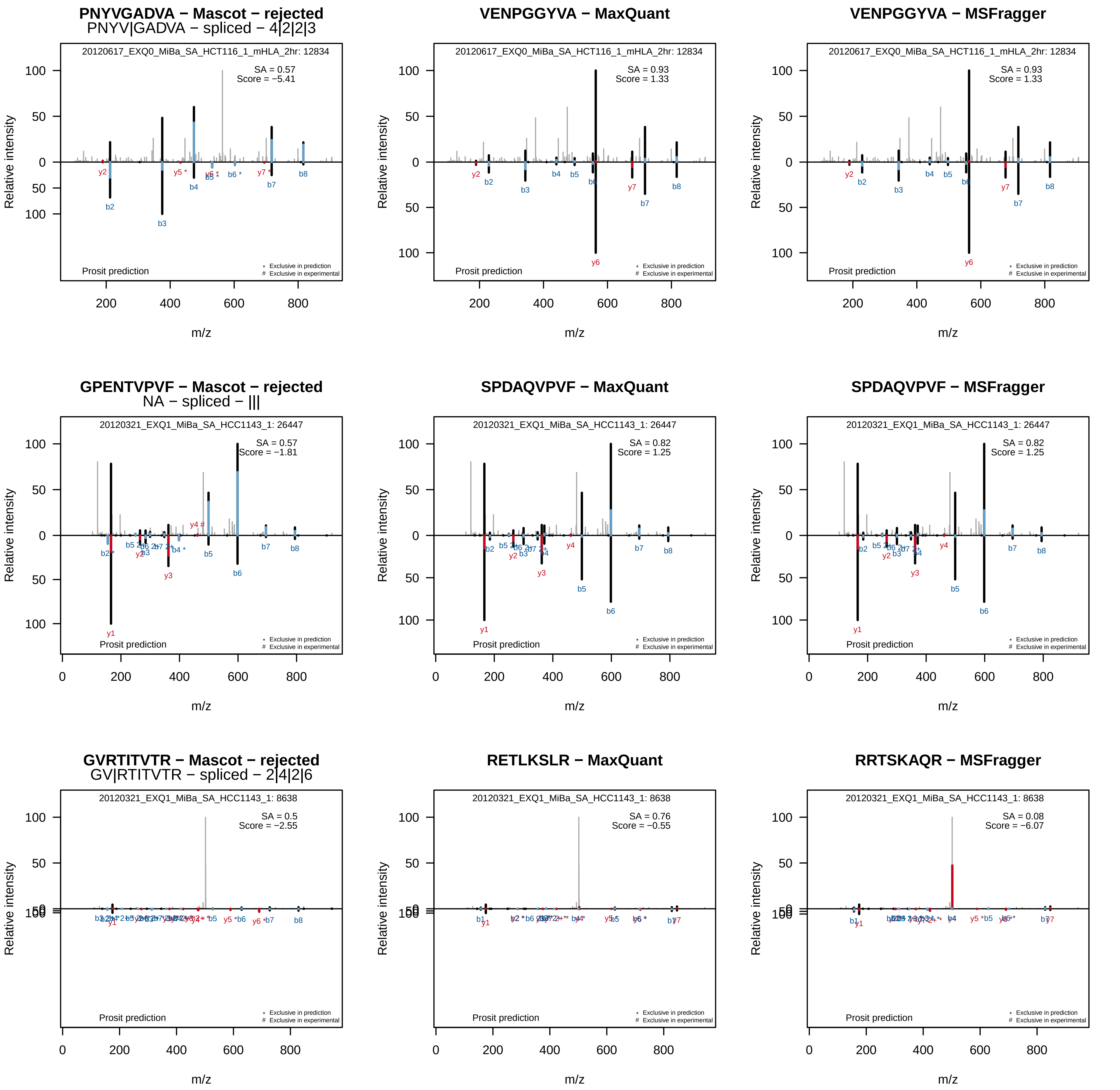
PNYVGADVA − Mascot − rejected
PNYV|GADVA − spliced − 4|2|2|3
20120617_EXQ0_MiBa_SA_HCT116_1_mHLA_2hr: 12834
SA = 0.57
Score = −5.41
VENPGGYVA − MaxQuant
20120617_EXQ0_MiBa_SA_HCT116_1_mHLA_2hr: 12834
SA = 0.93
Score = 1.33
VENPGGYVA − MSFragger
20120617_EXQ0_MiBa_SA_HCT116_1_mHLA_2hr: 12834
SA = 0.93
Score = 1.33
GPENTVPVF − Mascot − rejected
NA − spliced − |||
20120321_EXQ1_MiBa_SA_HCC1143_1: 26447
SA = 0.57
Score = −1.81
SPDAQVPVF − MaxQuant
20120321_EXQ1_MiBa_SA_HCC1143_1: 26447
SA = 0.82
Score = 1.25
SPDAQVPVF − MSFragger
20120321_EXQ1_MiBa_SA_HCC1143_1: 26447
SA = 0.82
Score = 1.25
GVRTITVTR − Mascot − rejected
GV|RTITVTR − spliced − 2|4|2|6
20120321_EXQ1_MiBa_SA_HCC1143_1: 8638
SA = 0.5
Score = −2.55
RETLKSLR − MaxQuant
20120321_EXQ1_MiBa_SA_HCC1143_1: 8638
SA = 0.76
Score = −0.55
RRTSKAQR − MSFragger
20120321_EXQ1_MiBa_SA_HCC1143_1: 8638
SA = 0.08
Score = −6.07
Relative intensity
m/z
Prosit prediction
⋆ Exclusive in prediction
Exclusive in experimental

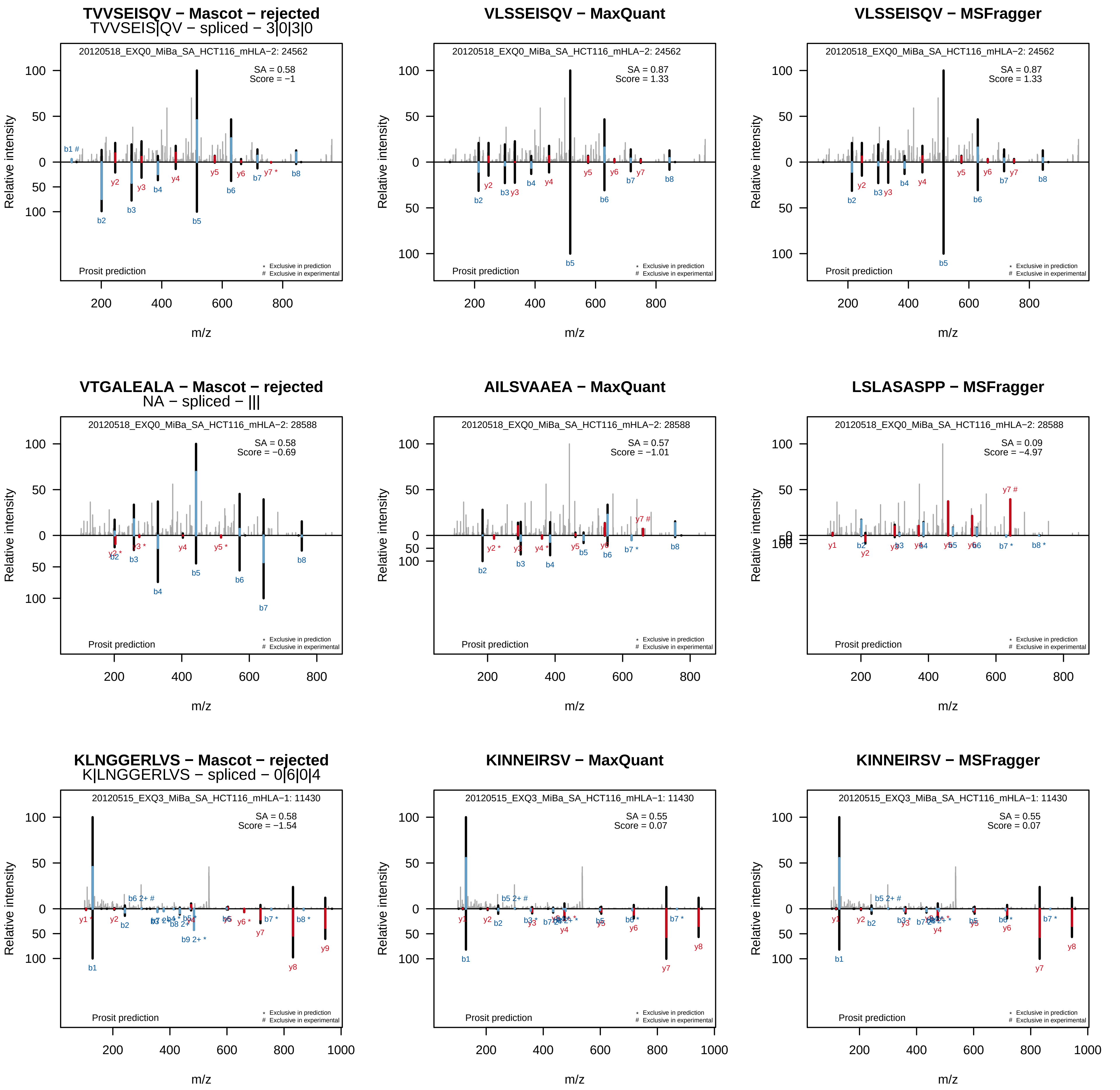
TVVSEISQV − Mascot − rejected
TVVSEIS|QV − spliced − 3|0|3|0
20120518_EXQ0_MiBa_SA_HCT116_mHLA−2: 24562
SA = 0.58
Score = −1
VLSSEISQV − MaxQuant
20120518_EXQ0_MiBa_SA_HCT116_mHLA−2: 24562
SA = 0.87
Score = 1.33
VLSSEISQV − MSFragger
20120518_EXQ0_MiBa_SA_HCT116_mHLA−2: 24562
SA = 0.87
Score = 1.33
VTGALEALA − Mascot − rejected
NA − spliced − |||
20120518_EXQ0_MiBa_SA_HCT116_mHLA−2: 28588
SA = 0.58
Score = −0.69
AILSVAAEA − MaxQuant
20120518_EXQ0_MiBa_SA_HCT116_mHLA−2: 28588
SA = 0.57
Score = −1.01
LSLASASPP − MSFragger
20120518_EXQ0_MiBa_SA_HCT116_mHLA−2: 28588
SA = 0.09
Score = −4.97
KLNGGERLVS − Mascot − rejected
K|LNGGERLVS − spliced − 0|6|0|4
20120515_EXQ3_MiBa_SA_HCT116_mHLA−1: 11430
SA = 0.58
Score = −1.54
KINNEIRSV − MaxQuant
20120515_EXQ3_MiBa_SA_HCT116_mHLA−1: 11430
SA = 0.55
Score = 0.07
KINNEIRSV − MSFragger
20120515_EXQ3_MiBa_SA_HCT116_mHLA−1: 11430
SA = 0.55
Score = 0.07
Relative intensity
m/z
Prosit prediction
⋆ Exclusive in prediction
Exclusive in experimental

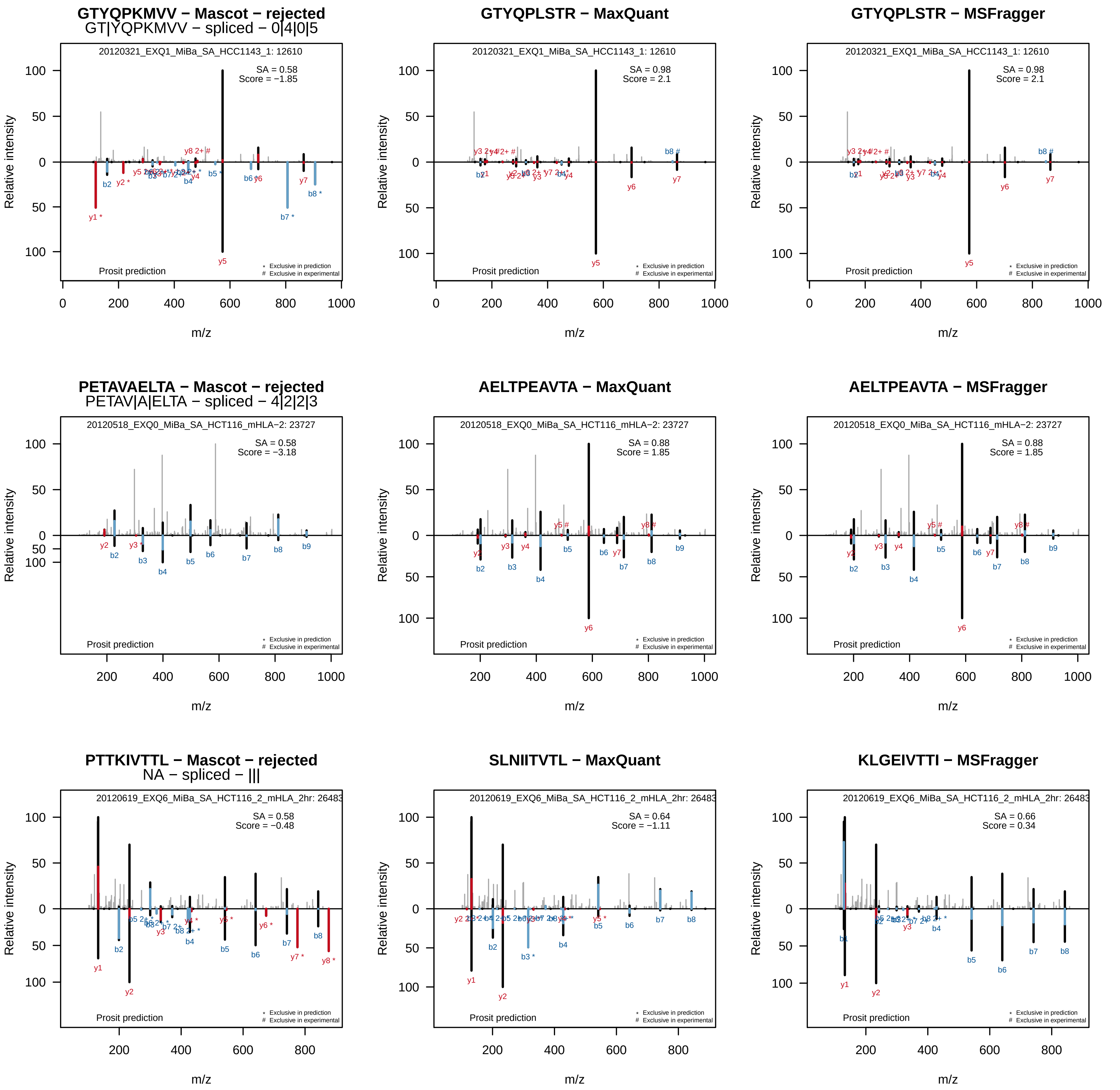
GTYQPKMVV − Mascot − rejected
GT|YQPKMVV − spliced − 0|4|0|5
20120321_EXQ1_MiBa_SA_HCC1143_1: 12610
SA = 0.58
Score = −1.85
GTYQPLSTR − MaxQuant
20120321_EXQ1_MiBa_SA_HCC1143_1: 12610
SA = 0.98
Score = 2.1
GTYQPLSTR − MSFragger
20120321_EXQ1_MiBa_SA_HCC1143_1: 12610
SA = 0.98
Score = 2.1
PETAVAELTA − Mascot − rejected
PETAV|A|ELTA − spliced − 4|2|2|3
20120518_EXQ0_MiBa_SA_HCT116_mHLA−2: 23727
SA = 0.58
Score = −3.18
AELTPEAVTA − MaxQuant
20120518_EXQ0_MiBa_SA_HCT116_mHLA−2: 23727
SA = 0.88
Score = 1.85
AELTPEAVTA − MSFragger
20120518_EXQ0_MiBa_SA_HCT116_mHLA−2: 23727
SA = 0.88
Score = 1.85
PTTKIVTTL − Mascot − rejected
NA − spliced − |||
20120619_EXQ6_MiBa_SA_HCT116_2_mHLA_2hr: 26483
SA = 0.58
Score = −0.48
SLNIITVTL − MaxQuant
20120619_EXQ6_MiBa_SA_HCT116_2_mHLA_2hr: 26483
SA = 0.64
Score = −1.11
KLGEIVTTI − MSFragger
20120619_EXQ6_MiBa_SA_HCT116_2_mHLA_2hr: 26483
SA = 0.66
Score = 0.34
Relative intensity
m/z
Prosit prediction
⋆ Exclusive in prediction
Exclusive in experimental

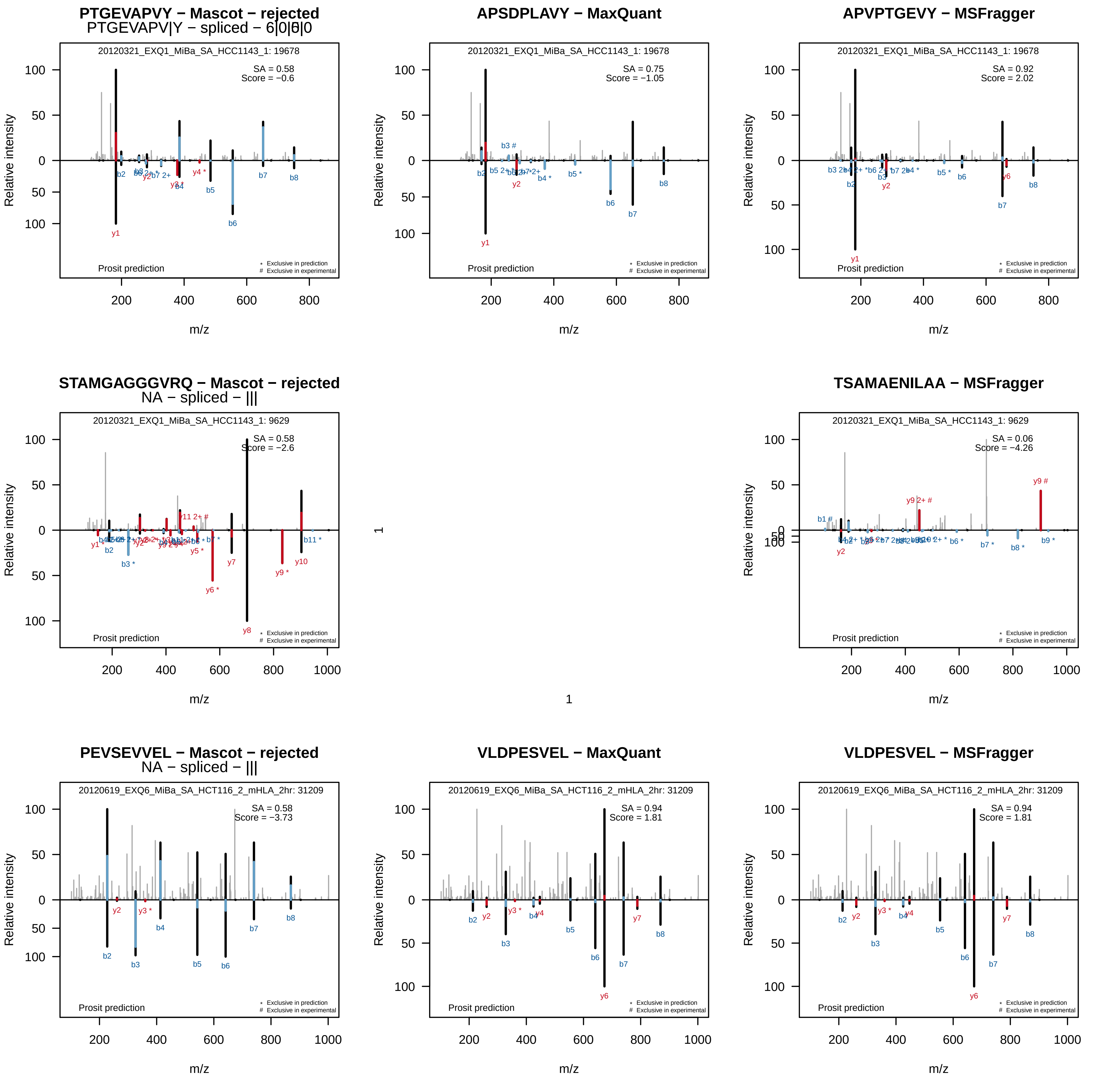

PTGEVAPVY − Mascot − rejected
PTGEVAPV|Y − spliced − 6|0|θ|0
20120321_EXQ1_MiBa_SA_HCC1143_1: 19678
SA = 0.58
Score = −0.6
Prosit prediction
⋆ Exclusive in prediction
Exclusive in experimental
Relative intensity
m/z
APSDPLAVY − MaxQuant
20120321_EXQ1_MiBa_SA_HCC1143_1: 19678
SA = 0.75
Score = −1.05
Prosit prediction
⋆ Exclusive in prediction
Exclusive in experimental
Relative intensity
m/z
APVPTGEVY − MSFragger
20120321_EXQ1_MiBa_SA_HCC1143_1: 19678
SA = 0.92
Score = 2.02
Prosit prediction
⋆ Exclusive in prediction
Exclusive in experimental
Relative intensity
m/z
STAMGAGGGVRQ − Mascot − rejected
NA − spliced − |||
20120321_EXQ1_MiBa_SA_HCC1143_1: 9629
SA = 0.58
Score = −2.6
Prosit prediction
⋆ Exclusive in prediction
Exclusive in experimental
Relative intensity
m/z
1
1
TSAMAENILAA − MSFragger
20120321_EXQ1_MiBa_SA_HCC1143_1: 9629
SA = 0.06
Score = −4.26
Prosit prediction
⋆ Exclusive in prediction
Exclusive in experimental
Relative intensity
m/z
PEVSEVVEL − Mascot − rejected
NA − spliced − |||
20120619_EXQ6_MiBa_SA_HCT116_2_mHLA_2hr: 31209
SA = 0.58
Score = −3.73
Prosit prediction
⋆ Exclusive in prediction
Exclusive in experimental
Relative intensity
m/z
VLDPESVEL − MaxQuant
20120619_EXQ6_MiBa_SA_HCT116_2_mHLA_2hr: 31209
SA = 0.94
Score = 1.81
Prosit prediction
⋆ Exclusive in prediction
Exclusive in experimental
Relative intensity
m/z
VLDPESVEL − MSFragger
20120619_EXQ6_MiBa_SA_HCT116_2_mHLA_2hr: 31209
SA = 0.94
Score = 1.81
Prosit prediction
⋆ Exclusive in prediction
Exclusive in experimental
Relative intensity
m/z

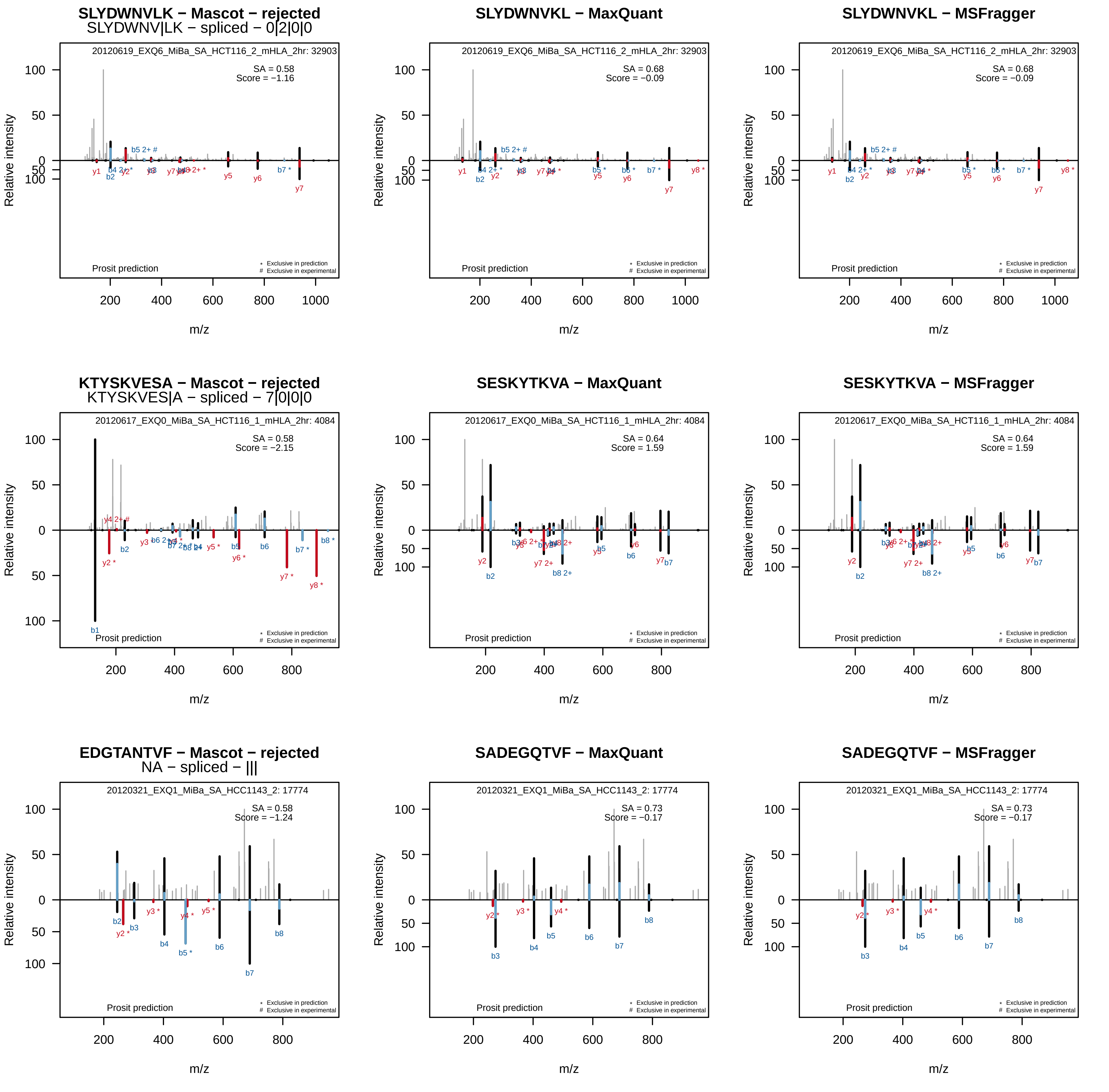

SLYDWNVLK − Mascot − rejected
SLYDWNV|LK − spliced − 0|2|0|0
20120619_EXQ6_MiBa_SA_HCT116_2_mHLA_2hr: 32903
SA = 0.58
Score = −1.16
SLYDWNVKL − MaxQuant
20120619_EXQ6_MiBa_SA_HCT116_2_mHLA_2hr: 32903
SA = 0.68
Score = −0.09
SLYDWNVKL − MSFragger
20120619_EXQ6_MiBa_SA_HCT116_2_mHLA_2hr: 32903
SA = 0.68
Score = −0.09
KTYSKVESA − Mascot − rejected
KTYSKVES|A − spliced − 7|0|0|0
20120617_EXQ0_MiBa_SA_HCT116_1_mHLA_2hr: 4084
SA = 0.58
Score = −2.15
SESKYTKVA − MaxQuant
20120617_EXQ0_MiBa_SA_HCT116_1_mHLA_2hr: 4084
SA = 0.64
Score = 1.59
SESKYTKVA − MSFragger
20120617_EXQ0_MiBa_SA_HCT116_1_mHLA_2hr: 4084
SA = 0.64
Score = 1.59
EDGTANTVF − Mascot − rejected
NA − spliced − |||
20120321_EXQ1_MiBa_SA_HCC1143_2: 17774
SA = 0.58
Score = −1.24
SADEGQTVF − MaxQuant
20120321_EXQ1_MiBa_SA_HCC1143_2: 17774
SA = 0.73
Score = −0.17
SADEGQTVF − MSFragger
20120321_EXQ1_MiBa_SA_HCC1143_2: 17774
SA = 0.73
Score = −0.17
Relative intensity
m/z
Prosit prediction
* Exclusive in prediction
# Exclusive in experimental

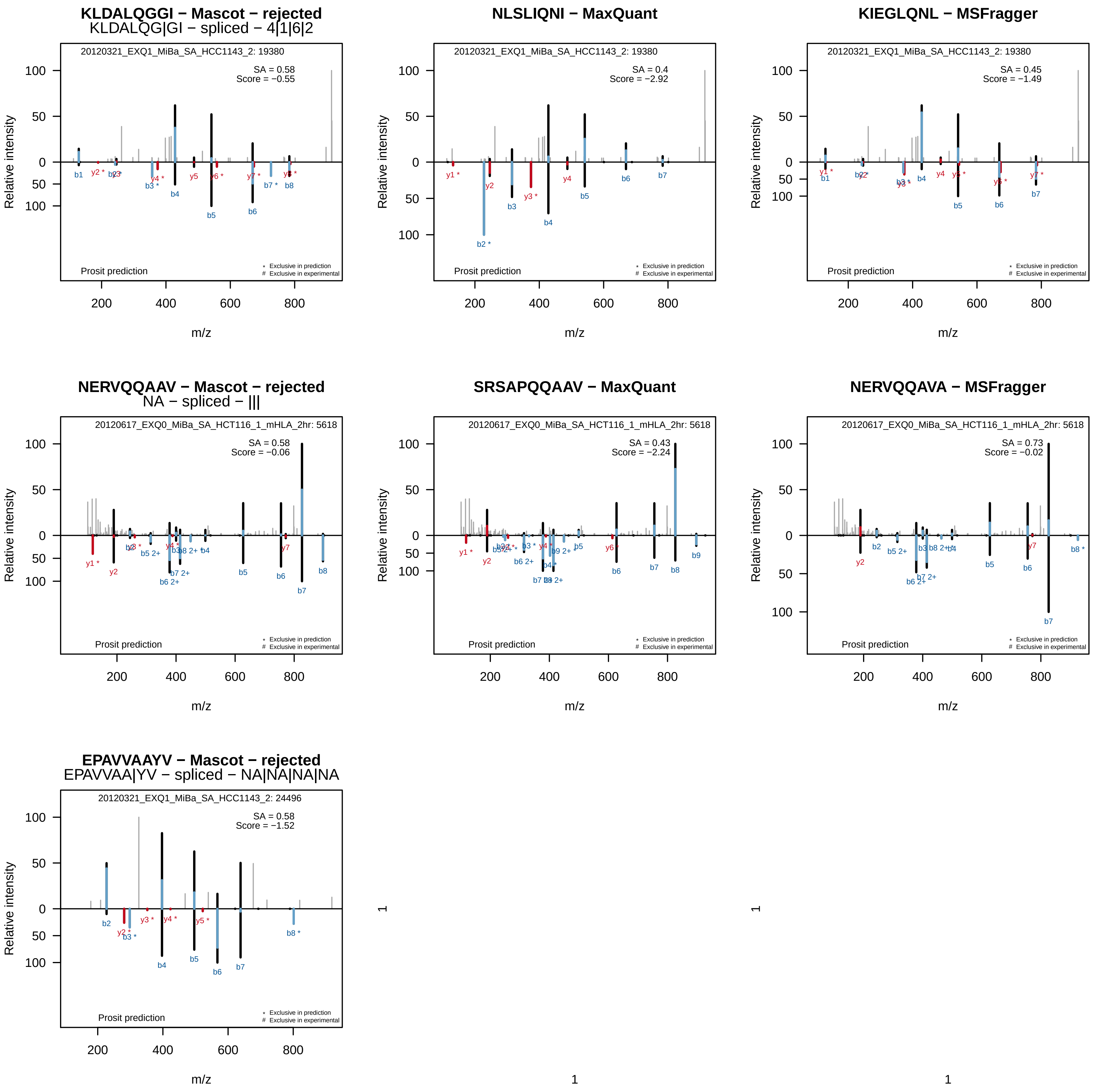

KLDALQGGI − Mascot − rejected
KLDALQG|GI − spliced − 4|1|6|2
20120321_EXQ1_MiBa_SA_HCC1143_2: 19380
SA = 0.58
Score = −0.55
NLSLIQNI − MaxQuant
20120321_EXQ1_MiBa_SA_HCC1143_2: 19380
SA = 0.4
Score = −2.92
KIEGLQNL − MSFragger
20120321_EXQ1_MiBa_SA_HCC1143_2: 19380
SA = 0.45
Score = −1.49
NERVQQAAV − Mascot − rejected
NA − spliced − |||
20120617_EXQ0_MiBa_SA_HCT116_1_mHLA_2hr: 5618
SA = 0.58
Score = −0.06
SRSAPQQAAV − MaxQuant
20120617_EXQ0_MiBa_SA_HCT116_1_mHLA_2hr: 5618
SA = 0.43
Score = −2.24
NERVQQAVA − MSFragger
20120617_EXQ0_MiBa_SA_HCT116_1_mHLA_2hr: 5618
SA = 0.73
Score = −0.02
EPAVVAAYV − Mascot − rejected
EPAVVAA|YV − spliced − NA|NA|NA|NA
20120321_EXQ1_MiBa_SA_HCC1143_2: 24496
SA = 0.58
Score = −1.52
Relative intensity
m/z
Prosit prediction
* Exclusive in prediction
# Exclusive in experimental

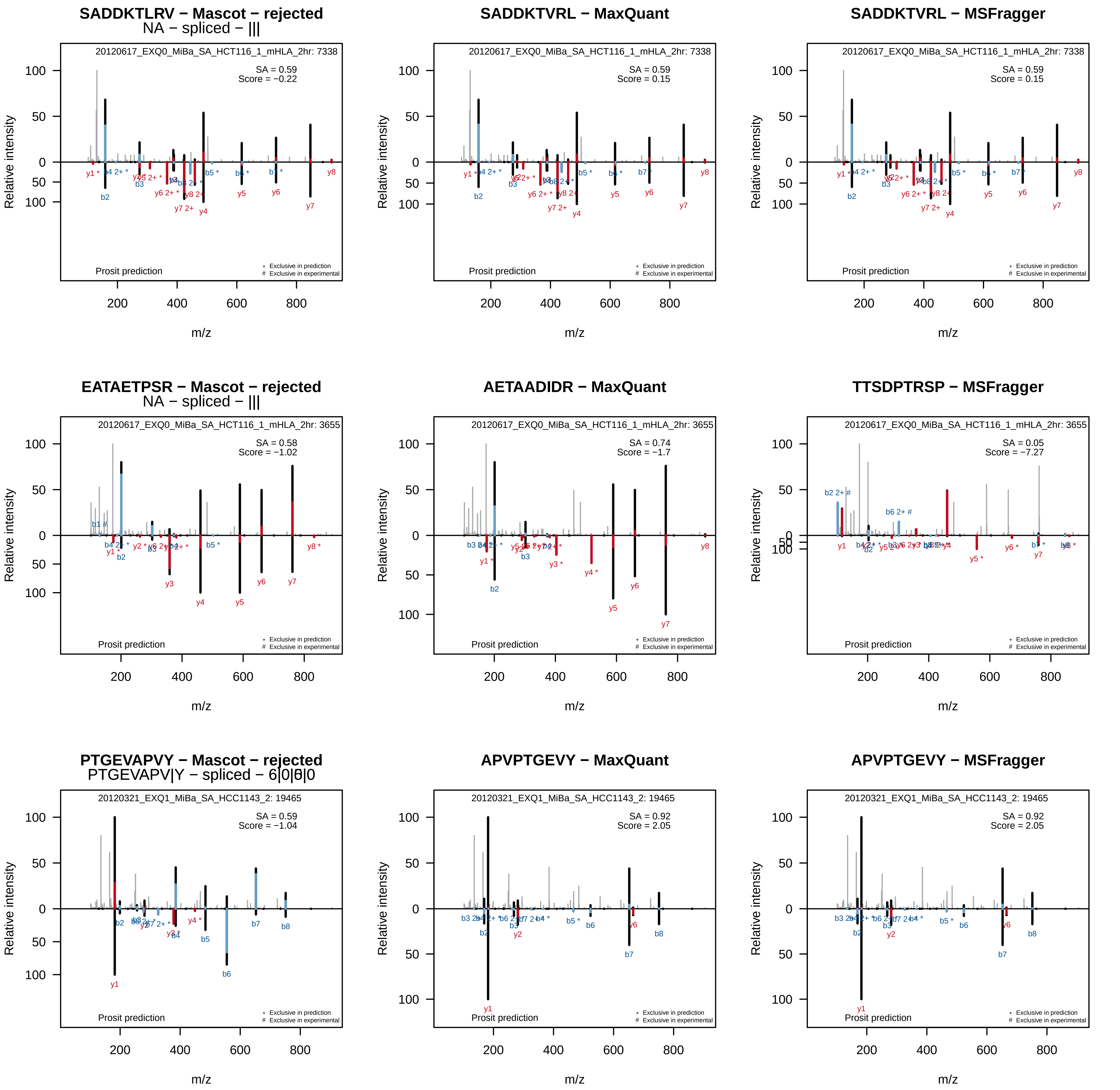
SADDKTLRV − Mascot − rejected
NA − spliced − |||
20120617_EXQ0_MiBa_SA_HCT116_1_mHLA_2hr: 7338
SA = 0.59
Score = −0.22
SADDKTVRL − MaxQuant
20120617_EXQ0_MiBa_SA_HCT116_1_mHLA_2hr: 7338
SA = 0.59
Score = 0.15
SADDKTVRL − MSFragger
20120617_EXQ0_MiBa_SA_HCT116_1_mHLA_2hr: 7338
SA = 0.59
Score = 0.15
EATAETPSR − Mascot − rejected
NA − spliced − |||
20120617_EXQ0_MiBa_SA_HCT116_1_mHLA_2hr: 3655
SA = 0.58
Score = −1.02
AETAADIDR − MaxQuant
20120617_EXQ0_MiBa_SA_HCT116_1_mHLA_2hr: 3655
SA = 0.74
Score = −1.7
TTSDPTRSP − MSFragger
20120617_EXQ0_MiBa_SA_HCT116_1_mHLA_2hr: 3655
SA = 0.05
Score = −7.27
PTGEVAPVY − Mascot − rejected
PTGEVAPV|Y − spliced − 6|0|6|0
20120321_EXQ1_MiBa_SA_HCC1143_2: 19465
SA = 0.59
Score = −1.04
APVPTGEVY − MaxQuant
20120321_EXQ1_MiBa_SA_HCC1143_2: 19465
SA = 0.92
Score = 2.05
APVPTGEVY − MSFragger
20120321_EXQ1_MiBa_SA_HCC1143_2: 19465
SA = 0.92
Score = 2.05
Relative intensity
m/z
Prosit prediction
* Exclusive in prediction
# Exclusive in experimental

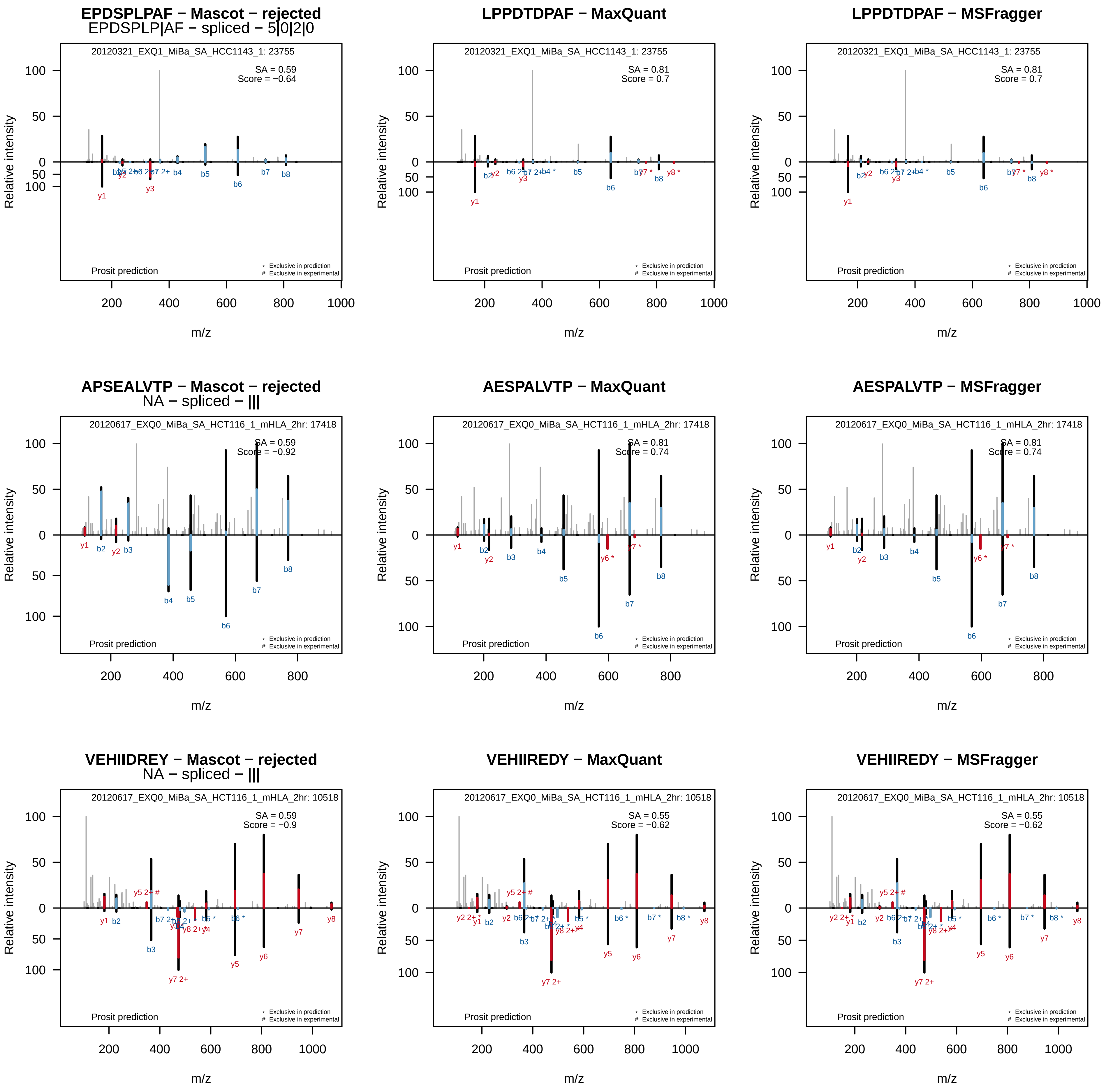
EPDSPLPAF − Mascot − rejected
EPDSPLP|AF − spliced − 5|0|2|0
20120321_EXQ1_MiBa_SA_HCC1143_1: 23755
SA = 0.59
Score = −0.64
LPPDTDPAF − MaxQuant
20120321_EXQ1_MiBa_SA_HCC1143_1: 23755
SA = 0.81
Score = 0.7
LPPDTDPAF − MSFragger
20120321_EXQ1_MiBa_SA_HCC1143_1: 23755
SA = 0.81
Score = 0.7
APSEALVTP − Mascot − rejected
NA − spliced − |||
20120617_EXQ0_MiBa_SA_HCT116_1_mHLA_2hr: 17418
SA = 0.59
Score = −0.92
AESPALVTP − MaxQuant
20120617_EXQ0_MiBa_SA_HCT116_1_mHLA_2hr: 17418
SA = 0.81
Score = 0.74
AESPALVTP − MSFragger
20120617_EXQ0_MiBa_SA_HCT116_1_mHLA_2hr: 17418
SA = 0.81
Score = 0.74
VEHIIDREY − Mascot − rejected
NA − spliced − |||
20120617_EXQ0_MiBa_SA_HCT116_1_mHLA_2hr: 10518
SA = 0.59
Score = −0.9
VEHIIREDY − MaxQuant
20120617_EXQ0_MiBa_SA_HCT116_1_mHLA_2hr: 10518
SA = 0.55
Score = −0.62
VEHIIREDY − MSFragger
20120617_EXQ0_MiBa_SA_HCT116_1_mHLA_2hr: 10518
SA = 0.55
Score = −0.62
Relative intensity
m/z
Prosit prediction
* Exclusive in prediction
# Exclusive in experimental

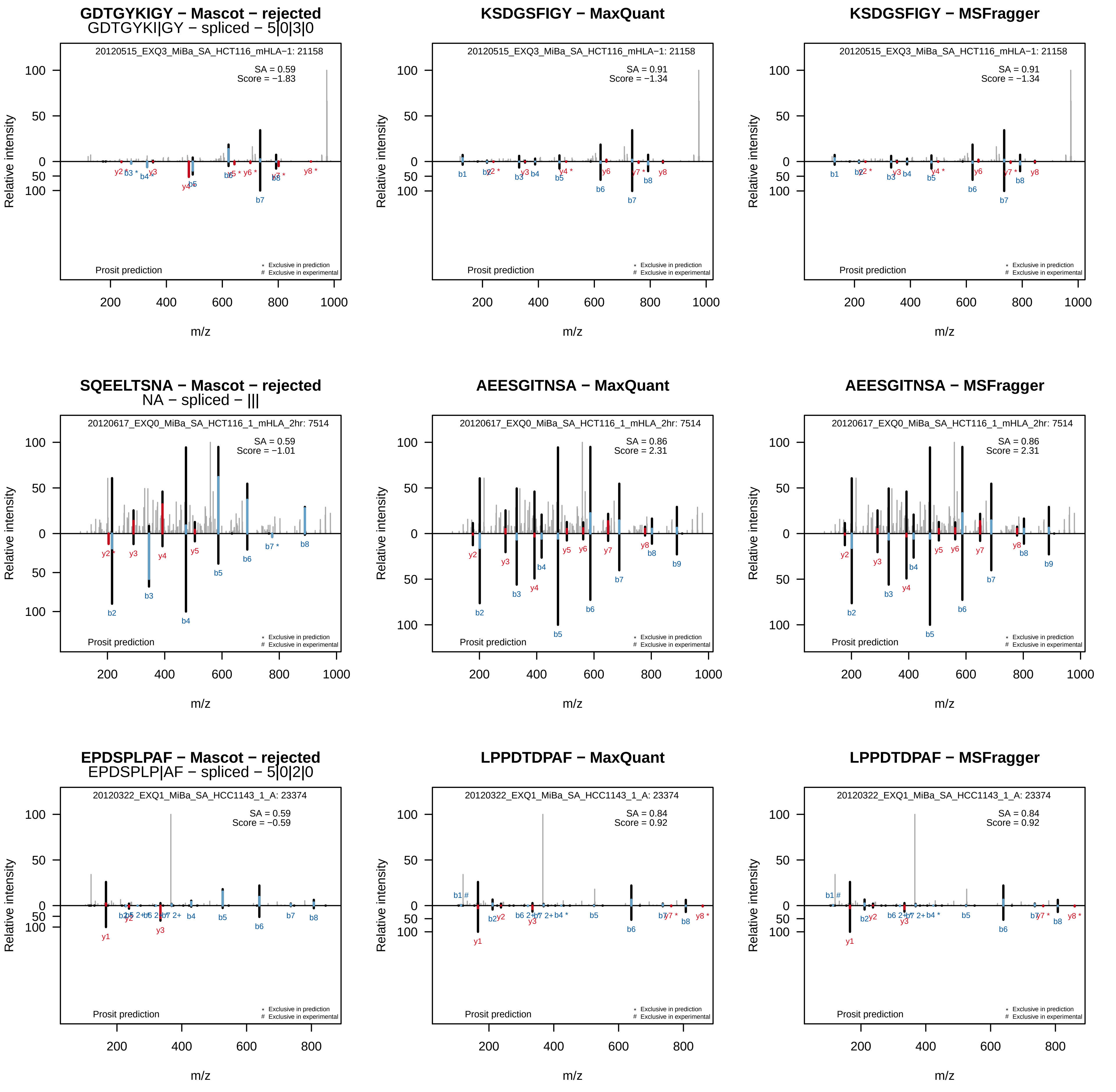

GDTGYKIGY − Mascot − rejected
GDTGYKI|GY − spliced − 5|0|3|0
20120515_EXQ3_MiBa_SA_HCT116_mHLA−1: 21158
SA = 0.59
Score = −1.83
KSDGSFIGY − MaxQuant
20120515_EXQ3_MiBa_SA_HCT116_mHLA−1: 21158
SA = 0.91
Score = −1.34
KSDGSFIGY − MSFragger
20120515_EXQ3_MiBa_SA_HCT116_mHLA−1: 21158
SA = 0.91
Score = −1.34
SQEELTSNA − Mascot − rejected
NA − spliced − |||
20120617_EXQ0_MiBa_SA_HCT116_1_mHLA_2hr: 7514
SA = 0.59
Score = −1.01
AEESGITNSA − MaxQuant
20120617_EXQ0_MiBa_SA_HCT116_1_mHLA_2hr: 7514
SA = 0.86
Score = 2.31
AEESGITNSA − MSFragger
20120617_EXQ0_MiBa_SA_HCT116_1_mHLA_2hr: 7514
SA = 0.86
Score = 2.31
EPDSPLPAF − Mascot − rejected
EPDSPLP|AF − spliced − 5|0|2|0
20120322_EXQ1_MiBa_SA_HCC1143_1_A: 23374
SA = 0.59
Score = −0.59
LPPDTDPAF − MaxQuant
20120322_EXQ1_MiBa_SA_HCC1143_1_A: 23374
SA = 0.84
Score = 0.92
LPPDTDPAF − MSFragger
20120322_EXQ1_MiBa_SA_HCC1143_1_A: 23374
SA = 0.84
Score = 0.92
Relative intensity
m/z
Prosit prediction
⋆ Exclusive in prediction
Exclusive in experimental

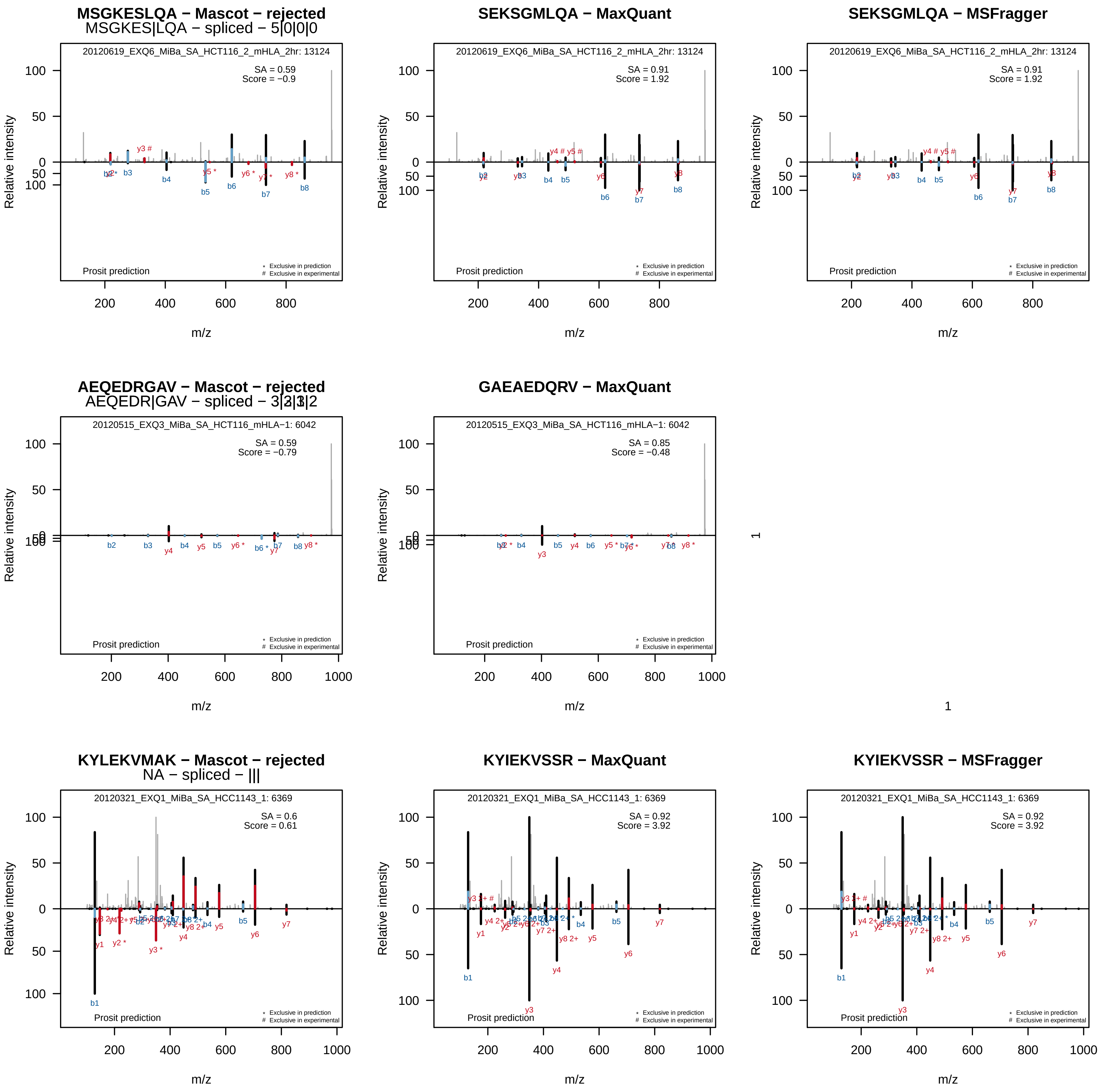
MSGKESLQA − Mascot − rejected
MSGKES|LQA − spliced − 5|0|0|0
20120619_EXQ6_MiBa_SA_HCT116_2_mHLA_2hr: 13124
SA = 0.59
Score = −0.9
Prosit prediction
⋆ Exclusive in prediction
Exclusive in experimental
Relative intensity
m/z
SEKSGMLQA − MaxQuant
20120619_EXQ6_MiBa_SA_HCT116_2_mHLA_2hr: 13124
SA = 0.91
Score = 1.92
Prosit prediction
SEKSGMLQA − MSFragger
20120619_EXQ6_MiBa_SA_HCT116_2_mHLA_2hr: 13124
SA = 0.91
Score = 1.92
Prosit prediction
AEQEDRGAV − Mascot − rejected
AEQEDR|GAV − spliced − 3|2|3|2
20120515_EXQ3_MiBa_SA_HCT116_mHLA−1: 6042
SA = 0.59
Score = −0.79
Prosit prediction
GAEAEDQRV − MaxQuant
20120515_EXQ3_MiBa_SA_HCT116_mHLA−1: 6042
SA = 0.85
Score = −0.48
Prosit prediction
KYLEKVMAK − Mascot − rejected
NA − spliced − |||
20120321_EXQ1_MiBa_SA_HCC1143_1: 6369
SA = 0.6
Score = 0.61
Prosit prediction
KYIEKVSSR − MaxQuant
20120321_EXQ1_MiBa_SA_HCC1143_1: 6369
SA = 0.92
Score = 3.92
Prosit prediction
KYIEKVSSR − MSFragger
20120321_EXQ1_MiBa_SA_HCC1143_1: 6369
SA = 0.92
Score = 3.92
Prosit prediction

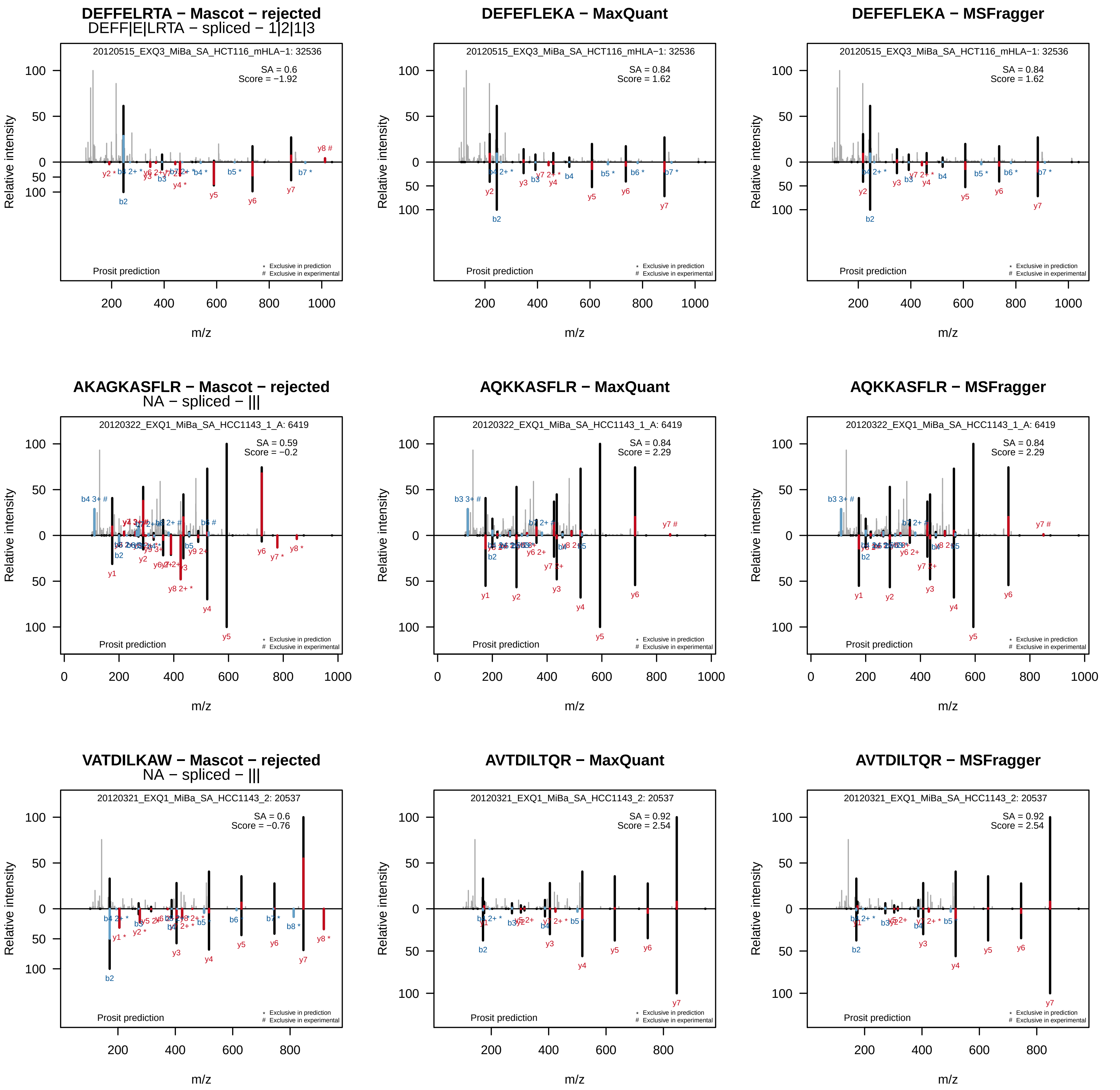
DEFFELRTA − Mascot − rejected
DEFF|E|LRTA − spliced − 1|2|1|3
20120515_EXQ3_MiBa_SA_HCT116_mHLA−1: 32536
SA = 0.6
Score = −1.92
DEFEFLEKA − MaxQuant
20120515_EXQ3_MiBa_SA_HCT116_mHLA−1: 32536
SA = 0.84
Score = 1.62
DEFEFLEKA − MSFragger
20120515_EXQ3_MiBa_SA_HCT116_mHLA−1: 32536
SA = 0.84
Score = 1.62
AKAGKASFLR − Mascot − rejected
NA − spliced − |||
20120322_EXQ1_MiBa_SA_HCC1143_1_A: 6419
SA = 0.59
Score = −0.2
AQKKASFLR − MaxQuant
20120322_EXQ1_MiBa_SA_HCC1143_1_A: 6419
SA = 0.84
Score = 2.29
AQKKASFLR − MSFragger
20120322_EXQ1_MiBa_SA_HCC1143_1_A: 6419
SA = 0.84
Score = 2.29
VATDILKAW − Mascot − rejected
NA − spliced − |||
20120321_EXQ1_MiBa_SA_HCC1143_2: 20537
SA = 0.6
Score = −0.76
AVTDILTQR − MaxQuant
20120321_EXQ1_MiBa_SA_HCC1143_2: 20537
SA = 0.92
Score = 2.54
AVTDILTQR − MSFragger
20120321_EXQ1_MiBa_SA_HCC1143_2: 20537
SA = 0.92
Score = 2.54
Relative intensity
m/z
Prosit prediction
* Exclusive in prediction
# Exclusive in experimental

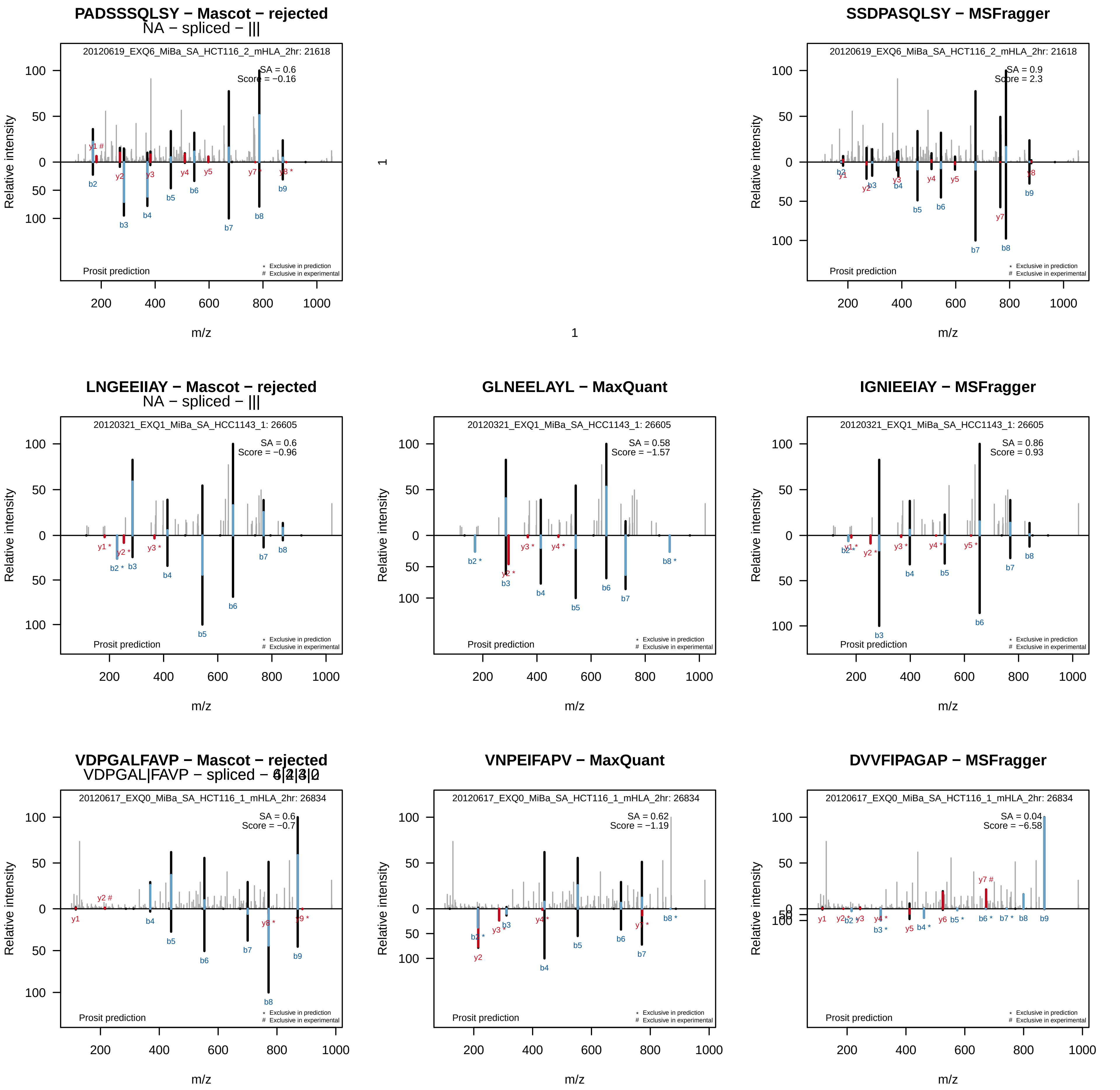
PADSSSQLSY − Mascot − rejected
NA − spliced − |||
20120619_EXQ6_MiBa_SA_HCT116_2_mHLA_2hr: 21618
SA = 0.6
Score = −0.16
SSDPASQLSY − MSFragger
20120619_EXQ6_MiBa_SA_HCT116_2_mHLA_2hr: 21618
SA = 0.9
Score = 2.3
LNGEEIIAY − Mascot − rejected
NA − spliced − |||
20120321_EXQ1_MiBa_SA_HCC1143_1: 26605
SA = 0.6
Score = −0.96
GLNEELAYL − MaxQuant
20120321_EXQ1_MiBa_SA_HCC1143_1: 26605
SA = 0.58
Score = −1.57
IGNIEEIAY − MSFragger
20120321_EXQ1_MiBa_SA_HCC1143_1: 26605
SA = 0.86
Score = 0.93
VDPGALFAVP − Mascot − rejected
VDPGAL|FAVP − spliced
20120617_EXQ0_MiBa_SA_HCT116_1_mHLA_2hr: 26834
SA = 0.6
Score = −0.7
VNPEIFAPV − MaxQuant
20120617_EXQ0_MiBa_SA_HCT116_1_mHLA_2hr: 26834
SA = 0.62
Score = −1.19
DVVFIPAGAP − MSFragger
20120617_EXQ0_MiBa_SA_HCT116_1_mHLA_2hr: 26834
SA = 0.04
Score = −6.58
Relative intensity
m/z
Prosit prediction
* Exclusive in prediction
# Exclusive in experimental

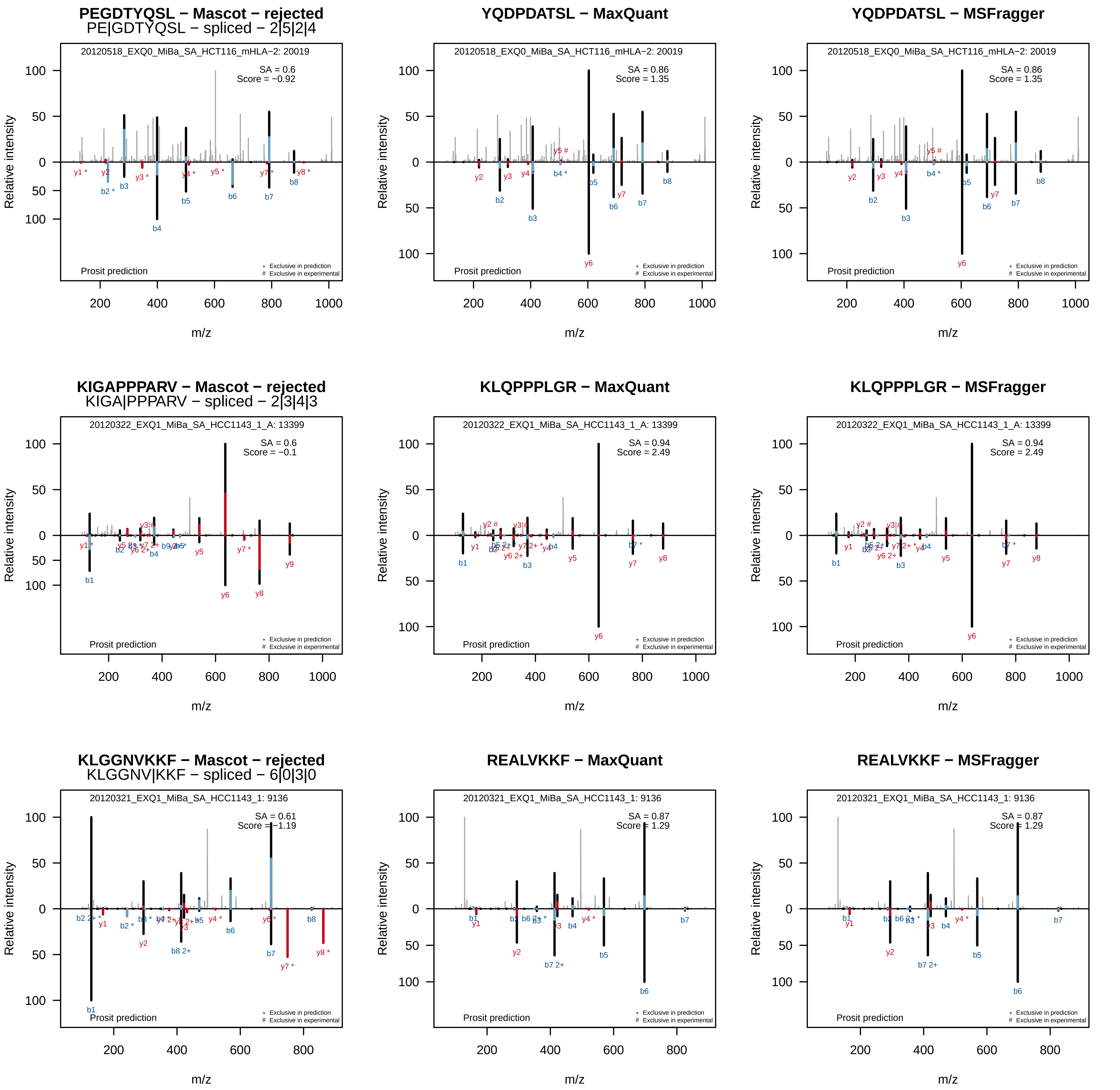
PEGDTYQSL − Mascot − rejected
PE|GDTYQSL − spliced − 2|5|2|4
20120518_EXQ0_MiBa_SA_HCT116_mHLA−2: 20019
SA = 0.6
Score = −0.92
YQDPDATSL − MaxQuant
20120518_EXQ0_MiBa_SA_HCT116_mHLA−2: 20019
SA = 0.86
Score = 1.35
YQDPDATSL − MSFragger
20120518_EXQ0_MiBa_SA_HCT116_mHLA−2: 20019
SA = 0.86
Score = 1.35
KIGAPPPARV − Mascot − rejected
KIGA|PPPARV − spliced − 2|3|4|3
20120322_EXQ1_MiBa_SA_HCC1143_1_A: 13399
SA = 0.6
Score = −0.1
KLQPPPLGR − MaxQuant
20120322_EXQ1_MiBa_SA_HCC1143_1_A: 13399
SA = 0.94
Score = 2.49
KLQPPPLGR − MSFragger
20120322_EXQ1_MiBa_SA_HCC1143_1_A: 13399
SA = 0.94
Score = 2.49
KLGGNVKKF − Mascot − rejected
KLGGNV|KKF − spliced − 6|0|3|0
20120321_EXQ1_MiBa_SA_HCC1143_1: 9136
SA = 0.61
Score = −1.19
REALVKKF − MaxQuant
20120321_EXQ1_MiBa_SA_HCC1143_1: 9136
SA = 0.87
Score = 1.29
REALVKKF − MSFragger
20120321_EXQ1_MiBa_SA_HCC1143_1: 9136
SA = 0.87
Score = 1.29
Relative intensity
m/z
Prosit prediction
⋆ Exclusive in prediction
Exclusive in experimental

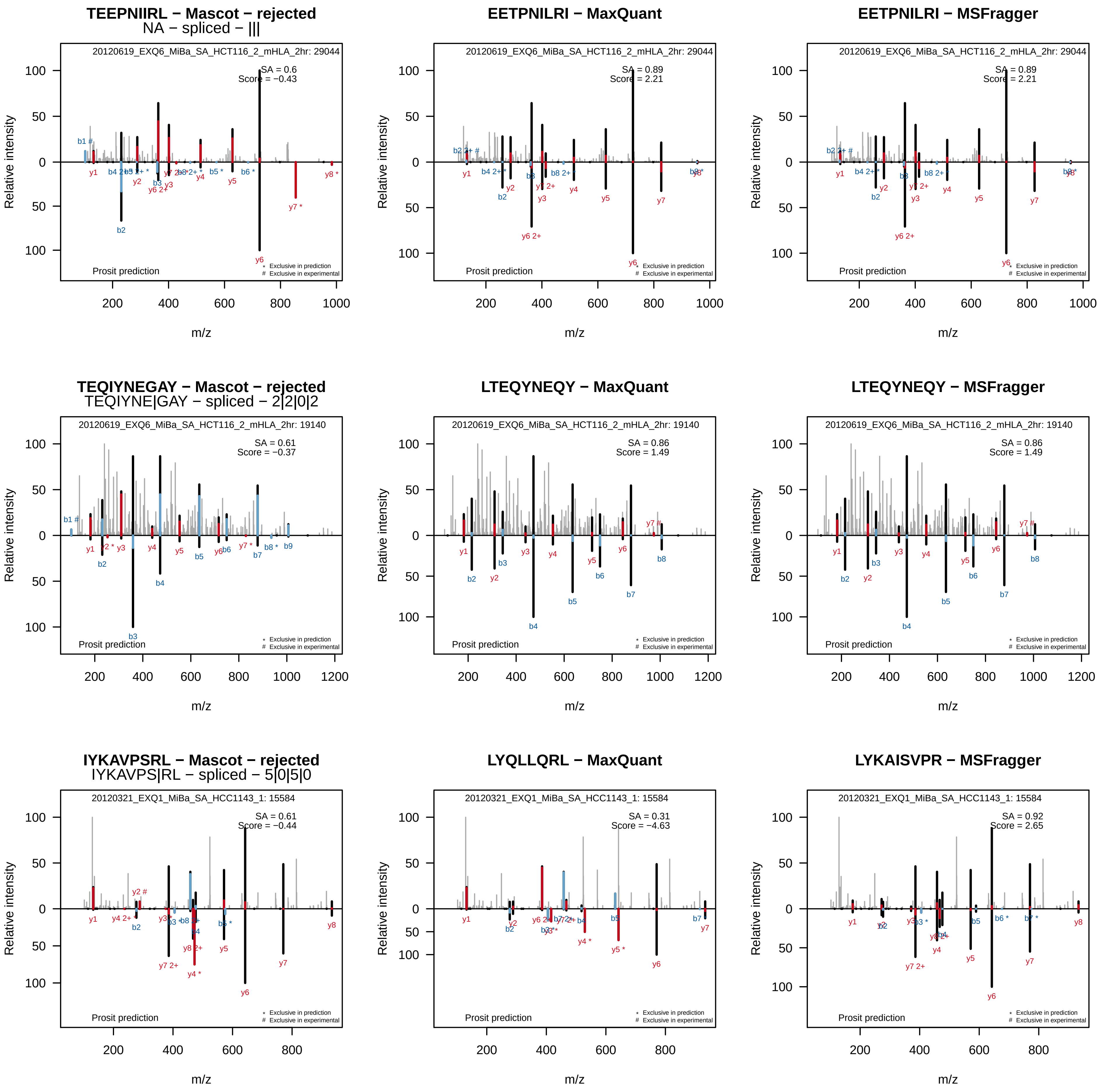

TEEPNIIRL − Mascot − rejected
NA − spliced − |||
20120619_EXQ6_MiBa_SA_HCT116_2_mHLA_2hr: 29044
SA = 0.6
Score = −0.43
EETPNILRI − MaxQuant
20120619_EXQ6_MiBa_SA_HCT116_2_mHLA_2hr: 29044
SA = 0.89
Score = 2.21
EETPNILRI − MSFragger
20120619_EXQ6_MiBa_SA_HCT116_2_mHLA_2hr: 29044
SA = 0.89
Score = 2.21
TEQIYNEGAY − Mascot − rejected
TEQIYNE|GAY − spliced − 2|2|0|2
20120619_EXQ6_MiBa_SA_HCT116_2_mHLA_2hr: 19140
SA = 0.61
Score = −0.37
LTEQYNEQY − MaxQuant
20120619_EXQ6_MiBa_SA_HCT116_2_mHLA_2hr: 19140
SA = 0.86
Score = 1.49
LTEQYNEQY − MSFragger
20120619_EXQ6_MiBa_SA_HCT116_2_mHLA_2hr: 19140
SA = 0.86
Score = 1.49
IYKAVPSRL − Mascot − rejected
IYKAVPS|RL − spliced − 5|0|5|0
20120321_EXQ1_MiBa_SA_HCC1143_1: 15584
SA = 0.61
Score = −0.44
LYQLLQRL − MaxQuant
20120321_EXQ1_MiBa_SA_HCC1143_1: 15584
SA = 0.31
Score = −4.63
LYKAISVPR − MSFragger
20120321_EXQ1_MiBa_SA_HCC1143_1: 15584
SA = 0.92
Score = 2.65
Relative intensity
m/z
Prosit prediction
* Exclusive in prediction
# Exclusive in experimental

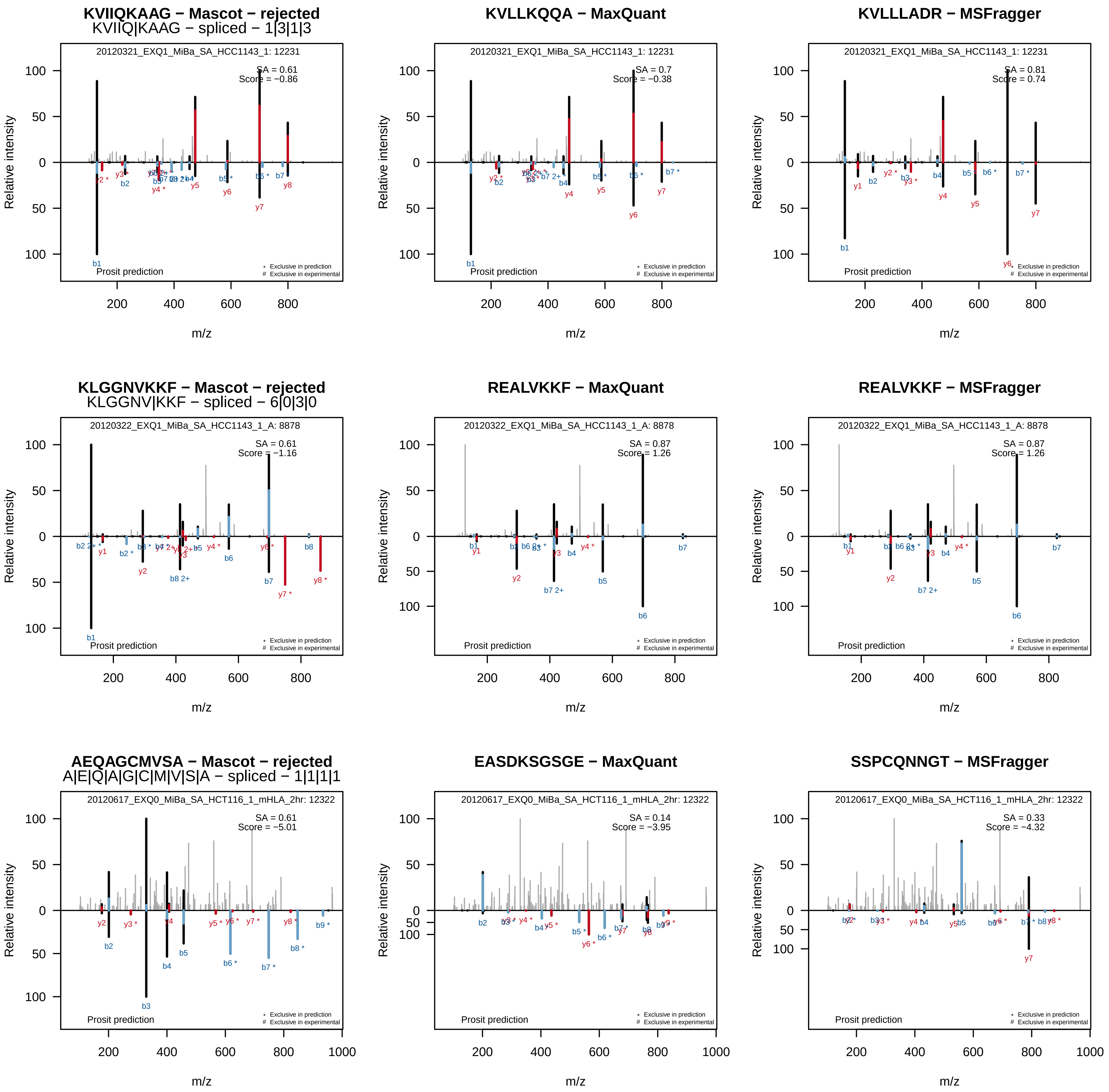

KVIIQKAAG − Mascot − rejected
KVIIQ|KAAG − spliced − 1|3|1|3
20120321_EXQ1_MiBa_SA_HCC1143_1: 12231
SA = 0.61
Score = −0.86
KVLLKQQA − MaxQuant
20120321_EXQ1_MiBa_SA_HCC1143_1: 12231
SA = 0.7
Score = −0.38
KVLLLADR − MSFragger
20120321_EXQ1_MiBa_SA_HCC1143_1: 12231
SA = 0.81
Score = 0.74
KLGGNVKKF − Mascot − rejected
KLGGNV|KKF − spliced − 6|0|3|0
20120322_EXQ1_MiBa_SA_HCC1143_1_A: 8878
SA = 0.61
Score = −1.16
REALVKKF − MaxQuant
20120322_EXQ1_MiBa_SA_HCC1143_1_A: 8878
SA = 0.87
Score = 1.26
REALVKKF − MSFragger
20120322_EXQ1_MiBa_SA_HCC1143_1_A: 8878
SA = 0.87
Score = 1.26
AEQAGCMVSA − Mascot − rejected
A|E|Q|A|G|C|M|V|S|A − spliced − 1|1|1|1
20120617_EXQ0_MiBa_SA_HCT116_1_mHLA_2hr: 12322
SA = 0.61
Score = −5.01
EASDKSGSGE − MaxQuant
20120617_EXQ0_MiBa_SA_HCT116_1_mHLA_2hr: 12322
SA = 0.14
Score = −3.95
SSPCQNNGT − MSFragger
20120617_EXQ0_MiBa_SA_HCT116_1_mHLA_2hr: 12322
SA = 0.33
Score = −4.32
Relative intensity
m/z
Prosit prediction
* Exclusive in prediction
# Exclusive in experimental

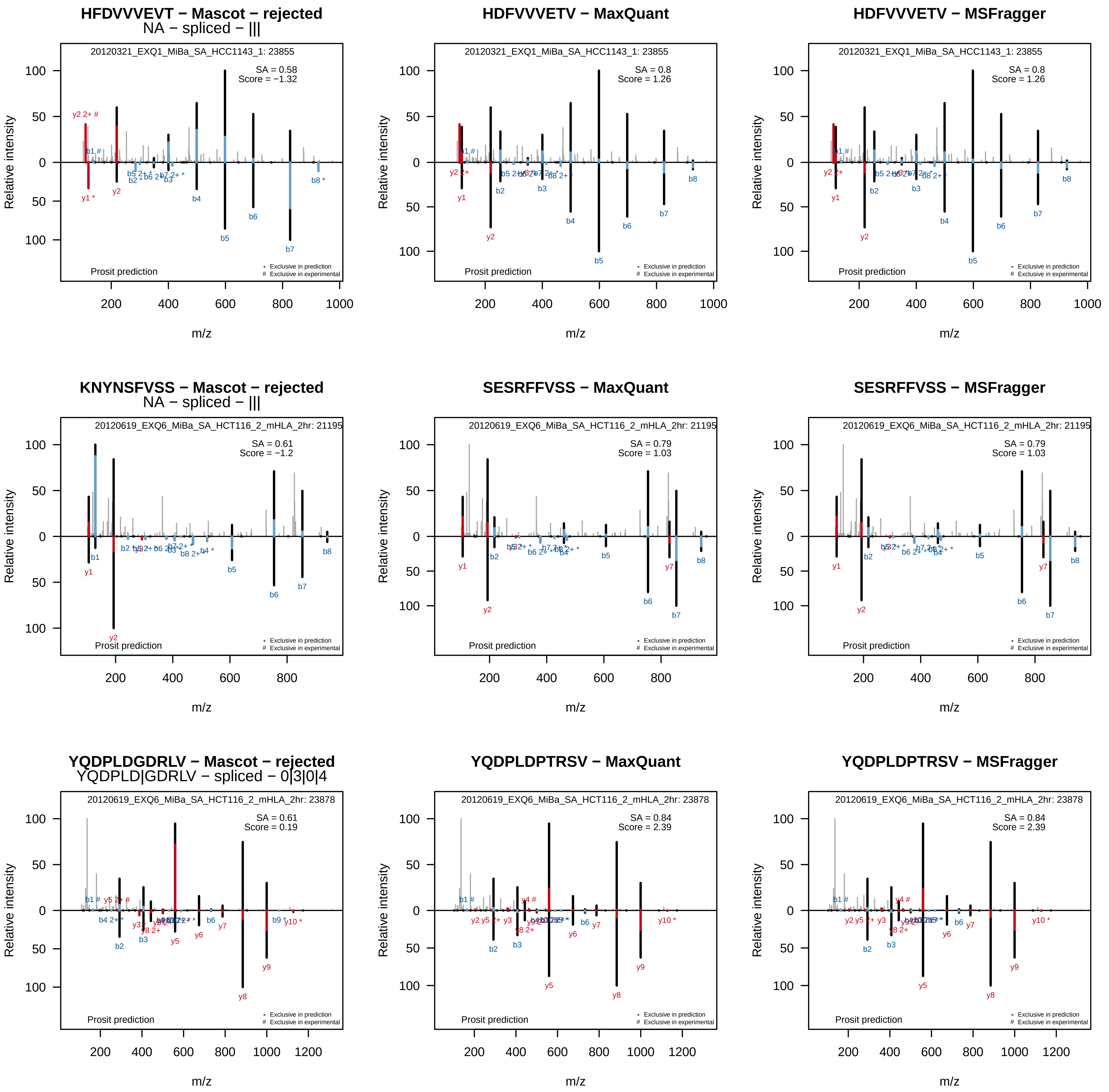
HFDVVVEVT − Mascot − rejected
NA − spliced − |||
20120321_EXQ1_MiBa_SA_HCC1143_1: 23855
SA = 0.58
Score = −1.32
HDFVVVETV − MaxQuant
20120321_EXQ1_MiBa_SA_HCC1143_1: 23855
SA = 0.8
Score = 1.26
HDFVVVETV − MSFragger
20120321_EXQ1_MiBa_SA_HCC1143_1: 23855
SA = 0.8
Score = 1.26
KNYNSFVSS − Mascot − rejected
NA − spliced − |||
20120619_EXQ6_MiBa_SA_HCT116_2_mHLA_2hr: 21195
SA = 0.61
Score = −1.2
SESRFFVSS − MaxQuant
20120619_EXQ6_MiBa_SA_HCT116_2_mHLA_2hr: 21195
SA = 0.79
Score = 1.03
SESRFFVSS − MSFragger
20120619_EXQ6_MiBa_SA_HCT116_2_mHLA_2hr: 21195
SA = 0.79
Score = 1.03
YQDPLDGDRLV − Mascot − rejected
YQDPLD|GDRLV − spliced − 0|3|0|4
20120619_EXQ6_MiBa_SA_HCT116_2_mHLA_2hr: 23878
SA = 0.61
Score = 0.19
YQDPLDPTRSV − MaxQuant
20120619_EXQ6_MiBa_SA_HCT116_2_mHLA_2hr: 23878
SA = 0.84
Score = 2.39
YQDPLDPTRSV − MSFragger
20120619_EXQ6_MiBa_SA_HCT116_2_mHLA_2hr: 23878
SA = 0.84
Score = 2.39
Relative intensity
m/z
Prosit prediction
⋆ Exclusive in prediction
Exclusive in experimental

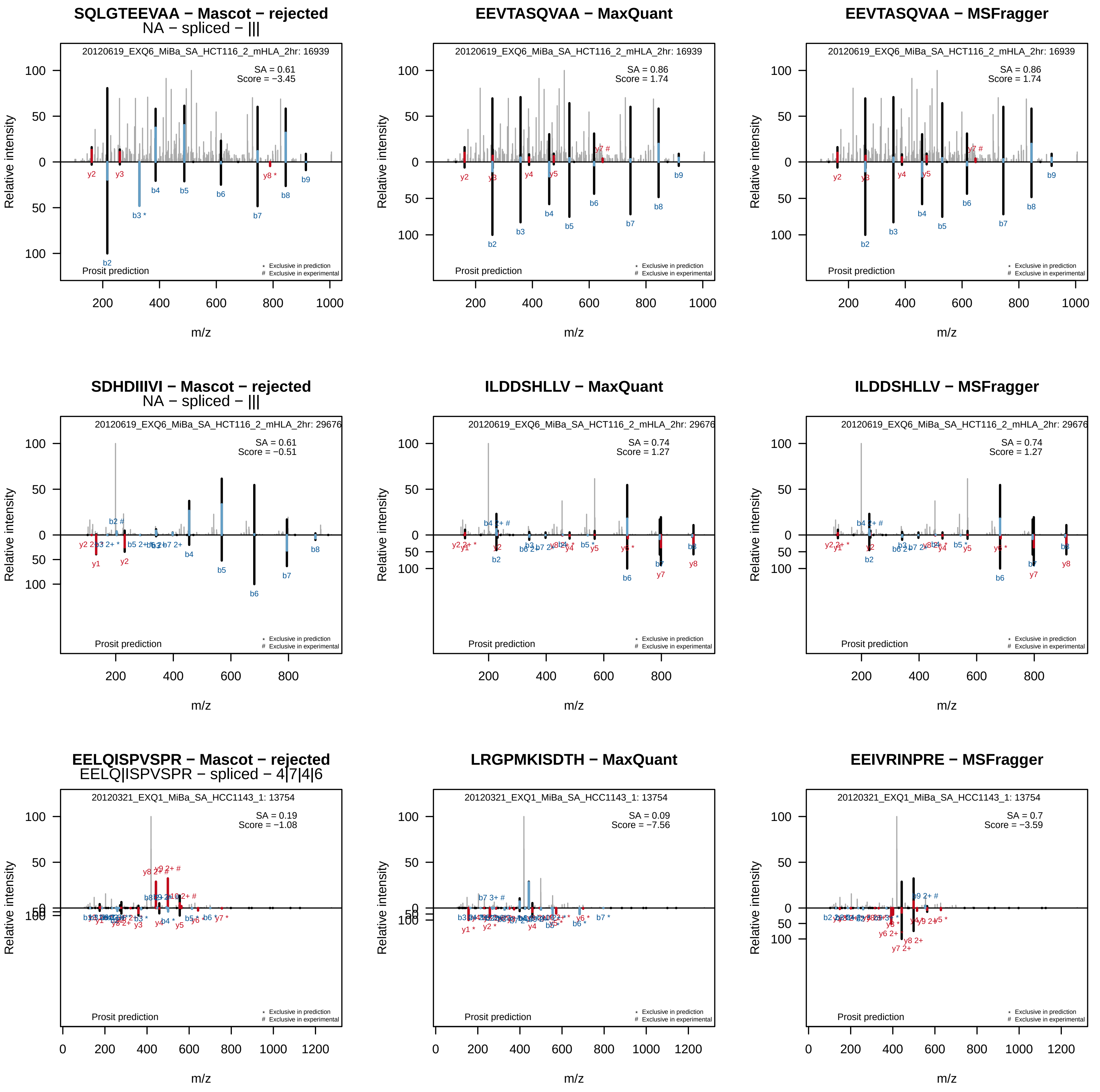
SQLGTEEVAA − Mascot − rejected
NA − spliced − |||
20120619_EXQ6_MiBa_SA_HCT116_2_mHLA_2hr: 16939
SA = 0.61
Score = −3.45
EEVTASQVAA − MaxQuant
20120619_EXQ6_MiBa_SA_HCT116_2_mHLA_2hr: 16939
SA = 0.86
Score = 1.74
EEVTASQVAA − MSFragger
20120619_EXQ6_MiBa_SA_HCT116_2_mHLA_2hr: 16939
SA = 0.86
Score = 1.74
SDHDIIIVI − Mascot − rejected
NA − spliced − |||
20120619_EXQ6_MiBa_SA_HCT116_2_mHLA_2hr: 29676
SA = 0.61
Score = −0.51
ILDDSHLLV − MaxQuant
20120619_EXQ6_MiBa_SA_HCT116_2_mHLA_2hr: 29676
SA = 0.74
Score = 1.27
ILDDSHLLV − MSFragger
20120619_EXQ6_MiBa_SA_HCT116_2_mHLA_2hr: 29676
SA = 0.74
Score = 1.27
EELQISPVSPR − Mascot − rejected
EELQ|ISPVSPR − spliced − 4|7|4|6
20120321_EXQ1_MiBa_SA_HCC1143_1: 13754
SA = 0.19
Score = −1.08
LRGPMKISDTH − MaxQuant
20120321_EXQ1_MiBa_SA_HCC1143_1: 13754
SA = 0.09
Score = −7.56
EEIVRINPRE − MSFragger
20120321_EXQ1_MiBa_SA_HCC1143_1: 13754
SA = 0.7
Score = −3.59
Relative intensity
m/z
Prosit prediction
⋆ Exclusive in prediction
Exclusive in experimental

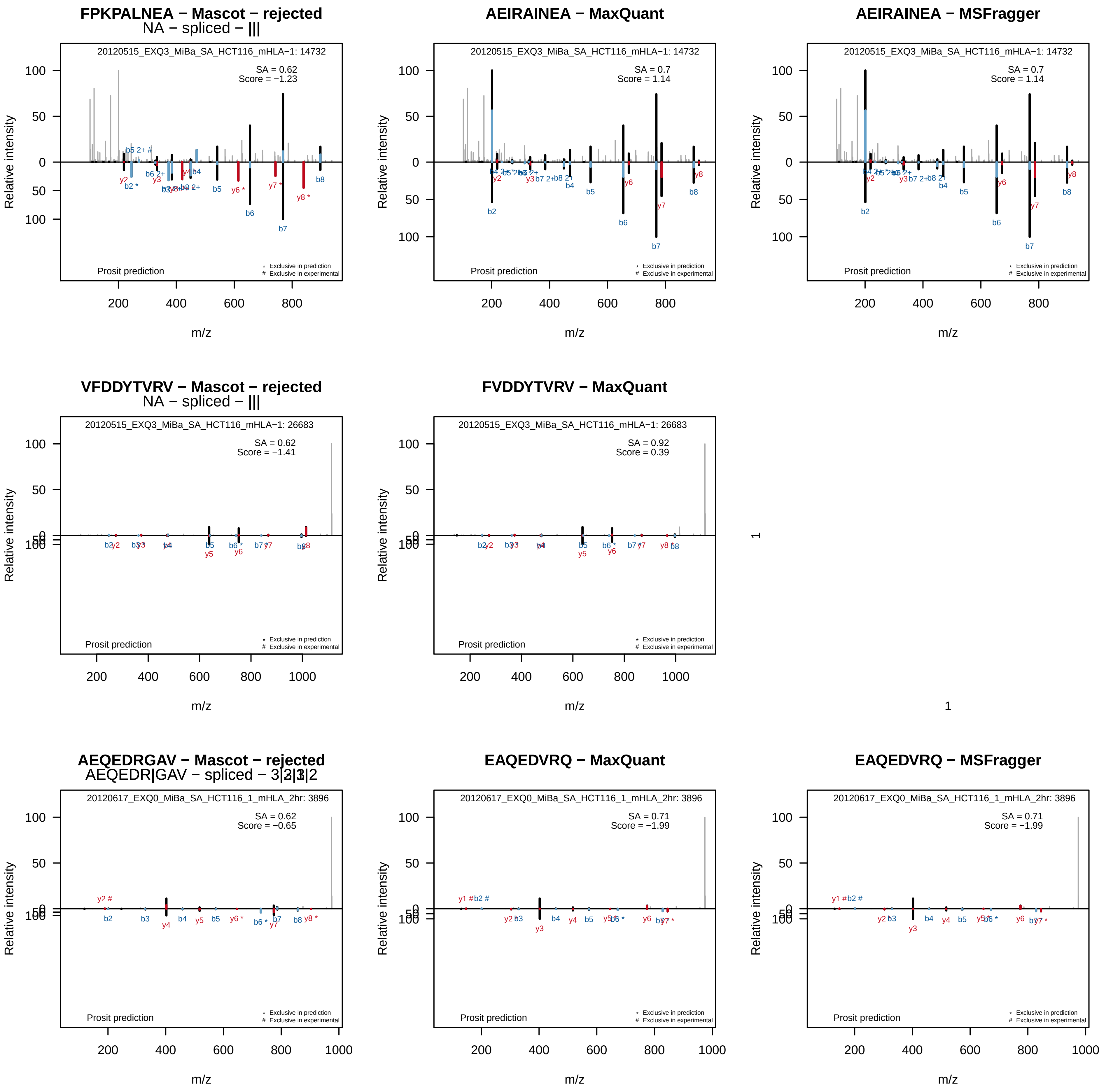

FPKPALNEA − Mascot − rejected
NA − spliced − |||
20120515_EXQ3_MiBa_SA_HCT116_mHLA−1: 14732
SA = 0.62
Score = −1.23
AEIRAINEA − MaxQuant
20120515_EXQ3_MiBa_SA_HCT116_mHLA−1: 14732
SA = 0.7
Score = 1.14
AEIRAINEA − MSFragger
20120515_EXQ3_MiBa_SA_HCT116_mHLA−1: 14732
SA = 0.7
Score = 1.14
VFDDYTVRV − Mascot − rejected
NA − spliced − |||
20120515_EXQ3_MiBa_SA_HCT116_mHLA−1: 26683
SA = 0.62
Score = −1.41
FVDDYTVRV − MaxQuant
20120515_EXQ3_MiBa_SA_HCT116_mHLA−1: 26683
SA = 0.92
Score = 0.39
AEQEDRGAV − Mascot − rejected
AEQEDR|GAV − spliced − 3|2|3|2
20120617_EXQ0_MiBa_SA_HCT116_1_mHLA_2hr: 3896
SA = 0.62
Score = −0.65
EAQEDVRQ − MaxQuant
20120617_EXQ0_MiBa_SA_HCT116_1_mHLA_2hr: 3896
SA = 0.71
Score = −1.99
EAQEDVRQ − MSFragger
20120617_EXQ0_MiBa_SA_HCT116_1_mHLA_2hr: 3896
SA = 0.71
Score = −1.99
Relative intensity
m/z
Prosit prediction
⋆ Exclusive in prediction
Exclusive in experimental

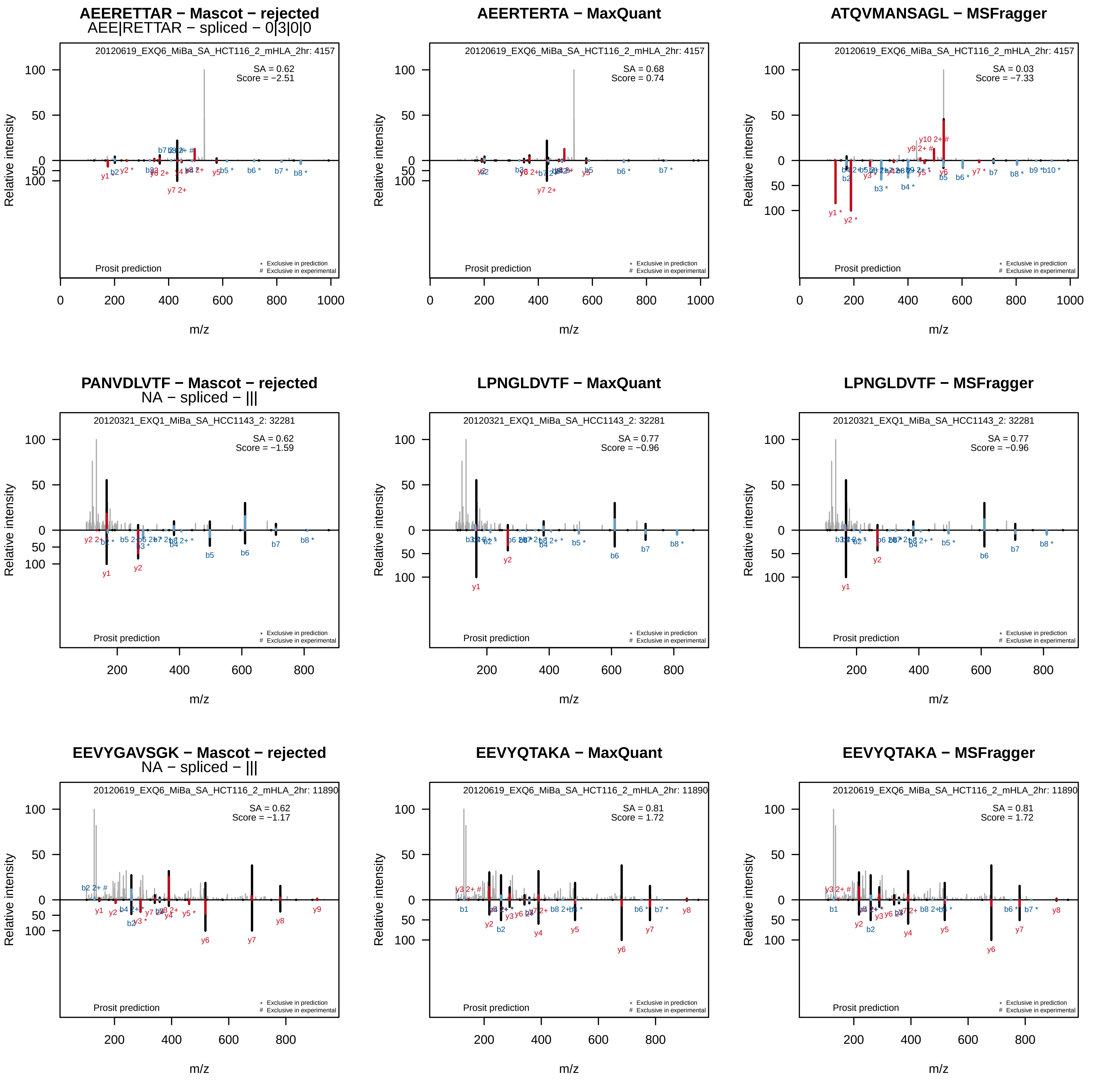

AEERETTAR − Mascot − rejected
AEE|RETTAR − spliced − 0|3|0|0
20120619_EXQ6_MiBa_SA_HCT116_2_mHLA_2hr: 4157
SA = 0.62
Score = −2.51
AEERTERTA − MaxQuant
20120619_EXQ6_MiBa_SA_HCT116_2_mHLA_2hr: 4157
SA = 0.68
Score = 0.74
ATQVMANSAGL − MSFragger
20120619_EXQ6_MiBa_SA_HCT116_2_mHLA_2hr: 4157
SA = 0.03
Score = −7.33
PANVDLVTF − Mascot − rejected
NA − spliced − |||
20120321_EXQ1_MiBa_SA_HCC1143_2: 32281
SA = 0.62
Score = −1.59
LPNGLDVTF − MaxQuant
20120321_EXQ1_MiBa_SA_HCC1143_2: 32281
SA = 0.77
Score = −0.96
LPNGLDVTF − MSFragger
20120321_EXQ1_MiBa_SA_HCC1143_2: 32281
SA = 0.77
Score = −0.96
EEVYGAVSGK − Mascot − rejected
NA − spliced − |||
20120619_EXQ6_MiBa_SA_HCT116_2_mHLA_2hr: 11890
SA = 0.62
Score = −1.17
EEVYQTAKA − MaxQuant
20120619_EXQ6_MiBa_SA_HCT116_2_mHLA_2hr: 11890
SA = 0.81
Score = 1.72
EEVYQTAKA − MSFragger
20120619_EXQ6_MiBa_SA_HCT116_2_mHLA_2hr: 11890
SA = 0.81
Score = 1.72
Relative intensity
m/z
Prosit prediction
* Exclusive in prediction
# Exclusive in experimental

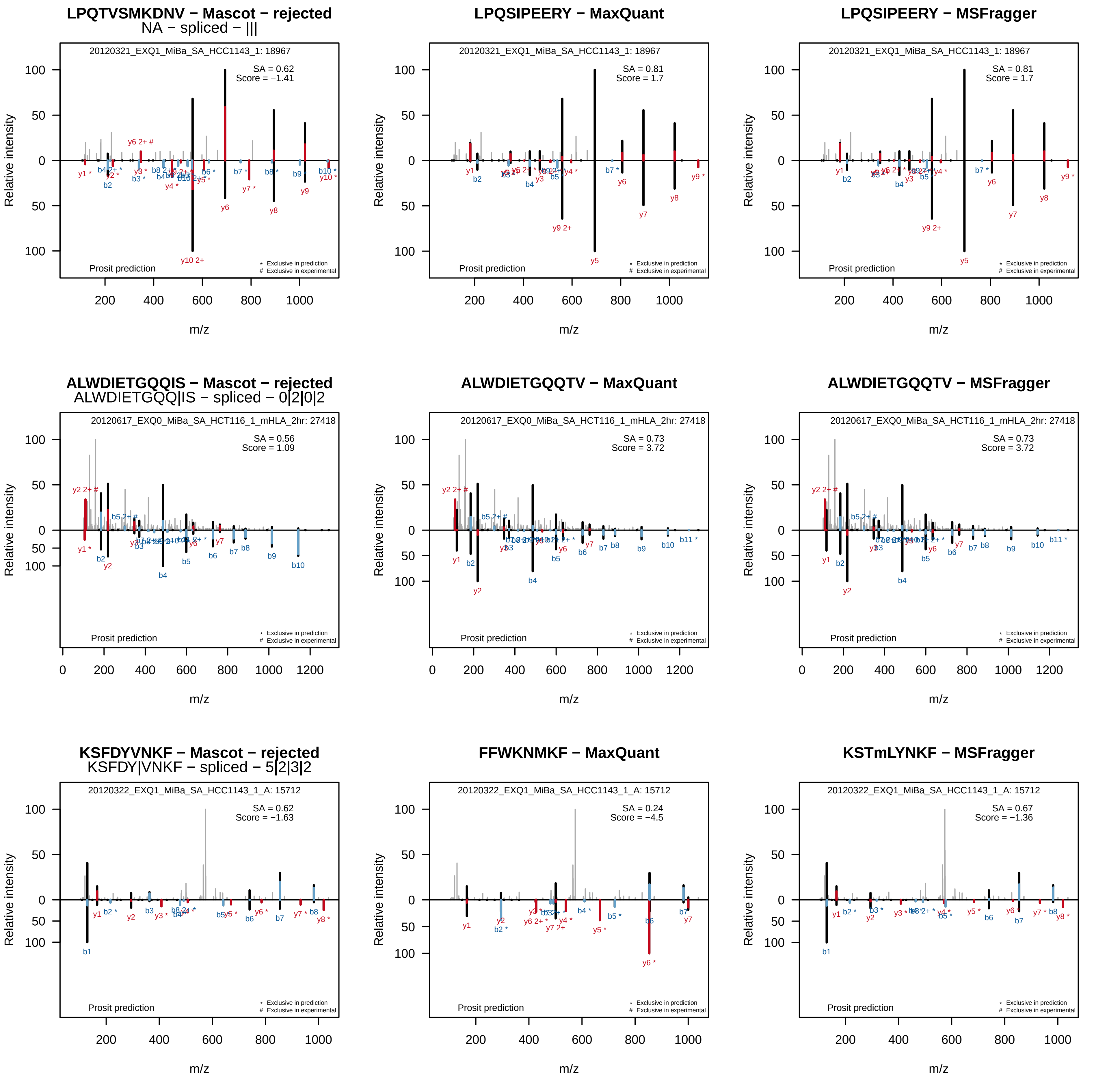

LPQTVSMKDNV − Mascot − rejected
NA − spliced − |||
20120321_EXQ1_MiBa_SA_HCC1143_1: 18967
SA = 0.62
Score = −1.41
LPQSIPEERY − MaxQuant
20120321_EXQ1_MiBa_SA_HCC1143_1: 18967
SA = 0.81
Score = 1.7
LPQSIPEERY − MSFragger
20120321_EXQ1_MiBa_SA_HCC1143_1: 18967
SA = 0.81
Score = 1.7
ALWDIETGQQIS − Mascot − rejected
ALWDIETGQQ|IS − spliced − 0|2|0|2
20120617_EXQ0_MiBa_SA_HCT116_1_mHLA_2hr: 27418
SA = 0.56
Score = 1.09
ALWDIETGQQTV − MaxQuant
20120617_EXQ0_MiBa_SA_HCT116_1_mHLA_2hr: 27418
SA = 0.73
Score = 3.72
ALWDIETGQQTV − MSFragger
20120617_EXQ0_MiBa_SA_HCT116_1_mHLA_2hr: 27418
SA = 0.73
Score = 3.72
KSFDYVNKF − Mascot − rejected
KSFDY|VNKF − spliced − 5|2|3|2
20120322_EXQ1_MiBa_SA_HCC1143_1_A: 15712
SA = 0.62
Score = −1.63
FFWKNMKF − MaxQuant
20120322_EXQ1_MiBa_SA_HCC1143_1_A: 15712
SA = 0.24
Score = −4.5
KSTmLYNKF − MSFragger
20120322_EXQ1_MiBa_SA_HCC1143_1_A: 15712
SA = 0.67
Score = −1.36
Relative intensity
m/z
Prosit prediction
* Exclusive in prediction
# Exclusive in experimental

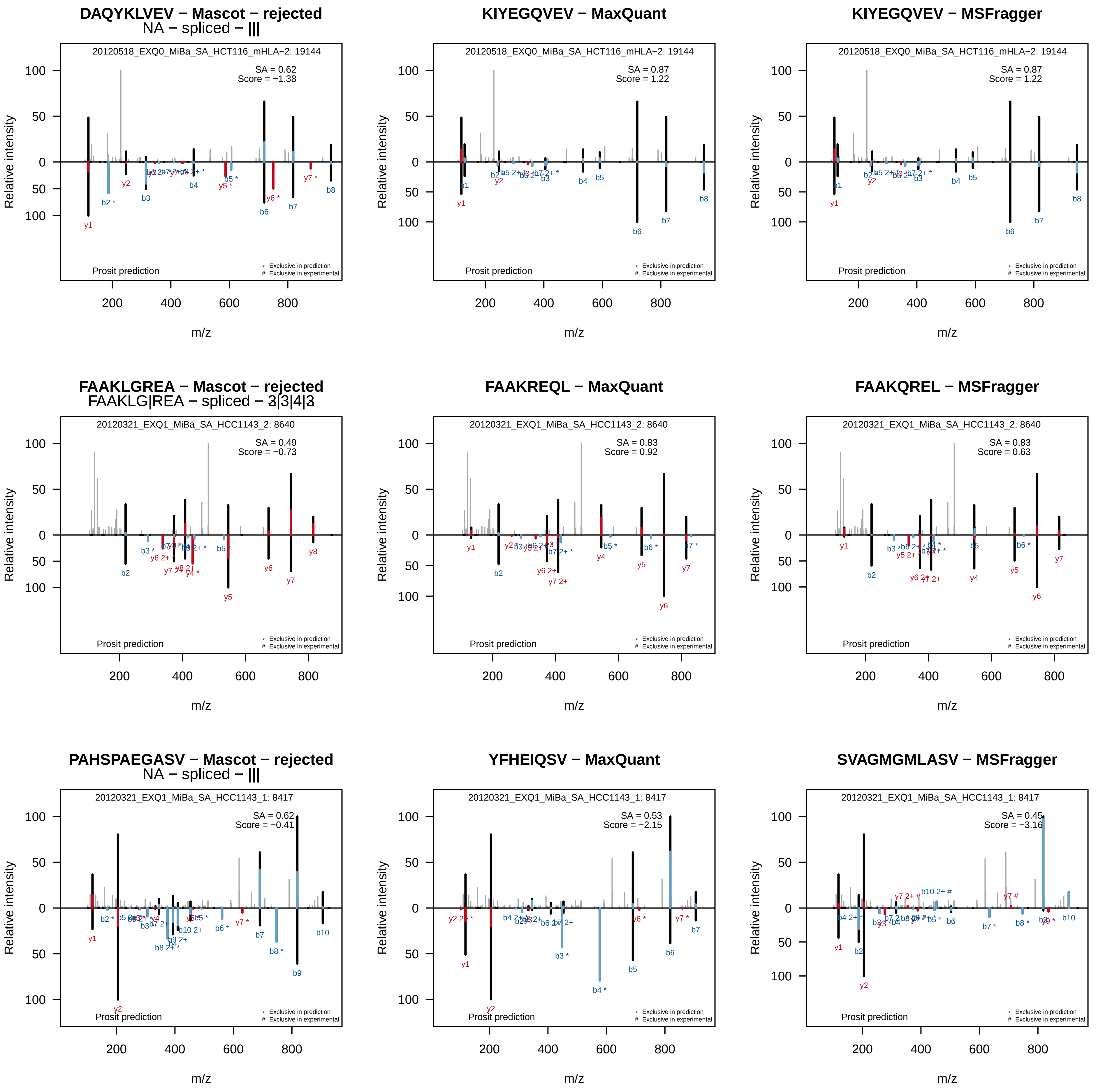

DAQYKLVEV − Mascot − rejected
NA − spliced − |||
20120518_EXQ0_MiBa_SA_HCT116_mHLA−2: 19144
SA = 0.62
Score = −1.38
KIYEGQVEV − MaxQuant
20120518_EXQ0_MiBa_SA_HCT116_mHLA−2: 19144
SA = 0.87
Score = 1.22
KIYEGQVEV − MSFragger
20120518_EXQ0_MiBa_SA_HCT116_mHLA−2: 19144
SA = 0.87
Score = 1.22
FAAKLGREA − Mascot − rejected
20120321_EXQ1_MiBa_SA_HCC1143_2: 8640
SA = 0.49
Score = −0.73
FAAKREQL − MaxQuant
20120321_EXQ1_MiBa_SA_HCC1143_2: 8640
SA = 0.83
Score = 0.92
FAAKQREL − MSFragger
20120321_EXQ1_MiBa_SA_HCC1143_2: 8640
SA = 0.83
Score = 0.63
PAHSPAEGASV − Mascot − rejected
NA − spliced − |||
20120321_EXQ1_MiBa_SA_HCC1143_1: 8417
SA = 0.62
Score = −0.41
YFHEIQSV − MaxQuant
20120321_EXQ1_MiBa_SA_HCC1143_1: 8417
SA = 0.53
Score = −2.15
SVAGMGMLASV − MSFragger
20120321_EXQ1_MiBa_SA_HCC1143_1: 8417
SA = 0.45
Score = −3.16
Relative intensity
m/z
Prosit prediction
* Exclusive in prediction
# Exclusive in experimental

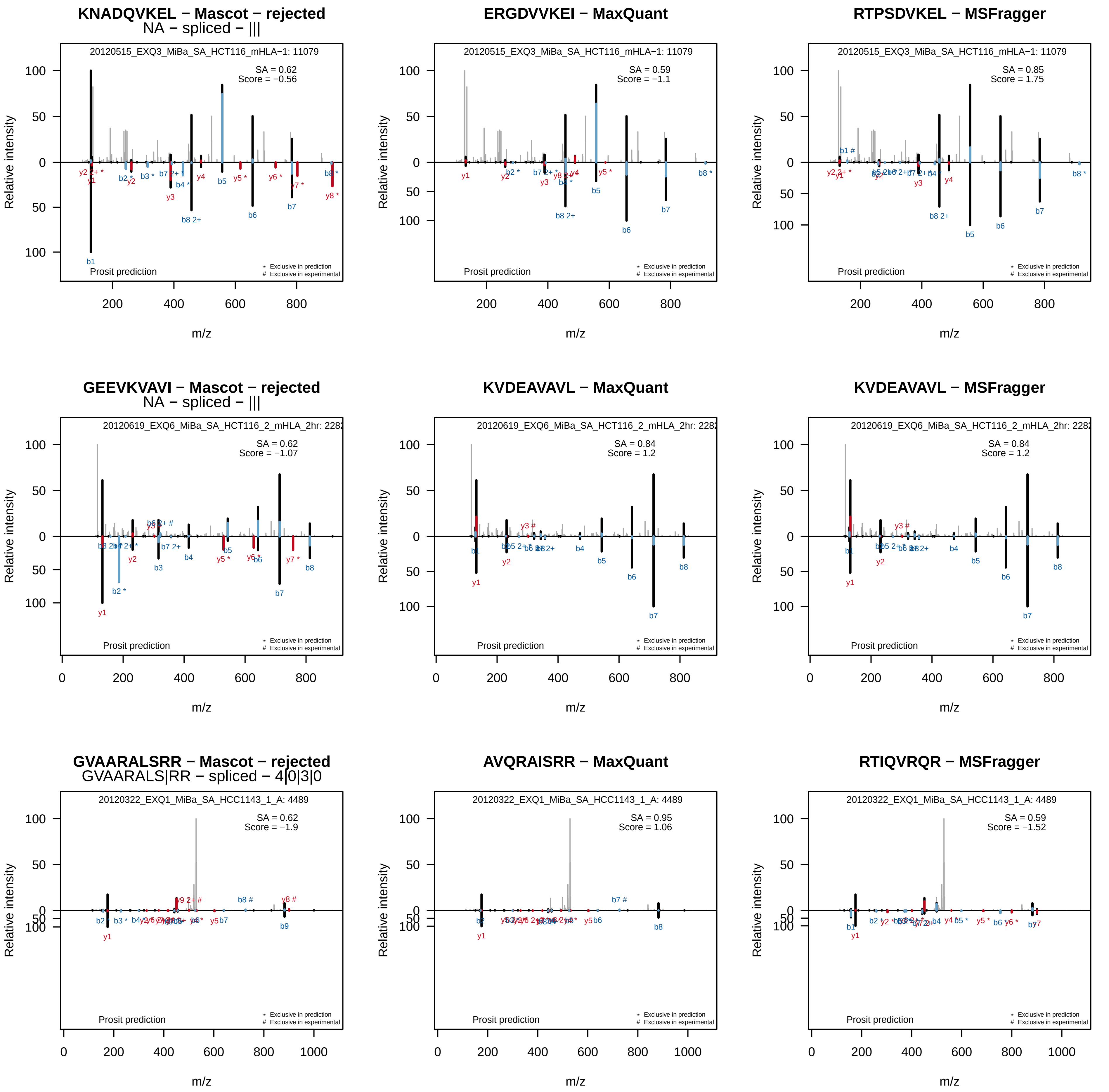

KNADQVKEL − Mascot − rejected
NA − spliced − |||
20120515_EXQ3_MiBa_SA_HCT116_mHLA−1: 11079
SA = 0.62
Score = −0.56
ERGDVVKEI − MaxQuant
20120515_EXQ3_MiBa_SA_HCT116_mHLA−1: 11079
SA = 0.59
Score = −1.1
RTPSDVKEL − MSFragger
20120515_EXQ3_MiBa_SA_HCT116_mHLA−1: 11079
SA = 0.85
Score = 1.75
GEEVKVAVI − Mascot − rejected
NA − spliced − |||
20120619_EXQ6_MiBa_SA_HCT116_2_mHLA_2hr: 2282
SA = 0.62
Score = −1.07
KVDEAVAVL − MaxQuant
20120619_EXQ6_MiBa_SA_HCT116_2_mHLA_2hr: 2282
SA = 0.84
Score = 1.2
KVDEAVAVL − MSFragger
20120619_EXQ6_MiBa_SA_HCT116_2_mHLA_2hr: 2282
SA = 0.84
Score = 1.2
GVAARALSRR − Mascot − rejected
GVAARALS|RR − spliced − 4|0|3|0
20120322_EXQ1_MiBa_SA_HCC1143_1_A: 4489
SA = 0.62
Score = −1.9
AVQRAISRR − MaxQuant
20120322_EXQ1_MiBa_SA_HCC1143_1_A: 4489
SA = 0.95
Score = 1.06
RTIQVRQR − MSFragger
20120322_EXQ1_MiBa_SA_HCC1143_1_A: 4489
SA = 0.59
Score = −1.52
Relative intensity
m/z
Prosit prediction
⋆ Exclusive in prediction
Exclusive in experimental

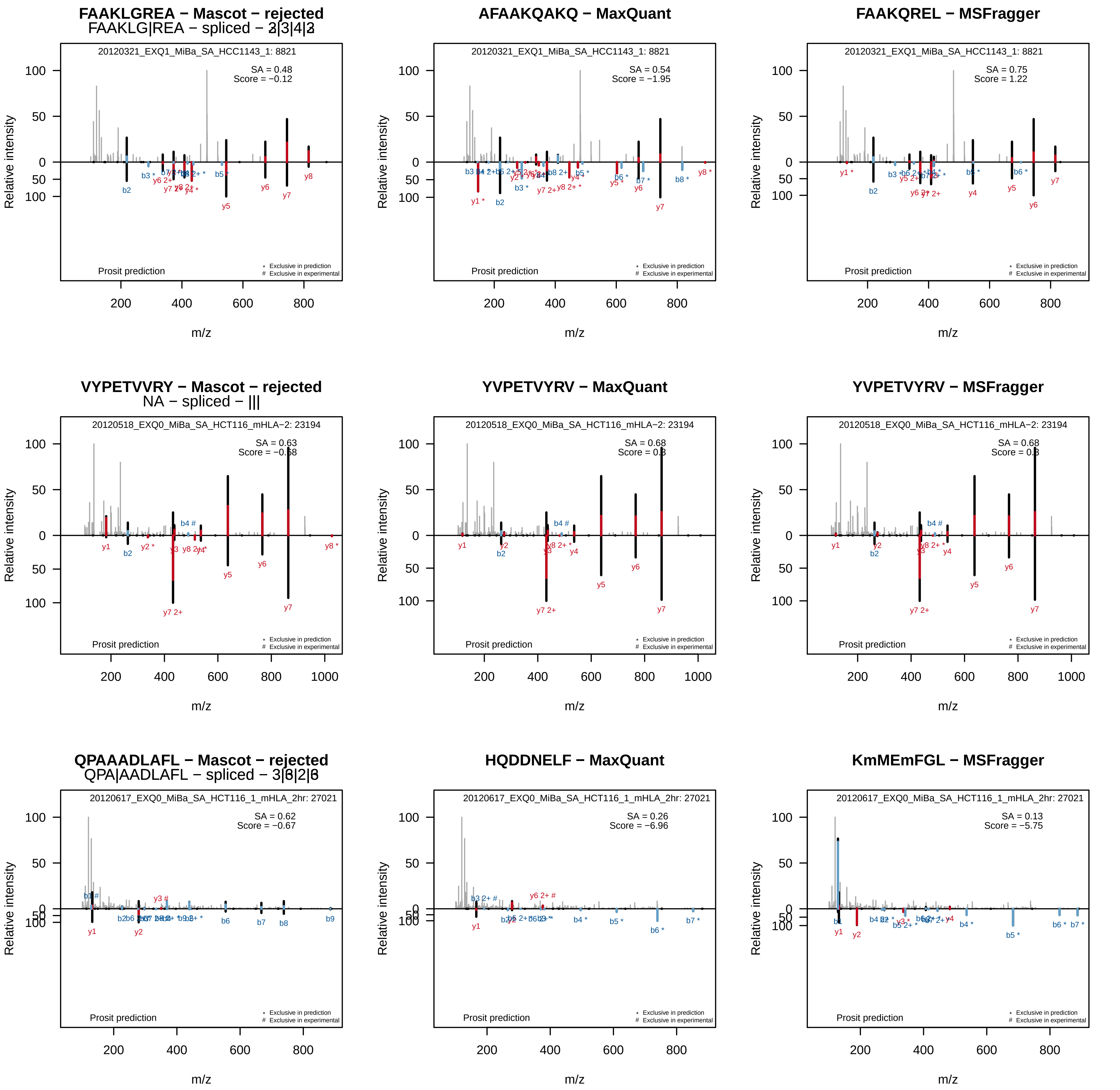

FAAKLGREA − Mascot − rejected
20120321_EXQ1_MiBa_SA_HCC1143_1: 8821
SA = 0.48
Score = −0.12
AFAAKQAKQ − MaxQuant
20120321_EXQ1_MiBa_SA_HCC1143_1: 8821
SA = 0.54
Score = −1.95
FAAKQREL − MSFragger
20120321_EXQ1_MiBa_SA_HCC1143_1: 8821
SA = 0.75
Score = 1.22
VYPETVVRY − Mascot − rejected
NA − spliced − |||
20120518_EXQ0_MiBa_SA_HCT116_mHLA−2: 23194
SA = 0.63
YVPETVYRV − MaxQuant
20120518_EXQ0_MiBa_SA_HCT116_mHLA−2: 23194
SA = 0.68
YVPETVYRV − MSFragger
20120518_EXQ0_MiBa_SA_HCT116_mHLA−2: 23194
SA = 0.68
QPAAADLAFL − Mascot − rejected
20120617_EXQ0_MiBa_SA_HCT116_1_mHLA_2hr: 27021
SA = 0.62
Score = −0.67
HQDDNELF − MaxQuant
20120617_EXQ0_MiBa_SA_HCT116_1_mHLA_2hr: 27021
SA = 0.26
Score = −6.96
KmMEmFGL − MSFragger
20120617_EXQ0_MiBa_SA_HCT116_1_mHLA_2hr: 27021
SA = 0.13
Score = −5.75
Relative intensity
m/z
Prosit prediction
⋆ Exclusive in prediction
Exclusive in experimental

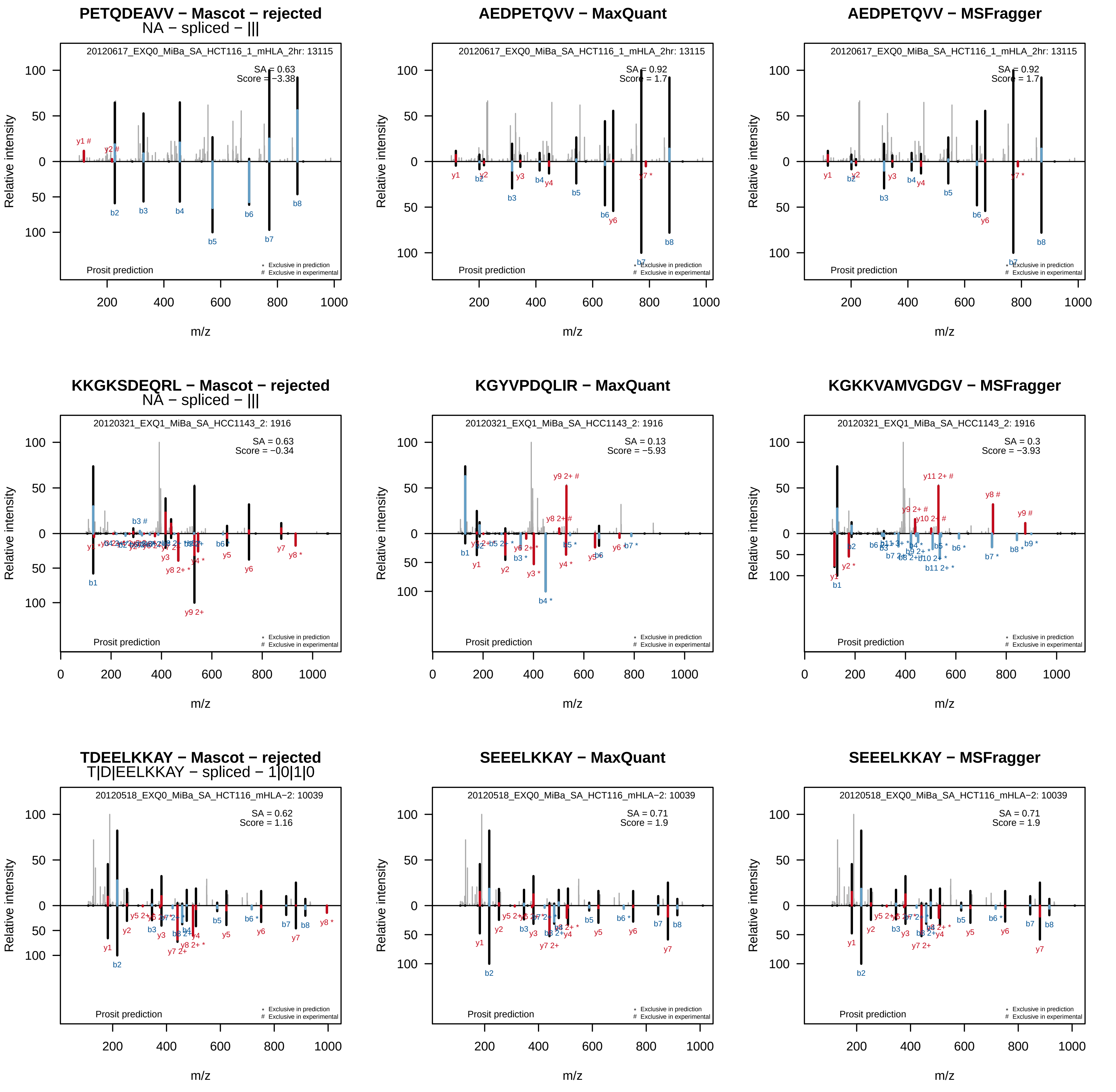
PETQDEAVV − Mascot − rejected
NA − spliced − |||
20120617_EXQ0_MiBa_SA_HCT116_1_mHLA_2hr: 13115
SA = 0.63
Score = −3.38
AEDPETQVV − MaxQuant
20120617_EXQ0_MiBa_SA_HCT116_1_mHLA_2hr: 13115
SA = 0.92
Score = 1.7
AEDPETQVV − MSFragger
20120617_EXQ0_MiBa_SA_HCT116_1_mHLA_2hr: 13115
SA = 0.92
Score = 1.7
KKGKSDEQRL − Mascot − rejected
NA − spliced − |||
20120321_EXQ1_MiBa_SA_HCC1143_2: 1916
SA = 0.63
Score = −0.34
KGYVPDQLIR − MaxQuant
20120321_EXQ1_MiBa_SA_HCC1143_2: 1916
SA = 0.13
Score = −5.93
KGKKVAMVGDGV − MSFragger
20120321_EXQ1_MiBa_SA_HCC1143_2: 1916
SA = 0.3
Score = −3.93
TDEELKKAY − Mascot − rejected
T|D|EELKKAY − spliced − 1|0|1|0
20120518_EXQ0_MiBa_SA_HCT116_mHLA−2: 10039
SA = 0.62
Score = 1.16
SEEELKKAY − MaxQuant
20120518_EXQ0_MiBa_SA_HCT116_mHLA−2: 10039
SA = 0.71
Score = 1.9
SEEELKKAY − MSFragger
20120518_EXQ0_MiBa_SA_HCT116_mHLA−2: 10039
SA = 0.71
Score = 1.9
Relative intensity
m/z
Prosit prediction
* Exclusive in prediction
# Exclusive in experimental

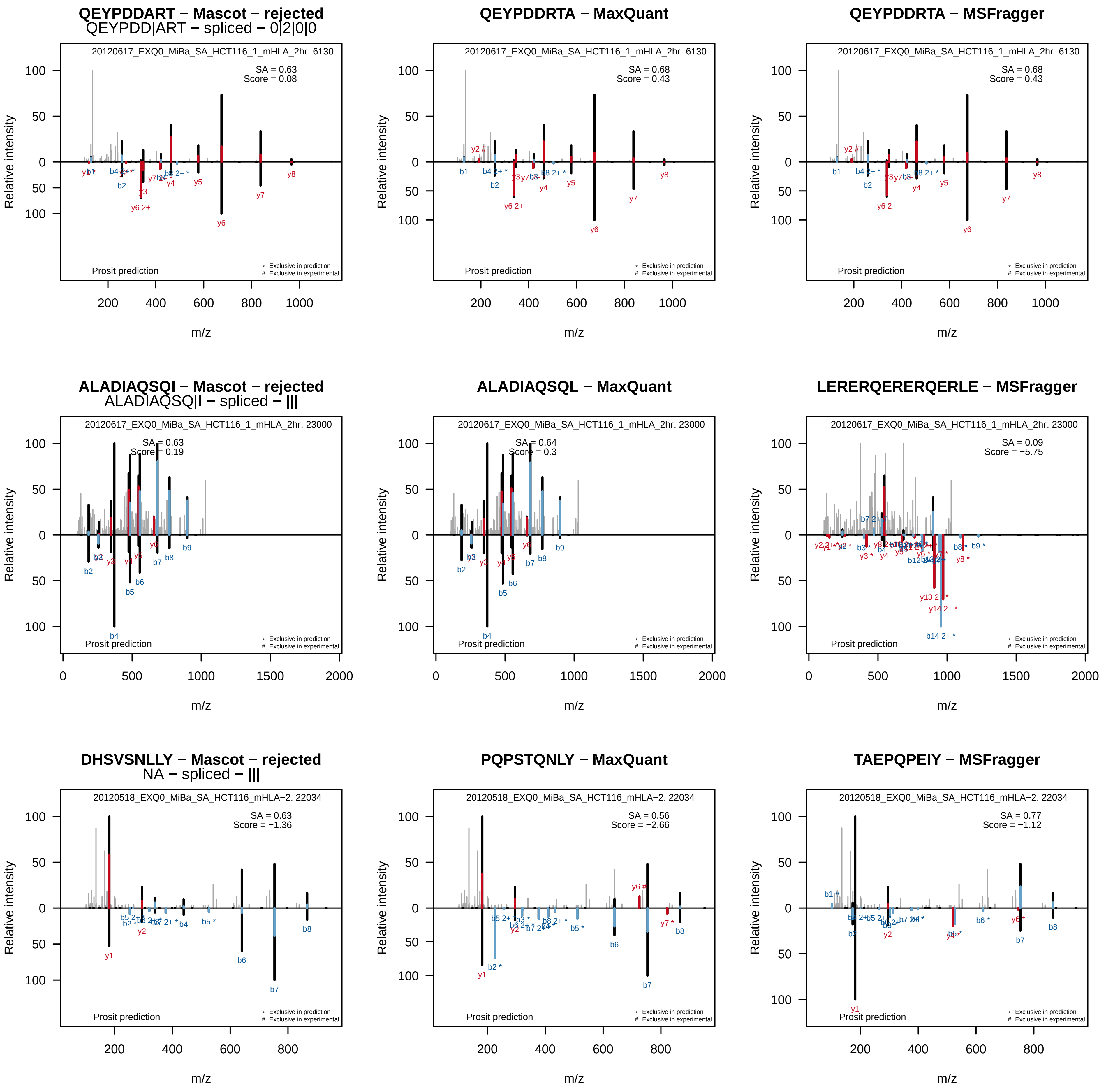
QEYPDDART − Mascot − rejected
QEYPDD|ART − spliced − 0|2|0|0
20120617_EXQ0_MiBa_SA_HCT116_1_mHLA_2hr: 6130
SA = 0.63
Score = 0.08
QEYPDDRTA − MaxQuant
20120617_EXQ0_MiBa_SA_HCT116_1_mHLA_2hr: 6130
SA = 0.68
Score = 0.43
QEYPDDRTA − MSFragger
20120617_EXQ0_MiBa_SA_HCT116_1_mHLA_2hr: 6130
SA = 0.68
Score = 0.43
ALADIAQSQI − Mascot − rejected
ALADIAQSQ|I − spliced − |||
20120617_EXQ0_MiBa_SA_HCT116_1_mHLA_2hr: 23000
SA = 0.63
Score = 0.19
ALADIAQSQL − MaxQuant
20120617_EXQ0_MiBa_SA_HCT116_1_mHLA_2hr: 23000
SA = 0.64
Score = 0.3
LERERQERERQERLE − MSFragger
20120617_EXQ0_MiBa_SA_HCT116_1_mHLA_2hr: 23000
SA = 0.09
Score = −5.75
DHSVSNLLY − Mascot − rejected
NA − spliced − |||
20120518_EXQ0_MiBa_SA_HCT116_mHLA−2: 22034
SA = 0.63
Score = −1.36
PQPSTQNLY − MaxQuant
20120518_EXQ0_MiBa_SA_HCT116_mHLA−2: 22034
SA = 0.56
Score = −2.66
TAEPQPEIY − MSFragger
20120518_EXQ0_MiBa_SA_HCT116_mHLA−2: 22034
SA = 0.77
Score = −1.12
Relative intensity
m/z
Prosit prediction
⋆ Exclusive in prediction
Exclusive in experimental

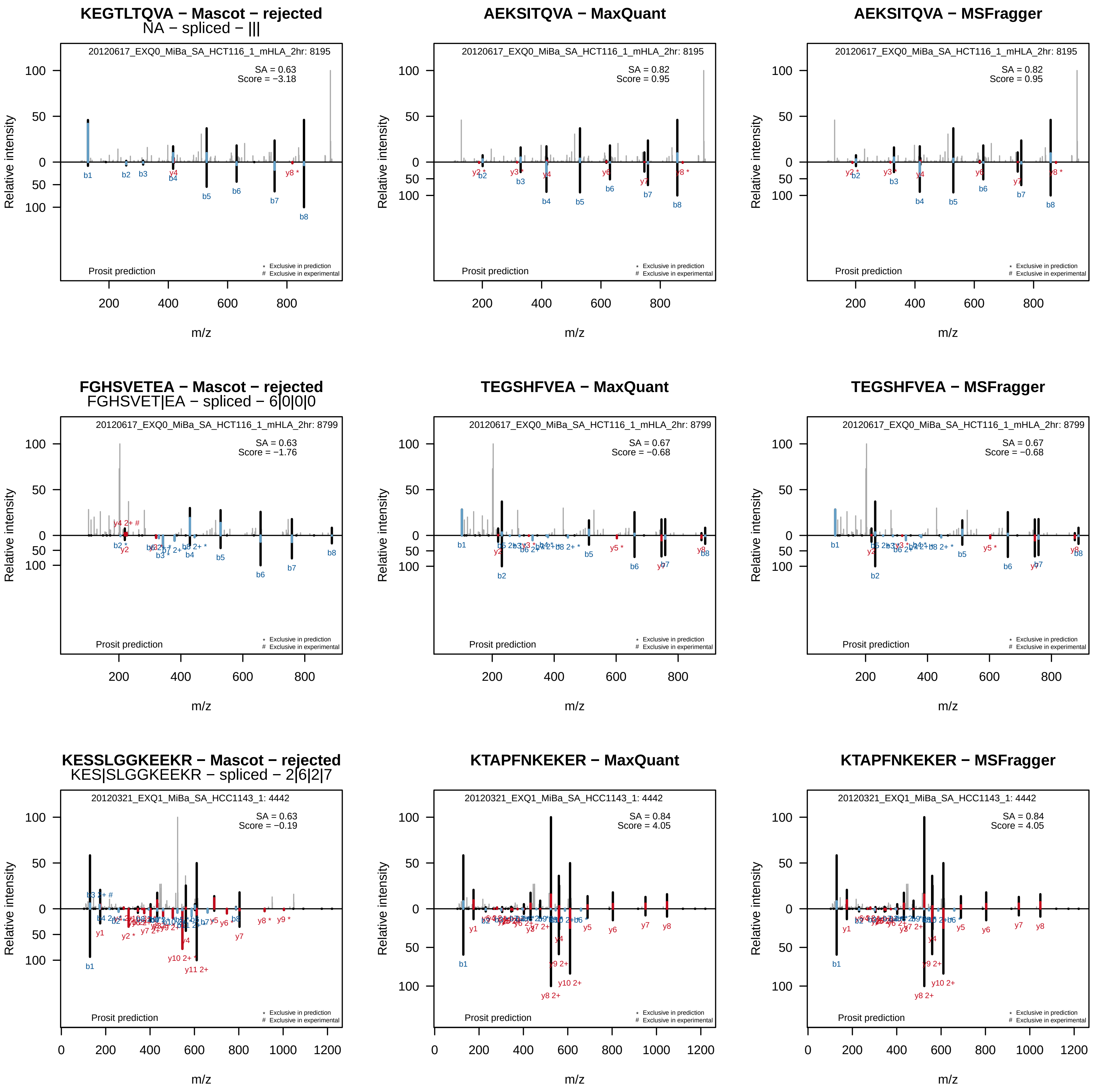
KEGTLTQVA − Mascot − rejected
NA − spliced − |||
20120617_EXQ0_MiBa_SA_HCT116_1_mHLA_2hr: 8195
SA = 0.63
Score = −3.18
Prosit prediction
⋆ Exclusive in prediction
Exclusive in experimental
AEKSITQVA − MaxQuant
20120617_EXQ0_MiBa_SA_HCT116_1_mHLA_2hr: 8195
SA = 0.82
Score = 0.95
Prosit prediction
⋆ Exclusive in prediction
Exclusive in experimental
AEKSITQVA − MSFragger
20120617_EXQ0_MiBa_SA_HCT116_1_mHLA_2hr: 8195
SA = 0.82
Score = 0.95
Prosit prediction
⋆ Exclusive in prediction
Exclusive in experimental
FGHSVETEA − Mascot − rejected
FGHSVET|EA − spliced − 6|0|0|0
20120617_EXQ0_MiBa_SA_HCT116_1_mHLA_2hr: 8799
SA = 0.63
Score = −1.76
Prosit prediction
⋆ Exclusive in prediction
Exclusive in experimental
TEGSHFVEA − MaxQuant
20120617_EXQ0_MiBa_SA_HCT116_1_mHLA_2hr: 8799
SA = 0.67
Score = −0.68
Prosit prediction
⋆ Exclusive in prediction
Exclusive in experimental
TEGSHFVEA − MSFragger
20120617_EXQ0_MiBa_SA_HCT116_1_mHLA_2hr: 8799
SA = 0.67
Score = −0.68
Prosit prediction
⋆ Exclusive in prediction
Exclusive in experimental
KESSLGGKEEKR − Mascot − rejected
KES|SLGGKEEKR − spliced − 2|6|2|7
20120321_EXQ1_MiBa_SA_HCC1143_1: 4442
SA = 0.63
Score = −0.19
Prosit prediction
⋆ Exclusive in prediction
Exclusive in experimental
KTAPFNKEKER − MaxQuant
20120321_EXQ1_MiBa_SA_HCC1143_1: 4442
SA = 0.84
Score = 4.05
Prosit prediction
⋆ Exclusive in prediction
Exclusive in experimental
KTAPFNKEKER − MSFragger
20120321_EXQ1_MiBa_SA_HCC1143_1: 4442
SA = 0.84
Score = 4.05
Prosit prediction
⋆ Exclusive in prediction
Exclusive in experimental
Relative intensity
m/z

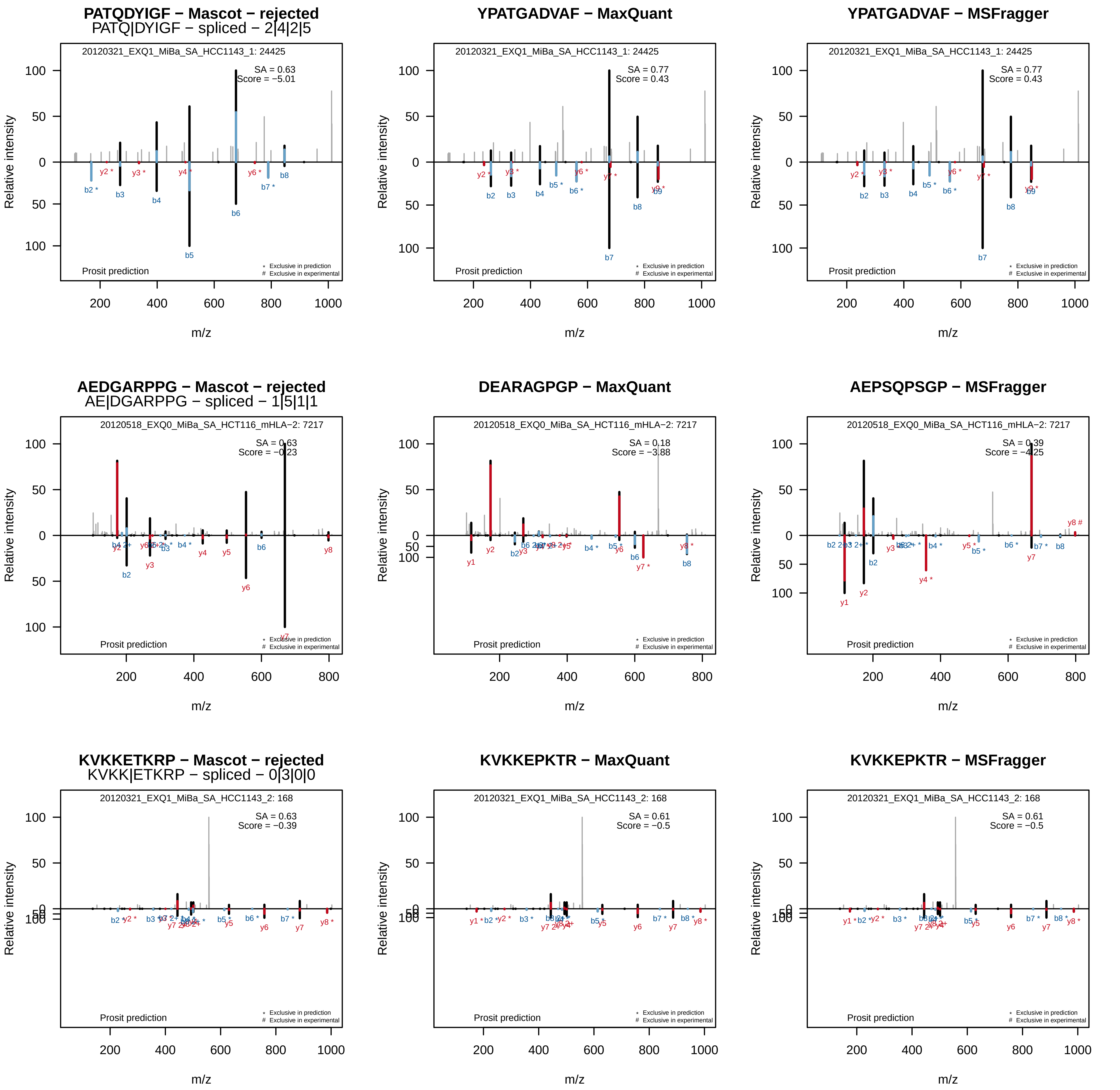
PATQDYIGF − Mascot − rejected
PATQ|DYIGF − spliced − 2|4|2|5
20120321_EXQ1_MiBa_SA_HCC1143_1: 24425
SA = 0.63
Score = −5.01
YPATGADVAF − MaxQuant
20120321_EXQ1_MiBa_SA_HCC1143_1: 24425
SA = 0.77
Score = 0.43
YPATGADVAF − MSFragger
20120321_EXQ1_MiBa_SA_HCC1143_1: 24425
SA = 0.77
Score = 0.43
AEDGARPPG − Mascot − rejected
AE|DGARPPG − spliced − 1|5|1|1
20120518_EXQ0_MiBa_SA_HCT116_mHLA−2: 7217
SA = 0.63
Score = −0.23
DEARAGPGP − MaxQuant
20120518_EXQ0_MiBa_SA_HCT116_mHLA−2: 7217
SA = 0.18
Score = −3.88
AEPSQPSGP − MSFragger
20120518_EXQ0_MiBa_SA_HCT116_mHLA−2: 7217
SA = 0.39
Score = −4.25
KVKKETKRP − Mascot − rejected
KVKK|ETKRP − spliced − 0|3|0|0
20120321_EXQ1_MiBa_SA_HCC1143_2: 168
SA = 0.63
Score = −0.39
KVKKEPKTR − MaxQuant
20120321_EXQ1_MiBa_SA_HCC1143_2: 168
SA = 0.61
Score = −0.5
KVKKEPKTR − MSFragger
20120321_EXQ1_MiBa_SA_HCC1143_2: 168
SA = 0.61
Score = −0.5
Relative intensity
m/z
Prosit prediction
⋆ Exclusive in prediction
Exclusive in experimental

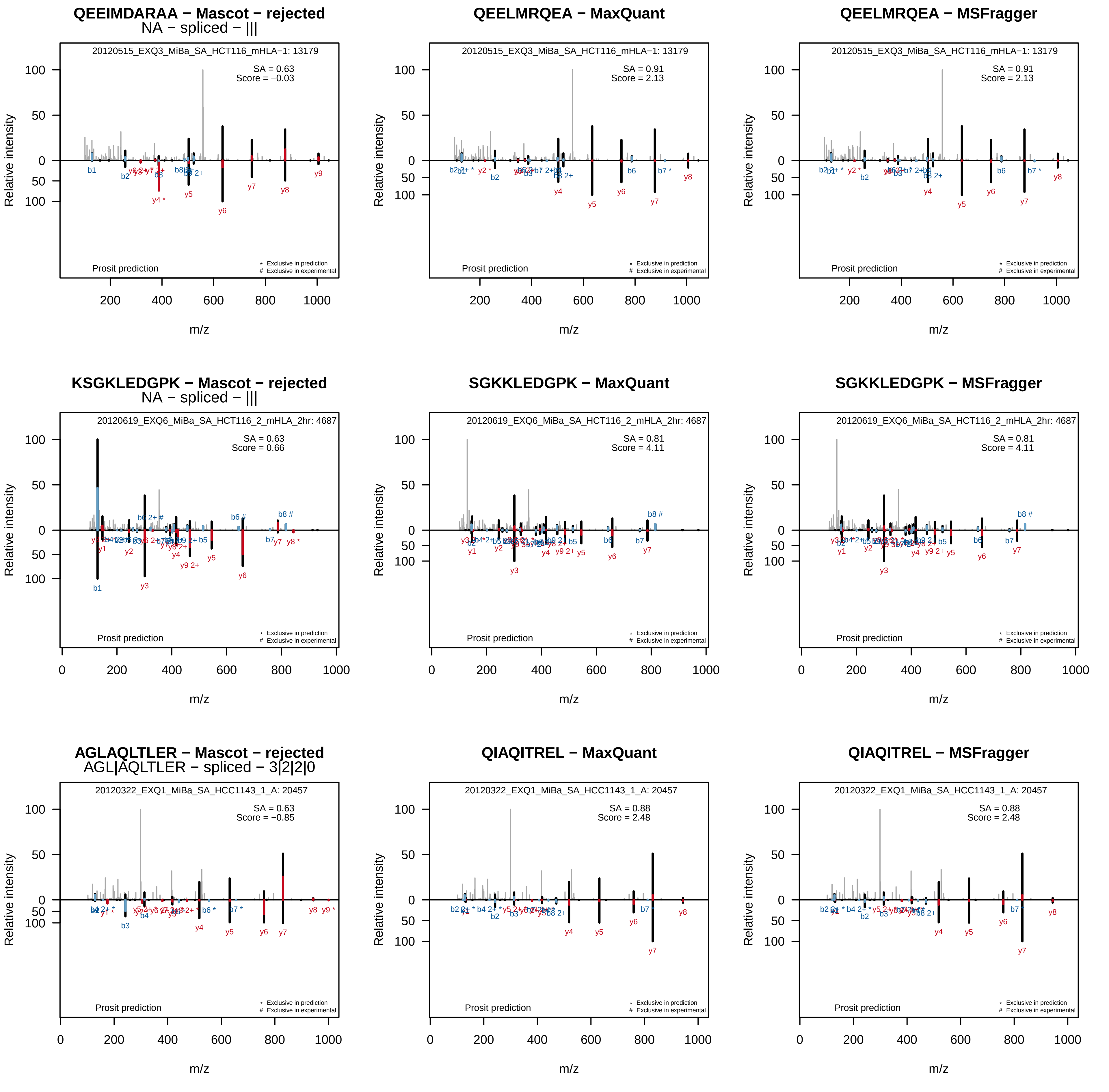

QEEIMDARAA − Mascot − rejected
NA − spliced − |||
20120515_EXQ3_MiBa_SA_HCT116_mHLA−1: 13179
SA = 0.63
Score = −0.03
QEELMRQEA − MaxQuant
20120515_EXQ3_MiBa_SA_HCT116_mHLA−1: 13179
SA = 0.91
Score = 2.13
QEELMRQEA − MSFragger
20120515_EXQ3_MiBa_SA_HCT116_mHLA−1: 13179
SA = 0.91
Score = 2.13
KSGKLEDGPK − Mascot − rejected
NA − spliced − |||
20120619_EXQ6_MiBa_SA_HCT116_2_mHLA_2hr: 4687
SA = 0.63
Score = 0.66
SGKKLEDGPK − MaxQuant
20120619_EXQ6_MiBa_SA_HCT116_2_mHLA_2hr: 4687
SA = 0.81
Score = 4.11
SGKKLEDGPK − MSFragger
20120619_EXQ6_MiBa_SA_HCT116_2_mHLA_2hr: 4687
SA = 0.81
Score = 4.11
AGLAQLTLER − Mascot − rejected
AGL|AQLTLER − spliced − 3|2|2|0
20120322_EXQ1_MiBa_SA_HCC1143_1_A: 20457
SA = 0.63
Score = −0.85
QIAQITREL − MaxQuant
20120322_EXQ1_MiBa_SA_HCC1143_1_A: 20457
SA = 0.88
Score = 2.48
QIAQITREL − MSFragger
20120322_EXQ1_MiBa_SA_HCC1143_1_A: 20457
SA = 0.88
Score = 2.48
Relative intensity
m/z
Prosit prediction
⋆ Exclusive in prediction
Exclusive in experimental

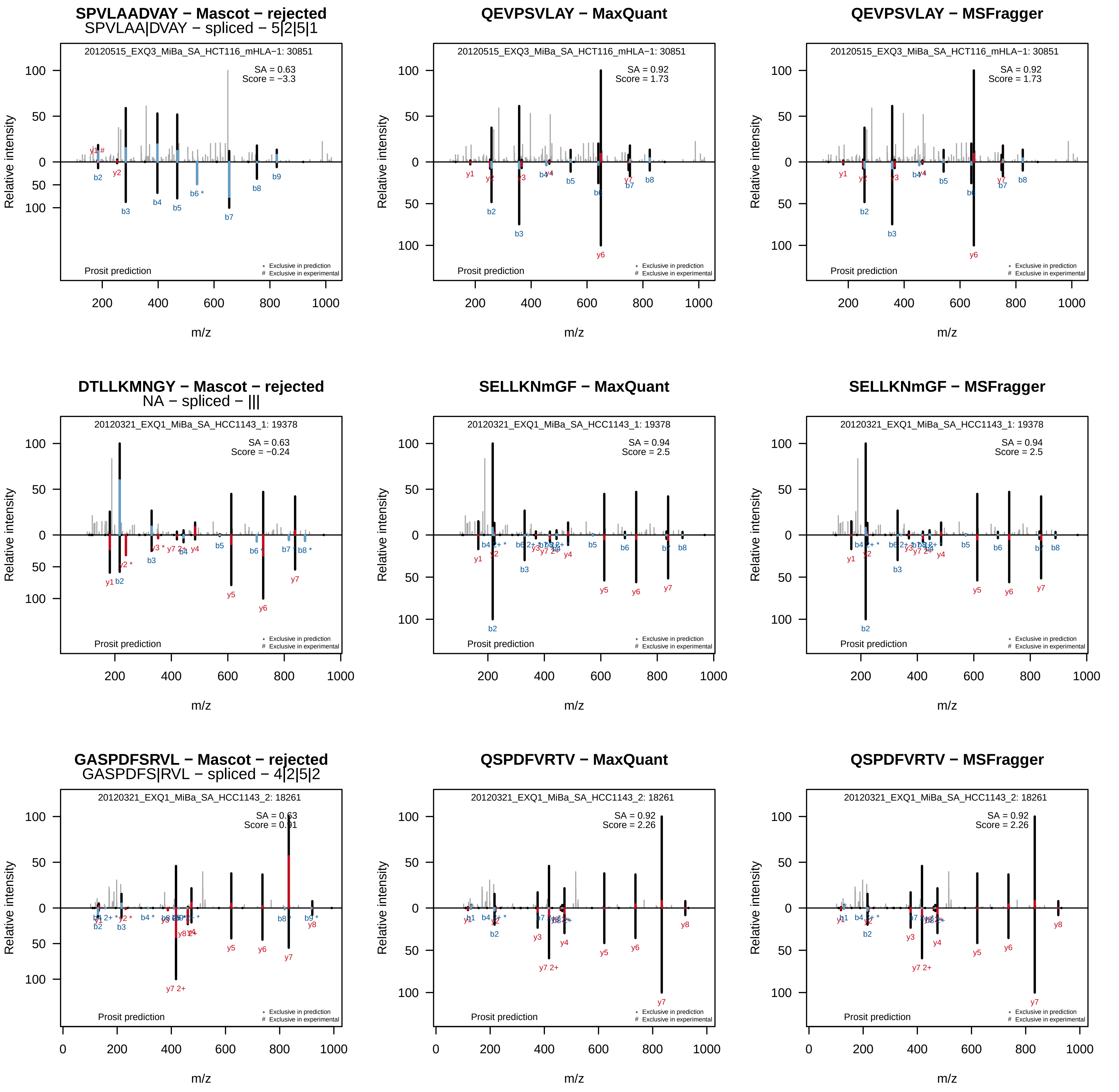
SPVLAADVAY − Mascot − rejected
SPVLAA|DVAY − spliced − 5|2|5|1
20120515_EXQ3_MiBa_SA_HCT116_mHLA−1: 30851
SA = 0.63
Score = −3.3
QEVPSVLAY − MaxQuant
20120515_EXQ3_MiBa_SA_HCT116_mHLA−1: 30851
SA = 0.92
Score = 1.73
QEVPSVLAY − MSFragger
20120515_EXQ3_MiBa_SA_HCT116_mHLA−1: 30851
SA = 0.92
Score = 1.73
DTLLKMNGY − Mascot − rejected
NA − spliced − |||
20120321_EXQ1_MiBa_SA_HCC1143_1: 19378
SA = 0.63
Score = −0.24
SELLKNmGF − MaxQuant
20120321_EXQ1_MiBa_SA_HCC1143_1: 19378
SA = 0.94
Score = 2.5
SELLKNmGF − MSFragger
20120321_EXQ1_MiBa_SA_HCC1143_1: 19378
SA = 0.94
Score = 2.5
GASPDFSRVL − Mascot − rejected
GASPDFS|RVL − spliced − 4|2|5|2
20120321_EXQ1_MiBa_SA_HCC1143_2: 18261
SA = 0.63
Score = 0.91
QSPDFVRTV − MaxQuant
20120321_EXQ1_MiBa_SA_HCC1143_2: 18261
SA = 0.92
Score = 2.26
QSPDFVRTV − MSFragger
20120321_EXQ1_MiBa_SA_HCC1143_2: 18261
SA = 0.92
Score = 2.26
Relative intensity
m/z
Prosit prediction
⋆ Exclusive in prediction
Exclusive in experimental

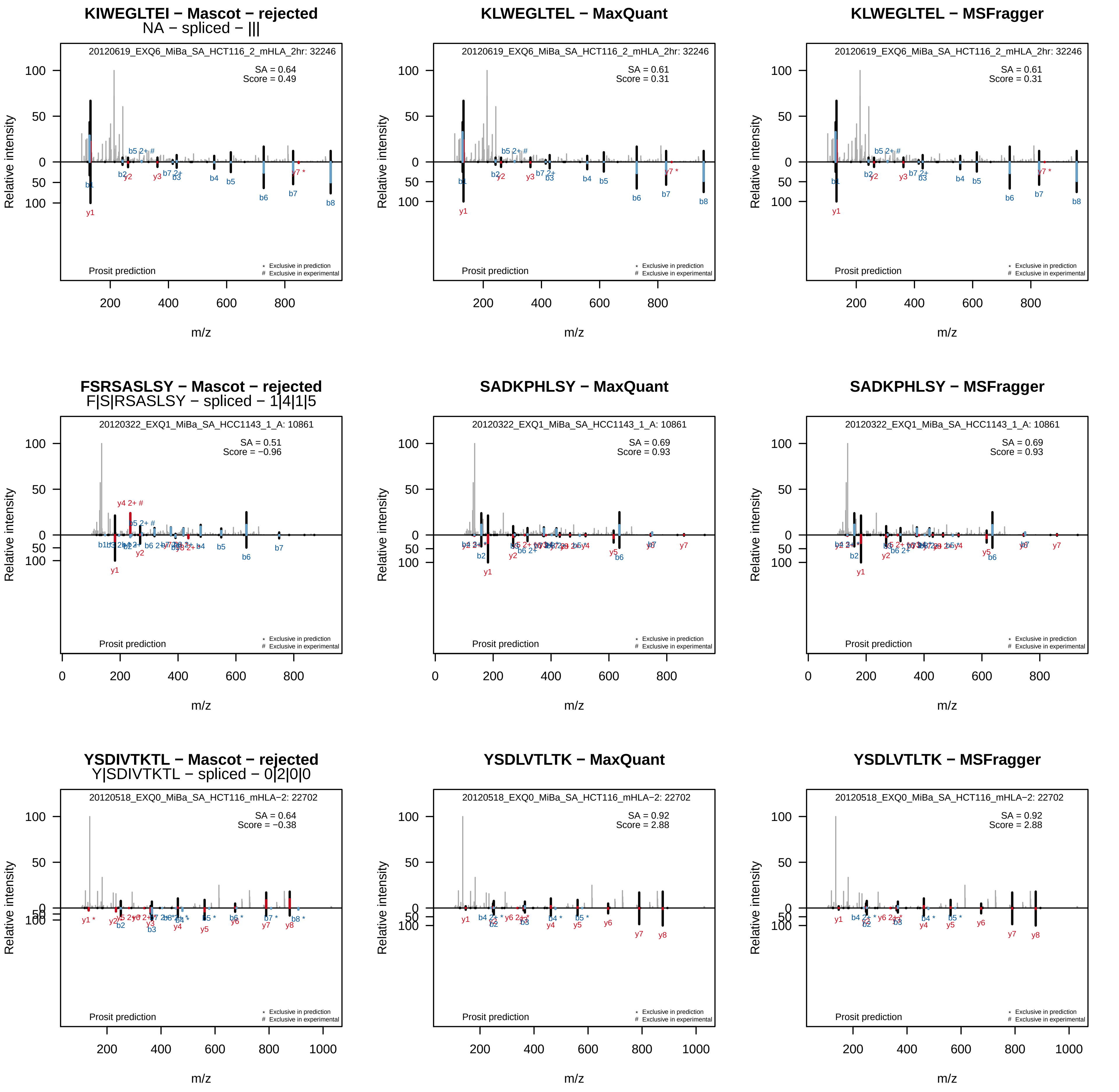
KIWEGLTEI − Mascot − rejected
NA − spliced − |||
20120619_EXQ6_MiBa_SA_HCT116_2_mHLA_2hr: 32246
SA = 0.64
Score = 0.49
KLWEGLTEL − MaxQuant
20120619_EXQ6_MiBa_SA_HCT116_2_mHLA_2hr: 32246
SA = 0.61
Score = 0.31
KLWEGLTEL − MSFragger
20120619_EXQ6_MiBa_SA_HCT116_2_mHLA_2hr: 32246
SA = 0.61
Score = 0.31
FSRSASLSY − Mascot − rejected
F|S|RSASLSY − spliced − 1|4|1|5
20120322_EXQ1_MiBa_SA_HCC1143_1_A: 10861
SA = 0.51
Score = −0.96
SADKPHLSY − MaxQuant
20120322_EXQ1_MiBa_SA_HCC1143_1_A: 10861
SA = 0.69
Score = 0.93
SADKPHLSY − MSFragger
20120322_EXQ1_MiBa_SA_HCC1143_1_A: 10861
SA = 0.69
Score = 0.93
YSDIVTKTL − Mascot − rejected
Y|SDIVTKTL − spliced − 0|2|0|0
20120518_EXQ0_MiBa_SA_HCT116_mHLA−2: 22702
SA = 0.64
Score = −0.38
YSDLVTLTK − MaxQuant
20120518_EXQ0_MiBa_SA_HCT116_mHLA−2: 22702
SA = 0.92
Score = 2.88
YSDLVTLTK − MSFragger
20120518_EXQ0_MiBa_SA_HCT116_mHLA−2: 22702
SA = 0.92
Score = 2.88
Relative intensity
m/z
Prosit prediction
* Exclusive in prediction
# Exclusive in experimental

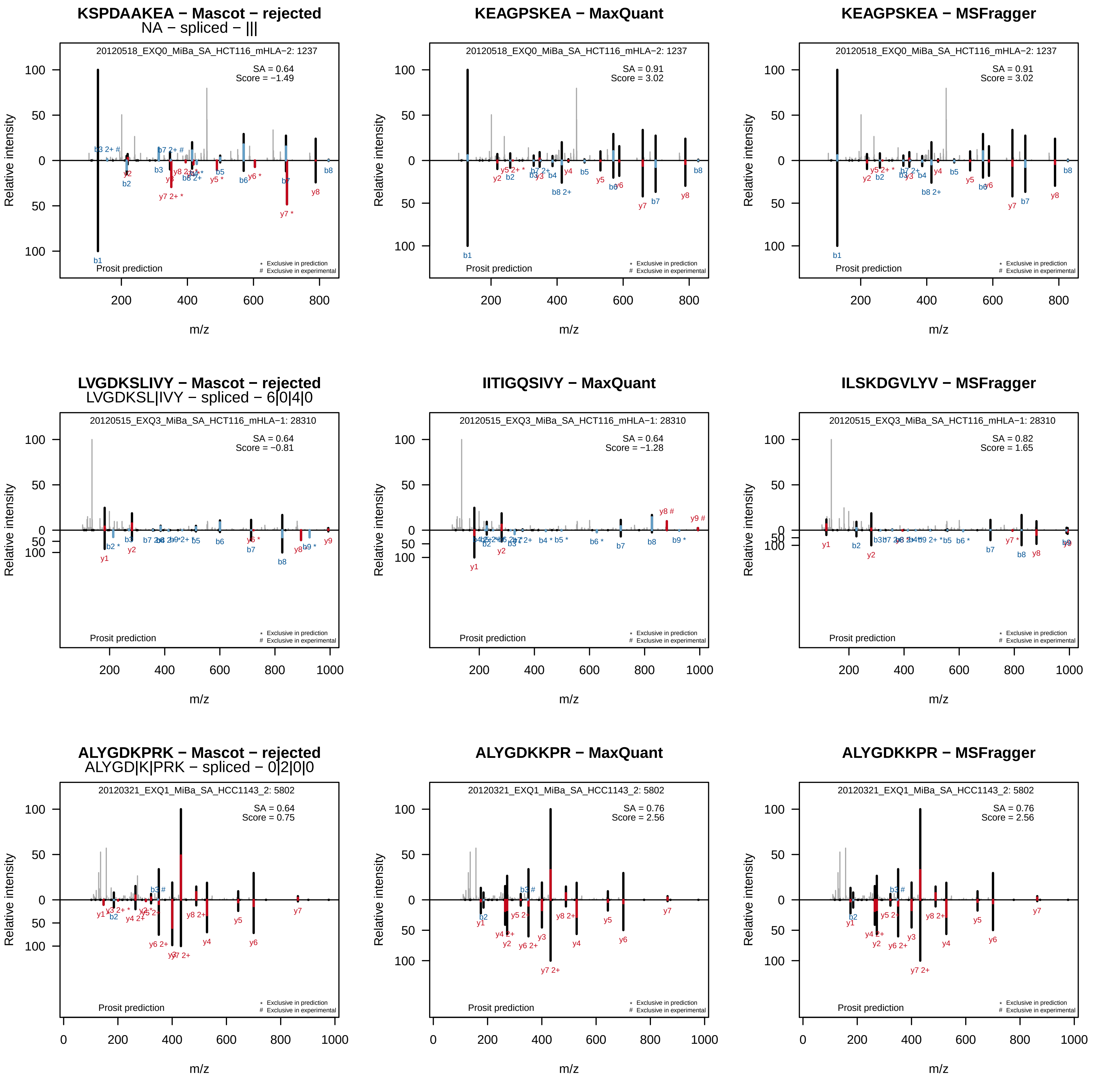

KSPDAAKEA − Mascot − rejected
NA − spliced − |||
20120518_EXQ0_MiBa_SA_HCT116_mHLA−2: 1237
SA = 0.64
Score = −1.49
KEAGPSKEA − MaxQuant
20120518_EXQ0_MiBa_SA_HCT116_mHLA−2: 1237
SA = 0.91
Score = 3.02
KEAGPSKEA − MSFragger
20120518_EXQ0_MiBa_SA_HCT116_mHLA−2: 1237
SA = 0.91
Score = 3.02
LVGDKSLIVY − Mascot − rejected
LVGDKSL|IVY − spliced − 6|0|4|0
20120515_EXQ3_MiBa_SA_HCT116_mHLA−1: 28310
SA = 0.64
Score = −0.81
IITIGQSIVY − MaxQuant
20120515_EXQ3_MiBa_SA_HCT116_mHLA−1: 28310
SA = 0.64
Score = −1.28
ILSKDGVLYV − MSFragger
20120515_EXQ3_MiBa_SA_HCT116_mHLA−1: 28310
SA = 0.82
Score = 1.65
ALYGDKPRK − Mascot − rejected
ALYGD|K|PRK − spliced − 0|2|0|0
20120321_EXQ1_MiBa_SA_HCC1143_2: 5802
SA = 0.64
Score = 0.75
ALYGDKKPR − MaxQuant
20120321_EXQ1_MiBa_SA_HCC1143_2: 5802
SA = 0.76
Score = 2.56
ALYGDKKPR − MSFragger
20120321_EXQ1_MiBa_SA_HCC1143_2: 5802
SA = 0.76
Score = 2.56
Relative intensity
m/z
Prosit prediction
⋆ Exclusive in prediction
Exclusive in experimental

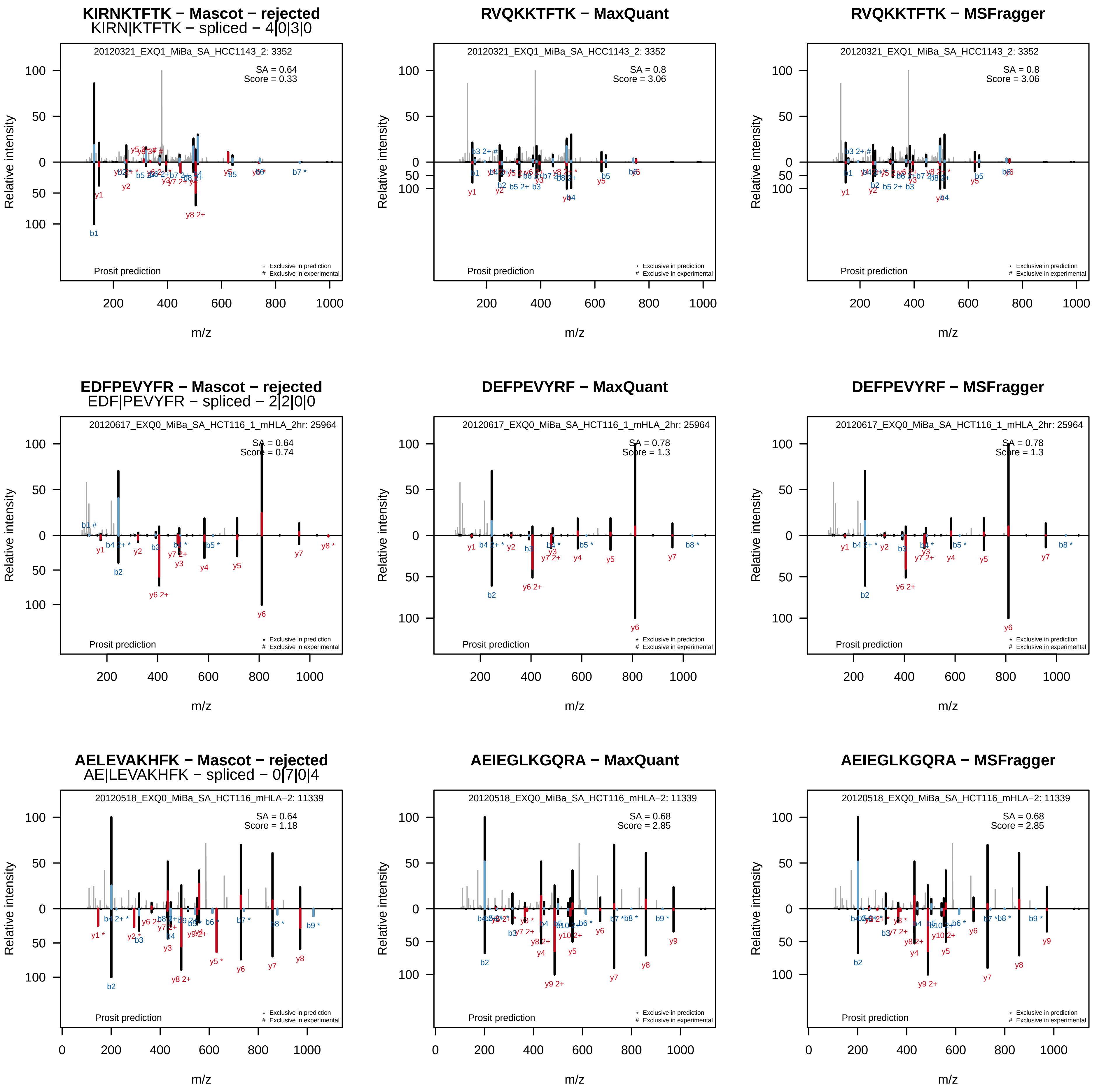
KIRNKTFTK − Mascot − rejected
KIRN|KTFTK − spliced − 4|0|3|0
20120321_EXQ1_MiBa_SA_HCC1143_2: 3352
SA = 0.64
Score = 0.33
RVQKKTFTK − MaxQuant
20120321_EXQ1_MiBa_SA_HCC1143_2: 3352
SA = 0.8
Score = 3.06
RVQKKTFTK − MSFragger
20120321_EXQ1_MiBa_SA_HCC1143_2: 3352
SA = 0.8
Score = 3.06
EDFPEVYFR − Mascot − rejected
EDF|PEVYFR − spliced − 2|2|0|0
20120617_EXQ0_MiBa_SA_HCT116_1_mHLA_2hr: 25964
SA = 0.64
Score = 0.74
DEFPEVYRF − MaxQuant
20120617_EXQ0_MiBa_SA_HCT116_1_mHLA_2hr: 25964
SA = 0.78
Score = 1.3
DEFPEVYRF − MSFragger
20120617_EXQ0_MiBa_SA_HCT116_1_mHLA_2hr: 25964
SA = 0.78
Score = 1.3
AELEVAKHFK − Mascot − rejected
AE|LEVAKHFK − spliced − 0|7|0|4
20120518_EXQ0_MiBa_SA_HCT116_mHLA−2: 11339
SA = 0.64
Score = 1.18
AEIEGLKGQRA − MaxQuant
20120518_EXQ0_MiBa_SA_HCT116_mHLA−2: 11339
SA = 0.68
Score = 2.85
AEIEGLKGQRA − MSFragger
20120518_EXQ0_MiBa_SA_HCT116_mHLA−2: 11339
SA = 0.68
Score = 2.85
Relative intensity
m/z
Prosit prediction
* Exclusive in prediction
# Exclusive in experimental

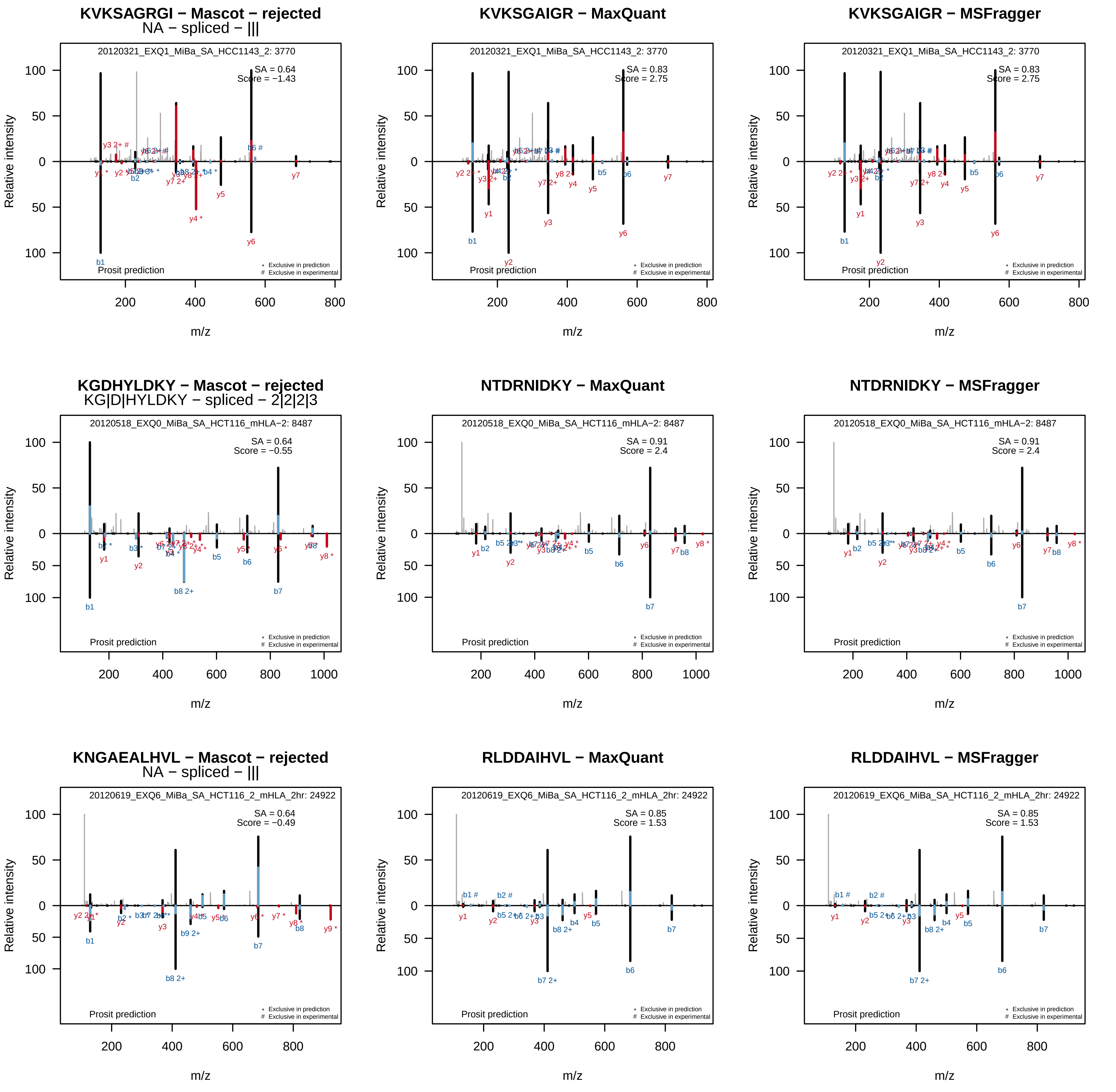

KVKSAGRGI − Mascot − rejected
NA − spliced − |||
20120321_EXQ1_MiBa_SA_HCC1143_2: 3770
SA = 0.64
Score = −1.43
KVKSGAIGR − MaxQuant
20120321_EXQ1_MiBa_SA_HCC1143_2: 3770
SA = 0.83
Score = 2.75
KVKSGAIGR − MSFragger
20120321_EXQ1_MiBa_SA_HCC1143_2: 3770
SA = 0.83
Score = 2.75
KGDHYLDKY − Mascot − rejected
KG|D|HYLDKY − spliced − 2|2|2|3
20120518_EXQ0_MiBa_SA_HCT116_mHLA−2: 8487
SA = 0.64
Score = −0.55
NTDRNIDKY − MaxQuant
20120518_EXQ0_MiBa_SA_HCT116_mHLA−2: 8487
SA = 0.91
Score = 2.4
NTDRNIDKY − MSFragger
20120518_EXQ0_MiBa_SA_HCT116_mHLA−2: 8487
SA = 0.91
Score = 2.4
KNGAEALHVL − Mascot − rejected
NA − spliced − |||
20120619_EXQ6_MiBa_SA_HCT116_2_mHLA_2hr: 24922
SA = 0.64
Score = −0.49
RLDDAIHVL − MaxQuant
20120619_EXQ6_MiBa_SA_HCT116_2_mHLA_2hr: 24922
SA = 0.85
Score = 1.53
RLDDAIHVL − MSFragger
20120619_EXQ6_MiBa_SA_HCT116_2_mHLA_2hr: 24922
SA = 0.85
Score = 1.53
Relative intensity
m/z
Prosit prediction
⋆ Exclusive in prediction
Exclusive in experimental

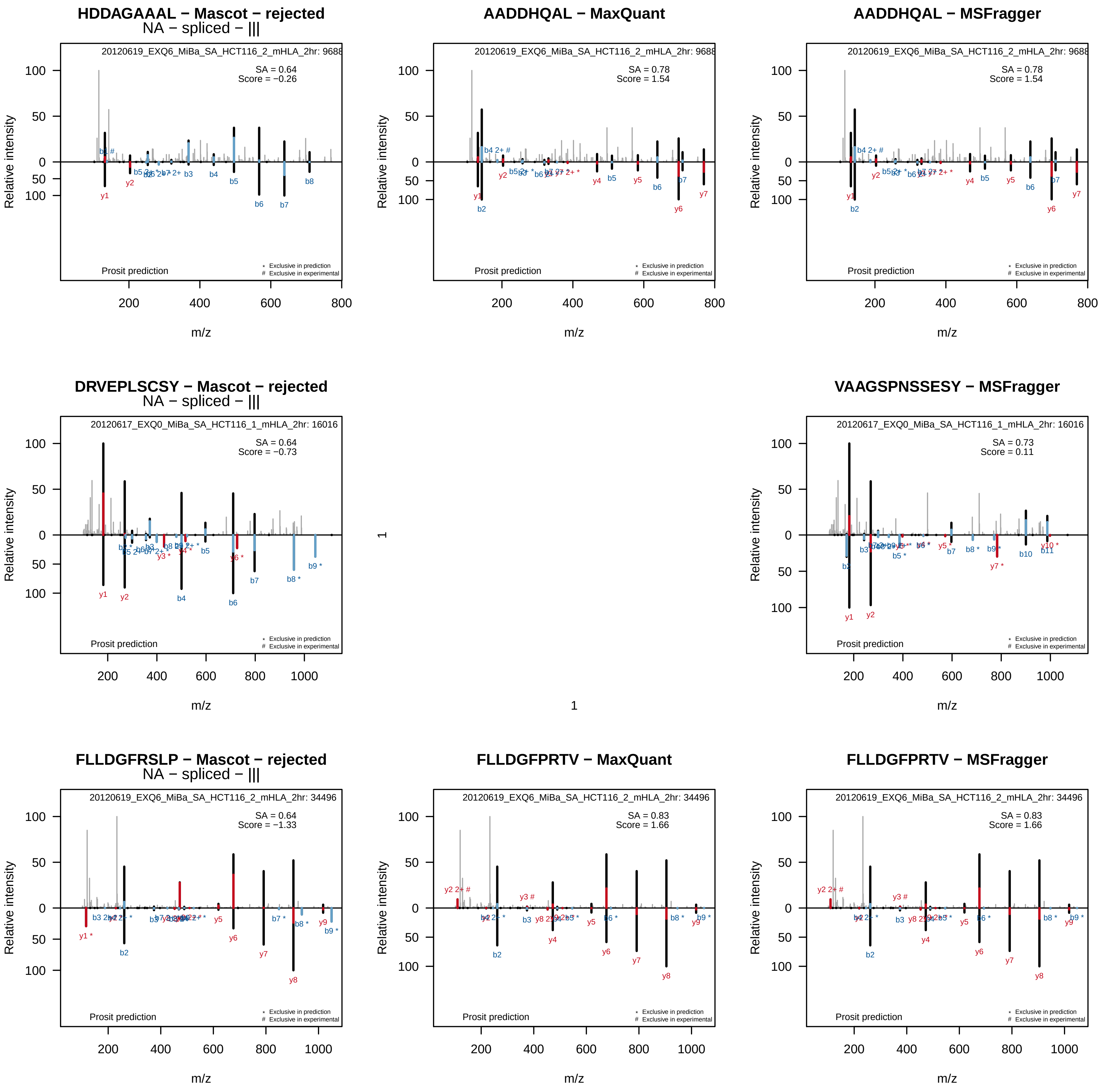
HDDAGAAAL − Mascot − rejected
NA − spliced − |||
20120619_EXQ6_MiBa_SA_HCT116_2_mHLA_2hr: 9688
SA = 0.64
Score = −0.26
AADDHQAL − MaxQuant
20120619_EXQ6_MiBa_SA_HCT116_2_mHLA_2hr: 9688
SA = 0.78
Score = 1.54
AADDHQAL − MSFragger
20120619_EXQ6_MiBa_SA_HCT116_2_mHLA_2hr: 9688
SA = 0.78
Score = 1.54
DRVEPLSCSY − Mascot − rejected
NA − spliced − |||
20120617_EXQ0_MiBa_SA_HCT116_1_mHLA_2hr: 16016
SA = 0.64
Score = −0.73
1
1
VAAGSPNSSESY − MSFragger
20120617_EXQ0_MiBa_SA_HCT116_1_mHLA_2hr: 16016
SA = 0.73
Score = 0.11
FLLDGFRSLP − Mascot − rejected
NA − spliced − |||
20120619_EXQ6_MiBa_SA_HCT116_2_mHLA_2hr: 34496
SA = 0.64
Score = −1.33
FLLDGFPRTV − MaxQuant
20120619_EXQ6_MiBa_SA_HCT116_2_mHLA_2hr: 34496
SA = 0.83
Score = 1.66
FLLDGFPRTV − MSFragger
20120619_EXQ6_MiBa_SA_HCT116_2_mHLA_2hr: 34496
SA = 0.83
Score = 1.66
Relative intensity
m/z
Prosit prediction
* Exclusive in prediction
# Exclusive in experimental

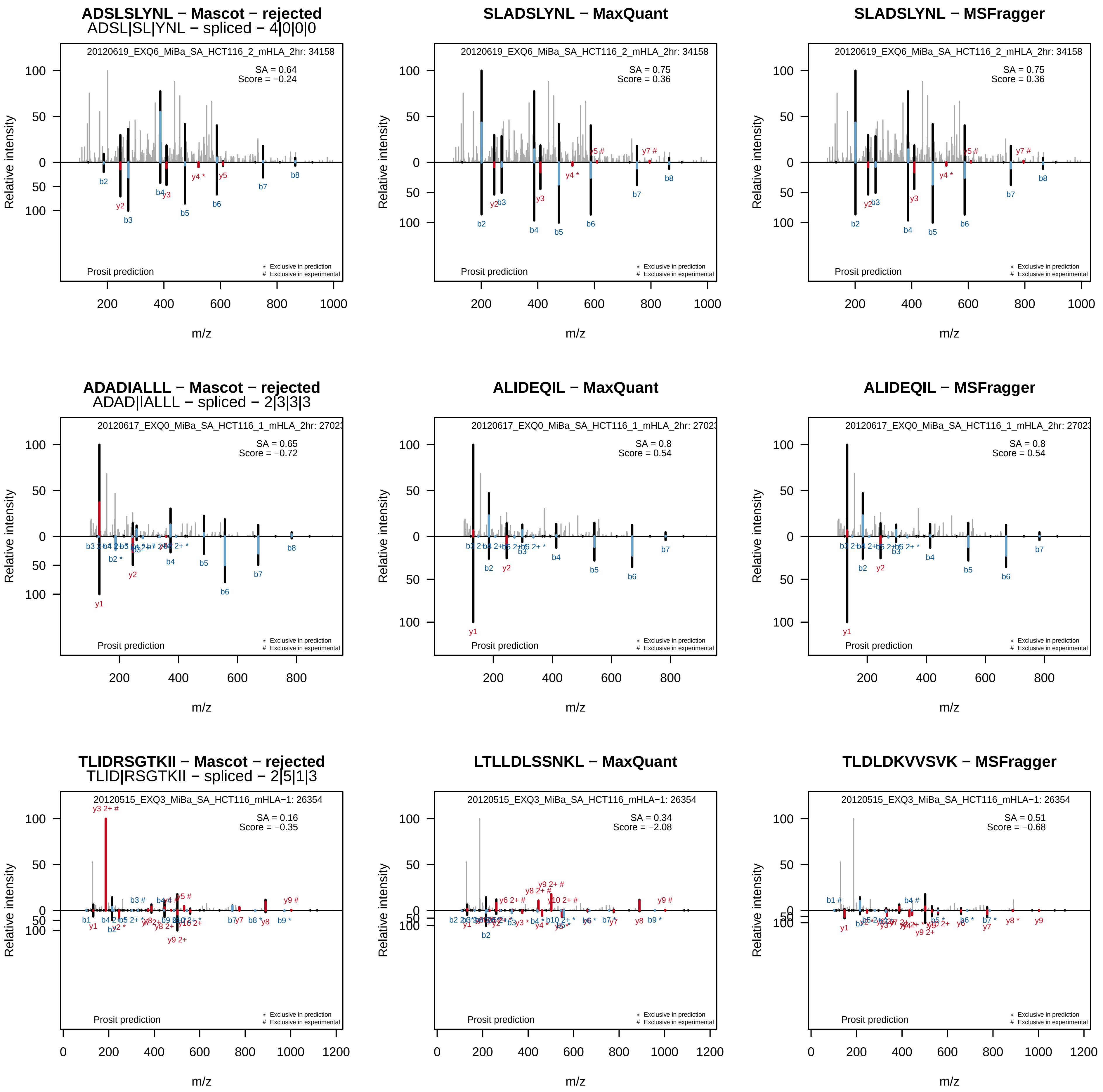

ADSLSLYNL − Mascot − rejected
ADSL|SL|YNL − spliced − 4|0|0|0
20120619_EXQ6_MiBa_SA_HCT116_2_mHLA_2hr: 34158
SA = 0.64
Score = −0.24
SLADSLYNL − MaxQuant
20120619_EXQ6_MiBa_SA_HCT116_2_mHLA_2hr: 34158
SA = 0.75
Score = 0.36
SLADSLYNL − MSFragger
20120619_EXQ6_MiBa_SA_HCT116_2_mHLA_2hr: 34158
SA = 0.75
Score = 0.36
ADADIALLL − Mascot − rejected
ADAD|IALLL − spliced − 2|3|3|3
20120617_EXQ0_MiBa_SA_HCT116_1_mHLA_2hr: 27023
SA = 0.65
Score = −0.72
ALIDEQIL − MaxQuant
20120617_EXQ0_MiBa_SA_HCT116_1_mHLA_2hr: 27023
SA = 0.8
Score = 0.54
ALIDEQIL − MSFragger
20120617_EXQ0_MiBa_SA_HCT116_1_mHLA_2hr: 27023
SA = 0.8
Score = 0.54
TLIDRSGTKII − Mascot − rejected
TLID|RSGTKII − spliced − 2|5|1|3
20120515_EXQ3_MiBa_SA_HCT116_mHLA−1: 26354
SA = 0.16
Score = −0.35
LTLLDLSSNKL − MaxQuant
20120515_EXQ3_MiBa_SA_HCT116_mHLA−1: 26354
SA = 0.34
Score = −2.08
TLDLDKVVSVK − MSFragger
20120515_EXQ3_MiBa_SA_HCT116_mHLA−1: 26354
SA = 0.51
Score = −0.68
Relative intensity
m/z
Prosit prediction
⋆ Exclusive in prediction
Exclusive in experimental

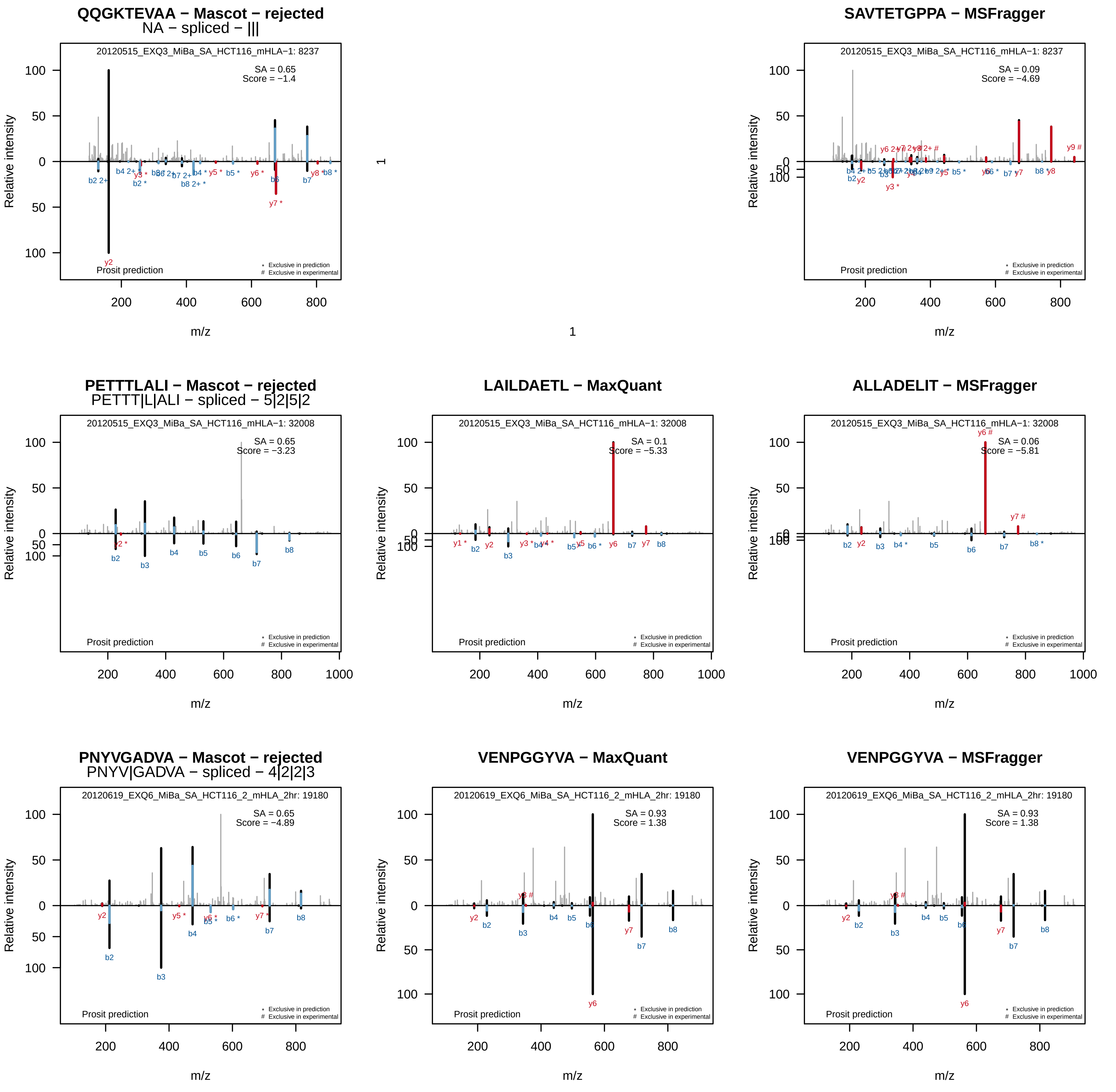

QQGKTEVAA − Mascot − rejected
NA − spliced − |||
20120515_EXQ3_MiBa_SA_HCT116_mHLA−1: 8237
SA = 0.65
Score = −1.4
Prosit prediction
SAVTETGPPA − MSFragger
20120515_EXQ3_MiBa_SA_HCT116_mHLA−1: 8237
SA = 0.09
Score = −4.69
PETTTLALI − Mascot − rejected
PETTT|L|ALI − spliced − 5|2|5|2
20120515_EXQ3_MiBa_SA_HCT116_mHLA−1: 32008
SA = 0.65
Score = −3.23
LAILDAETL − MaxQuant
20120515_EXQ3_MiBa_SA_HCT116_mHLA−1: 32008
SA = 0.1
Score = −5.33
ALLADELIT − MSFragger
20120515_EXQ3_MiBa_SA_HCT116_mHLA−1: 32008
SA = 0.06
Score = −5.81
PNYVGADVA − Mascot − rejected
PNYV|GADVA − spliced − 4|2|2|3
20120619_EXQ6_MiBa_SA_HCT116_2_mHLA_2hr: 19180
SA = 0.65
Score = −4.89
VENPGGYVA − MaxQuant
20120619_EXQ6_MiBa_SA_HCT116_2_mHLA_2hr: 19180
SA = 0.93
Score = 1.38
VENPGGYVA − MSFragger
20120619_EXQ6_MiBa_SA_HCT116_2_mHLA_2hr: 19180
SA = 0.93
Score = 1.38
Relative intensity
m/z
* Exclusive in prediction
# Exclusive in experimental

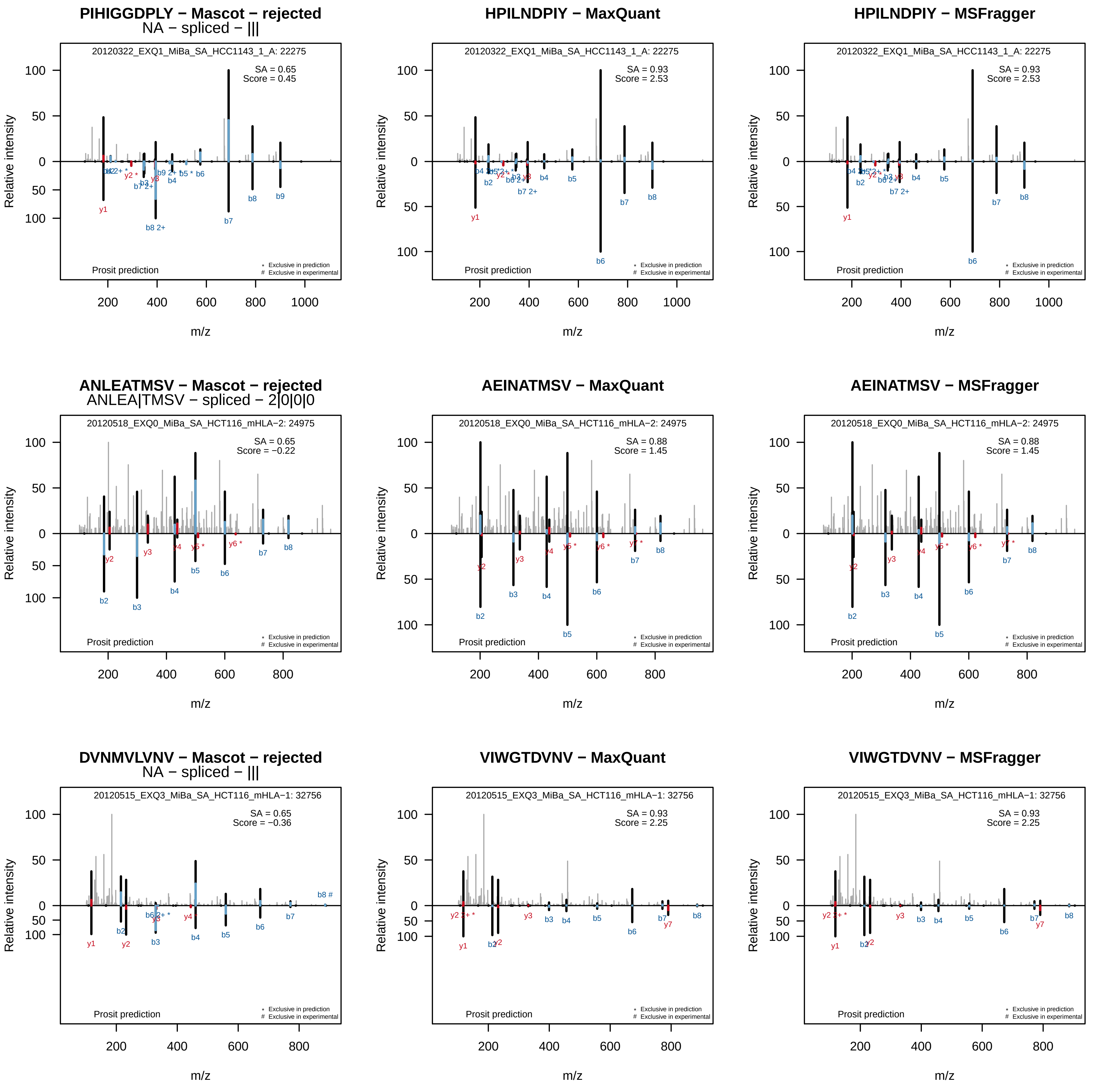

PIHIGGDPLY − Mascot − rejected
NA − spliced − |||
20120322_EXQ1_MiBa_SA_HCC1143_1_A: 22275
SA = 0.65
Score = 0.45
Prosit prediction
HPILNDPIY − MaxQuant
20120322_EXQ1_MiBa_SA_HCC1143_1_A: 22275
SA = 0.93
Score = 2.53
Prosit prediction
HPILNDPIY − MSFragger
20120322_EXQ1_MiBa_SA_HCC1143_1_A: 22275
SA = 0.93
Score = 2.53
Prosit prediction
ANLEATMSV − Mascot − rejected
ANLEA|TMSV − spliced − 2|0|0|0
20120518_EXQ0_MiBa_SA_HCT116_mHLA−2: 24975
SA = 0.65
Score = −0.22
Prosit prediction
AEINATMSV − MaxQuant
20120518_EXQ0_MiBa_SA_HCT116_mHLA−2: 24975
SA = 0.88
Score = 1.45
Prosit prediction
AEINATMSV − MSFragger
20120518_EXQ0_MiBa_SA_HCT116_mHLA−2: 24975
SA = 0.88
Score = 1.45
Prosit prediction
DVNMVLVNV − Mascot − rejected
NA − spliced − |||
20120515_EXQ3_MiBa_SA_HCT116_mHLA−1: 32756
SA = 0.65
Score = −0.36
Prosit prediction
VIWGTDVNV − MaxQuant
20120515_EXQ3_MiBa_SA_HCT116_mHLA−1: 32756
SA = 0.93
Score = 2.25
Prosit prediction
VIWGTDVNV − MSFragger
20120515_EXQ3_MiBa_SA_HCT116_mHLA−1: 32756
SA = 0.93
Score = 2.25
Prosit prediction
⋆ Exclusive in prediction
Exclusive in experimental
Relative intensity
m/z

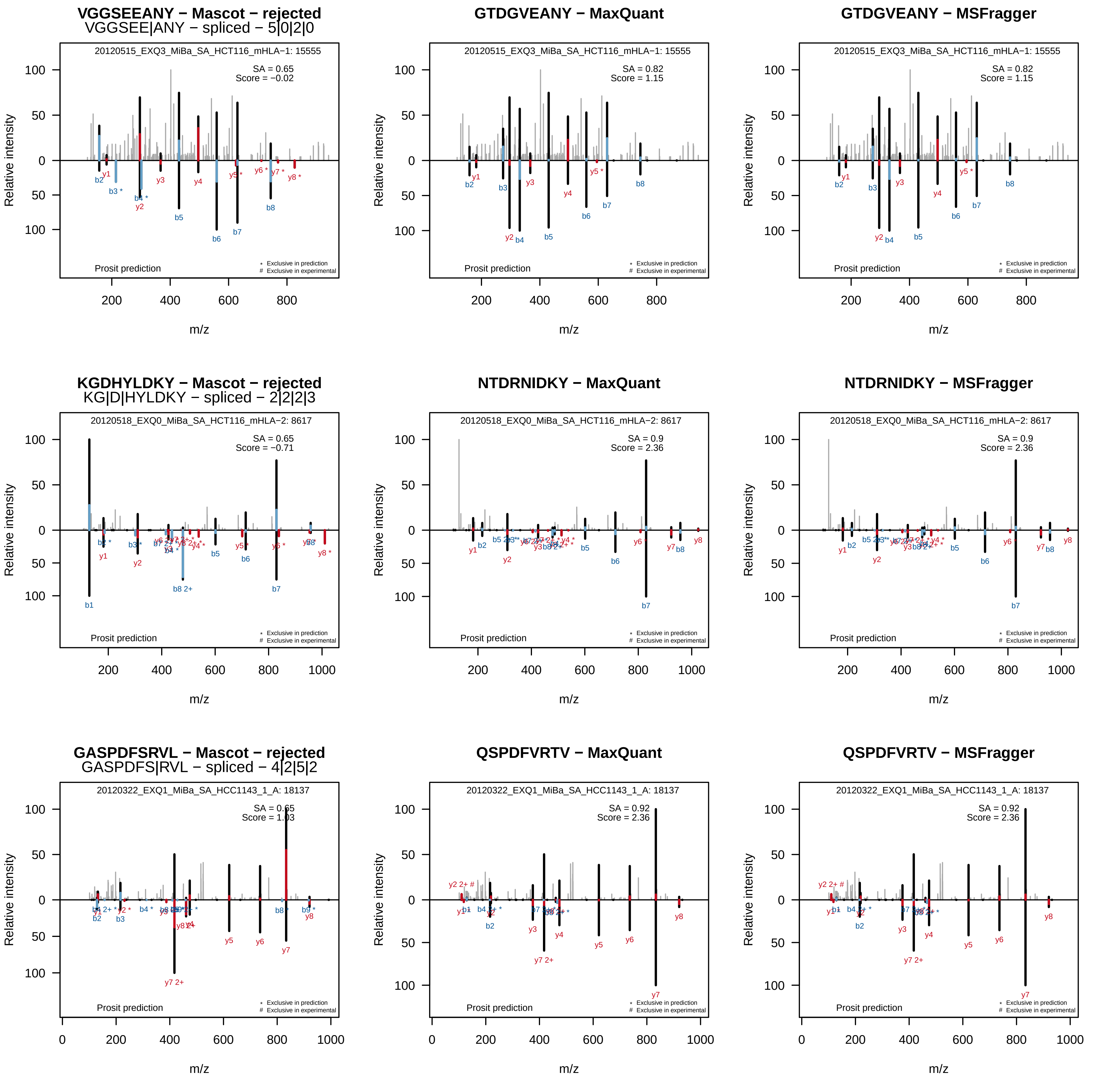
VGGSEEANY − Mascot − rejected
VGGSEE|ANY − spliced − 5|0|2|0
20120515_EXQ3_MiBa_SA_HCT116_mHLA−1: 15555
SA = 0.65
Score = −0.02
GTDGVEANY − MaxQuant
20120515_EXQ3_MiBa_SA_HCT116_mHLA−1: 15555
SA = 0.82
Score = 1.15
GTDGVEANY − MSFragger
20120515_EXQ3_MiBa_SA_HCT116_mHLA−1: 15555
SA = 0.82
Score = 1.15
KGDHYLDKY − Mascot − rejected
KG|D|HYLDKY − spliced − 2|2|2|3
20120518_EXQ0_MiBa_SA_HCT116_mHLA−2: 8617
SA = 0.65
Score = −0.71
NTDRNIDKY − MaxQuant
20120518_EXQ0_MiBa_SA_HCT116_mHLA−2: 8617
SA = 0.9
Score = 2.36
NTDRNIDKY − MSFragger
20120518_EXQ0_MiBa_SA_HCT116_mHLA−2: 8617
SA = 0.9
Score = 2.36
GASPDFSRVL − Mascot − rejected
GASPDFS|RVL − spliced − 4|2|5|2
20120322_EXQ1_MiBa_SA_HCC1143_1_A: 18137
SA = 0.65
Score = 1.03
QSPDFVRTV − MaxQuant
20120322_EXQ1_MiBa_SA_HCC1143_1_A: 18137
SA = 0.92
Score = 2.36
QSPDFVRTV − MSFragger
20120322_EXQ1_MiBa_SA_HCC1143_1_A: 18137
SA = 0.92
Score = 2.36
Relative intensity
m/z
Prosit prediction
* Exclusive in prediction
# Exclusive in experimental

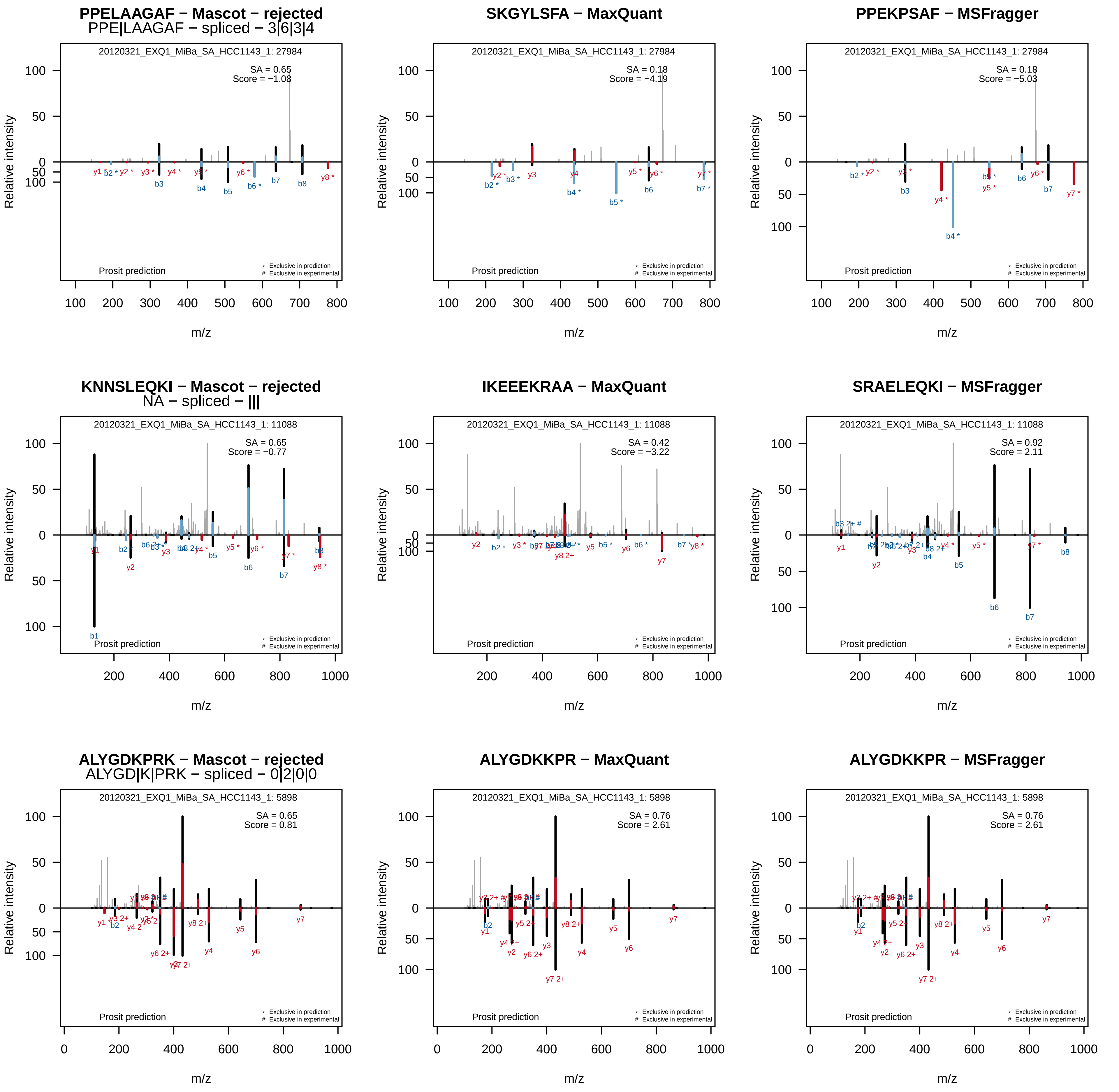
PPELAAGAF − Mascot − rejected
PPE|LAAGAF − spliced − 3|6|3|4
20120321_EXQ1_MiBa_SA_HCC1143_1: 27984
SA = 0.65
Score = −1.08
SKGYLSFA − MaxQuant
20120321_EXQ1_MiBa_SA_HCC1143_1: 27984
SA = 0.18
Score = −4.19
PPEKPSAF − MSFragger
20120321_EXQ1_MiBa_SA_HCC1143_1: 27984
SA = 0.18
Score = −5.03
KNNSLEQKI − Mascot − rejected
NA − spliced − |||
20120321_EXQ1_MiBa_SA_HCC1143_1: 11088
SA = 0.65
Score = −0.77
IKEEEKRAA − MaxQuant
20120321_EXQ1_MiBa_SA_HCC1143_1: 11088
SA = 0.42
Score = −3.22
SRAELEQKI − MSFragger
20120321_EXQ1_MiBa_SA_HCC1143_1: 11088
SA = 0.92
Score = 2.11
ALYGDKPRK − Mascot − rejected
ALYGD|K|PRK − spliced − 0|2|0|0
20120321_EXQ1_MiBa_SA_HCC1143_1: 5898
SA = 0.65
Score = 0.81
ALYGDKKPR − MaxQuant
20120321_EXQ1_MiBa_SA_HCC1143_1: 5898
SA = 0.76
Score = 2.61
ALYGDKKPR − MSFragger
20120321_EXQ1_MiBa_SA_HCC1143_1: 5898
SA = 0.76
Score = 2.61
Relative intensity
m/z
Prosit prediction
* Exclusive in prediction
# Exclusive in experimental

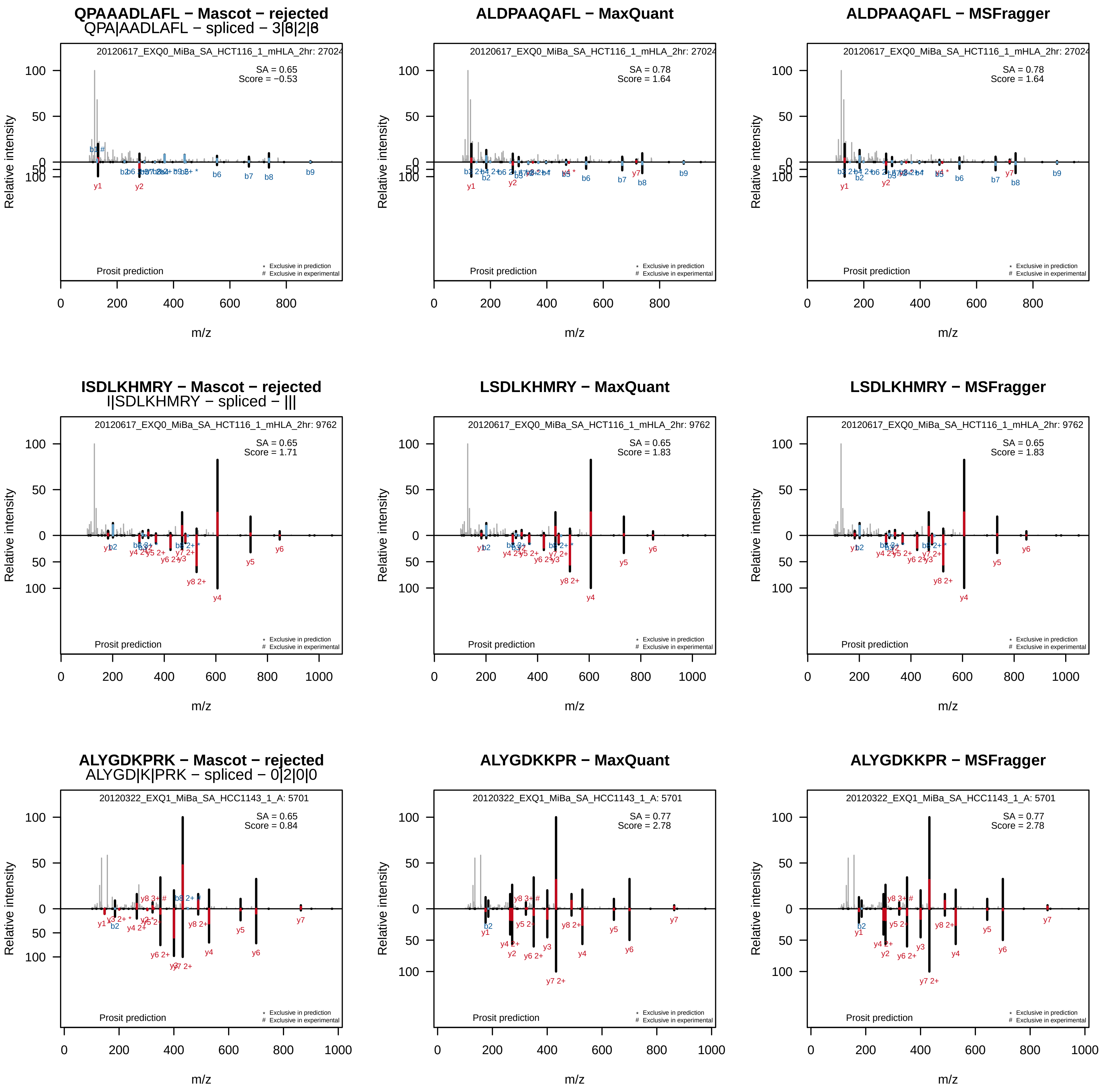

QPAAADLAFL − Mascot − rejected
QPA|AADLAFL − spliced − 3|3|2|3
20120617_EXQ0_MiBa_SA_HCT116_1_mHLA_2hr: 27024
SA = 0.65
Score = −0.53
ALDPAAQAFL − MaxQuant
20120617_EXQ0_MiBa_SA_HCT116_1_mHLA_2hr: 27024
SA = 0.78
Score = 1.64
ALDPAAQAFL − MSFragger
20120617_EXQ0_MiBa_SA_HCT116_1_mHLA_2hr: 27024
SA = 0.78
Score = 1.64
ISDLKHMRY − Mascot − rejected
I|SDLKHMRY − spliced − |||
20120617_EXQ0_MiBa_SA_HCT116_1_mHLA_2hr: 9762
SA = 0.65
Score = 1.71
LSDLKHMRY − MaxQuant
20120617_EXQ0_MiBa_SA_HCT116_1_mHLA_2hr: 9762
SA = 0.65
Score = 1.83
LSDLKHMRY − MSFragger
20120617_EXQ0_MiBa_SA_HCT116_1_mHLA_2hr: 9762
SA = 0.65
Score = 1.83
ALYGDKPRK − Mascot − rejected
ALYGD|K|PRK − spliced − 0|2|0|0
20120322_EXQ1_MiBa_SA_HCC1143_1_A: 5701
SA = 0.65
Score = 0.84
ALDYGDKKPR − MaxQuant
20120322_EXQ1_MiBa_SA_HCC1143_1_A: 5701
SA = 0.77
Score = 2.78
ALYGDKKPR − MSFragger
20120322_EXQ1_MiBa_SA_HCC1143_1_A: 5701
SA = 0.77
Score = 2.78
Relative intensity
m/z
Prosit prediction
⋆ Exclusive in prediction
Exclusive in experimental

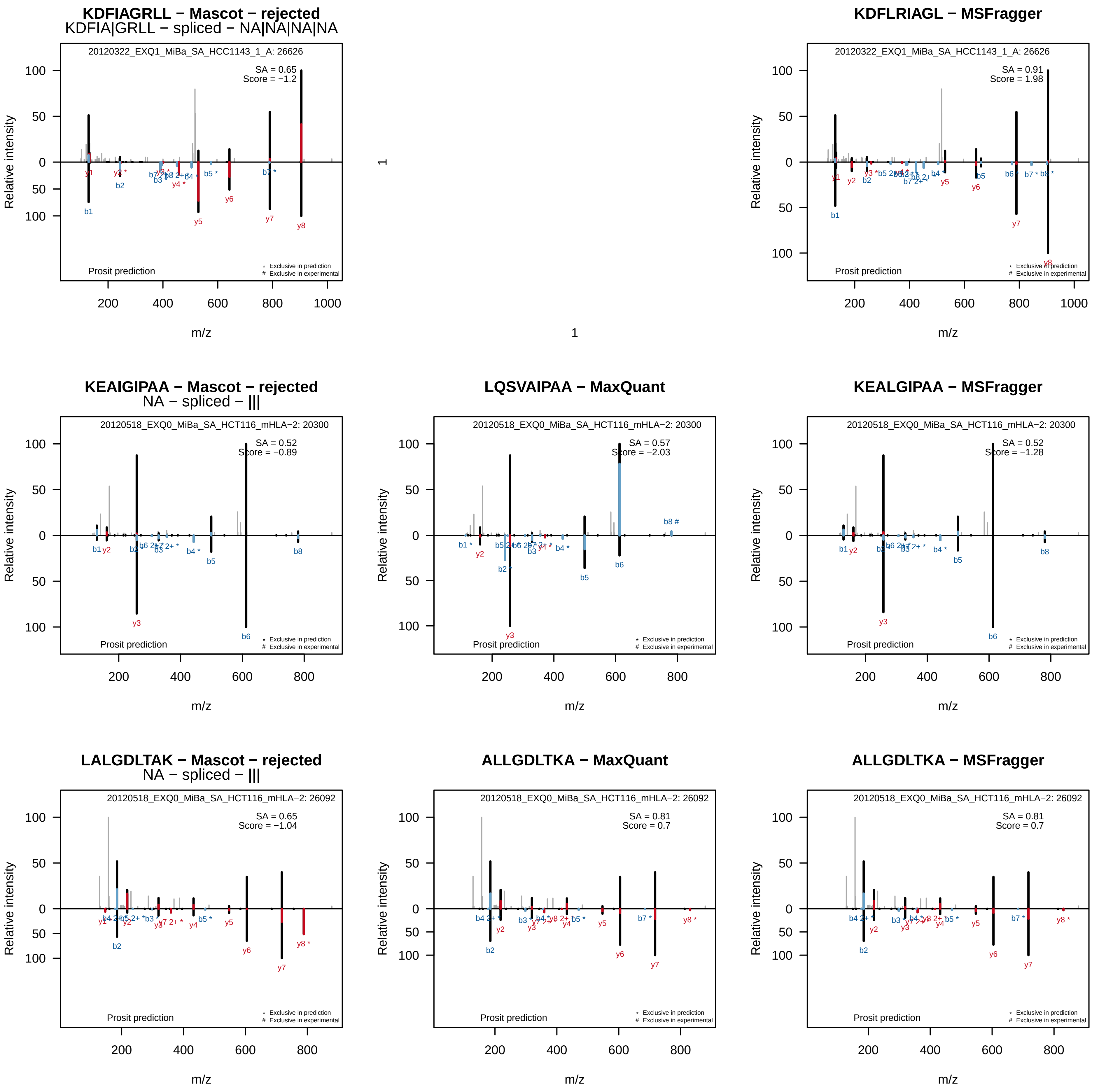
KDFIAGRLL − Mascot − rejected
KDFIA|GRLL − spliced − NA|NA|NA|NA
20120322_EXQ1_MiBa_SA_HCC1143_1_A: 26626
SA = 0.65
Score = −1.2
KDFLRIAGL − MSFragger
20120322_EXQ1_MiBa_SA_HCC1143_1_A: 26626
SA = 0.91
Score = 1.98
KEAIGIPAA − Mascot − rejected
NA − spliced − |||
20120518_EXQ0_MiBa_SA_HCT116_mHLA-2: 20300
SA = 0.52
Score = −0.89
LQSVAIPAA − MaxQuant
20120518_EXQ0_MiBa_SA_HCT116_mHLA-2: 20300
SA = 0.57
Score = −2.03
KEALGIPAA − MSFragger
20120518_EXQ0_MiBa_SA_HCT116_mHLA-2: 20300
SA = 0.52
Score = −1.28
LALGDLTAK − Mascot − rejected
NA − spliced − |||
20120518_EXQ0_MiBa_SA_HCT116_mHLA−2: 26092
SA = 0.65
Score = −1.04
ALLGDLTKA − MaxQuant
20120518_EXQ0_MiBa_SA_HCT116_mHLA−2: 26092
SA = 0.81
Score = 0.7
ALLGDLTKA − MSFragger
20120518_EXQ0_MiBa_SA_HCT116_mHLA−2: 26092
SA = 0.81
Score = 0.7
Relative intensity
m/z
Prosit prediction
* Exclusive in prediction
# Exclusive in experimental

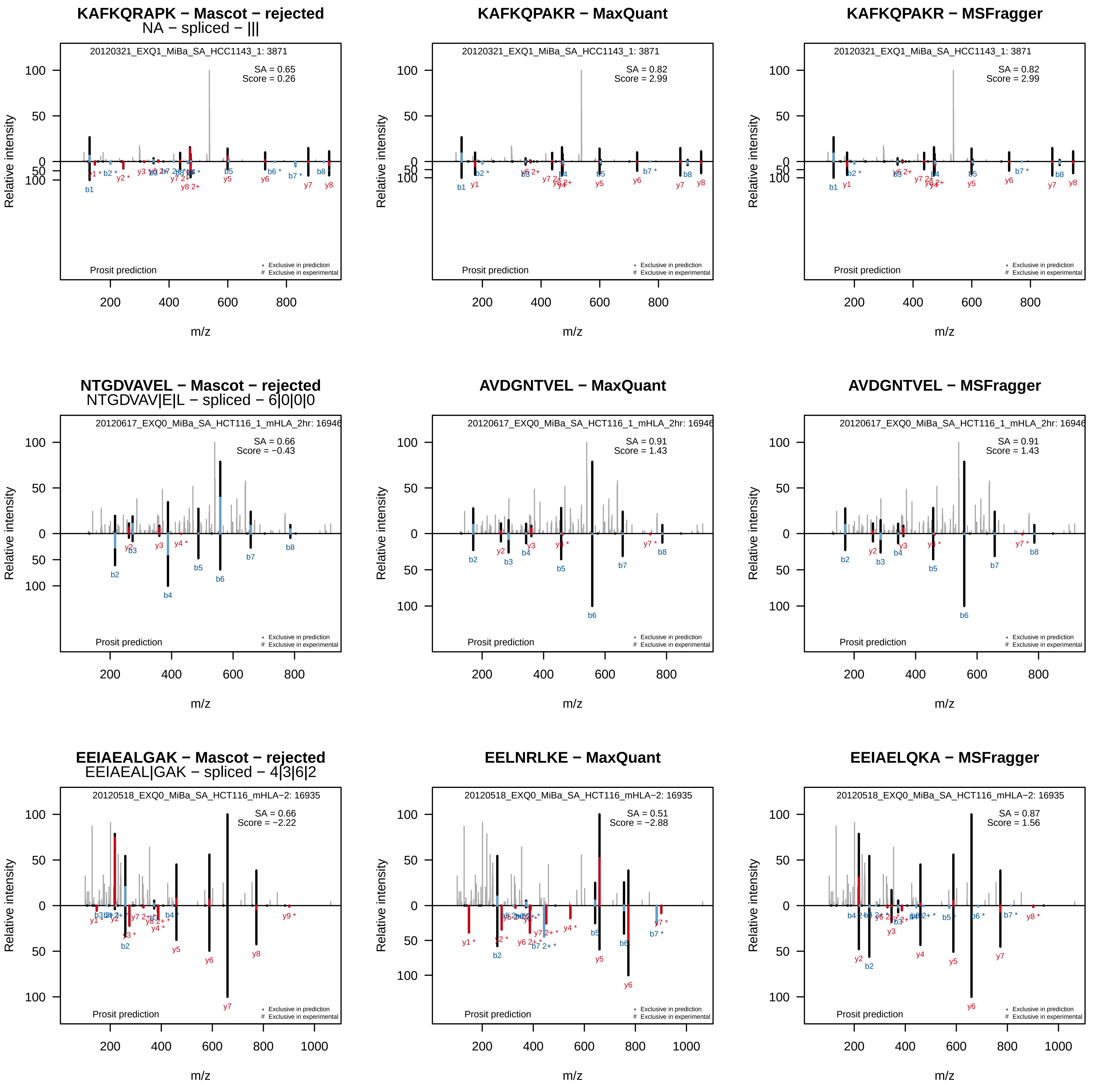

KAFKQRAPK − Mascot − rejected
NA − spliced − |||
20120321_EXQ1_MiBa_SA_HCC1143_1: 3871
SA = 0.65
Score = 0.26
KAFKQPAKR − MaxQuant
20120321_EXQ1_MiBa_SA_HCC1143_1: 3871
SA = 0.82
Score = 2.99
KAFKQPAKR − MSFragger
20120321_EXQ1_MiBa_SA_HCC1143_1: 3871
SA = 0.82
Score = 2.99
NTGDVAVEL − Mascot − rejected
NTGDVAV|E|L − spliced − 6|0|0|0
20120617_EXQ0_MiBa_SA_HCT116_1_mHLA_2hr: 16946
SA = 0.66
Score = −0.43
AVDGNTVEL − MaxQuant
20120617_EXQ0_MiBa_SA_HCT116_1_mHLA_2hr: 16946
SA = 0.91
Score = 1.43
AVDGNTVEL − MSFragger
20120617_EXQ0_MiBa_SA_HCT116_1_mHLA_2hr: 16946
SA = 0.91
Score = 1.43
EEIAEALGAK − Mascot − rejected
EEIAEAL|GAK − spliced − 4|3|6|2
20120518_EXQ0_MiBa_SA_HCT116_mHLA−2: 16935
SA = 0.66
Score = −2.22
EELNRLKE − MaxQuant
20120518_EXQ0_MiBa_SA_HCT116_mHLA−2: 16935
SA = 0.51
Score = −2.88
EEIAELQKA − MSFragger
20120518_EXQ0_MiBa_SA_HCT116_mHLA−2: 16935
SA = 0.87
Score = 1.56
Relative intensity
m/z
Prosit prediction
* Exclusive in prediction
# Exclusive in experimental

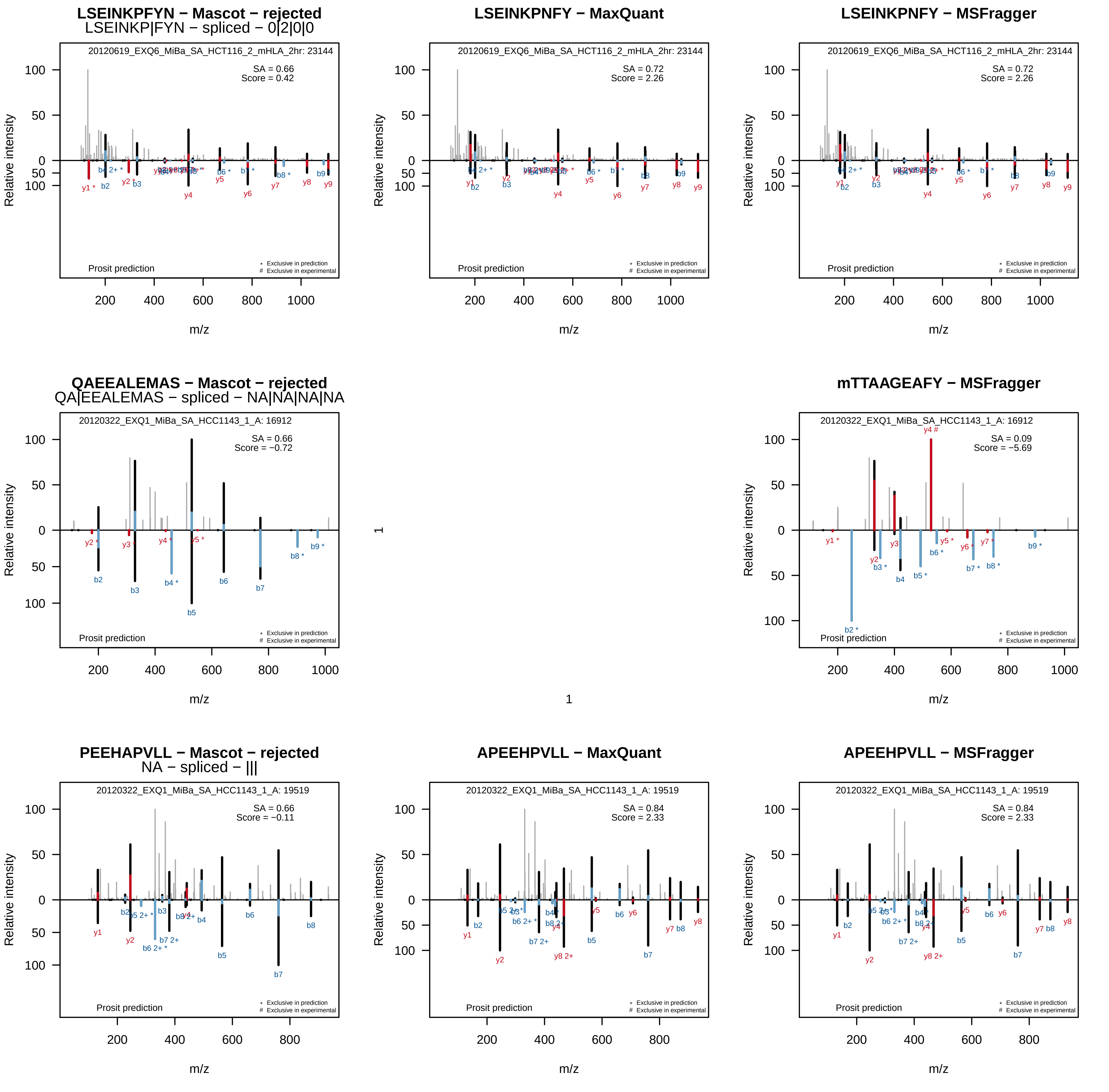

LSEINKPFYN − Mascot − rejected
LSEINKP|FYN − spliced − 0|2|0|0
20120619_EXQ6_MiBa_SA_HCT116_2_mHLA_2hr: 23144
SA = 0.66
Score = 0.42
LSEINKPNFY − MaxQuant
20120619_EXQ6_MiBa_SA_HCT116_2_mHLA_2hr: 23144
SA = 0.72
Score = 2.26
LSEINKPNFY − MSFragger
20120619_EXQ6_MiBa_SA_HCT116_2_mHLA_2hr: 23144
SA = 0.72
Score = 2.26
QAEEEALEMAS − Mascot − rejected
QA|EEEALEMAS − spliced − NA|NA|NA|NA
20120322_EXQ1_MiBa_SA_HCC1143_1_A: 16912
SA = 0.66
Score = −0.72
mTTAAGEAFY − MSFragger
20120322_EXQ1_MiBa_SA_HCC1143_1_A: 16912
SA = 0.09
Score = −5.69
PEEHAPVLL − Mascot − rejected
NA − spliced − |||
20120322_EXQ1_MiBa_SA_HCC1143_1_A: 19519
SA = 0.66
Score = −0.11
APEEHPVLL − MaxQuant
20120322_EXQ1_MiBa_SA_HCC1143_1_A: 19519
SA = 0.84
Score = 2.33
APEEHPVLL − MSFragger
20120322_EXQ1_MiBa_SA_HCC1143_1_A: 19519
SA = 0.84
Score = 2.33
Relative intensity
m/z
Prosit prediction
⋆ Exclusive in prediction
Exclusive in experimental

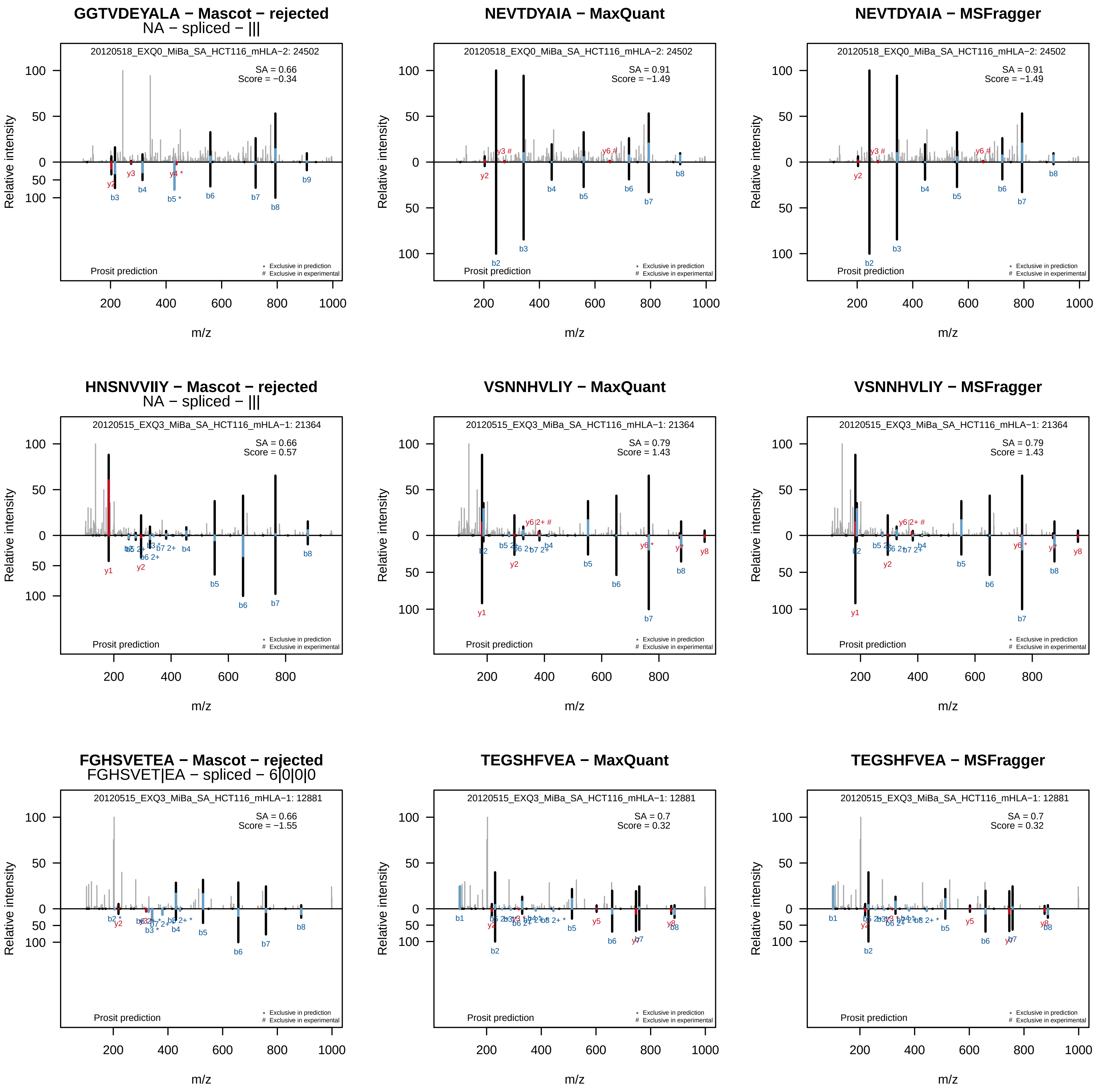
GGTVDEYALA − Mascot − rejected
NA − spliced − |||
20120518_EXQ0_MiBa_SA_HCT116_mHLA−2: 24502
SA = 0.66
Score = −0.34
NEVTDYAIA − MaxQuant
20120518_EXQ0_MiBa_SA_HCT116_mHLA−2: 24502
SA = 0.91
Score = −1.49
NEVTDYAIA − MSFragger
20120518_EXQ0_MiBa_SA_HCT116_mHLA−2: 24502
SA = 0.91
Score = −1.49
HNSNVVIIY − Mascot − rejected
NA − spliced − |||
20120515_EXQ3_MiBa_SA_HCT116_mHLA−1: 21364
SA = 0.66
Score = 0.57
VSNNHVLIY − MaxQuant
20120515_EXQ3_MiBa_SA_HCT116_mHLA−1: 21364
SA = 0.79
Score = 1.43
VSNNHVLIY − MSFragger
20120515_EXQ3_MiBa_SA_HCT116_mHLA−1: 21364
SA = 0.79
Score = 1.43
FGHSVETEA − Mascot − rejected
FGHSVET|EA − spliced − 6|0|0|0
20120515_EXQ3_MiBa_SA_HCT116_mHLA−1: 12881
SA = 0.66
Score = −1.55
TEGSHFVEA − MaxQuant
20120515_EXQ3_MiBa_SA_HCT116_mHLA−1: 12881
SA = 0.7
Score = 0.32
TEGSHFVEA − MSFragger
20120515_EXQ3_MiBa_SA_HCT116_mHLA−1: 12881
SA = 0.7
Score = 0.32
Relative intensity
m/z
Prosit prediction
⋆ Exclusive in prediction
Exclusive in experimental

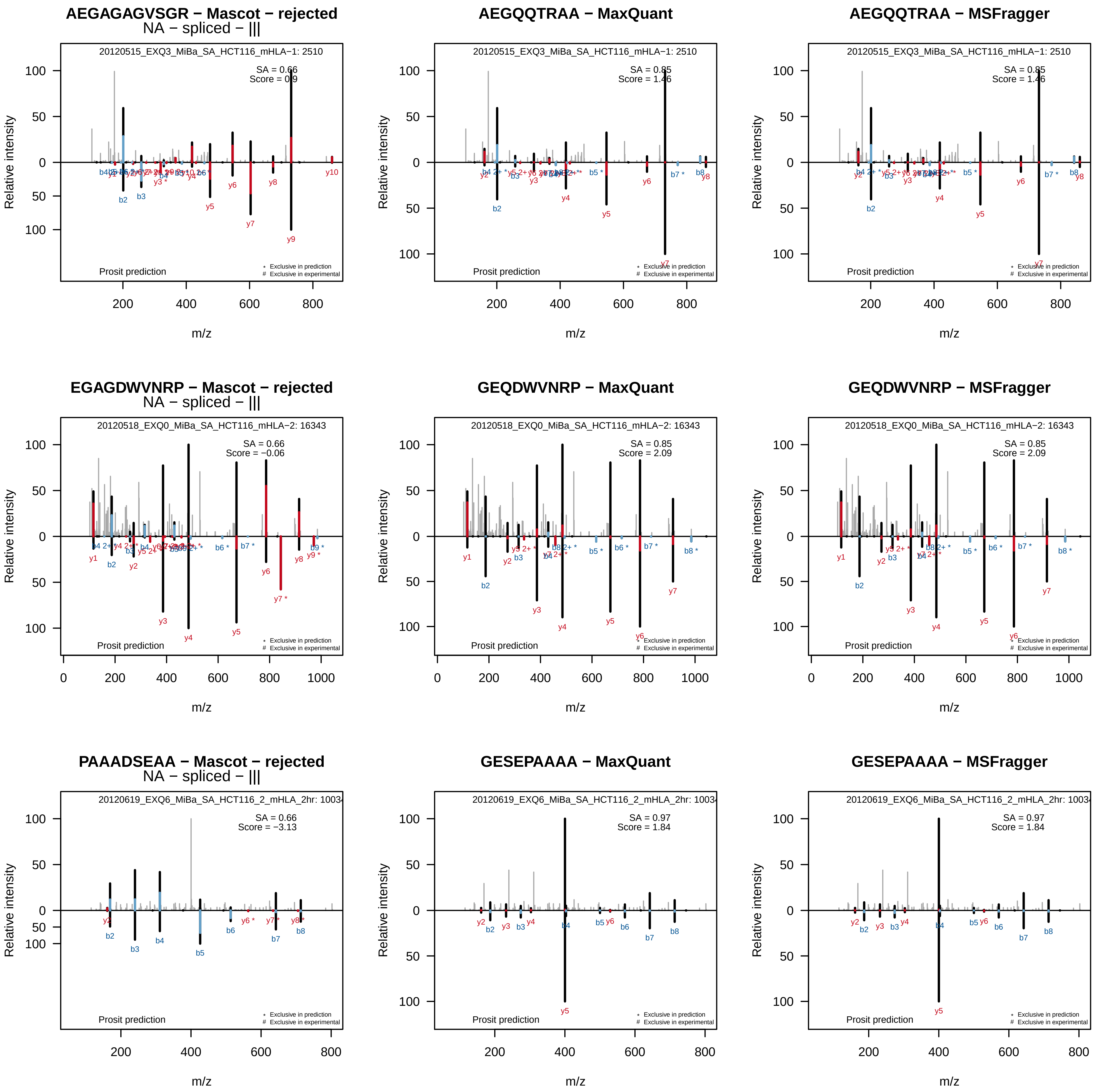
AEGAGAGVSGR − Mascot − rejected
NA − spliced − |||
20120515_EXQ3_MiBa_SA_HCT116_mHLA−1: 2510
SA = 0.66
Score = 0.9
AEGQQTRAA − MaxQuant
20120515_EXQ3_MiBa_SA_HCT116_mHLA−1: 2510
SA = 0.85
Score = 1.46
AEGQQTRAA − MSFragger
20120515_EXQ3_MiBa_SA_HCT116_mHLA−1: 2510
SA = 0.85
Score = 1.46
EGAGDWVNRP − Mascot − rejected
NA − spliced − |||
20120518_EXQ0_MiBa_SA_HCT116_mHLA−2: 16343
SA = 0.66
Score = −0.06
GEQDWVNRP − MaxQuant
20120518_EXQ0_MiBa_SA_HCT116_mHLA−2: 16343
SA = 0.85
Score = 2.09
GEQDWVNRP − MSFragger
20120518_EXQ0_MiBa_SA_HCT116_mHLA−2: 16343
SA = 0.85
Score = 2.09
PAAADSEAA − Mascot − rejected
NA − spliced − |||
20120619_EXQ6_MiBa_SA_HCT116_2_mHLA_2hr: 1003
SA = 0.66
Score = −3.13
GESEPAAAA − MaxQuant
20120619_EXQ6_MiBa_SA_HCT116_2_mHLA_2hr: 1003
SA = 0.97
Score = 1.84
GESEPAAAA − MSFragger
20120619_EXQ6_MiBa_SA_HCT116_2_mHLA_2hr: 1003
SA = 0.97
Score = 1.84
Relative intensity
m/z
Prosit prediction
* Exclusive in prediction
# Exclusive in experimental

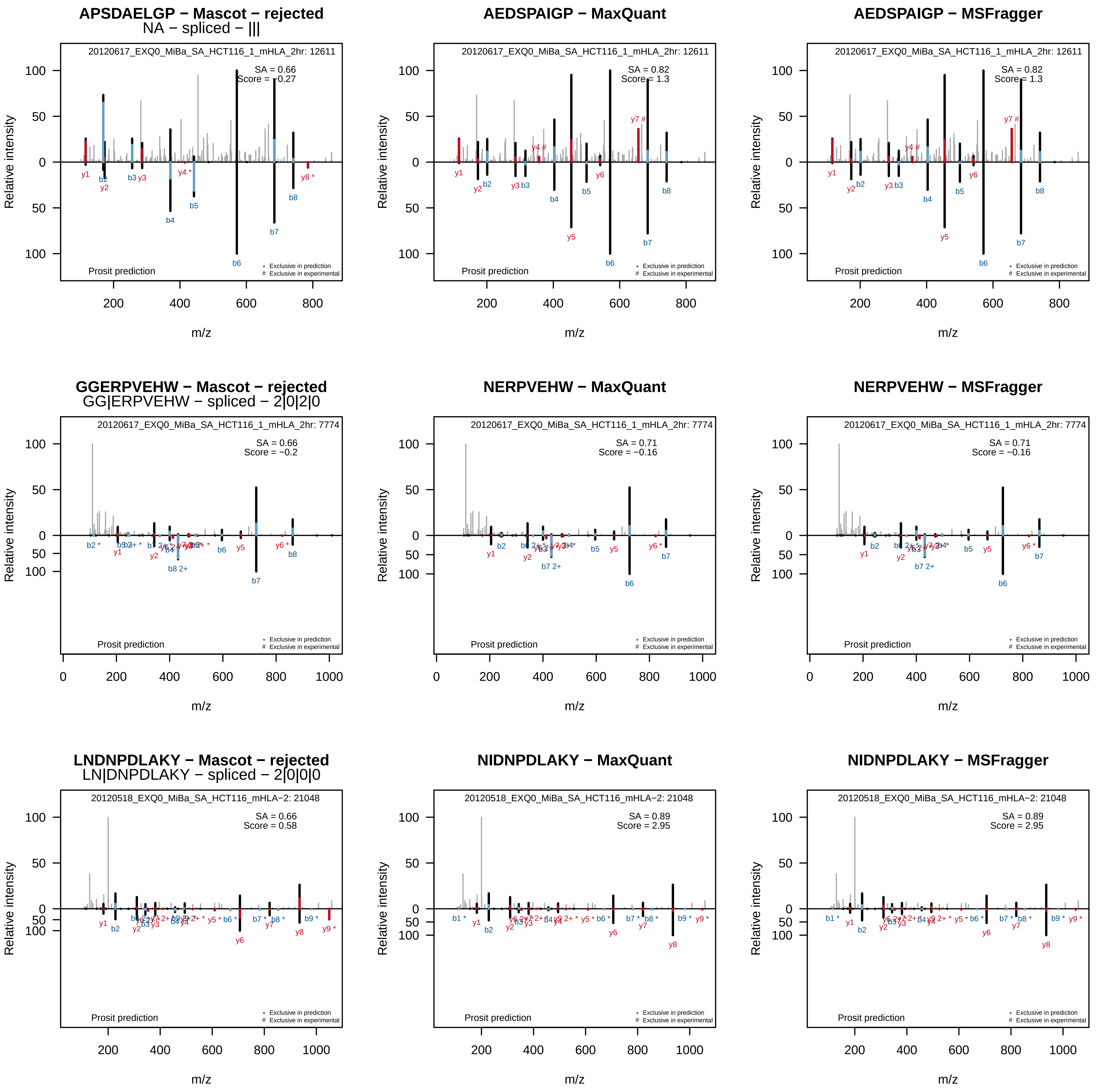

APSDAELGP − Mascot − rejected
NA − spliced − |||
20120617_EXQ0_MiBa_SA_HCT116_1_mHLA_2hr: 12611
SA = 0.66
Score = −0.27
AEDSPAIGP − MaxQuant
20120617_EXQ0_MiBa_SA_HCT116_1_mHLA_2hr: 12611
SA = 0.82
Score = 1.3
AEDSPAIGP − MSFragger
20120617_EXQ0_MiBa_SA_HCT116_1_mHLA_2hr: 12611
SA = 0.82
Score = 1.3
GGERPVEHW − Mascot − rejected
GG|ERPVEHW − spliced − 2|0|2|0
20120617_EXQ0_MiBa_SA_HCT116_1_mHLA_2hr: 7774
SA = 0.66
Score = −0.2
NERPVEHW − MaxQuant
20120617_EXQ0_MiBa_SA_HCT116_1_mHLA_2hr: 7774
SA = 0.71
Score = −0.16
NERPVEHW − MSFragger
20120617_EXQ0_MiBa_SA_HCT116_1_mHLA_2hr: 7774
SA = 0.71
Score = −0.16
LNDNPDLAKY − Mascot − rejected
LN|DNPDLAKY − spliced − 2|0|0|0
20120518_EXQ0_MiBa_SA_HCT116_mHLA−2: 21048
SA = 0.66
Score = 0.58
NIDNPDLAKY − MaxQuant
20120518_EXQ0_MiBa_SA_HCT116_mHLA−2: 21048
SA = 0.89
Score = 2.95
NIDNPDLAKY − MSFragger
20120518_EXQ0_MiBa_SA_HCT116_mHLA−2: 21048
SA = 0.89
Score = 2.95
Relative intensity
m/z
Prosit prediction
⋆ Exclusive in prediction
Exclusive in experimental

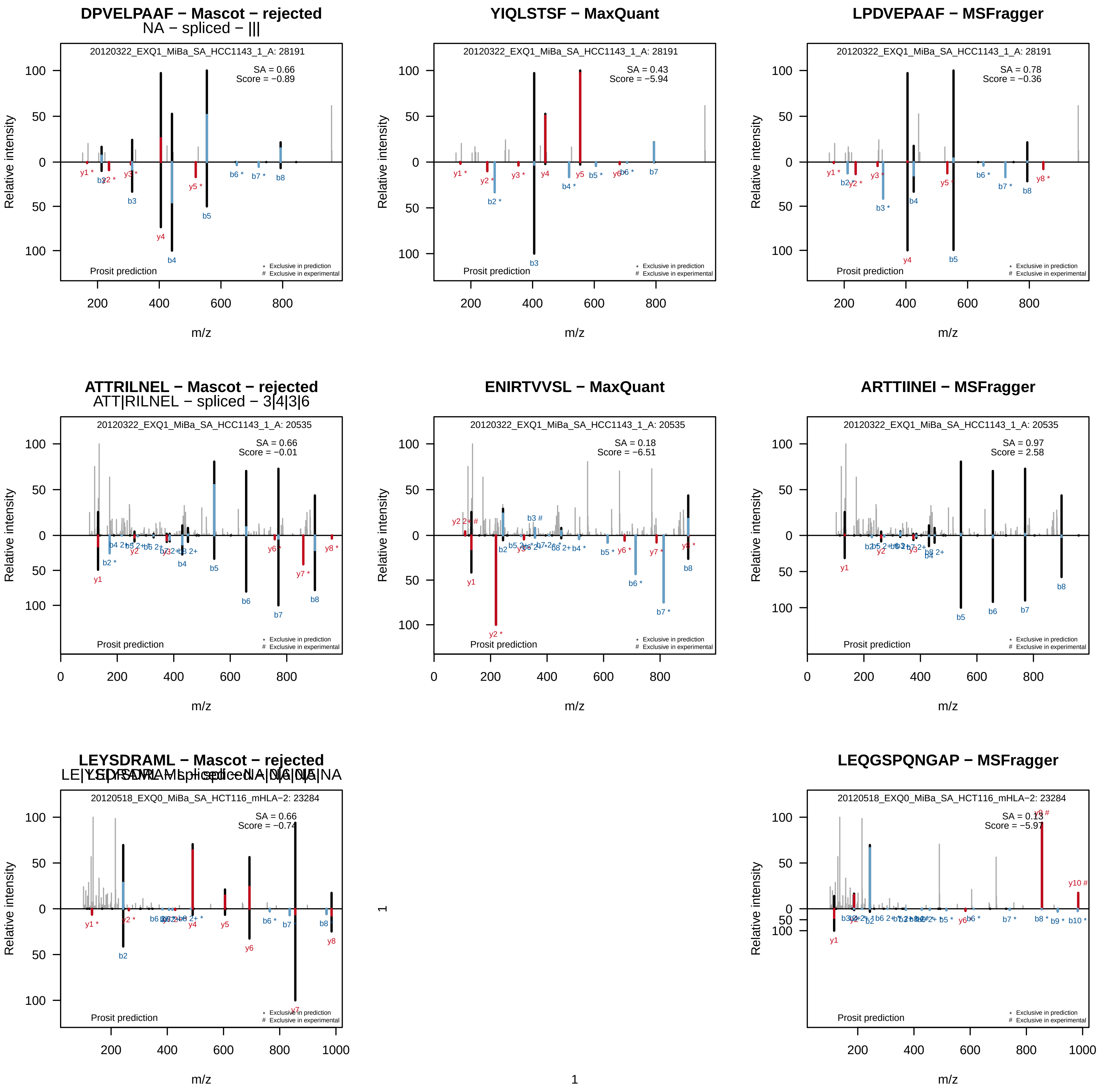

DPVELPAAF − Mascot − rejected
NA − spliced − |||
20120322_EXQ1_MiBa_SA_HCC1143_1_A: 28191
SA = 0.66
Score = −0.89
YIQLSTSF − MaxQuant
20120322_EXQ1_MiBa_SA_HCC1143_1_A: 28191
SA = 0.43
Score = −5.94
LPDVEPAAF − MSFragger
20120322_EXQ1_MiBa_SA_HCC1143_1_A: 28191
SA = 0.78
Score = −0.36
ATTRILNEL − Mascot − rejected
ATT|RILNEL − spliced − 3|4|3|6
20120322_EXQ1_MiBa_SA_HCC1143_1_A: 20535
SA = 0.66
Score = −0.01
ENIRTVVSL − MaxQuant
20120322_EXQ1_MiBa_SA_HCC1143_1_A: 20535
SA = 0.18
Score = −6.51
ARTTIINEI − MSFragger
20120322_EXQ1_MiBa_SA_HCC1143_1_A: 20535
SA = 0.97
Score = 2.58
LEYSDRAML − Mascot − rejected
20120518_EXQ0_MiBa_SA_HCT116_mHLA−2: 23284
SA = 0.66
LEQGSPQNGAP − MSFragger
20120518_EXQ0_MiBa_SA_HCT116_mHLA−2: 23284
SA = 0.13
Score = −5.97
Relative intensity
m/z
Prosit prediction
⋆ Exclusive in prediction
Exclusive in experimental

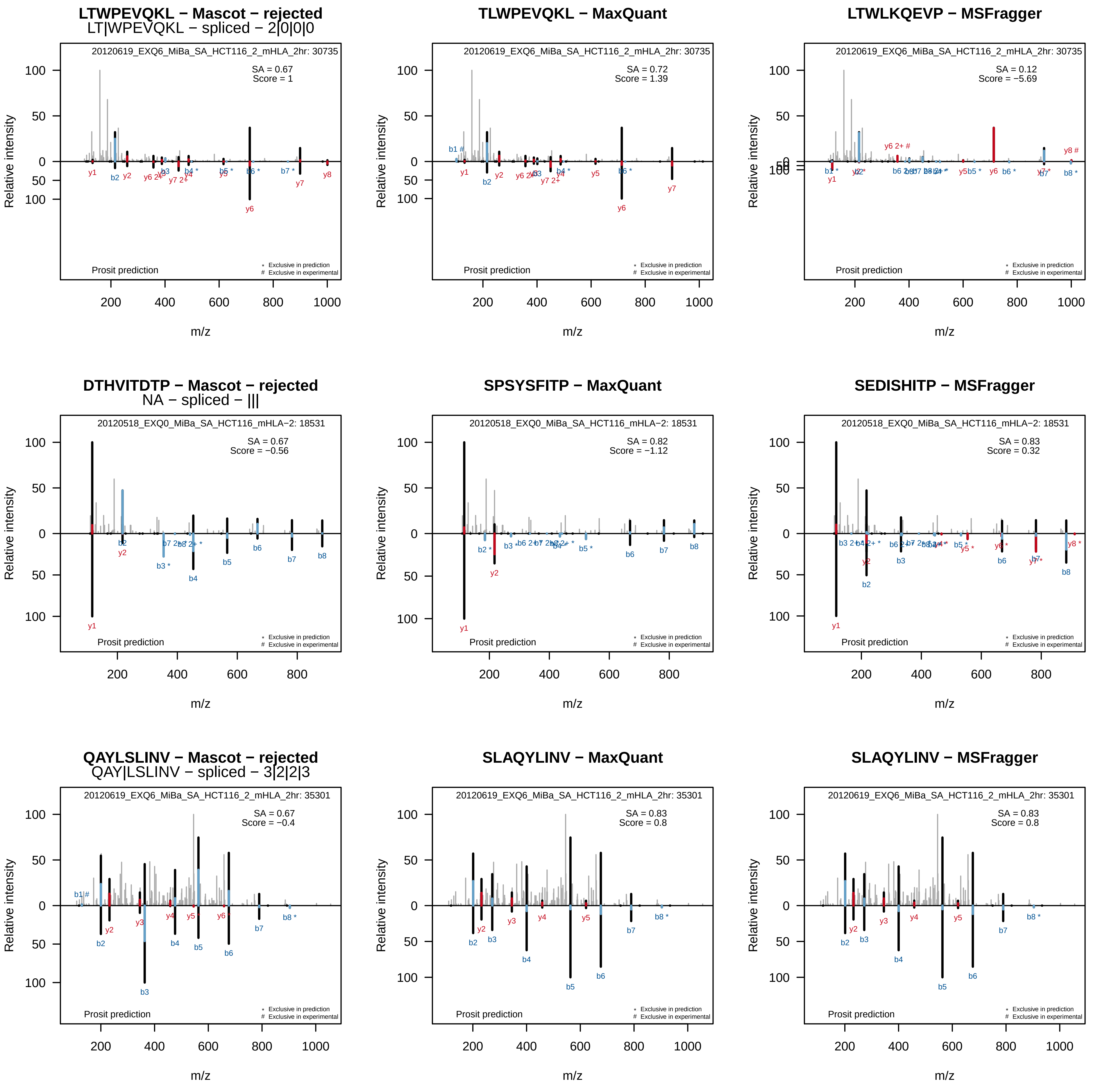
LTWPEVQKL − Mascot − rejected
LT|WPEVQKL − spliced − 2|0|0|0
20120619_EXQ6_MiBa_SA_HCT116_2_mHLA_2hr: 30735
SA = 0.67
Score = 1
TLWPEVQKL − MaxQuant
20120619_EXQ6_MiBa_SA_HCT116_2_mHLA_2hr: 30735
SA = 0.72
Score = 1.39
LTWLKQEVP − MSFragger
20120619_EXQ6_MiBa_SA_HCT116_2_mHLA_2hr: 30735
SA = 0.12
Score = −5.69
DTHVITDTP − Mascot − rejected
NA − spliced − |||
20120518_EXQ0_MiBa_SA_HCT116_mHLA−2: 18531
SA = 0.67
Score = −0.56
SPSYSFITP − MaxQuant
20120518_EXQ0_MiBa_SA_HCT116_mHLA−2: 18531
SA = 0.82
Score = −1.12
SEDISHITP − MSFragger
20120518_EXQ0_MiBa_SA_HCT116_mHLA−2: 18531
SA = 0.83
Score = 0.32
QAYLSLINV − Mascot − rejected
QAY|LSLINV − spliced − 3|2|2|3
20120619_EXQ6_MiBa_SA_HCT116_2_mHLA_2hr: 35301
SA = 0.67
Score = −0.4
SLAQYLINV − MaxQuant
20120619_EXQ6_MiBa_SA_HCT116_2_mHLA_2hr: 35301
SA = 0.83
Score = 0.8
SLAQYLINV − MSFragger
20120619_EXQ6_MiBa_SA_HCT116_2_mHLA_2hr: 35301
SA = 0.83
Score = 0.8
Relative intensity
m/z
Prosit prediction
* Exclusive in prediction
# Exclusive in experimental

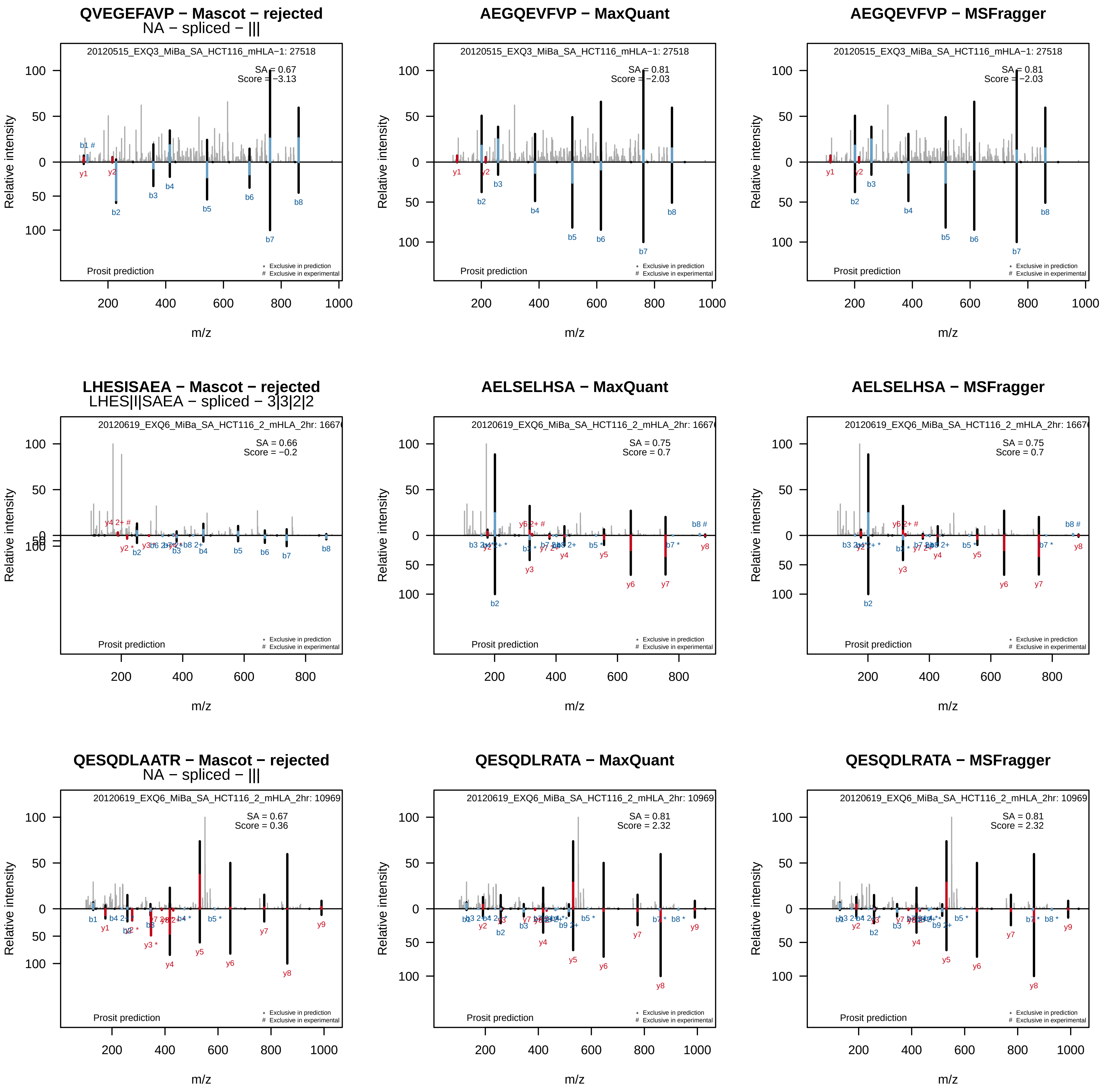

QVEGEFAVP − Mascot − rejected
NA − spliced − |||
20120515_EXQ3_MiBa_SA_HCT116_mHLA−1: 27518
SA = 0.67
Score = −3.13
AEGQEVFVP − MaxQuant
20120515_EXQ3_MiBa_SA_HCT116_mHLA−1: 27518
SA = 0.81
Score = −2.03
AEGQEVFVP − MSFragger
20120515_EXQ3_MiBa_SA_HCT116_mHLA−1: 27518
SA = 0.81
Score = −2.03
LHESISAEA − Mascot − rejected
LHES|I|SAEA − spliced − 3|3|2|2
20120619_EXQ6_MiBa_SA_HCT116_2_mHLA_2hr: 1667
SA = 0.66
Score = −0.2
AELSELHSA − MaxQuant
20120619_EXQ6_MiBa_SA_HCT116_2_mHLA_2hr: 1667
SA = 0.75
Score = 0.7
AELSELHSA − MSFragger
20120619_EXQ6_MiBa_SA_HCT116_2_mHLA_2hr: 1667
SA = 0.75
Score = 0.7
QESQDLAATR − Mascot − rejected
NA − spliced − |||
20120619_EXQ6_MiBa_SA_HCT116_2_mHLA_2hr: 10969
SA = 0.67
Score = 0.36
QESQDLRATA − MaxQuant
20120619_EXQ6_MiBa_SA_HCT116_2_mHLA_2hr: 10969
SA = 0.81
Score = 2.32
QESQDLRATA − MSFragger
20120619_EXQ6_MiBa_SA_HCT116_2_mHLA_2hr: 10969
SA = 0.81
Score = 2.32
Relative intensity
m/z
Prosit prediction
* Exclusive in prediction
# Exclusive in experimental

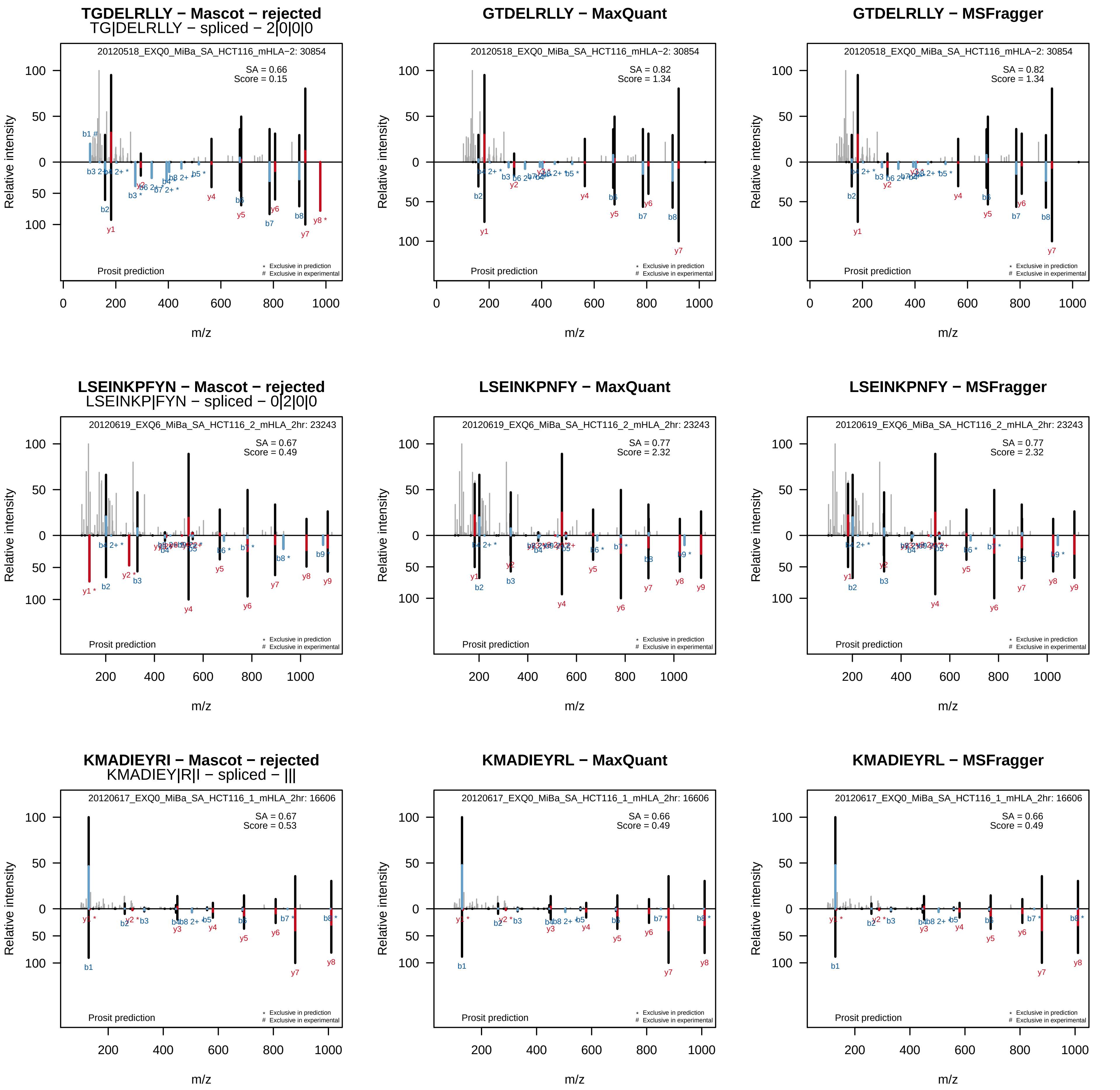
TGDELRLLY − Mascot − rejected
TG|DELRLLY − spliced − 2|0|0|0
20120518_EXQ0_MiBa_SA_HCT116_mHLA−2: 30854
SA = 0.66
Score = 0.15
GTDELRLLY − MaxQuant
20120518_EXQ0_MiBa_SA_HCT116_mHLA−2: 30854
SA = 0.82
Score = 1.34
GTDELRLLY − MSFragger
20120518_EXQ0_MiBa_SA_HCT116_mHLA−2: 30854
SA = 0.82
Score = 1.34
LSEINKPFYN − Mascot − rejected
LSEINKP|FYN − spliced − 0|2|0|0
20120619_EXQ6_MiBa_SA_HCT116_2_mHLA_2hr: 23243
SA = 0.67
Score = 0.49
LSEINKPNFY − MaxQuant
20120619_EXQ6_MiBa_SA_HCT116_2_mHLA_2hr: 23243
SA = 0.77
Score = 2.32
LSEINKPNFY − MSFragger
20120619_EXQ6_MiBa_SA_HCT116_2_mHLA_2hr: 23243
SA = 0.77
Score = 2.32
KMADIEYRI − Mascot − rejected
KMADIEY|R|I − spliced − |||
20120617_EXQ0_MiBa_SA_HCT116_1_mHLA_2hr: 16606
SA = 0.67
Score = 0.53
KMADIEYRL − MaxQuant
20120617_EXQ0_MiBa_SA_HCT116_1_mHLA_2hr: 16606
SA = 0.66
Score = 0.49
KMADIEYRL − MSFragger
20120617_EXQ0_MiBa_SA_HCT116_1_mHLA_2hr: 16606
SA = 0.66
Score = 0.49
Relative intensity
m/z
Prosit prediction
* Exclusive in prediction
# Exclusive in experimental

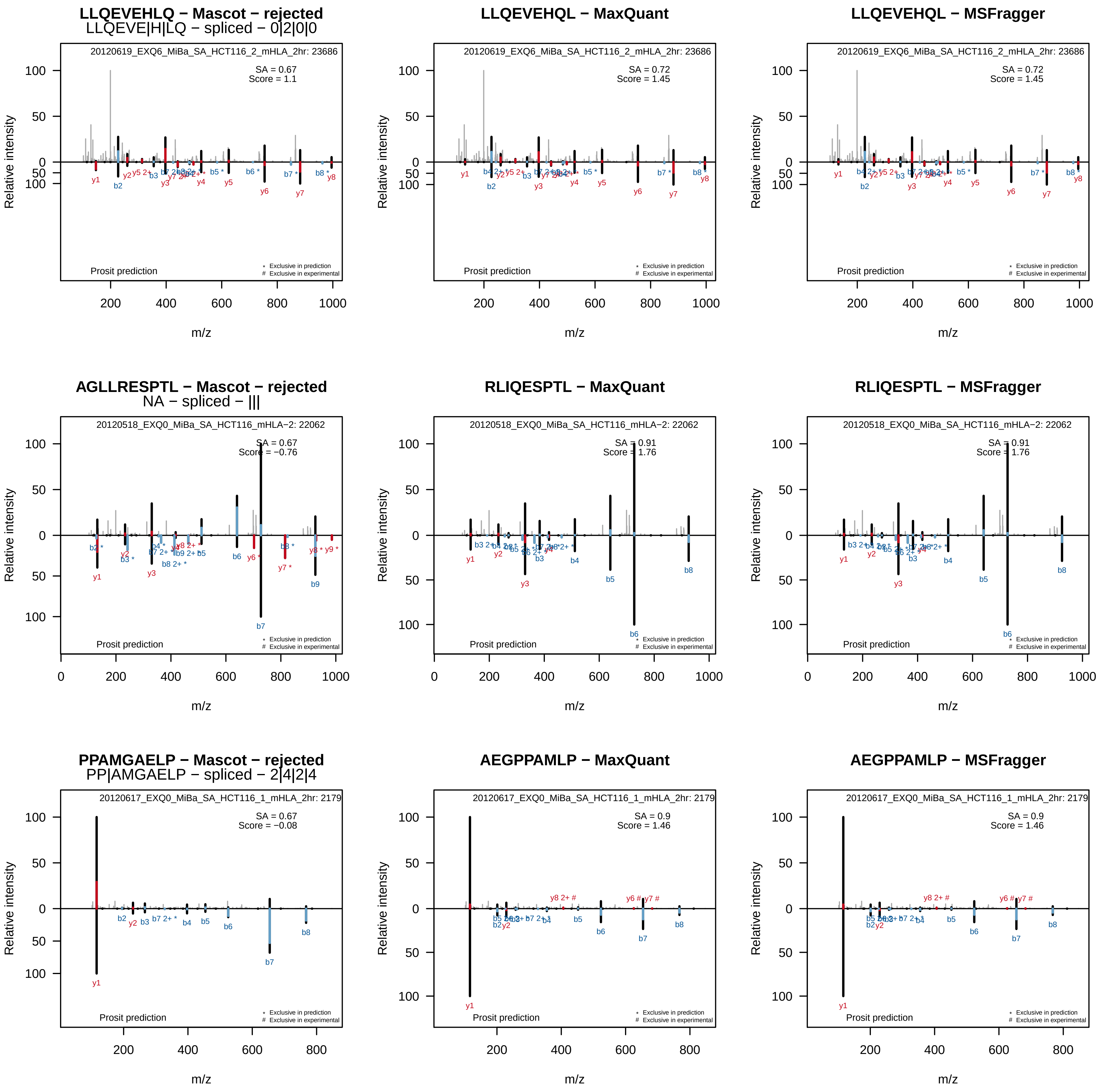

LLQEVEHLQ − Mascot − rejected
LLQEVE|H|LQ − spliced − 0|2|0|0
20120619_EXQ6_MiBa_SA_HCT116_2_mHLA_2hr: 23686
SA = 0.67
Score = 1.1
LLQEVEHQL − MaxQuant
20120619_EXQ6_MiBa_SA_HCT116_2_mHLA_2hr: 23686
SA = 0.72
Score = 1.45
LLQEVEHQL − MSFragger
20120619_EXQ6_MiBa_SA_HCT116_2_mHLA_2hr: 23686
SA = 0.72
Score = 1.45
AGLLRESPTL − Mascot − rejected
NA − spliced − |||
20120518_EXQ0_MiBa_SA_HCT116_mHLA−2: 22062
SA = 0.67
Score = −0.76
RLIQESPTL − MaxQuant
20120518_EXQ0_MiBa_SA_HCT116_mHLA−2: 22062
SA = 0.91
Score = 1.76
RLIQESPTL − MSFragger
20120518_EXQ0_MiBa_SA_HCT116_mHLA−2: 22062
SA = 0.91
Score = 1.76
PPAMGAELP − Mascot − rejected
PP|AMGAELP − spliced − 2|4|2|4
20120617_EXQ0_MiBa_SA_HCT116_1_mHLA_2hr: 2179
SA = 0.67
Score = −0.08
AEGPPAMLP − MaxQuant
20120617_EXQ0_MiBa_SA_HCT116_1_mHLA_2hr: 2179
SA = 0.9
Score = 1.46
AEGPPAMLP − MSFragger
20120617_EXQ0_MiBa_SA_HCT116_1_mHLA_2hr: 2179
SA = 0.9
Score = 1.46
Relative intensity
m/z
Prosit prediction
* Exclusive in prediction
# Exclusive in experimental

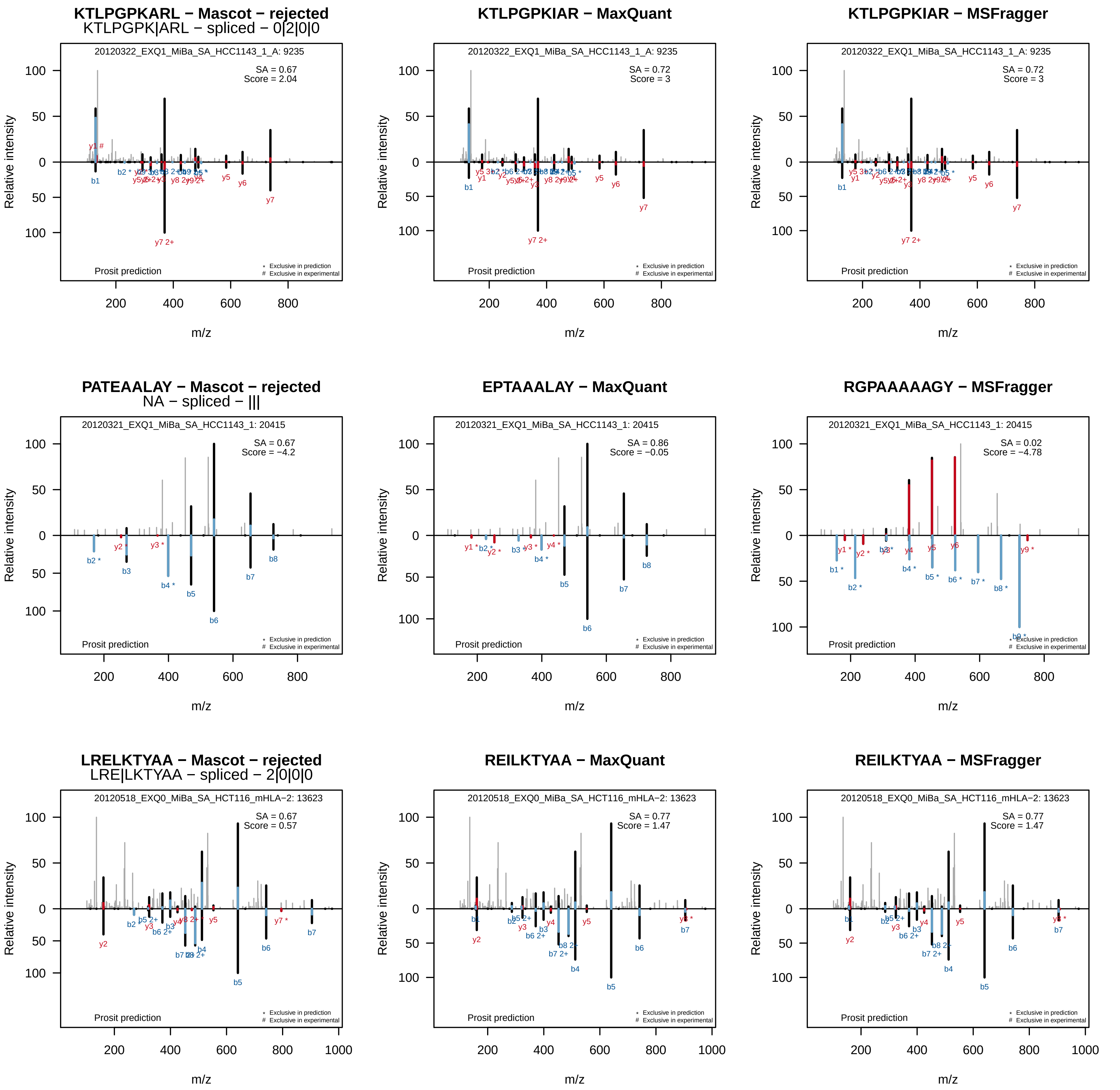
KTLPGPKARL − Mascot − rejected
KTLPGPK|ARL − spliced − 0|2|0|0
20120322_EXQ1_MiBa_SA_HCC1143_1_A: 9235
SA = 0.67
Score = 2.04
KTLPGPKIAR − MaxQuant
20120322_EXQ1_MiBa_SA_HCC1143_1_A: 9235
SA = 0.72
Score = 3
KTLPGPKIAR − MSFragger
20120322_EXQ1_MiBa_SA_HCC1143_1_A: 9235
SA = 0.72
Score = 3
PATEAALAY − Mascot − rejected
NA − spliced − |||
20120321_EXQ1_MiBa_SA_HCC1143_1: 20415
SA = 0.67
Score = −4.2
EPTAAALAY − MaxQuant
20120321_EXQ1_MiBa_SA_HCC1143_1: 20415
SA = 0.86
Score = −0.05
RGPAAAAAGY − MSFragger
20120321_EXQ1_MiBa_SA_HCC1143_1: 20415
SA = 0.02
Score = −4.78
LRELKTYAA − Mascot − rejected
LRE|LKTYAA − spliced − 2|0|0|0
20120518_EXQ0_MiBa_SA_HCT116_mHLA−2: 13623
SA = 0.67
Score = 0.57
REILKTYAA − MaxQuant
20120518_EXQ0_MiBa_SA_HCT116_mHLA−2: 13623
SA = 0.77
Score = 1.47
REILKTYAA − MSFragger
20120518_EXQ0_MiBa_SA_HCT116_mHLA−2: 13623
SA = 0.77
Score = 1.47
Relative intensity
m/z
Prosit prediction
⋆ Exclusive in prediction
Exclusive in experimental
y1 #
b1
y7 2+
y5
y6
y7
b2 *
b3
b4 *
b5
b6
b7
b8
y2 *
y3 *
y1 *
y4 *
b1 *
b2 *
b5 *
b6 *
b7 *
b8 *
b9 *
y9 *
y2
y3
y4
y5
y6
b6 2+
b7 2+
b8 2+
y7 *

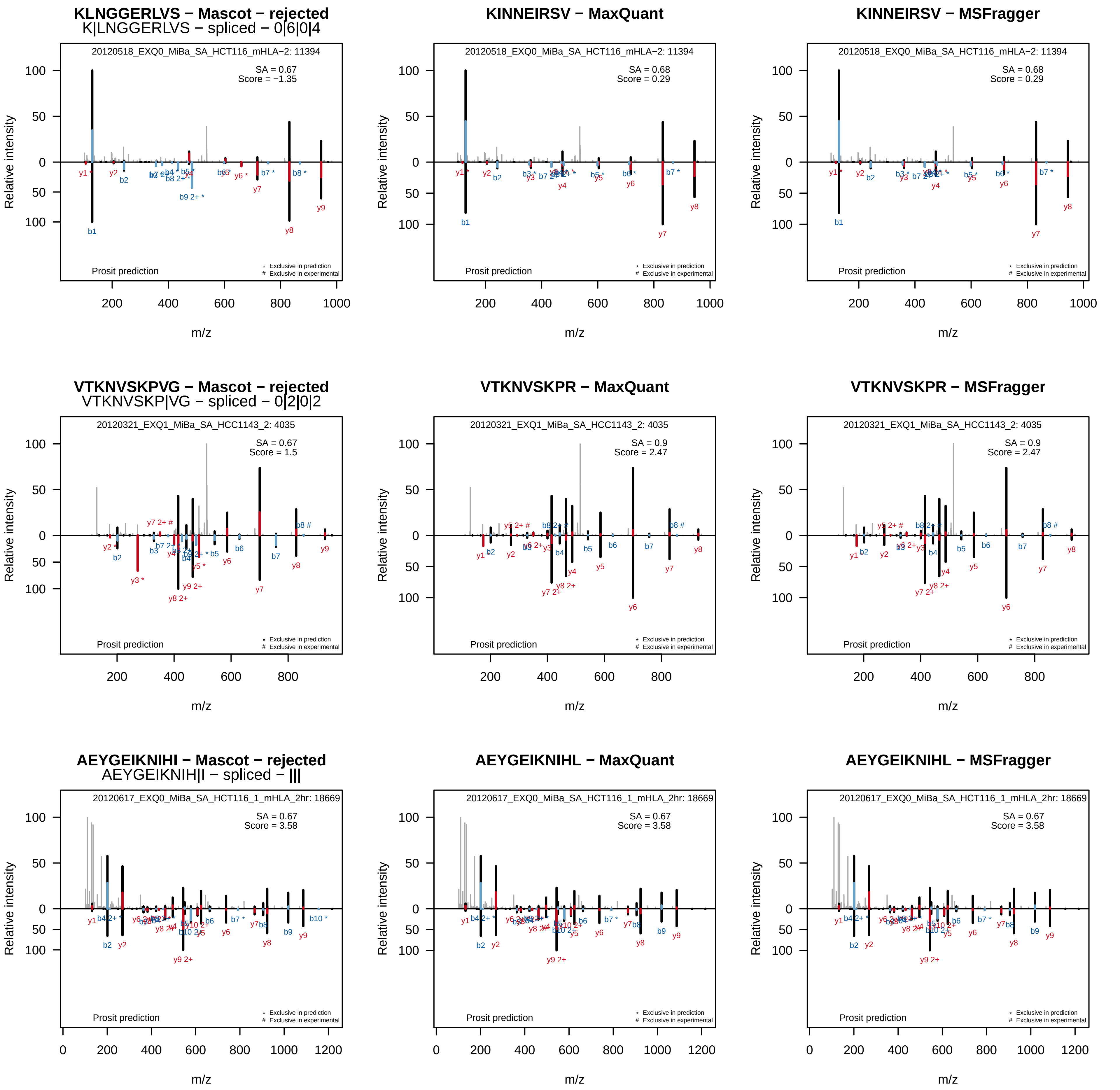
KLNGGERLVS − Mascot − rejected
K|LNGGERLVS − spliced − 0|6|0|4
20120518_EXQ0_MiBa_SA_HCT116_mHLA−2: 11394
SA = 0.67
Score = −1.35
KINNEIRSV − MaxQuant
20120518_EXQ0_MiBa_SA_HCT116_mHLA−2: 11394
SA = 0.68
Score = 0.29
KINNEIRSV − MSFragger
20120518_EXQ0_MiBa_SA_HCT116_mHLA−2: 11394
SA = 0.68
Score = 0.29
VTKNVSKPVG − Mascot − rejected
VTKNVSKP|VG − spliced − 0|2|0|2
20120321_EXQ1_MiBa_SA_HCC1143_2: 4035
SA = 0.67
Score = 1.5
VTKNVSKPR − MaxQuant
20120321_EXQ1_MiBa_SA_HCC1143_2: 4035
SA = 0.9
Score = 2.47
VTKNVSKPR − MSFragger
20120321_EXQ1_MiBa_SA_HCC1143_2: 4035
SA = 0.9
Score = 2.47
AEYGEIKNIHI − Mascot − rejected
AEYGEIKNIH|I − spliced − |||
20120617_EXQ0_MiBa_SA_HCT116_1_mHLA_2hr: 18669
SA = 0.67
Score = 3.58
AEYGEIKNIHL − MaxQuant
20120617_EXQ0_MiBa_SA_HCT116_1_mHLA_2hr: 18669
SA = 0.67
Score = 3.58
AEYGEIKNIHL − MSFragger
20120617_EXQ0_MiBa_SA_HCT116_1_mHLA_2hr: 18669
SA = 0.67
Score = 3.58
Relative intensity
m/z
Prosit prediction
* Exclusive in prediction
# Exclusive in experimental

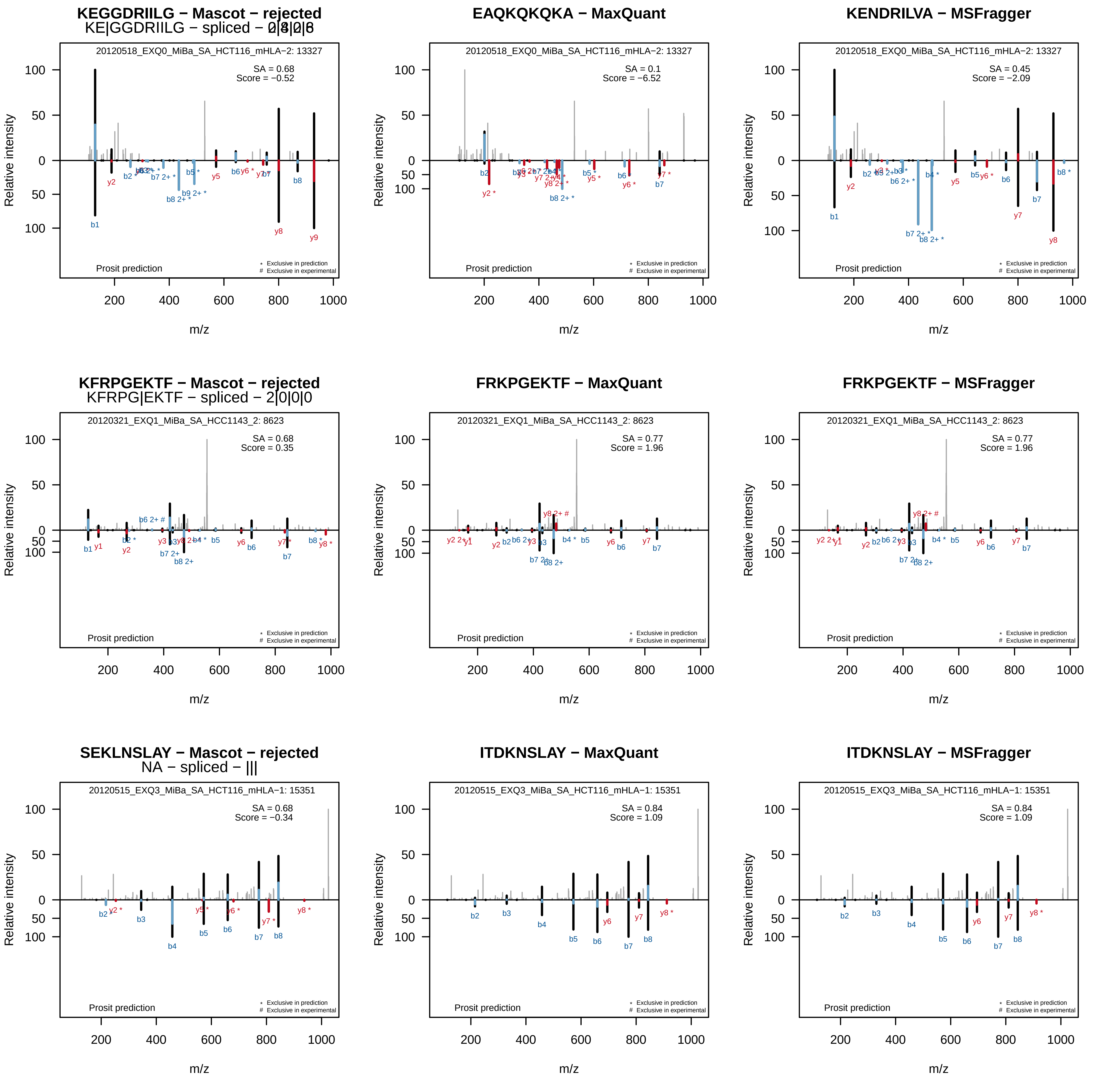

KEGGDRIILG − Mascot − rejected
20120518_EXQ0_MiBa_SA_HCT116_mHLA−2: 13327
SA = 0.68
Score = −0.52
EAQKQKQKA − MaxQuant
20120518_EXQ0_MiBa_SA_HCT116_mHLA−2: 13327
SA = 0.1
Score = −6.52
KENDRILVA − MSFragger
20120518_EXQ0_MiBa_SA_HCT116_mHLA−2: 13327
SA = 0.45
Score = −2.09
KFRPGEKTF − Mascot − rejected
KFRPG|EKTF − spliced − 2|0|0|0
20120321_EXQ1_MiBa_SA_HCC1143_2: 8623
SA = 0.68
Score = 0.35
FRKPGEKTF − MaxQuant
20120321_EXQ1_MiBa_SA_HCC1143_2: 8623
SA = 0.77
Score = 1.96
FRKPGEKTF − MSFragger
20120321_EXQ1_MiBa_SA_HCC1143_2: 8623
SA = 0.77
Score = 1.96
SEKLNSLAY − Mascot − rejected
NA − spliced − |||
20120515_EXQ3_MiBa_SA_HCT116_mHLA−1: 15351
SA = 0.68
Score = −0.34
ITDKNSLAY − MaxQuant
20120515_EXQ3_MiBa_SA_HCT116_mHLA−1: 15351
SA = 0.84
Score = 1.09
ITDKNSLAY − MSFragger
20120515_EXQ3_MiBa_SA_HCT116_mHLA−1: 15351
SA = 0.84
Score = 1.09
Relative intensity
m/z
Prosit prediction
⋆ Exclusive in prediction
Exclusive in experimental

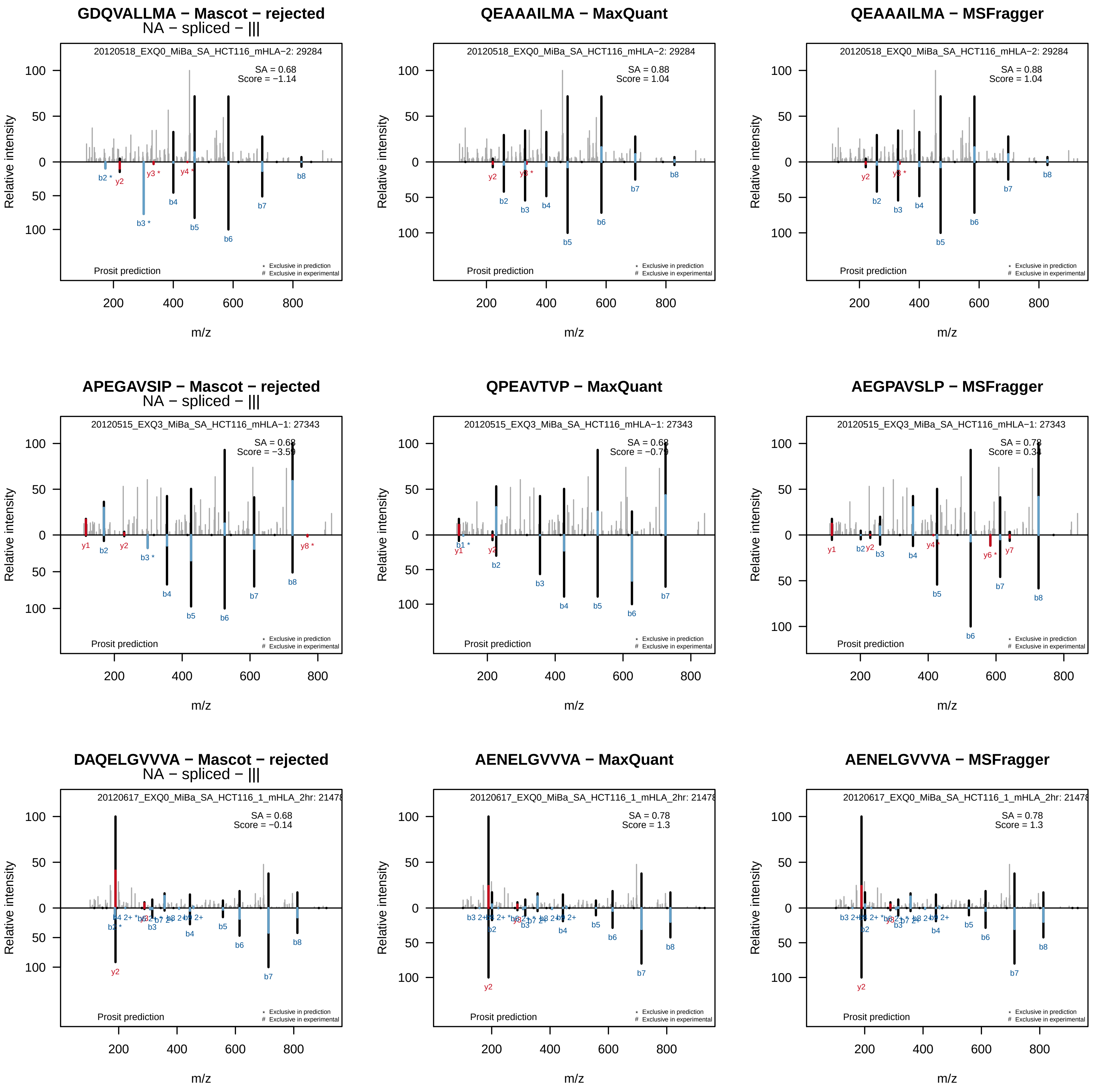
GDQVALLMA − Mascot − rejected
NA − spliced − |||
20120518_EXQ0_MiBa_SA_HCT116_mHLA−2: 29284
SA = 0.68
Score = −1.14
QEAAAILMA − MaxQuant
20120518_EXQ0_MiBa_SA_HCT116_mHLA−2: 29284
SA = 0.88
Score = 1.04
QEAAAILMA − MSFragger
20120518_EXQ0_MiBa_SA_HCT116_mHLA−2: 29284
SA = 0.88
Score = 1.04
APEGAVSIP − Mascot − rejected
NA − spliced − |||
20120515_EXQ3_MiBa_SA_HCT116_mHLA−1: 27343
SA = 0.63
Score = −3.59
QPEAVTVP − MaxQuant
20120515_EXQ3_MiBa_SA_HCT116_mHLA−1: 27343
SA = 0.63
Score = −0.79
AEGPAVSLP − MSFragger
20120515_EXQ3_MiBa_SA_HCT116_mHLA−1: 27343
SA = 0.73
Score = 0.34
DAQELGVVVA − Mascot − rejected
NA − spliced − |||
20120617_EXQ0_MiBa_SA_HCT116_1_mHLA_2hr: 21478
SA = 0.68
Score = −0.14
AENELGVVVA − MaxQuant
20120617_EXQ0_MiBa_SA_HCT116_1_mHLA_2hr: 21478
SA = 0.78
Score = 1.3
AENELGVVVA − MSFragger
20120617_EXQ0_MiBa_SA_HCT116_1_mHLA_2hr: 21478
SA = 0.78
Score = 1.3
Relative intensity
m/z
Prosit prediction
⋆ Exclusive in prediction
Exclusive in experimental

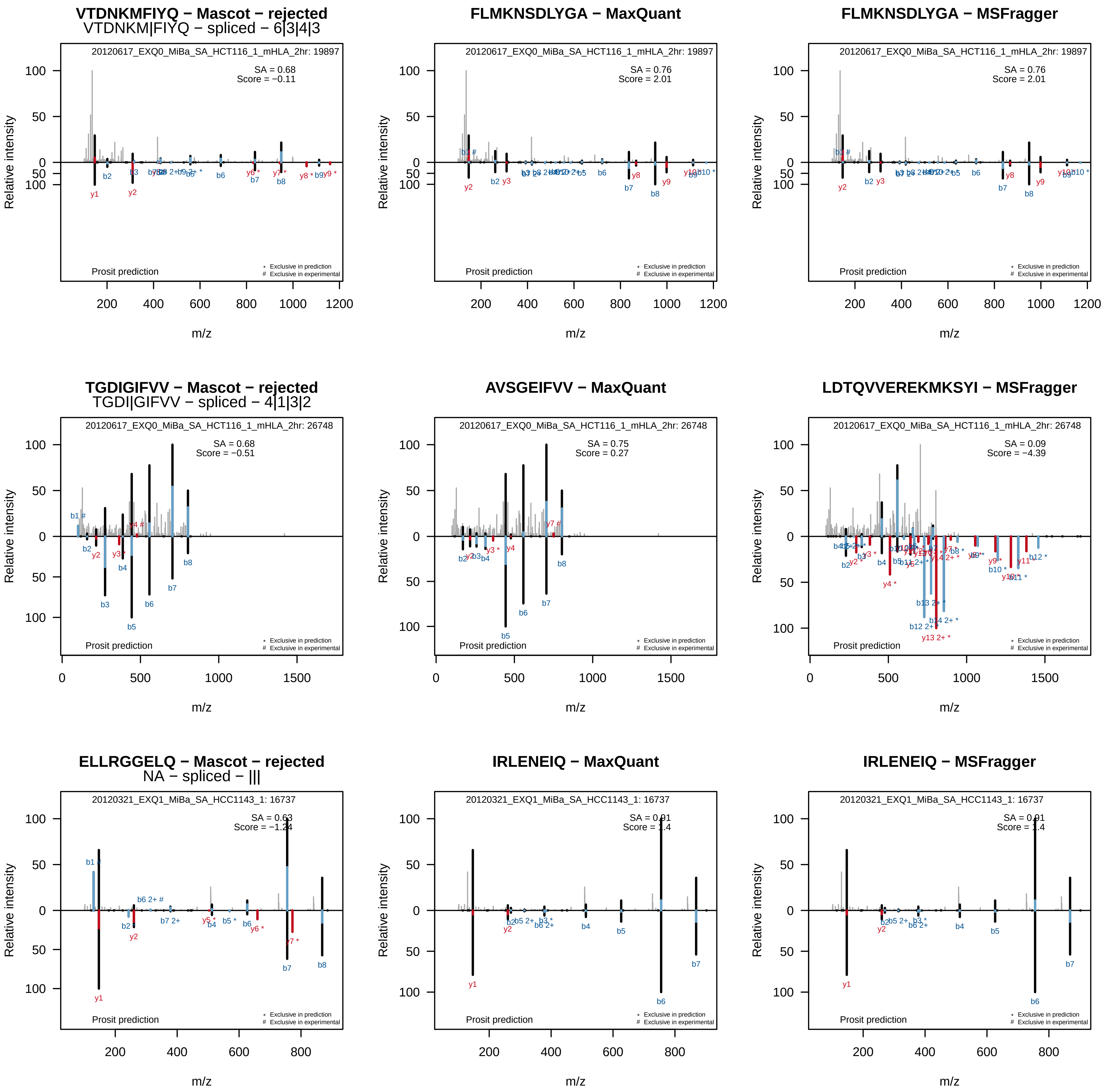

VTDNKMFIYQ − Mascot − rejected
VTDNKM|FIYQ − spliced − 6|3|4|3
20120617_EXQ0_MiBa_SA_HCT116_1_mHLA_2hr: 19897
SA = 0.68
Score = −0.11
FLMKNSDLYGA − MaxQuant
20120617_EXQ0_MiBa_SA_HCT116_1_mHLA_2hr: 19897
SA = 0.76
Score = 2.01
FLMKNSDLYGA − MSFragger
20120617_EXQ0_MiBa_SA_HCT116_1_mHLA_2hr: 19897
SA = 0.76
Score = 2.01
TGDIGIFVV − Mascot − rejected
TGDI|GIFVV − spliced − 4|1|3|2
20120617_EXQ0_MiBa_SA_HCT116_1_mHLA_2hr: 26748
SA = 0.68
Score = −0.51
AVSGEIFVV − MaxQuant
20120617_EXQ0_MiBa_SA_HCT116_1_mHLA_2hr: 26748
SA = 0.75
Score = 0.27
LDTQVVEREKMKSYI − MSFragger
20120617_EXQ0_MiBa_SA_HCT116_1_mHLA_2hr: 26748
SA = 0.09
Score = −4.39
ELLRGGELQ − Mascot − rejected
NA − spliced − |||
20120321_EXQ1_MiBa_SA_HCC1143_1: 16737
SA = 0.63
Score = −1.24
IRLENEIQ − MaxQuant
20120321_EXQ1_MiBa_SA_HCC1143_1: 16737
SA = 0.91
Score = 1.4
IRLENEIQ − MSFragger
20120321_EXQ1_MiBa_SA_HCC1143_1: 16737
SA = 0.91
Score = 1.4
Relative intensity
m/z
Prosit prediction
⋆ Exclusive in prediction
Exclusive in experimental

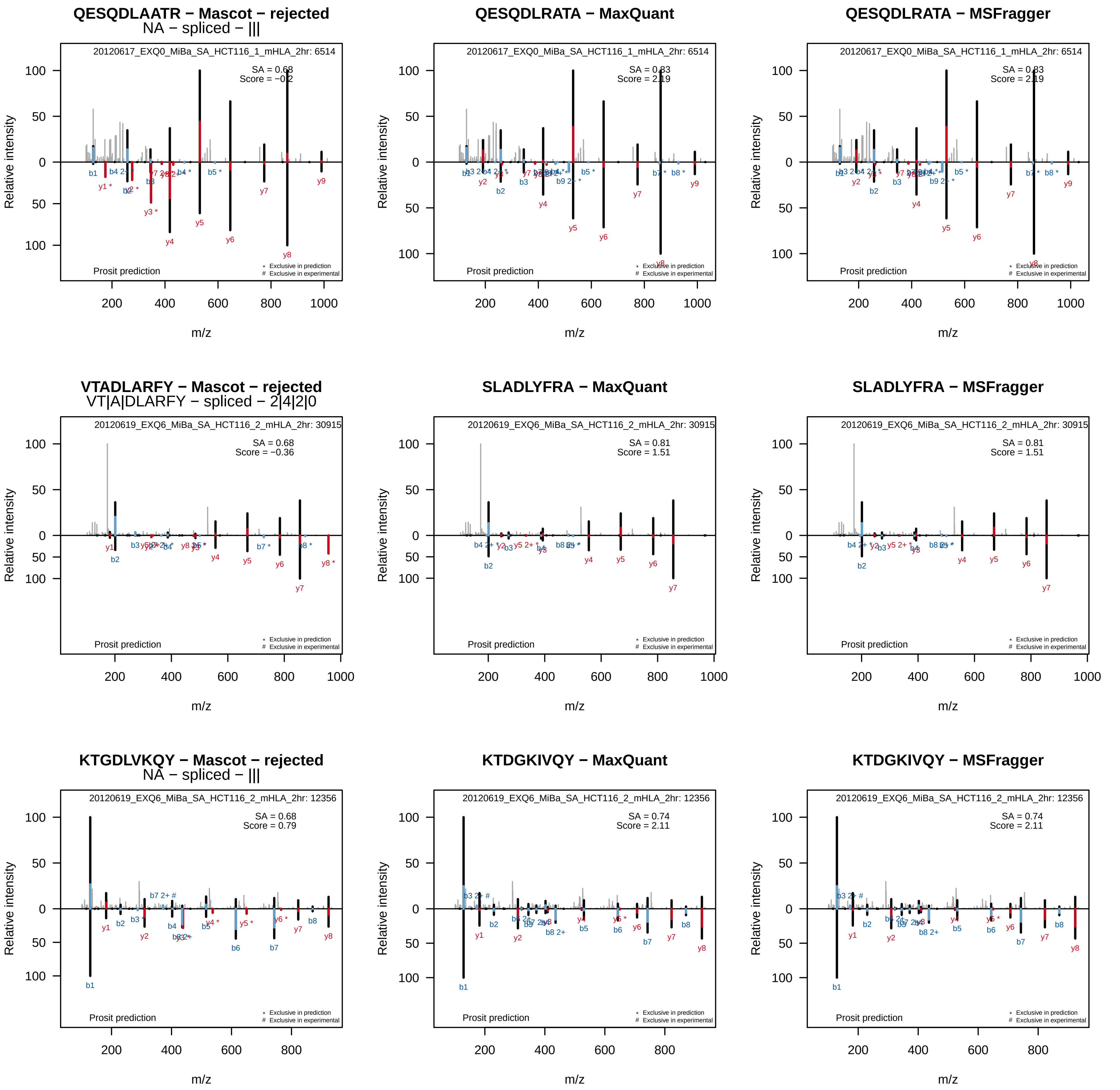
QESQDLAATR − Mascot − rejected
NA − spliced − |||
20120617_EXQ0_MiBa_SA_HCT116_1_mHLA_2hr: 6514
SA = 0.68
Score = −0.2
QESQDLRATA − MaxQuant
20120617_EXQ0_MiBa_SA_HCT116_1_mHLA_2hr: 6514
SA = 0.83
Score = 2.19
QESQDLRATA − MSFragger
20120617_EXQ0_MiBa_SA_HCT116_1_mHLA_2hr: 6514
SA = 0.83
Score = 2.19
VTADLARFY − Mascot − rejected
VT|A|DLARFY − spliced − 2|4|2|0
20120619_EXQ6_MiBa_SA_HCT116_2_mHLA_2hr: 30915
SA = 0.68
Score = −0.36
SLADLYFRA − MaxQuant
20120619_EXQ6_MiBa_SA_HCT116_2_mHLA_2hr: 30915
SA = 0.81
Score = 1.51
SLADLYFRA − MSFragger
20120619_EXQ6_MiBa_SA_HCT116_2_mHLA_2hr: 30915
SA = 0.81
Score = 1.51
KTGDLVKQY − Mascot − rejected
NA − spliced − |||
20120619_EXQ6_MiBa_SA_HCT116_2_mHLA_2hr: 12356
SA = 0.68
Score = 0.79
KTDGKIVQY − MaxQuant
20120619_EXQ6_MiBa_SA_HCT116_2_mHLA_2hr: 12356
SA = 0.74
Score = 2.11
KTDGKIVQY − MSFragger
20120619_EXQ6_MiBa_SA_HCT116_2_mHLA_2hr: 12356
SA = 0.74
Score = 2.11
Relative intensity
m/z
Prosit prediction
* Exclusive in prediction
# Exclusive in experimental

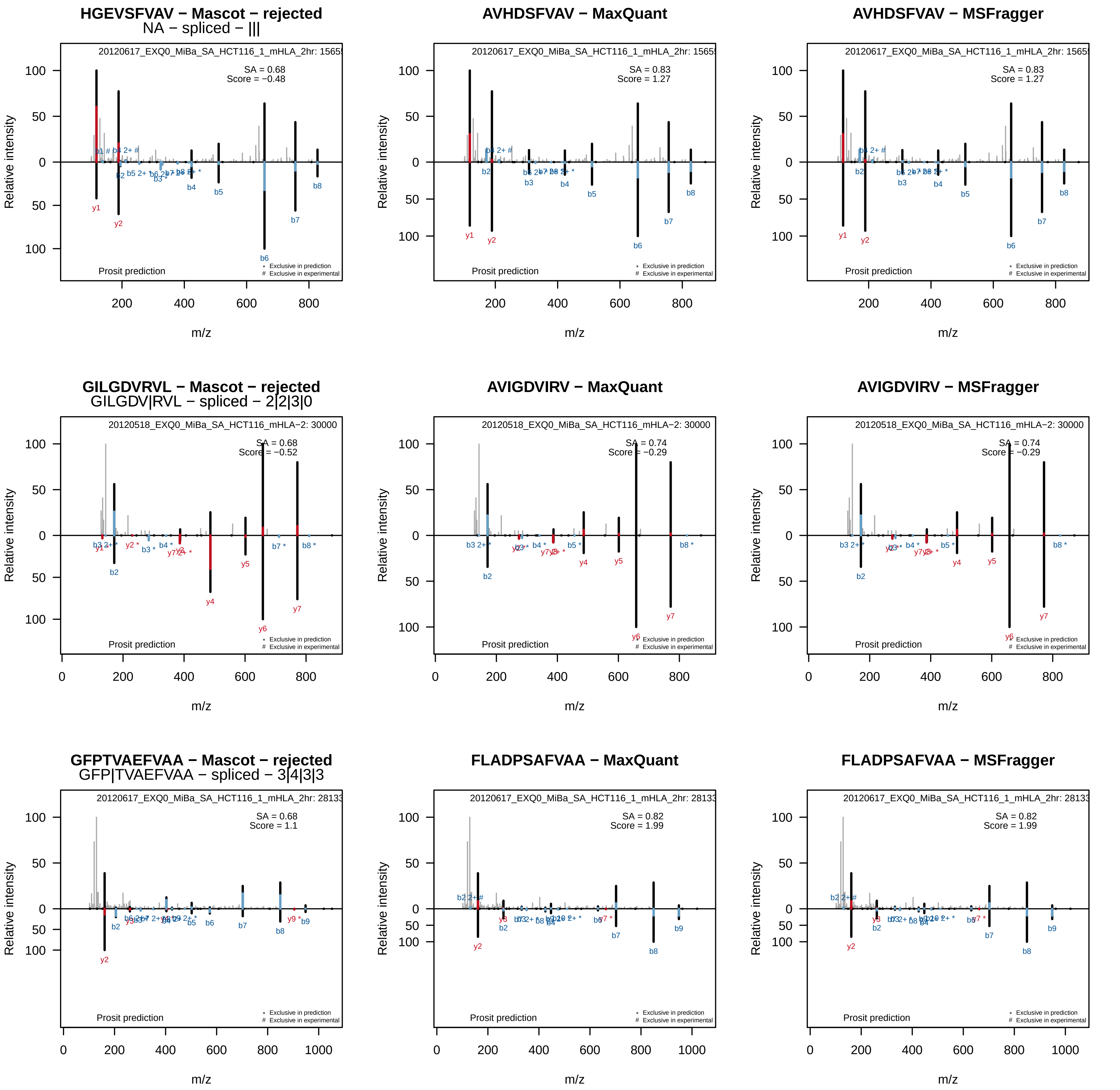
HGEVSFVAV − Mascot − rejected
NA − spliced − |||
AVHDSFVAV − MaxQuant
AVHDSFVAV − MSFragger
20120617_EXQ0_MiBa_SA_HCT116_1_mHLA_2hr: 1565
SA = 0.68
Score = −0.48
SA = 0.83
Score = 1.27
GILGDVRVL − Mascot − rejected
GILGDV|RVL − spliced − 2|2|3|0
AVIGDVIRV − MaxQuant
AVIGDVIRV − MSFragger
20120518_EXQ0_MiBa_SA_HCT116_mHLA−2: 30000
SA = 0.68
Score = −0.52
SA = 0.74
Score = −0.29
GFPTVAEFVAA − Mascot − rejected
GFP|TVAEFVAA − spliced − 3|4|3|3
FLADPSAFVAA − MaxQuant
FLADPSAFVAA − MSFragger
20120617_EXQ0_MiBa_SA_HCT116_1_mHLA_2hr: 28133
SA = 0.68
Score = 1.1
SA = 0.82
Score = 1.99
Relative intensity
m/z
Prosit prediction
* Exclusive in prediction
# Exclusive in experimental

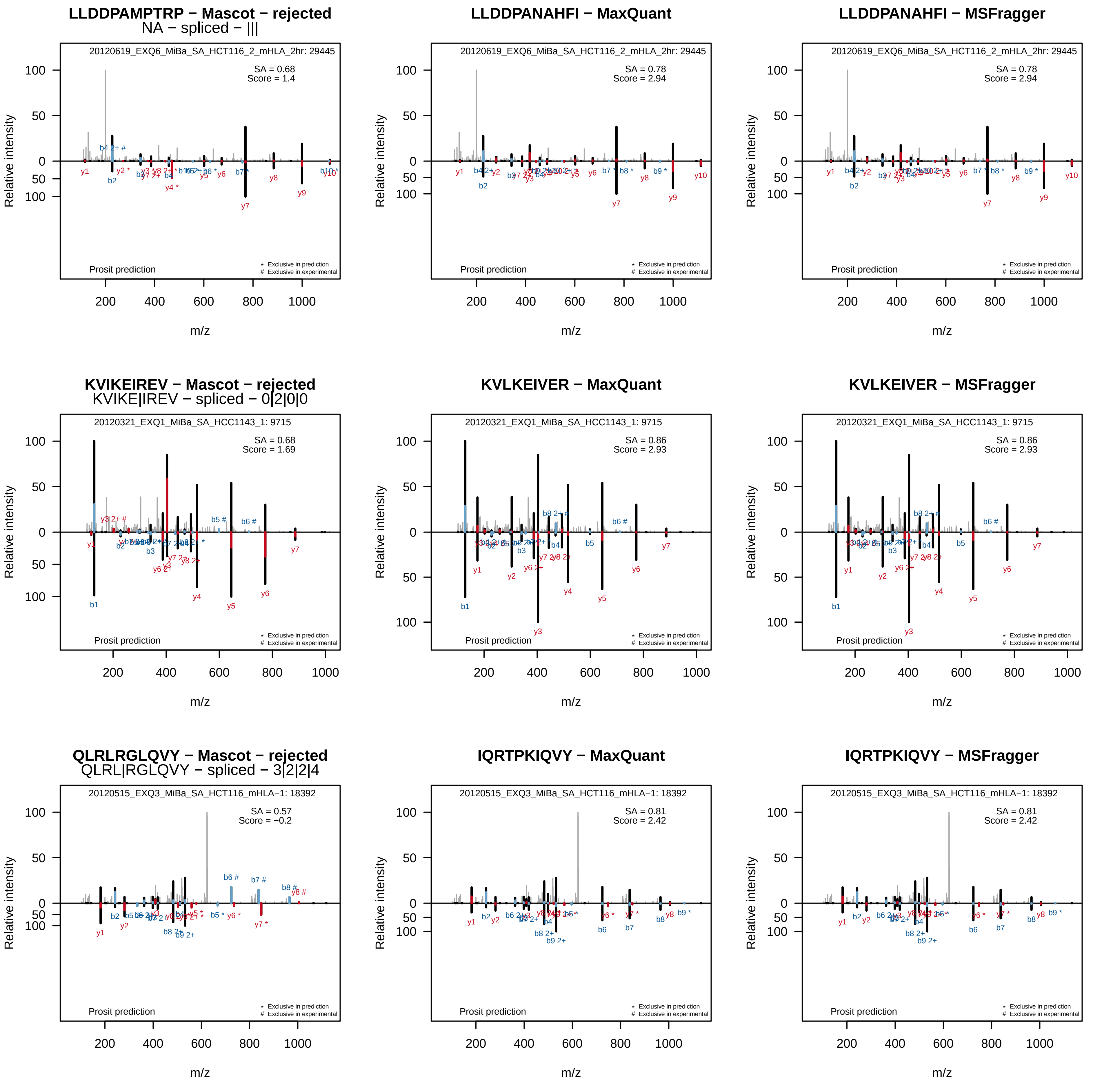
LLDDPAMPTRP − Mascot − rejected
NA − spliced − |||
20120619_EXQ6_MiBa_SA_HCT116_2_mHLA_2hr: 29445
SA = 0.68
Score = 1.4
LLDDPANAHFI − MaxQuant
20120619_EXQ6_MiBa_SA_HCT116_2_mHLA_2hr: 29445
SA = 0.78
Score = 2.94
LLDDPANAHFI − MSFragger
20120619_EXQ6_MiBa_SA_HCT116_2_mHLA_2hr: 29445
SA = 0.78
Score = 2.94
KVIKEIREV − Mascot − rejected
KVIKE|IREV − spliced − 0|2|0|0
20120321_EXQ1_MiBa_SA_HCC1143_1: 9715
SA = 0.68
Score = 1.69
KVLKEIVER − MaxQuant
20120321_EXQ1_MiBa_SA_HCC1143_1: 9715
SA = 0.86
Score = 2.93
KVLKEIVER − MSFragger
20120321_EXQ1_MiBa_SA_HCC1143_1: 9715
SA = 0.86
Score = 2.93
QLRLRGLQVY − Mascot − rejected
QLRL|RGLQVY − spliced − 3|2|2|4
20120515_EXQ3_MiBa_SA_HCT116_mHLA−1: 18392
SA = 0.57
Score = −0.2
IQRTPKIQVY − MaxQuant
20120515_EXQ3_MiBa_SA_HCT116_mHLA−1: 18392
SA = 0.81
Score = 2.42
IQRTPKIQVY − MSFragger
20120515_EXQ3_MiBa_SA_HCT116_mHLA−1: 18392
SA = 0.81
Score = 2.42
Relative intensity
m/z
Prosit prediction
* Exclusive in prediction
# Exclusive in experimental

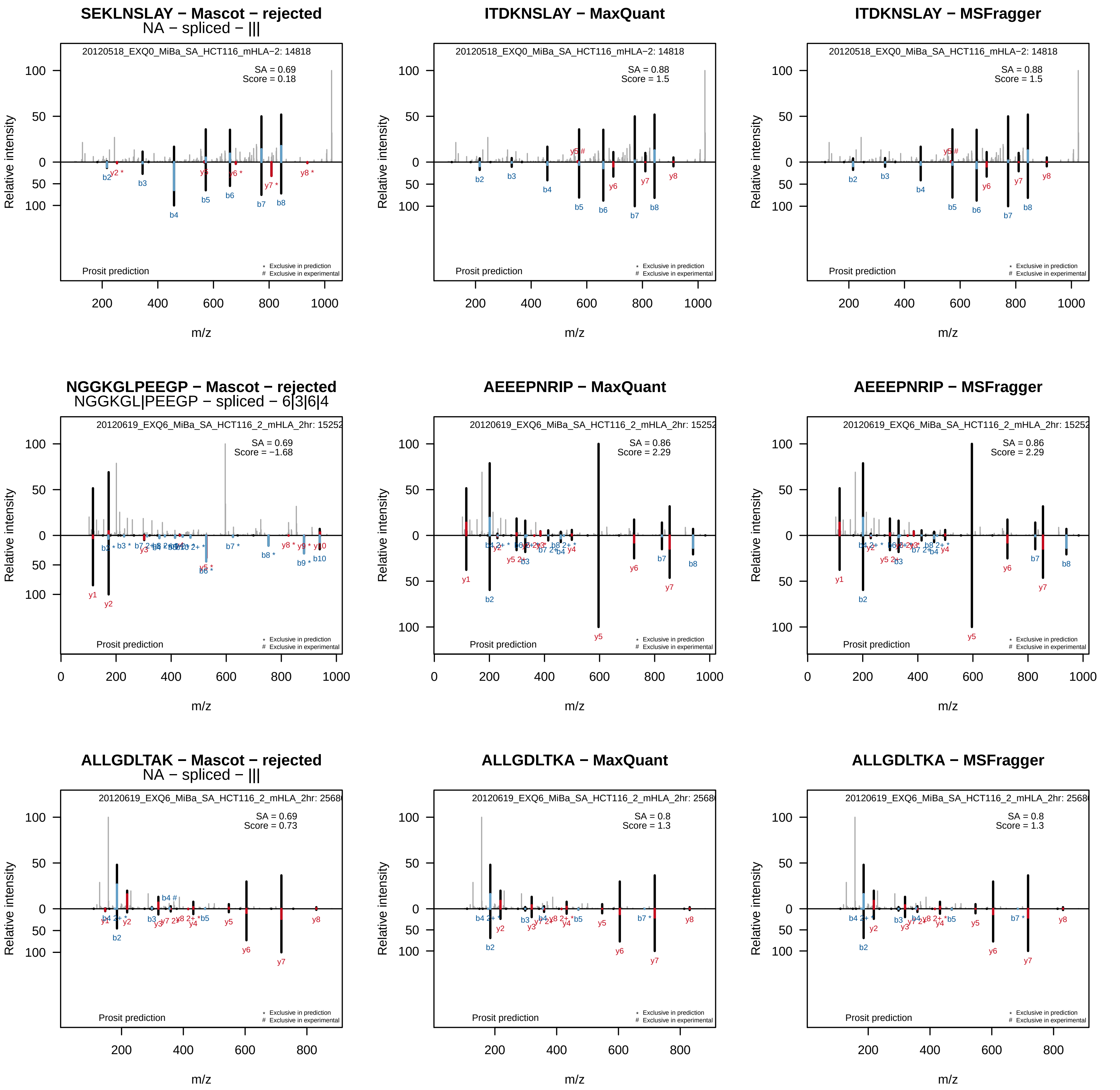
SEKLNSLAY − Mascot − rejected
NA − spliced − |||
20120518_EXQ0_MiBa_SA_HCT116_mHLA−2: 14818
SA = 0.69
Score = 0.18
Prosit prediction
⋆ Exclusive in prediction
Exclusive in experimental
ITDKNSLAY − MaxQuant
20120518_EXQ0_MiBa_SA_HCT116_mHLA−2: 14818
SA = 0.88
Score = 1.5
ITDKNSLAY − MSFragger
20120518_EXQ0_MiBa_SA_HCT116_mHLA−2: 14818
SA = 0.88
Score = 1.5
NGGKGLPEEGP − Mascot − rejected
NGGKGL|PEEGP − spliced − 6|3|6|4
20120619_EXQ6_MiBa_SA_HCT116_2_mHLA_2hr: 15252
SA = 0.69
Score = −1.68
AEEEPNRIP − MaxQuant
20120619_EXQ6_MiBa_SA_HCT116_2_mHLA_2hr: 15252
SA = 0.86
Score = 2.29
AEEEPNRIP − MSFragger
20120619_EXQ6_MiBa_SA_HCT116_2_mHLA_2hr: 15252
SA = 0.86
Score = 2.29
ALLGDLTAK − Mascot − rejected
NA − spliced − |||
20120619_EXQ6_MiBa_SA_HCT116_2_mHLA_2hr: 2568
SA = 0.69
Score = 0.73
ALLGDLTKA − MaxQuant
20120619_EXQ6_MiBa_SA_HCT116_2_mHLA_2hr: 2568
SA = 0.8
Score = 1.3
ALLGDLTKA − MSFragger
20120619_EXQ6_MiBa_SA_HCT116_2_mHLA_2hr: 2568
SA = 0.8
Score = 1.3
Relative intensity
m/z

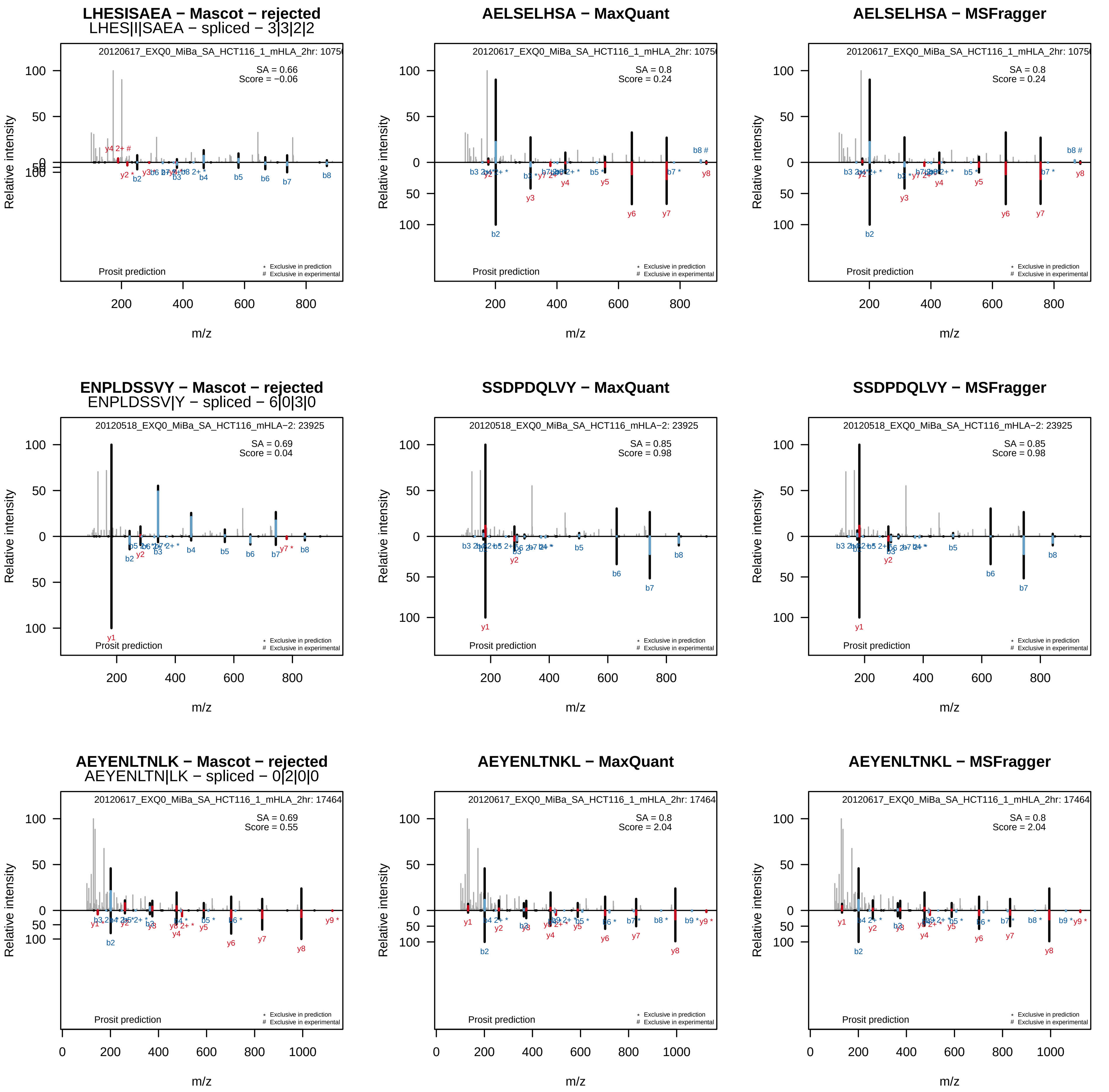

LHESISAEA − Mascot − rejected
LHES|I|SAEA − spliced − 3|3|2|2
20120617_EXQ0_MiBa_SA_HCT116_1_mHLA_2hr: 1075
SA = 0.66
Score = −0.06
AELSELHSA − MaxQuant
20120617_EXQ0_MiBa_SA_HCT116_1_mHLA_2hr: 1075
SA = 0.8
Score = 0.24
AELSELHSA − MSFragger
20120617_EXQ0_MiBa_SA_HCT116_1_mHLA_2hr: 1075
SA = 0.8
Score = 0.24
ENPLDSSVY − Mascot − rejected
ENPLDSSV|Y − spliced − 6|0|3|0
20120518_EXQ0_MiBa_SA_HCT116_mHLA−2: 23925
SA = 0.69
Score = 0.04
SSDPDQLVY − MaxQuant
20120518_EXQ0_MiBa_SA_HCT116_mHLA−2: 23925
SA = 0.85
Score = 0.98
SSDPDQLVY − MSFragger
20120518_EXQ0_MiBa_SA_HCT116_mHLA−2: 23925
SA = 0.85
Score = 0.98
AEYENLTNLK − Mascot − rejected
AEYENLTN|LK − spliced − 0|2|0|0
20120617_EXQ0_MiBa_SA_HCT116_1_mHLA_2hr: 17464
SA = 0.69
Score = 0.55
AEYENLTNKL − MaxQuant
20120617_EXQ0_MiBa_SA_HCT116_1_mHLA_2hr: 17464
SA = 0.8
Score = 2.04
AEYENLTNKL − MSFragger
20120617_EXQ0_MiBa_SA_HCT116_1_mHLA_2hr: 17464
SA = 0.8
Score = 2.04
Relative intensity
m/z
Prosit prediction
* Exclusive in prediction
# Exclusive in experimental

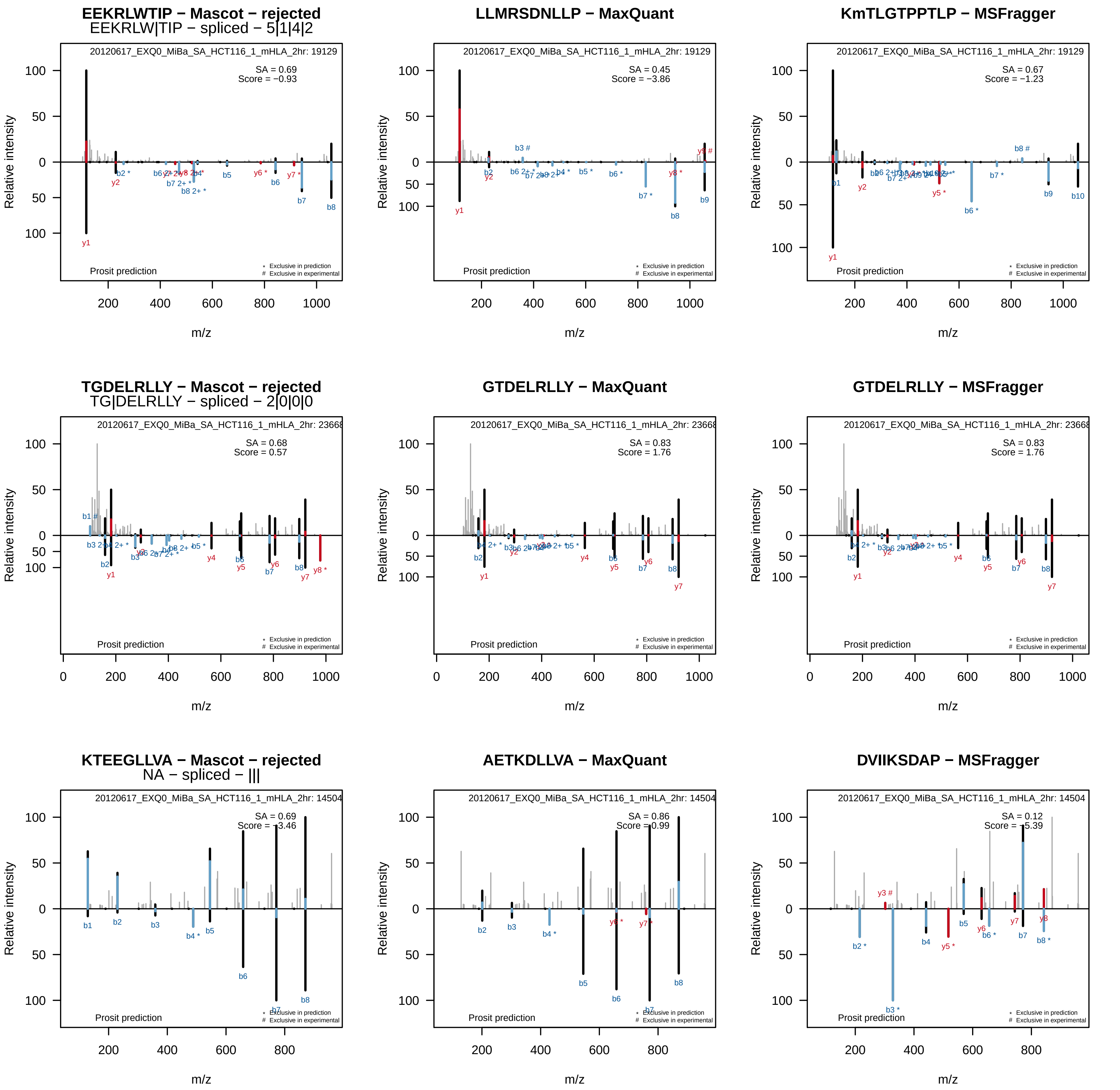

EEKRLWTIP − Mascot − rejected
EEKRLW|TIP − spliced − 5|1|4|2
20120617_EXQ0_MiBa_SA_HCT116_1_mHLA_2hr: 19129
SA = 0.69
Score = −0.93
LLMRSDNLLP − MaxQuant
20120617_EXQ0_MiBa_SA_HCT116_1_mHLA_2hr: 19129
SA = 0.45
Score = −3.86
KmTLGTPPTLP − MSFragger
20120617_EXQ0_MiBa_SA_HCT116_1_mHLA_2hr: 19129
SA = 0.67
Score = −1.23
TGDELRLLY − Mascot − rejected
TG|DELRLLY − spliced − 2|0|0|0
SA = 0.68
Score = 0.57
GTDELRLLY − MaxQuant
SA = 0.83
Score = 1.76
GTDELRLLY − MSFragger
SA = 0.83
Score = 1.76
KTEEGLLVA − Mascot − rejected
NA − spliced − |||
20120617_EXQ0_MiBa_SA_HCT116_1_mHLA_2hr: 14504
SA = 0.69
Score = 3.46
AETKDLLVA − MaxQuant
20120617_EXQ0_MiBa_SA_HCT116_1_mHLA_2hr: 14504
SA = 0.86
Score = 0.99
DVIIKSDAP − MSFragger
20120617_EXQ0_MiBa_SA_HCT116_1_mHLA_2hr: 14504
SA = 0.12
Score = 5.39
Relative intensity
m/z
Prosit prediction
* Exclusive in prediction
# Exclusive in experimental

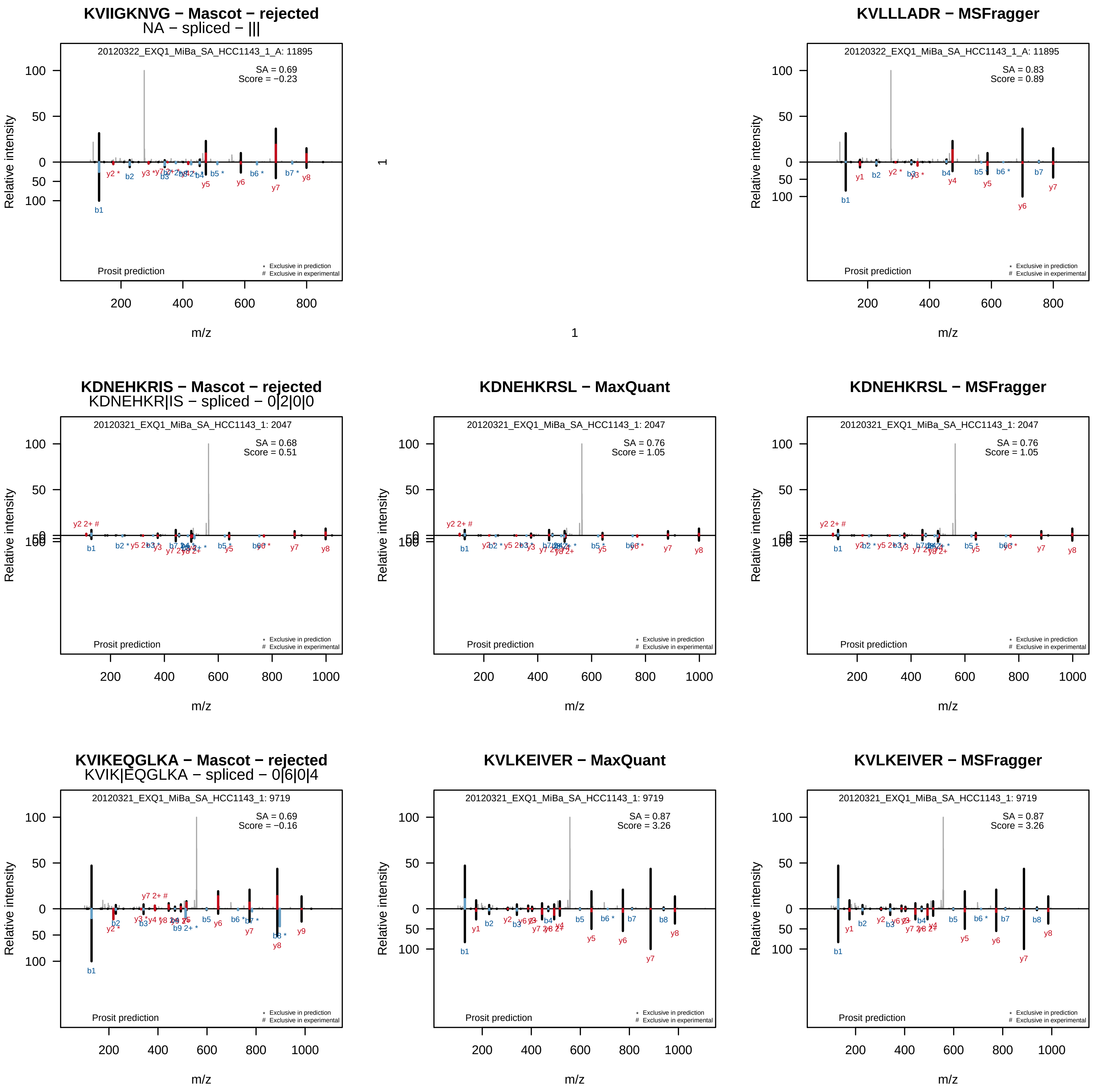
KVIIGKNVG − Mascot − rejected
NA − spliced − |||
20120322_EXQ1_MiBa_SA_HCC1143_1_A: 11895
SA = 0.69
Score = −0.23
KVLLLADR − MSFragger
20120322_EXQ1_MiBa_SA_HCC1143_1_A: 11895
SA = 0.83
Score = 0.89
KDNEHKRIS − Mascot − rejected
KDNEHKR|IS − spliced − 0|2|0|0
20120321_EXQ1_MiBa_SA_HCC1143_1: 2047
SA = 0.68
Score = 0.51
KDNEHKRSL − MaxQuant
20120321_EXQ1_MiBa_SA_HCC1143_1: 2047
SA = 0.76
Score = 1.05
KDNEHKRSL − MSFragger
20120321_EXQ1_MiBa_SA_HCC1143_1: 2047
SA = 0.76
Score = 1.05
KVIKEQGLKA − Mascot − rejected
KVIK|EQGLKA − spliced − 0|6|0|4
20120321_EXQ1_MiBa_SA_HCC1143_1: 9719
SA = 0.69
Score = −0.16
KVLKEIVER − MaxQuant
20120321_EXQ1_MiBa_SA_HCC1143_1: 9719
SA = 0.87
Score = 3.26
KVLKEIVER − MSFragger
20120321_EXQ1_MiBa_SA_HCC1143_1: 9719
SA = 0.87
Score = 3.26
Relative intensity
m/z
Prosit prediction
* Exclusive in prediction
# Exclusive in experimental

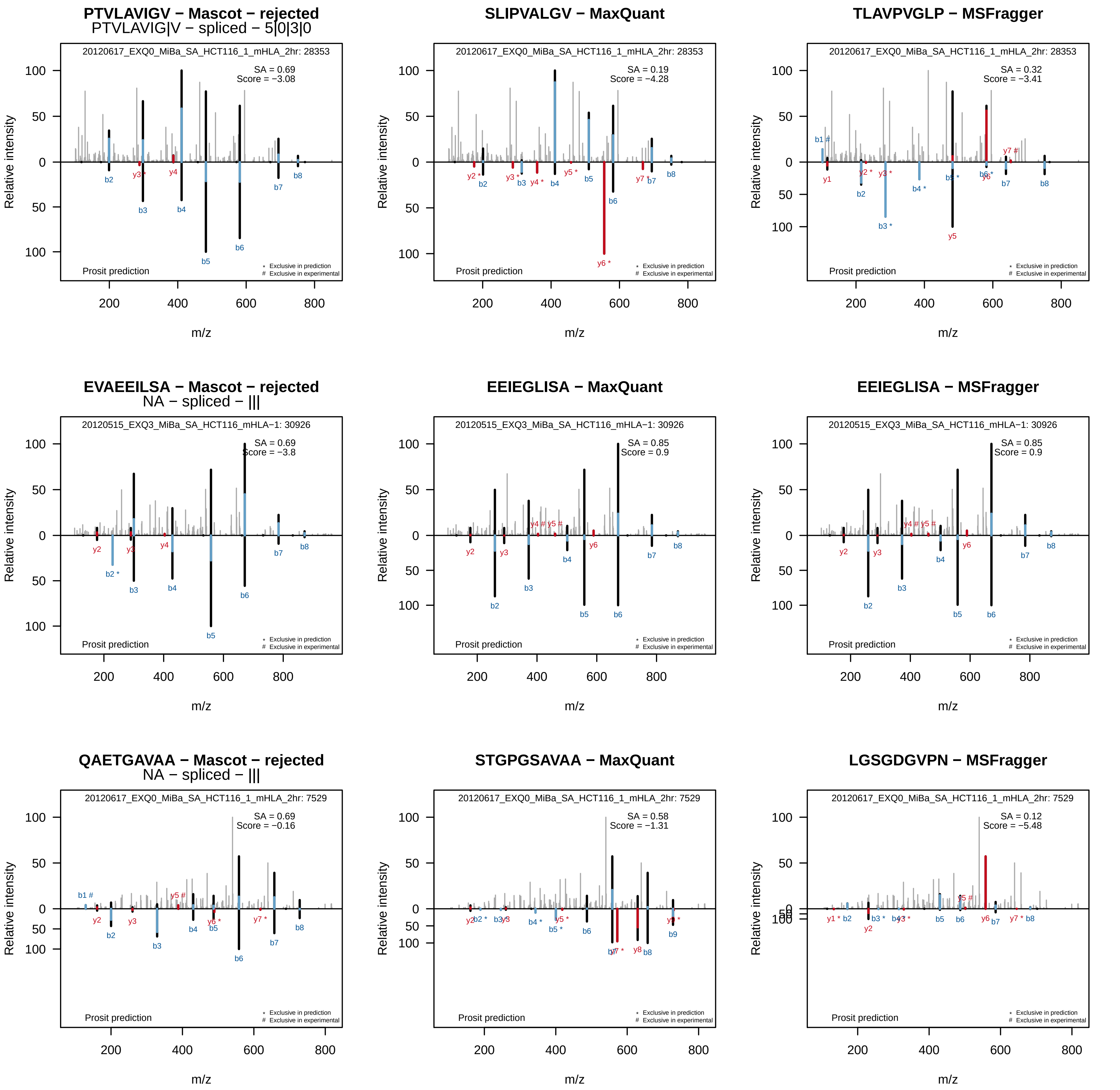

PTVLAVIGV − Mascot − rejected
PTVLAVIG|V − spliced − 5|0|3|0
20120617_EXQ0_MiBa_SA_HCT116_1_mHLA_2hr: 28353
SA = 0.69
Score = −3.08
Prosit prediction
SLIPVALGV − MaxQuant
20120617_EXQ0_MiBa_SA_HCT116_1_mHLA_2hr: 28353
SA = 0.19
Score = −4.28
Prosit prediction
TLAVPVGLP − MSFragger
20120617_EXQ0_MiBa_SA_HCT116_1_mHLA_2hr: 28353
SA = 0.32
Score = −3.41
Prosit prediction
EVAEEILSA − Mascot − rejected
NA − spliced − |||
20120515_EXQ3_MiBa_SA_HCT116_mHLA−1: 30926
SA = 0.69
Score = −3.8
Prosit prediction
EEIEGLISA − MaxQuant
20120515_EXQ3_MiBa_SA_HCT116_mHLA−1: 30926
SA = 0.85
Score = 0.9
Prosit prediction
EEIEGLISA − MSFragger
20120515_EXQ3_MiBa_SA_HCT116_mHLA−1: 30926
SA = 0.85
Score = 0.9
Prosit prediction
QAETGAVAA − Mascot − rejected
NA − spliced − |||
20120617_EXQ0_MiBa_SA_HCT116_1_mHLA_2hr: 7529
SA = 0.69
Score = −0.16
Prosit prediction
STGPGSAVAA − MaxQuant
20120617_EXQ0_MiBa_SA_HCT116_1_mHLA_2hr: 7529
SA = 0.58
Score = −1.31
Prosit prediction
LGSGDGVPN − MSFragger
20120617_EXQ0_MiBa_SA_HCT116_1_mHLA_2hr: 7529
SA = 0.12
Score = −5.48
Prosit prediction
* Exclusive in prediction
# Exclusive in experimental
Relative intensity
m/z

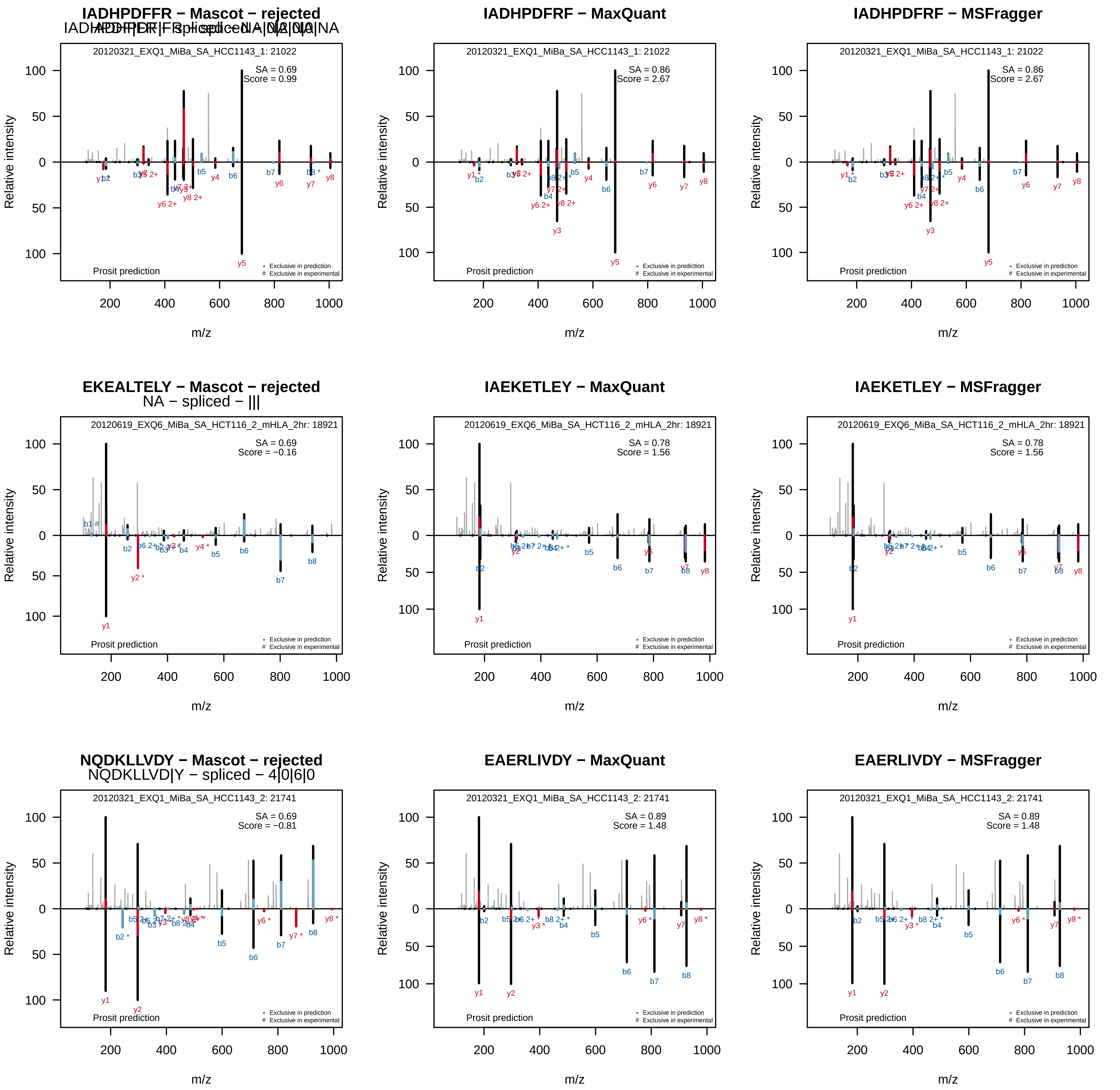

IADHPDFFR − Mascot − rejected
IADHPDFRF − MaxQuant
IADHPDFRF − MSFragger
20120321_EXQ1_MiBa_SA_HCC1143_1: 21022
SA = 0.69
Score = 0.99
SA = 0.86
Score = 2.67
EKEALTELY − Mascot − rejected
NA − spliced − |||
IAEKETLEY − MaxQuant
IAEKETLEY − MSFragger
20120619_EXQ6_MiBa_SA_HCT116_2_mHLA_2hr: 18921
SA = 0.69
Score = −0.16
SA = 0.78
Score = 1.56
NQDKLLVDY − Mascot − rejected
NQDKLLVD|Y − spliced − 4|0|6|0
EAERLIVDY − MaxQuant
EAERLIVDY − MSFragger
20120321_EXQ1_MiBa_SA_HCC1143_2: 21741
SA = 0.69
Score = −0.81
SA = 0.89
Score = 1.48
Prosit prediction
⋆ Exclusive in prediction
Exclusive in experimental
Relative intensity
m/z

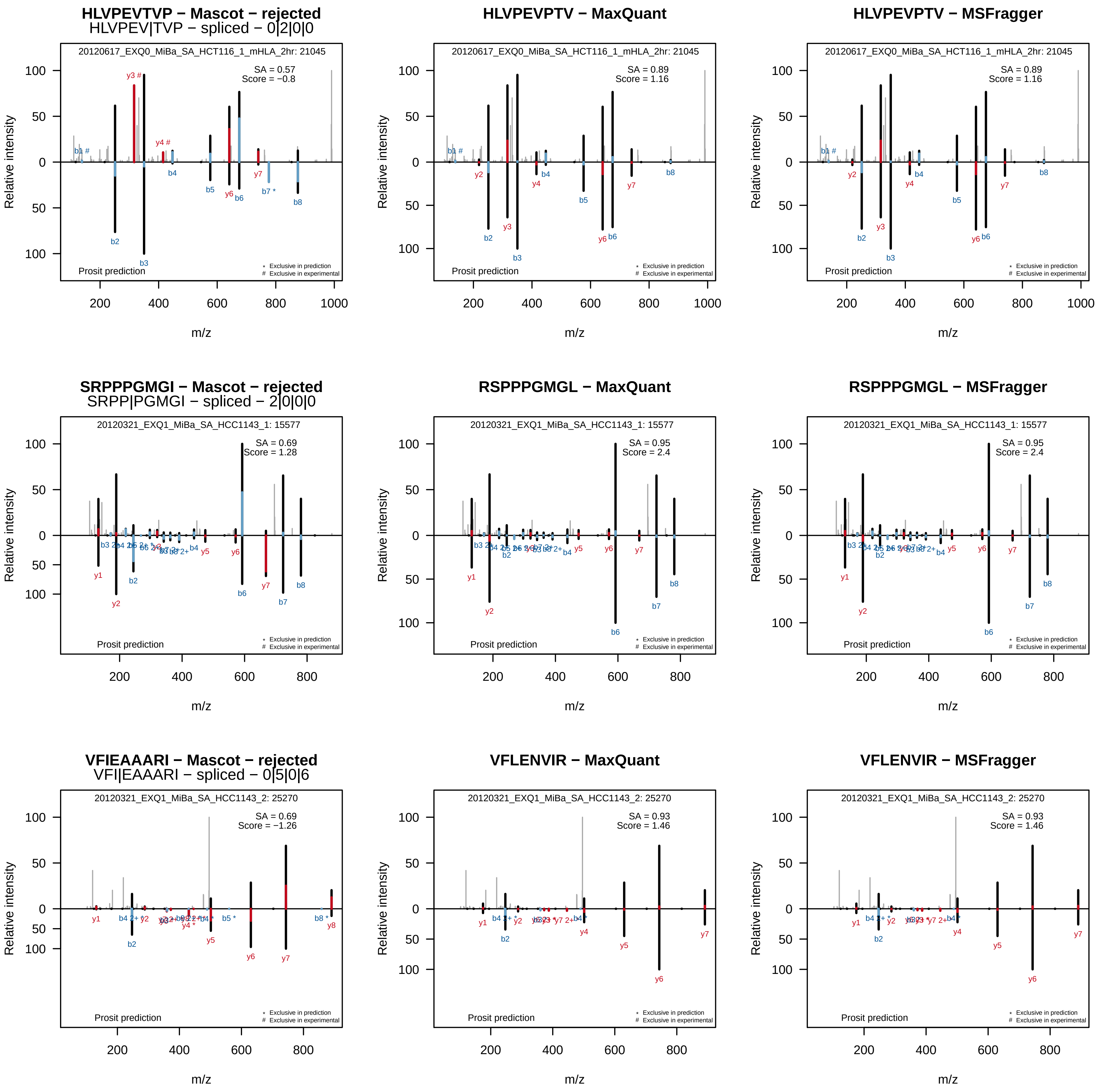
HLVPEVTVP − Mascot − rejected
HLVPEV|TVP − spliced − 0|2|0|0
20120617_EXQ0_MiBa_SA_HCT116_1_mHLA_2hr: 21045
SA = 0.57
Score = −0.8
HLVPEVPTV − MaxQuant
20120617_EXQ0_MiBa_SA_HCT116_1_mHLA_2hr: 21045
SA = 0.89
Score = 1.16
HLVPEVPTV − MSFragger
20120617_EXQ0_MiBa_SA_HCT116_1_mHLA_2hr: 21045
SA = 0.89
Score = 1.16
SRPPPGMGI − Mascot − rejected
SRPP|PGMGI − spliced − 2|0|0|0
20120321_EXQ1_MiBa_SA_HCC1143_1: 15577
SA = 0.69
Score = 1.28
RSPPPGMGL − MaxQuant
20120321_EXQ1_MiBa_SA_HCC1143_1: 15577
SA = 0.95
Score = 2.4
RSPPPGMGL − MSFragger
20120321_EXQ1_MiBa_SA_HCC1143_1: 15577
SA = 0.95
Score = 2.4
VFIEAAARI − Mascot − rejected
VFI|EAAARI − spliced − 0|5|0|6
20120321_EXQ1_MiBa_SA_HCC1143_2: 25270
SA = 0.69
Score = −1.26
VFLENVIR − MaxQuant
20120321_EXQ1_MiBa_SA_HCC1143_2: 25270
SA = 0.93
Score = 1.46
VFLENVIR − MSFragger
20120321_EXQ1_MiBa_SA_HCC1143_2: 25270
SA = 0.93
Score = 1.46
Relative intensity
m/z
Prosit prediction
⋆ Exclusive in prediction
Exclusive in experimental

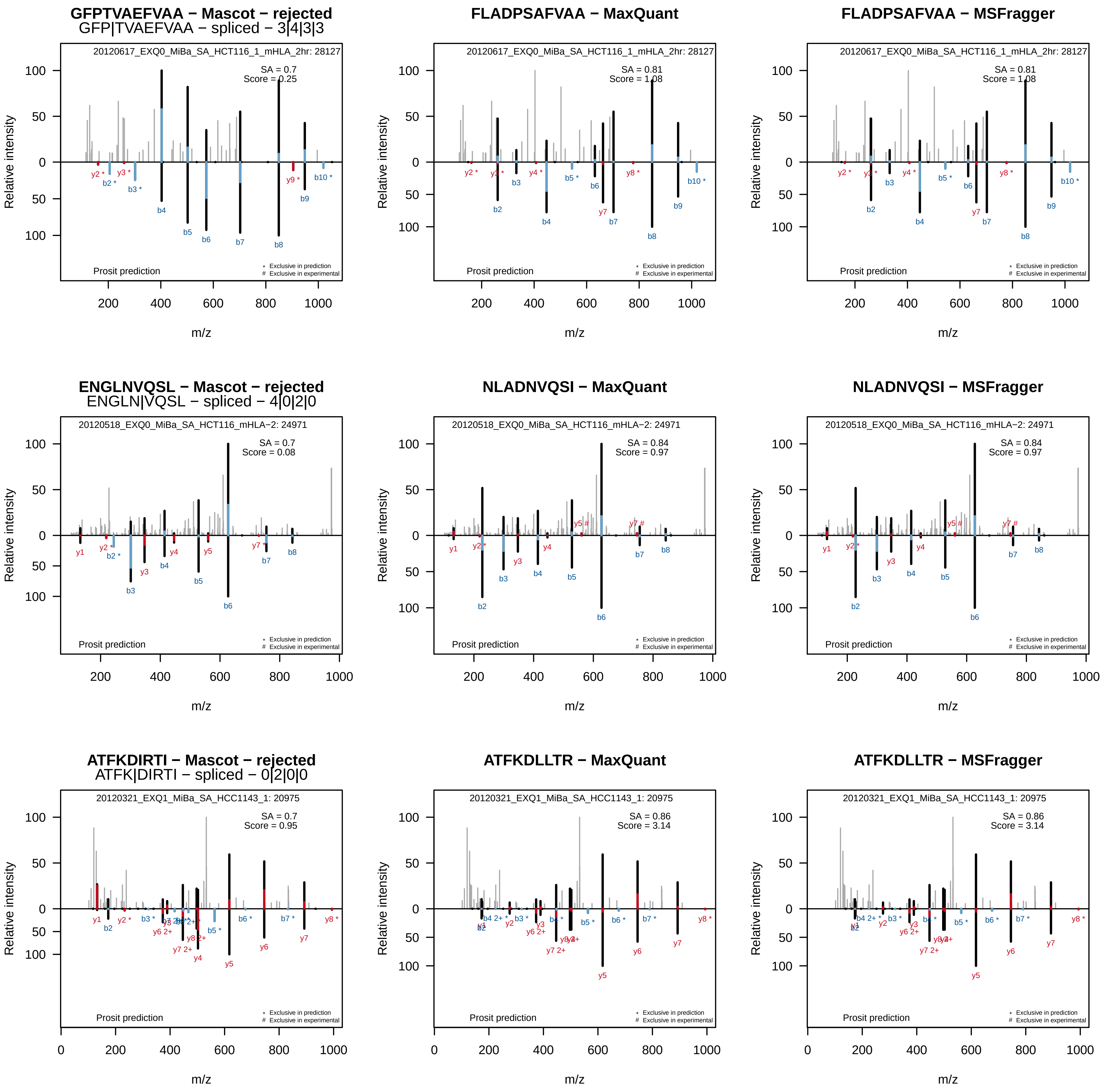

GFPTVAEFVAA − Mascot − rejected
GFP|TVAEFVAA − spliced − 3|4|3|3
20120617_EXQ0_MiBa_SA_HCT116_1_mHLA_2hr: 28127
SA = 0.7
Score = 0.25
FLADPSAFVAA − MaxQuant
SA = 0.81
Score = 1.08
FLADPSAFVAA − MSFragger
ENGLNVQSL − Mascot − rejected
ENGLN|VQSL − spliced − 4|0|2|0
20120518_EXQ0_MiBa_SA_HCT116_mHLA−2: 24971
SA = 0.7
Score = 0.08
NLADNVQSI − MaxQuant
SA = 0.84
Score = 0.97
NLADNVQSI − MSFragger
ATFKDIRTI − Mascot − rejected
ATFK|DIRTI − spliced − 0|2|0|0
20120321_EXQ1_MiBa_SA_HCC1143_1: 20975
SA = 0.7
Score = 0.95
ATFKDLLTR − MaxQuant
SA = 0.86
Score = 3.14
ATFKDLLTR − MSFragger
Relative intensity
m/z
Prosit prediction
* Exclusive in prediction
# Exclusive in experimental

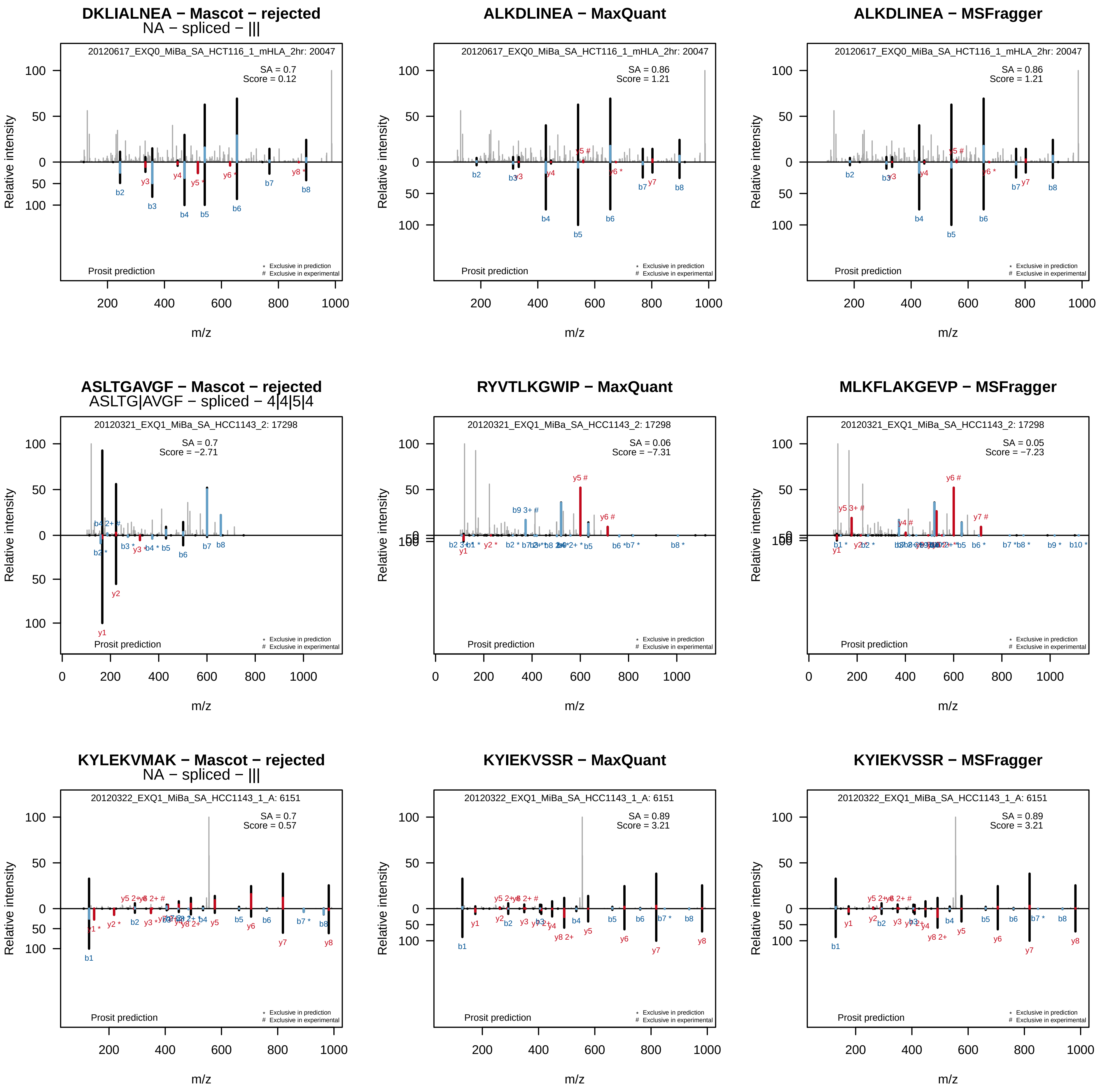

DKLIALNEA − Mascot − rejected
NA − spliced − |||
20120617_EXQ0_MiBa_SA_HCT116_1_mHLA_2hr: 20047
SA = 0.7
Score = 0.12
ALKDLINEA − MaxQuant
20120617_EXQ0_MiBa_SA_HCT116_1_mHLA_2hr: 20047
SA = 0.86
Score = 1.21
ALKDLINEA − MSFragger
20120617_EXQ0_MiBa_SA_HCT116_1_mHLA_2hr: 20047
SA = 0.86
Score = 1.21
ASLTGAVGF − Mascot − rejected
ASLTG|AVGF − spliced − 4|4|5|4
20120321_EXQ1_MiBa_SA_HCC1143_2: 17298
SA = 0.7
Score = −2.71
RYVTLKGWIP − MaxQuant
20120321_EXQ1_MiBa_SA_HCC1143_2: 17298
SA = 0.06
Score = −7.31
MLKFLAKGEVP − MSFragger
20120321_EXQ1_MiBa_SA_HCC1143_2: 17298
SA = 0.05
Score = −7.23
KYLEKVMAK − Mascot − rejected
NA − spliced − |||
20120322_EXQ1_MiBa_SA_HCC1143_1_A: 6151
SA = 0.7
Score = 0.57
KYIEKVSSR − MaxQuant
20120322_EXQ1_MiBa_SA_HCC1143_1_A: 6151
SA = 0.89
Score = 3.21
KYIEKVSSR − MSFragger
20120322_EXQ1_MiBa_SA_HCC1143_1_A: 6151
SA = 0.89
Score = 3.21
Relative intensity
m/z
Prosit prediction
* Exclusive in prediction
# Exclusive in experimental

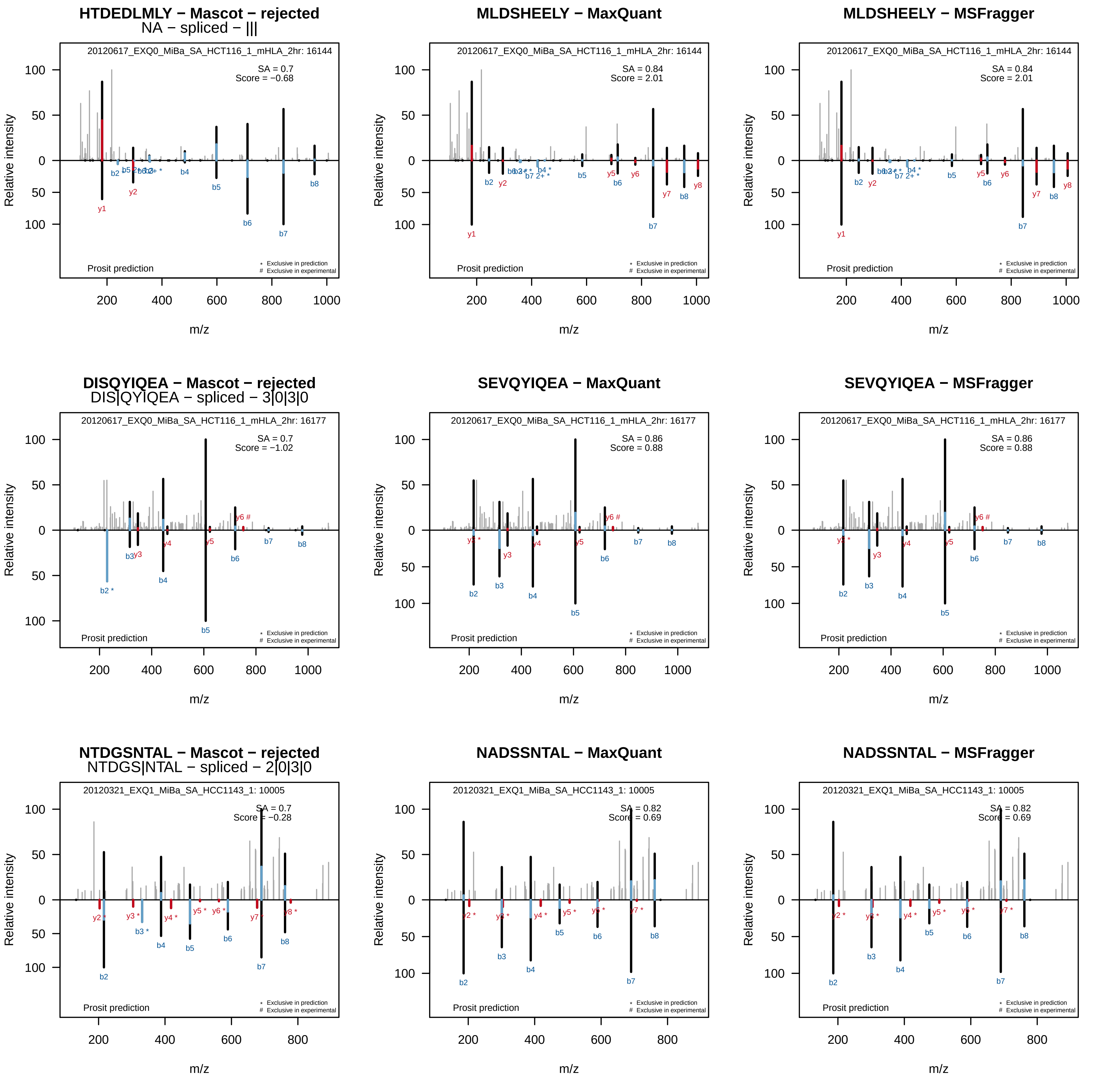
HTDEDLMLY − Mascot − rejected
NA − spliced − |||
20120617_EXQ0_MiBa_SA_HCT116_1_mHLA_2hr: 16144
SA = 0.7
Score = −0.68
MLDSHEELY − MaxQuant
20120617_EXQ0_MiBa_SA_HCT116_1_mHLA_2hr: 16144
SA = 0.84
Score = 2.01
MLDSHEELY − MSFragger
20120617_EXQ0_MiBa_SA_HCT116_1_mHLA_2hr: 16144
SA = 0.84
Score = 2.01
DISQYIQEA − Mascot − rejected
DIS|QYIQEA − spliced − 3|0|3|0
20120617_EXQ0_MiBa_SA_HCT116_1_mHLA_2hr: 16177
SA = 0.7
Score = −1.02
SEVQYIQEA − MaxQuant
20120617_EXQ0_MiBa_SA_HCT116_1_mHLA_2hr: 16177
SA = 0.86
Score = 0.88
SEVQYIQEA − MSFragger
20120617_EXQ0_MiBa_SA_HCT116_1_mHLA_2hr: 16177
SA = 0.86
Score = 0.88
NTDGSNTAL − Mascot − rejected
NTDGS|NTAL − spliced − 2|0|3|0
20120321_EXQ1_MiBa_SA_HCC1143_1: 10005
SA = 0.7
Score = −0.28
NADSSNTAL − MaxQuant
20120321_EXQ1_MiBa_SA_HCC1143_1: 10005
SA = 0.82
Score = 0.69
NADSSNTAL − MSFragger
20120321_EXQ1_MiBa_SA_HCC1143_1: 10005
SA = 0.82
Score = 0.69
Relative intensity
m/z
Prosit prediction
* Exclusive in prediction
# Exclusive in experimental

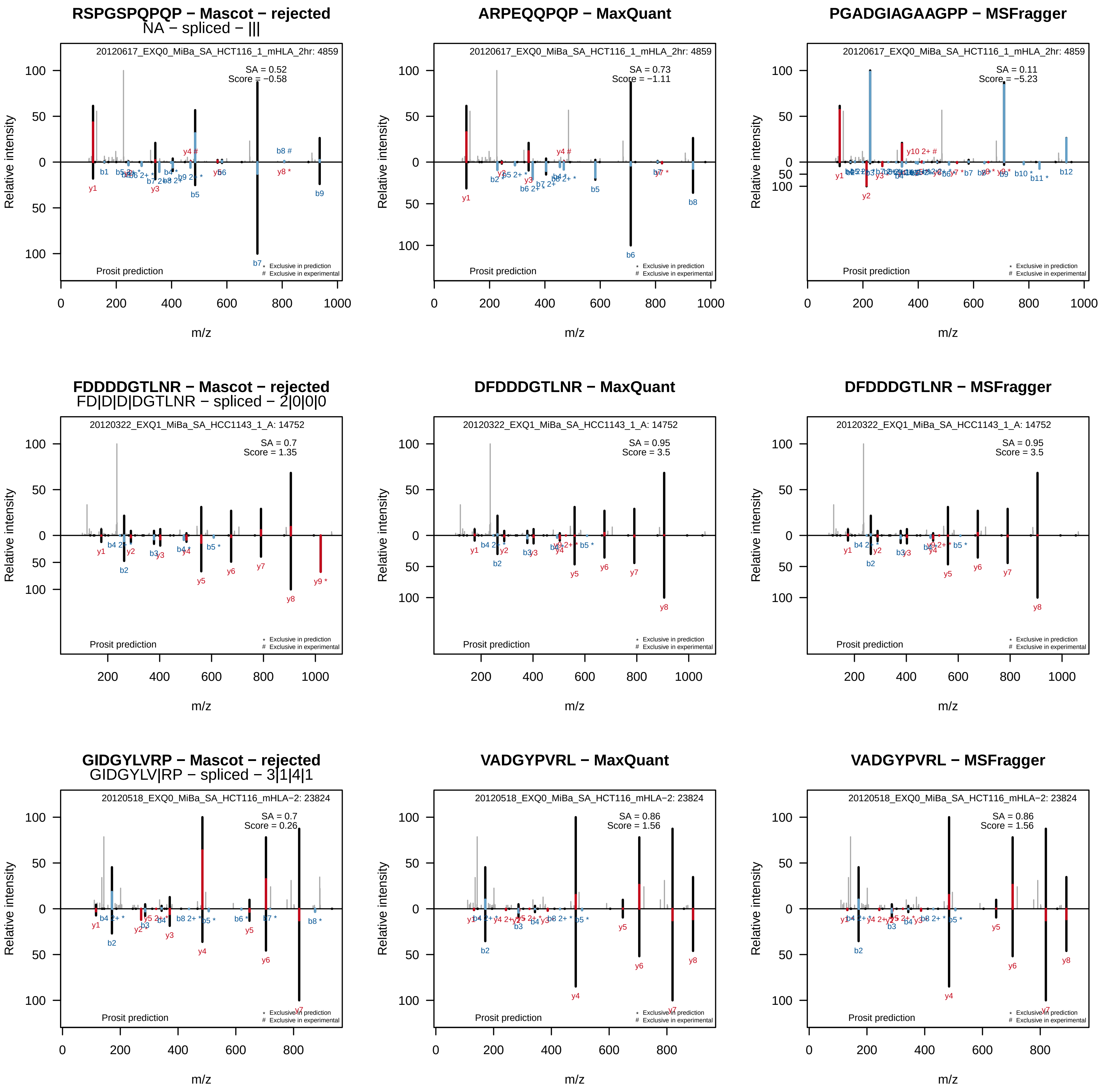
RSPGSPQPQP − Mascot − rejected
NA − spliced − |||
20120617_EXQ0_MiBa_SA_HCT116_1_mHLA_2hr: 4859
SA = 0.52
Score = −0.58
ARPEQQPQP − MaxQuant
20120617_EXQ0_MiBa_SA_HCT116_1_mHLA_2hr: 4859
SA = 0.73
Score = −1.11
PGADGIAGAAGPP − MSFragger
20120617_EXQ0_MiBa_SA_HCT116_1_mHLA_2hr: 4859
SA = 0.11
Score = −5.23
FDDDDGTLNR − Mascot − rejected
FD|D|D|DGTLNR − spliced − 2|0|0|0
20120322_EXQ1_MiBa_SA_HCC1143_1_A: 14752
SA = 0.7
Score = 1.35
DFDDDGTLNR − MaxQuant
20120322_EXQ1_MiBa_SA_HCC1143_1_A: 14752
SA = 0.95
Score = 3.5
DFDDDGTLNR − MSFragger
20120322_EXQ1_MiBa_SA_HCC1143_1_A: 14752
SA = 0.95
Score = 3.5
GIDGYLVRP − Mascot − rejected
GIDGYLV|RP − spliced − 3|1|4|1
20120518_EXQ0_MiBa_SA_HCT116_mHLA−2: 23824
SA = 0.7
Score = 0.26
VADGYPVRL − MaxQuant
20120518_EXQ0_MiBa_SA_HCT116_mHLA−2: 23824
SA = 0.86
Score = 1.56
VADGYPVRL − MSFragger
20120518_EXQ0_MiBa_SA_HCT116_mHLA−2: 23824
SA = 0.86
Score = 1.56
Relative intensity
m/z
Prosit prediction
⋆ Exclusive in prediction
Exclusive in experimental

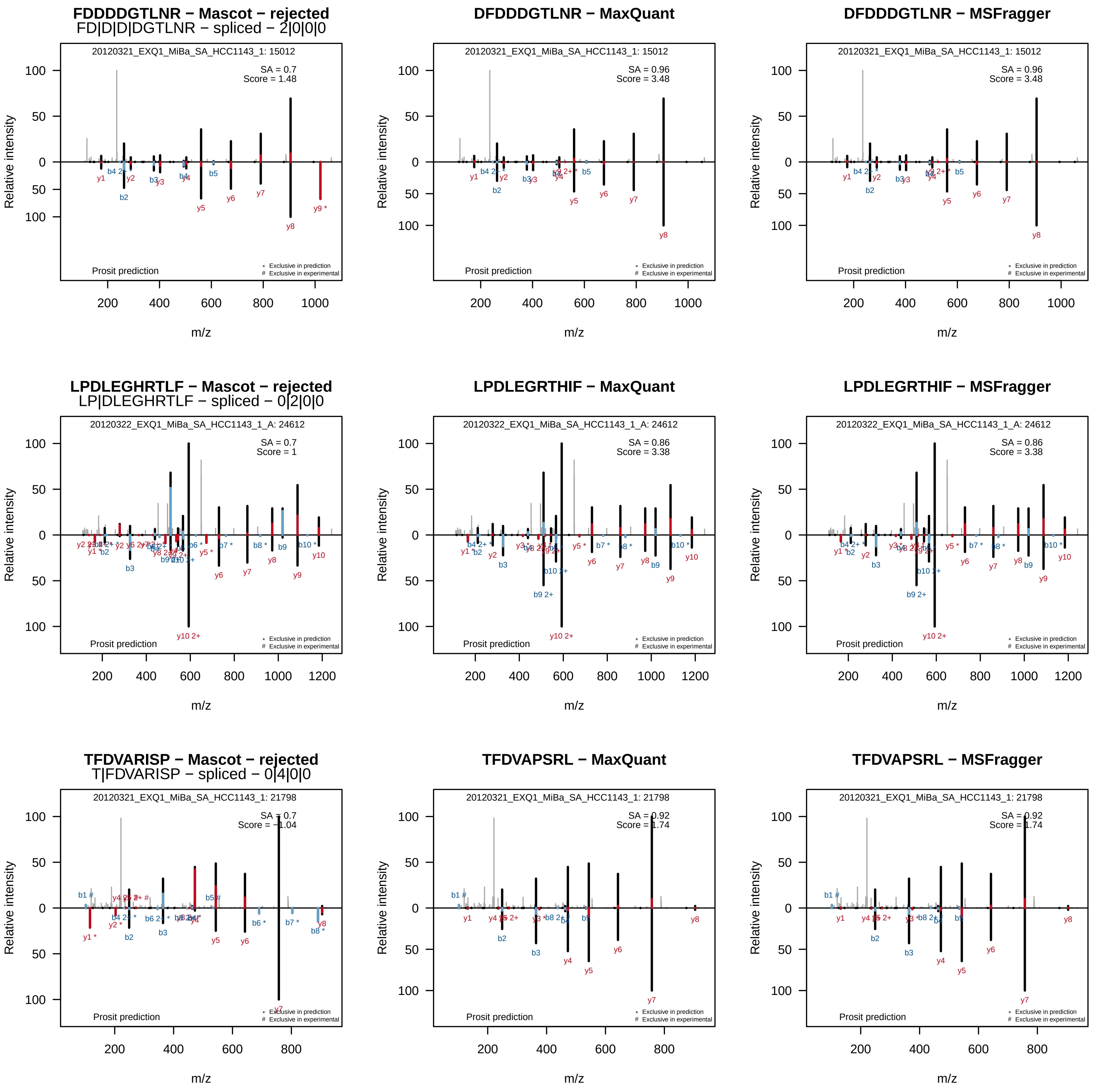
FDDDDGTLNR − Mascot − rejected
FD|D|D|DGTLNR − spliced − 2|0|0|0
DFDDDGTLNR − MaxQuant
DFDDDGTLNR − MSFragger
20120321_EXQ1_MiBa_SA_HCC1143_1: 15012
SA = 0.7
Score = 1.48
SA = 0.96
Score = 3.48
LPDLEGHRTLF − Mascot − rejected
LP|DLEGHRTLF − spliced − 0|2|0|0
LPDLEGRTHIF − MaxQuant
LPDLEGRTHIF − MSFragger
20120322_EXQ1_MiBa_SA_HCC1143_1_A: 24612
SA = 0.7
Score = 1
SA = 0.86
Score = 3.38
TFDVARISP − Mascot − rejected
T|FDVARISP − spliced − 0|4|0|0
TFDVAPSRL − MaxQuant
TFDVAPSRL − MSFragger
20120321_EXQ1_MiBa_SA_HCC1143_1: 21798
SA = 0.7
Score = −1.04
SA = 0.92
Score = 1.74
Relative intensity
m/z
Prosit prediction
⋆ Exclusive in prediction
Exclusive in experimental

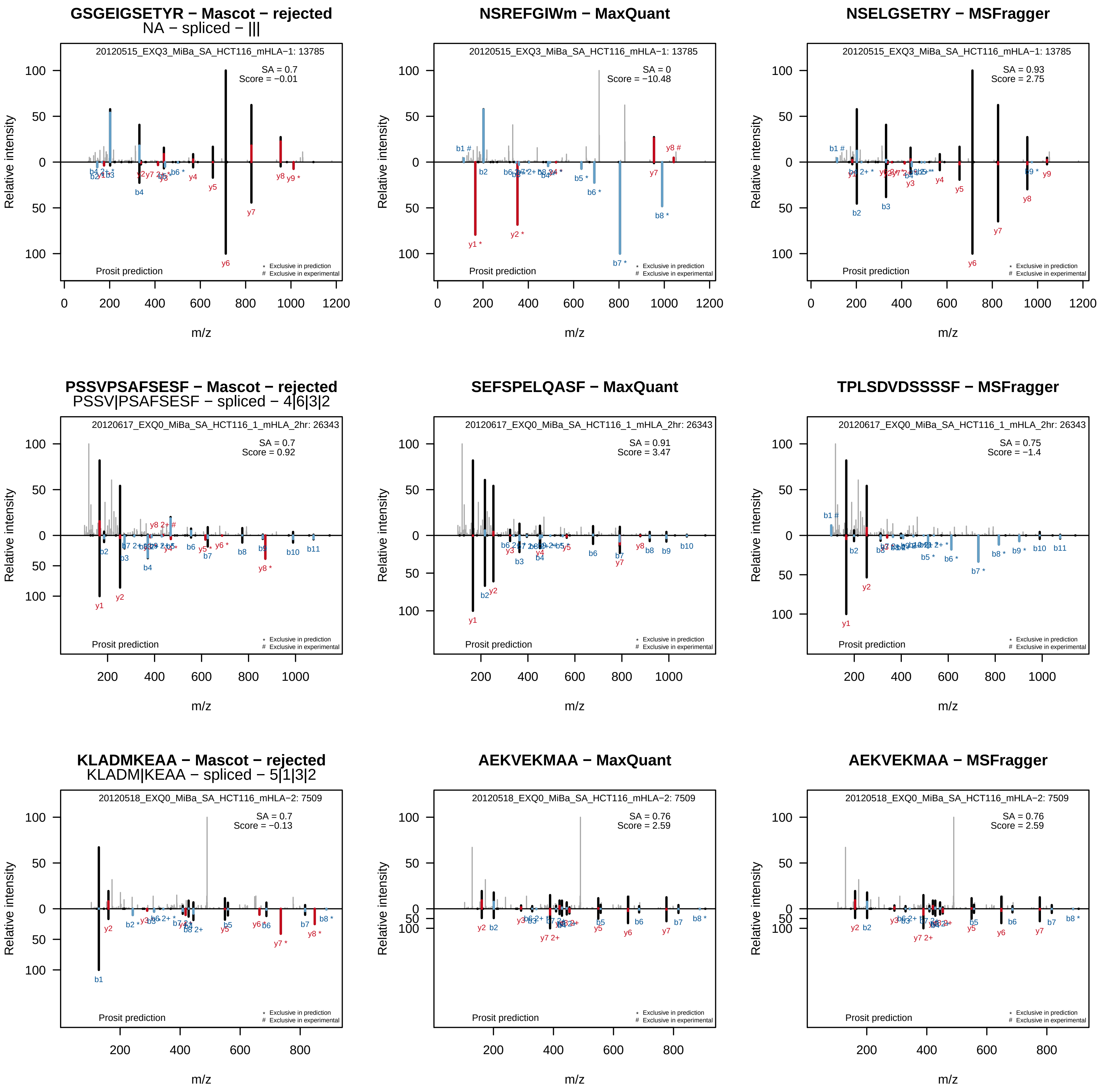
GSGEIGSETYR − Mascot − rejected
NA − spliced − |||
20120515_EXQ3_MiBa_SA_HCT116_mHLA−1: 13785
SA = 0.7
Score = −0.01
NSREFGIWm − MaxQuant
20120515_EXQ3_MiBa_SA_HCT116_mHLA−1: 13785
SA = 0
Score = −10.48
NSELGSETRY − MSFragger
20120515_EXQ3_MiBa_SA_HCT116_mHLA−1: 13785
SA = 0.93
Score = 2.75
PSSVPSAFSESF − Mascot − rejected
PSSV|PSAFSESF − spliced − 4|6|3|2
20120617_EXQ0_MiBa_SA_HCT116_1_mHLA_2hr: 26343
SA = 0.7
Score = 0.92
SEFSPELQASF − MaxQuant
20120617_EXQ0_MiBa_SA_HCT116_1_mHLA_2hr: 26343
SA = 0.91
Score = 3.47
TPLSDVDSSSSF − MSFragger
20120617_EXQ0_MiBa_SA_HCT116_1_mHLA_2hr: 26343
SA = 0.75
Score = −1.4
KLADMKEAA − Mascot − rejected
KLADM|KEAA − spliced − 5|1|3|2
20120518_EXQ0_MiBa_SA_HCT116_mHLA−2: 7509
SA = 0.7
Score = −0.13
AEKVEKMAA − MaxQuant
20120518_EXQ0_MiBa_SA_HCT116_mHLA−2: 7509
SA = 0.76
Score = 2.59
AEKVEKMAA − MSFragger
20120518_EXQ0_MiBa_SA_HCT116_mHLA−2: 7509
SA = 0.76
Score = 2.59
Relative intensity
m/z
Prosit prediction
* Exclusive in prediction
# Exclusive in experimental

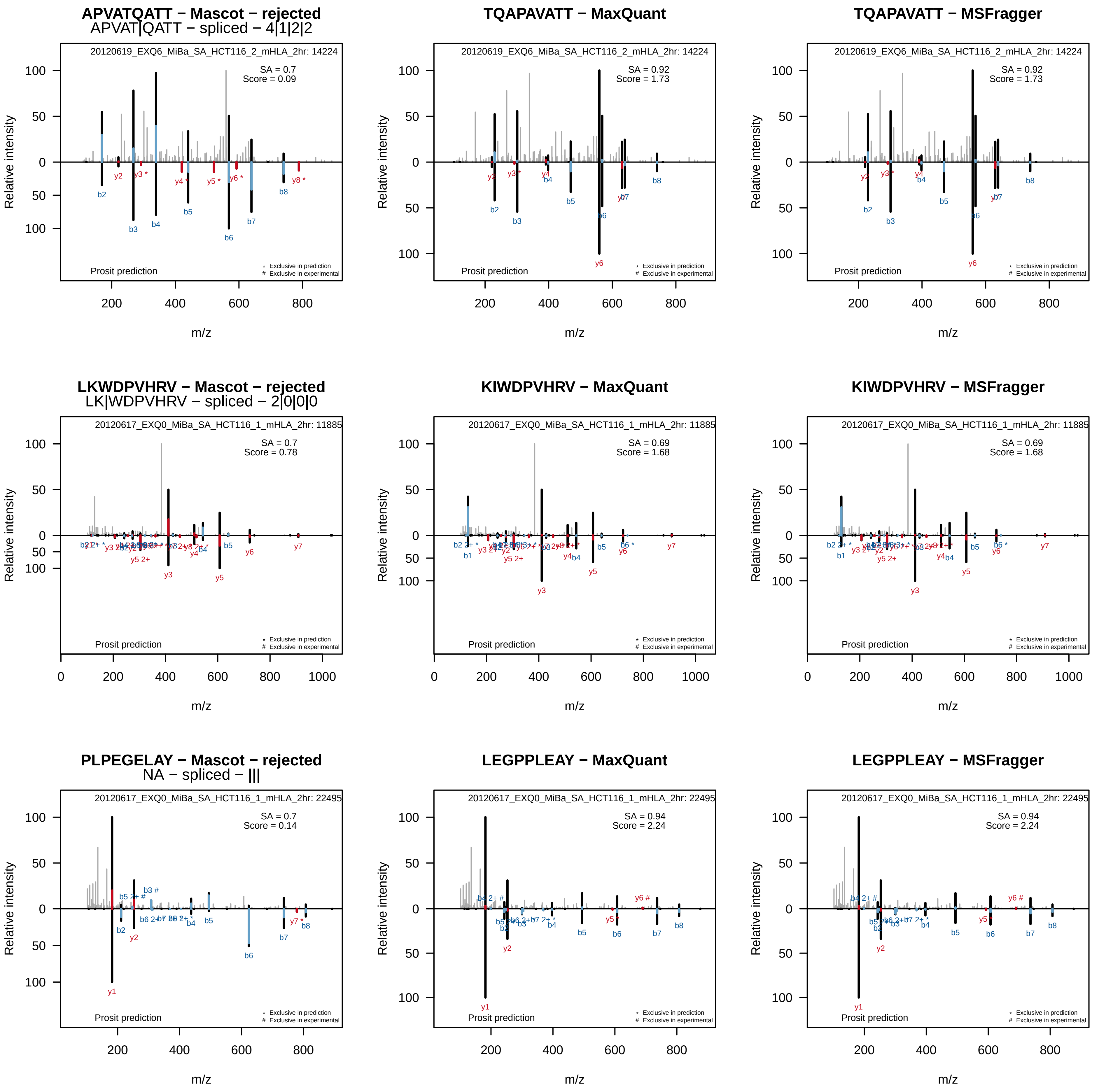
APVATQATT − Mascot − rejected
APVAT|QATT − spliced − 4|1|2|2
TQAPAVATT − MaxQuant
TQAPAVATT − MSFragger
20120619_EXQ6_MiBa_SA_HCT116_2_mHLA_2hr: 14224
SA = 0.7
Score = 0.09
SA = 0.92
Score = 1.73
LKWDPVHRV − Mascot − rejected
LK|WDPVHRV − spliced − 2|0|0|0
KIWDPVHRV − MaxQuant
KIWDPVHRV − MSFragger
20120617_EXQ0_MiBa_SA_HCT116_1_mHLA_2hr: 11885
SA = 0.7
Score = 0.78
SA = 0.69
Score = 1.68
PLPEGELAY − Mascot − rejected
NA − spliced − |||
LEGPPLEAY − MaxQuant
LEGPPLEAY − MSFragger
20120617_EXQ0_MiBa_SA_HCT116_1_mHLA_2hr: 22495
SA = 0.7
Score = 0.14
SA = 0.94
Score = 2.24
Relative intensity
m/z
Prosit prediction
⋆ Exclusive in prediction
Exclusive in experimental

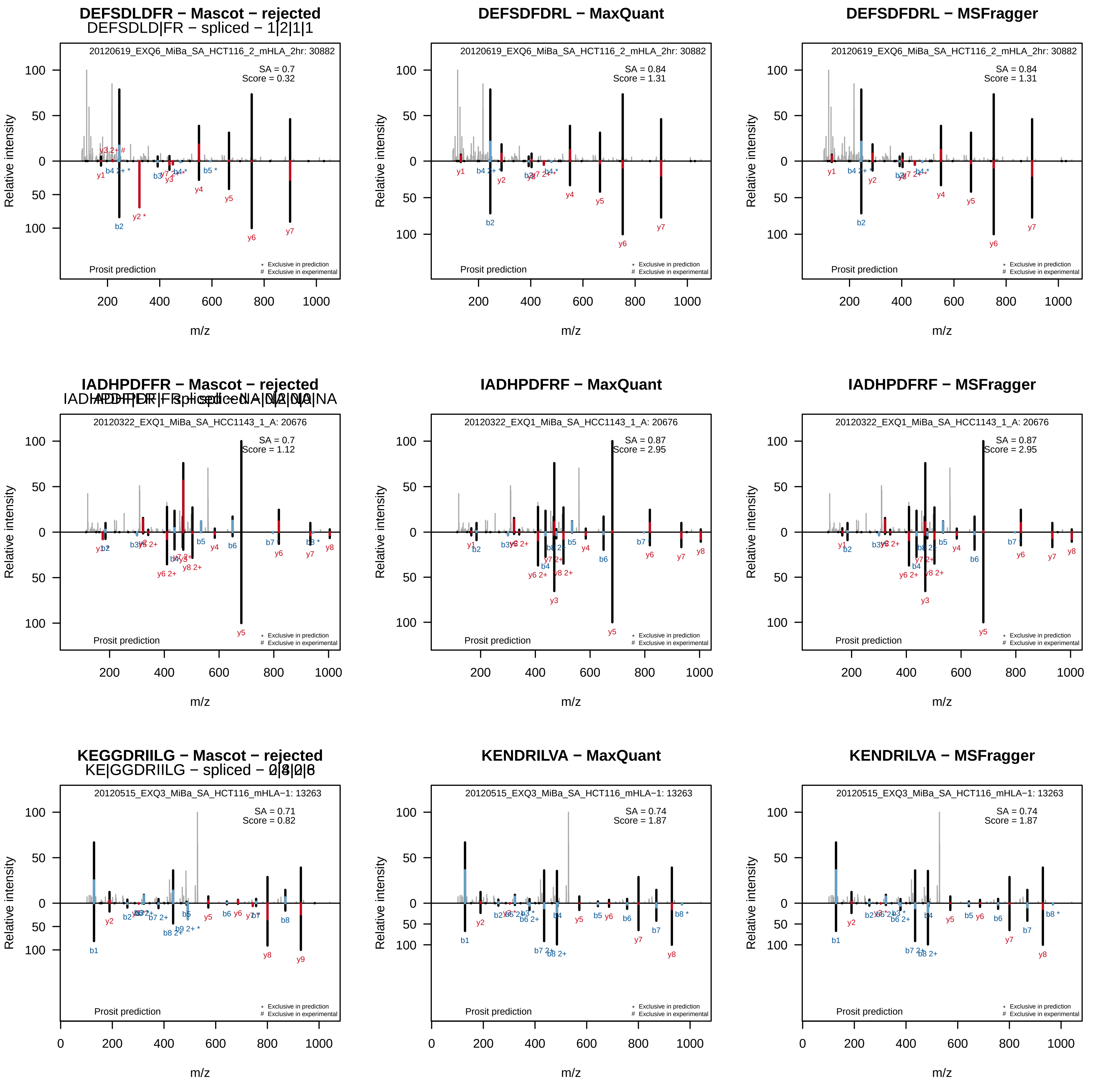

DEFSDLDFR − Mascot − rejected
DEFSDLD|FR − spliced − 1|2|1|1
20120619_EXQ6_MiBa_SA_HCT116_2_mHLA_2hr: 30882
SA = 0.7
Score = 0.32
DEFSDFDRL − MaxQuant
20120619_EXQ6_MiBa_SA_HCT116_2_mHLA_2hr: 30882
SA = 0.84
Score = 1.31
DEFSDFDRL − MSFragger
20120619_EXQ6_MiBa_SA_HCT116_2_mHLA_2hr: 30882
SA = 0.84
Score = 1.31
IADHPDFFR − Mascot − rejected
20120322_EXQ1_MiBa_SA_HCC1143_1_A: 20676
SA = 0.7
Score = 1.12
IADHPDFRF − MaxQuant
20120322_EXQ1_MiBa_SA_HCC1143_1_A: 20676
SA = 0.87
Score = 2.95
IADHPDFRF − MSFragger
20120322_EXQ1_MiBa_SA_HCC1143_1_A: 20676
SA = 0.87
Score = 2.95
KEGGDRIILG − Mascot − rejected
20120515_EXQ3_MiBa_SA_HCT116_mHLA−1: 13263
SA = 0.71
Score = 0.82
KENDRILVA − MaxQuant
20120515_EXQ3_MiBa_SA_HCT116_mHLA−1: 13263
SA = 0.74
Score = 1.87
KENDRILVA − MSFragger
20120515_EXQ3_MiBa_SA_HCT116_mHLA−1: 13263
SA = 0.74
Score = 1.87
Relative intensity
m/z
Prosit prediction
* Exclusive in prediction
# Exclusive in experimental

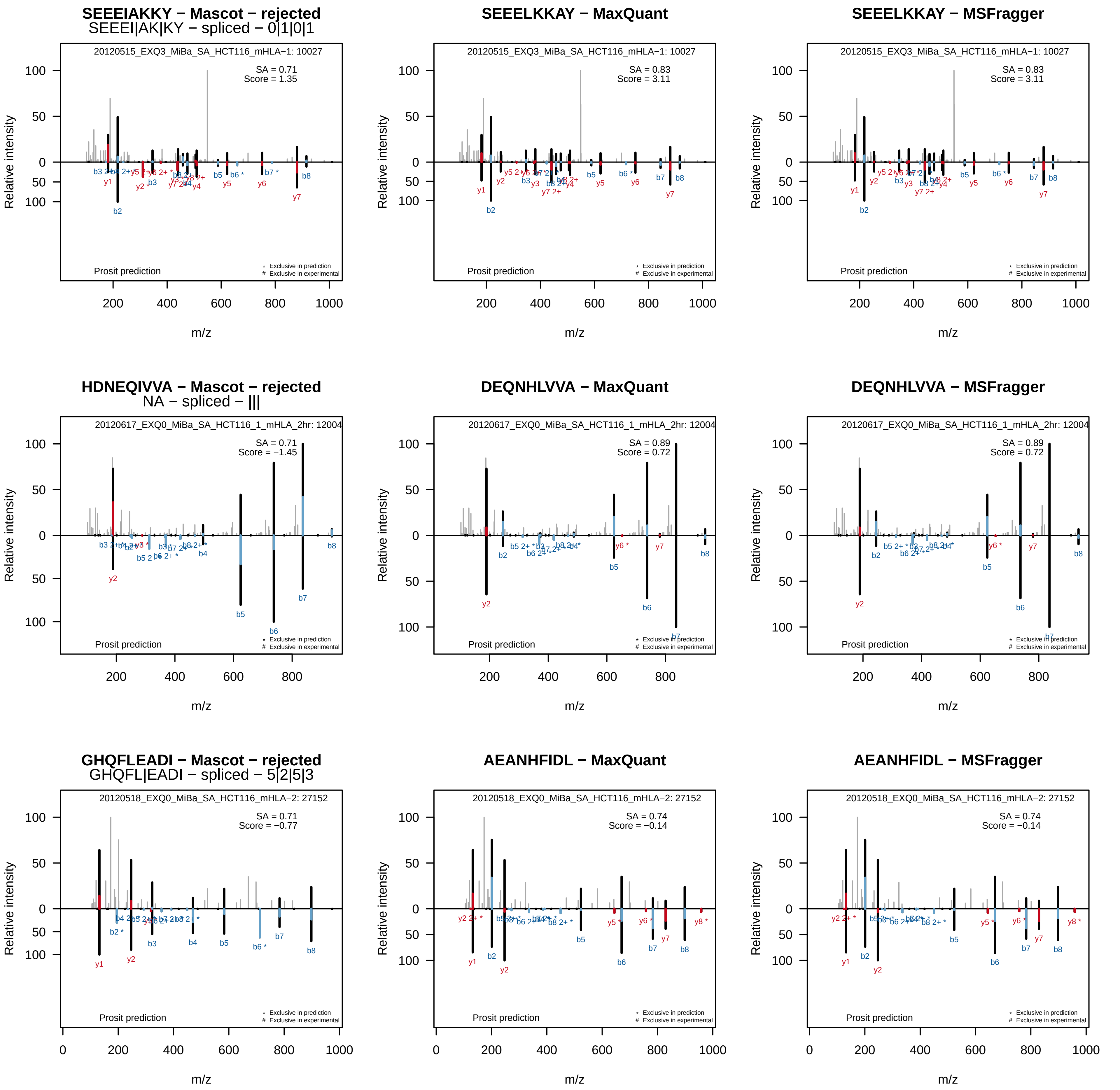

SEEEIAKKY − Mascot − rejected
SEEEI|AK|KY − spliced − 0|1|0|1
20120515_EXQ3_MiBa_SA_HCT116_mHLA−1: 10027
SA = 0.71
Score = 1.35
SEEELKKAY − MaxQuant
20120515_EXQ3_MiBa_SA_HCT116_mHLA−1: 10027
SA = 0.83
Score = 3.11
SEEELKKAY − MSFragger
20120515_EXQ3_MiBa_SA_HCT116_mHLA−1: 10027
SA = 0.83
Score = 3.11
HDNEQIVVA − Mascot − rejected
NA − spliced − |||
20120617_EXQ0_MiBa_SA_HCT116_1_mHLA_2hr: 12004
SA = 0.71
Score = −1.45
DEQNHLVVA − MaxQuant
20120617_EXQ0_MiBa_SA_HCT116_1_mHLA_2hr: 12004
SA = 0.89
Score = 0.72
DEQNHLVVA − MSFragger
20120617_EXQ0_MiBa_SA_HCT116_1_mHLA_2hr: 12004
SA = 0.89
Score = 0.72
GHQFLEADI − Mascot − rejected
GHQFL|EADI − spliced − 5|2|5|3
20120518_EXQ0_MiBa_SA_HCT116_mHLA−2: 27152
SA = 0.71
Score = −0.77
AEANHFIDL − MaxQuant
20120518_EXQ0_MiBa_SA_HCT116_mHLA−2: 27152
SA = 0.74
Score = −0.14
AEANHFIDL − MSFragger
20120518_EXQ0_MiBa_SA_HCT116_mHLA−2: 27152
SA = 0.74
Score = −0.14
Relative intensity
m/z
Prosit prediction
* Exclusive in prediction
# Exclusive in experimental

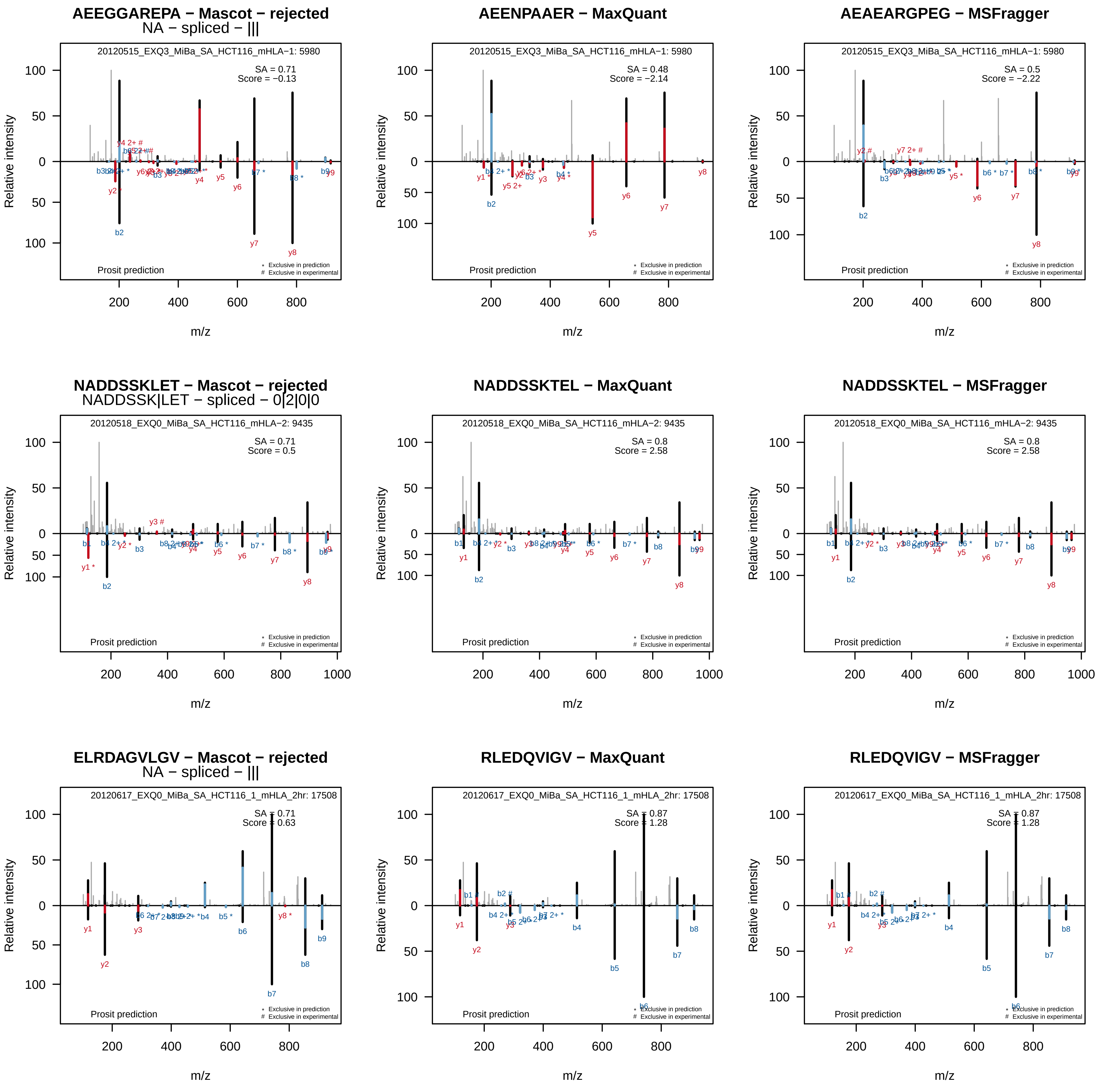
AEEGGAREPA − Mascot − rejected
NA − spliced − |||
20120515_EXQ3_MiBa_SA_HCT116_mHLA−1: 5980
SA = 0.71
Score = −0.13
AEENPAAER − MaxQuant
20120515_EXQ3_MiBa_SA_HCT116_mHLA−1: 5980
SA = 0.48
Score = −2.14
AEAEARGPEG − MSFragger
20120515_EXQ3_MiBa_SA_HCT116_mHLA−1: 5980
SA = 0.5
Score = −2.22
NADDSSKLET − Mascot − rejected
NADDSSK|LET − spliced − 0|2|0|0
20120518_EXQ0_MiBa_SA_HCT116_mHLA−2: 9435
SA = 0.71
Score = 0.5
NADDSSKTEL − MaxQuant
20120518_EXQ0_MiBa_SA_HCT116_mHLA−2: 9435
SA = 0.8
Score = 2.58
NADDSSKTEL − MSFragger
20120518_EXQ0_MiBa_SA_HCT116_mHLA−2: 9435
SA = 0.8
Score = 2.58
ELRDAGVLGV − Mascot − rejected
NA − spliced − |||
20120617_EXQ0_MiBa_SA_HCT116_1_mHLA_2hr: 17508
SA = 0.71
Score = 0.63
RLEDQVIGV − MaxQuant
20120617_EXQ0_MiBa_SA_HCT116_1_mHLA_2hr: 17508
SA = 0.87
Score = 1.28
RLEDQVIGV − MSFragger
20120617_EXQ0_MiBa_SA_HCT116_1_mHLA_2hr: 17508
SA = 0.87
Score = 1.28
Relative intensity
m/z
Prosit prediction
⋆ Exclusive in prediction
Exclusive in experimental

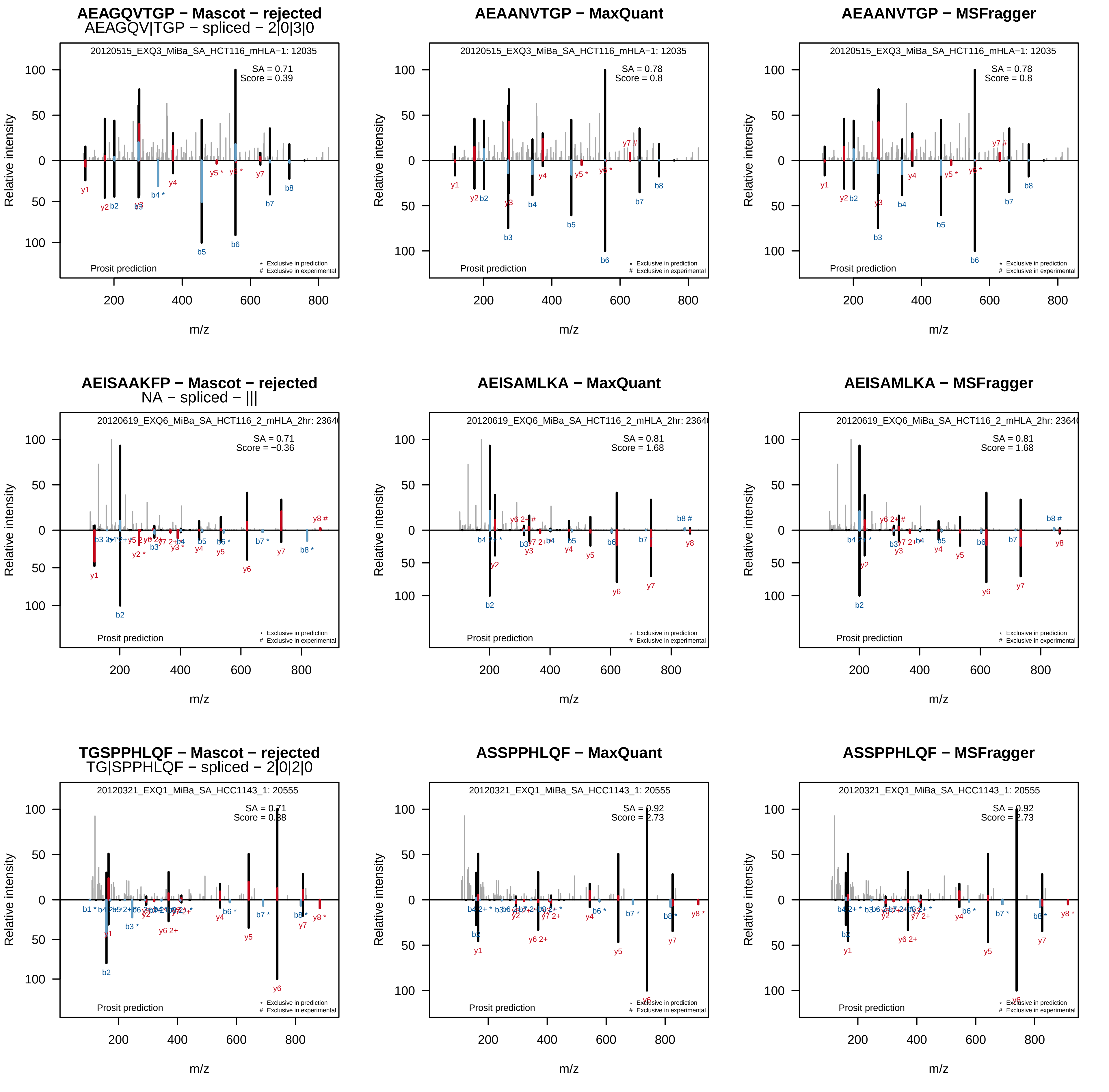

AEAGQVTGP − Mascot − rejected
AEAGQV|TGP − spliced − 2|0|3|0
20120515_EXQ3_MiBa_SA_HCT116_mHLA−1: 12035
SA = 0.71
Score = 0.39
AEAANVTGP − MaxQuant
20120515_EXQ3_MiBa_SA_HCT116_mHLA−1: 12035
SA = 0.78
Score = 0.8
AEAANVTGP − MSFragger
20120515_EXQ3_MiBa_SA_HCT116_mHLA−1: 12035
SA = 0.78
Score = 0.8
AEISAAKFP − Mascot − rejected
NA − spliced − |||
20120619_EXQ6_MiBa_SA_HCT116_2_mHLA_2hr: 23640
SA = 0.71
Score = −0.36
AEISAMLKA − MaxQuant
20120619_EXQ6_MiBa_SA_HCT116_2_mHLA_2hr: 23640
SA = 0.81
Score = 1.68
AEISAMLKA − MSFragger
20120619_EXQ6_MiBa_SA_HCT116_2_mHLA_2hr: 23640
SA = 0.81
Score = 1.68
TGSPPHLQF − Mascot − rejected
TG|SPPHLQF − spliced − 2|0|2|0
20120321_EXQ1_MiBa_SA_HCC1143_1: 20555
SA = 0.71
Score = 0.38
ASSPPHLQF − MaxQuant
20120321_EXQ1_MiBa_SA_HCC1143_1: 20555
SA = 0.92
Score = 2.73
ASSPPHLQF − MSFragger
20120321_EXQ1_MiBa_SA_HCC1143_1: 20555
SA = 0.92
Score = 2.73
Relative intensity
m/z
Prosit prediction
* Exclusive in prediction
# Exclusive in experimental

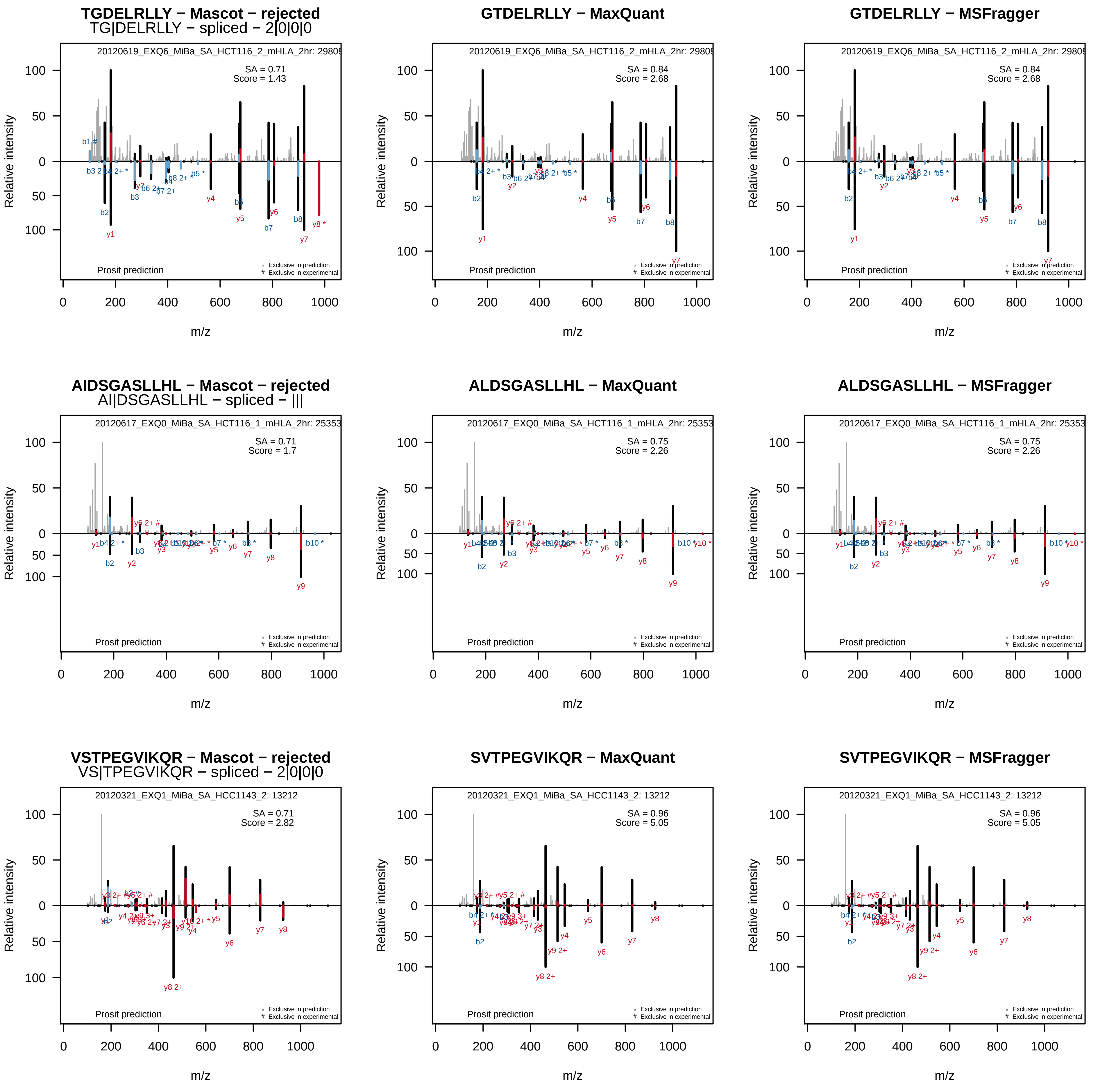

TGDELRLLY − Mascot − rejected
TG|DELRLLY − spliced − 2|0|0|0
20120619_EXQ6_MiBa_SA_HCT116_2_mHLA_2hr: 2980
SA = 0.71
Score = 1.43
GTDELRLLY − MaxQuant
20120619_EXQ6_MiBa_SA_HCT116_2_mHLA_2hr: 2980
SA = 0.84
Score = 2.68
GTDELRLLY − MSFragger
20120619_EXQ6_MiBa_SA_HCT116_2_mHLA_2hr: 2980
SA = 0.84
Score = 2.68
AIDSGASLLHL − Mascot − rejected
AI|DSGASLLHL − spliced − |||
20120617_EXQ0_MiBa_SA_HCT116_1_mHLA_2hr: 25353
SA = 0.71
Score = 1.7
ALDSGASLLHL − MaxQuant
20120617_EXQ0_MiBa_SA_HCT116_1_mHLA_2hr: 25353
SA = 0.75
Score = 2.26
ALDSGASLLHL − MSFragger
20120617_EXQ0_MiBa_SA_HCT116_1_mHLA_2hr: 25353
SA = 0.75
Score = 2.26
VSTPEGVIKQR − Mascot − rejected
VS|TPEGVIKQR − spliced − 2|0|0|0
20120321_EXQ1_MiBa_SA_HCC1143_2: 13212
SA = 0.71
Score = 2.82
SVTPEGVIKQR − MaxQuant
20120321_EXQ1_MiBa_SA_HCC1143_2: 13212
SA = 0.96
Score = 5.05
SVTPEGVIKQR − MSFragger
20120321_EXQ1_MiBa_SA_HCC1143_2: 13212
SA = 0.96
Score = 5.05
Relative intensity
m/z
Prosit prediction
* Exclusive in prediction
# Exclusive in experimental

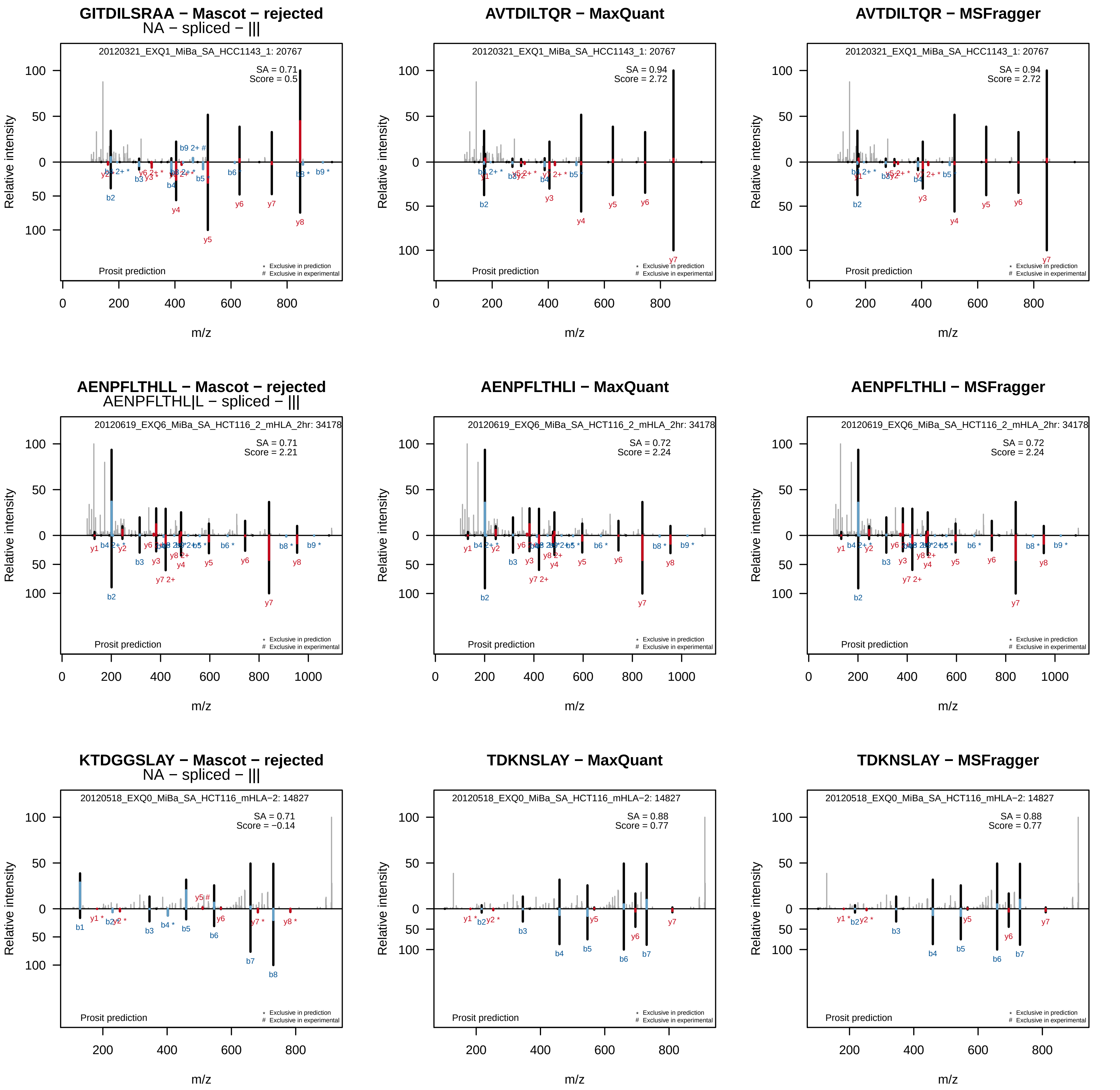

GITDILSRAA − Mascot − rejected
NA − spliced − |||
20120321_EXQ1_MiBa_SA_HCC1143_1: 20767
SA = 0.71
Score = 0.5
AVTDILTQR − MaxQuant
20120321_EXQ1_MiBa_SA_HCC1143_1: 20767
SA = 0.94
Score = 2.72
AVTDILTQR − MSFragger
20120321_EXQ1_MiBa_SA_HCC1143_1: 20767
SA = 0.94
Score = 2.72
AENPFLTHLL − Mascot − rejected
AENPFLTHL|L − spliced − |||
20120619_EXQ6_MiBa_SA_HCT116_2_mHLA_2hr: 34178
SA = 0.71
Score = 2.21
AENPFLTHLI − MaxQuant
20120619_EXQ6_MiBa_SA_HCT116_2_mHLA_2hr: 34178
SA = 0.72
Score = 2.24
AENPFLTHLI − MSFragger
20120619_EXQ6_MiBa_SA_HCT116_2_mHLA_2hr: 34178
SA = 0.72
Score = 2.24
KTDGGSLAY − Mascot − rejected
NA − spliced − |||
20120518_EXQ0_MiBa_SA_HCT116_mHLA−2: 14827
SA = 0.71
Score = −0.14
TDKNSLAY − MaxQuant
20120518_EXQ0_MiBa_SA_HCT116_mHLA−2: 14827
SA = 0.88
Score = 0.77
TDKNSLAY − MSFragger
20120518_EXQ0_MiBa_SA_HCT116_mHLA−2: 14827
SA = 0.88
Score = 0.77
Relative intensity
m/z
Prosit prediction
* Exclusive in prediction
# Exclusive in experimental

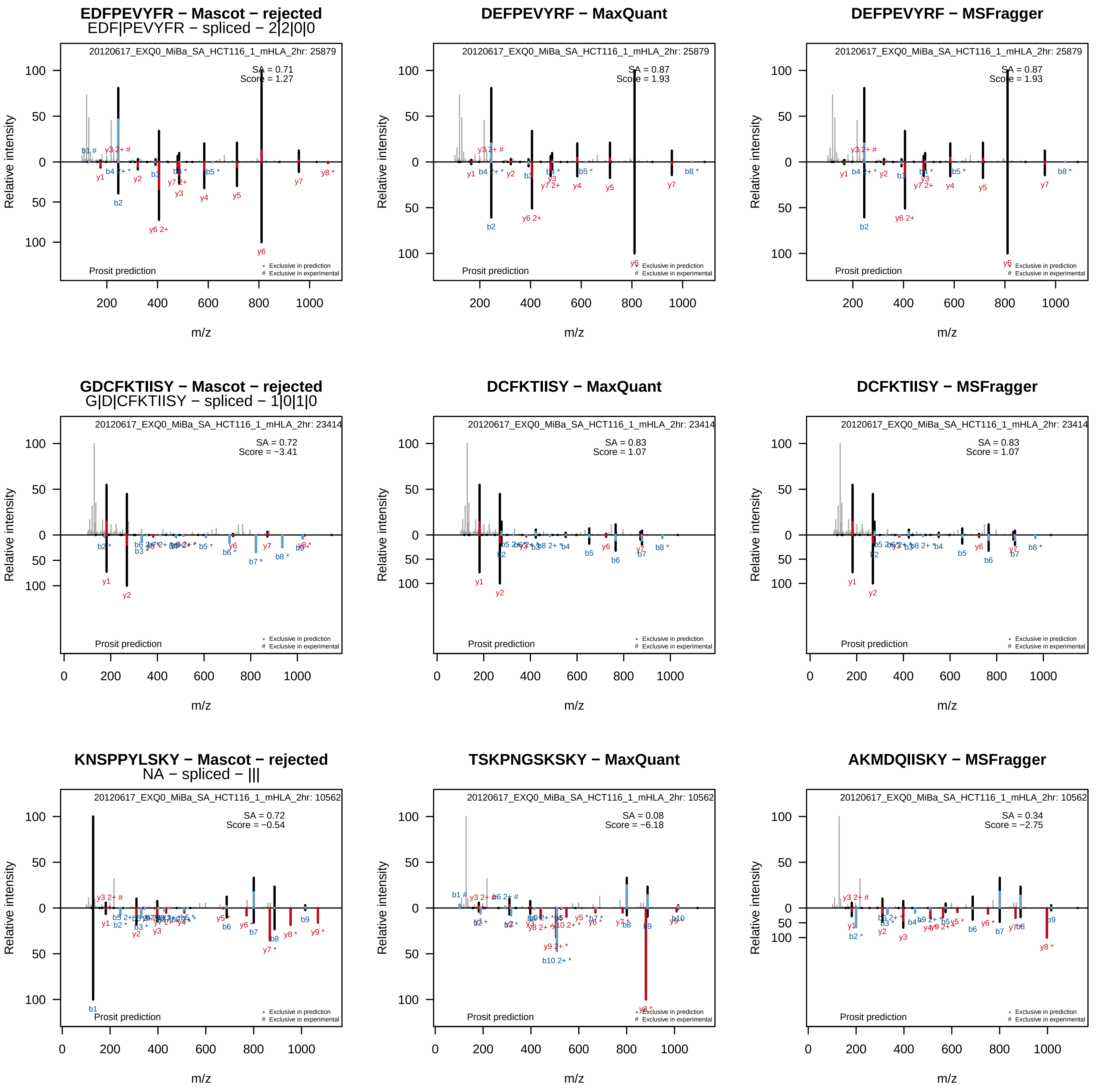
EDFPEVYFR − Mascot − rejected
EDF|PEVYFR − spliced − 2|2|0|0
20120617_EXQ0_MiBa_SA_HCT116_1_mHLA_2hr: 25879
SA = 0.71
Score = 1.27
DEFPEVYRF − MaxQuant
20120617_EXQ0_MiBa_SA_HCT116_1_mHLA_2hr: 25879
SA = 0.87
Score = 1.93
DEFPEVYRF − MSFragger
20120617_EXQ0_MiBa_SA_HCT116_1_mHLA_2hr: 25879
SA = 0.87
Score = 1.93
GDCFKTIISY − Mascot − rejected
G|D|CFKTIISY − spliced − 1|0|1|0
20120617_EXQ0_MiBa_SA_HCT116_1_mHLA_2hr: 23414
SA = 0.72
Score = −3.41
DCFKTIISY − MaxQuant
20120617_EXQ0_MiBa_SA_HCT116_1_mHLA_2hr: 23414
SA = 0.83
Score = 1.07
DCFKTIISY − MSFragger
20120617_EXQ0_MiBa_SA_HCT116_1_mHLA_2hr: 23414
SA = 0.83
Score = 1.07
KNSPPYLSKY − Mascot − rejected
NA − spliced − |||
20120617_EXQ0_MiBa_SA_HCT116_1_mHLA_2hr: 10562
SA = 0.72
Score = −0.54
TSKPNGSKSKY − MaxQuant
20120617_EXQ0_MiBa_SA_HCT116_1_mHLA_2hr: 10562
SA = 0.08
Score = −6.18
AKMDQIISKY − MSFragger
20120617_EXQ0_MiBa_SA_HCT116_1_mHLA_2hr: 10562
SA = 0.34
Score = −2.75
Relative intensity
m/z
Prosit prediction
* Exclusive in prediction
# Exclusive in experimental

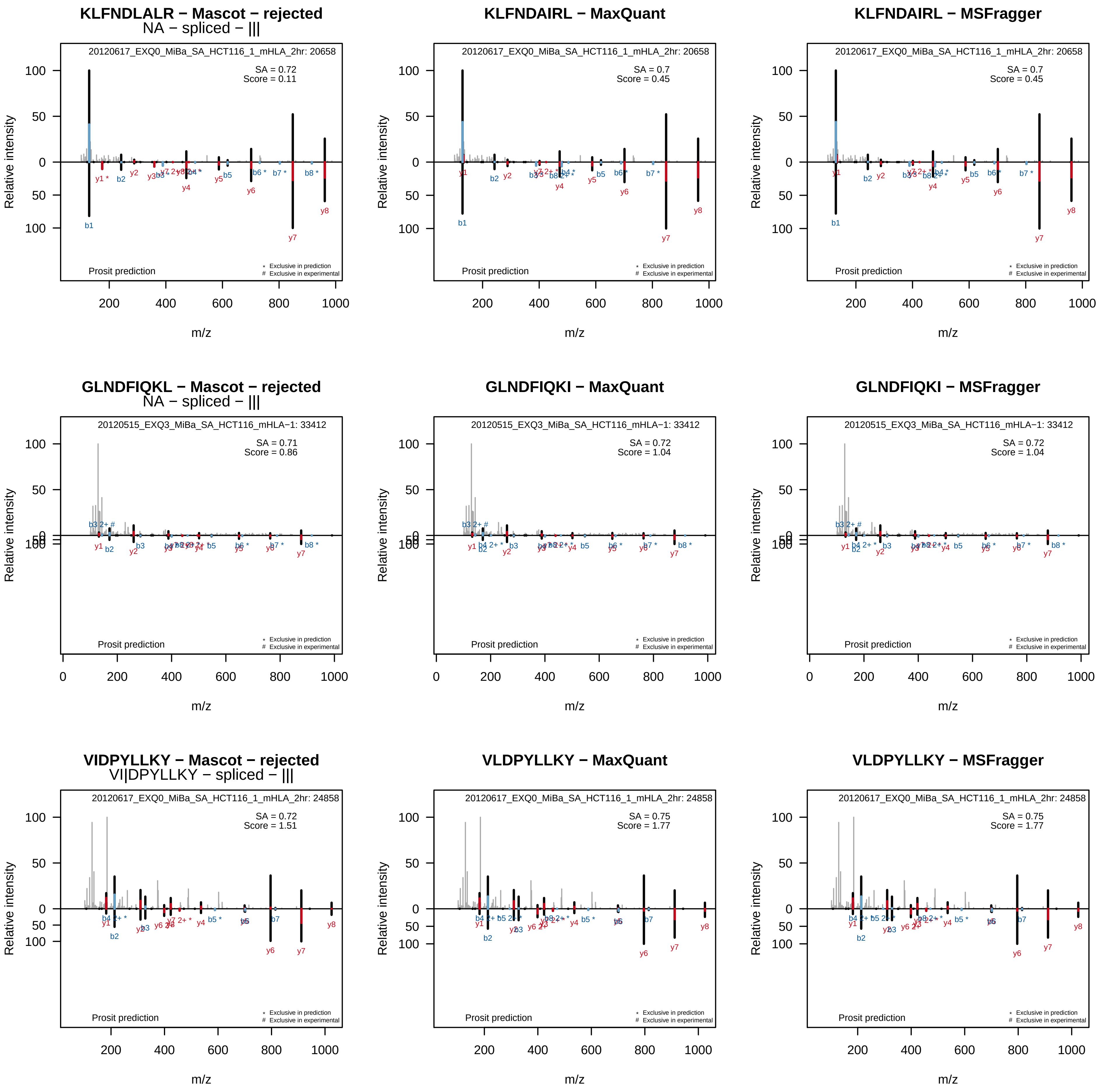
KLFNDLALR − Mascot − rejected
NA − spliced − |||
20120617_EXQ0_MiBa_SA_HCT116_1_mHLA_2hr: 20658
SA = 0.72
Score = 0.11
KLFNDAIRL − MaxQuant
20120617_EXQ0_MiBa_SA_HCT116_1_mHLA_2hr: 20658
SA = 0.7
Score = 0.45
KLFNDAIRL − MSFragger
20120617_EXQ0_MiBa_SA_HCT116_1_mHLA_2hr: 20658
SA = 0.7
Score = 0.45
GLNDFIQKL − Mascot − rejected
NA − spliced − |||
20120515_EXQ3_MiBa_SA_HCT116_mHLA−1: 33412
SA = 0.71
Score = 0.86
GLNDFIQKI − MaxQuant
20120515_EXQ3_MiBa_SA_HCT116_mHLA−1: 33412
SA = 0.72
Score = 1.04
GLNDFIQKI − MSFragger
20120515_EXQ3_MiBa_SA_HCT116_mHLA−1: 33412
SA = 0.72
Score = 1.04
VIDPYLLKY − Mascot − rejected
VI|DPYLLKY − spliced − |||
20120617_EXQ0_MiBa_SA_HCT116_1_mHLA_2hr: 24858
SA = 0.72
Score = 1.51
VLDPYLLKY − MaxQuant
20120617_EXQ0_MiBa_SA_HCT116_1_mHLA_2hr: 24858
SA = 0.75
Score = 1.77
VLDPYLLKY − MSFragger
20120617_EXQ0_MiBa_SA_HCT116_1_mHLA_2hr: 24858
SA = 0.75
Score = 1.77
Relative intensity
m/z
Prosit prediction
* Exclusive in prediction
# Exclusive in experimental

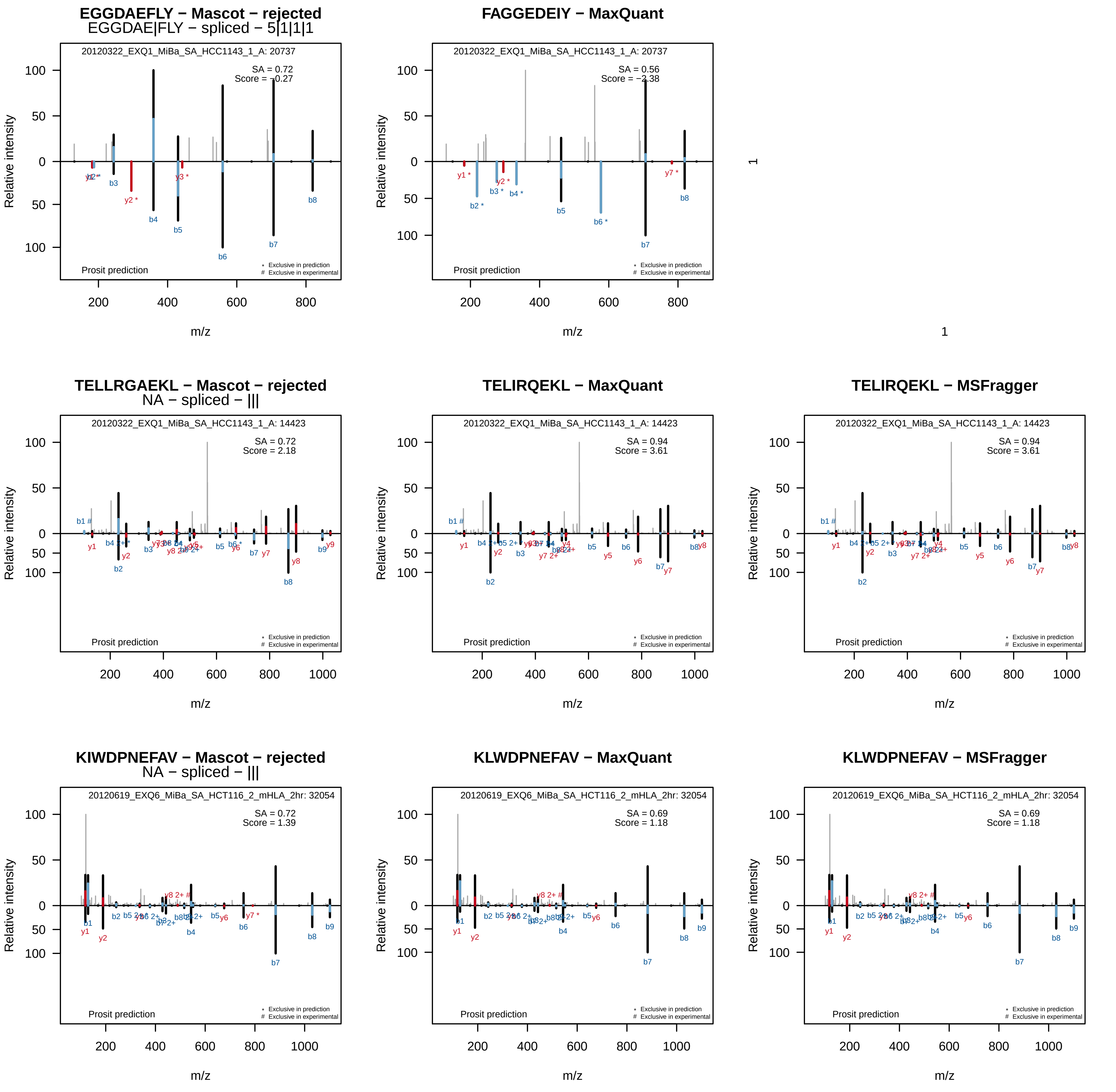
EGGDAEFLY − Mascot − rejected
EGGDAE|FLY − spliced − 5|1|1|1
20120322_EXQ1_MiBa_SA_HCC1143_1_A: 20737
SA = 0.72
Score = −0.27
FAGGEDEIY − MaxQuant
20120322_EXQ1_MiBa_SA_HCC1143_1_A: 20737
SA = 0.56
Score = −2.38
TELLRGAEKL − Mascot − rejected
NA − spliced − |||
20120322_EXQ1_MiBa_SA_HCC1143_1_A: 14423
SA = 0.72
Score = 2.18
TELIRQEKL − MaxQuant
20120322_EXQ1_MiBa_SA_HCC1143_1_A: 14423
SA = 0.94
Score = 3.61
TELIRQEKL − MSFragger
20120322_EXQ1_MiBa_SA_HCC1143_1_A: 14423
SA = 0.94
Score = 3.61
KIWDPNEFAV − Mascot − rejected
NA − spliced − |||
20120619_EXQ6_MiBa_SA_HCT116_2_mHLA_2hr: 32054
SA = 0.72
Score = 1.39
KLWDPNEFAV − MaxQuant
20120619_EXQ6_MiBa_SA_HCT116_2_mHLA_2hr: 32054
SA = 0.69
Score = 1.18
KLWDPNEFAV − MSFragger
20120619_EXQ6_MiBa_SA_HCT116_2_mHLA_2hr: 32054
SA = 0.69
Score = 1.18
Relative intensity
m/z
Prosit prediction
⋆ Exclusive in prediction
Exclusive in experimental

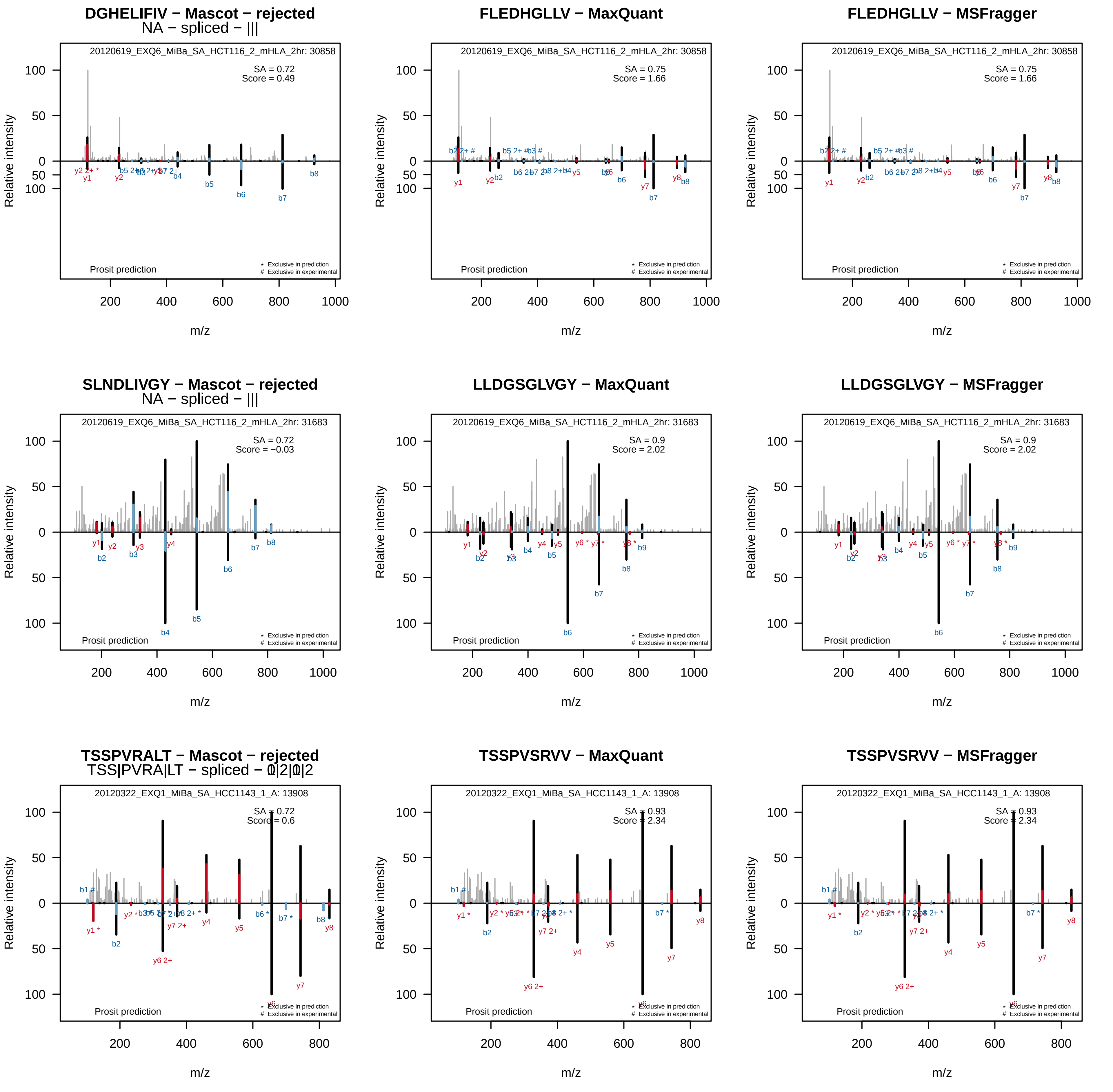
DGHELIFIV − Mascot − rejected
NA − spliced − |||
20120619_EXQ6_MiBa_SA_HCT116_2_mHLA_2hr: 30858
SA = 0.72
Score = 0.49
FLEDHGLLV − MaxQuant
20120619_EXQ6_MiBa_SA_HCT116_2_mHLA_2hr: 30858
SA = 0.75
Score = 1.66
FLEDHGLLV − MSFragger
20120619_EXQ6_MiBa_SA_HCT116_2_mHLA_2hr: 30858
SA = 0.75
Score = 1.66
SLNDLIVGY − Mascot − rejected
NA − spliced − |||
20120619_EXQ6_MiBa_SA_HCT116_2_mHLA_2hr: 31683
SA = 0.72
Score = −0.03
LLDGSGLVGY − MaxQuant
20120619_EXQ6_MiBa_SA_HCT116_2_mHLA_2hr: 31683
SA = 0.9
Score = 2.02
LLDGSGLVGY − MSFragger
20120619_EXQ6_MiBa_SA_HCT116_2_mHLA_2hr: 31683
SA = 0.9
Score = 2.02
TSSPVRALT − Mascot − rejected
TSS|PVRA|LT − spliced − 0|2|0|2
20120322_EXQ1_MiBa_SA_HCC1143_1_A: 13908
SA = 0.72
Score = 0.6
TSSPVSRVV − MaxQuant
20120322_EXQ1_MiBa_SA_HCC1143_1_A: 13908
SA = 0.93
Score = 2.34
TSSPVSRVV − MSFragger
20120322_EXQ1_MiBa_SA_HCC1143_1_A: 13908
SA = 0.93
Score = 2.34
Relative intensity
m/z
Prosit prediction
* Exclusive in prediction
# Exclusive in experimental

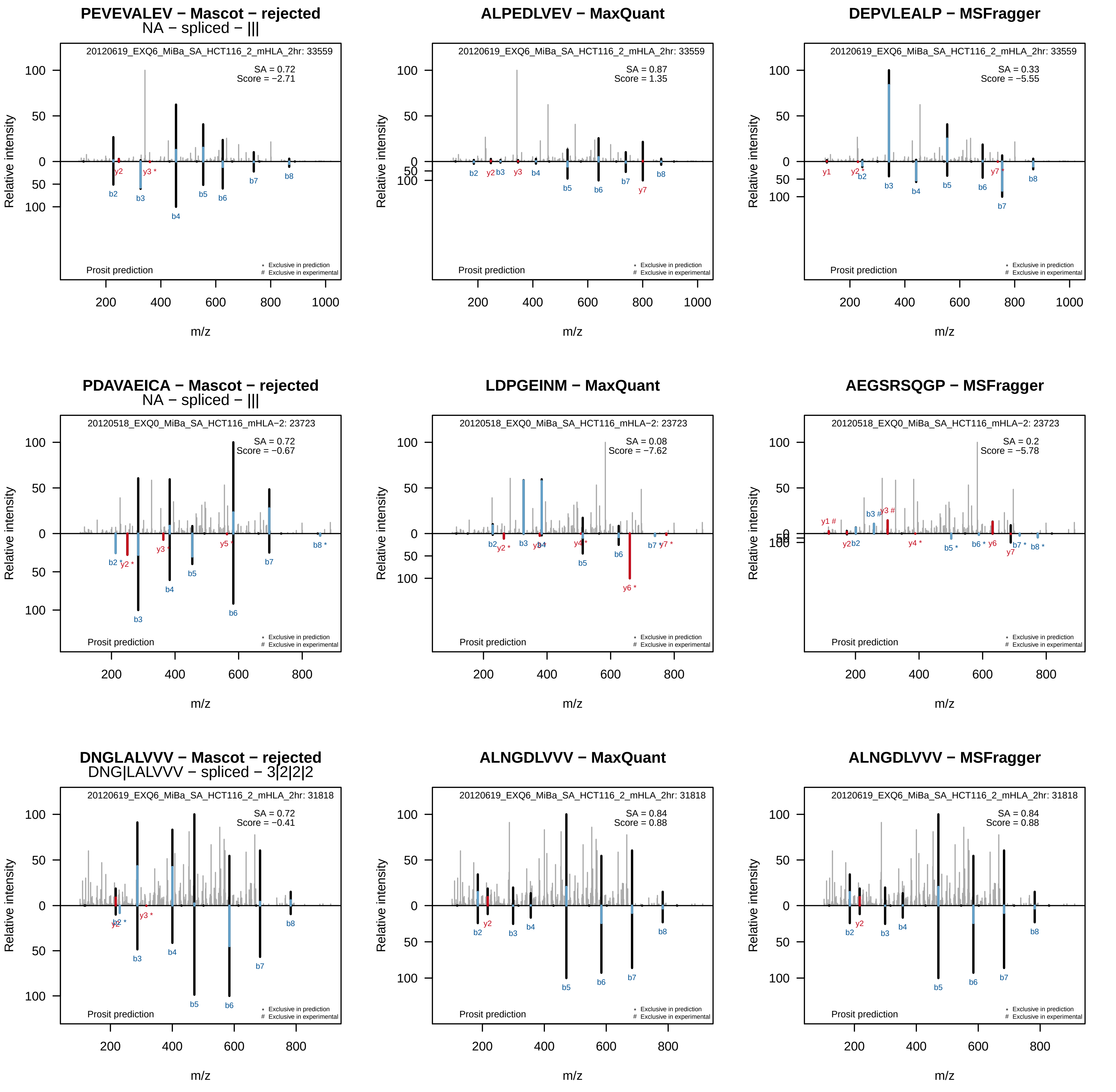
PEVEVALEV − Mascot − rejected
NA − spliced − |||
20120619_EXQ6_MiBa_SA_HCT116_2_mHLA_2hr: 33559
SA = 0.72
Score = −2.71
ALPEDLVEV − MaxQuant
20120619_EXQ6_MiBa_SA_HCT116_2_mHLA_2hr: 33559
SA = 0.87
Score = 1.35
DEPVLEALP − MSFragger
20120619_EXQ6_MiBa_SA_HCT116_2_mHLA_2hr: 33559
SA = 0.33
Score = −5.55
PDAVAEICA − Mascot − rejected
NA − spliced − |||
20120518_EXQ0_MiBa_SA_HCT116_mHLA−2: 23723
SA = 0.72
Score = −0.67
LDPGEINM − MaxQuant
20120518_EXQ0_MiBa_SA_HCT116_mHLA−2: 23723
SA = 0.08
Score = −7.62
AEGSRSQGP − MSFragger
20120518_EXQ0_MiBa_SA_HCT116_mHLA−2: 23723
SA = 0.2
Score = −5.78
DNGLALVVV − Mascot − rejected
DNG|LALVVV − spliced − 3|2|2|2
20120619_EXQ6_MiBa_SA_HCT116_2_mHLA_2hr: 31818
SA = 0.72
Score = −0.41
ALNGDLVVV − MaxQuant
20120619_EXQ6_MiBa_SA_HCT116_2_mHLA_2hr: 31818
SA = 0.84
Score = 0.88
ALNGDLVVV − MSFragger
20120619_EXQ6_MiBa_SA_HCT116_2_mHLA_2hr: 31818
SA = 0.84
Score = 0.88
Relative intensity
m/z
Prosit prediction
* Exclusive in prediction
# Exclusive in experimental

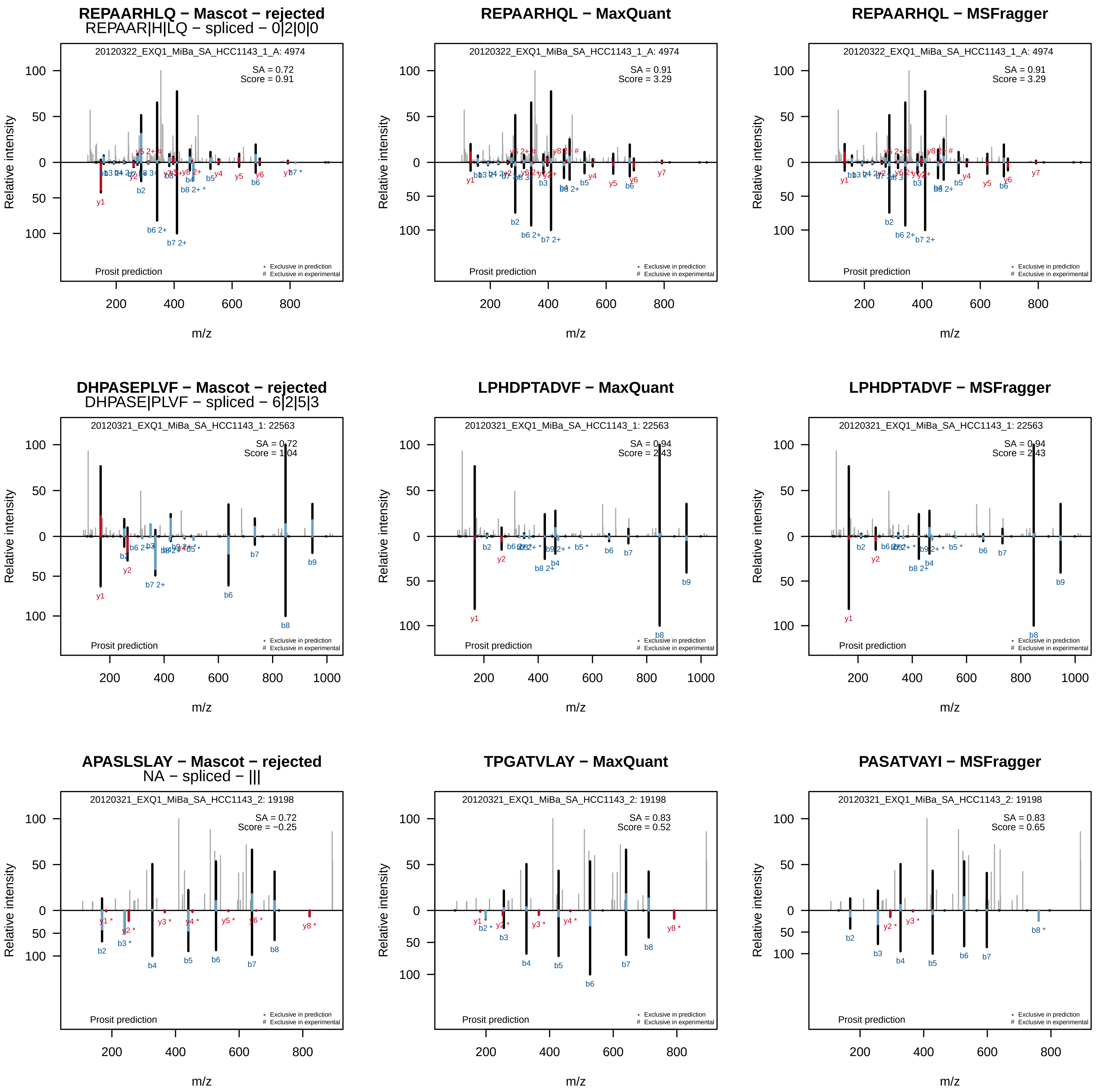
REPAARHLQ − Mascot − rejected
REPAAR|H|LQ − spliced − 0|2|0|0
20120322_EXQ1_MiBa_SA_HCC1143_1_A: 4974
SA = 0.72
Score = 0.91
REPAARHQL − MaxQuant
20120322_EXQ1_MiBa_SA_HCC1143_1_A: 4974
SA = 0.91
Score = 3.29
REPAARHQL − MSFragger
20120322_EXQ1_MiBa_SA_HCC1143_1_A: 4974
SA = 0.91
Score = 3.29
DHPASEPLVF − Mascot − rejected
DHPASE|PLVF − spliced − 6|2|5|3
20120321_EXQ1_MiBa_SA_HCC1143_1: 22563
SA = 0.72
Score = 1.04
LPHDPTADVF − MaxQuant
20120321_EXQ1_MiBa_SA_HCC1143_1: 22563
SA = 0.94
Score = 2.43
LPHDPTADVF − MSFragger
20120321_EXQ1_MiBa_SA_HCC1143_1: 22563
SA = 0.94
Score = 2.43
APASLSLAY − Mascot − rejected
NA − spliced − |||
20120321_EXQ1_MiBa_SA_HCC1143_2: 19198
SA = 0.72
Score = −0.25
TPGATVLAY − MaxQuant
20120321_EXQ1_MiBa_SA_HCC1143_2: 19198
SA = 0.83
Score = 0.52
PASATVAYI − MSFragger
20120321_EXQ1_MiBa_SA_HCC1143_2: 19198
SA = 0.83
Score = 0.65
Relative intensity
m/z
Prosit prediction
⋆ Exclusive in prediction
Exclusive in experimental

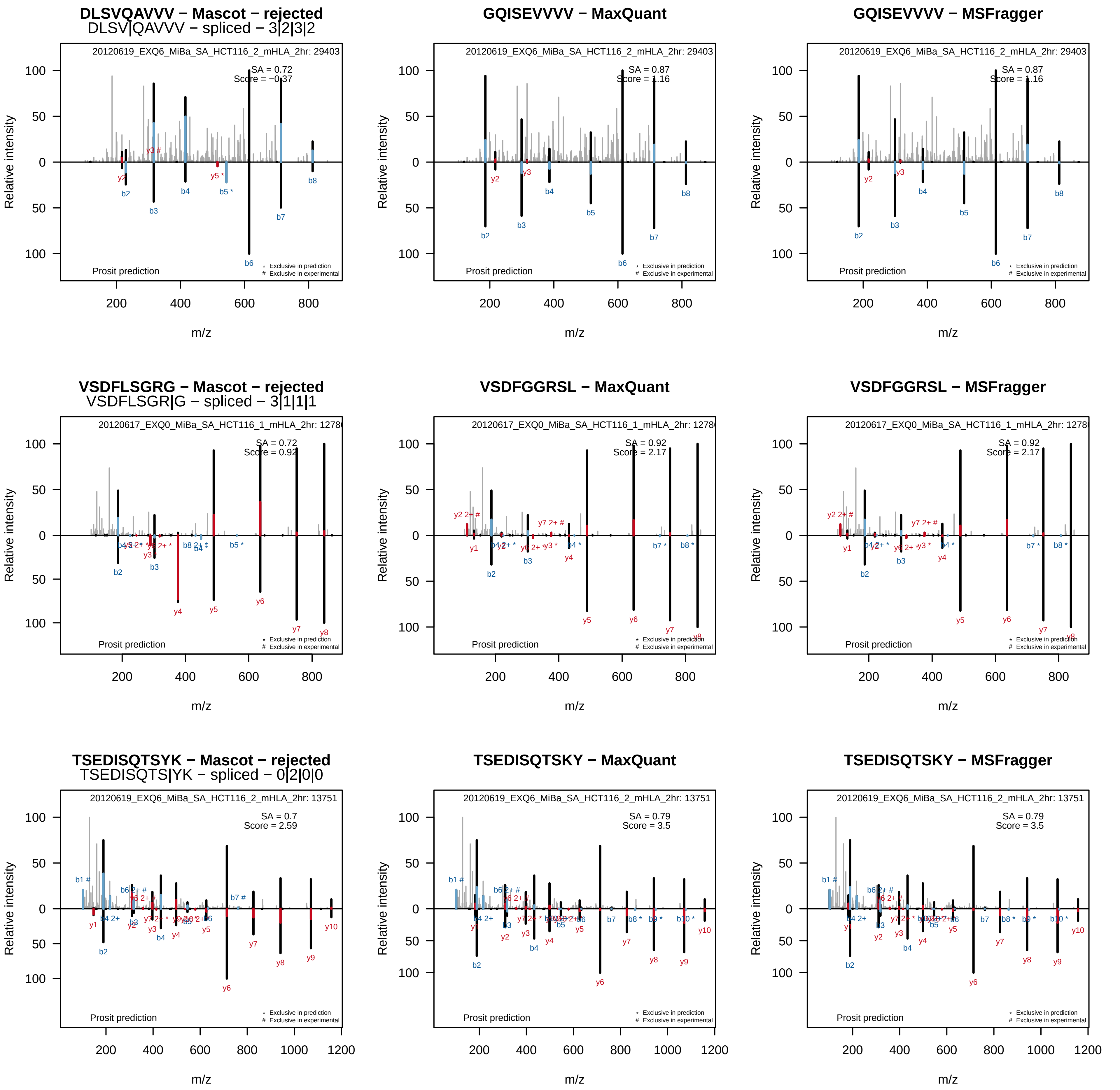
DLSVQAVVV − Mascot − rejected
DLSV|QAVVV − spliced − 3|2|3|2
20120619_EXQ6_MiBa_SA_HCT116_2_mHLA_2hr: 29403
SA = 0.72
Score = −0.37
GQISEVVVV − MaxQuant
20120619_EXQ6_MiBa_SA_HCT116_2_mHLA_2hr: 29403
SA = 0.87
Score = 1.16
GQISEVVVV − MSFragger
20120619_EXQ6_MiBa_SA_HCT116_2_mHLA_2hr: 29403
SA = 0.87
Score = 1.16
VSDFLSGRG − Mascot − rejected
VSDFLSGR|G − spliced − 3|1|1|1
SA = 0.72
Score = 0.92
VSDFGGRSL − MaxQuant
SA = 0.92
Score = 2.17
VSDFGGRSL − MSFragger
SA = 0.92
Score = 2.17
TSEDISQTSYK − Mascot − rejected
TSEDISQTS|YK − spliced − 0|2|0|0
20120619_EXQ6_MiBa_SA_HCT116_2_mHLA_2hr: 13751
SA = 0.7
Score = 2.59
TSEDISQTSKY − MaxQuant
20120619_EXQ6_MiBa_SA_HCT116_2_mHLA_2hr: 13751
SA = 0.79
Score = 3.5
TSEDISQTSKY − MSFragger
20120619_EXQ6_MiBa_SA_HCT116_2_mHLA_2hr: 13751
SA = 0.79
Score = 3.5
Relative intensity
m/z
Prosit prediction
⋆ Exclusive in prediction
Exclusive in experimental

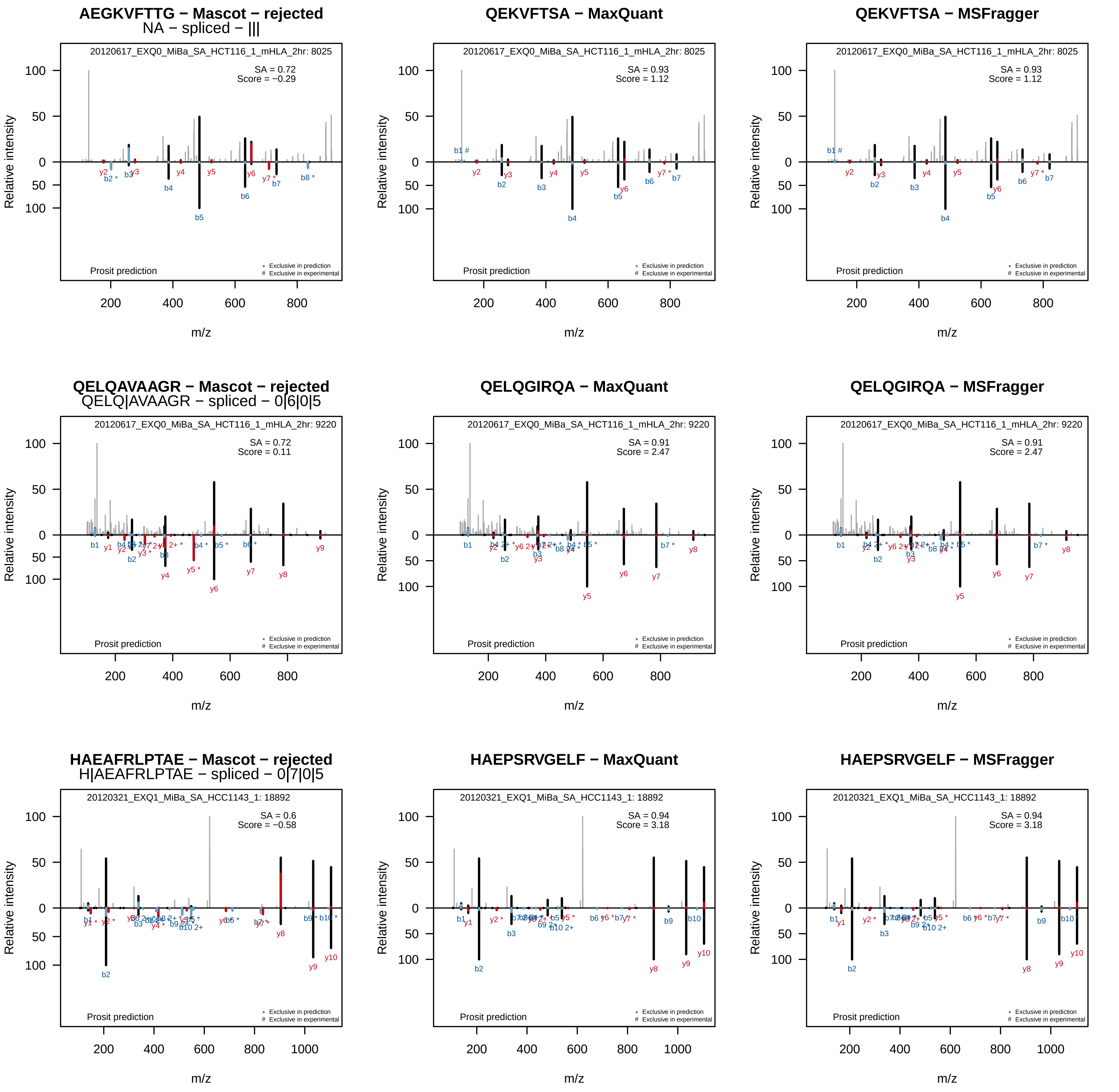
AEGKVFTTG − Mascot − rejected
NA − spliced − |||
20120617_EXQ0_MiBa_SA_HCT116_1_mHLA_2hr: 8025
SA = 0.72
Score = −0.29
QEKVFTSA − MaxQuant
20120617_EXQ0_MiBa_SA_HCT116_1_mHLA_2hr: 8025
SA = 0.93
Score = 1.12
QEKVFTSA − MSFragger
20120617_EXQ0_MiBa_SA_HCT116_1_mHLA_2hr: 8025
SA = 0.93
Score = 1.12
QELQAVAAGR − Mascot − rejected
QELQ|AVAAGR − spliced − 0|6|0|5
20120617_EXQ0_MiBa_SA_HCT116_1_mHLA_2hr: 9220
SA = 0.72
Score = 0.11
QELQGIRQA − MaxQuant
20120617_EXQ0_MiBa_SA_HCT116_1_mHLA_2hr: 9220
SA = 0.91
Score = 2.47
QELQGIRQA − MSFragger
20120617_EXQ0_MiBa_SA_HCT116_1_mHLA_2hr: 9220
SA = 0.91
Score = 2.47
HAEAFRLPTAE − Mascot − rejected
H|AEAFRLPTAE − spliced − 0|7|0|5
20120321_EXQ1_MiBa_SA_HCC1143_1: 18892
SA = 0.6
Score = −0.58
HAEPSRVGELF − MaxQuant
20120321_EXQ1_MiBa_SA_HCC1143_1: 18892
SA = 0.94
Score = 3.18
HAEPSRVGELF − MSFragger
20120321_EXQ1_MiBa_SA_HCC1143_1: 18892
SA = 0.94
Score = 3.18
Relative intensity
m/z
Prosit prediction
* Exclusive in prediction
# Exclusive in experimental

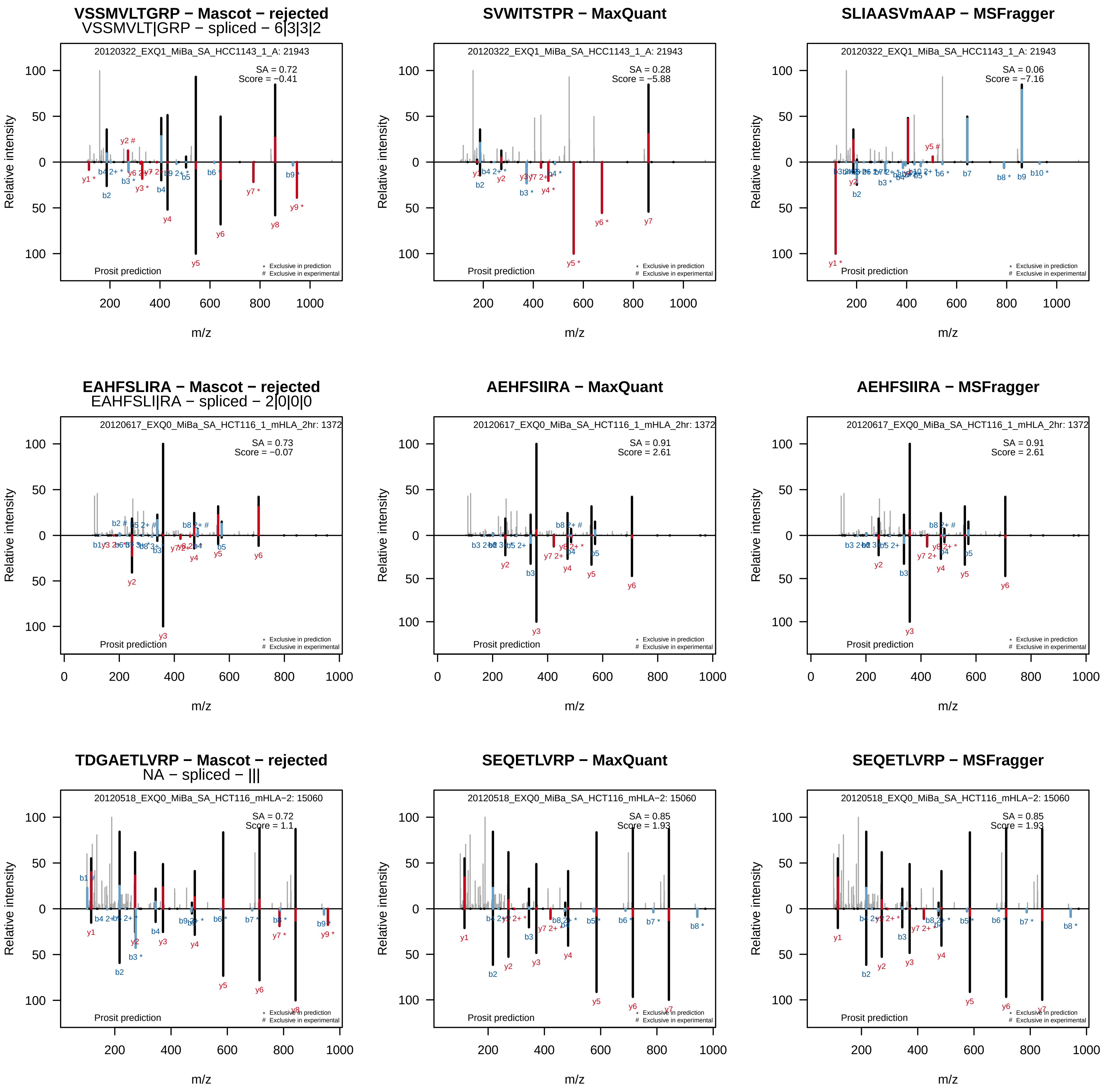

VSSMVLTGRP − Mascot − rejected
VSSMVLT|GRP − spliced − 6|3|3|2
20120322_EXQ1_MiBa_SA_HCC1143_1_A: 21943
SA = 0.72
Score = −0.41
SVWITSTPR − MaxQuant
20120322_EXQ1_MiBa_SA_HCC1143_1_A: 21943
SA = 0.28
Score = −5.88
SLIAASVmAAP − MSFragger
20120322_EXQ1_MiBa_SA_HCC1143_1_A: 21943
SA = 0.06
Score = −7.16
EAHFSLIRA − Mascot − rejected
EAHFSLI|RA − spliced − 2|0|0|0
20120617_EXQ0_MiBa_SA_HCT116_1_mHLA_2hr: 1372
SA = 0.73
Score = −0.07
AEHFSIIRA − MaxQuant
20120617_EXQ0_MiBa_SA_HCT116_1_mHLA_2hr: 1372
SA = 0.91
Score = 2.61
AEHFSIIRA − MSFragger
20120617_EXQ0_MiBa_SA_HCT116_1_mHLA_2hr: 1372
SA = 0.91
Score = 2.61
TDGAETLVRP − Mascot − rejected
NA − spliced − |||
20120518_EXQ0_MiBa_SA_HCT116_mHLA−2: 15060
SA = 0.72
Score = 1.1
SEQETLVRP − MaxQuant
20120518_EXQ0_MiBa_SA_HCT116_mHLA−2: 15060
SA = 0.85
Score = 1.93
SEQETLVRP − MSFragger
20120518_EXQ0_MiBa_SA_HCT116_mHLA−2: 15060
SA = 0.85
Score = 1.93
Relative intensity
m/z
Prosit prediction
* Exclusive in prediction
# Exclusive in experimental

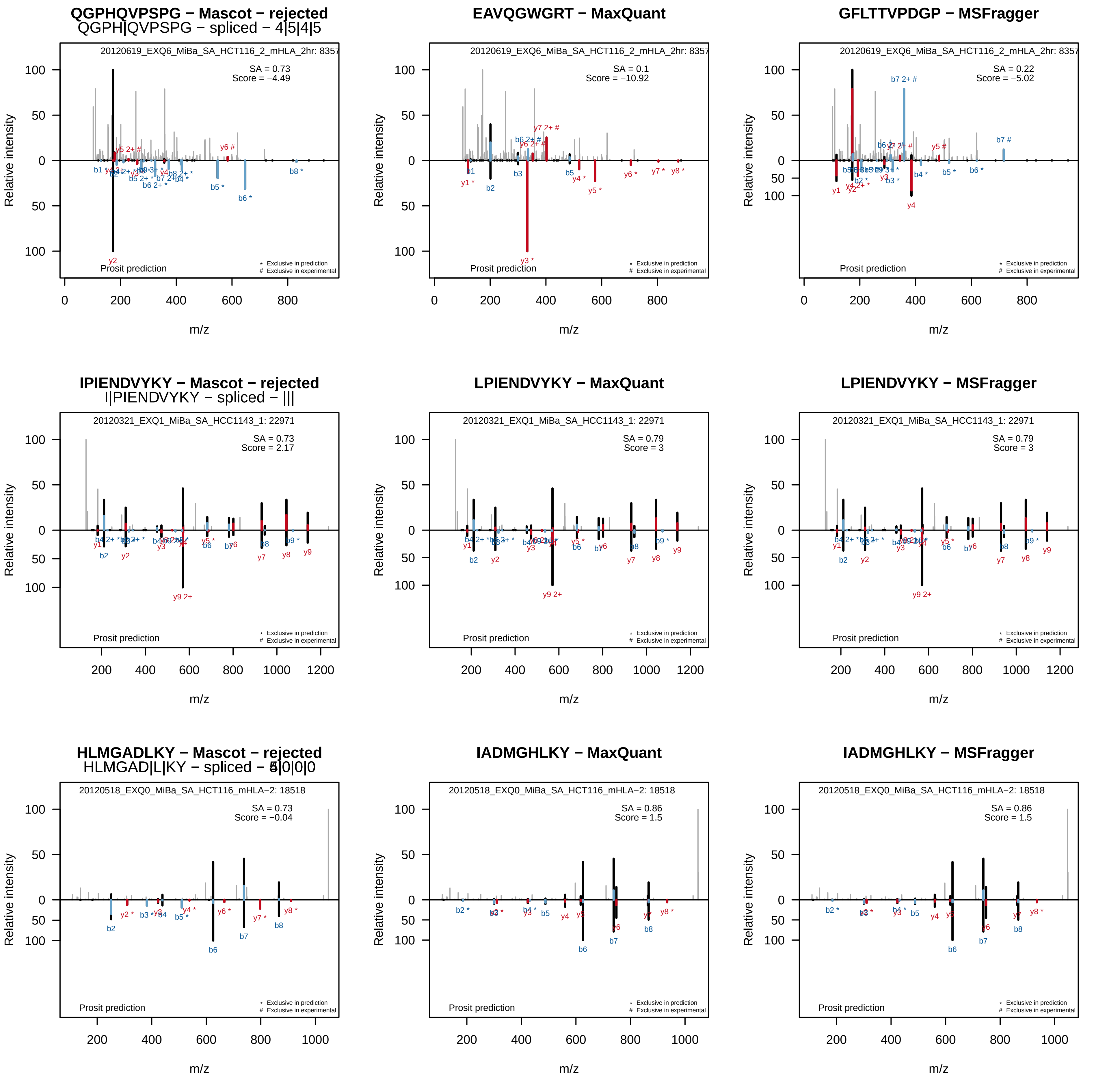
QGPHQVPSPG − Mascot − rejected
QGPH|QVPSPG − spliced − 4|5|4|5
20120619_EXQ6_MiBa_SA_HCT116_2_mHLA_2hr: 8357
SA = 0.73
Score = −4.49
EAVQGWGRT − MaxQuant
20120619_EXQ6_MiBa_SA_HCT116_2_mHLA_2hr: 8357
SA = 0.1
Score = −10.92
GFLTTVPDGP − MSFragger
20120619_EXQ6_MiBa_SA_HCT116_2_mHLA_2hr: 8357
SA = 0.22
Score = −5.02
IPIENDVYKY − Mascot − rejected
I|PIENDVYKY − spliced − |||
20120321_EXQ1_MiBa_SA_HCC1143_1: 22971
SA = 0.73
Score = 2.17
LPIENDVYKY − MaxQuant
20120321_EXQ1_MiBa_SA_HCC1143_1: 22971
SA = 0.79
Score = 3
LPIENDVYKY − MSFragger
20120321_EXQ1_MiBa_SA_HCC1143_1: 22971
SA = 0.79
Score = 3
HLMGADLKY − Mascot − rejected
HLMGAD|L|KY − spliced − 6|0|0|0
20120518_EXQ0_MiBa_SA_HCT116_mHLA−2: 18518
SA = 0.73
Score = −0.04
IADMGHLKY − MaxQuant
20120518_EXQ0_MiBa_SA_HCT116_mHLA−2: 18518
SA = 0.86
Score = 1.5
IADMGHLKY − MSFragger
20120518_EXQ0_MiBa_SA_HCT116_mHLA−2: 18518
SA = 0.86
Score = 1.5
Relative intensity
m/z
Prosit prediction
* Exclusive in prediction
# Exclusive in experimental

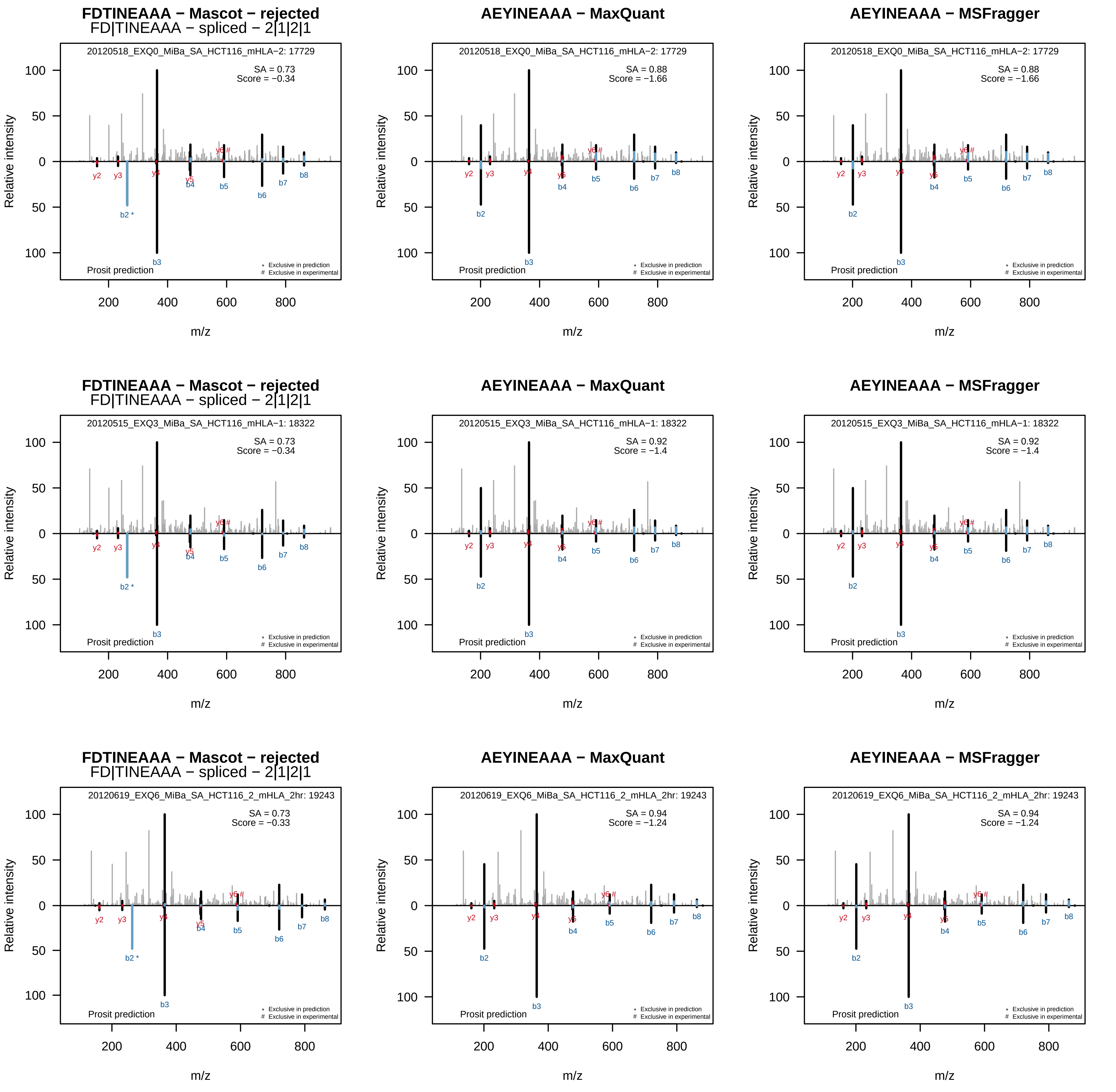

FDTINEAAA − Mascot − rejected
FD|TINEAAA − spliced − 2|1|2|1
20120518_EXQ0_MiBa_SA_HCT116_mHLA−2: 17729
SA = 0.73
Score = −0.34
AEYINEAAA − MaxQuant
20120518_EXQ0_MiBa_SA_HCT116_mHLA−2: 17729
SA = 0.88
Score = −1.66
AEYINEAAA − MSFragger
20120518_EXQ0_MiBa_SA_HCT116_mHLA−2: 17729
SA = 0.88
Score = −1.66
FDTINEAAA − Mascot − rejected
FD|TINEAAA − spliced − 2|1|2|1
20120515_EXQ3_MiBa_SA_HCT116_mHLA−1: 18322
SA = 0.73
Score = −0.34
AEYINEAAA − MaxQuant
20120515_EXQ3_MiBa_SA_HCT116_mHLA−1: 18322
SA = 0.92
Score = −1.4
AEYINEAAA − MSFragger
20120515_EXQ3_MiBa_SA_HCT116_mHLA−1: 18322
SA = 0.92
Score = −1.4
FDTINEAAA − Mascot − rejected
FD|TINEAAA − spliced − 2|1|2|1
20120619_EXQ6_MiBa_SA_HCT116_2_mHLA_2hr: 19243
SA = 0.73
Score = −0.33
AEYINEAAA − MaxQuant
20120619_EXQ6_MiBa_SA_HCT116_2_mHLA_2hr: 19243
SA = 0.94
Score = −1.24
AEYINEAAA − MSFragger
20120619_EXQ6_MiBa_SA_HCT116_2_mHLA_2hr: 19243
SA = 0.94
Score = −1.24
Relative intensity
m/z
Prosit prediction
⋆ Exclusive in prediction
Exclusive in experimental
y2 y3 y4 y5 y6 #
b2 * b3 b4 b5 b6 b7 b8

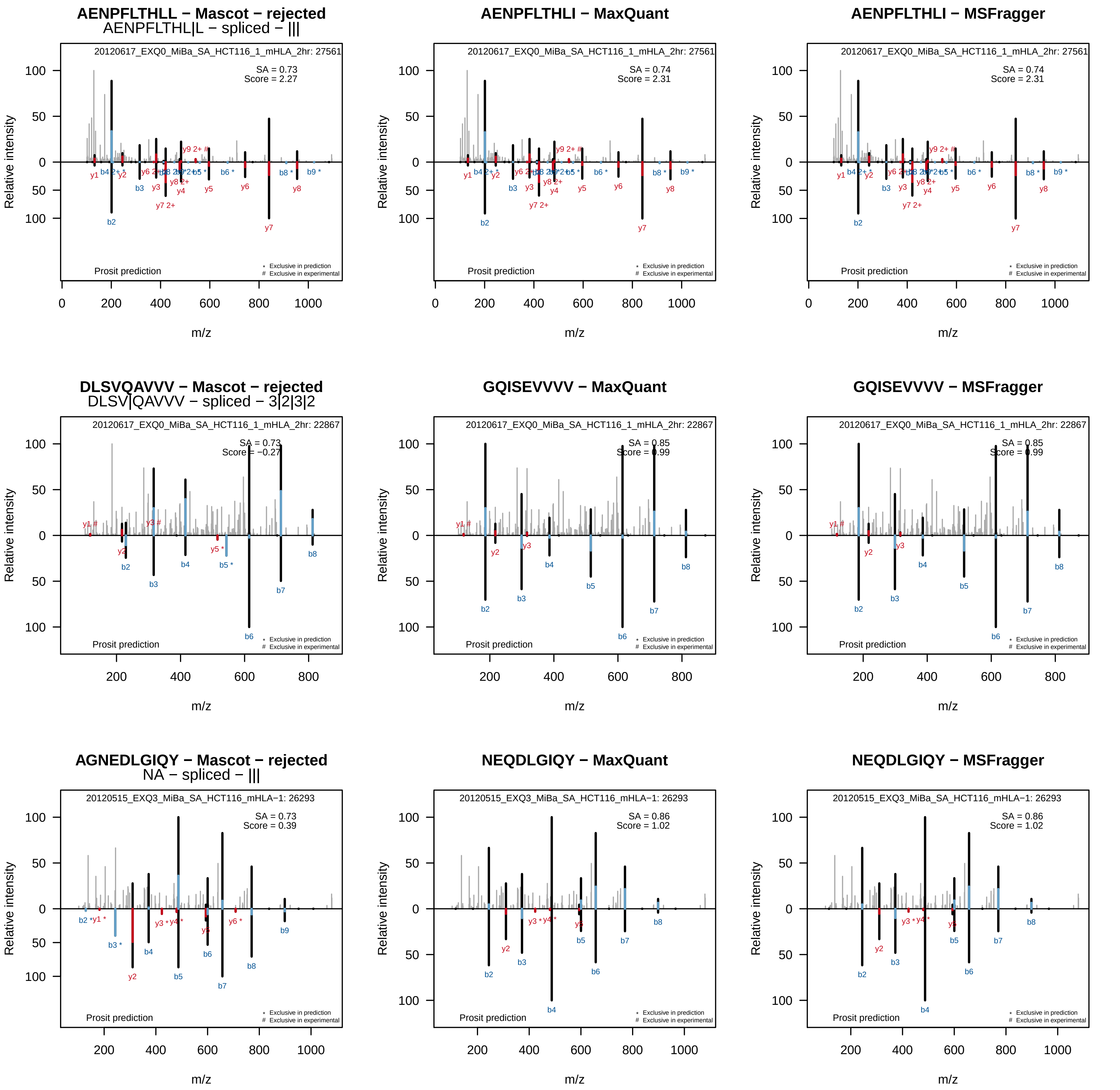
AENPFLTHLL − Mascot − rejected
AENPFLTHL|L − spliced − |||
20120617_EXQ0_MiBa_SA_HCT116_1_mHLA_2hr: 27561
SA = 0.73
Score = 2.27
AENPFLTHLI − MaxQuant
20120617_EXQ0_MiBa_SA_HCT116_1_mHLA_2hr: 27561
SA = 0.74
Score = 2.31
AENPFLTHLI − MSFragger
20120617_EXQ0_MiBa_SA_HCT116_1_mHLA_2hr: 27561
SA = 0.74
Score = 2.31
DLSVQAVVV − Mascot − rejected
DLSV|QAVVV − spliced − 3|2|3|2
20120617_EXQ0_MiBa_SA_HCT116_1_mHLA_2hr: 22867
SA = 0.73
Score = −0.27
GQISEVVVV − MaxQuant
20120617_EXQ0_MiBa_SA_HCT116_1_mHLA_2hr: 22867
SA = 0.85
Score = 0.99
GQISEVVVV − MSFragger
20120617_EXQ0_MiBa_SA_HCT116_1_mHLA_2hr: 22867
SA = 0.85
Score = 0.99
AGNEDLGIQY − Mascot − rejected
NA − spliced − |||
20120515_EXQ3_MiBa_SA_HCT116_mHLA−1: 26293
SA = 0.73
Score = 0.39
NEQDLGIQY − MaxQuant
20120515_EXQ3_MiBa_SA_HCT116_mHLA−1: 26293
SA = 0.86
Score = 1.02
NEQDLGIQY − MSFragger
20120515_EXQ3_MiBa_SA_HCT116_mHLA−1: 26293
SA = 0.86
Score = 1.02
Relative intensity
m/z
Prosit prediction
⋆ Exclusive in prediction
Exclusive in experimental

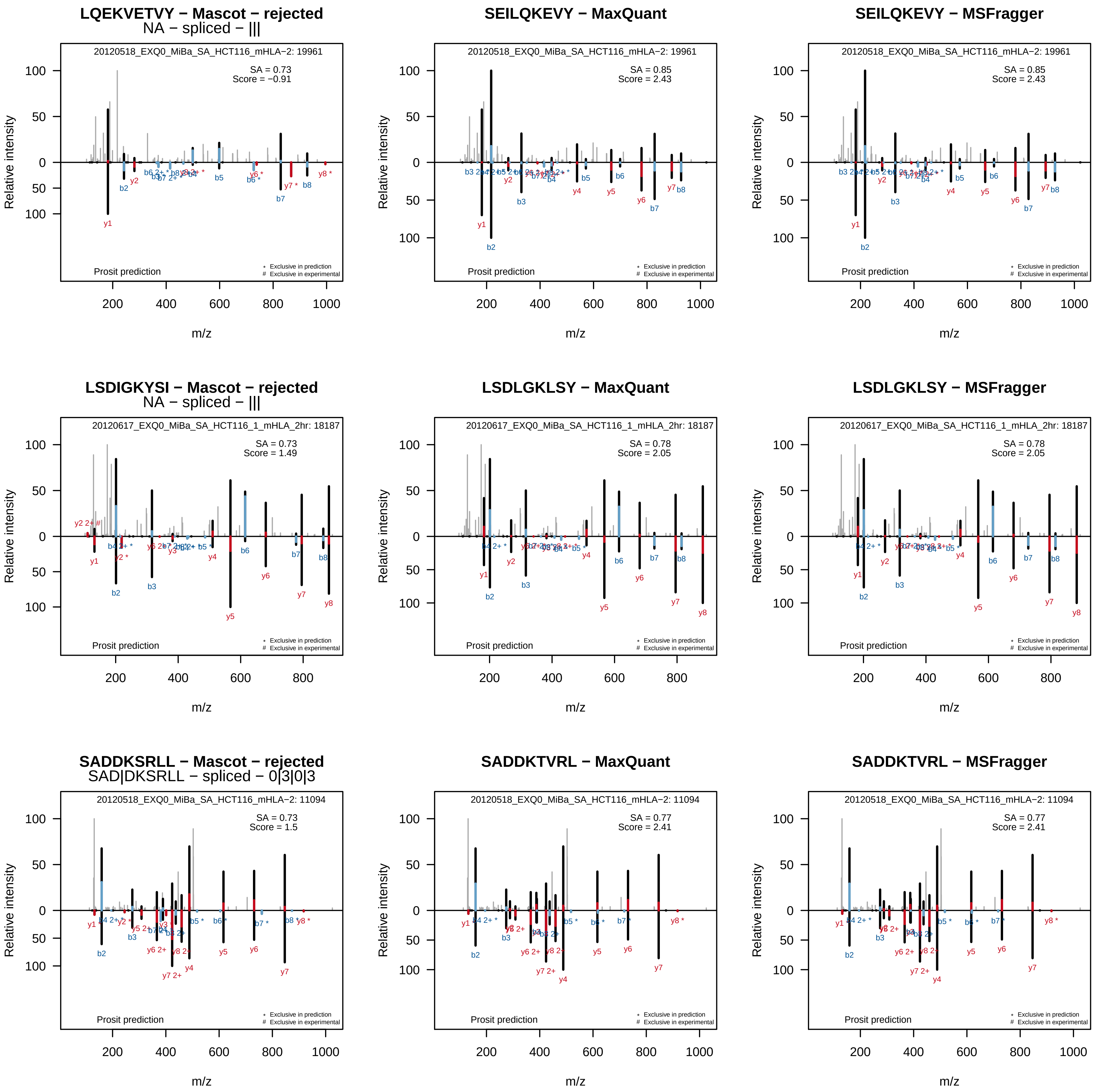
LQEKVETVY − Mascot − rejected
NA − spliced − |||
20120518_EXQ0_MiBa_SA_HCT116_mHLA−2: 19961
SA = 0.73
Score = −0.91
SEILQKEVY − MaxQuant
20120518_EXQ0_MiBa_SA_HCT116_mHLA−2: 19961
SA = 0.85
Score = 2.43
SEILQKEVY − MSFragger
20120518_EXQ0_MiBa_SA_HCT116_mHLA−2: 19961
SA = 0.85
Score = 2.43
LSDIGKYSI − Mascot − rejected
NA − spliced − |||
20120617_EXQ0_MiBa_SA_HCT116_1_mHLA_2hr: 18187
SA = 0.73
Score = 1.49
LSDLGKLSY − MaxQuant
20120617_EXQ0_MiBa_SA_HCT116_1_mHLA_2hr: 18187
SA = 0.78
Score = 2.05
LSDLGKLSY − MSFragger
20120617_EXQ0_MiBa_SA_HCT116_1_mHLA_2hr: 18187
SA = 0.78
Score = 2.05
SADDKSRLL − Mascot − rejected
SAD|DKSRLL − spliced − 0|3|0|3
20120518_EXQ0_MiBa_SA_HCT116_mHLA−2: 11094
SA = 0.73
Score = 1.5
SADDKTVRL − MaxQuant
20120518_EXQ0_MiBa_SA_HCT116_mHLA−2: 11094
SA = 0.77
Score = 2.41
SADDKTVRL − MSFragger
20120518_EXQ0_MiBa_SA_HCT116_mHLA−2: 11094
SA = 0.77
Score = 2.41
Relative intensity
m/z
Prosit prediction
* Exclusive in prediction
# Exclusive in experimental

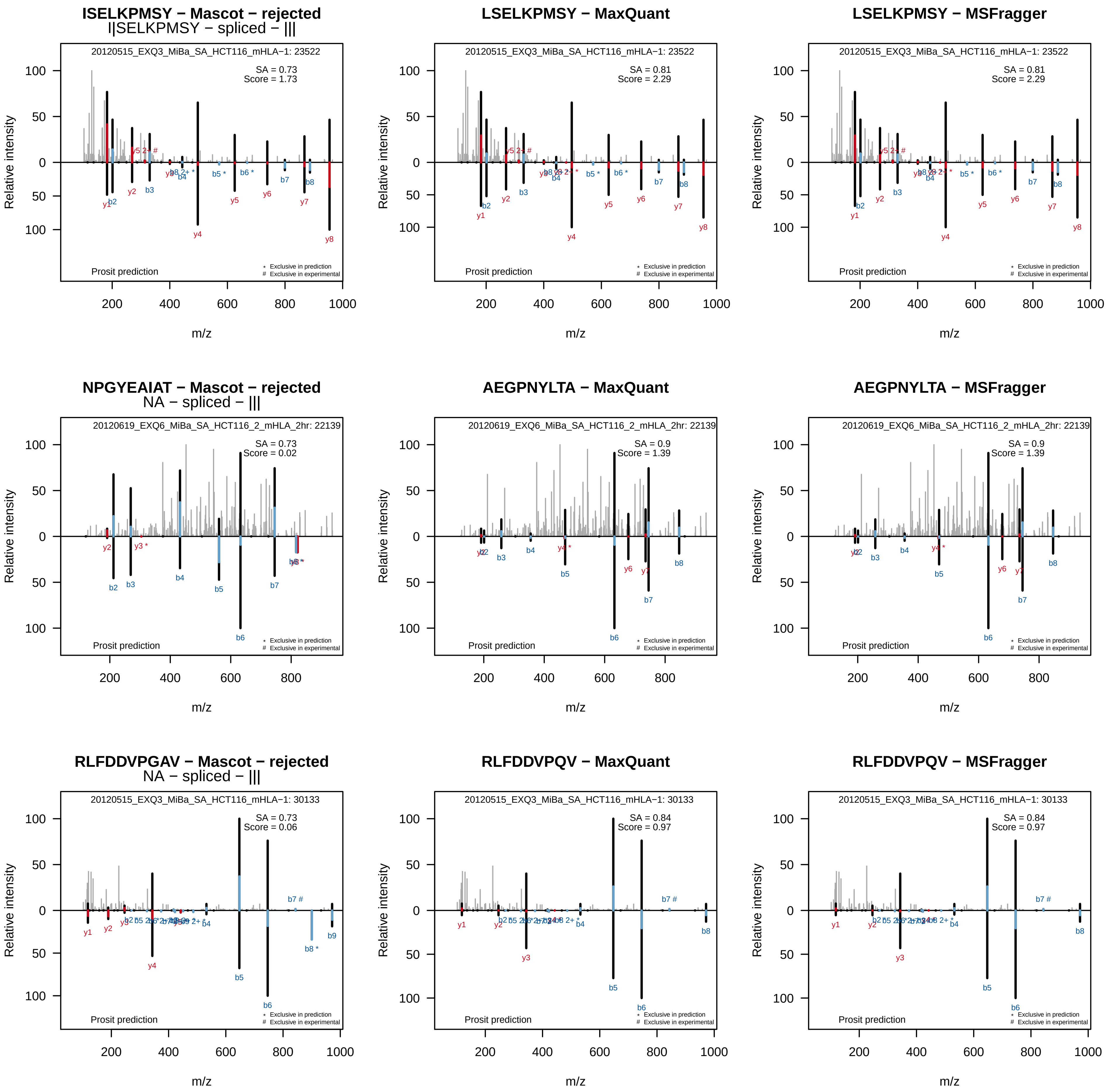

ISELKPMSY − Mascot − rejected
I|SELKPMSY − spliced − |||
20120515_EXQ3_MiBa_SA_HCT116_mHLA−1: 23522
SA = 0.73
Score = 1.73
LSELKPMSY − MaxQuant
20120515_EXQ3_MiBa_SA_HCT116_mHLA−1: 23522
SA = 0.81
Score = 2.29
LSELKPMSY − MSFragger
20120515_EXQ3_MiBa_SA_HCT116_mHLA−1: 23522
SA = 0.81
Score = 2.29
NPGYEAIAT − Mascot − rejected
NA − spliced − |||
20120619_EXQ6_MiBa_SA_HCT116_2_mHLA_2hr: 22139
SA = 0.73
Score = 0.02
AEGPNYLTA − MaxQuant
20120619_EXQ6_MiBa_SA_HCT116_2_mHLA_2hr: 22139
SA = 0.9
Score = 1.39
AEGPNYLTA − MSFragger
20120619_EXQ6_MiBa_SA_HCT116_2_mHLA_2hr: 22139
SA = 0.9
Score = 1.39
RLFDDVPGAV − Mascot − rejected
NA − spliced − |||
20120515_EXQ3_MiBa_SA_HCT116_mHLA−1: 30133
SA = 0.73
Score = 0.06
RLFDDVPQV − MaxQuant
20120515_EXQ3_MiBa_SA_HCT116_mHLA−1: 30133
SA = 0.84
Score = 0.97
RLFDDVPQV − MSFragger
20120515_EXQ3_MiBa_SA_HCT116_mHLA−1: 30133
SA = 0.84
Score = 0.97
Relative intensity
m/z
Prosit prediction
* Exclusive in prediction
# Exclusive in experimental

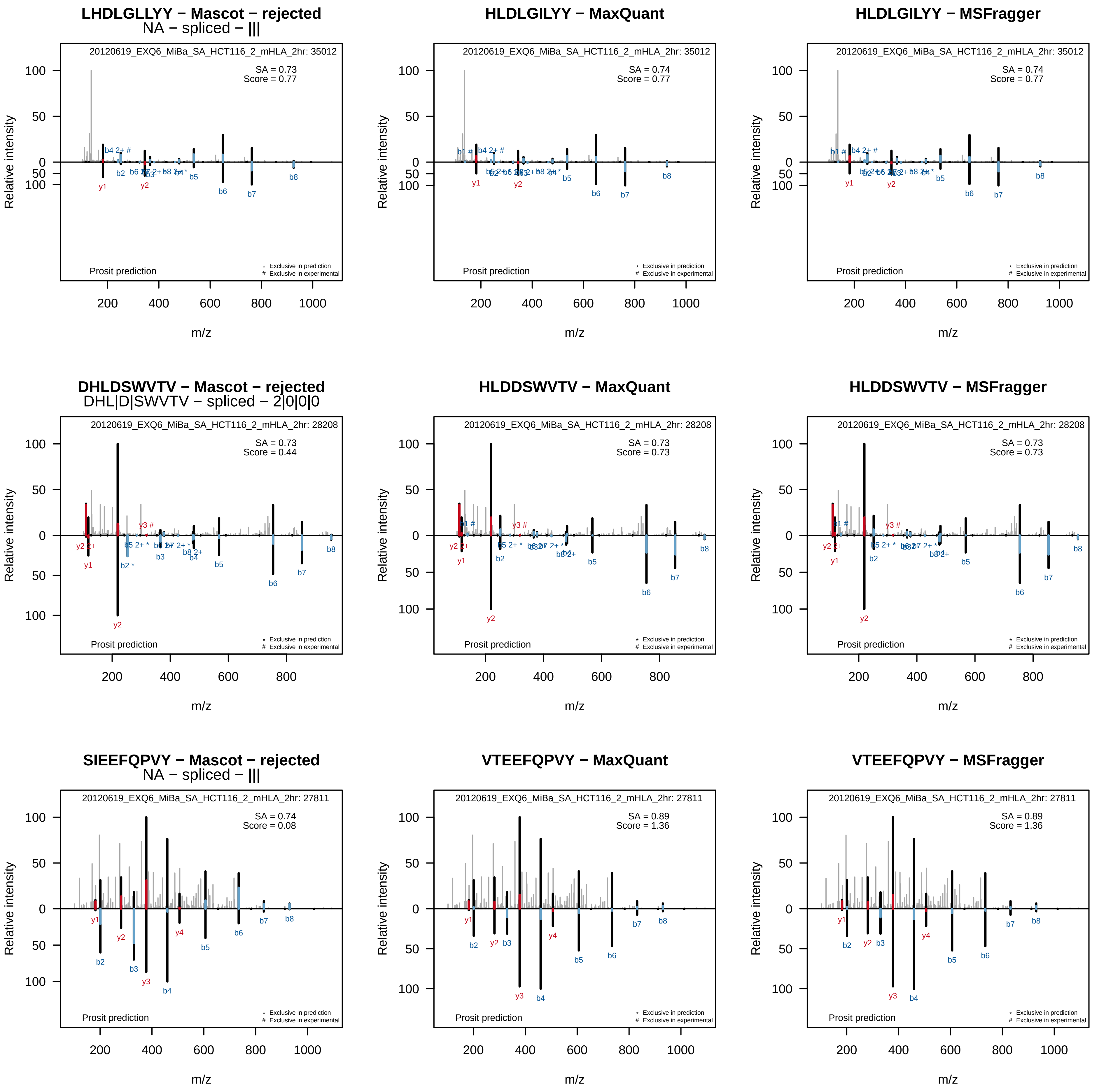
LHDLGLLYY − Mascot − rejected
NA − spliced − |||
20120619_EXQ6_MiBa_SA_HCT116_2_mHLA_2hr: 35012
SA = 0.73
Score = 0.77
HLDLGILYY − MaxQuant
20120619_EXQ6_MiBa_SA_HCT116_2_mHLA_2hr: 35012
SA = 0.74
Score = 0.77
HLDLGILYY − MSFragger
20120619_EXQ6_MiBa_SA_HCT116_2_mHLA_2hr: 35012
SA = 0.74
Score = 0.77
DHLDSWVTV − Mascot − rejected
DHL|D|SWVTV − spliced − 2|0|0|0
20120619_EXQ6_MiBa_SA_HCT116_2_mHLA_2hr: 28208
SA = 0.73
Score = 0.44
HLDDSWVTV − MaxQuant
20120619_EXQ6_MiBa_SA_HCT116_2_mHLA_2hr: 28208
SA = 0.73
Score = 0.73
HLDDSWVTV − MSFragger
20120619_EXQ6_MiBa_SA_HCT116_2_mHLA_2hr: 28208
SA = 0.73
Score = 0.73
SIEEFQPVY − Mascot − rejected
NA − spliced − |||
20120619_EXQ6_MiBa_SA_HCT116_2_mHLA_2hr: 27811
SA = 0.74
Score = 0.08
VTEEFQPVY − MaxQuant
20120619_EXQ6_MiBa_SA_HCT116_2_mHLA_2hr: 27811
SA = 0.89
Score = 1.36
VTEEFQPVY − MSFragger
20120619_EXQ6_MiBa_SA_HCT116_2_mHLA_2hr: 27811
SA = 0.89
Score = 1.36
Relative intensity
m/z
Prosit prediction
* Exclusive in prediction
# Exclusive in experimental

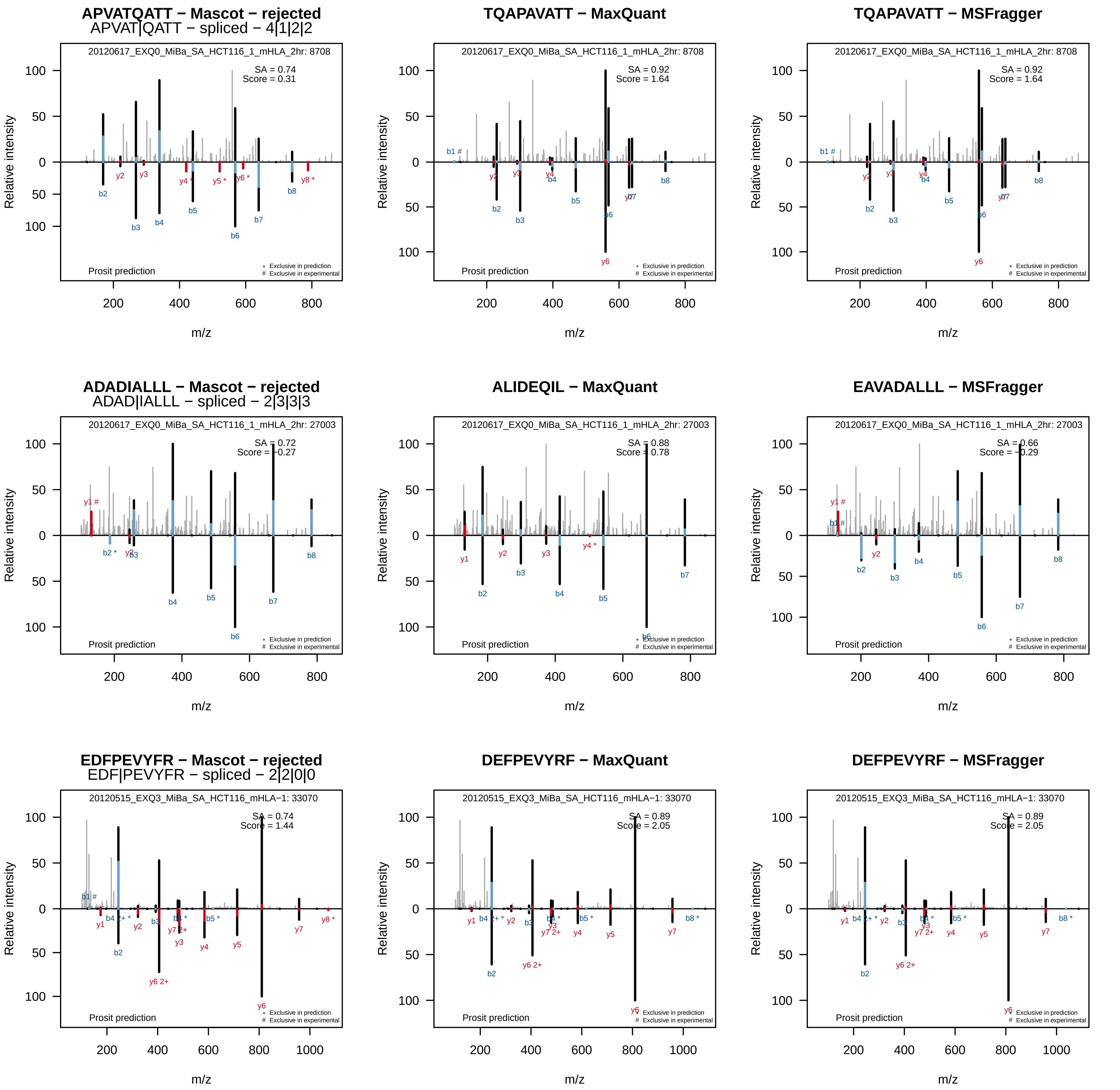

APVATQATT − Mascot − rejected
APVAT|QATT − spliced − 4|1|2|2
20120617_EXQ0_MiBa_SA_HCT116_1_mHLA_2hr: 8708
SA = 0.74
Score = 0.31
TQAPAVATT − MaxQuant
20120617_EXQ0_MiBa_SA_HCT116_1_mHLA_2hr: 8708
SA = 0.92
Score = 1.64
TQAPAVATT − MSFragger
20120617_EXQ0_MiBa_SA_HCT116_1_mHLA_2hr: 8708
SA = 0.92
Score = 1.64
ADADIALLL − Mascot − rejected
ADAD|IALLL − spliced − 2|3|3|3
20120617_EXQ0_MiBa_SA_HCT116_1_mHLA_2hr: 27003
SA = 0.72
Score = −0.27
ALIDEQIL − MaxQuant
20120617_EXQ0_MiBa_SA_HCT116_1_mHLA_2hr: 27003
SA = 0.88
Score = 0.78
EAVADALLL − MSFragger
20120617_EXQ0_MiBa_SA_HCT116_1_mHLA_2hr: 27003
SA = 0.66
Score = −0.29
EDFPEVYFR − Mascot − rejected
EDF|PEVYFR − spliced − 2|2|0|0
20120515_EXQ3_MiBa_SA_HCT116_mHLA−1: 33070
SA = 0.74
Score = 1.44
DEFPEVYRF − MaxQuant
20120515_EXQ3_MiBa_SA_HCT116_mHLA−1: 33070
SA = 0.89
Score = 2.05
DEFPEVYRF − MSFragger
20120515_EXQ3_MiBa_SA_HCT116_mHLA−1: 33070
SA = 0.89
Score = 2.05
Relative intensity
m/z
Prosit prediction
⋆ Exclusive in prediction
Exclusive in experimental

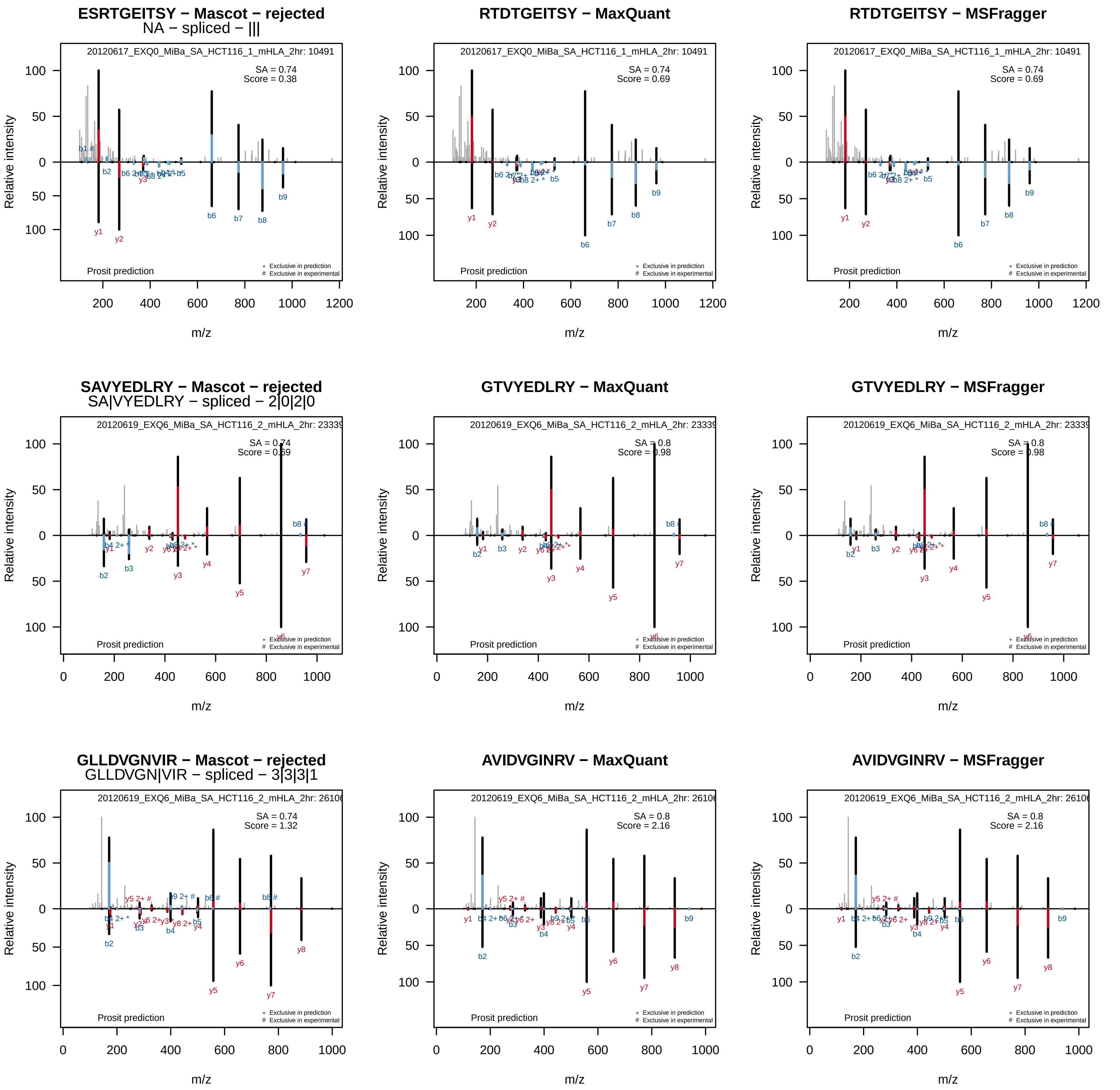

ESRTGEITSY − Mascot − rejected
NA − spliced − |||
20120617_EXQ0_MiBa_SA_HCT116_1_mHLA_2hr: 10491
SA = 0.74
Score = 0.38
Prosit prediction
Exclusive in prediction
Exclusive in experimental
Relative intensity
m/z
RTDTGEITSY − MaxQuant
20120617_EXQ0_MiBa_SA_HCT116_1_mHLA_2hr: 10491
SA = 0.74
Score = 0.69
Prosit prediction
RTDTGEITSY − MSFragger
20120617_EXQ0_MiBa_SA_HCT116_1_mHLA_2hr: 10491
SA = 0.74
Score = 0.69
Prosit prediction
SAVYYEDLRY − Mascot − rejected
SA|VYEDLRY − spliced − 2|0|2|0
20120619_EXQ6_MiBa_SA_HCT116_2_mHLA_2hr: 23339
SA = 0.74
Score = 0.59
Prosit prediction
GTVYEDLRY − MaxQuant
20120619_EXQ6_MiBa_SA_HCT116_2_mHLA_2hr: 23339
SA = 0.8
Score = 0.98
Prosit prediction
GTVYEDLRY − MSFragger
20120619_EXQ6_MiBa_SA_HCT116_2_mHLA_2hr: 23339
SA = 0.8
Score = 0.98
Prosit prediction
GLLDVGNVIR − Mascot − rejected
GLLDVGN|VIR − spliced − 3|3|3|1
20120619_EXQ6_MiBa_SA_HCT116_2_mHLA_2hr: 26106
SA = 0.74
Score = 1.32
Prosit prediction
AVIDVGINRV − MaxQuant
20120619_EXQ6_MiBa_SA_HCT116_2_mHLA_2hr: 26106
SA = 0.8
Score = 2.16
Prosit prediction
AVIDVGINRV − MSFragger
20120619_EXQ6_MiBa_SA_HCT116_2_mHLA_2hr: 26106
SA = 0.8
Score = 2.16
Prosit prediction

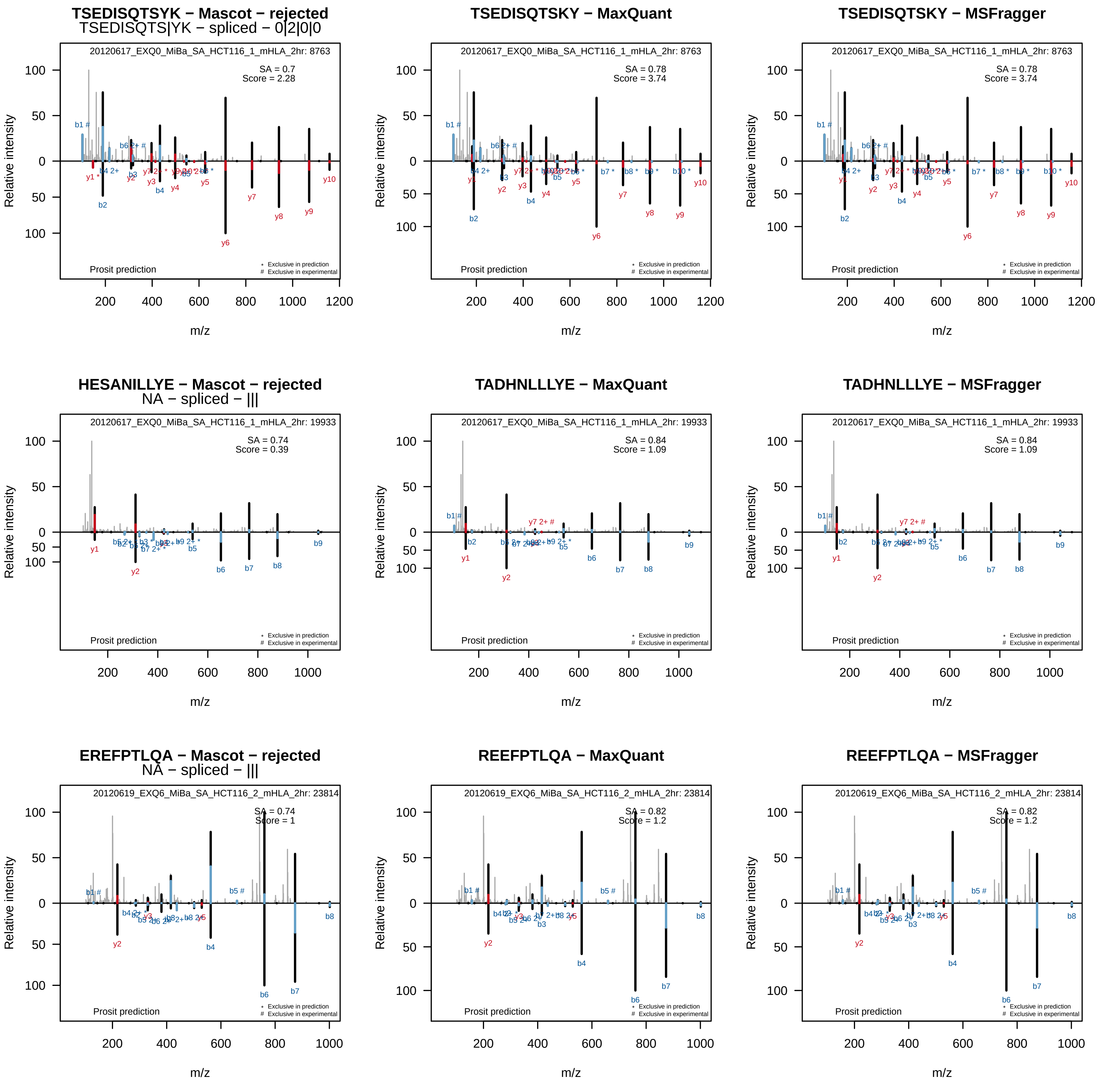

TSEDISQTSYK − Mascot − rejected
TSEDISQTS|YK − spliced − 0|2|0|0
20120617_EXQ0_MiBa_SA_HCT116_1_mHLA_2hr: 8763
SA = 0.7
Score = 2.28
TSEDISQTSKY − MaxQuant
20120617_EXQ0_MiBa_SA_HCT116_1_mHLA_2hr: 8763
SA = 0.78
Score = 3.74
TSEDISQTSKY − MSFragger
20120617_EXQ0_MiBa_SA_HCT116_1_mHLA_2hr: 8763
SA = 0.78
Score = 3.74
HESANILLYE − Mascot − rejected
NA − spliced − |||
20120617_EXQ0_MiBa_SA_HCT116_1_mHLA_2hr: 19933
SA = 0.74
Score = 0.39
TADHNLLLYE − MaxQuant
20120617_EXQ0_MiBa_SA_HCT116_1_mHLA_2hr: 19933
SA = 0.84
Score = 1.09
TADHNLLLYE − MSFragger
20120617_EXQ0_MiBa_SA_HCT116_1_mHLA_2hr: 19933
SA = 0.84
Score = 1.09
EREFPTLQA − Mascot − rejected
NA − spliced − |||
20120619_EXQ6_MiBa_SA_HCT116_2_mHLA_2hr: 23814
SA = 0.74
Score = 1
REEFPTLQA − MaxQuant
20120619_EXQ6_MiBa_SA_HCT116_2_mHLA_2hr: 23814
SA = 0.82
Score = 1.2
REEFPTLQA − MSFragger
20120619_EXQ6_MiBa_SA_HCT116_2_mHLA_2hr: 23814
SA = 0.82
Score = 1.2
Relative intensity
m/z
Prosit prediction
* Exclusive in prediction
# Exclusive in experimental

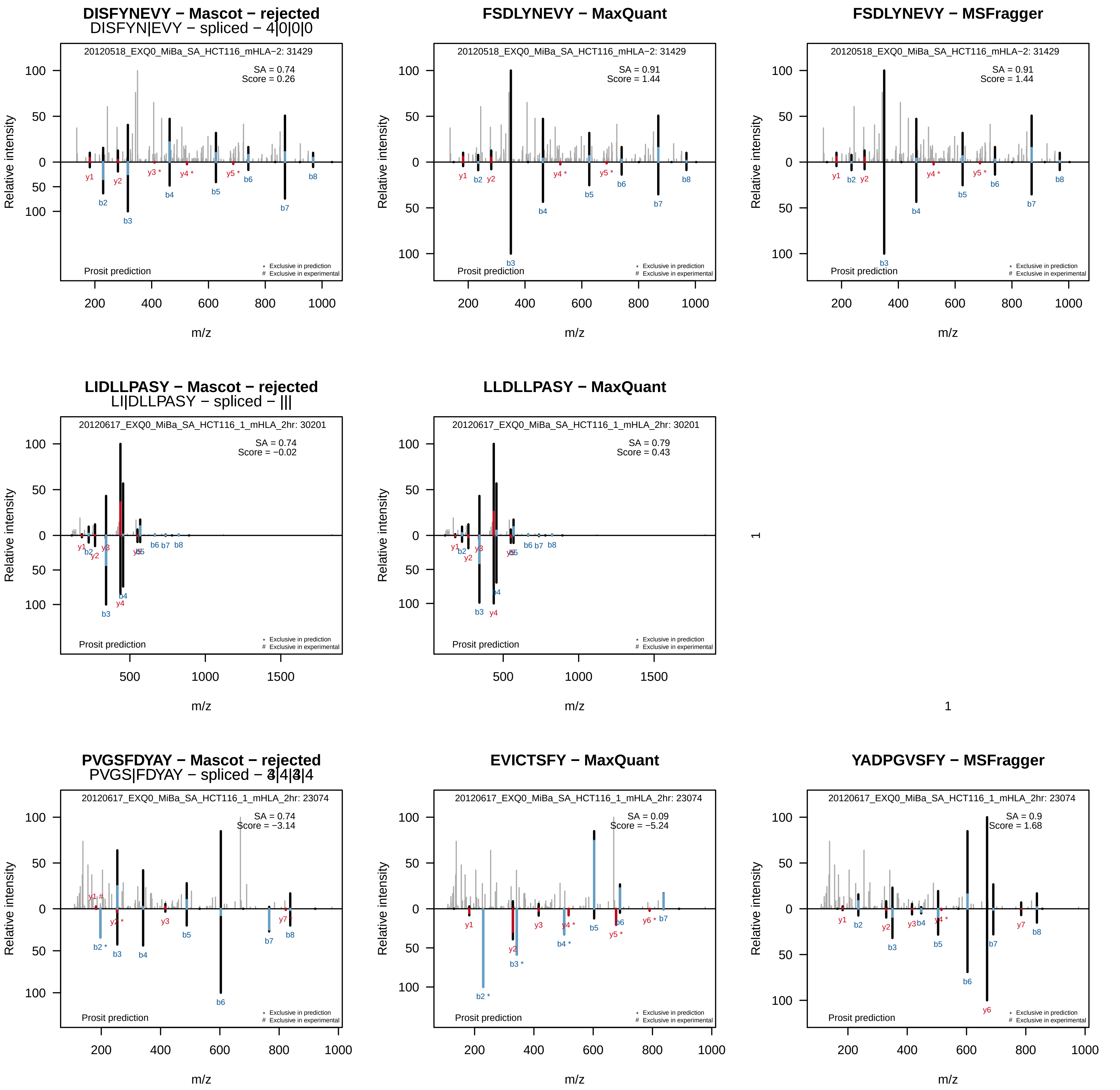

DISFYNEVY − Mascot − rejected
DISFYN|EVY − spliced − 4|0|0|0
20120518_EXQ0_MiBa_SA_HCT116_mHLA−2: 31429
SA = 0.74
Score = 0.26
FSDLYNEVY − MaxQuant
20120518_EXQ0_MiBa_SA_HCT116_mHLA−2: 31429
SA = 0.91
Score = 1.44
FSDLYNEVY − MSFragger
20120518_EXQ0_MiBa_SA_HCT116_mHLA−2: 31429
SA = 0.91
Score = 1.44
LIDLLPASY − Mascot − rejected
LI|DLLPASY − spliced − |||
20120617_EXQ0_MiBa_SA_HCT116_1_mHLA_2hr: 30201
SA = 0.74
Score = −0.02
LLDLLPASY − MaxQuant
20120617_EXQ0_MiBa_SA_HCT116_1_mHLA_2hr: 30201
SA = 0.79
Score = 0.43
PVGSFDYAY − Mascot − rejected
PVGS|FDYAY − spliced − 3|4|3|4
20120617_EXQ0_MiBa_SA_HCT116_1_mHLA_2hr: 23074
SA = 0.74
Score = −3.14
EVICTSFY − MaxQuant
20120617_EXQ0_MiBa_SA_HCT116_1_mHLA_2hr: 23074
SA = 0.09
Score = −5.24
YADPGVSFY − MSFragger
20120617_EXQ0_MiBa_SA_HCT116_1_mHLA_2hr: 23074
SA = 0.9
Score = 1.68
Relative intensity
m/z
Prosit prediction
⋆ Exclusive in prediction
Exclusive in experimental

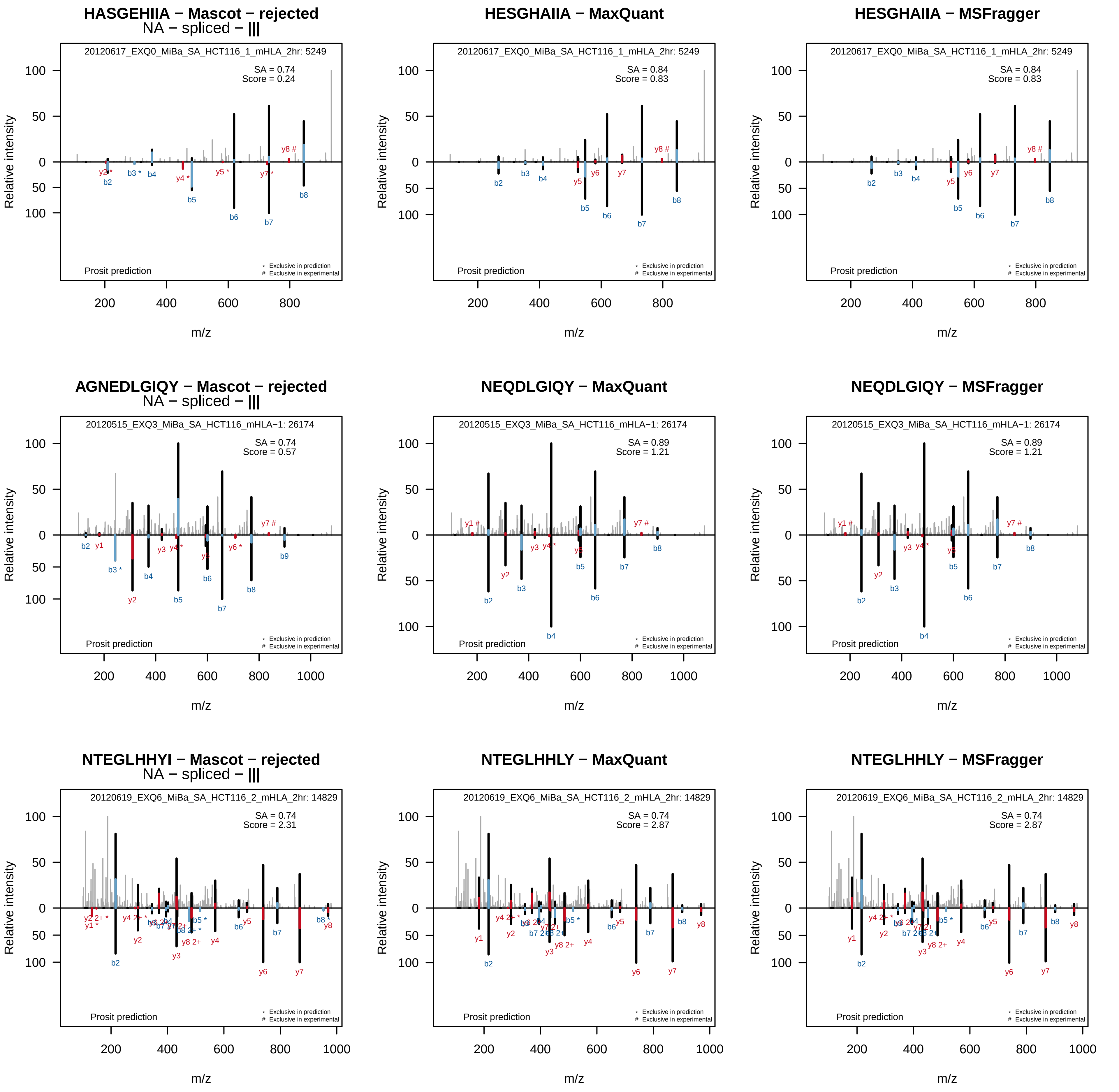

HASGEHIIA − Mascot − rejected
NA − spliced − |||
20120617_EXQ0_MiBa_SA_HCT116_1_mHLA_2hr: 5249
SA = 0.74
Score = 0.24
Prosit prediction
HESGHAIIA − MaxQuant
20120617_EXQ0_MiBa_SA_HCT116_1_mHLA_2hr: 5249
SA = 0.84
Score = 0.83
Prosit prediction
HESGHAIIA − MSFragger
20120617_EXQ0_MiBa_SA_HCT116_1_mHLA_2hr: 5249
SA = 0.84
Score = 0.83
Prosit prediction
AGNEDLGIQY − Mascot − rejected
NA − spliced − |||
20120515_EXQ3_MiBa_SA_HCT116_mHLA−1: 26174
SA = 0.74
Score = 0.57
Prosit prediction
NEQDLGIQY − MaxQuant
20120515_EXQ3_MiBa_SA_HCT116_mHLA−1: 26174
SA = 0.89
Score = 1.21
Prosit prediction
NEQDLGIQY − MSFragger
20120515_EXQ3_MiBa_SA_HCT116_mHLA−1: 26174
SA = 0.89
Score = 1.21
Prosit prediction
NTEGLHHYI − Mascot − rejected
NA − spliced − |||
20120619_EXQ6_MiBa_SA_HCT116_2_mHLA_2hr: 14829
SA = 0.74
Score = 2.31
Prosit prediction
NTEGLHHLY − MaxQuant
20120619_EXQ6_MiBa_SA_HCT116_2_mHLA_2hr: 14829
SA = 0.74
Score = 2.87
Prosit prediction
NTEGLHHLY − MSFragger
20120619_EXQ6_MiBa_SA_HCT116_2_mHLA_2hr: 14829
SA = 0.74
Score = 2.87
Prosit prediction
* Exclusive in prediction
# Exclusive in experimental
Relative intensity
m/z

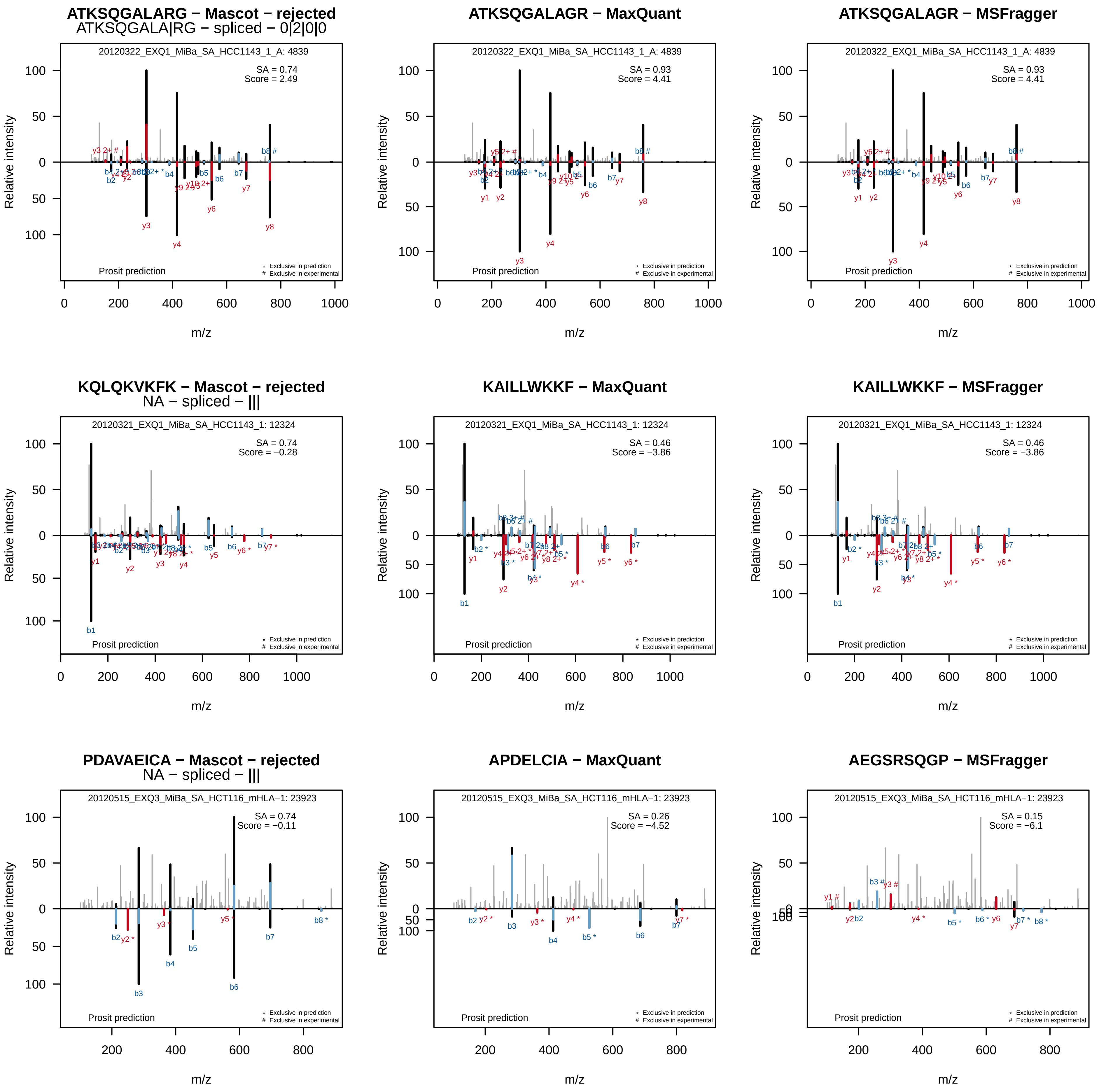
ATKSQGALARG − Mascot − rejected
ATKSQGALA|RG − spliced − 0|2|0|0
20120322_EXQ1_MiBa_SA_HCC1143_1_A: 4839
SA = 0.74
Score = 2.49
ATKSQGALAGR − MaxQuant
20120322_EXQ1_MiBa_SA_HCC1143_1_A: 4839
SA = 0.93
Score = 4.41
ATKSQGALAGR − MSFragger
20120322_EXQ1_MiBa_SA_HCC1143_1_A: 4839
SA = 0.93
Score = 4.41
KQLQKVKFK − Mascot − rejected
NA − spliced − |||
20120321_EXQ1_MiBa_SA_HCC1143_1: 12324
SA = 0.74
Score = −0.28
KAILLWKKF − MaxQuant
20120321_EXQ1_MiBa_SA_HCC1143_1: 12324
SA = 0.46
Score = −3.86
KAILLWKKF − MSFragger
20120321_EXQ1_MiBa_SA_HCC1143_1: 12324
SA = 0.46
Score = −3.86
PDAVAEICA − Mascot − rejected
NA − spliced − |||
20120515_EXQ3_MiBa_SA_HCT116_mHLA−1: 23923
SA = 0.74
Score = −0.11
APDELCIA − MaxQuant
20120515_EXQ3_MiBa_SA_HCT116_mHLA−1: 23923
SA = 0.26
Score = −4.52
AEGSRSQGP − MSFragger
20120515_EXQ3_MiBa_SA_HCT116_mHLA−1: 23923
SA = 0.15
Score = −6.1
Relative intensity
m/z
Prosit prediction
⋆ Exclusive in prediction
Exclusive in experimental

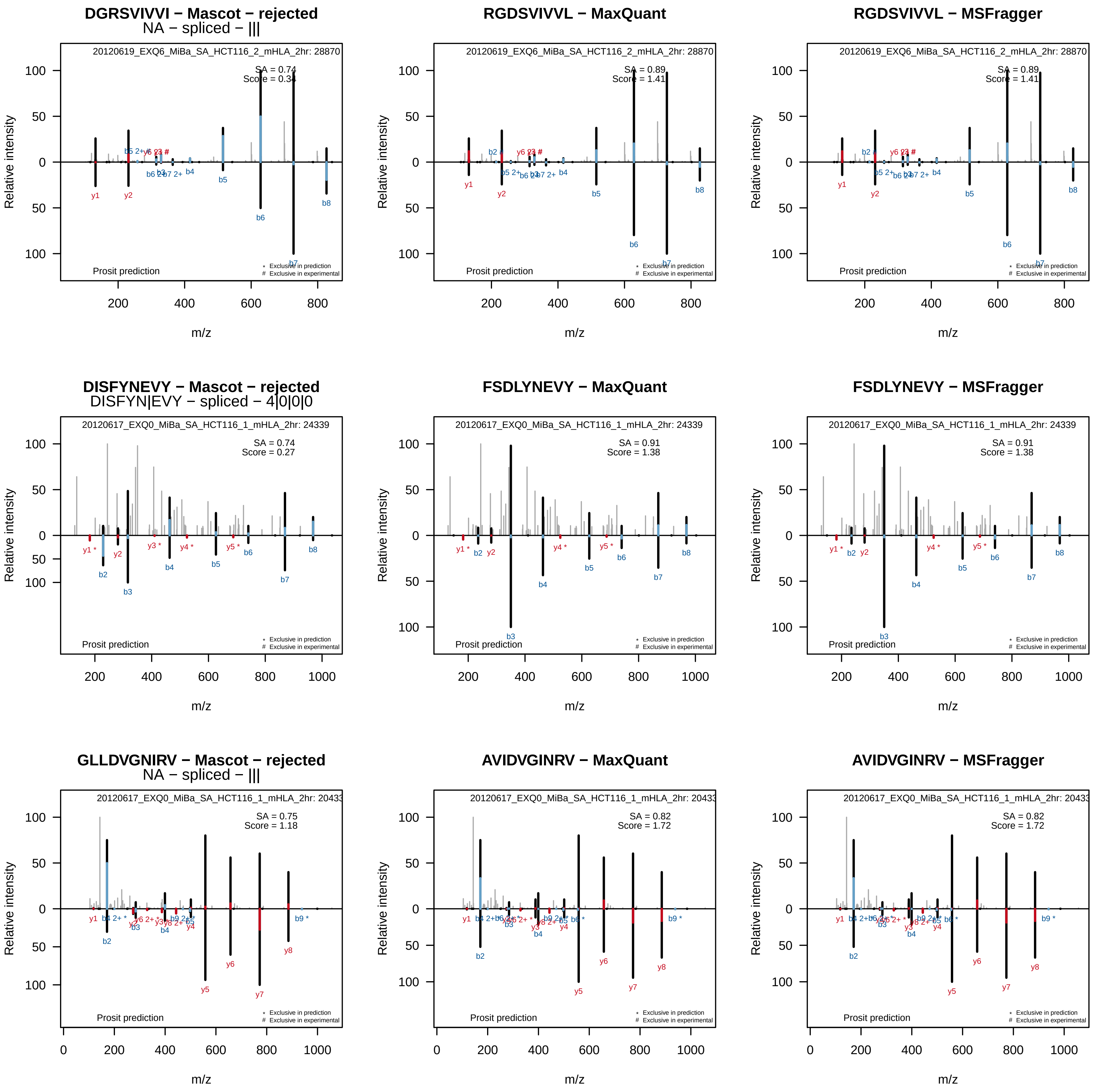

DGRSVIVVI − Mascot − rejected
NA − spliced − |||
20120619_EXQ6_MiBa_SA_HCT116_2_mHLA_2hr: 28870
SA = 0.74
Score = 0.34
RGDSVIVVL − MaxQuant
20120619_EXQ6_MiBa_SA_HCT116_2_mHLA_2hr: 28870
SA = 0.89
Score = 1.41
RGDSVIVVL − MSFragger
20120619_EXQ6_MiBa_SA_HCT116_2_mHLA_2hr: 28870
SA = 0.89
Score = 1.41
DISFYNEVY − Mascot − rejected
DISFYN|EVY − spliced − 4|0|0|0
20120617_EXQ0_MiBa_SA_HCT116_1_mHLA_2hr: 24339
SA = 0.74
Score = 0.27
FSDLYNEVY − MaxQuant
20120617_EXQ0_MiBa_SA_HCT116_1_mHLA_2hr: 24339
SA = 0.91
Score = 1.38
FSDLYNEVY − MSFragger
20120617_EXQ0_MiBa_SA_HCT116_1_mHLA_2hr: 24339
SA = 0.91
Score = 1.38
GLLDVGNIRV − Mascot − rejected
NA − spliced − |||
20120617_EXQ0_MiBa_SA_HCT116_1_mHLA_2hr: 20433
SA = 0.75
Score = 1.18
AVIDVGINRV − MaxQuant
20120617_EXQ0_MiBa_SA_HCT116_1_mHLA_2hr: 20433
SA = 0.82
Score = 1.72
AVIDVGINRV − MSFragger
20120617_EXQ0_MiBa_SA_HCT116_1_mHLA_2hr: 20433
SA = 0.82
Score = 1.72
Relative intensity
m/z
Prosit prediction
⋆ Exclusive in prediction
Exclusive in experimental

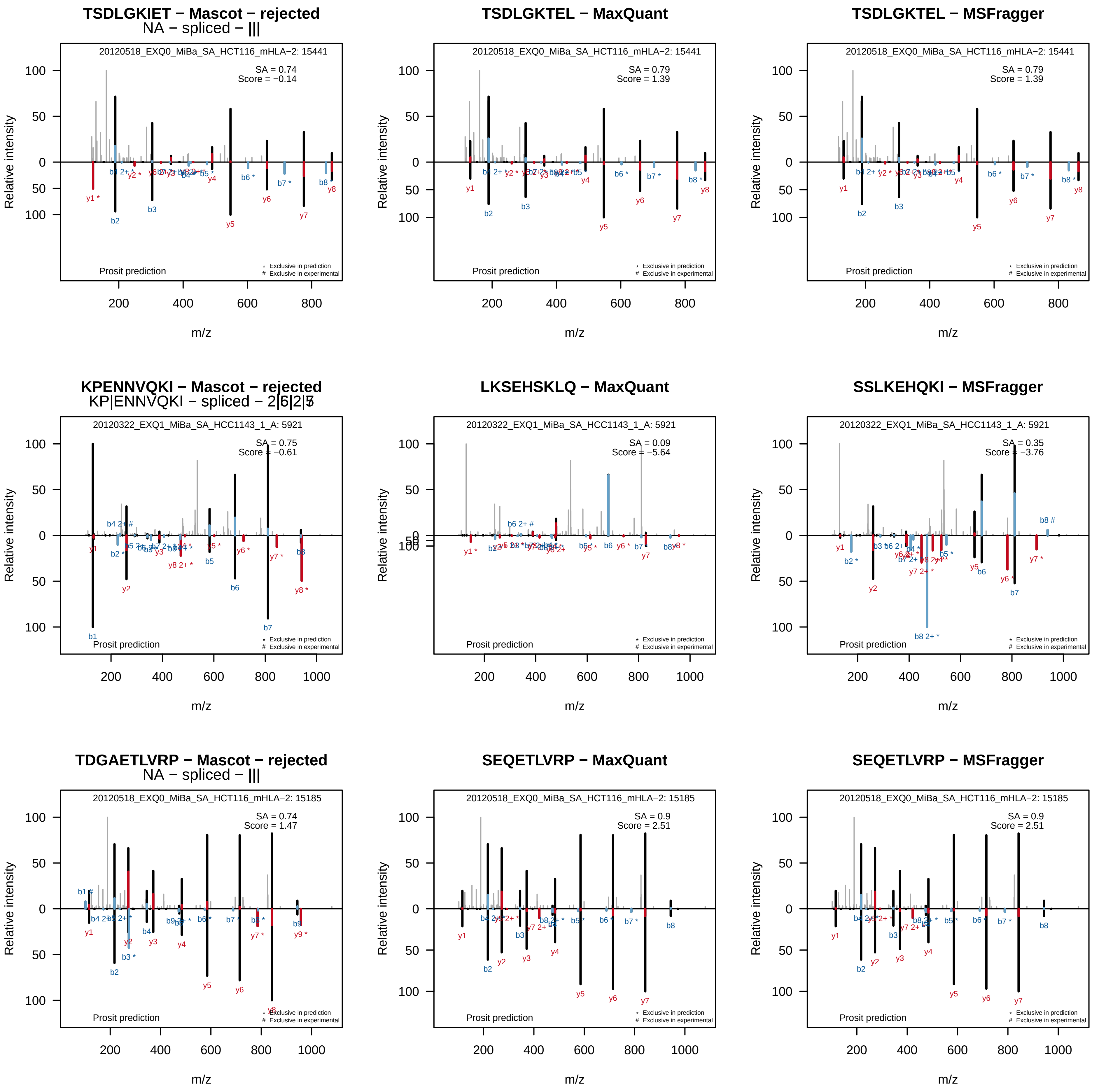
TSDLGKIET − Mascot − rejected
NA − spliced − |||
20120518_EXQ0_MiBa_SA_HCT116_mHLA−2: 15441
SA = 0.74
Score = −0.14
TSDLGKTEL − MaxQuant
20120518_EXQ0_MiBa_SA_HCT116_mHLA−2: 15441
SA = 0.79
Score = 1.39
TSDLGKTEL − MSFragger
20120518_EXQ0_MiBa_SA_HCT116_mHLA−2: 15441
SA = 0.79
Score = 1.39
KPENNVQKI − Mascot − rejected
20120322_EXQ1_MiBa_SA_HCC1143_1_A: 5921
SA = 0.75
LKSEHSKLQ − MaxQuant
20120322_EXQ1_MiBa_SA_HCC1143_1_A: 5921
SA = 0.09
Score = −5.64
SSLKEHQKI − MSFragger
20120322_EXQ1_MiBa_SA_HCC1143_1_A: 5921
SA = 0.35
TDGAETLVRP − Mascot − rejected
NA − spliced − |||
20120518_EXQ0_MiBa_SA_HCT116_mHLA−2: 15185
SA = 0.74
Score = 1.47
SEQETLVRP − MaxQuant
20120518_EXQ0_MiBa_SA_HCT116_mHLA−2: 15185
SA = 0.9
Score = 2.51
SEQETLVRP − MSFragger
20120518_EXQ0_MiBa_SA_HCT116_mHLA−2: 15185
SA = 0.9
Score = 2.51
Relative intensity
m/z
Prosit prediction
⋆ Exclusive in prediction
Exclusive in experimental

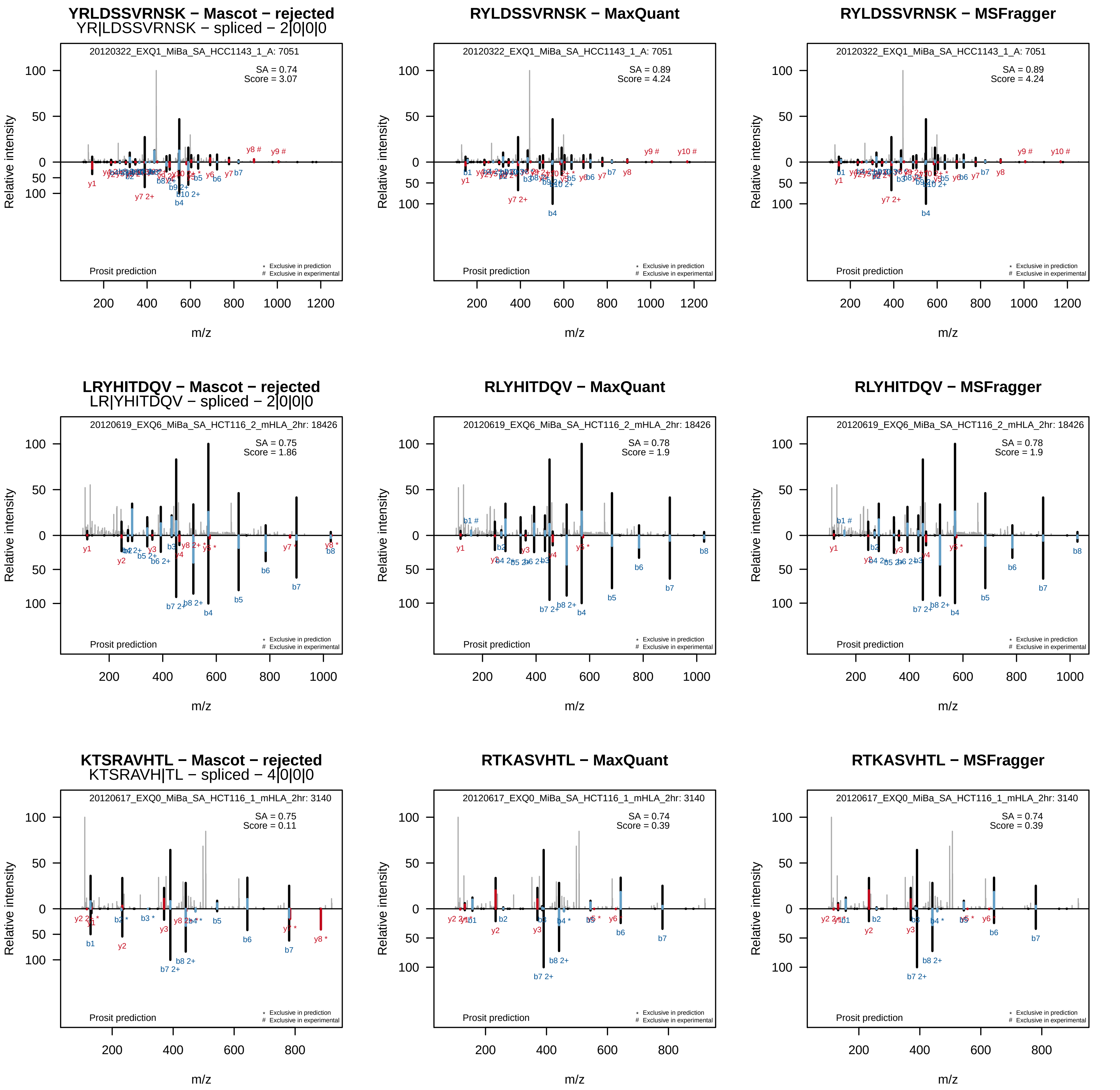
YRLDSSVRNSK − Mascot − rejected
YR|LDSSVRNSK − spliced − 2|0|0|0
20120322_EXQ1_MiBa_SA_HCC1143_1_A: 7051
SA = 0.74
Score = 3.07
RYLDSSVRNSK − MaxQuant
20120322_EXQ1_MiBa_SA_HCC1143_1_A: 7051
SA = 0.89
Score = 4.24
RYLDSSVRNSK − MSFragger
20120322_EXQ1_MiBa_SA_HCC1143_1_A: 7051
SA = 0.89
Score = 4.24
LRYHITDQV − Mascot − rejected
LR|YHITDQV − spliced − 2|0|0|0
20120619_EXQ6_MiBa_SA_HCT116_2_mHLA_2hr: 18426
SA = 0.75
Score = 1.86
RLYHITDQV − MaxQuant
20120619_EXQ6_MiBa_SA_HCT116_2_mHLA_2hr: 18426
SA = 0.78
Score = 1.9
RLYHITDQV − MSFragger
20120619_EXQ6_MiBa_SA_HCT116_2_mHLA_2hr: 18426
SA = 0.78
Score = 1.9
KTSRAVHTL − Mascot − rejected
KTSRAVH|TL − spliced − 4|0|0|0
20120617_EXQ0_MiBa_SA_HCT116_1_mHLA_2hr: 3140
SA = 0.75
Score = 0.11
RTKASVHTL − MaxQuant
20120617_EXQ0_MiBa_SA_HCT116_1_mHLA_2hr: 3140
SA = 0.74
Score = 0.39
RTKASVHTL − MSFragger
20120617_EXQ0_MiBa_SA_HCT116_1_mHLA_2hr: 3140
SA = 0.74
Score = 0.39
Relative intensity
m/z
Prosit prediction
* Exclusive in prediction
# Exclusive in experimental

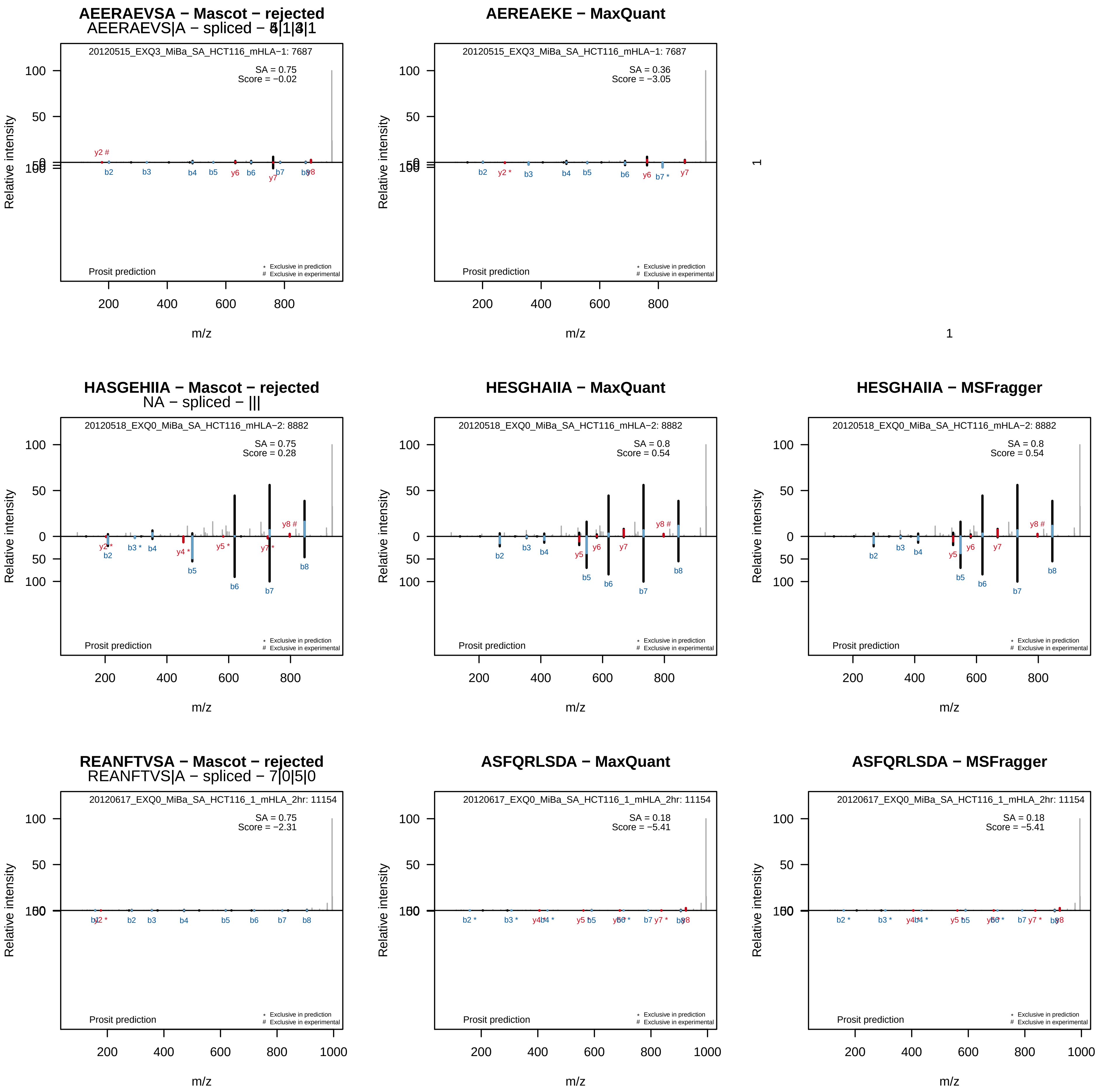
AEERAEVSA − Mascot − rejected
20120515_EXQ3_MiBa_SA_HCT116_mHLA−1: 7687
SA = 0.75
Score = −0.02
Prosit prediction
Exclusive in prediction
Exclusive in experimental
Relative intensity
m/z
AEREAEKE − MaxQuant
20120515_EXQ3_MiBa_SA_HCT116_mHLA−1: 7687
SA = 0.36
Score = −3.05
HASGEHIIA − Mascot − rejected
NA − spliced − |||
20120518_EXQ0_MiBa_SA_HCT116_mHLA−2: 8882
SA = 0.75
Score = 0.28
HESGHAIIA − MaxQuant
20120518_EXQ0_MiBa_SA_HCT116_mHLA−2: 8882
SA = 0.8
Score = 0.54
HESGHAIIA − MSFragger
20120518_EXQ0_MiBa_SA_HCT116_mHLA−2: 8882
SA = 0.8
Score = 0.54
REANFTVSA − Mascot − rejected
REANFTVS|A − spliced − 7|0|5|0
20120617_EXQ0_MiBa_SA_HCT116_1_mHLA_2hr: 11154
SA = 0.75
Score = −2.31
ASFQRLSDA − MaxQuant
20120617_EXQ0_MiBa_SA_HCT116_1_mHLA_2hr: 11154
SA = 0.18
Score = −5.41
ASFQRLSDA − MSFragger
20120617_EXQ0_MiBa_SA_HCT116_1_mHLA_2hr: 11154
SA = 0.18
Score = −5.41

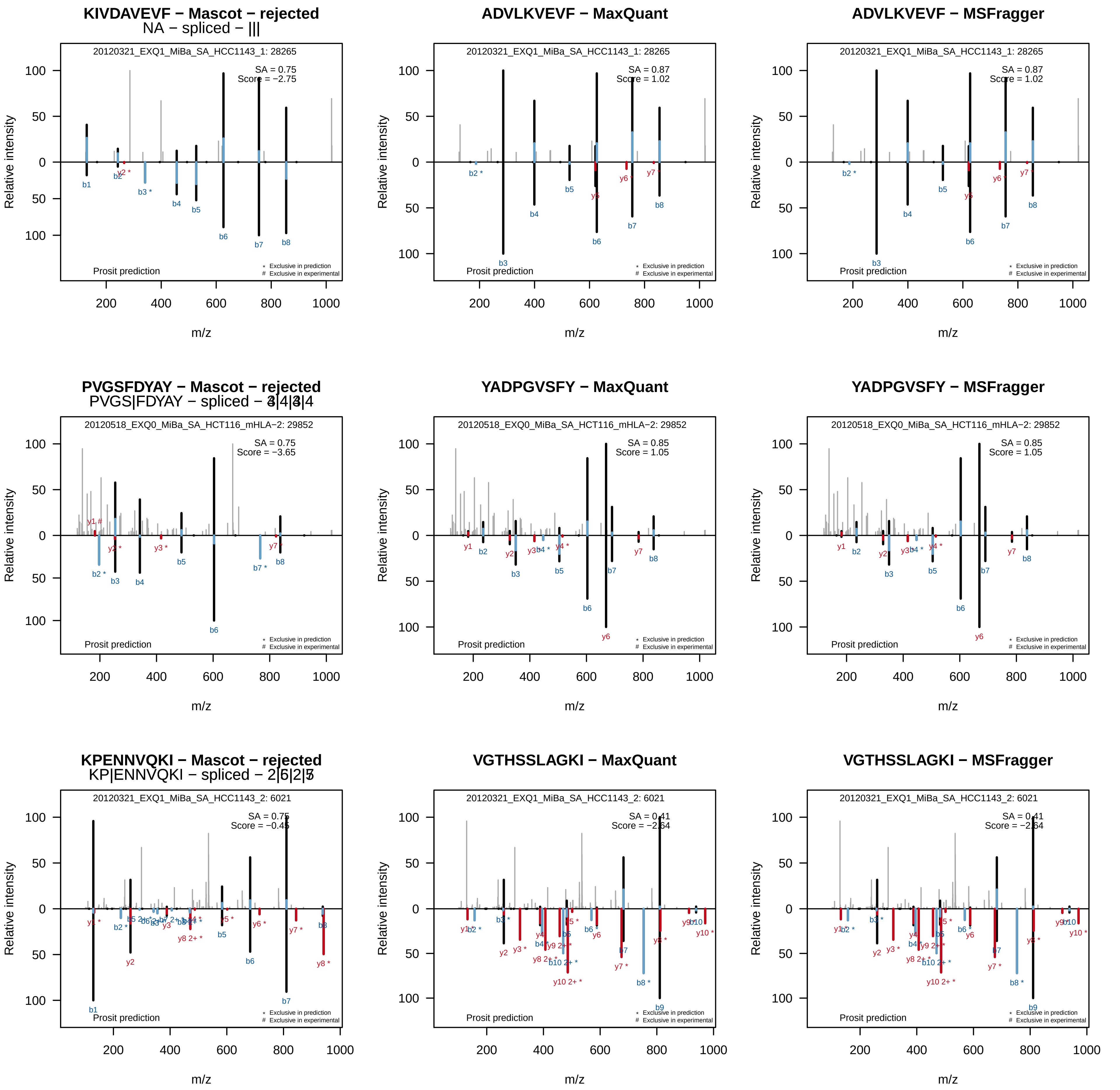
KIVDAVEVF − Mascot − rejected
NA − spliced − |||
20120321_EXQ1_MiBa_SA_HCC1143_1: 28265
SA = 0.75
Score = −2.75
ADVLKVEVF − MaxQuant
20120321_EXQ1_MiBa_SA_HCC1143_1: 28265
SA = 0.87
Score = 1.02
ADVLKVEVF − MSFragger
20120321_EXQ1_MiBa_SA_HCC1143_1: 28265
SA = 0.87
Score = 1.02
PVGSFDYAY − Mascot − rejected
PVGS|FDYAY − spliced − 4|4|4|4
20120518_EXQ0_MiBa_SA_HCT116_mHLA−2: 29852
SA = 0.75
Score = −3.65
YADPGVSFY − MaxQuant
20120518_EXQ0_MiBa_SA_HCT116_mHLA−2: 29852
SA = 0.85
Score = 1.05
YADPGVSFY − MSFragger
20120518_EXQ0_MiBa_SA_HCT116_mHLA−2: 29852
SA = 0.85
Score = 1.05
KPENNVQKI − Mascot − rejected
KP|ENNVQKI − spliced − 2|6|2|7
20120321_EXQ1_MiBa_SA_HCC1143_2: 6021
SA = 0.75
Score = −0.45
VGTHSSLAGKI − MaxQuant
20120321_EXQ1_MiBa_SA_HCC1143_2: 6021
SA = 0.41
Score = −2.64
VGTHSSLAGKI − MSFragger
20120321_EXQ1_MiBa_SA_HCC1143_2: 6021
SA = 0.41
Score = −2.64
Relative intensity
m/z
Prosit prediction
⋆ Exclusive in prediction
Exclusive in experimental

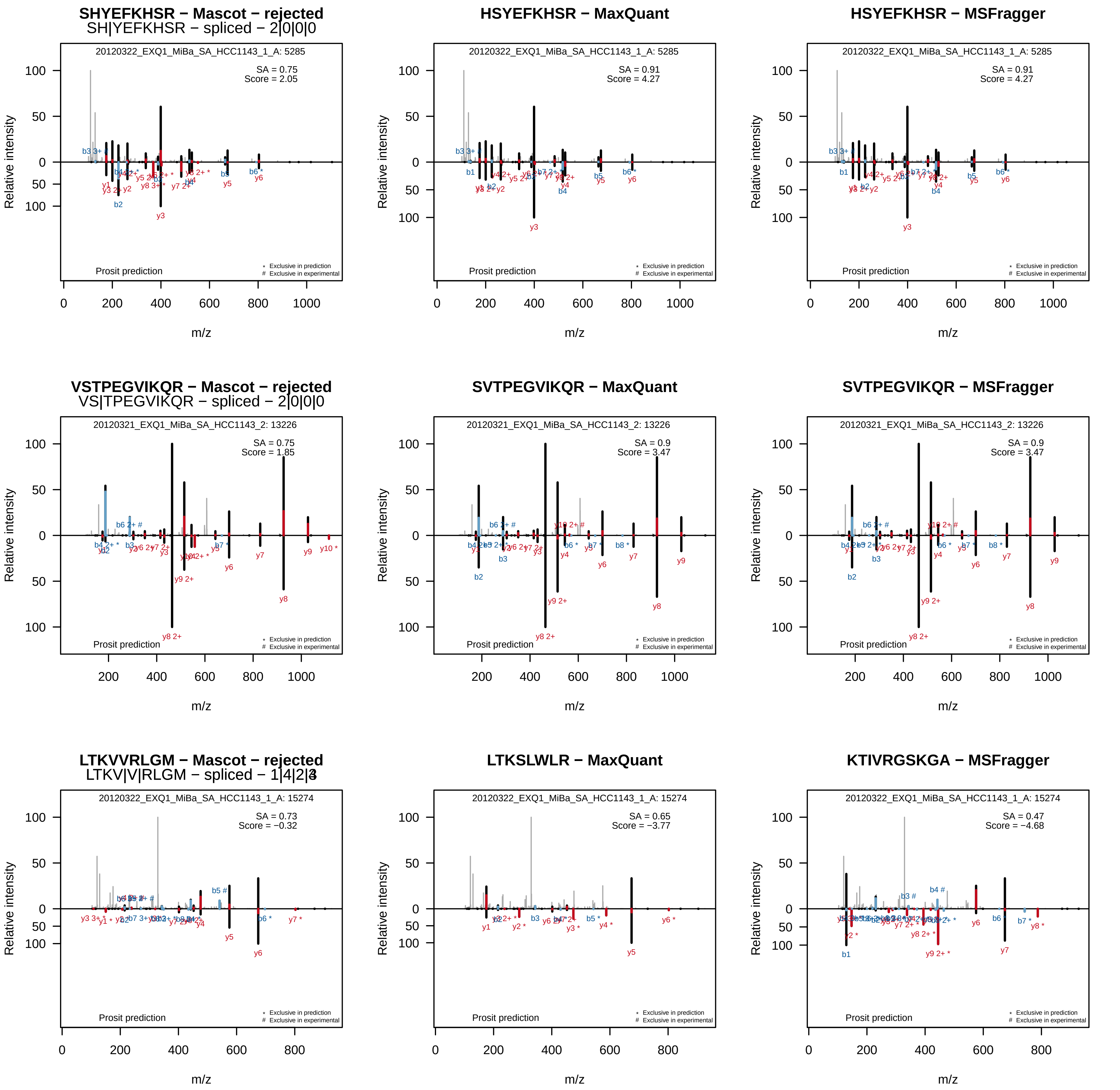
SHYEFKHSR − Mascot − rejected
SH|YEFKHSR − spliced − 2|0|0|0
20120322_EXQ1_MiBa_SA_HCC1143_1_A: 5285
SA = 0.75
Score = 2.05
HSYEFKHSR − MaxQuant
20120322_EXQ1_MiBa_SA_HCC1143_1_A: 5285
SA = 0.91
Score = 4.27
HSYEFKHSR − MSFragger
20120322_EXQ1_MiBa_SA_HCC1143_1_A: 5285
SA = 0.91
Score = 4.27
VSTPEGVIKQR − Mascot − rejected
VS|TPEGVIKQR − spliced − 2|0|0|0
20120321_EXQ1_MiBa_SA_HCC1143_2: 13226
SA = 0.75
Score = 1.85
SVTPEGVIKQR − MaxQuant
20120321_EXQ1_MiBa_SA_HCC1143_2: 13226
SA = 0.9
Score = 3.47
SVTPEGVIKQR − MSFragger
20120321_EXQ1_MiBa_SA_HCC1143_2: 13226
SA = 0.9
Score = 3.47
LTKVVRLGM − Mascot − rejected
LTKV|V|RLGM − spliced − 1|4|2|4
20120322_EXQ1_MiBa_SA_HCC1143_1_A: 15274
SA = 0.73
Score = −0.32
LTKSLWLR − MaxQuant
20120322_EXQ1_MiBa_SA_HCC1143_1_A: 15274
SA = 0.65
Score = −3.77
KTIVRGSKGA − MSFragger
20120322_EXQ1_MiBa_SA_HCC1143_1_A: 15274
SA = 0.47
Score = −4.68
Relative intensity
m/z
Prosit prediction
* Exclusive in prediction
# Exclusive in experimental

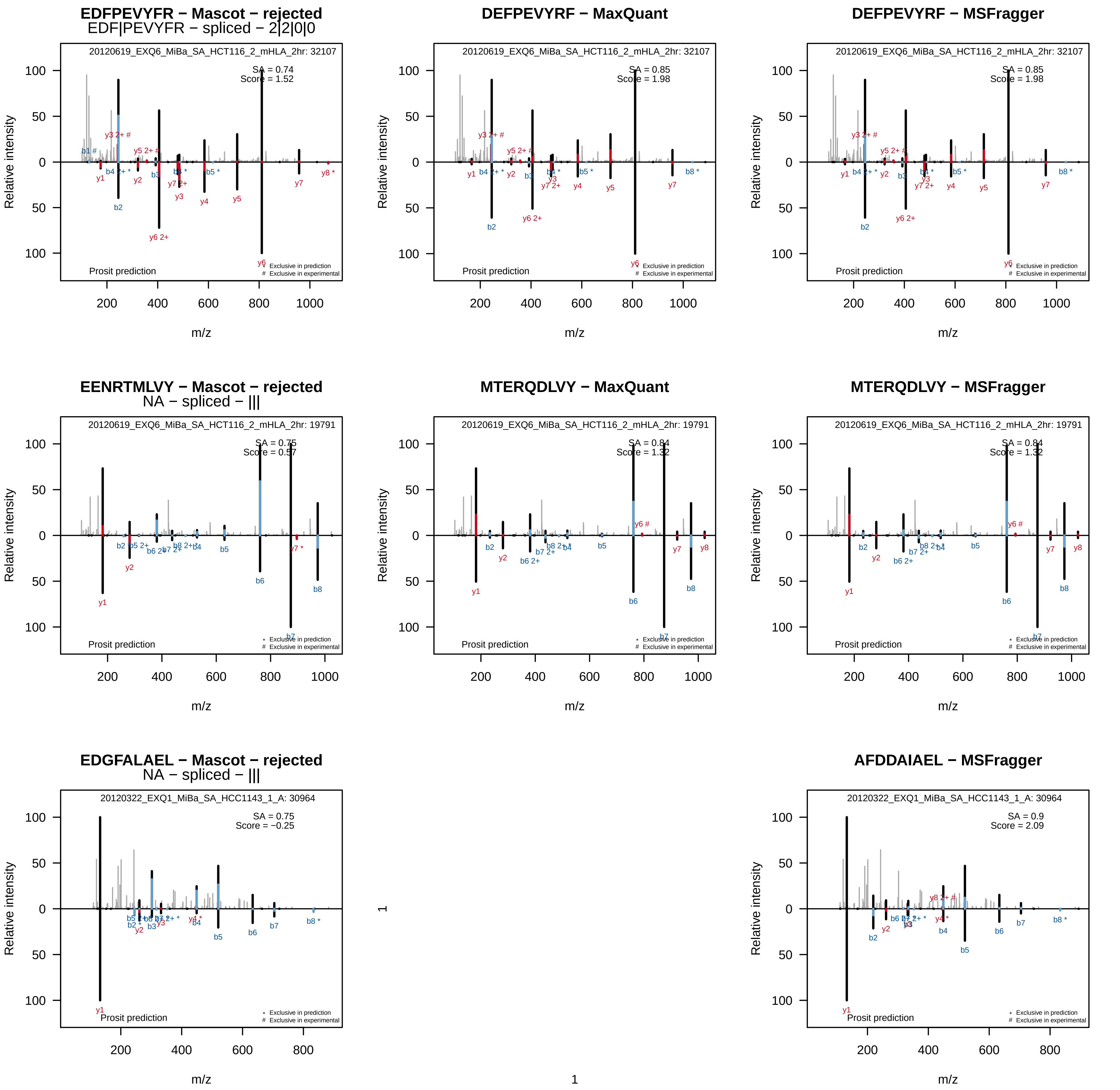
EDFPEVYFR − Mascot − rejected
EDF|PEVYFR − spliced − 2|2|0|0
20120619_EXQ6_MiBa_SA_HCT116_2_mHLA_2hr: 32107
SA = 0.74
Score = 1.52
DEFPEVYRF − MaxQuant
20120619_EXQ6_MiBa_SA_HCT116_2_mHLA_2hr: 32107
SA = 0.85
Score = 1.98
DEFPEVYRF − MSFragger
20120619_EXQ6_MiBa_SA_HCT116_2_mHLA_2hr: 32107
SA = 0.85
Score = 1.98
EENRTMLVY − Mascot − rejected
NA − spliced − |||
20120619_EXQ6_MiBa_SA_HCT116_2_mHLA_2hr: 19791
SA = 0.75
Score = 0.57
MTERQDLVY − MaxQuant
20120619_EXQ6_MiBa_SA_HCT116_2_mHLA_2hr: 19791
SA = 0.84
Score = 1.32
MTERQDLVY − MSFragger
20120619_EXQ6_MiBa_SA_HCT116_2_mHLA_2hr: 19791
SA = 0.84
Score = 1.32
EDGFALAEL − Mascot − rejected
NA − spliced − |||
20120322_EXQ1_MiBa_SA_HCC1143_1_A: 30964
SA = 0.75
Score = −0.25
AFDDAIAEL − MSFragger
20120322_EXQ1_MiBa_SA_HCC1143_1_A: 30964
SA = 0.9
Score = 2.09
Relative intensity
m/z
Prosit prediction
* Exclusive in prediction
# Exclusive in experimental
1

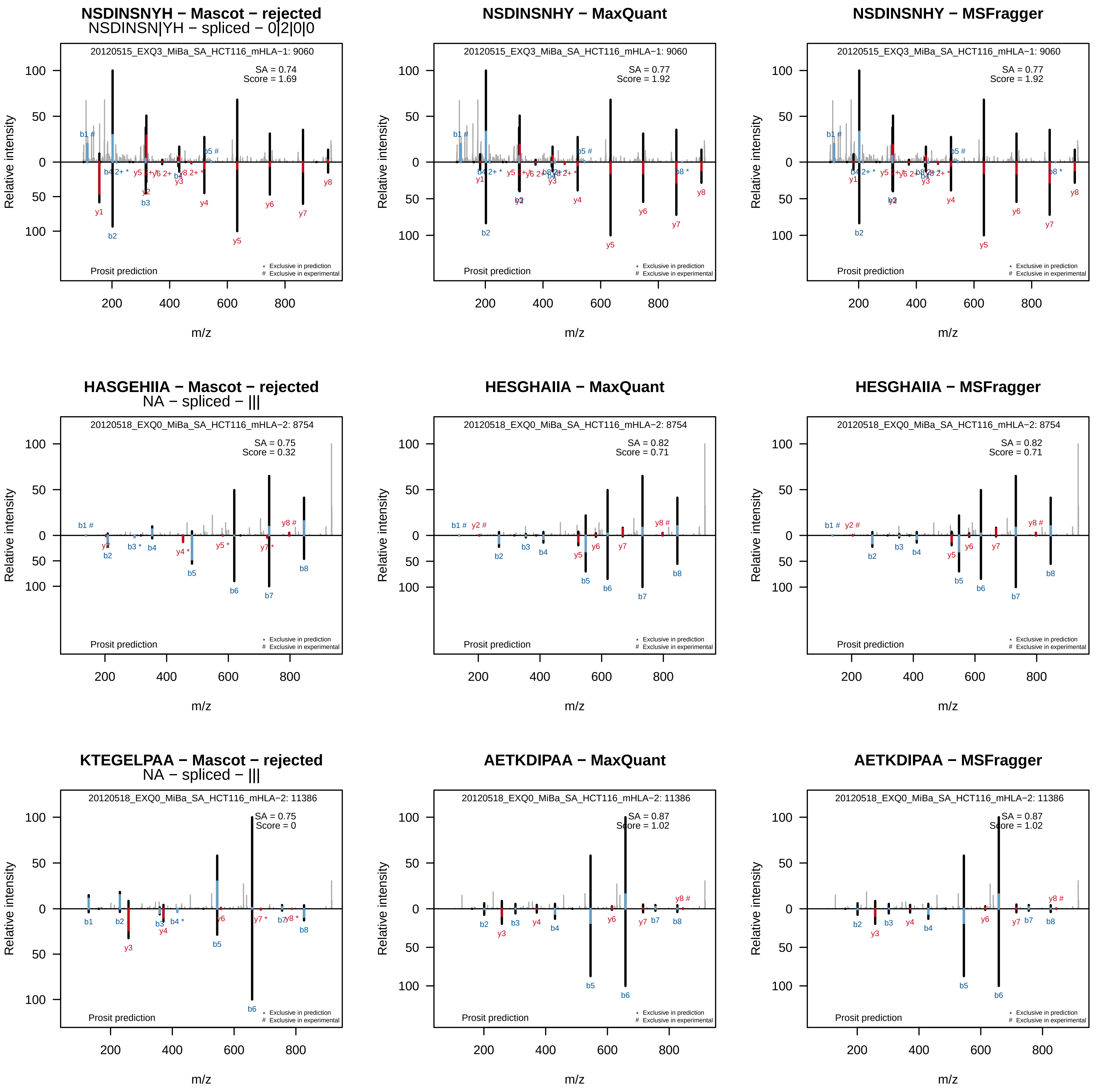

NSDINSNYH − Mascot − rejected
NSDINSN|YH − spliced − 0|2|0|0
20120515_EXQ3_MiBa_SA_HCT116_mHLA−1: 9060
SA = 0.74
Score = 1.69
NSDINSNHY − MaxQuant
20120515_EXQ3_MiBa_SA_HCT116_mHLA−1: 9060
SA = 0.77
Score = 1.92
NSDINSNHY − MSFragger
20120515_EXQ3_MiBa_SA_HCT116_mHLA−1: 9060
SA = 0.77
Score = 1.92
HASGEHIIA − Mascot − rejected
NA − spliced − |||
20120518_EXQ0_MiBa_SA_HCT116_mHLA−2: 8754
SA = 0.75
Score = 0.32
HESGHAIIA − MaxQuant
20120518_EXQ0_MiBa_SA_HCT116_mHLA−2: 8754
SA = 0.82
Score = 0.71
HESGHAIIA − MSFragger
20120518_EXQ0_MiBa_SA_HCT116_mHLA−2: 8754
SA = 0.82
Score = 0.71
KTEGELPAA − Mascot − rejected
NA − spliced − |||
20120518_EXQ0_MiBa_SA_HCT116_mHLA−2: 11386
SA = 0.75
Score = 0
AETKDIPAA − MaxQuant
20120518_EXQ0_MiBa_SA_HCT116_mHLA−2: 11386
SA = 0.87
Score = 1.02
AETKDIPAA − MSFragger
20120518_EXQ0_MiBa_SA_HCT116_mHLA−2: 11386
SA = 0.87
Score = 1.02
Prosit prediction
⋆ Exclusive in prediction
Exclusive in experimental
Relative intensity
m/z

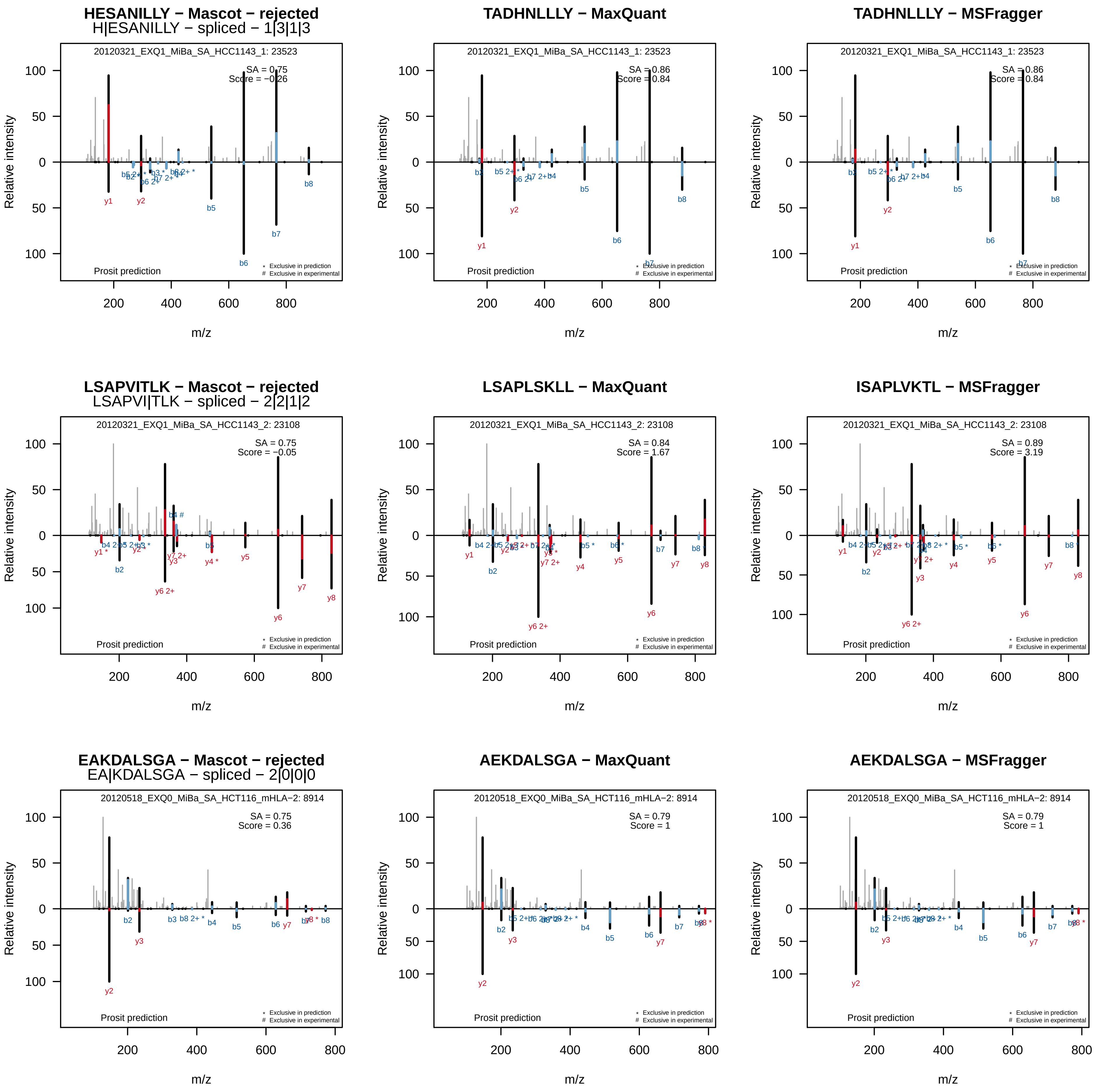

HESANILLY − Mascot − rejected
H|ESANILLY − spliced − 1|3|1|3
20120321_EXQ1_MiBa_SA_HCC1143_1: 23523
SA = 0.75
Score = −0.26
TADHNLLLY − MaxQuant
20120321_EXQ1_MiBa_SA_HCC1143_1: 23523
SA = 0.86
Score = 0.84
TADHNLLLY − MSFragger
20120321_EXQ1_MiBa_SA_HCC1143_1: 23523
SA = 0.86
Score = 0.84
LSAPVITLK − Mascot − rejected
LSAPVI|TLK − spliced − 2|2|1|2
20120321_EXQ1_MiBa_SA_HCC1143_2: 23108
SA = 0.75
Score = −0.05
LSAPLSKLL − MaxQuant
20120321_EXQ1_MiBa_SA_HCC1143_2: 23108
SA = 0.84
Score = 1.67
ISAPLVKTL − MSFragger
20120321_EXQ1_MiBa_SA_HCC1143_2: 23108
SA = 0.89
Score = 3.19
EAKDALSGA − Mascot − rejected
EA|KDALSGA − spliced − 2|0|0|0
20120518_EXQ0_MiBa_SA_HCT116_mHLA−2: 8914
SA = 0.75
Score = 0.36
AEKDALSGA − MaxQuant
20120518_EXQ0_MiBa_SA_HCT116_mHLA−2: 8914
SA = 0.79
Score = 1
AEKDALSGA − MSFragger
20120518_EXQ0_MiBa_SA_HCT116_mHLA−2: 8914
SA = 0.79
Score = 1
Relative intensity
m/z
Prosit prediction
⋆ Exclusive in prediction
Exclusive in experimental

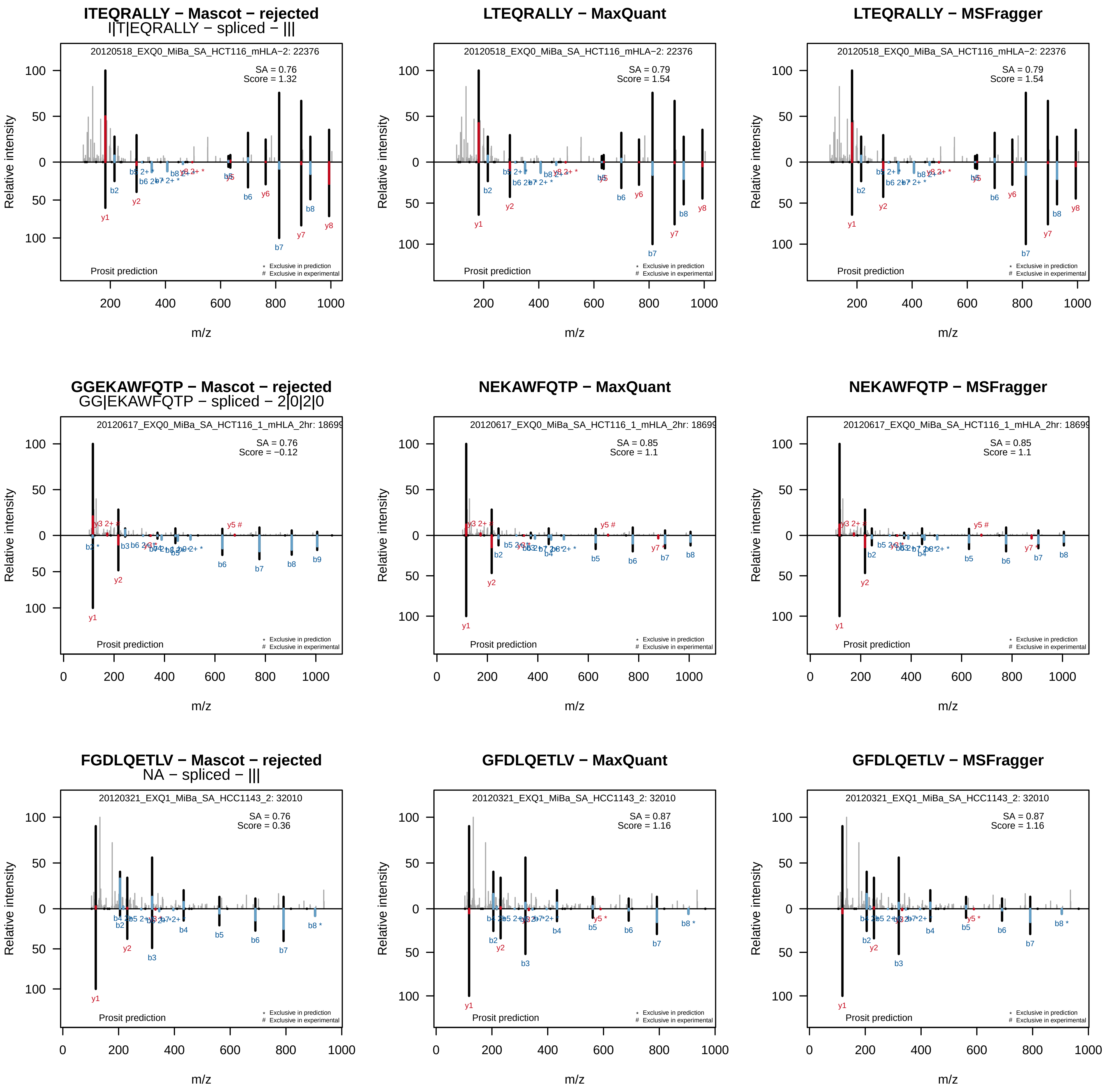

ITEQRALLY − Mascot − rejected
I|T|EQRALLY − spliced − |||
20120518_EXQ0_MiBa_SA_HCT116_mHLA−2: 22376
SA = 0.76
Score = 1.32
LTEQRALLY − MaxQuant
20120518_EXQ0_MiBa_SA_HCT116_mHLA−2: 22376
SA = 0.79
Score = 1.54
LTEQRALLY − MSFragger
20120518_EXQ0_MiBa_SA_HCT116_mHLA−2: 22376
SA = 0.79
Score = 1.54
GGEKAWFQTP − Mascot − rejected
GG|EKAWFQTP − spliced − 2|0|2|0
20120617_EXQ0_MiBa_SA_HCT116_1_mHLA_2hr: 18699
SA = 0.76
Score = −0.12
NEKAWFQTP − MaxQuant
20120617_EXQ0_MiBa_SA_HCT116_1_mHLA_2hr: 18699
SA = 0.85
Score = 1.1
NEKAWFQTP − MSFragger
20120617_EXQ0_MiBa_SA_HCT116_1_mHLA_2hr: 18699
SA = 0.85
Score = 1.1
FGDLQETLV − Mascot − rejected
NA − spliced − |||
20120321_EXQ1_MiBa_SA_HCC1143_2: 32010
SA = 0.76
Score = 0.36
GFDLQETLV − MaxQuant
20120321_EXQ1_MiBa_SA_HCC1143_2: 32010
SA = 0.87
Score = 1.16
GFDLQETLV − MSFragger
20120321_EXQ1_MiBa_SA_HCC1143_2: 32010
SA = 0.87
Score = 1.16
Relative intensity
m/z
Prosit prediction
* Exclusive in prediction
# Exclusive in experimental

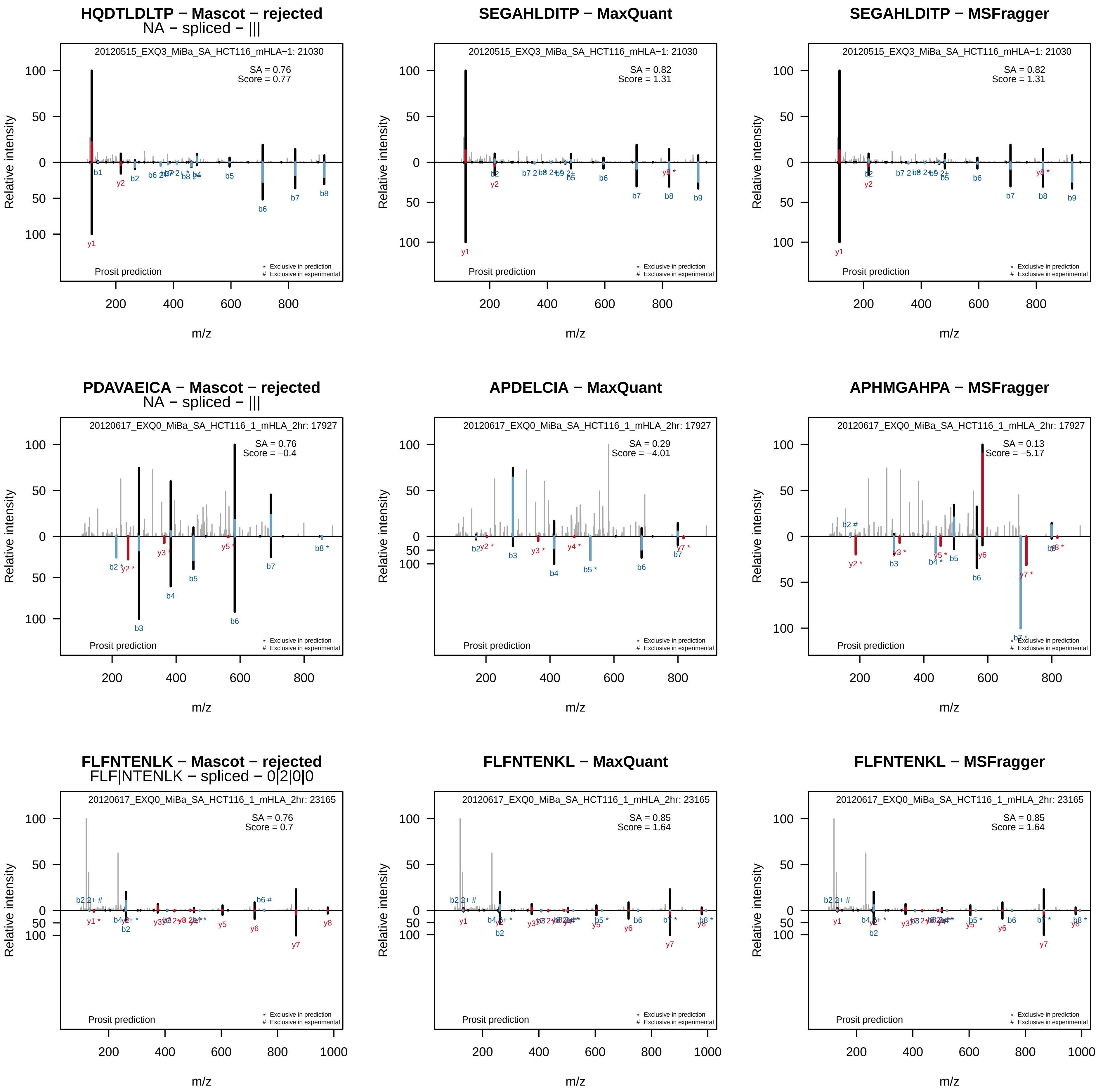
HQDTLDLTP − Mascot − rejected
NA − spliced − |||
20120515_EXQ3_MiBa_SA_HCT116_mHLA−1: 21030
SA = 0.76
Score = 0.77
SEGAHLDITP − MaxQuant
20120515_EXQ3_MiBa_SA_HCT116_mHLA−1: 21030
SA = 0.82
Score = 1.31
SEGAHLDITP − MSFragger
20120515_EXQ3_MiBa_SA_HCT116_mHLA−1: 21030
SA = 0.82
Score = 1.31
PDAVAEICA − Mascot − rejected
NA − spliced − |||
20120617_EXQ0_MiBa_SA_HCT116_1_mHLA_2hr: 17927
SA = 0.76
Score = −0.4
APDELCIA − MaxQuant
20120617_EXQ0_MiBa_SA_HCT116_1_mHLA_2hr: 17927
SA = 0.29
Score = −4.01
APHMGAHPA − MSFragger
20120617_EXQ0_MiBa_SA_HCT116_1_mHLA_2hr: 17927
SA = 0.13
Score = −5.17
FLFNTENLK − Mascot − rejected
FLF|NTENLK − spliced − 0|2|0|0
20120617_EXQ0_MiBa_SA_HCT116_1_mHLA_2hr: 23165
SA = 0.76
Score = 0.7
FLFNTENKL − MaxQuant
20120617_EXQ0_MiBa_SA_HCT116_1_mHLA_2hr: 23165
SA = 0.85
Score = 1.64
FLFNTENKL − MSFragger
20120617_EXQ0_MiBa_SA_HCT116_1_mHLA_2hr: 23165
SA = 0.85
Score = 1.64
Relative intensity
m/z
Prosit prediction
* Exclusive in prediction
# Exclusive in experimental

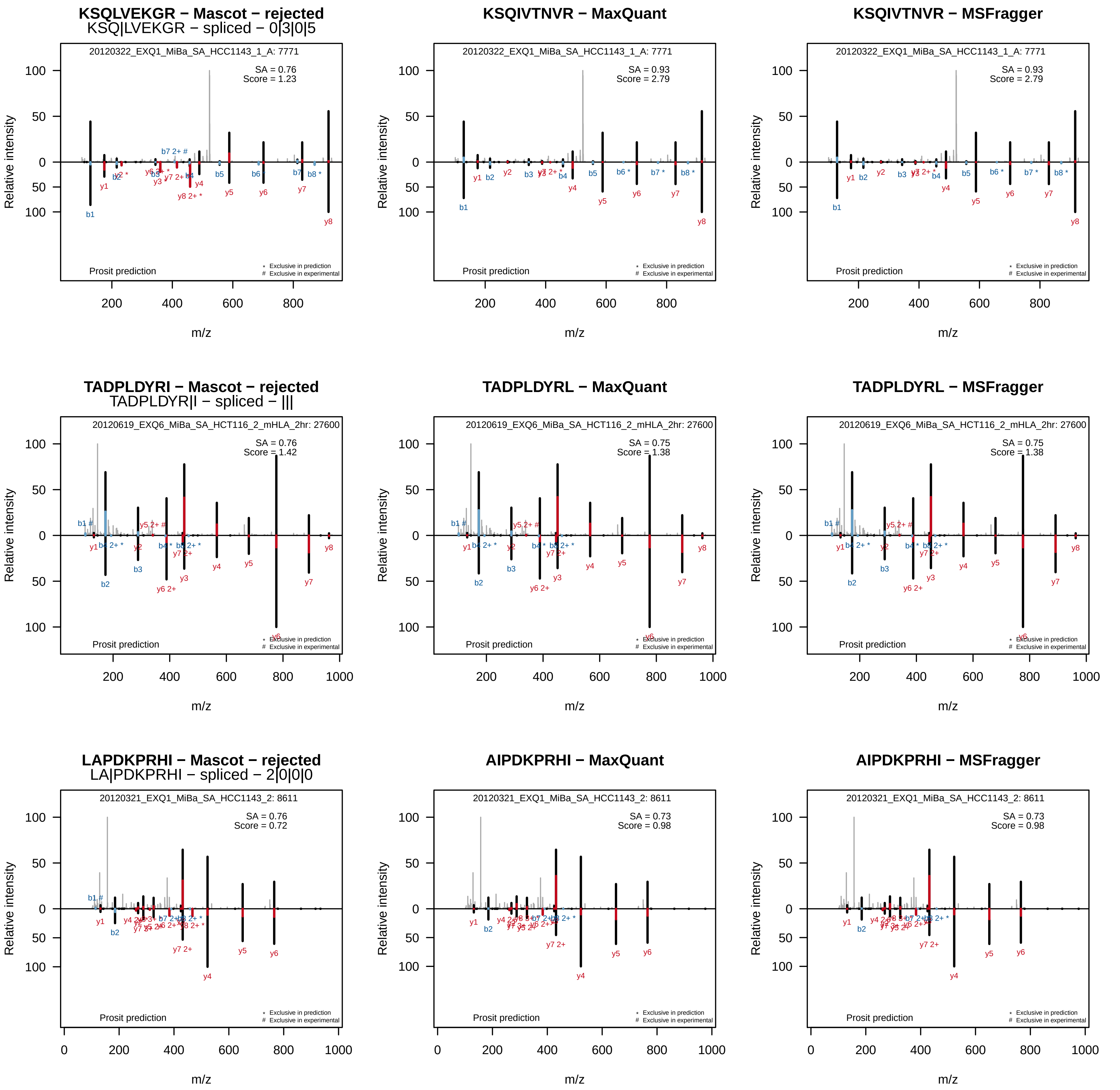

KSQLVEKGR − Mascot − rejected
KSQ|LVEKGR − spliced − 0|3|0|5
KSQIVTNVR − MaxQuant
KSQIVTNVR − MSFragger
20120322_EXQ1_MiBa_SA_HCC1143_1_A: 7771
SA = 0.76
Score = 1.23
SA = 0.93
Score = 2.79
TADPLDYRI − Mascot − rejected
TADPLDYR|I − spliced − |||
TADPLDYRL − MaxQuant
TADPLDYRL − MSFragger
20120619_EXQ6_MiBa_SA_HCT116_2_mHLA_2hr: 27600
SA = 0.76
Score = 1.42
SA = 0.75
Score = 1.38
LAPDKPRHI − Mascot − rejected
LA|PDKPRHI − spliced − 2|0|0|0
AIPDKPRHI − MaxQuant
AIPDKPRHI − MSFragger
20120321_EXQ1_MiBa_SA_HCC1143_2: 8611
SA = 0.76
Score = 0.72
SA = 0.73
Score = 0.98
Relative intensity
m/z
Prosit prediction
⋆ Exclusive in prediction
Exclusive in experimental

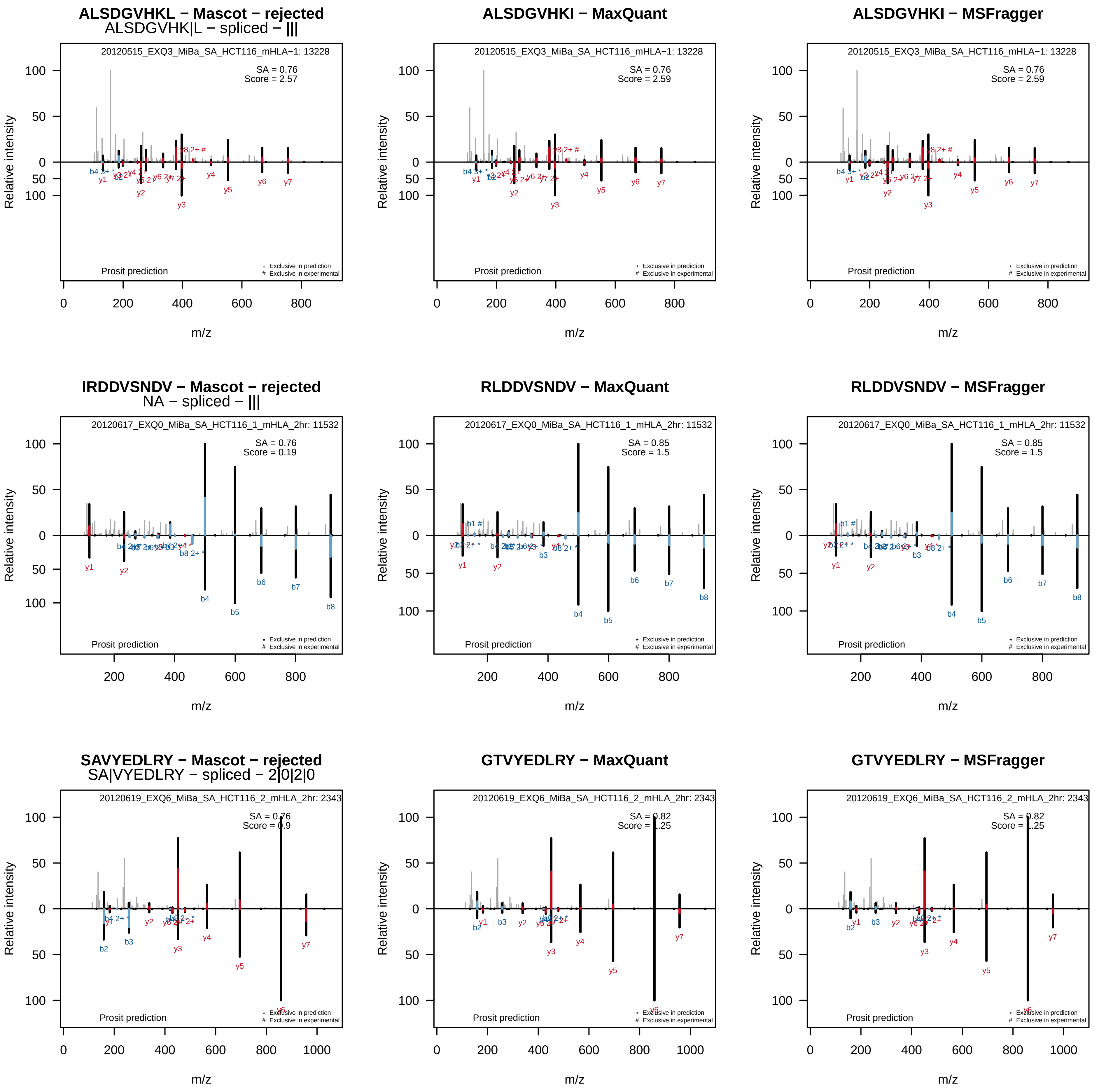
ALSDGVHKL − Mascot − rejected
ALSDGVHK|L − spliced − |||
20120515_EXQ3_MiBa_SA_HCT116_mHLA−1: 13228
SA = 0.76
Score = 2.57
ALSDGVHKI − MaxQuant
20120515_EXQ3_MiBa_SA_HCT116_mHLA−1: 13228
SA = 0.76
Score = 2.59
ALSDGVHKI − MSFragger
20120515_EXQ3_MiBa_SA_HCT116_mHLA−1: 13228
SA = 0.76
Score = 2.59
IRDDVSNDV − Mascot − rejected
NA − spliced − |||
20120617_EXQ0_MiBa_SA_HCT116_1_mHLA_2hr: 11532
SA = 0.76
Score = 0.19
RLDDVSNDV − MaxQuant
20120617_EXQ0_MiBa_SA_HCT116_1_mHLA_2hr: 11532
SA = 0.85
Score = 1.5
RLDDVSNDV − MSFragger
20120617_EXQ0_MiBa_SA_HCT116_1_mHLA_2hr: 11532
SA = 0.85
Score = 1.5
SAVYEDLRY − Mascot − rejected
SA|VYEDLRY − spliced − 2|0|2|0
20120619_EXQ6_MiBa_SA_HCT116_2_mHLA_2hr: 2343
SA = 0.76
Score = 0.9
GTVYEDLRY − MaxQuant
20120619_EXQ6_MiBa_SA_HCT116_2_mHLA_2hr: 2343
SA = 0.82
Score = 1.25
GTVYEDLRY − MSFragger
20120619_EXQ6_MiBa_SA_HCT116_2_mHLA_2hr: 2343
SA = 0.82
Score = 1.25
Relative intensity
m/z
Prosit prediction
* Exclusive in prediction
# Exclusive in experimental

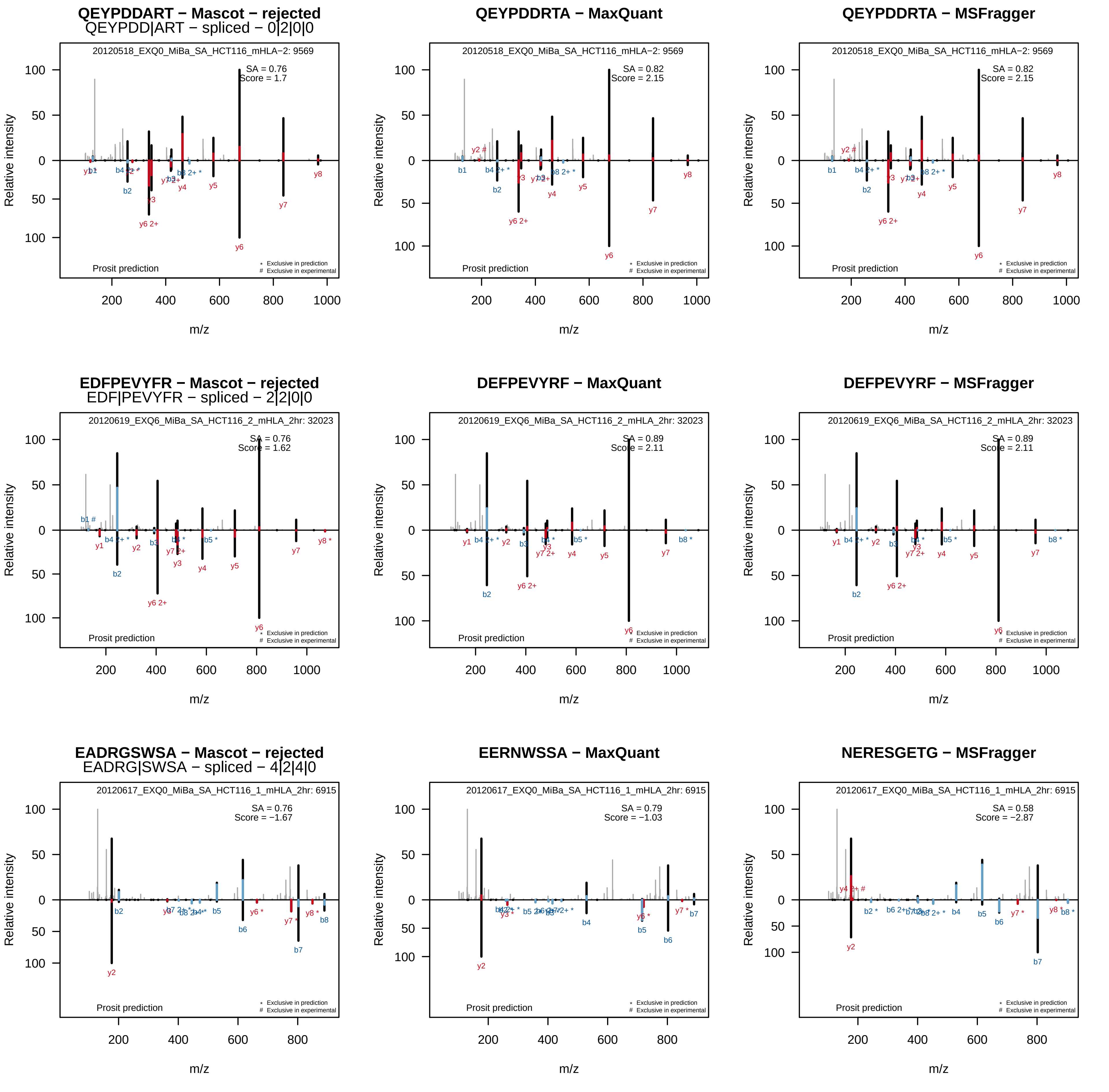
QEYPDDART − Mascot − rejected
QEYPDD|ART − spliced − 0|2|0|0
20120518_EXQ0_MiBa_SA_HCT116_mHLA−2: 9569
SA = 0.76
Score = 1.7
QEYPDDRTA − MaxQuant
20120518_EXQ0_MiBa_SA_HCT116_mHLA−2: 9569
SA = 0.82
Score = 2.15
QEYPDDRTA − MSFragger
20120518_EXQ0_MiBa_SA_HCT116_mHLA−2: 9569
SA = 0.82
Score = 2.15
EDFPEVYFR − Mascot − rejected
EDF|PEVYFR − spliced − 2|2|0|0
20120619_EXQ6_MiBa_SA_HCT116_2_mHLA_2hr: 32023
SA = 0.76
Score = 1.62
DEFPEVYRF − MaxQuant
20120619_EXQ6_MiBa_SA_HCT116_2_mHLA_2hr: 32023
SA = 0.89
Score = 2.11
DEFPEVYRF − MSFragger
20120619_EXQ6_MiBa_SA_HCT116_2_mHLA_2hr: 32023
SA = 0.89
Score = 2.11
EADRGSWSA − Mascot − rejected
EADRG|SWSA − spliced − 4|2|4|0
20120617_EXQ0_MiBa_SA_HCT116_1_mHLA_2hr: 6915
SA = 0.76
Score = −1.67
EERNWSSA − MaxQuant
20120617_EXQ0_MiBa_SA_HCT116_1_mHLA_2hr: 6915
SA = 0.79
Score = −1.03
NERESGETG − MSFragger
20120617_EXQ0_MiBa_SA_HCT116_1_mHLA_2hr: 6915
SA = 0.58
Score = −2.87
Relative intensity
m/z
Prosit prediction
* Exclusive in prediction
# Exclusive in experimental

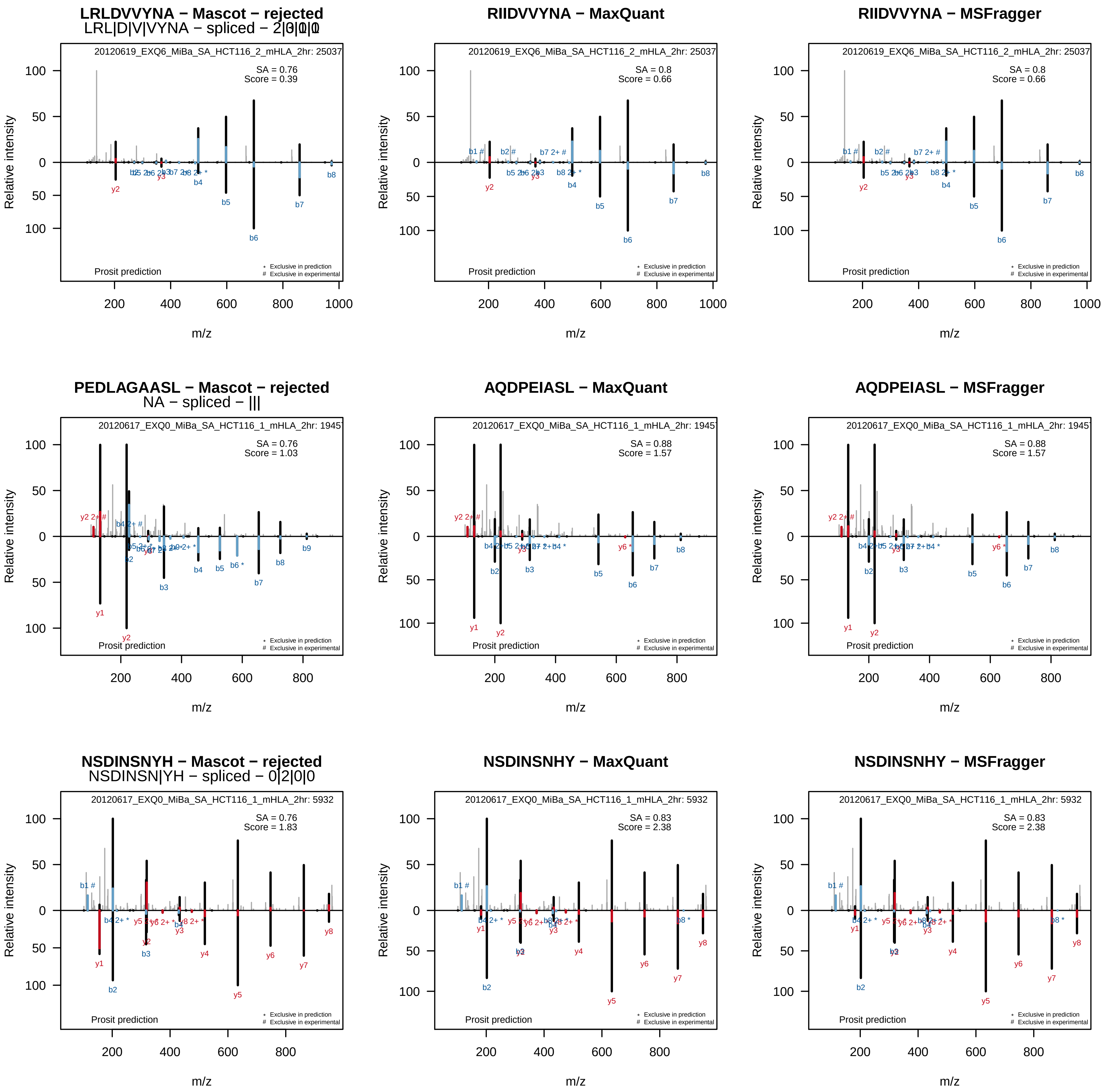
LRLDVVYNA − Mascot − rejected
LRL|D|V|VYNA − spliced − 2|3|0|1
20120619_EXQ6_MiBa_SA_HCT116_2_mHLA_2hr: 25037
SA = 0.76
Score = 0.39
RIIDVVYNA − MaxQuant
20120619_EXQ6_MiBa_SA_HCT116_2_mHLA_2hr: 25037
SA = 0.8
Score = 0.66
RIIDVVYNA − MSFragger
20120619_EXQ6_MiBa_SA_HCT116_2_mHLA_2hr: 25037
SA = 0.8
Score = 0.66
PEDLAGAASL − Mascot − rejected
NA − spliced − |||
20120617_EXQ0_MiBa_SA_HCT116_1_mHLA_2hr: 1945
SA = 0.76
Score = 1.03
AQDPEIASL − MaxQuant
20120617_EXQ0_MiBa_SA_HCT116_1_mHLA_2hr: 1945
SA = 0.88
Score = 1.57
AQDPEIASL − MSFragger
20120617_EXQ0_MiBa_SA_HCT116_1_mHLA_2hr: 1945
SA = 0.88
Score = 1.57
NSDINSNYH − Mascot − rejected
NSDINSN|YH − spliced − 0|2|0|0
20120617_EXQ0_MiBa_SA_HCT116_1_mHLA_2hr: 5932
SA = 0.76
Score = 1.83
NSDINSNHY − MaxQuant
20120617_EXQ0_MiBa_SA_HCT116_1_mHLA_2hr: 5932
SA = 0.83
Score = 2.38
NSDINSNHY − MSFragger
20120617_EXQ0_MiBa_SA_HCT116_1_mHLA_2hr: 5932
SA = 0.83
Score = 2.38
Relative intensity
m/z
Prosit prediction
* Exclusive in prediction
# Exclusive in experimental

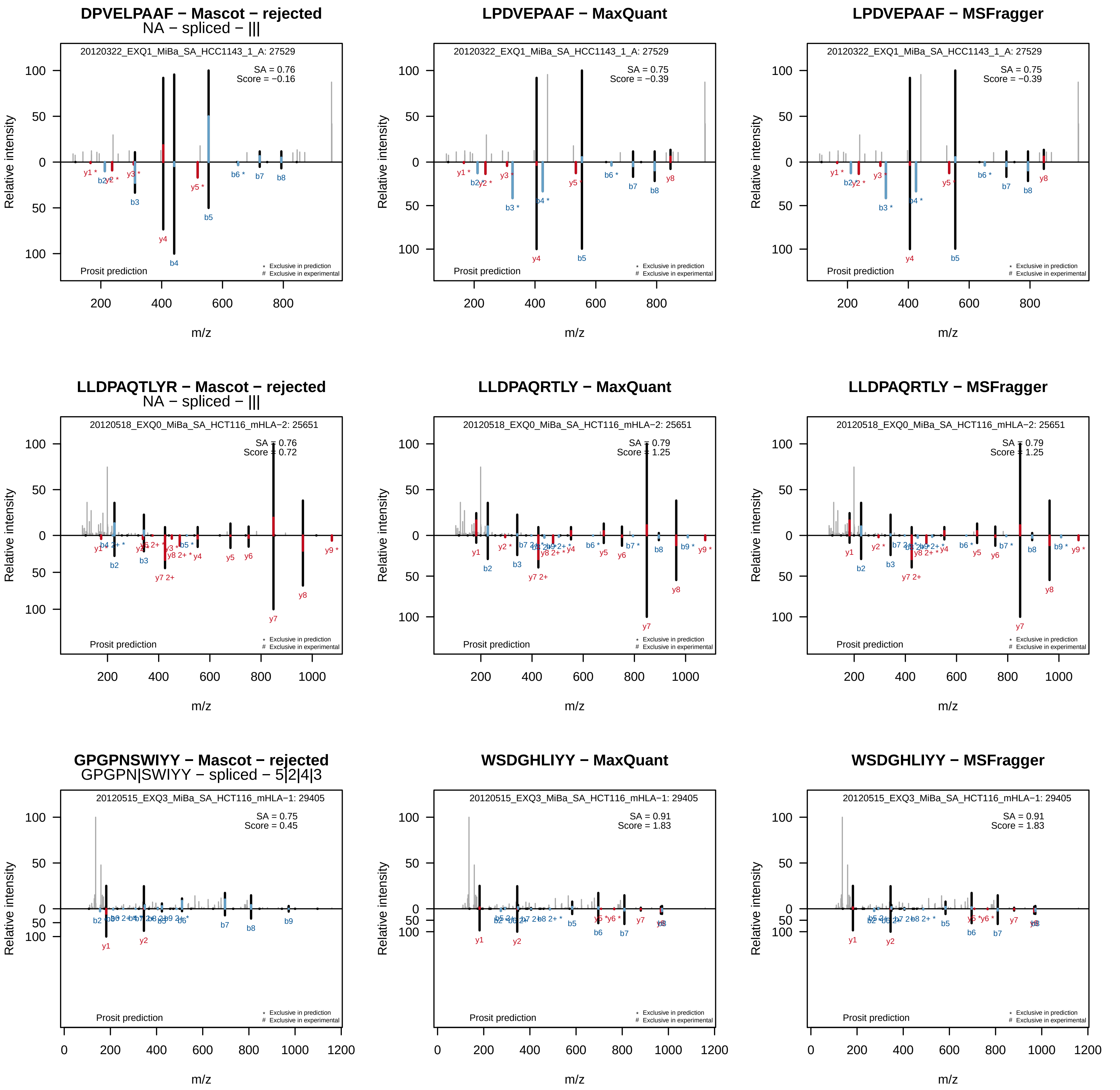
DPVELPAAF − Mascot − rejected
NA − spliced − |||
20120322_EXQ1_MiBa_SA_HCC1143_1_A: 27529
SA = 0.76
Score = −0.16
LPDVEPAAF − MaxQuant
20120322_EXQ1_MiBa_SA_HCC1143_1_A: 27529
SA = 0.75
Score = −0.39
LPDVEPAAF − MSFragger
20120322_EXQ1_MiBa_SA_HCC1143_1_A: 27529
SA = 0.75
Score = −0.39
LLDPAQTLYR − Mascot − rejected
NA − spliced − |||
20120518_EXQ0_MiBa_SA_HCT116_mHLA−2: 25651
SA = 0.76
Score = 0.72
LLDPAQRTLY − MaxQuant
20120518_EXQ0_MiBa_SA_HCT116_mHLA−2: 25651
SA = 0.79
Score = 1.25
LLDPAQRTLY − MSFragger
20120518_EXQ0_MiBa_SA_HCT116_mHLA−2: 25651
SA = 0.79
Score = 1.25
GPGPNSWIYY − Mascot − rejected
GPGPN|SWIYY − spliced − 5|2|4|3
20120515_EXQ3_MiBa_SA_HCT116_mHLA−1: 29405
SA = 0.75
Score = 0.45
WSDGHLIYY − MaxQuant
20120515_EXQ3_MiBa_SA_HCT116_mHLA−1: 29405
SA = 0.91
Score = 1.83
WSDGHLIYY − MSFragger
20120515_EXQ3_MiBa_SA_HCT116_mHLA−1: 29405
SA = 0.91
Score = 1.83
Relative intensity
m/z
Prosit prediction
* Exclusive in prediction
# Exclusive in experimental

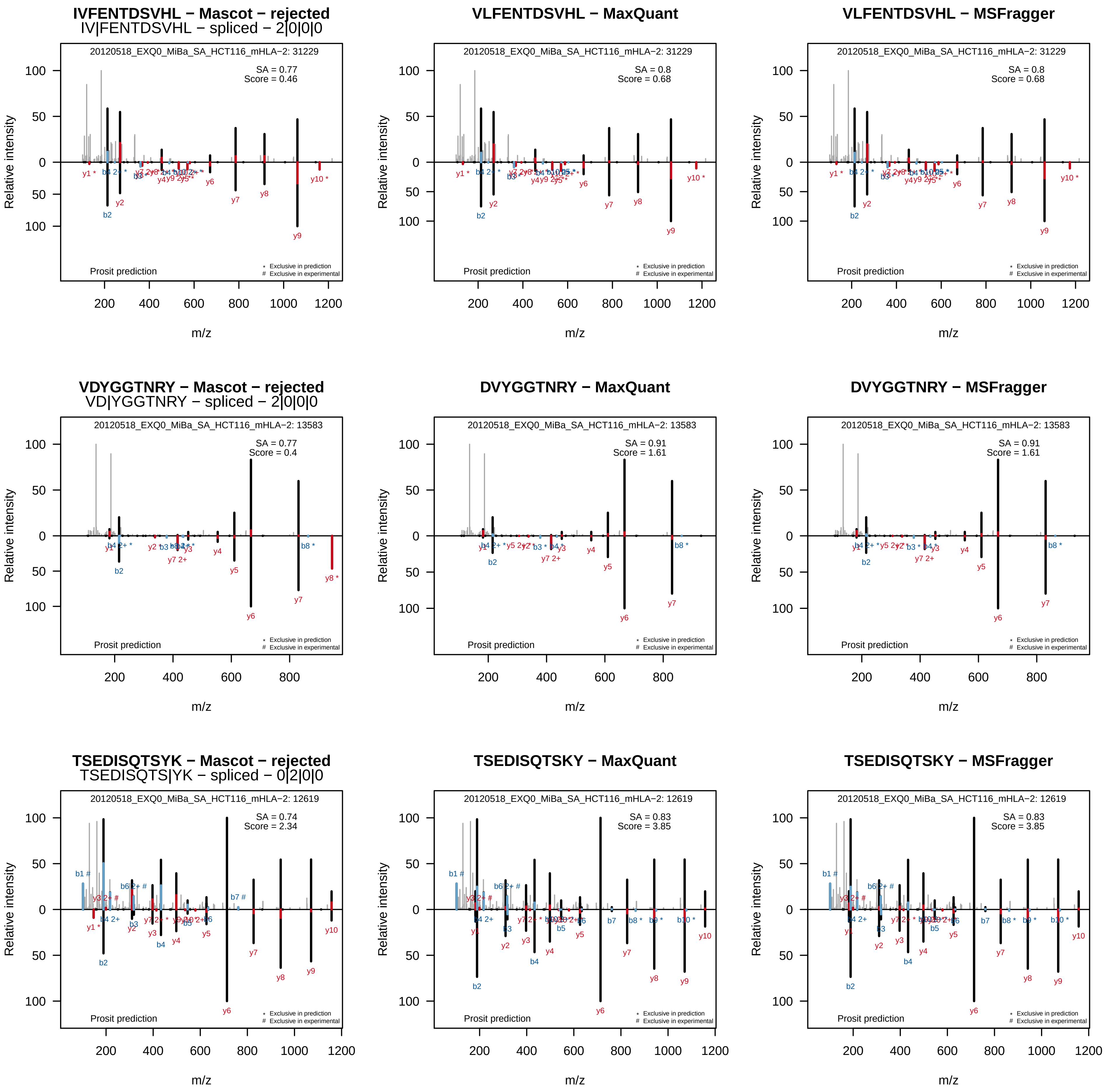
IVFENTDSVHL − Mascot − rejected
IV|FENTDSVHL − spliced − 2|0|0|0
20120518_EXQ0_MiBa_SA_HCT116_mHLA−2: 31229
SA = 0.77
Score = 0.46
VLFENTDSVHL − MaxQuant
20120518_EXQ0_MiBa_SA_HCT116_mHLA−2: 31229
SA = 0.8
Score = 0.68
VLFENTDSVHL − MSFragger
20120518_EXQ0_MiBa_SA_HCT116_mHLA−2: 31229
SA = 0.8
Score = 0.68
VDYGGTNRY − Mascot − rejected
VD|YGGTNRY − spliced − 2|0|0|0
20120518_EXQ0_MiBa_SA_HCT116_mHLA−2: 13583
SA = 0.77
Score = 0.4
DVYGGTNRY − MaxQuant
20120518_EXQ0_MiBa_SA_HCT116_mHLA−2: 13583
SA = 0.91
Score = 1.61
DVYGGTNRY − MSFragger
20120518_EXQ0_MiBa_SA_HCT116_mHLA−2: 13583
SA = 0.91
Score = 1.61
TSEDISQTSYK − Mascot − rejected
TSEDISQTS|YK − spliced − 0|2|0|0
20120518_EXQ0_MiBa_SA_HCT116_mHLA−2: 12619
SA = 0.74
Score = 2.34
TSEDISQTSKY − MaxQuant
20120518_EXQ0_MiBa_SA_HCT116_mHLA−2: 12619
SA = 0.83
Score = 3.85
TSEDISQTSKY − MSFragger
20120518_EXQ0_MiBa_SA_HCT116_mHLA−2: 12619
SA = 0.83
Score = 3.85
Relative intensity
m/z
Prosit prediction
* Exclusive in prediction
# Exclusive in experimental

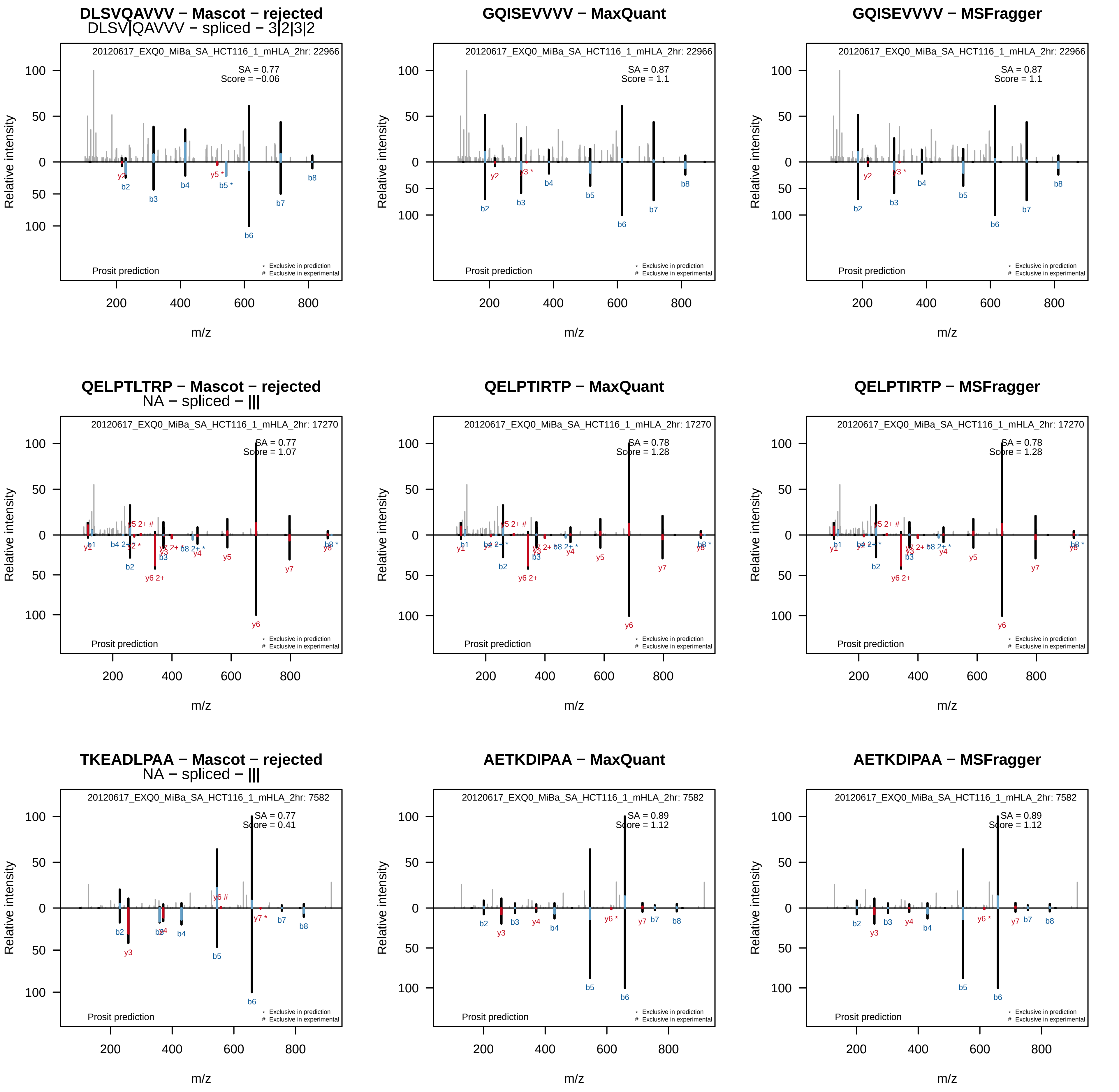

DLSVQAVVV − Mascot − rejected
DLSV|QAVVV − spliced − 3|2|3|2
20120617_EXQ0_MiBa_SA_HCT116_1_mHLA_2hr: 22966
SA = 0.77
Score = −0.06
GQISEVVVV − MaxQuant
20120617_EXQ0_MiBa_SA_HCT116_1_mHLA_2hr: 22966
SA = 0.87
Score = 1.1
GQISEVVVV − MSFragger
20120617_EXQ0_MiBa_SA_HCT116_1_mHLA_2hr: 22966
SA = 0.87
Score = 1.1
QELPTLTRP − Mascot − rejected
NA − spliced − |||
20120617_EXQ0_MiBa_SA_HCT116_1_mHLA_2hr: 17270
SA = 0.77
Score = 1.07
QELPTIRTP − MaxQuant
20120617_EXQ0_MiBa_SA_HCT116_1_mHLA_2hr: 17270
SA = 0.78
Score = 1.28
QELPTIRTP − MSFragger
20120617_EXQ0_MiBa_SA_HCT116_1_mHLA_2hr: 17270
SA = 0.78
Score = 1.28
TKEADLPAA − Mascot − rejected
NA − spliced − |||
20120617_EXQ0_MiBa_SA_HCT116_1_mHLA_2hr: 7582
SA = 0.77
Score = 0.41
AETKDIPAA − MaxQuant
20120617_EXQ0_MiBa_SA_HCT116_1_mHLA_2hr: 7582
SA = 0.89
Score = 1.12
AETKDIPAA − MSFragger
20120617_EXQ0_MiBa_SA_HCT116_1_mHLA_2hr: 7582
SA = 0.89
Score = 1.12
Relative intensity
m/z
Prosit prediction
⋆ Exclusive in prediction
Exclusive in experimental

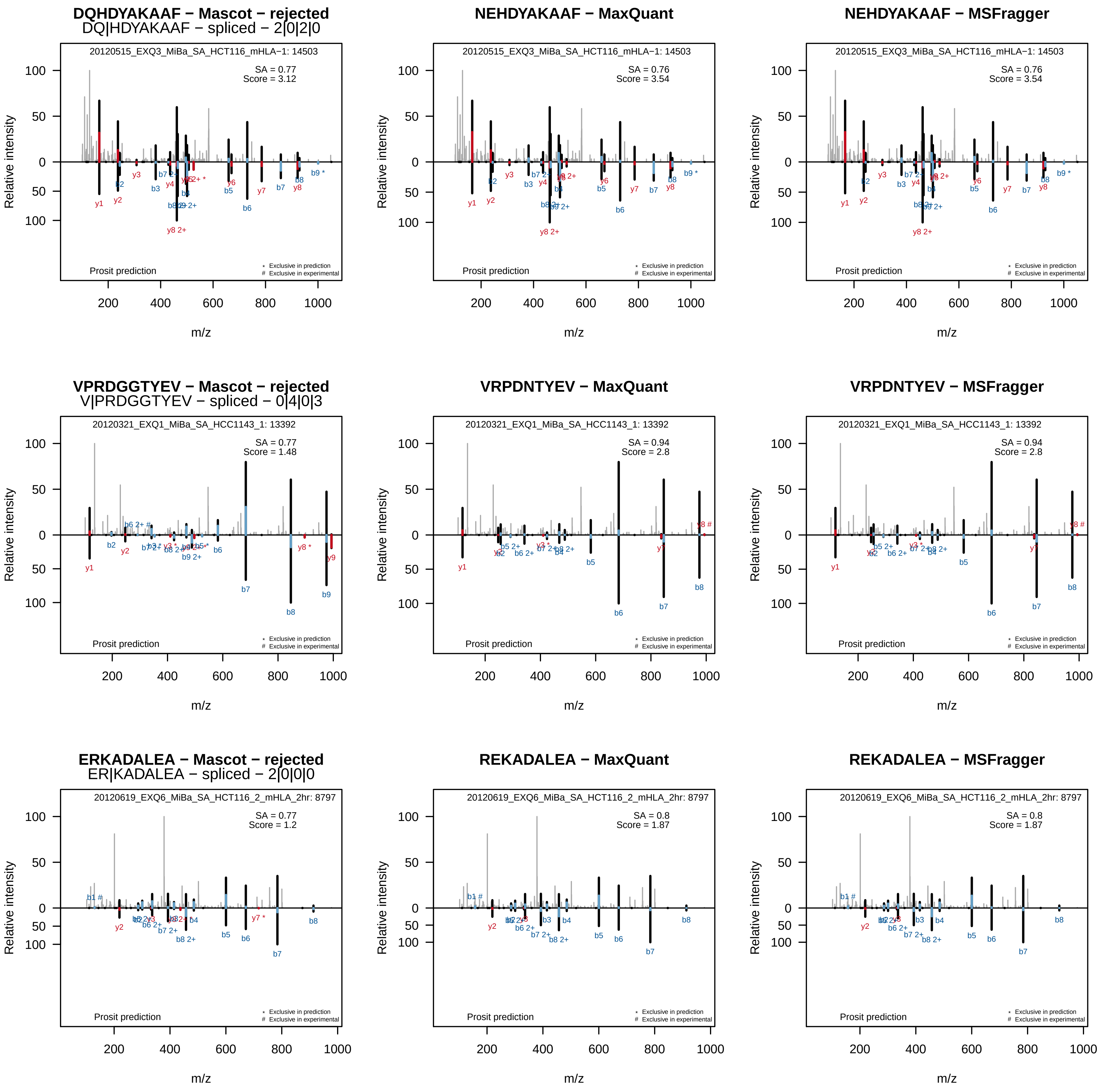
DQHDYAKAAF − Mascot − rejected
DQ|HDYAKAAF − spliced − 2|0|2|0
20120515_EXQ3_MiBa_SA_HCT116_mHLA−1: 14503
SA = 0.77
Score = 3.12
NEHDYAKAAF − MaxQuant
20120515_EXQ3_MiBa_SA_HCT116_mHLA−1: 14503
SA = 0.76
Score = 3.54
NEHDYAKAAF − MSFragger
20120515_EXQ3_MiBa_SA_HCT116_mHLA−1: 14503
SA = 0.76
Score = 3.54
VPRDGGTYEV − Mascot − rejected
V|PRDGGTYEV − spliced − 0|4|0|3
20120321_EXQ1_MiBa_SA_HCC1143_1: 13392
SA = 0.77
Score = 1.48
VRPDNTYEV − MaxQuant
20120321_EXQ1_MiBa_SA_HCC1143_1: 13392
SA = 0.94
Score = 2.8
VRPDNTYEV − MSFragger
20120321_EXQ1_MiBa_SA_HCC1143_1: 13392
SA = 0.94
Score = 2.8
ERKADALEA − Mascot − rejected
ER|KADALEA − spliced − 2|0|0|0
20120619_EXQ6_MiBa_SA_HCT116_2_mHLA_2hr: 8797
SA = 0.77
Score = 1.2
REKADALEA − MaxQuant
20120619_EXQ6_MiBa_SA_HCT116_2_mHLA_2hr: 8797
SA = 0.8
Score = 1.87
REKADALEA − MSFragger
20120619_EXQ6_MiBa_SA_HCT116_2_mHLA_2hr: 8797
SA = 0.8
Score = 1.87
Relative intensity
m/z
Prosit prediction
* Exclusive in prediction
# Exclusive in experimental

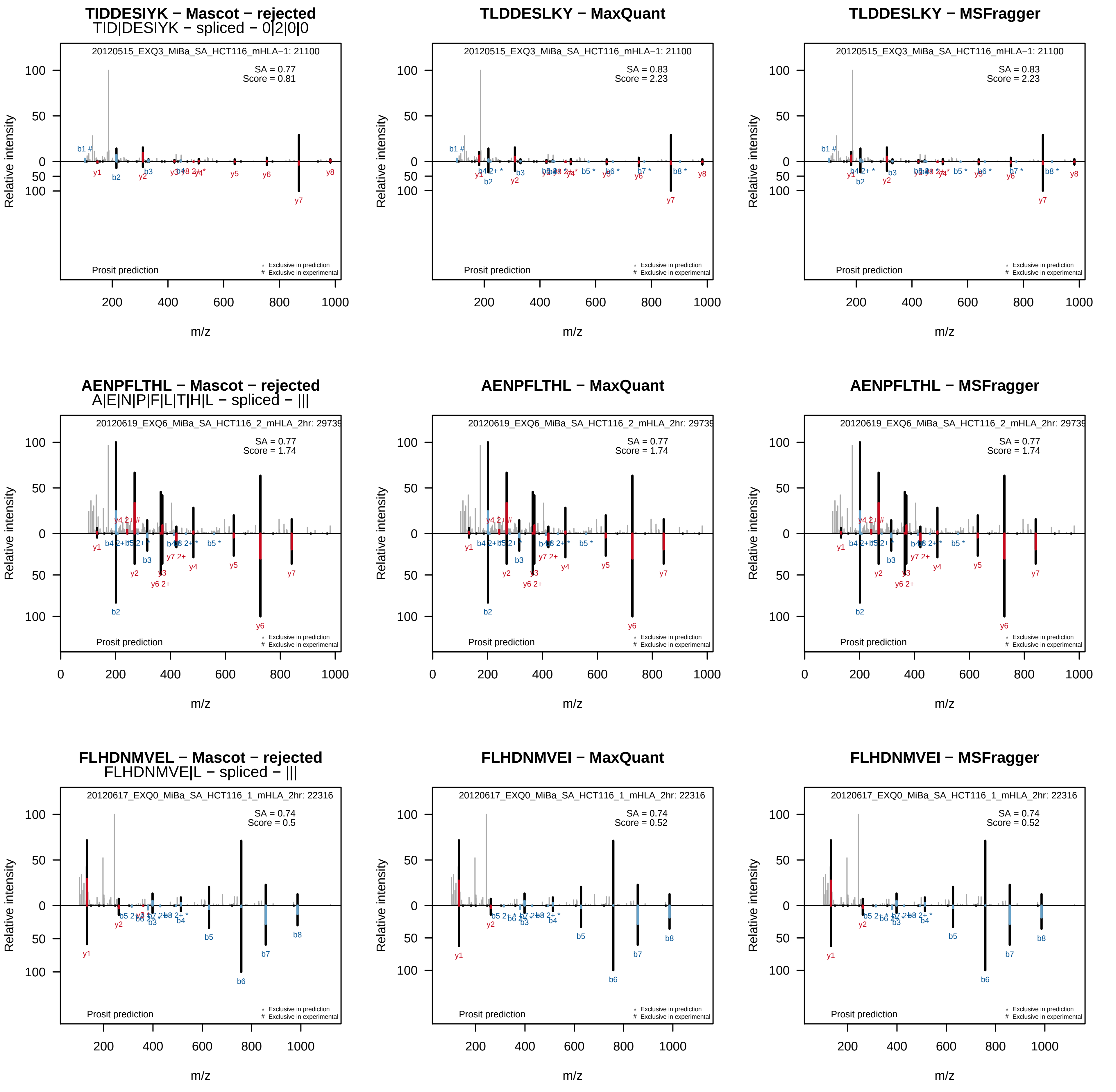
TIDDESIYK − Mascot − rejected
TID|DESIYK − spliced − 0|2|0|0
20120515_EXQ3_MiBa_SA_HCT116_mHLA−1: 21100
SA = 0.77
Score = 0.81
TLDDESLKY − MaxQuant
20120515_EXQ3_MiBa_SA_HCT116_mHLA−1: 21100
SA = 0.83
Score = 2.23
TLDDESLKY − MSFragger
20120515_EXQ3_MiBa_SA_HCT116_mHLA−1: 21100
SA = 0.83
Score = 2.23
AENPFLTHL − Mascot − rejected
A|E|N|P|F|L|T|H|L − spliced − |||
20120619_EXQ6_MiBa_SA_HCT116_2_mHLA_2hr: 29739
SA = 0.77
Score = 1.74
AENPFLTHL − MaxQuant
20120619_EXQ6_MiBa_SA_HCT116_2_mHLA_2hr: 29739
SA = 0.77
Score = 1.74
AENPFLTHL − MSFragger
20120619_EXQ6_MiBa_SA_HCT116_2_mHLA_2hr: 29739
SA = 0.77
Score = 1.74
FLHDNMVEL − Mascot − rejected
FLHDNMVE|L − spliced − |||
20120617_EXQ0_MiBa_SA_HCT116_1_mHLA_2hr: 22316
SA = 0.74
Score = 0.5
FLHDNMVEI − MaxQuant
20120617_EXQ0_MiBa_SA_HCT116_1_mHLA_2hr: 22316
SA = 0.74
Score = 0.52
FLHDNMVEI − MSFragger
20120617_EXQ0_MiBa_SA_HCT116_1_mHLA_2hr: 22316
SA = 0.74
Score = 0.52
Relative intensity
m/z
Prosit prediction
* Exclusive in prediction
# Exclusive in experimental

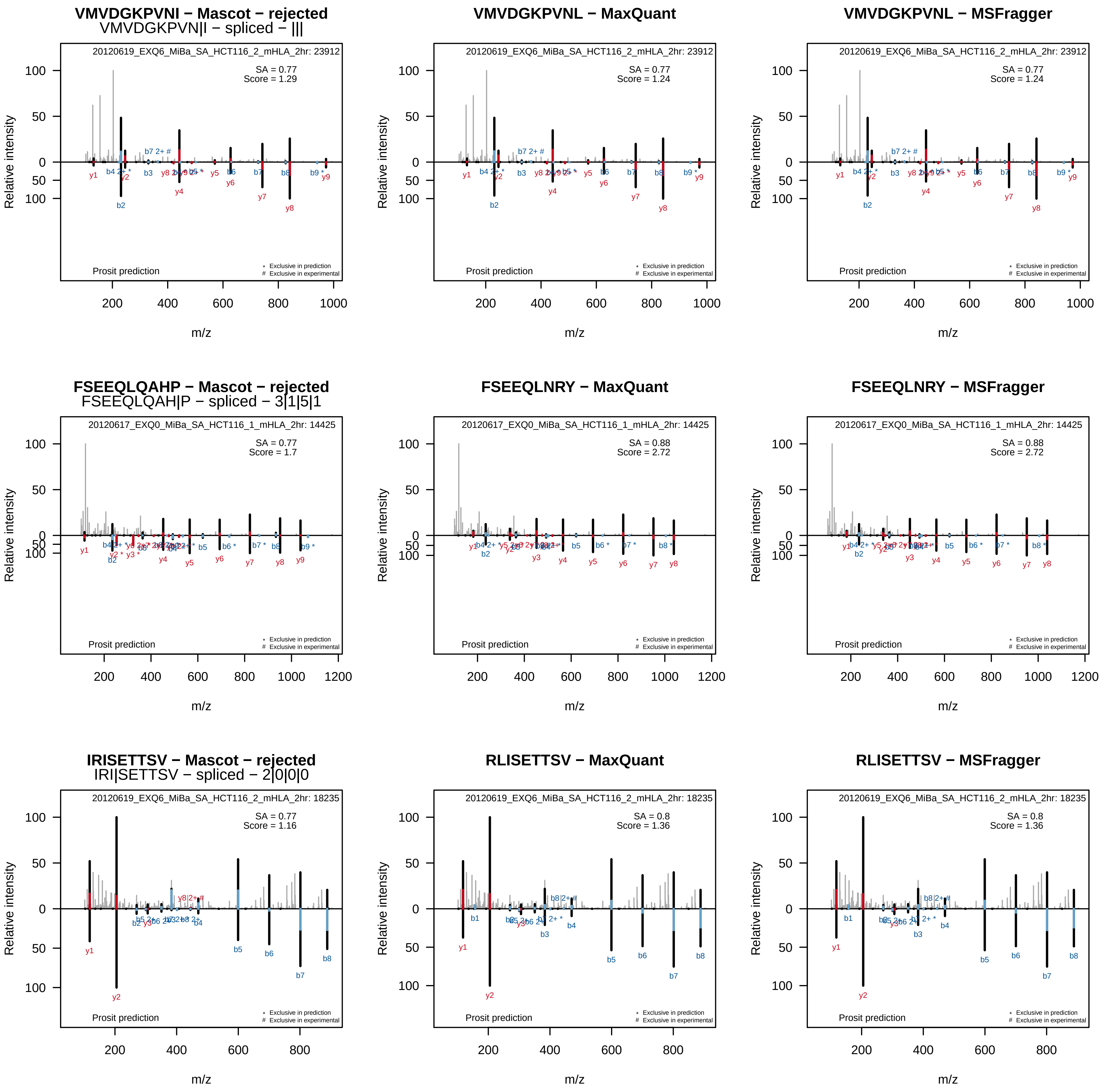
VMVDGKPVNI − Mascot − rejected
VMVDGKPVN|I − spliced − |||
VMVDGKPVNL − MaxQuant
VMVDGKPVNL − MSFragger
20120619_EXQ6_MiBa_SA_HCT116_2_mHLA_2hr: 23912
SA = 0.77
Score = 1.29
Score = 1.24
FSEEQLQAHP − Mascot − rejected
FSEEQLQAH|P − spliced − 3|1|5|1
FSEEQLNRY − MaxQuant
FSEEQLNRY − MSFragger
20120617_EXQ0_MiBa_SA_HCT116_1_mHLA_2hr: 14425
SA = 0.77
Score = 1.7
SA = 0.88
Score = 2.72
IRISETTSV − Mascot − rejected
IRI|SETTSV − spliced − 2|0|0|0
RLISETTSV − MaxQuant
RLISETTSV − MSFragger
20120619_EXQ6_MiBa_SA_HCT116_2_mHLA_2hr: 18235
SA = 0.77
Score = 1.16
SA = 0.8
Score = 1.36
Relative intensity
m/z
Prosit prediction
⋆ Exclusive in prediction
Exclusive in experimental

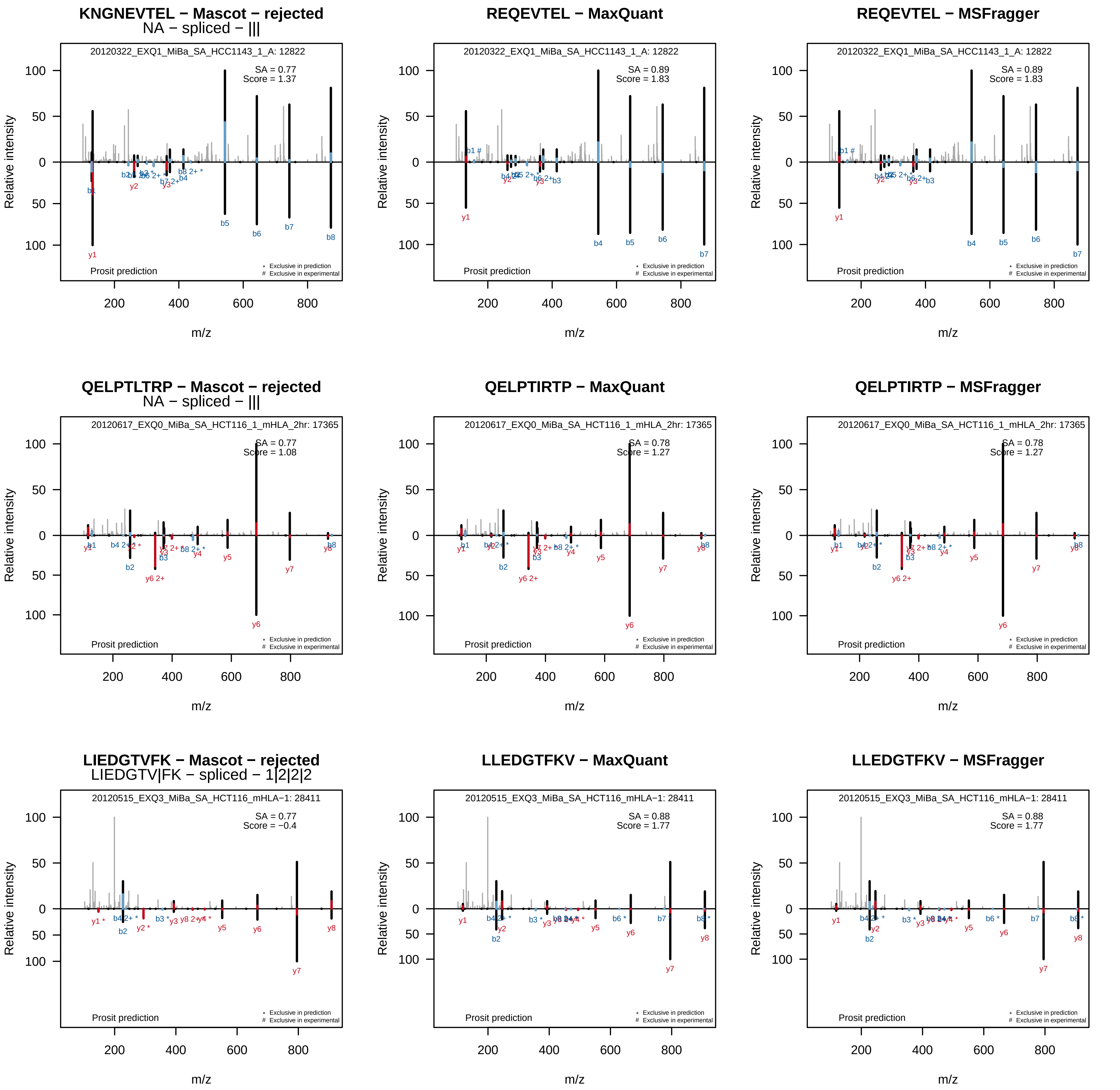

KNGNEVTEL − Mascot − rejected
NA − spliced − |||
20120322_EXQ1_MiBa_SA_HCC1143_1_A: 12822
SA = 0.77
Score = 1.37
REQEVTEL − MaxQuant
20120322_EXQ1_MiBa_SA_HCC1143_1_A: 12822
SA = 0.89
Score = 1.83
REQEVTEL − MSFragger
20120322_EXQ1_MiBa_SA_HCC1143_1_A: 12822
SA = 0.89
Score = 1.83
QELPTLTRP − Mascot − rejected
NA − spliced − |||
20120617_EXQ0_MiBa_SA_HCT116_1_mHLA_2hr: 17365
SA = 0.77
Score = 1.08
QELPTIRTP − MaxQuant
20120617_EXQ0_MiBa_SA_HCT116_1_mHLA_2hr: 17365
SA = 0.78
Score = 1.27
QELPTIRTP − MSFragger
20120617_EXQ0_MiBa_SA_HCT116_1_mHLA_2hr: 17365
SA = 0.78
Score = 1.27
LIEDGTVFK − Mascot − rejected
LIEDGTV|FK − spliced − 1|2|2|2
20120515_EXQ3_MiBa_SA_HCT116_mHLA−1: 28411
SA = 0.77
Score = −0.4
LLEDGTFKV − MaxQuant
20120515_EXQ3_MiBa_SA_HCT116_mHLA−1: 28411
SA = 0.88
Score = 1.77
LLEDGTFKV − MSFragger
20120515_EXQ3_MiBa_SA_HCT116_mHLA−1: 28411
SA = 0.88
Score = 1.77
Relative intensity
m/z
Prosit prediction
* Exclusive in prediction
# Exclusive in experimental

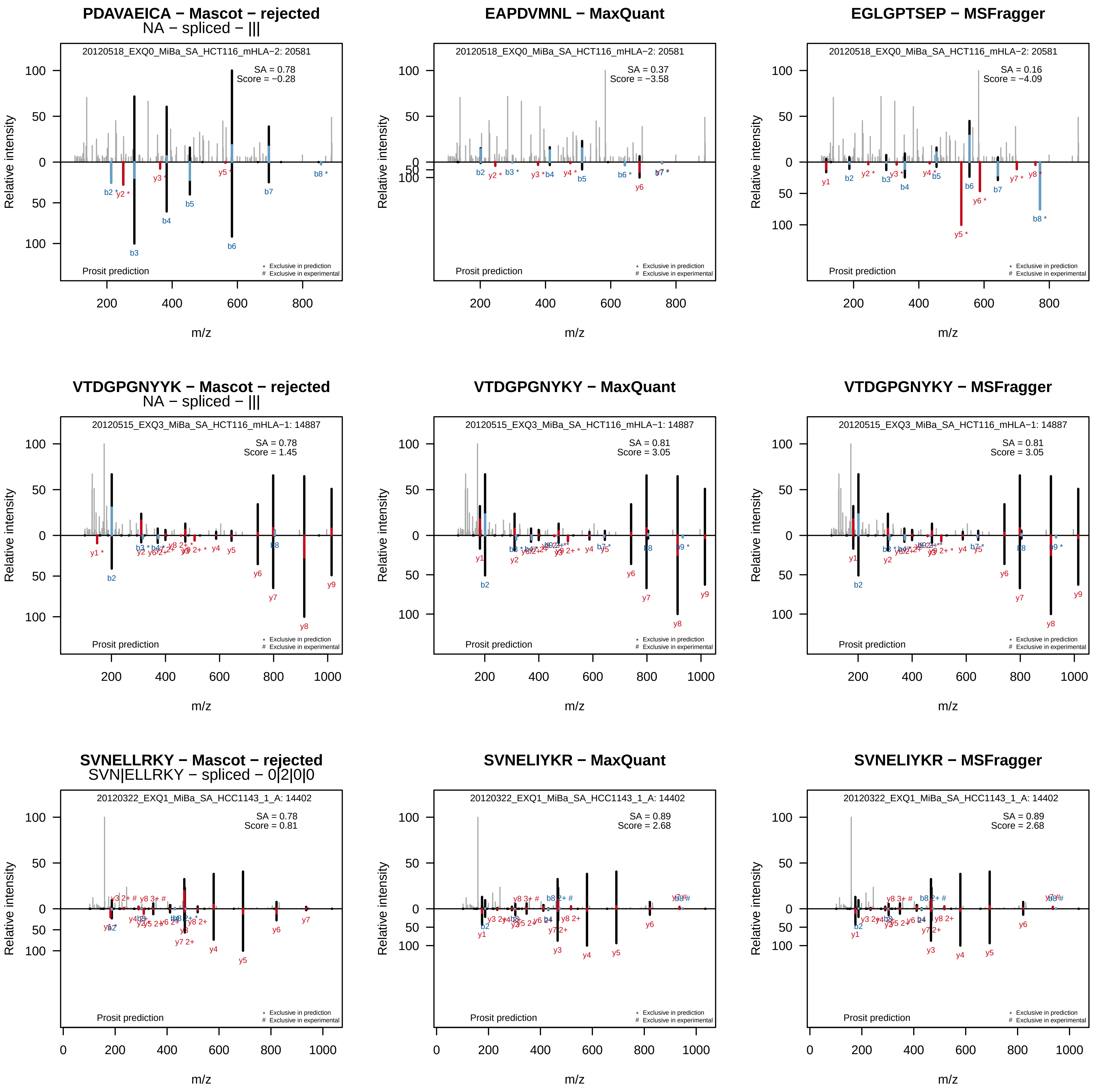

PDAVAEICA − Mascot − rejected
NA − spliced − |||
20120518_EXQ0_MiBa_SA_HCT116_mHLA−2: 20581
SA = 0.78
Score = −0.28
EAPDVMNL − MaxQuant
20120518_EXQ0_MiBa_SA_HCT116_mHLA−2: 20581
SA = 0.37
Score = −3.58
EGLGPTSEP − MSFragger
20120518_EXQ0_MiBa_SA_HCT116_mHLA−2: 20581
SA = 0.16
Score = −4.09
VTDGPGNYYK − Mascot − rejected
NA − spliced − |||
20120515_EXQ3_MiBa_SA_HCT116_mHLA−1: 14887
SA = 0.78
Score = 1.45
VTDGPGNYKY − MaxQuant
20120515_EXQ3_MiBa_SA_HCT116_mHLA−1: 14887
SA = 0.81
Score = 3.05
VTDGPGNYKY − MSFragger
20120515_EXQ3_MiBa_SA_HCT116_mHLA−1: 14887
SA = 0.81
Score = 3.05
SVNELLRKY − Mascot − rejected
SVN|ELLRKY − spliced − 0|2|0|0
20120322_EXQ1_MiBa_SA_HCC1143_1_A: 14402
SA = 0.78
Score = 0.81
SVNELIYKR − MaxQuant
20120322_EXQ1_MiBa_SA_HCC1143_1_A: 14402
SA = 0.89
Score = 2.68
SVNELIYKR − MSFragger
20120322_EXQ1_MiBa_SA_HCC1143_1_A: 14402
SA = 0.89
Score = 2.68
Relative intensity
m/z
Prosit prediction
* Exclusive in prediction
# Exclusive in experimental

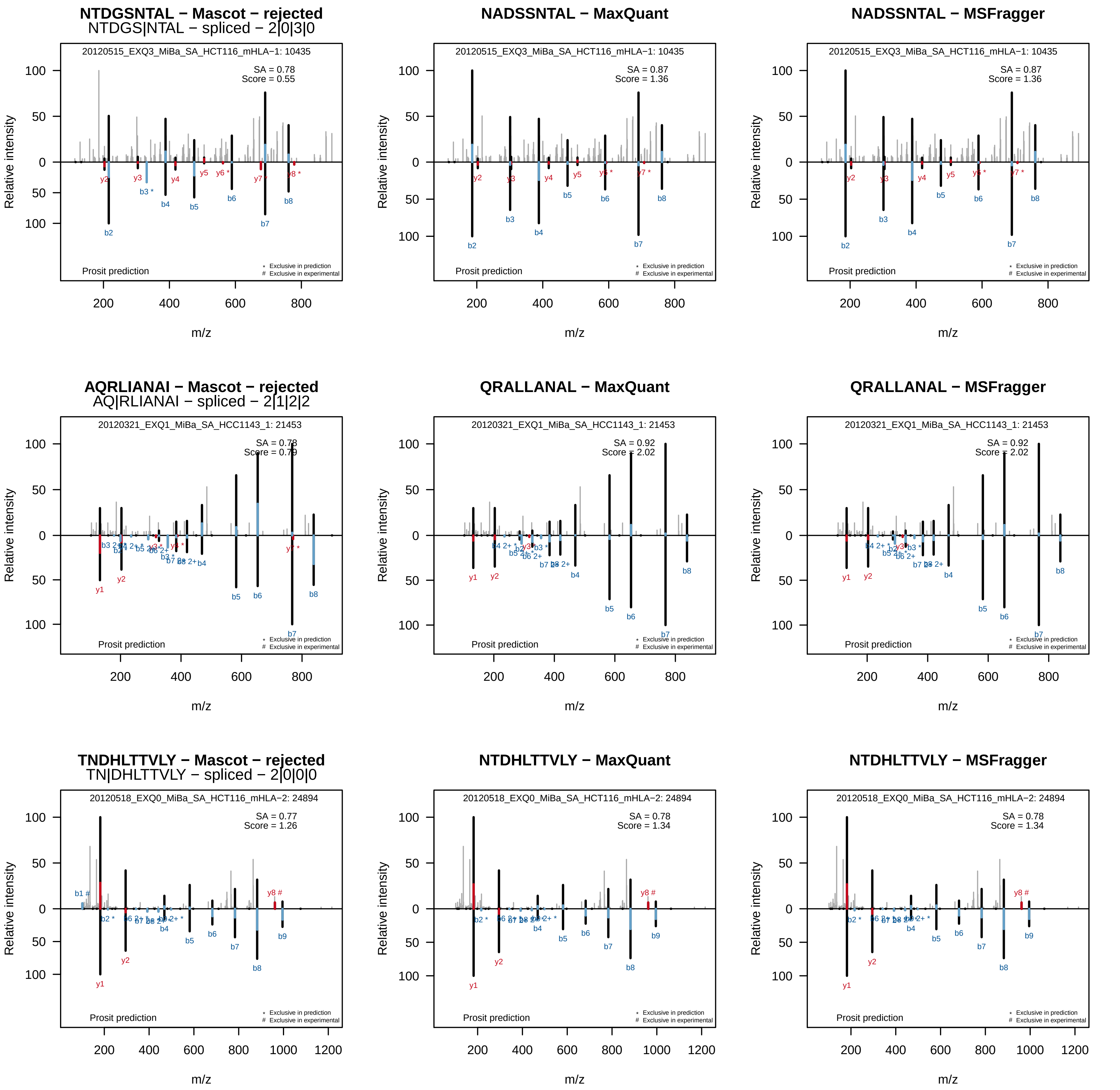

NTDGSNTAL − Mascot − rejected
NTDGS|NTAL − spliced − 2|0|3|0
20120515_EXQ3_MiBa_SA_HCT116_mHLA−1: 10435
SA = 0.78
Score = 0.55
NADSSNTAL − MaxQuant
20120515_EXQ3_MiBa_SA_HCT116_mHLA−1: 10435
SA = 0.87
Score = 1.36
NADSSNTAL − MSFragger
20120515_EXQ3_MiBa_SA_HCT116_mHLA−1: 10435
SA = 0.87
Score = 1.36
AQRLIANAI − Mascot − rejected
AQ|RLIANAI − spliced − 2|1|2|2
20120321_EXQ1_MiBa_SA_HCC1143_1: 21453
SA = 0.78
Score = 0.79
QRALLANAL − MaxQuant
20120321_EXQ1_MiBa_SA_HCC1143_1: 21453
SA = 0.92
Score = 2.02
QRALLANAL − MSFragger
20120321_EXQ1_MiBa_SA_HCC1143_1: 21453
SA = 0.92
Score = 2.02
TNDHLTTVLY − Mascot − rejected
TN|DHLTTVLY − spliced − 2|0|0|0
20120518_EXQ0_MiBa_SA_HCT116_mHLA−2: 24894
SA = 0.77
Score = 1.26
NTDHLTTVLY − MaxQuant
20120518_EXQ0_MiBa_SA_HCT116_mHLA−2: 24894
SA = 0.78
Score = 1.34
NTDHLTTVLY − MSFragger
20120518_EXQ0_MiBa_SA_HCT116_mHLA−2: 24894
SA = 0.78
Score = 1.34
Relative intensity
m/z
Prosit prediction
* Exclusive in prediction
# Exclusive in experimental

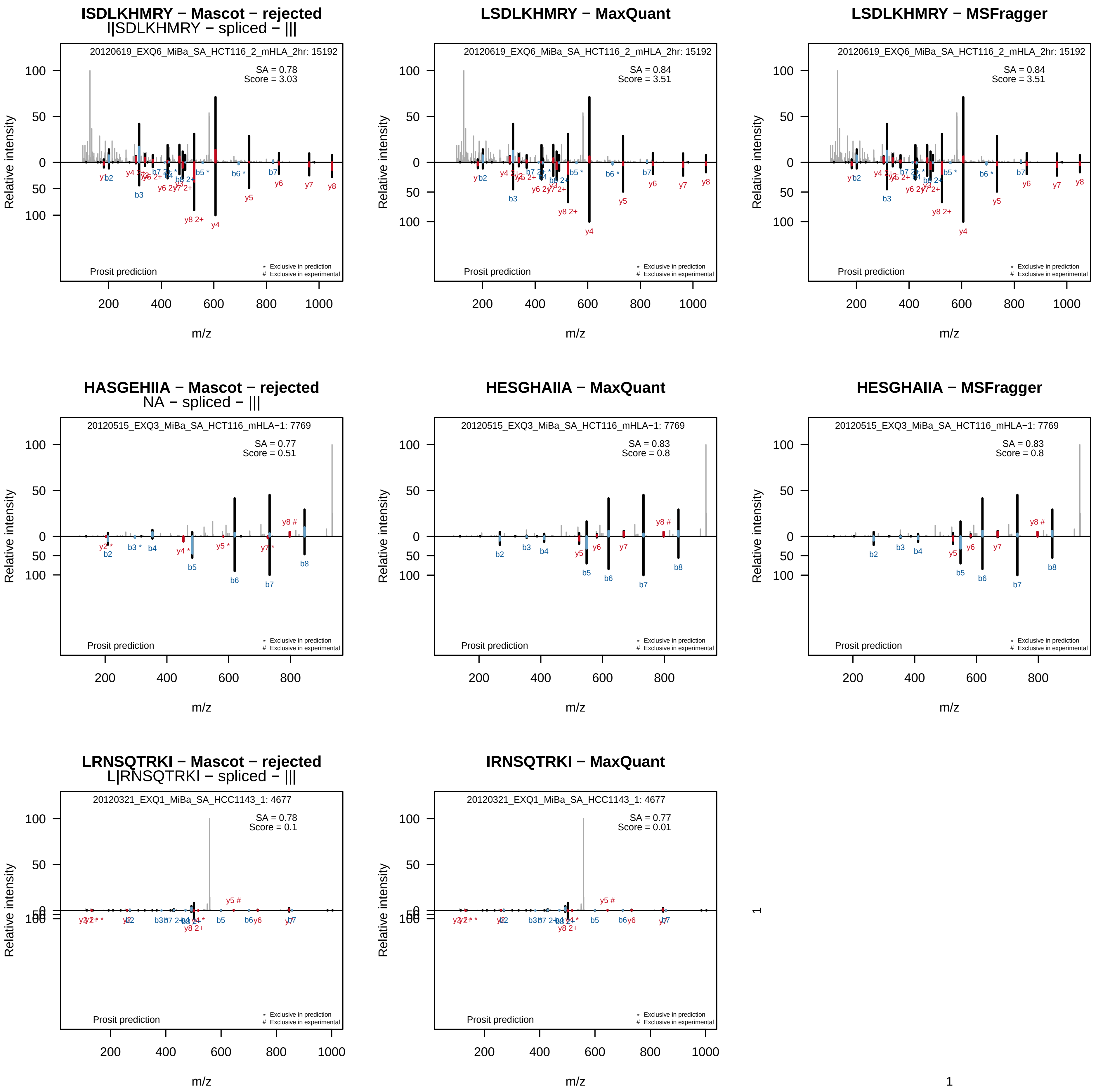
ISDLKHMRY − Mascot − rejected
I|SDLKHMRY − spliced − |||
20120619_EXQ6_MiBa_SA_HCT116_2_mHLA_2hr: 15192
SA = 0.78
Score = 3.03
Relative intensity
m/z
Prosit prediction
⋆ Exclusive in prediction
Exclusive in experimental
LSDLKHMRY − MaxQuant
20120619_EXQ6_MiBa_SA_HCT116_2_mHLA_2hr: 15192
SA = 0.84
Score = 3.51
LSDLKHMRY − MSFragger
20120619_EXQ6_MiBa_SA_HCT116_2_mHLA_2hr: 15192
SA = 0.84
Score = 3.51
HASGEHIIA − Mascot − rejected
NA − spliced − |||
20120515_EXQ3_MiBa_SA_HCT116_mHLA−1: 7769
SA = 0.77
Score = 0.51
HESGHAIIA − MaxQuant
20120515_EXQ3_MiBa_SA_HCT116_mHLA−1: 7769
SA = 0.83
Score = 0.8
HESGHAIIA − MSFragger
20120515_EXQ3_MiBa_SA_HCT116_mHLA−1: 7769
SA = 0.83
Score = 0.8
LRNSQTRKI − Mascot − rejected
L|RNSQTRKI − spliced − |||
20120321_EXQ1_MiBa_SA_HCC1143_1: 4677
SA = 0.78
Score = 0.1
IRNSQTRKI − MaxQuant
20120321_EXQ1_MiBa_SA_HCC1143_1: 4677
SA = 0.77
Score = 0.01
1
1

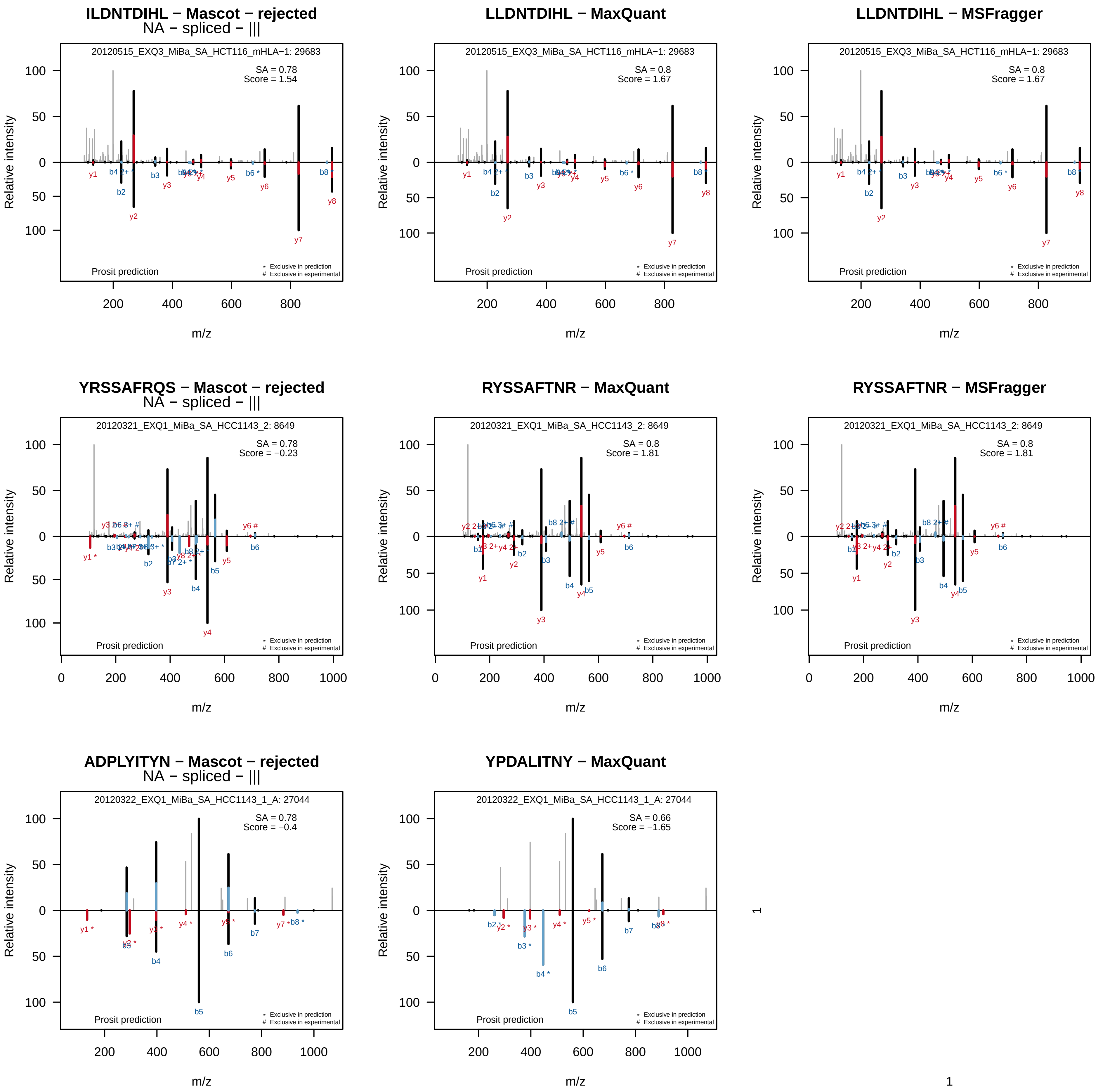
ILDNTDIHL − Mascot − rejected
NA − spliced − |||
20120515_EXQ3_MiBa_SA_HCT116_mHLA−1: 29683
SA = 0.78
Score = 1.54
LLDNTDIHL − MaxQuant
20120515_EXQ3_MiBa_SA_HCT116_mHLA−1: 29683
SA = 0.8
Score = 1.67
LLDNTDIHL − MSFragger
20120515_EXQ3_MiBa_SA_HCT116_mHLA−1: 29683
SA = 0.8
Score = 1.67
YRSSAFRQS − Mascot − rejected
NA − spliced − |||
20120321_EXQ1_MiBa_SA_HCC1143_2: 8649
SA = 0.78
Score = −0.23
RYSSAFTNR − MaxQuant
20120321_EXQ1_MiBa_SA_HCC1143_2: 8649
SA = 0.8
Score = 1.81
RYSSAFTNR − MSFragger
20120321_EXQ1_MiBa_SA_HCC1143_2: 8649
SA = 0.8
Score = 1.81
ADPLYITYN − Mascot − rejected
NA − spliced − |||
20120322_EXQ1_MiBa_SA_HCC1143_1_A: 27044
SA = 0.78
Score = −0.4
YPDALITNY − MaxQuant
20120322_EXQ1_MiBa_SA_HCC1143_1_A: 27044
SA = 0.66
Score = −1.65
Relative intensity
m/z
Prosit prediction
* Exclusive in prediction
# Exclusive in experimental
1
1

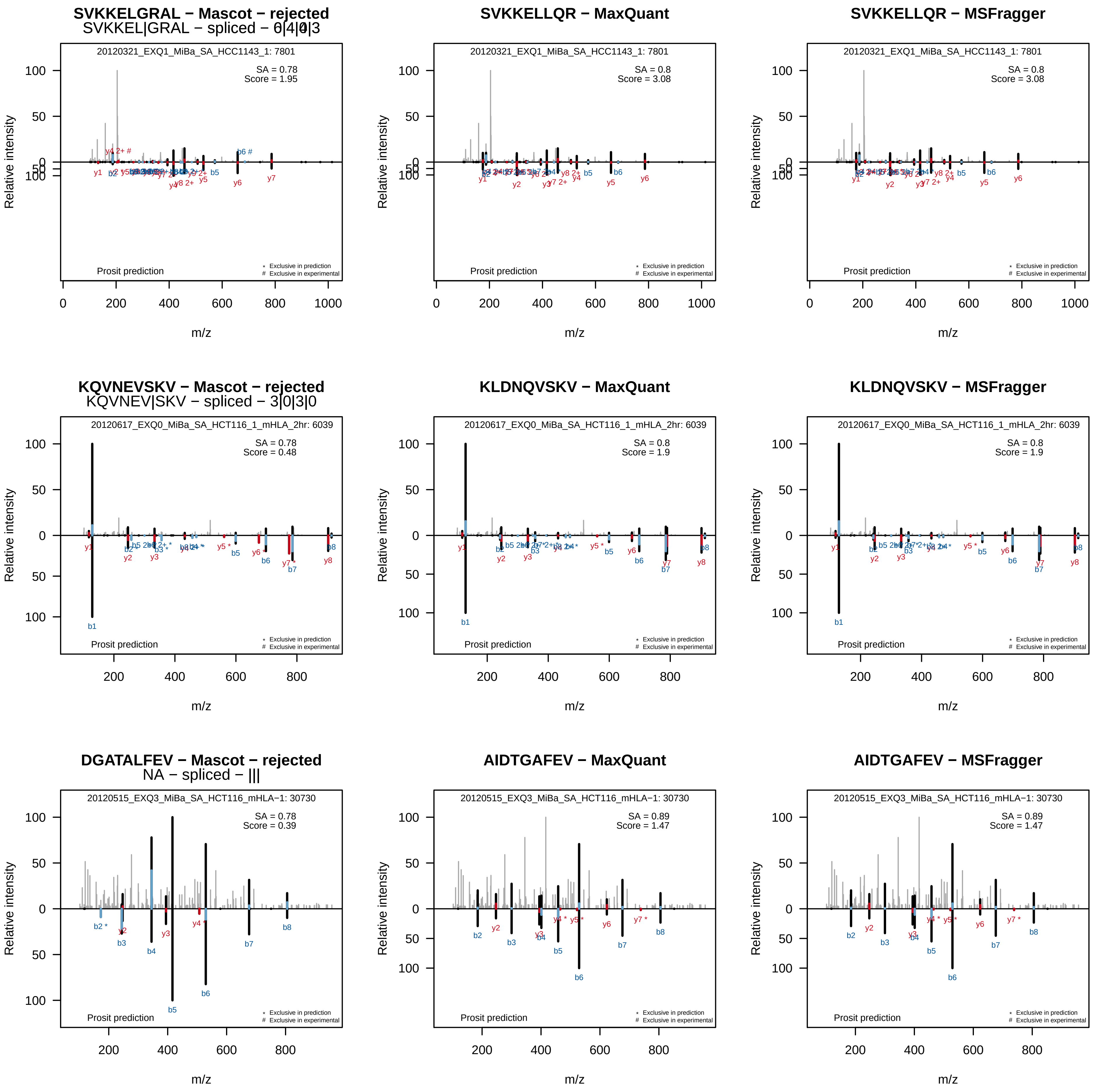
SVKKELGRAL − Mascot − rejected
SVKKEL|GRAL − spliced − 0|4|4|3
20120321_EXQ1_MiBa_SA_HCC1143_1: 7801
SA = 0.78
Score = 1.95
SVKKELLQR − MaxQuant
20120321_EXQ1_MiBa_SA_HCC1143_1: 7801
SA = 0.8
Score = 3.08
SVKKELLQR − MSFragger
20120321_EXQ1_MiBa_SA_HCC1143_1: 7801
SA = 0.8
Score = 3.08
KQVNEVSKV − Mascot − rejected
KQVNEV|SKV − spliced − 3|0|3|0
20120617_EXQ0_MiBa_SA_HCT116_1_mHLA_2hr: 6039
SA = 0.78
Score = 0.48
KLDNQVSKV − MaxQuant
20120617_EXQ0_MiBa_SA_HCT116_1_mHLA_2hr: 6039
SA = 0.8
Score = 1.9
KLDNQVSKV − MSFragger
20120617_EXQ0_MiBa_SA_HCT116_1_mHLA_2hr: 6039
SA = 0.8
Score = 1.9
DGATALFEV − Mascot − rejected
NA − spliced − |||
20120515_EXQ3_MiBa_SA_HCT116_mHLA−1: 30730
SA = 0.78
Score = 0.39
AIDTGAFEV − MaxQuant
20120515_EXQ3_MiBa_SA_HCT116_mHLA−1: 30730
SA = 0.89
Score = 1.47
AIDTGAFEV − MSFragger
20120515_EXQ3_MiBa_SA_HCT116_mHLA−1: 30730
SA = 0.89
Score = 1.47
Relative intensity
m/z
Prosit prediction
⋆ Exclusive in prediction
Exclusive in experimental

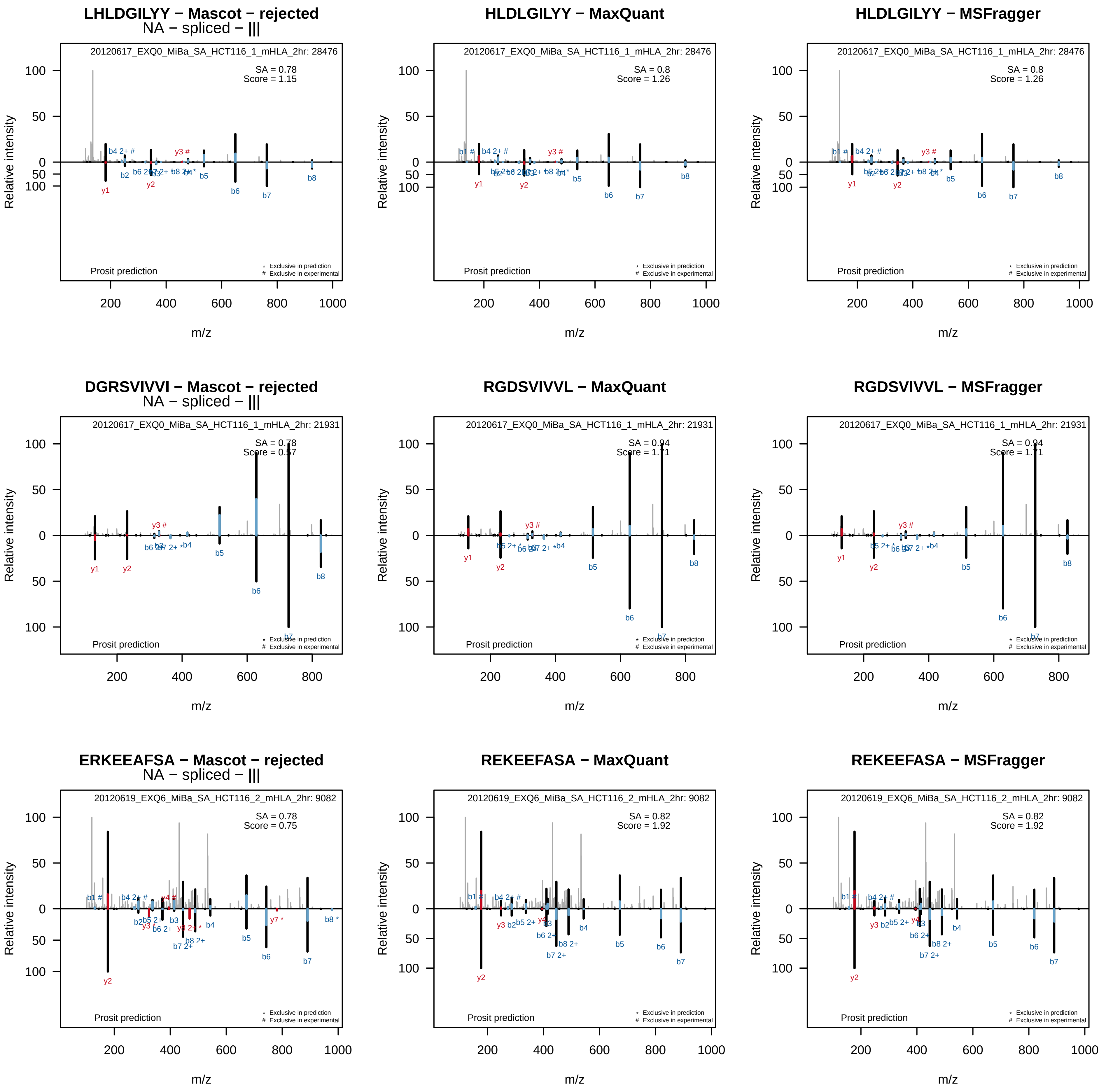

LHLDGILYY − Mascot − rejected
NA − spliced − |||
20120617_EXQ0_MiBa_SA_HCT116_1_mHLA_2hr: 28476
SA = 0.78
Score = 1.15
HLDLGILYY − MaxQuant
20120617_EXQ0_MiBa_SA_HCT116_1_mHLA_2hr: 28476
SA = 0.8
Score = 1.26
HLDLGILYY − MSFragger
20120617_EXQ0_MiBa_SA_HCT116_1_mHLA_2hr: 28476
SA = 0.8
Score = 1.26
DGRSVIVVI − Mascot − rejected
NA − spliced − |||
20120617_EXQ0_MiBa_SA_HCT116_1_mHLA_2hr: 21931
SA = 0.78
Score = 0.57
RGDSVIVVL − MaxQuant
20120617_EXQ0_MiBa_SA_HCT116_1_mHLA_2hr: 21931
SA = 0.94
Score = 1.71
RGDSVIVVL − MSFragger
20120617_EXQ0_MiBa_SA_HCT116_1_mHLA_2hr: 21931
SA = 0.94
Score = 1.71
ERKEEAFSA − Mascot − rejected
NA − spliced − |||
20120619_EXQ6_MiBa_SA_HCT116_2_mHLA_2hr: 9082
SA = 0.78
Score = 0.75
REKEEFASA − MaxQuant
20120619_EXQ6_MiBa_SA_HCT116_2_mHLA_2hr: 9082
SA = 0.82
Score = 1.92
REKEEFASA − MSFragger
20120619_EXQ6_MiBa_SA_HCT116_2_mHLA_2hr: 9082
SA = 0.82
Score = 1.92
Relative intensity
m/z
Prosit prediction
* Exclusive in prediction
# Exclusive in experimental

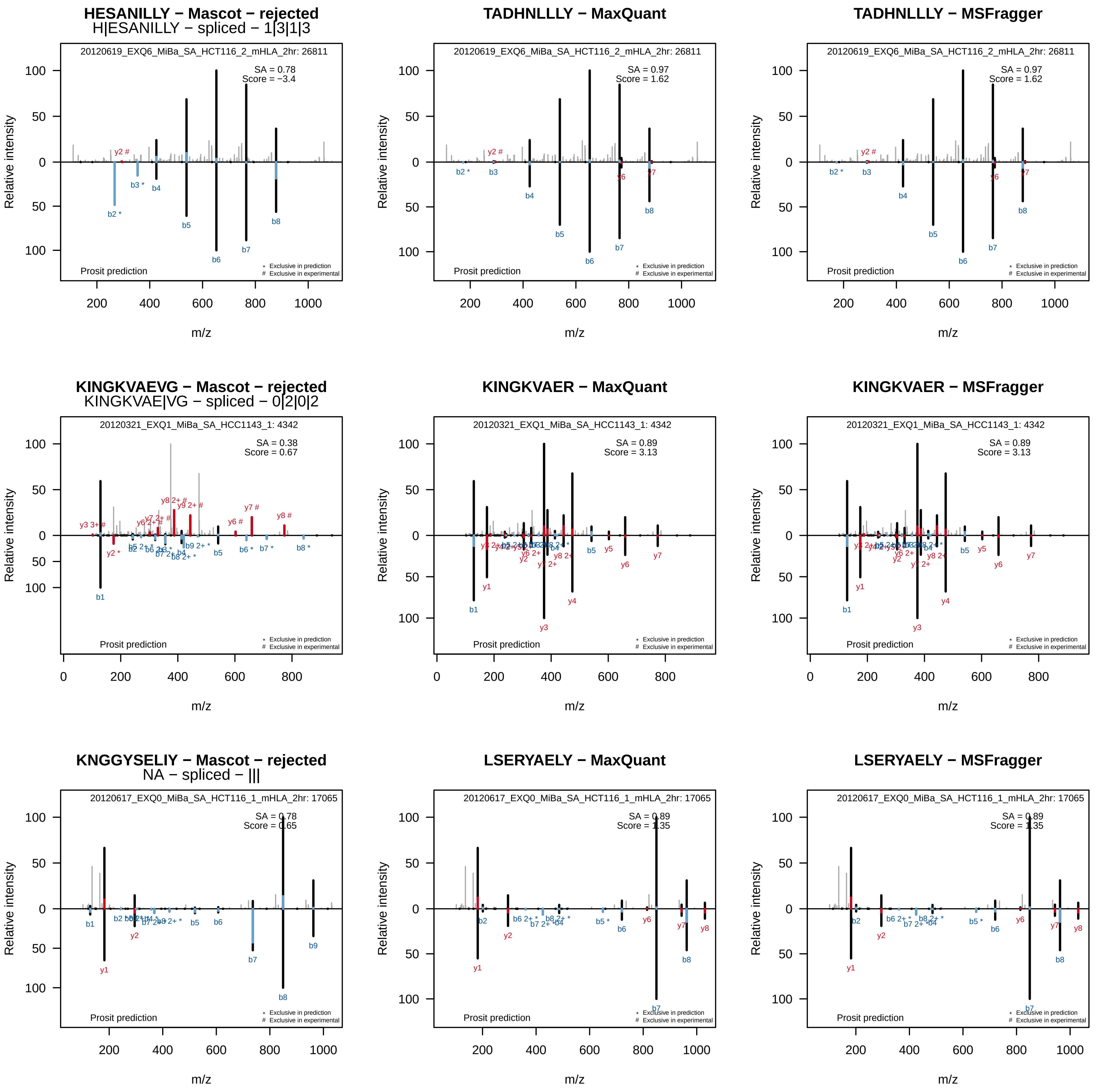

HESANILLY − Mascot − rejected
H|ESANILLY − spliced − 1|3|1|3
20120619_EXQ6_MiBa_SA_HCT116_2_mHLA_2hr: 26811
SA = 0.78
Score = −3.4
TADHNLLLY − MaxQuant
20120619_EXQ6_MiBa_SA_HCT116_2_mHLA_2hr: 26811
SA = 0.97
Score = 1.62
TADHNLLLY − MSFragger
20120619_EXQ6_MiBa_SA_HCT116_2_mHLA_2hr: 26811
SA = 0.97
Score = 1.62
KINGKVAEVG − Mascot − rejected
KINGKVAE|VG − spliced − 0|2|0|2
20120321_EXQ1_MiBa_SA_HCC1143_1: 4342
SA = 0.38
Score = 0.67
KINGKVAER − MaxQuant
20120321_EXQ1_MiBa_SA_HCC1143_1: 4342
SA = 0.89
Score = 3.13
KINGKVAER − MSFragger
20120321_EXQ1_MiBa_SA_HCC1143_1: 4342
SA = 0.89
Score = 3.13
KNGGYSELIY − Mascot − rejected
NA − spliced − |||
20120617_EXQ0_MiBa_SA_HCT116_1_mHLA_2hr: 17065
SA = 0.78
Score = 0.65
LSERYAELY − MaxQuant
20120617_EXQ0_MiBa_SA_HCT116_1_mHLA_2hr: 17065
SA = 0.89
Score = 1.35
LSERYAELY − MSFragger
20120617_EXQ0_MiBa_SA_HCT116_1_mHLA_2hr: 17065
SA = 0.89
Score = 1.35
Relative intensity
m/z
Prosit prediction
* Exclusive in prediction
# Exclusive in experimental

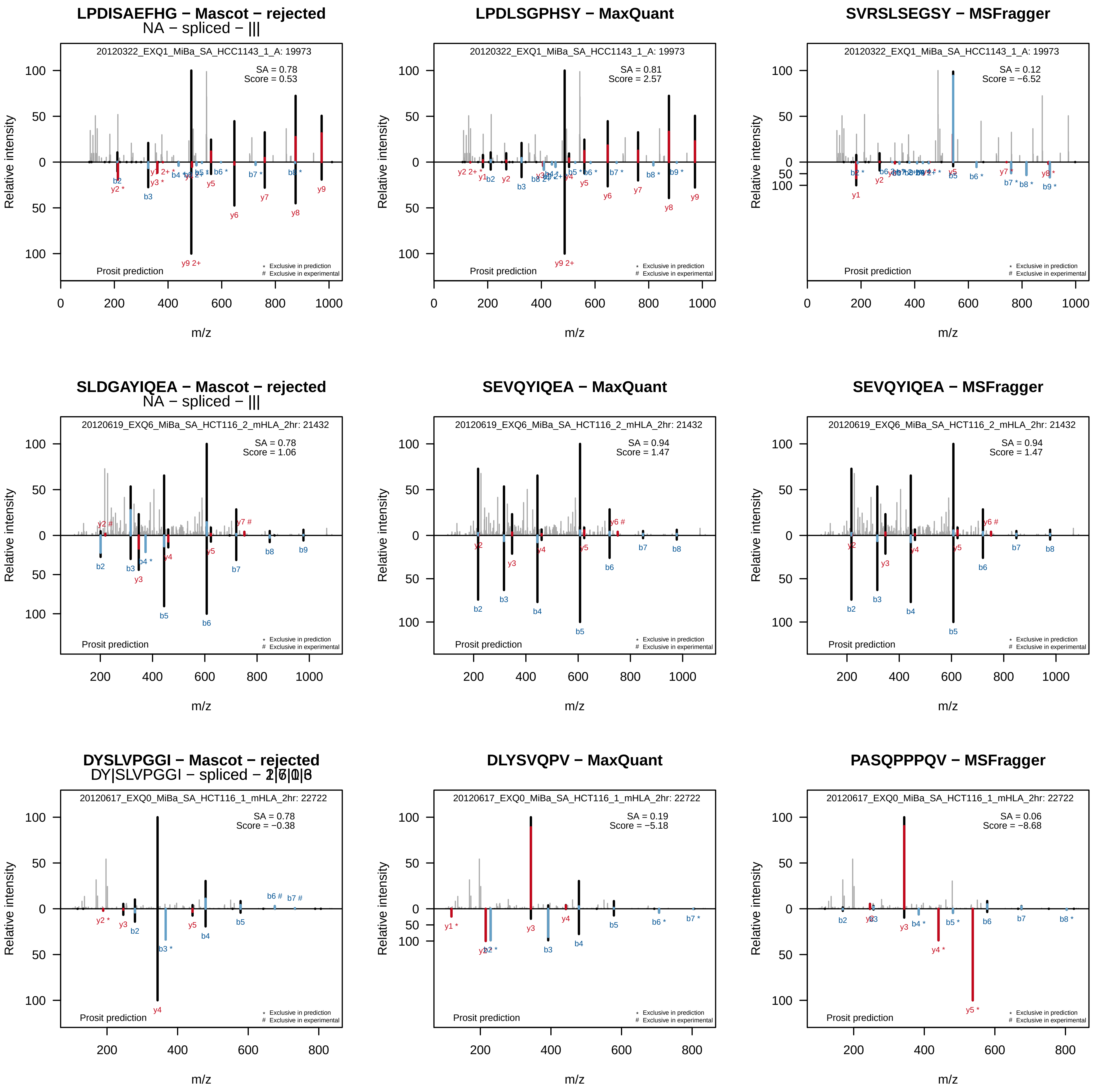

LPDISAEFHG − Mascot − rejected
NA − spliced − |||
20120322_EXQ1_MiBa_SA_HCC1143_1_A: 19973
SA = 0.78
Score = 0.53
LPDLSGPHSY − MaxQuant
20120322_EXQ1_MiBa_SA_HCC1143_1_A: 19973
SA = 0.81
Score = 2.57
SVRSLSEGSY − MSFragger
20120322_EXQ1_MiBa_SA_HCC1143_1_A: 19973
SA = 0.12
Score = −6.52
SLDGAYIQEA − Mascot − rejected
NA − spliced − |||
20120619_EXQ6_MiBa_SA_HCT116_2_mHLA_2hr: 21432
SA = 0.78
Score = 1.06
SEVQYIQEA − MaxQuant
20120619_EXQ6_MiBa_SA_HCT116_2_mHLA_2hr: 21432
SA = 0.94
Score = 1.47
SEVQYIQEA − MSFragger
20120619_EXQ6_MiBa_SA_HCT116_2_mHLA_2hr: 21432
SA = 0.94
Score = 1.47
DYSLVPGGI − Mascot − rejected
20120617_EXQ0_MiBa_SA_HCT116_1_mHLA_2hr: 22722
SA = 0.78
Score = −0.38
DLYSVQPV − MaxQuant
20120617_EXQ0_MiBa_SA_HCT116_1_mHLA_2hr: 22722
SA = 0.19
Score = −5.18
PASQPPPQV − MSFragger
20120617_EXQ0_MiBa_SA_HCT116_1_mHLA_2hr: 22722
SA = 0.06
Score = −8.68
Relative intensity
m/z
Prosit prediction
Exclusive in prediction
Exclusive in experimental

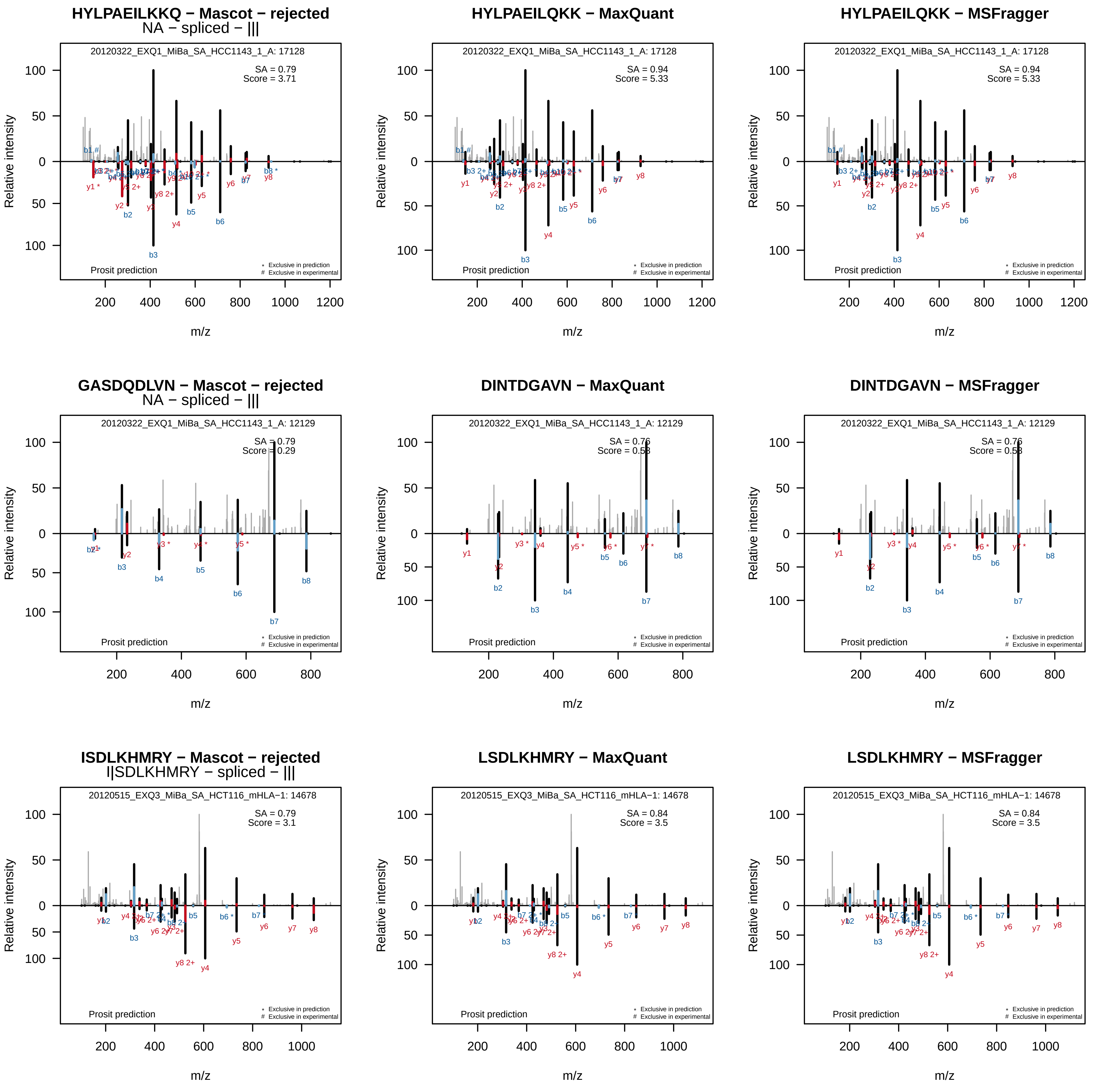
HYLPAEILKKQ − Mascot − rejected
NA − spliced − |||
20120322_EXQ1_MiBa_SA_HCC1143_1_A: 17128
SA = 0.79
Score = 3.71
HYLPAEILQKK − MaxQuant
20120322_EXQ1_MiBa_SA_HCC1143_1_A: 17128
SA = 0.94
Score = 5.33
HYLPAEILQKK − MSFragger
20120322_EXQ1_MiBa_SA_HCC1143_1_A: 17128
SA = 0.94
Score = 5.33
GASDQDLVN − Mascot − rejected
NA − spliced − |||
20120322_EXQ1_MiBa_SA_HCC1143_1_A: 12129
SA = 0.79
Score = 0.29
DINTDGAVN − MaxQuant
20120322_EXQ1_MiBa_SA_HCC1143_1_A: 12129
SA = 0.76
Score = 0.53
DINTDGAVN − MSFragger
20120322_EXQ1_MiBa_SA_HCC1143_1_A: 12129
SA = 0.76
Score = 0.53
ISDLKHMRY − Mascot − rejected
I|SDLKHMRY − spliced − |||
20120515_EXQ3_MiBa_SA_HCT116_mHLA−1: 14678
SA = 0.79
Score = 3.1
LSDLKHMRY − MaxQuant
20120515_EXQ3_MiBa_SA_HCT116_mHLA−1: 14678
SA = 0.84
Score = 3.5
LSDLKHMRY − MSFragger
20120515_EXQ3_MiBa_SA_HCT116_mHLA−1: 14678
SA = 0.84
Score = 3.5
Relative intensity
m/z
Prosit prediction
* Exclusive in prediction
# Exclusive in experimental

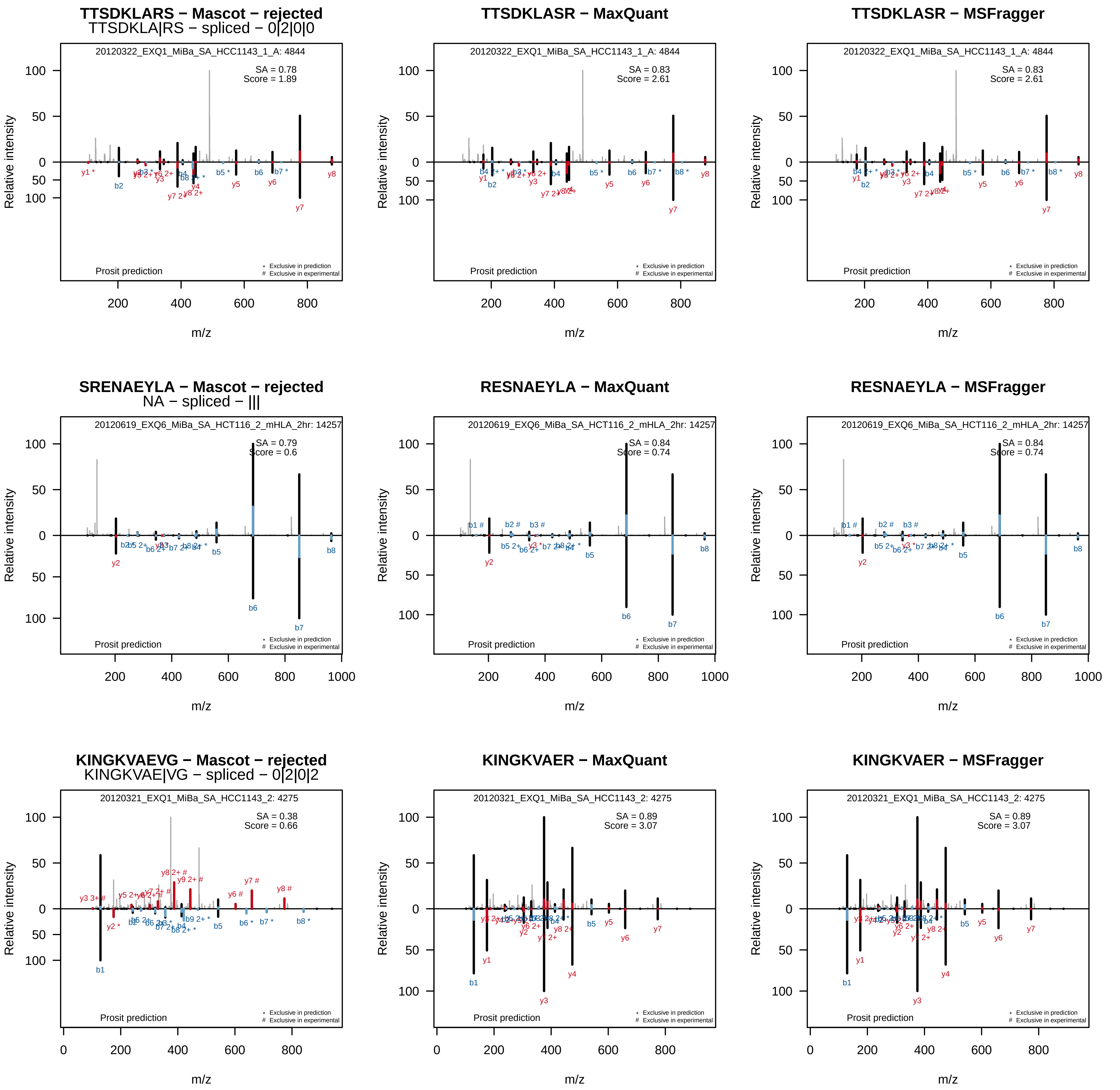

TTSDKLARS − Mascot − rejected
TTSDKLA|RS − spliced − 0|2|0|0
20120322_EXQ1_MiBa_SA_HCC1143_1_A: 4844
SA = 0.78
Score = 1.89
TTSDKLASR − MaxQuant
20120322_EXQ1_MiBa_SA_HCC1143_1_A: 4844
SA = 0.83
Score = 2.61
TTSDKLASR − MSFragger
20120322_EXQ1_MiBa_SA_HCC1143_1_A: 4844
SA = 0.83
Score = 2.61
SRENAEYLA − Mascot − rejected
NA − spliced − |||
20120619_EXQ6_MiBa_SA_HCT116_2_mHLA_2hr: 14257
SA = 0.79
Score = 0.6
RESNAEYLA − MaxQuant
20120619_EXQ6_MiBa_SA_HCT116_2_mHLA_2hr: 14257
SA = 0.84
Score = 0.74
RESNAEYLA − MSFragger
20120619_EXQ6_MiBa_SA_HCT116_2_mHLA_2hr: 14257
SA = 0.84
Score = 0.74
KINGKVAEVG − Mascot − rejected
KINGKVAE|VG − spliced − 0|2|0|2
20120321_EXQ1_MiBa_SA_HCC1143_2: 4275
SA = 0.38
Score = 0.66
KINGKVAER − MaxQuant
20120321_EXQ1_MiBa_SA_HCC1143_2: 4275
SA = 0.89
Score = 3.07
KINGKVAER − MSFragger
20120321_EXQ1_MiBa_SA_HCC1143_2: 4275
SA = 0.89
Score = 3.07
Relative intensity
m/z
Prosit prediction
* Exclusive in prediction
# Exclusive in experimental

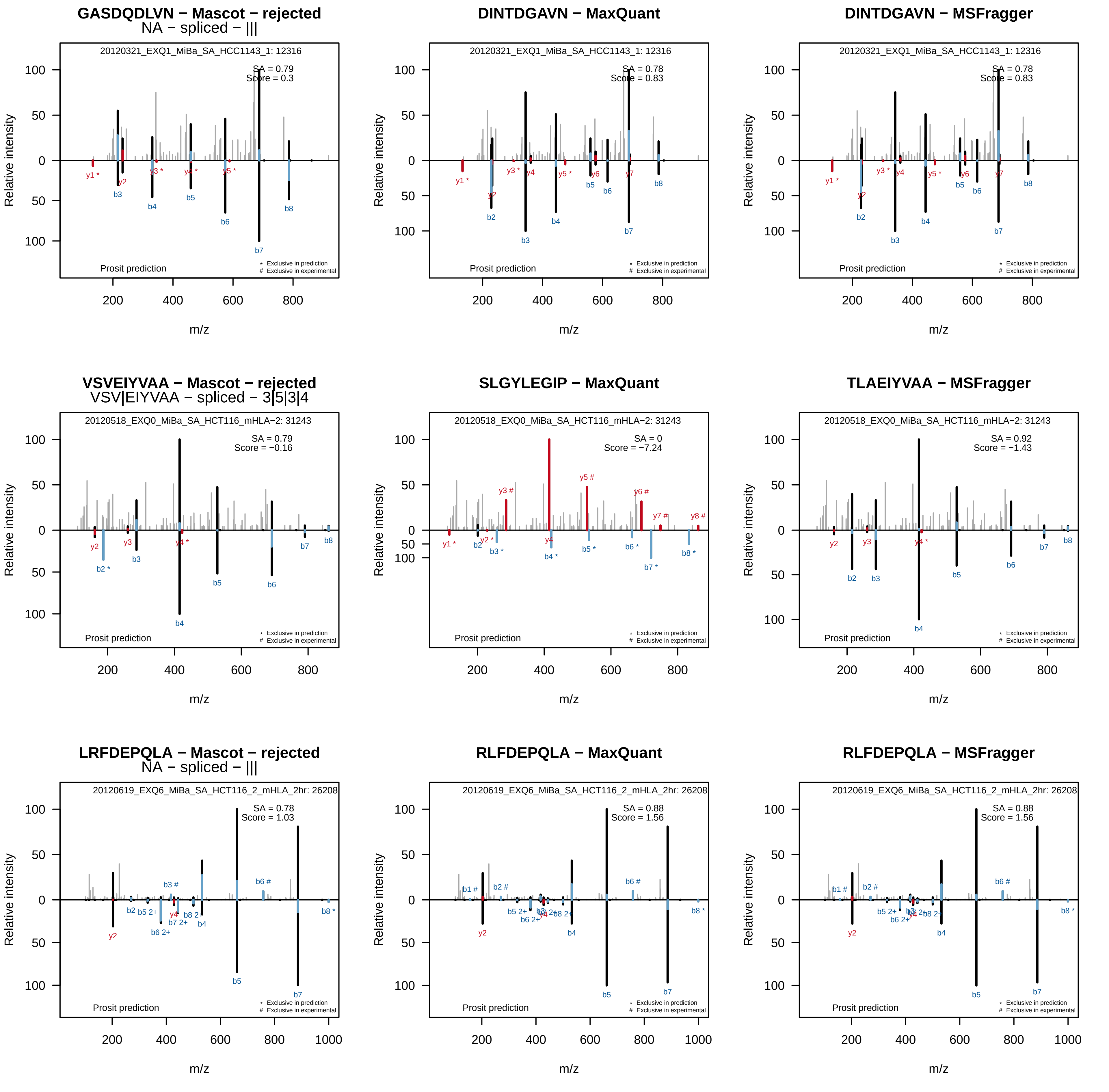
GASDQDLVN − Mascot − rejected
NA − spliced − |||
20120321_EXQ1_MiBa_SA_HCC1143_1: 12316
SA = 0.79
Score = 0.3
DINTDGAVN − MaxQuant
20120321_EXQ1_MiBa_SA_HCC1143_1: 12316
SA = 0.78
Score = 0.83
DINTDGAVN − MSFragger
20120321_EXQ1_MiBa_SA_HCC1143_1: 12316
SA = 0.78
Score = 0.83
VSVEIYVAA − Mascot − rejected
VSV|EIYVAA − spliced − 3|5|3|4
20120518_EXQ0_MiBa_SA_HCT116_mHLA−2: 31243
SA = 0.79
Score = −0.16
SLGYLEGIP − MaxQuant
20120518_EXQ0_MiBa_SA_HCT116_mHLA−2: 31243
SA = 0
Score = −7.24
TLAEIYVAA − MSFragger
20120518_EXQ0_MiBa_SA_HCT116_mHLA−2: 31243
SA = 0.92
Score = −1.43
LRFDEPQLA − Mascot − rejected
NA − spliced − |||
20120619_EXQ6_MiBa_SA_HCT116_2_mHLA_2hr: 26208
SA = 0.78
Score = 1.03
RLFDEPQLA − MaxQuant
20120619_EXQ6_MiBa_SA_HCT116_2_mHLA_2hr: 26208
SA = 0.88
Score = 1.56
RLFDEPQLA − MSFragger
20120619_EXQ6_MiBa_SA_HCT116_2_mHLA_2hr: 26208
SA = 0.88
Score = 1.56
Relative intensity
m/z
Prosit prediction
* Exclusive in prediction
# Exclusive in experimental

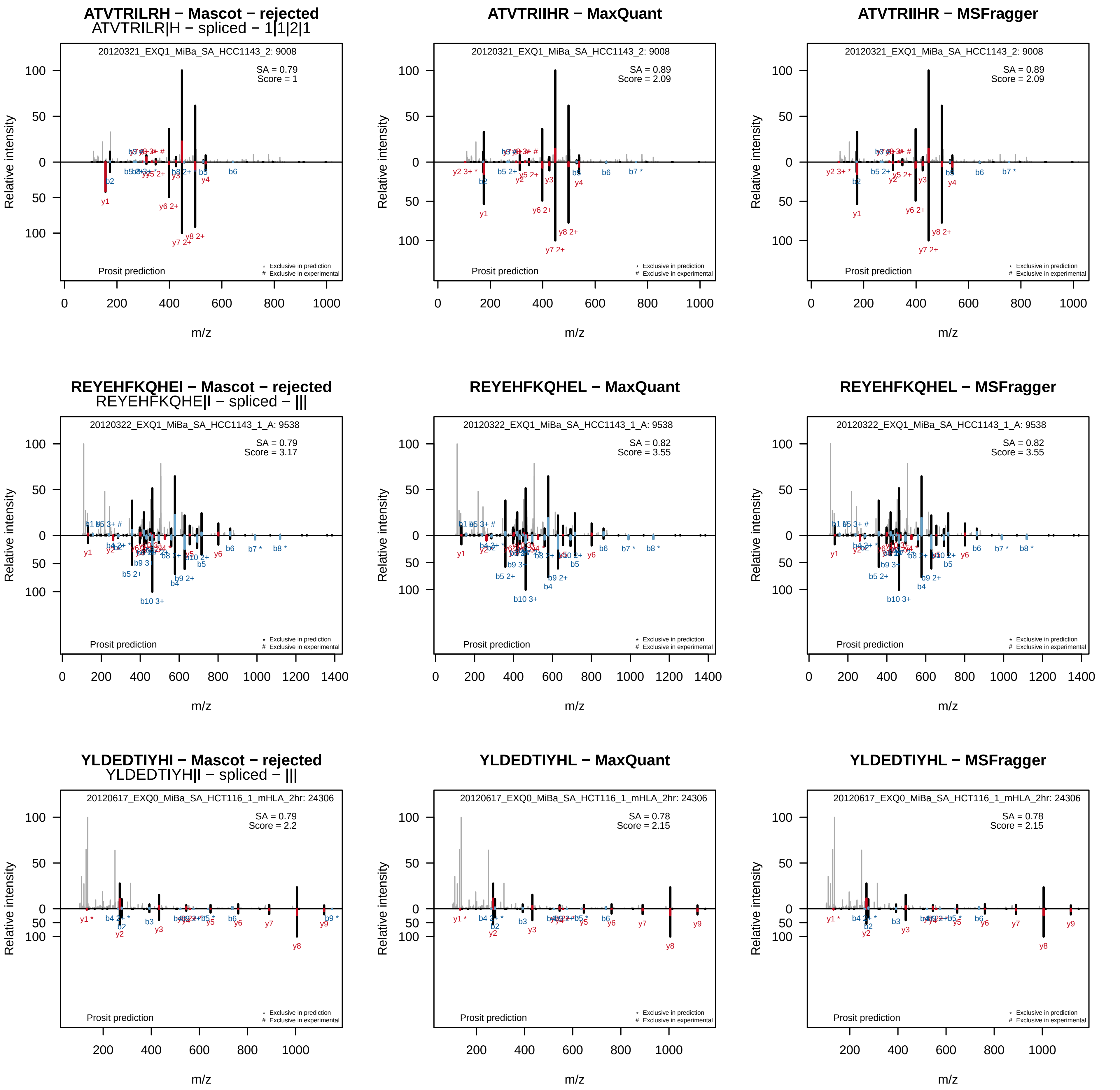

ATVTRILRH − Mascot − rejected
ATVTRILR|H − spliced − 1|1|2|1
20120321_EXQ1_MiBa_SA_HCC1143_2: 9008
SA = 0.79
Score = 1
ATVTRIIHR − MaxQuant
20120321_EXQ1_MiBa_SA_HCC1143_2: 9008
SA = 0.89
Score = 2.09
ATVTRIIHR − MSFragger
20120321_EXQ1_MiBa_SA_HCC1143_2: 9008
SA = 0.89
Score = 2.09
REYEHFKQHEI − Mascot − rejected
REYEHFKQHE|I − spliced − |||
20120322_EXQ1_MiBa_SA_HCC1143_1_A: 9538
SA = 0.79
Score = 3.17
REYEHFKQHEL − MaxQuant
20120322_EXQ1_MiBa_SA_HCC1143_1_A: 9538
SA = 0.82
Score = 3.55
REYEHFKQHEL − MSFragger
20120322_EXQ1_MiBa_SA_HCC1143_1_A: 9538
SA = 0.82
Score = 3.55
YLDEDTIYHI − Mascot − rejected
YLDEDTIYH|I − spliced − |||
20120617_EXQ0_MiBa_SA_HCT116_1_mHLA_2hr: 24306
SA = 0.79
Score = 2.2
YLDEDTIYHL − MaxQuant
20120617_EXQ0_MiBa_SA_HCT116_1_mHLA_2hr: 24306
SA = 0.78
Score = 2.15
YLDEDTIYHL − MSFragger
20120617_EXQ0_MiBa_SA_HCT116_1_mHLA_2hr: 24306
SA = 0.78
Score = 2.15
Relative intensity
m/z
Prosit prediction
* Exclusive in prediction
# Exclusive in experimental

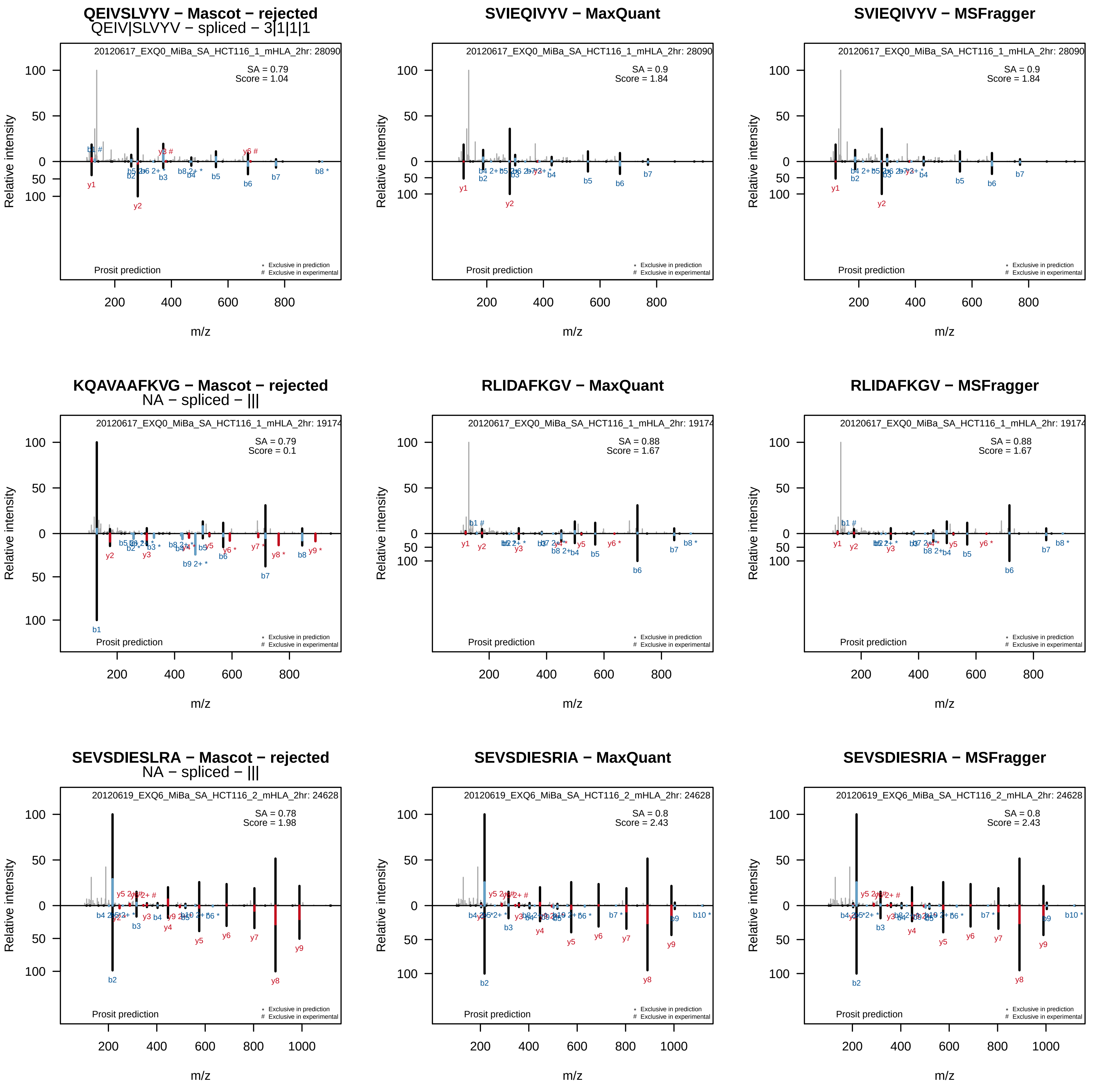

QEIVSLVYV − Mascot − rejected
QEIV|SLVYV − spliced − 3|1|1|1
20120617_EXQ0_MiBa_SA_HCT116_1_mHLA_2hr: 28090
SA = 0.79
Score = 1.04
SVIEQIVYV − MaxQuant
20120617_EXQ0_MiBa_SA_HCT116_1_mHLA_2hr: 28090
SA = 0.9
Score = 1.84
SVIEQIVYV − MSFragger
20120617_EXQ0_MiBa_SA_HCT116_1_mHLA_2hr: 28090
SA = 0.9
Score = 1.84
KQAVAAFKVG − Mascot − rejected
NA − spliced − |||
20120617_EXQ0_MiBa_SA_HCT116_1_mHLA_2hr: 19174
SA = 0.79
Score = 0.1
RLIDAFKGV − MaxQuant
20120617_EXQ0_MiBa_SA_HCT116_1_mHLA_2hr: 19174
SA = 0.88
Score = 1.67
RLIDAFKGV − MSFragger
20120617_EXQ0_MiBa_SA_HCT116_1_mHLA_2hr: 19174
SA = 0.88
Score = 1.67
SEVSDIESLRA − Mascot − rejected
NA − spliced − |||
20120619_EXQ6_MiBa_SA_HCT116_2_mHLA_2hr: 24628
SA = 0.78
Score = 1.98
SEVSDIESRIA − MaxQuant
20120619_EXQ6_MiBa_SA_HCT116_2_mHLA_2hr: 24628
SA = 0.8
Score = 2.43
SEVSDIESRIA − MSFragger
20120619_EXQ6_MiBa_SA_HCT116_2_mHLA_2hr: 24628
SA = 0.8
Score = 2.43
Relative intensity
m/z
Prosit prediction
* Exclusive in prediction
# Exclusive in experimental

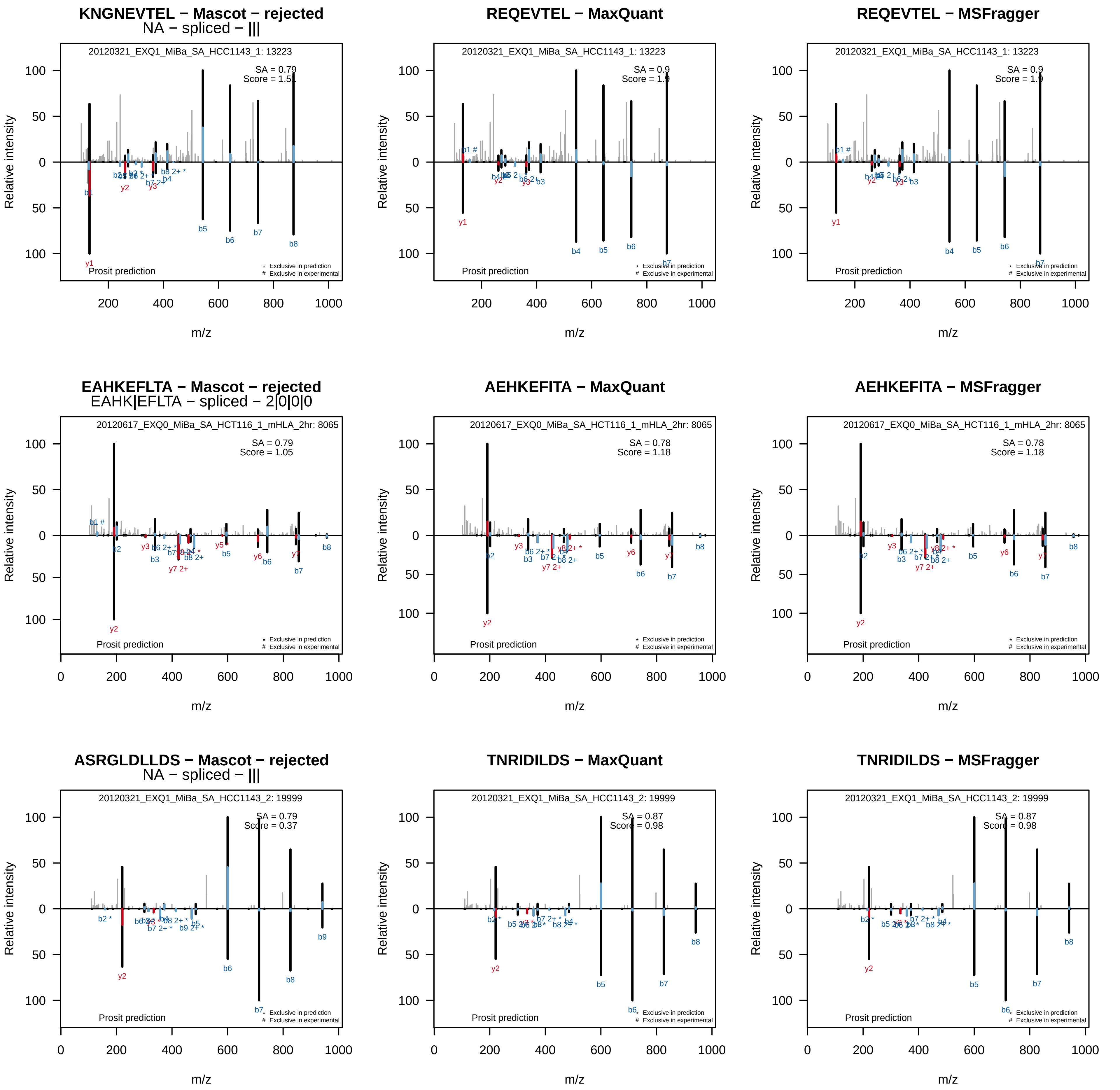

KNGNEVTEL − Mascot − rejected
NA − spliced − |||
20120321_EXQ1_MiBa_SA_HCC1143_1: 13223
SA = 0.79
Score = 1.51
REQEVTEL − MaxQuant
20120321_EXQ1_MiBa_SA_HCC1143_1: 13223
SA = 0.9
Score = 1.9
REQEVTEL − MSFragger
20120321_EXQ1_MiBa_SA_HCC1143_1: 13223
SA = 0.9
Score = 1.9
EAHKEFLTA − Mascot − rejected
EAHK|EFLTA − spliced − 2|0|0|0
20120617_EXQ0_MiBa_SA_HCT116_1_mHLA_2hr: 8065
SA = 0.79
Score = 1.05
AEHKEFITA − MaxQuant
20120617_EXQ0_MiBa_SA_HCT116_1_mHLA_2hr: 8065
SA = 0.78
Score = 1.18
AEHKEFITA − MSFragger
20120617_EXQ0_MiBa_SA_HCT116_1_mHLA_2hr: 8065
SA = 0.78
Score = 1.18
ASRGLDLLDS − Mascot − rejected
NA − spliced − |||
20120321_EXQ1_MiBa_SA_HCC1143_2: 19999
SA = 0.79
Score = 0.37
TNRIDILDS − MaxQuant
20120321_EXQ1_MiBa_SA_HCC1143_2: 19999
SA = 0.87
Score = 0.98
TNRIDILDS − MSFragger
20120321_EXQ1_MiBa_SA_HCC1143_2: 19999
SA = 0.87
Score = 0.98
Relative intensity
m/z
Prosit prediction
* Exclusive in prediction
# Exclusive in experimental

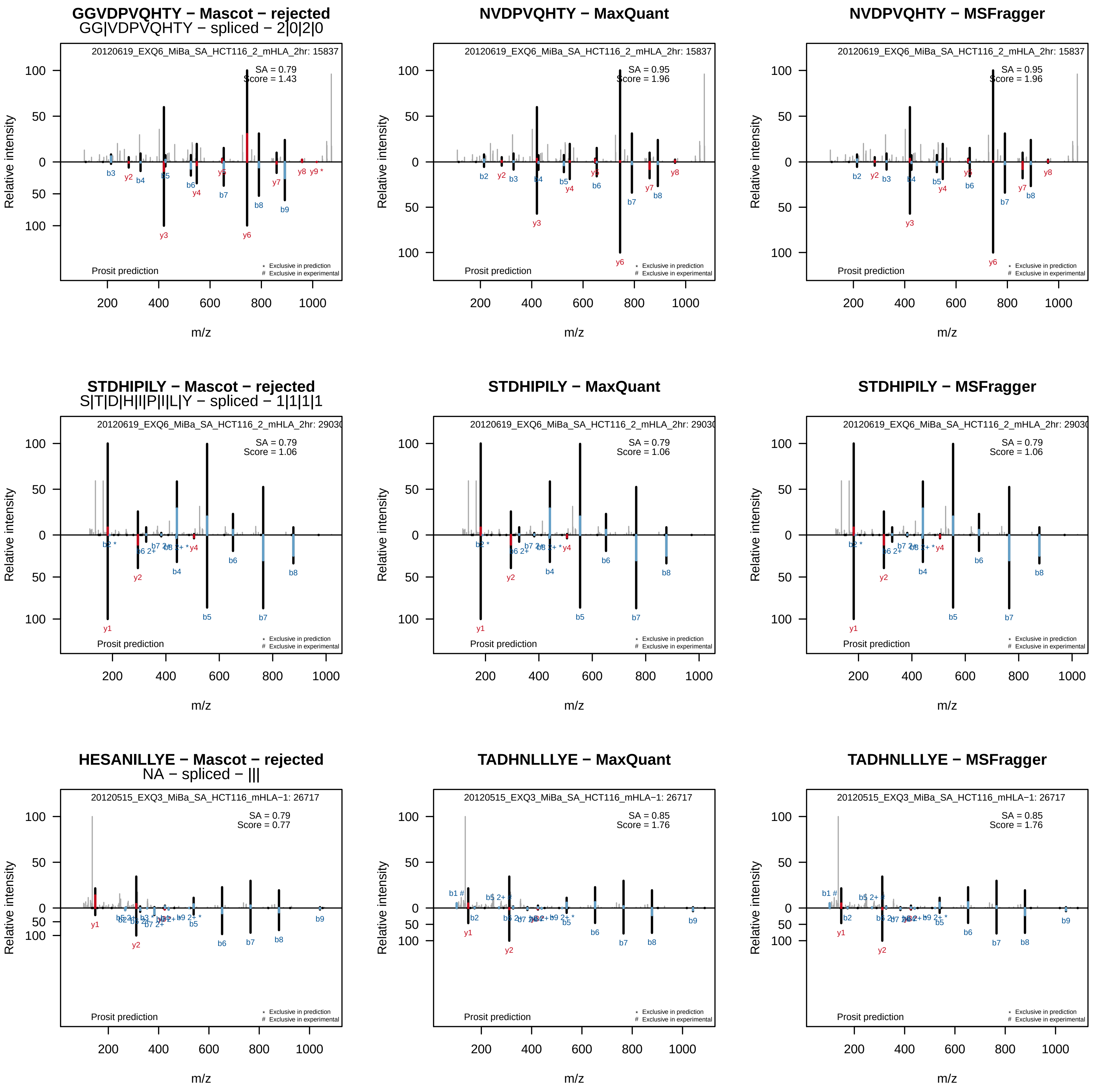
GGVDPVQHTY − Mascot − rejected
GG|VDPVQHTY − spliced − 2|0|2|0
20120619_EXQ6_MiBa_SA_HCT116_2_mHLA_2hr: 15837
SA = 0.79
Score = 1.43
Prosit prediction
⋆ Exclusive in prediction
Exclusive in experimental
NVDPVQHTY − MaxQuant
20120619_EXQ6_MiBa_SA_HCT116_2_mHLA_2hr: 15837
SA = 0.95
Score = 1.96
Prosit prediction
NVDPVQHTY − MSFragger
20120619_EXQ6_MiBa_SA_HCT116_2_mHLA_2hr: 15837
SA = 0.95
Score = 1.96
Prosit prediction
STDHIPILY − Mascot − rejected
S|T|D|H|I|P|I|L|Y − spliced − 1|1|1|1
SA = 0.79
Score = 1.06
Prosit prediction
STDHIPILY − MaxQuant
SA = 0.79
Score = 1.06
Prosit prediction
STDHIPILY − MSFragger
SA = 0.79
Score = 1.06
Prosit prediction
HESANILLYE − Mascot − rejected
NA − spliced − |||
20120515_EXQ3_MiBa_SA_HCT116_mHLA−1: 26717
SA = 0.79
Score = 0.77
Prosit prediction
TADHNLLLYE − MaxQuant
20120515_EXQ3_MiBa_SA_HCT116_mHLA−1: 26717
SA = 0.85
Score = 1.76
Prosit prediction
TADHNLLLYE − MSFragger
20120515_EXQ3_MiBa_SA_HCT116_mHLA−1: 26717
SA = 0.85
Score = 1.76
Prosit prediction
Relative intensity
m/z

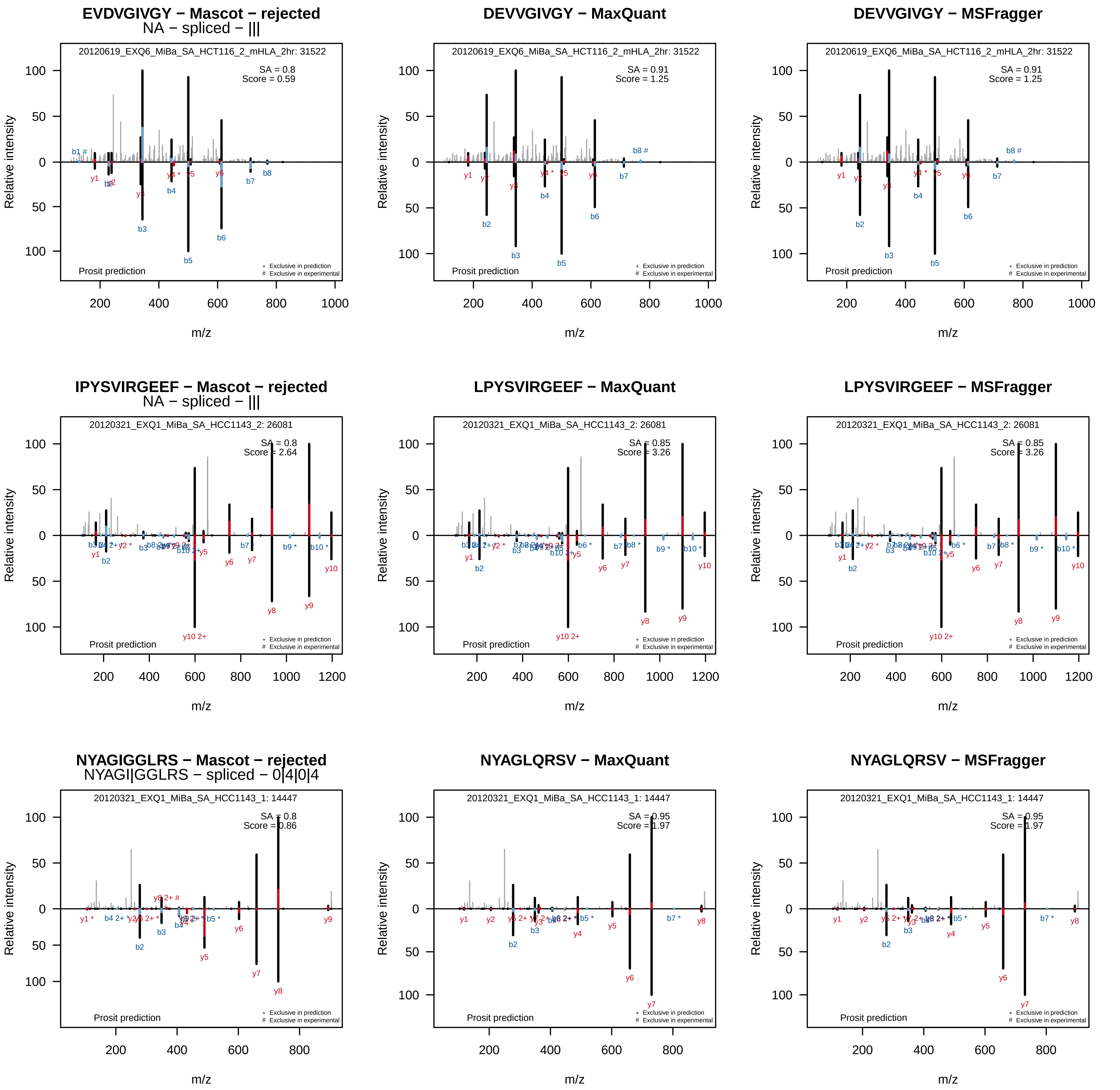

EVDVGIVGY − Mascot − rejected
NA − spliced − |||
20120619_EXQ6_MiBa_SA_HCT116_2_mHLA_2hr: 31522
SA = 0.8
Score = 0.59
DEVVGIVGY − MaxQuant
20120619_EXQ6_MiBa_SA_HCT116_2_mHLA_2hr: 31522
SA = 0.91
Score = 1.25
DEVVGIVGY − MSFragger
20120619_EXQ6_MiBa_SA_HCT116_2_mHLA_2hr: 31522
SA = 0.91
Score = 1.25
IPYSVIRGEEF − Mascot − rejected
NA − spliced − |||
20120321_EXQ1_MiBa_SA_HCC1143_2: 26081
SA = 0.8
Score = 2.64
LPYSVIRGEEF − MaxQuant
20120321_EXQ1_MiBa_SA_HCC1143_2: 26081
SA = 0.85
Score = 3.26
LPYSVIRGEEF − MSFragger
20120321_EXQ1_MiBa_SA_HCC1143_2: 26081
SA = 0.85
Score = 3.26
NYAGIGGLRS − Mascot − rejected
NYAGI|GGLRS − spliced − 0|4|0|4
20120321_EXQ1_MiBa_SA_HCC1143_1: 14447
SA = 0.8
Score = 0.86
NYAGLQRSV − MaxQuant
20120321_EXQ1_MiBa_SA_HCC1143_1: 14447
SA = 0.95
Score = 1.97
NYAGLQRSV − MSFragger
20120321_EXQ1_MiBa_SA_HCC1143_1: 14447
SA = 0.95
Score = 1.97
Relative intensity
m/z
Prosit prediction
⋆ Exclusive in prediction
Exclusive in experimental

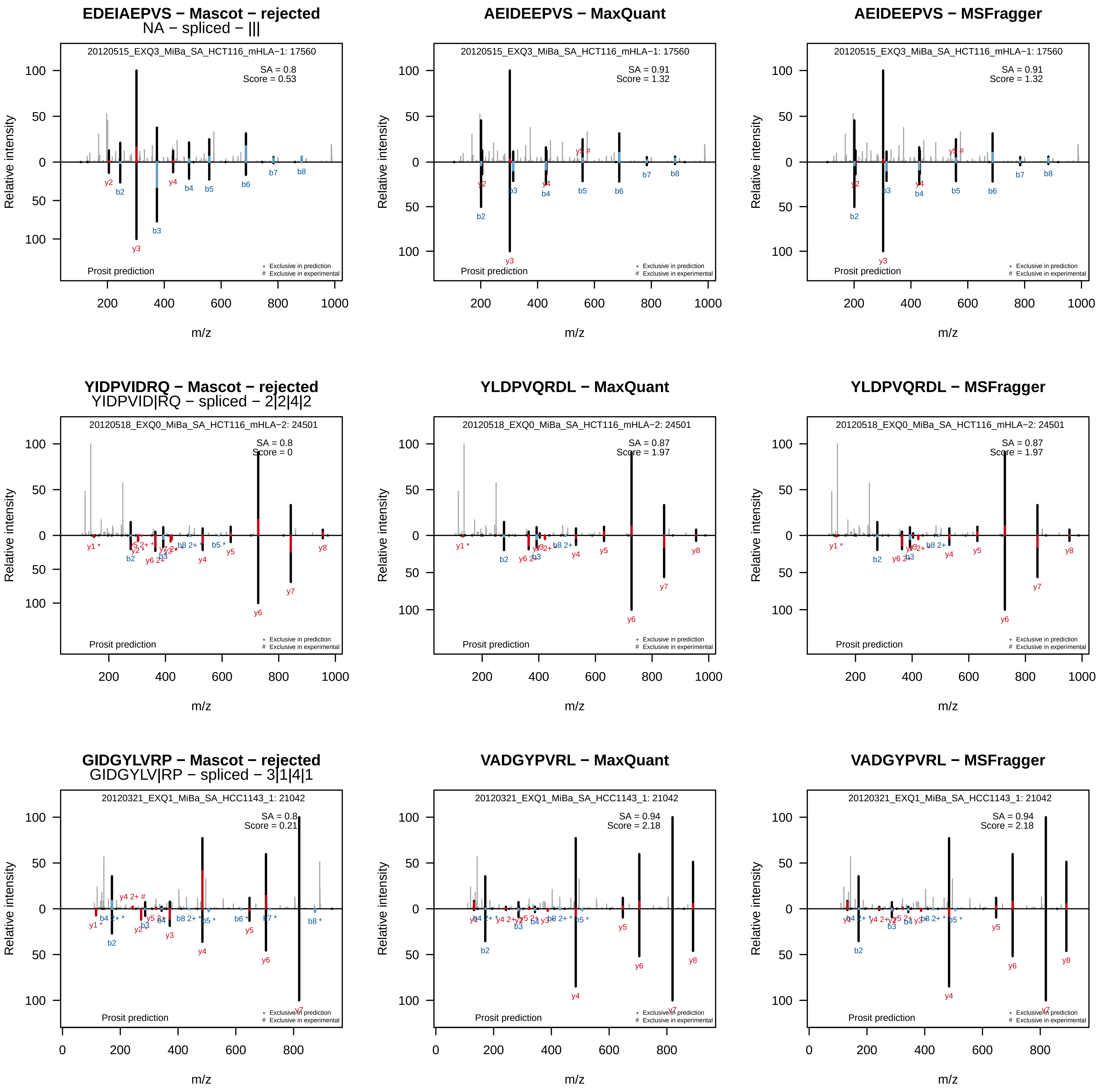
EDEIAEPVS − Mascot − rejected
NA − spliced − |||
20120515_EXQ3_MiBa_SA_HCT116_mHLA−1: 17560
SA = 0.8
Score = 0.53
AEIDEEPVS − MaxQuant
20120515_EXQ3_MiBa_SA_HCT116_mHLA−1: 17560
SA = 0.91
Score = 1.32
AEIDEEPVS − MSFragger
20120515_EXQ3_MiBa_SA_HCT116_mHLA−1: 17560
SA = 0.91
Score = 1.32
YIDPVIDRQ − Mascot − rejected
YIDPVID|RQ − spliced − 2|2|4|2
20120518_EXQ0_MiBa_SA_HCT116_mHLA−2: 24501
SA = 0.8
Score = 0
YLDPVQRDL − MaxQuant
20120518_EXQ0_MiBa_SA_HCT116_mHLA−2: 24501
SA = 0.87
Score = 1.97
YLDPVQRDL − MSFragger
20120518_EXQ0_MiBa_SA_HCT116_mHLA−2: 24501
SA = 0.87
Score = 1.97
GIDGYLVRP − Mascot − rejected
GIDGYLV|RP − spliced − 3|1|4|1
20120321_EXQ1_MiBa_SA_HCC1143_1: 21042
SA = 0.8
Score = 0.21
VADGYPVRL − MaxQuant
20120321_EXQ1_MiBa_SA_HCC1143_1: 21042
SA = 0.94
Score = 2.18
VADGYPVRL − MSFragger
20120321_EXQ1_MiBa_SA_HCC1143_1: 21042
SA = 0.94
Score = 2.18
Relative intensity
m/z
Prosit prediction
* Exclusive in prediction
# Exclusive in experimental

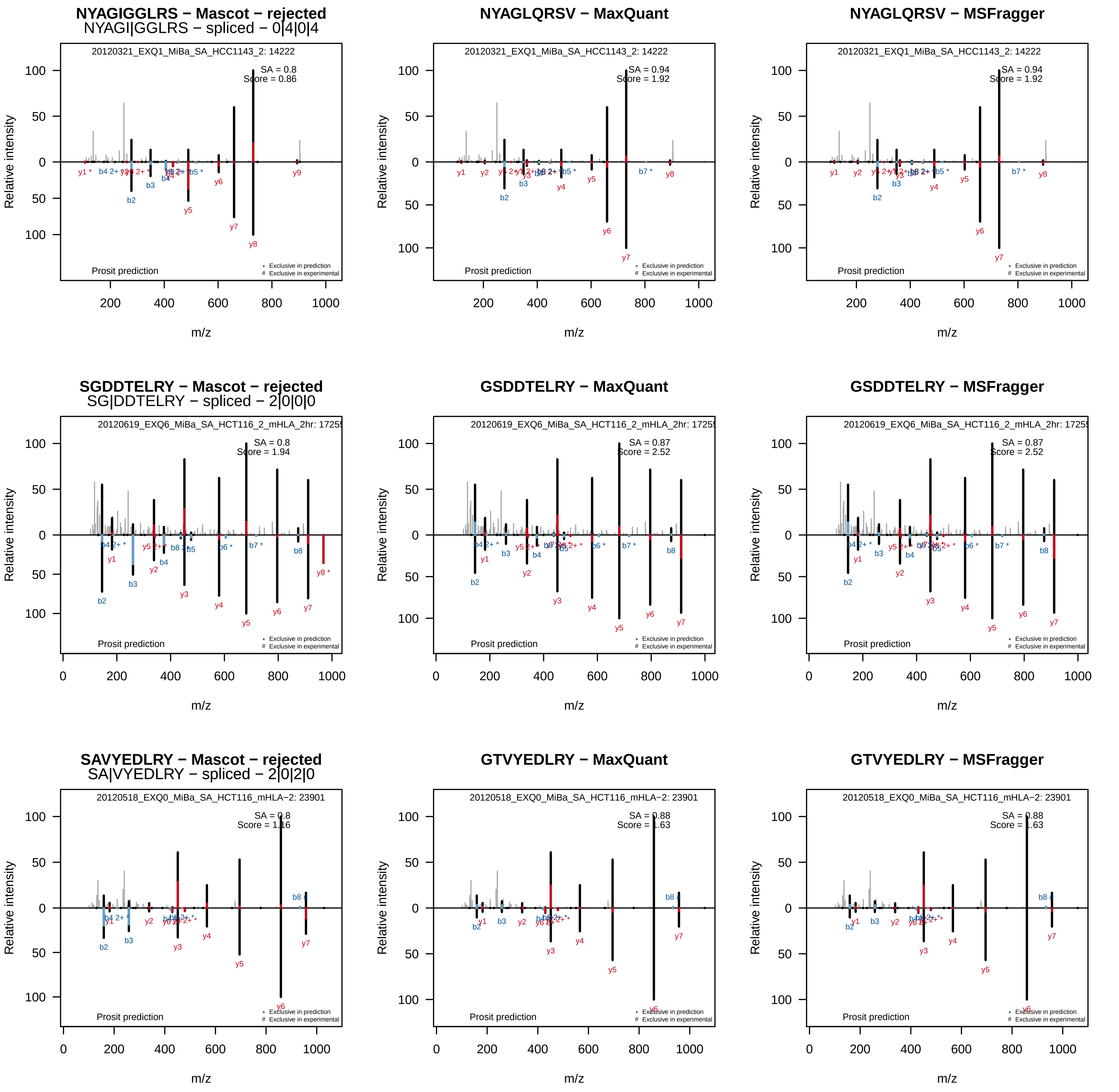
NYAGIGGLRS − Mascot − rejected
NYAGI|GGLRS − spliced − 0|4|0|4
20120321_EXQ1_MiBa_SA_HCC1143_2: 14222
SA = 0.8
Score = 0.86
NYAGLQRSV − MaxQuant
20120321_EXQ1_MiBa_SA_HCC1143_2: 14222
SA = 0.94
Score = 1.92
NYAGLQRSV − MSFragger
20120321_EXQ1_MiBa_SA_HCC1143_2: 14222
SA = 0.94
Score = 1.92
SGDDTELRY − Mascot − rejected
SG|DDTELRY − spliced − 2|0|0|0
20120619_EXQ6_MiBa_SA_HCT116_2_mHLA_2hr: 1725
SA = 0.8
Score = 1.94
GSDDTELRY − MaxQuant
20120619_EXQ6_MiBa_SA_HCT116_2_mHLA_2hr: 1725
SA = 0.87
Score = 2.52
GSDDTELRY − MSFragger
20120619_EXQ6_MiBa_SA_HCT116_2_mHLA_2hr: 1725
SA = 0.87
Score = 2.52
SAVYEDLRY − Mascot − rejected
SA|VYEDLRY − spliced − 2|0|2|0
20120518_EXQ0_MiBa_SA_HCT116_mHLA−2: 23901
SA = 0.8
Score = 1.16
GTVYEDLRY − MaxQuant
20120518_EXQ0_MiBa_SA_HCT116_mHLA−2: 23901
SA = 0.88
Score = 1.63
GTVYEDLRY − MSFragger
20120518_EXQ0_MiBa_SA_HCT116_mHLA−2: 23901
SA = 0.88
Score = 1.63
Relative intensity
m/z
Prosit prediction
⋆ Exclusive in prediction
Exclusive in experimental

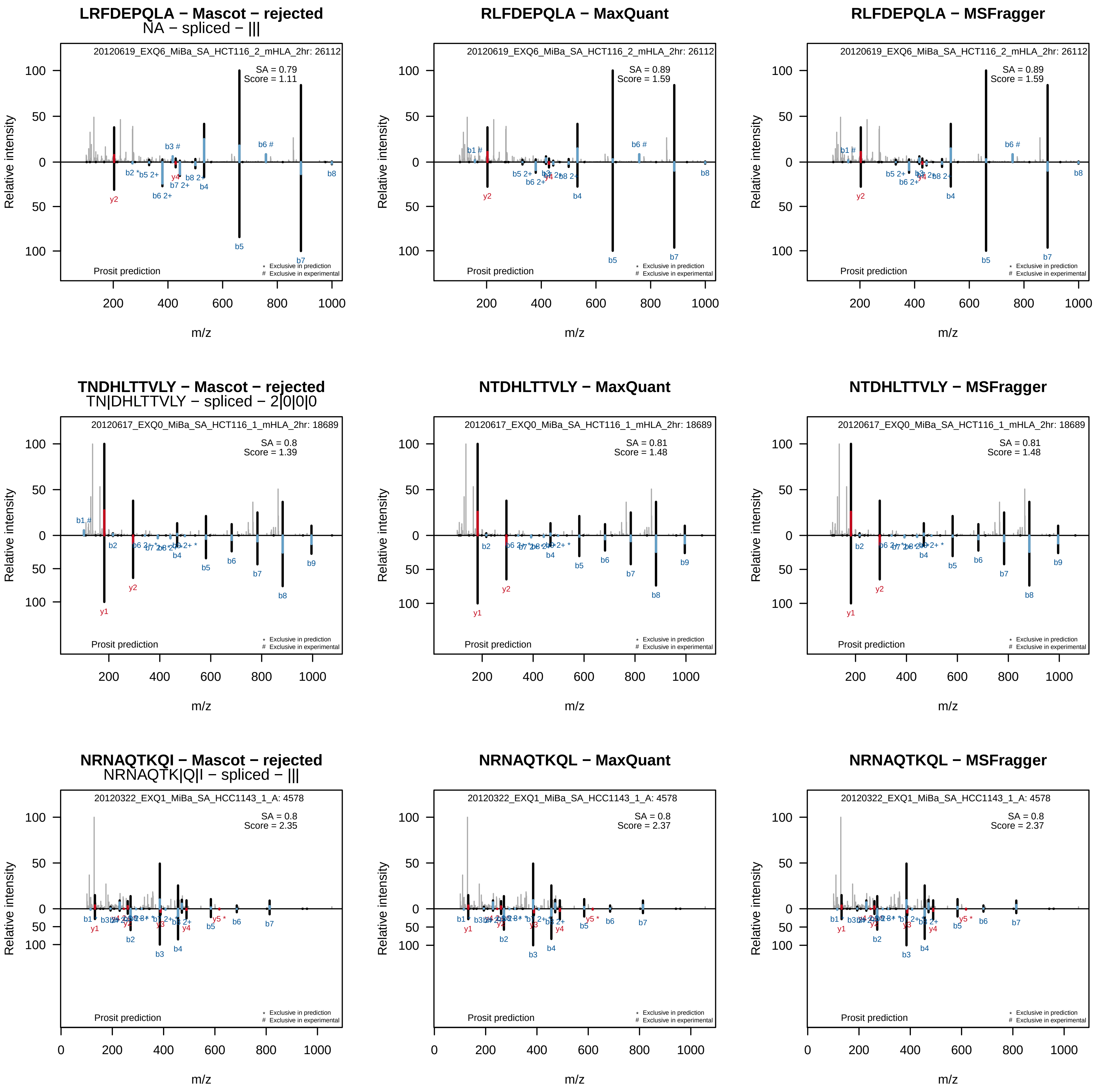
LRFDEPQLA − Mascot − rejected
NA − spliced − |||
20120619_EXQ6_MiBa_SA_HCT116_2_mHLA_2hr: 26112
SA = 0.79
Score = 1.11
RLFDEPQLA − MaxQuant
20120619_EXQ6_MiBa_SA_HCT116_2_mHLA_2hr: 26112
SA = 0.89
Score = 1.59
RLFDEPQLA − MSFragger
20120619_EXQ6_MiBa_SA_HCT116_2_mHLA_2hr: 26112
SA = 0.89
Score = 1.59
TNDHLTTVLY − Mascot − rejected
TN|DHLTTVLY − spliced − 2|0|0|0
20120617_EXQ0_MiBa_SA_HCT116_1_mHLA_2hr: 18689
SA = 0.8
Score = 1.39
NTDHLTTVLY − MaxQuant
20120617_EXQ0_MiBa_SA_HCT116_1_mHLA_2hr: 18689
SA = 0.81
Score = 1.48
NTDHLTTVLY − MSFragger
20120617_EXQ0_MiBa_SA_HCT116_1_mHLA_2hr: 18689
SA = 0.81
Score = 1.48
NRNAQTKQI − Mascot − rejected
NRNAQTK|Q|I − spliced − |||
20120322_EXQ1_MiBa_SA_HCC1143_1_A: 4578
SA = 0.8
Score = 2.35
NRNAQTKQL − MaxQuant
20120322_EXQ1_MiBa_SA_HCC1143_1_A: 4578
SA = 0.8
Score = 2.37
NRNAQTKQL − MSFragger
20120322_EXQ1_MiBa_SA_HCC1143_1_A: 4578
SA = 0.8
Score = 2.37
Relative intensity
m/z
Prosit prediction
⋆ Exclusive in prediction
Exclusive in experimental

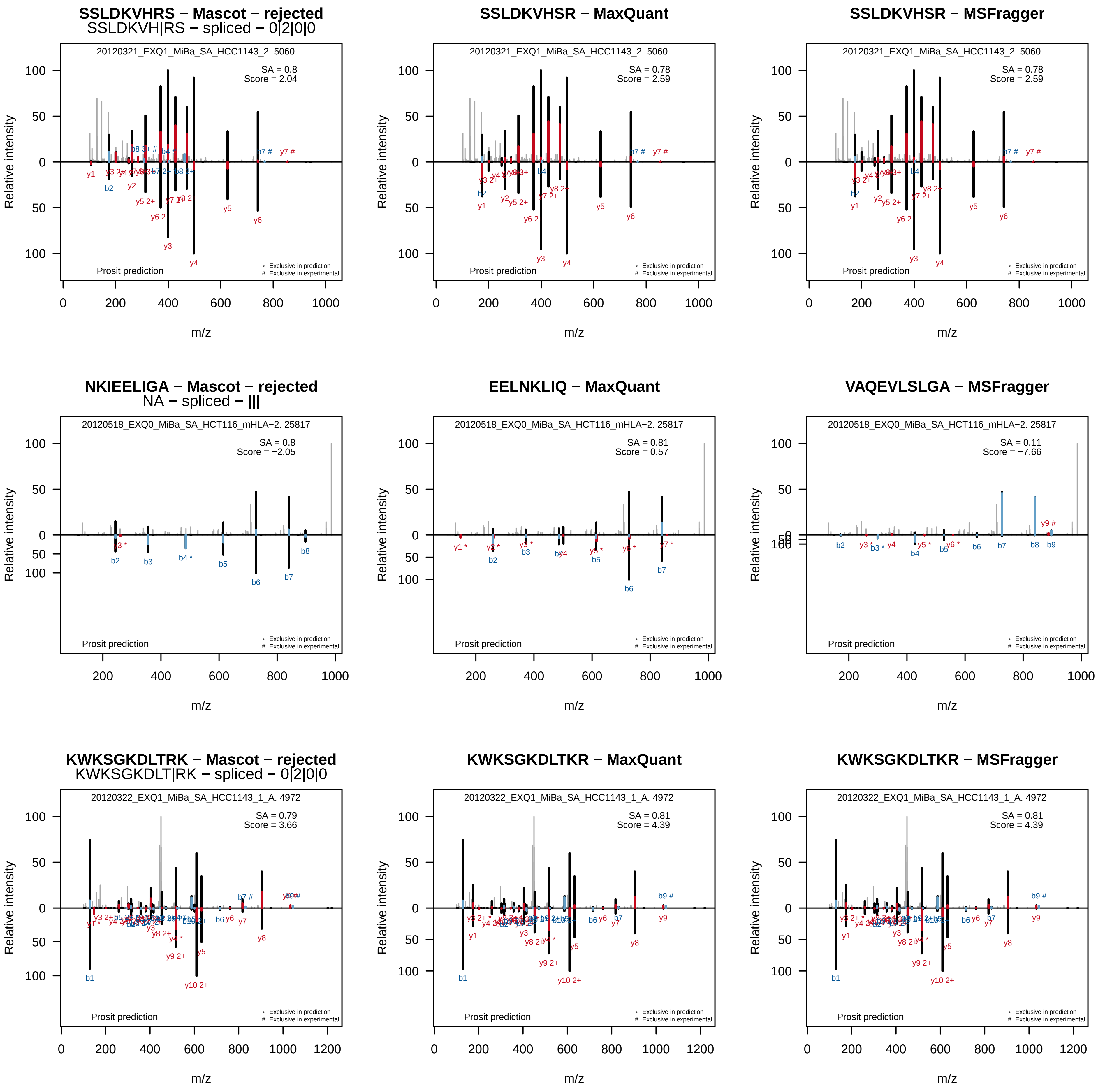
SSLDKVHRS − Mascot − rejected
SSLDKVH|RS − spliced − 0|2|0|0
20120321_EXQ1_MiBa_SA_HCC1143_2: 5060
SA = 0.8
Score = 2.04
SSLDKVHSR − MaxQuant
20120321_EXQ1_MiBa_SA_HCC1143_2: 5060
SA = 0.78
Score = 2.59
SSLDKVHSR − MSFragger
20120321_EXQ1_MiBa_SA_HCC1143_2: 5060
SA = 0.78
Score = 2.59
NKIEELIGA − Mascot − rejected
NA − spliced − |||
20120518_EXQ0_MiBa_SA_HCT116_mHLA−2: 25817
SA = 0.8
Score = −2.05
EELNKLIQ − MaxQuant
20120518_EXQ0_MiBa_SA_HCT116_mHLA−2: 25817
SA = 0.81
Score = 0.57
VAQEVLSLGA − MSFragger
20120518_EXQ0_MiBa_SA_HCT116_mHLA−2: 25817
SA = 0.11
Score = −7.66
KWKSGKDLTRK − Mascot − rejected
KWKSGKDLT|RK − spliced − 0|2|0|0
20120322_EXQ1_MiBa_SA_HCC1143_1_A: 4972
SA = 0.79
Score = 3.66
KWKSGKDLTKR − MaxQuant
20120322_EXQ1_MiBa_SA_HCC1143_1_A: 4972
SA = 0.81
Score = 4.39
KWKSGKDLTKR − MSFragger
20120322_EXQ1_MiBa_SA_HCC1143_1_A: 4972
SA = 0.81
Score = 4.39
Relative intensity
m/z
Prosit prediction
⋆ Exclusive in prediction
Exclusive in experimental

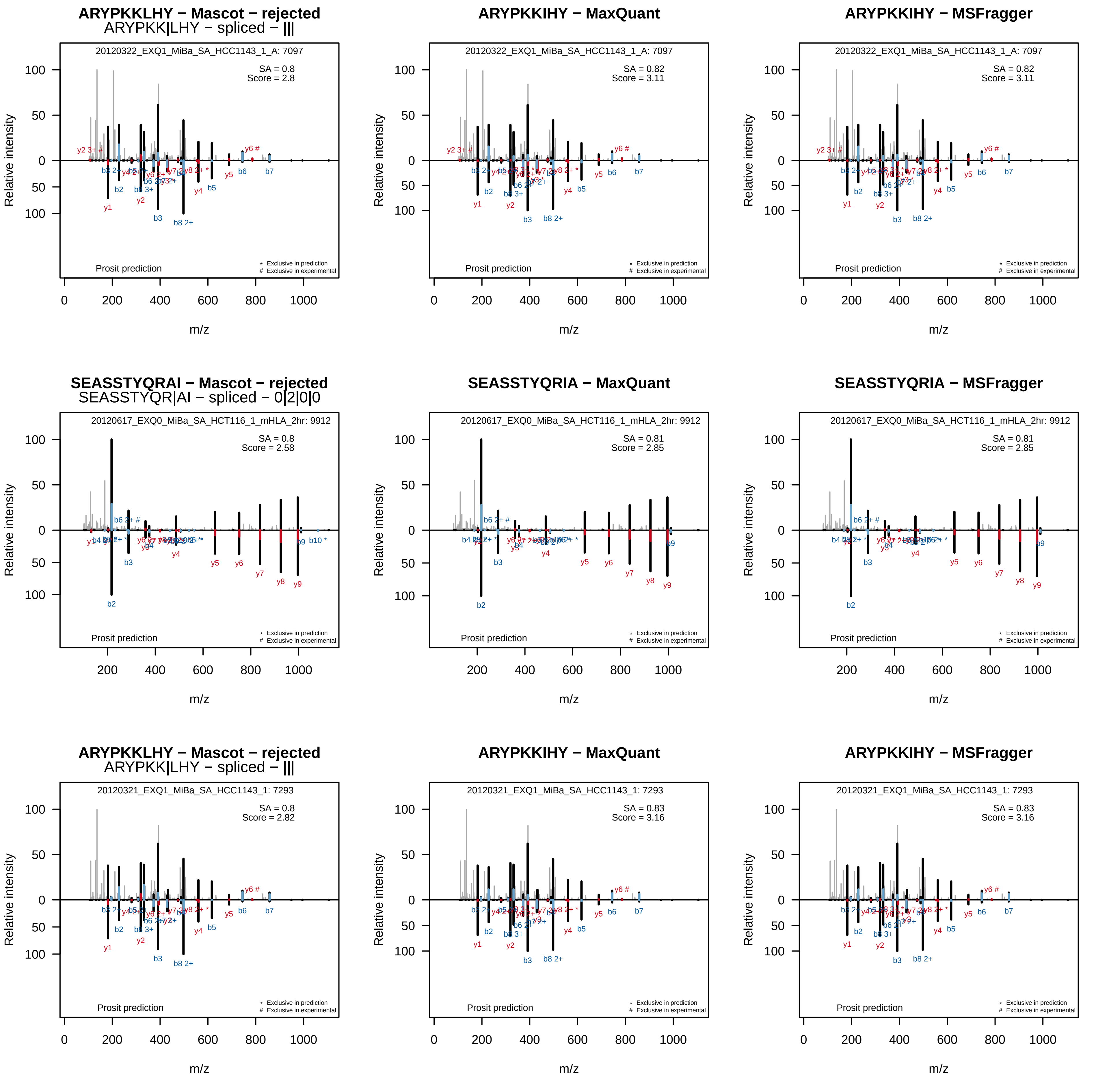

ARYPKKLHY − Mascot − rejected
ARYPKK|LHY − spliced − |||
20120322_EXQ1_MiBa_SA_HCC1143_1_A: 7097
SA = 0.8
Score = 2.8
ARYPKKIHY − MaxQuant
20120322_EXQ1_MiBa_SA_HCC1143_1_A: 7097
SA = 0.82
Score = 3.11
ARYPKKIHY − MSFragger
20120322_EXQ1_MiBa_SA_HCC1143_1_A: 7097
SA = 0.82
Score = 3.11
SEASSTYQRAI − Mascot − rejected
SEASSTYQR|AI − spliced − 0|2|0|0
20120617_EXQ0_MiBa_SA_HCT116_1_mHLA_2hr: 9912
SA = 0.8
Score = 2.58
SEASSTYQRIA − MaxQuant
20120617_EXQ0_MiBa_SA_HCT116_1_mHLA_2hr: 9912
SA = 0.81
Score = 2.85
SEASSTYQRIA − MSFragger
20120617_EXQ0_MiBa_SA_HCT116_1_mHLA_2hr: 9912
SA = 0.81
Score = 2.85
ARYPKKLHY − Mascot − rejected
ARYPKK|LHY − spliced − |||
20120321_EXQ1_MiBa_SA_HCC1143_1: 7293
SA = 0.8
Score = 2.82
ARYPKKIHY − MaxQuant
20120321_EXQ1_MiBa_SA_HCC1143_1: 7293
SA = 0.83
Score = 3.16
ARYPKKIHY − MSFragger
20120321_EXQ1_MiBa_SA_HCC1143_1: 7293
SA = 0.83
Score = 3.16
Relative intensity
m/z
Prosit prediction
* Exclusive in prediction
# Exclusive in experimental

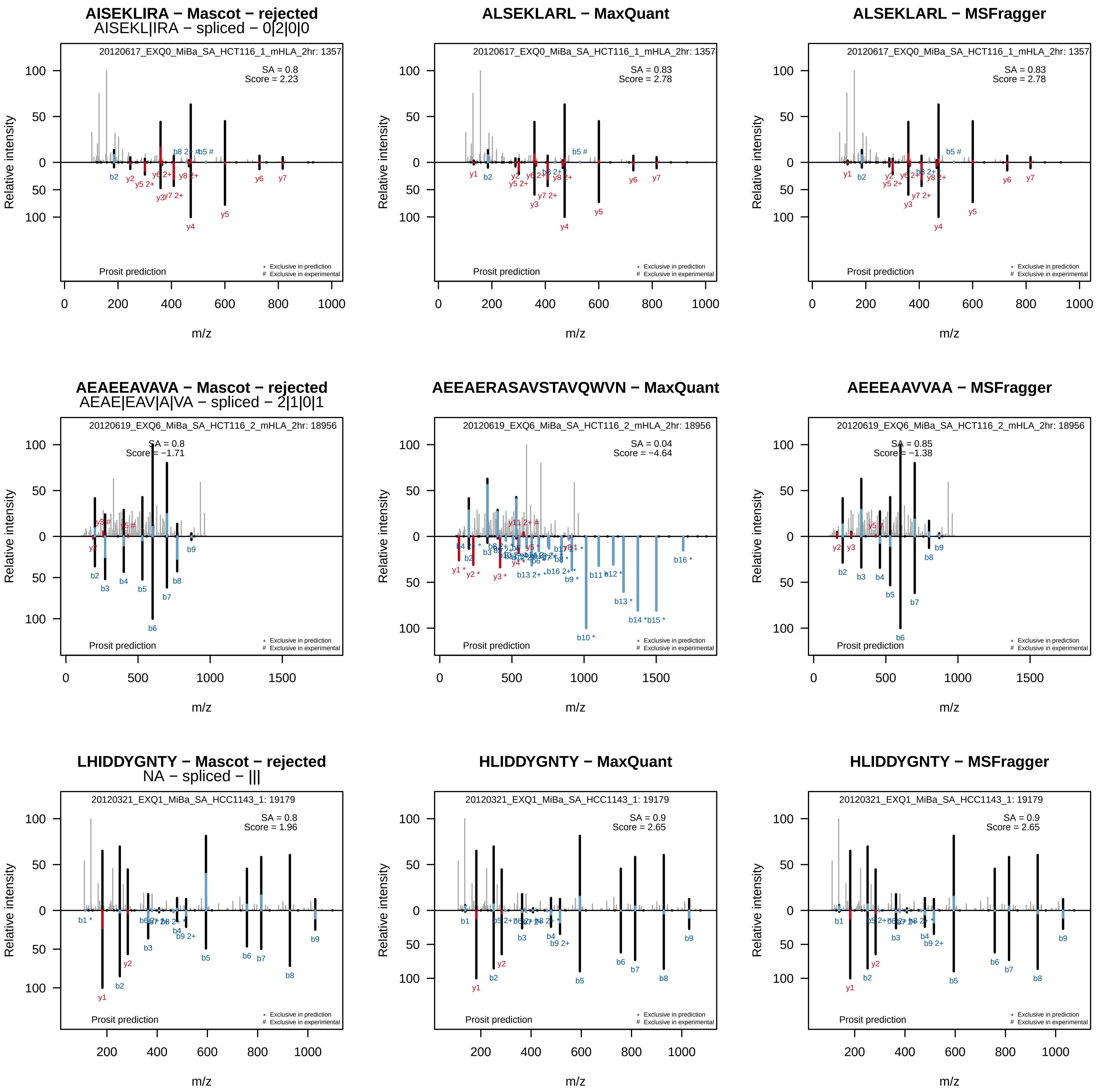
AISEKLIRA − Mascot − rejected
AISEKL|IRA − spliced − 0|2|0|0
20120617_EXQ0_MiBa_SA_HCT116_1_mHLA_2hr: 1357
SA = 0.8
Score = 2.23
ALSEKLARL − MaxQuant
20120617_EXQ0_MiBa_SA_HCT116_1_mHLA_2hr: 1357
SA = 0.83
Score = 2.78
ALSEKLARL − MSFragger
20120617_EXQ0_MiBa_SA_HCT116_1_mHLA_2hr: 1357
SA = 0.83
Score = 2.78
AEAEEAVAVA − Mascot − rejected
AEAE|EAV|A|VA − spliced − 2|1|0|1
20120619_EXQ6_MiBa_SA_HCT116_2_mHLA_2hr: 18956
SA = 0.8
Score = −1.71
AEEAERASAVSTAVQWVN − MaxQuant
20120619_EXQ6_MiBa_SA_HCT116_2_mHLA_2hr: 18956
SA = 0.04
Score = −4.64
AEEEAAVVAA − MSFragger
20120619_EXQ6_MiBa_SA_HCT116_2_mHLA_2hr: 18956
SA = 0.85
Score = −1.38
LHIDDYGNTY − Mascot − rejected
NA − spliced − |||
20120321_EXQ1_MiBa_SA_HCC1143_1: 19179
SA = 0.8
Score = 1.96
HLIDDYGNTY − MaxQuant
20120321_EXQ1_MiBa_SA_HCC1143_1: 19179
SA = 0.9
Score = 2.65
HLIDDYGNTY − MSFragger
20120321_EXQ1_MiBa_SA_HCC1143_1: 19179
SA = 0.9
Score = 2.65
Relative intensity
m/z
Prosit prediction
* Exclusive in prediction
# Exclusive in experimental

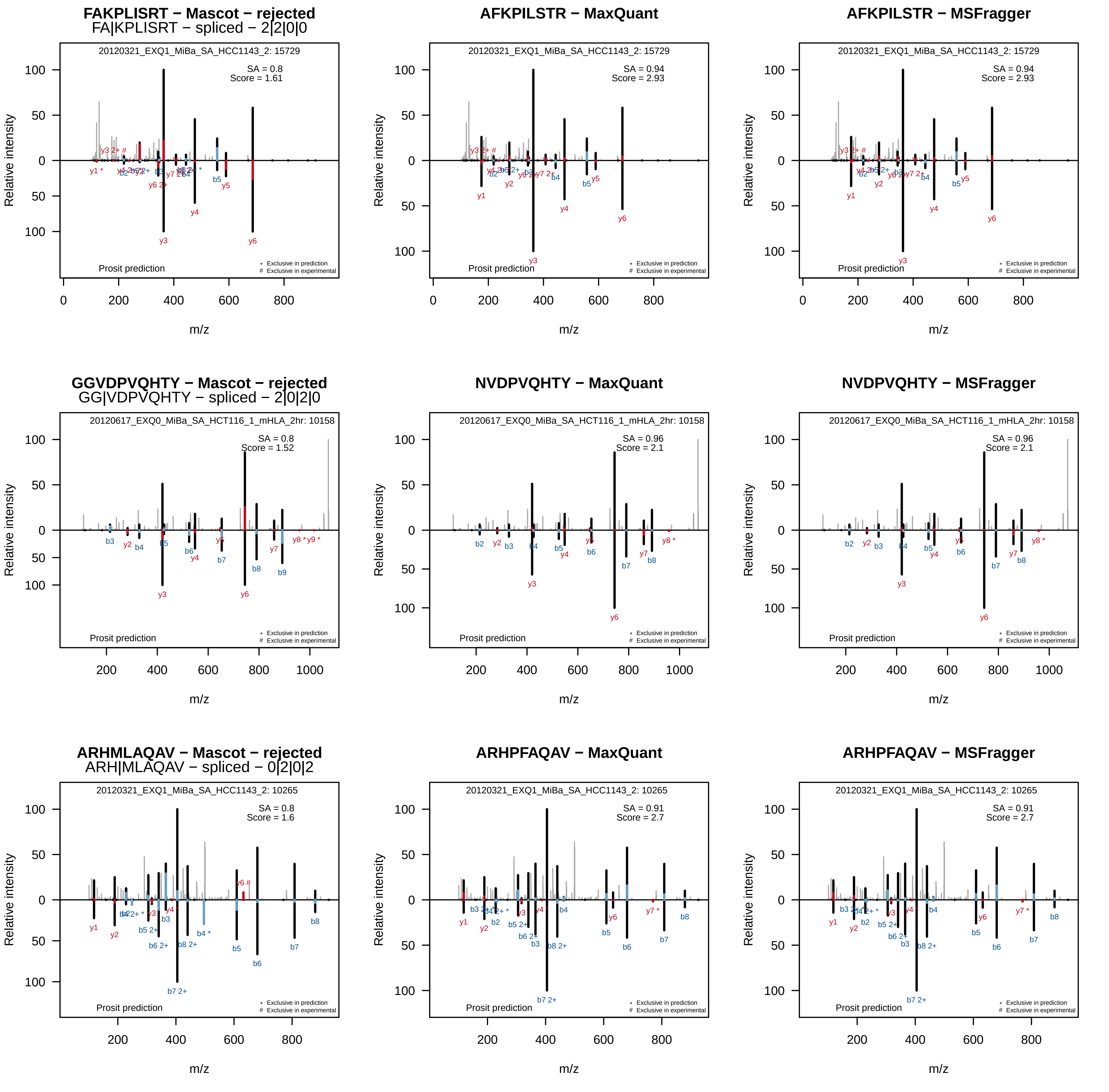

FAKPLISRT − Mascot − rejected
FA|KPLISRT − spliced − 2|2|0|0
20120321_EXQ1_MiBa_SA_HCC1143_2: 15729
SA = 0.8
Score = 1.61
AFKPILSTR − MaxQuant
20120321_EXQ1_MiBa_SA_HCC1143_2: 15729
SA = 0.94
Score = 2.93
AFKPILSTR − MSFragger
20120321_EXQ1_MiBa_SA_HCC1143_2: 15729
SA = 0.94
Score = 2.93
GGVDPVQHTY − Mascot − rejected
GG|VDPVQHTY − spliced − 2|0|2|0
20120617_EXQ0_MiBa_SA_HCT116_1_mHLA_2hr: 10158
SA = 0.8
Score = 1.52
NVDPVQHTY − MaxQuant
20120617_EXQ0_MiBa_SA_HCT116_1_mHLA_2hr: 10158
SA = 0.96
Score = 2.1
NVDPVQHTY − MSFragger
20120617_EXQ0_MiBa_SA_HCT116_1_mHLA_2hr: 10158
SA = 0.96
Score = 2.1
ARHMLAQAV − Mascot − rejected
ARH|MLAQAV − spliced − 0|2|0|2
20120321_EXQ1_MiBa_SA_HCC1143_2: 10265
SA = 0.8
Score = 1.6
ARHPFAQAV − MaxQuant
20120321_EXQ1_MiBa_SA_HCC1143_2: 10265
SA = 0.91
Score = 2.7
ARHPFAQAV − MSFragger
20120321_EXQ1_MiBa_SA_HCC1143_2: 10265
SA = 0.91
Score = 2.7
Relative intensity
m/z
Prosit prediction
* Exclusive in prediction
# Exclusive in experimental

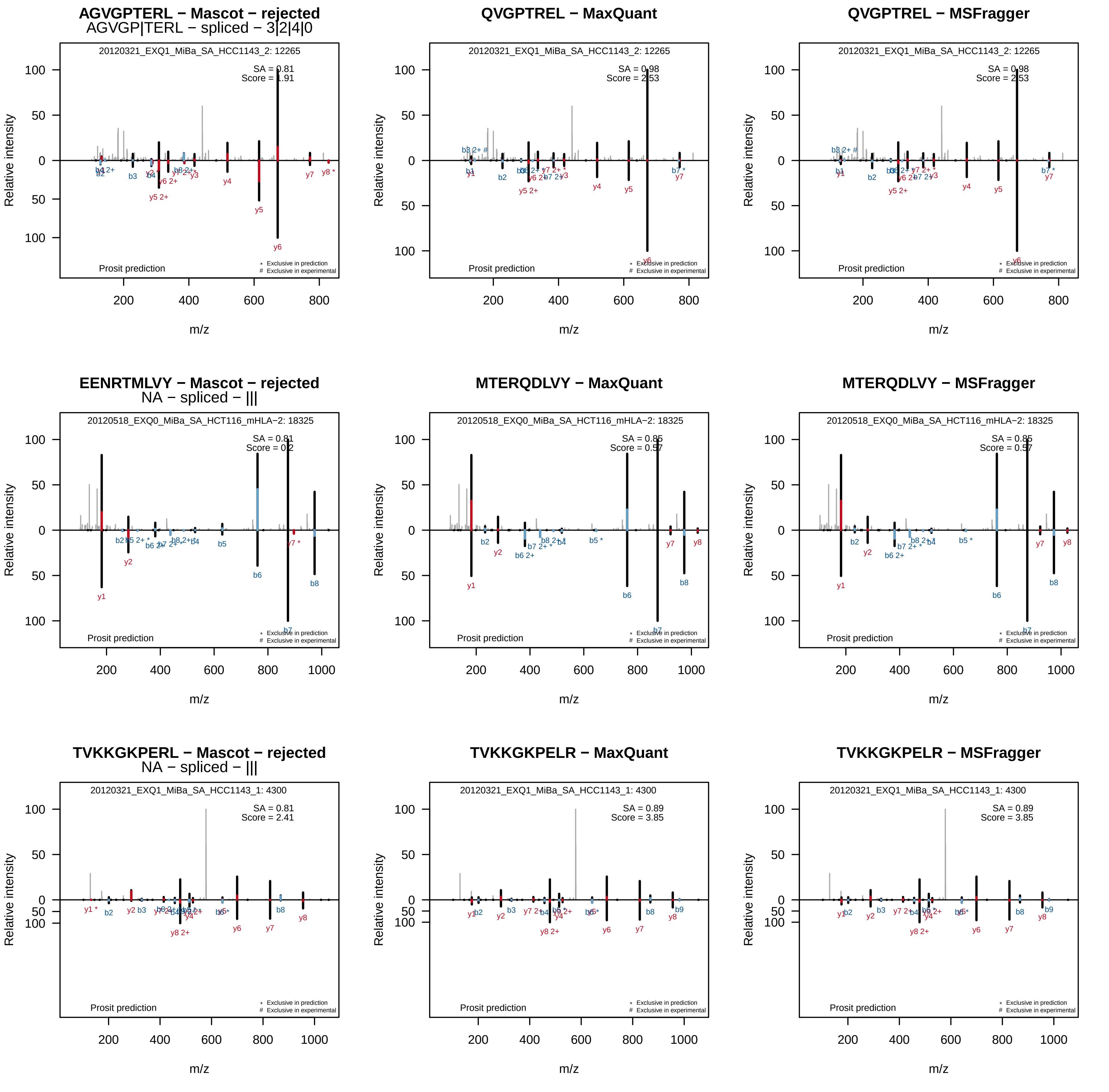
AGVGPTERL − Mascot − rejected
AGVGP|TERL − spliced − 3|2|4|0
20120321_EXQ1_MiBa_SA_HCC1143_2: 12265
SA = 0.81
Score = 1.91
QVGPTREL − MaxQuant
20120321_EXQ1_MiBa_SA_HCC1143_2: 12265
SA = 0.98
Score = 2.53
QVGPTREL − MSFragger
20120321_EXQ1_MiBa_SA_HCC1143_2: 12265
SA = 0.98
Score = 2.53
EENRTMLVY − Mascot − rejected
NA − spliced − |||
20120518_EXQ0_MiBa_SA_HCT116_mHLA−2: 18325
SA = 0.81
Score = 0.2
MTERQDLVY − MaxQuant
20120518_EXQ0_MiBa_SA_HCT116_mHLA−2: 18325
SA = 0.85
Score = 0.57
MTERQDLVY − MSFragger
20120518_EXQ0_MiBa_SA_HCT116_mHLA−2: 18325
SA = 0.85
Score = 0.57
TVKKGKPERL − Mascot − rejected
NA − spliced − |||
20120321_EXQ1_MiBa_SA_HCC1143_1: 4300
SA = 0.81
Score = 2.41
TVKKGKPELR − MaxQuant
20120321_EXQ1_MiBa_SA_HCC1143_1: 4300
SA = 0.89
Score = 3.85
TVKKGKPELR − MSFragger
20120321_EXQ1_MiBa_SA_HCC1143_1: 4300
SA = 0.89
Score = 3.85
Relative intensity
m/z
Prosit prediction
* Exclusive in prediction
# Exclusive in experimental

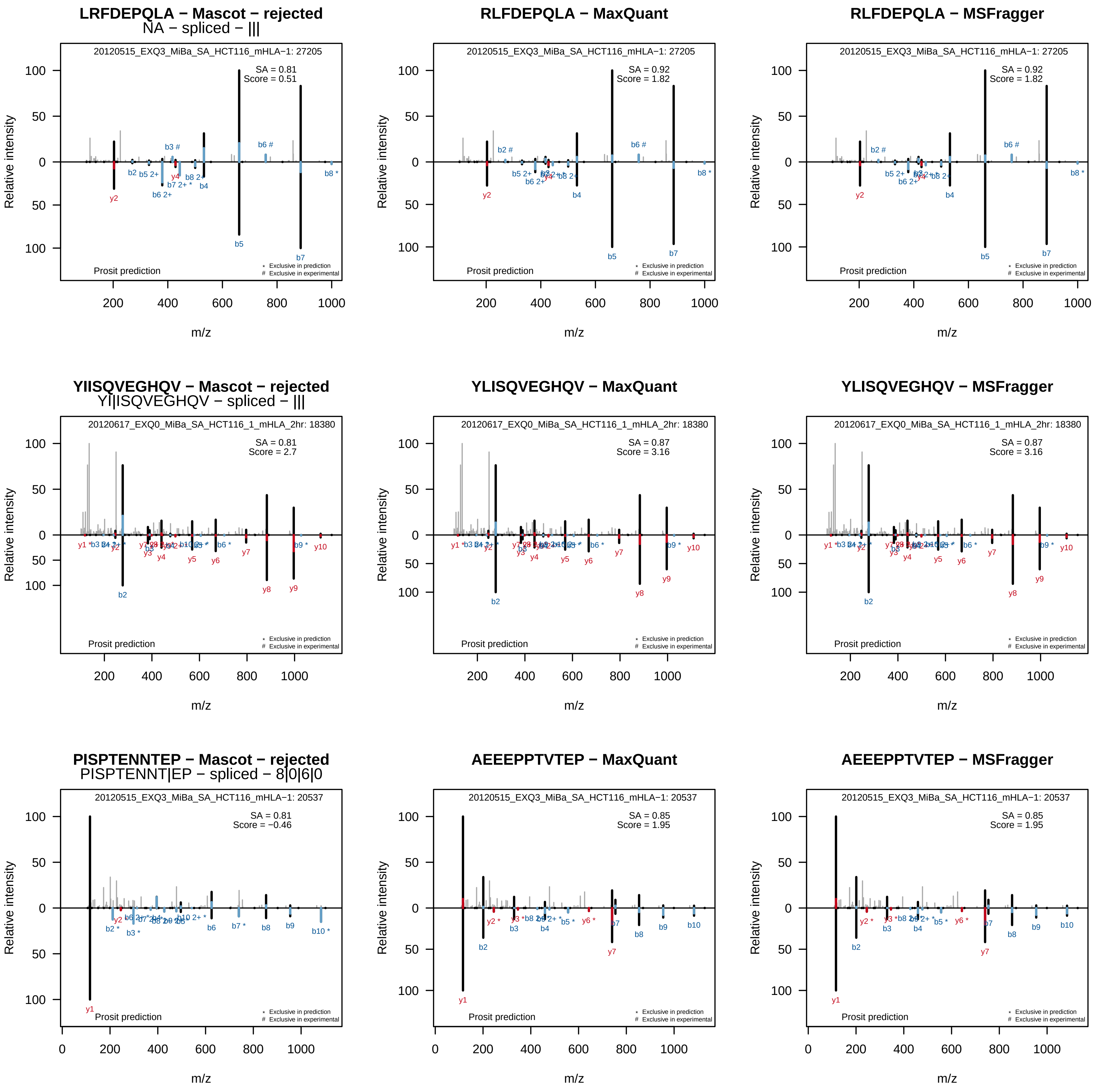
LRFDEPQLA − Mascot − rejected
NA − spliced − |||
20120515_EXQ3_MiBa_SA_HCT116_mHLA−1: 27205
SA = 0.81
Score = 0.51
RLFDEPQLA − MaxQuant
20120515_EXQ3_MiBa_SA_HCT116_mHLA−1: 27205
SA = 0.92
Score = 1.82
RLFDEPQLA − MSFragger
20120515_EXQ3_MiBa_SA_HCT116_mHLA−1: 27205
SA = 0.92
Score = 1.82
YIISQVEGHQV − Mascot − rejected
YI|ISQVEGHQV − spliced − |||
20120617_EXQ0_MiBa_SA_HCT116_1_mHLA_2hr: 18380
SA = 0.81
Score = 2.7
YLISQVEGHQV − MaxQuant
20120617_EXQ0_MiBa_SA_HCT116_1_mHLA_2hr: 18380
SA = 0.87
Score = 3.16
YLISQVEGHQV − MSFragger
20120617_EXQ0_MiBa_SA_HCT116_1_mHLA_2hr: 18380
SA = 0.87
Score = 3.16
PISPTENNTEP − Mascot − rejected
PISPTENNT|EP − spliced − 8|0|6|0
20120515_EXQ3_MiBa_SA_HCT116_mHLA−1: 20537
SA = 0.81
Score = −0.46
AEEEPPTVTEP − MaxQuant
20120515_EXQ3_MiBa_SA_HCT116_mHLA−1: 20537
SA = 0.85
Score = 1.95
AEEEPPTVTEP − MSFragger
20120515_EXQ3_MiBa_SA_HCT116_mHLA−1: 20537
SA = 0.85
Score = 1.95
Relative intensity
m/z
Prosit prediction
⋆ Exclusive in prediction
Exclusive in experimental

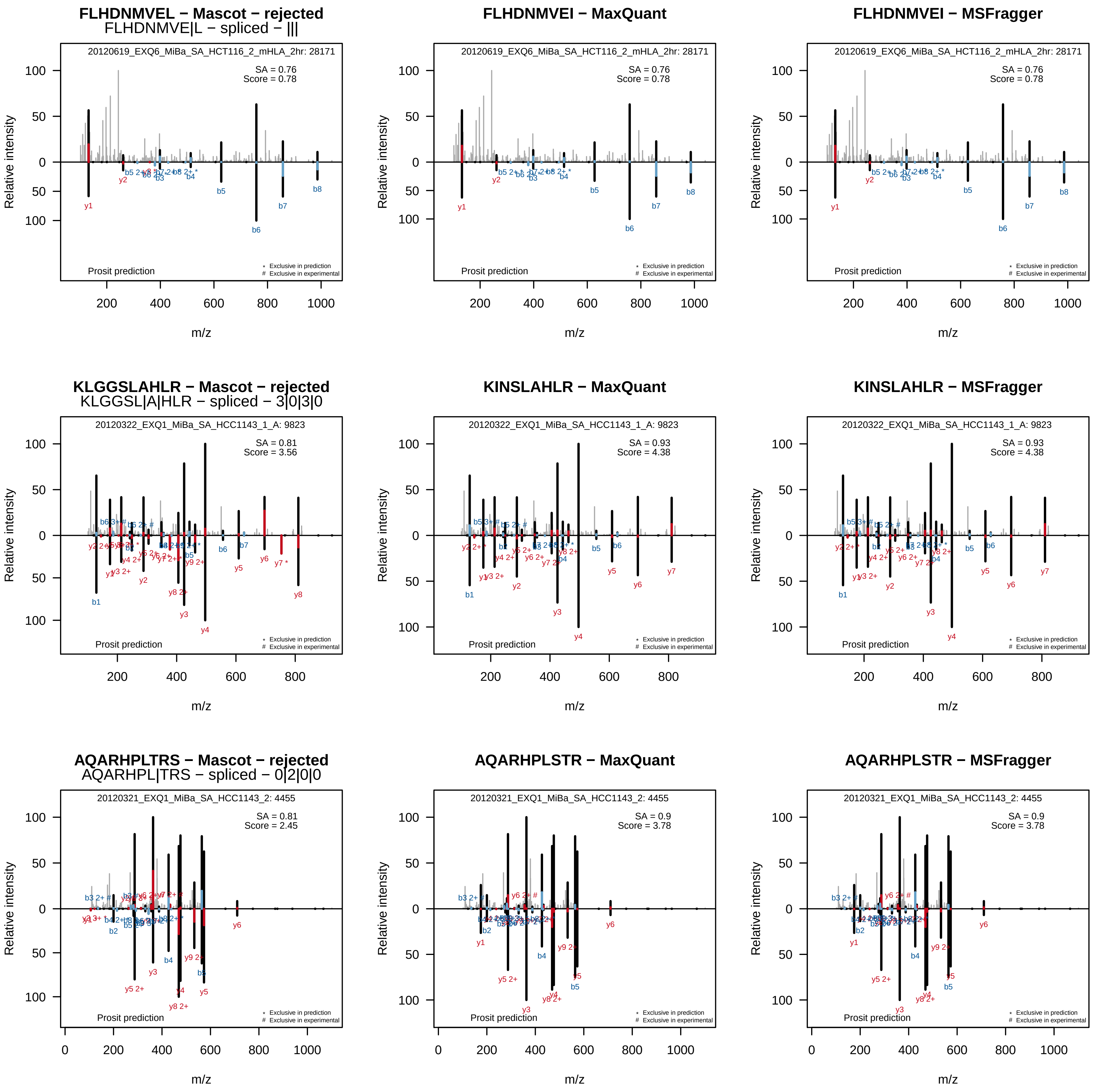

FLHDNMVEL − Mascot − rejected
FLHDNMVE|L − spliced − |||
20120619_EXQ6_MiBa_SA_HCT116_2_mHLA_2hr: 28171
SA = 0.76
Score = 0.78
FLHDNMVEI − MaxQuant
20120619_EXQ6_MiBa_SA_HCT116_2_mHLA_2hr: 28171
SA = 0.76
Score = 0.78
FLHDNMVEI − MSFragger
20120619_EXQ6_MiBa_SA_HCT116_2_mHLA_2hr: 28171
SA = 0.76
Score = 0.78
KLGGSLAHLR − Mascot − rejected
KLGGSL|A|HLR − spliced − 3|0|3|0
20120322_EXQ1_MiBa_SA_HCC1143_1_A: 9823
SA = 0.81
Score = 3.56
KINSLAHLR − MaxQuant
20120322_EXQ1_MiBa_SA_HCC1143_1_A: 9823
SA = 0.93
Score = 4.38
KINSLAHLR − MSFragger
20120322_EXQ1_MiBa_SA_HCC1143_1_A: 9823
SA = 0.93
Score = 4.38
AQARHPLTRS − Mascot − rejected
AQARHPL|TRS − spliced − 0|2|0|0
20120321_EXQ1_MiBa_SA_HCC1143_2: 4455
SA = 0.81
Score = 2.45
AQARHPLSTR − MaxQuant
20120321_EXQ1_MiBa_SA_HCC1143_2: 4455
SA = 0.9
Score = 3.78
AQARHPLSTR − MSFragger
20120321_EXQ1_MiBa_SA_HCC1143_2: 4455
SA = 0.9
Score = 3.78
Relative intensity
m/z
Prosit prediction
⋆ Exclusive in prediction
Exclusive in experimental

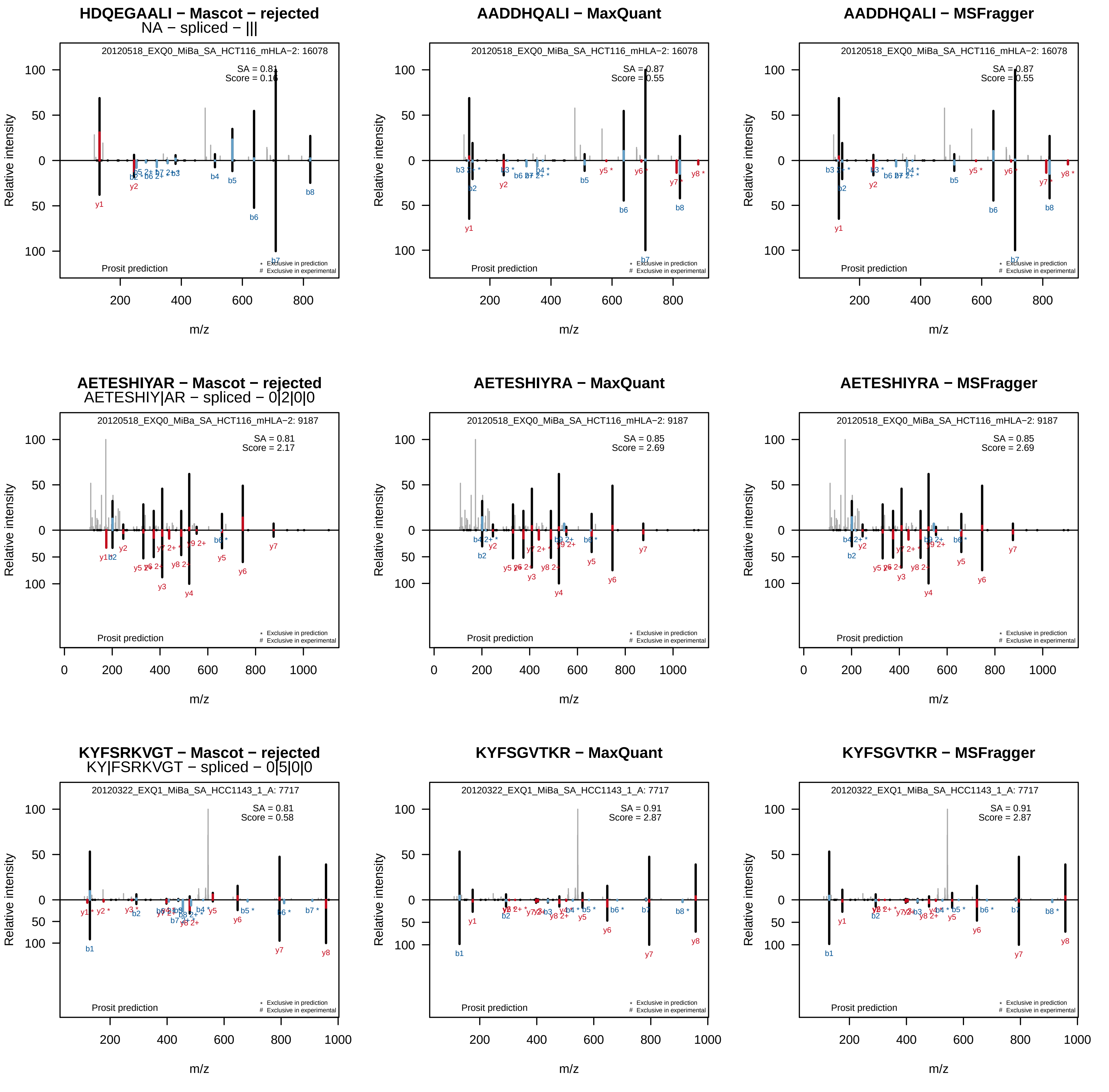

HDQEGAALI − Mascot − rejected
NA − spliced − |||
20120518_EXQ0_MiBa_SA_HCT116_mHLA−2: 16078
SA = 0.81
Score = 0.16
AADDHQALI − MaxQuant
20120518_EXQ0_MiBa_SA_HCT116_mHLA−2: 16078
SA = 0.87
Score = 0.55
AADDHQALI − MSFragger
20120518_EXQ0_MiBa_SA_HCT116_mHLA−2: 16078
SA = 0.87
Score = 0.55
AETESHIYAR − Mascot − rejected
AETESHIY|AR − spliced − 0|2|0|0
20120518_EXQ0_MiBa_SA_HCT116_mHLA−2: 9187
SA = 0.81
Score = 2.17
AETESHIYRA − MaxQuant
20120518_EXQ0_MiBa_SA_HCT116_mHLA−2: 9187
SA = 0.85
Score = 2.69
AETESHIYRA − MSFragger
20120518_EXQ0_MiBa_SA_HCT116_mHLA−2: 9187
SA = 0.85
Score = 2.69
KYFSRKVGT − Mascot − rejected
KY|FSRKVGT − spliced − 0|5|0|0
20120322_EXQ1_MiBa_SA_HCC1143_1_A: 7717
SA = 0.81
Score = 0.58
KYFSGVTKR − MaxQuant
20120322_EXQ1_MiBa_SA_HCC1143_1_A: 7717
SA = 0.91
Score = 2.87
KYFSGVTKR − MSFragger
20120322_EXQ1_MiBa_SA_HCC1143_1_A: 7717
SA = 0.91
Score = 2.87
Relative intensity
m/z
Prosit prediction
* Exclusive in prediction
# Exclusive in experimental

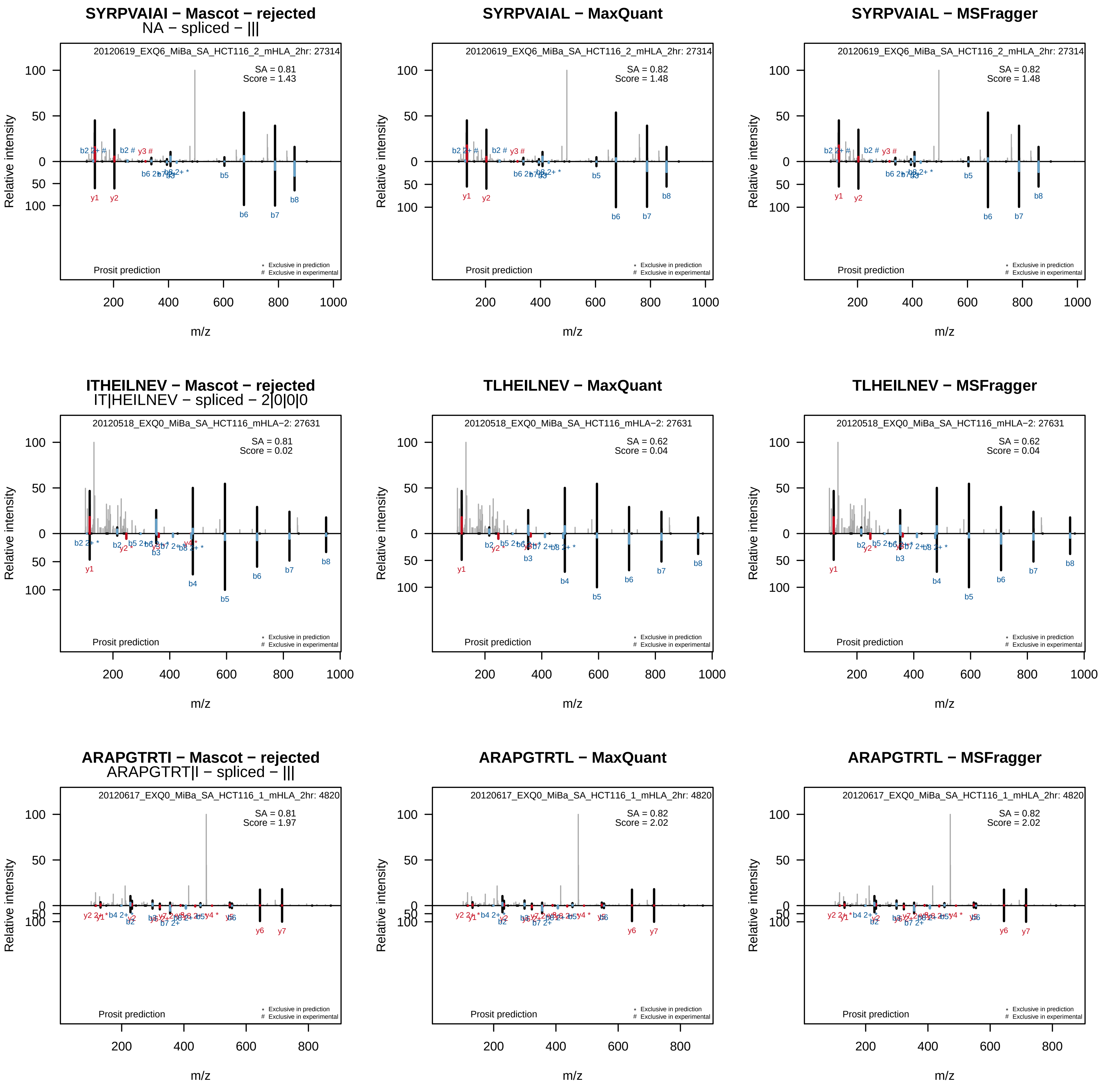
SYRPVAIAI − Mascot − rejected
NA − spliced − |||
20120619_EXQ6_MiBa_SA_HCT116_2_mHLA_2hr: 27314
SA = 0.81
Score = 1.43
SYRPVAIAL − MaxQuant
20120619_EXQ6_MiBa_SA_HCT116_2_mHLA_2hr: 27314
SA = 0.82
Score = 1.48
SYRPVAIAL − MSFragger
20120619_EXQ6_MiBa_SA_HCT116_2_mHLA_2hr: 27314
SA = 0.82
Score = 1.48
ITHEILNEV − Mascot − rejected
IT|HEILNEV − spliced − 2|0|0|0
20120518_EXQ0_MiBa_SA_HCT116_mHLA−2: 27631
SA = 0.81
Score = 0.02
TLHEILNEV − MaxQuant
20120518_EXQ0_MiBa_SA_HCT116_mHLA−2: 27631
SA = 0.62
Score = 0.04
TLHEILNEV − MSFragger
20120518_EXQ0_MiBa_SA_HCT116_mHLA−2: 27631
SA = 0.62
Score = 0.04
ARAPGTRTI − Mascot − rejected
ARAPGTRT|I − spliced − |||
20120617_EXQ0_MiBa_SA_HCT116_1_mHLA_2hr: 4820
SA = 0.81
Score = 1.97
ARAPGTRTL − MaxQuant
20120617_EXQ0_MiBa_SA_HCT116_1_mHLA_2hr: 4820
SA = 0.82
Score = 2.02
ARAPGTRTL − MSFragger
20120617_EXQ0_MiBa_SA_HCT116_1_mHLA_2hr: 4820
SA = 0.82
Score = 2.02
Relative intensity
m/z
Prosit prediction
* Exclusive in prediction
# Exclusive in experimental

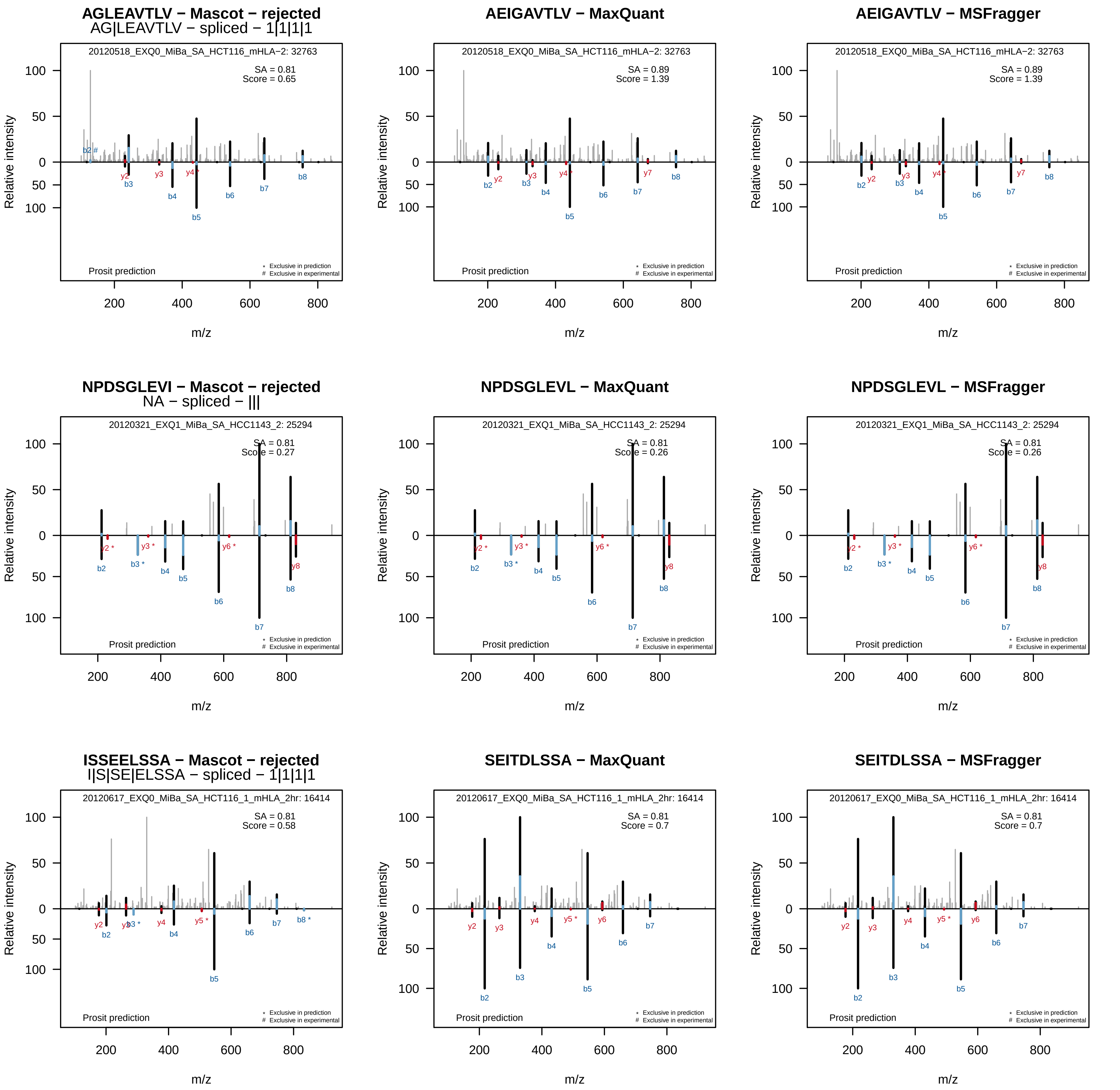
AGLEAVTLV − Mascot − rejected
AG|LEAVTLV − spliced − 1|1|1|1
20120518_EXQ0_MiBa_SA_HCT116_mHLA−2: 32763
SA = 0.81
Score = 0.65
AEIGAVTLV − MaxQuant
20120518_EXQ0_MiBa_SA_HCT116_mHLA−2: 32763
SA = 0.89
Score = 1.39
AEIGAVTLV − MSFragger
20120518_EXQ0_MiBa_SA_HCT116_mHLA−2: 32763
SA = 0.89
Score = 1.39
NPDSGLEVI − Mascot − rejected
NA − spliced − |||
20120321_EXQ1_MiBa_SA_HCC1143_2: 25294
SA = 0.81
Score = 0.27
NPDSGLEVL − MaxQuant
20120321_EXQ1_MiBa_SA_HCC1143_2: 25294
SA = 0.81
Score = 0.26
NPDSGLEVL − MSFragger
20120321_EXQ1_MiBa_SA_HCC1143_2: 25294
SA = 0.81
Score = 0.26
ISSEELSSA − Mascot − rejected
I|S|SE|ELSSA − spliced − 1|1|1|1
20120617_EXQ0_MiBa_SA_HCT116_1_mHLA_2hr: 16414
SA = 0.81
Score = 0.58
SEITDLSSA − MaxQuant
20120617_EXQ0_MiBa_SA_HCT116_1_mHLA_2hr: 16414
SA = 0.81
Score = 0.7
SEITDLSSA − MSFragger
20120617_EXQ0_MiBa_SA_HCT116_1_mHLA_2hr: 16414
SA = 0.81
Score = 0.7
Relative intensity
m/z
Prosit prediction
⋆ Exclusive in prediction
Exclusive in experimental

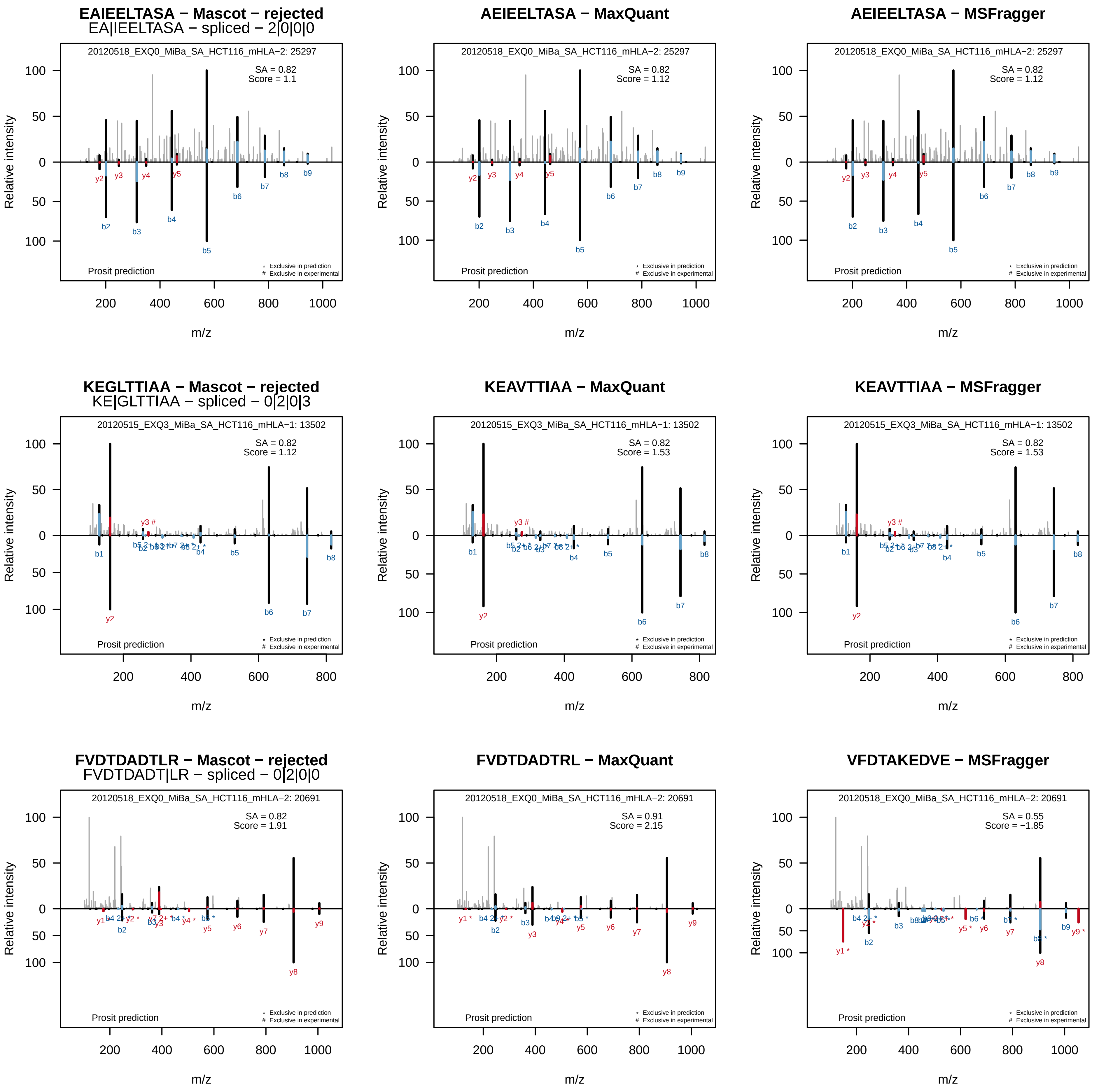

EAIEELTASA − Mascot − rejected
EA|IEELTASA − spliced − 2|0|0|0
20120518_EXQ0_MiBa_SA_HCT116_mHLA−2: 25297
SA = 0.82
Score = 1.1
AEIEELTASA − MaxQuant
20120518_EXQ0_MiBa_SA_HCT116_mHLA−2: 25297
SA = 0.82
Score = 1.12
AEIEELTASA − MSFragger
20120518_EXQ0_MiBa_SA_HCT116_mHLA−2: 25297
SA = 0.82
Score = 1.12
KEGLTTIAA − Mascot − rejected
KE|GLTTIAA − spliced − 0|2|0|3
20120515_EXQ3_MiBa_SA_HCT116_mHLA−1: 13502
SA = 0.82
Score = 1.12
KEAVTTIAA − MaxQuant
20120515_EXQ3_MiBa_SA_HCT116_mHLA−1: 13502
SA = 0.82
Score = 1.53
KEAVTTIAA − MSFragger
20120515_EXQ3_MiBa_SA_HCT116_mHLA−1: 13502
SA = 0.82
Score = 1.53
FVDTDADTLR − Mascot − rejected
FVDTDADT|LR − spliced − 0|2|0|0
20120518_EXQ0_MiBa_SA_HCT116_mHLA−2: 20691
SA = 0.82
Score = 1.91
FVDTDADTRL − MaxQuant
20120518_EXQ0_MiBa_SA_HCT116_mHLA−2: 20691
SA = 0.91
Score = 2.15
VFDTAKEDVE − MSFragger
20120518_EXQ0_MiBa_SA_HCT116_mHLA−2: 20691
SA = 0.55
Score = −1.85
Relative intensity
m/z
Prosit prediction
* Exclusive in prediction
# Exclusive in experimental

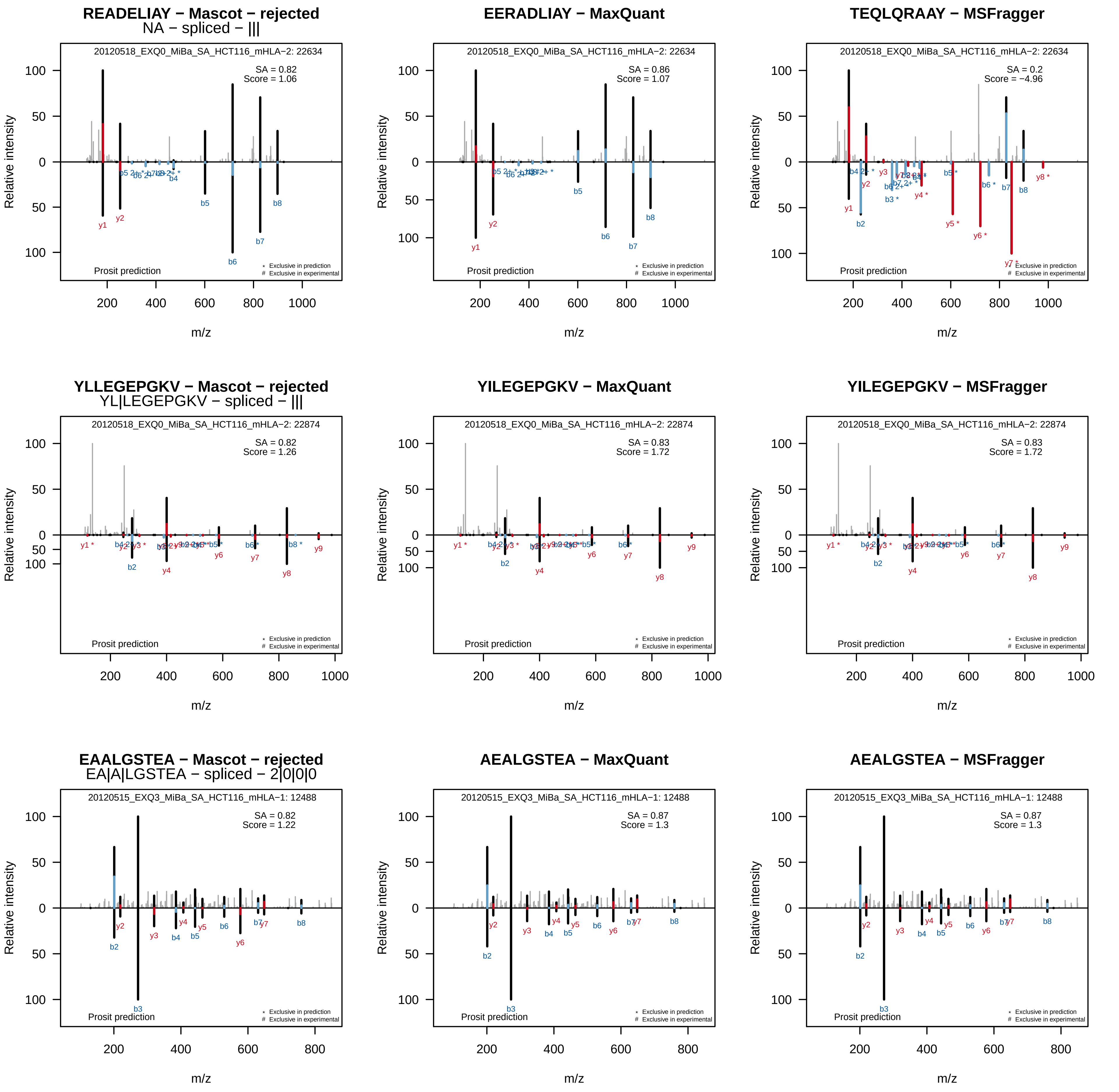

READEALIAY − Mascot − rejected
NA − spliced − |||
20120518_EXQ0_MiBa_SA_HCT116_mHLA−2: 22634
SA = 0.82
Score = 1.06
EERADLIAY − MaxQuant
20120518_EXQ0_MiBa_SA_HCT116_mHLA−2: 22634
SA = 0.86
Score = 1.07
TEQLQRAAY − MSFragger
20120518_EXQ0_MiBa_SA_HCT116_mHLA−2: 22634
SA = 0.2
Score = −4.96
YLLEGEPGKV − Mascot − rejected
YL|LEGEPGKV − spliced − |||
20120518_EXQ0_MiBa_SA_HCT116_mHLA−2: 22874
SA = 0.82
Score = 1.26
YILEGEPGKV − MaxQuant
20120518_EXQ0_MiBa_SA_HCT116_mHLA−2: 22874
SA = 0.83
Score = 1.72
YILEGEPGKV − MSFragger
20120518_EXQ0_MiBa_SA_HCT116_mHLA−2: 22874
SA = 0.83
Score = 1.72
EAALGSTEA − Mascot − rejected
EA|A|LGSTEA − spliced − 2|0|0|0
20120515_EXQ3_MiBa_SA_HCT116_mHLA−1: 12488
SA = 0.82
Score = 1.22
AEALGSTEA − MaxQuant
20120515_EXQ3_MiBa_SA_HCT116_mHLA−1: 12488
SA = 0.87
Score = 1.3
AEALGSTEA − MSFragger
20120515_EXQ3_MiBa_SA_HCT116_mHLA−1: 12488
SA = 0.87
Score = 1.3
Relative intensity
m/z
Prosit prediction
⋆ Exclusive in prediction
Exclusive in experimental

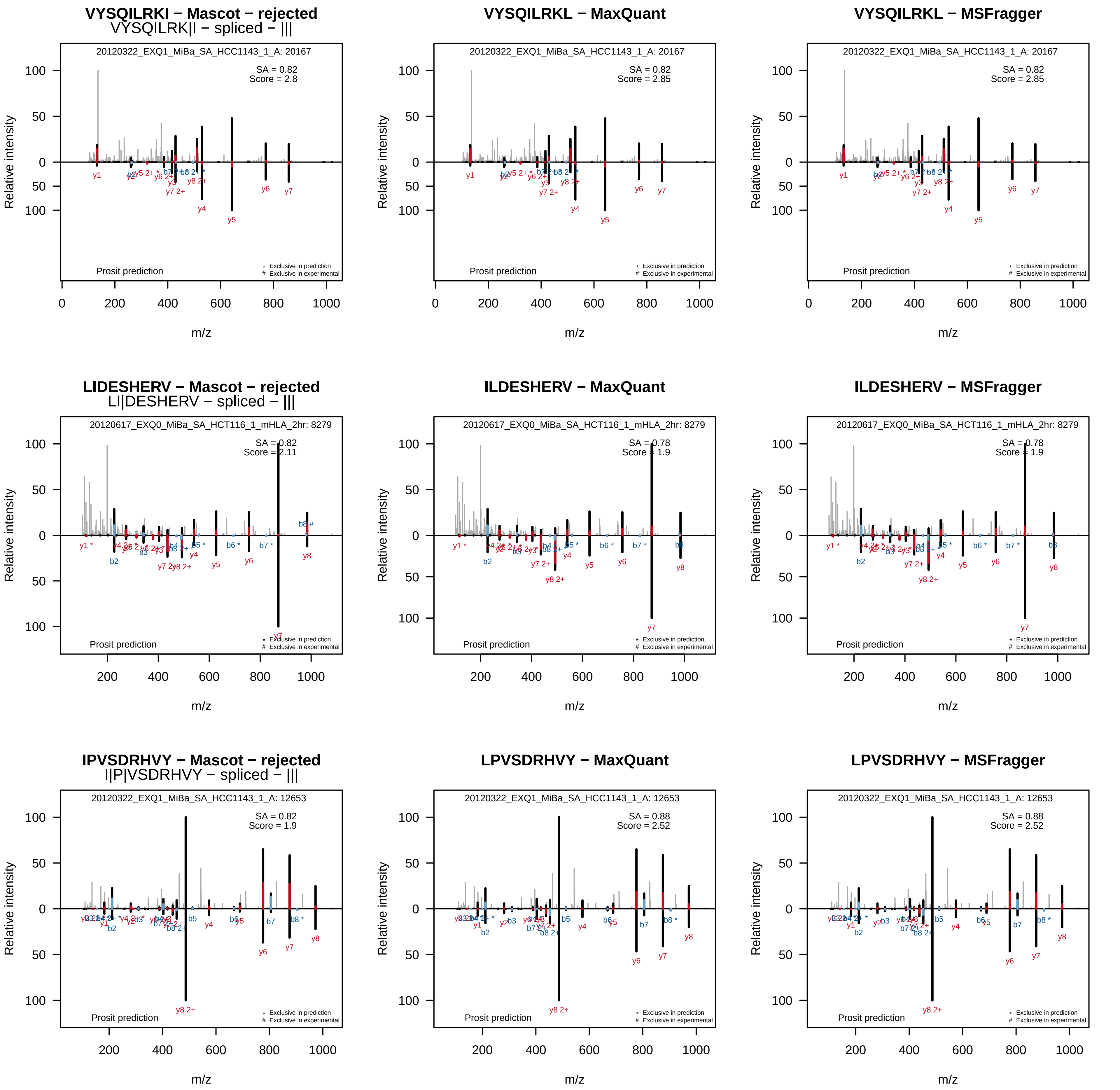
VYSQILRKI − Mascot − rejected
VYSQILRK|I − spliced − |||
20120322_EXQ1_MiBa_SA_HCC1143_1_A: 20167
SA = 0.82
Score = 2.8
VYSQILRKL − MaxQuant
20120322_EXQ1_MiBa_SA_HCC1143_1_A: 20167
SA = 0.82
Score = 2.85
VYSQILRKL − MSFragger
20120322_EXQ1_MiBa_SA_HCC1143_1_A: 20167
SA = 0.82
Score = 2.85
LIDESHERV − Mascot − rejected
LI|DESHERV − spliced − |||
20120617_EXQ0_MiBa_SA_HCT116_1_mHLA_2hr: 8279
SA = 0.82
Score = 2.11
ILDESHERV − MaxQuant
20120617_EXQ0_MiBa_SA_HCT116_1_mHLA_2hr: 8279
SA = 0.78
Score = 1.9
ILDESHERV − MSFragger
20120617_EXQ0_MiBa_SA_HCT116_1_mHLA_2hr: 8279
SA = 0.78
Score = 1.9
IPVSDRHVY − Mascot − rejected
I|P|VSDRHVY − spliced − |||
20120322_EXQ1_MiBa_SA_HCC1143_1_A: 12653
SA = 0.82
Score = 1.9
LPVSDRHVY − MaxQuant
20120322_EXQ1_MiBa_SA_HCC1143_1_A: 12653
SA = 0.88
Score = 2.52
LPVSDRHVY − MSFragger
20120322_EXQ1_MiBa_SA_HCC1143_1_A: 12653
SA = 0.88
Score = 2.52
Relative intensity
m/z
Prosit prediction
* Exclusive in prediction
# Exclusive in experimental

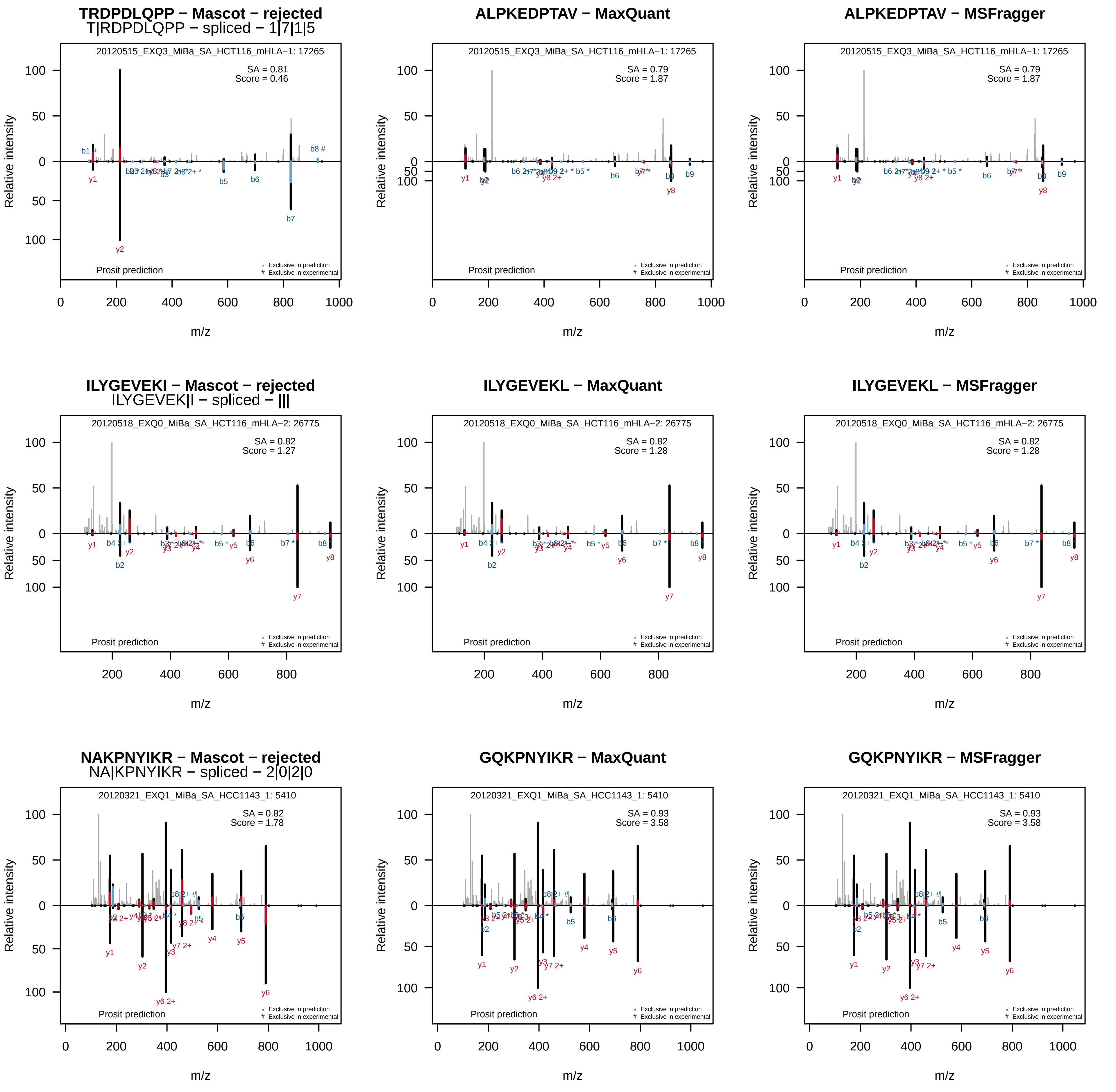
TRDPDLQPP − Mascot − rejected
T|RDPDLQPP − spliced − 1|7|1|5
20120515_EXQ3_MiBa_SA_HCT116_mHLA−1: 17265
SA = 0.81
Score = 0.46
ALPKEDPTAV − MaxQuant
20120515_EXQ3_MiBa_SA_HCT116_mHLA−1: 17265
SA = 0.79
Score = 1.87
ALPKEDPTAV − MSFragger
20120515_EXQ3_MiBa_SA_HCT116_mHLA−1: 17265
SA = 0.79
Score = 1.87
ILYGEVEKI − Mascot − rejected
ILYGEVEK|I − spliced − |||
20120518_EXQ0_MiBa_SA_HCT116_mHLA−2: 26775
SA = 0.82
Score = 1.27
ILYGEVEKL − MaxQuant
20120518_EXQ0_MiBa_SA_HCT116_mHLA−2: 26775
SA = 0.82
Score = 1.28
ILYGEVEKL − MSFragger
20120518_EXQ0_MiBa_SA_HCT116_mHLA−2: 26775
SA = 0.82
Score = 1.28
NAKPNYIKR − Mascot − rejected
NA|KPNYIKR − spliced − 2|0|2|0
20120321_EXQ1_MiBa_SA_HCC1143_1: 5410
SA = 0.82
Score = 1.78
GQKPNYIKR − MaxQuant
20120321_EXQ1_MiBa_SA_HCC1143_1: 5410
SA = 0.93
Score = 3.58
GQKPNYIKR − MSFragger
20120321_EXQ1_MiBa_SA_HCC1143_1: 5410
SA = 0.93
Score = 3.58
Relative intensity
m/z
Prosit prediction
⋆ Exclusive in prediction
Exclusive in experimental

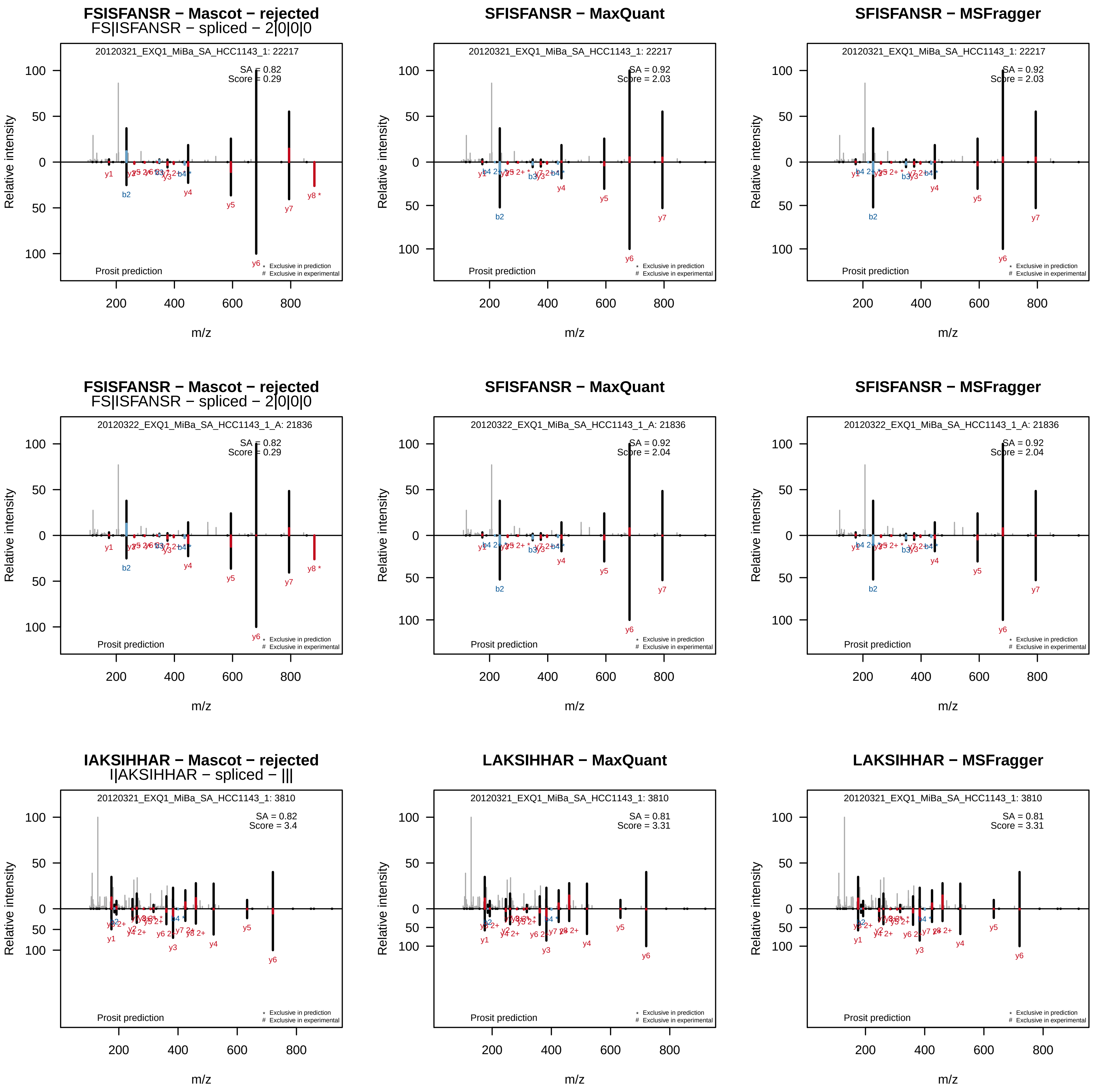
FSISFANSR − Mascot − rejected
FS|ISFANSR − spliced − 2|0|0|0
20120321_EXQ1_MiBa_SA_HCC1143_1: 22217
SA = 0.82
Score = 0.29
Prosit prediction
SFISFANSR − MaxQuant
20120321_EXQ1_MiBa_SA_HCC1143_1: 22217
SA = 0.92
Score = 2.03
Prosit prediction
SFISFANSR − MSFragger
20120321_EXQ1_MiBa_SA_HCC1143_1: 22217
SA = 0.92
Score = 2.03
Prosit prediction
FSISFANSR − Mascot − rejected
FS|ISFANSR − spliced − 2|0|0|0
20120322_EXQ1_MiBa_SA_HCC1143_1_A: 21836
SA = 0.82
Score = 0.29
Prosit prediction
SFISFANSR − MaxQuant
20120322_EXQ1_MiBa_SA_HCC1143_1_A: 21836
SA = 0.92
Score = 2.04
Prosit prediction
SFISFANSR − MSFragger
20120322_EXQ1_MiBa_SA_HCC1143_1_A: 21836
SA = 0.92
Score = 2.04
Prosit prediction
IAKSIHHAR − Mascot − rejected
I|AKSIHHAR − spliced − |||
20120321_EXQ1_MiBa_SA_HCC1143_1: 3810
SA = 0.82
Score = 3.4
Prosit prediction
LAKSIHHAR − MaxQuant
20120321_EXQ1_MiBa_SA_HCC1143_1: 3810
SA = 0.81
Score = 3.31
Prosit prediction
LAKSIHHAR − MSFragger
20120321_EXQ1_MiBa_SA_HCC1143_1: 3810
SA = 0.81
Score = 3.31
Prosit prediction
Relative intensity
m/z
* Exclusive in prediction
# Exclusive in experimental

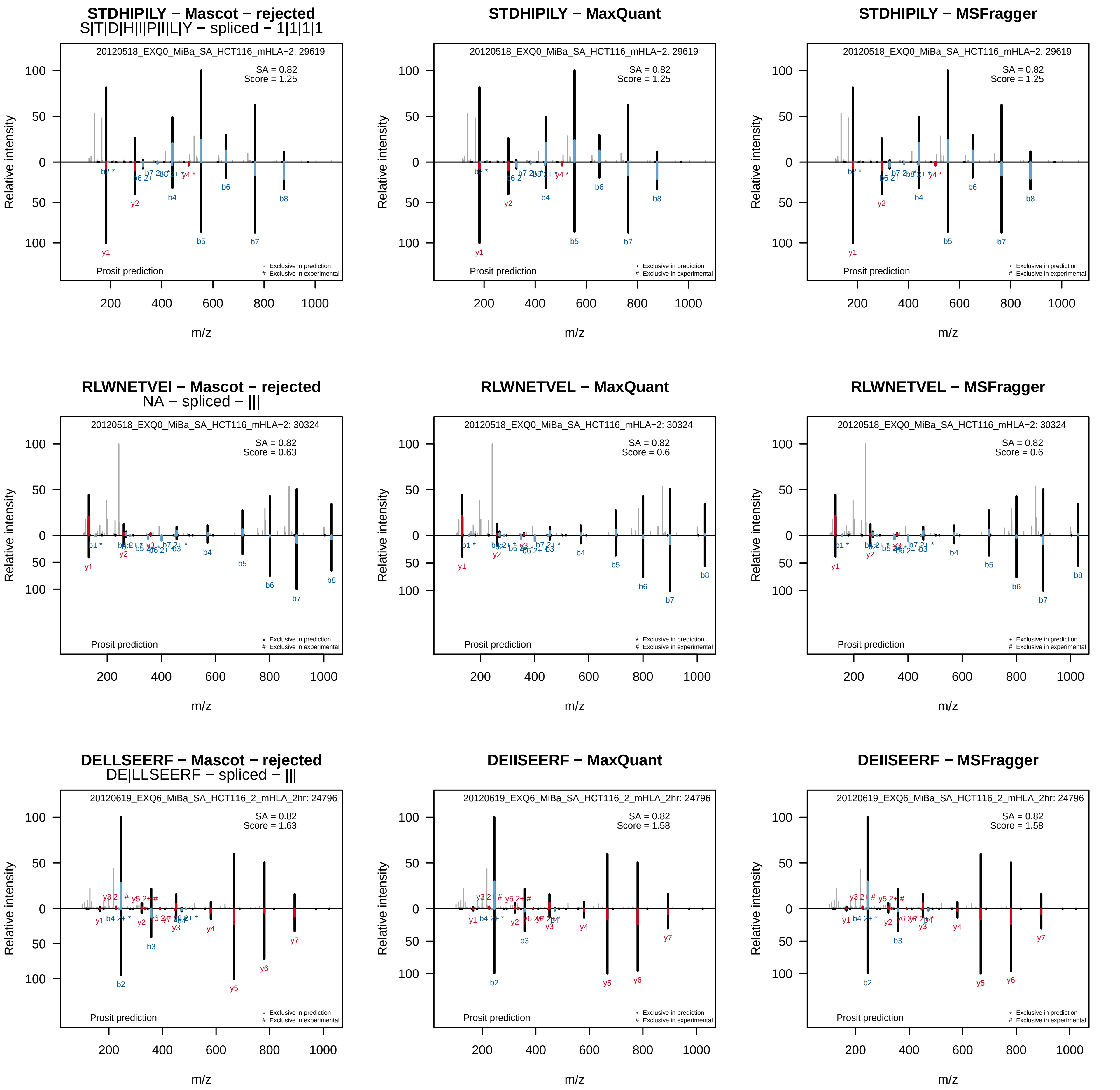
STDHIPILY − Mascot − rejected
S|T|D|H|I|P|I|L|Y − spliced − 1|1|1|1
STDHIPILY − MaxQuant
STDHIPILY − MSFragger
20120518_EXQ0_MiBa_SA_HCT116_mHLA−2: 29619
SA = 0.82
Score = 1.25
RLWNETVEI − Mascot − rejected
NA − spliced − |||
RLWNETVEL − MaxQuant
RLWNETVEL − MSFragger
20120518_EXQ0_MiBa_SA_HCT116_mHLA−2: 30324
SA = 0.82
Score = 0.63
Score = 0.6
DELLSEERF − Mascot − rejected
DE|LLSEERF − spliced − |||
DEIISEERF − MaxQuant
DEIISEERF − MSFragger
20120619_EXQ6_MiBa_SA_HCT116_2_mHLA_2hr: 24796
SA = 0.82
Score = 1.63
Score = 1.58
Relative intensity
m/z
Prosit prediction
⋆ Exclusive in prediction
Exclusive in experimental

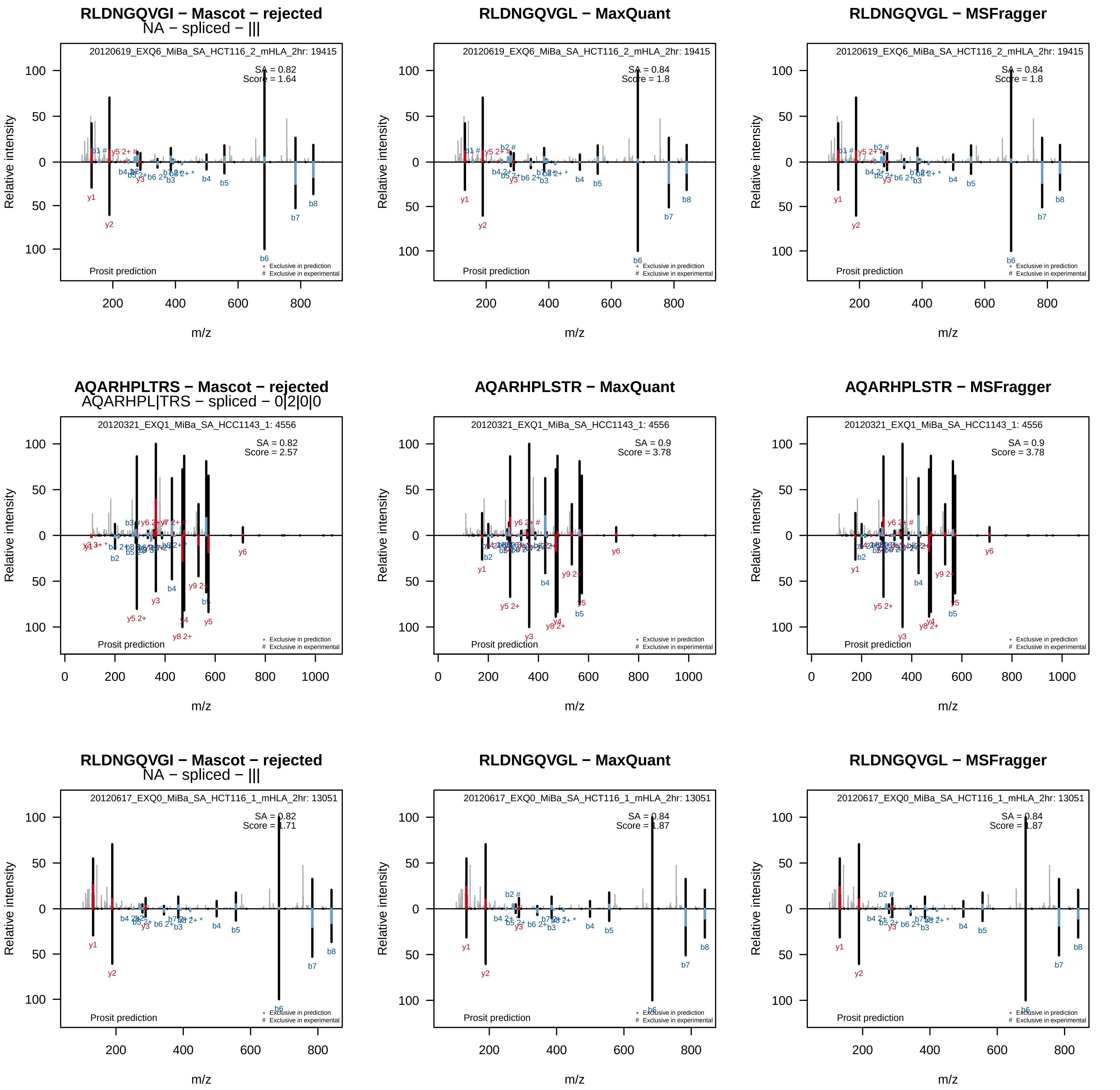

RLDNGQVGI − Mascot − rejected
NA − spliced − |||
20120619_EXQ6_MiBa_SA_HCT116_2_mHLA_2hr: 19415
SA = 0.82
Score = 1.64
Prosit prediction
Exclusive in prediction
Exclusive in experimental
Relative intensity
m/z
RLDNGQVGL − MaxQuant
20120619_EXQ6_MiBa_SA_HCT116_2_mHLA_2hr: 19415
SA = 0.84
Score = 1.8
RLDNGQVGL − MSFragger
20120619_EXQ6_MiBa_SA_HCT116_2_mHLA_2hr: 19415
SA = 0.84
Score = 1.8
AQARHPLTRS − Mascot − rejected
AQARHPL|TRS − spliced − 0|2|0|0
20120321_EXQ1_MiBa_SA_HCC1143_1: 4556
SA = 0.82
Score = 2.57
AQARHPLSTR − MaxQuant
20120321_EXQ1_MiBa_SA_HCC1143_1: 4556
SA = 0.9
Score = 3.78
AQARHPLSTR − MSFragger
20120321_EXQ1_MiBa_SA_HCC1143_1: 4556
SA = 0.9
Score = 3.78
RLDNGQVGI − Mascot − rejected
NA − spliced − |||
20120617_EXQ0_MiBa_SA_HCT116_1_mHLA_2hr: 13051
SA = 0.82
Score = 1.71
RLDNGQVGL − MaxQuant
20120617_EXQ0_MiBa_SA_HCT116_1_mHLA_2hr: 13051
SA = 0.84
Score = 1.87
RLDNGQVGL − MSFragger
20120617_EXQ0_MiBa_SA_HCT116_1_mHLA_2hr: 13051
SA = 0.84
Score = 1.87

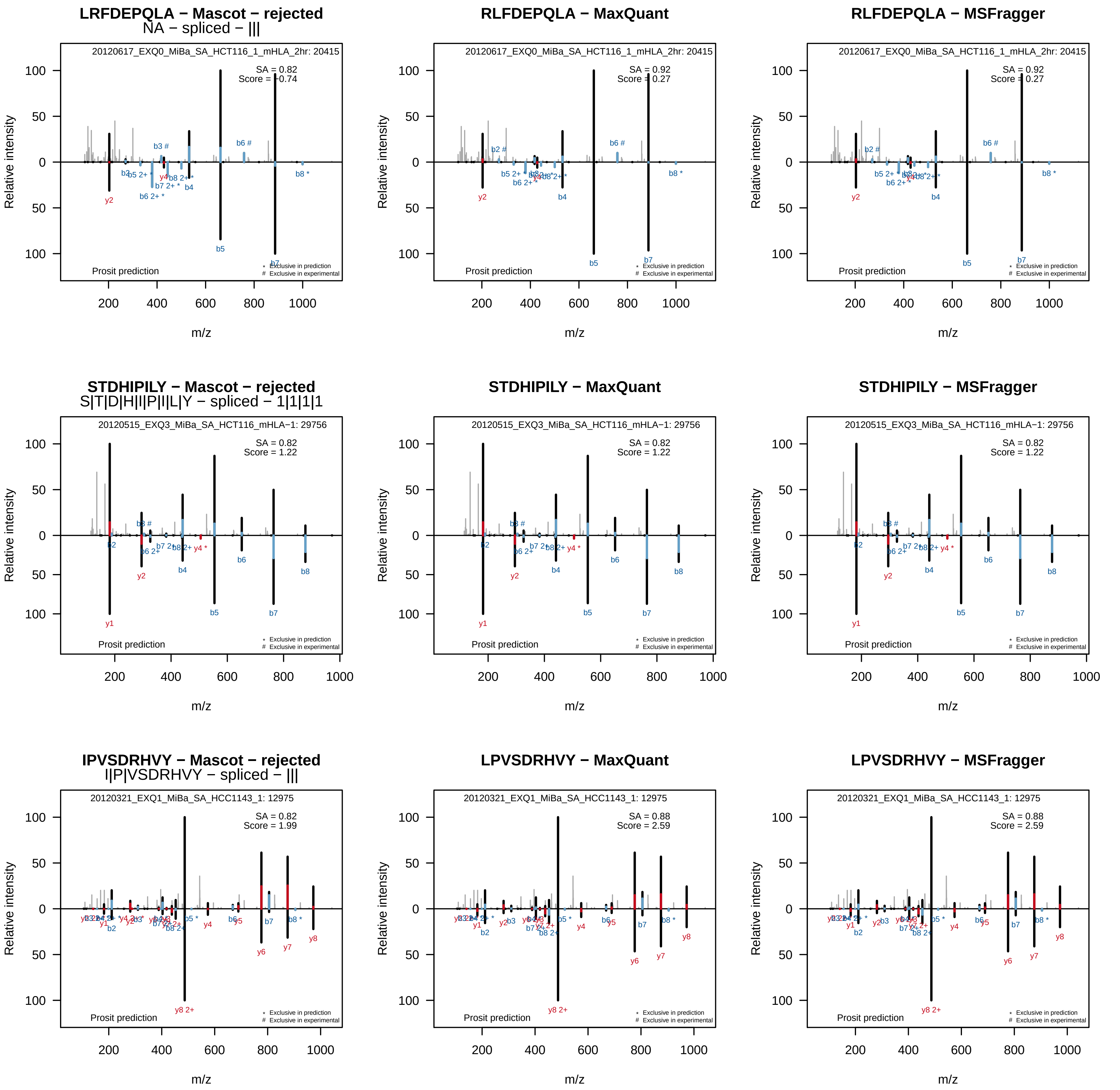

LRFDEPQLA − Mascot − rejected
NA − spliced − |||
20120617_EXQ0_MiBa_SA_HCT116_1_mHLA_2hr: 20415
SA = 0.82
Score = −0.74
RLFDEPQLA − MaxQuant
20120617_EXQ0_MiBa_SA_HCT116_1_mHLA_2hr: 20415
SA = 0.92
Score = 0.27
RLFDEPQLA − MSFragger
20120617_EXQ0_MiBa_SA_HCT116_1_mHLA_2hr: 20415
SA = 0.92
Score = 0.27
STDHIPILY − Mascot − rejected
S|T|D|H|I|P|I|L|Y − spliced − 1|1|1|1
20120515_EXQ3_MiBa_SA_HCT116_mHLA−1: 29756
SA = 0.82
Score = 1.22
STDHIPILY − MaxQuant
20120515_EXQ3_MiBa_SA_HCT116_mHLA−1: 29756
SA = 0.82
Score = 1.22
STDHIPILY − MSFragger
20120515_EXQ3_MiBa_SA_HCT116_mHLA−1: 29756
SA = 0.82
Score = 1.22
IPVSDRHVY − Mascot − rejected
I|P|VSDRHVY − spliced − |||
20120321_EXQ1_MiBa_SA_HCC1143_1: 12975
SA = 0.82
Score = 1.99
LPVSDRHVY − MaxQuant
20120321_EXQ1_MiBa_SA_HCC1143_1: 12975
SA = 0.88
Score = 2.59
LPVSDRHVY − MSFragger
20120321_EXQ1_MiBa_SA_HCC1143_1: 12975
SA = 0.88
Score = 2.59
Relative intensity
m/z
Prosit prediction
* Exclusive in prediction
# Exclusive in experimental

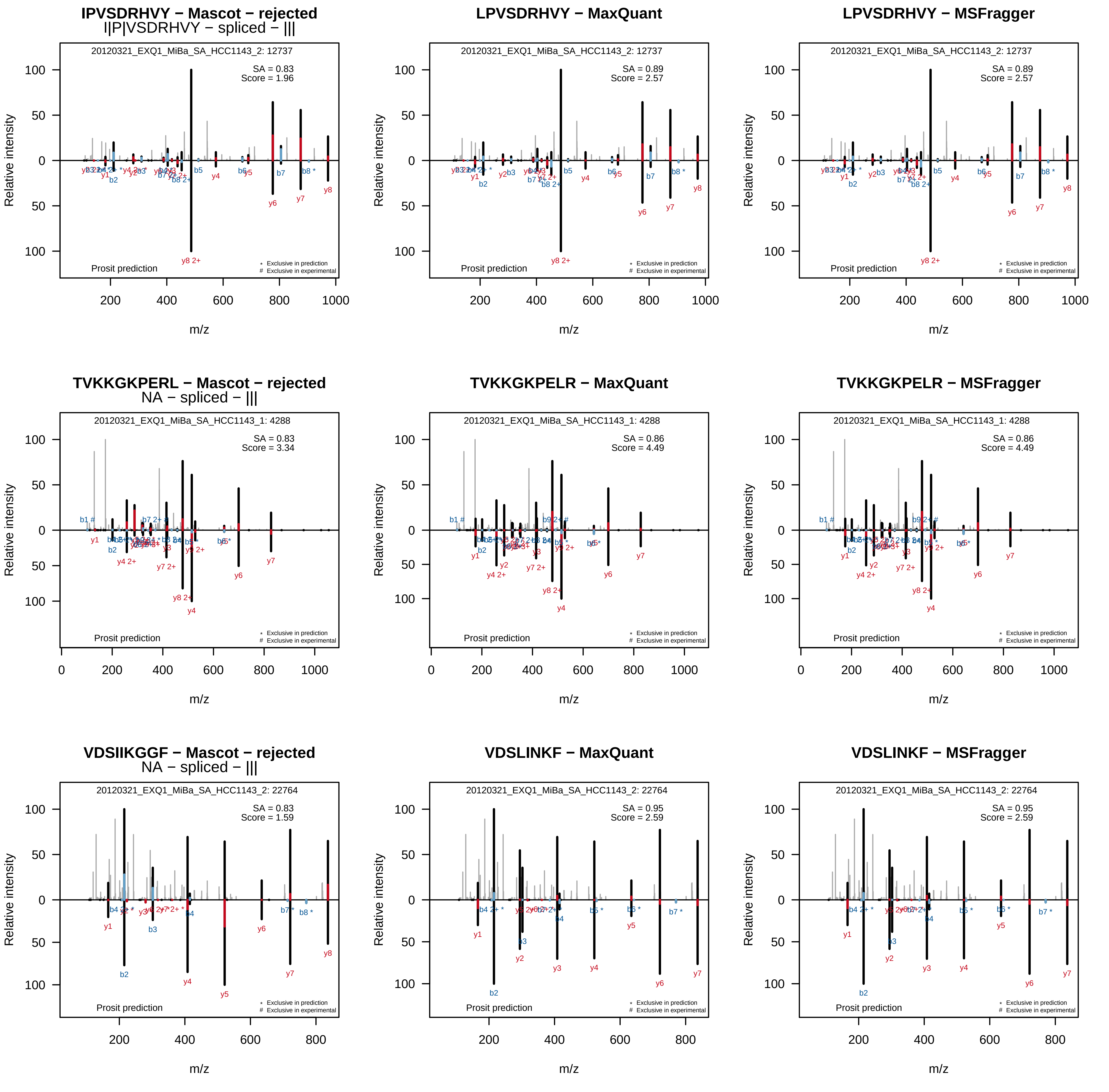

IPVSDRHVY − Mascot − rejected
I|P|VSDRHVY − spliced − |||
20120321_EXQ1_MiBa_SA_HCC1143_2: 12737
SA = 0.83
Score = 1.96
LPVSDRHVY − MaxQuant
20120321_EXQ1_MiBa_SA_HCC1143_2: 12737
SA = 0.89
Score = 2.57
LPVSDRHVY − MSFragger
20120321_EXQ1_MiBa_SA_HCC1143_2: 12737
SA = 0.89
Score = 2.57
TVKKGKPERL − Mascot − rejected
NA − spliced − |||
20120321_EXQ1_MiBa_SA_HCC1143_1: 4288
SA = 0.83
Score = 3.34
TVKKGKPELR − MaxQuant
20120321_EXQ1_MiBa_SA_HCC1143_1: 4288
SA = 0.86
Score = 4.49
TVKKGKPELR − MSFragger
20120321_EXQ1_MiBa_SA_HCC1143_1: 4288
SA = 0.86
Score = 4.49
VDSIIKGGF − Mascot − rejected
NA − spliced − |||
20120321_EXQ1_MiBa_SA_HCC1143_2: 22764
SA = 0.83
Score = 1.59
VDSLINKF − MaxQuant
20120321_EXQ1_MiBa_SA_HCC1143_2: 22764
SA = 0.95
Score = 2.59
VDSLINKF − MSFragger
20120321_EXQ1_MiBa_SA_HCC1143_2: 22764
SA = 0.95
Score = 2.59
Relative intensity
m/z
Prosit prediction
* Exclusive in prediction
# Exclusive in experimental

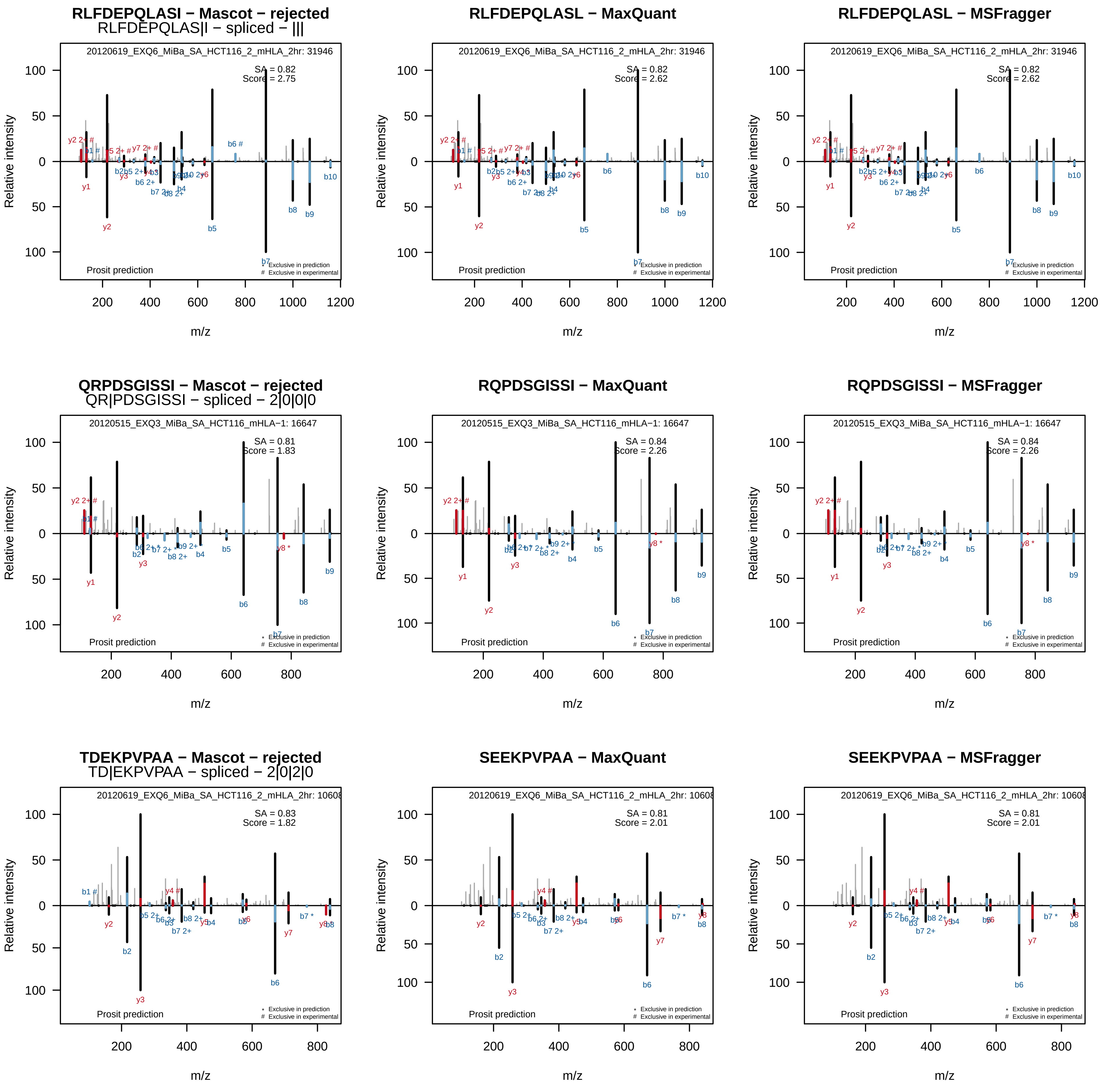
RLFDEPQLASI − Mascot − rejected
RLFDEPQLAS|I − spliced − |||
20120619_EXQ6_MiBa_SA_HCT116_2_mHLA_2hr: 31946
SA = 0.82
Score = 2.75
RLFDEPQLASL − MaxQuant
20120619_EXQ6_MiBa_SA_HCT116_2_mHLA_2hr: 31946
SA = 0.82
Score = 2.62
RLFDEPQLASL − MSFragger
20120619_EXQ6_MiBa_SA_HCT116_2_mHLA_2hr: 31946
SA = 0.82
Score = 2.62
QRPDSGISSI − Mascot − rejected
QR|PDSGISSI − spliced − 2|0|0|0
20120515_EXQ3_MiBa_SA_HCT116_mHLA−1: 16647
SA = 0.81
Score = 1.83
RQPDSGISSI − MaxQuant
20120515_EXQ3_MiBa_SA_HCT116_mHLA−1: 16647
SA = 0.84
Score = 2.26
RQPDSGISSI − MSFragger
20120515_EXQ3_MiBa_SA_HCT116_mHLA−1: 16647
SA = 0.84
Score = 2.26
TDEKPVPAA − Mascot − rejected
TD|EKPVPAA − spliced − 2|0|2|0
20120619_EXQ6_MiBa_SA_HCT116_2_mHLA_2hr: 10608
SA = 0.83
Score = 1.82
SEEKPVPAA − MaxQuant
20120619_EXQ6_MiBa_SA_HCT116_2_mHLA_2hr: 10608
SA = 0.81
Score = 2.01
SEEKPVPAA − MSFragger
20120619_EXQ6_MiBa_SA_HCT116_2_mHLA_2hr: 10608
SA = 0.81
Score = 2.01
Relative intensity
m/z
Prosit prediction
* Exclusive in prediction
# Exclusive in experimental

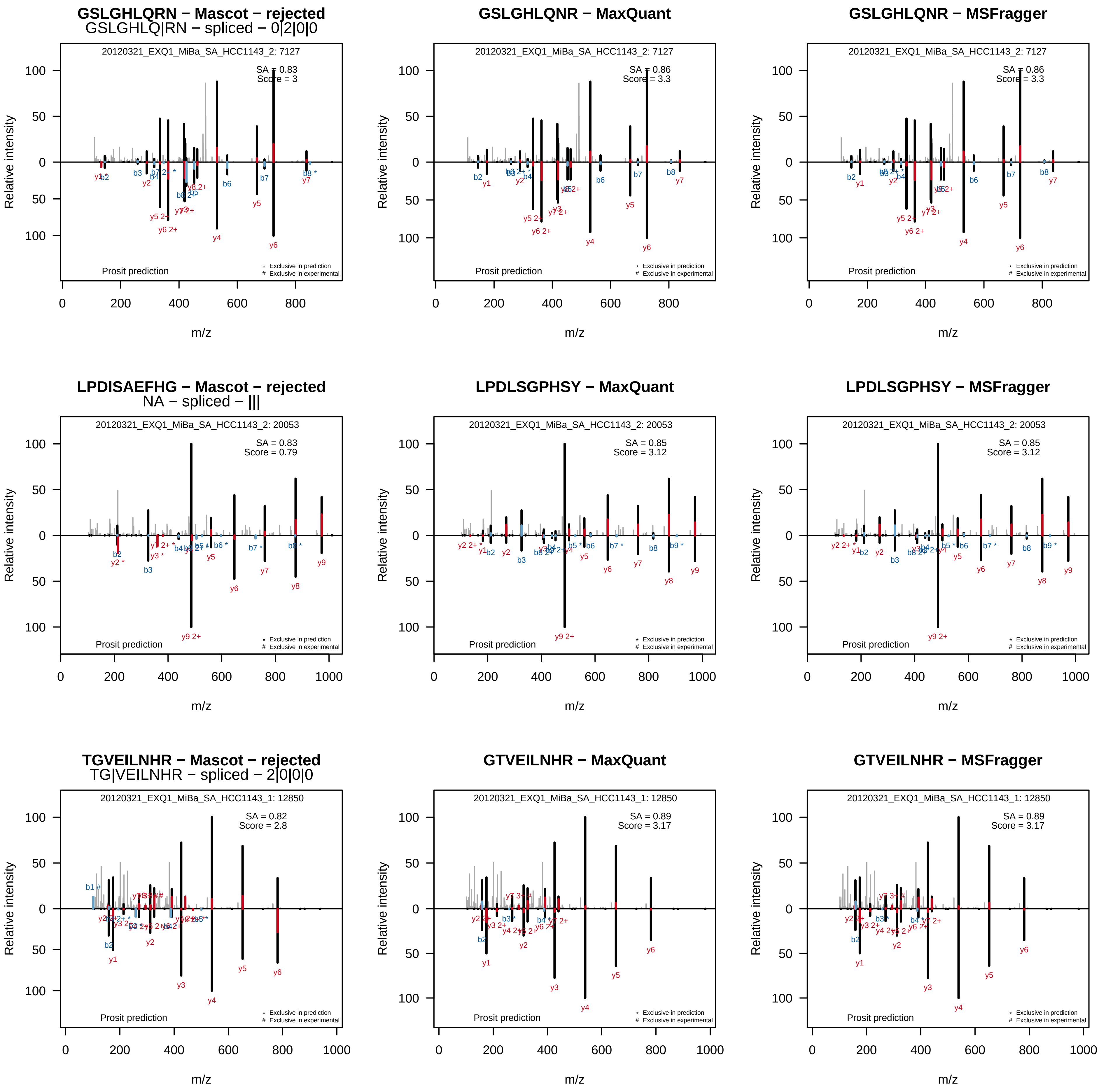
GSLGHLQRN − Mascot − rejected
GSLGHLQ|RN − spliced − 0|2|0|0
20120321_EXQ1_MiBa_SA_HCC1143_2: 7127
SA = 0.83
Score = 3
GSLGHLQNR − MaxQuant
20120321_EXQ1_MiBa_SA_HCC1143_2: 7127
SA = 0.86
Score = 3.3
GSLGHLQNR − MSFragger
20120321_EXQ1_MiBa_SA_HCC1143_2: 7127
SA = 0.86
Score = 3.3
LPDISAEFHG − Mascot − rejected
NA − spliced − |||
20120321_EXQ1_MiBa_SA_HCC1143_2: 20053
SA = 0.83
Score = 0.79
LPDLSGPHSY − MaxQuant
20120321_EXQ1_MiBa_SA_HCC1143_2: 20053
SA = 0.85
Score = 3.12
LPDLSGPHSY − MSFragger
20120321_EXQ1_MiBa_SA_HCC1143_2: 20053
SA = 0.85
Score = 3.12
TGVEILNHR − Mascot − rejected
TG|VEILNHR − spliced − 2|0|0|0
20120321_EXQ1_MiBa_SA_HCC1143_1: 12850
SA = 0.82
Score = 2.8
GTVEILNHR − MaxQuant
20120321_EXQ1_MiBa_SA_HCC1143_1: 12850
SA = 0.89
Score = 3.17
GTVEILNHR − MSFragger
20120321_EXQ1_MiBa_SA_HCC1143_1: 12850
SA = 0.89
Score = 3.17
Relative intensity
m/z
Prosit prediction
* Exclusive in prediction
# Exclusive in experimental

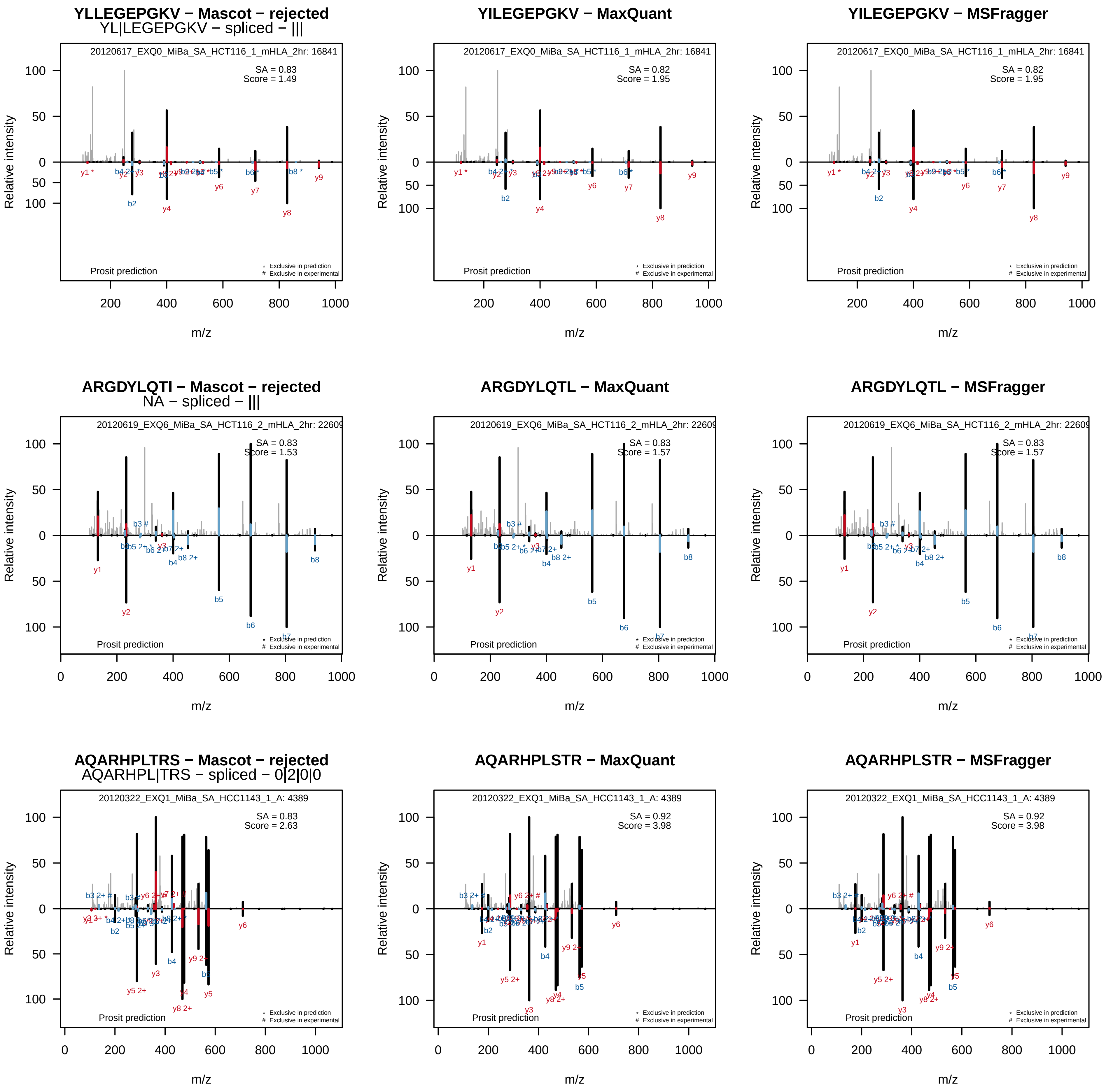

YLLEGEPGKV − Mascot − rejected
YL|LEGEPGKV − spliced − |||
YILEGEPGKV − MaxQuant
YILEGEPGKV − MSFragger
20120617_EXQ0_MiBa_SA_HCT116_1_mHLA_2hr: 16841
SA = 0.83
Score = 1.49
SA = 0.82
Score = 1.95
ARGDYLQTI − Mascot − rejected
NA − spliced − |||
ARGDYLQTL − MaxQuant
ARGDYLQTL − MSFragger
20120619_EXQ6_MiBa_SA_HCT116_2_mHLA_2hr: 22609
SA = 0.83
Score = 1.53
Score = 1.57
AQARHPLTRS − Mascot − rejected
AQARHPL|TRS − spliced − 0|2|0|0
AQARHPLSTR − MaxQuant
AQARHPLSTR − MSFragger
20120322_EXQ1_MiBa_SA_HCC1143_1_A: 4389
SA = 0.83
Score = 2.63
SA = 0.92
Score = 3.98
Relative intensity
m/z
Prosit prediction
* Exclusive in prediction
# Exclusive in experimental

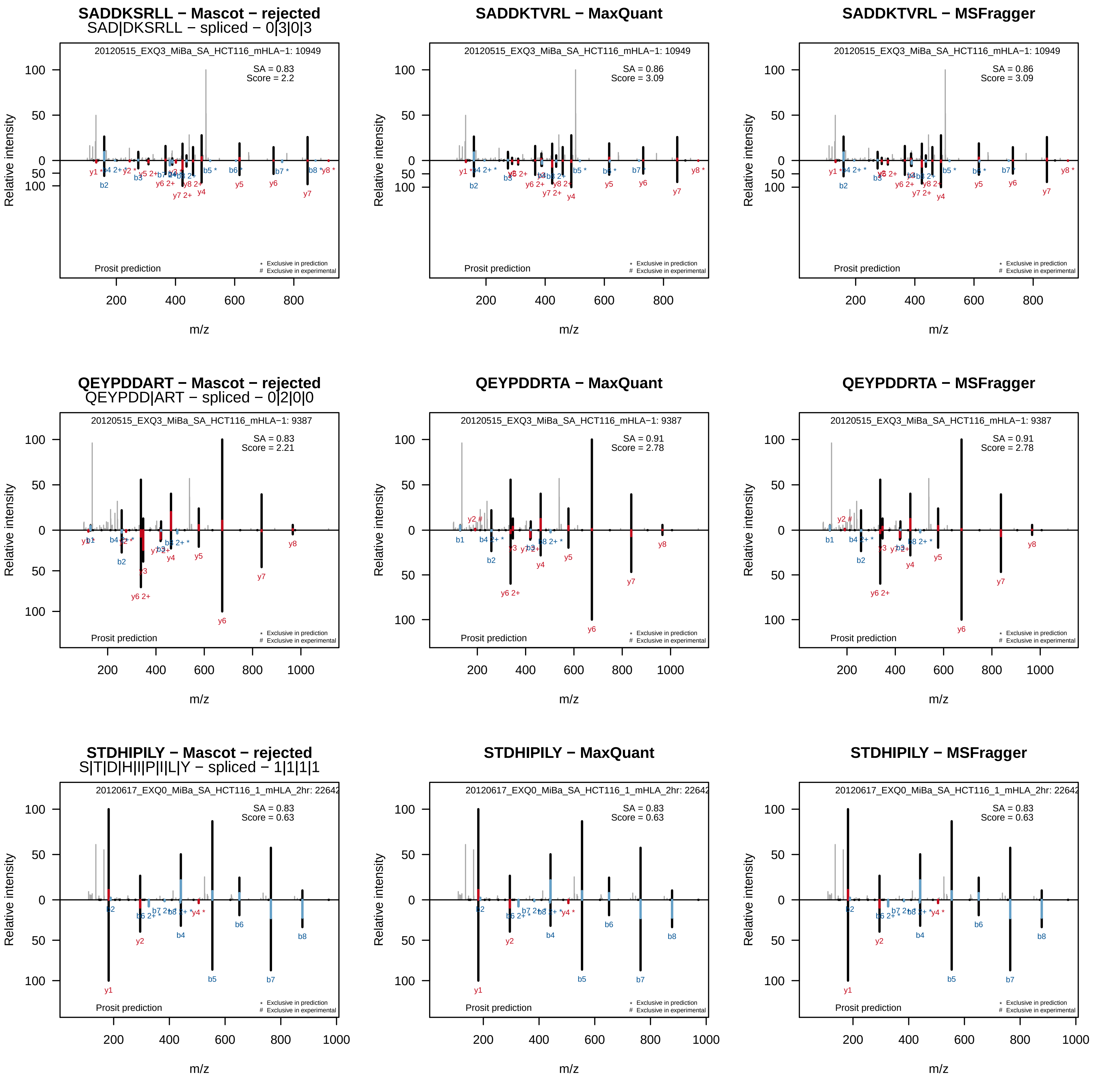
SADDKSRLL − Mascot − rejected
SAD|DKSRLL − spliced − 0|3|0|3
20120515_EXQ3_MiBa_SA_HCT116_mHLA−1: 10949
SA = 0.83
Score = 2.2
SADDKTVRL − MaxQuant
20120515_EXQ3_MiBa_SA_HCT116_mHLA−1: 10949
SA = 0.86
Score = 3.09
SADDKTVRL − MSFragger
20120515_EXQ3_MiBa_SA_HCT116_mHLA−1: 10949
SA = 0.86
Score = 3.09
QEYPDDART − Mascot − rejected
QEYPDD|ART − spliced − 0|2|0|0
20120515_EXQ3_MiBa_SA_HCT116_mHLA−1: 9387
SA = 0.83
Score = 2.21
QEYPDDRTA − MaxQuant
20120515_EXQ3_MiBa_SA_HCT116_mHLA−1: 9387
SA = 0.91
Score = 2.78
QEYPDDRTA − MSFragger
20120515_EXQ3_MiBa_SA_HCT116_mHLA−1: 9387
SA = 0.91
Score = 2.78
STDHIPILY − Mascot − rejected
S|T|D|H|I|P|I|L|Y − spliced − 1|1|1|1
20120617_EXQ0_MiBa_SA_HCT116_1_mHLA_2hr: 22642
SA = 0.83
Score = 0.63
STDHIPILY − MaxQuant
20120617_EXQ0_MiBa_SA_HCT116_1_mHLA_2hr: 22642
SA = 0.83
Score = 0.63
STDHIPILY − MSFragger
20120617_EXQ0_MiBa_SA_HCT116_1_mHLA_2hr: 22642
SA = 0.83
Score = 0.63
Relative intensity
m/z
Prosit prediction
⋆ Exclusive in prediction
Exclusive in experimental

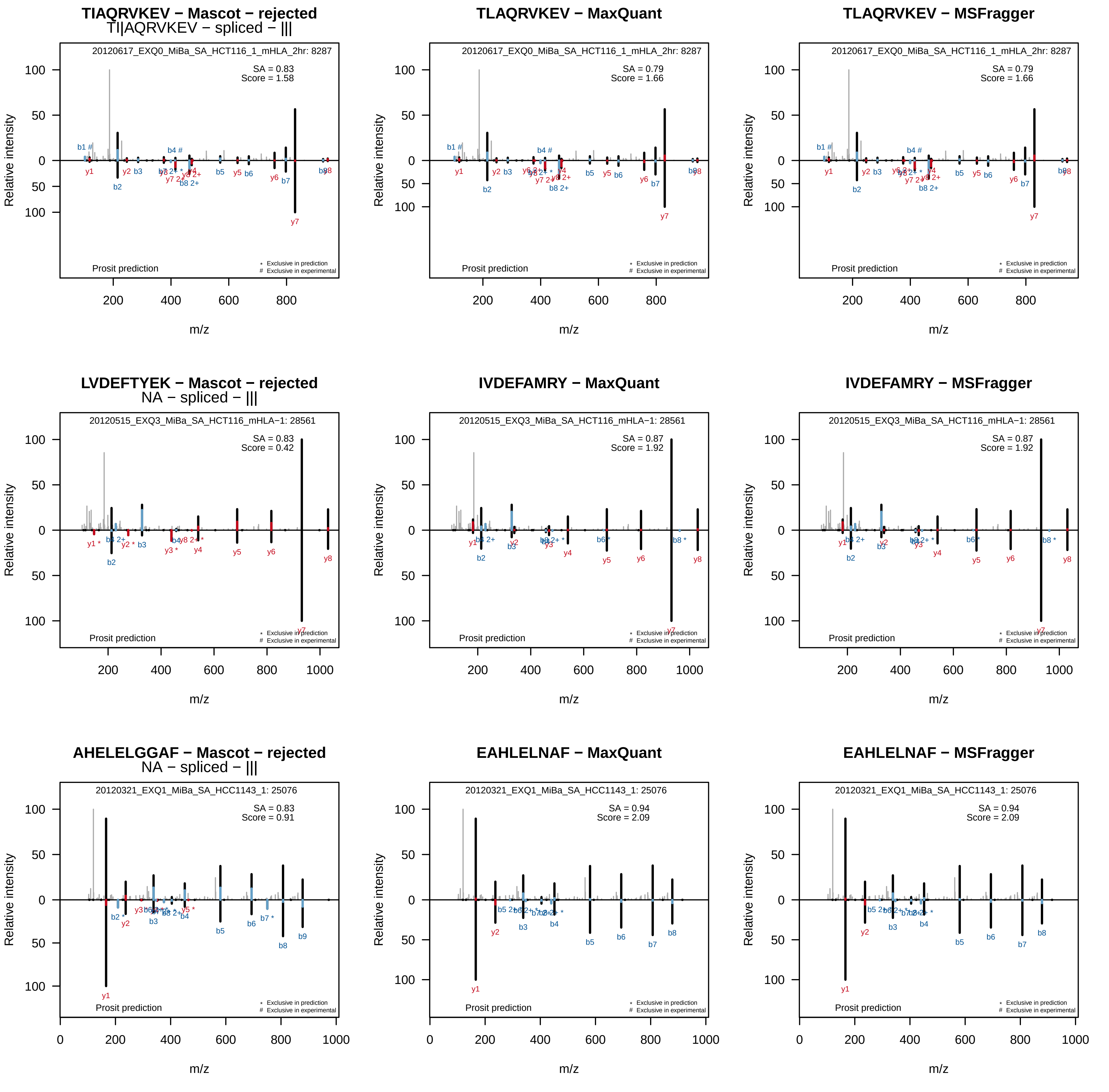

TIAQRVKEV − Mascot − rejected
TI|AQRVKEV − spliced − |||
20120617_EXQ0_MiBa_SA_HCT116_1_mHLA_2hr: 8287
SA = 0.83
Score = 1.58
TLAQRVKEV − MaxQuant
20120617_EXQ0_MiBa_SA_HCT116_1_mHLA_2hr: 8287
SA = 0.79
Score = 1.66
TLAQRVKEV − MSFragger
20120617_EXQ0_MiBa_SA_HCT116_1_mHLA_2hr: 8287
SA = 0.79
Score = 1.66
LVDEFTYEK − Mascot − rejected
NA − spliced − |||
20120515_EXQ3_MiBa_SA_HCT116_mHLA−1: 28561
SA = 0.83
Score = 0.42
IVDEFAMRY − MaxQuant
20120515_EXQ3_MiBa_SA_HCT116_mHLA−1: 28561
SA = 0.87
Score = 1.92
IVDEFAMRY − MSFragger
20120515_EXQ3_MiBa_SA_HCT116_mHLA−1: 28561
SA = 0.87
Score = 1.92
AHELELGGAF − Mascot − rejected
NA − spliced − |||
20120321_EXQ1_MiBa_SA_HCC1143_1: 25076
SA = 0.83
Score = 0.91
EAHLELNAF − MaxQuant
20120321_EXQ1_MiBa_SA_HCC1143_1: 25076
SA = 0.94
Score = 2.09
EAHLELNAF − MSFragger
20120321_EXQ1_MiBa_SA_HCC1143_1: 25076
SA = 0.94
Score = 2.09
Relative intensity
m/z
Prosit prediction
* Exclusive in prediction
# Exclusive in experimental

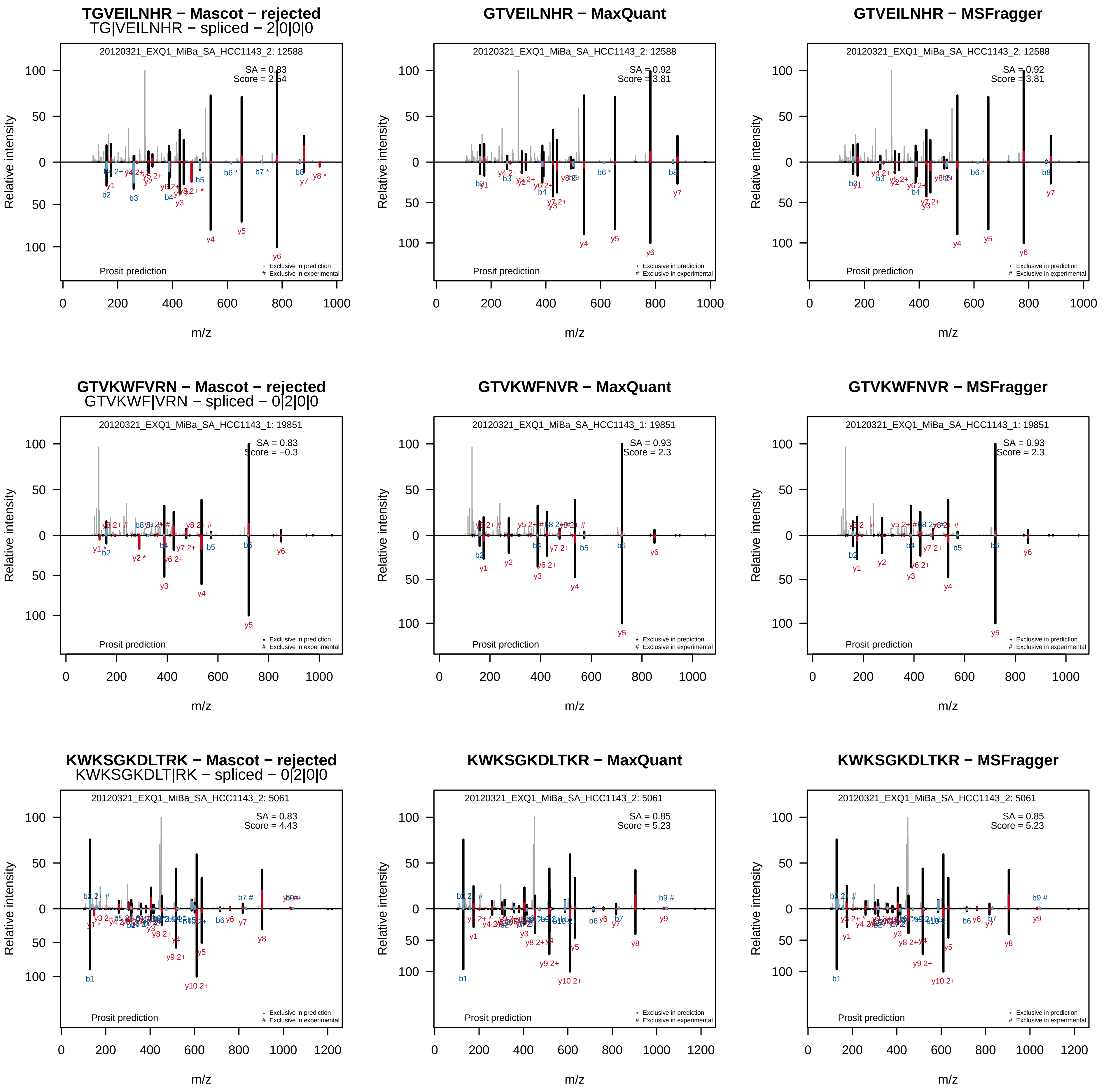
TGVEILNHR − Mascot − rejected
TG|VEILNHR − spliced − 2|0|0|0
20120321_EXQ1_MiBa_SA_HCC1143_2: 12588
SA = 0.83
Score = 2.54
GTVEILNHR − MaxQuant
20120321_EXQ1_MiBa_SA_HCC1143_2: 12588
SA = 0.92
Score = 3.81
GTVEILNHR − MSFragger
20120321_EXQ1_MiBa_SA_HCC1143_2: 12588
SA = 0.92
Score = 3.81
GTVKWFVRN − Mascot − rejected
GTVKWF|VRN − spliced − 0|2|0|0
20120321_EXQ1_MiBa_SA_HCC1143_1: 19851
SA = 0.83
Score = −0.3
GTVKWFNVR − MaxQuant
20120321_EXQ1_MiBa_SA_HCC1143_1: 19851
SA = 0.93
Score = 2.3
GTVKWFNVR − MSFragger
20120321_EXQ1_MiBa_SA_HCC1143_1: 19851
SA = 0.93
Score = 2.3
KWKSGKDLTRK − Mascot − rejected
KWKSGKDLT|RK − spliced − 0|2|0|0
20120321_EXQ1_MiBa_SA_HCC1143_2: 5061
SA = 0.83
Score = 4.43
KWKSGKDLTKR − MaxQuant
20120321_EXQ1_MiBa_SA_HCC1143_2: 5061
SA = 0.85
Score = 5.23
KWKSGKDLTKR − MSFragger
20120321_EXQ1_MiBa_SA_HCC1143_2: 5061
SA = 0.85
Score = 5.23
Relative intensity
m/z
Prosit prediction
⋆ Exclusive in prediction
Exclusive in experimental

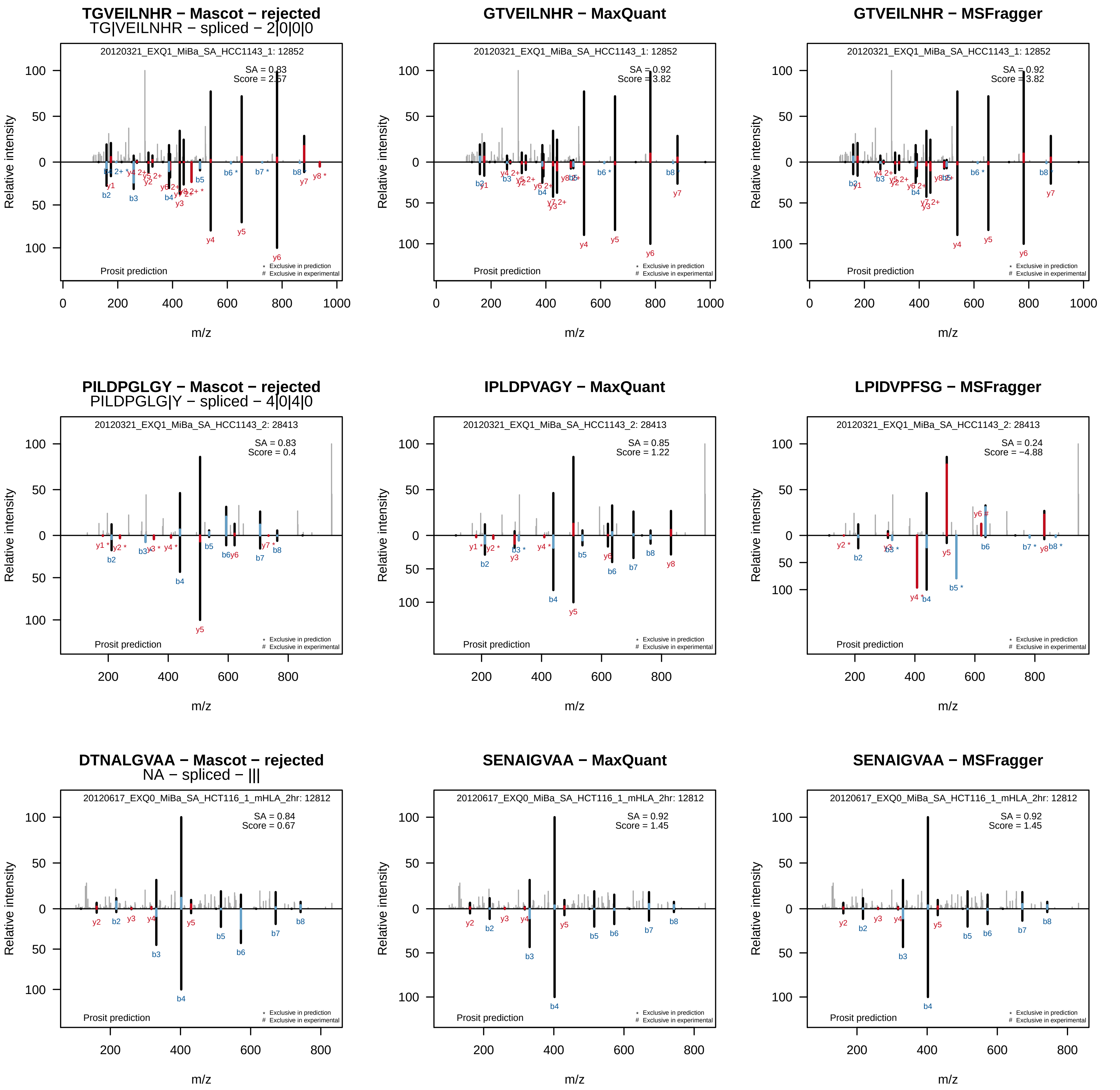

TGVEILNHR − Mascot − rejected
TG|VEILNHR − spliced − 2|0|0|0
20120321_EXQ1_MiBa_SA_HCC1143_1: 12852
SA = 0.83
Score = 2.57
GTVEILNHR − MaxQuant
20120321_EXQ1_MiBa_SA_HCC1143_1: 12852
SA = 0.92
Score = 3.82
GTVEILNHR − MSFragger
20120321_EXQ1_MiBa_SA_HCC1143_1: 12852
SA = 0.92
Score = 3.82
PILDPGLGY − Mascot − rejected
PILDPGLG|Y − spliced − 4|0|4|0
20120321_EXQ1_MiBa_SA_HCC1143_2: 28413
SA = 0.83
Score = 0.4
IPLDPVAGY − MaxQuant
20120321_EXQ1_MiBa_SA_HCC1143_2: 28413
SA = 0.85
Score = 1.22
LPIDVPFSG − MSFragger
20120321_EXQ1_MiBa_SA_HCC1143_2: 28413
SA = 0.24
Score = −4.88
DTNALGVAA − Mascot − rejected
NA − spliced − |||
20120617_EXQ0_MiBa_SA_HCT116_1_mHLA_2hr: 12812
SA = 0.84
Score = 0.67
SENAIGVAA − MaxQuant
20120617_EXQ0_MiBa_SA_HCT116_1_mHLA_2hr: 12812
SA = 0.92
Score = 1.45
SENAIGVAA − MSFragger
20120617_EXQ0_MiBa_SA_HCT116_1_mHLA_2hr: 12812
SA = 0.92
Score = 1.45
Relative intensity
m/z
Prosit prediction
⋆ Exclusive in prediction
Exclusive in experimental

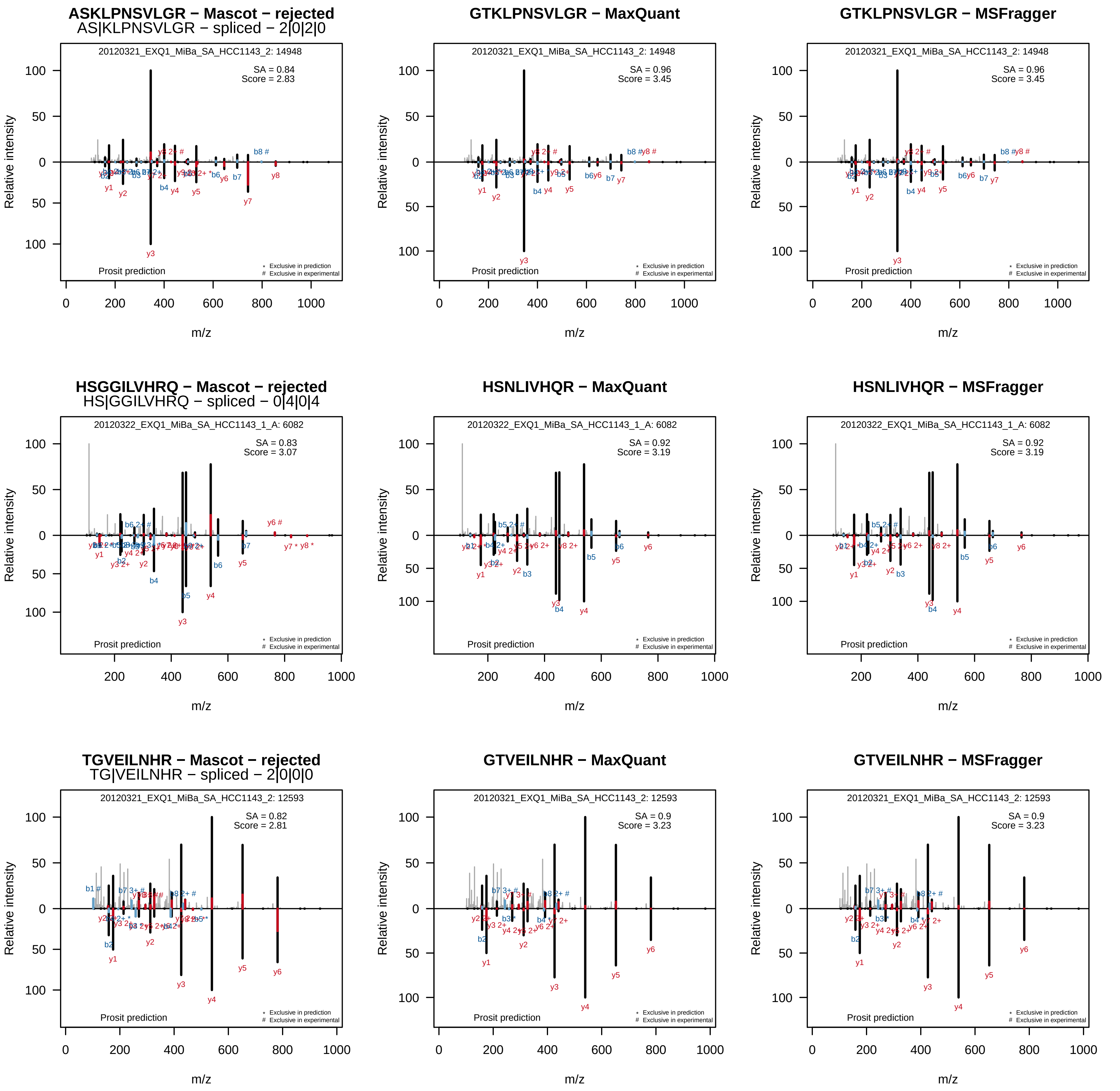
ASKLPNSVLGR − Mascot − rejected
AS|KLPNSVLGR − spliced − 2|0|2|0
20120321_EXQ1_MiBa_SA_HCC1143_2: 14948
SA = 0.84
Score = 2.83
GTKLPNSVLGR − MaxQuant
20120321_EXQ1_MiBa_SA_HCC1143_2: 14948
SA = 0.96
Score = 3.45
GTKLPNSVLGR − MSFragger
20120321_EXQ1_MiBa_SA_HCC1143_2: 14948
SA = 0.96
Score = 3.45
HSGGILVHRQ − Mascot − rejected
HS|GGILVHRQ − spliced − 0|4|0|4
20120322_EXQ1_MiBa_SA_HCC1143_1_A: 6082
SA = 0.83
Score = 3.07
HSNLIVHQR − MaxQuant
20120322_EXQ1_MiBa_SA_HCC1143_1_A: 6082
SA = 0.92
Score = 3.19
HSNLIVHQR − MSFragger
20120322_EXQ1_MiBa_SA_HCC1143_1_A: 6082
SA = 0.92
Score = 3.19
TGVEILNHR − Mascot − rejected
TG|VEILNHR − spliced − 2|0|0|0
20120321_EXQ1_MiBa_SA_HCC1143_2: 12593
SA = 0.82
Score = 2.81
GTVEILNHR − MaxQuant
20120321_EXQ1_MiBa_SA_HCC1143_2: 12593
SA = 0.9
Score = 3.23
GTVEILNHR − MSFragger
20120321_EXQ1_MiBa_SA_HCC1143_2: 12593
SA = 0.9
Score = 3.23
Relative intensity
m/z
Prosit prediction
* Exclusive in prediction
# Exclusive in experimental

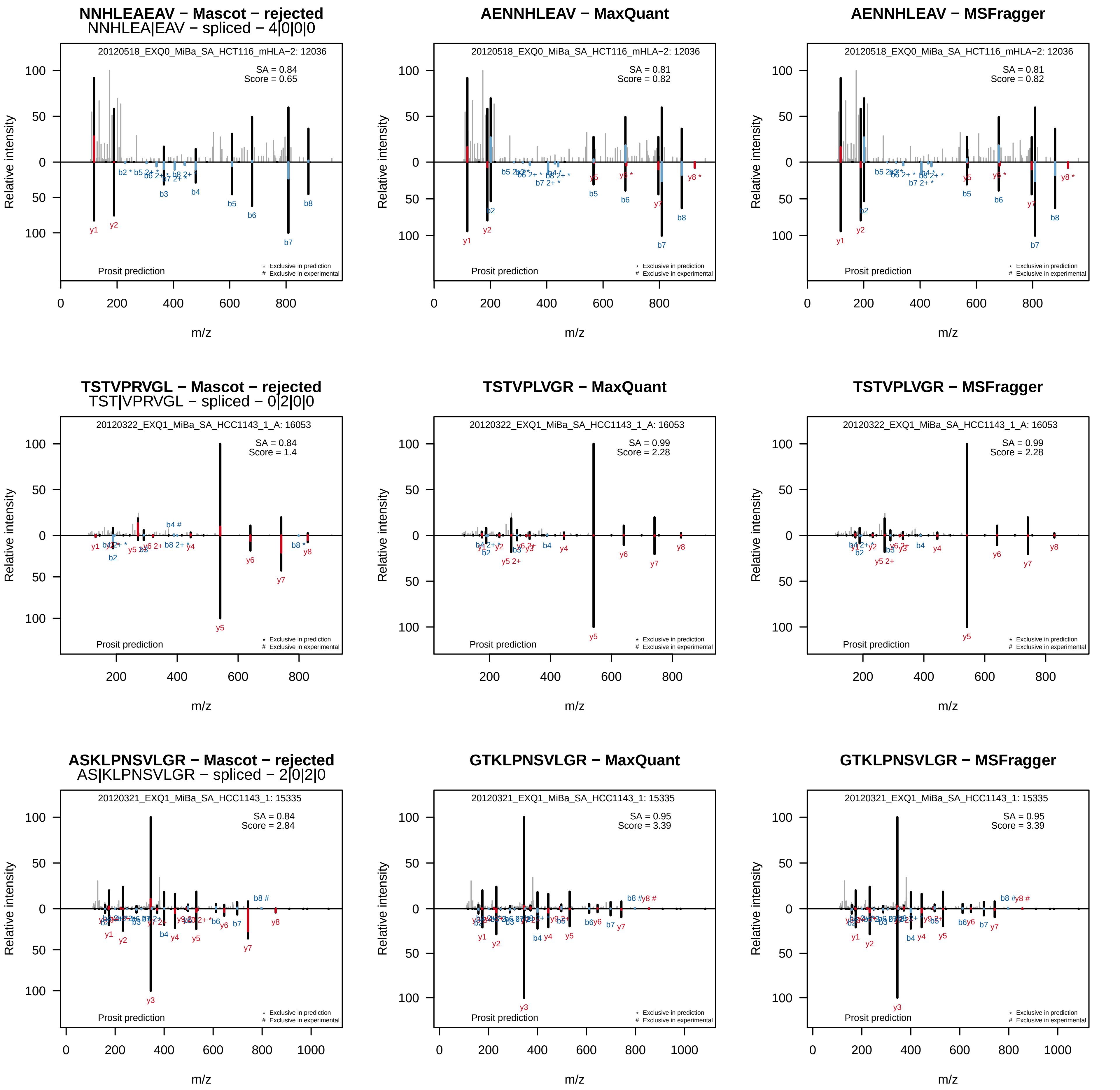

NNHLEAEAV − Mascot − rejected
NNHLEA|EAV − spliced − 4|0|0|0
20120518_EXQ0_MiBa_SA_HCT116_mHLA−2: 12036
SA = 0.84
Score = 0.65
Prosit prediction
AENNHLEAV − MaxQuant
20120518_EXQ0_MiBa_SA_HCT116_mHLA−2: 12036
SA = 0.81
Score = 0.82
Prosit prediction
AENNHLEAV − MSFragger
20120518_EXQ0_MiBa_SA_HCT116_mHLA−2: 12036
SA = 0.81
Score = 0.82
Prosit prediction
TSTVPRVGL − Mascot − rejected
TST|VPRVGL − spliced − 0|2|0|0
20120322_EXQ1_MiBa_SA_HCC1143_1_A: 16053
SA = 0.84
Score = 1.4
Prosit prediction
TSTVPLVGR − MaxQuant
20120322_EXQ1_MiBa_SA_HCC1143_1_A: 16053
SA = 0.99
Score = 2.28
Prosit prediction
TSTVPLVGR − MSFragger
20120322_EXQ1_MiBa_SA_HCC1143_1_A: 16053
SA = 0.99
Score = 2.28
Prosit prediction
ASKLPNSVLGR − Mascot − rejected
AS|KLPNSVLGR − spliced − 2|0|2|0
20120321_EXQ1_MiBa_SA_HCC1143_1: 15335
SA = 0.84
Score = 2.84
Prosit prediction
GTKLPNSVLGR − MaxQuant
20120321_EXQ1_MiBa_SA_HCC1143_1: 15335
SA = 0.95
Score = 3.39
Prosit prediction
GTKLPNSVLGR − MSFragger
20120321_EXQ1_MiBa_SA_HCC1143_1: 15335
SA = 0.95
Score = 3.39
Prosit prediction
Relative intensity
m/z
* Exclusive in prediction
# Exclusive in experimental

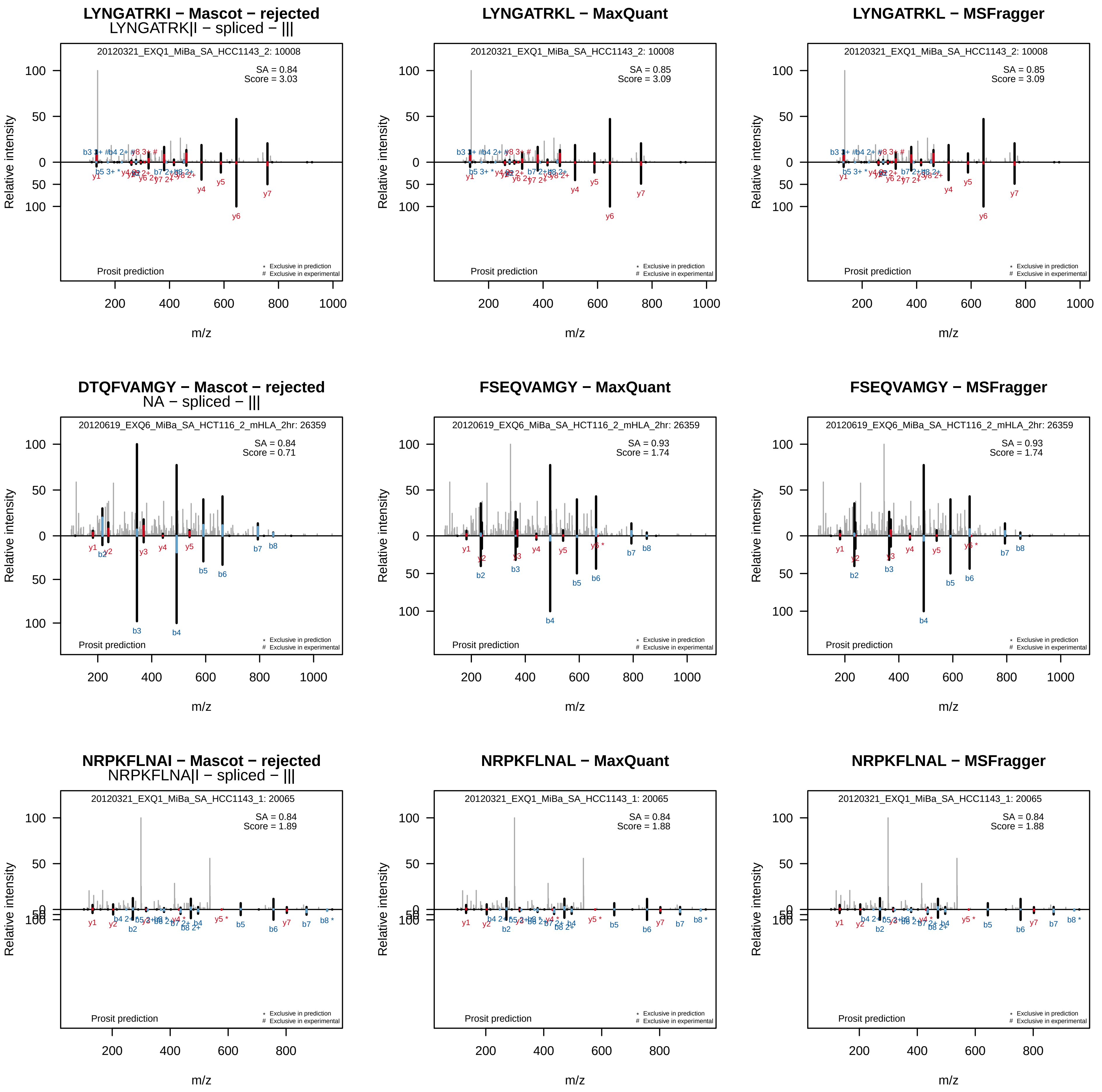

LYNGATRKI − Mascot − rejected
LYNGATRK|I − spliced − |||
20120321_EXQ1_MiBa_SA_HCC1143_2: 10008
SA = 0.84
Score = 3.03
LYNGATRKL − MaxQuant
20120321_EXQ1_MiBa_SA_HCC1143_2: 10008
SA = 0.85
Score = 3.09
LYNGATRKL − MSFragger
20120321_EXQ1_MiBa_SA_HCC1143_2: 10008
SA = 0.85
Score = 3.09
DTQFVAMGY − Mascot − rejected
NA − spliced − |||
20120619_EXQ6_MiBa_SA_HCT116_2_mHLA_2hr: 26359
SA = 0.84
Score = 0.71
FSEQVAMGY − MaxQuant
20120619_EXQ6_MiBa_SA_HCT116_2_mHLA_2hr: 26359
SA = 0.93
Score = 1.74
FSEQVAMGY − MSFragger
20120619_EXQ6_MiBa_SA_HCT116_2_mHLA_2hr: 26359
SA = 0.93
Score = 1.74
NRPKFLNAI − Mascot − rejected
NRPKFLNA|I − spliced − |||
20120321_EXQ1_MiBa_SA_HCC1143_1: 20065
SA = 0.84
Score = 1.89
NRPKFLNAL − MaxQuant
20120321_EXQ1_MiBa_SA_HCC1143_1: 20065
SA = 0.84
Score = 1.88
NRPKFLNAL − MSFragger
20120321_EXQ1_MiBa_SA_HCC1143_1: 20065
SA = 0.84
Score = 1.88
Relative intensity
m/z
Prosit prediction
* Exclusive in prediction
# Exclusive in experimental

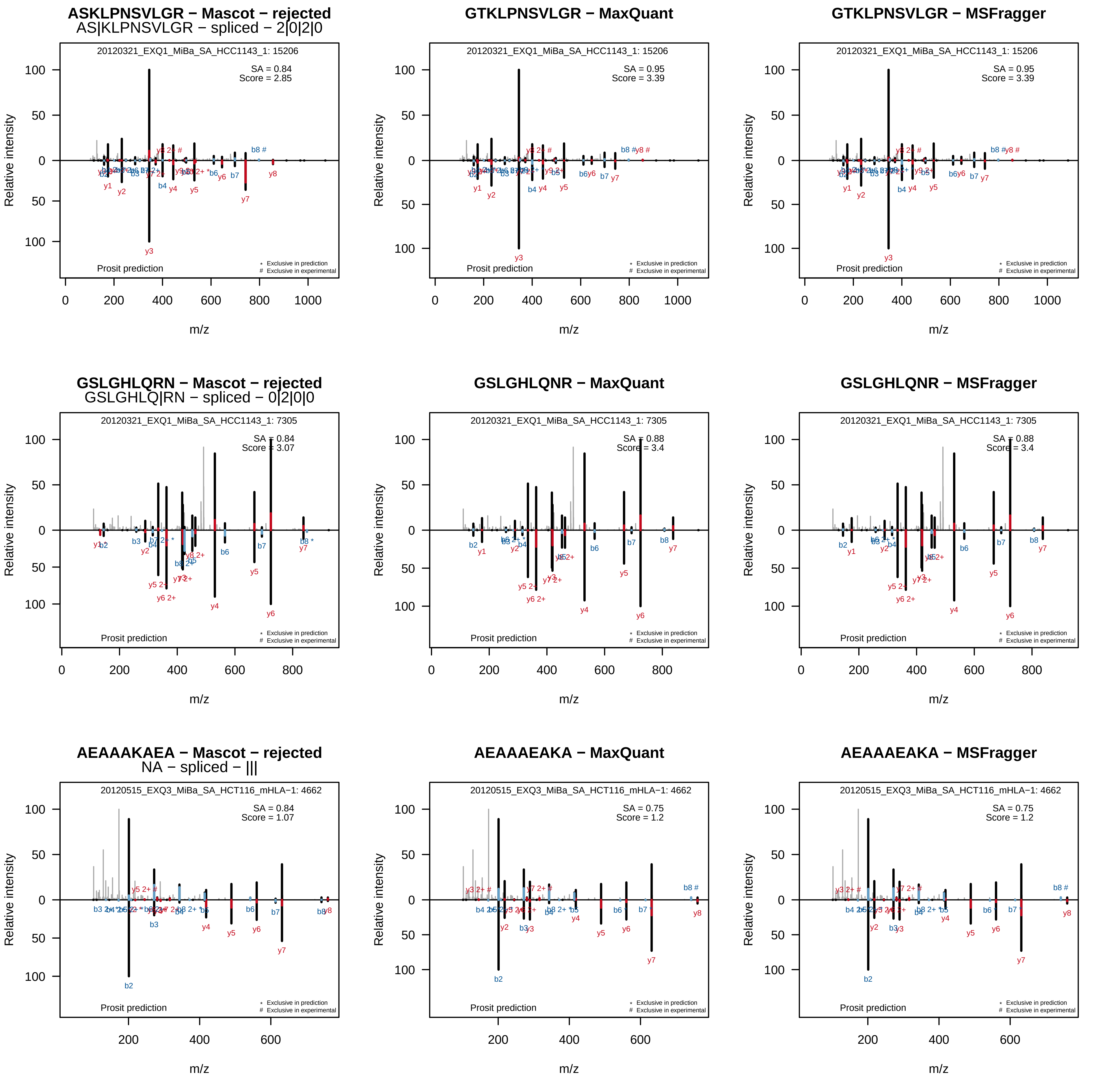
ASKLPNSVLGR − Mascot − rejected
AS|KLPNSVLGR − spliced − 2|0|2|0
20120321_EXQ1_MiBa_SA_HCC1143_1: 15206
SA = 0.84
Score = 2.85
GTKLPNSVLGR − MaxQuant
20120321_EXQ1_MiBa_SA_HCC1143_1: 15206
SA = 0.95
Score = 3.39
GTKLPNSVLGR − MSFragger
20120321_EXQ1_MiBa_SA_HCC1143_1: 15206
SA = 0.95
Score = 3.39
GSLGHLQRN − Mascot − rejected
GSLGHLQ|RN − spliced − 0|2|0|0
20120321_EXQ1_MiBa_SA_HCC1143_1: 7305
SA = 0.84
Score = 3.07
GSLGHLQNR − MaxQuant
20120321_EXQ1_MiBa_SA_HCC1143_1: 7305
SA = 0.88
Score = 3.4
GSLGHLQNR − MSFragger
20120321_EXQ1_MiBa_SA_HCC1143_1: 7305
SA = 0.88
Score = 3.4
AEAAAKAEA − Mascot − rejected
NA − spliced − |||
20120515_EXQ3_MiBa_SA_HCT116_mHLA−1: 4662
SA = 0.84
Score = 1.07
AEAAAEAKA − MaxQuant
20120515_EXQ3_MiBa_SA_HCT116_mHLA−1: 4662
SA = 0.75
Score = 1.2
AEAAAEAKA − MSFragger
20120515_EXQ3_MiBa_SA_HCT116_mHLA−1: 4662
SA = 0.75
Score = 1.2
Relative intensity
m/z
Prosit prediction
⋆ Exclusive in prediction
Exclusive in experimental

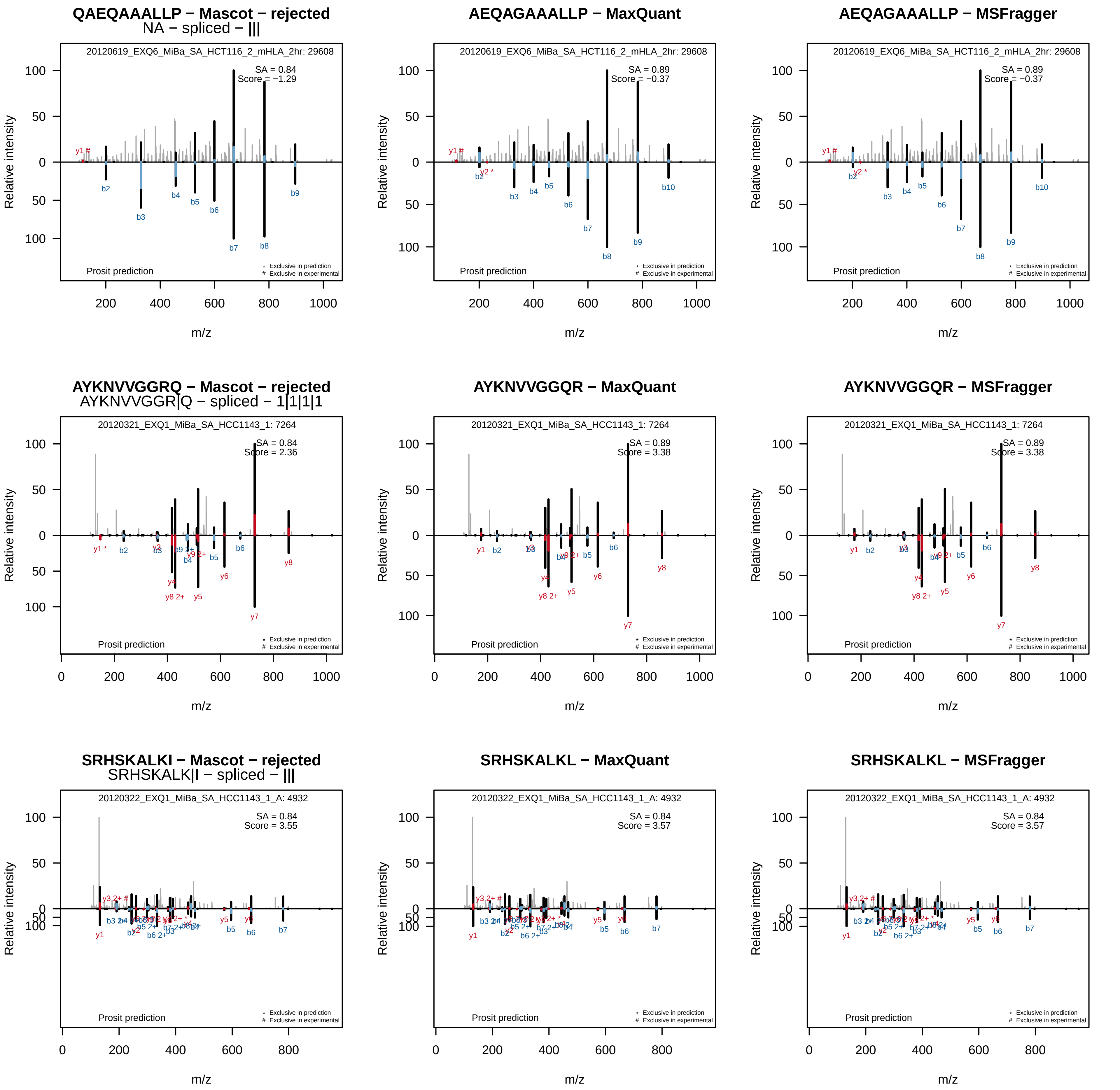
QAEQAAALLP − Mascot − rejected
NA − spliced − |||
20120619_EXQ6_MiBa_SA_HCT116_2_mHLA_2hr: 29608
SA = 0.84
Score = −1.29
AEQAGAAALLP − MaxQuant
20120619_EXQ6_MiBa_SA_HCT116_2_mHLA_2hr: 29608
SA = 0.89
Score = −0.37
AEQAGAAALLP − MSFragger
20120619_EXQ6_MiBa_SA_HCT116_2_mHLA_2hr: 29608
SA = 0.89
Score = −0.37
AYKNVVGGRQ − Mascot − rejected
AYKNVVGGR|Q − spliced − 1|1|1|1
20120321_EXQ1_MiBa_SA_HCC1143_1: 7264
SA = 0.84
Score = 2.36
AYKNVVGGQR − MaxQuant
20120321_EXQ1_MiBa_SA_HCC1143_1: 7264
SA = 0.89
Score = 3.38
AYKNVVGGQR − MSFragger
20120321_EXQ1_MiBa_SA_HCC1143_1: 7264
SA = 0.89
Score = 3.38
SRHSKALKI − Mascot − rejected
SRHSKALK|I − spliced − |||
20120322_EXQ1_MiBa_SA_HCC1143_1_A: 4932
SA = 0.84
Score = 3.55
SRHSKALKL − MaxQuant
20120322_EXQ1_MiBa_SA_HCC1143_1_A: 4932
SA = 0.84
Score = 3.57
SRHSKALKL − MSFragger
20120322_EXQ1_MiBa_SA_HCC1143_1_A: 4932
SA = 0.84
Score = 3.57
Relative intensity
m/z
Prosit prediction
⋆ Exclusive in prediction
Exclusive in experimental

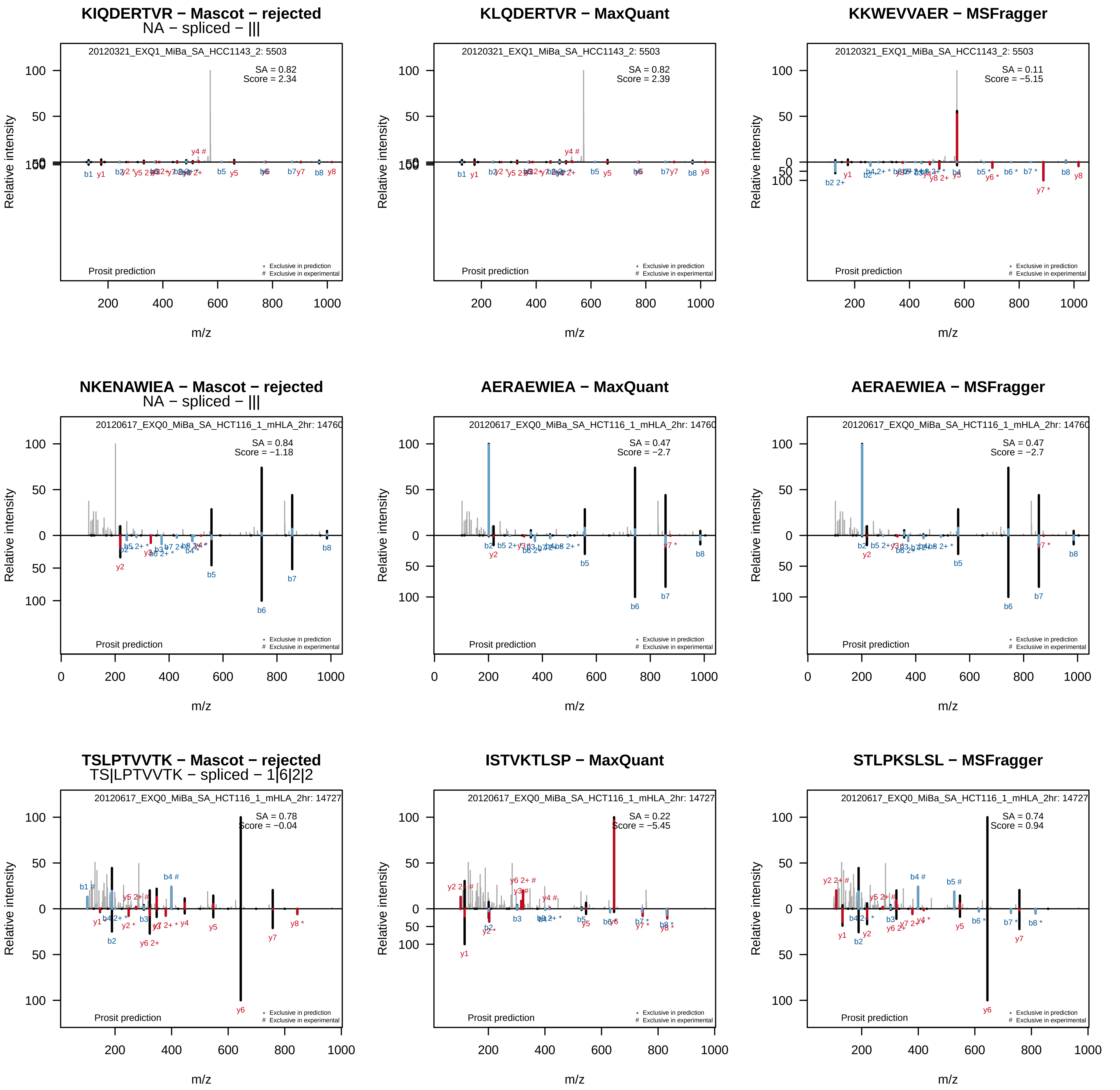

KIQDERTVR − Mascot − rejected
NA − spliced − |||
20120321_EXQ1_MiBa_SA_HCC1143_2: 5503
SA = 0.82
Score = 2.34
KLQDERTVR − MaxQuant
20120321_EXQ1_MiBa_SA_HCC1143_2: 5503
SA = 0.82
Score = 2.39
KKWEVVAER − MSFragger
20120321_EXQ1_MiBa_SA_HCC1143_2: 5503
SA = 0.11
Score = −5.15
NKENAWIEA − Mascot − rejected
NA − spliced − |||
20120617_EXQ0_MiBa_SA_HCT116_1_mHLA_2hr: 14760
SA = 0.84
Score = −1.18
AERAEWIEA − MaxQuant
20120617_EXQ0_MiBa_SA_HCT116_1_mHLA_2hr: 14760
SA = 0.47
Score = −2.7
AERAEWIEA − MSFragger
20120617_EXQ0_MiBa_SA_HCT116_1_mHLA_2hr: 14760
SA = 0.47
Score = −2.7
TSLPTVVTK − Mascot − rejected
TS|LPTVVTK − spliced − 1|6|2|2
20120617_EXQ0_MiBa_SA_HCT116_1_mHLA_2hr: 14727
SA = 0.78
Score = −0.04
ISTVKTLSP − MaxQuant
20120617_EXQ0_MiBa_SA_HCT116_1_mHLA_2hr: 14727
SA = 0.22
Score = −5.45
STLPKSLSL − MSFragger
20120617_EXQ0_MiBa_SA_HCT116_1_mHLA_2hr: 14727
SA = 0.74
Score = 0.94
Relative intensity
m/z
Prosit prediction
⋆ Exclusive in prediction
Exclusive in experimental

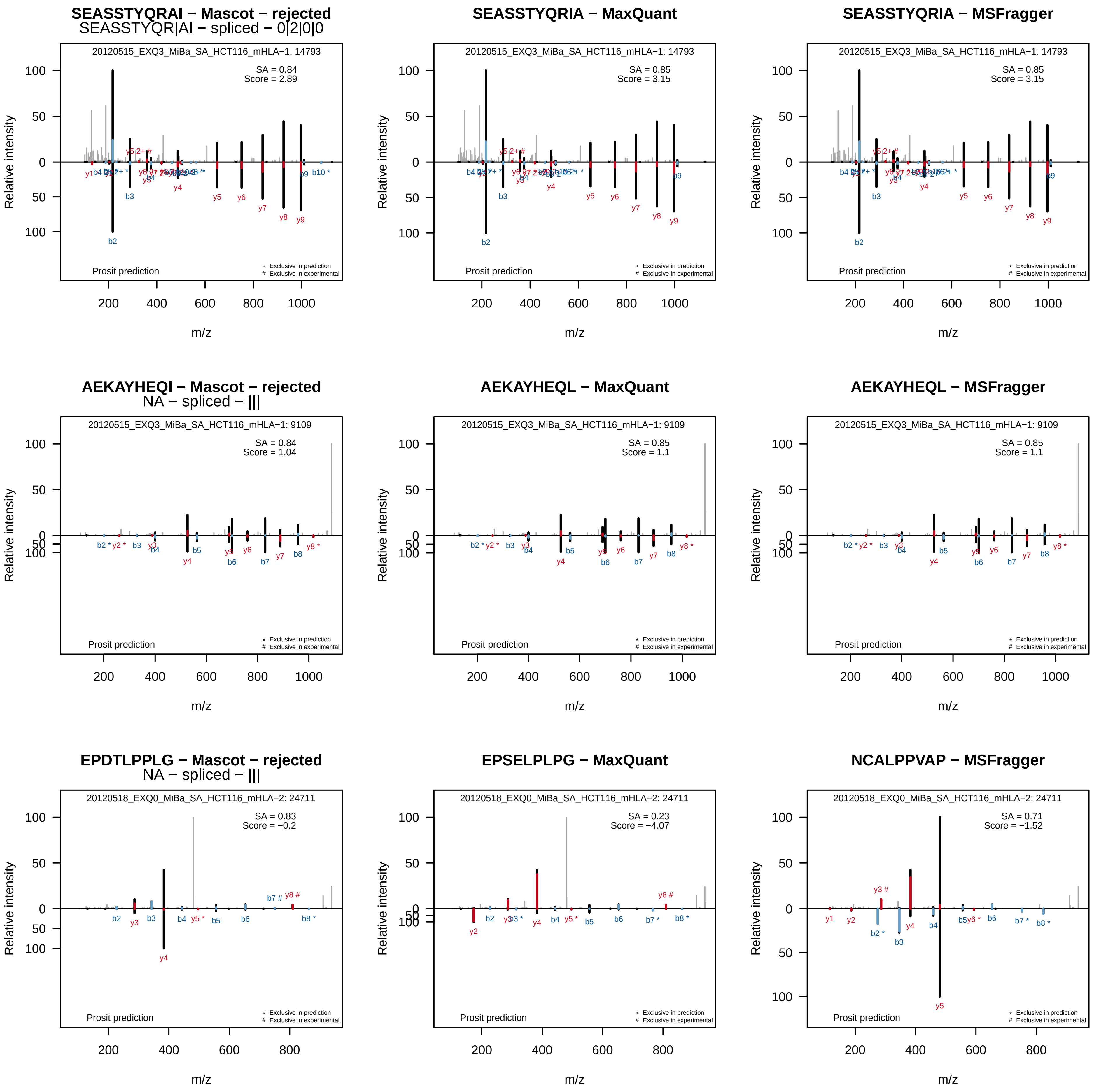

SEASSTYQRAI − Mascot − rejected
SEASSTYQR|AI − spliced − 0|2|0|0
20120515_EXQ3_MiBa_SA_HCT116_mHLA−1: 14793
SA = 0.84
Score = 2.89
SEASSTYQRIA − MaxQuant
20120515_EXQ3_MiBa_SA_HCT116_mHLA−1: 14793
SA = 0.85
Score = 3.15
SEASSTYQRIA − MSFragger
20120515_EXQ3_MiBa_SA_HCT116_mHLA−1: 14793
SA = 0.85
Score = 3.15
AEKAYHEQI − Mascot − rejected
NA − spliced − |||
20120515_EXQ3_MiBa_SA_HCT116_mHLA−1: 9109
SA = 0.84
Score = 1.04
AEKAYHEQL − MaxQuant
20120515_EXQ3_MiBa_SA_HCT116_mHLA−1: 9109
SA = 0.85
Score = 1.1
AEKAYHEQL − MSFragger
20120515_EXQ3_MiBa_SA_HCT116_mHLA−1: 9109
SA = 0.85
Score = 1.1
EPDTLPPLG − Mascot − rejected
NA − spliced − |||
20120518_EXQ0_MiBa_SA_HCT116_mHLA−2: 24711
SA = 0.83
Score = −0.2
EPSELPLPG − MaxQuant
20120518_EXQ0_MiBa_SA_HCT116_mHLA−2: 24711
SA = 0.23
Score = −4.07
NCALPPVAP − MSFragger
20120518_EXQ0_MiBa_SA_HCT116_mHLA−2: 24711
SA = 0.71
Score = −1.52
Relative intensity
m/z
Prosit prediction
⋆ Exclusive in prediction
Exclusive in experimental

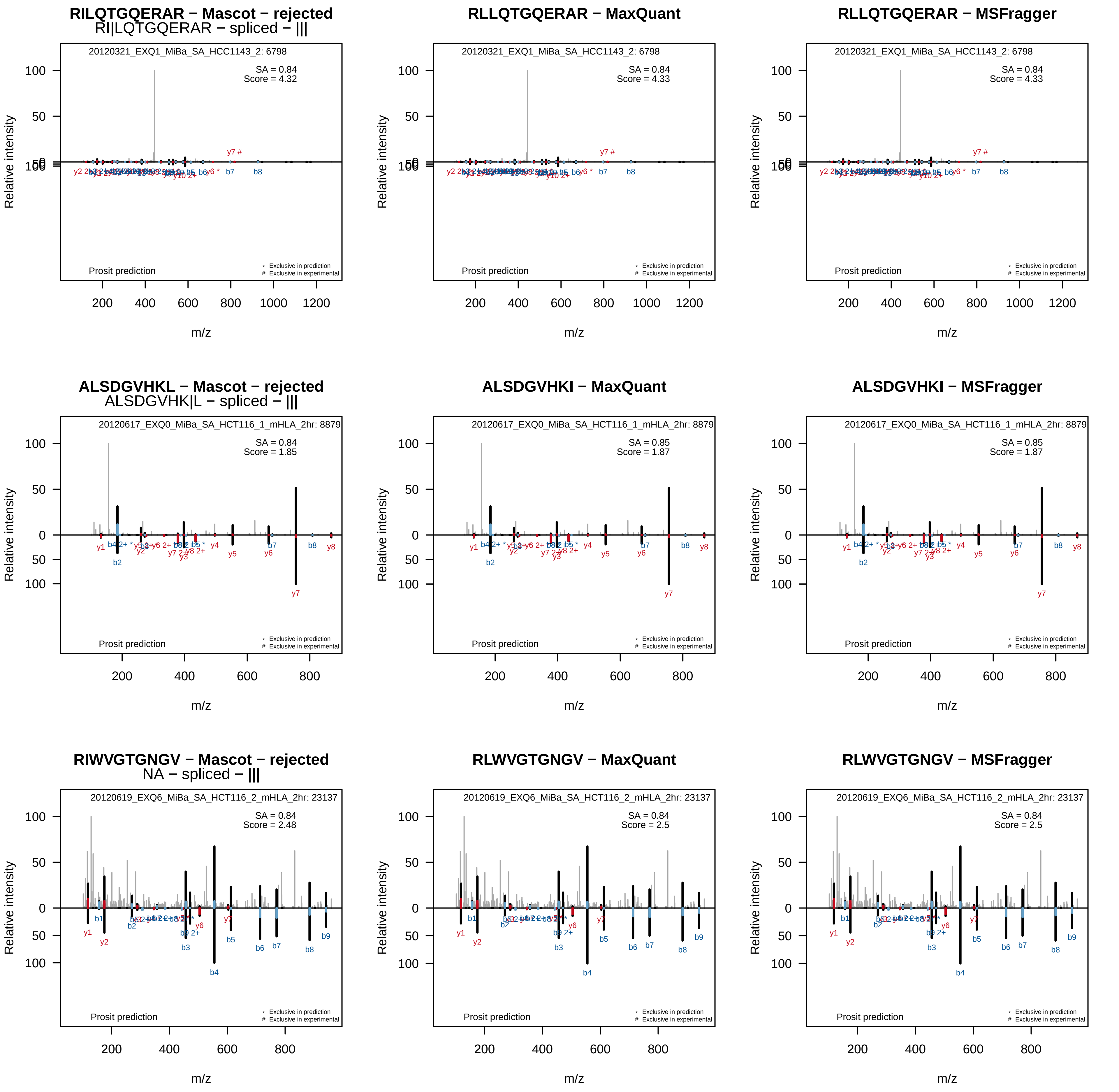

RILQTGQERAR − Mascot − rejected
RI|LQTGQERAR − spliced − |||
20120321_EXQ1_MiBa_SA_HCC1143_2: 6798
SA = 0.84
Score = 4.32
Prosit prediction
RLLQTGQERAR − MaxQuant
20120321_EXQ1_MiBa_SA_HCC1143_2: 6798
SA = 0.84
Score = 4.33
Prosit prediction
RLLQTGQERAR − MSFragger
20120321_EXQ1_MiBa_SA_HCC1143_2: 6798
SA = 0.84
Score = 4.33
Prosit prediction
ALSDGVHKL − Mascot − rejected
ALSDGVHK|L − spliced − |||
20120617_EXQ0_MiBa_SA_HCT116_1_mHLA_2hr: 8879
SA = 0.84
Score = 1.85
Prosit prediction
ALSDGVHKI − MaxQuant
20120617_EXQ0_MiBa_SA_HCT116_1_mHLA_2hr: 8879
SA = 0.85
Score = 1.87
Prosit prediction
ALSDGVHKI − MSFragger
20120617_EXQ0_MiBa_SA_HCT116_1_mHLA_2hr: 8879
SA = 0.85
Score = 1.87
Prosit prediction
RIWVGTGNGV − Mascot − rejected
NA − spliced − |||
20120619_EXQ6_MiBa_SA_HCT116_2_mHLA_2hr: 23137
SA = 0.84
Score = 2.48
Prosit prediction
RLWVGTGNGV − MaxQuant
20120619_EXQ6_MiBa_SA_HCT116_2_mHLA_2hr: 23137
SA = 0.84
Score = 2.5
Prosit prediction
RLWVGTGNGV − MSFragger
20120619_EXQ6_MiBa_SA_HCT116_2_mHLA_2hr: 23137
SA = 0.84
Score = 2.5
Prosit prediction
* Exclusive in prediction
# Exclusive in experimental
Relative intensity
m/z

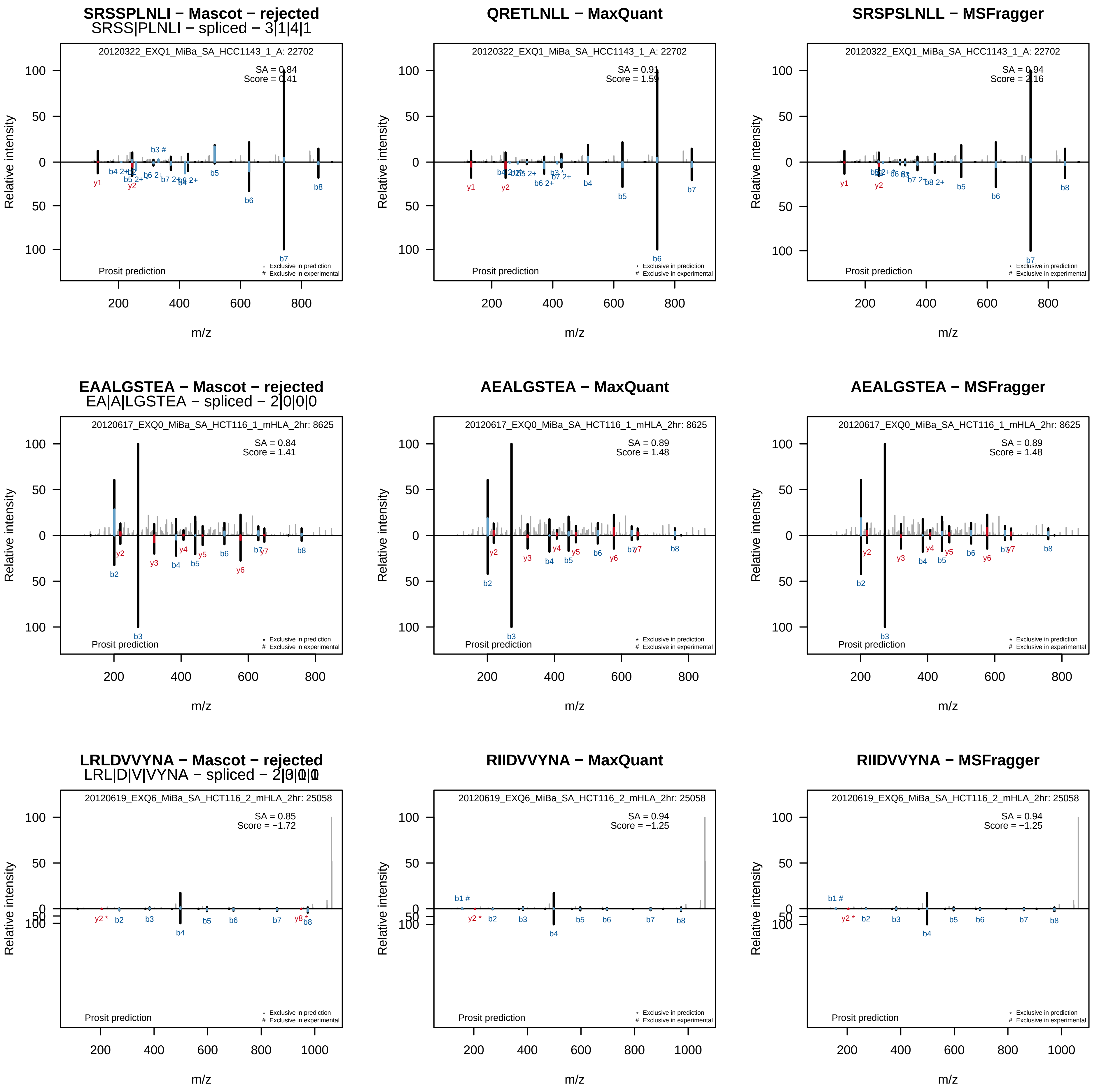
SRSSPLNLI − Mascot − rejected
SRSS|PLNLI − spliced − 3|1|4|1
20120322_EXQ1_MiBa_SA_HCC1143_1_A: 22702
SA = 0.84
Score = 0.41
QRETLNLL − MaxQuant
20120322_EXQ1_MiBa_SA_HCC1143_1_A: 22702
SA = 0.91
Score = 1.59
SRSPSLNLL − MSFragger
20120322_EXQ1_MiBa_SA_HCC1143_1_A: 22702
SA = 0.94
Score = 2.16
EAALGSTEA − Mascot − rejected
EA|A|LGSTEA − spliced − 2|0|0|0
20120617_EXQ0_MiBa_SA_HCT116_1_mHLA_2hr: 8625
SA = 0.84
Score = 1.41
AEALGSTEA − MaxQuant
20120617_EXQ0_MiBa_SA_HCT116_1_mHLA_2hr: 8625
SA = 0.89
Score = 1.48
AEALGSTEA − MSFragger
20120617_EXQ0_MiBa_SA_HCT116_1_mHLA_2hr: 8625
SA = 0.89
Score = 1.48
LRLDVVYNA − Mascot − rejected
20120619_EXQ6_MiBa_SA_HCT116_2_mHLA_2hr: 25058
SA = 0.85
Score = −1.72
RIIDVVYNA − MaxQuant
20120619_EXQ6_MiBa_SA_HCT116_2_mHLA_2hr: 25058
SA = 0.94
Score = −1.25
RIIDVVYNA − MSFragger
20120619_EXQ6_MiBa_SA_HCT116_2_mHLA_2hr: 25058
SA = 0.94
Score = −1.25
Relative intensity
m/z
Prosit prediction
⋆ Exclusive in prediction
Exclusive in experimental

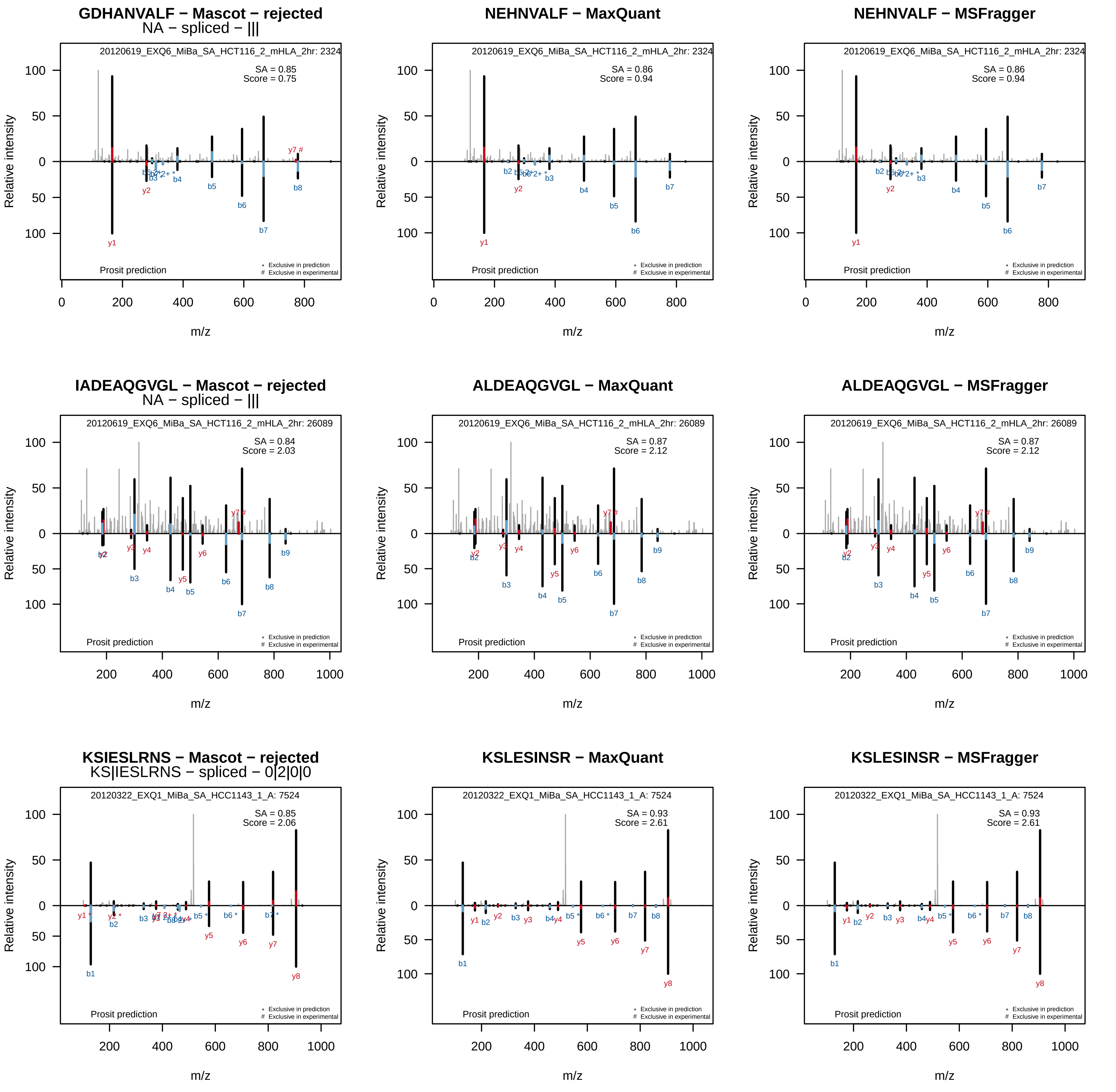
GDHANVALF − Mascot − rejected
NA − spliced − |||
20120619_EXQ6_MiBa_SA_HCT116_2_mHLA_2hr: 2324
SA = 0.85
Score = 0.75
Prosit prediction
⋆ Exclusive in prediction
Exclusive in experimental
Relative intensity
m/z
NEHNVALF − MaxQuant
20120619_EXQ6_MiBa_SA_HCT116_2_mHLA_2hr: 2324
SA = 0.86
Score = 0.94
NEHNVALF − MSFragger
20120619_EXQ6_MiBa_SA_HCT116_2_mHLA_2hr: 2324
SA = 0.86
Score = 0.94
IADEAQGVGL − Mascot − rejected
NA − spliced − |||
20120619_EXQ6_MiBa_SA_HCT116_2_mHLA_2hr: 26089
SA = 0.84
Score = 2.03
ALDEAQGVGL − MaxQuant
20120619_EXQ6_MiBa_SA_HCT116_2_mHLA_2hr: 26089
SA = 0.87
Score = 2.12
ALDEAQGVGL − MSFragger
20120619_EXQ6_MiBa_SA_HCT116_2_mHLA_2hr: 26089
SA = 0.87
Score = 2.12
KSIESLRNS − Mascot − rejected
KS|IESLRNS − spliced − 0|2|0|0
20120322_EXQ1_MiBa_SA_HCC1143_1_A: 7524
SA = 0.85
Score = 2.06
KSLESINSR − MaxQuant
20120322_EXQ1_MiBa_SA_HCC1143_1_A: 7524
SA = 0.93
Score = 2.61
KSLESINSR − MSFragger
20120322_EXQ1_MiBa_SA_HCC1143_1_A: 7524
SA = 0.93
Score = 2.61

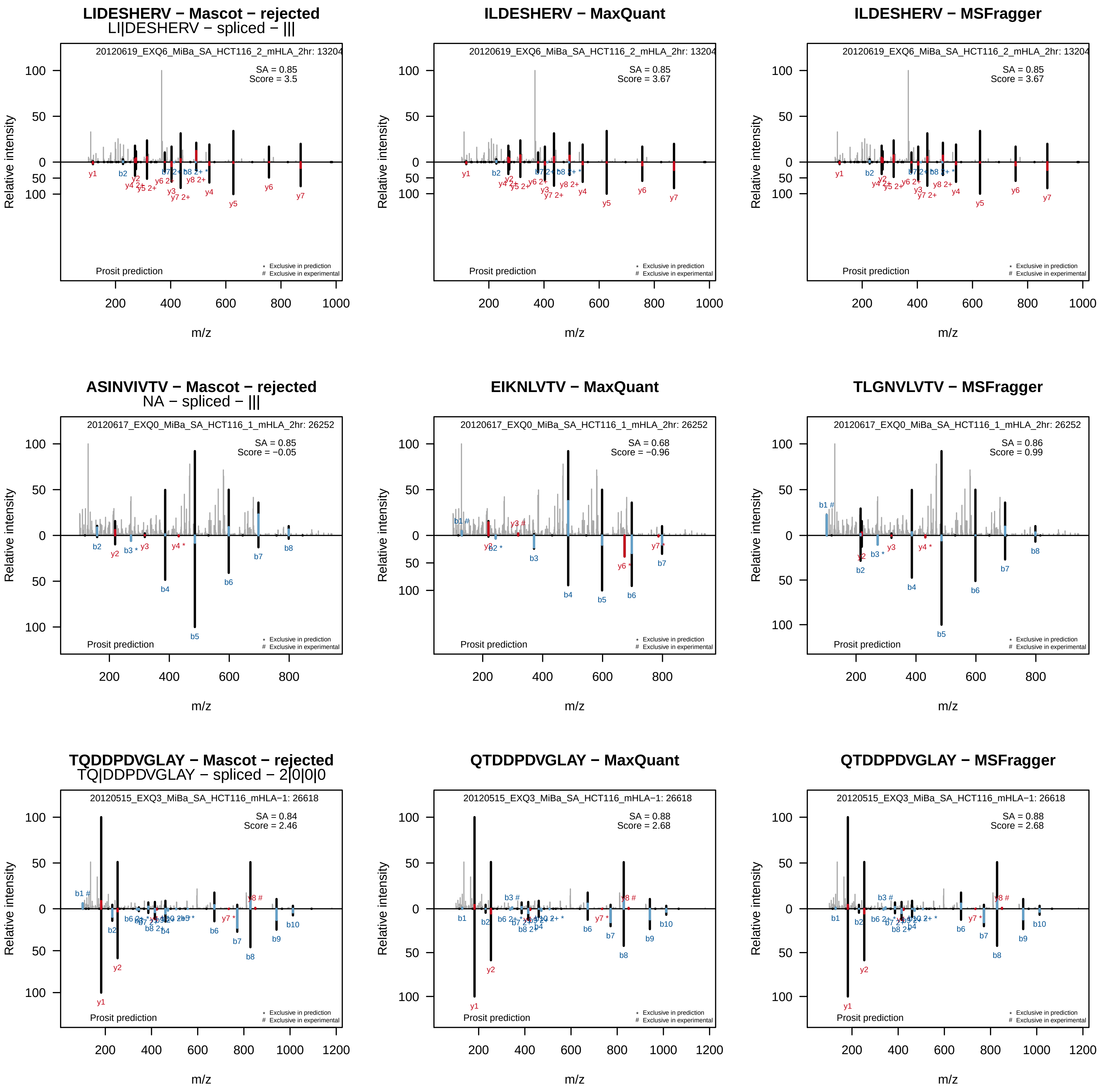

LIDESHERV − Mascot − rejected
LI|DESHERV − spliced − |||
20120619_EXQ6_MiBa_SA_HCT116_2_mHLA_2hr: 13204
SA = 0.85
Score = 3.5
ILDESHERV − MaxQuant
20120619_EXQ6_MiBa_SA_HCT116_2_mHLA_2hr: 13204
SA = 0.85
Score = 3.67
ILDESHERV − MSFragger
20120619_EXQ6_MiBa_SA_HCT116_2_mHLA_2hr: 13204
SA = 0.85
Score = 3.67
ASINVIVTV − Mascot − rejected
NA − spliced − |||
20120617_EXQ0_MiBa_SA_HCT116_1_mHLA_2hr: 26252
SA = 0.85
Score = −0.05
EIKNLVTV − MaxQuant
20120617_EXQ0_MiBa_SA_HCT116_1_mHLA_2hr: 26252
SA = 0.68
Score = −0.96
TLGNVLVTV − MSFragger
20120617_EXQ0_MiBa_SA_HCT116_1_mHLA_2hr: 26252
SA = 0.86
Score = 0.99
TQDDPDVGLAY − Mascot − rejected
TQ|DDPDVGLAY − spliced − 2|0|0|0
20120515_EXQ3_MiBa_SA_HCT116_mHLA−1: 26618
SA = 0.84
Score = 2.46
QTDDPDVGLAY − MaxQuant
20120515_EXQ3_MiBa_SA_HCT116_mHLA−1: 26618
SA = 0.88
Score = 2.68
QTDDPDVGLAY − MSFragger
20120515_EXQ3_MiBa_SA_HCT116_mHLA−1: 26618
SA = 0.88
Score = 2.68
Prosit prediction
⋆ Exclusive in prediction
Exclusive in experimental
Relative intensity
m/z

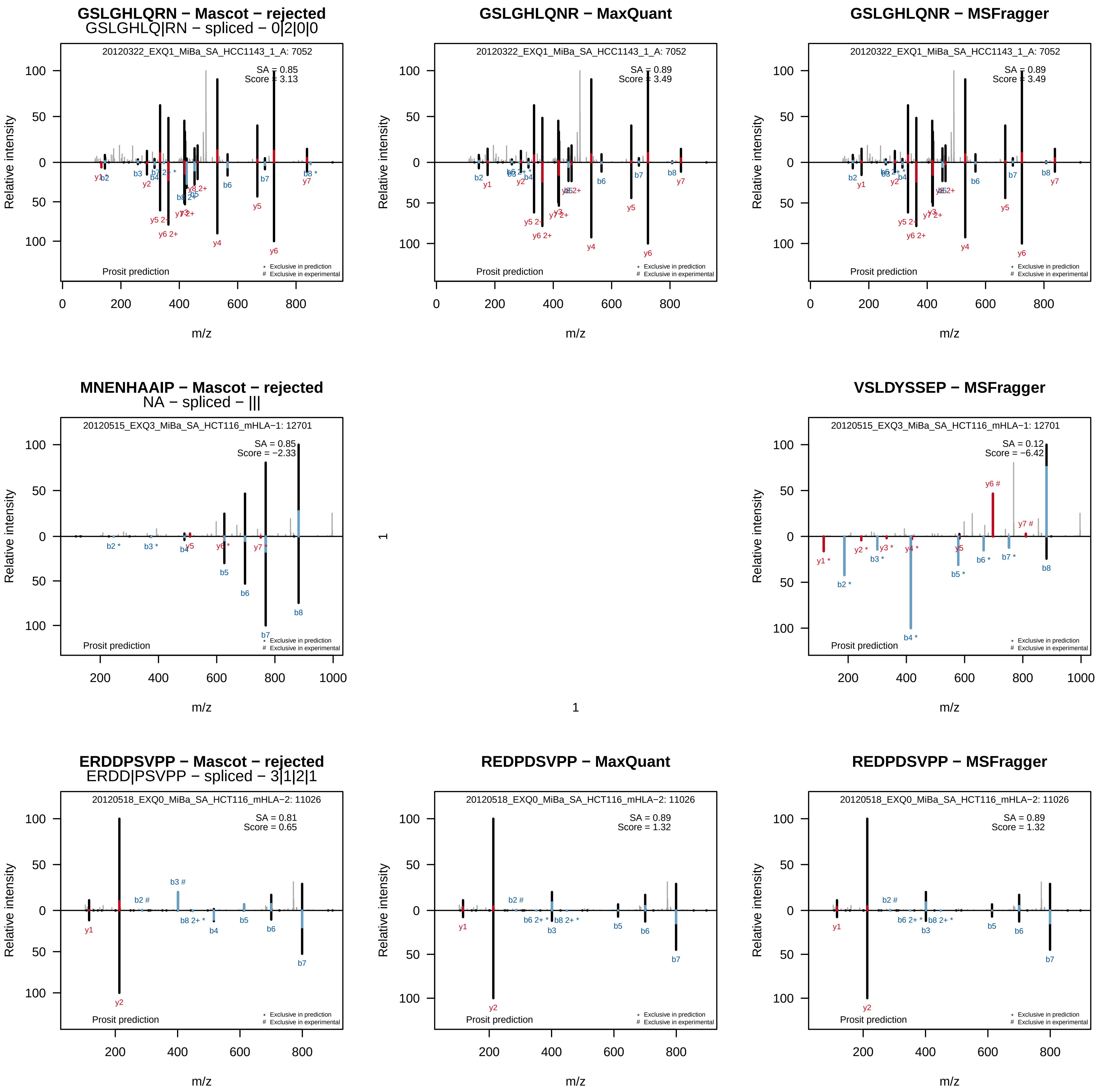
GSLGHLQRN − Mascot − rejected
GSLGHLQ|RN − spliced − 0|2|0|0
20120322_EXQ1_MiBa_SA_HCC1143_1_A: 7052
SA = 0.85
Score = 3.13
GSLGHLQNR − MaxQuant
20120322_EXQ1_MiBa_SA_HCC1143_1_A: 7052
SA = 0.89
Score = 3.49
GSLGHLQNR − MSFragger
20120322_EXQ1_MiBa_SA_HCC1143_1_A: 7052
SA = 0.89
Score = 3.49
MNENHAAIP − Mascot − rejected
NA − spliced − |||
20120515_EXQ3_MiBa_SA_HCT116_mHLA−1: 12701
SA = 0.85
Score = −2.33
VSLDYSSEP − MSFragger
20120515_EXQ3_MiBa_SA_HCT116_mHLA−1: 12701
SA = 0.12
Score = −6.42
ERDDPSVPP − Mascot − rejected
ERDD|PSVPP − spliced − 3|1|2|1
20120518_EXQ0_MiBa_SA_HCT116_mHLA−2: 11026
SA = 0.81
Score = 0.65
REDPDSVPP − MaxQuant
20120518_EXQ0_MiBa_SA_HCT116_mHLA−2: 11026
SA = 0.89
Score = 1.32
REDPDSVPP − MSFragger
20120518_EXQ0_MiBa_SA_HCT116_mHLA−2: 11026
SA = 0.89
Score = 1.32
Relative intensity
m/z
Prosit prediction
⋆ Exclusive in prediction
Exclusive in experimental
1

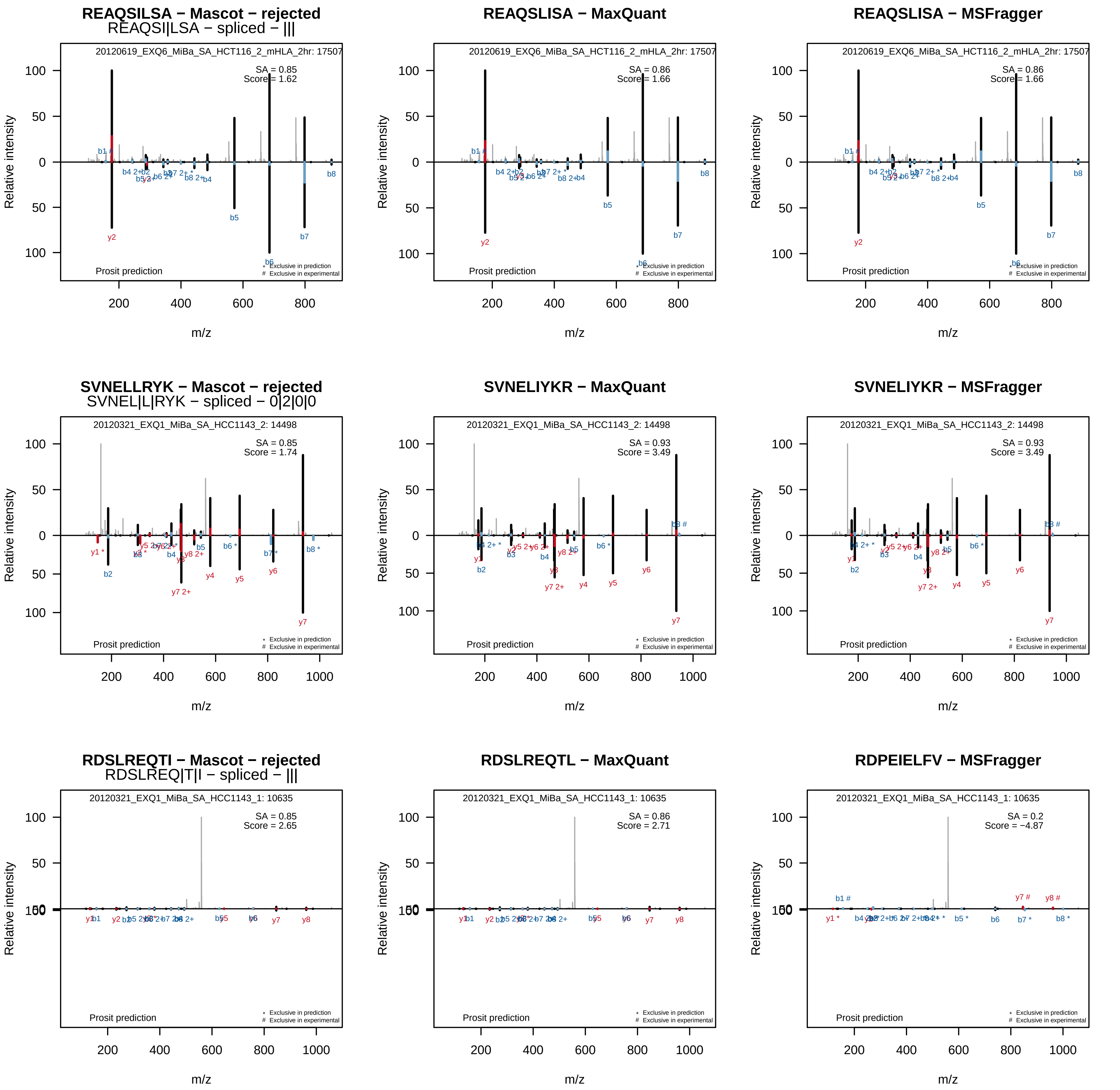
REAQSILSA − Mascot − rejected
REAQSI|LSA − spliced − |||
20120619_EXQ6_MiBa_SA_HCT116_2_mHLA_2hr: 17507
SA = 0.85
Score = 1.62
REAQSLISA − MaxQuant
20120619_EXQ6_MiBa_SA_HCT116_2_mHLA_2hr: 17507
SA = 0.86
Score = 1.66
REAQSLISA − MSFragger
20120619_EXQ6_MiBa_SA_HCT116_2_mHLA_2hr: 17507
SA = 0.86
Score = 1.66
SVNELLRYK − Mascot − rejected
SVNEL|L|RYK − spliced − 0|2|0|0
20120321_EXQ1_MiBa_SA_HCC1143_2: 14498
SA = 0.85
Score = 1.74
SVNELIYKR − MaxQuant
20120321_EXQ1_MiBa_SA_HCC1143_2: 14498
SA = 0.93
Score = 3.49
SVNELIYKR − MSFragger
20120321_EXQ1_MiBa_SA_HCC1143_2: 14498
SA = 0.93
Score = 3.49
RDSLREQTI − Mascot − rejected
RDSLREQ|T|I − spliced − |||
20120321_EXQ1_MiBa_SA_HCC1143_1: 10635
SA = 0.85
Score = 2.65
RDSLREQTL − MaxQuant
20120321_EXQ1_MiBa_SA_HCC1143_1: 10635
SA = 0.86
Score = 2.71
RDPEIELFV − MSFragger
20120321_EXQ1_MiBa_SA_HCC1143_1: 10635
SA = 0.2
Score = −4.87
Relative intensity
m/z
Prosit prediction
* Exclusive in prediction
# Exclusive in experimental

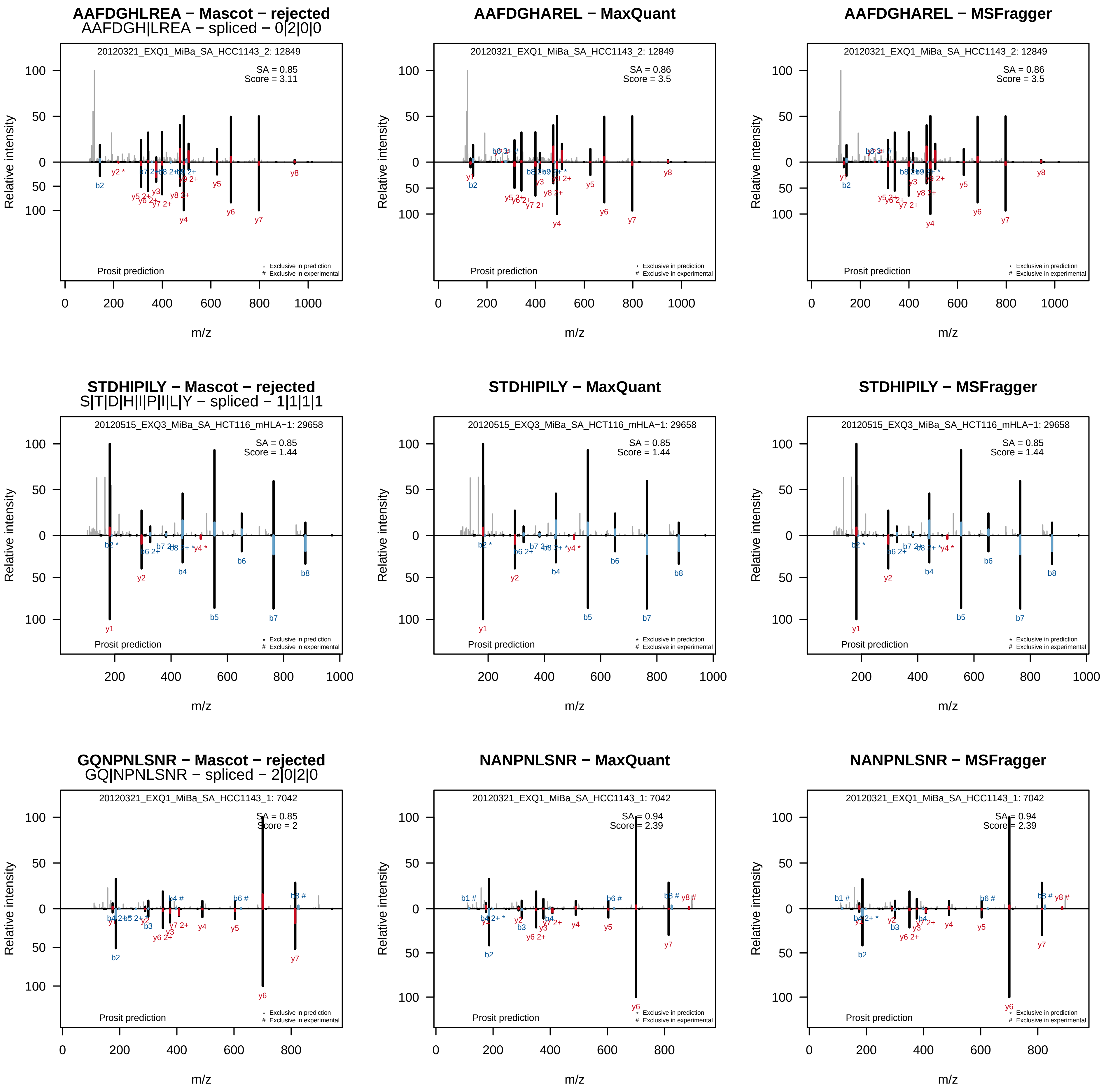

AAFDGHLREA − Mascot − rejected
AAFDGH|LREA − spliced − 0|2|0|0
20120321_EXQ1_MiBa_SA_HCC1143_2: 12849
SA = 0.85
Score = 3.11
AAFDGHAREL − MaxQuant
20120321_EXQ1_MiBa_SA_HCC1143_2: 12849
SA = 0.86
Score = 3.5
AAFDGHAREL − MSFragger
20120321_EXQ1_MiBa_SA_HCC1143_2: 12849
SA = 0.86
Score = 3.5
STDHIPILY − Mascot − rejected
S|T|D|H|I|P|I|L|Y − spliced − 1|1|1|1
20120515_EXQ3_MiBa_SA_HCT116_mHLA−1: 29658
SA = 0.85
Score = 1.44
STDHIPILY − MaxQuant
20120515_EXQ3_MiBa_SA_HCT116_mHLA−1: 29658
SA = 0.85
Score = 1.44
STDHIPILY − MSFragger
20120515_EXQ3_MiBa_SA_HCT116_mHLA−1: 29658
SA = 0.85
Score = 1.44
GQNPNLSNR − Mascot − rejected
GQ|NPNLSNR − spliced − 2|0|2|0
20120321_EXQ1_MiBa_SA_HCC1143_1: 7042
SA = 0.85
Score = 2
NANPNLSNR − MaxQuant
20120321_EXQ1_MiBa_SA_HCC1143_1: 7042
SA = 0.94
Score = 2.39
NANPNLSNR − MSFragger
20120321_EXQ1_MiBa_SA_HCC1143_1: 7042
SA = 0.94
Score = 2.39
Relative intensity
m/z
Prosit prediction
* Exclusive in prediction
# Exclusive in experimental

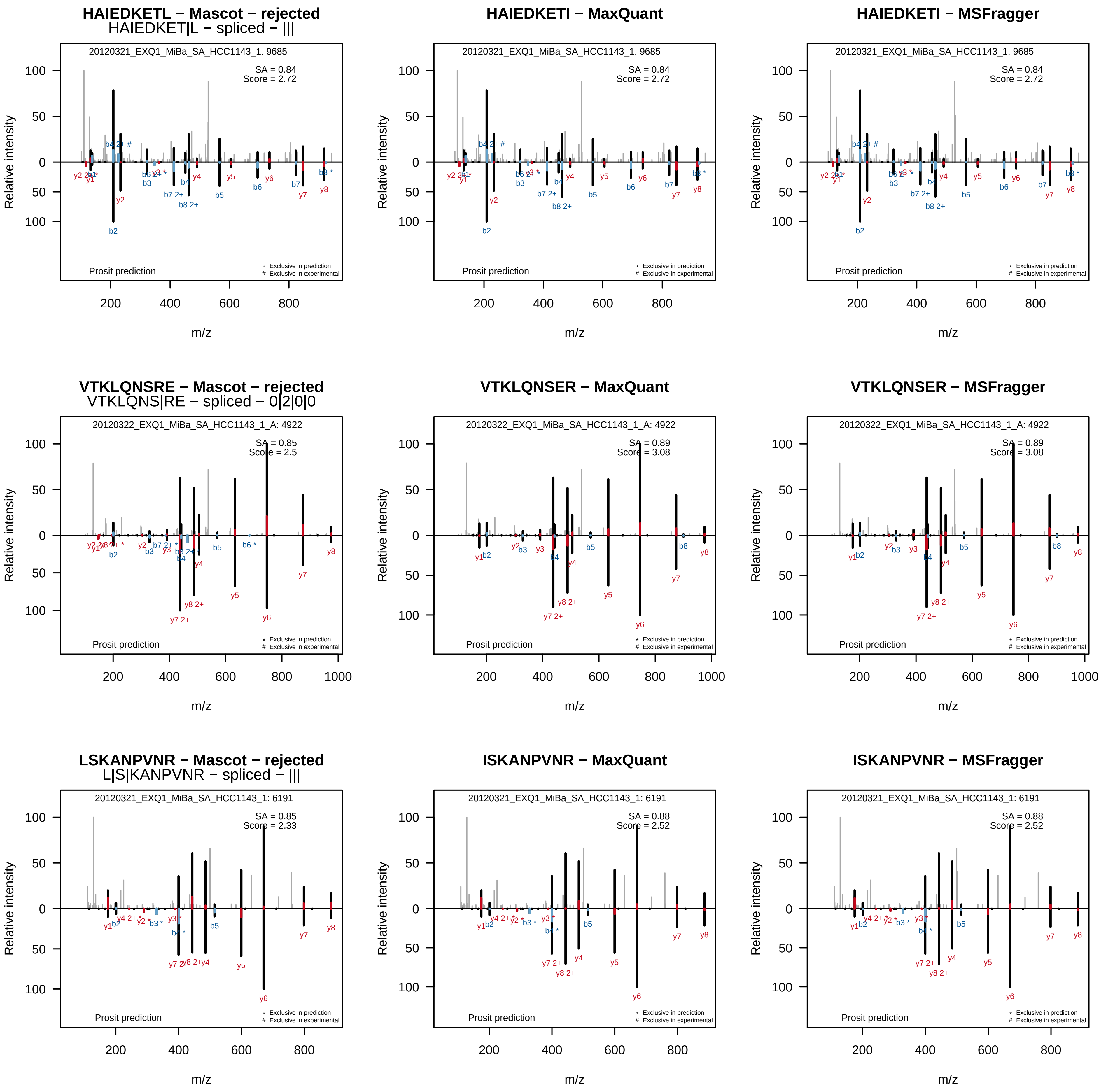

HAIEDKETL − Mascot − rejected
HAIEDKET|L − spliced − |||
20120321_EXQ1_MiBa_SA_HCC1143_1: 9685
SA = 0.84
Score = 2.72
HAIEDKETI − MaxQuant
20120321_EXQ1_MiBa_SA_HCC1143_1: 9685
SA = 0.84
Score = 2.72
HAIEDKETI − MSFragger
20120321_EXQ1_MiBa_SA_HCC1143_1: 9685
SA = 0.84
Score = 2.72
VTKLQNSRE − Mascot − rejected
VTKLQNS|RE − spliced − 0|2|0|0
20120322_EXQ1_MiBa_SA_HCC1143_1_A: 4922
SA = 0.85
Score = 2.5
VTKLQNSER − MaxQuant
20120322_EXQ1_MiBa_SA_HCC1143_1_A: 4922
SA = 0.89
Score = 3.08
VTKLQNSER − MSFragger
20120322_EXQ1_MiBa_SA_HCC1143_1_A: 4922
SA = 0.89
Score = 3.08
LSKANPVNR − Mascot − rejected
L|S|KANPVNR − spliced − |||
20120321_EXQ1_MiBa_SA_HCC1143_1: 6191
SA = 0.85
Score = 2.33
ISKANPVNR − MaxQuant
20120321_EXQ1_MiBa_SA_HCC1143_1: 6191
SA = 0.88
Score = 2.52
ISKANPVNR − MSFragger
20120321_EXQ1_MiBa_SA_HCC1143_1: 6191
SA = 0.88
Score = 2.52
Relative intensity
m/z
Prosit prediction
* Exclusive in prediction
# Exclusive in experimental

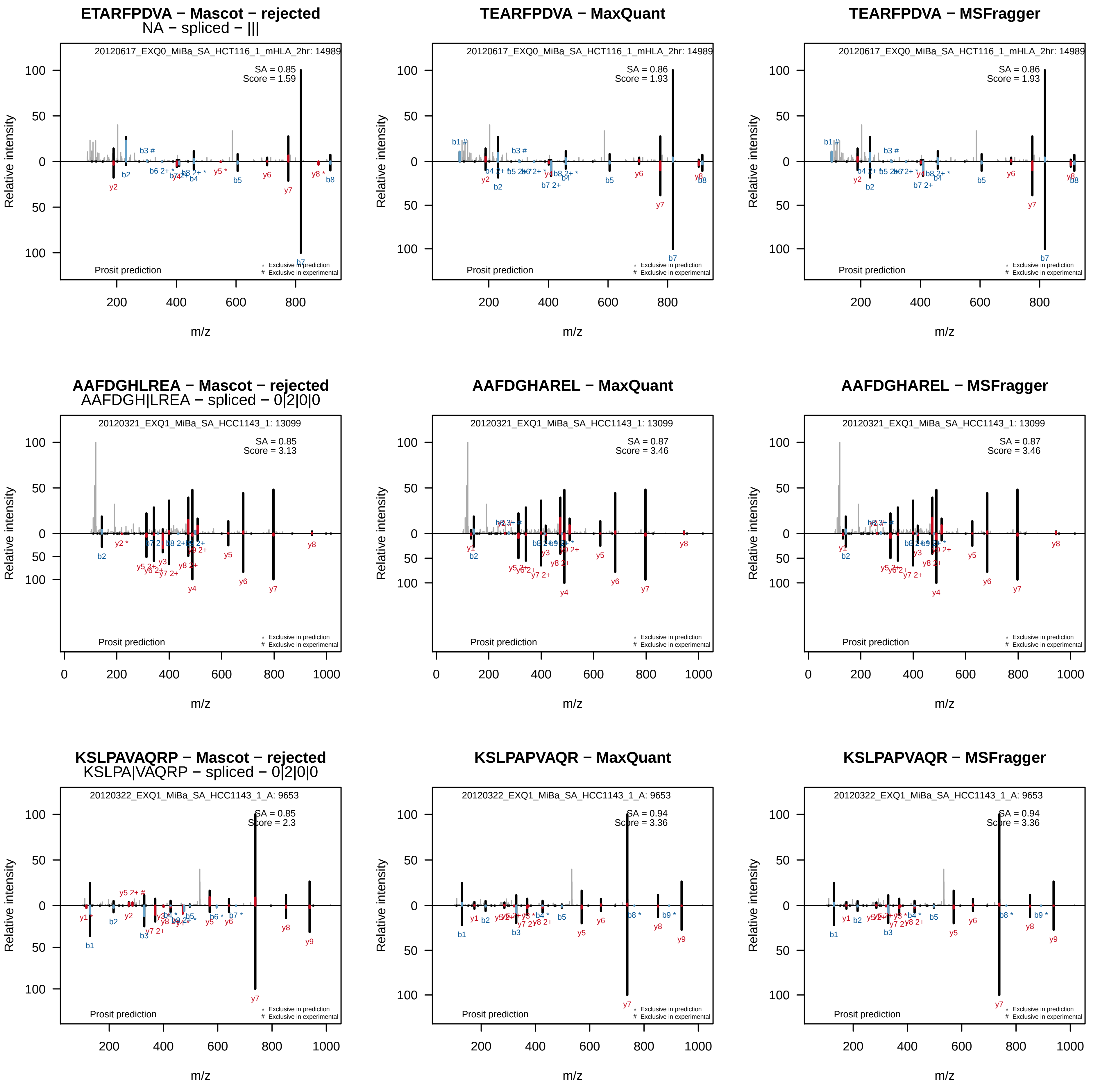

ETARFPDVA − Mascot − rejected
NA − spliced − |||
20120617_EXQ0_MiBa_SA_HCT116_1_mHLA_2hr: 14989
SA = 0.85
Score = 1.59
TEARFPDVA − MaxQuant
20120617_EXQ0_MiBa_SA_HCT116_1_mHLA_2hr: 14989
SA = 0.86
Score = 1.93
TEARFPDVA − MSFragger
20120617_EXQ0_MiBa_SA_HCT116_1_mHLA_2hr: 14989
SA = 0.86
Score = 1.93
AAFDGHLREA − Mascot − rejected
AAFDGH|LREA − spliced − 0|2|0|0
20120321_EXQ1_MiBa_SA_HCC1143_1: 13099
SA = 0.85
Score = 3.13
AAFDGHAREL − MaxQuant
20120321_EXQ1_MiBa_SA_HCC1143_1: 13099
SA = 0.87
Score = 3.46
AAFDGHAREL − MSFragger
20120321_EXQ1_MiBa_SA_HCC1143_1: 13099
SA = 0.87
Score = 3.46
KSLPAVAQRP − Mascot − rejected
KSLPA|VAQRP − spliced − 0|2|0|0
20120322_EXQ1_MiBa_SA_HCC1143_1_A: 9653
SA = 0.85
Score = 2.3
KSLPAPVAQR − MaxQuant
20120322_EXQ1_MiBa_SA_HCC1143_1_A: 9653
SA = 0.94
Score = 3.36
KSLPAPVAQR − MSFragger
20120322_EXQ1_MiBa_SA_HCC1143_1_A: 9653
SA = 0.94
Score = 3.36
Relative intensity
m/z
Prosit prediction
* Exclusive in prediction
# Exclusive in experimental

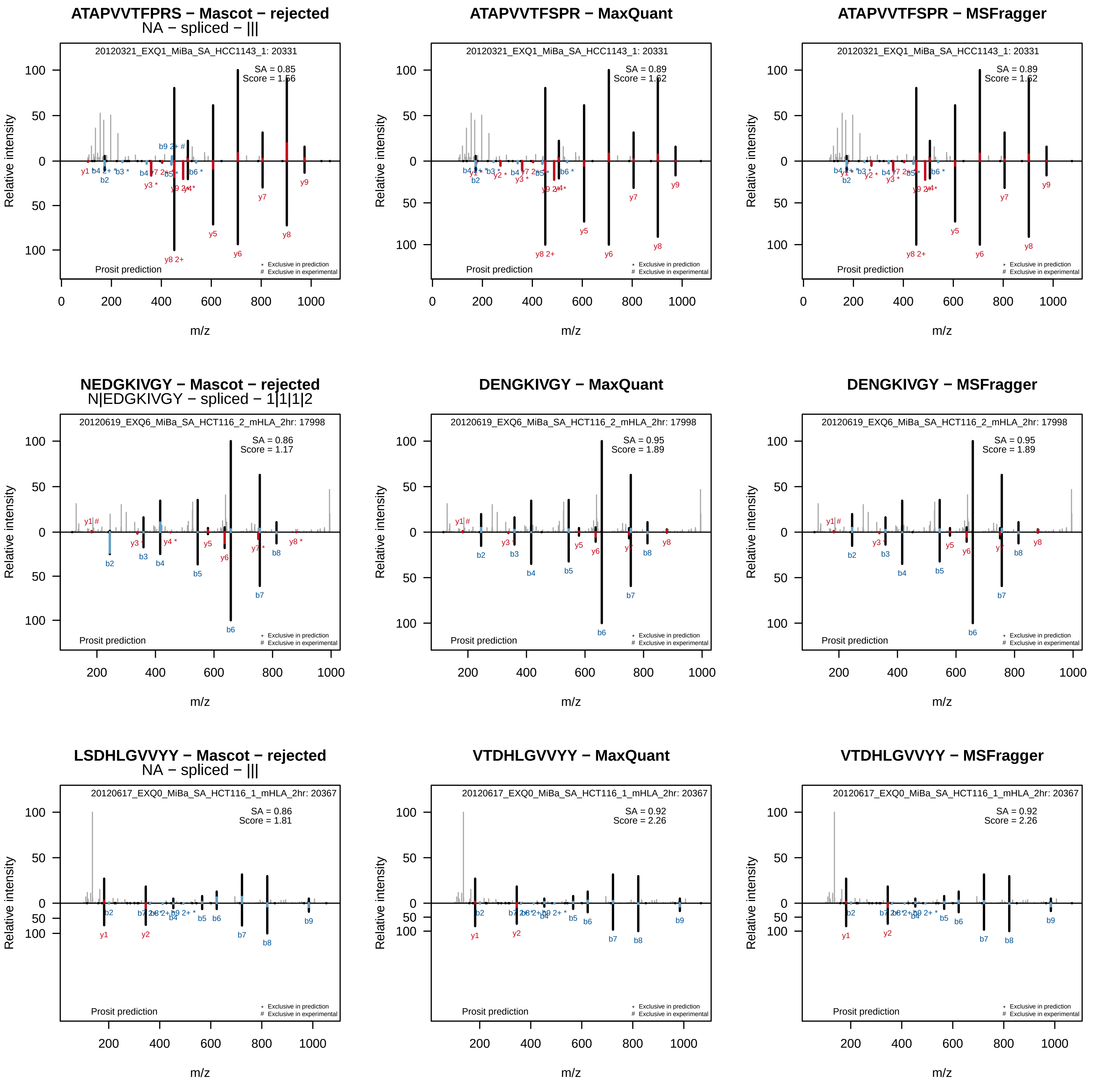

ATAPVVTFPRS − Mascot − rejected
NA − spliced − |||
20120321_EXQ1_MiBa_SA_HCC1143_1: 20331
SA = 0.85
Score = 1.56
ATAPVVTFSPR − MaxQuant
20120321_EXQ1_MiBa_SA_HCC1143_1: 20331
SA = 0.89
Score = 1.62
ATAPVVTFSPR − MSFragger
20120321_EXQ1_MiBa_SA_HCC1143_1: 20331
SA = 0.89
Score = 1.62
NEDGKIVGY − Mascot − rejected
N|EDGKIVGY − spliced − 1|1|1|2
20120619_EXQ6_MiBa_SA_HCT116_2_mHLA_2hr: 17998
SA = 0.86
Score = 1.17
DENGKIVGY − MaxQuant
20120619_EXQ6_MiBa_SA_HCT116_2_mHLA_2hr: 17998
SA = 0.95
Score = 1.89
DENGKIVGY − MSFragger
20120619_EXQ6_MiBa_SA_HCT116_2_mHLA_2hr: 17998
SA = 0.95
Score = 1.89
LSDHLGVVYY − Mascot − rejected
NA − spliced − |||
20120617_EXQ0_MiBa_SA_HCT116_1_mHLA_2hr: 20367
SA = 0.86
Score = 1.81
VTDHLGVVYY − MaxQuant
20120617_EXQ0_MiBa_SA_HCT116_1_mHLA_2hr: 20367
SA = 0.92
Score = 2.26
VTDHLGVVYY − MSFragger
20120617_EXQ0_MiBa_SA_HCT116_1_mHLA_2hr: 20367
SA = 0.92
Score = 2.26
Relative intensity
m/z
Prosit prediction
* Exclusive in prediction
# Exclusive in experimental

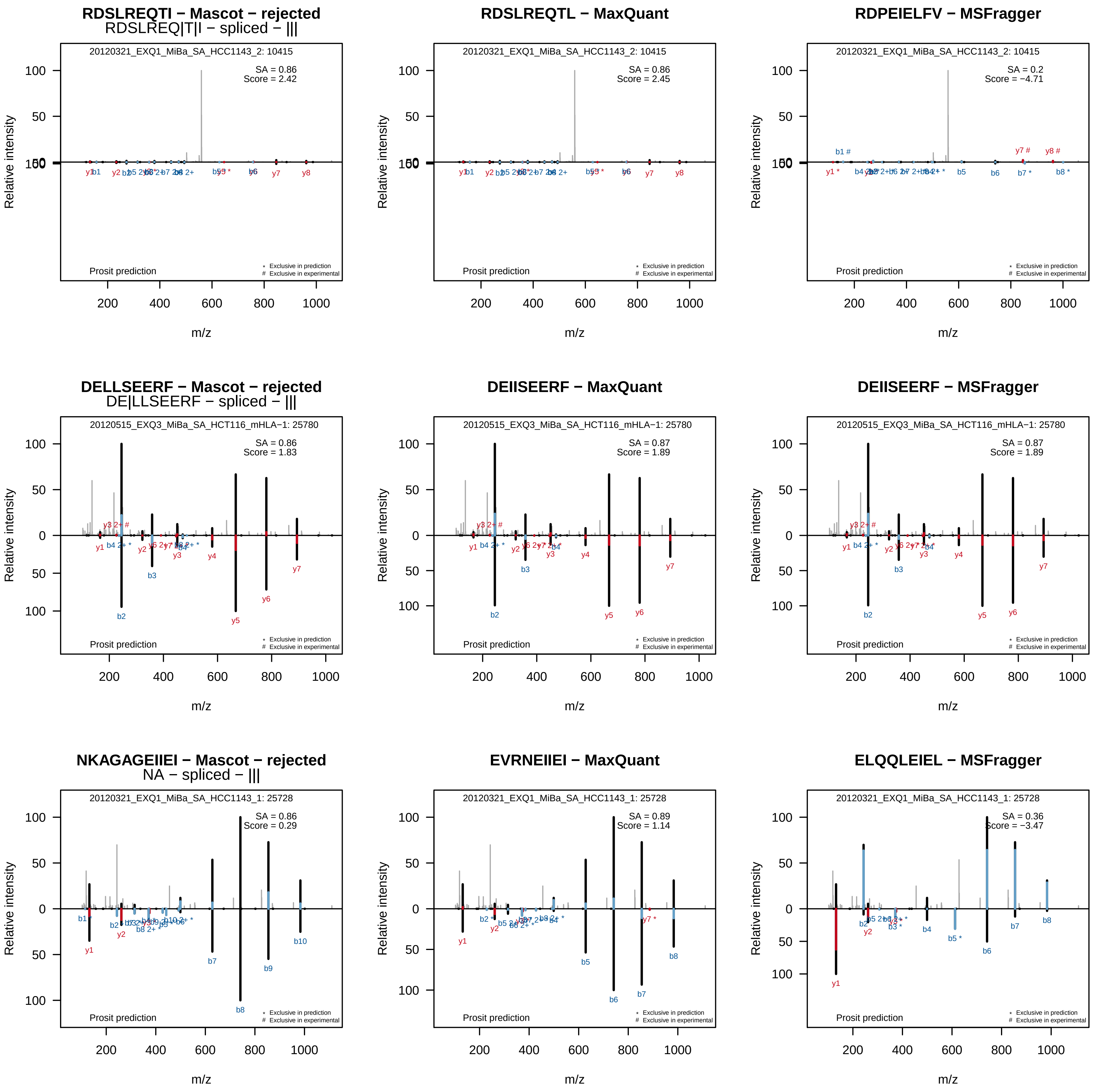

RDSLREQTI − Mascot − rejected
RDSLREQ|T|I − spliced − |||
20120321_EXQ1_MiBa_SA_HCC1143_2: 10415
SA = 0.86
Score = 2.42
RDSLREQTL − MaxQuant
20120321_EXQ1_MiBa_SA_HCC1143_2: 10415
SA = 0.86
Score = 2.45
RDPEIELFV − MSFragger
20120321_EXQ1_MiBa_SA_HCC1143_2: 10415
SA = 0.2
Score = −4.71
DELLSEERF − Mascot − rejected
DE|LLSEERF − spliced − |||
20120515_EXQ3_MiBa_SA_HCT116_mHLA−1: 25780
SA = 0.86
Score = 1.83
DEIISEERF − MaxQuant
20120515_EXQ3_MiBa_SA_HCT116_mHLA−1: 25780
SA = 0.87
Score = 1.89
DEIISEERF − MSFragger
20120515_EXQ3_MiBa_SA_HCT116_mHLA−1: 25780
SA = 0.87
Score = 1.89
NKAGAGEIIEI − Mascot − rejected
NA − spliced − |||
20120321_EXQ1_MiBa_SA_HCC1143_1: 25728
SA = 0.86
Score = 0.29
EVRNEIIEI − MaxQuant
20120321_EXQ1_MiBa_SA_HCC1143_1: 25728
SA = 0.89
Score = 1.14
ELQQLEIEL − MSFragger
20120321_EXQ1_MiBa_SA_HCC1143_1: 25728
SA = 0.36
Score = −3.47
Relative intensity
m/z
Prosit prediction
* Exclusive in prediction
# Exclusive in experimental

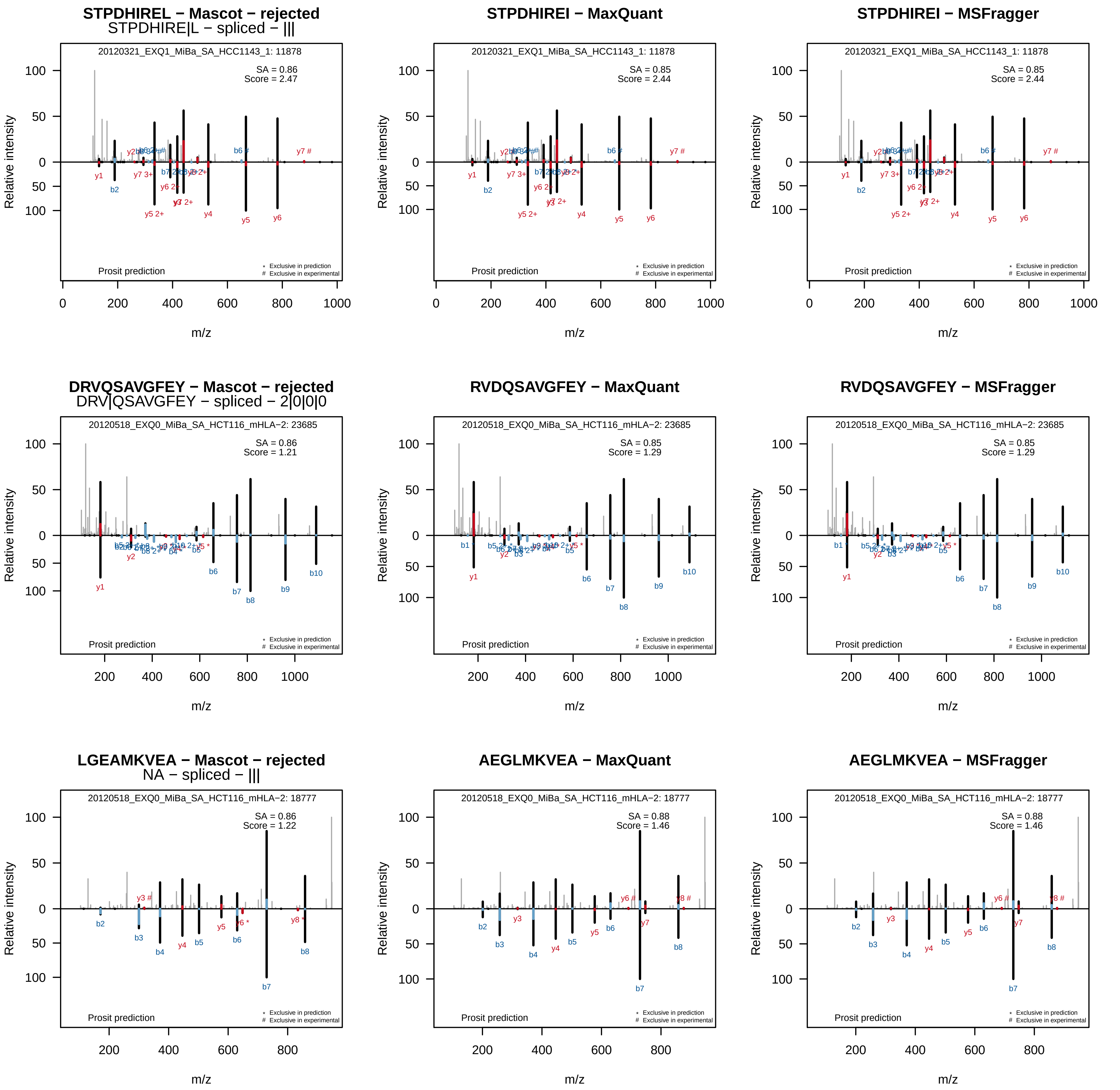

STPDHIREL − Mascot − rejected
STPDHIRE|L − spliced − |||
20120321_EXQ1_MiBa_SA_HCC1143_1: 11878
SA = 0.86
Score = 2.47
STPDHIREI − MaxQuant
20120321_EXQ1_MiBa_SA_HCC1143_1: 11878
SA = 0.85
Score = 2.44
STPDHIREI − MSFragger
20120321_EXQ1_MiBa_SA_HCC1143_1: 11878
SA = 0.85
Score = 2.44
DRVQSAVGFEY − Mascot − rejected
DRV|QSAVGFEY − spliced − 2|0|0|0
20120518_EXQ0_MiBa_SA_HCT116_mHLA−2: 23685
SA = 0.86
Score = 1.21
RVDQSAVGFEY − MaxQuant
20120518_EXQ0_MiBa_SA_HCT116_mHLA−2: 23685
SA = 0.85
Score = 1.29
RVDQSAVGFEY − MSFragger
20120518_EXQ0_MiBa_SA_HCT116_mHLA−2: 23685
SA = 0.85
Score = 1.29
LGEAMKVEA − Mascot − rejected
NA − spliced − |||
20120518_EXQ0_MiBa_SA_HCT116_mHLA−2: 18777
SA = 0.86
Score = 1.22
AEGLMKVEA − MaxQuant
20120518_EXQ0_MiBa_SA_HCT116_mHLA−2: 18777
SA = 0.88
Score = 1.46
AEGLMKVEA − MSFragger
20120518_EXQ0_MiBa_SA_HCT116_mHLA−2: 18777
SA = 0.88
Score = 1.46
Relative intensity
m/z
Prosit prediction
* Exclusive in prediction
# Exclusive in experimental

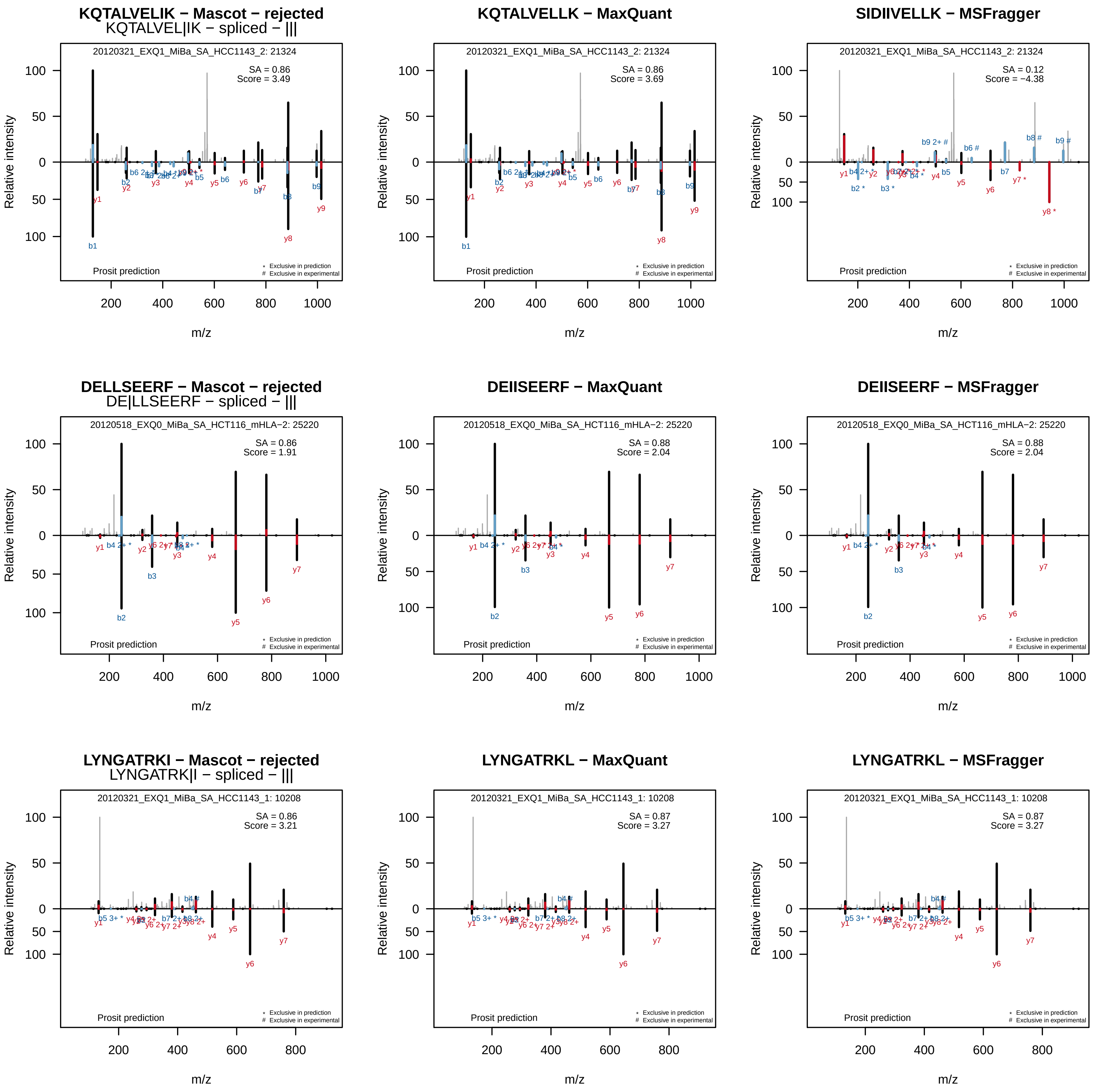
KQTALVELIK − Mascot − rejected
KQTALVEL|IK − spliced − |||
20120321_EXQ1_MiBa_SA_HCC1143_2: 21324
SA = 0.86
Score = 3.49
KQTALVELLK − MaxQuant
20120321_EXQ1_MiBa_SA_HCC1143_2: 21324
SA = 0.86
Score = 3.69
SIDIIVELLK − MSFragger
20120321_EXQ1_MiBa_SA_HCC1143_2: 21324
SA = 0.12
Score = −4.38
DELLSEERF − Mascot − rejected
DE|LLSEERF − spliced − |||
20120518_EXQ0_MiBa_SA_HCT116_mHLA−2: 25220
SA = 0.86
Score = 1.91
DEIISEERF − MaxQuant
20120518_EXQ0_MiBa_SA_HCT116_mHLA−2: 25220
SA = 0.88
Score = 2.04
DEIISEERF − MSFragger
20120518_EXQ0_MiBa_SA_HCT116_mHLA−2: 25220
SA = 0.88
Score = 2.04
LYNGATRKI − Mascot − rejected
LYNGATRK|I − spliced − |||
20120321_EXQ1_MiBa_SA_HCC1143_1: 10208
SA = 0.86
Score = 3.21
LYNGATRKL − MaxQuant
20120321_EXQ1_MiBa_SA_HCC1143_1: 10208
SA = 0.87
Score = 3.27
LYNGATRKL − MSFragger
20120321_EXQ1_MiBa_SA_HCC1143_1: 10208
SA = 0.87
Score = 3.27
Relative intensity
m/z
Prosit prediction
* Exclusive in prediction
# Exclusive in experimental

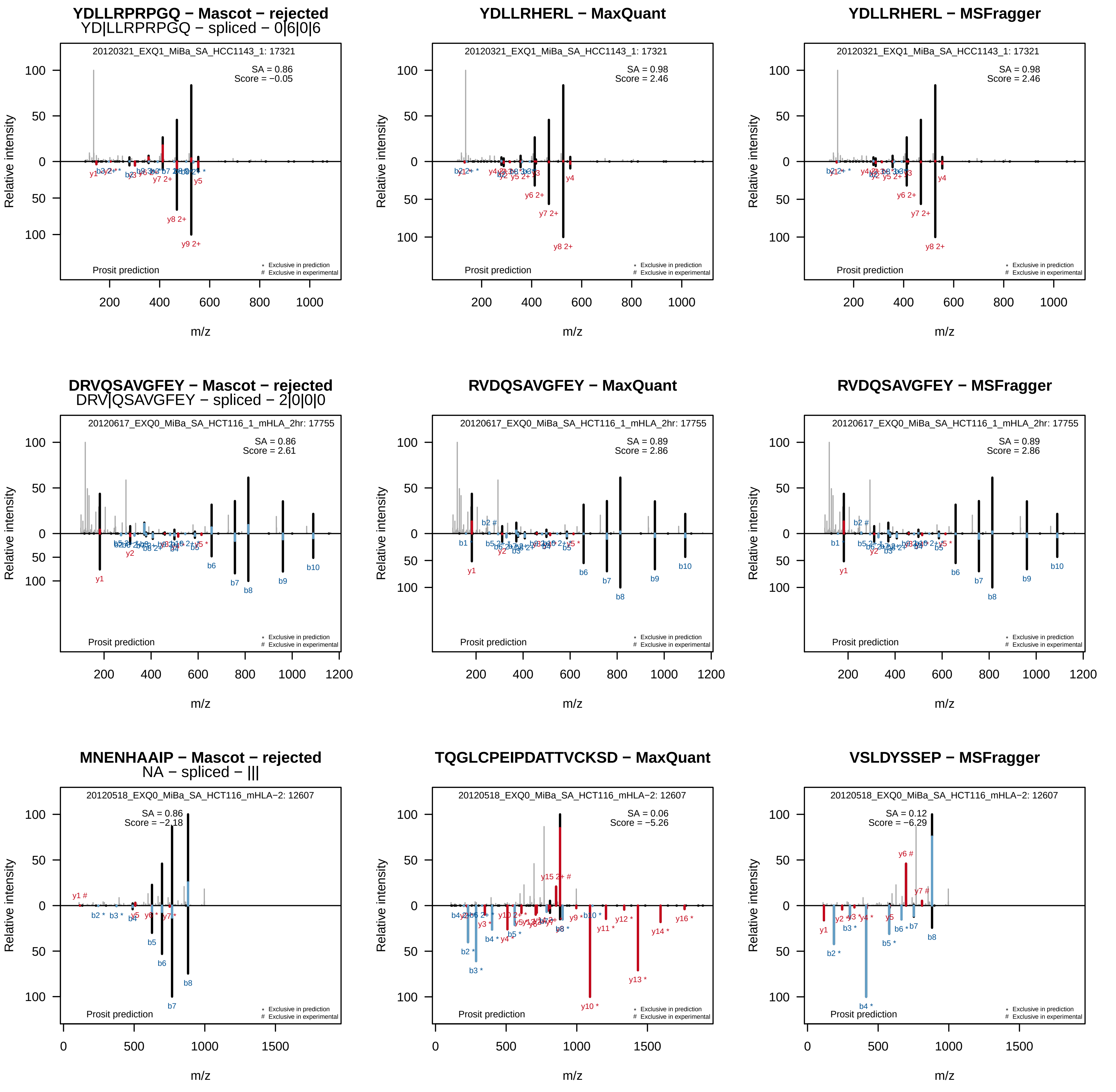

YDLLRPRPGQ − Mascot − rejected
YD|LLRPRPGQ − spliced − 0|6|0|6
20120321_EXQ1_MiBa_SA_HCC1143_1: 17321
SA = 0.86
Score = −0.05
YDLLRHERL − MaxQuant
20120321_EXQ1_MiBa_SA_HCC1143_1: 17321
SA = 0.98
Score = 2.46
YDLLRHERL − MSFragger
20120321_EXQ1_MiBa_SA_HCC1143_1: 17321
SA = 0.98
Score = 2.46
DRVQSAVGFEY − Mascot − rejected
DRV|QSAVGFEY − spliced − 2|0|0|0
20120617_EXQ0_MiBa_SA_HCT116_1_mHLA_2hr: 17755
SA = 0.86
Score = 2.61
RVDQSAVGFEY − MaxQuant
20120617_EXQ0_MiBa_SA_HCT116_1_mHLA_2hr: 17755
SA = 0.89
Score = 2.86
RVDQSAVGFEY − MSFragger
20120617_EXQ0_MiBa_SA_HCT116_1_mHLA_2hr: 17755
SA = 0.89
Score = 2.86
MNENHAAIP − Mascot − rejected
NA − spliced − |||
20120518_EXQ0_MiBa_SA_HCT116_mHLA−2: 12607
SA = 0.86
Score = −2.18
TQGLCPEIPDATTVCKSD − MaxQuant
20120518_EXQ0_MiBa_SA_HCT116_mHLA−2: 12607
SA = 0.06
Score = −5.26
VSLDYSSEP − MSFragger
20120518_EXQ0_MiBa_SA_HCT116_mHLA−2: 12607
SA = 0.12
Score = −6.29
Relative intensity
m/z
Prosit prediction
⋆ Exclusive in prediction
Exclusive in experimental

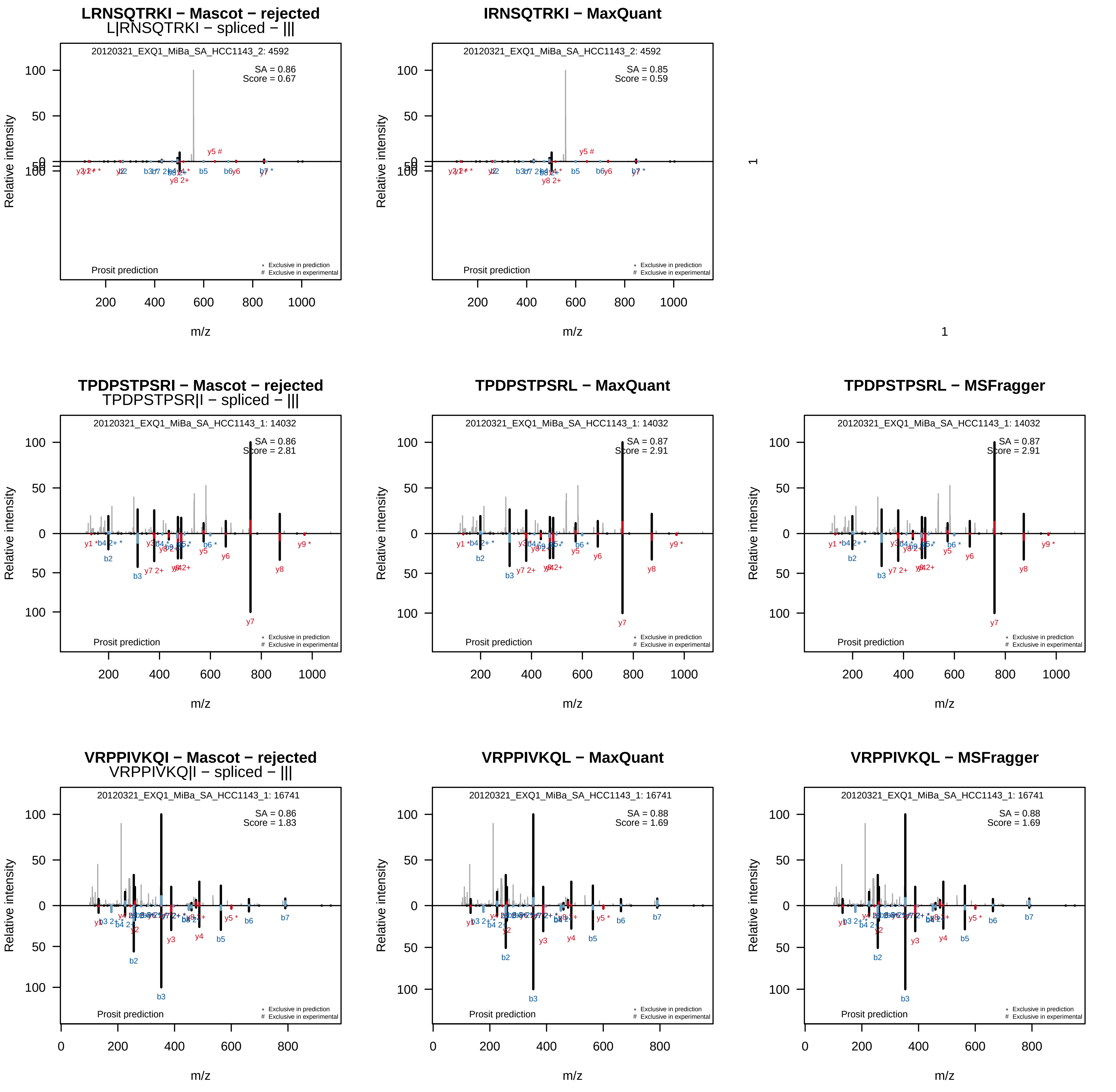

LRNSQTRKI − Mascot − rejected
L|RNSQTRKI − spliced − |||
20120321_EXQ1_MiBa_SA_HCC1143_2: 4592
SA = 0.86
Score = 0.67
IRNSQTRKI − MaxQuant
20120321_EXQ1_MiBa_SA_HCC1143_2: 4592
SA = 0.85
Score = 0.59
TPDPSTPSRI − Mascot − rejected
TPDPSTPSR|I − spliced − |||
20120321_EXQ1_MiBa_SA_HCC1143_1: 14032
SA = 0.86
Score = 2.81
TPDPSTPSRL − MaxQuant
20120321_EXQ1_MiBa_SA_HCC1143_1: 14032
SA = 0.87
Score = 2.91
TPDPSTPSRL − MSFragger
20120321_EXQ1_MiBa_SA_HCC1143_1: 14032
SA = 0.87
Score = 2.91
VRPPIVKQI − Mascot − rejected
VRPPIVKQ|I − spliced − |||
20120321_EXQ1_MiBa_SA_HCC1143_1: 16741
SA = 0.86
Score = 1.83
VRPPIVKQL − MaxQuant
20120321_EXQ1_MiBa_SA_HCC1143_1: 16741
SA = 0.88
Score = 1.69
VRPPIVKQL − MSFragger
20120321_EXQ1_MiBa_SA_HCC1143_1: 16741
SA = 0.88
Score = 1.69
Relative intensity
m/z
Prosit prediction
⋆ Exclusive in prediction
Exclusive in experimental

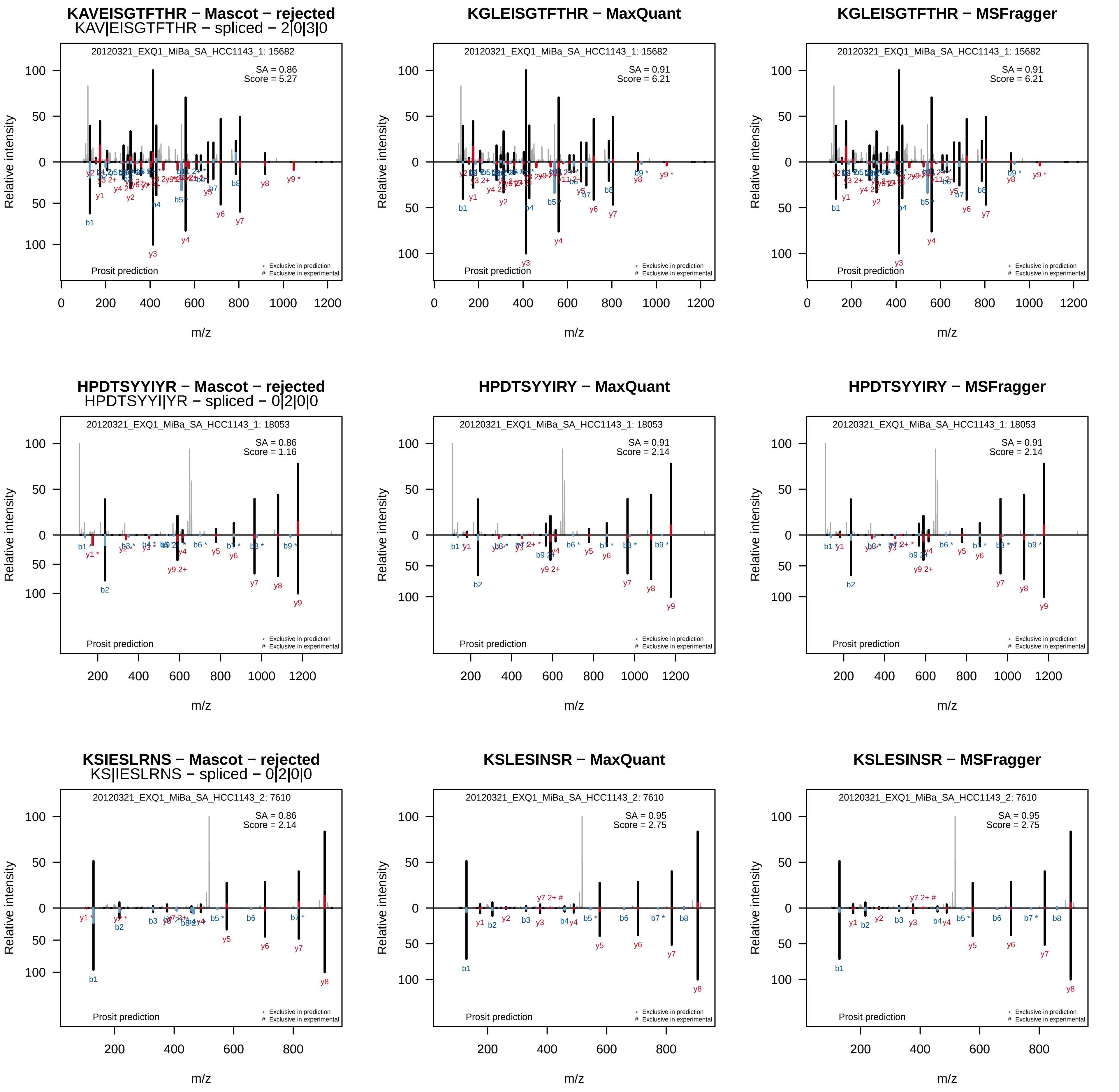

KAVEISGTFTHR − Mascot − rejected
KAV|EISGTFTHR − spliced − 2|0|3|0
20120321_EXQ1_MiBa_SA_HCC1143_1: 15682
SA = 0.86
Score = 5.27
KGLEISGTFTHR − MaxQuant
20120321_EXQ1_MiBa_SA_HCC1143_1: 15682
SA = 0.91
Score = 6.21
KGLEISGTFTHR − MSFragger
20120321_EXQ1_MiBa_SA_HCC1143_1: 15682
SA = 0.91
Score = 6.21
HPDTSYYIYR − Mascot − rejected
HPDTSYYI|YR − spliced − 0|2|0|0
20120321_EXQ1_MiBa_SA_HCC1143_1: 18053
SA = 0.86
Score = 1.16
HPDTSYYIRY − MaxQuant
20120321_EXQ1_MiBa_SA_HCC1143_1: 18053
SA = 0.91
Score = 2.14
HPDTSYYIRY − MSFragger
20120321_EXQ1_MiBa_SA_HCC1143_1: 18053
SA = 0.91
Score = 2.14
KSIESLRNS − Mascot − rejected
KS|IESLRNS − spliced − 0|2|0|0
20120321_EXQ1_MiBa_SA_HCC1143_2: 7610
SA = 0.86
Score = 2.14
KSLESINSR − MaxQuant
20120321_EXQ1_MiBa_SA_HCC1143_2: 7610
SA = 0.95
Score = 2.75
KSLESINSR − MSFragger
20120321_EXQ1_MiBa_SA_HCC1143_2: 7610
SA = 0.95
Score = 2.75
Relative intensity
m/z
Prosit prediction
* Exclusive in prediction
# Exclusive in experimental

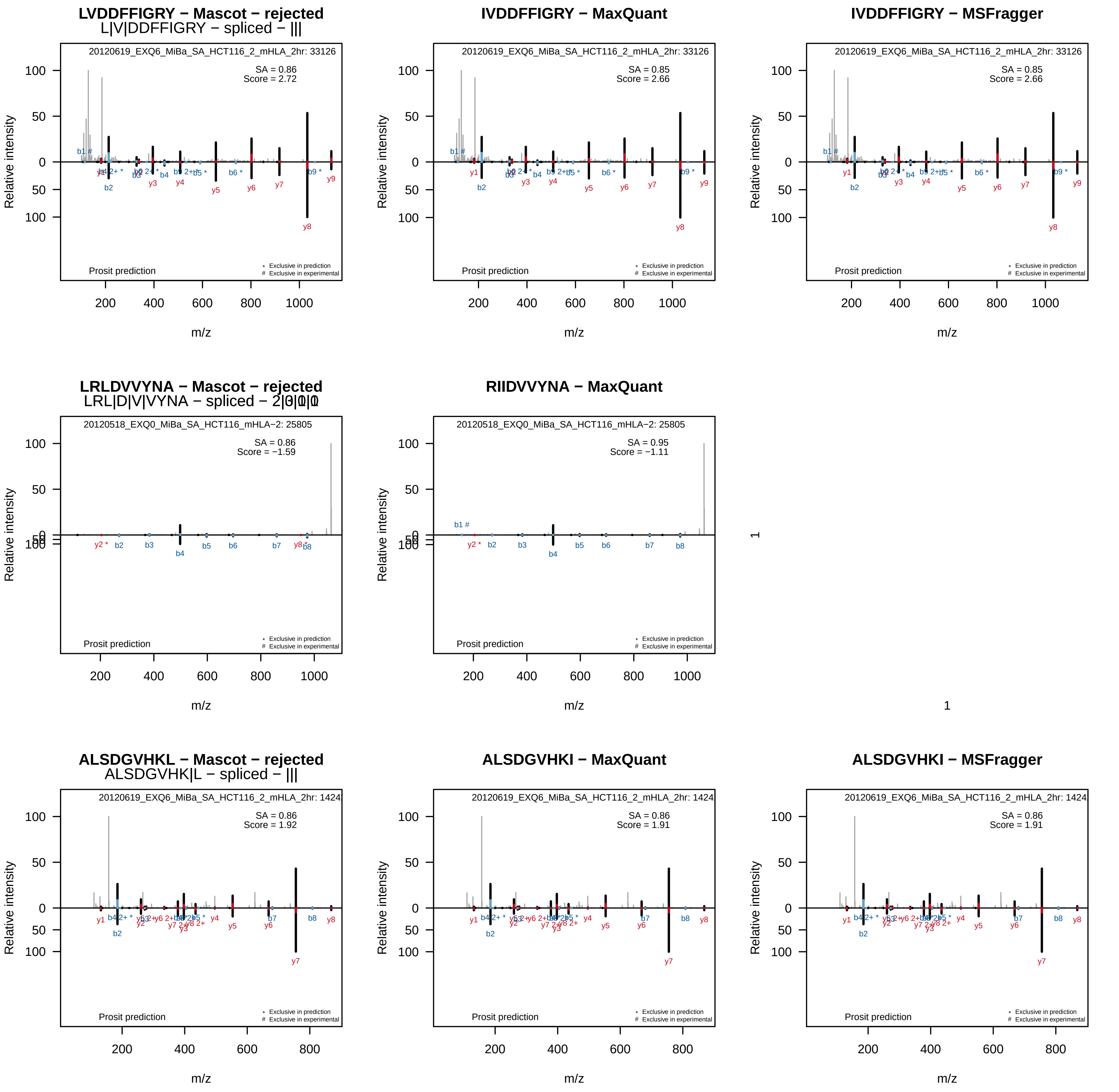

LVDDFFIGRY − Mascot − rejected
L|V|DDFFIGRY − spliced − |||
20120619_EXQ6_MiBa_SA_HCT116_2_mHLA_2hr: 33126
SA = 0.86
Score = 2.72
Prosit prediction
Exclusive in prediction
Exclusive in experimental
Relative intensity
m/z
IVDDFFIGRY − MaxQuant
20120619_EXQ6_MiBa_SA_HCT116_2_mHLA_2hr: 33126
SA = 0.85
Score = 2.66
Prosit prediction
IVDDFFIGRY − MSFragger
20120619_EXQ6_MiBa_SA_HCT116_2_mHLA_2hr: 33126
SA = 0.85
Score = 2.66
Prosit prediction
LRLDVVYNA − Mascot − rejected
RIIDVVYNA − MaxQuant
20120518_EXQ0_MiBa_SA_HCT116_mHLA−2: 25805
SA = 0.86
Score = −1.59
Prosit prediction
SA = 0.95
Score = −1.11
ALSDGVHKL − Mascot − rejected
ALSDGVHK|L − spliced − |||
SA = 0.86
Score = 1.92
Prosit prediction
ALSDGVHKI − MaxQuant
SA = 0.86
Score = 1.91
ALSDGVHKI − MSFragger
SA = 0.86
Score = 1.91
Prosit prediction

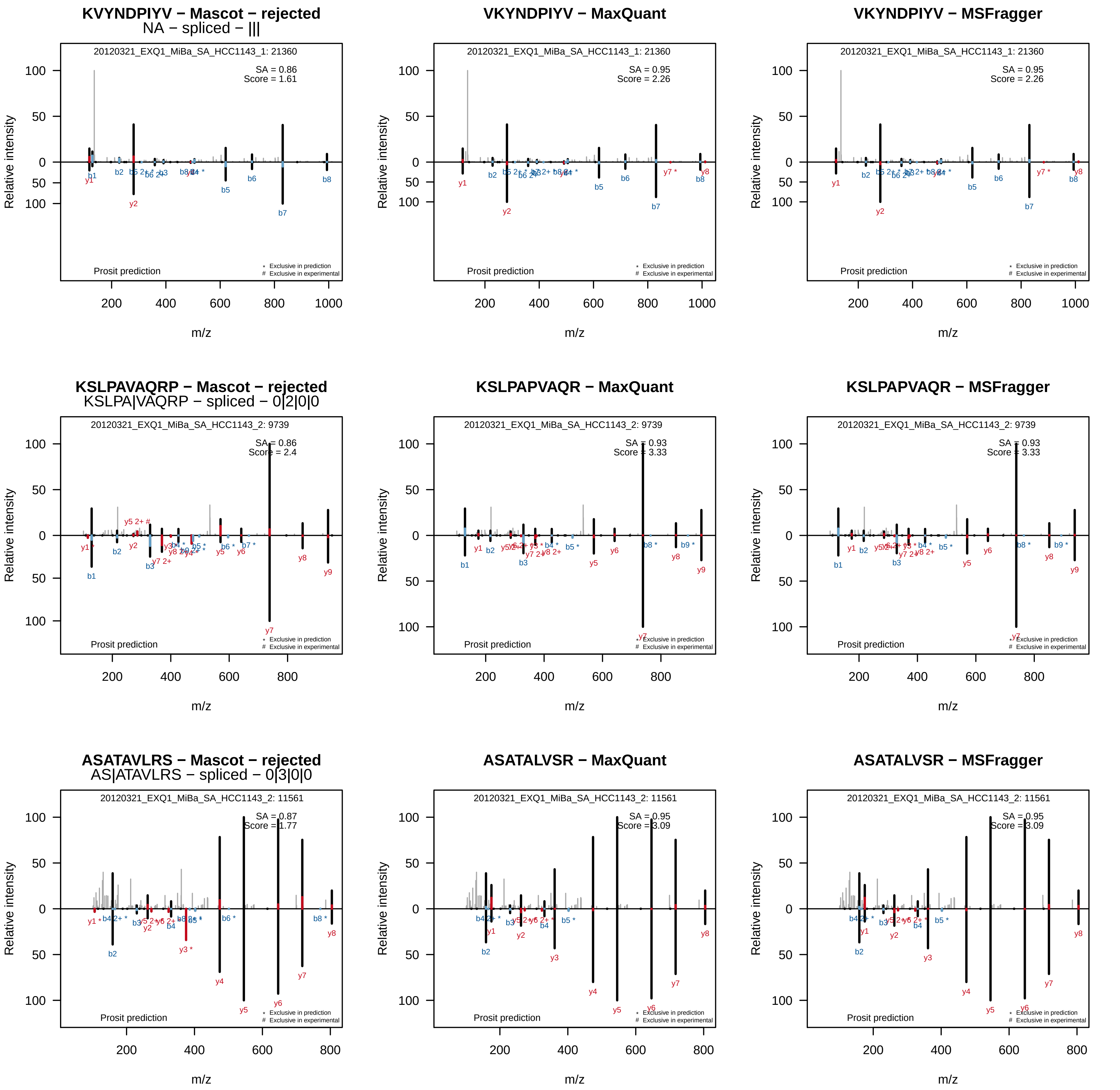
KVYNDPIYV − Mascot − rejected
NA − spliced − |||
20120321_EXQ1_MiBa_SA_HCC1143_1: 21360
SA = 0.86
Score = 1.61
VKYNDPIYV − MaxQuant
20120321_EXQ1_MiBa_SA_HCC1143_1: 21360
SA = 0.95
Score = 2.26
VKYNDPIYV − MSFragger
20120321_EXQ1_MiBa_SA_HCC1143_1: 21360
SA = 0.95
Score = 2.26
KSLPAVAQRP − Mascot − rejected
KSLPA|VAQRP − spliced − 0|2|0|0
20120321_EXQ1_MiBa_SA_HCC1143_2: 9739
SA = 0.86
Score = 2.4
KSLPAPVAQR − MaxQuant
20120321_EXQ1_MiBa_SA_HCC1143_2: 9739
SA = 0.93
Score = 3.33
KSLPAPVAQR − MSFragger
20120321_EXQ1_MiBa_SA_HCC1143_2: 9739
SA = 0.93
Score = 3.33
ASATAVLRS − Mascot − rejected
AS|ATAVLRS − spliced − 0|3|0|0
20120321_EXQ1_MiBa_SA_HCC1143_2: 11561
SA = 0.87
Score = 1.77
ASATALVSR − MaxQuant
20120321_EXQ1_MiBa_SA_HCC1143_2: 11561
SA = 0.95
Score = 3.09
ASATALVSR − MSFragger
20120321_EXQ1_MiBa_SA_HCC1143_2: 11561
SA = 0.95
Score = 3.09
Relative intensity
m/z
Prosit prediction
* Exclusive in prediction
# Exclusive in experimental

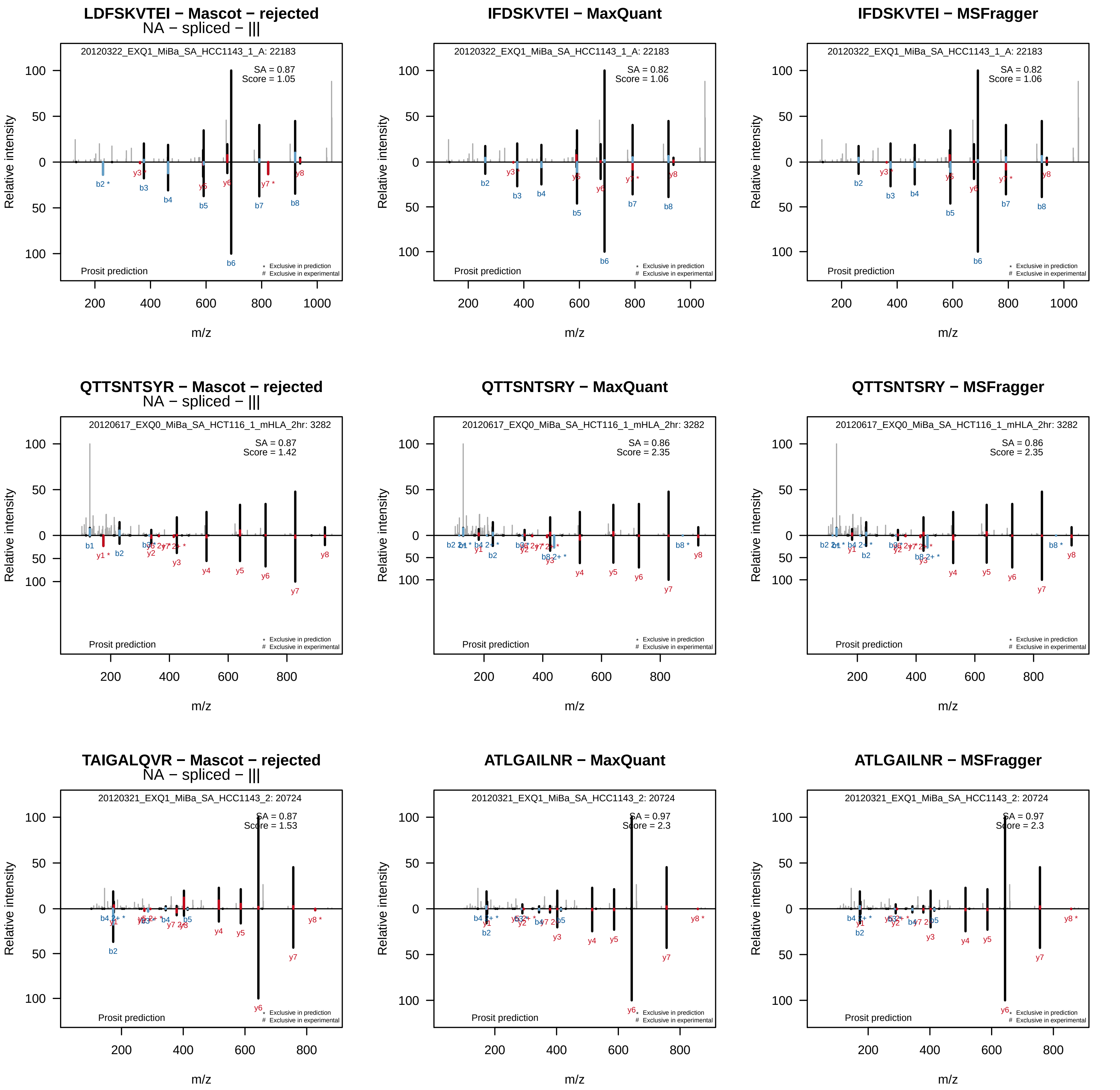
LDFSKVTEI − Mascot − rejected
NA − spliced − |||
20120322_EXQ1_MiBa_SA_HCC1143_1_A: 22183
SA = 0.87
Score = 1.05
IFDSKVTEI − MaxQuant
20120322_EXQ1_MiBa_SA_HCC1143_1_A: 22183
SA = 0.82
Score = 1.06
IFDSKVTEI − MSFragger
20120322_EXQ1_MiBa_SA_HCC1143_1_A: 22183
SA = 0.82
Score = 1.06
QTTSNTSYR − Mascot − rejected
NA − spliced − |||
20120617_EXQ0_MiBa_SA_HCT116_1_mHLA_2hr: 3282
SA = 0.87
Score = 1.42
QTTSNTSRY − MaxQuant
20120617_EXQ0_MiBa_SA_HCT116_1_mHLA_2hr: 3282
SA = 0.86
Score = 2.35
QTTSNTSRY − MSFragger
20120617_EXQ0_MiBa_SA_HCT116_1_mHLA_2hr: 3282
SA = 0.86
Score = 2.35
TAIGALQVR − Mascot − rejected
NA − spliced − |||
20120321_EXQ1_MiBa_SA_HCC1143_2: 20724
SA = 0.87
Score = 1.53
ATLGAILNR − MaxQuant
20120321_EXQ1_MiBa_SA_HCC1143_2: 20724
SA = 0.97
Score = 2.3
ATLGAILNR − MSFragger
20120321_EXQ1_MiBa_SA_HCC1143_2: 20724
SA = 0.97
Score = 2.3
Relative intensity
m/z
Prosit prediction
Exclusive in prediction
Exclusive in experimental

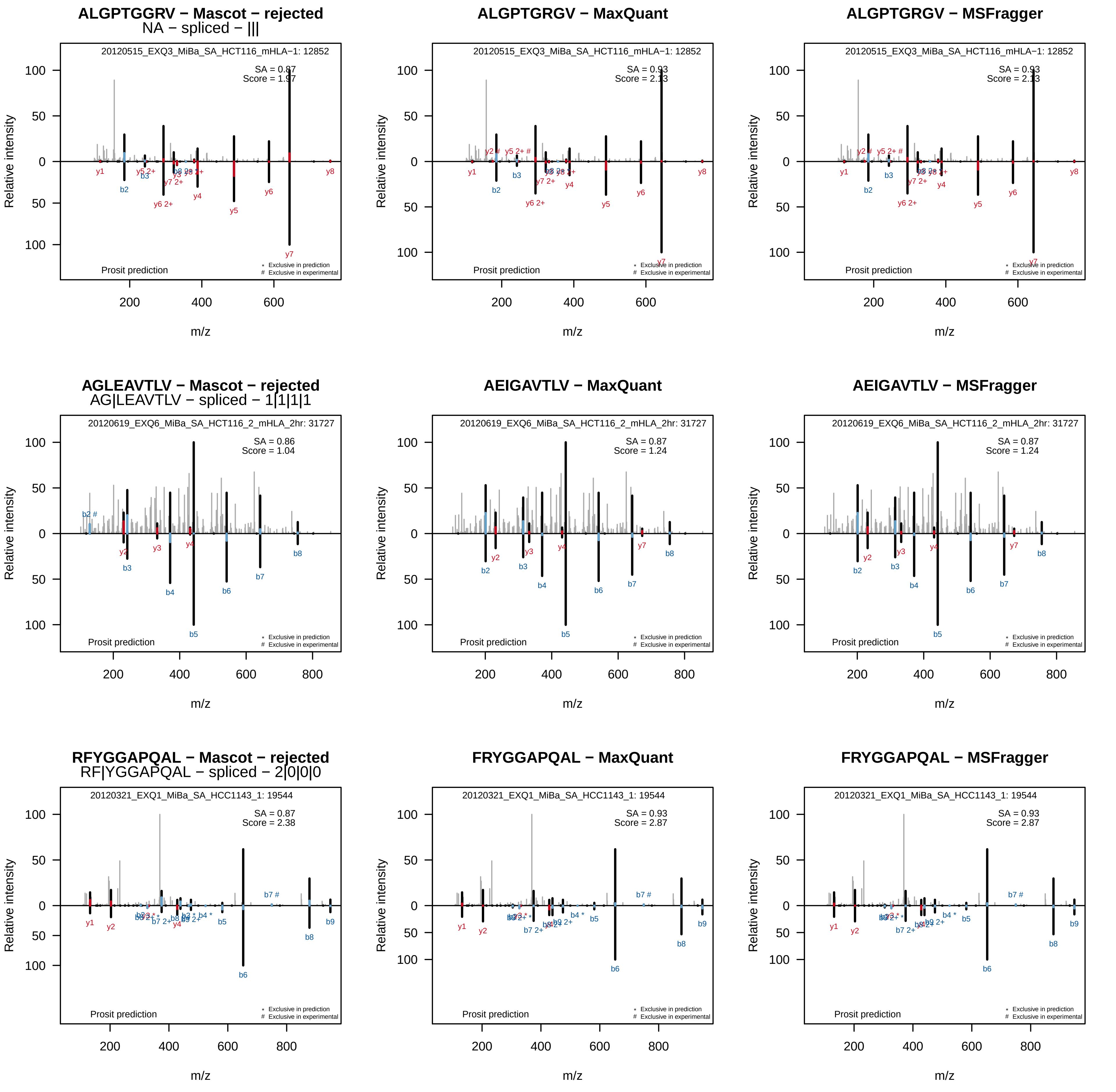
ALGPTGGRV − Mascot − rejected
NA − spliced − |||
20120515_EXQ3_MiBa_SA_HCT116_mHLA−1: 12852
SA = 0.87
Score = 1.97
ALGPTGRGV − MaxQuant
20120515_EXQ3_MiBa_SA_HCT116_mHLA−1: 12852
SA = 0.93
Score = 2.13
ALGPTGRGV − MSFragger
20120515_EXQ3_MiBa_SA_HCT116_mHLA−1: 12852
SA = 0.93
Score = 2.13
AGLEAVTLV − Mascot − rejected
AG|LEAVTLV − spliced − 1|1|1|1
20120619_EXQ6_MiBa_SA_HCT116_2_mHLA_2hr: 31727
SA = 0.86
Score = 1.04
AEIGAVTLV − MaxQuant
20120619_EXQ6_MiBa_SA_HCT116_2_mHLA_2hr: 31727
SA = 0.87
Score = 1.24
AEIGAVTLV − MSFragger
20120619_EXQ6_MiBa_SA_HCT116_2_mHLA_2hr: 31727
SA = 0.87
Score = 1.24
RFYGGAPQAL − Mascot − rejected
RF|YGGAPQAL − spliced − 2|0|0|0
20120321_EXQ1_MiBa_SA_HCC1143_1: 19544
SA = 0.87
Score = 2.38
FRYGGAPQAL − MaxQuant
20120321_EXQ1_MiBa_SA_HCC1143_1: 19544
SA = 0.93
Score = 2.87
FRYGGAPQAL − MSFragger
20120321_EXQ1_MiBa_SA_HCC1143_1: 19544
SA = 0.93
Score = 2.87
Relative intensity
m/z
Prosit prediction
* Exclusive in prediction
# Exclusive in experimental

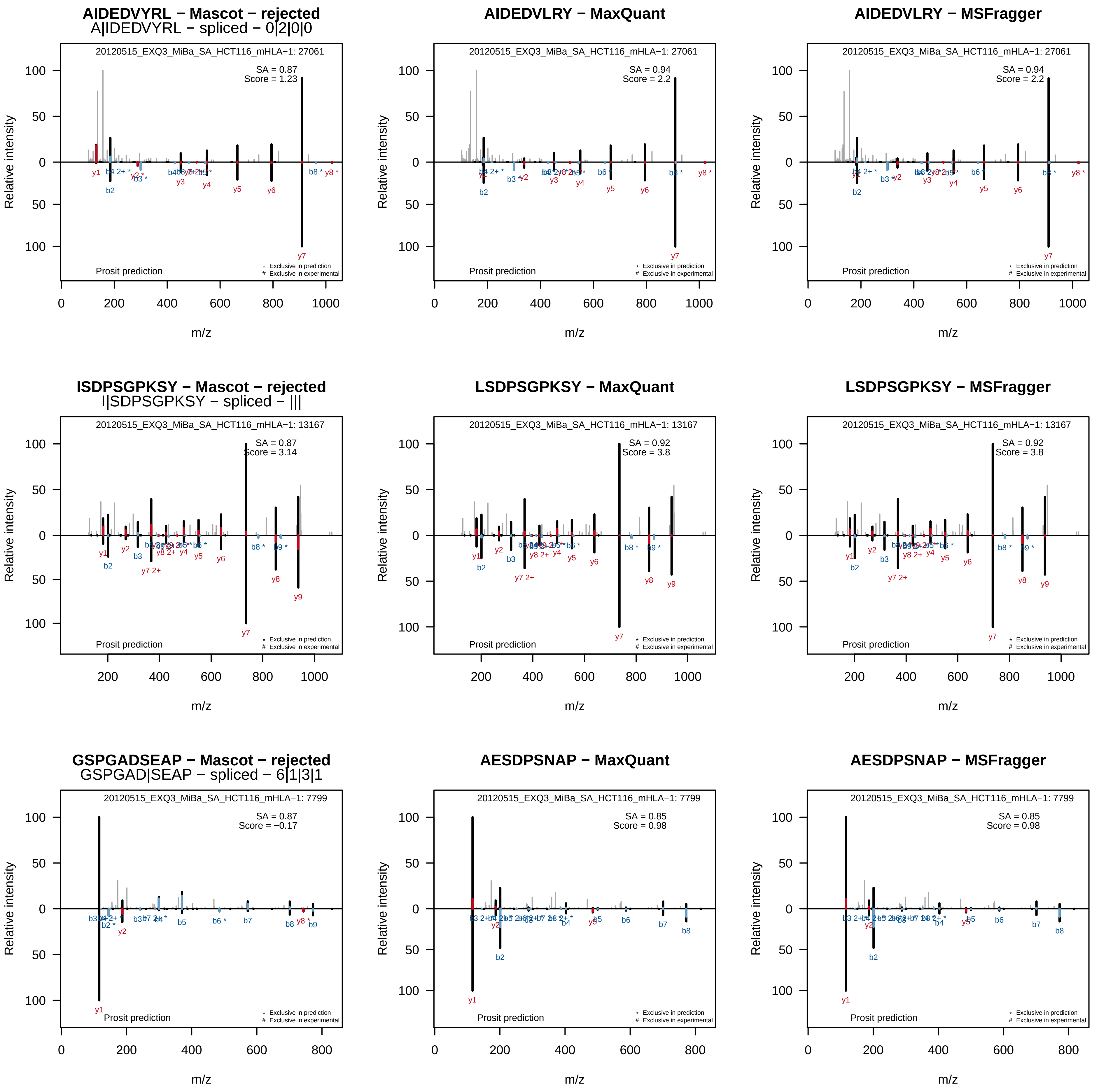

AIDEDVYRL − Mascot − rejected
A|IDEDVYRL − spliced − 0|2|0|0
20120515_EXQ3_MiBa_SA_HCT116_mHLA−1: 27061
SA = 0.87
Score = 1.23
AIDEDVLRY − MaxQuant
20120515_EXQ3_MiBa_SA_HCT116_mHLA−1: 27061
SA = 0.94
Score = 2.2
AIDEDVLRY − MSFragger
20120515_EXQ3_MiBa_SA_HCT116_mHLA−1: 27061
SA = 0.94
Score = 2.2
ISDPSGPKSY − Mascot − rejected
I|SDPSGPKSY − spliced − |||
20120515_EXQ3_MiBa_SA_HCT116_mHLA−1: 13167
SA = 0.87
Score = 3.14
LSDPSGPKSY − MaxQuant
20120515_EXQ3_MiBa_SA_HCT116_mHLA−1: 13167
SA = 0.92
Score = 3.8
LSDPSGPKSY − MSFragger
20120515_EXQ3_MiBa_SA_HCT116_mHLA−1: 13167
SA = 0.92
Score = 3.8
GSPGADSEAP − Mascot − rejected
GSPGAD|SEAP − spliced − 6|1|3|1
20120515_EXQ3_MiBa_SA_HCT116_mHLA−1: 7799
SA = 0.87
Score = −0.17
AESDPSNAP − MaxQuant
20120515_EXQ3_MiBa_SA_HCT116_mHLA−1: 7799
SA = 0.85
Score = 0.98
AESDPSNAP − MSFragger
20120515_EXQ3_MiBa_SA_HCT116_mHLA−1: 7799
SA = 0.85
Score = 0.98
Relative intensity
m/z
Prosit prediction
⋆ Exclusive in prediction
Exclusive in experimental

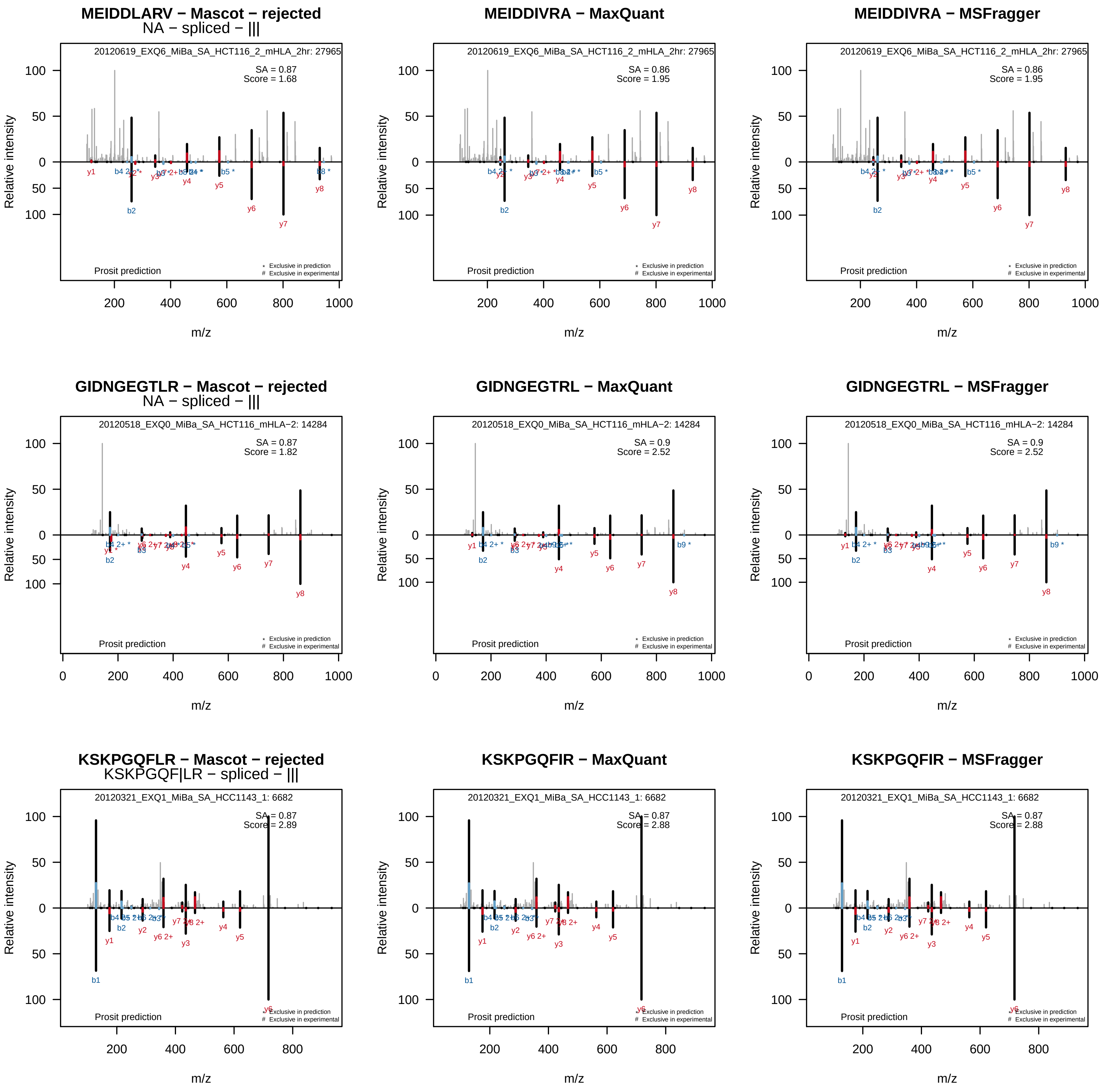

MEIDDLARV − Mascot − rejected
NA − spliced − |||
MEIDDIVRA − MaxQuant
MEIDDIVRA − MSFragger
20120619_EXQ6_MiBa_SA_HCT116_2_mHLA_2hr: 27965
SA = 0.87
Score = 1.68
SA = 0.86
Score = 1.95
GIDNGEGTLR − Mascot − rejected
NA − spliced − |||
GIDNGEGTRL − MaxQuant
GIDNGEGTRL − MSFragger
20120518_EXQ0_MiBa_SA_HCT116_mHLA−2: 14284
SA = 0.87
Score = 1.82
SA = 0.9
Score = 2.52
KSKPGQFLR − Mascot − rejected
KSKPGQF|LR − spliced − |||
KSKPGQFIR − MaxQuant
KSKPGQFIR − MSFragger
20120321_EXQ1_MiBa_SA_HCC1143_1: 6682
SA = 0.87
Score = 2.89
SA = 0.87
Score = 2.88
Relative intensity
m/z
Prosit prediction
Exclusive in prediction
Exclusive in experimental

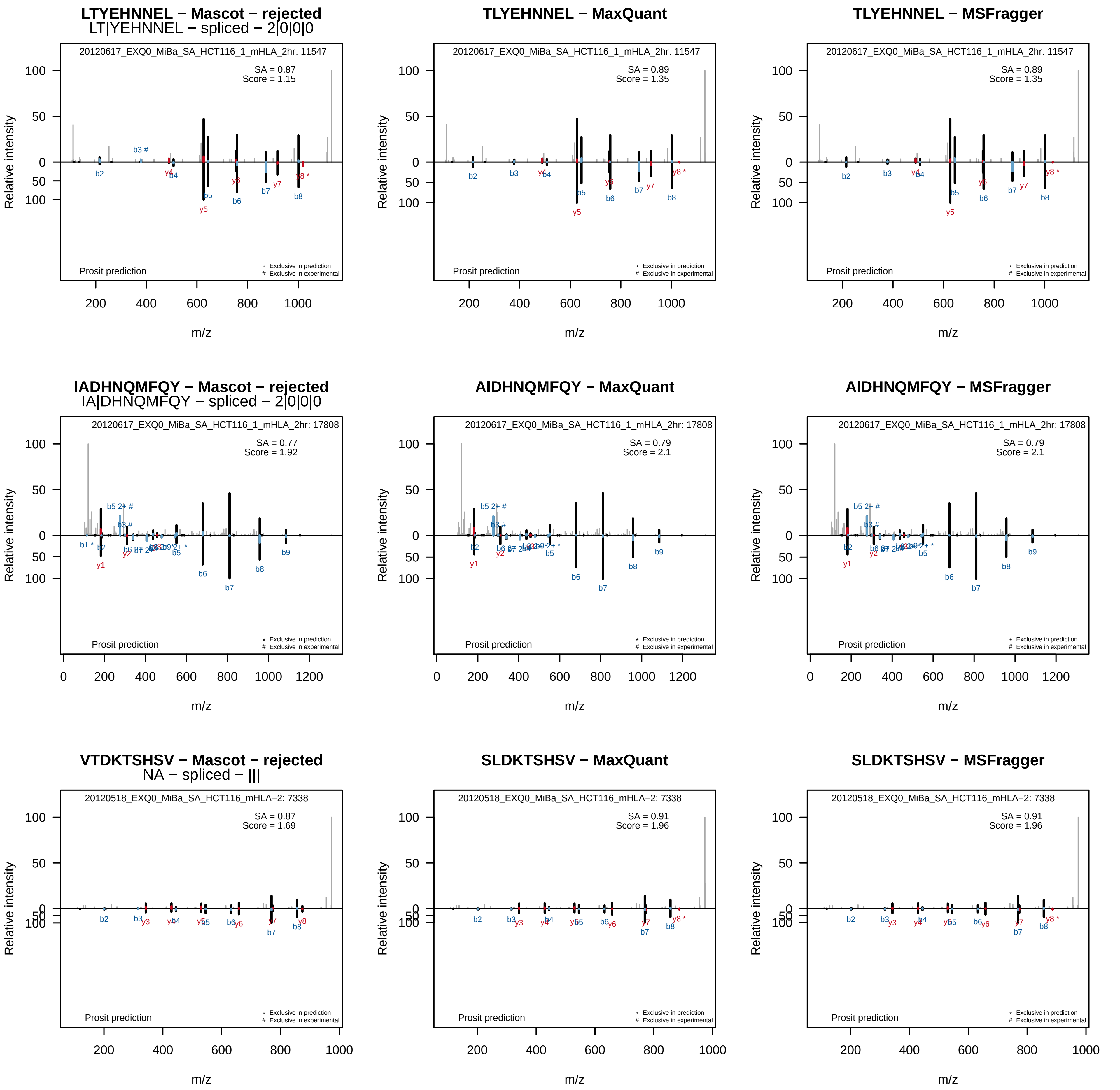

LTYEHNNEL − Mascot − rejected
LT|YEHNNEL − spliced − 2|0|0|0
20120617_EXQ0_MiBa_SA_HCT116_1_mHLA_2hr: 11547
SA = 0.87
Score = 1.15
Prosit prediction
⋆ Exclusive in prediction
Exclusive in experimental
TLYEHNNEL − MaxQuant
20120617_EXQ0_MiBa_SA_HCT116_1_mHLA_2hr: 11547
SA = 0.89
Score = 1.35
TLYEHNNEL − MSFragger
20120617_EXQ0_MiBa_SA_HCT116_1_mHLA_2hr: 11547
SA = 0.89
Score = 1.35
IADHNQMFQY − Mascot − rejected
IA|DHNQMFQY − spliced − 2|0|0|0
20120617_EXQ0_MiBa_SA_HCT116_1_mHLA_2hr: 17808
SA = 0.77
Score = 1.92
AIDHNQMFQY − MaxQuant
20120617_EXQ0_MiBa_SA_HCT116_1_mHLA_2hr: 17808
SA = 0.79
Score = 2.1
AIDHNQMFQY − MSFragger
20120617_EXQ0_MiBa_SA_HCT116_1_mHLA_2hr: 17808
SA = 0.79
Score = 2.1
VTDKTSHSV − Mascot − rejected
NA − spliced − |||
20120518_EXQ0_MiBa_SA_HCT116_mHLA−2: 7338
SA = 0.87
Score = 1.69
SLDKTSHSV − MaxQuant
20120518_EXQ0_MiBa_SA_HCT116_mHLA−2: 7338
SA = 0.91
Score = 1.96
SLDKTSHSV − MSFragger
20120518_EXQ0_MiBa_SA_HCT116_mHLA−2: 7338
SA = 0.91
Score = 1.96
Relative intensity
m/z

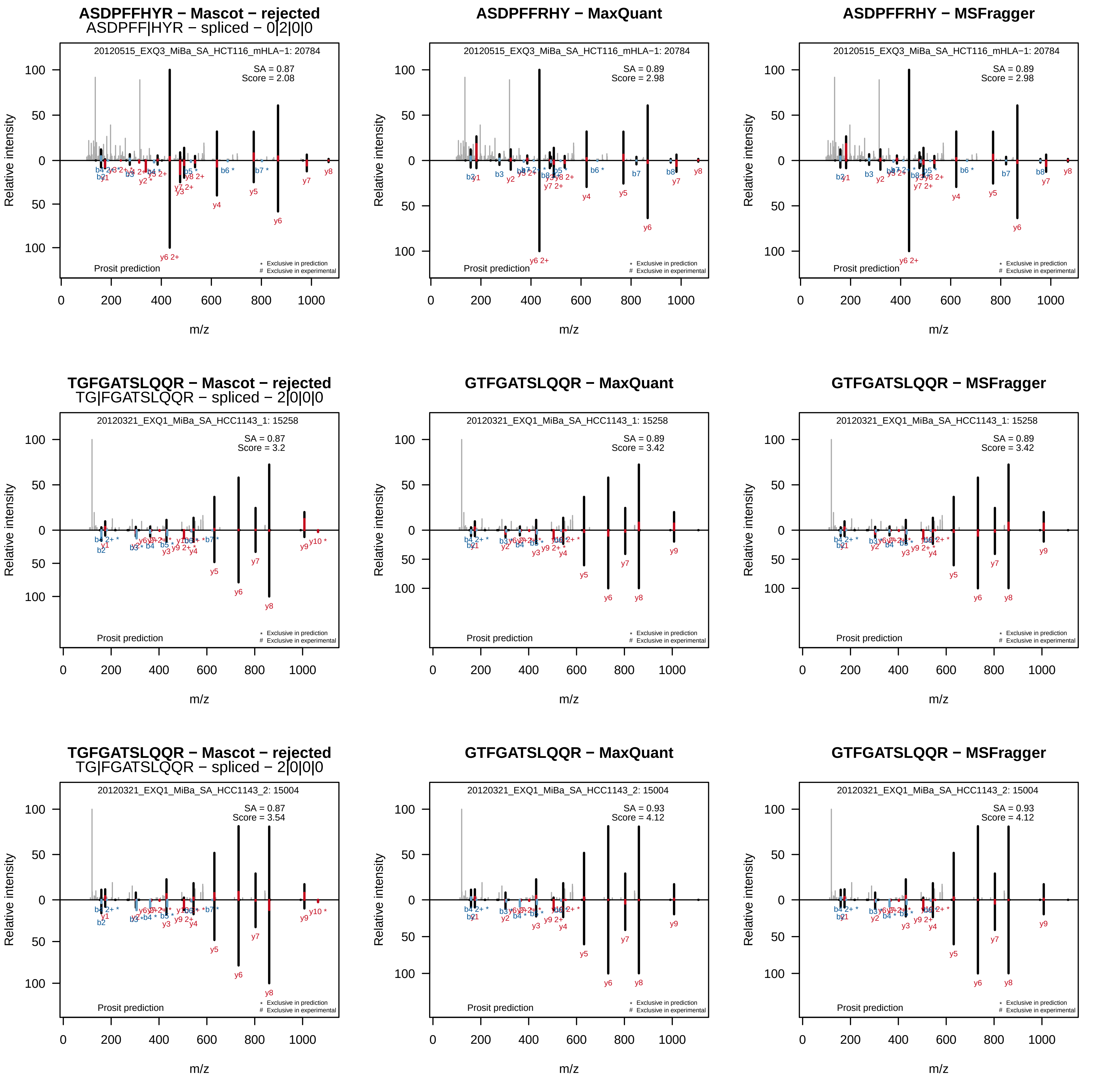

ASDPFFHYR − Mascot − rejected
ASDPFF|HYR − spliced − 0|2|0|0
20120515_EXQ3_MiBa_SA_HCT116_mHLA−1: 20784
SA = 0.87
Score = 2.08
ASDPFFRHY − MaxQuant
20120515_EXQ3_MiBa_SA_HCT116_mHLA−1: 20784
SA = 0.89
Score = 2.98
ASDPFFRHY − MSFragger
20120515_EXQ3_MiBa_SA_HCT116_mHLA−1: 20784
SA = 0.89
Score = 2.98
TGFGATSLQQR − Mascot − rejected
TG|FGATSLQQR − spliced − 2|0|0|0
20120321_EXQ1_MiBa_SA_HCC1143_1: 15258
SA = 0.87
Score = 3.2
GTFGATSLQQR − MaxQuant
20120321_EXQ1_MiBa_SA_HCC1143_1: 15258
SA = 0.89
Score = 3.42
GTFGATSLQQR − MSFragger
20120321_EXQ1_MiBa_SA_HCC1143_1: 15258
SA = 0.89
Score = 3.42
TGFGATSLQQR − Mascot − rejected
TG|FGATSLQQR − spliced − 2|0|0|0
20120321_EXQ1_MiBa_SA_HCC1143_2: 15004
SA = 0.87
Score = 3.54
GTFGATSLQQR − MaxQuant
20120321_EXQ1_MiBa_SA_HCC1143_2: 15004
SA = 0.93
Score = 4.12
GTFGATSLQQR − MSFragger
20120321_EXQ1_MiBa_SA_HCC1143_2: 15004
SA = 0.93
Score = 4.12
Relative intensity
m/z
Prosit prediction
* Exclusive in prediction
# Exclusive in experimental

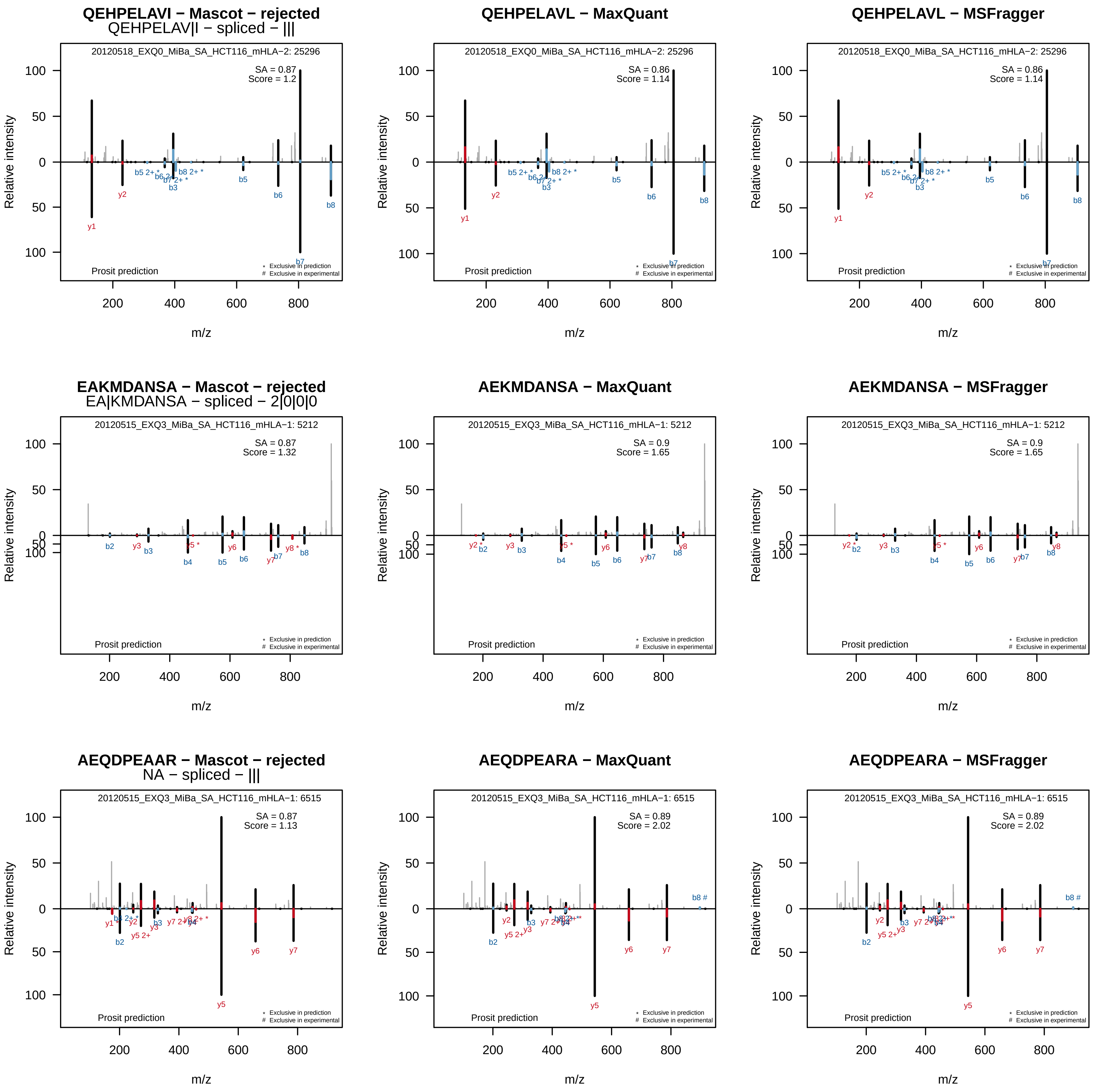

QEHPELAVI − Mascot − rejected
QEHPELAV|I − spliced − |||
20120518_EXQ0_MiBa_SA_HCT116_mHLA−2: 25296
SA = 0.87
Score = 1.2
QEHPELAVL − MaxQuant
20120518_EXQ0_MiBa_SA_HCT116_mHLA−2: 25296
SA = 0.86
Score = 1.14
QEHPELAVL − MSFragger
20120518_EXQ0_MiBa_SA_HCT116_mHLA−2: 25296
SA = 0.86
Score = 1.14
EAKMDANSA − Mascot − rejected
EA|KMDANSA − spliced − 2|0|0|0
20120515_EXQ3_MiBa_SA_HCT116_mHLA−1: 5212
SA = 0.87
Score = 1.32
AEKMDANSA − MaxQuant
20120515_EXQ3_MiBa_SA_HCT116_mHLA−1: 5212
SA = 0.9
Score = 1.65
AEKMDANSA − MSFragger
20120515_EXQ3_MiBa_SA_HCT116_mHLA−1: 5212
SA = 0.9
Score = 1.65
AEQDPEAAR − Mascot − rejected
NA − spliced − |||
20120515_EXQ3_MiBa_SA_HCT116_mHLA−1: 6515
SA = 0.87
Score = 1.13
AEQDPEARA − MaxQuant
20120515_EXQ3_MiBa_SA_HCT116_mHLA−1: 6515
SA = 0.89
Score = 2.02
AEQDPEARA − MSFragger
20120515_EXQ3_MiBa_SA_HCT116_mHLA−1: 6515
SA = 0.89
Score = 2.02
Relative intensity
m/z
Prosit prediction
⋆ Exclusive in prediction
Exclusive in experimental

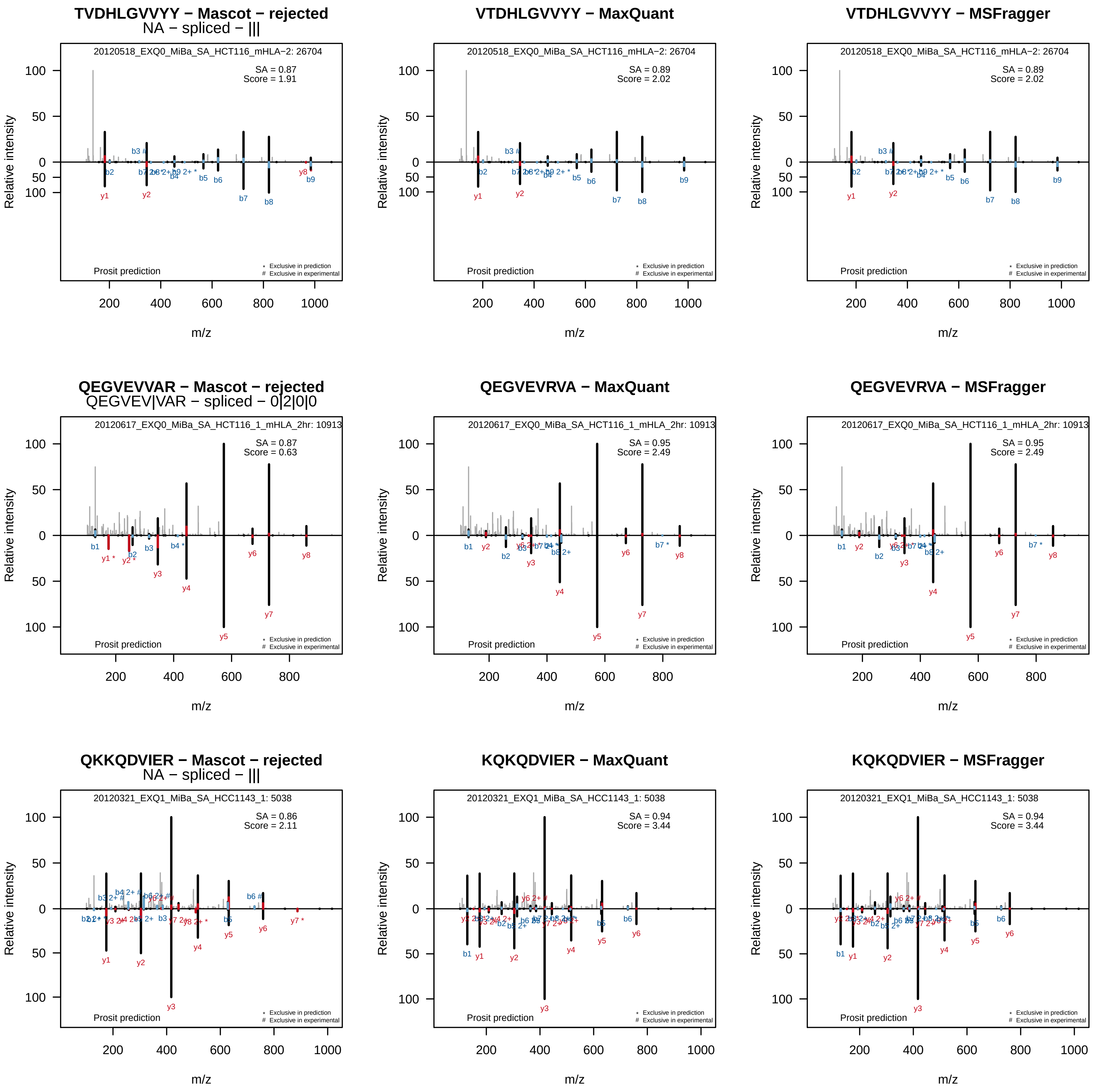
TVDHLGVVYY − Mascot − rejected
NA − spliced − |||
20120518_EXQ0_MiBa_SA_HCT116_mHLA−2: 26704
SA = 0.87
Score = 1.91
VTDHLGVVYY − MaxQuant
20120518_EXQ0_MiBa_SA_HCT116_mHLA−2: 26704
SA = 0.89
Score = 2.02
VTDHLGVVYY − MSFragger
20120518_EXQ0_MiBa_SA_HCT116_mHLA−2: 26704
SA = 0.89
Score = 2.02
QEGVEVVAR − Mascot − rejected
QEGVEV|VAR − spliced − 0|2|0|0
20120617_EXQ0_MiBa_SA_HCT116_1_mHLA_2hr: 10913
SA = 0.87
Score = 0.63
QEGVEVRVA − MaxQuant
20120617_EXQ0_MiBa_SA_HCT116_1_mHLA_2hr: 10913
SA = 0.95
Score = 2.49
QEGVEVRVA − MSFragger
20120617_EXQ0_MiBa_SA_HCT116_1_mHLA_2hr: 10913
SA = 0.95
Score = 2.49
QKKQDVIER − Mascot − rejected
NA − spliced − |||
20120321_EXQ1_MiBa_SA_HCC1143_1: 5038
SA = 0.86
Score = 2.11
KQKQDVIER − MaxQuant
20120321_EXQ1_MiBa_SA_HCC1143_1: 5038
SA = 0.94
Score = 3.44
KQKQDVIER − MSFragger
20120321_EXQ1_MiBa_SA_HCC1143_1: 5038
SA = 0.94
Score = 3.44
Relative intensity
m/z
Prosit prediction
⋆ Exclusive in prediction
Exclusive in experimental

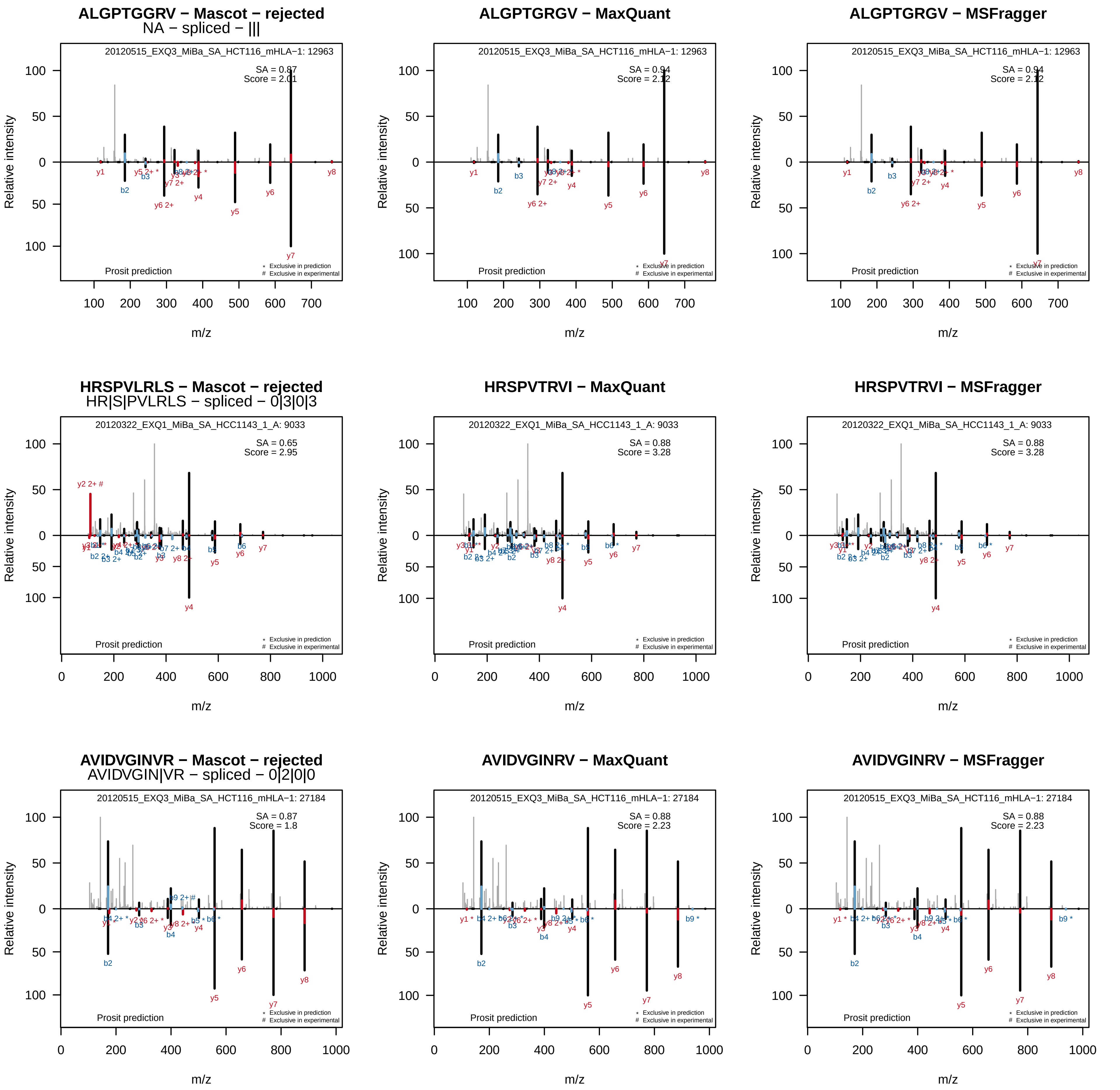

ALGPTGGRV − Mascot − rejected
NA − spliced − |||
20120515_EXQ3_MiBa_SA_HCT116_mHLA−1: 12963
SA = 0.87
Score = 2.01
ALGPTGRGV − MaxQuant
20120515_EXQ3_MiBa_SA_HCT116_mHLA−1: 12963
SA = 0.94
Score = 2.12
ALGPTGRGV − MSFragger
20120515_EXQ3_MiBa_SA_HCT116_mHLA−1: 12963
SA = 0.94
Score = 2.12
HRSPVLRLS − Mascot − rejected
HR|S|PVLRLS − spliced − 0|3|0|3
20120322_EXQ1_MiBa_SA_HCC1143_1_A: 9033
SA = 0.65
Score = 2.95
HRSPVTRVI − MaxQuant
20120322_EXQ1_MiBa_SA_HCC1143_1_A: 9033
SA = 0.88
Score = 3.28
HRSPVTRVI − MSFragger
20120322_EXQ1_MiBa_SA_HCC1143_1_A: 9033
SA = 0.88
Score = 3.28
AVIDVGINVR − Mascot − rejected
AVIDVGIN|VR − spliced − 0|2|0|0
20120515_EXQ3_MiBa_SA_HCT116_mHLA−1: 27184
SA = 0.87
Score = 1.8
AVIDVGINRV − MaxQuant
20120515_EXQ3_MiBa_SA_HCT116_mHLA−1: 27184
SA = 0.88
Score = 2.23
AVIDVGINRV − MSFragger
20120515_EXQ3_MiBa_SA_HCT116_mHLA−1: 27184
SA = 0.88
Score = 2.23
Relative intensity
m/z
Prosit prediction
* Exclusive in prediction
# Exclusive in experimental

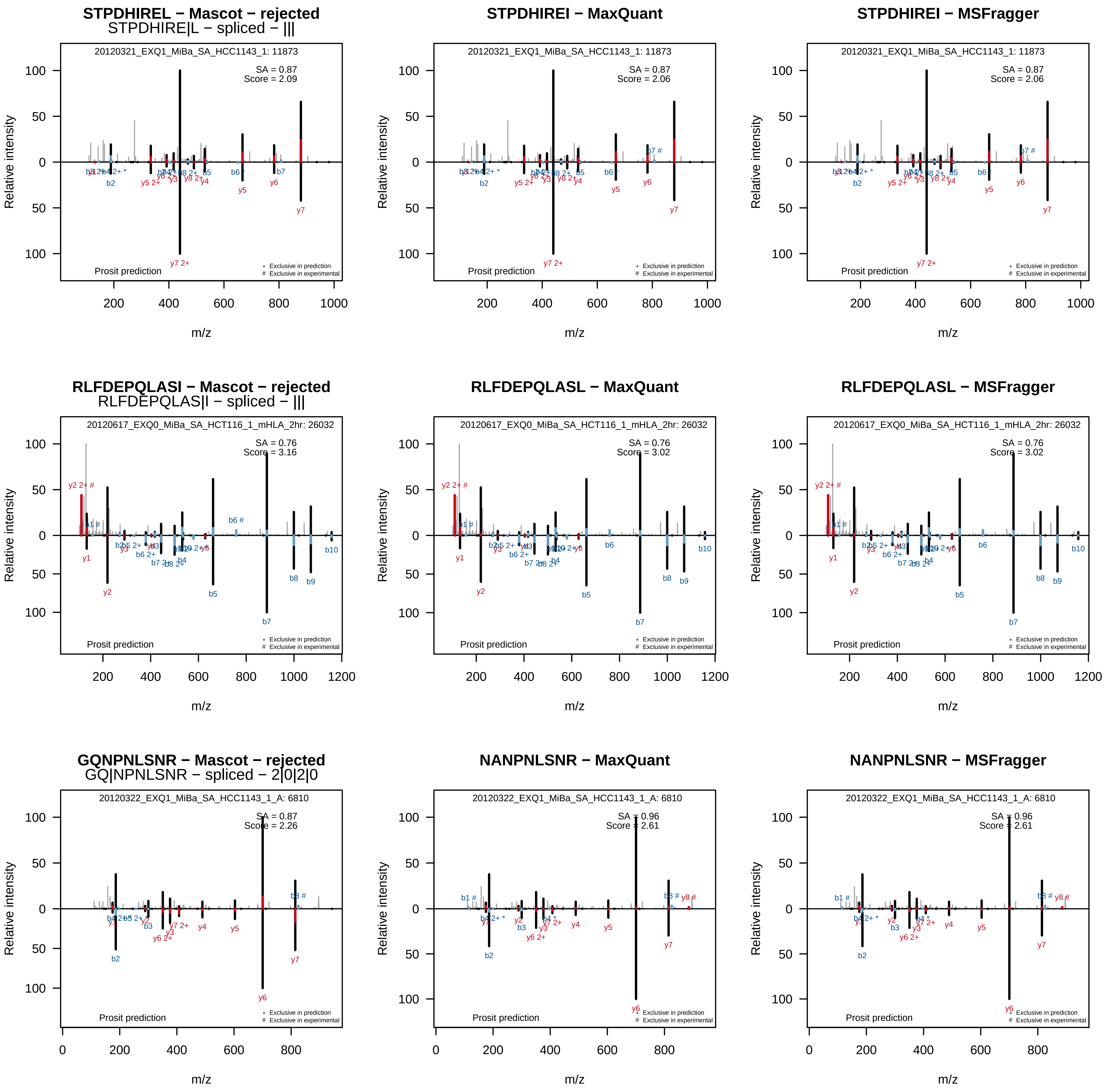

STPDHIREL − Mascot − rejected
STPDHIRE|L − spliced − |||
20120321_EXQ1_MiBa_SA_HCC1143_1: 11873
SA = 0.87
Score = 2.09
STPDHIREI − MaxQuant
20120321_EXQ1_MiBa_SA_HCC1143_1: 11873
SA = 0.87
Score = 2.06
STPDHIREI − MSFragger
20120321_EXQ1_MiBa_SA_HCC1143_1: 11873
SA = 0.87
Score = 2.06
RLFDEPQLASI − Mascot − rejected
RLFDEPQLAS|I − spliced − |||
20120617_EXQ0_MiBa_SA_HCT116_1_mHLA_2hr: 26032
SA = 0.76
Score = 3.16
RLFDEPQLASL − MaxQuant
20120617_EXQ0_MiBa_SA_HCT116_1_mHLA_2hr: 26032
SA = 0.76
Score = 3.02
RLFDEPQLASL − MSFragger
20120617_EXQ0_MiBa_SA_HCT116_1_mHLA_2hr: 26032
SA = 0.76
Score = 3.02
GQNPNLSNR − Mascot − rejected
GQ|NPNLSNR − spliced − 2|0|2|0
20120322_EXQ1_MiBa_SA_HCC1143_1_A: 6810
SA = 0.87
Score = 2.26
NANPNLSNR − MaxQuant
20120322_EXQ1_MiBa_SA_HCC1143_1_A: 6810
SA = 0.96
Score = 2.61
NANPNLSNR − MSFragger
20120322_EXQ1_MiBa_SA_HCC1143_1_A: 6810
SA = 0.96
Score = 2.61
Relative intensity
m/z
Prosit prediction
* Exclusive in prediction
# Exclusive in experimental

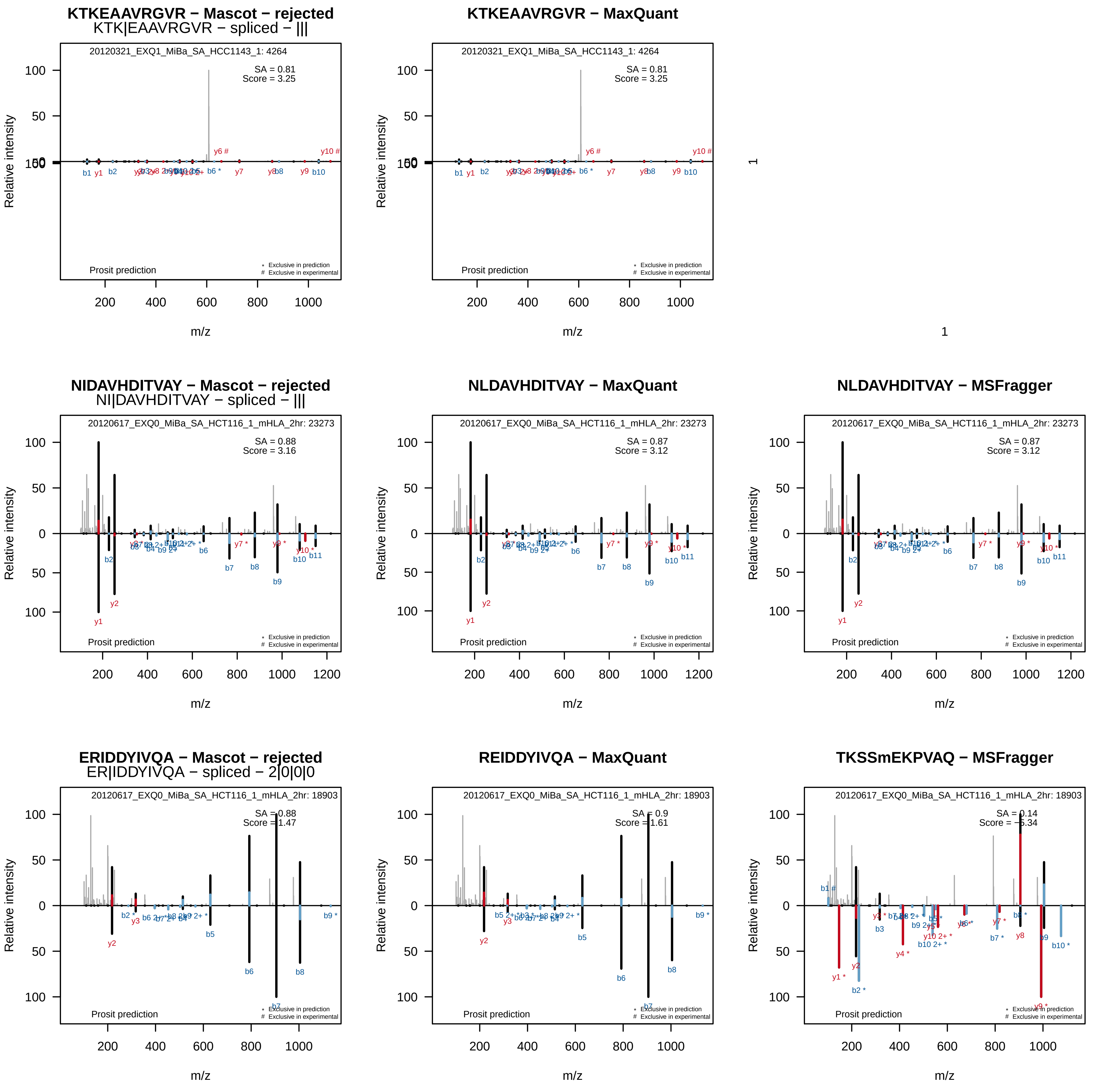
KTKEAAVRGVR − Mascot − rejected
KTK|EAAVRGVR − spliced − |||
20120321_EXQ1_MiBa_SA_HCC1143_1: 4264
SA = 0.81
Score = 3.25
Prosit prediction
Exclusive in prediction
Exclusive in experimental
KTKEAAVRGVR − MaxQuant
20120321_EXQ1_MiBa_SA_HCC1143_1: 4264
SA = 0.81
Score = 3.25
NIDAVHDITVAY − Mascot − rejected
NI|DAVHDITVAY − spliced − |||
20120617_EXQ0_MiBa_SA_HCT116_1_mHLA_2hr: 23273
SA = 0.88
Score = 3.16
NLDAVHDITVAY − MaxQuant
20120617_EXQ0_MiBa_SA_HCT116_1_mHLA_2hr: 23273
SA = 0.87
Score = 3.12
NLDAVHDITVAY − MSFragger
20120617_EXQ0_MiBa_SA_HCT116_1_mHLA_2hr: 23273
SA = 0.87
Score = 3.12
ERIDDYIVQA − Mascot − rejected
ER|IDDYIVQA − spliced − 2|0|0|0
20120617_EXQ0_MiBa_SA_HCT116_1_mHLA_2hr: 18903
SA = 0.88
Score = 1.47
REIDDYIVQA − MaxQuant
20120617_EXQ0_MiBa_SA_HCT116_1_mHLA_2hr: 18903
SA = 0.9
Score = 1.61
TKSSmEKPVAQ − MSFragger
20120617_EXQ0_MiBa_SA_HCT116_1_mHLA_2hr: 18903
SA = 0.14
Score = −5.34
Relative intensity
m/z

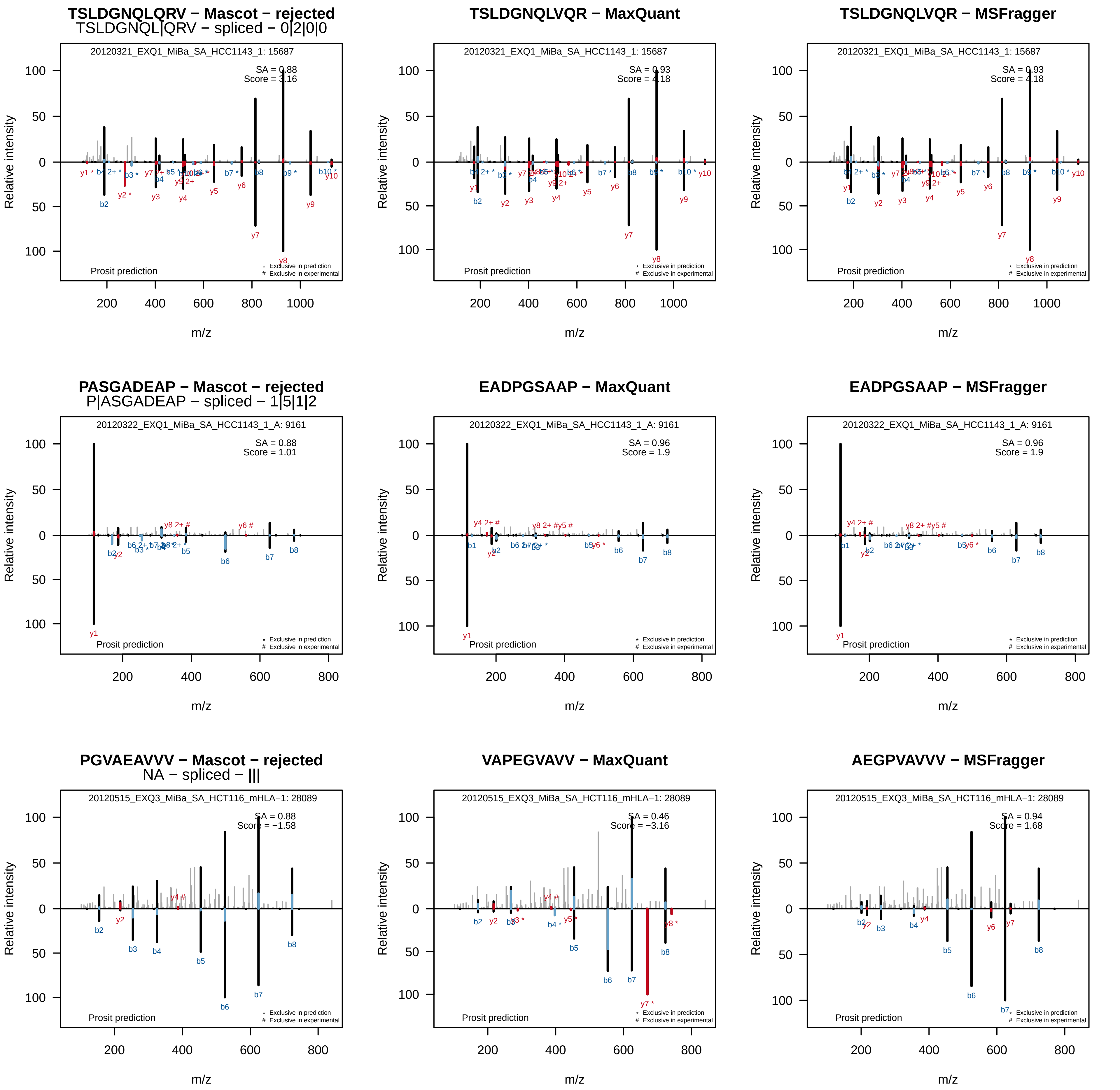

TSLDGNQLQRV − Mascot − rejected
TSLDGNQL|QRV − spliced − 0|2|0|0
20120321_EXQ1_MiBa_SA_HCC1143_1: 15687
SA = 0.88
Score = 3.16
TSLDGNQLVQR − MaxQuant
20120321_EXQ1_MiBa_SA_HCC1143_1: 15687
SA = 0.93
Score = 4.18
TSLDGNQLVQR − MSFragger
20120321_EXQ1_MiBa_SA_HCC1143_1: 15687
SA = 0.93
Score = 4.18
PASGADEAP − Mascot − rejected
P|ASGADEAP − spliced − 1|5|1|2
20120322_EXQ1_MiBa_SA_HCC1143_1_A: 9161
SA = 0.88
Score = 1.01
EADPGSAAP − MaxQuant
20120322_EXQ1_MiBa_SA_HCC1143_1_A: 9161
SA = 0.96
Score = 1.9
EADPGSAAP − MSFragger
20120322_EXQ1_MiBa_SA_HCC1143_1_A: 9161
SA = 0.96
Score = 1.9
PGVAEAVVV − Mascot − rejected
NA − spliced − |||
20120515_EXQ3_MiBa_SA_HCT116_mHLA−1: 28089
SA = 0.88
Score = −1.58
VAPEGVAVV − MaxQuant
20120515_EXQ3_MiBa_SA_HCT116_mHLA−1: 28089
SA = 0.46
Score = −3.16
AEGPVAVVV − MSFragger
20120515_EXQ3_MiBa_SA_HCT116_mHLA−1: 28089
SA = 0.94
Score = 1.68
Relative intensity
m/z
Prosit prediction
⋆ Exclusive in prediction
Exclusive in experimental

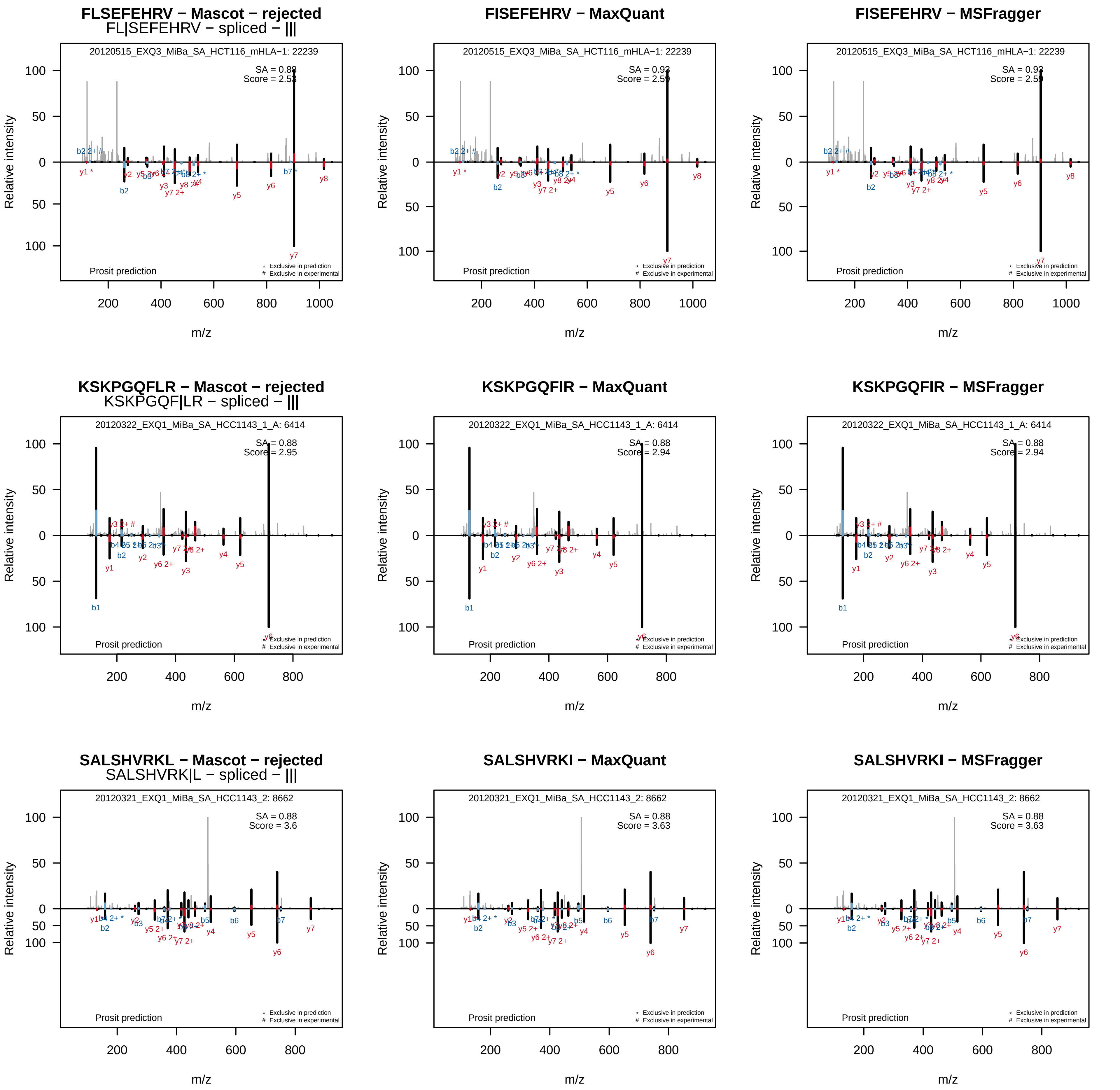
FLSEFEHRV − Mascot − rejected
FL|SEFEHRV − spliced − |||
20120515_EXQ3_MiBa_SA_HCT116_mHLA−1: 22239
SA = 0.88
Score = 2.53
FISEFEHRV − MaxQuant
20120515_EXQ3_MiBa_SA_HCT116_mHLA−1: 22239
SA = 0.92
Score = 2.59
FISEFEHRV − MSFragger
20120515_EXQ3_MiBa_SA_HCT116_mHLA−1: 22239
SA = 0.92
Score = 2.59
KSKPGQFLR − Mascot − rejected
KSKPGQF|LR − spliced − |||
20120322_EXQ1_MiBa_SA_HCC1143_1_A: 6414
SA = 0.88
Score = 2.95
KSKPGQFIR − MaxQuant
20120322_EXQ1_MiBa_SA_HCC1143_1_A: 6414
SA = 0.88
Score = 2.94
KSKPGQFIR − MSFragger
20120322_EXQ1_MiBa_SA_HCC1143_1_A: 6414
SA = 0.88
Score = 2.94
SALSHVRKL − Mascot − rejected
SALSHVRK|L − spliced − |||
20120321_EXQ1_MiBa_SA_HCC1143_2: 8662
SA = 0.88
Score = 3.6
SALSHVRKI − MaxQuant
20120321_EXQ1_MiBa_SA_HCC1143_2: 8662
SA = 0.88
Score = 3.63
SALSHVRKI − MSFragger
20120321_EXQ1_MiBa_SA_HCC1143_2: 8662
SA = 0.88
Score = 3.63
Relative intensity
m/z
Prosit prediction
⋆ Exclusive in prediction
Exclusive in experimental

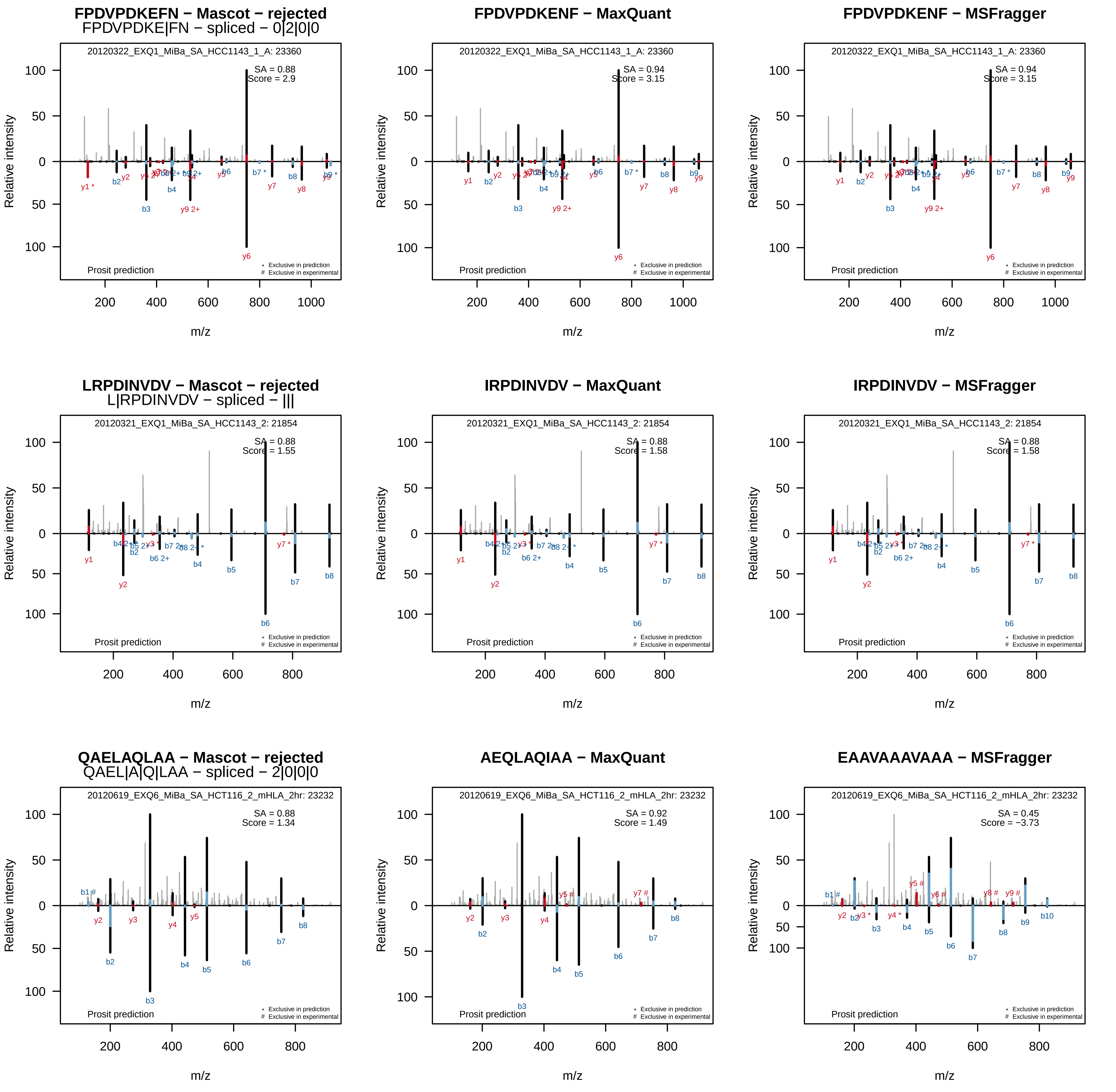

FPDVPDKEFN − Mascot − rejected
FPDVPDKE|FN − spliced − 0|2|0|0
20120322_EXQ1_MiBa_SA_HCC1143_1_A: 23360
SA = 0.88
Score = 2.9
FPDVPDKENF − MaxQuant
20120322_EXQ1_MiBa_SA_HCC1143_1_A: 23360
SA = 0.94
Score = 3.15
FPDVPDKENF − MSFragger
20120322_EXQ1_MiBa_SA_HCC1143_1_A: 23360
SA = 0.94
Score = 3.15
LRPDINVDV − Mascot − rejected
L|RPDINVDV − spliced − |||
20120321_EXQ1_MiBa_SA_HCC1143_2: 21854
SA = 0.88
Score = 1.55
IRPDINVDV − MaxQuant
20120321_EXQ1_MiBa_SA_HCC1143_2: 21854
SA = 0.88
Score = 1.58
IRPDINVDV − MSFragger
20120321_EXQ1_MiBa_SA_HCC1143_2: 21854
SA = 0.88
Score = 1.58
QAELAQLAA − Mascot − rejected
QAEL|A|Q|LAA − spliced − 2|0|0|0
20120619_EXQ6_MiBa_SA_HCT116_2_mHLA_2hr: 23232
SA = 0.88
Score = 1.34
AEQLAQIAA − MaxQuant
20120619_EXQ6_MiBa_SA_HCT116_2_mHLA_2hr: 23232
SA = 0.92
Score = 1.49
EAAVAAAVAAA − MSFragger
20120619_EXQ6_MiBa_SA_HCT116_2_mHLA_2hr: 23232
SA = 0.45
Score = −3.73
Relative intensity
m/z
Prosit prediction
⋆ Exclusive in prediction
Exclusive in experimental

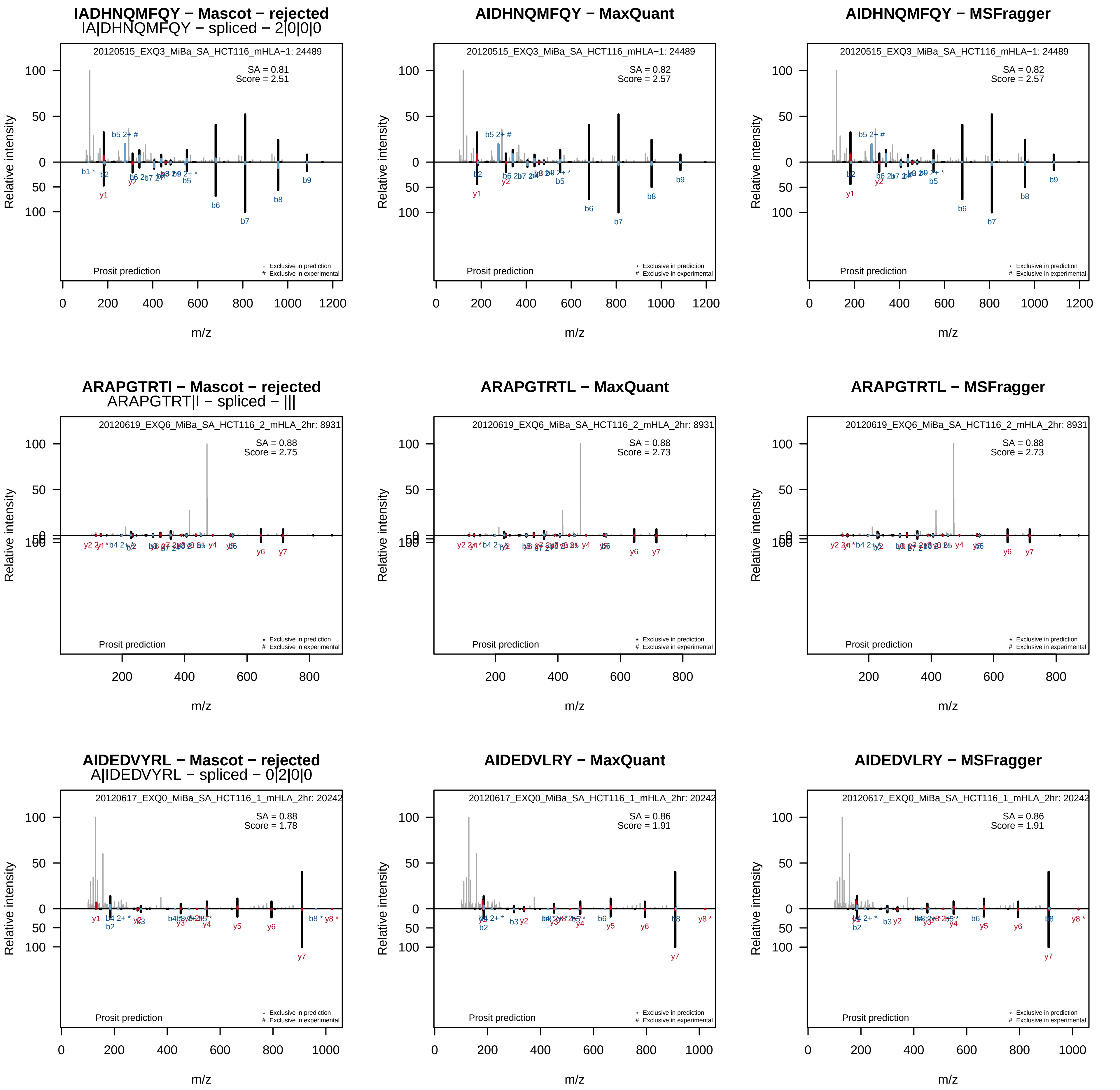
IADHNQMFQY − Mascot − rejected
IA|DHNQMFQY − spliced − 2|0|0|0
AIDHNQMFQY − MaxQuant
AIDHNQMFQY − MSFragger
20120515_EXQ3_MiBa_SA_HCT116_mHLA−1: 24489
SA = 0.81
Score = 2.51
SA = 0.82
Score = 2.57
ARAPGTRTI − Mascot − rejected
ARAPGTRT|I − spliced − |||
ARAPGTRTL − MaxQuant
ARAPGTRTL − MSFragger
20120619_EXQ6_MiBa_SA_HCT116_2_mHLA_2hr: 8931
SA = 0.88
Score = 2.75
Score = 2.73
AIDEDVYRL − Mascot − rejected
A|IDEDVYRL − spliced − 0|2|0|0
AIDEDVLRY − MaxQuant
AIDEDVLRY − MSFragger
20120617_EXQ0_MiBa_SA_HCT116_1_mHLA_2hr: 20242
SA = 0.88
Score = 1.78
SA = 0.86
Score = 1.91
Relative intensity
m/z
Prosit prediction
⋆ Exclusive in prediction
Exclusive in experimental

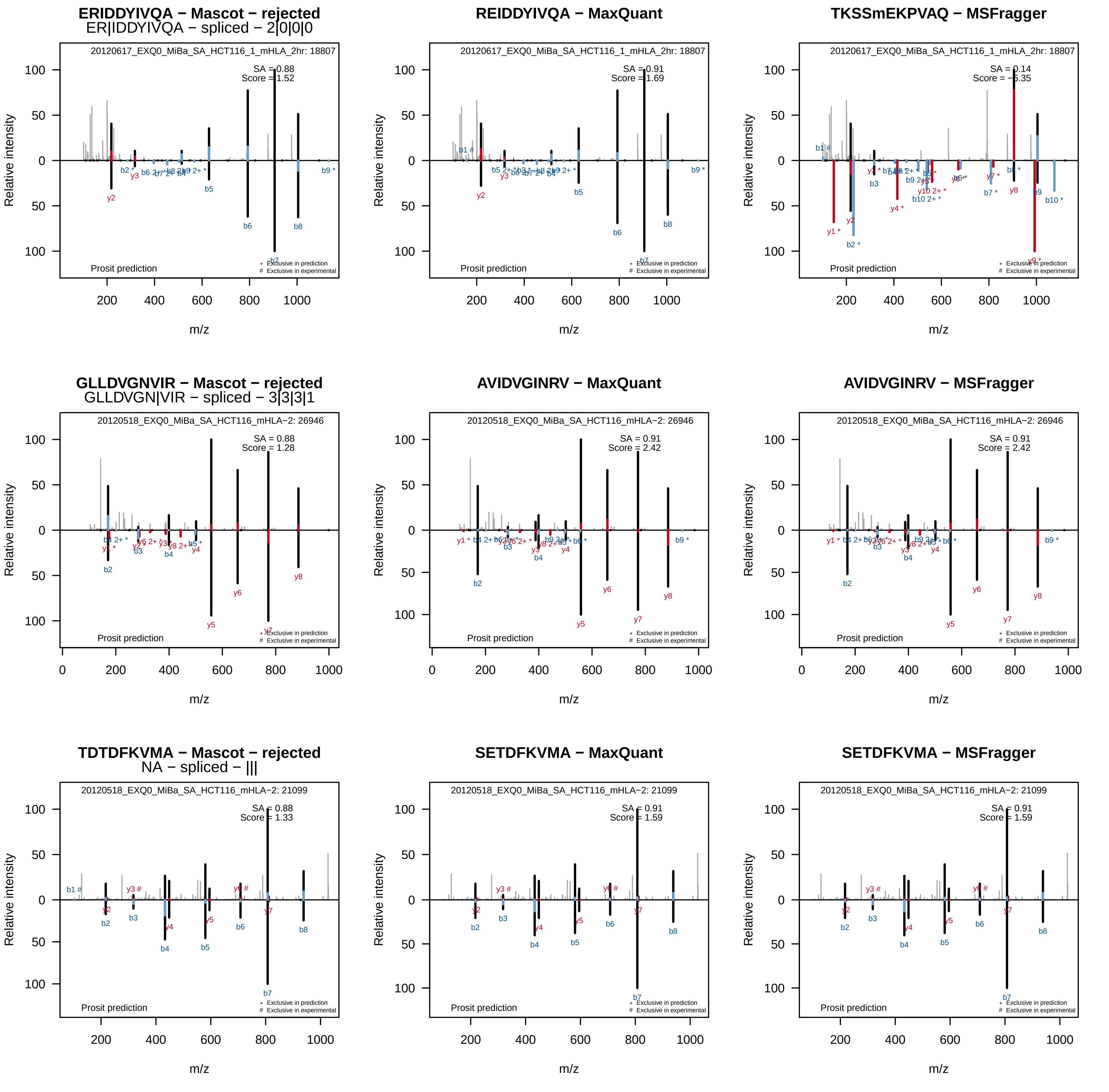
ERIDDYIVQA − Mascot − rejected
ER|IDDYIVQA − spliced − 2|0|0|0
20120617_EXQ0_MiBa_SA_HCT116_1_mHLA_2hr: 18807
SA = 0.88
Score = 1.52
Prosit prediction
⋆ Exclusive in prediction
Exclusive in experimental
Relative intensity
m/z
REIDDYIVQA − MaxQuant
20120617_EXQ0_MiBa_SA_HCT116_1_mHLA_2hr: 18807
SA = 0.91
Score = 1.69
Prosit prediction
TKSSmEKPVAQ − MSFragger
20120617_EXQ0_MiBa_SA_HCT116_1_mHLA_2hr: 18807
SA = 0.14
Score = −5.35
Prosit prediction
GLLDVGNVIR − Mascot − rejected
GLLDVGN|VIR − spliced − 3|3|3|1
20120518_EXQ0_MiBa_SA_HCT116_mHLA−2: 26946
SA = 0.88
Score = 1.28
Prosit prediction
AVIDVGINRV − MaxQuant
20120518_EXQ0_MiBa_SA_HCT116_mHLA−2: 26946
SA = 0.91
Score = 2.42
Prosit prediction
AVIDVGINRV − MSFragger
20120518_EXQ0_MiBa_SA_HCT116_mHLA−2: 26946
SA = 0.91
Score = 2.42
Prosit prediction
TDTDFKVMA − Mascot − rejected
NA − spliced − |||
20120518_EXQ0_MiBa_SA_HCT116_mHLA−2: 21099
SA = 0.88
Score = 1.33
Prosit prediction
SETDFKVMA − MaxQuant
20120518_EXQ0_MiBa_SA_HCT116_mHLA−2: 21099
SA = 0.91
Score = 1.59
Prosit prediction
SETDFKVMA − MSFragger
20120518_EXQ0_MiBa_SA_HCT116_mHLA−2: 21099
SA = 0.91
Score = 1.59
Prosit prediction

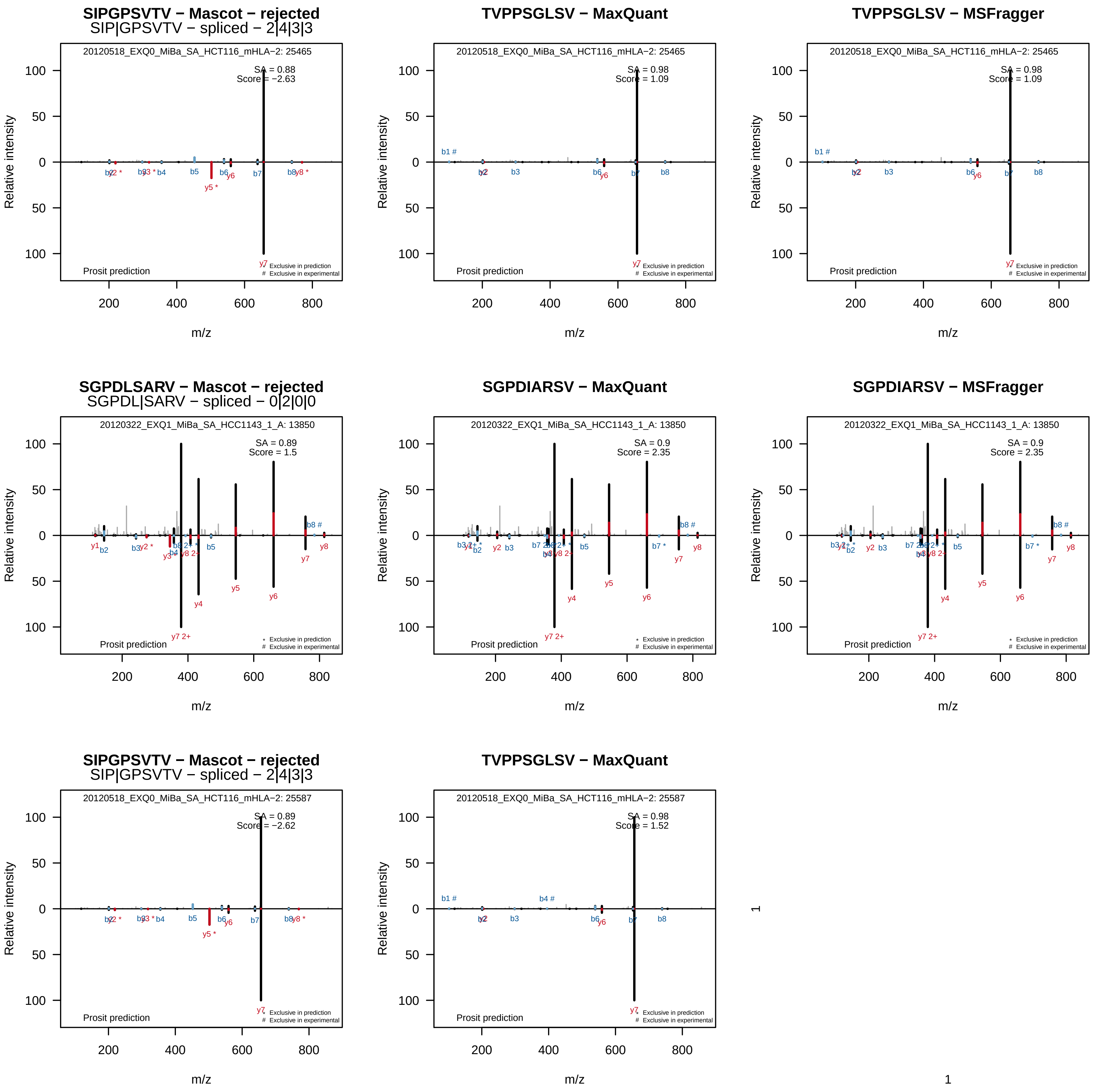

SIPGPSVTV − Mascot − rejected
SIP|GPSVTV − spliced − 2|4|3|3
20120518_EXQ0_MiBa_SA_HCT116_mHLA−2: 25465
SA = 0.88
Score = −2.63
Relative intensity
Prosit prediction
Exclusive in prediction
Exclusive in experimental
m/z
TVPPSGLSV − MaxQuant
20120518_EXQ0_MiBa_SA_HCT116_mHLA−2: 25465
SA = 0.98
Score = 1.09
TVPPSGLSV − MSFragger
20120518_EXQ0_MiBa_SA_HCT116_mHLA−2: 25465
SA = 0.98
Score = 1.09
SGPDLSARV − Mascot − rejected
SGPDL|SARV − spliced − 0|2|0|0
20120322_EXQ1_MiBa_SA_HCC1143_1_A: 13850
SA = 0.89
Score = 1.5
SGPDIARSV − MaxQuant
20120322_EXQ1_MiBa_SA_HCC1143_1_A: 13850
SA = 0.9
Score = 2.35
SGPDIARSV − MSFragger
20120322_EXQ1_MiBa_SA_HCC1143_1_A: 13850
SA = 0.9
Score = 2.35
SIPGPSVTV − Mascot − rejected
SIP|GPSVTV − spliced − 2|4|3|3
20120518_EXQ0_MiBa_SA_HCT116_mHLA−2: 25587
SA = 0.89
Score = −2.62
TVPPSGLSV − MaxQuant
20120518_EXQ0_MiBa_SA_HCT116_mHLA−2: 25587
SA = 0.98
Score = 1.52

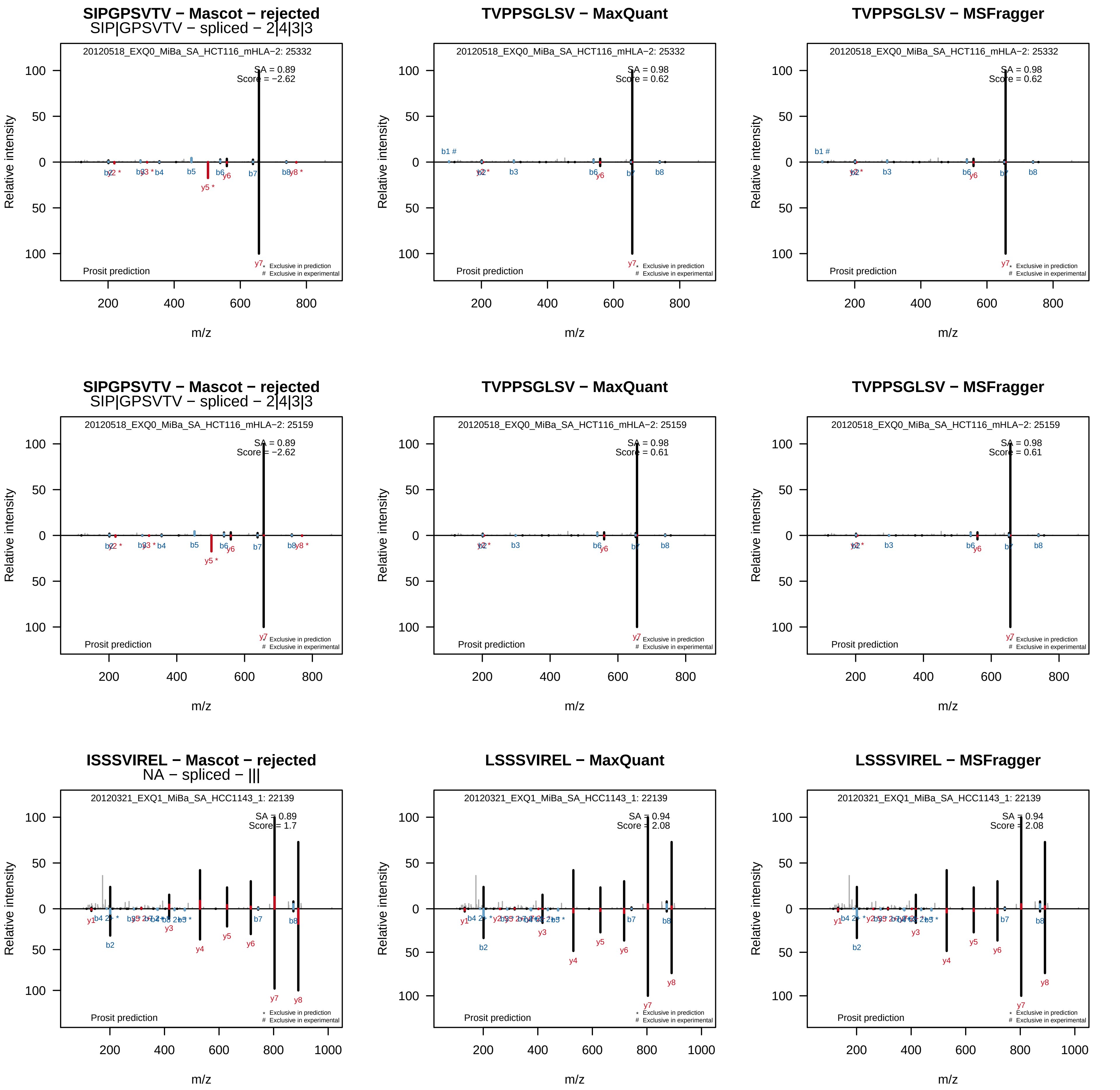
SIPGPSVTV − Mascot − rejected
SIP|GPSVTV − spliced − 2|4|3|3
20120518_EXQ0_MiBa_SA_HCT116_mHLA−2: 25332
SA = 0.89
Score = −2.62
Prosit prediction
Exclusive in prediction
Exclusive in experimental
Relative intensity
m/z
TVPPSGLSV − MaxQuant
20120518_EXQ0_MiBa_SA_HCT116_mHLA−2: 25332
SA = 0.98
Score = 0.62
Prosit prediction
TVPPSGLSV − MSFragger
20120518_EXQ0_MiBa_SA_HCT116_mHLA−2: 25332
SA = 0.98
Score = 0.62
SIPGPSVTV − Mascot − rejected
SIP|GPSVTV − spliced − 2|4|3|3
20120518_EXQ0_MiBa_SA_HCT116_mHLA−2: 25159
SA = 0.89
Score = −2.62
TVPPSGLSV − MaxQuant
20120518_EXQ0_MiBa_SA_HCT116_mHLA−2: 25159
SA = 0.98
Score = 0.61
TVPPSGLSV − MSFragger
20120518_EXQ0_MiBa_SA_HCT116_mHLA−2: 25159
SA = 0.98
Score = 0.61
ISSSVIREL − Mascot − rejected
NA − spliced − |||
20120321_EXQ1_MiBa_SA_HCC1143_1: 22139
SA = 0.89
Score = 1.7
LSSSVIREL − MaxQuant
20120321_EXQ1_MiBa_SA_HCC1143_1: 22139
SA = 0.94
Score = 2.08
LSSSVIREL − MSFragger
20120321_EXQ1_MiBa_SA_HCC1143_1: 22139
SA = 0.94
Score = 2.08

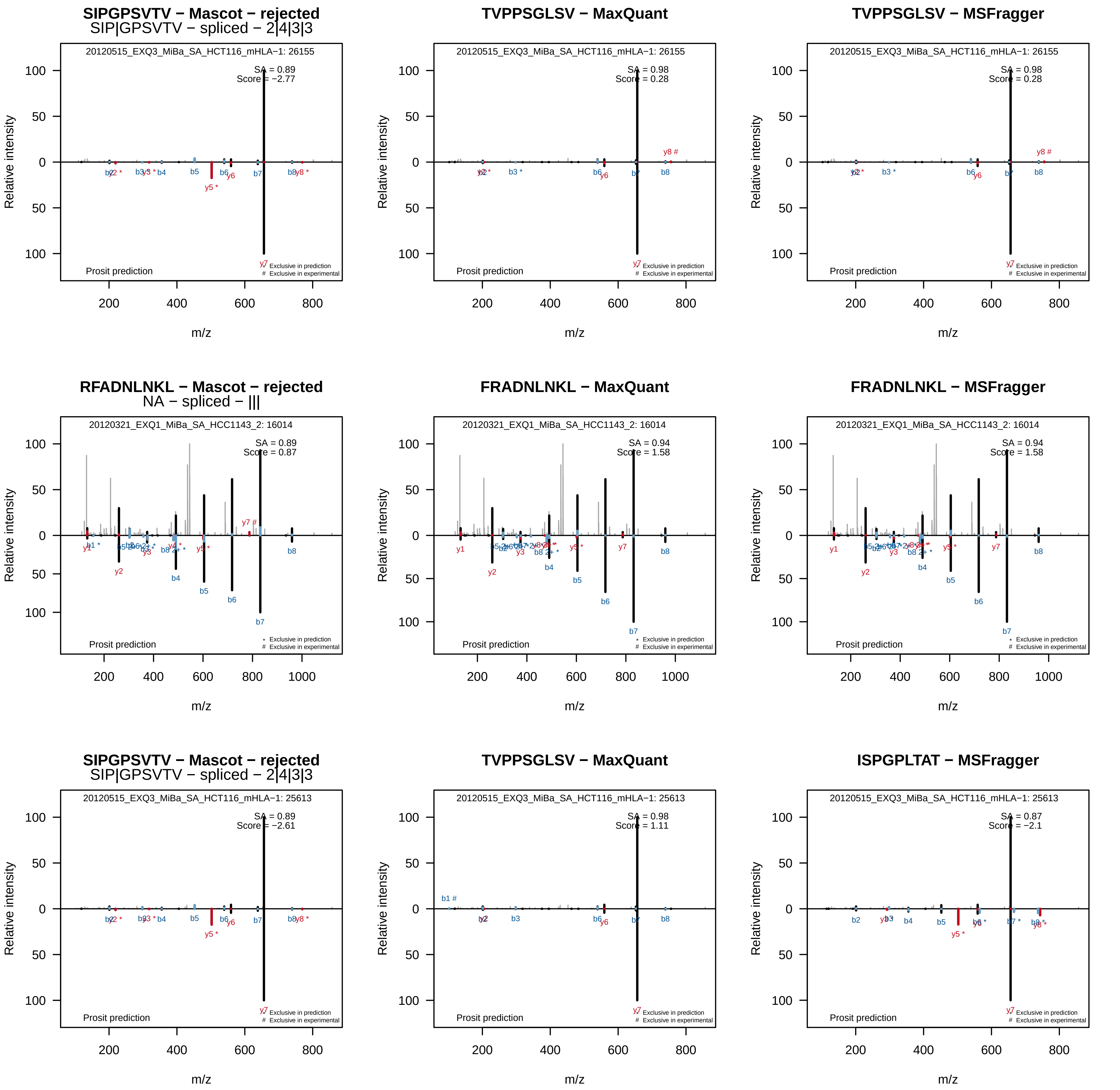
SIPGPSVTV − Mascot − rejected
SIP|GPSVTV − spliced − 2|4|3|3
20120515_EXQ3_MiBa_SA_HCT116_mHLA−1: 26155
SA = 0.89
Score = −2.77
TVPPSGLSV − MaxQuant
20120515_EXQ3_MiBa_SA_HCT116_mHLA−1: 26155
SA = 0.98
Score = 0.28
TVPPSGLSV − MSFragger
20120515_EXQ3_MiBa_SA_HCT116_mHLA−1: 26155
SA = 0.98
Score = 0.28
RFADNLNKL − Mascot − rejected
NA − spliced − |||
20120321_EXQ1_MiBa_SA_HCC1143_2: 16014
SA = 0.89
Score = 0.87
FRADNLNKL − MaxQuant
20120321_EXQ1_MiBa_SA_HCC1143_2: 16014
SA = 0.94
Score = 1.58
FRADNLNKL − MSFragger
20120321_EXQ1_MiBa_SA_HCC1143_2: 16014
SA = 0.94
Score = 1.58
SIPGPSVTV − Mascot − rejected
SIP|GPSVTV − spliced − 2|4|3|3
20120515_EXQ3_MiBa_SA_HCT116_mHLA−1: 25613
SA = 0.89
Score = −2.61
TVPPSGLSV − MaxQuant
20120515_EXQ3_MiBa_SA_HCT116_mHLA−1: 25613
SA = 0.98
Score = 1.11
ISPGPLTAT − MSFragger
20120515_EXQ3_MiBa_SA_HCT116_mHLA−1: 25613
SA = 0.87
Score = −2.1
Relative intensity
m/z
Prosit prediction
* Exclusive in prediction
# Exclusive in experimental

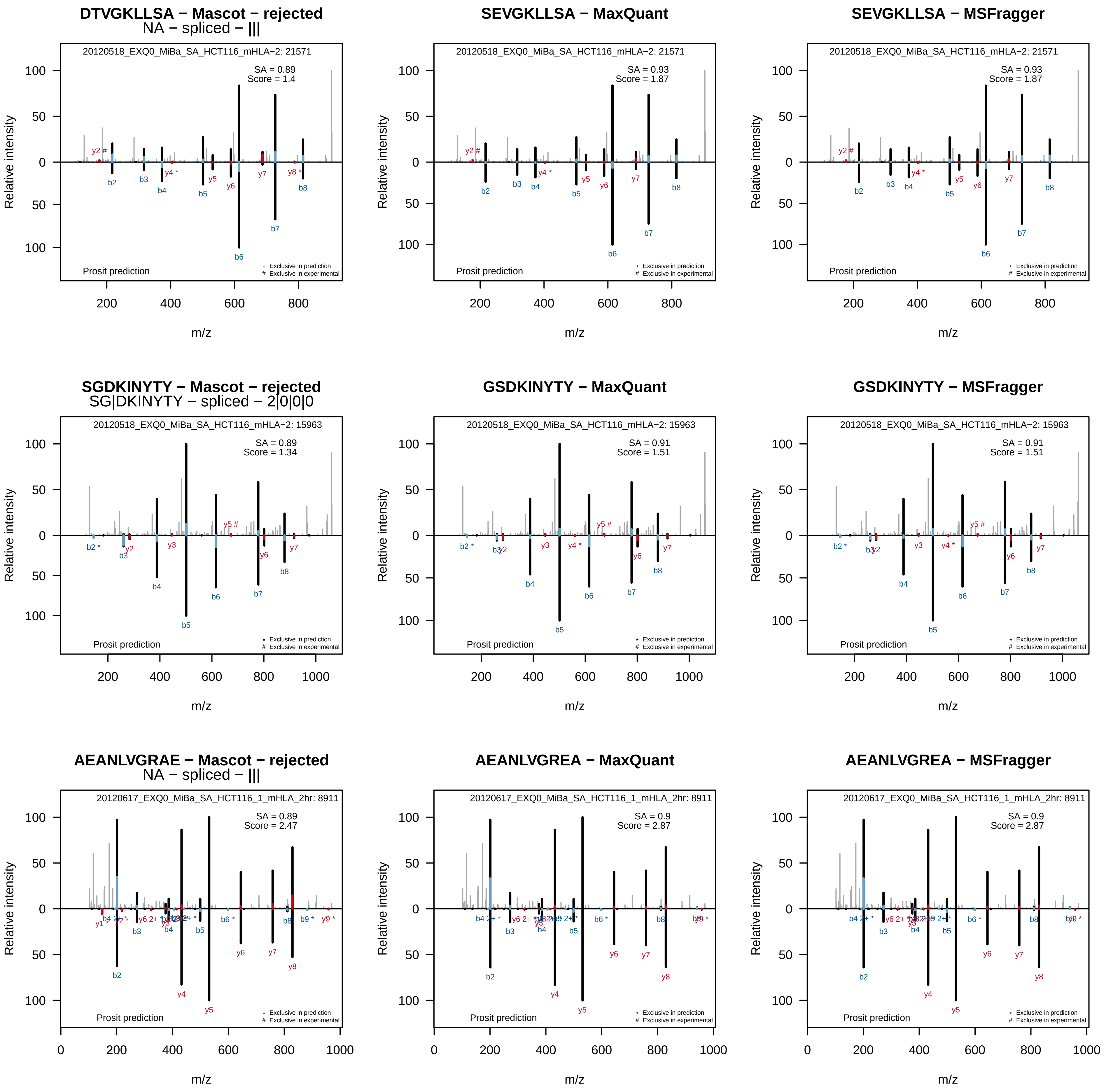
DTVGKLLSA − Mascot − rejected
NA − spliced − |||
20120518_EXQ0_MiBa_SA_HCT116_mHLA−2: 21571
SA = 0.89
Score = 1.4
SEVGKLLSA − MaxQuant
20120518_EXQ0_MiBa_SA_HCT116_mHLA−2: 21571
SA = 0.93
Score = 1.87
SEVGKLLSA − MSFragger
20120518_EXQ0_MiBa_SA_HCT116_mHLA−2: 21571
SA = 0.93
Score = 1.87
SGDKINYTY − Mascot − rejected
SG|DKINYTY − spliced − 2|0|0|0
20120518_EXQ0_MiBa_SA_HCT116_mHLA−2: 15963
SA = 0.89
Score = 1.34
GSDKINYTY − MaxQuant
20120518_EXQ0_MiBa_SA_HCT116_mHLA−2: 15963
SA = 0.91
Score = 1.51
GSDKINYTY − MSFragger
20120518_EXQ0_MiBa_SA_HCT116_mHLA−2: 15963
SA = 0.91
Score = 1.51
AEANLVGRAE − Mascot − rejected
NA − spliced − |||
20120617_EXQ0_MiBa_SA_HCT116_1_mHLA_2hr: 8911
SA = 0.89
Score = 2.47
AEANLVGREA − MaxQuant
20120617_EXQ0_MiBa_SA_HCT116_1_mHLA_2hr: 8911
SA = 0.9
Score = 2.87
AEANLVGREA − MSFragger
20120617_EXQ0_MiBa_SA_HCT116_1_mHLA_2hr: 8911
SA = 0.9
Score = 2.87
Relative intensity
m/z
Prosit prediction
* Exclusive in prediction
# Exclusive in experimental

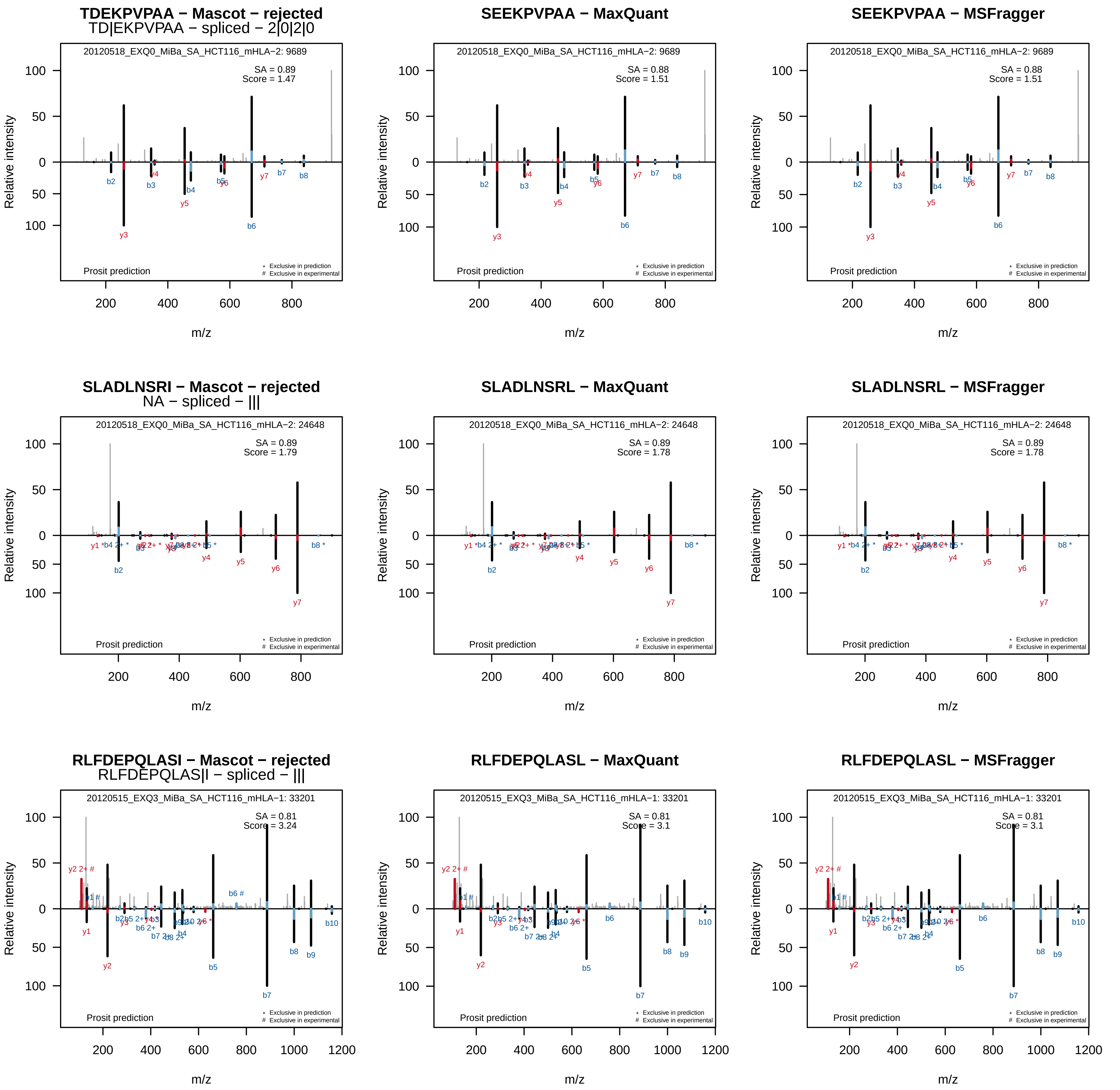

TDEKPVPAA − Mascot − rejected
TD|EKPVPAA − spliced − 2|0|2|0
20120518_EXQ0_MiBa_SA_HCT116_mHLA−2: 9689
SA = 0.89
Score = 1.47
Prosit prediction
⋆ Exclusive in prediction
Exclusive in experimental
SEEKPVPAA − MaxQuant
20120518_EXQ0_MiBa_SA_HCT116_mHLA−2: 9689
SA = 0.88
Score = 1.51
Prosit prediction
SEEKPVPAA − MSFragger
20120518_EXQ0_MiBa_SA_HCT116_mHLA−2: 9689
SA = 0.88
Score = 1.51
Prosit prediction
SLADLNSRI − Mascot − rejected
NA − spliced − |||
20120518_EXQ0_MiBa_SA_HCT116_mHLA−2: 24648
SA = 0.89
Score = 1.79
Prosit prediction
SLADLNSRL − MaxQuant
20120518_EXQ0_MiBa_SA_HCT116_mHLA−2: 24648
SA = 0.89
Score = 1.78
Prosit prediction
SLADLNSRL − MSFragger
20120518_EXQ0_MiBa_SA_HCT116_mHLA−2: 24648
SA = 0.89
Score = 1.78
Prosit prediction
RLFDEPQLASI − Mascot − rejected
RLFDEPQLAS|I − spliced − |||
20120515_EXQ3_MiBa_SA_HCT116_mHLA−1: 33201
SA = 0.81
Score = 3.24
Prosit prediction
RLFDEPQLASL − MaxQuant
20120515_EXQ3_MiBa_SA_HCT116_mHLA−1: 33201
SA = 0.81
Score = 3.1
Prosit prediction
RLFDEPQLASL − MSFragger
20120515_EXQ3_MiBa_SA_HCT116_mHLA−1: 33201
SA = 0.81
Score = 3.1
Prosit prediction
Relative intensity
m/z

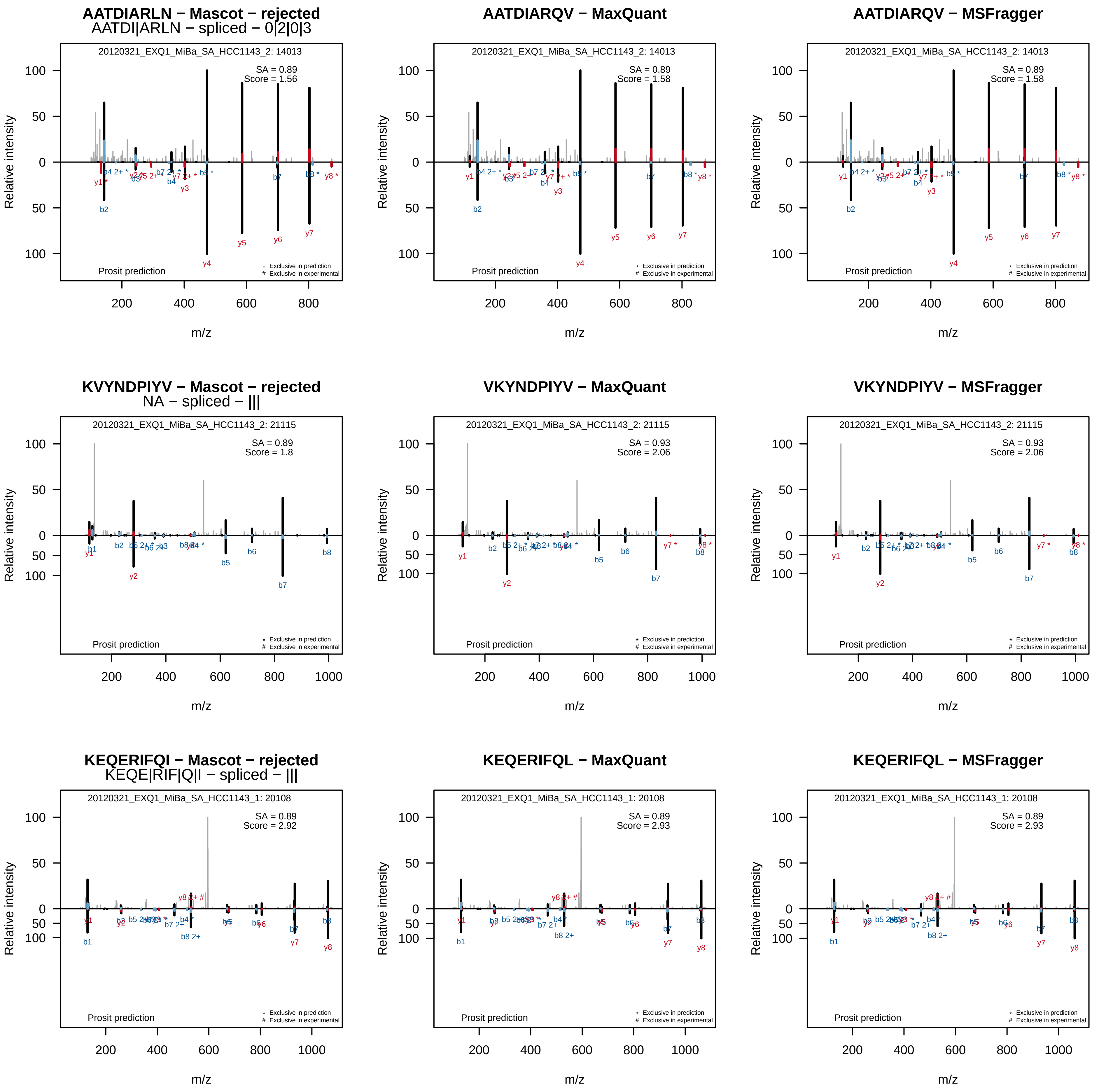

AATDIARLN − Mascot − rejected
AATDI|ARLN − spliced − 0|2|0|3
AATDIARQV − MaxQuant
AATDIARQV − MSFragger
20120321_EXQ1_MiBa_SA_HCC1143_2: 14013
SA = 0.89
Score = 1.56
Score = 1.58
KVYNDPIYV − Mascot − rejected
NA − spliced − |||
VKYNDPIYV − MaxQuant
VKYNDPIYV − MSFragger
20120321_EXQ1_MiBa_SA_HCC1143_2: 21115
SA = 0.89
Score = 1.8
SA = 0.93
Score = 2.06
KEQERIFQI − Mascot − rejected
KEQE|RIF|Q|I − spliced − |||
KEQERIFQL − MaxQuant
KEQERIFQL − MSFragger
20120321_EXQ1_MiBa_SA_HCC1143_1: 20108
SA = 0.89
Score = 2.92
Score = 2.93
Relative intensity
m/z
Prosit prediction
* Exclusive in prediction
# Exclusive in experimental

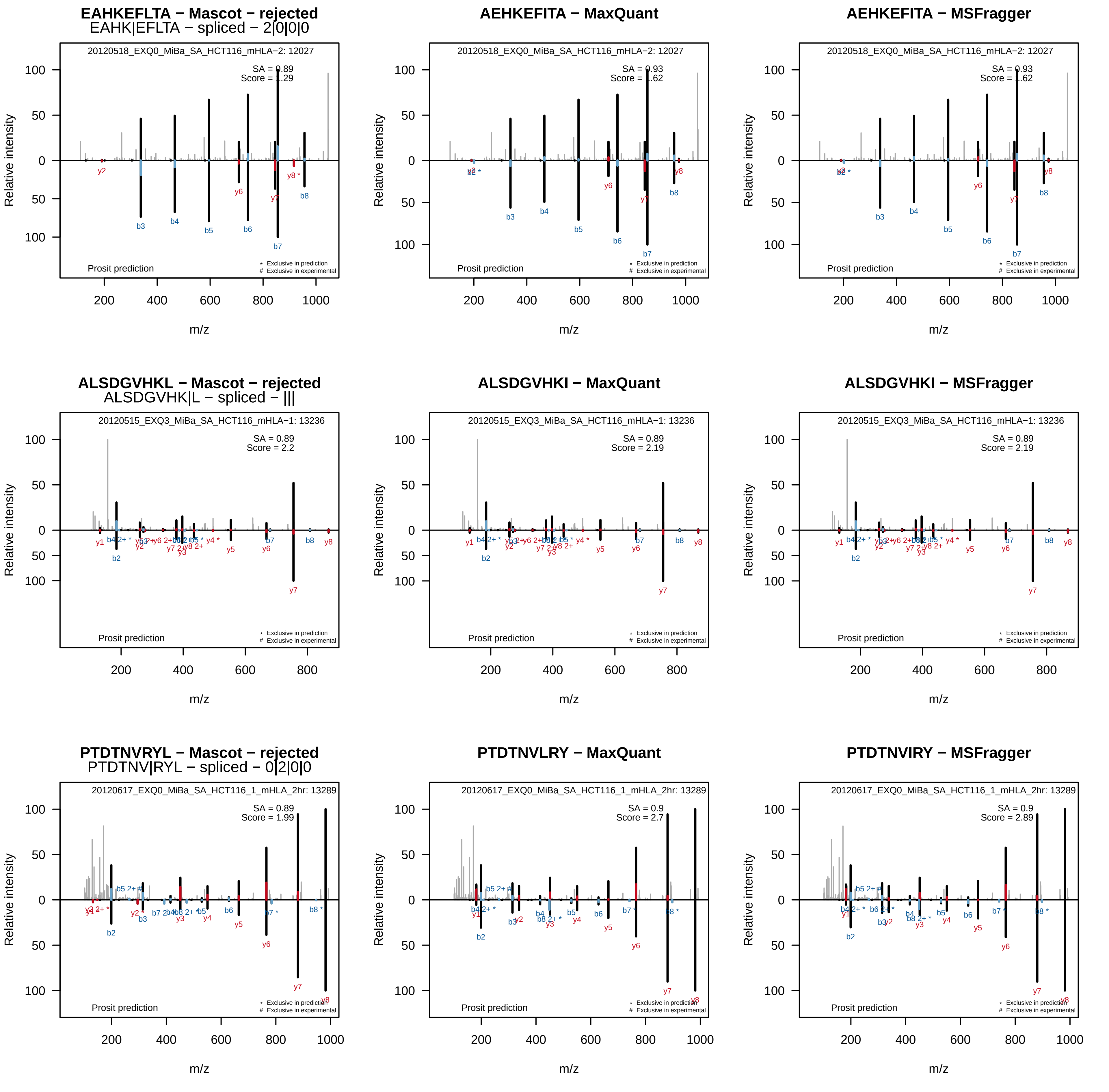

EAHKEFLTA − Mascot − rejected
EAHK|EFLTA − spliced − 2|0|0|0
AEHKEFITA − MaxQuant
AEHKEFITA − MSFragger
20120518_EXQ0_MiBa_SA_HCT116_mHLA−2: 12027
SA = 0.89
SA = 0.93
ALSDGVHKL − Mascot − rejected
ALSDGVHK|L − spliced − |||
ALSDGVHKI − MaxQuant
ALSDGVHKI − MSFragger
20120515_EXQ3_MiBa_SA_HCT116_mHLA−1: 13236
SA = 0.89
Score = 2.2
Score = 2.19
PTDTNVRYL − Mascot − rejected
PTDTNV|RYL − spliced − 0|2|0|0
PTDTNVLRY − MaxQuant
PTDTNVIRY − MSFragger
20120617_EXQ0_MiBa_SA_HCT116_1_mHLA_2hr: 13289
SA = 0.89
Score = 1.99
SA = 0.9
Score = 2.7
Score = 2.89
Relative intensity
m/z
Prosit prediction
⋆ Exclusive in prediction
Exclusive in experimental

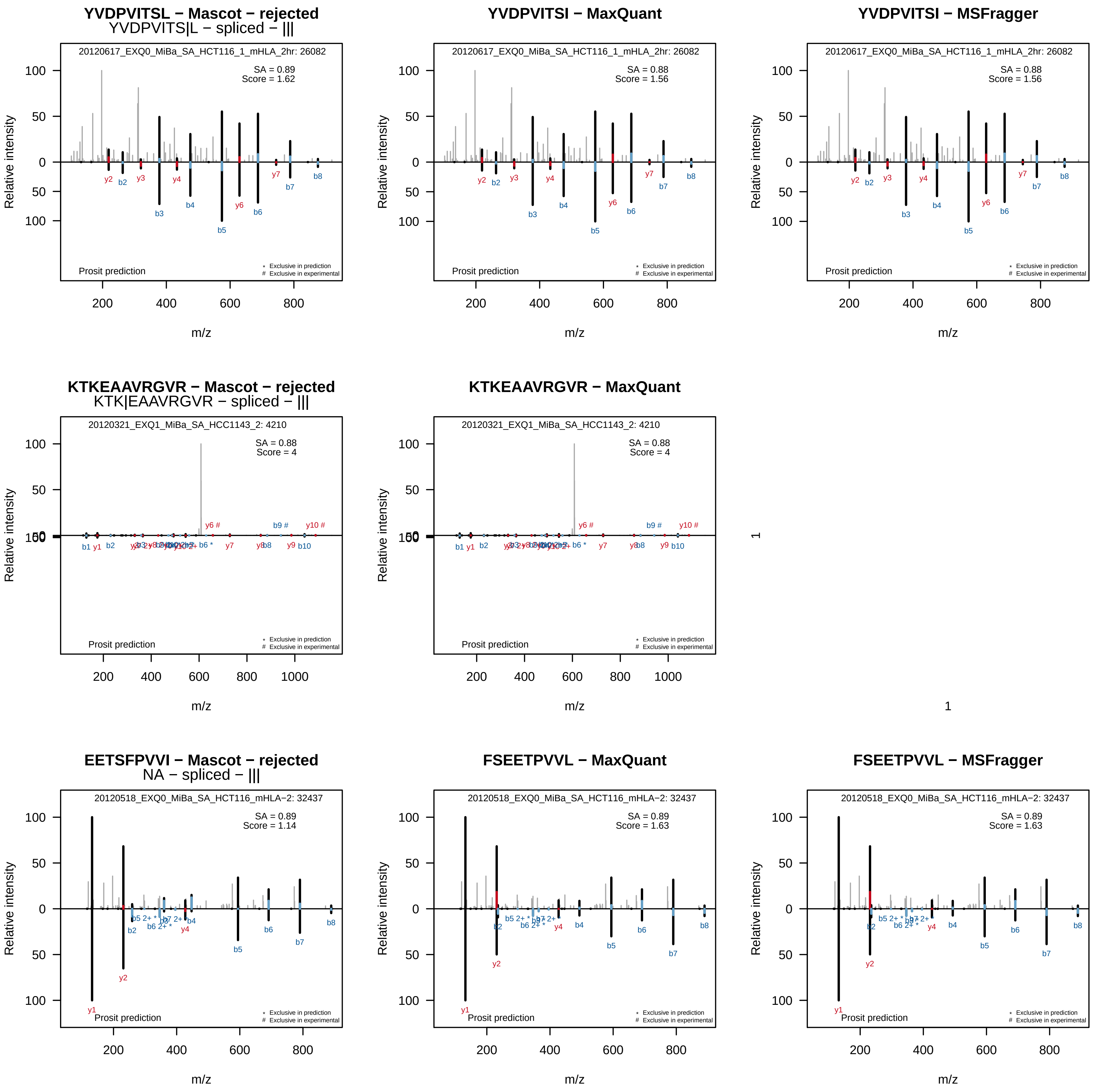

YVDPVITSL − Mascot − rejected
YVDPVITS|L − spliced − |||
20120617_EXQ0_MiBa_SA_HCT116_1_mHLA_2hr: 26082
SA = 0.89
Score = 1.62
YVDPVITSI − MaxQuant
20120617_EXQ0_MiBa_SA_HCT116_1_mHLA_2hr: 26082
SA = 0.88
Score = 1.56
YVDPVITSI − MSFragger
20120617_EXQ0_MiBa_SA_HCT116_1_mHLA_2hr: 26082
SA = 0.88
Score = 1.56
KTKEAAVRGVR − Mascot − rejected
KTK|EAAVRGVR − spliced − |||
20120321_EXQ1_MiBa_SA_HCC1143_2: 4210
SA = 0.88
Score = 4
KTKEAAVRGVR − MaxQuant
20120321_EXQ1_MiBa_SA_HCC1143_2: 4210
SA = 0.88
Score = 4
EETSFPVVI − Mascot − rejected
NA − spliced − |||
20120518_EXQ0_MiBa_SA_HCT116_mHLA−2: 32437
SA = 0.89
Score = 1.14
FSEETPVVL − MaxQuant
20120518_EXQ0_MiBa_SA_HCT116_mHLA−2: 32437
SA = 0.89
Score = 1.63
FSEETPVVL − MSFragger
20120518_EXQ0_MiBa_SA_HCT116_mHLA−2: 32437
SA = 0.89
Score = 1.63
Relative intensity
m/z
Prosit prediction
* Exclusive in prediction
# Exclusive in experimental

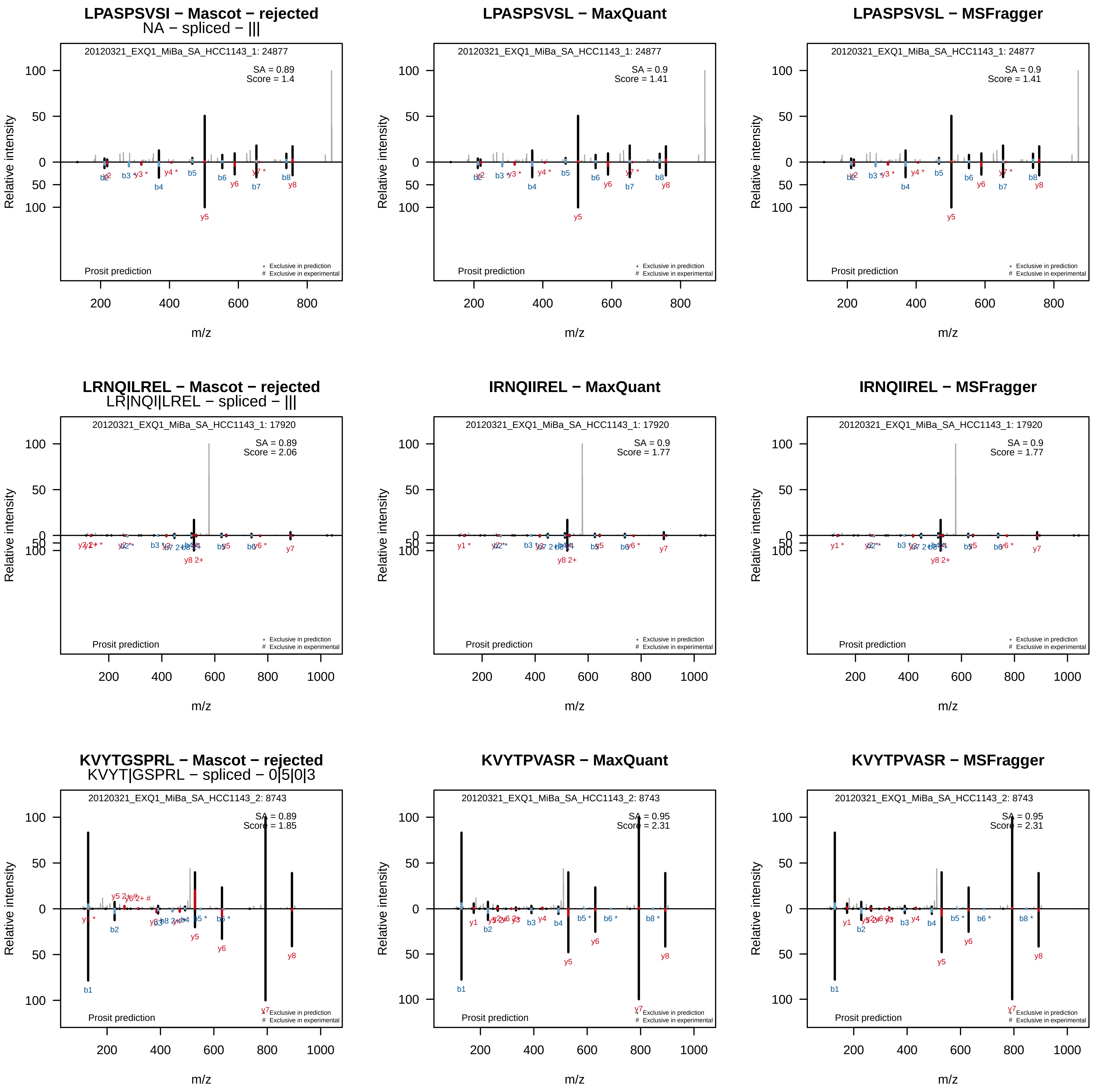

LPASPSVSI − Mascot − rejected
NA − spliced − |||
20120321_EXQ1_MiBa_SA_HCC1143_1: 24877
SA = 0.89
Score = 1.4
LPASPSVSL − MaxQuant
20120321_EXQ1_MiBa_SA_HCC1143_1: 24877
SA = 0.9
Score = 1.41
LPASPSVSL − MSFragger
20120321_EXQ1_MiBa_SA_HCC1143_1: 24877
SA = 0.9
Score = 1.41
LRNQILREL − Mascot − rejected
LR|NQI|LREL − spliced − |||
20120321_EXQ1_MiBa_SA_HCC1143_1: 17920
SA = 0.89
Score = 2.06
IRNQIIREL − MaxQuant
20120321_EXQ1_MiBa_SA_HCC1143_1: 17920
SA = 0.9
Score = 1.77
IRNQIIREL − MSFragger
20120321_EXQ1_MiBa_SA_HCC1143_1: 17920
SA = 0.9
Score = 1.77
KVYTGSPRL − Mascot − rejected
KVYT|GSPRL − spliced − 0|5|0|3
20120321_EXQ1_MiBa_SA_HCC1143_2: 8743
SA = 0.89
Score = 1.85
KVYTPVASR − MaxQuant
20120321_EXQ1_MiBa_SA_HCC1143_2: 8743
SA = 0.95
Score = 2.31
KVYTPVASR − MSFragger
20120321_EXQ1_MiBa_SA_HCC1143_2: 8743
SA = 0.95
Score = 2.31
Relative intensity
m/z
Prosit prediction
⋆ Exclusive in prediction
Exclusive in experimental

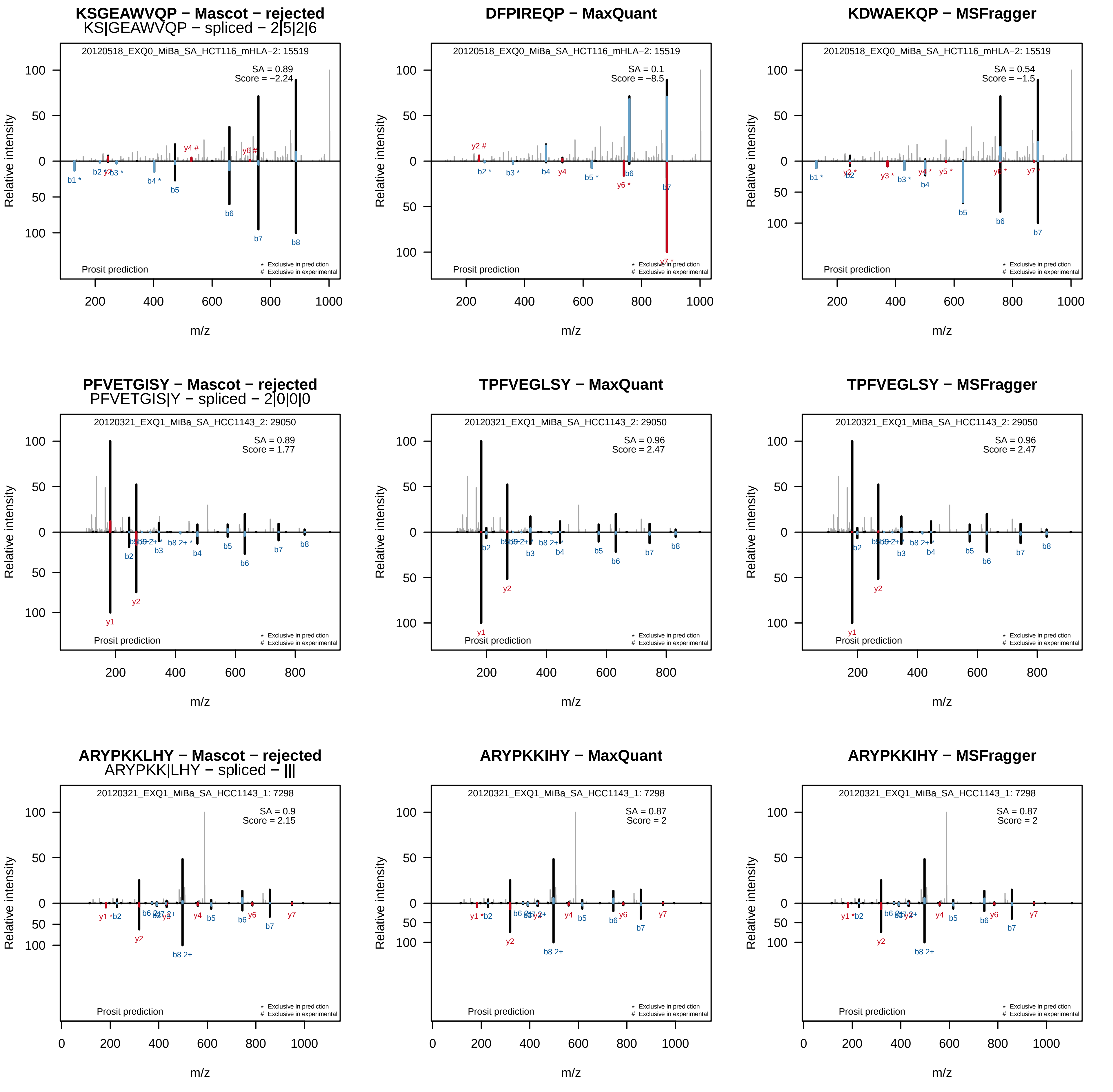
KSGEAWVQP − Mascot − rejected
KS|GEAWVQP − spliced − 2|5|2|6
20120518_EXQ0_MiBa_SA_HCT116_mHLA−2: 15519
SA = 0.89
Score = −2.24
DFPIREQP − MaxQuant
20120518_EXQ0_MiBa_SA_HCT116_mHLA−2: 15519
SA = 0.1
Score = −8.5
KDWAEKQP − MSFragger
20120518_EXQ0_MiBa_SA_HCT116_mHLA−2: 15519
SA = 0.54
Score = −1.5
PFVETGISY − Mascot − rejected
PFVETGIS|Y − spliced − 2|0|0|0
20120321_EXQ1_MiBa_SA_HCC1143_2: 29050
SA = 0.89
Score = 1.77
TPFVEGLSY − MaxQuant
20120321_EXQ1_MiBa_SA_HCC1143_2: 29050
SA = 0.96
Score = 2.47
TPFVEGLSY − MSFragger
20120321_EXQ1_MiBa_SA_HCC1143_2: 29050
SA = 0.96
Score = 2.47
ARYPKKLHY − Mascot − rejected
ARYPKK|LHY − spliced − |||
20120321_EXQ1_MiBa_SA_HCC1143_1: 7298
SA = 0.9
Score = 2.15
ARYPKKIHY − MaxQuant
20120321_EXQ1_MiBa_SA_HCC1143_1: 7298
SA = 0.87
Score = 2
ARYPKKIHY − MSFragger
20120321_EXQ1_MiBa_SA_HCC1143_1: 7298
SA = 0.87
Score = 2
Relative intensity
m/z
Prosit prediction
* Exclusive in prediction
# Exclusive in experimental

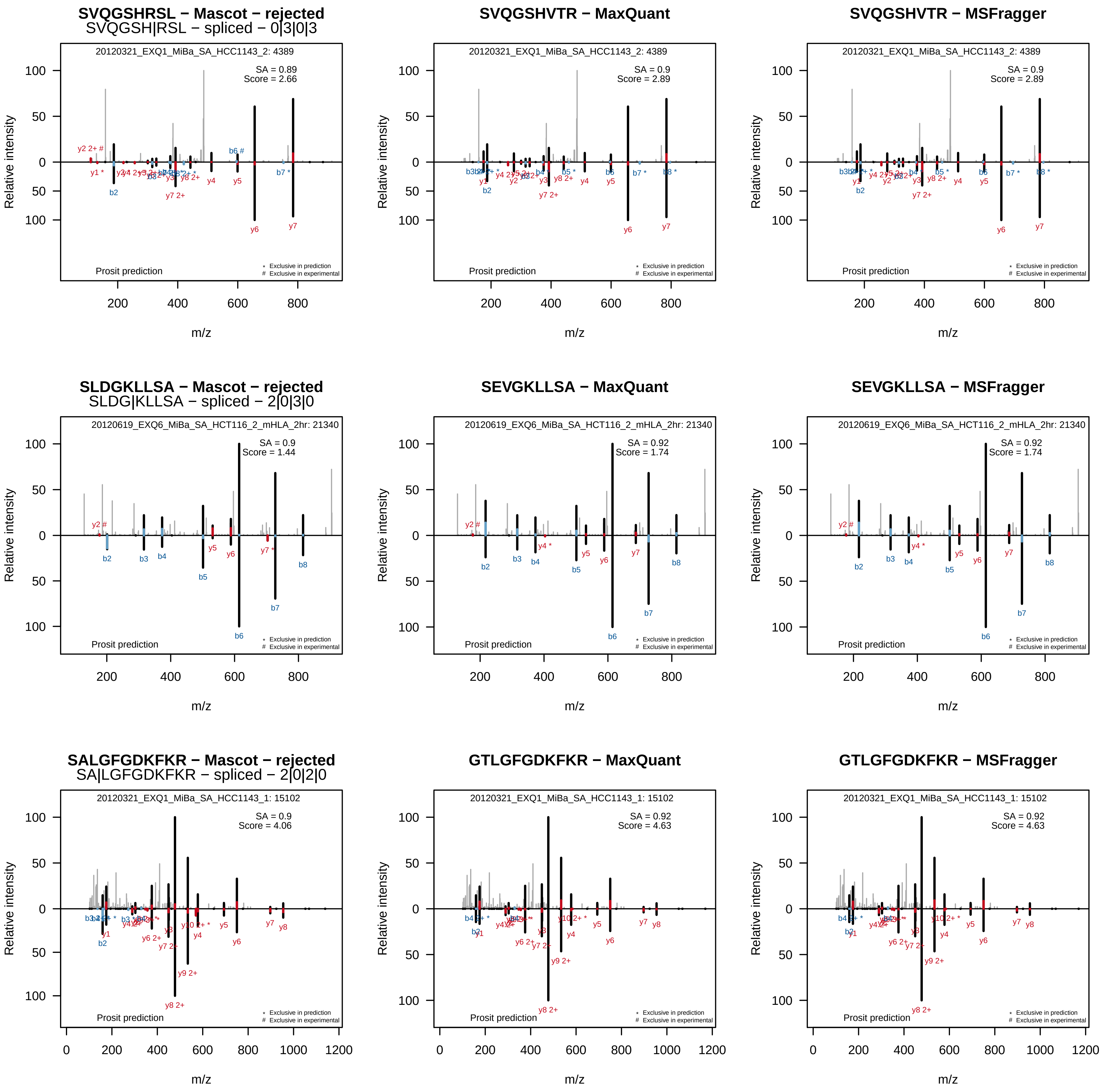
SVQGSHRSL − Mascot − rejected
SVQGSH|RSL − spliced − 0|3|0|3
20120321_EXQ1_MiBa_SA_HCC1143_2: 4389
SA = 0.89
Score = 2.66
SVQGSHVTR − MaxQuant
20120321_EXQ1_MiBa_SA_HCC1143_2: 4389
SA = 0.9
Score = 2.89
SVQGSHVTR − MSFragger
20120321_EXQ1_MiBa_SA_HCC1143_2: 4389
SA = 0.9
Score = 2.89
SLDGKLLSA − Mascot − rejected
SLDG|KLLSA − spliced − 2|0|3|0
20120619_EXQ6_MiBa_SA_HCT116_2_mHLA_2hr: 21340
SA = 0.9
Score = 1.44
SEVGKLLSA − MaxQuant
20120619_EXQ6_MiBa_SA_HCT116_2_mHLA_2hr: 21340
SA = 0.92
Score = 1.74
SEVGKLLSA − MSFragger
20120619_EXQ6_MiBa_SA_HCT116_2_mHLA_2hr: 21340
SA = 0.92
Score = 1.74
SALGFGDKFKR − Mascot − rejected
SA|LGFGDKFKR − spliced − 2|0|2|0
20120321_EXQ1_MiBa_SA_HCC1143_1: 15102
SA = 0.9
Score = 4.06
GTLGFGDKFKR − MaxQuant
20120321_EXQ1_MiBa_SA_HCC1143_1: 15102
SA = 0.92
Score = 4.63
GTLGFGDKFKR − MSFragger
20120321_EXQ1_MiBa_SA_HCC1143_1: 15102
SA = 0.92
Score = 4.63
Relative intensity
m/z
Prosit prediction
⋆ Exclusive in prediction
Exclusive in experimental

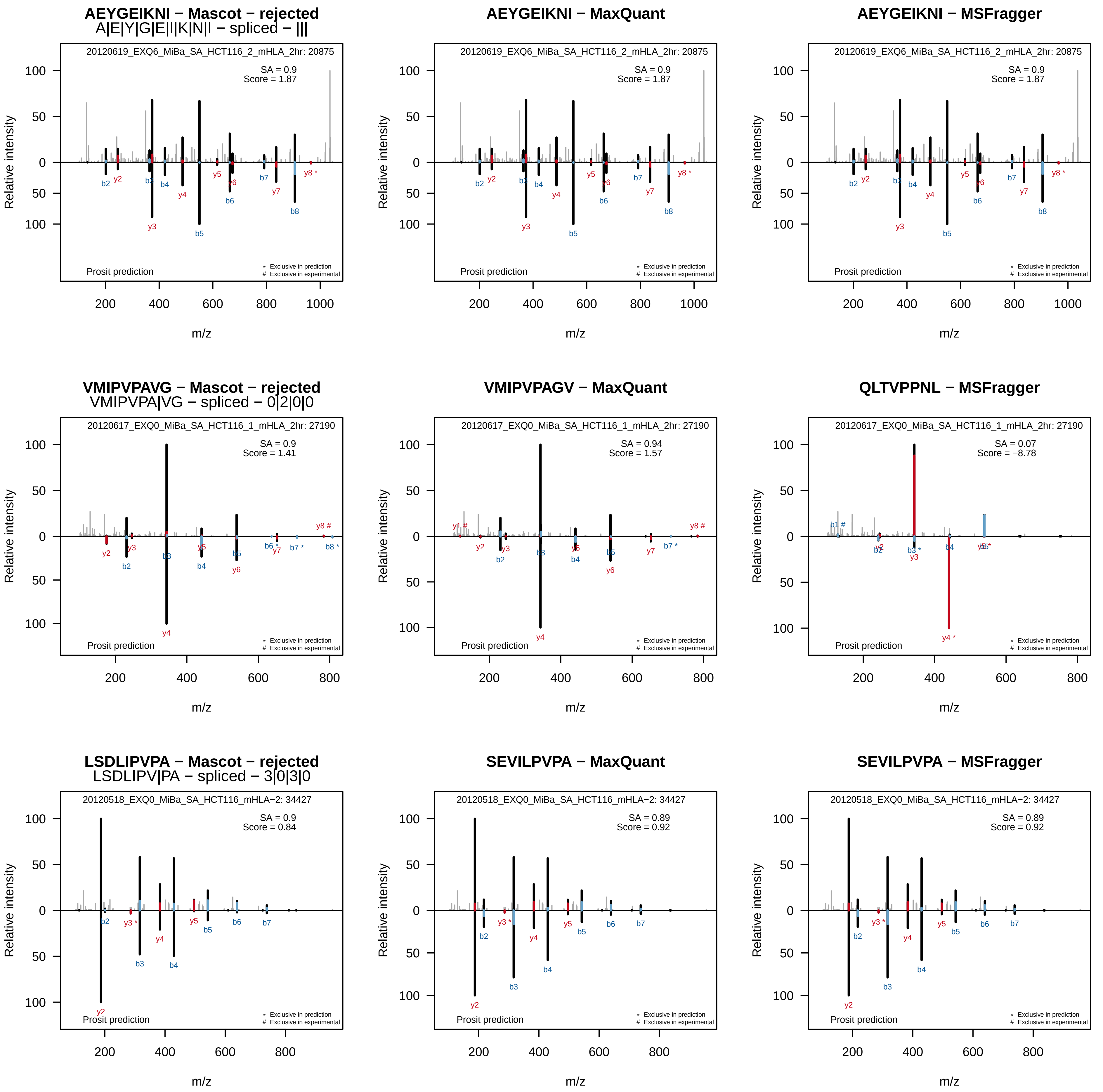

AEYGEIKNI − Mascot − rejected
A|E|Y|G|E|I|K|N|I − spliced − |||
20120619_EXQ6_MiBa_SA_HCT116_2_mHLA_2hr: 20875
SA = 0.9
Score = 1.87
AEYGEIKNI − MaxQuant
20120619_EXQ6_MiBa_SA_HCT116_2_mHLA_2hr: 20875
SA = 0.9
Score = 1.87
AEYGEIKNI − MSFragger
20120619_EXQ6_MiBa_SA_HCT116_2_mHLA_2hr: 20875
SA = 0.9
Score = 1.87
VMIPVPAVG − Mascot − rejected
VMIPVPA|VG − spliced − 0|2|0|0
20120617_EXQ0_MiBa_SA_HCT116_1_mHLA_2hr: 27190
SA = 0.9
Score = 1.41
VMIPVPAGV − MaxQuant
20120617_EXQ0_MiBa_SA_HCT116_1_mHLA_2hr: 27190
SA = 0.94
Score = 1.57
QLTVPPNL − MSFragger
20120617_EXQ0_MiBa_SA_HCT116_1_mHLA_2hr: 27190
SA = 0.07
Score = −8.78
LSDLIPVPA − Mascot − rejected
LSDLIPV|PA − spliced − 3|0|3|0
20120518_EXQ0_MiBa_SA_HCT116_mHLA−2: 34427
SA = 0.9
Score = 0.84
SEVILPVPA − MaxQuant
20120518_EXQ0_MiBa_SA_HCT116_mHLA−2: 34427
SA = 0.89
Score = 0.92
SEVILPVPA − MSFragger
20120518_EXQ0_MiBa_SA_HCT116_mHLA−2: 34427
SA = 0.89
Score = 0.92
Relative intensity
m/z
Prosit prediction
* Exclusive in prediction
# Exclusive in experimental

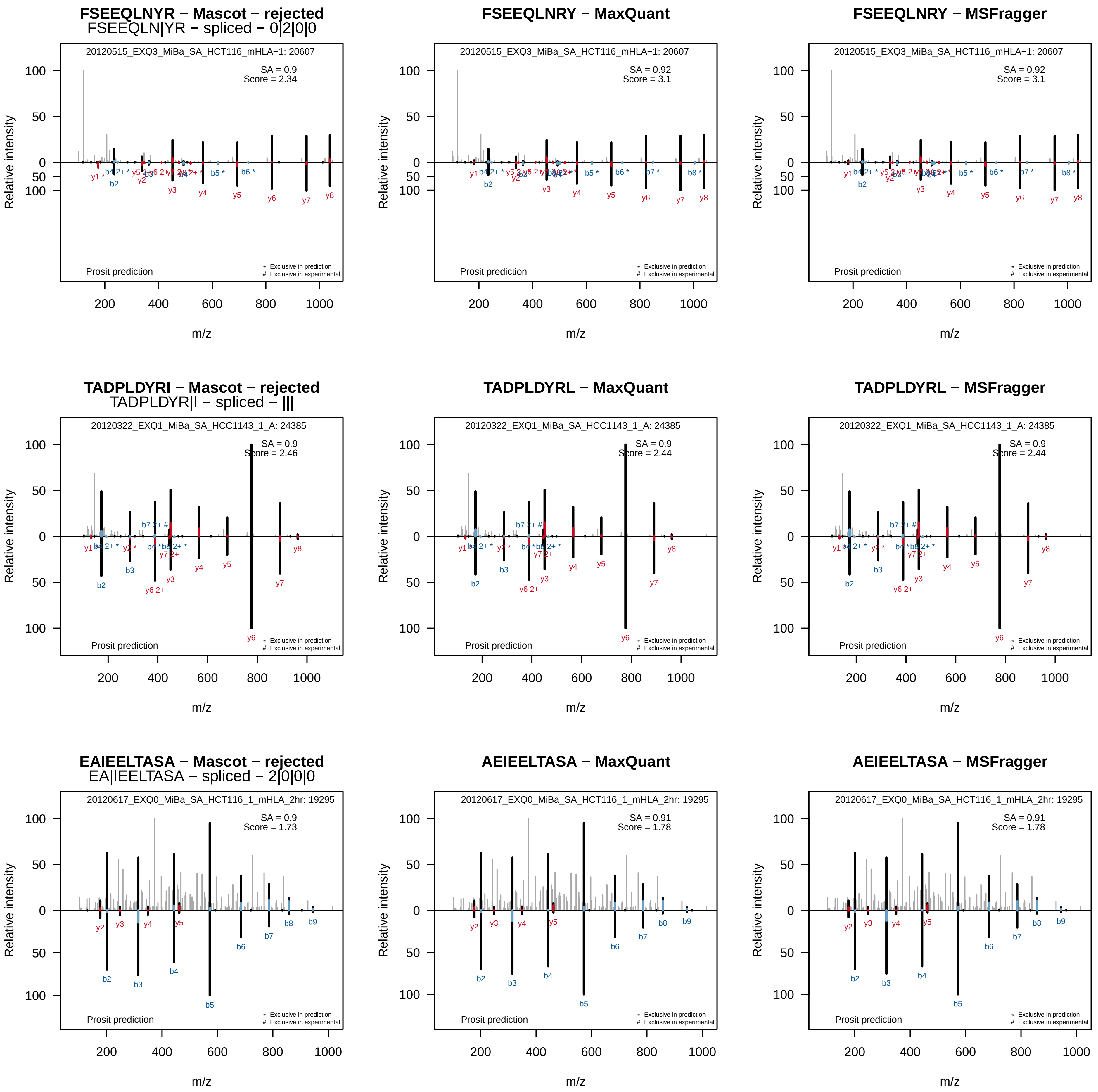
FSEEQLNYR − Mascot − rejected
FSEEQLN|YR − spliced − 0|2|0|0
20120515_EXQ3_MiBa_SA_HCT116_mHLA−1: 20607
SA = 0.9
Score = 2.34
FSEEQLNRY − MaxQuant
20120515_EXQ3_MiBa_SA_HCT116_mHLA−1: 20607
SA = 0.92
Score = 3.1
FSEEQLNRY − MSFragger
20120515_EXQ3_MiBa_SA_HCT116_mHLA−1: 20607
SA = 0.92
Score = 3.1
TADPLDYRI − Mascot − rejected
TADPLDYR|I − spliced − |||
20120322_EXQ1_MiBa_SA_HCC1143_1_A: 24385
SA = 0.9
Score = 2.46
TADPLDYRL − MaxQuant
20120322_EXQ1_MiBa_SA_HCC1143_1_A: 24385
SA = 0.9
Score = 2.44
TADPLDYRL − MSFragger
20120322_EXQ1_MiBa_SA_HCC1143_1_A: 24385
SA = 0.9
Score = 2.44
EAIEELTASA − Mascot − rejected
EA|IEELTASA − spliced − 2|0|0|0
20120617_EXQ0_MiBa_SA_HCT116_1_mHLA_2hr: 19295
SA = 0.9
Score = 1.73
AEIEELTASA − MaxQuant
20120617_EXQ0_MiBa_SA_HCT116_1_mHLA_2hr: 19295
SA = 0.91
Score = 1.78
AEIEELTASA − MSFragger
20120617_EXQ0_MiBa_SA_HCT116_1_mHLA_2hr: 19295
SA = 0.91
Score = 1.78
Relative intensity
m/z
Prosit prediction
⋆ Exclusive in prediction
Exclusive in experimental

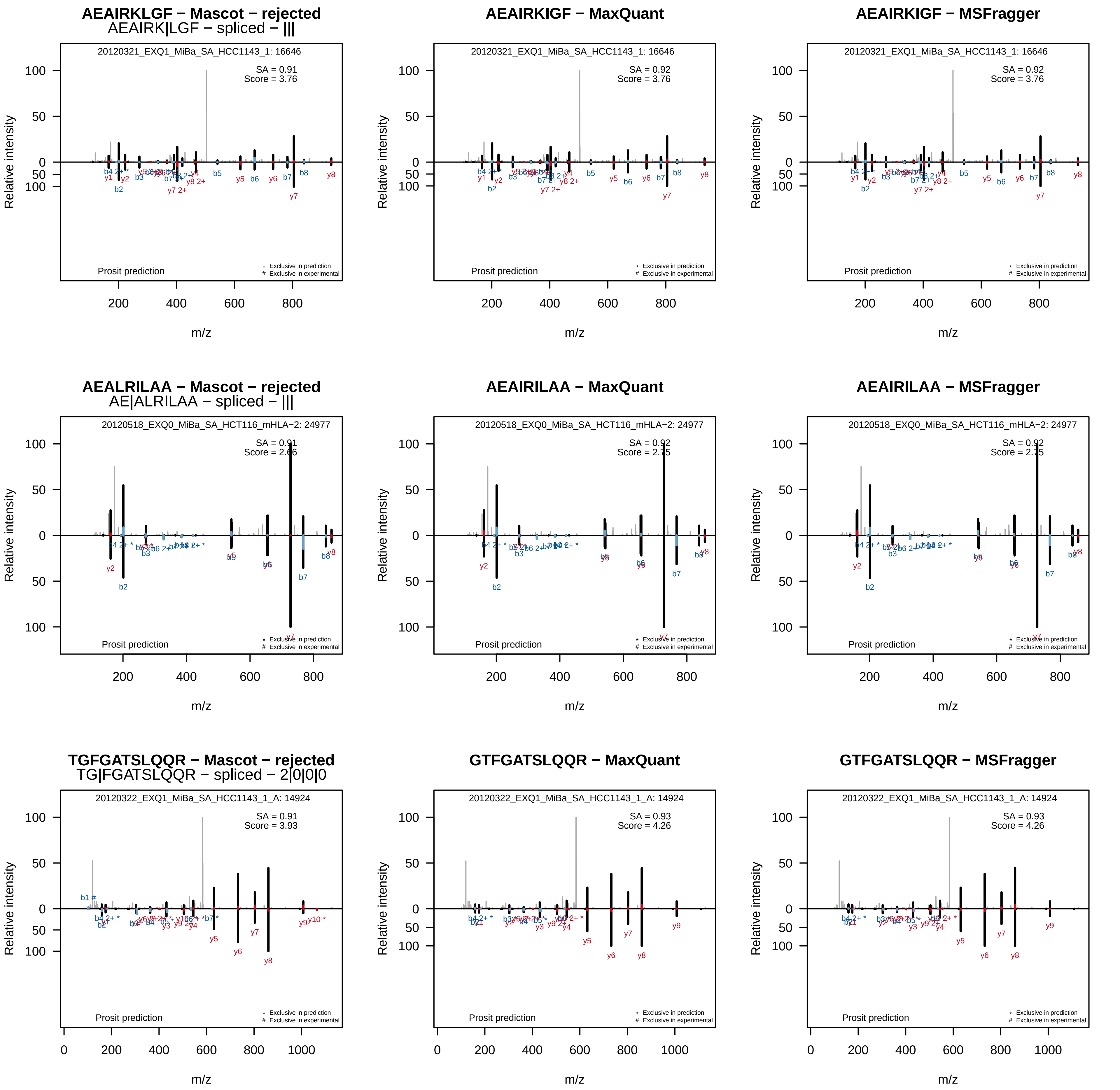
AEAIRKLGF − Mascot − rejected
AEAIRK|LGF − spliced − |||
AEAIRKIGF − MaxQuant
AEAIRKIGF − MSFragger
20120321_EXQ1_MiBa_SA_HCC1143_1: 16646
SA = 0.91
Score = 3.76
SA = 0.92
Score = 3.76
AEALRILAA − Mascot − rejected
AE|ALRILAA − spliced − |||
AEAIRILAA − MaxQuant
AEAIRILAA − MSFragger
20120518_EXQ0_MiBa_SA_HCT116_mHLA−2: 24977
SA = 0.91
Score = 2.66
SA = 0.92
Score = 2.75
TGFGATSLQQR − Mascot − rejected
TG|FGATSLQQR − spliced − 2|0|0|0
GTFGATSLQQR − MaxQuant
GTFGATSLQQR − MSFragger
20120322_EXQ1_MiBa_SA_HCC1143_1_A: 14924
SA = 0.91
Score = 3.93
SA = 0.93
Score = 4.26
Relative intensity
m/z
Prosit prediction
⋆ Exclusive in prediction
Exclusive in experimental

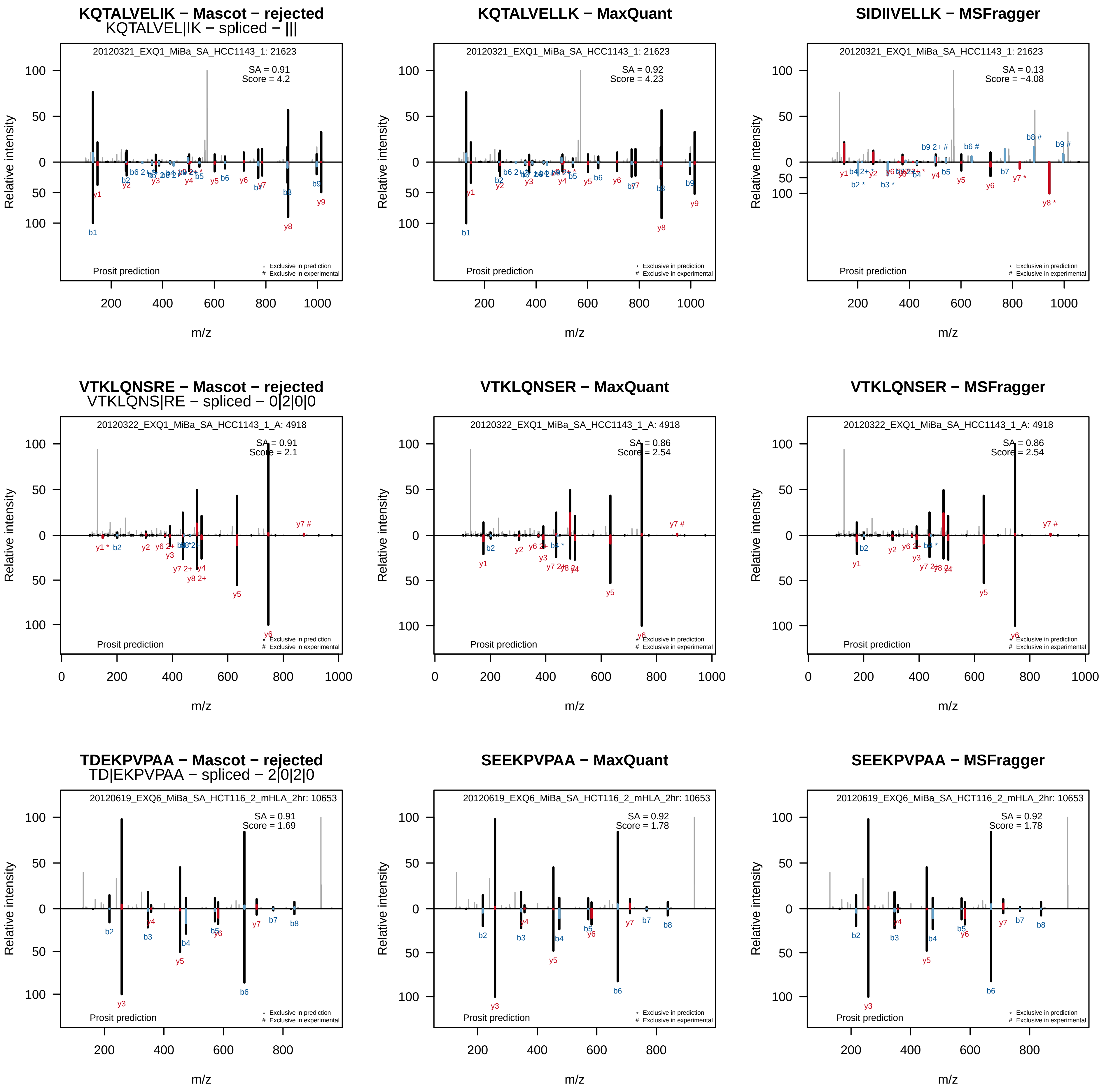

KQTALVELIK − Mascot − rejected
KQTALVEL|IK − spliced − |||
20120321_EXQ1_MiBa_SA_HCC1143_1: 21623
SA = 0.91
Score = 4.2
KQTALVELLK − MaxQuant
20120321_EXQ1_MiBa_SA_HCC1143_1: 21623
SA = 0.92
Score = 4.23
SIDIIVELLK − MSFragger
20120321_EXQ1_MiBa_SA_HCC1143_1: 21623
SA = 0.13
Score = −4.08
VTKLQNSRE − Mascot − rejected
VTKLQNS|RE − spliced − 0|2|0|0
20120322_EXQ1_MiBa_SA_HCC1143_1_A: 4918
SA = 0.91
Score = 2.1
VTKLQNSER − MaxQuant
20120322_EXQ1_MiBa_SA_HCC1143_1_A: 4918
SA = 0.86
Score = 2.54
VTKLQNSER − MSFragger
20120322_EXQ1_MiBa_SA_HCC1143_1_A: 4918
SA = 0.86
Score = 2.54
TDEKPVPAA − Mascot − rejected
TD|EKPVPAA − spliced − 2|0|2|0
20120619_EXQ6_MiBa_SA_HCT116_2_mHLA_2hr: 10653
SA = 0.91
Score = 1.69
SEEKPVPAA − MaxQuant
20120619_EXQ6_MiBa_SA_HCT116_2_mHLA_2hr: 10653
SA = 0.92
Score = 1.78
SEEKPVPAA − MSFragger
20120619_EXQ6_MiBa_SA_HCT116_2_mHLA_2hr: 10653
SA = 0.92
Score = 1.78
Relative intensity
m/z
Prosit prediction
* Exclusive in prediction
# Exclusive in experimental

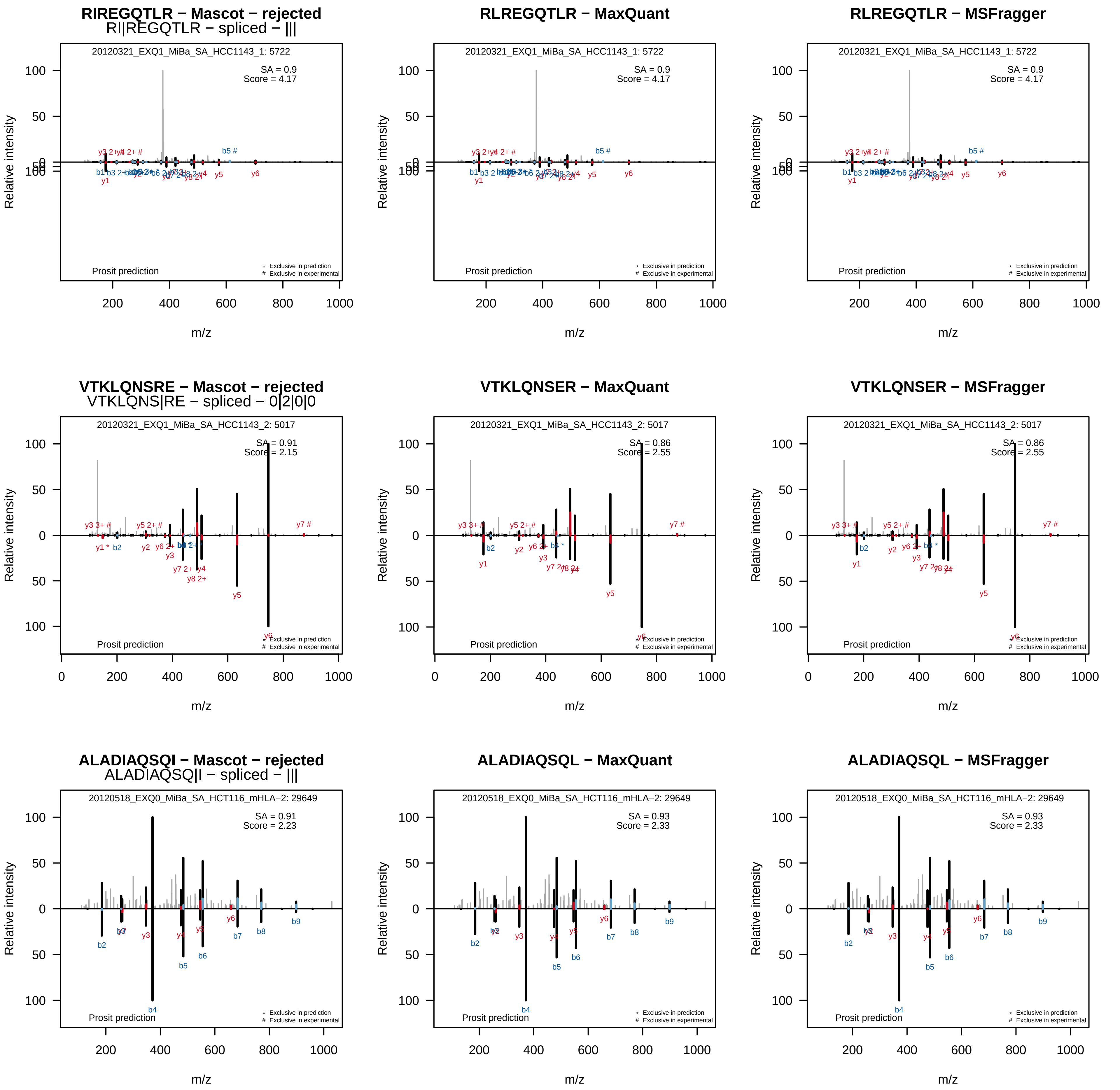

RIREGQTLR − Mascot − rejected
RI|REGQTLR − spliced − |||
20120321_EXQ1_MiBa_SA_HCC1143_1: 5722
SA = 0.9
Score = 4.17
RLREGQTLR − MaxQuant
20120321_EXQ1_MiBa_SA_HCC1143_1: 5722
SA = 0.9
Score = 4.17
RLREGQTLR − MSFragger
20120321_EXQ1_MiBa_SA_HCC1143_1: 5722
SA = 0.9
Score = 4.17
VTKLQNSRE − Mascot − rejected
VTKLQNS|RE − spliced − 0|2|0|0
20120321_EXQ1_MiBa_SA_HCC1143_2: 5017
SA = 0.91
Score = 2.15
VTKLQNSER − MaxQuant
20120321_EXQ1_MiBa_SA_HCC1143_2: 5017
SA = 0.86
Score = 2.55
VTKLQNSER − MSFragger
20120321_EXQ1_MiBa_SA_HCC1143_2: 5017
SA = 0.86
Score = 2.55
ALADIAQSQI − Mascot − rejected
ALADIAQSQ|I − spliced − |||
20120518_EXQ0_MiBa_SA_HCT116_mHLA−2: 29649
SA = 0.91
Score = 2.23
ALADIAQSQL − MaxQuant
20120518_EXQ0_MiBa_SA_HCT116_mHLA−2: 29649
SA = 0.93
Score = 2.33
ALADIAQSQL − MSFragger
20120518_EXQ0_MiBa_SA_HCT116_mHLA−2: 29649
SA = 0.93
Score = 2.33
Relative intensity
m/z
Prosit prediction
* Exclusive in prediction
# Exclusive in experimental

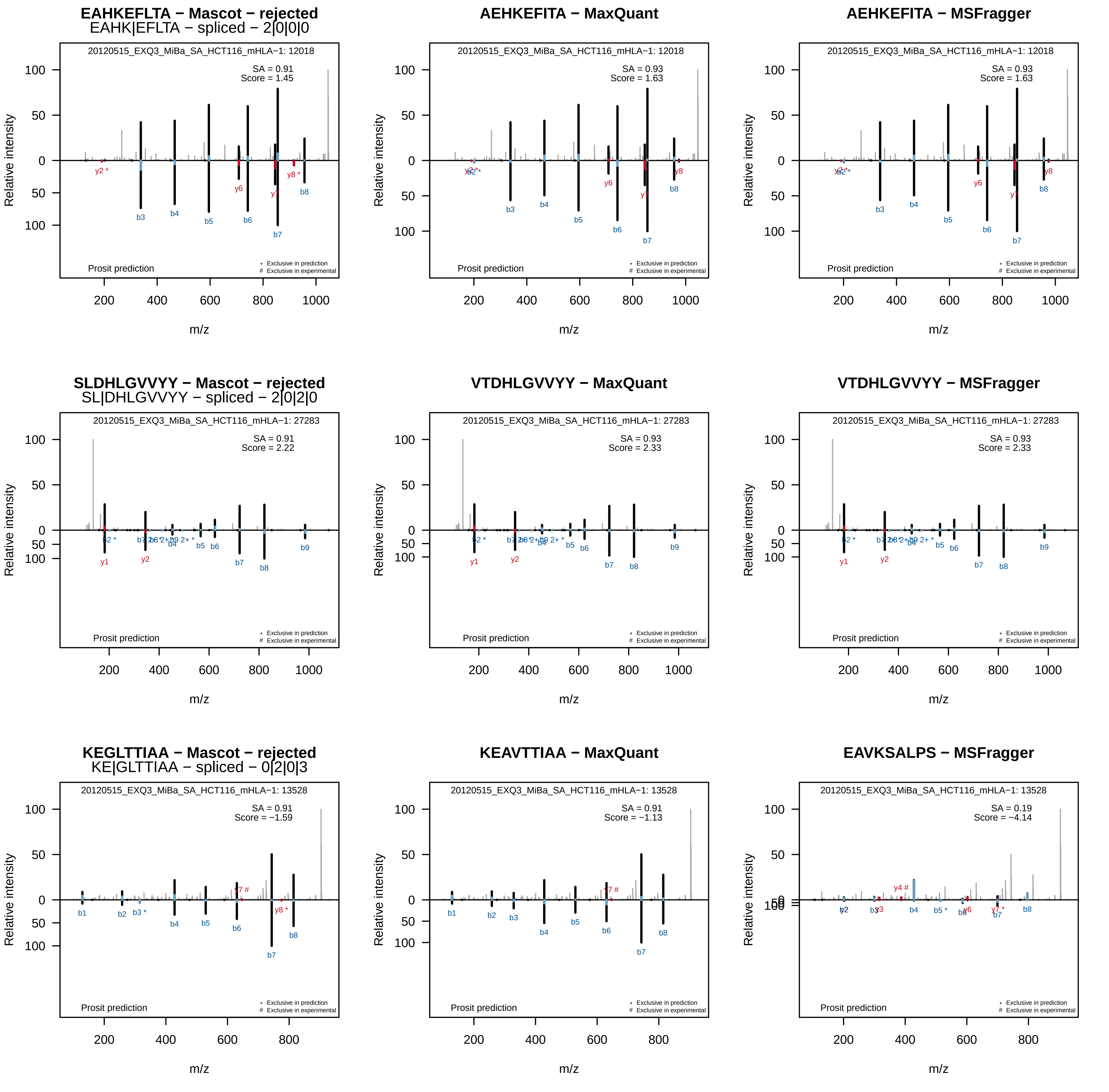

EAHKEFLTA − Mascot − rejected
EAHK|EFLTA − spliced − 2|0|0|0
20120515_EXQ3_MiBa_SA_HCT116_mHLA−1: 12018
SA = 0.91
Score = 1.45
AEHKEFITA − MaxQuant
20120515_EXQ3_MiBa_SA_HCT116_mHLA−1: 12018
SA = 0.93
Score = 1.63
AEHKEFITA − MSFragger
20120515_EXQ3_MiBa_SA_HCT116_mHLA−1: 12018
SA = 0.93
Score = 1.63
SLDHLGVVYY − Mascot − rejected
SL|DHLGVVYY − spliced − 2|0|2|0
20120515_EXQ3_MiBa_SA_HCT116_mHLA−1: 27283
SA = 0.91
Score = 2.22
VTDHLGVVYY − MaxQuant
20120515_EXQ3_MiBa_SA_HCT116_mHLA−1: 27283
SA = 0.93
Score = 2.33
VTDHLGVVYY − MSFragger
20120515_EXQ3_MiBa_SA_HCT116_mHLA−1: 27283
SA = 0.93
Score = 2.33
KEGLTTIAA − Mascot − rejected
KE|GLTTIAA − spliced − 0|2|0|3
20120515_EXQ3_MiBa_SA_HCT116_mHLA−1: 13528
SA = 0.91
Score = −1.59
KEAVTTIAA − MaxQuant
20120515_EXQ3_MiBa_SA_HCT116_mHLA−1: 13528
SA = 0.91
Score = −1.13
EAVKSALPS − MSFragger
20120515_EXQ3_MiBa_SA_HCT116_mHLA−1: 13528
SA = 0.19
Score = −4.14
Relative intensity
m/z
Prosit prediction
⋆ Exclusive in prediction
Exclusive in experimental

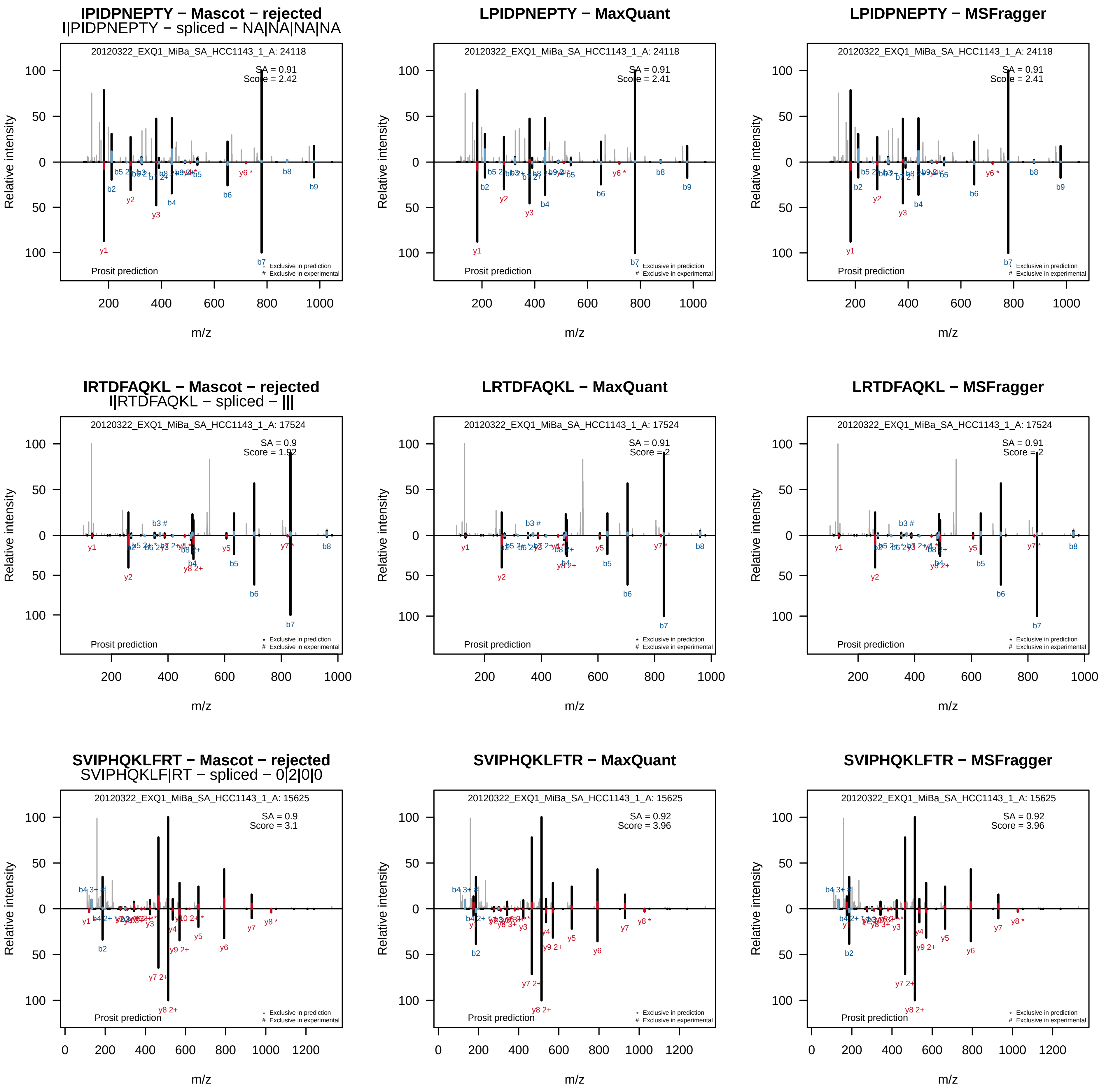
IPIDPNEPTY − Mascot − rejected
I|PIDPNEPTY − spliced − NA|NA|NA|NA
20120322_EXQ1_MiBa_SA_HCC1143_1_A: 24118
SA = 0.91
Score = 2.42
LPIDPNEPTY − MaxQuant
20120322_EXQ1_MiBa_SA_HCC1143_1_A: 24118
SA = 0.91
Score = 2.41
LPIDPNEPTY − MSFragger
20120322_EXQ1_MiBa_SA_HCC1143_1_A: 24118
SA = 0.91
Score = 2.41
IRTDFAQKL − Mascot − rejected
I|RTDFAQKL − spliced − |||
20120322_EXQ1_MiBa_SA_HCC1143_1_A: 17524
SA = 0.9
Score = 1.92
LRTDFAQKL − MaxQuant
20120322_EXQ1_MiBa_SA_HCC1143_1_A: 17524
SA = 0.91
Score = 2
LRTDFAQKL − MSFragger
20120322_EXQ1_MiBa_SA_HCC1143_1_A: 17524
SA = 0.91
Score = 2
SVIPHQKLFRT − Mascot − rejected
SVIPHQKLF|RT − spliced − 0|2|0|0
20120322_EXQ1_MiBa_SA_HCC1143_1_A: 15625
SA = 0.9
Score = 3.1
SVIPHQKLFTR − MaxQuant
20120322_EXQ1_MiBa_SA_HCC1143_1_A: 15625
SA = 0.92
Score = 3.96
SVIPHQKLFTR − MSFragger
20120322_EXQ1_MiBa_SA_HCC1143_1_A: 15625
SA = 0.92
Score = 3.96
Relative intensity
m/z
Prosit prediction
* Exclusive in prediction
# Exclusive in experimental

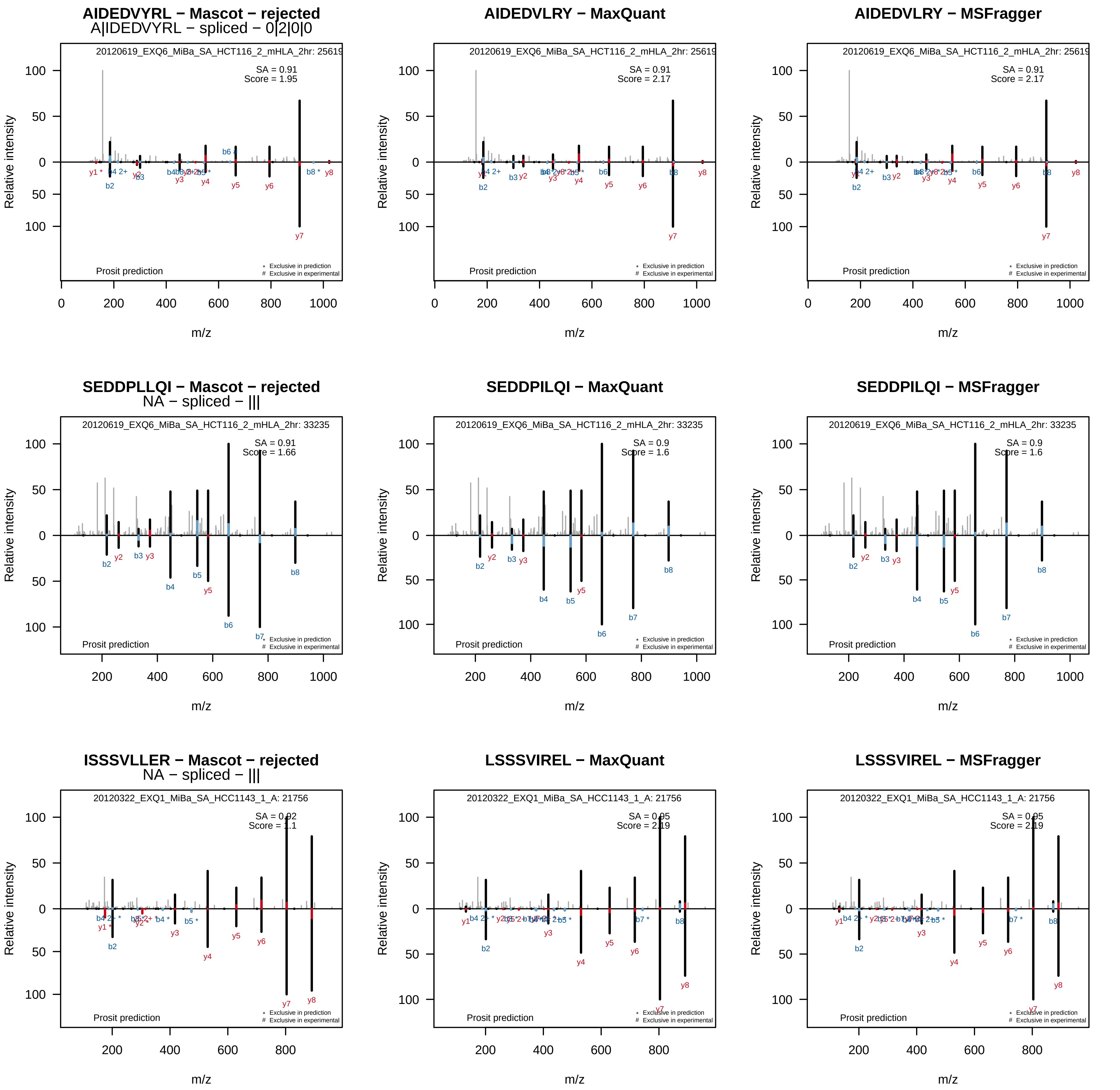

AIDEDVYRL − Mascot − rejected
A|IDEDVYRL − spliced − 0|2|0|0
20120619_EXQ6_MiBa_SA_HCT116_2_mHLA_2hr: 25619
SA = 0.91
Score = 1.95
AIDEDVLRY − MaxQuant
20120619_EXQ6_MiBa_SA_HCT116_2_mHLA_2hr: 25619
SA = 0.91
Score = 2.17
AIDEDVLRY − MSFragger
20120619_EXQ6_MiBa_SA_HCT116_2_mHLA_2hr: 25619
SA = 0.91
Score = 2.17
SEDDPLLQI − Mascot − rejected
NA − spliced − |||
20120619_EXQ6_MiBa_SA_HCT116_2_mHLA_2hr: 33235
SA = 0.91
Score = 1.66
SEDDPILQI − MaxQuant
20120619_EXQ6_MiBa_SA_HCT116_2_mHLA_2hr: 33235
SA = 0.9
Score = 1.6
SEDDPILQI − MSFragger
20120619_EXQ6_MiBa_SA_HCT116_2_mHLA_2hr: 33235
SA = 0.9
Score = 1.6
ISSSVLLER − Mascot − rejected
NA − spliced − |||
20120322_EXQ1_MiBa_SA_HCC1143_1_A: 21756
SA = 0.92
Score = 1.1
LSSSVIREL − MaxQuant
20120322_EXQ1_MiBa_SA_HCC1143_1_A: 21756
SA = 0.95
Score = 2.19
LSSSVIREL − MSFragger
20120322_EXQ1_MiBa_SA_HCC1143_1_A: 21756
SA = 0.95
Score = 2.19
Relative intensity
m/z
Prosit prediction
⋆ Exclusive in prediction
Exclusive in experimental

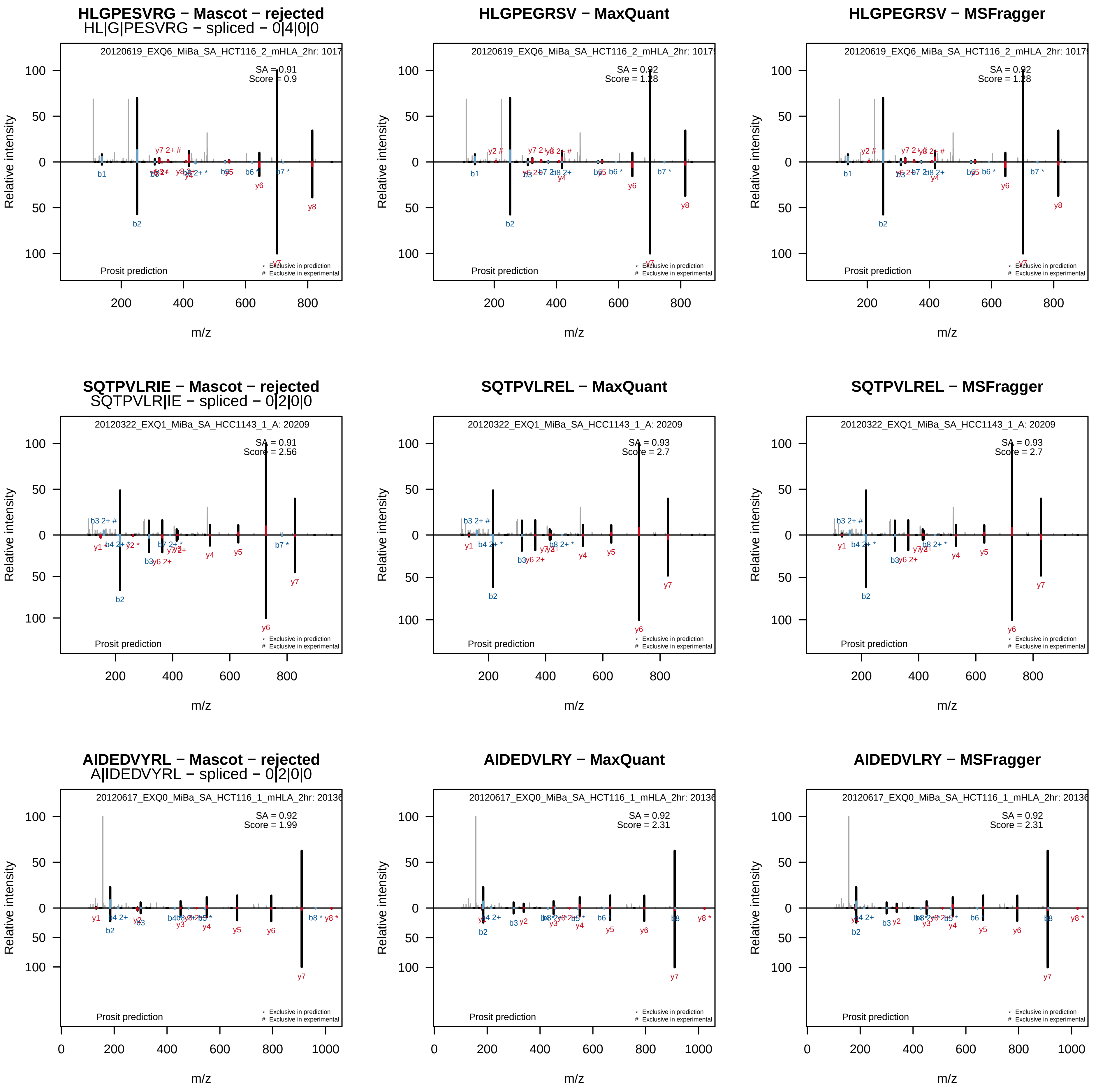

HLGPESVRG − Mascot − rejected
HL|G|PESVRG − spliced − 0|4|0|0
20120619_EXQ6_MiBa_SA_HCT116_2_mHLA_2hr: 1017
SA = 0.91
Score = 0.9
HLGPEGRSV − MaxQuant
20120619_EXQ6_MiBa_SA_HCT116_2_mHLA_2hr: 1017
SA = 0.92
Score = 1.28
HLGPEGRSV − MSFragger
20120619_EXQ6_MiBa_SA_HCT116_2_mHLA_2hr: 1017
SA = 0.92
Score = 1.28
SQTPVLRIE − Mascot − rejected
SQTPVLR|IE − spliced − 0|2|0|0
20120322_EXQ1_MiBa_SA_HCC1143_1_A: 20209
SA = 0.91
Score = 2.56
SQTPVLREL − MaxQuant
20120322_EXQ1_MiBa_SA_HCC1143_1_A: 20209
SA = 0.93
Score = 2.7
SQTPVLREL − MSFragger
20120322_EXQ1_MiBa_SA_HCC1143_1_A: 20209
SA = 0.93
Score = 2.7
AIDEDVYRL − Mascot − rejected
A|IDEDVYRL − spliced − 0|2|0|0
20120617_EXQ0_MiBa_SA_HCT116_1_mHLA_2hr: 20136
SA = 0.92
Score = 1.99
AIDEDVLRY − MaxQuant
20120617_EXQ0_MiBa_SA_HCT116_1_mHLA_2hr: 20136
SA = 0.92
Score = 2.31
AIDEDVLRY − MSFragger
20120617_EXQ0_MiBa_SA_HCT116_1_mHLA_2hr: 20136
SA = 0.92
Score = 2.31
Relative intensity
m/z
Prosit prediction
⋆ Exclusive in prediction
Exclusive in experimental

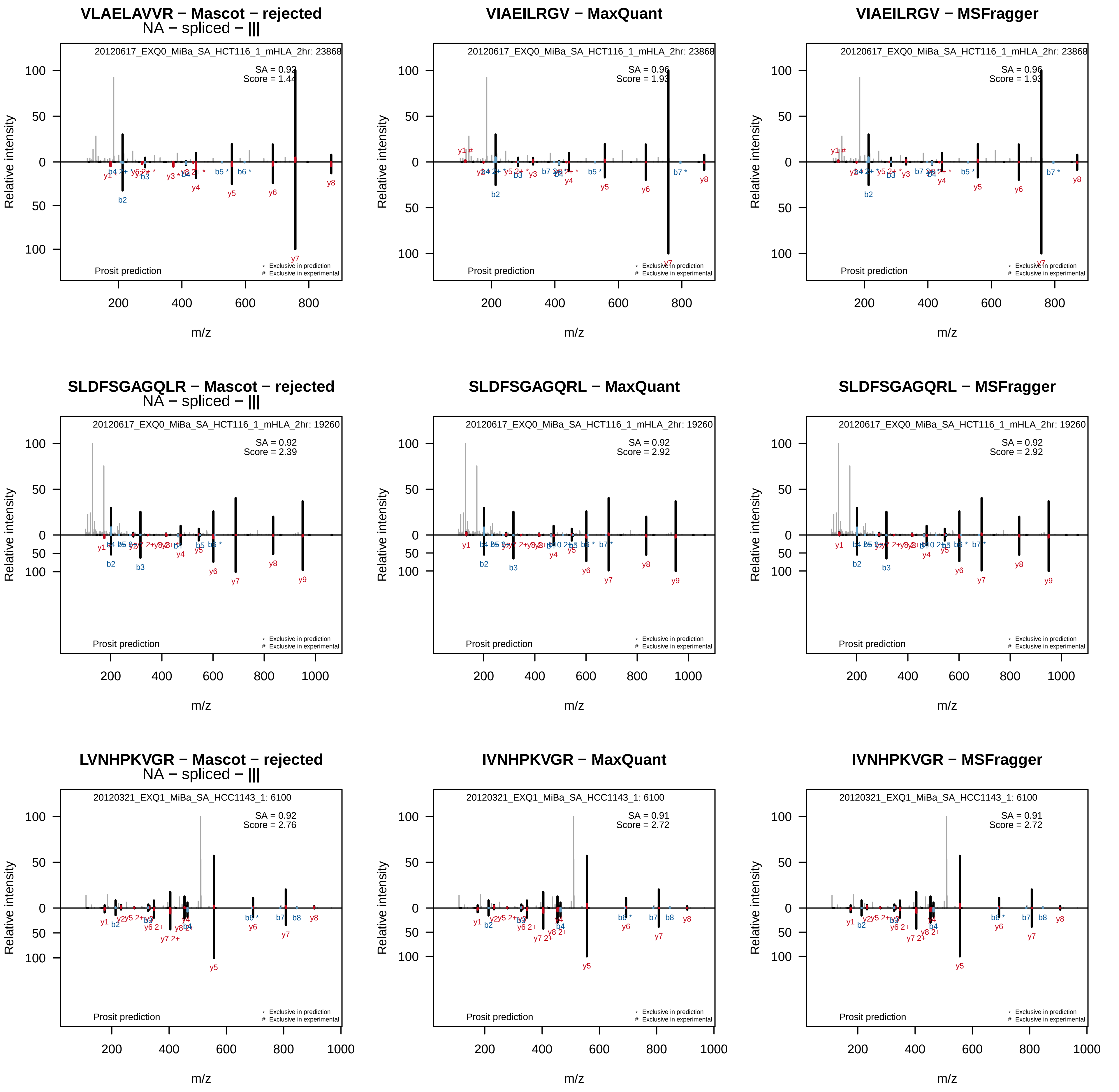

VLAELAVVR − Mascot − rejected
NA − spliced − |||
20120617_EXQ0_MiBa_SA_HCT116_1_mHLA_2hr: 23868
SA = 0.92
Score = 1.44
VIAEILRGV − MaxQuant
20120617_EXQ0_MiBa_SA_HCT116_1_mHLA_2hr: 23868
SA = 0.96
Score = 1.93
VIAEILRGV − MSFragger
20120617_EXQ0_MiBa_SA_HCT116_1_mHLA_2hr: 23868
SA = 0.96
Score = 1.93
SLDFSGAGQLR − Mascot − rejected
NA − spliced − |||
20120617_EXQ0_MiBa_SA_HCT116_1_mHLA_2hr: 19260
SA = 0.92
Score = 2.39
SLDFSGAGQRL − MaxQuant
20120617_EXQ0_MiBa_SA_HCT116_1_mHLA_2hr: 19260
SA = 0.92
Score = 2.92
SLDFSGAGQRL − MSFragger
20120617_EXQ0_MiBa_SA_HCT116_1_mHLA_2hr: 19260
SA = 0.92
Score = 2.92
LVNHPKVGR − Mascot − rejected
NA − spliced − |||
20120321_EXQ1_MiBa_SA_HCC1143_1: 6100
SA = 0.92
Score = 2.76
IVNHPKVGR − MaxQuant
20120321_EXQ1_MiBa_SA_HCC1143_1: 6100
SA = 0.91
Score = 2.72
IVNHPKVGR − MSFragger
20120321_EXQ1_MiBa_SA_HCC1143_1: 6100
SA = 0.91
Score = 2.72
Relative intensity
m/z
Prosit prediction
⋆ Exclusive in prediction
Exclusive in experimental

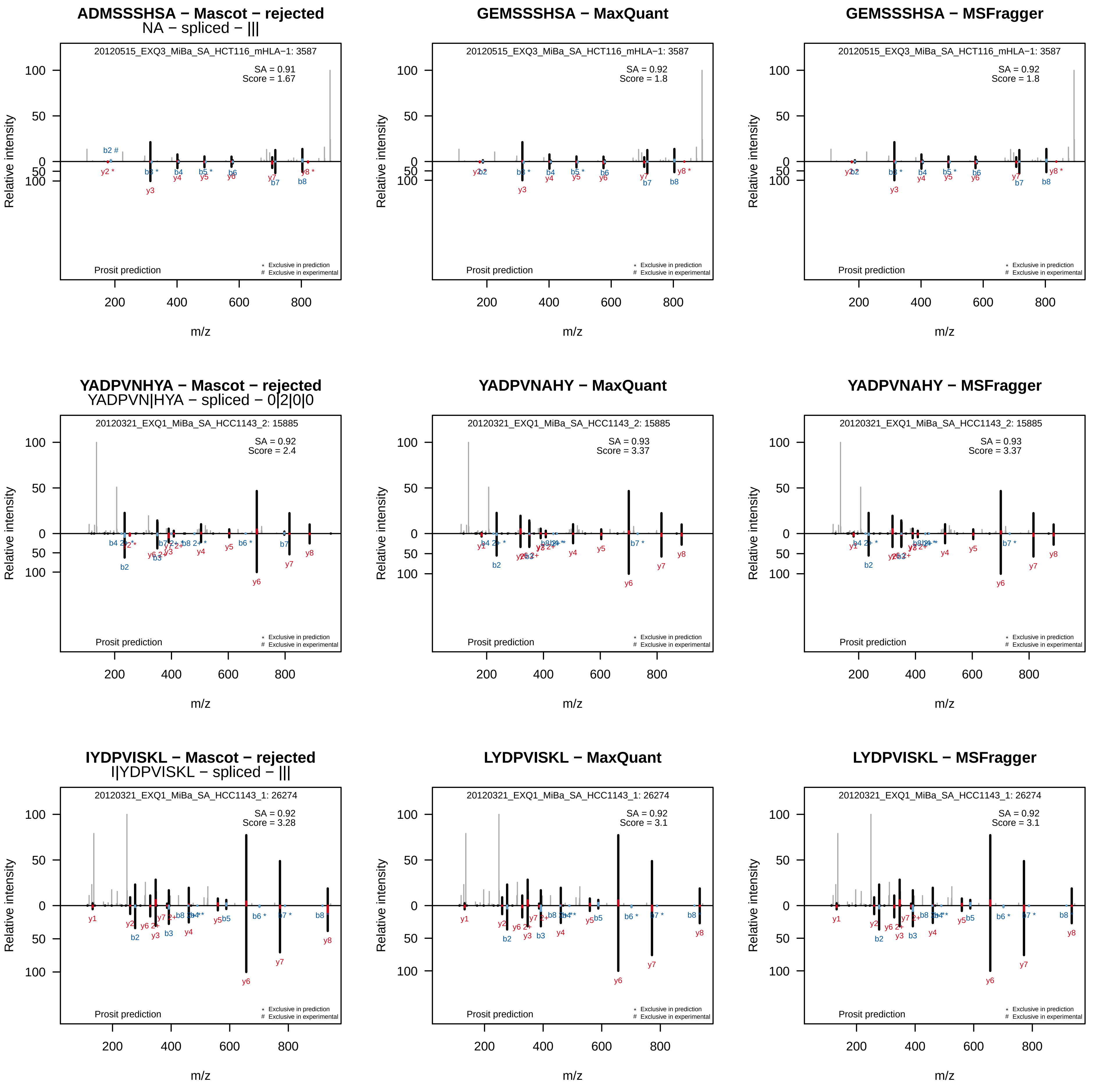
ADMSSSHSA − Mascot − rejected
NA − spliced − |||
20120515_EXQ3_MiBa_SA_HCT116_mHLA−1: 3587
SA = 0.91
Score = 1.67
GEMSSSHSA − MaxQuant
20120515_EXQ3_MiBa_SA_HCT116_mHLA−1: 3587
SA = 0.92
Score = 1.8
GEMSSSHSA − MSFragger
20120515_EXQ3_MiBa_SA_HCT116_mHLA−1: 3587
SA = 0.92
Score = 1.8
YADPVNHYA − Mascot − rejected
YADPVN|HYA − spliced − 0|2|0|0
20120321_EXQ1_MiBa_SA_HCC1143_2: 15885
SA = 0.92
Score = 2.4
YADPVNAHY − MaxQuant
20120321_EXQ1_MiBa_SA_HCC1143_2: 15885
SA = 0.93
Score = 3.37
YADPVNAHY − MSFragger
20120321_EXQ1_MiBa_SA_HCC1143_2: 15885
SA = 0.93
Score = 3.37
IYDPVISKL − Mascot − rejected
I|YDPVISKL − spliced − |||
20120321_EXQ1_MiBa_SA_HCC1143_1: 26274
SA = 0.92
Score = 3.28
LYDPVISKL − MaxQuant
20120321_EXQ1_MiBa_SA_HCC1143_1: 26274
SA = 0.92
Score = 3.1
LYDPVISKL − MSFragger
20120321_EXQ1_MiBa_SA_HCC1143_1: 26274
SA = 0.92
Score = 3.1
Relative intensity
m/z
Prosit prediction
* Exclusive in prediction
# Exclusive in experimental

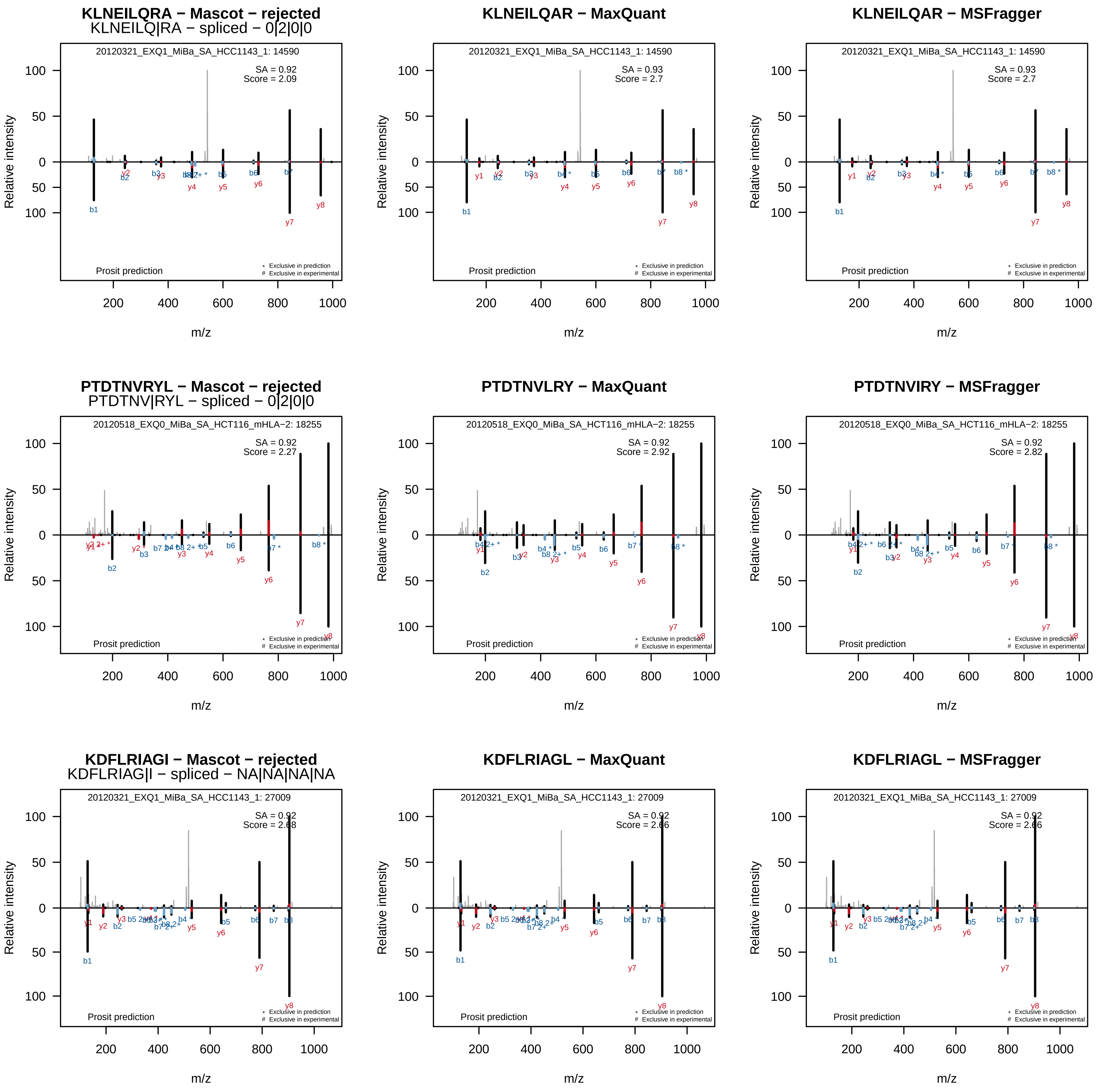

KLNEILQRA − Mascot − rejected
KLNEILQ|RA − spliced − 0|2|0|0
20120321_EXQ1_MiBa_SA_HCC1143_1: 14590
SA = 0.92
Score = 2.09
KLNEILQAR − MaxQuant
20120321_EXQ1_MiBa_SA_HCC1143_1: 14590
SA = 0.93
Score = 2.7
KLNEILQAR − MSFragger
20120321_EXQ1_MiBa_SA_HCC1143_1: 14590
SA = 0.93
Score = 2.7
PTDTNVRYL − Mascot − rejected
PTDTNV|RYL − spliced − 0|2|0|0
20120518_EXQ0_MiBa_SA_HCT116_mHLA−2: 18255
SA = 0.92
Score = 2.27
PTDTNVLRY − MaxQuant
20120518_EXQ0_MiBa_SA_HCT116_mHLA−2: 18255
SA = 0.92
Score = 2.92
PTDTNVIRY − MSFragger
20120518_EXQ0_MiBa_SA_HCT116_mHLA−2: 18255
SA = 0.92
Score = 2.82
KDFLRIAGI − Mascot − rejected
KDFLRIAG|I − spliced − NA|NA|NA|NA
20120321_EXQ1_MiBa_SA_HCC1143_1: 27009
SA = 0.92
Score = 2.68
KDFLRIAGL − MaxQuant
20120321_EXQ1_MiBa_SA_HCC1143_1: 27009
SA = 0.92
Score = 2.66
KDFLRIAGL − MSFragger
20120321_EXQ1_MiBa_SA_HCC1143_1: 27009
SA = 0.92
Score = 2.66
Relative intensity
m/z
Prosit prediction
* Exclusive in prediction
# Exclusive in experimental

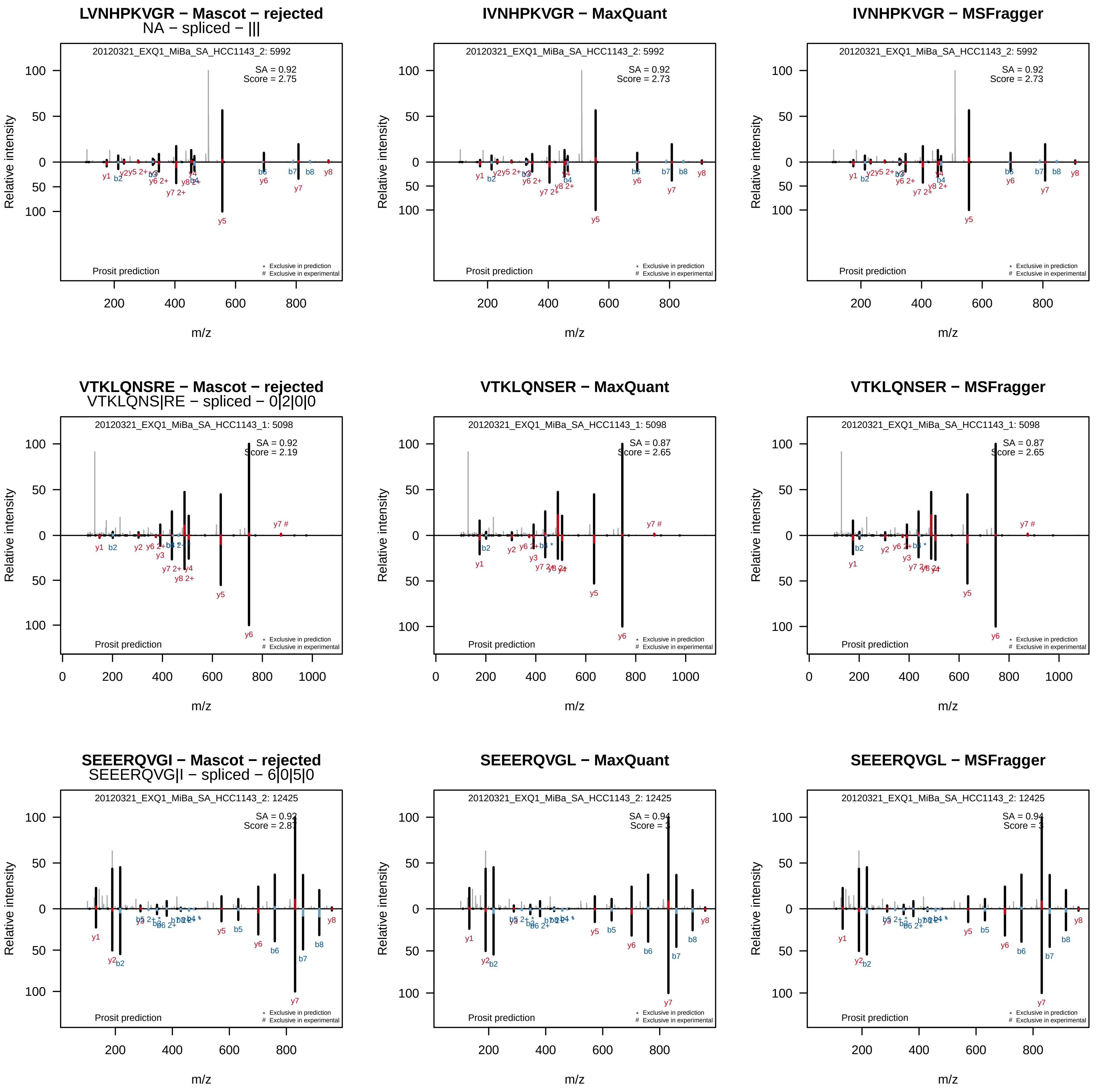
LVNHPKVGR − Mascot − rejected
NA − spliced − |||
20120321_EXQ1_MiBa_SA_HCC1143_2: 5992
SA = 0.92
Score = 2.75
IVNHPKVGR − MaxQuant
20120321_EXQ1_MiBa_SA_HCC1143_2: 5992
SA = 0.92
Score = 2.73
IVNHPKVGR − MSFragger
20120321_EXQ1_MiBa_SA_HCC1143_2: 5992
SA = 0.92
Score = 2.73
VTKLQNSRE − Mascot − rejected
VTKLQNS|RE − spliced − 0|2|0|0
20120321_EXQ1_MiBa_SA_HCC1143_1: 5098
SA = 0.92
Score = 2.19
VTKLQNSER − MaxQuant
20120321_EXQ1_MiBa_SA_HCC1143_1: 5098
SA = 0.87
Score = 2.65
VTKLQNSER − MSFragger
20120321_EXQ1_MiBa_SA_HCC1143_1: 5098
SA = 0.87
Score = 2.65
SEEERQVGI − Mascot − rejected
SEEERQVG|I − spliced − 6|0|5|0
20120321_EXQ1_MiBa_SA_HCC1143_2: 12425
SA = 0.92
Score = 2.87
SEEERQVGL − MaxQuant
20120321_EXQ1_MiBa_SA_HCC1143_2: 12425
SA = 0.94
Score = 3
SEEERQVGL − MSFragger
20120321_EXQ1_MiBa_SA_HCC1143_2: 12425
SA = 0.94
Score = 3
Relative intensity
m/z
Prosit prediction
⋆ Exclusive in prediction
Exclusive in experimental

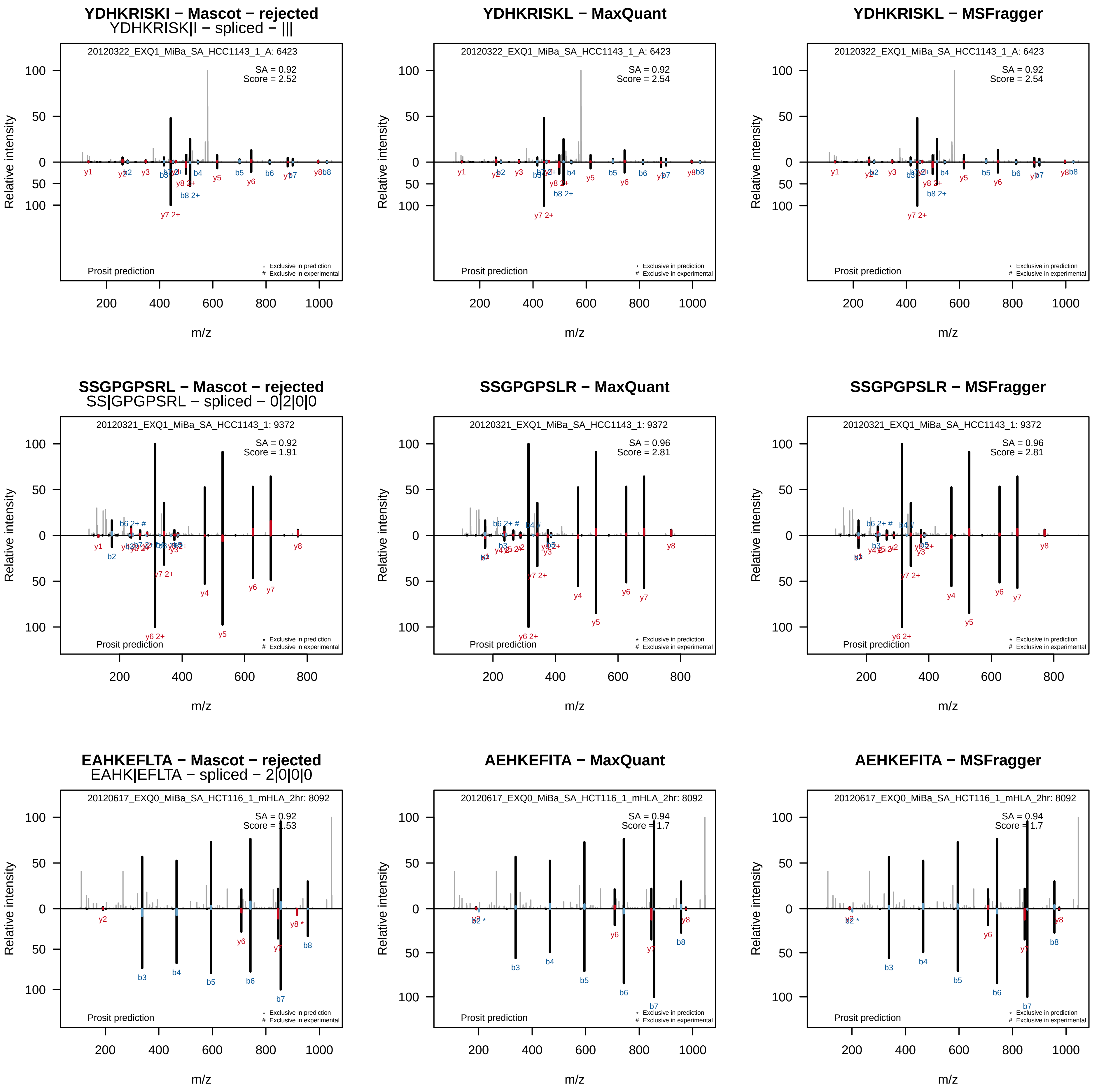
YDHKRISKI − Mascot − rejected
YDHKRISK|I − spliced − |||
20120322_EXQ1_MiBa_SA_HCC1143_1_A: 6423
SA = 0.92
Score = 2.52
YDHKRISKL − MaxQuant
20120322_EXQ1_MiBa_SA_HCC1143_1_A: 6423
SA = 0.92
Score = 2.54
YDHKRISKL − MSFragger
20120322_EXQ1_MiBa_SA_HCC1143_1_A: 6423
SA = 0.92
Score = 2.54
SSGPGPSRL − Mascot − rejected
SS|GPGPSRL − spliced − 0|2|0|0
20120321_EXQ1_MiBa_SA_HCC1143_1: 9372
SA = 0.92
Score = 1.91
SSGPGPSLR − MaxQuant
20120321_EXQ1_MiBa_SA_HCC1143_1: 9372
SA = 0.96
Score = 2.81
SSGPGPSLR − MSFragger
20120321_EXQ1_MiBa_SA_HCC1143_1: 9372
SA = 0.96
Score = 2.81
EAHKEFLTA − Mascot − rejected
EAHK|EFLTA − spliced − 2|0|0|0
20120617_EXQ0_MiBa_SA_HCT116_1_mHLA_2hr: 8092
SA = 0.92
Score = 1.53
AEHKEFITA − MaxQuant
20120617_EXQ0_MiBa_SA_HCT116_1_mHLA_2hr: 8092
SA = 0.94
Score = 1.7
AEHKEFITA − MSFragger
20120617_EXQ0_MiBa_SA_HCT116_1_mHLA_2hr: 8092
SA = 0.94
Score = 1.7
Relative intensity
m/z
Prosit prediction
* Exclusive in prediction
# Exclusive in experimental

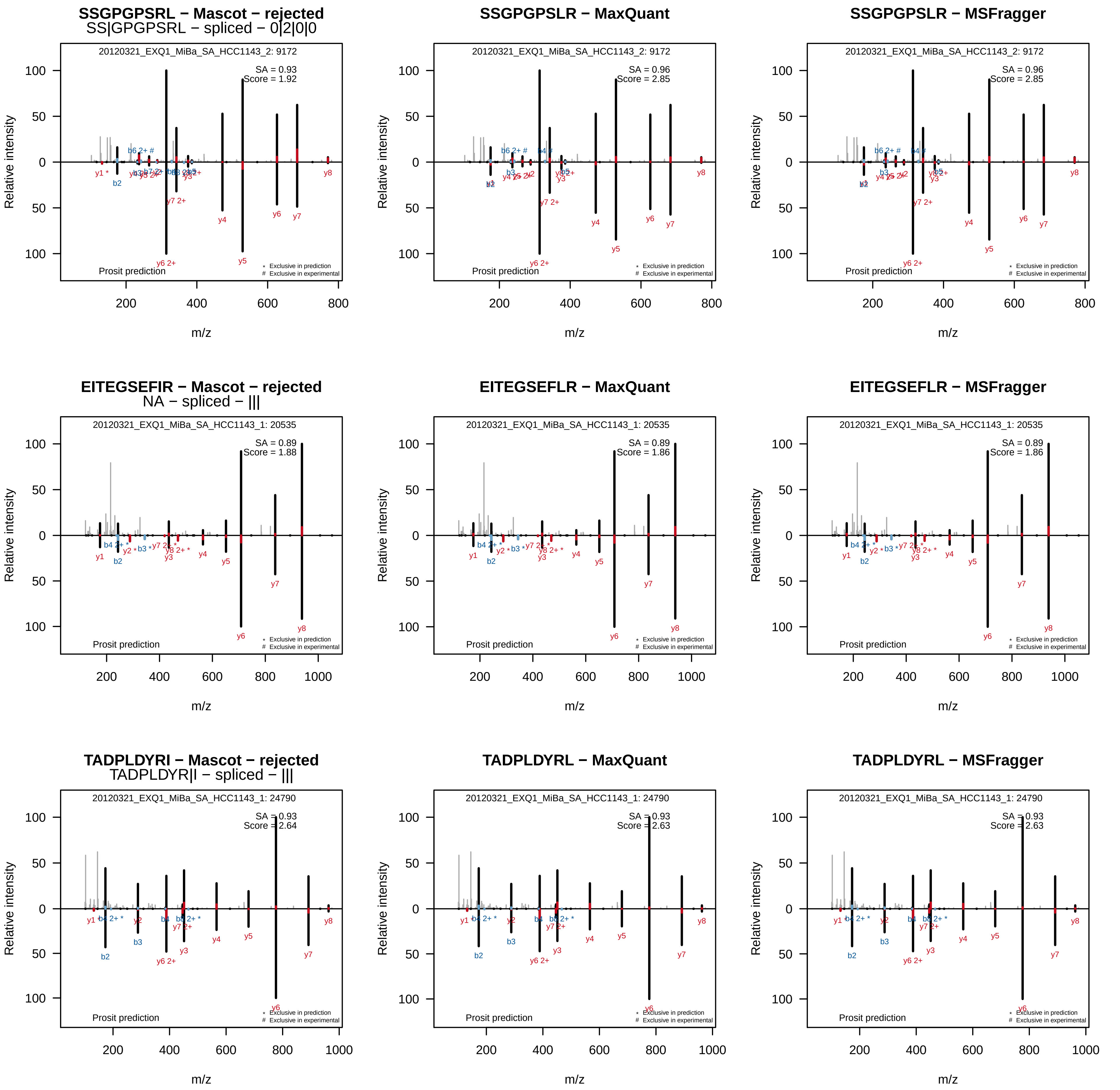

SSGPGPSRL − Mascot − rejected
SS|GPGPSRL − spliced − 0|2|0|0
SSGPGPSLR − MaxQuant
SSGPGPSLR − MSFragger
20120321_EXQ1_MiBa_SA_HCC1143_2: 9172
SA = 0.93
Score = 1.92
SA = 0.96
Score = 2.85
EITEGSEFIR − Mascot − rejected
NA − spliced − |||
EITEGSEFLR − MaxQuant
EITEGSEFLR − MSFragger
20120321_EXQ1_MiBa_SA_HCC1143_1: 20535
SA = 0.89
Score = 1.88
Score = 1.86
TADPLDYRI − Mascot − rejected
TADPLDYR|I − spliced − |||
TADPLDYRL − MaxQuant
TADPLDYRL − MSFragger
20120321_EXQ1_MiBa_SA_HCC1143_1: 24790
SA = 0.93
Score = 2.64
Score = 2.63
Relative intensity
m/z
Prosit prediction
⋆ Exclusive in prediction
Exclusive in experimental

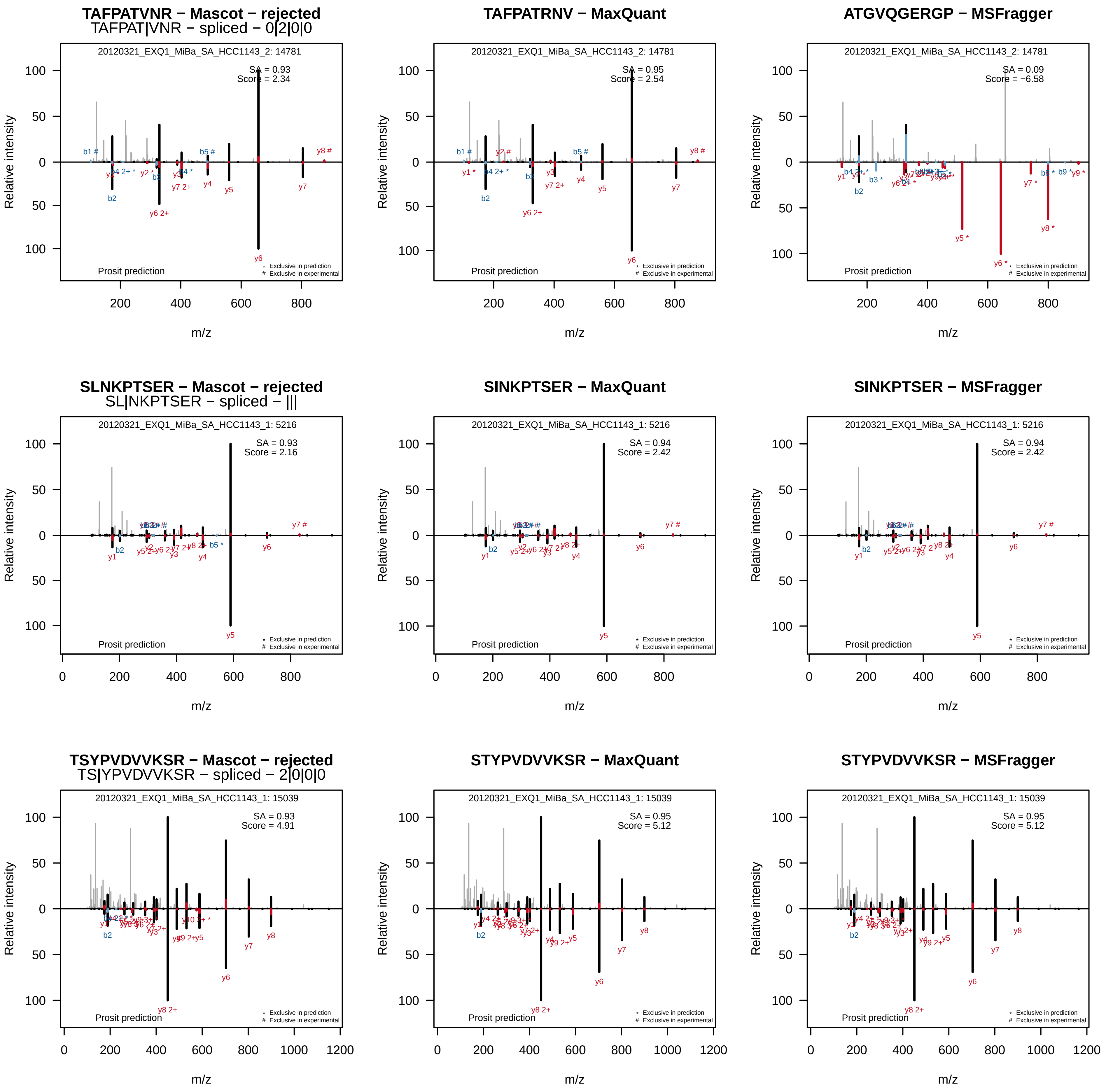
TAFPATVNR − Mascot − rejected
TAFPAT|VNR − spliced − 0|2|0|0
20120321_EXQ1_MiBa_SA_HCC1143_2: 14781
SA = 0.93
Score = 2.34
Prosit prediction
⋆ Exclusive in prediction
Exclusive in experimental
TAFPATRNV − MaxQuant
20120321_EXQ1_MiBa_SA_HCC1143_2: 14781
SA = 0.95
Score = 2.54
Prosit prediction
⋆ Exclusive in prediction
Exclusive in experimental
ATGVQGERGP − MSFragger
20120321_EXQ1_MiBa_SA_HCC1143_2: 14781
SA = 0.09
Score = −6.58
Prosit prediction
⋆ Exclusive in prediction
Exclusive in experimental
SLNKPTSER − Mascot − rejected
SL|NKPTSER − spliced − |||
20120321_EXQ1_MiBa_SA_HCC1143_1: 5216
SA = 0.93
Score = 2.16
Prosit prediction
⋆ Exclusive in prediction
Exclusive in experimental
SINKPTSER − MaxQuant
20120321_EXQ1_MiBa_SA_HCC1143_1: 5216
SA = 0.94
Score = 2.42
Prosit prediction
⋆ Exclusive in prediction
Exclusive in experimental
SINKPTSER − MSFragger
20120321_EXQ1_MiBa_SA_HCC1143_1: 5216
SA = 0.94
Score = 2.42
Prosit prediction
⋆ Exclusive in prediction
Exclusive in experimental
TSYPVDVVKSR − Mascot − rejected
TS|YPVDVVKSR − spliced − 2|0|0|0
20120321_EXQ1_MiBa_SA_HCC1143_1: 15039
SA = 0.93
Score = 4.91
Prosit prediction
⋆ Exclusive in prediction
Exclusive in experimental
STYPVDVVKSR − MaxQuant
20120321_EXQ1_MiBa_SA_HCC1143_1: 15039
SA = 0.95
Score = 5.12
Prosit prediction
⋆ Exclusive in prediction
Exclusive in experimental
STYPVDVVKSR − MSFragger
20120321_EXQ1_MiBa_SA_HCC1143_1: 15039
SA = 0.95
Score = 5.12
Prosit prediction
⋆ Exclusive in prediction
Exclusive in experimental
Relative intensity
m/z

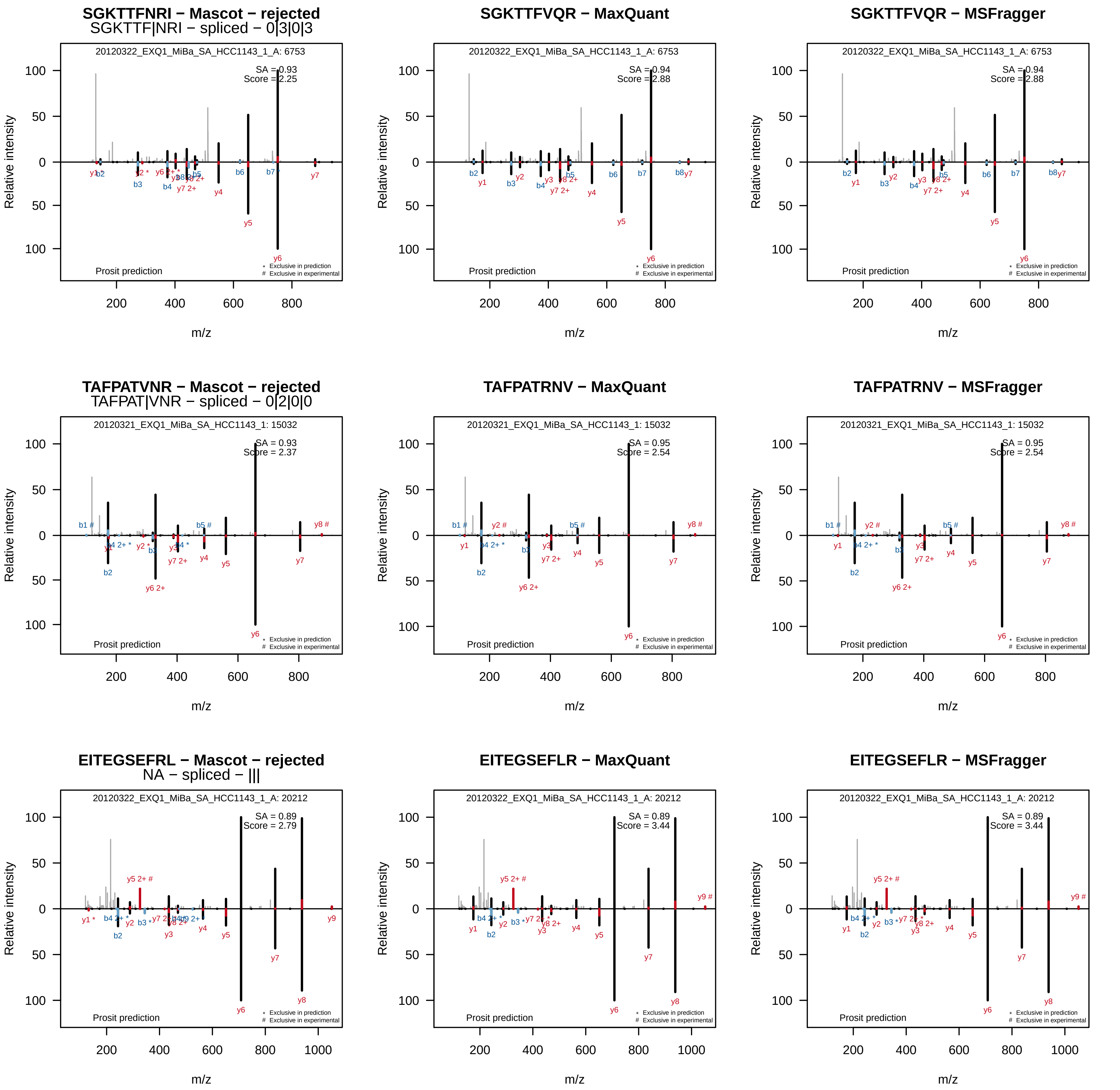

SGKTTFNRI − Mascot − rejected
SGKTTF|NRI − spliced − 0|3|0|3
20120322_EXQ1_MiBa_SA_HCC1143_1_A: 6753
SA = 0.93
Score = 2.25
SGKTTFVQR − MaxQuant
20120322_EXQ1_MiBa_SA_HCC1143_1_A: 6753
SA = 0.94
Score = 2.88
SGKTTFVQR − MSFragger
20120322_EXQ1_MiBa_SA_HCC1143_1_A: 6753
SA = 0.94
Score = 2.88
TAFPATVNR − Mascot − rejected
TAFPAT|VNR − spliced − 0|2|0|0
20120321_EXQ1_MiBa_SA_HCC1143_1: 15032
SA = 0.93
Score = 2.37
TAFPATRNV − MaxQuant
20120321_EXQ1_MiBa_SA_HCC1143_1: 15032
SA = 0.95
Score = 2.54
TAFPATRNV − MSFragger
20120321_EXQ1_MiBa_SA_HCC1143_1: 15032
SA = 0.95
Score = 2.54
EITEGSEFRL − Mascot − rejected
NA − spliced − |||
20120322_EXQ1_MiBa_SA_HCC1143_1_A: 20212
SA = 0.89
Score = 2.79
EITEGSEFLR − MaxQuant
20120322_EXQ1_MiBa_SA_HCC1143_1_A: 20212
SA = 0.89
Score = 3.44
EITEGSEFLR − MSFragger
20120322_EXQ1_MiBa_SA_HCC1143_1_A: 20212
SA = 0.89
Score = 3.44
Prosit prediction
⋆ Exclusive in prediction
Exclusive in experimental
Relative intensity
m/z

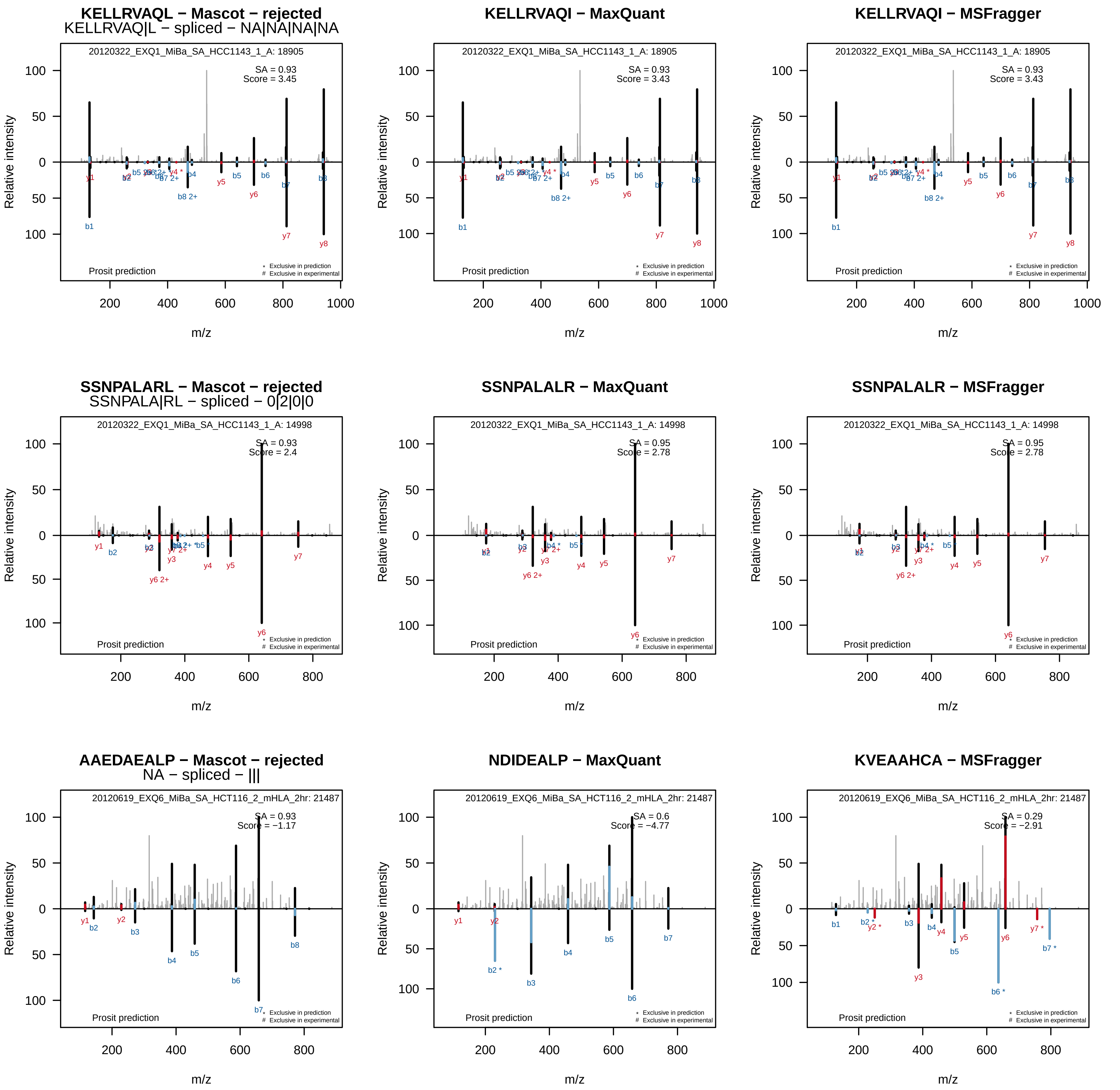
KELLRVAQL − Mascot − rejected
KELLRVAQ|L − spliced − NA|NA|NA|NA
KELLRVAQI − MaxQuant
KELLRVAQI − MSFragger
20120322_EXQ1_MiBa_SA_HCC1143_1_A: 18905
SA = 0.93
Score = 3.45
Score = 3.43
Score = 3.43
SSNPALARL − Mascot − rejected
SSNPALA|RL − spliced − 0|2|0|0
SSNPALALR − MaxQuant
SSNPALALR − MSFragger
20120322_EXQ1_MiBa_SA_HCC1143_1_A: 14998
SA = 0.93
Score = 2.4
SA = 0.95
Score = 2.78
SA = 0.95
Score = 2.78
AAEDAEALP − Mascot − rejected
NA − spliced − |||
NDIDEALP − MaxQuant
KVEAAHCA − MSFragger
20120619_EXQ6_MiBa_SA_HCT116_2_mHLA_2hr: 21487
SA = 0.93
Score = −1.17
SA = 0.6
Score = −4.77
SA = 0.29
Score = −2.91
Relative intensity
m/z
Prosit prediction
* Exclusive in prediction
# Exclusive in experimental

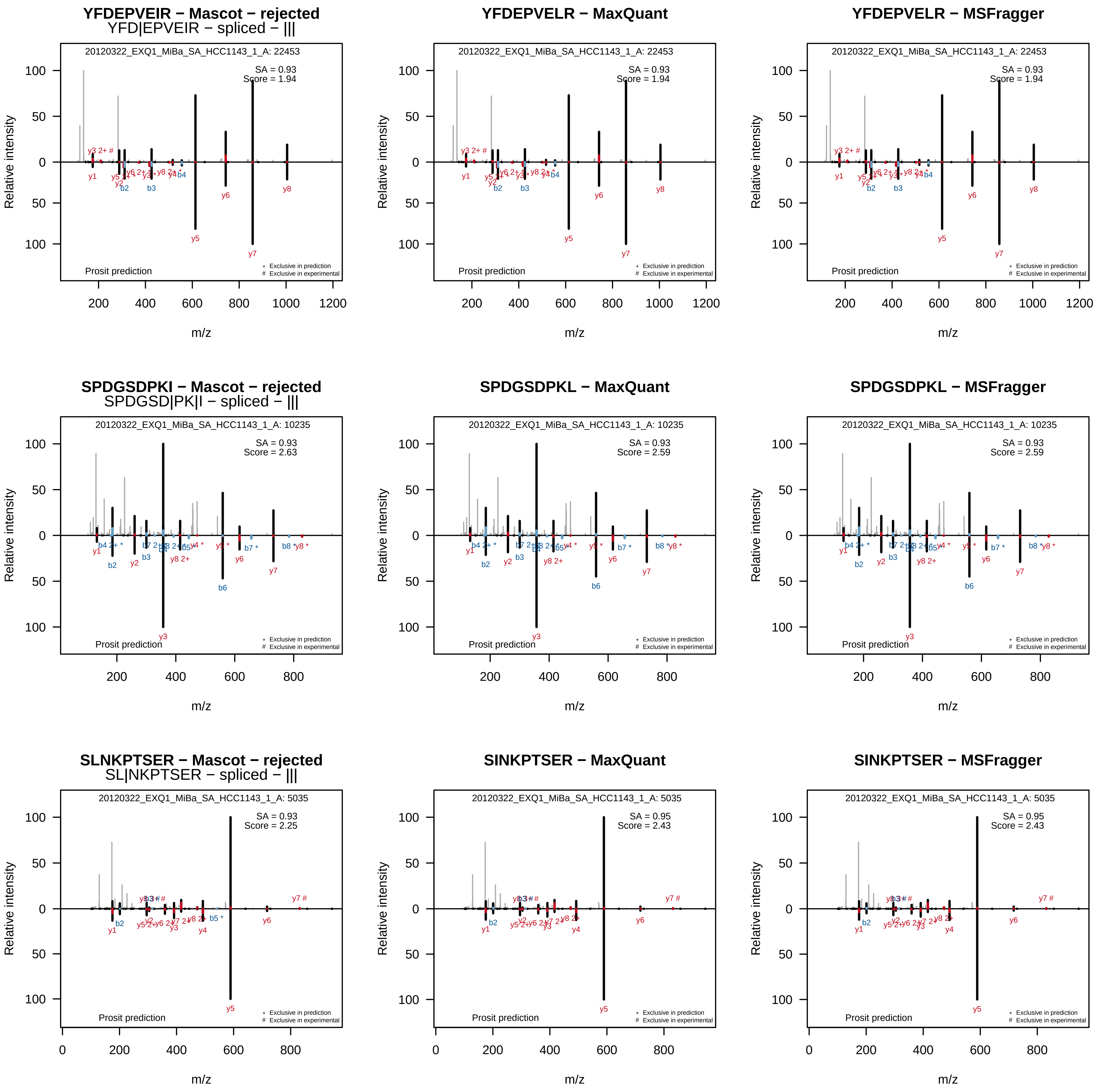
YFDEPVEIR − Mascot − rejected
YFD|EPVEIR − spliced − |||
20120322_EXQ1_MiBa_SA_HCC1143_1_A: 22453
SA = 0.93
Score = 1.94
YFDEPVELR − MaxQuant
20120322_EXQ1_MiBa_SA_HCC1143_1_A: 22453
SA = 0.93
Score = 1.94
YFDEPVELR − MSFragger
20120322_EXQ1_MiBa_SA_HCC1143_1_A: 22453
SA = 0.93
Score = 1.94
SPDGSDPKI − Mascot − rejected
SPDGSD|PK|I − spliced − |||
20120322_EXQ1_MiBa_SA_HCC1143_1_A: 10235
SA = 0.93
Score = 2.63
SPDGSDPKL − MaxQuant
20120322_EXQ1_MiBa_SA_HCC1143_1_A: 10235
SA = 0.93
Score = 2.59
SPDGSDPKL − MSFragger
20120322_EXQ1_MiBa_SA_HCC1143_1_A: 10235
SA = 0.93
Score = 2.59
SLNKPTSER − Mascot − rejected
SL|NKPTSER − spliced − |||
20120322_EXQ1_MiBa_SA_HCC1143_1_A: 5035
SA = 0.93
Score = 2.25
SINKPTSER − MaxQuant
20120322_EXQ1_MiBa_SA_HCC1143_1_A: 5035
SA = 0.95
Score = 2.43
SINKPTSER − MSFragger
20120322_EXQ1_MiBa_SA_HCC1143_1_A: 5035
SA = 0.95
Score = 2.43
Relative intensity
m/z
Prosit prediction
* Exclusive in prediction
# Exclusive in experimental

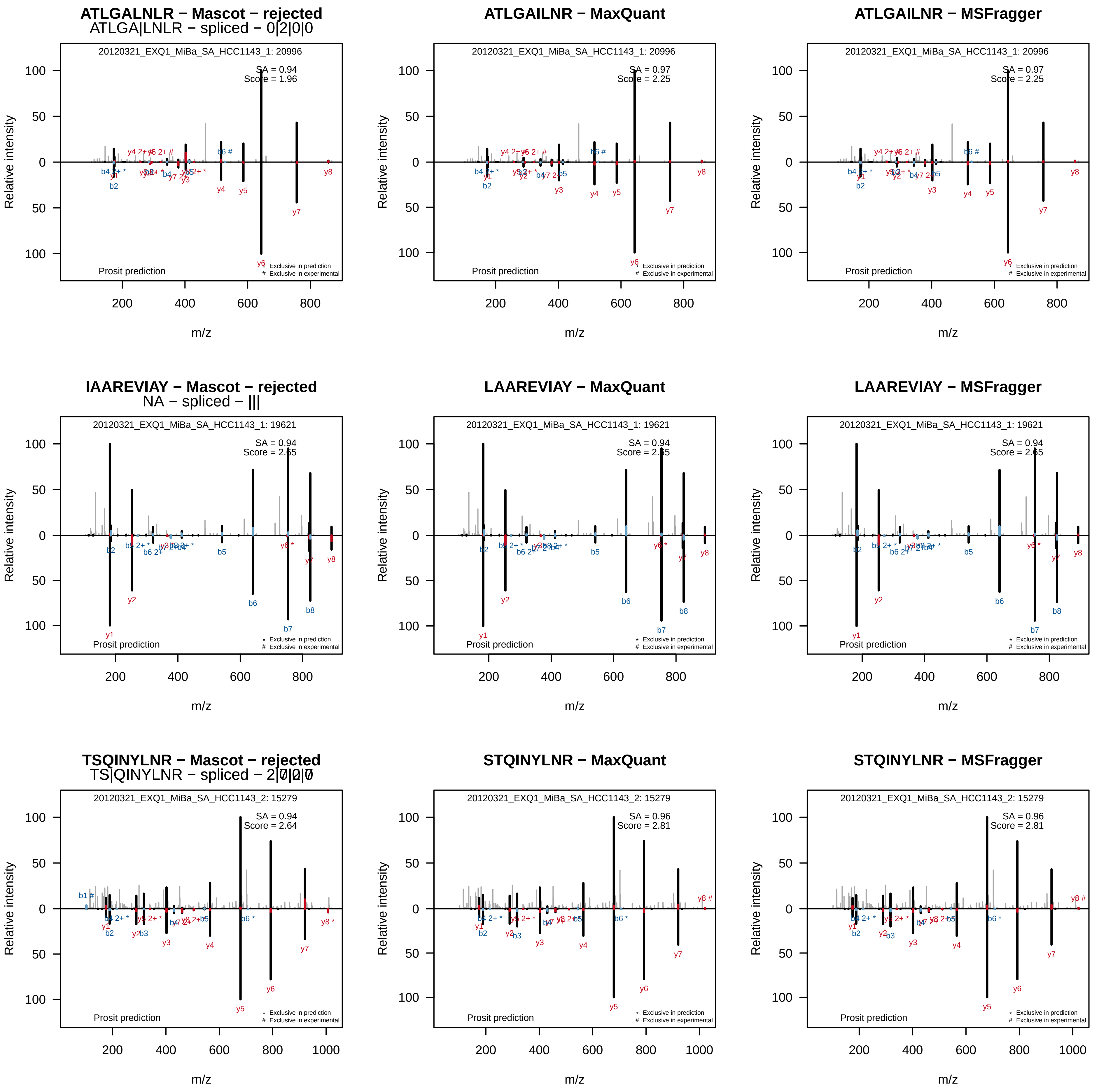

ATLGALNLR − Mascot − rejected
ATLGA|LNLR − spliced − 0|2|0|0
20120321_EXQ1_MiBa_SA_HCC1143_1: 20996
SA = 0.94
Score = 1.96
ATLGAILNR − MaxQuant
20120321_EXQ1_MiBa_SA_HCC1143_1: 20996
SA = 0.97
Score = 2.25
ATLGAILNR − MSFragger
20120321_EXQ1_MiBa_SA_HCC1143_1: 20996
SA = 0.97
Score = 2.25
IAAREVIAY − Mascot − rejected
NA − spliced − |||
20120321_EXQ1_MiBa_SA_HCC1143_1: 19621
SA = 0.94
Score = 2.65
LAAREVIAY − MaxQuant
20120321_EXQ1_MiBa_SA_HCC1143_1: 19621
SA = 0.94
Score = 2.65
LAAREVIAY − MSFragger
20120321_EXQ1_MiBa_SA_HCC1143_1: 19621
SA = 0.94
Score = 2.65
TSQINYLNR − Mascot − rejected
TS|QINYLNR − spliced − 2|0|2|0
20120321_EXQ1_MiBa_SA_HCC1143_2: 15279
SA = 0.94
Score = 2.64
STQINYLNR − MaxQuant
20120321_EXQ1_MiBa_SA_HCC1143_2: 15279
SA = 0.96
Score = 2.81
STQINYLNR − MSFragger
20120321_EXQ1_MiBa_SA_HCC1143_2: 15279
SA = 0.96
Score = 2.81
Relative intensity
m/z
Prosit prediction
* Exclusive in prediction
# Exclusive in experimental

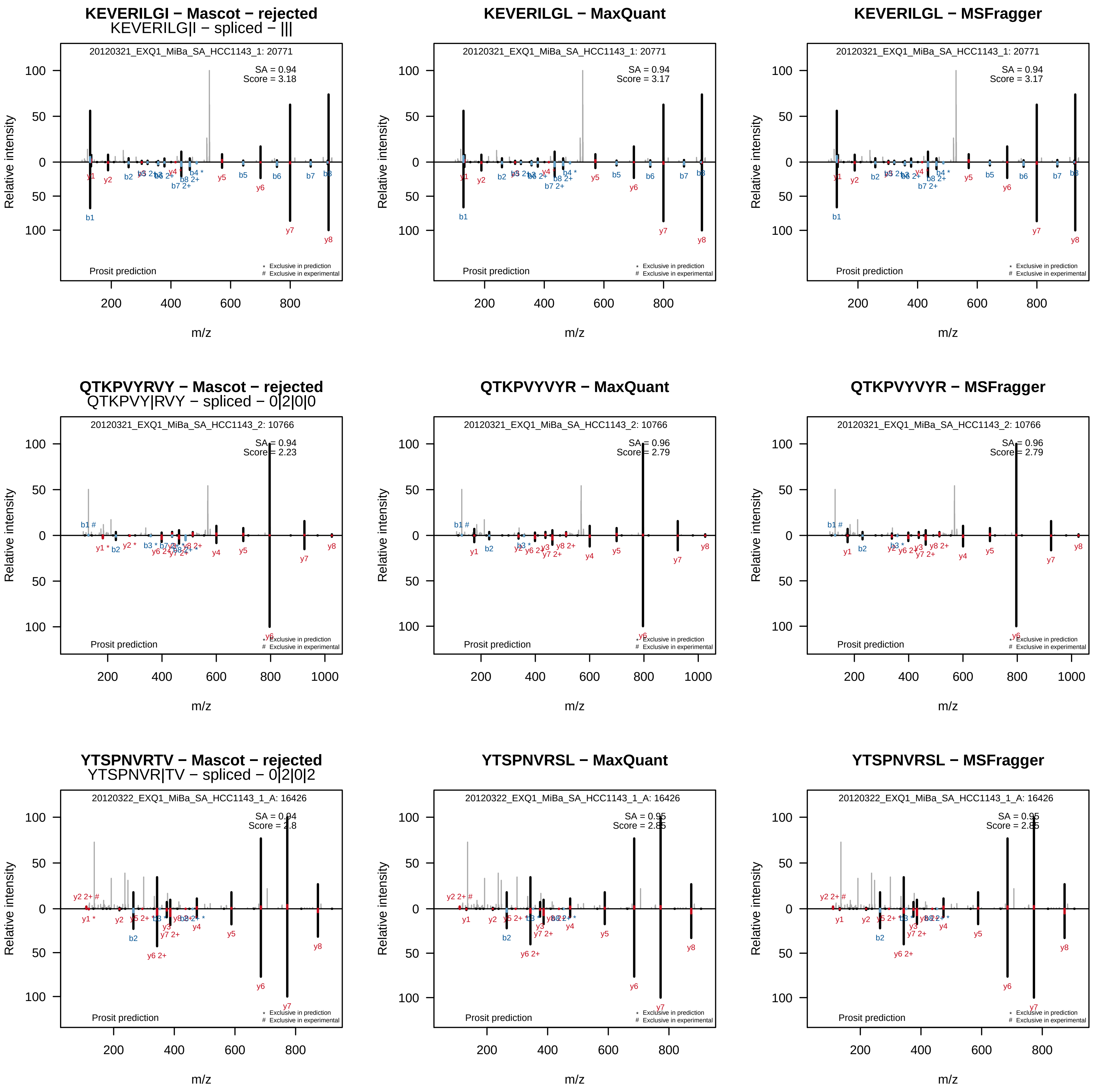

KEVERILGI − Mascot − rejected
KEVERILG|I − spliced − |||
20120321_EXQ1_MiBa_SA_HCC1143_1: 20771
SA = 0.94
Score = 3.18
KEVERILGL − MaxQuant
20120321_EXQ1_MiBa_SA_HCC1143_1: 20771
SA = 0.94
Score = 3.17
KEVERILGL − MSFragger
20120321_EXQ1_MiBa_SA_HCC1143_1: 20771
SA = 0.94
Score = 3.17
QTKPVYRVY − Mascot − rejected
QTKPVY|RVY − spliced − 0|2|0|0
20120321_EXQ1_MiBa_SA_HCC1143_2: 10766
SA = 0.94
Score = 2.23
QTKPVYVYR − MaxQuant
20120321_EXQ1_MiBa_SA_HCC1143_2: 10766
SA = 0.96
Score = 2.79
QTKPVYVYR − MSFragger
20120321_EXQ1_MiBa_SA_HCC1143_2: 10766
SA = 0.96
Score = 2.79
YTSPNVRTV − Mascot − rejected
YTSPNVR|TV − spliced − 0|2|0|2
20120322_EXQ1_MiBa_SA_HCC1143_1_A: 16426
SA = 0.94
Score = 2.8
YTSPNVRSL − MaxQuant
20120322_EXQ1_MiBa_SA_HCC1143_1_A: 16426
SA = 0.95
Score = 2.85
YTSPNVRSL − MSFragger
20120322_EXQ1_MiBa_SA_HCC1143_1_A: 16426
SA = 0.95
Score = 2.85
Relative intensity
m/z
Prosit prediction
* Exclusive in prediction
# Exclusive in experimental

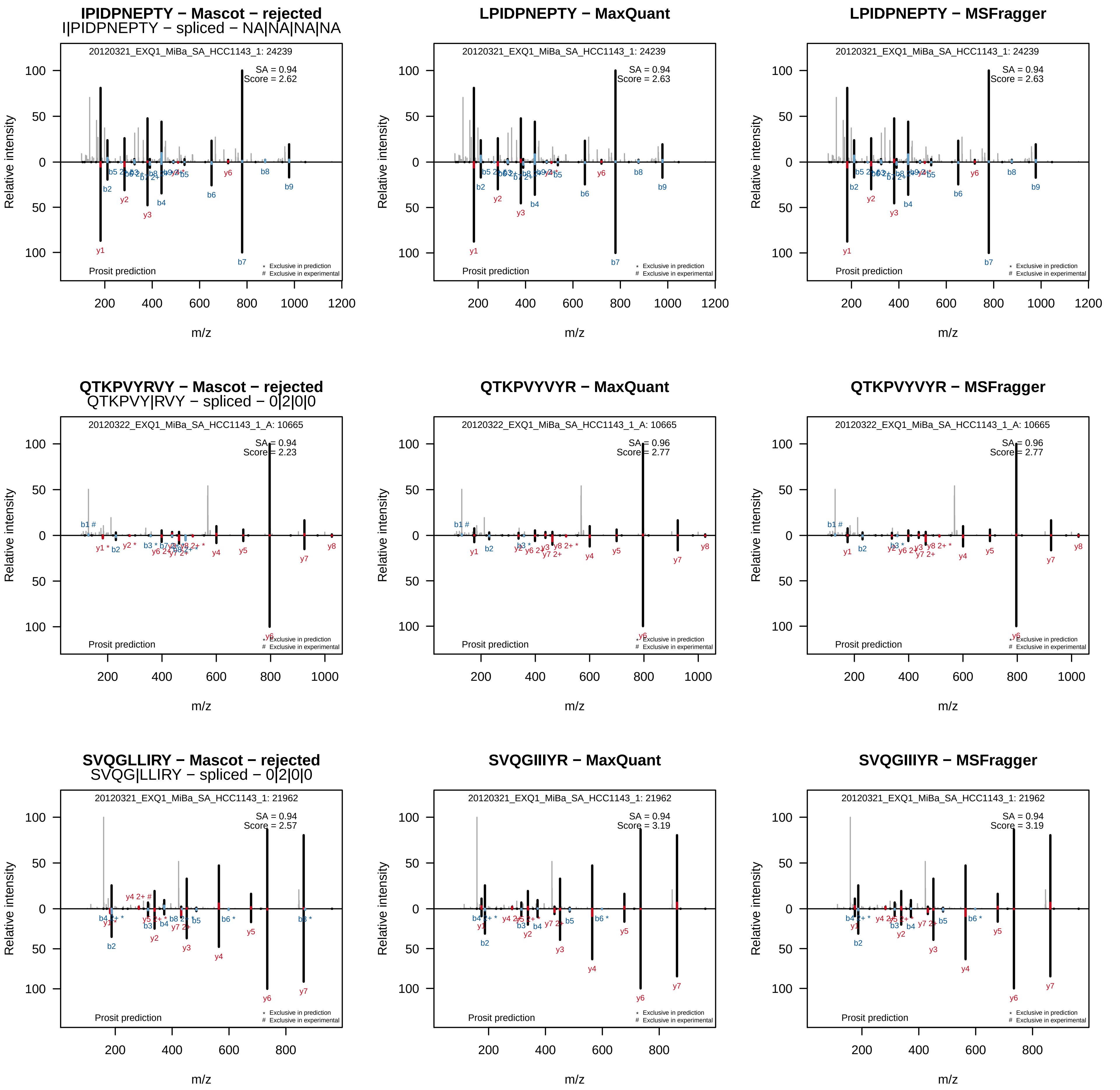

IPIDPNEPTY − Mascot − rejected
I|PIDPNEPTY − spliced − NA|NA|NA|NA
20120321_EXQ1_MiBa_SA_HCC1143_1: 24239
SA = 0.94
Score = 2.62
LPIDPNEPTY − MaxQuant
20120321_EXQ1_MiBa_SA_HCC1143_1: 24239
SA = 0.94
Score = 2.63
LPIDPNEPTY − MSFragger
20120321_EXQ1_MiBa_SA_HCC1143_1: 24239
SA = 0.94
Score = 2.63
QTKPVYRVY − Mascot − rejected
QTKPVY|RVY − spliced − 0|2|0|0
20120322_EXQ1_MiBa_SA_HCC1143_1_A: 10665
SA = 0.94
Score = 2.23
QTKPVYVYR − MaxQuant
20120322_EXQ1_MiBa_SA_HCC1143_1_A: 10665
SA = 0.96
Score = 2.77
QTKPVYVYR − MSFragger
20120322_EXQ1_MiBa_SA_HCC1143_1_A: 10665
SA = 0.96
Score = 2.77
SVQGLLIRY − Mascot − rejected
SVQG|LLIRY − spliced − 0|2|0|0
20120321_EXQ1_MiBa_SA_HCC1143_1: 21962
SA = 0.94
Score = 2.57
SVQGIIIYR − MaxQuant
20120321_EXQ1_MiBa_SA_HCC1143_1: 21962
SA = 0.94
Score = 3.19
SVQGIIIYR − MSFragger
20120321_EXQ1_MiBa_SA_HCC1143_1: 21962
SA = 0.94
Score = 3.19
Relative intensity
m/z
Prosit prediction
⋆ Exclusive in prediction
Exclusive in experimental

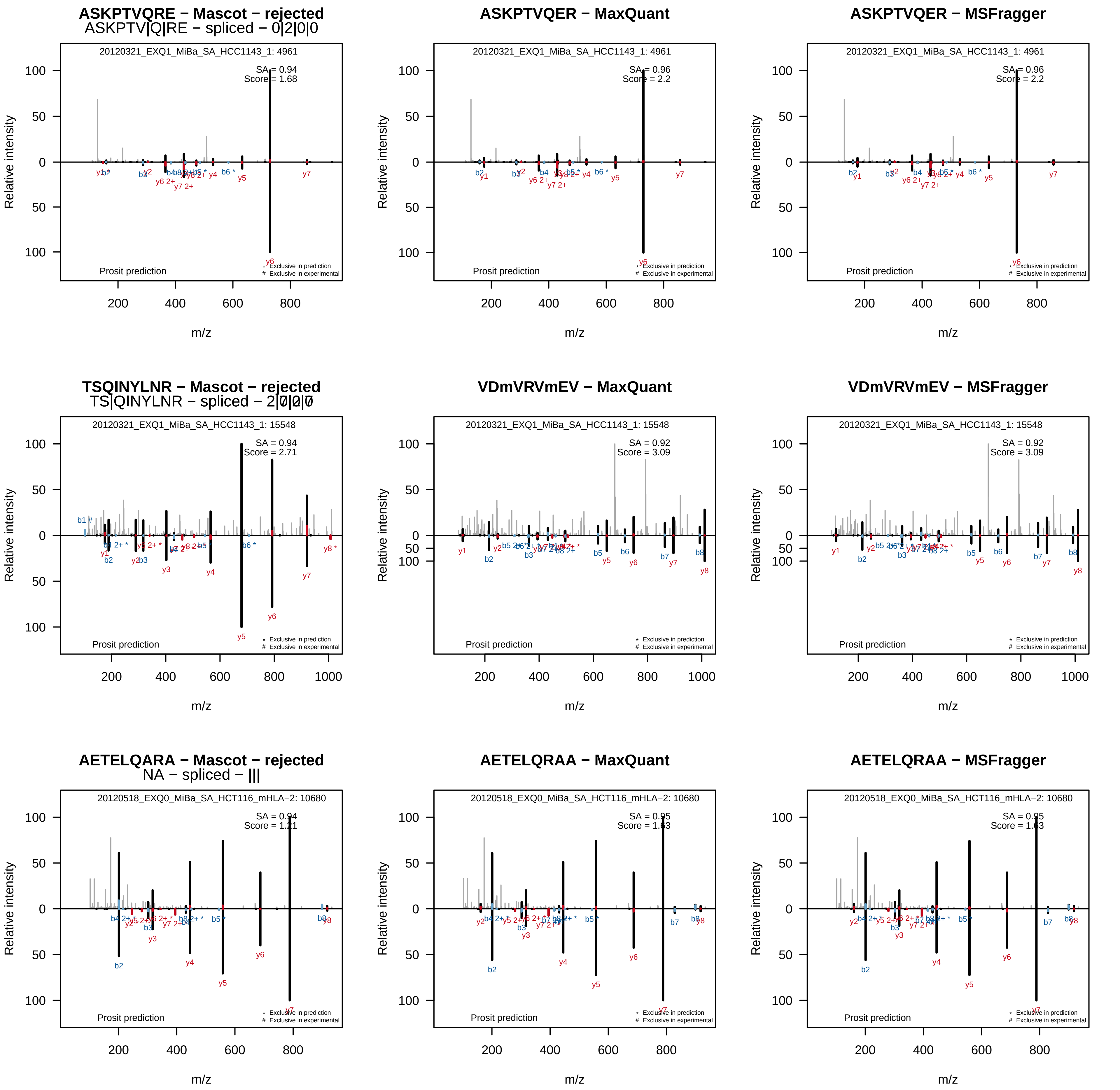
ASKPTVQRE − Mascot − rejected
ASKPTV|Q|RE − spliced − 0|2|0|0
20120321_EXQ1_MiBa_SA_HCC1143_1: 4961
SA = 0.94
Score = 1.68
ASKPTVQER − MaxQuant
20120321_EXQ1_MiBa_SA_HCC1143_1: 4961
SA = 0.96
Score = 2.2
ASKPTVQER − MSFragger
20120321_EXQ1_MiBa_SA_HCC1143_1: 4961
SA = 0.96
Score = 2.2
TSQINYLNR − Mascot − rejected
20120321_EXQ1_MiBa_SA_HCC1143_1: 15548
SA = 0.94
Score = 2.71
VDmVRVmEV − MaxQuant
20120321_EXQ1_MiBa_SA_HCC1143_1: 15548
SA = 0.92
Score = 3.09
VDmVRVmEV − MSFragger
20120321_EXQ1_MiBa_SA_HCC1143_1: 15548
SA = 0.92
Score = 3.09
AETELQARA − Mascot − rejected
NA − spliced − |||
20120518_EXQ0_MiBa_SA_HCT116_mHLA-2: 10680
SA = 0.94
Score = 1.21
AETELQRAA − MaxQuant
20120518_EXQ0_MiBa_SA_HCT116_mHLA-2: 10680
SA = 0.95
Score = 1.63
AETELQRAA − MSFragger
20120518_EXQ0_MiBa_SA_HCT116_mHLA-2: 10680
SA = 0.95
Score = 1.63
Prosit prediction
⋆ Exclusive in prediction
Exclusive in experimental
Relative intensity
m/z

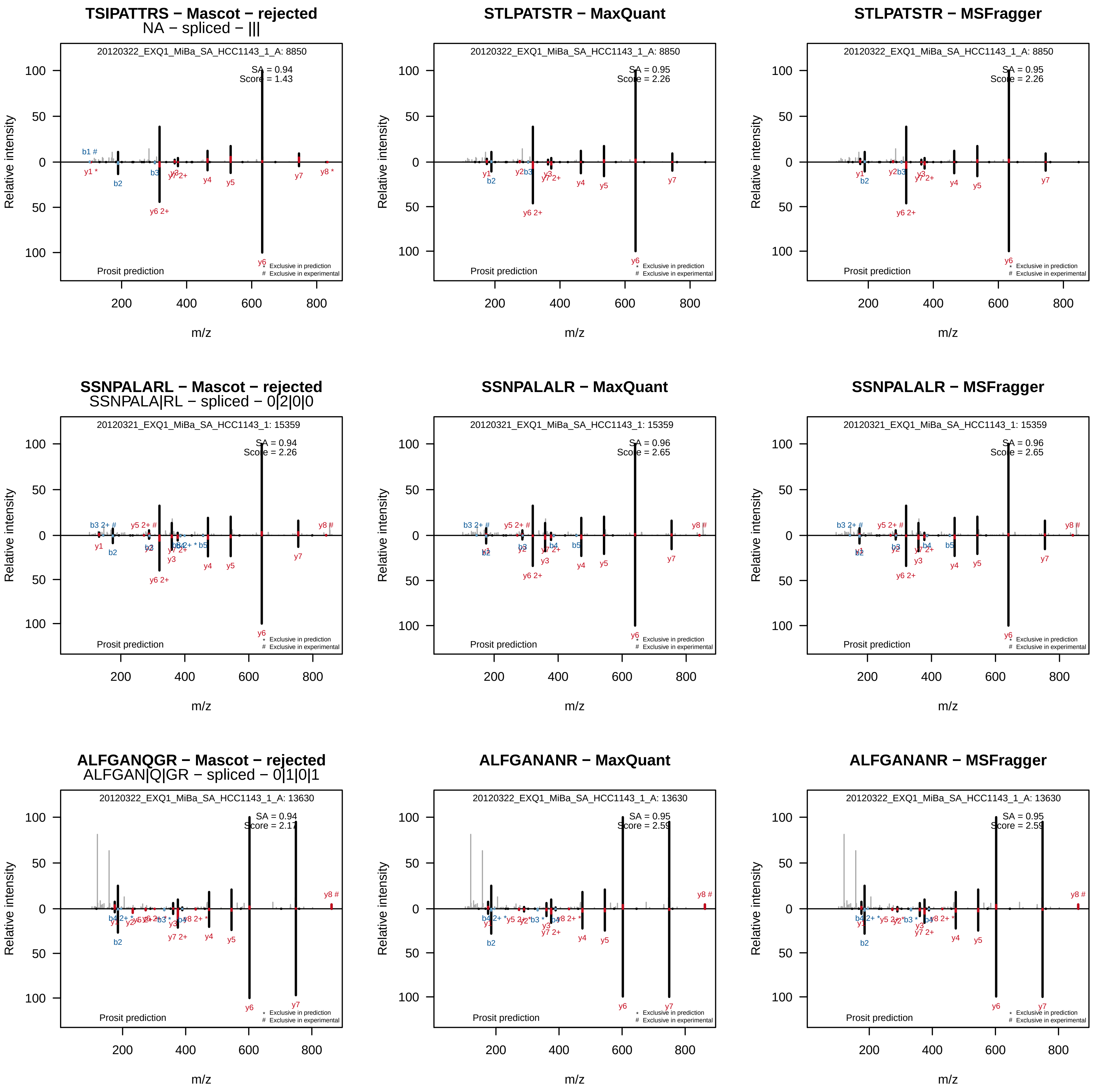

TSIPATTRS − Mascot − rejected
NA − spliced − |||
20120322_EXQ1_MiBa_SA_HCC1143_1_A: 8850
SA = 0.94
Score = 1.43
STLPATSTR − MaxQuant
20120322_EXQ1_MiBa_SA_HCC1143_1_A: 8850
SA = 0.95
Score = 2.26
STLPATSTR − MSFragger
20120322_EXQ1_MiBa_SA_HCC1143_1_A: 8850
SA = 0.95
Score = 2.26
SSNPALARL − Mascot − rejected
SSNPALA|RL − spliced − 0|2|0|0
20120321_EXQ1_MiBa_SA_HCC1143_1: 15359
SA = 0.94
Score = 2.26
SSNPALALR − MaxQuant
20120321_EXQ1_MiBa_SA_HCC1143_1: 15359
SA = 0.96
Score = 2.65
SSNPALALR − MSFragger
20120321_EXQ1_MiBa_SA_HCC1143_1: 15359
SA = 0.96
Score = 2.65
ALFGANQGR − Mascot − rejected
ALFGAN|Q|GR − spliced − 0|1|0|1
20120322_EXQ1_MiBa_SA_HCC1143_1_A: 13630
SA = 0.94
Score = 2.17
ALFGANANR − MaxQuant
20120322_EXQ1_MiBa_SA_HCC1143_1_A: 13630
SA = 0.95
Score = 2.59
ALFGANANR − MSFragger
20120322_EXQ1_MiBa_SA_HCC1143_1_A: 13630
SA = 0.95
Score = 2.59
Relative intensity
m/z
Prosit prediction
* Exclusive in prediction
# Exclusive in experimental

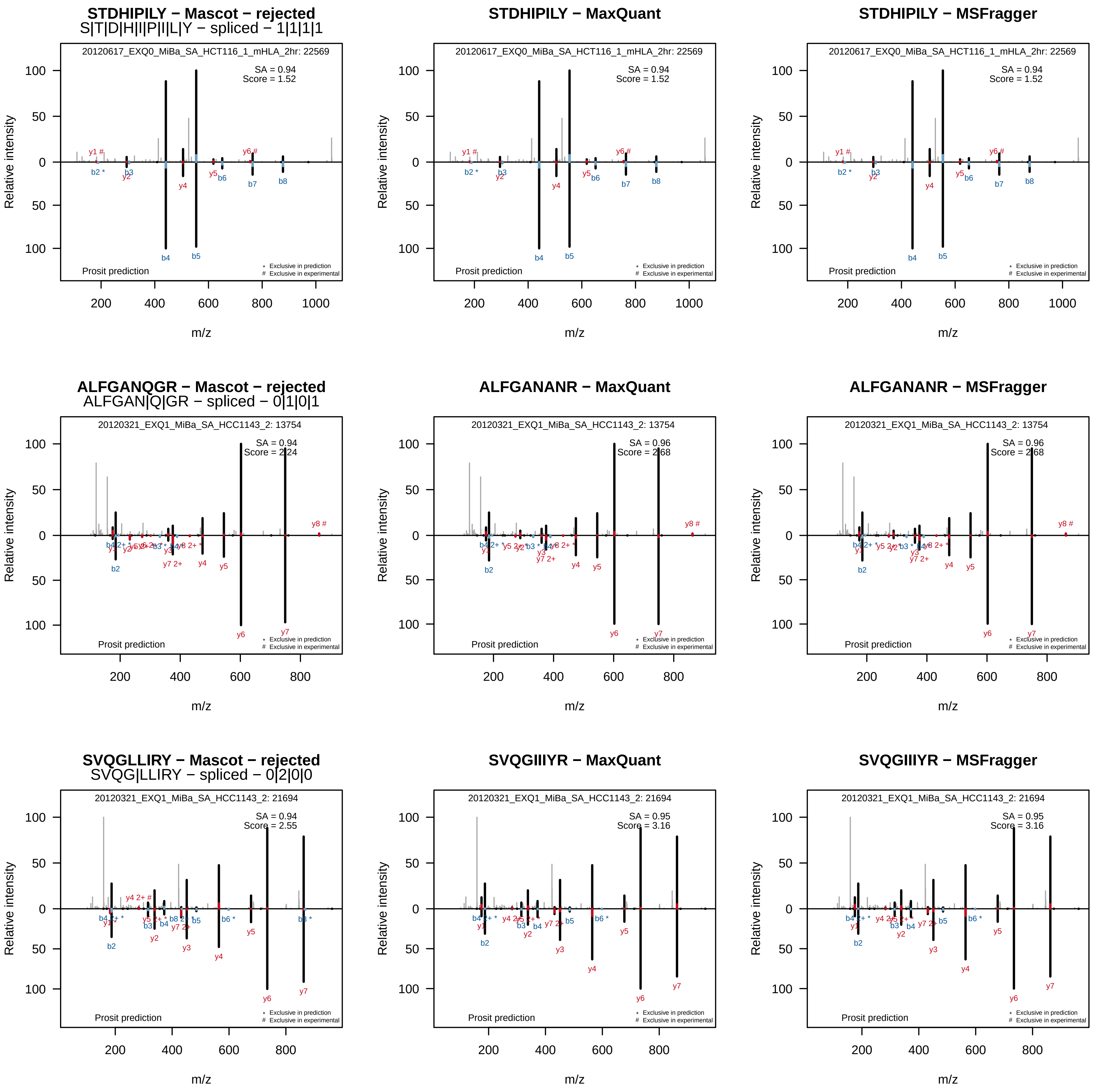
STDHIPILY − Mascot − rejected
S|T|D|H|I|P|I|L|Y − spliced − 1|1|1|1
STDHIPILY − MaxQuant
STDHIPILY − MSFragger
20120617_EXQ0_MiBa_SA_HCT116_1_mHLA_2hr: 22569
SA = 0.94
Score = 1.52
ALFGANQGR − Mascot − rejected
ALFGAN|Q|GR − spliced − 0|1|0|1
ALFGANANR − MaxQuant
ALFGANANR − MSFragger
20120321_EXQ1_MiBa_SA_HCC1143_2: 13754
SA = 0.94
Score = 2.24
SA = 0.96
Score = 2.68
SVQGLLIRY − Mascot − rejected
SVQG|LLIRY − spliced − 0|2|0|0
SVQGIIIYR − MaxQuant
SVQGIIIYR − MSFragger
20120321_EXQ1_MiBa_SA_HCC1143_2: 21694
SA = 0.94
Score = 2.55
SA = 0.95
Score = 3.16
Relative intensity
m/z
Prosit prediction
⋆ Exclusive in prediction
Exclusive in experimental

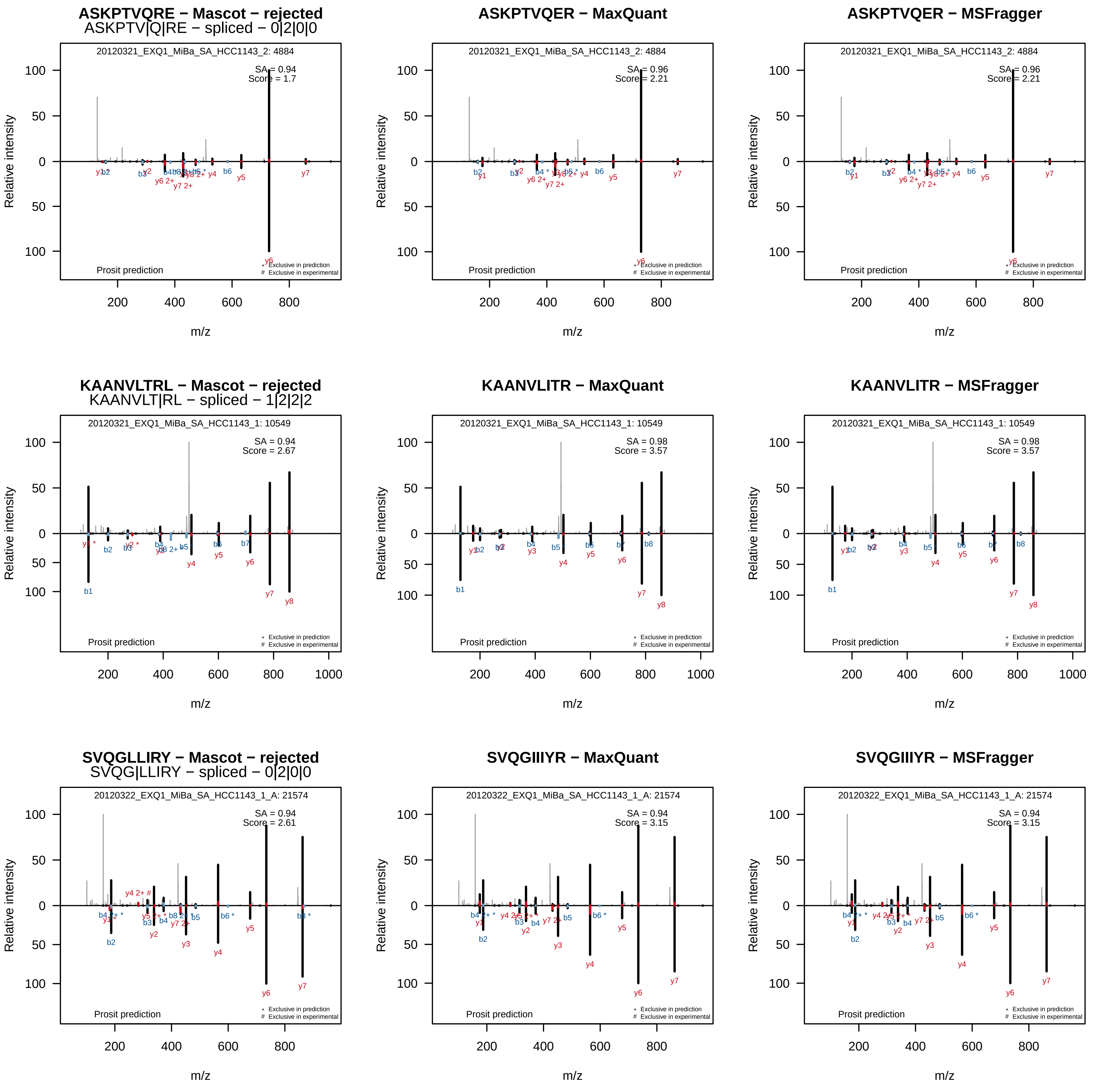
ASKPTVQRE − Mascot − rejected
ASKPTV|Q|RE − spliced − 0|2|0|0
ASKPTVQER − MaxQuant
ASKPTVQER − MSFragger
20120321_EXQ1_MiBa_SA_HCC1143_2: 4884
SA = 0.94
Score = 1.7
SA = 0.96
Score = 2.21
SA = 0.96
Score = 2.21
KAANVLTRL − Mascot − rejected
KAANVLT|RL − spliced − 1|2|2|2
KAANVLITR − MaxQuant
KAANVLITR − MSFragger
20120321_EXQ1_MiBa_SA_HCC1143_1: 10549
SA = 0.94
Score = 2.67
SA = 0.98
Score = 3.57
SA = 0.98
Score = 3.57
SVQGLLIRY − Mascot − rejected
SVQG|LLIRY − spliced − 0|2|0|0
SVQGIIIYR − MaxQuant
SVQGIIIYR − MSFragger
20120322_EXQ1_MiBa_SA_HCC1143_1_A: 21574
SA = 0.94
Score = 2.61
SA = 0.94
Score = 3.15
SA = 0.94
Score = 3.15
Relative intensity
m/z
Prosit prediction
* Exclusive in prediction
# Exclusive in experimental

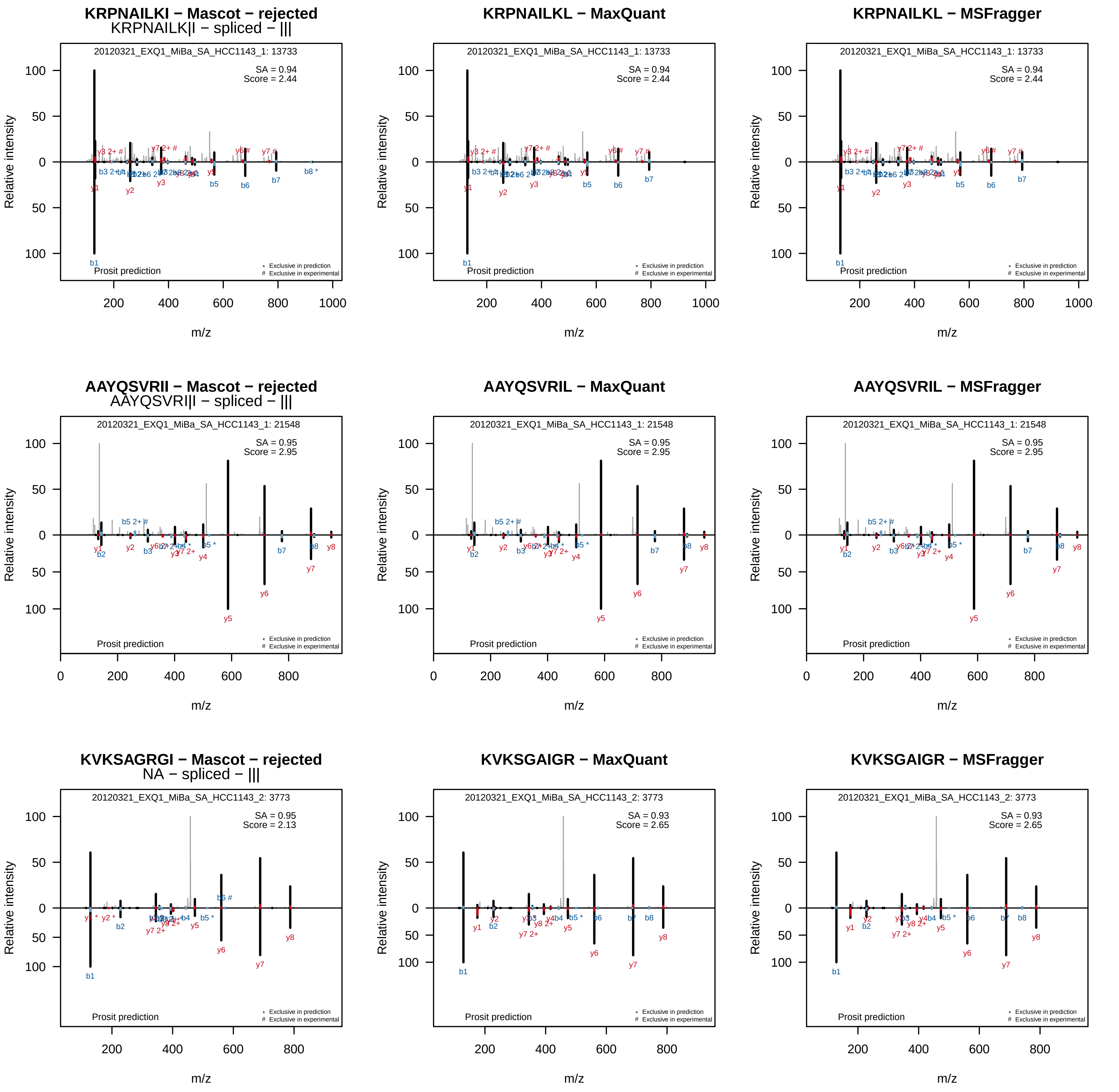

KRPNAILKI − Mascot − rejected
KRPNAILK|I − spliced − |||
20120321_EXQ1_MiBa_SA_HCC1143_1: 13733
SA = 0.94
Score = 2.44
Prosit prediction
* Exclusive in prediction
# Exclusive in experimental
Relative intensity
m/z
KRPNAILKL − MaxQuant
20120321_EXQ1_MiBa_SA_HCC1143_1: 13733
SA = 0.94
Score = 2.44
KRPNAILKL − MSFragger
20120321_EXQ1_MiBa_SA_HCC1143_1: 13733
SA = 0.94
Score = 2.44
AAYQSVRII − Mascot − rejected
AAYQSVRI|I − spliced − |||
20120321_EXQ1_MiBa_SA_HCC1143_1: 21548
SA = 0.95
Score = 2.95
AAYQSVRIL − MaxQuant
20120321_EXQ1_MiBa_SA_HCC1143_1: 21548
SA = 0.95
Score = 2.95
AAYQSVRIL − MSFragger
20120321_EXQ1_MiBa_SA_HCC1143_1: 21548
SA = 0.95
Score = 2.95
KVKSAGRGI − Mascot − rejected
NA − spliced − |||
20120321_EXQ1_MiBa_SA_HCC1143_2: 3773
SA = 0.95
Score = 2.13
KVKSGAIGR − MaxQuant
20120321_EXQ1_MiBa_SA_HCC1143_2: 3773
SA = 0.93
Score = 2.65
KVKSGAIGR − MSFragger
20120321_EXQ1_MiBa_SA_HCC1143_2: 3773
SA = 0.93
Score = 2.65

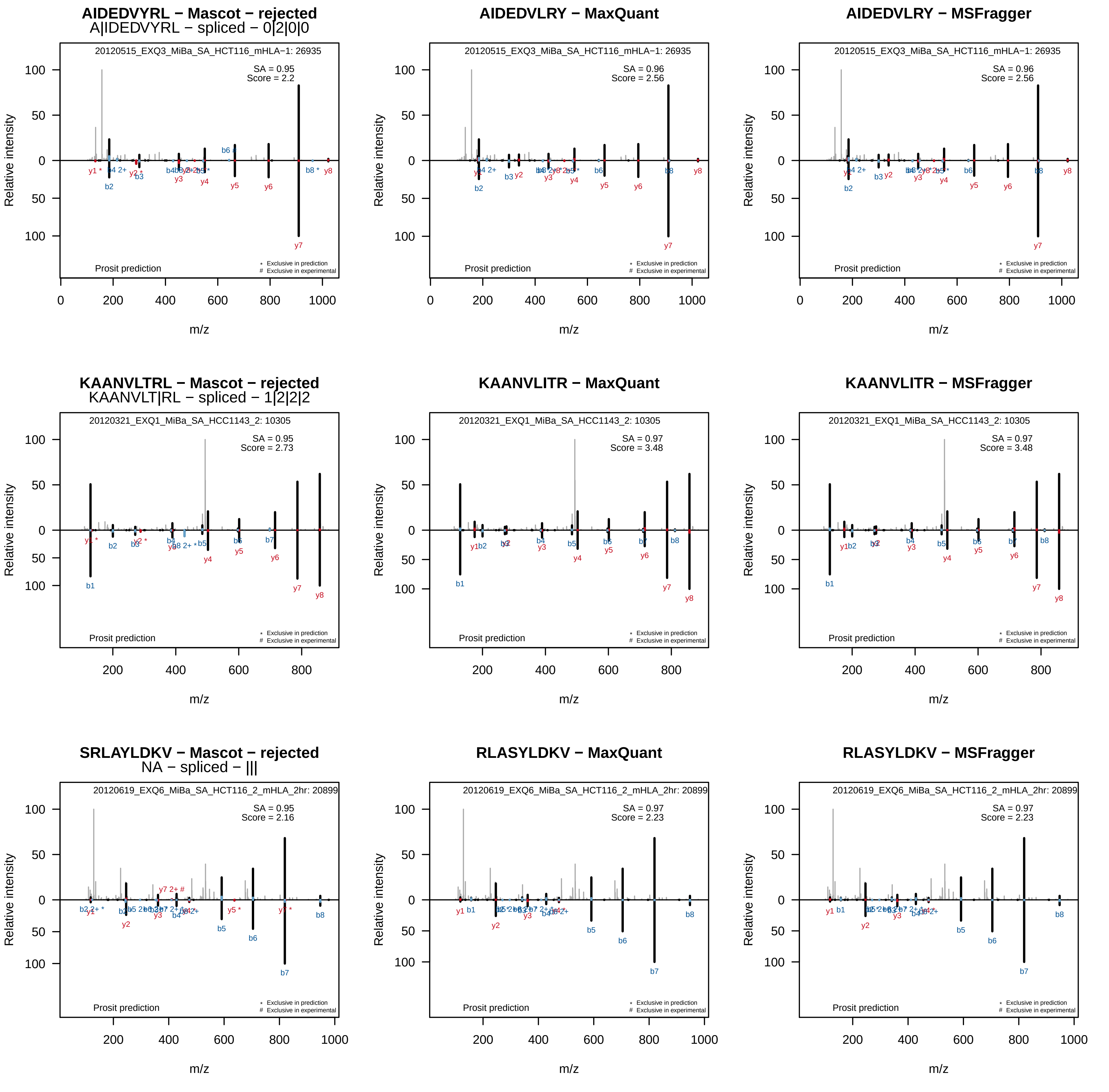

AIDEDVYRL − Mascot − rejected
A|IDEDVYRL − spliced − 0|2|0|0
20120515_EXQ3_MiBa_SA_HCT116_mHLA−1: 26935
SA = 0.95
Score = 2.2
AIDEDVLRY − MaxQuant
20120515_EXQ3_MiBa_SA_HCT116_mHLA−1: 26935
SA = 0.96
Score = 2.56
AIDEDVLRY − MSFragger
20120515_EXQ3_MiBa_SA_HCT116_mHLA−1: 26935
SA = 0.96
Score = 2.56
KAANVLTRL − Mascot − rejected
KAANVLT|RL − spliced − 1|2|2|2
20120321_EXQ1_MiBa_SA_HCC1143_2: 10305
SA = 0.95
Score = 2.73
KAANVLITR − MaxQuant
20120321_EXQ1_MiBa_SA_HCC1143_2: 10305
SA = 0.97
Score = 3.48
KAANVLITR − MSFragger
20120321_EXQ1_MiBa_SA_HCC1143_2: 10305
SA = 0.97
Score = 3.48
SRLAYLDKV − Mascot − rejected
NA − spliced − |||
20120619_EXQ6_MiBa_SA_HCT116_2_mHLA_2hr: 20899
SA = 0.95
Score = 2.16
RLASYLDKV − MaxQuant
20120619_EXQ6_MiBa_SA_HCT116_2_mHLA_2hr: 20899
SA = 0.97
Score = 2.23
RLASYLDKV − MSFragger
20120619_EXQ6_MiBa_SA_HCT116_2_mHLA_2hr: 20899
SA = 0.97
Score = 2.23
Relative intensity
m/z
Prosit prediction
* Exclusive in prediction
# Exclusive in experimental

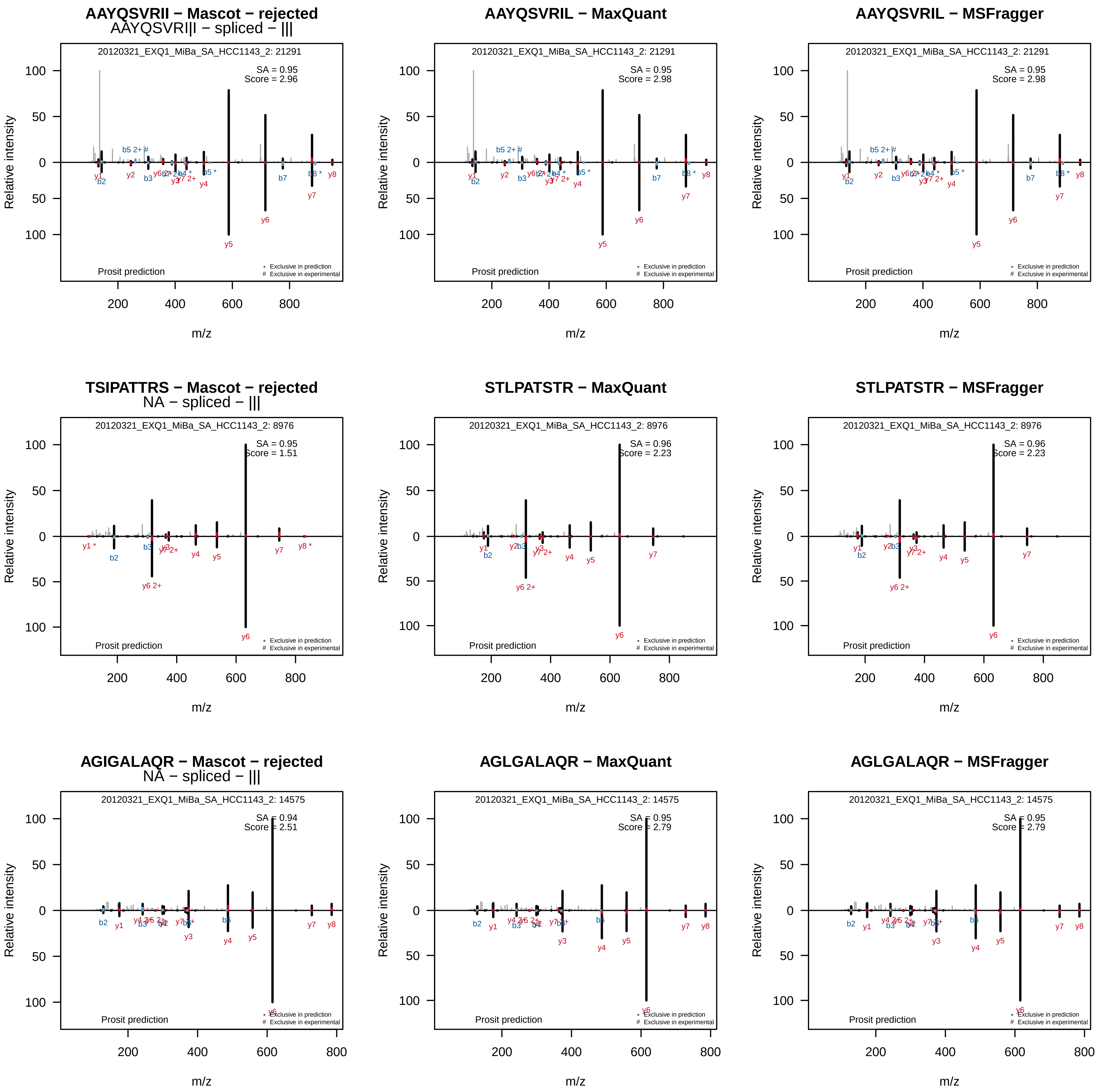
AAYQSVRII − Mascot − rejected
AAYQSVRI|I − spliced − |||
AAYQSVRIL − MaxQuant
AAYQSVRIL − MSFragger
20120321_EXQ1_MiBa_SA_HCC1143_2: 21291
SA = 0.95
Score = 2.96
SA = 0.95
Score = 2.98
SA = 0.95
Score = 2.98
TSIPATTRS − Mascot − rejected
NA − spliced − |||
STLPATSTR − MaxQuant
STLPATSTR − MSFragger
20120321_EXQ1_MiBa_SA_HCC1143_2: 8976
SA = 0.95
Score = 1.51
SA = 0.96
Score = 2.23
SA = 0.96
Score = 2.23
AGIGALAQR − Mascot − rejected
NA − spliced − |||
AGLGALAQR − MaxQuant
AGLGALAQR − MSFragger
20120321_EXQ1_MiBa_SA_HCC1143_2: 14575
SA = 0.94
Score = 2.51
SA = 0.95
Score = 2.79
SA = 0.95
Score = 2.79
Relative intensity
m/z
Prosit prediction
⋆ Exclusive in prediction
Exclusive in experimental

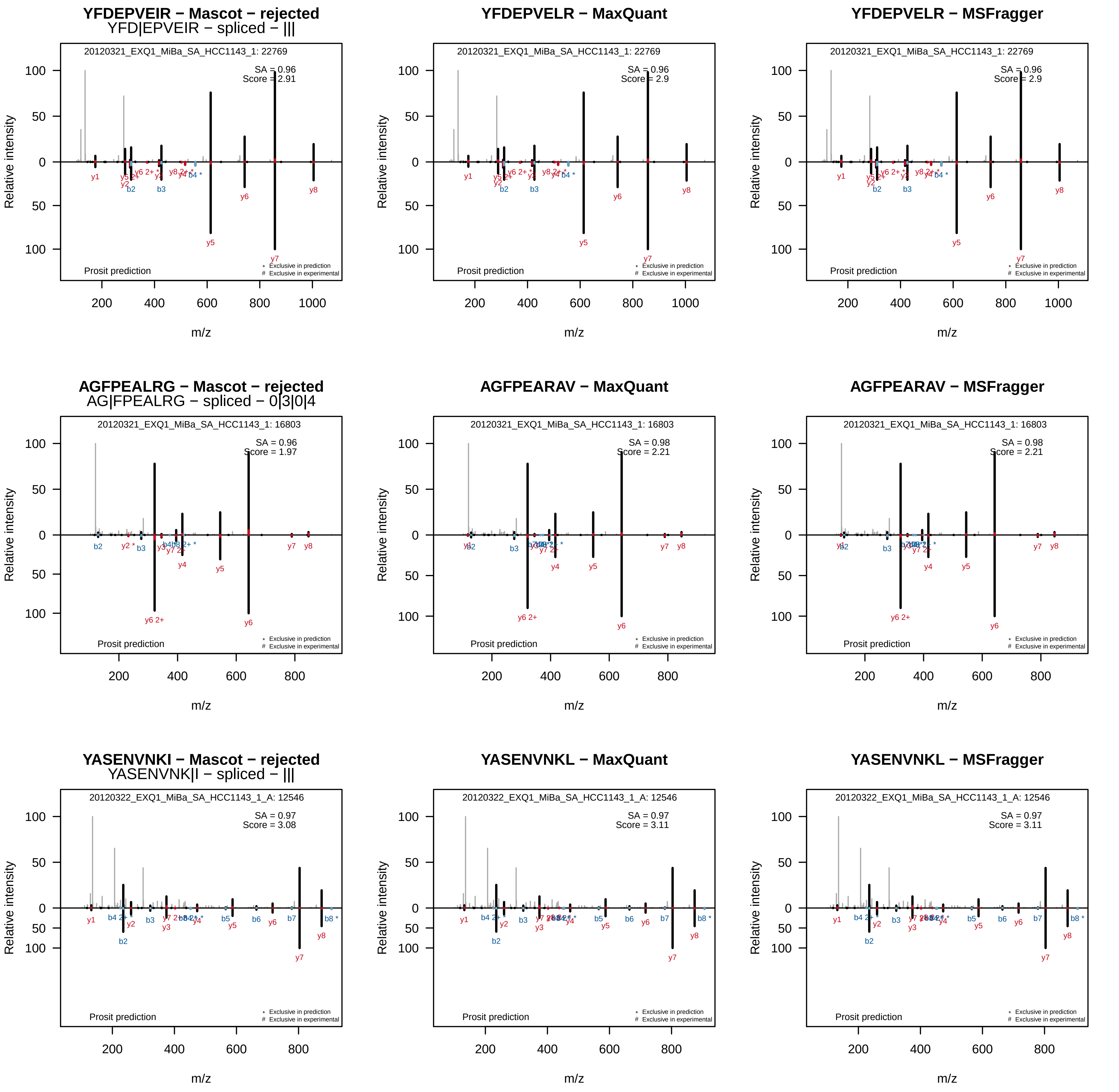
YFDEPVEIR − Mascot − rejected
YFD|EPVEIR − spliced − |||
YFDEPVELR − MaxQuant
YFDEPVELR − MSFragger
20120321_EXQ1_MiBa_SA_HCC1143_1: 22769
SA = 0.96
Score = 2.91
Score = 2.9
AGFPEALRG − Mascot − rejected
AG|FPEALRG − spliced − 0|3|0|4
AGFPEARAV − MaxQuant
AGFPEARAV − MSFragger
20120321_EXQ1_MiBa_SA_HCC1143_1: 16803
SA = 0.96
Score = 1.97
SA = 0.98
Score = 2.21
YASENVNKI − Mascot − rejected
YASENVNK|I − spliced − |||
YASENVNKL − MaxQuant
YASENVNKL − MSFragger
20120322_EXQ1_MiBa_SA_HCC1143_1_A: 12546
SA = 0.97
Score = 3.08
Score = 3.11
Relative intensity
m/z
Prosit prediction
Exclusive in prediction
Exclusive in experimental

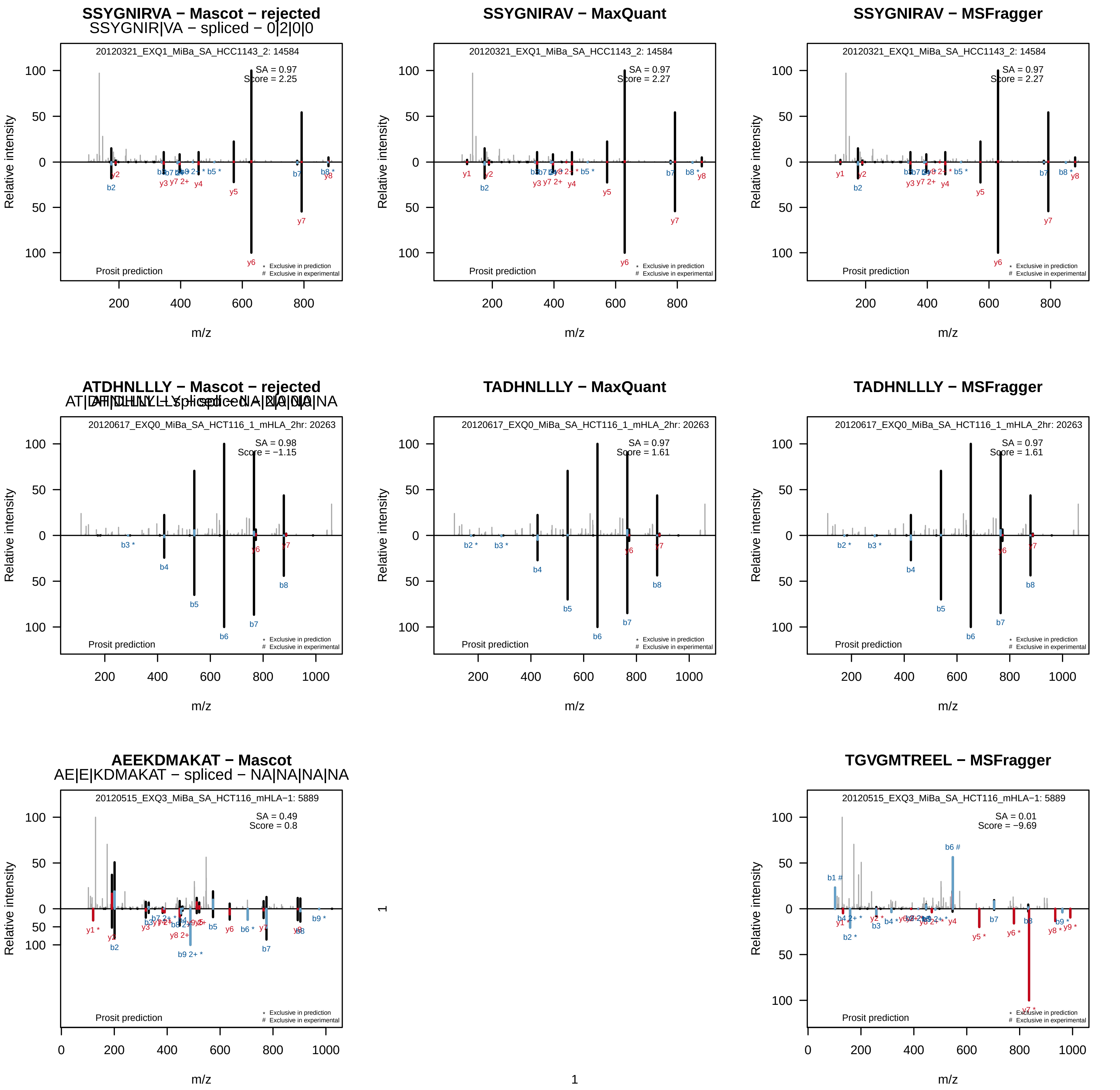
SSYGNIRVA − Mascot − rejected
SSYGNIR|VA − spliced − 0|2|0|0
20120321_EXQ1_MiBa_SA_HCC1143_2: 14584
SA = 0.97
Score = 2.25
SSYGNIRAV − MaxQuant
20120321_EXQ1_MiBa_SA_HCC1143_2: 14584
SA = 0.97
Score = 2.27
SSYGNIRAV − MSFragger
20120321_EXQ1_MiBa_SA_HCC1143_2: 14584
SA = 0.97
Score = 2.27
ATDHNLLLY − Mascot − rejected
20120617_EXQ0_MiBa_SA_HCT116_1_mHLA_2hr: 20263
SA = 0.98
Score = −1.15
TADHNLLLY − MaxQuant
20120617_EXQ0_MiBa_SA_HCT116_1_mHLA_2hr: 20263
SA = 0.97
Score = 1.61
TADHNLLLY − MSFragger
20120617_EXQ0_MiBa_SA_HCT116_1_mHLA_2hr: 20263
SA = 0.97
Score = 1.61
AEEKDMAKAT − Mascot
AE|E|KDMAKAT − spliced − NA|NA|NA|NA
20120515_EXQ3_MiBa_SA_HCT116_mHLA−1: 5889
SA = 0.49
Score = 0.8
TGVGMTREEL − MSFragger
20120515_EXQ3_MiBa_SA_HCT116_mHLA−1: 5889
SA = 0.01
Score = −9.69
Relative intensity
m/z
Prosit prediction
* Exclusive in prediction
# Exclusive in experimental

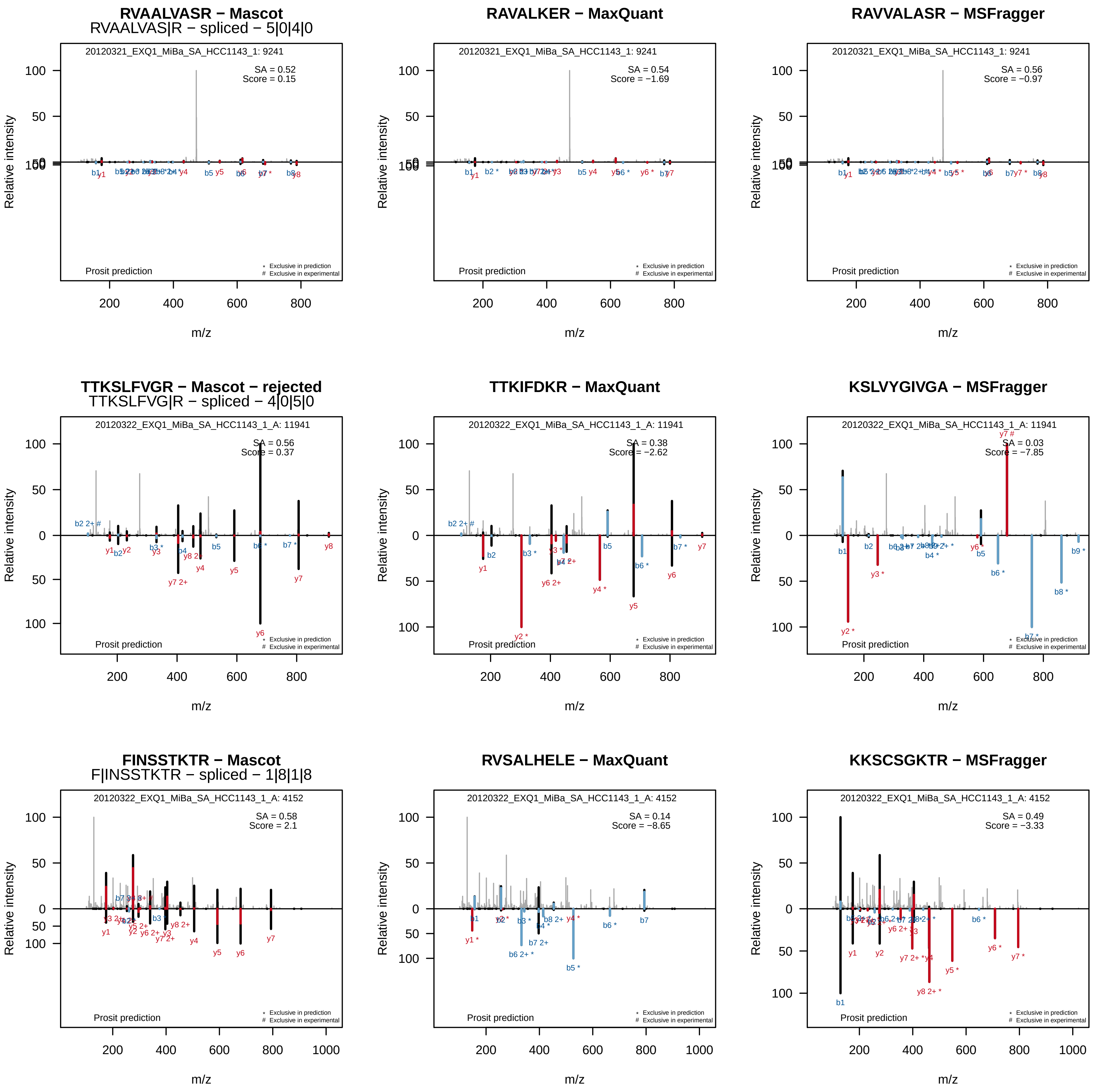
RVAALVASR − Mascot
RVAALVAS|R − spliced − 5|0|4|0
20120321_EXQ1_MiBa_SA_HCC1143_1: 9241
SA = 0.52
Score = 0.15
RAVALKER − MaxQuant
20120321_EXQ1_MiBa_SA_HCC1143_1: 9241
SA = 0.54
Score = −1.69
RAVVALASR − MSFragger
20120321_EXQ1_MiBa_SA_HCC1143_1: 9241
SA = 0.56
Score = −0.97
TTKSLFVGR − Mascot − rejected
TTKSLFVG|R − spliced − 4|0|5|0
20120322_EXQ1_MiBa_SA_HCC1143_1_A: 11941
SA = 0.56
Score = 0.37
TTKIFDKR − MaxQuant
20120322_EXQ1_MiBa_SA_HCC1143_1_A: 11941
SA = 0.38
Score = −2.62
KSLVYGIVGA − MSFragger
20120322_EXQ1_MiBa_SA_HCC1143_1_A: 11941
SA = 0.03
Score = −7.85
FINSSTKTR − Mascot
F|INSSTKTR − spliced − 1|8|1|8
20120322_EXQ1_MiBa_SA_HCC1143_1_A: 4152
SA = 0.58
Score = 2.1
RVSALHELE − MaxQuant
20120322_EXQ1_MiBa_SA_HCC1143_1_A: 4152
SA = 0.14
Score = −8.65
KKSCSGKTR − MSFragger
20120322_EXQ1_MiBa_SA_HCC1143_1_A: 4152
SA = 0.49
Score = −3.33
Relative intensity
m/z
Prosit prediction
* Exclusive in prediction
# Exclusive in experimental

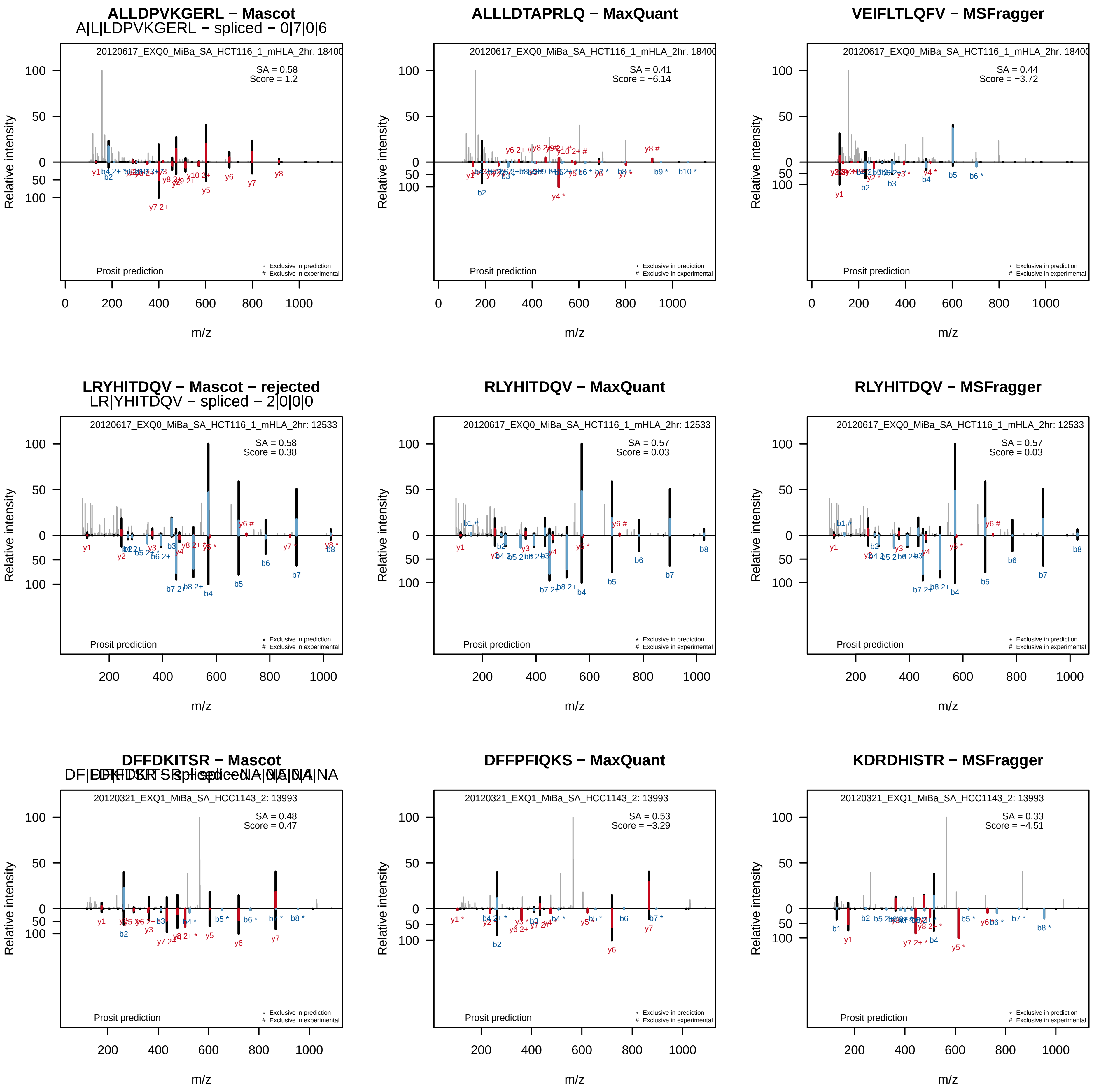
ALLDPVKGERL − Mascot
A|L|LDPVKGERL − spliced − 0|7|0|6
20120617_EXQ0_MiBa_SA_HCT116_1_mHLA_2hr: 18400
SA = 0.58
Score = 1.2
ALLLDTAPRLQ − MaxQuant
20120617_EXQ0_MiBa_SA_HCT116_1_mHLA_2hr: 18400
SA = 0.41
Score = −6.14
VEIFLTLQFV − MSFragger
20120617_EXQ0_MiBa_SA_HCT116_1_mHLA_2hr: 18400
SA = 0.44
Score = −3.72
LRYHITDQV − Mascot − rejected
LR|YHITDQV − spliced − 2|0|0|0
20120617_EXQ0_MiBa_SA_HCT116_1_mHLA_2hr: 12533
SA = 0.58
Score = 0.38
RLYHITDQV − MaxQuant
20120617_EXQ0_MiBa_SA_HCT116_1_mHLA_2hr: 12533
SA = 0.57
Score = 0.03
RLYHITDQV − MSFragger
20120617_EXQ0_MiBa_SA_HCT116_1_mHLA_2hr: 12533
SA = 0.57
Score = 0.03
DFFDKITSR − Mascot
20120321_EXQ1_MiBa_SA_HCC1143_2: 13993
SA = 0.48
Score = 0.47
DFFPFIQKS − MaxQuant
20120321_EXQ1_MiBa_SA_HCC1143_2: 13993
SA = 0.53
Score = −3.29
KDRDHISTR − MSFragger
20120321_EXQ1_MiBa_SA_HCC1143_2: 13993
SA = 0.33
Score = −4.51
Relative intensity
m/z
Prosit prediction
* Exclusive in prediction
# Exclusive in experimental

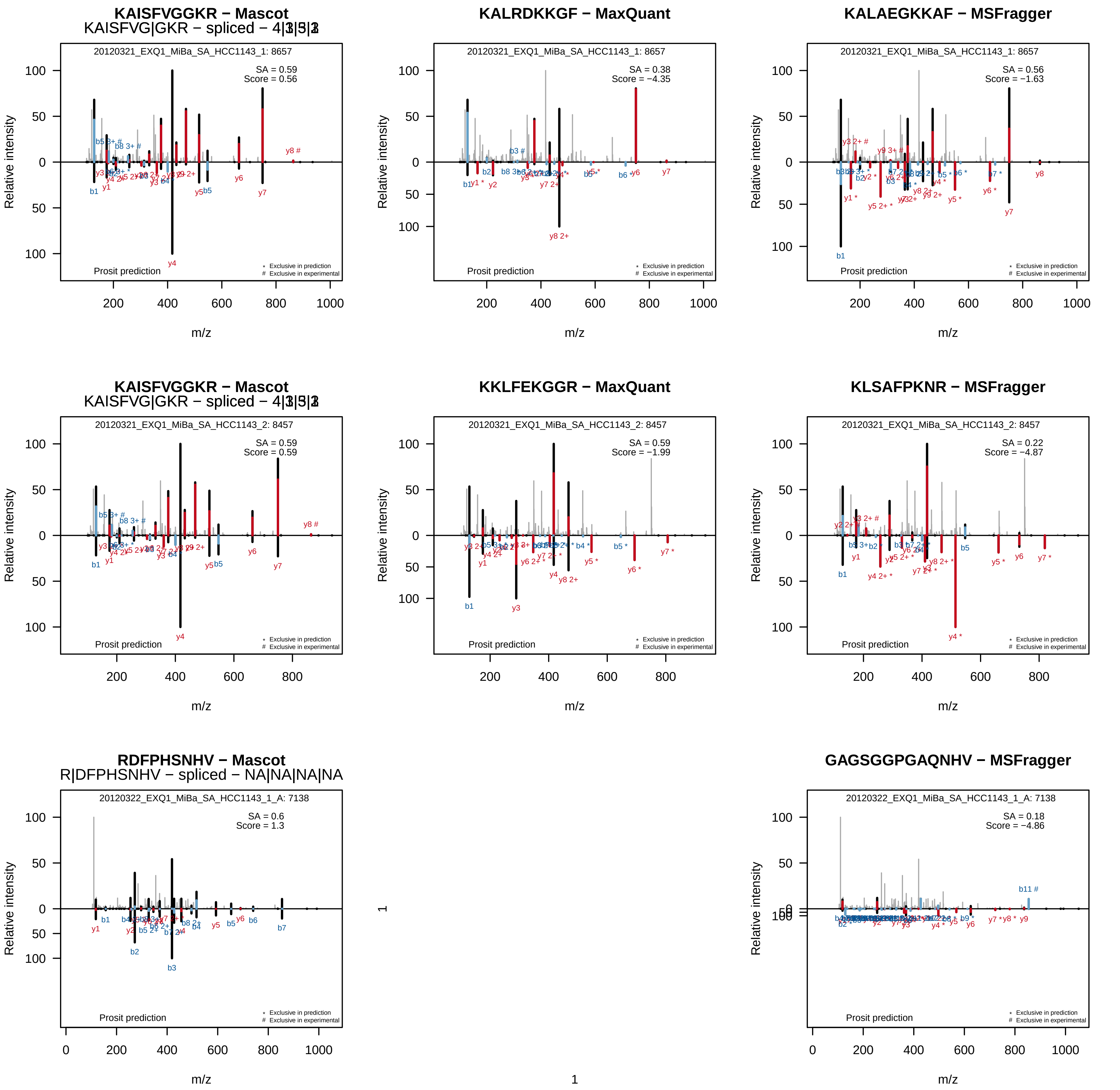
KAISFVGGKR − Mascot
KAISFVG|GKR − spliced − 4|3|5|2
20120321_EXQ1_MiBa_SA_HCC1143_1: 8657
SA = 0.59
Score = 0.56
KALRDKKGF − MaxQuant
20120321_EXQ1_MiBa_SA_HCC1143_1: 8657
SA = 0.38
Score = −4.35
KALAEGKKAF − MSFragger
20120321_EXQ1_MiBa_SA_HCC1143_1: 8657
SA = 0.56
Score = −1.63
KAISFVGGKR − Mascot
KAISFVG|GKR − spliced − 4|3|5|2
20120321_EXQ1_MiBa_SA_HCC1143_2: 8457
SA = 0.59
Score = 0.59
KKLFEKGGR − MaxQuant
20120321_EXQ1_MiBa_SA_HCC1143_2: 8457
SA = 0.59
Score = −1.99
KLSAFPKNR − MSFragger
20120321_EXQ1_MiBa_SA_HCC1143_2: 8457
SA = 0.22
Score = −4.87
RDFPHSNHV − Mascot
R|DFPHSNHV − spliced − NA|NA|NA|NA
20120322_EXQ1_MiBa_SA_HCC1143_1_A: 7138
SA = 0.6
Score = 1.3
GAGSGGPGAQNHV − MSFragger
20120322_EXQ1_MiBa_SA_HCC1143_1_A: 7138
SA = 0.18
Score = −4.86
Relative intensity
m/z
Prosit prediction
* Exclusive in prediction
# Exclusive in experimental

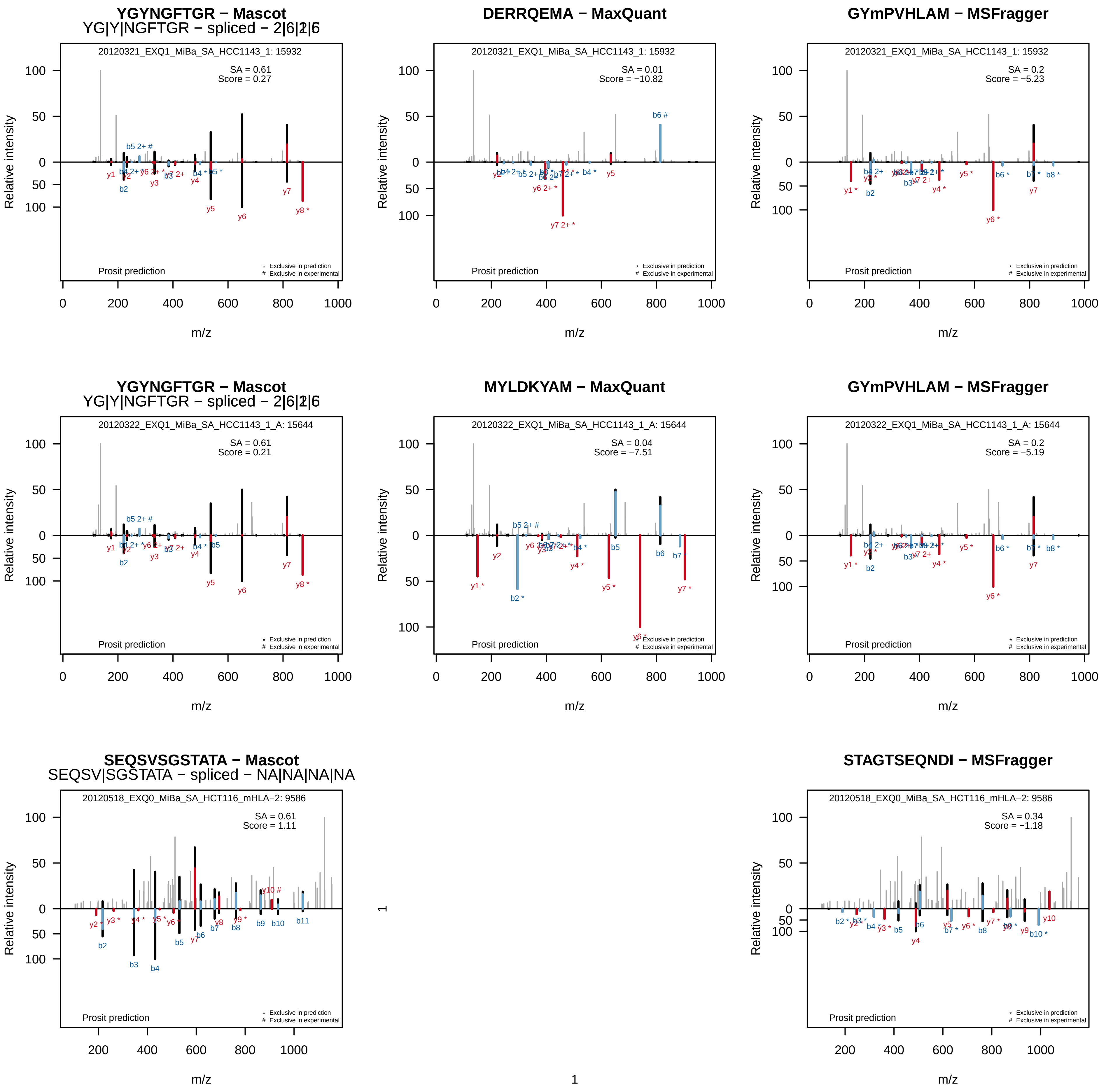
YGYNGFTGR − Mascot
YG|Y|NGFTGR − spliced − 2|6|2|6
20120321_EXQ1_MiBa_SA_HCC1143_1: 15932
SA = 0.61
Score = 0.27
DERRQEMA − MaxQuant
20120321_EXQ1_MiBa_SA_HCC1143_1: 15932
SA = 0.01
Score = −10.82
GYmPVHLAM − MSFragger
20120321_EXQ1_MiBa_SA_HCC1143_1: 15932
SA = 0.2
Score = −5.23
YGYNGFTGR − Mascot
YG|Y|NGFTGR − spliced − 2|6|2|6
20120322_EXQ1_MiBa_SA_HCC1143_1_A: 15644
SA = 0.61
Score = 0.21
MYLDKYAM − MaxQuant
20120322_EXQ1_MiBa_SA_HCC1143_1_A: 15644
SA = 0.04
Score = −7.51
GYmPVHLAM − MSFragger
20120322_EXQ1_MiBa_SA_HCC1143_1_A: 15644
SA = 0.2
Score = −5.19
SEQSVSGSTATA − Mascot
SEQSV|SGSTATA − spliced − NA|NA|NA|NA
20120518_EXQ0_MiBa_SA_HCT116_mHLA−2: 9586
SA = 0.61
Score = 1.11
STAGTSEQNDI − MSFragger
20120518_EXQ0_MiBa_SA_HCT116_mHLA−2: 9586
SA = 0.34
Score = −1.18
Relative intensity
m/z
Prosit prediction
⋆ Exclusive in prediction
Exclusive in experimental

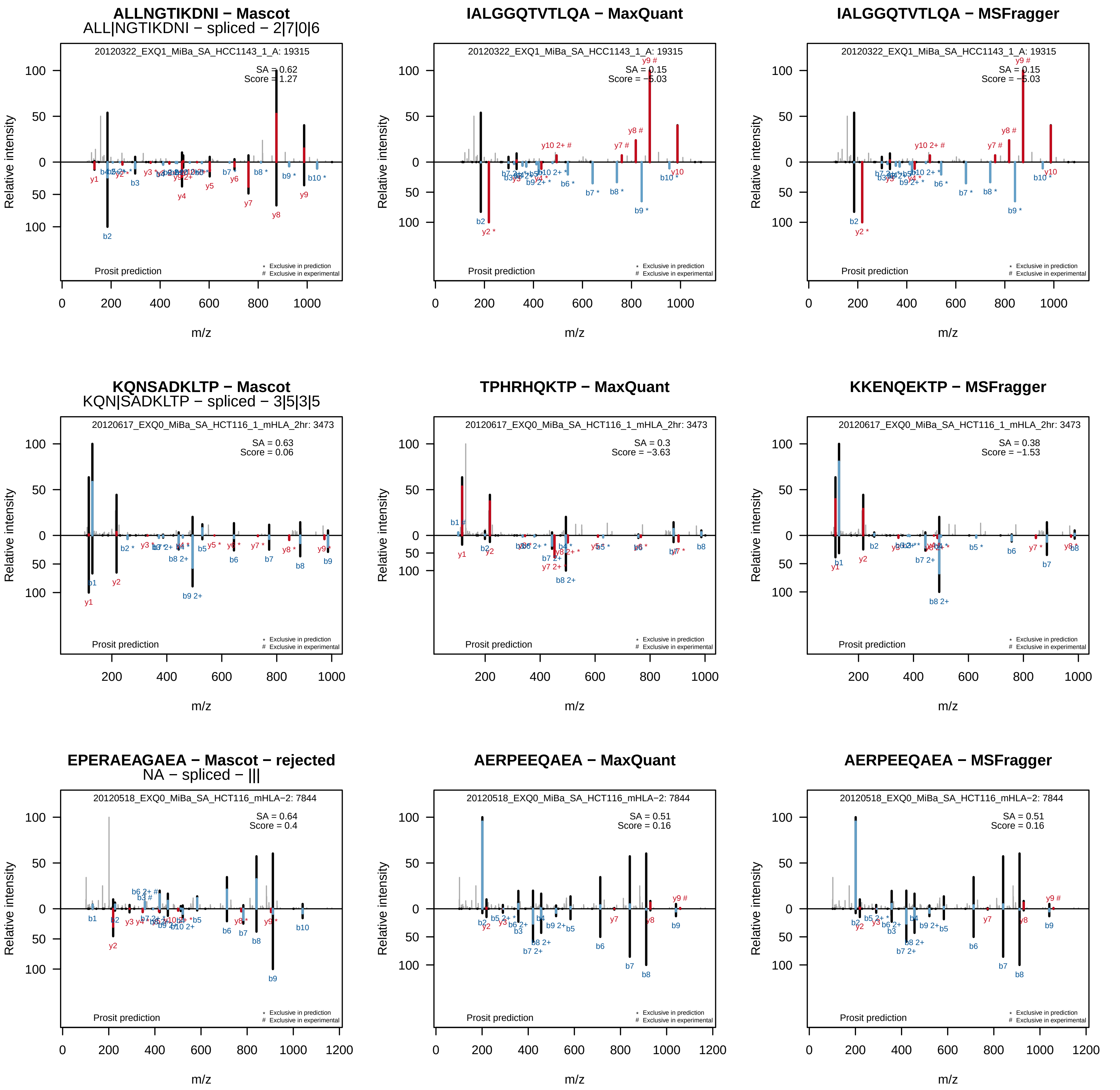

ALLNGTIKDNI − Mascot
ALL|NGTIKDNI − spliced − 2|7|0|6
20120322_EXQ1_MiBa_SA_HCC1143_1_A: 19315
SA = 0.62
Score = 1.27
IALGGQTVTLQA − MaxQuant
20120322_EXQ1_MiBa_SA_HCC1143_1_A: 19315
SA = 0.15
Score = −5.03
IALGGQTVTLQA − MSFragger
20120322_EXQ1_MiBa_SA_HCC1143_1_A: 19315
SA = 0.15
Score = −5.03
KQNSADKLTP − Mascot
KQN|SADKLTP − spliced − 3|5|3|5
20120617_EXQ0_MiBa_SA_HCT116_1_mHLA_2hr: 3473
SA = 0.63
Score = 0.06
TPHRHQKTP − MaxQuant
20120617_EXQ0_MiBa_SA_HCT116_1_mHLA_2hr: 3473
SA = 0.3
Score = −3.63
KKENQEKTP − MSFragger
20120617_EXQ0_MiBa_SA_HCT116_1_mHLA_2hr: 3473
SA = 0.38
Score = −1.53
EPERAEAGAEA − Mascot − rejected
NA − spliced − |||
20120518_EXQ0_MiBa_SA_HCT116_mHLA−2: 7844
SA = 0.64
Score = 0.4
AERPEEQAEA − MaxQuant
20120518_EXQ0_MiBa_SA_HCT116_mHLA−2: 7844
SA = 0.51
Score = 0.16
AERPEEQAEA − MSFragger
20120518_EXQ0_MiBa_SA_HCT116_mHLA−2: 7844
SA = 0.51
Score = 0.16
Relative intensity
m/z
Prosit prediction
* Exclusive in prediction
# Exclusive in experimental

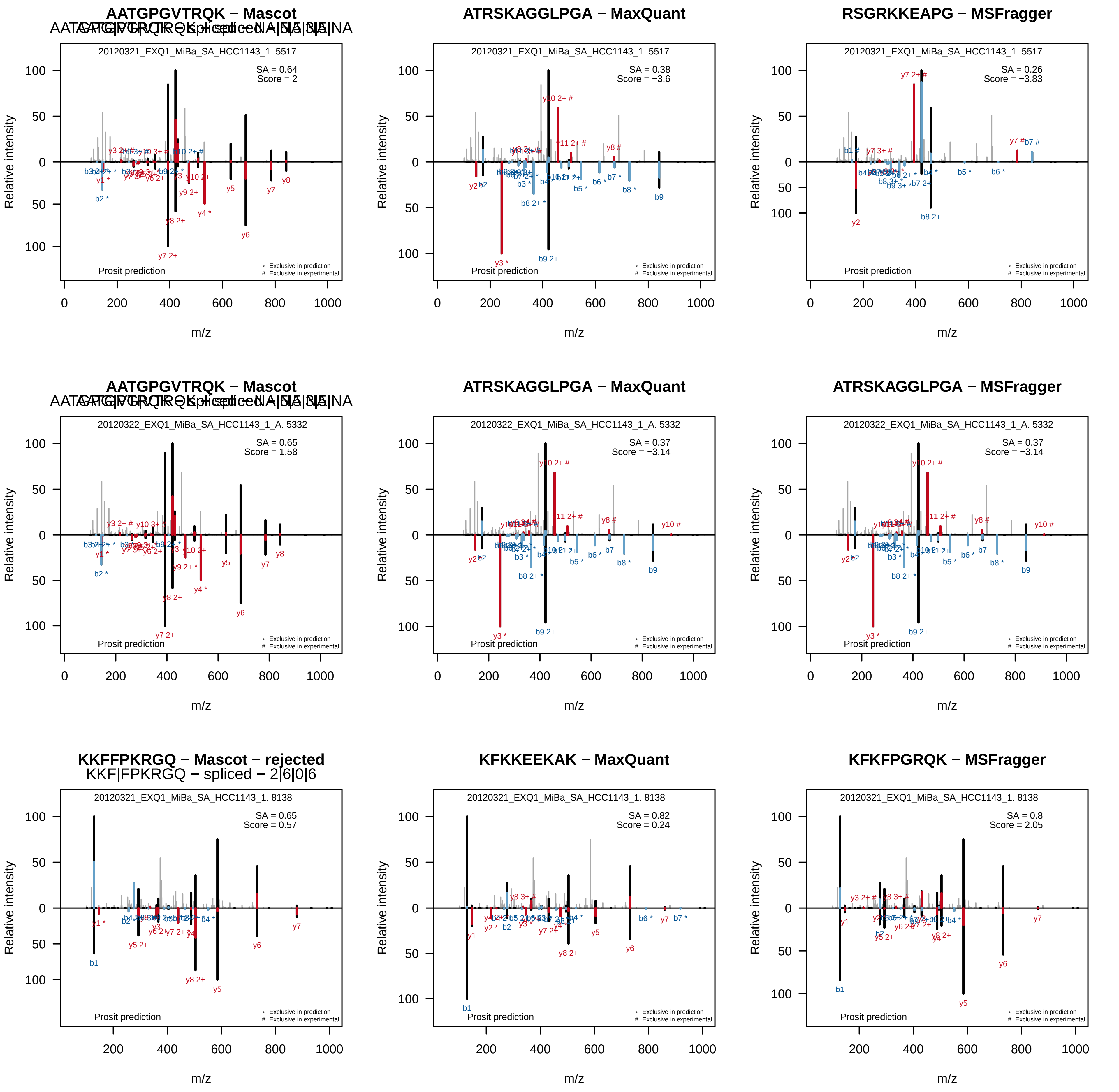

AATGPGVTRQK − Mascot
20120321_EXQ1_MiBa_SA_HCC1143_1: 5517
SA = 0.64
Score = 2
ATRSKAGGLPGA − MaxQuant
20120321_EXQ1_MiBa_SA_HCC1143_1: 5517
SA = 0.38
Score = −3.6
RSGRKKEAPG − MSFragger
20120321_EXQ1_MiBa_SA_HCC1143_1: 5517
SA = 0.26
Score = −3.83
AATGPGVTRQK − Mascot
20120322_EXQ1_MiBa_SA_HCC1143_1_A: 5332
SA = 0.65
Score = 1.58
ATRSKAGGLPGA − MaxQuant
20120322_EXQ1_MiBa_SA_HCC1143_1_A: 5332
SA = 0.37
Score = −3.14
ATRSKAGGLPGA − MSFragger
20120322_EXQ1_MiBa_SA_HCC1143_1_A: 5332
SA = 0.37
Score = −3.14
KKFFPKRGQ − Mascot − rejected
KKF|FPKRGQ − spliced − 2|6|0|6
20120321_EXQ1_MiBa_SA_HCC1143_1: 8138
SA = 0.65
Score = 0.57
KFKKEEKAK − MaxQuant
20120321_EXQ1_MiBa_SA_HCC1143_1: 8138
SA = 0.82
Score = 0.24
KFKFPGRQK − MSFragger
20120321_EXQ1_MiBa_SA_HCC1143_1: 8138
SA = 0.8
Score = 2.05
Relative intensity
m/z
Prosit prediction
* Exclusive in prediction
# Exclusive in experimental

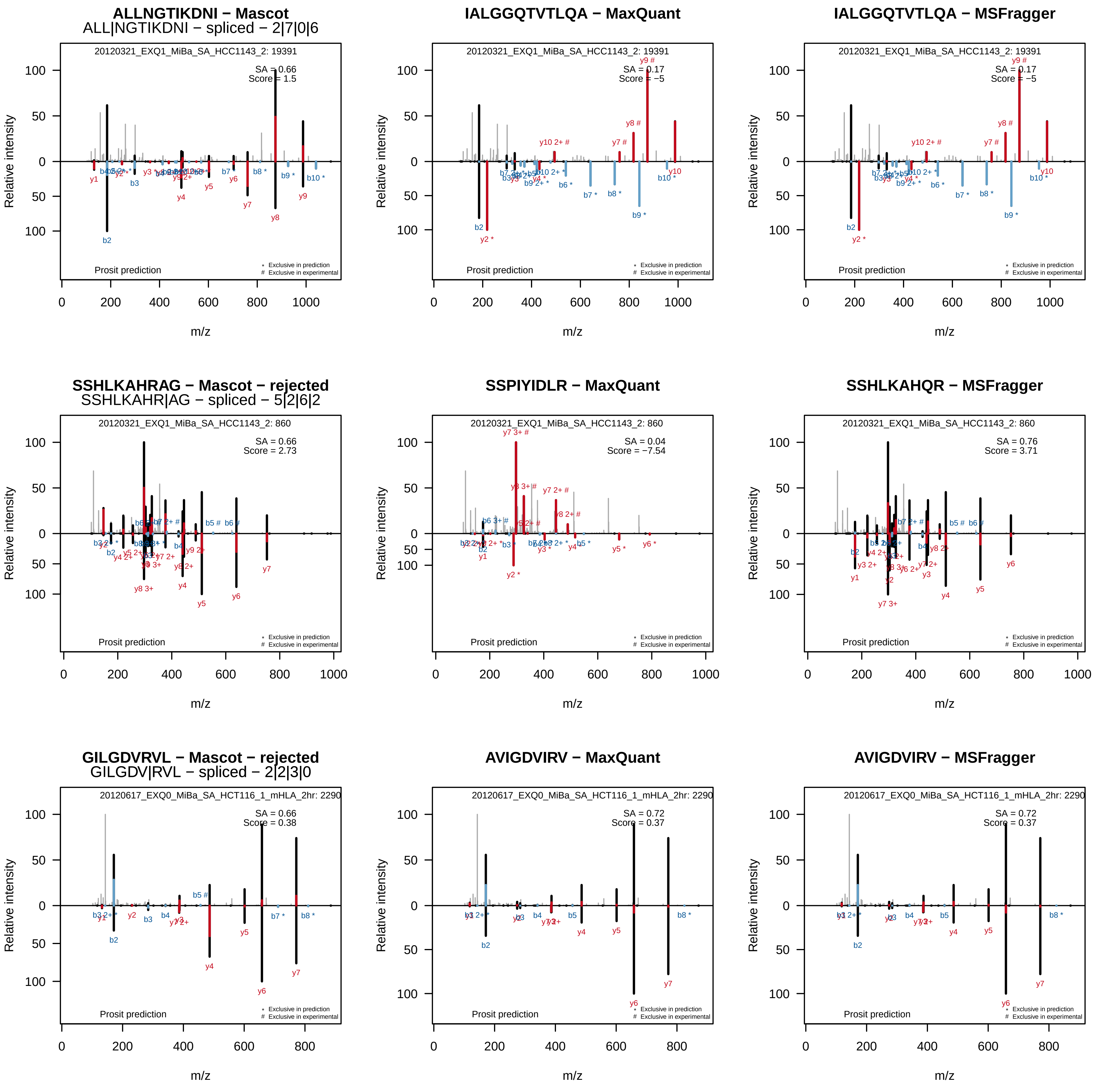
ALLNGTIKDNI − Mascot
ALL|NGTIKDNI − spliced − 2|7|0|6
20120321_EXQ1_MiBa_SA_HCC1143_2: 19391
SA = 0.66
Score = 1.5
IALGGQTVTLQA − MaxQuant
20120321_EXQ1_MiBa_SA_HCC1143_2: 19391
SA = 0.17
Score = −5
IALGGQTVTLQA − MSFragger
20120321_EXQ1_MiBa_SA_HCC1143_2: 19391
SA = 0.17
Score = −5
SSHLKAHRAG − Mascot − rejected
SSHLKAHR|AG − spliced − 5|2|6|2
20120321_EXQ1_MiBa_SA_HCC1143_2: 860
SA = 0.66
Score = 2.73
SSPIYIDLR − MaxQuant
20120321_EXQ1_MiBa_SA_HCC1143_2: 860
SA = 0.04
Score = −7.54
SSHLKAHQR − MSFragger
20120321_EXQ1_MiBa_SA_HCC1143_2: 860
SA = 0.76
Score = 3.71
GILGDVRVL − Mascot − rejected
GILGDV|RVL − spliced − 2|2|3|0
20120617_EXQ0_MiBa_SA_HCT116_1_mHLA_2hr: 2290
SA = 0.66
Score = 0.38
AVIGDVIRV − MaxQuant
20120617_EXQ0_MiBa_SA_HCT116_1_mHLA_2hr: 2290
SA = 0.72
Score = 0.37
AVIGDVIRV − MSFragger
20120617_EXQ0_MiBa_SA_HCT116_1_mHLA_2hr: 2290
SA = 0.72
Score = 0.37
Relative intensity
m/z
Prosit prediction
* Exclusive in prediction
# Exclusive in experimental

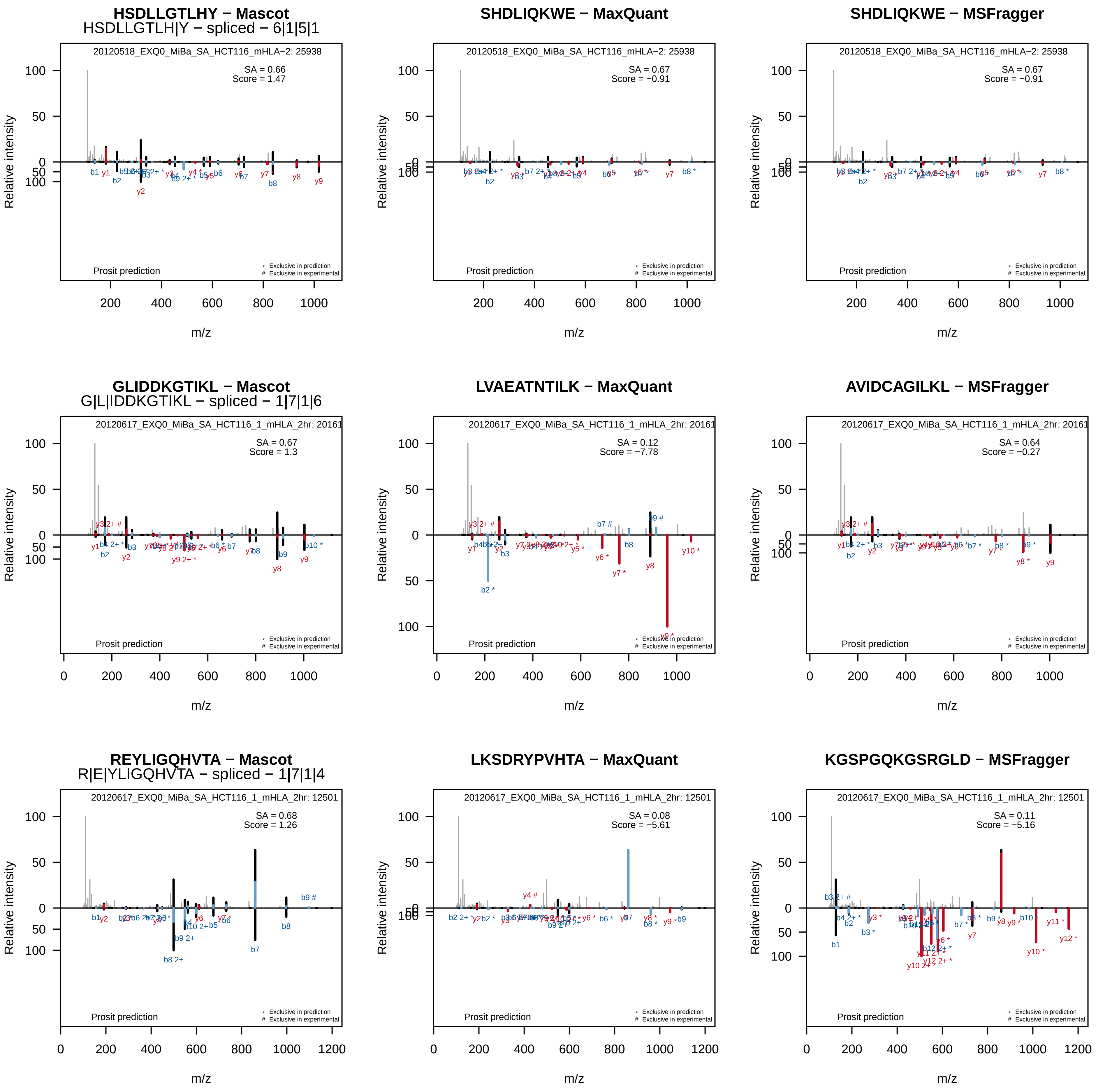

HSDLLGTLHY − Mascot
HSDLLGTLH|Y − spliced − 6|1|5|1
20120518_EXQ0_MiBa_SA_HCT116_mHLA−2: 25938
SA = 0.66
Score = 1.47
Prosit prediction
Exclusive in prediction
Exclusive in experimental
Relative intensity
m/z
SHDLIQKWE − MaxQuant
20120518_EXQ0_MiBa_SA_HCT116_mHLA−2: 25938
SA = 0.67
Score = −0.91
Prosit prediction
Exclusive in prediction
Exclusive in experimental
Relative intensity
m/z
SHDLIQKWE − MSFragger
20120518_EXQ0_MiBa_SA_HCT116_mHLA−2: 25938
SA = 0.67
Score = −0.91
Prosit prediction
Exclusive in prediction
Exclusive in experimental
Relative intensity
m/z
GLIDDKGTIKL − Mascot
G|L|IDDKGTIKL − spliced − 1|7|1|6
20120617_EXQ0_MiBa_SA_HCT116_1_mHLA_2hr: 2016
SA = 0.67
Score = 1.3
Prosit prediction
Exclusive in prediction
Exclusive in experimental
Relative intensity
m/z
LVAEATNTILK − MaxQuant
20120617_EXQ0_MiBa_SA_HCT116_1_mHLA_2hr: 2016
SA = 0.12
Score = −7.78
Prosit prediction
Exclusive in prediction
Exclusive in experimental
Relative intensity
m/z
AVIDCAGILKL − MSFragger
20120617_EXQ0_MiBa_SA_HCT116_1_mHLA_2hr: 2016
SA = 0.64
Score = −0.27
Prosit prediction
Exclusive in prediction
Exclusive in experimental
Relative intensity
m/z
REYLIGQHVTA − Mascot
R|E|YLIGQHVTA − spliced − 1|7|1|4
20120617_EXQ0_MiBa_SA_HCT116_1_mHLA_2hr: 12501
SA = 0.68
Score = 1.26
Prosit prediction
Exclusive in prediction
Exclusive in experimental
Relative intensity
m/z
LKSDRYPVHTA − MaxQuant
20120617_EXQ0_MiBa_SA_HCT116_1_mHLA_2hr: 12501
SA = 0.08
Score = −5.61
Prosit prediction
Exclusive in prediction
Exclusive in experimental
Relative intensity
m/z
KGSPGQKGSRGLD − MSFragger
20120617_EXQ0_MiBa_SA_HCT116_1_mHLA_2hr: 12501
SA = 0.11
Score = −5.16
Prosit prediction
Exclusive in prediction
Exclusive in experimental
Relative intensity
m/z

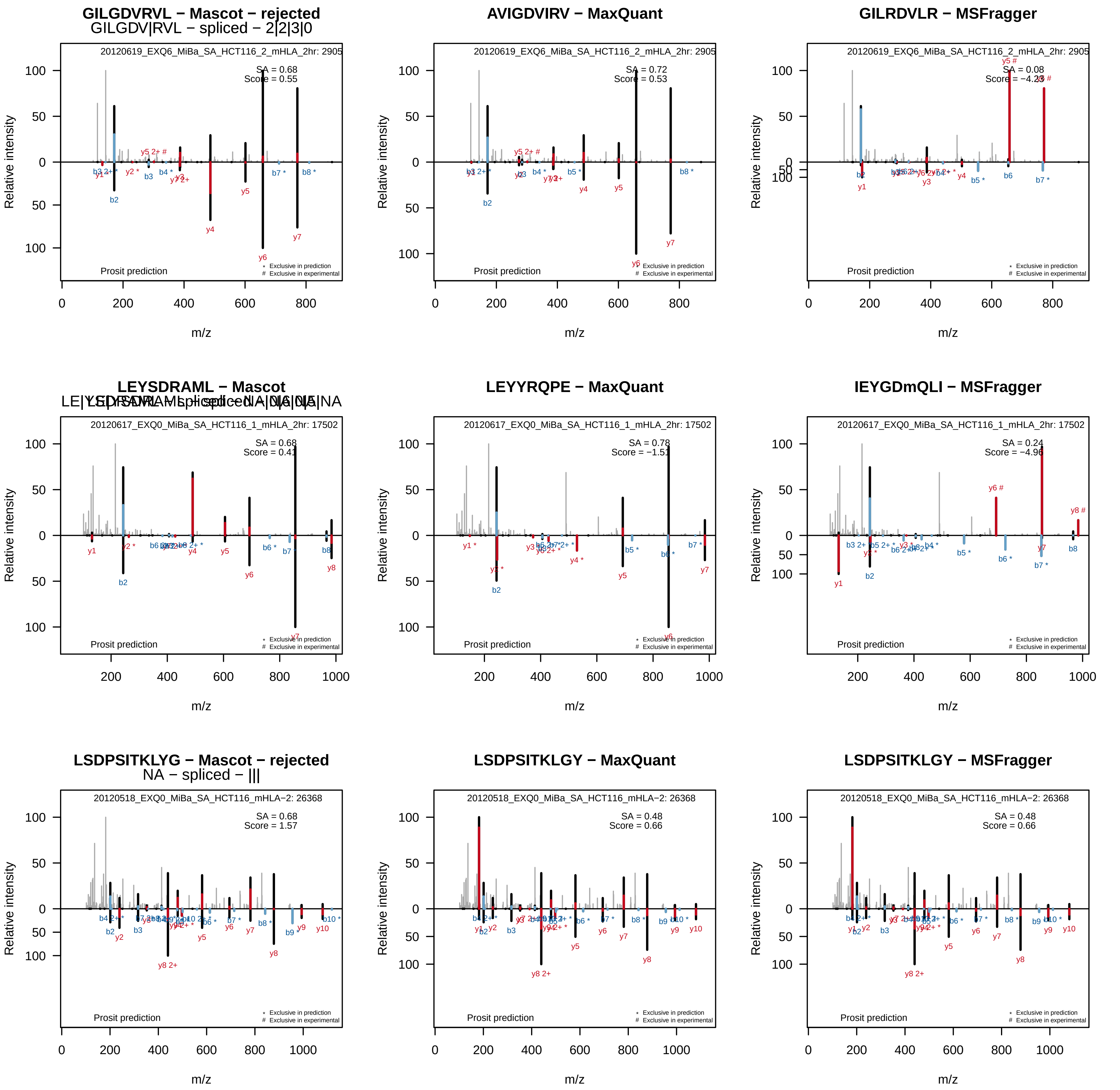
GILGDVRVL − Mascot − rejected
GILGDV|RVL − spliced − 2|2|3|0
20120619_EXQ6_MiBa_SA_HCT116_2_mHLA_2hr: 2905
SA = 0.68
Score = 0.55
AVIGDVIRV − MaxQuant
20120619_EXQ6_MiBa_SA_HCT116_2_mHLA_2hr: 2905
SA = 0.72
Score = 0.53
GILRDVLR − MSFragger
20120619_EXQ6_MiBa_SA_HCT116_2_mHLA_2hr: 2905
SA = 0.08
LEYSDRAML − Mascot
20120617_EXQ0_MiBa_SA_HCT116_1_mHLA_2hr: 17502
SA = 0.68
Score = 0.41
LEYYRQPE − MaxQuant
20120617_EXQ0_MiBa_SA_HCT116_1_mHLA_2hr: 17502
SA = 0.78
Score = −1.51
IEYGDmQLI − MSFragger
20120617_EXQ0_MiBa_SA_HCT116_1_mHLA_2hr: 17502
SA = 0.24
Score = −4.96
LSDPSITKLYG − Mascot − rejected
NA − spliced − |||
20120518_EXQ0_MiBa_SA_HCT116_mHLA−2: 26368
SA = 0.68
Score = 1.57
LSDPSITKLGY − MaxQuant
20120518_EXQ0_MiBa_SA_HCT116_mHLA−2: 26368
SA = 0.48
Score = 0.66
LSDPSITKLGY − MSFragger
20120518_EXQ0_MiBa_SA_HCT116_mHLA−2: 26368
SA = 0.48
Score = 0.66
Relative intensity
m/z
Prosit prediction
⋆ Exclusive in prediction
Exclusive in experimental

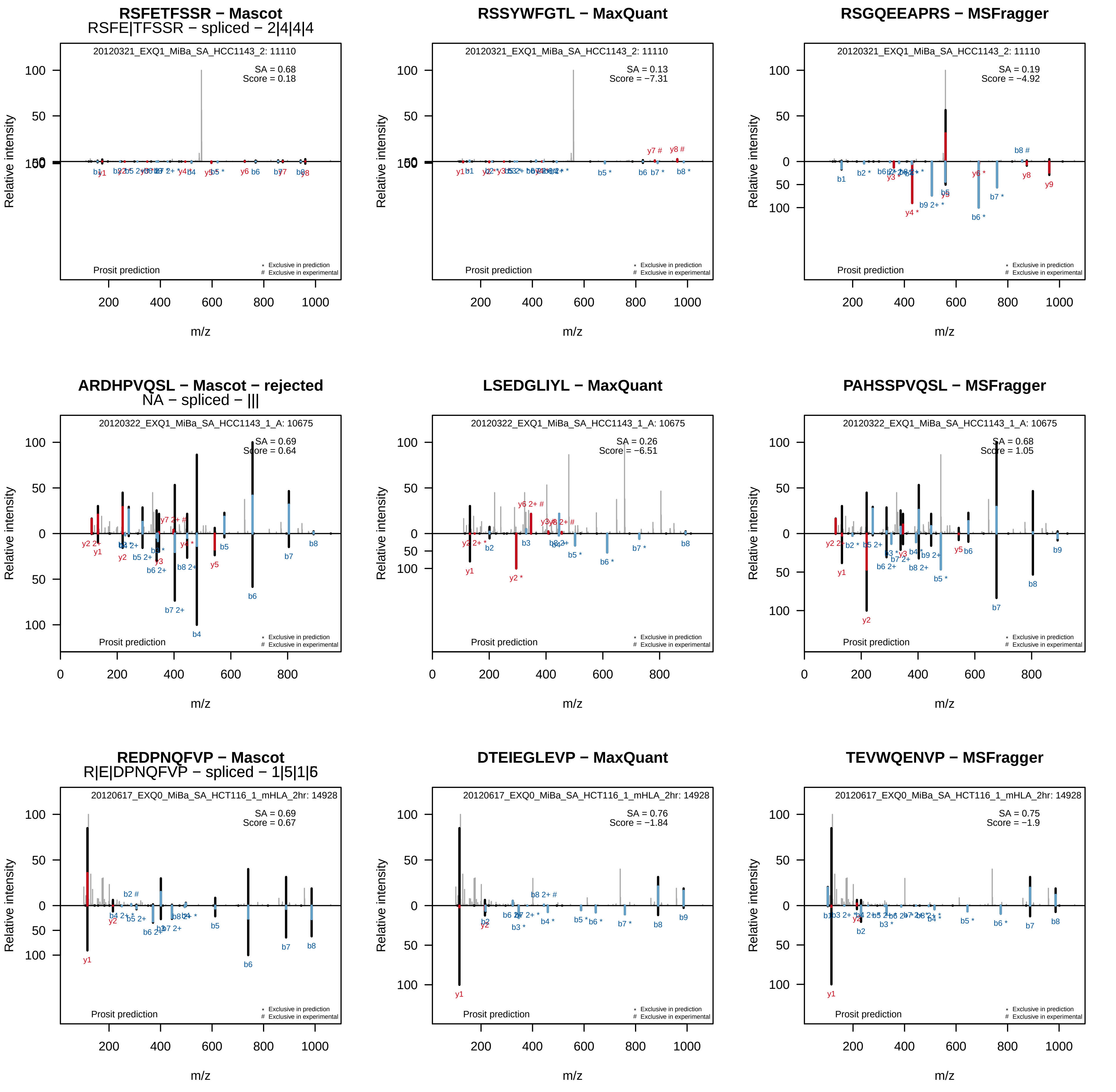
RSFETFSSR − Mascot
RSFE|TFSSR − spliced − 2|4|4|4
20120321_EXQ1_MiBa_SA_HCC1143_2: 11110
SA = 0.68
Score = 0.18
RSSYWFGTL − MaxQuant
20120321_EXQ1_MiBa_SA_HCC1143_2: 11110
SA = 0.13
Score = −7.31
RSGQEEAPRS − MSFragger
20120321_EXQ1_MiBa_SA_HCC1143_2: 11110
SA = 0.19
Score = −4.92
ARDHPVQSL − Mascot − rejected
NA − spliced − |||
20120322_EXQ1_MiBa_SA_HCC1143_1_A: 10675
SA = 0.69
Score = 0.64
LSEDGLIYL − MaxQuant
20120322_EXQ1_MiBa_SA_HCC1143_1_A: 10675
SA = 0.26
Score = −6.51
PAHSSPVQSL − MSFragger
20120322_EXQ1_MiBa_SA_HCC1143_1_A: 10675
SA = 0.68
Score = 1.05
REDPNQFVP − Mascot
R|E|DPNQFVP − spliced − 1|5|1|6
20120617_EXQ0_MiBa_SA_HCT116_1_mHLA_2hr: 14928
SA = 0.69
Score = 0.67
DTEIEGLEVP − MaxQuant
20120617_EXQ0_MiBa_SA_HCT116_1_mHLA_2hr: 14928
SA = 0.76
Score = −1.84
TEVWQENVP − MSFragger
20120617_EXQ0_MiBa_SA_HCT116_1_mHLA_2hr: 14928
SA = 0.75
Score = −1.9
Relative intensity
m/z
Prosit prediction
* Exclusive in prediction
# Exclusive in experimental

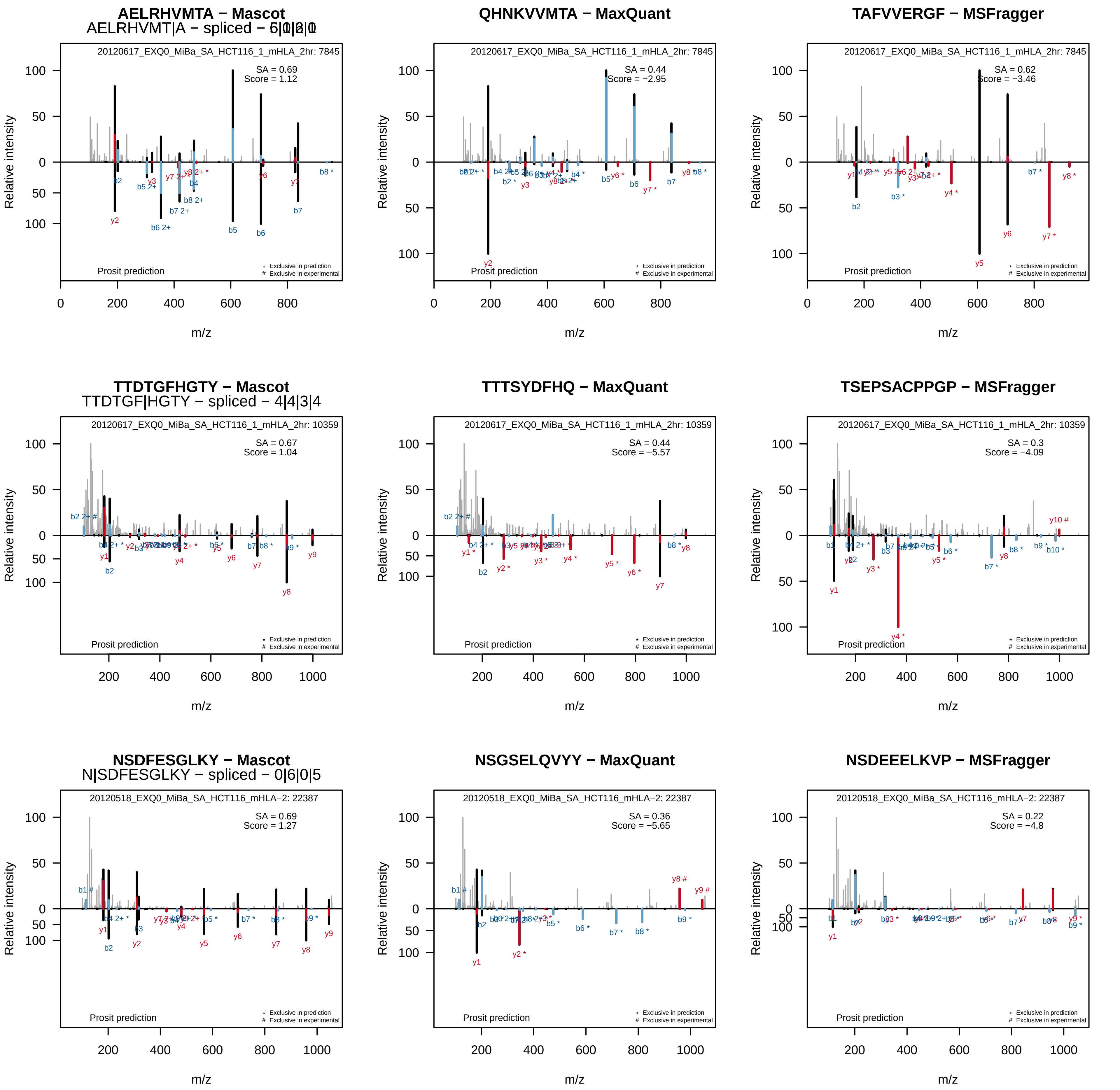

AELRHVMTA − Mascot
AELRHVMT|A − spliced − 6|1|2|1
20120617_EXQ0_MiBa_SA_HCT116_1_mHLA_2hr: 7845
SA = 0.69
Score = 1.12
QHNKVVMTA − MaxQuant
20120617_EXQ0_MiBa_SA_HCT116_1_mHLA_2hr: 7845
SA = 0.44
Score = −2.95
TAFVVERGF − MSFragger
20120617_EXQ0_MiBa_SA_HCT116_1_mHLA_2hr: 7845
SA = 0.62
Score = −3.46
TTDTGFHGTY − Mascot
TTDTGF|HGTY − spliced − 4|4|3|4
20120617_EXQ0_MiBa_SA_HCT116_1_mHLA_2hr: 10359
SA = 0.67
Score = 1.04
TTTSYDFHQ − MaxQuant
20120617_EXQ0_MiBa_SA_HCT116_1_mHLA_2hr: 10359
SA = 0.44
Score = −5.57
TSEPSACPPGP − MSFragger
20120617_EXQ0_MiBa_SA_HCT116_1_mHLA_2hr: 10359
SA = 0.3
Score = −4.09
NSDFESGLKY − Mascot
N|SDFESGLKY − spliced − 0|6|0|5
20120518_EXQ0_MiBa_SA_HCT116_mHLA−2: 22387
SA = 0.69
Score = 1.27
NSGSELQVYY − MaxQuant
20120518_EXQ0_MiBa_SA_HCT116_mHLA−2: 22387
SA = 0.36
Score = −5.65
NSDEEELKVP − MSFragger
20120518_EXQ0_MiBa_SA_HCT116_mHLA−2: 22387
SA = 0.22
Score = −4.8
Relative intensity
m/z
Prosit prediction
* Exclusive in prediction
# Exclusive in experimental

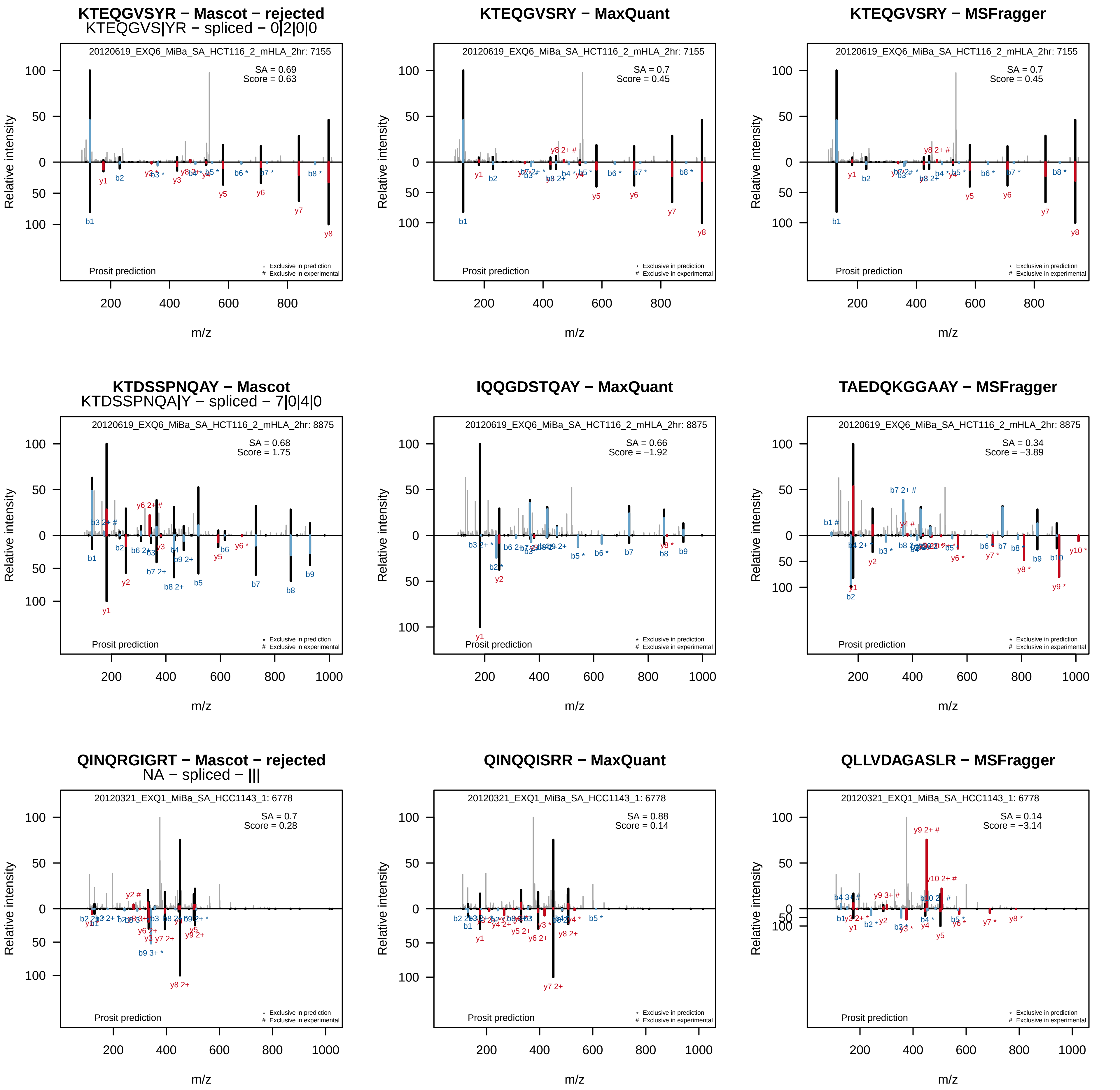
KTEQGVSYR − Mascot − rejected
KTEQGVS|YR − spliced − 0|2|0|0
20120619_EXQ6_MiBa_SA_HCT116_2_mHLA_2hr: 7155
SA = 0.69
Score = 0.63
KTEQGVSRY − MaxQuant
20120619_EXQ6_MiBa_SA_HCT116_2_mHLA_2hr: 7155
SA = 0.7
Score = 0.45
KTEQGVSRY − MSFragger
20120619_EXQ6_MiBa_SA_HCT116_2_mHLA_2hr: 7155
SA = 0.7
Score = 0.45
KTDSSPNQAY − Mascot
KTDSSPNQA|Y − spliced − 7|0|4|0
20120619_EXQ6_MiBa_SA_HCT116_2_mHLA_2hr: 8875
SA = 0.68
Score = 1.75
IQQGDSTQAY − MaxQuant
20120619_EXQ6_MiBa_SA_HCT116_2_mHLA_2hr: 8875
SA = 0.66
Score = −1.92
TAEDQKGGAAY − MSFragger
20120619_EXQ6_MiBa_SA_HCT116_2_mHLA_2hr: 8875
SA = 0.34
Score = −3.89
QINQRGIGRT − Mascot − rejected
NA − spliced − |||
20120321_EXQ1_MiBa_SA_HCC1143_1: 6778
SA = 0.7
Score = 0.28
QINQQISRR − MaxQuant
20120321_EXQ1_MiBa_SA_HCC1143_1: 6778
SA = 0.88
Score = 0.14
QLLVDAGASLR − MSFragger
20120321_EXQ1_MiBa_SA_HCC1143_1: 6778
SA = 0.14
Score = −3.14
Relative intensity
m/z
Prosit prediction
* Exclusive in prediction
# Exclusive in experimental

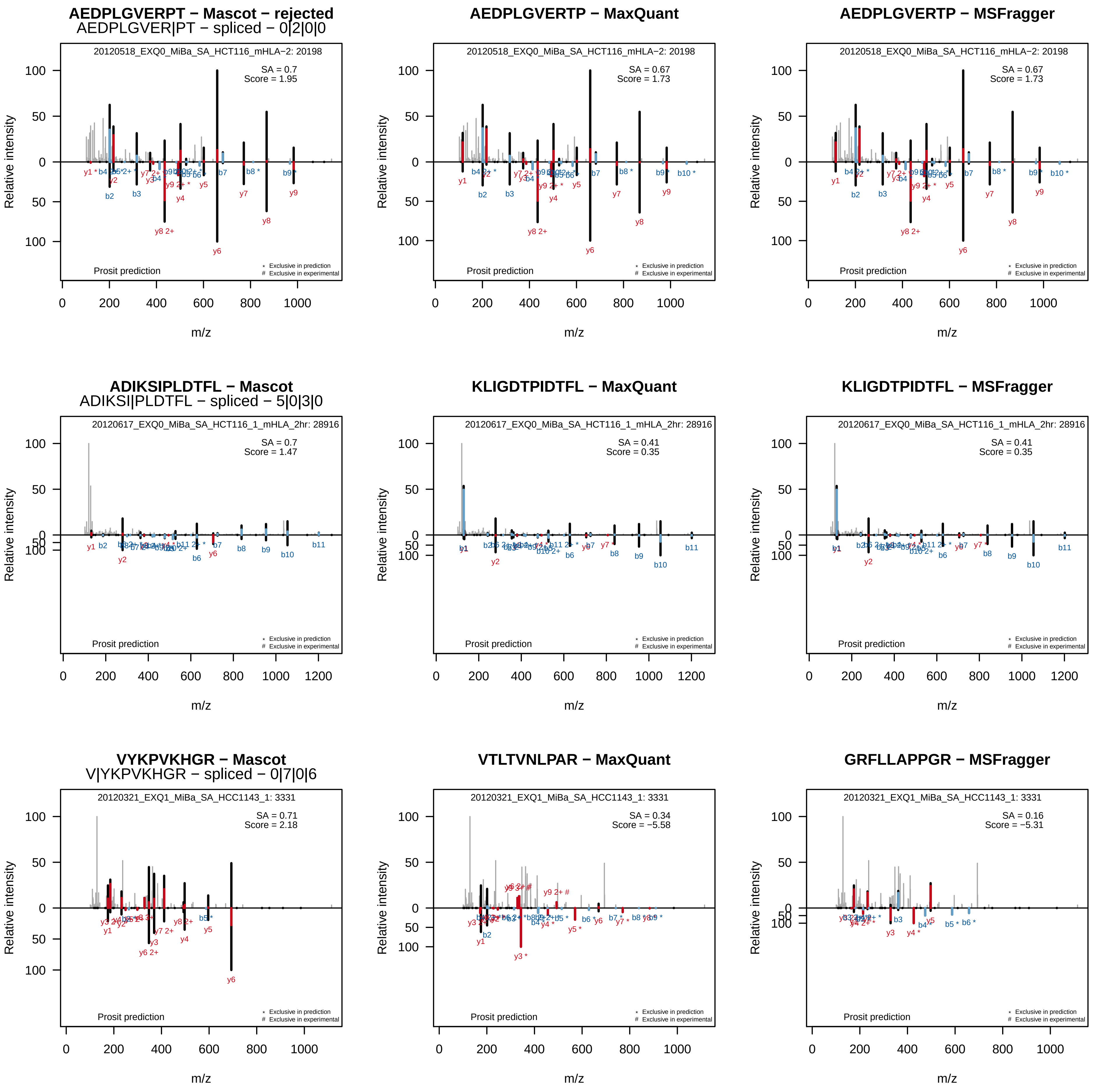

AEDPLGVERPT − Mascot − rejected
AEDPLGVER|PT − spliced − 0|2|0|0
20120518_EXQ0_MiBa_SA_HCT116_mHLA−2: 20198
SA = 0.7
Score = 1.95
AEDPLGVERTP − MaxQuant
20120518_EXQ0_MiBa_SA_HCT116_mHLA−2: 20198
SA = 0.67
Score = 1.73
AEDPLGVERTP − MSFragger
20120518_EXQ0_MiBa_SA_HCT116_mHLA−2: 20198
SA = 0.67
Score = 1.73
ADIKSIPLDTFL − Mascot
ADIKSI|PLDTFL − spliced − 5|0|3|0
20120617_EXQ0_MiBa_SA_HCT116_1_mHLA_2hr: 28916
SA = 0.7
Score = 1.47
KLIGDTPIDTFL − MaxQuant
20120617_EXQ0_MiBa_SA_HCT116_1_mHLA_2hr: 28916
SA = 0.41
Score = 0.35
KLIGDTPIDTFL − MSFragger
20120617_EXQ0_MiBa_SA_HCT116_1_mHLA_2hr: 28916
SA = 0.41
Score = 0.35
VYKPVKHGR − Mascot
V|YKPVKHGR − spliced − 0|7|0|6
20120321_EXQ1_MiBa_SA_HCC1143_1: 3331
SA = 0.71
Score = 2.18
VTLTVNLPAR − MaxQuant
20120321_EXQ1_MiBa_SA_HCC1143_1: 3331
SA = 0.34
Score = −5.58
GRFLLAPPGR − MSFragger
20120321_EXQ1_MiBa_SA_HCC1143_1: 3331
SA = 0.16
Score = −5.31
Relative intensity
m/z
Prosit prediction
* Exclusive in prediction
# Exclusive in experimental

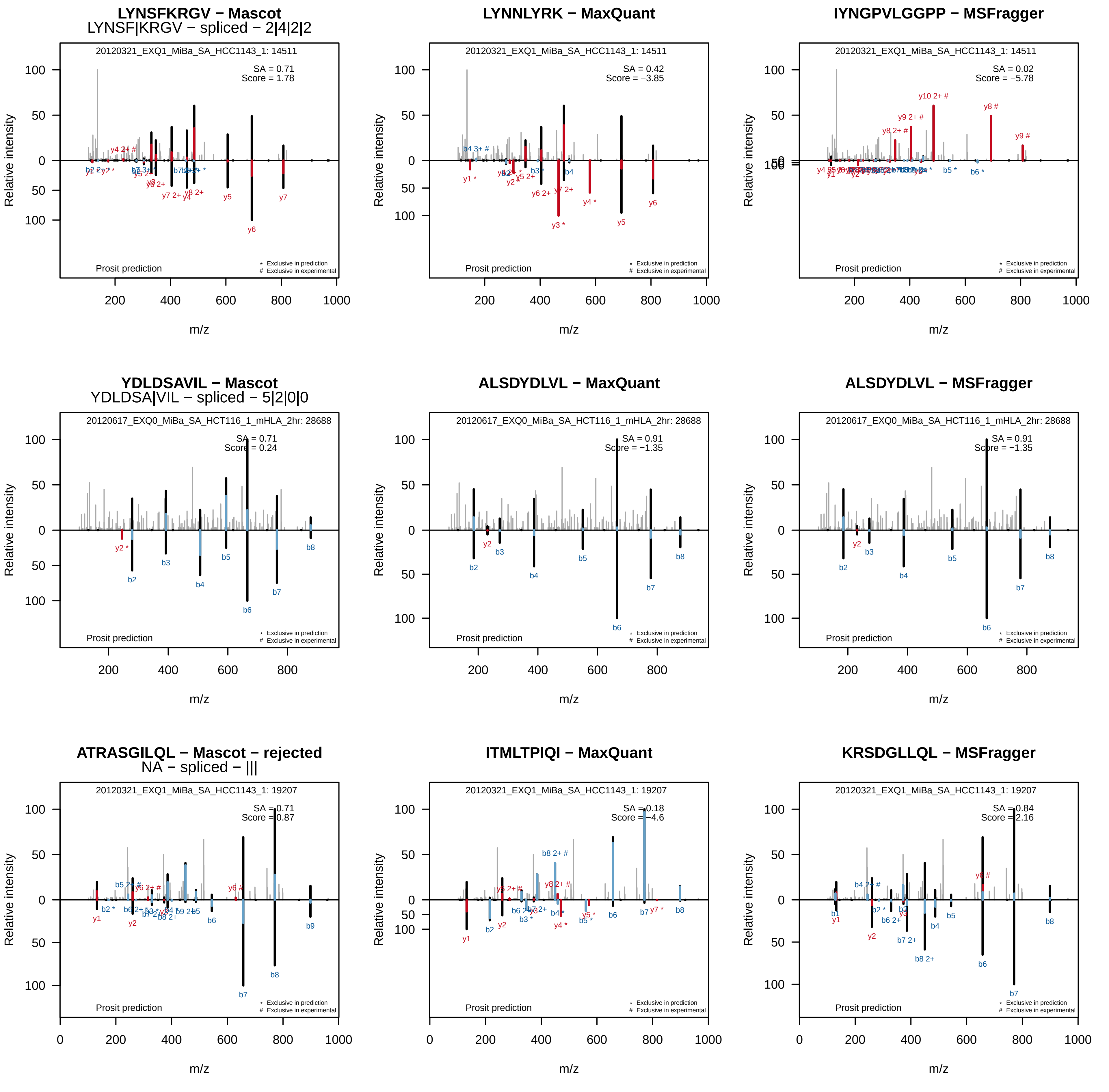

LYNSFKRGV − Mascot
LYNSF|KRGV − spliced − 2|4|2|2
20120321_EXQ1_MiBa_SA_HCC1143_1: 14511
SA = 0.71
Score = 1.78
Prosit prediction
* Exclusive in prediction
# Exclusive in experimental
LYNNLYRK − MaxQuant
20120321_EXQ1_MiBa_SA_HCC1143_1: 14511
SA = 0.42
Score = −3.85
Prosit prediction
* Exclusive in prediction
# Exclusive in experimental
IYNGPVLGGPP − MSFragger
20120321_EXQ1_MiBa_SA_HCC1143_1: 14511
SA = 0.02
Score = −5.78
Prosit prediction
* Exclusive in prediction
# Exclusive in experimental
YDLDSAVIL − Mascot
YDLDSA|VIL − spliced − 5|2|0|0
20120617_EXQ0_MiBa_SA_HCT116_1_mHLA_2hr: 28688
SA = 0.71
Score = 0.24
Prosit prediction
* Exclusive in prediction
# Exclusive in experimental
ALSDYDLVL − MaxQuant
20120617_EXQ0_MiBa_SA_HCT116_1_mHLA_2hr: 28688
SA = 0.91
Score = −1.35
Prosit prediction
* Exclusive in prediction
# Exclusive in experimental
ALSDYDLVL − MSFragger
20120617_EXQ0_MiBa_SA_HCT116_1_mHLA_2hr: 28688
SA = 0.91
Score = −1.35
Prosit prediction
* Exclusive in prediction
# Exclusive in experimental
ATRASGILQL − Mascot − rejected
NA − spliced − |||
20120321_EXQ1_MiBa_SA_HCC1143_1: 19207
SA = 0.71
Score = 0.87
Prosit prediction
* Exclusive in prediction
# Exclusive in experimental
ITMLTPIQI − MaxQuant
20120321_EXQ1_MiBa_SA_HCC1143_1: 19207
SA = 0.18
Score = −4.6
Prosit prediction
* Exclusive in prediction
# Exclusive in experimental
KRSDGLLQL − MSFragger
20120321_EXQ1_MiBa_SA_HCC1143_1: 19207
SA = 0.84
Score = 2.16
Prosit prediction
* Exclusive in prediction
# Exclusive in experimental
Relative intensity
m/z

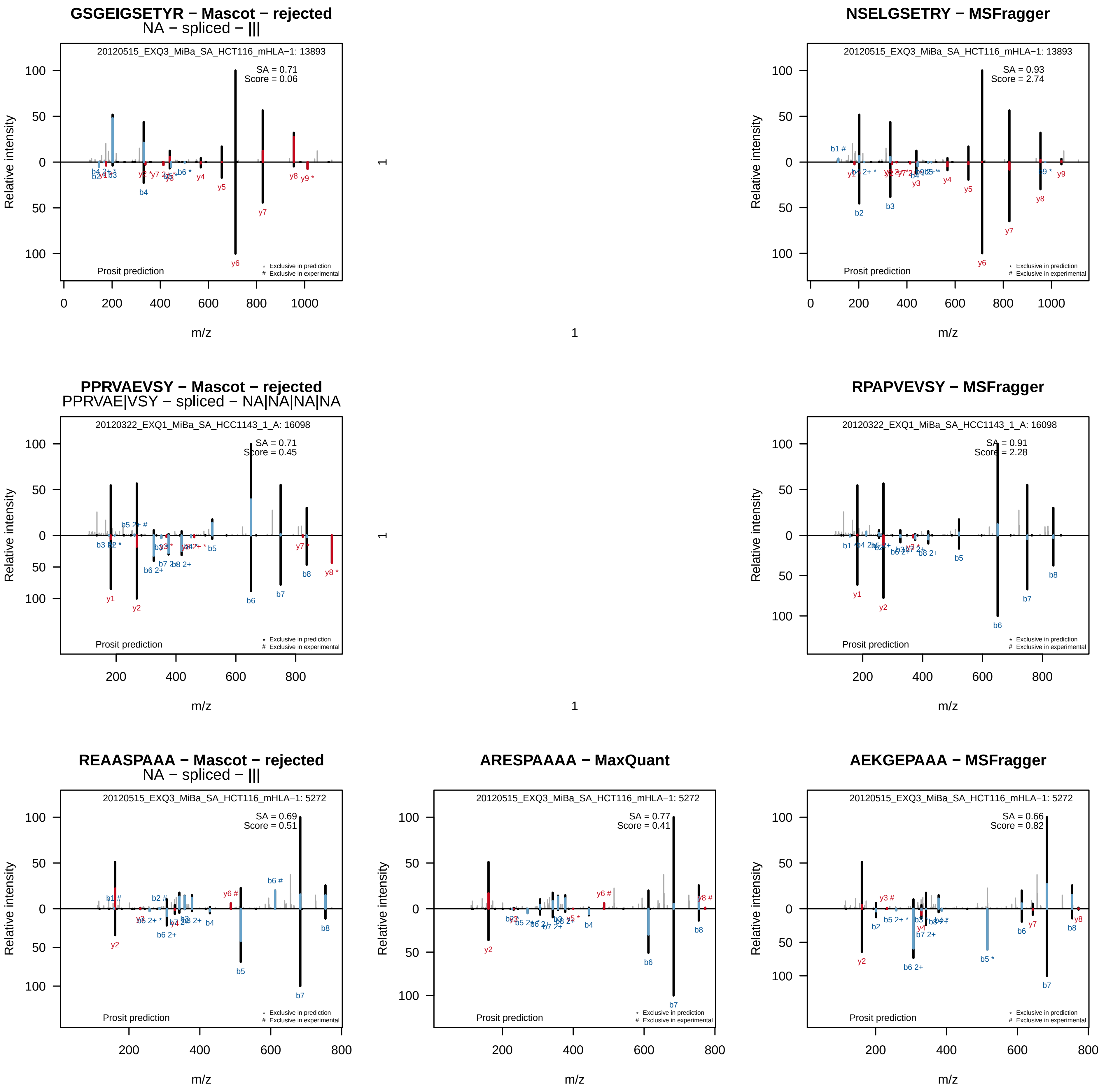
GSGEIGSETYR − Mascot − rejected
NA − spliced − |||
20120515_EXQ3_MiBa_SA_HCT116_mHLA−1: 13893
SA = 0.71
Score = 0.06
Prosit prediction
Exclusive in prediction
Exclusive in experimental
NSELGSETRY − MSFragger
20120515_EXQ3_MiBa_SA_HCT116_mHLA−1: 13893
SA = 0.93
Score = 2.74
PPRVAEVSY − Mascot − rejected
PPRVAE|VSY − spliced − NA|NA|NA|NA
20120322_EXQ1_MiBa_SA_HCC1143_1_A: 16098
SA = 0.71
Score = 0.45
RPAPVEVSY − MSFragger
20120322_EXQ1_MiBa_SA_HCC1143_1_A: 16098
SA = 0.91
Score = 2.28
REAASPAAA − Mascot − rejected
NA − spliced − |||
20120515_EXQ3_MiBa_SA_HCT116_mHLA−1: 5272
SA = 0.69
Score = 0.51
ARESPAAAA − MaxQuant
20120515_EXQ3_MiBa_SA_HCT116_mHLA−1: 5272
SA = 0.77
Score = 0.41
AEKGEPAAA − MSFragger
20120515_EXQ3_MiBa_SA_HCT116_mHLA−1: 5272
SA = 0.66
Score = 0.82
Relative intensity
m/z

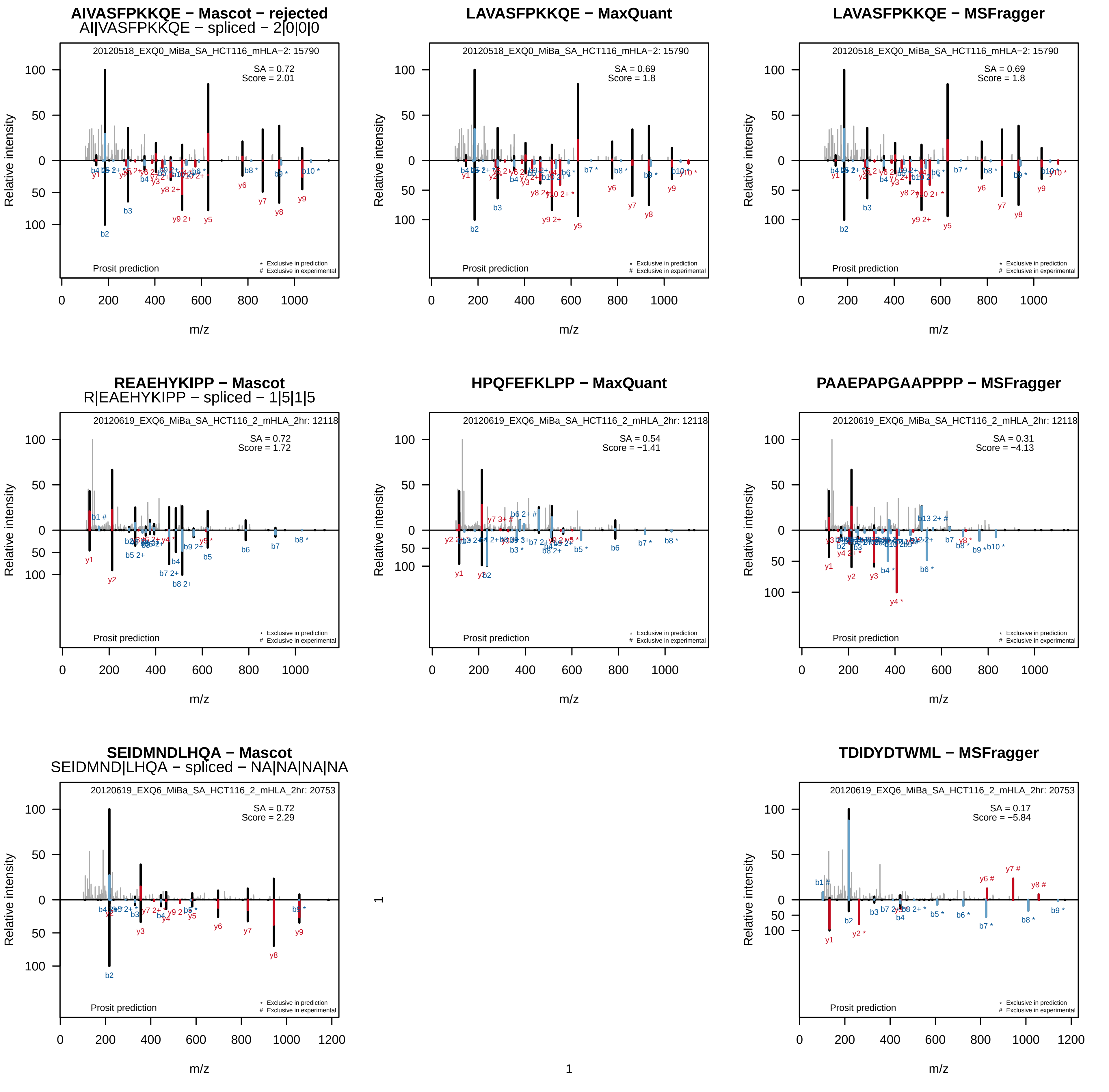
AIVASFPKKQE − Mascot − rejected
AI|VASFPKKQE − spliced − 2|0|0|0
20120518_EXQ0_MiBa_SA_HCT116_mHLA−2: 15790
SA = 0.72
Score = 2.01
LAVASFPKKQE − MaxQuant
20120518_EXQ0_MiBa_SA_HCT116_mHLA−2: 15790
SA = 0.69
Score = 1.8
LAVASFPKKQE − MSFragger
20120518_EXQ0_MiBa_SA_HCT116_mHLA−2: 15790
SA = 0.69
Score = 1.8
REAEHYKIPP − Mascot
R|EAEHYKIPP − spliced − 1|5|1|5
20120619_EXQ6_MiBa_SA_HCT116_2_mHLA_2hr: 12118
SA = 0.72
Score = 1.72
HPQFEFKLPP − MaxQuant
20120619_EXQ6_MiBa_SA_HCT116_2_mHLA_2hr: 12118
SA = 0.54
Score = −1.41
PAAEPAPGAAPPPP − MSFragger
20120619_EXQ6_MiBa_SA_HCT116_2_mHLA_2hr: 12118
SA = 0.31
Score = −4.13
SEIDMNDLHQA − Mascot
SEIDMND|LHQA − spliced − NA|NA|NA|NA
20120619_EXQ6_MiBa_SA_HCT116_2_mHLA_2hr: 20753
SA = 0.72
Score = 2.29
TDIDYDTWML − MSFragger
20120619_EXQ6_MiBa_SA_HCT116_2_mHLA_2hr: 20753
SA = 0.17
Score = −5.84
Relative intensity
m/z
Prosit prediction
* Exclusive in prediction
# Exclusive in experimental

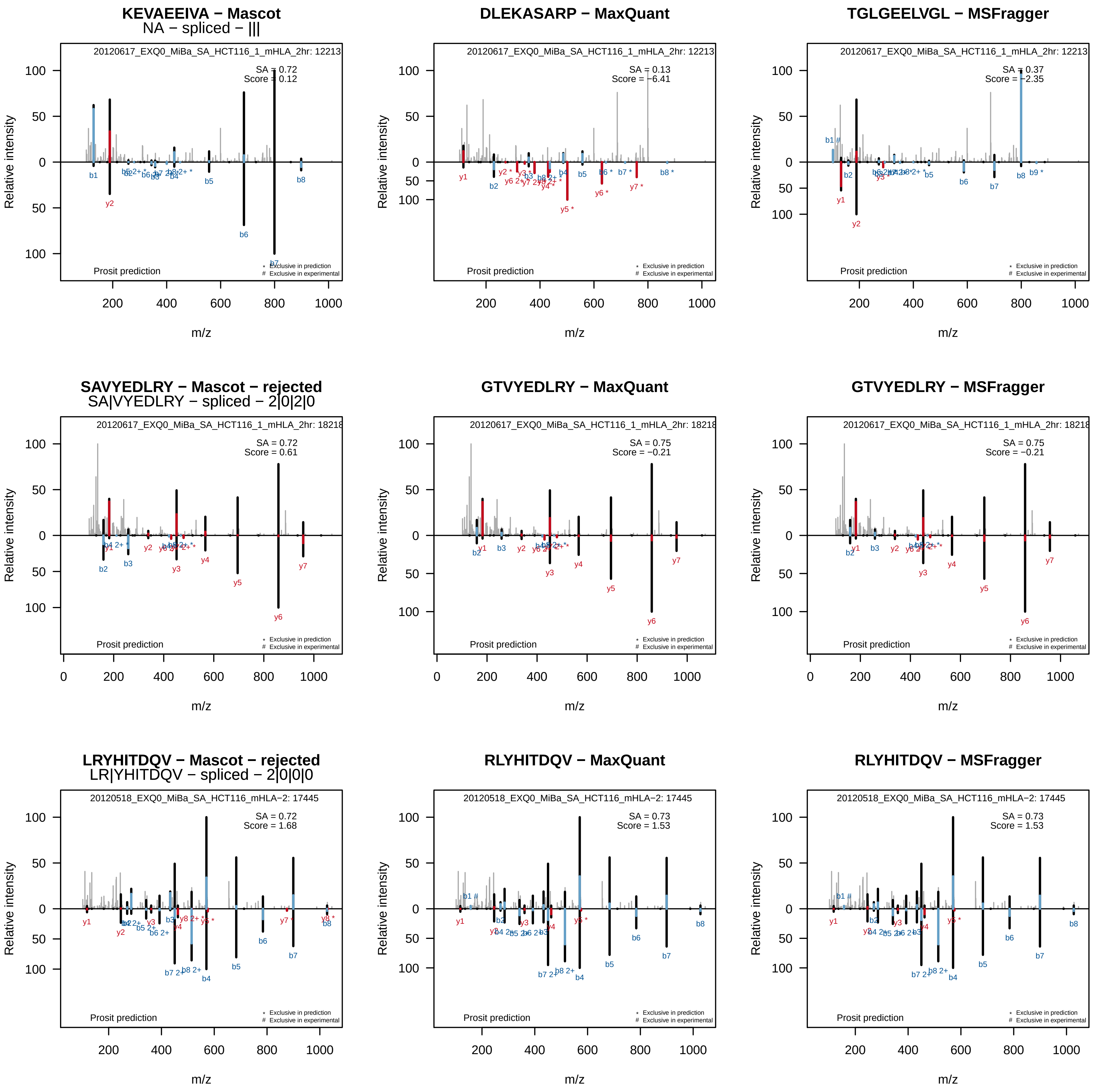
KEVAEEIVA − Mascot
NA − spliced − |||
20120617_EXQ0_MiBa_SA_HCT116_1_mHLA_2hr: 12213
SA = 0.72
Score = 0.12
DLEKASARP − MaxQuant
20120617_EXQ0_MiBa_SA_HCT116_1_mHLA_2hr: 12213
SA = 0.13
Score = −6.41
TGLGEELVGL − MSFragger
20120617_EXQ0_MiBa_SA_HCT116_1_mHLA_2hr: 12213
SA = 0.37
Score = −2.35
SAVYEDLRY − Mascot − rejected
SA|VYEDLRY − spliced − 2|0|2|0
20120617_EXQ0_MiBa_SA_HCT116_1_mHLA_2hr: 18218
SA = 0.72
Score = 0.61
GTVYEDLRY − MaxQuant
20120617_EXQ0_MiBa_SA_HCT116_1_mHLA_2hr: 18218
SA = 0.75
Score = −0.21
GTVYEDLRY − MSFragger
20120617_EXQ0_MiBa_SA_HCT116_1_mHLA_2hr: 18218
SA = 0.75
Score = −0.21
LRYHITDQV − Mascot − rejected
LR|YHITDQV − spliced − 2|0|0|0
20120518_EXQ0_MiBa_SA_HCT116_mHLA−2: 17445
SA = 0.72
Score = 1.68
RLYHITDQV − MaxQuant
20120518_EXQ0_MiBa_SA_HCT116_mHLA−2: 17445
SA = 0.73
Score = 1.53
RLYHITDQV − MSFragger
20120518_EXQ0_MiBa_SA_HCT116_mHLA−2: 17445
SA = 0.73
Score = 1.53
Relative intensity
m/z
Prosit prediction
* Exclusive in prediction
# Exclusive in experimental

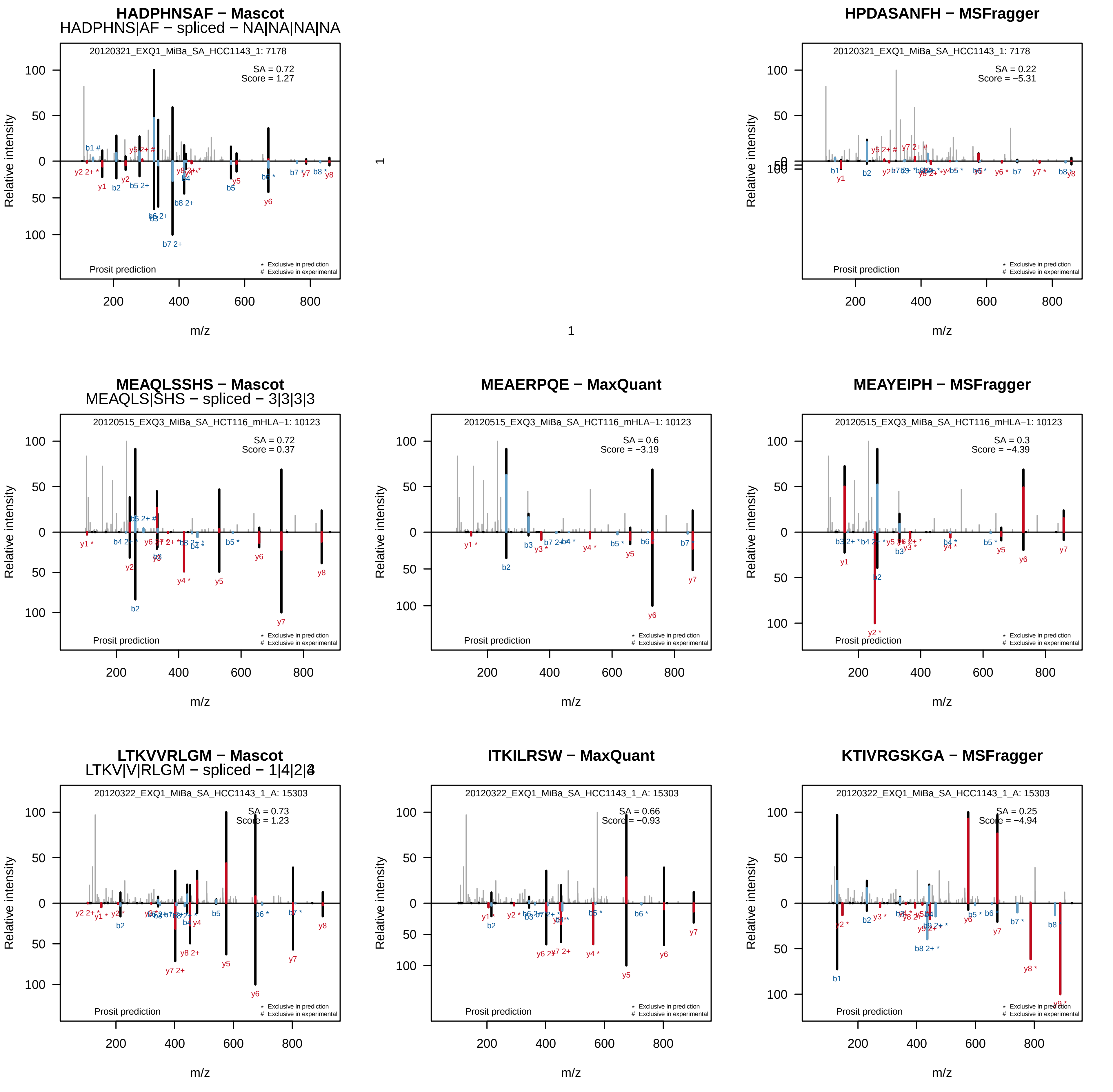

HADPHNSAF − Mascot
HADPHNS|AF − spliced − NA|NA|NA|NA
20120321_EXQ1_MiBa_SA_HCC1143_1: 7178
SA = 0.72
Score = 1.27
HPDASANFH − MSFragger
20120321_EXQ1_MiBa_SA_HCC1143_1: 7178
SA = 0.22
Score = −5.31
MEAQLSSHS − Mascot
MEAQLS|SHS − spliced − 3|3|3|3
20120515_EXQ3_MiBa_SA_HCT116_mHLA−1: 10123
SA = 0.72
Score = 0.37
MEAERPQE − MaxQuant
20120515_EXQ3_MiBa_SA_HCT116_mHLA−1: 10123
SA = 0.6
Score = −3.19
MEAYEIPH − MSFragger
20120515_EXQ3_MiBa_SA_HCT116_mHLA−1: 10123
SA = 0.3
Score = −4.39
LTKVVRLGM − Mascot
LTKV|V|RLGM − spliced − 1|4|2|4
20120322_EXQ1_MiBa_SA_HCC1143_1_A: 15303
SA = 0.73
Score = 1.23
ITKILRSW − MaxQuant
20120322_EXQ1_MiBa_SA_HCC1143_1_A: 15303
SA = 0.66
Score = −0.93
KTIVRGSKGA − MSFragger
20120322_EXQ1_MiBa_SA_HCC1143_1_A: 15303
SA = 0.25
Score = −4.94
Relative intensity
m/z
Prosit prediction
* Exclusive in prediction
# Exclusive in experimental

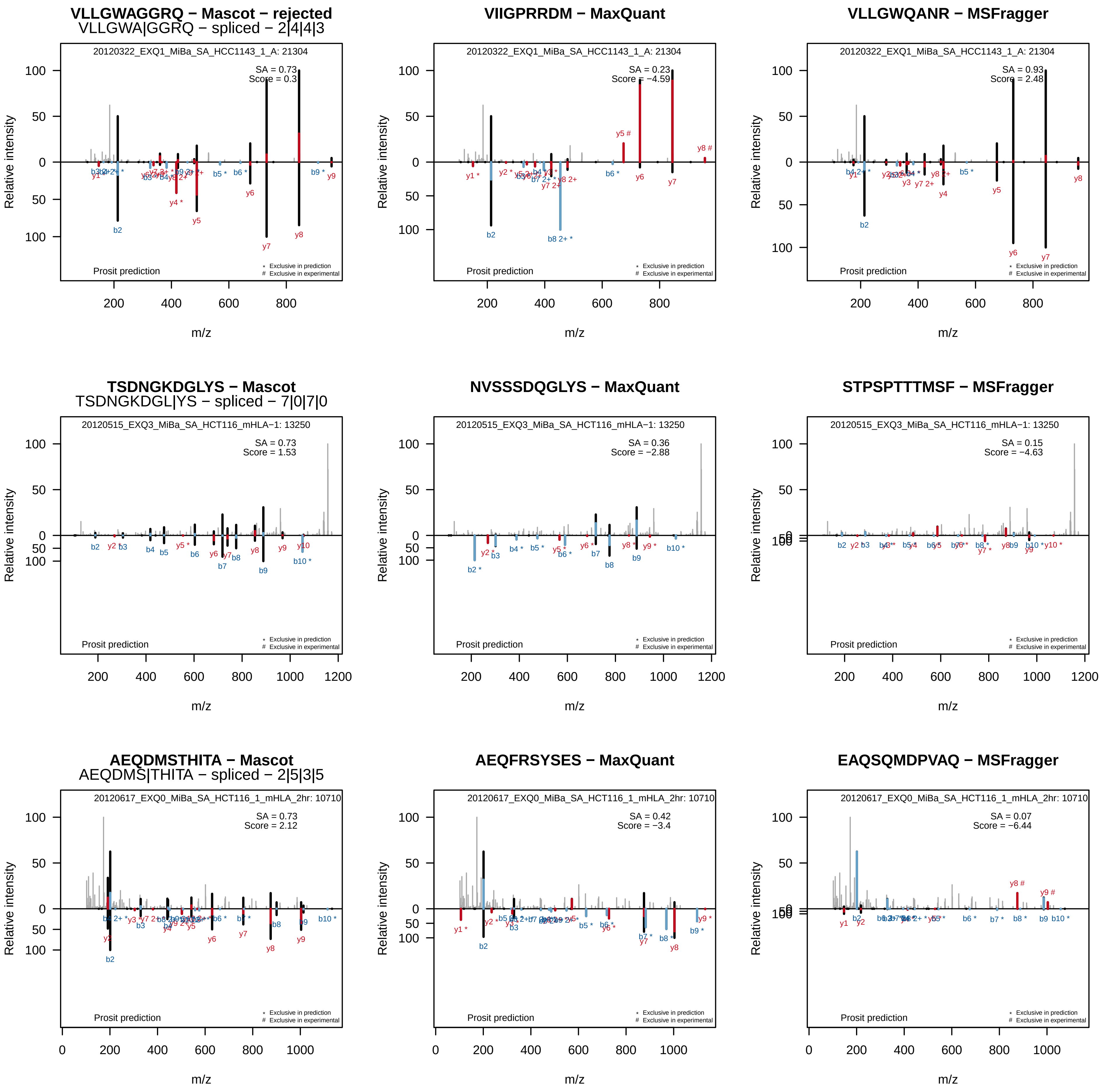

VLLGWAGGRQ − Mascot − rejected
VLLGWA|GGRQ − spliced − 2|4|4|3
20120322_EXQ1_MiBa_SA_HCC1143_1_A: 21304
SA = 0.73
Score = 0.3
Prosit prediction
⋆ Exclusive in prediction
Exclusive in experimental
VIIGPRRDM − MaxQuant
20120322_EXQ1_MiBa_SA_HCC1143_1_A: 21304
SA = 0.23
Score = −4.59
Prosit prediction
⋆ Exclusive in prediction
Exclusive in experimental
VLLGWQANR − MSFragger
20120322_EXQ1_MiBa_SA_HCC1143_1_A: 21304
SA = 0.93
Score = 2.48
Prosit prediction
⋆ Exclusive in prediction
Exclusive in experimental
TSDNGKDGLYS − Mascot
TSDNGKDGL|YS − spliced − 7|0|7|0
20120515_EXQ3_MiBa_SA_HCT116_mHLA−1: 13250
SA = 0.73
Score = 1.53
Prosit prediction
⋆ Exclusive in prediction
Exclusive in experimental
NVSSSDQGLYS − MaxQuant
20120515_EXQ3_MiBa_SA_HCT116_mHLA−1: 13250
SA = 0.36
Score = −2.88
Prosit prediction
⋆ Exclusive in prediction
Exclusive in experimental
STPSPTTTMSF − MSFragger
20120515_EXQ3_MiBa_SA_HCT116_mHLA−1: 13250
SA = 0.15
Score = −4.63
Prosit prediction
⋆ Exclusive in prediction
Exclusive in experimental
AEQDMSTHITA − Mascot
AEQDMS|THITA − spliced − 2|5|3|5
20120617_EXQ0_MiBa_SA_HCT116_1_mHLA_2hr: 10710
SA = 0.73
Score = 2.12
Prosit prediction
⋆ Exclusive in prediction
Exclusive in experimental
AEQFRSYSES − MaxQuant
20120617_EXQ0_MiBa_SA_HCT116_1_mHLA_2hr: 10710
SA = 0.42
Score = −3.4
Prosit prediction
⋆ Exclusive in prediction
Exclusive in experimental
EAQSQMDPVAQ − MSFragger
20120617_EXQ0_MiBa_SA_HCT116_1_mHLA_2hr: 10710
SA = 0.07
Score = −6.44
Prosit prediction
⋆ Exclusive in prediction
Exclusive in experimental
Relative intensity
m/z

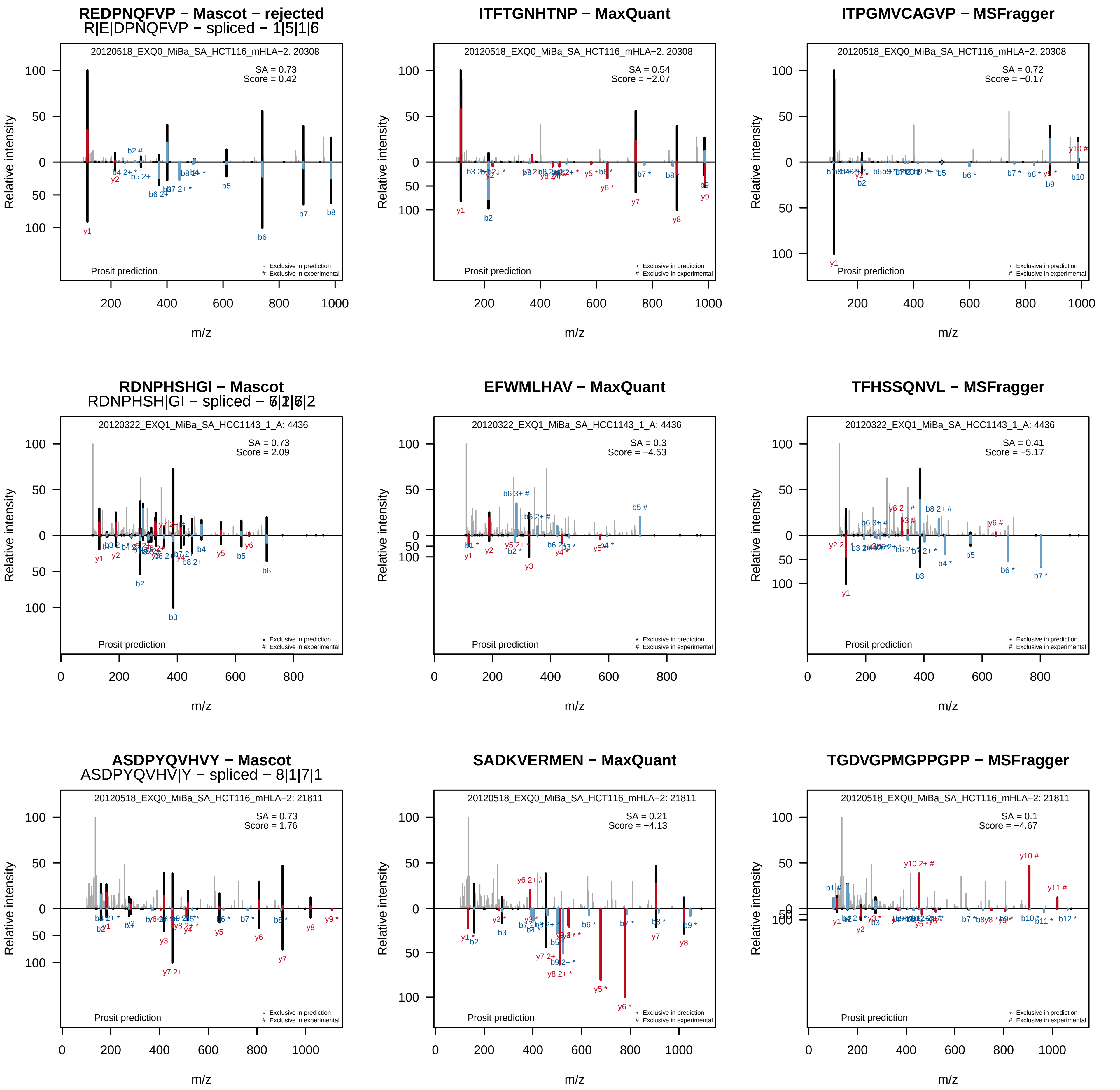

REDPNQFVP − Mascot − rejected
R|E|DPNQFVP − spliced − 1|5|1|6
20120518_EXQ0_MiBa_SA_HCT116_mHLA−2: 20308
SA = 0.73
Score = 0.42
ITFTGNHTNP − MaxQuant
20120518_EXQ0_MiBa_SA_HCT116_mHLA−2: 20308
SA = 0.54
Score = −2.07
ITPGMVCAGVP − MSFragger
20120518_EXQ0_MiBa_SA_HCT116_mHLA−2: 20308
SA = 0.72
Score = −0.17
RDNPHSHGI − Mascot
20120322_EXQ1_MiBa_SA_HCC1143_1_A: 4436
SA = 0.73
Score = 2.09
EFWMLHAV − MaxQuant
20120322_EXQ1_MiBa_SA_HCC1143_1_A: 4436
SA = 0.3
Score = −4.53
TFHSSQNVL − MSFragger
20120322_EXQ1_MiBa_SA_HCC1143_1_A: 4436
SA = 0.41
Score = −5.17
ASDPYQVHVY − Mascot
ASDPYQVHV|Y − spliced − 8|1|7|1
20120518_EXQ0_MiBa_SA_HCT116_mHLA−2: 21811
SA = 0.73
Score = 1.76
SADKVERMEN − MaxQuant
20120518_EXQ0_MiBa_SA_HCT116_mHLA−2: 21811
SA = 0.21
Score = −4.13
TGDVGPMGPPGPP − MSFragger
20120518_EXQ0_MiBa_SA_HCT116_mHLA−2: 21811
SA = 0.1
Score = −4.67
Relative intensity
m/z
Prosit prediction
⋆ Exclusive in prediction
Exclusive in experimental

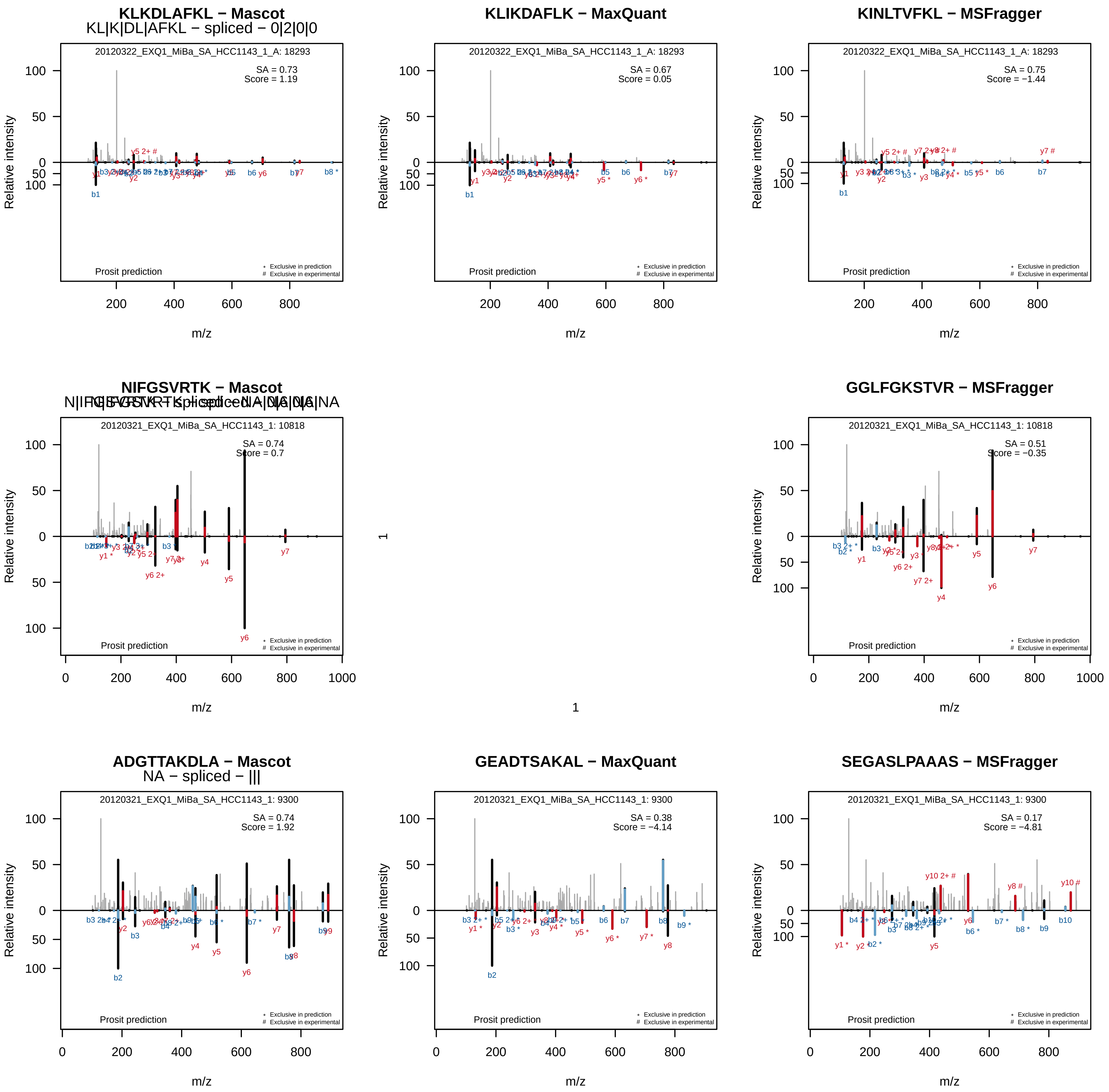

KLKDLAFKL − Mascot
KL|K|DL|AFKL − spliced − 0|2|0|0
20120322_EXQ1_MiBa_SA_HCC1143_1_A: 18293
SA = 0.73
Score = 1.19
KLIKDAFLK − MaxQuant
20120322_EXQ1_MiBa_SA_HCC1143_1_A: 18293
SA = 0.67
Score = 0.05
KINLTVFKL − MSFragger
20120322_EXQ1_MiBa_SA_HCC1143_1_A: 18293
SA = 0.75
Score = −1.44
NIFGSVRTK − Mascot
20120321_EXQ1_MiBa_SA_HCC1143_1: 10818
SA = 0.74
Score = 0.7
GGLFGKSTVR − MSFragger
20120321_EXQ1_MiBa_SA_HCC1143_1: 10818
SA = 0.51
Score = −0.35
ADGTTAKDLA − Mascot
NA − spliced − |||
20120321_EXQ1_MiBa_SA_HCC1143_1: 9300
SA = 0.74
Score = 1.92
GEADTSAKAL − MaxQuant
20120321_EXQ1_MiBa_SA_HCC1143_1: 9300
SA = 0.38
Score = −4.14
SEGASLPAAAS − MSFragger
20120321_EXQ1_MiBa_SA_HCC1143_1: 9300
SA = 0.17
Score = −4.81
Relative intensity
m/z
Prosit prediction
⋆ Exclusive in prediction
Exclusive in experimental

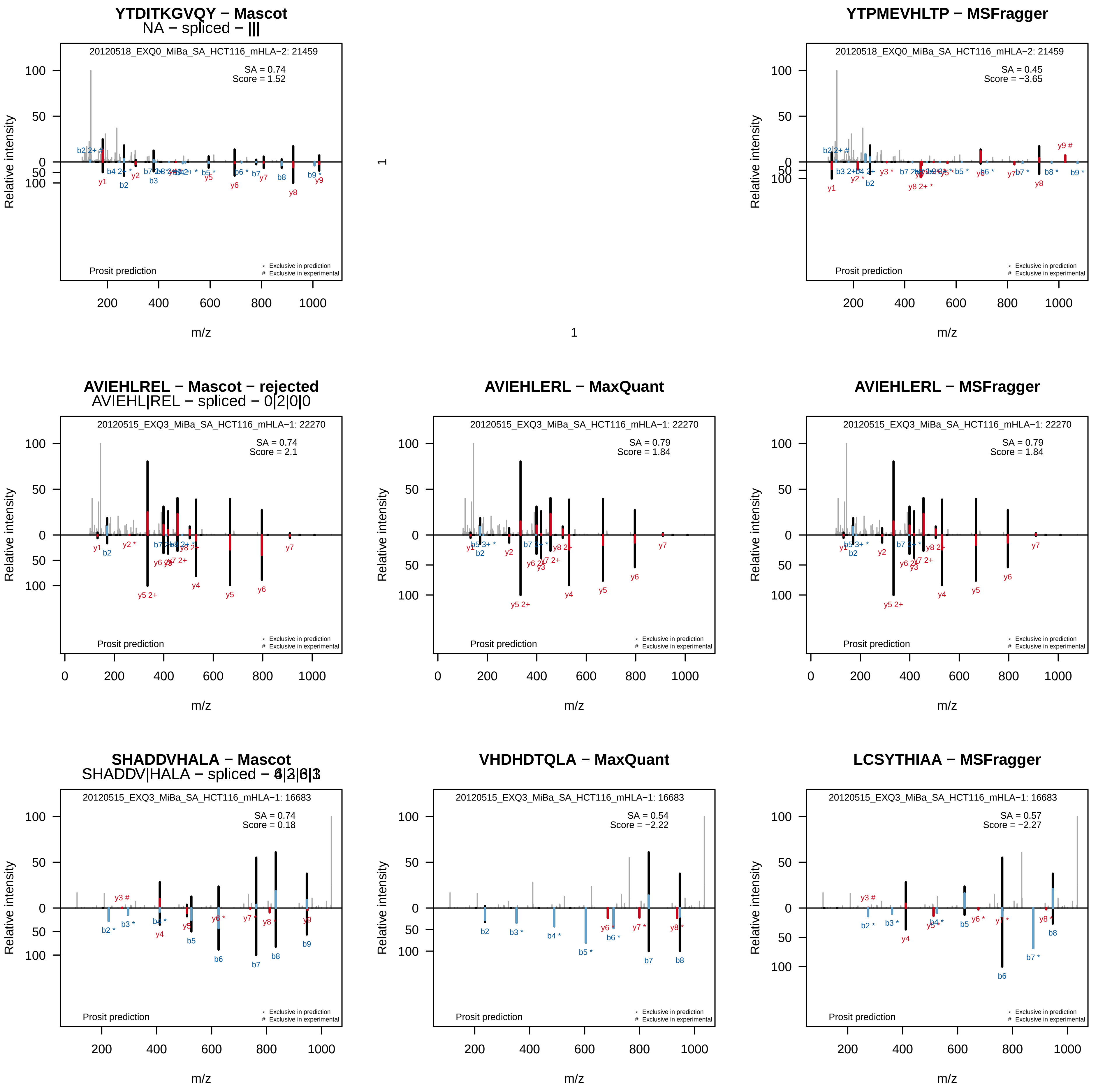
YTDITKGVQY − Mascot
NA − spliced − |||
20120518_EXQ0_MiBa_SA_HCT116_mHLA−2: 21459
SA = 0.74
Score = 1.52
Prosit prediction
YTPMEVHLTP − MSFragger
20120518_EXQ0_MiBa_SA_HCT116_mHLA−2: 21459
SA = 0.45
Score = −3.65
Prosit prediction
AVIEHLREL − Mascot − rejected
AVIEHL|REL − spliced − 0|2|0|0
20120515_EXQ3_MiBa_SA_HCT116_mHLA−1: 22270
SA = 0.74
Score = 2.1
Prosit prediction
AVIEHLERL − MaxQuant
20120515_EXQ3_MiBa_SA_HCT116_mHLA−1: 22270
SA = 0.79
Score = 1.84
Prosit prediction
AVIEHLERL − MSFragger
20120515_EXQ3_MiBa_SA_HCT116_mHLA−1: 22270
SA = 0.79
Score = 1.84
Prosit prediction
SHADDVHALA − Mascot
20120515_EXQ3_MiBa_SA_HCT116_mHLA−1: 16683
SA = 0.74
Score = 0.18
Prosit prediction
VHDHDTQLA − MaxQuant
20120515_EXQ3_MiBa_SA_HCT116_mHLA−1: 16683
SA = 0.54
Score = −2.22
Prosit prediction
LCSYTHIAA − MSFragger
20120515_EXQ3_MiBa_SA_HCT116_mHLA−1: 16683
SA = 0.57
Score = −2.27
Prosit prediction
Relative intensity
m/z

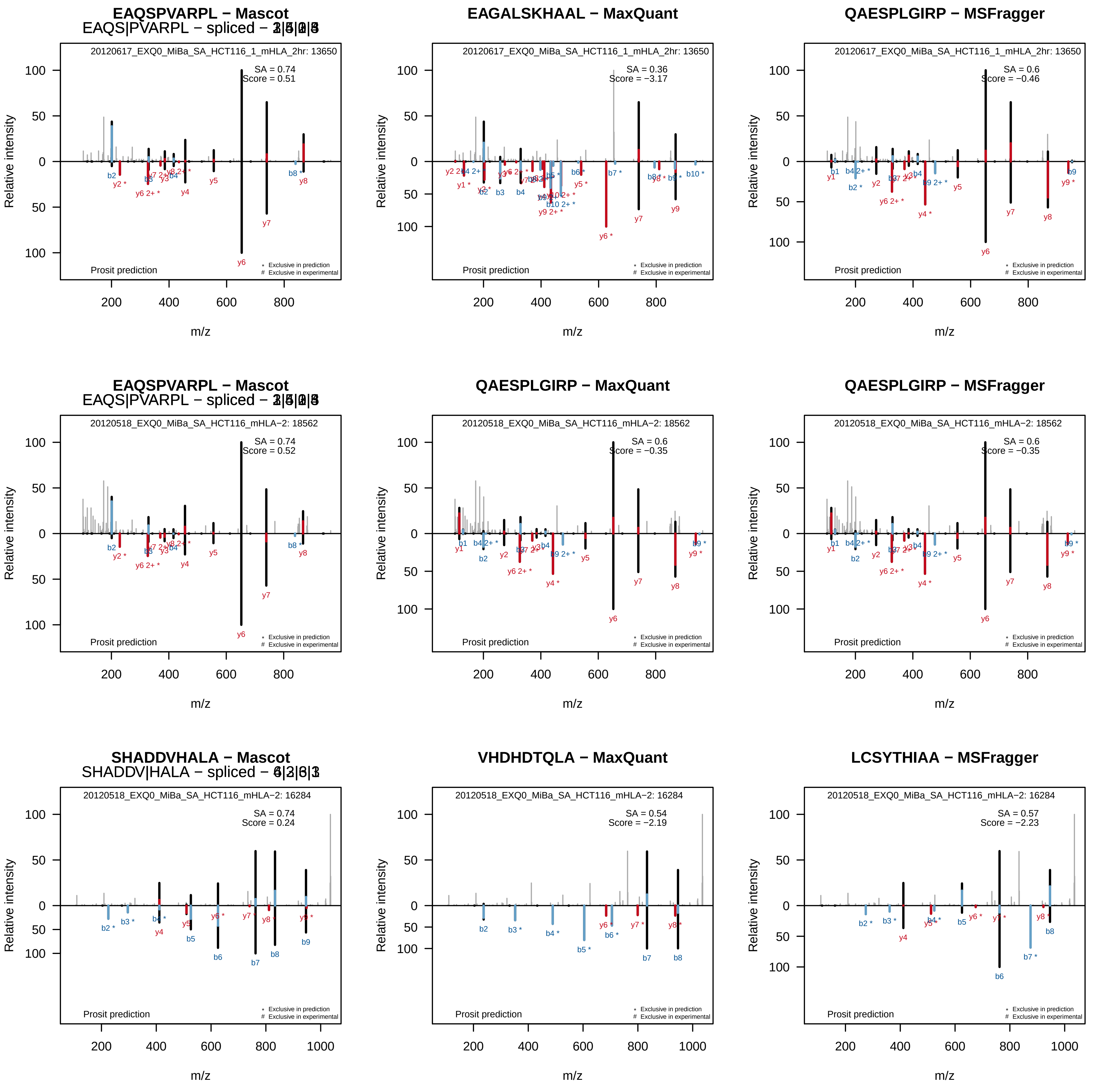
EAQSPVARPL − Mascot
20120617_EXQ0_MiBa_SA_HCT116_1_mHLA_2hr: 13650
SA = 0.74
Score = 0.51
EAGALSKHAAL − MaxQuant
20120617_EXQ0_MiBa_SA_HCT116_1_mHLA_2hr: 13650
SA = 0.36
Score = −3.17
QAESPLGIRP − MSFragger
20120617_EXQ0_MiBa_SA_HCT116_1_mHLA_2hr: 13650
SA = 0.6
Score = −0.46
EAQSPVARPL − Mascot
20120518_EXQ0_MiBa_SA_HCT116_mHLA−2: 18562
SA = 0.74
Score = 0.52
QAESPLGIRP − MaxQuant
20120518_EXQ0_MiBa_SA_HCT116_mHLA−2: 18562
SA = 0.6
Score = −0.35
QAESPLGIRP − MSFragger
20120518_EXQ0_MiBa_SA_HCT116_mHLA−2: 18562
SA = 0.6
Score = −0.35
SHADDVHALA − Mascot
20120518_EXQ0_MiBa_SA_HCT116_mHLA−2: 16284
SA = 0.74
Score = 0.24
VHDHDTQLA − MaxQuant
20120518_EXQ0_MiBa_SA_HCT116_mHLA−2: 16284
SA = 0.54
Score = −2.19
LCSYTHIAA − MSFragger
20120518_EXQ0_MiBa_SA_HCT116_mHLA−2: 16284
SA = 0.57
Score = −2.23
Relative intensity
m/z
Prosit prediction
⋆ Exclusive in prediction
Exclusive in experimental

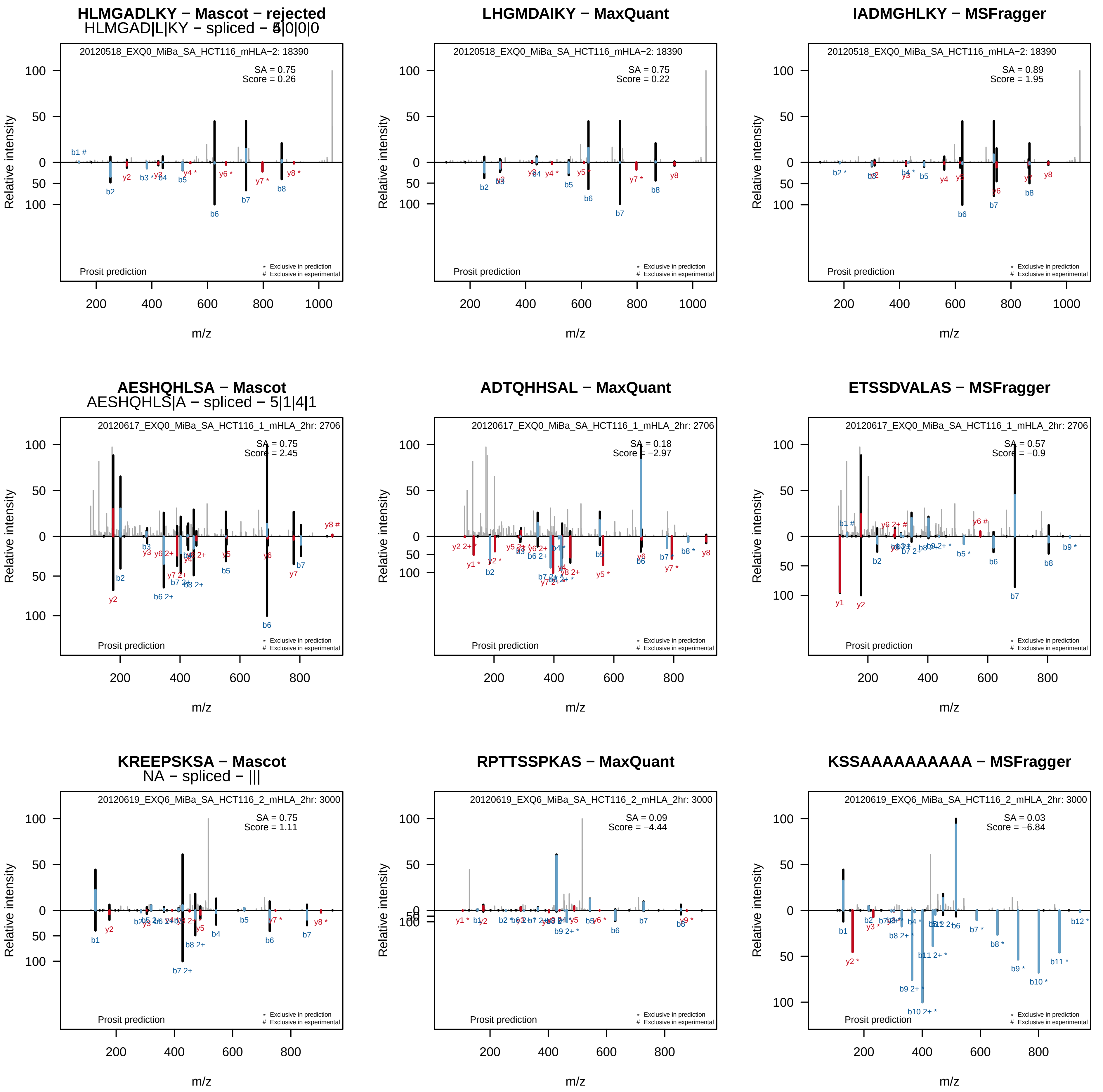

HLMGADLKY − Mascot − rejected
20120518_EXQ0_MiBa_SA_HCT116_mHLA−2: 18390
SA = 0.75
Score = 0.26
Prosit prediction
⋆ Exclusive in prediction
Exclusive in experimental
LHGMDAIKY − MaxQuant
20120518_EXQ0_MiBa_SA_HCT116_mHLA−2: 18390
SA = 0.75
Score = 0.22
IADMGHLKY − MSFragger
20120518_EXQ0_MiBa_SA_HCT116_mHLA−2: 18390
SA = 0.89
Score = 1.95
AESHQHLSA − Mascot
AESHQHLS|A − spliced − 5|1|4|1
20120617_EXQ0_MiBa_SA_HCT116_1_mHLA_2hr: 2706
SA = 0.75
Score = 2.45
ADTQHHSAL − MaxQuant
20120617_EXQ0_MiBa_SA_HCT116_1_mHLA_2hr: 2706
SA = 0.18
Score = −2.97
ETSSDVALAS − MSFragger
20120617_EXQ0_MiBa_SA_HCT116_1_mHLA_2hr: 2706
SA = 0.57
Score = −0.9
KREEPSKSA − Mascot
NA − spliced − |||
20120619_EXQ6_MiBa_SA_HCT116_2_mHLA_2hr: 3000
SA = 0.75
Score = 1.11
RPTTSSPKAS − MaxQuant
20120619_EXQ6_MiBa_SA_HCT116_2_mHLA_2hr: 3000
SA = 0.09
Score = −4.44
KSSAAAAAAAAAA − MSFragger
20120619_EXQ6_MiBa_SA_HCT116_2_mHLA_2hr: 3000
SA = 0.03
Score = −6.84
Relative intensity
m/z

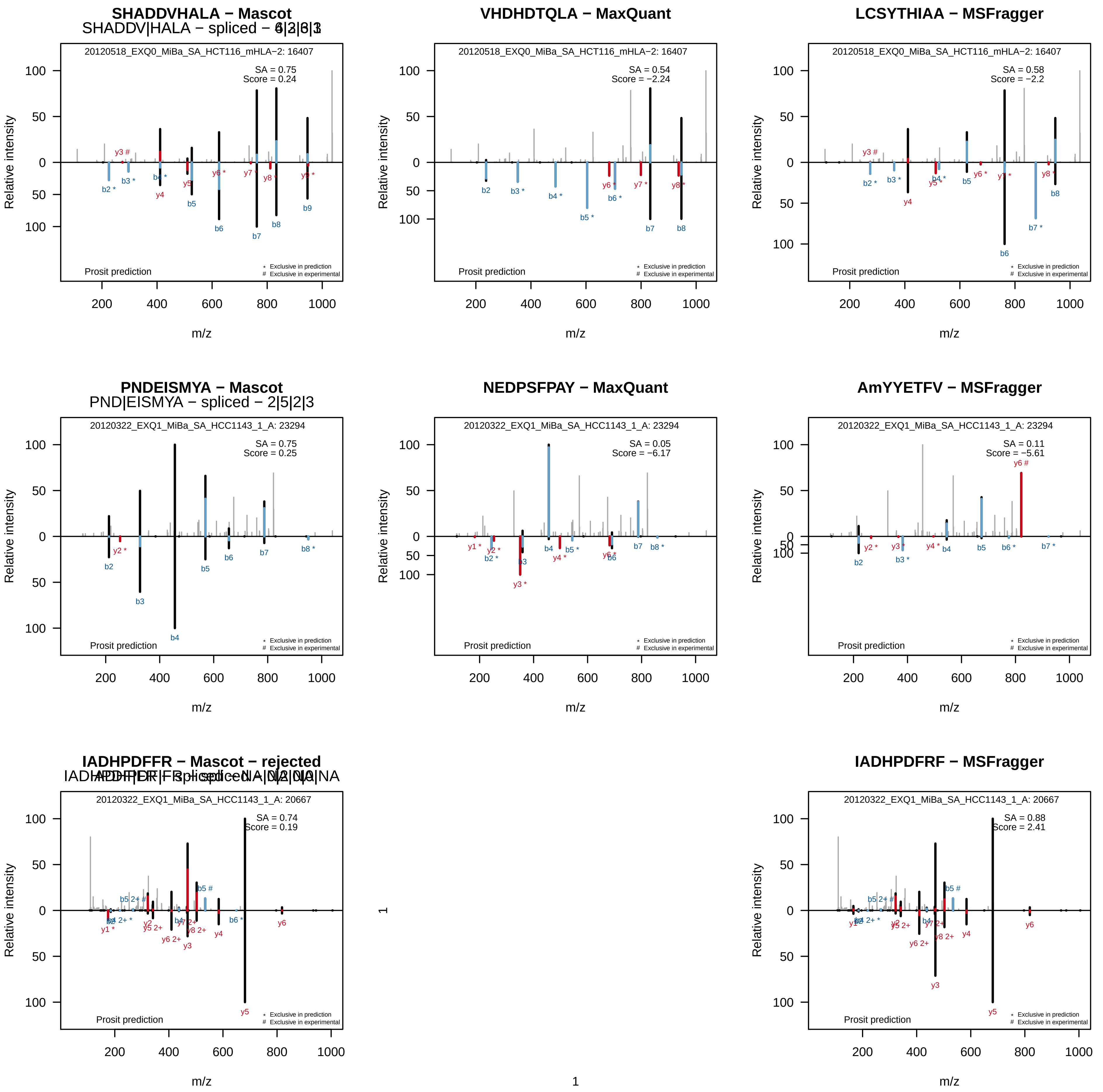

SHADDVHALA − Mascot
20120518_EXQ0_MiBa_SA_HCT116_mHLA−2: 16407
SA = 0.75
Score = 0.24
VHDHDTQLA − MaxQuant
20120518_EXQ0_MiBa_SA_HCT116_mHLA−2: 16407
SA = 0.54
Score = −2.24
LCSYTHIAA − MSFragger
20120518_EXQ0_MiBa_SA_HCT116_mHLA−2: 16407
SA = 0.58
Score = −2.2
PNDEISMYA − Mascot
PND|EISMYA − spliced − 2|5|2|3
20120322_EXQ1_MiBa_SA_HCC1143_1_A: 23294
SA = 0.75
Score = 0.25
NEDPSFPAY − MaxQuant
20120322_EXQ1_MiBa_SA_HCC1143_1_A: 23294
SA = 0.05
Score = −6.17
AmYYETFV − MSFragger
20120322_EXQ1_MiBa_SA_HCC1143_1_A: 23294
SA = 0.11
Score = −5.61
IADHPDFFR − Mascot − rejected
20120322_EXQ1_MiBa_SA_HCC1143_1_A: 20667
SA = 0.74
Score = 0.19
IADHPDFRF − MSFragger
20120322_EXQ1_MiBa_SA_HCC1143_1_A: 20667
SA = 0.88
Score = 2.41
Prosit prediction
* Exclusive in prediction
# Exclusive in experimental
Relative intensity
m/z

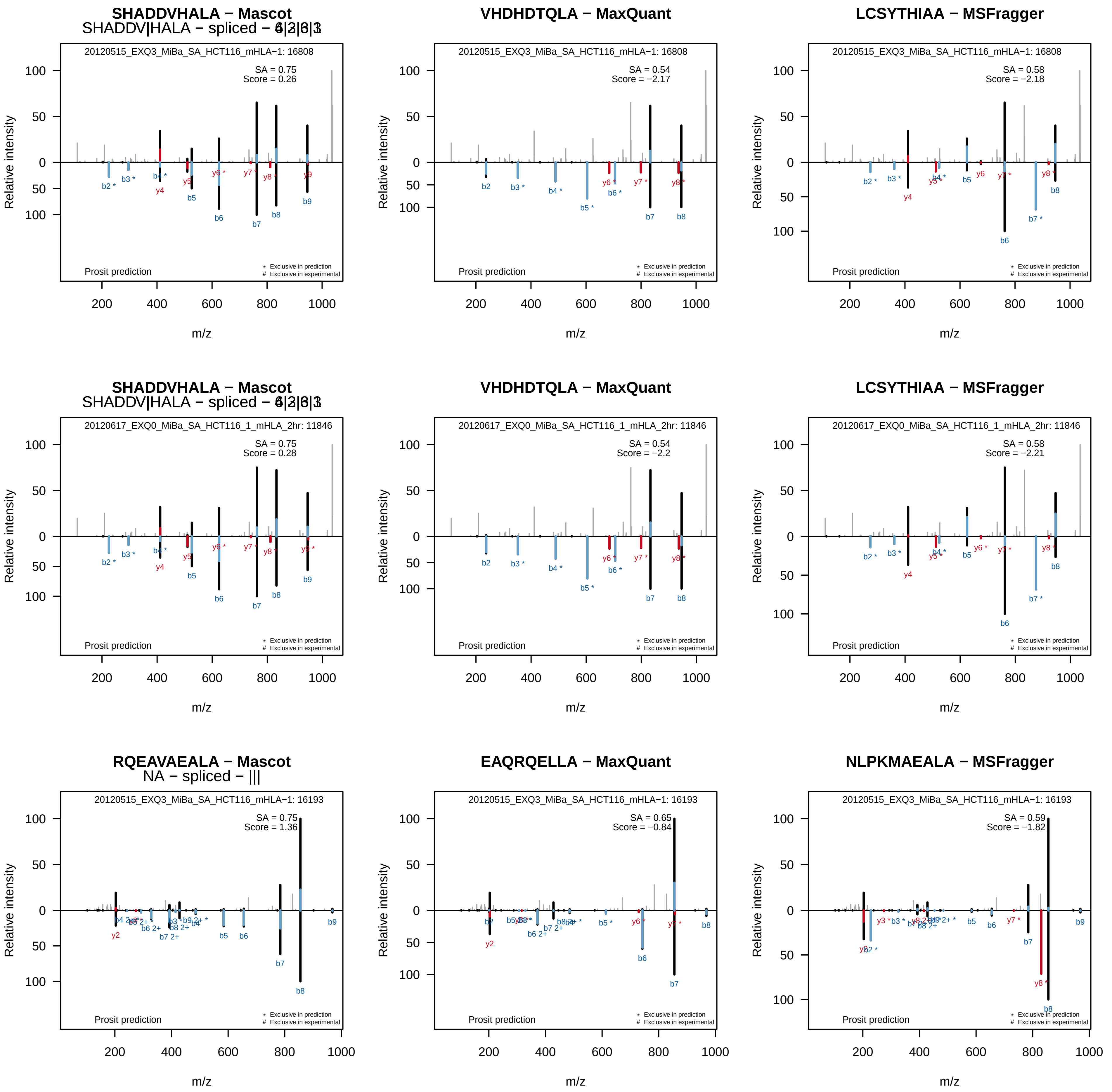

SHADDVHALA − Mascot
SHADDV|HALA − spliced −
20120515_EXQ3_MiBa_SA_HCT116_mHLA−1: 16808
SA = 0.75
Score = 0.26
VHDHDTQLA − MaxQuant
20120515_EXQ3_MiBa_SA_HCT116_mHLA−1: 16808
SA = 0.54
Score = −2.17
LCSYTHIAA − MSFragger
20120515_EXQ3_MiBa_SA_HCT116_mHLA−1: 16808
SA = 0.58
Score = −2.18
SHADDVHALA − Mascot
SHADDV|HALA − spliced −
20120617_EXQ0_MiBa_SA_HCT116_1_mHLA_2hr: 11846
SA = 0.75
Score = 0.28
VHDHDTQLA − MaxQuant
20120617_EXQ0_MiBa_SA_HCT116_1_mHLA_2hr: 11846
SA = 0.54
Score = −2.2
LCSYTHIAA − MSFragger
20120617_EXQ0_MiBa_SA_HCT116_1_mHLA_2hr: 11846
SA = 0.58
Score = −2.21
RQEAVAEALA − Mascot
NA − spliced − |||
20120515_EXQ3_MiBa_SA_HCT116_mHLA−1: 16193
SA = 0.75
Score = 1.36
EAQRQELLA − MaxQuant
20120515_EXQ3_MiBa_SA_HCT116_mHLA−1: 16193
SA = 0.65
Score = −0.84
NLPKMAEALA − MSFragger
20120515_EXQ3_MiBa_SA_HCT116_mHLA−1: 16193
SA = 0.59
Score = −1.82
Relative intensity
m/z
Prosit prediction
* Exclusive in prediction
# Exclusive in experimental

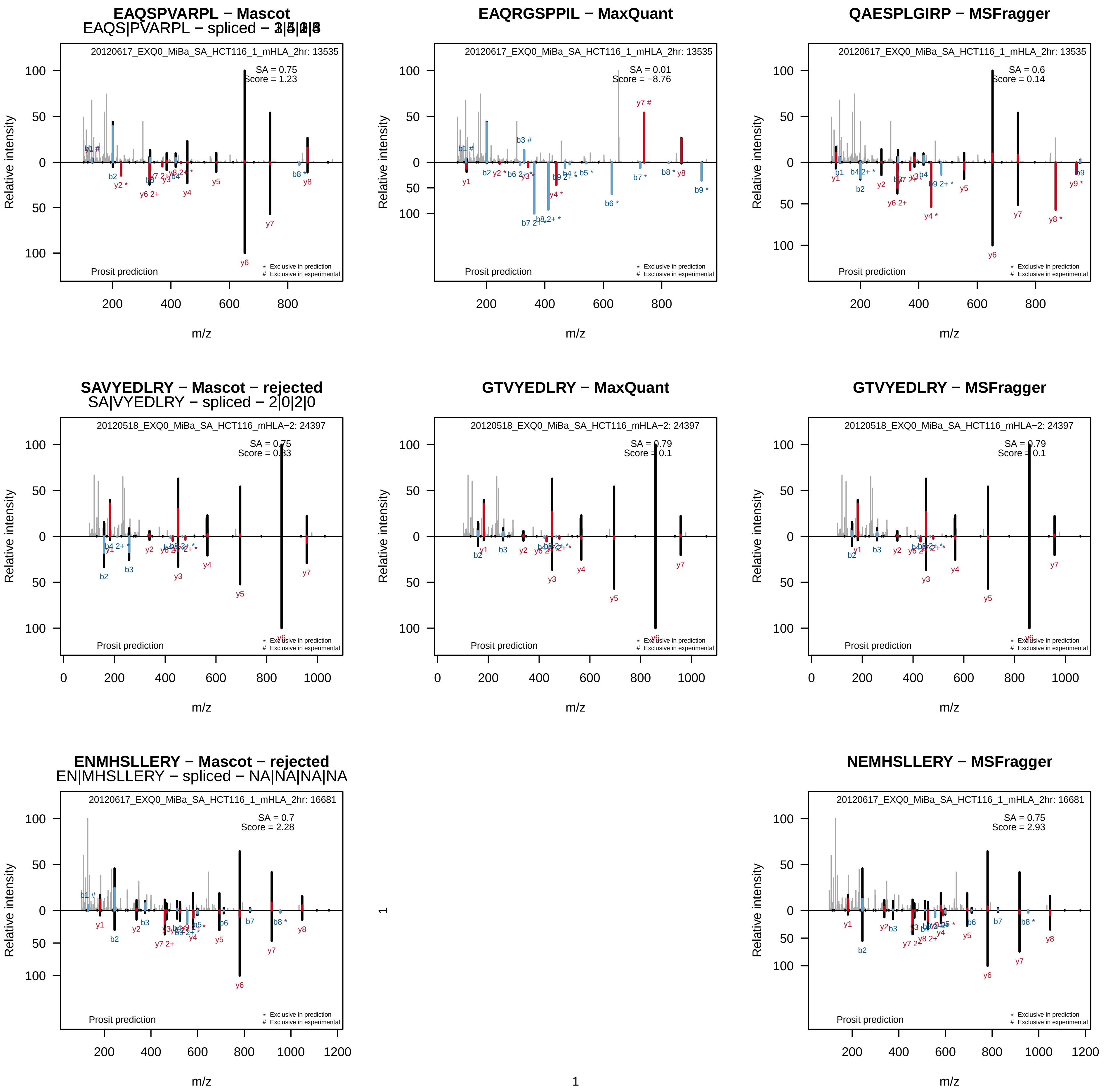

EAQSPVARPL − Mascot
EAQS|PVARPL − spliced
20120617_EXQ0_MiBa_SA_HCT116_1_mHLA_2hr: 13535
SA = 0.75
Score = 1.23
EAQRGSPPIL − MaxQuant
20120617_EXQ0_MiBa_SA_HCT116_1_mHLA_2hr: 13535
SA = 0.01
Score = −8.76
QAESPLGIRP − MSFragger
20120617_EXQ0_MiBa_SA_HCT116_1_mHLA_2hr: 13535
SA = 0.6
Score = 0.14
SAVYEDLRY − Mascot − rejected
SA|VYEDLRY − spliced − 2|0|2|0
20120518_EXQ0_MiBa_SA_HCT116_mHLA-2: 24397
SA = 0.75
Score = 0.33
GTVYEDLRY − MaxQuant
20120518_EXQ0_MiBa_SA_HCT116_mHLA-2: 24397
SA = 0.79
Score = 0.1
GTVYEDLRY − MSFragger
20120518_EXQ0_MiBa_SA_HCT116_mHLA-2: 24397
SA = 0.79
Score = 0.1
ENMHSLLERY − Mascot − rejected
EN|MHSLLERY − spliced − NA|NA|NA|NA
20120617_EXQ0_MiBa_SA_HCT116_1_mHLA_2hr: 16681
SA = 0.7
Score = 2.28
NEMHSLLERY − MSFragger
20120617_EXQ0_MiBa_SA_HCT116_1_mHLA_2hr: 16681
SA = 0.75
Score = 2.93
Relative intensity
m/z
Prosit prediction
* Exclusive in prediction
# Exclusive in experimental

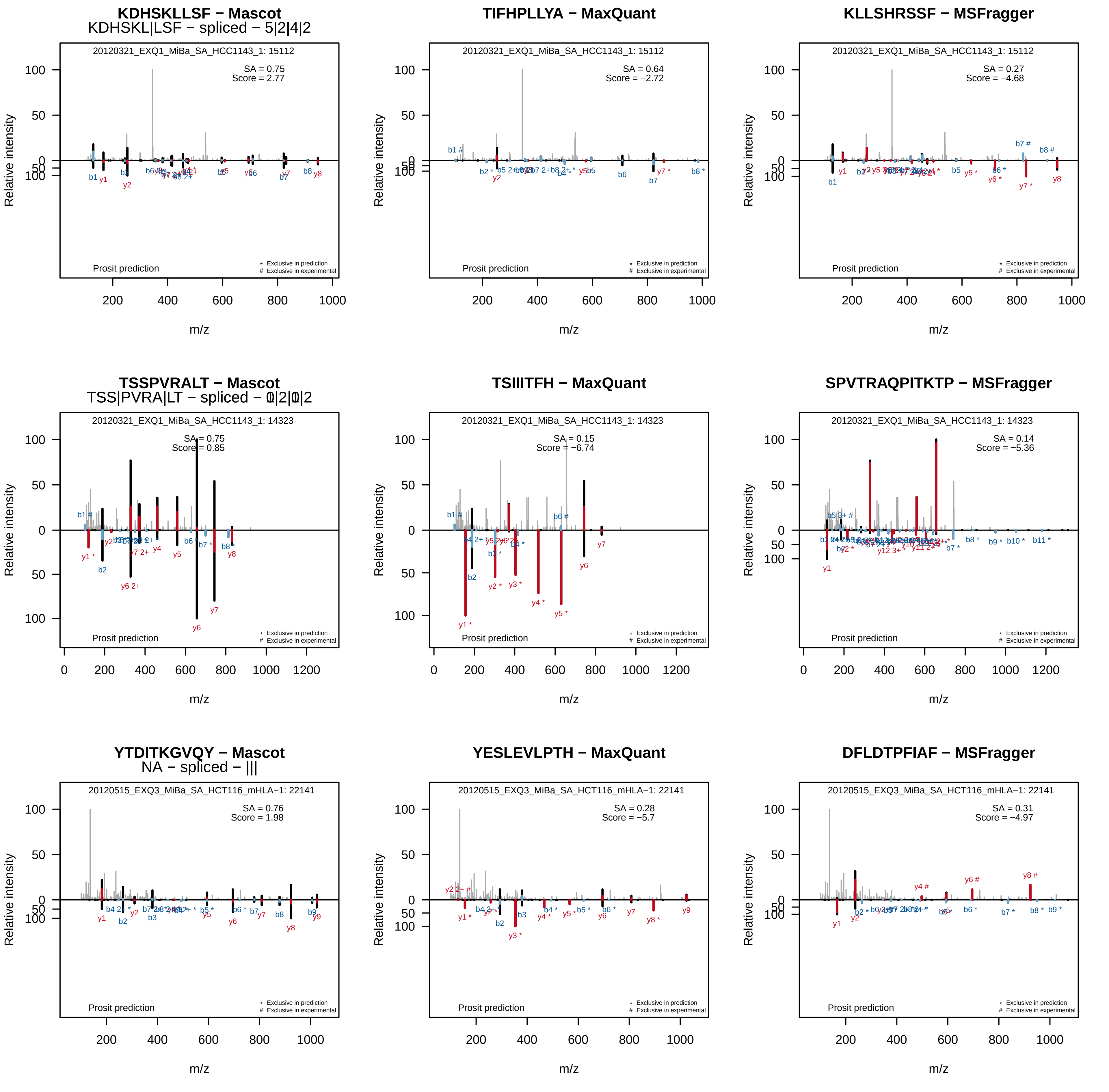

KDHSKLLSF − Mascot
KDHSKL|LSF − spliced − 5|2|4|2
20120321_EXQ1_MiBa_SA_HCC1143_1: 15112
SA = 0.75
Score = 2.77
TIFHPLLYA − MaxQuant
20120321_EXQ1_MiBa_SA_HCC1143_1: 15112
SA = 0.64
Score = −2.72
KLLSHRSSF − MSFragger
20120321_EXQ1_MiBa_SA_HCC1143_1: 15112
SA = 0.27
Score = −4.68
TSSPVRALT − Mascot
TSS|PVRA|LT − spliced − 1|2|1|2
20120321_EXQ1_MiBa_SA_HCC1143_1: 14323
SA = 0.75
Score = 0.85
TSIIITFH − MaxQuant
20120321_EXQ1_MiBa_SA_HCC1143_1: 14323
SA = 0.15
Score = −6.74
SPVTRAQPITKTP − MSFragger
20120321_EXQ1_MiBa_SA_HCC1143_1: 14323
SA = 0.14
Score = −5.36
YTDITKGVQY − Mascot
NA − spliced − |||
20120515_EXQ3_MiBa_SA_HCT116_mHLA−1: 22141
SA = 0.76
Score = 1.98
YESLEVLPTH − MaxQuant
20120515_EXQ3_MiBa_SA_HCT116_mHLA−1: 22141
SA = 0.28
Score = −5.7
DFLDTPFIAF − MSFragger
20120515_EXQ3_MiBa_SA_HCT116_mHLA−1: 22141
SA = 0.31
Score = −4.97
Relative intensity
m/z
Prosit prediction
* Exclusive in prediction
# Exclusive in experimental

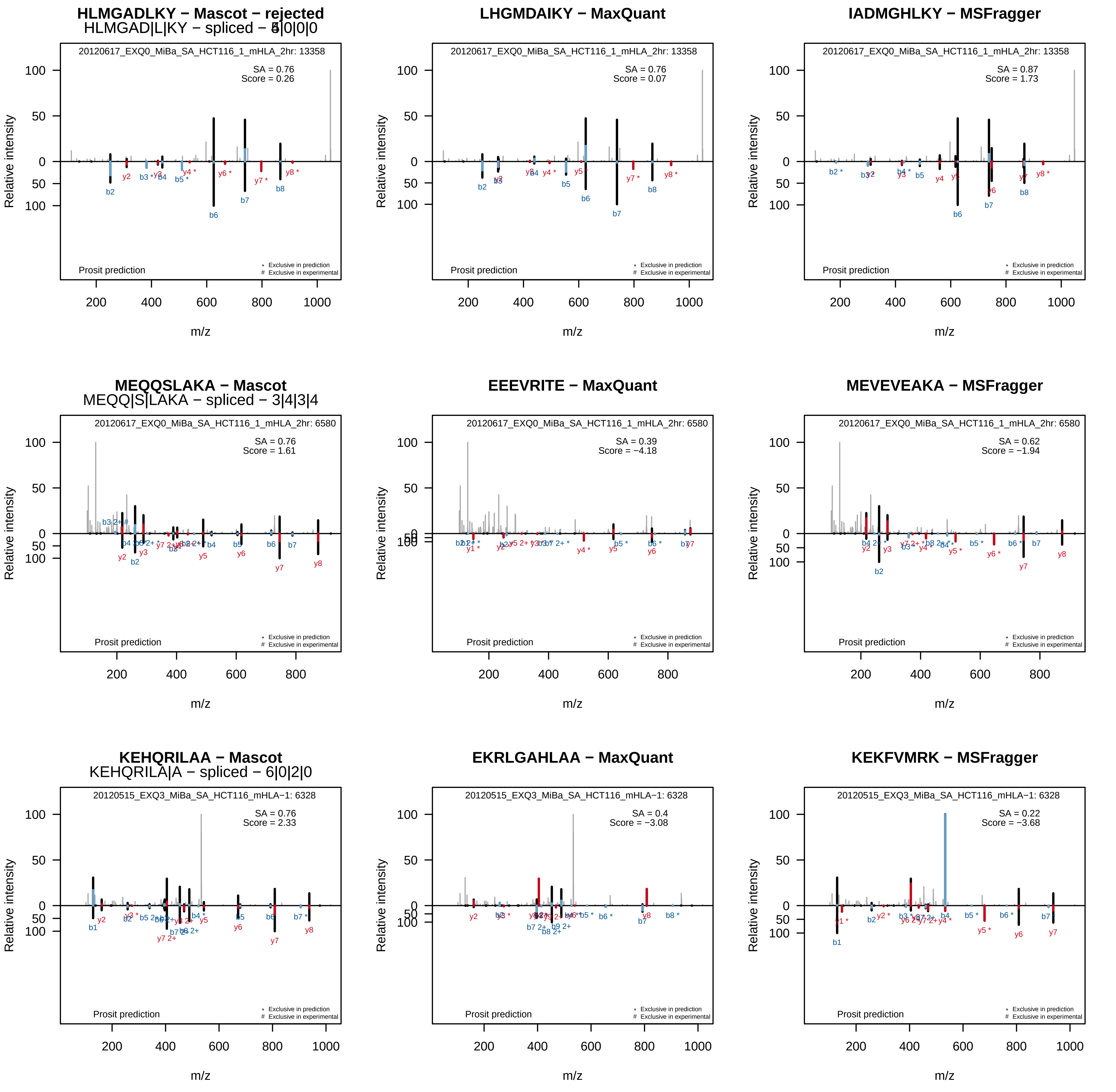
HLMGADLKY − Mascot − rejected
HLMGAD|L|KY − spliced − 6|0|0|0
20120617_EXQ0_MiBa_SA_HCT116_1_mHLA_2hr: 13358
SA = 0.76
Score = 0.26
LHGMDAIKY − MaxQuant
20120617_EXQ0_MiBa_SA_HCT116_1_mHLA_2hr: 13358
SA = 0.76
Score = 0.07
IADMGHLKY − MSFragger
20120617_EXQ0_MiBa_SA_HCT116_1_mHLA_2hr: 13358
SA = 0.87
Score = 1.73
MEQQSLAKA − Mascot
MEQQ|S|LAKA − spliced − 3|4|3|4
20120617_EXQ0_MiBa_SA_HCT116_1_mHLA_2hr: 6580
SA = 0.76
Score = 1.61
EEEVRITE − MaxQuant
20120617_EXQ0_MiBa_SA_HCT116_1_mHLA_2hr: 6580
SA = 0.39
Score = −4.18
MEVEVEAKA − MSFragger
20120617_EXQ0_MiBa_SA_HCT116_1_mHLA_2hr: 6580
SA = 0.62
Score = −1.94
KEHQRILAA − Mascot
KEHQRILA|A − spliced − 6|0|2|0
20120515_EXQ3_MiBa_SA_HCT116_mHLA−1: 6328
SA = 0.76
Score = 2.33
EKRLGAHLAA − MaxQuant
20120515_EXQ3_MiBa_SA_HCT116_mHLA−1: 6328
SA = 0.4
Score = −3.08
KEKFVMRK − MSFragger
20120515_EXQ3_MiBa_SA_HCT116_mHLA−1: 6328
SA = 0.22
Score = −3.68
Relative intensity
m/z
Prosit prediction
⋆ Exclusive in prediction
Exclusive in experimental

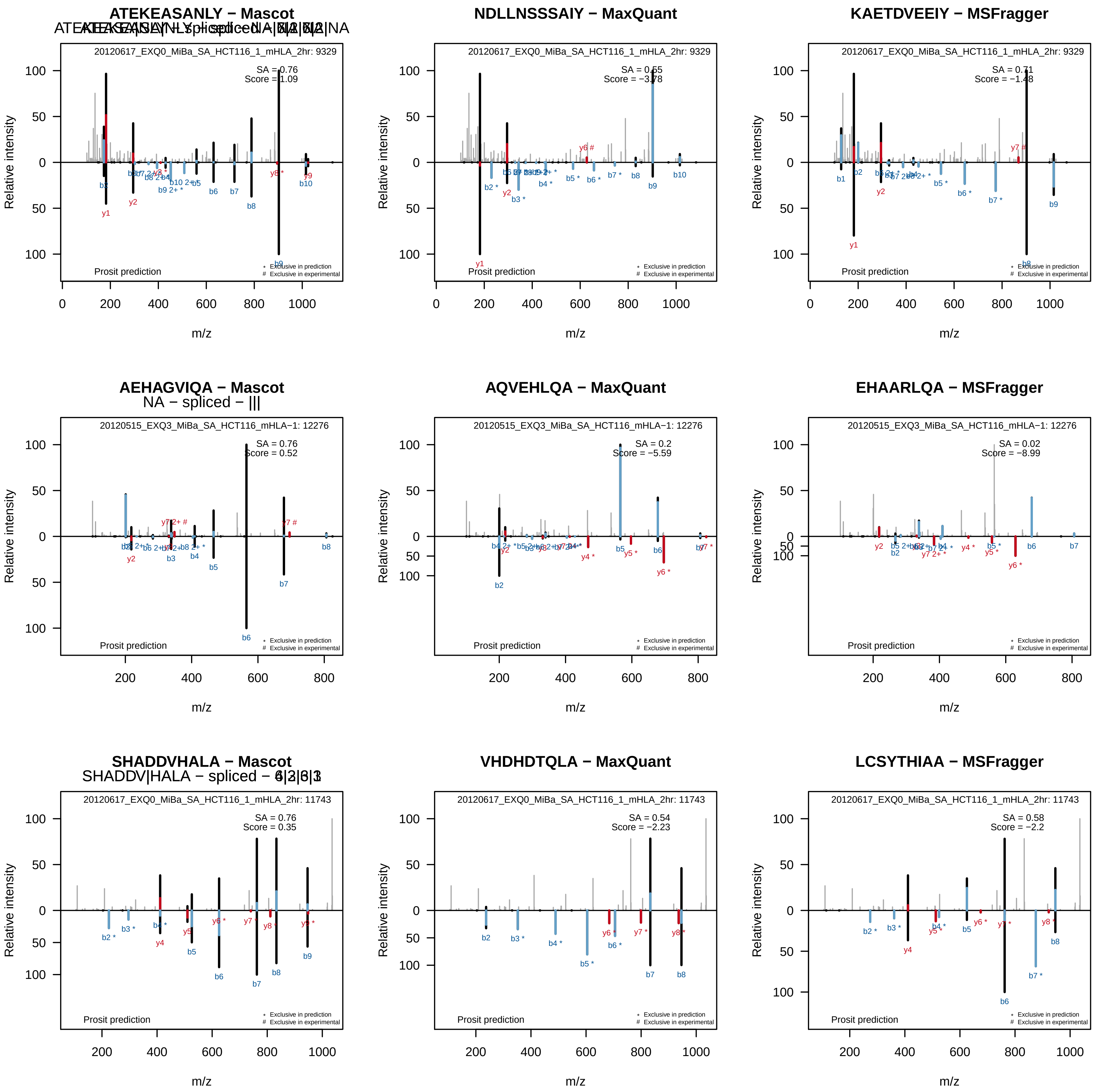
ATEKEASANLY − Mascot
20120617_EXQ0_MiBa_SA_HCT116_1_mHLA_2hr: 9329
SA = 0.76
Score = 1.09
NDLLNSSSAIY − MaxQuant
20120617_EXQ0_MiBa_SA_HCT116_1_mHLA_2hr: 9329
SA = 0.55
Score = −3.78
KAETDVEEIY − MSFragger
20120617_EXQ0_MiBa_SA_HCT116_1_mHLA_2hr: 9329
SA = 0.71
Score = −1.48
AEHAGVIQA − Mascot
NA − spliced − |||
20120515_EXQ3_MiBa_SA_HCT116_mHLA−1: 12276
SA = 0.76
Score = 0.52
AQVEHLQA − MaxQuant
20120515_EXQ3_MiBa_SA_HCT116_mHLA−1: 12276
SA = 0.2
Score = −5.59
EHAARLQA − MSFragger
20120515_EXQ3_MiBa_SA_HCT116_mHLA−1: 12276
SA = 0.02
Score = −8.99
SHADDVHALA − Mascot
20120617_EXQ0_MiBa_SA_HCT116_1_mHLA_2hr: 11743
SA = 0.76
Score = 0.35
VHDHDTQLA − MaxQuant
20120617_EXQ0_MiBa_SA_HCT116_1_mHLA_2hr: 11743
SA = 0.54
Score = −2.23
LCSYTHIAA − MSFragger
20120617_EXQ0_MiBa_SA_HCT116_1_mHLA_2hr: 11743
SA = 0.58
Score = −2.2
Relative intensity
m/z
Prosit prediction
* Exclusive in prediction
# Exclusive in experimental

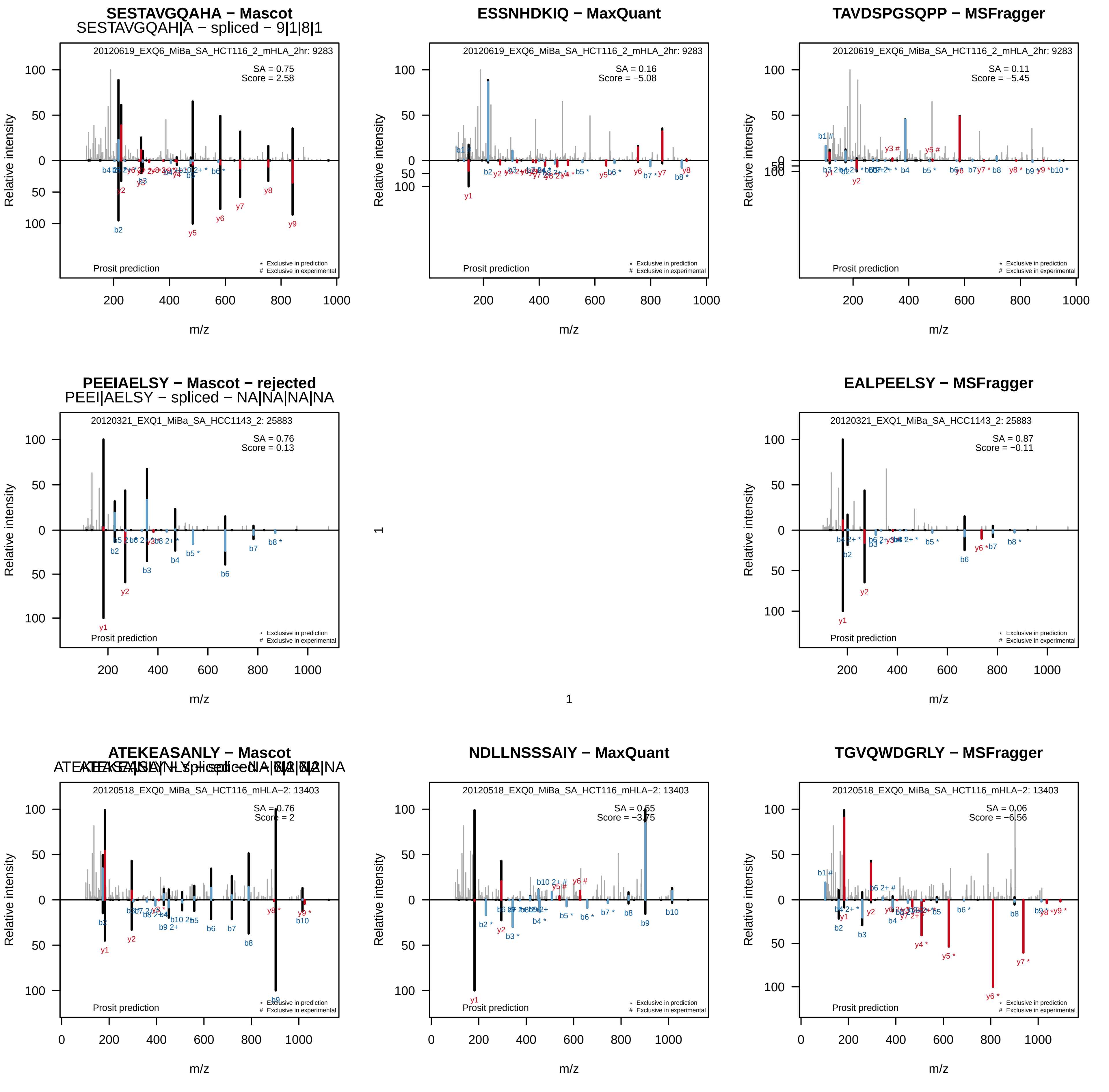
SESTAVGQAHA − Mascot
SESTAVGQAH|A − spliced − 9|1|8|1
20120619_EXQ6_MiBa_SA_HCT116_2_mHLA_2hr: 9283
SA = 0.75
Score = 2.58
Relative intensity
Prosit prediction
⋆ Exclusive in prediction
Exclusive in experimental
m/z
ESSNHDKIQ − MaxQuant
20120619_EXQ6_MiBa_SA_HCT116_2_mHLA_2hr: 9283
SA = 0.16
Score = −5.08
TAVDSPGSQPP − MSFragger
20120619_EXQ6_MiBa_SA_HCT116_2_mHLA_2hr: 9283
SA = 0.11
Score = −5.45
PEEIAELSY − Mascot − rejected
PEEI|AELSY − spliced − NA|NA|NA|NA
20120321_EXQ1_MiBa_SA_HCC1143_2: 25883
SA = 0.76
Score = 0.13
EALPEELSY − MSFragger
20120321_EXQ1_MiBa_SA_HCC1143_2: 25883
SA = 0.87
Score = −0.11
ATEKEASANLY − Mascot
20120518_EXQ0_MiBa_SA_HCT116_mHLA−2: 13403
SA = 0.76
Score = 2
NDLLNSSSAIY − MaxQuant
20120518_EXQ0_MiBa_SA_HCT116_mHLA−2: 13403
SA = 0.55
Score = −3.75
TGVQWDGRLY − MSFragger
20120518_EXQ0_MiBa_SA_HCT116_mHLA−2: 13403
SA = 0.06
Score = −6.56

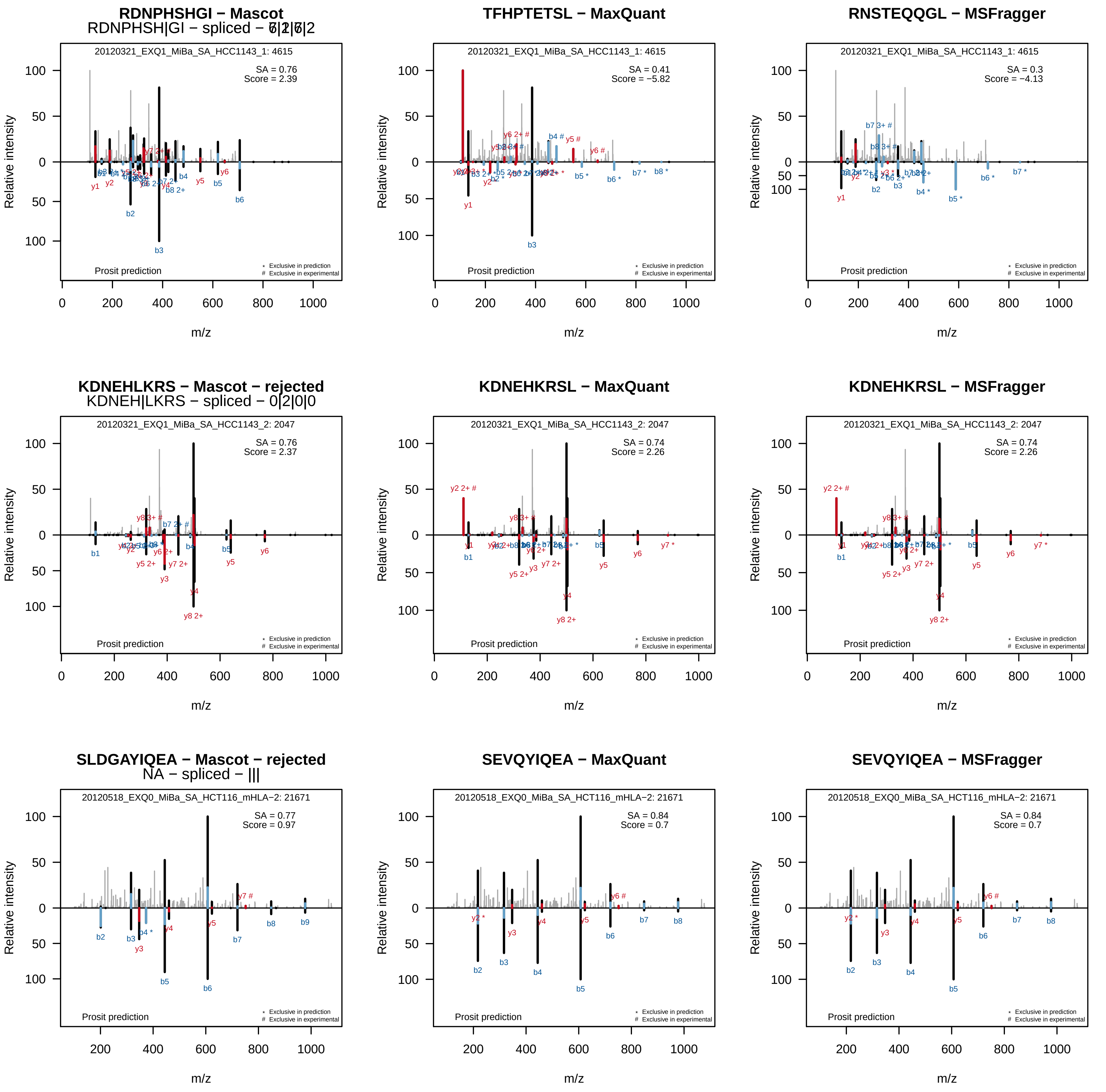
RDNPHSHGI − Mascot
RDNPHSH|GI − spliced
20120321_EXQ1_MiBa_SA_HCC1143_1: 4615
SA = 0.76
Score = 2.39
TFHPTETSL − MaxQuant
20120321_EXQ1_MiBa_SA_HCC1143_1: 4615
SA = 0.41
Score = −5.82
RNSTEQQGL − MSFragger
20120321_EXQ1_MiBa_SA_HCC1143_1: 4615
SA = 0.3
Score = −4.13
KDNEHLKRS − Mascot − rejected
KDNEH|LKRS − spliced − 0|2|0|0
20120321_EXQ1_MiBa_SA_HCC1143_2: 2047
SA = 0.76
Score = 2.37
KDNEHKRSL − MaxQuant
20120321_EXQ1_MiBa_SA_HCC1143_2: 2047
SA = 0.74
Score = 2.26
KDNEHKRSL − MSFragger
20120321_EXQ1_MiBa_SA_HCC1143_2: 2047
SA = 0.74
Score = 2.26
SLDGAYIQEA − Mascot − rejected
NA − spliced − |||
20120518_EXQ0_MiBa_SA_HCT116_mHLA−2: 21671
SA = 0.77
Score = 0.97
SEVQYIQEA − MaxQuant
20120518_EXQ0_MiBa_SA_HCT116_mHLA−2: 21671
SA = 0.84
Score = 0.7
SEVQYIQEA − MSFragger
20120518_EXQ0_MiBa_SA_HCT116_mHLA−2: 21671
SA = 0.84
Score = 0.7
Relative intensity
m/z
Prosit prediction
⋆ Exclusive in prediction
Exclusive in experimental

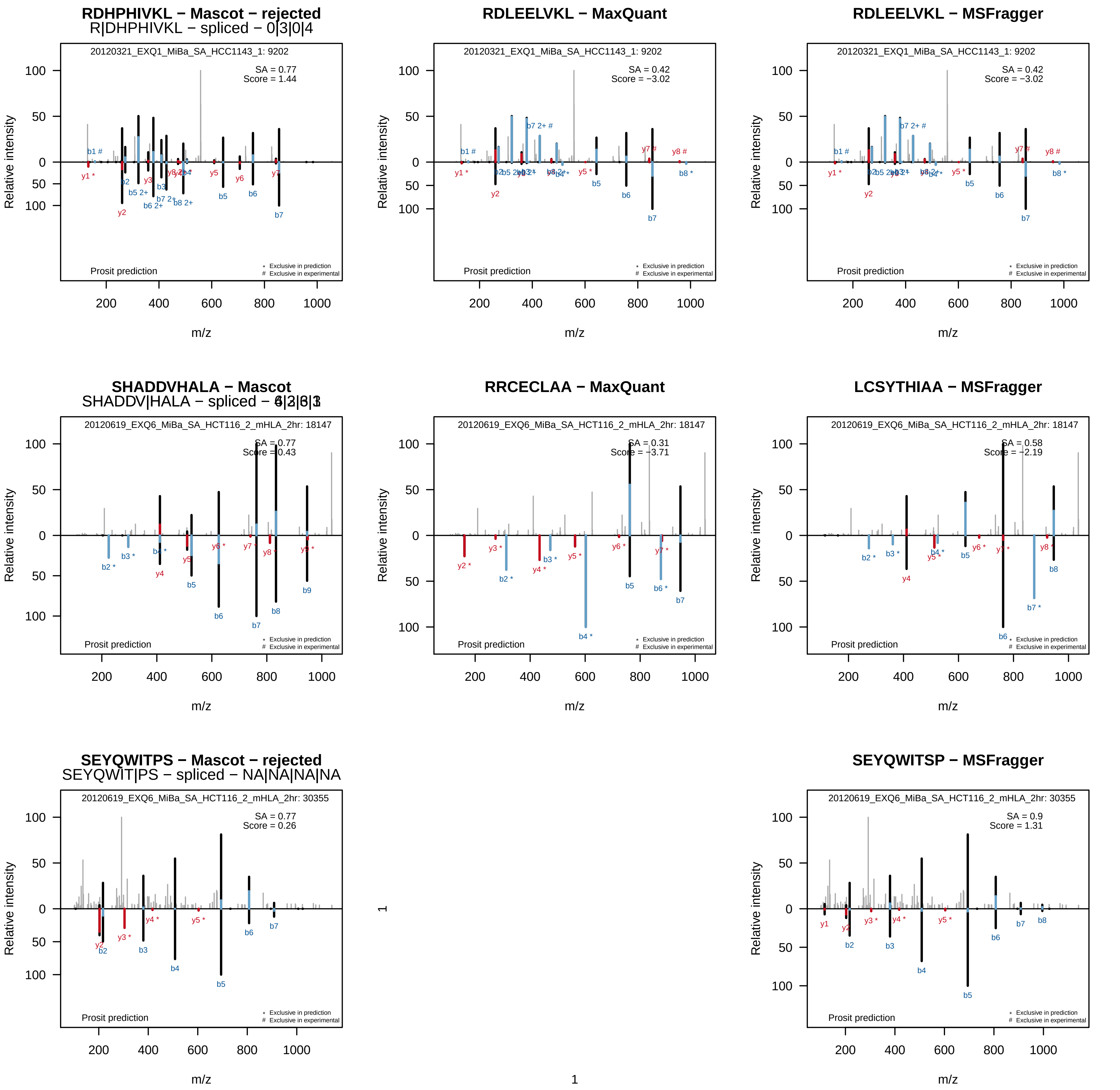
RDHPHIVKL − Mascot − rejected
R|DHPHIVKL − spliced − 0|3|0|4
20120321_EXQ1_MiBa_SA_HCC1143_1: 9202
SA = 0.77
Score = 1.44
RDLEELVKL − MaxQuant
20120321_EXQ1_MiBa_SA_HCC1143_1: 9202
SA = 0.42
Score = −3.02
RDLEELVKL − MSFragger
20120321_EXQ1_MiBa_SA_HCC1143_1: 9202
SA = 0.42
Score = −3.02
SHADDVHALA − Mascot
20120619_EXQ6_MiBa_SA_HCT116_2_mHLA_2hr: 18147
SA = 0.77
Score = 0.43
RRCECLAA − MaxQuant
20120619_EXQ6_MiBa_SA_HCT116_2_mHLA_2hr: 18147
SA = 0.31
Score = −3.71
LCSYTHIAA − MSFragger
20120619_EXQ6_MiBa_SA_HCT116_2_mHLA_2hr: 18147
SA = 0.58
Score = −2.19
SEYQWITPS − Mascot − rejected
SEYQWIT|PS − spliced − NA|NA|NA|NA
20120619_EXQ6_MiBa_SA_HCT116_2_mHLA_2hr: 30355
SA = 0.77
Score = 0.26
SEYQWITSP − MSFragger
20120619_EXQ6_MiBa_SA_HCT116_2_mHLA_2hr: 30355
SA = 0.9
Score = 1.31
Relative intensity
m/z
Prosit prediction
Exclusive in prediction
Exclusive in experimental

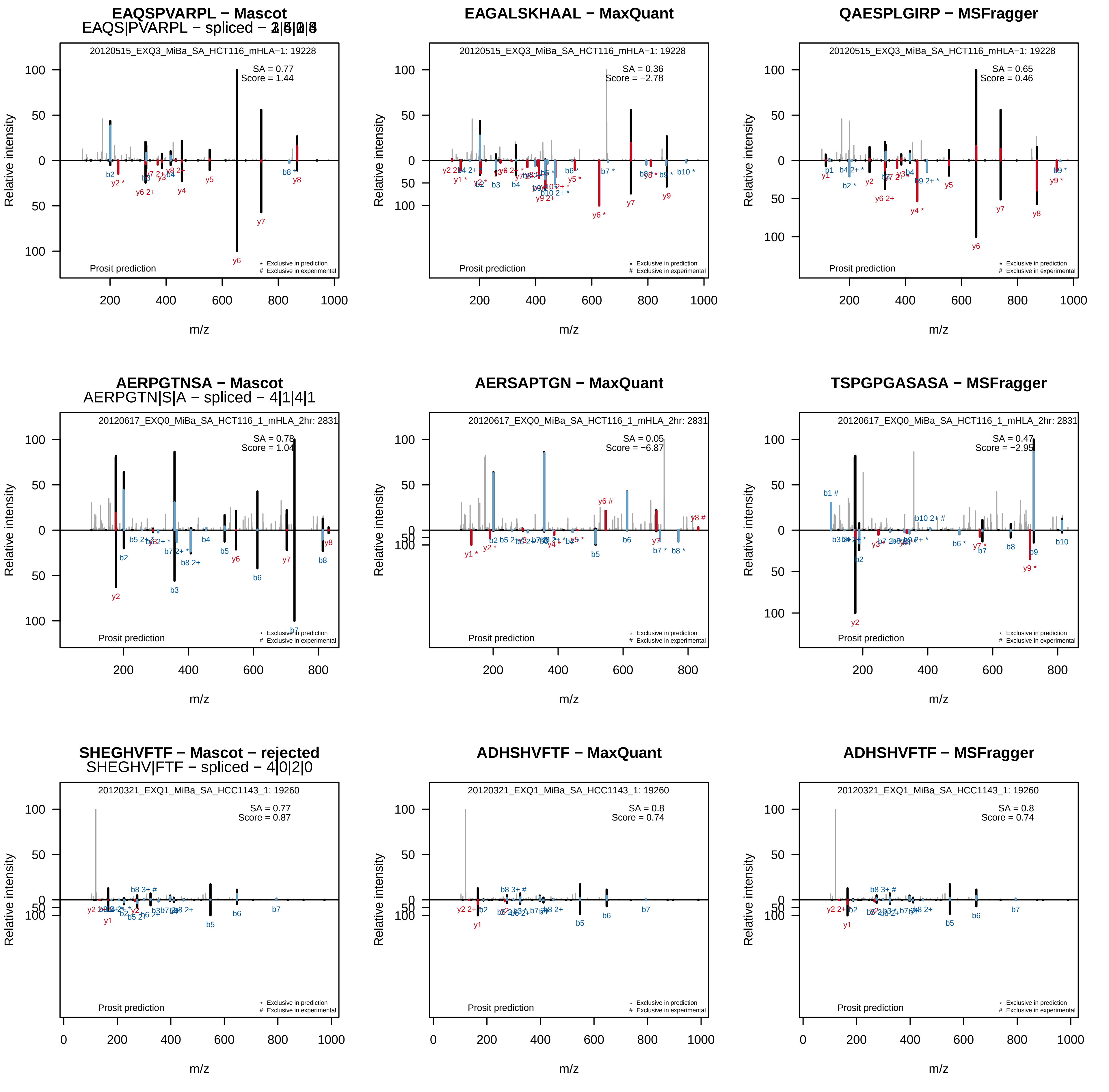
EAQSPVARPL − Mascot
EAQS|PVARPL − spliced
20120515_EXQ3_MiBa_SA_HCT116_mHLA−1: 19228
SA = 0.77
Score = 1.44
EAGALSKHAAL − MaxQuant
20120515_EXQ3_MiBa_SA_HCT116_mHLA−1: 19228
SA = 0.36
Score = −2.78
QAESPLGIRP − MSFragger
20120515_EXQ3_MiBa_SA_HCT116_mHLA−1: 19228
SA = 0.65
Score = 0.46
AERPGTNSA − Mascot
AERPGTN|S|A − spliced − 4|1|4|1
20120617_EXQ0_MiBa_SA_HCT116_1_mHLA_2hr: 2831
SA = 0.78
Score = 1.04
AERSAPTGN − MaxQuant
20120617_EXQ0_MiBa_SA_HCT116_1_mHLA_2hr: 2831
SA = 0.05
Score = −6.87
TSPGPGASASA − MSFragger
20120617_EXQ0_MiBa_SA_HCT116_1_mHLA_2hr: 2831
SA = 0.47
Score = −2.95
SHEGHVFTF − Mascot − rejected
SHEGHV|FTF − spliced − 4|0|2|0
20120321_EXQ1_MiBa_SA_HCC1143_1: 19260
SA = 0.77
Score = 0.87
ADHSHVFTF − MaxQuant
20120321_EXQ1_MiBa_SA_HCC1143_1: 19260
SA = 0.8
Score = 0.74
ADHSHVFTF − MSFragger
20120321_EXQ1_MiBa_SA_HCC1143_1: 19260
SA = 0.8
Score = 0.74
Relative intensity
m/z
Prosit prediction
* Exclusive in prediction
# Exclusive in experimental

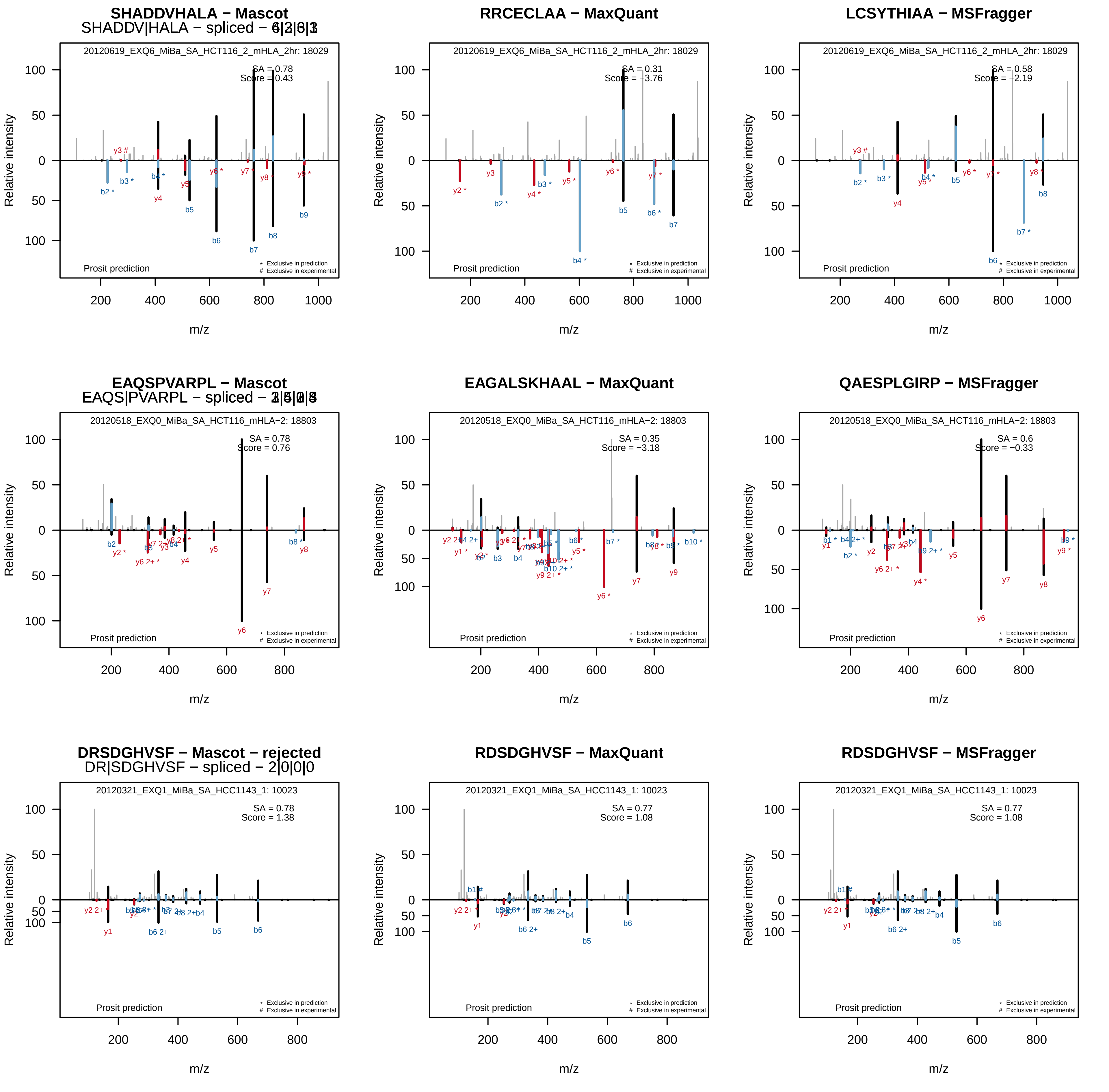

SHADDVHALA − Mascot
RRCECLAA − MaxQuant
LCSYTHIAA − MSFragger
20120619_EXQ6_MiBa_SA_HCT116_2_mHLA_2hr: 18029
SA = 0.78
Score = 0.43
SA = 0.31
Score = −3.76
SA = 0.58
Score = −2.19
EAQSPVARPL − Mascot
EAGALSKHAAL − MaxQuant
QAESPLGIRP − MSFragger
20120518_EXQ0_MiBa_SA_HCT116_mHLA−2: 18803
SA = 0.78
Score = 0.76
SA = 0.35
Score = −3.18
SA = 0.6
Score = −0.33
DRSDGHVSF − Mascot − rejected
DR|SDGHVSF − spliced − 2|0|0|0
RDSDGHVSF − MaxQuant
RDSDGHVSF − MSFragger
20120321_EXQ1_MiBa_SA_HCC1143_1: 10023
SA = 0.78
Score = 1.38
SA = 0.77
Score = 1.08
Relative intensity
m/z
Prosit prediction
* Exclusive in prediction
# Exclusive in experimental

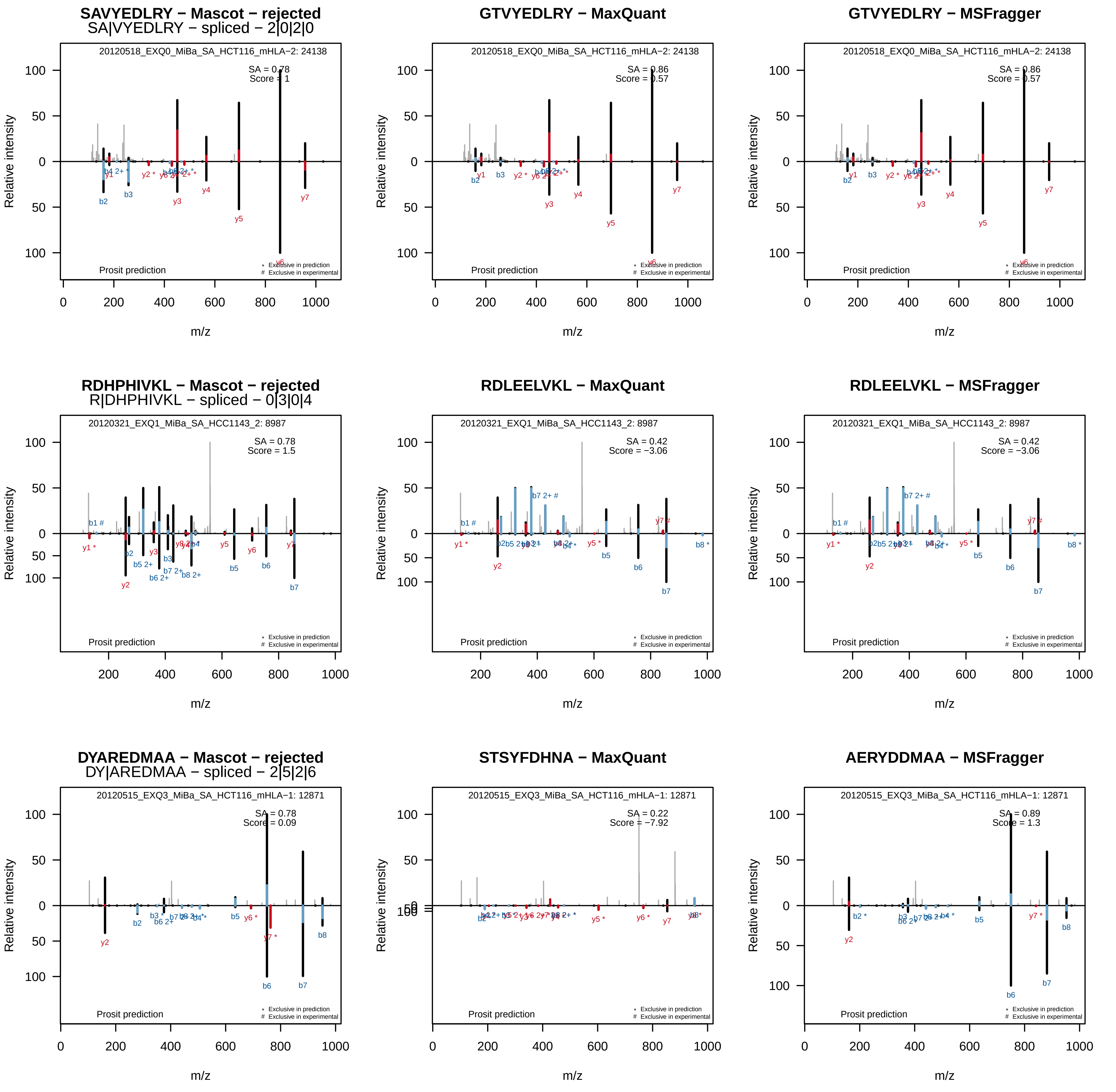
SAVYEDLRY − Mascot − rejected
SA|VYEDLRY − spliced − 2|0|2|0
20120518_EXQ0_MiBa_SA_HCT116_mHLA−2: 24138
SA = 0.78
Score = 1
GTVYEDLRY − MaxQuant
20120518_EXQ0_MiBa_SA_HCT116_mHLA−2: 24138
SA = 0.86
Score = 0.57
GTVYEDLRY − MSFragger
20120518_EXQ0_MiBa_SA_HCT116_mHLA−2: 24138
SA = 0.86
Score = 0.57
RDHPHIVKL − Mascot − rejected
R|DHPHIVKL − spliced − 0|3|0|4
20120321_EXQ1_MiBa_SA_HCC1143_2: 8987
SA = 0.78
Score = 1.5
RDLEELVKL − MaxQuant
20120321_EXQ1_MiBa_SA_HCC1143_2: 8987
SA = 0.42
Score = −3.06
RDLEELVKL − MSFragger
20120321_EXQ1_MiBa_SA_HCC1143_2: 8987
SA = 0.42
Score = −3.06
DYAREDMAA − Mascot − rejected
DY|AREDMAA − spliced − 2|5|2|6
20120515_EXQ3_MiBa_SA_HCT116_mHLA−1: 12871
SA = 0.78
Score = 0.09
STSYFDHNA − MaxQuant
20120515_EXQ3_MiBa_SA_HCT116_mHLA−1: 12871
SA = 0.22
Score = −7.92
AERYDDMAA − MSFragger
20120515_EXQ3_MiBa_SA_HCT116_mHLA−1: 12871
SA = 0.89
Score = 1.3
Relative intensity
m/z
Prosit prediction
* Exclusive in prediction
# Exclusive in experimental

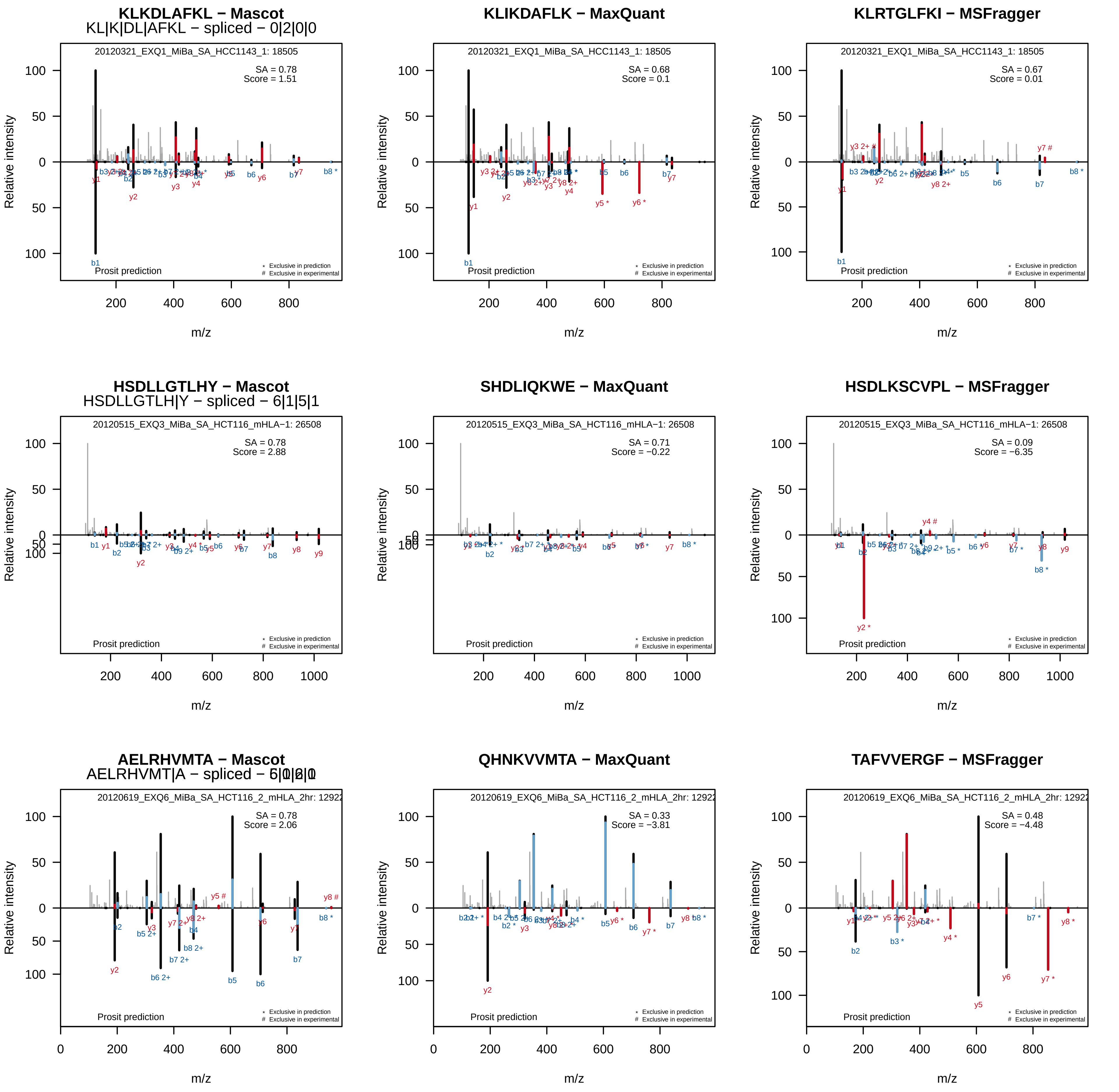
KLKDLAFKL − Mascot
KL|K|DL|AFKL − spliced − 0|2|0|0
20120321_EXQ1_MiBa_SA_HCC1143_1: 18505
SA = 0.78
Score = 1.51
KLIKDAFLK − MaxQuant
20120321_EXQ1_MiBa_SA_HCC1143_1: 18505
SA = 0.68
Score = 0.1
KLRTGLFKI − MSFragger
20120321_EXQ1_MiBa_SA_HCC1143_1: 18505
SA = 0.67
Score = 0.01
HSDLLGTLHY − Mascot
HSDLLGTLH|Y − spliced − 6|1|5|1
20120515_EXQ3_MiBa_SA_HCT116_mHLA−1: 26508
SA = 0.78
Score = 2.88
SHDLIQKWE − MaxQuant
20120515_EXQ3_MiBa_SA_HCT116_mHLA−1: 26508
SA = 0.71
Score = −0.22
HSDLKSCVPL − MSFragger
20120515_EXQ3_MiBa_SA_HCT116_mHLA−1: 26508
SA = 0.09
Score = −6.35
AELRHVMTA − Mascot
AELRHVMT|A − spliced − 6|1|2|1
20120619_EXQ6_MiBa_SA_HCT116_2_mHLA_2hr: 12922
SA = 0.78
Score = 2.06
QHNKVVMTA − MaxQuant
20120619_EXQ6_MiBa_SA_HCT116_2_mHLA_2hr: 12922
SA = 0.33
Score = −3.81
TAFVVERGF − MSFragger
20120619_EXQ6_MiBa_SA_HCT116_2_mHLA_2hr: 12922
SA = 0.48
Score = −4.48
Relative intensity
m/z
Prosit prediction
⋆ Exclusive in prediction
Exclusive in experimental

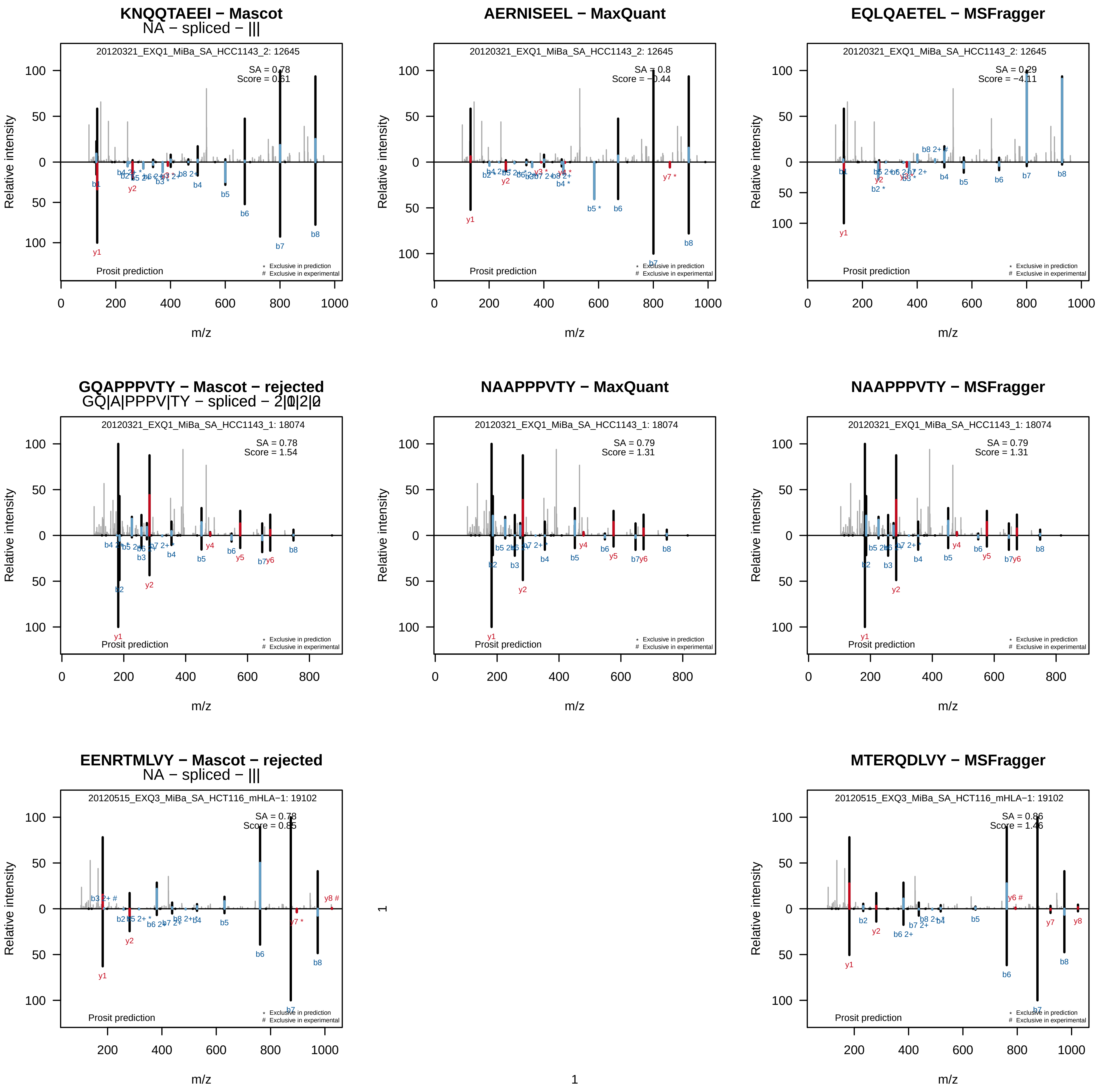
KNQQTAEEI − Mascot
NA − spliced − |||
20120321_EXQ1_MiBa_SA_HCC1143_2: 12645
AERNISEEL − MaxQuant
20120321_EXQ1_MiBa_SA_HCC1143_2: 12645
EQLQAETEL − MSFragger
20120321_EXQ1_MiBa_SA_HCC1143_2: 12645
GQAPPPVTY − Mascot − rejected
20120321_EXQ1_MiBa_SA_HCC1143_1: 18074
SA = 0.78
Score = 1.54
NAAPPPVTY − MaxQuant
20120321_EXQ1_MiBa_SA_HCC1143_1: 18074
SA = 0.79
Score = 1.31
NAAPPPVTY − MSFragger
20120321_EXQ1_MiBa_SA_HCC1143_1: 18074
SA = 0.79
Score = 1.31
EENRTMLVY − Mascot − rejected
NA − spliced − |||
20120515_EXQ3_MiBa_SA_HCT116_mHLA−1: 19102
MTERQDLVY − MSFragger
20120515_EXQ3_MiBa_SA_HCT116_mHLA−1: 19102
Relative intensity
m/z
Prosit prediction
* Exclusive in prediction
# Exclusive in experimental

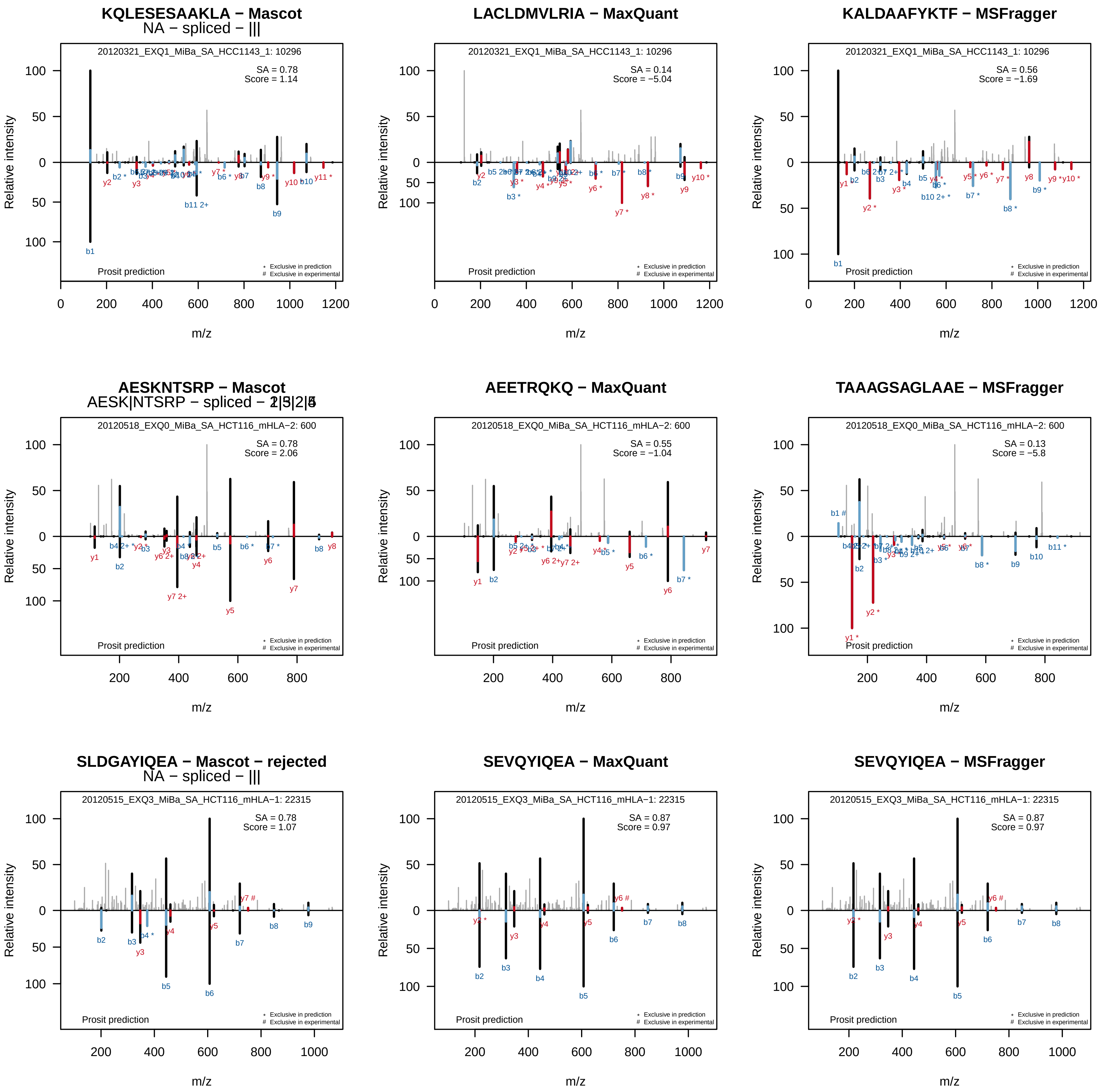

KQLESESAAKLA − Mascot
NA − spliced − |||
20120321_EXQ1_MiBa_SA_HCC1143_1: 10296
SA = 0.78
Score = 1.14
LACLDMVLRIA − MaxQuant
20120321_EXQ1_MiBa_SA_HCC1143_1: 10296
SA = 0.14
Score = −5.04
KALDAAFYKTF − MSFragger
20120321_EXQ1_MiBa_SA_HCC1143_1: 10296
SA = 0.56
Score = −1.69
AESKNTSRP − Mascot
20120518_EXQ0_MiBa_SA_HCT116_mHLA−2: 600
SA = 0.78
Score = 2.06
AEETRQKQ − MaxQuant
20120518_EXQ0_MiBa_SA_HCT116_mHLA−2: 600
SA = 0.55
Score = −1.04
TAAAGSAGLAAE − MSFragger
20120518_EXQ0_MiBa_SA_HCT116_mHLA−2: 600
SA = 0.13
Score = −5.8
SLDGAYIQEA − Mascot − rejected
NA − spliced − |||
20120515_EXQ3_MiBa_SA_HCT116_mHLA−1: 22315
SA = 0.78
Score = 1.07
SEVQYIQEA − MaxQuant
20120515_EXQ3_MiBa_SA_HCT116_mHLA−1: 22315
SA = 0.87
Score = 0.97
SEVQYIQEA − MSFragger
20120515_EXQ3_MiBa_SA_HCT116_mHLA−1: 22315
SA = 0.87
Score = 0.97
Relative intensity
m/z
Prosit prediction
⋆ Exclusive in prediction
Exclusive in experimental

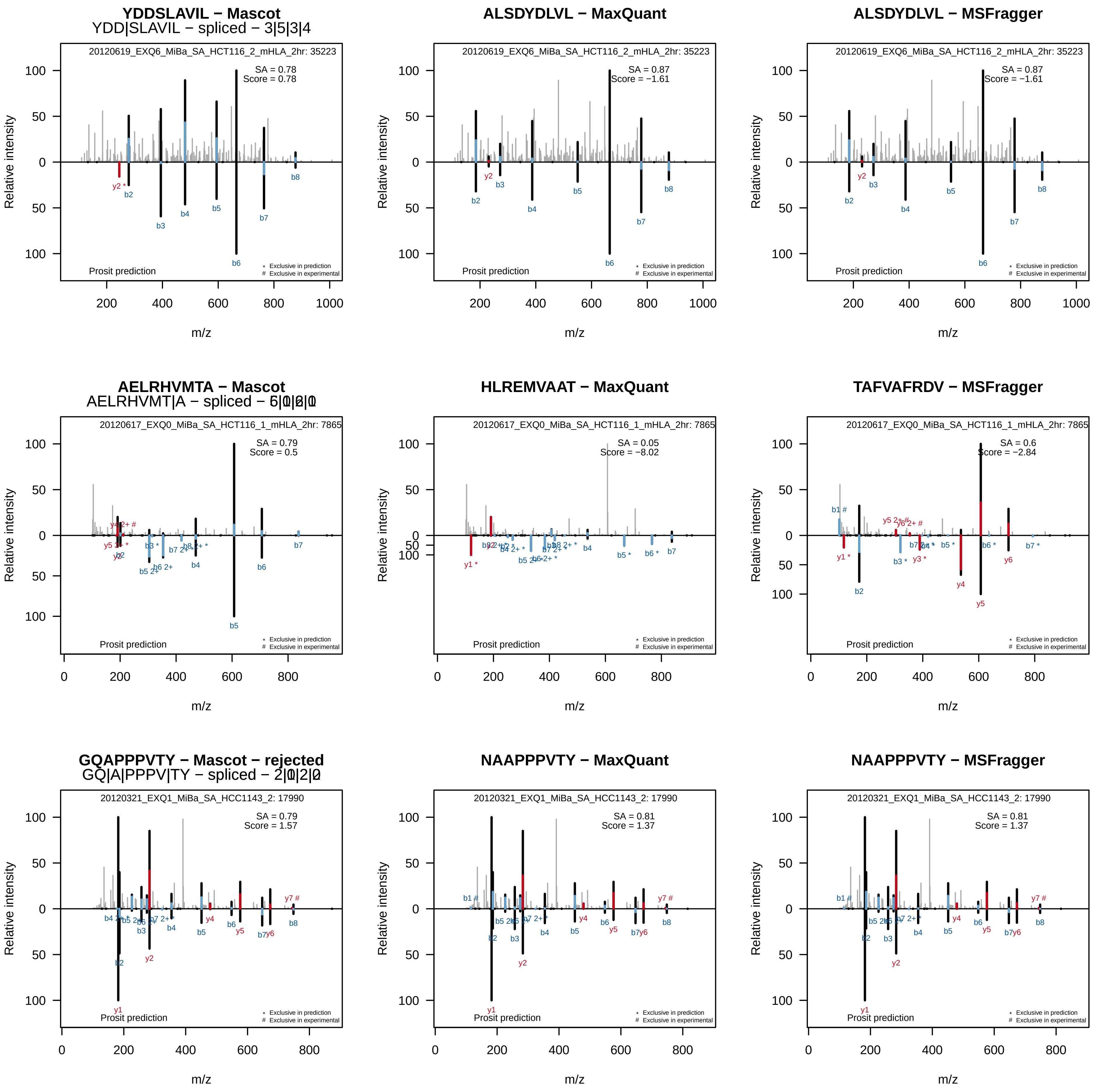

YDDSLAVIL − Mascot
YDD|SLAVIL − spliced − 3|5|3|4
20120619_EXQ6_MiBa_SA_HCT116_2_mHLA_2hr: 35223
SA = 0.78
Score = 0.78
ALSDYDLVL − MaxQuant
20120619_EXQ6_MiBa_SA_HCT116_2_mHLA_2hr: 35223
SA = 0.87
Score = −1.61
ALSDYDLVL − MSFragger
20120619_EXQ6_MiBa_SA_HCT116_2_mHLA_2hr: 35223
SA = 0.87
Score = −1.61
AELRHVMTA − Mascot
20120617_EXQ0_MiBa_SA_HCT116_1_mHLA_2hr: 7865
SA = 0.79
Score = 0.5
HLREMVAAT − MaxQuant
20120617_EXQ0_MiBa_SA_HCT116_1_mHLA_2hr: 7865
SA = 0.05
Score = −8.02
TAFVAFRDV − MSFragger
20120617_EXQ0_MiBa_SA_HCT116_1_mHLA_2hr: 7865
SA = 0.6
Score = −2.84
GQAPPPVTY − Mascot − rejected
20120321_EXQ1_MiBa_SA_HCC1143_2: 17990
SA = 0.79
Score = 1.57
NAAPPPVTY − MaxQuant
20120321_EXQ1_MiBa_SA_HCC1143_2: 17990
SA = 0.81
Score = 1.37
NAAPPPVTY − MSFragger
20120321_EXQ1_MiBa_SA_HCC1143_2: 17990
SA = 0.81
Score = 1.37
Relative intensity
m/z
Prosit prediction
* Exclusive in prediction
# Exclusive in experimental

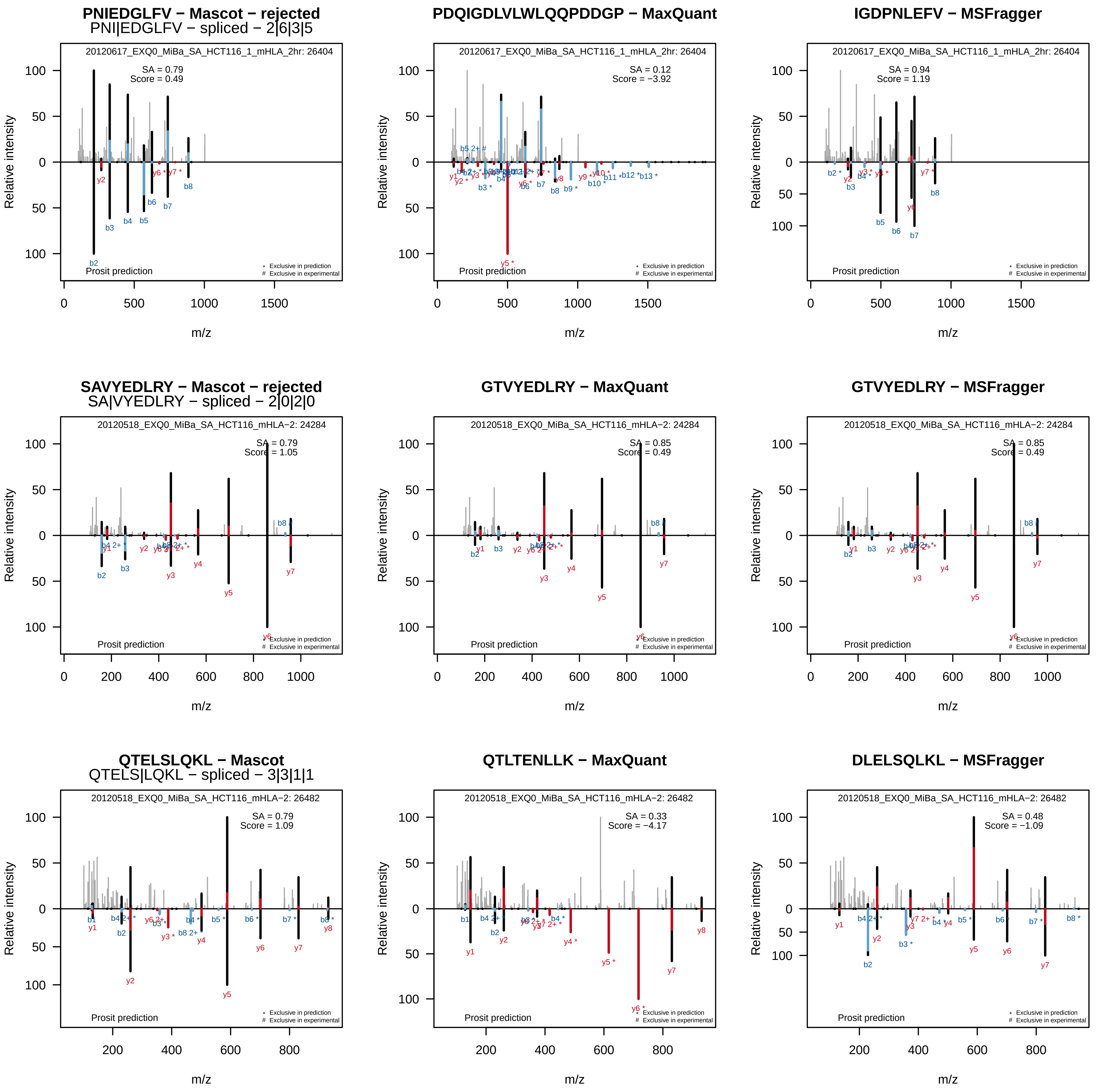
PNIEDGLFV − Mascot − rejected
PNI|EDGLFV − spliced − 2|6|3|5
20120617_EXQ0_MiBa_SA_HCT116_1_mHLA_2hr: 26404
SA = 0.79
Score = 0.49
PDQIGDLVLWLQQPDDGP − MaxQuant
20120617_EXQ0_MiBa_SA_HCT116_1_mHLA_2hr: 26404
SA = 0.12
Score = −3.92
IGDPNLEFV − MSFragger
20120617_EXQ0_MiBa_SA_HCT116_1_mHLA_2hr: 26404
SA = 0.94
Score = 1.19
SAVYEDLRY − Mascot − rejected
SA|VYEDLRY − spliced − 2|0|2|0
20120518_EXQ0_MiBa_SA_HCT116_mHLA−2: 24284
SA = 0.79
Score = 1.05
GTVYEDLRY − MaxQuant
20120518_EXQ0_MiBa_SA_HCT116_mHLA−2: 24284
SA = 0.85
Score = 0.49
GTVYEDLRY − MSFragger
20120518_EXQ0_MiBa_SA_HCT116_mHLA−2: 24284
SA = 0.85
Score = 0.49
QTELSLQKL − Mascot
QTELS|LQKL − spliced − 3|3|1|1
20120518_EXQ0_MiBa_SA_HCT116_mHLA−2: 26482
SA = 0.79
Score = 1.09
QTLTENLLK − MaxQuant
20120518_EXQ0_MiBa_SA_HCT116_mHLA−2: 26482
SA = 0.33
Score = −4.17
DLELSQLKL − MSFragger
20120518_EXQ0_MiBa_SA_HCT116_mHLA−2: 26482
SA = 0.48
Score = −1.09
Relative intensity
m/z
Prosit prediction
* Exclusive in prediction
# Exclusive in experimental

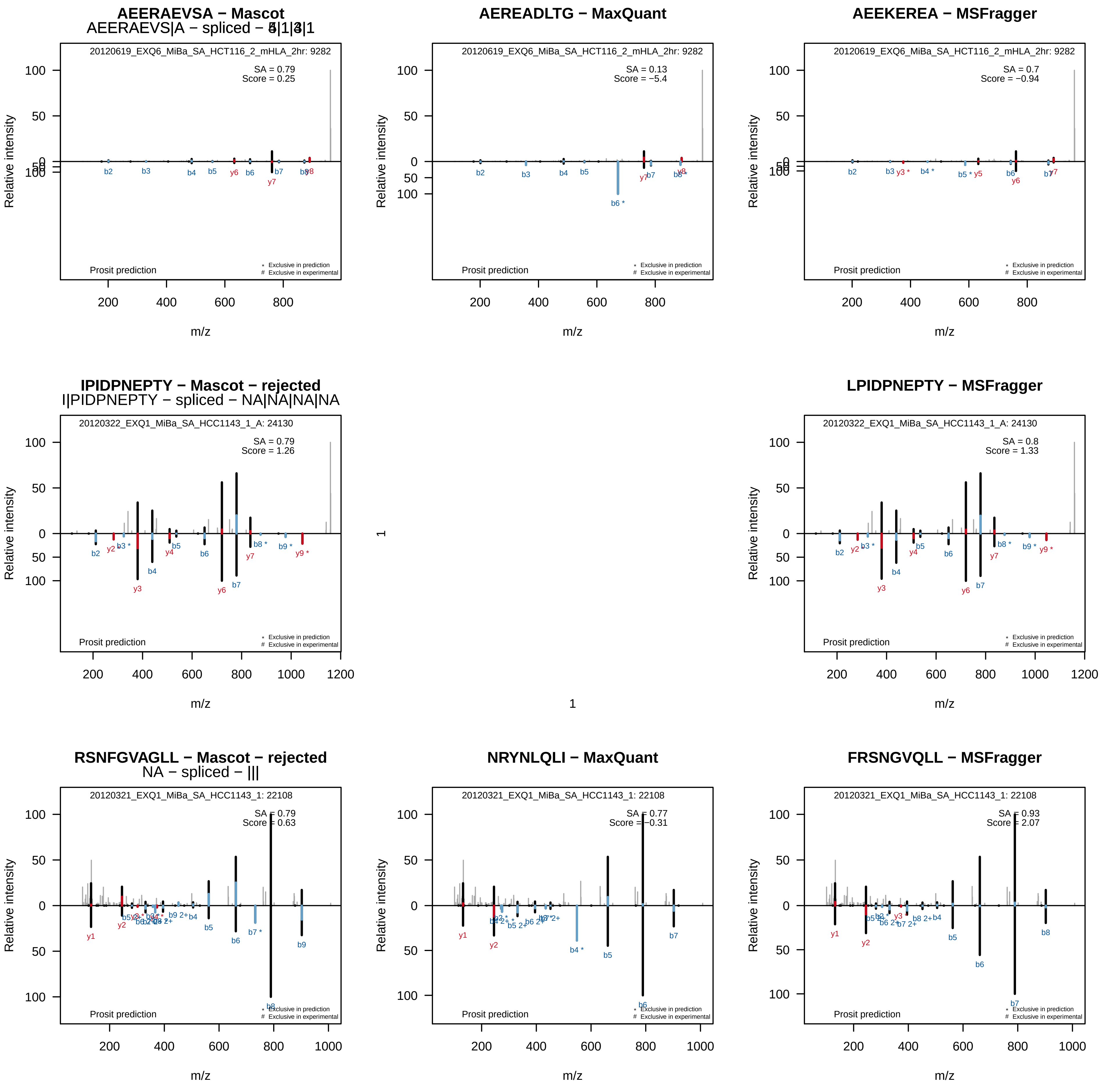

AEERAEVSA − Mascot
AEERAEVS|A − spliced − 4|1|3|1
20120619_EXQ6_MiBa_SA_HCT116_2_mHLA_2hr: 9282
SA = 0.79
Score = 0.25
AEREADLTG − MaxQuant
20120619_EXQ6_MiBa_SA_HCT116_2_mHLA_2hr: 9282
SA = 0.13
Score = −5.4
AEEKEREA − MSFragger
20120619_EXQ6_MiBa_SA_HCT116_2_mHLA_2hr: 9282
SA = 0.7
Score = −0.94
IPIDPNEPTY − Mascot − rejected
I|PIDPNEPTY − spliced − NA|NA|NA|NA
20120322_EXQ1_MiBa_SA_HCC1143_1_A: 24130
SA = 0.79
Score = 1.26
LPIDPNEPTY − MSFragger
20120322_EXQ1_MiBa_SA_HCC1143_1_A: 24130
SA = 0.8
Score = 1.33
RSNFGVAGLL − Mascot − rejected
NA − spliced − |||
20120321_EXQ1_MiBa_SA_HCC1143_1: 22108
SA = 0.79
Score = 0.63
NRYNLQLI − MaxQuant
20120321_EXQ1_MiBa_SA_HCC1143_1: 22108
SA = 0.77
Score = −0.31
FRSNGVQLL − MSFragger
20120321_EXQ1_MiBa_SA_HCC1143_1: 22108
SA = 0.93
Score = 2.07
Prosit prediction
⋆ Exclusive in prediction
Exclusive in experimental
Relative intensity
m/z

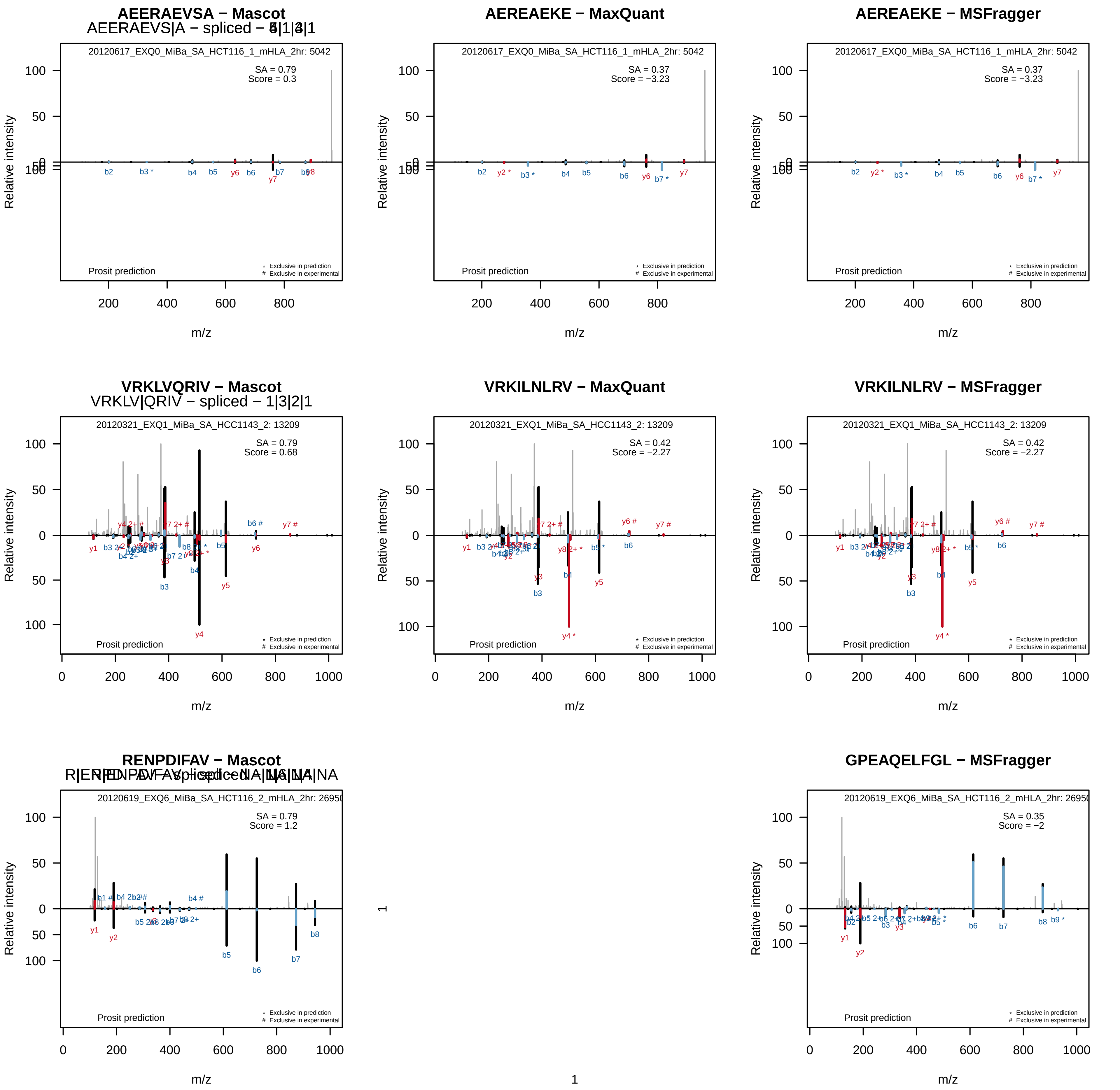
AEERAEVSA − Mascot
20120617_EXQ0_MiBa_SA_HCT116_1_mHLA_2hr: 5042
SA = 0.79
Score = 0.3
Prosit prediction
Exclusive in prediction
Exclusive in experimental
Relative intensity
m/z
AEREAEKE − MaxQuant
20120617_EXQ0_MiBa_SA_HCT116_1_mHLA_2hr: 5042
SA = 0.37
Score = −3.23
AEREAEKE − MSFragger
VRKLVQRIV − Mascot
VRKLV|QRIV − spliced − 1|3|2|1
20120321_EXQ1_MiBa_SA_HCC1143_2: 13209
SA = 0.79
Score = 0.68
VRKILNLRV − MaxQuant
SA = 0.42
Score = −2.27
VRKILNLRV − MSFragger
RENPDIFAV − Mascot
20120619_EXQ6_MiBa_SA_HCT116_2_mHLA_2hr: 26950
SA = 0.79
Score = 1.2
GPEAQELFGL − MSFragger
SA = 0.35
Score = −2

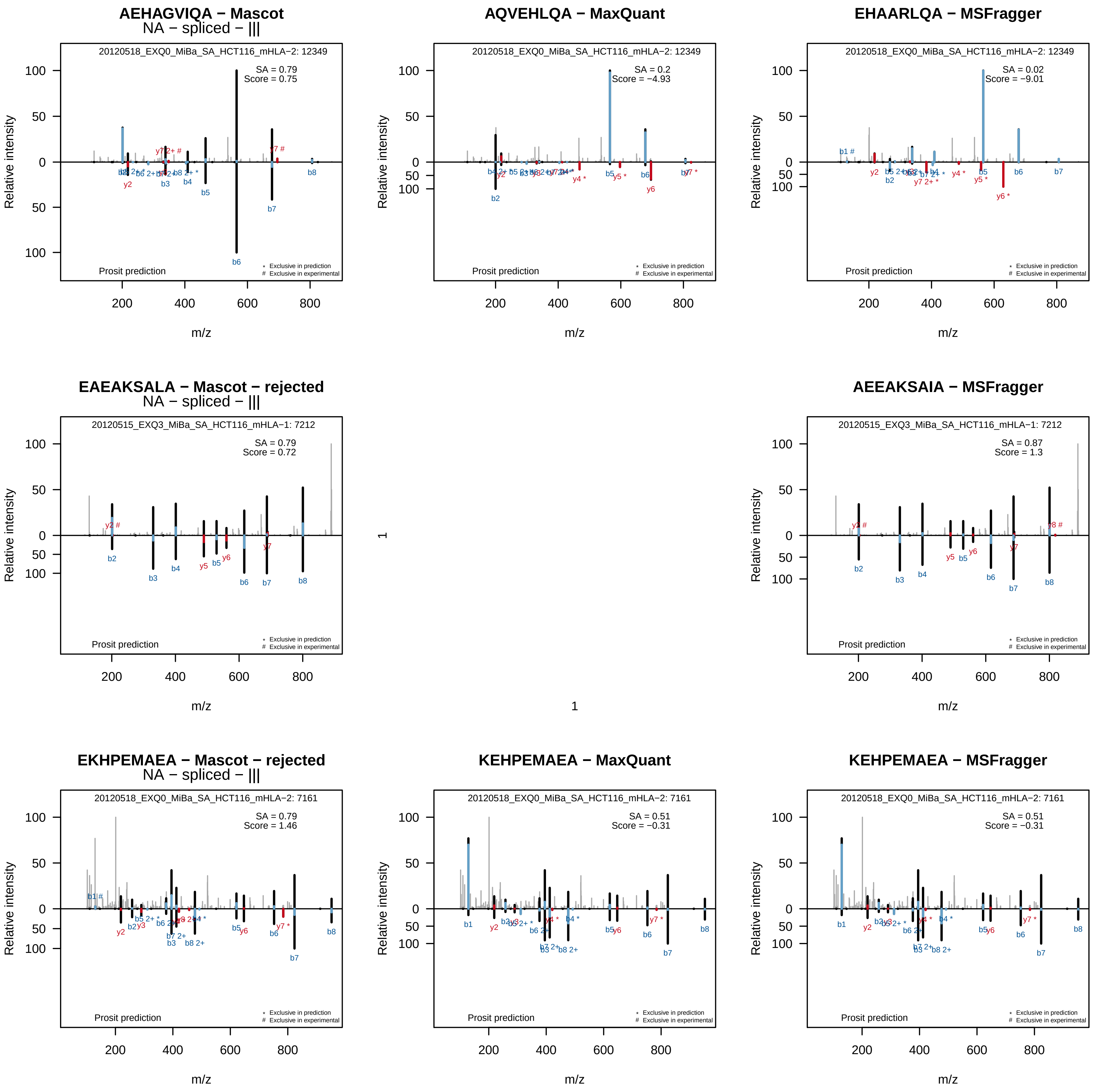

AEHAGVIQA − Mascot
NA − spliced − |||
20120518_EXQ0_MiBa_SA_HCT116_mHLA−2: 12349
SA = 0.79
Score = 0.75
Prosit prediction
⋆ Exclusive in prediction
Exclusive in experimental
AQVEHLQA − MaxQuant
20120518_EXQ0_MiBa_SA_HCT116_mHLA−2: 12349
SA = 0.2
Score = −4.93
EHAARLQA − MSFragger
20120518_EXQ0_MiBa_SA_HCT116_mHLA−2: 12349
SA = 0.02
Score = −9.01
EAEAKSALA − Mascot − rejected
NA − spliced − |||
20120515_EXQ3_MiBa_SA_HCT116_mHLA−1: 7212
SA = 0.79
Score = 0.72
AEEAKSAIA − MSFragger
20120515_EXQ3_MiBa_SA_HCT116_mHLA−1: 7212
SA = 0.87
Score = 1.3
EKHPEMAEA − Mascot − rejected
NA − spliced − |||
20120518_EXQ0_MiBa_SA_HCT116_mHLA−2: 7161
SA = 0.79
Score = 1.46
KEHPEMAEA − MaxQuant
20120518_EXQ0_MiBa_SA_HCT116_mHLA−2: 7161
SA = 0.51
Score = −0.31
KEHPEMAEA − MSFragger
20120518_EXQ0_MiBa_SA_HCT116_mHLA−2: 7161
SA = 0.51
Score = −0.31
Relative intensity
m/z
1

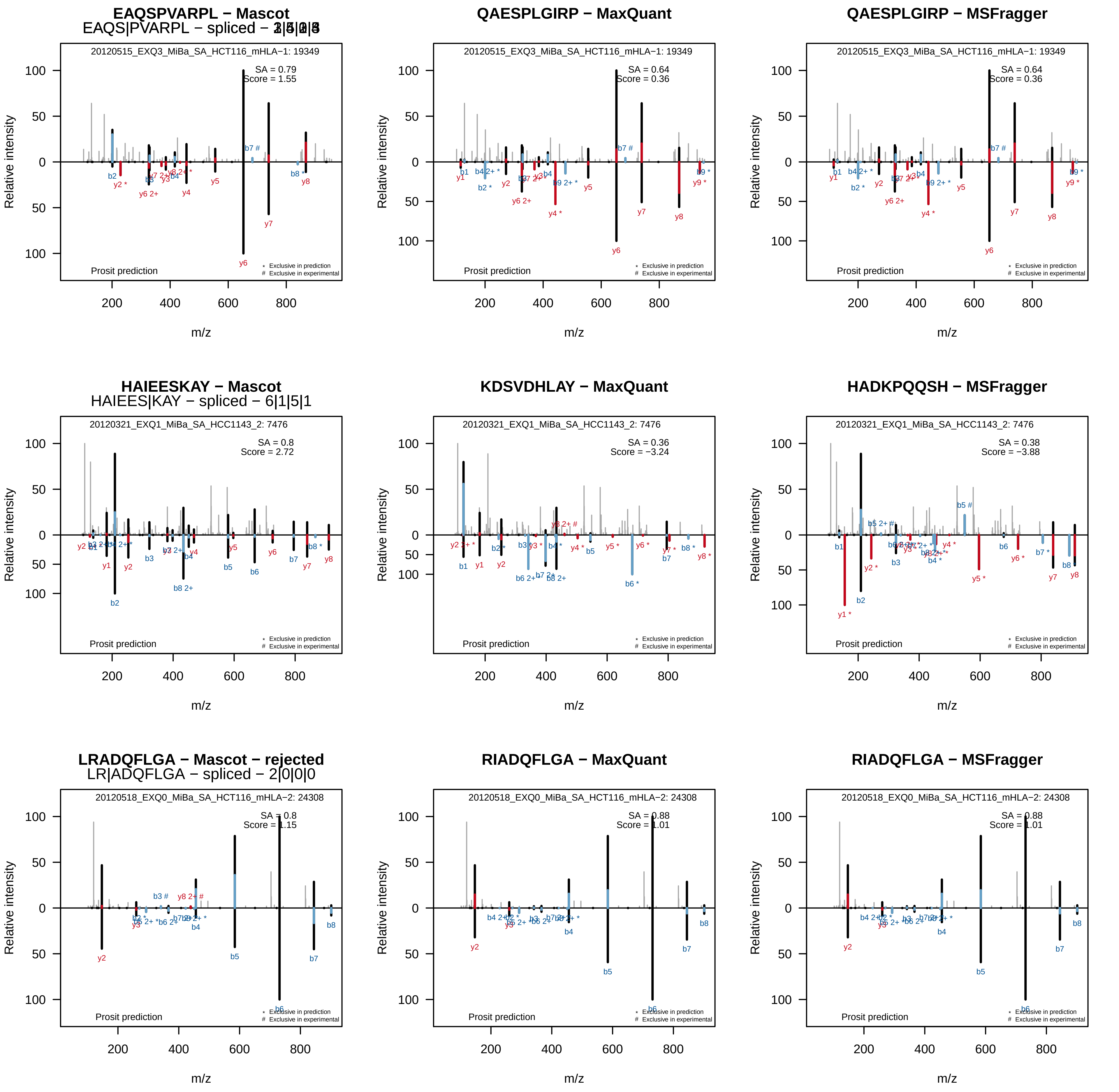

EAQSPVARPL − Mascot
EAQS|PVARPL − spliced
20120515_EXQ3_MiBa_SA_HCT116_mHLA−1: 19349
SA = 0.79
Score = 1.55
QAESPLGIRP − MaxQuant
20120515_EXQ3_MiBa_SA_HCT116_mHLA−1: 19349
SA = 0.64
Score = 0.36
QAESPLGIRP − MSFragger
20120515_EXQ3_MiBa_SA_HCT116_mHLA−1: 19349
SA = 0.64
Score = 0.36
HAIEESKAY − Mascot
HAIEES|KAY − spliced − 6|1|5|1
20120321_EXQ1_MiBa_SA_HCC1143_2: 7476
SA = 0.8
Score = 2.72
KDSVDHLAY − MaxQuant
20120321_EXQ1_MiBa_SA_HCC1143_2: 7476
SA = 0.36
Score = −3.24
HADKPQQSH − MSFragger
20120321_EXQ1_MiBa_SA_HCC1143_2: 7476
SA = 0.38
Score = −3.88
LRADQFLGA − Mascot − rejected
LR|ADQFLGA − spliced − 2|0|0|0
20120518_EXQ0_MiBa_SA_HCT116_mHLA−2: 24308
SA = 0.8
Score = 1.15
RIADQFLGA − MaxQuant
20120518_EXQ0_MiBa_SA_HCT116_mHLA−2: 24308
SA = 0.88
Score = 1.01
RIADQFLGA − MSFragger
20120518_EXQ0_MiBa_SA_HCT116_mHLA−2: 24308
SA = 0.88
Score = 1.01
Relative intensity
m/z
Prosit prediction
* Exclusive in prediction
# Exclusive in experimental

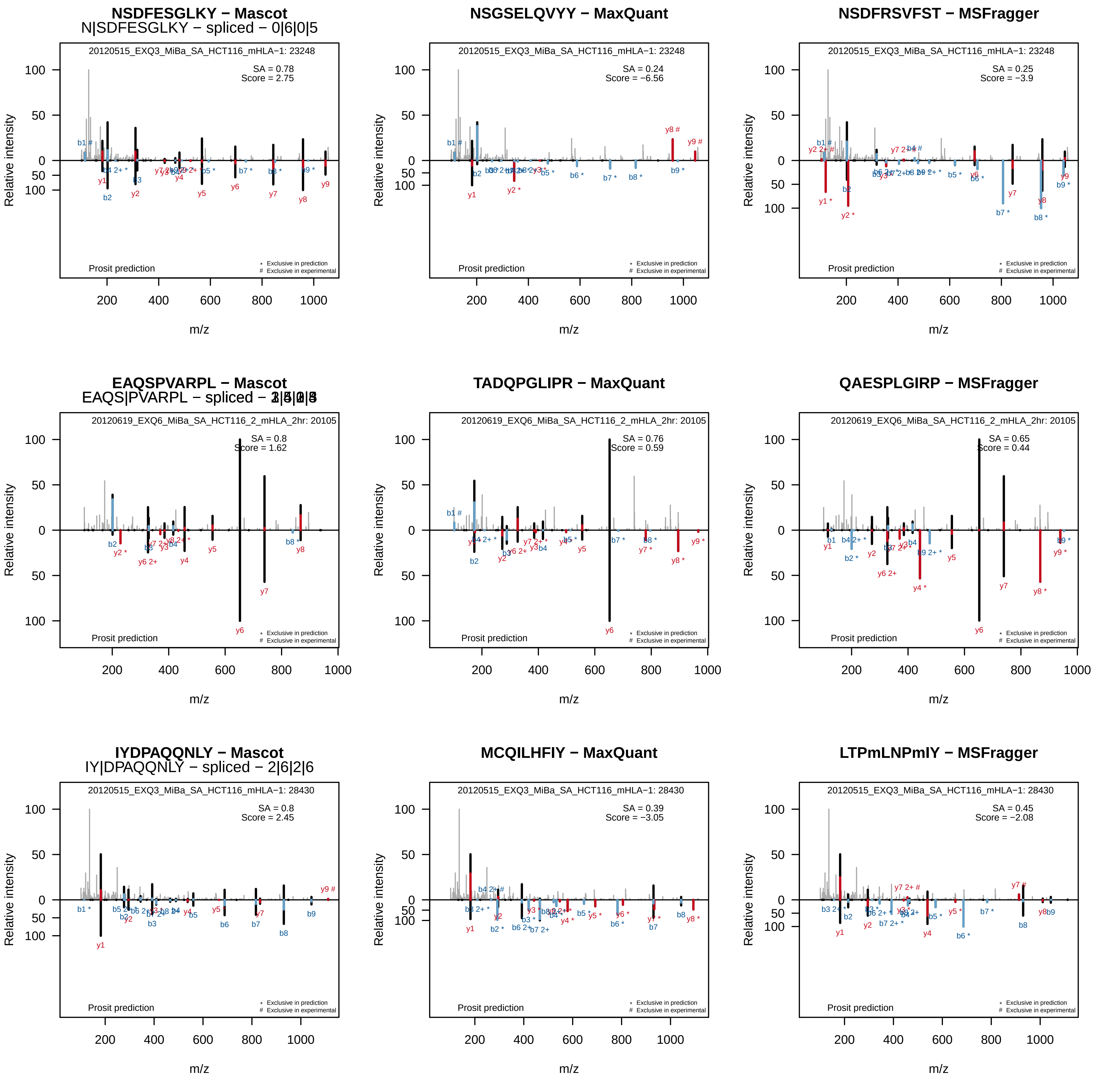

NSDFESGLKY − Mascot
N|SDFESGLKY − spliced − 0|6|0|5
20120515_EXQ3_MiBa_SA_HCT116_mHLA−1: 23248
SA = 0.78
Score = 2.75
NSGSELQVYY − MaxQuant
20120515_EXQ3_MiBa_SA_HCT116_mHLA−1: 23248
SA = 0.24
Score = −6.56
NSDFRSVFST − MSFragger
20120515_EXQ3_MiBa_SA_HCT116_mHLA−1: 23248
SA = 0.25
Score = −3.9
EAQSPVARPL − Mascot
EAQS|PVARPL − spliced −
20120619_EXQ6_MiBa_SA_HCT116_2_mHLA_2hr: 20105
SA = 0.8
Score = 1.62
TADQPGLIPR − MaxQuant
20120619_EXQ6_MiBa_SA_HCT116_2_mHLA_2hr: 20105
SA = 0.76
Score = 0.59
QAESPLGIRP − MSFragger
20120619_EXQ6_MiBa_SA_HCT116_2_mHLA_2hr: 20105
SA = 0.65
Score = 0.44
IYDPAQQNLY − Mascot
IY|DPAQQNLY − spliced − 2|6|2|6
20120515_EXQ3_MiBa_SA_HCT116_mHLA−1: 28430
SA = 0.8
Score = 2.45
MCQILHFIY − MaxQuant
20120515_EXQ3_MiBa_SA_HCT116_mHLA−1: 28430
SA = 0.39
Score = −3.05
LTPmLNPmIY − MSFragger
20120515_EXQ3_MiBa_SA_HCT116_mHLA−1: 28430
SA = 0.45
Score = −2.08
Relative intensity
m/z
Prosit prediction
* Exclusive in prediction
# Exclusive in experimental

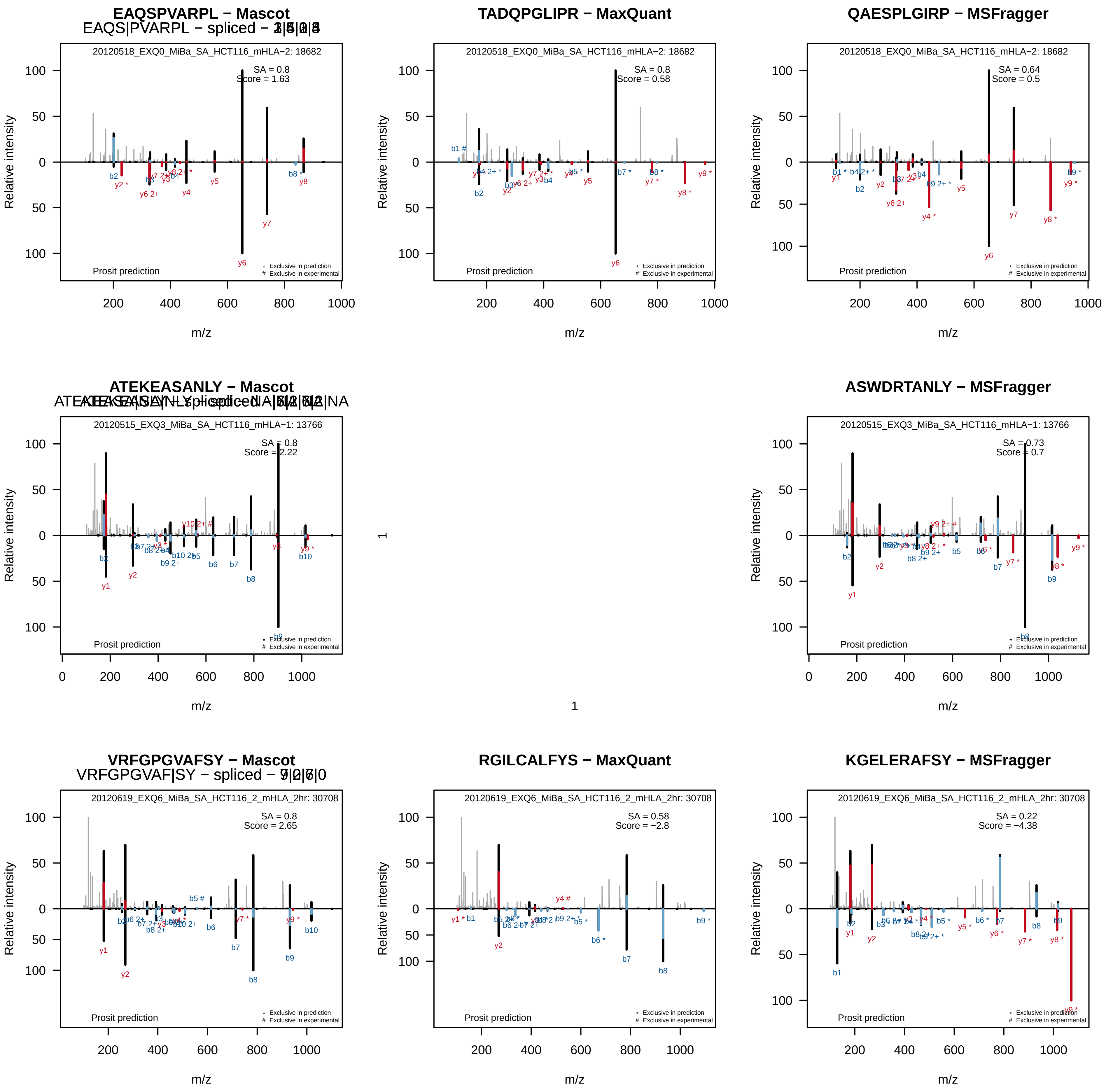

EAQSPVARPL − Mascot
EAQS|PVARPL − spliced
20120518_EXQ0_MiBa_SA_HCT116_mHLA−2: 18682
SA = 0.8
Score = 1.63
TADQPGLIPR − MaxQuant
20120518_EXQ0_MiBa_SA_HCT116_mHLA−2: 18682
SA = 0.8
Score = 0.58
QAESPLGIRP − MSFragger
20120518_EXQ0_MiBa_SA_HCT116_mHLA−2: 18682
SA = 0.64
Score = 0.5
ATEKEASANLY − Mascot
20120515_EXQ3_MiBa_SA_HCT116_mHLA−1: 13766
SA = 0.8
Score = 2.22
ASWDRTANLY − MSFragger
20120515_EXQ3_MiBa_SA_HCT116_mHLA−1: 13766
SA = 0.73
Score = 0.7
VRFGPGVAFSY − Mascot
VRFGPGVAF|SY − spliced
20120619_EXQ6_MiBa_SA_HCT116_2_mHLA_2hr: 30708
SA = 0.8
Score = 2.65
RGILCALFYS − MaxQuant
20120619_EXQ6_MiBa_SA_HCT116_2_mHLA_2hr: 30708
SA = 0.58
Score = −2.8
KGELERAFSY − MSFragger
20120619_EXQ6_MiBa_SA_HCT116_2_mHLA_2hr: 30708
SA = 0.22
Score = −4.38
Relative intensity
m/z
Prosit prediction
* Exclusive in prediction
# Exclusive in experimental

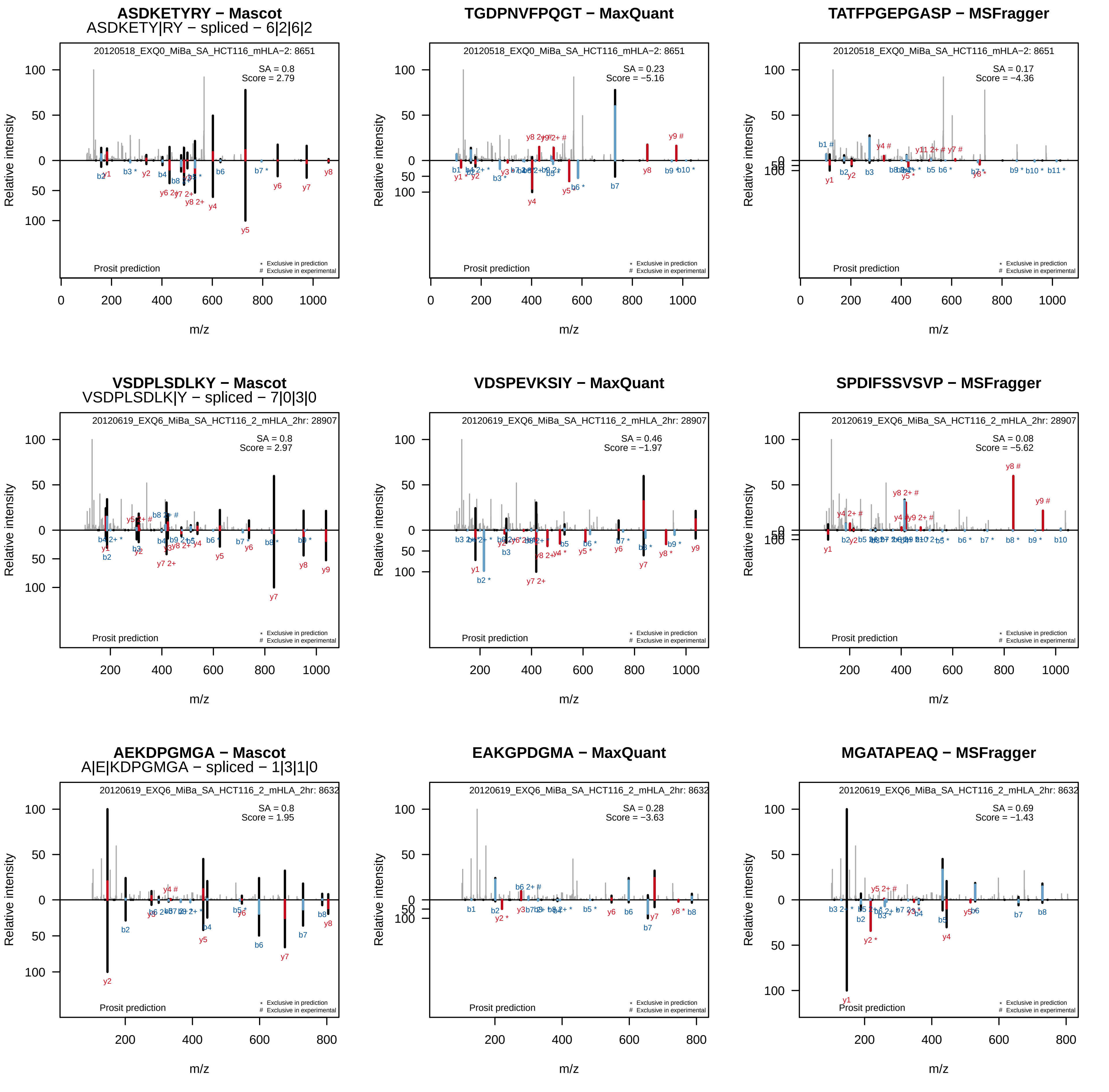

ASDKETYRY − Mascot
ASDKETY|RY − spliced − 6|2|6|2
20120518_EXQ0_MiBa_SA_HCT116_mHLA−2: 8651
SA = 0.8
Score = 2.79
TGDPNVFPQGT − MaxQuant
20120518_EXQ0_MiBa_SA_HCT116_mHLA−2: 8651
SA = 0.23
Score = −5.16
TATFPGEPGASP − MSFragger
20120518_EXQ0_MiBa_SA_HCT116_mHLA−2: 8651
SA = 0.17
Score = −4.36
VSDPLSDLKY − Mascot
VSDPLSDLK|Y − spliced − 7|0|3|0
20120619_EXQ6_MiBa_SA_HCT116_2_mHLA_2hr: 28907
SA = 0.8
Score = 2.97
VDSPEVKSIY − MaxQuant
20120619_EXQ6_MiBa_SA_HCT116_2_mHLA_2hr: 28907
SA = 0.46
Score = −1.97
SPDIFSSVSVP − MSFragger
20120619_EXQ6_MiBa_SA_HCT116_2_mHLA_2hr: 28907
SA = 0.08
Score = −5.62
AEKDPGMGA − Mascot
A|E|KDPGMGA − spliced − 1|3|1|0
20120619_EXQ6_MiBa_SA_HCT116_2_mHLA_2hr: 8632
SA = 0.8
Score = 1.95
EAKGPDGMA − MaxQuant
20120619_EXQ6_MiBa_SA_HCT116_2_mHLA_2hr: 8632
SA = 0.28
Score = −3.63
MGATAPEAQ − MSFragger
20120619_EXQ6_MiBa_SA_HCT116_2_mHLA_2hr: 8632
SA = 0.69
Score = −1.43
Relative intensity
m/z
Prosit prediction
* Exclusive in prediction
# Exclusive in experimental

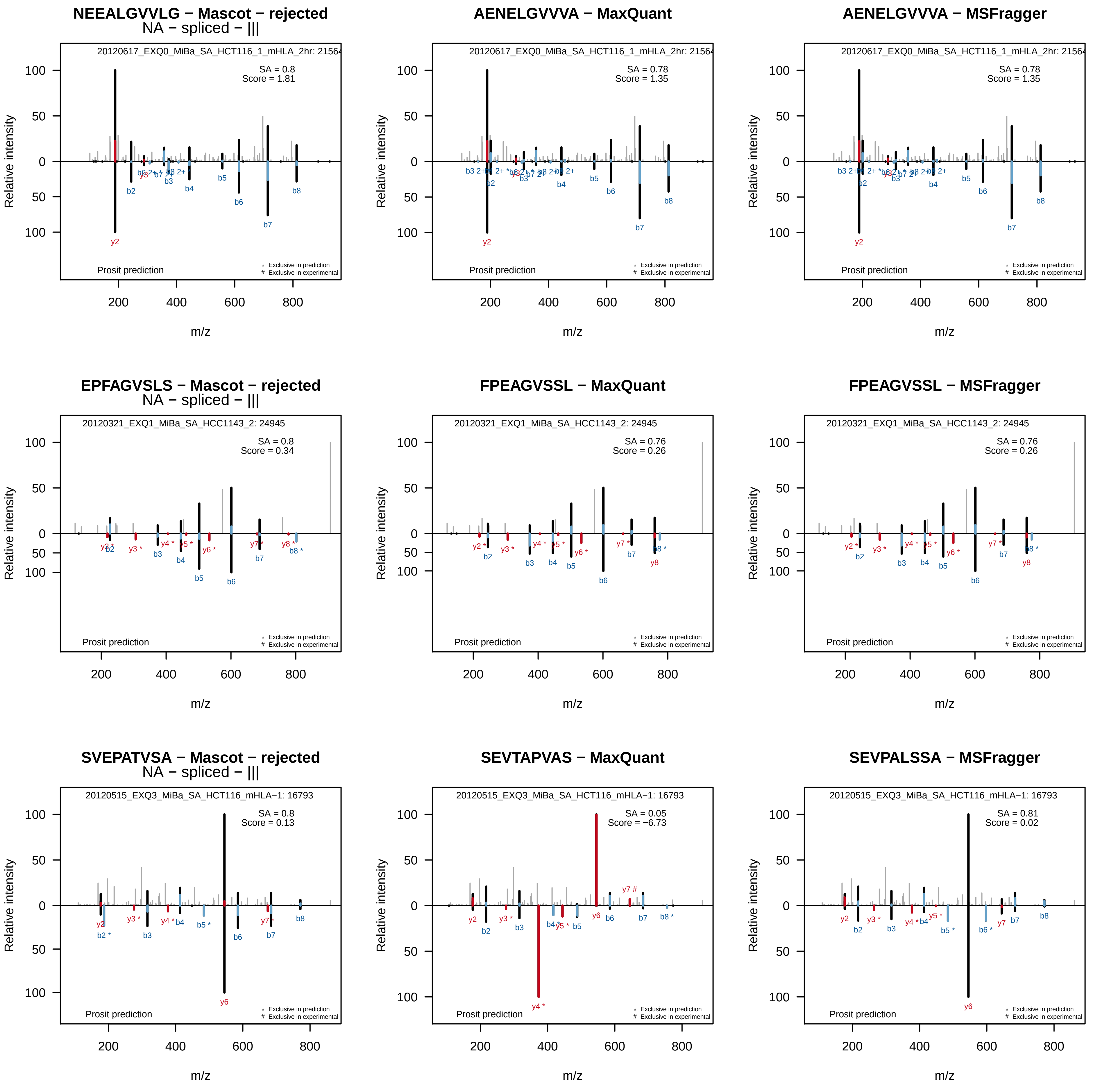
NEEALGVVLG − Mascot − rejected
NA − spliced − |||
20120617_EXQ0_MiBa_SA_HCT116_1_mHLA_2hr: 2156
SA = 0.8
Score = 1.81
AENELGVVVA − MaxQuant
20120617_EXQ0_MiBa_SA_HCT116_1_mHLA_2hr: 2156
SA = 0.78
Score = 1.35
AENELGVVVA − MSFragger
20120617_EXQ0_MiBa_SA_HCT116_1_mHLA_2hr: 2156
SA = 0.78
Score = 1.35
EPFAGVSLS − Mascot − rejected
NA − spliced − |||
20120321_EXQ1_MiBa_SA_HCC1143_2: 24945
SA = 0.8
Score = 0.34
FPEAGVSSL − MaxQuant
20120321_EXQ1_MiBa_SA_HCC1143_2: 24945
SA = 0.76
Score = 0.26
FPEAGVSSL − MSFragger
20120321_EXQ1_MiBa_SA_HCC1143_2: 24945
SA = 0.76
Score = 0.26
SVEPATVSA − Mascot − rejected
NA − spliced − |||
20120515_EXQ3_MiBa_SA_HCT116_mHLA−1: 16793
SA = 0.8
Score = 0.13
SEVTAPVAS − MaxQuant
20120515_EXQ3_MiBa_SA_HCT116_mHLA−1: 16793
SA = 0.05
Score = −6.73
SEVPALSSA − MSFragger
20120515_EXQ3_MiBa_SA_HCT116_mHLA−1: 16793
SA = 0.81
Score = 0.02
Relative intensity
m/z
Prosit prediction
* Exclusive in prediction
# Exclusive in experimental

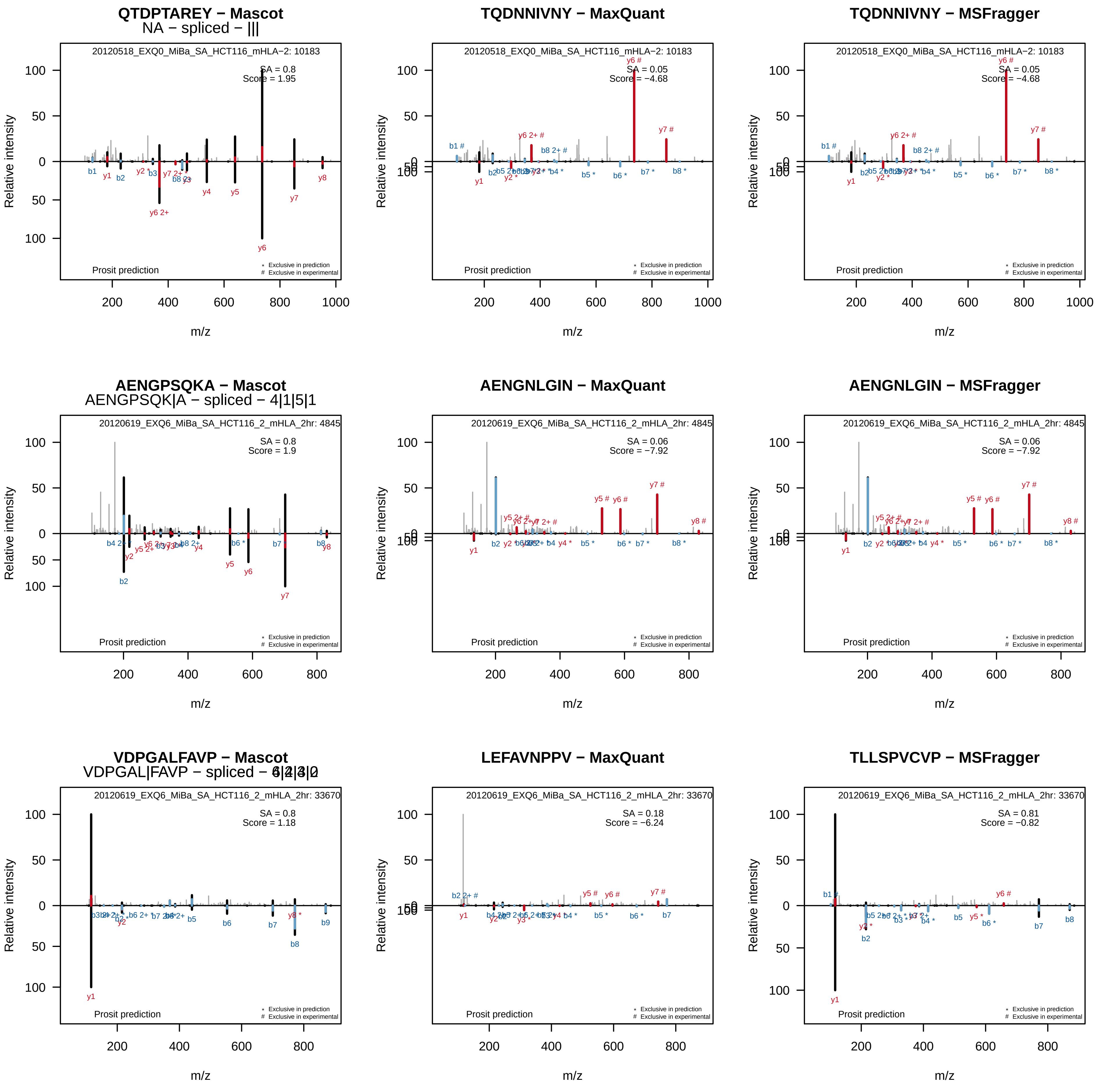

QTDPTAREY − Mascot
NA − spliced − |||
20120518_EXQ0_MiBa_SA_HCT116_mHLA−2: 10183
SA = 0.8
Score = 1.95
TQDNNIVNY − MaxQuant
20120518_EXQ0_MiBa_SA_HCT116_mHLA−2: 10183
SA = 0.05
Score = −4.68
TQDNNIVNY − MSFragger
20120518_EXQ0_MiBa_SA_HCT116_mHLA−2: 10183
SA = 0.05
Score = −4.68
AENGPSQKA − Mascot
AENGPSQK|A − spliced − 4|1|5|1
20120619_EXQ6_MiBa_SA_HCT116_2_mHLA_2hr: 4845
SA = 0.8
Score = 1.9
AENGNLGIN − MaxQuant
20120619_EXQ6_MiBa_SA_HCT116_2_mHLA_2hr: 4845
SA = 0.06
Score = −7.92
AENGNLGIN − MSFragger
20120619_EXQ6_MiBa_SA_HCT116_2_mHLA_2hr: 4845
SA = 0.06
Score = −7.92
VDPGALFAVP − Mascot
VDPGAL|FAVP − spliced
20120619_EXQ6_MiBa_SA_HCT116_2_mHLA_2hr: 33670
SA = 0.8
Score = 1.18
LEFAVNPPV − MaxQuant
20120619_EXQ6_MiBa_SA_HCT116_2_mHLA_2hr: 33670
SA = 0.18
Score = −6.24
TLLSPVCVP − MSFragger
20120619_EXQ6_MiBa_SA_HCT116_2_mHLA_2hr: 33670
SA = 0.81
Score = −0.82
Relative intensity
m/z
Prosit prediction
* Exclusive in prediction
# Exclusive in experimental

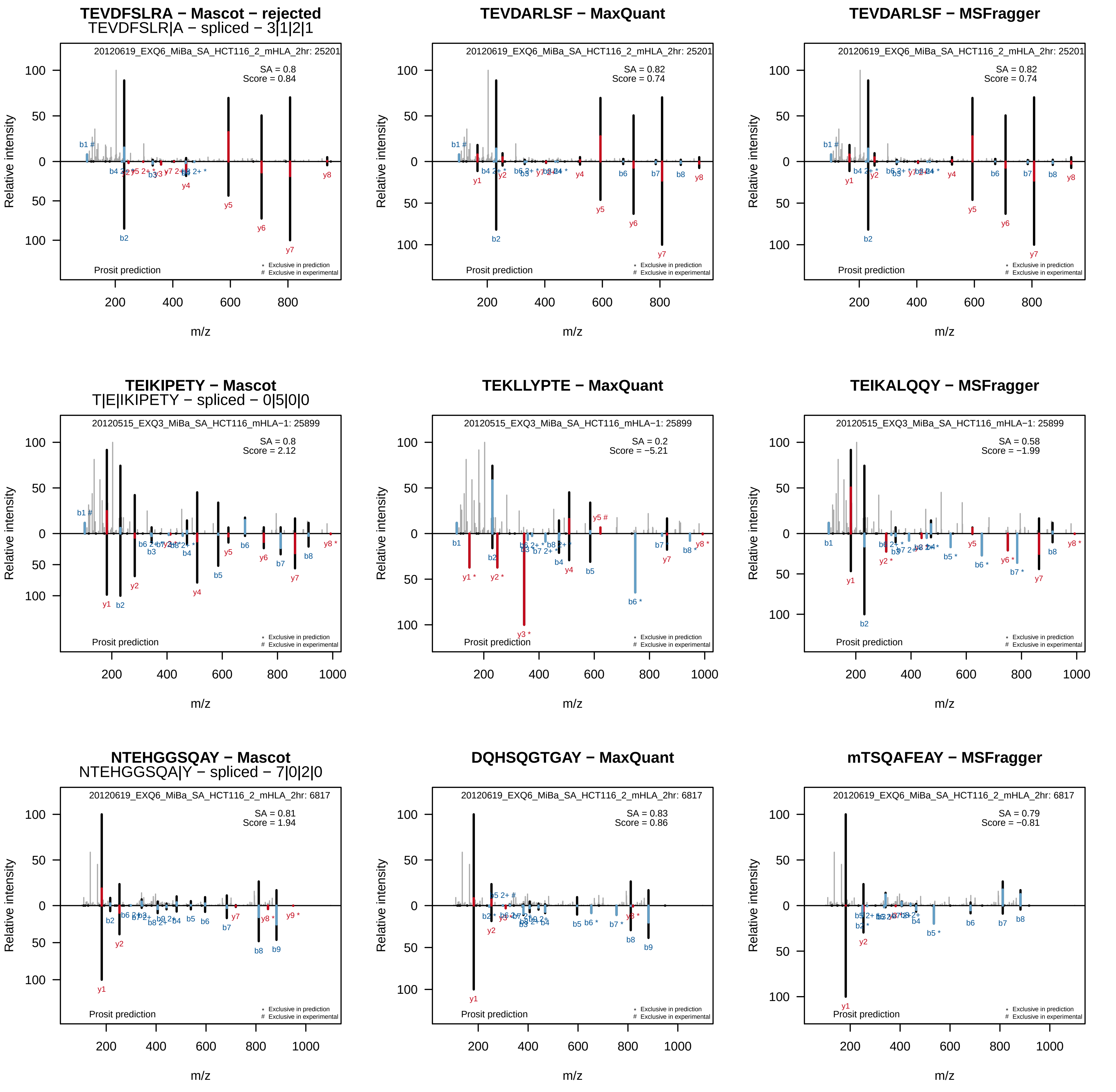
TEVDFSLRA − Mascot − rejected
TEVDFSLR|A − spliced − 3|1|2|1
20120619_EXQ6_MiBa_SA_HCT116_2_mHLA_2hr: 25201
SA = 0.8
Score = 0.84
Prosit prediction
TEVDARLSF − MaxQuant
20120619_EXQ6_MiBa_SA_HCT116_2_mHLA_2hr: 25201
SA = 0.82
Score = 0.74
Prosit prediction
TEVDARLSF − MSFragger
20120619_EXQ6_MiBa_SA_HCT116_2_mHLA_2hr: 25201
SA = 0.82
Score = 0.74
Prosit prediction
TEIKIPETY − Mascot
T|E|IKIPETY − spliced − 0|5|0|0
20120515_EXQ3_MiBa_SA_HCT116_mHLA−1: 25899
SA = 0.8
Score = 2.12
Prosit prediction
TEKLLYPTE − MaxQuant
20120515_EXQ3_MiBa_SA_HCT116_mHLA−1: 25899
SA = 0.2
Score = −5.21
Prosit prediction
TEIKALQQY − MSFragger
20120515_EXQ3_MiBa_SA_HCT116_mHLA−1: 25899
SA = 0.58
Score = −1.99
Prosit prediction
NTEHGGSQAY − Mascot
NTEHGGSQA|Y − spliced − 7|0|2|0
20120619_EXQ6_MiBa_SA_HCT116_2_mHLA_2hr: 6817
SA = 0.81
Score = 1.94
Prosit prediction
DQHSQGTGAY − MaxQuant
20120619_EXQ6_MiBa_SA_HCT116_2_mHLA_2hr: 6817
SA = 0.83
Score = 0.86
Prosit prediction
mTSQAFEAY − MSFragger
20120619_EXQ6_MiBa_SA_HCT116_2_mHLA_2hr: 6817
SA = 0.79
Score = −0.81
Prosit prediction
Relative intensity
m/z
* Exclusive in prediction
# Exclusive in experimental

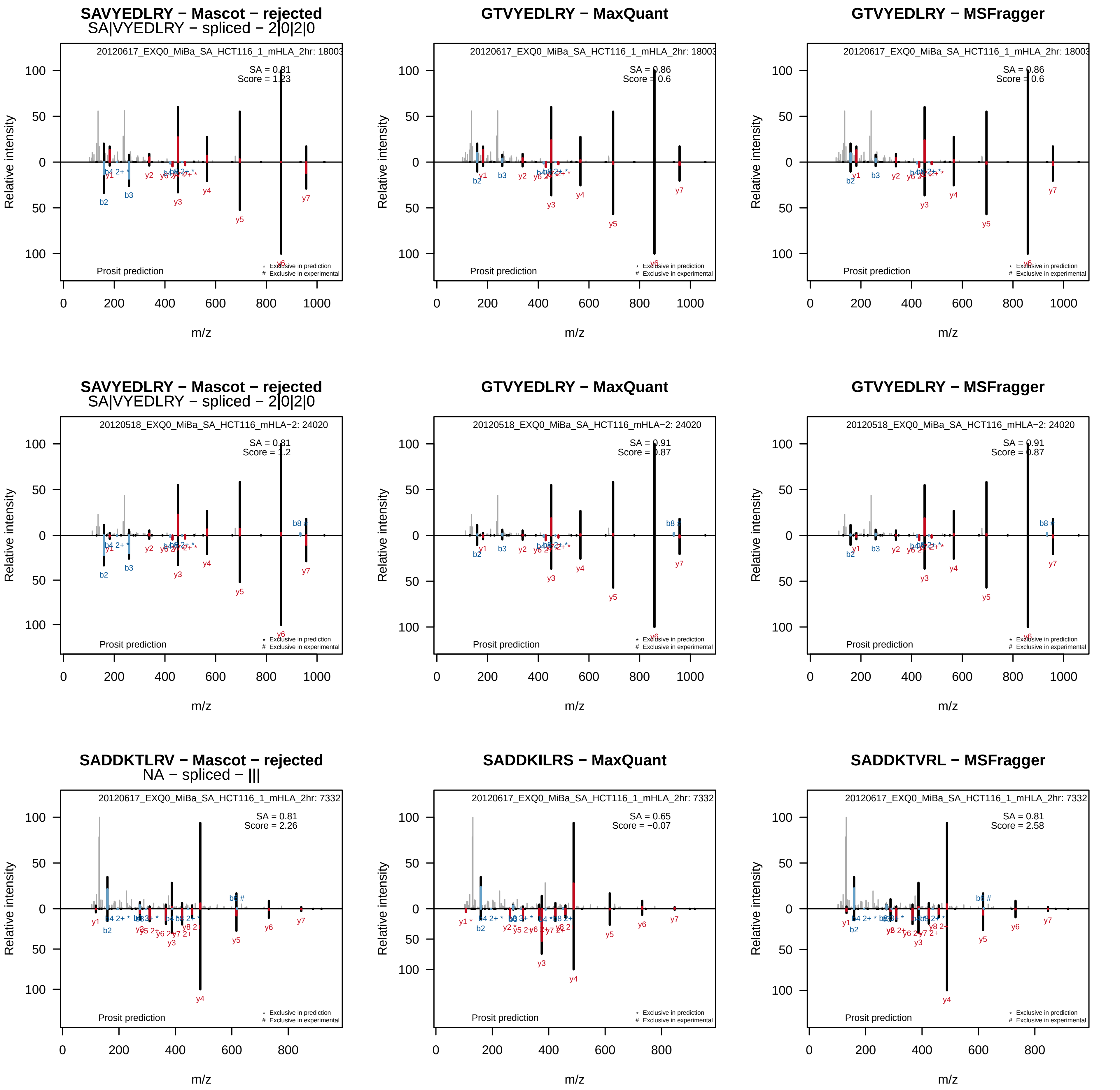

SAVYEDLRY − Mascot − rejected
SA|VYEDLRY − spliced − 2|0|2|0
20120617_EXQ0_MiBa_SA_HCT116_1_mHLA_2hr: 18003
SA = 0.81
Score = 1.23
GTVYEDLRY − MaxQuant
20120617_EXQ0_MiBa_SA_HCT116_1_mHLA_2hr: 18003
SA = 0.86
Score = 0.6
GTVYEDLRY − MSFragger
20120617_EXQ0_MiBa_SA_HCT116_1_mHLA_2hr: 18003
SA = 0.86
Score = 0.6
SAVYEDLRY − Mascot − rejected
SA|VYEDLRY − spliced − 2|0|2|0
20120518_EXQ0_MiBa_SA_HCT116_mHLA−2: 24020
SA = 0.81
Score = 1.2
GTVYEDLRY − MaxQuant
20120518_EXQ0_MiBa_SA_HCT116_mHLA−2: 24020
SA = 0.91
Score = 0.87
GTVYEDLRY − MSFragger
20120518_EXQ0_MiBa_SA_HCT116_mHLA−2: 24020
SA = 0.91
Score = 0.87
SADDKTLRV − Mascot − rejected
NA − spliced − |||
20120617_EXQ0_MiBa_SA_HCT116_1_mHLA_2hr: 7332
SA = 0.81
Score = 2.26
SADDKILRS − MaxQuant
20120617_EXQ0_MiBa_SA_HCT116_1_mHLA_2hr: 7332
SA = 0.65
Score = −0.07
SADDKTVRL − MSFragger
20120617_EXQ0_MiBa_SA_HCT116_1_mHLA_2hr: 7332
SA = 0.81
Score = 2.58
Relative intensity
m/z
Prosit prediction
Exclusive in prediction
Exclusive in experimental

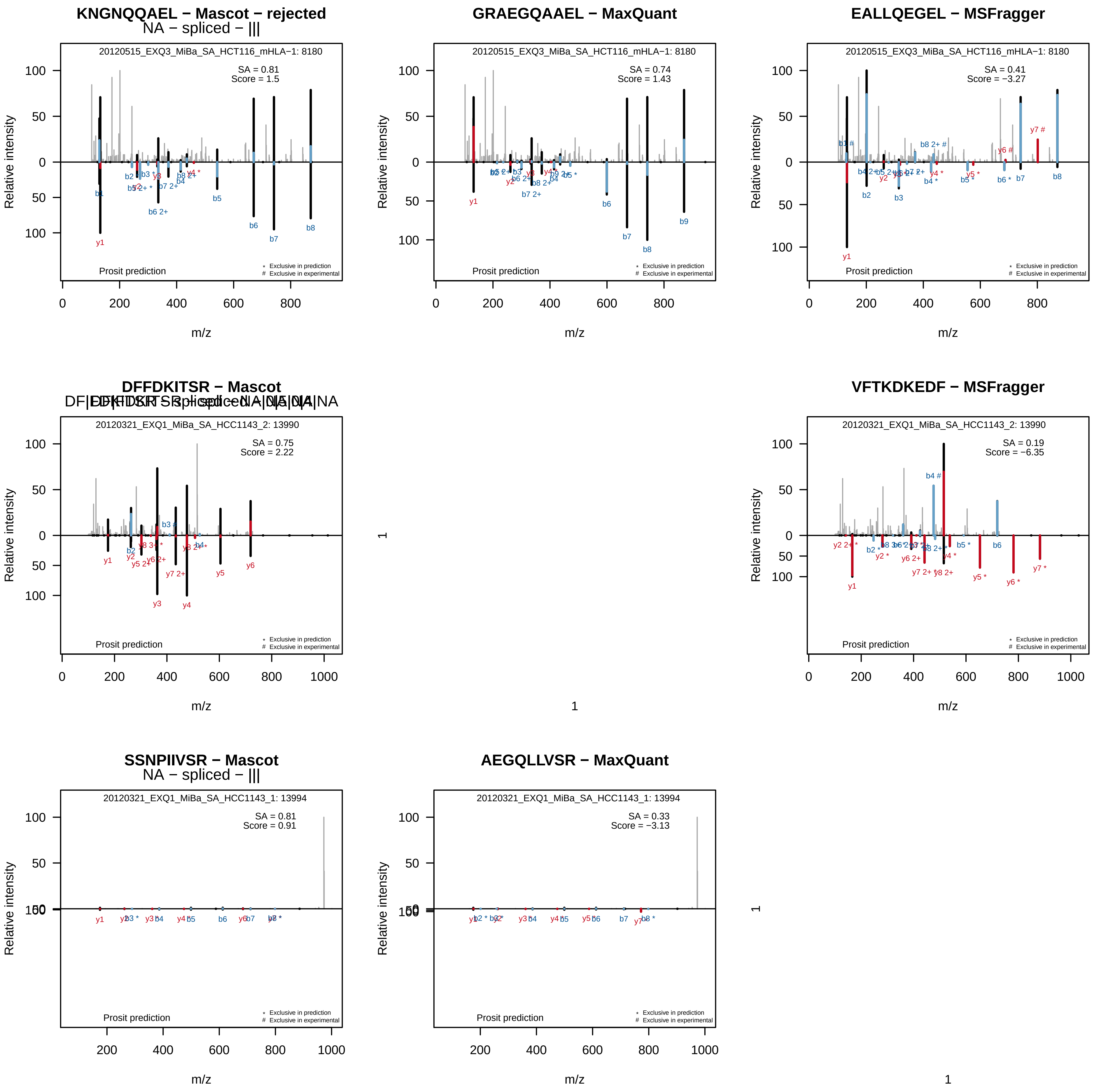

KNGNQQAEL − Mascot − rejected
NA − spliced − |||
20120515_EXQ3_MiBa_SA_HCT116_mHLA−1: 8180
SA = 0.81
Score = 1.5
Prosit prediction
GRAEGQAAEL − MaxQuant
20120515_EXQ3_MiBa_SA_HCT116_mHLA−1: 8180
SA = 0.74
Score = 1.43
Prosit prediction
EALLQEGEL − MSFragger
20120515_EXQ3_MiBa_SA_HCT116_mHLA−1: 8180
SA = 0.41
Score = −3.27
Prosit prediction
DFFDKITSR − Mascot
20120321_EXQ1_MiBa_SA_HCC1143_2: 13990
SA = 0.75
Score = 2.22
Prosit prediction
VFTKDKEDF − MSFragger
20120321_EXQ1_MiBa_SA_HCC1143_2: 13990
SA = 0.19
Score = −6.35
Prosit prediction
SSNPIIVSR − Mascot
NA − spliced − |||
20120321_EXQ1_MiBa_SA_HCC1143_1: 13994
SA = 0.81
Score = 0.91
Prosit prediction
AEGQLLVSR − MaxQuant
20120321_EXQ1_MiBa_SA_HCC1143_1: 13994
SA = 0.33
Score = −3.13
Prosit prediction
Relative intensity
m/z
⋆ Exclusive in prediction
Exclusive in experimental

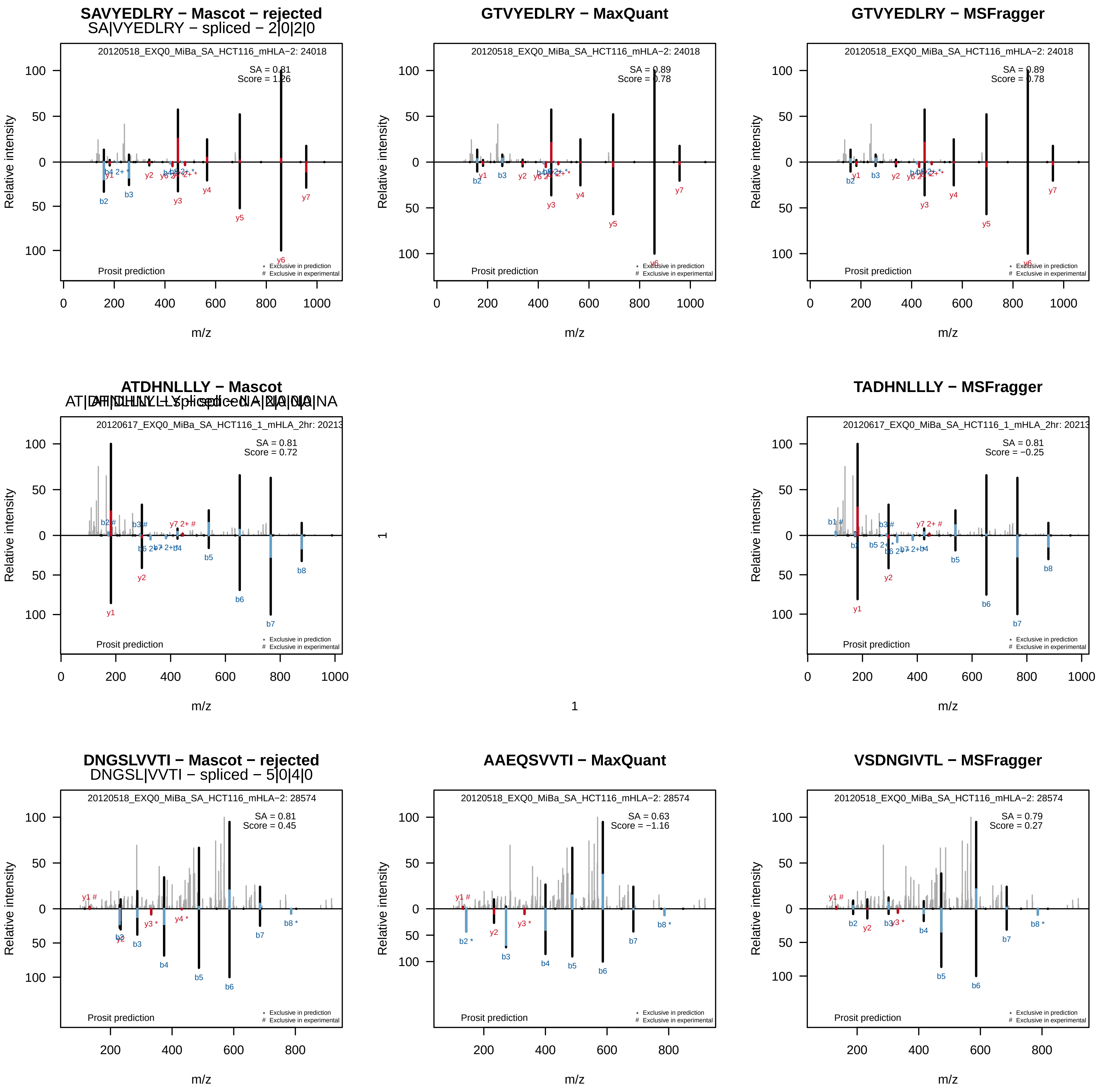
SAVYEDLRY − Mascot − rejected
SA|VYEDLRY − spliced − 2|0|2|0
20120518_EXQ0_MiBa_SA_HCT116_mHLA−2: 24018
SA = 0.81
Score = 1.26
GTVYEDLRY − MaxQuant
20120518_EXQ0_MiBa_SA_HCT116_mHLA−2: 24018
SA = 0.89
Score = 0.78
GTVYEDLRY − MSFragger
20120518_EXQ0_MiBa_SA_HCT116_mHLA−2: 24018
SA = 0.89
Score = 0.78
ATDHNLLLY − Mascot
20120617_EXQ0_MiBa_SA_HCT116_1_mHLA_2hr: 20213
SA = 0.81
Score = 0.72
TADHNLLLY − MSFragger
20120617_EXQ0_MiBa_SA_HCT116_1_mHLA_2hr: 20213
SA = 0.81
Score = −0.25
DNGSLVVTI − Mascot − rejected
DNGSL|VVTI − spliced − 5|0|4|0
20120518_EXQ0_MiBa_SA_HCT116_mHLA−2: 28574
SA = 0.81
Score = 0.45
AAEQSVVTI − MaxQuant
20120518_EXQ0_MiBa_SA_HCT116_mHLA−2: 28574
SA = 0.63
Score = −1.16
VSDNGIVTL − MSFragger
20120518_EXQ0_MiBa_SA_HCT116_mHLA−2: 28574
SA = 0.79
Score = 0.27
Relative intensity
m/z
Prosit prediction
⋆ Exclusive in prediction
Exclusive in experimental

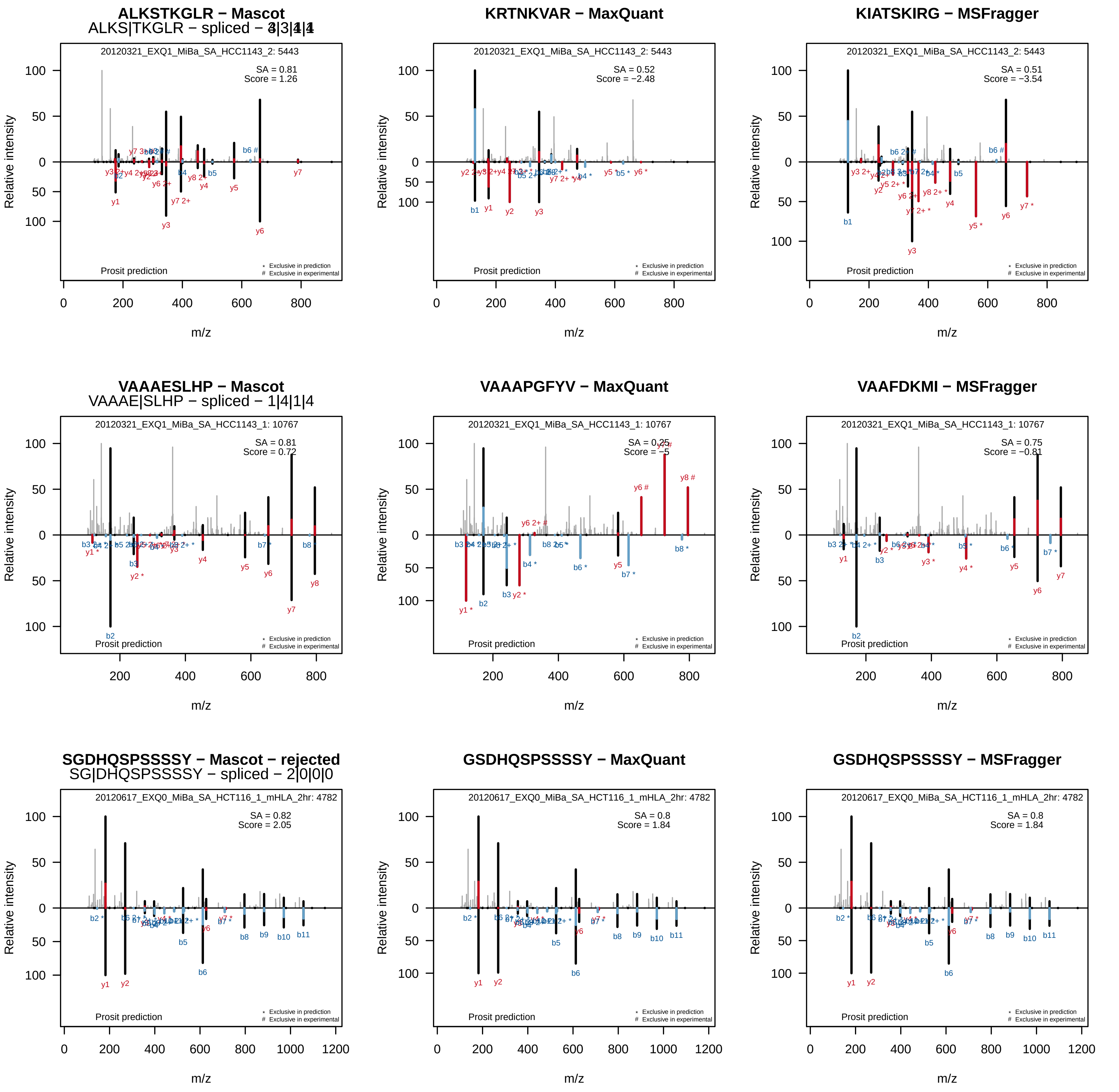
ALKSTKGLR − Mascot
ALKS|TKGLR − spliced
20120321_EXQ1_MiBa_SA_HCC1143_2: 5443
SA = 0.81
Score = 1.26
KRTNKVAR − MaxQuant
20120321_EXQ1_MiBa_SA_HCC1143_2: 5443
SA = 0.52
Score = −2.48
KIATSKIRG − MSFragger
20120321_EXQ1_MiBa_SA_HCC1143_2: 5443
SA = 0.51
Score = −3.54
VAAAESLHP − Mascot
VAAAE|SLHP − spliced − 1|4|1|4
20120321_EXQ1_MiBa_SA_HCC1143_1: 10767
SA = 0.81
Score = 0.72
VAAAPGFYV − MaxQuant
20120321_EXQ1_MiBa_SA_HCC1143_1: 10767
SA = 0.25
Score = −5
VAAFDKMI − MSFragger
20120321_EXQ1_MiBa_SA_HCC1143_1: 10767
SA = 0.75
Score = −0.81
SGDHQSPSSSSY − Mascot − rejected
SG|DHQSPSSSSY − spliced − 2|0|0|0
20120617_EXQ0_MiBa_SA_HCT116_1_mHLA_2hr: 4782
SA = 0.82
Score = 2.05
GSDHQSPSSSSY − MaxQuant
20120617_EXQ0_MiBa_SA_HCT116_1_mHLA_2hr: 4782
SA = 0.8
Score = 1.84
GSDHQSPSSSSY − MSFragger
20120617_EXQ0_MiBa_SA_HCT116_1_mHLA_2hr: 4782
SA = 0.8
Score = 1.84
Relative intensity
m/z
Prosit prediction
* Exclusive in prediction
# Exclusive in experimental

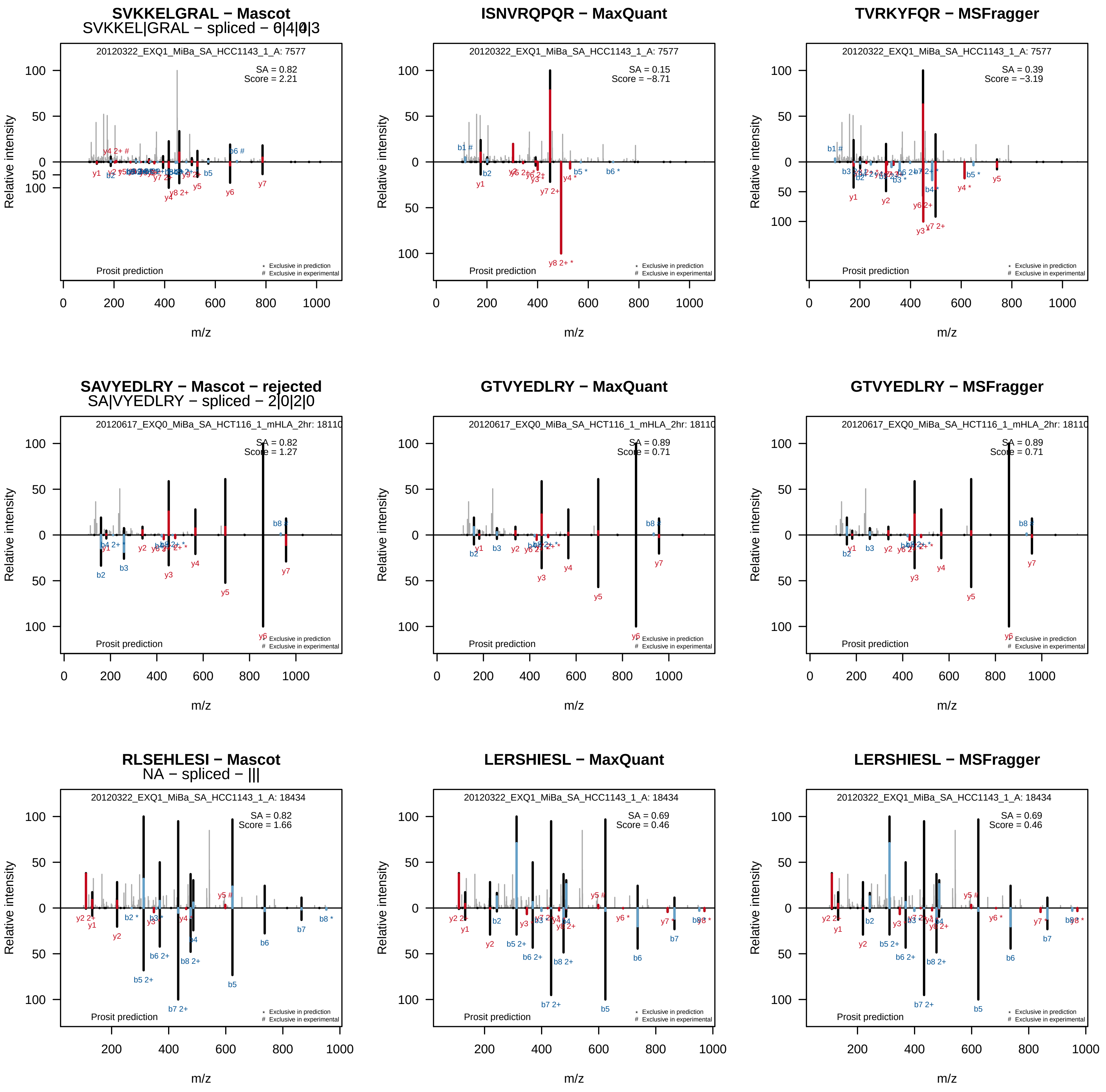

SVKKELGRAL − Mascot
SVKKEL|GRAL − spliced − 0|4|4|3
20120322_EXQ1_MiBa_SA_HCC1143_1_A: 7577
SA = 0.82
Score = 2.21
Prosit prediction
ISNVRQPQR − MaxQuant
20120322_EXQ1_MiBa_SA_HCC1143_1_A: 7577
SA = 0.15
Score = −8.71
Prosit prediction
TVRKYFQR − MSFragger
20120322_EXQ1_MiBa_SA_HCC1143_1_A: 7577
SA = 0.39
Score = −3.19
Prosit prediction
SAVYEDLRY − Mascot − rejected
SA|VYEDLRY − spliced − 2|0|2|0
20120617_EXQ0_MiBa_SA_HCT116_1_mHLA_2hr: 18110
SA = 0.82
Score = 1.27
Prosit prediction
GTVYEDLRY − MaxQuant
20120617_EXQ0_MiBa_SA_HCT116_1_mHLA_2hr: 18110
SA = 0.89
Score = 0.71
Prosit prediction
GTVYEDLRY − MSFragger
20120617_EXQ0_MiBa_SA_HCT116_1_mHLA_2hr: 18110
SA = 0.89
Score = 0.71
Prosit prediction
RLSEHLESI − Mascot
NA − spliced − |||
20120322_EXQ1_MiBa_SA_HCC1143_1_A: 18434
SA = 0.82
Score = 1.66
Prosit prediction
LERSHIESL − MaxQuant
20120322_EXQ1_MiBa_SA_HCC1143_1_A: 18434
SA = 0.69
Score = 0.46
Prosit prediction
LERSHIESL − MSFragger
20120322_EXQ1_MiBa_SA_HCC1143_1_A: 18434
SA = 0.69
Score = 0.46
Prosit prediction
* Exclusive in prediction
# Exclusive in experimental
Relative intensity
m/z

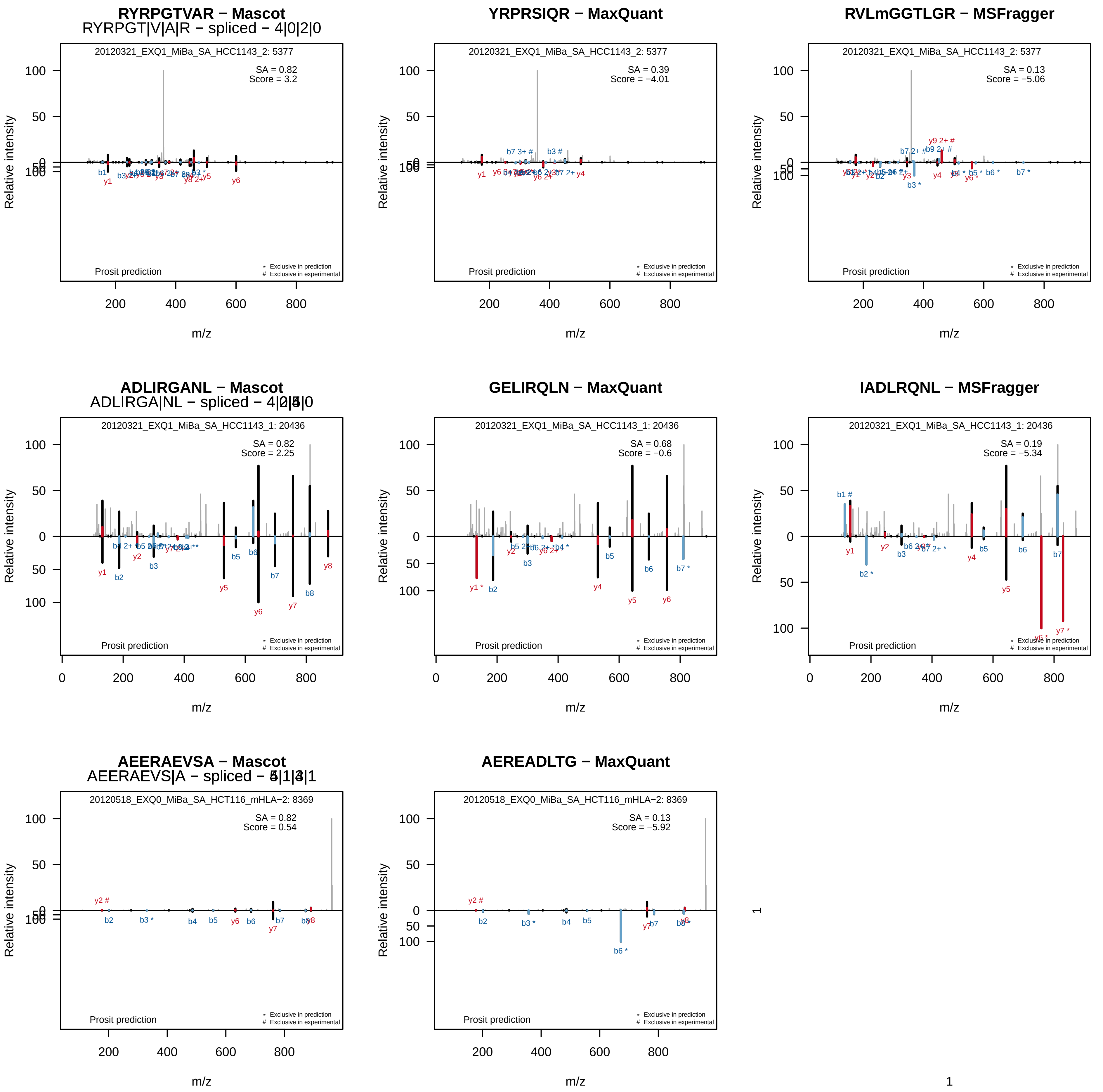

RYRPGTVAR − Mascot
RYRPGT|V|A|R − spliced − 4|0|2|0
20120321_EXQ1_MiBa_SA_HCC1143_2: 5377
SA = 0.82
Score = 3.2
YRPRSIQR − MaxQuant
20120321_EXQ1_MiBa_SA_HCC1143_2: 5377
SA = 0.39
Score = −4.01
RVLmGGTLGR − MSFragger
20120321_EXQ1_MiBa_SA_HCC1143_2: 5377
SA = 0.13
Score = −5.06
ADLIRGANL − Mascot
20120321_EXQ1_MiBa_SA_HCC1143_1: 20436
SA = 0.82
Score = 2.25
GELIRQLN − MaxQuant
20120321_EXQ1_MiBa_SA_HCC1143_1: 20436
SA = 0.68
Score = −0.6
IADLRQNL − MSFragger
20120321_EXQ1_MiBa_SA_HCC1143_1: 20436
SA = 0.19
Score = −5.34
AEERAEVSA − Mascot
20120518_EXQ0_MiBa_SA_HCT116_mHLA−2: 8369
SA = 0.82
Score = 0.54
AEREADLTG − MaxQuant
20120518_EXQ0_MiBa_SA_HCT116_mHLA−2: 8369
SA = 0.13
Score = −5.92
Relative intensity
m/z
Prosit prediction
* Exclusive in prediction
# Exclusive in experimental

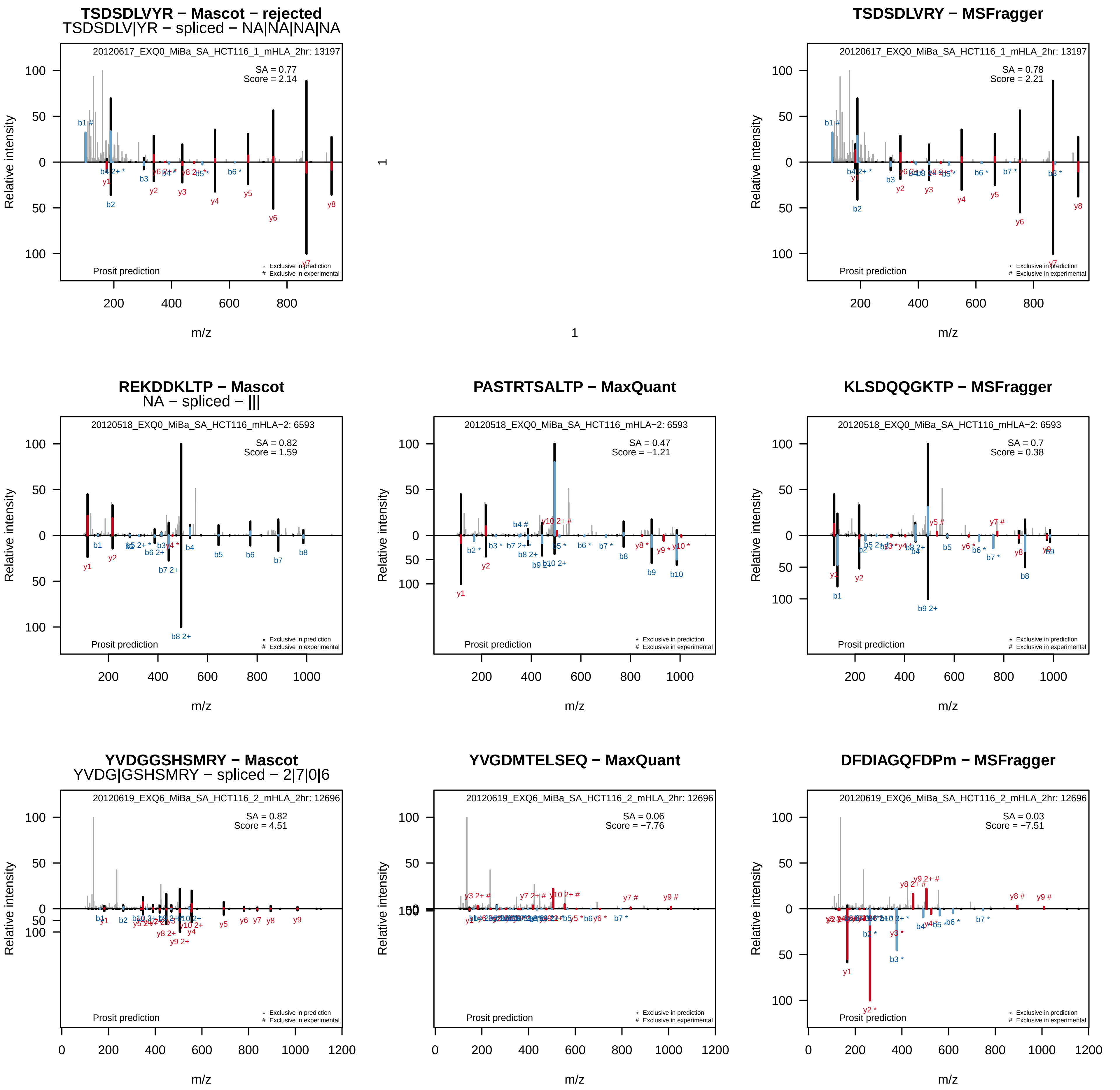

TSDSDLVYR − Mascot − rejected
TSDSDLV|YR − spliced − NA|NA|NA|NA
20120617_EXQ0_MiBa_SA_HCT116_1_mHLA_2hr: 13197
SA = 0.77
Score = 2.14
TSDSDLVRY − MSFragger
20120617_EXQ0_MiBa_SA_HCT116_1_mHLA_2hr: 13197
SA = 0.78
Score = 2.21
REKDDKLTP − Mascot
NA − spliced − |||
20120518_EXQ0_MiBa_SA_HCT116_mHLA−2: 6593
SA = 0.82
Score = 1.59
PASTRTSALTP − MaxQuant
20120518_EXQ0_MiBa_SA_HCT116_mHLA−2: 6593
SA = 0.47
Score = −1.21
KLSDQQGKTP − MSFragger
20120518_EXQ0_MiBa_SA_HCT116_mHLA−2: 6593
SA = 0.7
Score = 0.38
YVDGGSHSMRY − Mascot
YVDG|GSHSMRY − spliced − 2|7|0|6
20120619_EXQ6_MiBa_SA_HCT116_2_mHLA_2hr: 12696
SA = 0.82
Score = 4.51
YVGDMTELSEQ − MaxQuant
20120619_EXQ6_MiBa_SA_HCT116_2_mHLA_2hr: 12696
SA = 0.06
Score = −7.76
DFDIAGQFDPm − MSFragger
20120619_EXQ6_MiBa_SA_HCT116_2_mHLA_2hr: 12696
SA = 0.03
Score = −7.51
Relative intensity
m/z
Prosit prediction
* Exclusive in prediction
# Exclusive in experimental

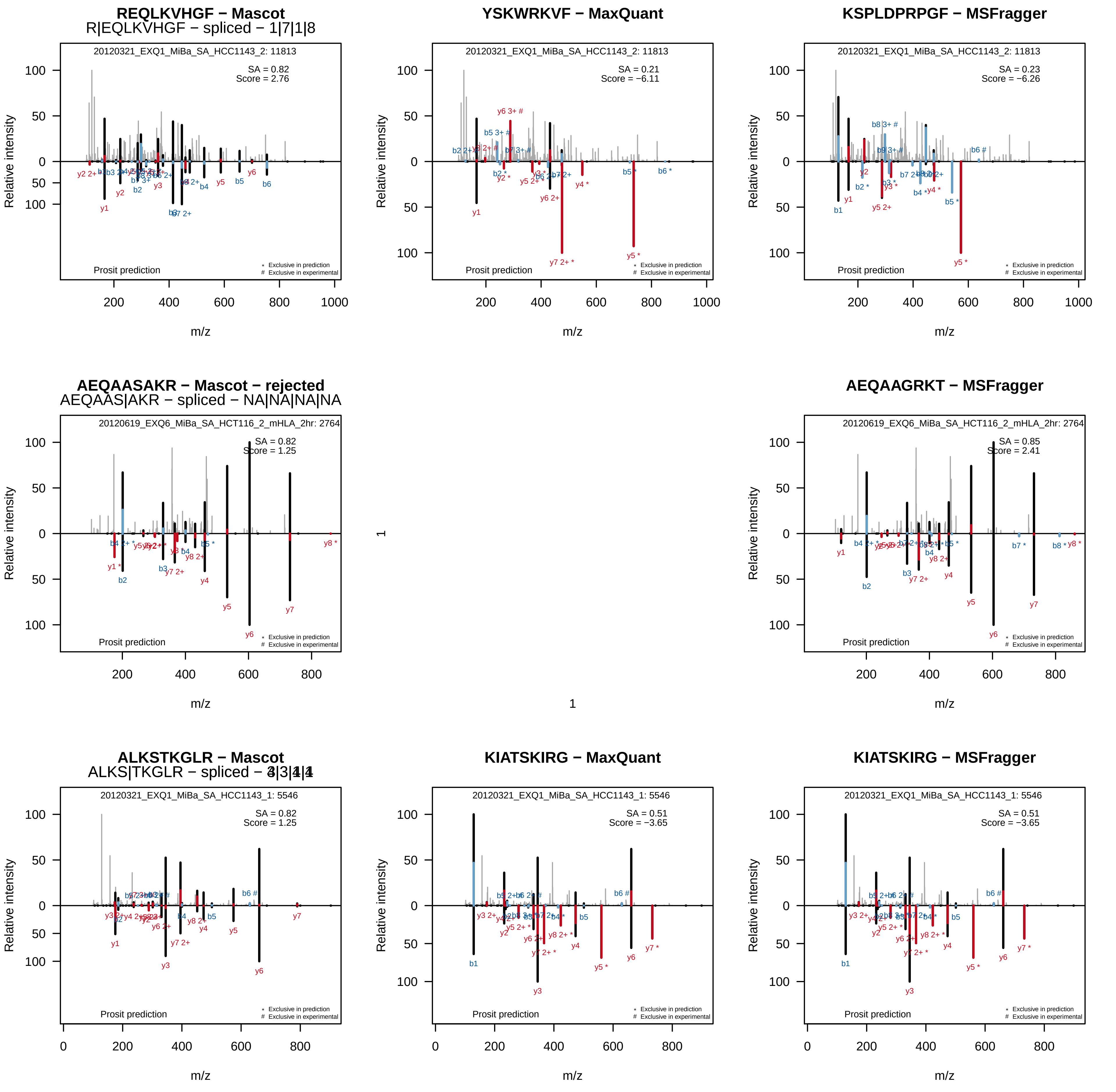
REQLKVHGF − Mascot
R|EQLKVHGF − spliced − 1|7|1|8
20120321_EXQ1_MiBa_SA_HCC1143_2: 11813
SA = 0.82
Score = 2.76
YSKWRKVF − MaxQuant
20120321_EXQ1_MiBa_SA_HCC1143_2: 11813
SA = 0.21
Score = −6.11
KSPLDPRPGF − MSFragger
20120321_EXQ1_MiBa_SA_HCC1143_2: 11813
SA = 0.23
Score = −6.26
AEQAASAKR − Mascot − rejected
AEQAAS|AKR − spliced − NA|NA|NA|NA
20120619_EXQ6_MiBa_SA_HCT116_2_mHLA_2hr: 2764
SA = 0.82
Score = 1.25
AEQAAGRKT − MSFragger
20120619_EXQ6_MiBa_SA_HCT116_2_mHLA_2hr: 2764
SA = 0.85
Score = 2.41
ALKSTKGLR − Mascot
20120321_EXQ1_MiBa_SA_HCC1143_1: 5546
SA = 0.82
Score = 1.25
KIATSKIRG − MaxQuant
20120321_EXQ1_MiBa_SA_HCC1143_1: 5546
SA = 0.51
Score = −3.65
KIATSKIRG − MSFragger
20120321_EXQ1_MiBa_SA_HCC1143_1: 5546
SA = 0.51
Score = −3.65
Relative intensity
m/z
Prosit prediction
* Exclusive in prediction
# Exclusive in experimental

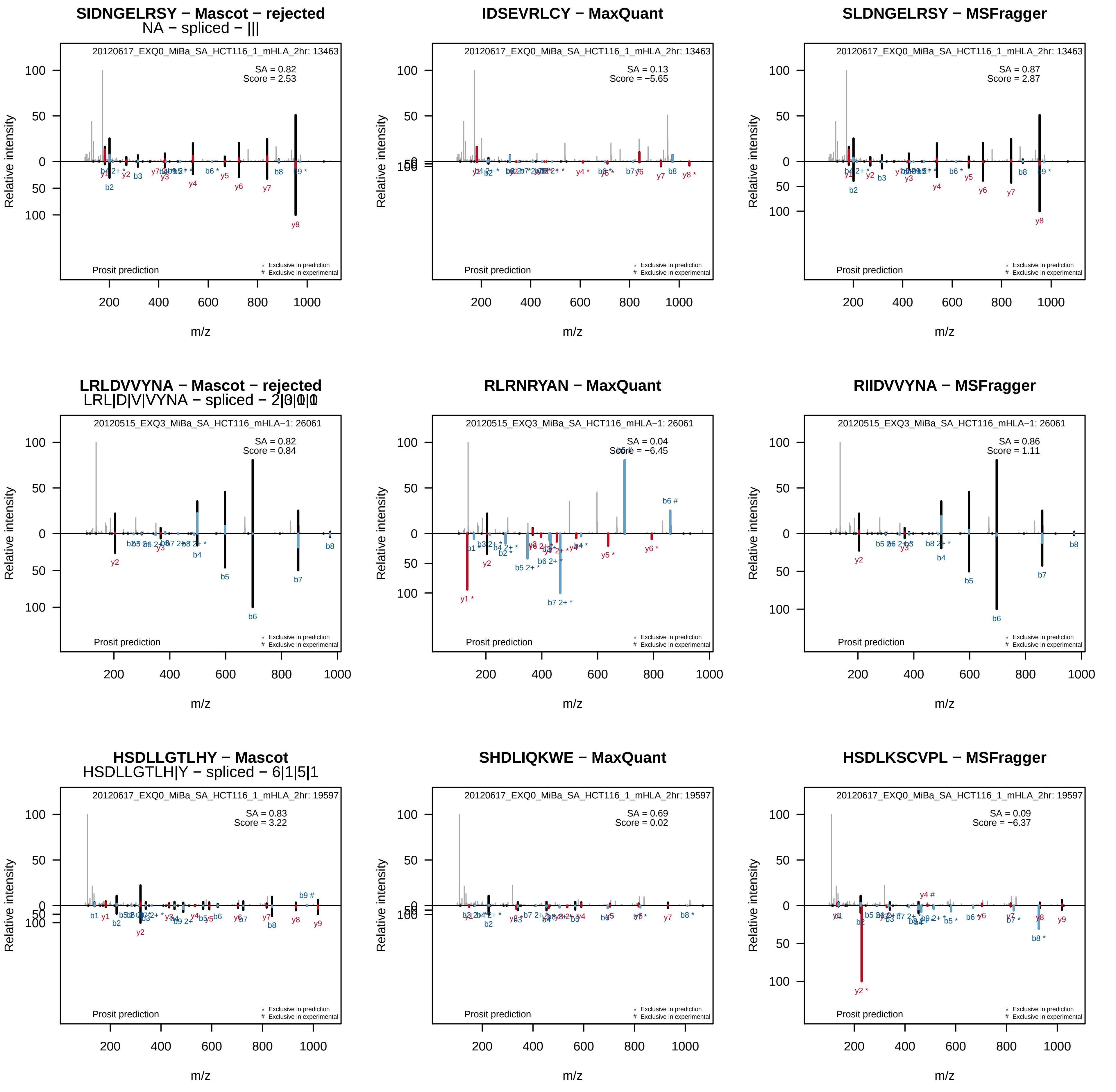

SIDNGELRSY − Mascot − rejected
NA − spliced − |||
20120617_EXQ0_MiBa_SA_HCT116_1_mHLA_2hr: 13463
SA = 0.82
Score = 2.53
IDSEVRLCY − MaxQuant
20120617_EXQ0_MiBa_SA_HCT116_1_mHLA_2hr: 13463
SA = 0.13
Score = −5.65
SLDNGELRSY − MSFragger
20120617_EXQ0_MiBa_SA_HCT116_1_mHLA_2hr: 13463
SA = 0.87
Score = 2.87
LRLDVVYNA − Mascot − rejected
20120515_EXQ3_MiBa_SA_HCT116_mHLA−1: 26061
SA = 0.82
Score = 0.84
RLRNRYAN − MaxQuant
20120515_EXQ3_MiBa_SA_HCT116_mHLA−1: 26061
SA = 0.04
Score = −6.45
RIIDVVYNA − MSFragger
20120515_EXQ3_MiBa_SA_HCT116_mHLA−1: 26061
SA = 0.86
Score = 1.11
HSDLLGTLHY − Mascot
HSDLLGTLH|Y − spliced − 6|1|5|1
20120617_EXQ0_MiBa_SA_HCT116_1_mHLA_2hr: 19597
SA = 0.83
Score = 3.22
SHDLIQKWE − MaxQuant
20120617_EXQ0_MiBa_SA_HCT116_1_mHLA_2hr: 19597
SA = 0.69
Score = 0.02
HSDLKSCVPL − MSFragger
20120617_EXQ0_MiBa_SA_HCT116_1_mHLA_2hr: 19597
SA = 0.09
Score = −6.37
Relative intensity
m/z
Prosit prediction
* Exclusive in prediction
# Exclusive in experimental

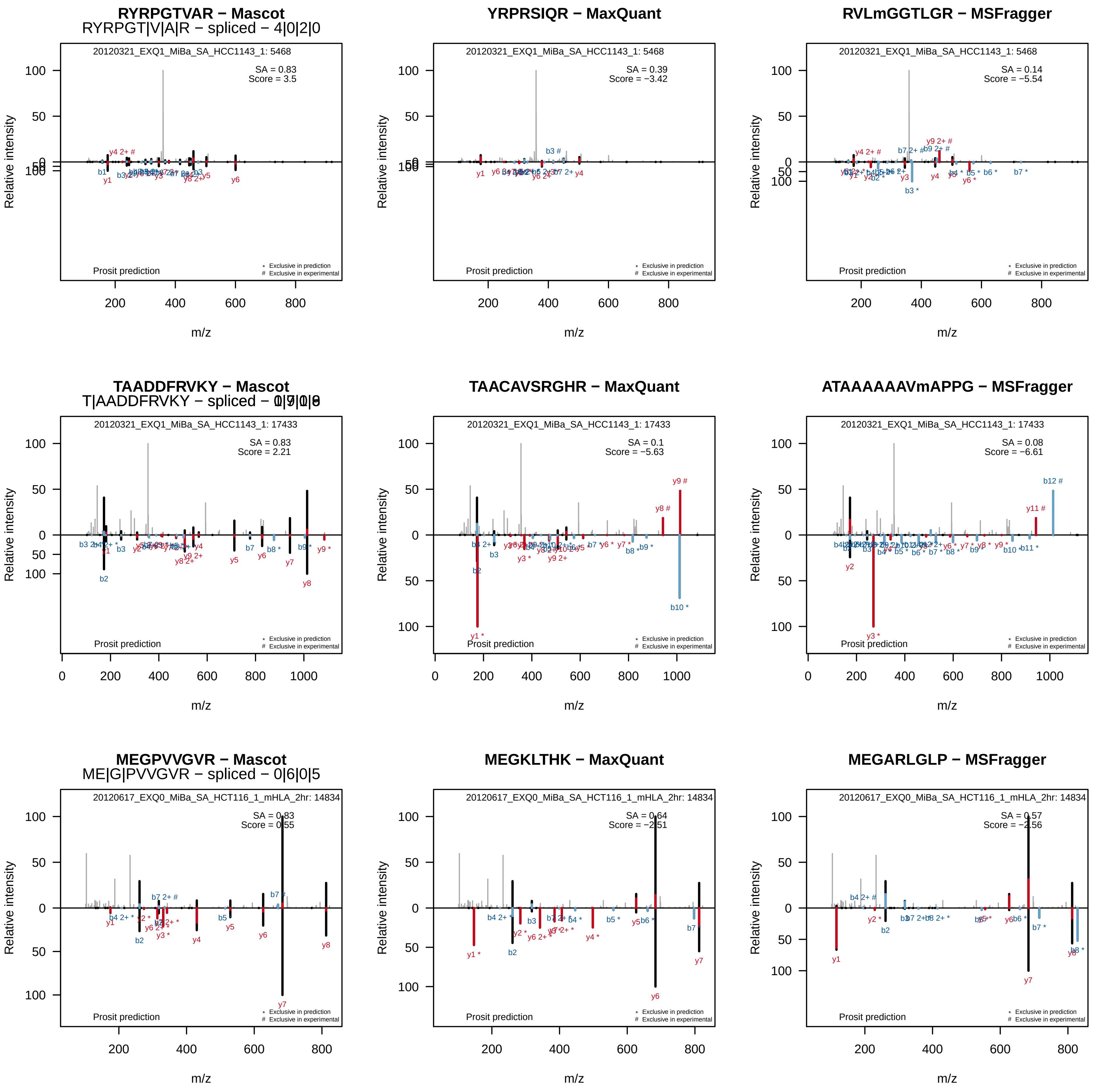
RYRPGTVAR − Mascot
RYRPGT|V|A|R − spliced − 4|0|2|0
20120321_EXQ1_MiBa_SA_HCC1143_1: 5468
SA = 0.83
Score = 3.5
YRPRSIQR − MaxQuant
20120321_EXQ1_MiBa_SA_HCC1143_1: 5468
SA = 0.39
Score = −3.42
RVLmGGTLGR − MSFragger
20120321_EXQ1_MiBa_SA_HCC1143_1: 5468
SA = 0.14
Score = −5.54
TAADDFRVKY − Mascot
20120321_EXQ1_MiBa_SA_HCC1143_1: 17433
SA = 0.83
Score = 2.21
TAACAVSRGHR − MaxQuant
20120321_EXQ1_MiBa_SA_HCC1143_1: 17433
SA = 0.1
Score = −5.63
ATAAAAAAVmAPPG − MSFragger
20120321_EXQ1_MiBa_SA_HCC1143_1: 17433
SA = 0.08
Score = −6.61
MEGPVVGVR − Mascot
ME|G|PVVGVR − spliced − 0|6|0|5
20120617_EXQ0_MiBa_SA_HCT116_1_mHLA_2hr: 14834
SA = 0.83
Score = 0.55
MEGKLTHK − MaxQuant
20120617_EXQ0_MiBa_SA_HCT116_1_mHLA_2hr: 14834
SA = 0.64
Score = −2.51
MEGARLGLP − MSFragger
20120617_EXQ0_MiBa_SA_HCT116_1_mHLA_2hr: 14834
SA = 0.57
Score = −2.56
Relative intensity
m/z
Prosit prediction
⋆ Exclusive in prediction
Exclusive in experimental

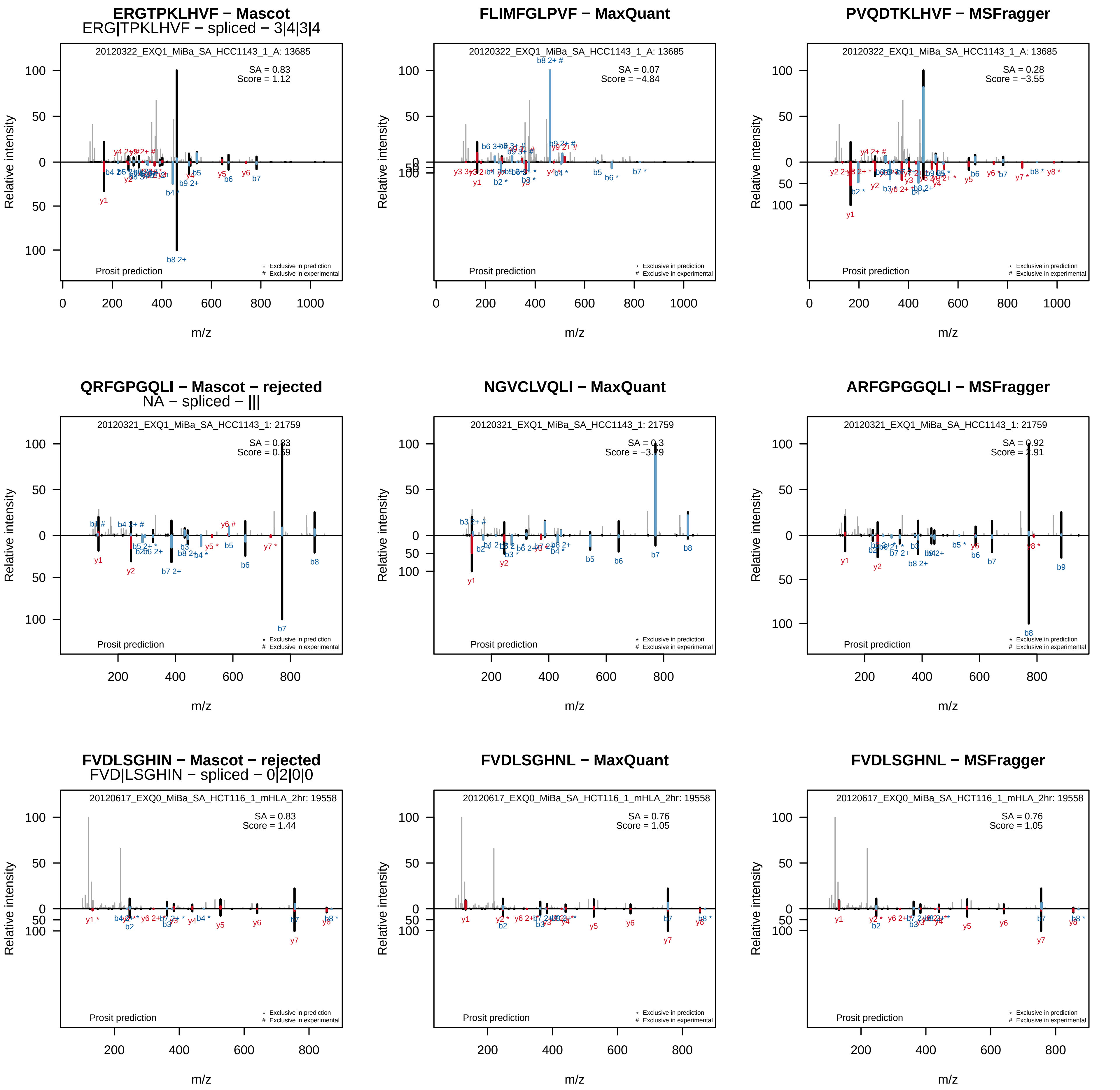

ERGTPKLHVF − Mascot
ERG|TPKLHVF − spliced − 3|4|3|4
20120322_EXQ1_MiBa_SA_HCC1143_1_A: 13685
SA = 0.83
Score = 1.12
FLIMFGLPVF − MaxQuant
20120322_EXQ1_MiBa_SA_HCC1143_1_A: 13685
SA = 0.07
Score = −4.84
PVQDTKLHVF − MSFragger
20120322_EXQ1_MiBa_SA_HCC1143_1_A: 13685
SA = 0.28
Score = −3.55
QRFGPGQLI − Mascot − rejected
NA − spliced − |||
20120321_EXQ1_MiBa_SA_HCC1143_1: 21759
SA = 0.83
Score = 0.59
NGVCLVQLI − MaxQuant
20120321_EXQ1_MiBa_SA_HCC1143_1: 21759
SA = 0.3
Score = −3.79
ARFGPGGQLI − MSFragger
20120321_EXQ1_MiBa_SA_HCC1143_1: 21759
SA = 0.92
Score = 2.91
FVDLSGHIN − Mascot − rejected
FVD|LSGHIN − spliced − 0|2|0|0
20120617_EXQ0_MiBa_SA_HCT116_1_mHLA_2hr: 19558
SA = 0.83
Score = 1.44
FVDLSGHNL − MaxQuant
20120617_EXQ0_MiBa_SA_HCT116_1_mHLA_2hr: 19558
SA = 0.76
Score = 1.05
FVDLSGHNL − MSFragger
20120617_EXQ0_MiBa_SA_HCT116_1_mHLA_2hr: 19558
SA = 0.76
Score = 1.05
Relative intensity
m/z
Prosit prediction
* Exclusive in prediction
# Exclusive in experimental

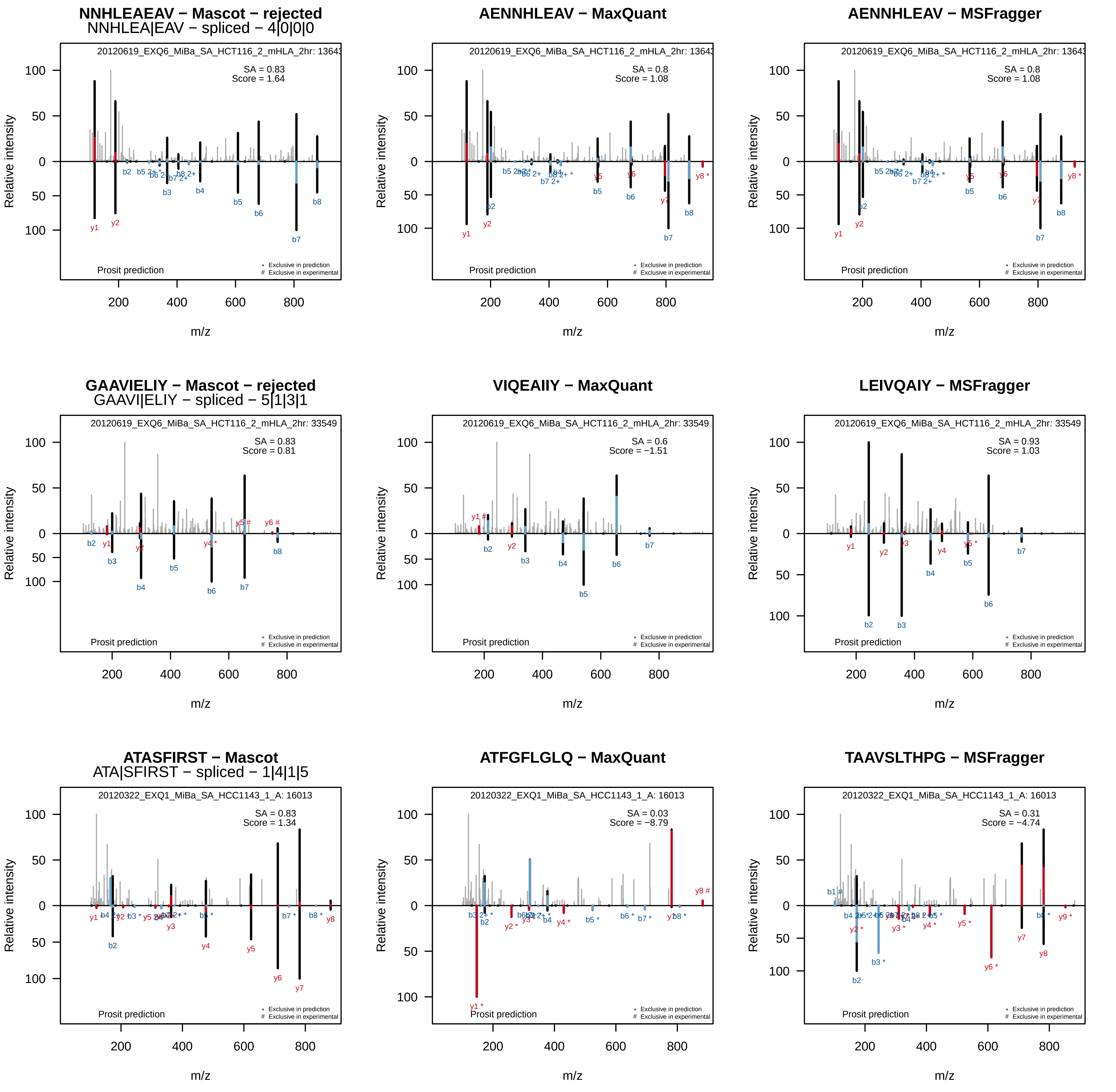
NNHLEAEAV − Mascot − rejected
NNHLEA|EAV − spliced − 4|0|0|0
20120619_EXQ6_MiBa_SA_HCT116_2_mHLA_2hr: 13643
SA = 0.83
Score = 1.64
AENNHLEAV − MaxQuant
20120619_EXQ6_MiBa_SA_HCT116_2_mHLA_2hr: 13643
SA = 0.8
Score = 1.08
AENNHLEAV − MSFragger
20120619_EXQ6_MiBa_SA_HCT116_2_mHLA_2hr: 13643
SA = 0.8
Score = 1.08
GAAVIELIY − Mascot − rejected
GAAVI|ELIY − spliced − 5|1|3|1
20120619_EXQ6_MiBa_SA_HCT116_2_mHLA_2hr: 33549
SA = 0.83
Score = 0.81
VIQEAIIY − MaxQuant
20120619_EXQ6_MiBa_SA_HCT116_2_mHLA_2hr: 33549
SA = 0.6
Score = −1.51
LEIVQAIY − MSFragger
20120619_EXQ6_MiBa_SA_HCT116_2_mHLA_2hr: 33549
SA = 0.93
Score = 1.03
ATASFIRST − Mascot
ATA|SFIRST − spliced − 1|4|1|5
20120322_EXQ1_MiBa_SA_HCC1143_1_A: 16013
SA = 0.83
Score = 1.34
ATFGFLGLQ − MaxQuant
20120322_EXQ1_MiBa_SA_HCC1143_1_A: 16013
SA = 0.03
Score = −8.79
TAAVSLTHPG − MSFragger
20120322_EXQ1_MiBa_SA_HCC1143_1_A: 16013
SA = 0.31
Score = −4.74
Relative intensity
m/z
Prosit prediction
* Exclusive in prediction
# Exclusive in experimental

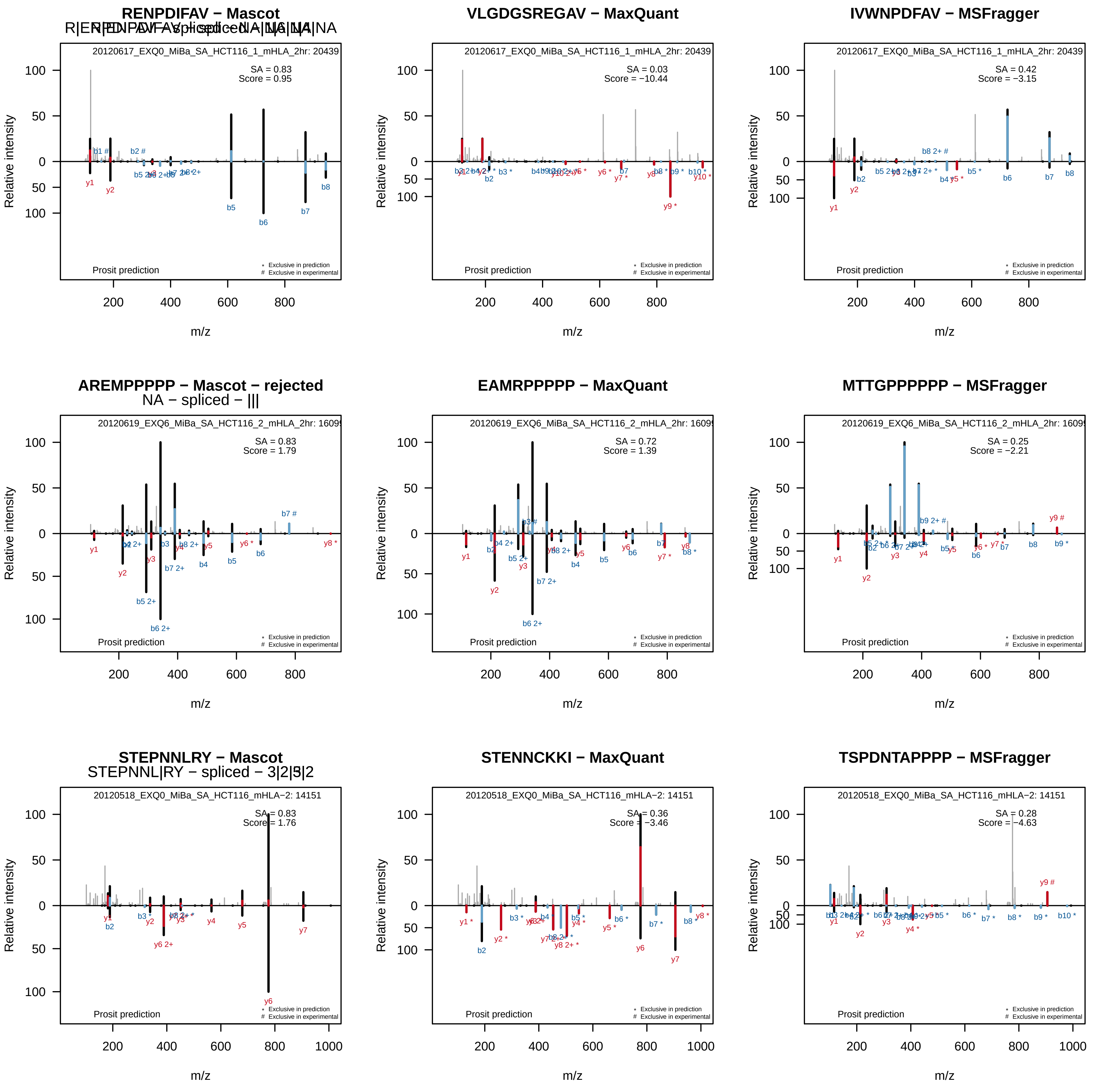

RENPDIFAV − Mascot
20120617_EXQ0_MiBa_SA_HCT116_1_mHLA_2hr: 20439
SA = 0.83
Score = 0.95
VLGDGSREGAV − MaxQuant
20120617_EXQ0_MiBa_SA_HCT116_1_mHLA_2hr: 20439
SA = 0.03
Score = −10.44
IVWNPDFAV − MSFragger
20120617_EXQ0_MiBa_SA_HCT116_1_mHLA_2hr: 20439
SA = 0.42
Score = −3.15
AREMPPPPP − Mascot − rejected
NA − spliced − |||
20120619_EXQ6_MiBa_SA_HCT116_2_mHLA_2hr: 1609
SA = 0.83
Score = 1.79
EAMRPPPPP − MaxQuant
20120619_EXQ6_MiBa_SA_HCT116_2_mHLA_2hr: 1609
SA = 0.72
Score = 1.39
MTTGPPPPPP − MSFragger
20120619_EXQ6_MiBa_SA_HCT116_2_mHLA_2hr: 1609
SA = 0.25
Score = −2.21
STEPNNLRY − Mascot
STEPNNL|RY − spliced − 3|2|5|2
20120518_EXQ0_MiBa_SA_HCT116_mHLA−2: 14151
SA = 0.83
Score = 1.76
STENNCKKI − MaxQuant
20120518_EXQ0_MiBa_SA_HCT116_mHLA−2: 14151
SA = 0.36
Score = −3.46
TSPDNTAPPPP − MSFragger
20120518_EXQ0_MiBa_SA_HCT116_mHLA−2: 14151
SA = 0.28
Score = −4.63
Relative intensity
m/z
Prosit prediction
* Exclusive in prediction
# Exclusive in experimental

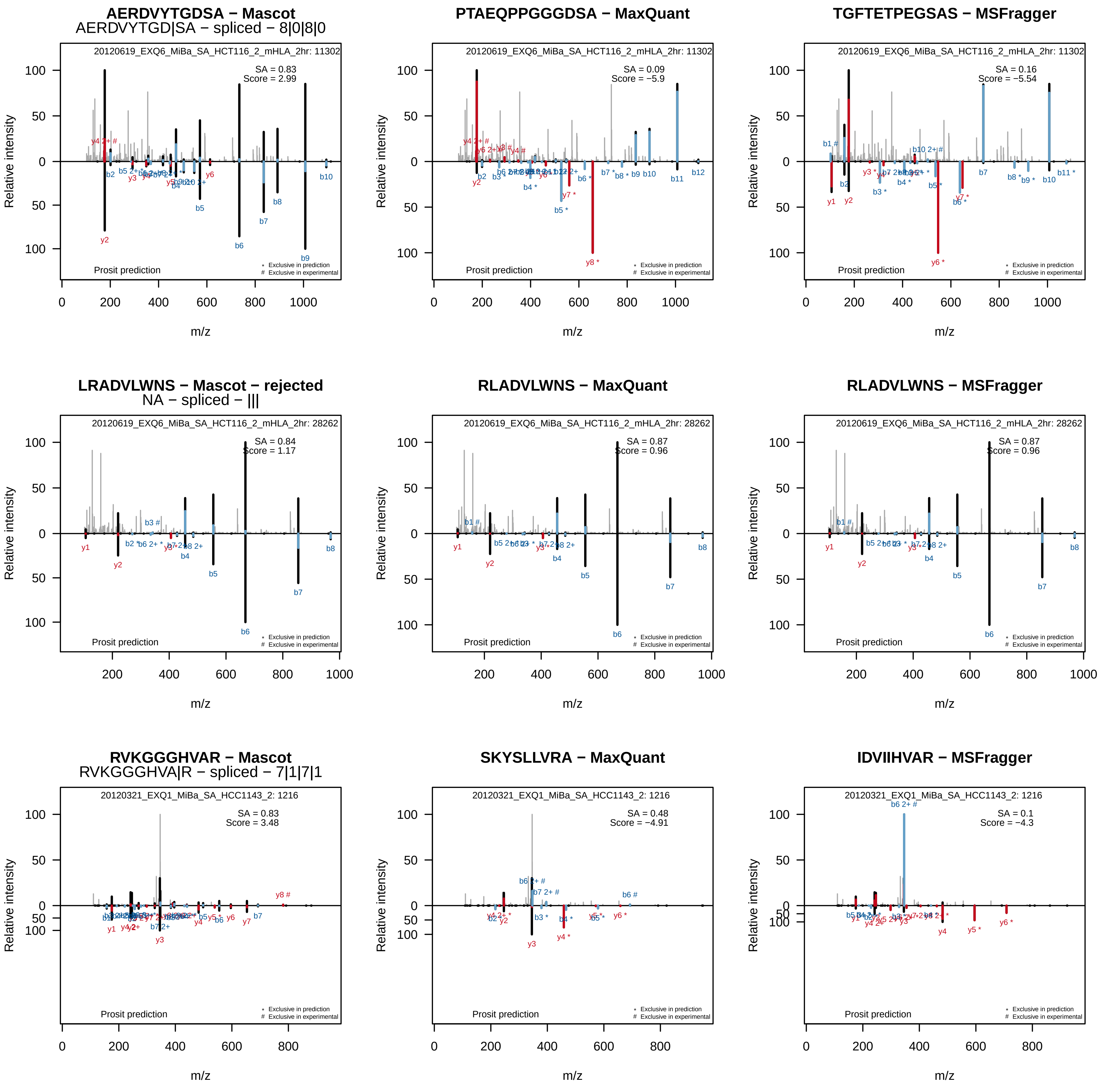
AERDVYTGDSA − Mascot
AERDVYTGD|SA − spliced − 8|0|8|0
20120619_EXQ6_MiBa_SA_HCT116_2_mHLA_2hr: 11302
SA = 0.83
Score = 2.99
Prosit prediction
PTAEQPPGGGDSA − MaxQuant
20120619_EXQ6_MiBa_SA_HCT116_2_mHLA_2hr: 11302
SA = 0.09
Score = −5.9
Prosit prediction
TGFTETPEGSAS − MSFragger
20120619_EXQ6_MiBa_SA_HCT116_2_mHLA_2hr: 11302
SA = 0.16
Score = −5.54
Prosit prediction
LRADVLWNS − Mascot − rejected
NA − spliced − |||
20120619_EXQ6_MiBa_SA_HCT116_2_mHLA_2hr: 28262
SA = 0.84
Score = 1.17
Prosit prediction
RLADVLWNS − MaxQuant
20120619_EXQ6_MiBa_SA_HCT116_2_mHLA_2hr: 28262
SA = 0.87
Score = 0.96
Prosit prediction
RLADVLWNS − MSFragger
20120619_EXQ6_MiBa_SA_HCT116_2_mHLA_2hr: 28262
SA = 0.87
Score = 0.96
Prosit prediction
RVKGGGHVAR − Mascot
RVKGGGHVA|R − spliced − 7|1|7|1
20120321_EXQ1_MiBa_SA_HCC1143_2: 1216
SA = 0.83
Score = 3.48
Prosit prediction
SKYSLLVRA − MaxQuant
20120321_EXQ1_MiBa_SA_HCC1143_2: 1216
SA = 0.48
Score = −4.91
Prosit prediction
IDVIIHVAR − MSFragger
20120321_EXQ1_MiBa_SA_HCC1143_2: 1216
SA = 0.1
Score = −4.3
Prosit prediction
* Exclusive in prediction
# Exclusive in experimental
Relative intensity
m/z

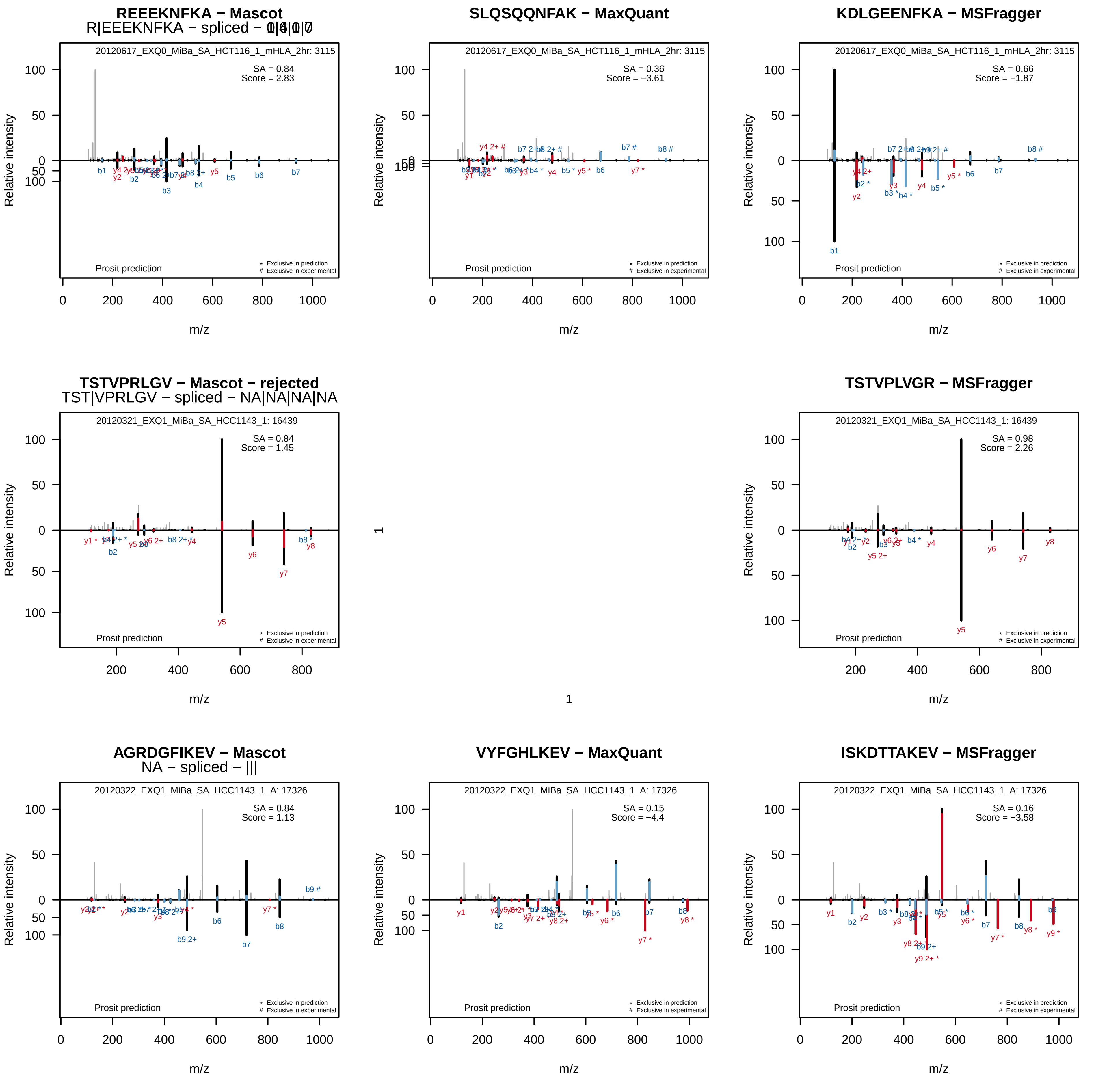
REEEKNFKA − Mascot
R|EEEKNFKA − spliced − 0|4|1|7
20120617_EXQ0_MiBa_SA_HCT116_1_mHLA_2hr: 3115
SA = 0.84
Score = 2.83
Prosit prediction
⋆ Exclusive in prediction
Exclusive in experimental
Relative intensity
m/z
SLQSQQNFAK − MaxQuant
20120617_EXQ0_MiBa_SA_HCT116_1_mHLA_2hr: 3115
SA = 0.36
Score = −3.61
Prosit prediction
⋆ Exclusive in prediction
Exclusive in experimental
Relative intensity
m/z
KDLGEENFKA − MSFragger
20120617_EXQ0_MiBa_SA_HCT116_1_mHLA_2hr: 3115
SA = 0.66
Score = −1.87
Prosit prediction
⋆ Exclusive in prediction
Exclusive in experimental
Relative intensity
m/z
TSTVPRLGV − Mascot − rejected
TST|VPRLGV − spliced − NA|NA|NA|NA
20120321_EXQ1_MiBa_SA_HCC1143_1: 16439
SA = 0.84
Score = 1.45
Prosit prediction
⋆ Exclusive in prediction
Exclusive in experimental
Relative intensity
m/z
1
1
TSTVPLVGR − MSFragger
20120321_EXQ1_MiBa_SA_HCC1143_1: 16439
SA = 0.98
Score = 2.26
Prosit prediction
⋆ Exclusive in prediction
Exclusive in experimental
Relative intensity
m/z
AGRDGFIKEV − Mascot
NA − spliced − |||
20120322_EXQ1_MiBa_SA_HCC1143_1_A: 17326
SA = 0.84
Score = 1.13
Prosit prediction
⋆ Exclusive in prediction
Exclusive in experimental
Relative intensity
m/z
VYFGHLKEV − MaxQuant
20120322_EXQ1_MiBa_SA_HCC1143_1_A: 17326
SA = 0.15
Score = −4.4
Prosit prediction
⋆ Exclusive in prediction
Exclusive in experimental
Relative intensity
m/z
ISKDTTAKEV − MSFragger
20120322_EXQ1_MiBa_SA_HCC1143_1_A: 17326
SA = 0.16
Score = −3.58
Prosit prediction
⋆ Exclusive in prediction
Exclusive in experimental
Relative intensity
m/z

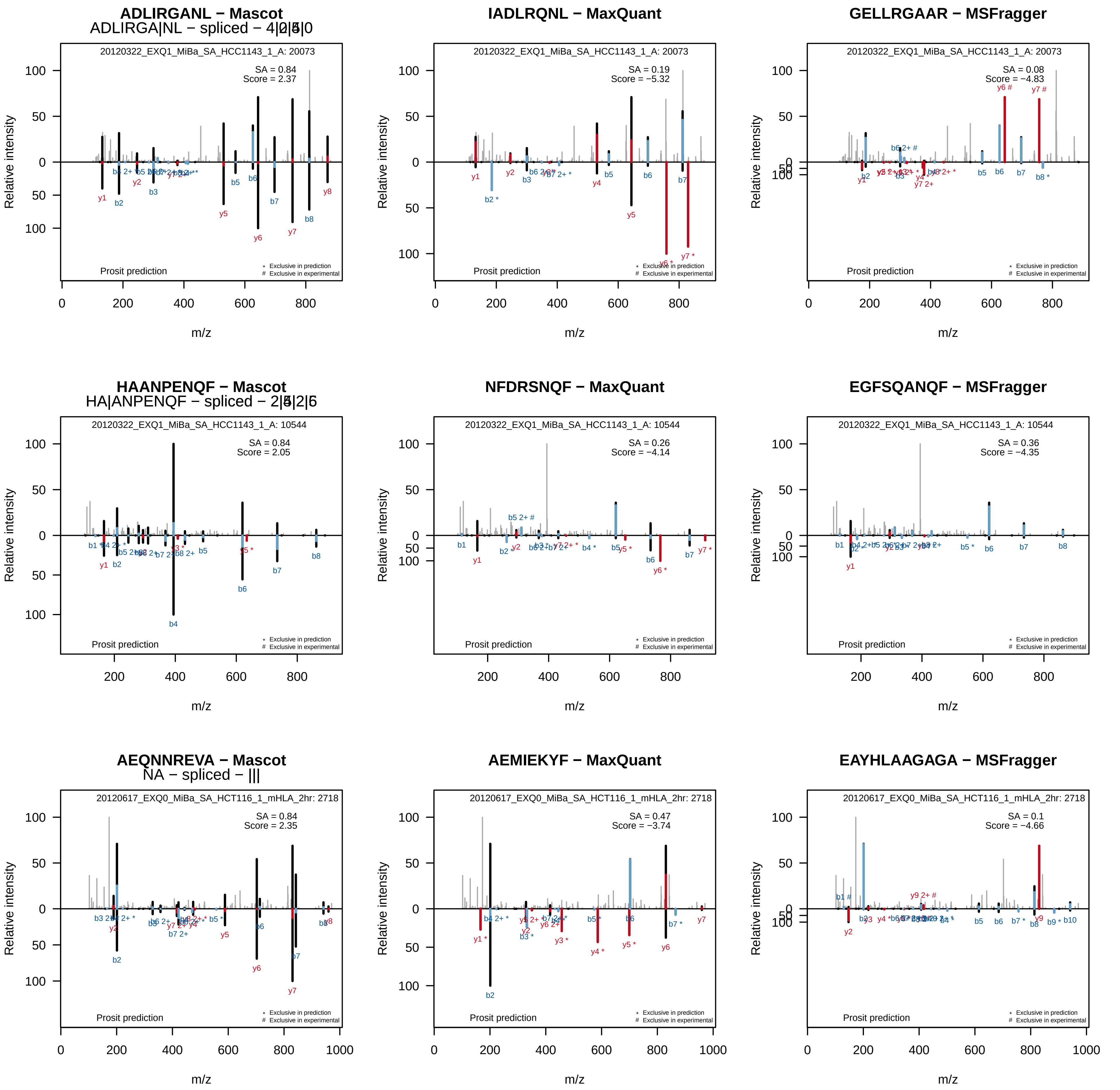
ADLIRGANL − Mascot
IADLRQNL − MaxQuant
GELLRGAAR − MSFragger
20120322_EXQ1_MiBa_SA_HCC1143_1_A: 20073
SA = 0.84
Score = 2.37
SA = 0.19
Score = −5.32
SA = 0.08
Score = −4.83
HAANPENQF − Mascot
NFDRSNQF − MaxQuant
EGFSQANQF − MSFragger
20120322_EXQ1_MiBa_SA_HCC1143_1_A: 10544
SA = 0.84
Score = 2.05
SA = 0.26
Score = −4.14
SA = 0.36
Score = −4.35
AEQNNREVA − Mascot
NA − spliced − |||
AEMIEKYF − MaxQuant
EAYHLAAGAGA − MSFragger
20120617_EXQ0_MiBa_SA_HCT116_1_mHLA_2hr: 2718
SA = 0.84
Score = 2.35
SA = 0.47
Score = −3.74
SA = 0.1
Score = −4.66
Prosit prediction
* Exclusive in prediction
# Exclusive in experimental
Relative intensity
m/z

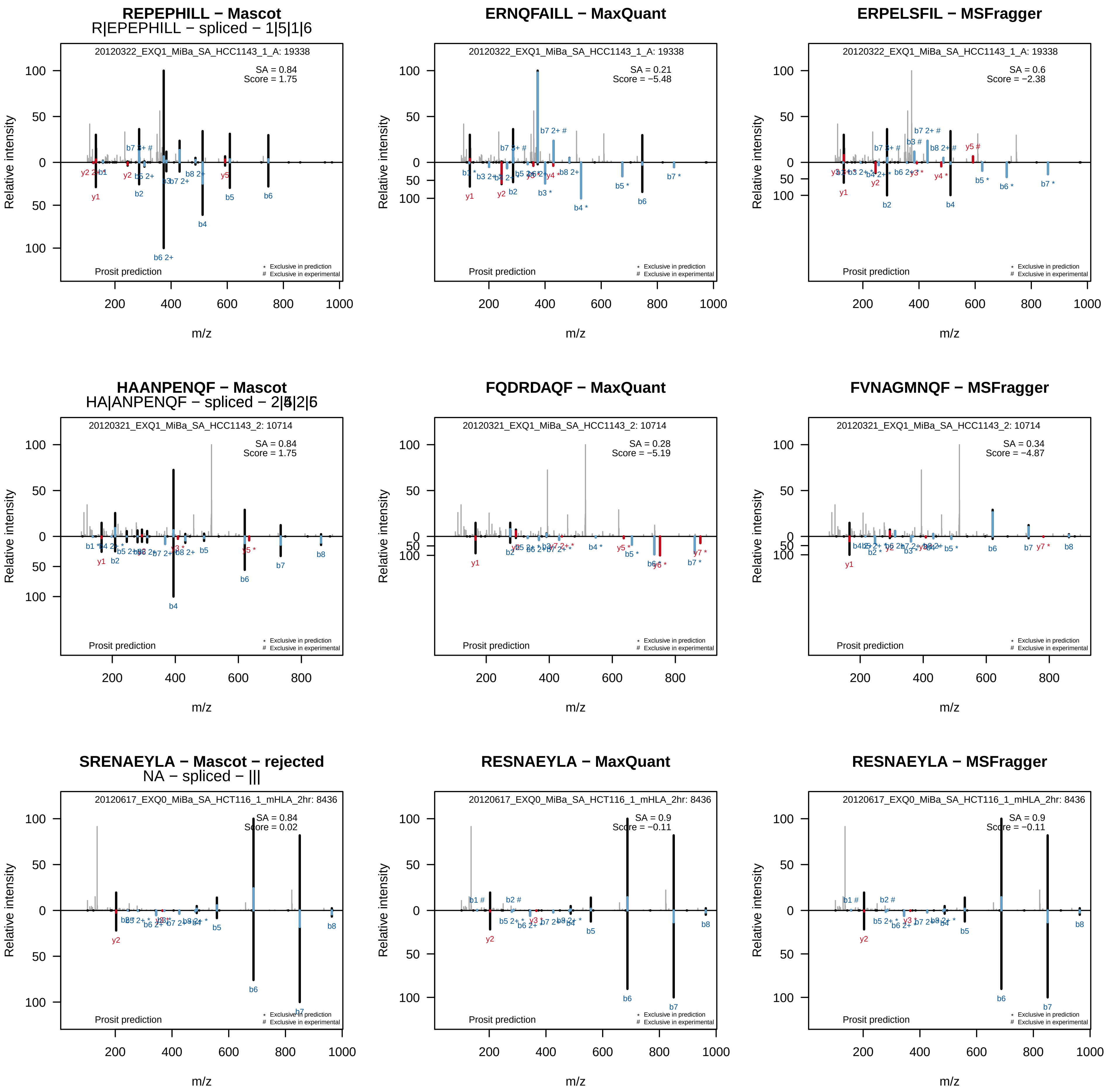
REPEPHILL − Mascot
R|EPEPHILL − spliced − 1|5|1|6
20120322_EXQ1_MiBa_SA_HCC1143_1_A: 19338
SA = 0.84
Score = 1.75
ERNQFAILL − MaxQuant
20120322_EXQ1_MiBa_SA_HCC1143_1_A: 19338
SA = 0.21
Score = −5.48
ERPELSFIL − MSFragger
20120322_EXQ1_MiBa_SA_HCC1143_1_A: 19338
SA = 0.6
Score = −2.38
HAANPENQF − Mascot
HA|ANPENQF − spliced − 2|5|2|6
20120321_EXQ1_MiBa_SA_HCC1143_2: 10714
SA = 0.84
Score = 1.75
FQDRDAQF − MaxQuant
20120321_EXQ1_MiBa_SA_HCC1143_2: 10714
SA = 0.28
Score = −5.19
FVNAGMNQF − MSFragger
20120321_EXQ1_MiBa_SA_HCC1143_2: 10714
SA = 0.34
Score = −4.87
SRENAEYLA − Mascot − rejected
NA − spliced − |||
20120617_EXQ0_MiBa_SA_HCT116_1_mHLA_2hr: 8436
SA = 0.84
Score = 0.02
RESNAEYLA − MaxQuant
20120617_EXQ0_MiBa_SA_HCT116_1_mHLA_2hr: 8436
SA = 0.9
Score = −0.11
RESNAEYLA − MSFragger
20120617_EXQ0_MiBa_SA_HCT116_1_mHLA_2hr: 8436
SA = 0.9
Score = −0.11
Relative intensity
m/z
Prosit prediction
⋆ Exclusive in prediction
Exclusive in experimental

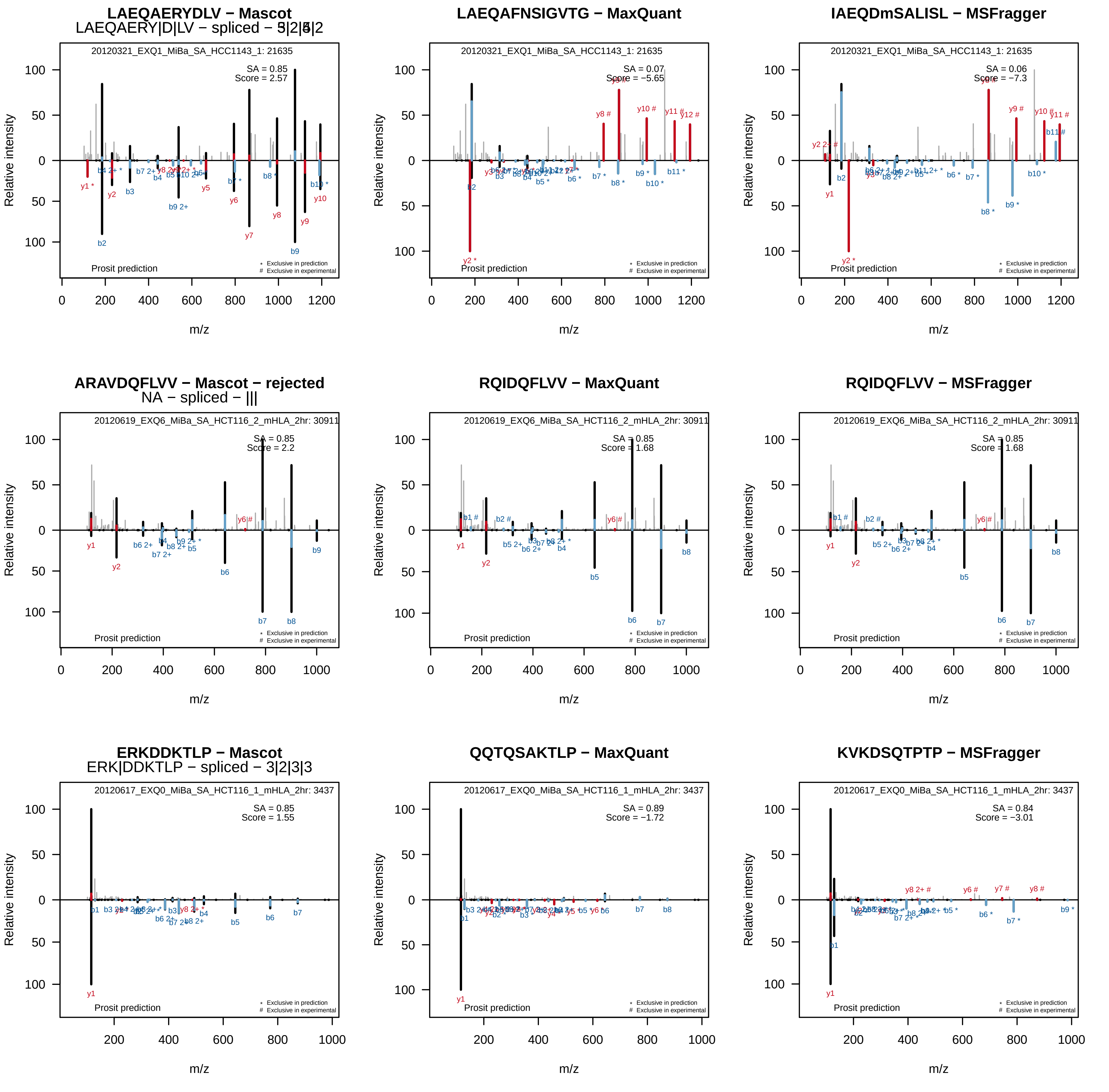
LAEQAERYDLV − Mascot
LAEQAERY|D|LV − spliced − 5|2|4|2
20120321_EXQ1_MiBa_SA_HCC1143_1: 21635
SA = 0.85
Score = 2.57
LAEQAFNSIGVTG − MaxQuant
20120321_EXQ1_MiBa_SA_HCC1143_1: 21635
SA = 0.07
Score = −5.65
IAEQDmSALISL − MSFragger
20120321_EXQ1_MiBa_SA_HCC1143_1: 21635
SA = 0.06
Score = −7.3
ARAVDQFLVV − Mascot − rejected
NA − spliced − |||
20120619_EXQ6_MiBa_SA_HCT116_2_mHLA_2hr: 30911
SA = 0.85
Score = 2.2
RQIDQFLVV − MaxQuant
20120619_EXQ6_MiBa_SA_HCT116_2_mHLA_2hr: 30911
SA = 0.85
Score = 1.68
RQIDQFLVV − MSFragger
20120619_EXQ6_MiBa_SA_HCT116_2_mHLA_2hr: 30911
SA = 0.85
Score = 1.68
ERKDDKTLP − Mascot
ERK|DDKTLP − spliced − 3|2|3|3
20120617_EXQ0_MiBa_SA_HCT116_1_mHLA_2hr: 3437
SA = 0.85
Score = 1.55
QQTQSAKTLP − MaxQuant
20120617_EXQ0_MiBa_SA_HCT116_1_mHLA_2hr: 3437
SA = 0.89
Score = −1.72
KVKDSQTPTP − MSFragger
20120617_EXQ0_MiBa_SA_HCT116_1_mHLA_2hr: 3437
SA = 0.84
Score = −3.01
Relative intensity
m/z
Prosit prediction
* Exclusive in prediction
# Exclusive in experimental

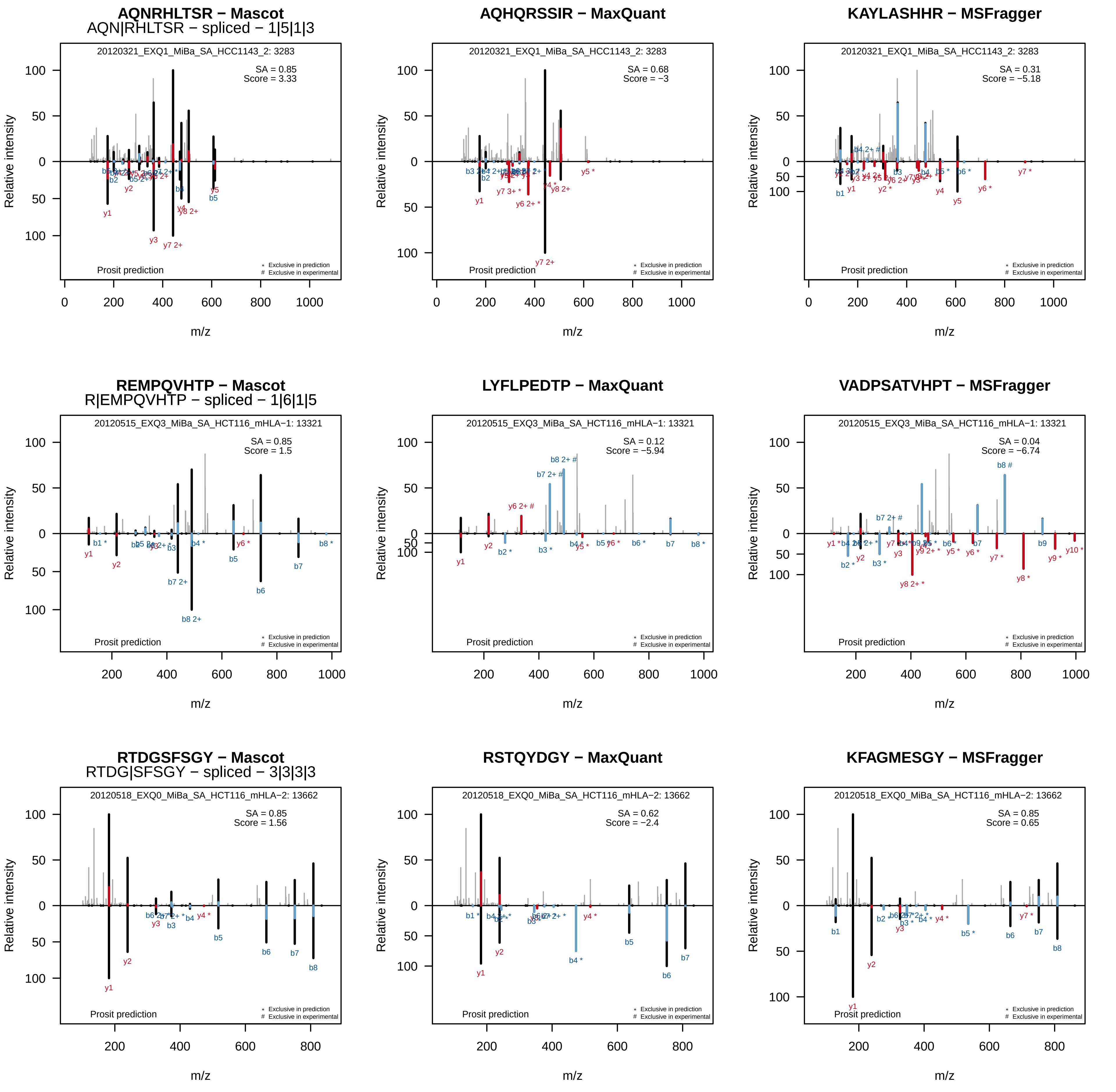
AQNRHLTSR − Mascot
AQN|RHLTSR − spliced − 1|5|1|3
20120321_EXQ1_MiBa_SA_HCC1143_2: 3283
SA = 0.85
Score = 3.33
AQHQRSSIR − MaxQuant
20120321_EXQ1_MiBa_SA_HCC1143_2: 3283
SA = 0.68
Score = −3
KAYLASHHR − MSFragger
20120321_EXQ1_MiBa_SA_HCC1143_2: 3283
SA = 0.31
Score = −5.18
REMPQVHTP − Mascot
R|EMPQVHTP − spliced − 1|6|1|5
20120515_EXQ3_MiBa_SA_HCT116_mHLA−1: 13321
SA = 0.85
Score = 1.5
LYFLPEDTP − MaxQuant
20120515_EXQ3_MiBa_SA_HCT116_mHLA−1: 13321
SA = 0.12
Score = −5.94
VADPSATVHPT − MSFragger
20120515_EXQ3_MiBa_SA_HCT116_mHLA−1: 13321
SA = 0.04
Score = −6.74
RTDGSFSGY − Mascot
RTDG|SFSGY − spliced − 3|3|3|3
20120518_EXQ0_MiBa_SA_HCT116_mHLA−2: 13662
SA = 0.85
Score = 1.56
RSTQYDGY − MaxQuant
20120518_EXQ0_MiBa_SA_HCT116_mHLA−2: 13662
SA = 0.62
Score = −2.4
KFAGMESGY − MSFragger
20120518_EXQ0_MiBa_SA_HCT116_mHLA−2: 13662
SA = 0.85
Score = 0.65
Relative intensity
m/z
Prosit prediction
* Exclusive in prediction
# Exclusive in experimental

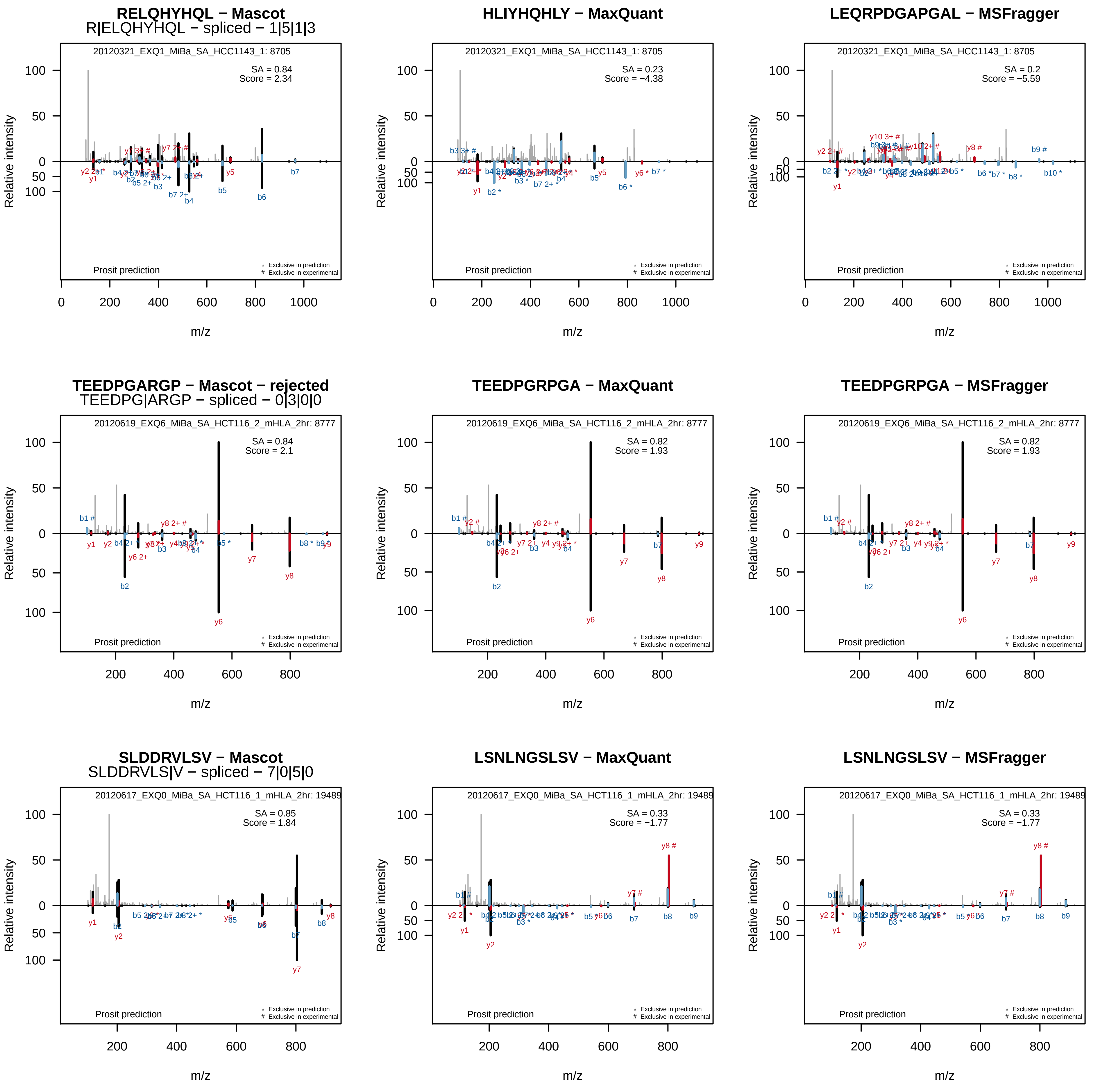
RELQHYHQL − Mascot
R|ELQHYHQL − spliced − 1|5|1|3
20120321_EXQ1_MiBa_SA_HCC1143_1: 8705
SA = 0.84
Score = 2.34
HLIYHQHLY − MaxQuant
20120321_EXQ1_MiBa_SA_HCC1143_1: 8705
SA = 0.23
Score = −4.38
LEQRPDGAPGAL − MSFragger
20120321_EXQ1_MiBa_SA_HCC1143_1: 8705
SA = 0.2
Score = −5.59
TEEDPGARGP − Mascot − rejected
TEEDPG|ARGP − spliced − 0|3|0|0
20120619_EXQ6_MiBa_SA_HCT116_2_mHLA_2hr: 8777
SA = 0.84
Score = 2.1
TEEDPGRPGA − MaxQuant
20120619_EXQ6_MiBa_SA_HCT116_2_mHLA_2hr: 8777
SA = 0.82
Score = 1.93
TEEDPGRPGA − MSFragger
20120619_EXQ6_MiBa_SA_HCT116_2_mHLA_2hr: 8777
SA = 0.82
Score = 1.93
SLDDRVLSV − Mascot
SLDDRVLS|V − spliced − 7|0|5|0
20120617_EXQ0_MiBa_SA_HCT116_1_mHLA_2hr: 19489
SA = 0.85
Score = 1.84
LSNLNGSLSV − MaxQuant
20120617_EXQ0_MiBa_SA_HCT116_1_mHLA_2hr: 19489
SA = 0.33
Score = −1.77
LSNLNGSLSV − MSFragger
20120617_EXQ0_MiBa_SA_HCT116_1_mHLA_2hr: 19489
SA = 0.33
Score = −1.77
Relative intensity
m/z
Prosit prediction
* Exclusive in prediction
# Exclusive in experimental

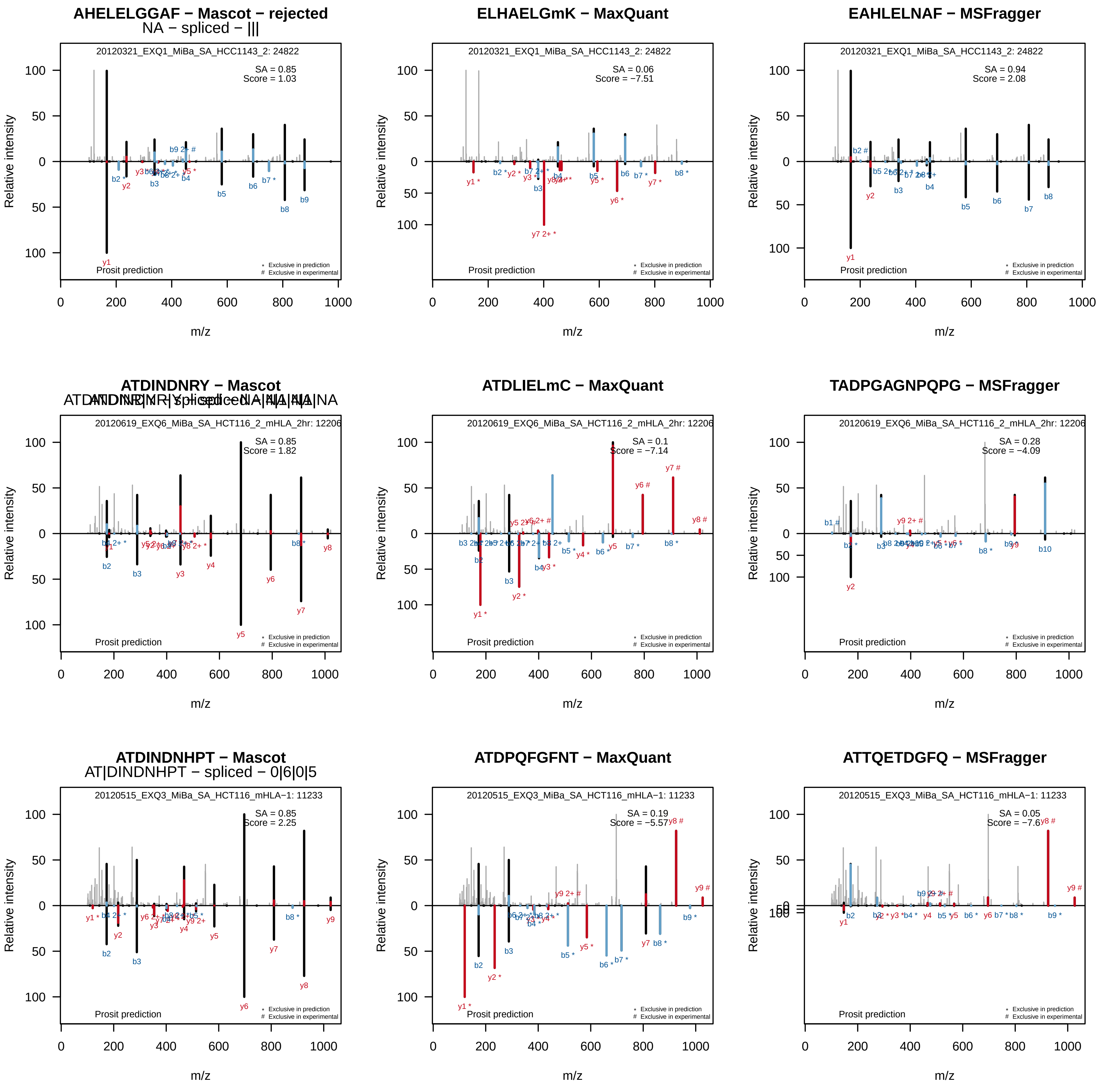
AHELELGGAF − Mascot − rejected
NA − spliced − |||
20120321_EXQ1_MiBa_SA_HCC1143_2: 24822
SA = 0.85
Score = 1.03
ELHAELGmK − MaxQuant
20120321_EXQ1_MiBa_SA_HCC1143_2: 24822
SA = 0.06
Score = −7.51
EAHLELNAF − MSFragger
20120321_EXQ1_MiBa_SA_HCC1143_2: 24822
SA = 0.94
Score = 2.08
ATDINDNRY − Mascot
20120619_EXQ6_MiBa_SA_HCT116_2_mHLA_2hr: 12206
SA = 0.85
Score = 1.82
ATDLIELmC − MaxQuant
20120619_EXQ6_MiBa_SA_HCT116_2_mHLA_2hr: 12206
SA = 0.1
Score = −7.14
TADPGAGNPQPG − MSFragger
20120619_EXQ6_MiBa_SA_HCT116_2_mHLA_2hr: 12206
SA = 0.28
Score = −4.09
ATDINDNHPT − Mascot
AT|DINDNHPT − spliced − 0|6|0|5
20120515_EXQ3_MiBa_SA_HCT116_mHLA−1: 11233
SA = 0.85
Score = 2.25
ATDPQFGFNT − MaxQuant
20120515_EXQ3_MiBa_SA_HCT116_mHLA−1: 11233
SA = 0.19
Score = −5.57
ATTQETDGFQ − MSFragger
20120515_EXQ3_MiBa_SA_HCT116_mHLA−1: 11233
SA = 0.05
Score = −7.6
Relative intensity
m/z
Prosit prediction
* Exclusive in prediction
# Exclusive in experimental

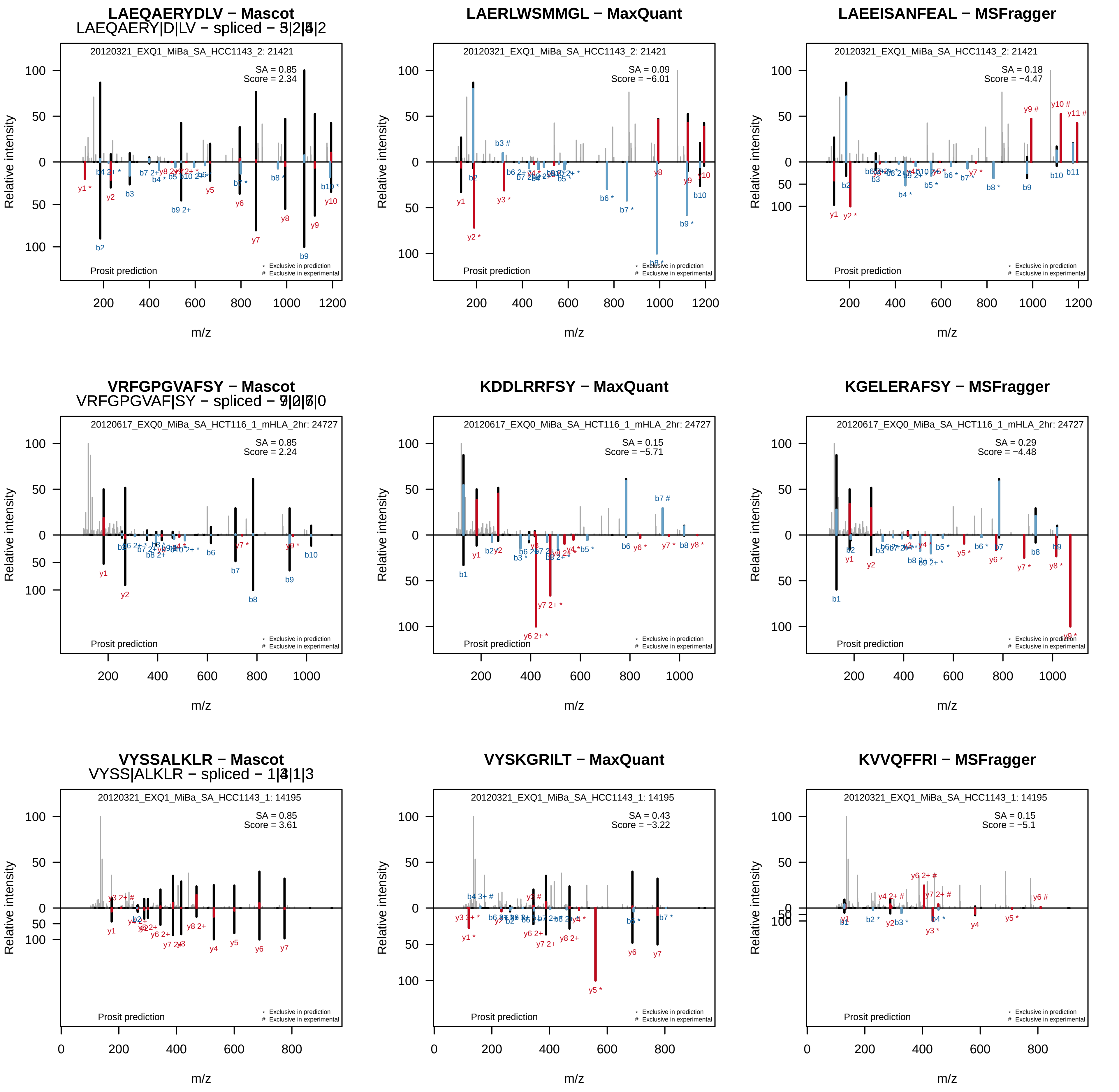

LAEQAERYDLV − Mascot
LAEQAERY|D|LV − spliced
20120321_EXQ1_MiBa_SA_HCC1143_2: 21421
SA = 0.85
Score = 2.34
LAERLWSMMGL − MaxQuant
20120321_EXQ1_MiBa_SA_HCC1143_2: 21421
SA = 0.09
Score = −6.01
LAEEISANFEAL − MSFragger
20120321_EXQ1_MiBa_SA_HCC1143_2: 21421
SA = 0.18
Score = −4.47
VRFGPGVAFSY − Mascot
VRFGPGVAF|SY − spliced
20120617_EXQ0_MiBa_SA_HCT116_1_mHLA_2hr: 24727
SA = 0.85
Score = 2.24
KDDLRRFSY − MaxQuant
20120617_EXQ0_MiBa_SA_HCT116_1_mHLA_2hr: 24727
SA = 0.15
Score = −5.71
KGELERAFSY − MSFragger
20120617_EXQ0_MiBa_SA_HCT116_1_mHLA_2hr: 24727
SA = 0.29
Score = −4.48
VYSSALKLR − Mascot
VYSS|ALKLR − spliced
20120321_EXQ1_MiBa_SA_HCC1143_1: 14195
SA = 0.85
Score = 3.61
VYSKGRILT − MaxQuant
20120321_EXQ1_MiBa_SA_HCC1143_1: 14195
SA = 0.43
Score = −3.22
KVVQFFRI − MSFragger
20120321_EXQ1_MiBa_SA_HCC1143_1: 14195
SA = 0.15
Score = −5.1
Relative intensity
m/z
Prosit prediction
* Exclusive in prediction
# Exclusive in experimental

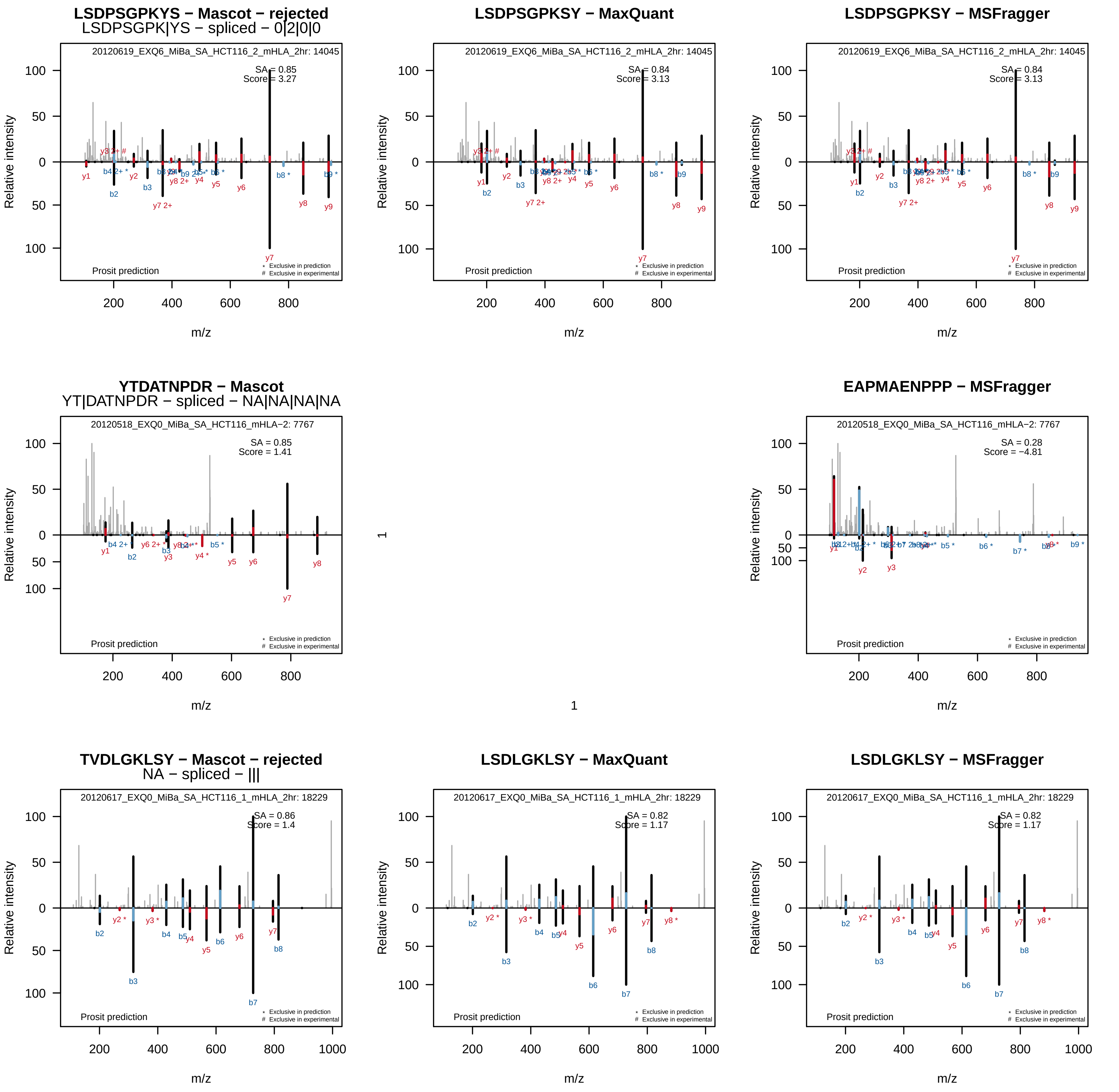
LSDPSGPKYS − Mascot − rejected
LSDPSGPK|YS − spliced − 0|2|0|0
20120619_EXQ6_MiBa_SA_HCT116_2_mHLA_2hr: 14045
SA = 0.85
Score = 3.27
Prosit prediction
LSDPSGPKSY − MaxQuant
20120619_EXQ6_MiBa_SA_HCT116_2_mHLA_2hr: 14045
SA = 0.84
Score = 3.13
Prosit prediction
LSDPSGPKSY − MSFragger
20120619_EXQ6_MiBa_SA_HCT116_2_mHLA_2hr: 14045
SA = 0.84
Score = 3.13
Prosit prediction
YTDATNPDR − Mascot
YT|DATNPDR − spliced − NA|NA|NA|NA
20120518_EXQ0_MiBa_SA_HCT116_mHLA−2: 7767
SA = 0.85
Score = 1.41
Prosit prediction
EAPMAENPPP − MSFragger
20120518_EXQ0_MiBa_SA_HCT116_mHLA−2: 7767
SA = 0.28
Score = −4.81
Prosit prediction
TVDLGKLSY − Mascot − rejected
NA − spliced − |||
20120617_EXQ0_MiBa_SA_HCT116_1_mHLA_2hr: 18229
SA = 0.86
Score = 1.4
Prosit prediction
LSDLGKLSY − MaxQuant
20120617_EXQ0_MiBa_SA_HCT116_1_mHLA_2hr: 18229
SA = 0.82
Score = 1.17
Prosit prediction
LSDLGKLSY − MSFragger
20120617_EXQ0_MiBa_SA_HCT116_1_mHLA_2hr: 18229
SA = 0.82
Score = 1.17
Prosit prediction
Relative intensity
m/z
* Exclusive in prediction
# Exclusive in experimental

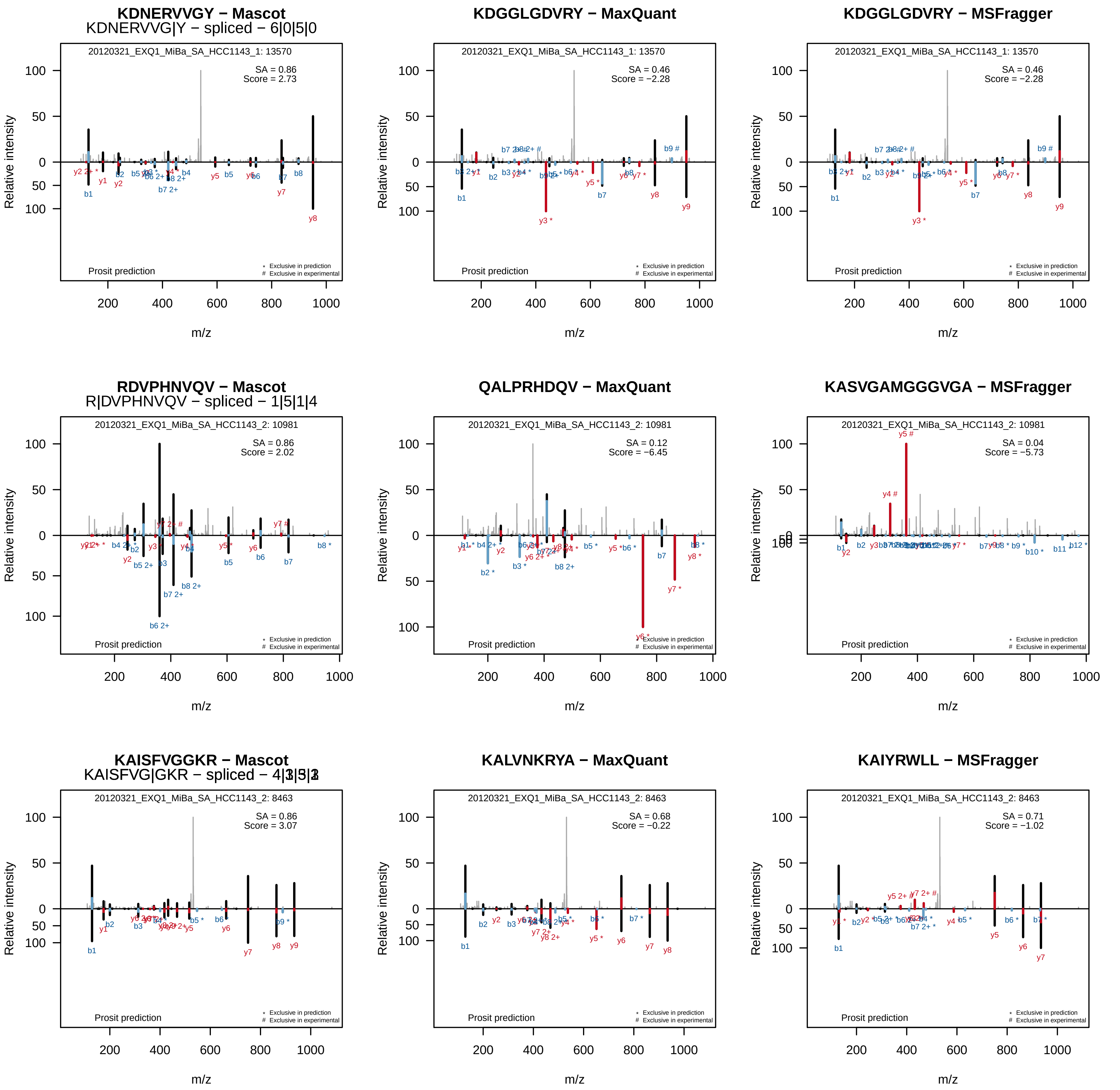

KDNERVVGY − Mascot
KDNERVVG|Y − spliced − 6|0|5|0
20120321_EXQ1_MiBa_SA_HCC1143_1: 13570
SA = 0.86
Score = 2.73
KDGGLGDVRY − MaxQuant
20120321_EXQ1_MiBa_SA_HCC1143_1: 13570
SA = 0.46
Score = −2.28
KDGGLGDVRY − MSFragger
20120321_EXQ1_MiBa_SA_HCC1143_1: 13570
SA = 0.46
Score = −2.28
RDVPHNVQV − Mascot
R|DVPHNVQV − spliced − 1|5|1|4
20120321_EXQ1_MiBa_SA_HCC1143_2: 10981
SA = 0.86
Score = 2.02
QALPRHDQV − MaxQuant
20120321_EXQ1_MiBa_SA_HCC1143_2: 10981
SA = 0.12
Score = −6.45
KASVGAMGGGVGA − MSFragger
20120321_EXQ1_MiBa_SA_HCC1143_2: 10981
SA = 0.04
Score = −5.73
KAISFVGGKR − Mascot
20120321_EXQ1_MiBa_SA_HCC1143_2: 8463
SA = 0.86
Score = 3.07
KALVNKRYA − MaxQuant
20120321_EXQ1_MiBa_SA_HCC1143_2: 8463
SA = 0.68
Score = −0.22
KAIYRWLL − MSFragger
20120321_EXQ1_MiBa_SA_HCC1143_2: 8463
SA = 0.71
Score = −1.02
Relative intensity
m/z
Prosit prediction
* Exclusive in prediction
# Exclusive in experimental

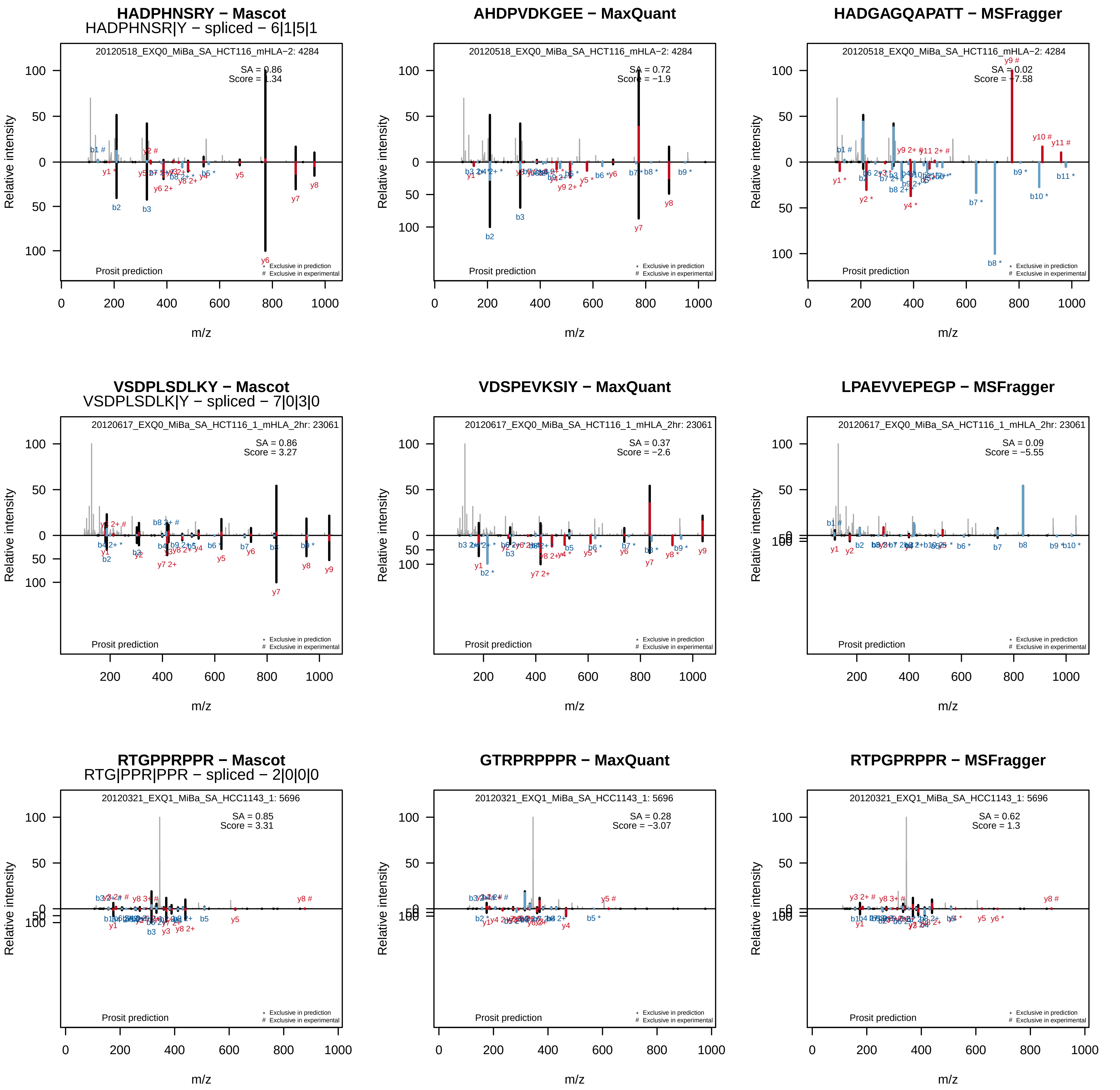
HADPHNSRY − Mascot
HADPHNSR|Y − spliced − 6|1|5|1
20120518_EXQ0_MiBa_SA_HCT116_mHLA−2: 4284
SA = 0.86
Score = 1.34
AHDPVDKGEE − MaxQuant
20120518_EXQ0_MiBa_SA_HCT116_mHLA−2: 4284
SA = 0.72
Score = −1.9
HADGAGQAPATT − MSFragger
20120518_EXQ0_MiBa_SA_HCT116_mHLA−2: 4284
SA = 0.02
Score = −7.58
VSDPLSDLKY − Mascot
VSDPLSDLK|Y − spliced − 7|0|3|0
20120617_EXQ0_MiBa_SA_HCT116_1_mHLA_2hr: 23061
SA = 0.86
Score = 3.27
VDSPEVKSIY − MaxQuant
20120617_EXQ0_MiBa_SA_HCT116_1_mHLA_2hr: 23061
SA = 0.37
Score = −2.6
LPAEVVEPEGP − MSFragger
20120617_EXQ0_MiBa_SA_HCT116_1_mHLA_2hr: 23061
SA = 0.09
Score = −5.55
RTGPPRPPR − Mascot
RTG|PPR|PPR − spliced − 2|0|0|0
20120321_EXQ1_MiBa_SA_HCC1143_1: 5696
SA = 0.85
Score = 3.31
GTRPRPPPR − MaxQuant
20120321_EXQ1_MiBa_SA_HCC1143_1: 5696
SA = 0.28
Score = −3.07
RTPGPRPPR − MSFragger
20120321_EXQ1_MiBa_SA_HCC1143_1: 5696
SA = 0.62
Score = 1.3
Relative intensity
m/z
Prosit prediction
* Exclusive in prediction
# Exclusive in experimental

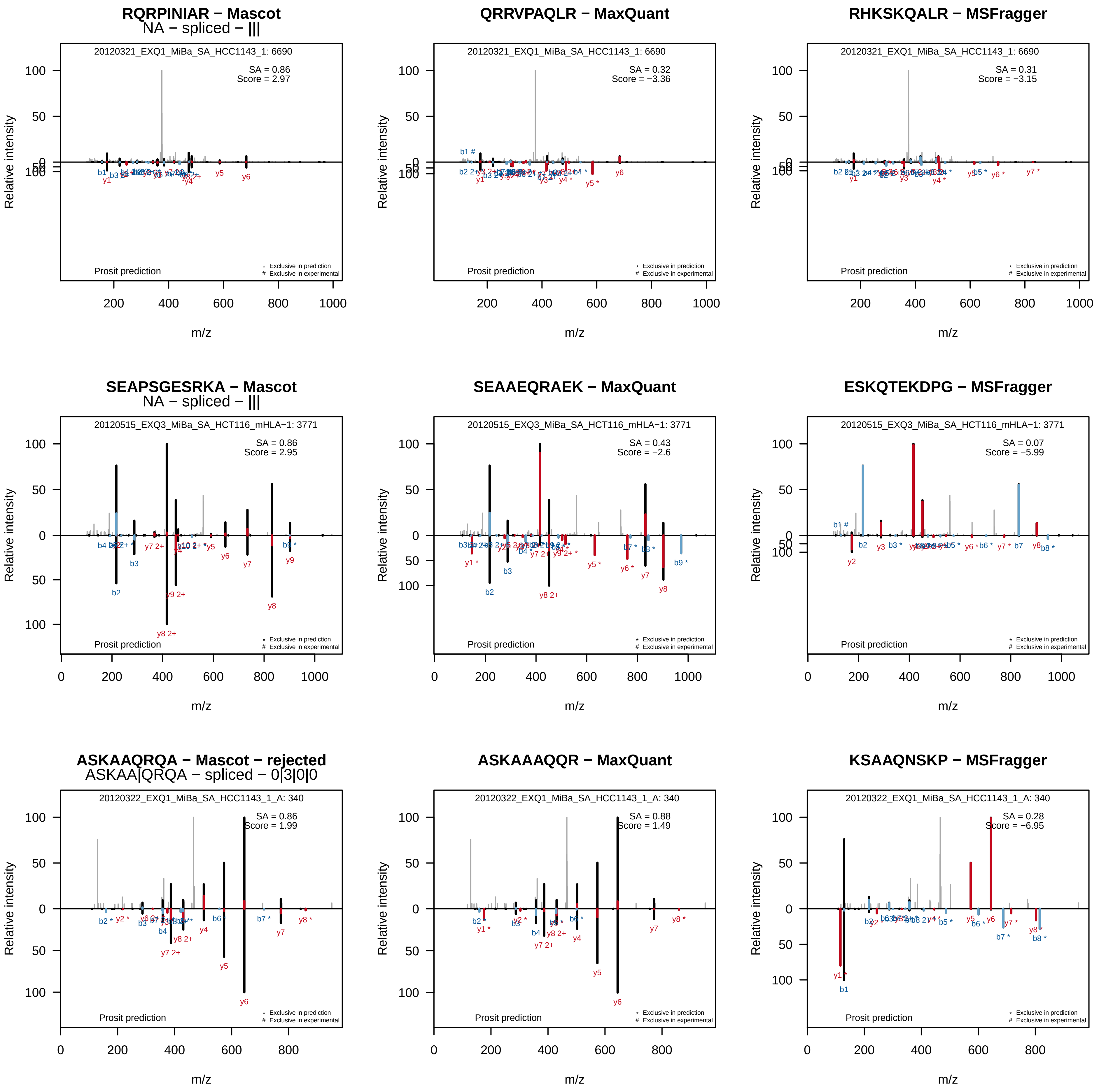
RQRPINIAR − Mascot
NA − spliced − |||
20120321_EXQ1_MiBa_SA_HCC1143_1: 6690
SA = 0.86
Score = 2.97
Prosit prediction
QRRVPAQLR − MaxQuant
20120321_EXQ1_MiBa_SA_HCC1143_1: 6690
SA = 0.32
Score = −3.36
Prosit prediction
RHKSKQALR − MSFragger
20120321_EXQ1_MiBa_SA_HCC1143_1: 6690
SA = 0.31
Score = −3.15
Prosit prediction
SEAPSGESRKA − Mascot
NA − spliced − |||
20120515_EXQ3_MiBa_SA_HCT116_mHLA−1: 3771
SA = 0.86
Score = 2.95
Prosit prediction
SEAAEQRAEK − MaxQuant
20120515_EXQ3_MiBa_SA_HCT116_mHLA−1: 3771
SA = 0.43
Score = −2.6
Prosit prediction
ESKQTEKDPG − MSFragger
20120515_EXQ3_MiBa_SA_HCT116_mHLA−1: 3771
SA = 0.07
Score = −5.99
Prosit prediction
ASKAAQRQA − Mascot − rejected
ASKAA|QRQA − spliced − 0|3|0|0
20120322_EXQ1_MiBa_SA_HCC1143_1_A: 340
SA = 0.86
Score = 1.99
Prosit prediction
ASKAAAQQR − MaxQuant
20120322_EXQ1_MiBa_SA_HCC1143_1_A: 340
SA = 0.88
Score = 1.49
Prosit prediction
KSAAQNSKP − MSFragger
20120322_EXQ1_MiBa_SA_HCC1143_1_A: 340
SA = 0.28
Score = −6.95
Prosit prediction
* Exclusive in prediction
# Exclusive in experimental
Relative intensity
m/z

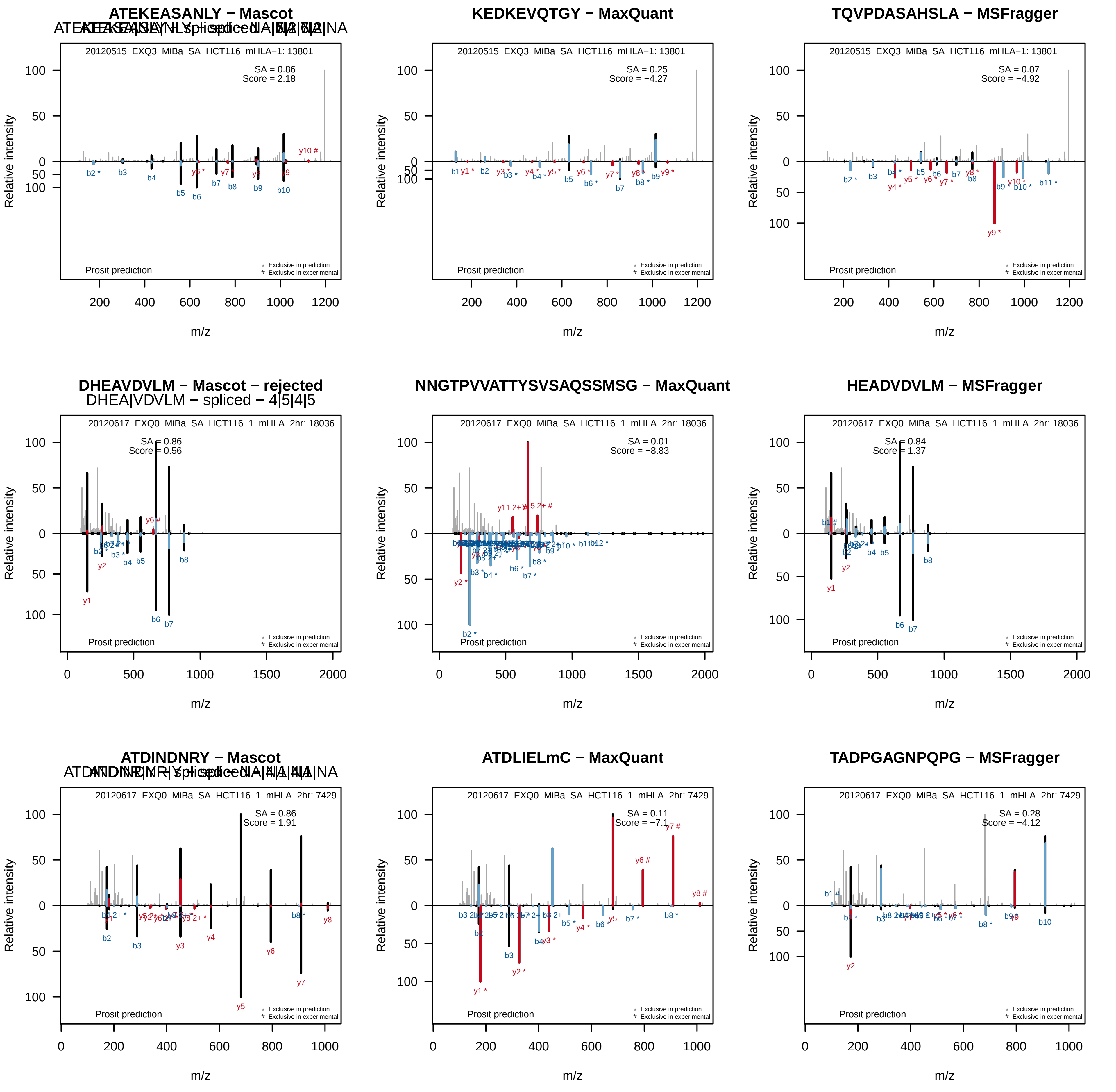

ATEKEASANLY − Mascot
KEDKEVQTGY − MaxQuant
TQVPDASAHSLA − MSFragger
20120515_EXQ3_MiBa_SA_HCT116_mHLA−1: 13801
SA = 0.86
Score = 2.18
SA = 0.25
Score = −4.27
SA = 0.07
Score = −4.92
DHEAVDVLM − Mascot − rejected
DHEA|VDVLM − spliced − 4|5|4|5
NNGTPVVATTYSVSAQSSMSG − MaxQuant
HEADVDVLM − MSFragger
20120617_EXQ0_MiBa_SA_HCT116_1_mHLA_2hr: 18036
SA = 0.86
Score = 0.56
SA = 0.01
Score = −8.83
SA = 0.84
Score = 1.37
ATDINDNRY − Mascot
ATDLIELmC − MaxQuant
TADPGAGNPQPG − MSFragger
20120617_EXQ0_MiBa_SA_HCT116_1_mHLA_2hr: 7429
SA = 0.86
Score = 1.91
SA = 0.11
Score = −7.1
SA = 0.28
Score = −4.12
Relative intensity
m/z
Prosit prediction
Exclusive in prediction
Exclusive in experimental

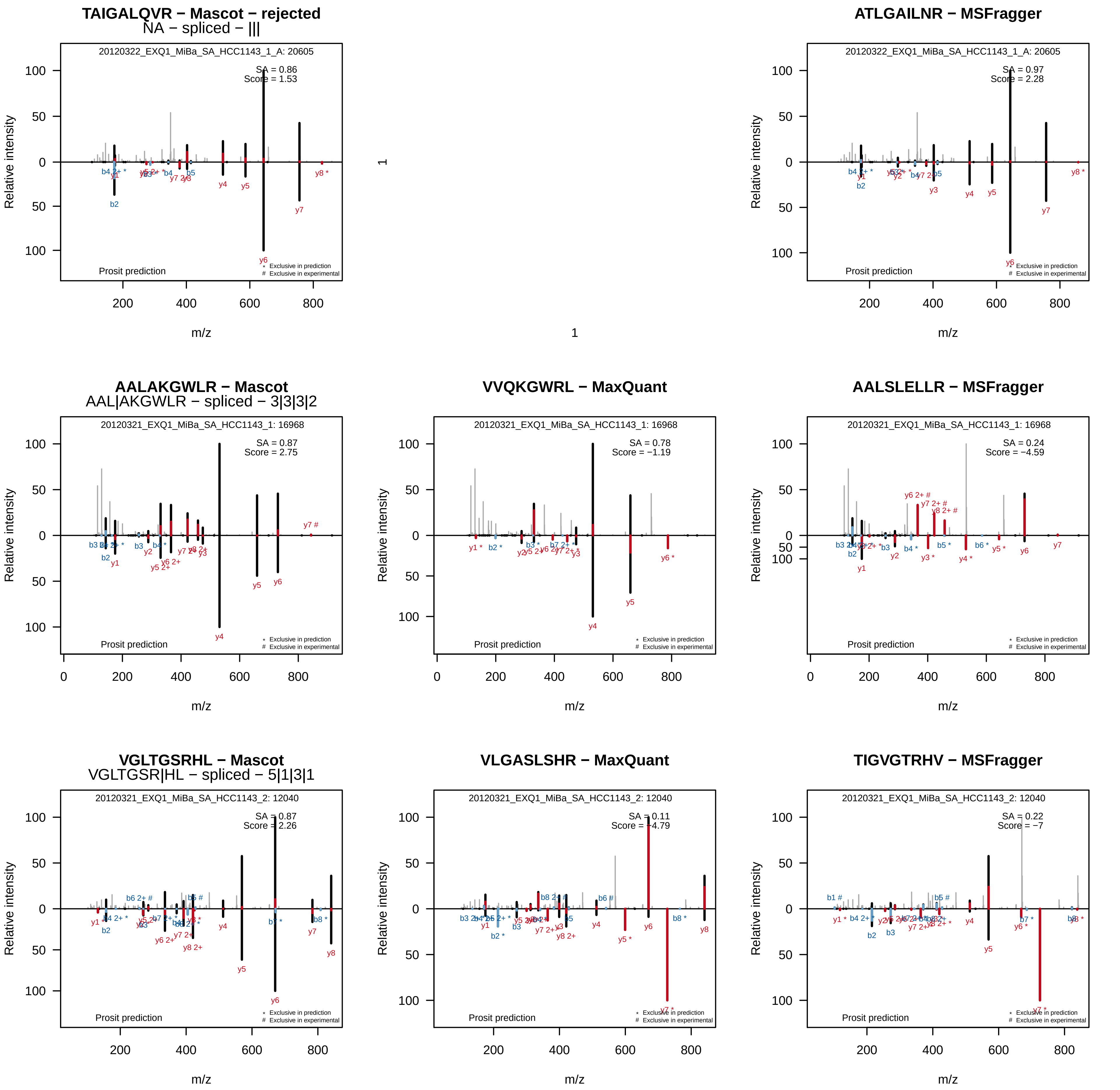

TAIGALQVR − Mascot − rejected
NA − spliced − |||
20120322_EXQ1_MiBa_SA_HCC1143_1_A: 20605
SA = 0.86
Score = 1.53
Prosit prediction
⋆ Exclusive in prediction
Exclusive in experimental
Relative intensity
m/z
1
ATLGAILNR − MSFragger
20120322_EXQ1_MiBa_SA_HCC1143_1_A: 20605
SA = 0.97
Score = 2.28
Prosit prediction
AALAKGWLR − Mascot
AAL|AKGWLR − spliced − 3|3|3|2
20120321_EXQ1_MiBa_SA_HCC1143_1: 16968
SA = 0.87
Score = 2.75
VVQKGWRL − MaxQuant
20120321_EXQ1_MiBa_SA_HCC1143_1: 16968
SA = 0.78
Score = −1.19
AALSLELLR − MSFragger
20120321_EXQ1_MiBa_SA_HCC1143_1: 16968
SA = 0.24
Score = −4.59
VGLTGSRHL − Mascot
VGLTGSR|HL − spliced − 5|1|3|1
20120321_EXQ1_MiBa_SA_HCC1143_2: 12040
SA = 0.87
Score = 2.26
VLGASLSHR − MaxQuant
20120321_EXQ1_MiBa_SA_HCC1143_2: 12040
SA = 0.11
Score = −4.79
TIGVGTRHV − MSFragger
20120321_EXQ1_MiBa_SA_HCC1143_2: 12040
SA = 0.22
Score = −7

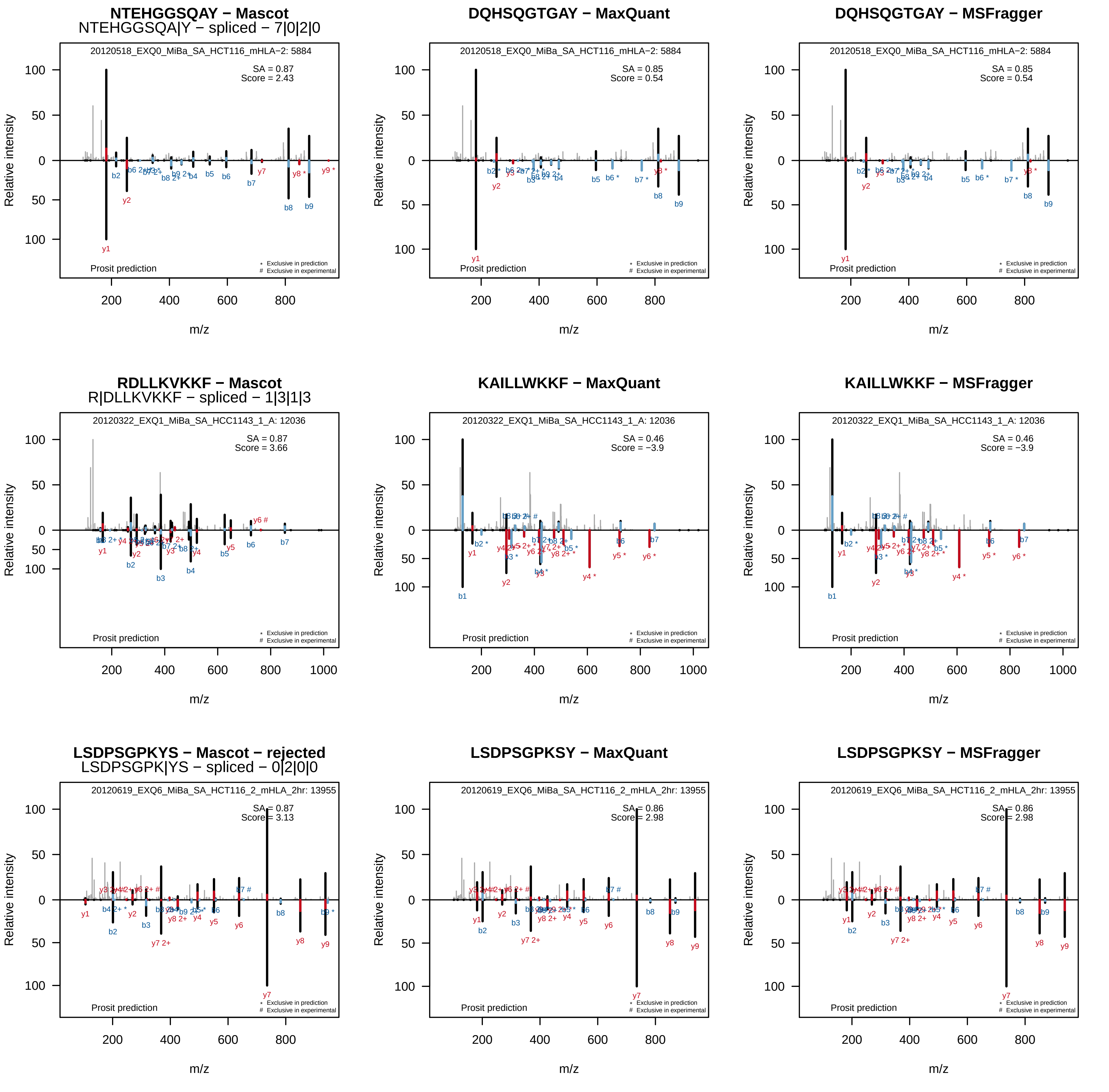

NTEHGGSQAY − Mascot
NTEHGGSQA|Y − spliced − 7|0|2|0
20120518_EXQ0_MiBa_SA_HCT116_mHLA−2: 5884
SA = 0.87
Score = 2.43
DQHSQGTGAY − MaxQuant
20120518_EXQ0_MiBa_SA_HCT116_mHLA−2: 5884
SA = 0.85
Score = 0.54
DQHSQGTGAY − MSFragger
20120518_EXQ0_MiBa_SA_HCT116_mHLA−2: 5884
SA = 0.85
Score = 0.54
RDLLKVKKF − Mascot
R|DLLKVKKF − spliced − 1|3|1|3
20120322_EXQ1_MiBa_SA_HCC1143_1_A: 12036
SA = 0.87
Score = 3.66
KAILLWKKF − MaxQuant
20120322_EXQ1_MiBa_SA_HCC1143_1_A: 12036
SA = 0.46
Score = −3.9
KAILLWKKF − MSFragger
20120322_EXQ1_MiBa_SA_HCC1143_1_A: 12036
SA = 0.46
Score = −3.9
LSDPSGPKYS − Mascot − rejected
LSDPSGPK|YS − spliced − 0|2|0|0
20120619_EXQ6_MiBa_SA_HCT116_2_mHLA_2hr: 13955
SA = 0.87
Score = 3.13
LSDPSGPKSY − MaxQuant
20120619_EXQ6_MiBa_SA_HCT116_2_mHLA_2hr: 13955
SA = 0.86
Score = 2.98
LSDPSGPKSY − MSFragger
20120619_EXQ6_MiBa_SA_HCT116_2_mHLA_2hr: 13955
SA = 0.86
Score = 2.98
Relative intensity
m/z
Prosit prediction
* Exclusive in prediction
# Exclusive in experimental

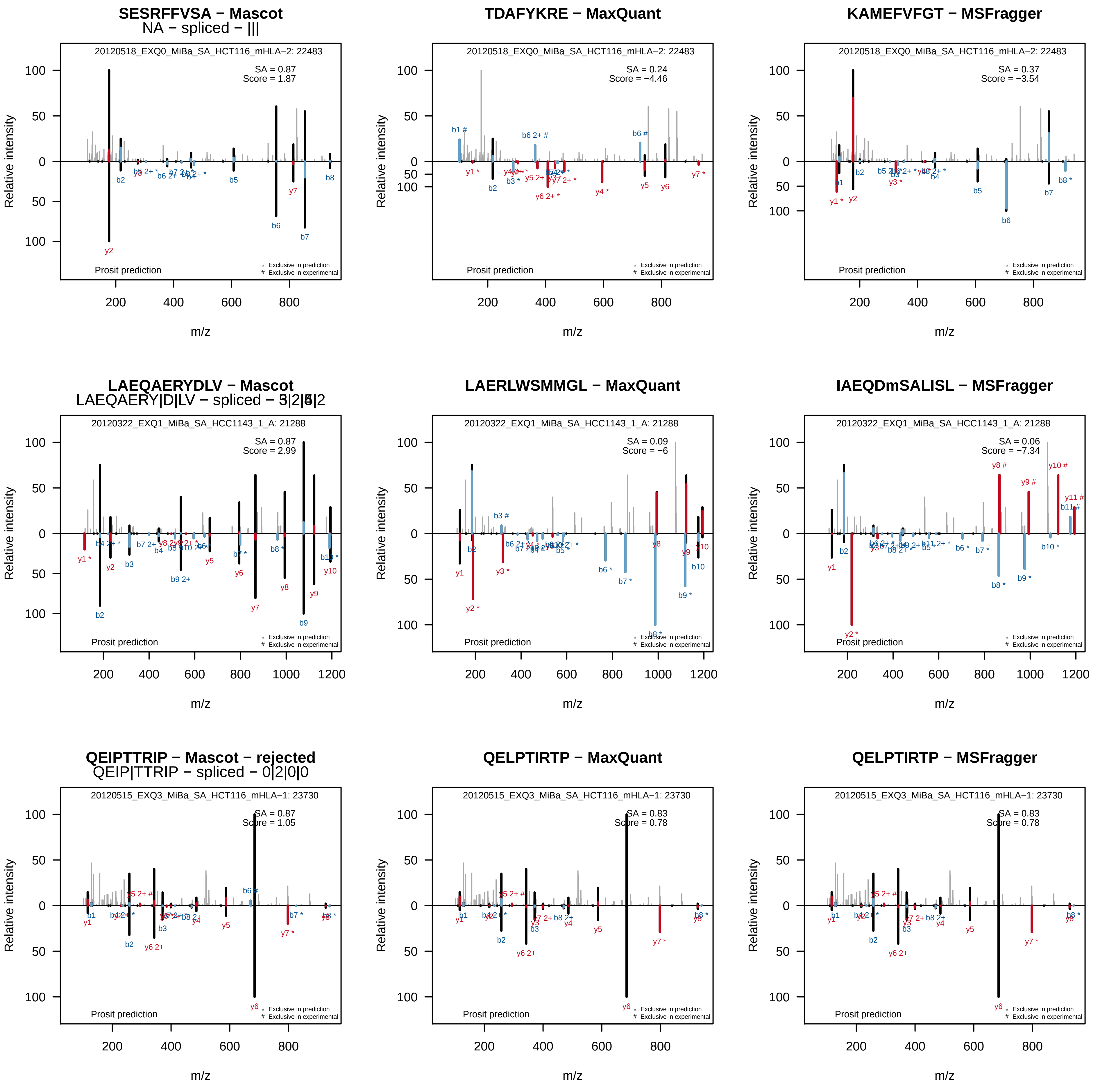
SESRFFVSA − Mascot
NA − spliced − |||
20120518_EXQ0_MiBa_SA_HCT116_mHLA−2: 22483
SA = 0.87
Score = 1.87
TDAFYKRE − MaxQuant
20120518_EXQ0_MiBa_SA_HCT116_mHLA−2: 22483
SA = 0.24
Score = −4.46
KAMEFVFGT − MSFragger
20120518_EXQ0_MiBa_SA_HCT116_mHLA−2: 22483
SA = 0.37
Score = −3.54
LAEQAERYDLV − Mascot
20120322_EXQ1_MiBa_SA_HCC1143_1_A: 21288
SA = 0.87
Score = 2.99
LAERLWSMMGL − MaxQuant
20120322_EXQ1_MiBa_SA_HCC1143_1_A: 21288
SA = 0.09
Score = −6
IAEQDmSALISL − MSFragger
20120322_EXQ1_MiBa_SA_HCC1143_1_A: 21288
SA = 0.06
Score = −7.34
QEIPTTRIP − Mascot − rejected
QEIP|TTRIP − spliced − 0|2|0|0
20120515_EXQ3_MiBa_SA_HCT116_mHLA−1: 23730
SA = 0.87
Score = 1.05
QELPTIRTP − MaxQuant
20120515_EXQ3_MiBa_SA_HCT116_mHLA−1: 23730
SA = 0.83
Score = 0.78
QELPTIRTP − MSFragger
20120515_EXQ3_MiBa_SA_HCT116_mHLA−1: 23730
SA = 0.83
Score = 0.78
Relative intensity
m/z
Prosit prediction
* Exclusive in prediction
# Exclusive in experimental

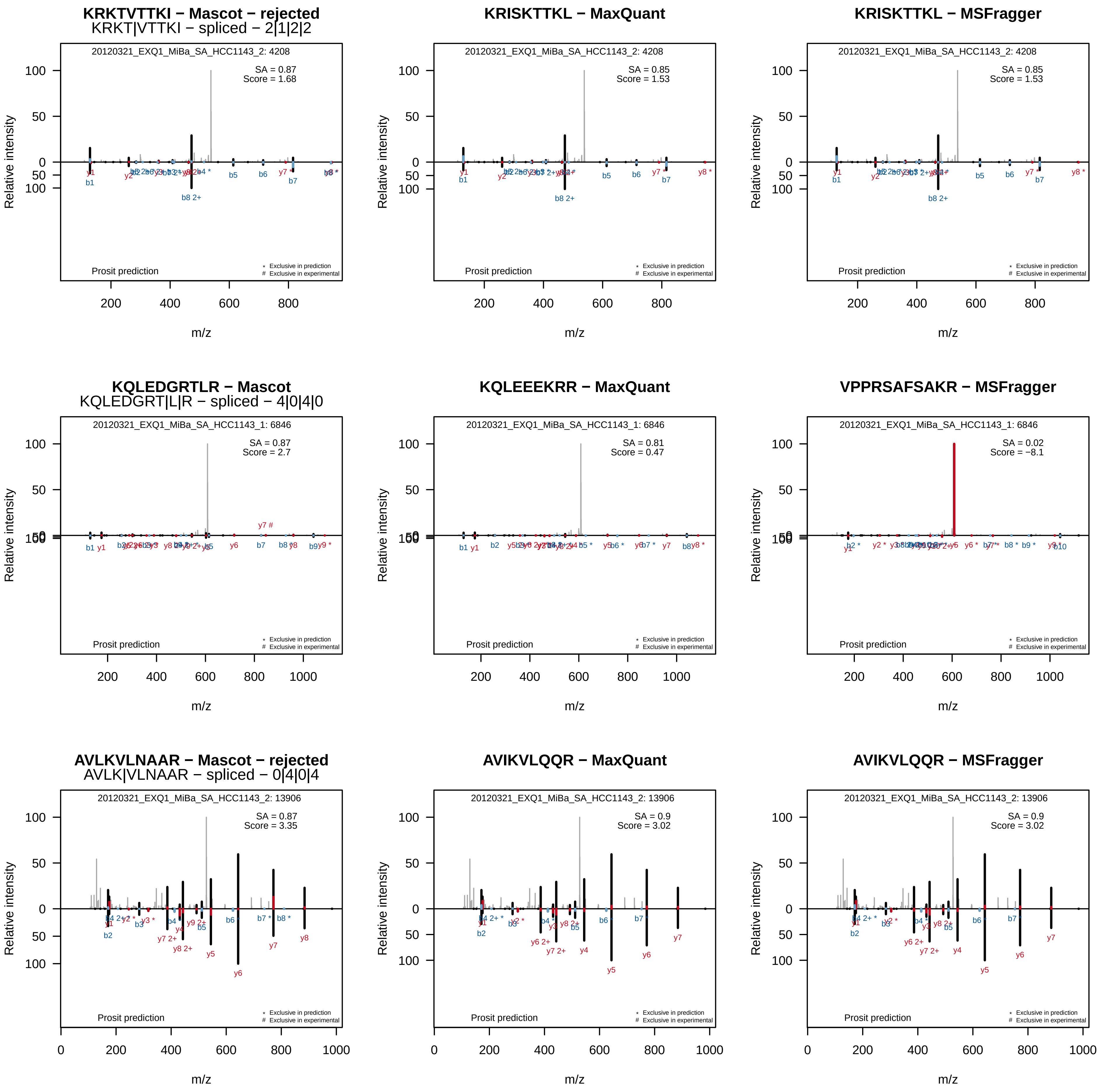

KRKTVTTKI − Mascot − rejected
KRKT|VTTKI − spliced − 2|1|2|2
20120321_EXQ1_MiBa_SA_HCC1143_2: 4208
SA = 0.87
Score = 1.68
KRISKTTKL − MaxQuant
20120321_EXQ1_MiBa_SA_HCC1143_2: 4208
SA = 0.85
Score = 1.53
KRISKTTKL − MSFragger
20120321_EXQ1_MiBa_SA_HCC1143_2: 4208
SA = 0.85
Score = 1.53
KQLEDGRTLR − Mascot
KQLEDGRT|L|R − spliced − 4|0|4|0
20120321_EXQ1_MiBa_SA_HCC1143_1: 6846
SA = 0.87
Score = 2.7
KQLEEEKRR − MaxQuant
20120321_EXQ1_MiBa_SA_HCC1143_1: 6846
SA = 0.81
Score = 0.47
VPPRSAFSAKR − MSFragger
20120321_EXQ1_MiBa_SA_HCC1143_1: 6846
SA = 0.02
Score = −8.1
AVLKVLNAAR − Mascot − rejected
AVLK|VLNAAR − spliced − 0|4|0|4
20120321_EXQ1_MiBa_SA_HCC1143_2: 13906
SA = 0.87
Score = 3.35
AVIKVLQQR − MaxQuant
20120321_EXQ1_MiBa_SA_HCC1143_2: 13906
SA = 0.9
Score = 3.02
AVIKVLQQR − MSFragger
20120321_EXQ1_MiBa_SA_HCC1143_2: 13906
SA = 0.9
Score = 3.02
Prosit prediction
* Exclusive in prediction
# Exclusive in experimental
Relative intensity
m/z

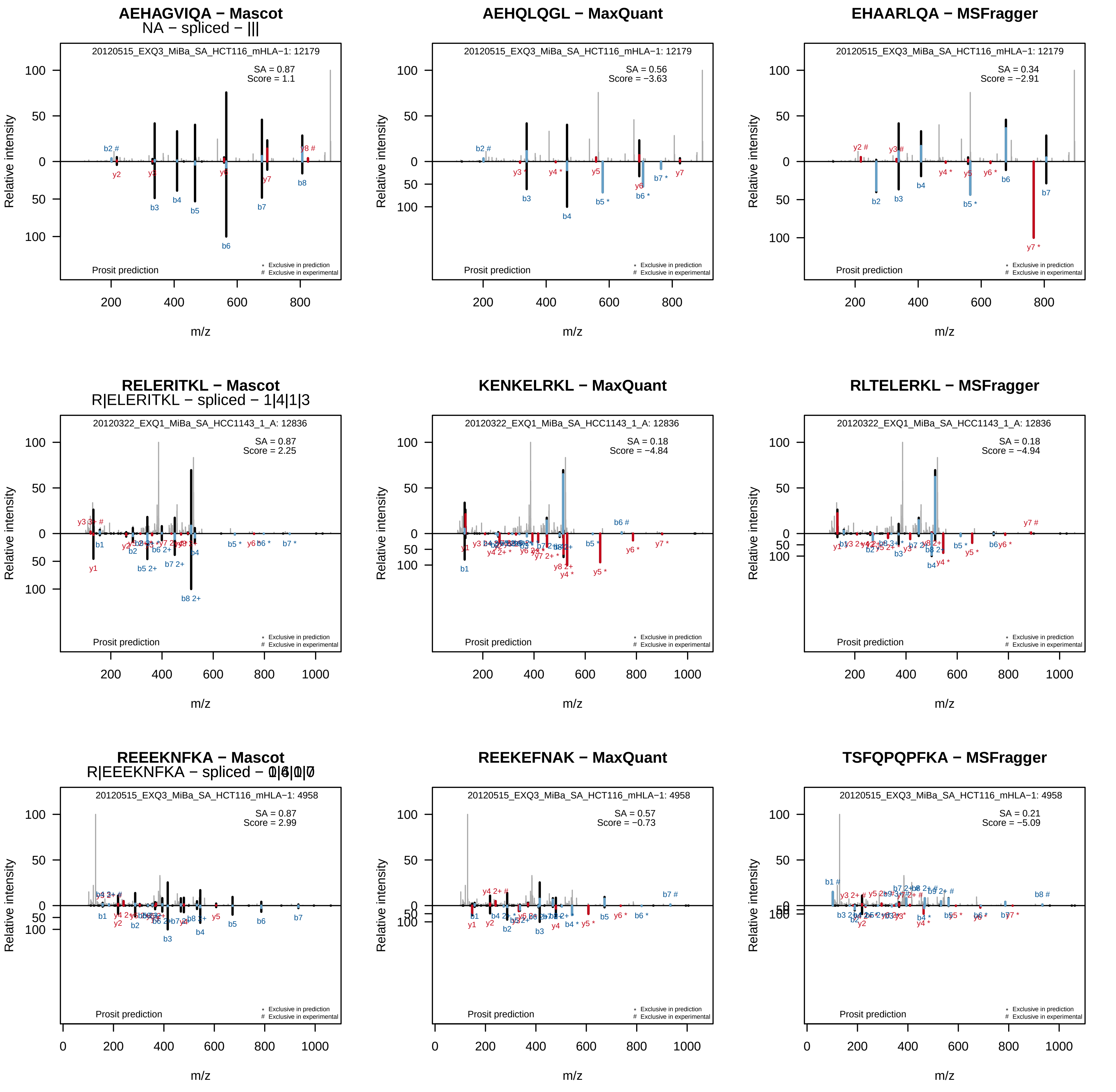
AEHAGVIQA − Mascot
NA − spliced − |||
20120515_EXQ3_MiBa_SA_HCT116_mHLA−1: 12179
SA = 0.87
Score = 1.1
AEHQLQGL − MaxQuant
20120515_EXQ3_MiBa_SA_HCT116_mHLA−1: 12179
SA = 0.56
Score = −3.63
EHAARLQA − MSFragger
20120515_EXQ3_MiBa_SA_HCT116_mHLA−1: 12179
SA = 0.34
Score = −2.91
RELERITKL − Mascot
R|ELERITKL − spliced − 1|4|1|3
20120322_EXQ1_MiBa_SA_HCC1143_1_A: 12836
SA = 0.87
Score = 2.25
KENKELRKL − MaxQuant
20120322_EXQ1_MiBa_SA_HCC1143_1_A: 12836
SA = 0.18
Score = −4.84
RLTELERKL − MSFragger
20120322_EXQ1_MiBa_SA_HCC1143_1_A: 12836
SA = 0.18
Score = −4.94
REEEKNFKA − Mascot
20120515_EXQ3_MiBa_SA_HCT116_mHLA−1: 4958
SA = 0.87
Score = 2.99
REEKEFNAK − MaxQuant
20120515_EXQ3_MiBa_SA_HCT116_mHLA−1: 4958
SA = 0.57
Score = −0.73
TSFQPQPFKA − MSFragger
20120515_EXQ3_MiBa_SA_HCT116_mHLA−1: 4958
SA = 0.21
Score = −5.09
Relative intensity
m/z
Prosit prediction
* Exclusive in prediction
# Exclusive in experimental

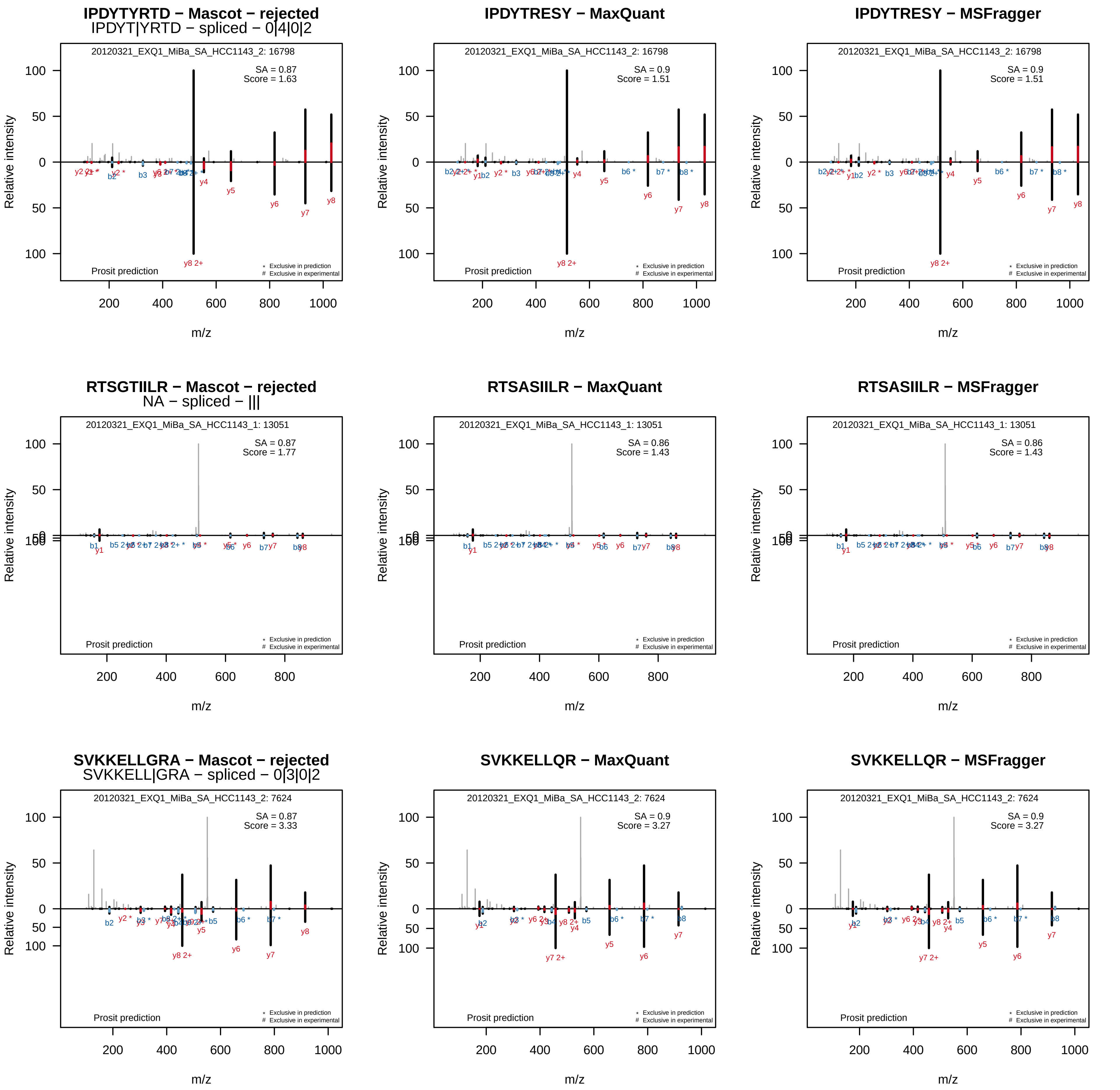

IPDYTYRTD − Mascot − rejected
IPDYT|YRTD − spliced − 0|4|0|2
20120321_EXQ1_MiBa_SA_HCC1143_2: 16798
SA = 0.87
Score = 1.63
IPDYTRESY − MaxQuant
20120321_EXQ1_MiBa_SA_HCC1143_2: 16798
SA = 0.9
Score = 1.51
IPDYTRESY − MSFragger
20120321_EXQ1_MiBa_SA_HCC1143_2: 16798
SA = 0.9
Score = 1.51
RTSGTIILR − Mascot − rejected
NA − spliced − |||
20120321_EXQ1_MiBa_SA_HCC1143_1: 13051
SA = 0.87
Score = 1.77
RTSASIILR − MaxQuant
20120321_EXQ1_MiBa_SA_HCC1143_1: 13051
SA = 0.86
Score = 1.43
RTSASIILR − MSFragger
20120321_EXQ1_MiBa_SA_HCC1143_1: 13051
SA = 0.86
Score = 1.43
SVKKELLGRA − Mascot − rejected
SVKKELL|GRA − spliced − 0|3|0|2
20120321_EXQ1_MiBa_SA_HCC1143_2: 7624
SA = 0.87
Score = 3.33
SVKKELLQR − MaxQuant
20120321_EXQ1_MiBa_SA_HCC1143_2: 7624
SA = 0.9
Score = 3.27
SVKKELLQR − MSFragger
20120321_EXQ1_MiBa_SA_HCC1143_2: 7624
SA = 0.9
Score = 3.27
Relative intensity
m/z
Prosit prediction
⋆ Exclusive in prediction
Exclusive in experimental

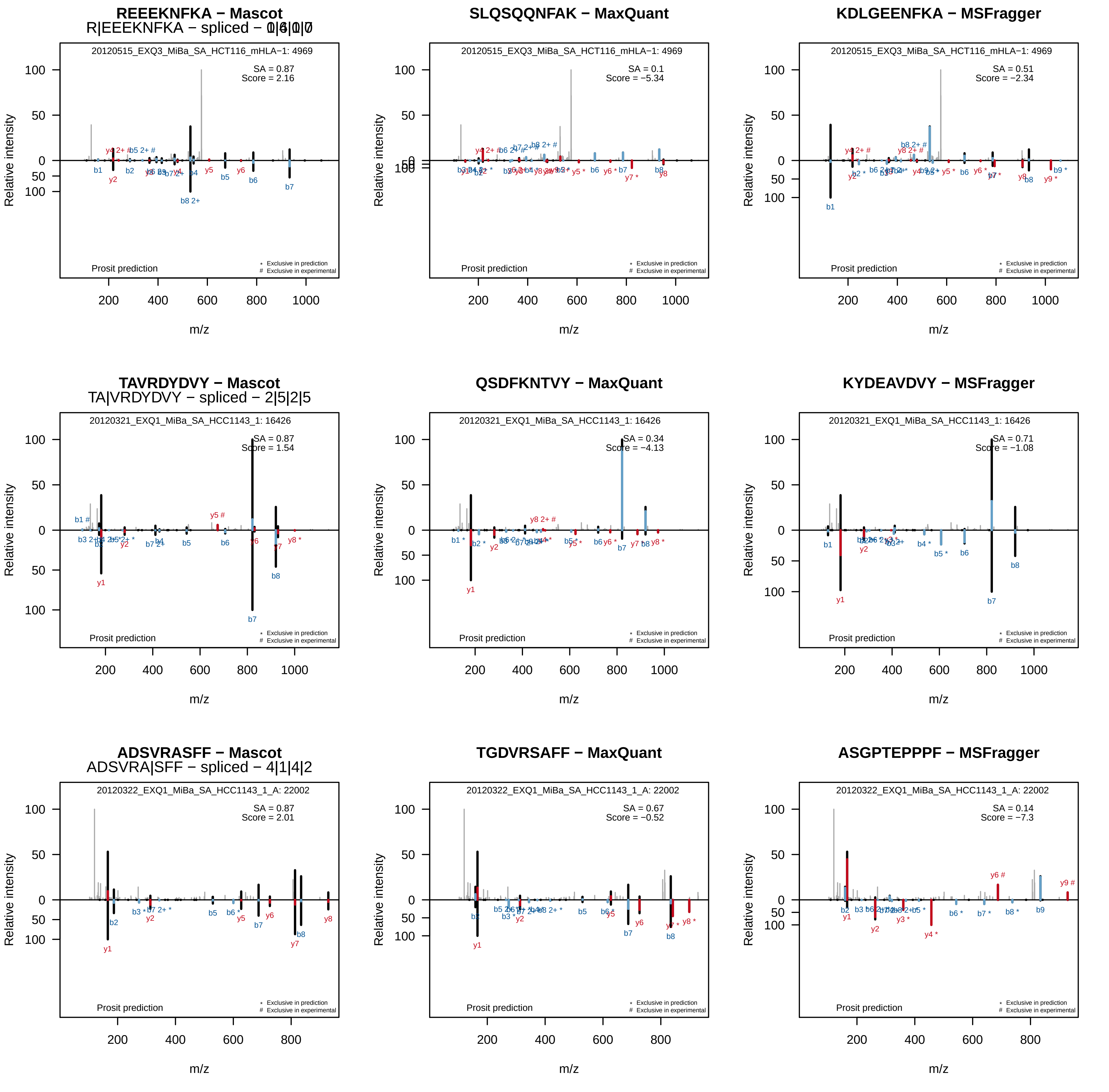
REEEKNFKA − Mascot
R|EEEKNFKA − spliced
20120515_EXQ3_MiBa_SA_HCT116_mHLA−1: 4969
SA = 0.87
Score = 2.16
SLQSQQNFAK − MaxQuant
20120515_EXQ3_MiBa_SA_HCT116_mHLA−1: 4969
SA = 0.1
Score = −5.34
KDLGEENFKA − MSFragger
20120515_EXQ3_MiBa_SA_HCT116_mHLA−1: 4969
SA = 0.51
Score = −2.34
TAVRDYDVY − Mascot
TA|VRDYDVY − spliced − 2|5|2|5
20120321_EXQ1_MiBa_SA_HCC1143_1: 16426
SA = 0.87
Score = 1.54
QSDFKNTVY − MaxQuant
20120321_EXQ1_MiBa_SA_HCC1143_1: 16426
SA = 0.34
Score = −4.13
KYDEAVDVY − MSFragger
20120321_EXQ1_MiBa_SA_HCC1143_1: 16426
SA = 0.71
Score = −1.08
ADSVRASFF − Mascot
ADSVRA|SFF − spliced − 4|1|4|2
20120322_EXQ1_MiBa_SA_HCC1143_1_A: 22002
SA = 0.87
Score = 2.01
TGDVRSAFF − MaxQuant
20120322_EXQ1_MiBa_SA_HCC1143_1_A: 22002
SA = 0.67
Score = −0.52
ASGPTEPPPF − MSFragger
20120322_EXQ1_MiBa_SA_HCC1143_1_A: 22002
SA = 0.14
Score = −7.3
Relative intensity
m/z
Prosit prediction
* Exclusive in prediction
# Exclusive in experimental

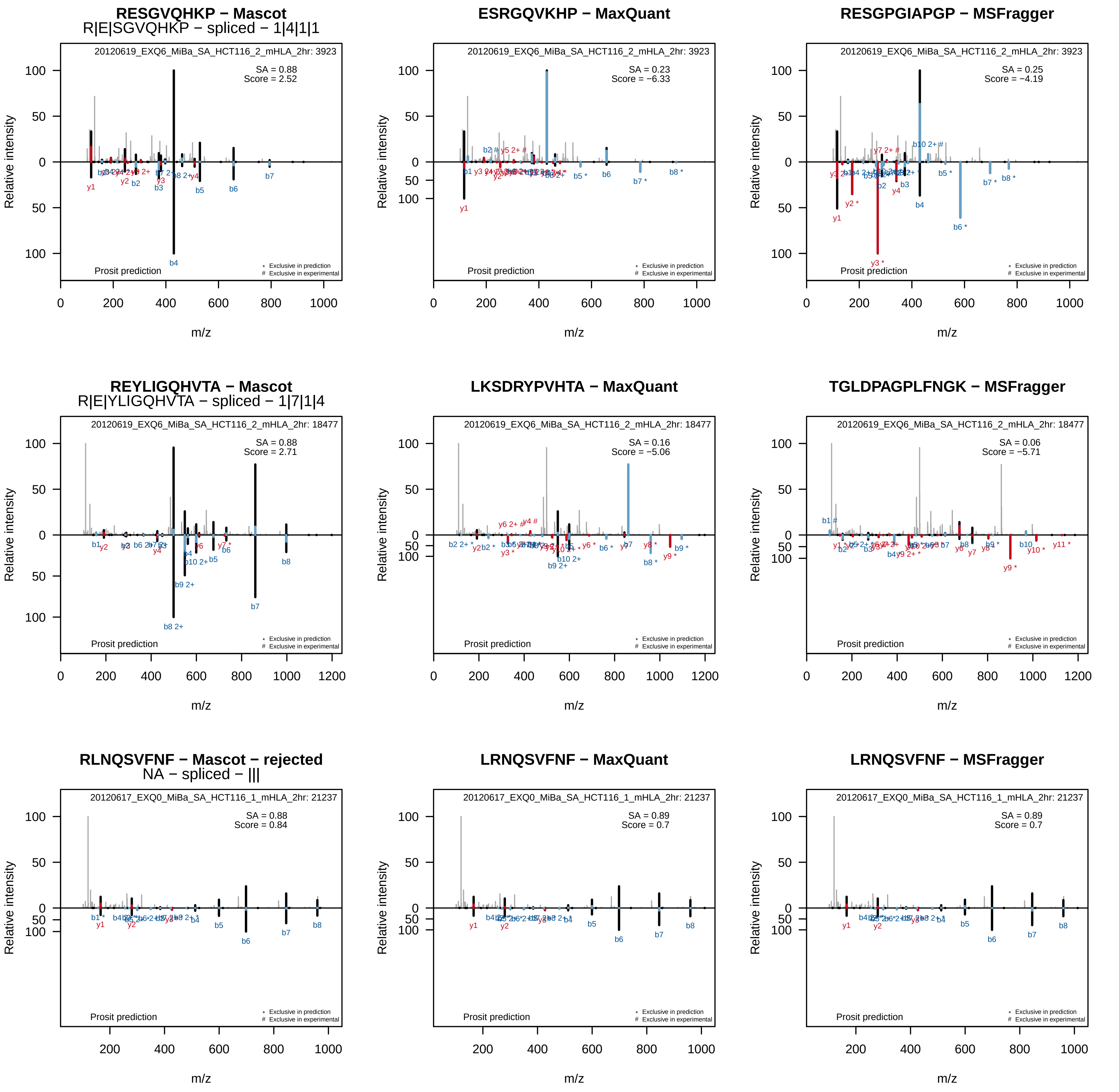
RESGVQHKP − Mascot
R|E|SGVQHKP − spliced − 1|4|1|1
20120619_EXQ6_MiBa_SA_HCT116_2_mHLA_2hr: 3923
SA = 0.88
Score = 2.52
ESRGQVKHP − MaxQuant
20120619_EXQ6_MiBa_SA_HCT116_2_mHLA_2hr: 3923
SA = 0.23
Score = −6.33
RESGPGIAPGP − MSFragger
20120619_EXQ6_MiBa_SA_HCT116_2_mHLA_2hr: 3923
SA = 0.25
Score = −4.19
REYLIGQHVTA − Mascot
R|E|YLIGQHVTA − spliced − 1|7|1|4
20120619_EXQ6_MiBa_SA_HCT116_2_mHLA_2hr: 18477
SA = 0.88
Score = 2.71
LKSDRYPVHTA − MaxQuant
20120619_EXQ6_MiBa_SA_HCT116_2_mHLA_2hr: 18477
SA = 0.16
Score = −5.06
TGLDPAGPLFNGK − MSFragger
20120619_EXQ6_MiBa_SA_HCT116_2_mHLA_2hr: 18477
SA = 0.06
Score = −5.71
RLNQSVFNF − Mascot − rejected
NA − spliced − |||
20120617_EXQ0_MiBa_SA_HCT116_1_mHLA_2hr: 21237
SA = 0.88
Score = 0.84
LRNQSVFNF − MaxQuant
20120617_EXQ0_MiBa_SA_HCT116_1_mHLA_2hr: 21237
SA = 0.89
Score = 0.7
LRNQSVFNF − MSFragger
20120617_EXQ0_MiBa_SA_HCT116_1_mHLA_2hr: 21237
SA = 0.89
Score = 0.7
Relative intensity
m/z
Prosit prediction
Exclusive in prediction
Exclusive in experimental

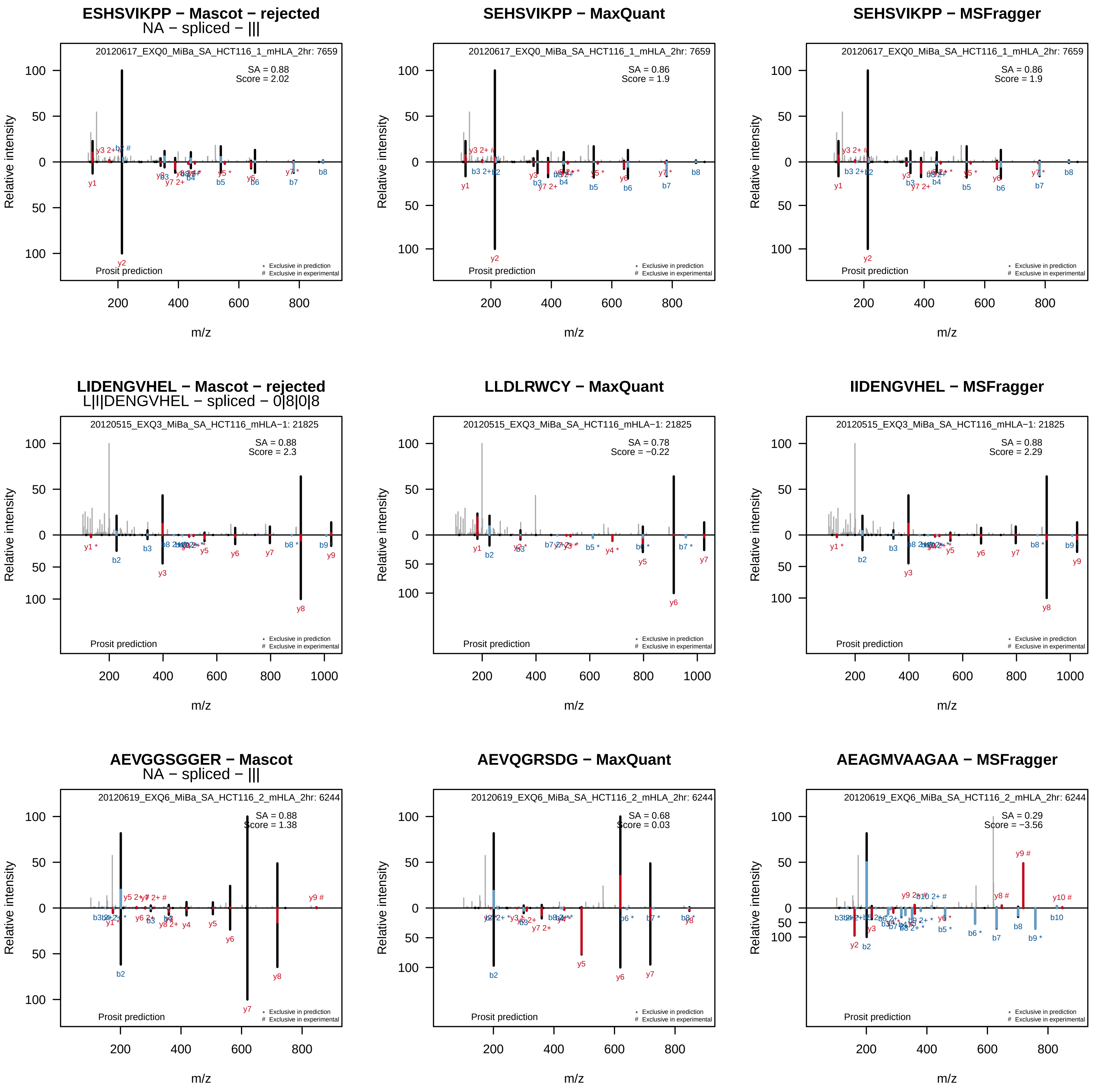
ESHSVIKPP − Mascot − rejected
NA − spliced − |||
20120617_EXQ0_MiBa_SA_HCT116_1_mHLA_2hr: 7659
SA = 0.88
Score = 2.02
SEHSVIKPP − MaxQuant
20120617_EXQ0_MiBa_SA_HCT116_1_mHLA_2hr: 7659
SA = 0.86
Score = 1.9
SEHSVIKPP − MSFragger
20120617_EXQ0_MiBa_SA_HCT116_1_mHLA_2hr: 7659
SA = 0.86
Score = 1.9
LIDENGVHEL − Mascot − rejected
L|I|DENGVHEL − spliced − 0|8|0|8
20120515_EXQ3_MiBa_SA_HCT116_mHLA−1: 21825
SA = 0.88
Score = 2.3
LLDLRWCY − MaxQuant
20120515_EXQ3_MiBa_SA_HCT116_mHLA−1: 21825
SA = 0.78
Score = −0.22
IIDENGVHEL − MSFragger
20120515_EXQ3_MiBa_SA_HCT116_mHLA−1: 21825
SA = 0.88
Score = 2.29
AEVGGSGGER − Mascot
NA − spliced − |||
20120619_EXQ6_MiBa_SA_HCT116_2_mHLA_2hr: 6244
SA = 0.88
Score = 1.38
AEVQGRSDG − MaxQuant
20120619_EXQ6_MiBa_SA_HCT116_2_mHLA_2hr: 6244
SA = 0.68
Score = 0.03
AEAGMVAAGAA − MSFragger
20120619_EXQ6_MiBa_SA_HCT116_2_mHLA_2hr: 6244
SA = 0.29
Score = −3.56
Relative intensity
m/z
Prosit prediction
⋆ Exclusive in prediction
Exclusive in experimental

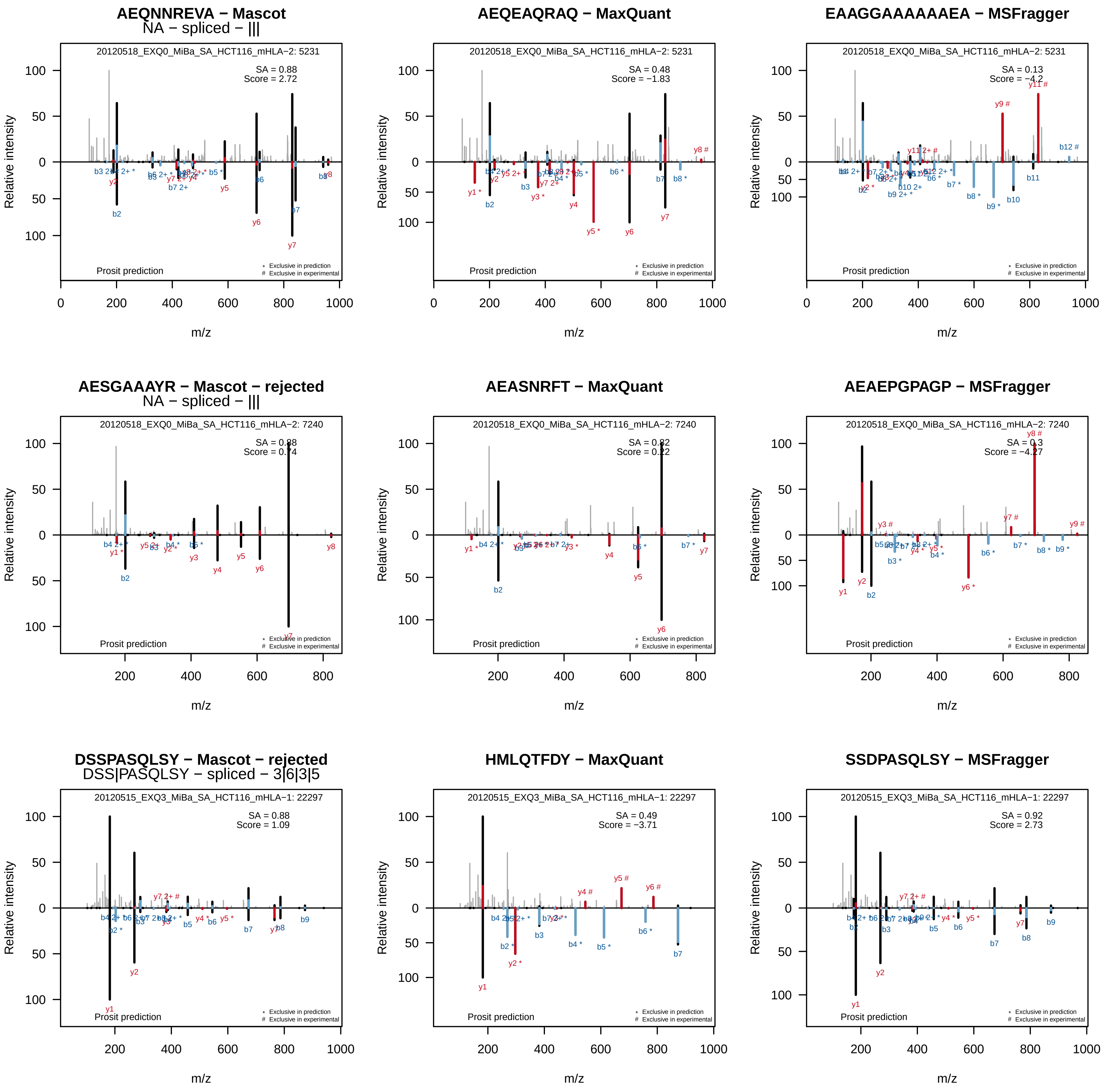
AEQNNREVA − Mascot
NA − spliced − |||
20120518_EXQ0_MiBa_SA_HCT116_mHLA−2: 5231
SA = 0.88
Score = 2.72
AEQEAQRAQ − MaxQuant
20120518_EXQ0_MiBa_SA_HCT116_mHLA−2: 5231
SA = 0.48
Score = −1.83
EAAGGAAAAAAEA − MSFragger
20120518_EXQ0_MiBa_SA_HCT116_mHLA−2: 5231
SA = 0.13
Score = −4.2
AESGAAAYR − Mascot − rejected
NA − spliced − |||
20120518_EXQ0_MiBa_SA_HCT116_mHLA−2: 7240
SA = 0.88
Score = 0.74
AEASNRFT − MaxQuant
20120518_EXQ0_MiBa_SA_HCT116_mHLA−2: 7240
SA = 0.82
Score = 0.22
AEAEPGPAGP − MSFragger
20120518_EXQ0_MiBa_SA_HCT116_mHLA−2: 7240
SA = 0.3
Score = −4.27
DSSPASQLSY − Mascot − rejected
DSS|PASQLSY − spliced − 3|6|3|5
20120515_EXQ3_MiBa_SA_HCT116_mHLA−1: 22297
SA = 0.88
Score = 1.09
HMLQTFDY − MaxQuant
20120515_EXQ3_MiBa_SA_HCT116_mHLA−1: 22297
SA = 0.49
Score = −3.71
SSDPASQLSY − MSFragger
20120515_EXQ3_MiBa_SA_HCT116_mHLA−1: 22297
SA = 0.92
Score = 2.73
Relative intensity
m/z
Prosit prediction
* Exclusive in prediction
# Exclusive in experimental

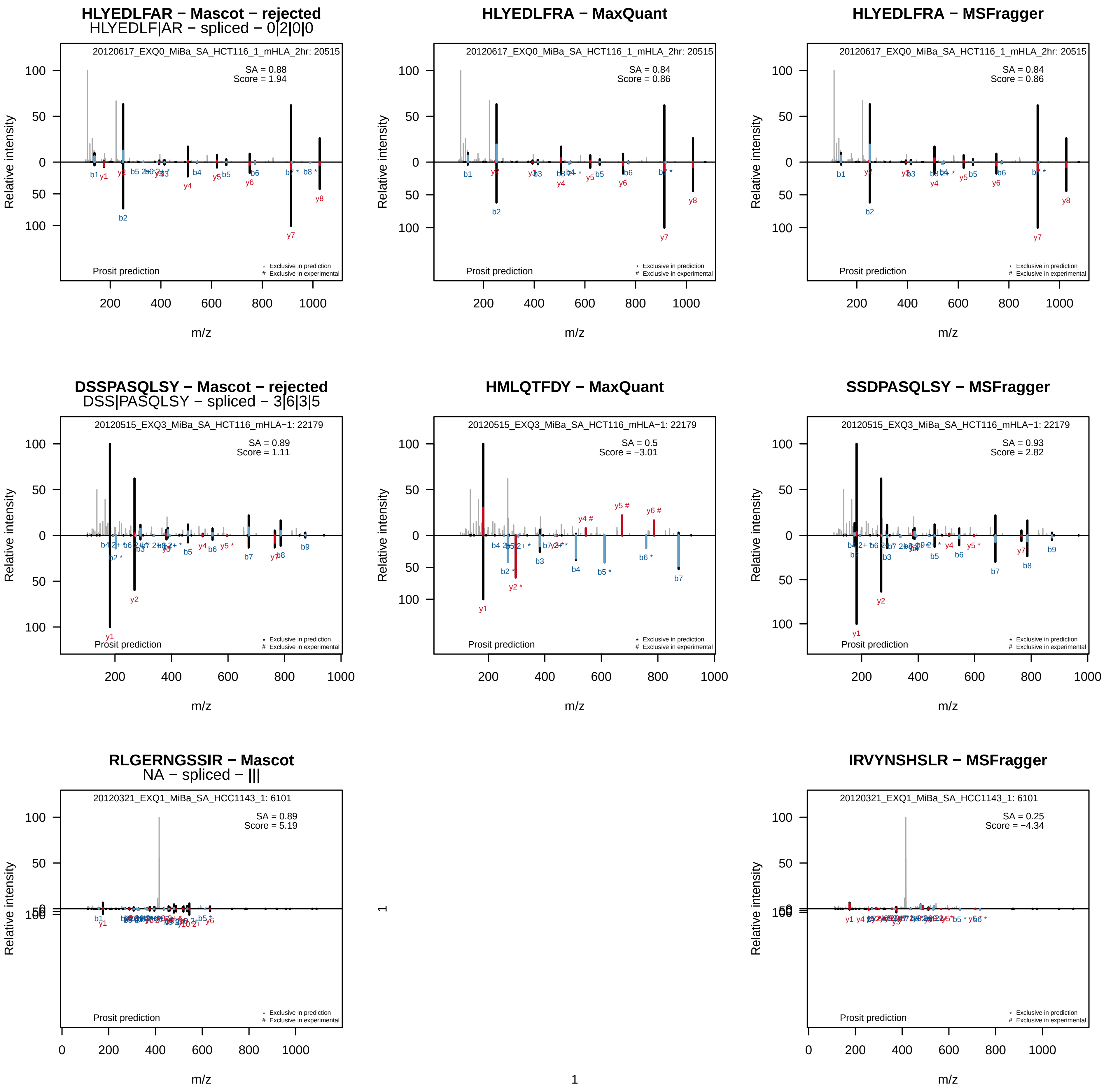

HLYEDLFAR − Mascot − rejected
HLYEDLF|AR − spliced − 0|2|0|0
20120617_EXQ0_MiBa_SA_HCT116_1_mHLA_2hr: 20515
SA = 0.88
Score = 1.94
HLYEDLFRA − MaxQuant
20120617_EXQ0_MiBa_SA_HCT116_1_mHLA_2hr: 20515
SA = 0.84
Score = 0.86
HLYEDLFRA − MSFragger
20120617_EXQ0_MiBa_SA_HCT116_1_mHLA_2hr: 20515
SA = 0.84
Score = 0.86
DSSPASQLSY − Mascot − rejected
DSS|PASQLSY − spliced − 3|6|3|5
20120515_EXQ3_MiBa_SA_HCT116_mHLA−1: 22179
SA = 0.89
Score = 1.11
HMLQTFDY − MaxQuant
20120515_EXQ3_MiBa_SA_HCT116_mHLA−1: 22179
SA = 0.5
Score = −3.01
SSDPASQLSY − MSFragger
20120515_EXQ3_MiBa_SA_HCT116_mHLA−1: 22179
SA = 0.93
Score = 2.82
RLGERNGSSIR − Mascot
NA − spliced − |||
20120321_EXQ1_MiBa_SA_HCC1143_1: 6101
SA = 0.89
Score = 5.19
IRVYNSHSLR − MSFragger
20120321_EXQ1_MiBa_SA_HCC1143_1: 6101
SA = 0.25
Score = −4.34
Relative intensity
m/z
Prosit prediction
⋆ Exclusive in prediction
Exclusive in experimental

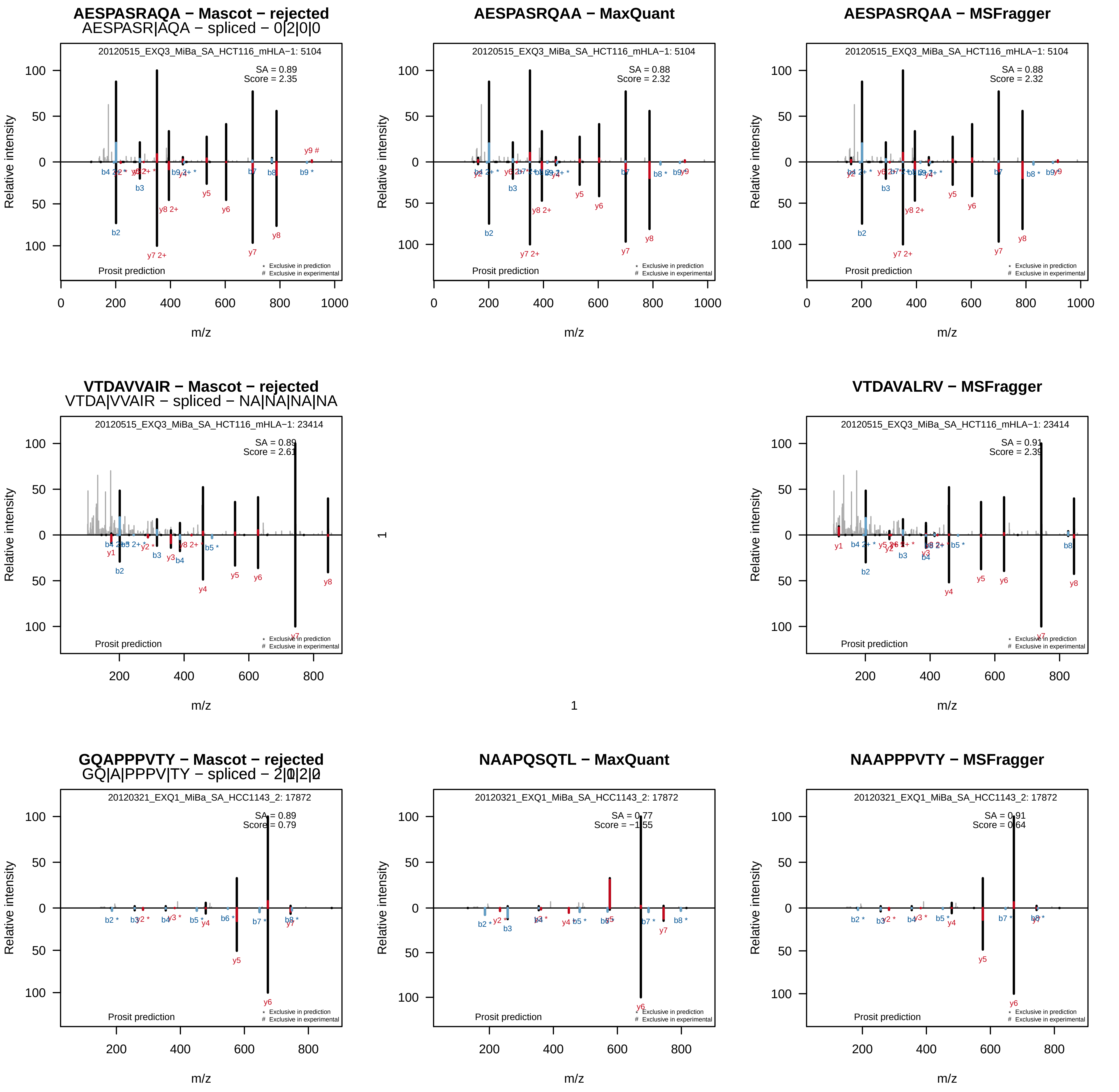
AESPASRAQA − Mascot − rejected
AESPASR|AQA − spliced − 0|2|0|0
20120515_EXQ3_MiBa_SA_HCT116_mHLA−1: 5104
SA = 0.89
Score = 2.35
AESPASRQAA − MaxQuant
20120515_EXQ3_MiBa_SA_HCT116_mHLA−1: 5104
SA = 0.88
Score = 2.32
AESPASRQAA − MSFragger
20120515_EXQ3_MiBa_SA_HCT116_mHLA−1: 5104
SA = 0.88
Score = 2.32
VTDAVVAIR − Mascot − rejected
VTDA|VVAIR − spliced − NA|NA|NA|NA
20120515_EXQ3_MiBa_SA_HCT116_mHLA−1: 23414
SA = 0.89
Score = 2.61
VTDAVALRV − MSFragger
20120515_EXQ3_MiBa_SA_HCT116_mHLA−1: 23414
SA = 0.91
Score = 2.39
GQAPPPVTY − Mascot − rejected
20120321_EXQ1_MiBa_SA_HCC1143_2: 17872
SA = 0.89
Score = 0.79
NAAPQSQTL − MaxQuant
20120321_EXQ1_MiBa_SA_HCC1143_2: 17872
SA = 0.77
Score = −1.55
NAAPPPVTY − MSFragger
20120321_EXQ1_MiBa_SA_HCC1143_2: 17872
SA = 0.91
Score = 0.64
Relative intensity
m/z
Prosit prediction
* Exclusive in prediction
# Exclusive in experimental

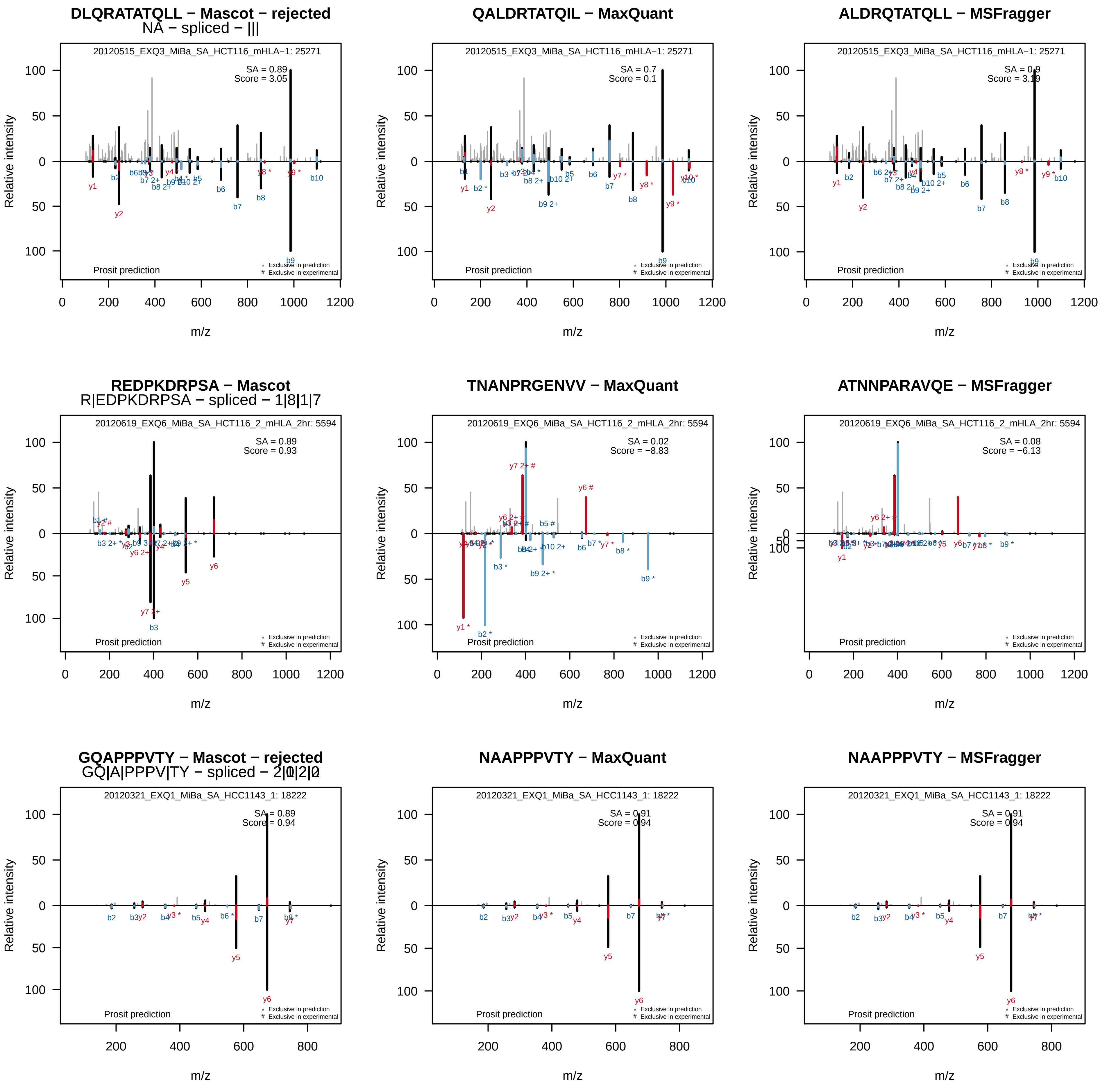
DLQRATATQLL − Mascot − rejected
NA − spliced − |||
20120515_EXQ3_MiBa_SA_HCT116_mHLA−1: 25271
SA = 0.89
Score = 3.05
QALDRTATQIL − MaxQuant
20120515_EXQ3_MiBa_SA_HCT116_mHLA−1: 25271
SA = 0.7
Score = 0.1
ALDRQTATQLL − MSFragger
20120515_EXQ3_MiBa_SA_HCT116_mHLA−1: 25271
SA = 0.9
Score = 3.19
REDPKDRPSA − Mascot
R|EDPKDRPSA − spliced − 1|8|1|7
20120619_EXQ6_MiBa_SA_HCT116_2_mHLA_2hr: 5594
SA = 0.89
Score = 0.93
TNANPRGENVV − MaxQuant
20120619_EXQ6_MiBa_SA_HCT116_2_mHLA_2hr: 5594
SA = 0.02
Score = −8.83
ATNNPARAVQE − MSFragger
20120619_EXQ6_MiBa_SA_HCT116_2_mHLA_2hr: 5594
SA = 0.08
Score = −6.13
GQAPPPVTY − Mascot − rejected
20120321_EXQ1_MiBa_SA_HCC1143_1: 18222
SA = 0.89
Score = 0.94
NAAPPPVTY − MaxQuant
20120321_EXQ1_MiBa_SA_HCC1143_1: 18222
SA = 0.91
Score = 0.94
NAAPPPVTY − MSFragger
20120321_EXQ1_MiBa_SA_HCC1143_1: 18222
SA = 0.91
Score = 0.94
Relative intensity
m/z
Prosit prediction
⋆ Exclusive in prediction
Exclusive in experimental

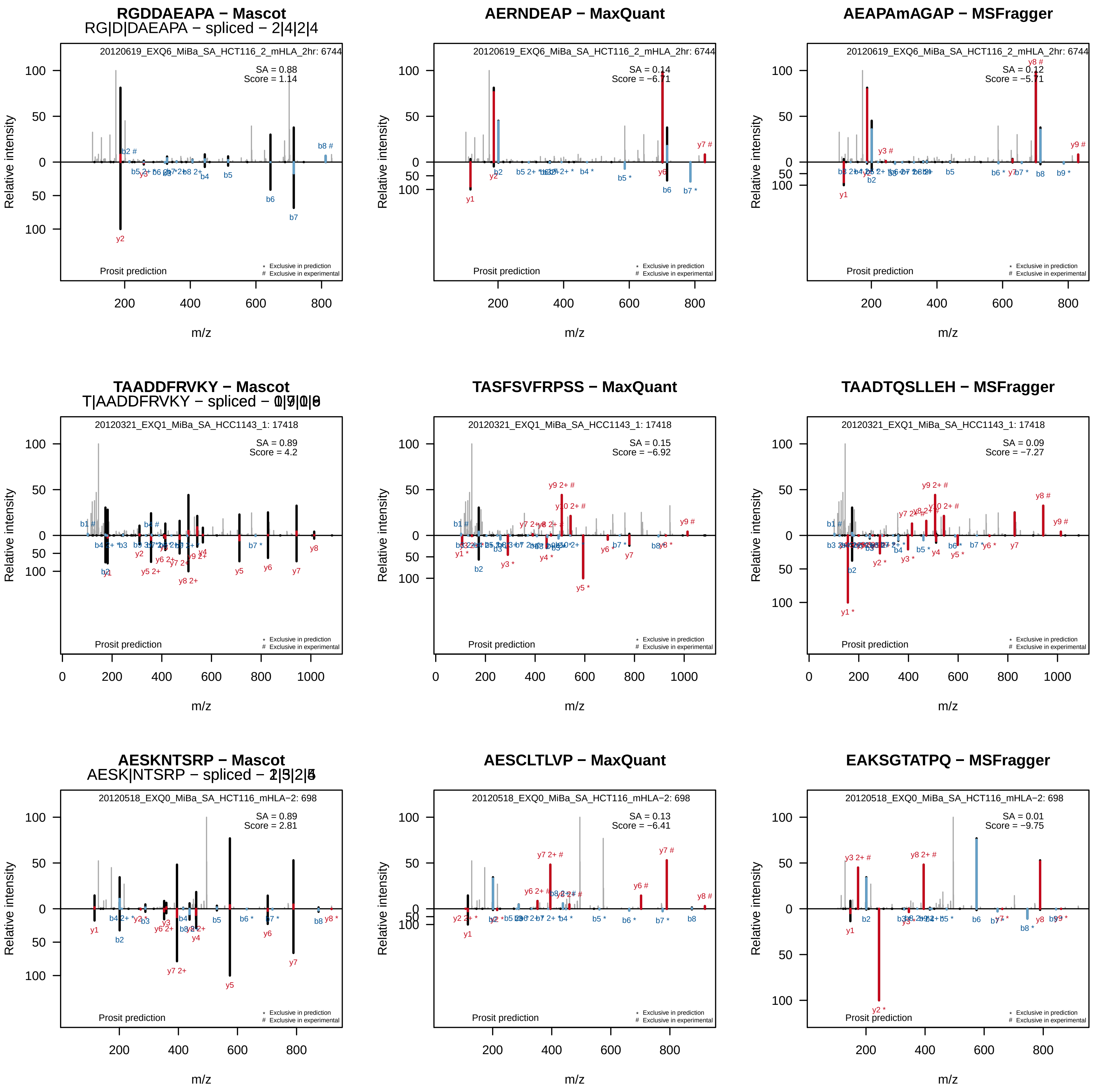

RGDDAEAPA − Mascot
RG|D|DAEAPA − spliced − 2|4|2|4
20120619_EXQ6_MiBa_SA_HCT116_2_mHLA_2hr: 6744
SA = 0.88
Score = 1.14
AERNDEAP − MaxQuant
20120619_EXQ6_MiBa_SA_HCT116_2_mHLA_2hr: 6744
SA = 0.14
Score = −6.71
AEAPAmAGAP − MSFragger
20120619_EXQ6_MiBa_SA_HCT116_2_mHLA_2hr: 6744
SA = 0.12
Score = −5.71
TAADDFRVKY − Mascot
20120321_EXQ1_MiBa_SA_HCC1143_1: 17418
SA = 0.89
Score = 4.2
TASFSVFRPSS − MaxQuant
20120321_EXQ1_MiBa_SA_HCC1143_1: 17418
SA = 0.15
Score = −6.92
TAADTQSLLEH − MSFragger
20120321_EXQ1_MiBa_SA_HCC1143_1: 17418
SA = 0.09
Score = −7.27
AESKNTSRP − Mascot
20120518_EXQ0_MiBa_SA_HCT116_mHLA−2: 698
SA = 0.89
Score = 2.81
AESCLTLVP − MaxQuant
20120518_EXQ0_MiBa_SA_HCT116_mHLA−2: 698
SA = 0.13
Score = −6.41
EAKSGTATPQ − MSFragger
20120518_EXQ0_MiBa_SA_HCT116_mHLA−2: 698
SA = 0.01
Score = −9.75
Relative intensity
m/z
Prosit prediction
⋆ Exclusive in prediction
Exclusive in experimental

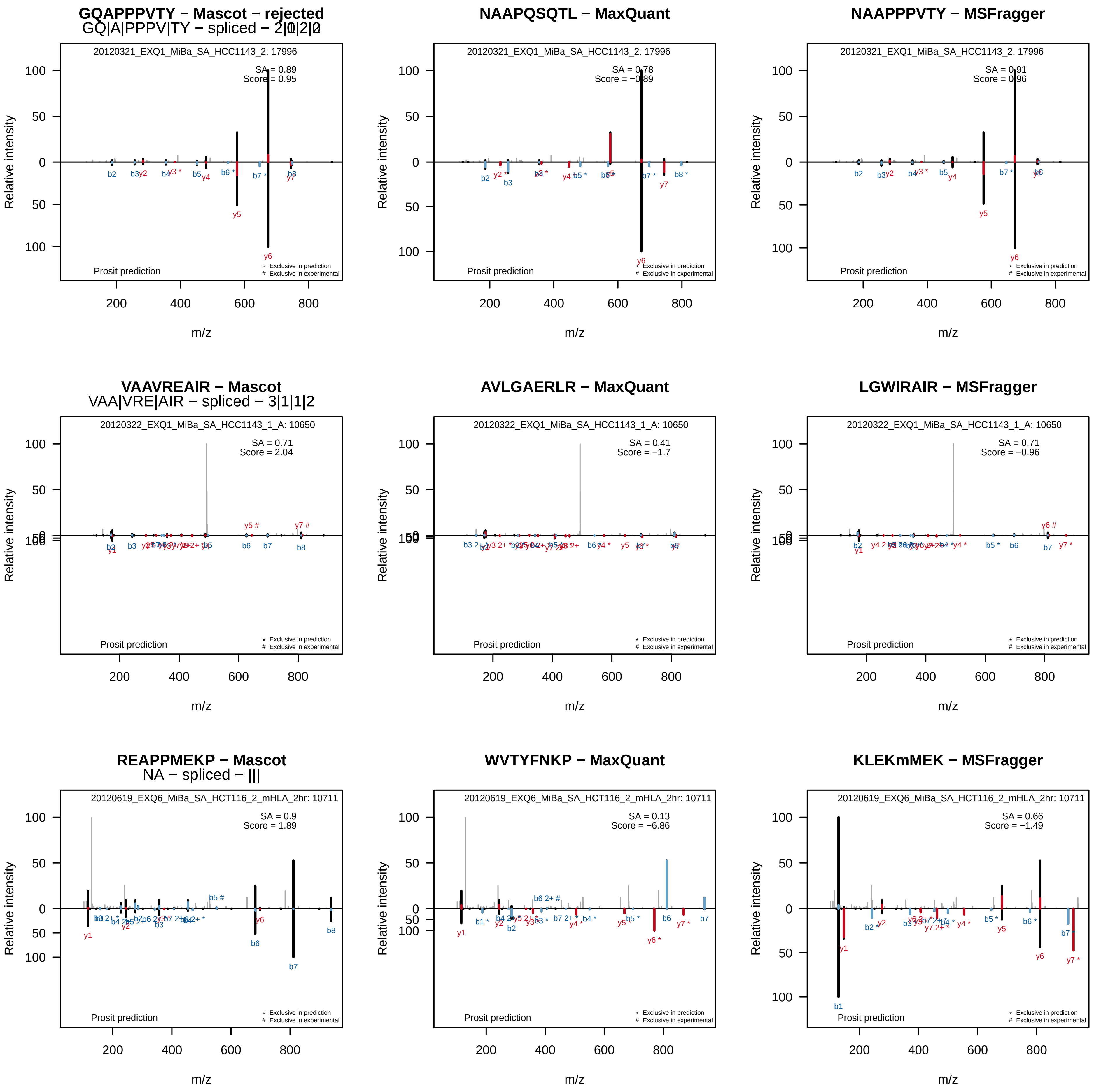

GQAPPPVTY − Mascot − rejected
20120321_EXQ1_MiBa_SA_HCC1143_2: 17996
SA = 0.89
Score = 0.95
Prosit prediction
NAAPQSQTL − MaxQuant
20120321_EXQ1_MiBa_SA_HCC1143_2: 17996
SA = 0.78
Score = −0.89
Prosit prediction
NAAPPPVTY − MSFragger
20120321_EXQ1_MiBa_SA_HCC1143_2: 17996
SA = 0.91
Score = 0.96
Prosit prediction
VAAVREAIR − Mascot
VAA|VRE|AIR − spliced − 3|1|1|2
20120322_EXQ1_MiBa_SA_HCC1143_1_A: 10650
SA = 0.71
Score = 2.04
Prosit prediction
AVLGAERLR − MaxQuant
20120322_EXQ1_MiBa_SA_HCC1143_1_A: 10650
SA = 0.41
Score = −1.7
Prosit prediction
LGWIRAIR − MSFragger
20120322_EXQ1_MiBa_SA_HCC1143_1_A: 10650
SA = 0.71
Score = −0.96
Prosit prediction
REAPPMEKP − Mascot
NA − spliced − |||
20120619_EXQ6_MiBa_SA_HCT116_2_mHLA_2hr: 10711
SA = 0.9
Score = 1.89
Prosit prediction
WVTYFNKP − MaxQuant
20120619_EXQ6_MiBa_SA_HCT116_2_mHLA_2hr: 10711
SA = 0.13
Score = −6.86
Prosit prediction
KLEKmMEK − MSFragger
20120619_EXQ6_MiBa_SA_HCT116_2_mHLA_2hr: 10711
SA = 0.66
Score = −1.49
Prosit prediction
Relative intensity
m/z
⋆ Exclusive in prediction
Exclusive in experimental

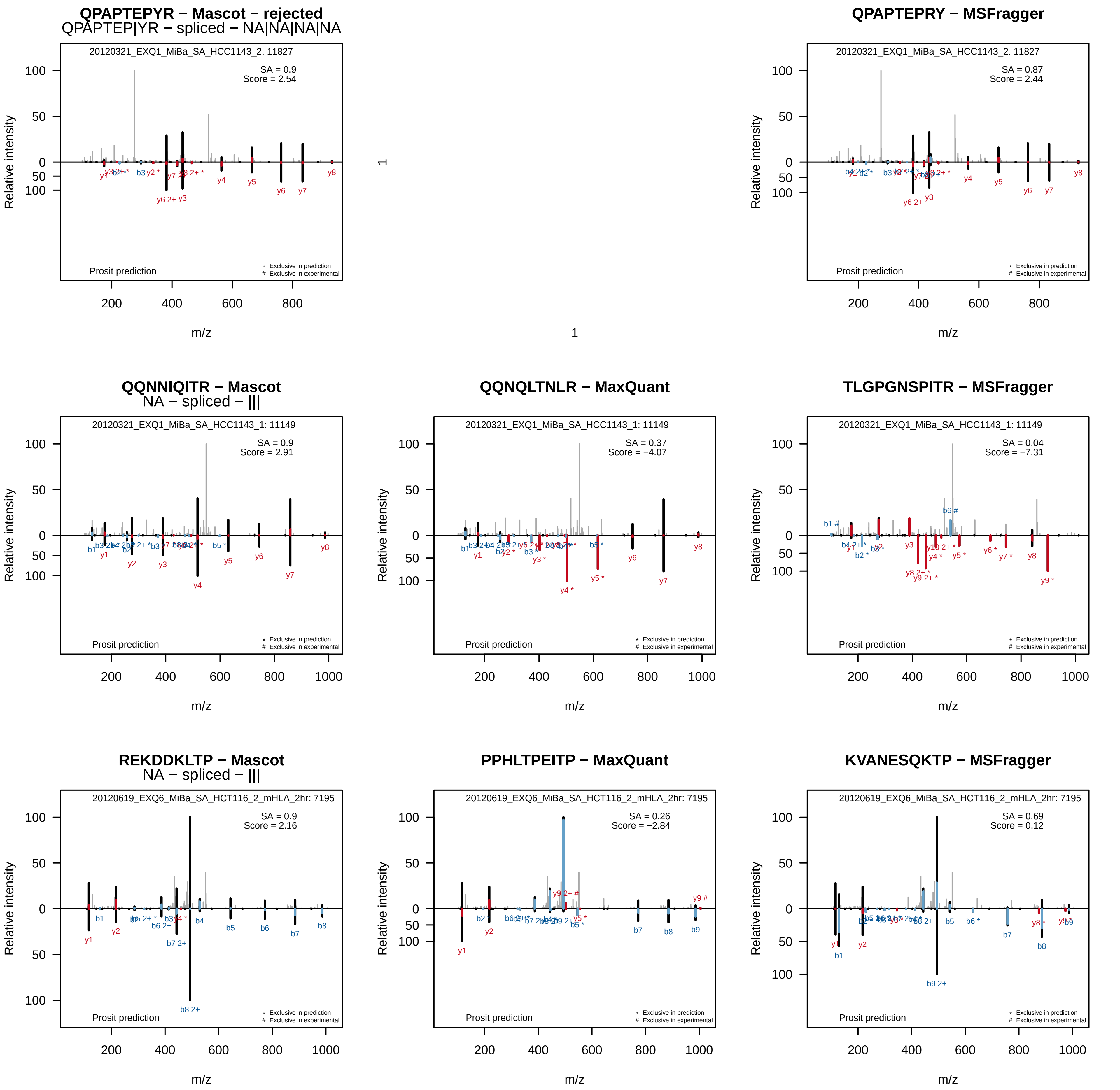
QPAPTEPYR − Mascot − rejected
QPAPTEP|YR − spliced − NA|NA|NA|NA
20120321_EXQ1_MiBa_SA_HCC1143_2: 11827
SA = 0.9
Score = 2.54
Prosit prediction
* Exclusive in prediction
# Exclusive in experimental
QPAPTEPRY − MSFragger
20120321_EXQ1_MiBa_SA_HCC1143_2: 11827
SA = 0.87
Score = 2.44
Prosit prediction
QQNNIQITR − Mascot
NA − spliced − |||
20120321_EXQ1_MiBa_SA_HCC1143_1: 11149
SA = 0.9
Score = 2.91
QQNQLTNLR − MaxQuant
20120321_EXQ1_MiBa_SA_HCC1143_1: 11149
SA = 0.37
Score = −4.07
TLGPGNSPITR − MSFragger
20120321_EXQ1_MiBa_SA_HCC1143_1: 11149
SA = 0.04
Score = −7.31
REKDDKLTP − Mascot
NA − spliced − |||
20120619_EXQ6_MiBa_SA_HCT116_2_mHLA_2hr: 7195
SA = 0.9
Score = 2.16
PPHLTPEITP − MaxQuant
20120619_EXQ6_MiBa_SA_HCT116_2_mHLA_2hr: 7195
SA = 0.26
Score = −2.84
KVANESQKTP − MSFragger
20120619_EXQ6_MiBa_SA_HCT116_2_mHLA_2hr: 7195
SA = 0.69
Score = 0.12
Relative intensity
m/z

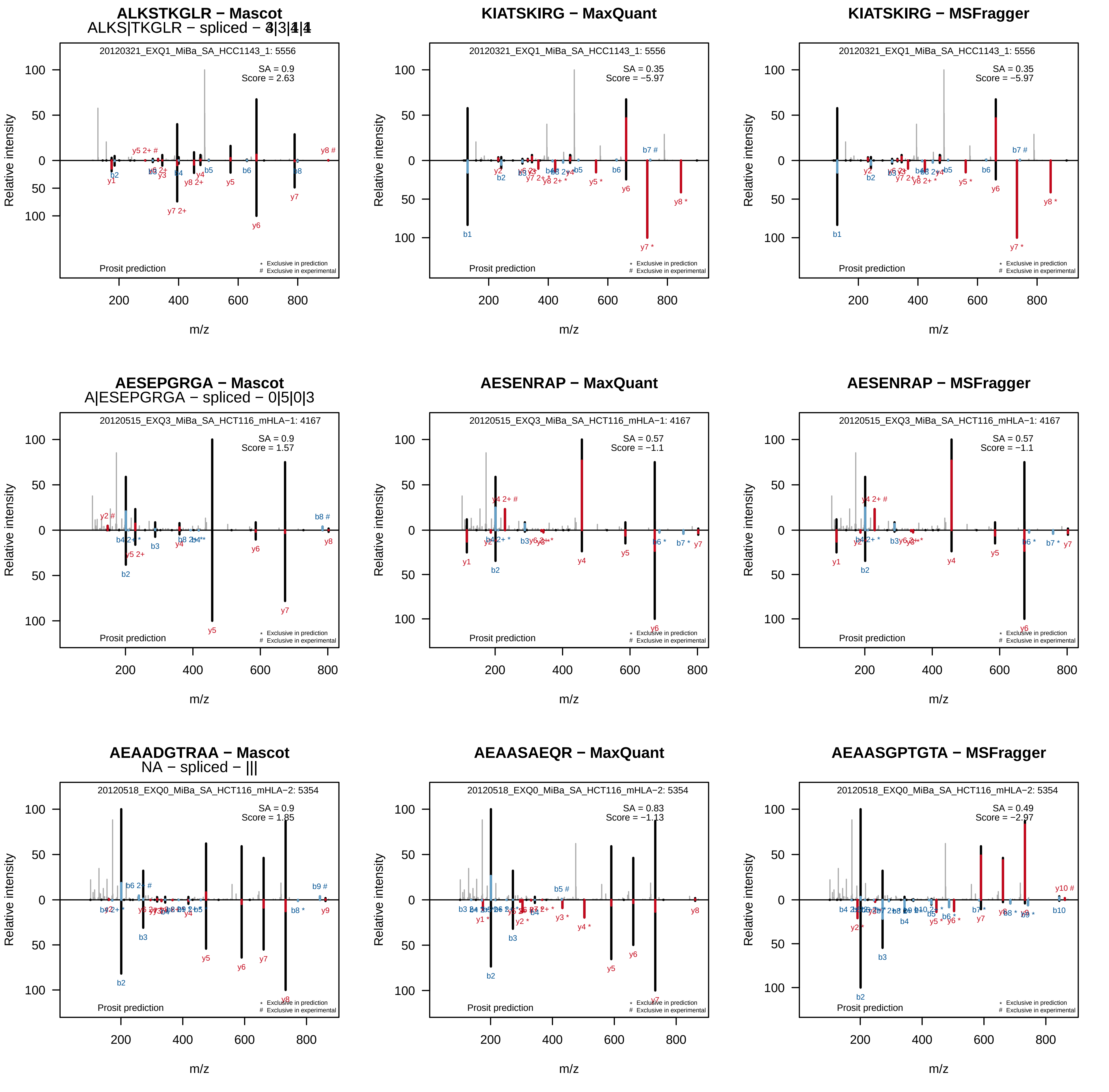
ALKSTKGLR − Mascot
ALKS|TKGLR − spliced −
20120321_EXQ1_MiBa_SA_HCC1143_1: 5556
SA = 0.9
Score = 2.63
KIATSKIRG − MaxQuant
20120321_EXQ1_MiBa_SA_HCC1143_1: 5556
SA = 0.35
Score = −5.97
KIATSKIRG − MSFragger
20120321_EXQ1_MiBa_SA_HCC1143_1: 5556
SA = 0.35
Score = −5.97
AESEPGRGA − Mascot
A|ESEPGRGA − spliced − 0|5|0|3
20120515_EXQ3_MiBa_SA_HCT116_mHLA−1: 4167
SA = 0.9
Score = 1.57
AESENRAP − MaxQuant
20120515_EXQ3_MiBa_SA_HCT116_mHLA−1: 4167
SA = 0.57
Score = −1.1
AESENRAP − MSFragger
20120515_EXQ3_MiBa_SA_HCT116_mHLA−1: 4167
SA = 0.57
Score = −1.1
AEAADGTRAA − Mascot
NA − spliced − |||
20120518_EXQ0_MiBa_SA_HCT116_mHLA−2: 5354
SA = 0.9
Score = 1.85
AEAASAEQR − MaxQuant
20120518_EXQ0_MiBa_SA_HCT116_mHLA−2: 5354
SA = 0.83
Score = −1.13
AEAASGPTGTA − MSFragger
20120518_EXQ0_MiBa_SA_HCT116_mHLA−2: 5354
SA = 0.49
Score = −2.97
Relative intensity
m/z
Prosit prediction
* Exclusive in prediction
# Exclusive in experimental

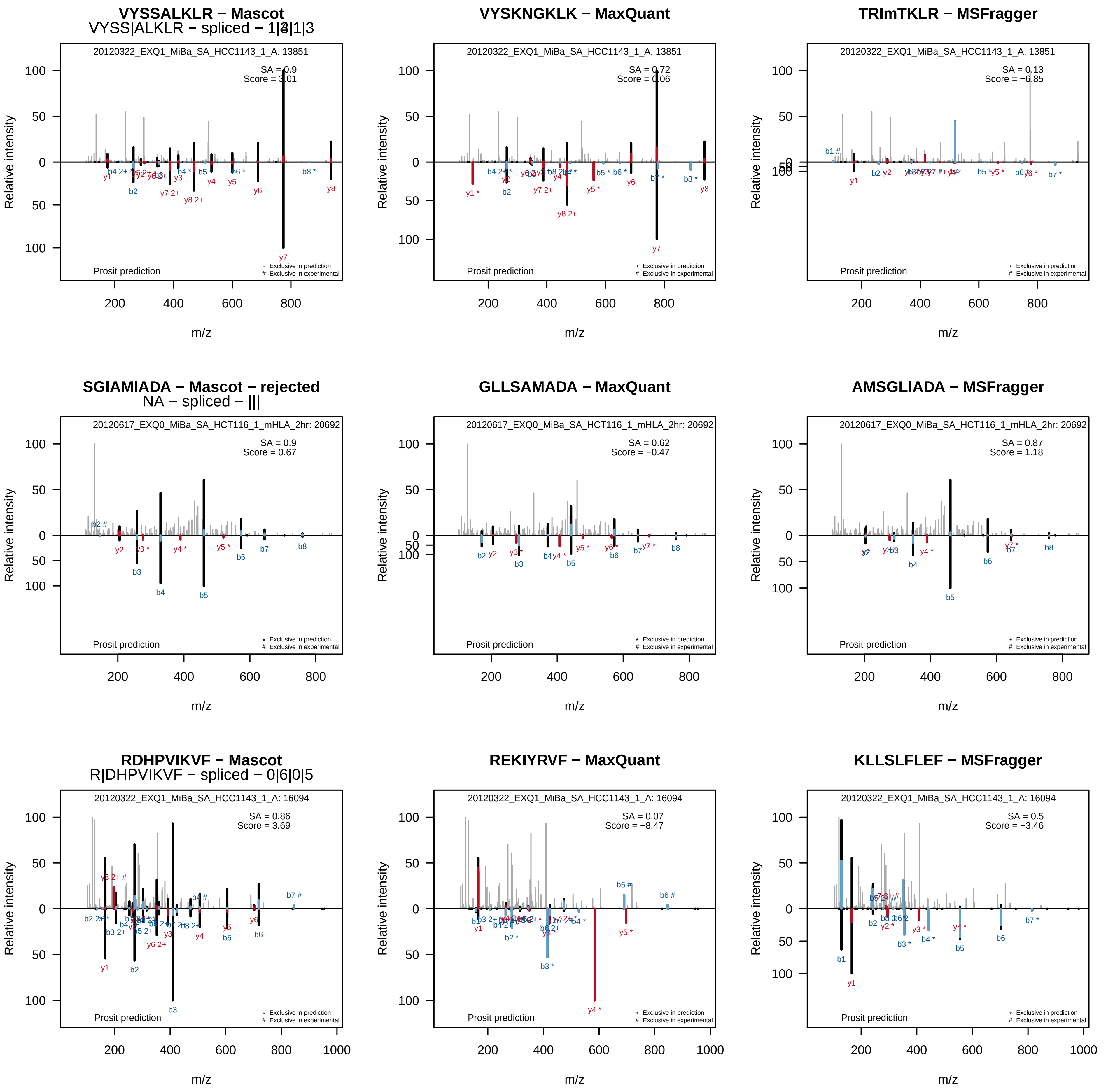
VYSSALKLR − Mascot
VYSS|ALKLR − spliced − 1|3|1|3
20120322_EXQ1_MiBa_SA_HCC1143_1_A: 13851
SA = 0.9
Score = 3.01
VYSKNGKLK − MaxQuant
20120322_EXQ1_MiBa_SA_HCC1143_1_A: 13851
SA = 0.72
Score = 0.06
TRImTKLR − MSFragger
20120322_EXQ1_MiBa_SA_HCC1143_1_A: 13851
SA = 0.13
Score = −6.85
SGIAMIADA − Mascot − rejected
NA − spliced − |||
20120617_EXQ0_MiBa_SA_HCT116_1_mHLA_2hr: 20692
SA = 0.9
Score = 0.67
GLLSAMADA − MaxQuant
20120617_EXQ0_MiBa_SA_HCT116_1_mHLA_2hr: 20692
SA = 0.62
Score = −0.47
AMSGLIADA − MSFragger
20120617_EXQ0_MiBa_SA_HCT116_1_mHLA_2hr: 20692
SA = 0.87
Score = 1.18
RDHPVIKVF − Mascot
R|DHPVIKVF − spliced − 0|6|0|5
20120322_EXQ1_MiBa_SA_HCC1143_1_A: 16094
SA = 0.86
Score = 3.69
REKIYRVF − MaxQuant
20120322_EXQ1_MiBa_SA_HCC1143_1_A: 16094
SA = 0.07
Score = −8.47
KLLSLFLEF − MSFragger
20120322_EXQ1_MiBa_SA_HCC1143_1_A: 16094
SA = 0.5
Score = −3.46
Relative intensity
m/z
Prosit prediction
⋆ Exclusive in prediction
Exclusive in experimental

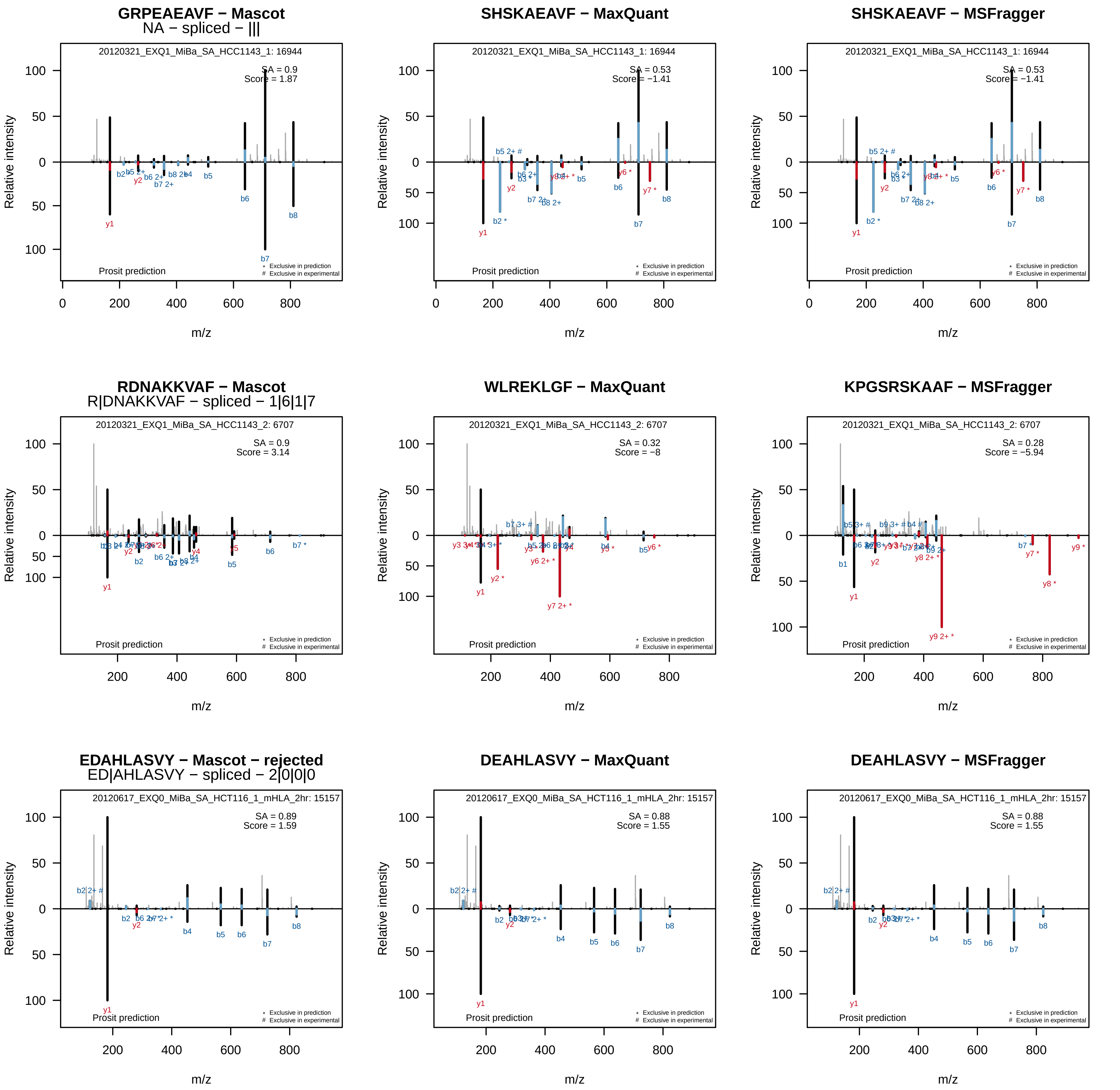
GRPEAEAVF − Mascot
NA − spliced − |||
20120321_EXQ1_MiBa_SA_HCC1143_1: 16944
SA = 0.9
Score = 1.87
SHSKAEAVF − MaxQuant
20120321_EXQ1_MiBa_SA_HCC1143_1: 16944
SA = 0.53
Score = −1.41
SHSKAEAVF − MSFragger
20120321_EXQ1_MiBa_SA_HCC1143_1: 16944
SA = 0.53
Score = −1.41
RDNAKKVAF − Mascot
R|DNAKKVAF − spliced − 1|6|1|7
20120321_EXQ1_MiBa_SA_HCC1143_2: 6707
SA = 0.9
Score = 3.14
WLREKLGF − MaxQuant
20120321_EXQ1_MiBa_SA_HCC1143_2: 6707
SA = 0.32
Score = −8
KPGSRSKAAF − MSFragger
20120321_EXQ1_MiBa_SA_HCC1143_2: 6707
SA = 0.28
Score = −5.94
EDAHLASVY − Mascot − rejected
ED|AHLASVY − spliced − 2|0|0|0
20120617_EXQ0_MiBa_SA_HCT116_1_mHLA_2hr: 15157
SA = 0.89
Score = 1.59
DEAHLASVY − MaxQuant
20120617_EXQ0_MiBa_SA_HCT116_1_mHLA_2hr: 15157
SA = 0.88
Score = 1.55
DEAHLASVY − MSFragger
20120617_EXQ0_MiBa_SA_HCT116_1_mHLA_2hr: 15157
SA = 0.88
Score = 1.55
Prosit prediction
* Exclusive in prediction
# Exclusive in experimental
Relative intensity
m/z

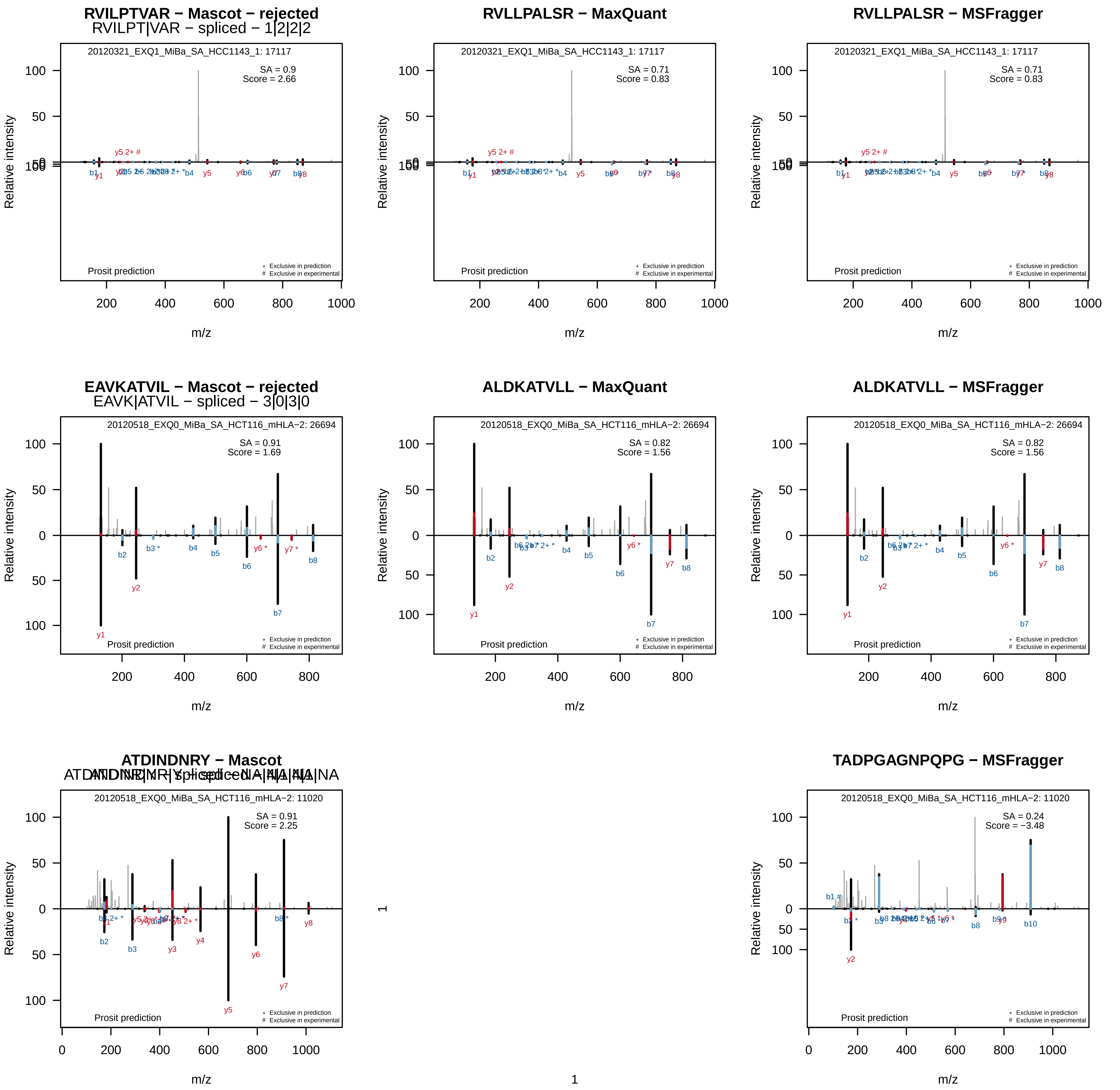
RVILPTVAR − Mascot − rejected
RVILPT|VAR − spliced − 1|2|2|2
20120321_EXQ1_MiBa_SA_HCC1143_1: 17117
SA = 0.9
Score = 2.66
RVLLPALSR − MaxQuant
20120321_EXQ1_MiBa_SA_HCC1143_1: 17117
SA = 0.71
Score = 0.83
RVLLPALSR − MSFragger
20120321_EXQ1_MiBa_SA_HCC1143_1: 17117
SA = 0.71
Score = 0.83
EAVKATVIL − Mascot − rejected
EAVK|ATVIL − spliced − 3|0|3|0
20120518_EXQ0_MiBa_SA_HCT116_mHLA−2: 26694
SA = 0.91
Score = 1.69
ALDKATVLL − MaxQuant
20120518_EXQ0_MiBa_SA_HCT116_mHLA−2: 26694
SA = 0.82
Score = 1.56
ALDKATVLL − MSFragger
20120518_EXQ0_MiBa_SA_HCT116_mHLA−2: 26694
SA = 0.82
Score = 1.56
ATDINDNRY − Mascot
20120518_EXQ0_MiBa_SA_HCT116_mHLA−2: 11020
SA = 0.91
Score = 2.25
TADPGAGNPQPG − MSFragger
20120518_EXQ0_MiBa_SA_HCT116_mHLA−2: 11020
SA = 0.24
Score = −3.48
Relative intensity
m/z
Prosit prediction
⋆ Exclusive in prediction
Exclusive in experimental
1

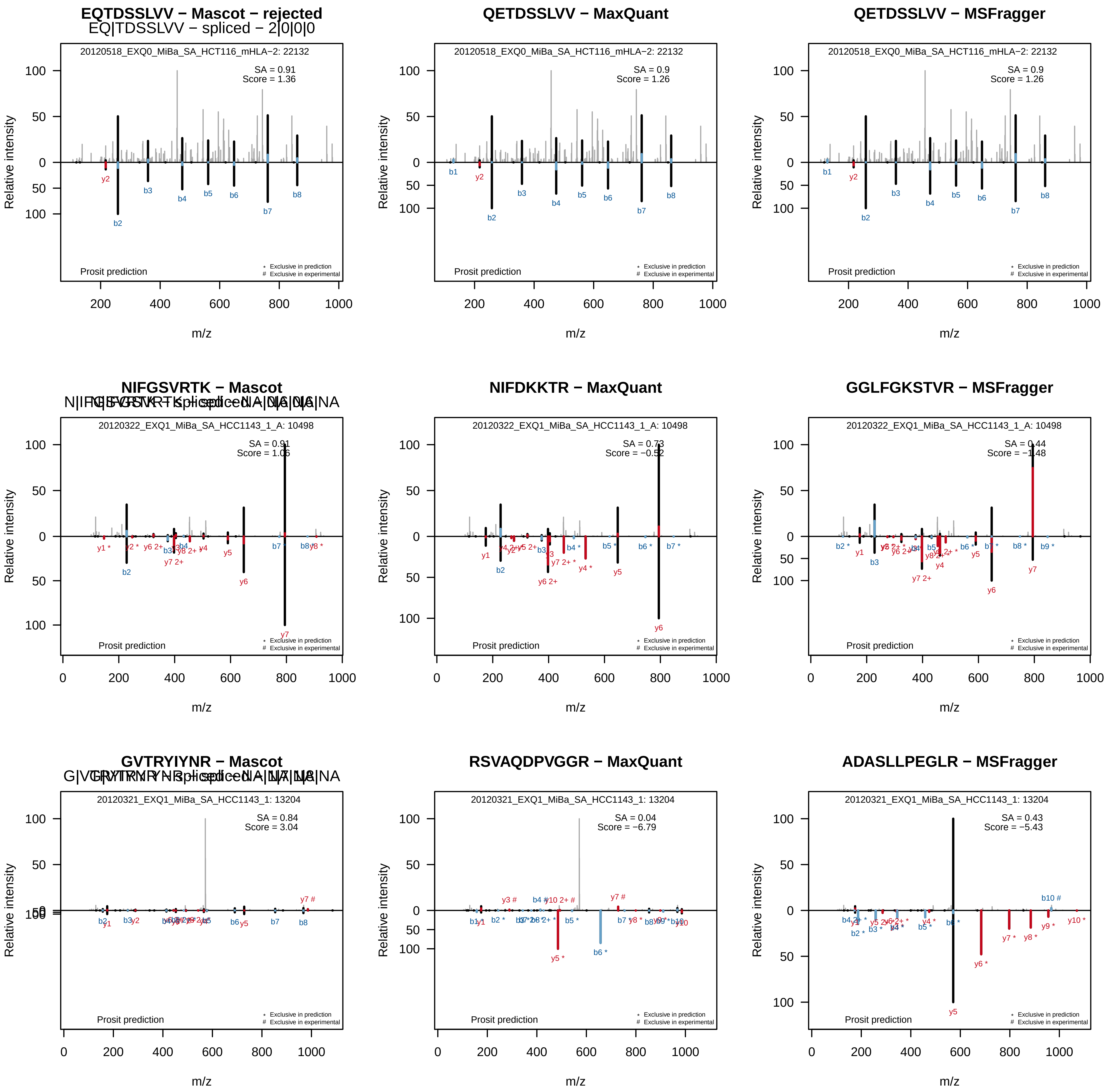

EQTDSSLVV − Mascot − rejected
EQ|TDSSLVV − spliced − 2|0|0|0
20120518_EXQ0_MiBa_SA_HCT116_mHLA−2: 22132
SA = 0.91
Score = 1.36
QETDSSLVV − MaxQuant
20120518_EXQ0_MiBa_SA_HCT116_mHLA−2: 22132
SA = 0.9
Score = 1.26
QETDSSLVV − MSFragger
20120518_EXQ0_MiBa_SA_HCT116_mHLA−2: 22132
SA = 0.9
Score = 1.26
NIFGSVRTK − Mascot
20120322_EXQ1_MiBa_SA_HCC1143_1_A: 10498
SA = 0.91
Score = 1.06
NIFDKKTR − MaxQuant
20120322_EXQ1_MiBa_SA_HCC1143_1_A: 10498
SA = 0.73
Score = −0.52
GGLFGKSTVR − MSFragger
20120322_EXQ1_MiBa_SA_HCC1143_1_A: 10498
SA = 0.44
Score = −1.48
GVTRYIYNR − Mascot
20120321_EXQ1_MiBa_SA_HCC1143_1: 13204
SA = 0.84
Score = 3.04
RSVAQDPVGGR − MaxQuant
20120321_EXQ1_MiBa_SA_HCC1143_1: 13204
SA = 0.04
Score = −6.79
ADASLLPEGLR − MSFragger
20120321_EXQ1_MiBa_SA_HCC1143_1: 13204
SA = 0.43
Score = −5.43
Prosit prediction
⋆ Exclusive in prediction
Exclusive in experimental
Relative intensity
m/z

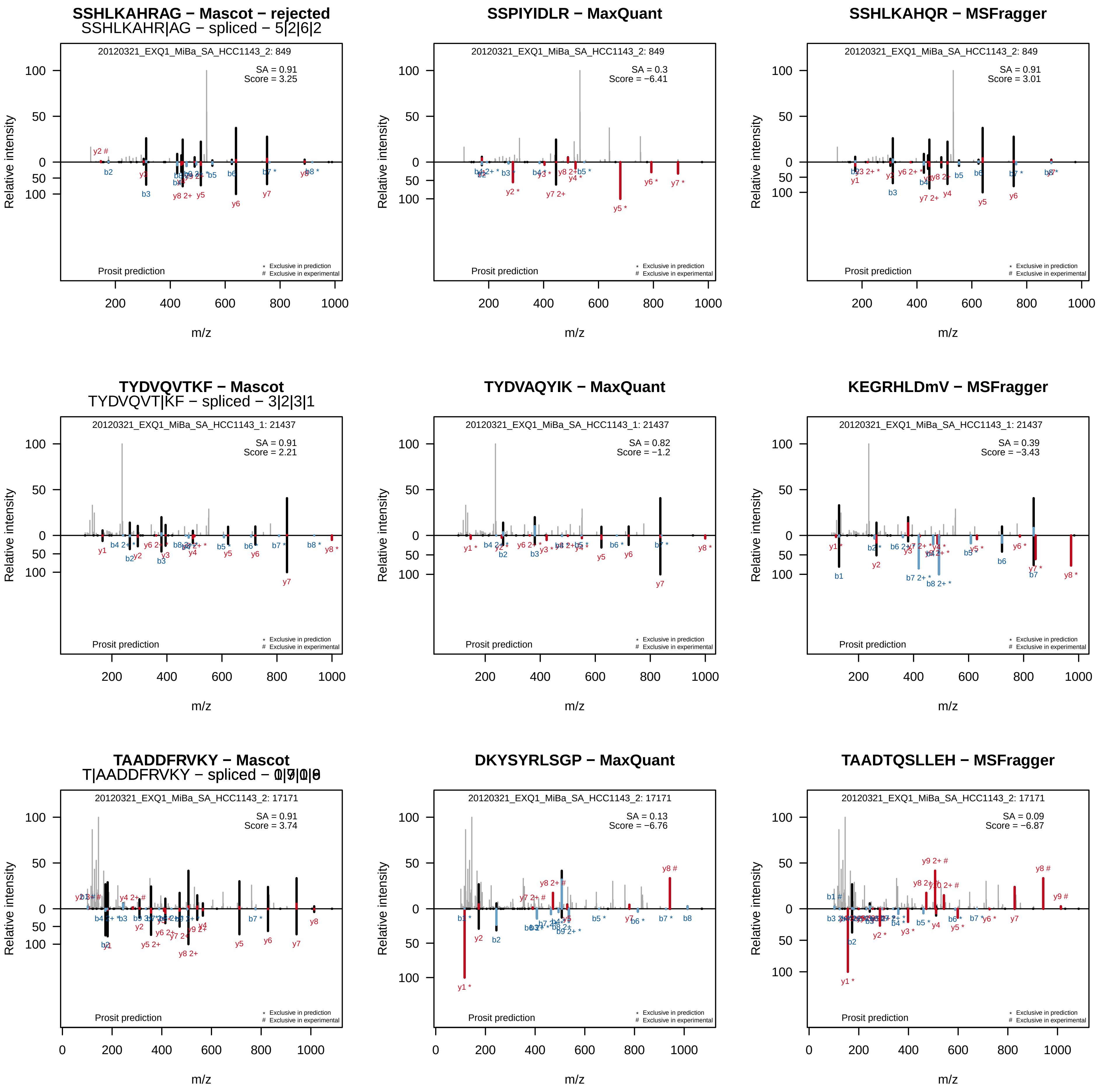
SSHLKAHRAG − Mascot − rejected
SSHLKAHR|AG − spliced − 5|2|6|2
20120321_EXQ1_MiBa_SA_HCC1143_2: 849
SA = 0.91
Score = 3.25
SSPIYIDLR − MaxQuant
20120321_EXQ1_MiBa_SA_HCC1143_2: 849
SA = 0.3
Score = −6.41
SSHLKAHQR − MSFragger
20120321_EXQ1_MiBa_SA_HCC1143_2: 849
SA = 0.91
Score = 3.01
TYDVQVTKF − Mascot
TYDVQVT|KF − spliced − 3|2|3|1
20120321_EXQ1_MiBa_SA_HCC1143_1: 21437
SA = 0.91
Score = 2.21
TYDVAQYIK − MaxQuant
20120321_EXQ1_MiBa_SA_HCC1143_1: 21437
SA = 0.82
Score = −1.2
KEGRHLDmV − MSFragger
20120321_EXQ1_MiBa_SA_HCC1143_1: 21437
SA = 0.39
Score = −3.43
TAADDFRVKY − Mascot
20120321_EXQ1_MiBa_SA_HCC1143_2: 17171
SA = 0.91
Score = 3.74
DKYSYRLSGP − MaxQuant
20120321_EXQ1_MiBa_SA_HCC1143_2: 17171
SA = 0.13
Score = −6.76
TAADTQSLLEH − MSFragger
20120321_EXQ1_MiBa_SA_HCC1143_2: 17171
SA = 0.09
Score = −6.87
Relative intensity
m/z
Prosit prediction
* Exclusive in prediction
# Exclusive in experimental

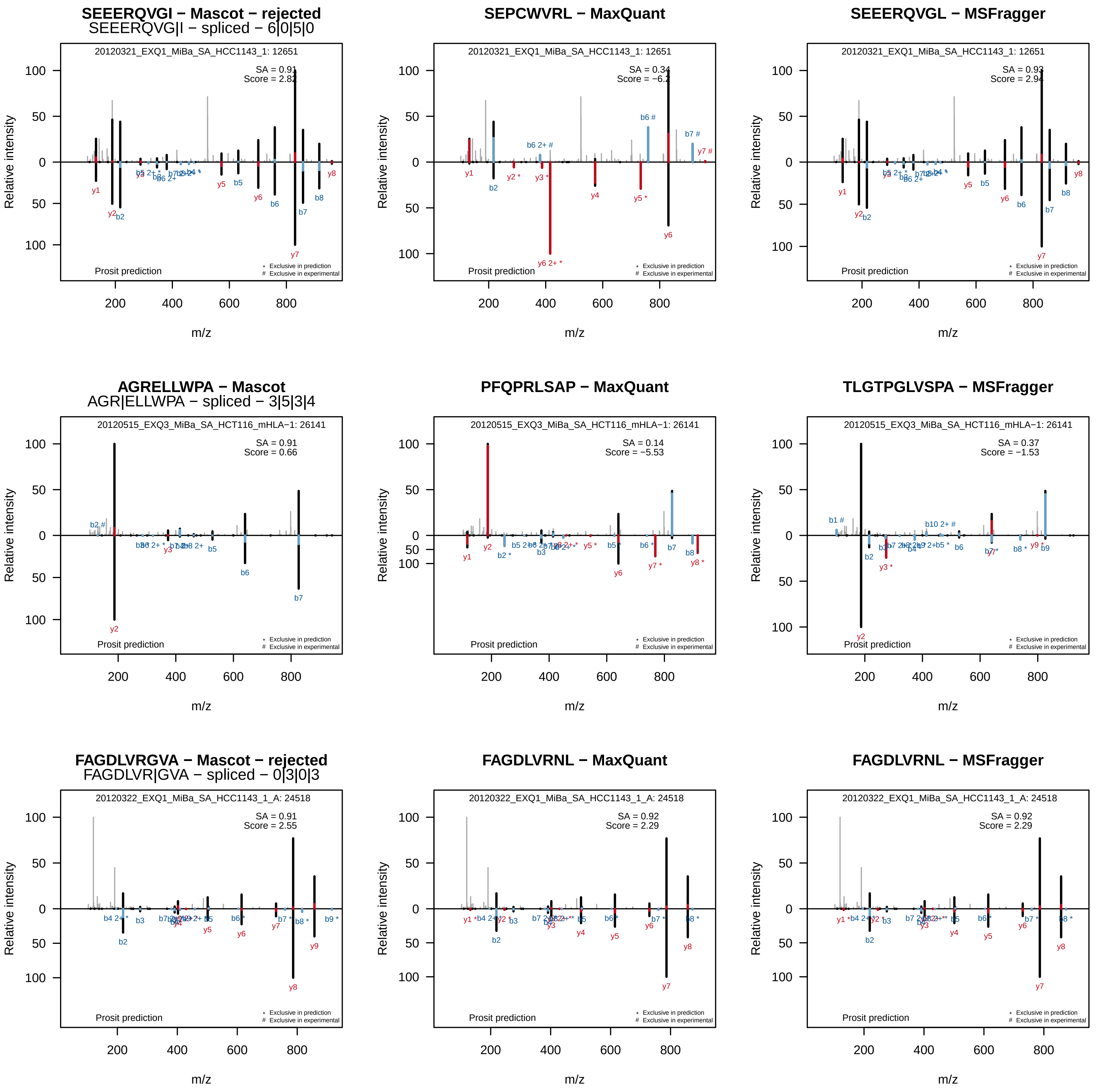
SEEERQVGI − Mascot − rejected
SEEERQVG|I − spliced − 6|0|5|0
20120321_EXQ1_MiBa_SA_HCC1143_1: 12651
SA = 0.91
Score = 2.82
SEPCWVRL − MaxQuant
20120321_EXQ1_MiBa_SA_HCC1143_1: 12651
SA = 0.34
Score = −6.2
SEEERQVGL − MSFragger
20120321_EXQ1_MiBa_SA_HCC1143_1: 12651
SA = 0.93
Score = 2.94
AGRELLWPA − Mascot
AGR|ELLWPA − spliced − 3|5|3|4
20120515_EXQ3_MiBa_SA_HCT116_mHLA−1: 26141
SA = 0.91
Score = 0.66
PFQPRLSAP − MaxQuant
20120515_EXQ3_MiBa_SA_HCT116_mHLA−1: 26141
SA = 0.14
Score = −5.53
TLGTPGLVSPA − MSFragger
20120515_EXQ3_MiBa_SA_HCT116_mHLA−1: 26141
SA = 0.37
Score = −1.53
FAGDLVRGVA − Mascot − rejected
FAGDLVR|GVA − spliced − 0|3|0|3
20120322_EXQ1_MiBa_SA_HCC1143_1_A: 24518
SA = 0.91
Score = 2.55
FAGDLVRNL − MaxQuant
20120322_EXQ1_MiBa_SA_HCC1143_1_A: 24518
SA = 0.92
Score = 2.29
FAGDLVRNL − MSFragger
20120322_EXQ1_MiBa_SA_HCC1143_1_A: 24518
SA = 0.92
Score = 2.29
Relative intensity
m/z
Prosit prediction
* Exclusive in prediction
# Exclusive in experimental

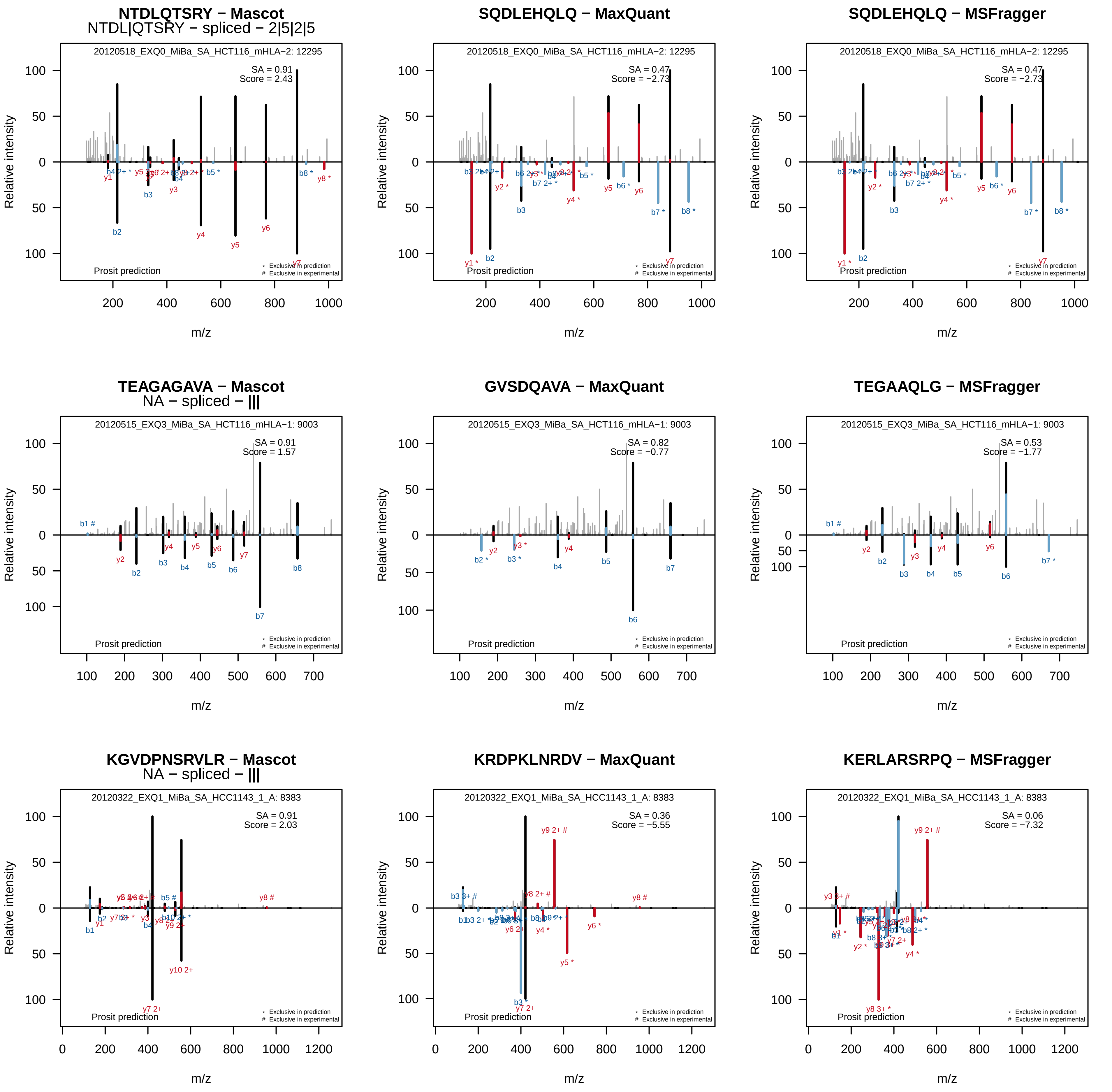

NTDLQTSRY − Mascot
NTDL|QTSRY − spliced − 2|5|2|5
20120518_EXQ0_MiBa_SA_HCT116_mHLA−2: 12295
SA = 0.91
Score = 2.43
SQDLEHQLQ − MaxQuant
20120518_EXQ0_MiBa_SA_HCT116_mHLA−2: 12295
SA = 0.47
Score = −2.73
SQDLEHQLQ − MSFragger
20120518_EXQ0_MiBa_SA_HCT116_mHLA−2: 12295
SA = 0.47
Score = −2.73
TEAGAGAVA − Mascot
NA − spliced − |||
20120515_EXQ3_MiBa_SA_HCT116_mHLA−1: 9003
SA = 0.91
Score = 1.57
GVSDQAVA − MaxQuant
20120515_EXQ3_MiBa_SA_HCT116_mHLA−1: 9003
SA = 0.82
Score = −0.77
TEGAAQLG − MSFragger
20120515_EXQ3_MiBa_SA_HCT116_mHLA−1: 9003
SA = 0.53
Score = −1.77
KGVDPNSRVLR − Mascot
NA − spliced − |||
20120322_EXQ1_MiBa_SA_HCC1143_1_A: 8383
SA = 0.91
Score = 2.03
KRDPKLNRDV − MaxQuant
20120322_EXQ1_MiBa_SA_HCC1143_1_A: 8383
SA = 0.36
Score = −5.55
KERLARSRPQ − MSFragger
20120322_EXQ1_MiBa_SA_HCC1143_1_A: 8383
SA = 0.06
Score = −7.32
Relative intensity
m/z
Prosit prediction
* Exclusive in prediction
# Exclusive in experimental

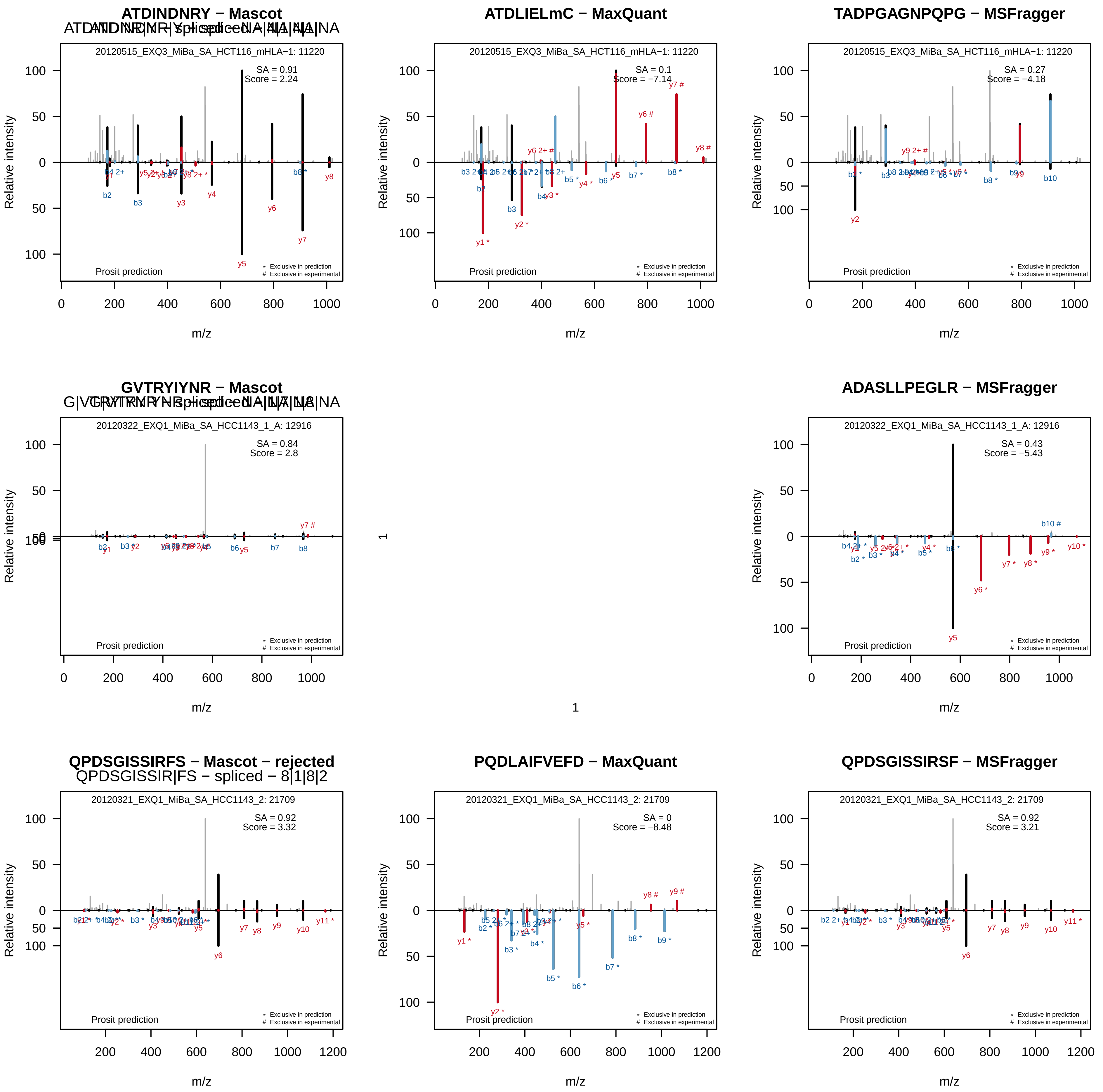
ATDINDNRY − Mascot
20120515_EXQ3_MiBa_SA_HCT116_mHLA−1: 11220
SA = 0.91
Score = 2.24
ATDLIELmC − MaxQuant
20120515_EXQ3_MiBa_SA_HCT116_mHLA−1: 11220
SA = 0.1
Score = −7.14
TADPGAGNPQPG − MSFragger
20120515_EXQ3_MiBa_SA_HCT116_mHLA−1: 11220
SA = 0.27
Score = −4.18
GVTRYIYNR − Mascot
20120322_EXQ1_MiBa_SA_HCC1143_1_A: 12916
SA = 0.84
Score = 2.8
ADASLLPEGLR − MSFragger
20120322_EXQ1_MiBa_SA_HCC1143_1_A: 12916
SA = 0.43
Score = −5.43
QPDSGISSIRFS − Mascot − rejected
QPDSGISSIR|FS − spliced − 8|1|8|2
20120321_EXQ1_MiBa_SA_HCC1143_2: 21709
SA = 0.92
Score = 3.32
PQDLAIFVEFD − MaxQuant
20120321_EXQ1_MiBa_SA_HCC1143_2: 21709
SA = 0
Score = −8.48
QPDSGISSIRSF − MSFragger
20120321_EXQ1_MiBa_SA_HCC1143_2: 21709
SA = 0.92
Score = 3.21
Relative intensity
m/z
Prosit prediction
⋆ Exclusive in prediction
Exclusive in experimental

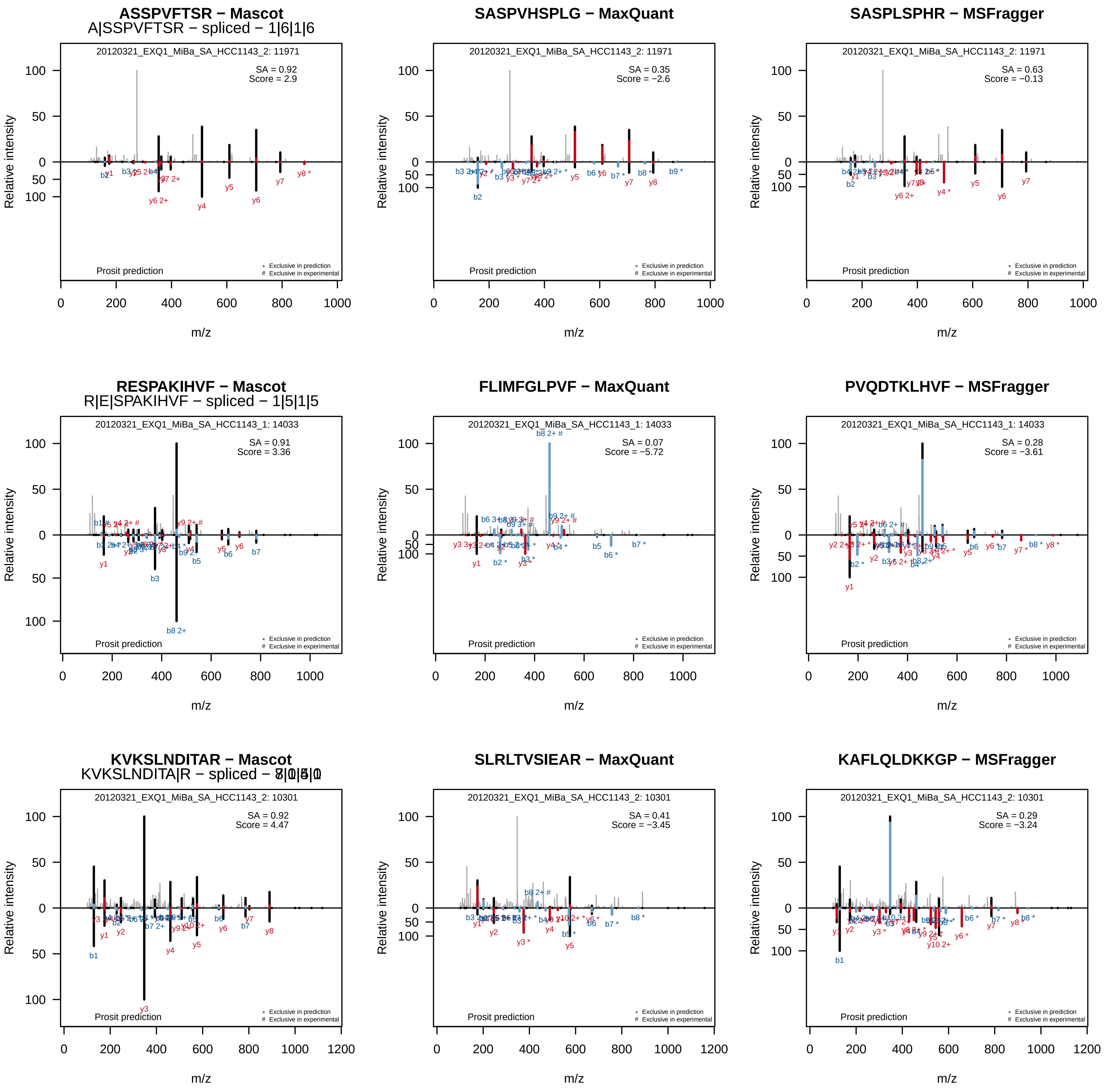

ASSPVFTSR − Mascot
A|SSPVFTSR − spliced − 1|6|1|6
20120321_EXQ1_MiBa_SA_HCC1143_2: 11971
SA = 0.92
Score = 2.9
SASPVHSPLG − MaxQuant
20120321_EXQ1_MiBa_SA_HCC1143_2: 11971
SA = 0.35
Score = −2.6
SASPLSPHR − MSFragger
20120321_EXQ1_MiBa_SA_HCC1143_2: 11971
SA = 0.63
Score = −0.13
RESPAKIHVF − Mascot
R|E|SPAKIHVF − spliced − 1|5|1|5
20120321_EXQ1_MiBa_SA_HCC1143_1: 14033
SA = 0.91
Score = 3.36
FLIMFGLPVF − MaxQuant
20120321_EXQ1_MiBa_SA_HCC1143_1: 14033
SA = 0.07
Score = −5.72
PVQDTKLHVF − MSFragger
20120321_EXQ1_MiBa_SA_HCC1143_1: 14033
SA = 0.28
Score = −3.61
KVKSLNDITAR − Mascot
KVKSLNDITA|R − spliced
20120321_EXQ1_MiBa_SA_HCC1143_2: 10301
SA = 0.92
Score = 4.47
SLRLTVSIEAR − MaxQuant
20120321_EXQ1_MiBa_SA_HCC1143_2: 10301
SA = 0.41
Score = −3.45
KAFLQLDKKGP − MSFragger
20120321_EXQ1_MiBa_SA_HCC1143_2: 10301
SA = 0.29
Score = −3.24
Prosit prediction
* Exclusive in prediction
# Exclusive in experimental
Relative intensity
m/z

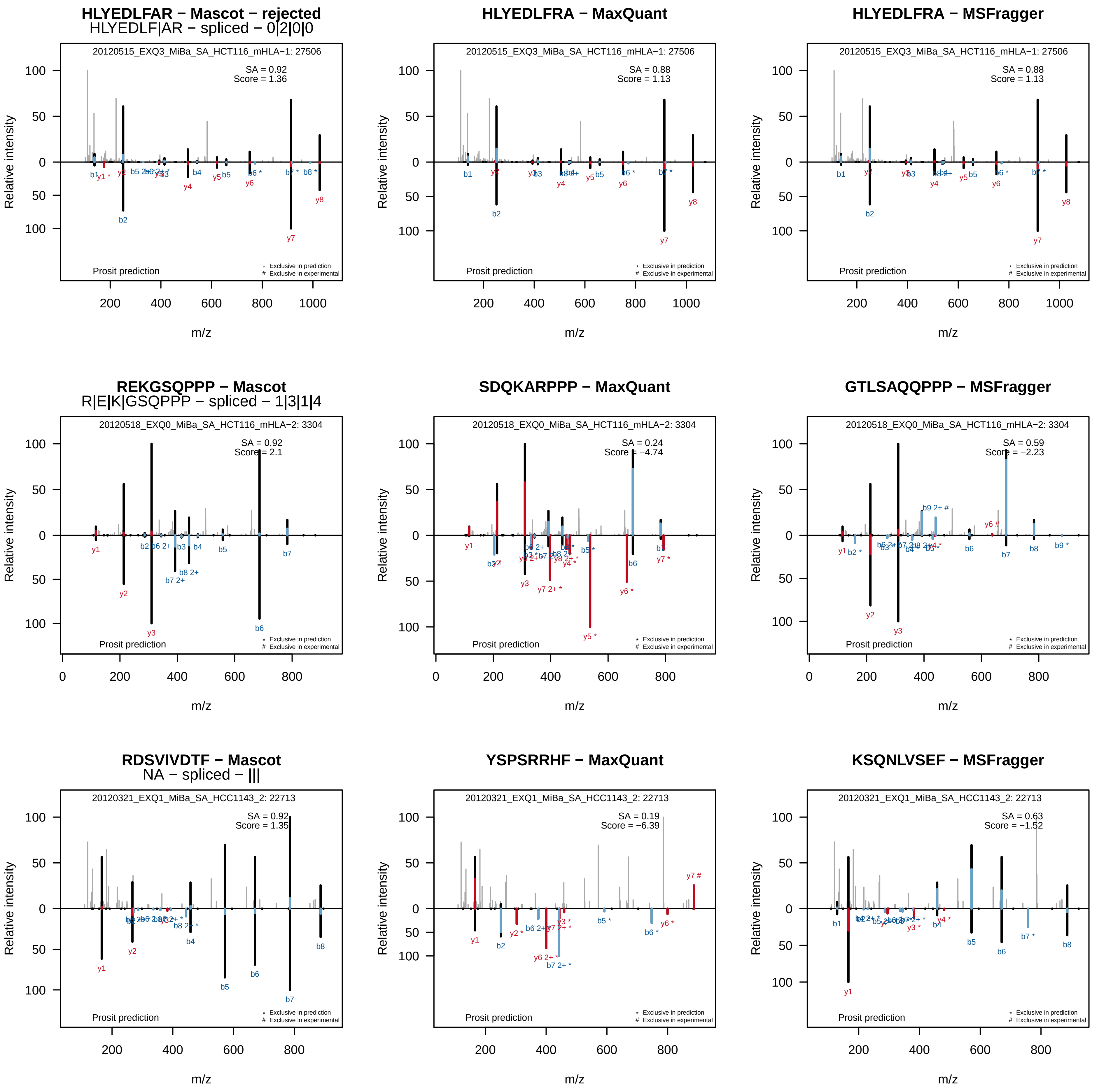
HLYEDLFAR − Mascot − rejected
HLYEDLF|AR − spliced − 0|2|0|0
20120515_EXQ3_MiBa_SA_HCT116_mHLA−1: 27506
SA = 0.92
Score = 1.36
HLYEDLFRA − MaxQuant
20120515_EXQ3_MiBa_SA_HCT116_mHLA−1: 27506
SA = 0.88
Score = 1.13
HLYEDLFRA − MSFragger
20120515_EXQ3_MiBa_SA_HCT116_mHLA−1: 27506
SA = 0.88
Score = 1.13
REKGSQPPP − Mascot
R|E|K|GSQPPP − spliced − 1|3|1|4
20120518_EXQ0_MiBa_SA_HCT116_mHLA−2: 3304
SA = 0.92
Score = 2.1
SDQKARPPP − MaxQuant
20120518_EXQ0_MiBa_SA_HCT116_mHLA−2: 3304
SA = 0.24
Score = −4.74
GTLSAQQPPP − MSFragger
20120518_EXQ0_MiBa_SA_HCT116_mHLA−2: 3304
SA = 0.59
Score = −2.23
RDSVIVDTF − Mascot
NA − spliced − |||
20120321_EXQ1_MiBa_SA_HCC1143_2: 22713
SA = 0.92
Score = 1.35
YSPSRRHF − MaxQuant
20120321_EXQ1_MiBa_SA_HCC1143_2: 22713
SA = 0.19
Score = −6.39
KSQNLVSEF − MSFragger
20120321_EXQ1_MiBa_SA_HCC1143_2: 22713
SA = 0.63
Score = −1.52
Relative intensity
m/z
Prosit prediction
* Exclusive in prediction
# Exclusive in experimental

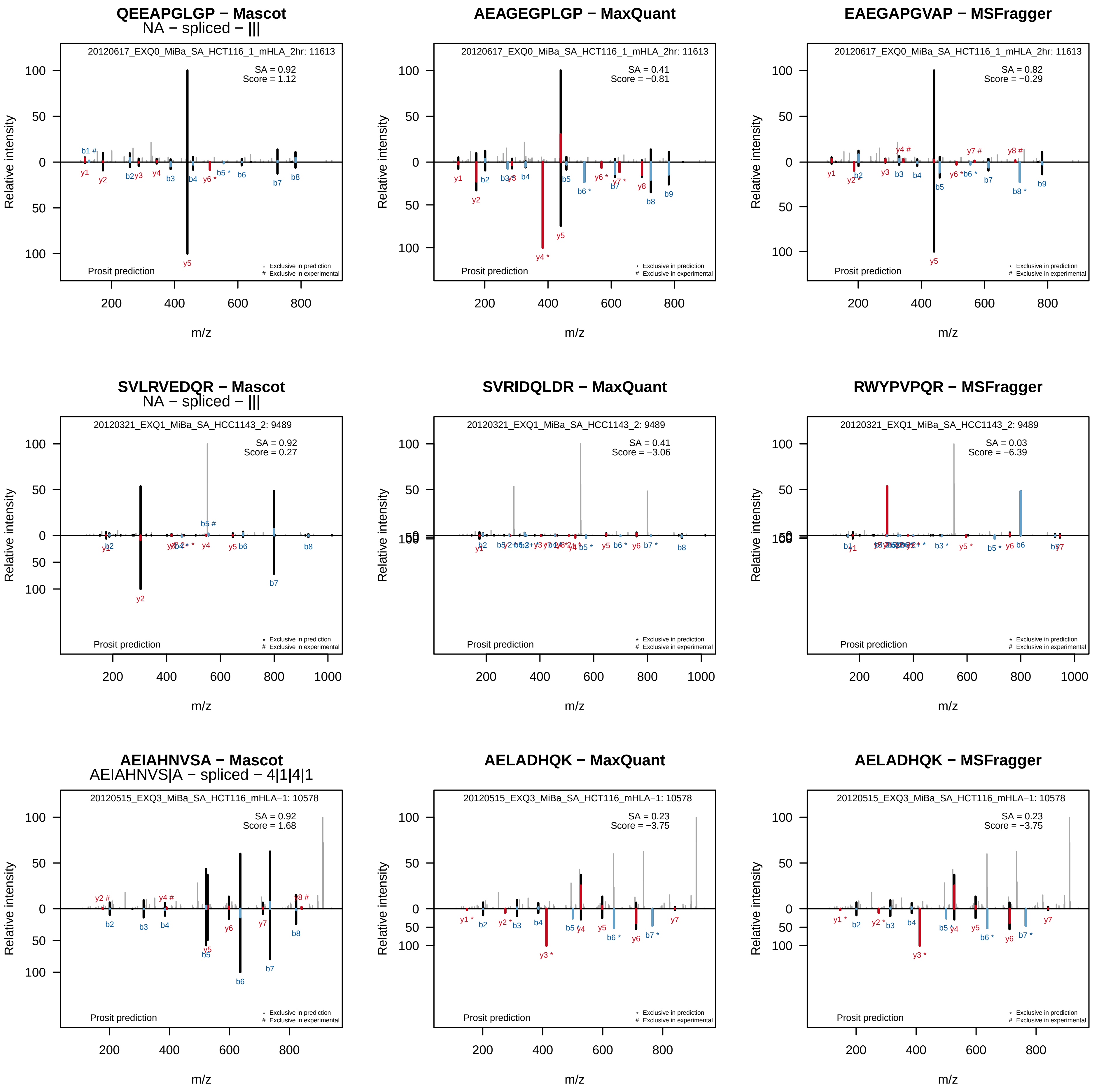

QEEAPGLGP − Mascot
NA − spliced − |||
20120617_EXQ0_MiBa_SA_HCT116_1_mHLA_2hr: 11613
SA = 0.92
Score = 1.12
AEAGEGPLGP − MaxQuant
20120617_EXQ0_MiBa_SA_HCT116_1_mHLA_2hr: 11613
SA = 0.41
Score = −0.81
EAEGAPGVAP − MSFragger
20120617_EXQ0_MiBa_SA_HCT116_1_mHLA_2hr: 11613
SA = 0.82
Score = −0.29
SVLRVEDQR − Mascot
NA − spliced − |||
20120321_EXQ1_MiBa_SA_HCC1143_2: 9489
SA = 0.92
Score = 0.27
SVRIDQLDR − MaxQuant
20120321_EXQ1_MiBa_SA_HCC1143_2: 9489
SA = 0.41
Score = −3.06
RWYPVPQR − MSFragger
20120321_EXQ1_MiBa_SA_HCC1143_2: 9489
SA = 0.03
Score = −6.39
AEIAHNVSA − Mascot
AEIAHNVS|A − spliced − 4|1|4|1
20120515_EXQ3_MiBa_SA_HCT116_mHLA−1: 10578
SA = 0.92
Score = 1.68
AELADHQK − MaxQuant
20120515_EXQ3_MiBa_SA_HCT116_mHLA−1: 10578
SA = 0.23
Score = −3.75
AELADHQK − MSFragger
20120515_EXQ3_MiBa_SA_HCT116_mHLA−1: 10578
SA = 0.23
Score = −3.75
Relative intensity
m/z
Prosit prediction
* Exclusive in prediction
# Exclusive in experimental

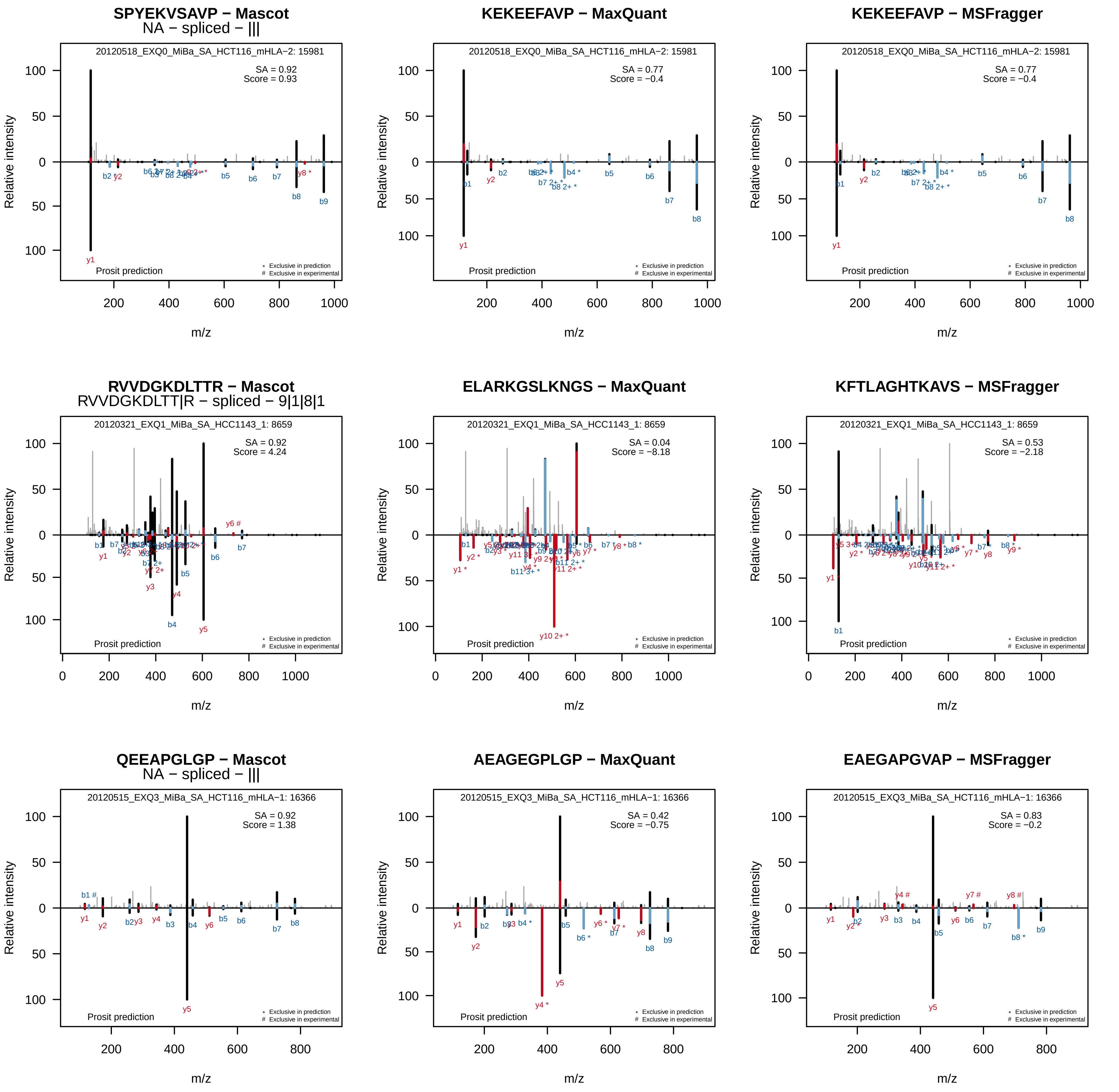
SPYEKVSAVP − Mascot
NA − spliced − |||
20120518_EXQ0_MiBa_SA_HCT116_mHLA−2: 15981
SA = 0.92
Score = 0.93
KEKEEFAVP − MaxQuant
20120518_EXQ0_MiBa_SA_HCT116_mHLA−2: 15981
SA = 0.77
Score = −0.4
KEKEEFAVP − MSFragger
20120518_EXQ0_MiBa_SA_HCT116_mHLA−2: 15981
SA = 0.77
Score = −0.4
RVVDGKDLTTR − Mascot
RVVDGKDLTT|R − spliced − 9|1|8|1
20120321_EXQ1_MiBa_SA_HCC1143_1: 8659
SA = 0.92
Score = 4.24
ELARKGSLKNGS − MaxQuant
20120321_EXQ1_MiBa_SA_HCC1143_1: 8659
SA = 0.04
Score = −8.18
KFTLAGHTKAVS − MSFragger
20120321_EXQ1_MiBa_SA_HCC1143_1: 8659
SA = 0.53
Score = −2.18
QEEAPGLGP − Mascot
NA − spliced − |||
20120515_EXQ3_MiBa_SA_HCT116_mHLA−1: 16366
SA = 0.92
Score = 1.38
AEAGEGPLGP − MaxQuant
20120515_EXQ3_MiBa_SA_HCT116_mHLA−1: 16366
SA = 0.42
Score = −0.75
EAEGAPGVAP − MSFragger
20120515_EXQ3_MiBa_SA_HCT116_mHLA−1: 16366
SA = 0.83
Score = −0.2
Relative intensity
Prosit prediction
* Exclusive in prediction
# Exclusive in experimental
m/z

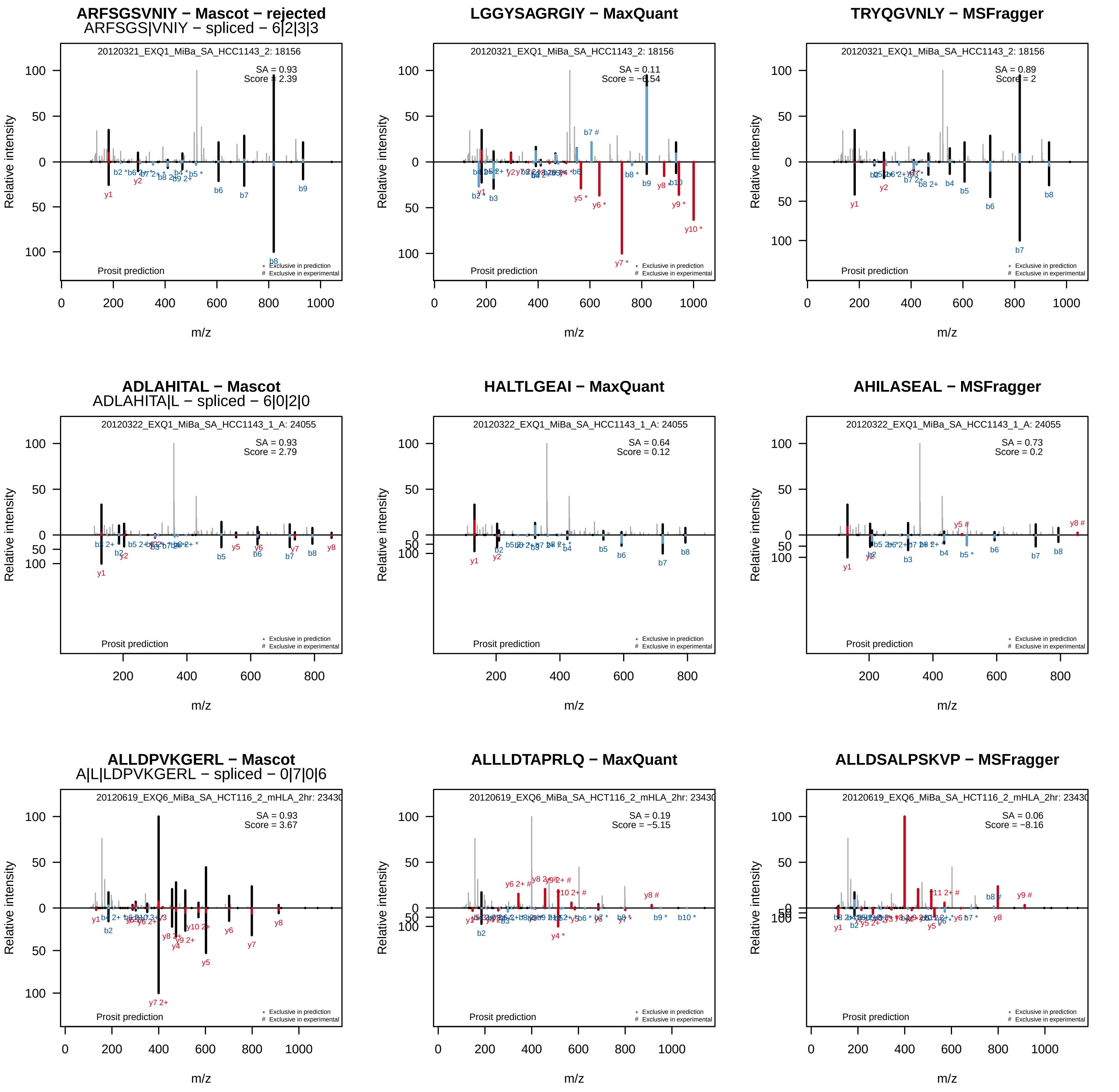

ARFSGSVNIY − Mascot − rejected
ARFSGS|VNIY − spliced − 6|2|3|3
20120321_EXQ1_MiBa_SA_HCC1143_2: 18156
SA = 0.93
Score = 2.39
LGGYSAGRGIY − MaxQuant
20120321_EXQ1_MiBa_SA_HCC1143_2: 18156
SA = 0.11
Score = −6.54
TRYQGVNLY − MSFragger
20120321_EXQ1_MiBa_SA_HCC1143_2: 18156
SA = 0.89
Score = 2
ADLAHITAL − Mascot
ADLAHITA|L − spliced − 6|0|2|0
20120322_EXQ1_MiBa_SA_HCC1143_1_A: 24055
SA = 0.93
Score = 2.79
HALTLGEAI − MaxQuant
20120322_EXQ1_MiBa_SA_HCC1143_1_A: 24055
SA = 0.64
Score = 0.12
AHILASEAL − MSFragger
20120322_EXQ1_MiBa_SA_HCC1143_1_A: 24055
SA = 0.73
Score = 0.2
ALLDPVKGERL − Mascot
A|L|LDPVKGERL − spliced − 0|7|0|6
20120619_EXQ6_MiBa_SA_HCT116_2_mHLA_2hr: 23430
SA = 0.93
Score = 3.67
ALLLDTAPRLQ − MaxQuant
20120619_EXQ6_MiBa_SA_HCT116_2_mHLA_2hr: 23430
SA = 0.19
Score = −5.15
ALLDSALPSKVP − MSFragger
20120619_EXQ6_MiBa_SA_HCT116_2_mHLA_2hr: 23430
SA = 0.06
Score = −8.16
Relative intensity
m/z
Prosit prediction
* Exclusive in prediction
# Exclusive in experimental

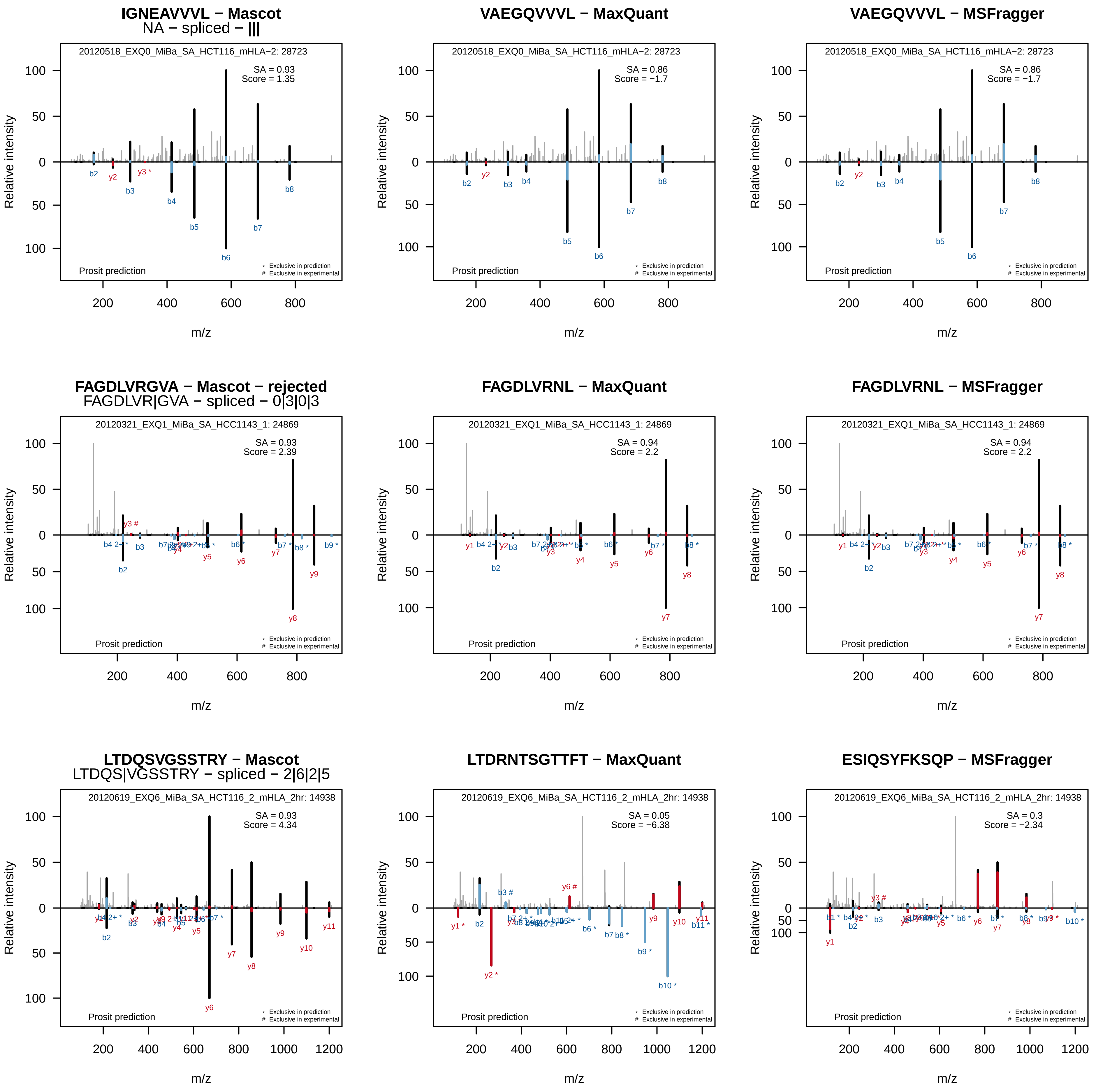

IGNEAVVVL − Mascot
NA − spliced − |||
20120518_EXQ0_MiBa_SA_HCT116_mHLA−2: 28723
SA = 0.93
Score = 1.35
b2 y2 y3 * b3 b4 b5 b6 b7 b8
VAEGQVVVL − MaxQuant
20120518_EXQ0_MiBa_SA_HCT116_mHLA−2: 28723
SA = 0.86
Score = −1.7
b2 y2 b3 b4 b5 b6 b7 b8
VAEGQVVVL − MSFragger
20120518_EXQ0_MiBa_SA_HCT116_mHLA−2: 28723
SA = 0.86
Score = −1.7
b2 y2 b3 b4 b5 b6 b7 b8
FAGDLVRGVA − Mascot − rejected
FAGDLVR|GVA − spliced − 0|3|0|3
20120321_EXQ1_MiBa_SA_HCC1143_1: 24869
SA = 0.93
Score = 2.39
y3 # b2 b3 y4 y5 y6 y7 y8 y9 b6 * b7 * b8 * b9 *
FAGDLVRNL − MaxQuant
20120321_EXQ1_MiBa_SA_HCC1143_1: 24869
SA = 0.94
Score = 2.2
y1 y2 b2 b3 y3 y4 y5 y6 y7 y8 b6 * b7 * b8 *
FAGDLVRNL − MSFragger
20120321_EXQ1_MiBa_SA_HCC1143_1: 24869
SA = 0.94
Score = 2.2
y1 y2 b2 b3 y3 y4 y5 y6 y7 y8 b6 * b7 * b8 *
LTDQSVGSSTRY − Mascot
LTDQS|VGSSTRY − spliced − 2|6|2|5
20120619_EXQ6_MiBa_SA_HCT116_2_mHLA_2hr: 14938
SA = 0.93
Score = 4.34
b2 y2 b3 b4 y4 b5 y5 y6 b7 * y7 y8 y9 y10 y11
LTDRNTSGTTFT − MaxQuant
20120619_EXQ6_MiBa_SA_HCT116_2_mHLA_2hr: 14938
SA = 0.05
Score = −6.38
b3 # y6 # y1 * b2 y2 * b6 * b7 b8 * b9 * b10 * y9 y10 y11 b11 *
ESIQSYFKSQP − MSFragger
20120619_EXQ6_MiBa_SA_HCT116_2_mHLA_2hr: 14938
SA = 0.3
Score = −2.34
y3 # b1 * b2 b3 y1 y5 b6 * y6 b7 * y7 b8 * y8 b9 * b10 *
Relative intensity
m/z
Prosit prediction
⋆ Exclusive in prediction
Exclusive in experimental

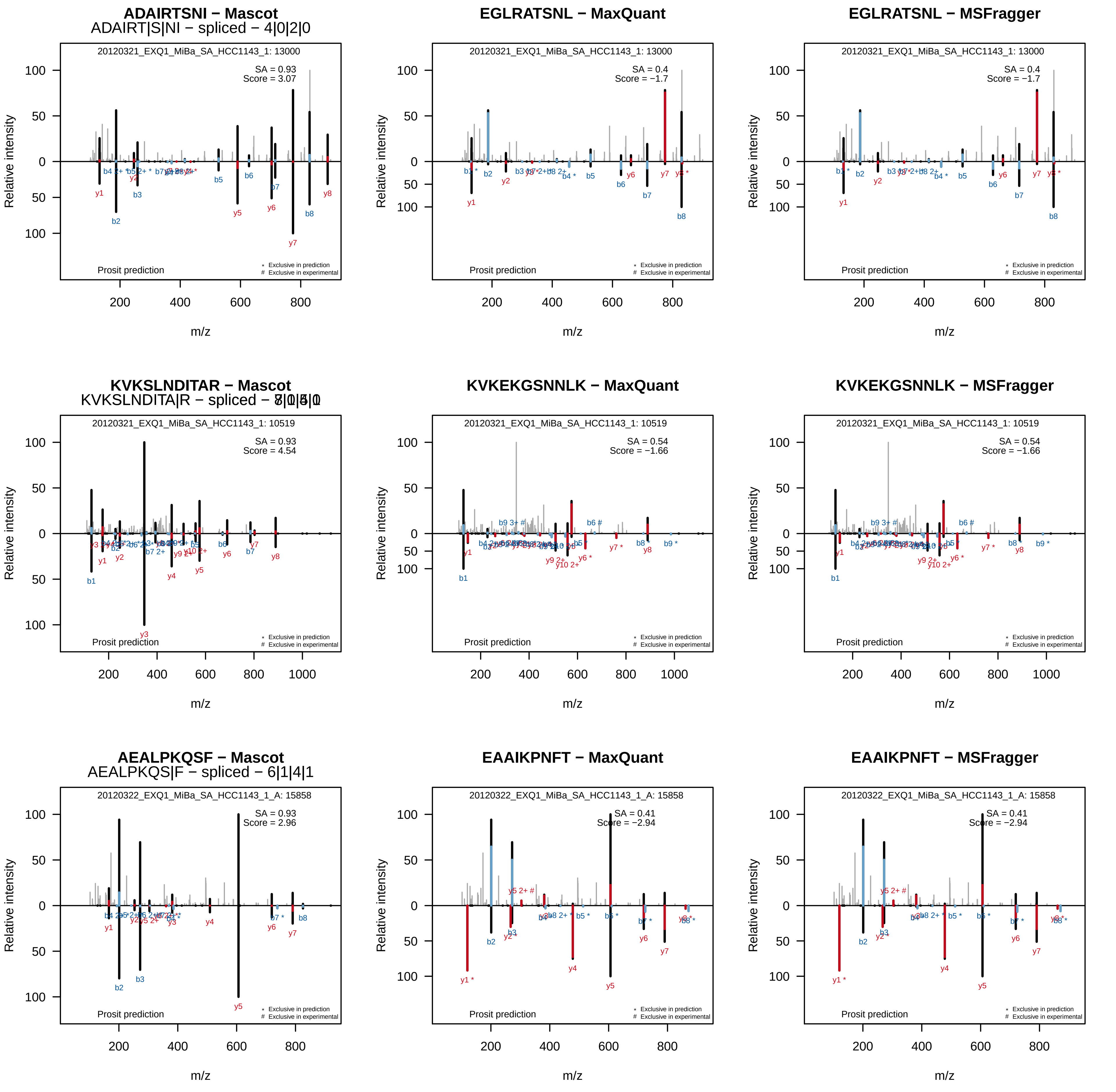
ADAIRTSNI − Mascot
ADAIRT|S|NI − spliced − 4|0|2|0
20120321_EXQ1_MiBa_SA_HCC1143_1: 13000
SA = 0.93
Score = 3.07
EGLRATSNL − MaxQuant
20120321_EXQ1_MiBa_SA_HCC1143_1: 13000
SA = 0.4
Score = −1.7
EGLRATSNL − MSFragger
20120321_EXQ1_MiBa_SA_HCC1143_1: 13000
SA = 0.4
Score = −1.7
KVKSLNDITAR − Mascot
20120321_EXQ1_MiBa_SA_HCC1143_1: 10519
SA = 0.93
Score = 4.54
KVKEKGSNNLK − MaxQuant
20120321_EXQ1_MiBa_SA_HCC1143_1: 10519
SA = 0.54
Score = −1.66
KVKEKGSNNLK − MSFragger
20120321_EXQ1_MiBa_SA_HCC1143_1: 10519
SA = 0.54
Score = −1.66
AEALPKQSF − Mascot
AEALPKQS|F − spliced − 6|1|4|1
20120322_EXQ1_MiBa_SA_HCC1143_1_A: 15858
SA = 0.93
Score = 2.96
EAAIKPNFT − MaxQuant
20120322_EXQ1_MiBa_SA_HCC1143_1_A: 15858
SA = 0.41
Score = −2.94
EAAIKPNFT − MSFragger
20120322_EXQ1_MiBa_SA_HCC1143_1_A: 15858
SA = 0.41
Score = −2.94
Relative intensity
m/z
Prosit prediction
* Exclusive in prediction
# Exclusive in experimental

TGDMLQLEY − Mascot − rejected
NA − spliced − |||

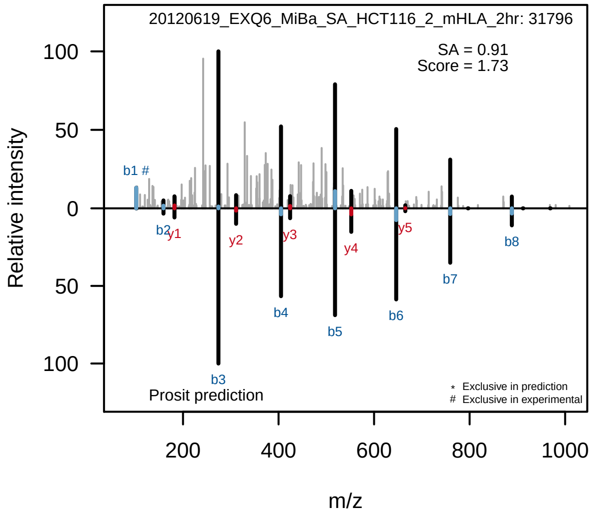

ASDMLQLEY − MSFragger

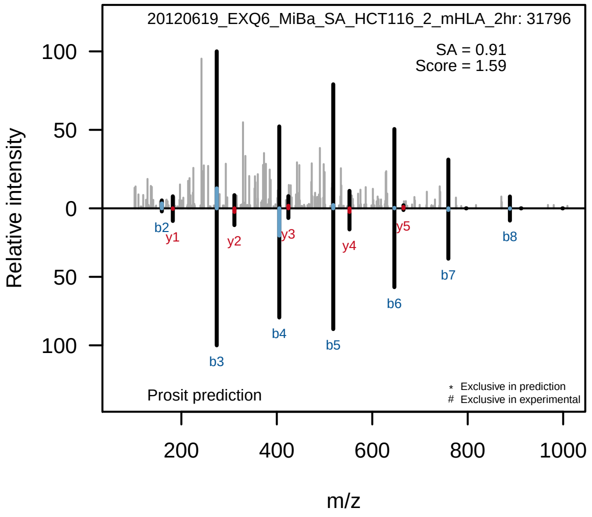

DEYGVSQAI − Mascot − rejected
NA − spliced − |||

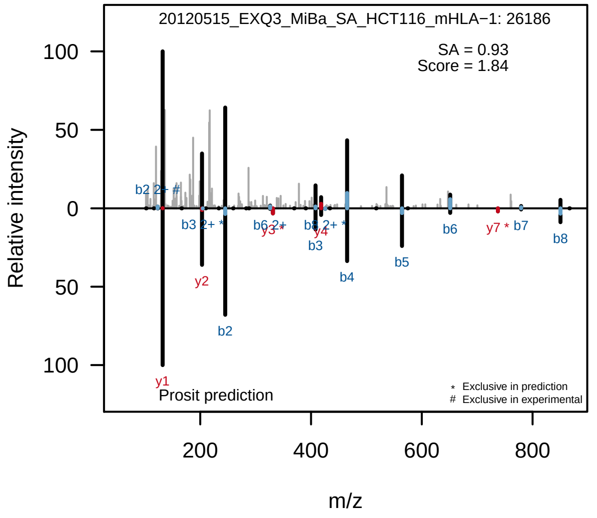

DEYGVSQAL − MSFragger

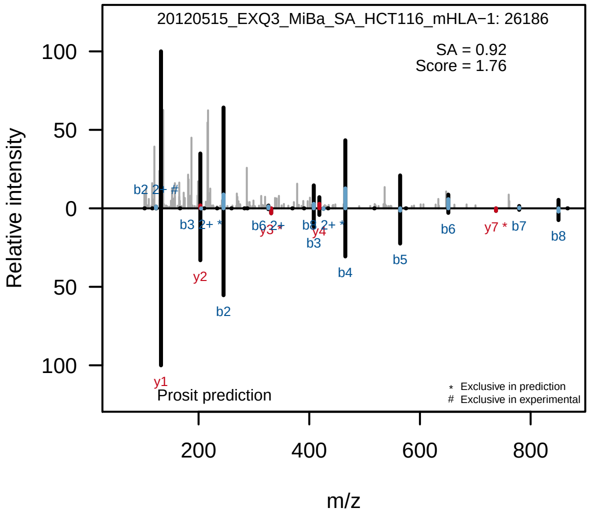

KDFLRIAGI − Mascot − rejected
KDFLRIAG|I − spliced − NA|NA|NA|NA

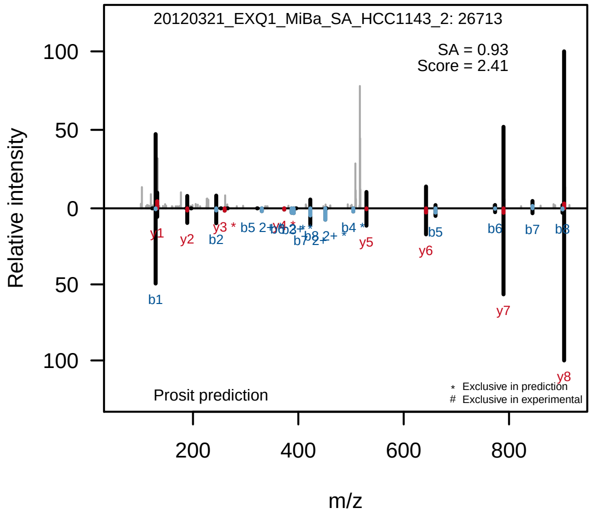

KDFLRIAGL − MSFragger

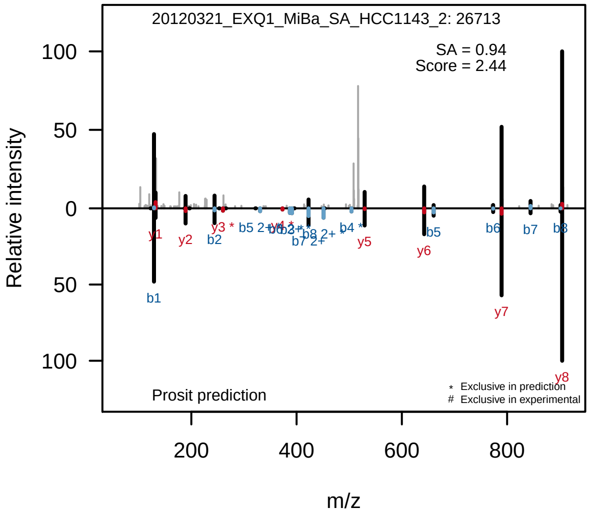

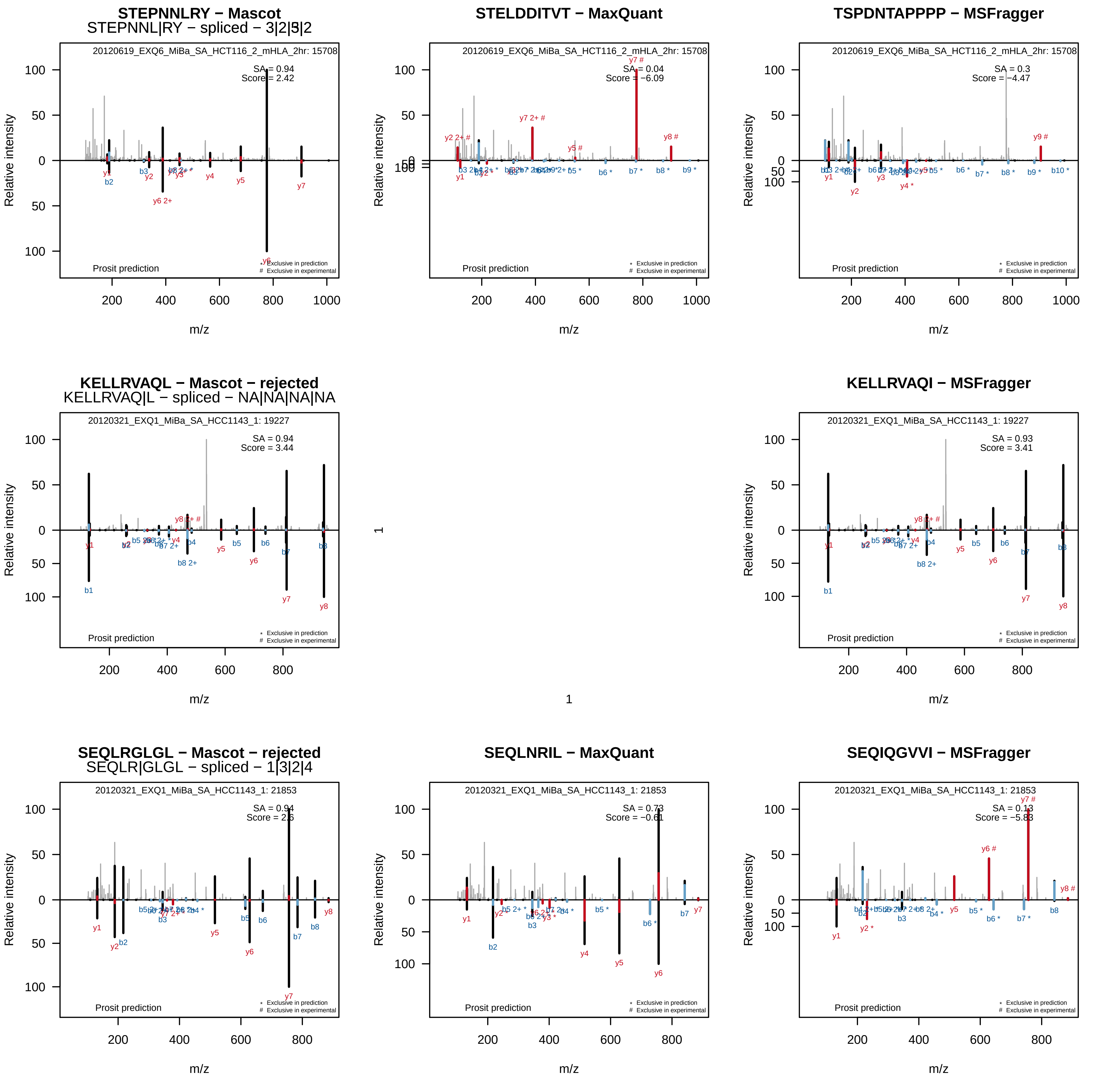

STEPNNLRY − Mascot
STEPNNL|RY − spliced − 3|2|3|2
20120619_EXQ6_MiBa_SA_HCT116_2_mHLA_2hr: 15708
SA = 0.94
Score = 2.42
STELDDITVT − MaxQuant
20120619_EXQ6_MiBa_SA_HCT116_2_mHLA_2hr: 15708
SA = 0.04
Score = −6.09
TSPDNTAPPPP − MSFragger
20120619_EXQ6_MiBa_SA_HCT116_2_mHLA_2hr: 15708
SA = 0.3
Score = −4.47
KELLRVAQL − Mascot − rejected
KELLRVAQ|L − spliced − NA|NA|NA|NA
20120321_EXQ1_MiBa_SA_HCC1143_1: 19227
SA = 0.94
Score = 3.44
KELLRVAQI − MSFragger
20120321_EXQ1_MiBa_SA_HCC1143_1: 19227
SA = 0.93
Score = 3.41
SEQLRGLGL − Mascot − rejected
SEQLR|GLGL − spliced − 1|3|2|4
20120321_EXQ1_MiBa_SA_HCC1143_1: 21853
SA = 0.94
Score = 2.6
SEQLNRIL − MaxQuant
20120321_EXQ1_MiBa_SA_HCC1143_1: 21853
SA = 0.73
Score = −0.61
SEQIQGVVI − MSFragger
20120321_EXQ1_MiBa_SA_HCC1143_1: 21853
SA = 0.13
Score = −5.83
Relative intensity
m/z
Prosit prediction
⋆ Exclusive in prediction
Exclusive in experimental

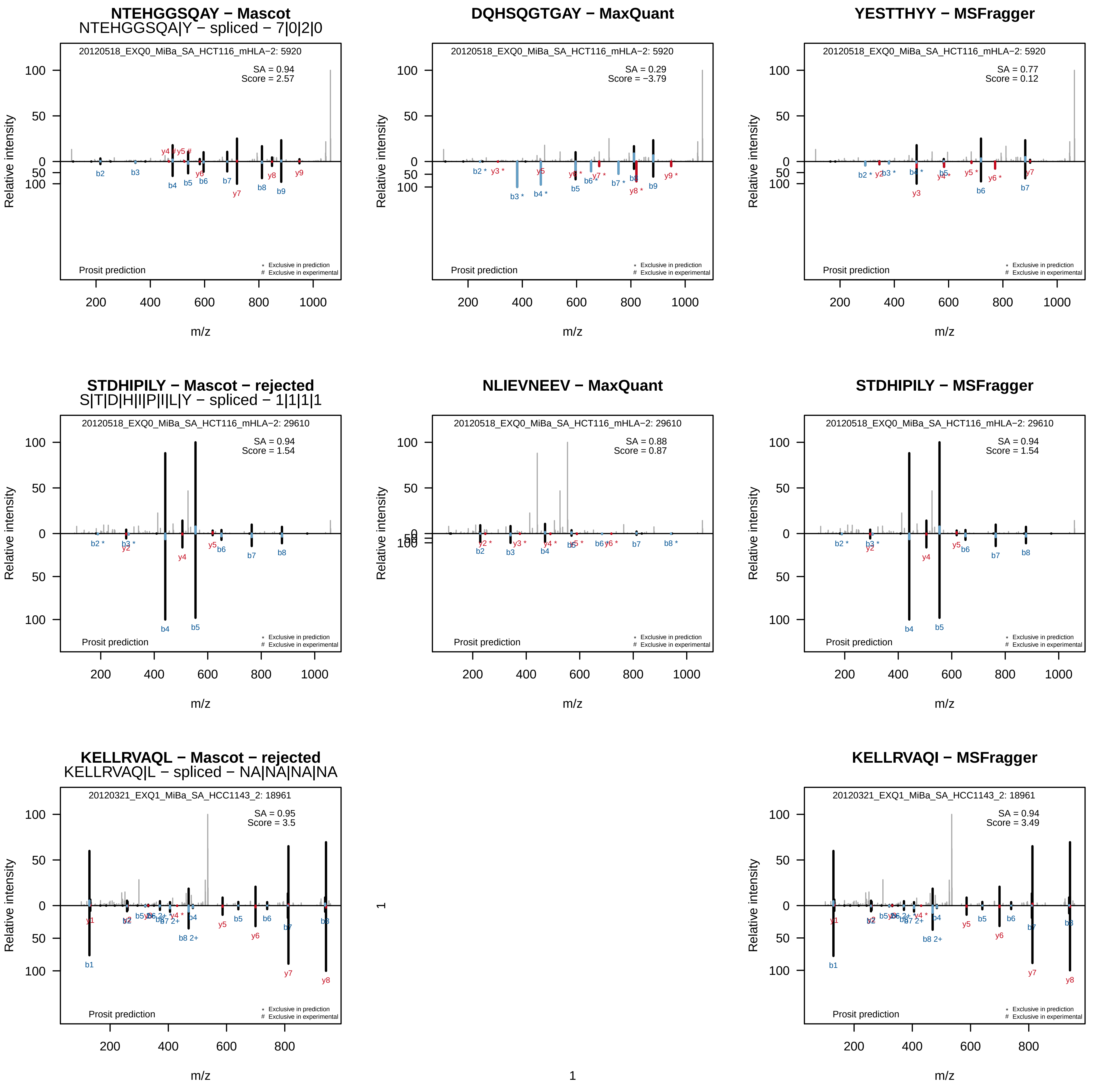
NTEHGGSQAY − Mascot
NTEHGGSQA|Y − spliced − 7|0|2|0
20120518_EXQ0_MiBa_SA_HCT116_mHLA−2: 5920
SA = 0.94
Score = 2.57
Prosit prediction
DQHSQGTGAY − MaxQuant
20120518_EXQ0_MiBa_SA_HCT116_mHLA−2: 5920
SA = 0.29
Score = −3.79
Prosit prediction
YESTTHYY − MSFragger
20120518_EXQ0_MiBa_SA_HCT116_mHLA−2: 5920
SA = 0.77
Score = 0.12
Prosit prediction
STDHIPILY − Mascot − rejected
S|T|D|H|I|P|I|L|Y − spliced − 1|1|1|1
20120518_EXQ0_MiBa_SA_HCT116_mHLA−2: 29610
SA = 0.94
Score = 1.54
Prosit prediction
NLIEVNEEV − MaxQuant
20120518_EXQ0_MiBa_SA_HCT116_mHLA−2: 29610
SA = 0.88
Score = 0.87
Prosit prediction
STDHIPILY − MSFragger
20120518_EXQ0_MiBa_SA_HCT116_mHLA−2: 29610
SA = 0.94
Score = 1.54
Prosit prediction
KELLRVAQL − Mascot − rejected
KELLRVAQ|L − spliced − NA|NA|NA|NA
20120321_EXQ1_MiBa_SA_HCC1143_2: 18961
SA = 0.95
Score = 3.5
Prosit prediction
KELLRVAQI − MSFragger
20120321_EXQ1_MiBa_SA_HCC1143_2: 18961
SA = 0.94
Score = 3.49
Prosit prediction
Relative intensity
m/z
⋆ Exclusive in prediction
Exclusive in experimental

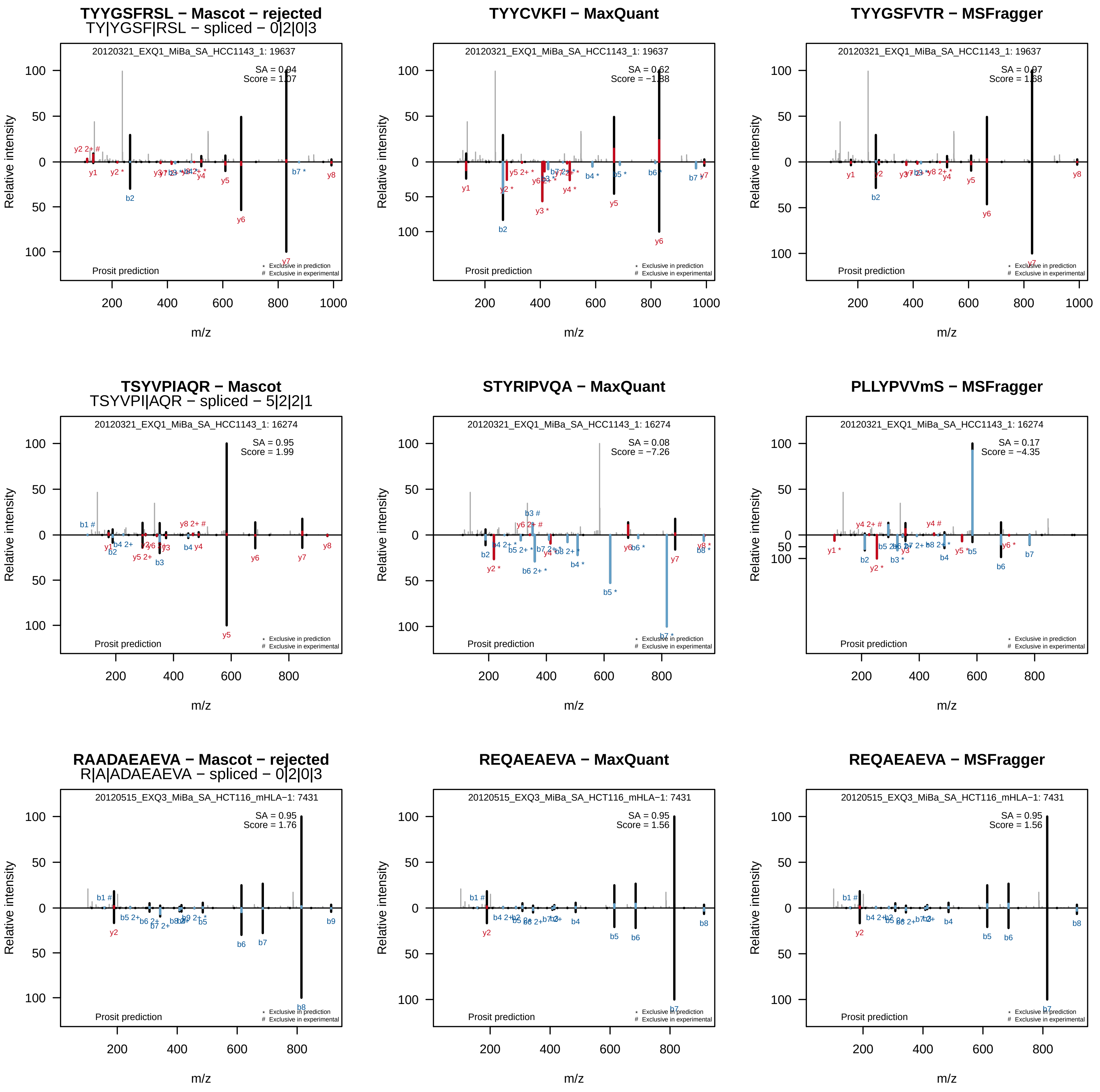
TYYGSFRSL − Mascot − rejected
TY|YGSF|RSL − spliced − 0|2|0|3
20120321_EXQ1_MiBa_SA_HCC1143_1: 19637
SA = 0.94
Score = 1.07
Prosit prediction
TYYCVKFI − MaxQuant
20120321_EXQ1_MiBa_SA_HCC1143_1: 19637
SA = 0.62
Score = −1.88
Prosit prediction
TYYGSFVTR − MSFragger
20120321_EXQ1_MiBa_SA_HCC1143_1: 19637
SA = 0.97
Score = 1.68
Prosit prediction
TSYVPIAQR − Mascot
TSYVPI|AQR − spliced − 5|2|2|1
20120321_EXQ1_MiBa_SA_HCC1143_1: 16274
SA = 0.95
Score = 1.99
Prosit prediction
STYRIPVQA − MaxQuant
20120321_EXQ1_MiBa_SA_HCC1143_1: 16274
SA = 0.08
Score = −7.26
Prosit prediction
PLLYPVVmS − MSFragger
20120321_EXQ1_MiBa_SA_HCC1143_1: 16274
SA = 0.17
Score = −4.35
Prosit prediction
RAADAEAEVA − Mascot − rejected
R|A|ADAEAEVA − spliced − 0|2|0|3
20120515_EXQ3_MiBa_SA_HCT116_mHLA−1: 7431
SA = 0.95
Score = 1.76
Prosit prediction
REQAEAEVA − MaxQuant
20120515_EXQ3_MiBa_SA_HCT116_mHLA−1: 7431
SA = 0.95
Score = 1.56
Prosit prediction
REQAEAEVA − MSFragger
20120515_EXQ3_MiBa_SA_HCT116_mHLA−1: 7431
SA = 0.95
Score = 1.56
Prosit prediction
⋆ Exclusive in prediction
Exclusive in experimental
Relative intensity
m/z

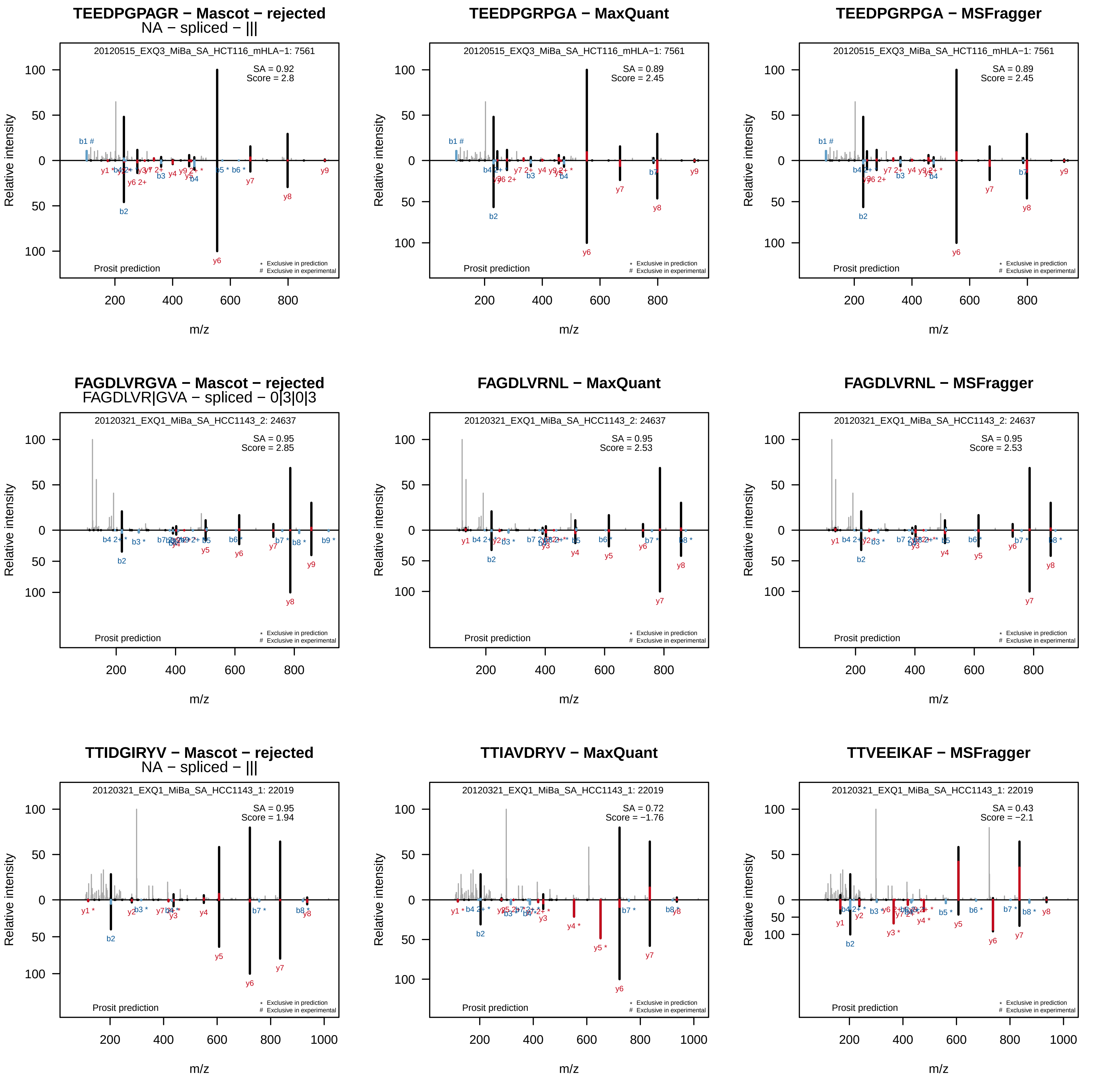

TEEDPGPAGR − Mascot − rejected
NA − spliced − |||
20120515_EXQ3_MiBa_SA_HCT116_mHLA−1: 7561
SA = 0.92
Score = 2.8
TEEDPGRPGA − MaxQuant
20120515_EXQ3_MiBa_SA_HCT116_mHLA−1: 7561
SA = 0.89
Score = 2.45
TEEDPGRPGA − MSFragger
20120515_EXQ3_MiBa_SA_HCT116_mHLA−1: 7561
SA = 0.89
Score = 2.45
FAGDLVRGVA − Mascot − rejected
FAGDLVR|GVA − spliced − 0|3|0|3
20120321_EXQ1_MiBa_SA_HCC1143_2: 24637
SA = 0.95
Score = 2.85
FAGDLVRNL − MaxQuant
20120321_EXQ1_MiBa_SA_HCC1143_2: 24637
SA = 0.95
Score = 2.53
FAGDLVRNL − MSFragger
20120321_EXQ1_MiBa_SA_HCC1143_2: 24637
SA = 0.95
Score = 2.53
TTIDGIRYV − Mascot − rejected
NA − spliced − |||
20120321_EXQ1_MiBa_SA_HCC1143_1: 22019
SA = 0.95
Score = 1.94
TTIAVDRYV − MaxQuant
20120321_EXQ1_MiBa_SA_HCC1143_1: 22019
SA = 0.72
Score = −1.76
TTVEEIKAF − MSFragger
20120321_EXQ1_MiBa_SA_HCC1143_1: 22019
SA = 0.43
Score = −2.1
Relative intensity
m/z
Prosit prediction
* Exclusive in prediction
# Exclusive in experimental

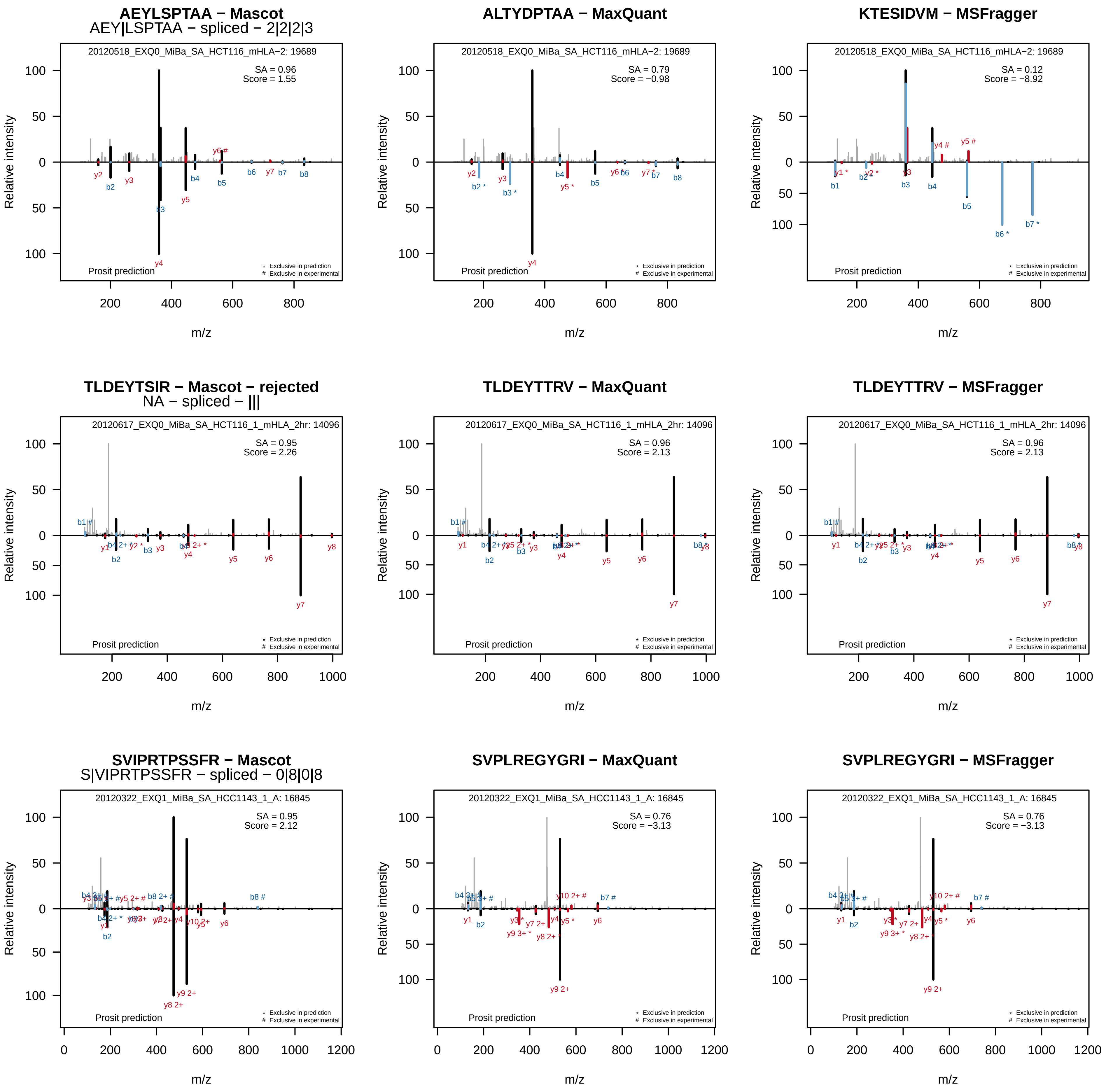
AEYLSPTAA − Mascot
AEY|LSPTAA − spliced − 2|2|2|3
20120518_EXQ0_MiBa_SA_HCT116_mHLA−2: 19689
SA = 0.96
Score = 1.55
ALTYDPTAA − MaxQuant
20120518_EXQ0_MiBa_SA_HCT116_mHLA−2: 19689
SA = 0.79
Score = −0.98
KTESIDVM − MSFragger
20120518_EXQ0_MiBa_SA_HCT116_mHLA−2: 19689
SA = 0.12
Score = −8.92
TLDEYTSIR − Mascot − rejected
NA − spliced − |||
20120617_EXQ0_MiBa_SA_HCT116_1_mHLA_2hr: 14096
SA = 0.95
Score = 2.26
TLDEYTTRV − MaxQuant
20120617_EXQ0_MiBa_SA_HCT116_1_mHLA_2hr: 14096
SA = 0.96
Score = 2.13
TLDEYTTRV − MSFragger
20120617_EXQ0_MiBa_SA_HCT116_1_mHLA_2hr: 14096
SA = 0.96
Score = 2.13
SVIPRTPSSFR − Mascot
S|VIPRTPSSFR − spliced − 0|8|0|8
20120322_EXQ1_MiBa_SA_HCC1143_1_A: 16845
SA = 0.95
Score = 2.12
SVPLREGYGRI − MaxQuant
20120322_EXQ1_MiBa_SA_HCC1143_1_A: 16845
SA = 0.76
Score = −3.13
SVPLREGYGRI − MSFragger
20120322_EXQ1_MiBa_SA_HCC1143_1_A: 16845
SA = 0.76
Score = −3.13
Relative intensity
m/z
Prosit prediction
* Exclusive in prediction
# Exclusive in experimental

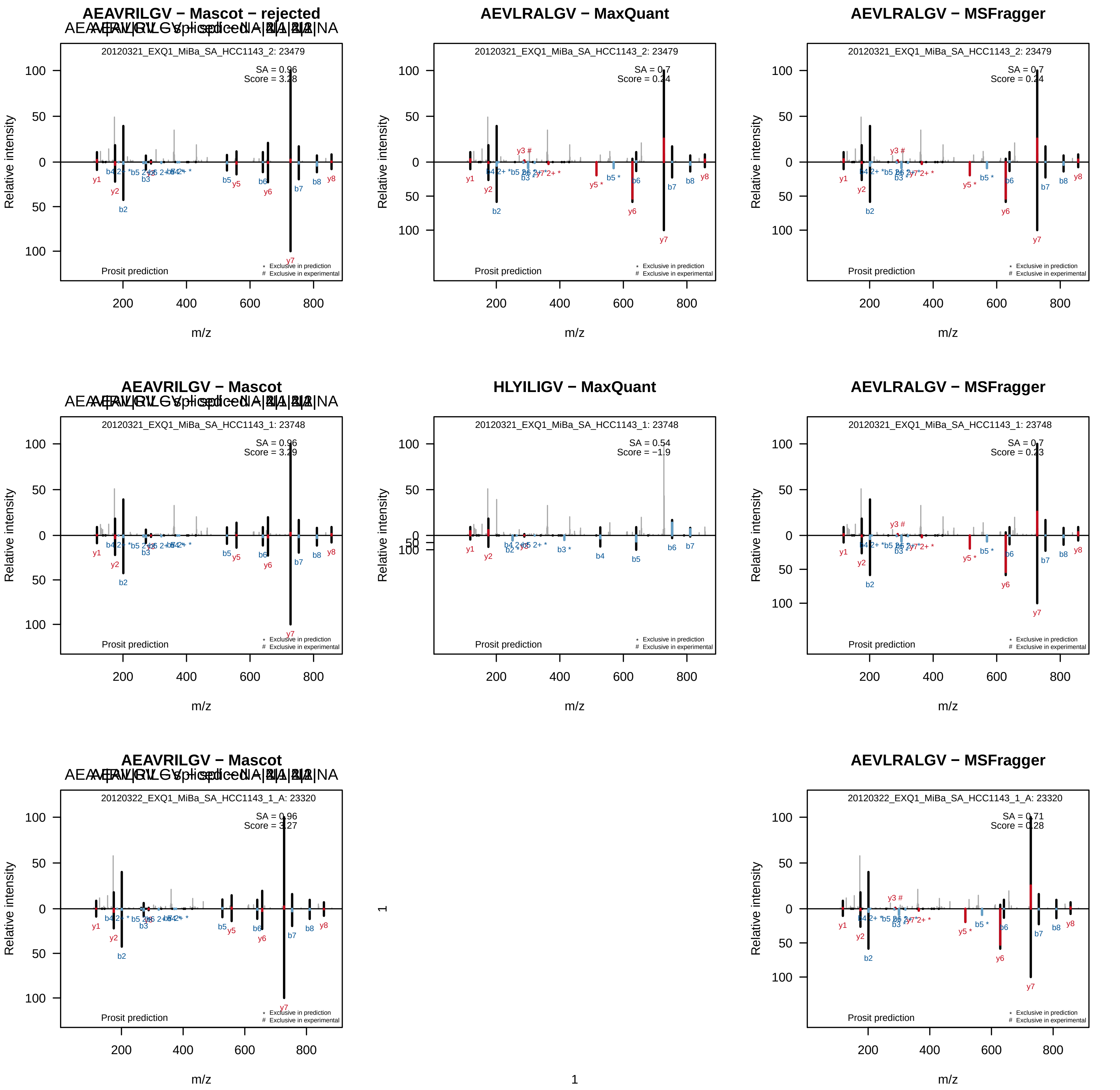
AEAVRILGV − Mascot − rejected
AEVLRALGV − MaxQuant
AEVLRALGV − MSFragger
AEAVRILGV − Mascot
HLYILIGV − MaxQuant
AEVLRALGV − MSFragger
AEAVRILGV − Mascot
AEVLRALGV − MSFragger
20120321_EXQ1_MiBa_SA_HCC1143_2: 23479
20120321_EXQ1_MiBa_SA_HCC1143_1: 23748
20120322_EXQ1_MiBa_SA_HCC1143_1_A: 23320
SA = 0.96
Score = 3.28
SA = 0.7
Score = 0.24
SA = 0.96
Score = 3.29
SA = 0.54
Score = −1.9
SA = 0.7
Score = 0.23
SA = 0.96
Score = 3.27
SA = 0.71
Score = 0.28
Relative intensity
m/z
Prosit prediction
Exclusive in prediction
Exclusive in experimental
1

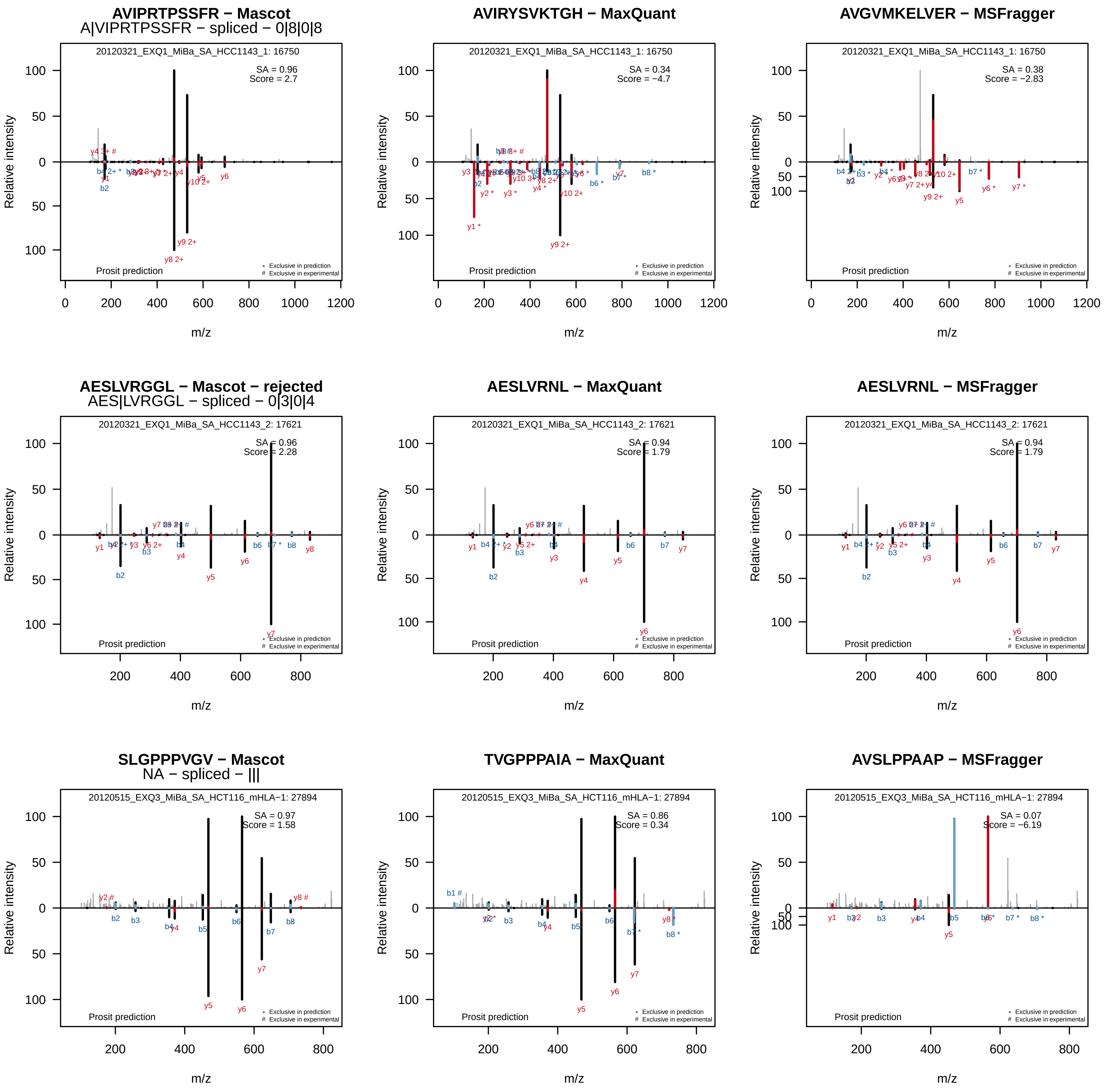

AVIPRTPSSFR − Mascot
A|VIPRTPSSFR − spliced − 0|8|0|8
20120321_EXQ1_MiBa_SA_HCC1143_1: 16750
SA = 0.96
Score = 2.7
Prosit prediction
⋆ Exclusive in prediction
Exclusive in experimental
Relative intensity
m/z
AVIRYSVKTGH − MaxQuant
20120321_EXQ1_MiBa_SA_HCC1143_1: 16750
SA = 0.34
Score = −4.7
Prosit prediction
AVGVMKELVER − MSFragger
20120321_EXQ1_MiBa_SA_HCC1143_1: 16750
SA = 0.38
Score = −2.83
Prosit prediction
AESLVRGGL − Mascot − rejected
AES|LVRGGL − spliced − 0|3|0|4
20120321_EXQ1_MiBa_SA_HCC1143_2: 17621
SA = 0.96
Score = 2.28
Prosit prediction
AESLVRNL − MaxQuant
20120321_EXQ1_MiBa_SA_HCC1143_2: 17621
SA = 0.94
Score = 1.79
Prosit prediction
AESLVRNL − MSFragger
20120321_EXQ1_MiBa_SA_HCC1143_2: 17621
SA = 0.94
Score = 1.79
Prosit prediction
SLGPPPVGV − Mascot
NA − spliced − |||
20120515_EXQ3_MiBa_SA_HCT116_mHLA−1: 27894
SA = 0.97
Score = 1.58
Prosit prediction
TVGPPPAIA − MaxQuant
20120515_EXQ3_MiBa_SA_HCT116_mHLA−1: 27894
SA = 0.86
Score = 0.34
Prosit prediction
AVSLPPAAP − MSFragger
20120515_EXQ3_MiBa_SA_HCT116_mHLA−1: 27894
SA = 0.07
Score = −6.19
Prosit prediction

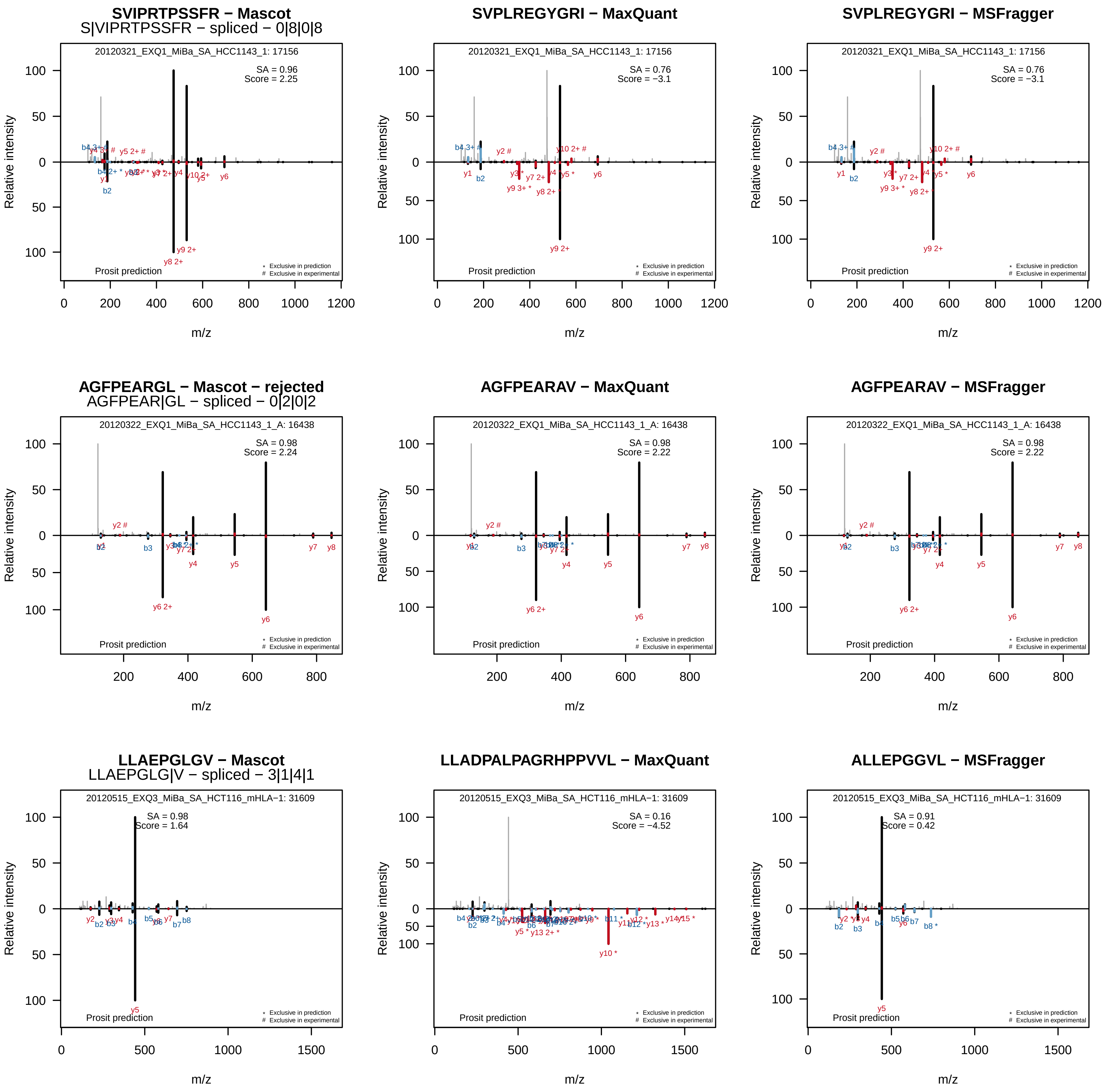
SVIPRTPSSFR − Mascot
S|VIPRTPSSFR − spliced − 0|8|0|8
20120321_EXQ1_MiBa_SA_HCC1143_1: 17156
SA = 0.96
Score = 2.25
SVPLREGYGRI − MaxQuant
20120321_EXQ1_MiBa_SA_HCC1143_1: 17156
SA = 0.76
Score = −3.1
SVPLREGYGRI − MSFragger
20120321_EXQ1_MiBa_SA_HCC1143_1: 17156
SA = 0.76
Score = −3.1
AGFPEARGL − Mascot − rejected
AGFPEAR|GL − spliced − 0|2|0|2
20120322_EXQ1_MiBa_SA_HCC1143_1_A: 16438
SA = 0.98
Score = 2.24
AGFPEARAV − MaxQuant
20120322_EXQ1_MiBa_SA_HCC1143_1_A: 16438
SA = 0.98
Score = 2.22
AGFPEARAV − MSFragger
20120322_EXQ1_MiBa_SA_HCC1143_1_A: 16438
SA = 0.98
Score = 2.22
LLAEPGLGV − Mascot
LLAEPGLG|V − spliced − 3|1|4|1
20120515_EXQ3_MiBa_SA_HCT116_mHLA−1: 31609
SA = 0.98
Score = 1.64
LLADPALPAGRHPPVVL − MaxQuant
20120515_EXQ3_MiBa_SA_HCT116_mHLA−1: 31609
SA = 0.16
Score = −4.52
ALLEPGGVL − MSFragger
20120515_EXQ3_MiBa_SA_HCT116_mHLA−1: 31609
SA = 0.91
Score = 0.42
Relative intensity
m/z
Prosit prediction
* Exclusive in prediction
# Exclusive in experimental

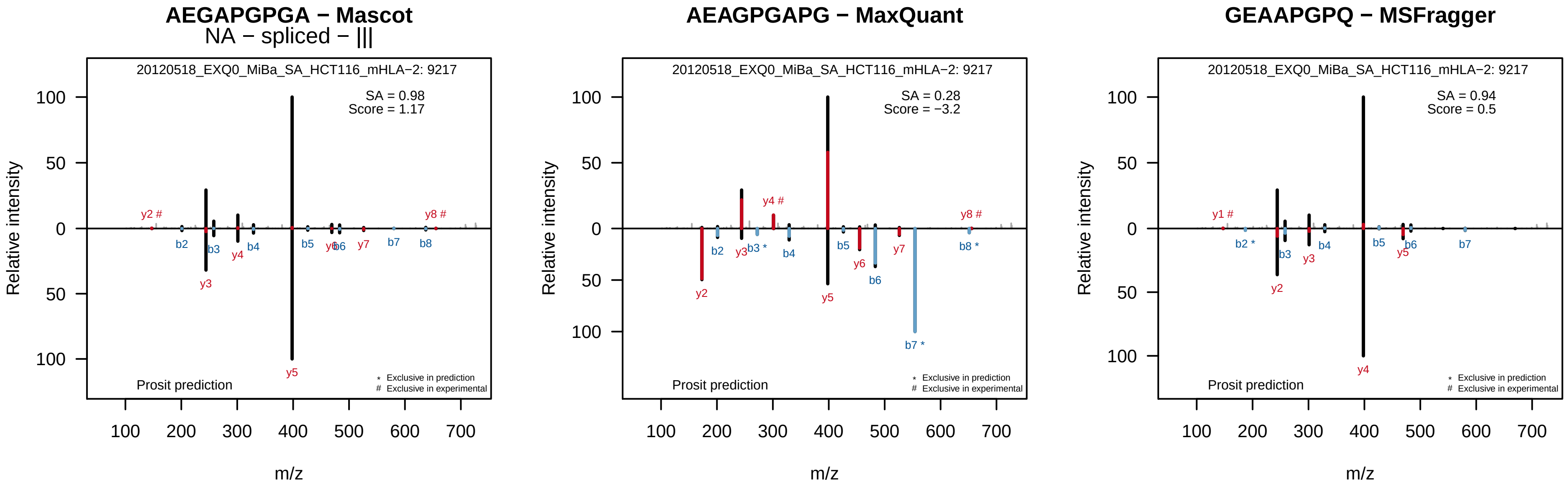
AEGAPGPGA − Mascot
NA − spliced − |||
20120518_EXQ0_MiBa_SA_HCT116_mHLA−2: 9217
SA = 0.98
Score = 1.17
Prosit prediction
⋆ Exclusive in prediction
Exclusive in experimental
Relative intensity
m/z
AEAGPGAPG − MaxQuant
20120518_EXQ0_MiBa_SA_HCT116_mHLA−2: 9217
SA = 0.28
Score = −3.2
Prosit prediction
⋆ Exclusive in prediction
Exclusive in experimental
Relative intensity
m/z
GEAAPGPQ − MSFragger
20120518_EXQ0_MiBa_SA_HCT116_mHLA−2: 9217
SA = 0.94
Score = 0.5
Prosit prediction
⋆ Exclusive in prediction
Exclusive in experimental
Relative intensity
m/z
